# Supplementary material for: Proteomic Analysis and Biochemical Characterization of the Nematocyst Extract of the Hydrozoan Velella velella
Source: Mar Drugs. 2024 Oct 12;22(10):468. doi: 10.3390/md22100468 (PMC11509761; doi:10.3390/md22100468)
Supplement: Supplementary file 1 [file marinedrugs-22-00468-s001.zip › Supplementary Figure S1.pdf]

Quantification of total proteins in the crude nematocyst extract

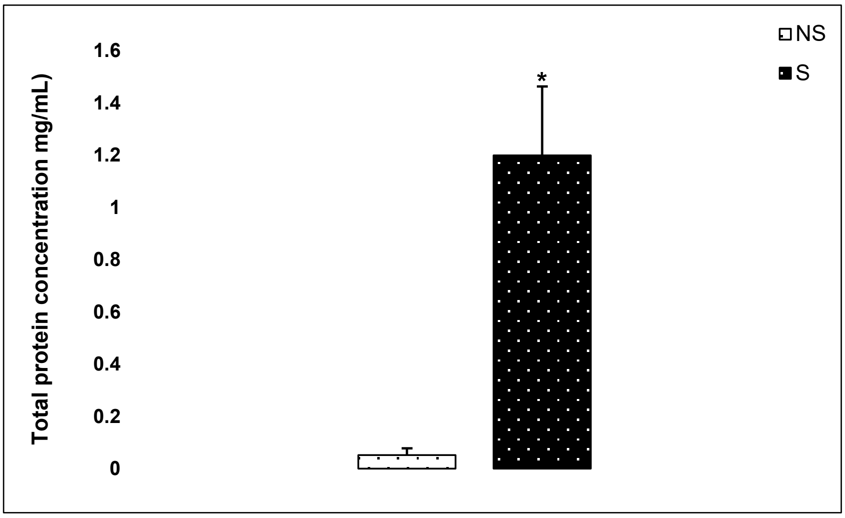

*Total protein concentration in the crude venom extract quantified before sonication (NS) and after sonication (S), expressed in mg/mL. Asterisks indicate a significant difference of the total protein concentration in the S sample versus the NS sample (one way Anova, * = $p < 0.05$).*

Collagenase activity evaluation

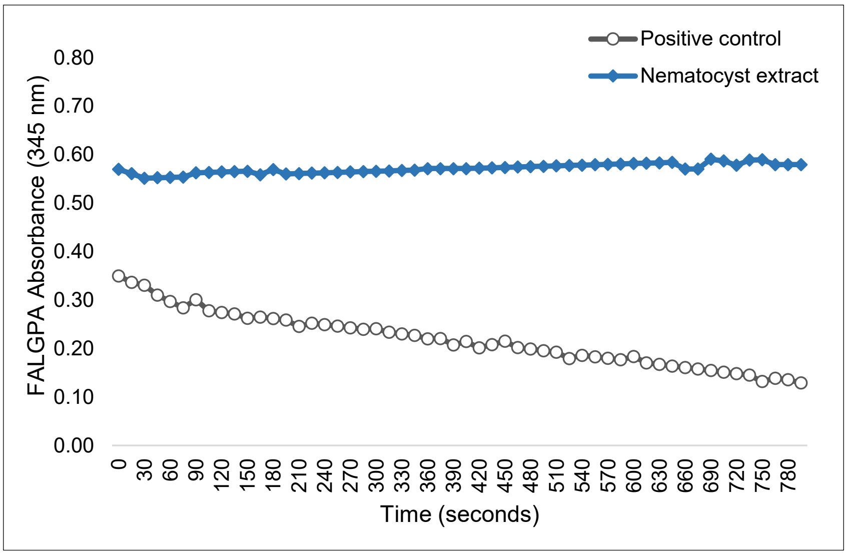

In vitro *collagenase activity. Enzymatic activity evaluated using FALGPA hydrolysis using 34 ng of crude nematocyst extract of* V. velella *sample (blue diamonds). Positive control was 0.04 U of commercial collagenase from* Clostridium histolyticum *(white circles).*

Zymographic analysis of active proteases

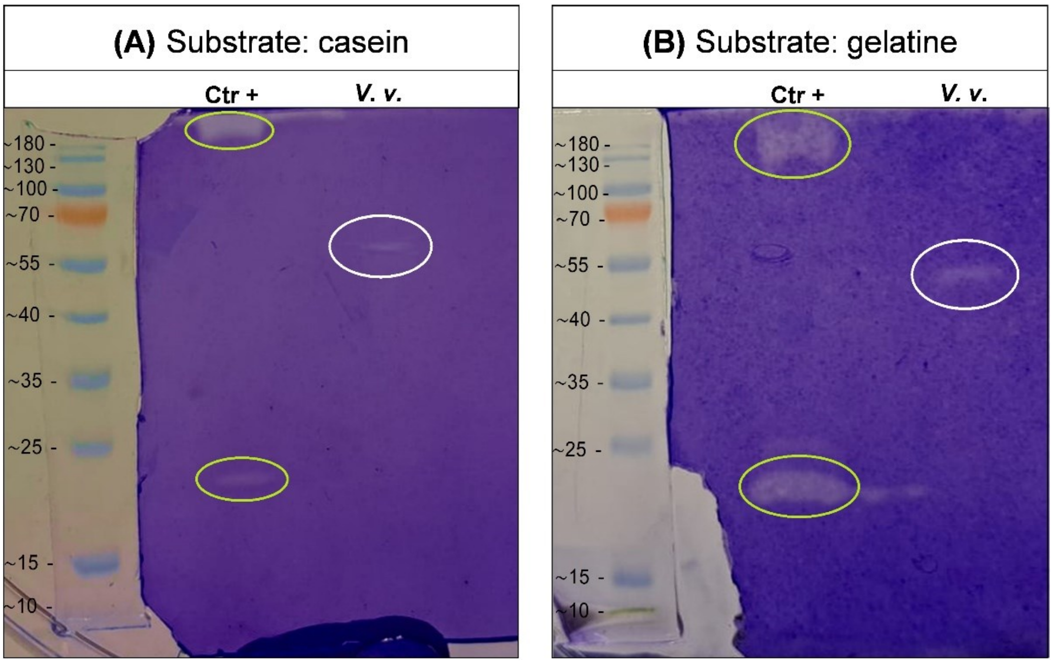

Zymography analysis of the V. velella crude venom extract using casein (panel A) and gelatin (panel B) as enzymatic substrates. The white bands indicating enzymatic activity are highlighted by green circles in the positive control (first lane of the gels), and by white circles for the crude venom samples (second lane of the gels).

Hyaluronidase activity assay

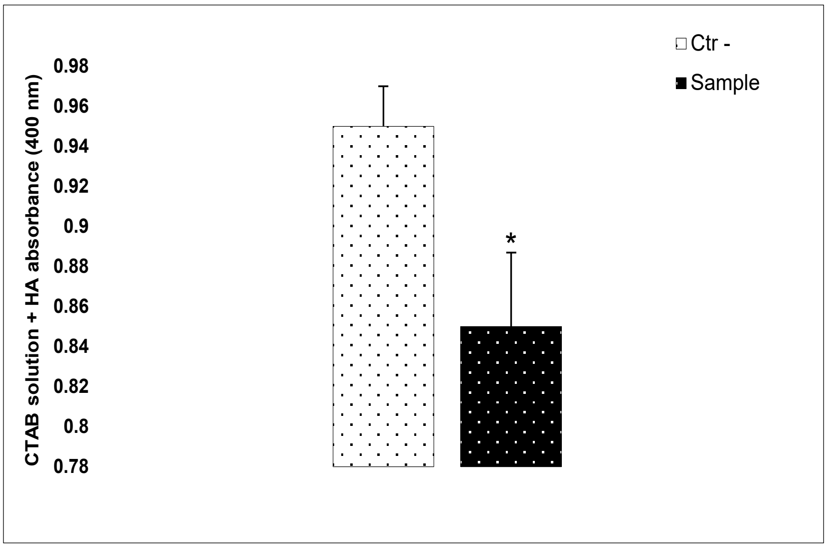

In vitro *hyaluronidase activity assay. The presence of enzymatic activity in* V. velella *venom (black column) was evaluated as reduction of 400 nm absorbance given by the turbidity of the undigested of hyaluronic acid (HA) in presence of a CTAB solution and compared with a negative control (Ctr -; white bars). Asterisks indicate a significant difference of the total protein concentration in the S sample versus the NS sample (one-way Anova, * = p < 0.05).*

DNase activity evaluation

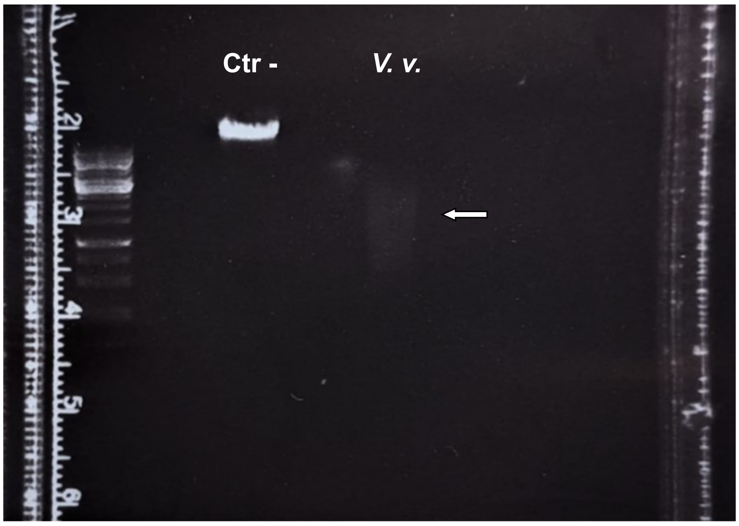

DNase activity of the V. velella *nematocyst crude extract by evaluation of the effect on* E. coli *genomic DNA integrity. (Ctr -)* E. coli *genomic DNA untreated sample, (V.v.)* E. coli *genomic DNA treated for 1 hour at 37 °C with 50 µg of nematocyst extract; blurred lane, highlighted by an arrow.*
