# Supplementary material for: Proteomic Analysis and Biochemical Characterization of the Nematocyst Extract of the Hydrozoan Velella velella
Source: Mar Drugs. 2024 Oct 12;22(10):468. doi: 10.3390/md22100468 (PMC11509761; doi:10.3390/md22100468)
Supplement: Supplementary file 1 [file marinedrugs-22-00468-s001.zip › Supplementary Figure S2.pdf]

## COMPLETENESS ASSESSMENT RESULTS:

| | |
|---|---|
| Total number of core genes queried | 954 |
| Number of core genes detected | |
| Complete | 750 (78.62%) |
| Complete + Partial | 859 (90.04%) |
| Number of missing core genes | 95 (9.96%) |
| Average number of orthologs per core genes | 1.38 |
| % of detected core genes that have more than 1 ortholog | 23.47 |
| Scores in BUSCO format | C:78.6%[S:60.2%,D:18.4%],F:11.4%,M:10.0% |

## LENGTH STATISTICS AND COMPOSITION:

| | |
|---|---|
| Number of sequences | 234123 |
| Total length (nt) | 115167979 |
| Longest sequence (nt) | 30058 |
| Shortest sequence (nt) | 188 |
| Mean sequence length (nt) | 492 |
| Median sequence length (nt) | 333 |
| N50 sequence length (nt) | 565 |
| L50 sequence count | 52502 |
| Number of sequences > 1K (nt) | 20150 (8.6% of total number) |
| Number of sequences > 10K (nt) | 18 (0.0% of total number) |
| Number of sequences > 100K (nt) | 0 (0.0% of total number) |
| Number of sequences > 1M (nt) | 0 (0.0% of total number) |
| Number of sequences > 10M (nt) | 0 (0.0% of total number) |
| Base composition (%) | A: 27.53 |
| | T: 27.89 |
| | G: 22.11 |
| | C: 22.47 |
| | N: 0.00 |
| | Other: 0.00 |
| Number of gaps (>=5 N's) | |
| GC-content (%) | 44.58 |
| Number of non-ACGTN (nt) | 0 |
