# Supplementary material for: Proteomic Analysis and Biochemical Characterization of the Nematocyst Extract of the Hydrozoan Velella velella
Source: Mar Drugs. 2024 Oct 12;22(10):468. doi: 10.3390/md22100468 (PMC11509761; doi:10.3390/md22100468)
Supplement: Supplementary file 1 [file marinedrugs-22-00468-s001.zip › Supplementary Figure S3.pdf]

Band 1

>TRINITY_DN244_c0_g1_i1.p1 type:complete len:3019 gc:universal TRINITY_DN244_c0_g1_i1:9748-692(-)

MDARGQRLLYCLLIFLSLATLLVSQQVEKCKRPVDLVFALDTSGSVKDSGFRLIQKFAKDILKTFDI
GVADSHVGVLTFSEQAEPQIKLTETFDKAELMQMIDSLVYQGYRTATDDALRVAKTDFFSTAGGA
RQGVPHVLVLLNDGKCTVCIEPVKEAAQKLKDMGVEIFTIGVTENIKKQELIDIASQPTAYHFFEL
DDFDQLVDVVGDIQTRSCEVKKGKCPIPSPTEQCSGNEINECTSDENCEGELKCCSDGCSRICKYP
LLNCFTPIDIAFALDSSSSIQPETFQEMKKFSNDIVDSFAVSQKKTRFAALVYSDRAEIGFNFVRFDD
ASSIKKAIQNLPKLDSKERIDYALELAKSDIFSLQGKVRTRRPMVLFVFYDGTVSSGMKDLSLVVEP
LKNYGVKIVSMGVGPETNYYQMRKISSNNSDVFRATNFKELLPEIYNIAKRACTEKPGRCDASLN
PLPKEACKNVKILNQCENDFDCFGDAKCCRKGCNNVCVIRGPVCPRKLDIALAVHQNMDESEF
AATKFYLQSVVDQFNVGPSQTHFSLLSFGQEPKVIFDFNSDQSSNEIVRRKIDNFQYQYGIGRISDV
LALSCDAVFCAAGGTRDDVPKMLVIVTSRTSPSSPSKLKEKLQTLRNRGVNILMIGVGDVDTSLLQ
AITSDDPLVEDRVLLSKRFDGIFQYTKDISDYACGMAPALPPPDNIKGDAKGECGAPKSCYKGPKG
SRGRPGTLGQQGESGDLGLVGPKGIQGRRGDKGPDGQMGSKGKRGPDGLIGLPGFPGYNGIPGV
PGIEGRRGRDGDCGLPGSSGEAGQAGLDGLSGPPGRAGPVGEKGEKGLPAPENPNIRIEPGDRGD
TGSVGLPGFKGIKGEEGFKGPSGDSGVDGIKGVDGEAGEMGDIGTYIYQKGPEGDRGARGPPGD
GDTQGATAGLGVQGEKGEKGMRGEAGDGSIFPGIKGRRGDQGPKGEEGSTGPAGPTGQRGPEG
EEGPKGIKGEIGDRGKDGTPGQPGQEGDLGEKGFDGLKGQDGDDAEGELGDSGGKGLKGENGL
AGSPGSIGTPGPPGSKGEPGDIQIGLSGDPGAIGPRGPRGDNGISGQPGMRGDSGPKGAKGNQGP
RGDRGRNANMLAGKDGMTGPPGPKGFTGPVGDPGFPGPPGPPSVVECVEGACPKGVPGAMGQ
IGKTGEPGPPGPPGPRGSKGVAGGPCPICPDGIQGDSGDIGQPGMPGKEGSRGRKGNIGDAGSKG
LQGRNGDDGDQGPVGKKGFIGDIGPVGENGVDALGDSVAPDGEKGSTGDTGQIGSRGPVGNDG
PKGFKGTIGERGRRGLQGEKGDMGLTGETGEDGSRGLEGAKGDLGDGLRGAKGDKGNIGQEGN
QGVVGDVGLQGPKGEQGAPPTEDELKNVKATKGSKGEIGSTGDAGEIGDIGPAGPRGPKGFDGA
AGPKGPSGVKGDRGNKGRAGDGEPGFQGFPGRPGKRGPPGDPGKPGKRGFSEPGDAGDRGDP
GKSGKKGADAPPPIGCFKFNEPTDIGFILDSSSSVDDDNFLRQLQFVKNIVRQYPVGPDQTQVGIIK
YGEKAVTGINFYDYVTEEGLFNAIDLLEHTKESESRLDLALKLARDELFTESVGARGFDAKKALVIL
GDGFISGGGTPSKTLMNNAMQYAEDLRGAGVSIFALAVGPEKNKALFHEFVSEKEYYLDIESYSQL
DNELGALQLSLQTGCISVGVPGRDGLPGYTGPKGQKGDDGPKGFSPQGPRGIKGIKGVRGTIGIG
VKGLPGDPGLQGPQGEKGRDGEDGSADPGPKGRPGDKGSRGDQIKGASGEDGDIGPIGPPGFDG
RKLCDNVLAVDLLFVLDTSSGVGVDNFNKMLKFIESIINVFDVDNQLTRVSVMTYDSDARLVIRFS
DYNDPDKLDAAIDNIPYANGDETRIDKALLLASSNAFDTSNGAREGVNKAVILMTDGQQSFAVD
QVALKDAVKPLVDANVLMYAIGIGSEISPVDLKTIAAERVLIADDFDGLSLKIEDQIVLLGRVGCQG
PPGKDGKPGANGEDGFPGLIGAKGQKGDRGEAGTEGLKGNKGSQGEAFPGLNRKGEKGDPGS
AGPLGLPGCEIKNQNEVIDLGFAIDASGSLYEEGFNREKEFIKTIVDRVGRVSEQGIRIGVIVYSDTAS
IEIRFDDHFNNEDLKNDIDNLPFFAKNTRMDLGLEEAKKMFGEQFGGRGSSKKVLILLADGQQTY
VPGVTEPSEVAKELAAAGVDIFAIGIGQEIDRTELESLISKPEHIFLASDISKLIDELTPEVTSALECEG
GPRGPRGRAGTDGPPGSKGLLGPIGDKGFPGEPGSAGFGPKGDDGENGTAGTIGDRGEKGFRGP
AGVPGKRGAVGPKGFPGPNGEKGDRGDEGFIGFEGARGLEGPKGQRGDSSDNGVAGVKGSTGD
NGFKGPKGDPGKLPPGNRIEDFKGETGGSGLEGDTGPKGKPGRALFEPGPQGLTGPKGEKGDQG
FFGMSGPIGNKGFQGLQGFRGDDGAAGPVGEDGFPGMKGLPSSSGDQGDKGKNGRKGESGEV
GQPGRVGEDGDQGEKGYAGFVGMPGKKGDQGEPGVQGPQQIRNEILDNIRPGPKGYPGSTGPA
GDEGEDGEVGEPGETVGAQGMNGDFGERGSQGPKGEQGDQGLQGMIGVIGDKGVKGRVGTG
GNPGTVGLRGLTGAPGEVIFGSEKGNKGDLGIIGLRGRAGQKGKAGAIGPRGRRGRNGAKGERG
PPGFPGLSGIEGDSGNEGDKGPRGDPGSVGNRGIKGVPGESGIAEGNYIVIHSQTHIIPDCPRDFKK
LWHGYSLMYVVGNGHSHSQDLGDAGSCSQKFSSKIFLFCEIIKVCKHASRNDFTYWLAGDVDHP
MMPVSNAAVEPFISRCAVCEAHSSHITVHSQDTYAPECPPNWSSLWSGYSFLMVAGAGKTGAGQ
SLSSSGSCLESFRPKPFVECQGARGTCHFFSDKHSFWLVTIDPNKQFEVMKPQTLIKDKNHDLTSR
VSRCRVCIRKTGTKNNTVEPNIATRIRRSIASWIL*

>TRINITY_DN1369_c0_g1_i1.p1 type:3prime_partial len:1577 gc:universal TRINITY_DN1369_c0_g1_i1:93-4820(+)
MRIQLTIFVTFSIILVSGQNSPPGICSTWDGSHYQTFDSYSFNYHGTCSYALALDCRPGSDDFAVHV
ENDEKCDNHGGTDCTRAVSLYVNDIEYKLGSNNTVLVDGKSNIVLPYFNEGVRVSKHAEYYFIDA
LEGKVVVKYDGRSSIYVQLDSSYQAGVCGLCGNFNGNKKDDLKLPGDKGLTKSLKEFGKSWEKP
RIGQVCKEVTEEHFDQCTNKSKMLVYYAEKECEILRTSSEFKKCHKIVDPTPFYEKCREDLCLCGS
GDKYCKCDVLEQYTRKCAHYGTVLEWRQQNLCYKNCTGNKVFSECGPACSSVCSAIGSKSVCK
DACIDGCHCPPHTSLSGDKCVPDDECPCEHNGDMYDHGDVINMKCKSCKCNGGKWKCNPLP
CDGEASVYGNGHFVSYDGKTYNYPGKCLYLYTYLSDRSFAVYLDRTSCDSLACHMNVMILFRGD
RIKLSASNSTLALEVNRNVKSFPYISSTGYRMYRFSTVFVRFEATNGIKVDWDTYSRLHVLMPHSL
KGKVFGLSGNFDFKTKNDFLTPRKDLAHSAIEFGNSWMMYSSRLNCSALAPDYRMTSCEANHQ
VAKYAESKCSILMSSLFRECHKVVDPFIYYENCKDDTCECNGDSKCYCSVIAAYSKACSSAGKPTE
GWRKYTPCEEKCSNGMVFTECGNSCLKTCDDQTDPNPFCEKECVEGCLCPVGQVLDKITEQCV
DIKHCGCTYGNRTISNGGTVAKDCNTCKCTKGVMVCTKKECRDKKAICSDGKEWSSCMSPCPK
TCQTKDDICPETGLDCIEGCQCKKGYIESANGTCVELSKCPCYHNGKEYQETQTISKDCNTCKCT
AGEWSCTEKLCDATCSVFGDPHYKTFDGKIFDFQGKCGHVMLTDKCANNDNATENIRISVKSST
CGSQGVTCTREITLNYHDTVVHMKRGARKIDIVSNKRQDHEKDQVKVYRHAGIYVHVLTPMGV
SILWDNATRLYITAPPSLSGKICGLCGNFDGSETNDFVTYQGDTAGNAVSFGDSWRTENNCPLTKE
IEDTCHNRKHRLDPSKRECAVIKSDVFKKCHTLVDPVPYYDRCVFDACGCDSIGDCDCLCAAIGA
YAKECIDEGVYINWRKGHNICEKEMCCPTNMTFVPKGTGCPKTCKYPNGLHHCPTKYVDGCQC
PEGLVQNNDANGDVTCVRPEECKVCKLDGKIYNEGEVVLYKCKECVCRDGKMTCSGNYCPTTL
PPTTTTESTTTPSTTPETTIPSTTTTESTTTPTTEPPTTTTTASTTTASTTTESTTTEATTTTKPTTTTTTEST
TTESTTTQSTTTPSTTPSTTMKPIDTCVYSNGTHNVTLERGDVIQVNECTKKTCVKDEVTGLHEIKV
TTTQCENSTCDTTCEASKSNRAILVKTPGECCPQCKKPEDLCDNPNEVPCNSTYCIPKSWFCDGN
KDCSNGRDEECCPNYCKVEGEIKKPGEVWTNKEKCLTCKCEGYYTKCRRMECPELDCPEERQVK
SEGECCPHCKPQTEPPTTTTEMTTTTTESTTTPATTPSTTSKETIIITEEPKSTTASPQEKCDCKVLQPV
GVSDGRIPDNQMTANSV
>TRINITY_DN1676_c0_g1_i12.p1 type:complete len:1631 gc:universal TRINITY_DN1676_c0_g1_i12:206-5098(+)
MLIITRTAKLLLLSLTYIYLTIAQDLTNATLNNGFEAGRAIKCKTDDGMVQRCMPVFENIAFGRDII
ASNTCGQTRQTYCVQTGTSGAVKICEVCDASDPDKAHPADLMTDLQFDDQPSWWQSETILDNK
SPVYLTLNLNKKYNVAFIRLRFHSVRPHSFAIYKKSTYDSNEQWLPYQFYSRTCQETYNVPNQQSV
SYSNQKLALCTDEASGMIPLSGGNVAFLTLQYRPGKRDFDNNPGLQDWVTVTALRFELDRVNTF
GDEVFGDPKVLRSYYFAISDITVGGRCKCNGHGSSCNNNNNEGTFQCVCEHNTAGKDCEKCLPI
YNDRPWRKASGRSANPCKACDCNGLADQCEFDQQLWVDSRYRSGGRCVNCKNNTDGINCEYC
KEHHFRSNVKEACKPCRCNPLGSRSLSCTTKGECACHPGVTGLKCDKCKEGYYGFSSSGCKACN
CDRDGSIGVNCDFTGQCNCKKKTVGKRCDQCAPLTFDLDRSNPKGCKECFCFGHGVSCDAIEGI
GVKEIFTTFEADFDGWKLQDESGASFDSNMEWDFRGQYISVKPPRNKQLYLVAPQRYLGNKLSSY
GKLFTFAYGIFLDPNDPLVSAGRDIIFEGNGLQAVYPINSQNNILPEARFYQFKYRLMEPVGMSTFD
FQKILVNLTAVKIRTAYMNERTGAIDNIKLYSTKFISINSPEQVTWQEICECEEGYTGDHCEKCDSGY
TRSGGDTSPFGECKKCFCNNHGNFCNPDTGICQCLHNTTGDSCDQCAPGFYGNPTAGTPEDCK
PCPCLYGNECILIGNSVKCTDCPEGHIGDLCELCDDGYFGDPNGTIGEPSRCQKCDCNGNIDPNAI
ANCDSLTGSCLKCINNTKNGPENKCELCADGFYGNSTIDPKGNCSACSCYEKGTMKPPFHDPKL
QLPCDTNGKCECLQNVIGDKCDMCPPGHWNVASGKGCELCNCNATGSKGSDCNLQTGQCEC
QDGVGGRKCDQCAPRFYGFSKDGCIACNCHPDGSKHQNCTQDGVCECLPGVLGIKCDQCPEN
KYNLSVGCIACPRCFDLIQVAVNKLRNKLKTFNITGNLTTGSIDIKDPEFEKLFNELKRNISELEQK
MKDLLEGDMAILEFYEVLVNQSSTLIEMIPGIQKMIKKSEGAAETGSVTISKAEEITEEIQNLLGQAE
KMLEAETGRIFKNVTSSSEDVSGLSKRMSEIAEEARKHADRQEQESKDIEFNTDMAYNNSKAAYE
QAKKARDTEEAISYLLDQMALGHDEALALSKNSTEIIAKANSTSIAALNDSDHLMMKAKEPLPN
LDIEAMKEKAKNIDAEGKATEFNATDLHEKNKDKITQLQNDDKEARELMERGTQLHNDSNALY
KIAKNASDEAKQAQEDGLKVAKDAQDMLKTLMEFENNIAKSKANATEALKLIGLIRRLISKANK
TTENASSNAIMASINATAALRLASAANKTTQDAMNEIESSRREVEKLLNETMDFMMKEVAQTKD

DIDDIEKSLEESEKLAKSNSMKLGDALNDSQAATDKASMVQNELLKSLDQIEDLMNEISNLDEIDF
ETLEDAEKRFEEAKDTIENKLKNEIEIMESKLSDQKKLLQQHELDLTPLKEQIMKNEELFKYLPKSC
FREKAKPEDIDK*
>TRINITY_DN1676_c0_g1_i6.p1 type:complete len:1631 gc:universal TRINITY_DN1676_c0_g1_i6:206-5098(+)
MLIITRTAKLLLLSLTYIYLTIAQDLTNATLNNGFEAGRAIKCKTDDGMVQRCMPVFENIAFGRDII
ASNTCGQTRQTYCVQTGTSGAVKICEVCDASDPDKAHPADLMTDLQFDDQPSWWQSETILDNK
SPVYLTLNLNKKYNVAFIRLRFHSVRPHSFAIYKKSTYDSNEQWLPYQFYSRTCQETYNVPNQQSV
SYSNQKLALCTDEASGMIPLSGGNVAFLTLQYRPGKRDFDNNPGLQDWVTVTALRFELDRVNTF
GDEVFGDPKVLRSYYFAISDITVGGRCKCNGHGSSCNNNNNEGTFQCVCEHNTAGKDCEKCLPI
YNDRPWRKASGRSANPCKACDCNGLADQCEFDQQLWVDSRYRSGGRCVNCKNNTDGINCEYC
KEHHFRSNVKEACKPCRCNPLGSRSLSCTTKGECACHPGVTGLKCDKCKEGYYGFSSSGCKACN
CDRDGSIGVNCDFTGQCNCKKKTVGKRCDQCAPLTFDLDRSNPKGCKECFCFGHGVSCDAIEGI
GVKEIFTTFEADFDGWKLQDESGASFDSNMEWDFRGQYISVKPPRNKQLYLVAPQRYLGNKLSSY
GKLFTFAYGIFLDPNDPLVSAGRDIIFEGNGLQAVYPINSQNNILPEARFYQFKYRLMEPVGMSTFD
FQKILVNLTAVKIRTAYMNERTGAIDNIKLYSTKFISINSPEQVTWQEICECEEGYTGDHCEKCDSGY
TRSGGDTSPFGECKKCFCNNHGNFCNPDTGICQCLHNTTGDSCDQCAPGFYGNPTAGTPEDCK
PCPCLYGNECILIGNSVKCTDCPEGHIGDLCELCDDGYFGDPNGTIGEPSRCQKCDCNGNIDPNAI
ANCDSLTGSCLKCINNTKNGPENKCELCADGFYGNSTIDPKGNCSACSCYEKGTMKPPFHDPKL
QLPCDTNGKCECLQNVIGDKCDMCPPGHWNVASGKGCELCNCNATGSKGSDCNLQTGQCEC
QDGVGGRKCDQCAPRFYGFSKDGCIACNCHPDGSKHQNCTQDGVCECLPGVLGIKCDQCPEN
KYNLSVGCIACPRCFDLIQVAVNKLRNKLKTFNITGNLTTGSIDIKDPEFEKLFNELKRNISELEQK
MKDLLEGDMAILEFYEVLVNQSSTLIEMIPGIQKMIKKSEGAAETGSVTISKAEEITEEIQNLLGQAE
KMLEAETGRIFKNVTSSSEDVSGLSKRMSEIAEEARKHADRQEQESKDIEFNTDMAYNNSKAAYE
QAKKARDTEEAISYLLDQMALGHDEALALSKNSTEIIAKANSTSIAALNDSDHLMMKAKEPLPN
LDIEAMKEKAKNIDAEGKATEFNATDLHEKNKDKITQLQNDDKEARELMERGTQLHNDSNALY
KIAKNASDEAKQAQEDGLKVAKDAQDMLKTLMEFENNIAKSKANATEALKLIGLIRRLISKANK
TTENASSNAIMASINATAALRLASAANKTTQDAMNEIESSRREVEKLLNETMDFMMKEVAQTKD
DIDDIEKSLEESEKLAKSNSMKLGDALNDSQAATDKASMVQNELLKSLDQIEDLMNEISNLDEIDF
ETLEDAEKRFEEAKDTIENKLKNEIEIMESKLSDQKKLLQQHELDLTPLKEQIMKNEELFKYLPKSC
FREKAKPEDIDK*
>TRINITY_DN262_c0_g1_i1.p1 type:complete len:1033 gc:universal TRINITY_DN262_c0_g1_i1:111-3209(+)
MADPSSLETKEKDGSYRVATKSGGKDKGKKSKKAAKKVNMDDLKKELEMTEHSCELETLVSQLE
TSLTQGLTKSIAARNLERDGPNALTPPKQTPEWVKFCKQLFGGFAMLLWVGAILCFFAYIIRSIREE
EPGMDELYLGIVLTVVVIITGCFSYYQESKSSKIMESFKKMVPQEAVVMRDGEKLNLNATECVVGD
LVFVKFGDRIPADLRIIESKGFKVDNSSLTGESEPQSRNSECSSDNPLETRNLAFFSTNAVEGTATGIV
VKTGDNTVMGRIANLASGLGSGKTPIALEIEHFIHIVTGVAVFLGVSFLIISLAMNYHWLESIIFLIGI
IVANVPEGLLATVTVCLTLTAKKMASKNCLVKHLEAVETLGSTSVICSDKTGTLTQNRMTVAHM
WFDNMVVEADTTEDQSGQAHDKGSTTWKALAKIGALCNRAEFRPNQEEEPILKRECNGDASET
AILKFVELSVGSVEDIRSKNKKIVEIPFNSTNKYQVSIHEQEDPDDPRYLLVMKGAPERILDRCSTIL
HEGVEKPLDEEWKENFNKAYVDLGGLGERVLGFCHYYLPADDFPKGFAFETEDPQNFPLEKLCF
VGLMSMIDPPRAAVPDAVSKCRHAGIKVIMVTGDHPITAKAIAKGVGIISEGNETVEDIAQRLNIP
KEEVNERDANAAVVHGSHLKDLPQEKLDDILKYHQEIVFARTSPQQKLIIVEGCQRMGAIVAVTG
DGVNDSPALKKADIGVAMGIAGSDVSKQAADMILLDDNFASIVTGVEEGRLIFDNLKKSIAYTLTS
NIPEISPFLMFILLAIPLPLGTVTILCIDLGTDLVPAISLAYEKAETDIMKRKPRDPVRDKLVNERLISL
AYGQIGMIQATAGFFTYFVIMAENGFLPGYLIGLRNDWDDKKNNAVMDSYGSEWTYNQRKNLE
LTCHTAFFTTIVVVQWADVIISKTRRLSLFQQGMTNWMLNFGIFFETALAAFLQYTPGLNTGLRLR
PMNFPWWFVGVPFSLLIFVYDELRRFLLRRNPGGWVEQETYY*
>TRINITY_DN15274_c0_g1_i1.p1 type:complete len:1822 gc:universal TRINITY_DN15274_c0_g1_i1:87-5552(+)

MECIRRSIIRLLLILGFYTLYISSQEECKRGGCFPATGDLLVGREKQLLANSTCGLNGPSKYCILSFLD
KTDKCFYCDSRRQVDERPDGYKNSHLPKYMVTREPSKRLQGWWQSVNGRQDVTIQLDLEAEFIF
THLIMTFKSFRPRAMYIERSSNYGNDWSDYRYFAYNCRKSFPNIPRGPQRKIDDVICEERYSKVEPS
TGGEVVMKVLDPIIQKKEDPYSLKVLNLLKLTNLRVHFKELHTFGDDILGDSPKVNQKYYYALYE
MVVRGSCSCYGHAEQCIPTKGTPANVNMVHGQCNCTHNTKGRNCEMCKDGYNDEPWRPAYQ
DQLNVCRKCNCNGHTDKCHFDPAVYKATGNTSGGVCDDCNHNTAGRQCELCKPLYYRDPYKN
IFDEDVCKPCDCDPSGSFGGGECETISDPNNSSALTVAGRCICKPNAQGLRCDSCKDGYWNLLES
NPDGCQSCNCDDKGTVELNKCDQQTGTCRCKRFVIGLTCSKCLEGFWNLGNTPEGCEPCNCDV
GGSLNAKCDDITGMCMCKPNIIGRKCDQVAPGSYFGNLDQVISEAENSLVRSDSSSVEIVREILNPT
DSRSFTGDGYLRVSDGTLVNFTNIRVPSTGFQTFVMRYSRPTPGSNNNVMVRITKTDGTILGGNGC
VQTNEREIILNGVLNDDNFLELGDMFCLEKDTDYSVQLVVSNRNSPNDGGVNRGVTNLDSIVM
MPNIDNLAIFQGREGMRLKSQYDLNNCRQYYLEPNGAANAPEICKKIQFSISAEINNGALKCNC
DPTGTNSSGSSDILTCQKSGGQCPCKPGVVGRRCEKCAPGYFGFDSNGCTACACNDTGSEKLFCN
GITGQCPCKNRIKGRQCNSCPSNHFGFPNCIACQCNEHSSTCDPISGECTNCKDNTAGPSCNLCA
EGYYGDATAGTEKDCQRCQCPGGSSLNQFSNTCHLKPDGKVVCNNCTEGYTGDQCDKCEDGY
YGNPLVPNGSCNKCDCNGNIDPFADGKCNAENGKCFNCINNSDGDHCENCKEGFFGNAKNQS
CRACVCNKLGTNSATSDSCDRETGQCECLPNVEGVECDRCKSGYYNIASGNGCVSCNCDKGGSL
SSHCNQLDGQCNCLNGYGGLDCTQCEDFYFGDPKQNTCQACGCNPVGSITLQCNRTTGVCMC
KDNVVGEKCDMCAPQTAGVMPQCEECHVCNDQWEDIVNDIEKNITTILTLKGSSPLNVSEIRAY
TNDIADLKNRLDKIEEDFGKQMVDPNKINRLRDKVYRFSYNLTGINRDADFYMEMVNETITRDE
KANDILDGLELRLATLQTNTEDFKNNVTKIVESNIGGALNSTRESLEKSRKAQESVNDSDLKIKES
KKMRKKIKKEIQNGDPEFEVMHNRSSEFVNNLADRENYLEGELEKLNRLLCGSDKRCGGCTKGG
CDTCGVSGENYTCDDGIKMMSMMASDKALEAKEAIDQKLENASELLENVNRAKQHVIQANISA
NMAEDKAEEVNKIGQAVLKNITTLIKDIQDFLATDFNHPNASKDLAMQTIALNISLTPDEVVELG
RKIRDAVKSLTGVDKILNESRNDYDTAIGLKEQALAAKERAIEVMNQTMAIIETLSQAEELQNSTL
AGVNVARADIEDVKDLLKKLKEKLDAADNDLQMADNSTASAEDKRNAILDQNLENREKLDLSE
KKIDEAEELGDKVQGSVDKMNEKYNATKAKLNEKEDMIEKVRARIKALADKTIKLYENADRKLR
RINKMKDSYIINEERAKDLYYEIRRLEIKEFFINTKLEGTAKCYSTCNPIIPESFCRDVYKNIAVKETN
AYAELEAEKASTTSLLI*
>TRINITY_DN319_c2_g1_i3.p1 type:complete len:2588 gc:universal TRINITY_DN319_c2_g1_i3:165-7928(+)
MVLLKGQLWILGFIFLTSTKYVMGDVPECSNNATVAVHCRFDEGNTCVDDPHPMHYVCVCNNL
GYMNDTDSVEGYSICKERDCSNSNETPTDLLKRICGVNTKTCTGKILQRECNCNTDSYRSKNGQY
CFFAPCQASPTIQQNCGPGNQCYGNAPNYICDCSARGYKRSAADNRHCELKSDEELTPIEEYCGK
GSLYANGTINNITCTCDTDKGFFPSATGETCISPSCYLNPQMQEKCGPENTCLGGKDDYVCLCSNP
NYKRNATDLQHCIRRTCADTSPAIDFKSICGEGATGCSGYVDKPLCSCDVSNHYVPAADGRSCIK
ATCKGNKKALEQCGQANSCTGSFDNFLCVCDANGYYLNTTNNTCVERECTDPDLVITDICGVGS
LSCNGKAGSPECICDKDKFYYASEDLRSCVKANCLSSETISKKCGKENSCYGSMESYVCNCNAPG
YLLNTTDNQGCYLGMCEDNGLEYDKVCGEGAISCNGTIKNLECNCGMNYVQSPDKKSCIRADC
RANQTMHEQCGEDNDCVGSLEDWMCICSTYGYKPDPMDMMKCKKVMCEEEVVNNVTVCGL
GTTGCKGHILKPECTCDIAADYVPNEDASRCIKGSCENNKMAMKDCGIANSCTGSYNNYVCVC
NGFGYKKLDDTHCVEQMCSDPDIDIRNVCGEGAHYCNGTVNQRVCSCNETEYFYPSADSKSCV
KATCAVSPMVMEQCGVENSCQGSQESFICNCNALGYLLNTTTNQHCYLGTCENGDFDLMQAC
GDGALYCNGTIKNLECQCDESKGYVQAPDKMSCIPNDCRGNLTMYRQCGQLNKCVGSIDDWR
CICDSPYYVAHSDIMKCKLLHCSDSEVDHVEVCGKGTIGCSGSTLHPTCHCNTTENYFASKDGMR
CVAGSCKTNQTAVKKCGNANSCAGSHSEWTCICDGIGYKHDPADPQNCLLKTCNDADIDIVATC
GVGAISCEGYQLMPKCECNDRDYYIPSDDRTSCELASCRANRTAMRACGEMNDCRGSFSSHICL
CHDPAYKSDPSDSRVCLEKTCEAGDFELVRVCGKGATGCSGSMKNIMCECDNAKGYVASADHT
QCIPSDCNNNVTMHLQCGEGNGCVGSLTDWYCVCSAPGYEPHASDHMKCHLKTCADPGVDH
VQICGMGATSCMGPVINPVCYCNESMYYIPSMDRTSCIKASCMSNKTAIAQCGSENSCSGSPDNF
VCLCQAYGYRQDTNNNQACIPSQCEDAGIDFVEICGQGGVGCSGSVLNPVCMCDHMKHFVPSP
DQNSCVLSTCRGNETAMTSCGEQNKCFGSSSSYICVCDAVGYKPHPTNLQECVLQTCEANELNP

VAVCGKGALSCKGTIQSLMCECDNSKGYVAAPGYKSCIPADCRANMTMHAQCGTGNRCRGSM
EDWSCVCDGIGSKPHPTDHMSCIAIDCSDPNVDIKSVCGYGATGCMGLARDPICHCDTSKYYVQ
SEDGRTCVPATCHTNKTAQMQCGPDNSCIGPPNDFICECTGFGYRHHETDKQKCVPIQCNETAL
FDIERVCGKGATGCRGNIRQPICDCDHAMYFHPSEDRRTCIEASCRRNSSIRADCGVENTCFGSTR
SWICDCNSYGSILNPSDVKKCMLATCEAGQLDPVAVCGKGALSCKGSMRDVQCTCNNELGYVH
SPDGKKCLPSDCSGNVTMHHQCGDANKCMGSLTDWVCVCDAPGYKPHAKDKMKCHQISCAD
NNVDPVDVCGIGATSCKGPITNPLCQCDTDNYYITSSDGASCIPATCKTNKTAIHQCGEANSCEG
PADNFICVCNAYGYKLDDNDHQRCVEISCDDPNAHESICGTGSTGCRGTARDPICECDHTQHFV
ASSDLTSCLLASCRANDTVMNHCGDGNTCFGSLTSHICVCEAFGYKLNPNNHKHCLEETCEDD
MLNPVAVCGKGAVSCSGPLKSVKCECDNNAGFFRSPGGMKCESTSCRQNPASQMMCGSQNQCY
GSYDDWYCDCSAPGYMANQTNAAECLKVMCDDMAVDVCGLGATGCTGHTLNPICTCDSSMG
YMMAKDKHSCVPMDCSMNPASSQCGYGNTCHGSYFSYVCVCSTAGTVMNKTNPMECINVACP
ADAVHVCGEGATNCSVTSTNNVECSCEEGYHPADDLHSCVLDDCRFNPKALKACGLLNKCVGS
YKSWKCNCHYPEFTQDPMDHQTCVVGPCMVEQCGKGNAPGSFQHYAVNCSGLYDDPICNCQP
EYTNDAKGGCIPKICSENPVAVQACGSGYCEGTYFKYTCKCDEPAFVQTPDKMQCVPAKCNIQQ
CGHGFDRGLYIDFAESCSGTYADPMCQCKTGFSGTNTSKCGPAIDGCKLDKCDPLATCQTLDVD
GNVKCTCKEGYSGSGMMGECYLDGGFSAYEGTKECSEVCGVNGVAEVTRFCNNPTADPKGKPC
NGTSPETIQVPCNRFACSSCTVRKTCGCGGLKPIFVEFYHEPNSTLEYFTDLKADDVQTALGFLTK
WKELSQQDLCLMYSKIELTVFVTLMGDSETQVSTMKSSLVSARDIMRKKLDCNINLGKKAQYYEA
YETIIKRHLAISNILKDLTIIKNSITKYMNNCDDSEIGMIRDVIGMM*
>TRINITY_DN319_c2_g1_i1.p1 type:complete len:2588 gc:universal TRINITY_DN319_c2_g1_i1:165-7928(+)
MVLLKGQLWILGFIFLTSTKYVMGDVPECSNNATVAVHCRFDEGNTCVDDPHPMHYVCVCNNL
GYMNDTDSVEGYSICKERDCSNSNETPTDLLKRICGVNTKTCTGKILQRECNCNTDSYRSKNGQY
CFFAPCQASPTIQQNCGPGNQCYGNAPNYICDCSARGYKRSAADNRHCELKSDEELTPIEEYCGK
GSLYANGTINNITCTCDTDKGFFPSATGETCISPSCYLNPQMQEKCGPENTCLGGKDDYVCLCSNP
NYKRNATDLQHCIRRTCADTSPAIDFKSICGEGATGCSGYVDKPLCSCDVSNHYVPAADGRSCIK
ATCKGNKKALEQCGQANSCTGSFDNFLCVCDANGYYLNTTNNTCVERECTDPDLVITDICGVGS
LSCNGKAGSPECICDKDKFYYASEDLRSCVKANCLSSETISKKCGKENSCYGSMESYVCNCNAPG
YLLNTTDNQGCYLGMCEDNGLEYDKVCGEGAISCNGTIKNLECNCGMNYVQSPDKKSCIRADC
RANQTMHEQCGEDNDCVGSLEDWMCICSTYGYKPDPMDMMKCKKVMCEEEVVNNVTVCGL
GTTGCKGHILKPECTCDIAADYVPNEDASRCIKGSCENNKMAMKDCGIANSCTGSYNNYVCVC
NGFGYKKLDDTHCVEQMCSDPDIDIRNVCGEGAHYCNGTVNQRVCSCNETEYFYPSADSKSCV
KATCAVSPMVMEQCGVENSCQGSQESFICNCNALGYLLNTTTNQHCYLGTCENGDFDLMQAC
GDGALYCNGTIKNLECQCDESKGYVQAPDKMSCIPNDCRGNLTMYRQCGQLNKCVGSIDDWR
CICDSPYYVAHSDIMKCKLLHCSDSEVDHVEVCGKGTIGCSGSTLHPTCHCNTTENYFASKDGMR
CVAGSCKTNQTAVKKCGNANSCAGSHSEWTCICDGIGYKHDPADPQNCLLKTCNDADIDIVATC
GVGAISCEGYQLMPKCECNDRDYYIPSDDRTSCELASCRANRTAMRACGEMNDCRGSFSSHICL
CHDPAYKSDPSDSRVCLEKTCEAGDFELVRVCGKGATGCSGSMKNIMCECDNAKGYVASADHT
QCIPSDCNNNVTMHLQCGEGNGCVGSLTDWYCVCSAPGYEPHASDHMKCHLKTCADPGVDH
VQICGMGATSCMGPVINPVCYCNESMYYIPSMDRTSCIKASCMSNKTAIAQCGSENSCSGSPDNF
VCLCQAYGYRQDTNNNQACIPSQCEDAGIDFVEICGQGGVGCSGSVLNPVCMCDHMKHFVPSP
DQNSCVLSTCRGNETAMTSCGEQNKCFGSSSSYICVCDAVGYKPHPTNLQECVLQTCEANELNP
VAVCGKGALSCKGTIQSLMCECDNSKGYVAAPGYKSCIPADCRANMTMHAQCGTGNRCRGSM
EDWSCVCDGIGSKPHPTDHMSCIAIDCSDPNVDIKSVCGYGATGCMGLARDPICHCDTSKYYVQ
SEDGRTCVPATCHTNKTAQMQCGPDNSCIGPPNDFICECTGFGYRHHETDKQKCVPIQCNETAL
FDIERVCGKGATGCRGNIRQPICDCDHAMYFHPSEDRRTCIEASCRRNSSIRADCGVENTCFGSTR
SWICDCNSYGSILNPSDVKKCMLATCEAGQLDPVAVCGKGALSCKGSMRDVQCTCNNELGYVH
SPDGKKCLPSDCSGNVTMHHQCGDANKCMGSLTDWVCVCDAPGYKPHAKDKMKCHQISCAD
NNVDPVDVCGIGATSCKGPITNPLCQCDTDNYYITSSDGASCIPATCKTNKTAIHQCGEANSCEG
PADNFICVCNAYGYKLDDNDHQRCVEISCDDPNAHESICGTGSTGCRGTARDPICECDHTQHFV
ASSDLTSCLLASCRANDTVMNHCGDGNTCFGSLTSHICVCEAFGYKLNPNNHKHCLEETCEDD

MLNPVAVCGKGAVSCSGPLKSVKCECDNNAGFFRSPGGMKCESTSCRQNPASQMMCGSQNQCY
GSYDDWYCDCSAPGYMANQTNAAECLKVMCDDMPVDVCGLGATGCTGHTLNPICTCDSSMG
YMMAKDKHSCVPMDCSMNPASSQCGYGNTCHGSYFSYVCVCSTAGTVMNKTNPMECINVACP
ADAVHVCGEGATNCSVTSTNNVECSCEEGYHPADDLHSCVLDDCRFNPKALKACGLLNKCVGS
YKSWKCNCHYPEFTQDPWDHQTCVVGPCMVEQCGKGNAPGSFQHYAVNCSGLYDDPICNCQP
EYTNDAKGGCIPKICSENPVAVQACGSGYCEGTYFKYTCKCDEPAFVQTPDKMQCVPAKCNIQQ
CGHGFDRGLYIDFAESCSGTYADPMCQCKTGFSGTNTSKCGPAIDGCKLDKCDPLATCQTLDVD
GNVKCTCKEGYSGSGMMGECYLDGGFSAYEGTKECSEVCGVNGVAEVTRFCNNPTADPKGKPC
NGTSPETIQVPCNRFACSSCTVRKTCGCGGLKPIFVEFYHEPNSTLEYFTDLKADDVQTALGFLTK
WKELSQQDLCLMYSKIELTVFVTLMGDSETQVSTMKSSLVSARDIMRKKLDCNINLGKKAQYYEA
YETIIKRHLAISNILKDLTIIKNSITKYMNNCDDSEIGMIRDVIGMM*
>TRINITY_DN428_c0_g1_i1.p1 type:complete len:1949 gc:universal TRINITY_DN428_c0_g1_i1:96-5942(+)
MADDADLKFLMVDRRQVSDPVAHAEWAAKKLVWVPSEELGFVAASVKEEKGDKIIAQIEGGKR
VTFHKDDIQRMNPPKFDKAEDMADLTCLNEASVLHNIKDRYFSDLIYTYSGLFCVVVNPYKRLPI
YTDKVINLYKGKKRHEVPPHVFSIADTAFRSMLQDRDNQSILCTGESGAGKTENTKKVIQYLTAIA
GRANQQKADEKSQGKLEVQLLQANPILEAFGNAKTVKNDNSSRFGKFIRINFDNSGFICGANIES
YLLEKARLVRQAPDERIFHIFYQLLLGASPEVKKQFLLLDTKSYNFLSNGHVQLPNMDDRQEFKV
TLEGMRDMGITQEELDPIFKVVSAVMLFGNMEFKQERKSDQATLPDNTIAQKICHLLGIQVTDFT
KSLLKPKVKVGREFTQKSQTKAQCEFAIEAITKALYERMFKWLVGRINKSLNRGKRDGASFIGILDI
AGFEIFQLNSFEQLCINYTNEKLQQLFNHTMFILEQEEYQKEGIDWKFIDFGLDLQPTIDLIDKPMG
VLSLLDEECWFPKATDKSYVEKLHKQQTKHPKYLKPDFRSNSDFCVIHYAGRVDYSAAQWLTKN
MDPLNDNVVSLLAHSKEAFIASIWKDTENVVGMSVTEGMDTTFGASRTRKGMFRTVSQLYKEQL
SGLMTTLHCTKPNFVRCIIPNYEKRAGKIDNHLVLDQLRCNGVLEGIRICRQGFPNRILFQEFRQR
YEILCPGIVPKGFMDGRKAAKQMIDNLEMDPNLYRIGQSKIFFRAGVLAHLEEERDIKLTEIIIQFQ
AFCRGNIARRNYRKRIQQLSAIRVIQRNGRSWLKLRNWQWWRLFTKVKPLLNVSRQEDELRSKE
EEFKKLQDKSDKLESLQKEQQQANEKLTEEKNTLAEQLQAEIELCQEAEEARNRLLQRKTELEDL
IQDFEMKLGEEEEKAVKYAEDKKKLQQGIQDLEESLEEEETQRQKLQLEKVQVEAKLKEMEDERQ
VAEDNNARLTRDKKQLEDRVAEFGQKLAQEEDRAKQLSKAKAKQEAFIAELEDQIAKGDKSTHE
LHKEKRKAEQDLSDLRAQLAQALQRIAELEDDLARREKELAEAIKRGDEEMAKRTTVEREKREL
QALLDELREDMESEKSAKDKVERQKRDLEQDLQELRSELEVTTDATVVQKEFQKKREEELMDLK
KQIEEEAKEHDQQMASLRTKHNGLVEDLNNQVDTFKRGKAALEKNKTALENENAEIAADLQRI
SQLKQEGDRKAKNLEQQLNEANSVRAVQDEKVSSLESEKAKYQKECDSLSTQIEEVETKAASLDR
AKQAAEAQLEDTQDQLAEETRQKLSYQTKLREASDEANRLQEQLEEEEDEKKAVQKVLSNLQV
QLDQAKKEIELKIQDFEQAEAARQKQKRENEELRGEVEKLTGDVSRLDKAKKKLQGDLDDVTVS
MERERNSANQMSSKQRKFDQLLAEEKANSQALGIERDNLEKLARQNETKALSLQNANEELEDR
LAEAERQRKQLQMELGDLVESKDDVGKSVHDLEKSKRLLEQQLAEQKTQVEEIEDELQACEDAK
LRLEVNMQAQKAQFDRELQAKEDAIEEGRRGLVKQLRELEAELEDERKLRQSASAAKRQLEGDV
KELEGQLDQANRIKEDGLKQLKKYQNQLKDVQRDLEDSRQSKDDLAAQVKDNEKKLKGLEAEF
LQMQEDLSAAERARRAIESERDELLEELNNNSALKNAVAEERKRYEAQLAALEDELEEERTQTEL
MQDKVKRSNLQLEQMNAELQEERSAAQSLDNTRMSLERQNKELQSKLADLESSLRSRQKNTVAT
LEAKINNLEIQLDQEAKERQQQNKALRRNEKRCKDLQMQIEEERSHTESYKQQLEKMNARVKN
MKRQLDESEEEVTRLNTAKRKLQRDFDENQESLESSQREANQLRQRLKGGASLSSSRSSGRGRRA
KDEDDDDDDANE*
>TRINITY_DN585_c0_g1_i5.p1 type:internal len:250 gc:universal TRINITY_DN585_c0_g1_i5:747-1(-)
KIQDKEGIPPDQQRLIFAGKQLEDGRTLSDYNIQKESTLHLVLRLRGGMQIFVKTLTGKTITLEVEPS
DTIENVKSKENVKSKIQDKEGIPPDQIQDKEGIPPDQQRLIFAGKQLEDGRTLSDYNIQKESTLHLV
LRLRGGMQIFVKTLTGKTITLEVEPSDTIENVKSKIQDKEGIPPDQQRLIFAGKQLEDGRTLSDYNIQ
KESTLHLVLRLRGGMQIFVKTLTGKTITLEVEPSDTIENVKSKIQD
>TRINITY_DN585_c0_g2_i1.p1 type:5prime_partial len:152 gc:universal TRINITY_DN585_c0_g2_i1:598-143(-)

LTGKTITLEVEPSDTIENVKSKIQDKEGIPPDQQRLIFAGKQLEDGRTLSDYNIQKESTLHLVLRLRG
GGKKRKKKNYTTPKKIKHKRKKVKLAVLKYYKVDENGKITRLRRECPSESCGAGIFMAQHFDRQ
YCGKCCLTYVFSKPGQDAK*
>TRINITY_DN554_c0_g1_i3.p2 type:complete len:268 gc:universal TRINITY_DN554_c0_g1_i3:1464-661(-)
MQIFVKTLTGKTITLEVEPSDTIENVKSKIQDKEGIPPDQQRLIFAGKQLEDGRTLSDYNIQKESTLH
LVLRLRGGMQIFVKTLTGKTITLEVEPSDTIENVKSKIQDKEGIPPDQQRLIFAGKQLEDGRTLSDY
NIQKESTLHLVLRLRGGMQIFVKTLTGKTITLEVEPSDTIENVLEVEPSDTIENVKSKIQDKEGIPPD
QQRLIFAGKQLEDGRTLSDYNIQKESTLHLVLRLRGGMQIFVTITRRDANFCQNLNWKNNNTRS*
>TRINITY_DN585_c0_g1_i8.p1 type:3prime_partial len:337 gc:universal TRINITY_DN585_c0_g1_i8:1008-1(-)
MQIFVKTLTGKTITLEVEPSDTIENVKSKIQDKEGIPPDQQRLIFAGKQLEDGRTLSDYNIQKESTLH
LVLRLRGGMQIFVKTLTGKTITLEVEPSDTIENVKSKIQDKEGIPPDQQRLIFAGKQLEDGRTLSDY
NIQKESTLHLVLRLRGGMQIFVKTLTGKTITLEVEPSDTIENVKSKIQDKEGIPPDQQRLIFAGKQLE
DGRTLSDYNIQKESTLHLVLRLRGGMQIFVKTLTGKTITLEVEPSDTIENVKSKIQDKEGIPPDQQR
LIFAGKQLEDGRTLSDYNIQKESTLHLVLRLRGGMQIFVKTLTGKTITLEVEPSDTIENVKSKIQD
>TRINITY_DN585_c0_g1_i2.p1 type:internal len:184 gc:universal TRINITY_DN585_c0_g1_i2:549-1(-)
KIQDKEGIPPDQQRLIFAGKQLEDGRTLSDYNIQKESTLHLVLRLRGGMQIFVKTLTGKTITLEVEPS
DTIENVKSKIQDKEGIPPDQQRLIFAGKQLEDGRTLSDYNIQKESTLHLVLRLRGGMQIFVKTLTGK
TITLEVEPSDTILRGGMQIFVKTLTGKTITLEVEPSDTIENVKSKIQD
>TRINITY_DN101_c0_g1_i1.p1 type:complete len:129 gc:universal TRINITY_DN101_c0_g1_i1:416-30(-)
MQIFVKTLTGKTITLEVEPSDTIENVKAKIQDKEGIPPDQQRLIFAGKQLEDGRTLSDYNIQKESTL
HLVLRLRGGVIEPSLRILAQKYNCDKMICRKCYARLHPRAKNCRKKKCGHSNNLRPKKKLK*
>TRINITY_DN585_c0_g1_i4.p1 type:complete len:232 gc:universal TRINITY_DN585_c0_g1_i4:847-152(-)
MQIFVKTLTGKTITLEVEPSDTIENVKSKIQDKEGIPPDQQRLIFAGKQLEDGRTLSDYNIQKESTLH
LVLRLRGGMQIFVKTLTGKTITLEVEPSDTIENVKSKIQDKEGIPPDQQRLIFAGKQLEDGRTLSDY
NIQKESTLHLVLRLRGGMQIFVKTLTGKTITLEVEPSDTIENVKSKIQDKEGIPPDQQRLIFGECKIK
NSRQGRNSTRPAKIDFCRKTIGRWKNIE*
>TRINITY_DN554_c0_g1_i5.p1 type:3prime_partial len:373 gc:universal TRINITY_DN554_c0_g1_i5:1118-3(-)
MQIFVKTLTGKTITLEVEPSDTIENVKSKIQDKEGIPPDQQRLIFAGKQLEDGRTLSDYNIQKESTLH
LVLRLRGGMQIFVKTLTGKTITLEVEPSDTIENVKSKIQDKEGIPPDQQRLIFAGKQLEDGRTLSDY
NIQKESTLHLVLRLRGGMQIFVKTLTGKTITLEVEPSDTIENVKSKIQDKEGIPPDQQRLIFAGKQLE
DGRTLSDYNIQKESTLHLVLRLRGGMQIFTIENVKSKIQDKEGIPPDQQRLIFAGKQLEDGRTLSDY
NIQKESTLHLVLRLRGGMQIFVKTLTGKTITLEVEPSDTIENVKSKIQDKEGIPPDQQRLIFAGKQLE
DGRTLSDYNIQKESTLHLVLRLRGGMQIFVKTLT
>TRINITY_DN40728_c1_g1_i1.p2 type:5prime_partial len:124 gc:universal TRINITY_DN40728_c1_g1_i1:1-372(+)
KTLTGKTITLDVEASDTIDNVKAKIQDKEGIPPDQQRLIFAGKQLEDGRTLSDYNIQKESTLHLVLR
LRGGVIEPSLAVFARKYNCDKMICRKCYARLPARAVNCRKKKCGHTNMLRPKKKLK*
>TRINITY_DN152_c0_g1_i15.p1 type:internal len:141 gc:universal TRINITY_DN152_c0_g1_i15:2-421(+)
FGSRNIGPSHHCSHAGVAQHHQFDRRPIAAMQIFVKTLTGKTITLDVEASDTIDNVKAKIQDKEGI
PPDQQRLIFAGKQLEDGRTLSDYNIQKESTLHLVLRLRGGMQIFVKTLTGKTITLDVEASDTIDNV
KAKIQDKE
>TRINITY_DN152_c0_g1_i2.p1 type:internal len:105 gc:universal TRINITY_DN152_c0_g1_i2:1-312(+)
QRLIFAGKQLEDGRTLSDYNIQKESTLHLVLRLRGGMQIFVKTLTGKTITLDVEASDTIDNVKAKIQ
DKEGIPPDQQRLIFAGKQLEDGRTLSDYNIQKESTLH
>TRINITY_DN152_c0_g1_i10.p1 type:internal len:180 gc:universal TRINITY_DN152_c0_g1_i10:1-537(+)

QRLIFAGKQLEDGRTLSDYNIQKERPDQQRLIFAGKQLEDGRTLSDYNIQKESTLHLVLRLRGGHA
PPCLAPARRHADLRQDADRHPARPAAPDLRREAARGWAHPFGLQHPEGIDAALGGKTITLDVE
ASDTIDNVKAKIQDKEGIPPDQQRLIFAGKQLEDGRTLSDYNIQKESTLH
>TRINITY_DN173718_c0_g1_i1.p1 type:3prime_partial len:112 gc:universal TRINITY_DN173718_c0_g1_i1:37-369(+)
MQIFVKTLTGKTITLEVEPSDSIENVKTKIQDKEGIPPDQQRLIFAGKQLEDGRTLSDYNIQKESTLH
LVLRLRGGAKKRKKKNYTTPKKIKHKRKKVKLAVLKYYKVDEN
>TRINITY_DN1580_c0_g1_i3.p1 type:5prime_partial len:179 gc:universal TRINITY_DN1580_c0_g1_i3:1-537(+)
RLIFAGKQLEDGRTLSDYNIQKESTLHLVLRLRGGRGGHCQVPCGIFDDPKLVADVKEAVATITKA
MAEITRLSAEALSPLSINQITRWVNTKEEHCGKIISLMSEYCMCQRVKPPSDPKSPFKGTATEKAED
QYIAALQSHHKVMLCAVKCKQTVDPANAEAMEAAVATMSKMYLPA*
>TRINITY_DN40728_c0_g1_i1.p1 type:internal len:105 gc:universal TRINITY_DN40728_c0_g1_i1:1-312(+)
HAVSGGGAGPESGPQRAQPALAVMQIFVKTLTGKTITLDVEASDTIDNVKAKIQDKEGIPPDQQR
LIFAGKQLEDGRTLSDYNIQKESTLHLVLRLRGGMQIFV
>TRINITY_DN1465_c1_g1_i1.p1 type:internal len:102 gc:universal TRINITY_DN1465_c1_g1_i1:2-304(+)
QRAAMQIFVKTLTGKTITLDVEASDTIDNVKAKIQDKEGIPPDQQRLIFAGKQLEDGRTLSDYNIQ
KESTLHLVLRLRGGVIEPSLAVLARKYNCDKMICR
>TRINITY_DN152_c0_g1_i9.p1 type:5prime_partial len:152 gc:universal TRINITY_DN152_c0_g1_i9:1-456(+)
QRLIFAGKQLEDGRTLSDYNIQKESTLHLVLRLRGGMQIFVKTLTGKTITLDVEASDTIDNVKAKIQ
DKEGIPPDQQRLIFAGKQLEDGRTLSDYNIQKESTLHLVLRLRGGHAPPCLAPARRHADLRQDAD
GEDHHARRRGERHDRQCQG*
>TRINITY_DN10545_c0_g1_i1.p3 type:5prime_partial len:162 gc:universal TRINITY_DN10545_c0_g1_i1:3-488(+)
QLEDGRTLSDYNIQKESTLHLVLRLRGGHCQVPCGIFDDPKLVADVKEAVSTITKAMTQIGELSAT
MNAININQMTRWVNTKEEHCSKIISLMSEYCLCQRVKPIGDPKCPFKSAEDYVAALESHHKVML
AAVKCKQNVDPAIGAALSEAVAEMSKMYMPA*
>TRINITY_DN103165_c0_g1_i1.p1 type:internal len:242 gc:universal TRINITY_DN103165_c0_g1_i1:723-1(-)
QLEDGRTLSDYNIQKESTLHLVLRLRGGMPIVGLGNFQEAGESEGASGKELPPWGPGGIYHEKQV
GSMCAVHCVNNMLQGPLFDYGDFGMVAQQLDQKERQLLGGSADLDYGNARADGFFNVQVIR
TVLERAGYTMEPMTNDAVQNGKMDTAKETAYILNKREHWFSVRRIGREWFDLNSCLKTPRHYT
DRDVRWHIGDGVKEGYSVFVVRGDFPRCALEEDSKKLVEAVQGCGRPDQGCS
>TRINITY_DN8227_c0_g1_i1.p1 type:internal len:135 gc:universal TRINITY_DN8227_c0_g1_i1:404-3(-)
GPGRAGGRWKPPGGGAAARHGAAMQIFVKTLTGKTITLDVEASDTIDNVKAKIQDKEGIPPDQ
QRLIFAGKQLEDGRTLSDYNIQKESTLHLVLRLRGGVIEPSLAVLARKYNCDKMICRKCYARLPAR
AVNC
>TRINITY_DN1030_c0_g1_i2.p1 type:complete len:1426 gc:universal TRINITY_DN1030_c0_g1_i2:4483-206(-)
MAKVRVLALLMLICVTASNAQDGEEVFPFDVQKGEKGQPGDDGEPGNNGAPGRQGSEGPQGP
DGDVGFDGEEGDEGEVGPPGPPGRPGTNRTSVVVVYGPPGPSGPQGPDGQIGPKGYQGETGEPG
SPGARGPRGDAGAVGEAGVPGKNGANGPKGEQGSIGPSGATGIPGLPGPSGQRGPKGSKGEVGE
LGPKGAKGDAGRNGDEGESGLPGRKGEPGPRGQNGTVGFTGPAGQEGPMGPIGPAGPDGPAGP
QGPRGADGPPGTAGSPGESGAPGLPGPVGQVGARGEPGFPGARGIPGDRGQAGPAGQDGVDG
AVGARGSPGSPGIAGAAGPPGTRGEPGKPGPRGLQGQQGEQGSTGVQGSVGGRGEKGPRGLTG
QAGVPGPPGDSGTDGRPGPPGPPGNNGQRGLAGLPGSPGLPGFPGIKGHVGERGFNGPRGEKGS
RGESGRPGQRGIPGVRGPPGDIGLPGIDGKDGNRGQPGKDGIPGPPGQPGPRGIPGSQGAAGSKG
DKGAKGRTGDPGFDGAPGDKGIPGQAGDQGVPGPRGRQGDRGEPGEQGARGGQGLPGPPGA

QGPQGRQGKRGSPGLPGARGNDGLPGRQGERGFPGESGNPGAPGRPGASGRKGPSGRPGQKGS
RGDPGMPGDTGPPGQVGRPGLPGAQGNPGAAGDQGEIGEAGERGRDGKPGPRGAMGQRGAP
GPRGNPGQRGDPGLPGDDGKPGSTGAPGAPGAPGEPGQSGPRGPTGRPGQAGAPGESGLKGFK
GNRGARGRPGRAGKKGADGLKGQRGSKGEQGLTGLQGAPGLPGNQGPAGPIGLPGEQGPQGP
PGPDGKPGSKGDKGRSGPDGRPGSPGRPGPDGSNGLNGRPGKDGSRGPQGRPGPRGKQGPTGY
VGAPGAPGVPGLIGPRGPMGDNGKDGKNGADGDPGEAGPQGAQGPQGEPGADGADGLPGRN
GNPGLPGPRGEKGDRGTKGGKGAKGNKGTAGPVGQPGARGDRGPAGTPGADGEKGADGIKGE
TGFNGAEGAVGDRGDTGDRGSKGDTGFMGFKGAKGPRGSKGTRGLPGYMGPQGDIGDKGPIGP
PGPTGPAGISGPVGLPGRKGPSGDPGPKGYAGKNGRQGPPGPPGPPGLAGPSMLPPWMGGGLA
DGESKGPVVEREEPDAPEDENEELPPNEVNPFYRVYQFYSSKKTTPTDNDVNVMENKFKTKVEEL
KTEFSRIDKPDGTKDYPARTCRDIHAYHPEAKSGLFWIDPNQGCKDDAIQVTCTFSVNEDDEPTV
TTCVDPAEKQSVPQRTWASKMHSRAQKWFDEDHELAKLKYNANLSQLRYLGLLSKTATQDVKV
HCKKYVVWHDKMNRNFDRAMRFMGTQEQEFLYKDVDSKSSKSRFVPEVITDGCSANSNSVAHT
HLRFTSRKFIRLPIVDFAPTYPESPKSEFGIELGPVCFKV*
>TRINITY_DN1030_c0_g1_i1.p1 type:complete len:1439 gc:universal TRINITY_DN1030_c0_g1_i1:4522-206(-)
MAKVRVLALLMLICVTASNTQDGVSVKIGDASIVSKEEVFPFDVQKGEKGQPGDDGEPGNNGAP
GRQGSEGPQGPDGDVGFDGEEGDEGEVGPPGPPGRPGTNRTSVVVVYGPPGPSGPQGPDGQIGP
KGYQGETGEPGSPGARGPRGDAGAVGEAGVPGKNGANGPKGEQGSIGPSGATGIPGLPGPSGQR
GPKGSKGEVGELGPKGAKGDAGRNGDEGESGLPGRKGEPGPRGQNGTVGFTGPAGQEGPMGPI
GPAGPDGPAGPQGPRGADGPPGTAGSPGESGAPGLPGPVGQVGARGEPGFPGARGIPGDRGQA
GPAGQDGVDGAVGARGSPGSPGIAGAAGPPGTRGEPGKPGPRGLQGQQGEQGSTGVQGSVGGR
GEKGPRGLTGQAGVPGPPGDSGTDGRPGPPGPPGNNGQRGLAGLPGSPGLPGFPGIKGHVGERG
FNGPRGEKGSRGESGRPGQRGIPGVRGPPGDIGLPGIDGKDGNRGQPGKDGIPGPPGQPGPRGIP
GSQGAAGSKGDKGAKGRTGDPGFDGAPGDKGIPGQAGDQGVPGPRGRQGDRGEPGEQGARG
GQGLPGPPGAQGPQGRQGKRGSPGLPGARGNDGLPGRQGERGFPGESGNPGAPGRPGASGRK
GPSGRPGQKGSRGDPGMPGDTGPPGQVGRPGLPGAQGNPGAAGDQGEIGEAGERGRDGKPGP
RGAMGQRGAPGPRGNPGQRGDPGLPGDDGKPGSTGAPGAPGAPGEPGQSGPRGPTGRPGQAG
APGESGLKGFKGNRGARGRPGRAGKKGADGLKGQRGSKGEQGLTGLQGAPGLPGNQGPAGPI
GLPGEQGPQGPPGPDGKPGSKGDKGRSGPDGRPGSPGRPGPDGSNGLNGRPGKDGSRGPQGRP
GPRGKQGPTGYVGAPGAPGVPGLIGPRGPMGDNGKDGKNGADGDPGEAGPQGAQGPQGEPG
ADGADGLPGRNGNPGLPGPRGEKGDRGTKGGKGAKGNKGTAGPVGQPGARGDRGPAGTPGA
DGEKGADGIKGETGFNGAEGAVGDRGDTGDRGSKGDTGFMGFKGAKGPRGSKGTRGLPGYMG
PQGDIGDKGPIGPPGPTGPAGISGPVGLPGRKGPSGDPGPKGYAGKNGRQGPPGPPGPPGLAGPS
MLPPWMGGGLADGESKGPVVEREEPDAPEDENEELPPNEVNPFYRVYQFYSSKKTTPTDNDVNV
MENKFKTKVEELKTEFSRIDKPDGTKDYPARTCRDIHAYHPEAKSGLFWIDPNQGCKDDAIQVTC
TFSVNEDDEPTVTTCVDPAEKQSVPQRTWASKMHSRAQKWFDEDHELAKLKYNANLSQLRYLG
LLSKTATQDVKVHCKKYVVWHDKMNRNFDRAMRFMGTQEQEFLYKDVDSKSSKSRFVPEVITD
GCSANSNSVAHTHLRFTSRKFIRLPIVDFAPTYPESPKSEFGIELGPVCFKV*
>TRINITY_DN982_c0_g1_i3.p1 type:complete len:9852 gc:universal TRINITY_DN982_c0_g1_i3:29697-142(-)
MSIKLCTFFVFFLIQCINSFPEIGLHLSLDRSYSFQGNGWKTVNGWEIKPRANGPYEIDAIKNISPEY
FPVPITGVYYITVSLPVEGASNGEFKSAIIVDGKNEEGRIGLMSSFQKTAFKNGASTLTFAGFVRLFA
DEKLTIEIYSDDINANYVVLESATFVFHFIGVTGSVPAYLAHTAQDQEYASDSIIKPWVTLGDSRVYE
SLSGFIKNEGVFTTICNGVHKMAVNIMIKANAGTYTLKALFQDEESATATVTINQPDSIATLNIFQN
FKWSEGMSITLQIHSSNGANFKVLKSSSWSMSFIGETTANFQEFSVFLMADQFLQPSQFPQRINIDL
NGVFQFLQSNPDQYNKGQLVSGADTKFKDNLDFIVDSTDLFYVSANIIIEGEAEDLEITIAVSQIDID
VLPEDGLSGRLTKISNSRRSISLSGIIFIREKQYLSFFVRSRDSSKSFTVSKDSFISVINMRYTVSSVSARQ
NENIDVTAGSWYDISGRFAEQKNGLYSFGKDFNINNGVFRTSHSGIHSVHANIQFSLDSDAFGDN
DDNKIVLRVVIDGQEDKISLMSIRKNPEKTATLKLFGSIYLRAGVTISFQVKSLMPNTVQYKILQRSS
ISISYIGPQWAVNSFHAVANQDLNYDGKPDDTRPDIPSPLNFWKTEAADEFISPFEVLESFDGTRYT
SVDDGLYVVSSLLILKNVDCDRSALFKLDFVTTGGSRASDKSNGFADSHFFIADSKGTEFTLSVTTA

MRLEVGFKVGLKLTVGSTSCRYTISKESSFSIAQWSKSSNTIEENRLKYIGVLARLSAPTEVSSPNTW
SNLGSNTMDINPGDKSRSSFRPGIFGGDDLAPITRTGKDFNGDVSDFTIKDPGIFFISGMITVTTTEN
ANEEFAATVQIDSEDIESGLYAAVINQGSTEMSLTFGGTIYLKSGARVRVVVKGGRTFNIAAGSSFSL
LKLQPDYKTPGIIVSIDEKITQAITRYEHKDLRADIHRTLTIGNLGFVESTGVYTIQVDGVYMVDYT
VILEATNPNAVFGAEILSDNTVVATGTSTFGNTKTIAITELVYFSKGAIVKAVLKSNTNGETLNNLQ
VDGGLTVYFLEPKEHVKDGHRSKQTSPQALPVRSFEKVTGFAAQSPDFAGGADSVDYTVPETGVY
LVAFTTRVNRVINGFKSRVLVNDNEFLNGNYQIVSAQNAPATLPLSIAMELTVNDVLSFQVMADG
SGMSLDGSTRSILKIDEVNTIRNVTEGLSAYKLRDQKVNGQQETIVEEWDTSVTEGTFLAKNVSFT
VDHSIVVVAAGVFQISVSVVVKNDRPSQSKFELRVLQGDDVKSHGLHSKVEIGGGREQTLTIGGSL
LLQKFDSLRIVVRDIADPLASYTIKAGSTVSVIIIDDNNGPIACEDEGLNMRRLPERIRKPTRESHTF
QCNVVSSLKAEKYTWFKDNVQLKEENQKNLVLNDLKLSDSGVYQCRVRFLELTGATNNATLSVY
DPYPKITSPKFIANVLENQDPPLTIEMFSLSAFKLSPEDEKASITTQIIAGDDQSFFTLSGPSKQTVTLS
TTKKLDYETQNEYNLTIKATNLEETALTTKGPYETTINYIVRVIDVNDHPPVFSQSIYSRSVPEDYPI
DGSIAKVSANDQDAGANVIYTLVGQVSGDLFKIDNTGLIQLRQNLDREIRDDHTIFVQASDGLFT
TVAEVVVTVTDVNDNFPNFMDTGFDISVSEATLTQSVILKVIATDPDLGTNSQLRYTIKSGNNENK
FSLNLLDGNLTLISKLDYEGTKIYNLEIDVQDSASPPKKSQNPLTVVIRVQDANDNNPTFTQSLYNT
RIAENTAVGTSVLKVSATDRDGEGINNPLTFSITDTDANEFFQVVSSGATAGEIRVRKSLNYDIRKD
YRFSVVAKDNNLGEPRYRTALVVINIDDTNNLRPTFEAGSASISISEGTSIGTSVHQVVAIDKDTVG
KLDYRIISGADGKFAINKETGDINLISAVDFESNKKTYTLEIQVNDGVRDALNPATVVINIQDFND
NQPEFIQKYYIFTALESISDSQAINVGNPVIARDADSTTNSQVDYSIQYDINNPSVPNEGDALFRIDS
NGLIFKKNGVILDFENVRYHTFTVVAIDRGTPKTLLGKAFVTINIQDVNDEAPSWSPAIYSVTVKES
AGINRDLVFLSTTDKDTSGAVTYSIVSGDSANQFQIFDNVIKIVKPLDFEKTETHTLKVSAQDDNG
NRAQLDATVTITVEEVNDNRPLVTNNNANSIQVSVPEDIPVNSLIVKLNGRDDDKGAPGQASNQ
FTFVSLDKTTDFTIDEVTGEIKNTVNLDYERQKSYSLVATIRDKGTPYLESKFLVDIIVENKNDERPIF
DPAIYTIKLSEKAPIDTVVAMLQATDLDTPKSQLRFSSVTTLDSHFSLDPITGIITLKTSFDKEVNGKD
GIFVISCTVNDGTSNSAQNAIVRVEVVDVNDNQPVFPTSEYFIQVNEGSSIGTILQNIAATDKDNT
NTHNQFTYTLEGKEAVDFRLNGNQLQTNKIFDYENKKSYSFDVLATNNLSPDKLVGKATVVVSIL
DQNDNNPVLSPSGVINLNISEGTIAPANIITIFATDKDSTINAEVQFTLTGDPTNTFTINQNTGEIML
AKSVDSFAKNEYTLSVTATDKGSPPRSSTATTLNISIKPVFVPGIEFENCPQIISFNENAAPNTFTKTL
TVKDRGSFNQVQFTVVSGQAGIQNLITVSQNGVITNGQRRADFEARKRHDFLVQAENERGAKAY
CHVTANINDVNEGPIFDSANYVFDVLEVIKINTVVFSVRATDPDFSSTTNGQLTYSLAGQEASQFSI
VPNNGQIKLAQPLDYEIKKQYQFNVIATDGGSLQTTSSVIVNVEDWNDNRPIFDQPQKLAINVPE
NSKPDNVFTVSATDKDSTTNGEIEYVGIYPTGIISIDKDTGTAKIIGDLNYEKRQKYDFVIVAKDKG
VPYLSNTREVSLSVLNLADEVPYFDTAAYTISISEKTSINTAVFRVMAKDNDLENFGKISSYEIISGNQ
LNRFRIDSNGNIIVQQSVVLENNNPNQYILTITAFDTQRTQSLNNTQVTINIIDLSDKQPFFIQNSYI
VEVQENNEVSKSLLSLEAQSNEPTFNPTYSIVQDPKNTDSQFFQIINGNTIVATTVFDYEKKRFYSFE
VQVIDTNNGKTNKALVVVRITDENDNKPFEVSGNLHFNITEHTLLGSTIGSLNGKDNDAGINKEF
EFSILEGNDDDKFMLLPTGQILLKNFVSVKEKDFYKIEVKITDKGTPSLFNIFTVRVTINSSQALVAS
NNPTIPEDKNTGEEITRVTAIDPNTGSSNGFIYSLVGTLSNSQFTIDAKTGAISLLASVDFETDRTHT
AVVKAEGPTSTGVTEVIVRVTDINDNKPIFDPTNFNLNINENFVVGQEIVLVRATDKDSSLNDNNV
IIYRIDSVNPSSGNDLFSVNPSTGRILLLKALDRENGDTSYVLTIAASNRGVSNKPDNTMTVTINIA
DVNDNFPIFEKSLYQGSVSEDANTGTSILTVRASDKDILLKNRNIRYSMTQPMAVQKFQIDPISGVV
SLKQEVDFETKQRYDFSVIASDTGDVAYSRTANVEITILDVPDVEPKFDPQRYEITIAESTLVSTSILK
LQVIDSVKNLNFNIKQGNSNSDFRLVASSGELFVNKQLDRERISRYQMTIEVSDSVNKAAIDAIVIV
NIADVNDNQPLFLQSGCYQANRAEGIETEQVVVNVQATDRDEGSNKEIVYSFSDACSVLDIDAN
TGAITTKQKFDYESARSHICTIKAEDKGTPKLSGFSCIILTVTDSNDLKPTFEVITSNARVLENIPVGS
VITRVLATDKDTLSSNKQFVYTIKTGNEDGRFTIDANGFIKLQQPLDYEKKTDYRLEIEAVDSTNQ
DMVSDPNAIVTVTVIEQNDNRPKFAKSYHRVNIEESTAIGTTVLKLSATDADAGVNEGLIRYVSLH
NTTVFNIDRTTGEITLATKLDYEVKKSYHLIAVVKDQGNPVLSARTLIDIFVNDVNDNPPVFVISSY
NNIVSEKAPIGQSIVTVSARDDDTGVNGQLKYKIRETQSMFRIDEDTGIIILNGDLNVENTNKLLYA
LTIDVVDSGVPPLQASNPATVSVQVSDVNDKQPVFALDRYQFEINENIEGNQIFGDSVTVRANDD
DLTGTNNQLTYRLVNTTMFDLVVTNQNVFIKTALGVSFDYETKKSYSFEIEAMDSAANKLYGRSLV

TVTVKDFNDNPPIITVPNIIVNISDATLIGSHIATIMAEDKDSGENGILIYTVTAGDSTNRFTMESDT
GSIILKNPVDSYTKDIYVLNVLVQDNGIPRRSATGTVTINVIPVDLTLLKFNTTCPFAISIPENQINSN
FLGLKALDAGEYVSKQLRYTINSDENDVNQIFALDSIKGEFKQLKEANYEERSRYDMTICATNERG
SKVCCHITIFITDINEPPSFIGLNNNNIYEVSISETTPIGTIIYRVLSKDQDLPGVLNGQTSYSLGQQNV
FIIDQMGQIRVNKTLDAETQLNYVFQVIVTDKNPTNPLQSAATIRVNILDYNDERPIIQNIPDITIDE
NSDNQVVRNIVVTDRDISNIGSNIELSIEPSNILTINNRQLRTVGSALDYEKVQRHTLYLTAKDKSFP
YLSSTTSFTVTINNLNDNKPKFPVNNYNLTVSEKTTVGTLILTLFAEDDDLEEFGEIANYEIVSGNQ
EDRFSIDSNGRLTLKKQLNWRNVKSYNLNIIARDKGLPSEQSQIPATVVINVADYSEEQPNFLQNL
YVKFIPETTPIDTLVLTVEATSGEPVYGPFKYTIVPKLPDTNNFQIFSNGEIKTINQFDYENQRNFTFD
VQVEDEKNGKTNRASVFIHITDVNDNIPQFTDLTRSNTQYNITEFTLPGSMITCLSATDRDHGKNA
EIRFRGIGGDGNGVFNVNLDGKVLLSQQIFYDKKNKYNLIVEAFDLGSPEQKAQLTLTIFLHKSYT
SVITTDVDINEDVETQTRVTRVSSIDPFTRSSNNFTYYLVGKSTTFFEINAQTGEIFTKATLDRETRDR
HVLTIQSRGPTTNGVGELVVNVKDINDNPPIFDPQVYQVTVSESANIGLSIVSVTATDKDIGVNGE
VTYSIEDGDQTIFSINPSTGSISLAGKLDFESKQSHTLTIKAVDRGNPPQDSVNKATVIVVVQDFND
NCPVFENSTYSLTIKEPGELGKEYLKLGITDNDNGRNQLFTCSIDEPLAEKYFEMINDCRLRLIKEL
DYEEFTSFRFVVKATDQGDVSCTTTTNMIVNVGDRADVIPFFSPLTYNISIPESTTVGTIVVSLKVGS
TFKGLLYSLFHKNTQDDLIKFGINKDTGEIFLKESVDREIQDKYEFTADVFDGSNNAVQRASVTINII
DVNDNAPFFNQPTCYQRDLTESTAIGTFVVDVSAADNDKGRNQQVTYSLLPSPSCTSFSVDETSG
RITTSQILDHEQQKLQHCLIKAKDNGTNSLSGYSCLTVNVVNVNDNKPAFIVNRFDVSVLETVPA
GTLVASLVARDVDNQGLITYSISNGNLNNQFTIDSSGKLRTRFPLDYENTKSYNLTIQASDNNGKFS
SPDDALVLITVLEVNDNRPQFLQSYYKVSIPEDTATGASILNVTAIDLDHGELTKSLFYAALENTTQ
FTVNANTGVISVAENLDYEIEDHYELVVIVQDRGTPHLSNRALVDIQITDVNDNAPMFVTTVYNR
ELSEKALIGAPVVKLNAMDADKSDKMTFTIDSVVQGAQGFFSINGDTIQLDKELDYEVPEQRTFV
LSVSVKDSRDRDGANKANVFINVRDINDREPIFDLNMYEVTVSENNVISNPIIEVTARDSDSTPEN
NNLLYSLVDGEDAASFSLLNNGQSAIITSKISFDYEKKKSYRFLVRASDKVRENFTAFSTVFVTVQD
TNDEAPVPEKDLIMVNISESTRVLSTIAIVFANDKDQGINGQLAFQIINHSEDKFEINNKGVIRLKST
LDFFQQNFYNFTVLVTDKGNPVRTSKPVTVLVYVYRTEIPGIIFEKNHYIFNINEKEIVSLVGTVNAI
DEGTYVSRQIVYSIVSGQNDIKDKFRAVNNNIIQDKEVNYKDQSEYNFVMKAINERGSIAFTHITIN
IMDVNEKPYFVGLNNKGRYEFSMSEIISVPSYIYAVRAKDDDFGLNGEVDYEIISNFRFGNFSIDQY
GRIQVLKDPDFEFTRSYEFQVRAKDRGTPSLESNLVSVVITVLDYNDNHPIFNIQEPLEISIKELVNN
TDVGTVTATDKDGSGLNRIVKYVAIYPSDHLTVNRDNGQIRVIEPFDYEIAHKHEFVVVAQDQGT
PYLNAYKVIKVEVLNENDEFPEFTVIEYNITISELTPIGSPVLFVHAQDKDRGPSGQVTNYKIKYVPP
SGGSGAGSPDKTFEITSDGQVVLKEKLEYKFQKYYQYRVTCTDNGSPPKNSINEAIIHLNIADASLE
QPIFSVNPAVVEVYENITLNSNIVKVEVDSIEPIKGPFEYSIIASEDSQPFMIDNSTGQITNKESFDYEI
KRKYVFLAEARDKHNNRTQRAMVVIFILDINDNIPTIETIAPQTFVQNISDTTYPGSVVAKLYSRDA
DSGENAMVDFTITGGDGENVFAINPNGYVTLIGPLDVRVKTQYTINVKVSDRGIPTLSNNRALTF
NIKKSKFIQNTVRFDRHHYKSNCIEENQINLNIIQLNATNSNPVAGSTIIYSLDGTLEERQPFIVNSQ
TGEISLSQTLDYEIKQEYDFVAVAVNNDGSRDVAVVTVCIKDLNDNSPIFEPQSYVVNVSESESVGY
LVYKTTVMDADKTDFNTTKSYDIIQGNVNDKFIIDSMGHIYIKSKIDYEALQVKHFDLRIRVNEGN
LVGDMVLRVNILDWNDVHPSFSTLNIYNFTIPEDKPVGEIIEILTANDGDATSPNNEVTYKLLTTED
DFILRRNDKGHGSLIVNKQLDYSKQNRYEYVALAEDKGYLNLLGTAIVIINIQDVNNHPPEFDDSE
YNTTVSEHAQIGTRVLQIHAHDKDTGLGEQIFFIYGDPTETFTISSQGFIILNKQLDYETQRDYIFDV
GARDGGNLVSLNNVTVHIKVSEVNDNEPIFDLSTYRNSYAEDTPLGTTLFTVRATDKDFIGNSIIYSI
SGANDDTSYVSVDQTTGDITLQRRMDYESKNYITFTVQAIDSNNRNKLKGSTHVIIDVTDVNDN
NPKFVFGSDLVYNVTVVENINVGTLVVVVDSQDADSGLNGQTRYHIVSGNNENKFFINELNGQI
NLINKLDALQTNKYVLTVMAKDLGNPSRSTNVTVTINVLRYFESTPMFLPSYAEVIYPENLALGSSI
YELNVTKTLPGSPMKLSFHSSSKFPEFTLQGNKIILSKEFDYEIKNRYELIIIATDEENDKAFAQVIVR
VQNLNDNIPYFTQNAYFGVVSELAFVGHSVLTITAKDDDKDFTELFYSIATQVPDNAFKLQDNIV
VVNGKLIRNKVYEFEVEAFDNTFTSKRATVSITVTPENLPSFVNATYIVTLQENYPISTNFLNISAGT
FSGITYVITSMKAKDMFNVDANGQISLTKSLDYEKQRQHVFPVTASVTSPITQVRVASVIVNVLNE
NDNAPVFVNGPYERNITEGHLVGTMVIRVDGHDDDSDKLTYSIVSGDDGKFSINSEGVISLISTLNF
DLKNKYVLNVRVSDGKFVTDTIVTINITPIVKFPPMFNQTLYLLSVKENTLVGTSILQVSTVNVIPPS

SFSINEQSGRDFFTIDANGVIRTNNALDYEAKRQHIFTLKVQDLRSMGHATVVVNIVNDDDTCPIL
TPATQTVQITEPVAPGTIVAAVQAKDIDSKSLNFTITGGNGLFRIDQHGGIKTINEVDISTSTEYELTV
TVDDGTCTKSVQVDVNVNPLAPCPDCNIYPFTQTIYEFSINENTVPASPIITVTTGQSLQRNFSIEDP
AALEYVTINRLTGEISVIKAFDYEATKHLSFNVMAKTQTTKNRNPFYVITSVSRVRITVRDLNDNCP
SIESPMYYEVSHSSPIVQDTVVTRILVEDPDTLKDHTYSISDNNFKIDSMGHIRTVQNIESFTAQFYN
LTVTVSDGQCSKTSNVGVNLAVNPTPMTYQFTSNGVYSKSVREDHNLQDVILSVALNQNYPRVFS
IVESDANDQFVIDSNSGALRLKTSLNYELKKRYDFTVMADIHGGVYKSYASVHIIVLDVNDECPIF
PVIGYNQRVSGTPVIGTIVEIIEANDADNATLEYSIVSGNEDGHFSLDNSGVVRVVKPFASSFSKTYG
LSVRATDNKCSADVPLTVQVSTCAYGEDYQFEESEYVYYLDENRNLGIVSNIKLKSTRAAFLTLSQ
NTHFTIDQNGDFRLIQTLNYETKKEYHLTLTANFTQDNVVTRIPIKVFVRDINDNCPAFSEELYAVQ
HTEPLREGLLIAKLSVIDDDTVQNFTYNIVSGDKRFSINENGEVVMNARLVTDTTLLSTTYKLEVSV
NDGKCAKNTKVDVFVYKTVVNSFLFPQPFYKFDISENQAIPFTIDRFVNVGGHEAVYGFLVSTDFF
QIDPKTAQLQLVKKLDFEVEKTHYVLISATYDDGTQTYTVVAIHVGDENDNAPQIFAHPPITRLVQ
NTPHNSIVTKIEVEDPDTSKEFTFKITNGNPLQNNDPDKALFRIESNGMIKTTRCISEDEIGNYVLTI
SVDDQVNTPTIFSNMQVQLKTTDDRSYVTNCGLGCPANDGPFSRYQFEQSLFIVNVTETAANPTV
LKRLTRPFVEKTTYTIGAGADGKFSIDQNGTLSLTGSLDYETKPVYYFIVSAVIPSQVVGRFITSKTAY
LVMVNDVDDSKPVIEGNGGTLAVSETSEIGQIVGTVRVKDSDSSSPLTYNVVNGSQYFNIDSAGNI
VIKDTLNAAGSPYTVTYTVSDGINPPVNGEFVVSISETNNNVPKFTQNIYEVSLSEDTATDTIAVTIL
ANDGDTNDKIAYSFLQSEGPFVINRDSGEIKLIGPLDYEAQKSYNMVVQATDSGGFANIAFVQIAV
GDVNDQVPVITPQTTPTQIHESTLVNSVVYRVIASDGDGNERLTYALANSNDFFTINSATGEITIKE
NLAKSNVSSHTLSIVVTDSSGKTSTQDVALNIRRSGTSQGCVKITTSQGQSSSPVINLPENTASGTTV
FKLVAGDASVFSNMRFTLNREANGVFAVTEQGELQLVAPLDYETKQFYSIIITAEDVTRGVSSQYNL
FVRISDVNEFTPVLETIAPVAVSELANIGDVIAQAKANDQDGDSQVTYSLGEFGNNDNKFTIDAAT
GNIILNKRLGSGAPHVVEVCASDGSIKKCQNVTFIVADTNDNPPSFQDLTGVGSIDLTNTSSYVVY
KPIVTDSDQTGTLSFSLKDPAPSQFSINSTTGEVKANKDIAKGTYTFGVCASDGRYTSCQSVTVVAS
DRSQSITPSFNQDQVGAIVDISASSGKQIIQVMASTLDKAKIEYELKNEADRNIFEINKDSGLITLKN
RTALTLGTIYTIPVKASRADDPLNFDEINIVISVGNQQLDSGSFIEQVLDTQHLVNFSLSTFDISKVSY
YIILGQQVGGDSKDDEKLPVSWYYATFGDESYRHRFYYIAKLDGSKFRNTGGSRKRRSIQDKDSKS
FTYLVGEENGCSKNTSMAAICNGPLKTGTEYRYKLRAYYDDGELRGIPVDQKPTEVKRTQAPQA
GKSIDEYREQDSYVITFWIIGLVIFGLLFWFSVLVIAILLCYKRRYEVTQAYGYSVQTRDKIDGPPTEY
SNTVYRFQNPSYPDNDTVKMKKVDASESRTKTEVQTTSMVVLDDYEQEQPQPDIPVQDYDAIEY
ETRSAQIKFHEESPEWESMDIQLRIDPTGNQDPVITRKSDSGSKDSDSDASKKYENTEYIEEKEDFV
NEKEDSLTRL
>TRINITY_DN11643_c0_g1_i1.p1 type:complete len:187 gc:universal TRINITY_DN11643_c0_g1_i1:1748-1188(-)
MPAKGKLTQEEEVLLQDFSRTVSKKSQALFYGNALMISVLPLWLFWKVHLMDPMSYAALFICGTL
SAAYLVAFAYKNTKFALKHKVAQKREVAITREVNTELDSRPETKKLSRKEKDDRILWKKNNVADT
EATTFSIFYTNTLYLFIIIIASFYVLRSFSPAVNFTLSTGMAAGTVALLSTGTQSS*
>TRINITY_DN477_c0_g1_i9.p1 type:complete len:306 gc:universal TRINITY_DN477_c0_g1_i9:1840-923(-)
MTRQCLGATLNENCGNAALVQFEVCNFQVPCPGLLTNWGPYTQCSATCQKDANSMPTQSRQR
FCTGGSFGGNCGGVQLLQTIGCNQLIICPVYGYWLQWSAFGECSKTCDQGTHVRYRNCTKSIPA
NAGNNCTGPAQEVKPCQVKPCPVTCPLTKKCDCRNMNYLAAAPGISQTVTNDVMGVAAFLDY
WTLDILKQICEGCRMTQLDQSKATVDSQILRTQAVLDRLNIEKRVYRDMITCNENPYITDDLFKVY
NDIWDRIVTTDGVMIELKASKMRFDTAITACRSYDWYHDLFGRFFQIKKSS*
>TRINITY_DN3795_c0_g1_i1.p1 type:5prime_partial len:867 gc:universal TRINITY_DN3795_c0_g1_i1:2719-119(-)
LEFIGKLKASFTGELEISMQMVGMIYEPFGVEKVAFGNLFMSLGVTIAAGLPSFQIGAKIMVGKGV
ANKGSSEADMFIFEGYFGFNPADPTDNYIYAKRSGDPLTLGNFIKNFEIDATLPQKIADSGFYGETIF
SFSAAGKEVEELGLEIQAGIYFKGALKILDFDIYAELNINTDPENLKIYANITLSPISWIGGLLRISRSA
SDDKNGPFLIVDIDAESVALEIQGFIYVLGISNEIAVMINETHFYFHIAGNLFSVLSASLTVEASYGVE
TLEDMSYSFRGCVTLDLRRIGEIVFQTMQDAQKAVSRAMAKAQQKIDDAKLAVANAFKKVKEW

KQGIENYKARLRARSAELERKKAELNYSCKDRCGKVCIGFLDWNPHCWKVWGHWCGCPKWN
SCKWKADDIICIAGCELKRAWGKFVTWAEQVGVQIMLGLSEVAQTLLSGLDFFEAIAQGVLSFAS
GFLSVVETFTNAVLNGIGEIVRAAANVIVVEHFCLSGHLRPVESACVACDAKIRIFNQPIEWKGEF
CIDLSFFQVIGHKTAVQHTPEIAEIDAKVQEFESEGNKVDAQVGDMKTEENKQNKEVGDKTSKN
VNDENFRARRSHVPTEEEAYYYRLAYDRLPEVNTRSFATEQSMKDGAPWLLTQTWTPSKRSLTPD
PSTLHRRILETDNEDHCKNVENLYSNYQNVAVGFGTFLKSADKEKAGFEAMRRSYIQGMNYLKN
EFEYGDKKHNVTDEEREHVKYWEEYVKNGLNSHIEKTESLLKTQRKSYIPDFRNQVNQIFKEQTK
STLPEYIKKMKSHVIGFYQKRSDIPKDHLEKQLKKWNILHQVLTDISSSETASYRDLSLQVASVNN
MLTDLRRSDIPCAA*
>TRINITY_DN2577_c1_g1_i1.p1 type:complete len:937 gc:universal TRINITY_DN2577_c1_g1_i1:3082-272(-)
MKTKLGQDTKLTIEGTFSRSGFTLAAGITTSLELAKDLSLTEAKLELEFTKDERSVALSCTLKYNNK
ELSGKEGLEFIGTLKVSFSGELEISMQMKGMVYEPFGVEKLAFGNIFLSLGVTIAGGLPSFQIGAKIM
IGKGVKNKGGEGVFIFEGYFGFNPADPTDNYIYAKRSGDPLTIGNFIKNFEIEATVPKKIANSGFYG
ETIISFSAAGKKVDELDLEIEAGFYFKGALKILDFDIYAELKVNPDPENRALFANITLSPLNWVMGL
LRISRSKDDDKNGPFLIVDIDSEKTAVQFEGFGYCLGIGAEIAVVVNETHFYFHFAGDLFTVLHASL
TVESAYGGDSISDMSFSFKGCVGLDLRKIADIVFKTMQDASKAVSLALGKARKKVENARGAVTAA
FDKVRKWRQGIESYKARLRARSAELERKKKELNYSCRDRCGKVCVGFLDWNPYCWKVWNTWH
GCPKWNPCRWKVDDIICIAGCELKKGWNKFVTWAEQVGIQIMLGLSEVAQFLLKGVEAFEKVAQ
GVLTFAVGVLNVVESFAKGVLAGVGAIVKAASNVIVIDRFCLSGNLKPVESACVGIDAKFSIFKVPI
EWKGEFCIDLNFFQIIGKKTAVKHTPAIAEIDSKVQEFESQGNEIDSKVGDMKDEESKQGNEVETE
TEKPYDINRRKRSHVPTAEEVYFHKLAYDRLPKVNTRSFETETSMKDGAPWILSKTWKPSKRDIVK
EESTLEKRILKTDSPDHCKNIENLYSNYQNIAEGFGTFLKNADKQKAGFQAMKRSYIDGMNYLKD
EFNYGDKRQNVTDEEREHVKYWEEYVNKGLTSHIEDTESLLKAQRKTYIPSFRKQVNQLFMEQT
KSTLPTYINKMKNTVIKSYHKRSDIPKEHMERQLRKWNNLHEALGEIASSETASYRDLSQHVASV
NYLLTNLRRSDIPCAA*
>TRINITY_DN2577_c1_g1_i2.p1 type:5prime_partial len:1087 gc:universal TRINITY_DN2577_c1_g1_i2:3532-272(-)
VTRKLYKEEEEEEGEGEEEEGEEGEKDGKEKNGKDGDKSKKKIVDGKKSSRVVKKASKTSNKKIV
QKGRTLKKKNQVKGEAKQKLAKGRALISKRSDGEEEGGEGEAEEEEEGGDFSLSHLSQVDLVRG
LQVSAEFALPGECKKDKLCEWMKTKLGQDTKLTIEGTFSRSGFTLAAGITTSLELAKDLSLTEAKL
ELEFTKDERSVALSCTLKYNNKELSGKEGLEFIGTLKVSFSGELEISMQMKGMVYEPFGVEKLAFG
NIFLSLGVTIAGGLPSFQIGAKIMIGKGVKNKGGEGVFIFEGYFGFNPADPTDNYIYAKRSGDPLTIG
NFIKNFEIEATVPKKIANSGFYGETIISFSAAGKKVDELDLEIEAGFYFKGALKILDFDIYAELKVNPD
PENRALFANITLSPLNWVMGLLRISRSKDDDKNGPFLIVDIDSEKTAVQFEGFGYCLGIGAEIAVVV
NETHFYFHFAGDLFTVLHASLTVESAYGGDSISDMSFSFKGCVGLDLRKIADIVFKTMQDASKAVS
LALGKARKKVENARGAVTAAFDKVRKWRQGIESYKARLRARSAELERKKKELNYSCRDRCGKV
CVGFLDWNPYCWKVWNTWHGCPKWNPCRWKVDDIICIAGCELKKGWNKFVTWAEQVGIQIM
LGLSEVAQFLLKGVEAFEKVAQGVLTFAVGVLNVVESFAKGVLAGVGAIVKAASNVIVIDRFCLSG
NLKPVESACVGIDAKFSIFKVPIEWKGEFCIDLNFFQIIGKKTAVKHTPAIAEIDSKVQEFESQGNEI
DSKVGDMKDEESKQGNEVETETEKPYDINRRKRSHVPTAEEVYFHKLAYDRLPKVNTRSFETETS
MKDGAPWILSKTWKPSKRDIVKEESTLEKRILKTDSPDHCKNIENLYSNYQNIAEGFGTFLKNAD
KQKAGFQAMKRSYIDGMNYLKDEFNYGDKRQNVTDEEREHVKYWEEYVNKGLTSHIEDTESLL
KAQRKTYIPSFRKQVNQLFMEQTKSTLPTYINKMKNTVIKSYHKRSDIPKEHMERQLRKWNNLH
EALGEIASSETASYRDLSQHVASVNYLLTNLRRSDIPCAA*
>TRINITY_DN286_c0_g1_i4.p1 type:complete len:224 gc:universal TRINITY_DN286_c0_g1_i4:40-711(+)
MIKVAIITIAIVACVLESTAAVRCYVGYSAADLPLAIGVRNIECGEEFNHCTTVKYRIGGITVTQGN
CAKRYGQAMCQFMRNSHKAMTDCEESLCKGHNCNSDVNDAEQVERKRVGPYENTLKCTTGL
QTKWPSIPFASANIPTAVEECFPGTKDCVTVEYDYIFQFESKIATLYNQFRLPTVQKMCNRPNVDQ
CYKWCTILSIHQNMDNCRVKCCQSDNCNA*

>TRINITY_DN286_c0_g1_i2.p1 type:3prime_partial len:106 gc:universal TRINITY_DN286_c0_g1_i2:40-354(+)
MIKVAIITIAIVACVLESTAAVRCYVGYSAADLPLAIGVRNIECGEEFNHCTTVKYRIGGITVTQGN
CAKRYGQAMCQFMRNPHKAMTECEESLCQGDNCNTAVN
>TRINITY_DN1505_c0_g1_i1.p1 type:complete len:154 gc:universal TRINITY_DN1505_c0_g1_i1:98-559(+)
MSLVIPEKFQHILRVMNTNIDGKTKIMYAMTSIKGVGRRFSNICLKKADIDMTKRAGELSDEEVER
LVTIMQNPRQYKIPDYFLNRKKDIKDGKFSQVLANGLDNKLREDLERLKKIRAHRGLRHYWGLR
VRGQHTKTTGRKGRTVGVSKKKN*
>TRINITY_DN1058_c3_g1_i1.p1 type:internal len:485 gc:universal TRINITY_DN1058_c3_g1_i1:1452-1(-)
IGSLTDWRCVCNAPGYYADANNSRRCRKEMCSENDIDAEKICGTGAISCVGPVVSPICFCDVKKN
VVSSTDGSRCIPASCKTNKTAITQCGNGNSCTGSSDNYICVCNSYGYKLDDNNNQACVEKTCSDS
DADFIEICGQGGLSCTGTMKKPVCECDHTKNFVPSDDRRSCVLASCRANPVVDAACGNMNTCY
GSESSWICLCNAPGSMLNPNNVRECLTGTCENGDIDVQTVCGRGALSCKGSVASLTCECNNHKG
YVQSPDRRSCLPSGCRQNTTMQEQCGEVNECIGSLTDWSCICSAPGYRPHDSDQMKCHRRMCY
DANVNPVEVCGVGAIGCNGSVVTPQCTCNTSAYYVPSLDGSMCIPAPCHSNKDALKKCGVGNS
CFGTTNNYVCVCDSFGYRLDDTDPHKCVEIQCDDDGVDIEKICGQGSTICDGTARKPFCQCDHA
SHFVPSLDSSSCLLASCRANGTVTDMCKDGNTCFGSM
>TRINITY_DN2971_c0_g1_i1.p1 type:complete len:239 gc:universal TRINITY_DN2971_c0_g1_i1:125-841(+)
MDAIFGERPISFKALTDFSKLEASTKKHLKNVYASLAISLTSAAVGAGLHVLTNILKGGILAGLGTI
GFMLAIAFTSNEPKNQLKRLGFLIGMAFCTGLSLGPLMDAVIQIEPTIISTAFFSSAMIFVCFSLCALL
VEDRSFLYMGGSLLSGMSIMMVMGLMNIFFQSQLIFDLNLYLGLFLFCGFILYDTQLIIEKRRNGDT
DFIWHSVDLFLDFINIFRRIMIILANKEKKNKRKN*
>TRINITY_DN8155_c0_g1_i1.p1 type:internal len:249 gc:universal TRINITY_DN8155_c0_g1_i1:744-1(-)
DPDSEECQEEEEEEGGEEEATEGEEGNSTSKTSEGGNKKKKKKTQKKKVGKVLGKGQRKLRNKR
KNLKKKNKSKPKKKGVNKKVALSKRSDDETTDDEEEEEEEEDSGDFSLSHLGSVDLVRGLYISAEF
AIPEDCKKDKLCEWLKNKVGAEAKLTIEGTFDTNGFKIAAGITMNLALSDDLFLTEAALELEFTKS
ERSVALACKLQYNNVELSGKEGLEFIGKLKASFTGELEISMQMVGMIYEPFG
>TRINITY_DN161_c0_g1_i2.p1 type:5prime_partial len:399 gc:universal TRINITY_DN161_c0_g1_i2:1683-487(-)
WNQEPLTCTYLYSVNSQLLKYKMADDDVAALVVDNGSGMCKAGFAGDDAPRAVFPSIVGRPRH
QGVMVGMGQKDSYVGDEAQSKRGILTLKYPIEHGIVTNWDDMEKIWHHTFYNELRVAPEEHPV
LLTEAPLNPKANREKMTQIMFETFNSPAMYVAIQAVLSLYASGRTTGIVLDSGDGVSHTVPIYEGY
ALPHAIIRLDLAGRDLTDYLMKILTERGYSFTTTAEREIVRDIKEKLCYVALDFEQEMQTAASSSSLE
KSYELPDGQVITIGNERFRCPETLFQPSFIGMESAGVHETTYNSIMKCDVDIRKDLYANTVLSGGTT
MFPGIADRMQKEISALAPPTMKIKIIAPPERKYSVWIGGSILASLSTFQQMWISKQEYDESGPSIVHR
KCF*
>TRINITY_DN161_c0_g1_i3.p1 type:5prime_partial len:399 gc:universal TRINITY_DN161_c0_g1_i3:1450-254(-)
WNQEPLTCTYLYSVNSQLLKYKMADDDVAALVVDNGSGMCKAGFAGDDAPRAVFPSIVGRPRH
QGVMVGMGQKDSYVGDEAQSKRGILTLKYPIEHGIVTNWDDMEKIWHHTFYNELRVAPEEHPV
LLTEAPLNPKANREKMTQIMFETFNSPAMYVAIQAVLSLYASGRTTGIVLDSGDGVSHTVPIYEGY
ALPHAIIRLDLAGRDLTDYLMKILTERGYSFTTTAEREIVRDIKEKLCYVALDFEQEMQTAASSSSLE
KSYELPDGQVITIGNERFRCPETLFQPSFIGMESAGIHETTYNSIMKCDVDIRKDLYANTVLSGGTT
MFPGIADRMQKEISALAPPTMKIKIIAPPERKYSVWIGGSILASLSTFQQMWISKEEYDESGPSIVHR
KCF*
>TRINITY_DN161_c0_g1_i1.p1 type:complete len:377 gc:universal TRINITY_DN161_c0_g1_i1:1384-254(-)

MADDDVAALVVDNGSGMCKAGFAGDDAPRAVFPSIVGRPRHQGVMVGMGQKDSYVGDEAQS
KRGILTLKYPIEHGIVTNWDDMEKIWHHTFYNELRVAPEEHPVLLTEAPLNPKANREKMTQIMFE
TFNSPAMYVAIQAVLSLYASGRTTGIVLDSGDGVSHTVPIYEGYALPHAIIRLDLAGRDLTDYLMKI
LTERGYSFTTTAEREIVRDIKEKLCYVALDFEQEMQTAASSSSLEKSYELPDGQVITIGNERFRCPETL
FQPSFIGMESAGIHETTYNSIMKCDVDIRKDLYANTVLSGGTTMFPGIADRMQKEISALAPPTMKI
KIIAPPERKYSVWIGGSILASLSTFQQMWISKEEYDESGPSIVHRKCF*
>TRINITY_DN1058_c0_g1_i1.p1 type:complete len:2639 gc:universal TRINITY_DN1058_c0_g1_i1:134-8050(+)
MVKYIIWILLLSIFKEAYGGDWAQWQNWSACQDSSGAPIGCGRGNQHRSRTCADNNCGAGAIIE
VRPCLDEICPTFNCQNSSEIAGMCKFDYSNSCVGDDNCHHCICKSEGFKVSGDQCYNYCVEVGC
DEEIEGVSQLKRYCGDQAISCTGTVLNRTCLCNPATSYPSKDGQYCYEATCKTSQEFMTNCGAEN
SCQGTNTSFVCECNGFGYGYEVVANVQNRKKCKLLSCEDGDLNPLEICGQGATACTGNVHNLT
CACNVTAGYIPSEDKKSCIPMPCNTDPAVGVQCLPRNTCEGSKIDWRCICSAYGFRNDPTDPKKCI
EAKCDDPNIDILGLCGTGVTSCTGFLSKRRCVCNTTENYIPTLDGSHCMPAPCNANENATKDCG
GENSCIGSFDSYMCVCNGYGFQKNATINKCEEVPCSSLPTAEVQRICGFGALSCDGTIRNPVCNC
NNDLQYYASEDMKSCIEASCSSKADYDSHCGNENSCVGNITHFECICETEGYKPDPSNKKKCILES
CELNEVDVLNVCGKGAISCKGNVRDLECVCDASKGYVQSPDTNKCEPSSCYGNATVSEQCGEEN
HCLGDLSDWICVCDAYGYKVNATNSMTCDKVMCGDTGADAVTVCGKETTSCSGHIAKPVCTC
NETAQYLPSPDSTRCVKASCKENDAARKACVPGNSCSGSYDEFICICDQFSYHQDPADNRKCIRE
KCEDLAADYDYEKICGVGGISCTGYVNQPVCVCNSTESYHPSNDRTSCVKASCSTSDAVRTQCGL
ENSCFGDIESFMCDCKAHGYKLEPINKTSCVLATCEDGDFSYHFQCGKGAVGCSGNINTLSCLCD
SAKGYVPSADMKRCVPNSCKDNPDVYIKCGMENTCLGSYADWFCVCSGAGYEANPADPKSCQL
MECSKDDAIHVCGYGAIDCKGPVLAPTCSCNYSDHYIASEDGTSCVPITCRKNDTARAQCGEENT
CKGHTDNFKCICDAYGFKADNVDHQKCVERQCTDGDADIIRICGHGGIRCTGPIRSAVCECNSTE
YYYPNADRSRCILASCKANETIMKSCGEFNSCEGNMESHICVCNGPGYKHHPSDDKQCVKETCE
MGEIDPIYLCGKGAVKCNGTYDIYTCECDNAMGYVQAPDGNSCKPFDCHANATMHKQCGDG
NRCIGSLTDWICVCDVAWYKPDPSNLMRCHEKTCKEADLELICGTGATGCSGSATNPTCECDSA
NYFVSSTDRRSCIKASCKTNITAVQQCGDGNSCYGTPDNYVCNCDAYGYRLDDNNNQACVEKK
CDDVGANYVEICGLGGIGCKGTVKNPVCECDQTKHYVASPDSTSCVLSNCRGNKTVMAACGNE
NKCFGSASSFICVCDGPGYKRNPSNLQECLPETCEAGEIKPHMVCGRGATACKGTMYHFECECN
NTLGFIRSPGHESCIPVNDCYANATMHEQCGEGNGCQGSVADWICKCDAPGYKPHKTNFQMC
HQRMCSDADIDLVEVCGVGVESCVGPIANPTCNCDTSKHYVLSADSTSCIPGSCKTNETIMAQCG
EGNSCSGTSENFVCICNGYGYRPHETDKQTCVLKKCSHSDVNAVDVCGQGALSCIGTMRDPICQ
CNNDMFFIPSDDSRSCVLASCSANSDVAVACGKVNGCFGSKSSWICDCSSPGTMLNPNNIRECLH
ETCESGKLDPIIVCGKGALGCNGTMSNVTCECDNSMGYVQSPNGMSCVASDCKTNSTMHELCG
DGNSCVGSLTDWMCVCDAPGYHPHPSDPMRCLQKMCSHANINPVDVCGIGATKCVGSITEPRC
TCDTAAYYVPSPDGTYCIPGSCQTNKTALEQCGNGNTCTGTADNFVCICNAPGYKLDDSNNKRC
IEISCNDADVDLVEICGQGSIGCIGTARQPVCQCDHSKHFVPSLESTSCQLASCRANDTIMDRCRM
GNTCFGSMTSHICVCEAKGFKVNPSDPTECVQETCEDKDIDALSACGKGATDCMGTLMNLTCQ
CDGASYVESPDKTSCLPVGCQQNVDVKKACGEGNTCEGSYDDWVCLCTNPEYIPHATDEKVCS
KKKCTDNPIGSCGIGATSCSGNVNIHKCTCNLTAGYMPSKDEQSCIPIDCKMNPKMNCGFGNTC
RGSFFSYVCECNTLGTMRNISNPKQCIEVDCSTDAVSICGVGATGCAVTPTNDVNCTCGEFYHPA
HDYHSCVLDDCKFNKLVPDACGVENECTGSISSWMCVCKGPKYIQDIKSTQLCAPGPCRIEQCGS
GKASGSFQYYAESCSGTYFEPTCNCKEGFEKDSYGGCIVKSCSRNNQSSVDCNDGAISCEGTYFKY
KCECRAPTHILNPANKRSCIPARCNPQLCGRGLVNGDYMDFASECSGSYTDFTCKCKYGLYGSGT
SKCSLEKRTCALSNDCHPNANCIDYDGHYFKCECKDGYSGDGYQCHRNGGLTPWSVVQPCDKT
CGTGYAQVMRTCDDPKPDTLGKKCNVPSPDVREVPCHQFACTACSVKKTCECENIRPYFVHEYV
EPDESLKYFTNLTAEDVEKALSAIEKWKLSVDEKEFCIKHTLPEIDGLISDINENLRSVDQTKLYTIT
ARDMMRNKLDCNVDLEKKDKYFRVYEAIVKRHFILKQVVSDLQAKEKLMVTYRSSCTKDSKVG
WLSSIFS*
>TRINITY_DN1058_c0_g1_i1.p2 type:complete len:117 gc:universal TRINITY_DN1058_c0_g1_i1:5843-5493(-)

MLIPMMVQNVCYGHIDTLVVLLFQYIQCFLVRKSPLSQHLHHCMKFQYIVYYCCHQACNQVHC
KCKQNYQLCRCMYFHFHIALMPFCWFGNFLVYNKFHLATVRSKLQCHMYIEVL*
>TRINITY_DN1058_c0_g1_i1.p3 type:complete len:115 gc:universal
TRINITY_DN1058_c0_g1_i1:1992-2336(+)
MQLQCVVKKLQVVRDILQNQYVHVMRQHNIYHHLIAPVVLRLPVKKMMLLGKHVFQATLALV
VMTNLYAYVINLAIIKILLTIENVSEKNAKILLLIMTMKKFVVWVVSVAQDM*
>TRINITY_DN1058_c0_g1_i1.p4 type:complete len:111 gc:universal
TRINITY_DN1058_c0_g1_i1:5265-4933(-)
MPFGDCTYPMLLSHSQVTLLIVPLHPNAPLPHTIIGSSFPLSQVSCKHSLILLGFSMVPGELQSHIQD
DLLPKHPLTFPQATATSELALQLARTQDRLSSDGIKNISLLH*
>TRINITY_DN1058_c0_g1_i1.p5 type:complete len:102 gc:universal
TRINITY_DN1058_c0_g1_i1:1240-935(-)
MTSIKSWYVVFCCITYASSLAQKSSTTSNSSATESQNVNIWIITFCFNTFLWISWIISKTICTTNTSPIYF
ASFACVSRKTLNTNCWICVTWHWYATFFIF*
>TRINITY_DN1058_c0_g1_i3.p1 type:complete len:2581 gc:universal
TRINITY_DN1058_c0_g1_i3:134-7876(+)
MVKYIIWILLLSIFKEAYGGDWAQWQNWSACQDSSGAPIGCGRGNQHRSRTCADNNCGAGAIIE
VRPCLDEICPTFNCQNSSEIAGMCKFDYSNSCVGDDNCHHCICKSEGFKVSGDQCYNYCVEVGC
DEEIEGVSQLKRYCGDQAISCTGTVLNRTCLCNPATSYPSKDGQYCYEATCKTSQEFMTNCGAEN
SCQGTNTSFVCECNGFGYGYEVVANVQNRKKCKLLSCEDGDLNPLEICGQGATACTGNVHNLT
CACNVTAGYIPSEDKKSCIPMPCNTDPAVGVQCLPRNTCEGSKIDWRCICSAYGFRNDPTDPKKCI
EAKCDDPNIDILGLCGTGVTSCTGFLSKRRCVCNTTENYIPTLDGSHCMPAPCNANENATKDCG
GENSCIGSFDSYMCVCNGYGFQKNATINKCEEVPCSSLPTAEVQRICGFGALSCDGTIRNPVCNC
NNDLQYYASEDMKSCIEASCSSKADYDSHCGNENSCVGNITHFECICETEGYKPDPSNKKKCILES
CELNEVDVLNVCGKGAISCKGNVRDLECVCDASKGYVQSPDTNKCEPSSCYGNATVSEQCGEEN
HCLGDLSDWICVCDAYGYKVNATNSMTCDKVMCGDTGADAVTVCGKETTSCSGHIAKPVCTC
NETAQYLPSPDSTRCVKASCKENDAARKACVPGNSCSGSYDEFICICDQFSYHQDPADNRKCIRE
KCEDLAADYDYEKICGVGGISCTGYVNQPVCVCNSTESYHPSNDRTSCVKASCSTSDAVRTQCGL
ENSCFGDIESFMCDCKAHGYKLEPINKTSCVLATCEDGDFSYHFQCGKGAVGCSGNINTLSCLCD
SAKGYVPSADMKRCVPNSCKDNPDVYIKCGMENTCLGSYADWFCVCSGAGYEANPADPKSCQL
MECSKDDAIHVCGYGAIDCKGPVLAPTCSCNYSDHYIASEDGTSCVPITCRKNDTARAQCGEENT
CKGHTDNFKCICDAYGFKADNVDHQKCVERQCTDGDADIIRICGHGGIRCTGPIRSAVCECNSTE
YYYPNADRSRCILASCKANETIMKSCGEFNSCEGNMESHICVCNGPGYKHHPSDDKQCVKETCE
MGEIDPIYLCGKGAVKCNGTYDIYTCECDNAMGYVQAPDGNSCKPFDCHANATMHKQCGDG
NRCIGSLTDWICVCDVAWYKPDPSNLMRCHEKTCKEADLELICGTGATGCSGSATNPTCECDSA
NYFVSSTDRRSCIKASCKTNITAVQQCGDGNSCYGTPDNYVCNCDAYGYRLDDNNNQACVEKK
CDDVGANYVEICGLGGIGCKGTVKNPVCECDQTKHYVASPDSTSCVLSNCRGNKTVMAACGNE
NKCFGSASSFICVCDGPGYKRNPSNLQECLPETCEAGEIKPHMVCGRGATACKGTMYHFECECN
NTLGFIRSPGHESCIPVNDCYANATMHEQCGEGNGCQGSVADWICKCDAPGYKPHKTNFQMC
HQRMCSDADIDLVEVCGVGVESCVGPIANPTCNCDTSKHYVLSADSTSCIPGSCKTNETIMAQCG
EGNSCSGTSENFVCICNGYGYRPHETDKQTCVLKKCSHSDVNAVDVCGQGALSCIGTMRDPICQ
CNNDMFFIPSDDSRSCVLASCSANSDVAVACGKVNGCFGSKSSWICDCSSPGTMLNPNNIRECLH
ETCESGKLDPIIVCGKGALGCNGTMSNVTCECDNSMGYVQSPNGMSCVASDCKTNSTMHELCG
DGNSCVGSLTDWMCVCDAPGYHPHPSDPMRCLQKMCSHANINPVDVCGIGATKCVGSITEPRC
TCDTAAYYVPSPDGTYCIPGSCQTNKTALEQCGNGNTCTGTADNFVCICNAPGYKLDDSNNKRC
IEISCNDADVDLVEICGQGSIGCIGTARQPVCQCDHSKHFVPSLESTSCQLASCRANDTIMDRCRM
GNTCFGSMTSHICVCEAKGFKVNPSDPTECVQETCEDKDIDALSACGKGATDCMGTLMNLTCQ
CDGASYVESPDKTSCLPVGCQQNVDVKKACGEGNTCEGSYDDWVCLCTNPEYIPHATDEKVCS
KKKCTDNPIGSCGIGATSCSGNVNIHKCTCNLTAGYMPSKDEQSCIPIDCKMNPKMNCGFGNTC
RGSFFSYVCECNTLGTMRNISNPKQCIEVDCSTDAVSICGVGATGCAVTPTNDVNCTCGEFYHPA
HDYHSCVLDDCKFNKLVPDACGVENECTGSISSWMCVCKGPKYIQDIKSTQLCAPGPCRIEQCGS
GKASGSFQYYAESCSGTYFEPTCNCKEGFEKDSYGGCIVKSCSRNNQSSVDCNDGAISCEGTYFKY

KCECRAPTHILNPANKRSCIPARCNPQLCGRGLVNGDYMDFASECSGSYTDFTCKCKYGLYGSGT
SKCSLEKRTCALSNDCHPNANCIDYDGHYFKCECKDGYSGDGYQCHRNGGLTPWSVVQPCDKT
CGTGYAQVMRTCDDPKPDTLGKKCNVPSPDVREVPCHQFACTGIKHTLPEIDGLISDINENLRSV
DQTKLYTITARDMMRNKLDCNVDLEKKDKYFRVYEAIVKRHFILKQVVSDLQAKEKLMVTYRSS
CTKDSKVGWLSSIFS*
>TRINITY_DN1058_c0_g1_i3.p1 type:complete len:2581 gc:universal TRINITY_DN1058_c0_g1_i3:134-7876(+)
MVKYIIWILLLLSIFKEAYGGDWAQWQNWSACQDSSGAPIGCGRGNQHRSRTCADNNCGAGAIIE
VRPCLDEICPTFNCQNSSEIAGMCKFDYSNSCVGDDNCHHCICKSEGFKVSGDQCYNYCVEVGC
DEEIEGVSQLKRYCGDQAISCTGTVLNRTCLCNPATSYPSKDGQYCYEATCKTSQEFMTNCGAEN
SCQGTNTSFVCECNGFGYGYEVVANVQNRKKCKLLSCEDGDLNPLEICGQGATACTGNVHNLT
CACNVTAGYIPSEDKKSCIPMPCNTDPAVGVQCLPRNTCEGSKIDWRCICSAYGFRNDPTDPKKCI
EAKCDDPNIDILGLCGTGVTSCTGFLSKRRCVCNTTENYIPTLDGSHCMPAPCNANENATKDCG
GENSCIGSFDSYMCVCNGYGFQKNATINKCEEVPCSSLPTAEVQRICGFGALSCDGTIRNPVCNC
NNDLQYYASEDMKSCIEASCSSKADYDSHCGNENSCVGNITHFECICETEGYKPDPSNKKKCILES
CELNEVDVLNVCGKGAISCKGNVRDLECVCDASKGYVQSPDTNKCEPSSCYGNATVSEQCGEEN
HCLGDLSDWICVCDAYGYKVNATNSMTCDKVMCGDTGADAVTVCGKETTSCSGHIAKPVCTC
NETAQYLPSPDSTRCVKASCKENDAARKACVPGNSCSGSYDEFICICDQFSYHQDPADNRKCIRE
KCEDLAADYDYEKICGVGGISCTGYVNQPVCVCNSTESYHPSNDRTSCVKASCSTSDAVRTQCGL
ENSCFGDIESFMCDCKAHGYKLEPINKTSCVLATCEDGDFSYHFQCGKGAVGCSGNINTLSCLCD
SAKGYVPSADMKRCVPNSCKDNPDVYIKCGMENTCLGSYADWFCVCSGAGYEANPADPKSCQL
MECSKDDAIHVCGYGAIDCKGPVLAPTCSCNYSDHYIASEDGTSCVPITCRKNDTARAQCGEENT
CKGHTDNFKCICDAYGFKADNVDHQKCVERQCTDGDADIIRICGHGGIRCTGPIRSAVCECNSTE
YYYPNADRSRCILASCKANETIMKSCGEFNSCEGNMESHICVCNGPGYKHHPSDDKQCVKETCE
MGEIDPIYLCGKGAVKCNGTYDIYTCECDNAMGYVQAPDGNSCKPFDCHANATMHKQCGDG
NRCIGSLTDWICVCDVAWYKPDPSNLMRCHEKTCKEADLELICGTGATGCSGSATNPTCECDSA
NYFVSSTDRRSCIKASCKTNITAVQQCGDGNSCYGTPDNYVCNCDAYGYRLDDNNNQACVEKK
CDDVGANYVEICGLGGIGCKGTVKNPVCECDQTKHYVASPDSTSCVLSNCRGNKTVMAACGNE
NKCFGSASSFICVCDGPGYKRNPSNLQECLPETCEAGEIKPHMVCGRGATACKGTMYHFECECN
NTLGFIRSPGHESCIPVNDCYANATMHEQCGEGNGCQGSVADWICKCDAPGYKPHKTNFQMC
HQRMCSDADIDLVEVCGVGVESCVGPIANPTCNCDTSKHYVLSADSTSCIPGSCKTNETIMAQCG
EGNSCSGTSENFVCICNGYGYRPHETDKQTCVLKKCSHSDVNAVDVCGQGALSCIGTMRDPICQ
CNNDMFFIPSDDSRSCVLASCSANSDVAVACGKVNGCFGSKSSWICDCSSPGTMLNPNNIRECLH
ETCESGKLDPIIVCGKGALGCNGTMSNVTCECDNSMGYVQSPNGMSCVASDCKTNSTMHELCG
DGNSCVGSLTDWMCVCDAPGYHPHPSDPMRCLQKMCSHANINPVDVCGIGATKCVGSITEPRC
TCDTAAYYVPSPDGTYCIPGSCQTNKTALEQCGNGNTCTGTADNFVCICNAPGYKLDDSNNKRC
IEISCNDADVDLVEICGQGSIGCIGTARQPVCQCDHSKHFVPSLESTSCQLASCRANDTIMDRCRM
GNTCFGSMTSHICVCEAKGFKVNPSDPTECVQETCEDKDIDALSACGKGATDCMGTLMNLTCQ
CDGASYVESPDKTSCLPVGCQQNVDVKKACGEGNTCEGSYDDWVCLCTNPEYIPHATDEKVCS
KKKCTDNPIGSCGIGATSCSGNVNIHKCTCNLTAGYMPSKDEQSCIPIDCKMNPKMNCGFGNTC
RGSFFSYVCECNTLGTMRNISNPKQCIEVDCSTDAVSICGVGATGCAVTPTNDVNCTCGEFYHPA
HDYHSCVLDDCKFNKLVPDACGVENECTGSISSWMCVCKGPKYIQDIKSTQLCAPGPCRIEQCGS
GKASGSFQYYAESCSGTYFEPTCNCKEGFEKDSYGGCIVKSCSRNNQSSVDCNDGAISCEGTYFKY
KCECRAPTHILNPANKRSCIPARCNPQLCGRGLVNGDYMDFASECSGSYTDFTCKCKYGLYGSGT
SKCSLEKRTCALSNDCHPNANCIDYDGHYFKCECKDGYSGDGYQCHRNGGLTPWSVVQPCDKT
CGTGYAQVMRTCDDPKPDTLGKKCNVPSPDVREVPCHQFACTGIKHTLPEIDGLISDINENLRSV
DQTKLYTITARDMMRNKLDCNVDLEKKDKYFRVYEAIVKRHFILKQVVSDLQAKEKLMVTYRSS
CTKDSKVGWLSSIFS*
>TRINITY_DN1058_c0_g1_i4.p1 type:complete len:723 gc:universal TRINITY_DN1058_c0_g1_i4:366-2534(+)
MDRCRMGNTCFGSMTSHICVCEAKGFKVNPSDPTECVQETCEDKDIDALSACGKGATDCMGTL
MNLTCQCDGASYVESPDKTSCLPVGCQQNVDVKKACGEGNTCEGSYDDWVCLCTNPEYIPHAT

DEKVCSKKKCTDNPIGSCGIGATSCSGNVNIHKCTCNLTAGYMPSKDEQSCIPIDCKMNPKMNC
GFGNTCRGSFFSYVCECNTLGTMRNISNPKQCIEVDCSTDAVSICGVGATGCAVTPTNDVNCTCG
EFYHPAHDYHSCVLDDCKFNKLVPDACGVENECTGSISSWMCVCKGPKYIQDIKSTQLCAPGPC
RIEQCGSGKASGSFQYYAESCSGTYFEPTCNCKEGFEKDSYGGCIVKSCSRNNQSSVDCNDGAISC
EGTYFKYKCECRAPTHILNPANKRSCIPARCNPQLCGRGLVNGDYMDFASECSGSYTDFTCKCKY
GLYGSGTSKCSLEKRTCALSNDCHPNANCIDYDGHYFKCECKDGYSGDGYQCHRNGGLTPWSV
VQPCDKTCGTGYAQVMRTCDDPKPDTLGKKCNVPSPDVREVPCHQFACTACSVKKTCECENIRP
YFVHEYVEPDESLKYFTNLTAEDVEKALSAIEKWKLSVDEKEFCIKHTLPEIDGLISDINENLRSVD
QTKLYTITARDMMRNKLDCNVDLEKKDKYFRVYEAIVKRHFILKQVVSDLQAKEKLMVTYRSSC
TKDSKVGWLSSIFS*
>TRINITY_DN1058_c0_g1_i2.p2 type:complete len:1300 gc:universal TRINITY_DN1058_c0_g1_i2:4090-7989(+)
MAACGNENKCFGSASSFICVCDGPGYKRNPSNLQECLPETCEAGEIKPHMVCGRGATACKGTM
YHFECECNNTLGFIRSPGHESCIPVNDCYANATMHEQCGEGNGCQGSVADWICKCDAPGYKPH
KTNFQMCHQRMCSDADIDLVEVCGVGVESCVGPIANPTCNCDTSKHYVLSADSTSCIPGSCKTN
ETIMAQCGEGNSCSGTSENFVCICNGYGYRPHETDKQTCVLKKCSHSDVNAVDVCGQGALSCIG
TMRDPICQCNNDMFFIPSDDSRSCVLASCSANSDVAVACGKVNGCFGSKSSWICDCSSPGTMLNP
NNIRECLHETCESGKLDPIIVCGKGALGCNGTMSNVTCECDNSMGYVQSPNGMSCVASDCKTNS
TMHELCGDGNSCVGSLTDWMCVCDAPGYHPHPSDPMRCLQKMCSHANINPVDVCGIGATKCV
GSITEPRCTCDTAAYYVPSPDGTYCIPGSCQTNKTALEQCGNGNTCTGTADNFVCICNAPGYKLD
DSNNKRCIEISCNDADVDLVEICGQGSIGCIGTARQPVCQCDHSKHFVPSLESTSCQLASCRANDT
IMDRCRMGNTCFGSMTSHICVCEAKGFKVNPSDPTECVQETCEDKDIDALSACGKGATDCMGTL
MNLTCQCDGASYVESPDKTSCLPVGCQQNVDVKKACGEGNTCEGSYDDWVCLCTNPEYIPHAT
DEKVCSKKKCTDNPIGSCGIGATSCSGNVNIHKCTCNLTAGYMPSKDEQSCIPIDCKMNPKMNC
GFGNTCRGSFFSYVCECNTLGTMRNISNPKQCIEVDCSTDAVSICGVGATGCAVTPTNDVNCTCG
EFYHPAHDYHSCVLDDCKFNKLVPDACGVENECTGSISSWMCVCKGPKYIQDIKSTQLCAPGPC
RIEQCGSGKASGSFQYYAESCSGTYFEPTCNCKEGFEKDSYGGCIVKSCSRNNQSSVDCNDGAISC
EGTYFKYKCECRAPTHILNPANKRSCIPARCNPQLCGRGLVNGDYMDFASECSGSYTDFTCKCKY
GLYGSGTSKCSLEKRTCALSNDCHPNANCIDYDGHYFKCECKDGYSGDGYQCHRNGGLTPWSV
VQPCDKTCGTGYAQVMRTCDDPKPDTLGKKCNVPSPDVREVPCHQFACTACSVKKTCECENIRP
YFVHEYVEPDESLKYFTNLTAEDVEKALSAIEKWKLSVDEKEFCIKHTLPEIDGLISDINENLRSVD
QTKLYTITARDMMRNKLDCNVDLEKKDKYFRVYEAIVKRHFILKQVVSDLQAKEKLMVTYRSSC
TKDSKVGWLSSIFS*
>TRINITY_DN691_c0_g1_i1.p1 type:complete len:1726 gc:universal TRINITY_DN691_c0_g1_i1:5662-485(-)
MMNLFRLIVLGVICFSYEGDAQQCGGSCSPCGNLCVGMKGDRGTAGLPGFRGPPGLPGFPGPEG
PIGQKGEKGPAGLNGMEGSQGARGKVGPPGLPGTPGRHGRPGNEGREGEAGLPGCNGTKGDR
GPEGPRGRDGIEGPQGLQGNVGPRGEPGDSTATKLKGQKGEPGSAGQQGEKGGRGMEGMKGD
TGKPGRNGRAGERGPKGEPGTSNVTIIGEKGSKGSVGPPGLPGRQCQVTGDKASNIAQGQKGNQ
GSKGEEGAKGEKGDAGLAGPQGEEGDIGPQGDRGDDGPQGPPGPEGEQGRSGIQGNKGQKGE
VGNNGKDGNDGQTGLTGQRGRQGTPGQKGEPGKDKKGLRGPEGAKGERGNDGEKGSDGPIGP
IGPRGPKGELGPLGMKGFKGSIGSAGPKGENGNPGDPGIKGRRGELGIKGDSGVKGAPGNPGISV
TGPDGMDGDTGPRGQSGGKGAVGFPGKPGPPGVVTIDGEEVLPGEPGPQGLKGFRGVQGDAGS
KGSRGEKGNACESSKCPAGPKGEKGLSGQDGLQGGDGEEGEKGEKGDKGDIGSRGDTGSRGEP
GPQGESGEKGEKGIQGPQGNSGKDIIVQGERGDKGTKGNAGTPGSIGFPGERGSPGESGDRGLSG
VNGLRGETGDRGARGVAGKPGVNGQKGQKGEIGSGARGFKGDKGEVGPDGPAGLRGPGGPVG
PKGDPFPTAVNGKTGEPGEQGAAGPKGAQGPVGPEGPRGFPGDIGPVGEPGDGEKGERGDDGA
RGERGEKGEPGQDGKDGDPGPKGEPGQEGKEGPPGMFKGNKGEKGATGLPGVDGLPGNSAPK
GEAGKDGQDGDPGPAGEKGDPGEAGSPGTEGPIGEDGPVGDKGEPGESRAGPKGNEGRAGAK
GENGRDGRAGRPGIGRPGDKGELGQKGEAVIGAKGSKGEQGLEGPPGPIGKPGVKGDAGVPGK
DGEAGGRGKDGERGLPGDKGDTGDIGFNGSPGAPGDKGEPGYSGQKGAKGMSGPRGPPGSTSN
VVGPKGNRGLPGMKGEPGQQGEKGLIGPQGSKGESGFGSEKGIKGSRGNPGAVGPSGEKGERGD

RGKDGLQGIAGPRGEEGNPGEKGSRGDQGPQGREGPSGELGPEGQKGDPGFAGEKGEKGDRGG
DGQKGDAGEPLRGEPGVAGPPGPTGPVGPPGKDYVFSGTAGELKGMKITKGDVGPDGVKGEKG
DRGDKGLDGRRGPTGPQGERGPKGEEGNMGPKGSQGRDGLTGEDGPKGSKGEPGLDGDVGEK
GEAGFQGLQGEKGNQGPQGLEGSTGMDGQKGQIGEKGVEGPPGPPCEPVNSVRGETGEEGPKG
DRGPKGQKGKPGPQSQAATPGEKGSKGEVGQPGPQGIKGEPGREGDFGPKGSPGAQGEKGDQG
QKGEPGQEGPEGLKGEPTRAFGPKGQKGETGLEGLRGPVGPKGEDGQTGPQGPDGPKGNEGEK
GDFGINGLNGAAGPSGPKGAIGLKGDSGPPGPTGAQGPRGSGGSPDFGFYYVKHSQTTIVPSCPA
GMKTLWTGYSFLYAQGNERAYGQDLGQPGSCLKRFSTMPYLFCDLQNQCTVASRNDYSFWLSTD
EPANMMKPAAGPEVEPYISRCAVCEAPANILAVHSQSVIVPDCPNGWENIWAGYSFLMYNSAGA
DGSGQLLASPGSCLEEFRANPFIECHGRGTCWYYGPTLSFWLSTIDDSNQFQIPKSETLKAGNLKT
RVSRCSVCMKTVRNGNFP*
>TRINITY_DN691_c0_g1_i2.p1 type:complete len:1673 gc:universal TRINITY_DN691_c0_g1_i2:5503-485(-)
MMNLFRLIVLGVICFSYEGDAQQCGGSCSPCGNLCVGMKGDRGTAGLPGFRGPPGLPGFPGPEG
PIGQKGEKGPAGLNGMEGSQGARGKVGPPGLPGTPGRHGRPGNEGREGEAGLPGCNGTKGDR
GPEGPRGRDGIEGPQGLQGNVGPRGEPGDSTATKLKGQKGEPGSAGQQGEKGGRGMEGMKGD
TGKPGRNGRAGERGPKGEPGTSNVTIIGEKGSKGSVGPPGLPGRQCQVTGDKASNIAQGQKGNQ
GSKGEEGAKGEKGDAGLAGPQGEEGDIGPQGDRGDDGPQGPPGPEGEQGRSGIQGNKGQKGE
VGNNGKDGNDGQTGLTGQRGRQGTPGQKGEPGKDKKGLRGPEGAKGERGNDGEKGSDGPIGP
IGPRGPKGELGPLGMKGFKGSIGSAGPKGENGNPGDPGIKGRRGELGIKGDSGVKGAPGNPGISV
TGPDGMDGDTGPRGQSGGKGAVGFPGKPGPPGVVTIDGEEVLPGEPGPQGLKGFRGVQGDAGS
KGSRGEKGNACESSKCPAGPKGEKGLSGQDGLQGGDGEEGEKGEKGDKGDIGSRGDTGSRGEP
GPQGESGEKGEKGIQGPQGNSGKDIIVQGERGDKGTKGNAGTPGSIGFPGERGSPGESGDRGLSG
VNGLRGETGDRGARGVAGKPGVNGQKGQKGEIGSGARGFKGDKGEVGPDGPAGLRGPGGPVG
PKGDPFPTAVNGKTGEPGEQGAAGPKGAQGPVGPEGPRGFPGDIGPVGEPGDGEKGERGDDGA
RGERGEKGEPGQDGKDGDPGPKGEPGQEGKEGPPGMFKGNKGEKGATGLPGVDGLPGNSAPK
GEAGKDGQDGDPGPAGEKGDPGEAGSPGTEGPIGEDGPVGDKGEPGESRAGPKGNEGRAGAK
GENGRDGRAGRPGIGRPGDKGELGQKGEAVIGAKGSKGEQGLEGPPGPIGKPGVKGDAGVPGK
DGEAGGRGKDGERGLPGDKGDTGDIGFNGSPGAPGDKGEPGYSGQKGAKGMSGPRGPPGSTSN
VVGPKGNRGLPGMKGEPGQQGEKGLIGPQGSKGESGFGSEKGIKGSRGNPGAVGPSGEKGERGD
RGKDGLQGIAGPRGEEGNPGEKGSRGDQGPQGREGPSGELGPEGQKGDPGFAGEKGEKGDRGG
DGQKGDAGEPLRGEPGVAGPPGPTGPQGERGPKGEEGNMGPKGSQGRDGLTGEDGPKGSKGEP
GLDGDVGEKGEAGFQGLQGEKGNQGPQGLEGSTGMDGQKGQIGEKGVEGPPGPPCEPVNSVR
GETGEEGPKGDRGPKGQKGKPGPQSQAATPGEKGSKGEVGQPGPQGIKGEPGREGDFGPKGSPG
AQGEKGDQGQKGEPGQEGPEGLKGEPTRAFGPKGQKGETGLEGLRGPVGPKGEDGQTGPQGP
DGPKGNEGEKGDFGINGLNGAAGPSGPKGAIGLKGDSGPPGPTGAQGPRGSGGSPDFGFYYVK
HSQTTIVPSCPAGMKTLWTGYSFLYAQGNERAYGQDLGQPGSCLKRFSTMPYLFCDLQNQCTVAS
RNDYSFWLSTDEPANMMKPAAGPEVEPYISRCAVCEAPANILAVHSQSVIVPDCPNGWENIWAG
YSFLMYNSAGADGSGQLLASPGSCLEEFRANPFIECHGRGTCWYYGPTLSFWLSTIDDSNQFQIPK
SETLKAGNLKTRVSRCSVCMKTVRNGNFP
>TRINITY_DN1616_c0_g1_i2.p1 type:3prime_partial len:3398 gc:universal TRINITY_DN1616_c0_g1_i2:218-10408(+)
MIIFLAFLAVAVGQEVLLPPYFDIAKGKNITATATCGEDYVAPKGSPGELFCTLADATGKFGIKGLK
CSHCVPGDYGDGDGKNARDHHIRNANDGSNRWWQSPPLSRGLQYRKINITMALGQSFHVAYVI
IKAANSPRPGVWALERSNDNGRTYTPWYYFAPTSSECKQYFGVDSYLPIVKDDTVICSTEYSKIPPF
KNGEIHISLINGRPGASNFSQSKTLQEWTTATNVRLRLLDIKTLLTDLLPLSLEDPTVTRRYYYSIKDI
TIGGRCMCNGFADECTPHTNIVGKYECNCQYNTEGPRCSRCKKGYVQKEWRQRTSTDKFECEKC
NCHGHSDECEYNEEINKRKLSLDVNGNYQGGGVCKNCQHNTLGINCEKCKPFFYQPANVPIDS
PNVCQPCNCDTQFSTGLCAEETGQCECKSNYVGRYCDQCNKGFHMFPVCKPCECDVNGSEDRE
CIPDQINGCKCKKGYAGRYCDECLPGYFGFPDCQPCACLTRYGAVNDNCERDNGQCSCKVGFK
GRRCEECAAGHYQFPFCKECGCDPEKTLDTVCDSGTGVCQCKSNFAGPKCESCSPGFYAHPSCFA
CGCSTAGSTSSVCDQNSGLCSCRPNYSGFKCDRCAEGYYGFPNCILCDCNPEGSRRVGICDRQDG

QCQCKPTYSGQKCDKCLPGFYGFPDCKRCNCNPAGTKPGPGGRPGDCSTSNNGQCVCKDNVQ
GLECNECKPGFFNLTESNRKGCSTCGCNGAGTNNGVKSCSVEGTCKCKPLVKQEDKCTECRDGY
YGLKRTNVFGCLGCNCEVGGSSSSVCSKDNGQCPCKDNIVGKRCESVAANSFYPDLCHIKAEME
EGKTTDGDVVRYDYQKEIFPDFSWKGFVAVNDQQPSVVVSINVPKTSEYQVKFRYALNQIQPVTA
TVTAFKGDSKIVGSINFASTQKGSGFVASLATVQDNLVQEMYFPLTEGQWNIKLEAPHSNLVVDY
AVLFPKEYAQGGALKKKVQNACDVMTDEKFCNLYRYIDVADKKDIIVLEAENPVKMRQQKPPVL
FNDTAVLSKLDFQAMVVLSPSNSRQLNHLFNVAKPGKYVVMLQYYHDSDTAKYLDVYVRSQIAV
QNGRALLQSCRYTFGCRQAVLNRGDNGVFVFNIKSTKNHLLTLLSRYAVVAVDSVMLVPYESWH
LDYSTPTLYCVTKDSKCTNMNYDVPAESRLVQANPLEQDLSEKPRYLIDRSVEVRYVKPGVNIINIR
DVKAGNYLLVAHYYQPNFPSFESRAAVYSGRTSGLRARFDFKFCPNLNGCRSPIRRPDSNSNLYVF
EEGEARIRFDIPNGLDAWIEYILMIPEGTNVSSLYTPRVLDYSDDFKKECSIDLNVCTSSNLSSFCKDS
IFTITTDFNNGSFPCKCNMDGSKSSSCQEFGGKCPCKDNVIGRECSRCKVGYYGFPNCKPCNCSN
CDEETGVCTCPRGVIGENCDQCAPGTFGFDPIIGCQKCNCNEIGTGSANAECNVTSGQCPCKEN
YSSRKCSACKPGYFNYPACTKCDCLDAGISAEKCDVLTGKCICQNNVIGLKCNECADGYFKTNDI
KGKQLCAKCWCNGRSTKSCTEAELFRATPITASKGWTVSVDTLNVIENGNGRFTISDVNTLPEPV
YWVAPNQFLGNRGLSYGSHFKYNFQYSVKQPSQNTNGDVVTDAVIKGDGYTIKSMRSVIEKEND
GYSASILMKETEWMLEDGSTIDRELFLAILSDIDTLQILATHGKNTISSTIYDISLETAGENGTDRVYG
IEKCFCPKEYADLSCSKCSKGYTRQKDGSCGKCQCLGKSNICDPQNGKCINCSGNTMGDRCEKC
KPGFYGDKCKACECPFSNPENSFANTCAINVNTSSLVCQCNAGYKGVRCDQCSDGYYGNPLQG
VTCKKCSCNGNINASFTGNCDNTTGDCLKCLYNSDGNQCQECRDGYYGDAVVEKNCTDCQCSI
CGTTTAPCNKATGQCQCKKNVEGSKCNQCKPNTWNFDACNGCEDCNCAEASETQQCDLKTG
QCKCPPTVFGRRCGFCKLGYYNYSSEGCQKCQCPDGKPCDVNTGKCCTGVLNEDCKLCPVGHII
EDGECVECDRCVQRTLQRVNTLIHNVTILEKHIIPAGGLNYGALETLGNRFSNLSNKGDEHISNL
DTLKKAIGFGEYRNHSGNMDYVRMYLVRDFKSCSFIVNIKTPPTSSAPATTMVTNTPGPDPIRDHL
SNKKISDSQCPPSLARVIDEGEKNIKLVEKKVVVLEKDAYESTENATATKKRAEDLLAKLKDFMVD
FKDVLSFANSTLKGNVVINIDGNRSLAEAEEMLKEIKDRNFNSTINRSEREKSAAINATIIAEQLMK
QASELANKAYKFNDTFKDFVKALRNLKMESELALLDANNATNSINLAKAVDFQSIIDNAQKLHS
QNNVTVTQAMEILQNAKSAYENAKDVFNKFHNQTGPLSDLMEELRNYVRKLEFDLQTTSPIVDD
SVKHAKRLRDSADRLKELLQDTKKFADAALRAAKVYQNIVDAINDALDAARMANETAHEADK
KVQQAQQDAVESSITSKVVDTEANDLTEEVKKTLKVDISDMKMRLKELRELRKRIQGRIDNVKED
VKSFAKEKEVGEKAMNISTQADGVMKVANKGNEASQDTNEKVEKDGEKVETLKVDISDINKAIK
DLASNVTLVEDTIPKIRSNIDEVNVKHEQIEEFIKTNVTNNIGYLKYLISNAHDILDQMQLSMKFNS
TSEVVLDAPKSVADPTINNKISLTFKINQGNGFLFIIGDFSGNSSGFMSLHVQNHTVVFDYKLNTG
DVQSLTNPKFIETDVNYDVMVTRNFATAVLNVQKSGNASTLMTAYQNRSNISPVLSLGQDTNVY
VGYHPSTPNQRPNFDGCINQFRFDEELMGLWHWKSAKNVGGCVQDFRSIEGQYSFDGDGFVEF
DLVSPELSGFRKIELRFATLEENGLIFLGTDPPTSLPSDIMFDIPLSNQIIAFNALEIKDGYLVSTLDLG
NGVQRLVHTNAGKVNDGKPHLFEMKVLISSLLIKFKLDKKKITEIAISPDTDIRLRLKEAFIGGLPQ
RAWLPNSNISTKGFTGAIGDAKYRGGEFKLVKRFNNIRKGFILPKGYSASFPEEKSYLRLDACNLK
QNSLFGFSFQTFEKNGIIFHSEEGSGSSRSSIEIILIDGQVKVDVSVGSQKRTLQTSVARGFSDGLWHT
LSLYFEETLQEADIVLLYLAVDKAYITGTVPKVDLKGSMYFGSKPSGTESGFNGNIRQVYCNKNEIN
LASGDVFEGGVVGSLKLLPPLKLADFTVVPTTLPPLTTSITTSESTTTETVMTTSTTSISGTTLEPPKET
TTNMAKTTENPTVKITSSEPIIETTMESTTVAKTTSAITMEESTTTPTSITTSTLETTISTLEPGSIEGSTT
TAE
>TRINITY_DN1616_c0_g1_i3.p1 type:complete len:3855 gc:universal
TRINITY_DN1616_c0_g1_i3:218-11782(+)
MIIFLAFLAVAVGQEVLLPPYFDIAKGKNITATATCGEDYVAPKGSPGELFCTLADATGKFGIKGLK
CSHCVPGDYGDGDGKNARDHHIRNANDGSNRWWQSPPLSRGLQYRKINITMALGQSFHVAYVI
IKAANSPRPGVWALERSNDNGRTYTPWYYFAPTSSECKQYFGVDSYLPIVKDDTVICSTEYSKIPPF
KNGEIHISLINGRPGASNFSQSKTLQEWTTATNVRLRLLDIKTLLTDLLPLSLEDPTVTRRYYYSIKDI
TIGGRCMCNGFADECTPHTNIVGKYECNCQYNTEGPRCSRCKKGYVQKEWRQRTSTDKFECEKC
NCHGHSDECEYNEEINKRKLSLDVNGNYQGGGVCKNCQHNTLGINCEKCKPFFYQPANVPIDS
PNVCQPCNCDTQFSTGLCAEETGQCECKSNYVGRYCDQCNKGFHMFPVCKPCECDVNGSEDRE

CIPDQINGCKCKKGYAGRYCDECLPGYFGFPDCQPCACLTRYGAVNDNCERDNGQCSCKVGFK
GRRCEECAAGHYQFPFCKECGCDPEKTLDTVCDSGTGVCQCKSNFAGPKCESCSPGFYAHPSCFA
CGCSTAGSTSSVCDQNSGLCSCRPNYSGFKCDRCAEGYYGFPNCILCDCNPEGSRRVGICDRQDG
QCQCKPTYSGQKCDKCLPGFYGFPDCKRCNCNPAGTKPGPGGRPGDCSTSNNGQCVCKDNVQ
GLECNECKPGFFNLTESNRKGCSTCGCNGAGTNNGVKSCSVEGTCKCKPLVKQEDKCTECRDGY
YGLKRTNVFGCLGCNCEVGGSSSSVCSKDNGQCPCKDNIVGKRCESVAANSFYPDLCHIKAEME
EGKTTDGDVVRYDYQKEIFPDFSWKGFVAVNDQQPSVVVSINVPKTSEYQVKFRYALNQIQPVTA
TVTAFKGDSKIVGSINFASTQKGSGFVASLATVQDNLVQEMYFPLTEGQWNIKLEAPHSNLVVDY
AVLFPKEYAQGGALKKKVQNACDVMTDEKFCNLYRYIDVADKKDIIVLEAENPVKMRQQKPPVL
FNDTAVLSKLDFQAMVVLSPSNSRQLNHLFNVAKPGKYVVMLQYYHDSDTAKYLDVYVRSQIAV
QNGRALLQSCRYTFGCRQAVLNRGDNGVFVFNIKSTKNHLLTLLSRYAVVAVDSVMLVPYESWH
LDYSTPTLYCVTKDSKCTNMNYDVPAESRLVQANPLEQDLSEKPRYLIDRSVEVRYVKPGVNIINIR
DVKAGNYLLVAHYYQPNFPSFESRAAVYSGRTSGLRARFDFKFCPNLNGCRSPIRRPDSNSNLYVF
EEGEARIRFDIPNGLDAWIEYILMIPEGTNVSSLYTPRVLDYSDDFKKECSIDLNVCTSSNLSSFCKDS
IFTITTDFNNGSFPCKCNMDGSKSSSCQEFGGKCPCKDNVIGRECSRCKVGYYGFPNCKPCNCSN
CDEETGVCTCPRGVIGENCDQCAPGTFGFDPIIGCQKCNCNEIGTGSANAECNVTSGQCPCKEN
YSSRKCSACKPGYFNYPACTKCDCLDAGISAEKCDVLTGKCICQNNVIGLKCNECADGYFKTNDI
KGKQLCAKCWCNGRSTKSCTEAELFRATPITASKGWTVSVDTLNVIENGNGRFTISDVNTLPEPV
YWVAPNQFLGNRGLSYGSHFKYNFQYSVKQPSQNTNGDVVTDAVIKGDGYTIKSMRSVIEKEND
GYSASILMKETEWMLEDGSTIDRELFLAILSDIDTLQILATHGKNTISSTIYDISLETAGENGTDRVYG
IEKCFCPKEYADLSCSKCSKGYTRQKDGSCGKCQCLGKSNICDPQNGKCINCSGNTMGDRCEKC
KPGFYGDKCKACECPFSNPENSFANTCAINVNTSSLVCQCNAGYKGVRCDQCSDGYYGNPLQG
VTCKKCSCNGNINASFTGNCDNTTGDCLKCLYNSDGNQCQECRDGYYGDAVVEKNCTDCQCSI
CGTTTAPCNKATGQCQCKKNVEGSKCNQCKPNTWNFDACNGCEDCNCAEASETQQCDLKTG
QCKCPPTVFGRRCGFCKLGYYNYSSEGCQKCQCPDGKPCDVNTGKCCTGVLNEDCKLCPVGHII
EDGECVECDRCVQRTLQRVNTLIHNVTILEKHIIPAGGLNYGALETLGNRFSNLSNKGDEHISNL
DTLKKAIGFGEYRNHSGNMDYVRMYLVRDFKSCSFIVNIKTPPTSSAPATTMVTNTPGPDPIRDHL
SNKKISDSQCPPSLARVIDEGEKNIKLVEKKVVVLEKDAYESTENATATKKRAEDLLAKLKDFMVD
FKDVLSFANSTLKGNVVINIDGNRSLAEAEEMLKEIKDRNFNSTINRSEREKSAAINATIIAEQLMK
QASELANKAYKFNDTFKDFVKALRNLKMESELALLDANNATNSINLAKAVDFQSIIDNAQKLHS
QNNVTVTQAMEILQNAKSAYENAKDVFNKFHNQTGPLSDLMEELRNYVRKLEFDLQTTSPIVDD
SVKHAKRLRDSADRLKELLQDTKKFADAALRAAKVYQNIVDAINDALDAARMANETAHEADK
KVQQAQQDAVESSITSKVVDTEANDLTEEVKKTLKVDISDMKMRLKELRELRKRIQGRIDNVKED
VKSFAKEKEVGEKAMNISTQADGVMKVANKGNEASQDTNEKVEKDGEKVETLKVDISDINKAIK
DLASNVTLVEDTIPKIRSNIDEVNVKHEQIEEFIKTNVTNNIGYLKYLISNAHDILDQMQLSMKFNS
TSEVVLDAPKSVADPTINNKISLTFKINQGNGFLFIIGDFSGNSSGFMSLHVQNHTVVFDYKLNTG
DVQSLTNPKFIETDVNYDVMVTRNFATAVLNVQKSGNASTLMTAYQNRSNISPVLSLGQDTNVY
VGYHPSTPNQRPNFDGCINQFRFDEELMGLWHWKSAKNVGGCVQDFRSIEGQYSFDGDGFVEF
DLVSPELSGFRKIELRFATLEENGLIFLGTDPPTSLPSDIMFDIPLSNQIIAFNALEIKDGYLVSTLDLG
NGVQRLVHTNAGKVNDGKPHLFEMKVLISSLLIKFKLDKKKITEIAISPDTDIRLRLKEAFIGGLPQ
RAWLPNSNISTKGFTGAIGDAKYRGGEFKLVKRFNNIRKGFILPKGYSASFPEEKSYLRLDACNLK
QNSLFGFSFQTFEKNGIIFHSEEGSGSSRSSIEIILIDGQVKVDVSVGSQKRTLQTSVARGFSDGLWHT
LSLYFEETLQEADIVLLYLAVDKAYITGTVPKVDLKGSMYFGSKPSGTESGFNGNIRQVYCNKNEIN
LASGDVFEGGVVGSLKLLPPLKLADFTVVPTTLPPLTTSITTSESTTTETVMTTSTTSISGTTLEPPKET
TTNMAKTTENPTVKITSSEPIIETTMESTTVAKTTSAITMEESTTTPTSITTSTLETTISTLEPGSIEGSTT
TAEPITTTAEAITTTATEPITTTEPITITQPITTVEVTTEPATTQESTTTPEPITTLETTTTPEPSTTPEPTTP
EELTTTAKPTTTIRVTTEPPKCQSNLEVYDEAYGFGMNEPSFIEFGVDGRKMLKSNQFEFLFYTAEN
DGMMISSEPSNLYDYEYVYMENGAISYAFNAGAGRVRVTSTKKYNDGKWHEVKISRSKESGTLTI
DGELIGTDSKSGKTAVNGIKKIVVGGSSVPYRNIPPKSQVGFKGGMRGFKLFNEALKPLKEIGITEA
YDELPIPEGVSFGPDGGYVALNFKSEYVVRTEYKLALDVRSVSSSGYIFFIGNYGADYLSLYIESDRK
VVAECNNGGGRFSVSIDVPTICNGEFHSILLEKKYKTLDLSVEGVQKSFTTTKGSTSADTGSNPPAF
GGFNGVGSRPKNLETFTGCITAVRIKDKAVDLTAPDVTIFGQVSNGCPSNIR

>TRINITY_DN1616_c0_g1_i7.p1 type:complete len:3855 gc:universal TRINITY_DN1616_c0_g1_i7:218-11782(+)
MIIFLAFLAVAVGQEVLLPPYFDIAKGKNITATATCGEDYVAPKGSPGELFCTLADATGKFGIKGLK
CSHCVPGDYGDGDGKNARDHHIRNANDGSNRWWQSPPLSRGLQYRKINITMALGQSFHVAYVI
IKAANSPRPGVWALERSNDNGRTYTPWYYFAPTSSECKQYFGVDSYLPIVKDDTVICSTEYSKIPPF
KNGEIHISLINGRPGASNFSQSKTLQEWTTATNVRLRLLDIKTLLTDLLPLSLEDPTVTRRYYYSIKDI
TIGGRCMCNGFADECTPHTNIVGKYECNCQYNTEGPRCSRCKKGYVQKEWRQRTSTDKFECEKC
NCHGHSDECEYNEEINKRKLSLDVNGNYQGGGVCKNCQHNTLGINCEKCKPFFYQPANVPIDS
PNVCQPCNCDTQFSTGLCAEETGQCECKSNYVGRYCDQCNKGFHMFPVCKPCECDVNGSEDRE
CIPDQINGCKCKKGYAGRYCDECLPGYFGFPDCQPCACLTRYGAVNDNCERDNGQCSCKVGFK
GRRCEECAAGHYQFPFCKECGCDPEKTLDTVCDSGTGVCQCKSNFAGPKCESCSPGFYAHPSCFA
CGCSTAGSTSSVCDQNSGLCSCRPNYSGFKCDRCAEGYYGFPNCILCDCNPEGSRRVGICDRQDG
QCQCKPTYSGQKCDKCLPGFYGFPDCKRCNCNPAGTKPGPGGRPGDCSTSNNGQCVCKDNVQ
GLECNECKPGFFNLTESNRKGCSTCGCNGAGTNNGVKSCSVEGTCKCKPLVKQEDKCTECRDGY
YGLKRTNVFGCLGCNCEVGGSSSSVCSKDNGQCPCKDNIVGKRCESVAANSFYPDLCHIKAEME
EGKTTDGDVVRYDYQKEIFPDFSWKGFVAVNDQQPSVVVSINVPKTSEYQVKFRYALNQIQPVTA
TVTAFKGDSKIVGSINFASTQKGSGFVASLATVQDNLVQEMYFPLTEGQWNIKLEAPHSNLVVDY
AVLFPKEYAQGGALKKKVQNACDVMTDEKFCNLYRYIDVADKKDIIVLEAENPVKMRQQKPPVL
FNDTAVLSKLDFQAMVVLSPSNSRQLNHLFNVAKPGKYVVMLQYYHDSDTAKYLDVYVRSQIAV
QNGRALLQSCRYTFGCRQAVLNRGDNGVFVFNIKSTKNHLLTLLSRYAVVAVDSVMLVPYESWH
LDYSTPTLYCVTKDSKCTNMNYDVPAESRLVQANPLEQDLSEKPRYLIDRSVEVRYVKPGVNIINIR
DVKAGNYLLVAHYYQPNFPSFESRAAVYSGRTSGLRARFDFKFCPNLNGCRSPIRRPDSNSNLYVF
EEGEARIRFDIPNGLDAWIEYILMIPEGTNVSSLYTPRVLDYSDDFKKECSIDLNVCTSSNLSSFCKDS
IFTITTDFNNGSFPCKCNMDGSKSSSCQEFGGKCPCKDNVIGRECSRCKVGYYGFPNCKPCNCSN
CDEETGVCTCPRGVIGENCDQCAPGTFGFDPIIGCQKCNCNEIGTGSANAECNVTSGQCPCKEN
YSSRKCSACKPGYFNYPACTKCDCLDAGISAEKCDVLTGKCICQNNVIGLKCNECADGYFKTNDI
KGKQLCAKCWCNGRSTKSCTEAELFRATPITASKGWTVSVDTLNVIENGNGRFTISDVNTLPEPV
YWVAPNQFLGNRGLSYGSHFKYNFQYSVKQPSQNTNGDVVTDAVIKGDGYTIKSMRSVIEKEND
GYSASILMKETEWMLEDGSTIDRELFLAILSDIDTLQILATHGKNTISSTIYDISLETAGENGTDRVYG
IEKCFCPKEYADLSCSKCSKGYTRQKDGSCGKCQCLGKSNICDPQNGKCINCSGNTMGDRCEKC
KPGFYGDKCKACECPFSNPENSFANTCAINVNTSSLVCQCNAGYKGVRCDQCSDGYYGNPLQG
VTCKKCSCNGNINASFTGNCDNTTGDCLKCLYNSDGNQCQECRDGYYGDAVVEKNCTDCQCSI
CGTTTAPCNKATGQCQCKKNVEGSKCNQCKPNTWNFDACNGCEDCNCAEASETQQCDLKTG
QCKCPPTVFGRRCGFCKLGYYNYSSEGCQKCQCPDGKPCDVNTGKCCTGVLNEDCKLCPVGHII
EDGECVECDRCVQRTLQRVNTLIHNVTILEKHIIPAGGLNYGALETLGNRFSNLSNKGDEHISNL
DTLKKAIGFGEYRNHSGNMDYVRMYLVRDFKSCSFIVNIKTPPTSSAPATTMVTNTPGPDPIRDHL
SNKKISDSQCPPSLARVIDEGEKNIKLVEKKVVVLEKDAYESTENATATKKRAEDLLAKLKDFMVD
FKDVLSFANSTLKGNVVINIDGNRSLAEAEEMLKEIKDRNFNSTINRSEREKSAAINATIIAEQLMK
QASELANKAYKFNDTFKDFVKALRNLKMESELALLDANNATNSINLAKAVDFQSIIDNAQKLHS
QNNVTVTQAMEILQNAKSAYENAKDVFNKFHNQTGPLSDLMEELRNYVRKLEFDLQTTSPIVDD
SVKHAKRLRDSADRLKELLQDTKKFADAALRAAKVYQNIVDAINDALDAARMANETAHEADK
KVQQAQQDAVESSITSKVVDTEANDLTEEVKKTLKVDISDMKMRLKELRELRKRIQGRIDNVKED
VKSFAKEKEVGEKAMNISTQADGVMKVANEGNKASQDTNGKVEKDGEKVETLKVDISDINKAI
KDLASNVTLVEDTIPKIRSNIDEVNVKHEQIEEFIKTNVTNNIGYLKYLISNAHDILDQMQLSMKF
NSTSEVVLDAPKSVADPTINNKISLTFKINQGNGFLFIIGDFSGNSSGFMSLHVQNHTVVFDYKLNT
GDVQSLTNPKFIETDVNYDVMVTRNFATAVLNVQKSGNASTLMTAYQNRSNISPVLSLGQDTNV
YVGYHPSTPNQRPNFDGCINQFRFDEELMGLWHWKSAKNVGGCVQDFRSIEGQYSFDGDGFVE
FDLVSPELSGFRKIELRFATLEENGLIFLGTDPPTSLPSDIMFDIPLSNQIIAFNALEIKDGYLVSTLDL
GNGVQRLVHTNAGKVNDGKPHLFEMKVLISSLLIKFKLDKKKITEIAISPDTDIRLRLKEAFIGGLP
QRAWLPNSNISTKGFTGAIGDAKYRGGEFKLVKRFNNIRKGFILPKGYSASFPEEKSYLRLDACNL
KQNSLFGFSFQTFEKNGIIFHSEEGSGSSRSSIEIILIDGQVKVDVSVGSQKRTLQTSVARGFSDGLWH
TLSLYFEETLQEADIVLLYLAVDKAYITGTVPKVDLKGSMYFGSKPSGTESGFNGNIRQVYCNKNEI

NLASGDVFEGGVVGSLKLLPPLKLADFTVVPTTLPPLTTSITTSESTTTETVMTTSTTSISGTTLEPPK
ETTTNMAKTTENPTVKITSSEPIIETTMESTTVAKTTSAITMEESTTTPTSITTSTLETTISTLEPGSIEGS
TTTAEPITTTAEAITTTATEPITTTEPITITQPITTVEVTTEPATTQESTTTPEPITTLETTTTPEPSTTPEPT
TPEELTTTAKPTTTIRVTTEPPKCQSNLEVYDEAYGFGMNEPSFIEFGVDGRKMLKSNQFEFLFYTA
ENDGMMISSEPSNLYDYEYVYMENGAISYAFNAGAGRVRVTSTKKYNDGKWHEVKISRSKESGTL
TIDGELIGTDSKSGKTAVNGIKKIVVGGSSVPYRNIPPKSQVGFKGGMRGFKLFNEALKPLKEIGITE
AYDELPIPEGVSFGPDGGYVALNFKSEYVVRTEYKLALDVRSVSSSGYIFFIGNYGADYLSLYIESDR
KVVAECNNGGGRFSVSIDVPTICNGEFHSILLEKKYKTLDLSVEGVQKSFTTTKGSTSADTGSNPPA
FGGFNGVGSRPKNLETFTGCITAVRIKDKAVDLTAPDVTIFGQVSNGCPSNIR*
>TRINITY_DN1616_c0_g1_i6.p1 type:complete len:3855 gc:universal TRINITY_DN1616_c0_g1_i6:218-11782(+)
MIIFLAFLAVAVGQEVLLPPYFDIAKGKNITATATCGEDYVAPKGSPGELFCTLADATGKFGIKGLK
CSHCVPGDYGDGDGKNARDHHIRNANDGSNRWWQSPPLSRGLQYRKINITMALGQSFHVAYVI
IKAANSPRPGVWALERSNDNGRTYTPWYYFAPTSSECKQYFGVDSYLPIVKDDTVICSTEYSKIPPF
KNGEIHISLINGRPGASNFSQSKTLQEWTTATNVRLRLLDIKTLLTDLLPLSLEDPTVTRRYYYSIKDI
TIGGRCMCNGFADECTPHTNIVGKYECNCQYNTEGPRCSRCKKGYVQKEWRQRTSTDKFECEKC
NCHGHSDECEYNEEINKRKLSLDVNGNYQGGGVCKNCQHNTLGINCEKCKPFFYQPANVPIDS
PNVCQPCNCDTQFSTGLCAEETGQCECKSNYVGRYCDQCNKGFHMFPVCKPCECDVNGSEDRE
CIPDQINGCKCKKGYAGRYCDECLPGYFGFPDCQPCACLTRYGAVNDNCERDNGQCSCKVGFK
GRRCEECAAGHYQFPFCKECGCDPEKTLDTVCDSGTGVCQCKSNFAGPKCESCSPGFYAHPSCFA
CGCSTAGSTSSVCDQNSGLCSCRPNYSGFKCDRCAEGYYGFPNCILCDCNPEGSRRVGICDRQDG
QCQCKPTYSGQKCDKCLPGFYGFPDCKRCNCNPAGTKPGPGGRPGDCSTSNNGQCVCKDNVQ
GLECNECKPGFFNLTESNRKGCSTCGCNGAGTNNGVKSCSVEGTCKCKPLVKQEDKCTECRDGY
YGLKRTNVFGCLGCNCEVGGSSSSVCSKDNGQCPCKDNIVGKRCESVAANSFYPDLCHIKAEME
EGKTTDGDVVRYDYQKEIFPDFSWKGFVAVNDQQPSVVVSINVPKTSEYQVKFRYALNQIQPVTA
TVTAFKGDSKIVGSINFASTQKGSGFVASLATVQDNLVQEMYFPLTEGQWNIKLEAPHSNLVVDY
AVLFPKEYAQGGALKKKVQNACDVMTDEKFCNLYRYIDVADKKDIIVLEAENPVKMRQQKPPVL
FNDTAVLSKLDFQAMVVLSPSNSRQLNHLFNVAKPGKYVVMLQYYHDSDTAKYLDVYVRSQIAV
QNGRALLQSCRYTFGCRQAVLNRGDNGVFVFNIKSTKNHLLTLLSRYAVVAVDSVMLVPYESWH
LDYSTPTLYCVTKDSKCTNMNYDVPAESRLVQANPLEQDLSEKPRYLIDRSVEVRYVKPGVNIINIR
DVKAGNYLLVAHYYQPNFPSFESRAAVYSGRTSGLRARFDFKFCPNLNGCRSPIRRPDSNSNLYVF
EEGEARIRFDIPNGLDAWIEYILMIPEGTNVSSLYTPRVLDYSDDFKKECSIDLNVCTSSNLSSFCKDS
IFTITTDFNNGSFPCKCNMDGSKSSSCQEFGGKCPCKDNVIGRECSRCKVGYYGFPNCKPCNCSN
CDEETGVCTCPRGVIGENCDQCAPGTFGFDPIIGCQKCNCNEIGTGSANAECNVTSGQCPCKEN
YSSRKCSACKPGYFNYPACTKCDCLDAGISAEKCDVLTGKCICQNNVIGLKCNECADGYFKTNDI
KGKQLCAKCWCNGRSTKSCTEAELFRATPITASKGWTVSVDTLNVIENGNGRFTISDVNTLPEPV
YWVAPNQFLGNRGLSYGSHFKYNFQYSVKQPSQNTNGDVVTDAVIKGDGYTIKSMRSVIEKEND
GYSASILMKETEWMLEDGSTIDRELFLAILSDIDTLQILATHGKNTISSTIYDISLETAGENGTDRVYG
IEKCFCPKEYADLSCSKCSKGYTRQKDGSCGKCQCLGKSNICDPQNGKCINCSGNTMGDRCEKC
KPGFYGDKCKACECPFSNPENSFANTCAINVNTSSLVCQCNAGYKGVRCDQCSDGYYGNPLQG
VTCKKCSCNGNINASFTGNCDNTTGDCLKCLYNSDGNQCQECRDGYYGDAVVEKNCTDCQCSI
CGTTTAPCNKATGQCQCKKNVEGSKCNQCKPNTWNFDACNGCEDCNCAEASETQQCDLKTG
QCKCPPTVFGRRCGFCKLGYYNYSSEGCQKCQCPDGKPCDVNTGKCCTGVLNEDCKLCPVGHII
EDGECVECDRCVQRTLQRVNTLIHNVTILEKHIIPAGGLNYGALETLGNRFSNLSNKGDEHISNL
DTLKKAIGFGEYRNHSGNMDYVRMYLVRDFKSCSFIVNIKTPPTSSAPATTMVTNTPGPDPIRDHL
SNKKISDSQCPPSLARVIDEGEKNIKLVEKKVVVLEKDAYESTENATATKKRAEDLLAKLKDFMVD
FKDVLSFANSTLKGNVVINIDGNRSLAEAEEMLKEIKDRNFNSTINRSEREKSAAINATIIAEQLMK
QASELANKAYKFNDTFKDFVKALRNLKMESELALLDANNATNSINLAKAVDFQSIIDNAQKLHS
QNNVTVTQAMEILQNAKSAYENAKDVFNKFHNQTGPLSDLMEELRNYVRKLEFDLQTTSPIVDD
SVKHAKRLRDSADRLKELLQDTKKFADAALRAAKVYQNIVDAINDALDAARMANETAHEADK
KVQQAQQDAVESSITSKVVDTEANDLTEEVKKTLKVDISDMKMRLKELRELRKRIQGRIDNVKED
VKSFAKEKEVGEKAMNISTQADGVMKVANKGNEASQDTNEKVEKDGEKVETLKVDISDINKAIK

DLASNVTLVEDTIPKIRSNIDEVNVKHEQIEEFIKTNVTNNIGYLKYLISNAHDILDQMQLSMKFNS
TSEVVLDAPKSVADPTINNKISLTFKINQGNGFLFIIGDFSGNSSGFMSLHVQNHTVVFDYKLNTG
DVQSLTNPKFIETDVNYDVMVTRNFATAVLNVQKSGNASTLMTAYQNRSNISPVLSLGQDTNVY
VGYHPSTPNQRPNFDGCINQFRFDEELMGLWHWKSAKNVGGCVQDFRSIEGQYSFDGDGFVEF
DLVSPELSGFRKIELRFATLEENGLIFLGTDPPTSLPSDIMFDIPLSNQIIAFNALEIKDGYLVSTLDLG
NGVQRLVHTNAGKVNDGKPHLFEMKVLISSLLIKFKLDKKKITEIAISPDTDIRLRLKEAFIGGLPQ
RAWLPNSNISTKGFTGAIGDAKYRGGEFKLVKRFNNIRKGFILPKGYSASFPEEKSYLRLDACNLK
QNSLFGFSFQTFEKNGIIFHSEEGSGSSRSSIEIILIDGQVKVDVSVGSQKRTLQTSVARGFSDGLWHT
LSLYFEETLQEADIVLLYLAVDKAYITGTVPKVDLKGSMYFGSKPSGTESGFNGNIRQVYCNKNEIN
LASGDVFEGGVVGSLKLLPPLKLADFTVVPTTLPPLTTSITTSESTTTETVMTTSTTSISGTTLEPPKET
TTNMAKTTENPTVKITSSEPIIETTMESTTVAKTTSAITMEESTTTPTSITTSTLETTISTLEPGSIEGSTT
TAESITTTAAPITTTATEPITTTESITITQPITTVEVTTEPATTQESTTTPEPITTLETTTTLEPSTTPEPTTP
EELTTTAKPTTTIRVTTEPPKCQSNLEVYDEAYGFGMNEPSFIEFGVDGRKMLKSNQFEFLFYTAEN
DGMMISSEPSNLYDYEYVYMENGAISYAFNAGAGRVRVTSTKKYNDGKWHEVKISRSKESGTLTI
DGELIGTDSKSGKTAVNGIKKIVVGGSSVPYRNIPPKSQVGFKGGMRGFKLFNEALKPLKEIGITEA
YDELPIPEGVSFGPDGGYVALNFKSEYVVRTEYKLALDVRSVSSSGYIFFIGNYGADYLSLYIESDRK
VVAECNNGGGRFSVSIDVPTICNGEFHSILLEKKYKTLDLSVEGVQKSFTTTKGSTSADTGSNPPAF
GGFNGVGSRPKNLETFTGCITAVRIKDKAVDLTAPDVTIFGQVSNGCPSNIR*
>TRINITY_DN8903_c0_g1_i1.p1 type:5prime_partial len:447 gc:universal TRINITY_DN8903_c0_g1_i1:1546-206(-)
DMATKACGLENMCVGSYKSWRCICQAPRYMQDPDNTQSCIHAPCRIEDCGSGYAPGSFQYYAES
CNGTFFNPICNCQEEYEKDMLGGCVPKSCRENPIAVAECGENGYCKGTYFKYECGCQGPKYVQN
PRNTRQCIEAPCNVQNCGHGFENELYVSFADSCIGKHADYTCQCKEGYTGTGTSRCIPDGKECGK
SYCHANATCAPLGKGEFDCTCKPGYSGDGLYCFLDGGWSDFVNTRECNASCGTHGYAEVTRYC
NNPVAERFGKQCGAKSPEQLEVPCNRFDCLGCTVKKDCRCTSVVKPLIVEGYNEPEETLKFFTDL
HPDDVKKAKDTLLKWKAFSNENLCRKYNKTTLHAKFFGVRDAAKEVNKMKSSYGSARDSMRI
KLDCNINIAKKTQYFGVYEMIVKRLFIMKQVVKDLHIIEDYVLKHMENCDDSPVGMIRNTFGK*
>TRINITY_DN11377_c0_g1_i1.p1 type:complete len:804 gc:universal TRINITY_DN11377_c0_g1_i1:3179-768(-)
MKQNWYNYFLLVTFAVCIIQYTNGQEERDSNPCVQATTCEACMQANPDCYWCRDVNYTEGEN
FSRDRCNLLIEFNDGCANLTSISSAFEPGQNSNFSDTVQVQPQTVTLDLRPGQPAVFNISVKPAPDF
PVDIYFLFDASFSMKDDLEKLEQLGAAIAQSINNITKNYRLGFGTFVDKKAVPFVEGPLFPTFPCSR
QVKVDNCAPVFPFRHTFSFSKNTTLFQQAVADQPLSGDLDRPEGSFDGLMQVVVCDQKIGWQPR
GKARRIVLLATDALPKIAGDGRLIGDLLQNDETCHLSPLNSDRSVLEYSESLTMDYPSISQLREAM
KKYEITPIIAVTEDVQDVYKDIANSWESLLAAFGVLNNDSSNIVDLVRENYKKISSTVRLLETKNEY
LDFEYKQLSPECQETLTSPNECEKVAIGQQVDFQVSVTLKKCPPEEENAANRGDIDIPGFGQVKIN
LNYLCTCPCSNNAIPNSVLCNTTGSLDCGICKCNQGFFGSKCECDETTVSRNDTLKCQSDQNDPS
FICSNQGICLCGVCDCFRVAAPRIISGKYCECKNFGCPKSRGQECGGDERGTCVCGQCKCKKEYA
GVDCGNFNCTEIEGPRCTKDGVQCSNRGFCDCTLGGCNCNQGYQGKYCEKCVTKNCKCNDLR
NCVLCKAYDRDDLNCTGCEYLDLQRVNDTQKTCTVQFNETCSAYYHYEIDPTQNQTQTQNVTV
YLEDDFFCVVPIVAGEPNSLWIILGIIAGIVLIGLLLLLIWKLLLILQGRREYNKFETSRTGSQWEQDQ
NPIYAPPDQKFDNPVFGGEF*
>TRINITY_DN1071_c0_g1_i3.p1 type:internal len:375 gc:universal TRINITY_DN1071_c0_g1_i3:1124-3(-)
DPKVAMRVEFYVCPPCGVTTPAITTTEQTTMPTTTTTESTTTPTTTPATTTESTTTTLPTTTTTTPTTT
QSTTSKETIIITEEPKPTTGLPQKKTTTTPRTTTESPTTTEATTTTPATTTEATTTTQATTTTTKATTTTP
ATTTTKATTTTKATTTTPATTTTKATTTTKATTTTPATTTTKATTTTEATTTPTTKPCECKVLQPVGV
SDGRIPDNQMTANSVLVNVSGPTFTGASQARLNNKPSKDGVGAWEPAQQEEIYLEVHFNKIEDV
REIRIQGHPTDKKFTKNFFLSTSQDGVNFEFTDEDLYEGNKDGNTIKSTKVSFKTKAVQIHPFNDVS
VGDPKVAMRVEFYVCPPCGVTTPAITTTEQTTMPT
>TRINITY_DN51907_c0_g1_i1.p1 type:3prime_partial len:421 gc:universal TRINITY_DN51907_c0_g1_i1:1262-3(-)

MITRGVTSITLELQDLQRNFNGSYKCIAKNLLGKREKILTLIVFDNPDPPINLKTESSTSTSILVTWES
PIFIGNSPITNYTVYYRLSNESIVINRTTTALYFNLTELVPAQEYIIKVMTSNRYFDSDFSGVIIRNTGE
AVPGEPTNFSAKHNNQSQQLYLNWDTPNPTNGMVSSYNYCYQMTGEKAWICKDTTDNSTTMI
NIKNLEKYTLYNVKVQARTSAGFGNFAVLNDIRTREEVPDNVCGIRKYIESRKIIVRWKPPLNKFG
ELRNYKVDFKNDDDSSDYFNICDTLETSCTIAKYILPFQKYVVRIETRNYLFSNSTTIAVTTQAEPPP
APSESLPAPSDDYVKEKHFTIKLVEFNESNGPIQKFDVIVVKRLENSSPVDSPSSYTNNDIASGVNG
LYIALRKNGAPKQNQEVQ
>TRINITY_DN4364_c0_g1_i1.p1 type:complete len:545 gc:universal
TRINITY_DN4364_c0_g1_i1:1962-328(-)
MEMSPPDNKSPAELSEAGTSYFDEKIPIPSEESEPTGKFKFSFKKLWAFTGPGFLMSIAYLDPGNIES
DLQQGAVAQYKLLWVLLVSTVIGLLMQVLAARLGTVSGKHLAEVCHERYPKVPRIILWLMVEMA
IIGSDMQEVIGTAVAFYLISNGKIPLYGGVLITICDTFVFLLLDKYGLRKLEVFFGFLITVMAVTFGYE
YVTVAPNQGQLLKGMFVPGCSDCNKDAATQAVGIVGAVIMPHNFYLHSALVKSRDVDRENRRS
VKEANIYYFIESAIALLVSFIINVFVVSVFAQAFYGKTYGDVYQNCLAHHNPHASYFNKTGDHMN
DPIEGDLFNGGIFLGCQFGSFAMYIWAIGLFASGQSSTMTGTYSGQFAMEGFLKLSWKRWKRVLLT
RSIAIIPTFALAFFQGIRNLTGMNDWLNVLQSLQLPFAVLPLLTFTNDITVMKEFKNPRSVKIITWIL
AIVIIGINMYFSALNLTNITMKILYLPAILFTIIWLTFYALMAYYALDLKFLDSISWLRRPKVSYEKFK
NYQTVGDDSRDT*
>TRINITY_DN11595_c0_g1_i1.p1 type:complete len:510 gc:universal
TRINITY_DN11595_c0_g1_i1:1768-239(-)
MLLLLIFCFVGHVYSFYVPAMAPHEFRKGDTVELKAVKMTSVKTQIPYDYYTLAFCDPAEKHYVP
ENLGEILRGDRIVNTPYNIKMDTEAKCQTVCPKKKISAKDSDVFIKRIREGYHVHMIIDNLPAATK
FQTEDGTPQFSHGFLLGAINNKRVFLYNHLQMIIYYNRDEKKDLYRVVGFEVYPTSVKNAESLDN
NQCKYPEKGQAEVLELNENAENEVQYSYSVQFKASTIRWASRWDNYLKMDNTQVHWFSIINSIV
IVLFLTGIMAIIIIRTLRRDIANYNKEDDMEDNIEETGWKLVHGDVFRPPRYPMLLSALIGSGVQLF
CMVLITIGFAALGMLSPASRGSLMTAIIFLYVFMGLFAGYYAGRLYKTLKGCHWKRAALMTGLLYP
GFVFSISFFLNFFLWGKESSAAIPFTTMIALFCLLFGISIPLVHLGYFFGYRKAPYTHPVRTNQIPRQV
PDQVWYMNPFVRIIIGGGDHFLCLDHVHYTYFSMQCSTLCQSWRLST*
>TRINITY_DN79126_c0_g1_i1.p1 type:complete len:115 gc:universal
TRINITY_DN79126_c0_g1_i1:397-53(-)
MVARLVLRRRLSYNTKSNRRRISKTPGGKLVYLYTKKAGATPKCGDCKCKLRGLKAVRPKKLAKL
SKTKKTVTRCYGGSRCGGCVRERIVRAFLIEEQKLVVRVLRAQQVKKSS*
>TRINITY_DN4595_c0_g1_i4.p1 type:5prime_partial len:1639 gc:universal
TRINITY_DN4595_c0_g1_i4:5857-941(-)
QCGVNTGTQVVYRRCNAALCKYQGNDYTRNQEVNIRTQACTVACDYTPWQNTQSCTKTCDGG
VLVQTRTERAFNKIETRNGNTPCNTQACAQELGPKQLRTCNVGQCGVNTGAQVVYRRCNAAL
CKYQGNDYTRNQEVNIRTQACTVACDYTPWQDTQSCTKTCGGGVLVQSRTERAFNKVETRNGN
TPCNTQVCSSELGPEQLRTCNVGQCGVNTGTQVVYRVCNAALCRYQGSDYTRNQEVNIRTQAC
TVACDYTPWQDTQSCTKSCDGGVLVQTRIERAFNRVETRDGTTPCNTRSCASDFSAERLRSCLNV
NQCGVNTGTQTVYIVCNGNFCRYQGREYNINEEVNVRQQNCTIACDYTAWEDTTICSKICDGGV
LEQRRLERAFNRLERRNGTTPCNTRACDSGFSAVLSRNCVNVNQCGVSNGTQLLYRVCNGEPCIL
QERSYKINDVVVVGSRACSVPCNYTEWRDTTSCSKTCGGGFFFETRTEQAFQKTESRLGKTLCNE
NSCVKDNFGNWTEWTACSKTCGMGMRYRSRECLKGFECIGLIEETETCKLGCRTETCPMGRPYP
GDCNFEDDCGKSWNQVNSIQRNAFFIVTSKDLAHQGLLPNMETTFNTKDCGRTMKLNSSVAGD
VNYLWTKRMMITDINTDVDDDVAAIDNYFKEENEQCVEFNFAVMANSLDKIGDMQVYLYYLEY
PNASLIYSKKGNTGLKWELVKRSIRVDTNKAFVILFQVNQGQSNVQYGDIGIDGFKVNPGNCQLK
GWSHWGEWKCSATCGFGTQTRMRHCLDKVNRTACVGMDRENKPCPMMKECPMNRDGNWG
QWTEFSKCSKTCDTGTYFRTRKCDSPSAKYNGSLCRVSQTKFNLPVVNDFEIDFKPCSVQQCNNT
FSQWTTWSSCSGTCGPGQRTRTRTCESGKRCSGIFEEIETCYMGCRLEKCPKFTAEIDCNFEDRNKS
DCQWKYYTVSGTSSWSFKTQPKNRLLSGPSGDSTIGCGYFLYYPSQTLSSAYAWGPNIYPTMDSYC
VKFKYSMVTSVNSSMGNLTVIKHFLNRPRKADTPLFFHSGVSQKQWNPMAIDITTDIQRSPFVIMF
KGQSDPNNAGLSDIAIDDYMLMAGKCNKSYSEWSEWSTCSRTCGVGQRTRTRVCLDDDIKVCK

MGDVATEGEACVKPLCAVPVDGNWGQWSAYSQCNARCNGTQKRERFCNNPAPSNGGRMCLL
GDTIFQLRNNYHAQIRPCSIDCGDNDTFSSYKRINDTCHFDCITGRVLQERECEQDTCFDGNTRFS
KGEKQYTQRACSIKEYCETSEYKCREFRSVCSTNTPSSCLDPQNRAVKSCECQVERGCSSMTNPGQ
TVKHKEFDVFYRPCRYVGVCFTDYETDAFSPYEKVNDTCHLSCSNGKVFEERECRQDNCIDGSNT
LTRGAKQYRETQCTTKEYCETSQYSCREISGPCSSNPPSSCQDPANKSLKVCTCQVTTGCASMLTA
GQAVSYLAEETFSRPCKYTGACNANGNTDKTFSPYERVNDTCHISCADGKVKQMRECKKDACV
DGGQNFPRGFKQFKDSSCTPNEFCETAQYSCRELSSACTSNPPSSCLVSTNKAVKMCDCNDPNGC
DSMITPGKKVNQGEKEFLQRPCRYTGVCFTDAETGT*
>TRINITY_DN140702_c0_g1_i1.p1 type:complete len:2036 gc:universal
TRINITY_DN140702_c0_g1_i1:94-6201(+)
MATSFKVSEIKCTELLNLEKKGAIDPLVEVEYLGLSKETENKDNDLNPSFGEVFDFDLSGKALGPE
DFLKITVKDYEKFGRNRVVGKADVYLQELLSNPSSTLSATLNDKNDRPTTGKITFKVDYIGPAGGG
AGGATGGGGDDRQAAERELEGDEDEGDPNAMPDGTPIPEGPAGDAMRAEKRKMKKNKKKRR
EHNLPDKPTDFQIRIKIIEGRQLPGGNINPTVRVHLGEETKQTRVKHSSNKPFYDETFFFNFNERPN
ELFDKLVTFEVHNSKNVLSEAMIGAFQFDVGTIYFEDSSHAIIRKWLLLTAPEDNDKKDGEETKGA
SLQPGGPPAGYLKITAIVLGPGDELPEKAKGSGKSEGGEEDIESNLLRPAGVTLRPATFLVKIYHAE
DIPQMDSAAFDGVKKLFSSGSAQKDHVDPYATFSFAGKTQKTEIKYGNNHPDFNQELRVAFKFPS
MCERLKLQMFDWDRIGNDDCIGTSFISLTAISGNGDEGFLPCFGPCYVNFYGSTREFSDLPDEHDN
LNKGRGEGCGYRGRALVEIDTRFGDHTAKPVQDIMAQELVRVQAYMRRRKYKLFASFLSANMIS
EADGLVEFEVSIGNYGNKLDPDTEPQSSSTPPTNPVYDGCAYYFLPWQESKPCCVVESHWEDVIFR
LEPINCLLKIIDKMEKNIKSVELGLKAKAQPAEVAALLIALLDQFISDVSMDLPKFAATDNGVNEL
DLKIQEIRSDELLNLKEEASTLREQATDVQEAMDTIDGYLKRMRDLCVEPQNSMPDVVIWMIAGS
KRVAYHRIPAYDIFYSENPLARGRKCASLQNFILKYPGKKGHDMEDHPEIPASVSVILWLGLEKHQ
AAWTDTEQMEGEYSVYAETYENQGKPPMFDWTAKIIKRPGFSDSEGKIRLNKEYYEPPEGWKWD
DPPDGDWFIDPEMSSNFDADAGHKKFMQDCFENESRVPAGNWGKSTPHYTNVQGDEIDHRDE
TSLPEGWSWTDDWKVDVNRACDEEGWEYCIEATGGGWGAVFRSYHFCRRRRWVRNRQLEADP
QKVMKQKKEEAKQGEGWEYAPVFGMKYHVKEHKFDFVRRRRWHRKMVCVKPGAVPMFKIPD
DDDKGKFHQIMPRIFLAYDEPHKWQFRAYLFQARDILGLDSEGTSDAYAKVCFQDQSQITDVIEET
LCPEFNQTLLFEDMQLYGSPSVFAENPPFVSIELFDKDKVGKDEFLGRVLAQPIVRLNGLKPPATRL
MWYDIHHGERTCGELLAAFELFLDEGADLPLPPPWLEKEKKFQVPSGIRPILVKTGVEILNWGVR
NMKKYQLASCTSPSVTYEIGREIITTDVIKNTKKNPNFLKNIFYKEVYLPKDPLYMPPIVIKVRDHR
TFGRKPVVGRHVIKSIDFVQPPKADPVVELPPADLVGEPEEKKDVTLDIEEKKKKKEIPVDDNLDW
WSKYFASADEIEKCGNYLKKGHKTIQIYDSELEEIKDFNGFSDFIVSYPLERGKEDDDEDDNVVGE
LKGLVKVYPLPQDPKDQTKIRYFTPKLKEKSKIVKCIVRVYVIRARDLQPKDPNGKADPFIVVQLG
KKKISTKDNYIPNEINPFFGSFFEMKADVPLDKDLKIKVMDYDFMSGDDLIGETMIDLENRFLSNC
RATCGLSPAYYTSGAYQWRDAKSPKEILDQVCRTKPQWLSNEQVVVNGQLVKIEDFEESPPTDPHI
GPVEQRLALHVLNNEDIIGHRLVPEHVESRSLKNPLQPSMDQGQLEIFVDIFPIANGVPGPPLDIAT
RKPKEYVLRVIIWNTAEVPPSETSITGEEMSDLYLKGWMEGQDDEKQKTDVHYRSMDGEGMFN
WRFVFPFKYMPEERVMVVEKKEHFWSLDTTKQTLPPRMNLQVWDNDLFSANDFIGNLELDLSK
LPRPKKAAKKATLAMLDTEGDDVMDLFQAKIAKGWWPMLSENEDGTRTMQGKVEMTLEIVTD
LEHEAKPAGQGQEEPNMNPKLDPPNRPETSFLWITSPWKTLKHIIWKNYKWYIIGFFLFIILILFIGL
VLYSVPGSIVHKIFGFQ*
>TRINITY_DN121418_c0_g1_i1.p1 type:complete len:259 gc:universal
TRINITY_DN121418_c0_g1_i1:1061-285(-)
MAENDIGDWFRSIPIISRWWFVLSIAFPLLGRFGLLPIYYMILDFNLVIYKFQIWRLFTSIVFFPLSPM
NGFHYLINLYFLYNYSTRLETGVFDGRPADYLFVLVFNSIALILLGFALNLYLLMDPLILSVLYIWCQ
LNKDTIVSFWFGSQFKAMYLPWVLAAFNMILGQGGLMEIIGICVGHLYFFLMFKYPQDFGGRRFI
STPSFFYNYLPNRRGGVAGFGQAPVGRRNPDEGRGRGGGGGFRGMFPGGGQRLGDQ*
>TRINITY_DN1936_c0_g1_i3.p1 type:internal len:1070 gc:universal
TRINITY_DN1936_c0_g1_i3:3208-2(-)
GEYKPTLGRCLSFWYHMYGSHVGELNVLVLQNKSRSAPIWNLKGNQGNVWRPSRVTIKSAVNH
NIIFEGRTGDGYMGDIAIDDVMMSEGPCTDYGNCDFERDLCGYTNKKNIDQFDWQLGSGGTSSV

TTGPSNDHSTQTALGQYMFVESSYPRLPHETAIIGSETIPVSGSGKCLQFWYHMYGEHIGSLEVNM
RVANDLSETIVWKLEGNQGNKWLQGQAPLLSRGQNMEVRFEGIRGTGYKGDIAIDDITVVDYSG
ACAITPKSAKPWGCNFELDFCKWQQSRADKFDWLRNQGATGSAGTGPKADHTTNTTQGYYAY
VETSFPRQPNDTAAIISPTIKGNGTKQTYCMWFAYHMYGPHVDTLKVYTRSQGSDKPVLWRRRGS
QGLNWNHAEVQVTVLYDTKVIIEAVRGKDYKGDIAIDDIDIWFGDCFSSGICTFEGADLCFWYVP
KNGQLYWQRASGATPSVKTGPKNDHTYGTSQGHYMYIETSNAPAKATAYMYSPWYYNTGQKC
VQFFYHMYGQQIGQLEVYIRYSNSRYLYRQFIRSGNQNNTWHMGQVTVKRNNVYFQIIVRGTKG
NGYQGDIAVDDFRIRDGACPAPGDCDFEHADFCSWMQEDNDDFDWIIGSGSTNSFLTGPSIDHT
TQSNGGYYAFIEASSPRQPGHKARFKSMVFAPTTGSGRCFSFWYHMRGYSIGNLTIYLRKVSNTKT
LSEEVMWLLGGNQGKAWNQGRFTLKSSTLHEVVIEGVRGSSYDGDIGIDDIEFTSGSCSFLPKGA
WPSGRSTPAPTQASTTLGPTIGNKGFDCNFDKEFCKTWMRNSTRTNFQWKRHQGSTSSVGTGPH
HDHTTNTDQGYYVYIESSSPAQPNDTAYLIGKDIPNNNGFCLSYWYHMYGPHVGSFRVYVVNKT
NAEARFVRVGTQGDKWKHGQLYISPKAGNPITIRFEGVRGSSFMGDIALDDISVRYGNQCPAPRE
CTFEHDNNGGSSSTCGWTQEKQKDSFDWSIGSGDTPSVLTGPNFDHTLETKQGHYFYIETSYPRK
FGENATIISQSYQQTLGKCFSFWYHMYGSTIGNLTLFLRRD
>TRINITY_DN1386_c0_g1_i1.p1 type:complete len:596 gc:universal TRINITY_DN1386_c0_g1_i1:2419-632(-)
MEYIEVKSFIVALVFVFSILGTAQSDLVINNVERTLDLSTQTAKYTTKIKFHNPGKKVEKSFLLALEK
DQEKHLSYLEILDKQDDNSLEYAAENVEGQSGRFFKVSFSKPLKENDDVTLHIETVFTHVLTPFPA
KIGQTEKQLVLYKGNTVFYSPYEIKEQSTTVKLASSAIESYSRLKPSRSSENTITYGPYKNSKPYRLHE
MKIHFENNSPFLVVNEMTRWIEISHWGNIAVEETYHMTHEGALLKGHFSRYDYQRSPTHSVIKSF
KTVLPAAARDVYYRDEIGNISTSNMLVNDDSVEIELRPRFPLFGGWQTRYMLGYNVPAYQYLYNK
GDNYILKMRLIDHVFDEFVIDDLNVKIILPEGSTNIEVVKPYELEEGKRDIHKTYLDTSGRTVIVLSK
KNVVENHIQDIQVHYTFKKLQLLQEPVICVAVFYILFLSVIILVRLDFSITKDAAKESRMKVASLLEE
LLSSCGRRGSLYYSYDSAIDKLKQNRNQNDFAVSLKKLNQDYNTLTQSINELCSLLIKEDPEIGDKV
NDLQKKENERKVIVDQLVTLATRVVTNKIGRQQYIESEKNSMAKREKLGEDIDNVLATL*
>TRINITY_DN2490_c0_g1_i1.p1 type:complete len:1044 gc:universal TRINITY_DN2490_c0_g1_i1:354-3485(+)
MDVNKKVNQAYIDDDDDITINFNPKSGDPNPQAQRFKISKLPSQSDSNNSMEDIEEADDVDGLE
KDNVQNTVSKHVQLTIPAEHYHNDHNDTRRASHTHTQTIGYMTHDAVPMSVFYRNQASMENA
ANGDMRPTLDQLHKGQGLDEARKRHWDALNEEDEQQDAERGTSNTKTKLGWIKGVLFPTLLN
IWGVILFLRMPWIVGQAGIGLSSVIVLLATVVTTVTALSMSAVCTNGEVKGGGAYFLISRSLGPQFG
GSIGIIFSVANAVAVALYLVGFGETVQALMARSDTYMVDATNDIRIIGLIALILIFVIAMIGLEWVIHT
QTFLLLLLVAAILCVAVGCVYPAPQESREKALAWGLPGYKSKSFADNFVPDYRDGEEFFGVFAIFFP
AATGIMAGSNLSGDLKDPQKAIPLGTLLAIGMTSTSYVALSWILGSFVMRDASGIIPVINVTVNGTT
MNSHTCEIGSCEYGSVNDLQIIEKISLWGPLVLIGIFAATLSSALASLVSAPKVFQAVCKDKIFPHIEL
FGKEYGKNGEPRNGYLLTLVISACFIAIGELNVIAPIISNFFLMAYALINYSVFAASIAKSPGWRPSFK
YYNKWCALVGFFLCIVIMFMINWWAALITIIVVAALYKYVDVRKPDINWGSSGQAHTYLKALRFT
RRLEDTDEHVKNFRLQVLCLTGQPSLRPNLVHFCSHITKHLGLLVCGEVNVTDKILSPSGNEGKW
LKKQKIKAFHQIIHSATLRNGAQSMLQSVGLGKLKPNTLIMGYLNRWQSIEVAKVDEYVHIIHDA
FHLRYGVGILRLQDGFDITDGVVDDQIENGVVENHEPDWYTEDWDENQKDTRGSDGDGSDQA
KEERQETFKPPDIVSAFPKKRKGYIDVWWLYDDGGLTILAPHLLALSSHWRGCKLRIFTPASDKKI
KANQIRMANLLKKFRIDFSSVVEFRGINKHPKEDSINQFKQLRRGEHLPQDGLLDKKTLRQIRLGE
LLHEHSSEAKLIVLTLPIPKEGMVTSLMYLSWLEVLSADLPPVLMLRGNQTSVLTFYS*
>TRINITY_DN17721_c0_g1_i2.p1 type:5prime_partial len:364 gc:universal TRINITY_DN17721_c0_g1_i2:2-1093(+)
FAPSSRRPAPSAAARRCTGAMQQYNSQTFKDVKPASAGPSAALQVGLFCFFVMTRAIHPTIIDASK
SVDPESGKKYFAYGEMTVVLGETIVTLVIAQLMALGLGGMKEWKAIWSPAPMKVFSFIGFVYALG
DFLEMASMGSLGGAAYQVLLQSKLLITALMMWGIKGAKQSSLQWNILILVMLSMCVYMIGGSSD
SGSGGGIPIMGVVNVLLKVTVSCFCAVLSDKYMKDFKSEPIYMQLVQFKCAWCATILALSFVDGK
TWQNGFFTGWNATTVGVLASFTVKGWSTMYLLAILDSVLKNIGEAVSVLVIYFMQVFLPVFEDTF
EVPKFLSVMVVILSVTAYVGSKSVVEKAAKYDKQSAGK*

>TRINITY_DN15584_c0_g1_i1.p1 type:complete len:275 gc:universal TRINITY_DN15584_c0_g1_i1:874-50(-)
MADSGSAGADRGGRGGFGRGGRGGRGRGRGRGRGRGKPEDKEWTPVTKLGRLVKDLKIKTLE
QIYLYSLPIKEFEIIDFFIGPSLKDEVLKIMPVQKQTRAGQRTRFKAFVAIGDYGGHVGLGVKCSKEV
ATAIRGAIIQAKLSIIPVRRGYWGNKIGKPHTVPCKVTGKCGSVTVRLIPAPRGTGIVAAGVSKKLL
QMAGIDDCYTSACGTTATVGNFAMATYMAIARTYSYLTPDMWAENQFQKTPYQEFTDYLAKNH
NVGGKPVAPATATA*
>TRINITY_DN1785_c0_g1_i1.p1 type:complete len:615 gc:universal TRINITY_DN1785_c0_g1_i1:2316-472(-)
MEKEKKISHDAGNVNLSYDHEKGANGKANTDIPLGDFHIAKNEETEKIKEKDPFDAVDDFKPAG
KPWIELTTGGKIKRVSVTVGKIVALLALLYFFICSLSFLSSAFRLLGGKTAGEAFKENDVISNPIAGL
MMGVVATVLLQSSSTTTSIVVTMVASGILEVDPAIPIIMGANIGTSVTNTIVAIGQIADKEQFRRAFA
GATVHDMFNLLCVLILLPLELVSGYLFRLTDAIISSLHLKDGDKSLKKEFLKAITKPFTNVIIQLDKKI
IEKVAQGDEEAVKKSLIKYWCDKGTKTTTFVNVTTLQNVTSTQNNITTYMMKNVTNLESKTETVF
KEPCEFLFHDTGMSDTAVGIIMLVISLTILCVCLVLIVKTLHSLLRGQIAIIIRKTFNSDFPKPFGFLTG
YVAILVGAGLTILVQSSSIFTSALTPLVGLGIVSLDRVYPLTLGANIGTTATGVLAALASSSSKLGSAL
QIALCHLFFNISGIAIWYPIPKLRRLPIKLAKKLGNTTAEYRWFAIAYLIFVFFLLPAFIFLLSLAGWK
VFVGIMVPIVTILLIIIIINVIQSKKPSLLPSKLQDWSWAPRACRSLEPYDRVACKIQKVLRIKKKKAT
VNA*
>TRINITY_DN2487_c1_g1_i3.p1 type:internal len:317 gc:universal TRINITY_DN2487_c1_g1_i3:1-948(+)
EINNGRLAMVAIIGMWFQNGLTGSAWGDWALYTDSPLRAAAPDFSKEIGACPPLGFWDPLGFSV
YKDPELAAAQFRRRRLVELQHGRVSMIACIGYIVPEYFRWPGFISPSEKLAFEDIPNGLGAAAKVPI
LGWGQMIGFAGAIEISNLSSPSSKTPGDYAGYGAFGLPFGKGIDDPAKREKSLLAEINNGRLAMVA
IIGMWFQNGLTGSAWGDWALYTDSPLRAAAPDFSKEIGACPPLGFWDPLGFSVYKDPELAAAQF
RRRRLVELQHGRVSMIACIGYIVPEYFRWPGFISPSEGLAFEDIPNGLGAAAKVP
>TRINITY_DN2433_c0_g1_i1.p1 type:5prime_partial len:267 gc:universal TRINITY_DN2433_c0_g1_i1:1-801(+)
LTFIESLRLRIDVYIVIKMQLISVIFGCLVVLLNLNNQYEASKKSSQRNDYYTYYKYYYYFKQPYHYK
PRNWPKVRGRCDVVADFKINGTNPIKEEAAKGRVVLLYLMKRTCLSCYAQLATLNDYAAYYRKV
NMPISIIALNHRKQPLPDTVLKKFPLVKFYDEPVDQNIFGKLKSKWRSTMVFDICGRQQYKFGYPY
SLLSYPFTRSGVMNTLNHHVSLCGKCNATLTTTPKETRPPTQVPRETRPKTTETPMELFLANNRLT
RV*
>TRINITY_DN17714_c0_g1_i1.p1 type:complete len:644 gc:universal TRINITY_DN17714_c0_g1_i1:102-2033(+)
MADVEQNGTYDDSNEKTKLHSNGTELEEKVEMNPPVVVEGEDRGGWGNKIEFLLASVGLAVGL
GNVWRFPYLCQQNGGGAFLIPYFIMMVIEGIPLFYIEFAIGQRFRRSAIGCWSKIHPALMGVGISCV
VISLLLCIYYIAVIAWCFYYFFVSFTSKLPWNIKNCPEYDRYKLLDNACKANGTATSCNATKTFSDC
CVHDPQLYYFYRKALNISPSIEDLGDGVQWKLFGCLVLSWIVVYACIVKGVKSSGKAVYFTATFPY
LILVILFFYGVTLEGADIGIVTLFKPDFSKLLEPSIWMDAATQMFFTLSLGFGALISFASYMPIKNNC
VRDAYTVVLINCGTSIFAGIVVFSILGHRELMLGEKADKVGGGPGLAFITFCDAFLQMPISPLWSILF
FTMLILLGIDSEFGTLEGLITPFYDMKWVKMRKELFTALVAFGMFLIGIGLVSSTGFYIFQIFDDFSVS
LGLLFIAFFQVIAVSWVYGNDKFANDIEYMTGSRPNLFWMICWKYISPLAIFVIFVSNCIKMASNSP
KYAAYVGCEFQSVDFSSVAKGIKGSIAKVDYPGWASIIIFIFVTASMVPIIIFLVKDFVSNRQGWIDGF
KRKLGNRYEYLPDPSVADPSRRKSPEEMEFQIQKEEELF*
>TRINITY_DN12627_c0_g1_i1.p1 type:5prime_partial len:1061 gc:universal TRINITY_DN12627_c0_g1_i1:3456-274(-)
YGENNRLKFNDARQYCQQWKDDQGDLVAIHSNYENSFIMSQLYDVNEPMYIGLTDSFSERTFLW
VDQSSVVYTNWYARQPSFVGNEDCVEMMPYFKTHGRWNDIGCSAERGFICQRTPSKTPLPTPEP
VKDSKPCPAGYRKFMKACYRVVHTPMTYDDAVKDCRTSVGGELGDLVSLHNQFEQSFVFSQFAK
FDFHSWIGLKKIKGEKEFKWSDETPYDYRYWLSLRYDNTVYNDDKCTTQEFSSKVDGKWKVTDC
TQKHPYMCKIERDFDQPHLTFIGNCTDGWTKFYDKCYKLQNYFSNWPAAREKCKGFGGNLVTIT

NSDIQKQMEYLARDAYRSVWIGLNDRAKEYVFEWSSGIPLLPDSYQNWGPNEPNDNSGTENCV
HMTTESSGGKWNDLSCSQRMPYICEHPLECNSALGMEDGSIPDTNINATSYVGTYFPKYARLNAK
SDNNRNTAWCTNITTGAESLTINLGEVMQITRISTQGLLDSSLGFSRGGYVVSYMLEYSNDGTTW
MTYISEDAYDQKPIALPGNIDASKIDGTTINLKAAYLRFIPKLYKNIICMRVELYGCKSVCSRALGM
TNFAISDKQLSVSSSQAGSEKAQLRPNIDGSWCAKDDDYSPFVRIDLPRLTKITRVTAISSSKVQYKI
AYSDDGELFTRFQTLTNEERVFSTVPSTHDLETAISAKHVKFDLLKKDQGKLCLKVELYGCLKDCD
AVLGMETKIIPDRLITASSAKPSNPARNARLSVGVYGDNSWCSSETDNAQYLQLDFVQEAHLTEIA
TQGFNGDSAKYVSKYNVQFSDDGDFWHQYSEELPGNSNGYSIQRNKLKEPIIARYIRINPTEWKN
GICMRVEFYGCPMKPFTAPKILTQKTYNGAIRILNEDFNENLLNANSIEYIELANKVETDITSLYLLE
ESFKDGFNSVTVNKFREGSVITEFTIKTHTSDASLDAAEPLRKAVQTGRVGTFDVDPNFFEIVTAST
DKSTATNDDSGGLSNGGKIALIVFFVILICIVLAGGVVYYLRSRRNGFRLQFQHRAFENPTHVDDL
QNDDMKVEFMNDDY*
>TRINITY_DN4927_c0_g1_i10.p1 type:3prime_partial len:1754 gc:universal TRINITY_DN4927_c0_g1_i10:5260-2(-)
MSNFKKLKLLLWKNYLLMKKRPRATIIEVTLPVSVVLILFIIRMTSVAPIDEEAKTYKPFNIDQQISIQ
MHMGAWKIAISPNTSFTQQIKEKLMKLDEIDEVKIFENEKQLVSTLVTDGTMPVLSQSYLCGVVFQ
DFNNNSKNIEYALRFPANPRTSMLGALAKWFTNFIFPYTFDGLGPRNPSKADPMYGGPPPYFAEG
FLKIQKIVDFSIIQMTNSSFDPSHFKVSMNRYPYPPHVKDPFIVILQNTFPLFLMLSLIFTALNMVKDI
VIEKERKLKESMKMMGLTGWLHWTAWFIRYFIPLLISISIITILLTVNFNGSNSKMLNHSDGSLIFVF
LVIYMLSVISFCFFISTCFYRASVAAAGIGVLWFLSYFPYFFWMNYYNLLPTIAKILSCLDFQLAMAF
GANLVGRFEGQGAGVQWSNLMEGVTVDDTFTFGHILGMLLLDTVLYGLMAWYIESVFPGEYGIP
QRWYFPFTAKYWSNGNRAERRSFSDEEYAMTGVSDFIERDPENLKVGISIDGLKKEFKTRKGIKVA
VDGVQLKFYQGQITSLLGHNGAGKTTTISMLTGLIPPTSGNARVGGYDIKNNMNGIRGSLGICPQ
HNVLFDALTVEEHLWFFTCLKGINDSLEIEKEVERMIESLGLIDKRYAPSKTLSGGMKRKLSVGIAL
VGNSKIVILDEPTSGMDVAARRSTWDLLQKERRDRTILLTTHFMDEADVLGDRIAILSKGQIKCCG
SSLFLKKRYGVGYHMVMVKEKGCDVEKVTDVLKKYVPTAEVESDVGLELSFALSSDYSHRFEELFS
EIEESRQKLKISSYGASVTTLEEVFMKVKEEDDLTDDTHQKLFRRLSGKSSPGKTNPGYDENNKAS
VNPQRLSQNQKNNSTSNPISWENAIGFTLKELDHMPNYSPQDGDVQLPSLTGSISKYNYANNMG
ITLFFQRWYAMFVKRLIHSKRHKISIVSQLLVPAINLMIALIVVRTMPKAQDSPPLAMNLNMFEKSE
VPLYIHEGTPLSEELSNSFQQYLGSLQQSSNVPQFVDSNMTKYLIEQEIDHFSQFNRHNLVGLEFKE
KYSRSYSTVWFNNEALHSPAVAMNTLSNTLLNLFTNNQEKKIITINEPLPTTASEGLLDLKLNPNG
FQIGFALCFGMSFLASSFIVFLVQERASKAKHVQFVSGVDPLSYWTSAYAWDVINFFLPTIVIVLIFFII
KVDAFQGVRIGYAMLVVLLYGWGIIPVMYIFSFLFNVASTAYTRMTMLNIITGLIMLLVVFIFNIPVV
NLLDEAKILSNIFIIFPNFSLGQALINIFNNYSIIEQYDNTFKLCVDTAHKMYPFLPIEQINKQCKTMI
EEFIPDNLYTRNYFSWDEPGIGRYLLAMAIEGFVYFAIVLMIEYGVFRPIGRCCRTIKRTATTVAISN
DINNEILDDDVKREQELVASKDYNGNEVLVLENLMKSFNTNQGELTAVNNISLRVPHGECFGLLG
QNGAGKTTTFKMLTGDETPTSGAAYVERFDVARELAEVRQRVGYCPQFDALIDLMTGRELLIMYS
RLRGVPEKHIQKLVEDLASNLGVFQYIDKCTKTYSGGNKRKLSTAIALAGDPPLIFLDEPSSGMDP
AARRNLWDSLSDAIAGGKSIVITSHSMEECEALCTRLAVMVNGQFKCLGSPQHLKAKFGDGYTLI
ARIGGINRNTSSLKEFIEKSFPGSILKDEHQGYVQYQLTDKTKTWANLFGIMEKAKETYNIEDYSIS
QATLEQIFLNFTRTQRVDDTR
>TRINITY_DN4927_c0_g1_i9.p1 type:3prime_partial len:1754 gc:universal TRINITY_DN4927_c0_g1_i9:5260-2(-)
MSNFKKLKLLLWKNYLLMKKRPRATIIEVTLPVSVVLILFIIRMTSVAPIDEEAKTYKPFNIDQQISIQ
MHMGAWKIAISPNTSFTQQIKEKLMKLDEIDEVKIFENEKQLVSTLVTDGTMPVLSQSYLCGVVFQ
DFNNNSKNIEYALRFPANPRTSMLGALAKWFTNFIFPYTFDGLGPRNPSKADPMYGGPPPYFAEG
FLKIQKIVDFSIIQMTNSSFDPSHFKVSMNRYPYPPHVKDPFIVILQNTFPLFLMLSLIFTALNMVKDI
VIEKERKLKESMKMMGLTGWLHWTAWFIRYFIPLLISISIITILLTVNFNGSNSKMLNHSDGSLIFVF
LVIYMLSVISFCFFISTCFYRASVAAAGIGVLWFLSYFPYFFWMNYYNLLPTIAKILSCLDFQLAMAF
GANLVGRFEGQGAGVQWSNLMEGVTVDDTFTFGHILGMLLLDTVLYGLMAWYIESVFPGEYGIP
QRWYFPFTAKYWSNGNRAERRSFSDEEYAMTGVSDFIERDPENLKVGISIDGLKKEFKTRKGIKVA
VDGVQLKFYQGQITSLLGHNGAGKTTTISMLTGLIPPTSGNARVGGYDIKNNMNGIRGSLGICPQ

HNVLFDALTVEEHLWFFTCLKGINDSLEIEKEVERMIESLGLIDKRYAPSKTLSGGMKRKLSVGIAL VGNSKIVILDEPTSGMDVAARRSTWDLLQKERRDRTILLTTHFMDEADVLGDRIAILSKGQIKCCG SSLFLKKRYGVGYHMVMVKEKGCDVEKVTDVLKKYVPTAEVESDVGLELSFALSSDYSHRFEELFS EIEESRQKLKISSYGASVTTLEEVFMKVKEEDDLTDDTHQKLFRRLSGKSSPGKTNPGYDENNKAS VNPQRLSQNQKNNSTSNPISWENAIGFTLKELDHMPNYSPQDGDVQLPSLTGSISKYNYANNMG ITLFFQRWYAMFVKRLIHSKRHKISIVSQLLVPAINLMIALIVVRTMPKAQDSPPLAMNLNMFEKSE VPLYIHEGTPLSEELSNSFQQYLGSLQQSSNVPQFVDSNMTKYLIEQEIDHFSQFNRHNLVGLEFKE KYSRSYSTVWFNNEALHSPAVAMNTLSNTLLNLFTNNQEKKIITINEPLPTTASEGLLDLKLNPNG FQIGFALCFGMSFLASSFIVFLVQERASKAKHVQFVSGVDPLSYWTSAYAWDVINFFLPTIVIVLIFFII KVDAFQGVRIGYAMLVVLLYGWGIIPVMYIFSFLFNVASTAYTRMTMLNIITGLIMLLVVFIFNIPVV NLLDEAKILSNIFIIFPNFSLGQALINIFNNYSIIEQYDNTFKLCVDTAHKMYPFLPIEQINKQCKTMI EEFIPDNLYTRNYFSWDEPGIGRYLLAMAIEGFVYFAIVLMIEYGVFRPIGRCCRTIKRTATTVAISN DINNEILDDDVKREQELVASKDYNGNEVLVLENLMKSFNTNQGELTAVNNISLRVPHGECFGLLG QNGAGKTTTFKMLTGDETPTSGAAYVERFDVARELAEVRQRVGYCPQFDALIDLMTGRELLIMYS RLRGVPEKHIQKLVEDLASNLGVFQYIDKCTKTYSGGNKRKLSTAIALAGDPPLIFLDEPSSGMDP AARRNLWDSLSDAIAGGKSIVITSHSMEECEALCTRLAVMVNGQFKCLGSPQHLKAKFGDGYTLI ARIGGINRNTSSLKEFIEKSFPGSILKDEHQGYVQYQLTDKTKTWANLFGIMEKAKETYNIEDYSIS QATLEQIFLNFTRTQRVDDTR

>TRINITY_DN598_c1_g1_i1.p1 type:5prime_partial len:745 gc:universal TRINITY_DN598_c1_g1_i1:2-2236(+)
RWSGCSKSCGVGVKTKSRECDNPRPSGGGRYCTGERTATEACKIQDCGVDGKFSHWSTWTACSK TCGGGAQKRARQCNSPPPSGDGKDCAGAREEFKGCSNDFCKGPTGVAKAQEIIERFLENFHKKT YPDREPSGKFLKAIFAIQKAVAEEFISVDPYVRSKTLEQRLKDLENFLKVATEGGSIDFARTLLGKGV FTFMKQKIKDGLPIDDIDKAAEGFLEKLRGDLGDKKMDEILSSQGDATLMFTIDTTGSMKQEINA AKAIAKQIISATRDYEVDYILSPFSDPGTGPVTHRSASERASFVSDIDRLRARGGGDCPELSFGGML NALNNDPQYGSPMFVFTDATAKDDTDANVASLKAAADATDATINFFTNLAGCNGRRDRKRGIE SYKEIASYTGGQVFPLKNEAELIKFTSYVKTSLQQDTVITSGGTLNSKSKRSLYSMQARHFMVDDGI SKLVISVGVQQLASQVALVDPNGKKVYPSTTLALSLIYQVSHPSPGQWKLDFPTSVGKYSYNVQAV SSSAIAFAHNFIYQQSVRKNSPPIPLANPLKGVKNMMVVRLGGTKNFDLNSIKIDLVDTSGNVLQ KGIGTDRLDSDEVRHATIDPPSGPFKVRLTGKTKSGYNFERVSQGISRPVSAAMRTNFAGYDFTAK LGKGPYAAQMLFINRGAHENFYFYAKANYGSIPNYKQTKRVTSQGFVTFTYKAPRQYNLKGKSD RITVTVKRQSTGEIVSHHTSILLVV*

>TRINITY_DN2963_c0_g1_i1.p1 type:complete len:116 gc:universal TRINITY_DN2963_c0_g1_i1:1072-725(-)
MSATGNLVTVLRKLYDEYNDKTPSRLKIVDGFLLYVMLTGIFQFLYCCLVGTFPFNSFLSGFISCVGT FILGVCLRVQTNPRNRTEFKGISPERAFADFIFANVVLHLVVMNFIG*

>TRINITY_DN1172_c0_g2_i2.p1 type:internal len:264 gc:universal TRINITY_DN1172_c0_g2_i2:790-2(-)
GDYGFKVLTSSDPAEKEKKLAAELANGRLAMVAIIGMFFQDGLTGSAWGDWALYTASPLRFFGG DSGPSYKAFDPATELGVQEPLGFWDPLGFTSDNDEAAFKRRRSVELKHGRVCMAASIGYIVPNYY TFPGFLSPSAGVKFADVPNGLAALGKVPALGWVQIIFFAGIVEGAFGFDDYKSGAPGDYGWKVLT SDDPAEKDKKLAAELANGRLAMVATIGMFFQDGLTGSAWGDWALYTASPLRAEPVSTAGVGAA TKGGTE

>TRINITY_DN1559_c0_g1_i1.p1 type:complete len:311 gc:universal TRINITY_DN1559_c0_g1_i1:221-1153(+)
MAEKKATFKLGFAENFALSGAAAVISKTAAAPIERVKLLVQNQDEMIKQGKLSEPYKGVIDCTTRT FKTEGLAPFWRGNLANCIRYFPTQALNFAFKDKIKAAFKVSKSDGYALGFGKNIASGGCAGALSL CFVYSLDFARTKLANDTKSSKKGGSREYNGLVDVYRKTLKSDGVAGLYRGFVLSCVGIFVYRGCYF GFYDTLKPVLLGADASLFMSFILGYGVTVTAGLISYPIDTIRRRMMMTSGQAVKYKGSIDCATQILK NEGAMSLMKGAGANILRGVAGAGVLAGFDKFKEVYIAMRKRQMGLE*

>TRINITY_DN33328_c0_g1_i1.p1 type:3prime_partial len:1422 gc:universal TRINITY_DN33328_c0_g1_i1:4264-2(-)

MLLKYFFLFLCLLLLNDIVYGKRRGGKKPARRPPPARRPPPSRKPVRRPPSRKPSPPSRAPSPPSPPSS
PPNRKPSSNKPANQASNDQGGPGKPASNAQGGGKAQELPDLNAILRAFAGKQTDSGSTQYEYG
GCVLDIGKDKCKIKFIPKKQSCHNKLALGKLFDKFVPKGKIKTLFKKYAPVDVFQVTRFEVDKCQ
GDLDFETEAKEAFNVINDKLKVNSGKLSVSFDYKKFMAAPSFDKLGIDIDGEVQLFGKKVPLTLK
KKRDSGEGIFSIKTASIKASDFAKLFSTKSVANAQSPPQTKMFTDAVIQNPSISGSRDDDGGFELVVK
GQVTGIKGIGAISLFVIIQKSSDSPVSVAIVADIKKIQPTVLLSSLTGKNLNSIPIIKDITANLAVEFANN
DMMMLKDPELNKLLGKYIANGKTIASGTKIKVELPIKEILTKVKTTASLSHVPSTIYMQMFLNGGK
VCFKFPDNIAMDLANVLVALLPKVPDLLFSKVLKTPPKIVIEKFDVNVATKYVDMVLEAPEEIVLG
SDLFKIKNAKFELKHEENKPWDFNIEATKQIGKTMIDINVKSEAKNYIFKATIDSLTTKELATTFGS
KATADGILTKMSFLNFGIRTLTVTAEFGDKFTLNIKGNPVLFNWEGAFFEGFIVGSGLNKKMAFGI
VVKNIRIDDIVFKLFKKRILQTTWMSELTATFLISNTDPDDNIDFTLPELKEHVKLKQGVFFKSELTI
PTDCRAEKICEVLRDKMKPGTKLYLEGEITLNGFKLTAGYKGHGLNFATDLTLTSAKLVLTVGTST
EFYLEGEVQMKNPNVKFIGKLGVGVTGIVELSLIMEGMWRTPFGLSYIAFGNLQLAVAIQPGVPVP
KLEFTAEMWVGKIGNNQEIKTKTAVGINPSNPLDMYFYGEINKLTLAEVAKAFEFKSTLPKVVQET
GFPEGLAAGYTTNPKGKTISHLKVNLKTGFFFRGAINILGYKLECEILVQIPKAIKVSVDMGPIKIAK
GLIKLAKGAKDLDNGPSLYVAISSNEVAVKIQAYAELLGLGAEVNIDVSDKGMKFFVYGNIFNLIE
ANVTVNAPYGNGDLAKSEFSVSSCISAGISDIQNTAFEEVDEGAKESQKIQKVADDEVKDASSWY
NSAMDKLLSYEKKLEEKTAIINRKSGKLEKLKEYLDASCVDHCPDVCQGGPVMRKRKGRFFNKK
PGWDDCKEMVKSIVCLGKCKGKKAYYQGKAAKKVVTMEIIEGSVKIAKKLIVIAKGFAERSKGL
MDLTQKAANKVVSLTKAGKDALGALAKYTKENLINIHLLCFDTTLAKAEGACFGVNVDLTVLG
NKKLQYEGEVCFNADFFKNMAKQVADKVFPGIISIKDKIMQAKNLFSDSDSKKKELEEYEKQSAQ
EEKNLEGQLERQNEDDDDDDDEEFNGKYKASFDP
>TRINITY_DN191687_c0_g1_i1.p1 type:internal len:241 gc:universal
TRINITY_DN191687_c0_g1_i1:1-720(+)
ILLPGPGGGPGRRPAPGARRLPRPLRRAQGRARPGGARVRRAGGGRRQVPDLPVRQPGGAHHA
GARGRRRCRGRVLLADAVAHAGLGRQVQPERPGRRVDHQGGGVRGGGGRRWPPVLRGARDG
GRPRHRDGEAPRWHGHVAGKSRGPRGPLRARQGRPQRGVQRRLGRAGAGRQGLPDAGDPGV
RPGLGVAGRARAVLATGVLRGRGQGRGHHVRLHDQEAVGVHAQGRRPGPGARGEE
>TRINITY_DN5554_c0_g1_i1.p1 type:complete len:316 gc:universal
TRINITY_DN5554_c0_g1_i1:223-1170(+)
MRIIIALLFCVVGYCYSADWGYEAGNPNVLEPKDWKQHFPKCGGRKQSPINIATRSAILSRLPKIIL
GEGWGTLLESADLSLANNGAKVSIKSSTPITTKQGGTIWNLAQFHLHWGKDSHSGSEHTINGMS
FPAEIHFVHFNSKYPTIGDAVPQPDGLMVLGFFIKVQENAVVPSILQKFFGSLDQIKFEDQKVKLDF
GGRALGDMLPPRGTSKYFNYPGSLTTPTCDESVNWNVMSTENAIVITEKQMTQLRSLSAGKKNE
AFKPQSGNFRPIQPRNGRTIRRNFRTATTKFRSVLRRYFPRIFSYFRVTPRFE*
>TRINITY_DN195348_c0_g1_i1.p1 type:internal len:311 gc:universal
TRINITY_DN195348_c0_g1_i1:1-930(+)
SGCNSIDLGLLVDFSSSKDYDSKQLTKFVREFIDKITNVFAFSATKSHLAYLPFSSRINENITGQWFD
NKYMKTIGDWDYQELTQYLDRVIVPYTPVTGYETGKANAHEAFQATNDKMFNALYGMRYNMT
KVLIVISDGNEDFKEKQNLVDRAIDQLKAKGVIIYTVSIGNNVNWDRVFRYASRESSIINGANFDD
VFNRFFDFAKMLCEDSKVPIPATRTTASTTTVSINTTKLTTTTTTELVPVTISTTTTMSTTLRPIQACY
EDIDLGFVVDFSKMNANDMKERQTANDIVKVIKKIASSFNIAANK
>TRINITY_DN113_c0_g1_i1.p1 type:complete len:1417 gc:universal
TRINITY_DN113_c0_g1_i1:4413-163(-)
MANWSLLYAALLFLYSVSAQEEERPITFIKGEPGPKGSRGMPGDDGSVGSTGRQGNEGRQGPRGD
TGPKGFKGEPGLIGPAGGASGGETKGPDPLAARVGVKGGTYGVNTVFDYQLGPPGPPGVPGNTG
GYGFKGDVGPVGVKGVPGLPGPPGSYGERGEPGEAGLDGVKGDRGDAGQTGPPGKKGVAGAV
GFPGLQGPFGYKGERGGAGPVGDEGVSGRKGEKGYAGEKGSRGDQGDAGFQGVQGPTGPIGPL
GNVGPRGPDGQRGIAGAIGAEGLKGAPGTRGPQGPKGQLGEPGQRGVKGVVGDKGPTGQQGS
QGPTGNPGDPGQPGKTGDKGSIGDVGLIGLTGPQGMQGERGTVGPRGPAGGAGKKGERGQAG
PIGATGEKGNVGDVGLDGARGAPGKSGIEGKVGQPGPQGEPGAVGLQGAQGPRGTDGQAGQA
GPKGNTGEGGFPGAAGGKGSKGLKGRGGDRGMQGEPGSAGKPGARGRDGKRGAAGEDGKNG

NPGQRGPQGSQGLTGAMGLVGAPGAPGDMGVAGLPGQQGRKGTRGLAGPTGQRGLRGEGGL
PGERGEQGAAGPQGTQGTQGSPGPQGRTGLDGKDGSPGDKGERGSMGTRGKQGEPGADGKQG
SRGTPGQQGIRGDSGDQGSPGLLGERGLAGQKGVAGRDGAPGMPGRTGRQGTIGATGAVGLRG
AKGGRGKDGETGEQGPQGQEGPTGPRGPRGASGEDGAPGQNGLPGLTGPMGEEGQPGRPGQP
GKQGPRGSQGEAGPVGPKGPLGARGDQGEQGPVGIEGVVGALGAVGSEGTKGPVGAMGSMGP
TGPTGPQGATGETGPIGPLGAQGRPGIAGNAGGKGPNGDPGNPGRPGSSGDQGPPGKDGSNGE
PGSPGKEGARGLIGRPGAEGIRGEAGTSGSTGRLGRKGELGPKGFPGKRGKDGTNGKRGEMGVS
GADGIRGSKGNSGKDGSAGIAGEDGVEGQIGEAGYQGSRGVRGDTGAPGDSGQPGDNGPNGA
KGSKGSSGRAGAQGVDGRLGARGDQGAEGARGKEGAAGAEGERGEKGKMGAPGIYGPAGDP
GFKGDEGEPGPVGPVGPHGPRGSPGEDGPSGKKGASGITGTNGRKGNRGQSGANGDTGTPGIPG
VPGSPGRPGPPGEIAGMSGAFARFFSAGGMGMKGPGNYYNNYYGQYRYYKAGNEKIRNQIKPH
LSTFKQYYELLKDVDDMMKATGTKSKPARSCKDLFTDYPNKITNDYWIDPNGGSTDDAVKVFCD
KMKNETCIFPKSYRAELENHYTGKDKFRFMYEDIKEDGEIEYAADITQTKILHLMSSKVRQEFSYN
CKNSLAVMKNNKDRYEYALKLKLDNEDIISMNMNTRSLDINILNDECWKKDNTVRTATYELISH
KPIKHQLPLLDVASFDIGGPNEEYGLTMGPVCFS
>TRINITY_DN761_c0_g1_i2.p1 type:complete len:830 gc:universal TRINITY_DN761_c0_g1_i2:87-2576(+)
MTSMFRSEEMTLCQLFLQSESAYACVSELGEIGVVEFRDLNPDVNAFQRKFVNEVRRCDEMERKL
RFMQKEIDKADIAIVDTGENPDAPHPREMIDLEAQFEQLENEMKDSNSNYEALQKSLLELLELRH
ILKKTQSFFEEEGYAETQARLQADHEPGVTGGGQLGFVAGVINRERIPTFERLLWRACRGNVFFK
QAEIDTALYDPGTGDLTHKCVFIVFFQGEQLKIRVKKICEGFRATLYPCPETPNERREMAIGVKTRIE
DLETVISQTQDHRHRLLQTIAKNIRVWLVKVKKIKAIYHTMNMFNLDVTQKCLIAECWAPVADL
DSIQAGLRRGTERSGAQVSSIVNRMSTKQDPPTFNRTNKFTKGFQAIIDAYGVASYQEVNPALYTVI
TFPFLFSVMFGDCGHGAIMALFALWLIMNERKIANWKAGGEMFDTVFQGRYIVFLMGLFSIYSGL
LYNDIFSKSLNIFGSAWVPTLNRTKYDYQPENYKEWSDGDRMLNPLDAPRETPYYFGIDPIWQLTS
NKLTFTNSLKMKLSVIFGVTHMIFGVILSLFNHLHFKQYVNIFCEFIPQLLFISCIFGYLVILIFYKWIA
FSIHSPSQPSILLGLLAMFLNFAKKKTDADRLYNGQDFLEPFLVVVAVICVPWMLLIKPFYLRWQH
NKKKQHGTNFARLHEDTAPLQDNEVNHHDDSGISSKNEEEEEFDFGEVFVHQCIHTIEYCLGCIS
NTASYLRLWALSLAHAQLSEVIWTMVFKIGLSMDGYVGSVAIFAVFAAWAILSVAILLIMEGLSAFL
HTLRLHWVEFNSKFYVGAGSKFVPFSFKDIMNDEAF*
>TRINITY_DN163801_c0_g1_i1.p1 type:complete len:298 gc:universal TRINITY_DN163801_c0_g1_i1:55-948(+)
MANPLKDIISRFSKGVPAGSSTGLGLLIGAGLAAYGIKESIYTVDGGHRAIIFSRIGGIQPGVYSEGL
HFRIPWFQYPIIYDIRARPRRIMSPTGSKDLQMVNIGLRVLARPLSSTLPHMYQRLGLDFDERVLPSI
CNEVLKSVVAQFNASQLITMRQEVSLMIRRDLTERAKDFDIILDDVSITDLSFSAQYTAAVESKQVA
QQEAQRAAFLVERAVQERQQKIVASEGEAKAATLLGEAMNENPGYLKLRRIRAAQKVARVIAQS
QNKVYLNANNLLLNIKDTDDIDDGLLSKKR*
>TRINITY_DN1739_c0_g1_i1.p1 type:complete len:400 gc:universal TRINITY_DN1739_c0_g1_i1:86-1285(+)
MAITDNIKDILSVKIKPRHDTYSDQYSRIFMVKIMMMSALLVGLNWYSDKIKCIIPDSISADGGFVG
AACWINGFYVYEKIRYHVNEVGYFGMPREIDHDGMFPDGQTCNSKDTLSSDPSMECQPMEKTFF
LQYQYMTFVMAAFALLYYAPYAIFRWINVDLVSLKGSIQDNSAEKIIGNYFNHRVNSPNKMRMRV
MGNIVVKILYIVVNIAAFLLLDSILNGGFTTYGTLWTQWAKLDNDLAYDYMGIRHSPKPANALLP
TFGFCEVYMSAKDIKTTVNNKFKFICEISQHILYQYVFIVVWFAMVFGIVLSIVGLLMQLIEHALTIT
CFLKQGDQARRMYSILTLRECEYLEYIRRKNIPLYNDVIKKLKEQFQDGFHKPRDHYNNDGLPKY
QDTQA*
>TRINITY_DN581_c1_g1_i1.p1 type:5prime_partial len:481 gc:universal TRINITY_DN581_c1_g1_i1:2139-697(-)
ETTVAVEQTTTTQAPVVTTTEKEVVTTKAVVTTAAPVEETTTSAPVSTAAKTEETTLAVKTTLAATT
KAEATTKSVVETTAAPIATTAAVIPTTKEEVVITTSTTASLKPTEFKVTLPSIGETLDIIEECPLILQCLP
NMMRVTFKKKDLEDVNFVRFNEISLIGNGGECEPNFNGTHFEFKVPLYGCGTRRRYINDAIIYSN
MIIDRIASIFIVRKNSLRIPITCYYPTSGKASASSNEIEQVANLLDQKDAYGDFTTYVKLFTDDTFSTSL

SGEKTSPDVAYQDRIYFEVGLNTKDERLSVFAENCSVTPTNNKHDPRRYFLIERGCKNDETVEFHA
STKHKQRISYEAFSFVGTTEPVTYLHCDLLVCDINATDKRCLNRECAYMNGKAKRRKKRAALPSA
LKPYSMEIHTAVNVLHTSLQKKAQKFAKQRQIMSPFMLYLVASIGSVATITLLIVAVISFKKRTVESA
TTTSDVQP
>TRINITY_DN11726_c0_g1_i1.p1 type:3prime_partial len:877 gc:universal TRINITY_DN11726_c0_g1_i1:2630-3(-)
MANDDKTGLLTSMEMADKKKASSMKNLMFDDGVRKIDYILAYQDLADDDEHKEEIEAHRALFL
KNLQSKGLEIEKAEHFKATKQSNGKEAYGTFQELEEEEENVVHYIKIHAPFDELCEIAEDMKTKLP
TALNDVNIKPWIEKTSVYRFFQSLDPFIVSDSHIKEEHNYFVAHFTKDRLNEFINHDKPNDFFSHSE
RSRLVYYVLNQTKFGEDELDLGIYSLTTKGIFIDAFPLHDGPVLENMDDEPVNDRQRLQKDWGC
MKRIFKYQPIEGIKNYFGEKTALYFSWLGFYTSLLIPAAIIGILCFIFGVATAWDYGAVKEVCDQVKN
NGSNLFYMCPLCDKLCSYYLLQDQGCMYSKVSHFFDNEATLFFSLVMAIWATLFLEFWKRKQVSL
AHEWHTMDFEEEEERPRPEYLARVTRLKKNPVTLKMEPYMPPGTKFSRIFGAFGVVAFFIVLVLA
AIVGVIVFRAAFYILLIRQDVETIRARSKIIVSACAACINLLAINILKFIYQRIALRLTLWENPRTNTDY
EDSFTIKMFWFQFVNTYASIFYVAFFKSEFFTGSPGQYKRFTAARFRFEGCSVQGCFLELTVQLIIIMV
GQQIIGNIMEIVLPFLKAKYNEYKNRNNVYVKSGEEPQWVSDFECQVQNQFSLFWQYLEIVLQYG
FVTMFVAAFPLAPFFALLNNMVEIRLDAINFIHNFRRPAAQRAEDIGAWYNILATLTMFSTIVNAF
VLAYTSELIPRMVYRYKYSATGSMYGFVDFSLAKFPVANFSSESRPDNPTKTVPVIQPTCRYPGYHE
HAFPFERSKEYWVITSARLAFILVFIVTVMAIRALIMYLVPDIPKDLELKIKREKLLAAESDRKFREK
TGKRKKRESVRSE
>TRINITY_DN77_c1_g1_i1.p1 type:3prime_partial len:1965 gc:universal TRINITY_DN77_c1_g1_i1:2-5893(+)
MGVQVYSLGIGSNYDIGELLDIASDDASVFRSQDVDELVSIVASITEQTCKGCTRAVDIHFALDASQ
NVGRENFQVIKNFIKAIGFKFIISETGSHMSASIFGEYGTLIFSMDQATQQEDFINEVDNVPYIGDFN
GNIDKALDMVSEDIFTFDGFSRQGVPKVLVVLTASNCDSCEKSVEEAVKPLKAQGVHVVAIPIGSN
ANMEEMDKMASYPTQDYVIPRASYEELLNGVFIQKVSEMVCSGRPGVCEEPPIEGECTEIINECTL
DVNCPANRKCCIKDCKASCEVPAAACITPIDLAFAFETTGFIEENFDEVKSFASSLLDHFRVEPAKT
HISISTYGESVNAKTAFYESYDKSALKQFIKDLEPDGGRQANIGDFLKTANDEIFNITNGIRQSSPR
MLVLFTNGVYSNDQIAKAQEESLKLKAEGQDVNILVINIGSIVEGNSVLRDVVSTPDFRNLHNVT
DLRRLVNFGFENKIAEAICASKVRKTRCKPRTPQACSVRDDLCQTDFDCPGYQECYSDGCRLRCQ
TCENRCGDKVDLLFMIDTSSRIGPEGFKNAKENIKKMLEWFTIEANNTNVAAITYSDNANYAMQ
LPAAGSNDPPQTLFDVQGRLDSIPYSGGSKSNLDKALKMAAAQVFYQNSLRLRKDSKKAIVIVTD
GVDDSTSRLEIASWPLRKQRVVVDGNDYAAIRLLAVTVGNNYNLDTLEKVVSPPIDKNIFLAADF
DDMYNGIRQLAEDSCKYTRQDVEPPVIAVKGDPGPKGEKGRQGNPGTNGTDGMDGIEGPKGER
GDDGPKGEKGDTGIAGPPGGDQGDLKGPDPMAEAVGIIEGTAVKGTRGVTYDLRDGPIGPPGPIG
GVGKTGLPGDSGEQGQKGRGGPPGLRGVFGGQGPKGNPGEDASPGLPGEPGPKGISGKKGPTGS
PGMPGPQGPFGYKGEQGIIGQPGDPGDGGKKGPKGYAGEKGDQGDIGDAGFPGPDGKVGPIGP
PGQRGLRGPEGSSGPIGKTGDIGNKGLPGPRGSRGLKGQSGNAGPRGGKGRTGDVGLVGEKGTQ
GSIGEAGQEGQPGVPGKEGPPGYPGPIGTPGPMGSRGDIGPQGEEGPKGSLGLKGNRGEAGRRG
LTGEKGETGDIGELGPKGAQGQQGKQGKTGKIGPRGQQGPLGFQGLQGQTGDPGQNGRQGPK
GNLGPTGEKGADGPKGSLGSAGLTGPKGLVGEPGNVGRPGLPGRDGKNGPQGANGKDGDPGA
SGPQGLQGSPGSMGTPGSTGDPGSPGDRGATGAVGDKGTRGDSGVRGEPGAFGQQGPPGLRGE
RGEKGPQGTNGVQGSPGPRGTPGLEGLDGIVGRKGTAGRPGNPGQSGEQGAVGSIGVPGPQGIS
GTPGERGDGGSDGGPGEQGPPGLQGSSGSPGRDGMPGRPGQQGTTGGKGKTGLRGPKGANGQ
NGVQGARGPDGLPGAPGLVGNRGTPGPNGIPGLDGNPGPAGKQGKQGKMGTPGMTGPAGLR
GPVGSPGLRGQKGPTGDRGDSGETGPIGVAGDIGPRGFTGELGLRGAIGEMGQIGETGPQGMPGS
QGTPGAAGLTGPAGPSGLPGKDGNKGPIGDPGNAGNPGRDGGPGSPGKDGTPGEPGTPGSQGK
QGLKGPFGPQGAPGEQGQRGLTGRQGSGGKDGPKGFPGNDGSPGISGKRGPRGKDGTPGSPGPI
GKDGRPGKPGINGTDGDPGNNGPKGDPGERGDSGDSGSLGASGDKGKTGSEGPAGFVGPRGSN
GKAGVPGKRGKRGDQGRDGPRGNSGPDGIDGSKGFKGNMGRFGIQGPPGDAGLKGALGPDGT
VGHTGPQGNTGDPGPDGPEGPSGSKGPAGNAGQIGNAGYPGSPGDGGIPGTAGLPGPPGPPGPP

GDMSGAFSGNYWDFVNAGGLQKGPRYYGGRSKRSLDQQDNSIEKVFKSNYETFKNFDTVWNQI
VDDLVIQKTLGSSKNPALTCGDLFKTNPITKSGDYWID
>TRINITY_DN31906_c0_g1_i1.p1 type:5prime_partial len:595 gc:universal TRINITY_DN31906_c0_g1_i1:1953-169(-)
AFGGDQFEPGQEEHLAKFFSIFYFTINFGSLISMLLTPILRGDVTCFGNDCYPLAFGVPAILMIISLLL
FLMGSKLYKKVPPEGNIVVEVSAAVGSAFKNRARGCGSGIKKDHWIEWASDNYTPVLKSDIKALF
KVLFMFLPLPVFWTLFDQQGSRWTLQAMEMNGDIGFLGTVKPDQMQALNPVFVMILIPFVETVI
YPIAKKCRLLTKPLQRMGFGMLLTALSFVMAGFLQIKMEGKSSVANLPATGYANYRIINTVPCSID
LYSNELKRKNMTIGYQEATDYFQVPVKFQKLVINSECPPYMNLETSVSVSEQKSYTLVIGTNHSTLV
SMQLTDDVTPLAHGHAKVRFVHAGGSVNDKVTVKLNKTLSIDLLPFEKPRYINIISKSYKIRVAPN
ASAKALALKNNVNFGNGGIYTIVIQPNKDIDKLKLFEYADVSPRPISILWQIPQYITITLGEVMFSIT
GLEFAYSQSPPSMKSCIMAAWLLTVSVGNLLVVILAGFKLTEDMAEEFFFFSVLLAVVTIIFAIMAWF
YKYVNYGASRDEADTTNIRDNENDDERTPLKKYSDDEKIYPDLKSSVEDEAPSTSKKESNL*
>TRINITY_DN5854_c0_g1_i1.p1 type:complete len:464 gc:universal TRINITY_DN5854_c0_g1_i1:1435-44(-)
MATNDFGGKLGQESIDAAWVILCAFIVFTMQSGFGLLESGLVSRKSEANVMVKNMIDVTVGGVIY
WFVGYGFSFGPNSGNANKMAGNGNFVLDVDHDDAFTYAHYFFQLSFATAATTIVSGAVAERVK
LPSYMIFAIINTGFIYVIPVHWVWGAGGWLNGKCFDFAGSCVVHMAGGISGLMGAIVLGPRYGR
FDGRAEYRMASPTNVVLGAFFLWWGWIGFNCGSTFAVSNGSWLTASKVAVVTMNGAMGGGVA
AIVLNFILQFKRSYLLDIPELVSGILGGLVSITACANVIRPWEALIVGFIGSLLTIGGGALLTKLKVDD
PVGCFSVHFISGFWSMVATGFFAEDNASDDLTGIFRGGNGKLLGWQLAACVTVTLWNGVLSGLIF
VVLKYTIGLRMDEEEEIRGSDEVEHNVKDLDFVLEGKHVLGKHVSGSVTYRNGHVNEKPQPKVV
NGTNNELVIMA*
>TRINITY_DN64_c0_g1_i1.p1 type:complete len:468 gc:universal TRINITY_DN64_c0_g1_i1:1555-152(-)
MPPKEAEKIHINIVVIGHVDSGKSTSTGHLIYKCGGIDKRAIEKFEKEAQEMGKGSFKYAWVLDKL
KAERERGITIDIALWKFKTGKYYVTIIDAPGHRDFIKNMITGTSQADCAVLIVASSTGEFEAGISKNG
QTREHALLAYTLGVKQLIVGVNKIDNTEPPYSEKRFDEIVKEVKTYVKKVGYDPKTVAFVPISGWH
GDNMLEASKNMGWYKGWTTERKETKTTTGKTLLEALDNILPPKRPTDRPLRLPLQDVYKIGGIG
TVPVGRVETGIIKPGMVVTFAPSNITTEVKSVEMHHEALLEAVPGDNVGFNIKNVSVKDIKRGNV
ASDSKNDPAKEAKSFYAQVIILNHPGEIHSGYQPVLDCHTAHVACKFSEIKQKIDRRSGKVLEEMP
KMVKSGDAAMIDLTPSKPMCVEAFSEYAPLGRFAVRDMRQTVAVGVIKSVTKSDGSGGKMTKSA
QKATGKKK*
>TRINITY_DN15109_c0_g2_i1.p1 type:3prime_partial len:209 gc:universal TRINITY_DN15109_c0_g2_i1:624-1(-)
MNSQLRNVLLWHEPRISGAIFVLGLFVLFALSIYTVLVVLTCIAIPVVVITVAIRVIFAIKSVIFKQPNE
NPFEYWLNEHWGIQDDKLTSINRRAADIVNNVVLYGKEVVLVKSVGESLMGLLFLYIWYVIGKNI
SGISLLFLGHLCAFTLPKIYDLKKKQINKFIKHAQNSYNECYQRILDNFPILNKYMKEQKNKEKPN
NNNNKEA
>TRINITY_DN7102_c0_g1_i1.p1 type:5prime_partial len:1263 gc:universal TRINITY_DN7102_c0_g1_i1:3-3791(+)
DKNELEEKVLPAVSVGTLFQYATGLDWFFMILGTFAAIGNGVAQPISFIVFGELINKFIISDSGEAIDL
EEEMTKFALYYVYIAIATIVLGYIQNTVWLLSSIRQAFEIRTKCFNSVLRQDIGWFDVIDAGEISTRM
TDDITKIQNGIGDKVGSLIQGISMLLGGFIIGFIFSYKLTLVIVAMTPLILITGFLTGKVVALITSDEQLS
YAKAGAIAEEVISSIRTVVAFGGEEKEIARYRTNLVDAEKSGVKKGISTGFAFGMFNAVIFGCCALG
FWYGGYLVAEDEITGGDLLIVFFSVLIGAAQLGQVGPNFEAFAVARGSARFIYDVIERVPAIDSMSD
SGLKPEIIGNVIFEDCEFEYPSRQEVKILKGLNLTVNCGQTVALVGESGCGKSTTIKLVQRFYDVSKG
KVLIDNTDIKEINVRHLRRHIGVVSQEPVLFDTTIADNIRMGNDEATLEMIEKAAVNANAHDFIKS
LPKGYETYVGEGGTQLSGGQKQRIAIARALVRNPKILLLDEATSALDTESEGIVQSALDKASEGRTT
IVIAHRLSTVKKADKIAVVVEGKIAEIGTHEELMEKHGEYFKLVMLQTFEEQEEDEGGDDTSLLSES
DREGLLRKQVLRTLSRMSSHDAEEDKKIEEKITKEFRRRSTFRKSAKDIRSLERQKSKINEEETDEDV
NIPSMWRILKLNLKEWPYMFVGFIFSALTGAVPVVFAVIMSEVLESFILTEQPQKMKDTSAFWSIMF

MVLGNVVGVSYFVAQYMFATAGEALTTRLREMAFRSLLRQEIGYFDLPQHNTGALTSRLASDASA
VQGATSTRLNTLTQVLVMGLSSLIIAFVFSWKLTLVILAFVPFLAFGGAVQTKLNTSFATDEQKKMG
EAGSIATESIMQIRTVASLGKEDYFYQRFLKLLLKPSQESRRSAHLFGLSFGFSMAIMFFNNAAAFR
MGGYLNVNDGLSLADMIKVIISIQFGSMTAGQIASFAPDYAKAKVAAGRILDLFDRVPPIDSYSQE
GIKDSLEGHVEFKDVKFHYPNRPSVPVLQGLDINIKKGQMLALVGSSGCGKSTSVSLLERFYDIYE
GALTLDGKAIKSFNVRHLRSELGIVQQEPTLFDCSIRDNILYGVDTSVRKVSQEEIENVCKSSNIHH
FITTLPQGYDTNVGDKGALISGGQKQRIAIARALIRNPKILLLDEATSALDSESEKVVQDALDVAM
KGRTAIVIAHRLSTIQQADLIAVIQSGKVVEMGTHQELLSKKGAYHILNQAQL*

Band 2
>TRINITY_DN982_c0_g1_i3.p1 type:complete len:9852 gc:universal TRINITY_DN982_c0_g1_i3:29697-142(-)
MSIKLCTFFVFFLIQCINSFPEIGLHLSLDRSYSFQGNGWKTVNGWEIKPRANGPYEIDAIKNISPEY
FPVPITGVYYITVSLPVEGASNGEFKSAIIVDGKNEEGRIGLMSSFQKTAFKNGASTLTFAGFVRLFA
DEKLTIEIYSDDINANYVVLESATFVFHFIGVTGSVPAYLAHTAQDQEYASDSIIKPWVTLGDSRVYE
SLSGFIKNEGVFTTICNGVHKMAVNIMIKANAGTYTLKALFQDEESATATVTINQPDSIATLNIFQN
FKWSEGMSITLQIHSSNGANFKVLKSSSWSMSFIGETTANFQEFSVFLMADQFLQPSQFPQRINIDL
NGVFQFLQSNPDQYNKGQLVSGADTKFKDNLDFIVDSTDLFYVSANIIIEGEAEDLEITIAVSQIDID
VLPEDGLSGRLTKISNSRRSISLSGIIFIREKQYLSFFVRSRDSSKSFTVSKDSFISVINMRYTVSSVSARQ
NENIDVTAGSWYDISGRFAEQKNGLYSFGKDFNINNGVFRTSHSGIHSVHANIQFSLDSDAFGDN
DDNKIVLRVVIDGQEDKISLMSIRKNPEKTATLKLFGSIYLRAGVTISFQVKSLMPNTVQYKILQRSS
ISISYIGPQWAVNSFHAVANQDLNYDGKPDDTRPDIPSPLNFWKTEAADEFISPFEVLESFDGTRYT
SVDDGLYVVSSLLILKNVDCDRSALFKLDFVTTGGSRASDKSNGFADSHFFIADSKGTEFTLSVTTA
MRLEVGFKVGLKLTVGSTSCRYTISKESSFSIAQWSKSSNTIEENRLKYIGVLARLSAPTEVSSPNTW
SNLGSNTMDINPGDKSRSSFRPGIFGGDDLAPITRTGKDFNGDVSDFTIKDPGIFFISGMITVTTTEN
ANEEFAATVQIDSEDIESGLYAAVINQGSTEMSLTFGGTIYLKSGARVRVVVKGGRTFNIAAGSSFSL
LKLQPDYKTPGIIVSIDEKITQAITRYEHKDLRADIHRTLTIGNLGFVESTGVYTIQVDGVYMVDYT
VILEATNPNAVFGAEILSDNTVVATGTSTFGNTKTIAITELVYFSKGAIVKAVLKSNTNGETLNNLQ
VDGGLTVYFLEPKEHVKDGHRSKQTSPQALPVRSFEKVTGFAAQSPDFAGGADSVDYTVPETGVY
LVAFTTRVNRVINGFKSRVLVNDNEFLNGNYQIVSAQNAPATLPLSIAMELTVNDVLSFQVMADG
SGMSLDGSTRSILKIDEVNTIRNVTEGLSAYKLRDQKVNGQQETIVEEWDTSVTEGTFLAKNVSFT
VDHSIVVVAAGVFQISVSVVVKNDRPSQSKFELRVLQGDDVKSHGLHSKVEIGGGREQTLTIGGSL
LLQKFDSLRIVVRDIADPLASYTIKAGSTVSVIIIDDNNGPIACEDEGLNMRRLPERIRKPTRESHTF
QCNVVSSLKAEKYTWFKDNVQLKEENQKNLVLNDLKLSDSGVYQCRVRFLELTGATNNATLSVY
DPYPKITSPKFIANVLENQDPPLTIEMFSLSAFKLSPEDEKASITTQIIAGDDQSFFTLSGPSKQTVTLS
TTKKLDYETQNEYNLTIKATNLEETALTTKGPYETTINYIVRVIDVNDHPPVFSQSIYSRSVPEDYPI
DGSIAKVSANDQDAGANVIYTLVGQVSGDLFKIDNTGLIQLRQNLDREIRDDHTIFVQASDGLFT
TVAEVVVTVTDVNDNFPNFMDTGFDISVSEATLTQSVILKVIATDPDLGTNSQLRYTIKSGNNENK
FSLNLLDGNLTLISKLDYEGTKIYNLEIDVQDSASPPKKSQNPLTVVIRVQDANDNNPTFTQSLYNT
RIAENTAVGTSVLKVSATDRDGEGINNPLTFSITDTDANEFFQVVSSGATAGEIRVRKSLNYDIRKD
YRFSVVAKDNNLGEPRYRTALVVINIDDTNNLRPTFEAGSASISISEGTSIGTSVHQVVAIDKDTVG
KLDYRIISGADGKFAINKETGDINLISAVDFESNKKTYTLEIQVNDGVRDALNPATVVINIQDFND
NQPEFIQKYYIFTALESISDSQAINVGNPVIARDADSTTNSQVDYSIQYDINNPSVPNEGDALFRIDS
NGLIFKKNGVILDFENVRYHTFTVVAIDRGTPKTLLGKAFVTINIQDVNDEAPSWSPAIYSVTVKES
AGINRDLVFLSTTDKDTSGAVTYSIVSGDSANQFQIFDNVIKIVKPLDFEKTETHTLKVSAQDDNG
NRAQLDATVTITVEEVNDNRPLVTNNNANSIQVSVPEDIPVNSLIVKLNGRDDDKGAPGQASNQ
FTFVSLDKTTDFTIDEVTGEIKNTVNLDYERQKSYSLVATIRDKGTPYLESKFLVDIIVENKNDERPIF
DPAIYTIKLSEKAPIDTVVAMLQATDLDTPKSQLRFSSVTTLDSHFSLDPITGIITLKTSFDKEVNGKD
GIFVISCTVNDGTSNSAQNAIVRVEVVDVNDNQPVFPTSEYFIQVNEGSSIGTILQNIAATDKDNT
NTHNQFTYTLEGKEAVDFRLNGNQLQTNKIFDYENKKSYSFDVLATNNLSPDKLVGKATVVVSIL
DQNDNNPVLSPSGVINLNISEGTIAPANIITIFATDKDSTINAEVQFTLTGDPTNTFTINQNTGEIML
AKSVDSFAKNEYTLSVTATDKGSPPRSSTATTLNISIKPVFVPGIEFENCPQIISFNENAAPNTFTKTL

TVKDRGSFNQVQFTVVSGQAGIQNLITVSQNGVITNGQRRADFEARKRHDFLVQAENERGAKAY
CHVTANINDVNEGPIFDSANYVFDVLEVIKINTVVFSVRATDPDFSSTTNGQLTYSLAGQEASQFSI
VPNNGQIKLAQPLDYEIKKQYQFNVIATDGGSLQTTSSVIVNVEDWNDNRPIFDQPQKLAINVPE
NSKPDNVFTVSATDKDSTTNGEIEYVGIYPTGIISIDKDTGTAKIIGDLNYEKRQKYDFVIVAKDKG
VPYLSNTREVSLSVLNLADEVPYFDTAAYTISISEKTSINTAVFRVMAKDNDLENFGKISSYEIISGNQ
LNRFRIDSNGNIIVQQSVVLENNNPNQYILTITAFDTQRTQSLNNTQVTINIIDLSDKQPFFIQNSYI
VEVQENNEVSKSLLSLEAQSNEPTFNPTYSIVQDPKNTDSQFFQIINGNTIVATTVFDYEKKRFYSFE
VQVIDTNNGKTNKALVVVRITDENDNKPFEVSGNLHFNITEHTLLGSTIGSLNGKDNDAGINKEF
EFSILEGNDDDKFMLLPTGQILLKNFVSVKEKDFYKIEVKITDKGTPSLFNIFTVRVTINSSQALVAS
NNPTIPEDKNTGEEITRVTAIDPNTGSSNGFIYSLVGTLSNSQFTIDAKTGAISLLASVDFETDRTHT
AVVKAEGPTSTGVTEVIVRVTDINDNKPIFDPTNFNLNINENFVVGQEIVLVRATDKDSSLNDNNV
IIYRIDSVNPSSGNDLFSVNPSTGRILLLKALDRENGDTSYVLTIAASNRGVSNKPDNTMTVTINIA
DVNDNFPIFEKSLYQGSVSEDANTGTSILTVRASDKDILLKNRNIRYSMTQPMAVQKFQIDPISGVV
SLKQEVDFETKQRYDFSVIASDTGDVAYSRTANVEITILDVPDVEPKFDPQRYEITIAESTLVSTSILK
LQVIDSVKNLNFNIKQGNSNSDFRLVASSGELFVNKQLDRERISRYQMTIEVSDSVNKAAIDAIVIV
NIADVNDNQPLFLQSGCYQANRAEGIETEQVVVNVQATDRDEGSNKEIVYSFSDACSVLDIDAN
TGAITTKQKFDYESARSHICTIKAEDKGTPKLSGFSCIILTVTDSNDLKPTFEVITSNARVLENIPVGS
VITRVLATDKDTLSSNKQFVYTIKTGNEDGRFTIDANGFIKLQQPLDYEKKTDYRLEIEAVDSTNQ
DMVSDPNAIVTVTVIEQNDNRPKFAKSYHRVNIEESTAIGTTVLKLSATDADAGVNEGLIRYVSLH
NTTVFNIDRTTGEITLATKLDYEVKKSYHLIAVVKDQGNPVLSARTLIDIFVNDVNDNPPVFVISSY
NNIVSEKAPIGQSIVTVSARDDDTGVNGQLKYKIRETQSMFRIDEDTGIIILNGDLNVENTNKLLYA
LTIDVVDSGVPPLQASNPATVSVQVSDVNDKQPVFALDRYQFEINENIEGNQIFGDSVTVRANDD
DLTGTNNQLTYRLVNTTMFDLVVTNQNVFIKTALGVSFDYETKKSYSFEIEAMDSAANKLYGRSLV
TVTVKDFNDNPPIITVPNIIVNISDATLIGSHIATIMAEDKDSGENGILIYTVTAGDSTNRFTMESDT
GSIILKNPVDSYTKDIYVLNVLVQDNGIPRRSATGTVTINVIPVDLTLLKFNTTCPFAISIPENQINSN
FLGLKALDAGEYVSKQLRYTINSDENDVNQIFALDSIKGEFKQLKEANYEERSRYDMTICATNERG
SKVCCHITIFITDINEPPSFIGLNNNNIYEVSISETTPIGTIIYRVLSKDQDLPGVLNGQTSYSLGQQNV
FIIDQMGQIRVNKTLDAETQLNYVFQVIVTDKNPTNPLQSAATIRVNILDYNDERPIIQNIPDITIDE
NSDNQVVRNIVVTDRDISNIGSNIELSIEPSNILTINNRQLRTVGSALDYEKVQRHTLYLTAKDKSFP
YLSSTTSFTVTINNLNDNKPKFPVNNYNLTVSEKTTVGTLILTLFAEDDDLEEFGEIANYEIVSGNQ
EDRFSIDSNGRLTLKKQLNWRNVKSYNLNIIARDKGLPSEQSQIPATVVINVADYSEEQPNFLQNL
YVKFIPETTPIDTLVLTVEATSGEPVYGPFKYTIVPKLPDTNNFQIFSNGEIKTINQFDYENQRNFTFD
VQVEDEKNGKTNRASVFIHITDVNDNIPQFTDLTRSNTQYNITEFTLPGSMITCLSATDRDHGKNA
EIRFRGIGGDGNGVFNVNLDGKVLLSQQIFYDKKNKYNLIVEAFDLGSPEQKAQLTLTIFLHKSYT
SVITTDVDINEDVETQTRVTRVSSIDPFTRSSNNFTYYLVGKSTTFFEINAQTGEIFTKATLDRETRDR
HVLTIQSRGPTTNGVGELVVNVKDINDNPPIFDPQVYQVTVSESANIGLSIVSVTATDKDIGVNGE
VTYSIEDGDQTIFSINPSTGSISLAGKLDFESKQSHTLTIKAVDRGNPPQDSVNKATVIVVVQDFND
NCPVFENSTYSLTIKEPGELGKEYLKLGITDNDNGRNQLFTCSIDEPLAEKYFEMINDCRLRLIKEL
DYEEFTSFRFVVKATDQGDVSCTTTTNMIVNVGDRADVIPFFSPLTYNISIPESTTVGTIVVSLKVGS
TFKGLLYSLFHKNTQDDLIKFGINKDTGEIFLKESVDREIQDKYEFTADVFDGSNNAVQRASVTINII
DVNDNAPFFNQPTCYQRDLTESTAIGTFVVDVSAADNDKGRNQQVTYSLLPSPSCTSFSVDETSG
RITTSQILDHEQQKLQHCLIKAKDNGTNSLSGYSCLTVNVVNVNDNKPAFIVNRFDVSVLETVPA
GTLVASLVARDVDNQGLITYSISNGNLNNQFTIDSSGKLRTRFPLDYENTKSYNLTIQASDNNGKFS
SPDDALVLITVLEVNDNRPQFLQSYYKVSIPEDTATGASILNVTAIDLDHGELTKSLFYAALENTTQ
FTVNANTGVISVAENLDYEIEDHYELVVIVQDRGTPHLSNRALVDIQITDVNDNAPMFVTTVYNR
ELSEKALIGAPVVKLNAMDADKSDKMTFTIDSVVQGAQGFFSINGDTIQLDKELDYEVPEQRTFV
LSVSVKDSRDRDGANKANVFINVRDINDREPIFDLNMYEVTVSENNVISNPIIEVTARDSDSTPEN
NNLLYSLVDGEDAASFSLLNNGQSAIITSKISFDYEKKKSYRFLVRASDKVRENFTAFSTVFVTVQD
TNDEAPVPEKDLIMVNISESTRVLSTIAIVFANDKDQGINGQLAFQIINHSEDKFEINNKGVIRLKST
LDFFQQNFYNFTVLVTDKGNPVRTSKPVTVLVYVYRTEIPGIIFEKNHYIFNINEKEIVSLVGTVNAI
DEGTYVSRQIVYSIVSGQNDIKDKFRAVNNNIIQDKEVNYKDQSEYNFVMKAINERGSIAFTHITIN
IMDVNEKPYFVGLNNKGRYEFSMSEIISVPSYIYAVRAKDDDFGLNGEVDYEIISNFRFGNFSIDQY

GRIQVLKDPDFEFTRSYEFQVRAKDRGTPSLESNLVSVVITVLDYNDNHPIFNIQEPLEISIKELVNN
TDVGTVTATDKDGSGLNRIVKYVAIYPSDHLTVNRDNGQIRVIEPFDYEIAHKHEFVVVAQDQGT
PYLNAYKVIKVEVLNENDEFPEFTVIEYNITISELTPIGSPVLFVHAQDKDRGPSGQVTNYKIKYVPP
SGGSGAGSPDKTFEITSDGQVVLKEKLEYKFQKYYQYRVTCTDNGSPPKNSINEAIIHLNIADASLE
QPIFSVNPAVVEVYENITLNSNIVKVEVDSIEPIKGPFEYSIIASEDSQPFMIDNSTGQITNKESFDYEI
KRKYVFLAEARDKHNNRTQRAMVVIFILDINDNIPTIETIAPQTFVQNISDTTYPGSVVAKLYSRDA
DSGENAMVDFTITGGDGENVFAINPNGYVTLIGPLDVRVKTQYTINVKVSDRGIPTLSNNRALTF
NIKKSKFIQNTVRFDRHHYKSNCIEENQINLNIIQLNATNSNPVAGSTIIYSLDGTLEERQPFIVNSQ
TGEISLSQTLDYEIKQEYDFVAVAVNNDGSRDVAVVTVCIKDLNDNSPIFEPQSYVVNVSESESVGY
LVYKTTVMDADKTDFNTTKSYDIIQGNVNDKFIIDSMGHIYIKSKIDYEALQVKHFDLRIRVNEGN
LVGDMVLRVNILDWNDVHPSFSTLNIYNFTIPEDKPVGEIIEILTANDGDATSPNNEVTYKLLTTED
DFILRRNDKGHGSLIVNKQLDYSKQNRYEYVALAEDKGYLNLLGTAIVIINIQDVNNHPPEFDDSE
YNTTVSEHAQIGTRVLQIHAHDKDTGLGEQIFFIYGDPTETFTISSQGFIILNKQLDYETQRDYIFDV
GARDGGNLVSLNNVTVHIKVSEVNDNEPIFDLSTYRNSYAEDTPLGTTLFTVRATDKDFIGNSIIYSI
SGANDDTSYVSVDQTTGDITLQRRMDYESKNYITFTVQAIDSNNRNKLKGSTHVIIDVTDVNDN
NPKFVFGSDLVYNVTVVENINVGTLVVVVDSQDADSGLNGQTRYHIVSGNNENKFFINELNGQI
NLINKLDALQTNKYVLTVMAKDLGNPSRSTNVTVTINVLRYFESTPMFLPSYAEVIYPENLALGSSI
YELNVTKTLPGSPMKLSFHSSSKFPEFTLQGNKIILSKEFDYEIKNRYELIIIATDEENDKAFAQVIVR
VQNLNDNIPYFTQNAYFGVVSELAFVGHSVLTITAKDDDKDFTELFYSIATQVPDNAFKLQDNIV
VVNGKLIRNKVYEFEVEAFDNTFTSKRATVSITVTPENLPSFVNATYIVTLQENYPISTNFLNISAGT
FSGITYVITSMKAKDMFNVDANGQISLTKSLDYEKQRQHVFPVTASVTSPITQVRVASVIVNVLNE
NDNAPVFVNGPYERNITEGHLVGTMVIRVDGHDDDSDKLTYSIVSGDDGKFSINSEGVISLISTLNF
DLKNKYVLNVRVSDGKFVTDTIVTINITPIVKFPPMFNQTLYLLSVKENTLVGTSILQVSTVNVIPPS
SFSINEQSGRDFFTIDANGVIRTNNALDYEAKRQHIFTLKVQDLRSMGHATVVVNIVNDDDTCPIL
TPATQTVQITEPVAPGTIVAAVQAKDIDSKSLNFTITGGNGLFRIDQHGGIKTINEVDISTSTEYELTV
TVDDGTCTKSVQVDVNVNPLAPCPDCNIYPFTQTIYEFSINENTVPASPIITVTTGQSLQRNFSIEDP
AALEYVTINRLTGEISVIKAFDYEATKHLSFNVMAKTQTTKNRNPFYVITSVSRVRITVRDLNDNCP
SIESPMYYEVSHSSPIVQDTVVTRILVEDPDTLKDHTYSISDNNFKIDSMGHIRTVQNIESFTAQFYN
LTVTVSDGQCSKTSNVGVNLAVNPTPMTYQFTSNGVYSKSVREDHNLQDVILSVALNQNYPRVFS
IVESDANDQFVIDSNSGALRLKTSLNYELKKRYDFTVMADIHGGVYKSYASVHIIVLDVNDECPIF
PVIGYNQRVSGTPVIGTIVEIIEANDADNATLEYSIVSGNEDGHFSLDNSGVVRVVKPFASSFSKTYG
LSVRATDNKCSADVPLTVQVSTCAYGEDYQFEESEYVVYYLDENRNLGIVSNIKLKSTRAAFLTLSQ
NTHFTIDQNGDFRLIQTLNYETKKEYHLTLTANFTQDNVVTRIPIKVFVRDINDNCPAFSEELYAVQ
HTEPLREGLLIAKLSVIDDDTVQNFTYNIVSGDKRFSINENGEVVMNARLVTDTTLLSTTYKLEVSV
NDGKCAKNTKVDVFVYKTVVNSFLFPQPFYKFDISENQAIPFTIDRFVNVGGHEAVYGFLVSTDFF
QIDPKTAQLQLVKKLDFEVEKTHYVLISATYDDGTQTYTVVAIHVGDENDNAPQIFAHPPITRLVQ
NTPHNSIVTKIEVEDPDTSKEFTFKITNGNPLQNNDPDKALFRIESNGMIKTTRCISEDEIGNYVLTI
SVDDQVNTPTIFSNMQVQLKTTDDRSYVTNCGLGCPANDGPFSRYQFEQSLFIVNVTETAANPTV
LKRLTRPFVEKTTYTIGAGADGKFSIDQNGTLSLTGSLDYETKPVYYFIVSAVIPSQVVGRFITSKTAY
LVMVNDVDDSKPVIEGNGGTLAVSETSEIGQIVGTVRVKDSDSSSPLTYNVVNGSQYFNIDSAGNI
VIKDTLNAAGSPYTVTYTVSDGINPPVNGEFVVSISETNNNVPKFTQNIYEVSLSEDTATDTIAVTIL
ANDGDTNDKIAYSFLQSEGPFVINRDSGEIKLIGPLDYEAQKSYNMVVQATDSGGFANIAFVQIAV
GDVNDQVPVITPQTTPTQIHESTLVNSVVYRVIASDGDGNERLTYALANSNDFFTINSATGEITIKE
NLAKSNVSSHTLSIVVTDSSGKTSTQDVALNIRRSGTSQGCVKITTSQGQSSSPVINLPENTASGTTV
FKLVAGDASVFSNMRFTLNREANGVFAVTEQGELQLVAPLDYETKQFYSIIITAEDVTRGVSSQYNL
FVRISDVNEFTPVLETIAPVAVSELANIGDVIAQAKANDQDGDSQVTYSLGEFGNNDNKFTIDAAT
GNIILNKRLGSGAPHVVEVCASDGSIKKCQNVTFIVADTNDNPPSFQDLTGVGSIDLTNTSSYVVY
KPIVTDSDQTGTLSFSLKDPAPSQFSINSTTGEVKANKDIAKGTYTFGVCASDGRYTSCQSVTVVAS
DRSQSITPSFNQDQVGAIVDISASSGKQIIQVMASTLDKAKIEYELKNEADRNIFEINKDSGLITLKN
RTALTLGTIYTIPVKASRADDPLNFDEINIVISVGNQQLDSGSFIEQVLDTQHLVNFSLSTFDISKVSY
YIILGQQVGGDSKDDEKLPVSWYYATFGDESYRHRFYYIAKLDGSKFRNTGGSRKRRSIQDKDSKS
FTYLVGEENGCSKNTSMAAICNGPLKTGTEYRYKLRAYYDDGELRGIPVDQKPTEVKRTQAPQA

GKSIDEYREQDSYVITFWIIGLVIFGLLFWFSVLVIAILLCYKRRYEVTQAYGYSVQTRDKIDGPPTEY
SNTVYRFQNPSYPDNDTVKMKKVDASESRTKTEVQTTSMVVLDDYEQEQPQPDIPVQDYDAIEY
ETRSAQIKFHEESPEWESMDIQLRIDPTGNQDPVITRKSDSGSKDSDSDASKKYENTEYIEEKEDFV
NEKEDSLTRL*
>TRINITY_DN1616_c0_g1_i3.p1 type:complete len:3855 gc:universal TRINITY_DN1616_c0_g1_i3:218-11782(+)
MIIFLAFLAVAVGQEVLLPPYFDIAKGKNITATATCGEDYVAPKGSPGELFCTLADATGKFGIKGLK
CSHCVPGDYGDGDGKNARDHHIRNANDGSNRWWQSPPLSRGLQYRKINITMALGQSFHVAYVI
IKAANSPRPGVWALERSNDNGRTYTPWYYFAPTSSECKQYFGVDSYLPIVKDDTVICSTEYSKIPPF
KNGEIHISLINGRPGASNFSQSKTLQEWTTATNVRLRLLDIKTLLTDLLPLSLEDPTVTRRYYYSIKDI
TIGGRCMCNGFADECTPHTNIVGKYECNCQYNTEGPRCSRCKKGYVQKEWRQRTSTDKFECEKC
NCHGHSDECEYNEEINKRKLSLDVNGNYQGGGVCKNCQHNTLGINCEKCKPFFYQPANVPIDS
PNVCQPCNCDTQFSTGLCAEETGQCECKSNYVGRYCDQCNKGFHMFPVCKPCECDVNGSEDRE
CIPDQINGCKCKKGYAGRYCDECLPGYFGFPDCQPCACLTRYGAVNDNCERDNGQCSCKVGFK
GRRCEECAAGHYQFPFCKECGCDPEKTLDTVCDSGTGVCQCKSNFAGPKCESCSPGFYAHPSCFA
CGCSTAGSTSSVCDQNSGLCSCRPNYSGFKCDRCAEGYYGFPNCILCDCNPEGSRRVGICDRQDG
QCQCKPTYSGQKCDKCLPGFYGFPDCKRCNCNPAGTKPGPGGRPGDCSTSNNGQCVCKDNVQ
GLECNECKPGFFNLTESNRKGCSTCGCNGAGTNNGVKSCSVEGTCKCKPLVKQEDKCTECRDGY
YGLKRTNVFGCLGCNCEVGGSSSSVCSKDNGQCPCKDNIVGKRCESVAANSFYPDLCHIKAEME
EGKTTDGDVVRYDYQKEIFPDFSWKGFVAVNDQQPSVVVSINVPKTSEYQVKFRYALNQIQPVTA
TVTAFKGDSKIVGSINFASTQKGSGFVASLATVQDNLVQEMYFPLTEGQWNIKLEAPHSNLVVDY
AVLFPKEYAQGGALKKKVQNACDVMTDEKFCNLYRYIDVADKKDIIVLEAENPVKMRQQKPPVL
FNDTAVLSKLDFQAMVVLSPSNSRQLNHLFNVAKPGKYVVMLQYYHDSDTAKYLDVYVRSQIAV
QNGRALLQSCRYTFGCRQAVLNRGDNGVFVFNIKSTKNHLLTLLSRYAVVAVDSVMLVPYESWH
LDYSTPTLYCVTKDSKCTNMNYDVPAESRLVQANPLEQDLSEKPRYLIDRSVEVRYVKPGVNIINIR
DVKAGNYLLVAHYYQPNFPSFESRAAVYSGRTSGLRARFDFKFCPNLNGCRSPIRRPDSNSNLYVF
EEGEARIRFDIPNGLDAWIEYILMIPEGTNVSSLYTPRVLDYSDDFKKECSIDLNVCTSSNLSSFCKDS
IFTITTDFNNGSFPCKCNMDGSKSSSCQEFGGKCPCKDNVIGRECSRCKVGYYGFPNCKPCNCSN
CDEETGVCTCPRGVIGENCDQCAPGTFGFDPIIGCQKCNCNEIGTGSANAECNVTSGQCPCKEN
YSSRKCSACKPGYFNYPACTKCDCLDAGISAEKCDVLTGKCICQNNVIGLKCNECADGYFKTNDI
KGKQLCAKCWCNGRSTKSCTEAELFRATPITASKGWTVSVDTLNVIENGNGRFTISDVNTLPEPV
YWVAPNQFLGNRGLSYGSHFKYNFQYSVKQPSQNTNGDVVTDAVIKGDGYTIKSMRSVIEKEND
GYSASILMKETEWMLEDGSTIDRELFLAILSDIDTLQILATHGKNTISSTIYDISLETAGENGTDRVYG
IEKCFCPKEYADLSCSKCSKGYTRQKDGSCGKCQCLGKSNICDPQNGKCINCSGNTMGDRCEKC
KPGFYGDKCKACECPFSNPENSFANTCAINVNTSSLVCQCNAGYKGVRCDQCSDGYYGNPLQG
VTCKKCSCNGNINASFTGNCDNTTGDCLKCLYNSDGNQCQECRDGYYGDAVVEKNCTDCQCSI
CGTTTAPCNKATGQCQCKKNVEGSKCNQCKPNTWNFDACNGCEDCNCAEASETQQCDLKTG
QCKCPPTVFGRRCGFCKLGYYNYSSEGCQKCQCPDGKPCDVNTGKCCTGVLNEDCKLCPVGHII
EDGECVECDRCVQRTLQRVNTLIHNVTILEKHIIPAGGLNYGALETLGNRFSNLSNKGDEHISNL
DTLKKAIGFGEYRNHSGNMDYVRMYLVRDFKSCSFIVNIKTPPTSSAPATTMVTNTPGPDPIRDHL
SNKKISDSQCPPSLARVIDEGEKNIKLVEKKVVVLEKDAYESTENATATKKRAEDLLAKLKDFMVD
FKDVLSFANSTLKGNVVINIDGNRSLAEAEEMLKEIKDRNFNSTINRSEREKSAAINATIIAEQLMK
QASELANKAYKFNDTFKDFVKALRNLKMESELALLDANNATNSINLAKAVDFQSIIDNAQKLHS
QNNVTVTQAMEILQNAKSAYENAKDVFNKFHNQTGPLSDLMEELRNYVRKLEFDLQTTSPIVDD
SVKHAKRLRDSADRLKELLQDTKKFADAALRAAKVYQNIVDAINDALDAARMANETAHEADK
KVQQAQQDAVESSITSKVVDTEANDLTEEVKKTLKVDISDMKMRLKELRELRKRIQGRIDNVKED
VKSFAKEKEVGEKAMNISTQADGVMKVANKGNEASQDTNEKVEKDGEKVETLKVDISDINKAIK
DLASNVTLVEDTIPKIRSNIDEVNVKHEQIEEFIKTNVTNNIGYLKYLISNAHDILDQMQLSMKFNS
TSEVVLDAPKSVADPTINNKISLTFKINQGNGFLFIIGDFSGNSSGFMSLHVQNHTVVFDYKLNTG
DVQSLTNPKFIETDVNYDVMVTRNFATAVLNVQKSGNASTLMTAYQNRSNISPVLSLGQDTNVY
VGYHPSTPNQRPNFDGCINQFRFDEELMGLWHWKSAKNVGGCVQDFRSIEGQYSFDGDGFVEF
DLVSPELSGFRKIELRFATLEENGLIFLGTDPPTSLPSDIMFDIPLSNQIIAFNALEIKDGYLVSTLDLG

NGVQRLVHTNAGKVNDGKPHLFEMKVLISSLLIKFKLDKKKITEIAISPDTDIRLRLKEAFIGGLPQ
RAWLPNSNISTKGFTGAIGDAKYRGGEFKLVKRFNNIRKGFILPKGYSASFPEEKSYLRLDACNLK
QNSLFGFSFQTFEKNGIIFHSEEGSGSSRSSIEIILIDGQVKVDVSVGSQKRTLQTSVARGFSDGLWHT
LSLYFEETLQEADIVLLYLAVDKAYITGTVPKVDLKGSMYFGSKPSGTESGFNGNIRQVYCNKNEIN
LASGDVFEGGVVGSLKLLPPLKLADFTVVPTTLPPLTTSITTSESTTTETVMTTSTTSISGTTLEPPKET
TTNMAKTTENPTVKITSSEPIIETTMESTTVAKTTSAITMEESTTTPTSITTSTLETTISTLEPGSIEGSTT
TAEPITTTAEAITTTATEPITTTEPITITQPITTVEVTTEPATTQESTTTPEPITTLETTTTPEPSTTPEPTTP
EELTTTAKPTTTIRVTTEPPKCQSNLEVYDEAYGFGMNEPSFIEFGVDGRKMLKSNQFEFLFYTAEN
DGMMISSEPSNLYDYEYVYMENGAISYAFNAGAGRVRVTSTKKYNDGKWHEVKISRSKESGTLTI
DGELIGTDSKSGKTAVNGIKKIVVGGSSVPYRNIPPKSQVGFKGGMRGFKLFNEALKPLKEIGITEA
YDELPIPEGVSFGPDGGYVALNFKSEYVVRTEYKLALDVRSVSSSGYIFFIGNYGADYLSLYIESDRK
VVAECNNGGGRFSVSIDVPTICNGEFHSILLEKKYKTLDLSVEGVQKSFTTTKGSTSADTGSNPPAF
GGFNGVGSRPKNLETFTGCITAVRIKDKAVDLTAPDVTIFGQVSNGCPSNIR*
>TRINITY_DN4781_c0_g1_i2.p1 type:5prime_partial len:3388 gc:universal TRINITY_DN4781_c0_g1_i2:10182-19(-)
QCIKKHMLCDMDRNCCDGSDEAAKLCKGYKTLDFETGLQQWIQFRNDDFDFDIQSGKTSSSGT
GPQTDHTLEEEHGKYIFIESSLRNENETANLGSPTLAATTSSLYASGDCSLRFWYHMYGSTIGNLTL
FLRRDNKDTLLWAKSKPEGNQWRPALVTVNSWYKPFQLIFVGTVGKSYFGDIAIDDLMMTDEKC
PSPGDCDFEDFDKCTYTQDLVEDDFDWIRGHGGTGSWRTGPSLDNTKKDNSGHYYFIEASAPRT
QGDRARLVSELFDPTSSTRGRCLQFYRHMYGTAVGTLNVYVKTGPGNSSDAESLVWTNAGNQG
NSWIQGQVPIYSSKPFRLVFEAVRGNGYAGDIAIDDINFNNNFAPCSFLPSNAAPPTLPPTTITPEDS
NCTFDKGLCRWKQMKSNDQFDWTLVSGSTGSVGTGPLGDHTSGKGSYIYIEASYPRKENDSAILQ
SPPIRPSLGSVCFKFAYHMHGAHVNALYIYKNVKGKRTMVWKRVGTQGNQWRNGQVNIRGVS
GIFNIEMKGIRGVDYQGDISLDDFVVTNGQCPTPSHCDFENNQCGWFNIGRGDDFNWKRSNGG
TPSLGTGPGTDHTTGVALGSYMFIETSYPRKYRDVANLYSPYYPGSKTGYCFNFWYHMYGSHIGSL
NLYQDTVKSTPIWNKTDNQGNMWKHGRVTVNSRAKFRLIFVGTVGKSFLGDIALDDVWVDDFP
CPAPGACDFESKSFCTWSQVPNKFNTSNYDDFDWELGNGPTPSFQTGPAVDHTKGTSVGTYAYV
EVSGLIKRGQKAQLMSEKFPPTNGKCMSFWYHMYGSGVGDLNIYLKYLGVKKSHLVKSISGQQG
NKWIQGEVGLHSQKDFNVIIEAVKGSSYAGDISLDDISILDGNCVGLCSSVQPVARVACGPQSVTS
ASCVVSYGCCYDNSVPNVPACFQHPSSCKAVPIIARQQCGYYSISKYFCARRGCCFDDSGAGGPP
CYHSLSKPTDFPTTRAPPTTQPPSKFDCNFEKNQCQYTNLRNDQFNWARHKGDTASFGTGPKFD
HTLRNQNGHYMFVETSFNKENDTANLGTPTIDFPTGGDDVCVRFWYHMYGQHVGAFKVYHKL
TTRVPTKPIWERIGSMVNKWVYAQLIVPRRPAFQIVFQGVRGKGYQGDIAVDDVGFVNGPCPPSL
QCDYQNGDLCGWAQDADDEFDWSVGNGKTMSTQTGPKADHTYETDFGKYLFIEGSSPRQPGD
KARIESFSHRPTKGMCFEFFYHMKGTGIGTLNIWIRRGKTMDSKPVWTLSGEQGDEWYRAKVTLV
EDTENWTVIMEGIRGADFNSDIAIDDVIFHQGDCAPQGDCDFENDDVSACGYENWPRNKVDWL
IYSGGTPSYNTGPMVDHTLGTKQGHYLYMETSSPAKKGDFGYFMSEKFRVNPNYKWCVDFYYH
MFGDGVGSFMVKMMYQTKYSRGRYLLQTLFKKEGNHDDIWQHGQVNITSSYDFQIVFQGVVGG
GITHRGDIAIDDITVKKGLCPAPKAPPKPNPCAIKCKDGTCVPSNKVCDFYPDCAQTSDNTDDTD
EKGCDGCDFEKGTCNWNDTSKGLFDWNRQNGGTSSYNTGPSVDHTKKNALGYYVYVEASKGQ
SSDLATLSSPVLNQASSTCKMTFWYHMYGTSIGTLNVYLVNGYRLTLHWTLSGNQGNLWKQGTV
YVNRQLKPYSVRFVAERSFSYRGDIAIDDIKFEGCELPRPAGFCNSRYQHRCAVTRACISYTRLCDY
TDDCGDGTDELNCRWTQYPYRCNFEYDLCYWRNLKDDDFDFTRQKGQTDSANTGPLFDHTTG
TSSGYYIFVETSWPRKTGDKARIGSGYLGPITRTYCILRFYYHMYGDHIGALSVKLRTCIGCPETQI
WRRNTTVGNYWVRHAMYLRSDKPFQVIFEAEKGSDYQGDLALDDISFDGCTPYQGTFPTVAPST
KPPPTTSPCPSGKFYCAKDAKCIKGSMKCDYIKDCSDGQDEQHCGPCDFEKDMCGYQDVSIGTL
EFKRTQPINDGSKNGPSVDHTTNSTKGYFAMAQKGSGNKGSPVSFLLSAQLSQTAPSCKVQLFYH
AKAGANYVRVSHRQPGSNGKTVISQVQSLSIRNMDKWQNDTVGVGQRPFGWQVLIGLLSEYAA
IDDIKFIDCFYPRPTGVCNRMYTQCDNGVCLDPEKMCDFSNDCGDNSDEKPSVCKNFKERCDFE
TDTCNWYQDQSDNFEWKRIKGGTPSGSTGTGTDHTTGTSQGYYMYIEASNPRKPNDTARLLSPV
FKPHGNKKCAMRFHYHMFGIHVDSLSVKMKFSQDDNSTMKTVWTKRGNQGNQWNTAKVYLL
SKVNFQVVMEARRGISFLGDIAIDDVSFSPDCLPDFKATLNPNPITKAPPGCKAGQFSCGDGQCI

DSDFFCNFKQDCKNNADEIRCPSSCDFEGGKSCNWFNAKFGDIMDWTVHQGKTPTNGTGPPYD
HTKGTKDGWYAYMEAHQRKASNFPNAHFVSPMYYQGAYDCKFSFWFNMYHYKISVQNDARL
NLLYRKNGKDTKIWSMARTTGNKWVQATVQLPRCPRDFRMIFEGHHYWATKTDAAIDDLKFM
CTEPKAPSSCANDEFQCTKTKQCIKKHMLCDMDRNCCDGSDEAAKLCKGYKTLDFETGLQQWI
QFRNDDFDFDIQSGKTSSSGTGPQTDHTLEEEHGKYIFIESSLRNENETANLGSPTLAATTSSLYASG
DCSLRFWYHMYGSHIGSLTVYTATTFGVPETQVWQLSGDQGNTWKRGEVKLDSNKDFQVIFEAK
MGTSYKGDISLDDISLTPGCVYNNAVLPGGPTPPPVDPCLPNFQCNEAGNCVSRAKVCDFVVDCS
DGSDESNATCGYPQGFEYGFGAWRNAHFDTYDFTLQQGATPSIQTGPTTDAKNDKKGYYAFLEV
SNRKRSRKAVLSSPTYAVAYSQCVLSFYYHMYGRETGILKVHLVTTNETTLLFELKGEQGNKWIKT
KAFVGRRYHPFYLNFTATVGSGYRGDIAIDEISFENCAHPQECTGVDPGKFRCKNGFCINQTEICN
AVDDCGDDSDEHVGSVCENYLGCTFENLNDPCNFTQAKSGDDFDWKLNRGWTGSYNTGPTRD
HTKGTSKGSYMYIEASGRVKGQKARLISLPVESRPGGNCQVRFFYHMFGKHINDLNVYILQSGTL
RLIKNANGDYGDMWREASLDLSKEKGVYRLVFEGVVGASYLSDIAIDDIIFNPDCMTNGSRVFPT
RIPCAANQFTCTNTRQCLSSSVRCNGVRDCRDGSDETGCKGPDTGKSSSVNTTTSIYASGVVGGLV
ILLIAVLIIYIVVKRKREKKLQLFSVFYDPTKQAENEKKQGKPSKNVTIKNEGVSNPIYDEQPMSEFS
MGELDTNMFTDEPYVVPAEPKGQTSSMANPLYQDPYMEDDDSPLSNF*
>TRINITY_DN262_c0_g1_i1.p1 type:complete len:1033 gc:universal TRINITY_DN262_c0_g1_i1:111-3209(+)
MADPSSLETKEKDGSYRVATKSGGKDKGKKSKKAAKKVNMDDLKKELEMTEHSCELETLVSQLE
TSLTQGLTKSIAARNLERDGPNALTPPKQTPEWVKFCKQLFGGFAMLLWVGAILCFFAYIIRSIREE
EPGMDELYLGIVLTVVVIITGCFSYYQESKSSKIMESFKKMVPQEAVVMRDGEKLNLNATECVVGD
LVFVKFGDRIPADLRIIESKGFKVDNSSLTGESEPQSRNSECSSDNPLETRNLAFFSTNAVEGTATGIV
VKTGDNTVMGRIANLASGLGSGKTPIALEIEHFIHIVTGVAVFLGVSFLIISLAMNYHWLESIIFLIGI
IVANVPEGLLATVTVCLTLTAKKMASKNCLVKHLEAVETLGSTSVICSDKTGTLTQNRMTVAHM
WFDNMVVEADTTEDQSGQAHDKGSTTWKALAKIGALCNRAEFRPNQEEEPILKRECNGDASET
AILKFVELSVGSVEDIRSKNKKIVEIPFNSTNKYQVSIHEQEDPDDPRYLLVMKGAPERILDRCSTIL
HEGVEKPLDEEWKENFNKAYVDLGGLGERVLGFCHYYLPADDFPKGFAFETEDPQNFPLEKLCF
VGLMSMIDPPRAAVPDAVSKCRHAGIKVIMVTGDHPITAKAIAKGVGIISEGNETVEDIAQRLNIP
KEEVNERDANAAVVHGSHLKDLPQEKLDDILKYHQEIVFARTSPQQKLIIVEGCQRMGAIVAVTG
DGVNDSPALKKADIGVAMGIAGSDVSKQAADMILLDDNFASIVTGVEEGRLIFDNLKKSIAYTLTS
NIPEISPFLMFILLAIPLPLGTVTILCIDLGTDLVPAISLAYEKAETDIMKRKPRDPVRDKLVNERLISL
AYGQIGMIQATAGFFTYFVIMAENGFLPGYLIGLRNDWDDKKNNAVMDSYGSEWTYNQRKNLE
LTCHTAFFTTIVVVQWADVIISKTRRLSLFQQGMTNWMLNFGIFFETALAAFLQYTPGLNTGLRLR
PMNFPWWFVGVPFSLLIFVYDELRRFLLRRNPGGWVEQETYY*
>TRINITY_DN4595_c0_g1_i4.p1 type:5prime_partial len:1639 gc:universal TRINITY_DN4595_c0_g1_i4:5857-941(-)
QCGVNTGTQVVYRRCNAALCKYQGNDYTRNQEVNIRTQACTVACDYTPWQNTQSCTKTCDGG
VLVQTRTERAFNKIETRNGNTPCNTQACAQELGPKQLRTCNVGQCGVNTGAQVVYRRCNAAL
CKYQGNDYTRNQEVNIRTQACTVACDYTPWQDTQSCTKTCGGGVLVQSRTERAFNKVETRNGN
TPCNTQVCSSELGPEQLRTCNVGQCGVNTGTQVVYRVCNAALCRYQGSDYTRNQEVNIRTQAC
TVACDYTPWQDTQSCTKSCDGGVLVQTRIERAFNRVETRDGTTPCNTRSCASDFSAERLRSCLNV
NQCGVNTGTQTVYIVCNGNFCRYQGREYNINEEVNVRQQNCTIACDYTAWEDTTICSKICDGGV
LEQRRLERAFNRLERRNGTTPCNTRACDSGFSAVLSRNCVNVNQCGVSNGTQLLYRVCNGEPCIL
QERSYKINDVVVVGSRACSVPCNYTEWRDTTSCSKTCGGGFFFETRTEQAFQKTESRLGKTLCNE
NSCVKDNFGNWTEWTACSKTCGMGMRYRSRECLKGFECIGLIEETETCKLGCRTETCPMGRPYP
GDCNFEDDCGKSWNQVNSIQRNAFFIVTSKDLAHQGLLPNMETTFNTKDCGRTMKLNSSVAGD
VNYLWTKRMMITDINTDVDDDVAAIDNYFKEENEQCVEFNFAVMANSLDKIGDMQVYLYYLEY
PNASLIYSKKGNTGLKWELVKRSIRVDTNKAFVILFQVNQGQSNVQYGDIGIDGFKVNPGNCQLK
GWSHWGEWKCSATCGFGTQTRMRHCLDKVNRTACVGMDRENKPCPMMKECPMNRDGNWG
QWTEFSKCSKTCDTGTYFRTRKCDSPSAKYNGSLCRVSQTKFNLPVVNDFEIDFKPCSVQQCNNT
FSQWTTWSSCSGTCGPGQRTRTRTCESGKRCSGIFEEIETCYMGCRLEKCPKFTAEIDCNFEDRNKS
DCQWKYYTVSGTSSWSFKTQPKNRLLSGPSGDSTIGCGYFLYYPSQTLSSAYAWGPNIYPTMDSYC

VKFKYSMVTSVNSSMGNLTVIKHFLNRPRKADTPLFFHSGVSQKQWNPMAIDITTDIQRSPFVIMF
KGQSDPNNAGLSDIAIDDYMLMAGKCNKSYSEWSEWSTCSRTCGVGQRTRTRVCLDDDIKVCK
MGDVATEGEACVKPLCAVPVDGNWGQWSAYSQCNARCNGTQKRERFCNNPAPSNGGRMCLL
GDTIFQLRNNYHAQIRPCSIDCGDNDTFSSYKRINDTCHFDCITGRVLQERECEQDTCFDGNTRFS
KGEKQYTQRACSIKEYCETSEYKCREFRSVCSTNTPSSCLDPQNRAVKSCECQVERGCSSMTNPGQ
TVKHKEFDVFYRPCRYVGVCFTDYETDAFSPYEKVNDTCHLSCSNGKVFEERECRQDNCIDGSNT
LTRGAKQYRETQCTTKEYCETSQYSCREISGPCSSNPPSSCQDPANKSLKVCTCQVTTGCASMLTA
GQAVSYLAEETFSRPCKYTGACNANGNTDKTFSPYERVNDTCHISCADGKVKQMRECKKDACV
DGGQNFPRGFKQFKDSSCTPNEFCETAQYSCRELSSACTSNPPSSCLVSTNKAVKMCDCNDPNGC
DSMITPGKKVNQGEKEFLQRPCRYTGVCFTDAETGT*

>TRINITY_DN244_c0_g1_i1.p1 type:complete len:3019 gc:universal TRINITY_DN244_c0_g1_i1:9748-692(-)
MDARGQRLLYCLLIFLSLATLLVSQQVEKCKRPVDLVFALDTSGSVKDSGFRLIQKFAKDILKTFDI
GVADSHVGVLTFSEQAEPQIKLTETFDKAELMQMIDSLVYQGYRTATDDALRVAKTDFFSTAGGA
RQGVPHVLVLLNDGKCTVCIEPVKEAAQKLKDMGVEIFTIGVTENIKKQELIDIASQPTAYHFFEL
DDFDQLVDVVGDIQTRSCEVKKGKCPIPSPTEQCSGNEINECTSDENCEGELKCCSDGCSRICKYP
LLNCFTPIDIAFALDSSSSIQPETFQEMKKFSNDIVDSFAVSQKKTRFAALVYSDRAEIGFNFVRFDD
ASSIKKAIQNLPKLDSKERIDYALELAKSDIFSLQGKVRTRRPMVLFVFYDGTVSSGMKDLSLVVEP
LKNYGVKIVSMGVGPETNYYQMRKISSNNSDVFRATNFKELLPEIYNIAKRACTEKPGRCDASLN
PLPKEACKNVKILNQCENDFDCFGDAKCCRKGCNNVCVIRGPVCPRKLDIALAVHQNMDESEF
AATKFYLQSVVDQFNVGPSQTHFSLLSFGQEPKVIFDFNSDQSSNEIVRRKIDNFQYQYGIGRISDV
LALSCDAVFCAAGGTRDDVPKMLVIVTSRTSPSSPSKLKEKLQTLRNRGVNILMIGVGDVDTSLLQ
AITSDDPLVEDRVLLSKRFDGIFQYTKDISDYACGMAPALPPPDNIKGDAKGECGAPKSCYKGPKG
SRGRPGTLGQQGESGDLGLVGPKGIQGRRGDKGPDGQMGSKGKRGPDGLIGLPGFPGYNGIPGV
PGIEGRRGRDGDCGLPGSSGEAGQAGLDGLSGPPGRAGPVGEKGEKGLPAPENPNIRIEPGDRGD
TGSVGLPGFKGIKGEEGFKGPSGDSGVDGIKGVDGEAGEMGDIGTYIYQKGPEGDRGARGPPGD
GDTQGATAGLGVQGEKGEKGMRGEAGDGSIFPGIKGRRGDQGPKGEEGSTGPAGPTGQRGPEG
EEGPKGIKGEIGDRGKDGTPGQPGQEGDLGEKGFDGLKGQDGDDAEGELGDSGGKGLKGENGL
AGSPGSIGTPGPPGSKGEPGDIQIGLSGDPGAIGPRGPRGDNGISGQPGMRGDSGPKGAKGNQGP
RGDRGRNANMLAGKDGMTGPPGPKGFTGPVGDPGFPGPPGPPSVVECVEGACPKGVPGAMGQ
IGKTGEPGPPGPPGPRGSKGVAGGPCPICPDGIQGDSGDIGQPGMPGKEGSRGRKGNIGDAGSKG
LQGRNGDDGDQGPVGKKGFIGDIGPVGENGVDALGDSVAPDGEKGSTGDTGQIGSRGPVGNDG
PKGFKGTIGERGRRGLQGEKGDMGLTGETGEDGSRGLEGAKGDLGDGLRGAKGDKGNIGQEGN
QGVVGDVGLQGPKGEQGAPPTEDELKNVKATKGSKGEIGSTGDAGEIGDIGPAGPRGPKGFDGA
AGPKGPSGVKGDRGNKGRAGDGEPGFQGFPGRPGKRGPPGDPGKPGKRGFSEPGDAGDRGDP
GKSGKKGADAPPPIGCFKFNEPTDIGFILDSSSSVDDDNFLRQLQFVKNIVRQYPVGPDQTQVGIIK
YGEKAVTGINFYDYVTEEGLFNAIDLLEHTKESESRLDLALKLARDELFTESVGARGFDAKKALVIL
GDGFISGGGTPSKTLMNNAMQYAEDLRGAGVSIFALAVGPEKNKALFHEFVSEKEYYLDIESYSQL
DNELGALQLSLQTGCISVGVPGRDGLPGYTGPKGQKGDDGPKGFSPQGPRGIKGIKGVRGTIGIG
VKGLPGDPGLQGPQGEKGRDGEDGSADPGPKGRPGDKGSRGDQIKGASGEDGDIGPIGPPGFDG
RKLCDNVLAVDLLFVLDTSSGVGVDNFNKMLKFIESIINVFDVDNQLTRVSVMTYDSDARLVIRFS
DYNDPDKLDAAIDNIPYANGDETRIDKALLLASSNAFDTSNGAREGVNKAVILMTDGQQSFAVD
QVALKDAVKPLVDANVLMYAIGIGSEISPVDLKTIAAERVLIADDFDGLSLKIEDQIVLLGRVGCQG
PPGKDGKPGANGEDGFPGLIGAKGQKGDRGEAGTEGLKGNKGSQGEAFPGLNRKGEKGDPGS
AGPLGLPGCEIKNQNEVIDLGFAIDASGSLYEEGFNREKEFIKTIVDRVGRVSEQGIRIGVIVYSDTAS
IEIRFDDHFNNEDLKNDIDNLPFFAKNTRMDLGLEEAKKMFGEQFGGRGSSKKVLILLADGQQTY
VPGVTEPSEVAKELAAAGVDIFAIGIGQEIDRTELESLISKPEHIFLASDISKLIDELTPEVTSALECEG
GPRGPRGRAGTDGPPGSKGLLGPIGDKGFPGEPGSAGFGPKGDDGENGTAGTIGDRGEKGFRGP
AGVPGKRGAVGPKGFPGPNGEKGDRGDEGFIGFEGARGLEGPKGQRGDSSDNGVAGVKGSTGD
NGFKGPKGDPGKLPPGNRIEDFKGETGGSGLEGDTGPKGKPGRALFEPGPQGLTGPKGEKGDQG
FFGMSGPIGNKGFQGLQGFRGDDGAAGPVGEDGFPGMKGLPSSSGDQGDKGKNGRKGESGEV

GQPGRVGEDGDQGEKGYAGFVGMPGKKGDQGEPGVQGPQQIRNEILDNIRPGPKGYPGSTGPA
GDEGEDGEVGEPGETVGAQGMNGDFGERGSQGPKGEQGDQGLQGMIGVIGDKGVKGRVGTG
GNPGTVGLRGLTGAPGEVIFGSEKGNKGDLGIIGLRGRAGQKGKAGAIGPRGRRGRNGAKGERG
PPGFPGLSGIEGDSGNEGDKGPRGDPGSVGNRGIKGVPGESGIAEGNYIVIHSQTHIIPDCPRDFKK
LWHGYSLMYVVGNGHSHSQDLGDAGSCSQKFSSKIFLFCEIIKVCKHASRNDFTYWLAGDVDHP
MMPVSNAAVEPFISRCAVCEAHSSHITVHSQDTYAPECPPNWSSLWSGYSFLMVAGAGKTGAGQ
SLSSSGSCLESFRPKPFVECQGARGTCHFFSDKHSFWLVTIDPNKQFEVMKPQTLIKDKNHDLTSR
VSRCRVCIRKTGTKNNTVEPNIATRIRRSIASWIL*
>TRINITY_DN1676_c0_g1_i12.p1 type:complete len:1631 gc:universal TRINITY_DN1676_c0_g1_i12:206-5098(+)
MLIITRTAKLLLLSLTYIYLTIAQDLTNATLNNGFEAGRAIKCKTDDGMVQRCMPVFENIAFGRDII
ASNTCGQTRQTYCVQTGTSGAVKICEVCDASDPDKAHPADLMTDLQFDDQPSWWQSETILDNK
SPVYLTLNLNKKYNVAFIRLRFHSVRPHSFAIYKKSTYDSNEQWLPYQFYSRTCQETYNVPNQQSV
SYSNQKLALCTDEASGMIPLSGGNVAFLTLQYRPGKRDFDNNPGLQDWVTVTALRFELDRVNTF
GDEVFGDPKVLRSYYFAISDITVGGRCKCNGHGSSCNNNNNEGTFQCVCEHNTAGKDCEKCLPI
YNDRPWRKASGRSANPCKACDCNGLADQCEFDQQLWVDSRYRSGGRCVNCKNNTDGINCEYC
KEHHFRSNVKEACKPCRCNPLGSRSLSCTTKGECACHPGVTGLKCDKCKEGYYGFSSSGCKACN
CDRDGSIGVNCDFTGQCNCKKKTVGKRCDQCAPLTFDLDRSNPKGCKECFCFGHGVSCDAIEGI
GVKEIFTTFEADFDGWKLQDESGASFDSNMEWDFRGQYISVKPPRNKQLYLVAPQRYLGNKLSSY
GKLFTFAYGIFLDPNDPLVSAGRDIIFEGNGLQAVYPINSQNNILPEARFYQFKYRLMEPVGMSTFD
FQKILVNLTAVKIRTAYMNERTGAIDNIKLYSTKFISINSPEQVTWQEICECEEGYTGDHCEKCDSGY
TRSGGDTSPFGECKKCFCNNHGNFCNPDTGICQCLHNTTGDSCDQCAPGFYGNPTAGTPEDCK
PCPCLYGNECILIGNSVKCTDCPEGHIGDLCELCDDGYFGDPNGTIGEPSRCQKCDCNGNIDPNAI
ANCDSLTGSCLKCINNTKNGPENKCELCADGFYGNSTIDPKGNCSACSCYEKGTMKPPFHDPKL
QLPCDTNGKCECLQNVIGDKCDMCPPGHWNVASGKGCELCNCNATGSKGSDCNLQTGQCEC
QDGVGGRKCDQCAPRFYGFSKDGCIACNCHPDGSKHQNCTQDGVCECLPGVLGIKCDQCPEN
KYNLSVGCIACPRCFDLIQVAVNKLRNKLKTFNITGNLTTGSIDIKDPEFEKLFNELKRNISELEQK
MKDLLEGDMAILEFYEVLVNQSSTLIEMIPGIQKMIKKSEGAAETGSVTISKAEEITEEIQNLLGQAE
KMLEAETGRIFKNVTSSSEDVSGLSKRMSEIAEEARKHADRQEQESKDIEFNTDMAYNNSKAAYE
QAKKARDTEEAISYLLDQMALGHDEALALSKNSTEIIAKANSTSIAALNDSDHLMMKAKEPLPN
LDIEAMKEKAKNIDAEGKATEFNATDLHEKNKDKITQLQNDDKEARELMERGTQLHNDSNALY
KIAKNASDEAKQAQEDGLKVAKDAQDMLKTLMEFENNIAKSKANATEALKLIGLIRRLISKANK
TTENASSNAIMASINATAALRLASAANKTTQDAMNEIESSRREVEKLLNETMDFMMKEVAQTKD
DIDDIEKSLEESEKLAKSNSMKLGDALNDSQAATDKASMVQNELLKSLDQIEDLMNEISNLDEIDF
ETLEDAEKRFEEAKDTIENKLKNEIEIMESKLSDQKKLLQQHELDLTPLKEQIMKNEELFKYLPKSC
FREKAKPEDIDK*
>TRINITY_DN6390_c0_g1_i1.p1 type:3prime_partial len:1941 gc:universal TRINITY_DN6390_c0_g1_i1:5820-1(-)
MVIAPKCKFSIFSYLVFGILSYNWNELDASTNIELVDGTGVLLQRDVDNVLNDAVVQKEYQQNIK
HKPVDKRCLGEPFYQGLCKEKLEDAIQQNITEGGCHNLLVKIQKDECSNGPKTLGGKGYCNFICS
QEICNSIYDIDKCVSKRLVHHFNKDKNECEEIMQADCGSESFTSSNQCQKRCIGNSDDKRETKVY
CDQFCLNGGKCIGEVYCQCPPAYHTATCGLSYCNPSCGVGGKCVKTGEGKYGCQCFPGYKGVK
CDKEDSKNMPVVKGLCLSAIKSVLTSPNTSFHDKPEEEKNYCLKYLFDLESNCDKQRPFVNYTEE
QCNHFCASDICDNIYSNDCGSIEERYGYVSSTGRCSKFLYNGCLGNTNNFKDKAACDTKYSPING
LMTSVALLRPLNHTCYIRGKTNFITFDGKVYDLLSGCSHYLVHDSDTTSDFNVILHNDPKVNYNE
KSKRKLEITADGKNVLLNQKVSSDRFFVQVDGRSVPLPYNDVPKIRQAGRYILVLTGDGVSIAFDG
YEDISITVNDYYRTGDGGDHRLNGLCGNLDGNSHNDWLDFTGELRENVRDFAKVYQTDRSTCK
MDNLSLKSLVYNPLHNDQEVFERAERHCDVINTDAFKPCHSVVSPEQYKVLCMQDYIKCNDDK
RDDCVCSSLSFYSRVCHKYGKDIKWRRANLCPITCQRNMKYYECGSACPKTCDNLITPTKCQTDC
VDGCHCPPDLYYDKSTENCVPRNLCSCYRDGIAYKHGSVRSFHCEECTCNGGSWECNKKDCEAT
CDIAGDPHIKTFDGLNYQMYGKCSYVLVDHCHYGNTGIKEFEVIVENKNCNDDALYTSCVRHLS
VNILQTNTRVSLDTAKDNNGTVLPVAFVNGKEGKEFRSKDYTITSVGGRNIIFQYKVNQLKVSWT

GHNAYVTVGHQFENQTCGLCGTYNFNKQDDFHTRSDTVEKSVTAFTQSWVYQSSDKVQDPSKC
HTAKWETSEKPCQIYTNKAQYAKTQCSIIIKPDGPFEDCHSIVPPGEYYSMCQDDACKCDDCYCH
IVAAYAKRCTDLNIRVGNWRDETLSCKQTCQAPKVLKQCNAIDSLDDDLSESLKCPSTCYELSQN
DTSICQSRGCIEGCYCPEGYFENEQNECVKQDQCSCYHDGKVYRHDEIRKDRCNECVCKGGRFE
CTDFDCVAHCASKGLVYSDCGKTCSNFNFNPQDSKCQAGCFCPAGKVLHENGTCVNPQTECQC
KEGDQFFDAGEVSPSDCSKVCNGDRSWKNLPGKDAKPQYDCSAFGQMHYRTLDGRMFKFSGG
LCPYVLLTDCRQDSATGFCDLTKANVNIHVRNLRCVNSYEAYMCKEVTLDLRLPNGEMAEFVLM
QNKIKMKTGLVTKVFDKGSYPQPKTAVVDGVEIFKSGLYLVVRAYHLNITVKYDRQNRVYVVPGR
EYKDAGKLAGLCGNYNGLDDDDFRRPDNSNAENELQFAKSWQDSNSACPDPSESDTCSSNKER
LPWAQKGCNVMKTGNFTACHKTVSPEPFIESCQQETCLCKSGGDCACFCSAVAAYVQECNRHG
VPIYWRRDGLCKLDCCDPCGHSYGYYTDNHVPNTCGVKDKVCKENDRCCKGAVVEGCGCPPG
TYDDGIKCQNETTTNVIPCSPSSSIVPSTSISVPSTIIEPVSSSSPSPMTSSVESQSPSPSSSVVSPTPSSSII
QSMSSSEIESISTTSSSFVSSIESSVQSSTSIVFPVVSSNSGESSVSSSMLPSTSWVGIHSVSSSMGESSVST
NVLPTDYLRTTDVFPLSSSEITYPSSSSSLEPHTPSSSILQESSSSVSKPVGISSVIEPEISSIRYSSMSSQN
VMSSSVSSSVSKPVGISSVIEPEISSIRYPSMSSGSVLESSVSTSFVLPSETVLSTSVFSTNTESPSSSMTLS
TSSSGFVFPSVTSTFQTSSKVLSSLPRSGPAENTVPLSTMFTKTV
>TRINITY_DN15274_c0_g1_i1.p1 type:complete len:1822 gc:universal
TRINITY_DN15274_c0_g1_i1:87-5552(+)
MECIRRSIIRLLLILGFYTLYISSQEECKRGGCFPATGDLLVGREKQLLANSTCGLNGPSKYCILSFLD
KTDKCFYCDSRRQVDERPDGYKNSHLPKYMVTREPSKRLQGWWQSVNGRQDVTIQLDLEAEFIF
THLIMTFKSFRPRAMYIERSSNYGNDWSDYRYFAYNCRKSFPNIPRGPQRKIDDVICEERYSKVEPS
TGGEVVMKVLDPIIQKKEDPYSLKVLNLLKLTNLRVHFKELHTFGDDILGDSPKVNQKYYYALYE
MVVRGSCSCYGHAEQCIPTKGTPANVNMVHGQCNCTHNTKGRNCEMCKDGYNDEPWRPAYQ
DQLNVCRKCNCNGHTDKCHFDPAVYKATGNTSGGVCDDCNHNTAGRQCELCKPLYYRDPYKN
IFDEDVCKPCDCDPSGSFGGGECETISDPNNSSALTVAGRCICKPNAQGLRCDSCKDGYWNLLES
NPDGCQSCNCDDKGTVELNKCDQQTGTCRCKRFVIGLTCSKCLEGFWNLGNTPEGCEPCNCDV
GGSLNAKCDDITGMCMCKPNIIGRKCDQVAPGSYFGNLDQVISEAENSLVRSDSSSVEIVREILNPT
DSRSFTGDGYLRVSDGTLVNFTNIRVPSTGFQTFVMRYSRPTPGSNNNVMVRITKTDGTILGGNGC
VQTNEREIILNGVLNDDNFLELGDMFCLEKDTDYSVQLVVSNRNSPNDGGVNRGVTNLDSIVM
MPNIDNLAIFQGREGMRLKSQYDLNNCRQYYLEPNGAANAPEICKKIQFSISAEINNGALKCNC
DPTGTNSSGSSDILTCQKSGGQCPCKPGVVGRRCEKCAPGYFGFDSNGCTACACNDTGSEKLFCN
GITGQCPCKNRIKGRQCNSCPSNHFGFPNCIACQCNEHSSTCDPISGECTNCKDNTAGPSCNLCA
EGYYGDATAGTEKDCQRCQCPGGSSLNQFSNTCHLKPDGKVVCNNCTEGYTGDQCDKCEDGY
YGNPLVPNGSCNKCDCNGNIDPFADGKCNAENGKCFNCINNSDGDHCENCKEGFFGNAKNQS
CRACVCNKLGTNSATSDSCDRETGQCECLPNVEGVECDRCKSGYYNIASGNGCVSCNCDKGGSL
SSHCNQLDGQCNCLNGYGGLDCTQCEDFYFGDPKQNTCQACGCNPVGSITLQCNRTTGVCMC
KDNVVGEKCDMCAPQTAGVMPQCEECHVCNDQWEDIVNDIEKNITTILTLKGSSPLNVSEIRAY
TNDIADLKNRLDKIEEDFGKQMVDPNKINRLRDKVYRFSYNLTGINRDADFYMEMVNETITRDE
KANDILDGLELRLATLQTNTEDFKNNVTKIVESNIGGALNSTRESLEKSRKAQESVNDSDLKIKES
KKMRKKIKKEIQNGDPEFEVMHNRSSEFVNNLADRENYLEGELEKLNRLLCGSDKRCGGCTKGG
CDTCGVSGENYTCDDGIKMMSMMASDKALEAKEAIDQKLENASELLENVNRAKQHVIQANISA
NMAEDKAEEVNKIGQAVLKNITTLIKDIQDFLATDFNHPNASKDLAMQTIALNISLTPDEVVELG
RKIRDAVKSLTGVDKILNESRNDYDTAIGLKEQALAAKERAIEVMNQTMAIIETLSQAEELQNSTL
AGVNVARADIEDVKDLLKKLKEKLDAADNDLQMADNSTASAEDKRNAILDQNLENREKLDLSE
KKIDEAEELGDKVQGSVDKMNEKYNATKAKLNEKEDMIEKVRARIKALADKTIKLYENADRKLR
RINKMKDSYIINEERAKDLYYEIRRLEIKEFFINTKLEGTAKCYSTCNPIIPESFCRDVYKNIAVKETN
AYAELEAEKASTTSLLI*
>TRINITY_DN1366_c0_g1_i1.p1 type:3prime_partial len:4702 gc:universal
TRINITY_DN1366_c0_g1_i1:236-14338(+)
MMEQDRQRPSRQEYNNRSLSRERIGSRDGSRDRNLSRDRDLSQTRDLSHTRDGSRDRSNLRTRDG
SQDRSFANRSDLYSSPYSSNSQAMPQTSATVEIENLELQATVSSQEELINQLAEDLRNVRKARDFSA
EVEILQKEQQCMDFKEQLEGKEMTIQHLKTELVQSQTVTEEVYKKYESIRSNRDAKDKSIRRYQQE

LHQVHDLLETAQKDKLACVDKMRQIQNEKSALEFDYQWLFDKIKNYEEHGEVFDMSSVSKSTTT
IKRTTTVYEDGQKIRVEDYQSDDSIMPLQNKFQQAQKGYRKAIGMQDDQLMRDKENLMNEFRS
MQSQMHDLKRIHENFMRESKEKDTLIERLKEAKMALDQELDHLTRENENLRQTNDKLENENSD
LRTELNSARNQVSKIEVQYDNCMKQRNYMKEQMEIITQNSAKGRDDYNSIEATMRDSQRKMDS
MQRDLTNKENLNKQLKNKIKILEEDFEQCRQDYNQCMVSRESLQQQKLDVERELKERHDRMDE
VETSYEDIQRRKADLQQELASLKKNLTRFINEEDSSNQMKSHLQNEVLAANKRISRLEAAYEASQ
QEKLELQQELAPATQRLSMMRAAYETLQQKKEQIQTDLIASQKRCAEYQAMNERLKQDRGEILN
EIQACKRRAAQADVDLDEAAKQYAILKGKLEDANRRAQSASEDLVTRQRTIEELRHKLEVSGRET
IQRESYYERIKQERRSLEDELRGCVTDLERVQNEARDVQRDNEKLRQDLGTAQQRLHETEAYWQ
KTKMENAKLQRDYANMERTVQELRDLSHKLQQDKEDCEKILYSTHKKYANLDKENERLGRENT
DLERELQSSKNKQSKLDAVIQDLKRKEAQLKAQLDDALKNRDELNRVKIVMNDLQKRLDGVTK
DGEQKDNLIAQLRNEKDNYKRDCDKFGRDYEGCRTMLETANREKDRLGKELNAANRRIADSD
VLVKRLQDENMRVRKDANSFEARIPILEDALQKSKANESKWALEANTYKTNVIRIEKELLVFKEE
HHSLVSHVSQNEDKNDGLAKKLDDALRKIFELEATNEMVSRENDELKRDIDNKTRQLEEQLTTV
ERANNDLKNQLNELRGRNKKQQDDYNRMQIDYNEFQSKWDGLSSDGQGKDRLIEQLRSELQN
STHEINQQHTDNENKKAYIERLTEEKQDLLNDVNTVNQRIYELEGLYKSSEDDKEGLQKEITELNR
KLNDVESDYDTLQRESDKEIRALKSKLGKVESQVDMILKDRDYIQDQLDAERLLSQKYKDEFERV
QREFLEHQKICERYADQILERDRIIDDLKNQMYKMETEIDTLKEQLRKAQTDQKVAEQQRDKAVE
KLNQAEMKIKDLENENDYLRKRLAELEREISVLEMKLNDAETENKNLRNKIKDLEDLIKTLRAKL
SNTETELQSIRHIISDRDVMIERLQKELDDANDQAHQLRRQVSELESQIELGDQERDDLRSRLANS
ERTIIELRKNLDKVIHDGQDLEAELAALKQKLIKMEPQMKQAQKQRDDYKTAYDNLKGKYQDL
KDELLANQRQLTDAKRAAEQYKKELRHKDHLLEEARGKILALAGQLQSARDELVKKQQENEQL
LDEKRLIQEELLRLRQKLGDLESANQILAMEKERLNEELISFNSKYTELRIKLEKSEQDRLESQRQA
MAYEQRLAKLEPQLQQILRENSELHLQVDRLADELRNALDENRHLKNDVEELQRIRDMQLKELY
EKDTIIEQLKAKLSNLENTVNHLQQENDKLHNLNDALEQEKHSLAHRLDNANSRIKELDDLYEQ
ERQKRDQLQNDLHDLQKKFVTLENAYKKSQERERDLKKKIDDLLGRIKDLEDQLAAEKKKNSDL
EAENDRLKDLIHDLEGVIDDLRRKLTEQENENDRLRAENDDLKQQIMNLQRRIDELEGVIAGLEQ
EKNFLEEELNSLRQTVNKLEAEIDNLTKQRDGLQGQLEDLRREIMRLKEVIMKLEGTNRELQRTID
ALKQEIYRKDRIIETLEAKRKQYELEIDNLKNEIIKRDVIIDSGDESKRDLEIQIKNYEGRIAIMDGRI
DKITLEKTEIERNYHNAVNRAKGLESQVLILNREKAELKGDIDQHFGKIRDDNEVVNKTQGELRE
KVRIIETYKKEIIEKTTIIERLTVIRQTVESERDQLKKDNATLKITVNQYKDNDKGMNDEIQKFTFKIT
TLEKDIINMKNERDEFKVKADNATSKLVEVETQLKRSIERETRLRGELDTLHNKYQNLETIYNELL
REKNDVDRKYKDLLQKHRELDSKYNKLLANFNDLKDQLQQANNKIADLKAKLDKEIYENRDL
RDQLDNLKNQYNKLRDDNQRLEQELADYKYKYEDAMKELAEKNDLIDELKAKIANQDRQINA
LKGTVNQLRADYERSMREQARLQDEIVKLQAVVADLEGKLHRLELENEKLRKENNELQAKIPAL
ENEIENLKDKLENLIKENRDLRNDNMNLKQENVQLEKEVDDLKDDIEKLKDEIRNKDNIIADLR
KVRQELEEEIDQLRHDVKQKEKEIDDLFNRIRDYESTIQILNTKLAEIEPAFEACKDRLRHQENQIQ
ELRNKITDLESALKQSQENEAELGNKLALTESKLNKITQLLDDIKRKNMELENENGHLKDELNKL
KDQNKKLENDNDRLSNELNECKAVIQRIEPMLDHLQKHKNQLEKELEDALNKLANLRNEHEA
HLREMKEKLHVIEVITRQLDEKDDLIEKLKQEKAALEKENESLNDALNKLHQTLDAVQRRKEEV
EKDFFEAKKRIAELENLLNALKEDKDDLERMLRDAKEKNADLDHALRNSQHNEAKLKGELDEA
RGRIQRQRNELAKLQIIVGDLRAANERLLRDNTEKQNVIDGLHSQIRGLESQNGSLKKENQKLRD
ALDKLQRELHNLEQENQQLKAKVFSLQLLNDSLREEKDRLYDNFQELEKDYNDLKDKYNQAVR
ENDRLKDELAFANKRIDELENMNNQLNNEIENLKNIIAGLKRQINDLQGVIDQLRRDKSDLEQTI
NIHSETTINQRPVFDESDYVPRSEYDELKALLEAANSTKEGAERDRDQYHQEHLELHKRISKFEAE
LEGATGKRDEYKRLLQEAERRIRELEGVNGRLEDREKELQKELLNALNEVSGLQQQIISLKVKLGK
FEADRVNYEDKISKLNNEINDLHQRLSQVLASIEEKDHIIEDMRQELADTQRRHAELDSQNQILR
DEIEKLHGIINDNERKIDDLEVKLQDQRNQNSQLKNQINELNARNNKLKDEINDLQRKVSDLER
DLDQAKRDNQEKQNVIDRLNQLKNQLENDLEALRQENAGMKSDLDRLRLDNRQLLQEKHDLE
MKIMGLESDKGTLEDNVERLLNEIENLRNKIQKLKDENDRLIIENKRISHELYKYNKKSGPSDYDS
LKDQRDKLQREIADLTKELRNYKTNITVLQNEKDDLYNRLKELERDLADRDHVIEELEREKDRLE
RELESARNRADKFELSNGQLENEKNRVQEELMDLHRKLTKLEGDYELACDKRDDLQKELDSAIA

RNKTLEDNYKRSQERENKLHQDLLAAQKKITILEHELQSLKDRVNSLERANKVLDDKKNELKDD
LIAAQKRISELEIMLEGADDRNKDLQNELDAMKRKNDELRETLRLTEHGKEDLINEIQNLTAKLS
KVENKLTQAENEKRYLEQQLEDAKQRTQKDRDEIMNLQKTIYELTRKCDSLTKDNANKQALIDD
LRAKKTTLEGEIESLKRDLQNNQYEIERVENDKKNLYDELENMKNKFNVQEQEYLVLQEEKEKLR
QDMIASHQSVCRLKENYENSLGDKGKLREELATFHRKIAELTTAYDSCRSEMNRYKGDCDNYKD
AYDDIKAKYDASQNEISKSRNDYEVCLQKYQKGEQAFTKSEADRERLQKEMLDVHRKSSEIAVLF
QTTKEEKRVLENQLSMLQDDLERYREGYETFNDKTNALQDEILVSHKNYTKIEHAYNRAVEREEK
LEKDCEEAKEIVANMEKDLDNARGKANELENELRKLQDKLRREQDEYEQNSEHAGKLQMTLRD
YESKLSQLRNVEDENKTLRDEINNLKSSINKVEMDYRSNHNDLTQVQDQYESSRNNINKLNNEIN
ELQRDIRQVTSENEKLQNDLQNAAQDKEQEGERLKSTIRMLEREIDSLKTQFGQTTEQLDIVSKDR
NVMHQEIQVILKKCVSIEMTYITKTEDSRFQTSGGDEYEKSINELKNHLDKSEREFDDREKKYKYL
EDQYVREKKDKSQLEKEIDEIQRRYDNMQHEIRVIEEDKKRLSDEYQDMFNKYNDLDRDFKNTQ
TKITTYETEINTYQTTINDMQYKFTNNEDQNKKNSQVYRDLQQKLDNATKENTTLKGELNDEKA
SYRNVKKDFERQVRELQRSSESYGKASHEKDTIIEDLKSKRQALESDIDQLRRDLNHGKDEYKRLQ
REFTQHKSDYQDQQRVITDLQQKVEEHKHSKITIEEEYKITVEKYIKEIRETHIKLSKVESELQTTRE
AQNLDSDERYKRLQNELQQERETTRKDIYGKNQEIEQFKSQISNQDQEIRRLRREIEELDKQLKEA
GSNYSSTILIQKQDAPKYSSTQVTESRISSSSAPRYSTEQEVLIDASDKDTEDEKKRYQSSFYRSTATIK
PGQSDYGSVDVVLQGSQDQGQDDKDYDYNRRATGLDTNAVNRSQ

>TRINITY_DN982_c0_g1_i1.p1 type:5prime_partial len:6412 gc:universal TRINITY_DN982_c0_g1_i1:19377-142(-)
KLDFETKQSHTLTIKAVDRGNPSQDSLNKATVIVVVQDFNDNCPLFSESIYNASVSESTAIGTTVLR
VSVTDKDVGNVNNEITCSIDEPLAKGFFSVNKINNNQFSVVLIKNLDYETHRGYRFVIKAKDNGE
VPCQLTTNMVVEVQDEPDVITNFKPFNYVISIPESTLIGTKLLELKVDSESSSITYTLQAKNQPGDLD
KFMLNGQSGELVLKGTVDFEKKSEYEFTATLNDGVNNGGNTANIVVNILDINDNPPVFSQSTCYI
RRMPENQQVNTTVTSVLATDSDQGINSNISYSLIKDSSCNNFEINEITGAISLTSSLNYEVRKEHYCII
KATDNGSPVLSSYSCLKVIVENVNDNSPVFLPKELEITVLESKPVNSFVIRVFATDKDEGNSITYSIV
GGNTQNAFAITPLTGHIILNQKLDYEIVKNYILEIQATDDGTPRRSSNLNAKVMVKVLEVNDNKP
KFNQQRYLRTIPEDTNIGSTIISVTATDDDAGLLTSKIQYIALENTNDFLIQPDTGDIVVSQNLDYER
QRTYQLVAVCSDSGTPPFTGRTLVDITITDVNDNAPILNPTLYNVLVSEKAPLGTSLIQVHAKDKDS
GINGGLKYQIVSGNDLNKFVINNSTGVITLQETLDREKYSSLYNLTVSVSDQATPSLNGNNAFVQI
RVLDVNDKQPVFVKSEYIFEISENQPLNSPIGLVQANDDDSTSPNNIIQYTIVPSKDSINFTMINNQI
LSNSVFDYEEQKSYEFEVQARDQSTGVNNEQLVGRTLVKVLIRDTNDNDPMIENQNIALNISDVT
LVGTLLFDIQATDKDNGENARLTYTISSGDTSTFRINENDGRFYLSKKLDFYNKNEYSLIVIVNDNG
SPQRNSVANVQINVYPIDQTLISFEPQCPIAFTIQENSLSSPMNIKAIDTGSYVSKTMRYMLKSNKSD
VSSMFQIVETNGVIIQNKAANYEERSRYDMTVCARNERGSEGCCPVTIYILDQNEQPFFDTDNDN
LDNGKYLVRVSETTPIGSVMFSVNTGDEDFGSLPNGQKTYSIKAKPGEEMLFLVDSYGRIITNTTLD
REKTPQYDFSVVVTDKGTPPRQAEAEVIVTVIDYNDQRPVFEIMSVRPVNENANNEVLGRVVAVD
IDSTSINNGIKYSIQPAGFLNISNTGTLTSVGSALDYEKNQKVTLYVTATDTGSPYLSATTSLQLDIINI
NDNKPYFLQNNYNLTVSEKTTVGTLVLTLFAEDDDLGEFGEIANYEIVSGNQEDRFSIDSNGRLTL
KNPLNWRNVKSYNLNIIARDKGLPSEQSQIPATVVINVADYSEEQPNFLQNLYVKFIPETTPIDTLV
LTVEATSGEPVYGPFKYTIVPKLPDTNNFQIFSNGEIKTINQFDYENQRNFTFDVQVEDEKNGKTN
RASVFIHITDVNDNIPQFTDLTRSNTQYNITEFTLPGSMITCLSATDRDHGKNAEIRFRGIGGDGNG
VFNVNLDGKVLLSQQIFYDKKNKYNLIVEAFDLGSPEQKAQLTLTIFLHKSYTSVITTDVDINEDVE
TQTRVTRVSSIDPFTRSSNNFTYYLVGKSTTFFEINAQTGEIFTKATLDRETRDRHVLTIQSRGPTTN
GVGELVVNVKDINDNPPIFDPQVYQVTVSESANIGLSIVSVTATDKDIGVNGEVTYSIEDGDQTIFSI
NPSTGSISLAGKLDFESKQSHTLTIKAVDRGNPPQDSVNKATVIVVVQDFNDNCPVFENSTYSLTIK
EPGELGKEYLKLGITDNDNGRNQLFTCSIDEPLAEKYFEMINDCRLRLIKELDYEEFTSFRFVVKAT
DQGDVSCTTTTNMIVNVGDRADVIPFFSPLTYNISIPESTTVGTIVVSLKVGSTFKGLLYSLFHKNTQ
DDLIKFGINKDTGEIFLKESVDREIQDKYEFTADVFDGSNNAVQRASVTINIIDVNDNAPFFNQPTC
YQRDLTESTAIGTFVVDVSAADNDKGRNQQVTYSLLPSPSCTSFSVDETSGRITTSQILDHEQQKLQ
HCLIKAKDNGTNSLSGYSCLTVNVVNVNDNKPAFIVNRFDVSVLETVPAGTLVASLVARDVDNQ

GLITYSISNGNLNNQFTIDSSGKLRTRFPLDYENTKSYNLTIQASDNNGKFSSPDDALVLITVLEVN
DNRPQFLQSYYKVSIPEDTATGASILNVTAIDLDHGELTKSLFYAALENTTQFTVNANTGVISVAEN
LDYEIEDHYELVVIVQDRGTPHLSNRALVDIQITDVNDNAPMFVTTVYNRELSEKALIGAPVVKL
NAMDADKSDKMTFTIDSVVQGAQGFFSINGDTIQLDKELDYEVPEQRTFVLSVSVKDSRDRDGA
NKANVFINVRDINDREPIFDLNMYEVTVSENNVISNPIIEVTARDSDSTPENNNLLYSLVDGEDAA
SFSLLNNGQSAIITSKISFDYEKKKSYRFLVRASDKVRENFTAFSTVFVTVQDTNDEAPVPEKDLIM
VNISESTRVLSTIAIVFANDKDQGINGQLAFQIINHSEDKFEINNKGVIRLKSTLDFFQQNFYNFTVL
VTDKGNPVRTSKPVTVLVYVYRTEIPGIIFEKNHYIFNINEKEIVSLVGTVNAIDEGTYVSRQIVYSIV
SGQNDIKDKFRAVNNNIIQDKEVNYKDQSEYNFVMKAINERGSIAFTHITINIMDVNEKPYFVGL
NNKGRYEFSMSEIISVPSYIYAVRAKDDDFGLNGEVDYEIISNFRFGNFSIDQYGRIQVLKDPDFEFT
RSYEFQVRAKDRGTPSLESNLVSVVITVLDYNDNHPIFNIQEPLEISIKELVNNTDVGTVTATDKDG
SGLNRIVKYVAIYPSDHLTVNRDNGQIRVIEPFDYEIAHKHEFVVVAQDQGTPYLNAYKVIKVEVL
NENDEFPEFTVIEYNITISELTPIGSPVLFVHAQDKDRGPSGQVTNYKIKYVPPSGGSGAGSPDKTFE
ITSDGQVVLKEKLEYKFQKYYQYRVTCTDNGSPPKNSINEAIIHLNIADASLEQPIFSVNPAVVEVY
ENITLNSNIVKVEVDSIEPIKGPFEYSIIASEDSQPFMIDNSTGQITNKESFDYEIKRKYVFLAEARDK
HNNRTQRAMVVIFILDINDNIPTIETIAPQTFVQNISDTTYPGSVVAKLYSRDADSGENAMVDFTIT
GGDGENVFAINPNGYVTLIGPLDVRVKTQYTINVKVSDRGIPTLSNNRALTFNIKKSKFIQNTVRF
DRHHYKSNCIEENQINLNIIQLNATNSNPVAGSTIIYSLDGTLEERQPFIVNSQTGEISLSQTLDYEIK
QEYDFVAVAVNNDGSRDVAVVTVCIKDLNDNSPIFEPQSYVVNVSESESVGYLVYKTTVMDADKT
DFNTTKSYDIIQGNVNDKFIIDSMGHIYIKSKIDYEALQVKHFDLRIRVNEGNLVGDMVLRVNILD
WNDVHPSFSTLNIYNFTIPEDKPVGEIIEILTANDGDATSPNNEVTYKLLTTEDDFILRRNDKGHGS
LIVNKQLDYSKQNRYEYVALAEDKGYLNLLGTAIVIINIQDVNNHPPEFDDSEYNTTVSEHAQIGT
RVLQIHAHDKDTGLGEQIFFIYGDPTETFTISSQGFIILNKQLDYETQRDYIFDVGARDGGNLVSLN
NVTVHIKVSEVNDNEPIFDLSTYRNSYAEDTPLGTTLFTVRATDKDFIGNSIIYSISGANDDTSYVSV
DQTTGDITLQRRMDYESKNYITFTVQAIDSNNRNKLKGSTHVIIDVTDVNDNNPKFVFGSDLVYN
VTVVENINVGTLVVVVDSQDADSGLNGQTRYHIVSGNNENKFFINELNGQINLINKLDALQTNK
YVLTVMAKDLGNPSRSTNVTVTINVLRYFESTPMFLPSYAEVIYPENLALGSSIYELNVTKTLPGSP
MKLSFHSSSKFPEFTLQGNKIILSKEFDYEIKNRYELIIIATDEENDKAFAQVIVRVQNLNDNIPYFTQ
NAYFGVVSELAFVGHSVLTITAKDDDKDFTELFYSIATQVPDNAFKLQDNIVVVNGKLIRNKVYEF
EVEAFDNTFTSKRATVSITVTPENLPSFVNATYIVTLQENYPISTNFLNISAGTFSGITYVITSMKAKD
MFNVDANGQISLTKSLDYEKQRQHVFPVTASVTSPITQVRVASVIVNVLNENDNAPVFVNGPYER
NITEGHLVGTMVIRVDGHDDDSDKLTYSIVSGDDGKFSINSEGVISLISTLNFDLKNKYVLNVRVSD
GKFVTDTIVTINITPIVKFPPMFNQTLYLLSVKENTLVGTSILQVSTVNVIPPSSFSINEQSGRDFFTID
ANGVIRTNNALDYEAKRQHIFTLKVQDLRSMGHATVVVNIVNDDDTCPILTPATQTVQITEPVAP
GTIVAAVQAKDIDSKSLNFTITGGNGLFRIDQHGGIKTINEVDISTSTEYELTVTVDDGTCTKSVQV
DVNVNPLAPCPDCNIYPFTQTIYEFSINENTVPASPIITVTTGQSLQRNFSIEDPAALEYVTINRLTGE
ISVIKAFDYEATKHLSFNVMAKTQTTKNRNPFYVITSVSRVRITVRDLNDNCPSIESPMYYEVSHSS
PIVQDTVVTRILVEDPDTLKDHTYSISDNNFKIDSMGHIRTVQNIESFTAQFYNLTVTVSDGQCSKT
SNVGVNLAVNPTPMTYQFTSNGVYSKSVREDHNLQDVILSVALNQNYPRVFSIVESDANDQFVID
SNSGALRLKTSLNYELKKRYDFTVMADIHGGVYKSYASVHIIVLDVNDECPIFPVIGYNQRVSGTP
VIGTIVEIIEANDADNATLEYSIVSGNEDGHFSLDNSGVVRVVKPFASSFSKTYGLSVRATDNKCSA
DVPLTVQVSTCAYGEDYQFEESEYVVYYLDENRNLGIVSNIKLKSTRAAFLTLSQNTHFTIDQNGDF
RLIQTLNYETKKEYHLTLTANFTQDNVVTRIPIKVFVRDINDNCPAFSEELYAVQHTEPLREGLLIA
KLSVIDDDTVQNFTYNIVSGDKRFSINENGEVVMNARLVTDTTLLSTTYKLEVSVNDGKCAKNTK
VDVFVYKTVVNSFLFPQPFYKFDISENQAIPFTIDRFVNVGGHEAVYGFLVSTDFFQIDPKTAQLQL
VKKLDFEVEKTHYVLISATYDDGTQTYTVVAIHVGDENDNAPQIFAHPPITRLVQNTPHNSIVTKI
EVEDPDTSKEFTFKITNGNPLQNNDPDKALFRIESNGMIKTTRCISEDEIGNYVLTISVDDQVNTPTI
FSNMQVQLKTTDDRSYVTNCGLGCPANDGPFSRYQFEQSLFIVNVTETAANPTVLKRLTRPFVEK
TTYTIGAGADGKFSIDQNGTLSLTGSLDYETKPVYYFIVSAVIPSQVVGRFITSKTAYLVMVNDVDD
SKPVIEGNGGTLAVSETSEIGQIVGTVRVKDSDSSSPLTYNVVNGSQYFNIDSAGNIVIKDTLNAAG
SPYTVTYTVSDGINPPVNGEFVVSISETNNNVPKFTQNIYEVSLSEDTATDTIAVTILANDGDTNDKI
AYSFLQSEGPFVINRDSGEIKLIGPLDYEAQKSYNMVVQATDSGGFANIAFVQIAVGDVNDQVPVI

TPQTTPTQIHESTLVNSVVYRVIASDGDGNERLTYALANSNDFFTINSATGEITIKENLAKSNVSSHT
LSIVVTDSSGKTSTQDVALNIRRSGTSQGCVKITTSQGQSSSPVINLPENTASGTTVFKLVAGDASVF
SNMRFTLNREANGVFAVTEQGELQLVAPLDYETKQFYSIIITAEDVTRGVSSQYNLFVRISDVNEFT
PVLETIAPVAVSELANIGDVIAQAKANDQDGDSQVTYSLGEFGNNDNKFTIDAATGNIILNKRLG
SGAPHVVEVCASDGSIKKCQNVTFIVADTNDNPPSFQDLTGVGSIDLTNTSSYVVYKPIVTDSDQT
GTLSFSLKDPAPSQFSINSTTGEVKANKDIAKGTYTFGVCASDGRYTSCQSVTVVASDRSQSITPSFN
QDQVGAIVDISASSGKQIIQVMASTLDKAKIEYELKNEADRNIFEINKDSGLITLKNRTALTLGTIYT
IPVKASRADDPLNFDEINIVISVGNQQLDSGSFIEQVLDTQHLVNFSLSTFDISKVSYYIILGQQVGG
DSKDDEKLPVSWYYATFGDESYRHRFYYIAKLDGSKFRNTGGSRKRRSIQDKDSKSFTYLVGEENG
CSKNTSMAAICNGPLKTGTEYRYKLRAYYDDGELRGIPVDQKPTEVKRTQAPQAGKSIDEYREQD
SYVITFWIIGLVIFGLLFWFSVLVIAILLCYKRRYEVTQAYGYSVQTRDKIDGPPTEYSNTVYRFQNPS
YPDNDTVKMKKVDASESRTKTEVQTTSMVVLDDYEQEQPQPDIPVQDYDAIEYETRSAQIKFHEE
SPEWESMDIQLRIDPTGNQDPVITRKSDSGSKDSDSDASKKYENTEYIEEKEDFVNEKEDSLTRL*
>TRINITY_DN319_c2_g1_i3.p1 type:complete len:2588 gc:universal TRINITY_DN319_c2_g1_i3:165-7928(+)
MVLLKGQLWILGFIFLTSTKYVMGDVPECSNNATVAVHCRFDEGNTCVDDPHPMHYVCVCNNL
GYMNDTDSVEGYSICKERDCSNSNETPTDLLKRICGVNTKTCTGKILQRECNCNTDSYRSKNGQY
CFFAPCQASPTIQQNCGPGNQCYGNAPNYICDCSARGYKRSAADNRHCELKSDEELTPIEEYCGK
GSLYANGTINNITCTCDTDKGFFPSATGETCISPSCYLNPQMQEKCGPENTCLGGKDDYVCLCSNP
NYKRNATDLQHCIRRTCADTSPAIDFKSICGEGATGCSGYVDKPLCSCDVSNHYVPAADGRSCIK
ATCKGNKKALEQCGQANSCTGSFDNFLCVCDANGYYLNTTNNTCVERECTDPDLVITDICGVGS
LSCNGKAGSPECICDKDKFYYASEDLRSCVKANCLSSETISKKCGKENSCYGSMESYVCNCNAPG
YLLNTTDNQGCYLGMCEDNGLEYDKVCGEGAISCNGTIKNLECNCGMNYVQSPDKKSCIRADC
RANQTMHEQCGEDNDCVGSLEDWMCICSTYGYKPDPMDMMKCKKVMCEEEVVNNVTVCGL
GTTGCKGHILKPECTCDIAADYVPNEDASRCIKGSCENNKMAMKDCGIANSCTGSYNNYVCVC
NGFGYKKLDDTHCVEQMCSDPDIDIRNVCGEGAHYCNGTVNQRVCSCNETEYFYPSADSKSCV
KATCAVSPMVMEQCGVENSCQGSQESFICNCNALGYLLNTTTNQHCYLGTCENGDFDLMQAC
GDGALYCNGTIKNLECQCDESKGYVQAPDKMSCIPNDCRGNLTMYRQCGQLNKCVGSIDDWR
CICDSPYYVAHSDIMKCKLLHCSDSEVDHVEVCGKGTIGCSGSTLHPTCHCNTTENYFASKDGMR
CVAGSCKTNQTAVKKCGNANSCAGSHSEWTCICDGIGYKHDPADPQNCLLKTCNDADIDIVATC
GVGAISCEGYQLMPKCECNDRDYYIPSDDRTSCELASCRANRTAMRACGEMNDCRGSFSSHICL
CHDPAYKSDPSDSRVCLEKTCEAGDFELVRVCGKGATGCSGSMKNIMCECDNAKGYVASADHT
QCIPSDCNNNVTMHLQCGEGNGCVGSLTDWYCVCSAPGYEPHASDHMKCHLKTCADPGVDH
VQICGMGATSCMGPVINPVCYCNESMYYIPSMDRTSCIKASCMSNKTAIAQCGSENSCSGSPDNF
VCLCQAYGYRQDTNNNQACIPSQCEDAGIDFVEICGQGGVGCSGSVLNPVCMCDHMKHFVPSP
DQNSCVLSTCRGNETAMTSCGEQNKCFGSSSSYICVCDAVGYKPHPTNLQECVLQTCEANELNP
VAVCGKGALSCKGTIQSLMCECDNSKGYVAAPGYKSCIPADCRANMTMHAQCGTGNRCRGSM
EDWSCVCDGIGSKPHPTDHMSCIAIDCSDPNVDIKSVCGYGATGCMGLARDPICHCDTSKYYVQ
SEDGRTCVPATCHTNKTAQMQCGPDNSCIGPPNDFICECTGFGYRHHETDKQKCVPIQCNETAL
FDIERVCGKGATGCRGNIRQPICDCDHAMYFHPSEDRRTCIEASCRRNSSIRADCGVENTCFGSTR
SWICDCNSYGSILNPSDVKKCMLATCEAGQLDPVAVCGKGALSCKGSMRDVQCTCNNELGYVH
SPDGKKCLPSDCSGNVTMHHQCGDANKCMGSLTDWVCVCDAPGYKPHAKDKMKCHQISCAD
NNVDPVDVCGIGATSCKGPITNPLCQCDTDNYYITSSDGASCIPATCKTNKTAIHQCGEANSCEG
PADNFICVCNAYGYKLDDNDHQRCVEISCDDPNAHESICGTGSTGCRGTARDPICECDHTQHFV
ASSDLTSCLLASCRANDTVMNHCGDGNTCFGSLTSHICVCEAFGYKLNPNNHKHCLEETCEDD
MLNPVAVCGKGAVSCSGPLKSVKCECDNNAGFFRSPGGMKCESTSCRQNPASQMMCGSQNQCY
GSYDDWYCDCSAPGYMANQTNAAECLKVMCDDMAVDVCGLGATGCTGHTLNPICTCDSSMG
YMMAKDKHSCVPMDCSMNPASSQCGYGNTCHGSYFSYVCVCSTAGTVMNKTNPMECINVACP
ADAVHVCGEGATNCSVTSTNNVECSCEEGYHPADDLHSCVLDDCRFNPKALKACGLLNKCVGS
YKSWKCNCHYPEFTQDPMDHQTCVVGPCMVEQCGKGNAPGSFQHYAVNCSGLYDDPICNCQP
EYTNDAKGGCIPKICSENPVAVQACGSGYCEGTYFKYTCKCDEPAFVQTPDKMQCVPAKCNIQQ
CGHGFDRGLYIDFAESCSGTYADPMCQCKTGFSGTNTSKCGPAIDGCKLDKCDPLATCQTLDVD

GNVKCTCKEGYSGSGMMGECYLDGGFSAYEGTKECSEVCGVNGVAEVTRFCNNPTADPKGKPC
NGTSPETIQVPCNRFACSSCTVRKTCGCGGLKPIFVEFYHEPNSTLEYFTDLKADDVQTALGFLTK
WKELSQQDLCLMYSKIELTVFVTLMGDSETQVSTMKSSLVSARDIMRKKLDCNINLGKKAQYYEA
YETIIKRHLAISNILKDLTIIKNSITKYMNNCDDSEIGMIRDVIGMM*
>TRINITY_DN1369_c0_g1_i1.p1 type:3prime_partial len:1577 gc:universal TRINITY_DN1369_c0_g1_i1:93-4820(+)
MRIQLTIFVTFSIILVSGQNSPPGICSTWDGSHYQTFDSYSFNYHGTCSYALALDCRPGSDDFAVHV
ENDEKCDNHGGTDCTRAVSLYVNDIEYKLGSNNTVLVDGKSNIVLPYFNEGVRVSKHAEYYFIDA
LEGKVVVKYDGRSSIYVQLDSSYQAGVCGLCGNFNGNKKDDLKLPGDKGLTKSLKEFGKSWEKP
RIGQVCKEVTEEHFDQCTNKSKMLVYYAEKECEILRTSSEFKKCHKIVDPTPFYEKCREDLCLCGS
GDKYCKCDVLEQYTRKCAHYGTVLEWRQQNLCYKNCTGNKVFSECGPACSSVCSAIGSKSVCK
DACIDGCHCPPHTSLSGDKCVPDDECPCEHNGDMYDHGDVINMKCKSCKCNGGKWKCNPLP
CDGEASVYGNGHFVSYDGKTYNYPGKCLYLYTYLSDRSFAVYLDRTSCDSLACHMNVMILFRGD
RIKLSASNSTLALEVNRNVKSFPYISSTGYRMYRFSTVFVRFEATNGIKVDWDTYSRLHVLMPHSL
KGKVFGLSGNFDFKTKNDFLTPRKDLAHSAIEFGNSWMMYSSRLNCSALAPDYRMTSCEANHQ
VAKYAESKCSILMSSLFRECHKVVDPFIYYENCKDDTCECNGDSKCYCSVIAAYSKACSSAGKPTE
GWRKYTPCEEKCSNGMVFTECGNSCLKTCDDQTDPNPFCEKECVEGCLCPVGQVLDKITEQCV
DIKHCGCTYGNRTISNGGTVAKDCNTCKCTKGVMVCTKKECRDKKAICSDGKEWSSCMSPCPK
TCQTKDDICPETGLDCIEGCQCKKGYIESANGTCVELSKCPCYHNGKEYQETQTISKDCNTCKCT
AGEWSCTEKLCDATCSVFGDPHYKTFDGKIFDFQGKCGHVMLTDKCANNDNATENIRISVKSST
CGSQGVTCTREITLNYHDTVVHMKRGARKIDIVSNKRQDHEKDQVKVYRHAGIYVHVLTPMGV
SILWDNATRLYITAPPSLSGKICGLCGNFDGSETNDFVTYQGDTAGNAVSFGDSWRTENNCPLTKE
IEDTCHNRKHRLDPSKRECAVIKSDVFKKCHTLVDPVPYYDRCVFDACGCDSIGDCDCLCAAIGA
YAKECIDEGVYINWRKGHNICEKEMCCPTNMTFVPKGTGCPKTCKYPNGLHHCPTKYVDGCQC
PEGLVQNNDANGDVTCVRPEECKVCKLDGKIYNEGEVVLYKCKECVCRDGKMTCSGNYCPTTL
PPTTTTESTTTPSTTPETTIPSTTTTESTTTPTTEPPTTTTTASTTASTTTESTTTEATTTTKPTTTTTEST
TTESTTTQSTTTPSTTPSTTMKPIDTCVYSNGTHNVTLERGDVIQVNECTKKTCVKDEVTGLHEIKV
TTTQCENSTCDTTCEASKSNRAILVKTPGECCPQCKKPEDLCDNPNEVPCNSTYCIPKSWFCDGN
KDCSNGRDEECCPNYCKVEGEIKKPGEVWTNKEKCLTCKCEGYYTKCRRMECPELDCPEERQVK
SEGECCPHCKPQTEPPTTTTEMTTTTTESTTTPATTPSTTSKETIIIITEEPKSTTASPQEKCDCKVLQPV
GVSDGRIPDNQMTANSV
>TRINITY_DN428_c0_g1_i1.p1 type:complete len:1949 gc:universal TRINITY_DN428_c0_g1_i1:96-5942(+)
MADDADLKFLMVDRRQVSDPVAHAEWAAKKLVWVPSEELGFVAASVKEEKGDKIIAQIEGGKR
VTFHKDDIQRMNPPKFDKAEDMADLTCLNEASVLHNIKDRYFSDLIYTYSGLFCVVVNPYKRLPI
YTDKVINLYKGKKRHEVPPHVFSIADTAFRSMLQDRDNQSILCTGESGAGKTENTKKVIQYLTAIA
GRANQQKADEKSQGKLEVQLLQANPILEAFGNAKTVKNDNSSRFGKFIRINFDNSGFICGANIES
YLLEKARLVRQAPDERIFHIFYQLLLGASPEVKKQFLLLDTKSYNFLSNGHVQLPNMDDRQEFKV
TLEGMRDMGITQEELDPIFKVVSAVMLFGNMEFKQERKSDQATLPDNTIAQKICHLLGIQVTDFT
KSLLKPKVKVGREFTQKSQTKAQCEFAIEAITKALYERMFKWLVGRINKSLNRGKRDGASFIGILDI
AGFEIFQLNSFEQLCINYTNEKLQQLFNHTMFILEQEEYQKEGIDWKFIDFGLDLQPTIDLIDKPMG
VLSLLDEECWFPKATDKSYVEKLHKQQTKHPKYLKPDFRSNSDFCVIHYAGRVDYSAAQWLTKN
MDPLNDNVVSLLAHSKEAFIASIWKDTENVVGMSVTEGMDTTFGASRTRKGMFRTVSQLYKEQL
SGLMTTLHCTKPNFVRCIIPNYEKRAGKIDNHLVLDQLRCNGVLEGIRICRQGFPNRILFQEFRQR
YEILCPGIVPKGFMDGRKAAKQMIDNLEMDPNLYRIGQSKIFFRAGVLAHLEEERDIKLTEIIIQFQ
AFCRGNIARRNYRKRIQQLSAIRVIQRNGRSWLKLRNWQWWRLFTKVKPLLNVSRQEDELRSKE
EEFKKLQDKSDKLESLQKEQQQANEKLTEEKNTLAEQLQAEIELCQEAEEARNRLLQRKTELEDL
IQDFEMKLGEEEEKAVKYAEDKKKLQQGIQDLEESLEEETQRQKLQLEKVQVEAKLKEMEDERQ
VAEDNNARLTRDKKQLEDRVAEFGQKLAQEEDRAKQLSKAKAKQEAFIAELEDQIAKGDKSTHE
LHKEKRKAEQDLSDLRAQLAQALQRIAELEDDLARREKELAEAIKRGDEEMAKRTTVEREKREL
QALLDELREDMESEKSAKDKVERQKRDLEQDLQELRSELEVTTDATVVQKEFQKKREEELMDLK
KQIEEEAKEHDQQMASLRTKHNGLVEDLNNQVDTFKRGKAALEKNKTALENENAEIAADLQRI

SQLKQEGDRKAKNLEQQLNEANSVRAVQDEKVSSLESEKAKYQKECDSLSTQIEEVETKAASLDR
AKQAAEAQLEDTQDQLAEETRQKLSYQTKLREASDEANRLQEQLEEEEDEKKAVQKVLSNLQV
QLDQAKKEIELKIQDFEQAEAARQKQKRENEELRGEVEKLTGDVSRLDKAKKKLQGDLDDVTVS
MERERNSANQMSSKQRKFDQLLAEEKANSQALGIERDNLEKLARQNETKALSLQNANEELEDR
LAEAERQRKQLQMELGDLVESKDDVGKSVHDLEKSKRLLEQQLAEQKTQVEEIEDELQACEDAK
LRLEVNMQAQKAQFDRELQAKEDAIEEGRRGLVKQLRELEAELEDERKLRQSASAAKRQLEGDV
KELEGQLDQANRIKEDGLKQLKKYQNQLKDVQRDLEDSRQSKDDLAAQVKDNEKKLKGLEAEF
LQMQEDLSAAERARRAIESERDELLEELNNNSALKNAVAEERKRYEAQLAALEDELEEERTQTEL
MQDKVKRSNLQLEQMNAELQEERSAAQSLDNTRMSLERQNKELQSKLADLESSLRSRQKNTVAT
LEAKINNLEIQLDQEAKERQQQNKALRRNEKRCKDLQMQIEEERSHTESYKQQLEKMNARVKN
MKRQLDESEEEVTRLNTAKRKLQRDFDENQESLESSQREANQLRQRLKGGASLSSSRSSGRGRRA
KDEDDDDDDANE*
>TRINITY_DN477_c0_g1_i9.p1 type:complete len:306 gc:universal TRINITY_DN477_c0_g1_i9:1840-923(-)
MTRQCLGATLNENCGNAALVQFEVCNFQVPCPGLLTNWGPYTQCSATCQKDANSMPTQSRQR
FCTGGSFGGNCGGVQLLQTIGCNQLIICPVYGYWLQWSAFGECSKTCDQGTHVRYRNCTKSIPA
NAGNNCTGPAQEVKPCQVKPCPVTCPLTKKCDCRNMNYLAAAPGISQTVTNDVMGVAAFLDY
WTLDILKQICEGCRMTQLDQSKATVDSQILRTQAVLDRLNIEKRVYRDMITCNENPYITDDLFKVY
NDIWDRIVTTDGVMIELKASKMRFDTAITACRSYDWYHDLFGRFFQIKKSS*

>TRINITY_DN11248_c0_g1_i1.p1 type:3prime_partial len:1700 gc:universal TRINITY_DN11248_c0_g1_i1:111-5207(+)
MEIRSLFVAFIYIVHSINAQCSSPWKSYKDSCYLFTAVSTVSPGHTWEDSRFICQRYGGDLLKLDND
NETKYINSQFDDLKIKYNHYWIGLNDIKKEGDWRWADQAPLNVTTQWRVSRGEPNNWQGNED
CIETTYDGQWNDNECSRMFGYICERSKDAVIPVTPTLPPFPTATDKPTTCEAGWIPWGTSCYGIIRG
LAKNFQDAQADCLRRFGDLVVINDQAENDFISNEISRSVGGLWIGLSDTYKEGTFYWVDKSELNF
TSWRAGEPSNDYGNEDCVEIYGRWGPGQWNDNHCTALRDYICEKPGGVHNCPRDWVSYKNY
CYQINAHPGQKVTWEDARKNCLDFNKAWRKSVNVAQADLVSITNLEEQKMLQKEFSKVNIKN
DNGFYYWIGLTDKNSTGGFYWTDGSTYKYSNWIGSPQFASQCVRSNVIGYQGGSWSKGDCSEAY
NYVCKVGRGQDRCYNPLGIQSFAVPNKAMTSNSVLGPSTPALAGRLYMKAGNSLDGAWCAGN
NTKTPYLQIDFGKMQKLTYMGHQGKSNSDAMVTSYYIKYSQDGIVWDTYGYIPPAQYYKFKIYIG
WRTNYEKVMNLPLPAPFIARYVRVYPLEWNIAPCMRIDFYGCQEEVDNQRCGLGWREDKKTKN
CYKFHNQITTWDSAQELCSIQGANLATIATPDEREFLSEQLGEFKSLVSEFWIGLNDLDYEGVFKW
VDGTPYQLSNWYYGNPKVRNEKSNCVVVRTTDSAWVNTECTVFRKFICKRNYISPYTPKESYNG
YVYSKTDYFWPLDSATDFEGSLPLTLNGNVDRVPFPIDYDVGVSRYALNVSGSSGFFKIDVANDVC
IHHPDADSCPHGITVSFVFKMSKKSSSKRQVIIDTTEKNKPLGYKVYTEFGSIYVNLRGHQKSYSGS
TVYNAHIFNHMTFTWSSLKGLSLYMNGKSRVFDLLGSYAKDYPKVDYGKSLLIGISGDSQDPAEL
MIRSLGIFKQVLDAKSIEAIYLGEFGTCKSHQFAHGSYCYEYNSTEVSWSNALSTCRDNGGDLISIH
SPVEQAFFNLKIAQYNYQRTNWIGLNAKSKNSFFKWADSSRLEYSYWAPLEPNNNNGRREDCVE
TFANGGWNDALCYLRNPFVCKYSRALHIMGSENTDDKEYKVFFSPYQTLTFGCPLGRSIEILTASY
GSVKDGCVKDVKDKVSNACVKEHCVRVQASDFFFNKINPCKDKNRFALKRLKVTYKCNLATPE
FDSTGCSNGKDSENSYRVFDKSNICYKLYRKPKTFKEAQKVCKDDNGDLVRINSRETQVYLTSLLH
TMYQKTDIWIGMMYNSTTQNYEWLDGSHVYYTNWVNRQPDPRNGDYVKVGLEEGAKNFLFW
KNGEANETLPFFCQRVKVGKTTQAPPTTQPRTGTCPKGWFTYNKNCYYVNTNRLSFPDAQAEC
QKYGKSSHLASVLSYEENRAVFYDVVNATVDFAWFGLTNEAIVTGFRWVDDQPVSFVNWYTNEP
HKINRHLCVGMGSGGKWIDLACYLKYPSICKLPLGEVIPTTTPLPTIPPTQASGLCEPDWIHYNGS
CYNFANRTSYADFHNARVTCNKMGAHVATIHYQKENDFISAQLKRMYIPNAYIGLTDFSIEGQW
QWEDESVVSYINFGEGEPKNWHGMEDCVVVKSIGEWNDIPCNLKRSFVCKKNNGTVVKTVNTT
AAPDSGYCPSGWKLYGNKCYYFRYGE
>TRINITY_DN5584_c0_g1_i1.p1 type:complete len:961 gc:universal TRINITY_DN5584_c0_g1_i1:2917-35(-)

MASEMSSPFDEEETLLDEKRSKSNDYEGGFCKKNWPVVITGFGVVVLICLGVVIGTAFKSDINSSN
SRLAREEKFPYKNIRLPGNVIPIKYRIYLHPNISDNEFGFSGNVRILVNVLNKTNSVLLHSKDLNILK
VKIFSNSAAFNQKSESLATEIPTQEFLHSKKLEMLHIALNENEYLDAGEYVIYISFKGSLSGGLEGFY
KSSYTTRSGEKRYLATSHFEPTAARAAFPCFDEPAMKANFIIIIVRDPQHVALSNMPIERNVTRRDG
KVTVHFQESVKMSTYLVAFVVCDFAYVSAKTKKNNIEVRVWAPKELIAQAEFAIQVAPNVLNYYE
EFFKVDFPLPKQDLIAIPDFGAGAMENWGLITYRLTSLLYDENESSSANKQWVAVVIAHELAHQW
FGNLVTMEWWNDLWLNEGFAAFVEYIGSDYHDKSWRMMDQFIIDDVQNSLALDSHANSHPISV
PVHDPAQINEIFDSISYDKGASIIRMMKNFIGSDAFHTGLTNYLTKYKFKNAKSDDLWACLTDAAK
DKDIDVKRVMDTWTLQMGYPVLNVKRDGEKVTLTQTHFLKDQDKPAKSIYNYKWDVPVTYFT
NGENTTQFDWFYKDNKSIVIKIDENADWFKINRDEIGFYRVNYDKDTWAALKAQLLNDHMKLS
ASDRSNLMDDAFELARAGQLDQIEALNMTEYLHKEKEYVPWITALSGIAFCGKMLQGREGYKYY
EKYIVQQVKPLVDELGWEDKGTHLQKYLRGSVLRSAVDHNYTKAKNQALEIFKDFKENNKAIKP
NIRSAVYLAGIKYGTKDDWDFMYNRYKTSQFPSEQRKIMFALTDTEDKNILKKYLSWTLDNDKIR
SQDSCGVIEHISMNPKGTEMAFKFVKKNWPEIYKRYGTSISLGKTIKRIMGKFKTTEKYREAKEFLD
THKLGSGRLAGKQALASVQNHINWLEKNEKKVVEWLKNNINNKKITK*
>TRINITY_DN3078_c0_g1_i1.p1 type:complete len:1109 gc:universal TRINITY_DN3078_c0_g1_i1:3628-302(-)
MKMRIYLFLILFATVYCRVVQKGPNDIPEETKNEIKNQEPKIKFEPEKDKKYFLAKDTTCAHDVDK
HCKNIRRENNFAVFVCLQDVIHKDEDDVAEECHETLWHYKYNLTRDPNFDQAAFKVCENVINEI
QECNDSELLKSPGFVTNCLIENRHAVSEAKCKSFLQKMSAIVFEDYRLIKGFYDSCVEDVKTLKCG
TVAKPTDEEYQHDQTEVISCLEEQLDLVSEKCQAQLVKVATMQADDYHLDRPLFYACQEARERF
CPEVKSGDGRIYKCLVEHKTNEMMLEKCRKKIEIREKLTQKDIKVNELLWKSCKADFTKYNCPKD
GISEHGAASSLLLCLQGKVAEGLPVDQSCQAEMRTFAIELFENYKISPVIVSKCDVEINQFCHHLIG
KTDDGDMMDCLMTQAATNNESVSKKCYDAVSDVIKETGAGNNYKIDRALYLACEPVITTLCQG
KEDAMILGCLMDNAHNSKMPEKCAAQVFHLQYFLSRDFRLDASLYKDCKKDAEEICSANDFNA
ESESKVPDNFVIACLYRNALDRKVDDRKVSQTCAEHVFRVMHQRATNIHLMPELERPCMADLG
KHCIDKGDKEGEEIDCLQTNYEKLSAKCQNAIKVFTIQEAKDFDLDKHLVDKCAPMVQQFCQRE
LEDGNGEKVLPCLVDHKNDIDMDHQCANAVEHWQLIEMKDFEFSPALTEMCLLDIRKHCQKSK
AKIEALHCLSENIVKDASSVTEKCRAQLKNELIEQSENIKLNPELYEACTKDIELFCRDVVPSGGQL
EECLKRNHKKLTDPKCKSLLFKEEKKESFDPELDYRLMHVCKPIIKRYCTQYHQPVDIMECLRQV
THDRDMSRECRAIITERQIEQAESFELDPELSKFCQDDVNRYCKEEIKHAEKGDDDGAIFGCLVNA
MIDNKPIKNDCEMYIRRREAEAAQELDLNPQFLLQCRDEITKICPNMDEDEIIECMKMNIPQIQSK
GCVQEVKKLIVEGIEDVHVDPHLQEQCARDIRTFCQSVPSKSGNVVLCLIDVHKAKNLHLQPNC
ENFLTKRLSLYNAAKVDYAVFDSISAVMNAVSESPNRNYVYMTLLLVLTILFVGGMLFGRYTKKIRS
EIKNR*
>TRINITY_DN140702_c0_g1_i1.p1 type:complete len:2036 gc:universal TRINITY_DN140702_c0_g1_i1:94-6201(+)
MATSFKVSEIKCTELLNLEKKGAIDPLVEVEYLGLSKETENKDNDLNPSFGEVFDFDLSGKALGPE
DFLKITVKDYEKFGRNRVVGKADVYLQELLSNPSSTLSATLNDKNDRPTTGKITFKVDYIGPAGGG
AGGATGGGGDDRQAAERELEGDEDEGDPNAMPDGTPIPEGPAGDAMRAEKRKMKKNKKKRR
EHNLPDKPTDFQIRIKIIEGRQLPGGNINPTVRVHLGEETKQTRVKHSSNKPFYDETFFFNFNERPN
ELFDKLVTFEVHNSKNVLSEAMIGAFQFDVGTIYFEDSSHAIIRKWLLLTAPEDNDKKDGEETKGA
SLQPGGPPAGYLKITAIVLGPGDELPEKAKGSGKSEGGEEDIESNLLRPAGVTLRPATFLVKIYHAE
DIPQMDSAAFDGVKKLFSSGSAQKDHVDPYATFSFAGKTQKTEIKYGNNHPDFNQELRVAFKFPS
MCERLKLQMFDWDRIGNDDCIGTSFISLTAISGNGDEGFLPCFGPCYVNFYGSTREFSDLPDEHDN
LNKGRGEGCGYRGRALVEIDTRFGDHTAKPVQDIMAQELVRVQAYMRRRKYKLFASFLSANMIS
EADGLVEFEVSIGNYGNKLDPDTEPQSSSTPPTNPVYDGCAYYFLPWQESKPCCVVESHWEDVIFR
LEPINCLLKIIDKMEKNIKSVELGLKAKAQPAEVAALLIALLDQFISDVSMDLPKFAATDNGVNEL
DLKIQEIRSDELLNLKEEASTLREQATDVQEAMDTIDGYLKRMRDLCVEPQNSMPDVVIWMIAGS
KRVAYHRIPAYDIFYSENPLARGRKCASLQNFILKYPGKKGHDMEDHPEIPASVSVILWLGLEKHQ
AAWTDTEQMEGEYSVYAETYENQGKPPMFDWTAKIIKRPGFSDSEGKIRLNKEYYEPPEGWKWD
DPPDGDWFIDPEMSSNFDADAGHKKFMQDCFENESRVPAGNWGKSTPHYTNVQGDEIDHRDE

TSLPEGWSWTDDWKVDVNRACDEEGWEYCIEATGGGWGAVFRSYHFCRRRRWVRNRQLEADP
QKVMKQKKEEAKQGEGWEYAPVFGMKYHVKEHKFDFVRRRRWHRKMVCVKPGAVPMFKIPD
DDDKGKFHQIMPRIFLAYDEPHKWQFRAYLFQARDILGLDSEGTSDAYAKVCFQDQSQITDVIEET
LCPEFNQTLLFEDMQLYGSPSVFAENPPFVSIELFDKDKVGKDEFLGRVLAQPIVRLNGLKPPATRL
MWYDIHHGERTCGELLAAFELFLDEGADLPLPPPWLEKEKKFQVPSGIRPILVKTGVEILNWGVR
NMKKYQLASCTSPSVTYEIGREIITTDVIKNTKKNPNFLKNIFYKEVYLPKDPLYMPPIVIKVRDHR
TFGRKPVVGRHVIKSIDFVQPPKADPVVELPPADLVGEPEEKKDVTLDIEEKKKKKEIPVDDNLDW
WSKYFASADEIEKCGNYLKKGHKTIQIYDSELEEIKDFNGFSDFIVSYPLERGKEDDDEDDNVVGE
LKGLVKVYPLPQDPKDQTKIRYFTPKLKEKSKIVKCIVRVYVIRARDLQPKDPNGKADPFIVVQLG
KKKISTKDNYIPNEINPFFGSFFEMKADVPLDKDLKIKVMDYDFMSGDDLIGETMIDLENRFLSNC
RATCGLSPAYYTSGAYQWRDAKSPKEILDQVCRTKPQWLSNEQVVVNGQLVKIEDFEESPPTDPHI
GPVEQRLALHVLNNEDIIGHRLVPEHVESRSLKNPLQPSMDQGQLEIFVDIFPIANGVPGPPLDIAT
RKPKEYVLRVIIWNTAEVPPSETSITGEEMSDLYLKGWMEGQDDEKQKTDVHYRSMDGEGMFN
WRFVFPFKYMPEERVMVVEKKEHFWSLDTTKQTLPPRMNLQVWDNDLFSANDFIGNLELDLSK
LPRPKKAAKKATLAMLDTEGDDVMDLFQAKIAKGWWPMLSENEDGTRTMQGKVEMTLEIVTD
LEHEAKPAGQGQEEPNMNPKLDPPNRPETSFLWITSPWKTLKHIIWKNYKWYIIGFFLFIILILFIGL
VLYSVPGSIVHKIFGFQ*
>TRINITY_DN1058_c0_g1_i1.p1 type:complete len:2639 gc:universal TRINITY_DN1058_c0_g1_i1:134-8050(+)
MVKYIIWILLLSIFKEAYGGDWAQWQNWSACQDSSGAPIGCGRGNQHRSRTCADNNCGAGAIIE
VRPCLDEICPTFNCQNSSEIAGMCKFDYSNSCVGDDNCHHCICKSEGFKVSGDQCYNYCVEVGC
DEEIEGVSQLKRYCGDQAISCTGTVLNRTCLCNPATSYPSKDGQYCYEATCKTSQEFMTNCGAEN
SCQGTNTSFVCECNGFGYGYEVVANVQNRKKCKLLSCEDGDLNPLEICGQGATACTGNVHNLT
CACNVTAGYIPSEDKKSCIPMPCNTDPAVGVQCLPRNTCEGSKIDWRCICSAYGFRNDPTDPKKCI
EAKCDDPNIDILGLCGTGVTSCTGFLSKRRCVCNTTENYIPTLDGSHCMPAPCNANENATKDCG
GENSCIGSFDSYMCVCNGYGFQKNATINKCEEVPCSSLPTAEVQRICGFGALSCDGTIRNPVCNC
NNDLQYYASEDMKSCIEASCSSKADYDSHCGNENSCVGNITHFECICETEGYKPDPSNKKKCILES
CELNEVDVLNVCGKGAISCKGNVRDLECVCDASKGYVQSPDTNKCEPSSCYGNATVSEQCGEEN
HCLGDLSDWICVCDAYGYKVNATNSMTCDKVMCGDTGADAVTVCGKETTSCSGHIAKPVCTC
NETAQYLPSPDSTRCVKASCKENDAARKACVPGNSCSGSYDEFICICDQFSYHQDPADNRKCIRE
KCEDLAADYDYEKICGVGGISCTGYVNQPVCVCNSTESYHPSNDRTSCVKASCSTSDAVRTQCGL
ENSCFGDIESFMCDCKAHGYKLEPINKTSCVLATCEDGDFSYHFQCGKGAVGCSGNINTLSCLCD
SAKGYVPSADMKRCVPNSCKDNPDVYIKCGMENTCLGSYADWFCVCSGAGYEANPADPKSCQL
MECSKDDAIHVCGYGAIDCKGPVLAPTCSCNYSDHYIASEDGTSCVPITCRKNDTARAQCGEENT
CKGHTDNFKCICDAYGFKADNVDHQKCVERQCTDGDADIIRICGHGGIRCTGPIRSAVCECNSTE
YYYPNADRSRCILASCKANETIMKSCGEFNSCEGNMESHICVCNGPGYKHHPSDDKQCVKETCE
MGEIDPIYLCGKGAVKCNGTYDIYTCECDNAMGYVQAPDGNSCKPFDCHANATMHKQCGDG
NRCIGSLTDWICVCDVAWYKPDPSNLMRCHEKTCKEADLELICGTGATGCSGSATNPTCECDSA
NYFVSSTDRRSCIKASCKTNITAVQQCGDGNSCYGTPDNYVCNCDAYGYRLDDNNNQACVEKK
CDDVGANYVEICGLGGIGCKGTVKNPVCECDQTKHYVASPDSTSCVLSNCRGNKTVMAACGNE
NKCFGSASSFICVCDGPGYKRNPSNLQECLPETCEAGEIKPHMVCGRGATACKGTMYHFECECN
NTLGFIRSPGHESCIPVNDCYANATMHEQCGEGNGCQGSVADWICKCDAPGYKPHKTNFQMC
HQRMCSDADIDLVEVCGVGVESCVGPIANPTCNCDTSKHYVLSADSTSCIPGSCKTNETIMAQCG
EGNSCSGTSENFVCICNGYGYRPHETDKQTCVLKKCSHSDVNAVDVCGQGALSCIGTMRDPICQ
CNNDMFFIPSDDSRSCVLASCSANSDVAVACGKVNGCFGSKSSWICDCSSPGTMLNPNNIRECLH
ETCESGKLDPIIVCGKGALGCNGTMSNVTCECDNSMGYVQSPNGMSCVASDCKTNSTMHELCG
DGNSCVGSLTDWMCVCDAPGYHPHPSDPMRCLQKMCSHANINPVDVCGIGATKCVGSITEPRC
TCDTAAYYVPSPDGTYCIPGSCQTNKTALEQCGNGNTCTGTADNFVCICNAPGYKLDDSNNKRC
IEISCNDADVDLVEICGQGSIGCIGTARQPVCQCDHSKHFVPSLESTSCQLASCRANDTIMDRCRM
GNTCFGSMTSHICVCEAKGFKVNPSDPTECVQETCEDKDIDALSACGKGATDCMGTLMNLTCQ
CDGASYVESPDKTSCLPVGCQQNVDVKKACGEGNTCEGSYDDWVCLCTNPEYIPHATDEKVCS
KKKCTDNPIGSCGIGATSCSGNVNIHKCTCNLTAGYMPSKDEQSCIPIDCKMNPKMNCGFGNTC

RGSFFSYVCECNTLGTMRNISNPKQCIEVDCSTDAVSICGVGATGCAVTPTNDVNCTCGEFYHPA
HDYHSCVLDDCKFNKLVPDACGVENECTGSISSWMCVCKGPKYIQDIKSTQLCAPGPCRIEQCGS
GKASGSFQYYAESCSGTYFEPTCNCKEGFEKDSYGGCIVKSCSRNNQSSVDCNDGAISCEGTYFKY
KCECRAPTHILNPANKRSCIPARCNPQLCGRGLVNGDYMDFASECSGSYTDFTCKCKYGLYGSGT
SKCSLEKRTCALSNDCHPNANCIDYDGHYFKCECKDGYSGDGYQCHRNGGLTPWSVVQPCDKT
CGTGYAQVMRTCDDPKPDTLGKKCNVPSPDVREVPCHQFACTACSVKKTCECENIRPYFVHEYV
EPDESLKYFTNLTAEDVEKALSAIEKWKLSVDEKEFCIKHTLPEIDGLISDINENLRSVDQTKLYTIT
ARDMMRNKLDCNVDLEKKDKYFRVYEAIVKRHFILKQVVSDLQAKEKLMVTYRSSCTKDSKVG
WLSSIFS*

>TRINITY_DN991_c0_g1_i2.p1 type:complete len:3229 gc:universal TRINITY_DN991_c0_g1_i2:278-9964(+)
MKKKMKVGDLLYSKTVMFLMLLFVISVSSEDLLDRNQNNAYFAKYYECPVGWVLRCEKCYLLVE
TAKTWTDARYYCKKHGGELVTVKDDSTNFFLARYVQQTNKKIHNVYIGLHDREKEGDFKWSDG
SEAIEKEFRWLPGEPHKSLTNENCVEMFTRSNQYLWNDAPCSDELSFICQMPACVQGSNRCDNG
KCVSKQAICDGVDDCGDSSDEKNCTKCGGYLNGMTGTFQSPNYPDKYGNNRRCSWVIEVPTSH
KIQLAFKDFGIETGFDYVEIFDGKTLAAKSFGRFNGDENPELIMSTSNQIRVTLVSDMTTSFKGFLA
TFKAIPDKCEFDLVIPKNGYEYIKSPMYPDNYPDNKKCIWNIRSEDPFGTLTMMFVELDTEENNDF
IEVRDGKGVKDLLLGRFSGENDVKFMSPIFSTAKHMFVKFQSNYMTSKKGFRAIIRSGCYAEINAS
QGMITSPGFPYQHYPKLIQCTWKINEPDNKAISFMFHRFDTELNLDVLYIKDKSGKIVGTYSGVLS
NNALQVQWILETSQSSLEVTFTSDVTYTMSGFNVTFSKGCPSISVLGGKVKGKSHYGGKISYECDE
GYHLRNGKTRTCLYSGKWSDPMPYCAKINCGHPGVVVDGGFQTYPKKDNFDVNTRLVYYCDA
GYRLIGNNERVCQNDSTWSGSQPECKLSFCDTLTTPKNGKIVGDDFTVGQSVTIKCNPGFNIVGV
TTLTCGKDGKWNADEPVCEAVTCPIIKPSDNMEVLNLPNNTDRFPYGAMFDIKCKNGFELNGEN
QIKCVAPGIWSPKLPKCYDVNECLQKSSACKGVGHQCINTIGSFKCECKNGYQKNGANTCQDID
ECKISNATVKCSQKCINQLGTYKCDCEEGYYLYSGDNSTVSRIELRTAVVNHTCYGVRCSVAPIPD
NGFVINYGRRHPVRLIYQCDRGFVIKGNEMVMCQKDGTWNREFPDCVHAGCQNVTVAKSATF
KVNGTKLSDGRYKLGTEVIYTCARGFKITSGNLRQECIQNTTHAFWSGELPVCERKSCPTLTAPVN
GKVSSMNNLFESEATFTCDDGFQLLGDAKLTCTESGKWSGATPVCAASSCPYPDVPKYGEFVGRL
ELGRRVIYSCYKTGYTLVGNKERTCLYKKGTGHYWTGDTPLCKDITPPVFTCTKQINVQLPRSENS
MLVKWDVPTATDSTGKRITVTVSPDIHPPAKLKAGQHNIVYTATDKAGNNATCTVVVNVQDKQI
PRIKCPPNVEIESDFPAKVTWDSGLFSDNVGISRVSFNPSNGSTFQSGKYHRVEMTVEDLQGNRDV
CVTEVYVKGCGCESNKSCNSIAPHNGGISTCHINMGITSCSLTCPKGLSFVQDNFKHDNVMCLPQ
GKWSEKDIPGCGERKSVTSCLNAVLEFEYNTIDTSKVDEDNLATYFNDLFKNFGNMKTTYCDAV
KFIPNEYELKSKNIELLFKMEPKGDILKDALKEVLEGCKTELENRFQSSKQDILNLVSHASVNLQA
KSIIKFDAASCCGDSKYYCCPKGHSVRAKGANVENIVCLPCSPGMFLNASSNHCTSCPKGYFTDT
SGNEACQKCPDNLNTLKHQSTKCTEKCPLGTYSSTGLQPTCRKCDYGEYQNEIGQTMCMVCPSS
TTTASRGSQKLEDCKAPCQLGHYSDTGLKPCVPCEKGTYQNETGRSVCKACPSGGIYAGIEGATK
EEECPSDNACDMKLCKNEAKCVPGVYSYQCLCKPGYKGKNCEIDIDECETKPCKNNGTCVDLV
NDYNCTCIPGLTGKSCETNVNDCKNDTCINAGVCEDLINDYKCWCPLGWEGKRCEKFINKCLSS
PCQHKSKCTPLLNAYKCDCTPGYKGHDCETDINECQSSPCYNNATCVDLLNDFECKCQPGFKGE
LCAIDINECVGSPCFNNATCIDGINGYRCECQPGFAGQNCSINVNECDTKPCLRNSTCIDLINKFK
CECEPGFYGDRCEFEINECEDEPCKNNATCIDQVNAFECLCPLGYKGVRCETEINECDVNPCKFG
TCIDLIGDYRCNCTPGYEGRNCDILIDNCVAKPCENNATCTNQVHDYSCKCLPGYEGKNCSIDTN
ECLPDPCNGNALNCTDIVNGYICYCKPGFRGTNCSEEINNCIPQPCMHNSTCKTIIETNDYQCNC
TAGYEGKQCENETNECLGSPCQNNATCIDKLATYECVCPTGFTGFNCETNIDDCKNVTCQNNG
MCFDKINDYYCRCHATHKGRHCEKKIPDEVDIKLPGGTGSVIIHSNISTVQEFSLSIWVRYLGSNSR
GDFLILANSNGKVMTSHFLKLSHQRVMVEFNGNMDIFSLDKMLNDGLWHHVFVSMSGLSSKIV
VALDSKVIIEETKSYLNGQSFFKPSGYLIIGKNVNRTGFFSPSGFAGEISQLAIWNRVLSPNEMNNLA
SNCSVDLKDSIIPWVGLLDNAGSKVELLERWTCGTNVCPAGFMGRFCEILIDKVAPTVASCPKSKK
VVSLLTSTRVNWPEPRFDDNVAVTEITRSHAPDQVFGWGDYVISYIAKDAFNNSATCVFELYVTPY
DCPDLEEPNHSASATGRWLWGLFFDFQCKPNYELAVPKPPFYSCGKMGTWSHGPPTYMDNPKN

LLPSCTGTARPIVIGTNAFTYSTTSKCDETFKAEFMKQFKRKVLEIHQYFKICGEKCDFANIRVKCT
DEDITTSRVKRDASLNEVKVYFDSKFNESLSNSFNDTQTLQAAIKNDKVDLSFTVGGETITSDKNN
VDIAEPKLACNPGWKFVKDKCVLCPAGTYRDSSMIKCELCEAGSYSSLGSATCVKCPHPKITYGT
GAKSMNECYENCSSGYYFNNGSCSKCQIGFYKSFKGLEECTQCPKGTTTTVIGASRLEDCSVECPL
GHELVGTECQPCMNGYYRSNKNQPECLPCPYGTTTYLVGSASNICYKKCELGFESSNDGKVCMP
CNTGYYREEEIQRKCLKCSGDKITLVYGAKSKNQCKEQTGTKFDVELRFTSLEWNNTLIDEDSAYF
RTTAYNIANKIAKIYENDKELLYIKIINFSEGSVIANMTISFEKEKKDPLEVLRKTVATGKLDGLSVDP
DSFKTDRCEFFDCPKDRVCWYNSTGRGCKCKPGFYETSDGKCFAGCEPDYCLNGGACVESDSGR
NCRCSKEYSGERCEKEEKDFSLALIIGLVVVAILILLLLCYCLRRRKRSGNVSLYKYWKSEDKEHGLS
SIPNTPIVQPVKGDNGDIAYAAENKLVEKDDEEQSESSDRK*
>TRINITY_DN1936_c0_g1_i3.p1 type:internal len:1070 gc:universal
TRINITY_DN1936_c0_g1_i3:3208-2(-)
GEYKPTLGRCLSFWYHMYGSHVGELNVLVLQNKSRSAPIWNLKGNQGNVWRPSRVTIKSAVNH
NIIFEGRTGDGYMGDIAIDDVMMSEGPCTDYGNCDFERDLCGYTNKKNIDQFDWQLGSGGTSSV
TTGPSNDHSTQTALGQYMFVESSYPRLPHETAIIGSETIPVSGSGKCLQFWYHMYGEHIGSLEVNM
RVANDLSETIVWKLEGNQGNKWLQGQAPLLSRGQNMEVRFEGIRGTGYKGDIAIDDITVVDYSG
ACAITPKSAKPWGCNFELDFCKWQQSRADKFDWLRNQGATGSAGTGPKADHTTNTTQGYYAY
VETSFPRQPNDTAAIISPTIKGNGTKQTYCMWFAYHMYGPHVDTLKVYTRSQGSDKPVLWRRRGS
QGLNWNHAEVQVTVLYDTKVIIEAVRGKDYKGDIAIDDIDIWFGDCFSSGICTFEGADLCFWYVP
KNGQLYWQRASGATPSVKTGPKNDHTYGTSQGHYMYIETSNAPAKATAYMYSPWYYNTGQKC
VQFFYHMYGQQIGQLEVYIRYSNSRYLYRQFIRSGNQNNTWHMGQVTVKRNNVYFQIIVRGTKG
NGYQGDIAVDDFRIRDGACPAPGDCDFEHADFCSWMQEDNDDFDWIIGSGSTNSFLTGPSIDHT
TQSNGGYYAFIEASSPRQPGHKARFKSMVFAPTTGSGRCFSFWYHMRGYSIGNLTIYLRKVSNTKT
LSEEVMWLLGGNQGKAWNQGRFTLKSSTLHEVVIEGVRGSSYDGDIGIDDIEFTSGSCSFLPKGA
WPSGRSTPAPTQASTTLGPTIGNKGFDCNFDKEFCKTWMRNSTRTNFQWKRHQGSTSSVGTGPH
HDHTTNTDQGYYVYIESSSPAQPNDTAYLIGKDIPNNNGFCLSYWYHMYGPHVGSFRVYVVNKT
NAEARFVRVGTQGDKWKHGQLYISPKAGNPITIRFEGVRGSSFMGDIALDDISVRYGNQCPAPRE
CTFEHDNNGGSSSTCGWTQEKQKDSFDWSIGSGDTPSVLTGPNFDHTLETKQGHYFYIETSYPRK
FGENATIISQSYQQTLGKCFSFWYHMYGSTIGNLTLFLRRD
>TRINITY_DN3034_c0_g1_i1.p1 type:complete len:1941 gc:universal
TRINITY_DN3034_c0_g1_i1:6289-467(-)
MAEEEDPMKYLQVDPAQALKDQSKTFDAKKWVWVPVIGEDGYAAAQVIKVDGDNVTVETNK
GHEMEVKKEKCDQMNPPKYEKCSDMASLTYLNEASVLYNLKARYTTGLIYTYSGLFCVAVNPYR
RLPIYSDQVVKLYRGKRRAEMPPHIFAIVDTAYQDMLTDHDNQSMLITGESGAGKTENTKKVIQYI
AKVAGVEKKKSDDGPVKRDLDEQVVEANPLLEAFGNAKTTRNNNSSRFGKFIRCHFSQTGKLAG
ADIESYLLEKNRVIHQASAERNYHIFYQLLYATTDEELQKLCLVTRRSEEFEFLKKGVPSVDAIDDN
EEFGYTTNAIEVLGFDEEEKQSMFKICAAILNFGNMKFKQKPRDEQAEVADPADGERVAYLLGLPI
GEFHKSLTKPKVKVGTEFVNKGQNVSQVLYSVAALSKALYERMFWWIVSRVNKALDTKERRSYFI
GVLDIAGFEIFEYNSFEQLCINLTNEKLQQFFNHHMFVLEQEEYKKEGIHWEFIDFGMDLEETIDLI
EKPMGVFAILEEECIVPKASDQTYLQKLHKQHAGKNGSYEKPTPKQTKSGGGDFILHHYAGSVGY
SVANWLEKNKDPINAHTAELFSKATVGLVSGLFADYNPDVTGGRRKGSAFQTVSFRHKEQLKNL
MTTLMSTSPHFVRCIIPNEKKTPGLVEGTLILHQLRCNGVLEGIRICRKGFPSRMLFQDFRQRYQIL
AASAIPSGFIDGKVAAEKLIDSLQLDQSEYRVGTTKVFFRAGIIGELEDMRDERLAKIITQFQAYCK
GHLARIEYKKMCEKRIGLAVIQRNVRKFLFLRNWQWWKLYCKVQPLLSIARAEEEMKEKEEELK
KALEKAKEDEEQRKKMEEQLTENIAEKEKLFADLQAETDRLINLEDKLMQIQQQKDRLEANLNE
TLEKLDGEEHSVSVLQEKMHDAEDRIDSLEAEASELKSNCSRLEVERANRDKQIDALNEDINKQD
ESIAKLGKEKKGLEDNIQELNETLQASEDKCSSLTKAKKKVEDALKESENSLSKEKDSKARLDKEK
RRVEGELKETNDKLSEAENELKNTQDTVYKREKAIKELEEARDNQDNQIKQLQKKIQELLQRIEE
LEEELEAERKQKQKSELSRKELESQIEELQDQLETAGGATHAQVEVSKKREQECSRLRKEIEDINA
ANDEALASTKHKFNAQLSEAQEELESVKKAKSKSDKERSQLAGDLEDVQGQLDQVKKQRASSD
KNNRMLEEQLAELRGKVSSLETDMGDSESRNLKFQNESATLLKQLEEAEHKLGLANKQNKQLQ
ASLGESKNVADDEARGKHDMQNKLQNALAQIEELQEQLEDEQTAKMDLQNKYSRANADAQQ

WKNKYDMEGASRVEELEDAKRKLAARVQEMEEALAAAESKAASMEKVKNRMNEEVEDLLLDL
EKAQAQAANLEKKQKKVDQQINEWKLKCDEVQEELEKSQRDNRSYSTELLKMRTANEDTVEKY
ESLKKENRALAAELQSVSEQLSEGGKNSLEVEKLKRKLGLENEELEVALEEAELALEQEEAKFLKI
QLELTQLKQSTDRRSAEKDEELETLRKNHARQLEALQNTIDAEQKGKAEAQKLRKKLEGDLADL
ENRLEGSDRNSADYLKTIKKLQAQIKELQAMIDDESRGRDDMRDTASRSEKRAMDLAVQLDEAR
VALEQSDRLRKLAENEKLENAERVAELQTMYNSIANAKRKAEGDYHSLQEEIEELENEAKMAEE
KANRAMNECARLSSELNGAQENASNAEKSKSLMARQITELQQQLEEAESMGGKSLKNQIRKLE
QKVMEVEGELDNEARKSTESIKIARKADKRVKELQFQLEDEHKAVERAQEVSDKLNQKMKKMR
LQLEESEQQSSSWQSKFKKAVVDLEEAEERCESAEQALQKARQRARSGASGMTTRGVSRARSRVR
SPQNDD*
>TRINITY_DN1936_c0_g2_i1.p1 type:3prime_partial len:1894 gc:universal TRINITY_DN1936_c0_g2_i1:5680-2(-)
MLSPAVTKVFIVLSVLGGLICQSCDFEVANACQWTQYAKDKFDWTRNFGNTGTGGTGPKTDHTS
EVKILKANTTGGNSNGAPCVFPFKFRKRTYTKCVRSMQRGKSWCATVPDMNKKRQWGYCMPT
GYYMYIEASSKRGNPRNKGDNAVLLSPVISKGPKCLQFWYHMYGPHIDTLNVYKKVSNNMTKL
WSLTGTQGNRWISANVTFNGGSQFQMAIEAIRGVEYKGDIAIDDITVDDGSCNADNGQKGNVTS
YGKCTFEKDKCGFKDGNTSTLTWTRHKGKTPSKGTGPLVDHTFGTGLAYYMYVEASNNPMQSA
RLYTTYTNLDSTGSCLSFWYHMFGPRTGELKVYINPPEIGKEIPSFSVGGEQGNLWIRGEVTVNTTT
ALVVFEGTTKYSYLGDLAIDDVSMERGKCEDPDSCSFQKDLCAWYNLPKSSGLDDFDWIVGAGV
TPSRFTGPTSDHNGDIAGKYLFMESSSPVKMGDKTWLSSELFNSTGKGKCFVFFYHMYGRDVGSL
NIYVNTSSGVELVWTQSRSKGKQWQNGQVDVERPGTYKVLVEGVVGKGYLGDIAIDDFKFKPGK
CQIVPSNASPKKPQTTMRPTTKRPTQPPTPILDCDFEFGVCSDWRQDYKNDTDNWKVGKGTTST
AGTGPSFDHTKGTIYGKYIYLEASYGRLNETARLISTSIPATGDTGGKCLTFWYHMYGPHINRLNIY
VMIGGHTGKPIWSRVGTQGNKWNKGQVTLVGRQPFQAVIEAVKGAKYQGDIAVDDVSLSSKKC
PQPGATTCTFENSQCGYIDDTSSDVQFQWTWQSQATSSLNTGPKFDHTLQSVSGFYMYTEASNA
KQGDKARLISPVNPGTSGSCLKFWYHMYGAGMGTLSVRLLVNNKLQSVPLWSETGNKGNEWK
VATTTIKTKDAYQVVFQGVIGTTYASDMAIDDVKLSDGECQSPGFCNFEDGLCTYANSKTDDFDW
LVGSSNTPSSFTGPKVDHTFGNASGSYLFLEVSKPIKAGDVAVLESQQFNAVGGKTRCLNFWYHM
YGSDIGTLQVIYKVFSGSQQERVLWKLTGQQQITEKDPWKYARVPVDMDADHMVMFKGIAGSG
YAGDIAIDDIQFTTLACRVEPSIANPTPTTPKPPTTFPKTAPPTAAPTPFSCNFEKDFCSLTSDSTAVF
NWTRSQGYTASQNTGPSTDHTYGRGFVKTYGPGSIRHAAGMCIHISMGGHTKPKDETALVLYNG
CGQDRTEFKYDNGLIKSTQYNMCFKLKGASTSAGTKVVISDTCTNKWKMLANGAFQDAKTNLC
LAPSGKLAYPQNNSPLIVSKDCKAKGVIFTFSPTMGFYALIEASAPQKPNDTARLISPVLNKPKCLS
FWYHMYGANINQLNVYTKSPNGVYGNPVWSKFGSHGDKWRPAHVYMNPKSSSYAYAIEGVRG
NGYEGDIAIDDVSVSDKCPAPVECTFEETNPVACGWTNLKTDDFDWSIGQGATSSQNTGPSNDH
TYNDQRGKYLYIETSSNLKAGAKALIQSPVYRGNGRQQGQCFQFWYHMYGASIGRLSLHLDQVG
STTKNRAWIQESDQGNRWSIGQITIKSQAAFQLTFEGIRGKSFTGDIALDDFRMFDGPCNNIATCD
FENEKCTWKNSYHKDDFDWIIGQGTTLSLNTGPVTDHSTNSGDGHYIFIDASRKKQGAKARLYSE
IIPRGFFTFIPYCIYFWYHMSGPDIGTLNLIAKHGKGKALEKVIWSLSGNQGSKWLQGTAPVTSSVD
FHLVFEAIVGDGYKGDITLDDIVYKLGLCSISPKAADPKLISTTSAPTPIPTTAVPAMYDCNFEKNSL
CQWKQDLTDQFNWTLVQGQTPTLGSGPSVDHTTGNKTGYYLFIETSNPRKAGDKARILSPSMDS
DKDHGHPHCLKFWFHMYGPHV
>TRINITY_DN161_c0_g1_i2.p1 type:5prime_partial len:399 gc:universal TRINITY_DN161_c0_g1_i2:1683-487(-)
WNQEPLTCTYLYSVNSQLLKYKMADDDVAALVVDNGSGMCKAGFAGDDAPRAVFPSIVGRPRH
QGVMVGMGQKDSYVGDEAQSKRGILTLKYPIEHGIVTNWDDMEKIWHHTFYNELRVAPEEHPV
LLTEAPLNPKANREKMTQIMFETFNSPAMYVAIQAVLSLYASGRTTGIVLDSGDGVSHTVPIYEGY
ALPHAIIRLDLAGRDLTDYLMKILTERGYSFTTTAEREIVRDIKEKLCYVALDFEQEMQTAASSSLE
KSYELPDGQVITIGNERFRCPETLFQPSFIGMESAGVHETTYNSIMKCDVDIRKDLYANTVLSGGTT
MFPGIADRMQKEISALAPPTMKIKIIAPPERKYSVWIGGSILASLSTFQQMWISKQEYDESGPSIVHR
KCF*

>TRINITY_DN691_c0_g1_i1.p1 type:complete len:1726 gc:universal TRINITY_DN691_c0_g1_i1:5662-485(-)
MMNLFRLIVLGVICFSYEGDAQQCGGSCSPCGNLCVGMKGDRGTAGLPGFRGPPGLPGFPGPEG
PIGQKGEKGPAGLNGMEGSQGARGKVGPPGLPGTPGRHGRPGNEGREGEAGLPGCNGTKGDR
GPEGPRGRDGIEGPQGLQGNVGPRGEPGDSTATKLKGQKGEPGSAGQQGEKGGRGMEGMKGD
TGKPGRNGRAGERGPKGEPGTSNVTIIGEKGSKGSVGPPGLPGRQCQVTGDKASNIAQGQKGNQ
GSKGEEGAKGEKGDAGLAGPQGEEGDIGPQGDRGDDGPQGPPGPEGEQGRSGIQGNKGQKGE
VGNNGKDGNDGQTGLTGQRGRQGTPGQKGEPGKDKKGLRGPEGAKGERGNDGEKGSDGPIGP
IGPRGPKGELGPLGMKGFKGSIGSAGPKGENGNPGDPGIKGRRGELGIKGDSGVKGAPGNPGISV
TGPDGMDGDTGPRGQSGGKGAVGFPGKPGPPGVVTIDGEEVLPGEPGPQGLKGFRGVQGDAGS
KGSRGEKGNACESSKCPAGPKGEKGLSGQDGLQGGDGEEGEKGEKGDKGDIGSRGDTGSRGEP
GPQGESGEKGEKGIQGPQGNSGKDIIVQGERGDKGTKGNAGTPGSIGFPGERGSPGESGDRGLSG
VNGLRGETGDRGARGVAGKPGVNGQKGQKGEIGSGARGFKGDKGEVGPDGPAGLRGPGGPVG
PKGDPFPTAVNGKTGEPGEQGAAGPKGAQGPVGPEGPRGFPGDIGPVGEPGDGEKGERGDDGA
RGERGEKGEPGQDGKDGDPGPKGEPGQEGKEGPPGMFKGNKGEKGATGLPGVDGLPGNSAPK
GEAGKDGQDGDPGPAGEKGDPGEAGSPGTEGPIGEDGPVGDKGEPGESRAGPKGNEGRAGAK
GENGRDGRAGRPGIGRPGDKGELGQKGEAVIGAKGSKGEQGLEGPPGPIGKPGVKGDAGVPGK
DGEAGGRGKDGERGLPGDKGDTGDIGFNGSPGAPGDKGEPGYSGQKGAKGMSGPRGPPGSTSN
VVGPKGNRGLPGMKGEPGQQGEKGLIGPQGSKGESGFGSEKGIKGSRGNPGAVGPSGEKGERGD
RGKDGLQGIAGPRGEEGNPGEKGSRGDQGPQGREGPSGELGPEGQKGDPGFAGEKGEKGDRGG
DGQKGDAGEPLRGEPGVAGPPGPTGPVGPPGKDYVFSGTAGELKGMKITKGDVGPDGVKGEKG
DRGDKGLDGRRGPTGPQGERGPKGEEGNMGPKGSQGRDGLTGEDGPKGSKGEPGLDGDVGEK
GEAGFQGLQGEKGNQGPQGLEGSTGMDGQKGQIGEKGVEGPPGPPCEPVNSVRGETGEEGPKG
DRGPKGQKGKPGPQSQAATPGEKGSKGEVGQPGPQGIKGEPGREGDFGPKGSPGAQGEKGDQG
QKGEPGQEGPEGLKGEPTRAFGPKGQKGETGLEGLRGPVGPKGEDGQTGPQGPDGPKGNEGEK
GDFGINGLNGAAGPSGPKGAIGLKGDSGPPGPTGAQGPRGSGGSPDFGFYYVKHSQTTIVPSCPA
GMKTLWTGYSFLYAQGNERAYGQDLGQPGSCLKRFSTMPYLFCDLQNQCTVASRNDYSFWLSTD
EPANMMKPAAGPEVEPYISRCAVCEAPANILAVHSQSVIVPDCPNGWENIWAGYSFLMYNSAGA
DGSGQLLASPGSCLEEFRANPFIECHGRGTCWYYGPTLSFWLSTIDDSNQFQIPKSETLKAGNLKT
RVSRCSVCMKTVRNGNFP*
>TRINITY_DN24268_c0_g1_i1.p1 type:3prime_partial len:2183 gc:universal TRINITY_DN24268_c0_g1_i1:122-6667(+)
MILLWCILLCLLNFTTVTCNDSNYNTGPNYNCKEDYDVSLRCQFDLGNSCVNNNGSDNSTLYKC
VCSHYGYEEKANNSLSTCQPISCTNINSVYPELIVRHCGLGVTECEGELVNRECTCNSSIHESSLDR
RYCFHASCKTNPQSAVKCGFQNQCYGTYNQYICACNAFGFIRKDNSNSECVLAKCEDNSLDPFI
ACGKGALSCNGTLVTLQCECDTARGFVASASGSSCIPPTCSISPTYLADCGEGNGCVGGYGAGNF
SCICSNPMYKRDPTDNRKCVKRTCDETPETPKMVCGEGALSCTGTVDAPICACNKSDNFVVTPD
ASGCVKATCSSNPAALAKCGEGNSCAGSYDEYICICGAFGYKYHATDGSCQETLCTENLEKACGI
GASSCIGNTTTRKCICNEADYYFQSEDQRSCVKAPCRTNDTIKEICGIENTCLGSEDSFICQCNIGFL
LDRTTNQACYLGTCENGDLDPVTLCGSGAMYCNGTMRNYTCGCDRNDGYVRSPDLTSCIPSDC
RGNSTMHEKCGVLNSCVGSLDDWMCDCHAYGYRGNPSNMQTCDKVMCGDPGAENVTICGLG
VTACDGHILNPVCKCDTTKNYIPSHDGTRCAPATCKNNKAALKACGIENSCSGSYDNYMCICNA
FGYRLDGQNNQCVEITCDDSDLDIRAVCGDGGRACNGTAKRPVCFCNETEHFVPSEDSRSCVKE
SCKASNEVSMKCGPENSCIGSKESFICVCNFEMGYRLNTATNQECYLGTCENGELDHLKHCGKG
SLSCKGNARNLTCECDKANGWVSSPNGMTCLPNDCRGNLTMHNECGKMNQCVGSIDDWRCI
CDGFGYETDPADIRKCRKKMCSDPDVDLLSLCGMGATGCTGPLINHRCLCNATEDFMTTLDGSK
CYNPKVTSCKSNATAIEQCGVGNSCAGPANDFICICDSHGYYPDMNNYQRCLLRKCDDPDADY
VEICGEGGIGCTGTMFNPVCKCNSAAWFYPVADRTSCVFASCRANQTVNTLCGLENECAGSMES
HVCICRDQGYKEHPSNSQVCLEKTCEENDFDLITVCGKGALSCSGTLKNLTCNCDTGNGYSQTSL
GNYCVPSDCQGNATTYDQCGPGNRCVGSLTDWRCLCDAPGYEPHPTDIAKCHQRSCGDATVD
HVMVCGIGATGCTGPILSPKCECNTTEYFAPTVDGTACIKASCKTNKTALEQCGLGNGCSGSHDN
FVCICDAYGYRLDDTNNQACVKSKCDDAGADYNKICGQGGIGCLGTVLNPVCECDFNKHFVPS

PDRTSCLLSDCRGNATALEACGKANTCFGSASSYICVCDYLGYKTNPANMQECLLQHCENGELD
PYNACGKGAVSCNGTIESLHCNCNYSMGYVASPDYSSCIPADCRANSTMYKQCGNGNGCVGSLT
DWMCVCDSYGYTPHASDMKKCHQQMCSDSGIDPVAVCGMGATSCMGAVSNPTCKCNTTSYYV
PSADGASCVPASCKTNKTALEQCGLENSCTGSATDFICICNAPNYRLDDNDNQKCVPKTCNDTN
RDLVEICGYGATDCKGTAMNPVCECNTTEYFVPSPDRRTCVTASCRTNAAVSIACGRQNSCFGSA
SSWICDCVAYGYMLNPNNVQECTLGTCESGYLDPLYVCGKGAVSCKGIWNNYTCECDNDKGYV
SSPDGSSCIQSSDCRANSTTYDQCGDANTCIGGMNDWVCVCDAPGYLPHPNDRQQCVQRMCS
DSDIDFVEVCGIGATGCTGSINSPVCQCNTSKYFTPSADGFSCIPASCKSNKTALQQCGEENTCHGS
ADNFVCVCNAFGYRLDDSNNQACVEISCMDPGVNYKEICGMGSTGCKGTARSPICECNSTQYFI
PSHDSKSCLLASCRANSNFTNSCGDGNSCIGSLTSHICVCDAAGFKLNPNNYRECIEETCEDDAIN
HINVCGKGAIGCKGTLNNITCHCDNDAGYYQSPDSKRCLSYACQQNGAAQDACGDGNKCVGS
YHDWFCECSAAGYEPHPTNSSICIKKKCMNDPINYCGLGALNCSGNTLNPICTCDSSAGYMAAG
NKHSCVPMDCTFNPNIDCGIGNTCKGSYFSYACVCSTPGTIRNMSNPMQCIDVTCPRDAVMACG
EGATSCEVTSTNHVICECGKGYHPGPYKNTCVLDDCRYNDMATKACGLENMCVGSYKSWRCIC
QAPRYMQD
>TRINITY_DN2577_c1_g1_i2.p1 type:5prime_partial len:1087 gc:universal TRINITY_DN2577_c1_g1_i2:3532-272(-)
VTRKLYKEEEEEEGEGEEEEGEEGEKDGKEKNGKDGDKSKKKIVDGKKSSRVVKKASKTSNKKIV
QKGRTLKKKNQVKGEAKQKLAKGRALISKRSDGEEEGGEGEAEEEEEGGDFSLSHLSQVDLVRG
LQVSAEFALPGECKKDKLCEWMKTKLGQDTKLTIEGTFSRSGFTLAAGITTSLELAKDLSLTEAKL
ELEFTKDERSVALSCTLKYNNKELSGKEGLEFIGTLKVSFSGELEISMQMKGMVYEPFGVEKLAFG
NIFLSLGVTIAGGLPSFQIGAKIMIGKGVKNKGGEGVFIFEGYFGFNPADPTDNYIYAKRSGDPLTIG
NFIKNFEIEATVPKKIANSGFYGETIISFSAAGKKVDELDLEIEAGFYFKGALKILDFDIYAELKVNPD
PENRALFANITLSPLNWVMGLLRISRSKDDDKNGPFLIVDIDSEKTAVQFEGFGYCLGIGAEIAVVV
NETHFYFHFAGDLFTVLHASLTVESAYGGDSISDMSFSFKGCVGLDLRKIADIVFKTMQDASKAVS
LALGKARKKVENARGAVTAAFDKVRKWRQGIESYKARLRARSAELERKKKELNYSCRDRCGKV
CVGFLDWNPYCWKVWNTWHGCPKWNPCRWKVDDIICIAGCELKKGWNKFVTWAEQVGIQIM
LGLSEVAQFLLKGVEAFEKVAQGVLTFAVGVLNVVESFAKGVLAGVGAIVKAASNVIVIDRFCLSG
NLKPVESACVGIDAKFSIFKVPIEWKGEFCIDLNFFQIIGKKTAVKHTPAIAEIDSKVQEFESQGNEI
DSKVGDMKDEESKQGNEVETETEKPYDINRRKRSHVPTAEEVYFHKLAYDRLPKVNTRSFETETS
MKDGAPWILSKTWKPSKRDIVKEESTLEKRILKTDSPDHCKNIENLYSNYQNIAEGFGTFLKNAD
KQKAGFQAMKRSYIDGMNYLKDEFNYGDKRQNVTDEEREHVKYWEEYVNKGLTSHIEDTESLL
KAQRKTYIPSFRKQVNQLFMEQTKSTLPTYINKMKNTVIKSYHKRSDIPKEHMERQLRKWNNLH
EALGEIASSETASYRDLSQHVASVNYLLTNLRRSDIPCAA*
>TRINITY_DN77_c1_g1_i1.p1 type:3prime_partial len:1965 gc:universal TRINITY_DN77_c1_g1_i1:2-5893(+)
MGVQVYSLGIGSNYDIGELLDIASDDASVFRSQDVDELVSIVASITEQTCKGCTRAVDIHFALDASQ
NVGRENFQVIKNFIKAIGFKFIISETGSHMSASIFGEYGTLIFSMDQATQQEDFINEVDNVPYIGDFN
GNIDKALDMVSEDIFTFDGFSRQGVPKVLVVLTASNCDSCEKSVEEAVKPLKAQGVHVVAIPIGSN
ANMEEMDKMASYPTQDYVIPRASYEELLNGVFIQKVSEMVCSGRPGVCEEPPIEGECTEIINECTL
DVNCPANRKCCIKDCKASCEVPAAACITPIDLAFAFETTGFIEENFDEVKSFASSLLDHFRVEPAKT
HISISTYGESVNAKTAFYESYDKSALKQFIKDLEPDGGRQANIGDFLKTANDEIFNITNGIRQSSPR
MLVLFTNGVYSNDQIAKAQEESLKLKAEGQDVNILVINIGSIVEGNSVLRDVVSTPDFRNLHNVT
DLRRLVNFGFENKIAEAICASKVRKTRCKPRTPQACSVRDDLCQTDFDCPGYQECYSDGCRLRCQ
TCENRCGDKVDLLFMIDTSSRIGPEGFKNAKENIKKMLEWFTIEANNTNVAAITYSDNANYAMQ
LPAAGSNDPPQTLFDVQGRLDSIPYSGGSKSNLDKALKMAAAQVFYQNSLRLRKDSKKAIVIVTD
GVDDSTSRLEIASWPLRKQRVVVDGNDYAAIRLLAVTVGNNYNLDTLEKVVSPPIDKNIFLAADF
DDMYNGIRQLAEDSCKYTRQDVEPPVIAVKGDPGPKGEKGRQGNPGTNGTDGMDGIEGPKGER
GDDGPKGEKGDTGIAGPPGGDQGDLKGPDPMAEAVGIIEGTAVKGTRGVTYDLRDGPIGPPGPIG
GVGKTGLPGDSGEQGQKGRGGPPGLRGVFGGQGPKGNPGEDASPGLPGEPGPKGISGKKGPTGS
PGMPGPQGPFGYKGEQGIIGQPGDPGDGGKKGPKGYAGEKGDQGDIGDAGFPGPDGKVGPIGP
PGQRGLRGPEGSSGPIGKTGDIGNKGLPGPRGSRGLKGQSGNAGPRGGKGRTGDVGLVGEKGTQ

GSIGEAGQEGQPGVPGKEGPPGYPGPIGTPGPMGSRGDIGPQGEEGPKGSLGLKGNRGEAGRRG
LTGEKGETGDIGELGPKGAQGQQGKQGKTGKIGPRGQQGPLGFQGLQGQTGDPGQNGRQGPK
GNLGPTGEKGADGPKGSLGSAGLTGPKGLVGEPGNVGRPGLPGRDGKNGPQGANGKDGDPGA
SGPQGLQGSPGSMGTPGSTGDPGSPGDRGATGAVGDKGTRGDSGVRGEPGAFGQQGPPGLRGE
RGEKGPQGTNGVQGSPGPRGTPGLEGLDGIVGRKGTAGRPGNPGQSGEQGAVGSIGVPGPQGIS
GTPGERGDGGSDGGPGEQGPPGLQGSSGSPGRDGMPGRPGQQGTTGGKGKTGLRGPKGANGQ
NGVQGARGPDGLPGAPGLVGNRGTPGPNGIPGLDGNPGPAGKQGKQGKMGTPGMTGPAGLR
GPVGSPGLRGQKGPTGDRGDSGETGPIGVAGDIGPRGFTGELGLRGAIGEMGQIGETGPQGMPGS
QGTPGAAGLTGPAGPSGLPGKDGNKGPIGDPGNAGNPGRDGGPGSPGKDGTPGEPGTPGSQGK
QGLKGPFGPQGAPGEQGQRGLTGRQGSGGKDGPKGFPGNDGSPGISGKRGPRGKDGTPGSPGPI
GKDGRPGKPGINGTDGDPGNNGPKGDPGERGDSGDSGSLGASGDKGKTGSEGPAGFVGPRGSN
GKAGVPGKRGKRGDQGRDGPRGNSGPDGIDGSKGFKGNMGRFGIQGPPGDAGLKGALGPDGT
VGHTGPQGNTGDPGPDGPEGPSGSKGPAGNAGQIGNAGYPGSPGDGGIPGTAGLPGPPGPPGPP
GDMSGAFSGNYWDFVNAGGLQKGPRYYGGRSKRSLDQQDNSIEKVFKSNYETFKNFDTVWNQI
VDDLVIQKTLGSSKNPALTCGDLFKTNPITKSGDYWID
>TRINITY_DN28257_c0_g1_i1.p1 type:3prime_partial len:1462 gc:universal TRINITY_DN28257_c0_g1_i1:4384-2(-)
MQNRSLCLLLLISLIVNVYGAFSEWSEWGACPRVCGISQKIRTRTCTRGGFCTGNLQEATHCCNDI
CMESSIQNDISNPSSSYSCHFDRKVTAQCQFDEGNTCIGESGFKGHHCICSAEGFEVTGTKCNDSC
QAVKCEDLTGPNLYKLHQHCGRHALSCTGTLPTRLCNCDTHSYLSSDGRFCYEATCQSTQEYRTR
CGVENKCVGTQDSFVCECDGAGYTTNVTDNSCVLMTCESGDLDPQSTCGFGATACTGNVLNLN
CTCGDGYVQAEDGQLCVAAPCTTNTTMQNNCGEENTCEGGLVDWKCICNTRGYTTDPTDVQT
CKKIMCEDNGFDSKTVCGPGSTSCVGHIVNPRCICDTTQNYVANINGTRCIKASCNADPSSRNKC
GAANSCFGTYGDFICICSAPGYKLDEGTGECVQKECSEAGIATFLCGVGASGCIGNQSNPLCVCK
ADEGYYPDSDGKTCVLAPCKSNPLYASQCGKENSCYGNKESFKCICDAPGFKEDPTNMKKCVLK
TCESGDLDLDFACGRGALSCKGTMKNVDCKCDNANGYVRAPEGNRCLKNDCYANVTSRELCG
TPNGCVGSIDDWMCNCNAYGYKPNTLDRMKCDKVMCGDPGVDHFTICGGGSTNCSGHITNPT
CVCNTTSNFVTSSDGFLQCVSVDCKTNPKAINDCGVENSCSGTYDNYVCICDAYGYRLDGINQA
CVKIKCDDPDVDYEKVCGVGGIGCNGTMESPSCICNASESFITTSDRRSCLKASCRGSEEAKSQCG
EANTCFGNADSFVCSCTGVGYKRDEMNLQSCSLETCEANELDHTIHCGKGSLGCTGNILNLTCIC
DNENGYVQSADGRKCVANDCKGNATTYEQCGDGNSCMGSLSEWFCVCIVPGYAPDENDPMKC
AKKMCSNSIAVDVCGPGATGCSGPIMRPKCECDKTMFYTPSRDGRQCLKESCEQNYASILQCGSS
NKCVGSADDFFCVCDGYGYKSNGTRDQLCIEKTCNDADADFVDICGTGGIGCTGTVLNPFCKC
NETEYYVPNRRSNHCVLASCRVNETILELCGRGNTCVGSLESHICQCDKPGYKYHDTNSQICLQE
TCEDGDLNPLRVCGKGAISCQGTLLNLTCECNNDMGYVQSPDATSCIAADCRSNASVPKQCGEL
NRCQGSLTNWVCLCDAPGYVADKSDFQKCHQKTCADPGVDPEKICGIGATGCTGPVATPICECN
TSAYYVPSADRYSCIPASCKSNKTAIKQCGEMNSCTGTSDNFICVCDGFGYELDAKDNQKCIKKTC
SDADADYIEICGQGGTKCQGSVGNHQCECDTKKYFVPSPNRKSCVFSNCRGNDTALRACGKGN
MCFGSTSSFICVCDAPGYKTNPSNLQECMKQTCEAGELNHMQVCGKGATGCKGTLDDLKCMC
DNNLGYAVSPDQTSCIPADCHANSTMVEECGQPNQCIGSLTDWRCVCNAPGY

>TRINITY_DN4815_c0_g1_i2.p1 type:complete len:638 gc:universal TRINITY_DN4815_c0_g1_i2:79-1992(+)
MVSSLLLVLICTVVYTAGISAFFQCDFEKVDICGFKSSSSNYTDTGEAPNWRRHTGTTPSAKDQNN
AHVTGPLEAQSGDYYMYFEASNLPSGASSTGTLTSRLFDATQFGVTDIVFHVSSKGESLSLLVLDFI
DYTTNDIFDEKVVFPRNKAAWQKYTFNLDEKIFKRKFYMQFIGYVREVNGKKDNGDWAIDNLRI
DGIAIVEKCENVKCNVNTNCKDTSNGPNCECLPGYSTKAGTSGKNTECIETCQAFTCNLNAECS
NTENGPDCKCESGYKAINGTSGKTTRCEETCQAFTCNLNAECSNTENGPDCKCESGYKAINGTS
GKTTRCEDDTCSNFQCSNNTNCENTVSGPNCTCKEGYRPVNGTRGRDTICEDINECAIKNICGGL
KVCRNVPGSHHCDCPPNYTFFAAENKCYRNGAFSQWRNVGSCSKTCGAGDQKQTRSCSNPSRE
APHGRDCPNEPTERAVACNSGPCLAGCPRNKTCTCENVRQYKTGEEPKKDFSSFNGLSITDGETS

RRYVHTYNREFIEKTIKDVNCNNNQLKSVYKNITDIIGILEGINKKYSSTKMEMQDIIDCNANIDIE
KNLWDVYDVLAEHSICNYQVQKDLEMKADRIQAEITRCNNRGWYSTILDAII*
>TRINITY_DN3795_c0_g1_i1.p1 type:5prime_partial len:867 gc:universal TRINITY_DN3795_c0_g1_i1:2719-119(-)
LEFIGKLKASFTGELEISMQMVGMIYEPFGVEKVAFGNLFMSLGVTIAAGLPSFQIGAKIMVGKGV
ANKGSSEADMFIFEGYFGFNPADPTDNYIYAKRSGDPLTLGNFIKNFEIDATLPQKIADSGFYGETIF
SFSAAGKEVEELGLEIQAGIYFKGALKILDFDIYAELNINTDPENLKIYANITLSPISWIGGLLRISRSA
SDDKNGPFLIVDIDAESVALEIQGFIYVLGISNEIAVMINETHFYFHIAGNLFSVLSASLTVEASYGVE
TLEDMSYSFRGCVTLDLRRIGEIVFQTMQDAQKAVSRAMAKAQQKIDDAKLAVANAFKKVKEW
KQGIENYKARLRARSAELERKKAELNYSCKDRCGKVCIGFLDWNPHCWKVWGHWCGCPKWN
SCKWKADDIICIAGCELKRAWGKFVTWAEQVGVQIMLGLSEVAQTLLSGLDFFEAIAQGVLSFAS
GFLSVVETFTNAVLNGIGEIVRAAANVIVVEHFCLSGHLRPVESACVACDAKIRIFNQPIEWKGEF
CIDLSFFQVIGHKTAVQHTPEIAEIDAKVQEFESEGNKVDAQVGDMKTEENKQNKEVGDKTSKN
VNDENFRARRSHVPTEEEAYYYRLAYDRLPEVNTRSFATEQSMKDGAPWLLTQTWTPSKRSLTPD
PSTLHRRILETDNEDHCKNVENLYSNYQNVAVGFGTFLKSADKEKAGFEAMRRSYIQGMNYLKN
EFEYGDKKHNVTDEEREHVKYWEEYVKNGLNSHIEKTESLLKTQRKSYIPDFRNQVNQIFKEQTK
STLPEYIKKMKSHVIGFYQKRSDIPKDHLEKQLKKWNILHQVLTDISSSETASYRDLSLQVASVNN
MLTDLRRSDIPCAA*
>TRINITY_DN8903_c0_g1_i1.p1 type:5prime_partial len:447 gc:universal TRINITY_DN8903_c0_g1_i1:1546-206(-)
DMATKACGLENMCVGSYKSWRCICQAPRYMQDPDNTQSCIHAPCRIEDCGSGYAPGSFQYYAES
CNGTFFNPICNCQEEYEKDMLGGCVPKSCRENPIAVAECGENGYCKGTYFKYECGCQGPKYVQN
PRNTRQCIEAPCNVQNCGHGFENELYVSFADSCIGKHADYTCQCKEGYTGTGTSRCIPDGKECGK
SYCHANATCAPLGKGEFDCTCKPGYSGDGLYCFLDGGWSDFVNTRECNASCGTHGYAEVTRYC
NNPVAERFGKQCGAKSPEQLEVPCNRFDCLGCTVKKDCRCTSVVKPLIVEGYNEPEETLKFFTDL
HPDDVKKAKDTLLKWKAFSNENLCRKYNKTTLHAKFFGVRDAAKEVNKMKSSYGSARDSMRI
KLDCNINIAKKTQYFGVYEMIVKRLFIMKQVVKDLHIIEDYVLKHMENCDDSPVGMIRNTFGK*
>TRINITY_DN160_c0_g1_i1.p1 type:complete len:1145 gc:universal TRINITY_DN160_c0_g1_i1:4810-1376(-)
MRLLAIIPLLVMLDIAKSDKLWECNFEENDCWAASSTRNHERNQLTLHWNRWKMRTPTGATAS
PVTGPMEAFEGKHYLYYEATESMDKLKMADGASKGVVTFKDINLMGRTQFILSFYYQVKGNDG
DFYDFDLMVSNTENTHYDLSMKSIDGKDWKHFKHIMKVEGAGVTSIDLKFTARSYGAFADYAID
NIVIEDYPDAGPCKDNRCLANEYCTITSSTQTVCVAKCTQQACGENAECILDSLAINKCKCRQGY
LPRVSNYGDKLNCYRPDASCDIEYNSLGQCRISTSIPIMYGHPSLWHGRYSPVSGNGYYVFKYDNT
ITLDAVSSQKLSFFYLVDNPNMINSLTVEYGSRKFEVPINREKTNIWTLYEVLILASTNQGVQIKASF
YAEGGVIGIDNIQFTQQNEVVVEKIYKESMMAATFEDGSVPTDLKVAMLFVTRYGKIDFGCLPGIE
LETRNGKLYCKDGVVQRQSACSQDSDCIGSNARCVLRNCVCNTNSYQISIGTYGEKATSCAVYPC
EINSPRCHSTAICSNIKTPGQNFYNDYTCKCGRSLNNVETIINPFIGGDKSTQCLAATPSDSYVCND
NNFKPSRTTEYCLKGNQATVEYSLRATNFGNYALFKHCDDPDDEYRSSFNDPGSFWRPIDLVAAA
NENLAVSVTYKTSLSNKFIKCNFEQARFGSKLTLAVNVNSNSRVLTFTNTQTSVNKQNPVYQGQV
FQSDNPTPGAEMSVTFTVTGNQAVDQNVDAILLDNVVCKGFDDVASVNTPCGNNACASNQYC
NPLLSNSEKECLCPTGQKDQNCPDVVPSGSVVSADGPKCIGHHEKYMNGACVCDDGFEKTGDT
CTVKINCGCNDGLCLSGDKCSCPLNLLKVNQACVRTNKDCAKDCVSTLGNGGYCDVTTGMCD
CLTGFVKSGRDDGSYRCDKHAVVQKINDPKGICSATCGTGFVNEIEVCVGATGTGFECMTNIVQG
TLGVCSLPACPATSSQCSIQKECACNNVRVPIGSDGKLRLDFDHEAKLNMIFRFLSKHNQKDLEFK
FREARCDNTKLDSIARTIEDDLKQLEFDTELQAAITTMKSILDCSPSGNDHNAAYWDVFDMLIELS
SYRPHAVSDLQLKKELVMHEQSRCGMRSNLHKVVDGYAFWNKK*
>TRINITY_DN77_c0_g2_i3.p1 type:internal len:1381 gc:universal TRINITY_DN77_c0_g2_i3:4141-2(-)
EPPTSTEPPTSTEPPTSTEGTTTSSGPPPLLFIDSEGSTTPSYPPPLIFTEPEGSSTSTELLSTTVSAPVLTT
SPPSQDLGDLGDVTTEPPTELASTTEQSPILMGDPGLKGEKGVKGFDAVNGTQGVKGDRGPKGD
RGENGTKGEKGDVGPPGPGGDFILGEIGAQKPSDPLSGAVGEIEGLAVPGPKGITYDVRSGPIGSIG
SVGKVGPIGLSGDDGKKGQIGQKGPSGPRGGFGNIGEKGKRGTDGVPGNPGEKGPKGTAGRKGE

LGGDGRPGVQGAFGHKGNDGTIGLDGETGDPGLKGAKGYAGEKGDQGDIGGAGLPGPKGEDG
PIGPPGQRGLRGPIGANGPLGDPGLAGDKGQQGTRGNPGYTGKPGESGPNGEKGLSGSIGPVGE
QGPIGSPGEQGRNGNDGTQGARGPRGLAGPDGPTGPQGPRGESGPQGSAGGPGNAGLKGVRG
KPGLRGLTGPKGESGDIGDPGARGPQGQVGKQGKTGPPGASGEPGLTGNQGPQGPGGDPGPNG
NKGLMGSQGPVGEKGSLGPKGSPGRAGLTGPKGLLGEQGSPGQPGLTGRDGKNGLQGVNGKD
GEPGASGPQGMQGAPGLIGTTGPPGDNGDFGDDGPAGADGDKGSRGDSGVRGPAGAPGDEGQ
TGMRGERGDKGPQGNPGSQGSPGPRGTPGLDGLEGAEGRKGTAGRNGNPGLTGAQGVVGNTG
LPGSQGSRGVPGEQGDSGSDGAPGEMGLPGLPGLKGSIGRQGLTGRRGQQGVPGPKGKYGLQG
EKGQKGEGGNAGSRGSEGLPGRAGRPGSRGEPGPVGSAGIDGNPGNSGEIGSPGRAGTKGMQG
PVGLRGPVGTSGLRGPKGPAGDKGDSGETGAIGIAGELGTPGVTGQSGPRGQIGEIGLRGENGPQ
GMPGSQGLSGPAGPTGPTGPIGLPGKDGNKGPVGEAGDPGKPGQAGNTGATGKDGLPGEPGAG
GNQGKEGPEGVKGTPGPIGAAGPRGLPGRQGFPGENGMAGFPGRDGSTGLAGNRGPRGEEGEQ
GTLGEKGVDGSPGSTGTNGGNGVPGDNGPKGYIGERGEVGPEGPEGELGEKGVKGEEGSEALKG
VRGPNGKSGASGKRGDRGDTGGNGPKGEDGSKGSVGLKGFKGNIGFTGFIGPIGAKGYKGDMG
PDGPEGHTGPQGNTGDPGVDGDPGAVGSKGPAGNPGQIGSEGNQGKPGDPGKPGEAGLSGPPG
SIGQPGDMTGAFAGMYWNYIHSAGMRSQRYYKGKNKEEVDEDSNSIDKLFKTTYETFQNFDFV
WNQVVDDLVVLKTLGTMKNPAVTCADLFKTNPYFQSGDYWIDPNEGSPDDAILVHCNATNYET
CIYPRWPSFEKDNWYEGEEDYLWAYKDRLLSDEEYISYSADI

>TRINITY_DN1795_c0_g1_i1.p1 type:5prime_partial len:3198 gc:universal TRINITY_DN1795_c0_g1_i1:9621-28(-)
CEKTCEKTVPFLIETSLNSFKEFDRNFCKTRWQVGVKVKAVSNICWTLTTAIHYMHSYQFDKSTFS
KDIEENVIEPVNDIVTKWKDIRKEMEDLFREQWKRNVKTINILKNYKKTTTKLQSAFSRALGSVKL
ESITEDKIIESAEQYLSERNTTVSLARYEVLNVAFINIISTIENLPNSIKRNQKANFAVDRGQANQVIS
TIDKIISDFPVSIKNICEDKNPENHFDYTAMSSLIKSTKEQYRNLMQKSNSISDIFLYSKRFSERAKSLL
KKANEIKASLFPCKLKSELIDSDAIKVNVEAFSELNSFIKNIQPLAKKSIKDFANNIKSIIGEFKNDITS
PVGELINQLNDYIDQSPAFNIPMVITETPQVGSLVLNSLEEYFKNTKQFFNAIEEVTKLCKGDCKLE
DVFGKSNLNEILEKLDQYLKPISKKASQFIKTIKFTPKGLPLFISSLEELRTSLQTFYASGTGYTKYGII
NLLKSFDKVVTSVQDVRSNLQTNYQYLTSKLNKNIKNFQIAMSNIKKSMNDLYKESKGKYQNIRD
TAIKIGQYEERLKNVVQKSKSIKNVIERRYEIVREGAEVINDVGSQFQYIYSSNLDKKLENYVDMEY
AMKVSTDINTVKENSEKILKQTNDIVKAVGIEIPKNVNVDIFTGDLSKMIDDMIKSTGNRKSYLAK
KLSSATNVVKSKVENVVVDIQKYVKNIVGKVKNYYNETYEKVKQKLEKYSKIYDEIEDTVKEIKN
KPIARLGEIKDATDEFVDSIDKYDLGMILSADPKLIPTKVNELKGLFTNFGSAMAEIDKILKNCSHC
DAINMFGYNYIKSLAVKLNGLGDKMFEKMERYGETIGDTIGEMQVVTKATQSIKTRFNKIFEEKKF
GANTFKELSDALFDSTSDINSMRNGSENVIKILFDKDVDLEVVSGTVNNIVFQLGNVMNKSHQVA
LKGEKVYEKAKEIKSRFTKIKQSASNLREGPLKARIDIARELATSTKSMLKELPTLFDLSKETLEEAG
INSDWLVGFGENINGLTKTLGNVITKTDRVLNAAGIAVEGFEDIRGDIPQIQEQFKTLSNAPWNEK
IPALKDLTASLDGVFQKTIDSTLNTTKALNISLTKNDLKSFADKIIGKERIERYQKLFKNVSTIMDKL
QEGPVPAIGKLTDKVEEFVAGLDDYEFGKMLLKNPSDVKQKFSEFKELAGTAGNILKNIGDITGV
CKACDANSIFGNGFVSSIATKIGTKFESVSKNISKVLDRVETGLGGWETIVGTANTISSNFKGLTEG
KITKEKFDQVSKALLKTSDDMKVFTNGSSKIFEALFNGNKDMDFLQEGFENLVNKVSDVFNKSSV
VLPKVGDVFESIQGVKGIFGKLKNNIEEISEGPIESRINVLKEITKGVRGIGKSLPVIFQQSQEVVTSL
GIDSNWFNDVSKNLMNVSNGISKVMNKTDIVLEGAGIVLTDVMEIKDIANEIGGDLNELSKAPLE
QKFKILESTFGKVDQILDQTDKIAGTIDETLTNVTNKDYNITSSIGKITGFASGALGNISDAVKKVSN
IYGDVMATVKSIKSGPIEKIGKLTDATRDFVKSIKNYDVADILLQTPKFAKERLGDFKEIIFESGDILK
NISGLITKHCKKCNLDRYFGKGFVNNVTSGIKSNLANITGKLENVLDKVEDSAGHLIDLVSSVKGI
KSQVDRLGEVDFSQDGFETISDVLLTSSSHLDDIKNSSSSFFQNLFGDDQDLNKLMGNYEGLLDKV
NGFVVKAGNFTKDLSETVGQFEDLKNQFDLAKSNFKNLTKGPLENRVDAVKKIATGFESIVRGIP
EILKTSKVSPEWLRSFGTKLEGVTSGISNVLNKTSVIADSVGQTIGHVGDVVDTAKLIGSEFERLGG
SESIGTKIDVALNIVTKVSNLTGQVNVVAKDVNNVFKNLTGKTLFKTDIFGEKTNKILGDISNGIGV
VADRYEQFRELSTTIQEAFDNIEDDPINFALNDLPKLFNQSVEFTKLIFDDVGNIAEKLGIEIDGME
GLDTDIVESAKSFFDFAQTTLKTIGSGAAFVNDFKNLLHSKNFKDSLQNLQKLQTSGVKFIENIDE

LGDKLFEDWDDIKGAFSETIDDISKSLGINLKEMGTKFGDGMKLAGDVFAIYSGLKELFNIKEINA
KTIIQAANNVIKVTNAAVNIANTYFDAGISTQGLEQAGQYINYVAAAYQMYEAVKLFKSWINDA
CDLTYEDVITKRNLTYSCAQPELQIEKVIVPIAQCNMINKEVIRGYGEPKSCCNKKECVFMQQVKC
LKENEVCSNSKLDLSKKLQISNYKLVPIYQSYLKSSSSMLVKQKEVKIMEELVNADTHKLKKYEAA
LFQSKKLVERIQSTNNVLEGQYKSISSLLQGGKAKIKVSKINFKSNLVYPDSRYVPLELTVTDEKQKS
SKVRVQFDFKKYSESLKVAAYTVLMSHVDHTPQSRYRRSVQYTSGTTQEVNELTHWTSSCIIYKET
FGTIEKLMSSLKTDMLKASDNVKSFGNENTNTASIAEEKLRNVINQASLESVNEIVRRWKYTSLM
VLSENVGSACSGFEDCFEAQLENMTRLYEPEMKGYDDVISKIELVQSNIMKIVKDEPRKRKTIISNY
ISSTIELLQDIDENTYFCDELPKFVKKMPLQQYAYYGETFQLECTVDGPASMQYIWRKNGTVMPTQ
TSFKLTLDNVDADDNGYYTCEGKSFTISKLSNRVLVQVNEKPRFISQPSDRLIPYPGGVKVTWVCN
GTAAPKNYFRWLFKPYQSDRVISITESSILTLQSVSQKNVGHYWCEISNGVTKISSRKAKLDVVRVL
PLKETASVNLDLKSMKKQTVCSLPTAKESDPLIDSVKVALQAKLTMFGSENAISVFYYPDRSTLNQ
AKLSVSVSSKPVKDIKANAVNLALAVSSERAQLQEKFTSFLSEIKKEDGVQVGYNNCHLNLTTLKL
AIDWKTDEFACPKGMGISEDNLKCIQCTPGTFDNRVGKCAQCPIGTYQPLPGQTKCIPCGEGKLT
PDEGTPDQHQCMSSKYECSDNMNMVILSDTSSGVKEEVWRTVKEFQKRVVSKLPLDIVKVSIGTY
GDNAKLHLSSKTFNSVEEFTKNIGGIRKSNGENRIDLGFDLAEKLSEAVSNEENKAIVFITSNAIEK
DALSQLERKAEELRLKGGEIFFITVGLKSDESLIYHSLASKPIDKHIFNVADSKDLMKWSEVTRRAF
C*
>TRINITY_DN61668_c0_g1_i1.p1 type:5prime_partial len:453 gc:universal TRINITY_DN61668_c0_g1_i1:2-1360(+)
FDGSDELGCVTVAPATTKVLHCDDDHAYCEDNLECYPKEGRCDNTYDCRDRSDEKNCGAIKTD
KPICFDFQCKSGKKECISNDKKCDGRRDCKDGEDELNCQKSDCLPFPFGFNCTRVKEQCYLHKIV
CDGSTNCRDETDEDKEICGVTKRVKNLRSKLDDLSTGGIQVRLFWDAPDVSLSKVKGYAVSFIDD
KFVQKALKVPLSPSRVTIPSLKRCRKYVFAVAVDHTDLKADKLLYESTEEIETPGRRSPNAPALISTH
GNKVLWKEDVGDCIDHYDIYNRFLSCMEMDSKTVVKNRLKSANVLFDIQSMLNSNSNDTKLYN
CSIYINYIRKSDGTPFNLQTDYFTFPHTHDHHHKTAASTGGKGKRNTLVWAMPVAVVVIVLCIGL
LLMVYKYRKLQNSFLAFAARGSYNRQVDDLDDEDDDNMAVAFHTGEDSPMINRFSDDEPLVVA*
>TRINITY_DN477_c0_g1_i7.p1 type:3prime_partial len:1130 gc:universal TRINITY_DN477_c0_g1_i7:3387-1(-)
MMGSIKVFVGLVLLSFTAIEIAQAQSGTVLSNKQFAFDFVVDGSTSIQDEQWNLLTDFVSRLAVKM
STLNNAKLSALQFSHTLNRILQCNQYANVEEARNTIKNMVQENGATGTLAAMLTSADNLMNVC
SAGDPNRQNVMIVIVDGNQNLEQKSHSEIVQDIKVNVKDKNILVLTLGVGPQVNRNEFGDYASD
SGKGFFVDDFTLLTDELVQRMYDELIRTYGDGPDYLTAWVNGPCSATCNPFPMANRPTLRQTRQ
CQEPNGGNCGGASLEQIINCNENIACAGETYLTAWVDSSACSLTCNPFPVSNQPTKRQTRECRQP
SGSCNGASLTQTINCNENVQCPGETYLTAWIDSSCSATCDTGSNNPVFTQTRQCRQTVGNCDGA
PLQQTRSCNVGVSCTAPTFLTAWSNATPCSATCNPFPMAVRPTLTQVRQCNEPNGGNCGGASLQ
QTVQCNLDVACAGETYLTNWVDLTACSATCDDASNRPVKQQERQCQQPGGNCDNLPLQQTVN
CNIGVSCTVSSSACSSKEISFTFVIDTSASVDNIGFFVLKDFMKKYMSQSTLGTDPRTSVAIVSYGAG
DAKVEVECDTITTQQSFNTTVDNLSQLFGIADDNSFNQALSTAFDASGRCARSSTSLNIIIMLSDGD
PPALENILTRDRIQSSSNYYFMFVAIQASDGAQSTFASLANQGRGLRNASSSFTDLYNFNTDLYYLE
TLSKACDVGGQWTEWSTSACSTTCGPGVVTRTRECRNRITGKLQQDCGPGPAQETQACNLQDC
QGTLTQWTSWSCPATCNINNDVNNPPLALRTRQCLGSTGQGNCNNADKTENEPCVRNVPCPG
AWTTWSVFSACSETCRSQSAFPTRRRTRQCIGGTLNFQCPPQIVNGISYREIWQEDCINVPNCPAK
FTQWTTWSCPVTCNVNNDRNNPPTATRTRTCDDSGTINGCANANILENQPCVQDVQCPGAWS
TWSAYGACSETCQSGNRSPTYTRTRTCLGGTLGFQCPPFITQIGGFQIFGTQTSNCNSEVKCPSKLT
QWTTWSCPVTCNNRNDVNNPPQARRTRRCDTSTGSGICESPIVEEFENCVANVPCPGRLTEFTYD
NCAHTCNVNNDVNNPPQRVGTRRCLDGTLG
>TRINITY_DN554_c0_g1_i4.p1 type:3prime_partial len:319 gc:universal TRINITY_DN554_c0_g1_i4:956-3(-)
MQIFVKTLTGKTITLEVEPSDTIENVKSKIQDKEGIPPDQQRLIFAGKQLEDGRTLSDYNIQKESTLH
LVLRLRGGMQIFVKTLTGKTITLEVEPSDTIENVKSKIQDKEGIPPDQQRLIFAGKQLEDGRTLSDY
NIQKESTLHLVLRLRGGMQIFVKTLTGKTITLEVEPSDTIENVKSKIQDKEGIPPDQQRLIFAGKQLE

DGRTLSDYNIQKESTLHLVLRLRGGMQIFKESTLHLVLRLRGGMQIFVKTLTGKTITLEVEPSDTIE NVKSKIQDKEGIPPDQQRLIFAGKQLEDGRTLSDYNIQKESTLHLVLR
>TRINITY_DN4494_c1_g1_i7.p1 type:internal len:536 gc:universal TRINITY_DN4494_c1_g1_i7:1607-3(-)
ELFSIKNGGRTGVPKLLILLTDGSQTQDIGAEDPAKIADELRADGINLIIIGIGQGVNQTELSNIAGG DDNVFSAASFDELIGTDFVSAIKSGSCEEAKKPPLPKLKRVLDVGFLLDSSDKVKDVYGIEKDFLST MASIFQIKTNGTRAGVISFGKDNEYNIRLNQYFDISKFKQAVDSLQYMGSGRDLKNALQYARTDM FTVGSGARVGMQNVMIIITGEKEVKDFKSDDVKYHANEIRSKGTAIIVVGIGNIKIEDLEAVAGDK KNVFIAPTPFNLTSYQFLNAVTVRLNEIDQKPSGPKCEAKVDVGFILDSSGSLRNEYGKEKDFLKTL ASTFGVSSNGSRAGVITFSYYSEHSIKLNDHSDISSFNQAVDNIPLMGATTRIDKALRLTQKELFNT RYGGRVGVPKLLILLTDGSQTQDAGAEDPAKIATELRADGINLLIIGIGKGVNQTELTNIAGGADN VFSAASFDELIGADFVGAIKNGSCAEAQKLEEPKCKAQVDVGFILDSSGSLRNEYGKEKDFLKTLA STF

>TRINITY_DN74720_c0_g1_i1.p1 type:internal len:124 gc:universal TRINITY_DN74720_c0_g1_i1:3-371(+)
FKRGGSNLNLALNLANRKMFNKDYGMRTDKKKVMLLYTDGEESFRSPSDTTTDEELKMLREKG VDIVVLAVGSKIYMDNVKKYAAKDSYIFNVSTFDQLKIRLNDLIQVMCEGNTLGFDVLIY
>TRINITY_DN113_c0_g1_i1.p1 type:complete len:1417 gc:universal TRINITY_DN113_c0_g1_i1:4413-163(-)
MANWSLLYAALLFLYSVSAQEEERPITFIKGEPGPKGSRGMPGDDGSVGSTGRQGNEGRQGPRGD TGPKGFKGEPGLIGPAGGASGGETKGPDPLAARVGVKGGTYGVNTVFDYQLGPPGPPGVPGNTG GYGFKGDVGPVGVKGVPGLPGPPGSYGERGEPGEAGLDGVKGDRGDAGQTGPPGKKGVAGAV GFPGLQGPFGYKGERGGAGPVGDEGVSGRKGEKGYAGEKGSRGDQGDAGFQGVQGPTGPIGPL GNVGPRGPDGQRGIAGAIGAEGLKGAPGTRGPQGPKGQLGEPGQRGVKGVVGDKGPTGQQGS QGPTGNPGDPGQPGKTGDKGSIGDVGLIGLTGPQGMQGERGTVGPRGPAGGAGKKGERGQAG PIGATGEKGNVGDVGLDGARGAPGKSGIEGKVGQPGPQGEPGAVGLQGAQGPRGTDGQAGQA GPKGNTGEGGFPGAAGGKGSKGLKGRGGDRGMQGEPGSAGKPGARGRDGKRGAAGEDGKNG NPGQRGPQGSQGLTGAMGLVGAPGAPGDMGVAGLPGQQGRKGTRGLAGPTGQRGLRGEGGL PGERGEQGAAGPQGTQGTQGSPGPQGRTGLDGKDGSPGDKGERGSMGTRGKQGEPGADGKQG SRGTPGQQGIRGDSGDQGSPGLLGERGLAGQKGVAGRDGAPGMPGRTGRQGTIGATGAVGLRG AKGGRGKDGETGEQGPQGQEGPTGPRGPRGASGEDGAPGQNGLPGLTGPMGEEGQPGRPGQP GKQGPRGSQGEAGPVGPKGPLGARGDQGEQGPVGIEGVVGALGAVGSEGTKGPVGAMGSMGP TGPTGPQGATGETGPIGPLGAQGRPGIAGNAGGKGPNGDPGNPGRPGSSGDQGPPGKDGSNGE PGSPGKEGARGLIGRPGAEGIRGEAGTSGSTGRLGRKGELGPKGFPGKRGKDGTNGKRGEMGVS GADGIRGSKGNSGKDGSAGIAGEDGVEGQIGEAGYQGSRGVRGDTGAPGDSGQPGDNGPNGA KGSKGSSGRAGAQGVDGRLGARGDQGAEGARGKEGAAGAEGERGEKGKMGAPGIYGPAGDP GFKGDEGEPGPVGPVGPHGPRGSPGEDGPSGKKGASGITGTNGRKGNRGQSGANGDTGTPGIPG VPGSPGRPGPPGEIAGMSGAFARFFSAGGMGMKGPGNYYNNYYGQYRYYKAGNEKIRNQIKPH LSTFKQYYELLKDVDDMMKATGTKSKPARSCKDLFTDYPNKITNDYWIDPNGGSTDDAVKVFCD KMKNETCIFPKSYRAELENHYTGKDKFRFMYEDIKEDGEIEYAADITQTKILHLMSSKVRQEFSYN CKNSLAVMKNNKDRYEYALKLKLDNEDIISMNMNTRSLDINILNDECWKKDNTVRTATYELISH KPIKHQLPLLDVASFDIGGPNEEYGLTMGPVCFS*
>TRINITY_DN25375_c0_g1_i7.p1 type:complete len:811 gc:universal TRINITY_DN25375_c0_g1_i7:112-2544(+)
MNVNEKMNIYFCIGTLLFQSLLVLKVEGIFNDCQTKGSCGKCISSSPECAWCEDIIYKRSLNSKSYY KKCDVLDNHKRQGCQNITNPPSSSTIDKDAELKGDTRVSPQEITVKLRPGQATTVDITVKTPKNFP IDLYYLMDLSLSMKDDLEQIKDLGDILGEEMKNVTTNFRLGFGGFVDKPINPYTTIKPDEIEAYIRD EDAQYTFTYKNYQPLDEDPSLFSKTLEQVNISFNIDGPESSLEALMQVIVCRNDIKWKDKRKARRI VIITTDADFHYSGDGLLGGQVIPNDAQCYLSKGNYTAWNQFDYPSLSQIRSVMIENQIVPIFAITGN FELYKKVAEFFGAGSGAVADRLSKNSDNVVPLIKEAYEKIAKIVNINSDAPKGIDMKYTAICGTGK NVEAREGTDFCDDVEIGETVVFKVEMTAKDCELVGEDLKFKIKTNFDDVNVAVKLLCECPCEQD

KSKIVIDSEHCNSRGNLTCGQCQCDLDFYRGRFCQCTVEEAKNQTLCRLPGEVGPVCSDQGFCEC
GECICDEPKLNRFGEIEYIYGDQCQCSNTSCGRTTNENLLCGGPSHGRCDCGECTCFSDWQGPTC
EVECVKSSAACIDEEGIECSGKGKCVCNRCVCDPGYIGDLCQECVSNCPDPCQDYRECVKCRVFK
MSDLSDEECKSECLKHNITSHIVTDKVPEDKRCIARDEDDCSFVFTFVKSNITDEVQLIVQQEKICP
AEPDILAIILGVIAGIVGIGLALLLIWKLLATIQDRREFAQFQKDRQDPKWDSGVNPIYQQATSTFQ
NPLYGNKGEETEKILDSQ*
>TRINITY_DN199_c0_g1_i1.p1 type:complete len:229 gc:universal TRINITY_DN199_c0_g1_i1:207-893(+)
MVQGSMTIVKYLMFFFNFIFWLSGLALIVIGAVIRDKYGDYFSYADSRFTNVAVFLIVVGVIVFIIGFL
GCCGAIKENYCMVTTFAVILGIIFILEIVAGAIGFAYKGQVEEKADKALVRASKNYNNDDEPGAKK
LLDWAQQRFECCEVASATGNGTCADKSPKSCHPDNDCNKTKYSQKCKEGFVEFMKDNLVTVGA
VALAIAFIQILGIVFACCLMKAIKGEYEVV*
>TRINITY_DN1043_c0_g1_i2.p1 type:complete len:700 gc:universal TRINITY_DN1043_c0_g1_i2:2410-311(-)
MKESYLKRFALLFSVLILFQEGGALLRENLIVNHRLRRNVYSEEKVTAVDLPQSREKRFVGTALIIIS
AASAGLDIYKQVRGCKDITPDLDDKIAEGKALQQSLLNLRDQVNLAWVPAESREEYTRRLASNM
IIIREQVSTFDSLQADLVKLLDQKLLDKLNNVTKHLENQIDRNNETEIGSFEDLEGKYMDTTTFALT
LTAKLLPVGFLLGTKFLLPKITARFSKGVAKAFAADNPSRVQKMLSSFAEKQGKLANWKAEKINSF
KVSLNSKWNTFKLKFPRITAAMNRMSNSKIGKALGKAMGKVKGIAVGVGKGAVVAFFIAFEVW
NFVQFHGACTEKRNKAIEGIATMNANKKNMTDLLTEIHDMKAKADEYYNLMKSNVTNDEFLN
FLEDIADLGENAKEQTKVIVDATNQIKQYKAEIKNADDSRTDALNTMLVTAFSNINFKLDCLRSK
LAIIKGVVDDCGIGLGVYDDIYARALAKQKKITETCIEGSYMDKNEIKGLMVAAMDLDKKQSICV
RNNPTKKQLVCEKWYDDVSDADILAANPDLTKDDVTYFISTCPPAPVNDQTKTTICVKKNNELA
TLQSIQQTYQKYDQTAINSAYEECQLSSISVTTICTAKKEGQTVVYAQQKFPVFPASMVTNVYNSC
KKETNITPADKMMICLLKKNGFALDSPVMANYVKNYDYATLEAFYNQCS*

>TRINITY_DN1910_c0_g1_i1.p1 type:complete len:447 gc:universal TRINITY_DN1910_c0_g1_i1:2183-843(-)
MREIVHLQAGQCGNQIGAKFWEVISDEHGIDATGTYHGDSDMQLERINVYYNEATGGKYVPRAI
LVDLEPGTMDSVRSGPFGQVFKPDNFIFGQSGAGNNWAKGHYTEGAELVDSVLDVVRKEAESCD
CLQGFQLTHSLGGGTGSGMGTLLISKVREEYPDRIMNSFSVVPSPKVSDTVVEPYNATLSVHQLVE
NTDETFCIDNEALYDICFRTLKLTTPTYGDLNHLVSATMSGVTTCLRFPGQLNADLRKLAVNMVP
FPRLHFFMPGFAPLTSRGSQQYRALTVPELTQQMFDSKNMMAACDPRHGRYLTVAAMFRGRMS
MKEVDEQMLNVQNKNSSYFVEWIPNNVKTAVCDIPPRGLKMAATFIGNSTAIQELFKRISEQFTA
MFRRKAFLHWYTGEGMDEMEFTEAESNMNDLVSEYQQYQDATAEDDGEYDEEEEEELED*
>TRINITY_DN761_c0_g1_i3.p1 type:complete len:845 gc:universal TRINITY_DN761_c0_g1_i3:129-2663(+)
MTSMFRSEEMTLCQLFLQSESAYACVSELGEIGVVEFRDLNPDVNAFQRKFVNEVRRCDEMERKL
RFMQKEIDKADIAIVDTGENPDAPHPREMIDLEAQFEQLENEMKDSNSNYEALQKSLLELLELRH
ILKKTQSFFEEAEHHVLQEQMGDITQANAEELSLLGDTDHSPAAPLGGRLGFVAGVINRERIPTFE
RLLWRACRGNVFFKQAEIDTALYDPGTGDLTHKCVFIVFFQGEQLKIRVKKICEGFRATLYPCPETP
NERREMAIGVKTRIEDLETVISQTQDHRHRLLQTIAKNIRVWLVKVKKIKAIYHTMNMFNLDVTQ
KCLIAECWAPVADLDSIQAGLRRGTERSGAQVSSIVNRMSTKQDPPTFNRTNKFTKGFQAIIDAYG
VASYQEVNPALYTVITFPFLFSVMFGDCGHGAIMALFALWLIMNERKIANWKAGGEMFDTVFQG
RYIVFLMGLFSIYSGLLYNDIFSKSLNIFGSAWVPTLNRTKYDYQPENYKEWSDGDRMLNPLDAPR
ETPYYFGIDPIWQLTSNKLTFTNSLKMKLSVIFGVTHMIFGVILSLFNHLHFKQYVNIFCEFIPQLLFI
SCIFGYLVILIFYKWIAFSIHSPSQPSILLGLLAMFLNFAKKKTDADRLYNGQDFLEPFLVVVAVICVP
WMLLIKPFYLRWQHNKKKQHGTNFARLHEDTAPLQDNEVNHHDDSGISSKNEEEEEFDFGEVF
VHQCIHTIEYCLGCISNTASYLRLWALSLAHAQLSEVIWTMVFKIGLSMDGYVGSVAIFAVFAAWA
ILSVAILLIMEGLSAFLHTLRLHWVEFNSKFYVGAGSKFVPFSFKDIMNDEAF*

>TRINITY_DN71276_c0_g1_i1.p1 type:5prime_partial len:1201 gc:universal TRINITY_DN71276_c0_g1_i1:1-3603(+)
LTFIESLRLSSIKNIFKMMISFETFVPSTTPYAEDYNTNPQVMMLSNKIKETIDTSLNPLASQIDRQKA
FQVQEEFKSSSPHCAEVGLYLYSVERKNSDAVRLCALKFVDHFIETKWNDLTYERKTFYKKESLKL
VEQMTFENETYLKEAAAQIVVEIIKRTWPQSWADLFKNLEELQAKGDAQLELVLIILMRLSEDVN
GLDERLTTQRKRDMMTTFQDNANELFEFFIKTIRDKVTALKTESSSLLIRLSEATLFTMMEFVSWA
HFDCLFNNEKIIFQILFWLLEVKYLRYYAAACLDNIFQRKGTPEKRIEFMVVLSDDVLNIVENVVRS
LKKTPENVLPEEGYKFLKKLAEMLVSFGKIQILSLWGDERGFNEKPVGYDRYLRLMLLLFQHESM
KLTYDVSKFWHALSIHPHGKLDDTFKELMPDLLDCAIMKIAKISDPKIPNNPSSHYSTMDFDDKE
NYAEYFANCRGHMTDVCRKITIISYTVVLDHCLAKIKQATSKPPPDVSGIPKNQIQITQHYLYWDA
LVHFLKNGFAGVFFRANKEISEDTISKCNEIITLLIHYKCEVPLFTIPVINLLESVFPIFKHSRDHLLPT
MQLLFSIIRLPQNDEDPLRDHVFKLKRHVCEAYAALCKEIPHCLVDYFKQLEEFAVYQMMHNASL
TGQEKCRLIEGVLRISKHVTVKEEQDHCINAIFDMIDSMFDKEPVRRACESPISLIQYIGLDQEGTV
AADSELRTRELLFAINLWTAACKALAMSKEEEVKDEITEEEMRTQIETYGWIKRMVTMLPKMLSL
LQSYSLIWQHQDKICDKRILVHKIITEINPTIQVFQPKCADEVNEKRGLGQYYLCTFYESAYTSLGSV
SKSVGPSLFSIGGLAEAMNEKLVQHWNNLPNYRLGRITRTFIKDICLACPKKYFETFIGPFVSQVLR
LYFERISNEWAKLAQLKENPREVTDKLQEQIEIAEERMIYLMSKDLLDLLLSLLTFKRKHLRSTTKP
ADGSLETFGELGHFLLDCEETNFIIVQIIFTSLTWPDTSAAIKVSQIILAVLKKIFTDTSKLNPELPKQL
LLLILQGLNIHGGDEFGRAALTNNVLQFYLHLRMQYRGIVQVMLMIPTCEEQHLREFDLAVFVQE
LPLTEQQQKKIFRRFVAGACGTVISKLHEKDVVIQDLPALMPRQKEKTLNKNGDADDDLGLCSLF
GV*
>TRINITY_DN28022_c0_g2_i1.p1 type:internal len:915 gc:universal TRINITY_DN28022_c0_g2_i1:1-2742(+)
GTCVPSNKVCDFYPDCAQTKDNTDNTDEDGCDGCDFESGWCKWNDTSTGIFDWKRQRGGTSSI
NTGPSTDHTTKNLRGYYIYVEASQGKSKNLASLSSPILNQASSRCHMTFWYHMYGTHIGTLNVYI
VNGYRLTRHWTLNGNQGKEWKQGTVYINRQQKAYSIRFVAERSYSDKGDIAIDDIQLTGCALPK
PALYCNTRFQHRCAATRACVSYSRLCDFTDDCGDGSDELNCRYTQYPYRCNFEYNLCSWKNLKD
DQFDFKREKGQTDSGGTGPAFDHTTGTTSGYYIYAEASRPRKMGDKARIGSGYLAPVTRTSCTLRF
YYHLYGDHIGGLSVKTRQCIGCPETQIWRRNTTVGNYWVRHRLFLRNNEPFQVIFEAEVGNGYQ
GDIGLDDVSFDGCAPYRGRFPTVGPSTIAPATTPVCAIGQLYCNKDAKCIKESMKCDYMEDCSDG
EDEQNCGSCDFENGMCGYQDVSTGRLEFKRTQPLNDGSNNGPSFDHTTNSTKGYFTMLQKGSG
NRDKLSYLISGKLSQTAPTCKVQIFYHANVGRNFIIIYHKQQDDNGKDIVSKLYSGVLKNADKWQ
NLTFALGQRPPGWQLKIGLMEIYTSIDDIQFKDCAFPRPTTGLCPRWKVLCDNGACLHPAKMCD
FVNDCGDNSDEKPYICNKNYKERCDFETDTCNWYQDQSDDFDWTRMKGRTPSGTTGAPVDHT
KGTSQGYYMYIETSGRRKLNDTARLLSTVLKPPSNRWCTMRFYYHMYGKHVDTLSVKIKLSQDD
NSTMTTLWQKKGNQGNQWNTGRVFLRYKTNFQVVIEAKRGISFLGDIAIDDISFSRACRPDFKAT
LSPDPITKTPPKGCKSGEFSCGDGQCIDASFVCNFKSDCKNNADEKRCPSSCDFEGGKSCNWFNA
QFADVMDWTVHQGKT
>TRINITY_DN1559_c0_g1_i1.p1 type:complete len:311 gc:universal TRINITY_DN1559_c0_g1_i1:221-1153(+)
MAEKKATFKLGFAENFALSGAAAVISKTAAAPIERVKLLVQNQDEMIKQGKLSEPYKGVIDCTTRT
FKTEGLAPFWRGNLANCIRYFPTQALNFAFKDKIKAAFKVSKSDGYALGFGKNIASGGCAGALSL
CFVYSLDFARTKLANDTKSSKKGGSREYNGLVDVYRKTLKSDGVAGLYRGFVLSCVGIFVYRGCYF
GFYDTLKPVLLGADASLFMSFILGYGVTVTAGLISYPIDTIRRRMMMTSGQAVKYKGSIDCATQILK
NEGAMSLMKGAGANILRGVAGAGVLAGFDKFKEVYIAMRKRQMGLE*

>TRINITY_DN64_c0_g1_i1.p1 type:complete len:468 gc:universal TRINITY_DN64_c0_g1_i1:1555-152(-)
MPPKEAEKIHINIVVIGHVDSGKSTSTGHLIYKCGGIDKRAIEKFEKEAQEMGKGSFKYAWVLDKL
KAERERGITIDIALWKFKTGKYYVTIIDAPGHRDFIKNMITGTSQADCAVLIVASSTGEFEAGISKNG
QTREHALLAYTLGVKQLIVGVNKIDNTEPPYSEKRFDEIVKEVKTYVKKVGYDPKTVAFVPISGWH
GDNMLEASKNMGWYKGWTTERKETKTTTGKTLLEALDNILPPKRPTDRPLRLPLQDVYKIGGIG

TVPVGRVETGIIKPGMVVTFAPSNITTEVKSVEMHHEALLEAVPGDNVGFNIKNVSVKDIKRGNV
ASDSKNDPAKEAKSFYAQVIILNHPGEIHSGYQPVLDCHTAHVACKFSEIKQKIDRRSGKVLEEMP
KMVKSGDAAMIDLTPSKPMCVEAFSEYAPLGRFAVRDMRQTVAVGVIKSVTKSDGSGGKMTKSA
QKATGKKK*
>TRINITY_DN1936_c0_g1_i4.p1 type:3prime_partial len:1762 gc:universal
TRINITY_DN1936_c0_g1_i4:5283-1(-)
MLSPAVTKVFIVLSVLGGLICQSCDFEVANACQWTQYAKDKFDWTRNFGNTGTGGTGPKTDHTS
EVKILKANTTGGNSNGAPCVFPFKFRKRTYTKCVRSMQRGKSWCATVPDMNKKRQWGYCMPT
GYYMYIEASSKRGNPRNKGDNAVLLSPVISKGPKCLQFWYHMYGPHIDTLNVYKMANDNMTK
VWKLSGTQGNKWSSVNISFDGSSSYQMAIEGVRGNYYRGDIAIDDINIIDGNCDGGDGSKVSASY
GKCTFEKNFCGYQNDTSADMIWRRNKGSTPTGGTGPSSDHTLGTKSGYYMYIEASTKKRYLAARI
SKTYTSLDSTGSCLTFWYHMFGPHTGTFNLYINPPVNSTNTPAFSVVGQQGNLWLKGDVTINTTS
ALVVIEGRLTYKSQFYKGDMAIDDISMTKGKCQNRDICTFQDDFCLWYNVPQAAGLDDFDWIRG
SGGTPTAFTGPTTDRNGNIIGRYIFIESSKPAIKGQKGWLSSELFSASGNGKCFTFYYHMYGGSIGTL
NVFVNTTSGLELVWTQSGSKGRQWKNGQVNVERPGSFKVLIEGIVGNGYRSDIAVDDFEFKSGKC
SIVPAEASPKFKLTTIAPTTIKPTIPKNLIIDCDFEFGICSNWKQDKKTDVVDWTLSRGRTQSRGTGP
RFDHTKSTSAGQYIYLEVSKRKMNDSARLISTFIPATGDTGGKCLTFWYHMYGPHINRLNIYVMIG
GHTGKPIWSRIGTQGNKWNKGQVTLVGRQPFQVVVEALSGISYKGDIAVDDISVSSTKCTQPSSTT
CSFEDSQCGYIDDTSSDVQFQWTRSSLSTSSSGTGPKTDHTMQSETGFYMYTEATRKKTGSKARLM
SPVNKATSGSCLQFWYHMFGADMGTLSIYLKVNNKLQSTPLWSEQGDKGNEWKVATATITTQDS
YQIVFEGKIGRGYRSDMAVDDIKLTDGNCPPPGFCDFETGYCTYFNSKNDKFDWLIGSSRTPSRFT
GPNFDHTYGNANGSYLFLEVSQPIKAGDIAILESQQFNAVGGKTRCLNFWYHMFGSDIGSLQVVY
KVFSGSQQERVLWNLTGQQQITEKDPWKYARVPVDMDADHMVMFKGIAGSGYKGDIAIDDVQF
TSLACTIDPPEANPNGKTTMKPPTTYPRTAPSTPVPTEFSCNFEKDLCKLTQDSKGTFNWTRIQGY
TGSPGTGPSTDHTYGRGFVPKYGPGSLRHAGGKCVHVLNGGFNKPKEGNPLVLYQGCGQTRLEF
VFKYGYLKLTRLNMCATLEGSSTSSGTKVVLSNTCMNKWKMLDNNTIQDVKTGLCLSPFKGSDY
PSNNSPLVVDKDCKPKSRIFTFSPTMGHYAYIETSPPRQPNDTARLISPILKTPRCLNFWYHMYGAH
VNMLNVYVKTSNGPYVSPAWAKYGSHGDTWKAAYIYMNMTGPYVYAFEGVRGIGWQGDIAID
DITLSESCPAPVECSFEETSPIMCGWTNLKNDDFDWSIGQGKTASTKTGPKNDHTYGTSQGHYMY
IETSNAPAKATAYMYSPWYYNTGQKCVQFFYHMYGQQIGQLEVYIRYSNSRYLYRQFIRSGNQNN
TWHMGQVTVKRNNVYFQIIVRGTKGNGYQGDIAVDDFRIRDGACPAPGDCDFEHADFCSWMQ
EDNDDFDWIIGSGSTNSFLTGPSIDHTTQSNGGYYAFIEASSPRQPGHKARFKSMVFAPTTGSGRCF
SFWYHMRGYSIGNLTIYLRKVSNTKTLSEEVMWLLGGNQGKAWNQGRFTLKSSTLHEVRFIYLRL
FHCKICLPCY
>TRINITY_DN2720_c0_g1_i6.p1 type:5prime_partial len:2710 gc:universal
TRINITY_DN2720_c0_g1_i6:2-8131(+)
NDGDDDDDDDDDDDDDDDDDENEGGDGNTDKEGGDGDEHCSDAFNDKCTNGYCRNIGG
QRRCVCKSGFKRDRNNFCADLDECTAYSGLCSGGRCINTHGSYKCDCPDGYMTSNDGFNCMDI
NECDNDTNICNQGTCRNYPGTYQCRCNKGYQSVGNVNCADINECVKEDTCPGKGICKNTDGS
YKCECPKGYKSNGENECNDINECKTIPNICSGGYCVNTGGSFQCICPDGYELAPDERSCKDKNEC
SYLPNVCHGGTCVNGPGSYRCQCRFGWTATEAGTGCIETRIDNCYAKFNGLQCSMPLMKQLTRY
KCCCGMENTGMRAFGTECKSCPKKGTDEYDLVCMNGPGVDENGSDIDECKNNAGMTVGLCD
NGRCVNMMKDFKCRCNTGYKNPVDTNKLCEDIDECSELDVCKGGTCTNTPGGYRCICQVGYRY
NRITMACEDENECLNNPCVGGTCKNLEGSFKCQCPPGSVYDASKRLCKDNRKGRCYAFVALGN
SCEQEIASFVSKSECCNSFGKGWGPSCESCNEQGSGLGCDAGYQREDGKCTDIDECKFLTTCQNG
RCVNTRGSFRCICPTGYELDPSGIACIDRRVGTCYRSVENGRCGMALADNFMKANCCCSAFSQA
WGSPCEVCPLKNSASYKELCPGGEGFHKPGEDMNECEVSGICKNGRCTNLFGSYKCECNSGFDM
ESSGTMCVDVNECRISGNSICGNGKCMNTYGSFRCDCDQGYKNDMMMEMCMDINECIESNPRP
CGGGVCINTQGSFKCDCPKGLKTMGNSIDCEDIDECLDPNICPNGVCQNFIGGHLCRCNNGYRS
SRDMLQCFDVDECLIQNGGCEYSCTNTDGSYRCGCQPGYELKNDRRMCKDTNECVKGKPCGEG
NQCNNLNGSYICTCNVGFRASPDGRACVDIDECSEDPNACRNGRCINTMGSFLCSCSLGYRLEVS
AMVCLDIDECKGKANGGCHPDAMCLNNIGSFDCKCRPGYDGNGRTCTDINECKLGLATCARD

ARCVNSVGSFSCKCKEGFNGDGEICLDKNECAEDKDLCRPGTCMNTIGGYKCDCTNGYKSTKD
GKKCVDINECETIEGICEHGTCVNNIGNFTCHCDLGFQLDKTGKTCVDVNECESTGMCPNGKCK
NMMGMYKCICDKSKGFMPNMNETACTDMRMGYCYSKFINEKQCGGAQLSPKQMTKQFCCCT
APDGTGWQDDSDETCEACPSMKTAEYKSLCPGGTSTHVNETTTMIKKHDGCKVLHNYCAGGN
CSDTGEQGVSCKCPVGTKFNVDYLACEDQNECLTDGICGLGGTCINTERSYQCKCDKGVTYDPV
GNICRDERNYTCYHASLNNGNCEIPLSIRFTIARCCCGNDRSRYGEDCRACPPSNSGTRQALCVG
KPEPPIVSTGQPPIGPTKPTNAPTTVGPDSTGTDGSGPDGTDGRGPDGTDGSRPDGSDGKSPDGSD
GKSPNGSDGKGPKGTDGKDPDGTDGKGPNGTDGLGPDGTDGKSPNGTDGKGSDGIDGKGPDG
TDGSSSNGIDGSGTDGTDGKGPDATDGTNPDGTDSTGPDGTDETDGTKVPKTTIDPRHDIDECKY
IKNSCGKGRCINTIGSYRCECDQGFEQTQSDGRTNCTDVDECESSPCQNGICNNMVGGFFCTCPE
GLKLDKTNLRCVDLDECSTERYCENGDCTNLPDGKGFICQCDRGHVKTVDQKSCEDLDECLMP
NICGNGQCINSKGSYRCECDQGFKLNEQGKCIERDECVEFPDLCRHGNCTNLFGSFMCTCNEGF
KQDATNQSCQDINECLQPGFCENGRCRNLIGGVLCTCDSGFEKTIDGRSCEDLNECEDPDNLCRI
GGTCVNIVGTYRCLCKQGFVTDLNAKQCIDIRRSFCFSTLDENQCTNANGLNITKSTCCCTMSAG
WGDPCELCPEKGTPEFEYYCPNGVGLERNKTDINECEANLGTCMNGKCVNTDGSFRCECNEGY
KLNPSDPRECIDQNECLVSNICGNGNCTNTVGSYECACEHGFQRGTNSPSCEDINECQEQKNICP
FRCYNLHGSFRCTCPRGYKLADDGKYCVDIDECENDFICPFKCKNIVAGFKCLCPEGYHRNPRTG
DCTEINECERNPNLCKPGGICRNIEGGYRCECMHPFMRSKNGRFCEDTRMGVCFSNVVDGLCQG
VAGRMKKVSIRDCCCTGGGAAFGSSCTPCPVLNSEAGRSLCTGYYNRSIDECKFIAGVCRNGKCI
DTAGGFMCMCNEGYQLTMDGKRCKDINECEDEFGAKLEDQCPGECKNTIGSYQCMCQKGFVI
NAQKTACVDENECLTGNHDCDHLCENTEGSYKCLCPEGYTMNGETCEDINECQVANICGLSTC
RNTDGGHECVCSRGLKFDEKRKSCVYITDGGDGGDENNGCINPNECGMCPPGYYPYHTISGGTQ
CVDINECRQKICGQANCMNNLGSYRCSCPYGLNYQNGRCQDGNECLRSPCSYGCTNNNGGFN
CQCPPGYYPVNGGHCVSTPGVACYACDTSDIPAEGVPLGGGLPQTAALYPSLGYQSAVGGQSVPL
INAPSNAQFYPNQPQQPQYNLNVPSYANNGYQQLNLGYQQNFNPYTMTSTFSYRNRRSIQSHIK
PVKVTMSRKSKAKISIVRIVSLLKNMTDHVNYKITHGNTTLFRLHNDNHGAIDLFNTHVLAVGH
YNLVIGGDIKNDSNILEDTLREIYYDSKKFLLKVTIRVL*
>TRINITY_DN138_c0_g1_i1.p1 type:complete len:401 gc:universal TRINITY_DN138_c0_g1_i1:157-1359(+)
MAARFLFITVILIVGNLIGDVCPERCERNWIKIGCFHDRIFPNRPYPFELVNHRDPINNHWDGNLI
NWRKYPDSIEKLACICANKTRTKGYQYFGLQFYGECWSGPSYNFHRDGPSTKCIGNDFKPCDDSS
KTECVGRAFTNYIYKLKDENPKVDGKWSEWEKWSPCSATCGYGERYRERFCSNPEPENGGQTCE
GEAREYEECNHGECQTTCDKEIDFGIALDYSSSVRQVNWNRTLDFVVELSKGFNISPEKVHFGVLS
FSHRTRFDFGLSDKTYWNSESFEKKIKSLEYRWGYTWTDKALKKVKQLIFCEKCGLRKDSLKYLLV
FTDGRSNNVKTDLKTVSDSIKDMGVNVISVGAGHTINHRELEEIATDDNHVFYVGGYAYLGDNM
NAIRRLTCG*
>TRINITY_DN113_c3_g1_i1.p1 type:3prime_partial len:1162 gc:universal TRINITY_DN113_c3_g1_i1:3485-3(-)
MEIVSRSRIAIFVLLGLLISVAESQFDPECPEIDPDVECSPPESTVNECLINNDCDVDKPLCCSNGCG
LKCTARERRSITPDGFTPEDEKLCRVPVDLGFIIDVSGSIGQTNFDKIKKFIEKIVDAFHIADDGTHV
GAVFYSDNASLAFDFNHLQGDQLNEMNVVREIEKIQVTDGQTRIDLGLELASRELFNPAAGMRI
HKPKIALVLTDGKQTRGELAPDAKDLHQASRLLKQTGVQVYSLGIGLDYDIGELLDIASDDASVF
RSQDFDELVSIVDILIEQTCKGCTRAVDVHFALDASANVGEENFVFMKDFVKAIGYNFIISETGAH
MSGSVFGDVGTLVFDKSQATEINEFENEVENMPYLGDATGNMDKVLKMISNEVFNFDGFTRQSS
PKVIVLLTSSNCDTCQESLEDLARPLKAEGIHIVAIPIGDPLFDEMQVLASHPIDQYAIPRATHQELL
NAEFIQKVSEMVCAGKPGICAEPPVKGECAEIINECSVDMRCPEDQKCCIKNCVASCVLPLAACIT
PIEIAFAFETTKYGIADFNNVKNFAINLLDHFKVGPSKAHVSLSTFSEDVTSLTKFIDDFDMESIKQSI
KDLKHDGGEESNLGNFLEIINTEVFSVRGGIRQQTPRVLVIFANGDINDEQAVKAQEEALHLKAD
GKDVKIIIVNIGSSTKTLDVLNEVATEGSFYEFFGSAELDSTAIETEISEKICSTKVRRSKCQRRIRSSCA
VKDDLCDSDFDCPGHQECFSDGCRLRCQTCEFRCKDKVDLLFVMDTSARIGRDNFERGKKNIKK
MLEWFTIDSNNTNVGVITYSDTANVAMPLPSRGNNNPAQSLIDVQDRLDSIPYSGGSVSHLDNAL
QLAAEKVFPDNGPNLRRDSKKAVVVITDSLDDSASQLELASWPLRRQRVQTEDGDASNIRILAFN

VGQELDLDLWGNIVSPPVEENVFYAADFDDMYNGIRQLAEDSCKYAAAITPPPIIKQKGEPGPPG
PPGIPGTNGTDGRNGTDGRRGPKGERGDTGLKGFKGEVGLVGPPGGGAEEQKGPDPLASAVGIV
NVTDLPGGGRIVFDLREGPVGQQGLTGIIGPKGPIGDTGEVGIKGRNGTTGPGGFFGLPGPKGQP
GTDGAKGLRGDPGLRGSAGRKGPTGAPGLTGPQGPFGYK

>TRINITY_DN7946_c0_g1_i1.p1 type:complete len:995 gc:universal TRINITY_DN7946_c0_g1_i1:83-3067(+)
MSIGFESSTSQLNVNDGITFKRRNSKLLRCFALIVVLLLILFIVFLAMYVRTDDEKNIIAAENEALKA
RISNVETTALPTSQEPATTSQQPTTPKIDPSLPYANVRLPQTIFPEHYDVNLNVYMNNRTVDGYVN
ITVTVKKPTKYFIIHAYNYTEVNGYLKNSMKQYMKTKKQFLYVPNQYYVIEVDEDVEPGTFYLDFE
FLYTLKKSLKGFYQSTYRNKENEEKLVASTQFQPVSARIAFPCFDEPAMRANFSMKITHTPGYFAL
ANMPNTTYRYLPERDMIYNEFETTPSMPTYLVAFAVCDFGSKTITANTRTPTKMTFYAPKDQVEQL
NYAMKVGKSVIEYFETYYNVSYPLPKADMIAIPDFAAGAMENWGLITYRTTALLFDEKASSTSNK
LRVATVVAHELAHQWFGNIVTMEWWSDLWLNEGFASYVEYIGMNSSEPTWNIIDFQVINDMAS
GLGLDSLVSSHPIYIPVEHPSEIDEIFDSISYSKGGSVLRMLDHFLSTDVFVKGLTRYLKKYSYGNAN
TKDLFDTLTKVSFENGDNLNVTKIMDSWVKQMGFPVVTVTIDDTTKKVGLTQERFYKAIKPDLTK
SKFNSSYNYKWYIPFYYVILDISDINDIKTVEANQTWLNLGNGEINYLPSLNLSNMVIKGNNDQY
GFYRVNYQEQTWDNIANILMNNHKAFSVKDRAGILSDAFALAESGRLNYCTAIKLFDYIDKEEEY
LPWKTAVDGISIISSGLTKTKPAKKYYKKFMLEKVTNLFEKLQFNKISDDMLTEKYLRNSIASLACG
SDHKGCLDNATKMFRQWREDDKPVPPDFRNLVYYYGVYYGGVEEWDFVFDKYLKSQIASEKIKL
LYALAGTRESWLLDRYLNYGLNDSIIKQQDVSYIFSYVANYNPNGRYLAWSFMKDNWNVIEERFV
ESFFTMRRIFSSVTSGFSTTYELEQVKEFVTNRVTNPGSAKQIIKQSEERIKSRISWIDTNEKTIEKCLK
DQLGL*
>TRINITY_DN661_c0_g1_i10.p1 type:complete len:253 gc:universal TRINITY_DN661_c0_g1_i10:1712-2470(+)
MQAVTNCMKGLFLIFNFVFWVTGLGLLITGILVKYTFSYMIKLSTNINYNLAPYIMIGCGVFIILIGF
MGCWASIKEHGWALKIYMFVLVLLFLVEIGGAIAGYVLKGKLSKGLKDGMDNAVTNYYTNEDSK
SAMDQVQEKIVKCCGVSNYTDYFNGKTCATVSKNETLRVPVTCCKSEDRSGCQYKDICKAAGDL
GIYENGCYDQLLKEAKEKFLIIGGVALGIAGFQILGVLCAYVLTKKFQSNSKYENF*
>TRINITY_DN118_c1_g1_i5.p1 type:5prime_partial len:203 gc:universal TRINITY_DN118_c1_g1_i5:620-12(-)
KVLFLCGFILLSGLAFAYEIEEIVEDAPKPEPEHVPEPEPEPVKEPEPEPEHKPEPEPEQCSTVKHQK
CSKADKVCSPEDSHCTFTQICTPFFVKKCCKVVSVEKKCVVVRNCIPVYSYEKVCEDVKRCPPRKR
CKNVCRQVDLGEHKVKSICEQECEVVEHPEPCKLVHTCFSKWVSKKDCTPRETCSDIEKRECREV
SSH*
>TRINITY_DN106_c0_g1_i9.p1 type:3prime_partial len:1309 gc:universal TRINITY_DN106_c0_g1_i9:137-4060(+)
MPSNSKLAVLLLILGIGITLSKAQDLDDEFLDRPGQCPVPPIEPNCDRERSPDESVQGCLMDMDCA
TGQKCCSDGCNLFCTSVNIPQPPQIVKGLPGDPGEPGIPGTSGADGDDGIDGVAGPIGNKGATGG
RGKKGRRGRPGPPGPPAPVDGSSLPEVGPIAGGDNKGPGPNAGAIGEEGMGADGFVTPKEGPAG
PAGPVGRLGPRGPYGERGDPGLPGPDGDMGPKGPDGPPGSVGENGRDGLNGDRGDPGQVGAI
GAQGVQGDQGPTGANGAAGYKGLKGDRGASGEKGAFGPDGEKGETGDNGLDGEVGPSGPQG
LQGSPGPRGPDGAVGPTGPIGARGPPGPTGLSGLPGAPGDEGAVGAVGEKGSLGPRGERGEQGE
RGPTGDAGAKGETGAMGLVGRDGTAGKKGALGSPGPQGLRGEAGPPGIQGLPGSDGAIGATGA
RGEPGEQGFDGAPGFKGTNGDSGPPGKPGEAGPQGKKGRSGRDGPAGPAGRDGDRGLQGIPGA
PGIPGRPGSKGSRGDRGTLGKQGTKGTAGAPGSPGETGPQGSRGAPGGIGKSGRPGKQGARGAS
GKDGRPGAPGQQGLRGKAGQLGLQGPKGAQGPSGGNGEPGIPGSKGQQGADGQKGSVGQPGS
PGASGATGERGTPGPPGDNGKTGPAGPIGEAGAPGRDGEPGKAGQRGDDGKAGDPGIRGPPGV
KGPNGLRGSPGLPGERGEQGSDGVPGNKGPVGPNGQPGPSGKPGSPGASGTKGPDGAKGQHGI
RGPKGLPGDNGKDGKRGKDGLPGPAGPGGETGPKGESGRPGSNGLPGKRGDPGQQGNRGPAG
MPGESGPQGLQGGDGPKGERGPRGDKGEPGPVGPSGEDGMSGPGGLDGSVGNKGGRGLSGPP
GGQGPPGMEGIRGRRGPPGNRGAVGALGPAGRDGPKGSSGEIGLPGRPGDNGAPGLPGKDGKN

GDPGEDGAPGIQGLMGAQGPLGPRGRPGRVGMPGFPGPQGNVGAKGEPGVPGVAGRKGKSGP
PGPQGMSGATGLPGQAGKDGASGIDGKPGTPGVRGAKGEAGQAGFTGSQGVTGMPGNPGAA
GAKGGRGDGGEPGIAGEGGNAGSPGQRGPNGNRGRKGYKGNKGNSGAKGDKGWRGMTGPT
GLPGPIGTQGDVGVDGPKGRGGPVGPPGPRGRSGREGAPGDEGMQGTDGLKGSVGNRGRDGD
DGINGPPGPPGPPGLPGGFGGGAGAINMFPPMGNVEKGPSYYGYQYYKSQDGKDFKTAQEEAK
NTVFAYTNKLDEVLQGLDKPDGSEVYPAKTCADLKLCHPDIKSGNYVLDPNLG
>TRINITY_DN12627_c0_g1_i1.p1 type:5prime_partial len:1061 gc:universal TRINITY_DN12627_c0_g1_i1:3456-274(-)
YGENNRLKFNDARQYCQQWKDDQGDLVAIHSNYENSFIMSQLYDVNEPMYIGLTDSFSERTFLW
VDQSSVVYTNWYARQPSFVGNEDCVEMMPYFKTHGRWNDIGCSAERGFICQRTPSKTPLPTPEP
VKDSKPCPAGYRKFMKACYRVVHTPMTYDDAVKDCRTSVGGELGDLVSLHNQFEQSFVFSQFAK
FDFHSWIGLKKIKGEKEFKWSDETPYDYRYWLSLRYDNTVYNDDKCTTQEFSSKVDGKWKVTDC
TQKHPYMCKIERDFDQPHLTFIGNCTDGWTKFYDKCYKLQNYFSNWPAAREKCKGFGGNLVTIT
NSDIQKQMEYLARDAYRSVWIGLNDRAKEYVFEWSSGIPLLPDSYQNWGPNEPNDNSGTENCV
HMTTESSGGKWNDLSCSQRMPYICEHPLECNSALGMEDGSIPDTNINATSYVGTYFPKYARLNAK
SDNNRNTAWCTNITTGAESLTINLGEVMQITRISTQGLLDSSLGFSRGGYVVSYMLEYSNDGTTW
MTYISEDAYDQKPIALPGNIDASKIDGTTINLKAAYLRFIPKLYKNIICMRVELYGCKSVCSRALGM
TNFAISDKQLSVSSSQAGSEKAQLRPNIDGSWCAKDDDYSPFVRIDLPRLTKITRVTAISSSKVQYKI
AYSDDGELFTRFQTLTNEERVFSTVPSTHDLETAISAKHVKFDLLKKDQGKLCLKVELYGCLKDCD
AVLGMETKIIPDRLITASSAKPSNPARNARLSVGVYGDNSWCSSETDNAQYLQLDFVQEAHLTEIA
TQGFNGDSAKYVSKYNVQFSDDGDFWHQYSEELPGNSNGYSIQRNKLKEPIIARYIRINPTEWKN
GICMRVEFYGCPMKPFTAPKILTQKTYNGAIRILNEDFNENLLNANSIEYIELANKVETDITSLYLLE
ESFKDGFNSVTVNKFREGSVITEFTIKTHTSDASLDAAEPLRKAVQTGRVGTFDVDPNFFEIVTAST
DKSTATNDDSGGLSNGGKIALIVFFVILICIVLAGGVVYYLRSRRNGFRLQFQHRAFENPTHVDDL
QNDDMKVEFMNDDY*

>TRINITY_DN195348_c0_g1_i1.p1 type:internal len:311 gc:universal TRINITY_DN195348_c0_g1_i1:1-930(+)
SGCNSIDLGLLVDFSSSKDYDSKQLTKFVREFIDKITNVFAFSATKSHLAYLPFSSRINENITGQWFD
NKYMKTIGDWDYQELTQYLDRVIVPYTPVTGYETGKANAHEAFQATNDKMFNALYGMRYNMT
KVLIVISDGNEDFKEKQNLVDRAIDQLKAKGVIIYTVSIGNNVNWDRVFRYASRESSIINGANFDD
VFNRFFDFAKMLCEDSKVPIPATRTTASTTTVSINTTKLTTTTTTELVPVTISTTTTMSTTLRPIQACY
EDIDLGFVVDFSKMNANDMKERQTANDIVKVIKKIASSFNIAANK
>TRINITY_DN1119_c0_g1_i1.p1 type:5prime_partial len:949 gc:universal TRINITY_DN1119_c0_g1_i1:2934-88(-)
FFFNPQTVMHEKKFSRMKFILLFSFFAISLGQNCNRMHKKIGCFWNDKNAKVLFNDRDSINNSTY
GALSDKLACKCNAVARSNGFKFFALRHFGICIGYNKITTGSKSIEHCIDTKSNGVCNLGDMNTCT
GTQNTADMMYQIVPEKCPSRHKKVGCYTEDRNATFLFNDRSNIDWPNFEKFLVELACKCNKVAL
EKGFNFFGIRFYGVCVGFKNISTEEKSAFHCKDPNYRPYRPCNMKDSNVCAGDHFLTDMIYKTER
QNVDKLFSEWSGWSTCSKSCGGGTQKRTRECKDKKSCSDAGGEEFKSCSNSFCKGSTGVAKAQE
VVETFLETFHKVEFPDIKPSGKYLKTIFEIQKAVGEEFNSVDPSVKGVPLEKRLTELEHFLKIAVEGG
SIDFARSTLGKGMFVFMKKKIKEGLPINDIDTAAKGFLNKLTGDIGSKKMNEILSSKGDATLMFTID
TTGSMAQEIAAAKAIAKQIISATREYEVDYILSPFNDPRTGPVRYRSTNDRANFVSDINKLLASGGG
DCPELSIKGMLNAFDKGPKYGSPMFVFTDASPKDATPSNMAALKAAASSNNAKITFFTNLKGCG
KELGIESYKEIASHTGGQVFPLKNEAELINFTEYVKSSLQQDTIIAAGGSLNSRISKRAAPRSHIFMV
DQSITRLIISVAFEKQNTGKHIKLLDPNNKNVDSSTMLAMSLIYDIKNPKVGLWGLSFPHIVGEFGY
NVQAVTENAIEFAHSFLHQHNVKGSDVTVSLENPIKGIRNMMVIRLGGADHIDLSTIKVHLVDHS
GNKLVSNVMTDRLDSDEIRNASIVPPTVQFKVLLTGKTKSGQQFQRMSQGSSTPVSAFMRTISAGY
DFTATMGKDWISIQIEIVNKGSAESFNFQAKTKYGTLKNPNQTKTVNKESMITFSYKPPNRHDLK
GKTDKVTVIAKRASGEKINHFISILLIK*
>TRINITY_DN193641_c0_g1_i1.p1 type:internal len:173 gc:universal TRINITY_DN193641_c0_g1_i1:2-517(+)

GSKQTGPTGDHTSGKGKYIFIEASSPRKPNDSAILQSPPIRPSYGAVCFKFAYHMHGAHVNALYIYK
NVNGKRTRVWKRTGTQGNQWRNGQANIRGLSGVFNIEVKGLRGVNYQGDISLDDFVVKDGKC
PRPKYCDFENGRCGWREAKNLDKFNWKRSNGGTPSKGTGPATD
>TRINITY_DN1821_c0_g1_i4.p1 type:complete len:637 gc:universal
TRINITY_DN1821_c0_g1_i4:304-2214(+)
MKLLIKFIVVFIFYDFALATTPEEFLKEYNAKIGQQGWPSSDASFAKATNISDENSEKAQEESKKYT
KIAQAWRANASLINLDGADADTKRQFKLLLTSATPKCESVIADISKVGSDLENIYSLGRIPNDRTKI
KTINSDKASLSLDEHLYLILANSRDPDELLYAWKGWRDAVGPKLKDKYTKYVELNNIGARENGFK
DKGEYKRKVYEVDNLKEIAEKFWDELKPFYQEVHAYVRYRLSNVYKDLVKDGEPIPAHLLGNM
WAQSWDNIFPLVKPFKDKPALDVTSTLKKKYSVKKMFEVAEEFFKSIGWEELPPSFWTKTLFEKPA
DRNVTCHASAWDFSIVKDGSKDVRVKMCTVINQEDFITIHHELGHIYYYLLYWDLPSVYRTGANP
GFHEAVGDTLVLSVQTPDHLHKIGLLEKVEDSKESDINFLLKTAMERIAFLPFGYLMDQWMWGV
YSGDILPNEYNKRWWEMRLKYQGIKPPVSRSENDFDPGAKYHIPADTPYIRYFFAHILQFTFHKYA
CKAAGNTKPLHKCSIYQSKEAGKALGDMLRLGKSKRWPLALKNLTGSEELSADPIKEYFQPLLVF
LKEQRKEHKYTLGWSGTDDNSNSSPNCQVSIALFIMCILFFLFGNKHF*
>TRINITY_DN5500_c0_g1_i2.p1 type:3prime_partial len:1183 gc:universal
TRINITY_DN5500_c0_g1_i2:3546-1(-)
MEIKNFLILLSLLSLTSQLKAGLVFNETGVDQPVDLFFVIDGSGSVGYELFDGQLQFVTELTDQLGG
VSPFNVHVAIAVITTSPYQAIGLTQYQVNSEFKQAVSEIFYPRGATRIDKALLAVERELLPTSRAGVP
KVLVILTDGIQARWPGVYNTHVIIERLFNAGVSPVVVGIGSDYRDLDRLNKLAIKENHHLNEPIEK
LFLTTDDMEVGSPALVQKAASTVKKILSNVARFGYEVRSDFAFLLDGSTYAEDQLKLMEGLASYFQ
INRELSRFAVASSQGGGRTYIEFNEAQLSRDFKAMLMKLNVDSESIDIGIAAKYAYEEIFRIEEGERYS
TPRTLVIFSGNAEAKDSTLFGKYYSLLKKANVNLVMIAVGGGKGRSNLRNFPVDYYLPDFNDEVS
FTEECNIIAREMHNYIIRFASDTQRSKKCLHDGDLFFSIDVSGSAGAYYQENLNIIEKIIASFEISNED
SRVGFAIDRDGNTQVTPLDKHNSLESLMRDFDSLPYYPRGRSDMVAKLKAAYNQFLTSSRIHFQDI
LFIFTDGYLNRKSESESKELIDKLKAFGVEVFVVSLGYDVNFPFLMTLSSDKGHFFHVNQLPKDSDS
FVNSIINKVCDYTETFQDKYEIPINVALVLDGSHCSEKFHEAQKTFSNVIIDGLDNSPLAKNYLFSYS
SGVQQLDSNSSLAINFGSDGNRLDLALNEVVKLFQSISSNEVNMAIFVTNAGLESEFNATMSQAR
KLRSLGVITMVICIGDNFNYDVIRTLASSRQLIYMIPSPSQLTDTKFLRHIISAAFEMAESMPPPRLEA
GKSWYPAFFGNWVDSNSLEGEVPDAPLKAYHFGPTVLWSKPIQQKNYIIESTIIPAGSESGRFGTCG
HIFKHKVGATKKEKYISKSWGYFLWSNSFLYITTDGTETSPRIEFDYSSRDKLNLKTIVDGSKVKIYI
DGVFIRSIELEGTEADSQSHMFVGVWCHRLSAVTVEDFKVSALSNPCTVNNGGCSQICSADPYEF
KCSCIPGYRLVEDESTCVAESTCPELITTTVITTTLSSSPPTTNQSKFISTTTKPDELIDTTATIISTRESDI
TTTLILSTKSTEKPIEKIEKTTTSAIGDILEQTTVAPIKTSTVFVPTTKEEVILTTKEMAETTLTVPTTASP
VEETTTVAPVSTAAKTEATTQAVITTLAATTK

>TRINITY_DN4770_c0_g1_i1.p1 type:5prime_partial len:1635 gc:universal
TRINITY_DN4770_c0_g1_i1:5012-108(-)
FMSDYGNSRIIKVDLKANKMNTFTVYRYISRTFFGIPISRERIGSRATGLAIDKIENRLYFCDDKVSSL
ESVRLDLTDHQVLYHNTKYSSGKFVLQTNKGEVVQPFNIEVFGDKMFWTDLYHGAIFSADKWSG
TNVQYVSGGLGRPKDLHVFYNKPKQGIKKIYLFNYLTDSSNKTALCFTSNKENVTEISDPPRCPKG
YIEKQFSSAFSNCSVTRGSDFVCKCENWMTCAQRSHAGVVACRSPSRPFMFFKVRECEQVVEAGF
CPLNITTSCSDDSKNCTVDDDCVSGEKCCYSKICERNRCHRKVSKRENPCLINNGGCSHFCLAVG
DSFRCDCPDTMELNETNPLMCQKIPPPTCDPPCLYVRNTTSIYRIPLNSTDGLLIRRRSVAQVLFHT
KSSIESFALDPIQKEIYWIDSSSGMINKSMLNGTNIKVVFSNRKEAAKFLAVDWTSDMLYWCDVDI
KAIMVGNTDGSAEKKLIQFDYKKKILGMTLDPVNGYIFWVLTSGSSELYGSSATIERSNLDGSGRK
TIVSKNVNYVKDLSIDSKRKRLLWVHYNPNIIESVALDGSDRKTEFALDNIASSMTLHDQLQVAYIS
QMQTIYAVKVGSPISNKSVIHINKQRGSLDQIDIHEFDISIAPRPCFAAKCSHMCLRSMDTTVCACP
TGMVLVNETKCIVAETGSEIIYFQNLDIKLVKYYNKEGYDMVTKLLTAPVNSSLDSAPLDYDSVEN
VVYFASGVTVYKLNIEDQNIEPVMTLDWIESKQIRFVKYDIISQLFAVMEYKSSKIFIVSTDGKIVSQY
NNFPSDMKQIEIEFFDGKLFYAVKDSITKLDILNDKKTNYNSENLEIESISIDVKDRHIYWSNIDGLV
KRSDFDFKNQAVINSDKTKHSISYIQILGNRLFYHSRWYGVIFSVNKNNFLDNKGITSAVKFNSKW

IAVKVSGDKDICNLPKDTGPCKALIPKWYYNKEKQQCEQFNYGGCLGNLNRFESQKECNDHCQ
SKNAGYCPYITNNSTTKCSLDKDCSLQEKCCGDKIKSCVKSVPKPDYPCRQNNGGCTHFCIGNSK
MFKCSCPSNMVLNMTDEKSCVKAVESGCMLPKDSGPCEALIPKWYYNQEKHKCEKFNYGGCLG
NKNRYDSEKECNYRCQDGCMLPKDSGPCEALIPKWYYNQEKHKCEKFNYGGCLGNKNRYDSE
KECNYRCQGKSSCTDNPCKNNASCISRSDGTFDRCMCYGRGFFGRYCEIDKETEAVTIRLQQNDE
VTKVTVTEIKDEIKKSFNIYCKENECKMEDGSASSVETRKKRKRRSSDKELTFESEDVDVLSFNEDN
GEVNVKFVIEKVQDGDYLVMSSSSVRSGFDEFGDSFGESLGLSYLGMTSDRQVAKSQSEDEDGNG
SVTIAVVIVLLLIIACVIIGFAAYKYRQRNAKGPSFGIPGFKKTQMSYRDLDKSSGEQTIPNDYHNPR
EFSNPVFEALETEKAEPMACSIDYSDNNKYKVGLEDSIVPATEKKPIVDFVEVNESGKLVSSNGEEI
PELLPPPYSSRPVTPKTQGTQQAYPPPYNSRNRPLRGTVLSTHGIGNTDATPNSFLPGDEDLFPSTL
SLQNSVSLEDSYGSELSDSLSSSMNRMLKSEANITAPKTIQIQDVKGNSEC*
>TRINITY_DN1071_c0_g1_i1.p1 type:internal len:660 gc:universal
TRINITY_DN1071_c0_g1_i1:1977-1(-)
DPKVAMRVEFYVCPPCGVTTPAITTTEQTTMPTTTTTESTTTPTTTPATTTESTTTTLPTTTTTTPTTT
QSTTSKETIIITEEPKPTTGLPQKKTTTTPRTTTESPTTTEATTTTPATTTEATTTTQATTTTTKATTTTP
ATTTTKATTTTEATTTPTTKPCECKVLQPVGVSDGRIPDNQMTANSVLVNVSGPTFTGASQARLN
NKPSKDGVGAWEPAQQEEIYLEVHFNKIEDVREIRIQGHPTDKKFTKNFFLSTSQDGVNFEFTDEN
LYEGNKDGNTIKSTKVSFKTKAVQIHPFNDVSVGDPKVAMRVEFYVCPPCGVTPPPTTTLPTTTTE
KPTTTAATTTEVVTKIITEPPKTTTAEPQTKCPPGQIEGNCSSEFDCHNRDICKPQITNGCCCPDGL
YKSYEICVKQEECGCIYNETKKNEGEVWYPNDYEVCKCLNNKQVCSERCPNTFKCAKHEKKIFK
KKADKCCDCVKNCQYCNHHGKYYGPNDTWTVSINDMECKTGRCVLDEEEECAEVEIDTVKCP
SECPKGKKRVGSADKCCECVCEECTVKSQDDCETNEEYVKAEGDCCGACLIKMTTEPKISGTTEI
KTFPSTTEESTTPKTFRPVTTATTRGTFPPLPPTTTTEKPTTTTEKPTTTTTTTEKPTTRGTFP
>TRINITY_DN14015_c0_g1_i3.p1 type:5prime_partial len:2566 gc:universal
TRINITY_DN14015_c0_g1_i3:2-7699(+)
PNEGVTIVFSNGDYELKKDYYAMIEAKSNGTLYKGKTVVHVTLTDKNDNPPRHQNLQVDIAISES
TKPGTIVTILYADDADDKQNGEIFFMLESASSDFSISSFNNTGVIRLSKELDNEQETSYKLMILLKDF
GSPSSKFSNMATVNIDVIDENDNTPLSEKTFDTIQVSRLKANGVIYQVKFTDADSGLNGQIEYDIEP
FKSFDDYFTINKTTGEVSLIKSLETTTEDNFWLFIIATDKGNPRRKGSFTLQVNVNEPSSLNPIFNPD
TYDIEIYENRLPNSLVARINVLNVDPNKDSLTFKIVSGNDGDRFLITNKGDLVLKQNVDFEQQNLY
NLVLSVRKNQNSVESVNQATVKIKVLDVNDNNPLFEKTDYTFIVTENTPINTFIGGVSAVDQDFGE
NSKVSYTIFDTTKTFIIDSSTGRITVVGQLNSFVENEYNFVVYAFDNGNPRRASSANVKVIVEDINE
APQFSPPSYNLNVSELLHPGDHIITPKVIDNDIGINSQVRLTLDASGNIDDTFLLENGKLKLNKQLD
FETRSTYDLTLVATDSGRLPLSTNAKVKINVMNVNEHRPIFNINPYMTEINEDQFSTPIFQVKASDK
DALSKVRYVIEDGLDYSLFSINQITGEITNSRLNANFKKEYSIQIKAFDLDNPQFYSHTVLVVKVIDI
NDNAPIFNKAQYSFTISDQTPVGTIIGKVFANDADTGFFGQVTYFLDNNFNGPFSLSGNDILINGD
LDFTKVSSYEFVIKARDSPGSSQGFRTTSTNISVNIVETNGTMPIFSREYYEVTIFSNISTQYTVVKVSA
TDNDGNEFGEIKYSLISDSSNLFQVDELIGIVSLKDGVQLQTRSFKYTNTLQVRATDGAGLFSTAYL
VIHVIPVSGNNPPVFLQIQYLSRVSYLTSKGESIVSVIATDPEGAAVSYEIKSGNELKKFGITNSGIIYL
EDQVENPEVNVYRLGIEAIDENGLRSDPDAQVIIQILYFNSTFQCKLIPTFSSNLYISQVEENKAPTII
TSVQANGSSQNDSIAYSIAYTKDVTQNDFEINSTNGVITSQRTFNYEMKNSYQFEVIAKDTNSLKE
GRTIIMVNIIDVNESPVFESSNIVRSLSQSSPVGTFVGLAETSDADKNDLITFSFVPANIGNIFQVDA
NGIIRTKSSLNAADSPYNFTLIASDKVGLTANKNIKVTVIKTNVPEFSQNAYLFSISESLNSVRIGQLS
TKQILEPVGYRIPNNNYNNLFIISDGGIISSSKGIDYESDDRKYNLCVEVFLQSNPSLFDTCDVTININ
DINDNRPVNKKDGQYTVEVSENVMVGKMIITLDVEDIDSGEKGFLNYLITSGNNGNEFRINNKG
EIYTLKPLDYEVKRSYSLTINVQDNGVPVKTIEAPLTVVINLVNENDNNPIFNPSSYTISISEQQALTT
ILKVTASDKDALIKSTYSILENEDTFSIDANGNINLVKAVDFEISSKYQFTVVANDGSRSGYAIVTVDI
TNINEKPSFGQNYAIEVSESTPVDTVILTVSATDVDIGVEGDLEYSIESGNDENTFSIQSNGQIKLLEE
LDYETKTEYTLSIKATDKGQPQLSATTIVNIKVRNINDNKPVFSKQKYELTVKENSQLSLTVSVTDA
DNLGNMKFSIPSSKAAGMFKIEINTLNTNEAVVSNIKPLDRETGAGHQFLIKVEDDVGNQGLSEF
VIVVEDENDNNPIFTSEEYNVTLFSKLNVNDAVVTVTATDADATYGVVSYAIISGNNDNMFRVNT
KGEILINKQNYDSRANYVLTIEASDNFETPKKSLTNTNVKIQIQSSGLTFEFAQDQYKYTKSEINIGD

TFGPITGTSSTSLDQIVYEIKDSSGTFEVTSNRNIKVLKKVTTDQNFCVAATTKNGGTLLDLTQIHIL
LAKTNENRPIFQFTNPKVVKISQNTLVNSVVAVTTASDSDTGSTITYSITEGGEFFNITNEGAITLKN
TLQTSTVNQFNVTIIANDGTFNSAPANVVINVKKENNFNPKLSFSSLNLSITEGNKVSQVLTDTDT
DADGDQLNFKIVGKTANNYFIINNTVLESKSPLDYEQVRSYEFCIEIKDSIDTNSRSVILPVSISVTNI
NDSNPVFTSKSNEVEVPYTWPPDITIIEASAEDSEGDRIEFSIVNSSFSSLFSIGKTSGVIKLNQRLKEA
KVYNLTVTATGVQSGKSSNDNFILTFTAVEAMSQTMPITFSDVTDNTMKVTLNAKEEFGEADYEK
MNTIYVYAQEYLPNLDEFVTRTLMEPVSYYDANTFESFRGRRFMIAKVDMERPTQRRKRRDTSLD
EKNGILTILIGNNQCANKYDPTIPCNGPLKAGQSYRYQLEGDLLNKRKLSSFTEPKTTTSAAQSVA
QFSASNYTFNIYYIIGLVMLGLLLAVSIWVIAVLLLYKRRGTKLMQLRESMLPPAINNAQTIANGKF
GSNARRYTNPVYGDYDDDRNFTSDFNLPRGQNNQELAQLKQQIIPIPNQNNGRANYNNRSEMV
NRVPRHFDNPGYREEERKPTYTATVMSSPTVPNPPPPPPPPPMENTRLPSSGTARSTNQPRLRRTSS
GRVIVQHLSGHQ*
>TRINITY_DN942_c0_g1_i5.p1 type:complete len:5116 gc:universal
TRINITY_DN942_c0_g1_i5:15664-317(-)
MKMKLALLVLFTGTTLAAPFWVLDDNLPRAEIKEVGCSGKRFQNGKSHVYEYEGVTYNTIEGST
DKKTGIKLTATATITPMSECKFMMQLKSVELMSKSMNQDTFTTNAESAKLKDELEKLMVGFTMK
GNQIVTIHASENEPTHVLNIKRSILSSLQLDESTEKETDINGICDVEIVTKSGKIVKSKTLSECTERAQ
NEIGFQSTSIKSATTLKPLDSTSKCEYVMEGDVIKSVVCEEKHIFKPFSAGYKTPSGAMTTVKQSLSF
KEYTVAAISAVPSDFTVETNLLFDHSTEEPTATSTINDQVEKVMLRLVDPAQKSDLSAKENSAQEFK
NLVTLFRRMDEKRMEATWTKYFDCIKSGVCTDANTRDLYRQYLLDGISHCGTSTCIAMVRNVIV
NGQITGERMNMFLRSIPFVTKTTMKMIGHVLEICEKNPSRQAYLTLGTLLVRHCKQNPEDCSENL
RNPVTMAEEHLVKKLGQECEGQDDHERVEELLRVLKAIGNAQRPARVQEKLMKCAEKSKHSNI
TTSIFDAFRGFSCDALKQGELLRVFEDINNEDETRIHAFITIMKCPTVEILEKVTMMLEKELSNQVA
SYMWSYLTNLMESSDPRHQVARDILTKMTAQKPLREVNLPVHQFSKAFEKSLHSEFLKSGLTTEG
HIIFNKDKYIPKMGMLNMKANMMGNSLDMLETKIGLDGIEPILETLVGPNGMLPKDLNIDLGSY
GKFNSEFLRKMLEKKNNKRKLFNDNLTKMHLKANKQFEEPCAHLSVKIMGQEVSVLSYDDILW
MIDQIDNMNAVQLLTNIAKGGHKTFSKSLMFLEMSHNVPTGMGLPLKLKLTGSAVGTVEFNGKF
DIRNLFWGTPAVNIKGHMKPSTVVELSGQMGVEGHYVSTGMFVKSSMFTSQIVKGAIEYKHGQIL
KINMDVPEEPLAAVKFSSSPFLFMNEESELVEGTSRFVNPEHCTKSALLGYGLCASMRLPIAFREYE
APYYPLSGPAHFAVRAIRGDEKLTTWQFLVELNKQSGTTFGNIEFSTPGAAYERKLNGKVSMKQE
GKMNMFTLSTEGYDRKASVELNFNKETYSTAIITKNNIFTTKDMTTKLAIFNEEIDSGRQYGIQAL
ATMDWYTFDHITKFVRKPNVYMFQTSTTYYPKKVVMGTIVYDTKENKLSTRIQANQFQQAYEFS
GKFSDDGQEKGVTVTGKCVTSGKSITLYGGIQNTKQTKQILFTADVLGYSAKQYIRYENNNDVHT
VSQFLSVNGKIISLNGELSSFTEMKRLKFVADIIGRKADTTFLLTNNKGEKSLSVDMNAMQKHAN
FKTGCYQAKDSNECKFNFKIGTKEGEILMKQIMRDGNVITTLETLFDKYVAGFRNVYSNKNANA
KSLCSSIFYGTEEKEHEPVNVCLKYNAASDEKKILAITFDIVDRNQQGEINMELTKQTEFVKMQTE
VVYNKNSLAKAMQSMKYSKDLNNEIKMEVNVGKYNAGFRWFTLKTTNGANVGIEGYAMDKKI
QFVNKYVKDVVSGTTESKVTSELYINDKLVPVTTETIFLNGKGIAGPKFQVNIGKHSVSYATLVTYG
KGVYGIDLEFLVAKESNMLFKLFNGAAASVTPKKMDFQYKFGFGINGKQYTYGGDMSYNNLGN
DEKMEHSVKLGLQYSINRRSSMTFVLSNDKTKAALNMEFEYIPTKSVSHSIEYTKNDRILQVSVEFL
PKMFYTFKSQLVKNEGYSLASSVTLNWKNYNRVLKMINSMVNNDKKFELSTVFGKDLTFTFTFG
KENQKSMTLSASGFKRSLKVIGTFVKPNVQLQVYKNGEKIMDVTTSFDGITNKFQFALTNGKDTY
LKMMKSYNEFVLASGKETYLKMKKKYNEFVVTSGKETYLKMMKTYNEFKNQYKFQINGKIWTK
VQEWYLKMTKMTKDNKITKFLAPFLDLTVKLDRETNDMKLVLSNGKQTFVKIVKSYNEAKNQH
DFQIFGMSKEWVDLMAKYNKRNKVLKVSFSFFGKELAMFTLNMKEKDTVSLTTGSDYWKKNIV
LTGSLDRSQKSIAGNFQYGKTKVQLKLRADWNNMILENAFTFNDNVAKIGLLREQNSMVFRVKL
SPTLMAQVVMEVKNDNIIKMTIQRSEGDKLIEETSMQFKLSKDLYNLAFKWNPETFKAVIRAVQN
MIQNGYYKDIKPFITNVFVKGQRMSRKALTTTVMTFTEKVKEAKTKLIDLITNFEASVDKINFVAIR
EQIGQLSLKTIQKLKELVKVTLKELMTKLENAKENIPELLKQYKQIRNKIEKISKDTAEVLPKFYKET
LEAMDKQYNKLNGYWKDVSKVVMSNFELVSKNLTDSTKPVVEKAIYLISNFKLRGKAVKEIVSIA
RELVEKYIQILIEQGKIIMANGKIQLENMKLKSMEHYKNLKLKGMEQYNNLKLKGMELYQKGKM
PYLDETVEGIIKISVEKYMDLKKKMTDFDFEKYIQSVRDQIMEKKINGKTIVEHLNTAKEEMKNLP

PALKENYVKLIELITKLTKALNTNLKEWKIFAKPLTNYMKLVALSTKKHFGPILTKSMDKILPLITGI
ELPDFKMMALEQWKQLENFMLPLIKPVGPLYENIKTQIRNIKVSGYPIGSFVDFNIEIAKTFIQTSLK
TTKQKLDENVKMVTEYANLIKKMTPEEIMNQITLKSTELTKEYKEKVLKVYEERQERLKVLVEKLK
ELYNEFKQQYERITAVPVQDHIDDMMFKSSKFVKGNLEEISKVVKQITEIEISKPTWDAWKQADV
MGHLDKYGINQKMIDFIKAVKEVDMKRIAQKTTRLIQDMISTIYSKAFVRFVQVYGKLEKVHDYL
RSIPKKEYEEWVKELKEFSLENKERVIEFMGKMYKQTQKKSLNLYTQSKFLYAIMHKIVLKNMEKI
NINLVKPSQELYSDIKYRSEQVYGDLEQPTIDVKNHYQKIINDFVEEQYTIVKGAVLRHYKSLEEYK
EIMYTKAQEKMEEVKKLIELKYNELLAKYGDMTYEEMYDNAYKYGKSQYKVVRKTVKRGYKKL
REQMEKMEERLNALYQLYSTKYSDLVNRIKTEIIPQTQEQYQKYRLIVINLIKKYQKVLMEKMAEL
KTRGMTIYNDLKTRGMAIYNDLKTKGMTIYTDNKDKTIKALFREVKQNAIKNINTVYVLVKNFG
DEKLKQVRTIIVTYYNKAKGVMVDVIIPEVKMELVSIINQTLRSSKVMTEEIMKAYTPHMVLAKNF
ANKGAILIIERGTVAMKASEKAIREFMDKAPAMMKDIVKKIKDQEYYNKIVNHEVVKKYIAKTQE
MIENLKTKVEQLKQDPTVVKYQKMFSDKVTELKERAKIVIDKLNQRIEELKNHPMTLKYQKIVTE
KMTELKKKAQLLLEDVKAKIEELRNNPSVLKYQNMMNEKIAELKAKVKMLVKELKKRIEDLRK
HPKTIEYQNMIKQKIVDLKKQISMYKQKIENLYQHPFVTKSMVTMKRIKRSALFTADKINVNLQP
KIKEASEMAQKRIEKVQEQAMSAVQFFKNEPEEAFWTGVSNLEKSGKDLYEQIKRLFNKELIKKQ
FDVLTTDAKMQWKIFLNDYFDDWSKEASRKMYEQVVTLVKNMNMKIIDMKMNMQKLPEVYK
PIIEDYKNQIMENYKNIVEYSLQNMIQGKVYLSNQWVELNNQWKACPYRLIMENPVWSEIGDEI
MQHEAMTAVKYVSNLGNLKFQAMKTILMKEYEIKKQLLNQKITELQVSLKKTVEDLRSKGLKNY
QLAKEKAEQMVKNIKEIAIKMDKFLEEKTIGEIVEITLEKYQMLITKVEFLRTQVVGLSQWYKEKA
TVIYNEKRKRAEKIFEEQKEKVLAVYQKYKAQVMPIYQKYEDKVKQYYNKYSSIIIEQYKMLLKKS
AQKYIQLRTKYEELDLKNKLNKLQKDAIDMYQNKKESALKLWEESGVPTKLVELKQMTIRKTIEA
LKQLPGQTKEQLTAVFDKYLNELKVQYKKYYSAGYTKLIKEYNDLKAKFEAKFELNRNLLIKLAK
PYTTPVVVAYKWVENEVAETALFVYRYHKLGERYNNGRELIKSEIERLTPILKNNFKKYNAEIRKY
VKDQADKYSAQALEMSKDLKQKAIVLSKDLSKKVVASLKTYSLMTGDKILRSIHSSMKCIDNVDF
GKVQKQISKKAQDFYREFTKYVIIDPSKGEILINIPHGDMVPSFTHQFEQIKLRARRSVQVLKENGK
VLMKKINNRMAELKVKIEKVRNKVMKKVMDNTVEVRRDLLSSYNINKRIFDRVYVKAQIIYKKS
LRKATNQYKVLKTKSQDYIRKATAQAKAYYEEYMTKAKDYIRKNTELAKVYYQKYMTIMKERYL
TVNKIIMDLYNTPPAELYKQLKESKASLKVIELIMHLYNTPTAELYKQLKASPTYLRIDRIIMDIYNT
PLSQRLEQLKTSSAYLKVNKMVMDIYNTQYMDIYNQRLEQIKNSPRYIKVKGMIIRLYNTPAKELL
SKLQYRGQKLYKIWFNKVKVMINKAIEKNAPLIKARLNAIKKELRPYTESLKTMYKKIKLGYSVKD
SFYPMYKQMKFATKFYRGEAMRQARKIKAQICKEDPKLCKAIKSSAKVHSFIINKYATRVNKVLNI
ALDKYQEGVAKTHRAYRHALNMIARPKSTYAAVAMVFKDHYVTFDGKSYEFDIPSSKCQYVLAH
DFQNADFTILKTQDAIKINNKDMKVVIFKNGKTMTVFPDKKISNGLPVQAGESKCIRQHNEIICN
FVEDKLRVVVDLENDITTVSISAWHHGKLQGLLGTNNRESFDDMKMSNGDLAKDQLQFTNSYE
MTGSPKCQLNKVAFKKLQCGKVTQKCKKLFENPKSELAGFFNIINAEPFIKACERDLVSCEKSKEA
KHCSSVNAYLSMIKSRGYPVELHSECMKEGERMTGDKWQQKQSNKEFDIVVLVSQRKTVNKKPI
MKVLQKLQSSFKSNSIDTRYALIGFGGKGVNEEAHVHALGKGASVFSNMNDLKRAITNTMVFNG
DEESNDAYHAIVEASLQRFRPGAHKIFVMFNSEPHTSHKYGATLKEAIFTLEKESHASLFVFDEINF
KSLNKKGLTIGQTNSKIYTTKNLKGLRLSGMELPKSEFKDMVESTNGGMFTNELMNKQNELATV
LFDAITDTVTQNQRQCLDCEKHTTWYGSTRQLCHPVAC*
>TRINITY_DN1189_c0_g1_i1.p1 type:5prime_partial len:449 gc:universal
TRINITY_DN1189_c0_g1_i1:1554-208(-)
VSAKSQVIAKMKFFLFSIAAILLVNSALADNAVQIPKTCEDVLAMETCDRLRAVAKKFHEKVGTV
NQAVTDAFKHHKTKAVEVLTYVKEYLVDKAVNFKCEDVLPADQCHNLLEFAKRLNVKASEASIA
LREAIADGSEQAKQLYQRAVSFLVNDVPNLKCEDFFKKDDTCDKIRNFATKVNAKAKDVTAAVK
KAIVENLENAKDGLKKAKQFLKAKISCDQVLNEKMCNTIQSIGDKFSVSMKKIDAALREAYANG
VRKWSELYEAAIKYIMDKWSIFGDDEPQRERREIQPENVKKAIKNLLDSLKVKSKKVRAAIMKLIE
SGKVKFGNLRKTVKQLLASLKGDKKSEDAVTAVYKRSVVDTLKETLQKLKEQIKDLAGKAKEQA
KAKIAELKELIKGALGSLDQDQSDELDQDFEMISDVLEAAADESFDEIANNALENHLGQYY*

>TRINITY_DN3182_c0_g1_i3.p1 type:complete len:784 gc:universal TRINITY_DN3182_c0_g1_i3:2869-518(-)
MDEFDLDRFSASDSCAKNRKKKSGNRSNSGILSLIATGTFVLLAFVGVLYVFKSDSKGSSFDELVTT
GGAVRRSVPDGVSNCSGVQLKQCNTKPCQSLRKYVLDSMANEKEMVDPCNNFYKFACSGWMQ
KNRIPKTSSTFSTFSKLNQKVESILHKIMKSKKKEVGMMKNVKNFYRSCENLAQIDKYGKKPMIN
LTNYLNSWDLAGDDSWNKNQWNFTATLLKIQTEFTSSGGPLVSVHVSDDPRLSTKHILEIDQAGP
SLPREIYLSKDPKKLKTLKAYTEYLEDVAYELCKDEPEKKCSKDQIKKRAKETIDFERQLAKISVAD
DVKQESWFKRMRLHELEKAVPDFPWDPYLNTIFKNITKIRKDQYIIVPALSYLKKMMGLVRNTSA
ETLSNYIVWSVIQDEVPYLSKPFLQIRMRYKEKVLGSKGLRKRWKTCVSYTNEYLGEILGKLYKDQ
NFKDSAKVTTEHMIKLIREAFQKNVNTLSWMDEATKEKVREKAGAMKDQVGYPSYIGNKTRFEE
KYKNLKIHADQLFQNRINMIKFAHMRMLNKICKEVDKEEWPMDPQTINAMYSFNQNGMIIPAG
ILQPPFFHGENLSMAMTYGAIGSILGHELTHGFDNTGRKFNKHGELKKQWWSNKSLAAFKEKSQ
CMIDQYNSYKVRGKFQINGKLTLGENIADNGGFKTALRGYNDWLCQNKADNLIEGLPFDNEQL
FFIAFAQAYCSNSRENEQYLATLNDRHSEEEFRVIGTLSNSKEFSRAFGCPIGSRMNPEKKCSVWV
NEETAK*
>TRINITY_DN984_c0_g1_i1.p1 type:complete len:1485 gc:universal TRINITY_DN984_c0_g1_i1:4558-104(-)
MVKSSWQQLLLLLILFCGFQSFEIDGEDKPGSCPPPLETLECNEVEPGELSCLTDSECSGTDKCCSD
GCNLICAAALPEIKGEMGAKGEPGPPGRNGTTGPQGPKGGAGIKGKKGKQGKQGNKGRRGRP
GPPGPPANDTEPGPDPLTEDDHGKGPGPGGILEDDLAEGGVAPGVLTTQDAPEGEKGARGRAGP
RGPYGERGNAGPIGDAGEVGDKGEQGPKGQPGPPGDDGQDGDRGAPGPIGEPGERGERGEIGK
QGRPGIGGYKGDKGHPGLAGPKGVGGEEGLKGDTGESGPVGEGGPAGPRGMPGKTGNQGPPG
PTGPTGMSGPQGLPGSAGLTGAPGSPGDQGPQGPKGDMGPVGQRGERGEQGERGLTGDNGPK
GERGLTGTAGRPGRDGAQGQTGTEGRPGLIGKRGPIGQPGSQGSEGPIGLPGKRGESGDIGLQGP
DGDKGQIGDSGAPGQTGGAGLPGRRGIPGREGPTGPQGETGRRGPPGLLGTPGSPGEMGSKGLR
GERGAPGVIGGKGATGAPGSAGETGAPGDRGLQGLQGKTGRPGKTGSSGIPGKDGRPGSAGPQ
GLRGVRGAVGLQGIKGSAGSNGLVGENGALGKKGDTGDPGLKGSPGAVGAPGKDGNVGERGV
PGAAGERGAQGPIGPIGQAGPPGKDGEPGKTGERGSDGKLGPQGLRGAPGLRGPNGNPGVAGQ
VGERGEIGADGAAGSKGPNGMLGTIGNPGAPGLAGSFGQKGPDGQKGAVGERGPKGFRGEDG
RDGERGGDGIAGPSGPQGVSGVKGEMGRTGANGIDGKRGLPGLMGPPGSTGPPGDEGAQGQD
GAKGIKGAMGPRGDKGPAGSIGEPGIDGGAGPIGSLGGSGPKGGRGSIGPSGEQGAQGLPGPTGR
RGVQGNRGSIGPVGLEGREGVKGSSGEPGMPGRTGENGVPGMPGKDGRNGDPGEDGIPGPMG
KPGMEGARGPRGQLGPGGLPGMPGLQGIQGVKGEPGLDGGDGLKGRKGSTGMQGVMGSSGLP
GKPGDDGAAGEDGIPGTPGDRGAKGDIGDVGFLGPIGPPGSPGFSGDIGEKGMIGAPGEQGNDG
EPGNPGEPGYRGLIGNPGAKGNKGGKGEVGDRGHKGYRGMRGARGPTGNVGDQGDDGLDGL
KGAKGPPGLPGPRGRTGREGPSGPIGDPGINGTKGFVGIRGNPGEDGSPGPAGPPGPTGNPGFPG
GLFGGALNYYPDQQQAEKGPLYGYHYGYNYRYYKSRREEDTDKEEIKAKNKKANRNVFRYAEE
LKMRMEAEIKPDGSEEFPAKTCKDIQMCFPDAKSGSYWLDTNGGSKTDAYVATCNFTDDVTETCI
APTVSSFDRKRWVTTGGKSQFTWFMKDLLEIDEGLTYTASVTQLKSLRLASTKMRQNVTYHCKNS
YAHHDQLENTAKTFVKLMSDDEDELHALGGRKERLDVLSDKCILKDDVWRKAVFEFSTKRTSKL
PIRDIAVFDVYDENQEFGIDIGPICFM*
>TRINITY_DN1613_c1_g1_i2.p1 type:internal len:1068 gc:universal TRINITY_DN1613_c1_g1_i2:3201-1(-)
TKSCGGGIQESTRSCSNPSPAHGGKPCSGELSKTRACNEHECPVDGGYTPWSAYGKCSKVCGTGV
QFSTRTCTDPKPKFGGEPCTGLTKKSRNCNTHPCPVNGNWGAFGSFTTCTRTCGGGIQKRYRKC
DSPAAAHGGKKCPGLSFQFKACNTVHCPVDGGYTQWSSFGSCTATCGGGSQTSTRTCTNPSPKY
GGEGCVGPSEKTRTCGTKPCPVNGGYTEWGEYSKCSEECGGGIKTKSRSCTNPAPAHGGKGCQG
PSSESDVCNEQPCPVDGNWSAFGPLSVCSRTCGGGTSLSKRTCTSPSPKYGGKSCSGSRYRIVACN
TRICPIHGGFTAWSNYGVCSEKCGGGVQTATRSCTNPRPLFGGKACVGSSRKSRICNSQPCPVNG
GFSDWSAYGKCSKSCGSGIQVSSRTCTNPAPAHGGKSCAGPLQQSRVCNTLPCPVNGNWGSFGR
FEECSVSCGGGVQKRFRPCNNPSPAHGGKACRGLSFEIQACNTKHCPIDGKYGSWSSYSKCSVTC
GGGTQVKTRKCNSPAPAYGGKNCVGVASHIQACSTHHCPVNGGLTSWSSFDECSEECGGGIQER

SRSCTNPAPAHGGKSCVGALVDSRKCNTHKCPVDGGFTKWSSYGSCSKSCGGGSQTSTRSCTLPK
PAYGGDACVGPTIKTRGCNSQACPVNGNWGGFGVYGTCSKSCGGGTQKRYRRCNNPAAAHGG
KQCSGLSYQIKACNTHHCPVNGNWGSWGSFATCSKPCGKGVQKRVRKCNNPSAAYNGKACPG
LDYQLQACNTHHCPVNGNWGAFGPYEACSKSCGGGTQKRHRRCNSPSPAYRGKTCSGSSYQQR
SCNTQHCPVHGKWGTWGSYGACSARCGGGIQKRYRSCNKPSAAYGGKACPGASYNIKSCNTQ
HCPVNGNWGSWGSYTTCTKSCGGGTQKKSRSCNNPAAKYGGKACSGSNYYLKTCNTHHCPVN
GNWGSWGSYTACTKSCGGGTQKKYRTCNSPTAKYGGKACSGSNYYLKTCNTHHCPVNGNWGS
WGSYTACTKSCGGGTQKKYRTCNSPTAKYGGKACSGSNYYLKTCNTHH
>TRINITY_DN2028_c0_g1_i1.p1 type:complete len:274 gc:universal
TRINITY_DN2028_c0_g1_i1:1608-787(-)
MGQSDGDDLTFGQKISREWRDFREFLYNSETGKVMGRSGTSWAKIGGFYLLFYGFLAGFFTALLFI
FYTTIDKPGIGGPKLIQYIENKPGLTRLDRSSKLQSYSASNNDRISSYKTVIDEFLDGYNTSDHSGAL
CGAAGTPKSDKPCIFDTTLLGECAPSKNTTYGLREKKPCIFIKINKVFGWTPADDGTGYLTLSCTG
HATVMPQGFPLTAFPYNGHKVYQSPIVAVKIDATKGKKNVDCTLKGKNIEVSNNFNPSRAFGKIR
IEGISATD*
>TRINITY_DN5633_c0_g1_i1.p1 type:complete len:848 gc:universal
TRINITY_DN5633_c0_g1_i1:2881-338(-)
MRRSNFLTIGIIAFFLIFSYVSADETSDEEVEPPKVDDDVGKSRDASKTDDEVVQREEEAINIDGLSV
AEMKQMRESAEKHAFQAEVSRMMKLIINSLYKNKEIFLRELISNSSDALDKIRFLSLTDTEALAAT
DELSVKIKADKENNVLHITDTGIGMTKDDLIKQLGTIAKSGTSEFFQKIQDAGTTDQASDLIGQFG
VGFYSAFLVADRVVVTSKNNDDKQHIWESDSVEFSISEDPRGDTLLRGTQISLYLKEEASDFLEPNT
LKDLIKKYSQFINFNIYLWSSKTEEVEEPIEEEPEKKEDEKKDDDKKEDADDKKDEEKKDDDEDD
AKVEEDKEEEKPKTKKVEKTVWDWELMNSSKPIWLRKPKEVTDDEYNEFYKAISKESDGPLTKT
HFVAEGEVTFKSVLFVPKNSPSDMFQDYGKRRESIKLYVRRVFITDDFQDMMPKYLSFIKGIVDSD
DLPLNVSREQLQQHKLLKVIKKKLVRKALDMIKKIPDEDYIEKFWKEFSTNIKLGVMEDHSNRTR
LAKLLRFRSSNDPEKLTSLTDYVERMKEKQETIFFMAGASLDEVKNSPFVERLLKKGYEVLYLIEPV
DEYCIQSLPEFDGKKFQNVAKEGLKIGNEAKEKARVDELEKEYEPLLKWLKEDALKDLIEKAAVSS
RLTESPCALVANSYGWSGNMERIMNSQAYAKSKDAGANFYATQKKLLEINVYHPLIQELQKRVKT
DKEDQTAKDLARVLFETATMRSGYTVKDSSDFAGRIERMLRLSMGVDVDAKVELPEEDDEEKEEE
KADDEGEEVNADGEEEESKEDIPEIKVDSKTVEPVAEHPTNEDAVENEAPAEGKTEL*

>TRINITY_DN598_c1_g1_i1.p1 type:5prime_partial len:745 gc:universal
TRINITY_DN598_c1_g1_i1:2-2236(+)
RWSGCSKSCGVGVKTKSRECDNPRPSGGGRYCTGERTATEACKIQDCGVDGKFSHWSTWTACSK
TCGGGAQKRARQCNSPPPSGDGKDCAGAREEFKGCSNDFCKGPTGVAKAQEIIERFLENFHKKT
YPDREPSGKFLKAIFAIQKAVAEEFISVDPYVRSKTLEQRLKDLENFLKVATEGGSIDFARTLLGKGV
FTFMKQKIKDGLPIDDIDKAAEGFLEKLRGDLGDKKMDEILSSQGDATLMFTIDTTGSMKQEINA
AKAIAKQIISATRDYEVDYILSPFSDPGTGPVTHRSASERASFVSDIDRLRARGGGDCPELSFGGML
NALNNDPQYGSPMFVFTDATAKDDTDANVASLKAAADATDATINFFTNLAGCNGRRDRKRGIE
SYKEIASYTGGQVFPLKNEAELIKFTSYVKTSLQQDTVITSGGTLNSKSKRSLYSMQARHFMVDDGI
SKLVISVGVQQLASQVALVDPNGKKVYPSTTLALSLIYQVSHPSPGQWKLDFPTSVGKYSYNVQAV
SSSAIAFAHNFIYQQSVRKNSPPIPLANPLKGVKNMMVVRLGGTKNFDLNSIKIDLVDTSGNVLQ
KGIGTDRLDSDEVRHATIDPPSGPFKVRLTGKTKSGYNFERVSQGISRPVSAAMRTNFAGYDFTAK
LGKGPYAAQMLFINRGAHENFYFYAKANYGSIPNYKQTKRVTSQGFVTFTYKAPRQYNLKGKSD
RITVTVKRQSTGEIVSHHTSILLVV*
>TRINITY_DN581_c1_g1_i1.p1 type:5prime_partial len:481 gc:universal
TRINITY_DN581_c1_g1_i1:2139-697(-)
ETTVAVEQTTTTQAPVVTTTEKEVVTTKAVVTTAAPVEETTTSAPVSTAAKTEETTLAVKTTLAATT
KAEATTKSVVETTAAPIATTAAVIPTTKEEVVITTSTTASLKPTEFKVTLPSIGETLDIIEECPLILQCLP
NMMRVTFKKKDLEDVNFVRFNEISLIGNGGECEPNFNGTHFEFKVPLYGCGTRRRYINDAIIYSN
MIIDRIASIFIVRKNSLRIPITCYYPTSGKASASSNEIEQVANLLDQKDAYGDFTTYVKLFTDDTFSTSL
SGEKTSPDVAYQDRIYFEVGLNTKDERLSVFAENCSVTPTNNKHDPRRYFLIERGCKNDETVEFHA

STKHKQRISYEAFSFVGTTEPVTYLHCDLLVCDINATDKRCLNRECAYMNGKAKRRKKRAALPSA
LKPYSMEIHTAVNVLHTSLQKKAQKFAKQRQIMSPFMLYLVASIGSVATITLLIVAVISFKKRTVESA
TTTSDVQP*
>TRINITY_DN106_c0_g1_i11.p1 type:internal len:621 gc:universal TRINITY_DN106_c0_g1_i11:2-1861(+)
RGPYGERGEPGLPGPEGDMGPKGPDGPPGSVGENGRDGLNGDRGDPGPVGAVGARGVQGDQG
PNGASGAAGYKGLKGDRGASGEKGAFGPDGEKGETGDNGLDGEVGPSGPQGLQGSPGPRGPD
GAVGPTGPIGARGPPGPTGLSGLPGAPGDEGAVGAVGEKGSLGPRGERGEQGERGPTGDAGAKG
ETGAMGLVGRDGTAGKKGALGSPGPQGLRGEAGPPGIQGLPGSDGAIGATGARGEPGEQGFDG
APGFKGTNGDSGPPGKPGEAGPQGKKGRSGRDGPAGPAGRDGDRGLQGIPGAPGIPGRPGSKGS
RGDRGTLGKQGTKGTAGAPGSPGETGPQGSRGAPGGIGKSGRPGKQGARGASGKDGRPGAPGQ
QGARGKDGQVGVGGPKGTRGAAGRNGEAGIPGAKGQQGEDGLKGSAGQPGSPGPSGPTGQSG
TPGATGAIGKTGPSGPIGAAGAPGRDGDPGKAGLRGDDGKAGDSGIRGIPGVKGPTGLRGQPGL
SGERGEQGADGAPGNKGPRGPNGQPGGSGQPGTPGAAGRKGPDGTKGPTGARGPKGLAGENG
KDGKPGKDGLPGANGPTGETGPKGESGRPGSDGLPGKRGEQGKQGNRGPAGMPG
>TRINITY_DN36_c0_g1_i16.p1 type:complete len:224 gc:universal TRINITY_DN36_c0_g1_i16:829-158(-)
MMIKTTIVAVVLAACVLQSFAGRCYVGYSAKGLPLAIGVRDVECGKGYDYCTTIKYRLGGIEVTQ
GNCARKYGQAMCQFMRNPHKAMTDCEETICQGDSCNKDVYDAEQAERKRVGPYKNTLKCTT
GLITKFPAIPFYTADVPNAIDECFPGTTECVTVTYDYLFKFETQIATKDNVFKLPCVQKMCNRHNI
DNCNKWCDILSIHQSMENCVAKCCKGDNCNA*
>TRINITY_DN495_c0_g1_i2.p1 type:complete len:655 gc:universal TRINITY_DN495_c0_g1_i2:152-2116(+)
MDFHWNTVIAGLIIVQVYSLAEYGTLNGKDSYRNAYPWLANDRNNNELGREDARSIYRSSDEID
AEYPQSDDEVDTNDIPKSYTSRDELDAEFAQRTYKFADDLDIENAQKSYASSDELDRDDEQRTNK
NTDDLDAEDAEREETINDSLDAENAQRRYTSIDELDGEDTQRVISKTITLPRLGAGRIYKSRFIHGP
YIRPKFVRPHKGIRLPHPNRIIIPGSWKRPAHGIFALKGKKAIFCEHKGIKRISCSNDKVIKVRSALY
GRTAGNVCGRSRVTNCRALNSFAIVQKKCNGHNSCDLNPSNSVFGDPCVGTVKYLNIEYFCVKR
TPPTINTPTLTPVTLVIPVQSSYQGDFGKSLTLKWNLGLTEKDRIIAVYLYDDIKPCKTKIIFSSTGFNT
PMLQESYAQLIFGNRISATISKSSYQLTIKGLKASDARSFTLEMFVSGPGRSSCKPILSVIKISKINNINS
DKVDGGYTGWSVYGPCSKACGGGVQERNRSCTNPAPANGGKACDGVPSETRACNTNQCPEAS
MSYASTKYPASNCIDGDINTRCHSKKGVNPWLKVDFHKKVLFKSVELIKYFKYVCCDAVIKISAIQ
SDGTKKLCATDDQAEKGVQDLEIELSDNCVGHSIELQLDTHGKKSALRLFEIKFNAE*

>TRINITY_DN3698_c0_g2_i3.p1 type:complete len:710 gc:universal TRINITY_DN3698_c0_g2_i3:78-2207(+)
MMSKILLQLQFLYFVAFSILDGSKIDLMIYKTIYNYSACVSLTNATHQIGCTSEIKGNQGVIHLVNSD
ADIDWIINHGPHQPYIPLLNTTMFNEIIMKRLMDSKKISGLLVMHERATENPSGGFSPDDRCPKDK
FGMYASDEHYGSCKKVKWNNLGNGMSFVFYGIPIFLLQSDEEIAFLIKCYEDYNKPVNNSARDYP
LCAAEMKDFMFAAKDTPTCRRKTEMPNPIQSTYCDPLGDWNIYGSLYPMVGELDKNQIIMLTAK
MDSTGFFHDFTPSVDNGVTGVVTLLSVAYALGKYKRENANASQVLKRPIVFTLFNGEDWDYIGSS
RMVWDMEHGRFPYEQANKDATSSSRSFDDLDLNYTSKLSLDNIGYFIEVSQVGKANHIFAHTDPL
SQKSSSVKNTVDHLLETFTDAAKESSLLFDKVDQSLKHPLPPASFQRFLRAKKDIPGIVLTDHKTQF
TNRYYNSRFDNAENLDLHLNLQNISYIKLHSPAITKLVNLSETVANAVFMLSSDGIKPIERITVPPN
VTAHLLYCLVYAANCPFFKEVLGKKTTALPKVPFSRYISVQQLDNPITQIFHRLLSYYTGDWLPEDE
CVKKRTRKKLPFVVYNYAIVRGSEENYTCYRASVYQTPAVSPAFELMDYASTEYSTWAESMWNSDI
GIQVYLKANPTIEGVILAAGFTMFMVSFIAVYFITKKSSILFTPNALVNG*
>TRINITY_DN10485_c0_g1_i1.p1 type:5prime_partial len:604 gc:universal TRINITY_DN10485_c0_g1_i1:2-1813(+)
NYSRLKSYFVAMKRTTKRIALAAAAVFLLVVIVVVVVVAVVVTRDDDEDEILTAKHEPVEAREYSK
ASVAADRLECSNIGRDMLKRNGSAIDAAVASLFCIGLLNMHSCGIGGGGFINFYKRSEKKAYIYDF
RETAPLSASENMYVNKSQERLSTVGGMSIAVPGNVKGLHYVWQKHGKLPWGDLLDPTIKIATDG

FKISKAVGDKIERESTNKLYLNEGFKEFLQKDDKTWYKTGEMLKATLLADTLKRIKANPHDFYNG
TMADEIVLDINEAGGSFSKKDLEDYKVTEREPVTTNVKGYTIYSMPPPGSGAVIEMTMNILEGFN
MSEANIKDNAELIKTTHKIIEALKFAFARRPLLGDPSFVADVSKVLTAMLNKTFAEELRHKIKDTPY
PNQTFYGGFYSQHQDPYGTSHLSILSEDGDAVGATDTINYGFGSGVRSTRNGIIYNNEMDDFSTPG
LQNLWGYLPAKSNFIKPGKRPLSSMSPTIVTNSDGDVVMVVGASGGSRIITATALTLIRTLYLNEEL
GRAVSEPRVHTHLLPYEVYINDPYTVSEELQDGLKDLGHKLVMSKYWAAIQAIYRKDISSPILAKC
DPRKGGLPAGY*
>TRINITY_DN8155_c0_g1_i1.p1 type:internal len:249 gc:universal TRINITY_DN8155_c0_g1_i1:744-1(-)
DPDSEECQEEEEEEGGEEEATEGEEGNSTSKTSEGGNKKKKKKTQKKKVGKVLGKGQRKLRNKR
KNLKKKNKSKPKKKGVNKKVALSKRSDDETTDDEEEEEEEEDSGDFSLSHLGSVDLVRGLYISAEF
AIPEDCKKDKLCEWLKNKVGAEAKLTIEGTFDTNGFKIAAGITMNLALSDDLFLTEAALELEFTKS
ERSVALACKLQYNNVELSGKEGLEFIGKLKASFTGELEISMQMVGMIYEPFG
>TRINITY_DN3655_c0_g1_i1.p1 type:internal len:536 gc:universal TRINITY_DN3655_c0_g1_i1:1605-1(-)
FWFHMYGPHVDTLNVYKKVSGSLQLVWTRVGDNGDQWKYAQVDMTSKYSYQAVIEGVRGSSY
KGDISIDDIVLADNACPPPLNCDFEHGKCGYVEDPLADFDWTRNKGETTSVGTGPPFDHTYQTN
QGYYMYAEASGRRKNGDKALLVGPVLPGSAPMCLEFWFHMRGWHIGQLNVYIKTSNGGKVLI
WRRSGNHGMQWKIGRVTVRPRSGRQIVFEAIRGLSFSGDIAIDDIKTVSGYCPPPGDCSFEKGMCS
WNNVKRTKLEQFDWILGSGDPYRFNDHTSGKLEGKYVYIESSAQGQGDRAWLISEDFYPTNGRC
VQFWAYMSGTNVGDLNVYIRDQNGTNSMIWTLSGQQGRPAKWFNGHAPIYSSGDYQIVFEAVR
GGGSFGFIGLDDILFTSFRCNNLPLNSVPPVTTKAPPTTLATTQPPTPAPNYYTCNFTYDYCNWHN
DSTAHFNWTRHQGSTASIQTGPSGDHTTGRSGWYIFLETSYPRKANDTARLISDDVPATSIMCLQF
WYHMYGDHIGNLTAYTKVGSNM
>TRINITY_DN1030_c0_g1_i2.p1 type:complete len:1426 gc:universal TRINITY_DN1030_c0_g1_i2:4483-206(-)
MAKVRVLALLMLICVTASNAQDGEEVFPFDVQKGEKGQPGDDGEPGNNGAPGRQGSEGPQGP
DGDVGFDGEEGDEGEVGPPGPPGRPGTNRTSVVVVYGPPGPSGPQGPDGQIGPKGYQGETGEPG
SPGARGPRGDAGAVGEAGVPGKNGANGPKGEQGSIGPSGATGIPGLPGPSGQRGPKGSKGEVGE
LGPKGAKGDAGRNGDEGESGLPGRKGEPGPRGQNGTVGFTGPAGQEGPMGPIGPAGPDGPAGP
QGPRGADGPPGTAGSPGESGAPGLPGPVGQVGARGEPGFPGARGIPGDRGQAGPAGQDGVDG
AVGARGSPGSPGIAGAAGPPGTRGEPGKPGPRGLQGQQGEQGSTGVQGSVGGRGEKGPRGLTG
QAGVPGPPGDSGTDGRPGPPGPPGNNGQRGLAGLPGSPGLPGFPGIKGHVGERGFNGPRGEKGS
RGESGRPGQRGIPGVRGPPGDIGLPGIDGKDGNRGQPGKDGIPGPPGQPGPRGIPGSQGAAGSKG
DKGAKGRTGDPGFDGAPGDKGIPGQAGDQGVPGPRGRQGDRGEPGEQGARGGQGLPGPPGA
QGPQGRQGKRGSPGLPGARGNDGLPGRQGERGFPGESGNPGAPGRPGASGRKGPSGRPGQKGS
RGDPGMPGDTGPPGQVGRPGLPGAQGNPGAAGDQGEIGEAGERGRDGKPGPRGAMGQRGAP
GPRGNPGQRGDPGLPGDDGKPGSTGAPGAPGAPGEPGQSGPRGPTGRPGQAGAPGESGLKGFK
GNRGARGRPGRAGKKGADGLKGQRGSKGEQGLTGLQGAPGLPGNQGPAGPIGLPGEQGPQGP
PGPDGKPGSKGDKGRSGPDGRPGSPGRPGPDGSNGLNGRPGKDGSRGPQGRPGPRGKQGPTGY
VGAPGAPGVPGLIGPRGPMGDNGKDGKNGADGDPGEAGPQGAQGPQGEPGADGADGLPGRN
GNPGLPGPRGEKGDRGTKGGKGAKGNKGTAGPVGQPGARGDRGPAGTPGADGEKGADGIKGE
TGFNGAEGAVGDRGDTGDRGSKGDTGFMGFKGAKGPRGSKGTRGLPGYMGPQGDIGDKGPIGP
PGPTGPAGISGPVGLPGRKGPSGDPGPKGYAGKNGRQGPPGPPGPPGLAGPSMLPPWMGGGLA
DGESKGPVVEREEPDAPEDENEELPPNEVNPFYRVYQFYSSKKTTPTDNDVNVMENKFKTKVEEL
KTEFSRIDKPDGTKDYPARTCRDIHAYHPEAKSGLFWIDPNQGCKDDAIQVTCTFSVNEDDEPTV
TTCVDPAEKQSVPQRTWASKMHSRAQKWFDEDHELAKLKYNANLSQLRYLGLLSKTATQDVKV
HCKKYVVWHDKMNRNFDRAMRFMGTQEQEFLYKDVDSKSSKSRFVPEVITDGCSANSNSVAHT
HLRFTSRKFIRLPIVDFAPTYPESPKSEFGIELGPVCFKV*

>TRINITY_DN6221_c0_g1_i8.p1 type:5prime_partial len:855 gc:universal TRINITY_DN6221_c0_g1_i8:1-2565(+)

ITSSSYTVPSTMVSETTPVSSSYPVMTESVSATSVSTLVKPASSSIGPITTSVKPLSSVSSLTNPATVSTST PSVSSSSAIKSVVSSAVVATPASSMTVYSSSSSVLKSSIAPETSTEQTVLVESSSSRPGESQVKSTISSSVE SPAIQSSRIISSVERSSSVITSSSYTAPSTMVSETTPVTSSYPVMTESVSASSVSTVVKSVSSNVGPITTSV KPLSSVSSLTNPATVSTSTPSVSSSSAIKSVVSSAVVATPASSMTVYSSSSSVLKSSIAPETSTEQTVLVES SSSRPGESQLLTTAARTRPSERTVLVSTSYPSSSLSSIEEFTIVKSSGKPVTFTFPPVPSKTSVNSISSSQS AQIQSSVIISTSFTVPATSAAVSSPAKLSSSEGPEFSSTASVSIVKSSVSSQIQSMKTSVPRPSLVSSATNP PTVSTSTPSVSSSPTKSSISTAFASTLASSMTKFSTFSNLPSSSITPETSTEQTIIVEPSSSTSEKPQMKTS TLRSIQSSVVESRKESSASSISRMTVASSSSPLITASSGMSSTILSSVSSMRPSASVVYSTVAPSLPSTTVY VSTVSTEVLTSIGSSRFTVQISSEVTVTTTLINPTPTPSSVVPTTSVNSCPACFHKGSYYAVNETWQDA TKCMNYTCVEVDNPCYPSNKSVQIEVSTPVCQTCPKGYKSVQNKNKCCPDCVPTENVPNMCN MKQYGNEYLSENDNKHGVCTSKQKYQVTGCGGFCESSVKAKLGAGIYSPTCQCCQPAQVKEHN VTMTCSDGFTYYTSFHQILNCQCRQQTCSSSYKTDDIDIVTEGNGHLREKRSILETFKADMNERKR HALLNDLAMIHVNKKRK*
>TRINITY_DN27058_c0_g1_i1.p1 type:internal len:188 gc:universal TRINITY_DN27058_c0_g1_i1:2-562(+)
FLENFLDVDYPMPKLDMFAIPDYASGATEHWGIITYREARLLYTPGESSLSNQRSTAGIIAHECAHL WFGNLVTCKWWNDLWVQEGMASYLEYYAQDYAHPEWNIIDSFIVNDWKTGISLDSLASSHPISQ PVDHPKQIKEIFDSITYNKGSSLFRMLDYYLGIDLFKAGLKRYLEKNKYKGATVKD
>TRINITY_DN149493_c0_g1_i1.p1 type:internal len:148 gc:universal TRINITY_DN149493_c0_g1_i1:2-442(+)
EVKKAGVNVTGKIAICKYGRGSRNAKTRFSEARDVIGLVLYSDPREHTYPGLEYPKGWMLNKDG VQRGTVNRLPGDALSIGFPSKDGYFRYGEDKYAGNPTIPVQPISYDTAEKILRWMKDDSVPLPEGF GGDLNVTYRLQSGAGRE
>TRINITY_DN4900_c0_g1_i1.p1 type:complete len:246 gc:universal TRINITY_DN4900_c0_g1_i1:153-890(+)
MAELTCGMKCLKMLLFAFNFIFWVCGIVVLGVGIWSRIESKDWDSLLGDDGTIVNAANIMIAAGI FVMIIGFLGCCGAMRENKVLLMLYAILLILIFVLEIAAGIFAYTKKDTVIEKFEENITKGVKNSYGKT DKASEAMTKAVDWFQENAECCGVDGPNDWKASDWGKSKNTTKIPGSCCEKVDADTCDIKDSY KKGCSEAGPQYIKDHMWQIGGVGVGIAFVQLFGIVSAICLCRAVNAETTA*
>TRINITY_DN8597_c0_g1_i1.p1 type:internal len:124 gc:universal TRINITY_DN8597_c0_g1_i1:369-1(-)
TCDGGVLVQRRTERAFNKVETRNGNTPCNTQACGGSDADFSEERVRACSNVNQCGVNTGTQTV YKVCNGNRCNFQGRDYQINEEVVIRVQSCSVVCDYSPWQDTQSCTKTCDGGVLIQSRTER

>TRINITY_DN187759_c0_g1_i1.p1 type:internal len:544 gc:universal TRINITY_DN187759_c0_g1_i1:1629-1(-)
FNKSPCTSCLCEPSGINCYTQPQCYLFAYPAVYQNKPILNPAEIVTSCMYPGFGGNSGHGCCFFPY KFMDQLQYTCLQRPDKSYWCSTTPNFDQDRKWAECGSCPKPPCPTKTPQVTTKAPCTGPSCNET IKTTITPQTTTPFTTTPASSTTLSPPLTTDETPICEEWGAWMKCSASCGGGYRDRYRHCHKEGEQPY LDQQTEACNTQPCPTSFPTPASSTVIKITPEGTTTTTTSSPSTKASSTSTTASTTKTTTTLSTTTLATITA ITTIPANTTPEIPVNCSKWSDWGKCSTTCGFGQKLRVKFCSLSQHTKEYRETKECYIEDCPPVCDP WSNYTACSKSCGGGAQKHTRKCIFNDNVHNEIKEFTETRYCNTQPCPPEYTEWSQWSQCSLSCN SGVQSRWREAMWEDLGNYINIPQYQTRECNTFKCPKESNCTAWSEFTECSKTCGGGLQERVRRCI ESKDDAFVDKETQVCNERSCVPSYCGEWSAWTNCTKPCDEGIKLRWRTCGGRYEEENSTCNEFP CYRPPGKMIKISKLLQ
>TRINITY_DN18_c0_g1_i1.p1 type:complete len:256 gc:universal TRINITY_DN18_c0_g1_i1:53-820(+)
MGIDIGLDEVKTKPFWIAALAEFQATMLFLICVTTVVLGWGKNDVSANNVEIGLGIGLAIASLAY AFGHISGGHINPAVSLGCVVGGRISIIRGVVYIVAQCIGAIIGSAITYACTEDSNRDKLGVTSLGPNV SVGQGFGLELIFTFLLVFFILSATDPKKNTEPYGTTLGIGIMIVVAHVSIIPFTGSSINPARSLGPAVMM NSWADHWIYWVAPFVGAIIAAVEYRFIFGVPENPRHDEERIPLAVESTTKYT*
>TRINITY_DN168145_c0_g1_i1.p1 type:internal len:186 gc:universal TRINITY_DN168145_c0_g1_i1:556-2(-)

RCGWREAKNLDKFNWKRSNGGTPSKGTGPATDHTTGVALGSYMFIETSRPRREGDNALLFGPKY
PGSKTGYCFNFWYHMFGSGIGSLNIYQDSIKSKPIWNRTDDQGNQWRHGRITVKSMRRFRLIFEGI
VGKNYRGDIAIDDVWIDDFPCPAPGACDFESKSFCTWSQVPNKFNTSNYDDFDWE
>TRINITY_DN1535_c0_g1_i1.p1 type:complete len:779 gc:universal TRINITY_DN1535_c0_g1_i1:86-2422(+)
MMKYFMLIVFTICLKNIHAQDKNLCTSAETCGKCLLKGGDCFWCIEEDFKFNRTRCNTEANLAA
QGCTDYGMGRGKKTNDADLDFTSVRQVKPQKVTLEIRPEEEQSFRIQVKSAKDYPVDLYYLMDL
SKSMENDLDKLQTLGTNIAAEIGNITKNYRLAFGSFVDKTVIPFVENDDANYKPCAECEPTFSFKH
VFDFSNDAKSFEKAVSEQKISGNLDTPEGGFDALMQVAVCEKEIGWNPKDKCRKIVVFVTDASPH
IAGDGRIGGIIRPNDGQCYLTEDGDIKMYTEATKMDYPSIGFLRSKLKDNAVIPLFAVTKNVTEVYK
SIAAELKDLQAVTGILEGDSSNIVEVIRDKVREISRTVTLTDDSSPDITVTYRVQNDPTKPNVCPSPV
APNRCEDVEIEQTVDFDVYVKASAKNCETKEKQDQTFTVNIPAFGKVEVNVKMICDCDCAKTSE
PDSPKCDGQGTFECASCKCNEGRYGDRCICDGNENKDMSLCKPNNVTDKICSGFGNCVCGVCQ
CNIRENPEERIDGNYCQCNNFNCPRHQNIVCGGPERGSCECGTCVCREGYLGENCGKTDCRIGV
KKCLDAKGQNCSLNGICECDRCLCDKGYEGKQCQTCVSCPGKCSEYKTCVLCKAFGETGEQCD
GCELIQHVLVNRTNEECVVPVDENCFVIYRFEIDPVSMNETLYIQRDKRCIIIVKTKPPILAIVLGTIG
GIVLLGLLLLLLWKLLITAYDNREFQKFEKDRMKSKWENDENPIYRPSKQQFQNPAYAGAN*
>TRINITY_DN10094_c0_g1_i1.p1 type:complete len:264 gc:universal TRINITY_DN10094_c0_g1_i1:1210-419(-)
MRHILKVFAVAFVLHCIDRTLSLPVSPVPPLLNKLLKDQNVTLGFILKGLQGPPGRDGAPGSSGLP
GPPGPPGWKGDPGTPGMNGAPGFMGNPGSPGAPGNDGLPGPPGLPGAPGWPGGSGMQGPPG
MPGLPGLPGSSGPSPIRYNGTVKCEEDTAWLRCNEYKHISIMSAFWGRRNFGLCTEHTGNLITNK
YCPTTPLFLTKVKDACEGTTMCEIRCTKFFFHDQSCLDVYKYLEVYYKCIEVINGHEVVNEDNLL
MANFLG*

>TRINITY_DN172571_c0_g1_i1.p1 type:complete len:104 gc:universal TRINITY_DN172571_c0_g1_i1:30-341(+)
MSGRGKGGKGLGKGGAKRHRKILRDNIQGITKPAIRRLARRGGVKRISGLIYEETRGVLKVFLENV
IRDAVTYTEHAKRKTVTAMDVVYALKRQGRTLYGFGG*
>TRINITY_DN2490_c0_g1_i1.p1 type:complete len:1044 gc:universal TRINITY_DN2490_c0_g1_i1:354-3485(+)
MDVNKKVNQAYIDDDDDITINFNPKSGDPNPQAQRFKISKLPSQSDSNNSMEDIEEADDVDGLE
KDNVQNTVSKHVQLTIPAEHYHNDHNDTRRASHTHTQTIGYMTHDAVPMSVFYRNQASMENA
ANGDMRPTLDQLHKGQGLDEARKRHWDALNEEDEQQDAERGTSNTKTKLGWIKGVLFPTLLN
IWGVILFLRMPWIVGQAGIGLSSVIVLLATVVTTVTALSMSAVCTNGEVKGGGAYFLISRSLGPQFG
GSIGIIFSVANAVAVALYLVGFGETVQALMARSDTYMVDATNDIRIIGLIALILIFVIAMIGLEWVIHT
QTFLLLLLVAAILCVAVGCVYPAPQESREKALAWGLPGYKSKSFADNFVPDYRDGEEFFGVFAIFFP
AATGIMAGSNLSGDLKDPQKAIPLGTLLAIGMTSTSYVALSWILGSFVMRDASGIIPVINVTVNGTT
MNSHTCEIGSCEYGSVNDLQIIEKISLWGPLVLIGIFAATLSSALASLVSAPKVFQAVCKDKIFPHIEL
FGKEYGKNGEPRNGYLLTLVISACFIAIGELNVIAPIISNFFLMAYALINYSVFAASIAKSPGWRPSFK
YYNKWCALVGFFLCIVIMFMINWWAALITIIVVAALYKYVDVRKPDINWGSSGQAHTYLKALRFT
RRLEDTDEHVKNFRLQVLCLTGQPSLRPNLVHFCSHITKHLGLLVCGEVNVTDKILSPSGNEGKW
LKKQKIKAFHQIIHSATLRNGAQSMLQSVGLGKLKPNTLIMGYLNRWQSIEVAKVDEYVHIIHDA
FHLRYGVGILRLQDGFDITDGVVDDQIENGVVENHEPDWYTEDWDENQKDTRGSDGDGSDQA
KEERQETFKPPDIVSAFPKKRKGYIDVWWLYDDGGLTILAPHLLALSSHWRGCKLRIFTPASDKKI
KANQIRMANLLKKFRIDFSSVVEFRGINKHPKEDSINQFKQLRRGEHLPQDGLLDKKTLRQIRLGE
LLHEHSSEAKLIVLTLPIPKEGMVTSLMYLSWLEVLSADLPPVLMLRGNQTSVLTFYS*
>TRINITY_DN6920_c0_g1_i3.p1 type:5prime_partial len:411 gc:universal TRINITY_DN6920_c0_g1_i3:2124-892(-)
CKRHQGKMSHRKFSAPRHGSLGFLPRKRCKRHQGKIKSFPKDDAAQPCHLTGFIGFKAGMTHIV
REVDRVGSKTHKKEVVEAVTIVETPPVVVVGIVGYVETPRGLRQLTTVWAEHLNEECLRRFYKNW
YKSQRKAFTKASKKWSDDDGKKSIERDLEKMKKYCTVIRVLVHTQQKLIKLKQKRAHIMELQIN

GGTVADKVDWAKKSLESKLSVSTVFGQDEMIDVIGITKGKGFRGVTFRWGTKKLPRKTHKGLRK
VACIGAWHPARVGFSIPRAGQCGYHHRVERNKKIYRIGRGIHTKDNKVIKNNAATDYDITNKTIT
PMGGFPHYGEVNEDFIMLKGCVPGPKKRVLTLRKSLLAQTSRKALEKITLKFIDTSSKFGHGRFQH
DQEKKNFMGLLKKDKERQAVEA*
>TRINITY_DN776_c0_g1_i1.p1 type:5prime_partial len:201 gc:universal TRINITY_DN776_c0_g1_i1:692-90(-)
RAGKTPHTASMVKNVCSKTYVTPRRPFEKARLDQELKLIGEYGLRNKREVWRVKYTLAKIRKRA
RDLLTLPEKDNRRLFEGNALLRRLVRIGVLDEGRMKLDYVLGLKVDDFLERRLQTQVFKLGLAKS
IHHARVLIRQRHIRVRKQLVNVPSFIVRLDSQKHIDFSTNSPYGGGRPGRVKRKNMKKGTGGGDG
DEEDED*
>TRINITY_DN2963_c0_g1_i1.p1 type:complete len:116 gc:universal TRINITY_DN2963_c0_g1_i1:1072-725(-)
MSATGNLVTVLRKLYDEYNDKTPSRLKIVDGFLLYVMLTGIFQFLYCCLVGTFPFNSFLSGFISCVGT
FILGVCLRVQTNPRNRTEFKGISPERAFADFIFANVVLHLVVMNFIG*

>TRINITY_DN61742_c0_g1_i1.p1 type:complete len:349 gc:universal TRINITY_DN61742_c0_g1_i1:1073-27(-)
MDNKTGFTFFVFTILLIGTILINHSRIEELKSHIETSKESVHKSRSKRDIKPDDMITLLQNADFKQYFQ
KEVNKTLKLYISSLPLKVFCGSECNINKANGSQQETFQLKIKRISLKVRPTPVLPIIQNNTMTCTAN
GNPKTLSGRWINLFTQQDIPFTKRISNDEVSITAGVEKQGQYQCLFTDGKDQVSAVTFVQKVSFNV
RYLSKKKASILLNELAKYGRVSDFKLCFSYTGSFRNYQNDINNDEVLVIIRNEQNEDLGFYQSGNY
FQLNSFLFKFNDTDNITIVASGVKQLSCFTSSTTCNANFEFKSESVSLRRYNTARNWKYSAESLQSK
GRVSRIEILKRQC*
>TRINITY_DN564_c0_g1_i1.p1 type:complete len:563 gc:universal TRINITY_DN564_c0_g1_i1:2018-330(-)
MKLLLFCAATLLVAGCVLADGVATQKKCEDFLPVDTCNQLRVIAQKFNEKAGAVNKAITDAFNK
HKTKAGEVLSYVKEYLVANAKNFSCASVLSTQQCQQLKDFAKLMHAKAGEASIAVREAIVNGA
KNSKVQFQKAMEFLKNDVSCEKILGLEQCEKLLESAKTLKENIKTVHQALVAALQKHLSKTKDIL
KSVRETLVEKAKNFNCTDVLTLEQCNRIKNIGQRLKLKSSEVQKAVKEAIANGAEKAKQIFSNAM
DFLLIDARNMTCEDLIKAETCQKIKDFAKKVHARASDVALAVREAIVQGAQSTQDTLKKAIEFLK
AEISCKDVFSIDTCDKIRKLADSFHVSLKTVNEAMREAIANGVTKVSDLYKAAVKYITEKWGGIFG
DEPQIEKRSVDEKLTNIGELLKQKITKMAMDLLDMLKVTNEKIREKVKELIENGKIKITNIKEKVN
DLLALVNSNDEDTMNDVNMIYKRSTEDSLVDLLKSIKGKSQEFIEKLLKLSKDKLEQIKEWIKQLK
QTDDSIPSDEMEDDLDLVHDVLKDGVNMAFADIVEDTIKNIE*
>TRINITY_DN4781_c0_g1_i3.p3 type:complete len:183 gc:universal TRINITY_DN4781_c0_g1_i3:2965-2417(-)
MCTEPKAPSSCANDEFQCTKTKQCIKKHMLNLTAYTKVGSKMTKLWTKSGTYGPRWRHATVDIS
SASTFKVVIEGTASSGYMGDIALDDLFLNNGACPPSGECSFEDPKLCGWTNMNGDNFDWTRDN
AGTSSSGTGPRFDHTYGTAQGFYVYIEASNPRSKGHKAWLKSGEYKPTLGRCLSV*
>TRINITY_DN18348_c0_g1_i1.p1 type:complete len:2565 gc:universal TRINITY_DN18348_c0_g1_i1:90-7784(+)
MSSSLIVVKVNLEKLEIVKTFQFEPTATFQDVMNLVRTKVPEAKPKANENYGIFHYDNNDPAKSQ
WLENERTLEFYGFGNGTEIIYRDKHRYLKVKMLDGSVKTVMVDDSQTVGVVLQTICEKVGITNTE
EYSLFIEEDKNEKKEEYSMGTLSRRDKSVKQSTLQRVEDRDKKKMETLKRKLHTDDETNWLGHD
KTLREAGVQDSHMVVLRRKYMYEQSVNIKNPVEINLLFVQSRDGILDGTYPCPMDEASKFAAYQ
VQIQYGDYDEARYKSGYIDMKEFLPKEFAKKKEAEKKIKEEHKQIIGMDALNAKYKYIQLCQNLP
TYGVTFFLVKEKMKGKNKLVPRLLGVNKESVLRVDEKTKQVLKVWPLTQVRRWAASPNSFTLDF
GDYTEGYYSVQTTEGEQISQLIAGYIDIILKKRRAHEVFDGMDDEESTMLETTVLPSRATVLQHQS
NKAQLPQSGSVAIPAVIRAGGQGPSSYEVGNMGDAGFTSSKGLTHSGHQAPMGHTARMDNFSP
GQKALFKNLDDGKQRLKVVVEDLSEEPTDIPDLGTDPQSLKWRDNQVEISKNNVANRLAAMSA
ATAAIISLTSGDPEAFNYTAVGAGVSTITSNLEDLVKGIRMLAALTDDDDKSKSLLDAARGLAAAF
SDLLNALKPGDGKPRQDLLAAAGNIGTLSADIIAALGDPDTDPELQELLLQLAKAVANATAALVL

NAKNVASVTEDPAAQDKIIDAAKDTAMTTSQLVACTKVLAPHINSPLCQEQMIEAAKLVANSVD
AVTGSCVDATDDDDLKDKIKKASQDVSDALNNLLQHIGDGSRVRPGGQYDSACDDILNATDKLF
NSMGNAGEMVKQAKVLAQATSQLVNGIKGDAEGEDDDDSQKRLLAAAKVLADATAKLVEAA
KGAAKNPNDSTEQEKLKKAAEDLCGATNAAASNALKKKLILRLEKAAKEAVASATQLVSASKA
AEPSNRNPASQQQMDESCKAVNDNSHDLIHSIRGSHNNPDSPAAQLGLITSSKNFVPPASKLVAD
SKAAVPTVGDNAVAYQLSNAGKNTQNALNELRVAAEKAAEVCGSLEIDNALAAVKDLEKELED
TKQSAADGTLKPFPNDNADDRALEVGATSKTVGATMAQLLTAAAQGNDSYTGIAARDTASALK
VLANAVRGVAATSDDPKDQQAILDAAKDVMQESGNLIEEAKNALEHPNDPDNQQKLAQVARG
VSNALNNVVNCLPGQRVVEDAIEDIANSSQVLASPHIRFPVTDEPFSQVQEKLNQSSLNLNAAAN
DVVHAAKGPTGHLAKASANFSHSYQDFQNSGLTFAGQVKDKGAQDQLINDLRKTSTVSSKLLL
ASKSLAADPGAPNAKNLLAAAAKGVTDAINQLLNQALTAAPGQKECDSAVRNVEAMAHVLD
NPVEPVNEASFFECLDTATEKSQELARSMQDITSSLKKDDVDTLAEAVGGASDAVCQLTESTAQA
AYLVAIADPSSTAGRAGLVDQSQFAKARDAIREACDGLLNPNASQQQVLSAATVIAKHTAGLCN
ACKAASTKTDNPVAKRHFVQSAKDVANNTANLVKFIKVYAGDMSDPNREKCAVATEPLLEAVD
NLVTYASSPEFASVPAKISAQAKAAQYPLIMSGKSMVTSSANYFLSTKQLAVNNKDQPTWQLFAQ
HSKALADAMKKIVTAMKENAPGQQECDEAADIINNAINKIDQASLAAISNNLQPTSEGSLKSIEE
KMALTCAQLKEAVPNTVEYAKSAPEKIGHSVIKLANLADPLADCAIGFASKLPGGSEKQKDLLDT
AKTVCESAQQLVLATKDAGGNPKAVNHHPAVHDAAEDLLEAIKDLTQQLEETASETGAVTALVD
SINRARSQVTMYVGDYRDKGVARVGEPVTMTFVACQDNMVKELKSIARGAQDMVGKANTKPK
ELGGLSQDMVKSYNVLTDDTKNALRLSNQPDIAERLKGSVGKLGDSCSNVVQAAGNVQSNPND
SYAKKDLSDKAKDVSEKVSYVLATLQAGSVGTQECINAISTLAGMIGDIDTTVMFAQAGTLNPEK
EGETFGDHREAILKTAKNLVEDTKNLVVAAQGSQEQLATSAQNVLSSMTKLIDNVKLGAAALGS
DDNEAQVMLLMAAKDVASALTELINSTKNSSGKSANDPAMDTLKGSAKTMVTSVSSLLKTVRSV
EDEASRGVRAIESTVDAVKQAVINLQVGEPERTATPEDLIRATKGVTLATAKAVAAGNSGKQEDIV
QAANVGRRYCTELLVVVKAAAFNADSESIKVATIVKGRDTAQAYCEVLDHVASVIIHPTPQKKQH
LTVLSKKVAYSVGELVKSAEDLKGADWVNPEDPNVIAENELLGAAASIEAAARKLAELKPRVRPK
QADESLNFEEQILEAAKSITAAAGALVKAATAAQRELVATGRAGVDTNDDGQWSQGLISAARMV
VGAIGSLCETANAAVQGNATEERIVASAKGVAASTAQLLLASRVKADANSVAQRNLQIAGNNVK
KASYQLVESASEQGNREMVIEVEVSQRKMKGIRQEREAENEVLRIERELEEYRMKLLQQRKQKYQ
KK*
>TRINITY_DN194261_c0_g1_i1.p1 type:internal len:124 gc:universal TRINITY_DN194261_c0_g1_i1:1-369(+)
ADANLIPINVLKFSKQVQAYLDAFDTAYSDMLQNQSISMGFAKFRMNALVQKAEQFQTKVMEL
MKKDVSPILLRMINDKIMNFERSFVLSGLSKDNPVIRHAVYATTYYKLKNTKFPGISEAI
>TRINITY_DN12542_c0_g1_i1.p1 type:internal len:226 gc:universal TRINITY_DN12542_c0_g1_i1:676-2(-)
FILDSSGSLRSDYGKEKDFLKTLAASFGVSSDGARAGVVTFSYNAEHSIKLNDHFDISNFNRAVDNI
PLMGSTTRIDKALRLSQKELFGLKNGGRAGVLKLLILLTDGSQTQDAGAEDPVKLADELRADGISI
LVVGIGAGVNLTELADIAGDDSRVFSAASFDELIGTDFVSAIKDGSCEEAKKKPEPKCEAKVDVGFI
LDSSGSLRNEYGKEKSFLKTLASTF
>TRINITY_DN624_c0_g1_i1.p1 type:complete len:884 gc:universal TRINITY_DN624_c0_g1_i1:218-2869(+)
MANQEEEEHFGYYYGEEEDRGLLDPAWEKQQKKTFTAWCNSHLRKVNVQIEELNVDFCDGLKL
MLLLEVISGERLPKPERGRLRFHLISNVNKALDFIASKGVKLVGIGAEEIVDGNLKMTLGMIWTIIL
RFAIQDITVDEFSAKEGLLLWCQRKTAPYKNVNVQNFHMSFKDGLAFCALIHRHRPDLIDYDKL
AKDDPMYNLNYAFDVAEKELDIPKMLDAEDMVNAVKPDERAVMTYVSCYYHAFSQSQQAETA
AKRILKALNVNQGNEKAIEDYENLASDLLDWLNNKIPTLDDRTTDSSLDTMQNKLNEFRDYKGT
EKPPKVDDKSKLENLFNTLQTKLRLSNRPLYMPAEGKMINDIDKRWKDLEQSEKDFQEWILNELR
KLERLDHLAMKFKHKCDIHEAWSTGQDDYLRSDDITDASLASLLASRKRHEAFESDLAAQQDRV
EQIVAIAQELNTLGYSKIDEINTRCKGICDGWDTLGELTTQRSEKLAKAIEIAERLDNLWLEFAKKA
SPFNNWMDGAKEDLMDMFNVHTIEEVNDLKDAQLKFKANMPAAQEEFNTIMGLQNAISSMSS
QPNPYTNTKAEDLQEKWQEVLELIPKRDSLLESETQKQQNNERLRVQFAQMANQVGAWIKQRM

GRMTDIGMNAQGTLERQLEELENLNAEINGFKQNVLDLDASARNVQEAMIFENPHTPYTMEVI
RIQFEQLLTGVTRNINEVENQILMRDSIGITEEQVKELRTSFNHFDKNSNGRLDYKEFKQCLVSLG
QLGDVEDKGEHTFNRIIAMVDPNETGYVSFQAFLDYMTREMADTDTADQVMESFRILAGDKPYI
TADELRRELPSEQAEYCIARMSAYNGPDGVPGALDYRSFSTALYGESDL*
>TRINITY_DN1058_c3_g1_i1.p1 type:internal len:485 gc:universal
TRINITY_DN1058_c3_g1_i1:1452-1(-)
IGSLTDWRCVCNAPGYYADANNSRRCRKEMCSENDIDAEKICGTGAISCVGPVVSPICFCDVKKN
VVSSTDGSRCIPASCKTNKTAITQCGNGNSCTGSSDNYICVCNSYGYKLDDNNNQACVEKTCSDS
DADFIEICGQGGLSCTGTMKKPVCECDHTKNFVPSDDRRSCVLASCRANPVVDAACGNMNTCY
GSESSWICLCNAPGSMLNPNNVRECLTGTCENGDIDVQTVCGRGALSCKGSVASLTCECNNHKG
YVQSPDRRSCLPSGCRQNTTMQEQCGEVNECIGSLTDWSCICSAPGYRPHDSDQMKCHRRMCY
DANVNPVEVCGVGAIGCNGSVVTPQCTCNTSAYYVPSLDGSMCIPAPCHSNKDALKKCGVGNS
CFGTTNNYVCVCDSFGYRLDDTDPHKCVEIQCDDDGVDIEKICGQGSTICDGTARKPFCQCDHA
SHFVPSLDSSSCLLASCRANGTVTDMCKDGNTCFGSM
>TRINITY_DN329_c0_g1_i2.p1 type:5prime_partial len:320 gc:universal
TRINITY_DN329_c0_g1_i2:1-960(+)
FNNSTHSPSLPTHVYLYCCRIKILILSFDPSTIEILKMAPPKFDDLGKEAKDLINKSFHFGVIKLEGKT
KAKNGVEFTTEGTHNTDTGTVNGSLETKFKYADYGLTFSEKCNTDNVISTKITIDNKLAKGLKVD
FDTTFAPVTGKKTAKVKSAYQHNNIHSTCDVDFDFAGPTIHGSAVFGYNGWHAGYQASYDTANS
KLIANNASLTYKEGDLVIHSGITDAVKYSGSVHHKVNSNLTAAALIQWASGTNNTSLTVAGKYAV
DNETFVKAKIDNNLRLGLSYAQKLRPGVELTMSSLVNAKSLEQGGHKLGLSLNFEA*
>TRINITY_DN11377_c0_g1_i1.p1 type:complete len:804 gc:universal
TRINITY_DN11377_c0_g1_i1:3179-768(-)
MKQNWYNYFLLVTFAVCIIQYTNGQEERDSNPCVQATTCEACMQANPDCYWCRDVNYTEGEN
FSRDRCNLLIEFNDGCANLTSISSAFEPGQNSNFSDTVQVQPQTVTLDLRPGQPAVFNISVKPAPDF
PVDIYFLFDASFSMKDDLEKLEQLGAAIAQSINNITKNYRLGFGTFVDKKAVPFVEGPLFPTFPCSR
QVKVDNCAPVFPFRHTFSFSKNTTLFQQAVADQPLSGDLDRPEGSFDGLMQVVVCDQKIGWQPR
GKARRIVLLATDALPKIAGDGRLIGDLLQNDETCHLSPLNSDRSVLEYSESLTMDYPSISQLREAM
KKYEITPIIAVTEDVQDVYKDIANSWESLLAAFGVLNNDSSNIVDLVRENYKKISSTVRLLETKNEY
LDFEYKQLSPECQETLTSPNECEKVAIGQQVDFQVSVTLKKCPPEEENAANRGDIDIPGFGQVKIN
LNYLCTCPCSNNAIPNSVLCNTTGSLDCGICKCNQGFFGSKCECDETTVSRNDTLKCQSDQNDPS
FICSNQGICLCGVCDCFRVAAPRIISGKYCECKNFGCPKSRGQECGGDERGTCVCGQCKCKKEYA
GVDCGNFNCTEIEGPRCTKDGVQCSNRGFCDCTLGGCNCNQGYQGKYCEKCVTKNCKCNDLR
NCVLCKAYDRDDLNCTGCEYLDLQRVNDTQKTCTVQFNETCSAYYHYEIDPTQNQTQTQNVTV
YLEDDFFCVVPIVAGEPNSLWIILGIIAGIVLIGLLLLLIWKLLLILQGRREYNKFETSRTGSQWEQDQ
NPIYAPPDQKFDNPVFGGEF*
>TRINITY_DN16079_c0_g2_i1.p1 type:5prime_partial len:396 gc:universal
TRINITY_DN16079_c0_g2_i1:1686-499(-)
GENETTIVSAITKQYENMLELRNISKEFEEIIKTLNIKERYRYFGPQKSFFDFENSKLFADELMTNINR
CEKENCSSFFSNHSVCMKHENYGSICQCNNYTYYDNETRKCEDIDECVVNNPCNLTTQYCVNVI
NYPAICGCKDGWGGEGCKIKRTCEDQRLICGVNSDCIIKENDLTSCVCHAGYYHGRFHECTKAS
NVLTAKGMKLKKNFTNELLNPESAAYKDLALEVEVTTKTELKKRIVDLADVLVNKFRRGSIEVDL
DIYLSGTSKPNEVLTELNDAIASGDMKGLDIDTQALASIEVTRTCALGNDECPTNSKCISGDGFDY
TCQCEDGYEKDGNECVKESNYKTTLAIVIPIVVVVLIILVGLFLYKCQTANRKSNEQNYGLEIRHKR
*
>TRINITY_DN9655_c0_g1_i1.p1 type:complete len:497 gc:universal
TRINITY_DN9655_c0_g1_i1:132-1622(+)
MYILVLFCGTLIYFNHFQFVRCQDLEGVFGKSLTLKWILNLNETIIAANLYHGTQISEDKKLFGSTG
SSNPTREQKPYSKTTFGNRISASISKLSYNVTINDLKYSDSGSFLLETYVQSASESYSKNEEVRSISDV
KGGPDICGNDIQKSYTYKEGETPKINITLCGVPKPAATFKFNTNGKNEPSFQMINNYTYVYNYQFP
KLNQNMCNKTLSFEAVGHDVDKTISRSSIIVLDEFIPTAPSNVRFSRIGNCTKVKWDPIDTGNCKV
DYVVEFFSNSILMSNKTIPVGNTLDICSPSVVNASGVIVFAIYMGRYNGEKSVLSPLLTTPPPTTTT

ATPETTPKGGGSFAADDDEGSSTPMIIGIVCGIFFLLLLVVLVAFLVKRKKKNDDDGTKLSNATED
MELKRPVSYQDEGDYASVGGIYAELGKGGRIGPKPVPIESNYSEVKIDESGYPAVGPTSDLSKERPN
YSTTVNRDRSRRHSLSADIEDNSEVIV*
>TRINITY_DN48767_c0_g1_i1.p1 type:complete len:533 gc:universal
TRINITY_DN48767_c0_g1_i1:2135-537(-)
MEQQELIGSASRENENPQNVEENRCNCSKRFWIGAIALLLLLIGITGVTVTCFVLEQKVLQYSHHY
GVVFDAGSGHTDITVYQWPSDQKDHGTAHTEQITFEKCSDTGISSYQTEPEQAGVMIRKCVEQVA
FKHLPHKDVKKTAVYVGATAGMRLLCERDKDSCDKILEAIRRVLNGFKFRQLKNRVRVLTGSEEG
TFGWITANLVNNALNLKVPAENNPKLIGALDMGGGSTQISFIPEHYNFPNKEDMSNLKLYGVNY
TVYTHSYLCYGFREAQRKLMAHLVEASNYSSKAIHPCWQKGYNETIPASWIWLSPCSKRPQASIFP
LKNSTMYKFVGNGTSDACENRIKGLFNKTNCEAPNCTFNGVYQPPLRGHFMAHSGFGHIGTFL
NITKGDMVEDFKTKGEKLCQEDWNKVVREHEDVKKKYLRNYCFEALYSYMFLTYGLNFPPNSN
DVHVTKTINGTEVGWSLGFMVNATNTIPIEAPSYRLTQQELITLLVVCFLIALVGLSMLIVCCVKRK
IERNLKYKYTLVN*
>TRINITY_DN1386_c0_g1_i1.p1 type:complete len:596 gc:universal
TRINITY_DN1386_c0_g1_i1:2419-632(-)
MEYIEVKSFIVALVFVFSILGTAQSDLVINNVERTLDLSTQTAKYTTKIKFHNPGKKVEKSFLLALEK
DQEKHLSYLEILDKQDDNSLEYAAENVEGQSGRFFKVSFSKPLKENDDVTLHIETVFTHVLTPFPA
KIGQTEKQLVLYKGNTVFYSPYEIKEQSTTVKLASSAIESYSRLKPSRSSENTITYGPYKNSKPYRLHE
MKIHFENNSPFLVVNEMTRWIEISHWGNIAVEETYHMTHEGALLKGHFSRYDYQRSPTHSVIKSF
KTVLPAAARDVYYRDEIGNISTSNMLVNDDSVEIELRPRFPLFGGWQTRYMLGYNVPAYQYLYNK
GDNYILKMRLIDHVFDEFVIDDLNVKIILPEGSTNIEVVKPYELEEGKRDIHKTYLDTSGRTVIVLSK
KNVVENHIQDIQVHYTFKKLQLLQEPVICVAVFYILFLSVIILVRLDFSITKDAAKESRMKVASLLEE
LLSSCGRRGSLYYSYDSAIDKLKQNRNQNDFAVSLKKLNQDYNTLTQSINELCSLLIKEDPEIGDKV
NDLQKKENERKVIVDQLVTLATRVVTNKIGRQQYIESEKNSMAKREKLGEDIDNVLATL*
>TRINITY_DN144214_c0_g1_i1.p1 type:internal len:154 gc:universal
TRINITY_DN144214_c0_g1_i1:461-3(-)
DSPLRAYESEPGVQAPVGFWDPLGLSTSGNEFDYKRRREVELKHGRVCMFATIGYIVPEYTRLPGL
LSPSYGVRFDDVPNGLGAFSKVPALGVCQIIIFAGLVEIQIYNDQVNGEPGNYGAGFLGIQSIGIMS
PGWKDPEARKKKLNAELANG
>TRINITY_DN11435_c0_g1_i1.p1 type:complete len:430 gc:universal
TRINITY_DN11435_c0_g1_i1:1616-327(-)
MGNIYTVGPNEALVVSGGCVGQRVKRTIVGGWAFAWACVSDVQSISLEVMTLNPRCEKVETAKG
VAVTVTGVAQVKVIKEDDLLKTACEQFLGKDPHEIEDILLQTLEGHLRAILGTLSVEAIYKDRDQF
AQLVREVASPDVGRMGIEILSFTIKDIVDEVSYLDSLGKTQTAEVKKNADVGVAEANRDAGIREAE
CDTQRQDVRFKADTTIADAARAYEMEKAEFDKEVQTKNAEAKLAYELQANKEQQRIRNEEIQIE
VIERRKLIEVEEKEIQRREQELQATVKLPAEAESYKVQTIAEANKSAKVYLAQAEAEKIKMIGSAQ
AAAIEAIGKAEAERMRQKAAAYKQYGDAALMSLILEAMPKIAAEVAAPLRKTEEIVLINDDNGN
KMSSEVTKLLGQLPPSVKALTGVDLSKALGKIPGAQVTA*
>TRINITY_DN144508_c0_g1_i1.p1 type:internal len:265 gc:universal
TRINITY_DN144508_c0_g1_i1:1-792(+)
PRRPNDTARLVSSDVPATSQMCFEFWYHMYGDHIGNLTVFKRIGKAMTMLWAKSGTQEPRWRH
ATVDITSASVFKVVVEGTASSGYMGDIALDDLFLTPGACPPSAECTFEDPQLCGWTNIAGDDFDW
TRGNAGTASWGTGPRTDHTYGTKQGFYMYIEASTPRSKGHKAWLKSEEYKPTLGRCLSFWYHMF
GLHIGELNVLISQNNSMSAPIWQMKGNQGRPWRPSRVTVKSSLNHNIVFEGRTGDGYRGDIAID
DVMISEGP
>TRINITY_DN3655_c0_g1_i2.p1 type:internal len:494 gc:universal
TRINITY_DN3655_c0_g1_i2:1481-3(-)
FWFHMYGPHVDTLNVYKKVSGSLQLVWTRVGDNGDQWKYAQVDMTSKYSYQAVIEGVRGSSY
KGDISIDDIVLADNACPPPLNCDFEHGKCGYVEDPLADFDWTRNKGETTSVGTGPPFDHTYQTN
QGYYMYAEASGRRKNGDKALLVGPVLPGSAPMCLEFWFHMRGWHIGQLNVYIKTSNGGKVLI
WRRSGNHGMQWKIARVTIRPRSSRQIVFEGVRGQTFSGDIAIDDIKTIDGYCPPPGDCSFENGMCS

WNNVKQSSLDQFDWVLGNYPARQGDHTSGTIAGKFVFARSSSKGKGERAWLISEDFYPTNGRCV
QFWALMSGISVGELNVYIRNQNGTNTLIWTLAGQQGRPPKWFNGHAPIYSTEDYQIVFETVRGG
GSYGSIALDDILFTNFKCKNLPSGSTPPVTTKPPPTTLATTQPPTPAPNDYTCNFTYDYCNWLNDS
TAHFNWTRHQGSTSSRKTGPTGDHTTGRSGWYIYLETSYPRRPND
>TRINITY_DN113_c2_g1_i1.p1 type:5prime_partial len:1207 gc:universal
TRINITY_DN113_c2_g1_i1:4425-805(-)
GPFGYKGEAGGPGVSGDAGESGKNGTKGYRGETGARGDIGDTGFPGPQGMVGPPGFVGKRGLP
GPDGKTGGIGPLGKQGNTGLPGSPGNSGLKGQTGNTGAIGPKGRVGLVGPVGLMGVKGASGEA
GLDGKPGMPGAEGPDGLSGPRGEPGPVGDSGETGPGGDIGPDGLVGLKGDVGKPGIRGLPGLKG
MAGDTGLVGAKGAAGPDGDQGATGKAGPRGEPGLVGIRGYPGAAGEPGPNGQTGLKGNQGRI
GETGADGLKGSKGTRGMTGPNGETGMIGLVGKPGVAGVDGRNGAPGMNGRNGARGVSGTAG
ADGQPGVMGVPGIPGDPGDMGDTGLTGANGNNGTRGDPGRRGPNGAIGERGSQGSRGDMGQ
RGNSGPTGFQGPPGPRGVAGLDGMDGKQGIKGSRGKSGNPGLAGEMGLPGNAGVVGQLGKRG
ASGQKGNSGSNGDNGEVGYPGKVGNVGDDGRPGTPGLNGRKGVTGETGKPGMRGPKGPSGD
DGSEGAQGTDGLPGPPGLTGSRGVAGLDGKPGDNGSPGAPGKMGAEGKPGAPGLVGQPGLRGP
VGAQGTRGVKGPTGDRGVSGDVGLEGFAGEMGQNGLRGAVGIIGQIGEIGTPGKPGPRGMMGA
QGLTGPSGPTGSTGIAGSPGRDGNKGPNGDQGDFGKVGKAGQSGSPGKDGLPGIPGKPGLQGEK
GFGGPLGQQGPEGEQGSRGTTGMQGMKGKLGPKGLSGLNGSPGKDGLRGPRGSRGTAGGIGPL
GVDGKPGTPGTNGTDGLPGDTGPKGTAGLRGEVGDFGAPGDVGENGATGPDGIIGAAGPRGRN
GDVGAPGSRGGAGDMGAEGPRGASGDDGRDGLKGLKGTMGRMGIPGPSGDLGEKGDNGPDG
RPGYTGPQGRYGDVGTDGDTGVQGMKGETGVSGPDGNKGPDGTSGDHGTPGTAGAPGPPGPP
GPPGDYSSFMSGGYMDFLRAGGFSKGPRYYSGKYKKSLNQESNTIEKVFQAKYQLFKEFDSVWN
QVVNDKITRKTSGSSKNPAVTCADLFKNNHHIGSGDYWVDPNEGSPDDALLVYCNAENNETCIY
PKSPSFDKYSTYMGEVDYTWVYSSRILTDEEYITYAADMVQLNMIRLMSDRVRQNITYHCKNSLS
DLQLKTDDDKTMHLDNLRRITVTMQNNCVVKDGTWRKSVFELNSKYLEQFPLQDIAYKIINSSD
EDFAIDIGPVCFS*
>TRINITY_DN66856_c0_g1_i1.p1 type:internal len:443 gc:universal
TRINITY_DN66856_c0_g1_i1:1327-2(-)
WSIGQGKTASTKTGPKNDHTYNDQKGKYLFMPTSNKKLRNGAKAQIQSPIYQVQPNGNSVKCF
QFWYHMYGSSIGSLNLYMKSQWLKRKLWTKVSYQGNLWWIGQVTVTSQTPYQLMFEGVRGSSY
TGDIALDDFRMLEGACNNLATCDFENDKCTWQNVLRSDDFDWLIGQGTTWTKWTGPLTDHST
DTGDGHYMFIDSSRKTKGAVARLYSERVQRSIFSFFPFCLSFWYHMQGPNIGTLNILTKTGRGKAN
EQLVWSLSGNQANSWKYATVSLKSRKGFIMVIEAIAGGGHKGDIAIDDIVFGRRFCAISPPEAKPT
VATTPGPSPPPTTPMPTVYDCNFEKKDICQWQQDVSDQFNWTLRQGATPTRGSGPTADHTTRNK
TGSYIFIETSNPRKAGDKARIISPPMDSDKYHGHPHCLQFWYHMFGSHIDTLNVYKKV
>TRINITY_DN6304_c0_g1_i1.p1 type:complete len:348 gc:universal
TRINITY_DN6304_c0_g1_i1:1626-583(-)
MGCCRKRAPEEEDQNKSNRPSDSKFKQQKLPAWQPILTASTVLPLFFFIGIVFLPIGITLLVTSNNIK
EKVIEYSTAENCMKCKAEISSPKAKPPSCECELTFELEEQWTGNVYFYYGLSNFYQNHRRYVRSRD
DNQLHGELVDSVNSDCKPFDKDGNGTIYAPCGAIANSMFNDTFTLVKEDSDQEVPLTSEGITWDS
DKNTKFKNPSNINDLNRYAKPKYWSKNITQLGGTDEKNRGFVNNDFIVWMRVAAFPNFRKLYR
KLDLMKNPSFRDRLPSGKYKLTIGYNFPVSSFGGEKQFILTTGSWMGGKNPFLGIAYIIVGCLCLIFG
FVFLVIHMKIRTRPLAS*
>TRINITY_DN2356_c0_g1_i1.p1 type:complete len:270 gc:universal
TRINITY_DN2356_c0_g1_i1:194-1003(+)
MGQGELLQKAEDLADQILRHSKHILPHLARFCLVSTFFEDGIRMWFQWSEQRDYFDHTWNCGW
FFATMFVVTNLVGQLAGCFMILARQKVEIAVGLLFGIIVLQTVAYQVFTDLKFFLRSLALAGGLILL
LAESRQESRSIFAGLPSLDNQNKPKTYMQLTGRVLLLLMYLTLLKFDMSLFHIVFNLLGTVLIMLVT
VGYKTKLSALVMVMFLMMINVYENAFWMIPQWKAMRDFLKYDFFQTMSVIGGLLLIVAFGPGG
ASLDEKKKW*
>TRINITY_DN7674_c0_g1_i1.p1 type:complete len:661 gc:universal
TRINITY_DN7674_c0_g1_i1:117-2099(+)

MDYYASEKKGLVVWDWLIIACYFVICLGLGIYSKFRRKQDESAESYFLAGRSMLWWAVGGSLYAS
NMGSEHFIGLAGSGAATGIGVVAYEWHASWILLVLGFFFVPVYIRSKIFTMPEYLRKRFQSKWMRI
YLTVVSLISYVTTKIAVDIYAGGLFLKTAVGLNIYVSSIILLSFTALYTLLGGLTAVIYTDVMQCVLMV
FGALALTIIGFDKIGGINALWDKYPSAFPLTTLATNGTIVSAALTTANTLNAANSVTSNSTAASYDA
HCYQVDPYWEHMFRPFDDGEFPWIGIMFSAPITGIWYWCTDQVIVQRTLGAKNLANARLGTIVA
GFLKLLPLYIMVMPGMIARVLFPNVIACGSPDTCKAACGNEYGCSNEAYPHLVLHILPQGLVGLM
LAVMIAALMSSLSSAFNSSSTIFTMDIYRVVRSKASNKELLIVGRLVVCVLVVIGVAWIPVVSRGHG
GQLFTYLQTIQSYLAPPTCAVFIVGMLWPRLTEAGALSSMLFGLLMAIIRLAFDVTVPKPHCGQVD
NRNFFAKLHFMYYALIMLLICVVMMVVISLFTQPVPMDMLGALTWPTLNKPRYKEGNQTDEFVL
STSNGDSNVKIKDASTFDPDQQDLIHDEDELAEIEEFENQSTIMKWGVRIASGCLLFTLFFLWIWY
R*
>TRINITY_DN738_c0_g1_i1.p1 type:3prime_partial len:338 gc:universal
TRINITY_DN738_c0_g1_i1:1011-1(-)
MVSNMLKLILLIGLISLTSGKTCRNIRKKIGCFMERGDTTVLFNDRNIINWQRYGEFSEKLACKCN
EAAKAKGYKYFALRFFAICVGLNTLTQNRIENEKCKDGNTVPFKPCNLDDDHDCAGGPNSEMV
YEIGRDGGYGEWGSFGLCSVTCGEGTSKRQRKCDNPTPLANGNKCDVLGSSNELKKCERKPCIV
VTDGGFGEWSSFSTCSKSCGGGVQYSVRKCDNPKPSNGGKNCVGDTRRTRQCSTNSCPPEKVDG
KFSKWSGWSRCSVTCGGGTRARARKCNNPSPSGGGRYCVGEKVEIQACSTKYCSVDGGFSPWSR
WSGCSKSCGVGTKTKS
>TRINITY_DN2796_c0_g1_i1.p1 type:5prime_partial len:521 gc:universal
TRINITY_DN2796_c0_g1_i1:1-1563(+)
LKQFRLKPVILARRPPTPVRLDSDGCQANFAMSVSLAKLRAAAKGGSAEAAVEEAIKAVEAKKFL
IECLKAREILDSRGNPTVEVDLTTQSGIFARAAVPSGASTGVHEACEKRDGDKSRYLGKGVTKAVH
MVNTVLAESLKGFDVSQQEALDKKMNELDGTPNKSKLGANAILGVSLAAAKAAAQGKGIPLYR
HIADLAGNAEEMVLPIPCFNVVNGGSHAGNKLAFQEYFIIPVGAASFKEAMRIGAECYHTLKGII
KKKFGGDATLIGDEGGFAPPCDARGGVELVMEAIEKAGYKDQCRIGMDVAASEFKVEGKDCYDL
GTWYPEAEKSDSLKMTGAQLAEFYAQLCKDFPIITIEDPFDQDDWEAWKLFTAKVGAGTQVVGD
DLTVTNVTRVQKAIDEKACNALLLKVNQIGSVTESIAAVKLCKGSDWGVMCSHRSGETEDVTIA
DLAVGLCTGQIKTGAPCRSERNAKYNQLLRIEEELGDKAVYAGASWRKPIWMGQTRAVKRGAE
QMNGGH*
>TRINITY_DN5_c0_g1_i15.p1 type:3prime_partial len:578 gc:universal
TRINITY_DN5_c0_g1_i15:1731-1(-)
MKALSGIVALTLFVSTVYSFQCDDVLYTRNCGGYTNAINSINGIYEVSNNRIADILNKLSQDFSTIFK
TFLPNLKDLKINGNVIRLDNNTKVIVNGNMTKEEALKAIGQLNHMMKVLKSFNLVGTTELQELN
KIITDLLDQALNQNLNAYLIIDKIDIDAIKGQIKNVLGSVSIITRTLIMNAIEAFDGKLQKVLGLIKPI
LEAINRRDIYDKIEAVVQLAVEAAKAKAGGQYAFEVDNQVEKAEINLIAFIDRLDQQAPIVKRGV
NDLWRKIQDALNGLNENAKKAATDLVNKYKPKIIESLDKLKKMVIDGAKKMIIELQGDIVKIITR
QNDKTLSKRSISDMWLKIKEAAKNLKSNIKDSVTDVLERYKPKVIDALNNVKKVVIEGAKKIVVE
VNNGIVKIITDGQTATSDDNLYVFEEFHHTAYVVIDDAAPSVYGFKDVWGKVIAKFTELGDKISAS
TRALIEKYKPKIINTLDNVKQFIIEEGKKIIIELQGDLIKIVVENMKGSVEKRAISDVWDKIKEAARN
LKSNIKDSVTDVLEKYKPKVIDALNNVKKVVIEGAKKIVVEVNNGIV
>TRINITY_DN75609_c0_g1_i1.p1 type:internal len:232 gc:universal
TRINITY_DN75609_c0_g1_i1:694-2(-)
ERTSDIVNEIFSRFNEYGFEPSLYKYDVLLSFPDPEKDNYVSLINSTGDKIFQNMRIEQVLEPSENDSS
ALPPYLAYGKSGEVKGDLVYVNYGRDEDFKYLVDNGIKLNGTIALIRYGKLGRGQKVTSAASFGC
VGVLLYGDPKDYSPGPTYPNGRWLPTDGVQRGSIIGYDKDVAEGDPMTKGYPALDWVYRTDVK
KVKGLPLIPALPIAAADAEKLLSMTSGMEVPKDW
>TRINITY_DN179_c0_g1_i1.p1 type:3prime_partial len:223 gc:universal
TRINITY_DN179_c0_g1_i1:84-749(+)
MLSNSKLAVFLLVLGIGIITLTKAQGQCPPPLDRDCNDERNPDDETDGCLIDNDCSSGRRCCSDGC
NLKCTEVQQPPDPEPGDPGEPGRRGETGRSGRPGFPGPEGPLGNPGPPGGRGKKGRRGRPGPPG

PPAPTGPGGEVGPLGTGGDGTKGPGPNAGALGEEGMGADGFVTPKEGPAGPAGPDGRQGPRGP
YGERGEPGLPGPEGDMGPKGPDGPPGSVG
>TRINITY_DN65215_c0_g1_i1.p1 type:5prime_partial len:383 gc:universal TRINITY_DN65215_c0_g1_i1:1336-188(-)
HYRKKTTMAAYDDDENFGTSDSYGNVGSFPKEFGYAEEQEELNNYSEEQKPKILLMGRRRSGKSS
ILKVVFHKMSPNETLFLESTNKIVKDDISNSSFVQFRIWDFPGQIDFFGHDFDSEQIFSGCGSLVFVI
DAQDDYLDALTKLHQTVTRAYKVNPNIKFEVFIHKVDGLTDDHKIETQRDIHQRATGELEDAGL
ENVQLSFYLTSIYDHSVFEAFSKVVQKLIIQLPTLENLLDILIQHSGIEKAFLFDVVSKIYIATDSAPVD
MQSYELCCDMIDVVIDISCIYGLKENGDANAFDTHSASIIRLNNSTVLYLREVNRYLALVCILREDN
FAKHGLIDYNFHCFRQAIQEVFEIRQQARDALLPKNGTILSKGESVVV*
>TRINITY_DN165469_c0_g1_i1.p1 type:complete len:2668 gc:universal TRINITY_DN165469_c0_g1_i1:41-8044(+)
MVEEDLNRSKELDIPKTLAYCFHNVTTVSLNRKQDIFRLLTQCIESQGCQDGHLKAVCKIISLTVM
RYRDSKSRQLVLKFVQKFLDIHELQAVKCLLTEIHILAKQLIKCGASLFSASCGLALLSWCSSISKKK
VQEASILKILYEVEAMCYSVICTCDSFRVKRLLKRAYLYLQDGKDAEQLENCLGVIISNSNCQLESIC
LVGFIFQYLTTKKEIQRVDEVKKRSLIDIIIKNYIQTKVKADYHYLKHAVPFLKYITHDDFKEILPAIE
KGLLRNPETVLQGVGYLLEGLSIDSSNYIEELSKCIAVHLISKNASLQADAVFAVSVIAKQCSDFSAI
NHLLKQLIKVLNESEGKLVFPEQKNAVLQAIKSVDCTSVTGSDHKENLATTLHDGLFPFILLEGHE
GVLTQALNVMKTWCKYFKNNLPKTFVDNIKKGLSLKVSTTVIQAHYLLVVSEALKGNTVSQASG
LLETLLQVIEKVKGQSHLINNVFLCISSSMVLLKMFLSDDVQVNAAKEDSFWNVILDPVKKNLVSE
KFLLSHQKEYFYHLSQCMNTMFLNYSSRFSQKLLEPWYRAFNVLMVYGDWKTKKSVKILLSKMF
DSGNYDTDEFQSRLFADLDSLLDQEMSKSSKIEDTEDILVVNHVVDAYLFLCIWNKKDINEDEVLA
VLRKCLIYVHHDTIYQHDSSLFSKIGSVLKKDVAKEFVEKYKDDLLKYFINLESLSKADNHSLKTLL
SLYPEALLDTLVDEIIKYFTKPDLINVTSQEYEIMKTPDGVAYDQSVFESAKAANQKSGNLKRESKA
YSYKDQKAEMELRKELEKKKGKSDKIELNKKQKEQLEVILTQEKAIRDKLKSLEQGVMKHASILSL
LLDMDSDLLCYHYLDLMNLLLCLCQSHIAAPHVIPIYTKLSEKLFEGDLKHLGQLVANVILRLKKP
VCELPMGWSVENLPEQAKRALNTLHTFVNERNVYLMMTYTRKKTFSVLVFSYCLPLLKSILKDQ
GALVKGSEEFRMKALQLIHIHAKLRSPMDMGSEKNDPELLPRKEMLQILSDVIVSCEGHNKEAKV
QQQATLAFDEVCKCASGRLGCAVATEEEINILLSNILSQCSILRRVSLKGLLEFVDILPTENVVIMTQ
LTRSVLIGRFDIVDENATLAKRLWDEADICIEPSMIQHLVENVTHPLDDVRLAASLALEFSLNERKE
VDIIPQLLDIYFEKLKIPPPIIDNLGRVVSQQFVDSWEARQGVGLALEKIAETVGEDKLGDLFSFFVP
KALGDRNESVRKQMLNASLQYVNLYGQKNMDMLLGVFQDFLDSAPDSKELDTIRQSVIILTGSIA
KHFDKTDPQIKSIFSKLMAALSTPSQSVQQAVANCLPPLCGAMKSEAPELFKNLLEQLLESESYGE
RRGAAFGLGGLVKGLGILSLKQQNVIPTLNDAIQNKKIWKHREGALFAFETLCTMLGRLFEPYVV
HLLPHLLLCFGDGNQYVREAADDTAKAVMKNLSNHGVKLVLPSLLKALEEDAWRTKTGSAELL
GAMSFCAPKQLSSCLPSIVPRLTEILADSHLKVQKAGQQALRQIGGVVRNPEIQEICPIILDALSDP
NKHTVACLQALLSTSFVHFIDAPSLALIMPTLEKALGQRPTETKKMAAQILGNMYALTDPKDLQP
YLQAVVPGLKKSLLDPSPEVRAVSARALGAIVKGMGEVDDLMPWLLETLTSEASSVDRSGAAQG
LSEVLFALGKDKLDKLMPDVIATSMKTELSPNIRDGYLMLYVYLPATFEDQFLDYIGLIIPAVLKGL
ADESEFVRDTSLKAGQRMINMYADSAIELLLPQLEAGLFDDHWRIRLSSVQLLGDLLFKLSGVTG
KQTTVSEEDDNFGTAYSSQVILETLGFDRRNRLFAGLYMGRSDVALGVRQAALHVWKVIVQNTA
KTLREILPVLFELLLGCLASQSYDKRQVAARALGDLVRKLGERILPEIIPILEAGLDHEDGDKRQGV
CIGLSEIMDSTSREMISMFEDSLINTIRKALVDELPNVREAAASSFENLHNTIGHRALEGVLPTVFQ
KLDDPQLSNYALDGLKQVMTVKSKSVLPFLVPKLTTPPVNTKALAILSSVAGEALIKHIEKILPAMI
QAIQVSQGTGLDGDSEFESTRTLILSVYDEVGVRTIVDELTTSSKSQMPGIRKVAASLLMVFCKETR
GDHVPYIQSLFRTIIQLMNDVDQDVVASAWGALDALVKRLDPSEQLQHLSSLRQAVRFIKDDIRN
EQLPGFCILKKGITPVLSIFREGILNGPQEIKEQAANGLGEIIKLTSPEALRPSVVHITGPLIRILGDRF
NSSVKVAILDTLGLLLQKVGALLKPFLPQLQTTFIKSLNDSALGVRQKAAWTLGLLTVLHTRVDSL
FTELKNGIKNNEDSAICETILGALRSITLKAGSKMSEGIRNTLYETLVELISSNEDGIRLKAAAALGA
MCQILPQDQIEQLLNDNLLDINQMLDWSICHGRAVALSYALYHAPSQVFDAVDEDDIVEAVVKY
STYDRVPICTYGVHSLGHLLMFSKDNADVAPQIVVSSIKKTLLNSSVDIRLASLSTIKHVSRNTPEV

MTFERIRYLIPEFISLLRDRNTSVRSSAEITLAFTLQMDKNDILFDECVNTISEASHFLEEFKKNTFSK
LNDSLATDKDDYFFAT*
>TRINITY_DN106952_c0_g1_i1.p1 type:3prime_partial len:135 gc:universal TRINITY_DN106952_c0_g1_i1:18-419(+)
MSDAAAPKGGKHAPKVAKKGEKRAGKKGGKIGGGSEKKRKRKRKETYAIYIYNVLRQVHPDVG
VSSKAMSIMNSFVNDIFERIASEASRLSLQNKKSTISSREIQTAVRLLLPGELAKHAVSEGTKAVTKY
TSS
>TRINITY_DN4481_c0_g1_i3.p1 type:internal len:369 gc:universal TRINITY_DN4481_c0_g1_i3:1-1104(+)
FGIDKVLTMLKFLLFLGFISITSGNTCRNLRKKIGCFIEDGGATSLFNDRNIINWRHYGEFSERLACK
CNEAAKSEGFKYFALRFYGICVGINELSQNRISLESCKDGYKLPFKPCNVYDDHDCVGGPSADMV
YEIGRHGGYGQWGSFGVCSTTCGKGTHTRQRLCNNPIPLGNGNKCDVLGSSIEEKECESKPCIVN
GRFSEWSSWNRCSVSCGGGSHARARKCINRSEGGKDCVGEKVQIEACNTKHCIVDGKYSQWSS
WTKCSKTCGEGIQERTRQCNNPRPSGGGKSCEGASEESRCCSNDFCKGPTGVAKAQETVERILGN
FHKKAYPDKKPSGKFLKAIFAIQKAVAEEFISVDPSVRGKTL
>TRINITY_DN17095_c0_g1_i1.p1 type:internal len:607 gc:universal TRINITY_DN17095_c0_g1_i1:1820-3(-)
CVEHAHCKRNEFHCIGTMNCILKSSLCNSVKDCQDGSDEGPKCTFSSCNDTCSYKCQKQPSGVG
ACICEPGKILADDGHSCIDENECDQVPSPCSQHCNNTEGSFMCSCTYGYILQDDKRGCKLNVSKE
EELLFVDVKEIKRYYMRSKLLQPVFTNLSRGVAFDFDSASGKLFYTDVTQNVIYSSYLDDFAKVVH
GEKRILDRGLAIPDGIAVDWICKVIYFAESAANRIDVIEFNGKNRASLISANLTTPRGLALDPRVGY
LFITDWGGKPKILRSQMDGSNLKVLITEKVGWPNGITIDYYDKTVYWVDARYDYIDAIDYNGNN
RRNIVKGKDLIPHPFSLSILDDDIYVTDWVLNGVLKISKLPGTKKVEQLVYNLSRPMDIHVISEKRQ
PYTDNPCDTRKKGNRCDQLCVIKSATESSCLCQARYILGSDGRTCEPANEFLLFARSWEVRGISLN
ASYLNDVMTPVLGLNSAVGTDFDALSEYVYFSDVKTDKIGRTFIGNDKGGKIEWIVEGNLDNPDG
LAVDWVGRNLYWTDSRISRQSEIGVSKLDGKFRKSLIQEPLGKPRGIVVYPEKGYLFYTDWMRPAK
LGRIDMDGSHLMILVN
>TRINITY_DN912_c0_g1_i2.p1 type:complete len:355 gc:universal TRINITY_DN912_c0_g1_i2:1586-522(-)
MGCTISQIDKEAVERSKMIDKNLKADNERSAREVKLLLLGAGESGKSTIVKQMKIIHESGYTSDEC
LQYKPVVYSNVIQSMLAIVRAMGTLKIEFVDPTKNDDALRLFSLAGSVEDGCFTPELMSIMKKLW
QDENVQHCFQRAREYQLNDSAGYYLTSLDRIGADDYVPTQQDVLRTRVKTTGIVETHFVFKDLH
FKMFDVGGQRSERKKWIHCFEGVTAIIFCVALSAYDLVLAEDEEINRMHESMKLFDSICNNKWFM
DTSIILFLNKKDLFAEKIKKSPLTVCFPEYMGANTYDEGGSYIQLQFENLNSRKGEKEIYTHFTCAT
DTNNIQFVFDAVTDVIIKNNLKDCGLF*
>TRINITY_DN59572_c0_g1_i1.p1 type:internal len:725 gc:universal TRINITY_DN59572_c0_g1_i1:3-2174(+)
FSSVLEYNIEDFELVNQYMFVTKNSSSHPGTKDLLVSYYRGDFQNTSFPMKNNGTSYIIADAHENE
TMVAVNHNGTANLYISNPEGTKYSLSLGNILYYNKDEGQKSYFEHSFVDLHRIKGISGIYIATSLTTG
AVGTRHLQSYITFNKGGYWSLLKAPEDTKNCSIWRGCSLHISQEFGYHNPLLNFSPITSKESAPGFL
IGTGNVGDSLKTKPNVYLSRDAGLSWRKVLQGHYYYNMLDHGGIIVAVEKFGLTETFTYSYDEGK
TWHSEVFSKERVRVYGLLTEPGEKSLIVTIYASYNKAHAWEVFEIDMTKVLGRKCSIDRDYNLWAV
ADQRNDTERCVLGKKQIFERRKAKSRCYNGYNYDRPINVSACLCTREDYQCDYGFKEKMILWFR
WDCVHDSDFEDYSKKPPSWCKPGLTYNISSGYVKAPGDACYGGEESKYVSTRKMCPIPKVDDYL
LYATRKSFFGYDFESKKSIEFKLDGYSNIVALEVAYDDGVLFFADKDRDSIVKIDTNNGNISTLLGFD
ESSTHIESMSYDWLSGNLYYCDSGNSTIGIINLAGKHKKEIMKFADADKPRAMMVHPTKGYLFWT
DWGAKPRIGRATMNGKNIKTIIDTEIYWPNGLTIDYDADLLYFADANKDRIEACGMDGENRRVIL
STGVYHPYAITIYNDRLYWDDWNQHSIFSIDLQGSGRETVVSYLAYAMDIKAFSKKRQKKGTNGG
T
>TRINITY_DN11522_c0_g1_i1.p1 type:5prime_partial len:849 gc:universal TRINITY_DN11522_c0_g1_i1:2860-314(-)

LGRECIMSGLPNVDIDSNKSDIGRIGLGNIRDPSISLTRGSKIDYNGYMGFSATSGYFGNDKHIHFA
GGSPRSNALKGRVIMYKDSAGYEISQYLDDPSDQIGTYFGSAMASADINGDGLADLVVGAPYYTG
TVSNEGIAYVYLNKNGIVELVQTLRQPEGKDVKENGMFGISIGSVGDLNNDGFADIAIGAPWGGE
DGKGIVFVYYGQRDPAQLKLMQTIKPSDLSGIDVDGFGYSFAEFYPYSGGPDQYGFIENDPDYNIY
PDLAVGAFKSNHAIVFKSRPVYTIVGRLSLGGVKRLDLVNKENLCPNGMKCLDKVQACLMFKEE
ESEGEVDYTLEVDSGRDSPKRAYLLDSDGKQVTKINKQTTVSKDEECDDYTIYVKNTTDVLSDLVI
KLSWKFVVPTTCDGVCPVANELCQFVDTKSIIFVKGCSDDGDDTCSSDLHLEIEKKEFTPKLVGTE
EIISAGATDGIILTFNIKNLGENAFDNSLKLEYPGKGDMKLVNIDDAKVISDYYGTPIWDSDVTREK
DVTTLSVMLSSPIVKGKPEQVQVKMSLNPIPKGTKFLKFKVYLETDSEETDTSNDFQEITIPVELVSN
LLVSGQSIPEILTWRKDVKDVQDKGAEITYNFLFQNQGPSNVDTSEAIILIPEKFKGSYFLNIYNYDV
KGSKSGSDPGSCRIEGELNPLKLDYYGRRIGNNTRKRRDTRGVPALECGADTTCREIICNFNIENR
NSATVTVKATLVDANFDSVIGAGREIHVTAEFSSDAVTPPSGLEFPVKSMVTTTVNSPYLTKTEGGE
SIPWWIILLCILAAIIIIGVIIFIMYKKGFFKRKKMADEDEALRNGDPEMDDD*
>TRINITY_DN75529_c0_g1_i1.p1 type:complete len:517 gc:universal TRINITY_DN75529_c0_g1_i1:94-1644(+)
MSSYHDVIISSIGDFGLAQTLIILAAAVPKLTAAWSILLMSFGGIETDWWRITKNLNSTGNSSDVIRL
FKDCKLKPGDTIEFDTSIKTVVNEWGLVCGKDWIPTTITTTQMFGVLIGAAVGGQLGEILGRRKTLI
IMLTLKILAVTIEGTAANWYVMIAAIFFVGISFGGFFVINNVYMLEFVGTKYRTPVGVIPFWGLGM
TLMGVLYKFSPNWRHICYVAVAIEIPCYILLCISPESIRWLFTQREFEEGKKKLVKLSKLNGRPLPDFS
NFGEVVNKEEQSRGASKKYTYLSLFKYKSTRIMSVILGYIWFCQSYTYYGLTLGVGHLVGDPAVNM
MIMGSTDVLCMAIVALISRCCGRKYTTIILMIITAFATLGITVAYFIKTEKFDLIMSILALSAQFTISGG
WMSVIVYTTELYPTVVRSVGYGFASICARVGSIVAPQINTMRKISIHLPYTVSGIMMLICCVLVLTFK
ETASSPLEDHVEKNAKEKDVELTDPTKVELTDNCYVVNEQAT*
>TRINITY_DN711_c0_g1_i2.p1 type:internal len:183 gc:universal TRINITY_DN711_c0_g1_i2:548-3(-)
KGESGRPGSDGLPGKRGEQGKQGNRGQAGVPGDSGPEGLQGGDGPKGSRGIRGNKGETGPDGP
AGEDGTPGMAGNLGSGGRKGARGISGGPGAQGPPGLVGMRGRRGAPGNRGPAGAVGPAGRD
GPKGNAGEVGAPGRPGANGAPGLPGKDGKNGDNGADGAAGVQGLMGAQGATGPRGSMG
>TRINITY_DN1783_c0_g1_i1.p2 type:5prime_partial len:473 gc:universal TRINITY_DN1783_c0_g1_i1:1460-42(-)
NDFGPSAWQLVTAGTYQFLGSLARVAFFYEMGKPKALVIGGQFSGTFTARDLKSKFHVTVVDCKE
YFEYTPGVLRAYVKPCHLDALTFTLQPVLERSMGVKFIWGEVKQLDGEKKCAAIKPMFADTTDIIS
FDYCVICSGCNFGPFKPMGESLWFPTVHEEERKISDWKHIDERFIEGRRRHVLEEFHKLKDLNTKK
AQILIVGAGFIGVEWATEIEYFFPDIKLTIIDALPRCLGPLPDSAAEYCSEYMHAAGIKEFYDCKYDA
KSPDFWKKVELPDGADVEYVCIGVKASNYFMPKETLSDKGPGGGGWIHFNKYMQVTKKDGTV
WGDGSVFAVGDCNYGCIGAPPNWEMPPVPKISYPGEEQALHAVVNIKKMYKGKTALQPTWWP
WGAGMFATSLGPHDACFVLAANENKGSGYMINWWIPAALQKEIIESTKINECKNGCIGILIWHF
VHHTPFNFWGRGPWMPSTH*
>TRINITY_DN98417_c0_g1_i1.p1 type:internal len:180 gc:universal TRINITY_DN98417_c0_g1_i1:2-538(+)
LLTGQVVTLGCEKAGPYWGPWGNWTSCSVTCGDGIMTRGRQCVGGNPGEGRCVPKNGNQQE
QSCNLGPCPVPPRPCDKDIDLGFIVDFSNTVDAGLHEKQLADKIKSNIMEIALLFDISSSGTHFSYIP
YSSNPGDYDPLQWFNNSVVKALESQDKSSQKFYLETVIKSDPEEEGFKRG
>TRINITY_DN38399_c0_g1_i1.p1 type:internal len:876 gc:universal TRINITY_DN38399_c0_g1_i1:2625-1(-)
GDGSNFYESPGLLDWMNVTNVHIRFSGLYRKFDYIDTRWHHYVVSEVSLIARCDCNGHGDGISC
PLNVVTGTRKCTCHDNTCGSHCERCCPAFNQYPWKRGTRSSWFSDNTTACERCNCLGHSDVCQ
YDQHAADNNLSLNINGEYKGGGVCMDCKHNTDGINCEKCKPYYYRPANVDPRSKDACPSCGC
SIAGTRAASSNIKFLECIKDDKTAKFYPGMKVGDCFCKQNVIGSKCDLCKDEFYRLEASNSLGCRR
CRCFLSGTVQRLNICHKDFFGQCPCKSNVKQRDCSICKDGYYDLRNDDESGCKWCDCDVGASIS
PYICDKITGVCTCRAHIHSRRCKKVDEGYYYPDMHYIFGERNIYLRANSRLTTKIDIPRDAMYQLF
VQFELRTRLSPMLQLELKLDGTTKFLLRSNNNLPYALIKVCDTGCFSYFRHDSTQNFFLNAGLYEV
RVVSTIANSKFLLYKFIAIPVEFITAAILVGELKESFLHSCDVTKNDMRLGTANETICLAGAFSMTVK

FEDGALPCNCNLIGSTSTSCNKYGGQCACKPGIGGRDCSYCKPEFYDYSRNGCKECGCKGRNKV
CDPVTGDCVCPPNSQGRVCDPCECNGNSKRCNRQGICFRCGFNTEGDHCERCKVGFYGNALKS
QCIECACHPLGSFNQSCDAVTGQCYCRPGVVGRTCSKCASNHYGLSVKGCKACNCNEFGSKSLQ
CSAIGQCTCLVAAGREKCDKCDPGFYGLPKMACKECSCNTTGSVLNTNCKNDEFGQCTCKNGV
EGRACNRCMKQHADFGNNGCRQCPLCTRKLQCHADEVARKLINVKTHLNLTEILIALQTNLIYT
QDTFQQIQVDAALFEHRLKQGKKDYTYLYDQKLMPWNK
>TRINITY_DN577_c0_g1_i1.p1 type:5prime_partial len:269 gc:universal TRINITY_DN577_c0_g1_i1:1086-280(-)
PCQNCADMAVGKNKRLSKGKKGSKKKIVDPFTKKDWYDVKSPSNFQIRNIGKTVVSRTVGTKIA
SDGLKGRVFEIAQADLNKDEVAFRKFKLICEDVQGRCVLTNFHGMTMTTDKLRSLVKKWQTCIE
AHVDVKTTDGYLLRLFAIGFTKKRQNSVKKTAYAKTTQVRAIRKKMVEIITREVSTKDLKDVVNK
LLPDSMGSDIEKASQGIYPLHDCYIRKVKCLKKPKFDVHKLMEMHESSGTTTTDATGGVVVERPD
NFEPPVLDSV*
>TRINITY_DN11595_c0_g1_i2.p1 type:complete len:615 gc:universal TRINITY_DN11595_c0_g1_i2:1952-108(-)
MLLLLIFCFVGHVYSFYVPAMAPHEFRKGDTVELKAVKMTSVKTQIPYDYYTLAFCDPAEKHYVP
ENLGEILRGDRIVNTPYNIKMDTEAKCQTVCPKKKISAKDSDVFIKRIREGYHVHMIIDNLPAATK
FQTEDGTPQFSHGFLLGAINNKRVFLYNHLQMIIYYNRDEKKDLYRVVGFEVYPTSVKNAESLDN
NQCKYPEKGQAEVLELNENAENEVQYSYSVQFKASTIRWASRWDNYLKMDNTQVHWFSIINSIV
IVLFLTGIMAIIIIRTLRRDIANYNKEDDMEDNIEETGWKLVHGDVFRPPRYPMLLSALIGSGVQLF
CMVLITIGFAALGMLSPASRGSLMTAIIFLYVFMGLFAGYYAGRLYKTLKGCHWKRAALMTGLLYP
GFVFSISFFLNFFLWGKESSAAIPFTTMIALFCLLFGISIPLVHLGYFFGYRKAPYTHPVRTNQIPRQV
PDQVWYMNPFVSVIMAGILPFGAIFIELFFILSALFENQFYYLFGFLFLVFIIIGICCSEIGIVIIYLQLCG
EDYHWWWRSFFMSGSCALYVFFYAVFYFVSKLEIVNVISGMVYFGYTIIMVFSFWVITGTVGFFATY
AFIRTIYSAVKID*
>TRINITY_DN3434_c0_g1_i1.p1 type:internal len:317 gc:universal TRINITY_DN3434_c0_g1_i1:1-948(+)
PAAGAPGRLKRHFASSEPQAIMGKVSQVIGAVVDVQFESALPPILNALEVQGFEHRLVLEVAQHL
GENMVRTIAMDGTDGLTRGQKVQDTGAPIKVPVGEQTLGRIINIIGEPIDELGPIETKKTYAIHRPT
PTFTEMNTTAQQLLTGIKVVDLLAPYAKGGKIGLFGGAGVGKTVVIMELIHNIALKHGGYSVFAG
VGERTREGNDLYHEMMASGVIKKDKAPGSKAALVYGQMNEPPGARARVALTGLTVAEYFRDEE
GQDVLLFVDNIFRFTQAGSEVSALLGRIPSAVGYQPTLATDLASLQERITTTQKGS
>TRINITY_DN15584_c0_g1_i1.p1 type:complete len:275 gc:universal TRINITY_DN15584_c0_g1_i1:874-50(-)
MADSGSAGADRGGRGGFGRGGRGGRGRGRGRGRGRGKPEDKEWTPVTKLGRLVKDLKIKTLE
QIYLYSLPIKEFEIIDFFIGPSLKDEVLKIMPVQKQTRAGQRTRFKAFVAIGDYGGHVGLGVKCSKEV
ATAIRGAIIQAKLSIIPVRRGYWGNKIGKPHTVPCKVTGKCGSVTVRLIPAPRGTGIVAAGVSKKLL
QMAGIDDCYTSACGTTATVGNFAMATYMAIARTYSYLTPDMWAENQFQKTPYQEFTDYLAKNH
NVGGKPVAPATATA*
>TRINITY_DN5115_c0_g1_i2.p1 type:3prime_partial len:180 gc:universal TRINITY_DN5115_c0_g1_i2:539-3(-)
MLQKTNDKLHLIIVILLSWSQVINGDGNVTFRFQRYYNPQQKNADGNSCDTFSGCDQIFKICLSL
VSLKQDACELLNFETEQYFKTLHIFREGGYLNRNKSAKNPIKAKFKGIYPGLVYTKIAIENYDATSS
IDVILKKSRTAFVNEKNKYFKYSENNKYTDFDYSIKYTCDMHYYRED
>TRINITY_DN557_c0_g1_i5.p1 type:complete len:571 gc:universal TRINITY_DN557_c0_g1_i5:26-1738(+)
MDKLRVIVALLLILQSTFAQDTSVAQTQACEKVLPSKVCEKLRETAGKLKEKVSVVTDAINAAAE
KHINNTAEIINFVNAYMAEKFNCTKVLAVDICNKLQTMGNNAKLKVIEVKEAIVEAIEDGANTSK
EIVQKSIEMMKRMVKCETFLGVQNCERLRYAATVFGESTEAVKDAIFTAVQTQYNNVNELLAEVQ
DVLRNAAKNFKCETALAPVACGKIRAIAKLLRLSTKTVQEAIERAVLNGLKTRDAIVNATLNFLQ
TKINCDTILSPTTCYNFMRVIGMLNEQKDLIQEALLSAAVQQLNNTKDVIAFVKNQIETAAKNFK
CTDVLPSRYCDLIKYVARRFQLRSVDLTLTIIEIMLNGTFDVKQIYDKTLGAMVEEAKDLKCEQLIH

EQACAILKAFASNFNLQMSQVTTAIEEAIVNGLNTTNDILKFSKEFLLELTSCDTFLSATICGTIERLS
NRLNVKFKSVINKLKEWYSNGVAIIQMPRKIIQWIWGQYFSQDEPQFDKRSVDEEKMALEDDFLG
MLDLTWEEMEKIIDETHKVVSKKKTEEIFASRWNGNRWNGNRWT*
>TRINITY_DN2885_c0_g1_i2.p1 type:complete len:114 gc:universal TRINITY_DN2885_c0_g1_i2:31-372(+)
MAQPVRLYSKGIFVGYQRGLRNQHEQTSLIKIEGVNTKADTSFYLGKRVAFVYRVSKKTVAKGDK
KPSRIRVVWGKVTRPHGASGVVRAKFRHNLPAKAIGGSVKVMLYPSRI*
>TRINITY_DN17714_c0_g1_i1.p1 type:complete len:644 gc:universal TRINITY_DN17714_c0_g1_i1:102-2033(+)
MADVEQNGTYDDSNEKTKLHSNGTELEEKVEMNPPVVVEGEDRGGWGNKIEFLLASVGLAVGL
GNVWRFPYLCQQNGGGAFLIPYFIMMVIEGIPLFYIEFAIGQRFRRSAIGCWSKIHPALMGVGISCV
VISLLLCIYYIAVIAWCFYYFFVSFTSKLPWNIKNCPEYDRYKLLDNACKANGTATSCNATKTFSDC
CVHDPQLYYFYRKALNISPSIEDLGDGVQWKLFGCLVLSWIVVYACIVKGVKSSGKAVYFTATFPY
LILVILFFYGVTLEGADIGIVTLFKPDFSKLLEPSIWMDAATQMFFTLSLGFGALISFASYMPIKNNC
VRDAYTVVLINCGTSIFAGIVVFSILGHRELMLGEKADKVGGGPGLAFITFCDAFLQMPISPLWSILF
FTMLILLGIDSEFGTLEGLITPFYDMKWVKMRKELFTALVAFGMFLIGIGLVSSTGFYIFQIFDDFSVS
LGLLFIAFFQVIAVSWVYGNDKFANDIEYMTGSRPNLFWMICWKYISPLAIFVIFVSNCIKMASNSP
KYAAYVGCEFQSVDFSSVAKGIKGSIAKVDYPGWASIIIFIFVTASMVPIIIFLVKDFVSNRQGWIDGF
KRKLGNRYEYLPDPSVADPSRRKSPEEMEFQIQKEEELF*
>TRINITY_DN250_c0_g1_i6.p1 type:complete len:452 gc:universal TRINITY_DN250_c0_g1_i6:131-1486(+)
MRECISVHVGQAGCQIGNACWELYCLEHGIQPDGQMPSDKTIGGGDDSFNTFFSETGAGKHVPR
AVFVDLEPTVVDEIRTGTYRQLFHPEQLITGKEDAANNYARGHYTVGKELVDQALDRIRKLADQ
CTGLQGFLIFHSFGGGTGSGFTSLLMERLSVDYGKKSKLEFSIYPAPQVSTAVVEPYNAVLTTHTTLE
HSDCAFMVDNEAIYDICRRNLDIERPTYTNLNRLIGQIVSSITASLRFDGALNVDLTEFQTNLVPYP
RIHFPLATYSPVISAEKAYHEQLTVSEITNACFEPANQMVKCDPRHGKYMACCLLYRGDVVPKDV
NAAIATIKTKRTIQFVDWCPTGFKVGINYQPPTVVPGGDLAKVQRAVCMLSNTTAVAEAWARLD
HKFDLMYAKRAFVHWYVGEGMEEGEFSEAREDMAALEKDYEEVGVDSVDAEDEEEGDEY*
>TRINITY_DN20282_c0_g1_i1.p1 type:internal len:202 gc:universal TRINITY_DN20282_c0_g1_i1:3-605(+)
EVPKDWVGDLNVTYRYGSLKNGLKADLRINNKLEVKRIRNVCGTILGDLEPDRYVLMGNHLDS
WVFGAVDASSGTTVMMEIARVLGEQLKKGWKPRRSIKMCGWNGEEFNLIGSVEYVQEFYNQLR
DEAIAYINLDSAVAGNYSLQTAASPLLHNLMYEVHRMVDDPYESNMSVFDKNRLVYKDKHDTE
NHKIYPLGAGSD
>TRINITY_DN6049_c0_g1_i1.p1 type:complete len:156 gc:universal TRINITY_DN6049_c0_g1_i1:1046-579(-)
MEKMANVVYSFENELFKSFMFYAAVLVLKMIAVQYVTIYFRLTRKVFSNEEDLPKNNAGDKIKIN
MSDPYVERARRAHLNDMENILPFLILALLYIATNPAFDTAILCFRIFTGVRVMHTIVYIGCIPQPARA
LTFFIGKLVNVYMAVMVVRATM*
>TRINITY_DN3332_c0_g1_i1.p1 type:complete len:336 gc:universal TRINITY_DN3332_c0_g1_i1:1457-450(-)
MKEFFILVIASCLVSKSYAYRSIGCFKHDSSNQYLLFSDKTEHRGEAFHSGVRIDWHHFQKYLLNAL
NRCEDAAAKQGKRFFSFRFFGICHGLNRLPRKKSQYCIHHSYEQCMKHHSKCVGSDEAEYVYRV
TKKITSGKVPGIKIYRNVFCEHKGSRVISCKTGEAIKVTQAYYGRTSKDHCGYRFYYNLNCEARRSE
AKIKATCNGRQSCQLNPHNSVYGDPCRFTTKYIDVEHICKPQRVRSRVACEHKGKRIISCGRKKIKI
TDAFYGRTARNVCGYRPYYNLRCKASRSEMKIKSICNGKHRCRLNPHNKVYGDPCHGTVKYISV
KYVCLR*
>TRINITY_DN8926_c0_g1_i1.p1 type:complete len:307 gc:universal TRINITY_DN8926_c0_g1_i1:1655-735(-)
MESITTFKKVLIFTTPIIYIIHLVLNALAAQGGSDNKLFPNSVSDISKTFQLELTPVGATFSIWGVIFT
WQLLWMVYTITTVCRSSPATNILSNKFYIAFIINIIFISAWLFTWAREEGVISFVVIVLGQIFLDITIGYA
LFDLKKFLDDQLPHGRAQIDIWLQRMLVQNGTLFYGTWTTVATLINLAVVLAYYIDLSTKTASLIS

LSLLGGIALSWFILENFAIKNYAQYTFSPYIVLIIALTGVYVNKESKGNAVGKLALALLIMSAVLLVIR
CIVIVVRHVKSKNKQTEDIMASAKYSSNMNI*
>TRINITY_DN163801_c0_g1_i1.p1 type:complete len:298 gc:universal TRINITY_DN163801_c0_g1_i1:55-948(+)
MANPLKDIISRFSKGVPAGSSTGLGLLIGAGLAAYGIKESIYTVDGGHRAIIFSRIGGIQPGVYSEGL
HFRIPWFQYPIIYDIRARPRRIMSPTGSKDLQMVNIGLRVLARPLSSTLPHMYQRLGLDFDERVLPSI
CNEVLKSVVAQFNASQLITMRQEVSLMIRRDLTERAKDFDIILDDVSITDLSFSAQYTAAVESKQVA
QQEAQRAAFLVERAVQERQQKIVASEGEAKAATLLGEAMNENPGYLKLRRIRAAQKVARVIAQS
QNKVYLNANNLLLNIKDTDDIDDGLLSKKR*
>TRINITY_DN124859_c0_g1_i1.p3 type:5prime_partial len:237 gc:universal TRINITY_DN124859_c0_g1_i1:884-174(-)
HRLEEDMRCVHHHEPRVRGPVRVAGQSEGLVPAGGDDGARLGHDHGDHVGFGRLQGLQGVG
EEDVHVVHDDAATDVEAAPLRFRFAQYQVGFGLRWCIEAEGARNARADPSDAGNQRHERTQV
GLAGRAALQRSAGRHLPRLRIARAGLREARGDDQRGPFGIRLPEGAALHRQMHQHLRDEDHA
ARQHVGRRNAGRQKHLLEDFGEGKDSLEGVWAGRHGESGAPGHQPKVHHHE*
>TRINITY_DN141338_c2_g1_i1.p1 type:complete len:720 gc:universal TRINITY_DN141338_c2_g1_i1:2850-691(-)
MSEADETETFAFQAEIAQLMSLIINTFYSNKEIFLRELISNSSDALDKIRYQSLTDSGVLESNKDMKIE
IIANKDEKTLTLFDTGIGMTKADLVNNLGTIAKSGTKAFMEALTAGADISMIGQFGVGFYSAYLVA
DKVEVVTKNNDDEQYIWCSSAGGSFTVKRDTINEPIGRGTKLVLYMKEDQLDFLDEKKLKDIIKK
HSQFIGYPIYLETQKTRDKEVSDDEADEEESTDKKEDEENEDKEDDGPKIEDVGDEEKEKEKKKKT
IKENYTEMEQINKTKPLWTRNPDDISTEEYAEFYKSLTNDWEEHLAVKHFSVEGQLEFRAILFVPK
RAPFDLFENKKQKNSIKLYVRRVFIMDNCEELMPEWLNFVKGVVDSEDLPLNISRETLQQSKILKV
IRKNLVKKCLELFAEISEDKDNYKKFYEQFSKNVKLGIHEDTQNRNKVAELLRYSSSASGDELTSFK
DYVSRMKENQKDIYYITGESKEIVAASAFVERVKKKGFEVLYLVDPIDEYAVQQLKEYDGKKLVCV
TKEGLELPEDEDEKKRQEELKAQFEDLCKVIKDILEKKVEKVVVSNRLVDSPCCIVTSTYGWSAN
MERIMKAQALRDTSTMGYMAAKKHLEINPEHQIMIALKKKIEADKNDKSIKDLIILLYETSLLSSG
FSLEDPQNHSSRIHRMIKLGLGVDDDGDVEEIETDDLPPLEGEGDDDEDKARMEEVD*
>TRINITY_DN2532_c2_g1_i1.p1 type:internal len:466 gc:universal TRINITY_DN2532_c2_g1_i1:1396-2(-)
GMAWPNGLSIDYSSNRVYWADAKVDKIESIGLNGEDRQVVVPLTSHNFGLAVGEDYIYWTDWL
AKSIIRASKKNVSERTIFRTNYGALMEIQIYDKTLQPVSNICNNSQCEQLCLIQPNKTYICACGDGF
KLRLADNRTCAESNKTSKGHMCRTGFFKCASSNRCIKDSWKCDGDKDCIDGSDESILLCVSKECS
PEEFQCSNKKCIPSEWKCDGVADCGDGGDEKLVECTKNPECNVTSQFKCDDGQCIDKKWQCDG
EDDCKDKSDEKLCEPTGCSILQFQCFTRNNEKRYGDLKTGECISQELACDQVKDCRDGSDESIEA
CLKVNTCQRSGNRKCDNGLCIKESYWCDKENDCGDWSDEKACYDPAIQKDLWCSSRPPNSNGT
ANDTEFCKCRPNEFQCNTTKKCIPLHTKCDYTVDCDDYSDEKNCTFTMCDELQYQCANRKCIDI
DHQCDDVDDCGDNSD
>TRINITY_DN1214_c0_g1_i1.p1 type:complete len:752 gc:universal TRINITY_DN1214_c0_g1_i1:19-2274(+)
MFIYHKILLSLILLLEIHYVTSQTTENRICQGKVRFLHCTEGTSMIIREAYFGRQYASFCKNRKFGTH
KYACKEEKVKEIISRKCDNQNSCVVGAIRRHLQQYYDICDGVSKYLYIKRSCINDDLKYIDTKENF
CESEERILSCGKNQVIEIKRVFWGRDENEDKCNGWYIQRFWANTCRSWYALGKVQEMCNGKRW
CNLVATKNIFGNPGCNSDTALSFKVTYKCVSGKTPVTTMATKKSTVQQTTTSTTMTTKTPNIAKTT
RKSTVRTTTTSRPITTKTPDTKNITMEPTTSTVYNTTTITQTPNITTTTTEATVPPTTPSGPIIRQLCEN
RNMILFCSNDKVIKIRYAFFGNKNAAICATKKVNTTSNTTNDFNCSTDALDYMKYFCQDQSFCYV
NAYAGNYGDPCPGVKKYLEFEYECAPKGNTTQPPIMVIPPVPIATLQPNMTKPSAKCGMNYVQS
GRVIGGTTPTKGQFPWQVAIYYKGDFICGGSLINSEWIVTAAHCFRSIITKDYQVILGEHDREILEGS
EVQHNVKGIFIHWKYNKDDRWSSSITHDYDIALVKLELPAMFNKLIAPICLPDDSYTKDNEECYIS
GWGKTTFPGQMTGILQHTKVSLVNKRTCQDRLRTAPGKPGDDITSNMFCAQGADKVSTGCHGD
SGGPYSCFSNNKWVLRGVVSWGSPRCNIKDMYLVLSNVQELEWWIDYMIDREANTPAPTMTTN
NSTVQRNSTSTPMITRTPDTKNITMEPTF*

>TRINITY_DN121236_c0_g1_i1.p1 type:internal len:568 gc:universal TRINITY_DN121236_c0_g1_i1:1701-1(-)
TCIMCAHDEYSPEHSSECIKRKSCTPSDYHQYWEPCKDDKTRKSFQWNQPKICNEYTGEKLPNSG
DFIECPPCNPGQYINGSECQFCPKGFYSNGKEACQRCPPETAPAPGLFYKFWNRIPKRPMHLFCL
ARPMSGCRTLVGWVVHDSYLATGLGHSDEAQLIMYIKIDRFIKKNSLFTMEFEISDCIGLTCRTKV
QTIDKHHSVQSIGVYEGNQPRTTIRYTRYNDVGTEYSILFEKKYDQDAQTYHYQSAQLKIYSIEITD
VENGGTRQCEACTTRGSGDCVTCEPGHYLSPNSQNCWKCPRGTYMPEHLQDAVGLQSCIQCPIG
STSEPGSTKCHSDCMLAFGEGVNRKYFNLTALAGPQFVRGPLLFTSKGYKFFHGFSISFCGHPYKE
SLCVDNITSTQGFHASICRSTFIPEEHKLLSTQSIIIGDTLQKINFISEESNANHTEQKQKRDIHRNEI
QYHFHAERITDVCNNGTSGVLRLICDPVASYSSKKPNIEFGNDECTSETCDGCNYAFTLKSPYACA
KCTKDDFEAITTSCNNGEKHIKYVWKEPRACVGGMEKPE
>TRINITY_DN4523_c0_g1_i1.p1 type:complete len:1645 gc:universal TRINITY_DN4523_c0_g1_i1:5332-398(-)
MEYDTDERQSAEEMDERRREQIAYEYLCHLEEARLWVEACINEELPATTEMEEGLRNGVILAKLA
HFFAPDVAPLRKIFDKDLTRYKERGLHFRHTDNIMYLIRSMEKIGLPKVFYPETTDIYDMKNMPRA
IYCIHALSLYLYKLGMAPQIQDLYGKATFTEEEINAMKQALDQYGIQMPSFGKIGGILANELSDND
AALHAAIVAINEAIDRHLPEETLTAVQNPAARMKDINVDQKKQYQDLLYESKLKKCQQAELKEK
KESDLDLYDKMLTQAEIQGHISKVNLEKYVELVNDLIEKRDENLLLKNLSSKEIALNNVQNQNGA
WYMQNLRDEKVSKAEQQGTPNAKLDHKEIQSAVNVSNSLADRVRNMESAVRQINNILENGTH
QELIQWLQRPEGLLPVVDASRPNLYMDNLKKSRMDKGGDLTHDDLTDDLKLLLAIATINQAIDM
NDENRLCDALENPEAKLADVDESLASRYLSHFVAVKKEKRDEVGVDHDDLNHVEIQALIEYVNT
EVQEENDLLSAISKINEAIKSMNSDDILSSLGITSAKLTNVRPKNAELYAIILKQAKEEKAKRSGDEN
TELWHEEIQQCVDKGNAIAEEAERLGTAVVAINEAVDKSDSADLLLALKAKSVALKSITPECADG
YHSELKEAKEKKDNVGWNGWTEYRVPQDYTYFFNYITKETRWSVPPDYQHDIGHLNREEIQVVI
SKVTANHDRDLFLKANEPSAVKIQAVWKGHKAKKEYEQRKHFILTQLPAIIKIQAHWRGYKARK
DYQKRLERLHGNEGAVIKIQSLVRMLVARKQYQDRLKYFKDHINEIVQIQAFLRANMAYHDYKM
LSKPQPPVKSVRKFLHLLEHDHIDFQEELELQKLKAQVVTDIRSLAQLESDLNTMDIKIGLLVRNRI
TLQDVVLHGKKLKRERSDSISASQGSSGLKSLSREKREQLQAYQHLFYLLQTNPTYLARLIFKMPQ
TKTTKFMESVVLTLYNYASNSREEYLLMKLFETALKEEIADKIDQIVEIVTGNPTVIKMVVHFNRGA
KGQTSLRDILAPLVKEVLTDKNLVINTSPVEVYKQWINQMETETGETTQLPYDVDNDFAMNQPE
VAKRVEVSMNALRVASDKFLTSFIKSIDKIPYGMRYVAMTLRESLKQKFPNAKEDDILKVVGNLIY
YRYMNPAIVAPDAFDIVDVSVEKGLTPDQRRNLGSIAKVLQYAASNKMFDRENSHLSPLNKYITSS
FARLKKFFVDASTVEDAEAHFNIDEYSDITMVSKPVVYMTVQEILDTHQLLSEHSNQITKDEDDPL
LELLDDLGEVPSVEDLVGSSSSSTGSNKETDDDDQRVAKLGRTEISLTLTNKFEVPEDDDSDMKAL
FVRTKKLIVEIMRVQQCETLIQVLETPATKQQEMDHQRFQKQRLDQEQKQKNSSNLRRSTSSVGD
HKLPLKEIKKKISRNLKALEQHGLVSNKNNYQDIVNAIAKDIRNQRKYRQRRKQELQKLKQTLE
NLTRKSTFYEEQVDYYNQYIKVCLDNLTKKKTGKVKKGKKQEEVINKGSVKYNAQKLAEKGVVL
EIEGLPKTQFKNVQFEISSSEDIGIFIVSAKFMGVAMEKVELVFQDLLQLQYDGVAVMKMFGKAKI
NVNLLVFLLNKKFYGK*
>TRINITY_DN8941_c0_g1_i12.p1 type:5prime_partial len:771 gc:universal TRINITY_DN8941_c0_g1_i12:2-2314(+)
ICGCECTSADGKTFQEHGPVVIATGGFGADFTDDSLLSKHRPDLSHLPTTNGDHCTGDGLKMSA
AVGADLVDLEWVQVHPTGLVHPDEPDAKVKFLAAEALRGVGGVLLDMEGHRFCNELGRRDYV
TGMMWKNKGVTMGSTSGFFLCLNGKASKEIEWHCKHYKGRGIMKSYKSMEEFAKEYGIPLANI
DATFKEYNALADKQTKDPEGGPYEAYGGGKSWDQWGKKFFHNLPMEVSDAFHVAIVTPVIHYC
MGGLEIDVDSAVCGADGKAIPGLYAAGEIAGGVHGNNRLGGNSLLDCVVFGRVAGQACCKYM
LGADMKDVSLADLSGGGLSGAVTSSKLSGGSYEDTMNKSAGAAGGAAAGGAVMNGGGGGGYS
LEEVAKHTSKTDCWVVVNGQVLDVTKFLGEHPGGELAILTFAGKDATEEFNMIHPPDVIPKYAPD
AIIGKVGTGAPAAPAAAAGGAAAPLLDKSGHGKARDWSKSEKNRKMRMEGEGKIPGWIGALC
YMVLGFMKEILFTIVPQKNVTLTNDRIGLTRSAMFLFIFIIIHAVGNLHVFLGPDDFNGYGYFYVRL
YWTGFGLQANIVEEYILLAALLHVFVALKRTWDISINYTIASGKLNLAISGITLLTFMVIHLFQRFG

ATQPFKLCPPPYLVNIGTVFHLRLNLFWVDTPGCTTAEVRDIYRMEFEVFKSLEWCLFYIFAVVVFS
THMCLGWAKAVPAPILDIPKRYHNKAIHVGYVMTAFIALIYVSFPVYTHLFPMSPGYISPEPALP*
>TRINITY_DN1339_c6_g1_i1.p1 type:internal len:570 gc:universal TRINITY_DN1339_c6_g1_i1:1-1707(+)
GMFFQDGLTGSAWGDWALYVDSPLRFFGGGEASTYKTFDPATELGVQEPVGFWDPLGFASDGDE
ATFKRRRSVEIKHGRVCMAASIGYIVPEYYKFPGFLSPSEGLQFADVPNGLAALSKVPALGWVQIIF
FAGIVEGAFGVTDDYRNGTPGDYGWKVITSDDPAEKEKKLSAELANGRLAMVAIIGMFFQDGLT
GSAWGDWALYTASPLRAFESELGVQAPVGFWDPAGYTNDGSMENFKRRRATEIKHGRVSMLAC
MGYMTPEITGKLPGYLSPSMKVQFADIPNGLGALSKVPGVGWAQIFAYCAYCELSQDQSAASPGG
AGDFGFKAITSSDPATLTRKLNAEIANGRLAMMAIIGMFFQDGLTGSAWGDWALYVDSPLRFFGG
GEASTYKTFDPATELGVQEPVGFWDPLGFASDGDEATFKRRRSVEIKHGRVCMAASIGYIVPEYYK
FPGFLSPSEGLQFADVPNGLAALSKVPALGWVQIIFFAGIVEGAFGVTDDYRNGTPGDYGWKVITS
DDPAEKEKKLSAELANGRLAMVAIIGMFFQDGLTGSAWGDWALYTASPL
>TRINITY_DN2357_c1_g1_i1.p1 type:complete len:1733 gc:universal TRINITY_DN2357_c1_g1_i1:5452-254(-)
MLKLFLFLIGAGHLSLAFAAICSETNGEGNKKNLKEPCFFNQLIDVKQWNMKRFMDQTDRFFDF
GVMRCSSYEVEGSGKMKLTCYHTKLFYIMKNIMSFGTTERTEFIFKFSPLTENDYKSTDVEISGKLS
QALRNTFIRATKPEGFQDFSLSTSAVFPQIDMETFIDQRKPDGFKTLTNNDKLENFPPKVAVNAEM
SGMFSALVTDTDKQAYEFLISFLGPMKEQQFYIFQSLTENINDELVIAFYKENPSNAGTTVRDIMDS
LRGVKWRGNKANEFVVSKDFAPIFEVQSSAMSISDGEIVNINSEALRNIAIKYVSPSSEAFILEGALI
YWHVDDSSSLKMSADQVPGVENPPTPTNETSTVPQENKIKRHTKPKTMKPLKREKHPATNKLTL
NAHIIHQNKIEIRSPDYLKGTYEKQLSTFLPYHYFKHDSIKNTLYPVKMIFDKITIGFKRKELRLYCNI
TQKYDLVQNFIEGEGIAVYAKAKLTMRKDADGDSEEIRFKDIGMSGTIEVLQGLVNVNLQYAKDG
TLYVVGLFDIANMTQFINATGMKHYLPENLRKTYPRLTNFAITRLKIKSKLNENVLRIIGRCLIFSEP
KTDDNLEAEKRDGIEDESPASLLYESKLRVVLTNTSGDLKYSVGIIGEDIPLQVAMRELFNATITGTS
WVKFKFFVMVMSNNNEINVAQAIDERLFNSFNSETGLQIKTVLLPPVEEDCKMSRKEKLDPLNKE
ALVKNEANGKETVEIHRSPFIVDYTCMFVKDQELLMENFEYATATVTPTSYEIKSLTQERFFDFQSD
PVVSVGNFGYTIKSVKGWDSIFFIEGEFRFMVKNDRLIFKGLITFDDMKGKLVLQLKQLHSGRQNF
GDAALINSGYSVIGKDDTFSKFHFDAFLYVGTVKTKLVNFRKNVEVIYDLRTDDIFMKGARNNNG
NIGATNKKRNSLLELFDAFGTDASSHVLLKELHAISLTWLIYQQNEEVVKTKHGIRRIQPGLNFYG
TLAGKGDRDLLFNMHYNKGMGALIADIELPNYKLPLRKDAKYHPFELQRSTKKQDLGGILFVKA
STTFTSAQAQGYLRVVGLEYDVEFPIMADGQFHMDLPYSNEIAKLFNEAEVRYRVSQDIGVTDFN
LVDPKIKIDLAPSLEKISRAIRHAIEAMIANVDEYLVNSGSFINFVTIVDGEISTMKEVINEIRRGMGK
LQSTLKQLVQQISSDCAEQCKDSKVFIYGYDFQGGKFVGNEDAHDVANVTCIAECEMIKPVKND
ERRSLLSSLKEALVQFKREKKRASIMQIMLDEHYNSLSKMNDAMHKNARAASLILSKTGNLKNP
KISVASLVLENQISVINFKGAGSKCLSGFKMAFSYNDVVGETDIDLCFNGEFDQFAAYAFVLSYYGE
DKYNQFGDLLDDAAELKQSLKTKTKIVTKLFKKIANARTTVELEEVQNEVEETILDYMDGLKKSQ
IPKHKIKAKKTKEAIGYITYGGKNVTLPGIDTEVLEALEIDQDYTIKNPKSKRVFMYSSPWAALNVL
HLHQNKSMESLPMITSIDNAGYCKRLTRLSSVLNSVRGSLSSYAASYVNAKKNFENVETNMKSGL
VELKKMHGSSIMHDDDKGNLQYWLTQYEKGVNRFSKLSNMKLHDHNNHGLARWKNEMDNS
LQHSGLGSIENVIEMLKKRSSSKRSNIDHDRWIRNASLMGKEISNLMKNSNKMRMADAELTVRS
MSQKFDNFFEALTKSCKASFKQF*
>TRINITY_DN7641_c0_g1_i1.p1 type:complete len:1239 gc:universal TRINITY_DN7641_c0_g1_i1:53-3769(+)
MAGTLLLLNVVLITLVLFFTINVSNGDIEVTKDAFKIYRQAHRQTQKNHIKLRFKTIHATGLIMFA
QGPNKKDYLSLELFRGKIRVSGDYGRGTDNKLRPIAGFYDMYLGKDLADNQWHTVEFIRNIRES
MIFIDRGTPNEKKNFMKSPPTYNELSVSSVTFGGFYSFAASELDSSKALARQGVHACFSEAIFSQTW
PSDNEVDKIDFLRTDIPVIGTINSFCSDTTPPYMPYFFPASSVHIALVGNYTIDSIKFKLKFRTYVSEQ
MIANYSTTNTGEKIMLGLDRKGRVEVRLDVQSNIIGGSNQQVIQTAKEEYHDGQWHEVSLVVDN
KLSADNKYIGQFTVDKKTRLFQITKKFEFDGYINIGFGFTGCMRDIEVNDDPVERVKKDPKKPTEY
YDLYDVGVIKGSCSLKDYCNPNPCQNGASCNQTEDNIMCDCKGTLYEGSTCHRPRFNYTCAGVR
KSGENRSDVHYIDFDGMGPMEPVLALCDLEKTVEATTTQINNTKFEDVDVATESGKNDVDIDYE

ASIIQMRELVLNSAFCEQYIKFTCKGTPIFRSPEGPPAVAWMGGDDILHYYWGGVPGRRGYCACG
LEEECHNKGKYCNCDSLDPFASIFDDGNLTIKQHLPVRQLKFDSVLKNSVEALLRVGHLRCSGFG
AHKIAATFRKPYSFLAVDHPDQPFDNIFAGELAFHFKTSMAYNYMVLAHAHGPFSGDYAKVMIW
SRTMVRVHLNFGFGDIKQDVDLAGTGRYLDDNDWHEFSMMFNLKELNVTLDGILMIQGLPLQD
DPVQFNVDDKSVYIGGSFHDPYGFVGCIRSFYVNGRIHDIYSYAEGQEDFGVYPGCGSACVILNRP
CNYGACIDEYDDFKCNCTVSPNDGKFCQNETFPKKFTGGQSITIDFTKLNGTKNQNIAQAIFVVG
FKTDQENVALAQLEGENMRHFSLALVNGYLTLFYTQKEKMAAQGGSLQDIETEILQISTRKLNNK
KHNIAKVHFSKEEIFLQVPNYDLVMGANVSERFIDLRNRNLGKKTDSFDKPVTFTVGQMKNIQID
PKSLTLPTSYTGCMSGAKIVFTPVATKEDRFPKAKAFDLFKLATESKKNDPALTGTFPATEPNGCSE
PLPTPGTLPSVGPSIQFNTRSPGLDNSVRVNTIDTLNRVVLFFVVLLIAVVILAFIYLLYKYVNRSHK
QYNRMRKTEKQKAYPIQQFTAKQRNYQPMDEPSYPSRSNPPPVEVHEPNPPLRQDDRDFYL*
>TRINITY_DN11643_c0_g1_i1.p1 type:complete len:187 gc:universal TRINITY_DN11643_c0_g1_i1:1748-1188(-)
MPAKGKLTQEEEVLLQDFSRTVSKKSQALFYGNALMISVLPLWLFWKVHLMDPMSYAALFICGTL
SAAYLVAFAYKNTKFALKHKVAQKREVAITREVNTELDSRPETKKLSRKEKDDRILWKKNNVADT
EATTFSIFYTNTLYLFIIIIASFYVLRSFSPAVNFTLSTGMAAGTVALLSTGTQSS*
>TRINITY_DN121418_c0_g1_i1.p1 type:complete len:259 gc:universal TRINITY_DN121418_c0_g1_i1:1061-285(-)
MAENDIGDWFRSIPIISRWWFVLSIAFPLLGRFGLLPIYYMILDFNLVIYKFQIWRLFTSIVFFPLSPM
NGFHYLINLYFLYNYSTRLETGVFDGRPADYLFVLVFNSIALILLGFALNLYLLMDPLILSVLYIWCQ
LNKDTIVSFWFGSQFKAMYLPWVLAAFNMILGQGGLMEIIGICVGHLYFFLMFKYPQDFGGRRFI
STPSFFYNYLPNRRGGVAGFGQAPVGRRNPDEGRGRGGGGGFRGMFPGGGQRLGDQ*
>TRINITY_DN57_c0_g1_i1.p1 type:complete len:712 gc:universal TRINITY_DN57_c0_g1_i1:140-2275(+)
MGHDGSTLPSFLPKMSFERQTTLLKLLILSIAAILSFSTRLFSVLRFESVIHEFDPYFNYRTTRFLAEEG
FYEFHNWFDDRAWYPLGRIIGGTIYPGLMITSAVLYHFMHFFHVTIEVRNVCVFLAPLFSSLTALVT
YQLTKELKDAGAGLIAASMIAIVPGYISRSVAGSYDNEGIAIFCMLLTYYLWIKSVKTGSLFWSTFCS
IAYFYMVSSWGGYVFLINLIPMHVFALILTGRFSHRIYVAYSTVYCIGTILSMQISFVGFQPVQTSEH
MAALGIFGLCQIHAFVDYLRYRLTPSYFEILFKSLVIISATIAGIIGAILTVTGKVAPWTGRFYSLLDPS
YAKNNIPIIASVSEHQPTTWSSFYFDLQMLVFMFPVGLYYCFAKLTDANIFIIIYGVTSIYFAGVMVR
LMLVLAPVACILAGIGISSVLTTYMRNIELSNDKPKPIKKAKQADPSYPIKSEVARCMVALITFFLITY
TFHCTWVTSEAYSSPSIVLSAKQSDGSRVIFDDFREAYYWLRQNTPEDAKVMSWWDYGYQITAM
ANRTILVDNNTWNNTHISRVGQAMASPEDKAYDIMRELDVDYVLVIFGGLTGYASDDINKFLWM
VRIGGSTDTGRHIKEHDYYTPAGEFRIDKDGSPVLLNCMMYKMCFYRFGSVYTEQGQPSGYDRVR
NAEIGNKEFELDVLEEAYTTEHWLVRIYKVKDLDNRGQ*
>TRINITY_DN36217_c0_g1_i1.p1 type:5prime_partial len:605 gc:universal TRINITY_DN36217_c0_g1_i1:2-1816(+)
YLTFIESLRLRKKIKTINKFWKKKMNDVAYSKDNEAIEIDNFASTERLADDDTDAKPRGQWGNKT
EFLLACIGYAVGLGNVWRFPYICQKNGGGAFLLPYFIMLIVEGIPLFYLELSLGQRLRLGPIPLWKK
VSPWANGVGYGMCIASFLSGCFYNVIIAWCLYYFFNSFQSPLPWNSCNSPVNSTEWMKCEEIGAT
QYYWYYEALQISPTINDLGGFAWKITLCLLTGWILVALGMWKGIQTTGKVVYFTATFPYIVLFIFIG
QGFTLEGYAEGLKFFFIPQWEKLADPTVWMEAAAQIFFSLSLAFGGLIAYASYNDEKNNTLQDALI
ISFTNCGTSVLAGISIFSILGYRATRKSKFCISELGGNISPIPRNCTVQHFLEQGSGGTGLAFIAFTEAIS
TLPVPTLFSLLFFLMLITLGMGSMFGSLEGLVTSLRDTPFFQKIRKELIIVIVAVPGFFAGFGFTQYSGE
YMVQLFNKFSIDIPILLIGFCELIFIPWVYGMDRFSADIEYMTGRKPPYAFIIMWKFVSPVALLVVLLL
SLYNMMVVTPLYEQWNGELARVSMTQYPGWALGVGMMVFTVSFLFVPAGIIFNIIRRKRKNYLL*
>TRINITY_DN26249_c0_g1_i1.p1 type:5prime_partial len:477 gc:universal TRINITY_DN26249_c0_g1_i1:1733-303(-)
QMKMAASNMAGHVVRNNLIFLLSFVIIIVAEEGRHARFEYKFSFKGPHLANKQGTVPFWNHYGS
AIPSDDQVRITPSMRDQRGGLWTKTKSSSEFWEVEIFFRITGRGRVGGDGLAMWFTEEAGIQGPVF
GSSDHWKGMGLFFDSFDNDGEQNNPYVMVMLNDGTKQYDHFKDGNSQQLGGCMRDFRNRP
HPIRAKIRYYNNILTVFYHSGMSERSDDYELCMRAENVVLPKEGFFGITAATGGLADDHDVIKFLT

HSLTPPLTDEQKQKILSEEQRKKYEEQYGEYTQKLETKKEEFQKEHPDKKAPEEKFESVYDRDVRL
IYENQNTIHHVVRALHEKVGELTNQLNNLHVTINNNNQLAQQQQPKGDGVTRQEVNTIINLQN
QVHNAIQELKQQSGGGGGDNTKTVQMEKTLQTVLEQVGHLVSKNNDRRPSCPTVSCVTPSILILF
LVAQVLLVAGYNFYKEHQNKAAKKFY*
>TRINITY_DN2113_c0_g1_i1.p1 type:complete len:1704 gc:universal TRINITY_DN2113_c0_g1_i1:5257-146(-)
MAQLLPIRFQEHLQLQNVGINGANIGFSTLTMESDKFICVREKVGEQSQVVIVDMADANNPTRR
PISADSAIMNPASKVIALKAAKTLQIFNIEMKSKMKAHNLTEDVIFWKWISVNTIGLVTETAVFHW
SMEGDSSPVKMFDRHQSLAGCQIINYKVDHSGQWLLLIGISAQQNRVVGAMQLYSVERKVSQPIE
GHAAVFSQFKVDGNPQTSNIFCFAVRGAAASKLHIIEVGTPPTGNQPFPKKAENIFFPPEADKDFP
VAMQASQKYGVVYLVTKYGYIHLYDIESAQCIYSNRISGDTIFVTASYEPTEGIIGVNRKGQVLSVSV
DEEKMVPFLIQVKQNGELARRLAERCNLPGADNLFLQAFNDKFASGNYQEAAKIAAKAPKGILR
TQQTIQRFQQLPSQQGQTSPLLQYFGILLDQGQLNKYESVELCRPVLMQNRKQLMEKWLKEDKL
DCSEELGDLAKQFDNTLALSVYLRANVPNKVILCFAETGQFQKIVLYAKKVGFEPDYVSLLRNVM
RVNPEAGANFSGMLIQDDEPLADLNQIVDVFMEFNLIPQCTSFLLDALKNNRPAEGPLQTRLLEM
NLLSAPQVADAILGNQMVTHYDRAHVAQLCEKAGLLQRALEHYSDIYDIKRAIVHTHLLNPEW
LVNYFGTLSVEDSIECLKAMLSHNIRQNLQICVQIASKYHEQLTTSSLIEIFESFKSFEGLFYFLGSIVN
FSQEEEVHFKYIQAACKTGQMKEVERICRESNNYEPERVKNFLKEAKLTDQLPLIIVCDRHDFVH
DLVLYLYKNNLQKYIEIYVQKVNPARLSIVVGGLLDVDCSEDVIKSLILSVKGQFSTDDLVAEVEKR
NRLKLLLPWLESRIHEGSEEPATHNALAKIYIDANNNPERFLRENQFYDSAVVGKYCEKRDPHLA
CVAYERGQCDQDLINVCNENSLFKSLARYLVRRRDAELWGTVLVEDNKYRRQLIDQVVQTALSET
QDPEDVSVTVKAFMTADLPNELIELLEKIVLENSVFSDHRNLQNLLILTAIKADRTRVMEYITRLD
NYDAPDIAGIAIVSELFEEAFAIFKKFEVNTSAIQVLIENIQNLDRSYEFAERCNDPAVWSILGKAQL
DQGLIKEAIDSYIKADDPSAYMEVVEASKNCGNFEDLVKFLQMARKKAKETFIDTELIFAFAHTGR
LADLEEFISSPNHANISQVGERCFEQKMFEAAKILFNNISNFARLASTLVHLGEYQNAVDGARKA
NSTRTWKEVCFACVDGQEFRLAQMCGLNIVVHADELDDLIRYYESRGYFEELISLMEAALGLERA
HMGMFTELSILYSKYKPTKMKEHLELFWSRVNIPKVLRACEQSHLWSELVFLYDKYEEYDNAVVT
MMKHPTVAWKESLFKDVITKVANIELYYKALQFYLDNKPLLINDLLVVLTPRMDHTRGVSFFQRA
CYLSLVKPYLRSVQNHNNKAINEALNSLLIDEEDYNGLRASIDAFDNFDNIALAQLLEKHELIEFR
RIGAYLYKGNNRWKQSVELCKKDHLYKDAMQFACESRDKAVAEDLLSWFLSIDKTDCFAATLYM
CYDLLEPDVVLELAWRNNMLDLAMPFMIQFMKDYYTKVDKLAESNAKRDEDKENAPPEAPIM
GVEQLMITAGPSSYVPQPNMYGMPPQGMAPQGMPPQGMPPQGMPPQGMPPQGMPPQQGFPQ
QGFYGGAM*
>TRINITY_DN39385_c0_g1_i1.p1 type:5prime_partial len:154 gc:universal TRINITY_DN39385_c0_g1_i1:2-463(+)
SNNITIKLSDNVTKINRSDGKEVAKIFLISDTHVYLLNSKTLQLIHQIEISKINKVTLTPYKDGIIVLH
VNQGSDKSLQKGDFILNSSNVIENVTRLAMLYESISQKNLDIDFSSNLCINTKGKNIDLVVQENDD
NNQLVFRKKGNQIMVSVQ*
>TRINITY_DN205_c0_g1_i3.p1 type:5prime_partial len:702 gc:universal TRINITY_DN205_c0_g1_i3:3651-1546(-)
SNMLKLILLSLLCTGTLASFKHHNELQTYTEEDAEDGVLCPGIKGQKGERGAIGHSGPQGNFGPQ
GFKGVPGGQGPPGKPGFEGPEGKRGETGERGERGENGLPGPQGPQGLRGKTGPQGKTGSIGVKG
ETGPMGPKGSRGYQGAKGPAGPKGENGDKGEVGTEGPTGEKGPRGLPGNIGPRGIDGVYGEPG
EKGEIGEQGQPGEKGKTGPRGNPGKNGSEGKKGETGPRGNTGAQGQQGEKGELGPQGFAGKN
GQKGNNGERGPEGKQGLQGLQGPQGEQGEMGLAGKAGKNGLPGPRGNPGLPGKKGIVGPIGK
MGEKGSRGQQGKRGFNGSKGDQGETGFKGDQGAPGKQGLQGPQGDKGPKGDKGVPGFNGV
NGFPGEKGKLGPVGKQGAPGLQGKAGDAGAPGLKGLQGEKGLPGKDGNDGLNGKDGIQGPP
GEKGNQGSNGSPGAAGPKGNQGKQGAPGVQGIKGTDGKNGRPGIKGEQGNQGEKGAQGQN
GKDGKSIPGPQGPQGAQGNDGPQGPRGFPGKNGKDGEKGNQGAKGENGKQGAPGDQGPQG
QTGDKGLTGSRGKQGIDGEKGTAGTDGKPGVQGPKGDKGAQGKQGSSGQDGESGSPGQQGDK
GSRGKDGSSNKLECVFVNGQGKYAQCPRSYQATSCSCSKDCTYRFMNQLCVCECPTYRGDAGAI
CCRMTGLSTKEVTKPDIS*

>TRINITY_DN2532_c1_g1_i1.p1 type:5prime_partial len:719 gc:universal TRINITY_DN2532_c1_g1_i1:3-2159(+)
DCGDNSDESHCDKPVKCDPRTSYKCQKSGKCIKYEKVCDGKIDCEEQDDEGPGCNINRCDNRN
GGCFQICHNTPNGSICKCRKGFYLLNDTYSCEDVNECEQSPEPCGQICINTKGSYRCKCTNGYTLR
PDGKRCRANGDKPVILFSDHKEVRQVDPITGETKTIVENLKGAIGVDFNWQKKMLYWSDVSEDK
IERMYFNGTGRETIINQGMNSPEGLAVDWVTNNIYWTDMRLDTIEAARPDGSMRSIIISDNLDAP
RALALDPSEGYIFWSDWGKNARIERANMDGGDRRVLSAEDVVWPNGLTVDLPTKKVFYIDARL
DKLSSMSYDGSNHKTVVRRGIINHPFAVSIFEEFVYFTDWSPGSIKRLSKLDGSLKLVYKKKLKKPM
DIQILQQMRQPNDESYINYCINHKCSHLCVLKPKGYSCKCPFGRQLVENSTRCESVKEVLLYARRQ
EIRGISLNPNDTVDKITPITGTQNAIGIDYHYKKQMIYWTDVSRDSISRVYLNGSNREDIIPSGLPSP
DGIAIDWIADNMYWTDAEYDKIEVSRLDGLYRKVVISTDLMQPRAISIYPQKGKLIWTDWKRKDI
EISLLDGSDRKVLVGDLGFPNGLSIDMDNDRVYWCDAQLDTISSVNLNGTDQRVLGISKTSIMHP
FGVAVFDGFVYWSDWGKRAISKVPIVGGPVIKLRENYLSLMGLRVYHKNKQKGTNMCANDTLC
QHL*
>TRINITY_DN100850_c0_g1_i1.p1 type:internal len:182 gc:universal TRINITY_DN100850_c0_g1_i1:543-1(-)
QDCFNPIQCEINGEWSGWSPWSECDRTCGDGNQYRNRSCTNPKPANRGKDCVGPSEEAKNCKQ
AECCDISYTNLGCFKDPGVEPRPLPELLFTDRDVLTLDSKRPLLGQDYQGDVTDLVCRCAKAAKN
KRYPIFAIQDFSDCLSGTQAHITYNNYGREDHFIYKPSPKPWIGCIDYDFNRC
>TRINITY_DN3655_c1_g1_i1.p1 type:internal len:191 gc:universal TRINITY_DN3655_c1_g1_i1:2-571(+)
TLNVYKKVGNALQVVWKRIGNNGDQWKYAQVDLTSKYKYQIVIEAIRGVKYQGDISIDDIVLAD
DSCPPPPTCDFEHGKCAYTDDPTANFNWRRNKGETASKGTGPPFDHTYQTNQGYYMYTEASGR
RKKGDKARLVGPVLPGSNPMCLEFWFHMKGWHIGQLNIYIKTPNGGKILIWRRKGNHGMQWKI
>TRINITY_DN909_c0_g1_i1.p1 type:5prime_partial len:313 gc:universal TRINITY_DN909_c0_g1_i1:1-939(+)
RRVLFRSLTFIESLRLSRLVNKKNKTFMEMSEKGGIYNDNEREMNFTGNSDYESGDLGFCGWFLTV
LSFIIAVCTFPLSLFFCIKIVQEYERAVIFRVGRLLSGGARGPGLFFILPCIDTYLKVDKRVISFDVPPQE
ILTRDSVTVSVDAVTYFKIQNPVASVCNVEDAQRSTKLLAQTTLRNELGTKNLSELLLEREAISRNI
SNILDQATDPWGVKVERVEVKDVRLPPMLQRAMAAEAESSREARAKVIAAEGEMNAARALKEA
SDVISESPQALQLRYLQTLTTISAEKNSTIIFPLPINMLQSMMNK*
>TRINITY_DN1774_c0_g1_i1.p1 type:3prime_partial len:208 gc:universal TRINITY_DN1774_c0_g1_i1:218-838(+)
MGISIGFEEIRNKNFWLAVLAEFEGTLLFLVCVTTVALGWGGNGTSANNVEIGIGIGLAIASLAQA
FGHVSGGHLNPAVSLGLALAGRVSIIRAVLYTIAQVLGGVAGSAITYGLTKSSNREAMGVVGLGSN
VTPVQGLILEAIFTFFLVFFVISITDPRKSVEAYGTTLGIGVMILVAHVCIIPFTGCGINPARAFGPSVV
RNIFN
>TRINITY_DN413_c0_g1_i4.p1 type:3prime_partial len:463 gc:universal TRINITY_DN413_c0_g1_i4:1388-3(-)
MVSLSVTCLSLLLFVTVNAEESDHCGYDEKSWYKAMGWCYHSGSRLLLGDFNGDKRTDFFCHD
SNGKNWVAVSNSDGSFGSTTWQDSKERWCASPNILLTGDFNGDGVTDILCHGTATGKKWIKYFN
KYGVPSGKSFYRAMSWCNHKGSQLYIGDFNGDKRDDLLCHDTSGRYWTSYAGTDESKVFHHTS
WYKALEWCRHATSKLYLGDFNGDKKTDVLCTDGKGYKWIMYAKTSGNLFTGTDWHTNLDWC
HESSTVFLRIGDVNGDGKDDLVCHSTAGNSWVAYTQPKGGFGNFCHLAKWCAAGKESFFLGYF
NQDSRADILCHDTKYGKKWIRYAECSFAEESDHCGYDGKSWHKAMRWCYHSGSRLLLGDFNG
DKRTDFLCHDSRGINWVAVSNSDGSFGSTTWHDSKERWCTSPNILLTGDFNGDGVTDMLCHDY
KTGKKWIKYFNKHGVPSGKSFYR
>TRINITY_DN101600_c0_g1_i1.p1 type:3prime_partial len:312 gc:universal TRINITY_DN101600_c0_g1_i1:935-3(-)
MEKSEKKRHFCCCACCTAAVVTAVVVLIGIMIACFGGASIPLFDDIYVSTIKKQMVLVKGSESYKN
WLSPPAPVYTKFYIFNYTNVDDYIAGKCKLNVTQRGPYSYREGRFNKVLYEDAEIITYLENKTLMF
DPETSCEGCTENDNMTVVNAAVITLMKVLSVKDPGFFVKIALGLLDKIFQADKDVALFINLSVHD

LLWGYHNPVLQRLGTIEKDLAKVSPVKLPFPDIDSFVALQYNGTKGADLTGNMTILTGNKNIDDV
QKYVAWRGIKEAPYWNDKYGRMLNGTDGTRFKPFLPSNERIYIFNSELC
>TRINITY_DN143676_c0_g1_i1.p1 type:internal len:124 gc:universal TRINITY_DN143676_c0_g1_i1:3-371(+)
QYLKMTRDEFNRYNVDMKVYFKLTFKLLLERYPFLETFTLQKLFDNFGTYRKERLELFGKLQTNV
LPLFCGIENKTYFFEMTYKELVEKCTFYDVDTEDEIFDESWKYVVSGKNENLEGLKSP
>TRINITY_DN33328_c0_g1_i1.p1 type:3prime_partial len:1422 gc:universal TRINITY_DN33328_c0_g1_i1:4264-2(-)
MLLKYFFLFLCLLLLNDIVYGKRRGGKKPARRPPPARRPPPSRKPVRRPPSRKPSPPSRAPSPPSPPSS
PPNRKPSSNKPANQASNDQGGPGKPASNAQGGGKAQELPDLNAILRAFAGKQTDSGSTQYEYG
GCVLDIGKDKCKIKFIPKKQSCHNKLALGKLFDKFVPKGKIKTLFKKYAPVDVFQVTRFEVDKCQ
GDLDFETEAKEAFNVINDKLKVNSGKLSVSFDYKKFMAAPSFDKLGIDIDGEVQLFGKKVPLTLK
KKRDSGEGIFSIKTASIKASDFAKLFSTKSVANAQSPPQTKMFTDAVIQNPSISGSRDDDGGFELVVK
GQVTGIKGIGAISLFVIIQKSSDSPVSVAIVADIKKIQPTVLLSSLTGKNLNSIPIIKDITANLAVEFANN
DMMMLKDPELNKLLGKYIANGKTIASGTKIKVELPIKEILTKVKTTASLSHVPSTIYMQMFLNGGK
VCFKFPDNIAMDLANVLVALLPKVPDLLFSKVLKTPPKIVIEKFDVNVATKYVDMVLEAPEEIVLG
SDLFKIKNAKFELKHEENKPWDFNIEATKQIGKTMIDINVKSEAKNYIFKATIDSLTTKELATTFGS
KATADGILTKMSFLNFGIRTLTVTAEFGDKFTLNIKGNPVLFNWEGAFFEGFIVGSGLNKKMAFGI
VVKNIRIDDIVFKLFKKRILQTTWMSELTATFLISNTDPDDNIDFTLPELKEHVKLKQGVFFKSELTI
PTDCRAEKICEVLRDKMKPGTKLYLEGEITLNGFKLTAGYKGHGLNFATDLTLTSAKLVLTVGTST
EFYLEGEVQMKNPNVKFIGKLGVGVTGIVELSLIMEGMWRTPFGLSYIAFGNLQLAVAIQPGVPVP
KLEFTAEMWVGKIGNNQEIKTKTAVGINPSNPLDMYFYGEINKLTLAEVAKAFEFKSTLPKVVQET
GFPEGLAAGYTTNPKGKTISHLKVNLKTGFFFRGAINILGYKLECEILVQIPKAIKVSVDMGPIKIAK
GLIKLAKGAKDLDNGPSLYVAISSNEVAVKIQAYAELLGLGAEVNIDVSDKGMKFFVYGNIFNLIE
ANVTVNAPYGNGDLAKSEFSVSSCISAGISDIQNTAFEEVDEGAKESQKIQKVADDEVKDASSWY
NSAMDKLLSYEKKLEEKTAIINRKSGKLEKLKEYLDASCVDHCPDVCQGGPVMRKRKGRFFNKK
PGWDDCKEMVKSIVCLGKCKGKKAYYQGKAAKKVVTMEIIEGSVKIAKKLIVIAKGFAERSKGL
MDLTQKAANKVVSLTKAGKDALGALAKYTKENLINIHLLCFDTTLAKAEGACFGVNVDLTVLG
NKKLQYEGEVCFNADFFKNMAKQVADKVFPGIISIKDKIMQAKNLFSDSDSKKKELEEYEKQSAQ
EEKNLEGQLERQNEDDDDDDDEEFNGKYKASFDP
>TRINITY_DN5554_c0_g1_i1.p1 type:complete len:316 gc:universal TRINITY_DN5554_c0_g1_i1:223-1170(+)
MRIIIALLFCVVGYCYSADWGYEAGNPNVLEPKDWKQHFPKCGGRKQSPINIATRSAILSRLPKIIL
GEGWGTLLESADLSLANNGAKVSIKSSTPITTKQGGTIWNLAQFHLHWGKDSHSGSEHTINGMS
FPAEIHFVHFNSKYPTIGDAVPQPDGLMVLGFFIKVQENAVVPSILQKFFGSLDQIKFEDQKVKLDF
GGRALGDMLPPRGTSKYFNYPGSLTTPTCDESVNWNVMSTENAIVITEKQMTQLRSLSAGKKNE
AFKPQSGNFRPIQPRNGRTIRRNFRTATTKFRSVLRRYFPRIFSYFRVTPRFE*
>TRINITY_DN2433_c0_g1_i2.p1 type:5prime_partial len:147 gc:universal TRINITY_DN2433_c0_g1_i2:1-441(+)
LTFIESLRLRKVNMPISIIALNHRKQPLPDTVLKKFPLVKFYDEPVDQNIFGKLKSKWRSTMVFDIC
GRQQYKFGYPYSLLSYPFTRSGVMNTLNHHVSLCGKCNATLTTTPKETRPPTQVPRETRPKTTETP
MELFLANNRLTRV*
>TRINITY_DN69484_c0_g1_i1.p1 type:internal len:142 gc:universal TRINITY_DN69484_c0_g1_i1:1-423(+)
SDNHAGKSIMKHFITSNVDSFWNSYDDSNISVTYDQVYHTNSTNHSTKLVTTSTCGHHLKLLILIT
THVSHVRQRELIRNTWGTDPKSDEKRWKTYFLIGRTKKIKDLNKIKLERDHFNDLVIGDILENFH
NLTYKVQMGF
>TRINITY_DN4513_c0_g1_i3.p1 type:5prime_partial len:116 gc:universal TRINITY_DN4513_c0_g1_i3:652-305(-)
KLQNDIEKLKTDLEAQKKEFEKLESDTKVSLEDKAKLSTENEKLQNDIGKLKTDLEAQKKDFAKL
EGDTKVSLEDKAKLSTENDKLKNDIEKLKTDLEAQKKDFEKLDGDTNIRK*

>TRINITY_DN31399_c0_g1_i1.p1 type:5prime_partial len:382 gc:universal TRINITY_DN31399_c0_g1_i1:2-1147(+)
YILFEQANILIDVFVSFQEPGTFSFNKPSNIVKESCGQAELEVERVNGADGKVEVVWKTKDQSALH
GKDFIGGEGTLVFEHGETLKSISIEIIDDHMFEKDETFMVELLEVKNEGAKFGRLKRTVVTIVSDDE
YQHMFDRVVNMAHMNLHQFQLGSSTWGQQFREAMNVNGGDLESASFMDYVMHFITFFWKIL
FALVPPCTWYGGWLSFFIILGMIGCLTAVVGDIATIFGCLVGLRKDVTAITFVALGTSLPDLFASRNA
AMNEKYADASIGNVTGSNGVNVFLGLGIPWLIASIYHEAKGKVFKTPTGSLATSVVTYSVCAIACI
LLLIIRRFVPFFGGGELGGPKGPMYASGIFMISLWFIYVLISALVSYGHIAAL*
>TRINITY_DN177106_c0_g1_i1.p1 type:internal len:139 gc:universal TRINITY_DN177106_c0_g1_i1:1-414(+)
NITVRARMQQQIIALNQLTNLAGEMQKYVEMATSNANAVLKKKYDLKQKIQLSINAAKEASELA
KTTRLLAFNLTFTLAPIFNRGKGVEEYASTCVDFIDVWLQNASKALIYSQNVLSGIALSIPDYAQVT
YLLIFVY
>TRINITY_DN2576_c3_g1_i1.p2 type:complete len:145 gc:universal TRINITY_DN2576_c3_g1_i1:807-373(-)
MMHKSSFLVFLVLGTFGTAIAIKCFDGEFIGGKIPSWVPSLPKPKSKECDSKFNACFTITMNKTFNV
MKFSKTFEMKKGLCGNDMAGCNVTCAKYTKAKSCEVSCCKKDDCNQQYFPTSDSIIKTSSIFVTV
AGIFYVFLNLLN*
>TRINITY_DN143583_c0_g1_i1.p1 type:internal len:103 gc:universal TRINITY_DN143583_c0_g1_i1:308-3(-)
SYPAQPNDTAYLIGSNIPNNNGFCISFWYHMYGPHVGSLNVYVVNSTAADLEFARIGTQGDKWK
HGQLYVAPSAGNPIAIRFEGVRGSSYLGDIALDDISVK
>TRINITY_DN3991_c0_g1_i1.p1 type:3prime_partial len:219 gc:universal TRINITY_DN3991_c0_g1_i1:84-737(+)
MTSWILNFLVLSIIALPCSSISNCKHKLEPVGCFKDRGSARAMPEQILNERDTTSKVYSKQLMSWK
NFHEWLPGFICRCAEVAARKGYSVIGIQFWGECWSGPNAESTYNRHGQVDSCINQNLKKNRAK
LSQCTAASGKHFTNYVYRLAPACHTQYKPVGCFQDNLQKPRPMPYYVSTDRETRLAIFSGKLLN
WKNWNTHMPDMICRCAAVAKSKGHQ
>TRINITY_DN72753_c0_g1_i1.p1 type:complete len:137 gc:universal TRINITY_DN72753_c0_g1_i1:95-505(+)
MARTKQTARKSTGGKAPRKQLATKAARKSAPSTGGVKKPHRYRPGTVALREIRRYQKSTELLIRK
LPFQRLVREIAQDFKTDLRFQSAAIGALQEAAEAYLVGLFEDTNLCAIHAKRVTIMPKDIQLARRIR
GERA*
>TRINITY_DN49617_c0_g1_i2.p1 type:internal len:100 gc:universal TRINITY_DN49617_c0_g1_i2:299-3(-)
CNRQCCGPARSFDLKILDNNNQEVIHLNRPLRCATCCFPCCLQELEVTSPPGTPIGYVIQKWSLCA
PKFEIQDAQRNPVLLIEGPICTCRCGDVEFKVL
>TRINITY_DN20671_c0_g1_i1.p1 type:5prime_partial len:1266 gc:universal TRINITY_DN20671_c0_g1_i1:4791-994(-)
KAYLKSRILLSSIGEVFSITMFEDYIYYTTWNPPYRIHRAHRWTGKETSVIKTSKDKFFGIQVVHHLR
QPTHKNPCENSGCKSLCMISPGGTSYKCGCADNVHLGSDSKTCFSNCTTGQFKCNNHICISKAW
YCDGEDDCGDNSDELNCKERKCKPDLFQCDNGDCTRRFYVCDGENDCRDNSDETDCHSRQCFS
FQYRCKNHNCILHNLKCDQKDDCGDNSDEEDCKKSTECSSSSFRCEDGECITKYWKCDGEYDCA
DRSDEKEELCKNTVCKPGFFKCHNSSKCISEHHVCDGDRDCSDNSDESIETCSNKTCTQSQFLCD
KKLCILNGWKCDGDQDCSDGTDEKDCKVPTACHSSEYQCKNGECINEQWRCDGDNDCGDNSD
EGHLANCTKSMCSRNNGFQCEKSAICIRRKWRCDGETDCRDGSDERDCSYGKRDCNPNEFTCK
DQSGCIRSDWVCDNTTDCLDESDEGYEFCSTHECNPEKQFRCKDDYTCIANYRVCDGQSDCND
GSDEKDCRPSCQWTDFKCGDGTCIDGAKRCNFYQDCKNNKDEEGCGRAENPITCNGTRGHRLC
NQLRCSPIQNNTYIKCHCDEGYKVVDSECVDVNECIEESSTCDHICVNLQGSYECKCAKGYKEIY
NFCKAKGDPSILMLPTADKIMILTHHSSPRPTASYFDPTDTSWPHMGSFDFFDFGYGSIMLVIADSK
RQHSLTGYLIKENFFRVGDPARRKRRTSEPIYEQSFKKTGMSPDHVSIDYINNNTYFADQNPNNRG
IFMMNLLNTLWIKKVIQLFRKVTGLTVNPDKGTLYWCEDAFEPIIYQANMDGTQVRSFVDNKLG

YPVSLVMDIPSQRLYWADYKKNSIESMSVDDTDEKTRMTVINQRSFPTLNRPISIDVFEGYVFGVM
NVTGTLFKVDKFGRVRRHTPKGSEKTKPKYEDYVLKADIQKITKLRIYQQNRQQAMASASPCSTA
YCPQLCVSLYEKPSKSLQAKCLCQSSGSNAVQSDCKKRNCDRSFCNGNGECREVNDRYTCSCDK
GFTPETNCKTGINDQCMCENGGECHKGMQCICPKGYHGKLCQHSCDEFMVRSNCVNGICHYD
DTNDKLMCRCKEGYSFSIEANLCEPVCNLTCPAKYTCVINRNGMPSCNCTTDDCETKTNPVGSIS
KTMTVVVPIIIVIIIVCIVIAVIILKRLRMRNKFAPYKMEIETENPAFEYRYFRNNVHDDSMPESDREF
FETKNFANPYAFEDQYEQSAFDDNYAEDAALVRDDDD*
>TRINITY_DN101992_c0_g1_i1.p1 type:internal len:139 gc:universal TRINITY_DN101992_c0_g1_i1:414-1(-)
AERYDHHSGIVESMDSNKISDNLKELTRLPHPPGRSRNKELGDMLADFWQNEGMDKIEKFKYNI
YLSYPEKPSTILLFGGAKTEKYTIEAEPPVDESEKGGTPVVPFNAFSANDIVKAELVFVNHCRESDF
DEVKKAG
>TRINITY_DN191650_c0_g1_i1.p1 type:complete len:239 gc:universal TRINITY_DN191650_c0_g1_i1:957-241(-)
MRGKGATCIRYLLFSFNVAFWLAGACAVAIGIWMLFDPGRFDDYLGKYSFVIPASILIGAGLFVFFV
GFCGCAGAFQEKKCLLGTYFTMLVLIFCVEIAAGVLAFLFRHKIKDEITKESQEVVKLKFGTGDPKI
DHAVNLVQQELKCCGAKSGDDYKTSRWKDNQLATKTVPESCCSDPSKCTKVTQTQYVYKQGCI
DELVEYTKENLMILGGIGIGLACIQLFGMCFACCLFFSIED*
>TRINITY_DN21554_c2_g1_i1.p1 type:5prime_partial len:348 gc:universal TRINITY_DN21554_c2_g1_i1:2-1045(+)
WYKANYGASGYYIVNYEDENWIQLLNIFNTDHLILSAVDRANILSDLTFIENSGRRRINLTLEFTKY
LKKERHYIPWEMGIKQVELIESVFRETEIGTCLKGYILDLTSHVLGELSLYKNHSFLEQQLINVLVRK
AVHYGKNDIIIQLQNLFLKWLIAEGGSELPSNLRSVILRVGVENGNSNIWEKLLSILKTTMSQNDR
GLIIDALASTKNSTILQRMTNKITGISLLGESGYIAGLAKVCSNKYGRDNAWRDIKNNWNWISER
NSADEIKYLVESCFSLGPNIDAAYLEINLEKLDDLLDNYEIGPFKRSLYQVKEKMMRTIEFRKRKDEI
AEWMEINMPGCFL*
>TRINITY_DN72625_c0_g1_i1.p1 type:complete len:1964 gc:universal TRINITY_DN72625_c0_g1_i1:173-6064(+)
MKLAFNIFTISLLIGNTIAQNNDVVQVCKSQADVVAVLDSSGSVGVQDYEELKKFVGFVANLLTIN
ENGNRLGVVTYSETAEVDFNLNEIYDSDQYGREALTRLSVRPREFKTNHYAGLKVGSDLLFNKAA
GARDNAAKFLIFTTDGRNNFDETNNRNRASQLATQLKADGVNIYVIKAEVMSDNNGRLDSRLYS
TDPNKFYRFINRFGEITNENFLDEVGLEFCELVQLPDNQQATVREDRNIGTLVTTIRAPGLNNPTFE
LIPQDNLPFIVDPLTGEIKTIGLLDFEQVQQYVFQVRVRATNPDVEKIITVTINVLDVNDNDPIPETA
NYRMSVAETTPLGQRILTVKANDPDGGTNGNVEYFLISGGDGKFEVDQATGEIRLIGQLNKRIKD
TYLLTLEARDMGIPTRKAPITVAIDVTSLNNFPPQFNEMSFVRTIPENFGAGTVINAAATDRDGDT
MVYSLIGENIPFSIDQNGLITVSGNIDFETIPNNVFQFQVQAEANGDLVTQDVIITITDINDNVPVYS
MTTYSGSINENSAAGTVVNFDNVIIATDADKTNANNMITYNIIDGNDGDRFQVTKNERGEAVISL
KSTPLDQETQGQYRLTLQAIDNGVDPRSLEMNSLLIIDVIDQNDNDPIPEESVYSIAVAETSPVGQSI
LTVRANDADSGMNGKVEYFLISGGDGKFQVDQATGQITLIGMLNKRVKESYTLNIEARDMGVPT
RNAPITVNIDVTSLNSFLPEFAGAGDAFERTIPENFGSDTVIKAAATDGDGDMVFYSLVGDNLPFA
IDQNGMITISGSIDFETIPNNPIRFQVKAEANGDMTLKDVVITITDVNDVTPEFTQQVYAGKIEENS
QTETVVRFDQPVIATDGDKSPANNVVTYRIVDGNVDNIFKIVKNAQGEAVISVNNAGLDREILNQ
YTLTIEAKDNGVDPGSLSSTTQVIIDIEDVNDIPPLINTSENKAVGEADFTATISEDSPIGTSVVKVEA
TDPDSNNDDLIFTIANDVPVPFTIDPKTGLITVSGPLDADDQDEFEIAVTVSDGVNNSTAIVKVNIA
DLNDNKPIFTTDTRDITIPLDTPNGQEIIKFTGSDADKTSPNNQFEFSLVDGDGKPFTITPDGSLIIN
GPLMPGDYMLTVKIMDKGTNPGPLSNVIIYNVKVVDTNDPPTFQNNEQTVTISENTADGTDVITF
AATDPNGDAITYEIVNPGSVPFEIDPITGVIRKMGLIDFESPVRSYTLDVKATDAKGKSTMGRVFVQ
ITDFNDNTPTFGPGNTDNFILTEGVYSNQLVSSFTATDLDATSPNNVFEYTLVGAPSMFLLTTNPNT
GAADLTVNGDIDAEQNMSFNFIIRATDKGNPALSMDYPVTITVGDINDNAPVFAANTPESIDLME
GDFNNQIVATFTANDDDTTAPNNLVDFELVGAPDFFKLIVNPETGTADLVIDGNVDIDGLDPTINF
IVRAKDGGNPALVKEFPLTVNIADINDNAPVFTLEAYNQVINENTPIGTKVVEVRATDSDSGPNG
DITYRIVDGDGTFGIDPKTGDITVLKNTDFESGVRSYEFTVEASDNGNPKQSATAKVKIDIRNLND

NDPIFTSIPITGNINENRPAGSVVQNIQPVNGILNQNNVKILREPPPGAQETEGVGFDAPFEIDPVT
GEVKSTRPFDFEEEKVFTMMVQAADGDNNPMLTTMTVMVNNLNDNDPIMKKANFEITLNQDT
PVGTFVLQVQATDIDQENQTKITYSIINIDGGNMNDFTINPDTGVITLNTDLSMNTVDIYNITIQAE
DQEIQGVIRRSTALAEIKILRPAEAEPDISTLCTNSCDIPEERLKCLDYSKFEITRKFNGTCYVDKLKA
AIADVKNNTMEIMNLNASFLECRNKVQNEITNPNTQFPVNGLFDLHRKCTVAIECKLEQLAFNL
MRKTTELTNNLKAEEEGFSMYDMSLSAFFQHISKMVSMSKSSRVD*
>TRINITY_DN1628_c0_g1_i2.p1 type:complete len:556 gc:universal TRINITY_DN1628_c0_g1_i2:81-1748(+)
MADINVAGLISIIIFYLAILLIGIFAAYVKNRKQAEAQTNQEKTNEVILAGRNIGLFIGCFTMTATWV
GGGYINGTAESVYTKGLVWAQAPWGYATSLAMGGIFFAKKMREAEYITMLDPLQHRYGKYMGA
VLYIPAFLGESFWSASILSALGATLSVILKLEMNLSVIVSACIAVGYTFFGGLYSVAYTDVVQLFCIGIG
LWLTVPFALTNNFVENISANNHLWLGTIKSNEAGKWIDYAFLLIGGGIPWQVYFQRVLSSKSPGR
AQVLSFVGSFGCFLMAIPAVLIGAAAKTADWDKVNITDADLVVNGTLTDARLVLPVVLQYLSPA
WVSFIGLGAISAAVMSSADSSVLSASSMFGYNIYKLLIRPKASPKEIVWVIRAGIVCVGIFATSIGLTV
KSIYILFHLCSDLVFVILFPQLVGAVHISFVNTYGSVAAYIVGLFFRLTGGEELIGFKPLIKYPFYNNN
HQNFPFRTLAMLLSFATLLGVSWLSNYLFDNGYVSEEKDYLGVLERRDKNKRRNAEDVLMTEVY
KKREEDDVHYTKNGKGMPNHEDM*
>TRINITY_DN21554_c1_g1_i1.p1 type:3prime_partial len:650 gc:universal TRINITY_DN21554_c1_g1_i1:1947-1(-)
MFPNNALKQQLFYHHYMFMTKKDDDNNIESETNDEEDNQEGEEDSPVNQLFPKKKSNVNSTS
NHYFTIPEFLSFGTMLLFIAGVALLASYQSFTKKKVDDIHFNVRKLMDIPENVIYDEVKLPKNIKPL
NYVIYLYPNLEFRNMTGVVKVTFQCIENSTDIVIHSSKHSVNRIELKNMSGGNIEITNIVRNEKRGY
LILSLKTALVSSLKYDLFLGFYKTLEYNPSGGVYLSQYLDVEMKTNLILTSMFEPTFARYAFPCFDEP
NLKAVFKIIVRHNSSDGYKAIANMPVEKRITVTETDVETHFEQTPLMSTYLIAITVCNYGYKETYSK
YGVPIRVYAPKDRLKEVDFAIKVAPLALDFYVDKLGMNYTLPKLDLISVTDLISSGEENWGLINLQ
EMYVLYNENITSSDWVPNIATSITHEISHLWFGDLVTPTWWRDMWLKESFAEYMEYLPINQYFPQ
FDLKTSFYKTNNVRAMTTDLGNYSHPLFTDETDPVILETYYDDITYLKGVAILSMLSDFLGDEDFF
KGINYYLKEHEYGNANVTQFWQAISAAQNNTNIAEMMNQWTVKTSFPLITLKIEKGKLYTKQER
FEFSDKVNNESSFWHVPLLVGFQDNDIIFRFHLKNLSDSYSLSSLQNKPSWYKANYG
>TRINITY_DN86076_c0_g1_i1.p1 type:internal len:210 gc:universal TRINITY_DN86076_c0_g1_i1:628-2(-)
IAQNAIHDSDWQVGHTKIFLKDAQDQELEIKRDRVVTAGVVLIQKTFRGHRYRKQYVKLRESTIV
LQKHWRAFTAKRRYKRMRVGFERLQAMVRSRQLHSSYKEMRVRIMAFQSRCRGYLARREFKIKL
GAIITIQAAFRMVLGKKKVMKIRQERMIQAEAERVRREEEARLRKKLGAAEAQRRANKAAEERA
NVLHMQMQQNALKEKQ
>TRINITY_DN16533_c0_g2_i1.p1 type:3prime_partial len:396 gc:universal TRINITY_DN16533_c0_g2_i1:1185-1(-)
MEEEKIEDTVEARLKKIRQRNIEELQNEYMKRCLHVDWKLGPLRIYLNPLTTIVSALVIWGLVIYCII
EKEEANSKFAILNEWITLRWTWLYVGTQDVWAVFVFWLYFSKYGSIKLGRDDEKPEFSDGTYFTM
LFSAGIGIGLFYFGVAEPIQHYSSPKGYKSNRYFNRYNMNVQAQDAINVTLFHWGVHGWIVYYVVI
GLLLGILCYRRGLPMTMRTCFYPIIGDKIFGIMGDIIDIFCIICTMFGVCTSLGLGVIQLNSGLNRLNS
DIAENNDVRVVIIWCVTAVATISVISGVKVGIRRLSEICFGLGCLILFICTFADDTWYLLNLFCQSTGY
YIQWIIQLGFHTDAFAQLGIAPDGKENPTWMRNWTIFYWGWWISWAPFVGMFIAKISPG
>TRINITY_DN33467_c0_g1_i1.p1 type:5prime_partial len:243 gc:universal TRINITY_DN33467_c0_g1_i1:788-60(-)
LGAGSDYYAFYKFVGIPSIDISYRQKDLDNKFNTSYYPMYHSLHDTIHWVENFVDKEYKIHLTAGK
VAIQYLLQLADLPLLPIRTKKYGEGVHRVVKKIVNKFNGTKVKGNESMHFLFTSGLLSMKMNEL
DDYLSSVKKDKPSEMKLRVLNDKLMQLEKGFTYPNGLPGKPQHRHYIMISSSSAVDSEYPFPGVC
DLLPKINETDTYKEIRKQISITASLLDGVKIHLQRYLKSLPYTPDVN*
>TRINITY_DN15542_c0_g1_i1.p1 type:internal len:195 gc:universal TRINITY_DN15542_c0_g1_i1:584-3(-)

GGISFYGSTRLLFLTKPQERFFSVMHCNFLSNQATVGFAIGLATKNTNKGILGPAMSYQVVIANSLF
KQNYMVLTEDKKVIGHGTIYGEEVTLNLRENNTFIENRNTTIVLDSSSLLFHGNSNSIFYRNTGVE
GGALGLYGTSWIALGKHSVISFKENHATVKGGAIYIRSNGPSRISFNSTLLTTGRCFIRYD
>TRINITY_DN107813_c0_g1_i1.p1 type:internal len:176 gc:universal TRINITY_DN107813_c0_g1_i1:525-1(-)
SRIDEILESEVPNVFLLTVVFFALNFLAATQDVAVDGWALTMLQKRNVAYASTCNSVGQTAGFFIG
NVVLLAFSSPEFSNTYIRSVPKEVGIITLSGFLLFWGIVFLITTTLVWIFKHEKEDTDVEDHGVVGTY
LMLVKILKKPAVQDLIIILMTVKIGFAAADAVTGLKLIEAG
>TRINITY_DN179_c0_g1_i2.p1 type:3prime_partial len:619 gc:universal TRINITY_DN179_c0_g1_i2:84-1937(+)
MLSNSKLAVFLLVLGIGIITLTKAQGQCPPPLDRDCNDERNPDDETDGCLIDNDCSSGRRCCSDGC
NLKCTEVQQPPDPEPGDPGEPGRRGETGRSGRPGFPGPEGPLGNPGPPGGRGKKGRRGRPGPPG
PVAGGEGAEVGPITDGSTKGPGPNAGALGEEGMGADGFVTPKEGPGGPAGPKGRLGPRGPYGE
RGEPGLPGPEGDMGLVGAAGPNGSPGENGRDGVNGERGDPGTVGGVGPRGSQGDQGPSGANG
AAGYKGLKGDQGQFGEKGGVGIDGERGEPGENGFDGSEGPTGQQGLQGPIGPRGPNGPRGLAG
ALGSRGLPGPPGLAGLLGLPGAPGSRGAKGENGALGARGEPGEQGERGISGDSGSKGEQGSVGP
RGVDGPAGPKGTMGLNGPQGPRGQSGPRGLRGLLGPDGEVGATGERGVPGDQGVVGPQGNKG
TNGDTGPPGKPGPAGAQGKRGRAGRDGPAGVSGPDGDRGQLGIPGASGVPGTPGPKGSRGDRG
TTGTEGIKGSAGAPGTAGETGFQGKRGGPGPAGKSGRSGKQGLPGAPGKDGRPGGPGQQGARG
VNGQVGLQGNKGAAGPSGGNGELGFPGTKGQQGEDGPVGSVGQPGAGG
>TRINITY_DN8008_c0_g1_i2.p1 type:5prime_partial len:1298 gc:universal TRINITY_DN8008_c0_g1_i2:4205-312(-)
IMSPKERTCPLTQYLFTPIYTGSCKISDAEQRANDRQVKSAVQMAGSAIGTVVGTIFGGGIGGAIGG
FIGGAIGSLIGGIIGLFSSCDKSYEVVTRSWVEERKCILLEPDVRSITRPYSECFDAKIKANTGFKVPY
TCNCKVNDCIVKSPSPDCLLQNQQCERKRKDFLERTKTVPPTFSKLYKSLKQCQDSVGASILKVQS
AKKTVQFYEKEHTRIKNMLLALEEENKFSKQALSDATSILQDDLCVSNAFMNNKNINELIDIKNV
QFDTLLPLVNNIRINAHVKVKGNDVLVPFVHELSNDNKVSLNAFGRKIIMNTICKKIRKRRSLDL
NEDEKNDLAFVPWINNVNGTATVADLSCVSLDKTLEFIRHTVKTLNKKSSYANDLLMQLQYSTN
KLNPSLYTAFTAYSGITTEKNDIIKANEALLMSANNELNKIQDQVSVSNVLDIWGNEAEIVTGFNN
VSVCLNYKDCIISAIDTLKNLPTMFRNSREAYMKEVMELEKELTKIYEEKNLIGLSAKAQSVERIVS
RIKYMSFHCSKRPIVTLKEVKDMDVEKGKNITLRCIVRSARAIKYYWQFNDDILDEENSHSLTIPVS
NETSGIYRCIAKNLVGNGTSNQIRITTYTKPSFIEEPKDQIYILPSHPQSNLVLTCNVTNNYDTTIAW
YYRSFTIGKRPNIINGQTSILLDLDGTKQENSGYYFCQAFNKHGRIKSREARVNILNSNIARLEVNIS
LDILQTDEISSNSFKKISGDMLSEIKDLGRTLTTTVIKSKTKTRVTFNLKVKPITDNVKSLSESFNISSR
MRQNLGSAAAVLLGKLSENSFKLPINDGEFAKVDNDTMTYGMKLNICQEGYKLHKNGFLCVQC
PFGTAGKDCKKCPLNTYADAPGLGICKKCETSKITEQIGSTSKSQCIGTTERKVDIIFAVDVSKRFLE
QINGLQKIRELLSKFIKTYSGTKYNTRFALLTYYKYVDVYLSLTEYYHSDDILGLLEILGEGSRMPDT
KLALVEIETMFDEESSSDRKKVAVFITNGGTSDSYYLMELFKYKRQAITLQNKGVHIISVGLGTVDH
RGLRALASYPKLENAIFPSSNSVAVQKMIYSIGNELKDNCYTPAVDFGIIYHPNHKESLVQITKEQF
LRNANWLSKLLLPIYRMFKQQSYYFMYKHSVPSNSFGSFSETGFQERIVSYLQQQTSGFDIYKKIST
AINVDMTEIYRSFSKVKVLIIITEEPLDEAVLKQYEISVHGEKIDIYNFVKDASSIEKFEGPFSDKPRA
HRLVYGIDEVNFLVNAINCNRTKICG*
>TRINITY_DN252_c0_g1_i1.p1 type:complete len:182 gc:universal TRINITY_DN252_c0_g1_i1:1409-864(-)
MASTLVILLICFVGGTFAQGCPRNKTCRCDNVIKYTTGNQPNKDLSSFPGLSLSDGPTARSYVQTY
TKMYIDDKIKEAKCDNNKLRAVDMDLNPIVMSLKTMNTNYVDKKTEMQDIIDCNPNLDIESDL
WDVYDVLADHCVCISQIHKDLKMKMEMVEAEMKRCDERGWYRSMLESFSIIS*
>TRINITY_DN95415_c0_g1_i1.p1 type:internal len:183 gc:universal TRINITY_DN95415_c0_g1_i1:546-1(-)
SCVQISAKMIEAMDPKVDPCKDFYQYACGGWLKDTILPDSSSRYSRFQQLSDKNNLALKQILADL
ISKKRNTTTKSLEKAVIYYKSCTNTKDIEKIGDKPHKELLKKIGSWAVIDKDFNESDWSPIDILATIH
RNISRKPVFNMVVDADIKNSSQNIVTFDQSGIIMDRESYLRNTSFHKRH

>TRINITY_DN794_c0_g1_i2.p1 type:complete len:250 gc:universal TRINITY_DN794_c0_g1_i2:72-821(+)
MGLTGCGAVTKWILVIFNSIFFILGIGVLGVGIWLKLEREDYEDISSYDFVTPSNIAIAIGVIVMVVSF
VGCVGAATENSCFLLLHFLLLFLIFVLQIIACVLAYVNRGKVEDGLESDFRKSIKEKYGEPEYEAVT
KVIDKFQNEFKCCGFSSYKDWRMSKYNKRTASIPKSCCIESQKESKLCGSPNPSGLLVYEKKGCYN
EVLDFLKENLLVVGAFALAMAIVQVFGLIFAIYLYILVVRANKGTFA*
>TRINITY_DN14729_c0_g1_i1.p1 type:complete len:698 gc:universal TRINITY_DN14729_c0_g1_i1:45-2138(+)
MKDVYIKRFAALFTVLLIFQECNGLLKNHLVQNVNEAKDVYHDGKSAAVAVSRQKRAIFGGILIG
LSIASAGIDIYKQVRGCKEVTPELEPKIEQAKRMQKNLISYKDSVNLAWVPAESKEVYAQRLASDM
VVLRESITFFKATLVDILKLLDQNILDEMNRLKTDLENDIENNKDVGVENFEQLEQKFFTKTQFAL
SLTFKLLPVAFLGGKFIYSKIVKNFARETASDWAQRNPGKVKKMLGNAAERAGKMKSWTKAKIN
SFKNGLKAKWGSFKAKYPRFTNGLNKLTSSRFGQFLSRNMGKIKAGCKALAKGLVVALFVGIEIW
NLVKFINGCKENLKKVNNAIESLSNGKKELDVIKSKIDRLQRDADKYWGLMKGNITSDGYLNVV
AEVAKLGENAKDQTTEIVKASKEIRRYVNEIKNAGVEKTDDLNKMVISAFRKINYKLDCLYSKTAI
VADLIDNCAIGWGKNEELYKRAKDKQKKITDDCIKGKYMDKNEVLRMMRGAMELKKKQTICIR
NNPAKKTIVCEKWYDDVSNSDILKVVSDLTLDDVKYMISVCPPAEVSQKLKDEICMKKNQDLITV
DEIRKQLHKYNQYKVAAAYFTCQLSSSSVNDICNSKKSGYDFNHVYKKYNVFSDKIVKTAFDGCK
KETNITPFDLQMICTLKNIAGLTIDSKGFDNYRAKYDLSALKRAYDQC*
>TRINITY_DN51907_c0_g1_i1.p1 type:3prime_partial len:421 gc:universal TRINITY_DN51907_c0_g1_i1:1262-3(-)
MITRGVTSITLELQDLQRNFNGSYKCIAKNLLGKREKILTLIVFDNPDPPINLKTESSTSTSILVTWES
PIFIGNSPITNYTVYYRLSNESIVINRTTTALYFNLTELVPAQEYIIKVMTSNRYFDSDFSGVIIRNTGE
AVPGEPTNFSAKHNNQSQQLYLNWDTPNPTNGMVSSYNYCYQMTGEKAWICKDTTDNSTTMI
NIKNLEKYTLYNVKVQARTSAGFGNFAVLNDIRTREEVPDNVCGIRKYIESRKIIVRWKPPLNKFG
ELRNYKVDFKNDDDSSDYFNICDTLETSCTIAKYILPFQKYVVRIETRNYLFSNSTTIAVTTQAEPPP
APSESLPAPSDDYVKEKHFTIKLVEFNESNGPIQKFDVIVVKRLENSSPVDSPSSYTNNDIASGVNG
LYIALRKNGAPKQNQEVQ
>TRINITY_DN1850_c0_g1_i3.p1 type:5prime_partial len:164 gc:universal TRINITY_DN1850_c0_g1_i3:659-168(-)
LSNVLDWLEERLELQSISDDILSKFVPAHVNIFYCFGGIVLITFILQVVLGFCLTLFYRPSVSEAFDSV
KVLILKVNFGWLIRSLHRWSSSLMVLAILLHISRVFLTGGFSKPRELIWVSGVLLSVASVSFGVTGYS
LPWDQLGFWASKIVTAVPESIDELFP*
>TRINITY_DN32804_c0_g1_i1.p1 type:5prime_partial len:746 gc:universal TRINITY_DN32804_c0_g1_i1:3-2240(+)
DNVTSNNNIEPSYKLIENLLHQKIERIFSSNDYEINNKQFRTPVGKFFKYDLVNQPQDIYKVVETGK
NELPSWLHFDYKKKRLLGVPTKQDISNGLKIEFHVKNNEALNGDRQPNHYITIDVYQPFRDPLKC
MNSEAASIASIIFNSDISEMDGLGRVELIKNIARAFGKRKKELFFEAGNRMDSTDSLYSANVQVAG
PGDKVVEDSSGFTVSFIIRCSSDITGSPEVASLKGLSQQGDFRSLSKESVLGWFVSTSKTNRVKRAVG
VGGTPGVKPTAVQGTPVLVPSSSISAKSSVMVKPTPSKSVEIVMSSTAVATTETPTTETPATETSSTEA
PATKPVTTTEEISTEITSTESSTTETTTPELSTTDSTTEPPTTMKATTTEDRSTPAVPSGNKPPVKSGEIG
IIYLFENQFFLERLELDLFSDPNGDELTVELRHIQNFEGGERETKVDKGNWIEYKNKMLYFSPSAK
HVGSHRFSLVATDPYYESVKALFEVRVQKDQSYKYVFNITLDMDYAALSNDVEAKFELLNTLANA
LDLESYNELRAVRILPGSVIVQFSPEELKDSDCNDPKIEAFKNKLKSSEFKEKFAPSYRVLDVGATER
QECVSQPIEDDSEGVLQHILIPIIIVVIVLIIILIVLCCVCRRKRKYNKAPINEDDKLYLNHKKPIIFQEE
YDEKESFFSLTPTTLPHEKPPVTNNHNEYSEGPGSSTTVSTESDEKALLAQGSPKESRQPFNSPPPY
GA*
>TRINITY_DN7849_c0_g1_i1.p1 type:complete len:251 gc:universal TRINITY_DN7849_c0_g1_i1:1417-665(-)
MGKRGCVTLSCVKTWFLVFAAILFILAAGLFGIGIWTLIAKNQYESLLGSVTYVTTVGLMLAGGLF
AMFVCIFGIIGAIKESRFMVLGFFLMIIIVCLMELIGGVLAYVYRGEVETEVKRSLNVTIATNYGLPG

NERKTIAIDNLQINYKCCGDVDYKSWERSDWYKQNITGNPVPDSCCITPSTLCGKRVHPSNIWRN
DLKPIGCFESLEGYLINHLFIIGVACLSSGLAQVICICLSMWLYRLIVEFR*
>TRINITY_DN99404_c0_g1_i1.p1 type:internal len:112 gc:universal TRINITY_DN99404_c0_g1_i1:2-334(+)
SNLEAITKSLEKQHKFYQSKLFKLQTDIHGVALEITKLKTSLHRTQISSSERRSSLLYINLRILKNKTDI
AIKGGLDVKEEAMTMLNTVQNFMSVATNAQNAANQALKKYY
>TRINITY_DN129928_c0_g1_i1.p1 type:internal len:154 gc:universal TRINITY_DN129928_c0_g1_i1:460-2(-)
YLGLFEGPAIIEPTTSTTTASPSTSYVSSPTASLNISRTSTVSSTNASVTPTPSSSSSTPTTSSVATTLAPQ
NYPREITFTFPNANCTVLFTPSTRASFLIDLRNKIANAVSVLSSTMKNFNFTCGSITVNYEQTHSGN
QTAEQASNNVKNAV
>TRINITY_DN62874_c0_g1_i1.p1 type:internal len:197 gc:universal TRINITY_DN62874_c0_g1_i1:2-589(+)
TNGAHWYNVPGGMQDFNYLISNCFEITLELSCCKYPNANSLQKHWNYNKNSLLKYLEQVHIGI
KGQVADSETGIGIPKAKISVEGINHDIKSVSNGDYWRLLLPGKYVVTVKAANYETIMKGGIIVSKE
KPTIVNFDMVRISSTSRPIRQTTIGHDTSQQTPIEISTSLNKKSSVPPSTDITNTATAPSVIKSTQN
>TRINITY_DN76789_c0_g1_i1.p1 type:internal len:381 gc:universal TRINITY_DN76789_c0_g1_i1:1140-1(-)
AKKNKQDIVKYDVNKEAKGLDFVGYNESSKYRDYGGLLIVLVISVILLVVGIILMLSAHIKTDSCEV
NTKTSKDKSVNKKASSDICSYSKEAQRINLHDFLHNVSEEYFNMFPEEIAWHPFADTQMIVKRFV
VHDPRAQNIKRITDKAKSLLDQGVGLKADLSKLKPREHKAYLQVLHFLESTFGASYLNYTAGLWL
MGPSGFCSEPICYVEYSIYNALGSMVIYKQKDIDNLMFHLKAFNESIFNYIDVLNSGIKKGMVRTK
VECVAGINALKGKHQNISEFGEEGIRVEWYGERFFVSSFYQNVTKELDAQSIKKYGKNVTQYIKDS
VIKYIGRSLVHLLKYLENDHIRYCRPNDIGPGGLSALPLDFVYLDGKKTNE
>TRINITY_DN4295_c0_g1_i1.p1 type:complete len:245 gc:universal TRINITY_DN4295_c0_g1_i1:1031-297(-)
MGENNEYFFTPLALLFMPEKCHNKFFVEFNILDAPCLKITISKLLGYAIILGSVLVKVPQIAKLLKA
GSGVGLSLLSIFMEMAAVTFTMAYSLQRGFPFSSWGEALFISIQNVIIVSLIYYYNKSTLGLIFFAPVFS
AITVTLCSTITPIYVLVKLQESNLIIMLISRFLQVFENYRNGHTGQLSMITILLVFFGGIARIFTTIQETG
DWLIASQYMLSSTMNAILLAQILWYWNAKVDEKKTQ*
>TRINITY_DN63421_c0_g1_i1.p1 type:internal len:278 gc:universal TRINITY_DN63421_c0_g1_i1:1-831(+)
QKKGTNGCTSTNAKDKCLFICLPLENGKHECACPDNLIKDGNTCKCPKNEVLHNGECKPKPGT
TCLSTQYACLSGECIPKHWKCDLENDCADGSDESARICSTCPPNRFRCSDGRCILSSYRCDGEFDC
TDKSDEANCTAPTASTCSSDNKIVCPNGRCINKNWLCDGDNDCQDNWDEDPKQCPNKNITTTV
APTFGPCPSNKFQCKVTGRCIPKDWRCDNIDDCADHSDEVNCDTKTCGPNEKSCDKNATCYLPF
QKCNGLADCFDGSDELGCVTV
>TRINITY_DN1280_c0_g1_i1.p1 type:complete len:426 gc:universal TRINITY_DN1280_c0_g1_i1:195-1472(+)
MGFETCGPNEAMVISGCGQERPLLISGGRKFVWEFFQKVQRISLNVMTLNVESQRVYTVQGVPIS
VTGIAQVKVQGQNEEMLHAACQQFLGKHENEIRNIALETLEGHQRAIMGTMTVEEIYQDRKKFS
KSVFEVASSDLVNMGISVISYTLKDIRDDEGYLKALGQRRIAEVQCEARIGQAMAKMQSGIKEAQ
AEEVRIKAEYENKTEVARSQRDFQLNKSAYDIEVNTKKATSELAFALQSAITNQKIKEEDMQIKVIE
RAQAINVQLQEIQRREKELEAQIRKPAEAERYKIEKIAEANRMKVVLEAEAEAESIRMKAEAEAE
VIQKKATAEAEQMSKKADAWRDYQEAAMVDMLLDTLPKVAAEIAAPLSKVKKVTMISSGSSDV
GAAKITGELLDIVIRMPQAMEQLTGVDITKTFRREK*
>TRINITY_DN1172_c0_g2_i2.p1 type:internal len:264 gc:universal TRINITY_DN1172_c0_g2_i2:790-2(-)
GDYGFKVLTSSDPAEKEKKLAAELANGRLAMVAIIGMFFQDGLTGSAWGDWALYTASPLRFFGG
DSGPSYKAFDPATELGVQEPLGFWDPLGFTSDNDEAAFKRRRSVELKHGRVCMAASIGYIVPNYY
TFPGFLSPSAGVKFADVPNGLAALGKVPALGWVQIIFFAGIVEGAFGFDDYKSGAPGDYGWKVLT

SDDPAEKDKKLAAELANGRLAMVATIGMFFQDGLTGSAWGDWALYTASPLRAEPVSTAGVGAA
TKGGTE
>TRINITY_DN172717_c0_g1_i1.p1 type:internal len:213 gc:universal TRINITY_DN172717_c0_g1_i1:1-636(+)
GDYYVVPKPISTCTKDNDNILRRDQIRKLPEWPGLKRSCIESSTSISSMPAPPRIPCMCYPGEMIDSS
DINNVLCRKCTLGHYSDGSGTACHICPTGYAALPTLQMYMLNNNYLDKDTLVQKCSGTLCKEG
DNFWESRGDYLATKRLPGTFDMSVSINNISITYYNARLIFNCSLDCRMTPSGDRLAKHEHCYLKIV
TYRNGEVLTNRDCVG
>TRINITY_DN45008_c0_g1_i1.p1 type:3prime_partial len:388 gc:universal TRINITY_DN45008_c0_g1_i1:1161-1(-)
MGLANWVHWTAWFIKNFVFLLLIMILATIMLKAGKIFEFSEGGIIFVFLLLYMLASIFSLFFVSTLFS
HPIRAMLFGATLWIATLAPYLSFASNEDFDALSNPLKAASCLLPNSCLGIMIRIISKRETSRIGVNWS
NFNKGTDIKGEDFTLGSAFGMLIIDCFLYALLTWYTENVFPGDYGIPRRFYFPFQPSYWCGKKTKA
CSPSDSDLQMHCKNKNNDYEDEPLGMEVGVHIENLRKVFKGPSSSKVAVDSLNLNILKGQITALL
GHNGAGKTTTMSMLTGLFPASNGTATINGKNISTEMDAIRDSLGLCPQHNVLFDRLTVTEHLDFF
TALKGVPKSQAKIEVQQMIDDIQLRDKANDASSMLSGGMKRKLCCAIALIGGSQT
>TRINITY_DN5397_c0_g1_i1.p1 type:internal len:161 gc:universal TRINITY_DN5397_c0_g1_i1:2-481(+)
CSVVCDYSPWQDTQSCTKTCDGGVLIQSRTERSFNKVESREGSTPCNTQACGSGLSEERFRKCNA
NQCGTNTGLQEVYKVCNAANCRFQGIDYTRNQEINIRTQACTTACDYTPWQDTTACSKSCNGG
VLVQSRTERTFNKIETRDGTTPCNTQACTQEL
>TRINITY_DN71928_c0_g1_i1.p1 type:complete len:202 gc:universal TRINITY_DN71928_c0_g1_i1:73-678(+)
MEKQHLLSRTISQVICFFGILQLLFATALGICGFVTLGLYKNINCFGIWAGLPMMVPALLSVMILGT
RHRLNAAAIFITNIVVLAITGYHAYLVYDETDFWISYREQASKQTPGAGNALINGCYNRGDHCVC
EDNRVYLEKNYVVQFCDQFRTGEQLFWTMFVLSLAGIVFSLLTWVLSFFTMFICTRKEEGNHRQK
DVYM*

Band 3
>TRINITY_DN982_c0_g1_i3.p1 type:complete len:9852 gc:universal TRINITY_DN982_c0_g1_i3:29697-142(-)
MSIKLCTFFVFFLIQCINSFPEIGLHLSLDRSYSFQGNGWKTVNGWEIKPRANGPYEIDAIKNISPEY
FPVPITGVYYITVSLPVEGASNGEFKSAIIVDGKNEEGRIGLMSSFQKTAFKNGASTLTFAGFVRLFA
DEKLTIEIYSDDINANYVVLESATFVFHFIGVTGSVPAYLAHTAQDQEYASDSIIKPWVTLGDSRVYE
SLSGFIKNEGVFTTICNGVHKMAVNIMIKANAGTYTLKALFQDEESATATVTINQPDSIATLNIFQN
FKWSEGMSITLQIHSSNGANFKVLKSSSWSMSFIGETTANFQEFSVFLMADQFLQPSQFPQRINIDL
NGVFQFLQSNPDQYNKGQLVSGADTKFKDNLDFIVDSTDLFYVSANIIIEGEAEDLEITIAVSQIDID
VLPEDGLSGRLTKISNSRRSISLSGIIFIREKQYLSFFVRSRDSSKSFTVSKDSFISVINMRYTVSSVSARQ
NENIDVTAGSWYDISGRFAEQKNGLYSFGKDFNINNGVFRTSHSGIHSVHANIQFSLDSDAFGDN
DDNKIVLRVVIDGQEDKISLMSIRKNPEKTATLKLFGSIYLRAGVTISFQVKSLMPNTVQYKILQRSS
ISISYIGPQWAVNSFHAVANQDLNYDGKPDDTRPDIPSPLNFWKTEAADEFISPFEVLESFDGTRYT
SVDDGLYVVSSLLILKNVDCDRSALFKLDFVTTGGSRASDKSNGFADSHFFIADSKGTEFTLSVTTA
MRLEVGFKVGLKLTVGSTSCRYTISKESSFSIAQWSKSSNTIEENRLKYIGVLARLSAPTEVSSPNTW
SNLGSNTMDINPGDKSRSSFRPGIFGGDDLAPITRTGKDFNGDVSDFTIKDPGIFFISGMITVTTTEN
ANEEFAATVQIDSEDIESGLYAAVINQGSTEMSLTFGGTIYLKSGARVRVVVKGGRTFNIAAGSSFSL
LKLQPDYKTPGIIVSIDEKITQAITRYEHKDLRADIHRTLTIGNLGFVESTGVYTIQVDGVYMVDYT
VILEATNPNAVFGAEILSDNTVVATGTSTFGNTKTIAITELVYFSKGAIVKAVLKSNTNGETLNNLQ
VDGGLTVYFLEPKEHVKDGHRSKQTSPQALPVRSFEKVTGFAAQSPDFAGGADSVDYTVPETGVY
LVAFTTRVNRVINGFKSRVLVNDNEFLNGNYQIVSAQNAPATLPLSIAMELTVNDVLSFQVMADG
SGMSLDGSTRSILKIDEVNTIRNVTEGLSAYKLRDQKVNGQQETIVEEWDTSVTEGTFLAKNVSFT
VDHSIVVVAAGVFQISVSVVVKNDRPSQSKFELRVLQGDDVKSHGLHSKVEIGGGREQTLTIGGSL
LLQKFDSLRIVVRDIADPLASYTIKAGSTVSVIIIDDNNGPIACEDEGLNMRRLPERIRKPTRESHTF

QCNVVSSLKAEKYTWFKDNVQLKEENQKNLVLNDLKLSDSGVYQCRVRFLELTGATNNATLSVY
DPYPKITSPKFIANVLENQDPPLTIEMFSLSAFKLSPEDEKASITTQIIAGDDQSFFTLSGPSKQTVTLS
TTKKLDYETQNEYNLTIKATNLEETALTTKGPYETTINYIVRVIDVNDHPPVFSQSIYSRSVPEDYPI
DGSIAKVSANDQDAGANVIYTLVGQVSGDLFKIDNTGLIQLRQNLDREIRDDHTIFVQASDGLFT
TVAEVVVTVTDVNDNFPNFMDTGFDISVSEATLTQSVILKVIATDPDLGTNSQLRYTIKSGNNENK
FSLNLLDGNLTLISKLDYEGTKIYNLEIDVQDSASPPKKSQNPLTVVIRVQDANDNNPTFTQSLYNT
RIAENTAVGTSVLKVSATDRDGEGINNPLTFSITDTDANEFFQVVSSGATAGEIRVRKSLNYDIRKD
YRFSVVAKDNNLGEPRYRTALVVINIDDTNNLRPTFEAGSASISISEGTSIGTSVHQVVAIDKDTVG
KLDYRIISGADGKFAINKETGDINLISAVDFESNKKTYTLEIQVNDGVRDALNPATVVINIQDFND
NQPEFIQKYYIFTALESISDSQAINVGNPVIARDADSTTNSQVDYSIQYDINNPSVPNEGDALFRIDS
NGLIFKKNGVILDFENVRYHTFTVVAIDRGTPKTLLGKAFVTINIQDVNDEAPSWSPAIYSVTVKES
AGINRDLVFLSTTDKDTSGAVTYSIVSGDSANQFQIFDNVIKIVKPLDFEKTETHTLKVSAQDDNG
NRAQLDATVTITVEEVNDNRPLVTNNNANSIQVSVPEDIPVNSLIVKLNGRDDDKGAPGQASNQ
FTFVSLDKTTDFTIDEVTGEIKNTVNLDYERQKSYSLVATIRDKGTPYLESKFLVDIIVENKNDERPIF
DPAIYTIKLSEKAPIDTVVAMLQATDLDTPKSQLRFSSVTTLDSHFSLDPITGIITLKTSFDKEVNGKD
GIFVISCTVNDGTSNSAQNAIVRVEVVDVNDNQPVFPTSEYFIQVNEGSSIGTILQNIAATDKDNT
NTHNQFTYTLEGKEAVDFRLNGNQLQTNKIFDYENKKSYSFDVLATNNLSPDKLVGKATVVVSIL
DQNDNNPVLSPSGVINLNISEGTIAPANIITIFATDKDSTINAEVQFTLTGDPTNTFTINQNTGEIML
AKSVDSFAKNEYTLSVTATDKGSPPRSSTATTLNISIKPVFVPGIEFENCPQIISFNENAAPNTFTKTL
TVKDRGSFNQVQFTVVSGQAGIQNLITVSQNGVITNGQRRADFEARKRHDFLVQAENERGAKAY
CHVTANINDVNEGPIFDSANYVFDVLEVIKINTVVFSVRATDPDFSSTTNGQLTYSLAGQEASQFSI
VPNNGQIKLAQPLDYEIKKQYQFNVIATDGGSLQTTSSVIVNVEDWNDNRPIFDQPQKLAINVPE
NSKPDNVFTVSATDKDSTTNGEIEYVGIYPTGIISIDKDTGTAKIIGDLNYEKRQKYDFVIVAKDKG
VPYLSNTREVSLSVLNLADEVPYFDTAAYTISISEKTSINTAVFRVMAKDNDLENFGKISSYEIISGNQ
LNRFRIDSNGNIIVQQSVVLENNNPNQYILTITAFDTQRTQSLNNTQVTINIIDLSDKQPFFIQNSYI
VEVQENNEVSKSLLSLEAQSNEPTFNPTYSIVQDPKNTDSQFFQIINGNTIVATTVFDYEKKRFYSFE
VQVIDTNNGKTNKALVVVRITDENDNKPFEVSGNLHFNITEHTLLGSTIGSLNGKDNDAGINKEF
EFSILEGNDDDKFMLLPTGQILLKNFVSVKEKDFYKIEVKITDKGTPSLFNIFTVRVTINSSQALVAS
NNPTIPEDKNTGEEITRVTAIDPNTGSSNGFIYSLVGTLSNSQFTIDAKTGAISLLASVDFETDRTHT
AVVKAEGPTSTGVTEVIVRVTDINDNKPIFDPTNFNLNINENFVVGQEIVLVRATDKDSSLNDNNV
IIYRIDSVNPSSGNDLFSVNPSTGRILLLKALDRENGDTSYVLTIAASNRGVSNKPDNTMTVTINIA
DVNDNFPIFEKSLYQGSVSEDANTGTSILTVRASDKDILLKNRNIRYSMTQPMAVQKFQIDPISGVV
SLKQEVDFETKQRYDFSVIASDTGDVAYSRTANVEITILDVPDVEPKFDPQRYEITIAESTLVSTSILK
LQVIDSVKNLNFNIKQGNSNSDFRLVASSGELFVNKQLDRERISRYQMTIEVSDSVNKAAIDAIVIV
NIADVNDNQPLFLQSGCYQANRAEGIETEQVVVNVQATDRDEGSNKEIVYSFSDACSVLDIDAN
TGAITTKQKFDYESARSHICTIKAEDKGTPKLSGFSCIILTVTDSNDLKPTFEVITSNARVLENIPVGS
VITRVLATDKDTLSSNKQFVYTIKTGNEDGRFTIDANGFIKLQQPLDYEKKTDYRLEIEAVDSTNQ
DMVSDPNAIVTVTVIEQNDNRPKFAKSYHRVNIEESTAIGTTVLKLSATDADAGVNEGLIRYVSLH
NTTVFNIDRTTGEITLATKLDYEVKKSYHLIAVVKDQGNPVLSARTLIDIFVNDVNDNPPVFVISSY
NNIVSEKAPIGQSIVTVSARDDDTGVNGQLKYKIRETQSMFRIDEDTGIIILNGDLNVENTNKLLYA
LTIDVVDSGVPPLQASNPATVSVQVSDVNDKQPVFALDRYQFEINENIEGNQIFGDSVTVRANDD
DLTGTNNQLTYRLVNTTMFDLVVTNQNVFIKTALGVSFDYETKKSYSFEIEAMDSAANKLYGRSLV
TVTVKDFNDNPPIITVPNIIVNISDATLIGSHIATIMAEDKDSGENGILIYTVTAGDSTNRFTMESDT
GSIILKNPVDSYTKDIYVLNVLVQDNGIPRRSATGTVTINVIPVDLTLLKFNTTCPFAISIPENQINSN
FLGLKALDAGEYVSKQLRYTINSDENDVNQIFALDSIKGEFKQLKEANYEERSRYDMTICATNERG
SKVCCHITIFITDINEPPSFIGLNNNNIYEVSISETTPIGTIIYRVLSKDQDLPGVLNGQTSYSLGQQNV
FIIDQMGQIRVNKTLDAETQLNYVFQVIVTDKNPTNPLQSAATIRVNILDYNDERPIIQNIPDITIDE
NSDNQVVRNIVVTDRDISNIGSNIELSIEPSNILTINNRQLRTVGSALDYEKVQRHTLYLTAKDKSFP
YLSSTTSFTVTINNLNDNKPKFPVNNYNLTVSEKTTVGTLILTLFAEDDDLEEFGEIANYEIVSGNQ
EDRFSIDSNGRLTLKKQLNWRNVKSYNLNIIARDKGLPSEQSQIPATVVINVADYSEEQPNFLQNL
YVKFIPETTPIDTLVLTVEATSGEPVYGPFKYTIVPKLPDTNNFQIFSNGEIKTINQFDYENQRNFTFD
VQVEDEKNGKTNRASVFIHITDVNDNIPQFTDLTRSNTQYNITEFTLPGSMITCLSATDRDHGKNA

EIRFRGIGGDGNGVFNVNLDGKVLLSQQIFYDKKNKYNLIVEAFDLGSPEQKAQLTLTIFLHKSYT
SVITTDVDINEDVETQTRVTRVSSIDPFTRSSNNFTYYLVGKSTTFFEINAQTGEIFTKATLDRETRDR
HVLTIQSRGPTTNGVGELVVNVKDINDNPPIFDPQVYQVTVSESANIGLSIVSVTATDKDIGVNGE
VTYSIEDGDQTIFSINPSTGSISLAGKLDFESKQSHTLTIKAVDRGNPPQDSVNKATVIVVVQDFND
NCPVFENSTYSLTIKEPGELGKEYLKLGITDNDNGRNQLFTCSIDEPLAEKYFEMINDCRLRLIKEL
DYEEFTSFRFVVKATDQGDVSCTTTTNMIVNVGDRADVIPFFSPLTYNISIPESTTVGTIVVSLKVGS
TFKGLLYSLFHKNTQDDLIKFGINKDTGEIFLKESVDREIQDKYEFTADVFDGSNNAVQRASVTINII
DVNDNAPFFNQPTCYQRDLTESTAIGTFVVDVSAADNDKGRNQQVTYSLLPSPSCTSFSVDETSG
RITTSQILDHEQQKLQHCLIKAKDNGTNSLSGYSCLTVNVVNVNDNKPAFIVNRFDVSVLETVPA
GTLVASLVARDVDNQGLITYSISNGNLNNQFTIDSSGKLRTRFPLDYENTKSYNLTIQASDNNGKFS
SPDDALVLITVLEVNDNRPQFLQSYYKVSIPEDTATGASILNVTAIDLDHGELTKSLFYAALENTTQ
FTVNANTGVISVAENLDYEIEDHYELVVIVQDRGTPHLSNRALVDIQITDVNDNAPMFVTTVYNR
ELSEKALIGAPVVKLNAMDADKSDKMTFTIDSVVQGAQGFFSINGDTIQLDKELDYEVPEQRTFV
LSVSVKDSRDRDGANKANVFINVRDINDREPIFDLNMYEVTVSENNVISNPIIEVTARDSDSTPEN
NNLLYSLVDGEDAASFSLLNNGQSAIITSKISFDYEKKKSYRFLVRASDKVRENFTAFSTVFVTVQD
TNDEAPVPEKDLIMVNISESTRVLSTIAIVFANDKDQGINGQLAFQIINHSEDKFEINNKGVIRLKST
LDFFQQNFYNFTVLVTDKGNPVRTSKPVTVLVYVYRTEIPGIIFEKNHYIFNINEKEIVSLVGTVNAI
DEGTYVSRQIVYSIVSGQNDIKDKFRAVNNNIIQDKEVNYKDQSEYNFVMKAINERGSIAFTHITIN
IMDVNEKPYFVGLNNKGRYEFSMSEIISVPSYIYAVRAKDDDFGLNGEVDYEIISNFRFGNFSIDQY
GRIQVLKDPDFEFTRSYEFQVRAKDRGTPSLESNLVSVVITVLDYNDNHPIFNIQEPLEISIKELVNN
TDVGTVTATDKDGSGLNRIVKYVAIYPSDHLTVNRDNGQIRVIEPFDYEIAHKHEFVVVAQDQGT
PYLNAYKVIKVEVLNENDEFPEFTVIEYNITISELTPIGSPVLFVHAQDKDRGPSGQVTNYKIKYVPP
SGGSGAGSPDKTFEITSDGQVVLKEKLEYKFQKYYQYRVTCTDNGSPPKNSINEAIIHLNIADASLE
QPIFSVNPAVVEVYENITLNSNIVKVEVDSIEPIKGPFEYSIIASEDSQPFMIDNSTGQITNKESFDYEI
KRKYVFLAEARDKHNNRTQRAMVVIFILDINDNIPTIETIAPQTFVQNISDTTYPGSVVAKLYSRDA
DSGENAMVDFTITGGDGENVFAINPNGYVTLIGPLDVRVKTQYTINVKVSDRGIPTLSNNRALTF
NIKKSKFIQNTVRFDRHHYKSNCIEENQINLNIIQLNATNSNPVAGSTIIYSLDGTLEERQPFIVNSQ
TGEISLSQTLDYEIKQEYDFVAVAVNNDGSRDVAVVTVCIKDLNDNSPIFEPQSYVVNVSESESVGY
LVYKTTVMDADKTDFNTTKSYDIIQGNVNDKFIIDSMGHIYIKSKIDYEALQVKHFDLRIRVNEGN
LVGDMVLRVNILDWNDVHPSFSTLNIYNFTIPEDKPVGEIIEILTANDGDATSPNNEVTYKLLTTED
DFILRRNDKGHGSLIVNKQLDYSKQNRYEYVALAEDKGYLNLLGTAIVIINIQDVNNHPPEFDDSE
YNTTVSEHAQIGTRVLQIHAHDKDTGLGEQIFFIYGDPTETFTISSQGFIILNKQLDYETQRDYIFDV
GARDGGNLVSLNNVTVHIKVSEVNDNEPIFDLSTYRNSYAEDTPLGTTLFTVRATDKDFIGNSIIYSI
SGANDDTSYVSVDQTTGDITLQRRMDYESKNYITFTVQAIDSNNRNKLKGSTHVIIDVTDVNDN
NPKFVFGSDLVYNVTVVENINVGTLVVVVDSQDADSGLNGQTRYHIVSGNNENKFFINELNGQI
NLINKLDALQTNKYVLTVMAKDLGNPSRSTNVTVTINVLRYFESTPMFLPSYAEVIYPENLALGSSI
YELNVTKTLPGSPMKLSFHSSSKFPEFTLQGNKIILSKEFDYEIKNRYELIIIATDEENDKAFAQVIVR
VQNLNDNIPYFTQNAYFGVVSELAFVGHSVLTITAKDDDKDFTELFYSIATQVPDNAFKLQDNIV
VVNGKLIRNKVYEFEVEAFDNTFTSKRATVSITVTPENLPSFVNATYIVTLQENYPISTNFLNISAGT
FSGITYVITSMKAKDMFNVDANGQISLTKSLDYEKQRQHVFPVTASVTSPITQVRVASVIVNVLNE
NDNAPVFVNGPYERNITEGHLVGTMVIRVDGHDDDSDKLTYSIVSGDDGKFSINSEGVISLISTLNF
DLKNKYVLNVRVSDGKFVTDTIVTINITPIVKFPPMFNQTLYLLSVKENTLVGTSILQVSTVNVIPPS
SFSINEQSGRDFFTIDANGVIRTNNALDYEAKRQHIFTLKVQDLRSMGHATVVVNIVNDDDTCPIL
TPATQTVQITEPVAPGTIVAAVQAKDIDSKSLNFTITGGNGLFRIDQHGGIKTINEVDISTSTEYELTV
TVDDGTCTKSVQVDVNVNPLAPCPDCNIYPFTQTIYEFSINENTVPASPIITVTTGQSLQRNFSIEDP
AALEYVTINRLTGEISVIKAFDYEATKHLSFNVMAKTQTTKNRNPFYVITSVSRVRITVRDLNDNCP
SIESPMYYEVSHSSPIVQDTVVTRILVEDPDTLKDHTYSISDNNFKIDSMGHIRTVQNIESFTAQFYN
LTVTVSDGQCSKTSNVGVNLAVNPTPMTYQFTSNGVYSKSVREDHNLQDVILSVALNQNYPRVFS
IVESDANDQFVIDSNSGALRLKTSLNYELKKRYDFTVMADIHGGVYKSYASVHIIVLDVNDECPIF
PVIGYNQRVSGTPVIGTIVEIIEANDADNATLEYSIVSGNEDGHFSLDNSGVVRVVKPFASSFSKTYG
LSVRATDNKCSADVPLTVQVSTCAYGEDYQFEESEYVYYLDENRNLGIVSNIKLKSTRAAFLTLSQ
NTHFTIDQNGDFRLIQTLNYETKKEYHLTLTANFTQDNVVTRIPIKVFVRDINDNCPAFSEELYAVQ

HTEPLREGLLIAKLSVIDDDTVQNFTYNIVSGDKRFSINENGEVVMNARLVTDTTLLSTTYKLEVSV
NDGKCAKNTKVDVFVYKTVVNSFLFPQPFYKFDISENQAIPFTIDRFVNVGGHEAVYGFLVSTDFF
QIDPKTAQLQLVKKLDFEVEKTHYVLISATYDDGTQTYTVVAIHVGDENDNAPQIFAHPPITRLVQ
NTPHNSIVTKIEVEDPDTSKEFTFKITNGNPLQNNDPDKALFRIESNGMIKTTRCISEDEIGNYVLTI
SVDDQVNTPTIFSNMQVQLKTTDDRSYVTNCGLGCPANDGPFSRYQFEQSLFIVNVTETAANPTV
LKRLTRPFVEKTTYTIGAGADGKFSIDQNGTLSLTGSLDYETKPVYYFIVSAVIPSQVVGRFITSKTAY
LVMVNDVDDSKPVIEGNGGTLAVSETSEIGQIVGTVRVKDSDSSSPLTYNVVNGSQYFNIDSAGNI
VIKDTLNAAGSPYTVTYTVSDGINPPVNGEFVVSISETNNNVPKFTQNIYEVSLSEDTATDTIAVTIL
ANDGDTNDKIAYSFLQSEGPFVINRDSGEIKLIGPLDYEAQKSYNMVVQATDSGGFANIAFVQIAV
GDVNDQVPVITPQTTPTQIHESTLVNSVVYRVIASDGDGNERLTYALANSNDFFTINSATGEITIKE
NLAKSNVSSHTLSIVVTDSSGKTSTQDVALNIRRSGTSQGCVKITTSQGQSSSPVINLPENTASGTTV
FKLVAGDASVFSNMRFTLNREANGVFAVTEQGELQLVAPLDYETKQFYSIIITAEDVTRGVSSQYNL
FVRISDVNEFTPVLETIAPVAVSELANIGDVIAQAKANDQDGDSQVTYSLGEFGNNDNKFTIDAAT
GNIILNKRLGSGAPHVVEVCASDGSIKKCQNVTFIVADTNDNPPSFQDLTGVGSIDLTNTSSYVVY
KPIVTDSDQTGTLSFSLKDPAPSQFSINSTTGEVKANKDIAKGTYTFGVCASDGRYTSCQSVTVVAS
DRSQSITPSFNQDQVGAIVDISASSGKQIIQVMASTLDKAKIEYELKNEADRNIFEINKDSGLITLKN
RTALTLGTIYTIPVKASRADDPLNFDEINIVISVGNQQLDSGSFIEQVLDTQHLVNFSLSTFDISKVSY
YIILGQQVGGDSKDDEKLPVSWYYATFGDESYRHRFYYIAKLDGSKFRNTGGSRKRRSIQDKDSKS
FTYLVGEENGCSKNTSMAAICNGPLKTGTEYRYKLRAYYDDGELRGIPVDQKPTEVKRTQAPQA
GKSIDEYREQDSYVITFWIIGLVIFGLLFWFSVLVIAILLCYKRRYEVTQAYGYSVQTRDKIDGPPTEY
SNTVYRFQNPSYPDNDTVKMKKVDASESRTKTEVQTTSMVVLDDYEQEQPQPDIPVQDYDAIEY
ETRSAQIKFHEESPEWESMDIQLRIDPTGNQDPVITRKSDSGSKDSDSDASKKYENTEYIEEKEDFV
NEKEDSLTRL*
>TRINITY_DN244_c0_g1_i1.p1 type:complete len:3019 gc:universal
TRINITY_DN244_c0_g1_i1:9748-692(-)
MDARGQRLLYCLLIFLSLATLLVSQQVEKCKRPVDLVFALDTSGSVKDSGFRLIQKFAKDILKTFDI
GVADSHVGVLTFSEQAEPQIKLTETFDKAELMQMIDSLVYQGYRTATDDALRVAKTDFFSTAGGA
RQGVPHVLVLLNDGKCTVCIEPVKEAAQKLKDMGVEIFTIGVTENIKKQELIDIASQPTAYHFFEL
DDFDQLVDVVGDIQTRSCEVKKGKCPIPSPTEQCSGNEINECTSDENCEGELKCCSDGCSRICKYP
LLNCFTPIDIAFALDSSSSIQPETFQEMKKFSNDIVDSFAVSQKKTRFAALVYSDRAEIGFNFVRFDD
ASSIKKAIQNLPKLDSKERIDYALELAKSDIFSLQGKVRTRRPMVLFVFYDGTVSSGMKDLSLVVEP
LKNYGVKIVSMGVGPETNYYQMRKISSNNSDVFRATNFKELLPEIYNIAKRACTEKPGRCDASLN
PLPKEACKNVKILNQCENDFDCFGDAKCCRKGCNNVCVIRGPVCPRKLDIALAVHQNMDESEF
AATKFYLQSVVDQFNVGPSQTHFSLLSFGQEPKVIFDFNSDQSSNEIVRRKIDNFQYQYGIGRISDV
LALSCDAVFCAAGGTRDDVPKMLVIVTSRTSPSSPSKLKEKLQTLRNRGVNILMIGVGDVDTSLLQ
AITSDDPLVEDRVLLSKRFDGIFQYTKDISDYACGMAPALPPPDNIKGDAKGECGAPKSCYKGPKG
SRGRPGTLGQQGESGDLGLVGPKGIQGRRGDKGPDGQMGSKGKRGPDGLIGLPGFPGYNGIPGV
PGIEGRRGRDGDCGLPGSSGEAGQAGLDGLSGPPGRAGPVGEKGEKGLPAPENPNIRIEPGDRGD
TGSVGLPGFKGIKGEEGFKGPSGDSGVDGIKGVDGEAGEMGDIGTYIYQKGPEGDRGARGPPGD
GDTQGATAGLGVQGEKGEKGMRGEAGDGSIFPGIKGRRGDQGPKGEEGSTGPAGPTGQRGPEG
EEGPKGIKGEIGDRGKDGTPGQPGQEGDLGEKGFDGLKGQDGDDAEGELGDSGGKGLKGENGL
AGSPGSIGTPGPPGSKGEPGDIQIGLSGDPGAIGPRGPRGDNGISGQPGMRGDSGPKGAKGNQGP
RGDRGRNANMLAGKDGMTGPPGPKGFTGPVGDPGFPGPPGPPSVVECVEGACPKGVPGAMGQ
IGKTGEPGPPGPPGPRGSKGVAGGPCPICPDGIQGDSGDIGQPGMPGKEGSRGRKGNIGDAGSKG
LQGRNGDDGDQGPVGKKGFIGDIGPVGENGVDALGDSVAPDGEKGSTGDTGQIGSRGPVGNDG
PKGFKGTIGERGRRGLQGEKGDMGLTGETGEDGSRGLEGAKGDLGDGLRGAKGDKGNIGQEGN
QGVVGDVGLQGPKGEQGAPPTEDELKNVKATKGSKGEIGSTGDAGEIGDIGPAGPRGPKGFDGA
AGPKGPSGVKGDRGNKGRAGDGEPGFQGFPGRPGKRGPPGDPGKPGKRGFSEPGDAGDRGDP
GKSGKKGADAPPPIGCFKFNEPTDIGFILDSSSSVDDDNFLRQLQFVKNIVRQYPVGPDQTQVGIIK
YGEKAVTGINFYDYVTEEGLFNAIDLLEHTKESESRLDLALKLARDELFTESVGARGFDAKKALVIL
GDGFISGGGTPSKTLMNNAMQYAEDLRGAGVSIFALAVGPEKNKALFHEFVSEKEYYLDIESYSQL
DNELGALQLSLQTGCISVGVPGRDGLPGYTGPKGQKGDDGPKGFSPQGPRGIKGIKGVRGTIGIG

VKGLPGDPGLQGPQGEKGRDGEDGSADPGPKGRPGDKGSRGDQIKGASGEDGDIGPIGPPGFDG
RKLCDNVLAVDLLFVLDTSSGVGVDNFNKMLKFIESIINVFDVDNQLTRVSVMTYDSDARLVIRFS
DYNDPDKLDAAIDNIPYANGDETRIDKALLLASSNAFDTSNGAREGVNKAVILMTDGQQSFAVD
QVALKDAVKPLVDANVLMYAIGIGSEISPVDLKTIAAERVLIADDFDGLSLKIEDQIVLLGRVGCQG
PPGKDGKPGANGEDGFPGLIGAKGQKGDRGEAGTEGLKGNKGSQGEAFPGLNRKGEKGDPGS
AGPLGLPGCEIKNQNEVIDLGFAIDASGSLYEEGFNREKEFIKTIVDRVGRVSEQGIRIGVIVYSDTAS
IEIRFDDHFNNEDLKNDIDNLPFFAKNTRMDLGLEEAKKMFGEQFGGRGSSKKVLILLADGQQTY
VPGVTEPSEVAKELAAAGVDIFAIGIGQEIDRTELESLISKPEHIFLASDISKLIDELTPEVTSALECEG
GPRGPRGRAGTDGPPGSKGLLGPIGDKGFPGEPGSAGFGPKGDDGENGTAGTIGDRGEKGFRGP
AGVPGKRGAVGPKGFPGPNGEKGDRGDEGFIGFEGARGLEGPKGQRGDSSDNGVAGVKGSTGD
NGFKGPKGDPGKLPPGNRIEDFKGETGGSGLEGDTGPKGKPGRALFEPGPQGLTGPKGEKGDQG
FFGMSGPIGNKGFQGLQGFRGDDGAAGPVGEDGFPGMKGLPSSSGDQGDKGKNGRKGESGEV
GQPGRVGEDGDQGEKGYAGFVGMPGKKGDQGEPGVQGPQQIRNEILDNIRPGPKGYPGSTGPA
GDEGEDGEVGEPGETVGAQGMNGDFGERGSQGPKGEQGDQGLQGMIGVIGDKGVKGRVGTG
GNPGTVGLRGLTGAPGEVIFGSEKGNKGDLGIIGLRGRAGQKGKAGAIGPRGRRGRNGAKGERG
PPGFPGLSGIEGDSGNEGDKGPRGDPGSVGNRGIKGVPGESGIAEGNYIVIHSQTHIIPDCPRDFKK
LWHGYSLMYVVGNGHSHSQDLGDAGSCSQKFSSKIFLFCEIIKVCKHASRNDFTYWLAGDVDHP
MMPVSNAAVEPFISRCAVCEAHSSHITVHSQDTYAPECPPNWSSLWSGYSFLMVAGAGKTGAGQ
SLSSSGSCLESFRPKPFVECQGARGTCHFFSDKHSFWLVTIDPNKQFEVMKPQTLIKDKNHDLTSR
VSRCRVCIRKTGTKNNTVEPNIATRIRRSIASWIL*
>TRINITY_DN262_c0_g1_i1.p1 type:complete len:1033 gc:universal TRINITY_DN262_c0_g1_i1:111-3209(+)
MADPSSLETKEKDGSYRVATKSGGKDKGKKSKKAAKKVNMDDLKKELEMTEHSCELETLVSQLE
TSLTQGLTKSIAARNLERDGPNALTPPKQTPEWVKFCKQLFGGFAMLLWVGAILCFFAYIIRSIREE
EPGMDELYLGIVLTVVVIITGCFSYYQESKSSKIMESFKKMVPQEAVVMRDGEKLNLNATECVVGD
LVFVKFGDRIPADLRIIESKGFKVDNSSLTGESEPQSRNSECSSDNPLETRNLAFFSTNAVEGTATGIV
VKTGDNTVMGRIANLASGLGSGKTPIALEIEHFIHIVTGVAVFLGVSFLIISLAMNYHWLESIIFLIGI
IVANVPEGLLATVTVCLTLTAKKMASKNCLVKHLEAVETLGSTSVICSDKTGTLTQNRMTVAHM
WFDNMVVEADTTEDQSGQAHDKGSTTWKALAKIGALCNRAEFRPNQEEEPILKRECNGDASET
AILKFVELSVGSVEDIRSKNKKIVEIPFNSTNKYQVSIHEQEDPDDPRYLLVMKGAPERILDRCSTIL
HEGVEKPLDEEWKENFNKAYVDLGGLGERVLGFCHYYLPADDFPKGFAFETEDPQNFPLEKLCF
VGLMSMIDPPRAAVPDAVSKCRHAGIKVIMVTGDHPITAKAIAKGVGIISEGNETVEDIAQRLNIP
KEEVNERDANAAVVHGSHLKDLPQEKLDDILKYHQEIVFARTSPQQKLIIVEGCQRMGAIVAVTG
DGVNDSPALKKADIGVAMGIAGSDVSKQAADMILLDDNFASIVTGVEEGRLIFDNLKKSIAYTLTS
NIPEISPFLMFILLAIPLPLGTVTILCIDLGTDLVPAISLAYEKAETDIMKRKPRDPVRDKLVNERLISL
AYGQIGMIQATAGFFTYFVIMAENGFLPGYLIGLRNDWDDKKNNAVMDSYGSEWTYNQRKNLE
LTCHTAFFTTIVVVQWADVIISKTRRLSLFQQGMTNWMLNFGIFFETALAAFLQYTPGLNTGLRLR
PMNFPWWFVGVPFSLLIFVYDELRRFLLRRNPGGWVEQETYY*
>TRINITY_DN982_c0_g1_i1.p1 type:5prime_partial len:6412 gc:universal TRINITY_DN982_c0_g1_i1:19377-142(-)
KLDFETKQSHTLTIKAVDRGNPSQDSLNKATVIVVVQDFNDNCPLFSESIYNASVSESTAIGTTVLR
VSVTDKDVGNVNNEITCSIDEPLAKGFFSVNKINNNQFSVVLIKNLDYETHRGYRFVIKAKDNGE
VPCQLTTNMVVEVQDEPDVITNFKPFNYVISIPESTLIGTKLLELKVDSESSSITYTLQAKNQPGDLD
KFMLNGQSGELVLKGTVDFEKKSEYEFTATLNDGVNNGGNTANIVVNILDINDNPPVFSQSTCYI
RRMPENQQVNTTVTSVLATDSDQGINSNISYSLIKDSSCNNFEINEITGAISLTSSLNYEVRKEHYCII
KATDNGSPVLSSYSCLKVIVENVNDNSPVFLPKELEITVLESKPVNSFVIRVFATDKDEGNSITYSIV
GGNTQNAFAITPLTGHIILNQKLDYEIVKNYILEIQATDDGTPRRSSNLNAKVMVKVLEVNDNKP
KFNQQRYLRTIPEDTNIGSTIISVTATDDDAGLLTSKIQYIALENTNDFLIQPDTGDIVVSQNLDYER
QRTYQLVAVCSDSGTPPFTGRTLVDITITDVNDNAPILNPTLYNVLVSEKAPLGTSLIQVHAKDKDS
GINGGLKYQIVSGNDLNKFVINNSTGVITLQETLDREKYSSLYNLTVSVSDQATPSLNGNNAFVQI
RVLDVNDKQPVFVKSEYIFEISENQPLNSPIGLVQANDDDSTSPNNIIQYTIVPSKDSINFTMINNQI
LSNSVFDYEEQKSYEFEVQARDQSTGVNNEQLVGRTLVKVLIRDTNDNDPMIENQNIALNISDVT

LVGTLLFDIQATDKDNGENARLTYTISSGDTSTFRINENDGRFYLSKKLDFYNKNEYSLIVIVNDNG
SPQRNSVANVQINVYPIDQTLISFEPQCPIAFTIQENSLSSPMNIKAIDTGSYVSKTMRYMLKSNKSD
VSSMFQIVETNGVIIQNKAANYEERSRYDMTVCARNERGSEGCCPVTIYILDQNEQPFFDTDNDN
LDNGKYLVRVSETTPIGSVMFSVNTGDEDFGSLPNGQKTYSIKAKPGEEMLFLVDSYGRIITNTTLD
REKTPQYDFSVVVTDKGTPPRQAEAEVIVTVIDYNDQRPVFEIMSVRPVNENANNEVLGRVVAVD
IDSTSINNGIKYSIQPAGFLNISNTGTLTSVGSALDYEKNQKVTLYVTATDTGSPYLSATTSLQLDIINI
NDNKPYFLQNNYNLTVSEKTTVGTLVLTLFAEDDDLGEFGEIANYEIVSGNQEDRFSIDSNGRLTL
KNPLNWRNVKSYNLNIIARDKGLPSEQSQIPATVVINVADYSEEQPNFLQNLYVKFIPETTPIDTLV
LTVEATSGEPVYGPFKYTIVPKLPDTNNFQIFSNGEIKTINQFDYENQRNFTFDVQVEDEKNGKTN
RASVFIHITDVNDNIPQFTDLTRSNTQYNITEFTLPGSMITCLSATDRDHGKNAEIRFRGIGGDGNG
VFNVNLDGKVLLSQQIFYDKKNKYNLIVEAFDLGSPEQKAQLTLTIFLHKSYTSVITTDVDINEDVE
TQTRVTRVSSIDPFTRSSNNFTYYLVGKSTTFFEINAQTGEIFTKATLDRETRDRHVLTIQSRGPTTN
GVGELVVNVKDINDNPPIFDPQVYQVTVSESANIGLSIVSVTATDKDIGVNGEVTYSIEDGDQTIFSI
NPSTGSISLAGKLDFESKQSHTLTIKAVDRGNPPQDSVNKATVIVVVQDFNDNCPVFENSTYSLTIK
EPGELGKEYLKLGITDNDNGRNQLFTCSIDEPLAEKYFEMINDCRLRLIKELDYEEFTSFRFVVKAT
DQGDVSCTTTTNMIVNVGDRADVIPFFSPLTYNISIPESTTVGTIVVSLKVGSTFKGLLYSLFHKNTQ
DDLIKFGINKDTGEIFLKESVDREIQDKYEFTADVFDGSNNAVQRASVTINIIDVNDNAPFFNQPTC
YQRDLTESTAIGTFVVDVSAADNDKGRNQQVTYSLLPSPSCTSFSVDETSGRITTSQILDHEQQKLQ
HCLIKAKDNGTNSLSGYSCLTVNVVNVNDNKPAFIVNRFDVSVLETVPAGTLVASLVARDVDNQ
GLITYSISNGNLNNQFTIDSSGKLRTRFPLDYENTKSYNLTIQASDNNGKFSSPDDALVLITVLEVN
DNRPQFLQSYYKVSIPEDTATGASILNVTAIDLDHGELTKSLFYAALENTTQFTVNANTGVISVAEN
LDYEIEDHYELVVIVQDRGTPHLSNRALVDIQITDVNDNAPMFVTTVYNRELSEKALIGAPVVKL
NAMDADKSDKMTFTIDSVVQGAQGFFSINGDTIQLDKELDYEVPEQRTFVLSVSVKDSRDRDGA
NKANVFINVRDINDREPIFDLNMYEVTVSENNVISNPIIEVTARDSDSTPENNNLLYSLVDGEDAA
SFSLLNNGQSAIITSKISFDYEKKKSYRFLVRASDKVRENFTAFSTVFVTVQDTNDEAPVPEKDLIM
VNISESTRVLSTIAIVFANDKDQGINGQLAFQIINHSEDKFEINNKGVIRLKSTLDFFQQNFYNFTVL
VTDKGNPVRTSKPVTVLVYVYRTEIPGIIFEKNHYIFNINEKEIVSLVGTVNAIDEGTYVSRQIVYSIV
SGQNDIKDKFRAVNNNIIQDKEVNYKDQSEYNFVMKAINERGSIAFTHITINIMDVNEKPYFVGL
NNKGRYEFSMSEIISVPSYIYAVRAKDDDFGLNGEVDYEIISNFRFGNFSIDQYGRIQVLKDPDFEFT
RSYEFQVRAKDRGTPSLESNLVSVVITVLDYNDNHPIFNIQEPLEISIKELVNNTDVGTVTATDKDG
SGLNRIVKYVAIYPSDHLTVNRDNGQIRVIEPFDYEIAHKHEFVVVAQDQGTPYLNAYKVIKVEVL
NENDEFPEFTVIEYNITISELTPIGSPVLFVHAQDKDRGPSGQVTNYKIKYVPPSGGSGAGSPDKTFE
ITSDGQVVLKEKLEYKFQKYYQYRVTCTDNGSPPKNSINEAIIHLNIADASLEQPIFSVNPAVVEVY
ENITLNSNIVKVEVDSIEPIKGPFEYSIIASEDSQPFMIDNSTGQITNKESFDYEIKRKYVFLAEARDK
HNNRTQRAMVVIFILDINDNIPTIETIAPQTFVQNISDTTYPGSVVAKLYSRDADSGENAMVDFTIT
GGDGENVFAINPNGYVTLIGPLDVRVKTQYTINVKVSDRGIPTLSNNRALTFNIKKSKFIQNTVRF
DRHHYKSNCIEENQINLNIIQLNATNSNPVAGSTIIYSLDGTLEERQPFIVNSQTGEISLSQTLDYEIK
QEYDFVAVAVNNDGSRDVAVVTVCIKDLNDNSPIFEPQSYVVNVSESESVGYLVYKTTVMDADKT
DFNTTKSYDIIQGNVNDKFIIDSMGHIYIKSKIDYEALQVKHFDLRIRVNEGNLVGDMVLRVNILD
WNDVHPSFSTLNIYNFTIPEDKPVGEIIEILTANDGDATSPNNEVTYKLLTTEDDFILRRNDKGHGS
LIVNKQLDYSKQNRYEYVALAEDKGYLNLLGTAIVIINIQDVNNHPPEFDDSEYNTTVSEHAQIGT
RVLQIHAHDKDTGLGEQIFFIYGDPTETFTISSQGFIILNKQLDYETQRDYIFDVGARDGGNLVSLN
NVTVHIKVSEVNDNEPIFDLSTYRNSYAEDTPLGTTLFTVRATDKDFIGNSIIYSISGANDDTSYVSV
DQTTGDITLQRRMDYESKNYITFTVQAIDSNNRNKLKGSTHVIIDVTDVNDNNPKFVFGSDLVYN
VTVVENINVGTLVVVVDSQDADSGLNGQTRYHIVSGNNENKFFINELNGQINLINKLDALQTNK
YVLTVMAKDLGNPSRSTNVTVTINVLRYFESTPMFLPSYAEVIYPENLALGSSIYELNVTKTLPGSP
MKLSFHSSSKFPEFTLQGNKIILSKEFDYEIKNRYELIIIATDEENDKAFAQVIVRVQNLNDNIPYFTQ
NAYFGVVSELAFVGHSVLTITAKDDDKDFTELFYSIATQVPDNAFKLQDNIVVVNGKLIRNKVYEF
EVEAFDNTFTSKRATVSITVTPENLPSFVNATYIVTLQENYPISTNFLNISAGTFSGITYVITSMKAKD
MFNVDANGQISLTKSLDYEKQRQHVFPVTASVTSPITQVRVASVIVNVLNENDNAPVFVNGPYER
NITEGHLVGTMVIRVDGHDDDSDKLTYSIVSGDDGKFSINSEGVISLISTLNFDLKNKYVLNVRVSD
GKFVTDTIVTINITPIVKFPPMFNQTLYLLSVKENTLVGTSILQVSTVNVIPPSSFSINEQSGRDFFTID

ANGVIRTNNALDYEAKRQHIFTLKVQDLRSMGHATVVVNIVNDDDTCPILTPATQTVQITEPVAP
GTIVAAVQAKDIDSKSLNFTITGGNGLFRIDQHGGIKTINEVDISTSTEYELTVTVDDGTCTKSVQV
DVNVNPLAPCPDCNIYPFTQTIYEFSINENTVPASPIITVTTGQSLQRNFSIEDPAALEYVTINRLTGE
ISVIKAFDYEATKHLSFNVMAKTQTTKNRNPFYVITSVSRVRITVRDLNDNCPSIESPMYYEVSHSS
PIVQDTVVTRILVEDPDTLKDHTYSISDNNFKIDSMGHIRTVQNIESFTAQFYNLTVTVSDGQCSKT
SNVGVNLAVNPTPMTYQFTSNGVYSKSVREDHNLQDVILSVALNQNYPRVFSIVESDANDQFVID
SNSGALRLKTSLNYELKKRYDFTVMADIHGGVYKSYASVHIIVLDVNDECPIFPVIGYNQRVSGTP
VIGTIVEIIEANDADNATLEYSIVSGNEDGHFSLDNSGVVRVVKPFASSFSKTYGLSVRATDNKCSA
DVPLTVQVSTCAYGEDYQFEESEYVYYLDENRNLGIVSNIKLKSTRAAFLTLSQNTHFTIDQNGDF
RLIQTLNYETKKEYHLTLTANFTQDNVVTRIPIKVFVRDINDNCPAFSEELYAVQHTEPLREGLLIA
KLSVIDDDTVQNFTYNIVSGDKRFSINENGEVVMNARLVTDTTLLSTTYKLEVSVNDGKCAKNTK
VDVFVYKTVVNSFLFPQPFYKFDISENQAIPFTIDRFVNVGGHEAVYGFLVSTDFFQIDPKTAQLQL
VKKLDFEVEKTHYVLISATYDDGTQTYTVVAIHVGDENDNAPQIFAHPPITRLVQNTPHNSIVTKI
EVEDPDTSKEFTFKITNGNPLQNNDPDKALFRIESNGMIKTTRCISEDEIGNYVLTISVDDQVNTPTI
FSNMQVQLKTTDDRSYVTNCGLGCPANDGPFSRYQFEQSLFIVNVTETAANPTVLKRLTRPFVEK
TTYTIGAGADGKFSIDQNGTLSLTGSLDYETKPVYYFIVSAVIPSQVVGRFITSKTAYLVMVNDVDD
SKPVIEGNGGTLAVSETSEIGQIVGTVRVKDSDSSSPLTYNVVNGSQYFNIDSAGNIVIKDTLNAAG
SPYTVTYTVSDGINPPVNGEFVVSISETNNNVPKFTQNIYEVSLSEDTATDTIAVTILANDGDTNDKI
AYSFLQSEGPFVINRDSGEIKLIGPLDYEAQKSYNMVVQATDSGGFANIAFVQIAVGDVNDQVPVI
TPQTTPTQIHESTLVNSVVYRVIASDGDGNERLTYALANSNDFFTINSATGEITIKENLAKSNVSSHT
LSIVVTDSSGKTSTQDVALNIRRSGTSQGCVKITTSQGQSSSPVINLPENTASGTTVFKLVAGDASVF
SNMRFTLNREANGVFAVTEQGELQLVAPLDYETKQFYSIIITAEDVTRGVSSQYNLFVRISDVNEFT
PVLETIAPVAVSELANIGDVIAQAKANDQDGDSQVTYSLGEFGNNDNKFTIDAATGNIILNKRLG
SGAPHVVEVCASDGSIKKCQNVTFIVADTNDNPPSFQDLTGVGSIDLTNTSSYVVVYKPIVTDSDQT
GTLSFSLKDPAPSQFSINSTTGEVKANKDIAKGTYTFGVCASDGRYTSCQSVTVVASDRSQSITPSFN
QDQVGAIVDISASSGKQIIQVMASTLDKAKIEYELKNEADRNIFEINKDSGLITLKNRTALTLGTIYT
IPVKASRADDPLNFDEINIVISVGNQQLDSGSFIEQVLDTQHLVNFSLSTFDISKVSYYIILGQQVGG
DSKDDEKLPVSWYYATFGDESYRHRFYYIAKLDGSKFRNTGGSRKRRSIQDKDSKSFTYLVGEENG
CSKNTSMAAICNGPLKTGTEYRYKLRAYYDDGELRGIPVDQKPTEVKRTQAPQAGKSIDEYREQD
SYVITFWIIGLVIFGLLFWFSVLVIAILLCYKRRYEVTQAYGYSVQTRDKIDGPPTEYSNTVYRFQNPS
YPDNDTVKMKKVDASESRTKTEVQTTSMVVLDDYEQEQPQPDIPVQDYDAIEYETRSAQIKFHEE
SPEWESMDIQLRIDPTGNQDPVITRKSDSGSKDSDSDASKKYENTEYIEEKEDFVNEKEDSLTRL*
>TRINITY_DN15274_c0_g1_i1.p1 type:complete len:1822 gc:universal TRINITY_DN15274_c0_g1_i1:87-5552(+)
MECIRRSIIRLLLILGFYTLYISSQEECKRGGCFPATGDLLVGREKQLLANSTCGLNGPSKYCILSFLD
KTDKCFYCDSRRQVDERPDGYKNSHLPKYMVTREPSKRLQGWWQSVNGRQDVTIQLDLEAEFIF
THLIMTFKSFRPRAMYIERSSNYGNDWSDYRYFAYNCRKSFPNIPRGPQRKIDDVICEERYSKVEPS
TGGEVVMKVLDPIIQKKEDPYSLKVLNLLKLTNLRVHFKELHTFGDDILGDSPKVNQKYYYALYE
MVVRGSCSCYGHAEQCIPTKGTPANVNMVHGQCNCTHNTKGRNCEMCKDGYNDEPWRPAYQ
DQLNVCRKCNCNGHTDKCHFDPAVYKATGNTSGGVCDDCNHNTAGRQCELCKPLYYRDPYKN
IFDEDVCKPCDCDPSGSFGGGECETISDPNNSSALTVAGRCICKPNAQGLRCDSCKDGYWNLLES
NPDGCQSCNCDDKGTVELNKCDQQTGTCRCKRFVIGLTCSKCLEGFWNLGNTPEGCEPCNCDV
GGSLNAKCDDITGMCMCKPNIIGRKCDQVAPGSYFGNLDQVISEAENSLVRSDSSSVEIVREILNPT
DSRSFTGDGYLRVSDGTLVNFTNIRVPSTGFQTFVMRYSRPTPGSNNNVMVRITKTDGTILGGNGC
VQTNEREIILNGVLNDDNFLELGDMFCLEKDTDYSVQLVVSNRNSPNDGGVNRGVTNLDSIVM
MPNIDNLAIFQGREGMRLKSQYDLNNCRQYYLEPNGAANAPEICKKIQFSISAEINNGALKCNC
DPTGTNSSGSSDILTCQKSGGQCPCKPGVVGRRCEKCAPGYFGFDSNGCTACACNDTGSEKLFCN
GITGQCPCKNRIKGRQCNSCPSNHFGFPNCIACQCNEHSSTCDPISGECTNCKDNTAGPSCNLCA
EGYYGDATAGTEKDCQRCQCPGGSSLNQFSNTCHLKPDGKVVCNNCTEGYTGDQCDKCEDGY
YGNPLVPNGSCNKCDCNGNIDPFADGKCNAENGKCFNCINNSDGDHCENCKEGFFGNAKNQS
CRACVCNKLGTNSATSDSCDRETGQCECLPNVEGVECDRCKSGYYNIASGNGCVSCNCDKGGSL
SSHCNQLDGQCNCLNGYGGLDCTQCEDFYFGDPKQNTCQACGCNPVGSITLQCNRTTGVCMC

KDNVVGEKCDMCAPQTAGVMPQCEECHVCNDQWEDIVNDIEKNITTILTLKGSSPLNVSEIRAY
TNDIADLKNRLDKIEEDFGKQMVDPNKINRLRDKVYRFSYNLTGINRDADFYMEMVNETITRDE
KANDILDGLELRLATLQTNTEDFKNNVTKIVESNIGGALNSTRESLEKSRKAQESVNDSDLKIKES
KKMRKKIKKEIQNGDPEFEVMHNRSSEFVNNLADRENYLEGELEKLNRLLCGSDKRCGGCTKGG
CDTCGVSGENYTCDDGIKMMSMMASDKALEAKEAIDQKLENASELLENVNRAKQHVIQANISA
NMAEDKAEEVNKIGQAVLKNITTLIKDIQDFLATDFNHPNASKDLAMQTIALNISLTPDEVVELG
RKIRDAVKSLTGVDKILNESRNDYDTAIGLKEQALAAKERAIEVMNQTMAIIETLSQAEELQNSTL
AGVNVARADIEDVKDLLKKLKEKLDAADNDLQMADNSTASAEDKRNAILDQNLENREKLDLSE
KKIDEAEELGDKVQGSVDKMNEKYNATKAKLNEKEDMIEKVRARIKALADKTIKLYENADRKLR
RINKMKDSYIINEERAKDLYYEIRRLEIKEFFINTKLEGTAKCYSTCNPIIPESFCRDVYKNIAVKETN
AYAELEAEKASTTSLLI*
>TRINITY_DN1616_c0_g1_i2.p1 type:3prime_partial len:3398 gc:universal TRINITY_DN1616_c0_g1_i2:218-10408(+)
MIIFLAFLAVAVGQEVLLPPYFDIAKGKNITATATCGEDYVAPKGSPGELFCTLADATGKFGIKGLK
CSHCVPGDYGDGDGKNARDHHIRNANDGSNRWWQSPPLSRGLQYRKINITMALGQSFHVAYVI
IKAANSPRPGVWALERSNDNGRTYTPWYYFAPTSSECKQYFGVDSYLPIVKDDTVICSTEYSKIPPF
KNGEIHISLINGRPGASNFSQSKTLQEWTTATNVRLRLLDIKTLLTDLLPLSLEDPTVTRRYYYSIKDI
TIGGRCMCNGFADECTPHTNIVGKYECNCQYNTEGPRCSRCKKGYVQKEWRQRTSTDKFECEKC
NCHGHSDECEYNEEINKRKLSLDVNGNYQGGGVCKNCQHNTLGINCEKCKPFFYQPANVPIDS
PNVCQPCNCDTQFSTGLCAEETGQCECKSNYVGRYCDQCNKGFHMFPVCKPCECDVNGSEDRE
CIPDQINGCKCKKGYAGRYCDECLPGYFGFPDCQPCACLTRYGAVNDNCERDNGQCSCKVGFK
GRRCEECAAGHYQFPFCKECGCDPEKTLDTVCDSGTGVCQCKSNFAGPKCESCSPGFYAHPSCFA
CGCSTAGSTSSVCDQNSGLCSCRPNYSGFKCDRCAEGYYGFPNCILCDCNPEGSRRVGICDRQDG
QCQCKPTYSGQKCDKCLPGFYGFPDCKRCNCNPAGTKPGPGGRPGDCSTSNNGQCVCKDNVQ
GLECNECKPGFFNLTESNRKGCSTCGCNGAGTNNGVKSCSVEGTCKCKPLVKQEDKCTECRDGY
YGLKRTNVFGCLGCNCEVGGSSSSVCSKDNGQCPCKDNIVGKRCESVAANSFYPDLCHIKAEME
EGKTTDGDVVRYDYQKEIFPDFSWKGFVAVNDQQPSVVVSINVPKTSEYQVKFRYALNQIQPVTA
TVTAFKGDSKIVGSINFASTQKGSGFVASLATVQDNLVQEMYFPLTEGQWNIKLEAPHSNLVVDY
AVLFPKEYAQGGALKKKVQNACDVMTDEKFCNLYRYIDVADKKDIIVLEAENPVKMRQQKPPVL
FNDTAVLSKLDFQAMVVLSPSNSRQLNHLFNVAKPGKYVVMLQYYHDSDTAKYLDVYVRSQIAV
QNGRALLQSCRYTFGCRQAVLNRGDNGVFVFNIKSTKNHLLTLLSRYAVVAVDSVMLVPYESWH
LDYSTPTLYCVTKDSKCTNMNYDVPAESRLVQANPLEQDLSEKPRYLIDRSVEVRYVKPGVNIINIR
DVKAGNYLLVAHYYQPNFPSFESRAAVYSGRTSGLRARFDFKFCPNLNGCRSPIRRPDSNSNLYVF
EEGEARIRFDIPNGLDAWIEYILMIPEGTNVSSLYTPRVLDYSDDFKKECSIDLNVCTSSNLSSFCKDS
IFTITTDFNNGSFPCKCNMDGSKSSSCQEFGGKCPCKDNVIGRECSRCKVGYYGFPNCKPCNCSN
CDEETGVCTCPRGVIGENCDQCAPGTFGFDPIIGCQKCNCNEIGTGSANAECNVTSGQCPCKEN
YSSRKCSACKPGYFNYPACTKCDCLDAGISAEKCDVLTGKCICQNNVIGLKCNECADGYFKTNDI
KGKQLCAKCWCNGRSTKSCTEAELFRATPITASKGWTVSVDTLNVIENGNGRFTISDVNTLPEPV
YWVAPNQFLGNRGLSYGSHFKYNFQYSVKQPSQNTNGDVVTDAVIKGDGYTIKSMRSVIEKEND
GYSASILMKETEWMLEDGSTIDRELFLAILSDIDTLQILATHGKNTISSTIYDISLETAGENGTDRVYG
IEKCFCPKEYADLSCSKCSKGYTRQKDGSCGKCQCLGKSNICDPQNGKCINCSGNTMGDRCEKC
KPGFYGDKCKACECPFSNPENSFANTCAINVNTSSLVCQCNAGYKGVRCDQCSDGYYGNPLQG
VTCKKCSCNGNINASFTGNCDNTTGDCLKCLYNSDGNQCQECRDGYYGDAVVEKNCTDCQCSI
CGTTTAPCNKATGQCQCKKNVEGSKCNQCKPNTWNFDACNGCEDCNCAEASETQQCDLKTG
QCKCPPTVFGRRCGFCKLGYYNYSSEGCQKCQCPDGKPCDVNTGKCCTGVLNEDCKLCPVGHII
EDGECVECDRCVQRTLQRVNTLIHNVTILEKHIIPAGGLNYGALETLGNRFSNLSNKGDEHISNL
DTLKKAIGFGEYRNHSGNMDYVRMYLVRDFKSCSFIVNIKTPPTSSAPATTMVTNTPGPDPIRDHL
SNKKISDSQCPPSLARVIDEGEKNIKLVEKKVVVLEKDAYESTENATATKKRAEDLLAKLKDFMVD
FKDVLSFANSTLKGNVVINIDGNRSLAEAEEMLKEIKDRNFNSTINRSEREKSAAINATIIAEQLMK
QASELANKAYKFNDTFKDFVKALRNLKMESELALLDANNATNSINLAKAVDFQSIIDNAQKLHS
QNNVTVTQAMEILQNAKSAYENAKDVFNKFHNQTGPLSDLMEELRNYVRKLEFDLQTTSPIVDD
SVKHAKRLRDSADRLKELLQDTKKFADAALRAAKVYQNIVDAINDALDAARMANETAHEADK

KVQQAQQDAVESSITSKVVDTEANDLTEEVKKTLKVDISDMKMRLKELRELRKRIQGRIDNVKED
VKSFAKEKEVGEKAMNISTQADGVMKVANKGNEASQDTNEKVEKDGEKVETLKVDISDINKAIK
DLASNVTLVEDTIPKIRSNIDEVNVKHEQIEEFIKTNVTNNIGYLKYLISNAHDILDQMQLSMKFNS
TSEVVLDAPKSVADPTINNKISLTFKINQGNGFLFIIGDFSGNSSGFMSLHVQNHTVVFDYKLNTG
DVQSLTNPKFIETDVNYDVMVTRNFATAVLNVQKSGNASTLMTAYQNRSNISPVLSLGQDTNVY
VGYHPSTPNQRPNFDGCINQFRFDEELMGLWHWKSAKNVGGCVQDFRSIEGQYSFDGDGFVEF
DLVSPELSGFRKIELRFATLEENGLIFLGTDPPTSLPSDIMFDIPLSNQIIAFNALEIKDGYLVSTLDLG
NGVQRLVHTNAGKVNDGKPHLFEMKVLISSLLIKFKLDKKKITEIAISPDTDIRLRLKEAFIGGLPQ
RAWLPNSNISTKGFTGAIGDAKYRGGEFKLVKRFNNIRKGFILPKGYSASFPEEKSYLRLDACNLK
QNSLFGFSFQTFEKNGIIFHSEEGSGSSRSSIEIILIDGQVKVDVSVGSQKRTLQTSVARGFSDGLWHT
LSLYFEETLQEADIVLLYLAVDKAYITGTVPKVDLKGSMYFGSKPSGTESGFNGNIRQVYCNKNEIN
LASGDVFEGGVVGSLKLLPPLKLADFTVVPTTLPPLTTSITTSESTTTETVMTTSTTSISGTTLEPPKET
TTNMAKTTENPTVKITSSEPIIETTMESTTVAKTTSAITMEESTTTPTSITTSTLETTISTLEPGSIEGSTT
TAE
>TRINITY_DN319_c2_g1_i3.p1 type:complete len:2588 gc:universal TRINITY_DN319_c2_g1_i3:165-7928(+)
MVLLKGQLWILGFIFLTSTKYVMGDVPECSNNATVAVHCRFDEGNTCVDDPHPMHYVCVCNNL
GYMNDTDSVEGYSICKERDCSNSNETPTDLLKRICGVNTKTCTGKILQRECNCNTDSYRSKNGQY
CFFAPCQASPTIQQNCGPGNQCYGNAPNYICDCSARGYKRSAADNRHCELKSDEELTPIEEYCGK
GSLYANGTINNITCTCDTDKGFFPSATGETCISPSCYLNPQMQEKCGPENTCLGGKDDYVCLCSNP
NYKRNATDLQHCIRRTCADTSPAIDFKSICGEGATGCSGYVDKPLCSCDVSNHYVPAADGRSCIK
ATCKGNKKALEQCGQANSCTGSFDNFLCVCDANGYYLNTTNNTCVERECTDPDLVITDICGVGS
LSCNGKAGSPECICDKDKFYYASEDLRSCVKANCLSSETISKKCGKENSCYGSMESYVCNCNAPG
YLLNTTDNQGCYLGMCEDNGLEYDKVCGEGAISCNGTIKNLECNCGMNYVQSPDKKSCIRADC
RANQTMHEQCGEDNDCVGSLEDWMCICSTYGYKPDPMDMMKCKKVMCEEEVVNNVTVCGL
GTTGCKGHILKPECTCDIAADYVPNEDASRCIKGSCENNKMAMKDCGIANSCTGSYNNYVCVC
NGFGYKKLDDTHCVEQMCSDPDIDIRNVCGEGAHYCNGTVNQRVCSCNETEYFYPSADSKSCV
KATCAVSPMVMEQCGVENSCQGSQESFICNCNALGYLLNTTTNQHCYLGTCENGDFDLMQAC
GDGALYCNGTIKNLECQCDESKGYVQAPDKMSCIPNDCRGNLTMYRQCGQLNKCVGSIDDWR
CICDSPYYVAHSDIMKCKLLHCSDSEVDHVEVCGKGTIGCSGSTLHPTCHCNTTENYFASKDGMR
CVAGSCKTNQTAVKKCGNANSCAGSHSEWTCICDGIGYKHDPADPQNCLLKTCNDADIDIVATC
GVGAISCEGYQLMPKCECNDRDYYIPSDDRTSCELASCRANRTAMRACGEMNDCRGSFSSHICL
CHDPAYKSDPSDSRVCLEKTCEAGDFELVRVCGKGATGCSGSMKNIMCECDNAKGYVASADHT
QCIPSDCNNNVTMHLQCGEGNGCVGSLTDWYCVCSAPGYEPHASDHMKCHLKTCADPGVDH
VQICGMGATSCMGPVINPVCYCNESMYYIPSMDRTSCIKASCMSNKTAIAQCGSENSCSGSPDNF
VCLCQAYGYRQDTNNNQACIPSQCEDAGIDFVEICGQGGVGCSGSVLNPVCMCDHMKHFVPSP
DQNSCVLSTCRGNETAMTSCGEQNKCFGSSSSYICVCDAVGYKPHPTNLQECVLQTCEANELNP
VAVCGKGALSCKGTIQSLMCECDNSKGYVAAPGYKSCIPADCRANMTMHAQCGTGNRCRGSM
EDWSCVCDGIGSKPHPTDHMSCIAIDCSDPNVDIKSVCGYGATGCMGLARDPICHCDTSKYYVQ
SEDGRTCVPATCHTNKTAQMQCGPDNSCIGPPNDFICECTGFGYRHHETDKQKCVPIQCNETAL
FDIERVCGKGATGCRGNIRQPICDCDHAMYFHPSEDRRTCIEASCRRNSSIRADCGVENTCFGSTR
SWICDCNSYGSILNPSDVKKCMLATCEAGQLDPVAVCGKGALSCKGSMRDVQCTCNNELGYVH
SPDGKKCLPSDCSGNVTMHHQCGDANKCMGSLTDWVCVCDAPGYKPHAKDKMKCHQISCAD
NNVDPVDVCGIGATSCKGPITNPLCQCDTDNYYITSSDGASCIPATCKTNKTAIHQCGEANSCEG
PADNFICVCNAYGYKLDDNDHQRCVEISCDDPNAHESICGTGSTGCRGTARDPICECDHTQHFV
ASSDLTSCLLASCRANDTVMNHCGDGNTCFGSLTSHICVCEAFGYKLNPNNHKHCLEETCEDD
MLNPVAVCGKGAVSCSGPLKSVKCECDNNAGFFRSPGGMKCESTSCRQNPASQMMCGSQNQCY
GSYDDWYCDCSAPGYMANQTNAAECLKVMCDDMAVDVCGLGATGCTGHTLNPICTCDSSMG
YMMAKDKHSCVPMDCSMNPASSQCGYGNTCHGSYFSYVCVCSTAGTVMNKTNPMECINVACP
ADAVHVCGEGATNCSVTSTNNVECSCEEGYHPADDLHSCVLDDCRFNPKALKACGLLNKCVGS
YKSWKCNCHYPEFTQDPMDHQTCVVGPCMVEQCGKGNAPGSFQHYAVNCSGLYDDPICNCQP
EYTNDAKGGCIPKICSENPVAVQACGSGYCEGTYFKYTCKCDEPAFVQTPDKMQCVPAKCNIQQ

CGHGFDRGLYIDFAESCSGTYADPMCQCKTGFSGTNTSKCGPAIDGCKLDKCDPLATCQTLDVD
GNVKCTCKEGYSGSGMMGECYLDGGFSAYEGTKECSEVCGVNGVAEVTRFCNNPTADPKGKPC
NGTSPETIQVPCNRFACSSCTVRKTCGCGGLKPIFVEFYHEPNSTLEYFTDLKADDVQTALGFLTK
WKELSQQDLCLMYSKIELTVFVTLMGDSETQVSTMKSSLVSARDIMRKKLDCNINLGKKAQYYEA
YETIIKRHLAISNILKDLTIIKNSITKYMNNCDDSEIGMIRDVIGMM*
>TRINITY_DN6390_c0_g1_i1.p1 type:3prime_partial len:1941 gc:universal TRINITY_DN6390_c0_g1_i1:5820-1(-)
MVIAPKCKFSIFSYLVFGILSYNWNELDASTNIELVDGTGVLLQRDVDNVLNDAVVQKEYQQNIK
HKPVDKRCLGEPFYQGLCKEKLEDAIQQNITEGGCHNLLVKIQKDECSNGPKTLGGKGYCNFICS
QEICNSIYDIDKCVSKRLVHHFNKDKNECEEIMQADCGSESFTSSNQCQKRCIGNSDDKRETKVY
CDQFCLNGGKCIGEVYCQCPPAYHTATCGLSYCNPSCGVGGKCVKTGEGKYGCQCFPGYKGVK
CDKEDSKNMPVVKGLCLSAIKSVLTSPNTSFHDKPEEEKNYCLKYLFDLESNCDKQRPFVNYTEE
QCNHFCASDICDNIYSNDCGSIEERYGYVSSTGRCSKFLYNGCLGNTNNFKDKAACDTKYSPING
LMTSVALLRPLNHTCYIRGKTNFITFDGKVYDLLSGCSHYLVHDSDTTSDFNVILHNDPKVNYNE
KSKRKLEITADGKNVLLNQKVSSDRFFVQVDGRSVPLPYNDVPKIRQAGRYILVLTGDGVSIAFDG
YEDISITVNDYYRTGDGGDHRLNGLCGNLDGNSHNDWLDFTGELRENVRDFAKVYQTDRSTCK
MDNLSLKSLVYNPLHNDQEVFERAERHCDVINTDAFKPCHSVVSPEQYKVLCMQDYIKCNDDK
RDDCVCSSLSFYSRVCHKYGKDIKWRRANLCPITCQRNMKYYECGSACPKTCDNLITPTKCQTDC
VDGCHCPPDLYYDKSTENCVPRNLCSCYRDGIAYKHGSVRSFHCEECTCNGGSWECNKKDCEAT
CDIAGDPHIKTFDGLNYQMYGKCSYVLVDHCHYGNTGIKEFEVIVENKNCNDDALYTSCVRHLS
VNILQTNTRVSLDTAKDNNGTVLPVAFVNGKEGKEFRSKDYTITSVGGRNIIFQYKVNQLKVSWT
GHNAYVTVGHQFENQTCGLCGTYNFNKQDDFHTRSDTVEKSVTAFTQSWVYQSSDKVQDPSKC
HTAKWETSEKPCQIYTNKAQYAKTQCSIIIKPDGPFEDCHSIVPPGEYYSMCQDDACKCDDCYCH
IVAAYAKRCTDLNIRVGNWRDETLSCKQTCQAPKVLKQCNAIDSLDDDLSESLKCPSTCYELSQN
DTSICQSRGCIEGCYCPEGYFENEQNECVKQDQCSCYHDGKVYRHDEIRKDRCNECVCKGGRFE
CTDFDCVAHCASKGLVYSDCGKTCSNFNFNPQDSKCQAGCFCPAGKVLHENGTCVNPQTECQC
KEGDQFFDAGEVSPSDCSKVCNGDRSWKNLPGKDAKPQYDCSAFGQMHYRTLDGRMFKFSGG
LCPYVLLTDCRQDSATGFCDLTKANVNIHVRNLRCVNSYEAYMCKEVTLDLRLPNGEMAEFVLM
QNKIKMKTGLVTKVFDKGSYPQPKTAVVDGVEIFKSGLYLVVRAYHLNITVKYDRQNRVYVVPGR
EYKDAGKLAGLCGNYNGLDDDDFRRPDNSNAENELQFAKSWQDSNSACPDPSESDTCSSNKER
LPWAQKGCNVMKTGNFTACHKTVSPEPFIESCQQETCLCKSGGDCACFCSAVAAYVQECNRHG
VPIYWRRDGLCKLDCCDPCGHSYGYYTDNHVPNTCGVKDKVCKENDRCCKGAVVEGCGCPPG
TYDDGIKCQNETTTNVIPCSPSSSIVPSTSISVPSTIIEPVSSSSPSPMTSSVESQSPSPSSSVVSPTPSSSII
QSMSSSEIESISTTSSSFVSSIESSVQSSTSIVFPVVSSNSGESSVSSSMLPSTSWVGIHSVSSSMGESSVST
NVLPTDYLRTTDVFPLSSSEITYPSSSSSLEPHTPSSSILQESSSSVSKPVGISSVIEPEISSIRYSSMSSQN
VMSSSVSSSVSKPVGISSVIEPEISSIRYPSMSSGSVLESSVSTSFVLPSETVLSTSVFSTNTESPSSSMTLS
TSSSGFVFPSVTSTFQTSSKVLSSLPRSGPAENTVPLSTMFTKTV
>TRINITY_DN477_c0_g1_i9.p1 type:complete len:306 gc:universal TRINITY_DN477_c0_g1_i9:1840-923(-)
MTRQCLGATLNENCGNAALVQFEVCNFQVPCPGLLTNWGPYTQCSATCQKDANSMPTQSRQR
FCTGGSFGGNCGGVQLLQTIGCNQLIICPVYGYWLQWSAFGECSKTCDQGTHVRYRNCTKSIPA
NAGNNCTGPAQEVKPCQVKPCPVTCPLTKKCDCRNMNYLAAAPGISQTVTNDVMGVAAFLDY
WTLDILKQICEGCRMTQLDQSKATVDSQILRTQAVLDRLNIEKRVYRDMITCNENPYITDDLFKVY
NDIWDRIVTTDGVMIELKASKMRFDTAITACRSYDWYHDLFGRFFQIKKSS*
>TRINITY_DN1366_c0_g1_i1.p1 type:3prime_partial len:4702 gc:universal TRINITY_DN1366_c0_g1_i1:236-14338(+)
MMEQDRQRPSRQEYNNRSLSRERIGSRDGSRDRNLSRDRDLSQTRDLSHTRDGSRDRSNLRTRDG
SQDRSFANRSDLYSSPYSSNSQAMPQTSATVEIENLELQATVSSQEELINQLAEDLRNVRKARDFSA
EVEILQKEQQCMDFKEQLEGKEMTIQHLKTELVQSQTVTEEVYKKYESIRSNRDAKDKSIRRYQQE
LHQVHDLLETAQKDKLACVDKMRQIQNEKSALEFDYQWLFDKIKNYEEHGEVFDMSSVSKSTTT
IKRTTTVYEDGQKIRVEDYQSDDSIMPLQNKFQQAQKGYRKAIGMQDDQLMRDKENLMNEFRS
MQSQMHDLKRIHENFMRESKEKDTLIERLKEAKMALDQELDHLTRENENLRQTNDKLENENSD

LRTELNSARNQVSKIEVQYDNCMKQRNYMKEQMEIITQNSAKGRDDYNSIEATMRDSQRKMDS
MQRDLTNKENLNKQLKNKIKILEEDFEQCRQDYNQCMVSRESLQQQKLDVERELKERHDRMDE
VETSYEDIQRRKADLQQELASLKKNLTRFINEEDSSNQMKSHLQNEVLAANKRISRLEAAYEASQ
QEKLELQQELAPATQRLSMMRAAYETLQQKKEQIQTDLIASQKRCAEYQAMNERLKQDRGEILN
EIQACKRRAAQADVDLDEAAKQYAILKGKLEDANRRAQSASEDLVTRQRTIEELRHKLEVSGRET
IQRESYYERIKQERRSLEDELRGCVTDLERVQNEARDVQRDNEKLRQDLGTAQQRLHETEAYWQ
KTKMENAKLQRDYANMERTVQELRDLSHKLQQDKEDCEKILYSTHKKYANLDKENERLGRENT
DLERELQSSKNKQSKLDAVIQDLKRKEAQLKAQLDDALKNRDELNRVKIVMNDLQKRLDGVTK
DGEQKDNLIAQLRNEKDNYKRDCDKFGRDYEGCRTMLETANREKDRLGKELNAANRRIADSD
VLVKRLQDENMRVRKDANSFEARIPILEDALQKSKANESKWALEANTYKTNVIRIEKELLVFKEE
HHSLVSHVSQNEDKNDGLAKKLDDALRKIFELEATNEMVSRENDELKRDIDNKTRQLEEQLTTV
ERANNDLKNQLNELRGRNKKQQDDYNRMQIDYNEFQSKWDGLSSDGQGKDRLIEQLRSELQN
STHEINQQHTDNENKKAYIERLTEEKQDLLNDVNTVNQRIYELEGLYKSSEDDKEGLQKEITELNR
KLNDVESDYDTLQRESDKEIRALKSKLGKVESQVDMILKDRDYIQDQLDAERLLSQKYKDEFERV
QREFLEHQKICERYADQILERDRIIDDLKNQMYKMETEIDTLKEQLRKAQTDQKVAEQQRDKAVE
KLNQAEMKIKDLENENDYLRKRLAELEREISVLEMKLNDAETENKNLRNKIKDLEDLIKTLRAKL
SNTETELQSIRHIISDRDVMIERLQKELDDANDQAHQLRRQVSELESQIELGDQERDDLRSRLANS
ERTIIELRKNLDKVIHDGQDLEAELAALKQKLIKMEPQMKQAQKQRDDYKTAYDNLKGKYQDL
KDELLANQRQLTDAKRAAEQYKKELRHKDHLLEEARGKILALAGQLQSARDELVKKQQENEQL
LDEKRLIQEELLRLRQKLGDLESANQILAMEKERLNEELISFNSKYTELRIKLEKSEQDRLESQRQA
MAYEQRLAKLEPQLQQILRENSELHLQVDRLADELRNALDENRHLKNDVEELQRIRDMQLKELY
EKDTIIEQLKAKLSNLENTVNHLQQENDKLHNLNDALEQEKHSLAHRLDNANSRIKELDDLYEQ
ERQKRDQLQNDLHDLQKKFVTLENAYKKSQERERDLKKKIDDLLGRIKDLEDQLAAEKKKNSDL
EAENDRLKDLIHDLEGVIDDLRRKLTEQENENDRLRAENDDLKQQIMNLQRRIDELEGVIAGLEQ
EKNFLEEELNSLRQTVNKLEAEIDNLTKQRDGLQGQLEDLRREIMRLKEVIMKLEGTNRELQRTID
ALKQEIYRKDRIIETLEAKRKQYELEIDNLKNEIIKRDVIIDSGDESKRDLEIQIKNYEGRIAIMDGRI
DKITLEKTEIERNYHNAVNRAKGLESQVLILNREKAELKGDIDQHFGKIRDDNEVVNKTQGELRE
KVRIIETYKKEIIEKTTIIERLTVIRQTVESERDQLKKDNATLKITVNQYKDNDKGMNDEIQKFTFKIT
TLEKDIINMKNERDEFKVKADNATSKLVEVETQLKRSIERETRLRGELDTLHNKYQNLETIYNELL
REKNDVDRKYKDLLQKHRELDSKYNKLLANFNDLKDQLQQANNKIADLKAKLDKEIYENRDL
RDQLDNLKNQYNKLRDDNQRLEQELADYKYKYEDAMKELAEKNDLIDELKAKIANQDRQINA
LKGTVNQLRADYERSMREQARLQDEIVKLQAVVADLEGKLHRLELENEKLRKENNELQAKIPAL
ENEIENLKDKLENLIKENRDLRNDNMNLKQENVQLEKEVDDLKDDIEKLKDEIRNKDNIIADLR
KVRQELEEEIDQLRHDVKQKEKEIDDLFNRIRDYESTIQILNTKLAEIEPAFEACKDRLRHQENQIQ
ELRNKITDLESALKQSQENEAELGNKLALTESKLNKITQLLDDIKRKNMELENENGHLKDELNKL
KDQNKKLENDNDRLSNELNECKAVIQRIEPMLDHLQKHKNQLEKELEDALNKLANLRNEHEA
HLREMKEKLHVIEVITRQLDEKDDLIEKLKQEKAALEKENESLNDALNKLHQTLDAVQRRKEEV
EKDFFEAKKRIAELENLLNALKEDKDDLERMLRDAKEKNADLDHALRNSQHNEAKLKGELDEA
RGRIQRQRNELAKLQIIVGDLRAANERLLRDNTEKQNVIDGLHSQIRGLESQNGSLKKENQKLRD
ALDKLQRELHNLEQENQQLKAKVFSLQLLNDSLREEKDRLYDNFQELEKDYNDLKDKYNQAVR
ENDRLKDELAFANKRIDELENMNNQLNNEIENLKNIIAGLKRQINDLQGVIDQLRRDKSDLEQTI
NIHSETTINQRPVFDESDYVPRSEYDELKALLEAANSTKEGAERDRDQYHQEHLELHKRISKFEAE
LEGATGKRDEYKRLLQEAERRIRELEGVNGRLEDREKELQKELLNALNEVSGLQQQIISLKVKLGK
FEADRVNYEDKISKLNNEINDLHQRLSQVLASIEEKDHIIEDMRQELADTQRRHAELDSQNQILR
DEIEKLHGIINDNERKIDDLEVKLQDQRNQNSQLKNQINELNARNNKLKDEINDLQRKVSDLER
DLDQAKRDNQEKQNVIDRLNQLKNQLENDLEALRQENAGMKSDLDRLRLDNRQLLQEKHDLE
MKIMGLESDKGTLEDNVERLLNEIENLRNKIQKLKDENDRLIIENKRISHELYKYNKKSGPSDYDS
LKDQRDKLQREIADLTKELRNYKTNITVLQNEKDDLYNRLKELERDLADRDHVIEELEREKDRLE
RELESARNRADKFELSNGQLENEKNRVQEELMDLHRKLTKLEGDYELACDKRDDLQKELDSAIA
RNKTLEDNYKRSQERENKLHQDLLAAQKKITILEHELQSLKDRVNSLERANKVLDDKKNELKDD
LIAAQKRISELEIMLEGADDRNKDLQNELDAMKRKNDELRETLRLTEHGKEDLINEIQNLTAKLS
KVENKLTQAENEKRYLEQQLEDAKQRTQKDRDEIMNLQKTIYELTRKCDSLTKDNANKQALIDD

LRAKKTTLEGEIESLKRDLQNNQYEIERVENDKKNLYDELENMKNKFNVQEQEYLVLQEEKEKLR
QDMIASHQSVCRLKENYENSLGDKGKLREELATFHRKIAELTTAYDSCRSEMNRYKGDCDNYKD
AYDDIKAKYDASQNEISKSRNDYEVCLQKYQKGEQAFTKSEADRERLQKEMLDVHRKSSEIAVLF
QTTKEEKRVLENQLSMLQDDLERYREGYETFNDKTNALQDEILVSHKNYTKIEHAYNRAVEREEK
LEKDCEEAKEIVANMEKDLDNARGKANELENELRKLQDKLRREQDEYEQNSEHAGKLQMTLRD
YESKLSQLRNVEDENKTLRDEINNLKSSINKVEMDYRSNHNDLTQVQDQYESSRNNINKLNNEIN
ELQRDIRQVTSENEKLQNDLQNAAQDKEQEGERLKSTIRMLEREIDSLKTQFGQTTEQLDIVSKDR
NVMHQEIQVILKKCVSIEMTYITKTEDSRFQTSGGDEYEKSINELKNHLDKSEREFDDREKKYKYL
EDQYVREKKDKSQLEKEIDEIQRRYDNMQHEIRVIEEDKKRLSDEYQDMFNKYNDLDRDFKNTQ
TKITTYETEINTYQTTINDMQYKFTNNEDQNKKNSQVYRDLQQKLDNATKENTTLKGELNDEKA
SYRNVKKDFERQVRELQRSSESYGKASHEKDTIIEDLKSKRQALESDIDQLRRDLNHGKDEYKRLQ
REFTQHKSDYQDQQRVITDLQQKVEEHKHSKITIEEEYKITVEKYIKEIRETHIKLSKVESELQTTRE
AQNLDSDERYKRLQNELQQERETTRKDIYGKNQEIEQFKSQISNQDQEIRRLRREIEELDKQLKEA
GSNYSSTILIQKQDAPKYSSTQVTESRISSSSAPRYSTEQEVLIDASDKDTEDEKKRYQSSFYRSTATIK
PGQSDYGSVDVVLQGSQDQGQDDKDYDYNRRATGLDTNAVNRSQ
>TRINITY_DN3078_c0_g1_i1.p1 type:complete len:1109 gc:universal TRINITY_DN3078_c0_g1_i1:3628-302(-)
MKMRIYLFLILFATVYCRVVQKGPNDIPEETKNEIKNQEPKIKFEPEKDKKYFLAKDTTCAHDVDK
HCKNIRRENNFAVFVCLQDVIHKDEDDVAEECHETLWHYKYNLTRDPNFDQAAFKVCENVINEI
QECNDSELLKSPGFVTNCLIENRHAVSEAKCKSFLQKMSAIVFEDYRLIKGFYDSCVEDVKTLKCG
TVAKPTDEEYQHDQTEVISCLEEQLDLVSEKCQAQLVKVATMQADDYHLDRPLFYACQEARERF
CPEVKSGDGRIYKCLVEHKTNEMMLEKCRKKIEIREKLTQKDIKVNELLWKSCKADFTKYNCPKD
GISEHGAASSLLLCLQGKVAEGLPVDQSCQAEMRTFAIELFENYKISPVIVSKCDVEINQFCHHLIG
KTDDGDMMDCLMTQAATNNESVSKKCYDAVSDVIKETGAGNNYKIDRALYLACEPVITTLCQG
KEDAMILGCLMDNAHNSKMPEKCAAQVFHLQYFLSRDFRLDASLYKDCKKDAEEICSANDFNA
ESESKVPDNFVIACLYRNALDRKVDDRKVSQTCAEHVFRVMHQRATNIHLMPELERPCMADLG
KHCIDKGDKEGEEIDCLQTNYEKLSAKCQNAIKVFTIQEAKDFDLDKHLVDKCAPMVQQFCQRE
LEDGNGEKVLPCLVDHKNDIDMDHQCANAVEHWQLIEMKDFEFSPALTEMCLLDIRKHCQKSK
AKIEALHCLSENIVKDASSVTEKCRAQLKNELIEQSENIKLNPELYEACTKDIELFCRDVVPSGGQL
EECLKRNHKKLTDPKCKSLLFKEEKKESFDPELDYRLMHVCKPIIKRYCTQYHQPVDIMECLRQV
THDRDMSRECRAIITERQIEQAESFELDPELSKFCQDDVNRYCKEEIKHAEKGDDDGAIFGCLVNA
MIDNKPIKNDCEMYIRRREAEAAQELDLNPQFLLQCRDEITKICPNMDEDEIIECMKMNIPQIQSK
GCVQEVKKLIVEGIEDVHVDPHLQEQCARDIRTFCQSVPSKSGNVVLCLIDVHKAKNLHLQPNC
ENFLTKRLSLYNAAKVDYAVFDSISAVMNAVSESPNRNYVYMTLLLVLTILFVGGMLFGRYTKKIRS
EIKNR*
>TRINITY_DN4494_c1_g1_i5.p1 type:internal len:439 gc:universal TRINITY_DN4494_c1_g1_i5:1316-3(-)
LTDGSQTQDVGAEDPAKIAAELRADGINLLIIGIGKGVNQTELTNIAGGADNVFSAASFDELIGTD
FVSAIKSGSCEEAKKPPLPKLKRVLDVGFLLDSSDKVKDVYGIEKDFLSTMASIFQIKTNGTRAGVIS
FGKDNEYNIRLNQYFDISKFKQAVDSLQYMGSGRDLKNALQYARTDMFTVGSGARVGMQNVMI
IITGEKEVKDFKSDDVKYHANEIRSKGTAIIVVGIGNIKIEDLLAVAGDKKNIFIAPTPFNLTSYQFLN
AVTVRLNEIDQKPSGPKCEAKVDVGFILDSSGSLRNEYGKEKDFLKTLASTFGVSSNGSRAGVITFS
YYSEHSIKLNDHSDISSFNQAVDNIPLMGSTTRIDKALRLTQKELFSIKNGGRAGVPKLLIMLTDGS
QTQDAGAEDPAKIANELRADGISLIVVGIGKGVNQTEL
>TRINITY_DN1535_c0_g1_i1.p1 type:complete len:779 gc:universal TRINITY_DN1535_c0_g1_i1:86-2422(+)
MMKYFMLIVFTICLKNIHAQDKNLCTSAETCGKCLLKGGDCFWCIEEDFKFNRTRCNTEANLAA
QGCTDYGMGRGKKTNDADLDFTSVRQVKPQKVTLEIRPEEEQSFRIQVKSAKDYPVDLYYLMDL
SKSMENDLDKLQTLGTNIAAEIGNITKNYRLAFGSFVDKTVIPFVENDDANYKPCAECEPTFSFKH
VFDFSNDAKSFEKAVSEQKISGNLDTPEGGFDALMQVAVCEKEIGWNPKDKCRKIVVFVTDASPH
IAGDGRIGGIIRPNDGQCYLTEDGDIKMYTEATKMDYPSIGFLRSKLKDNAVIPLFAVTKNVTEVYK
SIAAELKDLQAVTGILEGDSSNIVEVIRDKVREISRTVTLTDDSSPDITVTYRVQNDPTKPNVCPSPV

APNRCEDVEIEQTVDFDVYVKASAKNCETKEKQDQTFTVNIPAFGKVEVNVKMICDCDCAKTSE
PDSPKCDGQGTFECASCKCNEGRYGDRCICDGNENKDMSLCKPNNVTDKICSGFGNCVCGVCQ
CNIRENPEERIDGNYCQCNNFNCPRHQNIVCGGPERGSCECGTCVCREGYLGENCGKTDCRIGV
KKCLDAKGQNCSLNGICECDRCLCDKGYEGKQCQTCVSCPGKCSEYKTCVLCKAFGETGEQCD
GCELIQHVLVNRTNEECVVPVDENCFVIYRFEIDPVSMNETLYIQRDKRCIIIVKTKPPILAIVLGTIG
GIVLLGLLLLLLWKLLITAYDNREFQKFEKDRMKSKWENDENPIYRPSKQQFQNPAYAGAN*
>TRINITY_DN1043_c0_g1_i2.p1 type:complete len:700 gc:universal
TRINITY_DN1043_c0_g1_i2:2410-311(-)
MKESYLKRFALLFSVLILFQEGGALLRENLIVNHRLRRNVYSEEKVTAVDLPQSREKRFVGTALIIIS
AASAGLDIYKQVRGCKDITPDLDDKIAEGKALQQSLLNLRDQVNLAWVPAESREEYTRRLASNM
IIIREQVSTFDSLQADLVKLLDQKLLDKLNNVTKHLENQIDRNNETEIGSFEDLEGKYMDTTTFALT
LTAKLLPVGFLLGTKFLLPKITARFSKGVAKAFAADNPSRVQKMLSSFAEKQGKLANWKAEKINSF
KVSLNSKWNTFKLKFPRITAAMNRMSNSKIGKALGKAMGKVKGIAVGVGKGAVVAFFIAFEVW
NFVQFHGACTEKRNKAIEGIATMNANKKNMTDLLTEIHDMKAKADEYYNLMKSNVTNDEFLN
FLEDIADLGENAKEQTKVIVDATNQIKQYKAEIKNADDSRTDALNTMLVTAFSNINFKLDCLRSK
LAIIKGVVDDCGIGLGVYDDIYARALAKQKKITETCIEGSYMDKNEIKGLMVAAMDLDKKQSICV
RNNPTKKQLVCEKWYDDVSDADILAANPDLTKDDVTYFISTCPPAPVNDQTKTTICVKKNNELA
TLQSIQQTYQKYDQTAINSAYEECQLSSISVTTICTAKKEGQTVVYAQQKFPVFPASMVTNVYNSC
KKETNITPADKMMICLLKKNGFALDSPVMANYVKNYDYATLEAFYNQCS*
>TRINITY_DN161_c0_g1_i2.p1 type:5prime_partial len:399 gc:universal
TRINITY_DN161_c0_g1_i2:1683-487(-)
WNQEPLTCTYLYSVNSQLLKYKMADDDVAALVVDNGSGMCKAGFAGDDAPRAVFPSIVGRPRH
QGVMVGMGQKDSYVGDEAQSKRGILTLKYPIEHGIVTNWDDMEKIWHHTFYNELRVAPEEHPV
LLTEAPLNPKANREKMTQIMFETFNSPAMYVAIQAVLSLYASGRTTGIVLDSGDGVSHTVPIYEGY
ALPHAIIRLDLAGRDLTDYLMKILTERGYSFTTTAEREIVRDIKEKLCYVALDFEQEMQTAASSSSLE
KSYELPDGQVITIGNERFRCPETLFQPSFIGMESAGVHETTYNSIMKCDVDIRKDLYANTVLSGGTT
MFPGIADRMQKEISALAPPTMKIKIIAPPERKYSVWIGGSILASLSTFQQMWISKQEYDESGPSIVHR
KCF*
>TRINITY_DN691_c0_g1_i1.p1 type:complete len:1726 gc:universal
TRINITY_DN691_c0_g1_i1:5662-485(-)
MMNLFRLIVLGVICFSYEGDAQQCGGSCSPCGNLCVGMKGDRGTAGLPGFRGPPGLPGFPGPEG
PIGQKGEKGPAGLNGMEGSQGARGKVGPPGLPGTPGRHGRPGNEGREGEAGLPGCNGTKGDR
GPEGPRGRDGIEGPQGLQGNVGPRGEPGDSTATKLKGQKGEPGSAGQQGEKGGRGMEGMKGD
TGKPGRNGRAGERGPKGEPGTSNVTIIGEKGSKGSVGPPGLPGRQCQVTGDKASNIAQGQKGNQ
GSKGEEGAKGEKGDAGLAGPQGEEGDIGPQGDRGDDGPQGPPGPEGEQGRSGIQGNKGQKGE
VGNNGKDGNDGQTGLTGQRGRQGTPGQKGEPGKDKKGLRGPEGAKGERGNDGEKGSDGPIGP
IGPRGPKGELGPLGMKGFKGSIGSAGPKGENGNPGDPGIKGRRGELGIKGDSGVKGAPGNPGISV
TGPDGMDGDTGPRGQSGGKGAVGFPGKPGPPGVVTIDGEEVLPGEPGPQGLKGFRGVQGDAGS
KGSRGEKGNACESSKCPAGPKGEKGLSGQDGLQGGDGEEGEKGEKGDKGDIGSRGDTGSRGEP
GPQGESGEKGEKGIQGPQGNSGKDIIVQGERGDKGTKGNAGTPGSIGFPGERGSPGESGDRGLSG
VNGLRGETGDRGARGVAGKPGVNGQKGQKGEIGSGARGFKGDKGEVGPDGPAGLRGPGGPVG
PKGDPFPTAVNGKTGEPGEQGAAGPKGAQGPVGPEGPRGFPGDIGPVGEPGDGEKGERGDDGA
RGERGEKGEPGQDGKDGDPGPKGEPGQEGKEGPPGMFKGNKGEKGATGLPGVDGLPGNSAPK
GEAGKDGQDGDPGPAGEKGDPGEAGSPGTEGPIGEDGPVGDKGEPGESRAGPKGNEGRAGAK
GENGRDGRAGRPGIGRPGDKGELGQKGEAVIGAKGSKGEQGLEGPPGPIGKPGVKGDAGVPGK
DGEAGGRGKDGERGLPGDKGDTGDIGFNGSPGAPGDKGEPGYSGQKGAKGMSGPRGPPGSTSN
VVGPKGNRGLPGMKGEPGQQGEKGLIGPQGSKGESGFGSEKGIKGSRGNPGAVGPSGEKGERGD
RGKDGLQGIAGPRGEEGNPGEKGSRGDQGPQGREGPSGELGPEGQKGDPGFAGEKGEKGDRGG
DGQKGDAGEPLRGEPGVAGPPGPTGPVGPPGKDYVFSGTAGELKGMKITKGDVGPDGVKGEKG
DRGDKGLDGRRGPTGPQGERGPKGEEGNMGPKGSQGRDGLTGEDGPKGSKGEPGLDGDVGEK
GEAGFQGLQGEKGNQGPQGLEGSTGMDGQKGQIGEKGVEGPPGPPCEPVNSVRGETGEEGPKG
DRGPKGQKGKPGPQSQAATPGEKGSKGEVGQPGPQGIKGEPGREGDFGPKGSPGAQGEKGDQG

QKGEPGQEGPEGLKGEPTRAFGPKGQKGETGLEGLRGPVGPKGEDGQTGPQGPDGPKGNEGEK
GDFGINGLNGAAGPSGPKGAIGLKGDSGPPGPTGAQGPRGSGGSPDFGFYYVKHSQTTIVPSCPA
GMKTLWTGYSFLYAQGNERAYGQDLGQPGSCLKRFSTMPYLFCDLQNQCTVASRNDYSFWLSTD
EPANMMKPAAGPEVEPYISRCAVCEAPANILAVHSQSVIVPDCPNGWENIWAGYSFLMYNSAGA
DGSGQLLASPGSCLEEFRANPFIECHGRGTCWYYGPTLSFWLSTIDDSNQFQIPKSETLKAGNLKT
RVSRCSVCMKTVRNGNFP*
>TRINITY_DN477_c0_g1_i8.p1 type:complete len:985 gc:universal TRINITY_DN477_c0_g1_i8:3548-594(-)
MMGSIKVFVGLVLLSFTAIEIAQAQSGTVLSNKQFAFDFVVDGSTSIQDEQWNLLTDFVSRLAVKM
STLNNAKLSALQFSHTLNRILQCNQYANVEEARNTIKNMVQENGATGTLAAMLTSADNLMNVC
SAGDPNRQNVMIVIVDGNQNLEQKSHSEIVQDIKVNVKDKNILVLTLGVGPQVNRNEFGDYASD
SGKGFFVDDFTLLTDELVQRMYDELIRTYGDGPDYLTAWVNGPCSATCNPFPMANRPTLRQTRQ
CQEPNGGNCGGASLEQIINCNENIACAGETYLTAWVDSSACSLTCNPFPVSNQPTKRQTRECRQP
SGSCNGASLTQTINCNENVQCPGETYLTAWIDSSCSATCDTGSNNPVFTQTRQCRQTVGNCDGA
PLQQTRSCNVGVSCTAPTFLTAWSNATPCSATCNPFPMAVRPTLTQVRQCNEPNGGNCGGASLQ
QTVQCNLDVACAGETYLTNWVDLTACSATCDDASNRPVKQQERQCQQPGGNCDNLPLQQTVN
CNIGVSCTVSSSACSSKEISFTFVIDTSASVDNIGFFVLKDFMKKYMSQSTLGTDPRTSVAIVSYGAG
DAKVEVECDTITTQQSFNTTVDNLSQLFGIADDNSFNQALSTAFDASGRCARSSTSLNIIIMLSDGD
PPALENILTRDRIQSSSNYYFMFVAIQASDGAQSTFASLANQGRGLRNASSSFTDLYNFNTDLYYLE
TLSKACDVGGQWTEWSTSACSTTCGPGVVTRTRECRNRITGKLQQDCGPGPAQETQACNLQDC
QGTLTQWTSWSCPATCNINNDVNNPPLALRTRQCLGSTGQGNCNNADKTENEPCVRNVPCPG
AWTTWSVFSACSETCRSQSAFPTRRRTRQCIGGTLNFQCPPQIVNGISYREIWQEDCINVPNCPAK
FTQWTTWSCPVTCNVNNDRNNPPTATRTRTCDDSGTINGCANANILENQPCVQDVQCPGVRW
HTWRKLWWCQFNNISAL*
>TRINITY_DN140702_c0_g1_i1.p1 type:complete len:2036 gc:universal TRINITY_DN140702_c0_g1_i1:94-6201(+)
MATSFKVSEIKCTELLNLEKKGAIDPLVEVEYLGLSKETENKDNDLNPSFGEVFDFDLSGKALGPE
DFLKITVKDYEKFGRNRVVGKADVYLQELLSNPSSTLSATLNDKNDRPTTGKITFKVDYIGPAGGG
AGGATGGGGDDRQAAERELEGDEDEGDPNAMPDGTPIPEGPAGDAMRAEKRKMKKNKKKRR
EHNLPDKPTDFQIRIKIIEGRQLPGGNINPTVRVHLGEETKQTRVKHSSNKPFYDETFFFNFNERPN
ELFDKLVTFEVHNSKNVLSEAMIGAFQFDVGTIYFEDSSHAIIRKWLLLTAPEDNDKKDGEETKGA
SLQPGGPPAGYLKITAIVLGPGDELPEKAKGSGKSEGGEEDIESNLLRPAGVTLRPATFLVKIYHAE
DIPQMDSAAFDGVKKLFSSGSAQKDHVDPYATFSFAGKTQKTEIKYGNNHPDFNQELRVAFKFPS
MCERLKLQMFDWDRIGNDDCIGTSFISLTAISGNGDEGFLPCFGPCYVNFYGSTREFSDLPDEHDN
LNKGRGEGCGYRGRALVEIDTRFGDHTAKPVQDIMAQELVRVQAYMRRRKYKLFASFLSANMIS
EADGLVEFEVSIGNYGNKLDPDTEPQSSSTPPTNPVYDGCAYYFLPWQESKPCCVVESHWEDVIFR
LEPINCLLKIIDKMEKNIKSVELGLKAKAQPAEVAALLIALLDQFISDVSMDLPKFAATDNGVNEL
DLKIQEIRSDELLNLKEEASTLREQATDVQEAMDTIDGYLKRMRDLCVEPQNSMPDVVIWMIAGS
KRVAYHRIPAYDIFYSENPLARGRKCASLQNFILKYPGKKGHDMEDHPEIPASVSVILWLGLEKHQ
AAWTDTEQMEGEYSVYAETYENQGKPPMFDWTAKIIKRPGFSDSEGKIRLNKEYYEPPEGWKWD
DPPDGDWFIDPEMSSNFDADAGHKKFMQDCFENESRVPAGNWGKSTPHYTNVQGDEIDHRDE
TSLPEGWSWTDDWKVDVNRACDEEGWEYCIEATGGGWGAVFRSYHFCRRRRWVRNRQLEADP
QKVMKQKKEEAKQGEGWEYAPVFGMKYHVKEHKFDFVRRRRWHRKMVCVKPGAVPMFKIPD
DDDKGKFHQIMPRIFLAYDEPHKWQFRAYLFQARDILGLDSEGTSDAYAKVCFQDQSQITDVIEET
LCPEFNQTLLFEDMQLYGSPSVFAENPPFVSIELFDKDKVGKDEFLGRVLAQPIVRLNGLKPPATRL
MWYDIHHGERTCGELLAAFELFLDEGADLPLPPPWLEKEKKFQVPSGIRPILVKTGVEILNWGVR
NMKKYQLASCTSPSVTYEIGREIITTDVIKNTKKNPNFLKNIFYKEVYLPKDPLYMPPIVIKVRDHR
TFGRKPVVGRHVIKSIDFVQPPKADPVVELPPADLVGEPEEKKDVTLDIEEKKKKKEIPVDDNLDW
WSKYFASADEIEKCGNYLKKGHKTIQIYDSELEEIKDFNGFSDFIVSYPLERGKEDDDEDDNVVGE
LKGLVKVYPLPQDPKDQTKIRYFTPKLKEKSKIVKCIVRVYVIRARDLQPKDPNGKADPFIVVQLG
KKKISTKDNYIPNEINPFFGSFFEMKADVPLDKDLKIKVMDYDFMSGDDLIGETMIDLENRFLSNC
RATCGLSPAYYTSGAYQWRDAKSPKEILDQVCRTKPQWLSNEQVVVNGQLVKIEDFEESPPTDPHI

GPVEQRLALHVLNNEDIIGHRLVPEHVESRSLKNPLQPSMDQGQLEIFVDIFPIANGVPGPPLDIAT
RKPKEYVLRVIIWNTAEVPPSETSITGEEMSDLYLKGWMEGQDDEKQKTDVHYRSMDGEGMFN
WRFVFPFKYMPEERVMVVEKKEHFWSLDTTKQTLPPRMNLQVWDNDLFSANDFIGNLELDLSK
LPRPKKAAKKATLAMLDTEGDDVMDLFQAKIAKGWWPMLSENEDGTRTMQGKVEMTLEIVTD
LEHEAKPAGQGQEEPNMNPKLDPPNRPETSFLWITSPWKTLKHIIWKNYKWYIIGFFLFIILILFIGL
VLYSVPGSIVHKIFGFQ*
>TRINITY_DN2028_c0_g1_i1.p1 type:complete len:274 gc:universal TRINITY_DN2028_c0_g1_i1:1608-787(-)
MGQSDGDDLTFGQKISREWRDFREFLYNSETGKVMGRSGTSWAKIGGFYLLFYGFLAGFFTALLFI
FYTTIDKPGIGGPKLIQYIENKPGLTRLDRSSKLQSYSASNNDRISSYKTVIDEFLDGYNTSDHSGAL
CGAAGTPKSDKPCIFDTTLLGECAPSKNTTYGLREKKPCIFIKINKVFGWTPADDGTGYLTLSCTG
HATVMPQGFPLTAFPYNGHKVYQSPIVAVKIDATKGKKNVDCTLKGKNIEVSNNFNPSRAFGKIR
IEGISATD*
>TRINITY_DN428_c0_g1_i1.p1 type:complete len:1949 gc:universal TRINITY_DN428_c0_g1_i1:96-5942(+)
MADDADLKFLMVDRRQVSDPVAHAEWAAKKLVWVPSEELGFVAASVKEEKGDKIIAQIEGGKR
VTFHKDDIQRMNPPKFDKAEDMADLTCLNEASVLHNIKDRYFSDLIYTYSGLFCVVVNPYKRLPI
YTDKVINLYKGKKRHEVPPHVFSIADTAFRSMLQDRDNQSILCTGESGAGKTENTKKVIQYLTAIA
GRANQQKADEKSQGKLEVQLLQANPILEAFGNAKTVKNDNSSRFGKFIRINFDNSGFICGANIES
YLLEKARLVRQAPDERIFHIFYQLLLGASPEVKKQFLLLDTKSYNFLSNGHVQLPNMDDRQEFKV
TLEGMRDMGITQEELDPIFKVVSAVMLFGNMEFKQERKSDQATLPDNTIAQKICHLLGIQVTDFT
KSLLKPKVKVGREFTQKSQTKAQCEFAIEAITKALYERMFKWLVGRINKSLNRGKRDGASFIGILDI
AGFEIFQLNSFEQLCINYTNEKLQQLFNHTMFILEQEEYQKEGIDWKFIDFGLDLQPTIDLIDKPMG
VLSLLDEECWFPKATDKSYVEKLHKQQTKHPKYLKPDFRSNSDFCVIHYAGRVDYSAAQWLTKN
MDPLNDNVVSLLAHSKEAFIASIWKDTENVVGMSVTEGMDTTFGASRTRKGMFRTVSQLYKEQL
SGLMTTLHCTKPNFVRCIIPNYEKRAGKIDNHLVLDQLRCNGVLEGIRICRQGFPNRILFQEFRQR
YEILCPGIVPKGFMDGRKAAKQMIDNLEMDPNLYRIGQSKIFFRAGVLAHLEEERDIKLTEIIIQFQ
AFCRGNIARRNYRKRIQQLSAIRVIQRNGRSWLKLRNWQWWRLFTKVKPLLNVSRQEDELRSKE
EEFKKLQDKSDKLESLQKEQQQANEKLTEEKNTLAEQLQAEIELCQEAEEARNRLLQRKTELEDL
IQDFEMKLGEEEEKAVKYAEDKKKLQQGIQDLEESLEEEETQRQKLQLEKVQVEAKLKEMEDERQ
VAEDNNARLTRDKKQLEDRVAEFGQKLAQEEDRAKQLSKAKAKQEAFIAELEDQIAKGDKSTHE
LHKEKRKAEQDLSDLRAQLAQALQRIAELEDDLARREKELAEAIKRGDEEMAKRTTVEREKREL
QALLDELREDMESEKSAKDKVERQKRDLEQDLQELRSELEVTTDATVVQKEFQKKREEELMDLK
KQIEEEAKEHDQQMASLRTKHNGLVEDLNNQVDTFKRGKAALEKNKTALENENAEIAADLQRI
SQLKQEGDRKAKNLEQQLNEANSVRAVQDEKVSSLESEKAKYQKECDSLSTQIEEVETKAASLDR
AKQAAEAQLEDTQDQLAEETRQKLSYQTKLREASDEANRLQEQLEEEEDEKKAVQKVLSNLQV
QLDQAKKEIELKIQDFEQAEAARQKQKRENEELRGEVEKLTGDVSRLDKAKKKLQGDLDDVTVS
MERERNSANQMSSKQRKFDQLLAEEKANSQALGIERDNLEKLARQNETKALSLQNANEELEDR
LAEAERQRKQLQMELGDLVESKDDVGKSVHDLEKSKRLLEQQLAEQKTQVEEIEDELQACEDAK
LRLEVNMQAQKAQFDRELQAKEDAIEEGRRGLVKQLRELEAELEDERKLRQSASAAKRQLEGDV
KELEGQLDQANRIKEDGLKQLKKYQNQLKDVQRDLEDSRQSKDDLAAQVKDNEKKLKGLEAEF
LQMQEDLSAAERARRAIESERDELLEELNNNSALKNAVAEERKRYEAQLAALEDELEEERTQTEL
MQDKVKRSNLQLEQMNAELQEERSAAQSLDNTRMSLERQNKELQSKLADLESSLRSRQKNTVAT
LEAKINNLEIQLDQEAKERQQQNKALRRNEKRCKDLQMQIEEERSHTESYKQQLEKMNARVKN
MKRQLDESEEEVTRLNTAKRKLQRDFDENQESLESSQREANQLRQRLKGGASLSSSRSSGRGRRA
KDEDDDDDDANE*
>TRINITY_DN72625_c0_g1_i1.p1 type:complete len:1964 gc:universal TRINITY_DN72625_c0_g1_i1:173-6064(+)
MKLAFNIFTISLLIGNTIAQNNDVVQVCKSQADVVAVLDSSGSVGVQDYEELKKFVGFVANLLTIN
ENGNRLGVVTYSETAEVDFNLNEIYDSDQYGREALTRLSVRPREFKTNHYAGLKVGSDLLFNKAA
GARDNAAKFLIFTTDGRNNFDETNNRNRASQLATQLKADGVNIYVIKAEVMSDNNGRLDSRLYS
TDPNKFYRFINRFGEITNENFLDEVGLEFCELVQLPDNQQATVREDRNIGTLVTTIRAPGLNNPTFE

LIPQDNLPFIVDPLTGEIKTIGLLDFEQVQQYVFQVRVRATNPDVEKIITVTINVLDVNDNDPIPETA
NYRMSVAETTPLGQRILTVKANDPDGGTNGNVEYFLISGGDGKFEVDQATGEIRLIGQLNKRIKD
TYLLTLEARDMGIPTRKAPITVAIDVTSLNNFPPQFNEMSFVRTIPENFGAGTVINAAATDRDGDT
MVYSLIGENIPFSIDQNGLITVSGNIDFETIPNNVFQFQVQAEANGDLVTQDVIITITDINDNVPVYS
MTTYSGSINENSAAGTVVNFDNVIIATDADKTNANNMITYNIIDGNDGDRFQVTKNERGEAVISL
KSTPLDQETQGQYRLTLQAIDNGVDPRSLEMNSLLIIDVIDQNDNDPIPEESVYSIAVAETSPVGQSI
LTVRANDADSGMNGKVEYFLISGGDGKFQVDQATGQITLIGMLNKRVKESYTLNIEARDMGVPT
RNAPITVNIDVTSLNSFLPEFAGAGDAFERTIPENFGSDTVIKAAATDGDGDMVFYSLVGDNLPFA
IDQNGMITISGSIDFETIPNNPIRFQVKAEANGDMTLKDVVITITDVNDVTPEFTQQVYAGKIEENS
QTETVVRFDQPVIATDGDKSPANNVVTYRIVDGNVDNIFKIVKNAQGEAVISVNNAGLDREILNQ
YTLTIEAKDNGVDPGSLSSTTQVIIDIEDVNDIPPLINTSENKAVGEADFTATISEDSPIGTSVVKVEA
TDPDSNNDDLIFTIANDVPVPFTIDPKTGLITVSGPLDADDQDEFEIAVTVSDGVNNSTAIVKVNIA
DLNDNKPIFTTDTRDITIPLDTPNGQEIIKFTGSDADKTSPNNQFEFSLVDGDGKPFTITPDGSLIIN
GPLMPGDYMLTVKIMDKGTNPGPLSNVIIYNVKVVDTNDPPTFQNNEQTVTISENTADGTDVITF
AATDPNGDAITYEIVNPGSVPFEIDPITGVIRKMGLIDFESPVRSYTLDVKATDAKGKSTMGRVFVQ
ITDFNDNTPTFGPGNTDNFILTEGVYSNQLVSSFTATDLDATSPNNVFEYTLVGAPSMFLLTTNPNT
GAADLTVNGDIDAEQNMSFNFIIRATDKGNPALSMDYPVTITVGDINDNAPVFAANTPESIDLME
GDFNNQIVATFTANDDDTTAPNNLVDFELVGAPDFFKLIVNPETGTADLVIDGNVDIDGLDPTINF
IVRAKDGGNPALVKEFPLTVNIADINDNAPVFTLEAYNQVINENTPIGTKVVEVRATDSDSGPNG
DITYRIVDGDGTFGIDPKTGDITVLKNTDFESGVRSYEFTVEASDNGNPKQSATAKVKIDIRNLND
NDPIFTSIPITGNINENRPAGSVVQNIQPVNGILNQNNVKILREPPPGAQETEGVGFDAPFEIDPVT
GEVKSTRPFDFEEEKVFTMMVQAADGDNNPMLTTMTVMVNNLNDNDPIMKKANFEITLNQDT
PVGTFVLQVQATDIDQENQTKITYSIINIDGGNMNDFTINPDTGVITLNTDLSMNTVDIYNITIQAE
DQEIQGVIRRSTALAEIKILRPAEAEPDISTLCTNSCDIPEERLKCLDYSKFEITRKFNGTCYVDKLKA
AIADVKNNTMEIMNLNASFLECRNKVQNEITNPNTQFPVNGLFDLHRKCTVAIECKLEQLAFNL
MRKTTELTNNLKAEEEGFSMYDMSLSAFFQHISKMVSMSKSSRVD*
>TRINITY_DN12627_c0_g1_i1.p1 type:5prime_partial len:1061 gc:universal TRINITY_DN12627_c0_g1_i1:3456-274(-)
YGENNRLKFNDARQYCQQWKDDQGDLVAIHSNYENSFIMSQLYDVNEPMYIGLTDSFSERTFLW
VDQSSVVYTNWYARQPSFVGNEDCVEMMPYFKTHGRWNDIGCSAERGFICQRTPSKTPLPTPEP
VKDSKPCPAGYRKFMKACYRVVHTPMTYDDAVKDCRTSVGGELGDLVSLHNQFEQSFVFSQFAK
FDFHSWIGLKKIKGEKEFKWSDETPYDYRYWLSLRYDNTVYNDDKCTTQEFSSKVDGKWKVTDC
TQKHPYMCKIERDFDQPHLTFIGNCTDGWTKFYDKCYKLQNYFSNWPAAREKCKGFGGNLVTIT
NSDIQKQMEYLARDAYRSVWIGLNDRAKEYVFEWSSGIPLLPDSYQNWGPNEPNDNSGTENCV
HMTTESSGGKWNDLSCSQRMPYICEHPLECNSALGMEDGSIPDTNINATSYVGTYFPKYARLNAK
SDNNRNTAWCTNITTGAESLTINLGEVMQITRISTQGLLDSSLGFSRGGYVVSYMLEYSNDGTTW
MTYISEDAYDQKPIALPGNIDASKIDGTTINLKAAYLRFIPKLYKNIICMRVELYGCKSVCSRALGM
TNFAISDKQLSVSSSQAGSEKAQLRPNIDGSWCAKDDDYSPFVRIDLPRLTKITRVTAISSSKVQYKI
AYSDDGELFTRFQTLTNEERVFSTVPSTHDLETAISAKHVKFDLLKKDQGKLCLKVELYGCLKDCD
AVLGMETKIIPDRLITASSAKPSNPARNARLSVGVYGDNSWCSSETDNAQYLQLDFVQEAHLTEIA
TQGFNGDSAKYVSKYNVQFSDDGDFWHQYSEELPGNSNGYSIQRNKLKEPIIARYIRINPTEWKN
GICMRVEFYGCPMKPFTAPKILTQKTYNGAIRILNEDFNENLLNANSIEYIELANKVETDITSLYLLE
ESFKDGFNSVTVNKFREGSVITEFTIKTHTSDASLDAAEPLRKAVQTGRVGTFDVDPNFFEIVTAST
DKSTATNDDSGGLSNGGKIALIVFFVILICIVLAGGVVYYLRSRRNGFRLQFQHRAFENPTHVDDL
QNDDMKVEFMNDDY*
>TRINITY_DN598_c1_g1_i1.p1 type:5prime_partial len:745 gc:universal TRINITY_DN598_c1_g1_i1:2-2236(+)
RWSGCSKSCGVGVKTKSRECDNPRPSGGGRYCTGERTATEACKIQDCGVDGKFSHWSTWTACSK
TCGGGAQKRARQCNSPPPSGDGKDCAGAREEFKGCSNDFCKGPTGVAKAQEIIERFLENFHKKT
YPDREPSGKFLKAIFAIQKAVAEEFISVDPYVRSKTLEQRLKDLENFLKVATEGGSIDFARTLLGKGV
FTFMKQKIKDGLPIDDIDKAAEGFLEKLRGDLGDKKMDEILSSQGDATLMFTIDTTGSMKQEINA
AKAIAKQIISATRDYEVDYILSPFSDPGTGPVTHRSASERASFVSDIDRLRARGGGDCPELSFGGML

NALNNDPQYGSPMFVFTDATAKDDTDANVASLKAAADATDATINFFTNLAGCNGRRDRKRGIE
SYKEIASYTGGQVFPLKNEAELIKFTSYVKTSLQQDTVITSGGTLNSKSKRSLYSMQARHFMVDDGI
SKLVISVGVQQLASQVALVDPNGKKVYPSTTLALSLIYQVSHPSPGQWKLDFPTSVGKYSYNVQAV
SSSAIAFAHNFIYQQSVRKNSPPIPLANPLKGVKNMMVVRLGGTKNFDLNSIKIDLVDTSGNVLQ
KGIGTDRLDSDEVRHATIDPPSGPFKVRLTGKTKSGYNFERVSQGISRPVSAAMRTNFAGYDFTAK
LGKGPYAAQMLFINRGAHENFYFYAKANYGSIPNYKQTKRVTSQGFVTFTYKAPRQYNLKGKSD
RITVTVKRQSTGEIVSHHTSILLVV*
>TRINITY_DN761_c0_g1_i2.p1 type:complete len:830 gc:universal TRINITY_DN761_c0_g1_i2:87-2576(+)
MTSMFRSEEMTLCQLFLQSESAYACVSELGEIGVVEFRDLNPDVNAFQRKFVNEVRRCDEMERKL
RFMQKEIDKADIAIVDTGENPDAPHPREMIDLEAQFEQLENEMKDSNSNYEALQKSLLELLELRH
ILKKTQSFFEEEGYAETQARLQADHEPGVTGGGQLGFVAGVINRERIPTFERLLWRACRGNVFFK
QAEIDTALYDPGTGDLTHKCVFIVFFQGEQLKIRVKKICEGFRATLYPCPETPNERREMAIGVKTRIE
DLETVISQTQDHRHRLLQTIAKNIRVWLVKVKKIKAIYHTMNMFNLDVTQKCLIAECWAPVADL
DSIQAGLRRGTERSGAQVSSIVNRMSTKQDPPTFNRTNKFTKGFQAIIDAYGVASYQEVNPALYTVI
TFPFLFSVMFGDCGHGAIMALFALWLIMNERKIANWKAGGEMFDTVFQGRYIVFLMGLFSIYSGL
LYNDIFSKSLNIFGSAWVPTLNRTKYDYQPENYKEWSDGDRMLNPLDAPRETPYYFGIDPIWQLTS
NKLTFTNSLKMKLSVIFGVTHMIFGVILSLFNHLHFKQYVNIFCEFIPQLLFISCIFGYLVILIFYKWIA
FSIHSPSQPSILLGLLAMFLNFAKKKTDADRLYNGQDFLEPFLVVVAVICVPWMLLIKPFYLRWQH
NKKKQHGTNFARLHEDTAPLQDNEVNHHDDSGISSKNEEEEEFDFGEVFVHQCIHTIEYCLGCIS
NTASYLRLWALSLAHAQLSEVIWTMVFKIGLSMDGYVGSVAIFAVFAAWAILSVAILLIMEGLSAFL
HTLRLHWVEFNSKFYVGAGSKFVPFSFKDIMNDEAF*
>TRINITY_DN1936_c0_g2_i1.p1 type:3prime_partial len:1894 gc:universal TRINITY_DN1936_c0_g2_i1:5680-2(-)
MLSPAVTKVFIVLSVLGGLICQSCDFEVANACQWTQYAKDKFDWTRNFGNTGTGGTGPKTDHTS
EVKILKANTTGGNSNGAPCVFPFKFRKRTYTKCVRSMQRGKSWCATVPDMNKKRQWGYCMPT
GYYMYIEASSKRGNPRNKGDNAVLLSPVISKGPKCLQFWYHMYGPHIDTLNVYKKVSNNMTKL
WSLTGTQGNRWISANVTFNGGSQFQMAIEAIRGVEYKGDIAIDDITVDDGSCNADNGQKGNVTS
YGKCTFEKDKCGFKDGNTSTLTWTRHKGKTPSKGTGPLVDHTFGTGLAYYMYVEASNNPMQSA
RLYTTYTNLDSTGSCLSFWYHMFGPRTGELKVYINPPEIGKEIPSFSVGGEQGNLWIRGEVTVNTTT
ALVVFEGTTKYSYLGDLAIDDVSMERGKCEDPDSCSFQKDLCAWYNLPKSSGLDDFDWIVGAGV
TPSRFTGPTSDHNGDIAGKYLFMESSSPVKMGDKTWLSSELFNSTGKGKCFVFFYHMYGRDVGSL
NIYVNTSSGVELVWTQSRSKGKQWQNGQVDVERPGTYKVLVEGVVGKGYLGDIAIDDFKFKPGK
CQIVPSNASPKKPQTTMRPTTKRPTQPPTPILDCDFEFGVCSDWRQDYKNDTDNWKVGKGTTST
AGTGPSFDHTKGTIYGKYIYLEASYGRLNETARLISTSIPATGDTGGKCLTFWYHMYGPHINRLNIY
VMIGGHTGKPIWSRVGTQGNKWNKGQVTLVGRQPFQAVIEAVKGAKYQGDIAVDDVSLSSKKC
PQPGATTCTFENSQCGYIDDTSSDVQFQWTWQSQATSSLNTGPKFDHTLQSVSGFYMYTEASNA
KQGDKARLISPVNPGTSGSCLKFWYHMYGAGMGTLSVRLLVNNKLQSVPLWSETGNKGNEWK
VATTTIKTKDAYQVVFQGVIGTTYASDMAIDDVKLSDGECQSPGFCNFEDGLCTYANSKTDDFDW
LVGSSNTPSSFTGPKVDHTFGNASGSYLFLEVSKPIKAGDVAVLESQQFNAVGGKTRCLNFWYHM
YGSDIGTLQVIYKVFSGSQQERVLWKLTGQQQITEKDPWKYARVPVDMDADHMVMFKGIAGSG
YAGDIAIDDIQFTTLACRVEPSIANPTPTTPKPPTTFPKTAPPTAAPTPFSCNFEKDFCSLTSDSTAVF
NWTRSQGYTASQNTGPSTDHTYGRGFVKTYGPGSIRHAAGMCIHISMGGHTKPKDETALVLYNG
CGQDRTEFKYDNGLIKSTQYNMCFKLKGASTSAGTKVVISDTCTNKWKMLANGAFQDAKTNLC
LAPSGKLAYPQNNSPLIVSKDCKAKGVIFTFSPTMGFYALIEASAPQKPNDTARLISPVLNKPKCLS
FWYHMYGANINQLNVYTKSPNGVYGNPVWSKFGSHGDKWRPAHVYMNPKSSSYAYAIEGVRG
NGYEGDIAIDDVSVSDKCPAPVECTFEETNPVACGWTNLKTDDFDWSIGQGATSSQNTGPSNDH
TYNDQRGKYLYIETSSNLKAGAKALIQSPVYRGNGRQQGQCFQFWYHMYGASIGRLSLHLDQVG
STTKNRAWIQESDQGNRWSIGQITIKSQAAFQLTFEGIRGKSFTGDIALDDFRMFDGPCNNIATCD
FENEKCTWKNSYHKDDFDWIIGQGTTLSLNTGPVTDHSTNSGDGHYIFIDASRKKQGAKARLYSE
IIPRGFFTFIPYCIYFWYHMSGPDIGTLNLIAKHGKGKALEKVIWSLSGNQGSKWLQGTAPVTSSVD
FHLVFEAIVGDGYKGDITLDDIVYKLGLCSISPKAADPKLISTTSAPTPIPTTAVPAMYDCNFEKNSL

CQWKQDLTDQFNWTLVQGQTPTLGSGPSVDHTTGNKTGYYLFIETSNPRKAGDKARILSPSMDS
DKDHGHPHCLKFWFHMYGPHV
>TRINITY_DN624_c0_g1_i1.p1 type:complete len:884 gc:universal TRINITY_DN624_c0_g1_i1:218-2869(+)
MANQEEEEHFGYYYGEEEDRGLLDPAWEKQQKKTFTAWCNSHLRKVNVQIEELNVDFCDGLKL
MLLLEVISGERLPKPERGRLRFHLISNVNKALDFIASKGVKLVGIGAEEIVDGNLKMTLGMIWTIIL
RFAIQDITVDEFSAKEGLLLWCQRKTAPYKNVNVQNFHMSFKDGLAFCALIHRHRPDLIDYDKL
AKDDPMYNLNYAFDVAEKELDIPKMLDAEDMVNAVKPDERAVMTYVSCYYHAFSQSQQAETA
AKRILKALNVNQGNEKAIEDYENLASDLLDWLNNKIPTLDDRTTDSSLDTMQNKLNEFRDYKGT
EKPPKVDDKSKLENLFNTLQTKLRLSNRPLYMPAEGKMINDIDKRWKDLEQSEKDFQEWILNELR
KLERLDHLAMKFKHKCDIHEAWSTGQDDYLRSDDITDASLASLLASRKRHEAFESDLAAQQDRV
EQIVAIAQELNTLGYSKIDEINTRCKGICDGWDTLGELTTQRSEKLAKAIEIAERLDNLWLEFAKKA
SPFNNWMDGAKEDLMDMFNVHTIEEVNDLKDAQLKFKANMPAAQEEFNTIMGLQNAISSMSS
QPNPYTNTKAEDLQEKWQEVLELIPKRDSLLESETQKQQNNERLRVQFAQMANQVGAWIKQRM
GRMTDIGMNAQGTLERQLEELENLNAEINGFKQNVLDLDASARNVQEAMIFENPHTPYTMEVI
RIQFEQLLTGVTRNINEVENQILMRDSIGITEEQVKELRTSFNHFDKNSNGRLDYKEFKQCLVSLG
QLGDVEDKGEHTFNRIIAMVDPNETGYVSFQAFLDYMTREMADTDTADQVMESFRILAGDKPYI
TADELRRELPSEQAEYCIARMSAYNGPDGVPGALDYRSFSTALYGESDL*
>TRINITY_DN1058_c3_g1_i1.p1 type:internal len:485 gc:universal TRINITY_DN1058_c3_g1_i1:1452-1(-)
IGSLTDWRCVCNAPGYYADANNSRRCRKEMCSENDIDAEKICGTGAISCVGPVVSPICFCDVKKN
VVSSTDGSRCIPASCKTNKTAITQCGNGNSCTGSSDNYICVCNSYGYKLDDNNNQACVEKTCSDS
DADFIEICGQGGLSCTGTMKKPVCECDHTKNFVPSDDRRSCVLASCRANPVVDAACGNMNTCY
GSESSWICLCNAPGSMLNPNNVRECLTGTCENGDIDVQTVCGRGALSCKGSVASLTCECNNHKG
YVQSPDRRSCLPSGCRQNTTMQEQCGEVNECIGSLTDWSCICSAPGYRPHDSDQMKCHRRMCY
DANVNPVEVCGVGAIGCNGSVVTPQCTCNTSAYYVPSLDGSMCIPAPCHSNKDALKKCGVGNS
CFGTTNNYVCVCDSFGYRLDDTDPHKCVEIQCDDDGVDIEKICGQGSTICDGTARKPFCQCDHA
SHFVPSLDSSSCLLASCRANGTVTDMCKDGNTCFGSM
>TRINITY_DN1369_c0_g1_i1.p1 type:3prime_partial len:1577 gc:universal TRINITY_DN1369_c0_g1_i1:93-4820(+)
MRIQLTIFVTFSIILVSGQNSPPGICSTWDGSHYQTFDSYSFNYHGTCSYALALDCRPGSDDFAVHV
ENDEKCDNHGGTDCTRAVSLYVNDIEYKLGSNNTVLVDGKSNIVLPYFNEGVRVSKHAEYYFIDA
LEGKVVVKYDGRSSIYVQLDSSYQAGVCGLCGNFNGNKKDDLKLPGDKGLTKSLKEFGKSWEKP
RIGQVCKEVTEEHFDQCTNKSKMLVYYAEKECEILRTSSEFKKCHKIVDPTPFYEKCREDLCLCGS
GDKYCKCDVLEQYTRKCAHYGTVLEWRQQNLCYKNCTGNKVFSECGPACSSVCSAIGSKSVCK
DACIDGCHCPPHTSLSGDKCVPDDECPCEHNGDMYDHGDVINMKCKSCKCNGGKWKCNPLP
CDGEASVYGNGHFVSYDGKTYNYPGKCLYLYTYLSDRSFAVYLDRTSCDSLACHMNVMILFRGD
RIKLSASNSTLALEVNRNVKSFPYISSTGYRMYRFSTVFVRFEATNGIKVDWDTYSRLHVLMPHSL
KGKVFGLSGNFDFKTKNDFLTPRKDLAHSAIEFGNSWMMYSSRLNCSALAPDYRMTSCEANHQ
VAKYAESKCSILMSSLFRECHKVVDPFIYYENCKDDTCECNGDSKCYCSVIAAYSKACSSAGKPTE
GWRKYTPCEEKCSNGMVFTECGNSCLKTCDDQTDPNPFCEKECVEGCLCPVGQVLDKITEQCV
DIKHCGCTYGNRTISNGGTVAKDCNTCKCTKGVMVCTKKECRDKKAICSDGKEWSSCMSPCPK
TCQTKDDICPETGLDCIEGCQCKKGYIESANGTCVELSKCPCYHNGKEYQETQTISKDCNTCKCT
AGEWSCTEKLCDATCSVFGDPHYKTFDGKIFDFQGKCGHVMLTDKCANNDNATENIRISVKSST
CGSQGVTCTREITLNYHDTVVHMKRGARKIDIVSNKRQDHEKDQVKVYRHAGIYVHVLTPMGV
SILWDNATRLYITAPPSLSGKICGLCGNFDGSETNDFVTYQGDTAGNAVSFGDSWRTENNCPLTKE
IEDTCHNRKHRLDPSKRECAVIKSDVFKKCHTLVDPVPYYDRCVFDACGCDSIGDCDCLCAAIGA
YAKECIDEGVYINWRKGHNICEKEMCCPTNMTFVPKGTGCPKTCKYPNGLHHCPTKYVDGCQC
PEGLVQNNDANGDVTCVRPEECKVCKLDGKIYNEGEVVLYKCKECVCRDGKMTCSGNYCPTTL
PPTTTTESTTTPSTTPETTIPSTTTTESTTTPTTEPPTTTTTASTTTASTTTESTTTEATTTTKPTTTTTEST
TTESTTTQSTTTPSTTPSTTMKPIDTCVYSNGTHNVTLERGDVIQVNECTKKTCVKDEVTGLHEIKV
TTTQCENSTCDTTCEASKSNRAILVKTPGECCPQCKKPEDLCDNPNEVPCNSTYCIPKSWFCDGN

KDCSNGRDEECCPNYCKVEGEIKKPGEVWTNKEKCLTCKCEGYYTKCRRMECPELDCPEERQVK
SEGECCPHCKPQTEPPTTTTEMTTTTTESTTTPATTPSTTSKETIIITEEPKSTTASPQEKCDCKVLQPV
GVSDGRIPDNQMTANSV
>TRINITY_DN11248_c0_g1_i1.p1 type:3prime_partial len:1700 gc:universal
TRINITY_DN11248_c0_g1_i1:111-5207(+)
MEIRSLFVAFIYIVHSINAQCSSPWKSYKDSCYLFTAVSTVSPGHTWEDSRFICQRYGGDLLKLDND
NETKYINSQFDDLKIKYNHYWIGLNDIKKEGDWRWADQAPLNVTTQWRVSRGEPNNWQGNED
CIETTYDGQWNDNECSRMFGYICERSKDAVIPVTPTLPPFPTATDKPTTCEAGWIPWGTSCYGIIRG
LAKNFQDAQADCLRRFGDLVVINDQAENDFISNEISRSVGGLWIGLSDTYKEGTFYWVDKSELNF
TSWRAGEPSNDYGNEDCVEIYGRWGPGQWNDNHCTALRDYICEKPGGVHNCPRDWVSYKNY
CYQINAHPGQKVTWEDARKNCLDFNKAWRKSVNVAQADLVSITNLEEQKMLQKEFSKVNIKN
DNGFYYWIGLTDKNSTGGFYWTDGSTYKYSNWIGSPQFASQCVRSNVIGYQGGSWSKGDCSEAY
NYVCKVGRGQDRCYNPLGIQSFAVPNKAMTSNSVLGPSTPALAGRLYMKAGNSLDGAWCAGN
NTKTPYLQIDFGKMQKLTYMGHQGKSNSDAMVTSYYIKYSQDGIVWDTYGYIPPAQYYKFKIYIG
WRTNYEKVMNLPLPAPFIARYVRVYPLEWNIAPCMRIDFYGCQEEVDNQRCGLGWREDKKTKN
CYKFHNQITTWDSAQELCSIQGANLATIATPDEREFLSEQLGEFKSLVSEFWIGLNDLDYEGVFKW
VDGTPYQLSNWYYGNPKVRNEKSNCVVVRTTDSAWVNTECTVFRKFICKRNYISPYTPKESYNG
YVYSKTDYFWPLDSATDFEGSLPLTLNGNVDRVPFPIDYDVGVSRYALNVSGSSGFFKIDVANDVC
IHHPDADSCPHGITVSFVFKMSKKSSSKRQVIIDTTEKNKPLGYKVYTEFGSIYVNLRGHQKSYSGS
TVYNAHIFNHMTFTWSSLKGLSLYMNGKSRVFDLLGSYAKDYPKVDYGKSLLIGISGDSQDPAEL
MIRSLGIFKQVLDAKSIEAIYLGEFGTCKSHQFAHGSYCYEYNSTEVSWSNALSTCRDNGGDLISIH
SPVEQAFFNLKIAQYNYQRTNWIGLNAKSKNSFFKWADSSRLEYSYWAPLEPNNNNGRREDCVE
TFANGGWNDALCYLRNPFVCKYSRALHIMGSENTDDKEYKVFFSPYQTLTFGCPLGRSIEILTASY
GSVKDGCVKDVKDKVSNACVKEHCVRVQASDFFFNKINPCKDKNRFALKRLKVTYKCNLATPE
FDSTGCSNGKDSENSYRVFDKSNICYKLYRKPKTFKEAQKVCKDDNGDLVRINSRETQVYLTSLLH
TMYQKTDIWIGMMYNSTTQNYEWLDGSHVYYTNWVNRQPDPRNGDYVKVGLEEGAKNFLFW
KNGEANETLPFFCQRVKVGKTTQAPPTTQPRTGTCPKGWFTYNKNCYYVNTNRLSFPDAQAEC
QKYGKSSHLASVLSYEENRAVFYDVVNATVDFAWFGLTNEAIVTGFRWVDDQPVSFVNWYTNEP
HKINRHLCVGMGSGGKWIDLACYLKYPSICKLPLGEVIPTTTPLPTIPPTQASGLCEPDWIHYNGS
CYNFANRTSYADFHNARVTCNKMGAHVATIHYQKENDFISAQLKRMYIPNAYIGLTDFSIEGQW
QWEDESVVSYINFGEGEPKNWHGMEDCVVVKSIGEWNDIPCNLKRSFVCKKNNGTVVKTVNTT
AAPDSGYCPSGWKLYGNKCYYFRYGE
>TRINITY_DN10485_c0_g1_i1.p1 type:5prime_partial len:604 gc:universal
TRINITY_DN10485_c0_g1_i1:2-1813(+)
NYSRLKSYFVAMKRTTKRIALAAAAVFLLVVIVVVVVVAVVVTRDDDEDEILTAKHEPVEAREYSK
ASVAADRLECSNIGRDMLKRNGSAIDAAVASLFCIGLLNMHSCGIGGGGFINFYKRSEKKAYIYDF
RETAPLSASENMYVNKSQERLSTVGGMSIAVPGNVKGLHYVWQKHGKLPWGDLLDPTIKIATDG
FKISKAVGDKIERESTNKLYLNEGFKEFLQKDDKTWYKTGEMLKATLLADTLKRIKANPHDFYNG
TMADEIVLDINEAGGSFSKKDLEDYKVTEREPVTTNVKGYTIYSMPPPGSGAVIEMTMNILEGFN
MSEANIKDNAELIKTTHKIIEALKFAFARRPLLGDPSFVADVSKVLTAMLNKTFAEELRHKIKDTPY
PNQTFYGGFYSQHQDPYGTSHLSILSEDGDAVGATDTINYGFGSGVRSTRNGIIYNNEMDDFSTPG
LQNLWGYLPAKSNFIKPGKRPLSSMSPTIVTNSDGDVVMVVGASGGSRIITATALTLIRTLYLNEEL
GRAVSEPRVHTHLLPYEVYINDPYTVSEELQDGLKDLGHKLVMSKYWAAIQAIYRKDISSPILAKC
DPRKGGLPAGY*
>TRINITY_DN1058_c0_g1_i1.p1 type:complete len:2639 gc:universal
TRINITY_DN1058_c0_g1_i1:134-8050(+)
MVKYIIWILLLLSIFKEAYGGDWAQWQNWSACQDSSGAPIGCGRGNQHRSRTCADNNCGAGAIIE
VRPCLDEICPTFNCQNSSEIAGMCKFDYSNSCVGDDNCHHCICKSEGFKVSGDQCYNYCVEVGC
DEEIEGVSQLKRYCGDQAISCTGTVLNRTCLCNPATSYPSKDGQYCYEATCKTSQEFMTNCGAEN
SCQGTNTSFVCECNGFGYGYEVVANVQNRKKCKLLSCEDGDLNPLEICGQGATACTGNVHNLT
CACNVTAGYIPSEDKKSCIPMPCNTDPAVGVQCLPRNTCEGSKIDWRCICSAYGFRNDPTDPKKCI
EAKCDDPNIDILGLCGTGVTSCTGFLSKRRCVCNTTENYIPTLDGSHCMPAPCNANENATKDCG

GENSCIGSFDSYMCVCNGYGFQKNATINKCEEVPCSSLPTAEVQRICGFGALSCDGTIRNPVCNC
NNDLQYYASEDMKSCIEASCSSKADYDSHCGNENSCVGNITHFECICETEGYKPDPSNKKKCILES
CELNEVDVLNVCGKGAISCKGNVRDLECVCDASKGYVQSPDTNKCEPSSCYGNATVSEQCGEEN
HCLGDLSDWICVCDAYGYKVNATNSMTCDKVMCGDTGADAVTVCGKETTSCSGHIAKPVCTC
NETAQYLPSPDSTRCVKASCKENDAARKACVPGNSCSGSYDEFICICDQFSYHQDPADNRKCIRE
KCEDLAADYDYEKICGVGGISCTGYVNQPVCVCNSTESYHPSNDRTSCVKASCSTSDAVRTQCGL
ENSCFGDIESFMCDCKAHGYKLEPINKTSCVLATCEDGDFSYHFQCGKGAVGCSGNINTLSCLCD
SAKGYVPSADMKRCVPNSCKDNPDVYIKCGMENTCLGSYADWFCVCSGAGYEANPADPKSCQL
MECSKDDAIHVCGYGAIDCKGPVLAPTCSCNYSDHYIASEDGTSCVPITCRKNDTARAQCGEENT
CKGHTDNFKCICDAYGFKADNVDHQKCVERQCTDGDADIIRICGHGGIRCTGPIRSAVCECNSTE
YYYPNADRSRCILASCKANETIMKSCGEFNSCEGNMESHICVCNGPGYKHHPSDDKQCVKETCE
MGEIDPIYLCGKGAVKCNGTYDIYTCECDNAMGYVQAPDGNSCKPFDCHANATMHKQCGDG
NRCIGSLTDWICVCDVAWYKPDPSNLMRCHEKTCKEADLELICGTGATGCSGSATNPTCECDSA
NYFVSSTDRRSCIKASCKTNITAVQQCGDGNSCYGTPDNYVCNCDAYGYRLDDNNNQACVEKK
CDDVGANYVEICGLGGIGCKGTVKNPVCECDQTKHYVASPDSTSCVLSNCRGNKTVMAACGNE
NKCFGSASSFICVCDGPGYKRNPSNLQECLPETCEAGEIKPHMVCGRGATACKGTMYHFECECN
NTLGFIRSPGHESCIPVNDCYANATMHEQCGEGNGCQGSVADWICKCDAPGYKPHKTNFQMC
HQRMCSDADIDLVEVCGVGVESCVGPIANPTCNCDTSKHYVLSADSTSCIPGSCKTNETIMAQCG
EGNSCSGTSENFVCICNGYGYRPHETDKQTCVLKKCSHSDVNAVDVCGQGALSCIGTMRDPICQ
CNNDMFFIPSDDSRSCVLASCSANSDVAVACGKVNGCFGSKSSWICDCSSPGTMLNPNNIRECLH
ETCESGKLDPIIVCGKGALGCNGTMSNVTCECDNSMGYVQSPNGMSCVASDCKTNSTMHELCG
DGNSCVGSLTDWMCVCDAPGYHPHPSDPMRCLQKMCSHANINPVDVCGIGATKCVGSITEPRC
TCDTAAYYVPSPDGTYCIPGSCQTNKTALEQCGNGNTCTGTADNFVCICNAPGYKLDDSNNKRC
IEISCNDADVDLVEICGQGSIGCIGTARQPVCQCDHSKHFVPSLESTSCQLASCRANDTIMDRCRM
GNTCFGSMTSHICVCEAKGFKVNPSDPTECVQETCEDKDIDALSACGKGATDCMGTLMNLTCQ
CDGASYVESPDKTSCLPVGCQQNVDVKKACGEGNTCEGSYDDWVCLCTNPEYIPHATDEKVCS
KKKCTDNPIGSCGIGATSCSGNVNIHKCTCNLTAGYMPSKDEQSCIPIDCKMNPKMNCGFGNTC
RGSFFSYVCECNTLGTMRNISNPKQCIEVDCSTDAVSICGVGATGCAVTPTNDVNCTCGEFYHPA
HDYHSCVLDDCKFNKLVPDACGVENECTGSISSWMCVCKGPKYIQDIKSTQLCAPGPCRIEQCGS
GKASGSFQYYAESCSGTYFEPTCNCKEGFEKDSYGGCIVKSCSRNNQSSVDCNDGAISCEGTYFKY
KCECRAPTHILNPANKRSCIPARCNPQLCGRGLVNGDYMDFASECSGSYTDFTCKCKYGLYGSGT
SKCSLEKRTCALSNDCHPNANCIDYDGHYFKCECKDGYSGDGYQCHRNGGLTPWSVVQPCDKT
CGTGYAQVMRTCDDPKPDTLGKKCNVPSPDVREVPCHQFACTACSVKKTCECENIRPYFVHEYV
EPDESLKYFTNLTAEDVEKALSAIEKWKLSVDEKEFCIKHTLPEIDGLISDINENLRSVDQTKLYTIT
ARDMMRNKLDCNVDLEKKDKYFRVYEAIVKRHFILKQVVSDLQAKEKLMVTYRSSCTKDSKVG
WLSSIFS*
>TRINITY_DN1676_c0_g1_i12.p1 type:complete len:1631 gc:universal TRINITY_DN1676_c0_g1_i12:206-5098(+)
MLIITRTAKLLLLSLTYIYLTIAQDLTNATLNNGFEAGRAIKCKTDDGMVQRCMPVFENIAFGRDII
ASNTCGQTRQTYCVQTGTSGAVKICEVCDASDPDKAHPADLMTDLQFDDQPSWWQSETILDNK
SPVYLTLNLNKKYNVAFIRLRFHSVRPHSFAIYKKSTYDSNEQWLPYQFYSRTCQETYNVPNQQSV
SYSNQKLALCTDEASGMIPLSGGNVAFLTLQYRPGKRDFDNNPGLQDWVTVTALRFELDRVNTF
GDEVFGDPKVLRSYYFAISDITVGGRCKCNGHGSSCNNNNNEGTFQCVCEHNTAGKDCEKCLPI
YNDRPWRKASGRSANPCKACDCNGLADQCEFDQQLWVDSRYRSGGRCVNCKNNTDGINCEYC
KEHHFRSNVKEACKPCRCNPLGSRSLSCTTKGECACHPGVTGLKCDKCKEGYYGFSSSGCKACN
CDRDGSIGVNCDFTGQCNCKKKTVGKRCDQCAPLTFDLDRSNPKGCKECFCFGHGVSCDAIEGI
GVKEIFTTFEADFDGWKLQDESGASFDSNMEWDFRGQYISVKPPRNKQLYLVAPQRYLGNKLSSY
GKLFTFAYGIFLDPNDPLVSAGRDIIFEGNGLQAVYPINSQNNILPEARFYQFKYRLMEPVGMSTFD
FQKILVNLTAVKIRTAYMNERTGAIDNIKLYSTKFISINSPEQVTWQEICECEEGYTGDHCEKCDSGY
TRSGGDTSPFGECKKCFCNNHGNFCNPDTGICQCLHNTTGDSCDQCAPGFYGNPTAGTPEDCK
PCPCLYGNECILIGNSVKCTDCPEGHIGDLCELCDDGYFGDPNGTIGEPSRCQKCDCNGNIDPNAI
ANCDSLTGSCLKCINNTKNGPENKCELCADGFYGNSTIDPKGNCSACSCYEKGTMKPPFHDPKL

QLPCDTNGKCECLQNVIGDKCDMCPPGHWNVASGKGCELCNCNATGSKGSDCNLQTGQCEC
QDGVGGRKCDQCAPRFYGFSKDGCIACNCHPDGSKHQNCTQDGVCECLPGVLGIKCDQCPEN
KYNLSVGCIACPRCFDLIQVAVNKLRNKLKTFNITGNLTTGSIDIKDPEFEKLFNELKRNISELEQK
MKDLLEGDMAILEFYEVLVNQSSTLIEMIPGIQKMIKKSEGAAETGSVTISKAEEITEEIQNLLGQAE
KMLEAETGRIFKNVTSSSEDVSGLSKRMSEIAEEARKHADRQEQESKDIEFNTDMAYNNSKAAYE
QAKKARDTEEAISYLLDQMALGHDEALALSKNSTEIIAKANSTSIAALNDSDHLMMKAKEPLPN
LDIEAMKEKAKNIDAEGKATEFNATDLHEKNKDKITQLQNDDKEARELMERGTQLHNDSNALY
KIAKNASDEAKQAQEDGLKVAKDAQDMLKTLMEFENNIAKSKANATEALKLIGLIRRLISKANK
TTENASSNAIMASINATAALRLASAANKTTQDAMNEIESSRREVEKLLNETMDFMMKEVAQTKD
DIDDIEKSLEESEKLAKSNSMKLGDALNDSQAATDKASMVQNELLKSLDQIEDLMNEISNLDEIDF
ETLEDAEKRFEEAKDTIENKLKNEIEIMESKLSDQKKLLQQHELDLTPLKEQIMKNEELFKYLPKSC
FREKAKPEDIDK*
>TRINITY_DN991_c0_g1_i2.p1 type:complete len:3229 gc:universal TRINITY_DN991_c0_g1_i2:278-9964(+)
MKKKMKVGDLLYSKTVMFLMLLFVISVSSEDLLDRNQNNAYFAKYYECPVGWVLRCEKCYLLVE
TAKTWTDARYYCKKHGGELVTVKDDSTNFFLARYVQQTNKKIHNVYIGLHDREKEGDFKWSDG
SEAIEKEFRWLPGEPHKSLTNENCVEMFTRSNQYLWNDAPCSDELSFICQMPACVQGSNRCDNG
KCVSKQAICDGVDDCGDSSDEKNCTKCGGYLNGMTGTFQSPNYPDKYGNNRRCSWVIEVPTSH
KIQLAFKDFGIETGFDYVEIFDGKTLAAKSFGRFNGDENPELIMSTSNQIRVTLVSDMTTSFKGFLA
TFKAIPDKCEFDLVIPKNGYEYIKSPMYPDNYPDNKKCIWNIRSEDPFGTLTMMFVELDTEENNDF
IEVRDGKGVKDLLLGRFSGENDVKFMSPIFSTAKHMFVKFQSNYMTSKKGFRAIIRSGCYAEINAS
QGMITSPGFPYQHYPKLIQCTWKINEPDNKAISFMFHRFDTELNLDVLYIKDKSGKIVGTYSGVLS
NNALQVQWILETSQSSLEVTFTSDVTYTMSGFNVTFSKGCPSISVLGGKVKGKSHYGGKISYECDE
GYHLRNGKTRTCLYSGKWSDPMPYCAKINCGHPGVVVDGGFQTYPKKDNFDVNTRLVYYCDA
GYRLIGNNERVCQNDSTWSGSQPECKLSFCDTLTTPKNGKIVGDDFTVGQSVTIKCNPGFNIVGV
TTLTCGKDGKWNADEPVCEAVTCPIIKPSDNMEVLNLPNNTDRFPYGAMFDIKCKNGFELNGEN
QIKCVAPGIWSPKLPKCYDVNECLQKSSACKGVGHQCINTIGSFKCECKNGYQKNGANTCQDID
ECKISNATVKCSQKCINQLGTYKCDCEEGYYLYSGDNSTVSRIELRTAVVNHTCYGVRCSVAPIPD
NGFVINYGRRHPVRLIYQCDRGFVIKGNEMVMCQKDGTWNREFPDCVHAGCQNVTVAKSATF
KVNGTKLSDGRYKLGTEVIYTCARGFKITSGNLRQECIQNTTHAFWSGELPVCERKSCPTLTAPVN
GKVSSMNNLFESEATFTCDDGFQLLGDAKLTCTESGKWSGATPVCAASSCPYPDVPKYGEFVGRL
ELGRRVIYSCYKTGYTLVGNKERTCLYKKGTGHYWTGDTPLCKDITPPVFTCTKQINVQLPRSENS
MLVKWDVPTATDSTGKRITVTVSPDIHPPAKLKAGQHNIVYTATDKAGNNATCTVVVNVQDKQI
PRIKCPPNVEIESDFPAKVTWDSGLFSDNVGISRVSFNPSNGSTFQSGKYHRVEMTVEDLQGNRDV
CVTEVYVKGCGCESNKSCNSIAPHNGGISTCHINMGITSCSLTCPKGLSFVQDNFKHDNVMCLPQ
GKWSEKDIPGCGERKSVTSCLNAVLEFEYNTIDTSKVDEDNLATYFNDLFKNFGNMKTTYCDAV
KFIPNEYELKSKNIELLFKMEPKGDILKDALKEVLEGCKTELENRFQSSKQDILNLVSHASVNLQA
KSIIKFDAASCCGDSKYYCCPKGHSVRAKGANVENIVCLPCSPGMFLNASSNHCTSCPKGYFTDT
SGNEACQKCPDNLNTLKHQSTKCTEKCPLGTYSSTGLQPTCRKCDYGEYQNEIGQTMCMVCPSS
TTTASRGSQKLEDCKAPCQLGHYSDTGLKPCVPCEKGTYQNETGRSVCKACPSGGIYAGIEGATK
EEECPSDNACDMKLCKNEAKCVPGVYSYQCLCKPGYKGKNCEIDIDECETKPCKNNGTCVDLV
NDYNCTCIPGLTGKSCETNVNDCKNDTCINAGVCEDLINDYKCWCPLGWEGKRCEKFINKCLSS
PCQHKSKCTPLLNAYKCDCTPGYKGHDCETDINECQSSPCYNNATCVDLLNDFECKCQPGFKGE
LCAIDINECVGSPCFNNATCIDGINGYRCECQPGFAGQNCSINVNECDTKPCLRNSTCIDLINKFK
CECEPGFYGDRCEFEINECEDEPCKNNATCIDQVNAFECLCPLGYKGVRCETEINECDVNPCKFG
TCIDLIGDYRCNCTPGYEGRNCDILIDNCVAKPCENNATCTNQVHDYSCKCLPGYEGKNCSIDTN
ECLPDPCNGNALNCTDIVNGYICYCKPGFRGTNCSEEINNCIPQPCMHNSTCKTIIETNDYQCNC
TAGYEGKQCENETNECLGSPCQNNATCIDKLATYECVCPTGFTGFNCETNIDDCKNVTCQNNG
MCFDKINDYYCRCHATHKGRHCEKKIPDEVDIKLPGGTGSVIIHSNISTVQEFSLSIWVRYLGSNSR
GDFLILANSNGKVMTSHFLKLSHQRVMVEFNGNMDIFSLDKMLNDGLWHHVFVSMSGLSSKIV
VALDSKVIIEETKSYLNGQSFFKPSGYLIIGKNVNRTGFFSPSGFAGEISQLAIWNRVLSPNEMNNLA
SNCSVDLKDSIIPWVGLLDNAGSKVELLERWTCGTNVCPAGFMGRFCEILIDKVAPTVASCPKSKK

VVSLLTSTRVNWPEPRFDDNVAVTEITRSHAPDQVFGWGDYVISYIAKDAFNNSATCVFELYVTPY
DCPDLEEPNHSASATGRWLWGLFFDFQCKPNYELAVPKPPFYSCGKMGTWSHGPPTYMDNPKN
LLPSCTGTARPIVIGTNAFTYSTTSKCDETFKAEFMKQFKRKVLEIHQYFKICGEKCDFANIRVKCT
DEDITTSRVKRDASLNEVKVYFDSKFNESLSNSFNDTQTLQAAIKNDKVDLSFTVGGETITSDKNN
VDIAEPKLACNPGWKFVKDKCVLCPAGTYRDSSMIKCELCEAGSYSSLGSATCVKCPHPKITYGT
GAKSMNECYENCSSGYYFNNGSCSKCQIGFYKSFKGLEECTQCPKGTTTTVIGASRLEDCSVECPL
GHELVGTECQPCMNGYYRSNKNQPECLPCPYGTTTYLVGSASNICYKKCELGFESSNDGKVCMP
CNTGYYREEEIQRKCLKCSGDKITLVYGAKSKNQCKEQTGTKFDVELRFTSLEWNNTLIDEDSAYF
RTTAYNIANKIAKIYENDKELLYIKIINFSEGSVIANMTISFEKEKKDPLEVLRKTVATGKLDGLSVDP
DSFKTDRCEFFDCPKDRVCWYNSTGRGCKCKPGFYETSDGKCFAGCEPDYCLNGGACVESDSGR
NCRCSKEYSGERCEKEEKDFSLALIIGLVVVAILILLLLCYCLRRRKRSGNVSLYKYWKSEDKEHGLS
SIPNTPIVQPVKGDNGDIAYAAENKLVEKDDEEQSESSDRK*
>TRINITY_DN165469_c0_g1_i1.p1 type:complete len:2668 gc:universal TRINITY_DN165469_c0_g1_i1:41-8044(+)
MVEEDLNRSKELDIPKTLAYCFHNVTTVSLNRKQDIFRLLTQCIESQGCQDGHLKAVCKIISLTVM
RYRDSKSRQLVLKFVQKFLDIHELQAVKCLLTEIHILAKQLIKCGASLFSASCGLALLSWCSSISKKK
VQEASILKILYEVEAMCYSVICTCDSFRVKRLLKRAYLYLQDGKDAEQLENCLGVIISNSNCQLESIC
LVGFIFQYLTTKKEIQRVDEVKKRSLIDIIIKNYIQTKVKADYHYLKHAVPFLKYITHDDFKEILPAIE
KGLLRNPETVLQGVGYLLEGLSIDSSNYIEELSKCIAVHLISKNASLQADAVFAVSVIAKQCSDFSAI
NHLLKQLIKVLNESEGKLVFPEQKNAVLQAIKSVDCTSVTGSDHKENLATTLHDGLFPFILLEGHE
GVLTQALNVMKTWCKYFKNNLPKTFVDNIKKGLSLKVSTTVIQAHYLLVVSEALKGNTVSQASG
LLETLLQVIEKVKGQSHLINNVFLCISSSMVLLKMFLSDDVQVNAAKEDSFWNVILDPVKKNLVSE
KFLLSHQKEYFYHLSQCMNTMFLNYSSRFSQKLLEPWYRAFNVLMVYGDWKTKKSVKILLSKMF
DSGNYDTDEFQSRLFADLDSLLDQEMSKSSKIEDTEDILVVNHVVDAYLFLCIWNKKDINEDEVLA
VLRKCLIYVHHDTIYQHDSSLFSKIGSVLKKDVAKEFVEKYKDDLLKYFINLESLSKADNHSLKTLL
SLYPEALLDTLVDEIIKYFTKPDLINVTSQEYEIMKTPDGVAYDQSVFESAKAANQKSGNLKRESKA
YSYKDQKAEMELRKELEKKKGKSDKIELNKKQKEQLEVILTQEKAIRDKLKSLEQGVMKHASILSL
LLDMDSDLLCYHYLDLMNLLLCLCQSHIAAPHVIPIYTKLSEKLFEGDLKHLGQLVANVILRLKKP
VCELPMGWSVENLPEQAKRALNTLHTFVNERNVYLMMTYTRKKTFSVLVFSYCLPLLKSILKDQ
GALVKGSEEFRMKALQLIHIHAKLRSPMDMGSEKNDPELLPRKEMLQILSDVIVSCEGHNKEAKV
QQQATLAFDEVCKCASGRLGCAVATEEEINILLSNILSQCSILRRVSLKGLLEFVDILPTENVVIMTQ
LTRSVLIGRFDIVDENATLAKRLWDEADICIEPSMIQHLVENVTHPLDDVRLAASLALEFSLNERKE
VDIIPQLLDIYFEKLKIPPPIIDNLGRVVSQQFVDSWEARQGVGLALEKIAETVGEDKLGDLFSFFVP
KALGDRNESVRKQMLNASLQYVNLYGQKNMDMLLGVFQDFLDSAPDSKELDTIRQSVIILTGSIA
KHFDKTDPQIKSIFSKLMAALSTPSQSVQQAVANCLPPLCGAMKSEAPELFKNLLEQLLESESYGE
RRGAAFGLGGLVKGLGILSLKQQNVIPTLNDAIQNKKIWKHREGALFAFETLCTMLGRLFEPYVV
HLLPHLLLCFGDGNQYVREAADDTAKAVMKNLSNHGVKLVLPSLLKALEEDAWRTKTGSAELL
GAMSFCAPKQLSSCLPSIVPRLTEILADSHLKVQKAGQQALRQIGGVVRNPEIQEICPIILDALSDP
NKHTVACLQALLSTSFVHFIDAPSLALIMPTLEKALGQRPTETKKMAAQILGNMYALTDPKDLQP
YLQAVVPGLKKSLLDPSPEVRAVSARALGAIVKGMGEVDDLMPWLLETLTSEASSVDRSGAAQG
LSEVLFALGKDKLDKLMPDVIATSMKTELSPNIRDGYLMLYVYLPATFEDQFLDYIGLIIPAVLKGL
ADESEFVRDTSLKAGQRMINMYADSAIELLLPQLEAGLFDDHWRIRLSSVQLLGDLLFKLSGVTG
KQTTVSEEDDNFGTAYSSQVILETLGFDRRNRLFAGLYMGRSDVALGVRQAALHVWKVIVQNTA
KTLREILPVLFELLLGCLASQSYDKRQVAARALGDLVRKLGERILPEIIPILEAGLDHEDGDKRQGV
CIGLSEIMDSTSREMISMFEDSLINTIRKALVDELPNVREAAASSFENLHNTIGHRALEGVLPTVFQ
KLDDPQLSNYALDGLKQVMTVKSKSVLPFLVPKLTTPPVNTKALAILSSVAGEALIKHIEKILPAMI
QAIQVSQGTGLDGDSEFESTRTLILSVYDEVGVRTIVDELTTSSKSQMPGIRKVAASLLMVFCKETR
GDHVPYIQSLFRTIIQLMNDVDQDVVASAWGALDALVKRLDPSEQLQHLSSLRQAVRFIKDDIRN
EQLPGFCILKKGITPVLSIFREGILNGPQEIKEQAANGLGEIIKLTSPEALRPSVVHITGPLIRILGDRF
NSSVKVAILDTLGLLLQKVGALLKPFLPQLQTTFIKSLNDSALGVRQKAAWTLGLLTVLHTRVDSL
FTELKNGIKNNEDSAICETILGALRSITLKAGSKMSEGIRNTLYETLVELISSNEDGIRLKAAAALGA
MCQILPQDQIEQLLNDNLLDINQMLDWSICHGRAVALSYALYHAPSQVFDAVDEDDIVEAVVKY

STYDRVPICTYGVHSLGHLLMFSKDNADVAPQIVVSSIKKTLLNSSVDIRLASLSTIKHVSRNTPEV
MTFERIRYLIPEFISLLRDRNTSVRSSAEITLAFTLQMDKNDILFDECVNTISEASHFLEEFKKNTFSK
LNDSLATDKDDYFFAT*
>TRINITY_DN2003_c0_g1_i1.p1 type:complete len:370 gc:universal TRINITY_DN2003_c0_g1_i1:89-1198(+)
MGVINTLVVLTLALALEAADHMTPHEYEFWIKTATSCTQEIYEGDTSHSTYSDLKTKLEDGIKAAY
TNNTKYFSARVKSMQCTTKPHYVAAKIMLIMTGHFTFDEALADLKNEIMDNVLDGSALNLTDN
MLRASMDEGAKPKEKVTLKFSFTNELFCENMATLKVNLAKRVILSDGMKRIESNRIVIYEHECNE
EANKYKKAVMKFYIYDDNDFLTADAALTNLAYQVVDQLQYDGTAWKLGVIFHKHINDVDYESE
SEDEDFTEIEKTYIGFGAVVGLLLLIVIIVLFVSCICPNACGKQEDPYLIEDRPSRFTNVNTHRDAYSN
QVYSKEEYANGYEREVEMDTVLPHEEQTVFHGNQPNEGMKESV*
>TRINITY_DN984_c0_g1_i1.p1 type:complete len:1485 gc:universal TRINITY_DN984_c0_g1_i1:4558-104(-)
MVKSSWQQLLLLLILFCGFQSFEIDGEDKPGSCPPPLETLECNEVEPGELSCLTDSECSGTDKCCSD
GCNLICAAALPEIKGEMGAKGEPGPPGRNGTTGPQGPKGGAGIKGKKGKQGKQGNKGRRGRP
GPPGPPANDTEPGPDPLTEDDHGKGPGPGGILEDDLAEGGVAPGVLTTQDAPEGEKGARGRAGP
RGPYGERGNAGPIGDAGEVGDKGEQGPKGQPGPPGDDGQDGDRGAPGPIGEPGERGERGEIGK
QGRPGIGGYKGDKGHPGLAGPKGVGGEEGLKGDTGESGPVGEGGPAGPRGMPGKTGNQGPPG
PTGPTGMSGPQGLPGSAGLTGAPGSPGDQGPQGPKGDMGPVGQRGERGEQGERGLTGDNGPK
GERGLTGTAGRPGRDGAQGQTGTEGRPGLIGKRGPIGQPGSQGSEGPIGLPGKRGESGDIGLQGP
DGDKGQIGDSGAPGQTGGAGLPGRRGIPGREGPTGPQGETGRRGPPGLLGTPGSPGEMGSKGLR
GERGAPGVIGGKGATGAPGSAGETGAPGDRGLQGLQGKTGRPGKTGSSGIPGKDGRPGSAGPQ
GLRGVRGAVGLQGIKGSAGSNGLVGENGALGKKGDTGDPGLKGSPGAVGAPGKDGNVGERGV
PGAAGERGAQGPIGPIGQAGPPGKDGEPGKTGERGSDGKLGPQGLRGAPGLRGPNGNPGVAGQ
VGERGEIGADGAAGSKGPNGMLGTIGNPGAPGLAGSFGQKGPDGQKGAVGERGPKGFRGEDG
RDGERGGDGIAGPSGPQGVSGVKGEMGRTGANGIDGKRGLPGLMGPPGSTGPPGDEGAQGQD
GAKGIKGAMGPRGDKGPAGSIGEPGIDGGAGPIGSLGGSGPKGGRGSIGPSGEQGAQGLPGPTGR
RGVQGNRGSIGPVGLEGREGVKGSSGEPGMPGRTGENGVPGMPGKDGRNGDPGEDGIPGPMG
KPGMEGARGPRGQLGPGGLPGMPGLQGIQGVKGEPGLDGGDGLKGRKGSTGMQGVMGSSGLP
GKPGDDGAAGEDGIPGTPGDRGAKGDIGDVGFLGPIGPPGSPGFSGDIGEKGMIGAPGEQGNDG
EPGNPGEPGYRGLIGNPGAKGNKGGKGEVGDRGHKGYRGMRGARGPTGNVGDQGDDGLDGL
KGAKGPPGLPGPRGRTGREGPSGPIGDPGINGTKGFVGIRGNPGEDGSPGPAGPPGPTGNPGFPG
GLFGGALNYYPDQQQAEKGPLYGYHYGYNYRYYKSRREEDTDKEEIKAKNKKANRNVFRYAEE
LKMRMEAEIKPDGSEEFPAKTCKDIQMCFPDAKSGSYWLDTNGGSKTDAYVATCNFTDDVTETCI
APTVSSFDRKRWVTTGGKSQFTWFMKDLLEIDEGLTYTASVTQLKSLRLASTKMRQNVTYHCKNS
YAHHDQLENTAKTFVKLMSDDEDELHALGGRKERLDVLSDKCILKDDVWRKAVFEFSTKRTSKL
PIRDIAVFDVYDENQEFGIDIGPICFM*
>TRINITY_DN8903_c0_g1_i1.p1 type:5prime_partial len:447 gc:universal TRINITY_DN8903_c0_g1_i1:1546-206(-)
DMATKACGLENMCVGSYKSWRCICQAPRYMQDPDNTQSCIHAPCRIEDCGSGYAPGSFQYYAES
CNGTFFNPICNCQEEYEKDMLGGCVPKSCRENPIAVAECGENGYCKGTYFKYECGCQGPKYVQN
PRNTRQCIEAPCNVQNCGHGFENELYVSFADSCIGKHADYTCQCKEGYTGTGTSRCIPDGKECGK
SYCHANATCAPLGKGEFDCTCKPGYSGDGLYCFLDGGWSDFVNTRECNASCGTHGYAEVTRYC
NNPVAERFGKQCGAKSPEQLEVPCNRFDCLGCTVKKDCRCTSVVKPLIVEGYNEPEETLKFFTDL
HPDDVKKAKDTLLKWKAFSNENLCRKYNKTTLHAKFFGVRDAAKEVNKMKSSYGSARDSMRI
KLDCNINIAKKTQYFGVYEMIVKRLFIMKQVVKDLHIIEDYVLKHMENCDDSPVGMIRNTFGK*
>TRINITY_DN598_c1_g1_i2.p1 type:5prime_partial len:742 gc:universal TRINITY_DN598_c1_g1_i2:2-2227(+)
RWSGCSKSCGVGVKTKSRECDNPRPSGGGRYCTGERTATEACKIQDCGVDGKFSHWSTWTACSK
TCGGGAQKRARQCNSPPPSGDGKDCAGAREEFKGCSNDFCKGPTGVAKAQEIIERFLENFHKKT
YPDREPSGKFLKAIFAIQKAVAEEFISVDPSVRGKTLEQRLKDLEKFLKVATEGGSIDFARTLLGKGIF
TFMKKKIQEGLPIDDIDKAAEGFLEKLRGDVGEKKMNEILSSQGDATLMFTIDTTGSMKDEINAA

KAIAKQIISATRDYEVDYILSPFNDPRTGPVTHRSSSNRASFVTDIDRLRATGGGDCPELSFTGMLN
ALNQGPQYGSPMFVFTDASAKDDSASNIDSLKAAADLTDATINFFTNLNGCKGAKSVESYKEIAS
YTGGQVFPLKNEAELKKFTNYVKTSLQQDTVITSGGTLNSKSYKRSLYSMQAHDFMVDDGIIMIS
VAISVQKYASQVTILDPNGRRVNPSTTLSLSLVYQISHPPPGQWKINFPSSVGKYGYTVQAVSSSAIA
FAHNFIYQQSVRKNSPPIPLANPLKGVKNMMVVRLGGTKNFDLNSIKIDLVDTSGNVLQKGIGTD
RLDSDEVRHATIDPPSGPFKVRLTGKTKSGYNFERVSQGISRPVSAAMRTNFAGYDFTAKLGKGPY
AAQMLFINRGAHENFYFYAKANYGSIPNYKQTKRVTSQGFVTFTYKAPRQYNLKGKSDRITVTVK
RQSTGEIVSHHTSILLVV*
>TRINITY_DN69276_c0_g1_i1.p1 type:internal len:887 gc:universal TRINITY_DN69276_c0_g1_i1:2659-2(-)
FDRPKYQYSVFENANIGEAIDTVVVTQNTEINVNYSFYKPNSLFTINSMTGEISIAANLDYEEKTQH
FFHVEAKCISGSNIFTAYTSVKININDVNDEAPVFFGTNEAVVPENSLVGTPVLIVQASDADTGNN
GRVRYDIIDEVENLPLAINSDTGVIFVSGNLDYELKKSYSLPVSATDLGSPSRRAISMIKISVMDKND
NSPVFSKNYYELAIAEDQPSQLIACLAASDSDSDENGKLSYSIFLSDNSLAETFFINGTGCLSTLKKL
DRESKDSYSFTVVANDNGNPSLKGIAEVHVKVIDINDNIPIFSKSQYLLRISKNTLVGSTIISLDAVD
NDEGINGTVNYSITDGNIGSSFDIDSGGTVHLVRELSNTIAKYVLTIQAKDQGMVTSFLSTTKVEIE
VIGNSTSSGCYPLFTSSIYDVEISENIAINSNIITISANCPNNSNILYSLIESSINSGGKFAINGNGEIFVV
GTIDFEQTKFHSLIIKAYAESTPNMFSTSIVRIKLSDINDNRPIFSRTSMVIFLARNSKAKSIISTVYATD
KDSGSNQEISYQIQPNIQPSDLFEINALTGDVILSREILFKDIKNYRLQVLANDRGTPSLSSPQLTIIDI
KIYPDNMHTPQLQPSNHVKFPENSGINQLVYQAQASDLDIDLSIEALQNRPEAGNFGKFTYHLRN
TTGSQFFSISETNGRIENLISFDREKKESYDLIVEARDGGSPPRKSIQTVVVKISDVNEIPLFELSSYVF
YVSESSKIGLSIAQIMAKDNDAGQLGTLKYFIKGGNVGNKFSIKENNGILILSDFLNYMEQSVYNL
RIYAEDISNRPLGNNADVVINVLQVNASQIPVFDLSLYTYQVPEEQTSTLGTITARLGANRQTEIIYT
>TRINITY_DN22240_c0_g1_i1.p1 type:complete len:513 gc:universal TRINITY_DN22240_c0_g1_i1:155-1693(+)
MFRYKEKDDNKILQAYIKYLADVVLTLDHTKMTSKEIFSLANETFQFERELYKIHIPPEKLTTIDQTY
HKATLSYLQELVPSVDWVPYINHKLKRANKSVNKDEPILIDTLEYFKKLDEVLKKTPKRIMKNYLL
FRTADFYSVLLSDEFIKLKDPLYLAKFGVYKEPPRWKRCLYATEAKMGMALSSLYVKKKFTSGSYEE
ANKMVKAIREAFVSGFPDLKWMDKKTLEVAQEKANFIEQKIGYPKYILDSVKLDEKYKGLEFDG
GFFDNDFKGDEFIVANRYKHLHEPVNRDEWELTPMTVNAYYNPSENQIVFTAAIMQPPFFKFDYP
KSVNYGSMGAIIGHEITHGFDDNGRQYDKNGNFVSWWTDKSVKGFKELSKCFVEEYSKFTEAGL
HIKGNVTFGENLADTGGLKAAYKAYKRWVEENGEESLLPGLDKSNDEIFFISYSQSWCTEELDSRII
TQINTDVHSPAIERVRGALYNNEDFARIFKCPVGSPMNPKTTCSIWTDNSH*
>TRINITY_DN495_c0_g1_i5.p1 type:3prime_partial len:407 gc:universal TRINITY_DN495_c0_g1_i5:152-1369(+)
MDFHWNTVIAGLIIVQVYSLAEYGTLNGKDSYRNAYPWLANDRNNNELGREDARSIYRSSDEID
AEYPQSDDEVDTNDIPKSYTSRDELDAEFAQRTYKFADDLDIENAQKSYASSDELDRDDEQRTNK
NTDDLDAEDAEREETINDSLDAENAQRRYTSIDELDGEDTQRVISKTITLPRLGAGRIYKSRFIHGP
YIRPKFVRPHKGIRLPHPNRIIIPGSWKRPAHGIFALKGKKAIFCEHKGIKRISCSNDKVIKVRSALY
GRTAGNVCSISCSKDEEIKVKNALYGRTAGDVCGRSRVTNCRALNSFAIVQKKCNGHNSCDLNPS
NSVFGDPCVGTVKYLNIKYFCVKRITPTTTPNGKTAIFCEHKGIKSISCSKDEEIKVKNALYGRTAG
DVCGRSRVTN
>TRINITY_DN2576_c3_g1_i1.p2 type:complete len:145 gc:universal TRINITY_DN2576_c3_g1_i1:807-373(-)
MMHKSSFLVFLVLGTFGTAIAIKCFDGEFIGGKIPSWVPSLPKPKSKECDSKFNACFTITMNKTFNV
MKFSKTFEMKKGLCGNDMAGCNVTCAKYTKAKSCEVSCCKKDDCNQQYFPTSDSIIKTSSIFVTV
AGIFYVFLNLLN*
>TRINITY_DN10094_c0_g1_i1.p1 type:complete len:264 gc:universal TRINITY_DN10094_c0_g1_i1:1210-419(-)
MRHILKVFAVAFVLHCIDRTLSLPVSPVPPLLNKLLKDQNVTLGFILKGLQGPPGRDGAPGSSGLP
GPPGPPGWKGDPGTPGMNGAPGFMGNPGSPGAPGNDGLPGPPGLPGAPGWPGGSGMQGPPG
MPGLPGLPGSSGPSPIRYNGTVKCEEDTAWLRCNEYKHISIMSAFWGRRNFGLCTEHTGNLITNK

YCPTTPLFLTKVKDACEGTTMCEIRCTKFFFHDQSCLDVYKYLEVYYKCIEVINGHEVVNEDNLL
MANFLG*
>TRINITY_DN1071_c0_g1_i3.p1 type:internal len:375 gc:universal
TRINITY_DN1071_c0_g1_i3:1124-3(-)
DPKVAMRVEFYVCPPCGVTTPAITTTEQTTMPTTTTTESTTTPTTTPATTTESTTTTLPTTTTTTPTTT
QSTTSKETIIITEEPKPTTGLPQKKTTTTPRTTTESPTTTEATTTTPATTTEATTTTQATTTTTKATTTTP
ATTTTKATTTTKATTTTPATTTTKATTTTKATTTTPATTTTKATTTTEATTTPTTKPCECKVLQPVGV
SDGRIPDNQMTANSVLVNVSGPTFTGASQARLNNKPSKDGVGAWEPAQQEEIYLEVHFNKIEDV
REIRIQGHPTDKKFTKNFFLSTSQDGVNFEFTDEDLYEGNKDGNTIKSTKVSFKTKAVQIHPFNDVS
VGDPKVAMRVEFYVCPPCGVTTPAITTTEQTTMPT
>TRINITY_DN2217_c0_g1_i3.p1 type:complete len:566 gc:universal
TRINITY_DN2217_c0_g1_i3:1820-123(-)
MLRLVLIAISAAVAYGCSPLKHGCYAHSKSDAHLLHSDNSEDTHADIFSGTVIDWNNFGPYLQKA
ACRCNKAAAKAGYPFFSLRDFGECHGVGKGSTSFSALNLKKSVYCFGTDLGICDSSQHKHCAGA
EEGEFIYETKPEELTLLSEGKPSKQSSTYLGNEKTYGSYLAFDGELSQKVKRTCCHTKKSAAPWIQV
DLLQPSTIYSIKLANVLENYRNRLNNVKIYVSNNENFDGMKQLCASFVHMKTIVQTFTCQTPLTG
RYVRLVNNNNFLHICEMQVYGVQNSESITCPIEKGFCVKGNGHDQNSGVIKMNSVDGNTDNAQ
KECLELCQRNAKATGCEVIWNRHNRGCYIHTQEVARGNNVDRHFCWVFSKCTLNNVPPAVCPI
EKGYCVKGNGHDQNSGVIKMNSVDGNTENAQKECLELCQRNPKATGCEVIWNQHNRGCYIHT
QEVARGNNVDRHFCWVFSKCTLNNVPPAVCPIEKGYCVKGNGHDQNSGVIKMNSVDGNTENA
QKECLELCQRNPKATGCEVIWDRHNRGCYIHTQEIARGNNVARHFCWVFSKCNL*
>TRINITY_DN123121_c0_g1_i1.p1 type:internal len:127 gc:universal
TRINITY_DN123121_c0_g1_i1:3-380(+)
LNQKLCAQQVIHLEITDANDNFPVFSQSRLSLNVSEYTEVGQVIATVRANDLDERSQGNLLFSILK
DEDFFGILSNGDNGVIFLKQSLDAERRLTHQIEVTVSDREGKSSLQNMEIIVFVEDENDN
>TRINITY_DN17095_c0_g1_i1.p1 type:internal len:607 gc:universal
TRINITY_DN17095_c0_g1_i1:1820-3(-)
CVEHAHCKRNEFHCIGTMNCILKSSLCNSVKDCQDGSDEGPKCTFSSCNDTCSYKCQKQPSGVG
ACICEPGKILADDGHSCIDENECDQVPSPCSQHCNNTEGSFMCSCTYGYILQDDKRGCKLNVSKE
EELLFVDVKEIKRYYMRSKLLQPVFTNLSRGVAFDFDSASGKLFYTDVTQNVIYSSYLDDFAKVVH
GEKRILDRGLAIPDGIAVDWICKVIYFAESAANRIDVIEFNGKNRASLISANLTTPRGLALDPRVGY
LFITDWGGKPKILRSQMDGSNLKVLITEKVGWPNGITIDYYDKTVYWVDARYDYIDAIDYNGNN
RRNIVKGKDLIPHPFSLSILDDDIYVTDWVLNGVLKISKLPGTKKVEQLVYNLSRPMDIHVISEKRQ
PYTDNPCDTRKKGNRCDQLCVIKSATESSCLCQARYILGSDGRTCEPANEFLLFARSWEVRGISLN
ASYLNDVMTPVLGLNSAVGTDFDALSEYVYFSDVKTDKIGRTFIGNDKGGKIEWIVEGNLDNPDG
LAVDWVGRNLYWTDSRISRQSEIGVSKLDGKFRKSLIQEPLGKPRGIVVYPEKGYLFYTDWMRPAK
LGRIDMDGSHLMILVN
>TRINITY_DN30613_c0_g1_i1.p1 type:complete len:975 gc:universal
TRINITY_DN30613_c0_g1_i1:154-3078(+)
MPVFHTKTIEAILEPVAQQVSQLVVLHEEGEGGNAIPDLSGPVNAVSAAVSNLALVAKQTAASSK
DEILRKEMPPAYSKVEESSNQLVEASSLLKDDPFSSAGRKSLINGARGILNATSALLLAFDDGEVRKI
LNVCRQVLDYIQVAEVVEVLPDCVTFLKNLSPGLLSMAQKVDNRIPDLTHQTHCELLQKHIKSTK
ANAPLLINAMKAFIVANNKGVGRAEAQENRNFVISKLCDDIIEIMRVLQLKVDDEYDLEDPCNL
MRKNRHKIMEKMKMAKDWLGNPEADPNGTGERALKDMIKQARHVGALSNDPQIKNLCDELD
GMLDRLSQLRAQGLGNSPEAIQLAADIDKKLETLAKLVDDAIAREAAAGTRLPAVTTEGQYDQA
MRWIDDPRHDPSSVGKRAVDALLADGRRLAEQLMGPEKQELVGTINNVEKLSRQLQDLKAQGK
GNTPEAQRIAAQLKKELEKLKGLLQSGATKSVLDAFLDTSTPIKQLEKAAKAPEDAPNREQEFEE
KAGAFEYHTSKMIDAAEQTSIHGKNVSKQLVDNIQAAAKDLAELTPQVSHAGKLLLLNPRDKAV
ENHFNDLKQGWINKAEEVTALVDTATDTLDFVKASEESMKKEQAGALDGIDQMNLAMVVGKG
GNMARMANRVVQVAKGEADNSEDPEFKQAVSVAATELDKGIQPLVLSTKAVAMNPKMPPVRVE
FKATSDGLIGRVVDVRKAIEPEEVPPPLPPPPQLEEEPLPPPPPEDAFAKLQLKAKSKVAPEVPPPRP
PPPIVEGLIAADESRELHSMLRAPEADNRIAVAAHQLHVEASKWEEEGNKLIQAARKMAILFAK

MSKFISDEEYCETHSKRELIDTAKMITAAANELNKHARDIAVKCPDKRMKHDLLVTIDRIPTIATQ
LKILATVKATMFGANDPQADIEATEMLVGCAENLMGSARSVVKDCEAASIKIRTGQGQVMQWK
RETDYDYYS*
>TRINITY_DN2113_c0_g1_i2.p1 type:complete len:1704 gc:universal TRINITY_DN2113_c0_g1_i2:5257-146(-)
MAQLLPIRFQEHLQLQNVGINGANIGFSTLTMESDKFICVREKVGEQSQVVIVDMADANNPTRR
PISADSAIMNPASKVIALKAAKTLQIFNIEMKSKMKAHNLTEDVIFWKWISVNTIGLVTETAVFHW
SMEGDSSPVKMFDRHQSLAGCQIINYKVDHSGQWLLLIGISAQQNRVVGAMQLYSVERKVSQPIE
GHAAVFSQFKVDGNPQTSNIFCFAVRGAAASKLHIIEVGTPPTGNQPFPKKAENIFFPPEADKDFP
VAMQASQKYGVVYLVTKYGYIHLYDIESAQCIYSNRISGDTIFVTASYEPTEGIIGVNRKGQVLSVSV
DEEKMVPFLIQVKQNGELARRLAERCNLPGADNLFLQAFNDKFASGNYQEAAKIAAKAPKGILR
TQQTIQRFQQLPSQQGQTSPLLQYFGILLDQGQLNKYESVELCRPVLMQNRKQLMEKWLKEDKL
DCSEELGDLAKQFDNTLALSVYLRANVPNKVILCFAETGQFQKIVLYAKKVGFEPDYVSLLRNVM
RVNPEAGANFSGMLIQDDEPLADLNQIVDVFMEFNLIPQCTSFLLDALKNNRPAEGPLQTRLLEM
NLLSAPQVADAILGNQMVTHYDRAHVAQLCEKAGLLQRALEHYSDIYDIKRAIVHTHLLNPEW
LVNYFGTLSVEDSIECLKAMLSHNIRQNLQICVQIASKYHEQLTTSSLIEIFESFKSFEGLFYFLGSIVN
FSQEEEVHFKYIQAACKTGQMKEVERICRESNNYEPERVKNFLKEAKLTDQLPLIIVCDRHDFVH
DLVLYLYKNNLQKYIEIYVQKVNPARLSIVVGGLLDVDCSEDVIKSLILSVKGQFSTDDLVAEVEKR
NRLKLLLPWLESRIHEGSEEPATHNALAKIYIDANNNPERFLRENQFYDSAVVGKYCEKRDPHLA
CVAYERGQCDQDLINVCNENSLFKSLARYLVRRRDAELWGTVLVEDNKYRRQLIDQVVQTALSET
QDPEDVSVTVKAFMTADLPNELIELLEKIVLENSVFSDHRNLQNLLILTAIKADRTRVMEYITRLD
NYDAPDIAGIAIVSELFEEAFAIFKKFEVNTSAIQVLIENIQNLDRSYEFAERCNDPAVWSILGKAQL
DQGLIKEAIDSYIKADDPSAYMEVVEASKNCGNFEDLVKFLQMARKKAKETFIDTELIFAFAHTGR
LADLEEFISSPNHANISQVGERCFEQKMFEAAKILFNNISNFARLASTLVHLGEYQNAVDGARKA
NSTRTWKEVCFACVDGQEFRLAQMCGLNIVVHADELDDLIRYYESRGYFEELISLMEAALGLERA
HMGMFTELSILYSKYKPTKMKEHLELFWSRVNIPKVLRACEQSHLWSELVFLYDKYEEYDNAVVT
MMKHPTVAWKESLFKDVITKVANIELYYKALQFYLDNKPLLINDLLVVLTPRMDHTRGVSFFQRA
CYLSLVKPYLRSVQNHNNKAINEALNSLLIDEEDYNGLRASIDAFDNFDNIALAQLLEKHELIEFR
RIGAYLYKGNNRWKQSVELCKKDHLYKDAMQFACESRDKAVAEDLLSWFLSIDKTDCFAATLYM
CYDLLEPDVVLELAWRNNMLDLAMPFMIQFMKDYYTKVDKLAESNAKRDEDKENAPPEAPIM
GVEQLMITAGPSSYVPQPNMYGMPPQGMAPQGMPPQGMPPQGMPPQGMPPQGMPPQQGFPQ
QGFYGGAM*
>TRINITY_DN26392_c0_g1_i1.p1 type:internal len:469 gc:universal TRINITY_DN26392_c0_g1_i1:2-1405(+)
PDILIGASNSDKAYLFRSRPIITISDRLELNVTSVPLKKNCDNGDGRNYFCAVLKVYLSFQGISVSNK
FDLELSLTLDDDQVEKRAFIKTVKKNDTSLNSQFKRVLNNITVNTPVTEDFMVYFLVKADLDIQKP
IPIVSKYKKIEKSGGCGANPCPIFNIYGSIGSSIQVPYLRECGDDKKCTIDLVTTGSIELPGKEKEFEILY
GVTDEVYLNITVENLGEAAYLAYMKINASSDLDVISVNVNGGPNILWKSSNIDAETMQVEFSIAN
PIDKGGKYHILAKFSVGKQPRPSTPIYEFGMEAFALGEDKNMDNNRKTLTIGTKVETCIEVIGYSSV
PQVIYHSTAKQPPDVNATVNDIGDEILYQFLVQNKGNKPVDTLTVDIDIGAEKEKTTLMYISDLTV
DSVRCEEEKLNPKKFLIPDDISRTLNEESTKSSLKRKRRDVANDDGTEDKKGYKYSPLNCRTASC
>TRINITY_DN4815_c0_g1_i1.p1 type:complete len:595 gc:universal TRINITY_DN4815_c0_g1_i1:79-1863(+)
MVSSLLLVLICTVVYTAGISAFFQCDFEKVDICGFKSSSSNYTDTGEAPNWRRHTGTTPSAKDQNN
AHVTGPLEAQSGDYYMYFEASNLPSGASSTGTLTSRLFDATQFGVTDIVFHVSSKGESLSLLVLDFI
DYTTNDIFDEKVVFPRNKAAWQKYTFNLDEKIFKRKFYMQFIGYVREVNGKKDNGDWAIDNLRI
DGIAIVEKCENVKCNVNTNCKDTSNGPNCECLPGYSTKAGTSGKNTECIETCQAFTCNLNAECS
NTENGPDCKCESGYKAINGTSGKTTRCEDDTCSNFQCSNNTNCENTVSGPNCTCKEGYRPVNGT
RGRDTICEDINECAIKNICGGLKVCRNVPGSHHCDCPPNYTFFAAENKCYRNGAFSQWRNVGSC
SKTCGAGDQKQTRSCSNPSREAPHGRDCPNEPTERAVACNSGPCLAGCPRNKTCTCENVRQYKT
GEEPKKDFSSFNGLSITDGETSRRYVHTYNREFIEKTIKDVNCNNNQLKSVYKNITDIIGILEGINKK

YSSTKMEMQDIIDCNANIDIEKNLWDVYDVLAEHSICNYQVQKDLEMKADRIQAEITRCNNRG
WYSTILDAII*
>TRINITY_DN1030_c0_g1_i2.p1 type:complete len:1426 gc:universal
TRINITY_DN1030_c0_g1_i2:4483-206(-)
MAKVRVLALLMLICVTASNAQDGEEVFPFDVQKGEKGQPGDDGEPGNNGAPGRQGSEGPQGP
DGDVGFDGEEGDEGEVGPPGPPGRPGTNRTSVVVVYGPPGPSGPQGPDGQIGPKGYQGETGEPG
SPGARGPRGDAGAVGEAGVPGKNGANGPKGEQGSIGPSGATGIPGLPGPSGQRGPKGSKGEVGE
LGPKGAKGDAGRNGDEGESGLPGRKGEPGPRGQNGTVGFTGPAGQEGPMGPIGPAGPDGPAGP
QGPRGADGPPGTAGSPGESGAPGLPGPVGQVGARGEPGFPGARGIPGDRGQAGPAGQDGVDG
AVGARGSPGSPGIAGAAGPPGTRGEPGKPGPRGLQGQQGEQGSTGVQGSVGGRGEKGPRGLTG
QAGVPGPPGDSGTDGRPGPPGPPGNNGQRGLAGLPGSPGLPGFPGIKGHVGERGFNGPRGEKGS
RGESGRPGQRGIPGVRGPPGDIGLPGIDGKDGNRGQPGKDGIPGPPGQPGPRGIPGSQGAAGSKG
DKGAKGRTGDPGFDGAPGDKGIPGQAGDQGVPGPRGRQGDRGEPGEQGARGGQGLPGPPGA
QGPQGRQGKRGSPGLPGARGNDGLPGRQGERGFPGESGNPGAPGRPGASGRKGPSGRPGQKGS
RGDPGMPGDTGPPGQVGRPGLPGAQGNPGAAGDQGEIGEAGERGRDGKPGPRGAMGQRGAP
GPRGNPGQRGDPGLPGDDGKPGSTGAPGAPGAPGEPGQSGPRGPTGRPGQAGAPGESGLKGFK
GNRGARGRPGRAGKKGADGLKGQRGSKGEQGLTGLQGAPGLPGNQGPAGPIGLPGEQGPQGP
PGPDGKPGSKGDKGRSGPDGRPGSPGRPGPDGSNGLNGRPGKDGSRGPQGRPGPRGKQGPTGY
VGAPGAPGVPGLIGPRGPMGDNGKDGKNGADGDPGEAGPQGAQGPQGEPGADGADGLPGRN
GNPGLPGPRGEKGDRGTKGGKGAKGNKGTAGPVGQPGARGDRGPAGTPGADGEKGADGIKGE
TGFNGAEGAVGDRGDTGDRGSKGDTGFMGFKGAKGPRGSKGTRGLPGYMGPQGDIGDKGPIGP
PGPTGPAGISGPVGLPGRKGPSGDPGPKGYAGKNGRQGPPGPPGPPGLAGPSMLPPWMGGGLA
DGESKGPVVEREEPDAPEDENEELPPNEVNPFYRVYQFYSSKKTTPTDNDVNVMENKFKTKVEEL
KTEFSRIDKPDGTKDYPARTCRDIHAYHPEAKSGLFWIDPNQGCKDDAIQVTCTFSVNEDDEPTV
TTCVDPAEKQSVPQRTWASKMHSRAQKWFDEDHELAKLKYNANLSQLRYLGLLSKTATQDVKV
HCKKYVVWHDKMNRNFDRAMRFMGTQEQEFLYKDVDSKSSKSRFVPEVITDGCSANSNSVAHT
HLRFTSRKFIRLPIVDFAPTYPESPKSEFGIELGPVCFKV*
>TRINITY_DN13467_c0_g1_i1.p1 type:5prime_partial len:782 gc:universal
TRINITY_DN13467_c0_g1_i1:2472-127(-)
LSEIFTGTLYNMYQPPPGFCFDDFCTDPPIQDDPKLFDMNVQERVNLFVEETCKQAESYKTDHIILT
MGSDFQYENAHRWYKNLDKLIKYVNEDGRVNTFYSTPSRYLKALHEADKEWEVKTDDFFPYAH
CPHCYWTGYYTSRPAQKGYIRESNNILQACKQLEGMQKDNLHYPSSDFLRRAMALNQHHDAVS
GTEKQHVSNDYDKRLSMGRVECQDLMSRAISSKYSRSHNSNAAKQFHFQYCDYLNISVCRITETE
KSFVINVYNPLARPVATYLQIPIDSKDVIVLGSNGEPLKAQYNPISRQTSYLRKFHKGYAAFVLVFE
VCAPALGYATYFVNQTHDLKKKFKEFVPQPAYVSKVKVDNDFIENSKLRLEFSSESGRLVRMIRKD
RDLTLTVDQQFFWYEGSVGNDKSRQTSGAYIFRPNKTEPSPVCAENIAKVQVIKGPLVEEIRQTFGS
YVSQVIRLYKDAPYAEFEHTIGPIPIDDKKGKEIITHFSTNIESAGKFYTDANGREMKERVRDHRDT
WDYKVTEPVSGNYYPVNSRISIKDSSLQLTIMTDRSVGGTSFLDGDVELMLHRRLLRDDFLGVGEA
LNEPGVDGRGLITRGKFRVLLTTPEEAGRSHREQGELMLLQPLLSFSANPFTKEEWMEKFVPIYSG
LTKSLPENIHLLTMERIEKNLMIIRLEHFYEQNEDPKLSEPVTVELRGLFADFEIISLVEMNLSANQVI
NEKKMLKWKTTKVKKDKEEGASSSDDAGSKRFGEVFGILLKPMEIRTFIAEVAYK*
>TRINITY_DN1936_c0_g1_i4.p1 type:3prime_partial len:1762 gc:universal
TRINITY_DN1936_c0_g1_i4:5283-1(-)
MLSPAVTKVFIVLSVLGGLICQSCDFEVANACQWTQYAKDKFDWTRNFGNTGTGGTGPKTDHTS
EVKILKANTTGGNSNGAPCVFPFKFRKRTYTKCVRSMQRGKSWCATVPDMNKKRQWGYCMPT
GYYMYIEASSKRGNPRNKGDNAVLLSPVISKGPKCLQFWYHMYGPHIDTLNVYKMANDNMTK
VWKLSGTQGNKWSSVNISFDGSSSYQMAIEGVRGNYYRGDIAIDDINIIDGNCDGGDGSKVSASY
GKCTFEKNFCGYQNDTSADMIWRRNKGSTPTGGTGPSSDHTLGTKSGYYMYIEASTKKRYLAARI
SKTYTSLDSTGSCLTFWYHMFGPHTGTFNLYINPPVNSTNTPAFSVVGQQGNLWLKGDVTINTTS
ALVVIEGRLTYKSQFYKGDMAIDDISMTKGKCQNRDICTFQDDFCLWYNVPQAAGLDDFDWIRG
SGGTPTAFTGPTTDRNGNIIGRYIFIESSKPAIKGQKGWLSSELFSASGNGKCFTFYYHMYGGSIGTL
NVFVNTTSGLELVWTQSGSKGRQWKNGQVNVERPGSFKVLIEGIVGNGYRSDIAVDDFEFKSGKC

SIVPAEASPKFKLTTIAPTTIKPTIPKNLIIDCDFEFGICSNWKQDKKTDVVDWTLSRGRTQSRGTGP
RFDHTKSTSAGQYIYLEVSKRKMNDSARLISTFIPATGDTGGKCLTFWYHMYGPHINRLNIYVMIG
GHTGKPIWSRIGTQGNKWNKGQVTLVGRQPFQVVVEALSGISYKGDIAVDDISVSSTKCTQPSSTT
CSFEDSQCGYIDDTSSDVQFQWTRSSLSTSSSGTGPKTDHTMQSETGFYMYTEATRKKTGSKARLM
SPVNKATSGSCLQFWYHMFGADMGTLSIYLKVNNKLQSTPLWSEQGDKGNEWKVATATITTQDS
YQIVFEGKIGRGYRSDMAVDDIKLTDGNCPPPGFCDFETGYCTYFNSKNDKFDWLIGSSRTPSRFT
GPNFDHTYGNANGSYLFLEVSQPIKAGDIAILESQQFNAVGGKTRCLNFWYHMFGSDIGSLQVVY
KVFSGSQQERVLWNLTGQQQITEKDPWKYARVPVDMDADHMVMFKGIAGSGYKGDIAIDDVQF
TSLACTIDPPEANPNGKTTMKPPTTYPRTAPSTPVPTEFSCNFEKDLCKLTQDSKGTFNWTRIQGY
TGSPGTGPSTDHTYGRGFVPKYGPGSLRHAGGKCVHVLNGGFNKPKEGNPLVLYQGCGQTRLEF
VFKYGYLKLTRLNMCATLEGSSTSSGTKVVLSNTCMNKWKMLDNNTIQDVKTGLCLSPFKGSDY
PSNNSPLVVDKDCKPKSRIFTFSPTMGHYAYIETSPPRQPNDTARLISPILKTPRCLNFWYHMYGAH
VNMLNVYVKTSNGPYVSPAWAKYGSHGDTWKAAYIYMNMTGPYVYAFEGVRGIGWQGDIAID
DITLSESCPAPVECSFEETSPIMCGWTNLKNDDFDWSIGQGKTASTKTGPKNDHTYGTSQGHYMY
IETSNAPAKATAYMYSPWYYNTGQKCVQFFYHMYGQQIGQLEVYIRYSNSRYLYRQFIRSGNQNN
TWHMGQVTVKRNNVYFQIIVRGTKGNGYQGDIAVDDFRIRDGACPAPGDCDFEHADFCSWMQ
EDNDDFDWIIGSGSTNSFLTGPSIDHTTQSNGGYYAFIEASSPRQPGHKARFKSMVFAPTTGSGRCF
SFWYHMRGYSIGNLTIYLRKVSNTKTLSEEVMWLLGGNQGKAWNQGRFTLKSSTLHEVRFIYLRL
FHCKICLPCY
>TRINITY_DN61_c0_g1_i1.p1 type:complete len:625 gc:universal TRINITY_DN61_c0_g1_i1:2929-1055(-)
MRAVILLCIAMCTMVYADVYFHNIRGSNNRLKGQNRNRDNAYRMFDSQNNDRGGYNVGPTPM
YFYTGSILTLEWTNQHQCGGPNANCDLILQYMCSENLRDGLEERTIPERNKDCKDDDCNTDIRY
GMHEDYDYYMNCKNRQRNPGLFLADQELEGTSAKYTRQNPDGDRYGYECPEERDHYPYWLPT
PWIDIAIMTRDLRRCNYYRENSQNNNAKYYCKPPQKYLDELKKRDPPSNLPRIPISQDDCERFHY
PANDKTDRGVWTRHDSWNVDPPECIAAPPSRDNHNGNGEDGYFNSYNFTIPNAPHKNCAIRM
RYNISTSDYDVNLDADGNGDPARAINIHDLVGIDTKKEATERGYVFKDYPKVYPMKGEIGKKLGL
ALAINTAQYFRTFEDRSHRTSFKARTPELEGVEIRNLNVKGKRGNIVQVYPAVEYGFVPDRMLAE
VGDMIHIQWTGSDRNPRNNDGEGPAGEDRSNIAELDQPRVAQVVADYNEYGDLTRSYPAHLDN
ATFLGLSRDELISLALAKGDCEFDRQGARSRTPYFDMGPVALTKKGIYNYLCTINNNFTNRSQKGT
AIITEKSKAGGGSKATASASKSASFRSVNGKDQEKRKYHAYNYFEQYKKK*
>TRINITY_DN4595_c0_g1_i4.p1 type:5prime_partial len:1639 gc:universal TRINITY_DN4595_c0_g1_i4:5857-941(-)
QCGVNTGTQVVYRRCNAALCKYQGNDYTRNQEVNIRTQACTVACDYTPWQNTQSCTKTCDGG
VLVQTRTERAFNKIETRNGNTPCNTQACAQELGPKQLRTCNVGQCGVNTGAQVVYRRCNAAL
CKYQGNDYTRNQEVNIRTQACTVACDYTPWQDTQSCTKTCGGGVLVQSRTERAFNKVETRNGN
TPCNTQVCSSELGPEQLRTCNVGQCGVNTGTQVVYRVCNAALCRYQGSDYTRNQEVNIRTQAC
TVACDYTPWQDTQSCTKSCDGGVLVQTRIERAFNRVETRDGTTPCNTRSCASDFSAERLRSCLNV
NQCGVNTGTQTVYIVCNGNFCRYQGREYNINEEVNVRQQNCTIACDYTAWEDTTICSKICDGGV
LEQRRLERAFNRLERRNGTTPCNTRACDSGFSAVLSRNCVNVNQCGVSNGTQLLYRVCNGEPCIL
QERSYKINDVVVVGSRACSVPCNYTEWRDTTSCSKTCGGGFFFETRTEQAFQKTESRLGKTLCNE
NSCVKDNFGNWTEWTACSKTCGMGMRYRSRECLKGFECIGLIEETETCKLGCRTETCPMGRPYP
GDCNFEDDCGKSWNQVNSIQRNAFFIVTSKDLAHQGLLPNMETTFNTKDCGRTMKLNSSVAGD
VNYLWTKRMMITDINTDVDDDVAAIDNYFKEENEQCVEFNFAVMANSLDKIGDMQVYLYYLEY
PNASLIYSKKGNTGLKWELVKRSIRVDTNKAFVILFQVNQGQSNVQYGDIGIDGFKVNPGNCQLK
GWSHWGEWKCSATCGFGTQTRMRHCLDKVNRTACVGMDRENKPCPMMKECPMNRDGNWG
QWTEFSKCSKTCDTGTYFRTRKCDSPSAKYNGSLCRVSQTKFNLPVVNDFEIDFKPCSVQQCNNT
FSQWTTWSSCSGTCGPGQRTRTRTCESGKRCSGIFEEIETCYMGCRLEKCPKFTAEIDCNFEDRNKS
DCQWKYYTVSGTSSWSFKTQPKNRLLSGPSGDSTIGCGYFLYYPSQTLSSAYAWGPNIYPTMDSYC
VKFKYSMVTSVNSSMGNLTVIKHFLNRPRKADTPLFFHSGVSQKQWNPMAIDITTDIQRSPFVIMF
KGQSDPNNAGLSDIAIDDYMLMAGKCNKSYSEWSEWSTCSRTCGVGQRTRTRVCLDDDIKVCK
MGDVATEGEACVKPLCAVPVDGNWGQWSAYSQCNARCNGTQKRERFCNNPAPSNGGRMCLL

GDTIFQLRNNYHAQIRPCSIDCGDNDTFSSYKRINDTCHFDCITGRVLQERECEQDTCFDGNTRFS
KGEKQYTQRACSIKEYCETSEYKCREFRSVCSTNTPSSCLDPQNRAVKSCECQVERGCSSMTNPGQ
TVKHKEFDVFYRPCRYVGVCFTDYETDAFSPYEKVNDTCHLSCSNGKVFEERECRQDNCIDGSNT
LTRGAKQYRETQCTTKEYCETSQYSCREISGPCSSNPPSSCQDPANKSLKVCTCQVTTGCASMLTA
GQAVSYLAEETFSRPCKYTGACNANGNTDKTFSPYERVNDTCHISCADGKVKQMRECKKDACV
DGGQNFPRGFKQFKDSSCTPNEFCETAQYSCRELSSACTSNPPSSCLVSTNKAVKMCDCNDPNGC
DSMITPGKKVNQGEKEFLQRPCRYTGVCFTDAETGT*
>TRINITY_DN99404_c0_g1_i1.p1 type:internal len:112 gc:universal TRINITY_DN99404_c0_g1_i1:2-334(+)
SNLEAITKSLEKQHKFYQSKLFKLQTDIHGVALEITKLKTSLHRTQISSSERRSSLLYINLRILKNKTDI
AIKGGLDVKEEAMTMLNTVQNFMSVATNAQNAANQALKKYY
>TRINITY_DN942_c0_g1_i3.p1 type:complete len:5105 gc:universal TRINITY_DN942_c0_g1_i3:15631-317(-)
MKMKLALLVLFTGTTLAAPFWVLDDNLPRAEIKEVGCSGKRFQNGKSHVYEYEGVTYNTIEGST
DKKTGIKLTATATITPMSECKFMMQLKSVELMSKSMNQDTFTTNAESAKLKDELEKLMVGFTMK
GNQIVTIHASENEPTHVLNIKRSILSSLQLDESTEKETDINGICDVEIVTKSGKIVKSKTLSECTERAQ
NEIGFQSTSIKSATTLKPLDSTSKCEYVMEGDVIKSVVCEEKHIFKPFSAGYKTPSGAMTTVKQSLSF
KEYTVAAISAVPSDFTVETNLLFDHSTEEPTATSTINDQVEKVMLRLVDPAQKSDLSAKENSAQEFK
NLVTLFRRMDEKRMEATWTKYFDCIKSGVCTDANTRDLYRQYLLDGISHCGTSTCIAMVRNVIV
NGQITGERMNMFLRSIPFVTKTTMKMIGHVLEICEKNPSRQAYLTLGTLLVRHCKQNPEDCSENL
RNPVTMAEEHLVKKLGQECEGQDDHERVEELLRVLKAIGNAQRPARVQEKLMKCAEKSKHSNI
TTSIFDAFRGFSCDALKQGELLRVFEDINNEDETRIHAFITIMKCPTVEILEKVTMMLEKELSNQVA
SYMWSYLTNLMESSDPRHQVARDILTKMTAQKPLREVNLPVHQFSKAFEKSLHSEFLKSGLTTEG
HIIFNKDKYIPKMGMLNMKANMMGNSLDMLETKIGLDGIEPILETLVGPNGMLPKDLNIDLGSY
GKFNSEFLRKMLEKKNNKRKLFNDNLTKMHLKANKQFEEPCAHLSVKIMGQEVSVLSYDDILW
MIDQIDNMNAVQLLTNIAKGGHKTFSKSLMFLEMSHNVPTGMGLPLKLKLTGSAVGTVEFNGKF
DIRNLFWGTPAVNIKGHMKPSTVVELSGQMGVEGHYVSTGMFVKSSMFTSQIVKGAIEYKHGQIL
KINMDVPEEPLAAVKFSSSPFLFMNEESELVEGTSRFVNPEHCTKSALLGYGLCASMRLPIAFREYE
APYYPLSGPAHFAVRAIRGDEKLTTWQFLVELNKQSGTTFGNIEFSTPGAAYERKLNGKVSMKQE
GKMNMFTLSTEGYDRKASVELNFNKETYSTAIITKNNIFTTKDMTTKLAIFNEEIDSGRQYGIQAL
ATMDWYTFDHITKFVRKPNVYMFQTSTTYYPKKVVMGTIVYDTKENKLSTRIQANQFQQAYEFS
GKFSDDGQEKGVTVTGKCVTSGKSITLYGGIQNTKQTKQILFTADVLGYSAKQYIRYENNNDVHT
VSQFLSVNGKIISLNGELSSFTEMKRLKFVADIIGRKADTTFLLTNNKGEKSLSVDMNAMQKHAN
FKTGCYQAKDSNECKFNFKIGTKEGEILMKQIMRDGNVITTLETLFDKYVAGFRNVYSNKNANA
KSLCSSIFYGTEEKEHEPVNVCLKYNAASDEKKILAITFDIVDRNQQGEINMELTKQTEFVKMQTE
VVYNKNSLAKAMQSMKYSKDLNNEIKMEVNVGKYNAGFRWFTLKTTNGANVGIEGYAMDKKI
QFVNKYVKDVVSGTTESKVTSELYINDKLVPVTTETIFLNGKGIAGPKFQVNIGKHSVSYATLVTYG
KGVYGIDLEFLVAKESNMLFKLFNGAAASVTPKKMDFQYKFGFGINGKQYTYGGDMSYNNLGN
DEKMEHSVKLGLQYSINRRSSMTFVLSNDKTKAALNMEFEYIPTKSVSHSIEYTKNDRILQVSVEFL
PKMFYTFKSQLVKNEGYSLASSVTLNWKNYNRVLKMINSMVNNDKKFELSTVFGKDLTFTFTFG
KENQKSMTLSASGFKRSLKVIGTFVKPNVQLQVYKNGEKIMDVTTSFDGITNKFQFALTNGKDTY
LKMMKSYNEFVLASGKETYLKMKKKYNEFVVTSGKETYLKMMKTYNEFKNQYKFQINGKIWTK
VQEWYLKMTKMTKDNKITKFLAPFLDLTVKLDRETNDMKLVLSNGKQTFVKIVKSYNEAKNQH
DFQIFGMSKEWVDLMAKYNKRNKVLKVSFSFFGKELAMFTLNMKEKDTVSLTTGSDYWKKNIV
LTGSLDRSQKSIAGNFQYGKTKVQLKLRADWNNMILENAFTFNDNVAKIGLLREQNSMVFRVKL
SPTLMAQVVMEVKNDNIIKMTIQRSEGDKLIEETSMQFKLSKDLYNLAFKWNPETFKAVIRAVQN
MIQNGYYKDIKPFITNVFVKGQRMSRKALTTTVMTFTEKVKEAKTKLIDLITNFEASVDKINFVAIR
EQIGQLSLKTIQKLKELVKVTLKELMTKLENAKENIPELLKKYKQIRNKIEKLSKDTAEVLPKFYKE
TLEAMDKQYNKLNGYWKDVSKVVMSNFELVSKNLTDSTKPVVEKAIYLISNFKLRGKAVKEIVSI
ARELVEKYIQILIEQGKIIMANGKIQLENMKLKSMEHYKNLKLKGMEQYNNLKLKGMELYQKGK
MPYLDETVEGIIKISVEKYMDLKKKMTDFDFEKYIQSVRDQIMEKKINGKTIVEHLNTAKEEMKNL
PPALKENYVKLIELITKLTKALNTNLKEWKIFAKPLTNYMKLVALSTKKHFGPILTKSMDKILPLITG

IELPDFKMMALEQWKQLENFMLPLIKPVGPLYENIKTQIRNIKVSGYPIGSFVDFNIEIAKTFIQTSL
KTTKQKLDENVKMVTEYANLIKKMTPEEIMNQITLKSTELTKEYKEKVLKVYEERQERLKVLVEKL
KELYNEFKQQYERITAVPVQDHIDDMMFKSSKFVKGNLEEISKVVKQITEIEISKPTWDAWKQADV
MGHLDKYGINQKMIDFIKAVKEVDMKRIAQKTTRLIQDMISTIYSKAFVRFVQVYGKLEKVHDYL
RSIPKKEYEEWVKELKEFSLENKERVIEFMGKMYKQTQKKSLNLYTQSKFLYAIMHKIVLKNMEKI
NINLVKPSQELYSDIKYRSEQVYGDLEQPTIDVKNHYQKIINDFVEEQYTIVKGAVLRHYKSLEEYK
EIMYTKAQEKMEEVKKLIELKYNELLAKYGDMTYEEMYDNAYKYGKSQYKVVRKTVKRGYKKL
REQMEKMEERLNALYQLYSTKYSDLVNRIKTEIIPQTQEQYQKYRLIVINLIKKYQKVLMEKMAEL
KTRGMTIYNDLKTKGMTIYTDNKDKTIKALFREVKQNAIKNINTVYVLVKNFGDEKLKQVRTIIV
TYYNKAKGVMVDVIIPEVKMELVSIINQTLRSSKVMTEEIMKAYTPHMVLAKNFANKGAILIIERG
TVAMKASEKAIREFMDKAPAMMKDIVKKIKDQEYYNKIVNHEVVKKYIAKTQEMIENLKTKVEQ
LKQDPTVVKYQKMFSDKVTELKERAKIVIDKLNQRIEELKNHPMTLKYQKIVTEKMTELKKKAQ
LLLEDVKAKIEELRNNPSVLKYQNMMNEKIAELKAKVKMLVKELKKRIEDLRKHPKTIEYQNMIK
QKIVDLKKQISMYKQKIENLYQHPFVTKSMVTMKRIKRSALFTADKINVNLQPKIKEASEMAQKRI
EKVQEQAMSAVQFFKNEPEEAFWTGVSNLEKSGKDLYEQIKRLFNKELIKKQFDVLTTDAKMQW
KIFLNDYFDDWSKEASRKMYEQVVTLVKNMNMKIIDMKMNMQKLPEVYKPIIEDYKNQIMENY
KNIVEYSLQNMIQGKVYLSNQWVELNNQWKACPYRLIMENPVWSEIGDEIMQHEAMTAVKYVS
NLGNLKFQAMKTILMKEYEIKKQLLNQKITELQVSLKKTVEDLRSKGLKNYQLAKEKAEQMVKN
IKEIAIKMDKFLEEKTIGEIVEITLEKYQMLITKVEFLRTQVVGLSQWYKEKATVIYNEKRKRAEKIF
EEQKEKVLAVYQKYKAQVMPIYQKYEDKVKQYYNKYSSIIIEQYKMLLKKSAQKYIQLRTKYEEL
DLKNKLNKLQKDAIDMYQNKKESALKLWEESGVPTKLVELKQMTIRKTIEALKQLPGQTKEQLT
AVFDKYLNELKVQYKKYYSAGYTKLIKEYNDLKAKFEAKFELNRNLLIKLAKPYTTPVVVAYKWV
ENEVAETALFVYRYHKLGERYNNGRELIKSEIERLTPILKNNFKKYNAEIRKYVKDQADKYSAQAL
EMSKDLKQKAIVLSKDLSKKVVASLKTYSLMTGDKILRSIHSSMKCIDNVDFGKVQKQISKKAQDF
YREFTKYVIIDPSKGEILINIPHGDMVPSFTHQFEQIKLRARRSVQVLKENGKVLMKKINNRMAEL
KVKIEKVRNKVMKKVMDNTVEVRRDLLSSYNINKRIFDRVYVKAQIIYKKSLRKATNQYKVLKTK
SQDYIRKATAQAKAYYEEYMTKAKDYIRKNTELAKVYYQKYMTIMKERYLTVNKIIMDLYNTPPA
ELYKQLKESKASLKVIELIMHLYNTPTAELYKQLKASPTYLRIDRIIMDIYNTPLSQRLEQLKTSSAYL
KVNKMVMDIYNTQYMDIYNQRLEQIKNSPRYIKVKGMIIRLYNTPAKELLSKLQYRGQKLYKIWF
NKVKVMINKAIEKNAPLIKARLNAIKKELRPYTESLKTMYKKIKLGYSVKDSFYPMYKQMKFATK
FYRGEAMRQARKIKAQICKEDPKLCKAIKSSAKVHSFIINKYATRVNKVLNIALDKYQEGVAKTH
RAYRHALNMIARPKSTYAAVAMVFKDHYVTFDGKSYEFDIPSSKCQYVLAHDFQNADFTILKTQD
AIKINNKDMKVVIFKNGKTMTVFPDKKISNGLPVQAGESKCIRQHNEIICNFVEDKLRVVVDLEN
DITTVSISAWHHGKLQGLLGTNNRESFDDMKMSNGDLAKDQLQFTNSYEMTGSPKCQLNKVAF
KKLQCGKVTQKCKKLFENPKSELAGFFNIINAEPFIKACERDLVSCEKSKEAKHCSSVNAYLSMIKS
RGYPVELHSECMKEGERMTGDKWQQKQSNKEFDIVVLVSQRKTVNKKPIMKVLQKLQSSFKSNS
IDTRYALIGFGGKGVNEEAHVHALGKGASVFSNMNDLKRAITNTMVFNGDEESNDAYHAIVEAS
LQRFRPGAHKIFVMFNSEPHTSHKYGATLKEAIFTLEKESHASLFVFDEINFKSLNKKGLTIGQTNS
KIYTTKNLKGLRLSGMELPKSEFKDMVESTNGGMFTNELMNKQNELATVLFDAITDTVTQNQRQ
CLDCEKHTTWYGSTRQLCHPVAC*
>TRINITY_DN6542_c0_g1_i3.p1 type:5prime_partial len:545 gc:universal TRINITY_DN6542_c0_g1_i3:2-1636(+)
SSTTEAPATTAPTQSTTTTVATTATSAATTAAPEITSTASSTTEAPVTETPETTVITSKPSTTAAPTGYS
RVCYYTNWSQYRNGAGKFFPSDIDPFLCTHIAHSFAKIEGLNLAPYEWNDIDVLYPQMISLKLAN
PKLKILLAVGGWNHERQGVARFSDMVQTKSNRATFISNTIGFLKLHQFDGLDLDWEYPASHPGSR
PGDKQKFTELCKELLEAFVEDAKTTGTERLLLTSAVAAGTSTIATAYEISELTKYLDALHLMSYDLS
GGWNPTTGHHTAVNADSGNSVTNGLNAWISGGFERNKIVLGMGTYGRSFELSNPASNGIGAPAS
GYGSPGKYTKEKGFISYYEICEMKNAGMTVIRDPIAKAPYGVKGNQWIGYDDEESLRYKVQHFVV
KEQLAGAMFWALPLDDFKGQFCGKGNYPLMNAVKEELAKHENMATTTQSPAITAATTAAPEITS
TESSTTEAPATTAPTQSTTTTVATTATSAATTAAPEITSTASSTTEAPGNSNPNTISFHNCSNICYARS
HNSNARDNFNKKFNN*

>TRINITY_DN5584_c0_g1_i3.p1 type:complete len:962 gc:universal TRINITY_DN5584_c0_g1_i3:2920-35(-)
MASEMSSPFDEEETLLDEKRSKSNDYEGGFCKKNWPVVITGFGVVVLICLGVVIGTAFKSDINSSN
SRLAREEKFPYKNIRLPGNVIPIKYRIYLHPNISDNEFGFSGNVRILVNVLNKTNSVLLHSKDLNILK
VKIFSNSAAFNQKSESLATEIPTQEFLHSKKLEMLHIALNENEYLDAGEYVIYISFKGSLSGGLEGFY
KSSYTTRSGEKRYLATSHFEPTAARAAFPCFDEPAMKANFIIIIVRDPQHVALSNMPIERNVTRRDG
KVTVHFQESVKMSTYLVAFVVCDFAYVSAKTKKNNIEVRVWAPKELIAQAEFAIQVAPNILNYYEE
FFKVDFPLPKQDLIAIPDFGAGAMENWGLITYRLTSLLYDENESSSANKQWVAVVIAHELAHQWF
GNLVTMEWWNDLWLNEGFAAFVEYIGSDYHDKSWRMMDQFIIDDVQNSLALDSHANSHPISVP
VHDPAQINEIFDSISYDKGASIIRMMKNFIGSDAFHTGLTNYLTKYKFKNAKSDDLWACLTDAAK
DKDIDVKRVMDTWTLQMGYPVLNVKRDGEKVTLTQTHFLKDQDKPAKSIYNYKWDVPVTYFT
NGENTTQFDWFYKDNKSIVIKIDENADWFKINRDEIGFYRVNYDKDTWAALKAQLLNDHMKLS
ASDRSNLMDDAFELARAGQLDQIEALNMTEYLHKEKEYVPWITALSGIAFCGKMLQGREGYKYY
EKYIVQQVKPLVDELGWEDKGTHLQKYLRGSVLRSAVDHNYTKAKNQALEIFKDFKENNKAIKP
NIRSAVYLAGIKYGTKDDWDFMYNRYKTSQFPSEQRKIMFALTDTEDKNILKKYLSWTLDNDKIR
SQDSCGVIEHISMNPKGTEMAFKFVKKNWPEIYKRYGTSSFDMSSLIKTIIGTRKTKSGKEEAKEFL
DTHKLGSGRLAGKQALASVQNHINWLEKNEKKVVEWLKNNINNKKITK
>TRINITY_DN172571_c0_g1_i1.p1 type:complete len:104 gc:universal TRINITY_DN172571_c0_g1_i1:30-341(+)
MSGRGKGGKGLGKGGAKRHRKILRDNIQGITKPAIRRLARRGGVKRISGLIYEETRGVLKVFLENV
IRDAVTYTEHAKRKTVTAMDVVYALKRQGRTLYGFGG*
>TRINITY_DN71276_c0_g1_i1.p1 type:5prime_partial len:1201 gc:universal TRINITY_DN71276_c0_g1_i1:1-3603(+)
LTFIESLRLSSIKNIFKMMISFETFVPSTTPYAEDYNTNPQVMMLSNKIKETIDTSLNPLASQIDRQKA
FQVQEEFKSSSPHCAEVGLYLYSVERKNSDAVRLCALKFVDHFIETKWNDLTYERKTFYKKESLKL
VEQMTFENETYLKEAAAQIVVEIIKRTWPQSWADLFKNLEELQAKGDAQLELVLIILMRLSEDVN
GLDERLTTQRKRDMMTTFQDNANELFEFFIKTIRDKVTALKTESSSLLIRLSEATLFTMMEFVSWA
HFDCLFNNEKIIFQILFWLLEVKYLRYYAAACLDNIFQRKGTPEKRIEFMVVLSDDVLNIVENVVRS
LKKTPENVLPEEGYKFLKKLAEMLVSFGKIQILSLWGDERGFNEKPVGYDRYLRLMLLLFQHESM
KLTYDVSKFWHALSIHPHGKLDDTFKELMPDLLDCAIMKIAKISDPKIPNNPSSHYSTMDFDDKE
NYAEYFANCRGHMTDVCRKITIISYTVVLDHCLAKIKQATSKPPPDVSGIPKNQIQITQHYLYWDA
LVHFLKNGFAGVFFRANKEISEDTISKCNEIITLLIHYKCEVPLFTIPVINLLESVFPIFKHSRDHLLPT
MQLLFSIIRLPQNDEDPLRDHVFKLKRHVCEAYAALCKEIPHCLVDYFKQLEEFAVYQMMHNASL
TGQEKCRLIEGVLRISKHVTVKEEQDHCINAIFDMIDSMFDKEPVRRACESPISLIQYIGLDQEGTV
AADSELRTRELLFAINLWTAACKALAMSKEEEVKDEITEEEMRTQIETYGWIKRMVTMLPKMLSL
LQSYSLIWQHQDKICDKRILVHKIITEINPTIQVFQPKCADEVNEKRGLGQYYLCTFYESAYTSLGSV
SKSVGPSLFSIGGLAEAMNEKLVQHWNNLPNYRLGRITRTFIKDICLACPKKYFETFIGPFVSQVLR
LYFERISNEWAKLAQLKENPREVTDKLQEQIEIAEERMIYLMSKDLLDLLLSLLTFKRKHLRSTTKP
ADGSLETFGELGHFLLDCEETNFIIVQIIFTSLTWPDTSAAIKVSQIILAVLKKIFTDTSKLNPELPKQL
LLLILQGLNIHGGDEFGRAALTNNVLQFYLHLRMQYRGIVQVMLMIPTCEEQHLREFDLAVFVQE
LPLTEQQQKKIFRRFVAGACGTVISKLHEKDVVIQDLPALMPRQKEKTLNKNGDADDDLGLCSLF
GV*
>TRINITY_DN118_c1_g1_i3.p1 type:internal len:200 gc:universal TRINITY_DN118_c1_g1_i3:599-3(-)
KVLFLCGFILLSGLAFAYEIEEIVEDAPKPEPEHVPEPEPEPVKEPEPEPEHKPEPEPEQCSTVKHQK
CSKADKVCSPEDSHCTFTQICTPFFVKKCCKVVSVEKKCVVVRNCIPKFSYEKVCEDVKRCPPRKR
CKNVCRQVDLGEHKVKSICEQECEVVEHPEPCKLVHTCFSKWVSKKDCTPRETCSDIEKRECREV
>TRINITY_DN540_c0_g1_i1.p1 type:complete len:480 gc:universal TRINITY_DN540_c0_g1_i1:1503-64(-)
MATDKLEGLIARLEKVTGKLESVSVRGGSGGGAAVEDDPAWYDEYIAIHEGQWESFLKATKENG
DNDMVTLEQKLRAAFNAHKDLLTIAARNGKPSPKDLPELVKPLSAAIGEVQSFREKSRTSKKFNH
LSAVSEGVAFLGWVVSPSKPGQHVIDMKDSAEFYTNKILKEFKETDKVQVEWARGLRKMLMDFA
DHLKHCHPVHLLWNPDKPAATASTKASGGSGGPPPPPPPAAAPPPPPPVAAPSSAPSGGSEDSRA

ALFSALNQGSGVTAGLKKVSDDMKTHKNPALRNAQPVPGKTSSLNPRPYAPPQLKKFNAPVAEK
PPVFELQMKKWIIEHQKNNQSLVIDETNDKQTIYMYKCNNSVLQVKGKVNSITLDGCTKCGVVF
EDLISTCEFVNCQSIKAQANGKVPTISIDKTDGTQIYLNDKSLDTQIITAKSSEMNVCITKPDGDLN
EFPVPEQYKTVWNGTKFVTTIMDLNL*
>TRINITY_DN15584_c0_g1_i1.p1 type:complete len:275 gc:universal TRINITY_DN15584_c0_g1_i1:874-50(-)
MADSGSAGADRGGRGGFGRGGRGGRGRGRGRGRGRGKPEDKEWTPVTKLGRLVKDLKIKTLE
QIYLYSLPIKEFEIIDFFIGPSLKDEVLKIMPVQKQTRAGQRTRFKAFVAIGDYGGHVGLGVKCSKEV
ATAIRGAIIQAKLSIIPVRRGYWGNKIGKPHTVPCKVTGKCGSVTVRLIPAPRGTGIVAAGVSKKLL
QMAGIDDCYTSACGTTATVGNFAMATYMAIARTYSYLTPDMWAENQFQKTPYQEFTDYLAKNH
NVGGKPVAPATATA*
>TRINITY_DN101992_c0_g1_i1.p1 type:internal len:139 gc:universal TRINITY_DN101992_c0_g1_i1:414-1(-)
AERYDHHSGIVESMDSNKISDNLKELTRLPHPPGRSRNKELGDMLADFWQNEGMDKIEKFKYNI
YLSYPEKPSTILLFGGAKTEKYTIEAEPPVDESEKGGTPVVPFNAFSANDIVKAELVFVNHCRESDF
DEVKKAG
>TRINITY_DN413_c0_g1_i3.p1 type:complete len:350 gc:universal TRINITY_DN413_c0_g1_i3:1286-237(-)
MVSLSVTCLSLLLFVTVNAEESDHCGYDEKSWYKAMGWCYHSGSRLLLGDFNGDKRTDFFCHD
SNGKNWVAVSNSDGSFGSTTWQDSKERWCASPNILLTGDFNGDGVTDILCHGTATGKKWIKYFN
KYGVPSGKSFYRAMSWCNHKGSQLYIGDFNGDKRDDLLCHDTSGRYWTSYAGTDESKVFHHTS
WYKALEWCRHATSKLYLGDFNGDKKTDVLCTDGKGYKWIMYAKTSGNLFTGTDWHTNLDWC
HESSTVFLRIGDVNGDGKDDLVCHSTAGNSWVAYTQPKGGFGNFCHLAKWCAAGKESFFLGYF
NQDSRADILCHDTKYGKKWIRYAECSFAEESDHCG*
>TRINITY_DN97809_c0_g1_i1.p1 type:internal len:184 gc:universal TRINITY_DN97809_c0_g1_i1:2-550(+)
LGPDQVDVAGAGRDPRHLPRPLGRRLRVLDRHRGRVRQRHKAEADQDPKLETARLDVRRPGVQ
VRRVRHPRPSSRDQCHRDCHAPGRVEGRAQVDRGRRGLGRRRVEHEVHGAAQPAARGQEKGR
LRAQQILAVAAGQEEAVDVGARNRQGPPSALAAVSRAPHAQFARLAQRDRRVARAKGW
>TRINITY_DN1280_c0_g1_i1.p1 type:complete len:426 gc:universal TRINITY_DN1280_c0_g1_i1:195-1472(+)
MGFETCGPNEAMVISGCGQERPLLISGGRKFVWEFFQKVQRISLNVMTLNVESQRVYTVQGVPIS
VTGIAQVKVQGQNEEMLHAACQQFLGKHENEIRNIALETLEGHQRAIMGTMTVEEIYQDRKKFS
KSVFEVASSDLVNMGISVISYTLKDIRDDEGYLKALGQRRIAEVQCEARIGQAMAKMQSGIKEAQ
AEEVRIKAEYENKTEVARSQRDFQLNKSAYDIEVNTKKATSELAFALQSAITNQKIKEEDMQIKVIE
RAQAINVQLQEIQRREKELEAQIRKPAEAERYKIEKIAEANRMKVVLEAEAEAESIRMKAEAEAE
VIQKKATAEAEQMSKKADAWRDYQEAAMVDMLLDTLPKVAAEIAAPLSKVKKVTMISSGSSDV
GAAKITGELLDIVIRMPQAMEQLTGVDITKTFRREK*
>TRINITY_DN5633_c0_g1_i1.p1 type:complete len:848 gc:universal TRINITY_DN5633_c0_g1_i1:2881-338(-)
MRRSNFLTIGIIAFFLIFSYVSADETSDEEVEPPKVDDDVGKSRDASKTDDEVVQREEEAINIDGLSV
AEMKQMRESAEKHAFQAEVSRMMKLIINSLYKNKEIFLRELISNSSDALDKIRFLSLTDTEALAAT
DELSVKIKADKENNVLHITDTGIGMTKDDLIKQLGTIAKSGTSEFFQKIQDAGTTDQASDLIGQFG
VGFYSAFLVADRVVVTSKNNDDKQHIWESDSVEFSISEDPRGDTLLRGTQISLYLKEEASDFLEPNT
LKDLIKKYSQFINFNIYLWSSKTEEVEEPIEEEPEKKEDEKKDDDKKEDADDKKDEEKKDDDEDD
AKVEEDKEEEKPKTKKVEKTVWDWELMNSSKPIWLRKPKEVTDDEYNEFYKAISKESDGPLTKT
HFVAEGEVTFKSVLFVPKNSPSDMFQDYGKRRESIKLYVRRVFITDDFQDMMPKYLSFIKGIVDSD
DLPLNVSREQLQQHKLLKVIKKKLVRKALDMIKKIPDEDYIEKFWKEFSTNIKLGVMEDHSNRTR
LAKLLRFRSSNDPEKLTSLTDYVERMKEKQETIFFMAGASLDEVKNSPFVERLLKKGYEVLYLIEPV
DEYCIQSLPEFDGKKFQNVAKEGLKIGNEAKEKARVDELEKEYEPLLKWLKEDALKDLIEKAAVSS
RLTESPCALVANSYGWSGNMERIMNSQAYAKSKDAGANFYATQKKLLEINVYHPLIQELQKRVKT

DKEDQTAKDLARVLFETATMRSGYTVKDSSDFAGRIERMLRLSMGVDVDAKVELPEEDDEEKEEE
KADDEGEEVNADGEEEESKEDIPEIKVDSKTVEPVAEHPTNEDAVENEAPAEGKTEL*
>TRINITY_DN775_c0_g1_i1.p1 type:complete len:635 gc:universal TRINITY_DN775_c0_g1_i1:1927-23(-)
MKQMIFLLAVSLAILNFDGHVVDAITCKSNSVWSWECQNSRCVKVLASSNSDAIELNTCKLTCGK
YSMLFPKPSGEIQLGKKTVPFIPTQARLRSVKVKKSNPKATWLVQNAYSLFLNTLEHSYKRYSGSTS
RLYNNMCAQEAQKRVLVMDVILEDGDTKITLDTDESYDLTILADDKEITVSINAQNFFGARHAME
TVSQLAAFSESYNAMQIVSSAQVKDDKPSYAYRGLLLDTSRNYFSVDSILRLIRAMSYNKMNTLH
WHITDTHSFPIEIKSLPEMLRYGAYSSKRIYTHDDVRKIVSYASINGIRVLPEFDEPAHCGEGWQWG
PKAGLGNLAVCVNRQPWQKYCVEPPCGQLNPTNDNVYDVLGKIYKEYFQLFDPDIFHAGGDEI
NFNCWNTTTEITNWLKQNYGGVSEKNFMDMWGMFLDKSSSKIYEANNNKELPMILWTSKMTS
EEYLTQYMDPKKHIVQIWTASKDKELSNVIESGFRTILSTYDTLYLDCGYGNWLVEGNNWCSPYK
DWKLLYDNDPVKILESFNVTVTDKIRSTILGQEAAMWSEQVDEFSSEGKIWPRTAALAERLWTNP
NHNWRDAEYRLIYHRERMVERGVQADALQPLWCLQNAGNCYYDPAIKGEH*
>TRINITY_DN144508_c0_g1_i1.p1 type:internal len:265 gc:universal TRINITY_DN144508_c0_g1_i1:1-792(+)
PRRPNDTARLVSSDVPATSQMCFEFWYHMYGDHIGNLTVFKRIGKAMTMLWAKSGTQEPRWRH
ATVDITSASVFKVVVEGTASSGYMGDIALDDLFLTPGACPPSAECTFEDPQLCGWTNIAGDDFDW
TRGNAGTASWGTGPRTDHTYGTKQGFYMYIEASTPRSKGHKAWLKSEEYKPTLGRCLSFWYHMF
GLHIGELNVLISQNNSMSAPIWQMKGNQGRPWRPSRVTVKSSLNHNIVFEGRTGDGYRGDIAID
DVMISEGP
>TRINITY_DN71243_c0_g1_i1.p1 type:internal len:422 gc:universal TRINITY_DN71243_c0_g1_i1:1265-3(-)
PFVGIRIDSNTGSVFLAQHLDYETAQQHNVSIFAVEKDPPTRYVSSYFLINVLNVNEHSPQFQNNE
TLVITVNEDIQPGVIGSSPATDKDAGVFGNIDYKIIRFDDQLFDVTASGEIRLIGKLDYEKKRVHQFL
VEALDGGQPRRKSLRPVSVIVGDMNDNVPLFSQDVYNVSISDLTAIGSSLIKLSASDADDGLNGEII
YDISNGNFGTTFIIDKSTGVISLSRKQLLSELRKFTLKVSTRDKSPISPRNGETATVHVNFIEASANEP
FFSTTFQKLNVLENQAAGIKIGQSKVINSKMEAVYLIVSGSKTRITDYFGINYRTGEITAKQSFDRES
RDHYTFVVSARFIDDNSLTGYQTVDVSIIDENDNRPMFLGNISQTVSISSSFDGSRPVALLNAFDKD
LVSLDKIEMQLLNSQE
>TRINITY_DN278_c0_g1_i1.p1 type:complete len:470 gc:universal TRINITY_DN278_c0_g1_i1:3091-1682(-)
MAPSFNKTNGHMEENGGMIHANGKSSEKPINLGEQTYSYMTFKKMGLIMITLPLVFLLLVGLPSY
LYPVLVLLFFIPVFAGYIGLNNVIYCLYERHNQVSKIDNYIEFLDEDLKKKFTGRFIPIRDLYELYAEG
KLNFKGDVLQCLERRNEYVSYKLQLWHLKFFLSKLLPELLHARSQDKEQVCDHYDRGNDFYNA
YLGPLMVYTSGIRYSESDTLEDMQMNKLKAVCEKVQLKEGDRHLDIGCGWGTLVNYAAENYNS
QSEGVTLAKEQVCWAEEKAKSKEIMDRVNYLCMDYRDIPKKKYDKITCLEMSEHVGIKRYHHFL
KDVRNMLEDDGIFFLQIAGLRRAYQWEDFLWGFFMDTYIFPGADASLPLQFPIAELEAAGFEVES
VKTVGIHYSDTIKYWYHNWVKSDSHDKMIQKYGRRMVRVWDIFLAWSTIIARQGNSTCYQIVAH
KNFNHYPRRRFIQPKN*
>TRINITY_DN2963_c0_g1_i1.p1 type:complete len:116 gc:universal TRINITY_DN2963_c0_g1_i1:1072-725(-)
MSATGNLVTVLRKLYDEYNDKTPSRLKIVDGFLLYVMLTGIFQFLYCCLVGTFPFNSFLSGFISCVGT
FILGVCLRVQTNPRNRTEFKGISPERAFADFIFANVVLHLVVMNFIG*
>TRINITY_DN1559_c0_g1_i1.p1 type:complete len:311 gc:universal TRINITY_DN1559_c0_g1_i1:221-1153(+)
MAEKKATFKLGFAENFALSGAAAVISKTAAAPIERVKLLVQNQDEMIKQGKLSEPYKGVIDCTTRT
FKTEGLAPFWRGNLANCIRYFPTQALNFAFKDKIKAAFKVSKSDGYALGFGKNIASGGCAGALSL
CFVYSLDFARTKLANDTKSSKKGGSREYNGLVDVYRKTLKSDGVAGLYRGFVLSCVGIFVYRGCYF
GFYDTLKPVLLGADASLFMSFILGYGVTVTAGLISYPIDTIRRRMMMTSGQAVKYKGSIDCATQILK
NEGAMSLMKGAGANILRGVAGAGVLAGFDKFKEVYIAMRKRQMGLE*

>TRINITY_DN1682_c8_g1_i3.p1 type:internal len:117 gc:universal TRINITY_DN1682_c8_g1_i3:348-1(-)
VKEFLLSKKCIFSPILNLFMFMKKWTQFCSRLSLVKTTCKSDTKSCIETRFLYLIVIFITEYLGRSLKFC
KGAKFLEKLIIYQILVLGQLLKQSIFDKYDHKNQFCRSRTTKFKIQ
>TRINITY_DN195348_c0_g1_i1.p1 type:internal len:311 gc:universal TRINITY_DN195348_c0_g1_i1:1-930(+)
SGCNSIDLGLLVDFSSSKDYDSKQLTKFVREFIDKITNVFAFSATKSHLAYLPFSSRINENITGQWFD
NKYMKTIGDWDYQELTQYLDRVIVPYTPVTGYETGKANAHEAFQATNDKMFNALYGMRYNMT
KVLIVISDGNEDFKEKQNLVDRAIDQLKAKGVIIYTVSIGNNVNWDRVFRYASRESSIINGANFDD
VFNRFFDFAKMLCEDSKVPIPATRTTASTTTVSINTTKLTTTTTTELVPVTISTTTTMSTTLRPIQACY
EDIDLGFVVDFSKMNANDMKERQTANDIVKVIKKIASSFNIAANK
>TRINITY_DN113_c0_g1_i1.p1 type:complete len:1417 gc:universal TRINITY_DN113_c0_g1_i1:4413-163(-)
MANWSLLYAALLFLYSVSAQEEERPITFIKGEPGPKGSRGMPGDDGSVGSTGRQGNEGRQGPRGD
TGPKGFKGEPGLIGPAGGASGGETKGPDPLAARVGVKGGTYGVNTVFDYQLGPPGPPGVPGNTG
GYGFKGDVGPVGVKGVPGLPGPPGSYGERGEPGEAGLDGVKGDRGDAGQTGPPGKKGVAGAV
GFPGLQGPFGYKGERGGAGPVGDEGVSGRKGEKGYAGEKGSRGDQGDAGFQGVQGPTGPIGPL
GNVGPRGPDGQRGIAGAIGAEGLKGAPGTRGPQGPKGQLGEPGQRGVKGVVGDKGPTGQQGS
QGPTGNPGDPGQPGKTGDKGSIGDVGLIGLTGPQGMQGERGTVGPRGPAGGAGKKGERGQAG
PIGATGEKGNVGDVGLDGARGAPGKSGIEGKVGQPGPQGEPGAVGLQGAQGPRGTDGQAGQA
GPKGNTGEGGFPGAAGGKGSKGLKGRGGDRGMQGEPGSAGKPGARGRDGKRGAAGEDGKNG
NPGQRGPQGSQGLTGAMGLVGAPGAPGDMGVAGLPGQQGRKGTRGLAGPTGQRGLRGEGGL
PGERGEQGAAGPQGTQGTQGSPGPQGRTGLDGKDGSPGDKGERGSMGTRGKQGEPGADGKQG
SRGTPGQQGIRGDSGDQGSPGLLGERGLAGQKGVAGRDGAPGMPGRTGRQGTIGATGAVGLRG
AKGGRGKDGETGEQGPQGQEGPTGPRGPRGASGEDGAPGQNGLPGLTGPMGEEGQPGRPGQP
GKQGPRGSQGEAGPVGPKGPLGARGDQGEQGPVGIEGVVGALGAVGSEGTKGPVGAMGSMGP
TGPTGPQGATGETGPIGPLGAQGRPGIAGNAGGKGPNGDPGNPGRPGSSGDQGPPGKDGSNGE
PGSPGKEGARGLIGRPGAEGIRGEAGTSGSTGRLGRKGELGPKGFPGKRGKDGTNGKRGEMGVS
GADGIRGSKGNSGKDGSAGIAGEDGVEGQIGEAGYQGSRGVRGDTGAPGDSGQPGDNGPNGA
KGSKGSSGRAGAQGVDGRLGARGDQGAEGARGKEGAAGAEGERGEKGKMGAPGIYGPAGDP
GFKGDEGEPGPVGPVGPHGPRGSPGEDGPSGKKGASGITGTNGRKGNRGQSGANGDTGTPGIPG
VPGSPGRPGPPGEIAGMSGAFARFFSAGGMGMKGPGNYYNNYYGQYRYYKAGNEKIRNQIKPH
LSTFKQYYELLKDVDDMMKATGTKSKPARSCKDLFTDYPNKITNDYWIDPNGGSTDDAVKVFCD
KMKNETCIFPKSYRAELENHYTGKDKFRFMYEDIKEDGEIEYAADITQTKILHLMSSKVRQEFSYN
CKNSLAVMKNNKDRYEYALKLKLDNEDIISMNMNTRSLDINILNDECWKKDNTVRTATYELISH
KPIKHQLPLLDVASFDIGGPNEEYGLTMGPVCFS*
>TRINITY_DN4781_c0_g1_i3.p3 type:complete len:183 gc:universal TRINITY_DN4781_c0_g1_i3:2965-2417(-)
MCTEPKAPSSCANDEFQCTKTKQCIKKHMLNLTAYTKVGSKMTKLWTKSGTYGPRWRHATVDIS
SASTFKVVIEGTASSGYMGDIALDDLFLNNGACPPSGECSFEDPKLCGWTNMNGDNFDWTRDN
AGTSSSGTGPRFDHTYGTAQGFYVYIEASNPRSKGHKAWLKSGEYKPTLGRCLSV*
>TRINITY_DN113_c3_g1_i1.p1 type:3prime_partial len:1162 gc:universal TRINITY_DN113_c3_g1_i1:3485-3(-)
MEIVSRSRIAIFVLLGLLISVAESQFDPECPEIDPDVECSPPESTVNECLINNDCDVDKPLCCSNGCG
LKCTARERRSITPDGFTPEDEKLCRVPVDLGFIIDVSGSIGQTNFDKIKKFIEKIVDAFHIADDGTHV
GAVFYSDNASLAFDFNHLQGDQLNEMNVVREIEKIQVTDGQTRIDLGLELASRELFNPAAGMRI
HKPKIALVLTDGKQTRGELAPDAKDLHQASRLLKQTGVQVYSLGIGLDYDIGELLDIASDDASVF
RSQDFDELVSIVDILIEQTCKGCTRAVDVHFALDASANVGEENFVFMKDFVKAIGYNFIISETGAH
MSGSVFGDVGTLVFDKSQATEINEFENEVENMPYLGDATGNMDKVLKMISNEVFNFDGFTRQSS
PKVIVLLTSSNCDTCQESLEDLARPLKAEGIHIVAIPIGDPLFDEMQVLASHPIDQYAIPRATHQELL
NAEFIQKVSEMVCAGKPGICAEPPVKGECAEIINECSVDMRCPEDQKCCIKNCVASCVLPLAACIT
PIEIAFAFETTKYGIADFNNVKNFAINLLDHFKVGPSKAHVSLSTFSEDVTSLTKFIDDFDMESIKQSI

KDLKHDGGEESNLGNFLEIINTEVFSVRGGIRQQTPRVLVIFANGDINDEQAVKAQEEALHLKAD
GKDVKIIIVNIGSSTKTLDVLNEVATEGSFYEFFGSAELDSTAIETEISEKICSTKVRRSKCQRRIRSSCA
VKDDLCDSDFDCPGHQECFSDGCRLRCQTCEFRCKDKVDLLFVMDTSARIGRDNFERGKKNIKK
MLEWFTIDSNNTNVGVITYSDTANVAMPLPSRGNNNPAQSLIDVQDRLDSIPYSGGSVSHLDNAL
QLAAEKVFPDNGPNLRRDSKKAVVVITDSLDDSASQLELASWPLRRQRVQTEDGDASNIRILAFN
VGQELDLDLWGNIVSPPVEENVFYAADFDDMYNGIRQLAEDSCKYAAAITPPPIIKQKGEPGPPG
PPGIPGTNGTDGRNGTDGRRGPKGERGDTGLKGFKGEVGLVGPPGGGAEEQKGPDPLASAVGIV
NVTDLPGGGRIVFDLREGPVGQQGLTGIIGPKGPIGDTGEVGIKGRNGTTGPGGFFGLPGPKGQP
GTDGAKGLRGDPGLRGSAGRKGPTGAPGLTGPQGPFGYK
>TRINITY_DN61668_c0_g1_i1.p1 type:5prime_partial len:453 gc:universal TRINITY_DN61668_c0_g1_i1:2-1360(+)
FDGSDELGCVTVAPATTKVLHCDDDHAYCEDNLECYPKEGRCDNTYDCRDRSDEKNCGAIKTD
KPICFDFQCKSGKKECISNDKKCDGRRDCKDGEDELNCQKSDCLPFPFGFNCTRVKEQCYLHKIV
CDGSTNCRDETDEDKEICGVTKRVKNLRSKLDDLSTGGIQVRLFWDAPDVSLSKVKGYAVSFIDD
KFVQKALKVPLSPSRVTIPSLKRCRKYVFAVAVDHTDLKADKLLYESTEEIETPGRRSPNAPALISTH
GNKVLWKEDVGDCIDHYDIYNRFLSCMEMDSKTVVKNRLKSANVLFDIQSMLNSNSNDTKLYN
CSIYINYIRKSDGTPFNLQTDYFTFPHTHDHHHKTAASTGGKGKRNTLVWAMPVAVVVIVLCIGL
LLMVYKYRKLQNSFLAFAARGSYNRQVDDLDDEDDDNMAVAFHTGEDSPMINRFSDDEPLVVA*
>TRINITY_DN25375_c0_g1_i7.p1 type:complete len:811 gc:universal TRINITY_DN25375_c0_g1_i7:112-2544(+)
MNVNEKMNIYFCIGTLLFQSLLVLKVEGIFNDCQTKGSCGKCISSSPECAWCEDIIYKRSLNSKSYY
KKCDVLDNHKRQGCQNITNPPSSSTIDKDAELKGDTRVSPQEITVKLRPGQATTVDITVKTPKNFP
IDLYYLMDLSLSMKDDLEQIKDLGDILGEEMKNVTTNFRLGFGGFVDKPINPYTTIKPDEIEAYIRD
EDAQYTFTYKNYQPLDEDPSLFSKTLEQVNISFNIDGPESSLEALMQVIVCRNDIKWKDKRKARRI
VIITTDADFHYSGDGLLGGQVIPNDAQCYLSKGNYTAWNQFDYPSLSQIRSVMIENQIVPIFAITGN
FELYKKVAEFFGAGSGAVADRLSKNSDNVVPLIKEAYEKIAKIVNINSDAPKGIDMKYTAICGTGK
NVEAREGTDFCDDVEIGETVVFKVEMTAKDCELVGEDLKFKIKTNFDDVNVAVKLLCECPCEQD
KSKIVIDSEHCNSRGNLTCGQCQCDLDFYRGRFCQCTVEEAKNQTLCRLPGEVGPVCSDQGFCEC
GECICDEPKLNRFGEIEYIYGDQCQCSNTSCGRTTNENLLCGGPSHGRCDCGECTCFSDWQGPTC
EVECVKSSAACIDEEGIECSGKGKCVCNRCVCDPGYIGDLCQECVSNCPDPCQDYRECVKCRVFK
MSDLSDEECKSECLKHNITSHIVTDKVPEDKRCIARDEDDCSFVFTFVKSNITDEVQLIVQQEKICP
AEPDILAIILGVIAGIVGIGLALLLIWKLLATIQDRREFAQFQKDRQDPKWDSGVNPIYQQATSTFQ
NPLYGNKGEETEKILDSQ*
>TRINITY_DN77_c1_g1_i1.p1 type:3prime_partial len:1965 gc:universal TRINITY_DN77_c1_g1_i1:2-5893(+)
MGVQVYSLGIGSNYDIGELLDIASDDASVFRSQDVDELVSIVASITEQTCKGCTRAVDIHFALDASQ
NVGRENFQVIKNFIKAIGFKFIISETGSHMSASIFGEYGTLIFSMDQATQQEDFINEVDNVPYIGDFN
GNIDKALDMVSEDIFTFDGFSRQGVPKVLVVLTASNCDSCEKSVEEAVKPLKAQGVHVVAIPIGSN
ANMEEMDKMASYPTQDYVIPRASYEELLNGVFIQKVSEMVCSGRPGVCEEPPIEGECTEIINECTL
DVNCPANRKCCIKDCKASCEVPAAACITPIDLAFAFETTGFIEENFDEVKSFASSLLDHFRVEPAKT
HISISTYGESVNAKTAFYESYDKSALKQFIKDLEPDGGRQANIGDFLKTANDEIFNITNGIRQSSPR
MLVLFTNGVYSNDQIAKAQEESLKLKAEGQDVNILVINIGSIVEGNSVLRDVVSTPDFRNLHNVT
DLRRLVNFGFENKIAEAICASKVRKTRCKPRTPQACSVRDDLCQTDFDCPGYQECYSDGCRLRCQ
TCENRCGDKVDLLFMIDTSSRIGPEGFKNAKENIKKMLEWFTIEANNTNVAAITYSDNANYAMQ
LPAAGSNDPPQTLFDVQGRLDSIPYSGGSKSNLDKALKMAAAQVFYQNSLRLRKDSKKAIVIVTD
GVDDSTSRLEIASWPLRKQRVVVDGNDYAAIRLLAVTVGNNYNLDTLEKVVSPPIDKNIFLAADF
DDMYNGIRQLAEDSCKYTRQDVEPPVIAVKGDPGPKGEKGRQGNPGTNGTDGMDGIEGPKGER
GDDGPKGEKGDTGIAGPPGGDQGDLKGPDPMAEAVGIIEGTAVKGTRGVTYDLRDGPIGPPGPIG
GVGKTGLPGDSGEQGQKGRGGPPGLRGVFGGQGPKGNPGEDASPGLPGEPGPKGISGKKGPTGS
PGMPGPQGPFGYKGEQGIIGQPGDPGDGGKKGPKGYAGEKGDQGDIGDAGFPGPDGKVGPIGP
PGQRGLRGPEGSSGPIGKTGDIGNKGLPGPRGSRGLKGQSGNAGPRGGKGRTGDVGLVGEKGTQ
GSIGEAGQEGQPGVPGKEGPPGYPGPIGTPGPMGSRGDIGPQGEEGPKGSLGLKGNRGEAGRRG

LTGEKGETGDIGELGPKGAQGQQGKQGKTGKIGPRGQQGPLGFQGLQGQTGDPGQNGRQGPK
GNLGPTGEKGADGPKGSLGSAGLTGPKGLVGEPGNVGRPGLPGRDGKNGPQGANGKDGDPGA
SGPQGLQGSPGSMGTPGSTGDPGSPGDRGATGAVGDKGTRGDSGVRGEPGAFGQQGPPGLRGE
RGEKGPQGTNGVQGSPGPRGTPGLEGLDGIVGRKGTAGRPGNPGQSGEQGAVGSIGVPGPQGIS
GTPGERGDGGSDGGPGEQGPPGLQGSSGSPGRDGMPGRPGQQGTTGGKGKTGLRGPKGANGQ
NGVQGARGPDGLPGAPGLVGNRGTPGPNGIPGLDGNPGPAGKQGKQGKMGTPGMTGPAGLR
GPVGSPGLRGQKGPTGDRGDSGETGPIGVAGDIGPRGFTGELGLRGAIGEMGQIGETGPQGMPGS
QGTPGAAGLTGPAGPSGLPGKDGNKGPIGDPGNAGNPGRDGGPGSPGKDGTPGEPGTPGSQGK
QGLKGPFGPQGAPGEQGQRGLTGRQGSGGKDGPKGFPGNDGSPGISGKRGPRGKDGTPGSPGPI
GKDGRPGKPGINGTDGDPGNNGPKGDPGERGDSGDSGSLGASGDKGKTGSEGPAGFVGPRGSN
GKAGVPGKRGKRGDQGRDGPRGNSGPDGIDGSKGFKGNMGRFGIQGPPGDAGLKGALGPDGT
VGHTGPQGNTGDPGPDGPEGPSGSKGPAGNAGQIGNAGYPGSPGDGGIPGTAGLPGPPGPPGPP
GDMSGAFSGNYWDFVNAGGLQKGPRYYGGRSKRSLDQQDNSIEKVFKSNYETFKNFDTVWNQI
VDDLVIQKTLGSSKNPALTCGDLFKTNPITKSGDYWID
>TRINITY_DN64_c0_g1_i1.p1 type:complete len:468 gc:universal TRINITY_DN64_c0_g1_i1:1555-152(-)
MPPKEAEKIHINIVVIGHVDSGKSTSTGHLIYKCGGIDKRAIEKFEKEAQEMGKGSFKYAWVLDKL
KAERERGITIDIALWKFKTGKYYVTIIDAPGHRDFIKNMITGTSQADCAVLIVASSTGEFEAGISKNG
QTREHALLAYTLGVKQLIVGVNKIDNTEPPYSEKRFDEIVKEVKTYVKKVGYDPKTVAFVPISGWH
GDNMLEASKNMGWYKGWTTERKETKTTTGKTLLEALDNILPPKRPTDRPLRLPLQDVYKIGGIG
TVPVGRVETGIIKPGMVVTFAPSNITTEVKSVEMHHEALLEAVPGDNVGFNIKNVSVKDIKRGNV
ASDSKNDPAKEAKSFYAQVIILNHPGEIHSGYQPVLDCHTAHVACKFSEIKQKIDRRSGKVLEEMP
KMVKSGDAAMIDLTPSKPMCVEAFSEYAPLGRFAVRDMRQTVAVGVIKSVTKSDGSGGKMTKSA
QKATGKKK*
>TRINITY_DN55094_c0_g1_i1.p1 type:internal len:107 gc:universal TRINITY_DN55094_c0_g1_i1:319-2(-)
LTLDMLAQKPMNVIALIDEESRFPKGSDETMLNKLIRQHSKNPAFITVKSHAEKKFGIKHFAGSVY
YNTVGMLDKNRDTFSADLNHVIAQSRCKFLLHLFDKELKM
>TRINITY_DN247_c0_g1_i1.p1 type:complete len:373 gc:universal TRINITY_DN247_c0_g1_i1:38-1156(+)
MKLVLVIFVILVYTWNVSAGGKKCISNKDFMTPYPTNFWTEHYPKFVNNRPTIGIVAMEVLGQKM
LVNVPWSWNKDYFGSSFVKLVESAGGRAVPIKEDITEDDLDKLMRKLNGVVLPGGDADVGDSGY
ERISKQAIAFSKKMAKKNVTFPVLGICRGAQMMMMAEAGKDFLEVTDSLNLSIPLDFTSEQKESR
LFGNAPQGLIKSLSEDAITFNAHAMGIPTLNFYNNSGLQKAFRAISTNYDRNGTKFISTFEGRDAPI
YGLQWHPEKSLFVFNPVLAVDHSIKGVMASQYIANFFISEARKNNHTFRDRVEEEEHLLMSYYPS
YVGNITETPYEQIYLFDFVKPFQNPKYTPGKVNKNDDDNSGDDDDE*
>TRINITY_DN141561_c0_g1_i1.p1 type:internal len:248 gc:universal TRINITY_DN141561_c0_g1_i1:742-2(-)
RQWRKLEESESLFSCRPPVLLIVMIVFLFISYLLKETLSEECGPFGYKTDTLLSPKYWYFDTTNCPPIP
YGAQAGGENQTCRIYFNLCKVQLYQCSGTVCGRYLDDRDLVLLMGEYKYEPFSEGGDSFFVSYQ
GEEVACTKSKTEVKSKINTKMNFMCNHTAKWNNTIKRIHGGAQAPKPKSIIWNNKTCEYEISFDY
DEACIILPKGEKEGHDISVGSAILILFLPGVVLYFLLGALYNKMRGFKG

Band 4
>TRINITY_DN982_c0_g1_i3.p1 type:complete len:9852 gc:universal TRINITY_DN982_c0_g1_i3:29697-142(-)
MSIKLCTFFVFFLIQCINSFPEIGLHLSLDRSYSFQGNGWKTVNGWEIKPRANGPYEIDAIKNISPEY
FPVPITGVYYITVSLPVEGASNGEFKSAIIVDGKNEEGRIGLMSSFQKTAFKNGASTLTFAGFVRLFA
DEKLTIEIYSDDINANYVVLESATFVFHFIGVTGSVPAYLAHTAQDQEYASDSIIKPWVTLGDSRVYE
SLSGFIKNEGVFTTICNGVHKMAVNIMIKANAGTYTLKALFQDEESATATVTINQPDSIATLNIFQN
FKWSEGMSITLQIHSSNGANFKVLKSSSWSMSFIGETTANFQEFSVFLMADQFLQPSQFPQRINIDL
NGVFQFLQSNPDQYNKGQLVSGADTKFKDNLDFIVDSTDLFYVSANIIIEGEAEDLEITIAVSQIDID

VLPEDGLSGRLTKISNSRRSISLSGIIFIREKQYLSFFVRSRDSSKSFTVSKDSFISVINMRYTVSSVSARQ
NENIDVTAGSWYDISGRFAEQKNGLYSFGKDFNINNGVFRTSHSGIHSVHANIQFSLDSDAFGDN
DDNKIVLRVVIDGQEDKISLMSIRKNPEKTATLKLFGSIYLRAGVTISFQVKSLMPNTVQYKILQRSS
ISISYIGPQWAVNSFHAVANQDLNYDGKPDDTRPDIPSPLNFWKTEAADEFISPFEVLESFDGTRYT
SVDDGLYVVSSLLILKNVDCDRSALFKLDFVTTGGSRASDKSNGFADSHFFIADSKGTEFTLSVTTA
MRLEVGFKVGLKLTVGSTSCRYTISKESSFSIAQWSKSSNTIEENRLKYIGVLARLSAPTEVSSPNTW
SNLGSNTMDINPGDKSRSSFRPGIFGGDDLAPITRTGKDFNGDVSDFTIKDPGIFFISGMITVTTTEN
ANEEFAATVQIDSEDIESGLYAAVINQGSTEMSLTFGGTIYLKSGARVRVVVKGGRTFNIAAGSSFSL
LKLQPDYKTPGIIVSIDEKITQAITRYEHKDLRADIHRTLTIGNLGFVESTGVYTIQVDGVYMVDYT
VILEATNPNAVFGAEILSDNTVVATGTSTFGNTKTIAITELVYFSKGAIVKAVLKSNTNGETLNNLQ
VDGGLTVYFLEPKEHVKDGHRSKQTSPQALPVRSFEKVTGFAAQSPDFAGGADSVDYTVPETGVY
LVAFTTRVNRVINGFKSRVLVNDNEFLNGNYQIVSAQNAPATLPLSIAMELTVNDVLSFQVMADG
SGMSLDGSTRSILKIDEVNTIRNVTEGLSAYKLRDQKVNGQQETIVEEWDTSVTEGTFLAKNVSFT
VDHSIVVVAAGVFQISVSVVVKNDRPSQSKFELRVLQGDDVKSHGLHSKVEIGGGREQTLTIGGSL
LLQKFDSLRIVVRDIADPLASYTIKAGSTVSVIIIDDNNGPIACEDEGLNMRRLPERIRKPTRESHTF
QCNVVSSLKAEKYTWFKDNVQLKEENQKNLVLNDLKLSDSGVYQCRVRFLELTGATNNATLSVY
DPYPKITSPKFIANVLENQDPPLTIEMFSLSAFKLSPEDEKASITTQIIAGDDQSFFTLSGPSKQTVTLS
TTKKLDYETQNEYNLTIKATNLEETALTTKGPYETTINYIVRVIDVNDHPPVFSQSIYSRSVPEDYPI
DGSIAKVSANDQDAGANVIYTLVGQVSGDLFKIDNTGLIQLRQNLDREIRDDHTIFVQASDGLFT
TVAEVVVTVTDVNDNFPNFMDTGFDISVSEATLTQSVILKVIATDPDLGTNSQLRYTIKSGNNENK
FSLNLLDGNLTLISKLDYEGTKIYNLEIDVQDSASPPKKSQNPLTVVIRVQDANDNNPTFTQSLYNT
RIAENTAVGTSVLKVSATDRDGEGINNPLTFSITDTDANEFFQVVSSGATAGEIRVRKSLNYDIRKD
YRFSVVAKDNNLGEPRYRTALVVINIDDTNNLRPTFEAGSASISISEGTSIGTSVHQVVAIDKDTVG
KLDYRIISGADGKFAINKETGDINLISAVDFESNKKTYTLEIQVNDGVRDALNPATVVINIQDFND
NQPEFIQKYYIFTALESISDSQAINVGNPVIARDADSTTNSQVDYSIQYDINNPSVPNEGDALFRIDS
NGLIFKKNGVILDFENVRYHTFTVVAIDRGTPKTLLGKAFVTINIQDVNDEAPSWSPAIYSVTVKES
AGINRDLVFLSTTDKDTSGAVTYSIVSGDSANQFQIFDNVIKIVKPLDFEKTETHTLKVSAQDDNG
NRAQLDATVTITVEEVNDNRPLVTNNNANSIQVSVPEDIPVNSLIVKLNGRDDDKGAPGQASNQ
FTFVSLDKTTDFTIDEVTGEIKNTVNLDYERQKSYSLVATIRDKGTPYLESKFLVDIIVENKNDERPIF
DPAIYTIKLSEKAPIDTVVAMLQATDLDTPKSQLRFSSVTTLDSHFSLDPITGIITLKTSFDKEVNGKD
GIFVISCTVNDGTSNSAQNAIVRVEVVDVNDNQPVFPTSEYFIQVNEGSSIGTILQNIAATDKDNT
NTHNQFTYTLEGKEAVDFRLNGNQLQTNKIFDYENKKSYSFDVLATNNLSPDKLVGKATVVVSIL
DQNDNNPVLSPSGVINLNISEGTIAPANIITIFATDKDSTINAEVQFTLTGDPTNTFTINQNTGEIML
AKSVDSFAKNEYTLSVTATDKGSPPRSSTATTLNISIKPVFVPGIEFENCPQIISFNENAAPNTFTKTL
TVKDRGSFNQVQFTVVSGQAGIQNLITVSQNGVITNGQRRADFEARKRHDFLVQAENERGAKAY
CHVTANINDVNEGPIFDSANYVFDVLEVIKINTVVFSVRATDPDFSSTTNGQLTYSLAGQEASQFSI
VPNNGQIKLAQPLDYEIKKQYQFNVIATDGGSLQTTSSVIVNVEDWNDNRPIFDQPQKLAINVPE
NSKPDNVFTVSATDKDSTTNGEIEYVGIYPTGIISIDKDTGTAKIIGDLNYEKRQKYDFVIVAKDKG
VPYLSNTREVSLSVLNLADEVPYFDTAAYTISISEKTSINTAVFRVMAKDNDLENFGKISSYEIISGNQ
LNRFRIDSNGNIIVQQSVVLENNNPNQYILTITAFDTQRTQSLNNTQVTINIIDLSDKQPFFIQNSYI
VEVQENNEVSKSLLSLEAQSNEPTFNPTYSIVQDPKNTDSQFFQIINGNTIVATTVFDYEKKRFYSFE
VQVIDTNNGKTNKALVVVRITDENDNKPFEVSGNLHFNITEHTLLGSTIGSLNGKDNDAGINKEF
EFSILEGNDDDKFMLLPTGQILLKNFVSVKEKDFYKIEVKITDKGTPSLFNIFTVRVTINSSQALVAS
NNPTIPEDKNTGEEITRVTAIDPNTGSSNGFIYSLVGTLSNSQFTIDAKTGAISLLASVDFETDRTHT
AVVKAEGPTSTGVTEVIVRVTDINDNKPIFDPTNFNLNINENFVVGQEIVLVRATDKDSSLNDNNV
IIYRIDSVNPSSGNDLFSVNPSTGRILLLKALDRENGDTSYVLTIAASNRGVSNKPDNTMTVTINIA
DVNDNFPIFEKSLYQGSVSEDANTGTSILTVRASDKDILLKNRNIRYSMTQPMAVQKFQIDPISGVV
SLKQEVDFETKQRYDFSVIASDTGDVAYSRTANVEITILDVPDVEPKFDPQRYEITIAESTLVSTSILK
LQVIDSVKNLNFNIKQGNSNSDFRLVASSGELFVNKQLDRERISRYQMTIEVSDSVNKAAIDAIVIV
NIADVNDNQPLFLQSGCYQANRAEGIETEQVVVNVQATDRDEGSNKEIVYSFSDACSVLDIDAN
TGAITTKQKFDYESARSHICTIKAEDKGTPKLSGFSCIILTVTDSNDLKPTFEVITSNARVLENIPVGS
VITRVLATDKDTLSSNKQFVYTIKTGNEDGRFTIDANGFIKLQQPLDYEKKTDYRLEIEAVDSTNQ

DMVSDPNAIVTVTVIEQNDNRPKFAKSYHRVNIEESTAIGTTVLKLSATDADAGVNEGLIRYVSLH
NTTVFNIDRTTGEITLATKLDYEVKKSYHLIAVVKDQGNPVLSARTLIDIFVNDVNDNPPVFVISSY
NNIVSEKAPIGQSIVTVSARDDDTGVNGQLKYKIRETQSMFRIDEDTGIIILNGDLNVENTNKLLYA
LTIDVVDSGVPPLQASNPATVSVQVSDVNDKQPVFALDRYQFEINENIEGNQIFGDSVTVRANDD
DLTGTNNQLTYRLVNTTMFDLVVTNQNVFIKTALGVSFDYETKKSYSFEIEAMDSAANKLYGRSLV
TVTVKDFNDNPPIITVPNIIVNISDATLIGSHIATIMAEDKDSGENGILIYTVTAGDSTNRFTMESDT
GSIILKNPVDSYTKDIYVLNVLVQDNGIPRRSATGTVTINVIPVDLTLLKFNTTCPFAISIPENQINSN
FLGLKALDAGEYVSKQLRYTINSDENDVNQIFALDSIKGEFKQLKEANYEERSRYDMTICATNERG
SKVCCHITIFITDINEPPSFIGLNNNNIYEVSISETTPIGTIIYRVLSKDQDLPGVLNGQTSYSLGQQNV
FIIDQMGQIRVNKTLDAETQLNYVFQVIVTDKNPTNPLQSAATIRVNILDYNDERPIIQNIPDITIDE
NSDNQVVRNIVVTDRDISNIGSNIELSIEPSNILTINNRQLRTVGSALDYEKVQRHTLYLTAKDKSFP
YLSSTTSFTVTINNLNDNKPKFPVNNYNLTVSEKTTVGTLILTLFAEDDDLEEFGEIANYEIVSGNQ
EDRFSIDSNGRLTLKKQLNWRNVKSYNLNIIARDKGLPSEQSQIPATVVINVADYSEEQPNFLQNL
YVKFIPETTPIDTLVLTVEATSGEPVYGPFKYTIVPKLPDTNNFQIFSNGEIKTINQFDYENQRNFTFD
VQVEDEKNGKTNRASVFIHITDVNDNIPQFTDLTRSNTQYNITEFTLPGSMITCLSATDRDHGKNA
EIRFRGIGGDGNGVFNVNLDGKVLLSQQIFYDKKNKYNLIVEAFDLGSPEQKAQLTLTIFLHKSYT
SVITTDVDINEDVETQTRVTRVSSIDPFTRSSNNFTYYLVGKSTTFFEINAQTGEIFTKATLDRETRDR
HVLTIQSRGPTTNGVGELVVNVKDINDNPPIFDPQVYQVTVSESANIGLSIVSVTATDKDIGVNGE
VTYSIEDGDQTIFSINPSTGSISLAGKLDFESKQSHTLTIKAVDRGNPPQDSVNKATVIVVVQDFND
NCPVFENSTYSLTIKEPGELGKEYLKLGITDNDNGRNQLFTCSIDEPLAEKYFEMINDCRLRLIKEL
DYEEFTSFRFVVKATDQGDVSCTTTTNMIVNVGDRADVIPFFSPLTYNISIPESTTVGTIVVSLKVGS
TFKGLLYSLFHKNTQDDLIKFGINKDTGEIFLKESVDREIQDKYEFTADVFDGSNNAVQRASVTINII
DVNDNAPFFNQPTCYQRDLTESTAIGTFVVDVSAADNDKGRNQQVTYSLLPSPSCTSFSVDETSG
RITTSQILDHEQQKLQHCLIKAKDNGTNSLSGYSCLTVNVVNVNDNKPAFIVNRFDVSVLETVPA
GTLVASLVARDVDNQGLITYSISNGNLNNQFTIDSSGKLRTRFPLDYENTKSYNLTIQASDNNGKFS
SPDDALVLITVLEVNDNRPQFLQSYYKVSIPEDTATGASILNVTAIDLDHGELTKSLFYAALENTTQ
FTVNANTGVISVAENLDYEIEDHYELVVIVQDRGTPHLSNRALVDIQITDVNDNAPMFVTTVYNR
ELSEKALIGAPVVKLNAMDADKSDKMTFTIDSVVQGAQGFFSINGDTIQLDKELDYEVPEQRTFV
LSVSVKDSRDRDGANKANVFINVRDINDREPIFDLNMYEVTVSENNVISNPIIEVTARDSDSTPEN
NNLLYSLVDGEDAASFSLLNNGQSAIITSKISFDYEKKKSYRFLVRASDKVRENFTAFSTVFVTVQD
TNDEAPVPEKDLIMVNISESTRVLSTIAIVFANDKDQGINGQLAFQIINHSEDKFEINNKGVIRLKST
LDFFQQNFYNFTVLVTDKGNPVRTSKPVTVLVYVYRTEIPGIIFEKNHYIFNINEKEIVSLVGTVNAI
DEGTYVSRQIVYSIVSGQNDIKDKFRAVNNNIIQDKEVNYKDQSEYNFVMKAINERGSIAFTHITIN
IMDVNEKPYFVGLNNKGRYEFSMSEIISVPSYIYAVRAKDDDFGLNGEVDYEIISNFRFGNFSIDQY
GRIQVLKDPDFEFTRSYEFQVRAKDRGTPSLESNLVSVVITVLDYNDNHPIFNIQEPLEISIKELVNN
TDVGTVTATDKDGSGLNRIVKYVAIYPSDHLTVNRDNGQIRVIEPFDYEIAHKHEFVVVAQDQGT
PYLNAYKVIKVEVLNENDEFPEFTVIEYNITISELTPIGSPVLFVHAQDKDRGPSGQVTNYKIKYVPP
SGGSGAGSPDKTFEITSDGQVVLKEKLEYKFQKYYQYRVTCTDNGSPPKNSINEAIIHLNIADASLE
QPIFSVNPAVVEVYENITLNSNIVKVEVDSIEPIKGPFEYSIIASEDSQPFMIDNSTGQITNKESFDYEI
KRKYVFLAEARDKHNNRTQRAMVVIFILDINDNIPTIETIAPQTFVQNISDTTYPGSVVAKLYSRDA
DSGENAMVDFTITGGDGENVFAINPNGYVTLIGPLDVRVKTQYTINVKVSDRGIPTLSNNRALTF
NIKKSKFIQNTVRFDRHHYKSNCIEENQINLNIIQLNATNSNPVAGSTIIYSLDGTLEERQPFIVNSQ
TGEISLSQTLDYEIKQEYDFVAVAVNNDGSRDVAVVTVCIKDLNDNSPIFEPQSYVVNVSESESVGY
LVYKTTVMDADKTDFNTTKSYDIIQGNVNDKFIIDSMGHIYIKSKIDYEALQVKHFDLRIRVNEGN
LVGDMVLRVNILDWNDVHPSFSTLNIYNFTIPEDKPVGEIIEILTANDGDATSPNNEVTYKLLTTED
DFILRRNDKGHGSLIVNKQLDYSKQNRYEYVALAEDKGYLNLLGTAIVIINIQDVNNHPPEFDDSE
YNTTVSEHAQIGTRVLQIHAHDKDTGLGEQIFFIYGDPTETFTISSQGFIILNKQLDYETQRDYIFDV
GARDGGNLVSLNNVTVHIKVSEVNDNEPIFDLSTYRNSYAEDTPLGTTLFTVRATDKDFIGNSIIYSI
SGANDDTSYVSVDQTTGDITLQRRMDYESKNYITFTVQAIDSNNRNKLKGSTHVIIDVTDVNDN
NPKFVFGSDLVYNVTVVENINVGTLVVVVDSQDADSGLNGQTRYHIVSGNNENKFFINELNGQI
NLINKLDALQTNKYVLTVMAKDLGNPSRSTNVTVTINVLRYFESTPMFLPSYAEVIYPENLALGSSI
YELNVTKTLPGSPMKLSFHSSSKFPEFTLQGNKIILSKEFDYEIKNRYELIIIATDEENDKAFAQVIVR

VQNLNDNIPYFTQNAYFGVVSELAFVGHSVLTITAKDDDKDFTELFYSIATQVPDNAFKLQDNIV
VVNGKLIRNKVYEFEVEAFDNTFTSKRATVSITVTPENLPSFVNATYIVTLQENYPISTNFLNISAGT
FSGITYVITSMKAKDMFNVDANGQISLTKSLDYEKQRQHVFPVTASVTSPITQVRVASVIVNVLNE
NDNAPVFVNGPYERNITEGHLVGTMVIRVDGHDDDSDKLTYSIVSGDDGKFSINSEGVISLISTLNF
DLKNKYVLNVRVSDGKFVTDTIVTINITPIVKFPPMFNQTLYLLSVKENTLVGTSILQVSTVNVIPPS
SFSINEQSGRDFFTIDANGVIRTNNALDYEAKRQHIFTLKVQDLRSMGHATVVVNIVNDDDTCPIL
TPATQTVQITEPVAPGTIVAAVQAKDIDSKSLNFTITGGNGLFRIDQHGGIKTINEVDISTSTEYELTV
TVDDGTCTKSVQVDVNVNPLAPCPDCNIYPFTQTIYEFSINENTVPASPIITVTTGQSLQRNFSIEDP
AALEYVTINRLTGEISVIKAFDYEATKHLSFNVMAKTQTTKNRNPFYVITSVSRVRITVRDLNDNCP
SIESPMYYEVSHSSPIVQDTVVTRILVEDPDTLKDHTYSISDNNFKIDSMGHIRTVQNIESFTAQFYN
LTVTVSDGQCSKTSNVGVNLAVNPTPMTYQFTSNGVYSKSVREDHNLQDVILSVALNQNYPRVFS
IVESDANDQFVIDSNSGALRLKTSLNYELKKRYDFTVMADIHGGVYKSYASVHIIVLDVNDECPIF
PVIGYNQRVSGTPVIGTIVEIIEANDADNATLEYSIVSGNEDGHFSLDNSGVVRVVKPFASSFSKTYG
LSVRATDNKCSADVPLTVQVSTCAYGEDYQFEESEYVYYLDENRNLGIVSNIKLKSTRAAFLTLSQ
NTHFTIDQNGDFRLIQTLNYETKKEYHLTLTANFTQDNVVTRIPIKVFVRDINDNCPAFSEELYAVQ
HTEPLREGLLIAKLSVIDDDTVQNFTYNIVSGDKRFSINENGEVVMNARLVTDTTLLSTTYKLEVSV
NDGKCAKNTKVDVFVYKTVVNSFLFPQPFYKFDISENQAIPFTIDRFVNVGGHEAVYGFLVSTDFF
QIDPKTAQLQLVKKLDFEVEKTHYVLISATYDDGTQTYTVVAIHVGDENDNAPQIFAHPPITRLVQ
NTPHNSIVTKIEVEDPDTSKEFTFKITNGNPLQNNDPDKALFRIESNGMIKTTRCISEDEIGNYVLTI
SVDDQVNTPTIFSNMQVQLKTTDDRSYVTNCGLGCPANDGPFSRYQFEQSLFIVNVTETAANPTV
LKRLTRPFVEKTTYTIGAGADGKFSIDQNGTLSLTGSLDYETKPVYYFIVSAVIPSQVVGRFITSKTAY
LVMVNDVDDSKPVIEGNGGTLAVSETSEIGQIVGTVRVKDSDSSSPLTYNVVNGSQYFNIDSAGNI
VIKDTLNAAGSPYTVTYTVSDGINPPVNGEFVVSISETNNNVPKFTQNIYEVSLSEDTATDTIAVTIL
ANDGDTNDKIAYSFLQSEGPFVINRDSGEIKLIGPLDYEAQKSYNMVVQATDSGGFANIAFVQIAV
GDVNDQVPVITPQTTPTQIHESTLVNSVVYRVIASDGDGNERLTYALANSNDFFTINSATGEITIKE
NLAKSNVSSHTLSIVVTDSSGKTSTQDVALNIRRSGTSQGCVKITTSQGQSSSPVINLPENTASGTTV
FKLVAGDASVFSNMRFTLNREANGVFAVTEQGELQLVAPLDYETKQFYSIIITAEDVTRGVSSQYNL
FVRISDVNEFTPVLETIAPVAVSELANIGDVIAQAKANDQDGDSQVTYSLGEFGNNDNKFTIDAAT
GNIILNKRLGSGAPHVVEVCASDGSIKKCQNVTFIVADTNDNPPSFQDLTGVGSIDLTNTSSYVVY
KPIVTDSDQTGTLSFSLKDPAPSQFSINSTTGEVKANKDIAKGTYTFGVCASDGRYTSCQSVTVVAS
DRSQSITPSFNQDQVGAIVDISASSGKQIIQVMASTLDKAKIEYELKNEADRNIFEINKDSGLITLKN
RTALTLGTIYTIPVKASRADDPLNFDEINIVISVGNQQLDSGSFIEQVLDTQHLVNFSLSTFDISKVSY
YIILGQQVGGDSKDDEKLPVSWYYATFGDESYRHRFYYIAKLDGSKFRNTGGSRKRRSIQDKDSKS
FTYLVGEENGCSKNTSMAAICNGPLKTGTEYRYKLRAYYDDGELRGIPVDQKPTEVKRTQAPQA
GKSIDEYREQDSYVITFWIIGLVIFGLLFWFSVLVIAILLCYKRRYEVTQAYGYSVQTRDKIDGPPTEY
SNTVYRFQNPSYPDNDTVKMKKVDASESRTKTEVQTTSMVVLDDYEQEQPQPDIPVQDYDAIEY
ETRSAQIKFHEESPEWESMDIQLRIDPTGNQDPVITRKSDSGSKDSDSDASKKYENTEYIEEKEDFV
NEKEDSLTRL*
>TRINITY_DN982_c0_g1_i1.p1 type:5prime_partial len:6412 gc:universal TRINITY_DN982_c0_g1_i1:19377-142(-)
KLDFETKQSHTLTIKAVDRGNPSQDSLNKATVIVVVQDFNDNCPLFSESIYNASVSESTAIGTTVLR
VSVTDKDVGNVNNEITCSIDEPLAKGFFSVNKINNNQFSVVLIKNLDYETHRGYRFVIKAKDNGE
VPCQLTTNMVVEVQDEPDVITNFKPFNYVISIPESTLIGTKLLELKVDSESSSITYTLQAKNQPGDLD
KFMLNGQSGELVLKGTVDFEKKSEYEFTATLNDGVNNGGNTANIVVNILDINDNPPVFSQSTCYI
RRMPENQQVNTTVTSVLATDSDQGINSNISYSLIKDSSCNNFEINEITGAISLTSSLNYEVRKEHYCII
KATDNGSPVLSSYSCLKVIVENVNDNSPVFLPKELEITVLESKPVNSFVIRVFATDKDEGNSITYSIV
GGNTQNAFAITPLTGHIILNQKLDYEIVKNYILEIQATDDGTPRRSSNLNAKVMVKVLEVNDNKP
KFNQQRYLRTIPEDTNIGSTIISVTATDDDAGLLTSKIQYIALENTNDFLIQPDTGDIVVSQNLDYER
QRTYQLVAVCSDSGTPPFTGRTLVDITITDVNDNAPILNPTLYNVLVSEKAPLGTSLIQVHAKDKDS
GINGGLKYQIVSGNDLNKFVINNSTGVITLQETLDREKYSSLYNLTVSVSDQATPSLNGNNAFVQI
RVLDVNDKQPVFVKSEYIFEISENQPLNSPIGLVQANDDDSTSPNNIIQYTIVPSKDSINFTMINNQI
LSNSVFDYEEQKSYEFEVQARDQSTGVNNEQLVGRTLVKVLIRDTNDNDPMIENQNIALNISDVT

LVGTLLFDIQATDKDNGENARLTYTISSGDTSTFRINENDGRFYLSKKLDFYNKNEYSLIVIVNDNG
SPQRNSVANVQINVYPIDQTLISFEPQCPIAFTIQENSLSSPMNIKAIDTGSYVSKTMRYMLKSNKSD
VSSMFQIVETNGVIIQNKAANYEERSRYDMTVCARNERGSEGCCPVTIYILDQNEQPFFDTDNDN
LDNGKYLVRVSETTPIGSVMFSVNTGDEDFGSLPNGQKTYSIKAKPGEEMLFLVDSYGRIITNTTLD
REKTPQYDFSVVVTDKGTPPRQAEAEVIVTVIDYNDQRPVFEIMSVRPVNENANNEVLGRVVAVD
IDSTSINNGIKYSIQPAGFLNISNTGTLTSVGSALDYEKNQKVTLYVTATDTGSPYLSATTSLQLDIINI
NDNKPYFLQNNYNLTVSEKTTVGTLVLTLFAEDDDLGEFGEIANYEIVSGNQEDRFSIDSNGRLTL
KNPLNWRNVKSYNLNIIARDKGLPSEQSQIPATVVINVADYSEEQPNFLQNLYVKFIPETTPIDTLV
LTVEATSGEPVYGPFKYTIVPKLPDTNNFQIFSNGEIKTINQFDYENQRNFTFDVQVEDEKNGKTN
RASVFIHITDVNDNIPQFTDLTRSNTQYNITEFTLPGSMITCLSATDRDHGKNAEIRFRGIGGDGNG
VFNVNLDGKVLLSQQIFYDKKNKYNLIVEAFDLGSPEQKAQLTLTIFLHKSYTSVITTDVDINEDVE
TQTRVTRVSSIDPFTRSSNNFTYYLVGKSTTFFEINAQTGEIFTKATLDRETRDRHVLTIQSRGPTTN
GVGELVVNVKDINDNPPIFDPQVYQVTVSESANIGLSIVSVTATDKDIGVNGEVTYSIEDGDQTIFSI
NPSTGSISLAGKLDFESKQSHTLTIKAVDRGNPPQDSVNKATVIVVVQDFNDNCPVFENSTYSLTIK
EPGELGKEYLKLGITDNDNGRNQLFTCSIDEPLAEKYFEMINDCRLRLIKELDYEEFTSFRFVVKAT
DQGDVSCTTTTNMIVNVGDRADVIPFFSPLTYNISIPESTTVGTIVVSLKVGSTFKGLLYSLFHKNTQ
DDLIKFGINKDTGEIFLKESVDREIQDKYEFTADVFDGSNNAVQRASVTINIIDVNDNAPFFNQPTC
YQRDLTESTAIGTFVVDVSAADNDKGRNQQVTYSLLPSPSCTSFSVDETSGRITTSQILDHEQQKLQ
HCLIKAKDNGTNSLSGYSCLTVNVVNVNDNKPAFIVNRFDVSVLETVPAGTLVASLVARDVDNQ
GLITYSISNGNLNNQFTIDSSGKLRTRFPLDYENTKSYNLTIQASDNNGKFSSPDDALVLITVLEVN
DNRPQFLQSYYKVSIPEDTATGASILNVTAIDLDHGELTKSLFYAALENTTQFTVNANTGVISVAEN
LDYEIEDHYELVVIVQDRGTPHLSNRALVDIQITDVNDNAPMFVTTVYNRELSEKALIGAPVVKL
NAMDADKSDKMTFTIDSVVQGAQGFFSINGDTIQLDKELDYEVPEQRTFVLSVSVKDSRDRDGA
NKANVFINVRDINDREPIFDLNMYEVTVSENNVISNPIIEVTARDSDSTPENNNLLYSLVDGEDAA
SFSLLNNGQSAIITSKISFDYEKKKSYRFLVRASDKVRENFTAFSTVFVTVQDTNDEAPVPEKDLIM
VNISESTRVLSTIAIVFANDKDQGINGQLAFQIINHSEDKFEINNKGVIRLKSTLDFFQQNFYNFTVL
VTDKGNPVRTSKPVTVLVYVYRTEIPGIIFEKNHYIFNINEKEIVSLVGTVNAIDEGTYVSRQIVYSIV
SGQNDIKDKFRAVNNNIIQDKEVNYKDQSEYNFVMKAINERGSIAFTHITINIMDVNEKPYFVGL
NNKGRYEFSMSEIISVPSYIYAVRAKDDDFGLNGEVDYEIISNFRFGNFSIDQYGRIQVLKDPDFEFT
RSYEFQVRAKDRGTPSLESNLVSVVITVLDYNDNHPIFNIQEPLEISIKELVNNTDVGTVTATDKDG
SGLNRIVKYVAIYPSDHLTVNRDNGQIRVIEPFDYEIAHKHEFVVVAQDQGTPYLNAYKVIKVEVL
NENDEFPEFTVIEYNITISELTPIGSPVLFVHAQDKDRGPSGQVTNYKIKYVPPSGGSGAGSPDKTFE
ITSDGQVVLKEKLEYKFQKYYQYRVTCTDNGSPPKNSINEAIIHLNIADASLEQPIFSVNPAVVEVY
ENITLNSNIVKVEVDSIEPIKGPFEYSIIASEDSQPFMIDNSTGQITNKESFDYEIKRKYVFLAEARDK
HNNRTQRAMVVIFILDINDNIPTIETIAPQTFVQNISDTTYPGSVVAKLYSRDADSGENAMVDFTIT
GGDGENVFAINPNGYVTLIGPLDVRVKTQYTINVKVSDRGIPTLSNNRALTFNIKKSKFIQNTVRF
DRHHYKSNCIEENQINLNIIQLNATNSNPVAGSTIIYSLDGTLEERQPFIVNSQTGEISLSQTLDYEIK
QEYDFVAVAVNNDGSRDVAVVTVCIKDLNDNSPIFEPQSYVVNVSESESVGYLVYKTTVMDADKT
DFNTTKSYDIIQGNVNDKFIIDSMGHIYIKSKIDYEALQVKHFDLRIRVNEGNLVGDMVLRVNILD
WNDVHPSFSTLNIYNFTIPEDKPVGEIIEILTANDGDATSPNNEVTYKLLTTEDDFILRRNDKGHGS
LIVNKQLDYSKQNRYEYVALAEDKGYLNLLGTAIVIINIQDVNNHPPEFDDSEYNTTVSEHAQIGT
RVLQIHAHDKDTGLGEQIFFIYGDPTETFTISSQGFIILNKQLDYETQRDYIFDVGARDGGNLVSLN
NVTVHIKVSEVNDNEPIFDLSTYRNSYAEDTPLGTTLFTVRATDKDFIGNSIIYSISGANDDTSYVSV
DQTTGDITLQRRMDYESKNYITFTVQAIDSNNRNKLKGSTHVIIDVTDVNDNNPKFVFGSDLVYN
VTVVENINVGTLVVVVDSQDADSGLNGQTRYHIVSGNNENKFFINELNGQINLINKLDALQTNK
YVLTVMAKDLGNPSRSTNVTVTINVLRYFESTPMFLPSYAEVIYPENLALGSSIYELNVTKTLPGSP
MKLSFHSSSKFPEFTLQGNKIILSKEFDYEIKNRYELIIIATDEENDKAFAQVIVRVQNLNDNIPYFTQ
NAYFGVVSELAFVGHSVLTITAKDDDKDFTELFYSIATQVPDNAFKLQDNIVVVNGKLIRNKVYEF
EVEAFDNTFTSKRATVSITVTPENLPSFVNATYIVTLQENYPISTNFLNISAGTFSGITYVITSMKAKD
MFNVDANGQISLTKSLDYEKQRQHVFPVTASVTSPITQVRVASVIVNVLNENDNAPVFVNGPYER
NITEGHLVGTMVIRVDGHDDDSDKLTYSIVSGDDGKFSINSEGVISLISTLNFDLKNKYVLNVRVSD
GKFVTDTIVTINITPIVKFPPMFNQTLYLLSVKENTLVGTSILQVSTVNVIPPSSFSINEQSGRDFFTID

ANGVIRTNNALDYEAKRQHIFTLKVQDLRSMGHATVVVNIVNDDDTCPILTPATQTVQITEPVAP
GTIVAAVQAKDIDSKSLNFTITGGNGLFRIDQHGGIKTINEVDISTSTEYELTVTVDDGTCTKSVQV
DVNVNPLAPCPDCNIYPFTQTIYEFSINENTVPASPIITVTTGQSLQRNFSIEDPAALEYVTINRLTGE
ISVIKAFDYEATKHLSFNVMAKTQTTKNRNPFYVITSVSRVRITVRDLNDNCPSIESPMYYEVSHSS
PIVQDTVVTRILVEDPDTLKDHTYSISDNNFKIDSMGHIRTVQNIESFTAQFYNLTVTVSDGQCSKT
SNVGVNLAVNPTPMTYQFTSNGVYSKSVREDHNLQDVILSVALNQNYPRVFSIVESDANDQFVID
SNSGALRLKTSLNYELKKRYDFTVMADIHGGVYKSYASVHIIVLDVNDECPIFPVIGYNQRVSGTP
VIGTIVEIIEANDADNATLEYSIVSGNEDGHFSLDNSGVVRVVKPFASSFSKTYGLSVRATDNKCSA
DVPLTVQVSTCAYGEDYQFEESEYVYYLDENRNLGIVSNIKLKSTRAAFLTLSQNTHFTIDQNGDF
RLIQTLNYETKKEYHLTLTANFTQDNVVTRIPIKVFVRDINDNCPAFSEELYAVQHTEPLREGLLIA
KLSVIDDDTVQNFTYNIVSGDKRFSINENGEVVMNARLVTDTTLLSTTYKLEVSVNDGKCAKNTK
VDVFVYKTVVNSFLFPQPFYKFDISENQAIPFTIDRFVNVGGHEAVYGFLVSTDFFQIDPKTAQLQL
VKKLDFEVEKTHYVLISATYDDGTQTYTVVAIHVGDENDNAPQIFAHPPITRLVQNTPHNSIVTKI
EVEDPDTSKEFTFKITNGNPLQNNDPDKALFRIESNGMIKTTRCISEDEIGNYVLTISVDDQVNTPTI
FSNMQVQLKTTDDRSYVTNCGLGCPANDGPFSRYQFEQSLFIVNVTETAANPTVLKRLTRPFVEK
TTYTIGAGADGKFSIDQNGTLSLTGSLDYETKPVYYFIVSAVIPSQVVGRFITSKTAYLVMVNDVDD
SKPVIEGNGGTLAVSETSEIGQIVGTVRVKDSDSSSPLTYNVVNGSQYFNIDSAGNIVIKDTLNAAG
SPYTVTYTVSDGINPPVNGEFVVSISETNNNVPKFTQNIYEVSLSEDTATDTIAVTILANDGDTNDKI
AYSFLQSEGPFVINRDSGEIKLIGPLDYEAQKSYNMVVQATDSGGFANIAFVQIAVGDVNDQVPVI
TPQTTPTQIHESTLVNSVVYRVIASDGDGNERLTYALANSNDFFTINSATGEITIKENLAKSNVSSHT
LSIVVTDSSGKTSTQDVALNIRRSGTSQGCVKITTSQGQSSSPVINLPENTASGTTVFKLVAGDASVF
SNMRFTLNREANGVFAVTEQGELQLVAPLDYETKQFYSIIITAEDVTRGVSSQYNLFVRISDVNEFT
PVLETIAPVAVSELANIGDVIAQAKANDQDGDSQVTYSLGEFGNNDNKFTIDAATGNIILNKRLG
SGAPHVVEVCASDGSIKKCQNVTFIVADTNDNPPSFQDLTGVGSIDLTNTSSYVVVYKPIVTDSDQT
GTLSFSLKDPAPSQFSINSTTGEVKANKDIAKGTYTFGVCASDGRYTSCQSVTVVASDRSQSITPSFN
QDQVGAIVDISASSGKQIIQVMASTLDKAKIEYELKNEADRNIFEINKDSGLITLKNRTALTLGTIYT
IPVKASRADDPLNFDEINIVISVGNQQLDSGSFIEQVLDTQHLVNFSLSTFDISKVSYYIILGQQVGG
DSKDDEKLPVSWYYATFGDESYRHRFYYIAKLDGSKFRNTGGSRKRRSIQDKDSKSFTYLVGEENG
CSKNTSMAAICNGPLKTGTEYRYKLRAYYDDGELRGIPVDQKPTEVKRTQAPQAGKSIDEYREQD
SYVITFWIIGLVIFGLLFWFSVLVIAILLCYKRRYEVTQAYGYSVQTRDKIDGPPTEYSNTVYRFQNPS
YPDNDTVKMKKVDASESRTKTEVQTTSMVVLDDYEQEQPQPDIPVQDYDAIEYETRSAQIKFHEE
SPEWESMDIQLRIDPTGNQDPVITRKSDSGSKDSDSDASKKYENTEYIEEKEDFVNEKEDSLTRL*
>TRINITY_DN4595_c0_g1_i4.p1 type:5prime_partial len:1639 gc:universal TRINITY_DN4595_c0_g1_i4:5857-941(-)
QCGVNTGTQVVYRRCNAALCKYQGNDYTRNQEVNIRTQACTVACDYTPWQNTQSCTKTCDGG
VLVQTRTERAFNKIETRNGNTPCNTQACAQELGPKQLRTCNVGQCGVNTGAQVVYRRCNAAL
CKYQGNDYTRNQEVNIRTQACTVACDYTPWQDTQSCTKTCGGGVLVQSRTERAFNKVETRNGN
TPCNTQVCSSELGPEQLRTCNVGQCGVNTGTQVVYRVCNAALCRYQGSDYTRNQEVNIRTQAC
TVACDYTPWQDTQSCTKSCDGGVLVQTRIERAFNRVETRDGTTPCNTRSCASDFSAERLRSCLNV
NQCGVNTGTQTVYIVCNGNFCRYQGREYNINEEVNVRQQNCTIACDYTAWEDTTICSKICDGGV
LEQRRLERAFNRLERRNGTTPCNTRACDSGFSAVLSRNCVNVNQCGVSNGTQLLYRVCNGEPCIL
QERSYKINDVVVVGSRACSVPCNYTEWRDTTSCSKTCGGGFFFETRTEQAFQKTESRLGKTLCNE
NSCVKDNFGNWTEWTACSKTCGMGMRYRSRECLKGFECIGLIEETETCKLGCRTETCPMGRPYP
GDCNFEDDCGKSWNQVNSIQRNAFFIVTSKDLAHQGLLPNMETTFNTKDCGRTMKLNSSVAGD
VNYLWTKRMMITDINTDVDDDVAAIDNYFKEENEQCVEFNFAVMANSLDKIGDMQVYLYYLEY
PNASLIYSKKGNTGLKWELVKRSIRVDTNKAFVILFQVNQGQSNVQYGDIGIDGFKVNPGNCQLK
GWSHWGEWKCSATCGFGTQTRMRHCLDKVNRTACVGMDRENKPCPMMKECPMNRDGNWG
QWTEFSKCSKTCDTGTYFRTRKCDSPSAKYNGSLCRVSQTKFNLPVVNDFEIDFKPCSVQQCNNT
FSQWTTWSSCSGTCGPGQRTRTRTCESGKRCSGIFEEIETCYMGCRLEKCPKFTAEIDCNFEDRNKS
DCQWKYYTVSGTSSWSFKTQPKNRLLSGPSGDSTIGCGYFLYYPSQTLSSAYAWGPNIYPTMDSYC
VKFKYSMVTSVNSSMGNLTVIKHFLNRPRKADTPLFFHSGVSQKQWNPMAIDITTDIQRSPFVIMF
KGQSDPNNAGLSDIAIDDYMLMAGKCNKSYSEWSEWSTCSRTCGVGQRTRTRVCLDDDIKVCK

MGDVATEGEACVKPLCAVPVDGNWGQWSAYSQCNARCNGTQKRERFCNNPAPSNGGRMCLL
GDTIFQLRNNYHAQIRPCSIDCGDNDTFSSYKRINDTCHFDCITGRVLQERECEQDTCFDGNTRFS
KGEKQYTQRACSIKEYCETSEYKCREFRSVCSTNTPSSCLDPQNRAVKSCECQVERGCSSMTNPGQ
TVKHKEFDVFYRPCRYVGVCFTDYETDAFSPYEKVNDTCHLSCSNGKVFEERECRQDNCIDGSNT
LTRGAKQYRETQCTTKEYCETSQYSCREISGPCSSNPPSSCQDPANKSLKVCTCQVTTGCASMLTA
GQAVSYLAEETFSRPCKYTGACNANGNTDKTFSPYERVNDTCHISCADGKVKQMRECKKDACV
DGGQNFPRGFKQFKDSSCTPNEFCETAQYSCRELSSACTSNPPSSCLVSTNKAVKMCDCNDPNGC
DSMITPGKKVNQGEKEFLQRPCRYTGVCFTDAETGT*
>TRINITY_DN138_c0_g1_i1.p1 type:complete len:401 gc:universal TRINITY_DN138_c0_g1_i1:157-1359(+)
MAARFLFITVILIVGNLIGDVCPERCERNWIKIGCFHDRIFPNRPYPFELVNHRDPINNHWDGNLI
NWRKYPDSIEKLACICANKTRTKGYQYFGLQFYGECWSGPSYNFHRDGPSTKCIGNDFKPCDDSS
KTECVGRAFTNYIYKLKDENPKVDGKWSEWEKWSPCSATCGYGERYRERFCSNPEPENGGQTCE
GEAREYEECNHGECQTTCDKEIDFGIALDYSSSVRQVNWNRTLDFVVELSKGFNISPEKVHFGVLS
FSHRTRFDFGLSDKTYWNSESFEKKIKSLEYRWGYTWTDKALKKVKQLIFCEKCGLRKDSLKYLLV
FTDGRSNNVKTDLKTVSDSIKDMGVNVISVGAGHTINHRELEEIATDDNHVFYVGGYAYLGDNM
NAIRRLTCG*
>TRINITY_DN262_c0_g1_i1.p1 type:complete len:1033 gc:universal TRINITY_DN262_c0_g1_i1:111-3209(+)
MADPSSLETKEKDGSYRVATKSGGKDKGKKSKKAAKKVNMDDLKKELEMTEHSCELETLVSQLE
TSLTQGLTKSIAARNLERDGPNALTPPKQTPEWVKFCKQLFGGFAMLLWVGAILCFFAYIIRSIREE
EPGMDELYLGIVLTVVVIITGCFSYYQESKSSKIMESFKKMVPQEAVVMRDGEKLNLNATECVVGD
LVFVKFGDRIPADLRIIESKGFKVDNSSLTGESEPQSRNSECSSDNPLETRNLAFFSTNAVEGTATGIV
VKTGDNTVMGRIANLASGLGSGKTPIALEIEHFIHIVTGVAVFLGVSFLIISLAMNYHWLESIIFLIGI
IVANVPEGLLATVTVCLTLTAKKMASKNCLVKHLEAVETLGSTSVICSDKTGTLTQNRMTVAHM
WFDNMVVEADTTEDQSGQAHDKGSTTWKALAKIGALCNRAEFRPNQEEEPILKRECNGDASET
AILKFVELSVGSVEDIRSKNKKIVEIPFNSTNKYQVSIHEQEDPDDPRYLLVMKGAPERILDRCSTIL
HEGVEKPLDEEWKENFNKAYVDLGGLGERVLGFCHYYLPADDFPKGFAFETEDPQNFPLEKLCF
VGLMSMIDPPRAAVPDAVSKCRHAGIKVIMVTGDHPITAKAIAKGVGIISEGNETVEDIAQRLNIP
KEEVNERDANAAVVHGSHLKDLPQEKLDDILKYHQEIVFARTSPQQKLIIVEGCQRMGAIVAVTG
DGVNDSPALKKADIGVAMGIAGSDVSKQAADMILLDDNFASIVTGVEEGRLIFDNLKKSIAYTLTS
NIPEISPFLMFILLAIPLPLGTVTILCIDLGTDLVPAISLAYEKAETDIMKRKPRDPVRDKLVNERLISL
AYGQIGMIQATAGFFTYFVIMAENGFLPGYLIGLRNDWDDKKNNAVMDSYGSEWTYNQRKNLE
LTCHTAFFTTIVVVQWADVIISKTRRLSLFQQGMTNWMLNFGIFFETALAAFLQYTPGLNTGLRLR
PMNFPWWFVGVPFSLLIFVYDELRRFLLRRNPGGWVEQETYY*
>TRINITY_DN15274_c0_g1_i1.p1 type:complete len:1822 gc:universal TRINITY_DN15274_c0_g1_i1:87-5552(+)
MECIRRSIIRLLLILGFYTLYISSQEECKRGGCFPATGDLLVGREKQLLANSTCGLNGPSKYCILSFLD
KTDKCFYCDSRRQVDERPDGYKNSHLPKYMVTREPSKRLQGWWQSVNGRQDVTIQLDLEAEFIF
THLIMTFKSFRPRAMYIERSSNYGNDWSDYRYFAYNCRKSFPNIPRGPQRKIDDVICEERYSKVEPS
TGGEVVMKVLDPIIQKKEDPYSLKVLNLLKLTNLRVHFKELHTFGDDILGDSPKVNQKYYYALYE
MVVRGSCSCYGHAEQCIPTKGTPANVNMVHGQCNCTHNTKGRNCEMCKDGYNDEPWRPAYQ
DQLNVCRKCNCNGHTDKCHFDPAVYKATGNTSGGVCDDCNHNTAGRQCELCKPLYYRDPYKN
IFDEDVCKPCDCDPSGSFGGGECETISDPNNSSALTVAGRCICKPNAQGLRCDSCKDGYWNLLES
NPDGCQSCNCDDKGTVELNKCDQQTGTCRCKRFVIGLTCSKCLEGFWNLGNTPEGCEPCNCDV
GGSLNAKCDDITGMCMCKPNIIGRKCDQVAPGSYFGNLDQVISEAENSLVRSDSSSVEIVREILNPT
DSRSFTGDGYLRVSDGTLVNFTNIRVPSTGFQTFVMRYSRPTPGSNNNVMVRITKTDGTILGGNGC
VQTNEREIILNGVLNDDNFLELGDMFCLEKDTDYSVQLVVSNRNSPNDGGVNRGVTNLDSIVM
MPNIDNLAIFQGREGMRLKSQYDLNNCRQYYLEPNGAANAPEICKKIQFSISAEINNGALKCNC
DPTGTNSSGSSDILTCQKSGGQCPCKPGVVGRRCEKCAPGYFGFDSNGCTACACNDTGSEKLFCN
GITGQCPCKNRIKGRQCNSCPSNHFGFPNCIACQCNEHSSTCDPISGECTNCKDNTAGPSCNLCA
EGYYGDATAGTEKDCQRCQCPGGSSLNQFSNTCHLKPDGKVVCNNCTEGYTGDQCDKCEDGY

YGNPLVPNGSCNKCDCNGNIDPFADGKCNAENGKCFNCINNSDGDHCENCKEGFFGNAKNQS
CRACVCNKLGTNSATSDSCDRETGQCECLPNVEGVECDRCKSGYYNIASGNGCVSCNCDKGGSL
SSHCNQLDGQCNCLNGYGGLDCTQCEDFYFGDPKQNTCQACGCNPVGSITLQCNRTTGVCMC
KDNVVGEKCDMCAPQTAGVMPQCEECHVCNDQWEDIVNDIEKNITTILTLKGSSPLNVSEIRAY
TNDIADLKNRLDKIEEDFGKQMVDPNKINRLRDKVYRFSYNLTGINRDADFYMEMVNETITRDE
KANDILDGLELRLATLQTNTEDFKNNVTKIVESNIGGALNSTRESLEKSRKAQESVNDSDLKIKES
KKMRKKIKKEIQNGDPEFEVMHNRSSEFVNNLADRENYLEGELEKLNRLLCGSDKRCGGCTKGG
CDTCGVSGENYTCDDGIKMMSMMASDKALEAKEAIDQKLENASELLENVNRAKQHVIQANISA
NMAEDKAEEVNKIGQAVLKNITTLIKDIQDFLATDFNHPNASKDLAMQTIALNISLTPDEVVELG
RKIRDAVKSLTGVDKILNESRNDYDTAIGLKEQALAAKERAIEVMNQTMAIIETLSQAEELQNSTL
AGVNVARADIEDVKDLLKKLKEKLDAADNDLQMADNSTASAEDKRNAILDQNLENREKLDLSE
KKIDEAEELGDKVQGSVDKMNEKYNATKAKLNEKEDMIEKVRARIKALADKTIKLYENADRKLR
RINKMKDSYIINEERAKDLYYEIRRLEIKEFFINTKLEGTAKCYSTCNPIIPESFCRDVYKNIAVKETN
AYAELEAEKASTTSLLI*
>TRINITY_DN161_c0_g1_i2.p1 type:5prime_partial len:399 gc:universal TRINITY_DN161_c0_g1_i2:1683-487(-)
WNQEPLTCTYLYSVNSQLLKYKMADDDVAALVVDNGSGMCKAGFAGDDAPRAVFPSIVGRPRH
QGVMVGMGQKDSYVGDEAQSKRGILTLKYPIEHGIVTNWDDMEKIWHHTFYNELRVAPEEHPV
LLTEAPLNPKANREKMTQIMFETFNSPAMYVAIQAVLSLYASGRTTGIVLDSGDGVSHTVPIYEGY
ALPHAIIRLDLAGRDLTDYLMKILTERGYSFTTTAEREIVRDIKEKLCYVALDFEQEMQTAASSSLE
KSYELPDGQVITIGNERFRCPETLFQPSFIGMESAGVHETTYNSIMKCDVDIRKDLYANTVLSGGTT
MFPGIADRMQKEISALAPPTMKIKIIAPPERKYSVWIGGSILASLSTFQQMWISKQEYDESGPSIVHR
KCF*
>TRINITY_DN1616_c0_g1_i3.p1 type:complete len:3855 gc:universal TRINITY_DN1616_c0_g1_i3:218-11782(+)
MIIFLAFLAVAVGQEVLLPPYFDIAKGKNITATATCGEDYVAPKGSPGELFCTLADATGKFGIKGLK
CSHCVPGDYGDGDGKNARDHHIRNANDGSNRWWQSPPLSRGLQYRKINITMALGQSFHVAYVI
IKAANSPRPGVWALERSNDNGRTYTPWYYFAPTSSECKQYFGVDSYLPIVKDDTVICSTEYSKIPPF
KNGEIHISLINGRPGASNFSQSKTLQEWTTATNVRLRLLDIKTLLTDLLPLSLEDPTVTRRYYYSIKDI
TIGGRCMCNGFADECTPHTNIVGKYECNCQYNTEGPRCSRCKKGYVQKEWRQRTSTDKFECEKC
NCHGHSDECEYNEEINKRKLSLDVNGNYQGGGVCKNCQHNTLGINCEKCKPFFYQPANVPIDS
PNVCQPCNCDTQFSTGLCAEETGQCECKSNYVGRYCDQCNKGFHMFPVCKPCECDVNGSEDRE
CIPDQINGCKCKKGYAGRYCDECLPGYFGFPDCQPCACLTRYGAVNDNCERDNGQCSCKVGFK
GRRCEECAAGHYQFPFCKECGCDPEKTLDTVCDSGTGVCQCKSNFAGPKCESCSPGFYAHPSCFA
CGCSTAGSTSSVCDQNSGLCSCRPNYSGFKCDRCAEGYYGFPNCILCDCNPEGSRRVGICDRQDG
QCQCKPTYSGQKCDKCLPGFYGFPDCKRCNCNPAGTKPGPGGRPGDCSTSNNGQCVCKDNVQ
GLECNECKPGFFNLTESNRKGCSTCGCNGAGTNNGVKSCSVEGTCKCKPLVKQEDKCTECRDGY
YGLKRTNVFGCLGCNCEVGGSSSSVCSKDNGQCPCKDNIVGKRCESVAANSFYPDLCHIKAEME
EGKTTDGDVVRYDYQKEIFPDFSWKGFVAVNDQQPSVVVSINVPKTSEYQVKFRYALNQIQPVTA
TVTAFKGDSKIVGSINFASTQKGSGFVASLATVQDNLVQEMYFPLTEGQWNIKLEAPHSNLVVDY
AVLFPKEYAQGGALKKKVQNACDVMTDEKFCNLYRYIDVADKKDIIVLEAENPVKMRQQKPPVL
FNDTAVLSKLDFQAMVVLSPSNSRQLNHLFNVAKPGKYVVMLQYYHDSDTAKYLDVYVRSQIAV
QNGRALLQSCRYTFGCRQAVLNRGDNGVFVFNIKSTKNHLLTLLSRYAVVAVDSVMLVPYESWH
LDYSTPTLYCVTKDSKCTNMNYDVPAESRLVQANPLEQDLSEKPRYLIDRSVEVRYVKPGVNIINIR
DVKAGNYLLVAHYYQPNFPSFESRAAVYSGRTSGLRARFDFKFCPNLNGCRSPIRRPDSNSNLYVF
EEGEARIRFDIPNGLDAWIEYILMIPEGTNVSSLYTPRVLDYSDDFKKECSIDLNVCTSSNLSSFCKDS
IFTITTDFNNGSFPCKCNMDGSKSSSCQEFGGKCPCKDNVIGRECSRCKVGYYGFPNCKPCNCSN
CDEETGVCTCPRGVIGENCDQCAPGTFGFDPIIGCQKCNCNEIGTGSANAECNVTSGQCPCKEN
YSSRKCSACKPGYFNYPACTKCDCLDAGISAEKCDVLTGKCICQNNVIGLKCNECADGYFKTNDI
KGKQLCAKCWCNGRSTKSCTEAELFRATPITASKGWTVSVDTLNVIENGNGRFTISDVNTLPEPV
YWVAPNQFLGNRGLSYGSHFKYNFQYSVKQPSQNTNGDVVTDAVIKGDGYTIKSMRSVIEKEND
GYSASILMKETEWMLEDGSTIDRELFLAILSDIDTLQILATHGKNTISSTIYDISLETAGENGTDRVYG

IEKCFCPKEYADLSCSKCSKGYTRQKDGSCGKCQCLGKSNICDPQNGKCINCSGNTMGDRCEKC
KPGFYGDKCKACECPFSNPENSFANTCAINVNTSSLVCQCNAGYKGVRCDQCSDGYYGNPLQG
VTCKKCSCNGNINASFTGNCDNTTGDCLKCLYNSDGNQCQECRDGYYGDAVVEKNCTDCQCSI
CGTTTAPCNKATGQCQCKKNVEGSKCNQCKPNTWNFDACNGCEDCNCAEASETQQCDLKTG
QCKCPPTVFGRRCGFCKLGYYNYSSEGCQKCQCPDGKPCDVNTGKCCTGVLNEDCKLCPVGHII
EDGECVECDRCVQRTLQRVNTLIHNVTILEKHIIPAGGLNYGALETLGNRFSNLSNKGDEHISNL
DTLKKAIGFGEYRNHSGNMDYVRMYLVRDFKSCSFIVNIKTPPTSSAPATTMVTNTPGPDPIRDHL
SNKKISDSQCPPSLARVIDEGEKNIKLVEKKVVVLEKDAYESTENATATKKRAEDLLAKLKDFMVD
FKDVLSFANSTLKGNVVINIDGNRSLAEAEEMLKEIKDRNFNSTINRSEREKSAAINATIIAEQLMK
QASELANKAYKFNDTFKDFVKALRNLKMESELALLDANNATNSINLAKAVDFQSIIDNAQKLHS
QNNVTVTQAMEILQNAKSAYENAKDVFNKFHNQTGPLSDLMEELRNYVRKLEFDLQTTSPIVDD
SVKHAKRLRDSADRLKELLQDTKKFADAALRAAKVYQNIVDAINDALDAARMANETAHEADK
KVQQAQQDAVESSITSKVVDTEANDLTEEVKKTLKVDISDMKMRLKELRELRKRIQGRIDNVKED
VKSFAKEKEVGEKAMNISTQADGVMKVANKGNEASQDTNEKVEKDGEKVETLKVDISDINKAIK
DLASNVTLVEDTIPKIRSNIDEVNVKHEQIEEFIKTNVTNNIGYLKYLISNAHDILDQMQLSMKFNS
TSEVVLDAPKSVADPTINNKISLTFKINQGNGFLFIIGDFSGNSSGFMSLHVQNHTVVFDYKLNTG
DVQSLTNPKFIETDVNYDVMVTRNFATAVLNVQKSGNASTLMTAYQNRSNISPVLSLGQDTNVY
VGYHPSTPNQRPNFDGCINQFRFDEELMGLWHWKSAKNVGGCVQDFRSIEGQYSFDGDGFVEF
DLVSPELSGFRKIELRFATLEENGLIFLGTDPPTSLPSDIMFDIPLSNQIIAFNALEIKDGYLVSTLDLG
NGVQRLVHTNAGKVNDGKPHLFEMKVLISSLLIKFKLDKKKITEIAISPDTDIRLRLKEAFIGGLPQ
RAWLPNSNISTKGFTGAIGDAKYRGGEFKLVKRFNNIRKGFILPKGYSASFPEEKSYLRLDACNLK
QNSLFGFSFQTFEKNGIIFHSEEGSGSSRSSIEIILIDGQVKVDVSVGSQKRTLQTSVARGFSDGLWHT
LSLYFEETLQEADIVLLYLAVDKAYITGTVPKVDLKGSMYFGSKPSGTESGFNGNIRQVYCNKNEIN
LASGDVFEGGVVGSLKLLPPLKLADFTVVPTTLPPLTTSITTSESTTTETVMTTSTTSISGTTLEPPKET
TTNMAKTTENPTVKITSSEPIIETTMESTTVAKTTSAITMEESTTTPTSITTSTLETTISTLEPGSIEGSTT
TAEPITTTAEAITTTATEPITTTEPITITQPITTVEVTTEPATTQESTTTPEPITTLETTTTPEPSTTPEPTTP
EELTTTAKPTTTIRVTTEPPKCQSNLEVYDEAYGFGMNEPSFIEFGVDGRKMLKSNQFEFLFYTAEN
DGMMISSEPSNLYDYEYVYMENGAISYAFNAGAGRVRVTSTKKYNDGKWHEVKISRSKESGTLTI
DGELIGTDSKSGKTAVNGIKKIVVGGSSVPYRNIPPKSQVGFKGGMRGFKLFNEALKPLKEIGITEA
YDELPIPEGVSFGPDGGYVALNFKSEYVVRTEYKLALDVRSVSSSGYIFFIGNYGADYLSLYIESDRK
VVAECNNGGGRFSVSIDVPTICNGEFHSILLEKKYKTLDLSVEGVQKSFTTTKGSTSADTGSNPPAF
GGFNGVGSRPKNLETFTGCITAVRIKDKAVDLTAPDVTIFGQVSNGCPSNIR*
>TRINITY_DN1369_c0_g1_i1.p1 type:3prime_partial len:1577 gc:universal TRINITY_DN1369_c0_g1_i1:93-4820(+)
MRIQLTIFVTFSIILVSGQNSPPGICSTWDGSHYQTFDSYSFNYHGTCSYALALDCRPGSDDFAVHV
ENDEKCDNHGGTDCTRAVSLYVNDIEYKLGSNNTVLVDGKSNIVLPYFNEGVRVSKHAEYYFIDA
LEGKVVVKYDGRSSIYVQLDSSYQAGVCGLCGNFNGNKKDDLKLPGDKGLTKSLKEFGKSWEKP
RIGQVCKEVTEEHFDQCTNKSKMLVYYAEKECEILRTSSEFKKCHKIVDPTPFYEKCREDLCLCGS
GDKYCKCDVLEQYTRKCAHYGTVLEWRQQNLCYKNCTGNKVFSECGPACSSVCSAIGSKSVCK
DACIDGCHCPPHTSLSGDKCVPDDECPCEHNGDMYDHGDVINMKCKSCKCNGGKWKCNPLP
CDGEASVYGNGHFVSYDGKTYNYPGKCLYLYTYLSDRSFAVYLDRTSCDSLACHMNVMILFRGD
RIKLSASNSTLALEVNRNVKSFPYISSTGYRMYRFSTVFVRFEATNGIKVDWDTYSRLHVLMPHSL
KGKVFGLSGNFDFKTKNDFLTPRKDLAHSAIEFGNSWMMYSSRLNCSALAPDYRMTSCEANHQ
VAKYAESKCSILMSSLFRECHKVVDPFIYYENCKDDTCECNGDSKCYCSVIAAYSKACSSAGKPTE
GWRKYTPCEEKCSNGMVFTECGNSCLKTCDDQTDPNPFCEKECVEGCLCPVGQVLDKITEQCV
DIKHCGCTYGNRTISNGGTVAKDCNTCKCTKGVMVCTKKECRDKKAICSDGKEWSSCMSPCPK
TCQTKDDICPETGLDCIEGCQCKKGYIESANGTCVELSKCPCYHNGKEYQETQTISKDCNTCKCT
AGEWSCTEKLCDATCSVFGDPHYKTFDGKIFDFQGKCGHVMLTDKCANNDNATENIRISVKSST
CGSQGVTCTREITLNYHDTVVHMKRGARKIDIVSNKRQDHEKDQVKVYRHAGIYVHVLTPMGV
SILWDNATRLYITAPPSLSGKICGLCGNFDGSETNDFVTYQGDTAGNAVSFGDSWRTENNCPLTKE
IEDTCHNRKHRLDPSKRECAVIKSDVFKKCHTLVDPVPYYDRCVFDACGCDSIGDCDCLCAAIGA
YAKECIDEGVYINWRKGHNICEKEMCCPTNMTFVPKGTGCPKTCKYPNGLHHCPTKYVDGCQC

PEGLVQNNDANGDVTCVRPEECKVCKLDGKIYNEGEVVLYKCKECVCRDGKMTCSGNYCPTTL
PPTTTTESTTTPSTTPETTIPSTTTTESTTTPTTEPPTTTTTASTTTASTTTESTTTEATTTTKPTTTTTEST
TTESTTTQSTTTPSTTPSTTMKPIDTCVYSNGTHNVTLERGDVIQVNECTKKTCVKDEVTGLHEIKV
TTTQCENSTCDTTCEASKSNRAILVKTPGECCPQCKKPEDLCDNPNEVPCNSTYCIPKSWFCDGN
KDCSNGRDEECCPNYCKVEGEIKKPGEVWTNKEKCLTCKCEGYYTKCRRMECPELDCPEERQVK
SEGECCPHCKPQTEPPTTTTEMTTTTTESTTTPATTPSTTSKETIIIITEEPKSTTASPQEKCDCKVLQPV
GVSDGRIPDNQMTANSV
>TRINITY_DN140702_c0_g1_i1.p1 type:complete len:2036 gc:universal TRINITY_DN140702_c0_g1_i1:94-6201(+)
MATSFKVSEIKCTELLNLEKKGAIDPLVEVEYLGLSKETENKDNDLNPSFGEVFDFDLSGKALGPE
DFLKITVKDYEKFGRNRVVGKADVYLQELLSNPSSTLSATLNDKNDRPTTGKITFKVDYIGPAGGG
AGGATGGGGDDRQAAERELEGDEDEGDPNAMPDGTPIPEGPAGDAMRAEKRKMKKNKKKRR
EHNLPDKPTDFQIRIKIIEGRQLPGGNINPTVRVHLGEETKQTRVKHSSNKPFYDETFFFNFNERPN
ELFDKLVTFEVHNSKNVLSEAMIGAFQFDVGTIYFEDSSHAIIRKWLLLTAPEDNDKKDGEETKGA
SLQPGGPPAGYLKITAIVLGPGDELPEKAKGSGKSEGGEEDIESNLLRPAGVTLRPATFLVKIYHAE
DIPQMDSAAFDGVKKLFSSGSAQKDHVDPYATFSFAGKTQKTEIKYGNNHPDFNQELRVAFKFPS
MCERLKLQMFDWDRIGNDDCIGTSFISLTAISGNGDEGFLPCFGPCYVNFYGSTREFSDLPDEHDN
LNKGRGEGCGYRGRALVEIDTRFGDHTAKPVQDIMAQELVRVQAYMRRRKYKLFASFLSANMIS
EADGLVEFEVSIGNYGNKLDPDTEPQSSSTPPTNPVYDGCAYYFLPWQESKPCCVVESHWEDVIFR
LEPINCLLKIIDKMEKNIKSVELGLKAKAQPAEVAALLIALLDQFISDVSMDLPKFAATDNGVNEL
DLKIQEIRSDELLNLKEEASTLREQATDVQEAMDTIDGYLKRMRDLCVEPQNSMPDVVIWMIAGS
KRVAYHRIPAYDIFYSENPLARGRKCASLQNFILKYPGKKGHDMEDHPEIPASVSVILWLGLEKHQ
AAWTDTEQMEGEYSVYAETYENQGKPPMFDWTAKIIKRPGFSDSEGKIRLNKEYYEPPEGWKWD
DPPDGDWFIDPEMSSNFDADAGHKKFMQDCFENESRVPAGNWGKSTPHYTNVQGDEIDHRDE
TSLPEGWSWTDDWKVDVNRACDEEGWEYCIEATGGGWGAVFRSYHFCRRRRWVRNRQLEADP
QKVMKQKKEEAKQGEGWEYAPVFGMKYHVKEHKFDFVRRRRWHRKMVCVKPGAVPMFKIPD
DDDKGKFHQIMPRIFLAYDEPHKWQFRAYLFQARDILGLDSEGTSDAYAKVCFQDQSQITDVIEET
LCPEFNQTLLFEDMQLYGSPSVFAENPPFVSIELFDKDKVGKDEFLGRVLAQPIVRLNGLKPPATRL
MWYDIHHGERTCGELLAAFELFLDEGADLPLPPPWLEKEKKFQVPSGIRPILVKTGVEILNWGVR
NMKKYQLASCTSPSVTYEIGREIITTDVIKNTKKNPNFLKNIFYKEVYLPKDPLYMPPIVIKVRDHR
TFGRKPVVGRHVIKSIDFVQPPKADPVVELPPADLVGEPEEKKDVTLDIEEKKKKKEIPVDDNLDW
WSKYFASADEIEKCGNYLKKGHKTIQIYDSELEEIKDFNGFSDFIVSYPLERGKEDDDEDDNVVGE
LKGLVKVYPLPQDPKDQTKIRYFTPKLKEKSKIVKCIVRVYVIRARDLQPKDPNGKADPFIVVQLG
KKKISTKDNYIPNEINPFFGSFFEMKADVPLDKDLKIKVMDYDFMSGDDLIGETMIDLENRFLSNC
RATCGLSPAYYTSGAYQWRDAKSPKEILDQVCRTKPQWLSNEQVVVNGQLVKIEDFEESPPTDPHI
GPVEQRLALHVLNNEDIIGHRLVPEHVESRSLKNPLQPSMDQGQLEIFVDIFPIANGVPGPPLDIAT
RKPKEYVLRVIIWNTAEVPPSETSITGEEMSDLYLKGWMEGQDDEKQKTDVHYRSMDGEGMFN
WRFVFPFKYMPEERVMVVEKKEHFWSLDTTKQTLPPRMNLQVWDNDLFSANDFIGNLELDLSK
LPRPKKAAKKATLAMLDTEGDDVMDLFQAKIAKGWWPMLSENEDGTRTMQGKVEMTLEIVTD
LEHEAKPAGQGQEEPNMNPKLDPPNRPETSFLWITSPWKTLKHIIWKNYKWYIIGFFLFIILILFIGL
VLYSVPGSIVHKIFGFQ*
>TRINITY_DN428_c0_g1_i1.p1 type:complete len:1949 gc:universal TRINITY_DN428_c0_g1_i1:96-5942(+)
MADDADLKFLMVDRRQVSDPVAHAEWAAKKLVWVPSEELGFVAASVKEEKGDKIIAQIEGGKR
VTFHKDDIQRMNPPKFDKAEDMADLTCLNEASVLHNIKDRYFSDLIYTYSGLFCVVVNPYKRLPI
YTDKVINLYKGKKRHEVPPHVFSIADTAFRSMLQDRDNQSILCTGESGAGKTENTKKVIQYLTAIA
GRANQQKADEKSQGKLEVQLLQANPILEAFGNAKTVKNDNSSRFGKFIRINFDNSGFICGANIES
YLLEKARLVRQAPDERIFHIFYQLLLGASPEVKKQFLLLDTKSYNFLSNGHVQLPNMDDRQEFKV
TLEGMRDMGITQEELDPIFKVVSAVMLFGNMEFKQERKSDQATLPDNTIAQKICHLLGIQVTDFT
KSLLKPKVKVGREFTQKSQTKAQCEFAIEAITKALYERMFKWLVGRINKSLNRGKRDGASFIGILDI
AGFEIFQLNSFEQLCINYTNEKLQQLFNHTMFILEQEEYQKEGIDWKFIDFGLDLQPTIDLIDKPMG
VLSLLDEECWFPKATDKSYVEKLHKQQTKHPKYLKPDFRSNSDFCVIHYAGRVDYSAAQWLTKN

MDPLNDNVVSLLAHSKEAFIASIWKDTENVVGMSVTEGMDTTFGASRTRKGMFRTVSQLYKEQL
SGLMTTLHCTKPNFVRCIIPNYEKRAGKIDNHLVLDQLRCNGVLEGIRICRQGFPNRILFQEFRQR
YEILCPGIVPKGFMDGRKAAKQMIDNLEMDPNLYRIGQSKIFFRAGVLAHLEEERDIKLTEIIIQFQ
AFCRGNIARRNYRKRIQQLSAIRVIQRNGRSWLKLRNWQWWRLFTKVKPLLNVSRQEDELRSKE
EEFKKLQDKSDKLESLQKEQQQANEKLTEEKNTLAEQLQAEIELCQEAEEARNRLLQRKTELEDL
IQDFEMKLGEEEEKAVKYAEDKKKLQQGIQDLEESLEEEETQRQKLQLEKVQVEAKLKEMEDERQ
VAEDNNARLTRDKKQLEDRVAEFGQKLAQEEDRAKQLSKAKAKQEAFIAELEDQIAKGDKSTHE
LHKEKRKAEQDLSDLRAQLAQALQRIAELEDDLARREKELAEAIKRGDEEMAKRTTVEREKREL
QALLDELREDMESEKSAKDKVERQKRDLEQDLQELRSELEVTTDATVVQKEFQKKREEELMDLK
KQIEEEAKEHDQQMASLRTKHNGLVEDLNNQVDTFKRGKAALEKNKTALENENAEIAADLQRI
SQLKQEGDRKAKNLEQQLNEANSVRAVQDEKVSSLESEKAKYQKECDSLSTQIEEVETKAASLDR
AKQAAEAQLEDTQDQLAEETRQKLSYQTKLREASDEANRLQEQLEEEEDEKKAVQKVLSNLQV
QLDQAKKEIELKIQDFEQAEAARQKQKRENEELRGEVEKLTGDVSRLDKAKKKLQGDLDDVTVS
MERERNSANQMSSKQRKFDQLLAEEKANSQALGIERDNLEKLARQNETKALSLQNANEELEDR
LAEAERQRKQLQMELGDLVESKDDVGKSVHDLEKSKRLLEQQLAEQKTQVEEIEDELQACEDAK
LRLEVNMQAQKAQFDRELQAKEDAIEEGRRGLVKQLRELEAELEDERKLRQSASAAKRQLEGDV
KELEGQLDQANRIKEDGLKQLKKYQNQLKDVQRDLEDSRQSKDDLAAQVKDNEKKLKGLEAEF
LQMQEDLSAAERARRAIESERDELLEELNNNSALKNAVAEERKRYEAQLAALEDELEEERTQTEL
MQDKVKRSNLQLEQMNAELQEERSAAQSLDNTRMSLERQNKELQSKLADLESSLRSRQKNTVAT
LEAKINNLEIQLDQEAKERQQQNKALRRNEKRCKDLQMQIEEERSHTESYKQQLEKMNARVKN
MKRQLDESEEEVTRLNTAKRKLQRDFDENQESLESSQREANQLRQRLKGGASLSSSRSSGRGRRA
KDEDDDDDDANE*
>TRINITY_DN602_c0_g1_i3.p1 type:complete len:851 gc:universal TRINITY_DN602_c0_g1_i3:3046-494(-)
MIICRLHGLSLGIILIFSILYTSDARSRHAYGHKDASSPQTGKKRAESDAGAGTEAATGANTGAATA
SDSSSESQTFTNTKVNTRIATSSNARSGANAAAKVKTYAHANTETKVDTQQQKPTPPVPPEALTT
PPPGVTPPGPPGPPGSENNNTSSGSECKEKETKPPIIKVYCPNCPCPCPPPCPCKCPCIKVAPPPPAP
PPCICLPPCPPPCPPPPPPCPPPCPEPLPAPAPCLPPCAVPPCPCNIAPITLDPSLPCPMDCPLPPCAC
PFMKKDNITTTKTKRSVIRKRNLNIKRSHRVGTKRVNAGKSPNLAYAQPDSFEKAAVAADNKKCS
EIGVEILKKGGSAADAVVSTHCCVEIVNSHSTGLGGGGFMVYYDKKSGKSRAYDFRETLPKKYTD
TQNKTQGETILVPGVLRGLEAVWKDFGKLPWEELWKPCIKYAKEGFYVHEALAAGIVKKADYIM
ANLGLRELFAPDGELLQLGSLFKRPKLARTYEQIAKHGADLFYRGEMAKQIVNEIKEAGGVMQL
EDLSSYQVNIRETLKSNINGLKLLTSPAPSSGSLMALALKIMESFKWKPEDLLNNPSLVYHQIVEAM
KFAYAPTSYLGDPRFTNGTDKVEQYMVSPQTAKEMFKRIDNKSHPVEYYKPWSELFSAEKTGTSH
MSLLDQEGSAVSLTSSINAYFGSKLRSREFGFIYNNELADFSEFWPTVYNLTSDKKAPSKRPMSKVM
PGIFLKGKTVQGVWGAAGGFFIPSALTMSIANWLFFKDNLKVAITRPRLHCQLFPPTVVYEPTFPP
SLIPKLQEFGHKYVTNSTYDVSGQLNAIMGVVQAIVRLPNGSYSAEADYRKGGRPAGF*
>TRINITY_DN477_c0_g1_i7.p1 type:3prime_partial len:1130 gc:universal TRINITY_DN477_c0_g1_i7:3387-1(-)
MMGSIKVFVGLVLLSFTAIEIAQAQSGTVLSNKQFAFDFVVDGSTSIQDEQWNLLTDFVSRLAVKM
STLNNAKLSALQFSHTLNRILQCNQYANVEEARNTIKNMVQENGATGTLAAMLTSADNLMNVC
SAGDPNRQNVMIVIVDGNQNLEQKSHSEIVQDIKVNVKDKNILVLTLGVGPQVNRNEFGDYASD
SGKGFFVDDFTLLTDELVQRMYDELIRTYGDGPDYLTAWVNGPCSATCNPFPMANRPTLRQTRQ
CQEPNGGNCGGASLEQIINCNENIACAGETYLTAWVDSSACSLTCNPFPVSNQPTKRQTRECRQP
SGSCNGASLTQTINCNENVQCPGETYLTAWIDSSCSATCDTGSNNPVFTQTRQCRQTVGNCDGA
PLQQTRSCNVGVSCTAPTFLTAWSNATPCSATCNPFPMAVRPTLTQVRQCNEPNGGNCGGASLQ
QTVQCNLDVACAGETYLTNWVDLTACSATCDDASNRPVKQQERQCQQPGGNCDNLPLQQTVN
CNIGVSCTVSSSACSSKEISFTFVIDTSASVDNIGFFVLKDFMKKYMSQSTLGTDPRTSVAIVSYGAG
DAKVEVECDTITTQQSFNTTVDNLSQLFGIADDNSFNQALSTAFDASGRCARSSTSLNIIIMLSDGD
PPALENILTRDRIQSSSNYYFMFVAIQASDGAQSTFASLANQGRGLRNASSSFTDLYNFNTDLYYLE
TLSKACDVGGQWTEWSTSACSTTCGPGVVTRTRECRNRITGKLQQDCGPGPAQETQACNLQDC
QGTLTQWTSWSCPATCNINNDVNNPPLALRTRQCLGSTGQGNCNNADKTENEPCVRNVPCPG

AWTTWSVFSACSETCRSQSAFPTRRRTRQCIGGTLNFQCPPQIVNGISYREIWQEDCINVPNCPAK
FTQWTTWSCPVTCNVNNDRNNPPTATRTRTCDDSGTINGCANANILENQPCVQDVQCPGAWS
TWSAYGACSETCQSGNRSPTYTRTRTCLGGTLGFQCPPFITQIGGFQIFGTQTSNCNSEVKCPSKLT
QWTTWSCPVTCNNRNDVNNPPQARRTRRCDTSTGSGICESPIVEEFENCVANVPCPGRLTEFTYD
NCAHTCNVNNDVNNPPQRVGTRRCLDGTLG
>TRINITY_DN3078_c0_g1_i1.p1 type:complete len:1109 gc:universal TRINITY_DN3078_c0_g1_i1:3628-302(-)
MKMRIYLFLILFATVYCRVVQKGPNDIPEETKNEIKNQEPKIKFEPEKDKKYFLAKDTTCAHDVDK
HCKNIRRENNFAVFVCLQDVIHKDEDDVAEECHETLWHYKYNLTRDPNFDQAAFKVCENVINEI
QECNDSELLKSPGFVTNCLIENRHAVSEAKCKSFLQKMSAIVFEDYRLIKGFYDSCVEDVKTLKCG
TVAKPTDEEYQHDQTEVISCLEEQLDLVSEKCQAQLVKVATMQADDYHLDRPLFYACQEARERF
CPEVKSGDGRIYKCLVEHKTNEMMLEKCRKKIEIREKLTQKDIKVNELLWKSCKADFTKYNCPKD
GISEHGAASSLLLCLQGKVAEGLPVDQSCQAEMRTFAIELFENYKISPVIVSKCDVEINQFCHHLIG
KTDDGDMMDCLMTQAATNNESVSKKCYDAVSDVIKETGAGNNYKIDRALYLACEPVITTLCQG
KEDAMILGCLMDNAHNSKMPEKCAAQVFHLQYFLSRDFRLDASLYKDCKKDAEEICSANDFNA
ESESKVPDNFVIACLYRNALDRKVDDRKVSQTCAEHVFRVMHQRATNIHLMPELERPCMADLG
KHCIDKGDKEGEEIDCLQTNYEKLSAKCQNAIKVFTIQEAKDFDLDKHLVDKCAPMVQQFCQRE
LEDGNGEKVLPCLVDHKNDIDMDHQCANAVEHWQLIEMKDFEFSPALTEMCLLDIRKHCQKSK
AKIEALHCLSENIVKDASSVTEKCRAQLKNELIEQSENIKLNPELYEACTKDIELFCRDVVPSGGQL
EECLKRNHKKLTDPKCKSLLFKEEKKESFDPELDYRLMHVCKPIIKRYCTQYHQPVDIMECLRQV
THDRDMSRECRAIITERQIEQAESFELDPELSKFCQDDVNRYCKEEIKHAEKGDDDGAIFGCLVNA
MIDNKPIKNDCEMYIRRREAEAAQELDLNPQFLLQCRDEITKICPNMDEDEIIECMKMNIPQIQSK
GCVQEVKKLIVEGIEDVHVDPHLQEQCARDIRTFCQSVPSKSGNVVLCLIDVHKAKNLHLQPNC
ENFLTKRLSLYNAAKVDYAVFDSISAVMNAVSESPNRNYVYMTLLLVLTILFVGGMLFGRYTKKIRS
EIKNR*
>TRINITY_DN71276_c0_g1_i1.p1 type:5prime_partial len:1201 gc:universal TRINITY_DN71276_c0_g1_i1:1-3603(+)
LTFIESLRLSSIKNIFKMMISFETFVPSTTPYAEDYNTNPQVMMLSNKIKETIDTSLNPLASQIDRQKA
FQVQEEFKSSSPHCAEVGLYLYSVERKNSDAVRLCALKFVDHFIETKWNDLTYERKTFYKKESLKL
VEQMTFENETYLKEAAAQIVVEIIKRTWPQSWADLFKNLEELQAKGDAQLELVLIILMRLSEDVN
GLDERLTTQRKRDMMTTFQDNANELFEFFIKTIRDKVTALKTESSSLLIRLSEATLFTMMEFVSWA
HFDCLFNNEKIIFQILFWLLEVKYLRYYAAACLDNIFQRKGTPEKRIEFMVVLSDDVLNIVENVVRS
LKKTPENVLPEEGYKFLKKLAEMLVSFGKIQILSLWGDERGFNEKPVGYDRYLRLMLLLFQHESM
KLTYDVSKFWHALSIHPHGKLDDTFKELMPDLLDCAIMKIAKISDPKIPNNPSSHYSTMDFDDKE
NYAEYFANCRGHMTDVCRKITIISYTVVLDHCLAKIKQATSKPPPDVSGIPKNQIQITQHYLYWDA
LVHFLKNGFAGVFFRANKEISEDTISKCNEIITLLIHYKCEVPLFTIPVINLLESVFPIFKHSRDHLLPT
MQLLFSIIRLPQNDEDPLRDHVFKLKRHVCEAYAALCKEIPHCLVDYFKQLEEFAVYQMMHNASL
TGQEKCRLIEGVLRISKHVTVKEEQDHCINAIFDMIDSMFDKEPVRRACESPISLIQYIGLDQEGTV
AADSELRTRELLFAINLWTAACKALAMSKEEEVKDEITEEEMRTQIETYGWIKRMVTMLPKMLSL
LQSYSLIWQHQDKICDKRILVHKIITEINPTIQVFQPKCADEVNEKRGLGQYYLCTFYESAYTSLGSV
SKSVGPSLFSIGGLAEAMNEKLVQHWNNLPNYRLGRITRTFIKDICLACPKKYFETFIGPFVSQVLR
LYFERISNEWAKLAQLKENPREVTDKLQEQIEIAEERMIYLMSKDLLDLLLSLLTFKRKHLRSTTKP
ADGSLETFGELGHFLLDCEETNFIIVQIIFTSLTWPDTSAAIKVSQIILAVLKKIFTDTSKLNPELPKQL
LLLILQGLNIHGGDEFGRAALTNNVLQFYLHLRMQYRGIVQVMLMIPTCEEQHLREFDLAVFVQE
LPLTEQQQKKIFRRFVAGACGTVISKLHEKDVVIQDLPALMPRQKEKTLNKNGDADDDLGLCSLF
GV*
>TRINITY_DN72625_c0_g1_i1.p1 type:complete len:1964 gc:universal TRINITY_DN72625_c0_g1_i1:173-6064(+)
MKLAFNIFTISLLIGNTIAQNNDVVQVCKSQADVVAVLDSSGSVGVQDYEELKKFVGFVANLLTIN
ENGNRLGVVTYSETAEVDFNLNEIYDSDQYGREALTRLSVRPREFKTNHYAGLKVGSDLLFNKAA
GARDNAAKFLIFTTDGRNNFDETNNRNRASQLATQLKADGVNIYVIKAEVMSDNNGRLDSRLYS
TDPNKFYRFINRFGEITNENFLDEVGLEFCELVQLPDNQQATVREDRNIGTLVTTIRAPGLNNPTFE

LIPQDNLPFIVDPLTGEIKTIGLLDFEQVQQYVFQVRVRATNPDVEKIITVTINVLDVNDNDPIPETA
NYRMSVAETTPLGQRILTVKANDPDGGTNGNVEYFLISGGDGKFEVDQATGEIRLIGQLNKRIKD
TYLLTLEARDMGIPTRKAPITVAIDVTSLNNFPPQFNEMSFVRTIPENFGAGTVINAAATDRDGDT
MVYSLIGENIPFSIDQNGLITVSGNIDFETIPNNVFQFQVQAEANGDLVTQDVIITITDINDNVPVYS
MTTYSGSINENSAAGTVVNFDNVIIATDADKTNANNMITYNIIDGNDGDRFQVTKNERGEAVISL
KSTPLDQETQGQYRLTLQAIDNGVDPRSLEMNSLLIIDVIDQNDNDPIPEESVYSIAVAETSPVGQSI
LTVRANDADSGMNGKVEYFLISGGDGKFQVDQATGQITLIGMLNKRVKESYTLNIEARDMGVPT
RNAPITVNIDVTSLNSFLPEFAGAGDAFERTIPENFGSDTVIKAAATDGDGDMVFYSLVGDNLPFA
IDQNGMITISGSIDFETIPNNPIRFQVKAEANGDMTLKDVVITITDVNDVTPEFTQQVYAGKIEENS
QTETVVRFDQPVIATDGDKSPANNVVTYRIVDGNVDNIFKIVKNAQGEAVISVNNAGLDREILNQ
YTLTIEAKDNGVDPGSLSSTTQVIIDIEDVNDIPPLINTSENKAVGEADFTATISEDSPIGTSVVKVEA
TDPDSNNDDLIFTIANDVPVPFTIDPKTGLITVSGPLDADDQDEFEIAVTVSDGVNNSTAIVKVNIA
DLNDNKPIFTTDTRDITIPLDTPNGQEIIKFTGSDADKTSPNNQFEFSLVDGDGKPFTITPDGSLIIN
GPLMPGDYMLTVKIMDKGTNPGPLSNVIIYNVKVVDTNDPPTFQNNEQTVTISENTADGTDVITF
AATDPNGDAITYEIVNPGSVPFEIDPITGVIRKMGLIDFESPVRSYTLDVKATDAKGKSTMGRVFVQ
ITDFNDNTPTFGPGNTDNFILTEGVYSNQLVSSFTATDLDATSPNNVFEYTLVGAPSMFLLTTNPNT
GAADLTVNGDIDAEQNMSFNFIIRATDKGNPALSMDYPVTITVGDINDNAPVFAANTPESIDLME
GDFNNQIVATFTANDDDTTAPNNLVDFELVGAPDFFKLIVNPETGTADLVIDGNVDIDGLDPTINF
IVRAKDGGNPALVKEFPLTVNIADINDNAPVFTLEAYNQVINENTPIGTKVVEVRATDSDSGPNG
DITYRIVDGDGTFGIDPKTGDITVLKNTDFESGVRSYEFTVEASDNGNPKQSATAKVKIDIRNLND
NDPIFTSIPITGNINENRPAGSVVQNIQPVNGILNQNNVKILREPPPGAQETEGVGFDAPFEIDPVT
GEVKSTRPFDFEEEKVFTMMVQAADGDNNPMLTTMTVMVNNLNDNDPIMKKANFEITLNQDT
PVGTFVLQVQATDIDQENQTKITYSIINIDGGNMNDFTINPDTGVITLNTDLSMNTVDIYNITIQAE
DQEIQGVIRRSTALAEIKILRPAEAEPDISTLCTNSCDIPEERLKCLDYSKFEITRKFNGTCYVDKLKA
AIADVKNNTMEIMNLNASFLECRNKVQNEITNPNTQFPVNGLFDLHRKCTVAIECKLEQLAFNL
MRKTTELTNNLKAEEEGFSMYDMSLSAFFQHISKMVSMSKSSRVD*
>TRINITY_DN1119_c0_g1_i1.p1 type:5prime_partial len:949 gc:universal TRINITY_DN1119_c0_g1_i1:2934-88(-)
FFFNPQTVMHEKKFSRMKFILLFSFFAISLGQNCNRMHKKIGCFWNDKNAKVLFNDRDSINNSTY
GALSDKLACKCNAVARSNGFKFFALRHFGICIGYNKITTGSKSIEHCIDTKSNGVCNLGDMNTCT
GTQNTADMMYQIVPEKCPSRHKKVGCYTEDRNATFLFNDRSNIDWPNFEKFLVELACKCNKVAL
EKGFNFFGIRFYGVCVGFKNISTEEKSAFHCKDPNYRPYRPCNMKDSNVCAGDHFLTDMIYKTER
QNVDKLFSEWSGWSTCSKSCGGGTQKRTRECKDKKSCSDAGGEEFKSCSNSFCKGSTGVAKAQE
VVETFLETFHKVEFPDIKPSGKYLKTIFEIQKAVGEEFNSVDPSVKGVPLEKRLTELEHFLKIAVEGG
SIDFARSTLGKGMFVFMKKKIKEGLPINDIDTAAKGFLNKLTGDIGSKKMNEILSSKGDATLMFTID
TTGSMAQEIAAAKAIAKQIISATREYEVDYILSPFNDPRTGPVRYRSTNDRANFVSDINKLLASGGG
DCPELSIKGMLNAFDKGPKYGSPMFVFTDASPKDATPSNMAALKAAASSNNAKITFFTNLKGCG
KELGIESYKEIASHTGGQVFPLKNEAELINFTEYVKSSLQQDTIIAAGGSLNSRISKRAAPRSHIFMV
DQSITRLIISVAFEKQNTGKHIKLLDPNNKNVDSSTMLAMSLIYDIKNPKVGLWGLSFPHIVGEFGY
NVQAVTENAIEFAHSFLHQHNVKGSDVTVSLENPIKGIRNMMVIRLGGADHIDLSTIKVHLVDHS
GNKLVSNVMTDRLDSDEIRNASIVPPTVQFKVLLTGKTKSGQQFQRMSQGSSTPVSAFMRTISAGY
DFTATMGKDWISIQIEIVNKGSAESFNFQAKTKYGTLKNPNQTKTVNKESMITFSYKPPNRHDLK
GKTDKVTVIAKRASGEKINHFISILLIK*
>TRINITY_DN77_c1_g1_i1.p1 type:3prime_partial len:1965 gc:universal TRINITY_DN77_c1_g1_i1:2-5893(+)
MGVQVYSLGIGSNYDIGELLDIASDDASVFRSQDVDELVSIVASITEQTCKGCTRAVDIHFALDASQ
NVGRENFQVIKNFIKAIGFKFIISETGSHMSASIFGEYGTLIFSMDQATQQEDFINEVDNVPYIGDFN
GNIDKALDMVSEDIFTFDGFSRQGVPKVLVVLTASNCDSCEKSVEEAVKPLKAQGVHVVAIPIGSN
ANMEEMDKMASYPTQDYVIPRASYEELLNGVFIQKVSEMVCSGRPGVCEEPPIEGECTEIINECTL
DVNCPANRKCCIKDCKASCEVPAAACITPIDLAFAFETTGFIEENFDEVKSFASSLLDHFRVEPAKT
HISISTYGESVNAKTAFYESYDKSALKQFIKDLEPDGGRQANIGDFLKTANDEIFNITNGIRQSSPR
MLVLFTNGVYSNDQIAKAQEESLKLKAEGQDVNILVINIGSIVEGNSVLRDVVSTPDFRNLHNVT

DLRRLVNFGFENKIAEAICASKVRKTRCKPRTPQACSVRDDLCQTDFDCPGYQECYSDGCRLRCQ
TCENRCGDKVDLLFMIDTSSRIGPEGFKNAKENIKKMLEWFTIEANNTNVAAITYSDNANYAMQ
LPAAGSNDPPQTLFDVQGRLDSIPYSGGSKSNLDKALKMAAAQVFYQNSLRLRKDSKKAIVIVTD
GVDDSTSRLEIASWPLRKQRVVVDGNDYAAIRLLAVTVGNNYNLDTLEKVVSPPIDKNIFLAADF
DDMYNGIRQLAEDSCKYTRQDVEPPVIAVKGDPGPKGEKGRQGNPGTNGTDGMDGIEGPKGER
GDDGPKGEKGDTGIAGPPGGDQGDLKGPDPMAEAVGIIEGTAVKGTRGVTYDLRDGPIGPPGPIG
GVGKTGLPGDSGEQGQKGRGGPPGLRGVFGGQGPKGNPGEDASPGLPGEPGPKGISGKKGPTGS
PGMPGPQGPFGYKGEQGIIGQPGDPGDGGKKGPKGYAGEKGDQGDIGDAGFPGPDGKVGPIGP
PGQRGLRGPEGSSGPIGKTGDIGNKGLPGPRGSRGLKGQSGNAGPRGGKGRTGDVGLVGEKGTQ
GSIGEAGQEGQPGVPGKEGPPGYPGPIGTPGPMGSRGDIGPQGEEGPKGSLGLKGNRGEAGRRG
LTGEKGETGDIGELGPKGAQGQQGKQGKTGKIGPRGQQGPLGFQGLQGQTGDPGQNGRQGPK
GNLGPTGEKGADGPKGSLGSAGLTGPKGLVGEPGNVGRPGLPGRDGKNGPQGANGKDGDPGA
SGPQGLQGSPGSMGTPGSTGDPGSPGDRGATGAVGDKGTRGDSGVRGEPGAFGQQGPPGLRGE
RGEKGPQGTNGVQGSPGPRGTPGLEGLDGIVGRKGTAGRPGNPGQSGEQGAVGSIGVPGPQGIS
GTPGERGDGGSDGGPGEQGPPGLQGSSGSPGRDGMPGRPGQQGTTGGKGKTGLRGPKGANGQ
NGVQGARGPDGLPGAPGLVGNRGTPGPNGIPGLDGNPGPAGKQGKQGKMGTPGMTGPAGLR
GPVGSPGLRGQKGPTGDRGDSGETGPIGVAGDIGPRGFTGELGLRGAIGEMGQIGETGPQGMPGS
QGTPGAAGLTGPAGPSGLPGKDGNKGPIGDPGNAGNPGRDGGPGSPGKDGTPGEPGTPGSQGK
QGLKGPFGPQGAPGEQGQRGLTGRQGSGGKDGPKGFPGNDGSPGISGKRGPRGKDGTPGSPGPI
GKDGRPGKPGINGTDGDPGNNGPKGDPGERGDSGDSGSLGASGDKGKTGSEGPAGFVGPRGSN
GKAGVPGKRGKRGDQGRDGPRGNSGPDGIDGSKGFKGNMGRFGIQGPPGDAGLKGALGPDGT
VGHTGPQGNTGDPGPDGPEGPSGSKGPAGNAGQIGNAGYPGSPGDGGIPGTAGLPGPPGPPGPP
GDMSGAFSGNYWDFVNAGGLQKGPRYYGGRSKRSLDQQDNSIEKVFKSNYETFKNFDTVWNQI
VDDLVIQKTLGSSKNPALTCGDLFKTNPITKSGDYWID
>TRINITY_DN1653_c0_g1_i3.p1 type:complete len:1023 gc:universal TRINITY_DN1653_c0_g1_i3:180-3248(+)
MMRRSIKGSEDNWVEMQNDTFKNWVNANLKSRGLEVDNFETDFEDGVRLINLFEAVSKKSIGK
YAKQPKFANQKLENVSLVLRAMESDGVKLVSIDSDHVVNGNSKMILGLIWQLILRYQISGSDDKSF
KIALKKLLLIWLQHVIPEQGIRNLTTNWNNGIALCNLINVLKPGIFPEYNNLDKRTPVENLAKGM
QLAESELDIPQIISPENMASPNVDELSMMTYLSYFTQDGGIGESWTVDLVNFWNSSLSVTNFNTD
WNDGMRLGRLVETFLPGAMPIMESTDNLDNCRIAMDVAHEKLGISKIITAEEMSNPQIPELAVMS
YIVQFTKVKRVERASDKFSASGPGLVEPKIGIVNEFYVRSDAGYPLDGLQVTIIGPNNNYIPIEIDDS
DPQLRNYSYSCEEQGQYHINIIYEGEDIPRSPIIVTSQQDLNTIRVYGERLEDKHLYAELYERIGISVD
TSQSGDGRITSKTIGPDEKEIYSEVDAEGDGSYTVSFTPEVIGEYHTNVFWSGKPIHGSPFVVWVSD
PNKVFARGKCLYETRVGVESSFEVATNGAGPGHITGYAETNGVEYPINVRKREECLHDISFTANQV
GIYYVYINYNNVPIKDSPFQIKVGNPSLIEIEKHEEAIMESNQPQSFIVNVLKDAGFGDVLCVVRGP
AGEDVQCTIKDVNDSLKEIMFLPNYPGTYEVFVYYAGGLVERSPFIFAISEPKLAIYPENIRVSGNGL
SRAKLNELARFQVDTTNAGPGHLTVQIEDEKDDVEAGIRDNNNGTYDVTYTPKRSGKYRVIIKW
SDQHITDSPFEIIVDNRAEEVLVEGYGIKEGTAGQPVLFTLDGRNAGPGQLSCRCRAPSGKMTYVL
ISDNKDGTYTVDLNATEPGLHTVEVEWDNQAIPGSPFLVRIMQAPDVKKVRASGPGLRSGTLDSF
NGMFQVDSKGAGPGTLKVRIHGPKGAFKVEMSRENPKDRIINVRYNPTEPGTYTANIYWSDEHV
QGSPFEIFVGQNDQEVDRWDRNKEELSKSQVNVY*
>TRINITY_DN250_c0_g1_i3.p1 type:3prime_partial len:402 gc:universal TRINITY_DN250_c0_g1_i3:131-1333(+)
MRECISVHVGQAGCQIGNACWELYCLEHGIQPDGQMPSDKTIGGGDDSFNTFFSETGAGKHVPR
AVFVDLEPTVVDEIRTGTYRQLFHPEQLITGKEDAANNYARGHYTVGKELVDQVLDRIRKLADQC
TGLQGFLIFHSFGGGTGSGFTSLLMERLSVDYGKKSKLEFAIYPAPQVSTAVVEPYNSVLTTHTTLE
HSDCAFMVDNEAIYDICRRNLDIERPTYTNLNRLMGQIVSSITASLRFDGALNVDLTEFQTNLVPY
PRIHFPLATYSPVISAEKAYHEQLTVAEITNACFEPANQMVKCDPRHGKYMACCLLYRGDVVPKD
VNAAIATIKTKRTIQFVDWCPTGFKVGINYQPPTVVPGGDLAKVQRAVCMLSNTTAIAEAWARL
DHKFDLMYAK

>TRINITY_DN6390_c0_g1_i1.p1 type:3prime_partial len:1941 gc:universal TRINITY_DN6390_c0_g1_i1:5820-1(-)
MVIAPKCKFSIFSYLVFGILSYNWNELDASTNIELVDGTGVLLQRDVDNVLNDAVVQKEYQQNIK
HKPVDKRCLGEPFYQGLCKEKLEDAIQQNITEGGCHNLLVKIQKDECSNGPKTLGGKGYCNFICS
QEICNSIYDIDKCVSKRLVHHFNKDKNECEEIMQADCGSESFTSSNQCQKRCIGNSDDKRETKVY
CDQFCLNGGKCIGEVYCQCPPAYHTATCGLSYCNPSCGVGGKCVKTGEGKYGCQCFPGYKGVK
CDKEDSKNMPVVKGLCLSAIKSVLTSPNTSFHDKPEEEKNYCLKYLFDLESNCDKQRPFVNYTEE
QCNHFCASDICDNIYSNDCGSIEERYGYVSSTGRCSKFLYNGCLGNTNNFKDKAACDTKYSPING
LMTSVALLRPLNHTCYIRGKTNFITFDGKVYDLLSGCSHYLVHDSDTTSDFNVILHNDPKVNYNE
KSKRKLEITADGKNVLLNQKVSSDRFFVQVDGRSVPLPYNDVPKIRQAGRYILVLTGDGVSIAFDG
YEDISITVNDYYRTGDGGDHRLNGLCGNLDGNSHNDWLDFTGELRENVRDFAKVYQTDRSTCK
MDNLSLKSLVYNPLHNDQEVFERAERHCDVINTDAFKPCHSVVSPEQYKVLCMQDYIKCNDDK
RDDCVCSSLSFYSRVCHKYGKDIKWRRANLCPITCQRNMKYYECGSACPKTCDNLITPTKCQTDC
VDGCHCPPDLYYDKSTENCVPRNLCSCYRDGIAYKHGSVRSFHCEECTCNGGSWECNKKDCEAT
CDIAGDPHIKTFDGLNYQMYGKCSYVLVDHCHYGNTGIKEFEVIVENKNCNDDALYTSCVRHLS
VNILQTNTRVSLDTAKDNNGTVLPVAFVNGKEGKEFRSKDYTITSVGGRNIIFQYKVNQLKVSWT
GHNAYVTVGHQFENQTCGLCGTYNFNKQDDFHTRSDTVEKSVTAFTQSWVYQSSDKVQDPSKC
HTAKWETSEKPCQIYTNKAQYAKTQCSIIIKPDGPFEDCHSIVPPGEYYSMCQDDACKCDDCYCH
IVAAYAKRCTDLNIRVGNWRDETLSCKQTCQAPKVLKQCNAIDSLDDDLSESLKCPSTCYELSQN
DTSICQSRGCIEGCYCPEGYFENEQNECVKQDQCSCYHDGKVYRHDEIRKDRCNECVCKGGRFE
CTDFDCVAHCASKGLVYSDCGKTCSNFNFNPQDSKCQAGCFCPAGKVLHENGTCVNPQTECQC
KEGDQFFDAGEVSPSDCSKVCNGDRSWKNLPGKDAKPQYDCSAFGQMHYRTLDGRMFKFSGG
LCPYVLLTDCRQDSATGFCDLTKANVNIHVRNLRCVNSYEAYMCKEVTLDLRLPNGEMAEFVLM
QNKIKMKTGLVTKVFDKGSYPQPKTAVVDGVEIFKSGLYLVVRAYHLNITVKYDRQNRVYVVPGR
EYKDAGKLAGLCGNYNGLDDDDFRRPDNSNAENELQFAKSWQDSNSACPDPSESDTCSSNKER
LPWAQKGCNVMKTGNFTACHKTVSPEPFIESCQQETCLCKSGGDCACFCSAVAAYVQECNRHG
VPIYWRRDGLCKLDCCDPCGHSYGYYTDNHVPNTCGVKDKVCKENDRCCKGAVVEGCGCPPG
TYDDGIKCQNETTTNVIPCSPSSSIVPSTSISVPSTIIEPVSSSSPSPMTSSVESQSPSPSSSVVSPTPSSSII
QSMSSSEIESISTTSSSFVSSIESSVQSSTSIVFPVVSSNSGESSVSSSMLPSTSWVGIHSVSSSMGESSVST
NVLPTDYLRTTDVFPLSSSEITYPSSSSSLEPHTPSSSILQESSSSVSKPVGISSVIEPEISSIRYSSMSSQN
VMSSSVSSSVSKPVGISSVIEPEISSIRYPSMSSGSVLESSVSTSFVLPSETVLSTSVFSTNTESPSSSMTLS
TSSSGFVFPSVTSTFQTSSKVLSSLPRSGPAENTVPLSTMFTKTV
>TRINITY_DN319_c2_g1_i3.p1 type:complete len:2588 gc:universal TRINITY_DN319_c2_g1_i3:165-7928(+)
MVLLKGQLWILGFIFLTSTKYVMGDVPECSNNATVAVHCRFDEGNTCVDDPHPMHYVCVCNNL
GYMNDTDSVEGYSICKERDCSNSNETPTDLLKRICGVNTKTCTGKILQRECNCNTDSYRSKNGQY
CFFAPCQASPTIQQNCGPGNQCYGNAPNYICDCSARGYKRSAADNRHCELKSDEELTPIEEYCGK
GSLYANGTINNITCTCDTDKGFFPSATGETCISPSCYLNPQMQEKCGPENTCLGGKDDYVCLCSNP
NYKRNATDLQHCIRRTCADTSPAIDFKSICGEGATGCSGYVDKPLCSCDVSNHYVPAADGRSCIK
ATCKGNKKALEQCGQANSCTGSFDNFLCVCDANGYYLNTTNNTCVERECTDPDLVITDICGVGS
LSCNGKAGSPECICDKDKFYYASEDLRSCVKANCLSSETISKKCGKENSCYGSMESYVCNCNAPG
YLLNTTDNQGCYLGMCEDNGLEYDKVCGEGAISCNGTIKNLECNCGMNYVQSPDKKSCIRADC
RANQTMHEQCGEDNDCVGSLEDWMCICSTYGYKPDPMDMMKCKKVMCEEEVVNNVTVCGL
GTTGCKGHILKPECTCDIAADYVPNEDASRCIKGSCENNKMAMKDCGIANSCTGSYNNYVCVC
NGFGYKKLDDTHCVEQMCSDPDIDIRNVCGEGAHYCNGTVNQRVCSCNETEYFYPSADSKSCV
KATCAVSPMVMEQCGVENSCQGSQESFICNCNALGYLLNTTTNQHCYLGTCENGDFDLMQAC
GDGALYCNGTIKNLECQCDESKGYVQAPDKMSCIPNDCRGNLTMYRQCGQLNKCVGSIDDWR
CICDSPYYVAHSDIMKCKLLHCSDSEVDHVEVCGKGTIGCSGSTLHPTCHCNTTENYFASKDGMR
CVAGSCKTNQTAVKKCGNANSCAGSHSEWTCICDGIGYKHDPADPQNCLLKTCNDADIDIVATC
GVGAISCEGYQLMPKCECNDRDYYIPSDDRTSCELASCRANRTAMRACGEMNDCRGSFSSHICL
CHDPAYKSDPSDSRVCLEKTCEAGDFELVRVCGKGATGCSGSMKNIMCECDNAKGYVASADHT
QCIPSDCNNNVTMHLQCGEGNGCVGSLTDWYCVCSAPGYEPHASDHMKCHLKTCADPGVDH

VQICGMGATSCMGPVINPVCYCNESMYYIPSMDRTSCIKASCMSNKTAIAQCGSENSCSGSPDNF
VCLCQAYGYRQDTNNNQACIPSQCEDAGIDFVEICGQGGVGCSGSVLNPVCMCDHMKHFVPSP
DQNSCVLSTCRGNETAMTSCGEQNKCFGSSSSYICVCDAVGYKPHPTNLQECVLQTCEANELNP
VAVCGKGALSCKGTIQSLMCECDNSKGYVAAPGYKSCIPADCRANMTMHAQCGTGNRCRGSM
EDWSCVCDGIGSKPHPTDHMSCIAIDCSDPNVDIKSVCGYGATGCMGLARDPICHCDTSKYYVQ
SEDGRTCVPATCHTNKTAQMQCGPDNSCIGPPNDFICECTGFGYRHHETDKQKCVPIQCNETAL
FDIERVCGKGATGCRGNIRQPICDCDHAMYFHPSEDRRTCIEASCRRNSSIRADCGVENTCFGSTR
SWICDCNSYGSILNPSDVKKCMLATCEAGQLDPVAVCGKGALSCKGSMRDVQCTCNNELGYVH
SPDGKKCLPSDCSGNVTMHHQCGDANKCMGSLTDWVCVCDAPGYKPHAKDKMKCHQISCAD
NNVDPVDVCGIGATSCKGPITNPLCQCDTDNYYITSSDGASCIPATCKTNKTAIHQCGEANSCEG
PADNFICVCNAYGYKLDDNDHQRCVEISCDDPNAHESICGTGSTGCRGTARDPICECDHTQHFV
ASSDLTSCLLASCRANDTVMNHCGDGNTCFGSLTSHICVCEAFGYKLNPNNHKHCLEETCEDD
MLNPVAVCGKGAVSCSGPLKSVKCECDNNAGFFRSPGGMKCESTSCRQNPASQMMCGSQNQCY
GSYDDWYCDCSAPGYMANQTNAAECLKVMCDDMAVDVCGLGATGCTGHTLNPICTCDSSMG
YMMAKDKHSCVPMDCSMNPASSQCGYGNTCHGSYFSYVCVCSTAGTVMNKTNPMECINVACP
ADAVHVCGEGATNCSVTSTNNVECSCEEGYHPADDLHSCVLDDCRFNPKALKACGLLNKCVGS
YKSWKCNCHYPEFTQDPMDHQTCVVGPCMVEQCGKGNAPGSFQHYAVNCSGLYDDPICNCQP
EYTNDAKGGCIPKICSENPVAVQACGSGYCEGTYFKYTCKCDEPAFVQTPDKMQCVPAKCNIQQ
CGHGFDRGLYIDFAESCSGTYADPMCQCKTGFSGTNTSKCGPAIDGCKLDKCDPLATCQTLDVD
GNVKCTCKEGYSGSGMMGECYLDGGFSAYEGTKECSEVCGVNGVAEVTRFCNNPTADPKGKPC
NGTSPETIQVPCNRFACSSCTVRKTCGCGGLKPIFVEFYHEPNSTLEYFTDLKADDVQTALGFLTK
WKELSQQDLCLMYSKIELTVFVTLMGDSETQVSTMKSSLVSARDIMRKKLDCNINLGKKAQYYEA
YETIIKRHLAISNILKDLTIIKNSITKYMNNCDDSEIGMIRDVIGMM*
>TRINITY_DN477_c0_g1_i9.p1 type:complete len:306 gc:universal TRINITY_DN477_c0_g1_i9:1840-923(-)
MTRQCLGATLNENCGNAALVQFEVCNFQVPCPGLLTNWGPYTQCSATCQKDANSMPTQSRQR
FCTGGSFGGNCGGVQLLQTIGCNQLIICPVYGYWLQWSAFGECSKTCDQGTHVRYRNCTKSIPA
NAGNNCTGPAQEVKPCQVKPCPVTCPLTKKCDCRNMNYLAAAPGISQTVTNDVMGVAAFLDY
WTLDILKQICEGCRMTQLDQSKATVDSQILRTQAVLDRLNIEKRVYRDMITCNENPYITDDLFKVY
NDIWDRIVTTDGVMIELKASKMRFDTAITACRSYDWYHDLFGRFFQIKKSS*
>TRINITY_DN1280_c0_g1_i1.p1 type:complete len:426 gc:universal TRINITY_DN1280_c0_g1_i1:195-1472(+)
MGFETCGPNEAMVISGCGQERPLLISGGRKFVWEFFQKVQRISLNVMTLNVESQRVYTVQGVPIS
VTGIAQVKVQGQNEEMLHAACQQFLGKHENEIRNIALETLEGHQRAIMGTMTVEEIYQDRKKFS
KSVFEVASSDLVNMGISVISYTLKDIRDDEGYLKALGQRRIAEVQCEARIGQAMAKMQSGIKEAQ
AEEVRIKAEYENKTEVARSQRDFQLNKSAYDIEVNTKKATSELAFALQSAITNQKIKEEDMQIKVIE
RAQAINVQLQEIQRREKELEAQIRKPAEAERYKIEKIAEANRMKVVLEAEAEAESIRMKAEAEAE
VIQKKATAEAEQMSKKADAWRDYQEAAMVDMLLDTLPKVAAEIAAPLSKVKKVTMISSGSSDV
GAAKITGELLDIVIRMPQAMEQLTGVDITKTFRREK*
>TRINITY_DN36_c0_g1_i16.p1 type:complete len:224 gc:universal TRINITY_DN36_c0_g1_i16:829-158(-)
MMIKTTIVAVVLAACVLQSFAGRCYVGYSAKGLPLAIGVRDVECGKGYDYCTTIKYRLGGIEVTQ
GNCARKYGQAMCQFMRNPHKAMTDCEETICQGDSCNKDVYDAEQAERKRVGPYKNTLKCTT
GLITKFPAIPFYTADVPNAIDECFPGTTECVTVTYDYLFKFETQIATKDNVFKLPCVQKMCNRHNI
DNCNKWCDILSIHQSMENCVAKCCKGDNCNA*
>TRINITY_DN11248_c0_g1_i1.p1 type:3prime_partial len:1700 gc:universal TRINITY_DN11248_c0_g1_i1:111-5207(+)
MEIRSLFVAFIYIVHSINAQCSSPWKSYKDSCYLFTAVSTVSPGHTWEDSRFICQRYGGDLLKLDND
NETKYINSQFDDLKIKYNHYWIGLNDIKKEGDWRWADQAPLNVTTQWRVSRGEPNNWQGNED
CIETTYDGQWNDNECSRMFGYICERSKDAVIPVTPTLPPFPTATDKPTTCEAGWIPWGTSCYGIIRG
LAKNFQDAQADCLRRFGDLVVINDQAENDFISNEISRSVGGLWIGLSDTYKEGTFYWVDKSELNF
TSWRAGEPSNDYGNEDCVEIYGRWGPGQWNDNHCTALRDYICEKPGGVHNCPRDWVSYKNY

CYQINAHPGQKVTWEDARKNCLDFNKAWRKSVNVAQADLVSITNLEEQKMLQKEFSKVNIKN
DNGFYYWIGLTDKNSTGGFYWTDGSTYKYSNWIGSPQFASQCVRSNVIGYQGGSWSKGDCSEAY
NYVCKVGRGQDRCYNPLGIQSFAVPNKAMTSNSVLGPSTPALAGRLYMKAGNSLDGAWCAGN
NTKTPYLQIDFGKMQKLTYMGHQGKSNSDAMVTSYYIKYSQDGIVWDTYGYIPPAQYYKFKIYIG
WRTNYEKVMNLPLPAPFIARYVRVYPLEWNIAPCMRIDFYGCQEEVDNQRCGLGWREDKKTKN
CYKFHNQITTWDSAQELCSIQGANLATIATPDEREFLSEQLGEFKSLVSEFWIGLNDLDYEGVFKW
VDGTPYQLSNWYYGNPKVRNEKSNCVVVRTTDSAWVNTECTVFRKFICKRNYISPYTPKESYNG
YVYSKTDYFWPLDSATDFEGSLPLTLNGNVDRVPFPIDYDVGVSRYALNVSGSSGFFKIDVANDVC
IHHPDADSCPHGITVSFVFKMSKKSSSKRQVIIDTTEKNKPLGYKVYTEFGSIYVNLRGHQKSYSGS
TVYNAHIFNHMTFTWSSLKGLSLYMNGKSRVFDLLGSYAKDYPKVDYGKSLLIGISGDSQDPAEL
MIRSLGIFKQVLDAKSIEAIYLGEFGTCKSHQFAHGSYCYEYNSTEVSWSNALSTCRDNGGDLISIH
SPVEQAFFNLKIAQYNYQRTNWIGLNAKSKNSFFKWADSSRLEYSYWAPLEPNNNNGRREDCVE
TFANGGWNDALCYLRNPFVCKYSRALHIMGSENTDDKEYKVFFSPYQTLTFGCPLGRSIEILTASY
GSVKDGCVKDVKDKVSNACVKEHCVRVQASDFFFNKINPCKDKNRFALKRLKVTYKCNLATPE
FDSTGCSNGKDSENSYRVFDKSNICYKLYRKPKTFKEAQKVCKDDNGDLVRINSRETQVYLTSLLH
TMYQKTDIWIGMMYNSTTQNYEWLDGSHVYYTNWVNRQPDPRNGDYVKVGLEEGAKNFLFW
KNGEANETLPFFCQRVKVGKTTQAPPTTQPRTGTCPKGWFTYNKNCYYVNTNRLSFPDAQAEC
QKYGKSSHLASVLSYEENRAVFYDVVNATVDFAWFGLTNEAIVTGFRWVDDQPVSFVNWYTNEP
HKINRHLCVGMGSGGKWIDLACYLKYPSICKLPLGEVIPTTTPLPTIPPTQASGLCEPDWIHYNGS
CYNFANRTSYADFHNARVTCNKMGAHVATIHYQKENDFISAQLKRMYIPNAYIGLTDFSIEGQW
QWEDESVVSYINFGEGEPKNWHGMEDCVVVKSIGEWNDIPCNLKRSFVCKKNNGTVVKTVNTT
AAPDSGYCPSGWKLYGNKCYYFRYGE
>TRINITY_DN1676_c0_g1_i12.p1 type:complete len:1631 gc:universal
TRINITY_DN1676_c0_g1_i12:206-5098(+)
MLIITRTAKLLLLSLTYIYLTIAQDLTNATLNNGFEAGRAIKCKTDDGMVQRCMPVFENIAFGRDII
ASNTCGQTRQTYCVQTGTSGAVKICEVCDASDPDKAHPADLMTDLQFDDQPSWWQSETILDNK
SPVYLTLNLNKKYNVAFIRLRFHSVRPHSFAIYKKSTYDSNEQWLPYQFYSRTCQETYNVPNQQSV
SYSNQKLALCTDEASGMIPLSGGNVAFLTLQYRPGKRDFDNNPGLQDWVTVTALRFELDRVNTF
GDEVFGDPKVLRSYYFAISDITVGGRCKCNGHGSSCNNNNNEGTFQCVCEHNTAGKDCEKCLPI
YNDRPWRKASGRSANPCKACDCNGLADQCEFDQQLWVDSRYRSGGRCVNCKNNTDGINCEYC
KEHHFRSNVKEACKPCRCNPLGSRSLSCTTKGECACHPGVTGLKCDKCKEGYYGFSSSGCKACN
CDRDGSIGVNCDFTGQCNCKKKTVGKRCDQCAPLTFDLDRSNPKGCKECFCFGHGVSCDAIEGI
GVKEIFTTFEADFDGWKLQDESGASFDSNMEWDFRGQYISVKPPRNKQLYLVAPQRYLGNKLSSY
GKLFTFAYGIFLDPNDPLVSAGRDIIFEGNGLQAVYPINSQNNILPEARFYQFKYRLMEPVGMSTFD
FQKILVNLTAVKIRTAYMNERTGAIDNIKLYSTKFISINSPEQVTWQEICECEEGYTGDHCEKCDSGY
TRSGGDTSPFGECKKCFCNNHGNFCNPDTGICQCLHNTTGDSCDQCAPGFYGNPTAGTPEDCK
PCPCLYGNECILIGNSVKCTDCPEGHIGDLCELCDDGYFGDPNGTIGEPSRCQKCDCNGNIDPNAI
ANCDSLTGSCLKCINNTKNGPENKCELCADGFYGNSTIDPKGNCSACSCYEKGTMKPPFHDPKL
QLPCDTNGKCECLQNVIGDKCDMCPPGHWNVASGKGCELCNCNATGSKGSDCNLQTGQCEC
QDGVGGRKCDQCAPRFYGFSKDGCIACNCHPDGSKHQNCTQDGVCECLPGVLGIKCDQCPEN
KYNLSVGCIACPRCFDLIQVAVNKLRNKLKTFNITGNLTTGSIDIKDPEFEKLFNELKRNISELEQK
MKDLLEGDMAILEFYEVLVNQSSTLIEMIPGIQKMIKKSEGAAETGSVTISKAEEITEEIQNLLGQAE
KMLEAETGRIFKNVTSSSEDVSGLSKRMSEIAEEARKHADRQEQESKDIEFNTDMAYNNSKAAYE
QAKKARDTEEAISYLLDQMALGHDEALALSKNSTEIIAKANSTSIAALNDSDHLMMKAKEPLPN
LDIEAMKEKAKNIDAEGKATEFNATDLHEKNKDKITQLQNDDKEARELMERGTQLHNDSNALY
KIAKNASDEAKQAQEDGLKVAKDAQDMLKTLMEFENNIAKSKANATEALKLIGLIRRLISKANK
TTENASSNAIMASINATAALRLASAANKTTQDAMNEIESSRREVEKLLNETMDFMMKEVAQTKD
DIDDIEKSLEESEKLAKSNSMKLGDALNDSQAATDKASMVQNELLKSLDQIEDLMNEISNLDEIDF
ETLEDAEKRFEEAKDTIENKLKNEIEIMESKLSDQKKLLQQHELDLTPLKEQIMKNEELFKYLPKSC
FREKAKPEDIDK*
>TRINITY_DN691_c0_g1_i1.p1 type:complete len:1726 gc:universal
TRINITY_DN691_c0_g1_i1:5662-485(-)

MMNLFRLIVLGVICFSYEGDAQQCGGSCSPCGNLCVGMKGDRGTAGLPGFRGPPGLPGFPGPEG
PIGQKGEKGPAGLNGMEGSQGARGKVGPPGLPGTPGRHGRPGNEGREGEAGLPGCNGTKGDR
GPEGPRGRDGIEGPQGLQGNVGPRGEPGDSTATKLKGQKGEPGSAGQQGEKGGRGMEGMKGD
TGKPGRNGRAGERGPKGEPGTSNVTIIGEKGSKGSVGPPGLPGRQCQVTGDKASNIAQGQKGNQ
GSKGEEGAKGEKGDAGLAGPQGEEGDIGPQGDRGDDGPQGPPGPEGEQGRSGIQGNKGQKGE
VGNNGKDGNDGQTGLTGQRGRQGTPGQKGEPGKDKKGLRGPEGAKGERGNDGEKGSDGPIGP
IGPRGPKGELGPLGMKGFKGSIGSAGPKGENGNPGDPGIKGRRGELGIKGDSGVKGAPGNPGISV
TGPDGMDGDTGPRGQSGGKGAVGFPGKPGPPGVVTIDGEEVLPGEPGPQGLKGFRGVQGDAGS
KGSRGEKGNACESSKCPAGPKGEKGLSGQDGLQGGDGEEGEKGEKGDKGDIGSRGDTGSRGEP
GPQGESGEKGEKGIQGPQGNSGKDIIVQGERGDKGTKGNAGTPGSIGFPGERGSPGESGDRGLSG
VNGLRGETGDRGARGVAGKPGVNGQKGQKGEIGSGARGFKGDKGEVGPDGPAGLRGPGGPVG
PKGDPFPTAVNGKTGEPGEQGAAGPKGAQGPVGPEGPRGFPGDIGPVGEPGDGEKGERGDDGA
RGERGEKGEPGQDGKDGDPGPKGEPGQEGKEGPPGMFKGNKGEKGATGLPGVDGLPGNSAPK
GEAGKDGQDGDPGPAGEKGDPGEAGSPGTEGPIGEDGPVGDKGEPGESRAGPKGNEGRAGAK
GENGRDGRAGRPGIGRPGDKGELGQKGEAVIGAKGSKGEQGLEGPPGPIGKPGVKGDAGVPGK
DGEAGGRGKDGERGLPGDKGDTGDIGFNGSPGAPGDKGEPGYSGQKGAKGMSGPRGPPGSTSN
VVGPKGNRGLPGMKGEPGQQGEKGLIGPQGSKGESGFGSEKGIKGSRGNPGAVGPSGEKGERGD
RGKDGLQGIAGPRGEEGNPGEKGSRGDQGPQGREGPSGELGPEGQKGDPGFAGEKGEKGDRGG
DGQKGDAGEPLRGEPGVAGPPGPTGPVGPPGKDYVFSGTAGELKGMKITKGDVGPDGVKGEKG
DRGDKGLDGRRGPTGPQGERGPKGEEGNMGPKGSQGRDGLTGEDGPKGSKGEPGLDGDVGEK
GEAGFQGLQGEKGNQGPQGLEGSTGMDGQKGQIGEKGVEGPPGPPCEPVNSVRGETGEEGPKG
DRGPKGQKGKPGPQSQAATPGEKGSKGEVGQPGPQGIKGEPGREGDFGPKGSPGAQGEKGDQG
QKGEPGQEGPEGLKGEPTRAFGPKGQKGETGLEGLRGPVGPKGEDGQTGPQGPDGPKGNEGEK
GDFGINGLNGAAGPSGPKGAIGLKGDSGPPGPTGAQGPRGSGGSPDFGFYYVKHSQTTIVPSCPA
GMKTLWTGYSFLYAQGNERAYGQDLGQPGSCLKRFSTMPYLFCDLQNQCTVASRNDYSFWLSTD
EPANMMKPAAGPEVEPYISRCAVCEAPANILAVHSQSVIVPDCPNGWENIWAGYSFLMYNSAGA
DGSGQLLASPGSCLEEFRANPFIECHGRGTCWYYGPTLSFWLSTIDDSNQFQIPKSETLKAGNLKT
RVSRCSVCMKTVRNGNFP*
>TRINITY_DN4726_c0_g1_i1.p1 type:5prime_partial len:670 gc:universal TRINITY_DN4726_c0_g1_i1:2906-897(-)
DDGNETNRTIADDTTNNDKSNSLKRVQLPSLPAFSFTEAIDKVETMFKSSVQASPVATPQEKTKET
EKKKGTFSKLKTKARSIDNDKATSIKDGVTGAATGMIKFVKGIQAGKWTDIVQGSLDVIGSIAGM
LGPAGIMVSTVLSVLSTVIGLFGGPTGKKESQEGMMERVIKTALLDARNDELTSQVNGAKEIFNTIS
DTINIFREMEEKNLPDILSFYELSFEGIEIFGILEHYIKKWCAIGDVLSKAERDKLTDDAEKNKQSDK
RDMAARCLRITNLYCDLSIYRQILIADMAGVLSNANITTTSDAFLKLIEKERQKDGVILKFLWDPL
NFRDRRFVVSKYYSEIHNFPTITTYMNTVVKANKEFQEIVQNTKYGVGLMDKPNNEKFKKTLPM
ALECQKQELQGNCVQVPFSRIAFSHGTNPVYMYNSAGSIYVSDGFYLQVRINGTTNAEMKFYGPS
VYYIGIQKDNGNTFKYILTESSAKDFVRVCEHRNMAMNGKCLTLERRDYQFSELDRIGWNDKISS
LYVPEGIKLIAYEHSSFKAKVPIDYYGPATYNMISKEKTWSSIKMELTELRTTDMIKLCSNYKLGGIC
FYFEAKAGKFTKDDLISTKKDIHQIRSMEVPGNVTVIAYTDPMMQPKHFLAVYKGPKVVSETEQA
DPHLEIELKI*
>TRINITY_DN329_c0_g1_i2.p1 type:5prime_partial len:320 gc:universal TRINITY_DN329_c0_g1_i2:1-960(+)
FNNSTHSPSLPTHVYLYCCRIKILILSFDPSTIEILKMAPPKFDDLGKEAKDLINKSFHFGVIKLEGKT
KAKNGVEFTTEGTHNTDTGTVNGSLETKFKYADYGLTFSEKCNTDNVISTKITIDNKLAKGLKVD
FDTTFAPVTGKKTAKVKSAYQHNNIHSTCDVDFDFAGPTIHGSAVFGYNGWHAGYQASYDTANS
KLIANNASLTYKEGDLVIHSGITDAVKYSGSVHHKVNSNLTAAALIQWASGTNNTSLTVAGKYAV
DNETFVKAKIDNNLRLGLSYAQKLRPGVELTMSSLVNAKSLEQGGHKLGLSLNFEA*
>TRINITY_DN1936_c0_g1_i3.p1 type:internal len:1070 gc:universal TRINITY_DN1936_c0_g1_i3:3208-2(-)
GEYKPTLGRCLSFWYHMYGSHVGELNVLVLQNKSRSAPIWNLKGNQGNVWRPSRVTIKSAVNH
NIIFEGRTGDGYMGDIAIDDVMMSEGPCTDYGNCDFERDLCGYTNKKNIDQFDWQLGSGGTSSV

TTGPSNDHSTQTALGQYMFVESSYPRLPHETAIIGSETIPVSGSGKCLQFWYHMYGEHIGSLEVNM
RVANDLSETIVWKLEGNQGNKWLQGQAPLLSRGQNMEVRFEGIRGTGYKGDIAIDDITVVDYSG
ACAITPKSAKPWGCNFELDFCKWQQSRADKFDWLRNQGATGSAGTGPKADHTTNTTQGYYAY
VETSFPRQPNDTAAIISPTIKGNGTKQTYCMWFAYHMYGPHVDTLKVYTRSQGSDKPVLWRRRGS
QGLNWNHAEVQVTVLYDTKVIIEAVRGKDYKGDIAIDDIDIWFGDCFSSGICTFEGADLCFWYVP
KNGQLYWQRASGATPSVKTGPKNDHTYGTSQGHYMYIETSNAPAKATAYMYSPWYYNTGQKC
VQFFYHMYGQQIGQLEVYIRYSNSRYLYRQFIRSGNQNNTWHMGQVTVKRNNVYFQIIVRGTKG
NGYQGDIAVDDFRIRDGACPAPGDCDFEHADFCSWMQEDNDDFDWIIGSGSTNSFLTGPSIDHT
TQSNGGYYAFIEASSPRQPGHKARFKSMVFAPTTGSGRCFSFWYHMRGYSIGNLTIYLRKVSNTKT
LSEEVMWLLGGNQGKAWNQGRFTLKSSTLHEVVIEGVRGSSYDGDIGIDDIEFTSGSCSFLPKGA
WPSGRSTPAPTQASTTLGPTIGNKGFDCNFDKEFCKTWMRNSTRTNFQWKRHQGSTSSVGTGPH
HDHTTNTDQGYYVYIESSSPAQPNDTAYLIGKDIPNNNGFCLSYWYHMYGPHVGSFRVYVVNKT
NAEARFVRVGTQGDKWKHGQLYISPKAGNPITIRFEGVRGSSFMGDIALDDISVRYGNQCPAPRE
CTFEHDNNGGSSSTCGWTQEKQKDSFDWSIGSGDTPSVLTGPNFDHTLETKQGHYFYIETSYPRK
FGENATIISQSYQQTLGKCFSFWYHMYGSTIGNLTLFLRRD
>TRINITY_DN875_c0_g1_i1.p1 type:complete len:606 gc:universal TRINITY_DN875_c0_g1_i1:2207-390(-)
MLRLVLFAISASVVYGSCNPLKLGCFIHSGSDRNLLHSDNSEDIYTDFYSGTNIEWDKFSSYLQRAA
CRCDQAAANAGYRFFSLRDFGECHGIGKGASSFDALKLKKSMYCIAPNLGQCDISEHQHCTGAE
EGEFIYETKAQPAEKVIDGGYSSWSAWSVCSKECDQGLQERQRTCTNPEPKGKDAKDCSKLGKPS
ETRSCKIKDCPPSGETVIFCEHRGVKRIACENDQVVEILAANYGRTSTKHCVFGRTKVTNCRAAH
SMSIVKSQCDGFNYCDLNPHNDVFGNPCGFYTKYLEVKFRCVARKTSSRRAVLCEHRGSPPLSCP
NPDEVLSINKASYGRLSTHYCQYGHFKQTNCRALHSDALIKKTCNGLNSCSLNPSNTVYGNPCG
NVNKYISVEYTCKKTNAASRQAVFCEHQGGKTLKCANPDEVLSINKANYGRLSTDHCQFGHFK
QTNCRAEQSDALIKLACNGLNECSLNPSNDVYGNPCGNVRKYISVDYTCKKANAASLRAVFCE
HQGVKTLKCANPSQVLSITKAIYGRLSTDYCQLGLFKQTNCRAANSDGMIKKQCNGKNSCQLNP
SNAVYGDPCKYVRKYISVEYKCINK*
>TRINITY_DN2217_c0_g1_i2.p1 type:complete len:254 gc:universal TRINITY_DN2217_c0_g1_i2:884-123(-)
MNSVDGNTDNAQKECLELCQRNAKATGCEVIWNRHNRGCYIHTQEVARGNNVDRHFCWVFS
KCTLNNVPPAVCPIEKGYCVKGNGHDQNSGVIKMNSVDGNTENAQKECLELCQRNPKATGCEV
IWNQHNRGCYIHTQEVARGNNVDRHFCWVFSKCTLNNVPPAVCPIEKGYCVKGNGHDQNSGV
IKMNSVDGNTENAQKECLELCQRNPKATGCEVIWDRHNRGCYIHTQEIARGNNVARHFCWVFS
KCNL*
>TRINITY_DN941_c0_g1_i9.p1 type:complete len:3186 gc:universal TRINITY_DN941_c0_g1_i9:86-9643(+)
MADPAKIVDPIDDPTASDAEWKKIQQNTFTRWCNEHLKSSNIYIYNLETDLCDGLKLIALLQVLS
HKKIVRYNKKPTFRPQKIENIAIALKFIEKEQIKLVNIDASDILKGNLKLILGLIWTLILKYQISMSMV
ADDDDDPDQKTTPKQALMGWVKSKLPPELKCGNFNKDWNDGIALASLVDSTAPGLFPEWEDL
DPANKLDNAKEAMKRAEEWLGVPQVLTPEEITNPNVDELSVMTYVSYFPESKLKPGAPLKPKMN
NASKCSASGPGLQEHGVVVNKPAEFTVFTSGSSRGKLNVQVFGPGRKAIDCMVQDNHDNTYSC
QYTAPKTGVYDVHIRVGGHHIPKSPFRVNVASDLDTSKVTARGPGLEPHGLIVGRPASFEVIATDA
GSGELVIDVIDPKGNKKTELKVEARPDNVYFCTYMPVQVGRYVIVIKYGGKEVAKSPYHVSVTPA
ADRVKIYGPGLEPGLKTGKPASFTIDCSEAGEGGLDVKIVGPGKGDIKYEVKDNGDGTYTCTYKP
TRPGQYEITVKFDGGVVPKCPVKVSVGSTADPSKITAWGPGLERGVAGRPVEFYVNCKGAPVDNL
TVGVEGPGEAKVDMKTNSDGTVTVTYYPTKPGKYIIHVMYDEMDIKESPYSATISPRGDVTKVYA
EGPGLEAGNMAGKPAEFTVFTKGAGVKGNVQVTCVDENGKPCEVTVKDNGDGTFKVTYYPKTI
GKYVITISFMDEQIPKSPINVTIIPFSDPTKVKATGPGLQGGKTNKLAKFKIDASKAGEGGIGLTIEG
PNESEIKCDDNGDGTCNVEYMPTESGDYKINVLFADQHIPGSPFTAVIGDDFDASKVKADGPGLK
PGNKAGKPCDIAIDARKAGKAPLVVEVTDEVGNPVKVDLNEEEPGLFASSYHPEKEGPHTINITY
GGKHIPRSPFKVTVKPSSDASKVKVFGPGIKPTGVKSLEPTYFEVDAGEAGEGNVDIKIEPTAKGM
PPVKDIDVKPGKTPNSYIVTYIPPKDGEYNIVVTFAGVNVPKSPFKISVTPGSDASKCKASGPGLEE

PIVGKENVFQVETPPEAGLATLTCTIDTPSGRKLKADCKKNKDGNGYDVTWTPKEVGRHKVTVK
YGDKEVPESPFYVDVLGKPDPTKVKVYGPGLENAFVEKPAEFTIETRDAGPGNLGLTIEGPEEAKI
DCKDNGDGTCSVTYIPTKEGPHDINVLFDDQHVPGSPFKVAATEPIDASKVKCKGKGIGKSTPAS
YPAEFVVDATKAGKAPLEVDVVGPGDKKYPCDIVDNNDGTFNCAYVPEKKGRYEVTPKYGGTP
VPGAPFKVSGEPTGDASKVKCSGPGLDRPHIGHAAPFEVDTTKAGDGKLNVNAKGPDGKELPV
KMVEEEEGIFACEFVPEELGPHELEVLFGGQPVKGSTFKADATPAPELDKVRTPALEEILEASPCVD
QQFEAPVDCSEVPDNKGAKLSGKITTPAGKREKVDVTDNFDGTFALNYTPTEVGKYVLDVGYDG
IPIPGSPFEFEVTEGGPDKVKVFGKGIKKGVVAVPSRFTVDTKNAGPGDLSVAVEGPSKVDIECVDN
GDGTCEVTYVPVEPGEYDVIVNFDNQQVPGSPFKPQIVQDEPAIEVGRQSDLCLTPEGISAKDIPSL
KGVLQAPSGKEKPVDITEGPNDSFIASFVPDEPGKNLLHVKKRNKPIDGSPVPVMVREKPVIGEES
GIPMDLADCDLPKDLPKLNAVLTRPNGKEGPCGVEPGPDDTVAISFVPEEPGKHIIKVYKDGKQIE
DSPFVVMVEGSPDQYPKVGHPCDKDFYQPEPCRRGDISKMKGTLRRPSGKEEEVDVKLGPDGTIA
VSFVPTEAGEHLVSVKKDGRHVPNSPFSIMVIDEPTVPTVGSECSANFDIPELDLPKDLQYLKGTLT
RPNGVEEPLELSVGADNSLAINFVPKEPGKHLVSIKKRGKHVKDSPFEIMVEEGKPTDAPTGGPVV
GHDCGVNLDIPELSLPRDLKNLDAELTRPTGKKEPLKCDMGPEETLAVNFTPTEPGKHLIDVKKR
GRPVKGSPFEVMVGSTPVSEDQIPVSEEIMAQSAPDVESGLPGGLDSLDSGKDKPVTVGSPCDVNL
DIDGVDLPKDLDKLKAELTRPSGKKEPVKCEMADNGGLSLNFVPEETGKHLLEVKKNNRPVKGS
PFEIMVEESASPESKPTAGQKCDLDLDIPDIDLPRDLKDLKAQLTRPNGKQEPIECTCTPENTLGLE
FTPDEPGKHLIDVTKRGRPVKGSPFEVMVESPESKPTVGNNCGLDFDIPDIDLPKDLKVLKAELTR
PTGKKEPIECTATPENTLGLEFTPSEPGKHVIDVTKRGKPVQGSPFEVVVGEEEPASKVPTVGSECD
VNLEIPGLNLPRDFKYLDGTLKRPNKTSDEPLELTMNENGTIGVNFVPEAPGRHEISIKKHGKHVS
GSPFDVMVESAPSDQPEVGSPTTLGLDMVIPKEDLRLLKGTLVRPNGQEEPIEITLDDNGHPNVTF
TPQEVGNHVINIKKRGQHVPGSPINVMVGPAKKPTTCDVGFEIPGIKLPDDLKYLKGSVVSPSGTE
EPVELQAGQNNTIVVSFLPKEAGIHYIHIKKNGKPVGDSPYQVNVKPEDIAAGQPDASKVKCTGR
GLRHGLPGVKADFLVDTKDAGYGGLGLSVEGPSKVEINCVDNEDGTCAVDFTPAEAGQYLINVT
FADTHVPGSPFKCKVGGPGVEDDIVPMTDDGVECASSDMLAFPSSKARSPQDFVIKFSGSGDLKA
TVTRPSGVEDEAEVVETGPDTYTVRFVPRETGEHLVNVKSRRRHIPGSPFKVLVEAPAGGAASCKA
HGPGLEGGVAGQPCRFTVITRDAGPGGLAVAVEGPAKAEIQCHDNGDGSCDITWYPVESGEYML
HIRFADEAIPGSPFKAFVLPADEGKAMPMESFKDQVLRVGQQASFAVQMKGKKGKITASVQSPSG
VQIDCSAVELEEGNYAVRFVPRELGDHLVSVFLDGHHIPGSPFTVKVGGLDSDPSKVTAYGPGLKG
GVASQPAEFTVNALNAGSGALALSIDGPAKVKMHCVEQDDGTYKVIYNPTVPGVYEISIRFGGQH
IPGSAYKVRIVGSGDDAINIFGDASKCNASGPGLNQATIGQPATFKVDASNAGRGSIMVGVEGPVI
PAKEIIVKHTGNNVYSINYILEQSGDHILRVLWADNHIPGSPFHVNV*
>TRINITY_DN12627_c0_g1_i1.p1 type:5prime_partial len:1061 gc:universal TRINITY_DN12627_c0_g1_i1:3456-274(-)
YGENNRLKFNDARQYCQQWKDDQGDLVAIHSNYENSFIMSQLYDVNEPMYIGLTDSFSERTFLW
VDQSSVVYTNWYARQPSFVGNEDCVEMMPYFKTHGRWNDIGCSAERGFICQRTPSKTPLPTPEP
VKDSKPCPAGYRKFMKACYRVVHTPMTYDDAVKDCRTSVGGELGDLVSLHNQFEQSFVFSQFAK
FDFHSWIGLKKIKGEKEFKWSDETPYDYRYWLSLRYDNTVYNDDKCTTQEFSSKVDGKWKVTDC
TQKHPYMCKIERDFDQPHLTFIGNCTDGWTKFYDKCYKLQNYFSNWPAAREKCKGFGGNLVTIT
NSDIQKQMEYLARDAYRSVWIGLNDRAKEYVFEWSSGIPLLPDSYQNWGPNEPNDNSGTENCV
HMTTESSGGKWNDLSCSQRMPYICEHPLECNSALGMEDGSIPDTNINATSYVGTYFPKYARLNAK
SDNNRNTAWCTNITTGAESLTINLGEVMQITRISTQGLLDSSLGFSRGGYVVSYMLEYSNDGTTW
MTYISEDAYDQKPIALPGNIDASKIDGTTINLKAAYLRFIPKLYKNIICMRVELYGCKSVCSRALGM
TNFAISDKQLSVSSSQAGSEKAQLRPNIDGSWCAKDDDYSPFVRIDLPRLTKITRVTAISSSKVQYKI
AYSDDGELFTRFQTLTNEERVFSTVPSTHDLETAISAKHVKFDLLKKDQGKLCLKVELYGCLKDCD
AVLGMETKIIPDRLITASSAKPSNPARNARLSVGVYGDNSWCSSETDNAQYLQLDFVQEAHLTEIA
TQGFNGDSAKYVSKYNVQFSDDGDFWHQYSEELPGNSNGYSIQRNKLKEPIIARYIRINPTEWKN
GICMRVEFYGCPMKPFTAPKILTQKTYNGAIRILNEDFNENLLNANSIEYIELANKVETDITSLYLLE
ESFKDGFNSVTVNKFREGSVITEFTIKTHTSDASLDAAEPLRKAVQTGRVGTFDVDPNFFEIVTAST
DKSTATNDDSGGLSNGGKIALIVFFVILICIVLAGGVVYYLRSRRNGFRLQFQHRAFENPTHVDDL
QNDDMKVEFMNDDY*

>TRINITY_DN8597_c0_g1_i1.p1 type:internal len:124 gc:universal TRINITY_DN8597_c0_g1_i1:369-1(-)
TCDGGVLVQRRTERAFNKVETRNGNTPCNTQACGGSDADFSEERVRACSNVNQCGVNTGTQTV
YKVCNGNRCNFQGRDYQINEEVVIRVQSCSVVCDYSPWQDTQSCTKTCDGGVLIQSRTER
>TRINITY_DN113_c2_g1_i1.p1 type:5prime_partial len:1207 gc:universal TRINITY_DN113_c2_g1_i1:4425-805(-)
GPFGYKGEAGGPGVSGDAGESGKNGTKGYRGETGARGDIGDTGFPGPQGMVGPPGFVGKRGLP
GPDGKTGGIGPLGKQGNTGLPGSPGNSGLKGQTGNTGAIGPKGRVGLVGPVGLMGVKGASGEA
GLDGKPGMPGAEGPDGLSGPRGEPGPVGDSGETGPGGDIGPDGLVGLKGDVGKPGIRGLPGLKG
MAGDTGLVGAKGAAGPDGDQGATGKAGPRGEPGLVGIRGYPGAAGEPGPNGQTGLKGNQGRI
GETGADGLKGSKGTRGMTGPNGETGMIGLVGKPGVAGVDGRNGAPGMNGRNGARGVSGTAG
ADGQPGVMGVPGIPGDPGDMGDTGLTGANGNNGTRGDPGRRGPNGAIGERGSQGSRGDMGQ
RGNSGPTGFQGPPGPRGVAGLDGMDGKQGIKGSRGKSGNPGLAGEMGLPGNAGVVGQLGKRG
ASGQKGNSGSNGDNGEVGYPGKVGNVGDDGRPGTPGLNGRKGVTGETGKPGMRGPKGPSGD
DGSEGAQGTDGLPGPPGLTGSRGVAGLDGKPGDNGSPGAPGKMGAEGKPGAPGLVGQPGLRGP
VGAQGTRGVKGPTGDRGVSGDVGLEGFAGEMGQNGLRGAVGIIGQIGEIGTPGKPGPRGMMGA
QGLTGPSGPTGSTGIAGSPGRDGNKGPNGDQGDFGKVGKAGQSGSPGKDGLPGIPGKPGLQGEK
GFGGPLGQQGPEGEQGSRGTTGMQGMKGKLGPKGLSGLNGSPGKDGLRGPRGSRGTAGGIGPL
GVDGKPGTPGTNGTDGLPGDTGPKGTAGLRGEVGDFGAPGDVGENGATGPDGIIGAAGPRGRN
GDVGAPGSRGGAGDMGAEGPRGASGDDGRDGLKGLKGTMGRMGIPGPSGDLGEKGDNGPDG
RPGYTGPQGRYGDVGTDGDTGVQGMKGETGVSGPDGNKGPDGTSGDHGTPGTAGAPGPPGPP
GPPGDYSSFMSGGYMDFLRAGGFSKGPRYYSGKYKKSLNQESNTIEKVFQAKYQLFKEFDSVWN
QVVNDKITRKTSGSSKNPAVTCADLFKNNHHIGSGDYWVDPNEGSPDDALLVYCNAENNETCIY
PKSPSFDKYSTYMGEVDYTWVYSSRILTDEEYITYAADMVQLNMIRLMSDRVRQNITYHCKNSLS
DLQLKTDDDKTMHLDNLRRITVTMQNNCVVKDGTWRKSVFELNSKYLEQFPLQDIAYKIINSSD
EDFAIDIGPVCFS*
>TRINITY_DN113_c0_g1_i1.p1 type:complete len:1417 gc:universal TRINITY_DN113_c0_g1_i1:4413-163(-)
MANWSLLYAALLFLYSVSAQEEERPITFIKGEPGPKGSRGMPGDDGSVGSTGRQGNEGRQGPRGD
TGPKGFKGEPGLIGPAGGASGGETKGPDPLAARVGVKGGTYGVNTVFDYQLGPPGPPGVPGNTG
GYGFKGDVGPVGVKGVPGLPGPPGSYGERGEPGEAGLDGVKGDRGDAGQTGPPGKKGVAGAV
GFPGLQGPFGYKGERGGAGPVGDEGVSGRKGEKGYAGEKGSRGDQGDAGFQGVQGPTGPIGPL
GNVGPRGPDGQRGIAGAIGAEGLKGAPGTRGPQGPKGQLGEPGQRGVKGVVGDKGPTGQQGS
QGPTGNPGDPGQPGKTGDKGSIGDVGLIGLTGPQGMQGERGTVGPRGPAGGAGKKGERGQAG
PIGATGEKGNVGDVGLDGARGAPGKSGIEGKVGQPGPQGEPGAVGLQGAQGPRGTDGQAGQA
GPKGNTGEGGFPGAAGGKGSKGLKGRGGDRGMQGEPGSAGKPGARGRDGKRGAAGEDGKNG
NPGQRGPQGSQGLTGAMGLVGAPGAPGDMGVAGLPGQQGRKGTRGLAGPTGQRGLRGEGGL
PGERGEQGAAGPQGTQGTQGSPGPQGRTGLDGKDGSPGDKGERGSMGTRGKQGEPGADGKQG
SRGTPGQQGIRGDSGDQGSPGLLGERGLAGQKGVAGRDGAPGMPGRTGRQGTIGATGAVGLRG
AKGGRGKDGETGEQGPQGQEGPTGPRGPRGASGEDGAPGQNGLPGLTGPMGEEGQPGRPGQP
GKQGPRGSQGEAGPVGPKGPLGARGDQGEQGPVGIEGVVGALGAVGSEGTKGPVGAMGSMGP
TGPTGPQGATGETGPIGPLGAQGRPGIAGNAGGKGPNGDPGNPGRPGSSGDQGPPGKDGSNGE
PGSPGKEGARGLIGRPGAEGIRGEAGTSGSTGRLGRKGELGPKGFPGKRGKDGTNGKRGEMGVS
GADGIRGSKGNSGKDGSAGIAGEDGVEGQIGEAGYQGSRGVRGDTGAPGDSGQPGDNGPNGA
KGSKGSSGRAGAQGVDGRLGARGDQGAEGARGKEGAAGAEGERGEKGKMGAPGIYGPAGDP
GFKGDEGEPGPVGPVGPHGPRGSPGEDGPSGKKGASGITGTNGRKGNRGQSGANGDTGTPGIPG
VPGSPGRPGPPGEIAGMSGAFARFFSAGGMGMKGPGNYYNNYYGQYRYYKAGNEKIRNQIKPH
LSTFKQYYELLKDVDDMMKATGTKSKPARSCKDLFTDYPNKITNDYWIDPNGGSTDDAVKVFCD
KMKNETCIFPKSYRAELENHYTGKDKFRFMYEDIKEDGEIEYAADITQTKILHLMSSKVRQEFSYN
CKNSLAVMKNNKDRYEYALKLKLDNEDIISMNMNTRSLDINILNDECWKKDNTVRTATYELISH
KPIKHQLPLLDVASFDIGGPNEEYGLTMGPVCFS*

>TRINITY_DN199_c0_g1_i1.p1 type:complete len:229 gc:universal TRINITY_DN199_c0_g1_i1:207-893(+)
MVQGSMTIVKYLMFFFNFIFWLSGLALIVIGAVIRDKYGDYFSYADSRFTNVAVFLIVVGVIVFIIGFL
GCCGAIKENYCMVTTFAVILGIIFILEIVAGAIGFAYKGQVEEKADKALVRASKNYNNDDEPGAKK
LLDWAQQRFECCEVASATGNGTCADKSPKSCHPDNDCNKTKYSQKCKEGFVEFMKDNLVTVGA
VALAIAFIQILGIVFACCLMKAIKGEYEVV*
>TRINITY_DN55094_c0_g1_i1.p1 type:internal len:107 gc:universal TRINITY_DN55094_c0_g1_i1:319-2(-)
LTLDMLAQKPMNVIALIDEESRFPKGSDETMLNKLIRQHSKNPAFITVKSHAEKKFGIKHFAGSVY
YNTVGMLDKNRDTFSADLNHVIAQSRCKFLLHLFDKELKM
>TRINITY_DN8008_c0_g1_i2.p1 type:5prime_partial len:1298 gc:universal TRINITY_DN8008_c0_g1_i2:4205-312(-)
IMSPKERTCPLTQYLFTPIYTGSCKISDAEQRANDRQVKSAVQMAGSAIGTVVGTIFGGGIGGAIGG
FIGGAIGSLIGGIIGLFSSCDKSYEVVTRSWVEERKCILLEPDVRSITRPYSECFDAKIKANTGFKVPY
TCNCKVNDCIVKSPSPDCLLQNQQCERKRKDFLERTKTVPPTFSKLYKSLKQCQDSVGASILKVQS
AKKTVQFYEKEHTRIKNMLLALEEENKFSKQALSDATSILQDDLCVSNAFMNNKNINELIDIKNV
QFDTLLPLVNNIRINAHVKVKGNDVLVPFVHELSNDNKVSLNAFGRKIIMNTICKKIRKRRSLDL
NEDEKNDLAFVPWINNVNGTATVADLSCVSLDKTLEFIRHTVKTLNKKSSYANDLLMQLQYSTN
KLNPSLYTAFTAYSGITTEKNDIIKANEALLMSANNELNKIQDQVSVSNVLDIWGNEAEIVTGFNN
VSVCLNYKDCIISAIDTLKNLPTMFRNSREAYMKEVMELEKELTKIYEEKNLIGLSAKAQSVERIVS
RIKYMSFHCSKRPIVTLKEVKDMDVEKGKNITLRCIVRSARAIKYYWQFNDDILDEENSHSLTIPVS
NETSGIYRCIAKNLVGNGTSNQIRITTYTKPSFIEEPKDQIYILPSHPQSNLVLTCNVTNNYDTTIAW
YYRSFTIGKRPNIINGQTSILLDLDGTKQENSGYYFCQAFNKHGRIKSREARVNILNSNIARLEVNIS
LDILQTDEISSNSFKKISGDMLSEIKDLGRTLTTTVIKSKTKTRVTFNLKVKPITDNVKSLSESFNISSR
MRQNLGSAAAVLLGKLSENSFKLPINDGEFAKVDNDTMTYGMKLNICQEGYKLHKNGFLCVQC
PFGTAGKDCKKCPLNTYADAPGLGICKKCETSKITEQIGSTSKSQCIGTTERKVDIIFAVDVSKRFLE
QINGLQKIRELLSKFIKTYSGTKYNTRFALLTYYKYVDVYLSLTEYYHSDDILGLLEILGEGSRMPDT
KLALVEIETMFDEESSSDRKKVAVFITNGGTSDSYYLMELFKYKRQAITLQNKGVHIISVGLGTVDH
RGLRALASYPKLENAIFPSSNSVAVQKMIYSIGNELKDNCYTPAVDFGIIYHPNHKESLVQITKEQF
LRNANWLSKLLLPIYRMFKQQSYYFMYKHSVPSNSFGSFSETGFQERIVSYLQQQTSGFDIYKKIST
AINVDMTEIYRSFSKVKVLIIITEEPLDEAVLKQYEISVHGEKIDIYNFVKDASSIEKFEGPFSDKPRA
HRLVYGIDEVNFLVNAINCNRTKICG*
>TRINITY_DN1535_c0_g1_i1.p1 type:complete len:779 gc:universal TRINITY_DN1535_c0_g1_i1:86-2422(+)
MMKYFMLIVFTICLKNIHAQDKNLCTSAETCGKCLLKGGDCFWCIEEDFKFNRTRCNTEANLAA
QGCTDYGMGRGKKTNDADLDFTSVRQVKPQKVTLEIRPEEEQSFRIQVKSAKDYPVDLYYLMDL
SKSMENDLDKLQTLGTNIAAEIGNITKNYRLAFGSFVDKTVIPFVENDDANYKPCAECEPTFSFKH
VFDFSNDAKSFEKAVSEQKISGNLDTPEGGFDALMQVAVCEKEIGWNPKDKCRKIVVFVTDASPH
IAGDGRIGGIIRPNDGQCYLTEDGDIKMYTEATKMDYPSIGFLRSKLKDNAVIPLFAVTKNVTEVYK
SIAAELKDLQAVTGILEGDSSNIVEVIRDKVREISRTVTLTDDSSPDITVTYRVQNDPTKPNVCPSPV
APNRCEDVEIEQTVDFDVYVKASAKNCETKEKQDQTFTVNIPAFGKVEVNVKMICDCDCAKTSE
PDSPKCDGQGTFECASCKCNEGRYGDRCICDGNENKDMSLCKPNNVTDKICSGFGNCVCGVCQ
CNIRENPEERIDGNYCQCNNFNCPRHQNIVCGGPERGSCECGTCVCREGYLGENCGKTDCRIGV
KKCLDAKGQNCSLNGICECDRCLCDKGYEGKQCQTCVSCPGKCSEYKTCVLCKAFGETGEQCD
GCELIQHVLVNRTNEECVVPVDENCFVIYRFEIDPVSMNETLYIQRDKRCIIIVKTKPPILAIVLGTIG
GIVLLGLLLLLLWKLLITAYDNREFQKFEKDRMKSKWENDENPIYRPSKQQFQNPAYAGAN*
>TRINITY_DN1613_c1_g1_i2.p1 type:internal len:1068 gc:universal TRINITY_DN1613_c1_g1_i2:3201-1(-)
TKSCGGGIQESTRSCSNPSPAHGGKPCSGELSKTRACNEHECPVDGGYTPWSAYGKCSKVCGTGV
QFSTRTCTDPKPKFGGEPCTGLTKKSRNCNTHPCPVNGNWGAFGSFTTCTRTCGGGIQKRYRKC
DSPAAAHGGKKCPGLSFQFKACNTVHCPVDGGYTQWSSFGSCTATCGGGSQTSTRTCTNPSPKY
GGEGCVGPSEKTRTCGTKPCPVNGGYTEWGEYSKCSEECGGGIKTKSRSCTNPAPAHGGKGCQG

PSSESDVCNEQPCPVDGNWSAFGPLSVCSRTCGGGTSLSKRTCTSPSPKYGGKSCSGSRYRIVACN
TRICPIHGGFTAWSNYGVCSEKCGGGVQTATRSCTNPRPLFGGKACVGSSRKSRICNSQPCPVNG
GFSDWSAYGKCSKSCGSGIQVSSRTCTNPAPAHGGKSCAGPLQQSRVCNTLPCPVNGNWGSFGR
FEECSVSCGGGVQKRFRPCNNPSPAHGGKACRGLSFEIQACNTKHCPIDGKYGSWSSYSKCSVTC
GGGTQVKTRKCNSPAPAYGGKNCVGVASHIQACSTHHCPVNGGLTSWSSFDECSEECGGGIQER
SRSCTNPAPAHGGKSCVGALVDSRKCNTHKCPVDGGFTKWSSYGSCSKSCGGGSQTSTRSCTLPK
PAYGGDACVGPTIKTRGCNSQACPVNGNWGGFGVYGTCSKSCGGGTQKRYRRCNNPAAAHGG
KQCSGLSYQIKACNTHHCPVNGNWGSWGSFATCSKPCGKGVQKRVRKCNNPSAAYNGKACPG
LDYQLQACNTHHCPVNGNWGAFGPYEACSKSCGGGTQKRHRRCNSPSPAYRGKTCSGSSYQQR
SCNTQHCPVHGKWGTWGSYGACSARCGGGIQKRYRSCNKPSAAYGGKACPGASYNIKSCNTQ
HCPVNGNWGSWGSYTTCTKSCGGGTQKKSRSCNNPAAKYGGKACSGSNYYLKTCNTHHCPVN
GNWGSWGSYTACTKSCGGGTQKKYRTCNSPTAKYGGKACSGSNYYLKTCNTHHCPVNGNWGS
WGSYTACTKSCGGGTQKKYRTCNSPTAKYGGKACSGSNYYLKTCNTHH
>TRINITY_DN2113_c0_g1_i2.p1 type:complete len:1704 gc:universal TRINITY_DN2113_c0_g1_i2:5257-146(-)
MAQLLPIRFQEHLQLQNVGINGANIGFSTLTMESDKFICVREKVGEQSQVVIVDMADANNPTRR
PISADSAIMNPASKVIALKAAKTLQIFNIEMKSKMKAHNLTEDVIFWKWISVNTIGLVTETAVFHW
SMEGDSSPVKMFDRHQSLAGCQIINYKVDHSGQWLLLIGISAQQNRVVGAMQLYSVERKVSQPIE
GHAAVFSQFKVDGNPQTSNIFCFAVRGAAASKLHIIEVGTPPTGNQPFPKKAENIFFPPEADKDFP
VAMQASQKYGVVYLVTKYGYIHLYDIESAQCIYSNRISGDTIFVTASYEPTEGIIGVNRKGQVLSVSV
DEEKMVPFLIQVKQNGELARRLAERCNLPGADNLFLQAFNDKFASGNYQEAAKIAAKAPKGILR
TQQTIQRFQQLPSQQGQTSPLLQYFGILLDQGQLNKYESVELCRPVLMQNRKQLMEKWLKEDKL
DCSEELGDLAKQFDNTLALSVYLRANVPNKVILCFAETGQFQKIVLYAKKVGFEPDYVSLLRNVM
RVNPEAGANFSGMLIQDDEPLADLNQIVDVFMEFNLIPQCTSFLLDALKNNRPAEGPLQTRLLEM
NLLSAPQVADAILGNQMVTHYDRAHVAQLCEKAGLLQRALEHYSDIYDIKRAIVHTHLLNPEW
LVNYFGTLSVEDSIECLKAMLSHNIRQNLQICVQIASKYHEQLTTSSLIEIFESFKSFEGLFYFLGSIVN
FSQEEEVHFKYIQAACKTGQMKEVERICRESNNYEPERVKNFLKEAKLTDQLPLIIVCDRHDFVH
DLVLYLYKNNLQKYIEIYVQKVNPARLSIVVGGLLDVDCSEDVIKSLILSVKGQFSTDDLVAEVEKR
NRLKLLLPWLESRIHEGSEEPATHNALAKIYIDANNNPERFLRENQFYDSAVVGKYCEKRDPHLA
CVAYERGQCDQDLINVCNENSLFKSLARYLVRRRDAELWGTVLVEDNKYRRQLIDQVVQTALSET
QDPEDVSVTVKAFMTADLPNELIELLEKIVLENSVFSDHRNLQNLLILTAIKADRTRVMEYITRLD
NYDAPDIAGIAIVSELFEEAFAIFKKFEVNTSAIQVLIENIQNLDRSYEFAERCNDPAVWSILGKAQL
DQGLIKEAIDSYIKADDPSAYMEVVEASKNCGNFEDLVKFLQMARKKAKETFIDTELIFAFAHTGR
LADLEEFISSPNHANISQVGERCFEQKMFEAAKILFNNISNFARLASTLVHLGEYQNAVDGARKA
NSTRTWKEVCFACVDGQEFRLAQMCGLNIVVHADELDDLIRYYESRGYFEELISLMEAALGLERA
HMGMFTELSILYSKYKPTKMKEHLELFWSRVNIPKVLRACEQSHLWSELVFLYDKYEEYDNAVVT
MMKHPTVAWKESLFKDVITKVANIELYYKALQFYLDNKPLLINDLLVVLTPRMDHTRGVSFFQRA
CYLSLVKPYLRSVQNHNNKAINEALNSLLIDEEDYNGLRASIDAFDNFDNIALAQLLEKHELIEFR
RIGAYLYKGNNRWKQSVELCKKDHLYKDAMQFACESRDKAVAEDLLSWFLSIDKTDCFAATLYM
CYDLLEPDVVLELAWRNNMLDLAMPFMIQFMKDYYTKVDKLAESNAKRDEDKENAPPEAPIM
GVEQLMITAGPSSYVPQPNMYGMPPQGMAPQGMPPQGMPPQGMPPQGMPPQGMPPQQGFPQ
QGFYGGAM*
>TRINITY_DN5500_c0_g1_i2.p1 type:3prime_partial len:1183 gc:universal TRINITY_DN5500_c0_g1_i2:3546-1(-)
MEIKNFLILLSLLSLTSQLKAGLVFNETGVDQPVDLFFVIDGSGSVGYELFDGQLQFVTELTDQLGG
VSPFNVHVAIAVITTSPYQAIGLTQYQVNSEFKQAVSEIFYPRGATRIDKALLAVERELLPTSRAGVP
KVLVILTDGIQARWPGVYNTHVIIERLFNAGVSPVVVGIGSDYRDLDRLNKLAIKENHHLNEPIEK
LFLTTDDMEVGSPALVQKAASTVKKILSNVARFGYEVRSDFAFLLDGSTYAEDQLKLMEGLASYFQ
INRELSRFAVASSQGGGRTYIEFNEAQLSRDFKAMLMKLNVDSESIDIGIAAKYAYEEIFRIEEGERYS
TPRTLVIFSGNAEAKDSTLFGKYYSLLKKANVNLVMIAVGGGKGRSNLRNFPVDYYLPDFNDEVS
FTEECNIIAREMHNYIIRFASDTQRSKKCLHDGDLFFSIDVSGSAGAYYQENLNIIEKIIASFEISNED
SRVGFAIDRDGNTQVTPLDKHNSLESLMRDFDSLPYYPRGRSDMVAKLKAAYNQFLTSSRIHFQDI

LFIFTDGYLNRKSESESKELIDKLKAFGVEVFVVSLGYDVNFPFLMTLSSDKGHFFHVNQLPKDSDS
FVNSIINKVCDYTETFQDKYEIPINVALVLDGSHCSEKFHEAQKTFSNVIIDGLDNSPLAKNYLFSYS
SGVQQLDSNSSLAINFGSDGNRLDLALNEVVKLFQSISSNEVNMAIFVTNAGLESEFNATMSQAR
KLRSLGVITMVICIGDNFNYDVIRTLASSRQLIYMIPSPSQLTDTKFLRHIISAAFEMAESMPPPRLEA
GKSWYPAFFGNWVDSNSLEGEVPDAPLKAYHFGPTVLWSKPIQQKNYIIESTIIPAGSESGRFGTCG
HIFKHKVGATKKEKYISKSWGYFLWSNSFLYITTDGTETSPRIEFDYSSRDKLNLKTIVDGSKVKIYI
DGVFIRSIELEGTEADSQSHMFVGVWCHRLSAVTVEDFKVSALSNPCTVNNGGCSQICSADPYEF
KCSCIPGYRLVEDESTCVAESTCPELITTTVITTTLSSSPPTTNQSKFISTTTKPDELIDTTATIISTRESDI
TTTLILSTKSTEKPIEKIEKTTTSAIGDILEQTTVAPIKTSTVFVPTTKEEVILTTKEMAETTLTVPTTASP
VEETTTVAPVSTAAKTEATTQAVITTLAATTK
>TRINITY_DN4040_c0_g1_i1.p1 type:complete len:402 gc:universal TRINITY_DN4040_c0_g1_i1:1386-181(-)
MNIIICFIILLSLGSFATPHPYHMCKKRWEKVGCYHDPVAPNRPYPFQLVNHRDPTSNKWDGNLI
NWNRFSQSLHRLACICANRTRAEGYKYFGLQFYGECWSGPSLNFNKYGPSKECVGNLSQPCNNE
DTLRECVGKGHTNYIYKLLDEKPPIHGKWSEWGDWGACSKSCECGIKHRERKCNAPKPQNGGR
ECKGLSKEYSECNNGDCPKVCDKQVEIGILLDFSGSVRQVNWNATIDFVIRYSKGFMISPAKVHFG
VLSFSHFHRIDFKISNEKYWNRAAFEKKVKTLSYRFGYTNTDIALKAAKKEFFCDSCGNRKGALK
YLLVFTDGKSNGVTTELKVLSDSIKKMGVEIISVGTGYQINHEELEEIATDDSHVFYVKNYAYLMDS
MNAITKLTCGK*
>TRINITY_DN1320_c0_g2_i1.p1 type:complete len:260 gc:universal TRINITY_DN1320_c0_g2_i1:97-876(+)
MRCLSMIFAILLVYLFDQVSSRACRDYVNYQCRKYLCHVPKYHRFVNMYCKATCGLCPVIHGLQ
MSTMSTPTTTATTTTTTTTTTKTTTTTTTTTTTTTTTTTTTTTTTTTTTAATIVLLSGGKTISGTPEDAS
GPAVRCTDGDVTTLCITTQAIDAYFQVDLGSQKKVSRMEIKNYFHYENQLELKFLVSNDVTFATST
ICDTWDVRSVNEGDVQDKKRDCNSNVSSQTFRYARVQTVNNANEPEHLRVGEFEVYGVA*
>TRINITY_DN413_c0_g1_i3.p1 type:complete len:350 gc:universal TRINITY_DN413_c0_g1_i3:1286-237(-)
MVSLSVTCLSLLLFVTVNAEESDHCGYDEKSWYKAMGWCYHSGSRLLLGDFNGDKRTDFFCHD
SNGKNWVAVSNSDGSFGSTTWQDSKERWCASPNILLTGDFNGDGVTDILCHGTATGKKWIKYFN
KYGVPSGKSFYRAMSWCNHKGSQLYIGDFNGDKRDDLLCHDTSGRYWTSYAGTDESKVFHHTS
WYKALEWCRHATSKLYLGDFNGDKKTDVLCTDGKGYKWIMYAKTSGNLFTGTDWHTNLDWC
HESSTVFLRIGDVNGDGKDDLVCHSTAGNSWVAYTQPKGGFGNFCHLAKWCAAGKESFFLGYF
NQDSRADILCHDTKYGKKWIRYAECSFAEESDHCG*
>TRINITY_DN278_c0_g1_i1.p1 type:complete len:470 gc:universal TRINITY_DN278_c0_g1_i1:3091-1682(-)
MAPSFNKTNGHMEENGGMIHANGKSSEKPINLGEQTYSYMTFKKMGLIMITLPLVFLLLVGLPSY
LYPVLVLLFFIPVFAGYIGLNNVIYCLYERHNQVSKIDNYIEFLDEDLKKKFTGRFIPIRDLYELYAEG
KLNFKGDVLQCLERRNEYVSYKLQLWHLKFFLSKLLPELLHARSQDKEQVCDHYDRGNDFYNA
YLGPLMVYTSGIRYSESDTLEDMQMNKLKAVCEKVQLKEGDRHLDIGCGWGTLVNYAAENYNS
QSEGVTLAKEQVCWAEEKAKSKEIMDRVNYLCMDYRDIPKKKYDKITCLEMSEHVGIKRYHHFL
KDVRNMLEDDGIFFLQIAGLRRAYQWEDFLWGFFMDTYIFPGADASLPLQFPIAELEAAGFEVES
VKTVGIHYSDTIKYWYHNWVKSDSHDKMIQKYGRRMVRVWDIFLAWSTIIARQGNSTCYQIVAH
KNFNHYPRRRFIQPKN*
>TRINITY_DN598_c1_g1_i1.p1 type:5prime_partial len:745 gc:universal TRINITY_DN598_c1_g1_i1:2-2236(+)
RWSGCSKSCGVGVKTKSRECDNPRPSGGGRYCTGERTATEACKIQDCGVDGKFSHWSTWTACSK
TCGGGAQKRARQCNSPPPSGDGKDCAGAREEFKGCSNDFCKGPTGVAKAQEIIERFLENFHKKT
YPDREPSGKFLKAIFAIQKAVAEEFISVDPYVRSKTLEQRLKDLENFLKVATEGGSIDFARTLLGKGV
FTFMKQKIKDGLPIDDIDKAAEGFLEKLRGDLGDKKMDEILSSQGDATLMFTIDTTGSMKQEINA
AKAIAKQIISATRDYEVDYILSPFSDPGTGPVTHRSASERASFVSDIDRLRARGGGDCPELSFGGML
NALNNDPQYGSPMFVFTDATAKDDTDANVASLKAAADATDATINFFTNLAGCNGRRDRKRGIE
SYKEIASYTGGQVFPLKNEAELIKFTSYVKTSLQQDTVITSGGTLNSKSKRSLYSMQARHFMVDDGI

SKLVISVGVQQLASQVALVDPNGKKVYPSTTLALSLIYQVSHPSPGQWKLDFPTSVGKYSYNVQAV
SSSAIAFAHNFIYQQSVRKNSPPIPLANPLKGVKNMMVVRLGGTKNFDLNSIKIDLVDTSGNVLQ
KGIGTDRLDSDEVRHATIDPPSGPFKVRLTGKTKSGYNFERVSQGISRPVSAAMRTNFAGYDFTAK
LGKGPYAAQMLFINRGAHENFYFYAKANYGSIPNYKQTKRVTSQGFVTFTYKAPRQYNLKGKSD
RITVTVKRQSTGEIVSHHTSILLVV*
>TRINITY_DN113_c3_g1_i1.p1 type:3prime_partial len:1162 gc:universal TRINITY_DN113_c3_g1_i1:3485-3(-)
MEIVSRSRIAIFVLLGLLISVAESQFDPECPEIDPDVECSPPESTVNECLINNDCDVDKPLCCSNGCG
LKCTARERRSITPDGFTPEDEKLCRVPVDLGFIIDVSGSIGQTNFDKIKKFIEKIVDAFHIADDGTHV
GAVFYSDNASLAFDFNHLQGDQLNEMNVVREIEKIQVTDGQTRIDLGLELASRELFNPAAGMRI
HKPKIALVLTDGKQTRGELAPDAKDLHQASRLLKQTGVQVYSLGIGLDYDIGELLDIASDDASVF
RSQDFDELVSIVDILIEQTCKGCTRAVDVHFALDASANVGEENFVFMKDFVKAIGYNFIISETGAH
MSGSVFGDVGTLVFDKSQATEINEFENEVENMPYLGDATGNMDKVLKMISNEVFNFDGFTRQSS
PKVIVLLTSSNCDTCQESLEDLARPLKAEGIHIVAIPIGDPLFDEMQVLASHPIDQYAIPRATHQELL
NAEFIQKVSEMVCAGKPGICAEPPVKGECAEIINECSVDMRCPEDQKCCIKNCVASCVLPLAACIT
PIEIAFAFETTKYGIADFNNVKNFAINLLDHFKVGPSKAHVSLSTFSEDVTSLTKFIDDFDMESIKQSI
KDLKHDGGEESNLGNFLEIINTEVFSVRGGIRQQTPRVLVIFANGDINDEQAVKAQEEALHLKAD
GKDVKIIIVNIGSSTKTLDVLNEVATEGSFYEFFGSAELDSTAIETEISEKICSTKVRRSKCQRRIRSSCA
VKDDLCDSDFDCPGHQECFSDGCRLRCQTCEFRCKDKVDLLFVMDTSARIGRDNFERGKKNIKK
MLEWFTIDSNNTNVGVITYSDTANVAMPLPSRGNNNPAQSLIDVQDRLDSIPYSGGSVSHLDNAL
QLAAEKVFPDNGPNLRRDSKKAVVVITDSLDDSASQLELASWPLRRQRVQTEDGDASNIRILAFN
VGQELDLDLWGNIVSPPVEENVFYAADFDDMYNGIRQLAEDSCKYAAAITPPPIIKQKGEPGPPG
PPGIPGTNGTDGRNGTDGRRGPKGERGDTGLKGFKGEVGLVGPPGGGAEEQKGPDPLASAVGIV
NVTDLPGGGRIVFDLREGPVGQQGLTGIIGPKGPIGDTGEVGIKGRNGTTGPGGFFGLPGPKGQP
GTDGAKGLRGDPGLRGSAGRKGPTGAPGLTGPQGPFGYK
>TRINITY_DN165490_c0_g1_i1.p1 type:internal len:171 gc:universal TRINITY_DN165490_c0_g1_i1:2-511(+)
LKHDEKCFLAGWEGAIAAKHIGWWNKGRTLAKKAMQSLPPGPQHRAQRETASTLFLLLAEEEQ
EEKSRKVREMTRAKEAAPAPIDPKEHDWARSIAGQLNDALKCEDFRRPHHQLWKLVGPGLLKQ
DADAIFSEIRQLIWEKWNSVAWQQGYRTTWDIKARQKLCARLVN
>TRINITY_DN1505_c0_g1_i1.p1 type:complete len:154 gc:universal TRINITY_DN1505_c0_g1_i1:98-559(+)
MSLVIPEKFQHILRVMNTNIDGKTKIMYAMTSIKGVGRRFSNICLKKADIDMTKRAGELSDEEVER
LVTIMQNPRQYKIPDYFLNRKKDIKDGKFSQVLANGLDNKLREDLERLKKIRAHRGLRHYWGLR
VRGQHTKTTGRKGRTVGVSKKKN*
>TRINITY_DN1936_c0_g2_i1.p1 type:3prime_partial len:1894 gc:universal TRINITY_DN1936_c0_g2_i1:5680-2(-)
MLSPAVTKVFIVLSVLGGLICQSCDFEVANACQWTQYAKDKFDWTRNFGNTGTGGTGPKTDHTS
EVKILKANTTGGNSNGAPCVFPFKFRKRTYTKCVRSMQRGKSWCATVPDMNKKRQWGYCMPT
GYYMYIEASSKRGNPRNKGDNAVLLSPVISKGPKCLQFWYHMYGPHIDTLNVYKKVSNNMTKL
WSLTGTQGNRWISANVTFNGGSQFQMAIEAIRGVEYKGDIAIDDITVDDGSCNADNGQKGNVTS
YGKCTFEKDKCGFKDGNTSTLTWTRHKGKTPSKGTGPLVDHTFGTGLAYYMYVEASNNPMQSA
RLYTTYTNLDSTGSCLSFWYHMFGPRTGELKVYINPPEIGKEIPSFSVGGEQGNLWIRGEVTVNTTT
ALVVFEGTTKYSYLGDLAIDDVSMERGKCEDPDSCSFQKDLCAWYNLPKSSGLDDFDWIVGAGV
TPSRFTGPTSDHNGDIAGKYLFMESSSPVKMGDKTWLSSELFNSTGKGKCFVFFYHMYGRDVGSL
NIYVNTSSGVELVWTQSRSKGKQWQNGQVDVERPGTYKVLVEGVVGKGYLGDIAIDDFKFKPGK
CQIVPSNASPKKPQTTMRPTTKRPTQPPTPILDCDFEFGVCSDWRQDYKNDTDNWKVGKGTTST
AGTGPSFDHTKGTIYGKYIYLEASYGRLNETARLISTSIPATGDTGGKCLTFWYHMYGPHINRLNIY
VMIGGHTGKPIWSRVGTQGNKWNKGQVTLVGRQPFQAVIEAVKGAKYQGDIAVDDVSLSSKKC
PQPGATTCTFENSQCGYIDDTSSDVQFQWTWQSQATSSLNTGPKFDHTLQSVSGFYMYTEASNA
KQGDKARLISPVNPGTSGSCLKFWYHMYGAGMGTLSVRLLVNNKLQSVPLWSETGNKGNEWK
VATTTIKTKDAYQVVFQGVIGTTYASDMAIDDVKLSDGECQSPGFCNFEDGLCTYANSKTDDFDW

LVGSSNTPSSFTGPKVDHTFGNASGSYLFLEVSKPIKAGDVAVLESQQFNAVGGKTRCLNFWYHM
YGSDIGTLQVIYKVFSGSQQERVLWKLTGQQQITEKDPWKYARVPVDMDADHMVMFKGIAGSG
YAGDIAIDDIQFTTLACRVEPSIANPTPTTPKPPTTFPKTAPPTAAPTPFSCNFEKDFCSLTSDSTAVF
NWTRSQGYTASQNTGPSTDHTYGRGFVKTYGPGSIRHAAGMCIHISMGGHTKPKDETALVLYNG
CGQDRTEFKYDNGLIKSTQYNMCFKLKGASTSAGTKVVISDTCTNKWKMLANGAFQDAKTNLC
LAPSGKLAYPQNNSPLIVSKDCKAKGVIFTFSPTMGFYALIEASAPQKPNDTARLISPVLNKPKCLS
FWYHMYGANINQLNVYTKSPNGVYGNPVWSKFGSHGDKWRPAHVYMNPKSSSYAYAIEGVRG
NGYEGDIAIDDVSVSDKCPAPVECTFEETNPVACGWTNLKTDDFDWSIGQGATSSQNTGPSNDH
TYNDQRGKYLYIETSSNLKAGAKALIQSPVYRGNGRQQGQCFQFWYHMYGASIGRLSLHLDQVG
STTKNRAWIQESDQGNRWSIGQITIKSQAAFQLTFEGIRGKSFTGDIALDDFRMFDGPCNNIATCD
FENEKCTWKNSYHKDDFDWIIGQGTTLSLNTGPVTDHSTNSGDGHYIFIDASRKKQGAKARLYSE
IIPRGFFTFIPYCIYFWYHMSGPDIGTLNLIAKHGKGKALEKVIWSLSGNQGSKWLQGTAPVTSSVD
FHLVFEAIVGDGYKGDITLDDIVYKLGLCSISPKAADPKLISTTSAPTPIPTTAVPAMYDCNFEKNSL
CQWKQDLTDQFNWTLVQGQTPTLGSGPSVDHTTGNKTGYYLFIETSNPRKAGDKARILSPSMDS
DKDHGHPHCLKFWFHMYGPHV
>TRINITY_DN925_c0_g1_i1.p1 type:complete len:261 gc:universal TRINITY_DN925_c0_g1_i1:826-44(-)
MGRVIRGQRKGAGSIFKAHVKKRKGAAKLRSLDFAERNGYVKGVVKDIIHDPGRGAPLAIVAFR
DPYKFKLRKELFIATEGMHTGQFIYCGKKATLQIGNILPVGTMPEGTIICNLEEKSGDRGKLARAS
GDYATVVSHNRDSKVTRVKLPSGVKKAIPSFNRASVGIVAGGGRIDKPLLKAGRAYHKYKVKRNC
WPKVRGVAMNPVEHPHGGGNHQHIGFPSTVRRDTSAGRKVGLIAARRTGRIRGTAKVKDVGGD
KE*
>TRINITY_DN624_c0_g1_i1.p1 type:complete len:884 gc:universal TRINITY_DN624_c0_g1_i1:218-2869(+)
MANQEEEEHFGYYYGEEEDRGLLDPAWEKQQKKTFTAWCNSHLRKVNVQIEELNVDFCDGLKL
MLLLEVISGERLPKPERGRLRFHLISNVNKALDFIASKGVKLVGIGAEEIVDGNLKMTLGMIWTIIL
RFAIQDITVDEFSAKEGLLLWCQRKTAPYKNVNVQNFHMSFKDGLAFCALIHRHRPDLIDYDKL
AKDDPMYNLNYAFDVAEKELDIPKMLDAEDMVNAVKPDERAVMTYVSCYYHAFSQSQQAETA
AKRILKALNVNQGNEKAIEDYENLASDLLDWLNNKIPTLDDRTTDSSLDTMQNKLNEFRDYKGT
EKPPKVDDKSKLENLFNTLQTKLRLSNRPLYMPAEGKMINDIDKRWKDLEQSEKDFQEWILNELR
KLERLDHLAMKFKHKCDIHEAWSTGQDDYLRSDDITDASLASLLASRKRHEAFESDLAAQQDRV
EQIVAIAQELNTLGYSKIDEINTRCKGICDGWDTLGELTTQRSEKLAKAIEIAERLDNLWLEFAKKA
SPFNNWMDGAKEDLMDMFNVHTIEEVNDLKDAQLKFKANMPAAQEEFNTIMGLQNAISSMSS
QPNPYTNTKAEDLQEKWQEVLELIPKRDSLLESETQKQQNNERLRVQFAQMANQVGAWIKQRM
GRMTDIGMNAQGTLERQLEELENLNAEINGFKQNVLDLDASARNVQEAMIFENPHTPYTMEVI
RIQFEQLLTGVTRNINEVENQILMRDSIGITEEQVKELRTSFNHFDKNSNGRLDYKEFKQCLVSLG
QLGDVEDKGEHTFNRIIAMVDPNETGYVSFQAFLDYMTREMADTDTADQVMESFRILAGDKPYI
TADELRRELPSEQAEYCIARMSAYNGPDGVPGALDYRSFSTALYGESDL*
>TRINITY_DN495_c0_g1_i2.p1 type:complete len:655 gc:universal TRINITY_DN495_c0_g1_i2:152-2116(+)
MDFHWNTVIAGLIIVQVYSLAEYGTLNGKDSYRNAYPWLANDRNNNELGREDARSIYRSSDEID
AEYPQSDDEVDTNDIPKSYTSRDELDAEFAQRTYKFADDLDIENAQKSYASSDELDRDDEQRTNK
NTDDLDAEDAEREETINDSLDAENAQRRYTSIDELDGEDTQRVISKTITLPRLGAGRIYKSRFIHGP
YIRPKFVRPHKGIRLPHPNRIIIPGSWKRPAHGIFALKGKKAIFCEHKGIKRISCSNDKVIKVRSALY
GRTAGNVCGRSRVTNCRALNSFAIVQKKCNGHNSCDLNPSNSVFGDPCVGTVKYLNIEYFCVKR
TPPTINTPTLTPVTLVIPVQSSYQGDFGKSLTLKWNLGLTEKDRIIAVYLYDDIKPCKTKIIFSSTGFNT
PMLQESYAQLIFGNRISATISKSSYQLTIKGLKASDARSFTLEMFVSGPGRSSCKPILSVIKISKINNINS
DKVDGGYTGWSVYGPCSKACGGGVQERNRSCTNPAPANGGKACDGVPSETRACNTNQCPEAS
MSYASTKYPASNCIDGDINTRCHSKKGVNPWLKVDFHKKVLFKSVELIKYFKYVCCDAVIKISAIQ
SDGTKKLCATDDQAEKGVQDLEIELSDNCVGHSIELQLDTHGKKSALRLFEIKFNAE*
>TRINITY_DN10485_c0_g1_i1.p1 type:5prime_partial len:604 gc:universal TRINITY_DN10485_c0_g1_i1:2-1813(+)

NYSRLKSYFVAMKRTTKRIALAAAAVFLLVVIVVVVVVAVVVTRDDDEDEILTAKHEPVEAREYSK
ASVAADRLECSNIGRDMLKRNGSAIDAAVASLFCIGLLNMHSCGIGGGGFINFYKRSEKKAYIYDF
RETAPLSASENMYVNKSQERLSTVGGMSIAVPGNVKGLHYVWQKHGKLPWGDLLDPTIKIATDG
FKISKAVGDKIERESTNKLYLNEGFKEFLQKDDKTWYKTGEMLKATLLADTLKRIKANPHDFYNG
TMADEIVLDINEAGGSFSKKDLEDYKVTEREPVTTNVKGYTIYSMPPPGSGAVIEMTMNILEGFN
MSEANIKDNAELIKTTHKIIEALKFAFARRPLLGDPSFVADVSKVLTAMLNKTFAEELRHKIKDTPY
PNQTFYGGFYSQHQDPYGTSHLSILSEDGDAVGATDTINYGFGSGVRSTRNGIIYNNEMDDFSTPG
LQNLWGYLPAKSNFIKPGKRPLSSMSPTIVTNSDGDVVMVVGASGGSRIITATALTLIRTLYLNEEL
GRAVSEPRVHTHLLPYEVYINDPYTVSEELQDGLKDLGHKLVMSKYWAAIQAIYRKDISSPILAKC
DPRKGGLPAGY*
>TRINITY_DN585_c0_g1_i10.p1 type:3prime_partial len:236 gc:universal TRINITY_DN585_c0_g1_i10:705-1(-)
MQIFVKTLTGKTITLEVEPSDTIENVKSKIQDKEGIPPDQQRLIFAGKQLEDGRTLSDYNIQKESTLH
LVLRLRGGMQIFVKTLTGKTITLEVEPSDTIKIQDKEGIPPDQQRLIFAGKQLEDGRTLSDYNIQKES
TLHLVLRLRGGMQIENVKSKIQDKEGIPPDQQRLIFAGKQLEDGRTLSDYNIQKESTLHLVLRLRG
GMQIFVKTLTGKTITLEVEPSDTIENVKSKIQD
>TRINITY_DN12632_c1_g1_i1.p1 type:complete len:504 gc:universal TRINITY_DN12632_c1_g1_i1:165-1676(+)
MGNCLRKPKKQRQKEEKKLDATSLEPSLVEPNPQPPNSFPAPAPLGNSNGVQGATVFVALYDYD
ARINEDLSFRKGERLQIINTADGDWWYAKSLQTGREGYIPSTYVAPEKSYEAEDWYFGDIKRAEAE
KRLMMRSLPTGTFLVRKAESAPGNFSLSVRDGDSVKHYRIRKLDSGGYFITARAPFSSLHELVQHY
SRDADGLVCALNIPCPNNDKPLTMGVAKDVWEIPRESLRLTKKLGAGQFGEVWAGVWNNTTQV
AVKTLKEGTMSPAGFLEEASVMKNLRHKHLVQLYAICSDCEPIYIVTEFMCHGSLLDYLSKGEGQ
NLQLPTLIDMSAQVAAGMAFLEAQGYIHRDLAARNILVGENYVCKVADFGLARLIEDTEYTAHE
GAKFPIKWTAPEAALYNKFTIKSDVWSFGILMTEIVAKGRIPYPGMTNAETIHQVDRGYRMPQPP
GCPEPLYQILLNCWNKDPEARPTFEYLQLTLEDYFVSTEPGYRDLNQTDS*
>TRINITY_DN1058_c0_g1_i1.p1 type:complete len:2639 gc:universal TRINITY_DN1058_c0_g1_i1:134-8050(+)
MVKYIIWILLLSIFKEAYGGDWAQWQNWSACQDSSGAPIGCGRGNQHRSRTCADNNCGAGAIIE
VRPCLDEICPTFNCQNSSEIAGMCKFDYSNSCVGDDNCHHCICKSEGFKVSGDQCYNYCVEVGC
DEEIEGVSQLKRYCGDQAISCTGTVLNRTCLCNPATSYPSKDGQYCYEATCKTSQEFMTNCGAEN
SCQGTNTSFVCECNGFGYGYEVVANVQNRKKCKLLSCEDGDLNPLEICGQGATACTGNVHNLT
CACNVTAGYIPSEDKKSCIPMPCNTDPAVGVQCLPRNTCEGSKIDWRCICSAYGFRNDPTDPKKCI
EAKCDDPNIDILGLCGTGVTSCTGFLSKRRCVCNTTENYIPTLDGSHCMPAPCNANENATKDCG
GENSCIGSFDSYMCVCNGYGFQKNATINKCEEVPCSSLPTAEVQRICGFGALSCDGTIRNPVCNC
NNDLQYYASEDMKSCIEASCSSKADYDSHCGNENSCVGNITHFECICETEGYKPDPSNKKKCILES
CELNEVDVLNVCGKGAISCKGNVRDLECVCDASKGYVQSPDTNKCEPSSCYGNATVSEQCGEEN
HCLGDLSDWICVCDAYGYKVNATNSMTCDKVMCGDTGADAVTVCGKETTSCSGHIAKPVCTC
NETAQYLPSPDSTRCVKASCKENDAARKACVPGNSCSGSYDEFICICDQFSYHQDPADNRKCIRE
KCEDLAADYDYEKICGVGGISCTGYVNQPVCVCNSTESYHPSNDRTSCVKASCSTSDAVRTQCGL
ENSCFGDIESFMCDCKAHGYKLEPINKTSCVLATCEDGDFSYHFQCGKGAVGCSGNINTLSCLCD
SAKGYVPSADMKRCVPNSCKDNPDVYIKCGMENTCLGSYADWFCVCSGAGYEANPADPKSCQL
MECSKDDAIHVCGYGAIDCKGPVLAPTCSCNYSDHYIASEDGTSCVPITCRKNDTARAQCGEENT
CKGHTDNFKCICDAYGFKADNVDHQKCVERQCTDGDADIIRICGHGGIRCTGPIRSAVCECNSTE
YYYPNADRSRCILASCKANETIMKSCGEFNSCEGNMESHICVCNGPGYKHHPSDDKQCVKETCE
MGEIDPIYLCGKGAVKCNGTYDIYTCECDNAMGYVQAPDGNSCKPFDCHANATMHKQCGDG
NRCIGSLTDWICVCDVAWYKPDPSNLMRCHEKTCKEADLELICGTGATGCSGSATNPTCECDSA
NYFVSSTDRRSCIKASCKTNITAVQQCGDGNSCYGTPDNYVCNCDAYGYRLDDNNNQACVEKK
CDDVGANYVEICGLGGIGCKGTVKNPVCECDQTKHYVASPDSTSCVLSNCRGNKTVMAACGNE
NKCFGSASSFICVCDGPGYKRNPSNLQECLPETCEAGEIKPHMVCGRGATACKGTMYHFECECN
NTLGFIRSPGHESCIPVNDCYANATMHEQCGEGNGCQGSVADWICKCDAPGYKPHKTNFQMC
HQRMCSDADIDLVEVCGVGVESCVGPIANPTCNCDTSKHYVLSADSTSCIPGSCKTNETIMAQCG

EGNSCSGTSENFVCICNGYGYRPHETDKQTCVLKKCSHSDVNAVDVCGQGALSCIGTMRDPICQ
CNNDMFFIPSDDSRSCVLASCSANSDVAVACGKVNGCFGSKSSWICDCSSPGTMLNPNNIRECLH
ETCESGKLDPIIVCGKGALGCNGTMSNVTCECDNSMGYVQSPNGMSCVASDCKTNSTMHELCG
DGNSCVGSLTDWMCVCDAPGYHPHPSDPMRCLQKMCSHANINPVDVCGIGATKCVGSITEPRC
TCDTAAYYVPSPDGTYCIPGSCQTNKTALEQCGNGNTCTGTADNFVCICNAPGYKLDDSNNKRC
IEISCNDADVDLVEICGQGSIGCIGTARQPVCQCDHSKHFVPSLESTSCQLASCRANDTIMDRCRM
GNTCFGSMTSHICVCEAKGFKVNPSDPTECVQETCEDKDIDALSACGKGATDCMGTLMNLTCQ
CDGASYVESPDKTSCLPVGCQQNVDVKKACGEGNTCEGSYDDWVCLCTNPEYIPHATDEKVCS
KKKCTDNPIGSCGIGATSCSGNVNIHKCTCNLTAGYMPSKDEQSCIPIDCKMNPKMNCGFGNTC
RGSFFSYVCECNTLGTMRNISNPKQCIEVDCSTDAVSICGVGATGCAVTPTNDVNCTCGEFYHPA
HDYHSCVLDDCKFNKLVPDACGVENECTGSISSWMCVCKGPKYIQDIKSTQLCAPGPCRIEQCGS
GKASGSFQYYAESCSGTYFEPTCNCKEGFEKDSYGGCIVKSCSRNNQSSVDCNDGAISCEGTYFKY
KCECRAPTHILNPANKRSCIPARCNPQLCGRGLVNGDYMDFASECSGSYTDFTCKCKYGLYGSGT
SKCSLEKRTCALSNDCHPNANCIDYDGHYFKCECKDGYSGDGYQCHRNGGLTPWSVVQPCDKT
CGTGYAQVMRTCDDPKPDTLGKKCNVPSPDVREVPCHQFACTACSVKKTCECENIRPYFVHEYV
EPDESLKYFTNLTAEDVEKALSAIEKWKLSVDEKEFCIKHTLPEIDGLISDINENLRSVDQTKLYTIT
ARDMMRNKLDCNVDLEKKDKYFRVYEAIVKRHFILKQVVSDLQAKEKLMVTYRSSCTKDSKVG
WLSSIFS*
>TRINITY_DN65215_c0_g1_i1.p1 type:5prime_partial len:383 gc:universal TRINITY_DN65215_c0_g1_i1:1336-188(-)
HYRKKTTMAAYDDDENFGTSDSYGNVGSFPKEFGYAEEQEELNNYSEEQKPKILLMGRRRSGKSS
ILKVVFHKMSPNETLFLESTNKIVKDDISNSSFVQFRIWDFPGQIDFFGHDFDSEQIFSGCGSLVFVI
DAQDDYLDALTKLHQTVTRAYKVNPNIKFEVFIHKVDGLTDDHKIETQRDIHQRATGELEDAGL
ENVQLSFYLTSIYDHSVFEAFSKVVQKLIIQLPTLENLLDILIQHSGIEKAFLFDVVSKIYIATDSAPVD
MQSYELCCDMIDVVIDISCIYGLKENGDANAFDTHSASIIRLNNSTVLYLREVNRYLALVCILREDN
FAKHGLIDYNFHCFRQAIQEVFEIRQQARDALLPKNGTILSKGESVVV*
>TRINITY_DN165536_c0_g1_i1.p1 type:complete len:249 gc:universal TRINITY_DN165536_c0_g1_i1:851-105(-)
MSEQEISNLVEQAKLAEQAERYDDMVKYMKELTEKSTEGLSDEQRNLLSVAFKNVVGARRSAWR
IIHSLEEKKGNEGGNTELISSYRKEIEKELKERCNEVLSLIDKYLLPSAQKEGQKGTEGKVFYEKMK
GDYYRYLVEVTTGEDRANLSAQSKEAYDKATETATGLATTHPIKLGLALNFSVFHYEIMNDGKKA
CELAKKAFDDAIAELDSLKEDSYKDSTLIMQLLRDNLTLWTSENDNEENDAQ*
>TRINITY_DN165469_c0_g1_i1.p1 type:complete len:2668 gc:universal TRINITY_DN165469_c0_g1_i1:41-8044(+)
MVEEDLNRSKELDIPKTLAYCFHNVTTVSLNRKQDIFRLLTQCIESQGCQDGHLKAVCKIISLTVM
RYRDSKSRQLVLKFVQKFLDIHELQAVKCLLTEIHILAKQLIKCGASLFSASCGLALLSWCSSISKKK
VQEASILKILYEVEAMCYSVICTCDSFRVKRLLKRAYLYLQDGKDAEQLENCLGVIISNSNCQLESIC
LVGFIFQYLTTKKEIQRVDEVKKRSLIDIIIKNYIQTKVKADYHYLKHAVPFLKYITHDDFKEILPAIE
KGLLRNPETVLQGVGYLLEGLSIDSSNYIEELSKCIAVHLISKNASLQADAVFAVSVIAKQCSDFSAI
NHLLKQLIKVLNESEGKLVFPEQKNAVLQAIKSVDCTSVTGSDHKENLATTLHDGLFPFILLEGHE
GVLTQALNVMKTWCKYFKNNLPKTFVDNIKKGLSLKVSTTVIQAHYLLVVSEALKGNTVSQASG
LLETLLQVIEKVKGQSHLINNVFLCISSSMVLLKMFLSDDVQVNAAKEDSFWNVILDPVKKNLVSE
KFLLSHQKEYFYHLSQCMNTMFLNYSSRFSQKLLEPWYRAFNVLMVYGDWKTKKSVKILLSKMF
DSGNYDTDEFQSRLFADLDSLLDQEMSKSSKIEDTEDILVVNHVVDAYLFLCIWNKKDINEDEVLA
VLRKCLIYVHHDTIYQHDSSLFSKIGSVLKKDVAKEFVEKYKDDLLKYFINLESLSKADNHSLKTLL
SLYPEALLDTLVDEIIKYFTKPDLINVTSQEYEIMKTPDGVAYDQSVFESAKAANQKSGNLKRESKA
YSYKDQKAEMELRKELEKKKGKSDKIELNKKQKEQLEVILTQEKAIRDKLKSLEQGVMKHASILSL
LLDMDSDLLCYHYLDLMNLLLCLCQSHIAAPHVIPIYTKLSEKLFEGDLKHLGQLVANVILRLKKP
VCELPMGWSVENLPEQAKRALNTLHTFVNERNVYLMMTYTRKKTFSVLVFSYCLPLLKSILKDQ
GALVKGSEEFRMKALQLIHIHAKLRSPMDMGSEKNDPELLPRKEMLQILSDVIVSCEGHNKEAKV
QQQATLAFDEVCKCASGRLGCAVATEEEINILLSNILSQCSILRRVSLKGLLEFVDILPTENVVIMTQ
LTRSVLIGRFDIVDENATLAKRLWDEADICIEPSMIQHLVENVTHPLDDVRLAASLALEFSLNERKE

VDIIPQLLDIYFEKLKIPPPIIDNLGRVVSQQFVDSWEARQGVGLALEKIAETVGEDKLGDLFSFFVP
KALGDRNESVRKQMLNASLQYVNLYGQKNMDMLLGVFQDFLDSAPDSKELDTIRQSVIILTGSIA
KHFDKTDPQIKSIFSKLMAALSTPSQSVQQAVANCLPPLCGAMKSEAPELFKNLLEQLLESESYGE
RRGAAFGLGGLVKGLGILSLKQQNVIPTLNDAIQNKKIWKHREGALFAFETLCTMLGRLFEPYVV
HLLPHLLLCFGDGNQYVREAADDTAKAVMKNLSNHGVKLVLPSLLKALEEDAWRTKTGSAELL
GAMSFCAPKQLSSCLPSIVPRLTEILADSHLKVQKAGQQALRQIGGVVRNPEIQEICPIILDALSDP
NKHTVACLQALLSTSFVHFIDAPSLALIMPTLEKALGQRPTETKKMAAQILGNMYALTDPKDLQP
YLQAVVPGLKKSLLDPSPEVRAVSARALGAIVKGMGEVDDLMPWLLETLTSEASSVDRSGAAQG
LSEVLFALGKDKLDKLMPDVIATSMKTELSPNIRDGYLMLYVYLPATFEDQFLDYIGLIIPAVLKGL
ADESEFVRDTSLKAGQRMINMYADSAIELLLPQLEAGLFDDHWRIRLSSVQLLGDLLFKLSGVTG
KQTTVSEEDDNFGTAYSSQVILETLGFDRRNRLFAGLYMGRSDVALGVRQAALHVWKVIVQNTA
KTLREILPVLFELLLGCLASQSYDKRQVAARALGDLVRKLGERILPEIIPILEAGLDHEDGDKRQGV
CIGLSEIMDSTSREMISMFEDSLINTIRKALVDELPNVREAAASSFENLHNTIGHRALEGVLPTVFQ
KLDDPQLSNYALDGLKQVMTVKSKSVLPFLVPKLTTPPVNTKALAILSSVAGEALIKHIEKILPAMI
QAIQVSQGTGLDGDSEFESTRTLILSVYDEVGVRTIVDELTTSSKSQMPGIRKVAASLLMVFCKETR
GDHVPYIQSLFRTIIQLMNDVDQDVVASAWGALDALVKRLDPSEQLQHLSSLRQAVRFIKDDIRN
EQLPGFCILKKGITPVLSIFREGILNGPQEIKEQAANGLGEIIKLTSPEALRPSVVHITGPLIRILGDRF
NSSVKVAILDTLGLLLQKVGALLKPFLPQLQTTFIKSLNDSALGVRQKAAWTLGLLTVLHTRVDSL
FTELKNGIKNNEDSAICETILGALRSITLKAGSKMSEGIRNTLYETLVELISSNEDGIRLKAAAALGA
MCQILPQDQIEQLLNDNLLDINQMLDWSICHGRAVALSYALYHAPSQVFDAVDEDDIVEAVVKY
STYDRVPICTYGVHSLGHLLMFSKDNADVAPQIVVSSIKKTLLNSSVDIRLASLSTIKHVSRNTPEV
MTFERIRYLIPEFISLLRDRNTSVRSSAEITLAFTLQMDKNDILFDECVNTISEASHFLEEFKKNTFSK
LNDSLATDKDDYFFAT*
>TRINITY_DN2003_c0_g1_i1.p1 type:complete len:370 gc:universal TRINITY_DN2003_c0_g1_i1:89-1198(+)
MGVINTLVVLTLALALEAADHMTPHEYEFWIKTATSCTQEIYEGDTSHSTYSDLKTKLEDGIKAAY
TNNTKYFSARVKSMQCTTKPHYVAAKIMLIMTGHFTFDEALADLKNEIMDNVLDGSALNLTDN
MLRASMDEGAKPKEKVTLKFSFTNELFCENMATLKVNLAKRVILSDGMKRIESNRIVIYEHECNE
EANKYKKAVMKFYIYDDNDFLTADAALTNLAYQVVDQLQYDGTAWKLGVIFHKHINDVDYESE
SEDEDFTEIEKTYIGFGAVVGLLLLIVIIVLFVSCICPNACGKQEDPYLIEDRPSRFTNVNTHRDAYSN
QVYSKEEYANGYEREVEMDTVLPHEEQTVFHGNQPNEGMKESV*
>TRINITY_DN8903_c0_g1_i1.p1 type:5prime_partial len:447 gc:universal TRINITY_DN8903_c0_g1_i1:1546-206(-)
DMATKACGLENMCVGSYKSWRCICQAPRYMQDPDNTQSCIHAPCRIEDCGSGYAPGSFQYYAES
CNGTFFNPICNCQEEYEKDMLGGCVPKSCRENPIAVAECGENGYCKGTYFKYECGCQGPKYVQN
PRNTRQCIEAPCNVQNCGHGFENELYVSFADSCIGKHADYTCQCKEGYTGTGTSRCIPDGKECGK
SYCHANATCAPLGKGEFDCTCKPGYSGDGLYCFLDGGWSDFVNTRECNASCGTHGYAEVTRYC
NNPVAERFGKQCGAKSPEQLEVPCNRFDCLGCTVKKDCRCTSVVKPLIVEGYNEPEETLKFFTDL
HPDDVKKAKDTLLKWKAFSNENLCRKYNKTTLHAKFFGVRDAAKEVNKMKSSYGSARDSMRI
KLDCNINIAKKTQYFGVYEMIVKRLFIMKQVVKDLHIIEDYVLKHMENCDDSPVGMIRNTFGK*
>TRINITY_DN11377_c0_g1_i1.p1 type:complete len:804 gc:universal TRINITY_DN11377_c0_g1_i1:3179-768(-)
MKQNWYNYFLLVTFAVCIIQYTNGQEERDSNPCVQATTCEACMQANPDCYWCRDVNYTEGEN
FSRDRCNLLIEFNDGCANLTSISSAFEPGQNSNFSDTVQVQPQTVTLDLRPGQPAVFNISVKPAPDF
PVDIYFLFDASFSMKDDLEKLEQLGAAIAQSINNITKNYRLGFGTFVDKKAVPFVEGPLFPTFPCSR
QVKVDNCAPVFPFRHTFSFSKNTTLFQQAVADQPLSGDLDRPEGSFDGLMQVVVCDQKIGWQPR
GKARRIVLLATDALPKIAGDGRLIGDLLQNDETCHLSPLNSDRSVLEYSESLTMDYPSISQLREAM
KKYEITPIIAVTEDVQDVYKDIANSWESLLAAFGVLNNDSSNIVDLVRENYKKISSTVRLLETKNEY
LDFEYKQLSPECQETLTSPNECEKVAIGQQVDFQVSVTLKKCPPEEENAANRGDIDIPGFGQVKIN
LNYLCTCPCSNNAIPNSVLCNTTGSLDCGICKCNQGFFGSKCECDETTVSRNDTLKCQSDQNDPS
FICSNQGICLCGVCDCFRVAAPRIISGKYCECKNFGCPKSRGQECGGDERGTCVCGQCKCKKEYA
GVDCGNFNCTEIEGPRCTKDGVQCSNRGFCDCTLGGCNCNQGYQGKYCEKCVTKNCKCNDLR

NCVLCKAYDRDDLNCTGCEYLDLQRVNDTQKTCTVQFNETCSAYYHYEIDPTQNQTQTQNVTV YLEDDFFCVVPIVAGEPNSLWIILGIIAGIVLIGLLLLLIWKLLLILQGRREYNKFETSRTGSQWEQDQ NPIYAPPDQKFDNPVFGGEF*
>TRINITY_DN2472_c0_g1_i1.p1 type:complete len:344 gc:universal TRINITY_DN2472_c0_g1_i1:311-1342(+)
MSSGEIESLRQEAENLRNQIKDAQKVAADTSFSSVAQNVDGINRIQLRTRRTLRGHLAKIYAMHW ASDSRSLVSASQDGKLIVWDSYTTNKVHAIPLRSSWVMTCAYAQSGNYVACGGLDNICSIYSLKTR EGNVRVSRELPGHTGYLSCCRFLDDSQILTSSGDTTCCLWDIETGQQLTQFVGHTGDVMSLSLCN DSGRTFVSGACDASAKVWDIRDGMCRQTFIGHDSDINAVSYFPSGLSFGTGSDDATCRLFDIRSDQ ELMCYSHDNIICGITSVAFSKSGRLLLAGYDDFNCNIWDTIKGDRSGVLAGHDNRVSCLGVTDDG MAVGTGSWDSFLRIWN*
>TRINITY_DN123121_c0_g1_i1.p1 type:internal len:127 gc:universal TRINITY_DN123121_c0_g1_i1:3-380(+)
LNQKLCAQQVIHLEITDANDNFPVFSQSRLSLNVSEYTEVGQVIATVRANDLDERSQGNLLFSILK DEDFFGILSNGDNGVIFLKQSLDAERRLTHQIEVTVSDREGKSSLQNMEIIVFVEDENDN
>TRINITY_DN26392_c0_g1_i1.p1 type:internal len:469 gc:universal TRINITY_DN26392_c0_g1_i1:2-1405(+)
PDILIGASNSDKAYLFRSRPIITISDRLELNVTSVPLKKNCDNGDGRNYFCAVLKVYLSFQGISVSNK FDLELSLTLDDDQVEKRAFIKTVKKNDTSLNSQFKRVLNNITVNTPVTEDFMVYFLVKADLDIQKP IPIVSKYKKIEKSGGCGANPCPIFNIYGSIGSSIQVPYLRECGDDKKCTIDLVTTGSIELPGKEKEFEILY GVTDEVYLNITVENLGEAAYLAYMKINASSDLDVISVNVNGGPNILWKSSNIDAETMQVEFSIAN PIDKGGKYHILAKFSVGKQPRPSTPIYEFGMEAFALGEDKNMDNNRKTLTIGTKVETCIEVIGYSSV PQVIYHSTAKQPPDVNATVNDIGDEILYQFLVQNKGNKPVDTLTVDIDIGAEKEKTTLMYISDLTV DSVRCEEEKLNPKKFLIPDDISRTLNEESTKSSLKRKRRDVANDDGTEDKKGYKYSPLNCRTASC
>TRINITY_DN1030_c0_g1_i2.p1 type:complete len:1426 gc:universal TRINITY_DN1030_c0_g1_i2:4483-206(-)
MAKVRVLALLMLICVTASNAQDGEEVFPFDVQKGEKGQPGDDGEPGNNGAPGRQGSEGPQGP DGDVGFDGEEGDEGEVGPPGPPGRPGTNRTSVVVVYGPPGPSGPQGPDGQIGPKGYQGETGEPG SPGARGPRGDAGAVGEAGVPGKNGANGPKGEQGSIGPSGATGIPGLPGPSGQRGPKGSKGEVGE LGPKGAKGDAGRNGDEGESGLPGRKGEPGPRGQNGTVGFTGPAGQEGPMGPIGPAGPDGPAGP QGPRGADGPPGTAGSPGESGAPGLPGPVGQVGARGEPGFPGARGIPGDRGQAGPAGQDGVDG AVGARGSPGSPGIAGAAGPPGTRGEPGKPGPRGLQGQQGEQGSTGVQGSVGGRGEKGPRGLTG QAGVPGPPGDSGTDGRPGPPGPPGNNGQRGLAGLPGSPGLPGFPGIKGHVGERGFNGPRGEKGS RGESGRPGQRGIPGVRGPPGDIGLPGIDGKDGNRGQPGKDGIPGPPGQPGPRGIPGSQGAAGSKG DKGAKGRTGDPGFDGAPGDKGIPGQAGDQGVPGPRGRQGDRGEPGEQGARGGQGLPGPPGA QGPQGRQGKRGSPGLPGARGNDGLPGRQGERGFPGESGNPGAPGRPGASGRKGPSGRPGQKGS RGDPGMPGDTGPPGQVGRPGLPGAQGNPGAAGDQGEIGEAGERGRDGKPGPRGAMGQRGAP GPRGNPGQRGDPGLPGDDGKPGSTGAPGAPGAPGEPGQSGPRGPTGRPGQAGAPGESGLKGFK GNRGARGRPGRAGKKGADGLKGQRGSKGEQGLTGLQGAPGLPGNQGPAGPIGLPGEQGPQGP PGPDGKPGSKGDKGRSGPDGRPGSPGRPGPDGSNGLNGRPGKDGSRGPQGRPGPRGKQGPTGY VGAPGAPGVPGLIGPRGPMGDNGKDGKNGADGDPGEAGPQGAQGPQGEPGADGADGLPGRN GNPGLPGPRGEKGDRGTKGGKGAKGNKGTAGPVGQPGARGDRGPAGTPGADGEKGADGIKGE TGFNGAEGAVGDRGDTGDRGSKGDTGFMGFKGAKGPRGSKGTRGLPGYMGPQGDIGDKGPIGP PGPTGPAGISGPVGLPGRKGPSGDPGPKGYAGKNGRQGPPGPPGPPGLAGPSMLPPWMGGGLA DGESKGPVVEREEPDAPEDENEELPPNEVNPFYRVYQFYSSKKTTPTDNDVNVMENKFKTKVEEL KTEFSRIDKPDGTKDYPARTCRDIHAYHPEAKSGLFWIDPNQGCKDDAIQVTCTFSVNEDDEPTV TTCVDPAEKQSVPQRTWASKMHSRAQKWFDEDHELAKLKYNANLSQLRYLGLLSKTATQDVKV HCKKYVVWHDKMNRNFDRAMRFMGTQEQEFLYKDVDSKSSKSRFVPEVITDGCSANSNSVAHT HLRFTSRKFIRLPIVDFAPTYPESPKSEFGIELGPVCFKV*
>TRINITY_DN4494_c1_g1_i10.p1 type:5prime_partial len:462 gc:universal TRINITY_DN4494_c1_g1_i10:2120-735(-)

RIDKALRLTQKELFNTRYGGRVGVPKLLILLTDGSQTQDAGAEDPAKIAAELRADGINLLIIGIGKG
INQTELSNIAGGADNVFSAASFDELIGTDFIGAIKNGSCEGAKNPPNPLLKSVIDVAFLLDSSDNA
NDIFINEKEFLKRLASSLQIKSNGTRAGVVSFANQNNIDIKLNDYFDIAKFHPAVDQIQLLGSARDI
AKVLRFSQKSLFTETNGARIGMPKVLVLFTGEQQVADFKNDTLKTLAYQLRLQGMTILVIGIGNIK
EGDLVSVAGEKNNVFIAAKTDDLMGFKFLNATVARLDKIAQKKPEPKCEAKVDVGFILDSSGSLR
NEYGKEKDFLKTLASTFGVSSNGSRAGVITFSYYSEHSIKLNDHSDISSFNQAVDNIPLMGATTRID
KALRLTQKELFNTRYGGRVGVPKLLILLTDGSQTQDAGAEDPDTRCWCRRPCKNSYRTSSRWY*
>TRINITY_DN3347_c0_g1_i1.p1 type:5prime_partial len:924 gc:universal TRINITY_DN3347_c0_g1_i1:2-2773(+)
YGDGSDDDMDSIDEDEDEDEDDDDDEYSDDDDMSWKVRRASAKCLSAILGSRMDLLSEFYINVA
PKLIARFKEREENVKSDIFQAYISLLRQTKPVAMNDALEDKPDSPLSMLKQQVPLVVKALHKQLM
EKNIKTRQGCFNLFTELVGVLPSGLTEHIDKIMPGIHFCITDRNSTSNMKIDTLSFLGCILQHIKAEV
FYPHIHQFLPAVVECIHDPFYKITSEALTVAQNTIKVMRPTGTENYFDFKPYINDLYQSILHRLRATD
IDQEVKEKSITCMGQLLRNMGDNLSKQLPECLPIFLDRLRNEITRLTTVKSLTTIASSPLHIDLSILLL
DGIPLMATFLRKNHRGLKLHTLTCLDLLMQNYGNVMSTKHFEAVLDELSNLISETDLHVSQLSLN
LLTTMISKQQLLLKKVSSTVLSHIVILTRSPLLQGHALNSMVSFLTTLVRYSSSGLGYTDLLQMLTEV
IYGSTTENIHKSAYHSIARCASALTIACPSEAEAVVTKFIKDIKNSGTNDSIRLFSLLTIGEIGAHLDLS
SKAELKNVIVNSFSADSEDIKTAASFSLGHITIGNMDAYLPFILKEIESNAKRQYLLLHSLKEIIVCQS
TDPSSVKSLEQYVPSIWSLLLTHCENKEEGTRNVVAECLGKLTMVEPQKLLPELKSYLSSDLYSAQ
MRSTVLTAFKFTITDQPQSIDILLKDHLQAFLQPITDNDLNVRRVALTTFNSAAHNKPGLIRDLLTK
ILPHLYNETKVKKELIREVEMGPFKHTVDDGLDIRKAAFECMYTLLDSCLDRLDIFEFLNHVENGL
KDHYDIKMLTYLMLVRLSKLSPNAVFQHLERIIDPLRTTIQAKVKANSVKQEYEKQDELKRSALRA
VCALQKIPDAESHPILSDFVSQIRSNSDTKVMFESVQRDPSSTSLDKMDTAS*
>TRINITY_DN59572_c0_g1_i1.p1 type:internal len:725 gc:universal TRINITY_DN59572_c0_g1_i1:3-2174(+)
FSSVLEYNIEDFELVNQYMFVTKNSSSHPGTKDLLVSYYRGDFQNTSFPMKNNGTSYIIADAHENE
TMVAVNHNGTANLYISNPEGTKYSLSLGNILYYNKDEGQKSYFEHSFVDLHRIKGISGIYIATSLTTG
AVGTRHLQSYITFNKGGYWSLLKAPEDTKNCSIWRGCSLHISQEFGYHNPLLNFSPITSKESAPGFL
IGTGNVGDSLKTKPNVYLSRDAGLSWRKVLQGHYYYNMLDHGGIIVAVEKFGLTETFTYSYDEGK
TWHSEVFSKERVRVYGLLTEPGEKSLIVTIYASYNKAHAWEVFEIDMTKVLGRKCSIDRDYNLWAV
ADQRNDTERCVLGKKQIFERRKAKSRCYNGYNYDRPINVSACLCTREDYQCDYGFKEKMILWFR
WDCVHDSDFEDYSKKPPSWCKPGLTYNISSGYVKAPGDACYGGEESKYVSTRKMCPIPKVDDYL
LYATRKSFFGYDFESKKSIEFKLDGYSNIVALEVAYDDGVLFFADKDRDSIVKIDTNNGNISTLLGFD
ESSTHIESMSYDWLSGNLYYCDSGNSTIGIINLAGKHKKEIMKFADADKPRAMMVHPTKGYLFWT
DWGAKPRIGRATMNGKNIKTIIDTEIYWPNGLTIDYDADLLYFADANKDRIEACGMDGENRRVIL
STGVYHPYAITIYNDRLYWDDWNQHSIFSIDLQGSGRETVVSYLAYAMDIKAFSKKRQKKGTNGG
T
>TRINITY_DN160_c0_g1_i1.p1 type:complete len:1145 gc:universal TRINITY_DN160_c0_g1_i1:4810-1376(-)
MRLLAIIPLLVMLDIAKSDKLWECNFEENDCWAASSTRNHERNQLTLHWNRWKMRTPTGATAS
PVTGPMEAFEGKHYLYYEATESMDKLKMADGASKGVVTFKDINLMGRTQFILSFYYQVKGNDG
DFYDFDLMVSNTENTHYDLSMKSIDGKDWKHFKHIMKVEGAGVTSIDLKFTARSYGAFADYAID
NIVIEDYPDAGPCKDNRCLANEYCTITSSTQTVCVAKCTQQACGENAECILDSLAINKCKCRQGY
LPRVSNYGDKLNCYRPDASCDIEYNSLGQCRISTSIPIMYGHPSLWHGRYSPVSGNGYYVFKYDNT
ITLDAVSSQKLSFFYLVDNPNMINSLTVEYGSRKFEVPINREKTNIWTLYEVLILASTNQGVQIKASF
YAEGGVIGIDNIQFTQQNEVVVEKIYKESMMAATFEDGSVPTDLKVAMLFVTRYGKIDFGCLPGIE
LETRNGKLYCKDGVVQRQSACSQDSDCIGSNARCVLRNCVCNTNSYQISIGTYGEKATSCAVYPC
EINSPRCHSTAICSNIKTPGQNFYNDYTCKCGRSLNNVETIINPFIGGDKSTQCLAATPSDSYVCND
NNFKPSRTTEYCLKGNQATVEYSLRATNFGNYALFKHCDDPDDEYRSSFNDPGSFWRPIDLVAAA
NENLAVSVTYKTSLSNKFIKCNFEQARFGSKLTLAVNVNSNSRVLTFTNTQTSVNKQNPVYQGQV
FQSDNPTPGAEMSVTFTVTGNQAVDQNVDAILLDNVVCKGFDDVASVNTPCGNNACASNQYC
NPLLSNSEKECLCPTGQKDQNCPDVVPSGSVVSADGPKCIGHHEKYMNGACVCDDGFEKTGDT

CTVKINCGCNDGLCLSGDKCSCPLNLLKVNQACVRTNKDCAKDCVSTLGNGGYCDVTTGMCD
CLTGFVKSGRDDGSYRCDKHAVVQKINDPKGICSATCGTGFVNEIEVCVGATGTGFECMTNIVQG
TLGVCSLPACPATSSQCSIQKECACNNVRVPIGSDGKLRLDFDHEAKLNMIFRFLSKHNQKDLEFK
FREARCDNTKLDSIARTIEDDLKQLEFDTELQAAITTMKSILDCSPSGNDHNAAYWDVFDMLIELS
SYRPHAVSDLQLKKELVMHEQSRCGMRSNLHKVVDGYAFWNKK*
>TRINITY_DN11635_c0_g1_i1.p1 type:complete len:705 gc:universal
TRINITY_DN11635_c0_g1_i1:2148-34(-)
MREAYLKRFAVVLSLLLLLQESHALIESKILDDGVPEIIPETKTSSLQSLDKDQQREKRFIFTTILIVTT
VISIGAEIYKQARGCKDFTPDLEPTANKGKRLEAEIKKLQADVESAWKPVQTLQSYADKLDKNLV
KIREKINTYKILIKEILHILDRKALDEMNRVLRTLENDIDRGKEESVGSMEDFQAMVGSPLSFALSM
TAKTVTLGVAAHSLIAKSSAKVATETGATWAAKNPGRVSSMISKFAQAKGSVARWSASKIASFKSV
LSAKFAGNIGLKLAKFKSVFAKLGKTTIGKALGKAMSVTGKALVKTGAAIAAVILFAYDVWQFVK
FAQECHKKLREAREAVRTMEQSRIELTTIRLRITILAIQVTTAYNQMKASISSNDFKDFLREVIELGE
GAQVQTTDIKWAVNTLKVYIQYISYNNLYLTNWLNGYLLQAFDKINYKLGCLKSKLNIRMSVVN
NCEDGVDSFINLYNQAKNLERQTVTNDCIAGQYMSESEIRGMVQVAMEERGAQQICIRNNDDK
KKMACNNLQNQQSNSQNANSLGVSVADIVYFITQCETKDLKRTQQLSICSKKSEGNTIAQVKGSF
HSYYHSQVQKVYDDCDLADSQKISVCSLKENGYDINHVQNKYNLYPSLKVQAVYDGCQKRNYL
TPNEENQICVFQAAGMSPTNPLYAGFWKLFYTKVEKSVVDNRYNQPCP*
>TRINITY_DN48767_c0_g1_i1.p1 type:complete len:533 gc:universal
TRINITY_DN48767_c0_g1_i1:2135-537(-)
MEQQELIGSASRENENPQNVEENRCNCSKRFWIGAIALLLLLIGITGVTVTCFVLEQKVLQYSHHY
GVVFDAGSGHTDITVYQWPSDQKDHGTAHTEQITFEKCSDTGISSYQTEPEQAGVMIRKCVEQVA
FKHLPHKDVKKTAVYVGATAGMRLLCERDKDSCDKILEAIRRVLNGFKFRQLKNRVRVLTGSEEG
TFGWITANLVNNALNLKVPAENNPKLIGALDMGGGSTQISFIPEHYNFPNKEDMSNLKLYGVNY
TVYTHSYLCYGFREAQRKLMAHLVEASNYSSKAIHPCWQKGYNETIPASWIWLSPCSKRPQASIFP
LKNSTMYKFVGNGTSDACENRIKGLFNKTNCEAPNCTFNGVYQPPLRGHFMAHSGFGHIGTFL
NITKGDMVEDFKTKGEKLCQEDWNKVVREHEDVKKKYLRNYCFEALYSYMFLTYGLNFPPNSN
DVHVTKTINGTEVGWSLGFMVNATNTIPIEAPSYRLTQQELITLLVVCFLIALVGLSMLIVCCVKRK
IERNLKYKYTLVN*
>TRINITY_DN3016_c0_g1_i5.p1 type:complete len:406 gc:universal
TRINITY_DN3016_c0_g1_i5:2713-1496(-)
MAPPNLKKMIQVKIKPRQDSYSDQFSRILMVKMLMLTAAFTGITWMKDKVKCIVPKNHDADGG
FVSKSCWINGFYVFKGLKEKDTSYYYGIPKDVLSDGYDKYGALCSTKNQQGRLNQGCTPLEKTFF
LQYQWFPLVIAAIAFLYYLPYLMFRIVNTDLQEIKDIIKGRNANDVDAKAIVKRFFVRKNNSTSRL
KARVLLNVVVKVLYVVVNVLGMIFVDSSTNSEFMQYGANWSSWTALKNEDMHDYTNREHEKA
GHRLLPSFGLCQVYTTAQDIKTKVANDHTYICELSQHVIYQYILIVMWYMFVFGIIISIIGLIQHMVL
KTMAILSMNAEGDSVRKIYSDLTVREKEYLDFIRRKNLPVFTKVLDEISQLKGVSRSYATSESDRSSF
GATAPEKENMMME*
>TRINITY_DN79126_c0_g1_i1.p1 type:complete len:115 gc:universal
TRINITY_DN79126_c0_g1_i1:397-53(-)
MVARLVLRRRLSYNTKSNRRRISKTPGGKLVYLYTKKAGATPKCGDCKCKLRGLKAVRPKKLAKL
SKTKKTVTRCYGGSRCGGCVRERIVRAFLIEEQKLVVRVLRAQQVKKSS
>TRINITY_DN11522_c0_g1_i3.p1 type:5prime_partial len:855 gc:universal
TRINITY_DN11522_c0_g1_i3:2878-314(-)
FSAQLFKGSSKSILGGIPNIQLNRGRIGLGNIRDPSISLTRGSKIDYNGYMGFSATSGYFGNDKHIHF
AGGSPRSNALKGRVIMYKDSAGYEISQYLDDPSDQIGTYFGSAMASADINGDGLADLVVGAPYYT
GTVSNEGIAYVYLNKNGIVELVQTLRQPEGKDVKENGMFGISIGSVGDLNNDGFADIAIGAPWGG
EDGKGIVFVYYGQRDPAQLKLMQTIKPSDLSGIDVDGFGYSFAEFYPYSGGPDQYGFIENDPDYNI
YPDLAVGAFKSNHAIVFKSRPVYTIVGRLSLGGVKRLDLVNKENLCPNGMKCLDKVQACLMFKE
EESEGEVDYTLEVDSGRDSPKRAYLLDSDGKQVTKINKQTTVSKDEECDDYTIYVKNTTDVLSDLV
IKLSWKFVVPTTCDGVCPVANELCQFVDTKSIIFVKGCSDDGNDTCSSDLKIEKKEFPPKLVGNNR
LVGTEEIISAGATDGIILTFNIKNLGENAFDNSLKLEYPGKGDMKLVNIDDAKVISDYYGTPIWDSD

VTREKDVTTLSVMLSSPIVKGKPEQVQVKMSLNPIPKGTKFLKFKVYLETDSEETDTSNDFQEITIP
VELVSNLLVSGQSIPEILTWRKDVKDVQDKGAEITYNFLFQNQGPSNVDTSEAIILIPEKFKGSYFLN
IYNYDVKGSKSGSDPGSCRIEGELNPLKLDYYGRRIGNNTRKRRDTRGVPALECGADTTCREIICN
FNIENRNSATVTVKATLVDANFDSVIGAGREIHVTAEFSSDAVTPPSGLEFPVKSMVTTTVNSPYLT
KTEGGESIPWWIILLCILAAIIIIGVIIFIMYKKGFFKRKKMADEDEALRNGDPEMDDD*
>TRINITY_DN1194_c0_g1_i1.p1 type:complete len:555 gc:universal
TRINITY_DN1194_c0_g1_i1:101-1765(+)
MAARLGGGMMQPQIILLKEGTDSSQGIPQLISNINACEVVAEAIRTTLGPRGMDKLIVDKNGKSTI
SNDGATIVNLLDIVHPAARTLVDIAKSQDAEVGDGTTSVILLAVEFLHQIKSLVEENVHPQILIKGY
RKATKMALEKIKEINVQIKKDDQEEMRKLLQRCAETALSSKLVAGHKEFFGKMVCEAVMHLDEL
LPLNMIGIKKVKGGALEESQLITGVAFKKTFSYAGFEMQPKKYKDPKIALLNVELELKAEKENAEV
RVDNVEEYQKIVNAEWNILYEKLEKIVASGAKVVLSKLPIGDVATQYFADRDVFCAGRVVEEDLL
RTMKACGGSIQTSIHNLVDNVLGTCEDFEEVQIGCERYNLFQGCPRSKTVTLILRGGAEQFIEETER
SLHDAIMIVRRALKNDAVVAGGGAIEMELSKHLREHSRSIAGKEQLIIAAFAKALEIIPRQLCDNA
GFDSTTVLNKLRQKHAQGGKWFGVDILKEDIADNFEAFVWEPAIVRINALTAASEAACLVLSIDE
TVQNPKSDMSGDDNPITGKRGRMPQMGR*
>TRINITY_DN1783_c0_g1_i1.p2 type:5prime_partial len:473 gc:universal
TRINITY_DN1783_c0_g1_i1:1460-42(-)
NDFGPSAWQLVTAGTYQFLGSLARVAFFYEMGKPKALVIGGQFSGTFTARDLKSKFHVTVVDCKE
YFEYTPGVLRAYVKPCHLDALTFTLQPVLERSMGVKFIWGEVKQLDGEKKCAAIKPMFADTTDIIS
FDYCVICSGCNFGPFKPMGESLWFPTVHEEERKISDWKHIDERFIEGRRRHVLEEFHKLKDLNTKK
AQILIVGAGFIGVEWATEIEYFFPDIKLTIIDALPRCLGPLPDSAAEYCSEYMHAAGIKEFYDCKYDA
KSPDFWKKVELPDGADVEYVCIGVKASNYFMPKETLSDKGPGGGGWIHFNKYMQVTKKDGTV
WGDGSVFAVGDCNYGCIGAPPNWEMPPVPKISYPGEEQALHAVVNIKKMYKGKTALQPTWWP
WGAGMFATSLGPHDACFVLAANENKGSGYMINWWIPAALQKEIIESTKINECKNGCIGILIWHF
VHHTPFNFWGRGPWMPSTH*
>TRINITY_DN38399_c0_g1_i1.p1 type:internal len:876 gc:universal
TRINITY_DN38399_c0_g1_i1:2625-1(-)
GDGSNFYESPGLLDWMNVTNVHIRFSGLYRKFDYIDTRWHHYVVSEVSLIARCDCNGHGDGISC
PLNVVTGTRKCTCHDNTCGSHCERCCPAFNQYPWKRGTRSSWFSDNTTACERCNCLGHSDVCQ
YDQHAADNNLSLNINGEYKGGGVCMDCKHNTDGINCEKCKPYYYRPANVDPRSKDACPSCGC
SIAGTRAASSNIKFLECIKDDKTAKFYPGMKVGDCFCKQNVIGSKCDLCKDEFYRLEASNSLGCRR
CRCFLSGTVQRLNICHKDFFGQCPCKSNVKQRDCSICKDGYYDLRNDDESGCKWCDCDVGASIS
PYICDKITGVCTCRAHIHSRRCKKVDEGYYYPDMHYIFGERNIYLRANSRLTTKIDIPRDAMYQLF
VQFELRTRLSPMLQLELKLDGTTKFLLRSNNNLPYALIKVCDTGCFSYFRHDSTQNFFLNAGLYEV
RVVSTIANSKFLLYKFIAIPVEFITAAILVGELKESFLHSCDVTKNDMRLGTANETICLAGAFSMTVK
FEDGALPCNCNLIGSTSTSCNKYGGQCACKPGIGGRDCSYCKPEFYDYSRNGCKECGCKGRNKV
CDPVTGDCVCPPNSQGRVCDPCECNGNSKRCNRQGICFRCGFNTEGDHCERCKVGFYGNALKS
QCIECACHPLGSFNQSCDAVTGQCYCRPGVVGRTCSKCASNHYGLSVKGCKACNCNEFGSKSLQ
CSAIGQCTCLVAAGREKCDKCDPGFYGLPKMACKECSCNTTGSVLNTNCKNDEFGQCTCKNGV
EGRACNRCMKQHADFGNNGCRQCPLCTRKLQCHADEVARKLINVKTHLNLTEILIALQTNLIYT
QDTFQQIQVDAALFEHRLKQGKKDYTYLYDQKLMPWNK
>TRINITY_DN3991_c0_g1_i1.p1 type:3prime_partial len:219 gc:universal
TRINITY_DN3991_c0_g1_i1:84-737(+)
MTSWILNFLVLSIIALPCSSISNCKHKLEPVGCFKDRGSARAMPEQILNERDTTSKVYSKQLMSWK
NFHEWLPGFICRCAEVAARKGYSVIGIQFWGECWSGPNAESTYNRHGQVDSCINQNLKKNRAK
LSQCTAASGKHFTNYVYRLAPACHTQYKPVGCFQDNLQKPRPMPYYVSTDRETRLAIFSGKLLN
WKNWNTHMPDMICRCAAVAKSKGHQ
>TRINITY_DN5584_c0_g1_i3.p1 type:complete len:962 gc:universal
TRINITY_DN5584_c0_g1_i3:2920-35(-)
MASEMSSPFDEEETLLDEKRSKSNDYEGGFCKKNWPVVITGFGVVVLICLGVVIGTAFKSDINSSN
SRLAREEKFPYKNIRLPGNVIPIKYRIYLHPNISDNEFGFSGNVRILVNVLNKTNSVLLHSKDLNILK

VKIFSNSAAFNQKSESLATEIPTQEFLHSKKLEMLHIALNENEYLDAGEYVIYISFKGSLSGGLEGFY
KSSYTTRSGEKRYLATSHFEPTAARAAFPCFDEPAMKANFIIIIVRDPQHVALSNMPIERNVTRRDG
KVTVHFQESVKMSTYLVAFVVCDFAYVSAKTKKNNIEVRVWAPKELIAQAEFAIQVAPNILNYYEE
FFKVDFPLPKQDLIAIPDFGAGAMENWGLITYRLTSLLYDENESSSANKQWVAVVIAHELAHQWF
GNLVTMEWWNDLWLNEGFAAFVEYIGSDYHDKSWRMMDQFIIDDVQNSLALDSHANSHPISVP
VHDPAQINEIFDSISYDKGASIIRMMKNFIGSDAFHTGLTNYLTKYKFKNAKSDDLWACLTDAAK
DKDIDVKRVMDTWTLQMGYPVLNVKRDGEKVTLTQTHFLKDQDKPAKSIYNYKWDVPVTYFT
NGENTTQFDWFYKDNKSIVIKIDENADWFKINRDEIGFYRVNYDKDTWAALKAQLLNDHMKLS
ASDRSNLMDDAFELARAGQLDQIEALNMTEYLHKEKEYVPWITALSGIAFCGKMLQGREGYKYY
EKYIVQQVKPLVDELGWEDKGTHLQKYLRGSVLRSAVDHNYTKAKNQALEIFKDFKENNKAIKP
NIRSAVYLAGIKYGTKDDWDFMYNRYKTSQFPSEQRKIMFALTDTEDKNILKKYLSWTLDNDKIR
SQDSCGVIEHISMNPKGTEMAFKFVKKNWPEIYKRYGTSSFDMSSLIKTIIGTRKTKSGKEEAKEFL
DTHKLGSGRLAGKQALASVQNHINWLEKNEKKVVEWLKNNINNKKITK*
>TRINITY_DN941_c0_g1_i3.p1 type:internal len:363 gc:universal TRINITY_DN941_c0_g1_i3:3-1088(+)
LPDDLKYLKGSVVSPSGTEEPVELKPGQNNTILVSFFPKEAGIHYIHIKKNGKPVGDSPYQVTVKPE
DVAAAQPDASKVKCSGRDKPVTIGSPCDVNLDIDDIELPKDLDKLKADLTRPSGKKEPVKCEMA
DNGGLSLNFVPEETGKHLLEVKKNNRPVKGSPFEIMVEESASPESKPTAGQKCDLDLDIPDIDLPR
DLKDLKAQLTRPNGKQEPIECTCTPENTLGLEFTPDEPGKHLIDVTKRGRPVKGSPFEVMVESPES
KPTVGNNCGLDFDIPDIDLPKDLKVLKAELTRPTGKKEPIECTATPDNTLGLEFTPTETGKHIIDVT
KRGKPVKGSPFEVVVGSPESKPSVGSKCDLVV
>TRINITY_DN106_c0_g1_i2.p1 type:3prime_partial len:1309 gc:universal TRINITY_DN106_c0_g1_i2:137-4060(+)
MPSNSKLAVLLLILGIGITLSKAQDLDDEFLDRPGQCPVPPIEPNCDRERSPDESVQGCLMDMDCA
TGQKCCSDGCNLFCTSVNIPQPPQIVKGLPGDPGEPGIPGTSGADGDDGIDGVAGPIGNKGATGG
RGKKGRRGRPGPPGPPAPVDGSSLPEVGPIAGGDNKGPGPNAGAIGEEGMGADGFVTPKEGPAG
PAGPVGRLGPRGPYGERGDPGLPGPDGDMGPKGPDGPPGSVGENGRDGLNGDRGDPGQVGAI
GAQGVQGDQGPTGANGAAGYKGLKGDRGASGEKGAFGPDGEKGETGDNGLDGEVGPSGPQG
LQGSPGPRGPDGAVGPIGPIGARGLPGPPGLSGLPGAPGVEGAVGAVGEKGSLGPRGERGEQGER
GPTGDAGAKGETGAMGLVGRDGTAGKKGALGSPGPQGLRGEAGPPGIQGLPGSDGAIGATGAR
GEPGEQGFDGAPGFKGTNGDSGPPGKPGEAGPQGKKGRSGRDGPAGPAGRDGDRGLQGIPGAP
GIPGRPGSKGSRGDRGTLGKQGTKGTAGAPGSPGETGPQGSRGAPGGIGKSGRPGKQGARGASG
KDGRPGAPGQQGLRGKAGQLGLQGPKGAQGPSGGNGEPGIPGSKGQQGADGQKGSVGQPGSP
GASGATGERGTPGPPGDNGKTGPAGPIGEAGAPGRDGEPGKAGQRGDDGKAGDPGIRGPPGVK
GPNGLRGSPGLPGERGEQGSDGVPGNKGPVGPNGQPGPSGKPGSPGASGTKGPDGAKGKHGAR
GAKGYSGDNGKDGNRGKDGLAGLAGPAGATGSKGESGRPGANGLPGKRGDPGLQGNRGPAG
MPGDMGAQGVQGGDGQKGGRGPRGTKGEPGPTGPSGDDGMSGPGGLEGSLGNKGSRGLSGPS
GGQGRPGLEGMRGRRGPPGNRGSVGPLGPSGRGGQKGSLGTIGIPGKPGKNGAPGLPGKDGKN
GNPGDDGNPGMQGLMGPQGTVGLRGKPGHVGMPGFPGMQGNAGEKGAAGLPGINGSKGRT
GTTGPQGLTGPIGLPGQAGKDGVKGIDGTPGANGVRGPKGELGKAGFTGAVGITGMPGNPGAA
GAKGGRGDGGEPGIAGEGGNAGSPGQRGPNGNRGRKGYKGNKGNSGAKGDKGWRGMTGPT
GLPGPIGTQGDVGVDGPKGRGGPVGPPGPRGRSGREGAPGDEGMQGTDGLKGSVGNRGRDGD
DGINGPPGPPGPPGLPGGFGGGAGAINMFPPMGNVEKGPSYYGYQYYKSQDGKDFKTAQEEAK
NTVFAYTNKLDEVLQGLDKPDGSEVYPAKTCADLKLCHPDIKSGNYVLDPNLG
>TRINITY_DN1386_c0_g1_i1.p1 type:complete len:596 gc:universal TRINITY_DN1386_c0_g1_i1:2419-632(-)
MEYIEVKSFIVALVFVFSILGTAQSDLVINNVERTLDLSTQTAKYTTKIKFHNPGKKVEKSFLLALEK
DQEKHLSYLEILDKQDDNSLEYAAENVEGQSGRFFKVSFSKPLKENDDVTLHIETVFTHVLTPFPA
KIGQTEKQLVLYKGNTVFYSPYEIKEQSTTVKLASSAIESYSRLKPSRSSENTITYGPYKNSKPYRLHE
MKIHFENNSPFLVVNEMTRWIEISHWGNIAVEETYHMTHEGALLKGHFSRYDYQRSPTHSVIKSF
KTVLPAAARDVYYRDEIGNISTSNMLVNDDSVEIELRPRFPLFGGWQTRYMLGYNVPAYQYLYNK
GDNYILKMRLIDHVFDEFVIDDLNVKIILPEGSTNIEVVKPYELEEGKRDIHKTYLDTSGRTVIVLSK

KNVVENHIQDIQVHYTFKKLQLLQEPVICVAVFYILFLSVIILVRLDFSITKDAAKESRMKVASLLEE
LLSSCGRRGSLYYSYDSAIDKLKQNRNQNDFAVSLKKLNQDYNTLTQSINELCSLLIKEDPEIGDKV
NDLQKKENERKVIVDQLVTLATRVVTNKIGRQQYIESEKNSMAKREKLGEDIDNVLATL*
>TRINITY_DN6428_c0_g1_i2.p1 type:3prime_partial len:303 gc:universal TRINITY_DN6428_c0_g1_i2:906-1(-)
MLSSLKLERNKNLRYYRYRYSCCNIRVPSTSQKILNPFTLVVGGNAIYLDRQNVQCPGSAVITQLLL
RRYKSTIRYTYSCSTIPRKMECQNMKTPLNFDGKGDARYLDRHPVSCPNDYGLNKMRLQRGSNL
KTYAYHYRCCKPLYSVQFQENKVDGGFTNWSKYGTCTKSCGGGIQERSRSCENPKPLNGGKQCI
GVSVETRSCNTDPCPVDGGFSNWSNYGTCTKSCGGGIQERSRTCTNPKPMNGGKQCVGVSVETR
SCNTNTCLGPKQCKLKFTAYNKDGKGQVFYLDRHNVACKPDEM
>TRINITY_DN2390_c0_g1_i2.p1 type:complete len:467 gc:universal TRINITY_DN2390_c0_g1_i2:82-1482(+)
MSNVGIQNSDYAVYKDDVSYAEDNSKGIILSRLDGKLVEIRSYHINWESYHQGEMISNADYQFISH
YDRQNNDGKENLFGEDPEQCIMTLLSLINHVVKEHVIQYTLVLLDDIINRNKERVKKIHETVLKN
KFNIWPPFMSLLNRDDAVIMHMGSRLIAKLATYSRVRCPDSDLIYYLNWLKVQLCVPSNEYLQSV
AECLQWMMRIDDYRMPFVKADGINSVVSVLLSNLGFQLQYQLIFSIWMLSFQPKIANKIGENNVI
PVLADILRNSQKEKVTRIIIATFRNLLEKPSDCKMACVSLIQSKLVPVFNVLSSKTWGDEDIKEDIEY
IGEKLSTSLQDLSTWDEYVAEVKSGRLEWSPVHKQEKFWRENAIKIAENNYELLKILNKLLQYSN
DPLIQAVACHDLGEFIRHYPRGRPAFDALGCKEQIMIRMTHDDPQVKYEALLAVQKLLVHNWEF
LGKQIKIE*
>TRINITY_DN1071_c0_g1_i2.p1 type:internal len:339 gc:universal TRINITY_DN1071_c0_g1_i2:1016-3(-)
DPKVAMRVEFYVCPPCGVTTPAITTTEQTTMPTTTTTESTTTPTTTPATTTESTTTTLPTTTTTTPTTT
QSTTSKETIIITEEPKPTTGLPQKKTTTTPRTTTESPTTTEATTTTPATTTEATTTTQATTTTTKATTTTP
ATTTTKATTTTEATTTPTTKPCECKVLQPVGVSDGRIPDNQMTANSVLVNVSGPTFTGASQARLN
NKPSKDGVGAWEPAQQEEIYLEVHFNKIEDVREIRIQGHPTDKKFTKNFFLSTSQDGVNFEFTDED
LYEGNKDGNTIKSTKVSFKTKAVQIHPFNDVSVGDPKVAMRVEFYVCPPCGVTTPAITTTEQTTMP
T
>TRINITY_DN4815_c0_g1_i2.p1 type:complete len:638 gc:universal TRINITY_DN4815_c0_g1_i2:79-1992(+)
MVSSLLLVLICTVVYTAGISAFFQCDFEKVDICGFKSSSSNYTDTGEAPNWRRHTGTTPSAKDQNN
AHVTGPLEAQSGDYYMYFEASNLPSGASSTGTLTSRLFDATQFGVTDIVFHVSSKGESLSLLVLDFI
DYTTNDIFDEKVVFPRNKAAWQKYTFNLDEKIFKRKFYMQFIGYVREVNGKKDNGDWAIDNLRI
DGIAIVEKCENVKCNVNTNCKDTSNGPNCECLPGYSTKAGTSGKNTECIETCQAFTCNLNAECS
NTENGPDCKCESGYKAINGTSGKTTRCEETCQAFTCNLNAECSNTENGPDCKCESGYKAINGTS
GKTTRCEDDTCSNFQCSNNTNCENTVSGPNCTCKEGYRPVNGTRGRDTICEDINECAIKNICGGL
KVCRNVPGSHHCDCPPNYTFFAAENKCYRNGAFSQWRNVGSCSKTCGAGDQKQTRSCSNPSRE
APHGRDCPNEPTERAVACNSGPCLAGCPRNKTCTCENVRQYKTGEEPKKDFSSFNGLSITDGETS
RRYVHTYNREFIEKTIKDVNCNNNQLKSVYKNITDIIGILEGINKKYSSTKMEMQDIIDCNANIDIE
KNLWDVYDVLAEHSICNYQVQKDLEMKADRIQAEITRCNNRGWYSTILDAII*
>TRINITY_DN15584_c0_g1_i1.p1 type:complete len:275 gc:universal TRINITY_DN15584_c0_g1_i1:874-50(-)
MADSGSAGADRGGRGGFGRGGRGGRGRGRGRGRGRGKPEDKEWTPVTKLGRLVKDLKIKTLE
QIYLYSLPIKEFEIIDFFIGPSLKDEVLKIMPVQKQTRAGQRTRFKAFVAIGDYGGHVGLGVKCSKEV
ATAIRGAIIQAKLSIIPVRRGYWGNKIGKPHTVPCKVTGKCGSVTVRLIPAPRGTGIVAAGVSKKLL
QMAGIDDCYTSACGTTATVGNFAMATYMAIARTYSYLTPDMWAENQFQKTPYQEFTDYLAKNH
NVGGKPVAPATATA*
>TRINITY_DN187147_c0_g1_i1.p1 type:complete len:1223 gc:universal TRINITY_DN187147_c0_g1_i1:3708-40(-)
MLTKFETKSARVKGLTFHSKRPWVLASLHNGVIQLWDYRMCTLLERFDEHDGPVRGIDFHENQP
LFVSGGDDYKIKVWNYKQKKCIFTLLGHLDYIRTSFFHKEHPWIISCSDDQTIRIWNWQSRNCISV
LTGHNHYVMCAQFHAVEDYIVSASLDQTVRVWDISGLRKKYCAPGAKPKEDITAPKTPGQMDLF

GHSDAIVKHVLEGHDRGVNWVTFHPTMPLIVSAADDRQVKLWRMNDSKAWEVDTCRGHYNN
VSSVIFHPRLELILSNSEDKSIRVWDMSKRTGVQTFRREHDRFWILGAHPTLNLFAAGHDSGMIVF
KLQKERPAYAVHQNTLYYIKERYLRMYELGTSKDIPIMQLRKSSGRSTPLKLIFNPAERCILITYNND
TSAGESQVYELHTLPKDIRSTDPEQSEGRRAAGTAAVWVARNRFAVLDKQQNVVIKNLKNEVTK
KVQTPACDMLFYAGTGSLLLRDAEQVMLFDVQQKRVMATVKIAKAKNVIWSSDMSYAAILAKH
SIAICNRKMENLCTVHESVKVKSGGWDDSGVFVYTTSNHIKYTLTNGDAGIIRTLDLPIYITRVKGS
NVYCLDRECKTKVLNIDPTEFKFKLALVNHRYDEVLYMVRNAKLVGQSIISYLQKKGYPEVALHF
VKDEKTRFGLALECGNLEIALEAAKTLDDKACWEKLSEMALLQGNHQIVEMCYQRTKNFDRLS
FLYLTTGNSDKLRKMLKISEIRKDVSGHLHNALYCGDVEERIKVLKQVGQGSLAYLTAATHGLVEE
CEEIKATFDLNPEKLPPINPNAKLLRPAVPILKNEGNWPLLSVSKTLFEGAAAMKDAGTSNMAAA
VDMDDEGEGWGDSDLVLEEDAGFVDPEEKLDIDEEGGGWDVDDDDLELPPDLDVGPVSSGGED
YYVPPTRGNSQAQVWCTNSQIPVDHLVAGSFDSAARLLNEQLGVVSFEPYKQLFMLAYARGRVA
APGLPSTPPLFYYPHRNWKEAGAKAGLPAVAIKLNDLVQRLQTAYQLTTGGKFADAVERLHQILL
SVTLLSVESRQDVTEAQQLLGICREYILGLQMELNRKELPKQNAQQQKRICEMAAYFTHCNLQPV
HLVLTLRTALNLAFKIKNYKLASSFARRLLELGPKPEVSQQTRKILLACEKNPTNAVQFDYDELNP
FSICAVSYTPIYRGKPEEKCPLCQASYKPEYKGTICKICRVAEVGRPGSGLKISMGR*
>TRINITY_DN101992_c0_g1_i1.p1 type:internal len:139 gc:universal TRINITY_DN101992_c0_g1_i1:414-1(-)
AERYDHHSGIVESMDSNKISDNLKELTRLPHPPGRSRNKELGDMLADFWQNEGMDKIEKFKYNI
YLSYPEKPSTILLFGGAKTEKYTIEAEPPVDESEKGGTPVVPFNAFSANDIVKAELVFVNHCRESDF
DEVKKAG
>TRINITY_DN286_c0_g1_i2.p1 type:3prime_partial len:106 gc:universal TRINITY_DN286_c0_g1_i2:40-354(+)
MIKVAIITIAIVACVLESTAAVRCYVGYSAADLPLAIGVRNIECGEEFNHCTTVKYRIGGITVTQGN
CAKRYGQAMCQFMRNPHKAMTECEESLCQGDNCNTAVN
>TRINITY_DN4900_c0_g1_i1.p1 type:complete len:246 gc:universal TRINITY_DN4900_c0_g1_i1:153-890(+)
MAELTCGMKCLKMLLFAFNFIFWVCGIVVLGVGIWSRIESKDWDSLLGDDGTIVNAANIMIAAGI
FVMIIGFLGCCGAMRENKVLLMLYAILLILIFVLEIAAGIFAYTKKDTVIEKFEENITKGVKNSYGKT
DKASEAMTKAVDWFQENAECCGVDGPNDWKASDWGKSKNTTKIPGSCCEKVDADTCDIKDSY
KKGCSEAGPQYIKDHMWQIGGVGVGIAFVQLFGIVSAICLCRAVNAETTA*
>TRINITY_DN11435_c0_g1_i1.p1 type:complete len:430 gc:universal TRINITY_DN11435_c0_g1_i1:1616-327(-)
MGNIYTVGPNEALVVSGGCVGQRVKRTIVGGWAFAWACVSDVQSISLEVMTLNPRCEKVETAKG
VAVTVTGVAQVKVIKEDDLLKTACEQFLGKDPHEIEDILLQTLEGHLRAILGTLSVEAIYKDRDQF
AQLVREVASPDVGRMGIEILSFTIKDIVDEVSYLDSLGKTQTAEVKKNADVGVAEANRDAGIREAE
CDTQRQDVRFKADTTIADAARAYEMEKAEFDKEVQTKNAEAKLAYELQANKEQQRIRNEEIQIE
VIERRKLIEVEEKEIQRREQELQATVKLPAEAESYKVQTIAEANKSAKVYLAQAEAEKIKMIGSAQ
AAAIEAIGKAEAERMRQKAAAYKQYGDAALMSLILEAMPKIAAEVAAPLRKTEEIVLINDDNGN
KMSSEVTKLLGQLPPSVKALTGVDLSKALGKIPGAQVTA*
>TRINITY_DN2885_c0_g1_i2.p1 type:complete len:114 gc:universal TRINITY_DN2885_c0_g1_i2:31-372(+)
MAQPVRLYSKGIFVGYQRGLRNQHEQTSLIKIEGVNTKADTSFYLGKRVAFVYRVSKKTVAKGDK
KPSRIRVVWGKVTRPHGASGVVRAKFRHNLPAKAIGGSVKVMLYPSRI*
>TRINITY_DN6920_c0_g1_i2.p1 type:5prime_partial len:411 gc:universal TRINITY_DN6920_c0_g1_i2:2147-915(-)
CKRHQGKMSHRKFSAPRHGSLGFLPRKRCKRHQGKIKSFPKDDAAQPCHLTGFIGFKAGMTHIV
REVDRVGSKTHKKEVVEAVTIVETPPVVVVGIVGYVETPRGLRQLTTVWAEHLNEECLRRFYKNW
YKSQRKAFTKASKKWSDDDGKKSIERDLEKMKKYCTVIRVLVHTQQKLIKLKQKRAHIMELQIN
GGTVADKVDWAKKSLESKLSVSTVFGQDEMIDVIGITKGKGFRGVTFRWGTKKLPRKTHKGLRK
VACIGAWHPARVGFSIPRAGQCGYHHRVERNKKIYRIGRGIHTKDNKVIKNNAATDYDITNKTIT

PMGGFPHYGEVNEDFIMLKGCVPGPKKRVLTLRKSLLAQTSRKALEKITLKFIDTSSKFGHGRFQH
DQEKKNFMGLLKKDKERQAVEA*

>TRINITY_DN4781_c0_g1_i3.p1 type:5prime_partial len:1859 gc:universal TRINITY_DN4781_c0_g1_i3:8547-2971(-)

QCIKKHMLCDMDRNCCDGSDEAAKLCKGYKTLDFETGLQQWIQFRNDDFDFDIQSGKTSSSGT
GPQTDHTLEEEHGKYIFIESSLRNENETANLGSPTLAATTSSLYASGDCSLRFWYHMYGSTIGNLTL
FLRRDNKDTLLWAKSKPEGNQWRPALVTVNSWYKPFQLIFVGTVGKSYFGDIAIDDLMMTDEKC
PSPGDCDFEDFDKCTYTQDLVEDDFDWIRGHGGTGSWRTGPSLDNTKKDNSGHYYFIEASAPRT
QGDRARLVSELFDPTSSTRGRCLQFYRHMYGTAVGTLNVYVKTGPGNSSDAESLVWTNAGNQG
NSWIQGQVPIYSSKPFRLVFEAVRGNGYAGDIAIDDINFNNNFAPCSFLPSNAAPPTLPPTTITPEDS
NCTFDKGLCRWKQMKSNDQFDWTLVSGSTGSVGTGPLGDHTSGKGSYIYIEASYPRKENDSAILQ
SPPIRPSLGSVCFKFAYHMHGAHVNALYIYKNVKGKRTMVWKRVGTQGNQWRNGQVNIRGVS
GIFNIEMKGIRGVDYQGDISLDDFVVTNGQCPTPSHCDFENNQCGWFNIGRGDDFNWKRSNGG
TPSLGTGPGTDHTTGVALGSYMFIETSYPRKYRDVANLYSPYYPGSKTGYCFNFWYHMYGSHIGSL
NLYQDTVKSTPIWNKTDNQGNMWKHGRVTVNSRAKFRLIFVGTVGKSFLGDIALDDVWVDDFP
CPAPGACDFESKSFCTWSQVPNKFNTSNYDDFDWELGNGPTPSFQTGPAVDHTKGTSVGTYAYV
EVSGLIKRGQKAQLMSEKFPPTNGKCMSFWYHMYGSGVGDLNIYLKYLGVKKSHLVKSISGQQG
NKWIQGEVGLHSQKDFNVIIEAVKGSSYAGDISLDDISILDGNCVGLCSSVQPVARVACGPQSVTS
ASCVVSYGCCYDNSVPNVPACFQHPSSCKAVPIIARQQCGYYSISKYFCARRGCCFDDSGAGGPP
CYHSLSKPTDFPTTRAPPTTQPPSKFDCNFEKNQCQYTNLRNDQFNWARHKGDTASFGTGPKFD
HTLRNQNGHYMFVETSFNKENDTANLGTPTIDFPTGGDDVCVRFWYHMYGQHVGAFKVYHKL
TTRVPTKPIWERIGSMVNKWVYAQLIVPRRPAFQIVFQGVRGKGYQGDIAVDDVGFVNGPCPPSL
QCDYQNGDLCGWAQDADDEFDWSVGNGKTMSTQTGPKADHTYETDFGKYLFIEGSSPRQPGD
KARIESFSHRPTKGMCLEFFYHMKGEGIGSLNVWIRRGKTMDSKPIWTISGAQEDKWFRKKVSVIE
DTQNWSVIIEGIRGSSWASDIAIDDVVFHKGVCAPQGDCDFETDDLYSCGYRNWRRNKVDWLIY
SGSTPSFNTGPSFDHTFGTKQGHYLYMEASSPAKQGDYGLFYSELFRVNPNYKWCLDFYYHMYG
DGVGSLQVNKMYWPSYLKKRRPYFTTLFTKDGNHDDVWQHAQVNITTRNDFQIVFRGEIGGGT
SDKGDISIDDITVRQGLCAAPMPPPKPNPCAIKCKDGTCVPSNKVCDFYPDCAQTSDNTDDTDEK
GCDGCDFEKGTCNWNDTSKGLFDWNRQNGGTSSYNTGPSVDHTKKNALGYYVYVEASKGQSS
DLATLSSPVLNQASSTCKMTFWYHMYGTSIGTLNVYLVNGYRLTLHWTLSGNQGNLWKQGTVY
VNRQLKPYSVRFVAERSFSYRGDIAIDDIKFEGCELPRPAGFCNSRYQHRCAVTRACISYTRLCDYT
DDCGDGTDELNCRWTQYPYRCNFEYDLCYWRNLKDDDFDFTRQKGQTDSANTGPLFDHTTGT
SSGYYIFVETSWPRKTGDKARIGSGYLGPITRTYCILRFYYHMYGDHIGIIGQQKLMQLSMI*

>TRINITY_DN923_c0_g1_i1.p1 type:complete len:442 gc:universal TRINITY_DN923_c0_g1_i1:53-1378(+)

MAEKYDLTYKVGAYLDRHMVFPLLEFLSSKEIYDDTELLRGRLELLQGTNMLDFVKDEYQRLHQT
DEVPDELANKRQEVIRQMRELEAATESITAAFEDPDFQKEASSARDGAQLFKYLNETFGIRSENLD
TLYQYAKFQYECGNYNGASQYLYFYRVLVHDDDKNASAAIWGKLACEILTQNWDPALEEINRLK
EVIDGDHFSHPLQLLQQRTWLIHWSLFVFFNHAKGRDLLIDMFLYSPNYLNAIQTSCPHVLRYLTT
AVITNKRRKQILKDLVRVIQQESYTYRDPITEFLECLYVNFDFDGAQKKLLECETVLLNDFFLVACL
EDFIENARLFIFETFCRIHQCISIQMLAEKLNMTPEEAERWIVNLIRNARLDAKIDSKQGHVVMGT
QAAAVHQQVIERTRSIMFKTQAITTNIDKKLASLQKQPYTEPFGLAW*

>TRINITY_DN10094_c0_g1_i2.p1 type:complete len:264 gc:universal TRINITY_DN10094_c0_g1_i2:1210-419(-)

MRHILKVFAVAFVLHCIDRTLSLPVSPVPPLLNKLLKDQNVTLGFILKGLQGPPGRDGAPGSSGLP
GPPGPPGWKGDPGTPGMNGAPGFMGNPGSPGAPGNDGLPGPPGLPGAPGWPGGSGMQGPPG
MPGLPGLPGSSGPSPIRYNGTVKCEEDTAWLRCNEYKHISIMSAFWGRRNFGLCTEHTGNLITNK
YCPTTPLFLTKVKDACEGTTMCEIRCTKFFFHDQSCLDVYKYLEVYYKCIEVINGHEVVNEDNLL
MANFLG*

>TRINITY_DN79_c0_g1_i1.p1 type:5prime_partial len:478 gc:universal TRINITY_DN79_c0_g1_i1:2132-699(-)

VVSSESNVLVEFYAPWCGHCQSLAPEYAKAAKQLKESKSDIKLAKVDATVETKLAEKYEVQGFPTI
KFFKKGNALDYGGGRTDKEIVAWLNKKTGPPAKLLDNAADVKAFADERDVAILGFFNDKESSEL
AKAFLAVAEAVDDLEFAIAAPDASGDFAVTEDKIIVAKKFDDPQVVYSGEANIADIKKFVRAESIA
LVTEFTDEAAPKIFGGDIKNHLLLFISKKAENFAELKNTFQEAAKGFKGTVLFVYIDTDVEDNGRIL
EFFGLKAADAPTLRLIQLEGDMTKYVPETKDLDKDSVTSFVQNVIDGKIKPFLMSADVPEDWDK
NPVKVLVGKNFAEVALSKDKDVLVEFYAPWCGHCKQLAPIWDKLGEKYKDSENIVIAKMDSTAN
EVEEVKVSSFPTIKYFPKEGGVVDYNGGRTLDDFVKYLESGGKDVSGGADETGEDEDADEEEEGM
EEGMEEGDLKPEEPEVAKDEL*
>TRINITY_DN118327_c0_g1_i1.p1 type:5prime_partial len:702 gc:universal TRINITY_DN118327_c0_g1_i1:3-2108(+)
LFILMKSKMTEIYLKKFAVLFSILLIFQECNGFLKSQLANDHIVNDDDGKSTAVAVFRQKRGIFGAA
LIAISVASAGIDIYKQVAGCKHVTSDLVKKINQAKKMQRDLIAKRDRINLAWVPAQSKESYTQRLA
SDLIILKGSISLFDSLQVDLLKLLDQNLLDSINLLKRQLENDIEANKDLGIGSFEELEAKFYTKTQFA
LSLTFKLLPLGFLAGSYLLPKIAAKLAKTSAFRWSESNPGKIQKMYNKYADKKGQIKGWGKTKIQS
FKNGLRARWSALKAKFPRTRAAFNRLGNSRLGRFFSRNMGRIKTGVVRFAKGAVLGLFIGLEVW
SLVKFIKGCKDKLKKINEAIASLKNGEKKLNVVQSKINDLQNTADKYYGWMKNNLTSPDYLNYV
REIADLGDNAKDQTSEIVKATKEIKRYTNEIENADLQKTDKLNKMVISAFGKINYKLDCLRSKLAII
SDLVYSCSNGYGKFDDLYRRALDKQNKITSECIEGKYMDKKEVHELMKSAMQLKNKQTICIRNN
PTKKQFVCEKWYNDASNENILKSIPGLTLNDVNFFISVCPPADVTQKIKREICMEKNQNFITLKEIS
KKLSKYHQQNVVIAYNQCQLSTSSLKEICNAKKDGQDLDYVHKKYIVFPKNIVAKAYDGCKKET
NITPFDRQMICVIKSLGAGLDSNNFATFRKKYEFSAIKSVYDKC*
>TRINITY_DN3156_c0_g1_i1.p1 type:complete len:501 gc:universal TRINITY_DN3156_c0_g1_i1:39-1541(+)
MILKYLLCICLIIQTVRCGKPSLSDPLGLNDQYGSTSQSNFHFGAQGENYVLGSIWPKPQQETRED
VYFALDATKFEFKIVDKTSDVLEEAVIRYHDLVFPEKGARADENLDMITNLEIHVKNDIEPLSVESD
ESYTLVINAPTASLSSYTPWGALRGLETFSQAIHQNETGHYLAQKNKIVDYPRFHHRGFLIDTSRH
FVHLDVIYKFLDALAYSKFNVFHWHIVDDQSFPFVSQVFPSLHGQGAFNNKTHIYTPDDVNNVID
YARYRGIRVVPEFDTPGHTQSWYSIPGLLTPCYSGDKPNGNYGPIDPTVEKNYEFLREFFREVTMVF
PDKYLHMGGDEVSFSCWQSNPNIAKWMQDHDMGKNYSMLEQYYEQRLLDIVGDLNRGYVIW
QEVVDNQVKYYNCDPQAFNGTTEQKEMVIGGTACMWGEWVDGTNLLSRTWARGLSVGERLW
SAENVKDLNDATKRIWEHRCRYLRRGIPAENVVQSKYCVHEWRLINN*
>TRINITY_DN1936_c0_g1_i1.p1 type:3prime_partial len:1224 gc:universal TRINITY_DN1936_c0_g1_i1:3669-1(-)
MVCPAVITIFLFSVLGSSSSQNCDFEDVKSCKWTQYAADKFDWSRASGSTGSTGTGPVNDHTTLV
KTLKVNTTGGNANGASCVFPFIYQSKKYFKCLQYYNTEWCATVPNYDKQAKWGYCVPTGFYMF
IEASNPRKKGDNAILVSPFVPKGPKCLKFWYHMYGPHINRLNIYVMIGGHTGKPIWSRIGTQGNK
WNKGQVTLVGRQPFQVVVEALSGISYKGDIAVDDISVSSTKCTQPSSTTCSFEDSQCGYIDDTSSDV
QFQWTRSSLSTSSSGTGPKTDHTMQSETGFYMYTEATRKKTGSKARLMSPVNKATSGSCLQFWYH
MFGADMGTLSIYLKVNNKLQSTPLWSEQGDKGNEWKVATATITTQDSYQIVFEGKIGRGYRSDM
AVDDIKLTDGNCPPPGFCDFETGYCTYFNSKNDKFDWLIGSSRTPSRFTGPNFDHTYGNANGSYL
FLEVSQPIKAGDIAILESQQFNAVGGKTRCLNFWYHMFGSDIGSLQVVYKVFSGSQQERVLWNLT
GQQQITEKDPWKYARVPVDMDADHMVMFKGIAGSGYKGDIAIDDVQFTSLACTIDPPEANPNG
KTTMKPPTTYPRTAPSTPVPTEFSCNFEKDLCKLTQDSKGTFNWTRIQGYTGSPGTGPSTDHTYGR
GFVPKYGPGSLRHAGGKCVHVLNGGFNKPKEGNPLVLYQGCGQTRLEFVFKYGYLKLTRLNMC
ATLEGSSTSSGTKVVLSNTCMNKWKMLDNNTIQDVKTGLCLSPFKGSDYPSNNSPLVVDKDCKP
KSRIFTFSPTMGHYAYIETSPPRQPNDTARLISPILKTPRCLNFWYHMYGAHVNMLNVYVKTSNGP
YVSPAWAKYGSHGDTWKAAYIYMNMTGPYVYAFEGVRGIGWQGDIAIDDITLSESCPAPVECSFE
ETSPIMCGWTNLKNDDFDWSIGQGKTASTKTGPKNDHTYGTSQGHYMYIETSNAPAKATAYMYS
PWYYNTGQKCVQFFYHMYGQQIGQLEVYIRYSNSRYLYRQFIRSGNQNNTWHMGQVTVKRNN
VYFQIIVRGTKGNGYQGDIAVDDFRIRDGACPAPGDCDFEHADFCSWMQEDNDDFDWIIGSGST
NSFLTGPSIDHTTQSNGGYYAFIEASSPRQPGHKARFKSMVFAPTTGSGRCFSFWYHMRGYSIGNL
TIYLRKVSNTKTLSEEVMWLLGGNQGKAWNQGRFTLKSSTLHEVRFIYLRLFHCKICLPCY

>TRINITY_DN1559_c0_g1_i1.p1 type:complete len:311 gc:universal TRINITY_DN1559_c0_g1_i1:221-1153(+)
MAEKKATFKLGFAENFALSGAAAVISKTAAAPIERVKLLVQNQDEMIKQGKLSEPYKGVIDCTTRT
FKTEGLAPFWRGNLANCIRYFPTQALNFAFKDKIKAAFKVSKSDGYALGFGKNIASGGCAGALSL
CFVYSLDFARTKLANDTKSSKKGGSREYNGLVDVYRKTLKSDGVAGLYRGFVLSCVGIFVYRGCYF
GFYDTLKPVLLGADASLFMSFILGYGVTVTAGLISYPIDTIRRRMMMTSGQAVKYKGSIDCATQILK
NEGAMSLMKGAGANILRGVAGAGVLAGFDKFKEVYIAMRKRQMGLE*
>TRINITY_DN195348_c0_g1_i1.p1 type:internal len:311 gc:universal TRINITY_DN195348_c0_g1_i1:1-930(+)
SGCNSIDLGLLVDFSSSKDYDSKQLTKFVREFIDKITNVFAFSATKSHLAYLPFSSRINENITGQWFD
NKYMKTIGDWDYQELTQYLDRVIVPYTPVTGYETGKANAHEAFQATNDKMFNALYGMRYNMT
KVLIVISDGNEDFKEKQNLVDRAIDQLKAKGVIIYTVSIGNNVNWDRVFRYASRESSIINGANFDD
VFNRFFDFAKMLCEDSKVPIPATRTTASTTTVSINTTKLTTTTTTELVPVTISTTTTMSTTLRPIQACY
EDIDLGFVVDFSKMNANDMKERQTANDIVKVIKKIASSFNIAANK
>TRINITY_DN3034_c0_g1_i1.p1 type:complete len:1941 gc:universal TRINITY_DN3034_c0_g1_i1:6289-467(-)
MAEEEDPMKYLQVDPAQALKDQSKTFDAKKWVWVPVIGEDGYAAAQVIKVDGDNVTVETNK
GHEMEVKKEKCDQMNPPKYEKCSDMASLTYLNEASVLYNLKARYTTGLIYTYSGLFCVAVNPYR
RLPIYSDQVVKLYRGKRRAEMPPHIFAIVDTAYQDMLTDHDNQSMLITGESGAGKTENTKKVIQYI
AKVAGVEKKKSDDGPVKRDLDEQVVEANPLLEAFGNAKTTRNNNSSRFGKFIRCHFSQTGKLAG
ADIESYLLEKNRVIHQASAERNYHIFYQLLYATTDEELQKLCLVTRRSEEFEFLKKGVPSVDAIDDN
EEFGYTTNAIEVLGFDEEEKQSMFKICAAILNFGNMKFKQKPRDEQAEVADPADGERVAYLLGLPI
GEFHKSLTKPKVKVGTEFVNKGQNVSQVLYSVAALSKALYERMFWWIVSRVNKALDTKERRSYFI
GVLDIAGFEIFEYNSFEQLCINLTNEKLQQFFNHHMFVLEQEEYKKEGIHWEFIDFGMDLEETIDLI
EKPMGVFAILEEECIVPKASDQTYLQKLHKQHAGKNGSYEKPTPKQTKSGGGDFILHHYAGSVGY
SVANWLEKNKDPINAHTAELFSKATVGLVSGLFADYNPDVTGGRRKGSAFQTVSFRHKEQLKNL
MTTLMSTSPHFVRCIIPNEKKTPGLVEGTLILHQLRCNGVLEGIRICRKGFPSRMLFQDFRQRYQIL
AASAIPSGFIDGKVAAEKLIDSLQLDQSEYRVGTTKVFFRAGIIGELEDMRDERLAKIITQFQAYCK
GHLARIEYKKMCEKRIGLAVIQRNVRKFLFLRNWQWWKLYCKVQPLLSIARAEEEMKEKEEELK
KALEKAKEDEEQRKKMEEQLTENIAEKEKLFADLQAETDRLINLEDKLMQIQQQKDRLEANLNE
TLEKLDGEEHSVSVLQEKMHDAEDRIDSLEAEASELKSNCSRLEVERANRDKQIDALNEDINKQD
ESIAKLGKEKKGLEDNIQELNETLQASEDKCSSLTKAKKKVEDALKESENSLSKEKDSKARLDKEK
RRVEGELKETNDKLSEAENELKNTQDTVYKREKAIKELEEARDNQDNQIKQLQKKIQELLQRIEE
LEEELEAERKQKQKSELSRKELESQIEELQDQLETAGGATHAQVEVSKKREQECSRLRKEIEDINA
ANDEALASTKHKFNAQLSEAQEELESVKKAKSKSDKERSQLAGDLEDVQGQLDQVKKQRASSD
KNNRMLEEQLAELRGKVSSLETDMGDSESRNLKFQNESATLLKQLEEAEHKLGLANKQNKQLQ
ASLGESKNVADDEARGKHDMQNKLQNALAQIEELQEQLEDEQTAKMDLQNKYSRANADAQQ
WKNKYDMEGASRVEELEDAKRKLAARVQEMEEALAAAESKAASMEKVKNRMNEEVEDLLLDL
EKAQAQAANLEKKQKKVDQQINEWKLKCDEVQEELEKSQRDNRSYSTELLKMRTANEDTVEKY
ESLKKENRALAAELQSVSEQLSEGGKNSLEVEKLKRKLGLENEELEVALEEAELALEQEEAKFLKI
QLELTQLKQSTDRRSAEKDEELETLRKNHARQLEALQNTIDAEQKGKAEAQKLRKKLEGDLADL
ENRLEGSDRNSADYLKTIKKLQAQIKELQAMIDDESRGRDDMRDTASRSEKRAMDLAVQLDEAR
VALEQSDRLRKLAENEKLENAERVAELQTMYNSIANAKRKAEGDYHSLQEEIEELENEAKMAEE
KANRAMNECARLSSELNGAQENASNAEKSKSLMARQITELQQQLEEAESMGGKSLKNQIRKLE
QKVMEVEGELDNEARKSTESIKIARKADKRVKELQFQLEDEHKAVERAQEVSDKLNQKMKKMR
LQLEESEQQSSSWQSKFKKAVVDLEEAEERCESAEQALQKARQRARSGASGMTTRGVSRARSRVR
SPQNDD*
>TRINITY_DN5987_c0_g1_i4.p1 type:complete len:336 gc:universal TRINITY_DN5987_c0_g1_i4:136-1143(+)
MAKKNKVATVTDRETNTKPIVDQMKDTRNSTNSSKAKDNSKKNGGGGISWFNLFTFFIATSSLAV
NVALIRGLVPLPGCVGPQGKQGEQGLTGATGPAGKEGPQGVPGADGPRGFPGNDGMPGPEGKQ
GETGEQGPPGPQGVAGSKGDPGPQGKPGKQGEAGEQGPAGLQGAPGSKGEPGEIGEQGKQGPV

GPVGPKGERGNDGEQGLPGPVGPEGEPGPPGEQGPPGPAGLTGERGNQGEQGPAGPVGPKGDT
GKQGEQGPAGTDGQKGEIGNQGAQGPAGAVGPKGEIGNQGAQGPAGAVGPKGEIGNQGAQG
PAGVVGPKGNQGEQGAAGAS*
>TRINITY_DN360_c0_g1_i1.p1 type:5prime_partial len:476 gc:universal TRINITY_DN360_c0_g1_i1:2237-810(-)
FQELLNCKNSKQQTIRLPANRENLTMALPYSSAEDFYQSVVKRTPNEVEFHQAVKEVAFALWDFL
VKNPEYAKEGVLQRLTEPERTIMFRVPWVDDTGNIQVNRGYRCEFNSAIGPYKGGLRFHPTVNLG
ILKFLGFEQIFKNSLTSLPMGGGKGGSDFDPKGKSDAEVMRFCQSFMTELSRHIGPDTDVPAGDIG
VGGREIGYMFGQYKRLRNEFTGVLTGKGYNWGGSLIRPEATGYGCVYFTKEMLETKGHSFKDKIV
VISGSGNVSLFACEKCTELGAKVVTVSDSSGYVYDKDGIDKEKLAFLMDLKFTRRGRVIEYTQKYP
SATFHAGERPWGVKCDIALPSATQNEISGEEAATLANNGVLAVAEGANMPSTPEAIEVFLKHKVL
YGPGKAANAGGVAVSGLEMSQNSTRFQWSREEVDSKLHTIMVNIHKTCQKYGTDESGFTNYVIG
ANVGGFVKIADAMIAQGIV*
>TRINITY_DN761_c0_g1_i1.p1 type:complete len:830 gc:universal TRINITY_DN761_c0_g1_i1:129-2618(+)
MTSMFRSEEMTLCQLFLQSESAYACVSELGEIGVVEFRDLNPDVNAFQRKFVNEVRRCDEMERKL
RFMQKEIDKADIAIVDTGENPDAPHPREMIDLEAQFEQLENEMKDSNSNYEALQKSLLELLELRH
ILKKTQSFFEEEGYAETQARLQADHEPGVTGGGQLGFVAGVINRERIPTFERLLWRACRGNVFFK
QAEIDTALYDPGTGDLTHKCVFIVFFQGEQLKIRVKKICEGFRATLYPCPETPNERREMAIGVKTRIE
DLETVISQTQDHRHRLLQTIAKNIRVWLVKVKKIKAIYHTMNMFNLDVTQKCLIAECWAPVADL
DSIQAGLRRGTERSGAQVSSIVNRMSTKQDPPTFNRTNKFTKGFQAIIDAYGVASYQEVNPALYTVI
TFPFLFSVMFGDCGHGAIMALFALWLIMNERKIANWKAGGEMFDTVFQGRYIVFLMGLFSIYSGL
LYNDIFSKSLNIFGSAWVPTLNRTKYDYQPENYKEWSDGDRMLNPLDAPRETPYYFGIDPIWQLTS
NKLTFTNSLKMKLSVIFGVTHMIFGVILSLFNHLHFKQYVNIFCEFIPQLLFISCIFGYLVILIFYKWIA
FSIHSPSQPSILLGLLAMFLNFAKKKTDADRLYNGQDFLEPFLVVVAVICVPWMLLIKPFYLRWQH
NKKKQHGTNFARLHEDTAPLQDNEVNHHDDSGISSKNEEEEEFDFGEVFVHQCIHTIEYCLGCIS
NTASYLRLWALSLAHAQLSEVIWTMVFKIGLSMDGYVGSVAIFAVFAAWAILSVAILLIMEGLSAFL
HTLRLHWVEFNSKFYVGAGSKFVPFSFKDIMNDEAF*
>TRINITY_DN25375_c0_g1_i7.p1 type:complete len:811 gc:universal TRINITY_DN25375_c0_g1_i7:112-2544(+)
MNVNEKMNIYFCIGTLLFQSLLVLKVEGIFNDCQTKGSCGKCISSSPECAWCEDIIYKRSLNSKSYY
KKCDVLDNHKRQGCQNITNPPSSSTIDKDAELKGDTRVSPQEITVKLRPGQATTVDITVKTPKNFP
IDLYYLMDLSLSMKDDLEQIKDLGDILGEEMKNVTTNFRLGFGGFVDKPINPYTTIKPDEIEAYIRD
EDAQYTFTYKNYQPLDEDPSLFSKTLEQVNISFNIDGPESSLEALMQVIVCRNDIKWKDKRKARRI
VIITTDADFHYSGDGLLGGQVIPNDAQCYLSKGNYTAWNQFDYPSLSQIRSVMIENQIVPIFAITGN
FELYKKVAEFFGAGSGAVADRLSKNSDNVVPLIKEAYEKIAKIVNINSDAPKGIDMKYTAICGTGK
NVEAREGTDFCDDVEIGETVVFKVEMTAKDCELVGEDLKFKIKTNFDDVNVAVKLLCECPCEQD
KSKIVIDSEHCNSRGNLTCGQCQCDLDFYRGRFCQCTVEEAKNQTLCRLPGEVGPVCSDQGFCEC
GECICDEPKLNRFGEIEYIYGDQCQCSNTSCGRTTNENLLCGGPSHGRCDCGECTCFSDWQGPTC
EVECVKSSAACIDEEGIECSGKGKCVCNRCVCDPGYIGDLCQECVSNCPDPCQDYRECVKCRVFK
MSDLSDEECKSECLKHNITSHIVTDKVPEDKRCIARDEDDCSFVFTFVKSNITDEVQLIVQQEKICP
AEPDILAIILGVIAGIVGIGLALLLIWKLLATIQDRREFAQFQKDRQDPKWDSGVNPIYQQATSTFQ
NPLYGNKGEETEKILDSQ*
>TRINITY_DN141338_c2_g1_i1.p1 type:complete len:720 gc:universal TRINITY_DN141338_c2_g1_i1:2850-691(-)
MSEADETETFAFQAEIAQLMSLIINTFYSNKEIFLRELISNSSDALDKIRYQSLTDSGVLESNKDMKIE
IIANKDEKTLTLFDTGIGMTKADLVNNLGTIAKSGTKAFMEALTAGADISMIGQFGVGFYSAYLVA
DKVEVVTKNNDDEQYIWCSSAGGSFTVKRDTINEPIGRGTKLVLYMKEDQLDFLDEKKLKDIIKK
HSQFIGYPIYLETQKTRDKEVSDDEADEEESTDKKEDEENEDKEDDGPKIEDVGDEEKEKEKKKKT
IKENYTEMEQINKTKPLWTRNPDDISTEEYAEFYKSLTNDWEEHLAVKHFSVEGQLEFRAILFVPK
RAPFDLFENKKQKNSIKLYVRRVFIMDNCEELMPEWLNFVKGVVDSEDLPLNISRETLQQSKILKV
IRKNLVKKCLELFAEISEDKDNYKKFYEQFSKNVKLGIHEDTQNRNKVAELLRYSSSASGDELTSFK

DYVSRMKENQKDIYYITGESKEIVAASAFVERVKKKGFEVLYLVDPIDEYAVQQLKEYDGKKLVCV
TKEGLELPEDEDEKKRQEELKAQFEDLCKVIKDILEKKVEKVVVSNRLVDSPCCIVTSTYGWSAN
MERIMKAQALRDTSTMGYMAAKKHLEINPEHQIMIALKKKIEADKNDKSIKDLIILLYETSLLSSG
FSLEDPQNHSSRIHRMIKLGLGVDDDGDVEEIETDDLPPLEGEGDDDEDKARMEEVD*
>TRINITY_DN11096_c0_g1_i1.p1 type:complete len:557 gc:universal TRINITY_DN11096_c0_g1_i1:1858-188(-)
MLGDTEHFIELIRSMPSSVCILIVIPLVLLSAPTYSSHDFPVFRMQQYDLHGIKYGSRGAAVNLEARS
LTAKNLIRKCAIIKVTEFSMEVLQKALDNGLSALLIVLPPSLENLSDEQVKLLQNVEGDLMNAELII
PVYFAHETKRLRTLYEEITSAINGDAASSALKAMATVASANSFHFVTDCSESKAITDFPIVSFQGILS
GQGLEDQLPTVAVVAHYDSYGVVPTLAKGLDSSGSGVIALMEIARVFSMLYEKLASQPKYNILFLL
AGGGKFNFQGTKKWIEDSVESAEISLLAEADYVLCIDSIGNGNNLNLHVSKPPKEGAQAYTLVEH
LKEVSSKLYPDSSFNVIHKKVNLADEMLAWEHERFSLRRLPAGSLSYLDKPENTNRGSIFDRNININ
SLERNIKIVAEGLARHIFNVSSNNYGERFEIFTGEFSLDINHMKSWMDHLIQEPRSQQLISKDHKILS
GFEEVLSGYLKDVKRVVARAEKKDPEFVFYDVFNAKMNVYSVKPALFDLFLAIGIASYLALVYLVI
ENFNQFTEMLPKPVMNGKGVH*
>TRINITY_DN64_c0_g1_i1.p1 type:complete len:468 gc:universal TRINITY_DN64_c0_g1_i1:1555-152(-)
MPPKEAEKIHINIVVIGHVDSGKSTSTGHLIYKCGGIDKRAIEKFEKEAQEMGKGSFKYAWVLDKL
KAERERGITIDIALWKFKTGKYYVTIIDAPGHRDFIKNMITGTSQADCAVLIVASSTGEFEAGISKNG
QTREHALLAYTLGVKQLIVGVNKIDNTEPPYSEKRFDEIVKEVKTYVKKVGYDPKTVAFVPISGWH
GDNMLEASKNMGWYKGWTTERKETKTTTGKTLLEALDNILPPKRPTDRPLRLPLQDVYKIGGIG
TVPVGRVETGIIKPGMVVTFAPSNITTEVKSVEMHHEALLEAVPGDNVGFNIKNVSVKDIKRGNV
ASDSKNDPAKEAKSFYAQVIILNHPGEIHSGYQPVLDCHTAHVACKFSEIKQKIDRRSGKVLEEMP
KMVKSGDAAMIDLTPSKPMCVEAFSEYAPLGRFAVRDMRQTVAVGVIKSVTKSDGSGGKMTKSA
QKATGKKK*
>TRINITY_DN15151_c0_g1_i3.p1 type:internal len:560 gc:universal TRINITY_DN15151_c0_g1_i3:1-1677(+)
LDGAERPRILAAMLSTLRRAPRLAARAPLAAPAAARAFAAAKDIRHGSEARALMLEGCNRLAD
AVAVTMGPKGRNVVIEQPFGAPKVTKDGVTVAKSIDLKAAPMINVGAQLVKQVASKTNDVAGD
GTTTATVLARAIFREGCKAVAAGMNPMDIKRGMDAAAKVVLEDLAAQATTIESPTSIRNVATIAA
NGDAQIGEMITDAFEKVGKDGTITVQDGKTLEHELEVVEGMKLDRGFISPYFITNVKAQKVELEN
PLVLLYEKKISSVQAILPVLEQVAKVQKPLLLIAEDIDGEALAMLIVNKLRGGLKVAAVKAPGFGD
NRKEILKDLAVLTGAELLSDELGDKLEDLQITQLGTVGSATITKDDTVLLDGSGNKSAIDERCEAIR
DAIELSTSEYEKDKLKERLAKLSGGVAVIKVGGASEVEVNEVKDRLNDALCATKAALEEGIVPGG
GTALLYSAQKLEALTLDSLDQQVGVDAIKAALSQPCVQIAQNAGEEGAVVVRALLQEGNAARGF
NAATGQYVDMVEAGIIDPTKVVRTALADAVSVASLMTTTEA
>TRINITY_DN7102_c0_g1_i1.p1 type:5prime_partial len:1263 gc:universal TRINITY_DN7102_c0_g1_i1:3-3791(+)
DKNELEEKVLPAVSVGTLFQYATGLDWFFMILGTFAAIGNGVAQPISFIVFGELINKFIISDSGEAIDL
EEEMTKFALYYVYIAIATIVLGYIQNTVWLLSSIRQAFEIRTKCFNSVLRQDIGWFDVIDAGEISTRM
TDDITKIQNGIGDKVGSLIQGISMLLGGFIIGFIFSYKLTLVIVAMTPLILITGFLTGKVVALITSDEQLS
YAKAGAIAEEVISSIRTVVAFGGEEKEIARYRTNLVDAEKSGVKKGISTGFAFGMFNAVIFGCCALG
FWYGGYLVAEDEITGGDLLIVFFSVLIGAAQLGQVGPNFEAFAVARGSARFIYDVIERVPAIDSMSD
SGLKPEIIGNVIFEDCEFEYPSRQEVKILKGLNLTVNCGQTVALVGESGCGKSTTIKLVQRFYDVSKG
KVLIDNTDIKEINVRHLRRHIGVVSQEPVLFDTTIADNIRMGNDEATLEMIEKAAVNANAHDFIKS
LPKGYETYVGEGGTQLSGGQKQRIAIARALVRNPKILLLDEATSALDTESEGIVQSALDKASEGRTT
IVIAHRLSTVKKADKIAVVVEGKIAEIGTHEELMEKHGEYFKLVMLQTFEEQEEDEGGDDTSLLSES
DREGLLRKQVLRTLSRMSSHDAEEDKKIEEKITKEFRRRSTFRKSAKDIRSLERQKSKINEEETDEDV
NIPSMWRILKLNLKEWPYMFVGFIFSALTGAVPVVFAVIMSEVLESFILTEQPQKMKDTSAFWSIMF
MVLGNVVGVSYFVAQYMFATAGEALTTRLREMAFRSLLRQEIGYFDLPQHNTGALTSRLASDASA
VQGATSTRLNTLTQVLVMGLSSLIIAFVFSWKLTLVILAFVPFLAFGGAVQTKLNTSFATDEQKKMG
EAGSIATESIMQIRTVASLGKEDYFYQRFLKLLLKPSQESRRSAHLFGLSFGFSMAIMFFNNAAAFR

MGGYLNVNDGLSLADMIKVIISIQFGSMTAGQIASFAPDYAKAKVAAGRILDLFDRVPPIDSYSQE
GIKDSLEGHVEFKDVKFHYPNRPSVPVLQGLDINIKKGQMLALVGSSGCGKSTSVSLLERFYDIYE
GALTLDGKAIKSFNVRHLRSELGIVQQEPTLFDCSIRDNILYGVDTSVRKVSQEEIENVCKSSNIHH
FITTLPQGYDTNVGDKGALISGGQKQRIAIARALIRNPKILLLDEATSALDSESEKVVQDALDVAM
KGRTAIVIAHRLSTIQQADLIAVIQSGKVVEMGTHQELLSKKGAYHILNQAQL*
>TRINITY_DN34481_c0_g1_i1.p1 type:complete len:802 gc:universal TRINITY_DN34481_c0_g1_i1:2487-82(-)
MTTPHMSPQEEQEKLLEEALQIVKNQAFQMKRCLDKAKIMDALKHASNMLGELRTSLLSPKSYY
ELYMGVSDELRHLETFLLDEFDKGRKVNDLFELVQYAGNIVPRLYLLITVGIVYIKVGDVPKKDIIK
DLVEMCRGVQHPLRGLFLRNYLLQSTRGQLPDSETEESEGTIKDSIDFILLNFAEMNKLWVRMQH
QGHSKYKEKRERERQELRILVGTNLVRLSQLEGIDVETYKKDVLPGILEQCICCRDPIAQEYLMECII
QVFPDEFHLQTLNAFLKACAELHQDVNVKNIIIALIDRLAMFAHRGDGPGIPNDIRLFEIFSQQVA
HVIQSRSDMPTEDVVALQVSLINLALKCYPDRVDYVDKVLEYTEEIFSKLNLAYIDKNNAVSKELM
RLLRIPVDIYNNVLTILKLKHFAPLFDYFDYATRKEMAMYVINNALDNETLIPSQEEVDSVLQLAA
PLIMDQDDQPTEPEDPEDFAEEQGLMGRFVQLLYSDNPDQQYLILTTARKHFGNGGEQRIKLTLP
PLVFAAFRLAFIYRDAQEQDDKWDKKCQKIFQFCHQTICALSKAEYADLSLRLFLQGALAADQQ
KFTNAETVAYEFMSQAFAIYEDEISDSKAQLAAITLIIGTFEKTSCFTEENHEPLRTQCALAASKLLK
KPDQCRGVSVCSHLFWSGRVNEGESEEKDAASNECRDGKRVMECLKKSLRIANQCMDPTVQVQ
LFVEILNRYLYYFERGNEAVNSTILNQLIDKIREDLPSLEVNDEMEQINKHFQNTLSHIQSKKDSAD
ENEAAIYKDFIL*
>TRINITY_DN118520_c0_g1_i1.p1 type:complete len:581 gc:universal TRINITY_DN118520_c0_g1_i1:1895-153(-)
MYRLLSVIRKTTQTAKLRQGLSACISTTTHYCSPKEIKTGTDARALMLKGVDQLADTVAVTLGPK
GKNVMIEQSFGGPKITKDGVTVAKAIELPDKYMNIGAKLVQDVANNTNEEAGDGTTTATVLARA
IARSGFEAVSKGANPNEVRKGVMAAVEVVTQGLKDLARPVTTTEEIAQVASISANNDAAIGKLIS
DAMEKVGKDGVITVKDGKTVYDELEVIEGMKFDRGYISPYFINTAKGQKVEYQNVLVLICQSKIS
NVQDIVPVLELANQHKRPLLIIAEDVDGEALTALVINRLKIGLQVVAVKAPGFGDNRKNTLKDVA
ISTGAEVFGDDLNLKLDEVQLSDLGQIGELVVTKDDCLMMKGRGNTDLVNQRANHIHDEIKATN
SDYEREKFQERLAKLVGGVAVIKVGGSSEVEVNEKKDRVTDALNATRAAAAEGIVPGGGTALIRC
AKLLDTFQVPTEDQKWGVDIIRAALFKPCSTIVANTGIESAVVVEKVSSSKDPEYGYDAASGEFAN
LIKAGIIDPVKVVRIALSDAAGVASLLTTAEAVITEIPKEDPSMPPMGGGMGGMGGMGGMM*
>TRINITY_DN3271_c0_g1_i1.p1 type:complete len:399 gc:universal TRINITY_DN3271_c0_g1_i1:218-1414(+)
MTRIIWHKIKSELIMPYLDLDCKYYDLGLPYRDETNDQVTVDAAEAILKYNVGIKCATITPDEERV
EEFKLKYMWRSPNGTIRNILGGTVFREPILCDNIPRLVPGWTSPIVIGRHAHGDQYKAQDLVAPGP
GKFEMSFTPLEGSEKVTVNVFEFKENGGCMMGMYNTDDSIKDFAHSCFNYAIGKKWPLYMSTKN
TILKKYDGRFKDIFEEIFQDQYKSQFDALGIWYEHRLIDDMVAQCLKSSGAFVWACKNYDGDVQS
DTLAQGFGSLGLMTSVLLCPDGKTIESEAAHGTVTRHYREHQKGNATSTNPIASIYAWTRGLAHR
AKLDGNPELEKFSNSLELACVKAVEEGNMTKDLAGCIYGLKNVKREHYLLTNEYLDAVRDKLLQ
LWTPPSQV*
>TRINITY_DN1043_c0_g1_i2.p1 type:complete len:700 gc:universal TRINITY_DN1043_c0_g1_i2:2410-311(-)
MKESYLKRFALLFSVLILFQEGGALLRENLIVNHRLRRNVYSEEKVTAVDLPQSREKRFVGTALIIIS
AASAGLDIYKQVRGCKDITPDLDDKIAEGKALQQSLLNLRDQVNLAWVPAESREEYTRRLASNM
IIIREQVSTFDSLQADLVKLLDQKLLDKLNNVTKHLENQIDRNNETEIGSFEDLEGKYMDTTTFALT
LTAKLLPVGFLLGTKFLLPKITARFSKGVAKAFAADNPSRVQKMLSSFAEKQGKLANWKAEKINSF
KVSLNSKWNTFKLKFPRITAAMNRMSNSKIGKALGKAMGKVKGIAVGVGKGAVVAFFIAFEVW
NFVQFHGACTEKRNKAIEGIATMNANKKNMTDLLTEIHDMKAKADEYYNLMKSNVTNDEFLN
FLEDIADLGENAKEQTKVIVDATNQIKQYKAEIKNADDSRTDALNTMLVTAFSNINFKLDCLRSK
LAIIKGVVDDCGIGLGVYDDIYARALAKQKKITETCIEGSYMDKNEIKGLMVAAMDLDKKQSICV
RNNPTKKQLVCEKWYDDVSDADILAANPDLTKDDVTYFISTCPPAPVNDQTKTTICVKKNNELA

TLQSIQQTYQKYDQTAINSAYEECQLSSISVTTICTAKKEGQTVVYAQQKFPVFPASMVTNVYNSC
KKETNITPADKMMICLLKKNGFALDSPVMANYVKNYDYATLEAFYNQCS*
>TRINITY_DN31399_c0_g1_i2.p1 type:complete len:819 gc:universal
TRINITY_DN31399_c0_g1_i2:85-2541(+)
MAVNVSHVYTCPSDYLCSKAEQGTLLIPLINESEWGRGFRIFLYTLALLWCFMGIAIAADLFMCAI
EIITSKTKKIKVATEGDDKMGYEEIEIRVWNDTVANLTLMALGSSAPEILLSIIEIVGNNFESGELGPG
TIVGSAAFNLLVITACCVMAIPGGESKRINAFNVFLTTSFFAVFAYIWMFIVLTVSSKGILEVWEAAV
TLLLFGILVILAYIADKDLCVSVFTRKRRKKDIELDNFDLLNENVLKKHHPDLIEYMKEITANPSISE
QEAAKLIAIKIEASKRKNYGYYRVGALRSIGGGRKVVPAVTPKLQGIIDGGSTLNVNAETNVNDSV
PDNKATIEFTATAVSVMENNKVVQLGVERSGNMSSQVSVNFETIDGTAEAGSDYIAAKDTLVFEID
EIRKSIEIVIIDDYEWEPDETFFVKLHVPEGANDRVQIGHHSICEVTIINDDEPGTFSFNKPSNIVKES
CGQAELEVERVNGADGKVEVVWKTKDQSALHGKDFIGGEGTLVFEHGETLKSISIEIIDDHMFEK
DETFMVELLEVKNEGAKFGRLKRTVVTIVSDDEYQHMFDRVVNMAHMNLHQFQLGSSTWGQQ
FREAMNVNGGDLESASFMDYVMHFITFFWKILFALVPPCTWYGGWLSFFIILGMIGCLTAVVGDIA
TIFGCLVGLRKDVTAITFVALGTSLPDLFASRNAAMNEKYADASIGNVTGSNGVNVFLGLGIPWLI
ASIYHEAKGKVFKTPTGSLATSVVTYSVCAIACILLLIIRRFVPFFGGGELGGPKGPMYASGIFMISL
WFIYVLISALVSYGHIAAL*
>TRINITY_DN33065_c0_g2_i1.p1 type:3prime_partial len:804 gc:universal
TRINITY_DN33065_c0_g2_i1:2409-1(-)
MWQFVWLAVAITSQINGNVHHKHTKPRHQNLIDDIVPNDDFDLDYSHNIFIPFDAYDNYEKFLE
YAERSDLAKRGGVTSLPAIQSLPGDGSHTYAAPGVNCQVQLLNKKVEGTDKRVIERTSCAEGWTL
KNALGFFFAGVGHDLATKSAGLVHMNQLRFKMLIFEAEGYTFVADTDNEVYSNLQSKLSLKNAE
CRLTISNSYTFENSEIVLTGNHEVIEKITNAKKTLEVTFEKGKDTSSFDVKMKVPSMPLETFATIFLES
ETEFITADMGDTSYEINIDPIKAVIIESTSNSVTGVFDGSDGKFQLTGKFLSNDQNAFMGQATDIYLT
VNRKSLGSRTTYNLMLGFAKEVKVMDIIPKYVGKTEIQDEFDDNFLKTTIEKWHLLFTSKRIIVAD
GAEYKKTFANIFDADEYQEFKQGVGIKATVDLQKTLTACSSGSTVPDSLKSVKIKGLLGRRGLSMV
FPPGTSVPLIPLIKCKLRQIQDDLGFELPISLKGYEQKSKFKLEEIAYSSDSGAFKMSLLYENTITIYKD
VLEITNPQLTIEREGKDEPWQFTIAADVSIFQVDASVMISYSKTGYTVEVSSGNVPLTNLLRAFSFSD
KPAITFEIHDFKMQIEVKDKEEEEENEEEGEDEGNKNQLAIKRQDNKEEKKRDLKFSIAGKPVFN
GFDKVSIEALVWTSQTPAGVAVAVIMENIRLDEVLEKILDVPEVPMAWFSDMTLTMVISNVDAEG
CDNPAQSEDDCEEADEEEEEEEEGEGEEEEGEEGEKTQEGEEGEKGEEGGGGGPGGGPGGGGRG
PGGGRPRRG
>TRINITY_DN984_c0_g1_i1.p1 type:complete len:1485 gc:universal
TRINITY_DN984_c0_g1_i1:4558-104(-)
MVKSSWQQLLLLLILFCGFQSFEIDGEDKPGSCPPPLETLECNEVEPGELSCLTDSECSGTDKCCSD
GCNLICAAALPEIKGEMGAKGEPGPPGRNGTTGPQGPKGGAGIKGKKGKQGKQGNKGRRGRP
GPPGPPANDTEPGPDPLTEDDHGKGPGPGGILEDDLAEGGVAPGVLTTQDAPEGEKGARGRAGP
RGPYGERGNAGPIGDAGEVGDKGEQGPKGQPGPPGDDGQDGDRGAPGPIGEPGERGERGEIGK
QGRPGIGGYKGDKGHPGLAGPKGVGGEEGLKGDTGESGPVGEGGPAGPRGMPGKTGNQGPPG
PTGPTGMSGPQGLPGSAGLTGAPGSPGDQGPQGPKGDMGPVGQRGERGEQGERGLTGDNGPK
GERGLTGTAGRPGRDGAQGQTGTEGRPGLIGKRGPIGQPGSQGSEGPIGLPGKRGESGDIGLQGP
DGDKGQIGDSGAPGQTGGAGLPGRRGIPGREGPTGPQGETGRRGPPGLLGTPGSPGEMGSKGLR
GERGAPGVIGGKGATGAPGSAGETGAPGDRGLQGLQGKTGRPGKTGSSGIPGKDGRPGSAGPQ
GLRGVRGAVGLQGIKGSAGSNGLVGENGALGKKGDTGDPGLKGSPGAVGAPGKDGNVGERGV
PGAAGERGAQGPIGPIGQAGPPGKDGEPGKTGERGSDGKLGPQGLRGAPGLRGPNGNPGVAGQ
VGERGEIGADGAAGSKGPNGMLGTIGNPGAPGLAGSFGQKGPDGQKGAVGERGPKGFRGEDG
RDGERGGDGIAGPSGPQGVSGVKGEMGRTGANGIDGKRGLPGLMGPPGSTGPPGDEGAQGQD
GAKGIKGAMGPRGDKGPAGSIGEPGIDGGAGPIGSLGGSGPKGGRGSIGPSGEQGAQGLPGPTGR
RGVQGNRGSIGPVGLEGREGVKGSSGEPGMPGRTGENGVPGMPGKDGRNGDPGEDGIPGPMG
KPGMEGARGPRGQLGPGGLPGMPGLQGIQGVKGEPGLDGGDGLKGRKGSTGMQGVMGSSGLP
GKPGDDGAAGEDGIPGTPGDRGAKGDIGDVGFLGPIGPPGSPGFSGDIGEKGMIGAPGEQGNDG
EPGNPGEPGYRGLIGNPGAKGNKGGKGEVGDRGHKGYRGMRGARGPTGNVGDQGDDGLDGL

KGAKGPPGLPGPRGRTGREGPSGPIGDPGINGTKGFVGIRGNPGEDGSPGPAGPPGPTGNPGFPG
GLFGGALNYYPDQQQAEKGPLYGYHYGYNYRYYKSRREEDTDKEEIKAKNKKANRNVFRYAEE
LKMRMEAEIKPDGSEEFPAKTCKDIQMCFPDAKSGSYWLDTNGGSKTDAYVATCNFTDDVTETCI
APTVSSFDRKRWVTTGGKSQFTWFMKDLLEIDEGLTYTASVTQLKSLRLASTKMRQNVTYHCKNS
YAHHDQLENTAKTFVKLMSDDEDELHALGGRKERLDVLSDKCILKDDVWRKAVFEFSTKRTSKL
PIRDIAVFDVYDENQEFGIDIGPICFM*
>TRINITY_DN598_c1_g1_i2.p1 type:5prime_partial len:742 gc:universal TRINITY_DN598_c1_g1_i2:2-2227(+)
RWSGCSKSCGVGVKTKSRECDNPRPSGGGRYCTGERTATEACKIQDCGVDGKFSHWSTWTACSK
TCGGGAQKRARQCNSPPPSGDGKDCAGAREEFKGCSNDFCKGPTGVAKAQEIIERFLENFHKKT
YPDREPSGKFLKAIFAIQKAVAEEFISVDPSVRGKTLEQRLKDLEKFLKVATEGGSIDFARTLLGKGIF
TFMKKKIQEGLPIDDIDKAAEGFLEKLRGDVGEKKMNEILSSQGDATLMFTIDTTGSMKDEINAA
KAIAKQIISATRDYEVDYILSPFNDPRTGPVTHRSSSNRASFVTDIDRLRATGGGDCPELSFTGMLN
ALNQGPQYGSPMFVFTDASAKDDSASNIDSLKAAADLTDATINFFTNLNGCKGAKSVESYKEIAS
YTGGQVFPLKNEAELKKFTNYVKTSLQQDTVITSGGTLNSKSYKRSLYSMQAHDFMVDDGIIMIS
VAISVQKYASQVTILDPNGRRVNPSTTLSLSLVYQISHPPPGQWKINFPSSVGKYGYTVQAVSSSAIA
FAHNFIYQQSVRKNSPPIPLANPLKGVKNMMVVRLGGTKNFDLNSIKIDLVDTSGNVLQKGIGTD
RLDSDEVRHATIDPPSGPFKVRLTGKTKSGYNFERVSQGISRPVSAAMRTNFAGYDFTAKLGKGPY
AAQMLFINRGAHENFYFYAKANYGSIPNYKQTKRVTSQGFVTFTYKAPRQYNLKGKSDRITVTVK
RQSTGEIVSHHTSILLVV*
>TRINITY_DN22240_c0_g1_i1.p1 type:complete len:513 gc:universal TRINITY_DN22240_c0_g1_i1:155-1693(+)
MFRYKEKDDNKILQAYIKYLADVVLTLDHTKMTSKEIFSLANETFQFERELYKIHIPPEKLTTIDQTY
HKATLSYLQELVPSVDWVPYINHKLKRANKSVNKDEPILIDTLEYFKKLDEVLKKTPKRIMKNYLL
FRTADFYSVLLSDEFIKLKDPLYLAKFGVYKEPPRWKRCLYATEAKMGMALSSLYVKKKFTSGSYEE
ANKMVKAIREAFVSGFPDLKWMDKKTLEVAQEKANFIEQKIGYPKYILDSVKLDEKYKGLEFDG
GFFDNDFKGDEFIVANRYKHLHEPVNRDEWELTPMTVNAYYNPSENQIVFTAAIMQPPFFKFDYP
KSVNYGSMGAIIGHEITHGFDDNGRQYDKNGNFVSWWTDKSVKGFKELSKCFVEEYSKFTEAGL
HIKGNVTFGENLADTGGLKAAYKAYKRWVEENGEESLLPGLDKSNDEIFFISYSQSWCTEELDSRII
TQINTDVHSPAIERVRGALYNNEDFARIFKCPVGSPMNPKTTCSIWTDNSH*
>TRINITY_DN123_c0_g1_i1.p1 type:5prime_partial len:1231 gc:universal TRINITY_DN123_c0_g1_i1:2-3694(+)
RGGFRPFGTGLMGSPMLGPYAFGPARSGALGGFGIAPLVRPPTRGGAAGANRGSRSNSNRGGKK
TTPVKNNKSTPEAKKDNNINAKNDKKEDKKDEKKDDKKDEKKDDKKDDKKGGKTPTGKEIKV
EPKDDMKNIKKNFFKDPKVEPKNEKKPDTKAVTVKPEIKKESGPQNVTLDKFNSELHLSIDADGL
SAGNMKGEGFQYMWGGVRGTHGVKKGKVCYEVYLLENIKVHCPGENNPHVLRVGWSVDNSD
YQLGEDKLSWGYGGTAKSSYDSKFLDYGKKYTVGDTITCYIDFESNPKAIFFMLNGEYLGVAFRFG
NELGDKAVFPHITSKNMRFRVNFGKEKPKHHVTNGFRMMQDIPVDQLVLPSPSPKTPKEAEIIM
MVGLPACGKSFWCEKYAKENPDKKYYILGTNNIIERMKVSGLTRKRNYHGRWEMLIKRASLVLN
KLFTVAKNNPRNYVLDQTNVYFTARRRKMESFAGYKKIAAVIINKPDVLASRTEKQQKIEGKMVP
EGAVMEMKFNFTLPEKGPSFNEVWYLEETLPKAQELAANYRREGSNFKTKSKRKPESSPSQPESSP
SSEKKPKLDPKIISRGFDKPFSKPNTNSPQQSTFKAPSPAQNNANKTPNQDRFSKNEKSTSKVETNS
DNSRSKAGNFNKGHDGNKSNERPFFSNTPDRGNASNRGNDSNRGNSSDRGNAPNKGNSSDRG
NAPNKGNFADKGNAPNKGNSSDRGNAPNKGNASDSANKNSKNEKPKEESKKTPKRAIRGTLD
QLNSIINSEARKTENNSGNIRDSAQSGKNEKMGSLMDIDRTPQRESDSYQNLQSRNRDALSNQG
GKNKESQSAGLMDIRNVPQGNQDYRGKAGILDSSRNTSQDYRGKPGILDSSRNTPQDYRGKPGIL
DSSRNTARRNEYESPVGRGNNSYSDSPARGVSTPRGRASFNDSPARGARGGRGQSDISRSGSLAKS
QGSGGNRGGRGRGRSKHLGDVNKYDPVGNMGGYYSALKALPSVKDLIGNRSSFSSYSTRIDEPSR
FDQLYSSYEPYSFGRTQNANDSRSLNYSFGQTDSYSSLERDGGYRDSQNSKDFLGSYQQRKDSSYS
DNRGSQGGQRGYQDSSYNADGLDAKDQYQNDDYQESYSSQKDKSSYDQNTQSSRSYNQETSYE
ESSDYGRSRNQEQDEYARSGTYERGDTSYGTDDANRSSRRYEDDLQESEPMDGNYSTQDYGRSSQ
SSQRARIW*

>TRINITY_DN2357_c1_g1_i1.p1 type:complete len:1733 gc:universal TRINITY_DN2357_c1_g1_i1:5452-254(-)
MLKLFLFLIGAGHLSLAFAAICSETNGEGNKKNLKEPCFFNQLIDVKQWNMKRFMDQTDRFFDF
GVMRCSSYEVEGSGKMKLTCYHTKLFYIMKNIMSFGTTERTEFIFKFSPLTENDYKSTDVEISGKLS
QALRNTFIRATKPEGFQDFSLSTSAVFPQIDMETFIDQRKPDGFKTLTNNDKLENFPPKVAVNAEM
SGMFSALVTDTDKQAYEFLISFLGPMKEQQFYIFQSLTENINDELVIAFYKENPSNAGTTVRDIMDS
LRGVKWRGNKANEFVVSKDFAPIFEVQSSAMSISDGEIVNINSEALRNIAIKYVSPSSEAFILEGALI
YWHVDDSSSLKMSADQVPGVENPPTPTNETSTVPQENKIKRHTKPKTMKPLKREKHPATNKLTL
NAHIIHQNKIEIRSPDYLKGTYEKQLSTFLPYHYFKHDSIKNTLYPVKMIFDKITIGFKRKELRLYCNI
TQKYDLVQNFIEGEGIAVYAKAKLTMRKDADGDSEEIRFKDIGMSGTIEVLQGLVNVNLQYAKDG
TLYVVGLFDIANMTQFINATGMKHYLPENLRKTYPRLTNFAITRLKIKSKLNENVLRIIGRCLIFSEP
KTDDNLEAEKRDGIEDESPASLLYESKLRVVLTNTSGDLKYSVGIIGEDIPLQVAMRELFNATITGTS
WVKFKFFVMVMSNNNEINVAQAIDERLFNSFNSETGLQIKTVLLPPVEEDCKMSRKEKLDPLNKE
ALVKNEANGKETVEIHRSPFIVDYTCMFVKDQELLMENFEYATATVTPTSYEIKSLTQERFFDFQSD
PVVSVGNFGYTIKSVKGWDSIFFIEGEFRFMVKNDRLIFKGLITFDDMKGKLVLQLKQLHSGRQNF
GDAALINSGYSVIGKDDTFSKFHFDAFLYVGTVKTKLVNFRKNVEVIYDLRTDDIFMKGARNNNG
NIGATNKKRNSLLELFDAFGTDASSHVLLKELHAISLTWLIYQQNEEVVKTKHGIRRIQPGLNFYG
TLAGKGDRDLLFNMHYNKGMGALIADIELPNYKLPLRKDAKYHPFELQRSTKKQDLGGILFVKA
STTFTSAQAQGYLRVVGLEYDVEFPIMADGQFHMDLPYSNEIAKLFNEAEVRYRVSQDIGVTDFN
LVDPKIKIDLAPSLEKISRAIRHAIEAMIANVDEYLVNSGSFINFVTIVDGEISTMKEVINEIRRGMGK
LQSTLKQLVQQISSDCAEQCKDSKVFIYGYDFQGGKFVGNEDAHDVANVTCIAECEMIKPVKND
ERRSLLSSLKEALVQFKREKKRASIMQIMLDEHYNSLSKMNDAMHKNARAASLILSKTGNLKNP
KISVASLVLENQISVINFKGAGSKCLSGFKMAFSYNDVVGETDIDLCFNGEFDQFAAYAFVLSYYGE
DKYNQFGDLLDDAAELKQSLKTKTKIVTKLFKKIANARTTVELEEVQNEVEETILDYMDGLKKSQ
IPKHKIKAKKTKEAIGYITYGGKNVTLPGIDTEVLEALEIDQDYTIKNPKSKRVFMYSSPWAALNVL
HLHQNKSMESLPMITSIDNAGYCKRLTRLSSVLNSVRGSLSSYAASYVNAKKNFENVETNMKSGL
VELKKMHGSSIMHDDDKGNLQYWLTQYEKGVNRFSKLSNMKLHDHNNHGLARWKNEMDNS
LQHSGLGSIENVIEMLKKRSSSKRSNIDHDRWIRNASLMGKEISNLMKNSNKMRMADAELTVRS
MSQKFDNFFEALTKSCKASFKQF*
>TRINITY_DN495_c0_g1_i5.p1 type:3prime_partial len:407 gc:universal TRINITY_DN495_c0_g1_i5:152-1369(+)
MDFHWNTVIAGLIIVQVYSLAEYGTLNGKDSYRNAYPWLANDRNNNELGREDARSIYRSSDEID
AEYPQSDDEVDTNDIPKSYTSRDELDAEFAQRTYKFADDLDIENAQKSYASSDELDRDDEQRTNK
NTDDLDAEDAEREETINDSLDAENAQRRYTSIDELDGEDTQRVISKTITLPRLGAGRIYKSRFIHGP
YIRPKFVRPHKGIRLPHPNRIIIPGSWKRPAHGIFALKGKKAIFCEHKGIKRISCSNDKVIKVRSALY
GRTAGNVCSISCSKDEEIKVKNALYGRTAGDVCGRSRVTNCRALNSFAIVQKKCNGHNSCDLNPS
NSVFGDPCVGTVKYLNIKYFCVKRITPTTTPNGKTAIFCEHKGIKSISCSKDEEIKVKNALYGRTAG
DVCGRSRVTN
>TRINITY_DN135129_c0_g1_i1.p1 type:internal len:155 gc:universal TRINITY_DN135129_c0_g1_i1:1-462(+)
SSFSYMSTSPSRSMDISASSDEALTPGGTLIIRDNVDNRTSFYERLQEKDSFIAETSSALNTPQNSPRP
STTTPLPRTPQQDRTPPKRSPLVRMKSDSSDIEKRAKFSKDNVSSTFRPVSMLNLSVFASNRPKRLH
TGSESELSEHVPLSTHRR
>TRINITY_DN30613_c0_g1_i1.p1 type:complete len:975 gc:universal TRINITY_DN30613_c0_g1_i1:154-3078(+)
MPVFHTKTIEAILEPVAQQVSQLVVLHEEGEGGNAIPDLSGPVNAVSAAVSNLALVAKQTAASSK
DEILRKEMPPAYSKVEESSNQLVEASSLLKDDPFSSAGRKSLINGARGILNATSALLLAFDDGEVRKI
LNVCRQVLDYIQVAEVVEVLPDCVTFLKNLSPGLLSMAQKVDNRIPDLTHQTHCELLQKHIKSTK
ANAPLLINAMKAFIVANNKGVGRAEAQENRNFVISKLCDDIIEIMRVLQLKVDDEYDLEDPCNL
MRKNRHKIMEKMKMAKDWLGNPEADPNGTGERALKDMIKQARHVGALSNDPQIKNLCDELD
GMLDRLSQLRAQGLGNSPEAIQLAADIDKKLETLAKLVDDAIAREAAAGTRLPAVTTEGQYDQA
MRWIDDPRHDPSSVGKRAVDALLADGRRLAEQLMGPEKQELVGTINNVEKLSRQLQDLKAQGK

GNTPEAQRIAAQLKKELEKLKGLLQSGATKSVLDAFLDTSTPIKQLEKAAKAPEDAPNREQEFEE
KAGAFEYHTSKMIDAAEQTSIHGKNVSKQLVDNIQAAAKDLAELTPQVSHAGKLLLLNPRDKAV
ENHFNDLKQGWINKAEEVTALVDTATDTLDFVKASEESMKKEQAGALDGIDQMNLAMVVGKG
GNMARMANRVVQVAKGEADNSEDPEFKQAVSVAATELDKGIQPLVLSTKAVAMNPKMPPVRVE
FKATSDGLIGRVVDVRKAIEPEEVPPPLPPPPQLEEEPLPPPPPEDAFAKLQLKAKSKVAPEVPPPRP
PPPIVEGLIAADESRELHSMLRAPEADNRIAVAAHQLHVEASKWEEEGNKLIQAARKMAILFAK
MSKFISDEEYCETHSKRELIDTAKMITAAANELNKHARDIAVKCPDKRMKHDLLVTIDRIPTIATQ
LKILATVKATMFGANDPQADIEATEMLVGCAENLMGSARSVVKDCEAASIKIRTGQGQVMQWK
RETDYDYYS*
>TRINITY_DN4066_c0_g1_i1.p1 type:complete len:726 gc:universal TRINITY_DN4066_c0_g1_i1:143-2320(+)
MNTKFIFQIFIFLTLFFGQILTLLREPLNSFIRHHEKLNYDLENVHKRTRRALEISPFANVAIKFDAFG
NNLTLHLQRNHDIFAPDYEIVDGHGKRIDYDHSRFFQGEVEGFDKRSHCLGKIHDGRFEGTILLG
KEEYHVEPSERFFPKELIKGFHSVIYRMSDVEHSAKIAQPILPPRPTTLKDTKKHKETESFEDGKIRY
RRGTKNRHKNTCNLKLVADHMFMRKFVRRETAIDQMVMHYQAVEYIFRNQTFNTTDQFDSTFA
PEGIGFRIKEIHVLLEDTVPEPMKPDHISIYHLLELFSRDNHSSVCLSYLFTDRSFDNGVLGLAFIGYP
YGQAGGICDPYADFGGVKKTYNTGVLSFQLFNREAPRALTEITFAHELGHSFGAQHDPNTKECQ
PGDSKGGNYIMYDKGTPGFLEHNREFSKCSIAKMIPVIHAKGLEVTEGCFTQRYWAICGNGAHEE
GEICDCGTVETCVERCCTPYGAKTGTPCTLQNPAYECSPSRGPCCLNETCTIAREFLKCYDGTECM
ESRVCNGLREECPLAKLKANLTLCDKDRSVCINGFCQGSLCLRYGLEGCLCETKEDRCKVCCQYN
GQCTSSYDIPEMPNTTKTRGTPCNYYKGYCDAIGQCEAIDMEGPLRMLYITLFTEEGIKIWFSRYW
FVLLLGSFLILCMLAAFVYYCQHFTPSNNPVLQNKKPHKPLRRKRKTVSGGYTTGGRPLMAQSYA
*
>TRINITY_DN22492_c0_g1_i2.p1 type:complete len:1026 gc:universal TRINITY_DN22492_c0_g1_i2:39-3116(+)
MLQKTLIYTLLGCWITLLSHAAIVRKSGGDDFSLKVGKPQKPIGQPKLKDAKKEVKEEPTSDVIPQ
QDENNDTTDRQKHKEQVSPDAVESGMPPRYPLKAMSAEVSAHDNEIGGCRDHPRASRVCKNC
GNRNFAAEHESYTYSYCRKSCRHCDPELSSFHTFWRLRAASQFDPTWMVKEIEFYPKPTEYDNLA
TDPQRAYASSSYPGFKPSYAFDGNNKTSWYPSGWGLHDNNDDWIGFEFPVAVRVHAIRMVLDE
MHRTAAPQRIFVEASDKKDGPFTTKWKISNPTYEVDNVYKLLQCPVLWRKFESDEGSWCFRLIAK
FETWEVARNECTVEGGDLATIATEEEKRFLTDELNLGGFAWIGLNHKDQAYYWQDRANSSYKN
WKAGWSYSEVQKQNLNCVATDQDGFWYNFHCTNKYYFVCKKKLEPQAAPREDLILKAWPYEP
EIPPMIFLYPNITVPVINTKNLEPGVFPDENGKVPEEEEEETTADDLFYEQTVEHLKEKGLKPSEEIS
NDVDKEETDYTQQLNKIALKTQSRDDFRNETESDKLKFNSTEAVKQAKDLEEQKSVNLDQNFN
QYNSTDGVKQQSQPVVAAPSDGPSGNSGDNNTLPRIVNGKPVESPSVNPNNVGKPNTPVASPES
PENTDFAQPGDTTFTTPTPTVQSGDPSNAEKTNPQKATNNQQSGGQQPNQESGSNANQSQVQQ
QSQQQQPQQQQQQAQNNNNNQNGQQNASNQQNNNNNNNNQNNNNNQQQNNQQQN
NQNNQQQNNQNNQQQNNQNNQQQNGNNNGGNNNDLPNGLPPVMGGPAPQKIPEPPAPKE
GTKDNDSEKSVGGQADVMPDDAPSNDNDGGDETTTGNEENNDESNNNNNNNNNNMNNVNN
NNDNNQSRTLDKPLPNQGKNSSETVDKATNVGGTNKQENNDFMKLQKAKPGIRTHKIGHKKS
AGLKKTHMKNVVSPLKKVKTEKKINKQVKTVKKIKAVKVKHHKKALKKEVVIHKSEDDEEEGS
GSGDEGDQEDVDDILESITNGLW*
>TRINITY_DN61_c0_g1_i1.p1 type:complete len:625 gc:universal TRINITY_DN61_c0_g1_i1:2929-1055(-)
MRAVILLCIAMCTMVYADVYFHNIRGSNNRLKGQNRNRDNAYRMFDSQNNDRGGYNVGPTPM
YFYTGSILTLEWTNQHQCGGPNANCDLILQYMCSENLRDGLEERTIPERNKDCKDDDCNTDIRY
GMHEDYDYYMNCKNRQRNPGLFLADQELEGTSAKYTRQNPDGDRYGYECPEERDHYPYWLPT
PWIDIAIMTRDLRRCNYYRENSQNNNAKYYCKPPQKYLDELKKRDPPSNLPRIPISQDDCERFHY
PANDKTDRGVWTRHDSWNVDPPECIAAPPSRDNHNGNGEDGYFNSYNFTIPNAPHKNCAIRM
RYNISTSDYDVNLDADGNGDPARAINIHDLVGIDTKKEATERGYVFKDYPKVYPMKGEIGKKLGL
ALAINTAQYFRTFEDRSHRTSFKARTPELEGVEIRNLNVKGKRGNIVQVYPAVEYGFVPDRMLAE
VGDMIHIQWTGSDRNPRNNDGEGPAGEDRSNIAELDQPRVAQVVADYNEYGDLTRSYPAHLDN

ATFLGLSRDELISLALAKGDCEFDRQGARSRTPYFDMGPVALTKKGIYNYLCTINNNFTNRSQKGT
AIITEKSKAGGGSKATASASKSASFRSVNGKDQEKRKYHAYNYFEQYKKK*
>TRINITY_DN3036_c0_g1_i2.p2 type:internal len:548 gc:universal TRINITY_DN3036_c0_g1_i2:1643-3(-)
AFAGAARASPARQGSVARRALDQSSRYADLSLTEEDLIRNGKHVLVAYIMKPKAGYDYLATAAHF
AAESSTGTNVNVCTTDDFTKSVDALVYYIDPEEEEMKIAYPNLLFDRNIIDGRAMMCSFLTLTIGN
NQGMGDVEYGKIYDFFLPPEYLRLFDGPSCNVIDMWRILGRGTSNGGLVVGTIIKPKLGLQPKPFG
EACYAFWQGGDFIKNDEPQGNQVFCQMNECIPEVVKAMRAAVKETGVMKLFSANITADDPVE
MISRGKYVLSQFGPLSENCAFLVDGYVAGGTAITVARRNFPKQFLHYHRAGHGAVTSPQTQRGYT
AFVHTKISRVIGASGIHVGTMSFGKMEGDASDKNIAYMLQGDVADGPYYQQDWQGMKETTPIIS
GGMNALRLPAFFENLGHSNVILTAGGGAFGHKDGPKQGATSCRQGEEAWKLWKAGTYGDITLS
DGVIEYAKTHEEIKGAFLTFQKDADQIYPGWKEQLGYTGESSVQAASFDWKRKAGAAAFAGAAR
ASPARQGSVARRALDQSSRYADLSLTEEDLIR
>TRINITY_DN99404_c0_g1_i1.p1 type:internal len:112 gc:universal TRINITY_DN99404_c0_g1_i1:2-334(+)
SNLEAITKSLEKQHKFYQSKLFKLQTDIHGVALEITKLKTSLHRTQISSSERRSSLLYINLRILKNKTDI
AIKGGLDVKEEAMTMLNTVQNFMSVATNAQNAANQALKKYY
>TRINITY_DN1366_c0_g1_i1.p1 type:3prime_partial len:4702 gc:universal TRINITY_DN1366_c0_g1_i1:236-14338(+)
MMEQDRQRPSRQEYNNRSLSRERIGSRDGSRDRNLSRDRDLSQTRDLSHTRDGSRDRSNLRTRDG
SQDRSFANRSDLYSSPYSSNSQAMPQTSATVEIENLELQATVSSQEELINQLAEDLRNVRKARDFSA
EVEILQKEQQCMDFKEQLEGKEMTIQHLKTELVQSQTVTEEVYKKYESIRSNRDAKDKSIRRYQQE
LHQVHDLLETAQKDKLACVDKMRQIQNEKSALEFDYQWLFDKIKNYEEHGEVFDMSSVSKSTTT
IKRTTTVYEDGQKIRVEDYQSDDSIMPLQNKFQQAQKGYRKAIGMQDDQLMRDKENLMNEFRS
MQSQMHDLKRIHENFMRESKEKDTLIERLKEAKMALDQELDHLTRENENLRQTNDKLENENSD
LRTELNSARNQVSKIEVQYDNCMKQRNYMKEQMEIITQNSAKGRDDYNSIEATMRDSQRKMDS
MQRDLTNKENLNKQLKNKIKILEEDFEQCRQDYNQCMVSRESLQQQKLDVERELKERHDRMDE
VETSYEDIQRRKADLQQELASLKKNLTRFINEEDSSNQMKSHLQNEVLAANKRISRLEAAYEASQ
QEKLELQQELAPATQRLSMMRAAYETLQQKKEQIQTDLIASQKRCAEYQAMNERLKQDRGEILN
EIQACKRRAAQADVDLDEAAKQYAILKGKLEDANRRAQSASEDLVTRQRTIEELRHKLEVSGRET
IQRESYYERIKQERRSLEDELRGCVTDLERVQNEARDVQRDNEKLRQDLGTAQQRLHETEAYWQ
KTKMENAKLQRDYANMERTVQELRDLSHKLQQDKEDCEKILYSTHKKYANLDKENERLGRENT
DLERELQSSKNKQSKLDAVIQDLKRKEAQLKAQLDDALKNRDELNRVKIVMNDLQKRLDGVTK
DGEQKDNLIAQLRNEKDNYKRDCDKFGRDYEGCRTMLETANREKDRLGKELNAANRRIADSD
VLVKRLQDENMRVRKDANSFEARIPILEDALQKSKANESKWALEANTYKTNVIRIEKELLVFKEE
HHSLVSHVSQNEDKNDGLAKKLDDALRKIFELEATNEMVSRENDELKRDIDNKTRQLEEQLTTV
ERANNDLKNQLNELRGRNKKQQDDYNRMQIDYNEFQSKWDGLSSDGQGKDRLIEQLRSELQN
STHEINQQHTDNENKKAYIERLTEEKQDLLNDVNTVNQRIYELEGLYKSSEDDKEGLQKEITELNR
KLNDVESDYDTLQRESDKEIRALKSKLGKVESQVDMILKDRDYIQDQLDAERLLSQKYKDEFERV
QREFLEHQKICERYADQILERDRIIDDLKNQMYKMETEIDTLKEQLRKAQTDQKVAEQQRDKAVE
KLNQAEMKIKDLENENDYLRKRLAELEREISVLEMKLNDAETENKNLRNKIKDLEDLIKTLRAKL
SNTETELQSIRHIISDRDVMIERLQKELDDANDQAHQLRRQVSELESQIELGDQERDDLRSRLANS
ERTIIELRKNLDKVIHDGQDLEAELAALKQKLIKMEPQMKQAQKQRDDYKTAYDNLKGKYQDL
KDELLANQRQLTDAKRAAEQYKKELRHKDHLLEEARGKILALAGQLQSARDELVKKQQENEQL
LDEKRLIQEELLRLRQKLGDLESANQILAMEKERLNEELISFNSKYTELRIKLEKSEQDRLESQRQA
MAYEQRLAKLEPQLQQILRENSELHLQVDRLADELRNALDENRHLKNDVEELQRIRDMQLKELY
EKDTIIEQLKAKLSNLENTVNHLQQENDKLHNLNDALEQEKHSLAHRLDNANSRIKELDDLYEQ
ERQKRDQLQNDLHDLQKKFVTLENAYKKSQERERDLKKKIDDLLGRIKDLEDQLAAEKKKNSDL
EAENDRLKDLIHDLEGVIDDLRRKLTEQENENDRLRAENDDLKQQIMNLQRRIDELEGVIAGLEQ
EKNFLEEELNSLRQTVNKLEAEIDNLTKQRDGLQGQLEDLRREIMRLKEVIMKLEGTNRELQRTID
ALKQEIYRKDRIIETLEAKRKQYELEIDNLKNEIIKRDVIIDSGDESKRDLEIQIKNYEGRIAIMDGRI
DKITLEKTEIERNYHNAVNRAKGLESQVLILNREKAELKGDIDQHFGKIRDDNEVVNKTQGELRE

KVRIIETYKKEIIEKTTIIERLTVIRQTVESERDQLKKDNATLKITVNQYKDNDKGMNDEIQKFTFKIT
TLEKDIINMKNERDEFKVKADNATSKLVEVETQLKRSIERETRLRGELDTLHNKYQNLETIYNELL
REKNDVDRKYKDLLQKHRELDSKYNKLLANFNDLKDQLQQANNKIADLKAKLDKEIYENRDL
RDQLDNLKNQYNKLRDDNQRLEQELADYKYKYEDAMKELAEKNDLIDELKAKIANQDRQINA
LKGTVNQLRADYERSMREQARLQDEIVKLQAVVADLEGKLHRLELENEKLRKENNELQAKIPAL
ENEIENLKDKLENLIKENRDLRNDNMNLKQENVQLEKEVDDLKDDIEKLKDEIRNKDNIIADLR
KVRQELEEEIDQLRHDVKQKEKEIDDLFNRIRDYESTIQILNTKLAEIEPAFEACKDRLRHQENQIQ
ELRNKITDLESALKQSQENEAELGNKLALTESKLNKITQLLDDIKRKNMELENENGHLKDELNKL
KDQNKKLENDNDRLSNELNECKAVIQRIEPMLDHLQKHKNQLEKELEDALNKLANLRNEHEA
HLREMKEKLHVIEVITRQLDEKDDLIEKLKQEKAALEKENESLNDALNKLHQTLDAVQRRKEEV
EKDFFEAKKRIAELENLLNALKEDKDDLERMLRDAKEKNADLDHALRNSQHNEAKLKGELDEA
RGRIQRQRNELAKLQIIVGDLRAANERLLRDNTEKQNVIDGLHSQIRGLESQNGSLKKENQKLRD
ALDKLQRELHNLEQENQQLKAKVFSLQLLNDSLREEKDRLYDNFQELEKDYNDLKDKYNQAVR
ENDRLKDELAFANKRIDELENMNNQLNNEIENLKNIIAGLKRQINDLQGVIDQLRRDKSDLEQTI
NIHSETTINQRPVFDESDYVPRSEYDELKALLEAANSTKEGAERDRDQYHQEHLELHKRISKFEAE
LEGATGKRDEYKRLLQEAERRIRELEGVNGRLEDREKELQKELLNALNEVSGLQQQIISLKVKLGK
FEADRVNYEDKISKLNNEINDLHQRLSQVLASIEEKDHIIEDMRQELADTQRRHAELDSQNQILR
DEIEKLHGIINDNERKIDDLEVKLQDQRNQNSQLKNQINELNARNNKLKDEINDLQRKVSDLER
DLDQAKRDNQEKQNVIDRLNQLKNQLENDLEALRQENAGMKSDLDRLRLDNRQLLQEKHDLE
MKIMGLESDKGTLEDNVERLLNEIENLRNKIQKLKDENDRLIIENKRISHELYKYNKKSGPSDYDS
LKDQRDKLQREIADLTKELRNYKTNITVLQNEKDDLYNRLKELERDLADRDHVIEELEREKDRLE
RELESARNRADKFELSNGQLENEKNRVQEELMDLHRKLTKLEGDYELACDKRDDLQKELDSAIA
RNKTLEDNYKRSQERENKLHQDLLAAQKKITILEHELQSLKDRVNSLERANKVLDDKKNELKDD
LIAAQKRISELEIMLEGADDRNKDLQNELDAMKRKNDELRETLRLTEHGKEDLINEIQNLTAKLS
KVENKLTQAENEKRYLEQQLEDAKQRTQKDRDEIMNLQKTIYELTRKCDSLTKDNANKQALIDD
LRAKKTTLEGEIESLKRDLQNNQYEIERVENDKKNLYDELENMKNKFNVQEQEYLVLQEEKEKLR
QDMIASHQSVCRLKENYENSLGDKGKLREELATFHRKIAELTTAYDSCRSEMNRYKGDCDNYKD
AYDDIKAKYDASQNEISKSRNDYEVCLQKYQKGEQAFTKSEADRERLQKEMLDVHRKSSEIAVLF
QTTKEEKRVLENQLSMLQDDLERYREGYETFNDKTNALQDEILVSHKNYTKIEHAYNRAVEREEK
LEKDCEEAKEIVANMEKDLDNARGKANELENELRKLQDKLRREQDEYEQNSEHAGKLQMTLRD
YESKLSQLRNVEDENKTLRDEINNLKSSINKVEMDYRSNHNDLTQVQDQYESSRNNINKLNNEIN
ELQRDIRQVTSENEKLQNDLQNAAQDKEQEGERLKSTIRMLEREIDSLKTQFGQTTEQLDIVSKDR
NVMHQEIQVILKKCVSIEMTYITKTEDSRFQTSGGDEYEKSINELKNHLDKSEREFDDREKKYKYL
EDQYVREKKDKSQLEKEIDEIQRRYDNMQHEIRVIEEDKKRLSDEYQDMFNKYNDLDRDFKNTQ
TKITTYETEINTYQTTINDMQYKFTNNEDQNKKNSQVYRDLQQKLDNATKENTTLKGELNDEKA
SYRNVKKDFERQVRELQRSSESYGKASHEKDTIIEDLKSKRQALESDIDQLRRDLNHGKDEYKRLQ
REFTQHKSDYQDQQRVITDLQQKVEEHKHSKITIEEEYKITVEKYIKEIRETHIKLSKVESELQTTRE
AQNLDSDERYKRLQNELQQERETTRKDIYGKNQEIEQFKSQISNQDQEIRRLRREIEELDKQLKEA
GSNYSSTILIQKQDAPKYSSTQVTESRISSSSAPRYSTEQEVLIDASDKDTEDEKKRYQSSFYRSTATIK
PGQSDYGSVDVVLQGSQDQGQDDKDYDYNRRATGLDTNAVNRSQ
>TRINITY_DN2028_c0_g1_i1.p1 type:complete len:274 gc:universal TRINITY_DN2028_c0_g1_i1:1608-787(-)
MGQSDGDDLTFGQKISREWRDFREFLYNSETGKVMGRSGTSWAKIGGFYLLFYGFLAGFFTALLFI
FYTTIDKPGIGGPKLIQYIENKPGLTRLDRSSKLQSYSASNNDRISSYKTVIDEFLDGYNTSDHSGAL
CGAAGTPKSDKPCIFDTTLLGECAPSKNTTYGLREKKPCIFIKINKVFGWTPADDGTGYLTLSCTG
HATVMPQGFPLTAFPYNGHKVYQSPIVAVKIDATKGKKNVDCTLKGKNIEVSNNFNPSRAFGKIR
IEGISATD*
>TRINITY_DN36217_c0_g1_i1.p1 type:5prime_partial len:605 gc:universal TRINITY_DN36217_c0_g1_i1:2-1816(+)
YLTFIESLRLRKKIKTINKFWKKKMNDVAYSKDNEAIEIDNFASTERLADDDTDAKPRGQWGNKT
EFLLACIGYAVGLGNVWRFPYICQKNGGGAFLLPYFIMLIVEGIPLFYLELSLGQRLRLGPIPLWKK
VSPWANGVGYGMCIASFLSGCFYNVIIAWCLYYFFNSFQSPLPWNSCNSPVNSTEWMKCEEIGAT

QYYWYYEALQISPTINDLGGFAWKITLCLLTGWILVALGMWKGIQTTGKVVYFTATFPYIVLFIFIG
QGFTLEGYAEGLKFFFIPQWEKLADPTVWMEAAAQIFFSLSLAFGGLIAYASYNDEKNNTLQDALI
ISFTNCGTSVLAGISIFSILGYRATRKSKFCISELGGNISPIPRNCTVQHFLEQGSGGTGLAFIAFTEAIS
TLPVPTLFSLLFFLMLITLGMGSMFGSLEGLVTSLRDTPFFQKIRKELIIVIVAVPGFFAGFGFTQYSGE
YMVQLFNKFSIDIPILLIGFCELIFIPWVYGMDRFSADIEYMTGRKPPYAFIIMWKFVSPVALLVVLLL
SLYNMMVVTPLYEQWNGELARVSMTQYPGWALGVGMMVFTVSFLFVPAGIIFNIIRRKRKNYLL*
>TRINITY_DN26249_c0_g1_i1.p1 type:5prime_partial len:477 gc:universal TRINITY_DN26249_c0_g1_i1:1733-303(-)
QMKMAASNMAGHVVRNNLIFLLSFVIIIVAEEGRHARFEYKFSFKGPHLANKQGTVPFWNHYGS
AIPSDDQVRITPSMRDQRGGLWTKTKSSSEFWEVEIFFRITGRGRVGGDGLAMWFTEEAGIQGPVF
GSSDHWKGMGLFFDSFDNDGEQNNPYVMVMLNDGTKQYDHFKDGNSQQLGGCMRDFRNRP
HPIRAKIRYYNNILTVFYHSGMSERSDDYELCMRAENVVLPKEGFFGITAATGGLADDHDVIKFLT
HSLTPPLTDEQKQKILSEEQRKKYEEQYGEYTQKLETKKEEFQKEHPDKKAPEEKFESVYDRDVRL
IYENQNTIHHVVRALHEKVGELTNQLNNLHVTINNNNQLAQQQQPKGDGVTRQEVNTIINLQN
QVHNAIQELKQQSGGGGGDNTKTVQMEKTLQTVLEQVGHLVSKNNDRRPSCPTVSCVTPSILILF
LVAQVLLVAGYNFYKEHQNKAAKKFY*
>TRINITY_DN172571_c0_g1_i1.p1 type:complete len:104 gc:universal TRINITY_DN172571_c0_g1_i1:30-341(+)
MSGRGKGGKGLGKGGAKRHRKILRDNIQGITKPAIRRLARRGGVKRISGLIYEETRGVLKVFLENV
IRDAVTYTEHAKRKTVTAMDVVYALKRQGRTLYGFGG*
>TRINITY_DN1378_c0_g1_i1.p1 type:complete len:443 gc:universal TRINITY_DN1378_c0_g1_i1:2151-823(-)
MLRFAVLVVVASIGVSALYDSSDDVVELTQSNFNNHVIGSDDIWLVEFYAPWCGHCRNLAPAWK
QAATALKGIVKVGAVDMDQHQSVGGPYNVRGFPTIKVFGANKMSPSDYNGARSAQAIVDEAM
KVVQRVVSDRLGGRSSGGGGRRSGGSSGGSGNKDDVIELTDSNFEKEVLNTKDMVLVEFFAPWC
GHCQRLAPEWAKAATELKGKVKLAALDATVHGQTASRFQVQGYPTIKFFPAGDKDFSSAQDYQ
GGRTASDIVAWALAKHAENVDPPEILEITSNSVLTENCNEKPLCVISFLPDILDSMAAGRNGYISTL
KELGDKYKSKMWGWLWTAPGLHSNLEQALGVGGFGYPAMAVVNIRKKVYVLLRGSFGIDGIDE
LLKSIAVGRGRTEKLTQGLPTLKDVEAWDGKDGQLPEEEDIDLSDVELDDLDDEPKQEL*
>TRINITY_DN540_c0_g1_i1.p1 type:complete len:480 gc:universal TRINITY_DN540_c0_g1_i1:1503-64(-)
MATDKLEGLIARLEKVTGKLESVSVRGGSGGGAAVEDDPAWYDEYIAIHEGQWESFLKATKENG
DNDMVTLEQKLRAAFNAHKDLLTIAARNGKPSPKDLPELVKPLSAAIGEVQSFREKSRTSKKFNH
LSAVSEGVAFLGWVVSPSKPGQHVIDMKDSAEFYTNKILKEFKETDKVQVEWARGLRKMLMDFA
DHLKHCHPVHLLWNPDKPAATASTKASGGSGGPPPPPPPAAAPPPPPPVAAPSSAPSGGSEDSRA
ALFSALNQGSGVTAGLKKVSDDMKTHKNPALRNAQPVPGKTSSLNPRPYAPPQLKKFNAPVAEK
PPVFELQMKKWIIEHQKNNQSLVIDETNDKQTIYMYKCNNSVLQVKGKVNSITLDGCTKCGVVF
EDLISTCEFVNCQSIKAQANGKVPTISIDKTDGTQIYLNDKSLDTQIITAKSSEMNVCITKPDGDLN
EFPVPEQYKTVWNGTKFVTTIMDLNL*
>TRINITY_DN109148_c0_g1_i1.p2 type:internal len:131 gc:universal TRINITY_DN109148_c0_g1_i1:391-2(-)
PRVPAGVLRRDRRVPEAVRQGVDPGHLRPGRTLRDLPARHGLHPRAHFPGLLPRQPRGDQTGVP
QGAHLLGVLRQAVLGGHLVRQNPPRVAQALRRGGGGHPERGQHLRRGFRRPLHPPLALLLRVL
RGR
>TRINITY_DN97809_c0_g1_i1.p1 type:internal len:184 gc:universal TRINITY_DN97809_c0_g1_i1:2-550(+)
LGPDQVDVAGAGRDPRHLPRPLGRRLRVLDRHRGRVRQRHKAEADQDPKLETARLDVRRPGVQ
VRRVRHPRPSSRDQCHRDCHAPGRVEGRAQVDRGRRGLGRRRVEHEVHGAAQPAARGQEKGR
LRAQQILAVAAGQEEAVDVGARNRQGPPSALAAVSRAPHAQFARLAQRDRRVARAKGW
>TRINITY_DN773_c0_g1_i1.p1 type:complete len:440 gc:universal TRINITY_DN773_c0_g1_i1:1608-289(-)

MVSPPGPQDNKPPHKVADMSLADLGRRYIEMSENEMPGLMSMRKQYGEKKPLKGARVAGCLH
MTIQTAVLIETLTELGAEVQWSSCNIFSTQDHAAAAIAKTGVPVYAWKGETDEEYLWCIEQTLIFP
GGEPLNMILDDGGDLTNLVHEKYPELMKDIRGVSEETTTGVHNLYKMFKEGRLKCPAINVNDSV
TKSKFDNLYGCRESLVDGIKRATDVMIAGKICVVAGYGDVGKGCAEALRAFGARVMITEIDPINA
LQAAMAGYEVTTMDEACSKARMFVTSTGCSGIIEPHHFQQMPEDAIVCNIGHFDCEIDAKWLN
DNCERETVKPQVDRYTLPNGRHIILLAEGRLVNLGCAHGHPSFVMSNSFTNQVLAQIELWQNHK
NYEIGVVMLPKRLDEEVARLHLDHLSVKLTKLSKSQADYLNLPADGPFKPDHYRY*
>TRINITY_DN5633_c0_g1_i1.p1 type:complete len:848 gc:universal TRINITY_DN5633_c0_g1_i1:2881-338(-)
MRRSNFLTIGIIAFFLIFSYVSADETSDEEVEPPKVDDDVGKSRDASKTDDEVVQREEEAINIDGLSV
AEMKQMRESAEKHAFQAEVSRMMKLIINSLYKNKEIFLRELISNSSDALDKIRFLSLTDTEALAAT
DELSVKIKADKENNVLHITDTGIGMTKDDLIKQLGTIAKSGTSEFFQKIQDAGTTDQASDLIGQFG
VGFYSAFLVADRVVVTSKNNDDKQHIWESDSVEFSISEDPRGDTLLRGTQISLYLKEEASDFLEPNT
LKDLIKKYSQFINFNIYLWSSKTEEVEEPIEEEPEKKEDEKKDDDKKEDADDKKDEEKKDDDEDD
AKVEEDKEEEKPKTKKVEKTVWDWELMNSSKPIWLRKPKEVTDDEYNEFYKAISKESDGPLTKT
HFVAEGEVTFKSVLFVPKNSPSDMFQDYGKRRESIKLYVRRVFITDDFQDMMPKYLSFIKGIVDSD
DLPLNVSREQLQQHKLLKVIKKKLVRKALDMIKKIPDEDYIEKFWKEFSTNIKLGVMEDHSNRTR
LAKLLRFRSSNDPEKLTSLTDYVERMKEKQETIFFMAGASLDEVKNSPFVERLLKKGYEVLYLIEPV
DEYCIQSLPEFDGKKFQNVAKEGLKIGNEAKEKARVDELEKEYEPLLKWLKEDALKDLIEKAAVSS
RLTESPCALVANSYGWSGNMERIMNSQAYAKSKDAGANFYATQKKLLEINVYHPLIQELQKRVKT
DKEDQTAKDLARVLFETATMRSGYTVKDSSDFAGRIERMLRLSMGVDVDAKVELPEEDDEEKEEE
KADDEGEEVNADGEEEESKEDIPEIKVDSKTVEPVAEHPTNEDAVENEAPAEGKTEL*
>TRINITY_DN775_c0_g1_i1.p1 type:complete len:635 gc:universal TRINITY_DN775_c0_g1_i1:1927-23(-)
MKQMIFLLAVSLAILNFDGHVVDAITCKSNSVWSWECQNSRCVKVLASSNSDAIELNTCKLTCGK
YSMLFPKPSGEIQLGKKTVPFIPTQARLRSVKVKKSNPKATWLVQNAYSLFLNTLEHSYKRYSGSTS
RLYNNMCAQEAQKRVLVMDVILEDGDTKITLDTDESYDLTILADDKEITVSINAQNFFGARHAME
TVSQLAAFSESYNAMQIVSSAQVKDDKPSYAYRGLLLDTSRNYFSVDSILRLIRAMSYNKMNTLH
WHITDTHSFPIEIKSLPEMLRYGAYSSKRIYTHDDVRKIVSYASINGIRVLPEFDEPAHCGEGWQWG
PKAGLGNLAVCVNRQPWQKYCVEPPCGQLNPTNDNVYDVLGKIYKEYFQLFDPDIFHAGGDEI
NFNCWNTTTEITNWLKQNYGGVSEKNFMDMWGMFLDKSSSKIYEANNNKELPMILWTSKMTS
EEYLTQYMDPKKHIVQIWTASKDKELSNVIESGFRTILSTYDTLYLDCGYGNWLVEGNNWCSPYK
DWKLLYDNDPVKILESFNVTVTDKIRSTILGQEAAMWSEQVDEFSSEGKIWPRTAALAERLWTNP
NHNWRDAEYRLIYHRERMVERGVQADALQPLWCLQNAGNCYYDPAIKGEH*
>TRINITY_DN71243_c0_g1_i1.p1 type:internal len:422 gc:universal TRINITY_DN71243_c0_g1_i1:1265-3(-)
PFVGIRIDSNTGSVFLAQHLDYETAQQHNVSIFAVEKDPPTRYVSSYFLINVLNVNEHSPQFQNNE
TLVITVNEDIQPGVIGSSPATDKDAGVFGNIDYKIIRFDDQLFDVTASGEIRLIGKLDYEKKRVHQFL
VEALDGGQPRRKSLRPVSVIVGDMNDNVPLFSQDVYNVSISDLTAIGSSLIKLSASDADDGLNGEII
YDISNGNFGTTFIIDKSTGVISLSRKQLLSELRKFTLKVSTRDKSPISPRNGETATVHVNFIEASANEP
FFSTTFQKLNVLENQAAGIKIGQSKVINSKMEAVYLIVSGSKTRITDYFGINYRTGEITAKQSFDRES
RDHYTFVVSARFIDDNSLTGYQTVDVSIIDENDNRPMFLGNISQTVSISSSFDGSRPVALLNAFDKD
LVSLDKIEMQLLNSQE
>TRINITY_DN1172_c0_g2_i2.p1 type:internal len:264 gc:universal TRINITY_DN1172_c0_g2_i2:790-2(-)
GDYGFKVLTSSDPAEKEKKLAAELANGRLAMVAIIGMFFQDGLTGSAWGDWALYTASPLRFFGG
DSGPSYKAFDPATELGVQEPLGFWDPLGFTSDNDEAAFKRRRSVELKHGRVCMAASIGYIVPNYY
TFPGFLSPSAGVKFADVPNGLAALGKVPALGWVQIIFFAGIVEGAFGFDDYKSGAPGDYGWKVLT
SDDPAEKDKKLAAELANGRLAMVATIGMFFQDGLTGSAWGDWALYTASPLRAEPVSTAGVGAA
TKGGTE
>TRINITY_DN564_c0_g1_i1.p1 type:complete len:563 gc:universal TRINITY_DN564_c0_g1_i1:2018-330(-)

MKLLLFCAATLLVAGCVLADGVATQKKCEDFLPVDTCNQLRVIAQKFNEKAGAVNKAITDAFNK
HKTKAGEVLSYVKEYLVANAKNFSCASVLSTQQCQQLKDFAKLMHAKAGEASIAVREAIVNGA
KNSKVQFQKAMEFLKNDVSCEKILGLEQCEKLLESAKTLKENIKTVHQALVAALQKHLSKTKDIL
KSVRETLVEKAKNFNCTDVLTLEQCNRIKNIGQRLKLKSSEVQKAVKEAIANGAEKAKQIFSNAM
DFLLIDARNMTCEDLIKAETCQKIKDFAKKVHARASDVALAVREAIVQGAQSTQDTLKKAIEFLK
AEISCKDVFSIDTCDKIRKLADSFHVSLKTVNEAMREAIANGVTKVSDLYKAAVKYITEKWGGIFG
DEPQIEKRSVDEKLTNIGELLKQKITKMAMDLLDMLKVTNEKIREKVKELIENGKIKITNIKEKVN
DLLALVNSNDEDTMNDVNMIYKRSTEDSLVDLLKSIKGKSQEFIEKLLKLSKDKLEQIKEWIKQLK
QTDDSIPSDEMEDDLDLVHDVLKDGVNMAFADIVEDTIKNIE*
>TRINITY_DN14015_c0_g1_i3.p1 type:5prime_partial len:2566 gc:universal TRINITY_DN14015_c0_g1_i3:2-7699(+)
PNEGVTIVFSNGDYELKKDYYAMIEAKSNGTLYKGKTVVHVTLTDKNDNPPRHQNLQVDIAISES
TKPGTIVTILYADDADDKQNGEIFFMLESASSDFSISSFNNTGVIRLSKELDNEQETSYKLMILLKDF
GSPSSKFSNMATVNIDVIDENDNTPLSEKTFDTIQVSRLKANGVIYQVKFTDADSGLNGQIEYDIEP
FKSFDDYFTINKTTGEVSLIKSLETTTEDNFWLFIIATDKGNPRRKGSFTLQVNVNEPSSLNPIFNPD
TYDIEIYENRLPNSLVARINVLNVDPNKDSLTFKIVSGNDGDRFLITNKGDLVLKQNVDFEQQNLY
NLVLSVRKNQNSVESVNQATVKIKVLDVNDNNPLFEKTDYTFIVTENTPINTFIGGVSAVDQDFGE
NSKVSYTIFDTTKTFIIDSSTGRITVVGQLNSFVENEYNFVVYAFDNGNPRRASSANVKVIVEDINE
APQFSPPSYNLNVSELLHPGDHIITPKVIDNDIGINSQVRLTLDASGNIDDTFLLENGKLKLNKQLD
FETRSTYDLTLVATDSGRLPLSTNAKVKINVMNVNEHRPIFNINPYMTEINEDQFSTPIFQVKASDK
DALSKVRYVIEDGLDYSLFSINQITGEITNSRLNANFKKEYSIQIKAFDLDNPQFYSHTVLVVKVIDI
NDNAPIFNKAQYSFTISDQTPVGTIIGKVFANDADTGFFGQVTYFLDNNFNGPFSLSGNDILINGD
LDFTKVSSYEFVIKARDSPGSSQGFRTTSTNISVNIVETNGTMPIFSREYYEVTIFSNISTQYTVVKVSA
TDNDGNEFGEIKYSLISDSSNLFQVDELIGIVSLKDGVQLQTRSFKYTNTLQVRATDGAGLFSTAYL
VIHVIPVSGNNPPVFLQIQYLSRVSYLTSKGESIVSVIATDPEGAAVSYEIKSGNELKKFGITNSGIIYL
EDQVENPEVNVYRLGIEAIDENGLRSDPDAQVIIQILYFNSTFQCKLIPTFSSNLYISQVEENKAPTII
TSVQANGSSQNDSIAYSIAYTKDVTQNDFEINSTNGVITSQRTFNYEMKNSYQFEVIAKDTNSLKE
GRTIIMVNIIDVNESPVFESSNIVRSLSQSSPVGTFVGLAETSDADKNDLITFSFVPANIGNIFQVDA
NGIIRTKSSLNAADSPYNFTLIASDKVGLTANKNIKVTVIKTNVPEFSQNAYLFSISESLNSVRIGQLS
TKQILEPVGYRIPNNNYNNLFIISDGGIISSSKGIDYESDDRKYNLCVEVFLQSNPSLFDTCDVTININ
DINDNRPVNKKDGQYTVEVSENVMVGKMIITLDVEDIDSGEKGFLNYLITSGNNGNEFRINNKG
EIYTLKPLDYEVKRSYSLTINVQDNGVPVKTIEAPLTVVINLVNENDNNPIFNPSSYTISISEQQALTT
ILKVTASDKDALIKSTYSILENEDTFSIDANGNINLVKAVDFEISSKYQFTVVANDGSRSGYAIVTVDI
TNINEKPSFGQNYAIEVSESTPVDTVILTVSATDVDIGVEGDLEYSIESGNDENTFSIQSNGQIKLLEE
LDYETKTEYTLSIKATDKGQPQLSATTIVNIKVRNINDNKPVFSKQKYELTVKENSQLSLTVSVTDA
DNLGNMKFSIPSSKAAGMFKIEINTLNTNEAVVSNIKPLDRETGAGHQFLIKVEDDVGNQGLSEF
VIVVEDENDNNPIFTSEEYNVTLFSKLNVNDAVVTVTATDADATYGVVSYAIISGNNDNMFRVNT
KGEILINKQNYDSRANYVLTIEASDNFETPKKSLTNTNVKIQIQSSGLTFEFAQDQYKYTKSEINIGD
TFGPITGTSSTSLDQIVYEIKDSSGTFEVTSNRNIKVLKKVTTDQNFCVAATTKNGGTLLDLTQIHIL
LAKTNENRPIFQFTNPKVVKISQNTLVNSVVAVTTASDSDTGSTITYSITEGGEFFNITNEGAITLKN
TLQTSTVNQFNVTIIANDGTFNSAPANVVINVKKENNFNPKLSFSSLNLSITEGNKVSQVLTDTDT
DADGDQLNFKIVGKTANNYFIINNTVLESKSPLDYEQVRSYEFCIEIKDSIDTNSRSVILPVSISVTNI
NDSNPVFTSKSNEVEVPYTWPPDITIIEASAEDSEGDRIEFSIVNSSFSSLFSIGKTSGVIKLNQRLKEA
KVYNLTVTATGVQSGKSSNDNFILTFTAVEAMSQTMPITFSDVTDNTMKVTLNAKEEFGEADYEK
MNTIYVVYAQEYLPNLDEFVTRTLMEPVSYYDANTFESFRGRRFMIAKVDMERPTQRRKRRDTSLD
EKNGILTILIGNNQCANKYDPTIPCNGPLKAGQSYRYQLEGDLLNKRKLSSFTEPKTTTSAAQSVA
QFSASNYTFNIYYIIGLVMLGLLLAVSIWVIAVLLLYKRRGTKLMQLRESMLPPAINNAQTIANGKF
GSNARRYTNPVYGDYDDDRNFTSDFNLPRGQNNQELAQLKQQIIPIPNQNNGRANYNNRSEMV
NRVPRHFDNPGYREEERKPTYTATVMSSPTVPNPPPPPPPPPMENTRLPSSGTARSTNQPRLRRTSS
GRVIVQHLSGHQ*
>TRINITY_DN4781_c0_g1_i3.p3 type:complete len:183 gc:universal TRINITY_DN4781_c0_g1_i3:2965-2417(-)

MCTEPKAPSSCANDEFQCTKTKQCIKKHMLNLTAYTKVGSKMTKLWTKSGTYGPRWRHATVDIS
SASTFKVVIEGTASSGYMGDIALDDLFLNNGACPPSGECSFEDPKLCGWTNMNGDNFDWTRDN
AGTSSSGTGPRFDHTYGTAQGFYVYIEASNPRSKGHKAWLKSGEYKPTLGRCLSV*
>TRINITY_DN73457_c0_g1_i1.p1 type:complete len:467 gc:universal TRINITY_DN73457_c0_g1_i1:1435-35(-)
MMKILLLISVALTNLFCLSQAGEVCYPPFGCFNDEPPFNNALTQLPWHPDDVETKFFLYTRKTQD
KPERLSTYFKKTASYSHFNPNVSTVIFTHGYMEHGGRWYVPRLKDELLKLEDMNVIFVEWKKGA
FFPYHQAFGNARLVGRQIAYLLRVMRNDTGVNWDKVRIVGFSLGAHVVGYAGEALRKAGMLVP
RITGLDPAAPYYENTHIDVRLDPTDADLVDVWHTDGKTILIHGFGTIQRMGHIDFFPNGGFHQPG
CGKLDIDVVQYFACSHYRAVRYYIESINNACPYYAYPCENWKKFDQDQCTKCPDEGCQAMSYHL
KKPKKHIDRAFYLDTFTQFPFCGFHYEFTFYTNDAFLADLNAVVVVELFGEKDSEVLRLPHKYYES
GRTISMVTLGTKDLGKLKNMKIKFEAILDAWYLRALMIKPMYSQIEYTACYGIWLNSYKNKRDFK
IDYNPLNCPIAHPS*
>TRINITY_DN464_c0_g1_i4.p1 type:complete len:717 gc:universal TRINITY_DN464_c0_g1_i4:2167-17(-)
MLLHFGKSLHYLKFGTLFWRLKLVAIPRDTSNIINNIFLESKSQRYIRMSANILNEYSQNVGKMEDE
ISGLTSQLNALKRKLNGEIEDEDCSDQLKELRSKNQKLHYQKNIIQRALKKEQEKADSKMLNVQ
AALNVLFESAIRSAYPQIPNPPVALTIAHNPKFGDYQCNSAMAISKMLQKLTGEKIGPHVVAKTIV
ENIPSNSFIKKVDVAGPGFINLHLEKEFLKGVLEKVIKEKVYPPGIGNKKKVVIDFSSPNIAKEMHV
GHLRSTIIGESLSRLLEFVGHDVLRLNHVGDWGTQFGMLIAHLEDKYPDYKTVSPPIGDLLLFYKA
SKKRFDDEEDFKKRAYQRVVDLQGGNPDVRKAWQMICEVSRNEFQKIYDRLDVTLIERGESFYN
DLMSKVVENMKKSGICQKSDDGRWVAFAPNMKVPLIILKSDGGYTYDTSDMAALQQRLQEEHG
DWLIYVVDAGQGDHFKQIFGVGEAAGWYNSSITRVDHVAFGVVLGEDKKKFKTRSGDTVRLIDL
LDEGLKRSMEKLLEKGRDKELTKEELLAAQESVAYGCIKYADLSHNRVMDYVFSFDKMLDDKGN
TAVYLLYAYTRVRSIQRNAKKGRSEIEEYMKSNSLHFDHEKEWKLAKLLCRYPEIVVRCLDDLTLH
LLCEYLYELSSTLTEFYDACYCIEKNRETGEIISVDMNRIALLESVARIFEAGFHILGIKPISRM*
>TRINITY_DN67_c0_g1_i2.p1 type:5prime_partial len:474 gc:universal TRINITY_DN67_c0_g1_i2:1488-67(-)
VHREAPKKMKLLIAVLFALCAIACGENYMRVCYYTNWAQYRTRMEFFPEDLDANLCTHVVYAF
AKIGRGHKLTMYEWNDDKMYPRMMAMKKQNPNLKVLLAVGGWNHENGNGQGKFSKMVNS
NSNRRAFIDSSVALLRKWGFDGLDLDWEYPAGRPSGEKFPGDKQKFTQLCSELMSAFERDAGER
QTTRLMLTAAVAAGKGTIDAGYEVAKIGKILDILNLMAYDLHGAWDPVTGHHTAMATDGVDLP
NRVEFTIPYSVDYWIKKGFPAKKIALGMGTYGRAFRLKDKSQHGLGAPVTSGWPQPPKGQFTRE
AGFLAYYEICKMGLTIVKDNAVSAPYGYKGDIWVGFDDKESLIHKVNTQIKAKGLGGAMFWCLD
LDDFKGSQCGEGKYPLMGAVAKALGGYTPPPPPTPGPKPKSTRAPPGEKTDRPVKTEGPSGGVCR
AIGSWTGNANMNAWCVSNCARGNCPASVCKC*
>TRINITY_DN27379_c0_g1_i1.p1 type:complete len:583 gc:universal TRINITY_DN27379_c0_g1_i1:133-1881(+)
MFLFCFYLLTLLTTFTVKCHVKQAVPTFFHRRTPSGEILDRSSPDFNNHIKKPLGQAIDESDGSSYL
KLWPNPIKNGELVSVLWRNIKSPTEKDFIVYYCPYDDNPNHYLDLFHVTESLTWNQGYGHYQVR
VYNMRSQCIFKYYRHTDKESQMVGVSEKLNFVGGNNAPLQGHLALTGYPDEMRVIWNSAEINT
PPVVRYGLTKKLDMAEYEVEIRTYEANDMCTGPANTTDGFWHPGFIYDVVLNGLEAHTKYYYSY
GTEGHMSEIRNFTTQILPGDPTPFKFIVYGDMGVDSFPQGVTTSKQILDEIRENDIKLVFHNGDISY
ARGYAYIWEQWFHMIEPYATAVPYMIGIGNHEYDHRTGGEKDPSHAEGTGGFRPKWFNGGDDS
GGECGVPMYNRFHMPDNGHSIWWYSFEVGLVHLIMISTEHDFSPNSPMYNWLENDLRNVDRTK
TPWVIIAGHRAMYCSQSIASDYLVALNMQRLFEPLLYKYDVDLAFWAHYHSYERTCKVYQNKCR
PDGITHIVVGSAGKNRDPDVWYKKDWSVFHFADYGYGRVTVANKTHLGYEWIANRYDKVMDS
VWLVK*
>TRINITY_DN5397_c0_g1_i1.p1 type:internal len:161 gc:universal TRINITY_DN5397_c0_g1_i1:2-481(+)

CSVVCDYSPWQDTQSCTKTCDGGVLIQSRTERSFNKVESREGSTPCNTQACGSGLSEERFRKCNA
NQCGTNTGLQEVYKVCNAANCRFQGIDYTRNQEINIRTQACTTACDYTPWQDTTACSKSCNGG
VLVQSRTERTFNKIETRDGTTPCNTQACTQEL
>TRINITY_DN247_c0_g1_i1.p1 type:complete len:373 gc:universal TRINITY_DN247_c0_g1_i1:38-1156(+)
MKLVLVIFVILVYTWNVSAGGKKCISNKDFMTPYPTNFWTEHYPKFVNNRPTIGIVAMEVLGQKM
LVNVPWSWNKDYFGSSFVKLVESAGGRAVPIKEDITEDDLDKLMRKLNGVVLPGGDADVGDSGY
ERISKQAIAFSKKMAKKNVTFPVLGICRGAQMMMMAEAGKDFLEVTDSLNLSIPLDFTSEQKESR
LFGNAPQGLIKSLSEDAITFNAHAMGIPTLNFYNNSGLQKAFRAISTNYDRNGTKFISTFEGRDAPI
YGLQWHPEKSLFVFNPVLAVDHSIKGVMASQYIANFFISEARKNNHTFRDRVEEEEHLLMSYYPS
YVGNITETPYEQIYLFDFVKPFQNPKYTPGKVNKNDDDNSGDDDDE*
>TRINITY_DN17292_c0_g1_i1.p1 type:3prime_partial len:534 gc:universal TRINITY_DN17292_c0_g1_i1:38-1636(+)
MWKIYTIIFLALVSQAFSQDIVGCGGFVQSDVPIAFEKVEVKLYTKQGAYKFKTECAPNNGYFLVA
VYDKGDYVLKIDPPVGWGFEPKEISLNIDGKTDRCSKGEDLNFKFIGFRVSGKIGTVGKESGPSGV
TINLLDKDSKILSSNVTDEIGGFQYDNVMPGNYIAQASHPTWTLKQDKVHFTVENDNVETQTDI
VIAGYDVKGHVLSSGDPIQGVSFILFANGQIDKDAVSGCEKVPEDLLSKLKENGNEKPLCFVLSDK
DGLFSFPTVPTGNYILVPYYTTQNIVFDIVPSKLSFAVNFRSVMFKTPFQVYGFSVTGKVIDSKGNGI
PDVKVTATSQKGEKRSAVSNVDGHYLLENVTSNHYKFLLEKPHYHFIEESIHITPNTPNVKDIMST
RYSVCGKILAYQLPPGVLKVHQHKVLLTLFAEKVTSSNIKSTNPDMDGNFCFSTEPGKYKLEVHTT
ENERKLGFLLKPSHLTVDLVNEPILGLQFEQFRSVVTGKINCIEECKDLSVYMTPSNSPNDKQYGK
VEGSG

Band 5-6
>TRINITY_DN982_c0_g1_i1.p1 type:5prime_partial len:6412 gc:universal TRINITY_DN982_c0_g1_i1:19377-142(-)
KLDFETKQSHTLTIKAVDRGNPSQDSLNKATVIVVVQDFNDNCPLFSESIYNASVSESTAIGTTVLR
VSVTDKDVGNVNNEITCSIDEPLAKGFFSVNKINNNQFSVVLIKNLDYETHRGYRFVIKAKDNGE
VPCQLTTNMVVEVQDEPDVITNFKPFNYVISIPESTLIGTKLLELKVDSESSSITYTLQAKNQPGDLD
KFMLNGQSGELVLKGTVDFEKKSEYEFTATLNDGVNNGGNTANIVVNILDINDNPPVFSQSTCYI
RRMPENQQVNTTVTSVLATDSDQGINSNISYSLIKDSSCNNFEINEITGAISLTSSLNYEVRKEHYCII
KATDNGSPVLSSYSCLKVIVENVNDNSPVFLPKELEITVLESKPVNSFVIRVFATDKDEGNSITYSIV
GGNTQNAFAITPLTGHIILNQKLDYEIVKNYILEIQATDDGTPRRSSNLNAKVMVKVLEVNDNKP
KFNQQRYLRTIPEDTNIGSTIISVTATDDDAGLLTSKIQYIALENTNDFLIQPDTGDIVVSQNLDYER
QRTYQLVAVCSDSGTPPFTGRTLVDITITDVNDNAPILNPTLYNVLVSEKAPLGTSLIQVHAKDKDS
GINGGLKYQIVSGNDLNKFVINNSTGVITLQETLDREKYSSLYNLTVSVSDQATPSLNGNNAFVQI
RVLDVNDKQPVFVKSEYIFEISENQPLNSPIGLVQANDDDSTSPNNIIQYTIVPSKDSINFTMINNQI
LSNSVFDYEEQKSYEFEVQARDQSTGVNNEQLVGRTLVKVLIRDTNDNDPMIENQNIALNISDVT
LVGTLLFDIQATDKDNGENARLTYTISSGDTSTFRINENDGRFYLSKKLDFYNKNEYSLIVIVNDNG
SPQRNSVANVQINVYPIDQTLISFEPQCPIAFTIQENSLSSPMNIKAIDTGSYVSKTMRYMLKSNKSD
VSSMFQIVETNGVIIQNKAANYEERSRYDMTVCARNERGSEGCCPVTIYILDQNEQPFFDTDNDN
LDNGKYLVRVSETTPIGSVMFSVNTGDEDFGSLPNGQKTYSIKAKPGEEMLFLVDSYGRIITNTTLD
REKTPQYDFSVVVTDKGTPPRQAEAEVIVTVIDYNDQRPVFEIMSVRPVNENANNEVLGRVVAVD
IDSTSINNGIKYSIQPAGFLNISNTGTLTSVGSALDYEKNQKVTLYVTATDTGSPYLSATTSLQLDIINI
NDNKPYFLQNNYNLTVSEKTTVGTLVLTLFAEDDDLGEFGEIANYEIVSGNQEDRFSIDSNGRLTL
KNPLNWRNVKSYNLNIIARDKGLPSEQSQIPATVVINVADYSEEQPNFLQNLYVKFIPETTPIDTLV
LTVEATSGEPVYGPFKYTIVPKLPDTNNFQIFSNGEIKTINQFDYENQRNFTFDVQVEDEKNGKTN
RASVFIHITDVNDNIPQFTDLTRSNTQYNITEFTLPGSMITCLSATDRDHGKNAEIRFRGIGGDGNG
VFNVNLDGKVLLSQQIFYDKKNKYNLIVEAFDLGSPEQKAQLTLTIFLHKSYTSVITTDVDINEDVE
TQTRVTRVSSIDPFTRSSNNFTYYLVGKSTTFFEINAQTGEIFTKATLDRETRDRHVLTIQSRGPTTN
GVGELVVNVKDINDNPPIFDPQVYQVTVSESANIGLSIVSVTATDKDIGVNGEVTYSIEDGDQTIFSI
NPSTGSISLAGKLDFESKQSHTLTIKAVDRGNPPQDSVNKATVIVVVQDFNDNCPVFENSTYSLTIK

EPGELGKEYLKLGITDNDNGRNQLFTCSIDEPLAEKYFEMINDCRLRLIKELDYEEFTSFRFVVKAT
DQGDVSCTTTTNMIVNVGDRADVIPFFSPLTYNISIPESTTVGTIVVSLKVGSTFKGLLYSLFHKNTQ
DDLIKFGINKDTGEIFLKESVDREIQDKYEFTADVFDGSNNAVQRASVTINIIDVNDNAPFFNQPTC
YQRDLTESTAIGTFVVDVSAADNDKGRNQQVTYSLLPSPSCTSFSVDETSGRITTSQILDHEQQKLQ
HCLIKAKDNGTNSLSGYSCLTVNVVNVNDNKPAFIVNRFDVSVLETVPAGTLVASLVARDVDNQ
GLITYSISNGNLNNQFTIDSSGKLRTRFPLDYENTKSYNLTIQASDNNGKFSSPDDALVLITVLEVN
DNRPQFLQSYYKVSIPEDTATGASILNVTAIDLDHGELTKSLFYAALENTTQFTVNANTGVISVAEN
LDYEIEDHYELVVIVQDRGTPHLSNRALVDIQITDVNDNAPMFVTTVYNRELSEKALIGAPVVKL
NAMDADKSDKMTFTIDSVVQGAQGFFSINGDTIQLDKELDYEVPEQRTFVLSVSVKDSRDRDGA
NKANVFINVRDINDREPIFDLNMYEVTVSENNVISNPIIEVTARDSDSTPENNNLLYSLVDGEDAA
SFSLLNNGQSAIITSKISFDYEKKKSYRFLVRASDKVRENFTAFSTVFVTVQDTNDEAPVPEKDLIM
VNISESTRVLSTIAIVFANDKDQGINGQLAFQIINHSEDKFEINNKGVIRLKSTLDFFQQNFYNFTVL
VTDKGNPVRTSKPVTVLVYVYRTEIPGIIFEKNHYIFNINEKEIVSLVGTVNAIDEGTYVSRQIVYSIV
SGQNDIKDKFRAVNNNIIQDKEVNYKDQSEYNFVMKAINERGSIAFTHITINIMDVNEKPYFVGL
NNKGRYEFSMSEIISVPSYIYAVRAKDDDFGLNGEVDYEIISNFRFGNFSIDQYGRIQVLKDPDFEFT
RSYEFQVRAKDRGTPSLESNLVSVVITVLDYNDNHPIFNIQEPLEISIKELVNNTDVGTVTATDKDG
SGLNRIVKYVAIYPSDHLTVNRDNGQIRVIEPFDYEIAHKHEFVVVAQDQGTPYLNAYKVIKVEVL
NENDEFPEFTVIEYNITISELTPIGSPVLFVHAQDKDRGPSGQVTNYKIKYVPPSGGSGAGSPDKTFE
ITSDGQVVLKEKLEYKFQKYYQYRVTCTDNGSPPKNSINEAIIHLNIADASLEQPIFSVNPAVVEVY
ENITLNSNIVKVEVDSIEPIKGPFEYSIIASEDSQPFMIDNSTGQITNKESFDYEIKRKYVFLAEARDK
HNNRTQRAMVVIFILDINDNIPTIETIAPQTFVQNISDTTYPGSVVAKLYSRDADSGENAMVDFTIT
GGDGENVFAINPNGYVTLIGPLDVRVKTQYTINVKVSDRGIPTLSNNRALTFNIKKSKFIQNTVRF
DRHHYKSNCIEENQINLNIIQLNATNSNPVAGSTIIYSLDGTLEERQPFIVNSQTGEISLSQTLDYEIK
QEYDFVAVAVNNDGSRDVAVVTVCIKDLNDNSPIFEPQSYVVNVSESESVGYLVYKTTVMDADKT
DFNTTKSYDIIQGNVNDKFIIDSMGHIYIKSKIDYEALQVKHFDLRIRVNEGNLVGDMVLRVNILD
WNDVHPSFSTLNIYNFTIPEDKPVGEIIEILTANDGDATSPNNEVTYKLLTTEDDFILRRNDKGHGS
LIVNKQLDYSKQNRYEYVALAEDKGYLNLLGTAIVIINIQDVNNHPPEFDDSEYNTTVSEHAQIGT
RVLQIHAHDKDTGLGEQIFFIYGDPTETFTISSQGFIILNKQLDYETQRDYIFDVGARDGGNLVSLN
NVTVHIKVSEVNDNEPIFDLSTYRNSYAEDTPLGTTLFTVRATDKDFIGNSIIYSISGANDDTSYVSV
DQTTGDITLQRRMDYESKNYITFTVQAIDSNNRNKLKGSTHVIIDVTDVNDNNPKFVFGSDLVYN
VTVVENINVGTLVVVVDSQDADSGLNGQTRYHIVSGNNENKFFINELNGQINLINKLDALQTNK
YVLTVMAKDLGNPSRSTNVTVTINVLRYFESTPMFLPSYAEVIYPENLALGSSIYELNVTKTLPGSP
MKLSFHSSSKFPEFTLQGNKIILSKEFDYEIKNRYELIIIATDEENDKAFAQVIVRVQNLNDNIPYFTQ
NAYFGVVSELAFVGHSVLTITAKDDDKDFTELFYSIATQVPDNAFKLQDNIVVVNGKLIRNKVYEF
EVEAFDNTFTSKRATVSITVTPENLPSFVNATYIVTLQENYPISTNFLNISAGTFSGITYVITSMKAKD
MFNVDANGQISLTKSLDYEKQRQHVFPVTASVTSPITQVRVASVIVNVLNENDNAPVFVNGPYER
NITEGHLVGTMVIRVDGHDDDSDKLTYSIVSGDDGKFSINSEGVISLISTLNFDLKNKYVLNVRVSD
GKFVTDTIVTINITPIVKFPPMFNQTLYLLSVKENTLVGTSILQVSTVNVIPPSSFSINEQSGRDFFTID
ANGVIRTNNALDYEAKRQHIFTLKVQDLRSMGHATVVVNIVNDDDTCPILTPATQTVQITEPVAP
GTIVAAVQAKDIDSKSLNFTITGGNGLFRIDQHGGIKTINEVDISTSTEYELTVTVDDGTCTKSVQV
DVNVNPLAPCPDCNIYPFTQTIYEFSINENTVPASPIITVTTGQSLQRNFSIEDPAALEYVTINRLTGE
ISVIKAFDYEATKHLSFNVMAKTQTTKNRNPFYVITSVSRVRITVRDLNDNCPSIESPMYYEVSHSS
PIVQDTVVTRILVEDPDTLKDHTYSISDNNFKIDSMGHIRTVQNIESFTAQFYNLTVTVSDGQCSKT
SNVGVNLAVNPTPMTYQFTSNGVYSKSVREDHNLQDVILSVALNQNYPRVFSIVESDANDQFVID
SNSGALRLKTSLNYELKKRYDFTVMADIHGGVYKSYASVHIIVLDVNDECPIFPVIGYNQRVSGTP
VIGTIVEIIEANDADNATLEYSIVSGNEDGHFSLDNSGVVRVVKPFASSFSKTYGLSVRATDNKCSA
DVPLTVQVSTCAYGEDYQFEESEYVYYLDENRNLGIVSNIKLKSTRAAFLTLSQNTHFTIDQNGDF
RLIQTLNYETKKEYHLTLTANFTQDNVVTRIPIKVFVRDINDNCPAFSEELYAVQHTEPLREGLLIA
KLSVIDDDTVQNFTYNIVSGDKRFSINENGEVVMNARLVTDTTLLSTTYKLEVSVNDGKCAKNTK
VDVFVYKTVVNSFLFPQPFYKFDISENQAIPFTIDRFVNVGGHEAVYGFLVSTDFFQIDPKTAQLQL
VKKLDFEVEKTHYVLISATYDDGTQTYTVVAIHVGDENDNAPQIFAHPPITRLVQNTPHNSIVTKI
EVEDPDTSKEFTFKITNGNPLQNNDPDKALFRIESNGMIKTTRCISEDEIGNYVLTISVDDQVNTPTI

FSNMQVQLKTTDDRSYVTNCGLGCPANDGPFSRYQFEQSLFIVNVTETAANPTVLKRLTRPFVEK
TTYTIGAGADGKFSIDQNGTLSLTGSLDYETKPVYYFIVSAVIPSQVVGRFITSKTAYLVMVNDVDD
SKPVIEGNGGTLAVSETSEIGQIVGTVRVKDSDSSSPLTYNVVNGSQYFNIDSAGNIVIKDTLNAAG
SPYTVTYTVSDGINPPVNGEFVVSISETNNNVPKFTQNIYEVSLSEDTATDTIAVTILANDGDTNDKI
AYSFLQSEGPFVINRDSGEIKLIGPLDYEAQKSYNMVVQATDSGGFANIAFVQIAVGDVNDQVPVI
TPQTTPTQIHESTLVNSVVYRVIASDGDGNERLTYALANSNDFFTINSATGEITIKENLAKSNVSSHT
LSIVVTDSSGKTSTQDVALNIRRSGTSQGCVKITTSQGQSSSPVINLPENTASGTTVFKLVAGDASVF
SNMRFTLNREANGVFAVTEQGELQLVAPLDYETKQFYSIIITAEDVTRGVSSQYNLFVRISDVNEFT
PVLETIAPVAVSELANIGDVIAQAKANDQDGDSQVTYSLGEFGNNDNKFTIDAATGNIILNKRLG
SGAPHVVEVCASDGSIKKCQNVTFIVADTNDNPPSFQDLTGVGSIDLTNTSSYVVYKPIVTDSDQT
GTLSFSLKDPAPSQFSINSTTGEVKANKDIAKGTYTFGVCASDGRYTSCQSVTVVASDRSQSITPSFN
QDQVGAIVDISASSGKQIIQVMASTLDKAKIEYELKNEADRNIFEINKDSGLITLKNRTALTLGTIYT
IPVKASRADDPLNFDEINIVISVGNQQLDSGSFIEQVLDTQHLVNFSLSTFDISKVSYYIILGQQVGG
DSKDDEKLPVSWYYATFGDESYRHRFYYIAKLDGSKFRNTGGSRKRRSIQDKDSKSFTYLVGEENG
CSKNTSMAAICNGPLKTGTEYRYKLRAYYDDGELRGIPVDQKPTEVKRTQAPQAGKSIDEYREQD
SYVITFWIIGLVIFGLLFWFSVLVIAILLCYKRRYEVTQAYGYSVQTRDKIDGPPTEYSNTVYRFQNPS
YPDNDTVKMKKVDASESRTKTEVQTTSMVVLDDYEQEQPQPDIPVQDYDAIEYETRSAQIKFHEE
SPEWESMDIQLRIDPTGNQDPVITRKSDSGSKDSDSDASKKYENTEYIEEKEDFVNEKEDSLTRL*
>TRINITY_DN982_c0_g1_i3.p1 type:complete len:9852 gc:universal TRINITY_DN982_c0_g1_i3:29697-142(-)
MSIKLCTFFVFFLIQCINSFPEIGLHLSLDRSYSFQGNGWKTVNGWEIKPRANGPYEIDAIKNISPEY
FPVPITGVYYITVSLPVEGASNGEFKSAIIVDGKNEEGRIGLMSSFQKTAFKNGASTLTFAGFVRLFA
DEKLTIEIYSDDINANYVVLESATFVFHFIGVTGSVPAYLAHTAQDQEYASDSIIKPWVTLGDSRVYE
SLSGFIKNEGVFTTICNGVHKMAVNIMIKANAGTYTLKALFQDEESATATVTINQPDSIATLNIFQN
FKWSEGMSITLQIHSSNGANFKVLKSSSWSMSFIGETTANFQEFSVFLMADQFLQPSQFPQRINIDL
NGVFQFLQSNPDQYNKGQLVSGADTKFKDNLDFIVDSTDLFYVSANIIIEGEAEDLEITIAVSQIDID
VLPEDGLSGRLTKISNSRRSISLSGIIFIREKQYLSFFVRSRDSSKSFTVSKDSFISVINMRYTVSSVSARQ
NENIDVTAGSWYDISGRFAEQKNGLYSFGKDFNINNGVFRTSHSGIHSVHANIQFSLDSDAFGDN
DDNKIVLRVVIDGQEDKISLMSIRKNPEKTATLKLFGSIYLRAGVTISFQVKSLMPNTVQYKILQRSS
ISISYIGPQWAVNSFHAVANQDLNYDGKPDDTRPDIPSPLNFWKTEAADEFISPFEVLESFDGTRYT
SVDDGLYVVSSLLILKNVDCDRSALFKLDFVTTGGSRASDKSNGFADSHFFIADSKGTEFTLSVTTA
MRLEVGFKVGLKLTVGSTSCRYTISKESSFSIAQWSKSSNTIEENRLKYIGVLARLSAPTEVSSPNTW
SNLGSNTMDINPGDKSRSSFRPGIFGGDDLAPITRTGKDFNGDVSDFTIKDPGIFFISGMITVTTTEN
ANEEFAATVQIDSEDIESGLYAAVINQGSTEMSLTFGGTIYLKSGARVRVVVKGGRTFNIAAGSSFSL
LKLQPDYKTPGIIVSIDEKITQAITRYEHKDLRADIHRTLTIGNLGFVESTGVYTIQVDGVYMVDYT
VILEATNPNAVFGAEILSDNTVVATGTSTFGNTKTIAITELVYFSKGAIVKAVLKSNTNGETLNNLQ
VDGGLTVYFLEPKEHVKDGHRSKQTSPQALPVRSFEKVTGFAAQSPDFAGGADSVDYTVPETGVY
LVAFTTRVNRVINGFKSRVLVNDNEFLNGNYQIVSAQNAPATLPLSIAMELTVNDVLSFQVMADG
SGMSLDGSTRSILKIDEVNTIRNVTEGLSAYKLRDQKVNGQQETIVEEWDTSVTEGTFLAKNVSFT
VDHSIVVVAAGVFQISVSVVVKNDRPSQSKFELRVLQGDDVKSHGLHSKVEIGGGREQTLTIGGSL
LLQKFDSLRIVVRDIADPLASYTIKAGSTVSVIIIDDNNGPIACEDEGLNMRRLPERIRKPTRESHTF
QCNVVSSLKAEKYTWFKDNVQLKEENQKNLVLNDLKLSDSGVYQCRVRFLELTGATNNATLSVY
DPYPKITSPKFIANVLENQDPPLTIEMFSLSAFKLSPEDEKASITTQIIAGDDQSFFTLSGPSKQTVTLS
TTKKLDYETQNEYNLTIKATNLEETALTTKGPYETTINYIVRVIDVNDHPPVFSQSIYSRSVPEDYPI
DGSIAKVSANDQDAGANVIYTLVGQVSGDLFKIDNTGLIQLRQNLDREIRDDHTIFVQASDGLFT
TVAEVVVTVTDVNDNFPNFMDTGFDISVSEATLTQSVILKVIATDPDLGTNSQLRYTIKSGNNENK
FSLNLLDGNLTLISKLDYEGTKIYNLEIDVQDSASPPKKSQNPLTVVIRVQDANDNNPTFTQSLYNT
RIAENTAVGTSVLKVSATDRDGEGINNPLTFSITDTDANEFFQVVSSGATAGEIRVRKSLNYDIRKD
YRFSVVAKDNNLGEPRYRTALVVINIDDTNNLRPTFEAGSASISISEGTSIGTSVHQVVAIDKDTVG
KLDYRIISGADGKFAINKETGDINLISAVDFESNKKTYTLEIQVNDGVRDALNPATVVINIQDFND
NQPEFIQKYYIFTALESISDSQAINVGNPVIARDADSTTNSQVDYSIQYDINNPSVPNEGDALFRIDS
NGLIFKKNGVILDFENVRYHTFTVVAIDRGTPKTLLGKAFVTINIQDVNDEAPSWSPAIYSVTVKES

AGINRDLVFLSTTDKDTSGAVTYSIVSGDSANQFQIFDNVIKIVKPLDFEKTETHTLKVSAQDDNG
NRAQLDATVTITVEEVNDNRPLVTNNNANSIQVSVPEDIPVNSLIVKLNGRDDDKGAPGQASNQ
FTFVSLDKTTDFTIDEVTGEIKNTVNLDYERQKSYSLVATIRDKGTPYLESKFLVDIIVENKNDERPIF
DPAIYTIKLSEKAPIDTVVAMLQATDLDTPKSQLRFSSVTTLDSHFSLDPITGIITLKTSFDKEVNGKD
GIFVISCTVNDGTSNSAQNAIVRVEVVDVNDNQPVFPTSEYFIQVNEGSSIGTILQNIAATDKDNT
NTHNQFTYTLEGKEAVDFRLNGNQLQTNKIFDYENKKSYSFDVLATNNLSPDKLVGKATVVVSIL
DQNDNNPVLSPSGVINLNISEGTIAPANIITIFATDKDSTINAEVQFTLTGDPTNTFTINQNTGEIML
AKSVDSFAKNEYTLSVTATDKGSPPRSSTATTLNISIKPVFVPGIEFENCPQIISFNENAAPNTFTKTL
TVKDRGSFNQVQFTVVSGQAGIQNLITVSQNGVITNGQRRADFEARKRHDFLVQAENERGAKAY
CHVTANINDVNEGPIFDSANYVFDVLEVIKINTVVFSVRATDPDFSSTTNGQLTYSLAGQEASQFSI
VPNNGQIKLAQPLDYEIKKQYQFNVIATDGGSLQTTSSVIVNVEDWNDNRPIFDQPQKLAINVPE
NSKPDNVFTVSATDKDSTTNGEIEYVGIYPTGIISIDKDTGTAKIIGDLNYEKRQKYDFVIVAKDKG
VPYLSNTREVSLSVLNLADEVPYFDTAAYTISISEKTSINTAVFRVMAKDNDLENFGKISSYEIISGNQ
LNRFRIDSNGNIIVQQSVVLENNNPNQYILTITAFDTQRTQSLNNTQVTINIIDLSDKQPFFIQNSYI
VEVQENNEVSKSLLSLEAQSNEPTFNPTYSIVQDPKNTDSQFFQIINGNTIVATTVFDYEKKRFYSFE
VQVIDTNNGKTNKALVVVRITDENDNKPFEVSGNLHFNITEHTLLGSTIGSLNGKDNDAGINKEF
EFSILEGNDDDKFMLLPTGQILLKNFVSVKEKDFYKIEVKITDKGTPSLFNIFTVRVTINSSQALVAS
NNPTIPEDKNTGEEITRVTAIDPNTGSSNGFIYSLVGTLSNSQFTIDAKTGAISLLASVDFETDRTHT
AVVKAEGPTSTGVTEVIVRVTDINDNKPIFDPTNFNLNINENFVVGQEIVLVRATDKDSSLNDNNV
IIYRIDSVNPSSGNDLFSVNPSTGRILLLKALDRENGDTSYVLTIAASNRGVSNKPDNTMTVTINIA
DVNDNFPIFEKSLYQGSVSEDANTGTSILTVRASDKDILLKNRNIRYSMTQPMAVQKFQIDPISGVV
SLKQEVDFETKQRYDFSVIASDTGDVAYSRTANVEITILDVPDVEPKFDPQRYEITIAESTLVSTSILK
LQVIDSVKNLNFNIKQGNSNSDFRLVASSGELFVNKQLDRERISRYQMTIEVSDSVNKAAIDAIVIV
NIADVNDNQPLFLQSGCYQANRAEGIETEQVVVNVQATDRDEGSNKEIVYSFSDACSVLDIDAN
TGAITTKQKFDYESARSHICTIKAEDKGTPKLSGFSCIILTVTDSNDLKPTFEVITSNARVLENIPVGS
VITRVLATDKDTLSSNKQFVYTIKTGNEDGRFTIDANGFIKLQQPLDYEKKTDYRLEIEAVDSTNQ
DMVSDPNAIVTVTVIEQNDNRPKFAKSYHRVNIEESTAIGTTVLKLSATDADAGVNEGLIRYVSLH
NTTVFNIDRTTGEITLATKLDYEVKKSYHLIAVVKDQGNPVLSARTLIDIFVNDVNDNPPVFVISSY
NNIVSEKAPIGQSIVTVSARDDDTGVNGQLKYKIRETQSMFRIDEDTGIIILNGDLNVENTNKLLYA
LTIDVVDSGVPPLQASNPATVSVQVSDVNDKQPVFALDRYQFEINENIEGNQIFGDSVTVRANDD
DLTGTNNQLTYRLVNTTMFDLVVTNQNVFIKTALGVSFDYETKKSYSFEIEAMDSAANKLYGRSLV
TVTVKDFNDNPPIITVPNIIVNISDATLIGSHIATIMAEDKDSGENGILIYTVTAGDSTNRFTMESDT
GSIILKNPVDSYTKDIYVLNVLVQDNGIPRRSATGTVTINVIPVDLTLLKFNTTCPFAISIPENQINSN
FLGLKALDAGEYVSKQLRYTINSDENDVNQIFALDSIKGEFKQLKEANYEERSRYDMTICATNERG
SKVCCHITIFITDINEPPSFIGLNNNNIYEVSISETTPIGTIIYRVLSKDQDLPGVLNGQTSYSLGQQNV
FIIDQMGQIRVNKTLDAETQLNYVFQVIVTDKNPTNPLQSAATIRVNILDYNDERPIIQNIPDITIDE
NSDNQVVRNIVVTDRDISNIGSNIELSIEPSNILTINNRQLRTVGSALDYEKVQRHTLYLTAKDKSFP
YLSSTTSFTVTINNLNDNKPKFPVNNYNLTVSEKTTVGTLILTLFAEDDDLEEFGEIANYEIVSGNQ
EDRFSIDSNGRLTLKKQLNWRNVKSYNLNIIARDKGLPSEQSQIPATVVINVADYSEEQPNFLQNL
YVKFIPETTPIDTLVLTVEATSGEPVYGPFKYTIVPKLPDTNNFQIFSNGEIKTINQFDYENQRNFTFD
VQVEDEKNGKTNRASVFIHITDVNDNIPQFTDLTRSNTQYNITEFTLPGSMITCLSATDRDHGKNA
EIRFRGIGGDGNGVFNVNLDGKVLLSQQIFYDKKNKYNLIVEAFDLGSPEQKAQLTLTIFLHKSYT
SVITTDVDINEDVETQTRVTRVSSIDPFTRSSNNFTYYLVGKSTTFFEINAQTGEIFTKATLDRETRDR
HVLTIQSRGPTTNGVGELVVNVKDINDNPPIFDPQVYQVTVSESANIGLSIVSVTATDKDIGVNGE
VTYSIEDGDQTIFSINPSTGSISLAGKLDFESKQSHTLTIKAVDRGNPPQDSVNKATVIVVVQDFND
NCPVFENSTYSLTIKEPGELGKEYLKLGITDNDNGRNQLFTCSIDEPLAEKYFEMINDCRLRLIKEL
DYEEFTSFRFVVKATDQGDVSCTTTTNMIVNVGDRADVIPFFSPLTYNISIPESTTVGTIVVSLKVGS
TFKGLLYSLFHKNTQDDLIKFGINKDTGEIFLKESVDREIQDKYEFTADVFDGSNNAVQRASVTINII
DVNDNAPFFNQPTCYQRDLTESTAIGTFVVDVSAADNDKGRNQQVTYSLLPSPSCTSFSVDETSG
RITTSQILDHEQQKLQHCLIKAKDNGTNSLSGYSCLTVNVVNVNDNKPAFIVNRFDVSVLETVPA
GTLVASLVARDVDNQGLITYSISNGNLNNQFTIDSSGKLRTRFPLDYENTKSYNLTIQASDNNGKFS
SPDDALVLITVLEVNDNRPQFLQSYYKVSIPEDTATGASILNVTAIDLDHGELTKSLFYAALENTTQ

FTVNANTGVISVAENLDYEIEDHYELVVIVQDRGTPHLSNRALVDIQITDVNDNAPMFVTTVYNR
ELSEKALIGAPVVKLNAMDADKSDKMTFTIDSVVQGAQGFFSINGDTIQLDKELDYEVPEQRTFV
LSVSVKDSRDRDGANKANVFINVRDINDREPIFDLNMYEVTVSENNVISNPIIEVTARDSDSTPEN
NNLLYSLVDGEDAASFSLLNNGQSAIITSKISFDYEKKKSYRFLVRASDKVRENFTAFSTVFVTVQD
TNDEAPVPEKDLIMVNISESTRVLSTIAIVFANDKDQGINGQLAFQIINHSEDKFEINNKGVIRLKST
LDFFQQNFYNFTVLVTDKGNPVRTSKPVTVLVYVYRTEIPGIIFEKNHYIFNINEKEIVSLVGTVNAI
DEGTYVSRQIVYSIVSGQNDIKDKFRAVNNNIIQDKEVNYKDQSEYNFVMKAINERGSIAFTHITIN
IMDVNEKPYFVGLNNKGRYEFSMSEIISVPSYIYAVRAKDDDFGLNGEVDYEIISNFRFGNFSIDQY
GRIQVLKDPDFEFTRSYEFQVRAKDRGTPSLESNLVSVVITVLDYNDNHPIFNIQEPLEISIKELVNN
TDVGTVTATDKDGSGLNRIVKYVAIYPSDHLTVNRDNGQIRVIEPFDYEIAHKHEFVVVAQDQGT
PYLNAYKVIKVEVLNENDEFPEFTVIEYNITISELTPIGSPVLFVHAQDKDRGPSGQVTNYKIKYVPP
SGGSGAGSPDKTFEITSDGQVVLKEKLEYKFQKYYQYRVTCTDNGSPPKNSINEAIIHLNIADASLE
QPIFSVNPAVVEVYENITLNSNIVKVEVDSIEPIKGPFEYSIIASEDSQPFMIDNSTGQITNKESFDYEI
KRKYVFLAEARDKHNNRTQRAMVVIFILDINDNIPTIETIAPQTFVQNISDTTYPGSVVAKLYSRDA
DSGENAMVDFTITGGDGENVFAINPNGYVTLIGPLDVRVKTQYTINVKVSDRGIPTLSNNRALTF
NIKKSKFIQNTVRFDRHHYKSNCIEENQINLNIIQLNATNSNPVAGSTIIYSLDGTLEERQPFIVNSQ
TGEISLSQTLDYEIKQEYDFVAVAVNNDGSRDVAVVTVCIKDLNDNSPIFEPQSYVVNVSESESVGY
LVYKTTVMDADKTDFNTTKSYDIIQGNVNDKFIIDSMGHIYIKSKIDYEALQVKHFDLRIRVNEGN
LVGDMVLRVNILDWNDVHPSFSTLNIYNFTIPEDKPVGEIIEILTANDGDATSPNNEVTYKLLTTED
DFILRRNDKGHGSLIVNKQLDYSKQNRYEYVALAEDKGYLNLLGTAIVIINIQDVNNHPPEFDDSE
YNTTVSEHAQIGTRVLQIHAHDKDTGLGEQIFFIYGDPTETFTISSQGFIILNKQLDYETQRDYIFDV
GARDGGNLVSLNNVTVHIKVSEVNDNEPIFDLSTYRNSYAEDTPLGTTLFTVRATDKDFIGNSIIYSI
SGANDDTSYVSVDQTTGDITLQRRMDYESKNYITFTVQAIDSNNRNKLKGSTHVIIDVTDVNDN
NPKFVFGSDLVYNVTVVENINVGTLVVVVDSQDADSGLNGQTRYHIVSGNNENKFFINELNGQI
NLINKLDALQTNKYVLTVMAKDLGNPSRSTNVTVTINVLRYFESTPMFLPSYAEVIYPENLALGSSI
YELNVTKTLPGSPMKLSFHSSSKFPEFTLQGNKIILSKEFDYEIKNRYELIIIATDEENDKAFAQVIVR
VQNLNDNIPYFTQNAYFGVVSELAFVGHSVLTITAKDDDKDFTELFYSIATQVPDNAFKLQDNIV
VVNGKLIRNKVYEFEVEAFDNTFTSKRATVSITVTPENLPSFVNATYIVTLQENYPISTNFLNISAGT
FSGITYVITSMKAKDMFNVDANGQISLTKSLDYEKQRQHVFPVTASVTSPITQVRVASVIVNVLNE
NDNAPVFVNGPYERNITEGHLVGTMVIRVDGHDDDSDKLTYSIVSGDDGKFSINSEGVISLISTLNF
DLKNKYVLNVRVSDGKFVTDTIVTINITPIVKFPPMFNQTLYLLSVKENTLVGTSILQVSTVNVIPPS
SFSINEQSGRDFFTIDANGVIRTNNALDYEAKRQHIFTLKVQDLRSMGHATVVVNIVNDDDTCPIL
TPATQTVQITEPVAPGTIVAAVQAKDIDSKSLNFTITGGNGLFRIDQHGGIKTINEVDISTSTEYELTV
TVDDGTCTKSVQVDVNVNPLAPCPDCNIYPFTQTIYEFSINENTVPASPIITVTTGQSLQRNFSIEDP
AALEYVTINRLTGEISVIKAFDYEATKHLSFNVMAKTQTTKNRNPFYVITSVSRVRITVRDLNDNCP
SIESPMYYEVSHSSPIVQDTVVTRILVEDPDTLKDHTYSISDNNFKIDSMGHIRTVQNIESFTAQFYN
LTVTVSDGQCSKTSNVGVNLAVNPTPMTYQFTSNGVYSKSVREDHNLQDVILSVALNQNYPRVFS
IVESDANDQFVIDSNSGALRLKTSLNYELKKRYDFTVMADIHGGVYKSYASVHIIVLDVNDECPIF
PVIGYNQRVSGTPVIGTIVEIIEANDADNATLEYSIVSGNEDGHFSLDNSGVVRVVKPFASSFSKTYG
LSVRATDNKCSADVPLTVQVSTCAYGEDYQFEESEYVVYYLDENRNLGIVSNIKLKSTRAAFLTLSQ
NTHFTIDQNGDFRLIQTLNYETKKEYHLTLTANFTQDNVVTRIPIKVFVRDINDNCPAFSEELYAVQ
HTEPLREGLLIAKLSVIDDDTVQNFTYNIVSGDKRFSINENGEVVMNARLVTDTTLLSTTYKLEVSV
NDGKCAKNTKVDVFVYKTVVNSFLFPQPFYKFDISENQAIPFTIDRFVNVGGHEAVYGFLVSTDFF
QIDPKTAQLQLVKKLDFEVEKTHYVLISATYDDGTQTYTVVAIHVGDENDNAPQIFAHPPITRLVQ
NTPHNSIVTKIEVEDPDTSKEFTFKITNGNPLQNNDPDKALFRIESNGMIKTTRCISEDEIGNYVLTI
SVDDQVNTPTIFSNMQVQLKTTDDRSYVTNCGLGCPANDGPFSRYQFEQSLFIVNVTETAANPTV
LKRLTRPFVEKTTYTIGAGADGKFSIDQNGTLSLTGSLDYETKPVYYFIVSAVIPSQVVGRFITSKTAY
LVMVNDVDDSKPVIEGNGGTLAVSETSEIGQIVGTVRVKDSDSSSPLTYNVVNGSQYFNIDSAGNI
VIKDTLNAAGSPYTVTYTVSDGINPPVNGEFVVSISETNNNVPKFTQNIYEVSLSEDTATDTIAVTIL
ANDGDTNDKIAYSFLQSEGPFVINRDSGEIKLIGPLDYEAQKSYNMVVQATDSGGFANIAFVQIAV
GDVNDQVPVITPQTTPTQIHESTLVNSVVYRVIASDGDGNERLTYALANSNDFFTINSATGEITIKE
NLAKSNVSSHTLSIVVTDSSGKTSTQDVALNIRRSGTSQGCVKITTSQGQSSSPVINLPENTASGTTV

FKLVAGDASVFSNMRFTLNREANGVFAVTEQGELQLVAPLDYETKQFYSIIITAEDVTRGVSSQYNL
FVRISDVNEFTPVLETIAPVAVSELANIGDVIAQAKANDQDGDSQVTYSLGEFGNNDNKFTIDAAT
GNIILNKRLGSGAPHVVEVCASDGSIKKCQNVTFIVADTNDNPPSFQDLTGVGSIDLTNTSSYVVY
KPIVTDSDQTGTLSFSLKDPAPSQFSINSTTGEVKANKDIAKGTYTFGVCASDGRYTSCQSVTVVAS
DRSQSITPSFNQDQVGAIVDISASSGKQIIQVMASTLDKAKIEYELKNEADRNIFEINKDSGLITLKN
RTALTLGTIYTIPVKASRADDPLNFDEINIVISVGNQQLDSGSFIEQVLDTQHLVNFSLSTFDISKVSY
YIILGQQVGGDSKDDEKLPVSWYYATFGDESYRHRFYYIAKLDGSKFRNTGGSRKRRSIQDKDSKS
FTYLVGEENGCSKNTSMAAICNGPLKTGTEYRYKLRAYYDDGELRGIPVDQKPTEVKRTQAPQA
GKSIDEYREQDSYVITFWIIGLVIFGLLFWFSVLVIAILLCYKRRYEVTQAYGYSVQTRDKIDGPPTEY
SNTVYRFQNPSYPDNDTVKMKKVDASESRTKTEVQTTSMVVLDDYEQEQPQPDIPVQDYDAIEY
ETRSAQIKFHEESPEWESMDIQLRIDPTGNQDPVITRKSDSGSKDSDSDASKKYENTEYIEEKEDFV
NEKEDSLTRL*
>TRINITY_DN161_c0_g1_i2.p1 type:5prime_partial len:399 gc:universal
TRINITY_DN161_c0_g1_i2:1683-487(-)
WNQEPLTCTYLYSVNSQLLKYKMADDDVAALVVDNGSGMCKAGFAGDDAPRAVFPSIVGRPRH
QGVMVGMGQKDSYVGDEAQSKRGILTLKYPIEHGIVTNWDDMEKIWHHTFYNELRVAPEEHPV
LLTEAPLNPKANREKMTQIMFETFNSPAMYVAIQAVLSLYASGRTTGIVLDSGDGVSHTVPIYEGY
ALPHAIIRLDLAGRDLTDYLMKILTERGYSFTTTAEREIVRDIKEKLCYVALDFEQEMQTAASSSSLE
KSYELPDGQVITIGNERFRCPETLFQPSFIGMESAGVHETTYNSIMKCDVDIRKDLYANTVLSGGTT
MFPGIADRMQKEISALAPPTMKIKIIAPPERKYSVWIGGSILASLSTFQQMWISKQEYDESGPSIVHR
KCF*
>TRINITY_DN4595_c0_g1_i4.p1 type:5prime_partial len:1639 gc:universal
TRINITY_DN4595_c0_g1_i4:5857-941(-)
QCGVNTGTQVVYRRCNAALCKYQGNDYTRNQEVNIRTQACTVACDYTPWQNTQSCTKTCDGG
VLVQTRTERAFNKIETRNGNTPCNTQACAQELGPKQLRTCNVGQCGVNTGAQVVYRRCNAAL
CKYQGNDYTRNQEVNIRTQACTVACDYTPWQDTQSCTKTCGGGVLVQSRTERAFNKVETRNGN
TPCNTQVCSSELGPEQLRTCNVGQCGVNTGTQVVYRVCNAALCRYQGSDYTRNQEVNIRTQAC
TVACDYTPWQDTQSCTKSCDGGVLVQTRIERAFNRVETRDGTTPCNTRSCASDFSAERLRSCLNV
NQCGVNTGTQTVYIVCNGNFCRYQGREYNINEEVNVRQQNCTIACDYTAWEDTTICSKICDGGV
LEQRRLERAFNRLERRNGTTPCNTRACDSGFSAVLSRNCVNVNQCGVSNGTQLLYRVCNGEPCIL
QERSYKINDVVVVGSRACSVPCNYTEWRDTTSCSKTCGGGFFFETRTEQAFQKTESRLGKTLCNE
NSCVKDNFGNWTEWTACSKTCGMGMRYRSRECLKGFECIGLIEETETCKLGCRTETCPMGRPYP
GDCNFEDDCGKSWNQVNSIQRNAFFIVTSKDLAHQGLLPNMETTFNTKDCGRTMKLNSSVAGD
VNYLWTKRMMITDINTDVDDDVAAIDNYFKEENEQCVEFNFAVMANSLDKIGDMQVYLYYLEY
PNASLIYSKKGNTGLKWELVKRSIRVDTNKAFVILFQVNQGQSNVQYGDIGIDGFKVNPGNCQLK
GWSHWGEWKCSATCGFGTQTRMRHCLDKVNRTACVGMDRENKPCPMMKECPMNRDGNWG
QWTEFSKCSKTCDTGTYFRTRKCDSPSAKYNGSLCRVSQTKFNLPVVNDFEIDFKPCSVQQCNNT
FSQWTTWSSCSGTCGPGQRTRTRTCESGKRCSGIFEEIETCYMGCRLEKCPKFTAEIDCNFEDRNKS
DCQWKYYTVSGTSSWSFKTQPKNRLLSGPSGDSTIGCGYFLYYPSQTLSSAYAWGPNIYPTMDSYC
VKFKYSMVTSVNSSMGNLTVIKHFLNRPRKADTPLFFHSGVSQKQWNPMAIDITTDIQRSPFVIMF
KGQSDPNNAGLSDIAIDDYMLMAGKCNKSYSEWSEWSTCSRTCGVGQRTRTRVCLDDDIKVCK
MGDVATEGEACVKPLCAVPVDGNWGQWSAYSQCNARCNGTQKRERFCNNPAPSNGGRMCLL
GDTIFQLRNNYHAQIRPCSIDCGDNDTFSSYKRINDTCHFDCITGRVLQERECEQDTCFDGNTRFS
KGEKQYTQRACSIKEYCETSEYKCREFRSVCSTNTPSSCLDPQNRAVKSCECQVERGCSSMTNPGQ
TVKHKEFDVFYRPCRYVGVCFTDYETDAFSPYEKVNDTCHLSCSNGKVFEERECRQDNCIDGSNT
LTRGAKQYRETQCTTKEYCETSQYSCREISGPCSSNPPSSCQDPANKSLKVCTCQVTTGCASMLTA
GQAVSYLAEETFSRPCKYTGACNANGNTDKTFSPYERVNDTCHISCADGKVKQMRECKKDACV
DGGQNFPRGFKQFKDSSCTPNEFCETAQYSCRELSSACTSNPPSSCLVSTNKAVKMCDCNDPNGC
DSMITPGKKVNQGEKEFLQRPCRYTGVCFTDAETGT*
>TRINITY_DN15274_c0_g1_i1.p1 type:complete len:1822 gc:universal
TRINITY_DN15274_c0_g1_i1:87-5552(+)

MECIRRSIIRLLLILGFYTLYISSQEECKRGGCFPATGDLLVGREKQLLANSTCGLNGPSKYCILSFLD
KTDKCFYCDSRRQVDERPDGYKNSHLPKYMVTREPSKRLQGWWQSVNGRQDVTIQLDLEAEFIF
THLIMTFKSFRPRAMYIERSSNYGNDWSDYRYFAYNCRKSFPNIPRGPQRKIDDVICEERYSKVEPS
TGGEVVMKVLDPIIQKKEDPYSLKVLNLLKLTNLRVHFKELHTFGDDILGDSPKVNQKYYYALYE
MVVRGSCSCYGHAEQCIPTKGTPANVNMVHGQCNCTHNTKGRNCEMCKDGYNDEPWRPAYQ
DQLNVCRKCNCNGHTDKCHFDPAVYKATGNTSGGVCDDCNHNTAGRQCELCKPLYYRDPYKN
IFDEDVCKPCDCDPSGSFGGGECETISDPNNSSALTVAGRCICKPNAQGLRCDSCKDGYWNLLES
NPDGCQSCNCDDKGTVELNKCDQQTGTCRCKRFVIGLTCSKCLEGFWNLGNTPEGCEPCNCDV
GGSLNAKCDDITGMCMCKPNIIGRKCDQVAPGSYFGNLDQVISEAENSLVRSDSSSVEIVREILNPT
DSRSFTGDGYLRVSDGTLVNFTNIRVPSTGFQTFVMRYSRPTPGSNNNVMVRITKTDGTILGGNGC
VQTNEREIILNGVLNDDNFLELGDMFCLEKDTDYSVQLVVSNRNSPNDGGVNRGVTNLDSIVM
MPNIDNLAIFQGREGMRLKSQYDLNNCRQYYLEPNGAANAPEICKKIQFSISAEINNGALKCNC
DPTGTNSSGSSDILTCQKSGGQCPCKPGVVGRRCEKCAPGYFGFDSNGCTACACNDTGSEKLFCN
GITGQCPCKNRIKGRQCNSCPSNHFGFPNCIACQCNEHSSTCDPISGECTNCKDNTAGPSCNLCA
EGYYGDATAGTEKDCQRCQCPGGSSLNQFSNTCHLKPDGKVVCNNCTEGYTGDQCDKCEDGY
YGNPLVPNGSCNKCDCNGNIDPFADGKCNAENGKCFNCINNSDGDHCENCKEGFFGNAKNQS
CRACVCNKLGTNSATSDSCDRETGQCECLPNVEGVECDRCKSGYYNIASGNGCVSCNCDKGGSL
SSHCNQLDGQCNCLNGYGGLDCTQCEDFYFGDPKQNTCQACGCNPVGSITLQCNRTTGVCMC
KDNVVGEKCDMCAPQTAGVMPQCEECHVCNDQWEDIVNDIEKNITTILTLKGSSPLNVSEIRAY
TNDIADLKNRLDKIEEDFGKQMVDPNKINRLRDKVYRFSYNLTGINRDADFYMEMVNETITRDE
KANDILDGLELRLATLQTNTEDFKNNVTKIVESNIGGALNSTRESLEKSRKAQESVNDSDLKIKES
KKMRKKIKKEIQNGDPEFEVMHNRSSEFVNNLADRENYLEGELEKLNRLLCGSDKRCGGCTKGG
CDTCGVSGENYTCDDGIKMMSMMASDKALEAKEAIDQKLENASELLENVNRAKQHVIQANISA
NMAEDKAEEVNKIGQAVLKNITTLIKDIQDFLATDFNHPNASKDLAMQTIALNISLTPDEVVELG
RKIRDAVKSLTGVDKILNESRNDYDTAIGLKEQALAAKERAIEVMNQTMAIIETLSQAEELQNSTL
AGVNVARADIEDVKDLLKKLKEKLDAADNDLQMADNSTASAEDKRNAILDQNLENREKLDLSE
KKIDEAEELGDKVQGSVDKMNEKYNATKAKLNEKEDMIEKVRARIKALADKTIKLYENADRKLR
RINKMKDSYIINEERAKDLYYEIRRLEIKEFFINTKLEGTAKCYSTCNPIIPESFCRDVYKNIAVKETN
AYAELEAEKASTTSLLI*
>TRINITY_DN140702_c0_g1_i1.p1 type:complete len:2036 gc:universal TRINITY_DN140702_c0_g1_i1:94-6201(+)
MATSFKVSEIKCTELLNLEKKGAIDPLVEVEYLGLSKETENKDNDLNPSFGEVFDFDLSGKALGPE
DFLKITVKDYEKFGRNRVVGKADVYLQELLSNPSSTLSATLNDKNDRPTTGKITFKVDYIGPAGGG
AGGATGGGGDDRQAAERELEGDEDEGDPNAMPDGTPIPEGPAGDAMRAEKRKMKKNKKKRR
EHNLPDKPTDFQIRIKIIEGRQLPGGNINPTVRVHLGEETKQTRVKHSSNKPFYDETFFFNFNERPN
ELFDKLVTFEVHNSKNVLSEAMIGAFQFDVGTIYFEDSSHAIIRKWLLLTAPEDNDKKDGEETKGA
SLQPGGPPAGYLKITAIVLGPGDELPEKAKGSGKSEGGEEDIESNLLRPAGVTLRPATFLVKIYHAE
DIPQMDSAAFDGVKKLFSSGSAQKDHVDPYATFSFAGKTQKTEIKYGNNHPDFNQELRVAFKFPS
MCERLKLQMFDWDRIGNDDCIGTSFISLTAISGNGDEGFLPCFGPCYVNFYGSTREFSDLPDEHDN
LNKGRGEGCGYRGRALVEIDTRFGDHTAKPVQDIMAQELVRVQAYMRRRKYKLFASFLSANMIS
EADGLVEFEVSIGNYGNKLDPDTEPQSSSTPPTNPVYDGCAYYFLPWQESKPCCVVESHWEDVIFR
LEPINCLLKIIDKMEKNIKSVELGLKAKAQPAEVAALLIALLDQFISDVSMDLPKFAATDNGVNEL
DLKIQEIRSDELLNLKEEASTLREQATDVQEAMDTIDGYLKRMRDLCVEPQNSMPDVVIWMIAGS
KRVAYHRIPAYDIFYSENPLARGRKCASLQNFILKYPGKKGHDMEDHPEIPASVSVILWLGLEKHQ
AAWTDTEQMEGEYSVYAETYENQGKPPMFDWTAKIIKRPGFSDSEGKIRLNKEYYEPPEGWKWD
DPPDGDWFIDPEMSSNFDADAGHKKFMQDCFENESRVPAGNWGKSTPHYTNVQGDEIDHRDE
TSLPEGWSWTDDWKVDVNRACDEEGWEYCIEATGGGWGAVFRSYHFCRRRRWVRNRQLEADP
QKVMKQKKEEAKQGEGWEYAPVFGMKYHVKEHKFDFVRRRRWHRKMVCVKPGAVPMFKIPD
DDDKGKFHQIMPRIFLAYDEPHKWQFRAYLFQARDILGLDSEGTSDAYAKVCFQDQSQITDVIEET
LCPEFNQTLLFEDMQLYGSPSVFAENPPFVSIELFDKDKVGKDEFLGRVLAQPIVRLNGLKPPATRL
MWYDIHHGERTCGELLAAFELFLDEGADLPLPPPWLEKEKKFQVPSGIRPILVKTGVEILNWGVR
NMKKYQLASCTSPSVTYEIGREIITTDVIKNTKKNPNFLKNIFYKEVYLPKDPLYMPPIVIKVRDHR

TFGRKPVVGRHVIKSIDFVQPPKADPVVELPPADLVGEPEEKKDVTLDIEEKKKKKEIPVDDNLDW
WSKYFASADEIEKCGNYLKKGHKTIQIYDSELEEIKDFNGFSDFIVSYPLERGKEDDDEDDNVVGE
LKGLVKVYPLPQDPKDQTKIRYFTPKLKEKSKIVKCIVRVYVIRARDLQPKDPNGKADPFIVVQLG
KKKISTKDNYIPNEINPFFGSFFEMKADVPLDKDLKIKVMDYDFMSGDDLIGETMIDLENRFLSNC
RATCGLSPAYYTSGAYQWRDAKSPKEILDQVCRTKPQWLSNEQVVVNGQLVKIEDFEESPPTDPHI
GPVEQRLALHVLNNEDIIGHRLVPEHVESRSLKNPLQPSMDQGQLEIFVDIFPIANGVPGPPLDIAT
RKPKEYVLRVIIWNTAEVPPSETSITGEEMSDLYLKGWMEGQDDEKQKTDVHYRSMDGEGMFN
WRFVFPFKYMPEERVMVVEKKEHFWSLDTTKQTLPPRMNLQVWDNDLFSANDFIGNLELDLSK
LPRPKKAAKKATLAMLDTEGDDVMDLFQAKIAKGWWPMLSENEDGTRTMQGKVEMTLEIVTD
LEHEAKPAGQGQEEPNMNPKLDPPNRPETSFLWITSPWKTLKHIIWKNYKWYIIGFFLFIILILFIGL
VLYSVPGSIVHKIFGFQ*
>TRINITY_DN138_c0_g1_i1.p1 type:complete len:401 gc:universal TRINITY_DN138_c0_g1_i1:157-1359(+)
MAARFLFITVILIVGNLIGDVCPERCERNWIKIGCFHDRIFPNRPYPFELVNHRDPINNHWDGNLI
NWRKYPDSIEKLACICANKTRTKGYQYFGLQFYGECWSGPSYNFHRDGPSTKCIGNDFKPCDDSS
KTECVGRAFTNYIYKLKDENPKVDGKWSEWEKWSPCSATCGYGERYRERFCSNPEPENGGQTCE
GEAREYEECNHGECQTTCDKEIDFGIALDYSSSVRQVNWNRTLDFVVELSKGFNISPEKVHFGVLS
FSHRTRFDFGLSDKTYWNSESFEKKIKSLEYRWGYTWTDKALKKVKQLIFCEKCGLRKDSLKYLLV
FTDGRSNNVKTDLKTVSDSIKDMGVNVISVGAGHTINHRELEEIATDDNHVFYVGGYAYLGDNM
NAIRRLTCG*
>TRINITY_DN262_c0_g1_i1.p1 type:complete len:1033 gc:universal TRINITY_DN262_c0_g1_i1:111-3209(+)
MADPSSLETKEKDGSYRVATKSGGKDKGKKSKKAAKKVNMDDLKKELEMTEHSCELETLVSQLE
TSLTQGLTKSIAARNLERDGPNALTPPKQTPEWVKFCKQLFGGFAMLLWVGAILCFFAYIIRSIREE
EPGMDELYLGIVLTVVVIITGCFSYYQESKSSKIMESFKKMVPQEAVVMRDGEKLNLNATECVVGD
LVFVKFGDRIPADLRIIESKGFKVDNSSLTGESEPQSRNSECSSDNPLETRNLAFFSTNAVEGTATGIV
VKTGDNTVMGRIANLASGLGSGKTPIALEIEHFIHIVTGVAVFLGVSFLIISLAMNYHWLESIIFLIGI
IVANVPEGLLATVTVCLTLTAKKMASKNCLVKHLEAVETLGSTSVICSDKTGTLTQNRMTVAHM
WFDNMVVEADTTEDQSGQAHDKGSTTWKALAKIGALCNRAEFRPNQEEEPILKRECNGDASET
AILKFVELSVGSVEDIRSKNKKIVEIPFNSTNKYQVSIHEQEDPDDPRYLLVMKGAPERILDRCSTIL
HEGVEKPLDEEWKENFNKAYVDLGGLGERVLGFCHYYLPADDFPKGFAFETEDPQNFPLEKLCF
VGLMSMIDPPRAAVPDAVSKCRHAGIKVIMVTGDHPITAKAIAKGVGIISEGNETVEDIAQRLNIP
KEEVNERDANAAVVHGSHLKDLPQEKLDDILKYHQEIVFARTSPQQKLIIVEGCQRMGAIVAVTG
DGVNDSPALKKADIGVAMGIAGSDVSKQAADMILLDDNFASIVTGVEEGRLIFDNLKKSIAYTLTS
NIPEISPFLMFILLAIPLPLGTVTILCIDLGTDLVPAISLAYEKAETDIMKRKPRDPVRDKLVNERLISL
AYGQIGMIQATAGFFTYFVIMAENGFLPGYLIGLRNDWDDKKNNAVMDSYGSEWTYNQRKNLE
LTCHTAFFTTIVVVQWADVIISKTRRLSLFQQGMTNWMLNFGIFFETALAAFLQYTPGLNTGLRLR
PMNFPWWFVGVPFSLLIFVYDELRRFLLRRNPGGWVEQETYY*
>TRINITY_DN1616_c0_g1_i2.p1 type:3prime_partial len:3398 gc:universal TRINITY_DN1616_c0_g1_i2:218-10408(+)
MIIFLAFLAVAVGQEVLLPPYFDIAKGKNITATATCGEDYVAPKGSPGELFCTLADATGKFGIKGLK
CSHCVPGDYGDGDGKNARDHHIRNANDGSNRWWQSPPLSRGLQYRKINITMALGQSFHVAYVI
IKAANSPRPGVWALERSNDNGRTYTPWYYFAPTSSECKQYFGVDSYLPIVKDDTVICSTEYSKIPPF
KNGEIHISLINGRPGASNFSQSKTLQEWTTATNVRLRLLDIKTLLTDLLPLSLEDPTVTRRYYYSIKDI
TIGGRCMCNGFADECTPHTNIVGKYECNCQYNTEGPRCSRCKKGYVQKEWRQRTSTDKFECEKC
NCHGHSDECEYNEEINKRKLSLDVNGNYQGGGVCKNCQHNTLGINCEKCKPFFYQPANVPIDS
PNVCQPCNCDTQFSTGLCAEETGQCECKSNYVGRYCDQCNKGFHMFPVCKPCECDVNGSEDRE
CIPDQINGCKCKKGYAGRYCDECLPGYFGFPDCQPCACLTRYGAVNDNCERDNGQCSCKVGFK
GRRCEECAAGHYQFPFCKECGCDPEKTLDTVCDSGTGVCQCKSNFAGPKCESCSPGFYAHPSCFA
CGCSTAGSTSSVCDQNSGLCSCRPNYSGFKCDRCAEGYYGFPNCILCDCNPEGSRRVGICDRQDG
QCQCKPTYSGQKCDKCLPGFYGFPDCKRCNCNPAGTKPGPGGRPGDCSTSNNGQCVCKDNVQ
GLECNECKPGFFNLTESNRKGCSTCGCNGAGTNNGVKSCSVEGTCKCKPLVKQEDKCTECRDGY

YGLKRTNVFGCLGCNCEVGGSSSSVCSKDNGQCPCKDNIVGKRCESVAANSFYPDLCHIKAEME
EGKTTDGDVVRYDYQKEIFPDFSWKGFVAVNDQQPSVVVSINVPKTSEYQVKFRYALNQIQPVTA
TVTAFKGDSKIVGSINFASTQKGSGFVASLATVQDNLVQEMYFPLTEGQWNIKLEAPHSNLVVDY
AVLFPKEYAQGGALKKKVQNACDVMTDEKFCNLYRYIDVADKKDIIVLEAENPVKMRQQKPPVL
FNDTAVLSKLDFQAMVVLSPSNSRQLNHLFNVAKPGKYVVMLQYYHDSDTAKYLDVYVRSQIAV
QNGRALLQSCRYTFGCRQAVLNRGDNGVFVFNIKSTKNHLLTLLSRYAVVAVDSVMLVPYESWH
LDYSTPTLYCVTKDSKCTNMNYDVPAESRLVQANPLEQDLSEKPRYLIDRSVEVRYVKPGVNIINIR
DVKAGNYLLVAHYYQPNFPSFESRAAVYSGRTSGLRARFDFKFCPNLNGCRSPIRRPDSNSNLYVF
EEGEARIRFDIPNGLDAWIEYILMIPEGTNVSSLYTPRVLDYSDDFKKECSIDLNVCTSSNLSSFCKDS
IFTITTDFNNGSFPCKCNMDGSKSSSCQEFGGKCPCKDNVIGRECSRCKVGYYGFPNCKPCNCSN
CDEETGVCTCPRGVIGENCDQCAPGTFGFDPIIGCQKCNCNEIGTGSANAECNVTSGQCPCKEN
YSSRKCSACKPGYFNYPACTKCDCLDAGISAEKCDVLTGKCICQNNVIGLKCNECADGYFKTNDI
KGKQLCAKCWCNGRSTKSCTEAELFRATPITASKGWTVSVDTLNVIENGNGRFTISDVNTLPEPV
YWVAPNQFLGNRGLSYGSHFKYNFQYSVKQPSQNTNGDVVTDAVIKGDGYTIKSMRSVIEKEND
GYSASILMKETEWMLEDGSTIDRELFLAILSDIDTLQILATHGKNTISSTIYDISLETAGENGTDRVYG
IEKCFCPKEYADLSCSKCSKGYTRQKDGSCGKCQCLGKSNICDPQNGKCINCSGNTMGDRCEKC
KPGFYGDKCKACECPFSNPENSFANTCAINVNTSSLVCQCNAGYKGVRCDQCSDGYYGNPLQG
VTCKKCSCNGNINASFTGNCDNTTGDCLKCLYNSDGNQCQECRDGYYGDAVVEKNCTDCQCSI
CGTTTAPCNKATGQCQCKKNVEGSKCNQCKPNTWNFDACNGCEDCNCAEASETQQCDLKTG
QCKCPPTVFGRRCGFCKLGYYNYSSEGCQKCQCPDGKPCDVNTGKCCTGVLNEDCKLCPVGHII
EDGECVECDRCVQRTLQRVNTLIHNVTILEKHIIPAGGLNYGALETLGNRFSNLSNKGDEHISNL
DTLKKAIGFGEYRNHSGNMDYVRMYLVRDFKSCSFIVNIKTPPTSSAPATTMVTNTPGPDPIRDHL
SNKKISDSQCPPSLARVIDEGEKNIKLVEKKVVVLEKDAYESTENATATKKRAEDLLAKLKDFMVD
FKDVLSFANSTLKGNVVINIDGNRSLAEAEEMLKEIKDRNFNSTINRSEREKSAAINATIIAEQLMK
QASELANKAYKFNDTFKDFVKALRNLKMESELALLDANNATNSINLAKAVDFQSIIDNAQKLHS
QNNVTVTQAMEILQNAKSAYENAKDVFNKFHNQTGPLSDLMEELRNYVRKLEFDLQTTSPIVDD
SVKHAKRLRDSADRLKELLQDTKKFADAALRAAKVYQNIVDAINDALDAARMANETAHEADK
KVQQAQQDAVESSITSKVVDTEANDLTEEVKKTLKVDISDMKMRLKELRELRKRIQGRIDNVKED
VKSFAKEKEVGEKAMNISTQADGVMKVANKGNEASQDTNEKVEKDGEKVETLKVDISDINKAIK
DLASNVTLVEDTIPKIRSNIDEVNVKHEQIEEFIKTNVTNNIGYLKYLISNAHDILDQMQLSMKFNS
TSEVVLDAPKSVADPTINNKISLTFKINQGNGFLFIIGDFSGNSSGFMSLHVQNHTVVFDYKLNTG
DVQSLTNPKFIETDVNYDVMVTRNFATAVLNVQKSGNASTLMTAYQNRSNISPVLSLGQDTNVY
VGYHPSTPNQRPNFDGCINQFRFDEELMGLWHWKSAKNVGGCVQDFRSIEGQYSFDGDGFVEF
DLVSPELSGFRKIELRFATLEENGLIFLGTDPPTSLPSDIMFDIPLSNQIIAFNALEIKDGYLVSTLDLG
NGVQRLVHTNAGKVNDGKPHLFEMKVLISSLLIKFKLDKKKITEIAISPDTDIRLRLKEAFIGGLPQ
RAWLPNSNISTKGFTGAIGDAKYRGGEFKLVKRFNNIRKGFILPKGYSASFPEEKSYLRLDACNLK
QNSLFGFSFQTFEKNGIIFHSEEGSGSSRSSIEIILIDGQVKVDVSVGSQKRTLQTSVARGFSDGLWHT
LSLYFEETLQEADIVLLYLAVDKAYITGTVPKVDLKGSMYFGSKPSGTESGFNGNIRQVYCNKNEIN
LASGDVFEGGVVGSLKLLPPLKLADFTVVPTTLPPLTTSITTSESTTTETVMTTSTTSISGTTLEPPKET
TTNMAKTTENPTVKITSSEPIIETTMESTTVAKTTSAITMEESTTTPTSITTSTLETTISTLEPGSIEGSTT
TAE
>TRINITY_DN199_c0_g1_i1.p1 type:complete len:229 gc:universal TRINITY_DN199_c0_g1_i1:207-893(+)
MVQGSMTIVKYLMFFFNFIFWLSGLALIVIGAVIRDKYGDYFSYADSRFTNVAVFLIVVGVIVFIIGFL
GCCGAIKENYCMVTTFAVILGIIFILEIVAGAIGFAYKGQVEEKADKALVRASKNYNNDDEPGAKK
LLDWAQQRFECCEVASATGNGTCADKSPKSCHPDNDCNKTKYSQKCKEGFVEFMKDNLVTVGA
VALAIAFIQILGIVFACCLMKAIKGEYEVV*
>TRINITY_DN3078_c0_g1_i1.p1 type:complete len:1109 gc:universal TRINITY_DN3078_c0_g1_i1:3628-302(-)
MKMRIYLFLILFATVYCRVVQKGPNDIPEETKNEIKNQEPKIKFEPEKDKKYFLAKDTTCAHDVDK
HCKNIRRENNFAVFVCLQDVIHKDEDDVAEECHETLWHYKYNLTRDPNFDQAAFKVCENVINEI
QECNDSELLKSPGFVTNCLIENRHAVSEAKCKSFLQKMSAIVFEDYRLIKGFYDSCVEDVKTLKCG

TVAKPTDEEYQHDQTEVISCLEEQLDLVSEKCQAQLVKVATMQADDYHLDRPLFYACQEARERF
CPEVKSGDGRIYKCLVEHKTNEMMLEKCRKKIEIREKLTQKDIKVNELLWKSCKADFTKYNCPKD
GISEHGAASSLLLCLQGKVAEGLPVDQSCQAEMRTFAIELFENYKISPVIVSKCDVEINQFCHHLIG
KTDDGDMMDCLMTQAATNNESVSKKCYDAVSDVIKETGAGNNYKIDRALYLACEPVITTLCQG
KEDAMILGCLMDNAHNSKMPEKCAAQVFHLQYFLSRDFRLDASLYKDCKKDAEEICSANDFNA
ESESKVPDNFVIACLYRNALDRKVDDRKVSQTCAEHVFRVMHQRATNIHLMPELERPCMADLG
KHCIDKGDKEGEEIDCLQTNYEKLSAKCQNAIKVFTIQEAKDFDLDKHLVDKCAPMVQQFCQRE
LEDGNGEKVLPCLVDHKNDIDMDHQCANAVEHWQLIEMKDFEFSPALTEMCLLDIRKHCQKSK
AKIEALHCLSENIVKDASSVTEKCRAQLKNELIEQSENIKLNPELYEACTKDIELFCRDVVPSGGQL
EECLKRNHKKLTDPKCKSLLFKEEKKESFDPELDYRLMHVCKPIIKRYCTQYHQPVDIMECLRQV
THDRDMSRECRAIITERQIEQAESFELDPELSKFCQDDVNRYCKEEIKHAEKGDDDGAIFGCLVNA
MIDNKPIKNDCEMYIRRREAEAAQELDLNPQFLLQCRDEITKICPNMDEDEIIECMKMNIPQIQSK
GCVQEVKKLIVEGIEDVHVDPHLQEQCARDIRTFCQSVPSKSGNVVLCLIDVHKAKNLHLQPNC
ENFLTKRLSLYNAAKVDYAVFDSISAVMNAVSESPNRNYVYMTLLLVLTILFVGGMLFGRYTKKIRS
EIKNR*
>TRINITY_DN602_c0_g1_i3.p1 type:complete len:851 gc:universal TRINITY_DN602_c0_g1_i3:3046-494(-)
MIICRLHGLSLGIILIFSILYTSDARSRHAYGHKDASSPQTGKKRAESDAGAGTEAATGANTGAATA
SDSSSESQTFTNTKVNTRIATSSNARSGANAAAKVKTYAHANTETKVDTQQQKPTPPVPPEALTT
PPPGVTPPGPPGPPGSENNNTSSGSECKEKETKPPIIKVYCPNCPCPCPPPCPCKCPCIKVAPPPPAP
PPCICLPPCPPPCPPPPPPCPPPCPEPLPAPAPCLPPCAVPPCPCNIAPITLDPSLPCPMDCPLPPCAC
PFMKKDNITTTKTKRSVIRKRNLNIKRSHRVGTKRVNAGKSPNLAYAQPDSFEKAAVAADNKKCS
EIGVEILKKGGSAADAVVSTHCCVEIVNSHSTGLGGGGFMVYYDKKSGKSRAYDFRETLPKKYTD
TQNKTQGETILVPGVLRGLEAVWKDFGKLPWEELWKPCIKYAKEGFYVHEALAAGIVKKADYIM
ANLGLRELFAPDGELLQLGSLFKRPKLARTYEQIAKHGADLFYRGEMAKQIVNEIKEAGGVMQL
EDLSSYQVNIRETLKSNINGLKLLTSPAPSSGSLMALALKIMESFKWKPEDLLNNPSLVYHQIVEAM
KFAYAPTSYLGDPRFTNGTDKVEQYMVSPQTAKEMFKRIDNKSHPVEYYKPWSELFSAEKTGTSH
MSLLDQEGSAVSLTSSINAYFGSKLRSREFGFIYNNELADFSEFWPTVYNLTSDKKAPSKRPMSKVM
PGIFLKGKTVQGVWGAAGGFFIPSALTMSIANWLFFKDNLKVAITRPRLHCQLFPPTVVYEPTFPP
SLIPKLQEFGHKYVTNSTYDVSGQLNAIMGVVQAIVRLPNGSYSAEADYRKGGRPAGF*
>TRINITY_DN477_c0_g1_i7.p1 type:3prime_partial len:1130 gc:universal TRINITY_DN477_c0_g1_i7:3387-1(-)
MMGSIKVFVGLVLLSFTAIEIAQAQSGTVLSNKQFAFDFVVDGSTSIQDEQWNLLTDFVSRLAVKM
STLNNAKLSALQFSHTLNRILQCNQYANVEEARNTIKNMVQENGATGTLAAMLTSADNLMNVC
SAGDPNRQNVMIVIVDGNQNLEQKSHSEIVQDIKVNVKDKNILVLTLGVGPQVNRNEFGDYASD
SGKGFFVDDFTLLTDELVQRMYDELIRTYGDGPDYLTAWVNGPCSATCNPFPMANRPTLRQTRQ
CQEPNGGNCGGASLEQIINCNENIACAGETYLTAWVDSSACSLTCNPFPVSNQPTKRQTRECRQP
SGSCNGASLTQTINCNENVQCPGETYLTAWIDSSCSATCDTGSNNPVFTQTRQCRQTVGNCDGA
PLQQTRSCNVGVSCTAPTFLTAWSNATPCSATCNPFPMAVRPTLTQVRQCNEPNGGNCGGASLQ
QTVQCNLDVACAGETYLTNWVDLTACSATCDDASNRPVKQQERQCQQPGGNCDNLPLQQTVN
CNIGVSCTVSSSACSSKEISFTFVIDTSASVDNIGFFVLKDFMKKYMSQSTLGTDPRTSVAIVSYGAG
DAKVEVECDTITTQQSFNTTVDNLSQLFGIADDNSFNQALSTAFDASGRCARSSTSLNIIIMLSDGD
PPALENILTRDRIQSSSNYYFMFVAIQASDGAQSTFASLANQGRGLRNASSSFTDLYNFNTDLYYLE
TLSKACDVGGQWTEWSTSACSTTCGPGVVTRTRECRNRITGKLQQDCGPGPAQETQACNLQDC
QGTLTQWTSWSCPATCNINNDVNNPPLALRTRQCLGSTGQGNCNNADKTENEPCVRNVPCPG
AWTTWSVFSACSETCRSQSAFPTRRRTRQCIGGTLNFQCPPQIVNGISYREIWQEDCINVPNCPAK
FTQWTTWSCPVTCNVNNDRNNPPTATRTRTCDDSGTINGCANANILENQPCVQDVQCPGAWS
TWSAYGACSETCQSGNRSPTYTRTRTCLGGTLGFQCPPFITQIGGFQIFGTQTSNCNSEVKCPSKLT
QWTTWSCPVTCNNRNDVNNPPQARRTRRCDTSTGSGICESPIVEEFENCVANVPCPGRLTEFTYD
NCAHTCNVNNDVNNPPQRVGTRRCLDGTLG
>TRINITY_DN72625_c0_g1_i1.p1 type:complete len:1964 gc:universal TRINITY_DN72625_c0_g1_i1:173-6064(+)

MKLAFNIFTISLLIGNTIAQNNDVVQVCKSQADVVAVLDSSGSVGVQDYEELKKFVGFVANLLTIN
ENGNRLGVVTYSETAEVDFNLNEIYDSDQYGREALTRLSVRPREFKTNHYAGLKVGSDLLFNKAA
GARDNAAKFLIFTTDGRNNFDETNNRNRASQLATQLKADGVNIYVIKAEVMSDNNGRLDSRLYS
TDPNKFYRFINRFGEITNENFLDEVGLEFCELVQLPDNQQATVREDRNIGTLVTTIRAPGLNNPTFE
LIPQDNLPFIVDPLTGEIKTIGLLDFEQVQQYVFQVRVRATNPDVEKIITVTINVLDVNDNDPIPETA
NYRMSVAETTPLGQRILTVKANDPDGGTNGNVEYFLISGGDGKFEVDQATGEIRLIGQLNKRIKD
TYLLTLEARDMGIPTRKAPITVAIDVTSLNNFPPQFNEMSFVRTIPENFGAGTVINAAATDRDGDT
MVYSLIGENIPFSIDQNGLITVSGNIDFETIPNNVFQFQVQAEANGDLVTQDVIITITDINDNVPVYS
MTTYSGSINENSAAGTVVNFDNVIIATDADKTNANNMITYNIIDGNDGDRFQVTKNERGEAVISL
KSTPLDQETQGQYRLTLQAIDNGVDPRSLEMNSLLIIDVIDQNDNDPIPEESVYSIAVAETSPVGQSI
LTVRANDADSGMNGKVEYFLISGGDGKFQVDQATGQITLIGMLNKRVKESYTLNIEARDMGVPT
RNAPITVNIDVTSLNSFLPEFAGAGDAFERTIPENFGSDTVIKAAATDGDGDMVFYSLVGDNLPFA
IDQNGMITISGSIDFETIPNNPIRFQVKAEANGDMTLKDVVITITDVNDVTPEFTQQVYAGKIEENS
QTETVVRFDQPVIATDGDKSPANNVVTYRIVDGNVDNIFKIVKNAQGEAVISVNNAGLDREILNQ
YTLTIEAKDNGVDPGSLSSTTQVIIDIEDVNDIPPLINTSENKAVGEADFTATISEDSPIGTSVVKVEA
TDPDSNNDDLIFTIANDVPVPFTIDPKTGLITVSGPLDADDQDEFEIAVTVSDGVNNSTAIVKVNIA
DLNDNKPIFTTDTRDITIPLDTPNGQEIIKFTGSDADKTSPNNQFEFSLVDGDGKPFTITPDGSLIIN
GPLMPGDYMLTVKIMDKGTNPGPLSNVIIYNVKVVDTNDPPTFQNNEQTVTISENTADGTDVITF
AATDPNGDAITYEIVNPGSVPFEIDPITGVIRKMGLIDFESPVRSYTLDVKATDAKGKSTMGRVFVQ
ITDFNDNTPTFGPGNTDNFILTEGVYSNQLVSSFTATDLDATSPNNVFEYTLVGAPSMFLLTTNPNT
GAADLTVNGDIDAEQNMSFNFIIRATDKGNPALSMDYPVTITVGDINDNAPVFAANTPESIDLME
GDFNNQIVATFTANDDDTTAPNNLVDFELVGAPDFFKLIVNPETGTADLVIDGNVDIDGLDPTINF
IVRAKDGGNPALVKEFPLTVNIADINDNAPVFTLEAYNQVINENTPIGTKVVEVRATDSDSGPNG
DITYRIVDGDGTFGIDPKTGDITVLKNTDFESGVRSYEFTVEASDNGNPKQSATAKVKIDIRNLND
NDPIFTSIPITGNINENRPAGSVVQNIQPVNGILNQNNVKILREPPPGAQETEGVGFDAPFEIDPVT
GEVKSTRPFDFEEEKVFTMMVQAADGDNNPMLTTMTVMVNNLNDNDPIMKKANFEITLNQDT
PVGTFVLQVQATDIDQENQTKITYSIINIDGGNMNDFTINPDTGVITLNTDLSMNTVDIYNITIQAE
DQEIQGVIRRSTALAEIKILRPAEAEPDISTLCTNSCDIPEERLKCLDYSKFEITRKFNGTCYVDKLKA
AIADVKNNTMEIMNLNASFLECRNKVQNEITNPNTQFPVNGLFDLHRKCTVAIECKLEQLAFNL
MRKTTELTNNLKAEEEGFSMYDMSLSAFFQHISKMVSMSKSSRVD*
>TRINITY_DN250_c0_g1_i5.p1 type:complete len:452 gc:universal TRINITY_DN250_c0_g1_i5:131-1486(+)
MRECISVHVGQAGCQIGNACWELYCLEHGIQPDGQMPSDKTIGGGDDSFNTFFSETGAGKHVPR
AVFVDLEPTVVDEIRTGTYRQLFHPEQLITGKEDAANNYARGHYTVGKELVDQVLDRIRKLADQC
TGLQGFLIFHSFGGGTGSGFTSLLMERLSVDYGKKSKLEFAIYPAPQVSTAVVEPYNSVLTTHTTLE
HSDCAFMVDNEAIYDICRRNLDIERPTYTNLNRLIGQIVSSITASLRFDGALNVDLTEFQTNLVPYP
RIHFPLATYSPVISAEKAYHEQLTVSEITNACFEPANQMVKCDPRHGKYMACCLLYRGDVVPKDV
NAAIATIKTKRTIQFVDWCPTGFKVGINYQPPTVVPGGDLAKVQRAVCMLSNTTAIAEAWARLD
HKFDLMYAKRAFVHWYVGEGMEEGEFSEAREDMAALEKDYEEVGVDSVDAEDEEEGDEY*
>TRINITY_DN1119_c0_g1_i1.p1 type:5prime_partial len:949 gc:universal TRINITY_DN1119_c0_g1_i1:2934-88(-)
FFFNPQTVMHEKKFSRMKFILLFSFFAISLGQNCNRMHKKIGCFWNDKNAKVLFNDRDSINNSTY
GALSDKLACKCNAVARSNGFKFFALRHFGICIGYNKITTGSKSIEHCIDTKSNGVCNLGDMNTCT
GTQNTADMMYQIVPEKCPSRHKKVGCYTEDRNATFLFNDRSNIDWPNFEKFLVELACKCNKVAL
EKGFNFFGIRFYGVCVGFKNISTEEKSAFHCKDPNYRPYRPCNMKDSNVCAGDHFLTDMIYKTER
QNVDKLFSEWSGWSTCSKSCGGGTQKRTRECKDKKSCSDAGGEEFKSCSNSFCKGSTGVAKAQE
VVETFLETFHKVEFPDIKPSGKYLKTIFEIQKAVGEEFNSVDPSVKGVPLEKRLTELEHFLKIAVEGG
SIDFARSTLGKGMFVFMKKKIKEGLPINDIDTAAKGFLNKLTGDIGSKKMNEILSSKGDATLMFTID
TTGSMAQEIAAAKAIAKQIISATREYEVDYILSPFNDPRTGPVRYRSTNDRANFVSDINKLLASGGG
DCPELSIKGMLNAFDKGPKYGSPMFVFTDASPKDATPSNMAALKAAASSNNAKITFFTNLKGCG
KELGIESYKEIASHTGGQVFPLKNEAELINFTEYVKSSLQQDTIIAAGGSLNSRISKRAAPRSHIFMV
DQSITRLIISVAFEKQNTGKHIKLLDPNNKNVDSSTMLAMSLIYDIKNPKVGLWGLSFPHIVGEFGY

NVQAVTENAIEFAHSFLHQHNVKGSDVTVSLENPIKGIRNMMVIRLGGADHIDLSTIKVHLVDHS
GNKLVSNVMTDRLDSDEIRNASIVPPTVQFKVLLTGKTKSGQQFQRMSQGSSTPVSAFMRTISAGY
DFTATMGKDWISIQIEIVNKGSAESFNFQAKTKYGTLKNPNQTKTVNKESMITFSYKPPNRHDLK
GKTDKVTVIAKRASGEKINHFISILLIK*
>TRINITY_DN33065_c0_g2_i1.p1 type:3prime_partial len:804 gc:universal TRINITY_DN33065_c0_g2_i1:2409-1(-)
MWQFVWLAVAITSQINGNVHHKHTKPRHQNLIDDIVPNDDFDLDYSHNIFIPFDAYDNYEKFLE
YAERSDLAKRGGVTSLPAIQSLPGDGSHTYAAPGVNCQVQLLNKKVEGTDKRVIERTSCAEGWTL
KNALGFFFAGVGHDLATKSAGLVHMNQLRFKMLIFEAEGYTFVADTDNEVYSNLQSKLSLKNAE
CRLTISNSYTFENSEIVLTGNHEVIEKITNAKKTLEVTFEKGKDTSSFDVKMKVPSMPLETFATIFLES
ETEFITADMGDTSYEINIDPIKAVIIESTSNSVTGVFDGSDGKFQLTGKFLSNDQNAFMGQATDIYLT
VNRKSLGSRTTYNLMLGFAKEVKVMDIIPKYVGKTEIQDEFDDNFLKTTIEKWHLLFTSKRIIVAD
GAEYKKTFANIFDADEYQEFKQGVGIKATVDLQKTLTACSSGSTVPDSLKSVKIKGLLGRRGLSMV
FPPGTSVPLIPLIKCKLRQIQDDLGFELPISLKGYEQKSKFKLEEIAYSSDSGAFKMSLLYENTITIYKD
VLEITNPQLTIEREGKDEPWQFTIAADVSIFQVDASVMISYSKTGYTVEVSSGNVPLTNLLRAFSFSD
KPAITFEIHDFKMQIEVKDKEEEEENEEEGEDEGNKNQLAIKRQDNKEEKKRDLKFSIAGKPVFN
GFDKVSIEALVWTSQTPAGVAVAVIMENIRLDEVLEKILDVPEVPMAWFSDMTLTMVISNVDAEG
CDNPAQSEDDCEEADEEEEEEEEGEGEEEEGEEGEKTQEGEEGEKGEEGGGGGPGGGPGGGGRG
PGGGRPRRG
>TRINITY_DN244_c0_g1_i1.p1 type:complete len:3019 gc:universal TRINITY_DN244_c0_g1_i1:9748-692(-)
MDARGQRLLYCLLIFLSLATLLVSQQVEKCKRPVDLVFALDTSGSVKDSGFRLIQKFAKDILKTFDI
GVADSHVGVLTFSEQAEPQIKLTETFDKAELMQMIDSLVYQGYRTATDDALRVAKTDFFSTAGGA
RQGVPHVLVLLNDGKCTVCIEPVKEAAQKLKDMGVEIFTIGVTENIKKQELIDIASQPTAYHFFEL
DDFDQLVDVVGDIQTRSCEVKKGKCPIPSPTEQCSGNEINECTSDENCEGELKCCSDGCSRICKYP
LLNCFTPIDIAFALDSSSSIQPETFQEMKKFSNDIVDSFAVSQKKTRFAALVYSDRAEIGFNFVRFDD
ASSIKKAIQNLPKLDSKERIDYALELAKSDIFSLQGKVRTRRPMVLFVFYDGTVSSGMKDLSLVVEP
LKNYGVKIVSMGVGPETNYYQMRKISSNNSDVFRATNFKELLPEIYNIAKRACTEKPGRCDASLN
PLPKEACKNVKILNQCENDFDCFGDAKCCRKGCNNVCVIRGPVCPRKLDIALAVHQNMDESEF
AATKFYLQSVVDQFNVGPSQTHFSLLSFGQEPKVIFDFNSDQSSNEIVRRKIDNFQYQYGIGRISDV
LALSCDAVFCAAGGTRDDVPKMLVIVTSRTSPSSPSKLKEKLQTLRNRGVNILMIGVGDVDTSLLQ
AITSDDPLVEDRVLLSKRFDGIFQYTKDISDYACGMAPALPPPDNIKGDAKGECGAPKSCYKGPKG
SRGRPGTLGQQGESGDLGLVGPKGIQGRRGDKGPDGQMGSKGKRGPDGLIGLPGFPGYNGIPGV
PGIEGRRGRDGDCGLPGSSGEAGQAGLDGLSGPPGRAGPVGEKGEKGLPAPENPNIRIEPGDRGD
TGSVGLPGFKGIKGEEGFKGPSGDSGVDGIKGVDGEAGEMGDIGTYIYQKGPEGDRGARGPPGD
GDTQGATAGLGVQGEKGEKGMRGEAGDGSIFPGIKGRRGDQGPKGEEGSTGPAGPTGQRGPEG
EEGPKGIKGEIGDRGKDGTPGQPGQEGDLGEKGFDGLKGQDGDDAEGELGDSGGKGLKGENGL
AGSPGSIGTPGPPGSKGEPGDIQIGLSGDPGAIGPRGPRGDNGISGQPGMRGDSGPKGAKGNQGP
RGDRGRNANMLAGKDGMTGPPGPKGFTGPVGDPGFPGPPGPPSVVECVEGACPKGVPGAMGQ
IGKTGEPGPPGPPGPRGSKGVAGGPCPICPDGIQGDSGDIGQPGMPGKEGSRGRKGNIGDAGSKG
LQGRNGDDGDQGPVGKKGFIGDIGPVGENGVDALGDSVAPDGEKGSTGDTGQIGSRGPVGNDG
PKGFKGTIGERGRRGLQGEKGDMGLTGETGEDGSRGLEGAKGDLGDGLRGAKGDKGNIGQEGN
QGVVGDVGLQGPKGEQGAPPTEDELKNVKATKGSKGEIGSTGDAGEIGDIGPAGPRGPKGFDGA
AGPKGPSGVKGDRGNKGRAGDGEPGFQGFPGRPGKRGPPGDPGKPGKRGFSEPGDAGDRGDP
GKSGKKGADAPPPIGCFKFNEPTDIGFILDSSSSVDDDNFLRQLQFVKNIVRQYPVGPDQTQVGIIK
YGEKAVTGINFYDYVTEEGLFNAIDLLEHTKESESRLDLALKLARDELFTESVGARGFDAKKALVIL
GDGFISGGGTPSKTLMNNAMQYAEDLRGAGVSIFALAVGPEKNKALFHEFVSEKEYYLDIESYSQL
DNELGALQLSLQTGCISVGVPGRDGLPGYTGPKGQKGDDGPKGFSPQGPRGIKGIKGVRGTIGIG
VKGLPGDPGLQGPQGEKGRDGEDGSADPGPKGRPGDKGSRGDQIKGASGEDGDIGPIGPPGFDG
RKLCDNVLAVDLLFVLDTSSGVGVDNFNKMLKFIESIINVFDVDNQLTRVSVMTYDSDARLVIRFS
DYNDPDKLDAAIDNIPYANGDETRIDKALLLASSNAFDTSNGAREGVNKAVILMTDGQQSFAVD
QVALKDAVKPLVDANVLMYAIGIGSEISPVDLKTIAAERVLIADDFDGLSLKIEDQIVLLGRVGCQG

PPGKDGKPGANGEDGFPGLIGAKGQKGDRGEAGTEGLKGNKGSQGEAFPGLNRKGEKGDPGS
AGPLGLPGCEIKNQNEVIDLGFAIDASGSLYEEGFNREKEFIKTIVDRVGRVSEQGIRIGVIVYSDTAS
IEIRFDDHFNNEDLKNDIDNLPFFAKNTRMDLGLEEAKKMFGEQFGGRGSSKKVLILLADGQQTY
VPGVTEPSEVAKELAAAGVDIFAIGIGQEIDRTELESLISKPEHIFLASDISKLIDELTPEVTSALECEG
GPRGPRGRAGTDGPPGSKGLLGPIGDKGFPGEPGSAGFGPKGDDGENGTAGTIGDRGEKGFRGP
AGVPGKRGAVGPKGFPGPNGEKGDRGDEGFIGFEGARGLEGPKGQRGDSSDNGVAGVKGSTGD
NGFKGPKGDPGKLPPGNRIEDFKGETGGSGLEGDTGPKGKPGRALFEPGPQGLTGPKGEKGDQG
FFGMSGPIGNKGFQGLQGFRGDDGAAGPVGEDGFPGMKGLPSSSGDQGDKGKNGRKGESGEV
GQPGRVGEDGDQGEKGYAGFVGMPGKKGDQGEPGVQGPQQIRNEILDNIRPGPKGYPGSTGPA
GDEGEDGEVGEPGETVGAQGMNGDFGERGSQGPKGEQGDQGLQGMIGVIGDKGVKGRVGTG
GNPGTVGLRGLTGAPGEVIFGSEKGNKGDLGIIGLRGRAGQKGKAGAIGPRGRRGRNGAKGERG
PPGFPGLSGIEGDSGNEGDKGPRGDPGSVGNRGIKGVPGESGIAEGNYIVIHSQTHIIPDCPRDFKK
LWHGYSLMYVVGNGHSHSQDLGDAGSCSQKFSSKIFLFCEIIKVCKHASRNDFTYWLAGDVDHP
MMPVSNAAVEPFISRCAVCEAHSSHITVHSQDTYAPECPPNWSSLWSGYSFLMVAGAGKTGAGQ
SLSSSGSCLESFRPKPFVECQGARGTCHFFSDKHSFWLVTIDPNKQFEVMKPQTLIKDKNHDLTSR
VSRCRVCIRKTGTKNNTVEPNIATRIRRSIASWIL*
>TRINITY_DN2113_c0_g1_i2.p1 type:complete len:1704 gc:universal TRINITY_DN2113_c0_g1_i2:5257-146(-)
MAQLLPIRFQEHLQLQNVGINGANIGFSTLTMESDKFICVREKVGEQSQVVIVDMADANNPTRR
PISADSAIMNPASKVIALKAAKTLQIFNIEMKSKMKAHNLTEDVIFWKWISVNTIGLVTETAVFHW
SMEGDSSPVKMFDRHQSLAGCQIINYKVDHSGQWLLLIGISAQQNRVVGAMQLYSVERKVSQPIE
GHAAVFSQFKVDGNPQTSNIFCFAVRGAAASKLHIIEVGTPPTGNQPFPKKAENIFFPPEADKDFP
VAMQASQKYGVVYLVTKYGYIHLYDIESAQCIYSNRISGDTIFVTASYEPTEGIIGVNRKGQVLSVSV
DEEKMVPFLIQVKQNGELARRLAERCNLPGADNLFLQAFNDKFASGNYQEAAKIAAKAPKGILR
TQQTIQRFQQLPSQQGQTSPLLQYFGILLDQGQLNKYESVELCRPVLMQNRKQLMEKWLKEDKL
DCSEELGDLAKQFDNTLALSVYLRANVPNKVILCFAETGQFQKIVLYAKKVGFEPDYVSLLRNVM
RVNPEAGANFSGMLIQDDEPLADLNQIVDVFMEFNLIPQCTSFLLDALKNNRPAEGPLQTRLLEM
NLLSAPQVADAILGNQMVTHYDRAHVAQLCEKAGLLQRALEHYSDIYDIKRAIVHTHLLNPEW
LVNYFGTLSVEDSIECLKAMLSHNIRQNLQICVQIASKYHEQLTTSSLIEIFESFKSFEGLFYFLGSIVN
FSQEEEVHFKYIQAACKTGQMKEVERICRESNNYEPERVKNFLKEAKLTDQLPLIIVCDRHDFVH
DLVLYLYKNNLQKYIEIYVQKVNPARLSIVVGGLLDVDCSEDVIKSLILSVKGQFSTDDLVAEVEKR
NRLKLLLPWLESRIHEGSEEPATHNALAKIYIDANNNPERFLRENQFYDSAVVGKYCEKRDPHLA
CVAYERGQCDQDLINVCNENSLFKSLARYLVRRRDAELWGTVLVEDNKYRRQLIDQVVQTALSET
QDPEDVSVTVKAFMTADLPNELIELLEKIVLENSVFSDHRNLQNLLILTAIKADRTRVMEYITRLD
NYDAPDIAGIAIVSELFEEAFAIFKKFEVNTSAIQVLIENIQNLDRSYEFAERCNDPAVWSILGKAQL
DQGLIKEAIDSYIKADDPSAYMEVVEASKNCGNFEDLVKFLQMARKKAKETFIDTELIFAFAHTGR
LADLEEFISSPNHANISQVGERCFEQKMFEAAKILFNNISNFARLASTLVHLGEYQNAVDGARKA
NSTRTWKEVCFACVDGQEFRLAQMCGLNIVVHADELDDLIRYYESRGYFEELISLMEAALGLERA
HMGMFTELSILYSKYKPTKMKEHLELFWSRVNIPKVLRACEQSHLWSELVFLYDKYEEYDNAVVT
MMKHPTVAWKESLFKDVITKVANIELYYKALQFYLDNKPLLINDLLVVLTPRMDHTRGVSFFQRA
CYLSLVKPYLRSVQNHNNKAINEALNSLLIDEEDYNGLRASIDAFDNFDNIALAQLLEKHELIEFR
RIGAYLYKGNNRWKQSVELCKKDHLYKDAMQFACESRDKAVAEDLLSWFLSIDKTDCFAATLYM
CYDLLEPDVVLELAWRNNMLDLAMPFMIQFMKDYYTKVDKLAESNAKRDEDKENAPPEAPIM
GVEQLMITAGPSSYVPQPNMYGMPPQGMAPQGMPPQGMPPQGMPPQGMPPQGMPPQQGFPQ
QGFYGGAM*
>TRINITY_DN6390_c0_g1_i1.p1 type:3prime_partial len:1941 gc:universal TRINITY_DN6390_c0_g1_i1:5820-1(-)
MVIAPKCKFSIFSYLVFGILSYNWNELDASTNIELVDGTGVLLQRDVDNVLNDAVVQKEYQQNIK
HKPVDKRCLGEPFYQGLCKEKLEDAIQQNITEGGCHNLLVKIQKDECSNGPKTLGGKGYCNFICS
QEICNSIYDIDKCVSKRLVHHFNKDKNECEEIMQADCGSESFTSSNQCQKRCIGNSDDKRETKVY
CDQFCLNGGKCIGEVYCQCPPAYHTATCGLSYCNPSCGVGGKCVKTGEGKYGCQCFPGYKGVK
CDKEDSKNMPVVKGLCLSAIKSVLTSPNTSFHDKPEEEKNYCLKYLFDLESNCDKQRPFVNYTEE

QCNHFCASDICDNIYSNDCGSIEERYGYVSSTGRCSKFLYNGCLGNTNNFKDKAACDTKYSPING
LMTSVALLRPLNHTCYIRGKTNFITFDGKVYDLLSGCSHYLVHDSDTTSDFNVILHNDPKVNYNE
KSKRKLEITADGKNVLLNQKVSSDRFFVQVDGRSVPLPYNDVPKIRQAGRYILVLTGDGVSIAFDG
YEDISITVNDYYRTGDGGDHRLNGLCGNLDGNSHNDWLDFTGELRENVRDFAKVYQTDRSTCK
MDNLSLKSLVYNPLHNDQEVFERAERHCDVINTDAFKPCHSVVSPEQYKVLCMQDYIKCNDDK
RDDCVCSSLSFYSRVCHKYGKDIKWRRANLCPITCQRNMKYYECGSACPKTCDNLITPTKCQTDC
VDGCHCPPDLYYDKSTENCVPRNLCSCYRDGIAYKHGSVRSFHCEECTCNGGSWECNKKDCEAT
CDIAGDPHIKTFDGLNYQMYGKCSYVLVDHCHYGNTGIKEFEVIVENKNCNDDALYTSCVRHLS
VNILQTNTRVSLDTAKDNNGTVLPVAFVNGKEGKEFRSKDYTITSVGGRNIIFQYKVNQLKVSWT
GHNAYVTVGHQFENQTCGLCGTYNFNKQDDFHTRSDTVEKSVTAFTQSWVYQSSDKVQDPSKC
HTAKWETSEKPCQIYTNKAQYAKTQCSIIIKPDGPFEDCHSIVPPGEYYSMCQDDACKCDDCYCH
IVAAYAKRCTDLNIRVGNWRDETLSCKQTCQAPKVLKQCNAIDSLDDDLSESLKCPSTCYELSQN
DTSICQSRGCIEGCYCPEGYFENEQNECVKQDQCSCYHDGKVYRHDEIRKDRCNECVCKGGRFE
CTDFDCVAHCASKGLVYSDCGKTCSNFNFNPQDSKCQAGCFCPAGKVLHENGTCVNPQTECQC
KEGDQFFDAGEVSPSDCSKVCNGDRSWKNLPGKDAKPQYDCSAFGQMHYRTLDGRMFKFSGG
LCPYVLLTDCRQDSATGFCDLTKANVNIHVRNLRCVNSYEAYMCKEVTLDLRLPNGEMAEFVLM
QNKIKMKTGLVTKVFDKGSYPQPKTAVVDGVEIFKSGLYLVVRAYHLNITVKYDRQNRVYVVPGR
EYKDAGKLAGLCGNYNGLDDDDFRRPDNSNAENELQFAKSWQDSNSACPDPSESDTCSSNKER
LPWAQKGCNVMKTGNFTACHKTVSPEPFIESCQQETCLCKSGGDCACFCSAVAAYVQECNRHG
VPIYWRRDGLCKLDCCDPCGHSYGYYTDNHVPNTCGVKDKVCKENDRCCKGAVVEGCGCPPG
TYDDGIKCQNETTTNVIPCSPSSSIVPSTSISVPSTIIEPVSSSSPSPMTSSVESQSPSPSSSVVSPTPSSSII
QSMSSSEIESISTTSSSFVSSIESSVQSSTSIVFPVVSSNSGESSVSSSMLPSTSWVGIHSVSSSMGESSVST
NVLPTDYLRTTDVFPLSSSEITYPSSSSSLEPHTPSSSILQESSSSVSKPVGISSVIEPEISSIRYSSMSSQN
VMSSSVSSSVSKPVGISSVIEPEISSIRYPSMSSGSVLESSVSTSFVLPSETVLSTSVFSTNTESPSSSMTLS
TSSSGFVFPSVTSTFQTSSKVLSSLPRSGPAENTVPLSTMFTKTV
>TRINITY_DN319_c2_g1_i3.p1 type:complete len:2588 gc:universal TRINITY_DN319_c2_g1_i3:165-7928(+)
MVLLKGQLWILGFIFLTSTKYVMGDVPECSNNATVAVHCRFDEGNTCVDDPHPMHYVCVCNNL
GYMNDTDSVEGYSICKERDCSNSNETPTDLLKRICGVNTKTCTGKILQRECNCNTDSYRSKNGQY
CFFAPCQASPTIQQNCGPGNQCYGNAPNYICDCSARGYKRSAADNRHCELKSDEELTPIEEYCGK
GSLYANGTINNITCTCDTDKGFFPSATGETCISPSCYLNPQMQEKCGPENTCLGGKDDYVCLCSNP
NYKRNATDLQHCIRRTCADTSPAIDFKSICGEGATGCSGYVDKPLCSCDVSNHYVPAADGRSCIK
ATCKGNKKALEQCGQANSCTGSFDNFLCVCDANGYYLNTTNNTCVERECTDPDLVITDICGVGS
LSCNGKAGSPECICDKDKFYYASEDLRSCVKANCLSSETISKKCGKENSCYGSMESYVCNCNAPG
YLLNTTDNQGCYLGMCEDNGLEYDKVCGEGAISCNGTIKNLECNCGMNYVQSPDKKSCIRADC
RANQTMHEQCGEDNDCVGSLEDWMCICSTYGYKPDPMDMMKCKKVMCEEEVVNNVTVCGL
GTTGCKGHILKPECTCDIAADYVPNEDASRCIKGSCENNKMAMKDCGIANSCTGSYNNYVCVC
NGFGYKKLDDTHCVEQMCSDPDIDIRNVCGEGAHYCNGTVNQRVCSCNETEYFYPSADSKSCV
KATCAVSPMVMEQCGVENSCQGSQESFICNCNALGYLLNTTTNQHCYLGTCENGDFDLMQAC
GDGALYCNGTIKNLECQCDESKGYVQAPDKMSCIPNDCRGNLTMYRQCGQLNKCVGSIDDWR
CICDSPYYVAHSDIMKCKLLHCSDSEVDHVEVCGKGTIGCSGSTLHPTCHCNTTENYFASKDGMR
CVAGSCKTNQTAVKKCGNANSCAGSHSEWTCICDGIGYKHDPADPQNCLLKTCNDADIDIVATC
GVGAISCEGYQLMPKCECNDRDYYIPSDDRTSCELASCRANRTAMRACGEMNDCRGSFSSHICL
CHDPAYKSDPSDSRVCLEKTCEAGDFELVRVCGKGATGCSGSMKNIMCECDNAKGYVASADHT
QCIPSDCNNNVTMHLQCGEGNGCVGSLTDWYCVCSAPGYEPHASDHMKCHLKTCADPGVDH
VQICGMGATSCMGPVINPVCYCNESMYYIPSMDRTSCIKASCMSNKTAIAQCGSENSCSGSPDNF
VCLCQAYGYRQDTNNNQACIPSQCEDAGIDFVEICGQGGVGCSGSVLNPVCMCDHMKHFVPSP
DQNSCVLSTCRGNETAMTSCGEQNKCFGSSSSYICVCDAVGYKPHPTNLQECVLQTCEANELNP
VAVCGKGALSCKGTIQSLMCECDNSKGYVAAPGYKSCIPADCRANMTMHAQCGTGNRCRGSM
EDWSCVCDGIGSKPHPTDHMSCIAIDCSDPNVDIKSVCGYGATGCMGLARDPICHCDTSKYYVQ
SEDGRTCVPATCHTNKTAQMQCGPDNSCIGPPNDFICECTGFGYRHHETDKQKCVPIQCNETAL
FDIERVCGKGATGCRGNIRQPICDCDHAMYFHPSEDRRTCIEASCRRNSSIRADCGVENTCFGSTR

SWICDCNSYGSILNPSDVKKCMLATCEAGQLDPVAVCGKGALSCKGSMRDVQCTCNNELGYVH
SPDGKKCLPSDCSGNVTMHHQCGDANKCMGSLTDWVCVCDAPGYKPHAKDKMKCHQISCAD
NNVDPVDVCGIGATSCKGPITNPLCQCDTDNYYITSSDGASCIPATCKTNKTAIHQCGEANSCEG
PADNFICVCNAYGYKLDDNDHQRCVEISCDDPNAHESICGTGSTGCRGTARDPICECDHTQHFV
ASSDLTSCLLASCRANDTVMNHCGDGNTCFGSLTSHICVCEAFGYKLNPNNHKHCLEETCEDD
MLNPVAVCGKGAVSCSGPLKSVKCECDNNAGFFRSPGGMKCESTSCRQNPASQMMCGSQNQCY
GSYDDWYCDCSAPGYMANQTNAAECLKVMCDDMAVDVCGLGATGCTGHTLNPICTCDSSMG
YMMAKDKHSCVPMDCSMNPASSQCGYGNTCHGSYFSYVCVCSTAGTVMNKTNPMECINVACP
ADAVHVCGEGATNCSVTSTNNVECSCEEGYHPADDLHSCVLDDCRFNPKALKACGLLNKCVGS
YKSWKCNCHYPEFTQDPMDHQTCVVGPCMVEQCGKGNAPGSFQHYAVNCSGLYDDPICNCQP
EYTNDAKGGCIPKICSENPVAVQACGSGYCEGTYFKYTCKCDEPAFVQTPDKMQCVPAKCNIQQ
CGHGFDRGLYIDFAESCSGTYADPMCQCKTGFSGTNTSKCGPAIDGCKLDKCDPLATCQTLDVD
GNVKCTCKEGYSGSGMMGECYLDGGFSAYEGTKECSEVCGVNGVAEVTRFCNNPTADPKGKPC
NGTSPETIQVPCNRFACSSCTVRKTCGCGGLKPIFVEFYHEPNSTLEYFTDLKADDVQTALGFLTK
WKELSQQDLCLMYSKIELTVFVTLMGDSETQVSTMKSSLVSARDIMRKKLDCNINLGKKAQYYEA
YETIIKRHLAISNILKDLTIIKNSITKYMNNCDDSEIGMIRDVIGMM*
>TRINITY_DN477_c0_g1_i9.p1 type:complete len:306 gc:universal TRINITY_DN477_c0_g1_i9:1840-923(-)
MTRQCLGATLNENCGNAALVQFEVCNFQVPCPGLLTNWGPYTQCSATCQKDANSMPTQSRQR
FCTGGSFGGNCGGVQLLQTIGCNQLIICPVYGYWLQWSAFGECSKTCDQGTHVRYRNCTKSIPA
NAGNNCTGPAQEVKPCQVKPCPVTCPLTKKCDCRNMNYLAAAPGISQTVTNDVMGVAAFLDY
WTLDILKQICEGCRMTQLDQSKATVDSQILRTQAVLDRLNIEKRVYRDMITCNENPYITDDLFKVY
NDIWDRIVTTDGVMIELKASKMRFDTAITACRSYDWYHDLFGRFFQIKKSS*
>TRINITY_DN113_c3_g1_i1.p1 type:3prime_partial len:1162 gc:universal TRINITY_DN113_c3_g1_i1:3485-3(-)
MEIVSRSRIAIFVLLGLLISVAESQFDPECPEIDPDVECSPPESTVNECLINNDCDVDKPLCCSNGCG
LKCTARERRSITPDGFTPEDEKLCRVPVDLGFIIDVSGSIGQTNFDKIKKFIEKIVDAFHIADDGTHV
GAVFYSDNASLAFDFNHLQGDQLNEMNVVREIEKIQVTDGQTRIDLGLELASRELFNPAAGMRI
HKPKIALVLTDGKQTRGELAPDAKDLHQASRLLKQTGVQVYSLGIGLDYDIGELLDIASDDASVF
RSQDFDELVSIVDILIEQTCKGCTRAVDVHFALDASANVGEENFVFMKDFVKAIGYNFIISETGAH
MSGSVFGDVGTLVFDKSQATEINEFENEVENMPYLGDATGNMDKVLKMISNEVFNFDGFTRQSS
PKVIVLLTSSNCDTCQESLEDLARPLKAEGIHIVAIPIGDPLFDEMQVLASHPIDQYAIPRATHQELL
NAEFIQKVSEMVCAGKPGICAEPPVKGECAEIINECSVDMRCPEDQKCCIKNCVASCVLPLAACIT
PIEIAFAFETTKYGIADFNNVKNFAINLLDHFKVGPSKAHVSLSTFSEDVTSLTKFIDDFDMESIKQSI
KDLKHDGGEESNLGNFLEIINTEVFSVRGGIRQQTPRVLVIFANGDINDEQAVKAQEEALHLKAD
GKDVKIIIVNIGSSTKTLDVLNEVATEGSFYEFFGSAELDSTAIETEISEKICSTKVRRSKCQRRIRSSCA
VKDDLCDSDFDCPGHQECFSDGCRLRCQTCEFRCKDKVDLLFVMDTSARIGRDNFERGKKNIKK
MLEWFTIDSNNTNVGVITYSDTANVAMPLPSRGNNNPAQSLIDVQDRLDSIPYSGGSVSHLDNAL
QLAAEKVFPDNGPNLRRDSKKAVVVITDSLDDSASQLELASWPLRRQRVQTEDGDASNIRILAFN
VGQELDLDLWGNIVSPPVEENVFYAADFDDMYNGIRQLAEDSCKYAAAITPPPIIKQKGEPGPPG
PPGIPGTNGTDGRNGTDGRRGPKGERGDTGLKGFKGEVGLVGPPGGGAEEQKGPDPLASAVGIV
NVTDLPGGGRIVFDLREGPVGQQGLTGIIGPKGPIGDTGEVGIKGRNGTTGPGGFFGLPGPKGQP
GTDGAKGLRGDPGLRGSAGRKGPTGAPGLTGPQGPFGYK
>TRINITY_DN36_c0_g1_i16.p1 type:complete len:224 gc:universal TRINITY_DN36_c0_g1_i16:829-158(-)
MMIKTTIVAVVLAACVLQSFAGRCYVGYSAKGLPLAIGVRDVECGKGYDYCTTIKYRLGGIEVTQ
GNCARKYGQAMCQFMRNPHKAMTDCEETICQGDSCNKDVYDAEQAERKRVGPYKNTLKCTT
GLITKFPAIPFYTADVPNAIDECFPGTTECVTVTYDYLFKFETQIATKDNVFKLPCVQKMCNRHNI
DNCNKWCDILSIHQSMENCVAKCCKGDNCNA*
>TRINITY_DN624_c0_g1_i1.p1 type:complete len:884 gc:universal TRINITY_DN624_c0_g1_i1:218-2869(+)

MANQEEEEHFGYYYGEEEDRGLLDPAWEKQQKKTFTAWCNSHLRKVNVQIEELNVDFCDGLKL
MLLLEVISGERLPKPERGRLRFHLISNVNKALDFIASKGVKLVGIGAEEIVDGNLKMTLGMIWTIIL
RFAIQDITVDEFSAKEGLLLWCQRKTAPYKNVNVQNFHMSFKDGLAFCALIHRHRPDLIDYDKL
AKDDPMYNLNYAFDVAEKELDIPKMLDAEDMVNAVKPDERAVMTYVSCYYHAFSQSQQAETA
AKRILKALNVNQGNEKAIEDYENLASDLLDWLNNKIPTLDDRTTDSSLDTMQNKLNEFRDYKGT
EKPPKVDDKSKLENLFNTLQTKLRLSNRPLYMPAEGKMINDIDKRWKDLEQSEKDFQEWILNELR
KLERLDHLAMKFKHKCDIHEAWSTGQDDYLRSDDITDASLASLLASRKRHEAFESDLAAQQDRV
EQIVAIAQELNTLGYSKIDEINTRCKGICDGWDTLGELTTQRSEKLAKAIEIAERLDNLWLEFAKKA
SPFNNWMDGAKEDLMDMFNVHTIEEVNDLKDAQLKFKANMPAAQEEFNTIMGLQNAISSMSS
QPNPYTNTKAEDLQEKWQEVLELIPKRDSLLESETQKQQNNERLRVQFAQMANQVGAWIKQRM
GRMTDIGMNAQGTLERQLEELENLNAEINGFKQNVLDLDASARNVQEAMIFENPHTPYTMEVI
RIQFEQLLTGVTRNINEVENQILMRDSIGITEEQVKELRTSFNHFDKNSNGRLDYKEFKQCLVSLG
QLGDVEDKGEHTFNRIIAMVDPNETGYVSFQAFLDYMTREMADTDTADQVMESFRILAGDKPYI
TADELRRELPSEQAEYCIARMSAYNGPDGVPGALDYRSFSTALYGESDL*
>TRINITY_DN1369_c0_g1_i1.p1 type:3prime_partial len:1577 gc:universal TRINITY_DN1369_c0_g1_i1:93-4820(+)
MRIQLTIFVTFSIILVSGQNSPPGICSTWDGSHYQTFDSYSFNYHGTCSYALALDCRPGSDDFAVHV
ENDEKCDNHGGTDCTRAVSLYVNDIEYKLGSNNTVLVDGKSNIVLPYFNEGVRVSKHAEYYFIDA
LEGKVVVKYDGRSSIYVQLDSSYQAGVCGLCGNFNGNKKDDLKLPGDKGLTKSLKEFGKSWEKP
RIGQVCKEVTEEHFDQCTNKSKMLVYYAEKECEILRTSSEFKKCHKIVDPTPFYEKCREDLCLCGS
GDKYCKCDVLEQYTRKCAHYGTVLEWRQQNLCYKNCTGNKVFSECGPACSSVCSAIGSKSVCK
DACIDGCHCPPHTSLSGDKCVPDDECPCEHNGDMYDHGDVINMKCKSCKCNGGKWKCNPLP
CDGEASVYGNGHFVSYDGKTYNYPGKCLYLYTYLSDRSFAVYLDRTSCDSLACHMNVMILFRGD
RIKLSASNSTLALEVNRNVKSFPYISSTGYRMYRFSTVFVRFEATNGIKVDWDTYSRLHVLMPHSL
KGKVFGLSGNFDFKTKNDFLTPRKDLAHSAIEFGNSWMMYSSRLNCSALAPDYRMTSCEANHQ
VAKYAESKCSILMSSLFRECHKVVDPFIYYENCKDDTCECNGDSKCYCSVIAAYSKACSSAGKPTE
GWRKYTPCEEKCSNGMVFTECGNSCLKTCDDQTDPNPFCEKECVEGCLCPVGQVLDKITEQCV
DIKHCGCTYGNRTISNGGTVAKDCNTCKCTKGVMVCTKKECRDKKAICSDGKEWSSCMSPCPK
TCQTKDDICPETGLDCIEGCQCKKGYIESANGTCVELSKCPCYHNGKEYQETQTISKDCNTCKCT
AGEWSCTEKLCDATCSVFGDPHYKTFDGKIFDFQGKCGHVMLTDKCANNDNATENIRISVKSST
CGSQGVTCTREITLNYHDTVVHMKRGARKIDIVSNKRQDHEKDQVKVYRHAGIYVHVLTPMGV
SILWDNATRLYITAPPSLSGKICGLCGNFDGSETNDFVTYQGDTAGNAVSFGDSWRTENNCPLTKE
IEDTCHNRKHRLDPSKRECAVIKSDVFKKCHTLVDPVPYYDRCVFDACGCDSIGDCDCLCAAIGA
YAKECIDEGVYINWRKGHNICEKEMCCPTNMTFVPKGTGCPKTCKYPNGLHHCPTKYVDGCQC
PEGLVQNNDANGDVTCVRPEECKVCKLDGKIYNEGEVVLYKCKECVCRDGKMTCSGNYCPTTL
PPTTTTESTTTPSTTPETTIPSTTTTESTTTPTTEPPTTTTTASTTTASTTTESTTTEATTTTKPTTTTTEST
TTESTTTQSTTTPSTTPSTTMKPIDTCVYSNGTHNVTLERGDVIQVNECTKKTCVKDEVTGLHEIKV
TTTQCENSTCDTTCEASKSNRAILVKTPGECCPQCKKPEDLCDNPNEVPCNSTYCIPKSWFCDGN
KDCSNGRDEECCPNYCKVEGEIKKPGEVWTNKEKCLTCKCEGYYTKCRRMECPELDCPEERQVK
SEGECCPHCKPQTEPPTTTTEMTTTTTESTTTPATTPSTTSKETIIITEEPKSTTASPQEKCDCKVLQPV
GVSDGRIPDNQMTANSV
>TRINITY_DN1676_c0_g1_i12.p1 type:complete len:1631 gc:universal TRINITY_DN1676_c0_g1_i12:206-5098(+)
MLIITRTAKLLLLSLTYIYLTIAQDLTNATLNNGFEAGRAIKCKTDDGMVQRCMPVFENIAFGRDII
ASNTCGQTRQTYCVQTGTSGAVKICEVCDASDPDKAHPADLMTDLQFDDQPSWWQSETILDNK
SPVYLTLNLNKKYNVAFIRLRFHSVRPHSFAIYKKSTYDSNEQWLPYQFYSRTCQETYNVPNQQSV
SYSNQKLALCTDEASGMIPLSGGNVAFLTLQYRPGKRDFDNNPGLQDWVTVTALRFELDRVNTF
GDEVFGDPKVLRSYYFAISDITVGGRCKCNGHGSSCNNNNNEGTFQCVCEHNTAGKDCEKCLPI
YNDRPWRKASGRSANPCKACDCNGLADQCEFDQQLWVDSRYRSGGRCVNCKNNTDGINCEYC
KEHHFRSNVKEACKPCRCNPLGSRSLSCTTKGECACHPGVTGLKCDKCKEGYYGFSSSGCKACN
CDRDGSIGVNCDFTGQCNCKKKTVGKRCDQCAPLTFDLDRSNPKGCKECFCFGHGVSCDAIEGI
GVKEIFTTFEADFDGWKLQDESGASFDSNMEWDFRGQYISVKPPRNKQLYLVAPQRYLGNKLSSY

GKLFTFAYGIFLDPNDPLVSAGRDIIFEGNGLQAVYPINSQNNILPEARFYQFKYRLMEPVGMSTFD
FQKILVNLTAVKIRTAYMNERTGAIDNIKLYSTKFISINSPEQVTWQEICECEEGYTGDHCEKCDSGY
TRSGGDTSPFGECKKCFCNNHGNFCNPDTGICQCLHNTTGDSCDQCAPGFYGNPTAGTPEDCK
PCPCLYGNECILIGNSVKCTDCPEGHIGDLCELCDDGYFGDPNGTIGEPSRCQKCDCNGNIDPNAI
ANCDSLTGSCLKCINNTKNGPENKCELCADGFYGNSTIDPKGNCSACSCYEKGTMKPPFHDPKL
QLPCDTNGKCECLQNVIGDKCDMCPPGHWNVASGKGCELCNCNATGSKGSDCNLQTGQCEC
QDGVGGRKCDQCAPRFYGFSKDGCIACNCHPDGSKHQNCTQDGVCECLPGVLGIKCDQCPEN
KYNLSVGCIACPRCFDLIQVAVNKLRNKLKTFNITGNLTTGSIDIKDPEFEKLFNELKRNISELEQK
MKDLLEGDMAILEFYEVLVNQSSTLIEMIPGIQKMIKKSEGAAETGSVTISKAEEITEEIQNLLGQAE
KMLEAETGRIFKNVTSSSEDVSGLSKRMSEIAEEARKHADRQEQESKDIEFNTDMAYNNSKAAYE
QAKKARDTEEAISYLLDQMALGHDEALALSKNSTEIIAKANSTSIAALNDSDHLMMKAKEPLPN
LDIEAMKEKAKNIDAEGKATEFNATDLHEKNKDKITQLQNDDKEARELMERGTQLHNDSNALY
KIAKNASDEAKQAQEDGLKVAKDAQDMLKTLMEFENNIAKSKANATEALKLIGLIRRLISKANK
TTENASSNAIMASINATAALRLASAANKTTQDAMNEIESSRREVEKLLNETMDFMMKEVAQTKD
DIDDIEKSLEESEKLAKSNSMKLGDALNDSQAATDKASMVQNELLKSLDQIEDLMNEISNLDEIDF
ETLEDAEKRFEEAKDTIENKLKNEIEIMESKLSDQKKLLQQHELDLTPLKEQIMKNEELFKYLPKSC
FREKAKPEDIDK*
>TRINITY_DN123121_c0_g1_i1.p1 type:internal len:127 gc:universal
TRINITY_DN123121_c0_g1_i1:3-380(+)
LNQKLCAQQVIHLEITDANDNFPVFSQSRLSLNVSEYTEVGQVIATVRANDLDERSQGNLLFSILK
DEDFFGILSNGDNGVIFLKQSLDAERRLTHQIEVTVSDREGKSSLQNMEIIVFVEDENDN
>TRINITY_DN71276_c0_g1_i1.p1 type:5prime_partial len:1201 gc:universal
TRINITY_DN71276_c0_g1_i1:1-3603(+)
LTFIESLRLSSIKNIFKMMISFETFVPSTTPYAEDYNTNPQVMMLSNKIKETIDTSLNPLASQIDRQKA
FQVQEEFKSSSPHCAEVGLYLYSVERKNSDAVRLCALKFVDHFIETKWNDLTYERKTFYKKESLKL
VEQMTFENETYLKEAAAQIVVEIIKRTWPQSWADLFKNLEELQAKGDAQLELVLIILMRLSEDVN
GLDERLTTQRKRDMMTTFQDNANELFEFFIKTIRDKVTALKTESSSLLIRLSEATLFTMMEFVSWA
HFDCLFNNEKIIFQILFWLLEVKYLRYYAAACLDNIFQRKGTPEKRIEFMVVLSDDVLNIVENVVRS
LKKTPENVLPEEGYKFLKKLAEMLVSFGKIQILSLWGDERGFNEKPVGYDRYLRLMLLLFQHESM
KLTYDVSKFWHALSIHPHGKLDDTFKELMPDLLDCAIMKIAKISDPKIPNNPSSHYSTMDFDDKE
NYAEYFANCRGHMTDVCRKITIISYTVVLDHCLAKIKQATSKPPPDVSGIPKNQIQITQHYLYWDA
LVHFLKNGFAGVFFRANKEISEDTISKCNEIITLLIHYKCEVPLFTIPVINLLESVFPIFKHSRDHLLPT
MQLLFSIIRLPQNDEDPLRDHVFKLKRHVCEAYAALCKEIPHCLVDYFKQLEEFAVYQMMHNASL
TGQEKCRLIEGVLRISKHVTVKEEQDHCINAIFDMIDSMFDKEPVRRACESPISLIQYIGLDQEGTV
AADSELRTRELLFAINLWTAACKALAMSKEEEVKDEITEEEMRTQIETYGWIKRMVTMLPKMLSL
LQSYSLIWQHQDKICDKRILVHKIITEINPTIQVFQPKCADEVNEKRGLGQYYLCTFYESAYTSLGSV
SKSVGPSLFSIGGLAEAMNEKLVQHWNNLPNYRLGRITRTFIKDICLACPKKYFETFIGPFVSQVLR
LYFERISNEWAKLAQLKENPREVTDKLQEQIEIAEERMIYLMSKDLLDLLLSLLTFKRKHLRSTTKP
ADGSLETFGELGHFLLDCEETNFIIVQIIFTSLTWPDTSAAIKVSQIILAVLKKIFTDTSKLNPELPKQL
LLLILQGLNIHGGDEFGRAALTNNVLQFYLHLRMQYRGIVQVMLMIPTCEEQHLREFDLAVFVQE
LPLTEQQQKKIFRRFVAGACGTVISKLHEKDVVIQDLPALMPRQKEKTLNKNGDADDDLGLCSLF
GV*
>TRINITY_DN1386_c0_g1_i1.p1 type:complete len:596 gc:universal
TRINITY_DN1386_c0_g1_i1:2419-632(-)
MEYIEVKSFIVALVFVFSILGTAQSDLVINNVERTLDLSTQTAKYTTKIKFHNPGKKVEKSFLLALEK
DQEKHLSYLEILDKQDDNSLEYAAENVEGQSGRFFKVSFSKPLKENDDVTLHIETVFTHVLTPFPA
KIGQTEKQLVLYKGNTVFYSPYEIKEQSTTVKLASSAIESYSRLKPSRSSENTITYGPYKNSKPYRLHE
MKIHFENNSPFLVVNEMTRWIEISHWGNIAVEETYHMTHEGALLKGHFSRYDYQRSPTHSVIKSF
KTVLPAAARDVYYRDEIGNISTSNMLVNDDSVEIELRPRFPLFGGWQTRYMLGYNVPAYQYLYNK
GDNYILKMRLIDHVFDEFVIDDLNVKIILPEGSTNIEVVKPYELEEGKRDIHKTYLDTSGRTVIVLSK
KNVVENHIQDIQVHYTFKKLQLLQEPVICVAVFYILFLSVIILVRLDFSITKDAAKESRMKVASLLEE

LLSSCGRRGSLYYSYDSAIDKLKQNRNQNDFAVSLKKLNQDYNTLTQSINELCSLLIKEDPEIGDKV
NDLQKKENERKVIVDQLVTLATRVVTNKIGRQQYIESEKNSMAKREKLGEDIDNVLATL*
>TRINITY_DN428_c0_g1_i1.p1 type:complete len:1949 gc:universal TRINITY_DN428_c0_g1_i1:96-5942(+)
MADDADLKFLMVDRRQVSDPVAHAEWAAKKLVWVPSEELGFVAASVKEEKGDKIIAQIEGGKR
VTFHKDDIQRMNPPKFDKAEDMADLTCLNEASVLHNIKDRYFSDLIYTYSGLFCVVVNPYKRLPI
YTDKVINLYKGKKRHEVPPHVFSIADTAFRSMLQDRDNQSILCTGESGAGKTENTKKVIQYLTAIA
GRANQQKADEKSQGKLEVQLLQANPILEAFGNAKTVKNDNSSRFGKFIRINFDNSGFICGANIES
YLLEKARLVRQAPDERIFHIFYQLLLGASPEVKKQFLLLDTKSYNFLSNGHVQLPNMDDRQEFKV
TLEGMRDMGITQEELDPIFKVVSAVMLFGNMEFKQERKSDQATLPDNTIAQKICHLLGIQVTDFT
KSLLKPKVKVGREFTQKSQTKAQCEFAIEAITKALYERMFKWLVGRINKSLNRGKRDGASFIGILDI
AGFEIFQLNSFEQLCINYTNEKLQQLFNHTMFILEQEEYQKEGIDWKFIDFGLDLQPTIDLIDKPMG
VLSLLDEECWFPKATDKSYVEKLHKQQTKHPKYLKPDFRSNSDFCVIHYAGRVDYSAAQWLTKN
MDPLNDNVVSLLAHSKEAFIASIWKDTENVVGMSVTEGMDTTFGASRTRKGMFRTVSQLYKEQL
SGLMTTLHCTKPNFVRCIIPNYEKRAGKIDNHLVLDQLRCNGVLEGIRICRQGFPNRILFQEFRQR
YEILCPGIVPKGFMDGRKAAKQMIDNLEMDPNLYRIGQSKIFFRAGVLAHLEEERDIKLTEIIIQFQ
AFCRGNIARRNYRKRIQQLSAIRVIQRNGRSWLKLRNWQWWRLFTKVKPLLNVSRQEDELRSKE
EEFKKLQDKSDKLESLQKEQQQANEKLTEEKNTLAEQLQAEIELCQEAEEARNRLLQRKTELEDL
IQDFEMKLGEEEEKAVKYAEDKKKLQQGIQDLEESLEEEETQRQKLQLEKVQVEAKLKEMEDERQ
VAEDNNARLTRDKKQLEDRVAEFGQKLAQEEDRAKQLSKAKAKQEAFIAELEDQIAKGDKSTHE
LHKEKRKAEQDLSDLRAQLAQALQRIAELEDDLARREKELAEAIKRGDEEMAKRTTVEREKREL
QALLDELREDMESEKSAKDKVERQKRDLEQDLQELRSELEVTTDATVVQKEFQKKREEELMDLK
KQIEEEAKEHDQQMASLRTKHNGLVEDLNNQVDTFKRGKAALEKNKTALENENAEIAADLQRI
SQLKQEGDRKAKNLEQQLNEANSVRAVQDEKVSSLESEKAKYQKECDSLSTQIEEVETKAASLDR
AKQAAEAQLEDTQDQLAEETRQKLSYQTKLREASDEANRLQEQLEEEEDEKKAVQKVLSNLQV
QLDQAKKEIELKIQDFEQAEAARQKQKRENEELRGEVEKLTGDVSRLDKAKKKLQGDLDDVTVS
MERERNSANQMSSKQRKFDQLLAEEKANSQALGIERDNLEKLARQNETKALSLQNANEELEDR
LAEAERQRKQLQMELGDLVESKDDVGKSVHDLEKSKRLLEQQLAEQKTQVEEIEDELQACEDAK
LRLEVNMQAQKAQFDRELQAKEDAIEEGRRGLVKQLRELEAELEDERKLRQSASAAKRQLEGDV
KELEGQLDQANRIKEDGLKQLKKYQNQLKDVQRDLEDSRQSKDDLAAQVKDNEKKLKGLEAEF
LQMQEDLSAAERARRAIESERDELLEELNNNSALKNAVAEERKRYEAQLAALEDELEEERTQTEL
MQDKVKRSNLQLEQMNAELQEERSAAQSLDNTRMSLERQNKELQSKLADLESSLRSRQKNTVAT
LEAKINNLEIQLDQEAKERQQQNKALRRNEKRCKDLQMQIEEERSHTESYKQQLEKMNARVKN
MKRQLDESEEEVTRLNTAKRKLQRDFDENQESLESSQREANQLRQRLKGGASLSSSRSSGRGRRA
KDEDDDDDDANE*
>TRINITY_DN875_c0_g1_i1.p1 type:complete len:606 gc:universal TRINITY_DN875_c0_g1_i1:2207-390(-)
MLRLVLFAISASVVYGSCNPLKLGCFIHSGSDRNLLHSDNSEDIYTDFYSGTNIEWDKFSSYLQRAA
CRCDQAAANAGYRFFSLRDFGECHGIGKGASSFDALKLKKSMYCIAPNLGQCDISEHQHCTGAE
EGEFIYETKAQPAEKVIDGGYSSWSAWSVCSKECDQGLQERQRTCTNPEPKGKDAKDCSKLGKPS
ETRSCKIKDCPPSGETVIFCEHRGVKRIACENDQVVEILAANYGRTSTKHCVFGRTKVTNCRAAH
SMSIVKSQCDGFNYCDLNPHNDVFGNPCGFYTKYLEVKFRCVARKTSSRRAVLCEHRGSPPLSCP
NPDEVLSINKASYGRLSTHYCQYGHFKQTNCRALHSDALIKKTCNGLNSCSLNPSNTVYGNPCG
NVNKYISVEYTCKKTNAASRQAVFCEHQGGKTLKCANPDEVLSINKANYGRLSTDHCQFGHFK
QTNCRAEQSDALIKLACNGLNECSLNPSNDVYGNPCGNVRKYISVDYTCKKANAASLRAVFCE
HQGVKTLKCANPSQVLSITKAIYGRLSTDYCQLGLFKQTNCRAANSDGMIKKQCNGKNSCQLNP
SNAVYGDPCKYVRKYISVEYKCINK*
>TRINITY_DN8597_c0_g1_i1.p1 type:internal len:124 gc:universal TRINITY_DN8597_c0_g1_i1:369-1(-)
TCDGGVLVQRRTERAFNKVETRNGNTPCNTQACGGSDADFSEERVRACSNVNQCGVNTGTQTV
YKVCNGNRCNFQGRDYQINEEVVIRVQSCSVVCDYSPWQDTQSCTKTCDGGVLIQSRTER

>TRINITY_DN5987_c0_g1_i4.p1 type:complete len:336 gc:universal TRINITY_DN5987_c0_g1_i4:136-1143(+)
MAKKNKVATVTDRETNTKPIVDQMKDTRNSTNSSKAKDNSKKNGGGGISWFNLFTFFIATSSLAV
NVALIRGLVPLPGCVGPQGKQGEQGLTGATGPAGKEGPQGVPGADGPRGFPGNDGMPGPEGKQ
GETGEQGPPGPQGVAGSKGDPGPQGKPGKQGEAGEQGPAGLQGAPGSKGEPGEIGEQGKQGPV
GPVGPKGERGNDGEQGLPGPVGPEGEPGPPGEQGPPGPAGLTGERGNQGEQGPAGPVGPKGDT
GKQGEQGPAGTDGQKGEIGNQGAQGPAGAVGPKGEIGNQGAQGPAGAVGPKGEIGNQGAQG
PAGVVGPKGNQGEQGAAGAS*
>TRINITY_DN77_c1_g1_i1.p1 type:3prime_partial len:1965 gc:universal TRINITY_DN77_c1_g1_i1:2-5893(+)
MGVQVYSLGIGSNYDIGELLDIASDDASVFRSQDVDELVSIVASITEQTCKGCTRAVDIHFALDASQ
NVGRENFQVIKNFIKAIGFKFIISETGSHMSASIFGEYGTLIFSMDQATQQEDFINEVDNVPYIGDFN
GNIDKALDMVSEDIFTFDGFSRQGVPKVLVVLTASNCDSCEKSVEEAVKPLKAQGVHVVAIPIGSN
ANMEEMDKMASYPTQDYVIPRASYEELLNGVFIQKVSEMVCSGRPGVCEEPPIEGECTEIINECTL
DVNCPANRKCCIKDCKASCEVPAAACITPIDLAFAFETTGFIEENFDEVKSFASSLLDHFRVEPAKT
HISISTYGESVNAKTAFYESYDKSALKQFIKDLEPDGGRQANIGDFLKTANDEIFNITNGIRQSSPR
MLVLFTNGVYSNDQIAKAQEESLKLKAEGQDVNILVINIGSIVEGNSVLRDVVSTPDFRNLHNVT
DLRRLVNFGFENKIAEAICASKVRKTRCKPRTPQACSVRDDLCQTDFDCPGYQECYSDGCRLRCQ
TCENRCGDKVDLLFMIDTSSRIGPEGFKNAKENIKKMLEWFTIEANNTNVAAITYSDNANYAMQ
LPAAGSNDPPQTLFDVQGRLDSIPYSGGSKSNLDKALKMAAAQVFYQNSLRLRKDSKKAIVIVTD
GVDDSTSRLEIASWPLRKQRVVVDGNDYAAIRLLAVTVGNNYNLDTLEKVVSPPIDKNIFLAADF
DDMYNGIRQLAEDSCKYTRQDVEPPVIAVKGDPGPKGEKGRQGNPGTNGTDGMDGIEGPKGER
GDDGPKGEKGDTGIAGPPGGDQGDLKGPDPMAEAVGIIEGTAVKGTRGVTYDLRDGPIGPPGPIG
GVGKTGLPGDSGEQGQKGRGGPPGLRGVFGGQGPKGNPGEDASPGLPGEPGPKGISGKKGPTGS
PGMPGPQGPFGYKGEQGIIGQPGDPGDGGKKGPKGYAGEKGDQGDIGDAGFPGPDGKVGPIGP
PGQRGLRGPEGSSGPIGKTGDIGNKGLPGPRGSRGLKGQSGNAGPRGGKGRTGDVGLVGEKGTQ
GSIGEAGQEGQPGVPGKEGPPGYPGPIGTPGPMGSRGDIGPQGEEGPKGSLGLKGNRGEAGRRG
LTGEKGETGDIGELGPKGAQGQQGKQGKTGKIGPRGQQGPLGFQGLQGQTGDPGQNGRQGPK
GNLGPTGEKGADGPKGSLGSAGLTGPKGLVGEPGNVGRPGLPGRDGKNGPQGANGKDGDPGA
SGPQGLQGSPGSMGTPGSTGDPGSPGDRGATGAVGDKGTRGDSGVRGEPGAFGQQGPPGLRGE
RGEKGPQGTNGVQGSPGPRGTPGLEGLDGIVGRKGTAGRPGNPGQSGEQGAVGSIGVPGPQGIS
GTPGERGDGGSDGGPGEQGPPGLQGSSGSPGRDGMPGRPGQQGTTGGKGKTGLRGPKGANGQ
NGVQGARGPDGLPGAPGLVGNRGTPGPNGIPGLDGNPGPAGKQGKQGKMGTPGMTGPAGLR
GPVGSPGLRGQKGPTGDRGDSGETGPIGVAGDIGPRGFTGELGLRGAIGEMGQIGETGPQGMPGS
QGTPGAAGLTGPAGPSGLPGKDGNKGPIGDPGNAGNPGRDGGPGSPGKDGTPGEPGTPGSQGK
QGLKGPFGPQGAPGEQGQRGLTGRQGSGGKDGPKGFPGNDGSPGISGKRGPRGKDGTPGSPGPI
GKDGRPGKPGINGTDGDPGNNGPKGDPGERGDSGDSGSLGASGDKGKTGSEGPAGFVGPRGSN
GKAGVPGKRGKRGDQGRDGPRGNSGPDGIDGSKGFKGNMGRFGIQGPPGDAGLKGALGPDGT
VGHTGPQGNTGDPGPDGPEGPSGSKGPAGNAGQIGNAGYPGSPGDGGIPGTAGLPGPPGPPGPP
GDMSGAFSGNYWDFVNAGGLQKGPRYYGGRSKRSLDQQDNSIEKVFKSNYETFKNFDTVWNQI
VDDLVIQKTLGSSKNPALTCGDLFKTNPITKSGDYWID
>TRINITY_DN4781_c0_g1_i2.p1 type:5prime_partial len:3388 gc:universal TRINITY_DN4781_c0_g1_i2:10182-19(-)
QCIKKHMLCDMDRNCCDGSDEAAKLCKGYKTLDFETGLQQWIQFRNDDFDFDIQSGKTSSSGT
GPQTDHTLEEEHGKYIFIESSLRNENETANLGSPTLAATTSSLYASGDCSLRFWYHMYGSTIGNLTL
FLRRDNKDTLLWAKSKPEGNQWRPALVTVNSWYKPFQLIFVGTVGKSYFGDIAIDDLMMTDEKC
PSPGDCDFEDFDKCTYTQDLVEDDFDWIRGHGGTGSWRTGPSLDNTKKDNSGHYYFIEASAPRT
QGDRARLVSELFDPTSSTRGRCLQFYRHMYGTAVGTLNVYVKTGPGNSSDAESLVWTNAGNQG
NSWIQGQVPIYSSKPFRLVFEAVRGNGYAGDIAIDDINFNNNFAPCSFLPSNAAPPTLPPTTITPEDS
NCTFDKGLCRWKQMKSNDQFDWTLVSGSTGSVGTGPLGDHTSGKGSYIYIEASYPRKENDSAILQ
SPPIRPSLGSVCFKFAYHMHGAHVNALYIYKNVKGKRTMVWKRVGTQGNQWRNGQVNIRGVS
GIFNIEMKGIRGVDYQGDISLDDFVVTNGQCPTPSHCDFENNQCGWFNIGRGDDFNWKRSNGG

TPSLGTGPGTDHTTGVALGSYMFIETSYPRKYRDVANLYSPYYPGSKTGYCFNFWYHMYGSHIGSL
NLYQDTVKSTPIWNKTDNQGNMWKHGRVTVNSRAKFRLIFVGTVGKSFLGDIALDDVWVDDFP
CPAPGACDFESKSFCTWSQVPNKFNTSNYDDFDWELGNGPTPSFQTGPAVDHTKGTSVGTYAYV
EVSGLIKRGQKAQLMSEKFPPTNGKCMSFWYHMYGSGVGDLNIYLKYLGVKKSHLVKSISGQQG
NKWIQGEVGLHSQKDFNVIIEAVKGSSYAGDISLDDISILDGNCVGLCSSVQPVARVACGPQSVTS
ASCVVSYGCCYDNSVPNVPACFQHPSSCKAVPIIARQQCGYYSISKYFCARRGCCFDDSGAGGPP
CYHSLSKPTDFPTTRAPPTTQPPSKFDCNFEKNQCQYTNLRNDQFNWARHKGDTASFGTGPKFD
HTLRNQNGHYMFVETSFNKENDTANLGTPTIDFPTGGDDVCVRFWYHMYGQHVGAFKVYHKL
TTRVPTKPIWERIGSMVNKWVYAQLIVPRRPAFQIVFQGVRGKGYQGDIAVDDVGFVNGPCPPSL
QCDYQNGDLCGWAQDADDEFDWSVGNGKTMSTQTGPKADHTYETDFGKYLFIEGSSPRQPGD
KARIESFSHRPTKGMCFEFFYHMKGTGIGTLNIWIRRGKTMDSKPVWTLSGEQGDEWYRAKVTLV
EDTENWTVIMEGIRGADFNSDIAIDDVIFHQGDCAPQGDCDFENDDVSACGYENWPRNKVDWL
IYSGGTPSYNTGPMVDHTLGTKQGHYLYMETSSPAKKGDFGYFMSEKFRVNPNYKWCVDFYYH
MFGDGVGSFMVKMMYQTKYSRGRYLLQTLFKKEGNHDDIWQHGQVNITSSYDFQIVFQGVVGG
GITHRGDIAIDDITVKKGLCPAPKAPPKPNPCAIKCKDGTCVPSNKVCDFYPDCAQTSDNTDDTD
EKGCDGCDFEKGTCNWNDTSKGLFDWNRQNGGTSSYNTGPSVDHTKKNALGYYVYVEASKGQ
SSDLATLSSPVLNQASSTCKMTFWYHMYGTSIGTLNVYLVNGYRLTLHWTLSGNQGNLWKQGTV
YVNRQLKPYSVRFVAERSFSYRGDIAIDDIKFEGCELPRPAGFCNSRYQHRCAVTRACISYTRLCDY
TDDCGDGTDELNCRWTQYPYRCNFEYDLCYWRNLKDDDFDFTRQKGQTDSANTGPLFDHTTG
TSSGYYIFVETSWPRKTGDKARIGSGYLGPITRTYCILRFYYHMYGDHIGALSVKLRTCIGCPETQI
WRRNTTVGNYWVRHAMYLRSDKPFQVIFEAEKGSDYQGDLALDDISFDGCTPYQGTFPTVAPST
KPPPTTSPCPSGKFYCAKDAKCIKGSMKCDYIKDCSDGQDEQHCGPCDFEKDMCGYQDVSIGTL
EFKRTQPINDGSKNGPSVDHTTNSTKGYFAMAQKGSGNKGSPVSFLLSAQLSQTAPSCKVQLFYH
AKAGANYVRVSHRQPGSNGKTVISQVQSLSIRNMDKWQNDTVGVGQRPFGWQVLIGLLSEYAA
IDDIKFIDCFYPRPTGVCNRMYTQCDNGVCLDPEKMCDFSNDCGDNSDEKPSVCKNFKERCDFE
TDTCNWYQDQSDNFEWKRIKGGTPSGSTGTGTDHTTGTSQGYYMYIEASNPRKPNDTARLLSPV
FKPHGNKKCAMRFHYHMFGIHVDSLSVKMKFSQDDNSTMKTVWTKRGNQGNQWNTAKVYLL
SKVNFQVVMEARRGISFLGDIAIDDVSFSPDCLPDFKATLNPNPITKAPPPGCKAGQFSCGDGQCI
DSDFFCNFKQDCKNNADEIRCPSSCDFEGGKSCNWFNAKFGDIMDWTVHQGKTPTNGTGPPYD
HTKGTKDGWYAYMEAHQRKASNFPNAHFVSPMYYQGAYDCKFSFWFNMYHYKISVQNDARL
NLLYRKNGKDTKIWSMARTTGNKWVQATVQLPRCPRDFRMIFEGHHYWATKTDAAIDDLKFM
CTEPKAPSSCANDEFQCTKTKQCIKKHMLCDMDRNCCDGSDEAAKLCKGYKTLDFETGLQQWI
QFRNDDFDFDIQSGKTSSSGTGPQTDHTLEEEHGKYIFIESSLRNENETANLGSPTLAATTSSLYASG
DCSLRFWYHMYGSHIGSLTVYTATTFGVPETQVWQLSGDQGNTWKRGEVKLDSNKDFQVIFEAK
MGTSYKGDISLDDISLTPGCVYNNAVLPGGPTPPPVDPCLPNFQCNEAGNCVSRAKVCDFVVDCS
DGSDESNATCGYPQGFEYGFGAWRNAHFDTYDFTLQQGATPSIQTGPTTDAKNDKKGYYAFLEV
SNRKRSRKAVLSSPTYAVAYSQCVLSFYYHMYGRETGILKVHLVTTNETTLLFELKGEQGNKWIKT
KAFVGRRYHPFYLNFTATVGSGYRGDIAIDEISFENCAHPQECTGVDPGKFRCKNGFCINQTEICN
AVDDCGDDSDEHVGSVCENYLGCTFENLNDPCNFTQAKSGDDFDWKLNRGWTGSYNTGPTRD
HTKGTSKGSYMYIEASGRVKGQKARLISLPVESRPGGNCQVRFFYHMFGKHINDLNVYILQSGTL
RLIKNANGDYGDMWREASLDLSKEKGVYRLVFEGVVGASYLSDIAIDDIIFNPDCMTNGSRVFPT
RIPCAANQFTCTNTRQCLSSSVRCNGVRDCRDGSDETGCKGPDTGKSSSVNTTTSIYASGVVGGLV
ILLIAVLIIYIVVKRKREKKLQLFSVFYDPTKQAENEKKQGKPSKNVTIKNEGVSNPIYDEQPMSEFS
MGELDTNMFTDEPYVVPAEPKGQTSSMANPLYQDPYMEDDDSPLSNF*
>TRINITY_DN64_c0_g1_i1.p1 type:complete len:468 gc:universal TRINITY_DN64_c0_g1_i1:1555-152(-)
MPPKEAEKIHINIVVIGHVDSGKSTSTGHLIYKCGGIDKRAIEKFEKEAQEMGKGSFKYAWVLDKL
KAERERGITIDIALWKFKTGKYYVTIIDAPGHRDFIKNMITGTSQADCAVLIVASSTGEFEAGISKNG
QTREHALLAYTLGVKQLIVGVNKIDNTEPPYSEKRFDEIVKEVKTYVKKVGYDPKTVAFVPISGWH
GDNMLEASKNMGWYKGWTTERKETKTTTGKTLLEALDNILPPKRPTDRPLRLPLQDVYKIGGIG
TVPVGRVETGIIKPGMVVTFAPSNITTEVKSVEMHHEALLEAVPGDNVGFNIKNVSVKDIKRGNV
ASDSKNDPAKEAKSFYAQVIILNHPGEIHSGYQPVLDCHTAHVACKFSEIKQKIDRRSGKVLEEMP

KMVKSGDAAMIDLTPSKPMCVEAFSEYAPLGRFAVRDMRQTVAVGVIKSVTKSDGSGGKMTKSA
QKATGKKK*
>TRINITY_DN1653_c0_g1_i3.p1 type:complete len:1023 gc:universal TRINITY_DN1653_c0_g1_i3:180-3248(+)
MMRRSIKGSEDNWVEMQNDTFKNWVNANLKSRGLEVDNFETDFEDGVRLINLFEAVSKKSIGK
YAKQPKFANQKLENVSLVLRAMESDGVKLVSIDSDHVVNGNSKMILGLIWQLILRYQISGSDDKSF
KIALKKLLLIWLQHVIPEQGIRNLTTNWNNGIALCNLINVLKPGIFPEYNNLDKRTPVENLAKGM
QLAESELDIPQIISPENMASPNVDELSMMTYLSYFTQDGGIGESWTVDLVNFWNSSLSVTNFNTD
WNDGMRLGRLVETFLPGAMPIMESTDNLDNCRIAMDVAHEKLGISKIITAEEMSNPQIPELAVMS
YIVQFTKVKRVERASDKFSASGPGLVEPKIGIVNEFYVRSDAGYPLDGLQVTIIGPNNNYIPIEIDDS
DPQLRNYSYSCEEQGQYHINIIYEGEDIPRSPIIVTSQQDLNTIRVYGERLEDKHLYAELYERIGISVD
TSQSGDGRITSKTIGPDEKEIYSEVDAEGDGSYTVSFTPEVIGEYHTNVFWSGKPIHGSPFVVWVSD
PNKVFARGKCLYETRVGVESSFEVATNGAGPGHITGYAETNGVEYPINVRKREECLHDISFTANQV
GIYYVYINYNNVPIKDSPFQIKVGNPSLIEIEKHEEAIMESNQPQSFIVNVLKDAGFGDVLCVVRGP
AGEDVQCTIKDVNDSLKEIMFLPNYPGTYEVFVYYAGGLVERSPFIFAISEPKLAIYPENIRVSGNGL
SRAKLNELARFQVDTTNAGPGHLTVQIEDEKDDVEAGIRDNNNGTYDVTYTPKRSGKYRVIIKW
SDQHITDSPFEIIVDNRAEEVLVEGYGIKEGTAGQPVLFTLDGRNAGPGQLSCRCRAPSGKMTYVL
ISDNKDGTYTVDLNATEPGLHTVEVEWDNQAIPGSPFLVRIMQAPDVKKVRASGPGLRSGTLDSF
NGMFQVDSKGAGPGTLKVRIHGPKGAFKVEMSRENPKDRIINVRYNPTEPGTYTANIYWSDEHV
QGSPFEIFVGQNDQEVDRWDRNKEELSKSQVNVY*
>TRINITY_DN1535_c0_g1_i1.p1 type:complete len:779 gc:universal TRINITY_DN1535_c0_g1_i1:86-2422(+)
MMKYFMLIVFTICLKNIHAQDKNLCTSAETCGKCLLKGGDCFWCIEEDFKFNRTRCNTEANLAA
QGCTDYGMGRGKKTNDADLDFTSVRQVKPQKVTLEIRPEEEQSFRIQVKSAKDYPVDLYYLMDL
SKSMENDLDKLQTLGTNIAAEIGNITKNYRLAFGSFVDKTVIPFVENDDANYKPCAECEPTFSFKH
VFDFSNDAKSFEKAVSEQKISGNLDTPEGGFDALMQVAVCEKEIGWNPKDKCRKIVVFVTDASPH
IAGDGRIGGIIRPNDGQCYLTEDGDIKMYTEATKMDYPSIGFLRSKLKDNAVIPLFAVTKNVTEVYK
SIAAELKDLQAVTGILEGDSSNIVEVIRDKVREISRTVTLTDDSSPDITVTYRVQNDPTKPNVCPSPV
APNRCEDVEIEQTVDFDVYVKASAKNCETKEKQDQTFTVNIPAFGKVEVNVKMICDCDCAKTSE
PDSPKCDGQGTFECASCKCNEGRYGDRCICDGNENKDMSLCKPNNVTDKICSGFGNCVCGVCQ
CNIRENPEERIDGNYCQCNNFNCPRHQNIVCGGPERGSCECGTCVCREGYLGENCGKTDCRIGV
KKCLDAKGQNCSLNGICECDRCLCDKGYEGKQCQTCVSCPGKCSEYKTCVLCKAFGETGEQCD
GCELIQHVLVNRTNEECVVPVDENCFVIYRFEIDPVSMNETLYIQRDKRCIIIVKTKPPILAIVLGTIG
GIVLLGLLLLLLWKLLITAYDNREFQKFEKDRMKSKWENDENPIYRPSKQQFQNPAYAGAN*
>TRINITY_DN172571_c0_g1_i1.p1 type:complete len:104 gc:universal TRINITY_DN172571_c0_g1_i1:30-341(+)
MSGRGKGGKGLGKGGAKRHRKILRDNIQGITKPAIRRLARRGGVKRISGLIYEETRGVLKVFLENV
IRDAVTYTEHAKRKTVTAMDVVYALKRQGRTLYGFGG*
>TRINITY_DN413_c0_g1_i3.p1 type:complete len:350 gc:universal TRINITY_DN413_c0_g1_i3:1286-237(-)
MVSLSVTCLSLLLFVTVNAEESDHCGYDEKSWYKAMGWCYHSGSRLLLGDFNGDKRTDFFCHD
SNGKNWVAVSNSDGSFGSTTWQDSKERWCASPNILLTGDFNGDGVTDILCHGTATGKKWIKYFN
KYGVPSGKSFYRAMSWCNHKGSQLYIGDFNGDKRDDLLCHDTSGRYWTSYAGTDESKVFHHTS
WYKALEWCRHATSKLYLGDFNGDKKTDVLCTDGKGYKWIMYAKTSGNLFTGTDWHTNLDWC
HESSTVFLRIGDVNGDGKDDLVCHSTAGNSWVAYTQPKGGFGNFCHLAKWCAAGKESFFLGYF
NQDSRADILCHDTKYGKKWIRYAECSFAEESDHCG*
>TRINITY_DN1280_c0_g1_i1.p1 type:complete len:426 gc:universal TRINITY_DN1280_c0_g1_i1:195-1472(+)
MGFETCGPNEAMVISGCGQERPLLISGGRKFVWEFFQKVQRISLNVMTLNVESQRVYTVQGVPIS
VTGIAQVKVQGQNEEMLHAACQQFLGKHENEIRNIALETLEGHQRAIMGTMTVEEIYQDRKKFS
KSVFEVASSDLVNMGISVISYTLKDIRDDEGYLKALGQRRIAEVQCEARIGQAMAKMQSGIKEAQ
AEEVRIKAEYENKTEVARSQRDFQLNKSAYDIEVNTKKATSELAFALQSAITNQKIKEEDMQIKVIE

RAQAINVQLQEIQRREKELEAQIRKPAEAERYKIEKIAEANRMKVVLEAEAEAESIRMKAEAEAE
VIQKKATAEAEQMSKKADAWRDYQEAAMVDMLLDTLPKVAAEIAAPLSKVKKVTMISSGSSDV
GAAKITGELLDIVIRMPQAMEQLTGVDITKTFRREK*
>TRINITY_DN493_c0_g1_i1.p1 type:complete len:473 gc:universal TRINITY_DN493_c0_g1_i1:2681-1263(-)
MKCMKFISLMLITFTSILVAQDTGFIYYDDSSSSDIDSLTEPYGYQSVMDNREKSYEKPDMRYNFDK
PSASDIDSLNTPQYGYEPVMDDRERSYKRPDMRYNSDEPSARDIDSLNIEPYDYESLMDYRDTPSE
RPGMRFDFDESITGDIDSIDTPLDDYQSLMDHKRKPYARKRRSIRHRRFIGQRRWYGRRKFIRKNQ
RQRNGFRKINSQRKRVCSKGSNCRHKLEPMGCFKDIKSERAMTEEILNERDTASKVFNNRLINWR
QYHEWLPGFICRCAQAASERGYSVIGIQFWGECWSGPNSESTYNKHGQSDSCVNKNLQENRAN
LTQCAQAAGKDSTNYVYRIAPACNTKYEPVGCFQDDQKEPRPIPHYISTDRDSSLDIFSGKLLNW
KQWNTHMPDMICRCAALAKSKGHKMFAIQFWGECWSGPLSNDYKRNGASGECRGKDFKECSC
NSYYCVGKTHTNYVYRLS*
>TRINITY_DN1505_c0_g1_i1.p1 type:complete len:154 gc:universal TRINITY_DN1505_c0_g1_i1:98-559(+)
MSLVIPEKFQHILRVMNTNIDGKTKIMYAMTSIKGVGRRFSNICLKKADIDMTKRAGELSDEEVER
LVTIMQNPRQYKIPDYFLNRKKDIKDGKFSQVLANGLDNKLREDLERLKKIRAHRGLRHYWGLR
VRGQHTKTTGRKGRTVGVSKKKN*
>TRINITY_DN3448_c0_g1_i9.p3 type:internal len:102 gc:universal TRINITY_DN3448_c0_g1_i9:303-1(-)
HRGKIKSFPKDDASKAPHLTAFMSYKAGMTHIVRDVDRPGSKLHKKEVAEAVSVLEAPPMVVVG
FVGYVETPRGLRALTTVWAGHLSEECKRRFYKAWHRS
>TRINITY_DN11248_c0_g1_i1.p1 type:3prime_partial len:1700 gc:universal TRINITY_DN11248_c0_g1_i1:111-5207(+)
MEIRSLFVAFIYIVHSINAQCSSPWKSYKDSCYLFTAVSTVSPGHTWEDSRFICQRYGGDLLKLDND
NETKYINSQFDDLKIKYNHYWIGLNDIKKEGDWRWADQAPLNVTTQWRVSRGEPNNWQGNED
CIETTYDGQWNDNECSRMFGYICERSKDAVIPVTPTLPPFPTATDKPTTCEAGWIPWGTSCYGIIRG
LAKNFQDAQADCLRRFGDLVVINDQAENDFISNEISRSVGGLWIGLSDTYKEGTFYWVDKSELNF
TSWRAGEPSNDYGNEDCVEIYGRWGPGQWNDNHCTALRDYICEKPGGVHNCPRDWVSYKNY
CYQINAHPGQKVTWEDARKNCLDFNKAWRKSVNVAQADLVSITNLEEQKMLQKEFSKVNIKN
DNGFYYWIGLTDKNSTGGFYWTDGSTYKYSNWIGSPQFASQCVRSNVIGYQGGSWSKGDCSEAY
NYVCKVGRGQDRCYNPLGIQSFAVPNKAMTSNSVLGPSTPALAGRLYMKAGNSLDGAWCAGN
NTKTPYLQIDFGKMQKLTYMGHQGKSNSDAMVTSYYIKYSQDGIVWDTYGYIPPAQYYKFKIYIG
WRTNYEKVMNLPLPAPFIARYVRVYPLEWNIAPCMRIDFYGCQEEVDNQRCGLGWREDKKTKN
CYKFHNQITTWDSAQELCSIQGANLATIATPDEREFLSEQLGEFKSLVSEFWIGLNDLDYEGVFKW
VDGTPYQLSNWYYGNPKVRNEKSNCVVVRTTDSAWVNTECTVFRKFICKRNYISPYTPKESYNG
YVYSKTDYFWPLDSATDFEGSLPLTLNGNVDRVPFPIDYDVGVSRYALNVSGSSGFFKIDVANDVC
IHHPDADSCPHGITVSFVFKMSKKSSSKRQVIIDTTEKNKPLGYKVYTEFGSIYVNLRGHQKSYSGS
TVYNAHIFNHMTFTWSSLKGLSLYMNGKSRVFDLLGSYAKDYPKVDYGKSLLIGISGDSQDPAEL
MIRSLGIFKQVLDAKSIEAIYLGEFGTCKSHQFAHGSYCYEYNSTEVSWSNALSTCRDNGGDLISIH
SPVEQAFFNLKIAQYNYQRTNWIGLNAKSKNSFFKWADSSRLEYSYWAPLEPNNNNGRREDCVE
TFANGGWNDALCYLRNPFVCKYSRALHIMGSENTDDKEYKVFFSPYQTLTFGCPLGRSIEILTASY
GSVKDGCVKDVKDKVSNACVKEHCVRVQASDFFFNKINPCKDKNRFALKRLKVTYKCNLATPE
FDSTGCSNGKDSENSYRVFDKSNICYKLYRKPKTFKEAQKVCKDDNGDLVRINSRETQVYLTSLLH
TMYQKTDIWIGMMYNSTTQNYEWLDGSHVYYTNWVNRQPDPRNGDYVKVGLEEGAKNFLFW
KNGEANETLPFFCQRVKVGKTTQAPPTTQPRTGTCPKGWFTYNKNCYYVNTNRLSFPDAQAEC
QKYGKSSHLASVLSYEENRAVFYDVVNATVDFAWFGLTNEAIVTGFRWVDDQPVSFVNWYTNEP
HKINRHLCVGMGSGGKWIDLACYLKYPSICKLPLGEVIPTTTPLPTIPPTQASGLCEPDWIHYNGS
CYNFANRTSYADFHNARVTCNKMGAHVATIHYQKENDFISAQLKRMYIPNAYIGLTDFSIEGQW
QWEDESVVSYINFGEGEPKNWHGMEDCVVVKSIGEWNDIPCNLKRSFVCKKNNGTVVKTVNTT
AAPDSGYCPSGWKLYGNKCYYFRYGE

>TRINITY_DN9819_c0_g1_i7.p2 type:complete len:483 gc:universal TRINITY_DN9819_c0_g1_i7:1449-1(-)
MVHDLVLRQLGNGRKDAKGVARQQNDLLGMVLHLGWDPCVRDVLQRVRHPRILGDVHVFVI
DLASALVPNDVLQHRAETDRVVDFGLLLPREPDGLGVATALDVENTLRVPDVLIVADQVPLRVR
AERRLAGAAETEEDRDVVVLADVRRRVQGEGAVSRREIPLGHQRHQVVHDGEDALLHLAGILR
AEDDHLPPLEGHRDGRRRGHALRLAVGREDARVEDHERLLLGRRLEIHELFLGRGHQHVLHEER
VVRPGRDHADRHAVLGVPTAVAVHDVEALPGVEVIDSALAVGQEGLVLELDVYLAPPHVVRGRF
LEHHTLVQRRTASLFAALHGQGAGVDDGTAVLVPQRLLVQDPRWRVVVDLLHVQGQLVHLVH
DVLHVDPLGADLHGQRGLDLLGRPALELGLLKLLHQGGLHLVLARRRGAMADAARGERAPGR
ADDPPRRCQSGQHRCGASGGWRAGARWGPKRPSRGWELWNDT*
>TRINITY_DN585_c0_g1_i10.p1 type:3prime_partial len:236 gc:universal TRINITY_DN585_c0_g1_i10:705-1(-)
MQIFVKTLTGKTITLEVEPSDTIENVKSKIQDKEGIPPDQQRLIFAGKQLEDGRTLSDYNIQKESTLH
LVLRLRGGMQIFVKTLTGKTITLEVEPSDTIKIQDKEGIPPDQQRLIFAGKQLEDGRTLSDYNIQKES
TLHLVLRLRGGMQIENVKSKIQDKEGIPPDQQRLIFAGKQLEDGRTLSDYNIQKESTLHLVLRLRG
GMQIFVKTLTGKTITLEVEPSDTIENVKSKIQD
>TRINITY_DN1058_c0_g1_i1.p1 type:complete len:2639 gc:universal TRINITY_DN1058_c0_g1_i1:134-8050(+)
MVKYIIWILLLSIFKEAYGGDWAQWQNWSACQDSSGAPIGCGRGNQHRSRTCADNNCGAGAIIE
VRPCLDEICPTFNCQNSSEIAGMCKFDYSNSCVGDDNCHHCICKSEGFKVSGDQCYNYCVEVGC
DEEIEGVSQLKRYCGDQAISCTGTVLNRTCLCNPATSYPSKDGQYCYEATCKTSQEFMTNCGAEN
SCQGTNTSFVCECNGFGYGYEVVANVQNRKKCKLLSCEDGDLNPLEICGQGATACTGNVHNLT
CACNVTAGYIPSEDKKSCIPMPCNTDPAVGVQCLPRNTCEGSKIDWRCICSAYGFRNDPTDPKKCI
EAKCDDPNIDILGLCGTGVTSCTGFLSKRRCVCNTTENYIPTLDGSHCMPAPCNANENATKDCG
GENSCIGSFDSYMCVCNGYGFQKNATINKCEEVPCSSLPTAEVQRICGFGALSCDGTIRNPVCNC
NNDLQYYASEDMKSCIEASCSSKADYDSHCGNENSCVGNITHFECICETEGYKPDPSNKKKCILES
CELNEVDVLNVCGKGAISCKGNVRDLECVCDASKGYVQSPDTNKCEPSSCYGNATVSEQCGEEN
HCLGDLSDWICVCDAYGYKVNATNSMTCDKVMCGDTGADAVTVCGKETTSCSGHIAKPVCTC
NETAQYLPSPDSTRCVKASCKENDAARKACVPGNSCSGSYDEFICICDQFSYHQDPADNRKCIRE
KCEDLAADYDYEKICGVGGISCTGYVNQPVCVCNSTESYHPSNDRTSCVKASCSTSDAVRTQCGL
ENSCFGDIESFMCDCKAHGYKLEPINKTSCVLATCEDGDFSYHFQCGKGAVGCSGNINTLSCLCD
SAKGYVPSADMKRCVPNSCKDNPDVYIKCGMENTCLGSYADWFCVCSGAGYEANPADPKSCQL
MECSKDDAIHVCGYGAIDCKGPVLAPTCSCNYSDHYIASEDGTSCVPITCRKNDTARAQCGEENT
CKGHTDNFKCICDAYGFKADNVDHQKCVERQCTDGDADIIRICGHGGIRCTGPIRSAVCECNSTE
YYYPNADRSRCILASCKANETIMKSCGEFNSCEGNMESHICVCNGPGYKHHPSDDKQCVKETCE
MGEIDPIYLCGKGAVKCNGTYDIYTCECDNAMGYVQAPDGNSCKPFDCHANATMHKQCGDG
NRCIGSLTDWICVCDVAWYKPDPSNLMRCHEKTCKEADLELICGTGATGCSGSATNPTCECDSA
NYFVSSTDRRSCIKASCKTNITAVQQCGDGNSCYGTPDNYVCNCDAYGYRLDDNNNQACVEKK
CDDVGANYVEICGLGGIGCKGTVKNPVCECDQTKHYVASPDSTSCVLSNCRGNKTVMAACGNE
NKCFGSASSFICVCDGPGYKRNPSNLQECLPETCEAGEIKPHMVCGRGATACKGTMYHFECECN
NTLGFIRSPGHESCIPVNDCYANATMHEQCGEGNGCQGSVADWICKCDAPGYKPHKTNFQMC
HQRMCSDADIDLVEVCGVGVESCVGPIANPTCNCDTSKHYVLSADSTSCIPGSCKTNETIMAQCG
EGNSCSGTSENFVCICNGYGYRPHETDKQTCVLKKCSHSDVNAVDVCGQGALSCIGTMRDPICQ
CNNDMFFIPSDDSRSCVLASCSANSDVAVACGKVNGCFGSKSSWICDCSSPGTMLNPNNIRECLH
ETCESGKLDPIIVCGKGALGCNGTMSNVTCECDNSMGYVQSPNGMSCVASDCKTNSTMHELCG
DGNSCVGSLTDWMCVCDAPGYHPHPSDPMRCLQKMCSHANINPVDVCGIGATKCVGSITEPRC
TCDTAAYYVPSPDGTYCIPGSCQTNKTALEQCGNGNTCTGTADNFVCICNAPGYKLDDSNNKRC
IEISCNDADVDLVEICGQGSIGCIGTARQPVCQCDHSKHFVPSLESTSCQLASCRANDTIMDRCRM
GNTCFGSMTSHICVCEAKGFKVNPSDPTECVQETCEDKDIDALSACGKGATDCMGTLMNLTCQ
CDGASYVESPDKTSCLPVGCQQNVDVKKACGEGNTCEGSYDDWVCLCTNPEYIPHATDEKVCS
KKKCTDNPIGSCGIGATSCSGNVNIHKCTCNLTAGYMPSKDEQSCIPIDCKMNPKMNCGFGNTC
RGSFFSYVCECNTLGTMRNISNPKQCIEVDCSTDAVSICGVGATGCAVTPTNDVNCTCGEFYHPA

HDYHSCVLDDCKFNKLVPDACGVENECTGSISSWMCVCKGPKYIQDIKSTQLCAPGPCRIEQCGS
GKASGSFQYYAESCSGTYFEPTCNCKEGFEKDSYGGCIVKSCSRNNQSSVDCNDGAISCEGTYFKY
KCECRAPTHILNPANKRSCIPARCNPQLCGRGLVNGDYMDFASECSGSYTDFTCKCKYGLYGSGT
SKCSLEKRTCALSNDCHPNANCIDYDGHYFKCECKDGYSGDGYQCHRNGGLTPWSVVQPCDKT
CGTGYAQVMRTCDDPKPDTLGKKCNVPSPDVREVPCHQFACTACSVKKTCECENIRPYFVHEYV
EPDESLKYFTNLTAEDVEKALSAIEKWKLSVDEKEFCIKHTLPEIDGLISDINENLRSVDQTKLYTIT
ARDMMRNKLDCNVDLEKKDKYFRVYEAIVKRHFILKQVVSDLQAKEKLMVTYRSSCTKDSKVG
WLSSIFS*
>TRINITY_DN21998_c0_g1_i1.p1 type:5prime_partial len:507 gc:universal TRINITY_DN21998_c0_g1_i1:1561-41(-)
AFLKYLRDKMSKREFFYLFNIAVIGVAGIVFLAVVVLTYAGYIAPWSGRFYSLYDTGYAKIHIPIIASV
SEHQPTTWVSFFFDLHVLICVFPYGLWLCVKNINSERVFIVLYAVSAVYFAGVMVRLMLTLTPVVCI
LAAIGFSRTLDRYLNDQDEEQEKEPEQQEAEPKTNEGDNKKKKKESSKKENKLEAGMQDSTSMG
SHLKMLVVIVILMLLMLFTVHCTWVTSNAYSSPSIVLATSNYDGSRNILDDFREAYYWLSQNTPDD
ARIMSWWDYGYQIAGMANRTTLVDNNTWNNSHIALVGKAMSSNESAAYEIMRSLDVDYVLVIF
GGVIGYSGDDINKFLWMVRIAEGEHPKDIKESDYFTPQGEFRVDRGGSPTLLNCLMYKLCYYRFG
DLKLDYRAPSGYDRTRNTEIGNKNIKLTHLEEAYTTEHWLVRIYKVKPLETRTKSSMKQRNTSMK
KNKKKYYSKKTRSNKAGQIKDKLTIKKGTSAKSTKKTKKGKSKTKKY*
>TRINITY_DN167_c0_g1_i7.p1 type:internal len:492 gc:universal TRINITY_DN167_c0_g1_i7:2-1474(+)
PEEISSMVLSKMKETAENYLGETVSKAVITVPAYFNDSQRQATKDAGVIAGLDVLRVINEPTAAAI
AYGLDTKSSSAEKNILIYDLGGGTFDVSILNLDGGVFEVKSTAGDTHLGGEDFDNRLVNHFINEFK
RKHKKDISGNKRALRRLRTACEKAKRTLSSSTQASIEVDSLFEGIDFYTSITRARFEELNSDLFAKTM
EPVQKALNDASISKSEIDEVVLVGGSTRIPKIKKLLQNFFNGKELCNSLNPDEAVAYGAAIQAAVLS
GDKNEAVQDLLLLDVASLSLGIETAGGVMTKLIPRNTTIPASKSETFTTYADNQPGVSIQVYEGERA
MTKDNNLLGKFDLTGIPPAPRGVPQIEVTFDIDANGILNVTAKDKSTGKDKTITINNDKGRLTKDE
VERMVQEAEKYKEEDDKQRERINGKNQLENYIFQMKNTINDSNVKEKLDESDKTNISNKCEELIK
WLDNNQLAEKDEYEHKQKELEDICRP
>TRINITY_DN120478_c0_g1_i1.p1 type:complete len:544 gc:universal TRINITY_DN120478_c0_g1_i1:2674-1043(-)
MMRAPVMVLNSNTKRESGKKVQISNIEAAKTVADIIRTCLGPKAMLKMLMDPMGGIVMTNDG
NAILREIQVQHPAAKSMLEISRTQDEEVGDGTTSVVILAGEVLSVASQFIEDKIHPTLIIAAYRKALD
DMLDVLKEQISIPVDVDNDEEMINILKSCLGTKFVNQWSKLACNMALKASRTVSTEASGRREIDI
KRYAKVEKVPGGSLEESCVLDGIMLNKDILHPRMKRYIENPRILLLDCNLEFKKGESQTNIELSGET
DFNRILQLEEEFIKKMCDAIIKHNPTLVFTEKGVSDLAQHYLVKAGIAVIRRIKKTDNNRIARAVGA
TIVNRPEEIREEDLGLKCGLFEVKKIGDEYFSFLTKCEDPKACTILLRGASKDILNEVERNLQDAMH
VARNVMVDPRLVPGGGAAEMALSCALEEKAKSITGVVQWPYKAIAKAVEVIPKTLIQNCGVNAI
RTLTALRAKHAEGGNSSWGINGETGEIANMKDLGIWDCYSVKAQTLKTAVETAMLLLRIDDIVSG
SKKSKGDEGATSGGMPTM*
>TRINITY_DN691_c0_g1_i1.p1 type:complete len:1726 gc:universal TRINITY_DN691_c0_g1_i1:5662-485(-)
MMNLFRLIVLGVICFSYEGDAQQCGGSCSPCGNLCVGMKGDRGTAGLPGFRGPPGLPGFPGPEG
PIGQKGEKGPAGLNGMEGSQGARGKVGPPGLPGTPGRHGRPGNEGREGEAGLPGCNGTKGDR
GPEGPRGRDGIEGPQGLQGNVGPRGEPGDSTATKLKGQKGEPGSAGQQGEKGGRGMEGMKGD
TGKPGRNGRAGERGPKGEPGTSNVTIIGEKGSKGSVGPPGLPGRQCQVTGDKASNIAQGQKGNQ
GSKGEEGAKGEKGDAGLAGPQGEEGDIGPQGDRGDDGPQGPPGPEGEQGRSGIQGNKGQKGE
VGNNGKDGNDGQTGLTGQRGRQGTPGQKGEPGKDKKGLRGPEGAKGERGNDGEKGSDGPIGP
IGPRGPKGELGPLGMKGFKGSIGSAGPKGENGNPGDPGIKGRRGELGIKGDSGVKGAPGNPGISV
TGPDGMDGDTGPRGQSGGKGAVGFPGKPGPPGVVTIDGEEVLPGEPGPQGLKGFRGVQGDAGS
KGSRGEKGNACESSKCPAGPKGEKGLSGQDGLQGGDGEEGEKGEKGDKGDIGSRGDTGSRGEP
GPQGESGEKGEKGIQGPQGNSGKDIIVQGERGDKGTKGNAGTPGSIGFPGERGSPGESGDRGLSG
VNGLRGETGDRGARGVAGKPGVNGQKGQKGEIGSGARGFKGDKGEVGPDGPAGLRGPGGPVG

PKGDPFPTAVNGKTGEPGEQGAAGPKGAQGPVGPEGPRGFPGDIGPVGEPGDGEKGERGDDGA
RGERGEKGEPGQDGKDGDPGPKGEPGQEGKEGPPGMFKGNKGEKGATGLPGVDGLPGNSAPK
GEAGKDGQDGDPGPAGEKGDPGEAGSPGTEGPIGEDGPVGDKGEPGESRAGPKGNEGRAGAK
GENGRDGRAGRPGIGRPGDKGELGQKGEAVIGAKGSKGEQGLEGPPGPIGKPGVKGDAGVPGK
DGEAGGRGKDGERGLPGDKGDTGDIGFNGSPGAPGDKGEPGYSGQKGAKGMSGPRGPPGSTSN
VVGPKGNRGLPGMKGEPGQQGEKGLIGPQGSKGESGFGSEKGIKGSRGNPGAVGPSGEKGERGD
RGKDGLQGIAGPRGEEGNPGEKGSRGDQGPQGREGPSGELGPEGQKGDPGFAGEKGEKGDRGG
DGQKGDAGEPLRGEPGVAGPPGPTGPVGPPGKDYVFSGTAGELKGMKITKGDVGPDGVKGEKG
DRGDKGLDGRRGPTGPQGERGPKGEEGNMGPKGSQGRDGLTGEDGPKGSKGEPGLDGDVGEK
GEAGFQGLQGEKGNQGPQGLEGSTGMDGQKGQIGEKGVEGPPGPPCEPVNSVRGETGEEGPKG
DRGPKGQKGKPGPQSQAATPGEKGSKGEVGQPGPQGIKGEPGREGDFGPKGSPGAQGEKGDQG
QKGEPGQEGPEGLKGEPTRAFGPKGQKGETGLEGLRGPVGPKGEDGQTGPQGPDGPKGNEGEK
GDFGINGLNGAAGPSGPKGAIGLKGDSGPPGPTGAQGPRGSGGSPDFGFYYVKHSQTTIVPSCPA
GMKTLWTGYSFLYAQGNERAYGQDLGQPGSCLKRFSTMPYLFCDLQNQCTVASRNDYSFWLSTD
EPANMMKPAAGPEVEPYISRCAVCEAPANILAVHSQSVIVPDCPNGWENIWAGYSFLMYNSAGA
DGSGQLLASPGSCLEEFRANPFIECHGRGTCWYYGPTLSFWLSTIDDSNQFQIPKSETLKAGNLKT
RVSRCSVCMKTVRNGNFP*

>TRINITY_DN65215_c0_g1_i1.p1 type:5prime_partial len:383 gc:universal TRINITY_DN65215_c0_g1_i1:1336-188(-)
HYRKKTTMAAYDDDENFGTSDSYGNVGSFPKEFGYAEEQEELNNYSEEQKPKILLMGRRRSGKSS
ILKVVFHKMSPNETLFLESTNKIVKDDISNSSFVQFRIWDFPGQIDFFGHDFDSEQIFSGCGSLVFVI
DAQDDYLDALTKLHQTVTRAYKVNPNIKFEVFIHKVDGLTDDHKIETQRDIHQRATGELEDAGL
ENVQLSFYLTSIYDHSVFEAFSKVVQKLIIQLPTLENLLDILIQHSGIEKAFLFDVVSKIYIATDSAPVD
MQSYELCCDMIDVVIDISCIYGLKENGDANAFDTHSASIIRLNNSTVLYLREVNRYLALVCILREDN
FAKHGLIDYNFHCFRQAIQEVFEIRQQARDALLPKNGTILSKGESVVV*

>TRINITY_DN991_c0_g1_i2.p1 type:complete len:3229 gc:universal TRINITY_DN991_c0_g1_i2:278-9964(+)
MKKKMKVGDLLYSKTVMFLMLLFVISVSSEDLLDRNQNNAYFAKYYECPVGWVLRCEKCYLLVE
TAKTWTDARYYCKKHGGELVTVKDDSTNFFLARYVQQTNKKIHNVYIGLHDREKEGDFKWSDG
SEAIEKEFRWLPGEPHKSLTNENCVEMFTRSNQYLWNDAPCSDELSFICQMPACVQGSNRCDNG
KCVSKQAICDGVDDCGDSSDEKNCTKCGGYLNGMTGTFQSPNYPDKYGNNRRCSWVIEVPTSH
KIQLAFKDFGIETGFDYVEIFDGKTLAAKSFGRFNGDENPELIMSTSNQIRVTLVSDMTTSFKGFLA
TFKAIPDKCEFDLVIPKNGYEYIKSPMYPDNYPDNKKCIWNIRSEDPFGTLTMMFVELDTEENNDF
IEVRDGKGVKDLLLGRFSGENDVKFMSPIFSTAKHMFVKFQSNYMTSKKGFRAIIRSGCYAEINAS
QGMITSPGFPYQHYPKLIQCTWKINEPDNKAISFMFHRFDTELNLDVLYIKDKSGKIVGTYSGVLS
NNALQVQWILETSQSSLEVTFTSDVTYTMSGFNVTFSKGCPSISVLGGKVKGKSHYGGKISYECDE
GYHLRNGKTRTCLYSGKWSDPMPYCAKINCGHPGVVVDGGFQTYPKKDNFDVNTRLVYYCDA
GYRLIGNNERVCQNDSTWSGSQPECKLSFCDTLTTPKNGKIVGDDFTVGQSVTIKCNPGFNIVGV
TTLTCGKDGKWNADEPVCEAVTCPIIKPSDNMEVLNLPNNTDRFPYGAMFDIKCKNGFELNGEN
QIKCVAPGIWSPKLPKCYDVNECLQKSSACKGVGHQCINTIGSFKCECKNGYQKNGANTCQDID
ECKISNATVKCSQKCINQLGTYKCDCEEGYYLYSGDNSTVSRIELRTAVVNHTCYGVRCSVAPIPD
NGFVINYGRRHPVRLIYQCDRGFVIKGNEMVMCQKDGTWNREFPDCVHAGCQNVTVAKSATF
KVNGTKLSDGRYKLGTEVIYTCARGFKITSGNLRQECIQNTTHAFWSGELPVCERKSCPTLTAPVN
GKVSSMNNLFESEATFTCDDGFQLLGDAKLTCTESGKWSGATPVCAASSCPYPDVPKYGEFVGRL
ELGRRVIYSCYKTGYTLVGNKERTCLYKKGTGHYWTGDTPLCKDITPPVFTCTKQINVQLPRSENS
MLVKWDVPTATDSTGKRITVTVSPDIHPPAKLKAGQHNIVYTATDKAGNNATCTVVVNVQDKQI
PRIKCPPNVEIESDFPAKVTWDSGLFSDNVGISRVSFNPSNGSTFQSGKYHRVEMTVEDLQGNRDV
CVTEVYVKGCGCESNKSCNSIAPHNGGISTCHINMGITSCSLTCPKGLSFVQDNFKHDNVMCLPQ
GKWSEKDIPGCGERKSVTSCLNAVLEFEYNTIDTSKVDEDNLATYFNDLFKNFGNMKTTYCDAV
KFIPNEYELKSKNIELLFKMEPKGDILKDALKEVLEGCKTELENRFQSSKQDILNLVSHASVNLQA
KSIIKFDAASCCGDSKYYCCPKGHSVRAKGANVENIVCLPCSPGMFLNASSNHCTSCPKGYFTDT
SGNEACQKCPDNLNTLKHQSTKCTEKCPLGTYSSTGLQPTCRKCDYGEYQNEIGQTMCMVCPSS

TTTASRGSQKLEDCKAPCQLGHYSDTGLKPCVPCEKGTYQNETGRSVCKACPSGGIYAGIEGATK
EEECPSDNACDMKLCKNEAKCVPGVYSYQCLCKPGYKGKNCEIDIDECETKPCKNNGTCVDLV
NDYNCTCIPGLTGKSCETNVNDCKNDTCINAGVCEDLINDYKCWCPLGWEGKRCEKFINKCLSS
PCQHKSKCTPLLNAYKCDCTPGYKGHDCETDINECQSSPCYNNATCVDLLNDFECKCQPGFKGE
LCAIDINECVGSPCFNNATCIDGINGYRCECQPGFAGQNCSINVNECDTKPCLRNSTCIDLINKFK
CECEPGFYGDRCEFEINECEDEPCKNNATCIDQVNAFECLCPLGYKGVRCETEINECDVNPCKFG
TCIDLIGDYRCNCTPGYEGRNCDILIDNCVAKPCENNATCTNQVHDYSCKCLPGYEGKNCSIDTN
ECLPDPCNGNALNCTDIVNGYICYCKPGFRGTNCSEEINNCIPQPCMHNSTCKTIIETNDYQCNC
TAGYEGKQCENETNECLGSPCQNNATCIDKLATYECVCPTGFTGFNCETNIDDCKNVTCQNNG
MCFDKINDYYCRCHATHKGRHCEKKIPDEVDIKLPGGTGSVIIHSNISTVQEFSLSIWVRYLGSNSR
GDFLILANSNGKVMTSHFLKLSHQRVMVEFNGNMDIFSLDKMLNDGLWHHVFVSMSGLSSKIV
VALDSKVIIEETKSYLNGQSFFKPSGYLIIGKNVNRTGFFSPSGFAGEISQLAIWNRVLSPNEMNNLA
SNCSVDLKDSIIPWVGLLDNAGSKVELLERWTCGTNVCPAGFMGRFCEILIDKVAPTVASCPKSKK
VVSLLTSTRVNWPEPRFDDNVAVTEITRSHAPDQVFGWGDYVISYIAKDAFNNSATCVFELYVTPY
DCPDLEEPNHSASATGRWLWGLFFDFQCKPNYELAVPKPPFYSCGKMGTWSHGPPTYMDNPKN
LLPSCTGTARPIVIGTNAFTYSTTSKCDETFKAEFMKQFKRKVLEIHQYFKICGEKCDFANIRVKCT
DEDITTSRVKRDASLNEVKVYFDSKFNESLSNSFNDTQTLQAAIKNDKVDLSFTVGGETITSDKNN
VDIAEPKLACNPGWKFVKDKCVLCPAGTYRDSSMIKCELCEAGSYSSLGSATCVKCPHPKITYGT
GAKSMNECYENCSSGYYFNNGSCSKCQIGFYKSFKGLEECTQCPKGTTTTVIGASRLEDCSVECPL
GHELVGTECQPCMNGYYRSNKNQPECLPCPYGTTTYLVGSASNICYKKCELGFESSNDGKVCMP
CNTGYYREEEIQRKCLKCSGDKITLVYGAKSKNQCKEQTGTKFDVELRFTSLEWNNTLIDEDSAYF
RTTAYNIANKIAKIYENDKELLYIKIINFSEGSVIANMTISFEKEKKDPLEVLRKTVATGKLDGLSVDP
DSFKTDRCEFFDCPKDRVCWYNSTGRGCKCKPGFYETSDGKCFAGCEPDYCLNGGACVESDSGR
NCRCSKEYSGERCEKEEKDFSLALIIGLVVVAILILLLLCYCLRRRKRSGNVSLYKYWKSEDKEHGLS
SIPNTPIVQPVKGDNGDIAYAAENKLVEKDDEEQSESSDRK*
>TRINITY_DN165536_c0_g1_i1.p1 type:complete len:249 gc:universal TRINITY_DN165536_c0_g1_i1:851-105(-)
MSEQEISNLVEQAKLAEQAERYDDMVKYMKELTEKSTEGLSDEQRNLLSVAFKNVVGARRSAWR
IIHSLEEKKGNEGGNTELISSYRKEIEKELKERCNEVLSLIDKYLLPSAQKEGQKGTEGKVFYEKMK
GDYYRYLVEVTTGEDRANLSAQSKEAYDKATETATGLATTHPIKLGLALNFSVFHYEIMNDGKKA
CELAKKAFDDAIAELDSLKEDSYKDSTLIMQLLRDNLTLWTSENDNEENDAQ*
>TRINITY_DN329_c0_g1_i2.p1 type:5prime_partial len:320 gc:universal TRINITY_DN329_c0_g1_i2:1-960(+)
FNNSTHSPSLPTHVYLYCCRIKILILSFDPSTIEILKMAPPKFDDLGKEAKDLINKSFHFGVIKLEGKT
KAKNGVEFTTEGTHNTDTGTVNGSLETKFKYADYGLTFSEKCNTDNVISTKITIDNKLAKGLKVD
FDTTFAPVTGKKTAKVKSAYQHNNIHSTCDVDFDFAGPTIHGSAVFGYNGWHAGYQASYDTANS
KLIANNASLTYKEGDLVIHSGITDAVKYSGSVHHKVNSNLTAAALIQWASGTNNTSLTVAGKYAV
DNETFVKAKIDNNLRLGLSYAQKLRPGVELTMSSLVNAKSLEQGGHKLGLSLNFEA*
>TRINITY_DN2003_c0_g1_i1.p1 type:complete len:370 gc:universal TRINITY_DN2003_c0_g1_i1:89-1198(+)
MGVINTLVVLTLALALEAADHMTPHEYEFWIKTATSCTQEIYEGDTSHSTYSDLKTKLEDGIKAAY
TNNTKYFSARVKSMQCTTKPHYVAAKIMLIMTGHFTFDEALADLKNEIMDNVLDGSALNLTDN
MLRASMDEGAKPKEKVTLKFSFTNELFCENMATLKVNLAKRVILSDGMKRIESNRIVIYEHECNE
EANKYKKAVMKFYIYDDNDFLTADAALTNLAYQVVDQLQYDGTAWKLGVIFHKHINDVDYESE
SEDEDFTEIEKTYIGFGAVVGLLLLIVIIVLFVSCICPNACGKQEDPYLIEDRPSRFTNVNTHRDAYSN
QVYSKEEYANGYEREVEMDTVLPHEEQTVFHGNQPNEGMKESV*
>TRINITY_DN6188_c0_g1_i1.p1 type:complete len:566 gc:universal TRINITY_DN6188_c0_g1_i1:2302-605(-)
MVKAINVRVTTMDAELEFAIQPNTTGKQLFDQVVKTIGLREIWFFGLQYTDTKGYTAWLKLNKK
VNAQEVQKDVPLKFKFRAKFFPEDVAAELIQEVTQRLFFLQVKDGILTEEIYCPPETSVLLASYAVQ
VKYGDYTPEIHQKDFLIGDRLLPERVRGQHQMSKEQWEERIMSWWGEHKDMLREDAMMEYLKI
AQDLEMYGVNYFEIKNKRGTELYLGVDALGLNIYEHDDKLTPKIGFPWSEIRNISFNDKKFVIKPI

DRKAPDFLFYVPRLRINKRILSLCMGNHELYMRRRKPDTIEVQQMKAQAREEKQTKQLEKQELA
KEKQKRADAERQKIELEERLKKFELDAQEAQAALDKAKEEQEKLENEKCKAEEAAENVKKQKE
NAENERLKLEREAAAERIAKEEYEARTQKLEEESARIAAMVEEQNRKAKELEEQLEESQKREEEQ
KQELLRIISTPPAAMVQTDEQEDENMEHETNFESQDVEQMELNYTSHADKNERLRLQLKELGNE
LNLARDETKLTQEDILHEGNLKEGRDKYKTLRQIRSGNTKQRIDEFESM*
>TRINITY_DN69276_c0_g1_i1.p1 type:internal len:887 gc:universal TRINITY_DN69276_c0_g1_i1:2659-2(-)
FDRPKYQYSVFENANIGEAIDTVVVTQNTEINVNYSFYKPNSLFTINSMTGEISIAANLDYEEKTQH
FFHVEAKCISGSNIFTAYTSVKININDVNDEAPVFFGTNEAVVPENSLVGTPVLIVQASDADTGNN
GRVRYDIIDEVENLPLAINSDTGVIFVSGNLDYELKKSYSLPVSATDLGSPSRRAISMIKISVMDKND
NSPVFSKNYYELAIAEDQPSQLIACLAASDSDSDENGKLSYSIFLSDNSLAETFFINGTGCLSTLKKL
DRESKDSYSFTVVANDNGNPSLKGIAEVHVKVIDINDNIPIFSKSQYLLRISKNTLVGSTIISLDAVD
NDEGINGTVNYSITDGNIGSSFDIDSGGTVHLVRELSNTIAKYVLTIQAKDQGMVTSFLSTTKVEIE
VIGNSTSSGCYPLFTSSIYDVEISENIAINSNIITISANCPNNSNILYSLIESSINSGGKFAINGNGEIFVV
GTIDFEQTKFHSLIIKAYAESTPNMFSTSIVRIKLSDINDNRPIFSRTSMVIFLARNSKAKSIISTVYATD
KDSGSNQEISYQIQPNIQPSDLFEINALTGDVILSREILFKDIKNYRLQVLANDRGTPSLSSPQLTIIDI
KIYPDNMHTPQLQPSNHVKFPENSGINQLVYQAQASDLDIDLSIEALQNRPEAGNFGKFTYHLRN
TTGSQFFSISETNGRIENLISFDREKKESYDLIVEARDGGSPPRKSIQTVVVKISDVNEIPLFELSSYVF
YVSESSKIGLSIAQIMAKDNDAGQLGTLKYFIKGGNVGNKFSIKENNGILILSDFLNYMEQSVYNL
RIYAEDISNRPLGNNADVVINVLQVNASQIPVFDLSLYTYQVPEEQTSTLGTITARLGANRQTEIIYT
>TRINITY_DN65211_c0_g1_i2.p1 type:complete len:342 gc:universal TRINITY_DN65211_c0_g1_i2:1592-567(-)
MAVSQAPKRKEIYKYNAPWTVFGMNWSVRPDKKFRLALGSFIEEYSNKVQIVQLDEQRGEFAVK
AQFDHPYPTTKIIWIPDDKGHYPDLVATSGDYLRVWRVGEDEVRLEALLNNNKNSDYCAPLTSFD
WNEIDPTIVGTSSIDTTCTIWGLETGQPIGRVQGHVKTQLIAHDKEVYDIAFSRANGGRDQFASVG
GDGSVRLFDLRNLEHSTIIYEEPQHTGLLRLAWNKQDHNYLSTFAVESSKAIILDTRMPCTPVAELS
NHRASINGMAWAPHSSCHICTSGEDHQALIWDIQQMPRAIEDPILAYTAEGEINNIQWASTQPD
WIAICYNKNVEILRV*
>TRINITY_DN26392_c0_g1_i1.p1 type:internal len:469 gc:universal TRINITY_DN26392_c0_g1_i1:2-1405(+)
PDILIGASNSDKAYLFRSRPIITISDRLELNVTSVPLKKNCDNGDGRNYFCAVLKVYLSFQGISVSNK
FDLELSLTLDDDQVEKRAFIKTVKKNDTSLNSQFKRVLNNITVNTPVTEDFMVYFLVKADLDIQKP
IPIVSKYKKIEKSGGCGANPCPIFNIYGSIGSSIQVPYLRECGDDKKCTIDLVTTGSIELPGKEKEFEILY
GVTDEVYLNITVENLGEAAYLAYMKINASSDLDVISVNVNGGPNILWKSSNIDAETMQVEFSIAN
PIDKGGKYHILAKFSVGKQPRPSTPIYEFGMEAFALGEDKNMDNNRKTLTIGTKVETCIEVIGYSSV
PQVIYHSTAKQPPDVNATVNDIGDEILYQFLVQNKGNKPVDTLTVDIDIGAEKEKTTLMYISDLTV
DSVRCEEEKLNPKKFLIPDDISRTLNEESTKSSLKRKRRDVANDDGTEDKKGYKYSPLNCRTASC
>TRINITY_DN1030_c0_g1_i2.p1 type:complete len:1426 gc:universal TRINITY_DN1030_c0_g1_i2:4483-206(-)
MAKVRVLALLMLICVTASNAQDGEEVFPFDVQKGEKGQPGDDGEPGNNGAPGRQGSEGPQGP
DGDVGFDGEEGDEGEVGPPGPPGRPGTNRTSVVVVYGPPGPSGPQGPDGQIGPKGYQGETGEPG
SPGARGPRGDAGAVGEAGVPGKNGANGPKGEQGSIGPSGATGIPGLPGPSGQRGPKGSKGEVGE
LGPKGAKGDAGRNGDEGESGLPGRKGEPGPRGQNGTVGFTGPAGQEGPMGPIGPAGPDGPAGP
QGPRGADGPPGTAGSPGESGAPGLPGPVGQVGARGEPGFPGARGIPGDRGQAGPAGQDGVDG
AVGARGSPGSPGIAGAAGPPGTRGEPGKPGPRGLQGQQGEQGSTGVQGSVGGRGEKGPRGLTG
QAGVPGPPGDSGTDGRPGPPGPPGNNGQRGLAGLPGSPGLPGFPGIKGHVGERGFNGPRGEKGS
RGESGRPGQRGIPGVRGPPGDIGLPGIDGKDGNRGQPGKDGIPGPPGQPGPRGIPGSQGAAGSKG
DKGAKGRTGDPGFDGAPGDKGIPGQAGDQGVPGPRGRQGDRGEPGEQGARGGQGLPGPPGA
QGPQGRQGKRGSPGLPGARGNDGLPGRQGERGFPGESGNPGAPGRPGASGRKGPSGRPGQKGS
RGDPGMPGDTGPPGQVGRPGLPGAQGNPGAAGDQGEIGEAGERGRDGKPGPRGAMGQRGAP
GPRGNPGQRGDPGLPGDDGKPGSTGAPGAPGAPGEPGQSGPRGPTGRPGQAGAPGESGLKGFK
GNRGARGRPGRAGKKGADGLKGQRGSKGEQGLTGLQGAPGLPGNQGPAGPIGLPGEQGPQGP

PGPDGKPGSKGDKGRSGPDGRPGSPGRPGPDGSNGLNGRPGKDGSRGPQGRPGPRGKQGPTGY
VGAPGAPGVPGLIGPRGPMGDNGKDGKNGADGDPGEAGPQGAQGPQGEPGADGADGLPGRN
GNPGLPGPRGEKGDRGTKGGKGAKGNKGTAGPVGQPGARGDRGPAGTPGADGEKGADGIKGE
TGFNGAEGAVGDRGDTGDRGSKGDTGFMGFKGAKGPRGSKGTRGLPGYMGPQGDIGDKGPIGP
PGPTGPAGISGPVGLPGRKGPSGDPGPKGYAGKNGRQGPPGPPGPPGLAGPSMLPPWMGGGLA
DGESKGPVVEREEPDAPEDENEELPPNEVNPFYRVYQFYSSKKTTPTDNDVNVMENKFKTKVEEL
KTEFSRIDKPDGTKDYPARTCRDIHAYHPEAKSGLFWIDPNQGCKDDAIQVTCTFSVNEDDEPTV
TTCVDPAEKQSVPQRTWASKMHSRAQKWFDEDHELAKLKYNANLSQLRYLGLLSKTATQDVKV
HCKKYVVWHDKMNRNFDRAMRFMGTQEQEFLYKDVDSKSSKSRFVPEVITDGCSANSNSVAHT
HLRFTSRKFIRLPIVDFAPTYPESPKSEFGIELGPVCFKV*
>TRINITY_DN3347_c0_g1_i1.p1 type:5prime_partial len:924 gc:universal TRINITY_DN3347_c0_g1_i1:2-2773(+)
YGDGSDDDMDSIDEDEDEDEDDDDDEYSDDDDMSWKVRRASAKCLSAILGSRMDLLSEFYINVA
PKLIARFKEREENVKSDIFQAYISLLRQTKPVAMNDALEDKPDSPLSMLKQQVPLVVKALHKQLM
EKNIKTRQGCFNLFTELVGVLPSGLTEHIDKIMPGIHFCITDRNSTSNMKIDTLSFLGCILQHIKAEV
FYPHIHQFLPAVVECIHDPFYKITSEALTVAQNTIKVMRPTGTENYFDFKPYINDLYQSILHRLRATD
IDQEVKEKSITCMGQLLRNMGDNLSKQLPECLPIFLDRLRNEITRLTTVKSLTTIASSPLHIDLSILLL
DGIPLMATFLRKNHRGLKLHTLTCLDLLMQNYGNVMSTKHFEAVLDELSNLISETDLHVSQLSLN
LLTTMISKQQLLLKKVSSTVLSHIVILTRSPLLQGHALNSMVSFLTTLVRYSSSGLGYTDLLQMLTEV
IYGSTTENIHKSAYHSIARCASALTIACPSEAEAVVTKFIKDIKNSGTNDSIRLFSLLTIGEIGAHLDLS
SKAELKNVIVNSFSADSEDIKTAASFSLGHITIGNMDAYLPFILKEIESNAKRQYLLLHSLKEIIVCQS
TDPSSVKSLEQYVPSIWSLLLTHCENKEEGTRNVVAECLGKLTMVEPQKLLPELKSYLSSDLYSAQ
MRSTVLTAFKFTITDQPQSIDILLKDHLQAFLQPITDNDLNVRRVALTTFNSAAHNKPGLIRDLLTK
ILPHLYNETKVKKELIREVEMGPFKHTVDDGLDIRKAAFECMYTLLDSCLDRLDIFEFLNHVENGL
KDHYDIKMLTYLMLVRLSKLSPNAVFQHLERIIDPLRTTIQAKVKANSVKQEYEKQDELKRSALRA
VCALQKIPDAESHPILSDFVSQIRSNSDTKVMFESVQRDPSSTSLDKMDTAS*
>TRINITY_DN59572_c0_g1_i1.p1 type:internal len:725 gc:universal TRINITY_DN59572_c0_g1_i1:3-2174(+)
FSSVLEYNIEDFELVNQYMFVTKNSSSHPGTKDLLVSYYRGDFQNTSFPMKNNGTSYIIADAHENE
TMVAVNHNGTANLYISNPEGTKYSLSLGNILYYNKDEGQKSYFEHSFVDLHRIKGISGIYIATSLTTG
AVGTRHLQSYITFNKGGYWSLLKAPEDTKNCSIWRGCSLHISQEFGYHNPLLNFSPITSKESAPGFL
IGTGNVGDSLKTKPNVYLSRDAGLSWRKVLQGHYYYNMLDHGGIIVAVEKFGLTETFTYSYDEGK
TWHSEVFSKERVRVYGLLTEPGEKSLIVTIYASYNKAHAWEVFEIDMTKVLGRKCSIDRDYNLWAV
ADQRNDTERCVLGKKQIFERRKAKSRCYNGYNYDRPINVSACLCTREDYQCDYGFKEKMILWFR
WDCVHDSDFEDYSKKPPSWCKPGLTYNISSGYVKAPGDACYGGEESKYVSTRKMCPIPKVDDYL
LYATRKSFFGYDFESKKSIEFKLDGYSNIVALEVAYDDGVLFFADKDRDSIVKIDTNNGNISTLLGFD
ESSTHIESMSYDWLSGNLYYCDSGNSTIGIINLAGKHKKEIMKFADADKPRAMMVHPTKGYLFWT
DWGAKPRIGRATMNGKNIKTIIDTEIYWPNGLTIDYDADLLYFADANKDRIEACGMDGENRRVIL
STGVYHPYAITIYNDRLYWDDWNQHSIFSIDLQGSGRETVVSYLAYAMDIKAFSKKRQKKGTNGG
T
>TRINITY_DN160_c0_g1_i1.p1 type:complete len:1145 gc:universal TRINITY_DN160_c0_g1_i1:4810-1376(-)
MRLLAIIPLLVMLDIAKSDKLWECNFEENDCWAASSTRNHERNQLTLHWNRWKMRTPTGATAS
PVTGPMEAFEGKHYLYYEATESMDKLKMADGASKGVVTFKDINLMGRTQFILSFYYQVKGNDG
DFYDFDLMVSNTENTHYDLSMKSIDGKDWKHFKHIMKVEGAGVTSIDLKFTARSYGAFADYAID
NIVIEDYPDAGPCKDNRCLANEYCTITSSTQTVCVAKCTQQACGENAECILDSLAINKCKCRQGY
LPRVSNYGDKLNCYRPDASCDIEYNSLGQCRISTSIPIMYGHPSLWHGRYSPVSGNGYYVFKYDNT
ITLDAVSSQKLSFFYLVDNPNMINSLTVEYGSRKFEVPINREKTNIWTLYEVLILASTNQGVQIKASF
YAEGGVIGIDNIQFTQQNEVVVEKIYKESMMAATFEDGSVPTDLKVAMLFVTRYGKIDFGCLPGIE
LETRNGKLYCKDGVVQRQSACSQDSDCIGSNARCVLRNCVCNTNSYQISIGTYGEKATSCAVYPC
EINSPRCHSTAICSNIKTPGQNFYNDYTCKCGRSLNNVETIINPFIGGDKSTQCLAATPSDSYVCND
NNFKPSRTTEYCLKGNQATVEYSLRATNFGNYALFKHCDDPDDEYRSSFNDPGSFWRPIDLVAAA

NENLAVSVTYKTSLSNKFIKCNFEQARFGSKLTLAVNVNSNSRVLTFTNTQTSVNKQNPVYQGQV
FQSDNPTPGAEMSVTFTVTGNQAVDQNVDAILLDNVVCKGFDDVASVNTPCGNNACASNQYC
NPLLSNSEKECLCPTGQKDQNCPDVVPSGSVVSADGPKCIGHHEKYMNGACVCDDGFEKTGDT
CTVKINCGCNDGLCLSGDKCSCPLNLLKVNQACVRTNKDCAKDCVSTLGNGGYCDVTTGMCD
CLTGFVKSGRDDGSYRCDKHAVVQKINDPKGICSATCGTGFVNEIEVCVGATGTGFECMTNIVQG
TLGVCSLPACPATSSQCSIQKECACNNVRVPIGSDGKLRLDFDHEAKLNMIFRFLSKHNQKDLEFK
FREARCDNTKLDSIARTIEDDLKQLEFDTELQAAITTMKSILDCSPSGNDHNAAYWDVFDMLIELS
SYRPHAVSDLQLKKELVMHEQSRCGMRSNLHKVVDGYAFWNKK*
>TRINITY_DN15847_c0_g1_i2.p1 type:5prime_partial len:166 gc:universal TRINITY_DN15847_c0_g1_i2:3-500(+)
NRQGATQYKMSEQTERAFQKQPTIFQNKKRVVGKESKGKEQRFSKSVGLGFKTPREAIEGTYIDK
KCPFTGNVSIRGRILSGIVQSMKMKRTIIIRRDYLHFIKKYQRYEKRHKNLAAHMSPCFRDVQLGD
LVTVGQCRPLSKTVRFNVLKVNKSTSAKKSFEKF*
>TRINITY_DN3016_c0_g1_i5.p1 type:complete len:406 gc:universal TRINITY_DN3016_c0_g1_i5:2713-1496(-)
MAPPNLKKMIQVKIKPRQDSYSDQFSRILMVKMLMLTAAFTGITWMKDKVKCIVPKNHDADGG
FVSKSCWINGFYVFKGLKEKDTSYYYGIPKDVLSDGYDKYGALCSTKNQQGRLNQGCTPLEKTFF
LQYQWFPLVIAAIAFLYYLPYLMFRIVNTDLQEIKDIIKGRNANDVDAKAIVKRFFVRKNNSTSRL
KARVLLNVVVKVLYVVVNVLGMIFVDSSTNSEFMQYGANWSSWTALKNEDMHDYTNREHEKA
GHRLLPSFGLCQVYTTAQDIKTKVANDHTYICELSQHVIYQYILIVMWYMFVFGIIISIIGLIQHMVL
KTMAILSMNAEGDSVRKIYSDLTVREKEYLDFIRRKNLPVFTKVLDEISQLKGVSRSYATSESDRSSF
GATAPEKENMMME*
>TRINITY_DN11522_c0_g1_i3.p1 type:5prime_partial len:855 gc:universal TRINITY_DN11522_c0_g1_i3:2878-314(-)
FSAQLFKGSSKSILGGIPNIQLNRGRIGLGNIRDPSISLTRGSKIDYNGYMGFSATSGYFGNDKHIHF
AGGSPRSNALKGRVIMYKDSAGYEISQYLDDPSDQIGTYFGSAMASADINGDGLADLVVGAPYYT
GTVSNEGIAYVYLNKNGIVELVQTLRQPEGKDVKENGMFGISIGSVGDLNNDGFADIAIGAPWGG
EDGKGIVFVYYGQRDPAQLKLMQTIKPSDLSGIDVDGFGYSFAEFYPYSGGPDQYGFIENDPDYNI
YPDLAVGAFKSNHAIVFKSRPVYTIVGRLSLGGVKRLDLVNKENLCPNGMKCLDKVQACLMFKE
EESEGEVDYTLEVDSGRDSPKRAYLLDSDGKQVTKINKQTTVSKDEECDDYTIYVKNTTDVLSDLV
IKLSWKFVVPTTCDGVCPVANELCQFVDTKSIIFVKGCSDDGNDTCSSDLKIEKKEFPPKLVGNNR
LVGTEEIISAGATDGIILTFNIKNLGENAFDNSLKLEYPGKGDMKLVNIDDAKVISDYYGTPIWDSD
VTREKDVTTLSVMLSSPIVKGKPEQVQVKMSLNPIPKGTKFLKFKVYLETDSEETDTSNDFQEITIP
VELVSNLLVSGQSIPEILTWRKDVKDVQDKGAEITYNFLFQNQGPSNVDTSEAIILIPEKFKGSYFLN
IYNYDVKGSKSGSDPGSCRIEGELNPLKLDYYGRRIGNNTRKRRDTRGVPALECGADTTCREIICN
FNIENRNSATVTVKATLVDANFDSVIGAGREIHVTAEFSSDAVTPPSGLEFPVKSMVTTTVNSPYLT
KTEGGESIPWWIILLCILAAIIIIGVIIFIMYKKGFFKRKKMADEDEALRNGDPEMDDD*
>TRINITY_DN840_c0_g1_i1.p1 type:complete len:409 gc:universal TRINITY_DN840_c0_g1_i1:1437-211(-)
MAMVATDIKSMLSINIKSRHDSHTDQYNRIFMVKLLLVSSLIMGLSWFQDSINCMIPDTHGINGGF
VSATCWIQGVYVFKELQTRVDKVAYFGIPKDMNNDGMLPDGQLCTTRKRFDEEPNPLCKEMHK
TYFLQYQWMPFLIAALSILFYAPYVFFRTANTDLISLKNTLKTEEPDAEKIVKSYFNHRVNPQRTMY
LRLIFNLLVKILYLVANLVAFLGLDSLLNGEYVGYGNKWVSWSKMDNAVAYDYLGMRDFPKPGN
LLLPPFGYCEVYESAKDVKQSMANKHKFVCELSQNILYQYCLIVMWFALIFGIVISVLGIILLIIHYA
LNIMGIKSRGGAAKKLYKALTFRELEYLEFIRKRNILLFGEVLEQLRNEILGPNAPDDRAPLYPNLP
KDEYDAKRPGYQL*
>TRINITY_DN1936_c0_g1_i3.p1 type:internal len:1070 gc:universal TRINITY_DN1936_c0_g1_i3:3208-2(-)
GEYKPTLGRCLSFWYHMYGSHVGELNVLVLQNKSRSAPIWNLKGNQGNVWRPSRVTIKSAVNH
NIIFEGRTGDGYMGDIAIDDVMMSEGPCTDYGNCDFERDLCGYTNKKNIDQFDWQLGSGGTSSV
TTGPSNDHSTQTALGQYMFVESSYPRLPHETAIIGSETIPVSGSGKCLQFWYHMYGEHIGSLEVNM
RVANDLSETIVWKLEGNQGNKWLQGQAPLLSRGQNMEVRFEGIRGTGYKGDIAIDDITVVDYSG

ACAITPKSAKPWGCNFELDFCKWQQSRADKFDWLRNQGATGSAGTGPKADHTTNTTQGYYAY
VETSFPRQPNDTAAIISPTIKGNGTKQTYCMWFAYHMYGPHVDTLKVYTRSQGSDKPVLWRRRGS
QGLNWNHAEVQVTVLYDTKVIIEAVRGKDYKGDIAIDDIDIWFGDCFSSGICTFEGADLCFWYVP
KNGQLYWQRASGATPSVKTGPKNDHTYGTSQGHYMYIETSNAPAKATAYMYSPWYYNTGQKC
VQFFYHMYGQQIGQLEVYIRYSNSRYLYRQFIRSGNQNNTWHMGQVTVKRNNVYFQIIVRGTKG
NGYQGDIAVDDFRIRDGACPAPGDCDFEHADFCSWMQEDNDDFDWIIGSGSTNSFLTGPSIDHT
TQSNGGYYAFIEASSPRQPGHKARFKSMVFAPTTGSGRCFSFWYHMRGYSIGNLTIYLRKVSNTKT
LSEEVMWLLGGNQGKAWNQGRFTLKSSTLHEVVIEGVRGSSYDGDIGIDDIEFTSGSCSFLPKGA
WPSGRSTPAPTQASTTLGPTIGNKGFDCNFDKEFCKTWMRNSTRTNFQWKRHQGSTSSVGTGPH
HDHTTNTDQGYYVYIESSSPAQPNDTAYLIGKDIPNNNGFCLSYWYHMYGPHVGSFRVYVVNKT
NAEARFVRVGTQGDKWKHGQLYISPKAGNPITIRFEGVRGSSFMGDIALDDISVRYGNQCPAPRE
CTFEHDNNGGSSSTCGWTQEKQKDSFDWSIGSGDTPSVLTGPNFDHTLETKQGHYFYIETSYPRK
FGENATIISQSYQQTLGKCFSFWYHMYGSTIGNLTLFLRRD
>TRINITY_DN6542_c0_g1_i3.p1 type:5prime_partial len:545 gc:universal TRINITY_DN6542_c0_g1_i3:2-1636(+)
SSTTEAPATTAPTQSTTTTVATTATSAATTAAPEITSTASSTTEAPVTETPETTVITSKPSTTAAPTGYS
RVCYYTNWSQYRNGAGKFFPSDIDPFLCTHIAHSFAKIEGLNLAPYEWNDIDVLYPQMISLKLAN
PKLKILLAVGGWNHERQGVARFSDMVQTKSNRATFISNTIGFLKLHQFDGLDLDWEYPASHPGSR
PGDKQKFTELCKELLEAFVEDAKTTGTERLLLTSAVAAGTSTIATAYEISELTKYLDALHLMSYDLS
GGWNPTTGHHTAVNADSGNSVTNGLNAWISGGFERNKIVLGMGTYGRSFELSNPASNGIGAPAS
GYGSPGKYTKEKGFISYYEICEMKNAGMTVIRDPIAKAPYGVKGNQWIGYDDEESLRYKVQHFVV
KEQLAGAMFWALPLDDFKGQFCGKGNYPLMNAVKEELAKHENMATTTQSPAITAATTAAPEITS
TESSTTEAPATTAPTQSTTTTVATTATSAATTAAPEITSTASSTTEAPGNSNPNTISFHNCSNICYARS
HNSNARDNFNKKFNN*
>TRINITY_DN80458_c0_g1_i1.p1 type:5prime_partial len:157 gc:universal TRINITY_DN80458_c0_g1_i1:519-49(-)
SKSAKMGRMHANGKGISGSALPYRRSVPTWLKLTEEDVKDQIYKLAKKGLTPSQIGVILRDSHGV
AQVRYVTGNKILRILKAKGLAPSLPEDLHCLIKKAVAVRKHLERNRKDRDSKFRLILIEARIHRLAR
YYKVKRVLPPNWKYESSTASALVA*
>TRINITY_DN6428_c0_g1_i2.p1 type:3prime_partial len:303 gc:universal TRINITY_DN6428_c0_g1_i2:906-1(-)
MLSSLKLERNKNLRYYRYRYSCCNIRVPSTSQKILNPFTLVVGGNAIYLDRQNVQCPGSAVITQLLL
RRYKSTIRYTYSCSTIPRKMECQNMKTPLNFDGKGDARYLDRHPVSCPNDYGLNKMRLQRGSNL
KTYAYHYRCCKPLYSVQFQENKVDGGFTNWSKYGTCTKSCGGGIQERSRSCENPKPLNGGKQCI
GVSVETRSCNTDPCPVDGGFSNWSNYGTCTKSCGGGIQERSRTCTNPKPMNGGKQCVGVSVETR
SCNTNTCLGPKQCKLKFTAYNKDGKGQVFYLDRHNVACKPDEM
>TRINITY_DN1377_c0_g1_i1.p1 type:complete len:878 gc:universal TRINITY_DN1377_c0_g1_i1:68-2701(+)
MTGRARGRSRGRGRAAQDASQRPGGAQGGPPPQAAAPQQGGGGRGRTRGQPAPQQVAPQQR
PAAQTAVPPTARMAQMSVKDQKPATEERQPRRRRLDPAPDVLVTRPEGRTKEGTGGTPVNIVSNF
FKVIKMPNFKGLYQYNVSFDPEINSSKLKNALLYQMADVLGQTKCFDGMTLFLPKRLPDQITTRS
VTTHQGNAIVIKIMFTNVIPEISPQVLQMINIIFRRQLRCIKMELIGRNYFNSSRKIEVKAHKMEVW
PGFSTSILQYEKDVMLCADVQHKVVRQQSVLSMMYELHQKTREKNRFYELAAKKLIGETVITRYN
KKNYKIDDIEWNKNPNSTFDKNGTQITFKEYFEKTYDAKVGDDQQPLLVSLPKAKNRKSGIVGPI
LLLPEFCLISGLSDEIRSNFSIMKDLAQHTRINPAERAKNLEQFVQDLQTNEESKKELEQWNLKFES
KLLQMTGRAYGSEKMFQREDQFSYKIQDADWSRETRGKKLIHTVNMTDWLLVYYERDEPIATDF
KQTLVKVCGPMGMNVAEPQLCRLKDDTARSYFRALSEQIRPTTQIVVCIVPNNRKDRYDSIKKLC
CVEKPIPSQVIVSRTLSKKQMLMSVCTKIGIQLNCKLGGEIWAVDIPISKNPKDPPSLMVVGFDVYH
DSASKGKSIGGFIASTNRHLTKYYSRITQQQSHQEIADQLRVCMTGALKKFNELNGTMPSRIIVYR
DGVGDGQLRQVFDHELPQFKAAFNAISQGYDPKFTMIVVKKRISTRLFLSERGMKTNPLPGTVVD
TVVTRPEWYDFFLVSQSVRQGTVSPTHYNIIADTSGWKPDIFQRLTYKLCHLYYNWPGTIRVPAPC
QYAHKLAFLVGQSIHAEPDVQLADRLYYL*

>TRINITY_DN118_c1_g1_i3.p1 type:internal len:200 gc:universal TRINITY_DN118_c1_g1_i3:599-3(-)
KVLFLCGFILLSGLAFAYEIEEIVEDAPKPEPEHVPEPEPEPVKEPEPEPEHKPEPEPEQCSTVKHQK
CSKADKVCSPEDSHCTFTQICTPFFVKKCCKVVSVEKKCVVVRNCIPKFSYEKVCEDVKRCPPRKR
CKNVCRQVDLGEHKVKSICEQECEVVEHPEPCKLVHTCFSKWVSKKDCTPRETCSDIEKRECREV
>TRINITY_DN149493_c0_g1_i1.p1 type:internal len:148 gc:universal TRINITY_DN149493_c0_g1_i1:2-442(+)
EVKKAGVNVTGKIAICKYGRGSRNAKTRFSEARDVIGLVLYSDPREHTYPGLEYPKGWMLNKDG
VQRGTVNRLPGDALSIGFPSKDGYFRYGEDKYAGNPTIPVQPISYDTAEKILRWMKDDSVPLPEGF
GGDLNVTYRLQSGAGRE
>TRINITY_DN2217_c0_g1_i2.p1 type:complete len:254 gc:universal TRINITY_DN2217_c0_g1_i2:884-123(-)
MNSVDGNTDNAQKECLELCQRNAKATGCEVIWNRHNRGCYIHTQEVARGNNVDRHFCWVFS
KCTLNNVPPAVCPIEKGYCVKGNGHDQNSGVIKMNSVDGNTENAQKECLELCQRNPKATGCEV
IWNQHNRGCYIHTQEVARGNNVDRHFCWVFSKCTLNNVPPAVCPIEKGYCVKGNGHDQNSGV
IKMNSVDGNTENAQKECLELCQRNPKATGCEVIWDRHNRGCYIHTQEIARGNNVARHFCWVFS
KCNL*
>TRINITY_DN1071_c0_g1_i2.p1 type:internal len:339 gc:universal TRINITY_DN1071_c0_g1_i2:1016-3(-)
DPKVAMRVEFYVCPPCGVTTPAITTTEQTTMPTTTTTESTTTPTTTPATTTESTTTTLPTTTTTTPTTT
QSTTSKETIIIITEEPKPTTGLPQKKTTTTPRTTTESPTTTEATTTTPATTTEATTTTQATTTTTKATTTTP
ATTTTKATTTTEATTTPTTKPCECKVLQPVGVSDGRIPDNQMTANSVLVNVSGPTFTGASQARLN
NKPSKDGVGAWEPAQQEEIYLEVHFNKIEDVREIRIQGHPTDKKFTKNFFLSTSQDGVNFEFTDED
LYEGNKDGNTIKSTKVSFKTKAVQIHPFNDVSVGDPKVAMRVEFYVCPPCGVTTPAITTTEQTTMP
T
>TRINITY_DN77_c0_g2_i3.p1 type:internal len:1381 gc:universal TRINITY_DN77_c0_g2_i3:4141-2(-)
EPPTSTEPPTSTEPPTSTEGTTTSSGPPPLLFIDSEGSTTPSYPPPLIFTEPEGSSTSTELLSTTVSAPVLTT
SPPSQDLGDLGDVTTEPPTELASTTEQSPILMGDPGLKGEKGVKGFDAVNGTQGVKGDRGPKGD
RGENGTKGEKGDVGPPGPGGDFILGEIGAQKPSDPLSGAVGEIEGLAVPGPKGITYDVRSGPIGSIG
SVGKVGPIGLSGDDGKKGQIGQKGPSGPRGGFGNIGEKGKRGTDGVPGNPGEKGPKGTAGRKGE
LGGDGRPGVQGAFGHKGNDGTIGLDGETGDPGLKGAKGYAGEKGDQGDIGGAGLPGPKGEDG
PIGPPGQRGLRGPIGANGPLGDPGLAGDKGQQGTRGNPGYTGKPGESGPNGEKGLSGSIGPVGE
QGPIGSPGEQGRNGNDGTQGARGPRGLAGPDGPTGPQGPRGESGPQGSAGGPGNAGLKGVRG
KPGLRGLTGPKGESGDIGDPGARGPQGQVGKQGKTGPPGASGEPGLTGNQGPQGPGGDPGPNG
NKGLMGSQGPVGEKGSLGPKGSPGRAGLTGPKGLLGEQGSPGQPGLTGRDGKNGLQGVNGKD
GEPGASGPQGMQGAPGLIGTTGPPGDNGDFGDDGPAGADGDKGSRGDSGVRGPAGAPGDEGQ
TGMRGERGDKGPQGNPGSQGSPGPRGTPGLDGLEGAEGRKGTAGRNGNPGLTGAQGVVGNTG
LPGSQGSRGVPGEQGDSGSDGAPGEMGLPGLPGLKGSIGRQGLTGRRGQQGVPGPKGKYGLQG
EKGQKGEGGNAGSRGSEGLPGRAGRPGSRGEPGPVGSAGIDGNPGNSGEIGSPGRAGTKGMQG
PVGLRGPVGTSGLRGPKGPAGDKGDSGETGAIGIAGELGTPGVTGQSGPRGQIGEIGLRGENGPQ
GMPGSQGLSGPAGPTGPTGPIGLPGKDGNKGPVGEAGDPGKPGQAGNTGATGKDGLPGEPGAG
GNQGKEGPEGVKGTPGPIGAAGPRGLPGRQGFPGENGMAGFPGRDGSTGLAGNRGPRGEEGEQ
GTLGEKGVDGSPGSTGTNGGNGVPGDNGPKGYIGERGEVGPEGPEGELGEKGVKGEEGSEALKG
VRGPNGKSGASGKRGDRGDTGGNGPKGEDGSKGSVGLKGFKGNIGFTGFIGPIGAKGYKGDMG
PDGPEGHTGPQGNTGDPGVDGDPGAVGSKGPAGNPGQIGSEGNQGKPGDPGKPGEAGLSGPPG
SIGQPGDMTGAFAGMYWNYIHSAGMRSQRYYKGKNKEEVDEDSNSIDKLFKTTYETFQNFDFV
WNQVVDDLVVLKTLGTMKNPAVTCADLFKTNPYFQSGDYWIDPNEGSPDDAILVHCNATNYET
CIYPRWPSFEKDNWYEGEEDYLWAYKDRLLSDEEYISYSADI
>TRINITY_DN4815_c0_g1_i2.p1 type:complete len:638 gc:universal TRINITY_DN4815_c0_g1_i2:79-1992(+)
MVSSLLLVLICTVVYTAGISAFFQCDFEKVDICGFKSSSSNYTDTGEAPNWRRHTGTTPSAKDQNN
AHVTGPLEAQSGDYYMYFEASNLPSGASSTGTLTSRLFDATQFGVTDIVFHVSSKGESLSLLVLDFI
DYTTNDIFDEKVVFPRNKAAWQKYTFNLDEKIFKRKFYMQFIGYVREVNGKKDNGDWAIDNLRI

DGIAIVEKCENVKCNVNTNCKDTSNGPNCECLPGYSTKAGTSGKNTECIETCQAFTCNLNAECS
NTENGPDCKCESGYKAINGTSGKTTRCEETCQAFTCNLNAECSNTENGPDCKCESGYKAINGTS
GKTTRCEDDTCSNFQCSNNTNCENTVSGPNCTCKEGYRPVNGTRGRDTICEDINECAIKNICGGL
KVCRNVPGSHHCDCPPNYTFFAAENKCYRNGAFSQWRNVGSCSKTCGAGDQKQTRSCSNPSRE
APHGRDCPNEPTERAVACNSGPCLAGCPRNKTCTCENVRQYKTGEEPKKDFSSFNGLSITDGETS
RRYVHTYNREFIEKTIKDVNCNNNQLKSVYKNITDIIGILEGINKKYSSTKMEMQDIIDCNANIDIE
KNLWDVYDVLAEHSICNYQVQKDLEMKADRIQAEITRCNNRGWYSTILDAII*
>TRINITY_DN15584_c0_g1_i1.p1 type:complete len:275 gc:universal TRINITY_DN15584_c0_g1_i1:874-50(-)
MADSGSAGADRGGRGGFGRGGRGGRGRGRGRGRGRGKPEDKEWTPVTKLGRLVKDLKIKTLE
QIYLYSLPIKEFEIIDFFIGPSLKDEVLKIMPVQKQTRAGQRTRFKAFVAIGDYGGHVGLGVKCSKEV
ATAIRGAIIQAKLSIIPVRRGYWGNKIGKPHTVPCKVTGKCGSVTVRLIPAPRGTGIVAAGVSKKLL
QMAGIDDCYTSACGTTATVGNFAMATYMAIARTYSYLTPDMWAENQFQKTPYQEFTDYLAKNH
NVGGKPVAPATATA*
>TRINITY_DN187147_c0_g1_i1.p1 type:complete len:1223 gc:universal TRINITY_DN187147_c0_g1_i1:3708-40(-)
MLTKFETKSARVKGLTFHSKRPWVLASLHNGVIQLWDYRMCTLLERFDEHDGPVRGIDFHENQP
LFVSGGDDYKIKVWNYKQKKCIFTLLGHLDYIRTSFFHKEHPWIISCSDDQTIRIWNWQSRNCISV
LTGHNHYVMCAQFHAVEDYIVSASLDQTVRVWDISGLRKKYCAPGAKPKEDITAPKTPGQMDLF
GHSDAIVKHVLEGHDRGVNWVTFHPTMPLIVSAADDRQVKLWRMNDSKAWEVDTCRGHYNN
VSSVIFHPRLELILSNSEDKSIRVWDMSKRTGVQTFRREHDRFWILGAHPTLNLFAAGHDSGMIVF
KLQKERPAYAVHQNTLYYIKERYLRMYELGTSKDIPIMQLRKSSGRSTPLKLIFNPAERCILITYNND
TSAGESQVYELHTLPKDIRSTDPEQSEGRRAAGTAAVWVARNRFAVLDKQQNVVIKNLKNEVTK
KVQTPACDMLFYAGTGSLLLRDAEQVMLFDVQQKRVMATVKIAKAKNVIWSSDMSYAAILAKH
SIAICNRKMENLCTVHESVKVKSGGWDDSGVFVYTTSNHIKYTLTNGDAGIIRTLDLPIYITRVKGS
NVYCLDRECKTKVLNIDPTEFKFKLALVNHRYDEVLYMVRNAKLVGQSIISYLQKKGYPEVALHF
VKDEKTRFGLALECGNLEIALEAAKTLDDKACWEKLSEMALLQGNHQIVEMCYQRTKNFDRLS
FLYLTTGNSDKLRKMLKISEIRKDVSGHLHNALYCGDVEERIKVLKQVGQGSLAYLTAATHGLVEE
CEEIKATFDLNPEKLPPINPNAKLLRPAVPILKNEGNWPLLSVSKTLFEGAAAMKDAGTSNMAAA
VDMDDEGEGWGDSDLVLEEDAGFVDPEEKLDIDEEGGGWDVDDDDLELPPDLDVGPVSSGGED
YYVPPTRGNSQAQVWCTNSQIPVDHLVAGSFDSAARLLNEQLGVVSFEPYKQLFMLAYARGRVA
APGLPSTPPLFYYPHRNWKEAGAKAGLPAVAIKLNDLVQRLQTAYQLTTGGKFADAVERLHQILL
SVTLLSVESRQDVTEAQQLLGICREYILGLQMELNRKELPKQNAQQQKRICEMAAYFTHCNLQPV
HLVLTLRTALNLAFKIKNYKLASSFARRLLELGPKPEVSQQTRKILLACEKNPTNAVQFDYDELNP
FSICAVSYTPIYRGKPEEKCPLCQASYKPEYKGTICKICRVAEVGRPGSGLKISMGR*
>TRINITY_DN101992_c0_g1_i1.p1 type:internal len:139 gc:universal TRINITY_DN101992_c0_g1_i1:414-1(-)
AERYDHHSGIVESMDSNKISDNLKELTRLPHPPGRSRNKELGDMLADFWQNEGMDKIEKFKYNI
YLSYPEKPSTILLFGGAKTEKYTIEAEPPVDESEKGGTPVVPFNAFSANDIVKAELVFVNHCRESDF
DEVKKAG
>TRINITY_DN2885_c0_g1_i2.p1 type:complete len:114 gc:universal TRINITY_DN2885_c0_g1_i2:31-372(+)
MAQPVRLYSKGIFVGYQRGLRNQHEQTSLIKIEGVNTKADTSFYLGKRVAFVYRVSKKTVAKGDK
KPSRIRVVWGKVTRPHGASGVVRAKFRHNLPAKAIGGSVKVMLYPSRI*
>TRINITY_DN79_c0_g1_i1.p1 type:5prime_partial len:478 gc:universal TRINITY_DN79_c0_g1_i1:2132-699(-)
VVSSESNVLVEFYAPWCGHCQSLAPEYAKAAKQLKESKSDIKLAKVDATVETKLAEKYEVQGFPTI
KFFKKGNALDYGGGRTDKEIVAWLNKKTGPPAKLLDNAADVKAFADERDVAILGFFNDKESSEL
AKAFLAVAEAVDDLEFAIAAPDASGDFAVTEDKIIVAKKFDDPQVVYSGEANIADIKKFVRAESIA
LVTEFTDEAAPKIFGGDIKNHLLLFISKKAENFAELKNTFQEAAKGFKGTVLFVYIDTDVEDNGRIL
EFFGLKAADAPTLRLIQLEGDMTKYVPETKDLDKDSVTSFVQNVIDGKIKPFLMSADVPEDWDK
NPVKVLVGKNFAEVALSKDKDVLVEFYAPWCGHCKQLAPIWDKLGEKYKDSENIVIAKMDSTAN

EVEEVKVSSFPTIKYFPKEGGVVDYNGGRTLDDFVKYLESGGKDVSGGADETGEDEDADEEEEGM EEGMEEGDLKPEEPEVAKDEL*
>TRINITY_DN12627_c0_g1_i1.p1 type:5prime_partial len:1061 gc:universal TRINITY_DN12627_c0_g1_i1:3456-274(-)
YGENNRLKFNDARQYCQQWKDDQGDLVAIHSNYENSFIMSQLYDVNEPMYIGLTDSFSERTFLW VDQSSVVYTNWYARQPSFVGNEDCVEMMPYFKTHGRWNDIGCSAERGFICQRTPSKTPLPTPEP VKDSKPCPAGYRKFMKACYRVVHTPMTYDDAVKDCRTSVGGELGDLVSLHNQFEQSFVFSQFAK FDFHSWIGLKKIKGEKEFKWSDETPYDYRYWLSLRYDNTVYNDDKCTTQEFSSKVDGKWKVTDC TQKHPYMCKIERDFDQPHLTFIGNCTDGWTKFYDKCYKLQNYFSNWPAAREKCKGFGGNLVTIT NSDIQKQMEYLARDAYRSVWIGLNDRAKEYVFEWSSGIPLLPDSYQNWGPNEPNDNSGTENCV HMTTESSGGKWNDLSCSQRMPYICEHPLECNSALGMEDGSIPDTNINATSYVGTYFPKYARLNAK SDNNRNTAWCTNITTGAESLTINLGEVMQITRISTQGLLDSSLGFSRGGYVVSYMLEYSNDGTTW MTYISEDAYDQKPIALPGNIDASKIDGTTINLKAAYLRFIPKLYKNIICMRVELYGCKSVCSRALGM TNFAISDKQLSVSSSQAGSEKAQLRPNIDGSWCAKDDDYSPFVRIDLPRLTKITRVTAISSSKVQYKI AYSDDGELFTRFQTLTNEERVFSTVPSTHDLETAISAKHVKFDLLKKDQGKLCLKVELYGCLKDCD AVLGMETKIIPDRLITASSAKPSNPARNARLSVGVYGDNSWCSSETDNAQYLQLDFVQEAHLTEIA TQGFNGDSAKYVSKYNVQFSDDGDFWHQYSEELPGNSNGYSIQRNKLKEPIIARYIRINPTEWKN GICMRVEFYGCPMKPFTAPKILTQKTYNGAIRILNEDFNENLLNANSIEYIELANKVETDITSLYLLE ESFKDGFNSVTVNKFREGSVITEFTIKTHTSDASLDAAEPLRKAVQTGRVGTFDVDPNFFEIVTAST DKSTATNDDSGGLSNGGKIALIVFFVILICIVLAGGVVYYLRSRRNGFRLQFQHRAFENPTHVDDL QNDDMKVEFMNDDY*
>TRINITY_DN1936_c0_g1_i1.p1 type:3prime_partial len:1224 gc:universal TRINITY_DN1936_c0_g1_i1:3669-1(-)
MVCPAVITIFLFSVLGSSSSQNCDFEDVKSCKWTQYAADKFDWSRASGSTGSTGTGPVNDHTTLV KTLKVNTTGGNANGASCVFPFIYQSKKYFKCLQYYNTEWCATVPNYDKQAKWGYCVPTGFYMF IEASNPRKKGDNAILVSPFVPKGPKCLKFWYHMYGPHINRLNIYVMIGGHTGKPIWSRIGTQGNK WNKGQVTLVGRQPFQVVVEALSGISYKGDIAVDDISVSSTKCTQPSSTTCSFEDSQCGYIDDTSSDV QFQWTRSSLSTSSSGTGPKTDHTMQSETGFYMYTEATRKKTGSKARLMSPVNKATSGSCLQFWYH MFGADMGTLSIYLKVNNKLQSTPLWSEQGDKGNEWKVATATITTQDSYQIVFEGKIGRGYRSDM AVDDIKLTDGNCPPPGFCDFETGYCTYFNSKNDKFDWLIGSSRTPSRFTGPNFDHTYGNANGSYL FLEVSQPIKAGDIAILESQQFNAVGGKTRCLNFWYHMFGSDIGSLQVVYKVFSGSQQERVLWNLT GQQQITEKDPWKYARVPVDMDADHMVMFKGIAGSGYKGDIAIDDVQFTSLACTIDPPEANPNG KTTMKPPTTYPRTAPSTPVPTEFSCNFEKDLCKLTQDSKGTFNWTRIQGYTGSPGTGPSTDHTYGR GFVPKYGPGSLRHAGGKCVHVLNGGFNKPKEGNPLVLYQGCGQTRLEFVFKYGYLKLTRLNMC ATLEGSSTSSGTKVVLSNTCMNKWKMLDNNTIQDVKTGLCLSPFKGSDYPSNNSPLVVDKDCKP KSRIFTFSPTMGHYAYIETSPPRQPNDTARLISPILKTPRCLNFWYHMYGAHVNMLNVYVKTSNGP YVSPAWAKYGSHGDTWKAAYIYMNMTGPYVYAFEGVRGIGWQGDIAIDDITLSESCPAPVECSFE ETSPIMCGWTNLKNDDFDWSIGQGKTASTKTGPKNDHTYGTSQGHYMYIETSNAPAKATAYMYS PWYYNTGQKCVQFFYHMYGQQIGQLEVYIRYSNSRYLYRQFIRSGNQNNTWHMGQVTVKRNN VYFQIIVRGTKGNGYQGDIAVDDFRIRDGACPAPGDCDFEHADFCSWMQEDNDDFDWIIGSGST NSFLTGPSIDHTTQSNGGYYAFIEASSPRQPGHKARFKSMVFAPTTGSGRCFSFWYHMRGYSIGNL TIYLRKVSNTKTLSEEVMWLLGGNQGKAWNQGRFTLKSSTLHEVRFIYLRLFHCKICLPCY
>TRINITY_DN144508_c0_g1_i1.p1 type:internal len:265 gc:universal TRINITY_DN144508_c0_g1_i1:1-792(+)
PRRPNDTARLVSSDVPATSQMCFEFWYHMYGDHIGNLTVFKRIGKAMTMLWAKSGTQEPRWRH ATVDITSASVFKVVVEGTASSGYMGDIALDDLFLTPGACPPSAECTFEDPQLCGWTNIAGDDFDW TRGNAGTASWGTGPRTDHTYGTKQGFYMYIEASTPRSKGHKAWLKSEEYKPTLGRCLSFWYHMF GLHIGELNVLISQNNSMSAPIWQMKGNQGRPWRPSRVTVKSSLNHNIVFEGRTGDGYRGDIAID DVMISEGP
>TRINITY_DN2626_c0_g1_i2.p1 type:internal len:219 gc:universal TRINITY_DN2626_c0_g1_i2:2-655(+)

SSDRGSSPFLIPGRQPAARTRSRAMPPAQRRVLVIGGSFAGLCVARDLKDHYLVTIVDAKEFFEYTP
GVLRAYVKPKHLDALTFTLYPVIEGRMGCKFIWGEVTELNGADRTCTIQPMFTQEQEVIDFDYCVI
AAGCNFGVFHKWGESLWFPTIHAAARPEGSWPHLDERYLEGRRRHILEEYASIGALNDKGASILV
VGAGFIGVEWITELQHFFPK
>TRINITY_DN1559_c0_g1_i1.p1 type:complete len:311 gc:universal
TRINITY_DN1559_c0_g1_i1:221-1153(+)
MAEKKATFKLGFAENFALSGAAAVISKTAAAPIERVKLLVQNQDEMIKQGKLSEPYKGVIDCTTRT
FKTEGLAPFWRGNLANCIRYFPTQALNFAFKDKIKAAFKVSKSDGYALGFGKNIASGGCAGALSL
CFVYSLDFARTKLANDTKSSKKGGSREYNGLVDVYRKTLKSDGVAGLYRGFVLSCVGIFVYRGCYF
GFYDTLKPVLLGADASLFMSFILGYGVTVTAGLISYPIDTIRRRMMMTSGQAVKYKGSIDCATQILK
NEGAMSLMKGAGANILRGVAGAGVLAGFDKFKEVYIAMRKRQMGLE*
>TRINITY_DN3986_c0_g1_i1.p1 type:complete len:446 gc:universal
TRINITY_DN3986_c0_g1_i1:252-1589(+)
MREIVHLQAGQCGNQIGAKFWEVISDEHGIDPTGTYHGDSDLQLERINVYYNEATGGKYVPRAV
LVDLEPGTMDSVRSGPFGQIFRPDNFVFGQSGAGNNWAKGHYTEGAELVDSVLDVVRKEAESCD
CLQGFQLTHSLGGGTGSGMGTLLISKIREEYPDRIMNTFSVVPSPKVSDTVVEPYNATLSVHQLVE
NTDETFCIDNEALYDICFRTLKLTTPTYGDLNHLVSATMSGVTTCLRFPGQLNADLRKLAVNMVP
FPRLHFFMPGFAPLTSRGSQQYRALTVPELTQQMFDAKNMMAACDPRHGRYLTVAAMFRGRMS
MKEVDEQMLNVQNKNSSYFVEWIPNNVKTAVCDIPPRGLKMSATFIGNSTAIQELFKRISEQFTA
MFRRKAFLHWYTGEGMDEMEFTEAESNMNDLVSEYQQYQDATAEDEGEFDEEEEEEDA*
>TRINITY_DN195348_c0_g1_i1.p1 type:internal len:311 gc:universal
TRINITY_DN195348_c0_g1_i1:1-930(+)
SGCNSIDLGLLVDFSSSKDYDSKQLTKFVREFIDKITNVFAFSATKSHLAYLPFSSRINENITGQWFD
NKYMKTIGDWDYQELTQYLDRVIVPYTPVTGYETGKANAHEAFQATNDKMFNALYGMRYNMT
KVLIVISDGNEDFKEKQNLVDRAIDQLKAKGVIIYTVSIGNNVNWDRVFRYASRESSIINGANFDD
VFNRFFDFAKMLCEDSKVPIPATRTTASTTTVSINTTKLTTTTTTELVPVTISTTTTMSTTLRPIQACY
EDIDLGFVVDFSKMNANDMKERQTANDIVKVIKKIASSFNIAANK
>TRINITY_DN3034_c0_g1_i1.p1 type:complete len:1941 gc:universal
TRINITY_DN3034_c0_g1_i1:6289-467(-)
MAEEEDPMKYLQVDPAQALKDQSKTFDAKKWVWVPVIGEDGYAAAQVIKVDGDNVTVETNK
GHEMEVKKEKCDQMNPPKYEKCSDMASLTYLNEASVLYNLKARYTTGLIYTYSGLFCVAVNPYR
RLPIYSDQVVKLYRGKRRAEMPPHIFAIVDTAYQDMLTDHDNQSMLITGESGAGKTENTKKVIQYI
AKVAGVEKKKSDDGPVKRDLDEQVVEANPLLEAFGNAKTTRNNNSSRFGKFIRCHFSQTGKLAG
ADIESYLLEKNRVIHQASAERNYHIFYQLLYATTDEELQKLCLVTRRSEEFEFLKKGVPSVDAIDDN
EEFGYTTNAIEVLGFDEEEKQSMFKICAAILNFGNMKFKQKPRDEQAEVADPADGERVAYLLGLPI
GEFHKSLTKPKVKVGTEFVNKGQNVSQVLYSVAALSKALYERMFWWIVSRVNKALDTKERRSYFI
GVLDIAGFEIFEYNSFEQLCINLTNEKLQQFFNHHMFVLEQEEYKKEGIHWEFIDFGMDLEETIDLI
EKPMGVFAILEEECIVPKASDQTYLQKLHKQHAGKNGSYEKPTPKQTKSGGGDFILHHYAGSVGY
SVANWLEKNKDPINAHTAELFSKATVGLVSGLFADYNPDVTGGRRKGSAFQTVSFRHKEQLKNL
MTTLMSTSPHFVRCIIPNEKKTPGLVEGTLILHQLRCNGVLEGIRICRKGFPSRMLFQDFRQRYQIL
AASAIPSGFIDGKVAAEKLIDSLQLDQSEYRVGTTKVFFRAGIIGELEDMRDERLAKIITQFQAYCK
GHLARIEYKKMCEKRIGLAVIQRNVRKFLFLRNWQWWKLYCKVQPLLSIARAEEEMKEKEEELK
KALEKAKEDEEQRKKMEEQLTENIAEKEKLFADLQAETDRLINLEDKLMQIQQQKDRLEANLNE
TLEKLDGEEHSVSVLQEKMHDAEDRIDSLEAEASELKSNCSRLEVERANRDKQIDALNEDINKQD
ESIAKLGKEKKGLEDNIQELNETLQASEDKCSSLTKAKKKVEDALKESENSLSKEKDSKARLDKEK
RRVEGELKETNDKLSEAENELKNTQDTVYKREKAIKELEEARDNQDNQIKQLQKKIQELLQRIEE
LEEELEAERKQKQKSELSRKELESQIEELQDQLETAGGATHAQVEVSKKREQECSRLRKEIEDINA
ANDEALASTKHKFNAQLSEAQEELESVKKAKSKSDKERSQLAGDLEDVQGQLDQVKKQRASSD
KNNRMLEEQLAELRGKVSSLETDMGDSESRNLKFQNESATLLKQLEEAEHKLGLANKQNKQLQ
ASLGESKNVADDEARGKHDMQNKLQNALAQIEELQEQLEDEQTAKMDLQNKYSRANADAQQ
WKNKYDMEGASRVEELEDAKRKLAARVQEMEEALAAAESKAASMEKVKNRMNEEVEDLLLDL
EKAQAQAANLEKKQKKVDQQINEWKLKCDEVQEELEKSQRDNRSYSTELLKMRTANEDTVEKY

ESLKKENRALAAELQSVSEQLSEGGKNSLEVEKLKRKLGLENEELEVALEEAELALEQEEAKFLKI
QLELTQLKQSTDRRSAEKDEELETLRKNHARQLEALQNTIDAEQKGKAEAQKLRKKLEGDLADL
ENRLEGSDRNSADYLKTIKKLQAQIKELQAMIDDESRGRDDMRDTASRSEKRAMDLAVQLDEAR
VALEQSDRLRKLAENEKLENAERVAELQTMYNSIANAKRKAEGDYHSLQEEIEELENEAKMAEE
KANRAMNECARLSSELNGAQENASNAEKSKSLMARQITELQQQLEEAESMGGKSLKNQIRKLE
QKVMEVEGELDNEARKSTESIKIARKADKRVKELQFQLEDEHKAVERAQEVSDKLNQKMKKMR
LQLEESEQQSSSWQSKFKKAVVDLEEAEERCESAEQALQKARQRARSGASGMTTRGVSRARSRVR
SPQNDD*
>TRINITY_DN1704_c0_g1_i1.p1 type:5prime_partial len:190 gc:universal
TRINITY_DN1704_c0_g1_i1:1-570(+)
SLPSSQIPIMAPTKEEKKVDKVNPMKEIRIQKLCINICVGESGDRLVRAGKVLEALTGQTPVFSKAR
YTVRSFGIRRNEKIAVHCTVRGAKAEEILEKGLKVKEYELRRGNFADTGNFGFGIAEHIDLGIKYDP
SIGIYGMDFYVVLARPGYNIAYRRRKKSRVGFQHRLTKVDAMKWFQQKYDGILLK*
>TRINITY_DN163801_c0_g1_i1.p1 type:complete len:298 gc:universal
TRINITY_DN163801_c0_g1_i1:55-948(+)
MANPLKDIISRFSKGVPAGSSTGLGLLIGAGLAAYGIKESIYTVDGGHRAIIFSRIGGIQPGVYSEGL
HFRIPWFQYPIIYDIRARPRRIMSPTGSKDLQMVNIGLRVLARPLSSTLPHMYQRLGLDFDERVLPSI
CNEVLKSVVAQFNASQLITMRQEVSLMIRRDLTERAKDFDIILDDVSITDLSFSAQYTAAVESKQVA
QQEAQRAAFLVERAVQERQQKIVASEGEAKAATLLGEAMNENPGYLKLRRIRAAQKVARVIAQS
QNKVYLNANNLLLNIKDTDDIDDGLLSKKR*
>TRINITY_DN761_c0_g1_i1.p1 type:complete len:830 gc:universal TRINITY_DN761_c0_g1_i1:129-2618(+)
MTSMFRSEEMTLCQLFLQSESAYACVSELGEIGVVEFRDLNPDVNAFQRKFVNEVRRCDEMERKL
RFMQKEIDKADIAIVDTGENPDAPHPREMIDLEAQFEQLENEMKDSNSNYEALQKSLLELLELRH
ILKKTQSFFEEEGYAETQARLQADHEPGVTGGGQLGFVAGVINRERIPTFERLLWRACRGNVFFK
QAEIDTALYDPGTGDLTHKCVFIVFFQGEQLKIRVKKICEGFRATLYPCPETPNERREMAIGVKTRIE
DLETVISQTQDHRHRLLQTIAKNIRVWLVKVKKIKAIYHTMNMFNLDVTQKCLIAECWAPVADL
DSIQAGLRRGTERSGAQVSSIVNRMSTKQDPPTFNRTNKFTKGFQAIIDAYGVASYQEVNPALYTVI
TFPFLFSVMFGDCGHGAIMALFALWLIMNERKIANWKAGGEMFDTVFQGRYIVFLMGLFSIYSGL
LYNDIFSKSLNIFGSAWVPTLNRTKYDYQPENYKEWSDGDRMLNPLDAPRETPYYFGIDPIWQLTS
NKLTFTNSLKMKLSVIFGVTHMIFGVILSLFNHLHFKQYVNIFCEFIPQLLFISCIFGYLVILIFYKWIA
FSIHSPSQPSILLGLLAMFLNFAKKKTDADRLYNGQDFLEPFLVVVAVICVPWMLLIKPFYLRWQH
NKKKQHGTNFARLHEDTAPLQDNEVNHHDDSGISSKNEEEEEFDFGEVFVHQCIHTIEYCLGCIS
NTASYLRLWALSLAHAQLSEVIWTMVFKIGLSMDGYVGSVAIFAVFAAWAILSVAILLIMEGLSAFL
HTLRLHWVEFNSKFYVGAGSKFVPFSFKDIMNDEAF*
>TRINITY_DN1739_c0_g1_i1.p1 type:complete len:400 gc:universal TRINITY_DN1739_c0_g1_i1:86-1285(+)
MAITDNIKDILSVKIKPRHDTYSDQYSRIFMVKIMMMSALLVGLNWYSDKIKCIIPDSISADGGFVG
AACWINGFYVYEKIRYHVNEVGYFGMPREIDHDGMFPDGQTCNSKDTLSSDPSMECQPMEKTFF
LQYQYMTFVMAAFALLYYAPYAIFRWINVDLVSLKGSIQDNSAEKIIGNYFNHRVNSPNKMRMRV
MGNIVVKILYIVVNIAAFLLLDSILNGGFTTYGTLWTQWAKLDNDLAYDYMGIRHSPKPANALLP
TFGFCEVYMSAKDIKTTVNNKFKFICEISQHILYQYVFIVVWFAMVFGIVLSIVGLLMQLIEHALTIT
CFLKQGDQARRMYSILTLRECEYLEYIRRKNIPLYNDVIKKLKEQFQDGFHKPRDHYNNDGLPKY
QDTQA*
>TRINITY_DN141338_c2_g1_i1.p1 type:complete len:720 gc:universal
TRINITY_DN141338_c2_g1_i1:2850-691(-)
MSEADETETFAFQAEIAQLMSLIINTFYSNKEIFLRELISNSSDALDKIRYQSLTDSGVLESNKDMKIE
IIANKDEKTLTLFDTGIGMTKADLVNNLGTIAKSGTKAFMEALTAGADISMIGQFGVGFYSAYLVA
DKVEVVTKNNDDEQYIWCSSAGGSFTVKRDTINEPIGRGTKLVLYMKEDQLDFLDEKKLKDIIKK
HSQFIGYPIYLETQKTRDKEVSDDEADEEESTDKKEDEENEDKEDDGPKIEDVGDEEKEKEKKKKT
IKENYTEMEQINKTKPLWTRNPDDISTEEYAEFYKSLTNDWEEHLAVKHFSVEGQLEFRAILFVPK
RAPFDLFENKKQKNSIKLYVRRVFIMDNCEELMPEWLNFVKGVVDSEDLPLNISRETLQQSKILKV

IRKNLVKKCLELFAEISEDKDNYKKFYEQFSKNVKLGIHEDTQNRNKVAELLRYSSSASGDELTSFK
DYVSRMKENQKDIYYITGESKEIVAASAFVERVKKKGFEVLYLVDPIDEYAVQQLKEYDGKKLVCV
TKEGLELPEDEDEKKRQEELKAQFEDLCKVIKDILEKKVEKVVVSNRLVDSPCCIVTSTYGWSAN
MERIMKAQALRDTSTMGYMAAKKHLEINPEHQIMIALKKKIEADKNDKSIKDLIILLYETSLLSSG
FSLEDPQNHSSRIHRMIKLGLGVDDDGDVEEIETDDLPPLEGEGDDDEDKARMEEVD*
>TRINITY_DN118520_c0_g1_i1.p1 type:complete len:581 gc:universal TRINITY_DN118520_c0_g1_i1:1895-153(-)
MYRLLSVIRKTTQTAKLRQGLSACISTTTHYCSPKEIKTGTDARALMLKGVDQLADTVAVTLGPK
GKNVMIEQSFGGPKITKDGVTVAKAIELPDKYMNIGAKLVQDVANNTNEEAGDGTTTATVLARA
IARSGFEAVSKGANPNEVRKGVMAAVEVVTQGLKDLARPVTTTEEIAQVASISANNDAAIGKLIS
DAMEKVGKDGVITVKDGKTVYDELEVIEGMKFDRGYISPYFINTAKGQKVEYQNVLVLICQSKIS
NVQDIVPVLELANQHKRPLLIIAEDVDGEALTALVINRLKIGLQVVAVKAPGFGDNRKNTLKDVA
ISTGAEVFGDDLNLKLDEVQLSDLGQIGELVVTKDDCLMMKGRGNTDLVNQRANHIHDEIKATN
SDYEREKFQERLAKLVGGVAVIKVGGSSEVEVNEKKDRVTDALNATRAAAAEGIVPGGGTALIRC
AKLLDTFQVPTEDQKWGVDIIRAALFKPCSTIVANTGIESAVVVEKVSSSKDPEYGYDAASGEFAN
LIKAGIIDPVKVVRIALSDAAGVASLLTTAEAVITEIPKEDPSMPPMGGGMGGMGGMGGMM*
>TRINITY_DN1043_c0_g1_i2.p1 type:complete len:700 gc:universal TRINITY_DN1043_c0_g1_i2:2410-311(-)
MKESYLKRFALLFSVLILFQEGGALLRENLIVNHRLRRNVYSEEKVTAVDLPQSREKRFVGTALIIIS
AASAGLDIYKQVRGCKDITPDLDDKIAEGKALQQSLLNLRDQVNLAWVPAESREEYTRRLASNM
IIIREQVSTFDSLQADLVKLLDQKLLDKLNNVTKHLENQIDRNNETEIGSFEDLEGKYMDTTTFALT
LTAKLLPVGFLLGTKFLLPKITARFSKGVAKAFAADNPSRVQKMLSSFAEKQGKLANWKAEKINSF
KVSLNSKWNTFKLKFPRITAAMNRMSNSKIGKALGKAMGKVKGIAVGVGKGAVVAFFIAFEVW
NFVQFHGACTEKRNKAIEGIATMNANKKNMTDLLTEIHDMKAKADEYYNLMKSNVTNDEFLN
FLEDIADLGENAKEQTKVIVDATNQIKQYKAEIKNADDSRTDALNTMLVTAFSNINFKLDCLRSK
LAIIKGVVDDCGIGLGVYDDIYARALAKQKKITETCIEGSYMDKNEIKGLMVAAMDLDKKQSICV
RNNPTKKQLVCEKWYDDVSDADILAANPDLTKDDVTYFISTCPPAPVNDQTKTTICVKKNNELA
TLQSIQQTYQKYDQTAINSAYEECQLSSISVTTICTAKKEGQTVVYAQQKFPVFPASMVTNVYNSC
KKETNITPADKMMICLLKKNGFALDSPVMANYVKNYDYATLEAFYNQCS*
>TRINITY_DN108993_c0_g1_i1.p1 type:3prime_partial len:180 gc:universal TRINITY_DN108993_c0_g1_i1:538-2(-)
MGNTATAKKGDSENAVKAFLTQAQEEFKKKWDNPSHNTASLDEFERIKTLGTGSFGRVMLVRHR
ENGKYYAMKILDKQKVVKLKQVEHTLNEKRILQAIDFPFLVRLDWHFKDNSNLYMVLEFVPGGE
MFSHLRRIGRFSEPHSRFYAAQIVLAFEYLHSLDLIYRDLKPENLLIDEKG
>TRINITY_DN1613_c1_g1_i2.p1 type:internal len:1068 gc:universal TRINITY_DN1613_c1_g1_i2:3201-1(-)
TKSCGGGIQESTRSCSNPSPAHGGKPCSGELSKTRACNEHECPVDGGYTPWSAYGKCSKVCGTGV
QFSTRTCTDPKPKFGGEPCTGLTKKSRNCNTHPCPVNGNWGAFGSFTTCTRTCGGGIQKRYRKC
DSPAAAHGGKKCPGLSFQFKACNTVHCPVDGGYTQWSSFGSCTATCGGGSQTSTRTCTNPSPKY
GGEGCVGPSEKTRTCGTKPCPVNGGYTEWGEYSKCSEECGGGIKTKSRSCTNPAPAHGGKGCQG
PSSESDVCNEQPCPVDGNWSAFGPLSVCSRTCGGGTSLSKRTCTSPSPKYGGKSCSGSRYRIVACN
TRICPIHGGFTAWSNYGVCSEKCGGGVQTATRSCTNPRPLFGGKACVGSSRKSRICNSQPCPVNG
GFSDWSAYGKCSKSCGSGIQVSSRTCTNPAPAHGGKSCAGPLQQSRVCNTLPCPVNGNWGSFGR
FEECSVSCGGGVQKRFRPCNNPSPAHGGKACRGLSFEIQACNTKHCPIDGKYGSWSSYSKCSVTC
GGGTQVKTRKCNSPAPAYGGKNCVGVASHIQACSTHHCPVNGGLTSWSSFDECSEECGGGIQER
SRSCTNPAPAHGGKSCVGALVDSRKCNTHKCPVDGGFTKWSSYGSCSKSCGGGSQTSTRSCTLPK
PAYGGDACVGPTIKTRGCNSQACPVNGNWGGFGVYGTCSKSCGGGTQKRYRRCNNPAAAHGG
KQCSGLSYQIKACNTHHCPVNGNWGSWGSFATCSKPCGKGVQKRVRKCNNPSAAYNGKACPG
LDYQLQACNTHHCPVNGNWGAFGPYEACSKSCGGGTQKRHRRCNSPSPAYRGKTCSGSSYQQR
SCNTQHCPVHGKWGTWGSYGACSARCGGGIQKRYRSCNKPSAAYGGKACPGASYNIKSCNTQ
HCPVNGNWGSWGSYTTCTKSCGGGTQKKSRSCNNPAAKYGGKACSGSNYYLKTCNTHHCPVN

GNWGSWGSYTACTKSCGGGTQKKYRTCNSPTAKYGGKACSGSNYYLKTCNTHHCPVNGNWGS
WGSYTACTKSCGGGTQKKYRTCNSPTAKYGGKACSGSNYYLKTCNTHH
>TRINITY_DN598_c1_g1_i2.p1 type:5prime_partial len:742 gc:universal TRINITY_DN598_c1_g1_i2:2-2227(+)
RWSGCSKSCGVGVKTKSRECDNPRPSGGGRYCTGERTATEACKIQDCGVDGKFSHWSTWTACSK
TCGGGAQKRARQCNSPPPSGDGKDCAGAREEFKGCSNDFCKGPTGVAKAQEIIERFLENFHKKT
YPDREPSGKFLKAIFAIQKAVAEEFISVDPSVRGKTLEQRLKDLEKFLKVATEGGSIDFARTLLGKGIF
TFMKKKIQEGLPIDDIDKAAEGFLEKLRGDVGEKKMNEILSSQGDATLMFTIDTTGSMKDEINAA
KAIAKQIISATRDYEVDYILSPFNDPRTGPVTHRSSSNRASFVTDIDRLRATGGGDCPELSFTGMLN
ALNQGPQYGSPMFVFTDASAKDDSASNIDSLKAAADLTDATINFFTNLNGCKGAKSVESYKEIAS
YTGGQVFPLKNEAELKKFTNYVKTSLQQDTVITSGGTLNSKSYKRSLYSMQAHDFMVDDGIIMIS
VAISVQKYASQVTILDPNGRRVNPSTTLSLSLVYQISHPPPGQWKINFPSSVGKYGYTVQAVSSSAIA
FAHNFIYQQSVRKNSPPIPLANPLKGVKNMMVVRLGGTKNFDLNSIKIDLVDTSGNVLQKGIGTD
RLDSDEVRHATIDPPSGPFKVRLTGKTKSGYNFERVSQGISRPVSAAMRTNFAGYDFTAKLGKGPY
AAQMLFINRGAHENFYFYAKANYGSIPNYKQTKRVTSQGFVTFTYKAPRQYNLKGKSDRITVTVK
RQSTGEIVSHHTSILLVV*
>TRINITY_DN5001_c0_g1_i1.p1 type:complete len:1575 gc:universal TRINITY_DN5001_c0_g1_i1:40-4764(+)
MHAGLTSVFLTKRFISHFVLQKQIRSTFPVIQTFYRGFTSNMIEVNADGKNPASVVLLAVEHLKKH
EIQVTVKDNNKTFIKVSNTIVEGENDVSRFLARKYPEVGLYGKSGIESSQIDSWLDFSCLYIKGSQDL
LPSLKELNRAIGQADYLVGPKLTVADLSVWSALHGNSKWKNLLDSGKAGEPVSKWFKTCMQQG
TFKVPTESSPNKIKSKDKGSNPKNSKSNMEEGGKFIDLPGAKIGEVVVRFPPEASGYLHIGHAKAA
LLNQYYKDHYKGKLIMRFDDTNPAKENSHYEKIILEDIKLLEMTPDIYSRTSDYFEEMFVYAEQLIK
QGDAYIDDTPADLMKTLREQRLPSPKRESSIECNLKMFDEMKKGTPEGLKCCLRAKIDYKSDNGC
LRDPVFYRCRDEEHVQHGNKYKAYPTYDFACPIVDSVEGVTHAMRTTEYLDRDVQYAWVIEKLG
IRKVDIYSYSRLNLINTVLSKRKLTYLVDEGFVEGWDDPRFPTVRGVLRRGMTIEGLREFIAAQGSS
KSNVHMEWDKIWAFNKKVIDPIAPRYNALLKEEVVPVTVHGVEESTAQYPQHPQNPGSEKKTVY
YSNKVFVEGADANLMKEGDLVTFINWGNLRIAKVNKSDDKVTSLEANLELDNKDFKKTLKVTW
LAEHSEAPFIPTKCGFFENIIEKADMTREDDFKQHIRKDTKKVSDMMGDQCLANLKKGDIIQLQR
RGYFICDEPYQPPSRHSCKESPCILFHIPDGSTAGMMNQGKSKNTAGGKTSSSTKSSDNKNKGSSK
KQNKEKSPPADTGTIVEHTAVQQELFDSIVVQGDKIRNLKAQKATKDEITAEVATLKELKSKYKTS
TGKEFVNPTAATAAAPSKQPSKQPSKQQAASLSTSATVNHTPEQQALFDKIVEQGNKIRTLKAEK
ASKDQITSEVNVLKDLKSQYKSSTGSEFSNPSANNQSPTKKSKEKMAPVPVATPEAVTVESPEVEA
VKQKIVAQGDKIRELKSKKIEKAELQPEIDNLLKFKAEYKELTGSEYKPPGGGGNQKKGKEKKQN
EPKKAPEKKVTQQEAADGSAKKITRLGLEAKKEESLSDWYQQVITKAELIEYYDVSGCYILRPMAY
GIWERIKAFFDTRIKSIGVENCYFPMFVSQAALEKEKEHIADFAPEVAWVTKSGQSELAEPIAIRPTS
ETVMYPAYAKWIQSHRDLPLRLNQWCNIVRWEFKHPQPFLRTREFLWQEGHTAFATREEACEEV
HIILDWYEKVYTDLLAIPVVKGRKTEKEKFAGGDFTTTVEAFIAASGRAIQGATSHHLGQNFSKMF
DIVIEDPNNPDSKDKIHVHQNSWGLTTRTIGVMVMVHGDNKGLVIPPNVSNVQVVIVPVGVTAN
TSDDQRKLIKDTSLSYRDLLLDSGVRVKVDDRENYSPGWKFNHWELKGVPIRIELGPRDIEKNEYV
AVRRDTGEKISMKRESASTDLSNLLKTIQEDMYKSACKQLNDNLTVVKSWEEFLSMLDQNKLIQA
PFCGAIPCEEKIKKDSARDDGTADAGAPSMGAKSLCIPFNQPGKVDGLKCVCPCCSKDAVSYTLF
GRSY*
>TRINITY_DN785_c0_g1_i1.p1 type:complete len:289 gc:universal TRINITY_DN785_c0_g1_i1:1062-196(-)
MIIESKMLSSIMLKMIFVVVFLCGITLSEGIEKCDLQLEPIGCFKDDVWKRTMPEQILNERDPLNKV
YNNRMINWKKFDTYLPGFICRCAEAAFKKGYRVIGMQFWGECWSGPEAHLHFDSLGAADTCSN
QKFEREYDNITQCVSAIGMDSTNYVYRIAPTVCDTHYEPVGCFKDEQKVPRPMPHYISTDRDYSL
KIFSGKTIDWRNWNTYSPEMICRCATLAKSKGHALFSIQFWGECWSGPETNGYMRDGVTTDCRG
KDFEMCPCNGYHCVGKAYTNYVYRLGHHH*
>TRINITY_DN11097_c0_g1_i1.p1 type:complete len:730 gc:universal TRINITY_DN11097_c0_g1_i1:2348-159(-)

MVRPQGVTQEHSSKPSTSERNSFVFSALSKSGCFETLPSIYDSIIIQRSHWLHTEGFSMRTSSTGSQQ
TSRRAFYEELQAFSSTIPFYESLSWALLSLSGCLNPVSTPNIREEILADLEGRSVDESREVLLVFATLFD
VYKSYQNSIVVEISAILPGGILRCISLGQTEGFNSYNTVGLINYQPIVYPVGRNVLGRLFNVTGSCID
LYNEVTMDMPFVRSVSTEETHTPQKTTLIKTGANADTSSDSDCVKANALFKECTSHINVKETSLD
HLISLYCGCYMGMTSAPIAPISRLPIEAESSRAPEHMVQKLQECMGTYSGSADPSTPNQIGSKKQPF
WLSKEALESGVSPIHSTPLPISKLRVDLSLFETGIKVVDLLTPYKKGGKIGLFGGAGVGKTVVIMEFI
RNLAIEHKGISIFCGVGERTREGNDLYSEMAEAGIICKDYTTMKPSDPSSNGGPTGSIDQHSVTFTR
SSVTLVYGQMNESPGCRMRVAHAGLTVAEFFRDVFKQDVLVFVDNVFRFLQAGSELSTLLGRMPS
AVGYQPTLATEMGAFQERIIAALWGSITSIQAIYVPADDLTDPAPVTIFTHLDAVSSLSRELAAKGIY
PAVDPFNSTSRMLDPQCLTSEHFRVALEVQQILQRYRELQDVIAILGIEELSEEDLKTVERARKIERF
LSQPFSVAEVFTKIKGIFVPLEATIRSFAMVLSGALDDVPEGNFYMKGGLDTLKL*
>TRINITY_DN61_c0_g1_i1.p1 type:complete len:625 gc:universal TRINITY_DN61_c0_g1_i1:2929-1055(-)
MRAVILLCIAMCTMVYADVYFHNIRGSNNRLKGQNRNRDNAYRMFDSQNNDRGGYNVGPTPM
YFYTGSILTLEWTNQHQCGGPNANCDLILQYMCSENLRDGLEERTIPERNKDCKDDDCNTDIRY
GMHEDYDYYMNCKNRQRNPGLFLADQELEGTSAKYTRQNPDGDRYGYECPEERDHYPYWLPT
PWIDIAIMTRDLRRCNYYRENSQNNNAKYYCKPPQKYLDELKKRDPPSNLPRIPISQDDCERFHY
PANDKTDRGVWTRHDSWNVDPPECIAAPPSRDNHNGNGEDGYFNSYNFTIPNAPHKNCAIRM
RYNISTSDYDVNLDADGNGDPARAINIHDLVGIDTKKEATERGYVFKDYPKVYPMKGEIGKKLGL
ALAINTAQYFRTFEDRSHRTSFKARTPELEGVEIRNLNVKGKRGNIVQVYPAVEYGFVPDRMLAE
VGDMIHIQWTGSDRNPRNNDGEGPAGEDRSNIAELDQPRVAQVVADYNEYGDLTRSYPAHLDN
ATFLGLSRDELISLALAKGDCEFDRQGARSRTPYFDMGPVALTKKGIYNYLCTINNNFTNRSQKGT
AIITEKSKAGGGSKATASASKSASFRSVNGKDQEKRKYHAYNYFEQYKKK*
>TRINITY_DN2028_c0_g1_i1.p1 type:complete len:274 gc:universal TRINITY_DN2028_c0_g1_i1:1608-787(-)
MGQSDGDDLTFGQKISREWRDFREFLYNSETGKVMGRSGTSWAKIGGFYLLFYGFLAGFFTALLFI
FYTTIDKPGIGGPKLIQYIENKPGLTRLDRSSKLQSYSASNNDRISSYKTVIDEFLDGYNTSDHSGAL
CGAAGTPKSDKPCIFDTTLLGECAPSKNTTYGLREKKPCIFIKINKVFGWTPADDGTGYLTLSCTG
HATVMPQGFPLTAFPYNGHKVYQSPIVAVKIDATKGKKNVDCTLKGKNIEVSNNFNPSRAFGKIR
IEGISATD*
>TRINITY_DN26249_c0_g1_i1.p1 type:5prime_partial len:477 gc:universal TRINITY_DN26249_c0_g1_i1:1733-303(-)
QMKMAASNMAGHVVRNNLIFLLSFVIIIVAEEGRHARFEYKFSFKGPHLANKQGTVPFWNHYGS
AIPSDDQVRITPSMRDQRGGLWTKTKSSSEFWEVEIFFRITGRGRVGGDGLAMWFTEEAGIQGPVF
GSSDHWKGMGLFFDSFDNDGEQNNPYVMVMLNDGTKQYDHFKDGNSQQLGGCMRDFRNRP
HPIRAKIRYYNNILTVFYHSGMSERSDDYELCMRAENVVLPKEGFFGITAATGGLADDHDVIKFLT
HSLTPPLTDEQKQKILSEEQRKKYEEQYGEYTQKLETKKEEFQKEHPDKKAPEEKFESVYDRDVRL
IYENQNTIHHVVRALHEKVGELTNQLNNLHVTINNNNQLAQQQQPKGDGVTRQEVNTIINLQN
QVHNAIQELKQQSGGGGGDNTKTVQMEKTLQTVLEQVGHLVSKNNDRRPSCPTVSCVTPSILILF
LVAQVLLVAGYNFYKEHQNKAAKKFY*
>TRINITY_DN17117_c0_g1_i1.p1 type:5prime_partial len:345 gc:universal TRINITY_DN17117_c0_g1_i1:1183-149(-)
KCYKHYKVFTKMNLKVILYLFALYLVSLYAQIDLSECNGIPGACELSLNQVTLPGAHNAGAGFDG
VLYYHGLYAAAASCWYRNQGENIYDLLNDGVRYFDIDLCYEERSGYEKGAWACHQDAYGGPMS
KFLGQVDKWINEDDNRNDVVVLHFNRDHEAGDEVQKKIGKSIIKLLEDLWKPSSGNIRAGNVV
MQSHLYAKLKNSVQENKRIYVFLHHRLQTTRRSYLFHENKVGYTWKSMGFVGSNKCKQLADDIS
DECPKQTGRPFVRLDLYLTWGLCVHDLAGYCNDKIEYTANRCYEKIENASKRIGETVNFIVIDYAD
SAAGKKQVEVAKKLQCLILKNLG*
>TRINITY_DN17358_c0_g1_i1.p1 type:3prime_partial len:125 gc:universal TRINITY_DN17358_c0_g1_i1:42-413(+)
MSSFLVVPLKKTASVDFIKPLSNYIKNTFSGDVLNEVKDSLAELNQLRANAVVRSLDKHETSLEVL
QRYHDQLSTMEAKLPITEDQIRLSLTWYDAFDKGSIFGSTKCALSSGAYERFGVLFNI

>TRINITY_DN7299_c0_g1_i1.p2 type:5prime_partial len:345 gc:universal TRINITY_DN7299_c0_g1_i1:1201-167(-)
WRLGGGGAAYAVKGAWQGEPTALRGAVKGDFGTVVAGLEPAVAFPPVRYSREGVSLSVEDGHL
NAEYAAKLDEDTMFNLRVDDEKAWVASLAGHDASLRVRGEGADLDRLSWDASQESSVEDVGD
VRVEFNSDKAYNLTVTRESLATVAGAELDAKVRATNAGVTGRFGLRRQLPRGAALTYTVENPVG
VYDLGKSTHIGRLSAPVAGGEAALRVEGDASAQDYEGSYRRGLAGGQADLRLSHKDGALGYNV
SYARGLGEALPVDAAAQVGVDEEGAYTRLTAGRSLGHGLAARYEALARLGFGEEAQQHFRQAV
RLSNKLGHAELSHGGGEGARLRVGYEFNA*
>TRINITY_DN5500_c0_g1_i2.p1 type:3prime_partial len:1183 gc:universal TRINITY_DN5500_c0_g1_i2:3546-1(-)
MEIKNFLILLSLLSLTSQLKAGLVFNETGVDQPVDLFFVIDGSGSVGYELFDGQLQFVTELTDQLGG
VSPFNVHVAIAVITTSPYQAIGLTQYQVNSEFKQAVSEIFYPRGATRIDKALLAVERELLPTSRAGVP
KVLVILTDGIQARWPGVYNTHVIIERLFNAGVSPVVVGIGSDYRDLDRLNKLAIKENHHLNEPIEK
LFLTTDDMEVGSPALVQKAASTVKKILSNVARFGYEVRSDFAFLLDGSTYAEDQLKLMEGLASYFQ
INRELSRFAVASSQGGGRTYIEFNEAQLSRDFKAMLMKLNVDSESIDIGIAAKYAYEEIFRIEEGERYS
TPRTLVIFSGNAEAKDSTLFGKYYSLLKKANVNLVMIAVGGGKGRSNLRNFPVDYYLPDFNDEVS
FTEECNIIAREMHNYIIRFASDTQRSKKCLHDGDLFFSIDVSGSAGAYYQENLNIIEKIIASFEISNED
SRVGFAIDRDGNTQVTPLDKHNSLESLMRDFDSLPYYPRGRSDMVAKLKAAYNQFLTSSRIHFQDI
LFIFTDGYLNRKSESESKELIDKLKAFGVEVFVVSLGYDVNFPFLMTLSSDKGHFFHVNQLPKDSDS
FVNSIINKVCDYTETFQDKYEIPINVALVLDGSHCSEKFHEAQKTFSNVIIDGLDNSPLAKNYLFSYS
SGVQQLDSNSSLAINFGSDGNRLDLALNEVVKLFQSISSNEVNMAIFVTNAGLESEFNATMSQAR
KLRSLGVITMVICIGDNFNYDVIRTLASSRQLIYMIPSPSQLTDTKFLRHIISAAFEMAESMPPPRLEA
GKSWYPAFFGNWVDSNSLEGEVPDAPLKAYHFGPTVLWSKPIQQKNYIIESTIIPAGSESGRFGTCG
HIFKHKVGATKKEKYISKSWGYFLWSNSFLYITTDGTETSPRIEFDYSSRDKLNLKTIVDGSKVKIYI
DGVFIRSIELEGTEADSQSHMFVGVWCHRLSAVTVEDFKVSALSNPCTVNNGGCSQICSADPYEF
KCSCIPGYRLVEDESTCVAESTCPELITTTVITTTLSSSPPTTNQSKFISTTTKPDELIDTTATIISTRESDI
TTTLILSTKSTEKPIEKIEKTTTSAIGDILEQTTVAPIKTSTVFVPTTKEEVILTTKEMAETTLTVPTTASP
VEETTTVAPVSTAAKTEATTQAVITTLAATTK
>TRINITY_DN61707_c0_g1_i1.p1 type:3prime_partial len:417 gc:universal TRINITY_DN61707_c0_g1_i1:1250-3(-)
MNVIAAIIRIAILALVFQHEAEGKVYYIRTDADSGRCTYQGYIPGGKEINISPIKAPTAQLETNVLA
NIAGFGGKAGTGVMHLSKFTETIGKIAPKLGPALGVFGVAFGVVADFVKPSPADILAATNEAIGKL
TEEVNDRFTDMKSYVDLELMNVVKERINVEYKHMFTMFSNCIKDVSLERVLECQEDANQYITSGS
GKFMLFNMDDYSDSSPPTANEIKKMELTFLPFREYVSLNLMQLQVLTDTYKDLQNSVKDGPLIYR
KHLSELSEEAAKLIVYARNMFKWIDKTYDEFSRQRCKETKTYWSRCTTEGSWSGNTACYATAQCQ
FDITIPDNKKCKTKCTVRVDGKKIYDRYDFKNKNYGYEEAGQLFAGGKLDKKASSYLNQQAADV
KNYWQAEVLSSIPVWEEMKKRADD
>TRINITY_DN1668_c0_g1_i1.p1 type:complete len:220 gc:universal TRINITY_DN1668_c0_g1_i1:748-89(-)
MTGMMWKVIAIAAFVGMAYTDDLNPLTYLPDGVETSHEAGESLSNMTITNETIVQEKTKGFGFL
DMTSNAQYSKGLRGSYLPRYLSRTLKPFAAISANMSKVVDGLQEYIKDLIGKDHEDHFFALVGKE
QYGQTRAIFLKFNQREDGRYNLKKLSFRGTFKLAADLVITRYTNQETGEINDVVSYVPKEKVSKD
DVKDLLNLMVPQLAEVMKGFAPKEL*
>TRINITY_DN97809_c0_g1_i1.p1 type:internal len:184 gc:universal TRINITY_DN97809_c0_g1_i1:2-550(+)
LGPDQVDVAGAGRDPRHLPRPLGRRLRVLDRHRGRVRQRHKAEADQDPKLETARLDVRRPGVQ
VRRVRHPRPSSRDQCHRDCHAPGRVEGRAQVDRGRRGLGRRRVEHEVHGAAQPAARGQEKGR
LRAQQILAVAAGQEEAVDVGARNRQGPPSALAAVSRAPHAQFARLAQRDRRVARAKGW
>TRINITY_DN773_c0_g1_i1.p1 type:complete len:440 gc:universal TRINITY_DN773_c0_g1_i1:1608-289(-)
MVSPPGPQDNKPPHKVADMSLADLGRRYIEMSENEMPGLMSMRKQYGEKKPLKGARVAGCLH
MTIQTAVLIETLTELGAEVQWSSCNIFSTQDHAAAAIAKTGVPVYAWKGETDEEYLWCIEQTLIFP

GGEPLNMILDDGGDLTNLVHEKYPELMKDIRGVSEETTTGVHNLYKMFKEGRLKCPAINVNDSV
TKSKFDNLYGCRESLVDGIKRATDVMIAGKICVVAGYGDVGKGCAEALRAFGARVMITEIDPINA
LQAAMAGYEVTTMDEACSKARMFVTSTGCSGIIEPHHFQQMPEDAIVCNIGHFDCEIDAKWLN
DNCERETVKPQVDRYTLPNGRHIILLAEGRLVNLGCAHGHPSFVMSNSFTNQVLAQIELWQNHK
NYEIGVVMLPKRLDEEVARLHLDHLSVKLTKLSKSQADYLNLPADGPFKPDHYRY*
>TRINITY_DN5633_c0_g1_i1.p1 type:complete len:848 gc:universal TRINITY_DN5633_c0_g1_i1:2881-338(-)
MRRSNFLTIGIIAFFLIFSYVSADETSDEEVEPPKVDDDVGKSRDASKTDDEVVQREEEAINIDGLSV
AEMKQMRESAEKHAFQAEVSRMMKLIINSLYKNKEIFLRELISNSSDALDKIRFLSLTDTEALAAT
DELSVKIKADKENNVLHITDTGIGMTKDDLIKQLGTIAKSGTSEFFQKIQDAGTTDQASDLIGQFG
VGFYSAFLVADRVVVTSKNNDDKQHIWESDSVEFSISEDPRGDTLLRGTQISLYLKEEASDFLEPNT
LKDLIKKYSQFINFNIYLWSSKTEEVEEPIEEEPEKKEDEKKDDDKKEDADDKKDEEKKDDDEDD
AKVEEDKEEEKPKTKKVEKTVWDWELMNSSKPIWLRKPKEVTDDEYNEFYKAISKESDGPLTKT
HFVAEGEVTFKSVLFVPKNSPSDMFQDYGKRRESIKLYVRRVFITDDFQDMMPKYLSFIKGIVDSD
DLPLNVSREQLQQHKLLKVIKKKLVRKALDMIKKIPDEDYIEKFWKEFSTNIKLGVMEDHSNRTR
LAKLLRFRSSNDPEKLTSLTDYVERMKEKQETIFFMAGASLDEVKNSPFVERLLKKGYEVLYLIEPV
DEYCIQSLPEFDGKKFQNVAKEGLKIGNEAKEKARVDELEKEYEPLLKWLKEDALKDLIEKAAVSS
RLTESPCALVANSYGWSGNMERIMNSQAYAKSKDAGANFYATQKKLLEINVYHPLIQELQKRVKT
DKEDQTAKDLARVLFETATMRSGYTVKDSSDFAGRIERMLRLSMGVDVDAKVELPEEDDEEKEEE
KADDEGEEVNADGEEEESKEDIPEIKVDSKTVEPVAEHPTNEDAVENEAPAEGKTEL*
>TRINITY_DN775_c0_g1_i1.p1 type:complete len:635 gc:universal TRINITY_DN775_c0_g1_i1:1927-23(-)
MKQMIFLLAVSLAILNFDGHVVDAITCKSNSVWSWECQNSRCVKVLASSNSDAIELNTCKLTCGK
YSMLFPKPSGEIQLGKKTVPFIPTQARLRSVKVKKSNPKATWLVQNAYSLFLNTLEHSYKRYSGSTS
RLYNNMCAQEAQKRVLVMDVILEDGDTKITLDTDESYDLTILADDKEITVSINAQNFFGARHAME
TVSQLAAFSESYNAMQIVSSAQVKDDKPSYAYRGLLLDTSRNYFSVDSILRLIRAMSYNKMNTLH
WHITDTHSFPIEIKSLPEMLRYGAYSSKRIYTHDDVRKIVSYASINGIRVLPEFDEPAHCGEGWQWG
PKAGLGNLAVCVNRQPWQKYCVEPPCGQLNPTNDNVYDVLGKIYKEYFQLFDPDIFHAGGDEI
NFNCWNTTTEITNWLKQNYGGVSEKNFMDMWGMFLDKSSSKIYEANNNKELPMILWTSKMTS
EEYLTQYMDPKKHIVQIWTASKDKELSNVIESGFRTILSTYDTLYLDCGYGNWLVEGNNWCSPYK
DWKLLYDNDPVKILESFNVTVTDKIRSTILGQEAAMWSEQVDEFSSEGKIWPRTAALAERLWTNP
NHNWRDAEYRLIYHRERMVERGVQADALQPLWCLQNAGNCYYDPAIKGEH*
>TRINITY_DN71243_c0_g1_i1.p1 type:internal len:422 gc:universal TRINITY_DN71243_c0_g1_i1:1265-3(-)
PFVGIRIDSNTGSVFLAQHLDYETAQQHNVSIFAVEKDPPTRYVSSYFLINVLNVNEHSPQFQNNE
TLVITVNEDIQPGVIGSSPATDKDAGVFGNIDYKIIRFDDQLFDVTASGEIRLIGKLDYEKKRVHQFL
VEALDGGQPRRKSLRPVSVIVGDMNDNVPLFSQDVYNVSISDLTAIGSSLIKLSASDADDGLNGEII
YDISNGNFGTTFIIDKSTGVISLSRKQLLSELRKFTLKVSTRDKSPISPRNGETATVHVNFIEASANEP
FFSTTFQKLNVLENQAAGIKIGQSKVINSKMEAVYLIVSGSKTRITDYFGINYRTGEITAKQSFDRES
RDHYTFVVSARFIDDNSLTGYQTVDVSIIDENDNRPMFLGNISQTVSISSSFDGSRPVALLNAFDKD
LVSLDKIEMQLLNSQE
>TRINITY_DN33065_c0_g1_i1.p1 type:3prime_partial len:663 gc:universal TRINITY_DN33065_c0_g1_i1:1986-1(-)
MWWFVWLAVAITINVDGKKASKHKSLLEDVDLDEDFDLDYSHNIFIPLDAYENYEKFLEYAQGT
DLAKRSGVSPLPVIQSLPADGVHTYSAPGINCQVELLNKEVEGTDKRVIERKTCPEGWTLKDAMK
YFFADVGQDLQSDSAGLVHIEQLKFKMLVFEPSGGFSFVADTDGEDYSNLQSKLSLSNVDFKLTM
GASYSYESSEISITGNHEVIERSTNEAKQIEVTFEKGKDTSNFVVKMNIPSMKLETFATVFLASETEF
VTSEIGDVTFNINIDPIKAVTIESTSNSVNGVFDTVEGKFQLTAKFTSDDTNAFDGEATDIYLTVNR
NSLGAPTHYNLMLGYAKTINVMDVVRKYIGKPEIQEQFDDKFLKNTIEKWYLMFTSSRIKLAADD
DYQNMFSDIFTDDDYKEFKKGVGIKAPVNLQETLTACGGGTVPDTLSDIKINGLLTKSGLSLKFPS
NTAVPLFALMKCKLRKIQSDLGLDIPDYLKKIEEATNFKLEEFSYDHESGEFKMSLLYSNTITIYKDV
LEITNPQLTIEREGTDEPWQFTIAADIMLFGVEASVSVQYSKTGFTVEVSSDVIPLSKLLTAFSFGIEP

PFTFEIHDFKMQLVVSDKEEEEGEEGEKTQEGEEGEKGEEGGGGGPGGGPGGGGRGPGGGRPRR
G
>TRINITY_DN598_c1_g1_i1.p1 type:5prime_partial len:745 gc:universal TRINITY_DN598_c1_g1_i1:2-2236(+)
RWSGCSKSCGVGVKTKSRECDNPRPSGGGRYCTGERTATEACKIQDCGVDGKFSHWSTWTACSK
TCGGGAQKRARQCNSPPPSGDGKDCAGAREEFKGCSNDFCKGPTGVAKAQEIIERFLENFHKKT
YPDREPSGKFLKAIFAIQKAVAEEFISVDPYVRSKTLEQRLKDLENFLKVATEGGSIDFARTLLGKGV
FTFMKQKIKDGLPIDDIDKAAEGFLEKLRGDLGDKKMDEILSSQGDATLMFTIDTTGSMKQEINA
AKAIAKQIISATRDYEVDYILSPFSDPGTGPVTHRSASERASFVSDIDRLRARGGGDCPELSFGGML
NALNNDPQYGSPMFVFTDATAKDDTDANVASLKAAADATDATINFFTNLAGCNGRRDRKRGIE
SYKEIASYTGGQVFPLKNEAELIKFTSYVKTSLQQDTVITSGGTLNSKSKRSLYSMQARHFMVDDGI
SKLVISVGVQQLASQVALVDPNGKKVYPSTTLALSLIYQVSHPSPGQWKLDFPTSVGKYSYNVQAV
SSSAIAFAHNFIYQQSVRKNSPPIPLANPLKGVKNMMVVRLGGTKNFDLNSIKIDLVDTSGNVLQ
KGIGTDRLDSDEVRHATIDPPSGPFKVRLTGKTKSGYNFERVSQGISRPVSAAMRTNFAGYDFTAK
LGKGPYAAQMLFINRGAHENFYFYAKANYGSIPNYKQTKRVTSQGFVTFTYKAPRQYNLKGKSD
RITVTVKRQSTGEIVSHHTSILLVV*
>TRINITY_DN128406_c0_g1_i1.p1 type:5prime_partial len:150 gc:universal TRINITY_DN128406_c0_g1_i1:474-25(-)
FGSTRKMSQTQSVQVFGRKKTATAVAHCKRGNGLLKINGCPLHLVEPSTLRYKVQEPILLLGKERF
EGVDIRVRVKGGGVVSRIYAIRQAIAKSLVAYYQKYVDEVSKKEIRDILVQYDRSLLVADPRRSEPKK
FGGPGARARYQKSYR*
>TRINITY_DN564_c0_g1_i1.p1 type:complete len:563 gc:universal TRINITY_DN564_c0_g1_i1:2018-330(-)
MKLLLFCAATLLVAGCVLADGVATQKKCEDFLPVDTCNQLRVIAQKFNEKAGAVNKAITDAFNK
HKTKAGEVLSYVKEYLVANAKNFSCASVLSTQQCQQLKDFAKLMHAKAGEASIAVREAIVNGA
KNSKVQFQKAMEFLKNDVSCEKILGLEQCEKLLESAKTLKENIKTVHQALVAALQKHLSKTKDIL
KSVRETLVEKAKNFNCTDVLTLEQCNRIKNIGQRLKLKSSEVQKAVKEAIANGAEKAKQIFSNAM
DFLLIDARNMTCEDLIKAETCQKIKDFAKKVHARASDVALAVREAIVQGAQSTQDTLKKAIEFLK
AEISCKDVFSIDTCDKIRKLADSFHVSLKTVNEAMREAIANGVTKVSDLYKAAVKYITEKWGGIFG
DEPQIEKRSVDEKLTNIGELLKQKITKMAMDLLDMLKVTNEKIREKVKELIENGKIKITNIKEKVN
DLLALVNSNDEDTMNDVNMIYKRSTEDSLVDLLKSIKGKSQEFIEKLLKLSKDKLEQIKEWIKQLK
QTDDSIPSDEMEDDLDLVHDVLKDGVNMAFADIVEDTIKNIE*
>TRINITY_DN14015_c0_g1_i3.p1 type:5prime_partial len:2566 gc:universal TRINITY_DN14015_c0_g1_i3:2-7699(+)
PNEGVTIVFSNGDYELKKDYYAMIEAKSNGTLYKGKTVVHVTLTDKNDNPPRHQNLQVDIAISES
TKPGTIVTILYADDADDKQNGEIFFMLESASSDFSISSFNNTGVIRLSKELDNEQETSYKLMILLKDF
GSPSSKFSNMATVNIDVIDENDNTPLSEKTFDTIQVSRLKANGVIYQVKFTDADSGLNGQIEYDIEP
FKSFDDYFTINKTTGEVSLIKSLETTTEDNFWLFIIATDKGNPRRKGSFTLQVNVNEPSSLNPIFNPD
TYDIEIYENRLPNSLVARINVLNVDPNKDSLTFKIVSGNDGDRFLITNKGDLVLKQNVDFEQQNLY
NLVLSVRKNQNSVESVNQATVKIKVLDVNDNNPLFEKTDYTFIVTENTPINTFIGGVSAVDQDFGE
NSKVSYTIFDTTKTFIIDSSTGRITVVGQLNSFVENEYNFVVYAFDNGNPRRASSANVKVIVEDINE
APQFSPPSYNLNVSELLHPGDHIITPKVIDNDIGINSQVRLTLDASGNIDDTFLLENGKLKLNKQLD
FETRSTYDLTLVATDSGRLPLSTNAKVKINVMNVNEHRPIFNINPYMTEINEDQFSTPIFQVKASDK
DALSKVRYVIEDGLDYSLFSINQITGEITNSRLNANFKKEYSIQIKAFDLDNPQFYSHTVLVVKVIDI
NDNAPIFNKAQYSFTISDQTPVGTIIGKVFANDADTGFFGQVTYFLDNNFNGPFSLSGNDILINGD
LDFTKVSSYEFVIKARDSPGSSQGFRTTSTNISVNIVETNGTMPIFSREYYEVTIFSNISTQYTVVKVSA
TDNDGNEFGEIKYSLISDSSNLFQVDELIGIVSLKDGVQLQTRSFKYTNTLQVRATDGAGLFSTAYL
VIHVIPVSGNNPPVFLQIQYLSRVSYLTSKGESIVSVIATDPEGAAVSYEIKSGNELKKFGITNSGIIYL
EDQVENPEVNVYRLGIEAIDENGLRSDPDAQVIIQILYFNSTFQCKLIPTFSSNLYISQVEENKAPTII
TSVQANGSSQNDSIAYSIAYTKDVTQNDFEINSTNGVITSQRTFNYEMKNSYQFEVIAKDTNSLKE
GRTIIMVNIIDVNESPVFESSNIVRSLSQSSPVGTFVGLAETSDADKNDLITFSFVPANIGNIFQVDA
NGIIRTKSSLNAADSPYNFTLIASDKVGLTANKNIKVTVIKTNVPEFSQNAYLFSISESLNSVRIGQLS

TKQILEPVGYRIPNNNYNNLFIISDGGIISSSKGIDYESDDRKYNLCVEVFLQSNPSLFDTCDVTININ
DINDNRPVNKKDGQYTVEVSENVMVGKMIITLDVEDIDSGEKGFLNYLITSGNNGNEFRINNKG
EIYTLKPLDYEVKRSYSLTINVQDNGVPVKTIEAPLTVVINLVNENDNNPIFNPSSYTISISEQQALTT
ILKVTASDKDALIKSTYSILENEDTFSIDANGNINLVKAVDFEISSKYQFTVVANDGSRSGYAIVTVDI
TNINEKPSFGQNYAIEVSESTPVDTVILTVSATDVDIGVEGDLEYSIESGNDENTFSIQSNGQIKLLEE
LDYETKTEYTLSIKATDKGQPQLSATTIVNIKVRNINDNKPVFSKQKYELTVKENSQLSLTVSVTDA
DNLGNMKFSIPSSKAAGMFKIEINTLNTNEAVVSNIKPLDRETGAGHQFLIKVEDDVGNQGLSEF
VIVVEDENDNNPIFTSEEYNVTLFSKLNVNDAVVTVTATDADATYGVVSYAIISGNNDNMFRVNT
KGEILINKQNYDSRANYVLTIEASDNFETPKKSLTNTNVKIQIQSSGLTFEFAQDQYKYTKSEINIGD
TFGPITGTSSTSLDQIVYEIKDSSGTFEVTSNRNIKVLKKVTTDQNFCVAATTKNGGTLLDLTQIHIL
LAKTNENRPIFQFTNPKVVKISQNTLVNSVVAVTTASDSDTGSTITYSITEGGEFFNITNEGAITLKN
TLQTSTVNQFNVTIIANDGTFNSAPANVVINVKKENNFNPKLSFSSLNLSITEGNKVSQVLTDTDT
DADGDQLNFKIVGKTANNYFIINNTVLESKSPLDYEQVRSYEFCIEIKDSIDTNSRSVILPVSISVTNI
NDSNPVFTSKSNEVEVPYTWPPDITIIEASAEDSEGDRIEFSIVNSSFSSLFSIGKTSGVIKLNQRLKEA
KVYNLTVTATGVQSGKSSNDNFILTFTAVEAMSQTMPITFSDVTDNTMKVTLNAKEEFGEADYEK
MNTIYVYAQEYLPNLDEFVTRTLMEPVSYYDANTFESFRGRRFMIAKVDMERPTQRRKRRDTSLD
EKNGILTILIGNNQCANKYDPTIPCNGPLKAGQSYRYQLEGDLLNKRKLSSFTEPKTTTSAAQSVA
QFSASNYTFNIYYIIGLVMLGLLLAVSIWVIAVLLLYKRRGTKLMQLRESMLPPAINNAQTIANGKF
GSNARRYTNPVYGDYDDDRNFTSDFNLPRGQNNQELAQLKQQIIPIPNQNNGRANYNNRSEMV
NRVPRHFDNPGYREEERKPTYTATVMSSPTVPNPPPPPPPPPMENTRLPSSGTARSTNQPRLRRTSS
GRVIVQHLSGHQ*
>TRINITY_DN4781_c0_g1_i3.p3 type:complete len:183 gc:universal TRINITY_DN4781_c0_g1_i3:2965-2417(-)
MCTEPKAPSSCANDEFQCTKTKQCIKKHMLNLTAYTKVGSKMTKLWTKSGTYGPRWRHATVDIS
SASTFKVVIEGTASSGYMGDIALDDLFLNNGACPPSGECSFEDPKLCGWTNMNGDNFDWTRDN
AGTSSSGTGPRFDHTYGTAQGFYVYIEASNPRSKGHKAWLKSGEYKPTLGRCLSV*
>TRINITY_DN3052_c0_g1_i1.p1 type:complete len:362 gc:universal TRINITY_DN3052_c0_g1_i1:45-1130(+)
MAHLSQEQRNELKKNAESIVAPGKGILAADESTGTVGKRFAGINVENNEENRRLYRQLLFTADKS
ISNYLGGIILFHETLYHKDDNGKPLVELIKELGINPGIKVDKGIVPLHGTNNEGTTQGLDGLAERC
KQYKQDGCTFAKFRCVIKINTPLGVPTDIAIQENAQVLARYANICQANGLVPIVEPEILIDGDHNL
ATSQRVTERVLACVYKTLVDHHVYLEGTLLKPNMVTPGQSCPEKASAEEVGVATVKALRRTVPA
AVPGITFLSGGQSEVEATKHLDAMNKTDLARPWALSFSYGRALQASVLKAWGGKKENVAEAQK
VLLHRAHLNSLASLGKYEGENASGATGDSLFVANHQY*
>TRINITY_DN5397_c0_g1_i1.p1 type:internal len:161 gc:universal TRINITY_DN5397_c0_g1_i1:2-481(+)
CSVVCDYSPWQDTQSCTKTCDGGVLIQSRTERSFNKVESREGSTPCNTQACGSGLSEERFRKCNA
NQCGTNTGLQEVYKVCNAANCRFQGIDYTRNQEINIRTQACTTACDYTPWQDTTACSKSCNGG
VLVQSRTERTFNKIETRDGTTPCNTQACTQEL
>TRINITY_DN247_c0_g1_i1.p1 type:complete len:373 gc:universal TRINITY_DN247_c0_g1_i1:38-1156(+)
MKLVLVIFVILVYTWNVSAGGKKCISNKDFMTPYPTNFWTEHYPKFVNNRPTIGIVAMEVLGQKM
LVNVPWSWNKDYFGSSFVKLVESAGGRAVPIKEDITEDDLDKLMRKLNGVVLPGGDADVGDSGY
ERISKQAIAFSKKMAKKNVTFPVLGICRGAQMMMMAEAGKDFLEVTDSLNLSIPLDFTSEQKESR
LFGNAPQGLIKSLSEDAITFNAHAMGIPTLNFYNNSGLQKAFRAISTNYDRNGTKFISTFEGRDAPI
YGLQWHPEKSLFVFNPVLAVDHSIKGVMASQYIANFFISEARKNNHTFRDRVEEEEHLLMSYYPS
YVGNITETPYEQIYLFDFVKPFQNPKYTPGKVNKNDDDNSGDDDDE*
>TRINITY_DN5153_c0_g1_i1.p1 type:complete len:862 gc:universal TRINITY_DN5153_c0_g1_i1:61-2646(+)
MSEKKEFHRLSRSVRPIHYNITLKPDLENFTFAGNEDIDLEIKKETKHIKVNSLDIKVSSAVLTVDSQ
DIEPSDIRYKEDEETVTFKFSNPIPVGNAKLSLKFTGELNDKMKGFYRSKYTSASGEEKHMAVTQFE
PTDARRAFPCWDEPAVKATFDITMIAPKDRVVLSNMDVKSEKDHAEDDSFKEVAFNTTPIMSTYL

VAFVIGEFDFIEETLENGVLARVYTPLGKTEQGTFALKITSKTLPFYEEYFGIKYPLPKIDLIAIPDFAA
GAMENWGLVTYRETALLVDEKESSASSKQWVAIVVGHELAHQWFGNLTTMEWWTHLWLNEGF
ASWIEYLCVDYCCPEYDIWTQFVTNDFTRALELDALSNSHPIEVKVGHPSEVDEIFDAISYSKGSCV
IRMLHDFVGVGDFKRGLNSYLTTYKYANGETEDLWYHLEKASGKPVANVMATWTKQMGYPVLS
VSAEYVDTQCILKISQKKFTADGSADPNDQRWAIPINIMTNDNPKFSTLMTDPEMVLFMENEPES
NYIKLNPGQIGFYRTCYSSDLITKLVPVINSLSPVDRLGLESDLFALSIAGVSKTVDFLNILKGYKDE
MNYTVWTDIDSNISSLGVILQNTDNFELFKEYVIKLYKPISEALGWEPKDDDDHLTALLRNLILRRL
GNYGDADTLAEGQRKFKAHVDGTELVNADIRSAVYSMVSKHGDEETVDQFINLYRNTTHSEEKT
RILTYIGASYKSEVIEKSLKFSISDDVRSQDTVFALCSSTGSLNGRRLTWNFVKENWDDLFKRYQGG
FLLSRLISRTISNFATVEEANDVKTFFEEHKAPAAERCIKQGIESIQLNNAWLTRDGESINQWLFQE
N*

Band 7
>TRINITY_DN982_c0_g1_i3.p1 type:complete len:9852 gc:universal TRINITY_DN982_c0_g1_i3:29697-142(-)
MSIKLCTFFVFFLIQCINSFPEIGLHLSLDRSYSFQGNGWKTVNGWEIKPRANGPYEIDAIKNISPEY
FPVPITGVYYITVSLPVEGASNGEFKSAIIVDGKNEEGRIGLMSSFQKTAFKNGASTLTFAGFVRLFA
DEKLTIEIYSDDINANYVVLESATFVFHFIGVTGSVPAYLAHTAQDQEYASDSIIKPWVTLGDSRVYE
SLSGFIKNEGVFTTICNGVHKMAVNIMIKANAGTYTLKALFQDEESATATVTINQPDSIATLNIFQN
FKWSEGMSITLQIHSSNGANFKVLKSSSWSMSFIGETTANFQEFSVFLMADQFLQPSQFPQRINIDL
NGVFQFLQSNPDQYNKGQLVSGADTKFKDNLDFIVDSTDLFYVSANIIIEGEAEDLEITIAVSQIDID
VLPEDGLSGRLTKISNSRRSISLSGIIFIREKQYLSFFVRSRDSSKSFTVSKDSFISVINMRYTVSSVSARQ
NENIDVTAGSWYDISGRFAEQKNGLYSFGKDFNINNGVFRTSHSGIHSVHANIQFSLDSDAFGDN
DDNKIVLRVVIDGQEDKISLMSIRKNPEKTATLKLFGSIYLRAGVTISFQVKSLMPNTVQYKILQRSS
ISISYIGPQWAVNSFHAVANQDLNYDGKPDDTRPDIPSPLNFWKTEAADEFISPFEVLESFDGTRYT
SVDDGLYVVSSLLILKNVDCDRSALFKLDFVTTGGSRASDKSNGFADSHFFIADSKGTEFTLSVTTA
MRLEVGFKVGLKLTVGSTSCRYTISKESSFSIAQWSKSSNTIEENRLKYIGVLARLSAPTEVSSPNTW
SNLGSNTMDINPGDKSRSSFRPGIFGGDDLAPITRTGKDFNGDVSDFTIKDPGIFFISGMITVTTTEN
ANEEFAATVQIDSEDIESGLYAAVINQGSTEMSLTFGGTIYLKSGARVRVVVKGGRTFNIAAGSSFSL
LKLQPDYKTPGIIVSIDEKITQAITRYEHKDLRADIHRTLTIGNLGFVESTGVYTIQVDGVYMVDYT
VILEATNPNAVFGAEILSDNTVVATGTSTFGNTKTIAITELVYFSKGAIVKAVLKSNTNGETLNNLQ
VDGGLTVYFLEPKEHVKDGHRSKQTSPQALPVRSFEKVTGFAAQSPDFAGGADSVDYTVPETGVY
LVAFTTRVNRVINGFKSRVLVNDNEFLNGNYQIVSAQNAPATLPLSIAMELTVNDVLSFQVMADG
SGMSLDGSTRSILKIDEVNTIRNVTEGLSAYKLRDQKVNGQQETIVEEWDTSVTEGTFLAKNVSFT
VDHSIVVVAAGVFQISVSVVVKNDRPSQSKFELRVLQGDDVKSHGLHSKVEIGGGREQTLTIGGSL
LLQKFDSLRIVVRDIADPLASYTIKAGSTVSVIIIDDNNGPIACEDEGLNMRRLPERIRKPTRESHTF
QCNVVSSLKAEKYTWFKDNVQLKEENQKNLVLNDLKLSDSGVYQCRVRFLELTGATNNATLSVY
DPYPKITSPKFIANVLENQDPPLTIEMFSLSAFKLSPEDEKASITTQIIAGDDQSFFTLSGPSKQTVTLS
TTKKLDYETQNEYNLTIKATNLEETALTTKGPYETTINYIVRVIDVNDHPPVFSQSIYSRSVPEDYPI
DGSIAKVSANDQDAGANVIYTLVGQVSGDLFKIDNTGLIQLRQNLDREIRDDHTIFVQASDGLFT
TVAEVVVTVTDVNDNFPNFMDTGFDISVSEATLTQSVILKVIATDPDLGTNSQLRYTIKSGNNENK
FSLNLLDGNLTLISKLDYEGTKIYNLEIDVQDSASPPKKSQNPLTVVIRVQDANDNNPTFTQSLYNT
RIAENTAVGTSVLKVSATDRDGEGINNPLTFSITDTDANEFFQVVSSGATAGEIRVRKSLNYDIRKD
YRFSVVAKDNNLGEPRYRTALVVINIDDTNNLRPTFEAGSASISISEGTSIGTSVHQVVAIDKDTVG
KLDYRIISGADGKFAINKETGDINLISAVDFESNKKTYTLEIQVNDGVRDALNPATVVINIQDFND
NQPEFIQKYYIFTALESISDSQAINVGNPVIARDADSTTNSQVDYSIQYDINNPSVPNEGDALFRIDS
NGLIFKKNGVILDFENVRYHTFTVVAIDRGTPKTLLGKAFVTINIQDVNDEAPSWSPAIYSVTVKES
AGINRDLVFLSTTDKDTSGAVTYSIVSGDSANQFQIFDNVIKIVKPLDFEKTETHTLKVSAQDDNG
NRAQLDATVTITVEEVNDNRPLVTNNNANSIQVSVPEDIPVNSLIVKLNGRDDDKGAPGQASNQ
FTFVSLDKTTDFTIDEVTGEIKNTVNLDYERQKSYSLVATIRDKGTPYLESKFLVDIIVENKNDERPIF
DPAIYTIKLSEKAPIDTVVAMLQATDLDTPKSQLRFSSVTTLDSHFSLDPITGIITLKTSFDKEVNGKD
GIFVISCTVNDGTSNSAQNAIVRVEVVDVNDNQPVFPTSEYFIQVNEGSSIGTILQNIAATDKDNT

NTHNQFTYTLEGKEAVDFRLNGNQLQTNKIFDYENKKSYSFDVLATNNLSPDKLVGKATVVVSIL
DQNDNNPVLSPSGVINLNISEGTIAPANIITIFATDKDSTINAEVQFTLTGDPTNTFTINQNTGEIML
AKSVDSFAKNEYTLSVTATDKGSPPRSSTATTLNISIKPVFVPGIEFENCPQIISFNENAAPNTFTKTL
TVKDRGSFNQVQFTVVSGQAGIQNLITVSQNGVITNGQRRADFEARKRHDFLVQAENERGAKAY
CHVTANINDVNEGPIFDSANYVFDVLEVIKINTVVFSVRATDPDFSSTTNGQLTYSLAGQEASQFSI
VPNNGQIKLAQPLDYEIKKQYQFNVIATDGGSLQTTSSVIVNVEDWNDNRPIFDQPQKLAINVPE
NSKPDNVFTVSATDKDSTTNGEIEYVGIYPTGIISIDKDTGTAKIIGDLNYEKRQKYDFVIVAKDKG
VPYLSNTREVSLSVLNLADEVPYFDTAAYTISISEKTSINTAVFRVMAKDNDLENFGKISSYEIISGNQ
LNRFRIDSNGNIIVQQSVVLENNNPNQYILTITAFDTQRTQSLNNTQVTINIIDLSDKQPFFIQNSYI
VEVQENNEVSKSLLSLEAQSNEPTFNPTYSIVQDPKNTDSQFFQIINGNTIVATTVFDYEKKRFYSFE
VQVIDTNNGKTNKALVVVRITDENDNKPFEVSGNLHFNITEHTLLGSTIGSLNGKDNDAGINKEF
EFSILEGNDDDKFMLLPTGQILLKNFVSVKEKDFYKIEVKITDKGTPSLFNIFTVRVTINSSQALVAS
NNPTIPEDKNTGEEITRVTAIDPNTGSSNGFIYSLVGTLSNSQFTIDAKTGAISLLASVDFETDRTHT
AVVKAEGPTSTGVTEVIVRVTDINDNKPIFDPTNFNLNINENFVVGQEIVLVRATDKDSSLNDNNV
IIYRIDSVNPSSGNDLFSVNPSTGRILLLKALDRENGDTSYVLTIAASNRGVSNKPDNTMTVTINIA
DVNDNFPIFEKSLYQGSVSEDANTGTSILTVRASDKDILLKNRNIRYSMTQPMAVQKFQIDPISGVV
SLKQEVDFETKQRYDFSVIASDTGDVAYSRTANVEITILDVPDVEPKFDPQRYEITIAESTLVSTSILK
LQVIDSVKNLNFNIKQGNSNSDFRLVASSGELFVNKQLDRERISRYQMTIEVSDSVNKAAIDAIVIV
NIADVNDNQPLFLQSGCYQANRAEGIETEQVVVNVQATDRDEGSNKEIVYSFSDACSVLDIDAN
TGAITTKQKFDYESARSHICTIKAEDKGTPKLSGFSCIILTVTDSNDLKPTFEVITSNARVLENIPVGS
VITRVLATDKDTLSSNKQFVYTIKTGNEDGRFTIDANGFIKLQQPLDYEKKTDYRLEIEAVDSTNQ
DMVSDPNAIVTVTVIEQNDNRPKFAKSYHRVNIEESTAIGTTVLKLSATDADAGVNEGLIRYVSLH
NTTVFNIDRTTGEITLATKLDYEVKKSYHLIAVVKDQGNPVLSARTLIDIFVNDVNDNPPVFVISSY
NNIVSEKAPIGQSIVTVSARDDDTGVNGQLKYKIRETQSMFRIDEDTGIIILNGDLNVENTNKLLYA
LTIDVVDSGVPPLQASNPATVSVQVSDVNDKQPVFALDRYQFEINENIEGNQIFGDSVTVRANDD
DLTGTNNQLTYRLVNTTMFDLVVTNQNVFIKTALGVSFDYETKKSYSFEIEAMDSAANKLYGRSLV
TVTVKDFNDNPPIITVPNIIVNISDATLIGSHIATIMAEDKDSGENGILIYTVTAGDSTNRFTMESDT
GSIILKNPVDSYTKDIYVLNVLVQDNGIPRRSATGTVTINVIPVDLTLLKFNTTCPFAISIPENQINSN
FLGLKALDAGEYVSKQLRYTINSDENDVNQIFALDSIKGEFKQLKEANYEERSRYDMTICATNERG
SKVCCHITIFITDINEPPSFIGLNNNNIYEVSISETTPIGTIIYRVLSKDQDLPGVLNGQTSYSLGQQNV
FIIDQMGQIRVNKTLDAETQLNYVFQVIVTDKNPTNPLQSAATIRVNILDYNDERPIIQNIPDITIDE
NSDNQVVRNIVVTDRDISNIGSNIELSIEPSNILTINNRQLRTVGSALDYEKVQRHTLYLTAKDKSFP
YLSSTTSFTVTINNLNDNKPKFPVNNYNLTVSEKTTVGTLILTLFAEDDDLEEFGEIANYEIVSGNQ
EDRFSIDSNGRLTLKKQLNWRNVKSYNLNIIARDKGLPSEQSQIPATVVINVADYSEEQPNFLQNL
YVKFIPETTPIDTLVLTVEATSGEPVYGPFKYTIVPKLPDTNNFQIFSNGEIKTINQFDYENQRNFTFD
VQVEDEKNGKTNRASVFIHITDVNDNIPQFTDLTRSNTQYNITEFTLPGSMITCLSATDRDHGKNA
EIRFRGIGGDGNGVFNVNLDGKVLLSQQIFYDKKNKYNLIVEAFDLGSPEQKAQLTLTIFLHKSYT
SVITTDVDINEDVETQTRVTRVSSIDPFTRSSNNFTYYLVGKSTTFFEINAQTGEIFTKATLDRETRDR
HVLTIQSRGPTTNGVGELVVNVKDINDNPPIFDPQVYQVTVSESANIGLSIVSVTATDKDIGVNGE
VTYSIEDGDQTIFSINPSTGSISLAGKLDFESKQSHTLTIKAVDRGNPPQDSVNKATVIVVVQDFND
NCPVFENSTYSLTIKEPGELGKEYLKLGITDNDNGRNQLFTCSIDEPLAEKYFEMINDCRLRLIKEL
DYEEFTSFRFVVKATDQGDVSCTTTTNMIVNVGDRADVIPFFSPLTYNISIPESTTVGTIVVSLKVGS
TFKGLLYSLFHKNTQDDLIKFGINKDTGEIFLKESVDREIQDKYEFTADVFDGSNNAVQRASVTINII
DVNDNAPFFNQPTCYQRDLTESTAIGTFVVDVSAADNDKGRNQQVTYSLLPSPSCTSFSVDETSG
RITTSQILDHEQQKLQHCLIKAKDNGTNSLSGYSCLTVNVVNVNDNKPAFIVNRFDVSVLETVPA
GTLVASLVARDVDNQGLITYSISNGNLNNQFTIDSSGKLRTRFPLDYENTKSYNLTIQASDNNGKFS
SPDDALVLITVLEVNDNRPQFLQSYYKVSIPEDTATGASILNVTAIDLDHGELTKSLFYAALENTTQ
FTVNANTGVISVAENLDYEIEDHYELVVIVQDRGTPHLSNRALVDIQITDVNDNAPMFVTTVYNR
ELSEKALIGAPVVKLNAMDADKSDKMTFTIDSVVQGAQGFFSINGDTIQLDKELDYEVPEQRTFV
LSVSVKDSRDRDGANKANVFINVRDINDREPIFDLNMYEVTVSENNVISNPIIEVTARDSDSTPEN
NNLLYSLVDGEDAASFSLLNNGQSAIITSKISFDYEKKKSYRFLVRASDKVRENFTAFSTVFVTVQD
TNDEAPVPEKDLIMVNISESTRVLSTIAIVFANDKDQGINGQLAFQIINHSEDKFEINNKGVIRLKST

LDFFQQNFYNFTVLVTDKGNPVRTSKPVTVLVYVYRTEIPGIIFEKNHYIFNINEKEIVSLVGTVNAI
DEGTYVSRQIVYSIVSGQNDIKDKFRAVNNNIIQDKEVNYKDQSEYNFVMKAINERGSIAFTHITIN
IMDVNEKPYFVGLNNKGRYEFSMSEIISVPSYIYAVRAKDDDFGLNGEVDYEIISNFRFGNFSIDQY
GRIQVLKDPDFEFTRSYEFQVRAKDRGTPSLESNLVSVVITVLDYNDNHPIFNIQEPLEISIKELVNN
TDVGTVTATDKDGSGLNRIVKYVAIYPSDHLTVNRDNGQIRVIEPFDYEIAHKHEFVVVAQDQGT
PYLNAYKVIKVEVLNENDEFPEFTVIEYNITISELTPIGSPVLFVHAQDKDRGPSGQVTNYKIKYVPP
SGGSGAGSPDKTFEITSDGQVVLKEKLEYKFQKYYQYRVTCTDNGSPPKNSINEAIIHLNIADASLE
QPIFSVNPAVVEVYENITLNSNIVKVEVDSIEPIKGPFEYSIIASEDSQPFMIDNSTGQITNKESFDYEI
KRKYVFLAEARDKHNNRTQRAMVVIFILDINDNIPTIETIAPQTFVQNISDTTYPGSVVAKLYSRDA
DSGENAMVDFTITGGDGENVFAINPNGYVTLIGPLDVRVKTQYTINVKVSDRGIPTLSNNRALTF
NIKKSKFIQNTVRFDRHHYKSNCIEENQINLNIIQLNATNSNPVAGSTIIYSLDGTLEERQPFIVNSQ
TGEISLSQTLDYEIKQEYDFVAVAVNNDGSRDVAVVTVCIKDLNDNSPIFEPQSYVVNVSESESVGY
LVYKTTVMDADKTDFNTTKSYDIIQGNVNDKFIIDSMGHIYIKSKIDYEALQVKHFDLRIRVNEGN
LVGDMVLRVNILDWNDVHPSFSTLNIYNFTIPEDKPVGEIIEILTANDGDATSPNNEVTYKLLTTED
DFILRRNDKGHGSLIVNKQLDYSKQNRYEYVALAEDKGYLNLLGTAIVIINIQDVNNHPPEFDDSE
YNTTVSEHAQIGTRVLQIHAHDKDTGLGEQIFFIYGDPTETFTISSQGFIILNKQLDYETQRDYIFDV
GARDGGNLVSLNNVTVHIKVSEVNDNEPIFDLSTYRNSYAEDTPLGTTLFTVRATDKDFIGNSIIYSI
SGANDDTSYVSVDQTTGDITLQRRMDYESKNYITFTVQAIDSNNRNKLKGSTHVIIDVTDVNDN
NPKFVFGSDLVYNVTVVENINVGTLVVVVDSQDADSGLNGQTRYHIVSGNNENKFFINELNGQI
NLINKLDALQTNKYVLTVMAKDLGNPSRSTNVTVTINVLRYFESTPMFLPSYAEVIYPENLALGSSI
YELNVTKTLPGSPMKLSFHSSSKFPEFTLQGNKIILSKEFDYEIKNRYELIIIATDEENDKAFAQVIVR
VQNLNDNIPYFTQNAYFGVVSELAFVGHSVLTITAKDDDKDFTELFYSIATQVPDNAFKLQDNIV
VVNGKLIRNKVYEFEVEAFDNTFTSKRATVSITVTPENLPSFVNATYIVTLQENYPISTNFLNISAGT
FSGITYVITSMKAKDMFNVDANGQISLTKSLDYEKQRQHVFPVTASVTSPITQVRVASVIVNVLNE
NDNAPVFVNGPYERNITEGHLVGTMVIRVDGHDDDSDKLTYSIVSGDDGKFSINSEGVISLISTLNF
DLKNKYVLNVRVSDGKFVTDTIVTINITPIVKFPPMFNQTLYLLSVKENTLVGTSILQVSTVNVIPPS
SFSINEQSGRDFFTIDANGVIRTNNALDYEAKRQHIFTLKVQDLRSMGHATVVVNIVNDDDTCPIL
TPATQTVQITEPVAPGTIVAAVQAKDIDSKSLNFTITGGNGLFRIDQHGGIKTINEVDISTSTEYELTV
TVDDGTCTKSVQVDVNVNPLAPCPDCNIYPFTQTIYEFSINENTVPASPIITVTTGQSLQRNFSIEDP
AALEYVTINRLTGEISVIKAFDYEATKHLSFNVMAKTQTTKNRNPFYVITSVSRVRITVRDLNDNCP
SIESPMYYEVSHSSPIVQDTVVTRILVEDPDTLKDHTYSISDNNFKIDSMGHIRTVQNIESFTAQFYN
LTVTVSDGQCSKTSNVGVNLAVNPTPMTYQFTSNGVYSKSVREDHNLQDVILSVALNQNYPRVFS
IVESDANDQFVIDSNSGALRLKTSLNYELKKRYDFTVMADIHGGVYKSYASVHIIVLDVNDECPIF
PVIGYNQRVSGTPVIGTIVEIIEANDADNATLEYSIVSGNEDGHFSLDNSGVVRVVKPFASSFSKTYG
LSVRATDNKCSADVPLTVQVSTCAYGEDYQFEESEYVVYYLDENRNLGIVSNIKLKSTRAAFLTLSQ
NTHFTIDQNGDFRLIQTLNYETKKEYHLTLTANFTQDNVVTRIPIKVFVRDINDNCPAFSEELYAVQ
HTEPLREGLLIAKLSVIDDDTVQNFTYNIVSGDKRFSINENGEVVMNARLVTDTTLLSTTYKLEVSV
NDGKCAKNTKVDVFVYKTVVNSFLFPQPFYKFDISENQAIPFTIDRFVNVGGHEAVYGFLVSTDFF
QIDPKTAQLQLVKKLDFEVEKTHYVLISATYDDGTQTYTVVAIHVGDENDNAPQIFAHPPITRLVQ
NTPHNSIVTKIEVEDPDTSKEFTFKITNGNPLQNNDPDKALFRIESNGMIKTTRCISEDEIGNYVLTI
SVDDQVNTPTIFSNMQVQLKTTDDRSYVTNCGLGCPANDGPFSRYQFEQSLFIVNVTETAANPTV
LKRLTRPFVEKTTYTIGAGADGKFSIDQNGTLSLTGSLDYETKPVYYFIVSAVIPSQVVGRFITSKTAY
LVMVNDVDDSKPVIEGNGGTLAVSETSEIGQIVGTVRVKDSDSSSPLTYNVVNGSQYFNIDSAGNI
VIKDTLNAAGSPYTVTYTVSDGINPPVNGEFVVSISETNNNVPKFTQNIYEVSLSEDTATDTIAVTIL
ANDGDTNDKIAYSFLQSEGPFVINRDSGEIKLIGPLDYEAQKSYNMVVQATDSGGFANIAFVQIAV
GDVNDQVPVITPQTTPTQIHESTLVNSVVYRVIASDGDGNERLTYALANSNDFFTINSATGEITIKE
NLAKSNVSSHTLSIVVTDSSGKTSTQDVALNIRRSGTSQGCVKITTSQGQSSSPVINLPENTASGTTV
FKLVAGDASVFSNMRFTLNREANGVFAVTEQGELQLVAPLDYETKQFYSIIITAEDVTRGVSSQYNL
FVRISDVNEFTPVLETIAPVAVSELANIGDVIAQAKANDQDGDSQVTYSLGEFGNNDNKFTIDAAT
GNIILNKRLGSGAPHVVEVCASDGSIKKCQNVTFIVADTNDNPPSFQDLTGVGSIDLTNTSSYVVY
KPIVTDSDQTGTLSFSLKDPAPSQFSINSTTGEVKANKDIAKGTYTFGVCASDGRYTSCQSVTVVAS
DRSQSITPSFNQDQVGAIVDISASSGKQIIQVMASTLDKAKIEYELKNEADRNIFEINKDSGLITLKN

RTALTLGTIYTIPVKASRADDPLNFDEINIVISVGNQQLDSGSFIEQVLDTQHLVNFSLSTFDISKVSY
YIILGQQVGGDSKDDEKLPVSWYYATFGDESYRHRFYYIAKLDGSKFRNTGGSRKRRSIQDKDSKS
FTYLVGEENGCSKNTSMAAICNGPLKTGTEYRYKLRAYYDDGELRGIPVDQKPTEVKRTQAPQA
GKSIDEYREQDSYVITFWIIGLVIFGLLFWFSVLVIAILLCYKRRYEVTQAYGYSVQTRDKIDGPPTEY
SNTVYRFQNPSYPDNDTVKMKKVDASESRTKTEVQTTSMVVLDDYEQEQPQPDIPVQDYDAIEY
ETRSAQIKFHEESPEWESMDIQLRIDPTGNQDPVITRKSDSGSKDSDSDASKKYENTEYIEEKEDFV
NEKEDSLTRL*
>TRINITY_DN329_c0_g1_i2.p1 type:5prime_partial len:320 gc:universal TRINITY_DN329_c0_g1_i2:1-960(+)
FNNSTHSPSLPTHVYLYCCRIKILILSFDPSTIEILKMAPPKFDDLGKEAKDLINKSFHFGVIKLEGKT
KAKNGVEFTTEGTHNTDTGTVNGSLETKFKYADYGLTFSEKCNTDNVISTKITIDNKLAKGLKVD
FDTTFAPVTGKKTAKVKSAYQHNNIHSTCDVDFDFAGPTIHGSAVFGYNGWHAGYQASYDTANS
KLIANNASLTYKEGDLVIHSGITDAVKYSGSVHHKVNSNLTAAALIQWASGTNNTSLTVAGKYAV
DNETFVKAKIDNNLRLGLSYAQKLRPGVELTMSSLVNAKSLEQGGHKLGLSLNFEA*
>TRINITY_DN262_c0_g1_i1.p1 type:complete len:1033 gc:universal TRINITY_DN262_c0_g1_i1:111-3209(+)
MADPSSLETKEKDGSYRVATKSGGKDKGKKSKKAAKKVNMDDLKKELEMTEHSCELETLVSQLE
TSLTQGLTKSIAARNLERDGPNALTPPKQTPEWVKFCKQLFGGFAMLLWVGAILCFFAYIIRSIREE
EPGMDELYLGIVLTVVVIITGCFSYYQESKSSKIMESFKKMVPQEAVVMRDGEKLNLNATECVVGD
LVFVKFGDRIPADLRIIESKGFKVDNSSLTGESEPQSRNSECSSDNPLETRNLAFFSTNAVEGTATGIV
VKTGDNTVMGRIANLASGLGSGKTPIALEIEHFIHIVTGVAVFLGVSFLIISLAMNYHWLESIIFLIGI
IVANVPEGLLATVTVCLTLTAKKMASKNCLVKHLEAVETLGSTSVICSDKTGTLTQNRMTVAHM
WFDNMVVEADTTEDQSGQAHDKGSTTWKALAKIGALCNRAEFRPNQEEEPILKRECNGDASET
AILKFVELSVGSVEDIRSKNKKIVEIPFNSTNKYQVSIHEQEDPDDPRYLLVMKGAPERILDRCSTIL
HEGVEKPLDEEWKENFNKAYVDLGGLGERVLGFCHYYLPADDFPKGFAFETEDPQNFPLEKLCF
VGLMSMIDPPRAAVPDAVSKCRHAGIKVIMVTGDHPITAKAIAKGVGIISEGNETVEDIAQRLNIP
KEEVNERDANAAVVHGSHLKDLPQEKLDDILKYHQEIVFARTSPQQKLIIVEGCQRMGAIVAVTG
DGVNDSPALKKADIGVAMGIAGSDVSKQAADMILLDDNFASIVTGVEEGRLIFDNLKKSIAYTLTS
NIPEISPFLMFILLAIPLPLGTVTILCIDLGTDLVPAISLAYEKAETDIMKRKPRDPVRDKLVNERLISL
AYGQIGMIQATAGFFTYFVIMAENGFLPGYLIGLRNDWDDKKNNAVMDSYGSEWTYNQRKNLE
LTCHTAFFTTIVVVQWADVIISKTRRLSLFQQGMTNWMLNFGIFFETALAAFLQYTPGLNTGLRLR
PMNFPWWFVGVPFSLLIFVYDELRRFLLRRNPGGWVEQETYY*
>TRINITY_DN160_c0_g1_i1.p1 type:complete len:1145 gc:universal TRINITY_DN160_c0_g1_i1:4810-1376(-)
MRLLAIIPLLVMLDIAKSDKLWECNFEENDCWAASSTRNHERNQLTLHWNRWKMRTPTGATAS
PVTGPMEAFEGKHYLYYEATESMDKLKMADGASKGVVTFKDINLMGRTQFILSFYYQVKGNDG
DFYDFDLMVSNTENTHYDLSMKSIDGKDWKHFKHIMKVEGAGVTSIDLKFTARSYGAFADYAID
NIVIEDYPDAGPCKDNRCLANEYCTITSSTQTVCVAKCTQQACGENAECILDSLAINKCKCRQGY
LPRVSNYGDKLNCYRPDASCDIEYNSLGQCRISTSIPIMYGHPSLWHGRYSPVSGNGYYVFKYDNT
ITLDAVSSQKLSFFYLVDNPNMINSLTVEYGSRKFEVPINREKTNIWTLYEVLILASTNQGVQIKASF
YAEGGVIGIDNIQFTQQNEVVVEKIYKESMMAATFEDGSVPTDLKVAMLFVTRYGKIDFGCLPGIE
LETRNGKLYCKDGVVQRQSACSQDSDCIGSNARCVLRNCVCNTNSYQISIGTYGEKATSCAVYPC
EINSPRCHSTAICSNIKTPGQNFYNDYTCKCGRSLNNVETIINPFIGGDKSTQCLAATPSDSYVCND
NNFKPSRTTEYCLKGNQATVEYSLRATNFGNYALFKHCDDPDDEYRSSFNDPGSFWRPIDLVAAA
NENLAVSVTYKTSLSNKFIKCNFEQARFGSKLTLAVNVNSNSRVLTFTNTQTSVNKQNPVYQGQV
FQSDNPTPGAEMSVTFTVTGNQAVDQNVDAILLDNVVCKGFDDVASVNTPCGNNACASNQYC
NPLLSNSEKECLCPTGQKDQNCPDVVPSGSVVSADGPKCIGHHEKYMNGACVCDDGFEKTGDT
CTVKINCGCNDGLCLSGDKCSCPLNLLKVNQACVRTNKDCAKDCVSTLGNGGYCDVTTGMCD
CLTGFVKSGRDDGSYRCDKHAVVQKINDPKGICSATCGTGFVNEIEVCVGATGTGFECMTNIVQG
TLGVCSLPACPATSSQCSIQKECACNNVRVPIGSDGKLRLDFDHEAKLNMIFRFLSKHNQKDLEFK
FREARCDNTKLDSIARTIEDDLKQLEFDTELQAAITTMKSILDCSPSGNDHNAAYWDVFDMLIELS
SYRPHAVSDLQLKKELVMHEQSRCGMRSNLHKVVDGYAFWNKK*

>TRINITY_DN982_c0_g1_i1.p1 type:5prime_partial len:6412 gc:universal TRINITY_DN982_c0_g1_i1:19377-142(-)
KLDFETKQSHTLTIKAVDRGNPSQDSLNKATVIVVVQDFNDNCPLFSESIYNASVSESTAIGTTVLR
VSVTDKDVGNVNNEITCSIDEPLAKGFFSVNKINNNQFSVVLIKNLDYETHRGYRFVIKAKDNGE
VPCQLTTNMVVEVQDEPDVITNFKPFNYVISIPESTLIGTKLLELKVDSESSSITYTLQAKNQPGDLD
KFMLNGQSGELVLKGTVDFEKKSEYEFTATLNDGVNNGGNTANIVVNILDINDNPPVFSQSTCYI
RRMPENQQVNTTVTSVLATDSDQGINSNISYSLIKDSSCNNFEINEITGAISLTSSLNYEVRKEHYCII
KATDNGSPVLSSYSCLKVIVENVNDNSPVFLPKELEITVLESKPVNSFVIRVFATDKDEGNSITYSIV
GGNTQNAFAITPLTGHIILNQKLDYEIVKNYILEIQATDDGTPRRSSNLNAKVMVKVLEVNDNKP
KFNQQRYLRTIPEDTNIGSTIISVTATDDDAGLLTSKIQYIALENTNDFLIQPDTGDIVVSQNLDYER
QRTYQLVAVCSDSGTPPFTGRTLVDITITDVNDNAPILNPTLYNVLVSEKAPLGTSLIQVHAKDKDS
GINGGLKYQIVSGNDLNKFVINNSTGVITLQETLDREKYSSLYNLTVSVSDQATPSLNGNNAFVQI
RVLDVNDKQPVFVKSEYIFEISENQPLNSPIGLVQANDDDSTSPNNIIQYTIVPSKDSINFTMINNQI
LSNSVFDYEEQKSYEFEVQARDQSTGVNNEQLVGRTLVKVLIRDTNDNDPMIENQNIALNISDVT
LVGTLLFDIQATDKDNGENARLTYTISSGDTSTFRINENDGRFYLSKKLDFYNKNEYSLIVIVNDNG
SPQRNSVANVQINVYPIDQTLISFEPQCPIAFTIQENSLSSPMNIKAIDTGSYVSKTMRYMLKSNKSD
VSSMFQIVETNGVIIQNKAANYEERSRYDMTVCARNERGSEGCCPVTIYILDQNEQPFFDTDNDN
LDNGKYLVRVSETTPIGSVMFSVNTGDEDFGSLPNGQKTYSIKAKPGEEMLFLVDSYGRIITNTTLD
REKTPQYDFSVVVTDKGTPPRQAEAEVIVTVIDYNDQRPVFEIMSVRPVNENANNEVLGRVVAVD
IDSTSINNGIKYSIQPAGFLNISNTGTLTSVGSALDYEKNQKVTLYVTATDTGSPYLSATTSLQLDIINI
NDNKPYFLQNNYNLTVSEKTTVGTLVLTLFAEDDDLGEFGEIANYEIVSGNQEDRFSIDSNGRLTL
KNPLNWRNVKSYNLNIIARDKGLPSEQSQIPATVVINVADYSEEQPNFLQNLYVKFIPETTPIDTLV
LTVEATSGEPVYGPFKYTIVPKLPDTNNFQIFSNGEIKTINQFDYENQRNFTFDVQVEDEKNGKTN
RASVFIHITDVNDNIPQFTDLTRSNTQYNITEFTLPGSMITCLSATDRDHGKNAEIRFRGIGGDGNG
VFNVNLDGKVLLSQQIFYDKKNKYNLIVEAFDLGSPEQKAQLTLTIFLHKSYTSVITTDVDINEDVE
TQTRVTRVSSIDPFTRSSNNFTYYLVGKSTTFFEINAQTGEIFTKATLDRETRDRHVLTIQSRGPTTN
GVGELVVNVKDINDNPPIFDPQVYQVTVSESANIGLSIVSVTATDKDIGVNGEVTYSIEDGDQTIFSI
NPSTGSISLAGKLDFESKQSHTLTIKAVDRGNPPQDSVNKATVIVVVQDFNDNCPVFENSTYSLTIK
EPGELGKEYLKLGITDNDNGRNQLFTCSIDEPLAEKYFEMINDCRLRLIKELDYEEFTSFRFVVKAT
DQGDVSCTTTTNMIVNVGDRADVIPFFSPLTYNISIPESTTVGTIVVSLKVGSTFKGLLYSLFHKNTQ
DDLIKFGINKDTGEIFLKESVDREIQDKYEFTADVFDGSNNAVQRASVTINIIDVNDNAPFFNQPTC
YQRDLTESTAIGTFVVDVSAADNDKGRNQQVTYSLLPSPSCTSFSVDETSGRITTSQILDHEQQKLQ
HCLIKAKDNGTNSLSGYSCLTVNVVNVNDNKPAFIVNRFDVSVLETVPAGTLVASLVARDVDNQ
GLITYSISNGNLNNQFTIDSSGKLRTRFPLDYENTKSYNLTIQASDNNGKFSSPDDALVLITVLEVN
DNRPQFLQSYYKVSIPEDTATGASILNVTAIDLDHGELTKSLFYAALENTTQFTVNANTGVISVAEN
LDYEIEDHYELVVIVQDRGTPHLSNRALVDIQITDVNDNAPMFVTTVYNRELSEKALIGAPVVKL
NAMDADKSDKMTFTIDSVVQGAQGFFSINGDTIQLDKELDYEVPEQRTFVLSVSVKDSRDRDGA
NKANVFINVRDINDREPIFDLNMYEVTVSENNVISNPIIEVTARDSDSTPENNNLLYSLVDGEDAA
SFSLLNNGQSAIITSKISFDYEKKKSYRFLVRASDKVRENFTAFSTVFVTVQDTNDEAPVPEKDLIM
VNISESTRVLSTIAIVFANDKDQGINGQLAFQIINHSEDKFEINNKGVIRLKSTLDFFQQNFYNFTVL
VTDKGNPVRTSKPVTVLVYVYRTEIPGIIFEKNHYIFNINEKEIVSLVGTVNAIDEGTYVSRQIVYSIV
SGQNDIKDKFRAVNNNIIQDKEVNYKDQSEYNFVMKAINERGSIAFTHITINIMDVNEKPYFVGL
NNKGRYEFSMSEIISVPSYIYAVRAKDDDFGLNGEVDYEIISNFRFGNFSIDQYGRIQVLKDPDFEFT
RSYEFQVRAKDRGTPSLESNLVSVVITVLDYNDNHPIFNIQEPLEISIKELVNNTDVGTVTATDKDG
SGLNRIVKYVAIYPSDHLTVNRDNGQIRVIEPFDYEIAHKHEFVVVAQDQGTPYLNAYKVIKVEVL
NENDEFPEFTVIEYNITISELTPIGSPVLFVHAQDKDRGPSGQVTNYKIKYVPPSGGSGAGSPDKTFE
ITSDGQVVLKEKLEYKFQKYYQYRVTCTDNGSPPKNSINEAIIHLNIADASLEQPIFSVNPAVVEVY
ENITLNSNIVKVEVDSIEPIKGPFEYSIIASEDSQPFMIDNSTGQITNKESFDYEIKRKYVFLAEARDK
HNNRTQRAMVVIFILDINDNIPTIETIAPQTFVQNISDTTYPGSVVAKLYSRDADSGENAMVDFTIT
GGDGENVFAINPNGYVTLIGPLDVRVKTQYTINVKVSDRGIPTLSNNRALTFNIKKSKFIQNTVRF
DRHHYKSNCIEENQINLNIIQLNATNSNPVAGSTIIYSLDGTLEERQPFIVNSQTGEISLSQTLDYEIK
QEYDFVAVAVNNDGSRDVAVVTVCIKDLNDNSPIFEPQSYVVNVSESESVGYLVYKTTVMDADKT

DFNTTKSYDIIQGNVNDKFIIDSMGHIYIKSKIDYEALQVKHFDLRIRVNEGNLVGDMVLRVNILD
WNDVHPSFSTLNIYNFTIPEDKPVGEIIEILTANDGDATSPNNEVTYKLLTTEDDFILRRNDKGHGS
LIVNKQLDYSKQNRYEYVALAEDKGYLNLLGTAIVIINIQDVNNHPPEFDDSEYNTTVSEHAQIGT
RVLQIHAHDKDTGLGEQIFFIYGDPTETFTISSQGFIILNKQLDYETQRDYIFDVGARDGGNLVSLN
NVTVHIKVSEVNDNEPIFDLSTYRNSYAEDTPLGTTLFTVRATDKDFIGNSIIYSISGANDDTSYVSV
DQTTGDITLQRRMDYESKNYITFTVQAIDSNNRNKLKGSTHVIIDVTDVNDNNPKFVFGSDLVYN
VTVVENINVGTLVVVVDSQDADSGLNGQTRYHIVSGNNENKFFINELNGQINLINKLDALQTNK
YVLTVMAKDLGNPSRSTNVTVTINVLRYFESTPMFLPSYAEVIYPENLALGSSIYELNVTKTLPGSP
MKLSFHSSSKFPEFTLQGNKIILSKEFDYEIKNRYELIIIATDEENDKAFAQVIVRVQNLNDNIPYFTQ
NAYFGVVSELAFVGHSVLTITAKDDDKDFTELFYSIATQVPDNAFKLQDNIVVVNGKLIRNKVYEF
EVEAFDNTFTSKRATVSITVTPENLPSFVNATYIVTLQENYPISTNFLNISAGTFSGITYVITSMKAKD
MFNVDANGQISLTKSLDYEKQRQHVFPVTASVTSPITQVRVASVIVNVLNENDNAPVFVNGPYER
NITEGHLVGTMVIRVDGHDDDSDKLTYSIVSGDDGKFSINSEGVISLISTLNFDLKNKYVLNVRVSD
GKFVTDTIVTINITPIVKFPPMFNQTLYLLSVKENTLVGTSILQVSTVNVIPPSSFSINEQSGRDFFTID
ANGVIRTNNALDYEAKRQHIFTLKVQDLRSMGHATVVVNIVNDDDTCPILTPATQTVQITEPVAP
GTIVAAVQAKDIDSKSLNFTITGGNGLFRIDQHGGIKTINEVDISTSTEYELTVTVDDGTCTKSVQV
DVNVNPLAPCPDCNIYPFTQTIYEFSINENTVPASPIITVTTGQSLQRNFSIEDPAALEYVTINRLTGE
ISVIKAFDYEATKHLSFNVMAKTQTTKNRNPFYVITSVSRVRITVRDLNDNCPSIESPMYYEVSHSS
PIVQDTVVTRILVEDPDTLKDHTYSISDNNFKIDSMGHIRTVQNIESFTAQFYNLTVTVSDGQCSKT
SNVGVNLAVNPTPMTYQFTSNGVYSKSVREDHNLQDVILSVALNQNYPRVFSIVESDANDQFVID
SNSGALRLKTSLNYELKKRYDFTVMADIHGGVYKSYASVHIIVLDVNDECPIFPVIGYNQRVSGTP
VIGTIVEIIEANDADNATLEYSIVSGNEDGHFSLDNSGVVRVVKPFASSFSKTYGLSVRATDNKCSA
DVPLTVQVSTCAYGEDYQFEESEYVYYLDENRNLGIVSNIKLKSTRAAFLTLSQNTHFTIDQNGDF
RLIQTLNYETKKEYHLTLTANFTQDNVVTRIPIKVFVRDINDNCPAFSEELYAVQHTEPLREGLLIA
KLSVIDDDTVQNFTYNIVSGDKRFSINENGEVVMNARLVTDTTLLSTTYKLEVSVNDGKCAKNTK
VDVFVYKTVVNSFLFPQPFYKFDISENQAIPFTIDRFVNVGGHEAVYGFLVSTDFFQIDPKTAQLQL
VKKLDFEVEKTHYVLISATYDDGTQTYTVVAIHVGDENDNAPQIFAHPPITRLVQNTPHNSIVTKI
EVEDPDTSKEFTFKITNGNPLQNNDPDKALFRIESNGMIKTTRCISEDEIGNYVLTISVDDQVNTPTI
FSNMQVQLKTTDDRSYVTNCGLGCPANDGPFSRYQFEQSLFIVNVTETAANPTVLKRLTRPFVEK
TTYTIGAGADGKFSIDQNGTLSLTGSLDYETKPVYYFIVSAVIPSQVVGRFITSKTAYLVMVNDVDD
SKPVIEGNGGTLAVSETSEIGQIVGTVRVKDSDSSSPLTYNVVNGSQYFNIDSAGNIVIKDTLNAAG
SPYTVTYTVSDGINPPVNGEFVVSISETNNNVPKFTQNIYEVSLSEDTATDTIAVTILANDGDTNDKI
AYSFLQSEGPFVINRDSGEIKLIGPLDYEAQKSYNMVVQATDSGGFANIAFVQIAVGDVNDQVPVI
TPQTTPTQIHESTLVNSVVYRVIASDGDGNERLTYALANSNDFFTINSATGEITIKENLAKSNVSSHT
LSIVVTDSSGKTSTQDVALNIRRSGTSQGCVKITTSQGQSSSPVINLPENTASGTTVFKLVAGDASVF
SNMRFTLNREANGVFAVTEQGELQLVAPLDYETKQFYSIIITAEDVTRGVSSQYNLFVRISDVNEFT
PVLETIAPVAVSELANIGDVIAQAKANDQDGDSQVTYSLGEFGNNDNKFTIDAATGNIILNKRLG
SGAPHVVEVCASDGSIKKCQNVTFIVADTNDNPPSFQDLTGVGSIDLTNTSSYVVYKPIVTDSDQT
GTLSFSLKDPAPSQFSINSTTGEVKANKDIAKGTYTFGVCASDGRYTSCQSVTVVASDRSQSITPSFN
QDQVGAIVDISASSGKQIIQVMASTLDKAKIEYELKNEADRNIFEINKDSGLITLKNRTALTLGTIYT
IPVKASRADDPLNFDEINIVISVGNQQLDSGSFIEQVLDTQHLVNFSLSTFDISKVSYYIILGQQVGG
DSKDDEKLPVSWYYATFGDESYRHRFYYIAKLDGSKFRNTGGSRKRRSIQDKDSKSFTYLVGEENG
CSKNTSMAAICNGPLKTGTEYRYKLRAYYDDGELRGIPVDQKPTEVKRTQAPQAGKSIDEYREQD
SYVITFWIIGLVIFGLLFWFSVLVIAILLCYKRRYEVTQAYGYSVQTRDKIDGPPTEYSNTVYRFQNPS
YPDNDTVKMKKVDASESRTKTEVQTTSMVVLDDYEQEQPQPDIPVQDYDAIEYETRSAQIKFHEE
SPEWESMDIQLRIDPTGNQDPVITRKSDSGSKDSDSDASKKYENTEYIEEKEDFVNEKEDSLTRL*
>TRINITY_DN1369_c0_g1_i1.p1 type:3prime_partial len:1577 gc:universal TRINITY_DN1369_c0_g1_i1:93-4820(+)
MRIQLTIFVTFSIILVSGQNSPPGICSTWDGSHYQTFDSYSFNYHGTCSYALALDCRPGSDDFAVHV
ENDEKCDNHGGTDCTRAVSLYVNDIEYKLGSNNTVLVDGKSNIVLPYFNEGVRVSKHAEYYFIDA
LEGKVVVKYDGRSSIYVQLDSSYQAGVCGLCGNFNGNKKDDLKLPGDKGLTKSLKEFGKSWEKP
RIGQVCKEVTEEHFDQCTNKSKMLVYYAEKECEILRTSSEFKKCHKIVDPTPFYEKCREDLCLCGS

GDKYCKCDVLEQYTRKCAHYGTVLEWRQQNLCYKNCTGNKVFSECGPACSSVCSAIGSKSVCK
DACIDGCHCPPHTSLSGDKCVPDDECPCEHNGDMYDHGDVINMKCKSCKCNGGKWKCNPLP
CDGEASVYGNGHFVSYDGKTYNYPGKCLYLYTYLSDRSFAVYLDRTSCDSLACHMNVMILFRGD
RIKLSASNSTLALEVNRNVKSFPYISSTGYRMYRFSTVFVRFEATNGIKVDWDTYSRLHVLMPHSL
KGKVFGLSGNFDFKTKNDFLTPRKDLAHSAIEFGNSWMMYSSRLNCSALAPDYRMTSCEANHQ
VAKYAESKCSILMSSLFRECHKVVDPFIYYENCKDDTCECNGDSKCYCSVIAAYSKACSSAGKPTE
GWRKYTPCEEKCSNGMVFTECGNSCLKTCDDQTDPNPFCEKECVEGCLCPVGQVLDKITEQCV
DIKHCGCTYGNRTISNGGTVAKDCNTCKCTKGVMVCTKKECRDKKAICSDGKEWSSCMSPCPK
TCQTKDDICPETGLDCIEGCQCKKGYIESANGTCVELSKCPCYHNGKEYQETQTISKDCNTCKCT
AGEWSCTEKLCDATCSVFGDPHYKTFDGKIFDFQGKCGHVMLTDKCANNDNATENIRISVKSST
CGSQGVTCTREITLNYHDTVVHMKRGARKIDIVSNKRQDHEKDQVKVYRHAGIYVHVLTPMGV
SILWDNATRLYITAPPSLSGKICGLCGNFDGSETNDFVTYQGDTAGNAVSFGDSWRTENNCPLTKE
IEDTCHNRKHRLDPSKRECAVIKSDVFKKCHTLVDPVPYYDRCVFDACGCDSIGDCDCLCAAIGA
YAKECIDEGVYINWRKGHNICEKEMCCPTNMTFVPKGTGCPKTCKYPNGLHHCPTKYVDGCQC
PEGLVQNNDANGDVTCVRPEECKVCKLDGKIYNEGEVVLYKCKECVCRDGKMTCSGNYCPTTL
PPTTTTESTTTPSTTPETTIPSTTTTESTTTPTTEPPTTTTTASTTTASTTTESTTTEATTTTKPTTTTTEST
TTESTTTQSTTTPSTTPSTTMKPIDTCVYSNGTHNVTLERGDVIQVNECTKKTCVKDEVTGLHEIKV
TTTQCENSTCDTTCEASKSNRAILVKTPGECCPQCKKPEDLCDNPNEVPCNSTYCIPKSWFCDGN
KDCSNGRDEECCPNYCKVEGEIKKPGEVWTNKEKCLTCKCEGYYTKCRRMECPELDCPEERQVK
SEGECCPHCKPQTEPPTTTTEMTTTTTESTTTPATTPSTTSKETIIIITEEPKSTTASPQEKCDCKVLQPV
GVSDGRIPDNQMTANSV
>TRINITY_DN3991_c0_g1_i1.p1 type:3prime_partial len:219 gc:universal TRINITY_DN3991_c0_g1_i1:84-737(+)
MTSWILNFLVLSIIALPCSSISNCKHKLEPVGCFKDRGSARAMPEQILNERDTTSKVYSKQLMSWK
NFHEWLPGFICRCAEVAARKGYSVIGIQFWGECWSGPNAESTYNRHGQVDSCINQNLKKNRAK
LSQCTAASGKHFTNYVYRLAPACHTQYKPVGCFQDNLQKPRPMPYYVSTDRETRLAIFSGKLLN
WKNWNTHMPDMICRCAAVAKSKGHQ
>TRINITY_DN240_c0_g1_i1.p1 type:5prime_partial len:397 gc:universal TRINITY_DN240_c0_g1_i1:1518-328(-)
LKAAAAFSGVSPFALVQPRARVPSRSRVAMVRRSPLPVLGLAGAACFFARSPCFIPGGPLRRQPWA
AAAGSAAVLGATPALADPIGDAAKKLADASYPFAKEVDWNSGIYLQAPGKLAPLEALKAIDKMI
VLGSQADPKLLKAAADAHHKAISSASGPTGMTSKADWEAVNAALGRVFASVPESSVMDVYNAV
AKITDPGVPAYLKSKVNGADAEKAYAAFLEFKDVVKANQVSSAAAAPVPAAGGDKIGAAAQAL
ADQSYPFLKSVDWTSDLYLKPLPGVSAAKALKAVDKAIVMGSAMDGNLLKAAASAHHQAIGSI
DAAGVTSAADYASVTAALGRAIASAPTSKVMDVYNAFAGIVQPSVPNTLFSKVDPLAANAAASA
FYEFKDVVKAAQR*
>TRINITY_DN4595_c0_g1_i4.p1 type:5prime_partial len:1639 gc:universal TRINITY_DN4595_c0_g1_i4:5857-941(-)
QCGVNTGTQVVYRRCNAALCKYQGNDYTRNQEVNIRTQACTVACDYTPWQNTQSCTKTCDGG
VLVQTRTERAFNKIETRNGNTPCNTQACAQELGPKQLRTCNVGQCGVNTGAQVVYRRCNAAL
CKYQGNDYTRNQEVNIRTQACTVACDYTPWQDTQSCTKTCGGGVLVQSRTERAFNKVETRNGN
TPCNTQVCSSELGPEQLRTCNVGQCGVNTGTQVVYRVCNAALCRYQGSDYTRNQEVNIRTQAC
TVACDYTPWQDTQSCTKSCDGGVLVQTRIERAFNRVETRDGTTPCNTRSCASDFSAERLRSCLNV
NQCGVNTGTQTVYIVCNGNFCRYQGREYNINEEVNVRQQNCTIACDYTAWEDTTICSKICDGGV
LEQRRLERAFNRLERRNGTTPCNTRACDSGFSAVLSRNCVNVNQCGVSNGTQLLYRVCNGEPCIL
QERSYKINDVVVVGSRACSVPCNYTEWRDTTSCSKTCGGGFFFETRTEQAFQKTESRLGKTLCNE
NSCVKDNFGNWTEWTACSKTCGMGMRYRSRECLKGFECIGLIEETETCKLGCRTETCPMGRPYP
GDCNFEDDCGKSWNQVNSIQRNAFFIVTSKDLAHQGLLPNMETTFNTKDCGRTMKLNSSVAGD
VNYLWTKRMMITDINTDVDDDVAAIDNYFKEENEQCVEFNFAVMANSLDKIGDMQVYLYYLEY
PNASLIYSKKGNTGLKWELVKRSIRVDTNKAFVILFQVNQGQSNVQYGDIGIDGFKVNPGNCQLK
GWSHWGEWKCSATCGFGTQTRMRHCLDKVNRTACVGMDRENKPCPMMKECPMNRDGNWG
QWTEFSKCSKTCDTGTYFRTRKCDSPSAKYNGSLCRVSQTKFNLPVVNDFEIDFKPCSVQQCNNT

FSQWTTWSSCSGTCGPGQRTRTRTCESGKRCSGIFEEIETCYMGCRLEKCPKFTAEIDCNFEDRNKS
DCQWKYYTVSGTSSWSFKTQPKNRLLSGPSGDSTIGCGYFLYYPSQTLSSAYAWGPNIYPTMDSYC
VKFKYSMVTSVNSSMGNLTVIKHFLNRPRKADTPLFFHSGVSQKQWNPMAIDITTDIQRSPFVIMF
KGQSDPNNAGLSDIAIDDYMLMAGKCNKSYSEWSEWSTCSRTCGVGQRTRTRVCLDDDIKVCK
MGDVATEGEACVKPLCAVPVDGNWGQWSAYSQCNARCNGTQKRERFCNNPAPSNGGRMCLL
GDTIFQLRNNYHAQIRPCSIDCGDNDTFSSYKRINDTCHFDCITGRVLQERECEQDTCFDGNTRFS
KGEKQYTQRACSIKEYCETSEYKCREFRSVCSTNTPSSCLDPQNRAVKSCECQVERGCSSMTNPGQ
TVKHKEFDVFYRPCRYVGVCFTDYETDAFSPYEKVNDTCHLSCSNGKVFEERECRQDNCIDGSNT
LTRGAKQYRETQCTTKEYCETSQYSCREISGPCSSNPPSSCQDPANKSLKVCTCQVTTGCASMLTA
GQAVSYLAEETFSRPCKYTGACNANGNTDKTFSPYERVNDTCHISCADGKVKQMRECKKDACV
DGGQNFPRGFKQFKDSSCTPNEFCETAQYSCRELSSACTSNPPSSCLVSTNKAVKMCDCNDPNGC
DSMITPGKKVNQGEKEFLQRPCRYTGVCFTDAETGT*
>TRINITY_DN138_c0_g1_i1.p1 type:complete len:401 gc:universal TRINITY_DN138_c0_g1_i1:157-1359(+)
MAARFLFITVILIVGNLIGDVCPERCERNWIKIGCFHDRIFPNRPYPFELVNHRDPINNHWDGNLI
NWRKYPDSIEKLACICANKTRTKGYQYFGLQFYGECWSGPSYNFHRDGPSTKCIGNDFKPCDDSS
KTECVGRAFTNYIYKLKDENPKVDGKWSEWEKWSPCSATCGYGERYRERFCSNPEPENGGQTCE
GEAREYEECNHGECQTTCDKEIDFGIALDYSSSVRQVNWNRTLDFVVELSKGFNISPEKVHFGVLS
FSHRTRFDFGLSDKTYWNSESFEKKIKSLEYRWGYTWTDKALKKVKQLIFCEKCGLRKDSLKYLLV
FTDGRSNNVKTDLKTVSDSIKDMGVNVISVGAGHTINHRELEEIATDDNHVFYVGGYAYLGDNM
NAIRRLTCG*
>TRINITY_DN161_c0_g1_i3.p1 type:5prime_partial len:399 gc:universal TRINITY_DN161_c0_g1_i3:1450-254(-)
WNQEPLTCTYLYSVNSQLLKYKMADDDVAALVVDNGSGMCKAGFAGDDAPRAVFPSIVGRPRH
QGVMVGMGQKDSYVGDEAQSKRGILTLKYPIEHGIVTNWDDMEKIWHHTFYNELRVAPEEHPV
LLTEAPLNPKANREKMTQIMFETFNSPAMYVAIQAVLSLYASGRTTGIVLDSGDGVSHTVPIYEGY
ALPHAIIRLDLAGRDLTDYLMKILTERGYSFTTTAEREIVRDIKEKLCYVALDFEQEMQTAASSSSLE
KSYELPDGQVITIGNERFRCPETLFQPSFIGMESAGIHETTYNSIMKCDVDIRKDLYANTVLSGGTT
MFPGIADRMQKEISALAPPTMKIKIIAPPERKYSVWIGGSILASLSTFQQMWISKEEYDESGPSIVHR
KCF*
>TRINITY_DN3190_c1_g1_i1.p1 type:complete len:315 gc:universal TRINITY_DN3190_c1_g1_i1:68-1012(+)
MATVVDYPDFDAQADAETLRKAMKGLGTDEAAITFILGSRSNKQRQEIKTQFKQMFGKDLIDDL
KSELGGHYEDVVVALMKKPDEYDASELHNAMSGLGTHENVLIEILCSRSNAEIKAIKETYKKKYK
ADLEKDLQGETSGSFKRLLTSLVQGARLEDEPADHASAQADAHALFKAGESQWGTDESRFNAIL
ASKSFAQLEMIFHEYQLISNKTLEKAIKSEFSGDIQNGLLAIGKCAQSRPAFFAERLHKSMRGLGTT
DSTLIRVMVSRCEVDMVQIKQAFERNYGKTLNSYIADDCSGDYKKILLKLCGPE*
>TRINITY_DN1616_c0_g1_i2.p1 type:3prime_partial len:3398 gc:universal TRINITY_DN1616_c0_g1_i2:218-10408(+)
MIIFLAFLAVAVGQEVLLPPYFDIAKGKNITATATCGEDYVAPKGSPGELFCTLADATGKFGIKGLK
CSHCVPGDYGDGDGKNARDHHIRNANDGSNRWWQSPPLSRGLQYRKINITMALGQSFHVAYVI
IKAANSPRPGVWALERSNDNGRTYTPWYYFAPTSSECKQYFGVDSYLPIVKDDTVICSTEYSKIPPF
KNGEIHISLINGRPGASNFSQSKTLQEWTTATNVRLRLLDIKTLLTDLLPLSLEDPTVTRRYYYSIKDI
TIGGRCMCNGFADECTPHTNIVGKYECNCQYNTEGPRCSRCKKGYVQKEWRQRTSTDKFECEKC
NCHGHSDECEYNEEINKRKLSLDVNGNYQGGGVCKNCQHNTLGINCEKCKPFFYQPANVPIDS
PNVCQPCNCDTQFSTGLCAEETGQCECKSNYVGRYCDQCNKGFHMFPVCKPCECDVNGSEDRE
CIPDQINGCKCKKGYAGRYCDECLPGYFGFPDCQPCACLTRYGAVNDNCERDNGQCSCKVGFK
GRRCEECAAGHYQFPFCKECGCDPEKTLDTVCDSGTGVCQCKSNFAGPKCESCSPGFYAHPSCFA
CGCSTAGSTSSVCDQNSGLCSCRPNYSGFKCDRCAEGYYGFPNCILCDCNPEGSRRVGICDRQDG
QCQCKPTYSGQKCDKCLPGFYGFPDCKRCNCNPAGTKPGPGGRPGDCSTSNNGQCVCKDNVQ
GLECNECKPGFFNLTESNRKGCSTCGCNGAGTNNGVKSCSVEGTCKCKPLVKQEDKCTECRDGY
YGLKRTNVFGCLGCNCEVGGSSSSVCSKDNGQCPCKDNIVGKRCESVAANSFYPDLCHIKAEME

EGKTTDGDVVRYDYQKEIFPDFSWKGFVAVNDQQPSVVVSINVPKTSEYQVKFRYALNQIQPVTA
TVTAFKGDSKIVGSINFASTQKGSGFVASLATVQDNLVQEMYFPLTEGQWNIKLEAPHSNLVVDY
AVLFPKEYAQGGALKKKVQNACDVMTDEKFCNLYRYIDVADKKDIIVLEAENPVKMRQQKPPVL
FNDTAVLSKLDFQAMVVLSPSNSRQLNHLFNVAKPGKYVVMLQYYHDSDTAKYLDVYVRSQIAV
QNGRALLQSCRYTFGCRQAVLNRGDNGVFVFNIKSTKNHLLTLLSRYAVVAVDSVMLVPYESWH
LDYSTPTLYCVTKDSKCTNMNYDVPAESRLVQANPLEQDLSEKPRYLIDRSVEVRYVKPGVNIINIR
DVKAGNYLLVAHYYQPNFPSFESRAAVYSGRTSGLRARFDFKFCPNLNGCRSPIRRPDSNSNLYVF
EEGEARIRFDIPNGLDAWIEYILMIPEGTNVSSLYTPRVLDYSDDFKKECSIDLNVCTSSNLSSFCKDS
IFTITTDFNNGSFPCKCNMDGSKSSSCQEFGGKCPCKDNVIGRECSRCKVGYYGFPNCKPCNCSN
CDEETGVCTCPRGVIGENCDQCAPGTFGFDPIIGCQKCNCNEIGTGSANAECNVTSGQCPCKEN
YSSRKCSACKPGYFNYPACTKCDCLDAGISAEKCDVLTGKCICQNNVIGLKCNECADGYFKTNDI
KGKQLCAKCWCNGRSTKSCTEAELFRATPITASKGWTVSVDTLNVIENGNGRFTISDVNTLPEPV
YWVAPNQFLGNRGLSYGSHFKYNFQYSVKQPSQNTNGDVVTDAVIKGDGYTIKSMRSVIEKEND
GYSASILMKETEWMLEDGSTIDRELFLAILSDIDTLQILATHGKNTISSTIYDISLETAGENGTDRVYG
IEKCFCPKEYADLSCSKCSKGYTRQKDGSCGKCQCLGKSNICDPQNGKCINCSGNTMGDRCEKC
KPGFYGDKCKACECPFSNPENSFANTCAINVNTSSLVCQCNAGYKGVRCDQCSDGYYGNPLQG
VTCKKCSCNGNINASFTGNCDNTTGDCLKCLYNSDGNQCQECRDGYYGDAVVEKNCTDCQCSI
CGTTTAPCNKATGQCQCKKNVEGSKCNQCKPNTWNFDACNGCEDCNCAEASETQQCDLKTG
QCKCPPTVFGRRCGFCKLGYYNYSSEGCQKCQCPDGKPCDVNTGKCCTGVLNEDCKLCPVGHII
EDGECVECDRCVQRTLQRVNTLIHNVTILEKHIIPAGGLNYGALETLGNRFSNLSNKGDEHISNL
DTLKKAIGFGEYRNHSGNMDYVRMYLVRDFKSCSFIVNIKTPPTSSAPATTMVTNTPGPDPIRDHL
SNKKISDSQCPPSLARVIDEGEKNIKLVEKKVVVLEKDAYESTENATATKKRAEDLLAKLKDFMVD
FKDVLSFANSTLKGNVVINIDGNRSLAEAEEMLKEIKDRNFNSTINRSEREKSAAINATIIAEQLMK
QASELANKAYKFNDTFKDFVKALRNLKMESELALLDANNATNSINLAKAVDFQSIIDNAQKLHS
QNNVTVTQAMEILQNAKSAYENAKDVFNKFHNQTGPLSDLMEELRNYVRKLEFDLQTTSPIVDD
SVKHAKRLRDSADRLKELLQDTKKFADAALRAAKVYQNIVDAINDALDAARMANETAHEADK
KVQQAQQDAVESSITSKVVDTEANDLTEEVKKTLKVDISDMKMRLKELRELRKRIQGRIDNVKED
VKSFAKEKEVGEKAMNISTQADGVMKVANKGNEASQDTNEKVEKDGEKVETLKVDISDINKAIK
DLASNVTLVEDTIPKIRSNIDEVNVKHEQIEEFIKTNVTNNIGYLKYLISNAHDILDQMQLSMKFNS
TSEVVLDAPKSVADPTINNKISLTFKINQGNGFLFIIGDFSGNSSGFMSLHVQNHTVVFDYKLNTG
DVQSLTNPKFIETDVNYDVMVTRNFATAVLNVQKSGNASTLMTAYQNRSNISPVLSLGQDTNVY
VGYHPSTPNQRPNFDGCINQFRFDEELMGLWHWKSAKNVGGCVQDFRSIEGQYSFDGDGFVEF
DLVSPELSGFRKIELRFATLEENGLIFLGTDPPTSLPSDIMFDIPLSNQIIAFNALEIKDGYLVSTLDLG
NGVQRLVHTNAGKVNDGKPHLFEMKVLISSLLIKFKLDKKKITEIAISPDTDIRLRLKEAFIGGLPQ
RAWLPNSNISTKGFTGAIGDAKYRGGEFKLVKRFNNIRKGFILPKGYSASFPEEKSYLRLDACNLK
QNSLFGFSFQTFEKNGIIFHSEEGSGSSRSSIEIILIDGQVKVDVSVGSQKRTLQTSVARGFSDGLWHT
LSLYFEETLQEADIVLLYLAVDKAYITGTVPKVDLKGSMYFGSKPSGTESGFNGNIRQVYCNKNEIN
LASGDVFEGGVVGSLKLLPPLKLADFTVVPTTLPPLTTSITTSESTTTETVMTTSTTSISGTTLEPPKET
TTNMAKTTENPTVKITSSEPIIETTMESTTVAKTTSAITMEESTTTPTSITTSTLETTISTLEPGSIEGSTT
TAE
>TRINITY_DN72625_c0_g1_i1.p1 type:complete len:1964 gc:universal TRINITY_DN72625_c0_g1_i1:173-6064(+)
MKLAFNIFTISLLIGNTIAQNNDVVQVCKSQADVVAVLDSSGSVGVQDYEELKKFVGFVANLLTIN
ENGNRLGVVTYSETAEVDFNLNEIYDSDQYGREALTRLSVRPREFKTNHYAGLKVGSDLLFNKAA
GARDNAAKFLIFTTDGRNNFDETNNRNRASQLATQLKADGVNIYVIKAEVMSDNNGRLDSRLYS
TDPNKFYRFINRFGEITNENFLDEVGLEFCELVQLPDNQQATVREDRNIGTLVTTIRAPGLNNPTFE
LIPQDNLPFIVDPLTGEIKTIGLLDFEQVQQYVFQVRVRATNPDVEKIITVTINVLDVNDNDPIPETA
NYRMSVAETTPLGQRILTVKANDPDGGTNGNVEYFLISGGDGKFEVDQATGEIRLIGQLNKRIKD
TYLLTLEARDMGIPTRKAPITVAIDVTSLNNFPPQFNEMSFVRTIPENFGAGTVINAAATDRDGDT
MVYSLIGENIPFSIDQNGLITVSGNIDFETIPNNVFQFQVQAEANGDLVTQDVIITITDINDNVPVYS
MTTYSGSINENSAAGTVVNFDNVIIATDADKTNANNMITYNIIDGNDGDRFQVTKNERGEAVISL
KSTPLDQETQGQYRLTLQAIDNGVDPRSLEMNSLLIIDVIDQNDNDPIPEESVYSIAVAETSPVGQSI

LTVRANDADSGMNGKVEYFLISGGDGKFQVDQATGQITLIGMLNKRVKESYTLNIEARDMGVPT
RNAPITVNIDVTSLNSFLPEFAGAGDAFERTIPENFGSDTVIKAAATDGDGDMVFYSLVGDNLPFA
IDQNGMITISGSIDFETIPNNPIRFQVKAEANGDMTLKDVVITITDVNDVTPEFTQQVYAGKIEENS
QTETVVRFDQPVIATDGDKSPANNVVTYRIVDGNVDNIFKIVKNAQGEAVISVNNAGLDREILNQ
YTLTIEAKDNGVDPGSLSSTTQVIIDIEDVNDIPPLINTSENKAVGEADFTATISEDSPIGTSVVKVEA
TDPDSNNDDLIFTIANDVPVPFTIDPKTGLITVSGPLDADDQDEFEIAVTVSDGVNNSTAIVKVNIA
DLNDNKPIFTTDTRDITIPLDTPNGQEIIKFTGSDADKTSPNNQFEFSLVDGDGKPFTITPDGSLIIN
GPLMPGDYMLTVKIMDKGTNPGPLSNVIIYNVKVVDTNDPPTFQNNEQTVTISENTADGTDVITF
AATDPNGDAITYEIVNPGSVPFEIDPITGVIRKMGLIDFESPVRSYTLDVKATDAKGKSTMGRVFVQ
ITDFNDNTPTFGPGNTDNFILTEGVYSNQLVSSFTATDLDATSPNNVFEYTLVGAPSMFLLTTNPNT
GAADLTVNGDIDAEQNMSFNFIIRATDKGNPALSMDYPVTITVGDINDNAPVFAANTPESIDLME
GDFNNQIVATFTANDDDTTAPNNLVDFELVGAPDFFKLIVNPETGTADLVIDGNVDIDGLDPTINF
IVRAKDGGNPALVKEFPLTVNIADINDNAPVFTLEAYNQVINENTPIGTKVVEVRATDSDSGPNG
DITYRIVDGDGTFGIDPKTGDITVLKNTDFESGVRSYEFTVEASDNGNPKQSATAKVKIDIRNLND
NDPIFTSIPITGNINENRPAGSVVQNIQPVNGILNQNNVKILREPPPGAQETEGVGFDAPFEIDPVT
GEVKSTRPFDFEEEKVFTMMVQAADGDNNPMLTTMTVMVNNLNDNDPIMKKANFEITLNQDT
PVGTFVLQVQATDIDQENQTKITYSIINIDGGNMNDFTINPDTGVITLNTDLSMNTVDIYNITIQAE
DQEIQGVIRRSTALAEIKILRPAEAEPDISTLCTNSCDIPEERLKCLDYSKFEITRKFNGTCYVDKLKA
AIADVKNNTMEIMNLNASFLECRNKVQNEITNPNTQFPVNGLFDLHRKCTVAIECKLEQLAFNL
MRKTTELTNNLKAEEEGFSMYDMSLSAFFQHISKMVSMSKSSRVD*
>TRINITY_DN15274_c0_g1_i1.p1 type:complete len:1822 gc:universal TRINITY_DN15274_c0_g1_i1:87-5552(+)
MECIRRSIIRLLLILGFYTLYISSQEECKRGGCFPATGDLLVGREKQLLANSTCGLNGPSKYCILSFLD
KTDKCFYCDSRRQVDERPDGYKNSHLPKYMVTREPSKRLQGWWQSVNGRQDVTIQLDLEAEFIF
THLIMTFKSFRPRAMYIERSSNYGNDWSDYRYFAYNCRKSFPNIPRGPQRKIDDVICEERYSKVEPS
TGGEVVMKVLDPIIQKKEDPYSLKVLNLLKLTNLRVHFKELHTFGDDILGDSPKVNQKYYYALYE
MVVRGSCSCYGHAEQCIPTKGTPANVNMVHGQCNCTHNTKGRNCEMCKDGYNDEPWRPAYQ
DQLNVCRKCNCNGHTDKCHFDPAVYKATGNTSGGVCDDCNHNTAGRQCELCKPLYYRDPYKN
IFDEDVCKPCDCDPSGSFGGGECETISDPNNSSALTVAGRCICKPNAQGLRCDSCKDGYWNLLES
NPDGCQSCNCDDKGTVELNKCDQQTGTCRCKRFVIGLTCSKCLEGFWNLGNTPEGCEPCNCDV
GGSLNAKCDDITGMCMCKPNIIGRKCDQVAPGSYFGNLDQVISEAENSLVRSDSSSVEIVREILNPT
DSRSFTGDGYLRVSDGTLVNFTNIRVPSTGFQTFVMRYSRPTPGSNNNVMVRITKTDGTILGGNGC
VQTNEREIILNGVLNDDNFLELGDMFCLEKDTDYSVQLVVSNRNSPNDGGVNRGVTNLDSIVM
MPNIDNLAIFQGREGMRLKSQYDLNNCRQYYLEPNGAANAPEICKKIQFSISAEINNGALKCNC
DPTGTNSSGSSDILTCQKSGGQCPCKPGVVGRRCEKCAPGYFGFDSNGCTACACNDTGSEKLFCN
GITGQCPCKNRIKGRQCNSCPSNHFGFPNCIACQCNEHSSTCDPISGECTNCKDNTAGPSCNLCA
EGYYGDATAGTEKDCQRCQCPGGSSLNQFSNTCHLKPDGKVVCNNCTEGYTGDQCDKCEDGY
YGNPLVPNGSCNKCDCNGNIDPFADGKCNAENGKCFNCINNSDGDHCENCKEGFFGNAKNQS
CRACVCNKLGTNSATSDSCDRETGQCECLPNVEGVECDRCKSGYYNIASGNGCVSCNCDKGGSL
SSHCNQLDGQCNCLNGYGGLDCTQCEDFYFGDPKQNTCQACGCNPVGSITLQCNRTTGVCMC
KDNVVGEKCDMCAPQTAGVMPQCEECHVCNDQWEDIVNDIEKNITTILTLKGSSPLNVSEIRAY
TNDIADLKNRLDKIEEDFGKQMVDPNKINRLRDKVYRFSYNLTGINRDADFYMEMVNETITRDE
KANDILDGLELRLATLQTNTEDFKNNVTKIVESNIGGALNSTRESLEKSRKAQESVNDSDLKIKES
KKMRKKIKKEIQNGDPEFEVMHNRSSEFVNNLADRENYLEGELEKLNRLLCGSDKRCGGCTKGG
CDTCGVSGENYTCDDGIKMMSMMASDKALEAKEAIDQKLENASELLENVNRAKQHVIQANISA
NMAEDKAEEVNKIGQAVLKNITTLIKDIQDFLATDFNHPNASKDLAMQTIALNISLTPDEVVELG
RKIRDAVKSLTGVDKILNESRNDYDTAIGLKEQALAAKERAIEVMNQTMAIIETLSQAEELQNSTL
AGVNVARADIEDVKDLLKKLKEKLDAADNDLQMADNSTASAEDKRNAILDQNLENREKLDLSE
KKIDEAEELGDKVQGSVDKMNEKYNATKAKLNEKEDMIEKVRARIKALADKTIKLYENADRKLR
RINKMKDSYIINEERAKDLYYEIRRLEIKEFFINTKLEGTAKCYSTCNPIIPESFCRDVYKNIAVKETN
AYAELEAEKASTTSLLI*

>TRINITY_DN477_c0_g1_i7.p1 type:3prime_partial len:1130 gc:universal TRINITY_DN477_c0_g1_i7:3387-1(-)
MMGSIKVFVGLVLLSFTAIEIAQAQSGTVLSNKQFAFDFVVDGSTSIQDEQWNLLTDFVSRLAVKM
STLNNAKLSALQFSHTLNRILQCNQYANVEEARNTIKNMVQENGATGTLAAMLTSADNLMNVC
SAGDPNRQNVMIVIVDGNQNLEQKSHSEIVQDIKVNVKDKNILVLTLGVGPQVNRNEFGDYASD
SGKGFFVDDFTLLTDELVQRMYDELIRTYGDGPDYLTAWVNGPCSATCNPFPMANRPTLRQTRQ
CQEPNGGNCGGASLEQIINCNENIACAGETYLTAWVDSSACSLTCNPFPVSNQPTKRQTRECRQP
SGSCNGASLTQTINCNENVQCPGETYLTAWIDSSCSATCDTGSNNPVFTQTRQCRQTVGNCDGA
PLQQTRSCNVGVSCTAPTFLTAWSNATPCSATCNPFPMAVRPTLTQVRQCNEPNGGNCGGASLQ
QTVQCNLDVACAGETYLTNWVDLTACSATCDDASNRPVKQQERQCQQPGGNCDNLPLQQTVN
CNIGVSCTVSSSACSSKEISFTFVIDTSASVDNIGFFVLKDFMKKYMSQSTLGTDPRTSVAIVSYGAG
DAKVEVECDTITTQQSFNTTVDNLSQLFGIADDNSFNQALSTAFDASGRCARSSTSLNIIIMLSDGD
PPALENILTRDRIQSSSNYYFMFVAIQASDGAQSTFASLANQGRGLRNASSSFTDLYNFNTDLYYLE
TLSKACDVGGQWTEWSTSACSTTCGPGVVTRTRECRNRITGKLQQDCGPGPAQETQACNLQDC
QGTLTQWTSWSCPATCNINNDVNNPPLALRTRQCLGSTGQGNCNNADKTENEPCVRNVPCPG
AWTTWSVFSACSETCRSQSAFPTRRRTRQCIGGTLNFQCPPQIVNGISYREIWQEDCINVPNCPAK
FTQWTTWSCPVTCNVNNDRNNPPTATRTRTCDDSGTINGCANANILENQPCVQDVQCPGAWS
TWSAYGACSETCQSGNRSPTYTRTRTCLGGTLGFQCPPFITQIGGFQIFGTQTSNCNSEVKCPSKLT
QWTTWSCPVTCNNRNDVNNPPQARRTRRCDTSTGSGICESPIVEEFENCVANVPCPGRLTEFTYD
NCAHTCNVNNDVNNPPQRVGTRRCLDGTLG
>TRINITY_DN477_c0_g1_i7.p1 type:3prime_partial len:1130 gc:universal TRINITY_DN477_c0_g1_i7:3387-1(-)
MMGSIKVFVGLVLLSFTAIEIAQAQSGTVLSNKQFAFDFVVDGSTSIQDEQWNLLTDFVSRLAVKM
STLNNAKLSALQFSHTLNRILQCNQYANVEEARNTIKNMVQENGATGTLAAMLTSADNLMNVC
SAGDPNRQNVMIVIVDGNQNLEQKSHSEIVQDIKVNVKDKNILVLTLGVGPQVNRNEFGDYASD
SGKGFFVDDFTLLTDELVQRMYDELIRTYGDGPDYLTAWVNGPCSATCNPFPMANRPTLRQTRQ
CQEPNGGNCGGASLEQIINCNENIACAGETYLTAWVDSSACSLTCNPFPVSNQPTKRQTRECRQP
SGSCNGASLTQTINCNENVQCPGETYLTAWIDSSCSATCDTGSNNPVFTQTRQCRQTVGNCDGA
PLQQTRSCNVGVSCTAPTFLTAWSNATPCSATCNPFPMAVRPTLTQVRQCNEPNGGNCGGASLQ
QTVQCNLDVACAGETYLTNWVDLTACSATCDDASNRPVKQQERQCQQPGGNCDNLPLQQTVN
CNIGVSCTVSSSACSSKEISFTFVIDTSASVDNIGFFVLKDFMKKYMSQSTLGTDPRTSVAIVSYGAG
DAKVEVECDTITTQQSFNTTVDNLSQLFGIADDNSFNQALSTAFDASGRCARSSTSLNIIIMLSDGD
PPALENILTRDRIQSSSNYYFMFVAIQASDGAQSTFASLANQGRGLRNASSSFTDLYNFNTDLYYLE
TLSKACDVGGQWTEWSTSACSTTCGPGVVTRTRECRNRITGKLQQDCGPGPAQETQACNLQDC
QGTLTQWTSWSCPATCNINNDVNNPPLALRTRQCLGSTGQGNCNNADKTENEPCVRNVPCPG
AWTTWSVFSACSETCRSQSAFPTRRRTRQCIGGTLNFQCPPQIVNGISYREIWQEDCINVPNCPAK
FTQWTTWSCPVTCNVNNDRNNPPTATRTRTCDDSGTINGCANANILENQPCVQDVQCPGAWS
TWSAYGACSETCQSGNRSPTYTRTRTCLGGTLGFQCPPFITQIGGFQIFGTQTSNCNSEVKCPSKLT
QWTTWSCPVTCNNRNDVNNPPQARRTRRCDTSTGSGICESPIVEEFENCVANVPCPGRLTEFTYD
NCAHTCNVNNDVNNPPQRVGTRRCLDGTLG
>TRINITY_DN5818_c1_g1_i1.p1 type:complete len:282 gc:universal TRINITY_DN5818_c1_g1_i1:49-894(+)
MHPPNSAPVKSEYTNVEIPERDTDSYGWCALLLTILSFVIVVCTFPLSLLFCLKIVQEYERAVIFRVG
RIRRGGAKGPGLFFVLPCIDNYIKVDLRVVSFDVPPQEVLTRDSVTVSVDAVTYFRISSPIDSVCNIE
DATRSTKLLAQTTLRNELGTKNLSEVLLEREAISRNIQRILDAATDPWGVKVERVEVKDVRLPMM
LQRAMAAEAEASREARAKVISAEGEMNAARALKEASEIISESPQALQLRYLQTLQTISAEKNSTIIF
PLPIEMLSKAISS*
>TRINITY_DN15584_c0_g1_i1.p1 type:complete len:275 gc:universal TRINITY_DN15584_c0_g1_i1:874-50(-)
MADSGSAGADRGGRGGFGRGGRGGRGRGRGRGRGRGKPEDKEWTPVTKLGRLVKDLKIKTLE
QIYLYSLPIKEFEIIDFFIGPSLKDEVLKIMPVQKQTRAGQRTRFKAFVAIGDYGGHVGLGVKCSKEV
ATAIRGAIIQAKLSIIPVRRGYWGNKIGKPHTVPCKVTGKCGSVTVRLIPAPRGTGIVAAGVSKKLL

QMAGIDDCYTSACGTTATVGNFAMATYMAIARTYSYLTPDMWAENQFQKTPYQEFTDYLAKNH
NVGGKPVAPATATA*
>TRINITY_DN11435_c0_g1_i1.p1 type:complete len:430 gc:universal TRINITY_DN11435_c0_g1_i1:1616-327(-)
MGNIYTVGPNEALVVSGGCVGQRVKRTIVGGWAFAWACVSDVQSISLEVMTLNPRCEKVETAKG
VAVTVTGVAQVKVIKEDDLLKTACEQFLGKDPHEIEDILLQTLEGHLRAILGTLSVEAIYKDRDQF
AQLVREVASPDVGRMGIEILSFTIKDIVDEVSYLDSLGKTQTAEVKKNADVGVAEANRDAGIREAE
CDTQRQDVRFKADTTIADAARAYEMEKAEFDKEVQTKNAEAKLAYELQANKEQQRIRNEEIQIE
VIERRKLIEVEEKEIQRREQELQATVKLPAEAESYKVQTIAEANKSAKVYLAQAEAEKIKMIGSAQ
AAAIEAIGKAEAERMRQKAAAYKQYGDAALMSLILEAMPKIAAEVAAPLRKTEEIVLINDDNGN
KMSSEVTKLLGQLPPSVKALTGVDLSKALGKIPGAQVTA*
>TRINITY_DN3078_c0_g1_i1.p1 type:complete len:1109 gc:universal TRINITY_DN3078_c0_g1_i1:3628-302(-)
MKMRIYLFLILFATVYCRVVQKGPNDIPEETKNEIKNQEPKIKFEPEKDKKYFLAKDTTCAHDVDK
HCKNIRRENNFAVFVCLQDVIHKDEDDVAEECHETLWHYKYNLTRDPNFDQAAFKVCENVINEI
QECNDSELLKSPGFVTNCLIENRHAVSEAKCKSFLQKMSAIVFEDYRLIKGFYDSCVEDVKTLKCG
TVAKPTDEEYQHDQTEVISCLEEQLDLVSEKCQAQLVKVATMQADDYHLDRPLFYACQEARERF
CPEVKSGDGRIYKCLVEHKTNEMMLEKCRKKIEIREKLTQKDIKVNELLWKSCKADFTKYNCPKD
GISEHGAASSLLLCLQGKVAEGLPVDQSCQAEMRTFAIELFENYKISPVIVSKCDVEINQFCHHLIG
KTDDGDMMDCLMTQAATNNESVSKKCYDAVSDVIKETGAGNNYKIDRALYLACEPVITTLCQG
KEDAMILGCLMDNAHNSKMPEKCAAQVFHLQYFLSRDFRLDASLYKDCKKDAEEICSANDFNA
ESESKVPDNFVIACLYRNALDRKVDDRKVSQTCAEHVFRVMHQRATNIHLMPELERPCMADLG
KHCIDKGDKEGEEIDCLQTNYEKLSAKCQNAIKVFTIQEAKDFDLDKHLVDKCAPMVQQFCQRE
LEDGNGEKVLPCLVDHKNDIDMDHQCANAVEHWQLIEMKDFEFSPALTEMCLLDIRKHCQKSK
AKIEALHCLSENIVKDASSVTEKCRAQLKNELIEQSENIKLNPELYEACTKDIELFCRDVVPSGGQL
EECLKRNHKKLTDPKCKSLLFKEEKKESFDPELDYRLMHVCKPIIKRYCTQYHQPVDIMECLRQV
THDRDMSRECRAIITERQIEQAESFELDPELSKFCQDDVNRYCKEEIKHAEKGDDDGAIFGCLVNA
MIDNKPIKNDCEMYIRRREAEAAQELDLNPQFLLQCRDEITKICPNMDEDEIIECMKMNIPQIQSK
GCVQEVKKLIVEGIEDVHVDPHLQEQCARDIRTFCQSVPSKSGNVVLCLIDVHKAKNLHLQPNC
ENFLTKRLSLYNAAKVDYAVFDSISAVMNAVSESPNRNYVYMTLLLVLTILFVGGMLFGRYTKKIRS
EIKNR*
>TRINITY_DN2113_c0_g1_i2.p1 type:complete len:1704 gc:universal TRINITY_DN2113_c0_g1_i2:5257-146(-)
MAQLLPIRFQEHLQLQNVGINGANIGFSTLTMESDKFICVREKVGEQSQVVIVDMADANNPTRR
PISADSAIMNPASKVIALKAAKTLQIFNIEMKSKMKAHNLTEDVIFWKWISVNTIGLVTETAVFHW
SMEGDSSPVKMFDRHQSLAGCQIINYKVDHSGQWLLLIGISAQQNRVVGAMQLYSVERKVSQPIE
GHAAVFSQFKVDGNPQTSNIFCFAVRGAAASKLHIIEVGTPPTGNQPFPKKAENIFFPPEADKDFP
VAMQASQKYGVVYLVTKYGYIHLYDIESAQCIYSNRISGDTIFVTASYEPTEGIIGVNRKGQVLSVSV
DEEKMVPFLIQVKQNGELARRLAERCNLPGADNLFLQAFNDKFASGNYQEAAKIAAKAPKGILR
TQQTIQRFQQLPSQQGQTSPLLQYFGILLDQGQLNKYESVELCRPVLMQNRKQLMEKWLKEDKL
DCSEELGDLAKQFDNTLALSVYLRANVPNKVILCFAETGQFQKIVLYAKKVGFEPDYVSLLRNVM
RVNPEAGANFSGMLIQDDEPLADLNQIVDVFMEFNLIPQCTSFLLDALKNNRPAEGPLQTRLLEM
NLLSAPQVADAILGNQMVTHYDRAHVAQLCEKAGLLQRALEHYSDIYDIKRAIVHTHLLNPEW
LVNYFGTLSVEDSIECLKAMLSHNIRQNLQICVQIASKYHEQLTTSSLIEIFESFKSFEGLFYFLGSIVN
FSQEEEVHFKYIQAACKTGQMKEVERICRESNNYEPERVKNFLKEAKLTDQLPLIIVCDRHDFVH
DLVLYLYKNNLQKYIEIYVQKVNPARLSIVVGGLLDVDCSEDVIKSLILSVKGQFSTDDLVAEVEKR
NRLKLLLPWLESRIHEGSEEPATHNALAKIYIDANNNPERFLRENQFYDSAVVGKYCEKRDPHLA
CVAYERGQCDQDLINVCNENSLFKSLARYLVRRRDAELWGTVLVEDNKYRRQLIDQVVQTALSET
QDPEDVSVTVKAFMTADLPNELIELLEKIVLENSVFSDHRNLQNLLILTAIKADRTRVMEYITRLD
NYDAPDIAGIAIVSELFEEAFAIFKKFEVNTSAIQVLIENIQNLDRSYEFAERCNDPAVWSILGKAQL
DQGLIKEAIDSYIKADDPSAYMEVVEASKNCGNFEDLVKFLQMARKKAKETFIDTELIFAFAHTGR
LADLEEFISSPNHANISQVGERCFEQKMFEAAKILFNNISNFARLASTLVHLGEYQNAVDGARKA

NSTRTWKEVCFACVDGQEFRLAQMCGLNIVVHADELDDLIRYYESRGYFEELISLMEAALGLERA
HMGMFTELSILYSKYKPTKMKEHLELFWSRVNIPKVLRACEQSHLWSELVFLYDKYEEYDNAVVT
MMKHPTVAWKESLFKDVITKVANIELYYKALQFYLDNKPLLINDLLVVLTPRMDHTRGVSFFQRA
CYLSLVKPYLRSVQNHNNKAINEALNSLLIDEEDYNGLRASIDAFDNFDNIALAQLLEKHELIEFR
RIGAYLYKGNNRWKQSVELCKKDHLYKDAMQFACESRDKAVAEDLLSWFLSIDKTDCFAATLYM
CYDLLEPDVVLELAWRNNMLDLAMPFMIQFMKDYYTKVDKLAESNAKRDEDKENAPPEAPIM
GVEQLMITAGPSSYVPQPNMYGMPPQGMAPQGMPPQGMPPQGMPPQGMPPQGMPPQQGFPQ
QGFYGGAM*
>TRINITY_DN6390_c0_g1_i1.p1 type:3prime_partial len:1941 gc:universal TRINITY_DN6390_c0_g1_i1:5820-1(-)
MVIAPKCKFSIFSYLVFGILSYNWNELDASTNIELVDGTGVLLQRDVDNVLNDAVVQKEYQQNIK
HKPVDKRCLGEPFYQGLCKEKLEDAIQQNITEGGCHNLLVKIQKDECSNGPKTLGGKGYCNFICS
QEICNSIYDIDKCVSKRLVHHFNKDKNECEEIMQADCGSESFTSSNQCQKRCIGNSDDKRETKVY
CDQFCLNGGKCIGEVYCQCPPAYHTATCGLSYCNPSCGVGGKCVKTGEGKYGCQCFPGYKGVK
CDKEDSKNMPVVKGLCLSAIKSVLTSPNTSFHDKPEEEKNYCLKYLFDLESNCDKQRPFVNYTEE
QCNHFCASDICDNIYSNDCGSIEERYGYVSSTGRCSKFLYNGCLGNTNNFKDKAACDTKYSPING
LMTSVALLRPLNHTCYIRGKTNFITFDGKVYDLLSGCSHYLVHDSDTTSDFNVILHNDPKVNYNE
KSKRKLEITADGKNVLLNQKVSSDRFFVQVDGRSVPLPYNDVPKIRQAGRYILVLTGDGVSIAFDG
YEDISITVNDYYRTGDGGDHRLNGLCGNLDGNSHNDWLDFTGELRENVRDFAKVYQTDRSTCK
MDNLSLKSLVYNPLHNDQEVFERAERHCDVINTDAFKPCHSVVSPEQYKVLCMQDYIKCNDDK
RDDCVCSSLSFYSRVCHKYGKDIKWRRANLCPITCQRNMKYYECGSACPKTCDNLITPTKCQTDC
VDGCHCPPDLYYDKSTENCVPRNLCSCYRDGIAYKHGSVRSFHCEECTCNGGSWECNKKDCEAT
CDIAGDPHIKTFDGLNYQMYGKCSYVLVDHCHYGNTGIKEFEVIVENKNCNDDALYTSCVRHLS
VNILQTNTRVSLDTAKDNNGTVLPVAFVNGKEGKEFRSKDYTITSVGGRNIIFQYKVNQLKVSWT
GHNAYVTVGHQFENQTCGLCGTYNFNKQDDFHTRSDTVEKSVTAFTQSWVYQSSDKVQDPSKC
HTAKWETSEKPCQIYTNKAQYAKTQCSIIIKPDGPFEDCHSIVPPGEYYSMCQDDACKCDDCYCH
IVAAYAKRCTDLNIRVGNWRDETLSCKQTCQAPKVLKQCNAIDSLDDDLSESLKCPSTCYELSQN
DTSICQSRGCIEGCYCPEGYFENEQNECVKQDQCSCYHDGKVYRHDEIRKDRCNECVCKGGRFE
CTDFDCVAHCASKGLVYSDCGKTCSNFNFNPQDSKCQAGCFCPAGKVLHENGTCVNPQTECQC
KEGDQFFDAGEVSPSDCSKVCNGDRSWKNLPGKDAKPQYDCSAFGQMHYRTLDGRMFKFSGG
LCPYVLLTDCRQDSATGFCDLTKANVNIHVRNLRCVNSYEAYMCKEVTLDLRLPNGEMAEFVLM
QNKIKMKTGLVTKVFDKGSYPQPKTAVVDGVEIFKSGLYLVVRAYHLNITVKYDRQNRVYVVPGR
EYKDAGKLAGLCGNYNGLDDDDFRRPDNSNAENELQFAKSWQDSNSACPDPSESDTCSSNKER
LPWAQKGCNVMKTGNFTACHKTVSPEPFIESCQQETCLCKSGGDCACFCSAVAAYVQECNRHG
VPIYWRRDGLCKLDCCDPCGHSYGYYTDNHVPNTCGVKDKVCKENDRCCKGAVVEGCGCPPG
TYDDGIKCQNETTTNVIPCSPSSSIVPSTSISVPSTIIEPVSSSSPSPMTSSVESQSPSPSSSVVSPTPSSSII
QSMSSSEIESISTTSSSFVSSIESSVQSSTSIVFPVVSSNSGESSVSSSMLPSTSWVGIHSVSSSMGESSVST
NVLPTDYLRTTDVFPLSSSEITYPSSSSSLEPHTPSSSILQESSSSVSKPVGISSVIEPEISSIRYSSMSSQN
VMSSSVSSSVSKPVGISSVIEPEISSIRYPSMSSGSVLESSVSTSFVLPSETVLSTSVFSTNTESPSSSMTLS
TSSSGFVFPSVTSTFQTSSKVLSSLPRSGPAENTVPLSTMFTKTV
>TRINITY_DN691_c0_g1_i1.p1 type:complete len:1726 gc:universal TRINITY_DN691_c0_g1_i1:5662-485(-)
MMNLFRLIVLGVICFSYEGDAQQCGGSCSPCGNLCVGMKGDRGTAGLPGFRGPPGLPGFPGPEG
PIGQKGEKGPAGLNGMEGSQGARGKVGPPGLPGTPGRHGRPGNEGREGEAGLPGCNGTKGDR
GPEGPRGRDGIEGPQGLQGNVGPRGEPGDSTATKLKGQKGEPGSAGQQGEKGGRGMEGMKGD
TGKPGRNGRAGERGPKGEPGTSNVTIIGEKGSKGSVGPPGLPGRQCQVTGDKASNIAQGQKGNQ
GSKGEEGAKGEKGDAGLAGPQGEEGDIGPQGDRGDDGPQGPPGPEGEQGRSGIQGNKGQKGE
VGNNGKDGNDGQTGLTGQRGRQGTPGQKGEPGKDKKGLRGPEGAKGERGNDGEKGSDGPIGP
IGPRGPKGELGPLGMKGFKGSIGSAGPKGENGNPGDPGIKGRRGELGIKGDSGVKGAPGNPGISV
TGPDGMDGDTGPRGQSGGKGAVGFPGKPGPPGVVTIDGEEVLPGEPGPQGLKGFRGVQGDAGS
KGSRGEKGNACESSKCPAGPKGEKGLSGQDGLQGGDGEEGEKGEKGDKGDIGSRGDTGSRGEP
GPQGESGEKGEKGIQGPQGNSGKDIIVQGERGDKGTKGNAGTPGSIGFPGERGSPGESGDRGLSG

VNGLRGETGDRGARGVAGKPGVNGQKGQKGEIGSGARGFKGDKGEVGPDGPAGLRGPGGPVG
PKGDPFPTAVNGKTGEPGEQGAAGPKGAQGPVGPEGPRGFPGDIGPVGEPGDGEKGERGDDGA
RGERGEKGEPGQDGKDGDPGPKGEPGQEGKEGPPGMFKGNKGEKGATGLPGVDGLPGNSAPK
GEAGKDGQDGDPGPAGEKGDPGEAGSPGTEGPIGEDGPVGDKGEPGESRAGPKGNEGRAGAK
GENGRDGRAGRPGIGRPGDKGELGQKGEAVIGAKGSKGEQGLEGPPGPIGKPGVKGDAGVPGK
DGEAGGRGKDGERGLPGDKGDTGDIGFNGSPGAPGDKGEPGYSGQKGAKGMSGPRGPPGSTSN
VVGPKGNRGLPGMKGEPGQQGEKGLIGPQGSKGESGFGSEKGIKGSRGNPGAVGPSGEKGERGD
RGKDGLQGIAGPRGEEGNPGEKGSRGDQGPQGREGPSGELGPEGQKGDPGFAGEKGEKGDRGG
DGQKGDAGEPLRGEPGVAGPPGPTGPVGPPGKDYVFSGTAGELKGMKITKGDVGPDGVKGEKG
DRGDKGLDGRRGPTGPQGERGPKGEEGNMGPKGSQGRDGLTGEDGPKGSKGEPGLDGDVGEK
GEAGFQGLQGEKGNQGPQGLEGSTGMDGQKGQIGEKGVEGPPGPPCEPVNSVRGETGEEGPKG
DRGPKGQKGKPGPQSQAATPGEKGSKGEVGQPGPQGIKGEPGREGDFGPKGSPGAQGEKGDQG
QKGEPGQEGPEGLKGEPTRAFGPKGQKGETGLEGLRGPVGPKGEDGQTGPQGPDGPKGNEGEK
GDFGINGLNGAAGPSGPKGAIGLKGDSGPPGPTGAQGPRGSGGSPDFGFYYVKHSQTTIVPSCPA
GMKTLWTGYSFLYAQGNERAYGQDLGQPGSCLKRFSTMPYLFCDLQNQCTVASRNDYSFWLSTD
EPANMMKPAAGPEVEPYISRCAVCEAPANILAVHSQSVIVPDCPNGWENIWAGYSFLMYNSAGA
DGSGQLLASPGSCLEEFRANPFIECHGRGTCWYYGPTLSFWLSTIDDSNQFQIPKSETLKAGNLKT
RVSRCSVCMKTVRNGNFP*
>TRINITY_DN1386_c0_g1_i1.p1 type:complete len:596 gc:universal TRINITY_DN1386_c0_g1_i1:2419-632(-)
MEYIEVKSFIVALVFVFSILGTAQSDLVINNVERTLDLSTQTAKYTTKIKFHNPGKKVEKSFLLALEK
DQEKHLSYLEILDKQDDNSLEYAAENVEGQSGRFFKVSFSKPLKENDDVTLHIETVFTHVLTPFPA
KIGQTEKQLVLYKGNTVFYSPYEIKEQSTTVKLASSAIESYSRLKPSRSSENTITYGPYKNSKPYRLHE
MKIHFENNSPFLVVNEMTRWIEISHWGNIAVEETYHMTHEGALLKGHFSRYDYQRSPTHSVIKSF
KTVLPAAARDVYYRDEIGNISTSNMLVNDDSVEIELRPRFPLFGGWQTRYMLGYNVPAYQYLYNK
GDNYILKMRLIDHVFDEFVIDDLNVKIILPEGSTNIEVVKPYELEEGKRDIHKTYLDTSGRTVIVLSK
KNVVENHIQDIQVHYTFKKLQLLQEPVICVAVFYILFLSVIILVRLDFSITKDAAKESRMKVASLLEE
LLSSCGRRGSLYYSYDSAIDKLKQNRNQNDFAVSLKKLNQDYNTLTQSINELCSLLIKEDPEIGDKV
NDLQKKENERKVIVDQLVTLATRVVTNKIGRQQYIESEKNSMAKREKLGEDIDNVLATL*
>TRINITY_DN428_c0_g1_i1.p1 type:complete len:1949 gc:universal TRINITY_DN428_c0_g1_i1:96-5942(+)
MADDADLKFLMVDRRQVSDPVAHAEWAAKKLVWVPSEELGFVAASVKEEKGDKIIAQIEGGKR
VTFHKDDIQRMNPPKFDKAEDMADLTCLNEASVLHNIKDRYFSDLIYTYSGLFCVVVNPYKRLPI
YTDKVINLYKGKKRHEVPPHVFSIADTAFRSMLQDRDNQSILCTGESGAGKTENTKKVIQYLTAIA
GRANQQKADEKSQGKLEVQLLQANPILEAFGNAKTVKNDNSSRFGKFIRINFDNSGFICGANIES
YLLEKARLVRQAPDERIFHIFYQLLLGASPEVKKQFLLLDTKSYNFLSNGHVQLPNMDDRQEFKV
TLEGMRDMGITQEELDPIFKVVSAVMLFGNMEFKQERKSDQATLPDNTIAQKICHLLGIQVTDFT
KSLLKPKVKVGREFTQKSQTKAQCEFAIEAITKALYERMFKWLVGRINKSLNRGKRDGASFIGILDI
AGFEIFQLNSFEQLCINYTNEKLQQLFNHTMFILEQEEYQKEGIDWKFIDFGLDLQPTIDLIDKPMG
VLSLLDEECWFPKATDKSYVEKLHKQQTKHPKYLKPDFRSNSDFCVIHYAGRVDYSAAQWLTKN
MDPLNDNVVSLLAHSKEAFIASIWKDTENVVGMSVTEGMDTTFGASRTRKGMFRTVSQLYKEQL
SGLMTTLHCTKPNFVRCIIPNYEKRAGKIDNHLVLDQLRCNGVLEGIRICRQGFPNRILFQEFRQR
YEILCPGIVPKGFMDGRKAAKQMIDNLEMDPNLYRIGQSKIFFRAGVLAHLEEERDIKLTEIIIQFQ
AFCRGNIARRNYRKRIQQLSAIRVIQRNGRSWLKLRNWQWWRLFTKVKPLLNVSRQEDELRSKE
EEFKKLQDKSDKLESLQKEQQQANEKLTEEKNTLAEQLQAEIELCQEAEEARNRLLQRKTELEDL
IQDFEMKLGEEEEKAVKYAEDKKKLQQGIQDLEESLEEETQRQKLQLEKVQVEAKLKEMEDERQ
VAEDNNARLTRDKKQLEDRVAEFGQKLAQEEDRAKQLSKAKAKQEAFIAELEDQIAKGDKSTHE
LHKEKRKAEQDLSDLRAQLAQALQRIAELEDDLARREKELAEAIKRGDEEMAKRTTVEREKREL
QALLDELREDMESEKSAKDKVERQKRDLEQDLQELRSELEVTTDATVVQKEFQKKREEELMDLK
KQIEEEAKEHDQQMASLRTKHNGLVEDLNNQVDTFKRGKAALEKNKTALENENAEIAADLQRI
SQLKQEGDRKAKNLEQQLNEANSVRAVQDEKVSSLESEKAKYQKECDSLSTQIEEVETKAASLDR
AKQAAEAQLEDTQDQLAEETRQKLSYQTKLREASDEANRLQEQLEEEEDEKKAVQKVLSNLQV

QLDQAKKEIELKIQDFEQAEAARQKQKRENEELRGEVEKLTGDVSRLDKAKKKLQGDLDDVTVS
MERERNSANQMSSKQRKFDQLLAEEKANSQALGIERDNLEKLARQNETKALSLQNANEELEDR
LAEAERQRKQLQMELGDLVESKDDVGKSVHDLEKSKRLLEQQLAEQKTQVEEIEDELQACEDAK
LRLEVNMQAQKAQFDRELQAKEDAIEEGRRGLVKQLRELEAELEDERKLRQSASAAKRQLEGDV
KELEGQLDQANRIKEDGLKQLKKYQNQLKDVQRDLEDSRQSKDDLAAQVKDNEKKLKGLEAEF
LQMQEDLSAAERARRAIESERDELLEELNNNSALKNAVAEERKRYEAQLAALEDELEEERTQTEL
MQDKVKRSNLQLEQMNAELQEERSAAQSLDNTRMSLERQNKELQSKLADLESSLRSRQKNTVAT
LEAKINNLEIQLDQEAKERQQQNKALRRNEKRCKDLQMQIEEERSHTESYKQQLEKMNARVKN
MKRQLDESEEEVTRLNTAKRKLQRDFDENQESLESSQREANQLRQRLKGGASLSSSRSSGRGRRA
KDEDDDDDDANE*
>TRINITY_DN1199_c0_g1_i2.p1 type:complete len:296 gc:universal TRINITY_DN1199_c0_g1_i2:164-1051(+)
MDGLNDAATKAKTIYGGNLLFSAAMTAVAHPMTYVKVLIQIGHEPLPPQQVTTLFGKKVMRLP
NFFQYAGHIKKIDGWFGLYRGLGPRVVHNICSSAITNSVTNKIKGNSRDEARPNDKSVEGFLRETG
NLAIAKTAGIVVSYPFHLISIRMMVQFVGREGHYRNIFTSVQEIYREEGILGFFSGLIPHLVGELFAL
WMFKTLNFLVQNYLISEEYSQVAEIKSYSQGVSQYIASMVTYPFQLVSNIMSVNDSRLVAGNPPLM
PIYDSWTDCWIELGKKGLRNRGSTLFRRNVLT*
>TRINITY_DN3986_c0_g1_i1.p1 type:complete len:446 gc:universal TRINITY_DN3986_c0_g1_i1:252-1589(+)
MREIVHLQAGQCGNQIGAKFWEVISDEHGIDPTGTYHGDSDLQLERINVYYNEATGGKYVPRAV
LVDLEPGTMDSVRSGPFGQIFRPDNFVFGQSGAGNNWAKGHYTEGAELVDSVLDVVRKEAESCD
CLQGFQLTHSLGGGTGSGMGTLLISKIREEYPDRIMNTFSVVPSPKVSDTVVEPYNATLSVHQLVE
NTDETFCIDNEALYDICFRTLKLTTPTYGDLNHLVSATMSGVTTCLRFPGQLNADLRKLAVNMVP
FPRLHFFMPGFAPLTSRGSQQYRALTVPELTQQMFDAKNMMAACDPRHGRYLTVAAMFRGRMS
MKEVDEQMLNVQNKNSSYFVEWIPNNVKTAVCDIPPRGLKMSATFIGNSTAIQELFKRISEQFTA
MFRRKAFLHWYTGEGMDEMEFTEAESNMNDLVSEYQQYQDATAEDEGEFDEEEEEEDA*
>TRINITY_DN199_c0_g1_i1.p1 type:complete len:229 gc:universal TRINITY_DN199_c0_g1_i1:207-893(+)
MVQGSMTIVKYLMFFFNFIFWLSGLALIVIGAVIRDKYGDYFSYADSRFTNVAVFLIVVGVIVFIIGFL
GCCGAIKENYCMVTTFAVILGIIFILEIVAGAIGFAYKGQVEEKADKALVRASKNYNNDDEPGAKK
LLDWAQQRFECCEVASATGNGTCADKSPKSCHPDNDCNKTKYSQKCKEGFVEFMKDNLVTVGA
VALAIAFIQILGIVFACCLMKAIKGEYEVV*
>TRINITY_DN64_c0_g1_i1.p1 type:complete len:468 gc:universal TRINITY_DN64_c0_g1_i1:1555-152(-)
MPPKEAEKIHINIVVIGHVDSGKSTSTGHLIYKCGGIDKRAIEKFEKEAQEMGKGSFKYAWVLDKL
KAERERGITIDIALWKFKTGKYYVTIIDAPGHRDFIKNMITGTSQADCAVLIVASSTGEFEAGISKNG
QTREHALLAYTLGVKQLIVGVNKIDNTEPPYSEKRFDEIVKEVKTYVKKVGYDPKTVAFVPISGWH
GDNMLEASKNMGWYKGWTTERKETKTTTGKTLLEALDNILPPKRPTDRPLRLPLQDVYKIGGIG
TVPVGRVETGIIKPGMVVTFAPSNITTEVKSVEMHHEALLEAVPGDNVGFNIKNVSVKDIKRGNV
ASDSKNDPAKEAKSFYAQVIILNHPGEIHSGYQPVLDCHTAHVACKFSEIKQKIDRRSGKVLEEMP
KMVKSGDAAMIDLTPSKPMCVEAFSEYAPLGRFAVRDMRQTVAVGVIKSVTKSDGSGGKMTKSA
QKATGKKK*
>TRINITY_DN244_c0_g1_i1.p1 type:complete len:3019 gc:universal TRINITY_DN244_c0_g1_i1:9748-692(-)
MDARGQRLLYCLLIFLSLATLLVSQQVEKCKRPVDLVFALDTSGSVKDSGFRLIQKFAKDILKTFDI
GVADSHVGVLTFSEQAEPQIKLTETFDKAELMQMIDSLVYQGYRTATDDALRVAKTDFFSTAGGA
RQGVPHVLVLLNDGKCTVCIEPVKEAAQKLKDMGVEIFTIGVTENIKKQELIDIASQPTAYHFFEL
DDFDQLVDVVGDIQTRSCEVKKGKCPIPSPTEQCSGNEINECTSDENCEGELKCCSDGCSRICKYP
LLNCFTPIDIAFALDSSSSIQPETFQEMKKFSNDIVDSFAVSQKKTRFAALVYSDRAEIGFNFVRFDD
ASSIKKAIQNLPKLDSKERIDYALELAKSDIFSLQGKVRTRRPMVLFVFYDGTVSSGMKDLSLVVEP
LKNYGVKIVSMGVGPETNYYQMRKISSNNSDVFRATNFKELLPEIYNIAKRACTEKPGRCDASLN
PLPKEACKNVKILNQCENDFDCFGDAKCCRKGCNNVCVIRGPVCPRKLDIALAVHQNMDESEF

AATKFYLQSVVDQFNVGPSQTHFSLLSFGQEPKVIFDFNSDQSSNEIVRRKIDNFQYQYGIGRISDV
LALSCDAVFCAAGGTRDDVPKMLVIVTSRTSPSSPSKLKEKLQTLRNRGVNILMIGVGDVDTSLLQ
AITSDDPLVEDRVLLSKRFDGIFQYTKDISDYACGMAPALPPPDNIKGDAKGECGAPKSCYKGPKG
SRGRPGTLGQQGESGDLGLVGPKGIQGRRGDKGPDGQMGSKGKRGPDGLIGLPGFPGYNGIPGV
PGIEGRRGRDGDCGLPGSSGEAGQAGLDGLSGPPGRAGPVGEKGEKGLPAPENPNIRIEPGDRGD
TGSVGLPGFKGIKGEEGFKGPSGDSGVDGIKGVDGEAGEMGDIGTYIYQKGPEGDRGARGPPGD
GDTQGATAGLGVQGEKGEKGMRGEAGDGSIFPGIKGRRGDQGPKGEEGSTGPAGPTGQRGPEG
EEGPKGIKGEIGDRGKDGTPGQPGQEGDLGEKGFDGLKGQDGDDAEGELGDSGGKGLKGENGL
AGSPGSIGTPGPPGSKGEPGDIQIGLSGDPGAIGPRGPRGDNGISGQPGMRGDSGPKGAKGNQGP
RGDRGRNANMLAGKDGMTGPPGPKGFTGPVGDPGFPGPPGPPSVVECVEGACPKGVPGAMGQ
IGKTGEPGPPGPPGPRGSKGVAGGPCPICPDGIQGDSGDIGQPGMPGKEGSRGRKGNIGDAGSKG
LQGRNGDDGDQGPVGKKGFIGDIGPVGENGVDALGDSVAPDGEKGSTGDTGQIGSRGPVGNDG
PKGFKGTIGERGRRGLQGEKGDMGLTGETGEDGSRGLEGAKGDLGDGLRGAKGDKGNIGQEGN
QGVVGDVGLQGPKGEQGAPPTEDELKNVKATKGSKGEIGSTGDAGEIGDIGPAGPRGPKGFDGA
AGPKGPSGVKGDRGNKGRAGDGEPGFQGFPGRPGKRGPPGDPGKPGKRGFSEPGDAGDRGDP
GKSGKKGADAPPPIGCFKFNEPTDIGFILDSSSSVDDDNFLRQLQFVKNIVRQYPVGPDQTQVGIIK
YGEKAVTGINFYDYVTEEGLFNAIDLLEHTKESESRLDLALKLARDELFTESVGARGFDAKKALVIL
GDGFISGGGTPSKTLMNNAMQYAEDLRGAGVSIFALAVGPEKNKALFHEFVSEKEYYLDIESYSQL
DNELGALQLSLQTGCISVGVPGRDGLPGYTGPKGQKGDDGPKGFSPQGPRGIKGIKGVRGTIGIG
VKGLPGDPGLQGPQGEKGRDGEDGSADPGPKGRPGDKGSRGDQIKGASGEDGDIGPIGPPGFDG
RKLCDNVLAVDLLFVLDTSSGVGVDNFNKMLKFIESIINVFDVDNQLTRVSVMTYDSDARLVIRFS
DYNDPDKLDAAIDNIPYANGDETRIDKALLLASSNAFDTSNGAREGVNKAVILMTDGQQSFAVD
QVALKDAVKPLVDANVLMYAIGIGSEISPVDLKTIAAERVLIADDFDGLSLKIEDQIVLLGRVGCQG
PPGKDGKPGANGEDGFPGLIGAKGQKGDRGEAGTEGLKGNKGSQGEAFPGLNRKGEKGDPGS
AGPLGLPGCEIKNQNEVIDLGFAIDASGSLYEEGFNREKEFIKTIVDRVGRVSEQGIRIGVIVYSDTAS
IEIRFDDHFNNEDLKNDIDNLPFFAKNTRMDLGLEEAKKMFGEQFGGRGSSKKVLILLADGQQTY
VPGVTEPSEVAKELAAAGVDIFAIGIGQEIDRTELESLISKPEHIFLASDISKLIDELTPEVTSALECEG
GPRGPRGRAGTDGPPGSKGLLGPIGDKGFPGEPGSAGFGPKGDDGENGTAGTIGDRGEKGFRGP
AGVPGKRGAVGPKGFPGPNGEKGDRGDEGFIGFEGARGLEGPKGQRGDSSDNGVAGVKGSTGD
NGFKGPKGDPGKLPPGNRIEDFKGETGGSGLEGDTGPKGKPGRALFEPGPQGLTGPKGEKGDQG
FFGMSGPIGNKGFQGLQGFRGDDGAAGPVGEDGFPGMKGLPSSSGDQGDKGKNGRKGESGEV
GQPGRVGEDGDQGEKGYAGFVGMPGKKGDQGEPGVQGPQQIRNEILDNIRPGPKGYPGSTGPA
GDEGEDGEVGEPGETVGAQGMNGDFGERGSQGPKGEQGDQGLQGMIGVIGDKGVKGRVGTG
GNPGTVGLRGLTGAPGEVIFGSEKGNKGDLGIIGLRGRAGQKGKAGAIGPRGRRGRNGAKGERG
PPGFPGLSGIEGDSGNEGDKGPRGDPGSVGNRGIKGVPGESGIAEGNYIVIHSQTHIIPDCPRDFKK
LWHGYSLMYVVGNGHSHSQDLGDAGSCSQKFSSKIFLFCEIIKVCKHASRNDFTYWLAGDVDHP
MMPVSNAAVEPFISRCAVCEAHSSHITVHSQDTYAPECPPNWSSLWSGYSFLMVAGAGKTGAGQ
SLSSSGSCLESFRPKPFVECQGARGTCHFFSDKHSFWLVTIDPNKQFEVMKPQTLIKDKNHDLTSR
VSRCRVCIRKTGTKNNTVEPNIATRIRRSIASWIL*
>TRINITY_DN250_c0_g1_i3.p1 type:3prime_partial len:402 gc:universal
TRINITY_DN250_c0_g1_i3:131-1333(+)
MRECISVHVGQAGCQIGNACWELYCLEHGIQPDGQMPSDKTIGGGDDSFNTFFSETGAGKHVPR
AVFVDLEPTVVDEIRTGTYRQLFHPEQLITGKEDAANNYARGHYTVGKELVDQVLDRIRKLADQC
TGLQGFLIFHSFGGGTGSGFTSLLMERLSVDYGKKSKLEFAIYPAPQVSTAVVEPYNSVLTTHTTLE
HSDCAFMVDNEAIYDICRRNLDIERPTYTNLNRLMGQIVSSITASLRFDGALNVDLTEFQTNLVPY
PRIHFPLATYSPVISAEKAYHEQLTVAEITNACFEPANQMVKCDPRHGKYMACCLLYRGDVVPKD
VNAAIATIKTKRTIQFVDWCPTGFKVGINYQPPTVVPGGDLAKVQRAVCMLSNTTAIAEAWARL
DHKFDLMYAK
>TRINITY_DN3036_c0_g1_i2.p2 type:internal len:548 gc:universal
TRINITY_DN3036_c0_g1_i2:1643-3(-)
AFAGAARASPARQGSVARRALDQSSRYADLSLTEEDLIRNGKHVLVAYIMKPKAGYDYLATAAHF
AAESSTGTNVNVCTTDDFTKSVDALVYYIDPEEEEMKIAYPNLLFDRNIIDGRAMMCSFLTLTIGN

NQGMGDVEYGKIYDFFLPPEYLRLFDGPSCNVIDMWRILGRGTSNGGLVVGTIIKPKLGLQPKPFG
EACYAFWQGGDFIKNDEPQGNQVFCQMNECIPEVVKAMRAAVKETGVMKLFSANITADDPVE
MISRGKYVLSQFGPLSENCAFLVDGYVAGGTAITVARRNFPKQFLHYHRAGHGAVTSPQTQRGYT
AFVHTKISRVIGASGIHVGTMSFGKMEGDASDKNIAYMLQGDVADGPYYQQDWQGMKETTPIIS
GGMNALRLPAFFENLGHSNVILTAGGGAFGHKDGPKQGATSCRQGEEAWKLWKAGTYGDITLS
DGVIEYAKTHEEIKGAFLTFQKDADQIYPGWKEQLGYTGESSVQAASFDWKRKAGAAAFAGAAR
ASPARQGSVARRALDQSSRYADLSLTEEDLIR
>TRINITY_DN73067_c0_g1_i1.p1 type:complete len:831 gc:universal TRINITY_DN73067_c0_g1_i1:3714-1222(-)
MHWFVILLLFGVIFVTVSNYEIDVLSTFELSEDIAGISPLDNKYQAYQFAADGKELLLDDSIKTEIVR
GVTNFKDFFIAVNIRHDKQKPSSLLSLQTKTTGKQKFTLWINAEEEKIGIRSHSKESKKGKKNLTFR
KVPLNTGQWHRIVLNFKKIDTDEPVVDLYVDCKFHDSKTFPFSIGHALIEDALISELRLGQLKHLP
EKDPIRFIGAMQDFRLVSNRDVHWYNRPQHCYNMMIEVKGMKAYHEPVSTISHDVDNMAAAM
KNMQKQLKLQLDEIRDIKNWLQQCTACKTAAENVLGGEIPKACSQNRCNENANCIESNGGSAF
RCECKAGYAGNGEKCGLDSDSDGVPDNKLDCLGKECSQDNCPGFPNAGQEDADEDGSGDACD
IDDDNDGVLDGDDNCQFLSNRDQKDSDADNRGDACDNCPFESNYNQLDSNNDGLGDACTRD
ADGDGIRDKSDNCPRVYNRDQIDRDSDGIGDACDNCIDIYNPIQKDVDNDNLGDACDSNVDID
NDGIQDNKDNCLYIANANQQDIDNDGIGDACDPDDDNDNIIDENDNCRLIYNPDQSDSDGDG
QGDACEDDADKDETVDLKDACPENPEIQNTNFTNYKHIMLDPEGTSQVDPEWEILNKGAEIKQ
WRNSDPGIAIGDKVFGGVDYSGTFYVHDDKDDDFAGFVFSFQSNSKFYALMWKKDKQTYWFET
PYKVTGHAGIQLKVVTSSTGPGEHMRNALWSSISIPGQTKIIWSDPSKEGWKPKTSYRWTLMHRP
SEGLVRITIKEGSQVRADTGYLIDKTYKGGKLGVLVFSQKEIVFSALQYRCTEQIPADYNPNSPN*
>TRINITY_DN477_c0_g1_i9.p1 type:complete len:306 gc:universal TRINITY_DN477_c0_g1_i9:1840-923(-)
MTRQCLGATLNENCGNAALVQFEVCNFQVPCPGLLTNWGPYTQCSATCQKDANSMPTQSRQR
FCTGGSFGGNCGGVQLLQTIGCNQLIICPVYGYWLQWSAFGECSKTCDQGTHVRYRNCTKSIPA
NAGNNCTGPAQEVKPCQVKPCPVTCPLTKKCDCRNMNYLAAAPGISQTVTNDVMGVAAFLDY
WTLDILKQICEGCRMTQLDQSKATVDSQILRTQAVLDRLNIEKRVYRDMITCNENPYITDDLFKVY
NDIWDRIVTTDGVMIELKASKMRFDTAITACRSYDWYHDLFGRFFQIKKSS*
>TRINITY_DN1280_c0_g1_i1.p1 type:complete len:426 gc:universal TRINITY_DN1280_c0_g1_i1:195-1472(+)
MGFETCGPNEAMVISGCGQERPLLISGGRKFVWEFFQKVQRISLNVMTLNVESQRVYTVQGVPIS
VTGIAQVKVQGQNEEMLHAACQQFLGKHENEIRNIALETLEGHQRAIMGTMTVEEIYQDRKKFS
KSVFEVASSDLVNMGISVISYTLKDIRDDEGYLKALGQRRIAEVQCEARIGQAMAKMQSGIKEAQ
AEEVRIKAEYENKTEVARSQRDFQLNKSAYDIEVNTKKATSELAFALQSAITNQKIKEEDMQIKVIE
RAQAINVQLQEIQRREKELEAQIRKPAEAERYKIEKIAEANRMKVVLEAEAEAESIRMKAEAEAE
VIQKKATAEAEQMSKKADAWRDYQEAAMVDMLLDTLPKVAAEIAAPLSKVKKVTMISSGSSDV
GAAKITGELLDIVIRMPQAMEQLTGVDITKTFRREK*
>TRINITY_DN909_c0_g1_i1.p1 type:5prime_partial len:313 gc:universal TRINITY_DN909_c0_g1_i1:1-939(+)
RRVLFRSLTFIESLRLSRLVNKKNKTFMEMSEKGGIYNDNEREMNFTGNSDYESGDLGFCGWFLTV
LSFIIAVCTFPLSLFFCIKIVQEYERAVIFRVGRLLSGGARGPGLFFILPCIDTYLKVDKRVISFDVPPQE
ILTRDSVTVSVDAVTYFKIQNPVASVCNVEDAQRSTKLLAQTTLRNELGTKNLSELLLEREAISRNI
SNILDQATDPWGVKVERVEVKDVRLPPMLQRAMAAEAESSREARAKVIAAEGEMNAARALKEA
SDVISESPQALQLRYLQTLTTISAEKNSTIIFPLPINMLQSMMNK*
>TRINITY_DN413_c0_g1_i4.p1 type:3prime_partial len:463 gc:universal TRINITY_DN413_c0_g1_i4:1388-3(-)
MVSLSVTCLSLLLFVTVNAEESDHCGYDEKSWYKAMGWCYHSGSRLLLGDFNGDKRTDFFCHD
SNGKNWVAVSNSDGSFGSTTWQDSKERWCASPNILLTGDFNGDGVTDILCHGTATGKKWIKYFN
KYGVPSGKSFYRAMSWCNHKGSQLYIGDFNGDKRDDLLCHDTSGRYWTSYAGTDESKVFHHTS
WYKALEWCRHATSKLYLGDFNGDKKTDVLCTDGKGYKWIMYAKTSGNLFTGTDWHTNLDWC
HESSTVFLRIGDVNGDGKDDLVCHSTAGNSWVAYTQPKGGFGNFCHLAKWCAAGKESFFLGYF

NQDSRADILCHDTKYGKKWIRYAECSFAEESDHCGYDGKSWHKAMRWCYHSGSRLLLGDFNG
DKRTDFLCHDSRGINWVAVSNSDGSFGSTTWHDSKERWCTSPNILLTGDFNGDGVTDMLCHDY
KTGKKWIKYFNKHGVPSGKSFYR
>TRINITY_DN113_c3_g1_i1.p1 type:3prime_partial len:1162 gc:universal TRINITY_DN113_c3_g1_i1:3485-3(-)
MEIVSRSRIAIFVLLGLLISVAESQFDPECPEIDPDVECSPPESTVNECLINNDCDVDKPLCCSNGCG
LKCTARERRSITPDGFTPEDEKLCRVPVDLGFIIDVSGSIGQTNFDKIKKFIEKIVDAFHIADDGTHV
GAVFYSDNASLAFDFNHLQGDQLNEMNVVREIEKIQVTDGQTRIDLGLELASRELFNPAAGMRI
HKPKIALVLTDGKQTRGELAPDAKDLHQASRLLKQTGVQVYSLGIGLDYDIGELLDIASDDASVF
RSQDFDELVSIVDILIEQTCKGCTRAVDVHFALDASANVGEENFVFMKDFVKAIGYNFIISETGAH
MSGSVFGDVGTLVFDKSQATEINEFENEVENMPYLGDATGNMDKVLKMISNEVFNFDGFTRQSS
PKVIVLLTSSNCDTCQESLEDLARPLKAEGIHIVAIPIGDPLFDEMQVLASHPIDQYAIPRATHQELL
NAEFIQKVSEMVCAGKPGICAEPPVKGECAEIINECSVDMRCPEDQKCCIKNCVASCVLPLAACIT
PIEIAFAFETTKYGIADFNNVKNFAINLLDHFKVGPSKAHVSLSTFSEDVTSLTKFIDDFDMESIKQSI
KDLKHDGGEESNLGNFLEIINTEVFSVRGGIRQQTPRVLVIFANGDINDEQAVKAQEEALHLKAD
GKDVKIIIVNIGSSTKTLDVLNEVATEGSFYEFFGSAELDSTAIETEISEKICSTKVRRSKCQRRIRSSCA
VKDDLCDSDFDCPGHQECFSDGCRLRCQTCEFRCKDKVDLLFVMDTSARIGRDNFERGKKNIKK
MLEWFTIDSNNTNVGVITYSDTANVAMPLPSRGNNNPAQSLIDVQDRLDSIPYSGGSVSHLDNAL
QLAAEKVFPDNGPNLRRDSKKAVVVITDSLDDSASQLELASWPLRRQRVQTEDGDASNIRILAFN
VGQELDLDLWGNIVSPPVEENVFYAADFDDMYNGIRQLAEDSCKYAAAITPPPIIKQKGEPGPPG
PPGIPGTNGTDGRNGTDGRRGPKGERGDTGLKGFKGEVGLVGPPGGGAEEQKGPDPLASAVGIV
NVTDLPGGGRIVFDLREGPVGQQGLTGIIGPKGPIGDTGEVGIKGRNGTTGPGGFFGLPGPKGQP
GTDGAKGLRGDPGLRGSAGRKGPTGAPGLTGPQGPFGYK
>TRINITY_DN1189_c0_g1_i1.p1 type:5prime_partial len:449 gc:universal TRINITY_DN1189_c0_g1_i1:1554-208(-)
VSAKSQVIAKMKFFLFSIAAILLVNSALADNAVQIPKTCEDVLAMETCDRLRAVAKKFHEKVGTV
NQAVTDAFKHHKTKAVEVLTYVKEYLVDKAVNFKCEDVLPADQCHNLLEFAKRLNVKASEASIA
LREAIADGSEQAKQLYQRAVSFLVNDVPNLKCEDFFKKDDTCDKIRNFATKVNAKAKDVTAAVK
KAIVENLENAKDGLKKAKQFLKAKISCDQVLNEKMCNTIQSIGDKFSVSMKKIDAALREAYANG
VRKWSELYEAAIKYIMDKWSIFGDDEPQRERREIQPENVKKAIKNLLDSLKVKSKKVRAAIMKLIE
SGKVKFGNLRKTVKQLLASLKGDKKSEDAVTAVYKRSVVDTLKETLQKLKEQIKDLAGKAKEQA
KAKIAELKELIKGALGSLDQDQSDELDQDFEMISDVLEAAADESFDEIANNALENHLGQYY*
>TRINITY_DN36_c0_g1_i16.p1 type:complete len:224 gc:universal TRINITY_DN36_c0_g1_i16:829-158(-)
MMIKTTIVAVVLAACVLQSFAGRCYVGYSAKGLPLAIGVRDVECGKGYDYCTTIKYRLGGIEVTQ
GNCARKYGQAMCQFMRNPHKAMTDCEETICQGDSCNKDVYDAEQAERKRVGPYKNTLKCTT
GLITKFPAIPFYTADVPNAIDECFPGTTECVTVTYDYLFKFETQIATKDNVFKLPCVQKMCNRHNI
DNCNKWCDILSIHQSMENCVAKCCKGDNCNA*
>TRINITY_DN2636_c0_g1_i1.p1 type:complete len:314 gc:universal TRINITY_DN2636_c0_g1_i1:986-45(-)
MSAIAVVVIVIAAVLLHFSVHKIEEGHVAVYYRGGALLSSVSGPGYHLMAPMLTKYKSVQTTLQT
DEVKNVPCGTSGGVVIYFDRIEVVNLLSVDKVHDIVKNFTADYDKALIFNKIHHELNQFCSVKSL
QEVYIDLFDQIDENLKRALQQDLNEMAPGLKILAVRVTKPKIPEQIRKNYELMEAEKTKLLIRIQH
QKVVEKEAETERKLAVIQAEKQSQVAGIQYEQKIMEKESMKRMSEIEDLTHLAKRKAMADAEFY
THQKQAESNKMKLTPEYLELMRIEAISKNNKVYYGSNIPNIFLNEGGLSQKTK*
>TRINITY_DN495_c0_g1_i2.p1 type:complete len:655 gc:universal TRINITY_DN495_c0_g1_i2:152-2116(+)
MDFHWNTVIAGLIIVQVYSLAEYGTLNGKDSYRNAYPWLANDRNNNELGREDARSIYRSSDEID
AEYPQSDDEVDTNDIPKSYTSRDELDAEFAQRTYKFADDLDIENAQKSYASSDELDRDDEQRTNK
NTDDLDAEDAEREETINDSLDAENAQRRYTSIDELDGEDTQRVISKTITLPRLGAGRIYKSRFIHGP
YIRPKFVRPHKGIRLPHPNRIIIPGSWKRPAHGIFALKGKKAIFCEHKGIKRISCSNDKVIKVRSALY
GRTAGNVCGRSRVTNCRALNSFAIVQKKCNGHNSCDLNPSNSVFGDPCVGTVKYLNIEYFCVKR

TPPTINTPTLTPVTLVIPVQSSYQGDFGKSLTLKWNLGLTEKDRIIAVYLYDDIKPCKTKIIFSSTGFNT
PMLQESYAQLIFGNRISATISKSSYQLTIKGLKASDARSFTLEMFVSGPGRSSCKPILSVIKISKINNINS
DKVDGGYTGWSVYGPCSKACGGGVQERNRSCTNPAPANGGKACDGVPSETRACNTNQCPEAS
MSYASTKYPASNCIDGDINTRCHSKKGVNPWLKVDFHKKVLFKSVELIKYFKYVCCDAVIKISAIQ
SDGTKKLCATDDQAEKGVQDLEIELSDNCVGHSIELQLDTHGKKSALRLFEIKFNAE*
>TRINITY_DN3545_c0_g1_i2.p1 type:5prime_partial len:236 gc:universal TRINITY_DN3545_c0_g1_i2:3-710(+)
SGPGRSSCRPILSVIKISKINNIDSGKVEGGYTMWSVYGSCSKSCGGGAQKRVRSCTNPAPANGGK
HCDGVSFETRECNINQCPVDGGYTMWSVYGSCSKSCGGGAQKRVRSCTNPAPANGGKPCDGVS
FETRECNINQCPVCPEIVGTYDISSRHHYGGAKYGTVVFKKLSNSKTVEAYYTINDGRRGVLTCKIE
SAQPDQCSGHGYYGKYFTYYKNRCQIIWSNDVIYTKQNC*
>TRINITY_DN120478_c0_g1_i1.p1 type:complete len:544 gc:universal TRINITY_DN120478_c0_g1_i1:2674-1043(-)
MMRAPVMVLNSNTKRESGKKVQISNIEAAKTVADIIRTCLGPKAMLKMLMDPMGGIVMTNDG
NAILREIQVQHPAAKSMLEISRTQDEEVGDGTTSVVILAGEVLSVASQFIEDKIHPTLIIAAYRKALD
DMLDVLKEQISIPVDVDNDEEMINILKSCLGTKFVNQWSKLACNMALKASRTVSTEASGRREIDI
KRYAKVEKVPGGSLEESCVLDGIMLNKDILHPRMKRYIENPRILLLDCNLEFKKGESQTNIELSGET
DFNRILQLEEEFIKKMCDAIIKHNPTLVFTEKGVSDLAQHYLVKAGIAVIRRIKKTDNNRIARAVGA
TIVNRPEEIREEDLGLKCGLFEVKKIGDEYFSFLTKCEDPKACTILLRGASKDILNEVERNLQDAMH
VARNVMVDPRLVPGGGAAEMALSCALEEKAKSITGVVQWPYKAIAKAVEVIPKTLIQNCGVNAI
RTLTALRAKHAEGGNSSWGINGETGEIANMKDLGIWDCYSVKAQTLKTAVETAMLLLRIDDIVSG
SKKSKGDEGATSGGMPTM*
>TRINITY_DN165536_c0_g1_i1.p1 type:complete len:249 gc:universal TRINITY_DN165536_c0_g1_i1:851-105(-)
MSEQEISNLVEQAKLAEQAERYDDMVKYMKELTEKSTEGLSDEQRNLLSVAFKNVVGARRSAWR
IIHSLEEKKGNEGGNTELISSYRKEIEKELKERCNEVLSLIDKYLLPSAQKEGQKGTEGKVFYEKMK
GDYYRYLVEVTTGEDRANLSAQSKEAYDKATETATGLATTHPIKLGLALNFSVFHYEIMNDGKKA
CELAKKAFDDAIAELDSLKEDSYKDSTLIMQLLRDNLTLWTSENDNEENDAQ*
>TRINITY_DN8903_c0_g1_i1.p1 type:5prime_partial len:447 gc:universal TRINITY_DN8903_c0_g1_i1:1546-206(-)
DMATKACGLENMCVGSYKSWRCICQAPRYMQDPDNTQSCIHAPCRIEDCGSGYAPGSFQYYAES
CNGTFFNPICNCQEEYEKDMLGGCVPKSCRENPIAVAECGENGYCKGTYFKYECGCQGPKYVQN
PRNTRQCIEAPCNVQNCGHGFENELYVSFADSCIGKHADYTCQCKEGYTGTGTSRCIPDGKECGK
SYCHANATCAPLGKGEFDCTCKPGYSGDGLYCFLDGGWSDFVNTRECNASCGTHGYAEVTRYC
NNPVAERFGKQCGAKSPEQLEVPCNRFDCLGCTVKKDCRCTSVVKPLIVEGYNEPEETLKFFTDL
HPDDVKKAKDTLLKWKAFSNENLCRKYNKTTLHAKFFGVRDAAKEVNKMKSSYGSARDSMRI
KLDCNINIAKKTQYFGVYEMIVKRLFIMKQVVKDLHIIEDYVLKHMENCDDSPVGMIRNTFGK*
>TRINITY_DN2472_c0_g1_i1.p1 type:complete len:344 gc:universal TRINITY_DN2472_c0_g1_i1:311-1342(+)
MSSGEIESLRQEAENLRNQIKDAQKVAADTSFSSVAQNVDGINRIQLRTRRTLRGHLAKIYAMHW
ASDSRSLVSASQDGKLIVWDSYTTNKVHAIPLRSSWVMTCAYAQSGNYVACGGLDNICSIYSLKTR
EGNVRVSRELPGHTGYLSCCRFLDDSQILTSSGDTTCCLWDIETGQQLTQFVGHTGDVMSLSLCN
DSGRTFVSGACDASAKVWDIRDGMCRQTFIGHDSDINAVSYFPSGLSFGTGSDDATCRLFDIRSDQ
ELMCYSHDNIICGITSVAFSKSGRLLLAGYDDFNCNIWDTIKGDRSGVLAGHDNRVSCLGVTDDG
MAVGTGSWDSFLRIWN*
>TRINITY_DN11522_c0_g1_i3.p1 type:5prime_partial len:855 gc:universal TRINITY_DN11522_c0_g1_i3:2878-314(-)
FSAQLFKGSSKSILGGIPNIQLNRGRIGLGNIRDPSISLTRGSKIDYNGYMGFSATSGYFGNDKHIHF
AGGSPRSNALKGRVIMYKDSAGYEISQYLDDPSDQIGTYFGSAMASADINGDGLADLVVGAPYYT
GTVSNEGIAYVYLNKNGIVELVQTLRQPEGKDVKENGMFGISIGSVGDLNNDGFADIAIGAPWGG
EDGKGIVFVYYGQRDPAQLKLMQTIKPSDLSGIDVDGFGYSFAEFYPYSGGPDQYGFIENDPDYNI
YPDLAVGAFKSNHAIVFKSRPVYTIVGRLSLGGVKRLDLVNKENLCPNGMKCLDKVQACLMFKE

EESEGEVDYTLEVDSGRDSPKRAYLLDSDGKQVTKINKQTTVSKDEECDDYTIYVKNTTDVLSDLV
IKLSWKFVVPTTCDGVCPVANELCQFVDTKSIIFVKGCSDDGNDTCSSDLKIEKKEFPPKLVGNNR
LVGTEEIISAGATDGIILTFNIKNLGENAFDNSLKLEYPGKGDMKLVNIDDAKVISDYYGTPIWDSD
VTREKDVTTLSVMLSSPIVKGKPEQVQVKMSLNPIPKGTKFLKFKVYLETDSEETDTSNDFQEITIP
VELVSNLLVSGQSIPEILTWRKDVKDVQDKGAEITYNFLFQNQGPSNVDTSEAIILIPEKFKGSYFLN
IYNYDVKGSKSGSDPGSCRIEGELNPLKLDYYGRRIGNNTRKRRDTRGVPALECGADTTCREIICN
FNIENRNSATVTVKATLVDANFDSVIGAGREIHVTAEFSSDAVTPPSGLEFPVKSMVTTTVNSPYLT
KTEGGESIPWWIILLCILAAIIIIGVIIFIMYKKGFFKRKKMADEDEALRNGDPEMDDD*
>TRINITY_DN602_c0_g1_i3.p1 type:complete len:851 gc:universal TRINITY_DN602_c0_g1_i3:3046-494(-)
MIICRLHGLSLGIILIFSILYTSDARSRHAYGHKDASSPQTGKKRAESDAGAGTEAATGANTGAATA
SDSSSESQTFTNTKVNTRIATSSNARSGANAAAKVKTYAHANTETKVDTQQQKPTPPVPPEALTT
PPPGVTPPGPPGPPGSENNNTSSGSECKEKETKPPIIKVYCPNCPCPCPPPCPCKCPCIKVAPPPPAP
PPCICLPPCPPPCPPPPPPCPPPCPEPLPAPAPCLPPCAVPPCPCNIAPITLDPSLPCPMDCPLPPCAC
PFMKKDNITTTKTKRSVIRKRNLNIKRSHRVGTKRVNAGKSPNLAYAQPDSFEKAAVAADNKKCS
EIGVEILKKGGSAADAVVSTHCCVEIVNSHSTGLGGGGFMVYYDKKSGKSRAYDFRETLPKKYTD
TQNKTQGETILVPGVLRGLEAVWKDFGKLPWEELWKPCIKYAKEGFYVHEALAAGIVKKADYIM
ANLGLRELFAPDGELLQLGSLFKRPKLARTYEQIAKHGADLFYRGEMAKQIVNEIKEAGGVMQL
EDLSSYQVNIRETLKSNINGLKLLTSPAPSSGSLMALALKIMESFKWKPEDLLNNPSLVYHQIVEAM
KFAYAPTSYLGDPRFTNGTDKVEQYMVSPQTAKEMFKRIDNKSHPVEYYKPWSELFSAEKTGTSH
MSLLDQEGSAVSLTSSINAYFGSKLRSREFGFIYNNELADFSEFWPTVYNLTSDKKAPSKRPMSKVM
PGIFLKGKTVQGVWGAAGGFFIPSALTMSIANWLFFKDNLKVAITRPRLHCQLFPPTVVYEPTFPP
SLIPKLQEFGHKYVTNSTYDVSGQLNAIMGVVQAIVRLPNGSYSAEADYRKGGRPAGF*
>TRINITY_DN166344_c0_g1_i1.p1 type:complete len:360 gc:universal TRINITY_DN166344_c0_g1_i1:93-1172(+)
MQKPKKYDWKDSNMALFGSDTEKKVKKESAESEPAWQGAGADVGVQVWRINKFKVEHWDKE
QYGEFYNGDSYIVLNTYKEPDTDELKYDVHFWIGKYSTQDEYGTAAYKTVELDTFLDDKPIQHRE
VQGHESSKFKTYFPTLTLLKGGADTGFKRVLPEAYEPRLFHVKKQNRTQITCTQISLKRGNLKSED
VFIIDLGDKIYQYNGSSCSADEKYKATQEVARLKSVRGKARAETIEERTVSDEHPALMVLKDGSSK
EKSTVEGEKKMLRVSDADGSIGMDEVASGDDISKDKLTSDDVYIINTGSHVYCWIGSGASIDERKN
GLSYASNYLNKTETPWLPISVVGQGKEDSQFQKAF*
>TRINITY_DN118_c1_g1_i5.p1 type:5prime_partial len:203 gc:universal TRINITY_DN118_c1_g1_i5:620-12(-)
KVLFLCGFILLSGLAFAYEIEEIVEDAPKPEPEHVPEPEPEPVKEPEPEPEHKPEPEPEQCSTVKHQK
CSKADKVCSPEDSHCTFTQICTPFFVKKCCKVVSVEKKCVVVRNCIPVYSYEKVCEDVKRCPPRKR
CKNVCRQVDLGEHKVKSICEQECEVVEHPEPCKLVHTCFSKWVSKKDCTPRETCSDIEKRECREV
SSH*
>TRINITY_DN1936_c0_g1_i3.p1 type:internal len:1070 gc:universal TRINITY_DN1936_c0_g1_i3:3208-2(-)
GEYKPTLGRCLSFWYHMYGSHVGELNVLVLQNKSRSAPIWNLKGNQGNVWRPSRVTIKSAVNH
NIIFEGRTGDGYMGDIAIDDVMMSEGPCTDYGNCDFERDLCGYTNKKNIDQFDWQLGSGGTSSV
TTGPSNDHSTQTALGQYMFVESSYPRLPHETAIIGSETIPVSGSGKCLQFWYHMYGEHIGSLEVNM
RVANDLSETIVWKLEGNQGNKWLQGQAPLLSRGQNMEVRFEGIRGTGYKGDIAIDDITVVDYSG
ACAITPKSAKPWGCNFELDFCKWQQSRADKFDWLRNQGATGSAGTGPKADHTTNTTQGYYAY
VETSFPRQPNDTAAIISPTIKGNGTKQTYCMWFAYHMYGPHVDTLKVYTRSQGSDKPVLWRRRGS
QGLNWNHAEVQVTVLYDTKVIIEAVRGKDYKGDIAIDDIDIWFGDCFSSGICTFEGADLCFWYVP
KNGQLYWQRASGATPSVKTGPKNDHTYGTSQGHYMYIETSNAPAKATAYMYSPWYYNTGQKC
VQFFYHMYGQQIGQLEVYIRYSNSRYLYRQFIRSGNQNNTWHMGQVTVKRNNVYFQIIVRGTKG
NGYQGDIAVDDFRIRDGACPAPGDCDFEHADFCSWMQEDNDDFDWIIGSGSTNSFLTGPSIDHT
TQSNGGYYAFIEASSPRQPGHKARFKSMVFAPTTGSGRCFSFWYHMRGYSIGNLTIYLRKVSNTKT
LSEEVMWLLGGNQGKAWNQGRFTLKSSTLHEVVIEGVRGSSYDGDIGIDDIEFTSGSCSFLPKGA
WPSGRSTPAPTQASTTLGPTIGNKGFDCNFDKEFCKTWMRNSTRTNFQWKRHQGSTSSVGTGPH

HDHTTNTDQGYYVYIESSSPAQPNDTAYLIGKDIPNNNGFCLSYWYHMYGPHVGSFRVYVVNKT
NAEARFVRVGTQGDKWKHGQLYISPKAGNPITIRFEGVRGSSFMGDIALDDISVRYGNQCPAPRE
CTFEHDNNGGSSSTCGWTQEKQKDSFDWSIGSGDTPSVLTGPNFDHTLETKQGHYFYIETSYPRK
FGENATIISQSYQQTLGKCFSFWYHMYGSTIGNLTLFLRRD
>TRINITY_DN942_c0_g1_i2.p1 type:complete len:5116 gc:universal TRINITY_DN942_c0_g1_i2:15664-317(-)
MKMKLALLVLFTGTTLAAPFWVLDDNLPRAEIKEVGCSGKRFQNGKSHVYEYEGVTYNTIEGST
DKKTGIKLTATATITPMSECKFMMQLKSVELMSKSMNQDTFTTNAESAKLKDELEKLMVGFTMK
GNQIVTIHASENEPTHVLNIKRSILSSLQLDESTEKETDINGICDVEIVTKSGKIVKSKTLSECTERAQ
NEIGFQSTSIKSATTLKPLDSTSKCEYVMEGDVIKSVVCEEKHIFKPFSAGYKTPSGAMTTVKQSLSF
KEYTVAAISAVPSDFTVETNLLFDHSTEEPTATSTINDQVEKVMLRLVDPAQKSDLSAKENSAQEFK
NLVTLFRRMDEKRMEATWTKYFDCIKSGVCTDANTRDLYRQYLLDGISHCGTSTCIAMVRNVIV
NGQITGERMNMFLRSIPFVTKTTMKMIGHVLEICEKNPSRQAYLTLGTLLVRHCKQNPEDCSENL
RNPVTMAEEHLVKKLGQECEGQDDHERVEELLRVLKAIGNAQRPARVQEKLMKCAEKSKHSNI
TTSIFDAFRGFSCDALKQGELLRVFEDINNEDETRIHAFITIMKCPTVEILEKVTMMLEKELSNQVA
SYMWSYLTNLMESSDPRHQVARDILTKMTAQKPLREVNLPVHQFSKAFEKSLHSEFLKSGLTTEG
HIIFNKDKYIPKMGMLNMKANMMGNSLDMLETKIGLDGIEPILETLVGPNGMLPKDLNIDLGSY
GKFNSEFLRKMLEKKNNKRKLFNDNLTKMHLKANKQFEEPCAHLSVKIMGQEVSVLSYDDILW
MIDQIDNMNAVQLLTNIAKGGHKTFSKSLMFLEMSHNVPTGMGLPLKLKLTGSAVGTVEFNGKF
DIRNLFWGTPAVNIKGHMKPSTVVELSGQMGVEGHYVSTGMFVKSSMFTSQIVKGAIEYKHGQIL
KINMDVPEEPLAAVKFSSSPFLFMNEESELVEGTSRFVNPEHCTKSALLGYGLCASMRLPIAFREYE
APYYPLSGPAHFAVRAIRGDEKLTTWQFLVELNKQSGTTFGNIEFSTPGAAYERKLNGKVSMKQE
GKMNMFTLSTEGYDRKASVELNFNKETYSTAIITKNNIFTTKDMTTKLAIFNEEIDSGRQYGIQAL
ATMDWYTFDHITKFVRKPNVYMFQTSTTYYPKKVVMGTIVYDTKENKLSTRIQANQFQQAYEFS
GKFSDDGQEKGVTVTGKCVTSGKSITLYGGIQNTKQTKQILFTADVLGYSAKQYIRYENNNDVHT
VSQFLSVNGKIISLNGELSSFTEMKRLKFVADIIGRKADTTFLLTNNKGEKSLSVDMNAMQKHAN
FKTGCYQAKDSNECKFNFKIGTKEGEILMKQIMRDGNVITTLETLFDKYVAGFRNVYSNKNANA
KSLCSSIFYGTEEKEHEPVNVCLKYNAASDEKKILAITFDIVDRNQQGEINMELTKQTEFVKMQTE
VVYNKNSLAKAMQSMKYSKDLNNEIKMEVNVGKYNAGFRWFTLKTTNGANVGIEGYAMDKKI
QFVNKYVKDVVSGTTESKVTSELYINDKLVPVTTETIFLNGKGIAGPKFQVNIGKHSVSYATLVTYG
KGVYGIDLEFLVAKESNMLFKLFNGAAASVTPKKMDFQYKFGFGINGKQYTYGGDMSYNNLGN
DEKMEHSVKLGLQYSINRRSSMTFVLSNDKTKAALNMEFEYIPTKSVSHSIEYTKNDRILQVSVEFL
PKMFYTFKSQLVKNEGYSLASSVTLNWKNYNRVLKMINSMVNNDKKFELSTVFGKDLTFTFTFG
KENQKSMTLSASGFKRSLKVIGTFVKPNVQLQVYKNGEKIMDVTTSFDGITNKFQFALTNGKDTY
LKMMKSYNEFVLASGKETYLKMKKKYNEFVVTSGKETYLKMMKTYNEFKNQYKFQINGKIWTK
VQEWYLKMTKMTKDNKITKFLAPFLDLTVKLDRETNDMKLVLSNGKQTFVKIVKSYNEAKNQH
DFQIFGMSKEWVDLMAKYNKRNKVLKVSFSFFGKELAMFTLNMKEKDTVSLTTGSDYWKKNIV
LTGSLDRSQKSIAGNFQYGKTKVQLKLRADWNNMILENAFTFNDNVAKIGLLREQNSMVFRVKL
SPTLMAQVVMEVKNDNIIKMTIQRSEGDKLIEETSMQFKLSKDLYNLAFKWNPETFKAVIRAVQN
MIQNGYYKDIKPFITNVFVKGQRMSRKALTTTVMTFTEKVKEAKTKLIDLITNFEASVDKINFVAIR
EQIGQLSLKTIQKLKELVKVTLKELMTKLENAKENIPELLKKYKQIRNKIEKLSKDTAEVLPKFYKE
TLEAMDKQYNKLNGYWKDVSKVVMSNFELVSKNLTDSTKPVVEKAIYLISNFKLRGKAVKEIVSI
ARELVEKYIQILIEQGKIIMANGKIQLENMKLKSMEHYKNLKLKGMEQYNNLKLKGMELYQKGK
MPYLDETVEGIIKISVEKYMDLKKKMTDFDFEKYIQSVRDQIMEKKINGKTIVEHLNTAKEEMKNL
PPALKENYVKLIELITKLTKALNTNLKEWKIFAKPLTNYMKLVALSTKKHFGPILTKSMDKILPLITG
IELPDFKMMALEQWKQLENFMLPLIKPVGPLYENIKTQIRNIKVSGYPIGSFVDFNIEIAKTFIQTSL
KTTKQKLDENVKMVTEYANLIKKMTPEEIMNQITLKSTELTKEYKEKVLKVYEERQERLKVLVEKL
KELYNEFKQQYERITAVPVQDHIDDMMFKSSKFVKGNLEEISKVVKQITEIEISKPTWDAWKQADV
MGHLDKYGINQKMIDFIKAVKEVDMKRIAQKTTRLIQDMISTIYSKAFVRFVQVYGKLEKVHDYL
RSIPKKEYEEWVKELKEFSLENKERVIEFMGKMYKQTQKKSLNLYTQSKFLYAIMHKIVLKNMEKI
NINLVKPSQELYSDIKYRSEQVYGDLEQPTIDVKNHYQKIINDFVEEQYTIVKGAVLRHYKSLEEYK
EIMYTKAQEKMEEVKKLIELKYNELLAKYGDMTYEEMYDNAYKYGKSQYKVVRKTVKRGYKKL

REQMEKMEERLNALYQLYSTKYSDLVNRIKTEIIPQTQEQYQKYRLIVINLIKKYQKVLMEKMAEL
KTRGMTIYNDLKTRGMAIYNDLKTKGMTIYTDNKDKTIKALFREVKQNAIKNINTVYVLVKNFG
DEKLKQVRTIIVTYYNKAKGVMVDVIIPEVKMELVSIINQTLRSSKVMTEEIMKAYTPHMVLAKNF
ANKGAILIIERGTVAMKASEKAIREFMDKAPAMMKDIVKKIKDQEYYNKIVNHEVVKKYIAKTQE
MIENLKTKVEQLKQDPTVVKYQKMFSDKVTELKERAKIVIDKLNQRIEELKNHPMTLKYQKIVTE
KMTELKKKAQLLLEDVKAKIEELRNNPSVLKYQNMMNEKIAELKAKVKMLVKELKKRIEDLRK
HPKTIEYQNMIKQKIVDLKKQISMYKQKIENLYQHPFVTKSMVTMKRIKRSALFTADKINVNLQP
KIKEASEMAQKRIEKVQEQAMSAVQFFKNEPEEAFWTGVSNLEKSGKDLYEQIKRLFNKELIKKQ
FDVLTTDAKMQWKIFLNDYFDDWSKEASRKMYEQVVTLVKNMNMKIIDMKMNMQKLPEVYK
PIIEDYKNQIMENYKNIVEYSLQNMIQGKVYLSNQWVELNNQWKACPYRLIMENPVWSEIGDEI
MQHEAMTAVKYVSNLGNLKFQAMKTILMKEYEIKKQLLNQKITELQVSLKKTVEDLRSKGLKNY
QLAKEKAEQMVKNIKEIAIKMDKFLEEKTIGEIVEITLEKYQMLITKVEFLRTQVVGLSQWYKEKA
TVIYNEKRKRAEKIFEEQKEKVLAVYQKYKAQVMPIYQKYEDKVKQYYNKYSSIIIEQYKMLLKKS
AQKYIQLRTKYEELDLKNKLNKLQKDAIDMYQNKKESALKLWEESGVPTKLVELKQMTIRKTIEA
LKQLPGQTKEQLTAVFDKYLNELKVQYKKYYSAGYTKLIKEYNDLKAKFEAKFELNRNLLIKLAK
PYTTPVVVAYKWVENEVAETALFVYRYHKLGERYNNGRELIKSEIERLTPILKNNFKKYNAEIRKY
VKDQADKYSAQALEMSKDLKQKAIVLSKDLSKKVVASLKTYSLMTGDKILRSIHSSMKCIDNVDF
GKVQKQISKKAQDFYREFTKYVIIDPSKGEILINIPHGDMVPSFTHQFEQIKLRARRSVQVLKENGK
VLMKKINNRMAELKVKIEKVRNKVMKKVMDNTVEVRRDLLSSYNINKRIFDRVYVKAQIIYKKS
LRKATNQYKVLKTKSQDYIRKATAQAKAYYEEYMTKAKDYIRKNTELAKVYYQKYMTIMKERYL
TVNKIIMDLYNTPPAELYKQLKESKASLKVIELIMHLYNTPTAELYKQLKASPTYLRIDRIIMDIYNT
PLSQRLEQLKTSSAYLKVNKMVMDIYNTQYMDIYNQRLEQIKNSPRYIKVKGMIIRLYNTPAKELL
SKLQYRGQKLYKIWFNKVKVMINKAIEKNAPLIKARLNAIKKELRPYTESLKTMYKKIKLGYSVKD
SFYPMYKQMKFATKFYRGEAMRQARKIKAQICKEDPKLCKAIKSSAKVHSFIINKYATRVNKVLNI
ALDKYQEGVAKTHRAYRHALNMIARPKSTYAAVAMVFKDHYVTFDGKSYEFDIPSSKCQYVLAH
DFQNADFTILKTQDAIKINNKDMKVVIFKNGKTMTVFPDKKISNGLPVQAGESKCIRQHNEIICN
FVEDKLRVVVDLENDITTVSISAWHHGKLQGLLGTNNRESFDDMKMSNGDLAKDQLQFTNSYE
MTGSPKCQLNKVAFKKLQCGKVTQKCKKLFENPKSELAGFFNIINAEPFIKACERDLVSCEKSKEA
KHCSSVNAYLSMIKSRGYPVELHSECMKEGERMTGDKWQQKQSNKEFDIVVLVSQRKTVNKKPI
MKVLQKLQSSFKSNSIDTRYALIGFGGKGVNEEAHVHALGKGASVFSNMNDLKRAITNTMVFNG
DEESNDAYHAIVEASLQRFRPGAHKIFVMFNSEPHTSHKYGATLKEAIFTLEKESHASLFVFDEINF
KSLNKKGLTIGQTNSKIYTTKNLKGLRLSGMELPKSEFKDMVESTNGGMFTNELMNKQNELATV
LFDAITDTVTQNQRQCLDCEKHTTWYGSTRQLCHPVAC*
>TRINITY_DN875_c0_g1_i1.p1 type:complete len:606 gc:universal TRINITY_DN875_c0_g1_i1:2207-390(-)
MLRLVLFAISASVVYGSCNPLKLGCFIHSGSDRNLLHSDNSEDIYTDFYSGTNIEWDKFSSYLQRAA
CRCDQAAANAGYRFFSLRDFGECHGIGKGASSFDALKLKKSMYCIAPNLGQCDISEHQHCTGAE
EGEFIYETKAQPAEKVIDGGYSSWSAWSVCSKECDQGLQERQRTCTNPEPKGKDAKDCSKLGKPS
ETRSCKIKDCPPSGETVIFCEHRGVKRIACENDQVVEILAANYGRTSTKHCVFGRTKVTNCRAAH
SMSIVKSQCDGFNYCDLNPHNDVFGNPCGFYTKYLEVKFRCVARKTSSRRAVLCEHRGSPPLSCP
NPDEVLSINKASYGRLSTHYCQYGHFKQTNCRALHSDALIKKTCNGLNSCSLNPSNTVYGNPCG
NVNKYISVEYTCKKTNAASRQAVFCEHQGGKTLKCANPDEVLSINKANYGRLSTDHCQFGHFK
QTNCRAEQSDALIKLACNGLNECSLNPSNDVYGNPCGNVRKYISVDYTCKKANAASLRAVFCE
HQGVKTLKCANPSQVLSITKAIYGRLSTDYCQLGLFKQTNCRAANSDGMIKKQCNGKNSCQLNP
SNAVYGDPCKYVRKYISVEYKCINK*
>TRINITY_DN8341_c0_g1_i1.p1 type:complete len:368 gc:universal TRINITY_DN8341_c0_g1_i1:1281-178(-)
MFRILILLAICLTTGNCKVRTLSLAKGKLRGSVKVINEETDQHTRSSYPHNNNTETETKKICDQLK
KFTPNLCSIYSAKTKCPVHCALPPALVKRELDCGKKFSQAESRKADKRIFGGKESQIGEWPWMVTI
MTKSTVPVKYCSGAILSKDFILTAAHCFKEPGLKDPTKWQAISGNDHLVYDNKREAKHDVRKIMI
HEDFGSPSEPSFSDIAVVQLGKSLDLKQENSNRLCLPDIDHTTVPGMTCYVAGWGKASSNETSTAL

ILRQGALKLIEKYVCKLPNVIPGGIKDRELCAGWEDSRTDVCKGDSGGSLSCEIEGKWYAIGIISRG
YKCGTQRGERNPFAALADVRTFKEWIVNAVLNDSGAL*
>TRINITY_DN941_c0_g1_i9.p1 type:complete len:3186 gc:universal TRINITY_DN941_c0_g1_i9:86-9643(+)
MADPAKIVDPIDDPTASDAEWKKIQQNTFTRWCNEHLKSSNIYIYNLETDLCDGLKLIALLQVLS
HKKIVRYNKKPTFRPQKIENIAIALKFIEKEQIKLVNIDASDILKGNLKLILGLIWTLILKYQISMSMV
ADDDDDPDQKTTPKQALMGWVKSKLPPELKCGNFNKDWNDGIALASLVDSTAPGLFPEWEDL
DPANKLDNAKEAMKRAEEWLGVPQVLTPEEITNPNVDELSVMTYVSYFPESKLKPGAPLKPKMN
NASKCSASGPGLQEHGVVVNKPAEFTVFTSGSSRGKLNVQVFGPGRKAIDCMVQDNHDNTYSC
QYTAPKTGVYDVHIRVGGHHIPKSPFRVNVASDLDTSKVTARGPGLEPHGLIVGRPASFEVIATDA
GSGELVIDVIDPKGNKKTELKVEARPDNVYFCTYMPVQVGRYVIVIKYGGKEVAKSPYHVSVTPA
ADRVKIYGPGLEPGLKTGKPASFTIDCSEAGEGGLDVKIVGPGKGDIKYEVKDNGDGTYTCTYKP
TRPGQYEITVKFDGGVVPKCPVKVSVGSTADPSKITAWGPGLERGVAGRPVEFYVNCKGAPVDNL
TVGVEGPGEAKVDMKTNSDGTVTVTYYPTKPGKYIIHVMYDEMDIKESPYSATISPRGDVTKVYA
EGPGLEAGNMAGKPAEFTVFTKGAGVKGNVQVTCVDENGKPCEVTVKDNGDGTFKVTYYPKTI
GKYVITISFMDEQIPKSPINVTIIPFSDPTKVKATGPGLQGGKTNKLAKFKIDASKAGEGGIGLTIEG
PNESEIKCDDNGDGTCNVEYMPTESGDYKINVLFADQHIPGSPFTAVIGDDFDASKVKADGPGLK
PGNKAGKPCDIAIDARKAGKAPLVVEVTDEVGNPVKVDLNEEEPGLFASSYHPEKEGPHTINITY
GGKHIPRSPFKVTVKPSSDASKVKVFGPGIKPTGVKSLEPTYFEVDAGEAGEGNVDIKIEPTAKGM
PPVKDIDVKPGKTPNSYIVTYIPPKDGEYNIVVTFAGVNVPKSPFKISVTPGSDASKCKASGPGLEE
PIVGKENVFQVETPPEAGLATLTCTIDTPSGRKLKADCKKNKDGNGYDVTWTPKEVGRHKVTVK
YGDKEVPESPFYVDVLGKPDPTKVKVYGPGLENAFVEKPAEFTIETRDAGPGNLGLTIEGPEEAKI
DCKDNGDGTCSVTYIPTKEGPHDINVLFDDQHVPGSPFKVAATEPIDASKVKCKGKGIGKSTPAS
YPAEFVVDATKAGKAPLEVDVVGPGDKKYPCDIVDNNDGTFNCAYVPEKKGRYEVTPKYGGTP
VPGAPFKVSGEPTGDASKVKCSGPGLDRPHIGHAAPFEVDTTKAGDGKLNVNAKGPDGKELPV
KMVEEEEGIFACEFVPEELGPHELEVLFGGQPVKGSTFKADATPAPELDKVRTPALEEILEASPCVD
QQFEAPVDCSEVPDNKGAKLSGKITTPAGKREKVDVTDNFDGTFALNYTPTEVGKYVLDVGYDG
IPIPGSPFEFEVTEGGPDKVKVFGKGIKKGVVAVPSRFTVDTKNAGPGDLSVAVEGPSKVDIECVDN
GDGTCEVTYVPVEPGEYDVIVNFDNQQVPGSPFKPQIVQDEPAIEVGRQSDLCLTPEGISAKDIPSL
KGVLQAPSGKEKPVDITEGPNDSFIASFVPDEPGKNLLHVKKRNKPIDGSPVPVMVREKPVIGEES
GIPMDLADCDLPKDLPKLNAVLTRPNGKEGPCGVEPGPDDTVAISFVPEEPGKHIIKVYKDGKQIE
DSPFVVMVEGSPDQYPKVGHPCDKDFYQPEPCRRGDISKMKGTLRRPSGKEEEVDVKLGPDGTIA
VSFVPTEAGEHLVSVKKDGRHVPNSPFSIMVIDEPTVPTVGSECSANFDIPELDLPKDLQYLKGTLT
RPNGVEEPLELSVGADNSLAINFVPKEPGKHLVSIKKRGKHVKDSPFEIMVEEGKPTDAPTGGPVV
GHDCGVNLDIPELSLPRDLKNLDAELTRPTGKKEPLKCDMGPEETLAVNFTPTEPGKHLIDVKKR
GRPVKGSPFEVMVGSTPVSEDQIPVSEEIMAQSAPDVESGLPGGLDSLDSGKDKPVTVGSPCDVNL
DIDGVDLPKDLDKLKAELTRPSGKKEPVKCEMADNGGLSLNFVPEETGKHLLEVKKNNRPVKGS
PFEIMVEESASPESKPTAGQKCDLDLDIPDIDLPRDLKDLKAQLTRPNGKQEPIECTCTPENTLGLE
FTPDEPGKHLIDVTKRGRPVKGSPFEVMVESPESKPTVGNNCGLDFDIPDIDLPKDLKVLKAELTR
PTGKKEPIECTATPENTLGLEFTPSEPGKHVIDVTKRGKPVQGSPFEVVVGEEEPASKVPTVGSECD
VNLEIPGLNLPRDFKYLDGTLKRPNKTSDEPLELTMNENGTIGVNFVPEAPGRHEISIKKHGKHVS
GSPFDVMVESAPSDQPEVGSPTTLGLDMVIPKEDLRLLKGTLVRPNGQEEPIEITLDDNGHPNVTF
TPQEVGNHVINIKKRGQHVPGSPINVMVGPAKKPTTCDVGFEIPGIKLPDDLKYLKGSVVSPSGTE
EPVELQAGQNNTIVVSFLPKEAGIHYIHIKKNGKPVGDSPYQVNVKPEDIAAGQPDASKVKCTGR
GLRHGLPGVKADFLVDTKDAGYGGLGLSVEGPSKVEINCVDNEDGTCAVDFTPAEAGQYLINVT
FADTHVPGSPFKCKVGGPGVEDDIVPMTDDGVECASSDMLAFPSSKARSPQDFVIKFSGSGDLKA
TVTRPSGVEDEAEVVETGPDTYTVRFVPRETGEHLVNVKSRRRHIPGSPFKVLVEAPAGGAASCKA
HGPGLEGGVAGQPCRFTVITRDAGPGGLAVAVEGPAKAEIQCHDNGDGSCDITWYPVESGEYML
HIRFADEAIPGSPFKAFVLPADEGKAMPMESFKDQVLRVGQQASFAVQMKGKKGKITASVQSPSG
VQIDCSAVELEEGNYAVRFVPRELGDHLVSVFLDGHHIPGSPFTVKVGGLDSDPSKVTAYGPGLKG
GVASQPAEFTVNALNAGSGALALSIDGPAKVKMHCVEQDDGTYKVIYNPTVPGVYEISIRFGGQH

IPGSAYKVRIVGSGDDAINIFGDASKCNASGPGLNQATIGQPATFKVDASNAGRGSIMVGVEGPVI
PAKEIIVKHTGNNVYSINYILEQSGDHILRVLWADNHIPGSPFHVNV*
>TRINITY_DN6920_c0_g1_i2.p1 type:5prime_partial len:411 gc:universal TRINITY_DN6920_c0_g1_i2:2147-915(-)
CKRHQGKMSHRKFSAPRHGSLGFLPRKRCKRHQGKIKSFPKDDAAQPCHLTGFIGFKAGMTHIV
REVDRVGSKTHKKEVVEAVTIVETPPVVVVGIVGYVETPRGLRQLTTVWAEHLNEECLRRFYKNW
YKSQRKAFTKASKKWSDDDGKKSIERDLEKMKKYCTVIRVLVHTQQKLIKLKQKRAHIMELQIN
GGTVADKVDWAKKSLESKLSVSTVFGQDEMIDVIGITKGKGFRGVTFRWGTKKLPRKTHKGLRK
VACIGAWHPARVGFSIPRAGQCGYHHRVERNKKIYRIGRGIHTKDNKVIKNNAATDYDITNKTIT
PMGGFPHYGEVNEDFIMLKGCVPGPKKRVLTLRKSLLAQTSRKALEKITLKFIDTSSKFGHGRFQH
DQEKKNFMGLLKKDKERQAVEA*
>TRINITY_DN144508_c0_g1_i1.p1 type:internal len:265 gc:universal TRINITY_DN144508_c0_g1_i1:1-792(+)
PRRPNDTARLVSSDVPATSQMCFEFWYHMYGDHIGNLTVFKRIGKAMTMLWAKSGTQEPRWRH
ATVDITSASVFKVVVEGTASSGYMGDIALDDLFLTPGACPPSAECTFEDPQLCGWTNIAGDDFDW
TRGNAGTASWGTGPRTDHTYGTKQGFYMYIEASTPRSKGHKAWLKSEEYKPTLGRCLSFWYHMF
GLHIGELNVLISQNNSMSAPIWQMKGNQGRPWRPSRVTVKSSLNHNIVFEGRTGDGYRGDIAID
DVMISEGP
>TRINITY_DN8597_c0_g1_i1.p1 type:internal len:124 gc:universal TRINITY_DN8597_c0_g1_i1:369-1(-)
TCDGGVLVQRRTERAFNKVETRNGNTPCNTQACGGSDADFSEERVRACSNVNQCGVNTGTQTV
YKVCNGNRCNFQGRDYQINEEVVIRVQSCSVVCDYSPWQDTQSCTKTCDGGVLIQSRTER
>TRINITY_DN163801_c0_g1_i1.p1 type:complete len:298 gc:universal TRINITY_DN163801_c0_g1_i1:55-948(+)
MANPLKDIISRFSKGVPAGSSTGLGLLIGAGLAAYGIKESIYTVDGGHRAIIFSRIGGIQPGVYSEGL
HFRIPWFQYPIIYDIRARPRRIMSPTGSKDLQMVNIGLRVLARPLSSTLPHMYQRLGLDFDERVLPSI
CNEVLKSVVAQFNASQLITMRQEVSLMIRRDLTERAKDFDIILDDVSITDLSFSAQYTAAVESKQVA
QQEAQRAAFLVERAVQERQQKIVASEGEAKAATLLGEAMNENPGYLKLRRIRAAQKVARVIAQS
QNKVYLNANNLLLNIKDTDDIDDGLLSKKR*
>TRINITY_DN360_c0_g1_i1.p1 type:5prime_partial len:476 gc:universal TRINITY_DN360_c0_g1_i1:2237-810(-)
FQELLNCKNSKQQTIRLPANRENLTMALPYSSAEDFYQSVVKRTPNEVEFHQAVKEVAFALWDFL
VKNPEYAKEGVLQRLTEPERTIMFRVPWVDDTGNIQVNRGYRCEFNSAIGPYKGGLRFHPTVNLG
ILKFLGFEQIFKNSLTSLPMGGGKGGSDFDPKGKSDAEVMRFCQSFMTELSRHIGPDTDVPAGDIG
VGGREIGYMFGQYKRLRNEFTGVLTGKGYNWGGSLIRPEATGYGCVYFTKEMLETKGHSFKDKIV
VISGSGNVSLFACEKCTELGAKVVTVSDSSGYVYDKDGIDKEKLAFLMDLKFTRRGRVIEYTQKYP
SATFHAGERPWGVKCDIALPSATQNEISGEEAATLANNGVLAVAEGANMPSTPEAIEVFLKHKVL
YGPGKAANAGGVAVSGLEMSQNSTRFQWSREEVDSKLHTIMVNIHKTCQKYGTDESGFTNYVIG
ANVGGFVKIADAMIAQGIV*
>TRINITY_DN141338_c2_g1_i1.p1 type:complete len:720 gc:universal TRINITY_DN141338_c2_g1_i1:2850-691(-)
MSEADETETFAFQAEIAQLMSLIINTFYSNKEIFLRELISNSSDALDKIRYQSLTDSGVLESNKDMKIE
IIANKDEKTLTLFDTGIGMTKADLVNNLGTIAKSGTKAFMEALTAGADISMIGQFGVGFYSAYLVA
DKVEVVTKNNDDEQYIWCSSAGGSFTVKRDTINEPIGRGTKLVLYMKEDQLDFLDEKKLKDIIKK
HSQFIGYPIYLETQKTRDKEVSDDEADEEESTDKKEDEENEDKEDDGPKIEDVGDEEKEKEKKKKT
IKENYTEMEQINKTKPLWTRNPDDISTEEYAEFYKSLTNDWEEHLAVKHFSVEGQLEFRAILFVPK
RAPFDLFENKKQKNSIKLYVRRVFIMDNCEELMPEWLNFVKGVVDSEDLPLNISRETLQQSKILKV
IRKNLVKKCLELFAEISEDKDNYKKFYEQFSKNVKLGIHEDTQNRNKVAELLRYSSSASGDELTSFK
DYVSRMKENQKDIYYITGESKEIVAASAFVERVKKKGFEVLYLVDPIDEYAVQQLKEYDGKKLVCV
TKEGLELPEDEDEKKRQEELKAQFEDLCKVIKDILEKKVEKVVVSNRLVDSPCCIVTSTYGWSAN
MERIMKAQALRDTSTMGYMAAKKHLEINPEHQIMIALKKKIEADKNDKSIKDLIILLYETSLLSSG
FSLEDPQNHSSRIHRMIKLGLGVDDDGDVEEIETDDLPPLEGEGDDDEDKARMEEVD*

>TRINITY_DN495_c0_g1_i5.p1 type:3prime_partial len:407 gc:universal TRINITY_DN495_c0_g1_i5:152-1369(+)
MDFHWNTVIAGLIIVQVYSLAEYGTLNGKDSYRNAYPWLANDRNNNELGREDARSIYRSSDEID
AEYPQSDDEVDTNDIPKSYTSRDELDAEFAQRTYKFADDLDIENAQKSYASSDELDRDDEQRTNK
NTDDLDAEDAEREETINDSLDAENAQRRYTSIDELDGEDTQRVISKTITLPRLGAGRIYKSRFIHGP
YIRPKFVRPHKGIRLPHPNRIIIPGSWKRPAHGIFALKGKKAIFCEHKGIKRISCSNDKVIKVRSALY
GRTAGNVCSISCSKDEEIKVKNALYGRTAGDVCGRSRVTNCRALNSFAIVQKKCNGHNSCDLNPS
NSVFGDPCVGTVKYLNIKYFCVKRITPTTTPNGKTAIFCEHKGIKSISCSKDEEIKVKNALYGRTAG
DVCGRSRVTN
>TRINITY_DN785_c0_g1_i1.p1 type:complete len:289 gc:universal TRINITY_DN785_c0_g1_i1:1062-196(-)
MIIESKMLSSIMLKMIFVVVFLCGITLSEGIEKCDLQLEPIGCFKDDVWKRTMPEQILNERDPLNKV
YNNRMINWKKFDTYLPGFICRCAEAAFKKGYRVIGMQFWGECWSGPEAHLHFDSLGAADTCSN
QKFEREYDNITQCVSAIGMDSTNYVYRIAPTVCDTHYEPVGCFKDEQKVPRPMPHYISTDRDYSL
KIFSGKTIDWRNWNTYSPEMICRCATLAKSKGHALFSIQFWGECWSGPETNGYMRDGVTTDCRG
KDFEMCPCNGYHCVGKAYTNYVYRLGHHH*
>TRINITY_DN7659_c0_g1_i2.p1 type:complete len:359 gc:universal TRINITY_DN7659_c0_g1_i2:82-1158(+)
MASAATDNRKVALITGVTGQDGSYLAEFLLDKGYEVHGIIRRSSSFNTGRIQHLYKDRTAHLEGN
LHLHYGDLTDSSCLLHVVSKAQPTEIYNLGAQSHVKVSFDLAEYTADVDAMGTLRLLEAVKICGL
MHKVKFYQASTSELYGKVHEIPQTETTPFHPRSPYGAAKLYAYWIVQNYRESYNLFGCNGILFNH
ESPRRGETFVTRKITRAVAKIHLGLQEELVLGNIDSVRDWGHARDYVEAMWLMLQQEQPETYVIA
TGETHTVREFVEKSFGLIDVKIRWEGTGENEIGYDAASNKVRVRISSKYYRPAEVEYLHGNPAKAE
KELGWKRKVSFDELVKEMIDSDIALMKKDPEA*
>TRINITY_DN140702_c0_g1_i1.p1 type:complete len:2036 gc:universal TRINITY_DN140702_c0_g1_i1:94-6201(+)
MATSFKVSEIKCTELLNLEKKGAIDPLVEVEYLGLSKETENKDNDLNPSFGEVFDFDLSGKALGPE
DFLKITVKDYEKFGRNRVVGKADVYLQELLSNPSSTLSATLNDKNDRPTTGKITFKVDYIGPAGGG
AGGATGGGGDDRQAAERELEGDEDEGDPNAMPDGTPIPEGPAGDAMRAEKRKMKKNKKKRR
EHNLPDKPTDFQIRIKIIEGRQLPGGNINPTVRVHLGEETKQTRVKHSSNKPFYDETFFFNFNERPN
ELFDKLVTFEVHNSKNVLSEAMIGAFQFDVGTIYFEDSSHAIIRKWLLLTAPEDNDKKDGEETKGA
SLQPGGPPAGYLKITAIVLGPGDELPEKAKGSGKSEGGEEDIESNLLRPAGVTLRPATFLVKIYHAE
DIPQMDSAAFDGVKKLFSSGSAQKDHVDPYATFSFAGKTQKTEIKYGNNHPDFNQELRVAFKFPS
MCERLKLQMFDWDRIGNDDCIGTSFISLTAISGNGDEGFLPCFGPCYVNFYGSTREFSDLPDEHDN
LNKGRGEGCGYRGRALVEIDTRFGDHTAKPVQDIMAQELVRVQAYMRRRKYKLFASFLSANMIS
EADGLVEFEVSIGNYGNKLDPDTEPQSSSTPPTNPVYDGCAYYFLPWQESKPCCVVESHWEDVIFR
LEPINCLLKIIDKMEKNIKSVELGLKAKAQPAEVAALLIALLDQFISDVSMDLPKFAATDNGVNEL
DLKIQEIRSDELLNLKEEASTLREQATDVQEAMDTIDGYLKRMRDLCVEPQNSMPDVVIWMIAGS
KRVAYHRIPAYDIFYSENPLARGRKCASLQNFILKYPGKKGHDMEDHPEIPASVSVILWLGLEKHQ
AAWTDTEQMEGEYSVYAETYENQGKPPMFDWTAKIIKRPGFSDSEGKIRLNKEYYEPPEGWKWD
DPPDGDWFIDPEMSSNFDADAGHKKFMQDCFENESRVPAGNWGKSTPHYTNVQGDEIDHRDE
TSLPEGWSWTDDWKVDVNRACDEEGWEYCIEATGGGWGAVFRSYHFCRRRRWVRNRQLEADP
QKVMKQKKEEAKQGEGWEYAPVFGMKYHVKEHKFDFVRRRRWHRKMVCVKPGAVPMFKIPD
DDDKGKFHQIMPRIFLAYDEPHKWQFRAYLFQARDILGLDSEGTSDAYAKVCFQDQSQITDVIEET
LCPEFNQTLLFEDMQLYGSPSVFAENPPFVSIELFDKDKVGKDEFLGRVLAQPIVRLNGLKPPATRL
MWYDIHHGERTCGELLAAFELFLDEGADLPLPPPWLEKEKKFQVPSGIRPILVKTGVEILNWGVR
NMKKYQLASCTSPSVTYEIGREIITTDVIKNTKKNPNFLKNIFYKEVYLPKDPLYMPPIVIKVRDHR
TFGRKPVVGRHVIKSIDFVQPPKADPVVELPPADLVGEPEEKKDVTLDIEEKKKKKEIPVDDNLDW
WSKYFASADEIEKCGNYLKKGHKTIQIYDSELEEIKDFNGFSDFIVSYPLERGKEDDDEDDNVVGE
LKGLVKVYPLPQDPKDQTKIRYFTPKLKEKSKIVKCIVRVYVIRARDLQPKDPNGKADPFIVVQLG
KKKISTKDNYIPNEINPFFGSFFEMKADVPLDKDLKIKVMDYDFMSGDDLIGETMIDLENRFLSNC
RATCGLSPAYYTSGAYQWRDAKSPKEILDQVCRTKPQWLSNEQVVVNGQLVKIEDFEESPPTDPHI

GPVEQRLALHVLNNEDIIGHRLVPEHVESRSLKNPLQPSMDQGQLEIFVDIFPIANGVPGPPLDIAT
RKPKEYVLRVIIWNTAEVPPSETSITGEEMSDLYLKGWMEGQDDEKQKTDVHYRSMDGEGMFN
WRFVFPFKYMPEERVMVVEKKEHFWSLDTTKQTLPPRMNLQVWDNDLFSANDFIGNLELDLSK
LPRPKKAAKKATLAMLDTEGDDVMDLFQAKIAKGWWPMLSENEDGTRTMQGKVEMTLEIVTD
LEHEAKPAGQGQEEPNMNPKLDPPNRPETSFLWITSPWKTLKHIIWKNYKWYIIGFFLFIILILFIGL
VLYSVPGSIVHKIFGFQ*
>TRINITY_DN57_c0_g1_i1.p1 type:complete len:712 gc:universal TRINITY_DN57_c0_g1_i1:140-2275(+)
MGHDGSTLPSFLPKMSFERQTTLLKLLILSIAAILSFSTRLFSVLRFESVIHEFDPYFNYRTTRFLAEEG
FYEFHNWFDDRAWYPLGRIIGGTIYPGLMITSAVLYHFMHFFHVTIEVRNVCVFLAPLFSSLTALVT
YQLTKELKDAGAGLIAASMIAIVPGYISRSVAGSYDNEGIAIFCMLLTYYLWIKSVKTGSLFWSTFCS
IAYFYMVSSWGGYVFLINLIPMHVFALILTGRFSHRIYVAYSTVYCIGTILSMQISFVGFQPVQTSEH
MAALGIFGLCQIHAFVDYLRYRLTPSYFEILFKSLVIISATIAGIIGAILTVTGKVAPWTGRFYSLLDPS
YAKNNIPIIASVSEHQPTTWSSFYFDLQMLVFMFPVGLYYCFAKLTDANIFIIIYGVTSIYFAGVMVR
LMLVLAPVACILAGIGISSVLTTYMRNIELSNDKPKPIKKAKQADPSYPIKSEVARCMVALITFFLITY
TFHCTWVTSEAYSSPSIVLSAKQSDGSRVIFDDFREAYYWLRQNTPEDAKVMSWWDYGYQITAM
ANRTILVDNNTWNNTHISRVGQAMASPEDKAYDIMRELDVDYVLVIFGGLTGYASDDINKFLWM
VRIGGSTDTGRHIKEHDYYTPAGEFRIDKDGSPVLLNCMMYKMCFYRFGSVYTEQGQPSGYDRVR
NAEIGNKEFELDVLEEAYTTEHWLVRIYKVKDLDNRGQ*
>TRINITY_DN140696_c0_g1_i1.p1 type:complete len:260 gc:universal TRINITY_DN140696_c0_g1_i1:43-822(+)
MPRGPKKHLKRLNAPKHWMLDKLSGNFAPRPSSGPHKLRESLPLVIFLRNRLKYALNYTEVMKI
MKQRLIKVDGKVRTDICYPAGFMDVITIEKTGEFFRLLYDVKGRFVCHRISSDESLYKLGKVKRVAF
GKNSVPYLVTHDARTINYPDPSVNVNDTVVIDIKTGKINDFIKFDTGNTVMVIGGRNTGRVGIITH
RERHASSYDIVHIKDAAGNQFATRLSNIFLVGKGNKAMVTLPRGKGVRLTIAEERDRRLASK*
>TRINITY_DN98968_c0_g1_i1.p1 type:3prime_partial len:424 gc:universal TRINITY_DN98968_c0_g1_i1:1269-1(-)
MEYKNILTSCIFLISVITNTQQTSYVNYFNDQSDLLRSFNIPVKPERSFNTGLPASYMKYARTNDFY
HLYARLFNENQDGKQMAFLTKPISFPSNIETVCVYFEYWMHNLGNTQLYIVGDTTDSTQMQNIN
VNNLVNTENRKERLVQTANEPNSCWREVNSCFNVKGKTKFTLHIQLVPPSTGIVSTNGAIALDSVI
VTTDKRIESTACTRRVGRRIKRDVSFTNLVSNPAAIPGMNLIFHNYDASKTTQDFECGDDLGLCGN
KYLGWTLTNQCSSSCGNGFIIESRTYRGQAIIAQYRQNFQQACNRGSCSTGPPQPPGNNLGPEQL
ATCSVDQCGVNTGTQVVFRVCNAALCQYQGTDYSLNQQVTIRVQSCTVACDYTAWQDTSACSK
TCDGGVLVQRRTERAFNKVETRNGNTPCNTQA
>TRINITY_DN319_c2_g1_i3.p1 type:complete len:2588 gc:universal TRINITY_DN319_c2_g1_i3:165-7928(+)
MVLLKGQLWILGFIFLTSTKYVMGDVPECSNNATVAVHCRFDEGNTCVDDPHPMHYVCVCNNL
GYMNDTDSVEGYSICKERDCSNSNETPTDLLKRICGVNTKTCTGKILQRECNCNTDSYRSKNGQY
CFFAPCQASPTIQQNCGPGNQCYGNAPNYICDCSARGYKRSAADNRHCELKSDEELTPIEEYCGK
GSLYANGTINNITCTCDTDKGFFPSATGETCISPSCYLNPQMQEKCGPENTCLGGKDDYVCLCSNP
NYKRNATDLQHCIRRTCADTSPAIDFKSICGEGATGCSGYVDKPLCSCDVSNHYVPAADGRSCIK
ATCKGNKKALEQCGQANSCTGSFDNFLCVCDANGYYLNTTNNTCVERECTDPDLVITDICGVGS
LSCNGKAGSPECICDKDKFYYASEDLRSCVKANCLSSETISKKCGKENSCYGSMESYVCNCNAPG
YLLNTTDNQGCYLGMCEDNGLEYDKVCGEGAISCNGTIKNLECNCGMNYVQSPDKKSCIRADC
RANQTMHEQCGEDNDCVGSLEDWMCICSTYGYKPDPMDMMKCKKVMCEEEVVNNVTVCGL
GTTGCKGHILKPECTCDIAADYVPNEDASRCIKGSCENNKMAMKDCGIANSCTGSYNNYVCVC
NGFGYKKLDDTHCVEQMCSDPDIDIRNVCGEGAHYCNGTVNQRVCSCNETEYFYPSADSKSCV
KATCAVSPMVMEQCGVENSCQGSQESFICNCNALGYLLNTTTNQHCYLGTCENGDFDLMQAC
GDGALYCNGTIKNLECQCDESKGYVQAPDKMSCIPNDCRGNLTMYRQCGQLNKCVGSIDDWR
CICDSPYYVAHSDIMKCKLLHCSDSEVDHVEVCGKGTIGCSGSTLHPTCHCNTTENYFASKDGMR
CVAGSCKTNQTAVKKCGNANSCAGSHSEWTCICDGIGYKHDPADPQNCLLKTCNDADIDIVATC
GVGAISCEGYQLMPKCECNDRDYYIPSDDRTSCELASCRANRTAMRACGEMNDCRGSFSSHICL

CHDPAYKSDPSDSRVCLEKTCEAGDFELVRVCGKGATGCSGSMKNIMCECDNAKGYVASADHT
QCIPSDCNNNVTMHLQCGEGNGCVGSLTDWYCVCSAPGYEPHASDHMKCHLKTCADPGVDH
VQICGMGATSCMGPVINPVCYCNESMYYIPSMDRTSCIKASCMSNKTAIAQCGSENSCSGSPDNF
VCLCQAYGYRQDTNNNQACIPSQCEDAGIDFVEICGQGGVGCSGSVLNPVCMCDHMKHFVPSP
DQNSCVLSTCRGNETAMTSCGEQNKCFGSSSSYICVCDAVGYKPHPTNLQECVLQTCEANELNP
VAVCGKGALSCKGTIQSLMCECDNSKGYVAAPGYKSCIPADCRANMTMHAQCGTGNRCRGSM
EDWSCVCDGIGSKPHPTDHMSCIAIDCSDPNVDIKSVCGYGATGCMGLARDPICHCDTSKYYVQ
SEDGRTCVPATCHTNKTAQMQCGPDNSCIGPPNDFICECTGFGYRHHETDKQKCVPIQCNETAL
FDIERVCGKGATGCRGNIRQPICDCDHAMYFHPSEDRRTCIEASCRRNSSIRADCGVENTCFGSTR
SWICDCNSYGSILNPSDVKKCMLATCEAGQLDPVAVCGKGALSCKGSMRDVQCTCNNELGYVH
SPDGKKCLPSDCSGNVTMHHQCGDANKCMGSLTDWVCVCDAPGYKPHAKDKMKCHQISCAD
NNVDPVDVCGIGATSCKGPITNPLCQCDTDNYYITSSDGASCIPATCKTNKTAIHQCGEANSCEG
PADNFICVCNAYGYKLDDNDHQRCVEISCDDPNAHESICGTGSTGCRGTARDPICECDHTQHFV
ASSDLTSCLLASCRANDTVMNHCGDGNTCFGSLTSHICVCEAFGYKLNPNNHKHCLEETCEDD
MLNPVAVCGKGAVSCSGPLKSVKCECDNNAGFFRSPGGMKCESTSCRQNPASQMMCGSQNQCY
GSYDDWYCDCSAPGYMANQTNAAECLKVMCDDMAVDVCGLGATGCTGHTLNPICTCDSSMG
YMMAKDKHSCVPMDCSMNPASSQCGYGNTCHGSYFSYVCVCSTAGTVMNKTNPMECINVACP
ADAVHVCGEGATNCSVTSTNNVECSCEEGYHPADDLHSCVLDDCRFNPKALKACGLLNKCVGS
YKSWKCNCHYPEFTQDPMDHQTCVVGPCMVEQCGKGNAPGSFQHYAVNCSGLYDDPICNCQP
EYTNDAKGGCIPKICSENPVAVQACGSGYCEGTYFKYTCKCDEPAFVQTPDKMQCVPAKCNIQQ
CGHGFDRGLYIDFAESCSGTYADPMCQCKTGFSGTNTSKCGPAIDGCKLDKCDPLATCQTLDVD
GNVKCTCKEGYSGSGMMGECYLDGGFSAYEGTKECSEVCGVNGVAEVTRFCNNPTADPKGKPC
NGTSPETIQVPCNRFACSSCTVRKTCGCGGLKPIFVEFYHEPNSTLEYFTDLKADDVQTALGFLTK
WKELSQQDLCLMYSKIELTVFVTLMGDSETQVSTMKSSLVSARDIMRKKLDCNINLGKKAQYYEA
YETIIKRHLAISNILKDLTIIKNSITKYMNNCDDSEIGMIRDVIGMM*
>TRINITY_DN773_c0_g1_i1.p1 type:complete len:440 gc:universal TRINITY_DN773_c0_g1_i1:1608-289(-)
MVSPPGPQDNKPPHKVADMSLADLGRRYIEMSENEMPGLMSMRKQYGEKKPLKGARVAGCLH
MTIQTAVLIETLTELGAEVQWSSCNIFSTQDHAAAAIAKTGVPVYAWKGETDEEYLWCIEQTLIFP
GGEPLNMILDDGGDLTNLVHEKYPELMKDIRGVSEETTTGVHNLYKMFKEGRLKCPAINVNDSV
TKSKFDNLYGCRESLVDGIKRATDVMIAGKICVVAGYGDVGKGCAEALRAFGARVMITEIDPINA
LQAAMAGYEVTTMDEACSKARMFVTSTGCSGIIEPHHFQQMPEDAIVCNIGHFDCEIDAKWLN
DNCERETVKPQVDRYTLPNGRHIILLAEGRLVNLGCAHGHPSFVMSNSFTNQVLAQIELWQNHK
NYEIGVVMLPKRLDEEVARLHLDHLSVKLTKLSKSQADYLNLPADGPFKPDHYRY*
>TRINITY_DN5633_c0_g1_i1.p1 type:complete len:848 gc:universal TRINITY_DN5633_c0_g1_i1:2881-338(-)
MRRSNFLTIGIIAFFLIFSYVSADETSDEEVEPPKVDDDVGKSRDASKTDDEVVQREEEAINIDGLSV
AEMKQMRESAEKHAFQAEVSRMMKLIINSLYKNKEIFLRELISNSSDALDKIRFLSLTDTEALAAT
DELSVKIKADKENNVLHITDTGIGMTKDDLIKQLGTIAKSGTSEFFQKIQDAGTTDQASDLIGQFG
VGFYSAFLVADRVVVTSKNNDDKQHIWESDSVEFSISEDPRGDTLLRGTQISLYLKEEASDFLEPNT
LKDLIKKYSQFINFNIYLWSSKTEEVEEPIEEEPEKKEDEKKDDDKKEDADDKKDEEKKDDDEDD
AKVEEDKEEEKPKTKKVEKTVWDWELMNSSKPIWLRKPKEVTDDEYNEFYKAISKESDGPLTKT
HFVAEGEVTFKSVLFVPKNSPSDMFQDYGKRRESIKLYVRRVFITDDFQDMMPKYLSFIKGIVDSD
DLPLNVSREQLQQHKLLKVIKKKLVRKALDMIKKIPDEDYIEKFWKEFSTNIKLGVMEDHSNRTR
LAKLLRFRSSNDPEKLTSLTDYVERMKEKQETIFFMAGASLDEVKNSPFVERLLKKGYEVLYLIEPV
DEYCIQSLPEFDGKKFQNVAKEGLKIGNEAKEKARVDELEKEYEPLLKWLKEDALKDLIEKAAVSS
RLTESPCALVANSYGWSGNMERIMNSQAYAKSKDAGANFYATQKKLLEINVYHPLIQELQKRVKT
DKEDQTAKDLARVLFETATMRSGYTVKDSSDFAGRIERMLRLSMGVDVDAKVELPEEDDEEKEEE
KADDEGEEVNADGEEEESKEDIPEIKVDSKTVEPVAEHPTNEDAVENEAPAEGKTEL*
>TRINITY_DN1355_c0_g1_i1.p1 type:complete len:333 gc:universal TRINITY_DN1355_c0_g1_i1:132-1130(+)

MGDGENSVLNIDSIITRLLEARGRPGKTVQMSEAEVRGLCVKSRELFLSQPILLELEAPLKICGDIH
GQYSDLLRLFEYGGFPPEANYLFLGDYVDRGKQSLETICLLLAYKIKYPENFFLLRGNHECASINRI
YGFYDECKRRFNIKIWKTFTDCFNCLPIAAIVDEKIFCCHGGLSPDLQSMEQIRRIMRPTDVPDTGL
LCDLLWSDPDKEIQGWGENDRGVSFTFGVDVVARFLNRHDLDLICRAHQVVEDGYEFFAKRQLV
TLFSAPNYCGEFDNAGGMMSVDDTLMCSFQILKPAEKKAKYQYGGLNAGRPITPPRNQQQNKK
AKGK*
>TRINITY_DN1505_c0_g1_i1.p1 type:complete len:154 gc:universal TRINITY_DN1505_c0_g1_i1:98-559(+)
MSLVIPEKFQHILRVMNTNIDGKTKIMYAMTSIKGVGRRFSNICLKKADIDMTKRAGELSDEEVER
LVTIMQNPRQYKIPDYFLNRKKDIKDGKFSQVLANGLDNKLREDLERLKKIRAHRGLRHYWGLR
VRGQHTKTTGRKGRTVGVSKKKN*
>TRINITY_DN925_c0_g1_i1.p1 type:complete len:261 gc:universal TRINITY_DN925_c0_g1_i1:826-44(-)
MGRVIRGQRKGAGSIFKAHVKKRKGAAKLRSLDFAERNGYVKGVVKDIIHDPGRGAPLAIVAFR
DPYKFKLRKELFIATEGMHTGQFIYCGKKATLQIGNILPVGTMPEGTIICNLEEKSGDRGKLARAS
GDYATVVSHNRDSKVTRVKLPSGVKKAIPSFNRASVGIVAGGGRIDKPLLKAGRAYHKYKVKRNC
WPKVRGVAMNPVEHPHGGGNHQHIGFPSTVRRDTSAGRKVGLIAARRTGRIRGTAKVKDVGGD
KE*
>TRINITY_DN1819_c0_g1_i5.p1 type:complete len:432 gc:universal TRINITY_DN1819_c0_g1_i5:79-1374(+)
MILQLLLILSIWNVAEGLDNGLALTPPMGWLQWERFRCITDCKKYPKQCISDSLFKRTADRLVSDG
YRDVGYEYVNIDDCWAELQRTKKGEMVANHERFSQGIKALADYIHSQDLKMGIYTDFGTKTCG
GYPGSIFYMKEDTMSFADWGIDMIKVDGCYVCGDLYADGYKAFGFHMNQTARPMLYSCSWPAY
LNDKGKPYKEIAEHCNIWRNWADIQDSWDSVLSIINYNGEHQDEIVPYVGPGNFNDIDMLLIGD
FGLSQEQCKTQFALWAILAAPLFISADLDTMQDWQKEILLNKEIIDVNQDVLGKMGKRYYNENN
QQTWMRPLSNGEMAVAMMSTRTDIPVQMGFTLQQLSLNGIYNVRDLYTHEDKGNITQGYEAEV
NPTGVVMLKLTPVKSEEVEPAVIHSGYENAEVNEIKEKIEEMYEYIIY*
>TRINITY_DN624_c0_g1_i1.p1 type:complete len:884 gc:universal TRINITY_DN624_c0_g1_i1:218-2869(+)
MANQEEEEHFGYYYGEEEDRGLLDPAWEKQQKKTFTAWCNSHLRKVNVQIEELNVDFCDGLKL
MLLLEVISGERLPKPERGRLRFHLISNVNKALDFIASKGVKLVGIGAEEIVDGNLKMTLGMIWTIIL
RFAIQDITVDEFSAKEGLLLWCQRKTAPYKNVNVQNFHMSFKDGLAFCALIHRHRPDLIDYDKL
AKDDPMYNLNYAFDVAEKELDIPKMLDAEDMVNAVKPDERAVMTYVSCYYHAFSQSQQAETA
AKRILKALNVNQGNEKAIEDYENLASDLLDWLNNKIPTLDDRTTDSSLDTMQNKLNEFRDYKGT
EKPPKVDDKSKLENLFNTLQTKLRLSNRPLYMPAEGKMINDIDKRWKDLEQSEKDFQEWILNELR
KLERLDHLAMKFKHKCDIHEAWSTGQDDYLRSDDITDASLASLLASRKRHEAFESDLAAQQDRV
EQIVAIAQELNTLGYSKIDEINTRCKGICDGWDTLGELTTQRSEKLAKAIEIAERLDNLWLEFAKKA
SPFNNWMDGAKEDLMDMFNVHTIEEVNDLKDAQLKFKANMPAAQEEFNTIMGLQNAISSMSS
QPNPYTNTKAEDLQEKWQEVLELIPKRDSLLESETQKQQNNERLRVQFAQMANQVGAWIKQRM
GRMTDIGMNAQGTLERQLEELENLNAEINGFKQNVLDLDASARNVQEAMIFENPHTPYTMEVI
RIQFEQLLTGVTRNINEVENQILMRDSIGITEEQVKELRTSFNHFDKNSNGRLDYKEFKQCLVSLG
QLGDVEDKGEHTFNRIIAMVDPNETGYVSFQAFLDYMTREMADTDTADQVMESFRILAGDKPYI
TADELRRELPSEQAEYCIARMSAYNGPDGVPGALDYRSFSTALYGESDL*
>TRINITY_DN11248_c0_g1_i1.p1 type:3prime_partial len:1700 gc:universal TRINITY_DN11248_c0_g1_i1:111-5207(+)
MEIRSLFVAFIYIVHSINAQCSSPWKSYKDSCYLFTAVSTVSPGHTWEDSRFICQRYGGDLLKLDND
NETKYINSQFDDLKIKYNHYWIGLNDIKKEGDWRWADQAPLNVTTQWRVSRGEPNNWQGNED
CIETTYDGQWNDNECSRMFGYICERSKDAVIPVTPTLPPFPTATDKPTTCEAGWIPWGTSCYGIIRG
LAKNFQDAQADCLRRFGDLVVINDQAENDFISNEISRSVGGLWIGLSDTYKEGTFYWVDKSELNF
TSWRAGEPSNDYGNEDCVEIYGRWGPGQWNDNHCTALRDYICEKPGGVHNCPRDWVSYKNY
CYQINAHPGQKVTWEDARKNCLDFNKAWRKSVNVAQADLVSITNLEEQKMLQKEFSKVNIKN
DNGFYYWIGLTDKNSTGGFYWTDGSTYKYSNWIGSPQFASQCVRSNVIGYQGGSWSKGDCSEAY

NYVCKVGRGQDRCYNPLGIQSFAVPNKAMTSNSVLGPSTPALAGRLYMKAGNSLDGAWCAGN
NTKTPYLQIDFGKMQKLTYMGHQGKSNSDAMVTSYYIKYSQDGIVWDTYGYIPPAQYYKFKIYIG
WRTNYEKVMNLPLPAPFIARYVRVYPLEWNIAPCMRIDFYGCQEEVDNQRCGLGWREDKKTKN
CYKFHNQITTWDSAQELCSIQGANLATIATPDEREFLSEQLGEFKSLVSEFWIGLNDLDYEGVFKW
VDGTPYQLSNWYYGNPKVRNEKSNCVVVRTTDSAWVNTECTVFRKFICKRNYISPYTPKESYNG
YVYSKTDYFWPLDSATDFEGSLPLTLNGNVDRVPFPIDYDVGVSRYALNVSGSSGFFKIDVANDVC
IHHPDADSCPHGITVSFVFKMSKKSSSKRQVIIDTTEKNKPLGYKVYTEFGSIYVNLRGHQKSYSGS
TVYNAHIFNHMTFTWSSLKGLSLYMNGKSRVFDLLGSYAKDYPKVDYGKSLLIGISGDSQDPAEL
MIRSLGIFKQVLDAKSIEAIYLGEFGTCKSHQFAHGSYCYEYNSTEVSWSNALSTCRDNGGDLISIH
SPVEQAFFNLKIAQYNYQRTNWIGLNAKSKNSFFKWADSSRLEYSYWAPLEPNNNNGRREDCVE
TFANGGWNDALCYLRNPFVCKYSRALHIMGSENTDDKEYKVFFSPYQTLTFGCPLGRSIEILTASY
GSVKDGCVKDVKDKVSNACVKEHCVRVQASDFFFNKINPCKDKNRFALKRLKVTYKCNLATPE
FDSTGCSNGKDSENSYRVFDKSNICYKLYRKPKTFKEAQKVCKDDNGDLVRINSRETQVYLTSLLH
TMYQKTDIWIGMMYNSTTQNYEWLDGSHVYYTNWVNRQPDPRNGDYVKVGLEEGAKNFLFW
KNGEANETLPFFCQRVKVGKTTQAPPTTQPRTGTCPKGWFTYNKNCYYVNTNRLSFPDAQAEC
QKYGKSSHLASVLSYEENRAVFYDVVNATVDFAWFGLTNEAIVTGFRWVDDQPVSFVNWYTNEP
HKINRHLCVGMGSGGKWIDLACYLKYPSICKLPLGEVIPTTTPLPTIPPTQASGLCEPDWIHYNGS
CYNFANRTSYADFHNARVTCNKMGAHVATIHYQKENDFISAQLKRMYIPNAYIGLTDFSIEGQW
QWEDESVVSYINFGEGEPKNWHGMEDCVVVKSIGEWNDIPCNLKRSFVCKKNNGTVVKTVNTT
AAPDSGYCPSGWKLYGNKCYYFRYGE
>TRINITY_DN151_c0_g1_i4.p1 type:complete len:317 gc:universal TRINITY_DN151_c0_g1_i4:106-1056(+)
MQLANIIVSSFCFFGLAYSADWGYVEGTVEGPSNWKNDFPKCGGKQQSPIDIVTSDVEYSELPKIE
MGTNWDMLLADTNMTIHNNGHAVGCDIIGPRPITTRQGDKTWNLAQFHIHWGANSVTGSEHT
IDGKHYPAEIHFVHYQSTYNSLGEALPHSDGLMVLGFFIKVDDSALISSTTQLIFRFFKEIQYKGDTK
LLFTIRRSLFDIFHSKSTSKYFHYPGSLTTPTCDESVTWNVMHPENALIITSDMMKVLQGLSGKENG
EPVKSMSGNYRPVLPLNGRVVKRNFDESSASTIIVSLMSVTIPMLTIALIISN*
>TRINITY_DN6397_c0_g2_i4.p1 type:internal len:115 gc:universal TRINITY_DN6397_c0_g2_i4:343-2(-)
LTSSDQAELTKKLSAEVANGRLAMMAIIGMFFQDGLTGSAWGDWSLYTASPLRAFENELGVQDP
VGFWDPAGFTADGNVENFRRRRQTELKHGRVSMLATMGYITPELTGKLPG
>TRINITY_DN123301_c0_g1_i1.p1 type:complete len:272 gc:universal TRINITY_DN123301_c0_g1_i1:90-905(+)
MAQSASYQKMDVKKEEYHAVSGASYGEEKIPQAITSQPTAVSGVESYVLDLDASQVATWTEQDVI
NNFVERVGLGYANELFMANKINGKSLMLLREDHLKEMGIQAIGDRVFLLDMIQMLKKKKKEMQ
TTAALWSGETPADGCEYSDSCGHCIRRYLCPCCISRTYWKVTGQGVFYRKVPPCRQYFGTVSTDY
MDYRFFKDLELKEEHQCCCFCPRYRLELYVDDKDHNASAKSDEANPSFPIPHELLHPEAPKVEKII
RNAWNEARLVSE*
>TRINITY_DN585_c0_g1_i10.p1 type:3prime_partial len:236 gc:universal TRINITY_DN585_c0_g1_i10:705-1(-)
MQIFVKTLTGKTITLEVEPSDTIENVKSKIQDKEGIPPDQQRLIFAGKQLEDGRTLSDYNIQKESTLH
LVLRLRGGMQIFVKTLTGKTITLEVEPSDTIKIQDKEGIPPDQQRLIFAGKQLEDGRTLSDYNIQKES
TLHLVLRLRGGMQIENVKSKIQDKEGIPPDQQRLIFAGKQLEDGRTLSDYNIQKESTLHLVLRLRG
GMQIFVKTLTGKTITLEVEPSDTIENVKSKIQD
>TRINITY_DN3589_c0_g1_i1.p1 type:3prime_partial len:548 gc:universal TRINITY_DN3589_c0_g1_i1:63-1703(+)
MDVTKVETKPFEGQKPGTSGLRKPVMTFFQENYTENFIQSVFNAVGPQLYGRTLIVGGDGRFFMS
EAIKVIVQMAAANKVGKLIIGLNGILSTPAVSCLIRKRKAIGGIILTASHNPGGKDGDFGIKYNTSN
GGPAPETITNDIYNETTKITEYELCKDVTVDLSKSGYSNFDVNGKKFYVEVIDSVKDYLGMMKRVF
DFKLLKEFLSQEDFKFVASGMNGVTGPYLKRIFCVELGAPETSLMNAEPLEDFGGEHPDPNMTYA
ASFVDKMRTGEYNIGAAFDGDGDRNMILGHKGFFVNPSDSVAVIAAHHSIIPYFKKTGLKGLARS
MPTSGALDRVAEKLEIKLYEVPTGWKFFGNLMDSGKISICGEESFGTGSYHVREKDGIWAFLAWLS

ILASTKKSVEQILEDHWATYGRNFFTRYDYENVEDESGNKMMTKLRELICTNELKGQQLGAYVVD
TLDEFKYVDPVDESVTEKQGVRLLFTDGSRIVFRLSGTGSQGTTIRLYIESYVTDAIKIKENTQSALK
PLIDIALKISNLPSLTGREK
>TRINITY_DN384_c0_g1_i2.p1 type:complete len:314 gc:universal TRINITY_DN384_c0_g1_i2:1018-77(-)
MRIAVALFLVFYLYPTEACKDYLNPTVCRIGVKQKGDCEYYKRLCMKTCGGCGGVIRPTQGPGPG
PVIPAGDCGVSSFQGGRIISGTTAREGAWPWQILLKANGRAGCGGSLISANWVLTAAHCITGGSYS
VVLGEHDRRRRSGHEQEIRVSRAIVHPRWNKRSFEYDYALLRLSQPARMNQYVKPVCLPTGEPRP
GQSCYITGWGKTSSRGQMHYILQQAPMQVIDSNTCNRKNYRAIRIAIRPSMVCGGGSGRISGCQG
DSGGPFVCNEGGKFVLHGDVSHGSRECSSSKTYTVFGRVNHVRSWIRQTANV*
>TRINITY_DN146208_c0_g1_i1.p1 type:5prime_partial len:284 gc:universal TRINITY_DN146208_c0_g1_i1:876-25(-)
TDSHIRTTSAFIPCAKPSKMPPKTKKIKVKKVAAAPFQTSKPAPKKSVNVLIERRPRNFGIGADVQP
KRDLTRFVRWPKYVRLQRQKRILYQRLKVPPSINQFTQTLDQQTATQLFKLLHKYRPETKAEKKQ
RLTQLAETKAAGKQESSRKKPVTIKYGINHITNLVESKRAQLVVIAHDVDPIEIVVWLPALCRKMG
VPYCIVKGKARLGKLVHKKTATAVCLTAVRQEDRHSLEKVSDAVRTNYNERFDEIRRHWGGGIM
GGKSQAKVAKAERAKAKELRV*
>TRINITY_DN852_c0_g1_i1.p1 type:complete len:589 gc:universal TRINITY_DN852_c0_g1_i1:92-1858(+)
MAATENQDDSLYPIAVLIDELRNEDVQLRLNSIKKLSTIALALGVERTKTELIPFLTDTIYDEDEVLL
ALAEQLAGFSPLVGGPEHVHCLLPPLESLASVEETVVRDKAVESLRQVANDHSEQDLENHFVPLV
KRLAQGDWFTSRTSACGLFASCYPRVGSNTRGELRGAFKTLCCDDTPMVRRAAAGKLGEFSKTV
EMEYLKSDLIPSFTSLANDDQDSVRTLTVEACVSIAGILPDADKETLVLPILRNAAQDKSWRVRYM
VAQKVVDLQKALGPDLSKQDLVPAYAALLKDCEAEVKTASAERVKDFSENLPEDIREDAIMKNLL
PCVKDLVIDPNMHVKSALAGVIMGLAPMLGKDNTIEHLLPLFLTMLKDEYPDVRLNIISNLDSVN
KVIGIHQLSQSLLPAIMELAEDPKWRVRLAIIEYMPLLAGQLGVQFFDEKLSSLCMGWLVDHVYAI
REAAAINLKNLVEKFGLEWAQKNVIPQVLQMAKGANYLHRMTTLFAINTMIDVCGPDITTKLILP
TVISLSKDNVANVRFNVAKTLQKIAPLIDVQIIQRDVKPCLNQLNNDADVDVKYFASVALSACG*
>TRINITY_DN1058_c0_g1_i1.p1 type:complete len:2639 gc:universal TRINITY_DN1058_c0_g1_i1:134-8050(+)
MVKYIIWILLLSIFKEAYGGDWAQWQNWSACQDSSGAPIGCGRGNQHRSRTCADNNCGAGAIIE
VRPCLDEICPTFNCQNSSEIAGMCKFDYSNSCVGDDNCHHCICKSEGFKVSGDQCYNYCVEVGC
DEEIEGVSQLKRYCGDQAISCTGTVLNRTCLCNPATSYPSKDGQYCYEATCKTSQEFMTNCGAEN
SCQGTNTSFVCECNGFGYGYEVVANVQNRKKCKLLSCEDGDLNPLEICGQGATACTGNVHNLT
CACNVTAGYIPSEDKKSCIPMPCNTDPAVGVQCLPRNTCEGSKIDWRCICSAYGFRNDPTDPKKCI
EAKCDDPNIDILGLCGTGVTSCTGFLSKRRCVCNTTENYIPTLDGSHCMPAPCNANENATKDCG
GENSCIGSFDSYMCVCNGYGFQKNATINKCEEVPCSSLPTAEVQRICGFGALSCDGTIRNPVCNC
NNDLQYYASEDMKSCIEASCSSKADYDSHCGNENSCVGNITHFECICETEGYKPDPSNKKKCILES
CELNEVDVLNVCGKGAISCKGNVRDLECVCDASKGYVQSPDTNKCEPSSCYGNATVSEQCGEEN
HCLGDLSDWICVCDAYGYKVNATNSMTCDKVMCGDTGADAVTVCGKETTSCSGHIAKPVCTC
NETAQYLPSPDSTRCVKASCKENDAARKACVPGNSCSGSYDEFICICDQFSYHQDPADNRKCIRE
KCEDLAADYDYEKICGVGGISCTGYVNQPVCVCNSTESYHPSNDRTSCVKASCSTSDAVRTQCGL
ENSCFGDIESFMCDCKAHGYKLEPINKTSCVLATCEDGDFSYHFQCGKGAVGCSGNINTLSCLCD
SAKGYVPSADMKRCVPNSCKDNPDVYIKCGMENTCLGSYADWFCVCSGAGYEANPADPKSCQL
MECSKDDAIHVCGYGAIDCKGPVLAPTCSCNYSDHYIASEDGTSCVPITCRKNDTARAQCGEENT
CKGHTDNFKCICDAYGFKADNVDHQKCVERQCTDGDADIIRICGHGGIRCTGPIRSAVCECNSTE
YYYPNADRSRCILASCKANETIMKSCGEFNSCEGNMESHICVCNGPGYKHHPSDDKQCVKETCE
MGEIDPIYLCGKGAVKCNGTYDIYTCECDNAMGYVQAPDGNSCKPFDCHANATMHKQCGDG
NRCIGSLTDWICVCDVAWYKPDPSNLMRCHEKTCKEADLELICGTGATGCSGSATNPTCECDSA
NYFVSSTDRRSCIKASCKTNITAVQQCGDGNSCYGTPDNYVCNCDAYGYRLDDNNNQACVEKK
CDDVGANYVEICGLGGIGCKGTVKNPVCECDQTKHYVASPDSTSCVLSNCRGNKTVMAACGNE
NKCFGSASSFICVCDGPGYKRNPSNLQECLPETCEAGEIKPHMVCGRGATACKGTMYHFECECN

NTLGFIRSPGHESCIPVNDCYANATMHEQCGEGNGCQGSVADWICKCDAPGYKPHKTNFQMC
HQRMCSDADIDLVEVCGVGVESCVGPIANPTCNCDTSKHYVLSADSTSCIPGSCKTNETIMAQCG
EGNSCSGTSENFVCICNGYGYRPHETDKQTCVLKKCSHSDVNAVDVCGQGALSCIGTMRDPICQ
CNNDMFFIPSDDSRSCVLASCSANSDVAVACGKVNGCFGSKSSWICDCSSPGTMLNPNNIRECLH
ETCESGKLDPIIVCGKGALGCNGTMSNVTCECDNSMGYVQSPNGMSCVASDCKTNSTMHELCG
DGNSCVGSLTDWMCVCDAPGYHPHPSDPMRCLQKMCSHANINPVDVCGIGATKCVGSITEPRC
TCDTAAYYVPSPDGTYCIPGSCQTNKTALEQCGNGNTCTGTADNFVCICNAPGYKLDDSNNKRC
IEISCNDADVDLVEICGQGSIGCIGTARQPVCQCDHSKHFVPSLESTSCQLASCRANDTIMDRCRM
GNTCFGSMTSHICVCEAKGFKVNPSDPTECVQETCEDKDIDALSACGKGATDCMGTLMNLTCQ
CDGASYVESPDKTSCLPVGCQQNVDVKKACGEGNTCEGSYDDWVCLCTNPEYIPHATDEKVCS
KKKCTDNPIGSCGIGATSCSGNVNIHKCTCNLTAGYMPSKDEQSCIPIDCKMNPKMNCGFGNTC
RGSFFSYVCECNTLGTMRNISNPKQCIEVDCSTDAVSICGVGATGCAVTPTNDVNCTCGEFYHPA
HDYHSCVLDDCKFNKLVPDACGVENECTGSISSWMCVCKGPKYIQDIKSTQLCAPGPCRIEQCGS
GKASGSFQYYAESCSGTYFEPTCNCKEGFEKDSYGGCIVKSCSRNNQSSVDCNDGAISCEGTYFKY
KCECRAPTHILNPANKRSCIPARCNPQLCGRGLVNGDYMDFASECSGSYTDFTCKCKYGLYGSGT
SKCSLEKRTCALSNDCHPNANCIDYDGHYFKCECKDGYSGDGYQCHRNGGLTPWSVVQPCDKT
CGTGYAQVMRTCDDPKPDTLGKKCNVPSPDVREVPCHQFACTACSVKKTCECENIRPYFVHEYV
EPDESLKYFTNLTAEDVEKALSAIEKWKLSVDEKEFCIKHTLPEIDGLISDINENLRSVDQTKLYTIT
ARDMMRNKLDCNVDLEKKDKYFRVYEAIVKRHFILKQVVSDLQAKEKLMVTYRSSCTKDSKVG
WLSSIFS*
>TRINITY_DN4726_c0_g1_i1.p1 type:5prime_partial len:670 gc:universal TRINITY_DN4726_c0_g1_i1:2906-897(-)
DDGNETNRTIADDTTNNDKSNSLKRVQLPSLPAFSFTEAIDKVETMFKSSVQASPVATPQEKTKET
EKKKGTFSKLKTKARSIDNDKATSIKDGVTGAATGMIKFVKGIQAGKWTDIVQGSLDVIGSIAGM
LGPAGIMVSTVLSVLSTVIGLFGGPTGKKESQEGMMERVIKTALLDARNDELTSQVNGAKEIFNTIS
DTINIFREMEEKNLPDILSFYELSFEGIEIFGILEHYIKKWCAIGDVLSKAERDKLTDDAEKNKQSDK
RDMAARCLRITNLYCDLSIYRQILIADMAGVLSNANITTTSDAFLKLIEKERQKDGVILKFLWDPL
NFRDRRFVVSKYYSEIHNFPTITTYMNTVVKANKEFQEIVQNTKYGVGLMDKPNNEKFKKTLPM
ALECQKQELQGNCVQVPFSRIAFSHGTNPVYMYNSAGSIYVSDGFYLQVRINGTTNAEMKFYGPS
VYYIGIQKDNGNTFKYILTESSAKDFVRVCEHRNMAMNGKCLTLERRDYQFSELDRIGWNDKISS
LYVPEGIKLIAYEHSSFKAKVPIDYYGPATYNMISKEKTWSSIKMELTELRTTDMIKLCSNYKLGGIC
FYFEAKAGKFTKDDLISTKKDIHQIRSMEVPGNVTVIAYTDPMMQPKHFLAVYKGPKVVSETEQA
DPHLEIELKI*
>TRINITY_DN803_c0_g1_i1.p1 type:complete len:330 gc:universal TRINITY_DN803_c0_g1_i1:1049-60(-)
MKFLLISIVVLSFASTVENRPNKRFLKCDDVLATTVCDKFRNAASKVQLTANNVEDFVKDAYDKG
LTKAEDIKSYVEKQLEEKLLSKSCEELTGGKAFCDQLEKLGLFLRKTKDDLKPYIVKAILKGKQTA
DSIKQEVEDFLKNDLMNMECEDVLTTDQCNRMKEVGAALKLKKDEIKQAMIDSAIKGFNKIEDG
IKDTFENMKKMAEKFDCEDLLNPDVCVSIRHYSQKLRIGMPKMMRAVRTAIVMGYKVGKGFTK
VVFKLADAFIDCEDFLSAESCGKIKEAAAKIGAKIPEVNAAIKDAVIKGMTQAETIYKNTLDYLKN
IFGKK*
>TRINITY_DN13813_c0_g1_i6.p2 type:5prime_partial len:370 gc:universal TRINITY_DN13813_c0_g1_i6:1389-280(-)
GRGSLGMSDCAPPTDGGPTKLGACNPRARIHCADAAGDKNWSWCGHKAKEKRRRASGAPSA
NPFDSSAEERLAKLFERPVRPGEAVPQLVRLDDQRWRAMEHRRPHEAVQAVLAQGRAEGRDGP
RMRALRAIEVLYRRAGARVDEVDAAEGAESAPARHDVVLLHEARDLLLHDRLDDLLRIANELAL
HHELQALVAGGQRHRVRMERGAPAQRLCLEVLLDVAPHRHHGQGQIRARQPLGTRHNVGHD
VVMLEAPELARAPEAHHDLVANHQDTVLVAQSPHTLHVPLRCLVHAARPDHRLEHDCRDGPG
PLASDLLLQMRQRLRRAFLLRSAEREAVGVRGVHRLHETNRRAGALDEAAAEVARGCQ*
>TRINITY_DN69276_c0_g1_i1.p1 type:internal len:887 gc:universal TRINITY_DN69276_c0_g1_i1:2659-2(-)

FDRPKYQYSVFENANIGEAIDTVVVTQNTEINVNYSFYKPNSLFTINSMTGEISIAANLDYEEKTQH
FFHVEAKCISGSNIFTAYTSVKININDVNDEAPVFFGTNEAVVPENSLVGTPVLIVQASDADTGNN
GRVRYDIIDEVENLPLAINSDTGVIFVSGNLDYELKKSYSLPVSATDLGSPSRRAISMIKISVMDKND
NSPVFSKNYYELAIAEDQPSQLIACLAASDSDSDENGKLSYSIFLSDNSLAETFFINGTGCLSTLKKL
DRESKDSYSFTVVANDNGNPSLKGIAEVHVKVIDINDNIPIFSKSQYLLRISKNTLVGSTIISLDAVD
NDEGINGTVNYSITDGNIGSSFDIDSGGTVHLVRELSNTIAKYVLTIQAKDQGMVTSFLSTTKVEIE
VIGNSTSSGCYPLFTSSIYDVEISENIAINSNIITISANCPNNSNILYSLIESSINSGGKFAINGNGEIFVV
GTIDFEQTKFHSLIIKAYAESTPNMFSTSIVRIKLSDINDNRPIFSRTSMVIFLARNSKAKSIISTVYATD
KDSGSNQEISYQIQPNIQPSDLFEINALTGDVILSREILFKDIKNYRLQVLANDRGTPSLSSPQLTIIDI
KIYPDNMHTPQLQPSNHVKFPENSGINQLVYQAQASDLDIDLSIEALQNRPEAGNFGKFTYHLRN
TTGSQFFSISETNGRIENLISFDREKKESYDLIVEARDGGSPPRKSIQTVVVKISDVNEIPLFELSSYVF
YVSESSKIGLSIAQIMAKDNDAGQLGTLKYFIKGGNVGNKFSIKENNGILILSDFLNYMEQSVYNL
RIYAEDISNRPLGNNADVVINVLQVNASQIPVFDLSLYTYQVPEEQTSTLGTITARLGANRQTEIIYT
>TRINITY_DN876_c0_g1_i2.p1 type:complete len:225 gc:universal TRINITY_DN876_c0_g1_i2:1136-462(-)
MLLSIVITLFSSTLVASSVQRDCSWMKRRLISLEPRFASASEYEGQINVIFPYSKVLNSLINYQIKIEMN
AFYSYLSMSHFFGQEDNYLEGFAKFYHKAAMEELEHAEKFMTYQAKRGGRVNLHEISRPDQKW
ENAYHTLKASLTLERNVTKEVLCLHHFAEKFEDVDLVNFLEGEVIPEQYASMKEFQSNIKTLSRMS
SGKGEDDFMNYGVAEARFDKELLGK*
>TRINITY_DN16079_c0_g2_i1.p1 type:5prime_partial len:396 gc:universal TRINITY_DN16079_c0_g2_i1:1686-499(-)
GENETTIVSAITKQYENMLELRNISKEFEEIIKTLNIKERYRYFGPQKSFFDFENSKLFADELMTNINR
CEKENCSSFFSNHSVCMKHENYGSICQCNNYTYYDNETRKCEDIDECVVNNPCNLTTQYCVNVI
NYPAICGCKDGWGGEGCKIKRTCEDQRLICGVNSDCIIKENDLTSCVCHAGYYHGRFHECTKAS
NVLTAKGMKLKKNFTNELLNPESAAYKDLALEVEVTTKTELKKRIVDLADVLVNKFRRGSIEVDL
DIYLSGTSKPNEVLTELNDAIASGDMKGLDIDTQALASIEVTRTCALGNDECPTNSKCISGDGFDY
TCQCEDGYEKDGNECVKESNYKTTLAIVIPIVVVVLIILVGLFLYKCQTANRKSNEQNYGLEIRHKR
*
>TRINITY_DN140996_c0_g1_i1.p1 type:internal len:109 gc:universal TRINITY_DN140996_c0_g1_i1:326-3(-)
VPHQPEQCVTVKHQSCSKPDRVCLPHDHHCTFSQLCRPFNVRKCCTVVSVERRCQFVNRCVPKY
SYTRVCRHVQQCPPKCRNVCRQVDLGEHQVKSICEQKCDDSAHR
>TRINITY_DN1030_c0_g1_i2.p1 type:complete len:1426 gc:universal TRINITY_DN1030_c0_g1_i2:4483-206(-)
MAKVRVLALLMLICVTASNAQDGEEVFPFDVQKGEKGQPGDDGEPGNNGAPGRQGSEGPQGP
DGDVGFDGEEGDEGEVGPPGPPGRPGTNRTSVVVVYGPPGPSGPQGPDGQIGPKGYQGETGEPG
SPGARGPRGDAGAVGEAGVPGKNGANGPKGEQGSIGPSGATGIPGLPGPSGQRGPKGSKGEVGE
LGPKGAKGDAGRNGDEGESGLPGRKGEPGPRGQNGTVGFTGPAGQEGPMGPIGPAGPDGPAGP
QGPRGADGPPGTAGSPGESGAPGLPGPVGQVGARGEPGFPGARGIPGDRGQAGPAGQDGVDG
AVGARGSPGSPGIAGAAGPPGTRGEPGKPGPRGLQGQQGEQGSTGVQGSVGGRGEKGPRGLTG
QAGVPGPPGDSGTDGRPGPPGPPGNNGQRGLAGLPGSPGLPGFPGIKGHVGERGFNGPRGEKGS
RGESGRPGQRGIPGVRGPPGDIGLPGIDGKDGNRGQPGKDGIPGPPGQPGPRGIPGSQGAAGSKG
DKGAKGRTGDPGFDGAPGDKGIPGQAGDQGVPGPRGRQGDRGEPGEQGARGGQGLPGPPGA
QGPQGRQGKRGSPGLPGARGNDGLPGRQGERGFPGESGNPGAPGRPGASGRKGPSGRPGQKGS
RGDPGMPGDTGPPGQVGRPGLPGAQGNPGAAGDQGEIGEAGERGRDGKPGPRGAMGQRGAP
GPRGNPGQRGDPGLPGDDGKPGSTGAPGAPGAPGEPGQSGPRGPTGRPGQAGAPGESGLKGFK
GNRGARGRPGRAGKKGADGLKGQRGSKGEQGLTGLQGAPGLPGNQGPAGPIGLPGEQGPQGP
PGPDGKPGSKGDKGRSGPDGRPGSPGRPGPDGSNGLNGRPGKDGSRGPQGRPGPRGKQGPTGY
VGAPGAPGVPGLIGPRGPMGDNGKDGKNGADGDPGEAGPQGAQGPQGEPGADGADGLPGRN
GNPGLPGPRGEKGDRGTKGGKGAKGNKGTAGPVGQPGARGDRGPAGTPGADGEKGADGIKGE
TGFNGAEGAVGDRGDTGDRGSKGDTGFMGFKGAKGPRGSKGTRGLPGYMGPQGDIGDKGPIGP
PGPTGPAGISGPVGLPGRKGPSGDPGPKGYAGKNGRQGPPGPPGPPGLAGPSMLPPWMGGGLA

DGESKGPVVEREEPDAPEDENEELPPNEVNPFYRVYQFYSSKKTTPTDNDVNVMENKFKTKVEEL
KTEFSRIDKPDGTKDYPARTCRDIHAYHPEAKSGLFWIDPNQGCKDDAIQVTCTFSVNEDDEPTV
TTCVDPAEKQSVPQRTWASKMHSRAQKWFDEDHELAKLKYNANLSQLRYLGLLSKTATQDVKV
HCKKYVVWHDKMNRNFDRAMRFMGTQEQEFLYKDVDSKSSKSRFVPEVITDGCSANSNSVAHT
HLRFTSRKFIRLPIVDFAPTYPESPKSEFGIELGPVCFKV*
>TRINITY_DN1194_c0_g1_i1.p1 type:complete len:555 gc:universal TRINITY_DN1194_c0_g1_i1:101-1765(+)
MAARLGGGMMQPQIILLKEGTDSSQGIPQLISNINACEVVAEAIRTTLGPRGMDKLIVDKNGKSTI
SNDGATIVNLLDIVHPAARTLVDIAKSQDAEVGDGTTSVILLAVEFLHQIKSLVEENVHPQILIKGY
RKATKMALEKIKEINVQIKKDDQEEMRKLLQRCAETALSSKLVAGHKEFFGKMVCEAVMHLDEL
LPLNMIGIKKVKGGALEESQLITGVAFKKTFSYAGFEMQPKKYKDPKIALLNVELELKAEKENAEV
RVDNVEEYQKIVNAEWNILYEKLEKIVASGAKVVLSKLPIGDVATQYFADRDVFCAGRVVEEDLL
RTMKACGGSIQTSIHNLVDNVLGTCEDFEEVQIGCERYNLFQGCPRSKTVTLILRGGAEQFIEETER
SLHDAIMIVRRALKNDAVVAGGGAIEMELSKHLREHSRSIAGKEQLIIAAFAKALEIIPRQLCDNA
GFDSTTVLNKLRQKHAQGGKWFGVDILKEDIADNFEAFVWEPAIVRINALTAASEAACLVLSIDE
TVQNPKSDMSGDDNPITGKRGRMPQMGR*
>TRINITY_DN79_c1_g1_i1.p1 type:5prime_partial len:450 gc:universal TRINITY_DN79_c1_g1_i1:3268-1919(-)
EFYAPWCGHCKRLAPEYETAATKLKKNDPPVPLAKVDCVGEGKETCGKHGVSGYPTLKIFRGGQ
VSQDYDGPRDGAGIVNYMKKNSGPSSVEIKDNEHLEKKLNEAEDPLVVGYFKEENDLYKRFMKA
ADKQRASFAFAHSLNGDVNSKQGHENVIVLHRPNHMHSKHEDPKEFLSNENANTNQIIDFIKEK
QNGLVGQLTAANQESFTSPLLVTVYYKVDWKRNPKGSRYWRNRVLRVAKKFVGAATFAIAAKSE
FARQIGDWGFDVDNDDVYAIGTNAKKQVFKMEDKFSVNNLEKFVNEFKDGLLKPYIKSEPLPEN
NDGPVKVVVGENFDKIVNDPTKDVLIEFYAPWCGHCKSLEPKYTELGEKLTNNKDIVIAKMDAT
ANDVPHPYEVQGFPTIFWAPMGSKDNPKKYEGGREVSNFVDYIKREATNPVEIESKKKKKKKEL
*
>TRINITY_DN310_c0_g1_i1.p1 type:complete len:298 gc:universal TRINITY_DN310_c0_g1_i1:1091-198(-)
MFKIFNIYLILAVLILPTTVLFSGQAYAKEDAVEDEVVTDDVENDEDSKVETEEEVKEETAPSGKDA
EDEEEKTADGIRTSPDAETAVLFPDYPDKELPAGKPLHVLVGFNNKGSSEFVVDSIDASFRYPQDYS
YHIQNFTGTTFNRVVKPGEEATFRYSFHPHESYGGRPFGLTVMILYKDVDGNQYASGVFNETVTFK
EVDESFDGETFFLYVLLVAVALLVVFGLNYAFSNSKLKKAVTSKPTTEYGTQNGRHDVDYEWLPKE
TTTDFSKGSPSPRRSPRQRRVKRNAGSSGDE*
>TRINITY_DN2637_c0_g1_i7.p2 type:internal len:233 gc:universal TRINITY_DN2637_c0_g1_i7:3-698(+)
GYCARDALVACRAQLSDAGVPSAEETDAHIEELKAAAQRLVAGAAAAVVFATTAAGDAFAYPIF
AQQNYKEPRDATGKIACANCHLASKLIEAKMPSEVLPDTIFKATIEIPAKYEKRYQPIADGSKAKM
NVGAIAIMPEGWKLAPRDRLPKSLKKEMKGLAWAPYSKEQPNILVAGPVPGERYETMVLPILAPD
PATNPNVVFDKASIFFGGNRGRGQVYPEGNQSNNNQF
>TRINITY_DN5584_c0_g1_i3.p1 type:complete len:962 gc:universal TRINITY_DN5584_c0_g1_i3:2920-35(-)
MASEMSSPFDEEETLLDEKRSKSNDYEGGFCKKNWPVVITGFGVVVLICLGVVIGTAFKSDINSSN
SRLAREEKFPYKNIRLPGNVIPIKYRIYLHPNISDNEFGFSGNVRILVNVLNKTNSVLLHSKDLNILK
VKIFSNSAAFNQKSESLATEIPTQEFLHSKKLEMLHIALNENEYLDAGEYVIYISFKGSLSGGLEGFY
KSSYTTRSGEKRYLATSHFEPTAARAAFPCFDEPAMKANFIIIIVRDPQHVALSNMPIERNVTRRDG
KVTVHFQESVKMSTYLVAFVVCDFAYVSAKTKKNNIEVRVWAPKELIAQAEFAIQVAPNILNYYEE
FFKVDFPLPKQDLIAIPDFGAGAMENWGLITYRLTSLLYDENESSSANKQWVAVVIAHELAHQWF
GNLVTMEWWNDLWLNEGFAAFVEYIGSDYHDKSWRMMDQFIIDDVQNSLALDSHANSHPISVP
VHDPAQINEIFDSISYDKGASIIRMMKNFIGSDAFHTGLTNYLTKYKFKNAKSDDLWACLTDAAK
DKDIDVKRVMDTWTLQMGYPVLNVKRDGEKVTLTQTHFLKDQDKPAKSIYNYKWDVPVTYFT
NGENTTQFDWFYKDNKSIVIKIDENADWFKINRDEIGFYRVNYDKDTWAALKAQLLNDHMKLS
ASDRSNLMDDAFELARAGQLDQIEALNMTEYLHKEKEYVPWITALSGIAFCGKMLQGREGYKYY

EKYIVQQVKPLVDELGWEDKGTHLQKYLRGSVLRSAVDHNYTKAKNQALEIFKDFKENNKAIKP
NIRSAVYLAGIKYGTKDDWDFMYNRYKTSQFPSEQRKIMFALTDTEDKNILKKYLSWTLDNDKIR
SQDSCGVIEHISMNPKGTEMAFKFVKKNWPEIYKRYGTSSFDMSSLIKTIIGTRKTKSGKEEAKEFL
DTHKLGSGRLAGKQALASVQNHINWLEKNEKKVVEWLKNNINNKKITK*
>TRINITY_DN1821_c0_g1_i6.p1 type:complete len:638 gc:universal
TRINITY_DN1821_c0_g1_i6:297-2210(+)
MMKLLIKFIVVFIFYDFALATTPEEFLKEYNAKIGQQGWPSSDASFAKATNISDENSEKAQEESKKY
TKIAQAWRANASLINLDGADADTKRQFKLLLTSATPKCESVIADISKVGSDLENIYSLGRIPNDRTK
IKTINSDKASLSLDEHLYLILANSRDPDELLYAWKGWRDAVGPKLKDKYTKYVELNNIGARENGF
KDKGEYKRKVYEVDNLKEIAEKFWDELKPFYQEVHAYVRYRLSNVYKDLVKDGEPIPAHLLGNM
WAQSWDNIFPLVKPFKDKPALDVTSTLKKKYSVKKMFEVAEEFFKSIGWEELPPSFWTKTLFEKPA
DRNVTCHASAWDFSIVKDGSKDVRVKMCTVINQEDFITIHHELGHIYYYLLYWDLPSVYRTGANP
GFHEAVGDTLVLSVQTPDHLHKIGLLEKVEDSKESDINFLLKTAMERIAFLPFGYLMDQWMWGV
YSGDILPNEYNKRWWEMRLKYQGIKPPVSRSENDFDPGAKYHIPADTPYIRYFFAHILQFTFHKYA
CKAAGNTKPLHKCSIYQSKEAGKALGDMLRLGKSKRWPLALKNLTGSEELSADPIKEYFQPLLVF
LKEQRKEHKYTLGWSGTDDNSNSSPNCQVSIALFIMCILFFLFGNKHF*
>TRINITY_DN71276_c0_g1_i1.p1 type:5prime_partial len:1201 gc:universal
TRINITY_DN71276_c0_g1_i1:1-3603(+)
LTFIESLRLSSIKNIFKMMISFETFVPSTTPYAEDYNTNPQVMMLSNKIKETIDTSLNPLASQIDRQKA
FQVQEEFKSSSPHCAEVGLYLYSVERKNSDAVRLCALKFVDHFIETKWNDLTYERKTFYKKESLKL
VEQMTFENETYLKEAAAQIVVEIIKRTWPQSWADLFKNLEELQAKGDAQLELVLIILMRLSEDVN
GLDERLTTQRKRDMMTTFQDNANELFEFFIKTIRDKVTALKTESSSLLIRLSEATLFTMMEFVSWA
HFDCLFNNEKIIFQILFWLLEVKYLRYYAAACLDNIFQRKGTPEKRIEFMVVLSDDVLNIVENVVRS
LKKTPENVLPEEGYKFLKKLAEMLVSFGKIQILSLWGDERGFNEKPVGYDRYLRLMLLLFQHESM
KLTYDVSKFWHALSIHPHGKLDDTFKELMPDLLDCAIMKIAKISDPKIPNNPSSHYSTMDFDDKE
NYAEYFANCRGHMTDVCRKITIISYTVVLDHCLAKIKQATSKPPPDVSGIPKNQIQITQHYLYWDA
LVHFLKNGFAGVFFRANKEISEDTISKCNEIITLLIHYKCEVPLFTIPVINLLESVFPIFKHSRDHLLPT
MQLLFSIIRLPQNDEDPLRDHVFKLKRHVCEAYAALCKEIPHCLVDYFKQLEEFAVYQMMHNASL
TGQEKCRLIEGVLRISKHVTVKEEQDHCINAIFDMIDSMFDKEPVRRACESPISLIQYIGLDQEGTV
AADSELRTRELLFAINLWTAACKALAMSKEEEVKDEITEEEMRTQIETYGWIKRMVTMLPKMLSL
LQSYSLIWQHQDKICDKRILVHKIITEINPTIQVFQPKCADEVNEKRGLGQYYLCTFYESAYTSLGSV
SKSVGPSLFSIGGLAEAMNEKLVQHWNNLPNYRLGRITRTFIKDICLACPKKYFETFIGPFVSQVLR
LYFERISNEWAKLAQLKENPREVTDKLQEQIEIAEERMIYLMSKDLLDLLLSLLTFKRKHLRSTTKP
ADGSLETFGELGHFLLDCEETNFIIVQIIFTSLTWPDTSAAIKVSQIILAVLKKIFTDTSKLNPELPKQL
LLLILQGLNIHGGDEFGRAALTNNVLQFYLHLRMQYRGIVQVMLMIPTCEEQHLREFDLAVFVQE
LPLTEQQQKKIFRRFVAGACGTVISKLHEKDVVIQDLPALMPRQKEKTLNKNGDADDDLGLCSLF
GV*
>TRINITY_DN6428_c0_g1_i2.p1 type:3prime_partial len:303 gc:universal
TRINITY_DN6428_c0_g1_i2:906-1(-)
MLSSLKLERNKNLRYYRYRYSCCNIRVPSTSQKILNPFTLVVGGNAIYLDRQNVQCPGSAVITQLLL
RRYKSTIRYTYSCSTIPRKMECQNMKTPLNFDGKGDARYLDRHPVSCPNDYGLNKMRLQRGSNL
KTYAYHYRCCKPLYSVQFQENKVDGGFTNWSKYGTCTKSCGGGIQERSRSCENPKPLNGGKQCI
GVSVETRSCNTDPCPVDGGFSNWSNYGTCTKSCGGGIQERSRTCTNPKPMNGGKQCVGVSVETR
SCNTNTCLGPKQCKLKFTAYNKDGKGQVFYLDRHNVACKPDEM
>TRINITY_DN1272_c0_g1_i1.p1 type:complete len:262 gc:universal
TRINITY_DN1272_c0_g1_i1:957-172(-)
MRCQLFLLVACMAIATCSAYDWRNKVQNMLKYAKVNIFDRAAYILGDKRQHQLGKPWFCHKL
DCPAFQVVGNYTNKKDGFEERCYPETAWVMTSKEVDANENDIETRDMFMKLFKYIQGENEKQE
KIEMTVPVFTYSQLKKDDPSKRYISMHFFVPPSQEKNIPKPTRDDVTIMDVKADCYYVRSFGGYV
MSFGSKIMNEEKKIHAALEKNNLDFEARKTMAAGYDSPFRLFNRHNEVWVAKRKTMDSGCCTP
RKSTNVMY*

>TRINITY_DN1377_c0_g1_i1.p1 type:complete len:878 gc:universal TRINITY_DN1377_c0_g1_i1:68-2701(+)
MTGRARGRSRGRGRAAQDASQRPGGAQGGPPPQAAAPQQGGGGRGRTRGQPAPQQVAPQQR PAAQTAVPPTARMAQMSVKDQKPATEERQPRRRRLDPAPDVLVTRPEGRTKEGTGGTPVNIVSNF FKVIKMPNFKGLYQYNVSFDPEINSSKLKNALLYQMADVLGQTKCFDGMTLFLPKRLPDQITTRS VTTHQGNAIVIKIMFTNVIPEISPQVLQMINIIFRRQLRCIKMELIGRNYFNSSRKIEVKAHKMEVW PGFSTSILQYEKDVMLCADVQHKVVRQQSVLSMMYELHQKTREKNRFYELAAKKLIGETVITRYN KKNYKIDDIEWNKNPNSTFDKNGTQITFKEYFEKTYDAKVGDDQQPLLVSLPKAKNRKSGIVGPI LLLPEFCLISGLSDEIRSNFSIMKDLAQHTRINPAERAKNLEQFVQDLQTNEESKKELEQWNLKFES KLLQMTGRAYGSEKMFQREDQFSYKIQDADWSRETRGKKLIHTVNMTDWLLVYYERDEPIATDF KQTLVKVCGPMGMNVAEPQLCRLKDDTARSYFRALSEQIRPTTQIVVCIVPNNRKDRYDSIKKLC CVEKPIPSQVIVSRTLSKKQMLMSVCTKIGIQLNCKLGGEIWAVDIPISKNPKDPPSLMVVGFDVYH DSASKGKSIGGFIASTNRHLTKYYSRITQQQSHQEIADQLRVCMTGALKKFNELNGTMPSRIIVYR DGVGDGQLRQVFDHELPQFKAAFNAISQGYDPKFTMIVVKKRISTRLFLSERGMKTNPLPGTVVD TVVTRPEWYDFFLVSQSVRQGTVSPTHYNIIADTSGWKPDIFQRLTYKLCHLYYNWPGTIRVPAPC QYAHKLAFLVGQSIHAEPDVQLADRLYYL*
>TRINITY_DN16120_c0_g1_i2.p1 type:5prime_partial len:232 gc:universal TRINITY_DN16120_c0_g1_i2:734-39(-)
GFFLKGLNGPPGPPGPPGPSGEPGPPGLPGAPGAPGHVGEDGAPGPMGPPGPPGPGGPPGFPGE KGDPGVAGLPGSAGLPGAPGLPGPQGPPGIAGPEPITPNFTVICEGEKGWVQCKQYELIKITKAF WGRDDYETCPKVPAGLTAGRLCETNTANTEAKVNGQCKNEQGCEVVASNIFFDDNSCGNVYKY LKIGYECIPDESNAVDVLREGGRRKRKRATKDKRSFRDS*
>TRINITY_DN2217_c0_g1_i2.p1 type:complete len:254 gc:universal TRINITY_DN2217_c0_g1_i2:884-123(-)
MNSVDGNTDNAQKECLELCQRNAKATGCEVIWNRHNRGCYIHTQEVARGNNVDRHFCWVFS KCTLNNVPPAVCPIEKGYCVKGNGHDQNSGVIKMNSVDGNTENAQKECLELCQRNPKATGCEV IWNQHNRGCYIHTQEVARGNNVDRHFCWVFSKCTLNNVPPAVCPIEKGYCVKGNGHDQNSGV IKMNSVDGNTENAQKECLELCQRNPKATGCEVIWDRHNRGCYIHTQEIARGNNVARHFCWVFS KCNL*
>TRINITY_DN730_c0_g2_i3.p1 type:complete len:235 gc:universal TRINITY_DN730_c0_g2_i3:1019-315(-)
MPKKDRTELNRELEAFAVQIPSANSGFRMQLGETFIQNVKDGKIKGFEYKVRDPYRNDLFHDTSR IDLYTDYELIMDNLYVKNRSSYKVEVEDEKGLLAFKEKLLSKPFMCHAKLHLMLKEDLTQKLSND FFVTVFPENPDVKAAIEKGFQHALDCVKNKKHYKVWFTPEIWFWYKDFVTNATKTQFEFGDLRY SKIELNDKKGFFSSSKDLNCPIKPSIIELVTTEAIMPDGY*
>TRINITY_DN187147_c0_g1_i1.p1 type:complete len:1223 gc:universal TRINITY_DN187147_c0_g1_i1:3708-40(-)
MLTKFETKSARVKGLTFHSKRPWVLASLHNGVIQLWDYRMCTLLERFDEHDGPVRGIDFHENQP LFVSGGDDYKIKVWNYKQKKCIFTLLGHLDYIRTSFFHKEHPWIISCSDDQTIRIWNWQSRNCISV LTGHNHYVMCAQFHAVEDYIVSASLDQTVRVWDISGLRKKYCAPGAKPKEDITAPKTPGQMDLF GHSDAIVKHVLEGHDRGVNWVTFHPTMPLIVSAADDRQVKLWRMNDSKAWEVDTCRGHYNN VSSVIFHPRLELILSNSEDKSIRVWDMSKRTGVQTFRREHDRFWILGAHPTLNLFAAGHDSGMIVF KLQKERPAYAVHQNTLYYIKERYLRMYELGTSKDIPIMQLRKSSGRSTPLKLIFNPAERCILITYNND TSAGESQVYELHTLPKDIRSTDPEQSEGRRAAGTAAVWVARNRFAVLDKQQNVVIKNLKNEVTK KVQTPACDMLFYAGTGSLLLRDAEQVMLFDVQQKRVMATVKIAKAKNVIWSSDMSYAAILAKH SIAICNRKMENLCTVHESVKVKSGGWDDSGVFVYTTSNHIKYTLTNGDAGIIRTLDLPIYITRVKGS NVYCLDRECKTKVLNIDPTEFKFKLALVNHRYDEVLYMVRNAKLVGQSIISYLQKKGYPEVALHF VKDEKTRFGLALECGNLEIALEAAKTLDDKACWEKLSEMALLQGNHQIVEMCYQRTKNFDRLS FLYLTTGNSDKLRKMLKISEIRKDVSGHLHNALYCGDVEERIKVLKQVGQGSLAYLTAATHGLVEE CEEIKATFDLNPEKLPPINPNAKLLRPAVPILKNEGNWPLLSVSKTLFEGAAAMKDAGTSNMAAA VDMDDEGEGWGDSDLVLEEDAGFVDPEEKLDIDEEGGGWDVDDDDLELPPDLDVGPVSSGGED YYVPPTRGNSQAQVWCTNSQIPVDHLVAGSFDSAARLLNEQLGVVSFEPYKQLFMLAYARGRVA

APGLPSTPPLFYYPHRNWKEAGAKAGLPAVAIKLNDLVQRLQTAYQLTTGGKFADAVERLHQILL
SVTLLSVESRQDVTEAQQLLGICREYILGLQMELNRKELPKQNAQQQKRICEMAAYFTHCNLQPV
HLVLTLRTALNLAFKIKNYKLASSFARRLLELGPKPEVSQQTRKILLACEKNPTNAVQFDYDELNP
FSICAVSYTPIYRGKPEEKCPLCQASYKPEYKGTICKICRVAEVGRPGSGLKISMGR*
>TRINITY_DN101992_c0_g1_i1.p1 type:internal len:139 gc:universal TRINITY_DN101992_c0_g1_i1:414-1(-)
AERYDHHSGIVESMDSNKISDNLKELTRLPHPPGRSRNKELGDMLADFWQNEGMDKIEKFKYNI
YLSYPEKPSTILLFGGAKTEKYTIEAEPPVDESEKGGTPVVPFNAFSANDIVKAELVFVNHCRESDF
DEVKKAG
>TRINITY_DN2885_c0_g1_i2.p1 type:complete len:114 gc:universal TRINITY_DN2885_c0_g1_i2:31-372(+)
MAQPVRLYSKGIFVGYQRGLRNQHEQTSLIKIEGVNTKADTSFYLGKRVAFVYRVSKKTVAKGDK
KPSRIRVVWGKVTRPHGASGVVRAKFRHNLPAKAIGGSVKVMLYPSRI*
>TRINITY_DN923_c0_g1_i1.p1 type:complete len:442 gc:universal TRINITY_DN923_c0_g1_i1:53-1378(+)
MAEKYDLTYKVGAYLDRHMVFPLLEFLSSKEIYDDTELLRGRLELLQGTNMLDFVKDEYQRLHQT
DEVPDELANKRQEVIRQMRELEAATESITAAFEDPDFQKEASSARDGAQLFKYLNETFGIRSENLD
TLYQYAKFQYECGNYNGASQYLYFYRVLVHDDDKNASAAIWGKLACEILTQNWDPALEEINRLK
EVIDGDHFSHPLQLLQQRTWLIHWSLFVFFNHAKGRDLLIDMFLYSPNYLNAIQTSCPHVLRYLTT
AVITNKRRKQILKDLVRVIQQESYTYRDPITEFLECLYVNFDFDGAQKKLLECETVLLNDFFLVACL
EDFIENARLFIFETFCRIHQCISIQMLAEKLNMTPEEAERWIVNLIRNARLDAKIDSKQGHVVMGT
QAAAVHQQVIERTRSIMFKTQAITTNIDKKLASLQKQPYTEPFGLAW*
>TRINITY_DN32270_c0_g1_i1.p2 type:5prime_partial len:309 gc:universal TRINITY_DN32270_c0_g1_i1:1-927(+)
AMSPEGGAPPAAPGAEPPLQFEMFSREWQQLSQQDNFDGFRIEAVTQVTKYLQAAHTLFLGTQL
RECGYIYQFGPVFQSEGGRTVMVARAGLDGGVSGRIIQKFGTGFEVKASSQTNLKNQQQNIHDA
AAEYTGKDWTCGAKLAWQGAWLAGGSFTQRLLPSLQLGGDLTCVAVNNLMTIWQLSARWTEG
KNVFTAGLNRQPDPKSPVGANLHEIRLQYVRKLSDRLSLGTEFKLSHPDKDSGLSMGYEYAFRNA
RVQGLLETDGRVSCCVSDYQGLGFSGMIDYVQGNYKFGVLMHVMPPEQGQPPQ*
>TRINITY_DN1936_c0_g1_i1.p1 type:3prime_partial len:1224 gc:universal TRINITY_DN1936_c0_g1_i1:3669-1(-)
MVCPAVITIFLFSVLGSSSSQNCDFEDVKSCKWTQYAADKFDWSRASGSTGSTGTGPVNDHTTLV
KTLKVNTTGGNANGASCVFPFIYQSKKYFKCLQYYNTEWCATVPNYDKQAKWGYCVPTGFYMF
IEASNPRKKGDNAILVSPFVPKGPKCLKFWYHMYGPHINRLNIYVMIGGHTGKPIWSRIGTQGNK
WNKGQVTLVGRQPFQVVVEALSGISYKGDIAVDDISVSSTKCTQPSSTTCSFEDSQCGYIDDTSSDV
QFQWTRSSLSTSSSGTGPKTDHTMQSETGFYMYTEATRKKTGSKARLMSPVNKATSGSCLQFWYH
MFGADMGTLSIYLKVNNKLQSTPLWSEQGDKGNEWKVATATITTQDSYQIVFEGKIGRGYRSDM
AVDDIKLTDGNCPPPGFCDFETGYCTYFNSKNDKFDWLIGSSRTPSRFTGPNFDHTYGNANGSYL
FLEVSQPIKAGDIAILESQQFNAVGGKTRCLNFWYHMFGSDIGSLQVVYKVFSGSQQERVLWNLT
GQQQITEKDPWKYARVPVDMDADHMVMFKGIAGSGYKGDIAIDDVQFTSLACTIDPPEANPNG
KTTMKPPTTYPRTAPSTPVPTEFSCNFEKDLCKLTQDSKGTFNWTRIQGYTGSPGTGPSTDHTYGR
GFVPKYGPGSLRHAGGKCVHVLNGGFNKPKEGNPLVLYQGCGQTRLEFVFKYGYLKLTRLNMC
ATLEGSSTSSGTKVVLSNTCMNKWKMLDNNTIQDVKTGLCLSPFKGSDYPSNNSPLVVDKDCKP
KSRIFTFSPTMGHYAYIETSPPRQPNDTARLISPILKTPRCLNFWYHMYGAHVNMLNVYVKTSNGP
YVSPAWAKYGSHGDTWKAAYIYMNMTGPYVYAFEGVRGIGWQGDIAIDDITLSESCPAPVECSFE
ETSPIMCGWTNLKNDDFDWSIGQGKTASTKTGPKNDHTYGTSQGHYMYIETSNAPAKATAYMYS
PWYYNTGQKCVQFFYHMYGQQIGQLEVYIRYSNSRYLYRQFIRSGNQNNTWHMGQVTVKRNN
VYFQIIVRGTKGNGYQGDIAVDDFRIRDGACPAPGDCDFEHADFCSWMQEDNDDFDWIIGSGST
NSFLTGPSIDHTTQSNGGYYAFIEASSPRQPGHKARFKSMVFAPTTGSGRCFSFWYHMRGYSIGNL
TIYLRKVSNTKTLSEEVMWLLGGNQGKAWNQGRFTLKSSTLHEVRFIYLRLFHCKICLPCY
>TRINITY_DN81944_c0_g1_i1.p1 type:5prime_partial len:222 gc:universal TRINITY_DN81944_c0_g1_i1:3-668(+)

VHQVIARYSINMLPQILSTFVILTVSTALADKPWFCHDLDCPSYKVAGAFTEKKDGFEVRCYPKTV
WAMTQAEFSAKSSSYSTGSLFMKLFKYISGNNKEGKKVAMTAPVFTYSDLKKGDNSTRVIKMHFF
VPPAQESTIPAPSGDDIEIVTVPADCYYVHSFGGFVFSMGRKIRRETGNLVKSLEKAGIKSYDLSKSM
YAGYDSPFRLFGRHNEVWLAKM*
>TRINITY_DN1559_c0_g1_i1.p1 type:complete len:311 gc:universal
TRINITY_DN1559_c0_g1_i1:221-1153(+)
MAEKKATFKLGFAENFALSGAAAVISKTAAAPIERVKLLVQNQDEMIKQGKLSEPYKGVIDCTTRT
FKTEGLAPFWRGNLANCIRYFPTQALNFAFKDKIKAAFKVSKSDGYALGFGKNIASGGCAGALSL
CFVYSLDFARTKLANDTKSSKKGGSREYNGLVDVYRKTLKSDGVAGLYRGFVLSCVGIFVYRGCYF
GFYDTLKPVLLGADASLFMSFILGYGVTVTAGLISYPIDTIRRRMMMTSGQAVKYKGSIDCATQILK
NEGAMSLMKGAGANILRGVAGAGVLAGFDKFKEVYIAMRKRQMGLE*
>TRINITY_DN144181_c0_g1_i1.p1 type:5prime_partial len:309 gc:universal
TRINITY_DN144181_c0_g1_i1:2-928(+)
INVLAKRFDVKVMQIDVTFPINSEKEVLELFENTLKENKHIKIAVFDHISSATAVKMPIHDIGRLCR
KYEVISIVDAAHAPGQIILDLDNIPVDIYVGNLHKWYYTPKSCAIMHVNPKLHDVMQPTVVSHG
CYGNFHWRFFKQATKNYSAQMTAGFAVQYMEKLGGIRHLHEYITPFVEWAVELFKRDWGTKELS
IPDSMKAPYMRVVYLPDIFTQVYGTDAAGADRLIIDLIKKYNLSMFVNPAQGKLATRVSAQIFSLK
SEYYYASRCIMDEAKILQEGVNVKIMNEAKILQEYENIKIKIKDEAKPL*
>TRINITY_DN3034_c0_g1_i1.p1 type:complete len:1941 gc:universal
TRINITY_DN3034_c0_g1_i1:6289-467(-)
MAEEEDPMKYLQVDPAQALKDQSKTFDAKKWVWVPVIGEDGYAAAQVIKVDGDNVTVETNK
GHEMEVKKEKCDQMNPPKYEKCSDMASLTYLNEASVLYNLKARYTTGLIYTYSGLFCVAVNPYR
RLPIYSDQVVKLYRGKRRAEMPPHIFAIVDTAYQDMLTDHDNQSMLITGESGAGKTENTKKVIQYI
AKVAGVEKKKSDDGPVKRDLDEQVVEANPLLEAFGNAKTTRNNNSSRFGKFIRCHFSQTGKLAG
ADIESYLLEKNRVIHQASAERNYHIFYQLLYATTDEELQKLCLVTRRSEEFEFLKKGVPSVDAIDDN
EEFGYTTNAIEVLGFDEEEKQSMFKICAAILNFGNMKFKQKPRDEQAEVADPADGERVAYLLGLPI
GEFHKSLTKPKVKVGTEFVNKGQNVSQVLYSVAALSKALYERMFWWIVSRVNKALDTKERRSYFI
GVLDIAGFEIFEYNSFEQLCINLTNEKLQQFFNHHMFVLEQEEYKKEGIHWEFIDFGMDLEETIDLI
EKPMGVFAILEEECIVPKASDQTYLQKLHKQHAGKNGSYEKPTPKQTKSGGGDFILHHYAGSVGY
SVANWLEKNKDPINAHTAELFSKATVGLVSGLFADYNPDVTGGRRKGSAFQTVSFRHKEQLKNL
MTTLMSTSPHFVRCIIPNEKKTPGLVEGTLILHQLRCNGVLEGIRICRKGFPSRMLFQDFRQRYQIL
AASAIPSGFIDGKVAAEKLIDSLQLDQSEYRVGTTKVFFRAGIIGELEDMRDERLAKIITQFQAYCK
GHLARIEYKKMCEKRIGLAVIQRNVRKFLFLRNWQWWKLYCKVQPLLSIARAEEEMKEKEEELK
KALEKAKEDEEQRKKMEEQLTENIAEKEKLFADLQAETDRLINLEDKLMQIQQQKDRLEANLNE
TLEKLDGEEHSVSVLQEKMHDAEDRIDSLEAEASELKSNCSRLEVERANRDKQIDALNEDINKQD
ESIAKLGKEKKGLEDNIQELNETLQASEDKCSSLTKAKKKVEDALKESENSLSKEKDSKARLDKEK
RRVEGELKETNDKLSEAENELKNTQDTVYKREKAIKELEEARDNQDNQIKQLQKKIQELLQRIEE
LEEELEAERKQKQKSELSRKELESQIEELQDQLETAGGATHAQVEVSKKREQECSRLRKEIEDINA
ANDEALASTKHKFNAQLSEAQEELESVKKAKSKSDKERSQLAGDLEDVQGQLDQVKKQRASSD
KNNRMLEEQLAELRGKVSSLETDMGDSESRNLKFQNESATLLKQLEEAEHKLGLANKQNKQLQ
ASLGESKNVADDEARGKHDMQNKLQNALAQIEELQEQLEDEQTAKMDLQNKYSRANADAQQ
WKNKYDMEGASRVEELEDAKRKLAARVQEMEEALAAAESKAASMEKVKNRMNEEVEDLLLDL
EKAQAQAANLEKKQKKVDQQINEWKLKCDEVQEELEKSQRDNRSYSTELLKMRTANEDTVEKY
ESLKKENRALAAELQSVSEQLSEGGKNSLEVEKLKRKLGLENEELEVALEEAELALEQEEAKFLKI
QLELTQLKQSTDRRSAEKDEELETLRKNHARQLEALQNTIDAEQKGKAEAQKLRKKLEGDLADL
ENRLEGSDRNSADYLKTIKKLQAQIKELQAMIDDESRGRDDMRDTASRSEKRAMDLAVQLDEAR
VALEQSDRLRKLAENEKLENAERVAELQTMYNSIANAKRKAEGDYHSLQEEIEELENEAKMAEE
KANRAMNECARLSSELNGAQENASNAEKSKSLMARQITELQQQLEEAESMGGKSLKNQIRKLE
QKVMEVEGELDNEARKSTESIKIARKADKRVKELQFQLEDEHKAVERAQEVSDKLNQKMKKMR
LQLEESEQQSSSWQSKFKKAVVDLEEAEERCESAEQALQKARQRARSGASGMTTRGVSRARSRVR
SPQNDD*

>TRINITY_DN7504_c0_g1_i1.p1 type:complete len:414 gc:universal TRINITY_DN7504_c0_g1_i1:87-1328(+)
MAKIPGGIVVEMDGDEMTRVIWDMIKTKLIFPFVDLDIKSYDLSIQNRDNTDDKVTVDAAEAML
KYNVGIKCATITPDEKRVEEFNLKKMWKSPNGTIRNILGGTVFREAIICKNIPKLVPGWTKPIVIGR
HAHADQYKATDFLVPEAGTLEMKFTPKGQTEPTISYVVNNFAGAGVAMGMYNTDKSIRDFAHSS
FQCALEKNYPLYLSTKNTILKKYDGRFKDIFQEIYDNEYKGKYEAKGIWYEHRLIDDMVAYALKVE
GGFVWACKNYDGDVQSDTVAQGYGSLGMMTSVLMCPDGKTIEAEAAHGTVTRHYRLHQKGQ
ETSTNPVASIFAWTRGLAHRGKLDGNERLMNFAKLLEEVCVATIENGSMTKDLAICIKGMKNVTR
ADYLNTTEFLQKIADNLTTKLNLSHL*
>TRINITY_DN1119_c0_g1_i1.p1 type:5prime_partial len:949 gc:universal TRINITY_DN1119_c0_g1_i1:2934-88(-)
FFFNPQTVMHEKKFSRMKFILLFSFFAISLGQNCNRMHKKIGCFWNDKNAKVLFNDRDSINNSTY
GALSDKLACKCNAVARSNGFKFFALRHFGICIGYNKITTGSKSIEHCIDTKSNGVCNLGDMNTCT
GTQNTADMMYQIVPEKCPSRHKKVGCYTEDRNATFLFNDRSNIDWPNFEKFLVELACKCNKVAL
EKGFNFFGIRFYGVCVGFKNISTEEKSAFHCKDPNYRPYRPCNMKDSNVCAGDHFLTDMIYKTER
QNVDKLFSEWSGWSTCSKSCGGGTQKRTRECKDKKSCSDAGGEEFKSCSNSFCKGSTGVAKAQE
VVETFLETFHKVEFPDIKPSGKYLKTIFEIQKAVGEEFNSVDPSVKGVPLEKRLTELEHFLKIAVEGG
SIDFARSTLGKGMFVFMKKKIKEGLPINDIDTAAKGFLNKLTGDIGSKKMNEILSSKGDATLMFTID
TTGSMAQEIAAAKAIAKQIISATREYEVDYILSPFNDPRTGPVRYRSTNDRANFVSDINKLLASGGG
DCPELSIKGMLNAFDKGPKYGSPMFVFTDASPKDATPSNMAALKAAASSNNAKITFFTNLKGCG
KELGIESYKEIASHTGGQVFPLKNEAELINFTEYVKSSLQQDTIIAAGGSLNSRISKRAAPRSHIFMV
DQSITRLIISVAFEKQNTGKHIKLLDPNNKNVDSSTMLAMSLIYDIKNPKVGLWGLSFPHIVGEFGY
NVQAVTENAIEFAHSFLHQHNVKGSDVTVSLENPIKGIRNMMVIRLGGADHIDLSTIKVHLVDHS
GNKLVSNVMTDRLDSDEIRNASIVPPTVQFKVLLTGKTKSGQQFQRMSQGSSTPVSAFMRTISAGY
DFTATMGKDWISIQIEIVNKGSAESFNFQAKTKYGTLKNPNQTKTVNKESMITFSYKPPNRHDLK
GKTDKVTVIAKRASGEKINHFISILLIK*
>TRINITY_DN761_c0_g1_i1.p1 type:complete len:830 gc:universal TRINITY_DN761_c0_g1_i1:129-2618(+)
MTSMFRSEEMTLCQLFLQSESAYACVSELGEIGVVEFRDLNPDVNAFQRKFVNEVRRCDEMERKL
RFMQKEIDKADIAIVDTGENPDAPHPREMIDLEAQFEQLENEMKDSNSNYEALQKSLLELLELRH
ILKKTQSFFEEEGYAETQARLQADHEPGVTGGGQLGFVAGVINRERIPTFERLLWRACRGNVFFK
QAEIDTALYDPGTGDLTHKCVFIVFFQGEQLKIRVKKICEGFRATLYPCPETPNERREMAIGVKTRIE
DLETVISQTQDHRHRLLQTIAKNIRVWLVKVKKIKAIYHTMNMFNLDVTQKCLIAECWAPVADL
DSIQAGLRRGTERSGAQVSSIVNRMSTKQDPPTFNRTNKFTKGFQAIIDAYGVASYQEVNPALYTVI
TFPFLFSVMFGDCGHGAIMALFALWLIMNERKIANWKAGGEMFDTVFQGRYIVFLMGLFSIYSGL
LYNDIFSKSLNIFGSAWVPTLNRTKYDYQPENYKEWSDGDRMLNPLDAPRETPYYFGIDPIWQLTS
NKLTFTNSLKMKLSVIFGVTHMIFGVILSLFNHLHFKQYVNIFCEFIPQLLFISCIFGYLVILIFYKWIA
FSIHSPSQPSILLGLLAMFLNFAKKKTDADRLYNGQDFLEPFLVVVAVICVPWMLLIKPFYLRWQH
NKKKQHGTNFARLHEDTAPLQDNEVNHHDDSGISSKNEEEEEFDFGEVFVHQCIHTIEYCLGCIS
NTASYLRLWALSLAHAQLSEVIWTMVFKIGLSMDGYVGSVAIFAVFAAWAILSVAILLIMEGLSAFL
HTLRLHWVEFNSKFYVGAGSKFVPFSFKDIMNDEAF*
>TRINITY_DN25375_c0_g1_i7.p1 type:complete len:811 gc:universal TRINITY_DN25375_c0_g1_i7:112-2544(+)
MNVNEKMNIYFCIGTLLFQSLLVLKVEGIFNDCQTKGSCGKCISSSPECAWCEDIIYKRSLNSKSYY
KKCDVLDNHKRQGCQNITNPPSSSTIDKDAELKGDTRVSPQEITVKLRPGQATTVDITVKTPKNFP
IDLYYLMDLSLSMKDDLEQIKDLGDILGEEMKNVTTNFRLGFGGFVDKPINPYTTIKPDEIEAYIRD
EDAQYTFTYKNYQPLDEDPSLFSKTLEQVNISFNIDGPESSLEALMQVIVCRNDIKWKDKRKARRI
VIITTDADFHYSGDGLLGGQVIPNDAQCYLSKGNYTAWNQFDYPSLSQIRSVMIENQIVPIFAITGN
FELYKKVAEFFGAGSGAVADRLSKNSDNVVPLIKEAYEKIAKIVNINSDAPKGIDMKYTAICGTGK
NVEAREGTDFCDDVEIGETVVFKVEMTAKDCELVGEDLKFKIKTNFDDVNVAVKLLCECPCEQD
KSKIVIDSEHCNSRGNLTCGQCQCDLDFYRGRFCQCTVEEAKNQTLCRLPGEVGPVCSDQGFCEC
GECICDEPKLNRFGEIEYIYGDQCQCSNTSCGRTTNENLLCGGPSHGRCDCGECTCFSDWQGPTC

EVECVKSSAACIDEEGIECSGKGKCVCNRCVCDPGYIGDLCQECVSNCPDPCQDYRECVKCRVFK
MSDLSDEECKSECLKHNITSHIVTDKVPEDKRCIARDEDDCSFVFTFVKSNITDEVQLIVQQEKICP
AEPDILAIILGVIAGIVGIGLALLLIWKLLATIQDRREFAQFQKDRQDPKWDSGVNPIYQQATSTFQ
NPLYGNKGEETEKILDSQ*
>TRINITY_DN124859_c0_g1_i1.p3 type:5prime_partial len:237 gc:universal TRINITY_DN124859_c0_g1_i1:884-174(-)
HRLEEDMRCVHHHEPRVRGPVRVAGQSEGLVPAGGDDGARLGHDHGDHVGFGRLQGLQGVG
EEDVHVVHDDAATDVEAAPLRFRFAQYQVGFGLRWCIEAEGARNARADPSDAGNQRHERTQV
GLAGRAALQRSAGRHLPRLRIARAGLREARGDDQRGPFGIRLPEGAALHRQMHQHLRDEDHA
ARQHVGRRNAGRQKHLLEDFGEGKDSLEGVWAGRHGESGAPGHQPKVHHHE*
>TRINITY_DN77_c1_g1_i1.p1 type:3prime_partial len:1965 gc:universal TRINITY_DN77_c1_g1_i1:2-5893(+)
MGVQVYSLGIGSNYDIGELLDIASDDASVFRSQDVDELVSIVASITEQTCKGCTRAVDIHFALDASQ
NVGRENFQVIKNFIKAIGFKFIISETGSHMSASIFGEYGTLIFSMDQATQQEDFINEVDNVPYIGDFN
GNIDKALDMVSEDIFTFDGFSRQGVPKVLVVLTASNCDSCEKSVEEAVKPLKAQGVHVVAIPIGSN
ANMEEMDKMASYPTQDYVIPRASYEELLNGVFIQKVSEMVCSGRPGVCEEPPIEGECTEIINECTL
DVNCPANRKCCIKDCKASCEVPAAACITPIDLAFAFETTGFIEENFDEVKSFASSLLDHFRVEPAKT
HISISTYGESVNAKTAFYESYDKSALKQFIKDLEPDGGRQANIGDFLKTANDEIFNITNGIRQSSPR
MLVLFTNGVYSNDQIAKAQEESLKLKAEGQDVNILVINIGSIVEGNSVLRDVVSTPDFRNLHNVT
DLRRLVNFGFENKIAEAICASKVRKTRCKPRTPQACSVRDDLCQTDFDCPGYQECYSDGCRLRCQ
TCENRCGDKVDLLFMIDTSSRIGPEGFKNAKENIKKMLEWFTIEANNTNVAAITYSDNANYAMQ
LPAAGSNDPPQTLFDVQGRLDSIPYSGGSKSNLDKALKMAAAQVFYQNSLRLRKDSKKAIVIVTD
GVDDSTSRLEIASWPLRKQRVVVDGNDYAAIRLLAVTVGNNYNLDTLEKVVSPPIDKNIFLAADF
DDMYNGIRQLAEDSCKYTRQDVEPPVIAVKGDPGPKGEKGRQGNPGTNGTDGMDGIEGPKGER
GDDGPKGEKGDTGIAGPPGGDQGDLKGPDPMAEAVGIIEGTAVKGTRGVTYDLRDGPIGPPGPIG
GVGKTGLPGDSGEQGQKGRGGPPGLRGVFGGQGPKGNPGEDASPGLPGEPGPKGISGKKGPTGS
PGMPGPQGPFGYKGEQGIIGQPGDPGDGGKKGPKGYAGEKGDQGDIGDAGFPGPDGKVGPIGP
PGQRGLRGPEGSSGPIGKTGDIGNKGLPGPRGSRGLKGQSGNAGPRGGKGRTGDVGLVGEKGTQ
GSIGEAGQEGQPGVPGKEGPPGYPGPIGTPGPMGSRGDIGPQGEEGPKGSLGLKGNRGEAGRRG
LTGEKGETGDIGELGPKGAQGQQGKQGKTGKIGPRGQQGPLGFQGLQGQTGDPGQNGRQGPK
GNLGPTGEKGADGPKGSLGSAGLTGPKGLVGEPGNVGRPGLPGRDGKNGPQGANGKDGDPGA
SGPQGLQGSPGSMGTPGSTGDPGSPGDRGATGAVGDKGTRGDSGVRGEPGAFGQQGPPGLRGE
RGEKGPQGTNGVQGSPGPRGTPGLEGLDGIVGRKGTAGRPGNPGQSGEQGAVGSIGVPGPQGIS
GTPGERGDGGSDGGPGEQGPPGLQGSSGSPGRDGMPGRPGQQGTTGGKGKTGLRGPKGANGQ
NGVQGARGPDGLPGAPGLVGNRGTPGPNGIPGLDGNPGPAGKQGKQGKMGTPGMTGPAGLR
GPVGSPGLRGQKGPTGDRGDSGETGPIGVAGDIGPRGFTGELGLRGAIGEMGQIGETGPQGMPGS
QGTPGAAGLTGPAGPSGLPGKDGNKGPIGDPGNAGNPGRDGGPGSPGKDGTPGEPGTPGSQGK
QGLKGPFGPQGAPGEQGQRGLTGRQGSGGKDGPKGFPGNDGSPGISGKRGPRGKDGTPGSPGPI
GKDGRPGKPGINGTDGDPGNNGPKGDPGERGDSGDSGSLGASGDKGKTGSEGPAGFVGPRGSN
GKAGVPGKRGKRGDQGRDGPRGNSGPDGIDGSKGFKGNMGRFGIQGPPGDAGLKGALGPDGT
VGHTGPQGNTGDPGPDGPEGPSGSKGPAGNAGQIGNAGYPGSPGDGGIPGTAGLPGPPGPPGPP
GDMSGAFSGNYWDFVNAGGLQKGPRYYGGRSKRSLDQQDNSIEKVFKSNYETFKNFDTVWNQI
VDDLVIQKTLGSSKNPALTCGDLFKTNPITKSGDYWID
>TRINITY_DN3452_c0_g1_i1.p1 type:complete len:466 gc:universal TRINITY_DN3452_c0_g1_i1:139-1536(+)
MDVACFRFRMILSIISFTLLTFDLPAVNSEALHLTEENYQDTLDKNKLVLVNFYADWCMFSQQLAP
IFHQTADIIHEEYSNVKLGRVDCEEQQKIALDNMVSKYPTIKIFRNGKALRKEYRGQRSVDAFAQF
IKEHLKPVVQDITTSPNDMKINENRNTIVGYLESKDSPGYQSFAKIAEDLGETTDFFALLGDASHK
ERAVGESVVFKPKKSYGESESTYSGSVSNLDRLKAWATEKGNPLVREITFENGEELTEEGLPFLLLFY
KPGDTTSIQRFKNGVMRELLPERPRINFLIADGTTFSHPLHHLGKSLDDLPLICIDSFRHMYLFKKF
DEVDVPGKLIAFINDLHSGKLHQDFHNPSPDDQAAQGHHHGGQAQIDNNAQQPPQEQQPDQ

KQPDQKGEDTIQNTDQPPSGSGNKGSGKKPASGSGSGQSPPETVFHKLAPSENRYTLLHWRDYS
GNYRDEL*
>TRINITY_DN15151_c0_g1_i3.p1 type:internal len:560 gc:universal TRINITY_DN15151_c0_g1_i3:1-1677(+)
LDGAERPRILAAMLSTLRRAPRLAARAPLAAPAAARAFAAAKDIRHGSEARALMLEGCNRLAD
AVAVTMGPKGRNVVIEQPFGAPKVTKDGVTVAKSIDLKAAPMINVGAQLVKQVASKTNDVAGD
GTTTATVLARAIFREGCKAVAAGMNPMDIKRGMDAAAKVVLEDLAAQATTIESPTSIRNVATIAA
NGDAQIGEMITDAFEKVGKDGTITVQDGKTLEHELEVVEGMKLDRGFISPYFITNVKAQKVELEN
PLVLLYEKKISSVQAILPVLEQVAKVQKPLLLIAEDIDGEALAMLIVNKLRGGLKVAAVKAPGFGD
NRKEILKDLAVLTGAELLSDELGDKLEDLQITQLGTVGSATITKDDTVLLDGSGNKSAIDERCEAIR
DAIELSTSEYEKDKLKERLAKLSGGVAVIKVGGASEVEVNEVKDRLNDALCATKAALEEGIVPGG
GTALLYSAQKLEALTLDSLDQQVGVDAIKAALSQPCVQIAQNAGEEGAVVVRALLQEGNAARGF
NAATGQYVDMVEAGIIDPTKVVRTALADAVSVASLMTTTEA
>TRINITY_DN38657_c0_g1_i4.p1 type:3prime_partial len:302 gc:universal TRINITY_DN38657_c0_g1_i4:181-1083(+)
MAIDNSRFRSPLPFILILGVIGSIILYLFISYRNENTILKDKVEYYVYKSTSQGQALTKTKNSLDTCIAQ
EQSFQQGIQNLQVQVKDKEKEIGTLKGDIDACNLRRDLGNKFYTNVKQILGKLEALQELEKGQS
RIPDEAYDLLKDEIGRIKENGEKVKILEKEKKIVEEKLLKQEETGKSLETQMKDYQSQFEELTEKNR
KLSAQIKVVENDIAKDKARGFQVKQISNETNVQKIILGDAPASDHDAPASDNDAPASNGAPASD
DAPASENSDDDAPASENSDDDAPASENSDDDAPASE
>TRINITY_DN9413_c0_g1_i1.p1 type:complete len:419 gc:universal TRINITY_DN9413_c0_g1_i1:1414-158(-)
MKCEILLIFYVFFGSANSRKSAMFQDQFTSASFWNRLKANATLYMSPQNSAYSTGRGRSGYSTDTD
MCDRVTGVPRHLTFITRYNHYRKYTHAYNIPIISTSRVQDAALKRACYVLKFLLADRKVLRDSLYR
NFGRVGIMAVNEGTTSIPEHRWLGPSWDQRARGLGGTLSVPISTGGEENLLCWKSRDRYPREDIF
LHEFSHGIQEVAVRGGGIPGYFSRLTSAYNYARSRGLWRSTYAMSTVQEYFAEGVQSFFNVNDYSR
TPNGIHGPVNTRSKLRTYDPTLFRLIKEVFPCMNIIKDRCDKSSTTASFRMNCDGSRPVRTVRPPSG
KTTTTTPSTKSGCQDLKPASCVGWKVYCSNPSHLKSMQYYCKKTCGFCGRPSNCNDNNTLCGS
WASRNECTRNPKYMLVNCKKSCNRC*
>TRINITY_DN119684_c0_g1_i1.p1 type:complete len:662 gc:universal TRINITY_DN119684_c0_g1_i1:2182-197(-)
MKLEIILFTLLVALTVHCSEKKDDSKKDVGTVIGIDLGTTYSCVGVFKNGRVEIIPNDQGNRITPSYV
AFTEEGERLIGDAAKNQLTSNPENTVFDAKRLIGREWGDKAVVHDMKFFPFKVVEKGKKPHVQ
VNVKENEKKTFAAEEISAMVLVKMKEIAEAYLGKKVTHAVVTVPAYFNDAQRQATKDAGVIAGL
NVMRIINEPTAAAIAYGLDKKEGEKNILVFDLGGGTFDVSLLTIDNGVFEVVATNGDTHLGGEDF
DQRVMDHFIKLYKKKKSKDIRKDNRAVQKLRREVEKAKRALSTQHQARIEVESLFDGEDFSETLT
RAKFEELNSDLFKSTMKPVQKVIDDSGLKKKEIHEIVLVGGSTRIPKVQQLVKDFFGGKEPSRGINP
DEAVAYGAAVQGGVLGGEEDTGEIVLLDVNPLTMGIETVGGVMTKLIGRNTVVPTKKSQIFSTAA
DNQQTVTIQVFEGERPMTKDNHLLGKFDLNGIPPAPRGVPQIEVTFEIDVNGILRVSAEDKGTGK
KEKITITNDQNRLSPEDIERMVNDAEKFADEDKKVKEKVDAKNEFESYAYSLKNQISEKDKLGGK
LSEEDKGVLEKAVDEAISWMDSHQDASVDELKEAKKKLEDVATPIVSKIYQQQGGAPPPPGGEG
GEEKEDKEEL*
>TRINITY_DN1335_c0_g1_i1.p1 type:5prime_partial len:651 gc:universal TRINITY_DN1335_c0_g1_i1:2201-249(-)
LSRKSYTMKAACLFLSALSAIFCINASTLTKTLTHLEQQKLVKSLDKSYNDLESLHYAAVSLNALGK
SLSNTDQHCQKALDKVNQENTESVFRFSEVMKVLKCKKVPSLKLQTLINDNTDSVGYSHVIAAM
VNLGQKVDAETLKKFVSVAKDNDNPASAAETFYAASLLPNSEVMKPIYSMVEDIIAQADEVNGDI
LQYEGGLGVTSGVVRGIILLGEKNGAPLLKVGQSVKFAQYLINRKFVYKVKDIYHLVVALNALSA
NQQQVPLVVSVHASSSITRDNNMLKVRVTNVLDQSIPDVSVTAESFSTSDDSVTLFENRPFSKSSDK
DDYLVLEGEVGVGYIAAHSFGLDIMAADPSKGLYNCKVNVKSPKNSKRYAMGGAFQIPVKVLAR
VMVEDVEVAAGDKDHTLTRTTQLAYPNKLSSKIEADYHQKIVMTFNLKDVHTKELVTAHQTFIR
LVHEKTGQEIFFVAEPDEKDNYKFTLDVGATAKDSFNSLSGQYKMSLIVGDATMQVPINWDLGD

VVLTFAGEPKKTKRQERITQPKPTIDHMFRVPEKRPPKVVSTAFTILVLSPLLIMFAMWVKIGANIS NFTFSLSTLLFHVGLCGIFVLYYFFWTKLNMFETLKLLAVVGGMTFLGGNKMLADMAAAKYRS*
>TRINITY_DN941_c0_g1_i10.p1 type:5prime_partial len:114 gc:universal TRINITY_DN941_c0_g1_i10:3-344(+)
IDVTKRGKPVKGSPFEVVVGSPESKPSVGSKCDLDFDIPDIDLPKDLKVLKADLTRPTGKKEPIECT VTPENTLGLEFTPSEPGKHYKARKTSKRQSIRSCGRKPRIKTFSWK*
>TRINITY_DN34481_c0_g1_i1.p1 type:complete len:802 gc:universal TRINITY_DN34481_c0_g1_i1:2487-82(-)
MTTPHMSPQEEQEKLLEEALQIVKNQAFQMKRCLDKAKIMDALKHASNMLGELRTSLLSPKSYY ELYMGVSDELRHLETFLLDEFDKGRKVNDLFELVQYAGNIVPRLYLLITVGIVYIKVGDVPKKDIIK DLVEMCRGVQHPLRGLFLRNYLLQSTRGQLPDSETEESEGTIKDSIDFILLNFAEMNKLWVRMQH QGHSKYKEKRERERQELRILVGTNLVRLSQLEGIDVETYKKDVLPGILEQCICCRDPIAQEYLMECII QVFPDEFHLQTLNAFLKACAELHQDVNVKNIIIALIDRLAMFAHRGDGPGIPNDIRLFEIFSQQVA HVIQSRSDMPTEDVVALQVSLINLALKCYPDRVDYVDKVLEYTEEIFSKLNLAYIDKNNAVSKELM RLLRIPVDIYNNVLTILKLKHFAPLFDYFDYATRKEMAMYVINNALDNETLIPSQEEVDSVLQLAA PLIMDQDDQPTEPEDPEDFAEEQGLMGRFVQLLYSDNPDQQYLILTTARKHFGNGGEQRIKLTLP PLVFAAFRLAFIYRDAQEQDDKWDKKCQKIFQFCHQTICALSKAEYADLSLRLFLQGALAADQQ KFTNAETVAYEFMSQAFAIYEDEISDSKAQLAAITLIIGTFEKTSCFTEENHEPLRTQCALAASKLLK KPDQCRGVSVCSHLFWSGRVNEGESEEKDAASNECRDGKRVMECLKKSLRIANQCMDPTVQVQ LFVEILNRYLYYFERGNEAVNSTILNQLIDKIREDLPSLEVNDEMEQINKHFQNTLSHIQSKKDSAD ENEAAIYKDFIL*
>TRINITY_DN8008_c0_g1_i2.p1 type:5prime_partial len:1298 gc:universal TRINITY_DN8008_c0_g1_i2:4205-312(-)
IMSPKERTCPLTQYLFTPIYTGSCKISDAEQRANDRQVKSAVQMAGSAIGTVVGTIFGGGIGGAIGG FIGGAIGSLIGGIIGLFSSCDKSYEVVTRSWVEERKCILLEPDVRSITRPYSECFDAKIKANTGFKVPY TCNCKVNDCIVKSPSPDCLLQNQQCERKRKDFLERTKTVPPTFSKLYKSLKQCQDSVGASILKVQS AKKTVQFYEKEHTRIKNMLLALEEENKFSKQALSDATSILQDDLCVSNAFMNNKNINELIDIKNV QFDTLLPLVNNIRINAHVKVKGNDVLVPFVHELSNDNKVSLNAFGRKIIMNTICKKIRKRRSLDL NEDEKNDLAFVPWINNVNGTATVADLSCVSLDKTLEFIRHTVKTLNKKSSYANDLLMQLQYSTN KLNPSLYTAFTAYSGITTEKNDIIKANEALLMSANNELNKIQDQVSVSNVLDIWGNEAEIVTGFNN VSVCLNYKDCIISAIDTLKNLPTMFRNSREAYMKEVMELEKELTKIYEEKNLIGLSAKAQSVERIVS RIKYMSFHCSKRPIVTLKEVKDMDVEKGKNITLRCIVRSARAIKYYWQFNDDILDEENSHSLTIPVS NETSGIYRCIAKNLVGNGTSNQIRITTYTKPSFIEEPKDQIYILPSHPQSNLVLTCNVTNNYDTTIAW YYRSFTIGKRPNIINGQTSILLDLDGTKQENSGYYFCQAFNKHGRIKSREARVNILNSNIARLEVNIS LDILQTDEISSNSFKKISGDMLSEIKDLGRTLTTTVIKSKTKTRVTFNLKVKPITDNVKSLSESFNISSR MRQNLGSAAAVLLGKLSENSFKLPINDGEFAKVDNDTMTYGMKLNICQEGYKLHKNGFLCVQC PFGTAGKDCKKCPLNTYADAPGLGICKKCETSKITEQIGSTSKSQCIGTTERKVDIIFAVDVSKRFLE QINGLQKIRELLSKFIKTYSGTKYNTRFALLTYYKYVDVYLSLTEYYHSDDILGLLEILGEGSRMPDT KLALVEIETMFDEESSSDRKKVAVFITNGGTSDSYYLMELFKYKRQAITLQNKGVHIISVGLGTVDH RGLRALASYPKLENAIFPSSNSVAVQKMIYSIGNELKDNCYTPAVDFGIIYHPNHKESLVQITKEQF LRNANWLSKLLLPIYRMFKQQSYYFMYKHSVPSNSFGSFSETGFQERIVSYLQQQTSGFDIYKKIST AINVDMTEIYRSFSKVKVLIIITEEPLDEAVLKQYEISVHGEKIDIYNFVKDASSIEKFEGPFSDKPRA HRLVYGIDEVNFLVNAINCNRTKICG*
>TRINITY_DN1320_c0_g1_i1.p1 type:complete len:229 gc:universal TRINITY_DN1320_c0_g1_i1:105-791(+)
MRCLSIILTILLVHLFDQASSRSCRDYLNNQCKKYLCNVPKYHHFIKSFCKATCGLCPVIHGLQMS TMSTPTTTTTTATTTKAPEIVLLSSGKTITGNAFDTPGPANQCNDGNAASLCITLQNETAFIQVDLG SAKKISRMELKKYYHYENQLEINFLVSNDATFSASTVCDIWNVRSVNEGDIQDKRRDCNSNASSQ TFRYARVQTVNNKGAREHLRVGEFEVYGVL*
>TRINITY_DN945_c0_g1_i1.p1 type:complete len:468 gc:universal TRINITY_DN945_c0_g1_i1:151-1554(+)

MKLPQSFSHIIRNGTKTHNVVRSMFTRCQSTQVQTTDLKARLEEIIPDKRKEVAEFRKEHGNTKIG
EVTVGMAYGGMRGIKGLVTETSVLDPEEGIRFRGYTIPECQEVLPSAPGGEEPLPEGIFWLLCTGEIP
NKAQVDALSKEWAARAELPQHVVMMLKNFPEDLHPMTQFLTAIAALNKDSKFAKAYADGVPK
TDYWMHTFEDSMDLIAKLPVLAACIYRNLYRDGKVPPINPEKDWGANLADMLGYDNPKFTELM
RLYLTIHSDHEGGNVSAHATHLIGSALSDPYLSLAAGICGLAGPLHGLANQEVLVWLHKLTSEIGF
DVSKEQMQEYIRETVKTSVVPGYGHAVLRKTDPRYTCQREFALKHLPDDPLFKVVGRLYEVVPDE
LLALGKVANPWPNVDAHSGVLLQHYGMTEMNYYTVLFGVSRALGVLSSLVWDRAFGLPIERPK
SMSTAGLKKLVGSS*
>TRINITY_DN1653_c0_g1_i3.p1 type:complete len:1023 gc:universal TRINITY_DN1653_c0_g1_i3:180-3248(+)
MMRRSIKGSEDNWVEMQNDTFKNWVNANLKSRGLEVDNFETDFEDGVRLINLFEAVSKKSIGK
YAKQPKFANQKLENVSLVLRAMESDGVKLVSIDSDHVVNGNSKMILGLIWQLILRYQISGSDDKSF
KIALKKLLLIWLQHVIPEQGIRNLTTNWNNGIALCNLINVLKPGIFPEYNNLDKRTPVENLAKGM
QLAESELDIPQIISPENMASPNVDELSMMTYLSYFTQDGGIGESWTVDLVNFWNSSLSVTNFNTD
WNDGMRLGRLVETFLPGAMPIMESTDNLDNCRIAMDVAHEKLGISKIITAEEMSNPQIPELAVMS
YIVQFTKVKRVERASDKFSASGPGLVEPKIGIVNEFYVRSDAGYPLDGLQVTIIGPNNNYIPIEIDDS
DPQLRNYSYSCEEQGQYHINIIYEGEDIPRSPIIVTSQQDLNTIRVYGERLEDKHLYAELYERIGISVD
TSQSGDGRITSKTIGPDEKEIYSEVDAEGDGSYTVSFTPEVIGEYHTNVFWSGKPIHGSPFVVWVSD
PNKVFARGKCLYETRVGVESSFEVATNGAGPGHITGYAETNGVEYPINVRKREECLHDISFTANQV
GIYYVYINYNNVPIKDSPFQIKVGNPSLIEIEKHEEAIMESNQPQSFIVNVLKDAGFGDVLCVVRGP
AGEDVQCTIKDVNDSLKEIMFLPNYPGTYEVFVYYAGGLVERSPFIFAISEPKLAIYPENIRVSGNGL
SRAKLNELARFQVDTTNAGPGHLTVQIEDEKDDVEAGIRDNNNGTYDVTYTPKRSGKYRVIIKW
SDQHITDSPFEIIVDNRAEEVLVEGYGIKEGTAGQPVLFTLDGRNAGPGQLSCRCRAPSGKMTYVL
ISDNKDGTYTVDLNATEPGLHTVEVEWDNQAIPGSPFLVRIMQAPDVKKVRASGPGLRSGTLDSF
NGMFQVDSKGAGPGTLKVRIHGPKGAFKVEMSRENPKDRIINVRYNPTEPGTYTANIYWSDEHV
QGSPFEIFVGQNDQEVDRWDRNKEELSKSQVNVY*
>TRINITY_DN286_c0_g1_i4.p1 type:complete len:224 gc:universal TRINITY_DN286_c0_g1_i4:40-711(+)
MIKVAIITIAIVACVLESTAAVRCYVGYSAADLPLAIGVRNIECGEEFNHCTTVKYRIGGITVTQGN
CAKRYGQAMCQFMRNSHKAMTDCEESLCKGHNCNSDVNDAEQVERKRVGPYENTLKCTTGL
QTKWPSIPFASANIPTAVEECFPGTKDCVTVEYDYIFQFESKIATLYNQFRLPTVQKMCNRPNVDQ
CYKWCTILSIHQNMDNCRVKCCQSDNCNA*
>TRINITY_DN14781_c0_g1_i1.p1 type:complete len:1181 gc:universal TRINITY_DN14781_c0_g1_i1:4332-790(-)
MVKMNSCILRSILITCFIATLQLQHVAATPVSPTVKPDGYATSASNSKLDVFATTPLCPNKMDIVLA
IDSSGSVTSVWSEMIDDAQIFTSNFQIGESMTRMAIIDFSAVSNVYTPLDANNTGESIYQKLEKMEN
QQQNGESWIELGFDRANDMFRSANPPRDDVRKVLVMYTDGDLTSGDHDSLKDMIKSQQSNNIE
HYIVKIKNGTQKVGMEMMKSSGCKVFQMQEPPAFGQPSVEDAVCFGSGGQPPIPVVQTTPPTPPI
SGSEKSTETTLSPGIKTTATSISIKNPREICLQKADTGVCYAYIQRYFYNASSEKCELFIYGGCGGNEN
NFGSKKDCYGQCEGVKLPTVIPTPPLSTSSTMHPPINKKCLTERSEAEALIRTKVPTLVQVFVPECD
EDGNYKAIQHHEGTNIYWCVDPETGDVVNGSKTEGSPPNCNITSPDESSQILSYPTPPPVEVNKLV
MTVPPIGAKTLPPKSEPLIGPQPTPEPEPMPAPSTDYEHPKNPCGEENDFLKQRNNFDRIKSDEEFY
FDCDGRDLYKPLQKRIRDNERWCVNQKTGAIIPGTKRTGFQPDPVCDGERAASGIQTTPNPDLFP
MPEPKDIPTPKPNDIFYPKNGCPEENDFISQRETTSHSKNGMILDKKYVFYYQCERNGDYKPMQR
NAGTGERWCVNTKNGNIIEGTSRPSGQQDPMCEEGMKSNVGITEVAGVGEIGEDIFNPKSSCDVE
NERFEEREKEISNKGDQVTLFKYGCDHKGNYKPLQYDGFKQVQWCVNPITGSLIKGKKLPTGQPI
DCSQENNLKSSESQNSSSTTNQAISSSSSIDQNNQNDKYHPNSFNCNNENAKTPPYKLGAMRYQ
CEDNGDFKPLQSHGSTGERWCVNDKSGNIIQGTLRMAGQPDPECLVNMNAKITTNDRNDTNIP
DMNGQQIQPITETLPFNQISPINMTIRKQNGSDNDDLMRTETASNVAKETESRLYGNHNITHSPPA
ISPEGEELLSLSNECEYKIGEKYGPQSNLKKLGCFNDIQQPPRPFPIMLLDETNVTVDVPYSKDEWV
KFIKGFVCKCAKETRNRGFFTFGIQNYAQCYSGPDEGRYAEDREAAEGMCINEDQEPCKTNDKQ

FCAGISKQDPHTNKTMHANFVYMIMTETHQEIKYIEYDRKKRSVNKRGKNHHVFNQRLVQRYR
QK*
>TRINITY_DN118520_c0_g1_i1.p1 type:complete len:581 gc:universal TRINITY_DN118520_c0_g1_i1:1895-153(-)
MYRLLSVIRKTTQTAKLRQGLSACISTTTHYCSPKEIKTGTDARALMLKGVDQLADTVAVTLGPK
GKNVMIEQSFGGPKITKDGVTVAKAIELPDKYMNIGAKLVQDVANNTNEEAGDGTTTATVLARA
IARSGFEAVSKGANPNEVRKGVMAAVEVVTQGLKDLARPVTTTEEIAQVASISANNDAAIGKLIS
DAMEKVGKDGVITVKDGKTVYDELEVIEGMKFDRGYISPYFINTAKGQKVEYQNVLVLICQSKIS
NVQDIVPVLELANQHKRPLLIIAEDVDGEALTALVINRLKIGLQVVAVKAPGFGDNRKNTLKDVA
ISTGAEVFGDDLNLKLDEVQLSDLGQIGELVVTKDDCLMMKGRGNTDLVNQRANHIHDEIKATN
SDYEREKFQERLAKLVGGVAVIKVGGSSEVEVNEKKDRVTDALNATRAAAAEGIVPGGGTALIRC
AKLLDTFQVPTEDQKWGVDIIRAALFKPCSTIVANTGIESAVVVEKVSSSKDPEYGYDAASGEFAN
LIKAGIIDPVKVVRIALSDAAGVASLLTTAEAVITEIPKEDPSMPPMGGGMGGMGGMGGMM*
>TRINITY_DN3271_c0_g1_i1.p1 type:complete len:399 gc:universal TRINITY_DN3271_c0_g1_i1:218-1414(+)
MTRIIWHKIKSELIMPYLDLDCKYYDLGLPYRDETNDQVTVDAAEAILKYNVGIKCATITPDEERV
EEFKLKYMWRSPNGTIRNILGGTVFREPILCDNIPRLVPGWTSPIVIGRHAHGDQYKAQDLVAPGP
GKFEMSFTPLEGSEKVTVNVFEFKENGGCMMGMYNTDDSIKDFAHSCFNYAIGKKWPLYMSTKN
TILKKYDGRFKDIFEEIFQDQYKSQFDALGIWYEHRLIDDMVAQCLKSSGAFVWACKNYDGDVQS
DTLAQGFGSLGLMTSVLLCPDGKTIESEAAHGTVTRHYREHQKGNATSTNPIASIYAWTRGLAHR
AKLDGNPELEKFSNSLELACVKAVEEGNMTKDLAGCIYGLKNVKREHYLLTNEYLDAVRDKLLQ
LWTPPSQV*
>TRINITY_DN1043_c0_g1_i2.p1 type:complete len:700 gc:universal TRINITY_DN1043_c0_g1_i2:2410-311(-)
MKESYLKRFALLFSVLILFQEGGALLRENLIVNHRLRRNVYSEEKVTAVDLPQSREKRFVGTALIIIS
AASAGLDIYKQVRGCKDITPDLDDKIAEGKALQQSLLNLRDQVNLAWVPAESREEYTRRLASNM
IIIREQVSTFDSLQADLVKLLDQKLLDKLNNVTKHLENQIDRNNETEIGSFEDLEGKYMDTTTFALT
LTAKLLPVGFLLGTKFLLPKITARFSKGVAKAFAADNPSRVQKMLSSFAEKQGKLANWKAEKINSF
KVSLNSKWNTFKLKFPRITAAMNRMSNSKIGKALGKAMGKVKGIAVGVGKGAVVAFFIAFEVW
NFVQFHGACTEKRNKAIEGIATMNANKKNMTDLLTEIHDMKAKADEYYNLMKSNVTNDEFLN
FLEDIADLGENAKEQTKVIVDATNQIKQYKAEIKNADDSRTDALNTMLVTAFSNINFKLDCLRSK
LAIIKGVVDDCGIGLGVYDDIYARALAKQKKITETCIEGSYMDKNEIKGLMVAAMDLDKKQSICV
RNNPTKKQLVCEKWYDDVSDADILAANPDLTKDDVTYFISTCPPAPVNDQTKTTICVKKNNELA
TLQSIQQTYQKYDQTAINSAYEECQLSSISVTTICTAKKEGQTVVYAQQKFPVFPASMVTNVYNSC
KKETNITPADKMMICLLKKNGFALDSPVMANYVKNYDYATLEAFYNQCS*
>TRINITY_DN729_c0_g1_i1.p1 type:5prime_partial len:723 gc:universal TRINITY_DN729_c0_g1_i1:2179-11(-)
YFGHFPTRKMPDGVEVLVKNFLEKNVPSIDGELLEYIVDVTASSADDFESTEDIYDAVGAFLHEGT
ENENEKEIKKLCTGIIKLLKRDTISNENENVKILDAPIHLGKIAKEESNKSTEQNSIWLKQANDTSE
LKTVNLKKLEKAESKIKSKLEKRDKDGLPNGDLNCAGPTTNQVIQKKDLKSDQSGKLVDIRIENF
DLAYGSKILMQNCNLVMAFGRRYGFVGRNGMGKTTLLRAISRRELFIPSHVTVLHVEQEVIGDDT
LAVDSVLEADTVRASLLKEEAELAKNIEKSTNNAAAARLSEIYAKLSEIEADKAPAKASLILFGLGF
STEMQKMTTKEFSGGWRMRLALARALFTKPDLLLLDEPTNMLDVKAILWLENYLQTWSTTLLVV
SHDRMFLDAVATDIIFLHSKSLDCYKGNYTQFEKTREEKLKNQQREYEAQKQYRDHLQAYVDRW
RVNANRAAQAQSKLKILEKLPDLKPVEIDPEVVLRLPECDRVNATMLRFDEVTFGYTKDRNIYEK
VDLTANMDCRIAVLGENGSGKTTLLKILLGELEPTDGVKHVHRNLRMGYFSQHHVDSLSMNQTS
LGLFRDRFPGKQDVEYRTHLGRYGVSGELAMRPITSLSGGQKSRVAFAVMTWHEPNFFILDEPTN
HLDIETVEALGKALNKFNGGLILVSHNEQLIQMIAKETWLCGEKTVKKIEGGFPAYKKALEEEFRR
INAT*
>TRINITY_DN1907_c0_g1_i2.p1 type:complete len:210 gc:universal TRINITY_DN1907_c0_g1_i2:53-682(+)

MDMDTTFPLVISLLFSILCCSFAVELTFELPDNARECFHEEIHNATKCTLEFQVVTGGHYDVDVSLK
DPHGKVLYDEKKKQYDSHTFEATVPGEYVFCFSNEFSSFTHKTVYFDFQSGDEPPLAPGMGKHHT
ALTLLESSAVTIHESLKVIIDYQTHHRLREGTSRDMAEYLNERVQYWSIGQSVIVVAMSIIQVYILRN
FFSEKRDRL*
>TRINITY_DN31399_c0_g1_i2.p1 type:complete len:819 gc:universal TRINITY_DN31399_c0_g1_i2:85-2541(+)
MAVNVSHVYTCPSDYLCSKAEQGTLLIPLINESEWGRGFRIFLYTLALLWCFMGIAIAADLFMCAI
EIITSKTKKIKVATEGDDKMGYEEIEIRVWNDTVANLTLMALGSSAPEILLSIIEIVGNNFESGELGPG
TIVGSAAFNLLVITACCVMAIPGGESKRINAFNVFLTTSFFAVFAYIWMFIVLTVSSKGILEVWEAAV
TLLLFGILVILAYIADKDLCVSVFTRKRRKKDIELDNFDLLNENVLKKHHPDLIEYMKEITANPSISE
QEAAKLIAIKIEASKRKNYGYYRVGALRSIGGGRKVVPAVTPKLQGIIDGGSTLNVNAETNVNDSV
PDNKATIEFTATAVSVMENNKVVQLGVERSGNMSSQVSVNFETIDGTAEAGSDYIAAKDTLVFEID
EIRKSIEIVIIDDYEWEPDETFFVKLHVPEGANDRVQIGHHSICEVTIINDDEPGTFSFNKPSNIVKES
CGQAELEVERVNGADGKVEVVWKTKDQSALHGKDFIGGEGTLVFEHGETLKSISIEIIDDHMFEK
DETFMVELLEVKNEGAKFGRLKRTVVTIVSDDEYQHMFDRVVNMAHMNLHQFQLGSSTWGQQ
FREAMNVNGGDLESASFMDYVMHFITFFWKILFALVPPCTWYGGWLSFFIILGMIGCLTAVVGDIA
TIFGCLVGLRKDVTAITFVALGTSLPDLFASRNAAMNEKYADASIGNVTGSNGVNVFLGLGIPWLI
ASIYHEAKGKVFKTPTGSLATSVVTYSVCAIACILLLIIRRFVPFFGGGELGGPKGPMYASGIFMISL
WFIYVLISALVSYGHIAAL*
>TRINITY_DN1613_c1_g1_i2.p1 type:internal len:1068 gc:universal TRINITY_DN1613_c1_g1_i2:3201-1(-)
TKSCGGGIQESTRSCSNPSPAHGGKPCSGELSKTRACNEHECPVDGGYTPWSAYGKCSKVCGTGV
QFSTRTCTDPKPKFGGEPCTGLTKKSRNCNTHPCPVNGNWGAFGSFTTCTRTCGGGIQKRYRKC
DSPAAAHGGKKCPGLSFQFKACNTVHCPVDGGYTQWSSFGSCTATCGGGSQTSTRTCTNPSPKY
GGEGCVGPSEKTRTCGTKPCPVNGGYTEWGEYSKCSEECGGGIKTKSRSCTNPAPAHGGKGCQG
PSSESDVCNEQPCPVDGNWSAFGPLSVCSRTCGGGTSLSKRTCTSPSPKYGGKSCSGSRYRIVACN
TRICPIHGGFTAWSNYGVCSEKCGGGVQTATRSCTNPRPLFGGKACVGSSRKSRICNSQPCPVNG
GFSDWSAYGKCSKSCGSGIQVSSRTCTNPAPAHGGKSCAGPLQQSRVCNTLPCPVNGNWGSFGR
FEECSVSCGGGVQKRFRPCNNPSPAHGGKACRGLSFEIQACNTKHCPIDGKYGSWSSYSKCSVTC
GGGTQVKTRKCNSPAPAYGGKNCVGVASHIQACSTHHCPVNGGLTSWSSFDECSEECGGGIQER
SRSCTNPAPAHGGKSCVGALVDSRKCNTHKCPVDGGFTKWSSYGSCSKSCGGGSQTSTRSCTLPK
PAYGGDACVGPTIKTRGCNSQACPVNGNWGGFGVYGTCSKSCGGGTQKRYRRCNNPAAAHGG
KQCSGLSYQIKACNTHHCPVNGNWGSWGSFATCSKPCGKGVQKRVRKCNNPSAAYNGKACPG
LDYQLQACNTHHCPVNGNWGAFGPYEACSKSCGGGTQKRHRRCNSPSPAYRGKTCSGSSYQQR
SCNTQHCPVHGKWGTWGSYGACSARCGGGIQKRYRSCNKPSAAYGGKACPGASYNIKSCNTQ
HCPVNGNWGSWGSYTTCTKSCGGGTQKKSRSCNNPAAKYGGKACSGSNYYLKTCNTHHCPVN
GNWGSWGSYTACTKSCGGGTQKKYRTCNSPTAKYGGKACSGSNYYLKTCNTHHCPVNGNWGS
WGSYTACTKSCGGGTQKKYRTCNSPTAKYGGKACSGSNYYLKTCNTHH
>TRINITY_DN3180_c0_g1_i1.p1 type:complete len:1889 gc:universal TRINITY_DN3180_c0_g1_i1:5857-191(-)
MGYLRVTWICVITLSTIVLSEFIFDDDEFGEKRTFGDPDSLVKRSSSGSTKKHVSTFTRTQRFSGGGST
CNYRVIKRSGRQYVVFRSSRHTRVANGCTKIHVQSFLNNHFGGKNLLTTFPNLNGDNFEVVQMM
YSLETRVAFMEAISKTSMAFFGGGNLEFGKSGDVPLTLKLQWTEDHDSSAWDPSDIQVELETTKV
VLSAASFHFKAKKSYHNPDHFMIEKFDTQGVGNFRVSAYTSAIAGLDNTAFGADFSQDKYGALT
WNDFRGYGFFDPAENFQLVLHPSTYVRNSRLFNNEETSQFIILSNPVSDDTTYVTLMAQFGQSTNI
KDTLNGIMTSVNADIGIIKRINSRAFCLTVSSGEYDFLFTPPYTKDRNLMNPCFPLEYQDARDNQN
DLSKQKKKMTADINIHVTINFKENVGSCNDDSSIDTSSLPPKMTINGNENWKGFSWSFPEQAKGP
MMAIMKCMWQNSAAFQGLPYSLPFGDSEVVSISDFLDSPTEKTIGLKITPPEQWRTIPGIDLTFNYL
EFTASYNIPDQTWELSGTADMRIDKFEVFLKLEYSRTTVEVEDDDDDDSSNEDDGEGPDKKAKKK
SDKKKKQSKNSKSSKSESKLMAKLRKRKSMDALLKSLPDTFQDDDFNNNHHVFYDNLIIPRSLPV
KNNQSANVSLNKDAGTNIVPAAVKNSSLATAATTSKRTTEVEKQWTFTILMPTLLVKDIYTIFSPQ
STLPAEYPKVFNNGIKNLQISWAWGNEFEVRFRGQLVIMGWDKILLETVFWKQMPSRSLKFGIGIY

FQKIDVDAILKHIFQDESISVPWLSNLDCKLVIGNSEGFKDVSKKKKKIKKIKKAKSNKKVSKKQR
KTSKSKSKTSAALTRSTRGMEEYIYADLAEKRQADDDDDGDDDDAEPDVKFTYGSLADNALIAG
AGFEMTWYRPTEEEKCLGNTLCKFIYEKVQSNTQFVLAGQVDTGGITISAEVKDAGIVLATDLKL
ENAKLEIDGRAGGGIKLSCQMIYTNSEIATNPLTFEGAIEIGFAGELTFRFTMGGTIDSAFGYNRIAL
GNILLGATIDFKAASFTIEFGVTVLMGLLNEDPITIRGYFGLSPTNWIDSWVYVKASKLTIGSLGKAF
DFSVPDTFKDEGFPEGFQFSLSRKVVNIESLGIEIPSGLYLVGKFKFKFFELSADCTIDPANKAFDVKL
EMNKQNWIGGMITISSYTDENKGPMFHIKVDENTPFYANIDGKLTVLGLSQEILVRIDENSGALIH
LRGTPFPFIFDAALDINAALDPNTKSFNGGTFKITVSSSEFIPTLARKIRDWLEGLKSEGTQAKQDG
MTAITKGEEDSETITGKKSLWDSALDTWKSNLQRRESELAAKQRAITGALQACIDECELVCVPFFD
WEEDCFTIFGWSAPCPWPNSCAKKVPNYFCYAGCALDQLGSAFLTWVESTLTKIGLALQSTAVFL
TDMVEGIAAALTKLGEYIVAAKDWLINQFQAALNWVADLLTKQIVLRKLVLEATVQPPFKYCFG
YDIDITLFTWHITDGGHVCLDTLIRDLIYSLFGYNQDSKKALGMKDQIEEAKSKTLSIKEAAEELEE
SAETTQAEVDMQTAKTFEEELKRKRTMPTKEMFKFIDEKSKNTKYSKAHQKIMDTPHGMPAYQI
HNNHMLENLEKRSPWVVVRSFKDGNRNLEPKRIADHEIVKRSLSFGDGSKPCSRLRRSLATYDTI
VNVLIRSARNLENMEKNHFAHKEFHGHGIRKLDKRIAELELKNMTDEQREDAYFWYDKANEGY
EDFHNKVRRNIDYHDNYALPLLHNQLNYILKKSGKEGGFLKRVEDMHSEALHDFKRSIKNEEDR
ADGQELLSKIKKSMLAIVTDENTPLSRYTRSINGMKNDITRMNKMVLAC*
>TRINITY_DN598_c1_g1_i2.p1 type:5prime_partial len:742 gc:universal TRINITY_DN598_c1_g1_i2:2-2227(+)
RWSGCSKSCGVGVKTKSRECDNPRPSGGGRYCTGERTATEACKIQDCGVDGKFSHWSTWTACSK
TCGGGAQKRARQCNSPPPSGDGKDCAGAREEFKGCSNDFCKGPTGVAKAQEIIERFLENFHKKT
YPDREPSGKFLKAIFAIQKAVAEEFISVDPSVRGKTLEQRLKDLEKFLKVATEGGSIDFARTLLGKGIF
TFMKKKIQEGLPIDDIDKAAEGFLEKLRGDVGEKKMNEILSSQGDATLMFTIDTTGSMKDEINAA
KAIAKQIISATRDYEVDYILSPFNDPRTGPVTHRSSSNRASFVTDIDRLRATGGGDCPELSFTGMLN
ALNQGPQYGSPMFVFTDASAKDDSASNIDSLKAAADLTDATINFFTNLNGCKGAKSVESYKEIAS
YTGGQVFPLKNEAELKKFTNYVKTSLQQDTVITSGGTLNSKSYKRSLYSMQAHDFMVDDGIIMIS
VAISVQKYASQVTILDPNGRRVNPSTTLSLSLVYQISHPPPGQWKINFPSSVGKYGYTVQAVSSSAIA
FAHNFIYQQSVRKNSPPIPLANPLKGVKNMMVVRLGGTKNFDLNSIKIDLVDTSGNVLQKGIGTD
RLDSDEVRHATIDPPSGPFKVRLTGKTKSGYNFERVSQGISRPVSAAMRTNFAGYDFTAKLGKGPY
AAQMLFINRGAHENFYFYAKANYGSIPNYKQTKRVTSQGFVTFTYKAPRQYNLKGKSDRITVTVK
RQSTGEIVSHHTSILLVV*
>TRINITY_DN29322_c0_g1_i1.p1 type:5prime_partial len:776 gc:universal TRINITY_DN29322_c0_g1_i1:2-2329(+)
LTFIESLRLRIMSNIMKYILLLLALLLFVSAEEEKTAVTEREFPNYDIFRAIQKFSIKHEVDMNNFRHL
FLEKGNSNNTMDFSKVKNYNNFDREILKTLFHMYKSKDAAPPQVSTSCQMSILEFVKPFMNESSI
TPALIFKLLGSPLGKLLDSWGKPGSRILVGNIGWLGGYDECQVIQSTRYCLTNFTFLTMFSGGQYGS
CFPKTCTESDVLVIHKYLETTINPSLPGYLAFDQTICEEPTEYDTGTIITIVIFSIILLLCLVGSLTEILQD
LFTPLKEPEPKLGEANGAFQTNSEDVYLKHDVDDNVVLVGPKIIPQRGNAFTNFLLCFSILKNTRT
IFSTKLPKGAIGSINGIRTISMTWVILGHTFAFIQYTIAFDNRLLFTDHLRKLSFMAVGNAYVAVDTF
FMLSGLLVAYLMLKQIDKNGVMTLKQYLMYLLHRYIRLTPSLIALILFYVNINPLILSGPTAVYYQK
NPTGADPCIDHWWTNLLYINNVYPNDLGKECVGWTWYLANDTQFYIISPLLLIIMYQLERRISEK
YRFFAVFGFIGSVCSIFFIILAVLIAVNDYPTFVTGLFDPSNPRMKNAGGINKLYIKPYARVPAYLAGL
ILGYILSRKMEFRKRHRTVANIIGWCISIAVALSIIYGPYHVIRKSDHYFMTDVENVFFGTFHRFAFV
ACVGWVVYSCHNKMGLGLVNDFLSWKAWIPLSRLTYGAYLIHPVVIFFTNQTQENAYHFQLSNS
ATLFVSWVTLSYGLSYIICVFIEQPTKNLEMMIFK*
>TRINITY_DN123_c0_g1_i1.p1 type:5prime_partial len:1231 gc:universal TRINITY_DN123_c0_g1_i1:2-3694(+)
RGGFRPFGTGLMGSPMLGPYAFGPARSGALGGFGIAPLVRPPTRGGAAGANRGSRSNSNRGGKK
TTPVKNNKSTPEAKKDNNINAKNDKKEDKKDEKKDDKKDEKKDDKKDDKKGGKTPTGKEIKV
EPKDDMKNIKKNFFKDPKVEPKNEKKPDTKAVTVKPEIKKESGPQNVTLDKFNSELHLSIDADGL
SAGNMKGEGFQYMWGGVRGTHGVKKGKVCYEVYLLENIKVHCPGENNPHVLRVGWSVDNSD
YQLGEDKLSWGYGGTAKSSYDSKFLDYGKKYTVGDTITCYIDFESNPKAIFFMLNGEYLGVAFRFG

NELGDKAVFPHITSKNMRFRVNFGKEKPKHHVTNGFRMMQDIPVDQLVLPSPSPKTPKEAEIIM
MVGLPACGKSFWCEKYAKENPDKKYYILGTNNIIERMKVSGLTRKRNYHGRWEMLIKRASLVLN
KLFTVAKNNPRNYVLDQTNVYFTARRRKMESFAGYKKIAAVIINKPDVLASRTEKQQKIEGKMVP
EGAVMEMKFNFTLPEKGPSFNEVWYLEETLPKAQELAANYRREGSNFKTKSKRKPESSPSQPESSP
SSEKKPKLDPKIISRGFDKPFSKPNTNSPQQSTFKAPSPAQNNANKTPNQDRFSKNEKSTSKVETNS
DNSRSKAGNFNKGHDGNKSNERPFFSNTPDRGNASNRGNDSNRGNSSDRGNAPNKGNSSDRG
NAPNKGNFADKGNAPNKGNSSDRGNAPNKGNASDSANKNSKNEKPKEESKKTPKRAIRGTLD
QLNSIINSEARKTENNSGNIRDSAQSGKNEKMGSLMDIDRTPQRESDSYQNLQSRNRDALSNQG
GKNKESQSAGLMDIRNVPQGNQDYRGKAGILDSSRNTSQDYRGKPGILDSSRNTPQDYRGKPGIL
DSSRNTARRNEYESPVGRGNNSYSDSPARGVSTPRGRASFNDSPARGARGGRGQSDISRSGSLAKS
QGSGGNRGGRGRGRSKHLGDVNKYDPVGNMGGYYSALKALPSVKDLIGNRSSFSSYSTRIDEPSR
FDQLYSSYEPYSFGRTQNANDSRSLNYSFGQTDSYSSLERDGGYRDSQNSKDFLGSYQQRKDSSYS
DNRGSQGGQRGYQDSSYNADGLDAKDQYQNDDYQESYSSQKDKSSYDQNTQSSRSYNQETSYE
ESSDYGRSRNQEQDEYARSGTYERGDTSYGTDDANRSSRRYEDDLQESEPMDGNYSTQDYGRSSQ
SSQRARIW*
>TRINITY_DN2357_c1_g1_i1.p1 type:complete len:1733 gc:universal TRINITY_DN2357_c1_g1_i1:5452-254(-)
MLKLFLFLIGAGHLSLAFAAICSETNGEGNKKNLKEPCFFNQLIDVKQWNMKRFMDQTDRFFDF
GVMRCSSYEVEGSGKMKLTCYHTKLFYIMKNIMSFGTTERTEFIFKFSPLTENDYKSTDVEISGKLS
QALRNTFIRATKPEGFQDFSLSTSAVFPQIDMETFIDQRKPDGFKTLTNNDKLENFPPKVAVNAEM
SGMFSALVTDTDKQAYEFLISFLGPMKEQQFYIFQSLTENINDELVIAFYKENPSNAGTTVRDIMDS
LRGVKWRGNKANEFVVSKDFAPIFEVQSSAMSISDGEIVNINSEALRNIAIKYVSPSSEAFILEGALI
YWHVDDSSSLKMSADQVPGVENPPTPTNETSTVPQENKIKRHTKPKTMKPLKREKHPATNKLTL
NAHIIHQNKIEIRSPDYLKGTYEKQLSTFLPYHYFKHDSIKNTLYPVKMIFDKITIGFKRKELRLYCNI
TQKYDLVQNFIEGEGIAVYAKAKLTMRKDADGDSEEIRFKDIGMSGTIEVLQGLVNVNLQYAKDG
TLYVVGLFDIANMTQFINATGMKHYLPENLRKTYPRLTNFAITRLKIKSKLNENVLRIIGRCLIFSEP
KTDDNLEAEKRDGIEDESPASLLYESKLRVVLTNTSGDLKYSVGIIGEDIPLQVAMRELFNATITGTS
WVKFKFFVMVMSNNNEINVAQAIDERLFNSFNSETGLQIKTVLLPPVEEDCKMSRKEKLDPLNKE
ALVKNEANGKETVEIHRSPFIVDYTCMFVKDQELLMENFEYATATVTPTSYEIKSLTQERFFDFQSD
PVVSVGNFGYTIKSVKGWDSIFFIEGEFRFMVKNDRLIFKGLITFDDMKGKLVLQLKQLHSGRQNF
GDAALINSGYSVIGKDDTFSKFHFDAFLYVGTVKTKLVNFRKNVEVIYDLRTDDIFMKGARNNNG
NIGATNKKRNSLLELFDAFGTDASSHVLLKELHAISLTWLIYQQNEEVVKTKHGIRRIQPGLNFYG
TLAGKGDRDLLFNMHYNKGMGALIADIELPNYKLPLRKDAKYHPFELQRSTKKQDLGGILFVKA
STTFTSAQAQGYLRVVGLEYDVEFPIMADGQFHMDLPYSNEIAKLFNEAEVRYRVSQDIGVTDFN
LVDPKIKIDLAPSLEKISRAIRHAIEAMIANVDEYLVNSGSFINFVTIVDGEISTMKEVINEIRRGMGK
LQSTLKQLVQQISSDCAEQCKDSKVFIYGYDFQGGKFVGNEDAHDVANVTCIAECEMIKPVKND
ERRSLLSSLKEALVQFKREKKRASIMQIMLDEHYNSLSKMNDAMHKNARAASLILSKTGNLKNP
KISVASLVLENQISVINFKGAGSKCLSGFKMAFSYNDVVGETDIDLCFNGEFDQFAAYAFVLSYYGE
DKYNQFGDLLDDAAELKQSLKTKTKIVTKLFKKIANARTTVELEEVQNEVEETILDYMDGLKKSQ
IPKHKIKAKKTKEAIGYITYGGKNVTLPGIDTEVLEALEIDQDYTIKNPKSKRVFMYSSPWAALNVL
HLHQNKSMESLPMITSIDNAGYCKRLTRLSSVLNSVRGSLSSYAASYVNAKKNFENVETNMKSGL
VELKKMHGSSIMHDDDKGNLQYWLTQYEKGVNRFSKLSNMKLHDHNNHGLARWKNEMDNS
LQHSGLGSIENVIEMLKKRSSSKRSNIDHDRWIRNASLMGKEISNLMKNSNKMRMADAELTVRS
MSQKFDNFFEALTKSCKASFKQF*
>TRINITY_DN135129_c0_g1_i1.p1 type:internal len:155 gc:universal TRINITY_DN135129_c0_g1_i1:1-462(+)
SSFSYMSTSPSRSMDISASSDEALTPGGTLIIRDNVDNRTSFYERLQEKDSFIAETSSALNTPQNSPRP
STTTPLPRTPQQDRTPPKRSPLVRMKSDSSDIEKRAKFSKDNVSSTFRPVSMLNLSVFASNRPKRLH
TGSESELSEHVPLSTHR
>TRINITY_DN1614_c0_g1_i1.p1 type:complete len:218 gc:universal TRINITY_DN1614_c0_g1_i1:56-709(+)

MHKTKICIWLVVRYRSNPFRGYNRRPDLKSAGAKMTKYSTEPENPSKSCKARGSYLRVHFKNTHE
TAQAIKKMHIRKAYRYLKDVVDMKQIIPFRRFNGGVGRKAQAKAHGCSQGRWPKKSAQFLLQL
LKNAESNADLKGLDVDSLVIDHIQVNRAPYMRRRTYRAHGRINPYMSSPCHIEMILAEKEQVVPR
AEEEVEVKKVSKKKLAREKLKSRE*
>TRINITY_DN1143_c0_g1_i1.p1 type:complete len:629 gc:universal TRINITY_DN1143_c0_g1_i1:2539-653(-)
MLRSRTIITNTLPKLKSINRLEPSFSCISNARCFSVSAANKDASESFAMGIFTGQANVSQIFPYPDVLT
DDQREFLSSLVDPVEKFMSEQNNAFNNDALEEIESETLEGLKEMGAFGLQVPAELDGVGLTNTQY
ARLVEVVGRYDLGVGIVLGAHQSIGFKGILLVGNEEQKKKYLPSLATGEKFAAFCLTEPSSGSDAA
SIRTNAKLSDDGKHWILNGGKLWISNGGIADVFTVFAQTPVLDEKSGETKNKVTAFIVERGFGGIS
SGPPEKKMGIKCSNTAEVHFEDVKIPVENILGGVGQGFKVAMHILNNGRFGMAAALSGTQKGLI
AQAIDHAASRTQFGQKINNFGVIQEKIARMAVRHYVTESMAYVICGMMDKGFKDFQLEAAISKI
YASEAAWEVADETIQVLGGMGYMKDCGAERVMRDLRIFRIFEGTNDILRLFVSLTGLQGAGKELR
KLQKSLSNPLSNMGTLLEQGLLRSKRAAGISSGPDLSSNVHPDLRKEADLASKGIGKFGSTSEALL
VKYKKSIIDQQMVCSRLSDSAIDLFGMVCVLSRATKSLNNNVPNAVHEALIAKTFCSEAYKRINTN
LESCKNEHNMATDKLKAQITSDITNHMGVVTTHPLGF*
>TRINITY_DN5229_c0_g1_i1.p1 type:complete len:728 gc:universal TRINITY_DN5229_c0_g1_i1:2363-180(-)
MFKSLTISISVLTFILLFQEGSSSPVVAANSTNNAANATILIEEDESNKRQETEPKSFSDDIEDVIKIKD
SMENAGLIRDNTGAVNKAKTSRIAGKLREKARSIDSDSADSATEGVSGAGEGLLTLVEGIKGGNW
VDIVTGGLDMISSVLGMLGPMSVIASSVLSLFSMAFGLFGGATGGEESTEDMMTRVIEKALLEAKA
EELRSEVNGIKRLLQSFSSSIANFRETNSLTQEQALTLYDQGFRGVAQFGVLEHYIEKWCAVGDPN
TYDTPVKKGELNDMVSRCLDMVNLYCDLSVFRQIMLADMASLIAEVGIEGTPEVFMHLLRKEQK
SDKKLLKFLMDPANNRDKRFVITKYFTSLTKYTTITAYLKKLSTLDLSDVETKKSARAEISKDEKFFD
KKFTLVCTDIELRGNCFQFSELGAKRDASLKGWKKKVVSLYVSDGVEVQGFHRPDLQGSLFGPYY
GPAVYGKVSGSKDWESMNILASETSALDLLRVCENENMDTEGVCEAIETKNYPNLGAMDKRGT
AMYYVSTIRKKAKCRYSKCDVWNTGKWWCERSKRRDWEECLIGRYWSERIKSMTIPKGYKIYGY
SETDYGGVEYGPLYGPSTVLKVHPSAAWKSLKMVKTTQSTTKMVQMCSEEGLKGFCDYFIANSG
QTDLSNVHGVNWDSDKRIKSMMVPGSITVYTYSEKSYGGMEMGPYDGPVTITKVDGADGEEQIK
SFKIMRRY*
>TRINITY_DN11097_c0_g1_i1.p1 type:complete len:730 gc:universal TRINITY_DN11097_c0_g1_i1:2348-159(-)
MVRPQGVTQEHSSKPSTSERNSFVFSALSKSGCFETLPSIYDSIIIQRSHWLHTEGFSMRTSSTGSQQ
TSRRAFYEELQAFSSTIPFYESLSWALLSLSGCLNPVSTPNIREEILADLEGRSVDESREVLLVFATLFD
VYKSYQNSIVVEISAILPGGILRCISLGQTEGFNSYNTVGLINYQPIVYPVGRNVLGRLFNVTGSCID
LYNEVTMDMPFVRSVSTEETHTPQKTTLIKTGANADTSSDSDCVKANALFKECTSHINVKETSLD
HLISLYCGCYMGMTSAPIAPISRLPIEAESSRAPEHMVQKLQECMGTYSGSADPSTPNQIGSKKQPF
WLSKEALESGVSPIHSTPLPISKLRVDLSLFETGIKVVDLLTPYKKGGKIGLFGGAGVGKTVVIMEFI
RNLAIEHKGISIFCGVGERTREGNDLYSEMAEAGIICKDYTTMKPSDPSSNGGPTGSIDQHSVTFTR
SSVTLVYGQMNESPGCRMRVAHAGLTVAEFFRDVFKQDVLVFVDNVFRFLQAGSELSTLLGRMPS
AVGYQPTLATEMGAFQERIIAALWGSITSIQAIYVPADDLTDPAPVTIFTHLDAVSSLSRELAAKGIY
PAVDPFNSTSRMLDPQCLTSEHFRVALEVQQILQRYRELQDVIAILGIEELSEEDLKTVERARKIERF
LSQPFSVAEVFTKIKGIFVPLEATIRSFAMVLSGALDDVPEGNFYMKGGLDTLKL*
>TRINITY_DN61_c0_g1_i1.p1 type:complete len:625 gc:universal TRINITY_DN61_c0_g1_i1:2929-1055(-)
MRAVILLCIAMCTMVYADVYFHNIRGSNNRLKGQNRNRDNAYRMFDSQNNDRGGYNVGPTPM
YFYTGSILTLEWTNQHQCGGPNANCDLILQYMCSENLRDGLEERTIPERNKDCKDDDCNTDIRY
GMHEDYDYYMNCKNRQRNPGLFLADQELEGTSAKYTRQNPDGDRYGYECPEERDHYPYWLPT
PWIDIAIMTRDLRRCNYYRENSQNNNAKYYCKPPQKYLDELKKRDPPSNLPRIPISQDDCERFHY
PANDKTDRGVWTRHDSWNVDPPECIAAPPSRDNHNGNGEDGYFNSYNFTIPNAPHKNCAIRM
RYNISTSDYDVNLDADGNGDPARAINIHDLVGIDTKKEATERGYVFKDYPKVYPMKGEIGKKLGL
ALAINTAQYFRTFEDRSHRTSFKARTPELEGVEIRNLNVKGKRGNIVQVYPAVEYGFVPDRMLAE

VGDMIHIQWTGSDRNPRNNDGEGPAGEDRSNIAELDQPRVAQVVADYNEYGDLTRSYPAHLDN
ATFLGLSRDELISLALAKGDCEFDRQGARSRTPYFDMGPVALTKKGIYNYLCTINNNFTNRSQKGT
AIITEKSKAGGGSKATASASKSASFRSVNGKDQEKRKYHAYNYFEQYKKK*
>TRINITY_DN1366_c0_g1_i1.p1 type:3prime_partial len:4702 gc:universal
TRINITY_DN1366_c0_g1_i1:236-14338(+)
MMEQDRQRPSRQEYNNRSLSRERIGSRDGSRDRNLSRDRDLSQTRDLSHTRDGSRDRSNLRTRDG
SQDRSFANRSDLYSSPYSSNSQAMPQTSATVEIENLELQATVSSQEELINQLAEDLRNVRKARDFSA
EVEILQKEQQCMDFKEQLEGKEMTIQHLKTELVQSQTVTEEVYKKYESIRSNRDAKDKSIRRYQQE
LHQVHDLLETAQKDKLACVDKMRQIQNEKSALEFDYQWLFDKIKNYEEHGEVFDMSSVSKSTTT
IKRTTTVYEDGQKIRVEDYQSDDSIMPLQNKFQQAQKGYRKAIGMQDDQLMRDKENLMNEFRS
MQSQMHDLKRIHENFMRESKEKDTLIERLKEAKMALDQELDHLTRENENLRQTNDKLENENSD
LRTELNSARNQVSKIEVQYDNCMKQRNYMKEQMEIITQNSAKGRDDYNSIEATMRDSQRKMDS
MQRDLTNKENLNKQLKNKIKILEEDFEQCRQDYNQCMVSRESLQQQKLDVERELKERHDRMDE
VETSYEDIQRRKADLQQELASLKKNLTRFINEEDSSNQMKSHLQNEVLAANKRISRLEAAYEASQ
QEKLELQQELAPATQRLSMMRAAYETLQQKKEQIQTDLIASQKRCAEYQAMNERLKQDRGEILN
EIQACKRRAAQADVDLDEAAKQYAILKGKLEDANRRAQSASEDLVTRQRTIEELRHKLEVSGRET
IQRESYYERIKQERRSLEDELRGCVTDLERVQNEARDVQRDNEKLRQDLGTAQQRLHETEAYWQ
KTKMENAKLQRDYANMERTVQELRDLSHKLQQDKEDCEKILYSTHKKYANLDKENERLGRENT
DLERELQSSKNKQSKLDAVIQDLKRKEAQLKAQLDDALKNRDELNRVKIVMNDLQKRLDGVTK
DGEQKDNLIAQLRNEKDNYKRDCDKFGRDYEGCRTMLETANREKDRLGKELNAANRRIADSD
VLVKRLQDENMRVRKDANSFEARIPILEDALQKSKANESKWALEANTYKTNVIRIEKELLVFKEE
HHSLVSHVSQNEDKNDGLAKKLDDALRKIFELEATNEMVSRENDELKRDIDNKTRQLEEQLTTV
ERANNDLKNQLNELRGRNKKQQDDYNRMQIDYNEFQSKWDGLSSDGQGKDRLIEQLRSELQN
STHEINQQHTDNENKKAYIERLTEEKQDLLNDVNTVNQRIYELEGLYKSSEDDKEGLQKEITELNR
KLNDVESDYDTLQRESDKEIRALKSKLGKVESQVDMILKDRDYIQDQLDAERLLSQKYKDEFERV
QREFLEHQKICERYADQILERDRIIDDLKNQMYKMETEIDTLKEQLRKAQTDQKVAEQQRDKAVE
KLNQAEMKIKDLENENDYLRKRLAELEREISVLEMKLNDAETENKNLRNKIKDLEDLIKTLRAKL
SNTETELQSIRHIISDRDVMIERLQKELDDANDQAHQLRRQVSELESQIELGDQERDDLRSRLANS
ERTIIELRKNLDKVIHDGQDLEAELAALKQKLIKMEPQMKQAQKQRDDYKTAYDNLKGKYQDL
KDELLANQRQLTDAKRAAEQYKKELRHKDHLLEEARGKILALAGQLQSARDELVKKQQENEQL
LDEKRLIQEELLRLRQKLGDLESANQILAMEKERLNEELISFNSKYTELRIKLEKSEQDRLESQRQA
MAYEQRLAKLEPQLQQILRENSELHLQVDRLADELRNALDENRHLKNDVEELQRIRDMQLKELY
EKDTIIEQLKAKLSNLENTVNHLQQENDKLHNLNDALEQEKHSLAHRLDNANSRIKELDDLYEQ
ERQKRDQLQNDLHDLQKKFVTLENAYKKSQERERDLKKKIDDLLGRIKDLEDQLAAEKKKNSDL
EAENDRLKDLIHDLEGVIDDLRRKLTEQENENDRLRAENDDLKQQIMNLQRRIDELEGVIAGLEQ
EKNFLEEELNSLRQTVNKLEAEIDNLTKQRDGLQGQLEDLRREIMRLKEVIMKLEGTNRELQRTID
ALKQEIYRKDRIIETLEAKRKQYELEIDNLKNEIIKRDVIIDSGDESKRDLEIQIKNYEGRIAIMDGRI
DKITLEKTEIERNYHNAVNRAKGLESQVLILNREKAELKGDIDQHFGKIRDDNEVVNKTQGELRE
KVRIIETYKKEIIEKTTIIERLTVIRQTVESERDQLKKDNATLKITVNQYKDNDKGMNDEIQKFTFKIT
TLEKDIINMKNERDEFKVKADNATSKLVEVETQLKRSIERETRLRGELDTLHNKYQNLETIYNELL
REKNDVDRKYKDLLQKHRELDSKYNKLLANFNDLKDQLQQANNKIADLKAKLDKEIYENRDL
RDQLDNLKNQYNKLRDDNQRLEQELADYKYKYEDAMKELAEKNDLIDELKAKIANQDRQINA
LKGTVNQLRADYERSMREQARLQDEIVKLQAVVADLEGKLHRLELENEKLRKENNELQAKIPAL
ENEIENLKDKLENLIKENRDLRNDNMNLKQENVQLEKEVDDLKDDIEKLKDEIRNKDNIIADLR
KVRQELEEEIDQLRHDVKQKEKEIDDLFNRIRDYESTIQILNTKLAEIEPAFEACKDRLRHQENQIQ
ELRNKITDLESALKQSQENEAELGNKLALTESKLNKITQLLDDIKRKNMELENENGHLKDELNKL
KDQNKKLENDNDRLSNELNECKAVIQRIEPMLDHLQKHKNQLEKELEDALNKLANLRNEHEA
HLREMKEKLHVIEVITRQLDEKDDLIEKLKQEKAALEKENESLNDALNKLHQTLDAVQRRKEEV
EKDFFEAKKRIAELENLLNALKEDKDDLERMLRDAKEKNADLDHALRNSQHNEAKLKGELDEA
RGRIQRQRNELAKLQIIVGDLRAANERLLRDNTEKQNVIDGLHSQIRGLESQNGSLKKENQKLRD
ALDKLQRELHNLEQENQQLKAKVFSLQLLNDSLREEKDRLYDNFQELEKDYNDLKDKYNQAVR
ENDRLKDELAFANKRIDELENMNNQLNNEIENLKNIIAGLKRQINDLQGVIDQLRRDKSDLEQTI

NIHSETTINQRPVFDESDYVPRSEYDELKALLEAANSTKEGAERDRDQYHQEHLELHKRISKFEAE
LEGATGKRDEYKRLLQEAERRIRELEGVNGRLEDREKELQKELLNALNEVSGLQQQIISLKVKLGK
FEADRVNYEDKISKLNNEINDLHQRLSQVLASIEEKDHIIEDMRQELADTQRRHAELDSQNQILR
DEIEKLHGIINDNERKIDDLEVKLQDQRNQNSQLKNQINELNARNNKLKDEINDLQRKVSDLER
DLDQAKRDNQEKQNVIDRLNQLKNQLENDLEALRQENAGMKSDLDRLRLDNRQLLQEKHDLE
MKIMGLESDKGTLEDNVERLLNEIENLRNKIQKLKDENDRLIIENKRISHELYKYNKKSGPSDYDS
LKDQRDKLQREIADLTKELRNYKTNITVLQNEKDDLYNRLKELERDLADRDHVIEELEREKDRLE
RELESARNRADKFELSNGQLENEKNRVQEELMDLHRKLTKLEGDYELACDKRDDLQKELDSAIA
RNKTLEDNYKRSQERENKLHQDLLAAQKKITILEHELQSLKDRVNSLERANKVLDDKKNELKDD
LIAAQKRISELEIMLEGADDRNKDLQNELDAMKRKNDELRETLRLTEHGKEDLINEIQNLTAKLS
KVENKLTQAENEKRYLEQQLEDAKQRTQKDRDEIMNLQKTIYELTRKCDSLTKDNANKQALIDD
LRAKKTTLEGEIESLKRDLQNNQYEIERVENDKKNLYDELENMKNKFNVQEQEYLVLQEEKEKLR
QDMIASHQSVCRLKENYENSLGDKGKLREELATFHRKIAELTTAYDSCRSEMNRYKGDCDNYKD
AYDDIKAKYDASQNEISKSRNDYEVCLQKYQKGEQAFTKSEADRERLQKEMLDVHRKSSEIAVLF
QTTKEEKRVLENQLSMLQDDLERYREGYETFNDKTNALQDEILVSHKNYTKIEHAYNRAVEREEK
LEKDCEEAKEIVANMEKDLDNARGKANELENELRKLQDKLRREQDEYEQNSEHAGKLQMTLRD
YESKLSQLRNVEDENKTLRDEINNLKSSINKVEMDYRSNHNDLTQVQDQYESSRNNINKLNNEIN
ELQRDIRQVTSENEKLQNDLQNAAQDKEQEGERLKSTIRMLEREIDSLKTQFGQTTEQLDIVSKDR
NVMHQEIQVILKKCVSIEMTYITKTEDSRFQTSGGDEYEKSINELKNHLDKSEREFDDREKKYKYL
EDQYVREKKDKSQLEKEIDEIQRRYDNMQHEIRVIEEDKKRLSDEYQDMFNKYNDLDRDFKNTQ
TKITTYETEINTYQTTINDMQYKFTNNEDQNKKNSQVYRDLQQKLDNATKENTTLKGELNDEKA
SYRNVKKDFERQVRELQRSSESYGKASHEKDTIIEDLKSKRQALESDIDQLRRDLNHGKDEYKRLQ
REFTQHKSDYQDQQRVITDLQQKVEEHKHSKITIEEEYKITVEKYIKEIRETHIKLSKVESELQTTRE
AQNLDSDERYKRLQNELQQERETTRKDIYGKNQEIEQFKSQISNQDQEIRRLRREIEELDKQLKEA
GSNYSSTILIQKQDAPKYSSTQVTESRISSSSAPRYSTEQEVLIDASDKDTEDEKKRYQSSFYRSTATIK
PGQSDYGSVDVVLQGSQDQGQDDKDYDYNRRATGLDTNAVNRSQ
>TRINITY_DN2028_c0_g1_i1.p1 type:complete len:274 gc:universal TRINITY_DN2028_c0_g1_i1:1608-787(-)
MGQSDGDDLTFGQKISREWRDFREFLYNSETGKVMGRSGTSWAKIGGFYLLFYGFLAGFFTALLFI
FYTTIDKPGIGGPKLIQYIENKPGLTRLDRSSKLQSYSASNNDRISSYKTVIDEFLDGYNTSDHSGAL
CGAAGTPKSDKPCIFDTTLLGECAPSKNTTYGLREKKPCIFIKINKVFGWTPADDGTGYLTLSCTG
HATVMPQGFPLTAFPYNGHKVYQSPIVAVKIDATKGKKNVDCTLKGKNIEVSNNFNPSRAFGKIR
IEGISATD*
>TRINITY_DN26249_c0_g1_i1.p1 type:5prime_partial len:477 gc:universal TRINITY_DN26249_c0_g1_i1:1733-303(-)
QMKMAASNMAGHVVRNNLIFLLSFVIIIVAEEGRHARFEYKFSFKGPHLANKQGTVPFWNHYGS
AIPSDDQVRITPSMRDQRGGLWTKTKSSSEFWEVEIFFRITGRGRVGGDGLAMWFTEEAGIQGPVF
GSSDHWKGMGLFFDSFDNDGEQNNPYVMVMLNDGTKQYDHFKDGNSQQLGGCMRDFRNRP
HPIRAKIRYYNNILTVFYHSGMSERSDDYELCMRAENVVLPKEGFFGITAATGGLADDHDVIKFLT
HSLTPPLTDEQKQKILSEEQRKKYEEQYGEYTQKLETKKEEFQKEHPDKKAPEEKFESVYDRDVRL
IYENQNTIHHVVRALHEKVGELTNQLNNLHVTINNNNQLAQQQQPKGDGVTRQEVNTIINLQN
QVHNAIQELKQQSGGGGGDNTKTVQMEKTLQTVLEQVGHLVSKNNDRRPSCPTVSCVTPSILILF
LVAQVLLVAGYNFYKEHQNKAAKKFY*
>TRINITY_DN172571_c0_g1_i1.p1 type:complete len:104 gc:universal TRINITY_DN172571_c0_g1_i1:30-341(+)
MSGRGKGGKGLGKGGAKRHRKILRDNIQGITKPAIRRLARRGGVKRISGLIYEETRGVLKVFLENV
IRDAVTYTEHAKRKTVTAMDVVYALKRQGRTLYGFGG*
>TRINITY_DN8707_c0_g1_i2.p1 type:complete len:564 gc:universal TRINITY_DN8707_c0_g1_i2:2751-1060(-)
MSGFVRQSKYRHVFGSPARKDGCYEGFRVTNCAFDGNFIAANKHFIAVCVEVGGGGAFIIVPVGS
TGRLDVNIPKVSAHKEYILDLQWNPYNDFMLASCSEDGTIKIWEIPEKGLFMNIGDEKALLSLEYH
EKRCTQVLWHPSASNVLMSVSQEPKVCVWNLDEGSVEVEILEHPELIYNATWSQKGDKIGTTCK

DKKIRIFDAREGTLLVQGMGHEGAKPSRVIFTFQDKYLFTTGFSKMSERQYACWNAETIEQLGLEE
LDTANGCLVPTYDADSQLVFLNAKGDSNIRYYELVDGDPYYFYLSTYQSSAPQRGFGFIPKRCINV
NANEISKGYKLHGTKTGYIEPVSFTVPRKSETFQQDLYPDAIGDEAALEPDDWFSGKNAEPKRIA
MSTFFKGKTKPTGAAGGGLKKGGLKGLKAKKEAKTAAKSAAKSEPAKPAPVKTESKDKVDAAP
PAAAAVEQKSSSTAVAAPAAAAPVVVSGVDEKVVKELQNEIKSLKDGDKKLQKEIISLKDKLKDY
DTLRSDIKLLCDAVKKNDERITALESLVAEETDEEETGEGGDE*
>TRINITY_DN1378_c0_g1_i1.p1 type:complete len:443 gc:universal TRINITY_DN1378_c0_g1_i1:2151-823(-)
MLRFAVLVVVASIGVSALYDSSDDVVELTQSNFNNHVIGSDDIWLVEFYAPWCGHCRNLAPAWK
QAATALKGIVKVGAVDMDQHQSVGGPYNVRGFPTIKVFGANKMSPSDYNGARSAQAIVDEAM
KVVQRVVSDRLGGRSSGGGGRRSGGSSGGSGNKDDVIELTDSNFEKEVLNTKDMVLVEFFAPWC
GHCQRLAPEWAKAATELKGKVKLAALDATVHGQTASRFQVQGYPTIKFFPAGDKDFSSAQDYQ
GGRTASDIVAWALAKHAENVDPPEILEITSNSVLTENCNEKPLCVISFLPDILDSMAAGRNGYISTL
KELGDKYKSKMWGWLWTAPGLHSNLEQALGVGGFGYPAMAVVNIRKKVYVLLRGSFGIDGIDE
LLKSIAVGRGRTEKLTQGLPTLKDVEAWDGKDGQLPEEEDIDLSDVELDDLDDEPKQEL*
>TRINITY_DN86511_c0_g1_i1.p1 type:internal len:143 gc:universal TRINITY_DN86511_c0_g1_i1:427-2(-)
VLLLEATKVSCDKIFSGKFPIPPVDKDTFVTLCKLCSCNILLLTHDGYYRQVDGLAMGSQPAPLFA
NIWLSDFDDRVHCNSKFFPRYMDDILTITTTENIDKKVSEINDLHVKLQFTHEIEENNRIPFLDMLII
RNNTTLNS
>TRINITY_DN74934_c0_g1_i1.p1 type:5prime_partial len:359 gc:universal TRINITY_DN74934_c0_g1_i1:1215-139(-)
QPNTFDRVCYGYIPIFGCLYLCGLRAAEEMAKLQNDKEFAKICADRFALGSKNLDSLCFTNGKWY
THVPDPNHMTDVMSSCTLVGALYGQWWAHYLNLGYLLPEDHIKSHLKYTYERNYVDSYTKDIIQ
SPRRFCDSRDNGWTIGVWDKGETPAHPLLYSSEASWSGIIYPFAALLLHEGLQYEGLKVLEVTRYL
YDGTRRSPWNEIECGDHYARPMSGFALFKFASGQSWSIDNNGGVHLSFNPILKQQEFKSFFILNSC
WGSYEQNDDTFKIIVSYGKLSLASFKLPSNLFVNKPKFEEIHVKIQRCLKSENICDDDIFIYQKVMN
FKLIDELMLKFEEPLEINSDEVLCVSVTYG*
>TRINITY_DN1668_c0_g1_i1.p1 type:complete len:220 gc:universal TRINITY_DN1668_c0_g1_i1:748-89(-)
MTGMMWKVIAIAAFVGMAYTDDLNPLTYLPDGVETSHEAGESLSNMTITNETIVQEKTKGFGFL
DMTSNAQYSKGLRGSYLPRYLSRTLKPFAAISANMSKVVDGLQEYIKDLIGKDHEDHFFALVGKE
QYGQTRAIFLKFNQREDGRYNLKKLSFRGTFKLAADLVITRYTNQETGEINDVVSYVPKEKVSKD
DVKDLLNLMVPQLAEVMKGFAPKEL*
>TRINITY_DN97639_c0_g1_i1.p1 type:internal len:298 gc:universal TRINITY_DN97639_c0_g1_i1:891-1(-)
RGSAGARLYPGTRVLLHSLKAGKALNGKEGTCENWDATIGRMHVQLVEGGQVKALRPLNLQKL
KVPEPPDDQAEDTDDDPVYTRVKEVFASCDTDGNGIIDAQEFSACLHKLGLGDRCLPAFLNAIDK
SGDREIQYEEFCDWALAPASRKTQKAAINVYWPATKGQAEIIGGDDSEPEDDEEYTLEDVERSVGE
LPTGWPSHGVKLLNNMRARFPGYPIEGIVWKMQEENFIGGNVIRAIRKTGVREVETVPPSAVRVG
DPGDFPAGYRVRATPLKVYREGSRNWSFQNMRDEKLDP
>TRINITY_DN116_c0_g1_i1.p1 type:complete len:297 gc:universal TRINITY_DN116_c0_g1_i1:72-962(+)
MGFVKAIKNKQYFKRFQVKFKRRREGKTDYYARKRLVTQDKNKYNTPKYRLIVRFTNKDIVCQIA
YAKIAGDVIVCAAYSHELPRYGVKVGLTNYAAAYCTGLLVARRLLHKLNLHETYSGQTEVDGDEY
NVESVDDGPAAFRCYLDIGLNRTTTGARVFGALKGAVDGGLEIPHSVSRFPGFDSESDDFNAEVH
RNHIFGKNVGDYMRALQDEDEDAFKRQFSKYIKEGVKPDNIEGMYKKCHAAIRADPAHKPSGK
EKFKGKRYGRKKMSFAQKMDSVRQKKEAFKRKMEQDED*
>TRINITY_DN97809_c0_g1_i1.p1 type:internal len:184 gc:universal TRINITY_DN97809_c0_g1_i1:2-550(+)

LGPDQVDVAGAGRDPRHLPRPLGRRLRVLDRHRGRVRQRHKAEADQDPKLETARLDVRRPGVQ
VRRVRHPRPSSRDQCHRDCHAPGRVEGRAQVDRGRRGLGRRRVEHEVHGAAQPAARGQEKGR
LRAQQILAVAAGQEEAVDVGARNRQGPPSALAAVSRAPHAQFARLAQRDRRVARAKGW
>TRINITY_DN374_c0_g1_i1.p1 type:5prime_partial len:290 gc:universal TRINITY_DN374_c0_g1_i1:1-870(+)
FHSGVNIDWNNFKKYLKDALCRCEKAAAAKGKKYFSFRYFGICHGLDSLPKESSEYCFHHTFEQ
CMNNHNECVGADEAEYIYKVGVQIPTNTKPALKVQRKVFCEHRGSKTVTCGAGETIKVTKAYYG
RTSSSHCGYRGFYNLNCKGSTSYSKIKSACDGKQTCRLNPHNNVFGDPCKYTVKYIDIEYVCKPP
VKKAIFCEHRGTKSITCNEGEKIEVVHGYYGRQSSAHCGFRNFYNTRCKASSSVGKIKNKCDGHR
SCNLLPANSVYGDPCVRTVKYIEVDYRCIKN*
>TRINITY_DN58867_c0_g1_i2.p1 type:complete len:354 gc:universal TRINITY_DN58867_c0_g1_i2:80-1141(+)
MVSLYGDMMFNIENGYLEGLVRGFRSGILNRTDYLNLIQCETLEDLKLHLQSTDYGNFLSNEAGP
LTVSVIDEKLREKLVIEFDHLRIQAVEPLSQFMDYITYSYMIDNIILLITGTLHQRSISELIPKCHPLGR
FPEMASINIASTPAELYNAVLVDTPLAPFFVDCISEQDLDEMNIEIIRNSLYKSYLESFSRFCDSLGGT
NSEVMKTILQFEADRRAIMITINSFGTELNKDDRSKLYPKCGKLYPDGLARLARAEDYDQVRQVA
DCYAEYQPLFEGTGTNPGDKTLEDKFFEYEVKLNVNSFMQQFHFGVFYAYVKLKEQECRNIVWIA
ECISQRNKSKIDNYINIFE*
>TRINITY_DN33065_c0_g1_i1.p1 type:3prime_partial len:663 gc:universal TRINITY_DN33065_c0_g1_i1:1986-1(-)
MWWFVWLAVAITINVDGKKASKHKSLLEDVDLDEDFDLDYSHNIFIPLDAYENYEKFLEYAQGT
DLAKRSGVSPLPVIQSLPADGVHTYSAPGINCQVELLNKEVEGTDKRVIERKTCPEGWTLKDAMK
YFFADVGQDLQSDSAGLVHIEQLKFKMLVFEPSGGFSFVADTDGEDYSNLQSKLSLSNVDFKLTM
GASYSYESSEISITGNHEVIERSTNEAKQIEVTFEKGKDTSNFVVKMNIPSMKLETFATVFLASETEF
VTSEIGDVTFNINIDPIKAVTIESTSNSVNGVFDTVEGKFQLTAKFTSDDTNAFDGEATDIYLTVNR
NSLGAPTHYNLMLGYAKTINVMDVVRKYIGKPEIQEQFDDKFLKNTIEKWYLMFTSSRIKLAADD
DYQNMFSDIFTDDDYKEFKKGVGIKAPVNLQETLTACGGGTVPDTLSDIKINGLLTKSGLSLKFPS
NTAVPLFALMKCKLRKIQSDLGLDIPDYLKKIEEATNFKLEEFSYDHESGEFKMSLLYSNTITIYKDV
LEITNPQLTIEREGTDEPWQFTIAADIMLFGVEASVSVQYSKTGFTVEVSSDVIPLSKLLTAFSFGIEP
PFTFEIHDFKMQLVVSDKEEEEGEEGEKTQEGEEGEKGEEGGGGGPGGGPGGGGRGPGGGRPRR
G
>TRINITY_DN1172_c0_g2_i2.p1 type:internal len:264 gc:universal TRINITY_DN1172_c0_g2_i2:790-2(-)
GDYGFKVLTSSDPAEKEKKLAAELANGRLAMVAIIGMFFQDGLTGSAWGDWALYTASPLRFFGG
DSGPSYKAFDPATELGVQEPLGFWDPLGFTSDNDEAAFKRRRSVELKHGRVCMAASIGYIVPNYY
TFPGFLSPSAGVKFADVPNGLAALGKVPALGWVQIIFFAGIVEGAFGFDDYKSGAPGDYGWKVLT
SDDPAEKDKKLAAELANGRLAMVATIGMFFQDGLTGSAWGDWALYTASPLRAEPVSTAGVGAA
TKGGTE
>TRINITY_DN2963_c0_g1_i1.p1 type:complete len:116 gc:universal TRINITY_DN2963_c0_g1_i1:1072-725(-)
MSATGNLVTVLRKLYDEYNDKTPSRLKIVDGFLLYVMLTGIFQFLYCCLVGTFPFNSFLSGFISCVGT
FILGVCLRVQTNPRNRTEFKGISPERAFADFIFANVVLHLVVMNFIG*
>TRINITY_DN128406_c0_g1_i1.p1 type:5prime_partial len:150 gc:universal TRINITY_DN128406_c0_g1_i1:474-25(-)
FGSTRKMSQTQSVQVFGRKKTATAVAHCKRGNGLLKINGCPLHLVEPSTLRYKVQEPILLLGKERF
EGVDIRVRVKGGGVVSRIYAIRQAIAKSLVAYYQKYVDEVSKKEIRDILVQYDRSLLVADPRRSEPKK
FGGPGARARYQKSYR*
>TRINITY_DN4781_c0_g1_i3.p3 type:complete len:183 gc:universal TRINITY_DN4781_c0_g1_i3:2965-2417(-)
MCTEPKAPSSCANDEFQCTKTKQCIKKHMLNLTAYTKVGSKMTKLWTKSGTYGPRWRHATVDIS
SASTFKVVIEGTASSGYMGDIALDDLFLNNGACPPSGECSFEDPKLCGWTNMNGDNFDWTRDN
AGTSSSGTGPRFDHTYGTAQGFYVYIEASNPRSKGHKAWLKSGEYKPTLGRCLSV*

>TRINITY_DN464_c0_g1_i4.p1 type:complete len:717 gc:universal TRINITY_DN464_c0_g1_i4:2167-17(-)
MLLHFGKSLHYLKFGTLFWRLKLVAIPRDTSNIINNIFLESKSQRYIRMSANILNEYSQNVGKMEDE
ISGLTSQLNALKRKLNGEIEDEDCSDQLKELRSKNQKLHYQKNIIQRALKKEQEKADSKMLNVQ
AALNVLFESAIRSAYPQIPNPPVALTIAHNPKFGDYQCNSAMAISKMLQKLTGEKIGPHVVAKTIV
ENIPSNSFIKKVDVAGPGFINLHLEKEFLKGVLEKVIKEKVYPPGIGNKKKVVIDFSSPNIAKEMHV
GHLRSTIIGESLSRLLEFVGHDVLRLNHVGDWGTQFGMLIAHLEDKYPDYKTVSPPIGDLLLFYKA
SKKRFDDEEDFKKRAYQRVVDLQGGNPDVRKAWQMICEVSRNEFQKIYDRLDVTLIERGESFYN
DLMSKVVENMKKSGICQKSDDGRWVAFAPNMKVPLIILKSDGGYTYDTSDMAALQQRLQEEHG
DWLIYVVDAGQGDHFKQIFGVGEAAGWYNSSITRVDHVAFGVVLGEDKKKFKTRSGDTVRLIDL
LDEGLKRSMEKLLEKGRDKELTKEELLAAQESVAYGCIKYADLSHNRVMDYVFSFDKMLDDKGN
TAVYLLYAYTRVRSIQRNAKKGRSEIEEYMKSNSLHFDHEKEWKLAKLLCRYPEIVVRCLDDLTLH
LLCEYLYELSSTLTEFYDACYCIEKNRETGEIISVDMNRIALLESVARIFEAGFHILGIKPISRM*
>TRINITY_DN61668_c0_g1_i1.p1 type:5prime_partial len:453 gc:universal TRINITY_DN61668_c0_g1_i1:2-1360(+)
FDGSDELGCVTVAPATTKVLHCDDDHAYCEDNLECYPKEGRCDNTYDCRDRSDEKNCGAIKTD
KPICFDFQCKSGKKECISNDKKCDGRRDCKDGEDELNCQKSDCLPFPFGFNCTRVKEQCYLHKIV
CDGSTNCRDETDEDKEICGVTKRVKNLRSKLDDLSTGGIQVRLFWDAPDVSLSKVKGYAVSFIDD
KFVQKALKVPLSPSRVTIPSLKRCRKYVFAVAVDHTDLKADKLLYESTEEIETPGRRSPNAPALISTH
GNKVLWKEDVGDCIDHYDIYNRFLSCMEMDSKTVVKNRLKSANVLFDIQSMLNSNSNDTKLYN
CSIYINYIRKSDGTPFNLQTDYFTFPHTHDHHHKTAASTGGKGKRNTLVWAMPVAVVVIVLCIGL
LLMVYKYRKLQNSFLAFAARGSYNRQVDDLDDEDDDNMAVAFHTGEDSPMINRFSDDEPLVVA*
>TRINITY_DN1091_c0_g1_i1.p1 type:complete len:212 gc:universal TRINITY_DN1091_c0_g1_i1:200-835(+)
MKLILICLFAVLVYSIHCDQPSDPVWPNLFQQSFREKFYYPVIGTHYTNGTYYYDYKNLRYRLDRD
NGRYDRYCGLNGIRAFQNTPCTHLVLDGTRWLVYPEKKECCECCHAANGCGVLKPTWLTGAEF
VSKSNGIYKWNQKGLQDNFYSERVTDRIMTEIDQVPNDTQYYDPDSFKTKIENIEDVFKLPSYCKR
GTKCSFFSACRAVGFF*
>TRINITY_DN3202_c2_g1_i1.p1 type:5prime_partial len:263 gc:universal TRINITY_DN3202_c2_g1_i1:949-161(-)
WSQGNRGCYIHTQEVARGNNARRHMCWVFSKCSKNPDVNLKSCPIESGLCVKENGLDQNSGV
RKLHHYDVTTPYAEADCFKRCSTYRYGEITGCEVIWNQGNRGCYLHSQPISRGNGIARHMCWVF
SKCTTSKVPATTAPKALSMTDTYFGKTELDPIIGVKRIGCYRMHRHRFRKKVNMKGSMDPLSFIDR
VKQCRDIVNNKEAEFEIFGVSRYGSQCDFYLPAEVKNEKPKPDSEDCHFDTMGGDTAYYVYKLCK
DEKQC*
>TRINITY_DN3052_c0_g1_i1.p1 type:complete len:362 gc:universal TRINITY_DN3052_c0_g1_i1:45-1130(+)
MAHLSQEQRNELKKNAESIVAPGKGILAADESTGTVGKRFAGINVENNEENRRLYRQLLFTADKS
ISNYLGGIILFHETLYHKDDNGKPLVELIKELGINPGIKVDKGIVPLHGTNNEGTTQGLDGLAERC
KQYKQDGCTFAKFRCVIKINTPLGVPTDIAIQENAQVLARYANICQANGLVPIVEPEILIDGDHNL
ATSQRVTERVLACVYKTLVDHHVYLEGTLLKPNMVTPGQSCPEKASAEEVGVATVKALRRTVPA
AVPGITFLSGGQSEVEATKHLDAMNKTDLARPWALSFSYGRALQASVLKAWGGKKENVAEAQK
VLLHRAHLNSLASLGKYEGENASGATGDSLFVANHQY*
>TRINITY_DN5397_c0_g1_i1.p1 type:internal len:161 gc:universal TRINITY_DN5397_c0_g1_i1:2-481(+)
CSVVCDYSPWQDTQSCTKTCDGGVLIQSRTERSFNKVESREGSTPCNTQACGSGLSEERFRKCNA
NQCGTNTGLQEVYKVCNAANCRFQGIDYTRNQEINIRTQACTTACDYTPWQDTTACSKSCNGG
VLVQSRTERTFNKIETRDGTTPCNTQACTQEL
>TRINITY_DN493_c0_g1_i1.p1 type:complete len:473 gc:universal TRINITY_DN493_c0_g1_i1:2681-1263(-)
MKCMKFISLMLITFTSILVAQDTGFIYYDDSSSSDIDSLTEPYGYQSVMDNREKSYEKPDMRYNFDK
PSASDIDSLNTPQYGYEPVMDDRERSYKRPDMRYNSDEPSARDIDSLNIEPYDYESLMDYRDTPSE

RPGMRFDFDESITGDIDSIDTPLDDYQSLMDHKRKPYARKRRSIRHRRFIGQRRWYGRRKFIRKNQ
RQRNGFRKINSQRKRVCSKGSNCRHKLEPMGCFKDIKSERAMTEEILNERDTASKVFNNRLINWR
QYHEWLPGFICRCAQAASERGYSVIGIQFWGECWSGPNSESTYNKHGQSDSCVNKNLQENRAN
LTQCAQAAGKDSTNYVYRIAPACNTKYEPVGCFQDDQKEPRPIPHYISTDRDSSLDIFSGKLLNW
KQWNTHMPDMICRCAALAKSKGHKMFAIQFWGECWSGPLSNDYKRNGASGECRGKDFKECSC
NSYYCVGKTHTNYVYRLS*

Band 8

>TRINITY_DN982_c0_g1_i3.p1 type:complete len:9852 gc:universal TRINITY_DN982_c0_g1_i3:29697-142(-)
MSIKLCTFFVFFLIQCINSFPEIGLHLSLDRSYSFQGNGWKTVNGWEIKPRANGPYEIDAIKNISPEY
FPVPITGVYYITVSLPVEGASNGEFKSAIIVDGKNEEGRIGLMSSFQKTAFKNGASTLTFAGFVRLFA
DEKLTIEIYSDDINANYVVLESATFVFHFIGVTGSVPAYLAHTAQDQEYASDSIIKPWVTLGDSRVYE
SLSGFIKNEGVFTTICNGVHKMAVNIMIKANAGTYTLKALFQDEESATATVTINQPDSIATLNIFQN
FKWSEGMSITLQIHSSNGANFKVLKSSSWSMSFIGETTANFQEFSVFLMADQFLQPSQFPQRINIDL
NGVFQFLQSNPDQYNKGQLVSGADTKFKDNLDFIVDSTDLFYVSANIIIEGEAEDLEITIAVSQIDID
VLPEDGLSGRLTKISNSRRSISLSGIIFIREKQYLSFFVRSRDSSKSFTVSKDSFISVINMRYTVSSVSARQ
NENIDVTAGSWYDISGRFAEQKNGLYSFGKDFNINNGVFRTSHSGIHSVHANIQFSLDSDAFGDN
DDNKIVLRVVIDGQEDKISLMSIRKNPEKTATLKLFGSIYLRAGVTISFQVKSLMPNTVQYKILQRSS
ISISYIGPQWAVNSFHAVANQDLNYDGKPDDTRPDIPSPLNFWKTEAADEFISPFEVLESFDGTRYT
SVDDGLYVVSSLLILKNVDCDRSALFKLDFVTTGGSRASDKSNGFADSHFFIADSKGTEFTLSVTTA
MRLEVGFKVGLKLTVGSTSCRYTISKESSFSIAQWSKSSNTIEENRLKYIGVLARLSAPTEVSSPNTW
SNLGSNTMDINPGDKSRSSFRPGIFGGDDLAPITRTGKDFNGDVSDFTIKDPGIFFISGMITVTTTEN
ANEEFAATVQIDSEDIESGLYAAVINQGSTEMSLTFGGTIYLKSGARVRVVVKGGRTFNIAAGSSFSL
LKLQPDYKTPGIIVSIDEKITQAITRYEHKDLRADIHRTLTIGNLGFVESTGVYTIQVDGVYMVDYT
VILEATNPNAVFGAEILSDNTVVATGTSTFGNTKTIAITELVYFSKGAIVKAVLKSNTNGETLNNLQ
VDGGLTVYFLEPKEHVKDGHRSKQTSPQALPVRSFEKVTGFAAQSPDFAGGADSVDYTVPETGVY
LVAFTTRVNRVINGFKSRVLVNDNEFLNGNYQIVSAQNAPATLPLSIAMELTVNDVLSFQVMADG
SGMSLDGSTRSILKIDEVNTIRNVTEGLSAYKLRDQKVNGQQETIVEEWDTSVTEGTFLAKNVSFT
VDHSIVVVAAGVFQISVSVVVKNDRPSQSKFELRVLQGDDVKSHGLHSKVEIGGGREQTLTIGGSL
LLQKFDSLRIVVRDIADPLASYTIKAGSTVSVIIIDDNNGPIACEDEGLNMRRLPERIRKPTRESHTF
QCNVVSSLKAEKYTWFKDNVQLKEENQKNLVLNDLKLSDSGVYQCRVRFLELTGATNNATLSVY
DPYPKITSPKFIANVLENQDPPLTIEMFSLSAFKLSPEDEKASITTQIIAGDDQSFFTLSGPSKQTVTLS
TTKKLDYETQNEYNLTIKATNLEETALTTKGPYETTINYIVRVIDVNDHPPVFSQSIYSRSVPEDYPI
DGSIAKVSANDQDAGANVIYTLVGQVSGDLFKIDNTGLIQLRQNLDREIRDDHTIFVQASDGLFT
TVAEVVVTVTDVNDNFPNFMDTGFDISVSEATLTQSVILKVIATDPDLGTNSQLRYTIKSGNNENK
FSLNLLDGNLTLISKLDYEGTKIYNLEIDVQDSASPPKKSQNPLTVVIRVQDANDNNPTFTQSLYNT
RIAENTAVGTSVLKVSATDRDGEGINNPLTFSITDTDANEFFQVVSSGATAGEIRVRKSLNYDIRKD
YRFSVVAKDNNLGEPRYRTALVVINIDDTNNLRPTFEAGSASISISEGTSIGTSVHQVVAIDKDTVG
KLDYRIISGADGKFAINKETGDINLISAVDFESNKKTYTLEIQVNDGVRDALNPATVVINIQDFND
NQPEFIQKYYIFTALESISDSQAINVGNPVIARDADSTTNSQVDYSIQYDINNPSVPNEGDALFRIDS
NGLIFKKNGVILDFENVRYHTFTVVAIDRGTPKTLLGKAFVTINIQDVNDEAPSWSPAIYSVTVKES
AGINRDLVFLSTTDKDTSGAVTYSIVSGDSANQFQIFDNVIKIVKPLDFEKTETHTLKVSAQDDNG
NRAQLDATVTITVEEVNDNRPLVTNNNANSIQVSVPEDIPVNSLIVKLNGRDDDKGAPGQASNQ
FTFVSLDKTTDFTIDEVTGEIKNTVNLDYERQKSYSLVATIRDKGTPYLESKFLVDIIVENKNDERPIF
DPAIYTIKLSEKAPIDTVVAMLQATDLDTPKSQLRFSSVTTLDSHFSLDPITGIITLKTSFDKEVNGKD
GIFVISCTVNDGTSNSAQNAIVRVEVVDVNDNQPVFPTSEYFIQVNEGSSIGTILQNIAATDKDNT
NTHNQFTYTLEGKEAVDFRLNGNQLQTNKIFDYENKKSYSFDVLATNNLSPDKLVGKATVVVSIL
DQNDNNPVLSPSGVINLNISEGTIAPANIITIFATDKDSTINAEVQFTLTGDPTNTFTINQNTGEIML
AKSVDSFAKNEYTLSVTATDKGSPPRSSTATTLNISIKPVFVPGIEFENCPQIISFNENAAPNTFTKTL
TVKDRGSFNQVQFTVVSGQAGIQNLITVSQNGVITNGQRRADFEARKRHDFLVQAENERGAKAY

CHVTANINDVNEGPIFDSANYVFDVLEVIKINTVVFSVRATDPDFSSTTNGQLTYSLAGQEASQFSI
VPNNGQIKLAQPLDYEIKKQYQFNVIATDGGSLQTTSSVIVNVEDWNDNRPIFDQPQKLAINVPE
NSKPDNVFTVSATDKDSTTNGEIEYVGIYPTGIISIDKDTGTAKIIGDLNYEKRQKYDFVIVAKDKG
VPYLSNTREVSLSVLNLADEVPYFDTAAYTISISEKTSINTAVFRVMAKDNDLENFGKISSYEIISGNQ
LNRFRIDSNGNIIVQQSVVLENNNPNQYILTITAFDTQRTQSLNNTQVTINIIDLSDKQPFFIQNSYI
VEVQENNEVSKSLLSLEAQSNEPTFNPTYSIVQDPKNTDSQFFQIINGNTIVATTVFDYEKKRFYSFE
VQVIDTNNGKTNKALVVVRITDENDNKPFEVSGNLHFNITEHTLLGSTIGSLNGKDNDAGINKEF
EFSILEGNDDDKFMLLPTGQILLKNFVSVKEKDFYKIEVKITDKGTPSLFNIFTVRVTINSSQALVAS
NNPTIPEDKNTGEEITRVTAIDPNTGSSNGFIYSLVGTLSNSQFTIDAKTGAISLLASVDFETDRTHT
AVVKAEGPTSTGVTEVIVRVTDINDNKPIFDPTNFNLNINENFVVGQEIVLVRATDKDSSLNDNNV
IIYRIDSVNPSSGNDLFSVNPSTGRILLLKALDRENGDTSYVLTIAASNRGVSNKPDNTMTVTINIA
DVNDNFPIFEKSLYQGSVSEDANTGTSILTVRASDKDILLKNRNIRYSMTQPMAVQKFQIDPISGVV
SLKQEVDFETKQRYDFSVIASDTGDVAYSRTANVEITILDVPDVEPKFDPQRYEITIAESTLVSTSILK
LQVIDSVKNLNFNIKQGNSNSDFRLVASSGELFVNKQLDRERISRYQMTIEVSDSVNKAAIDAIVIV
NIADVNDNQPLFLQSGCYQANRAEGIETEQVVVNVQATDRDEGSNKEIVYSFSDACSVLDIDAN
TGAITTKQKFDYESARSHICTIKAEDKGTPKLSGFSCIILTVTDSNDLKPTFEVITSNARVLENIPVGS
VITRVLATDKDTLSSNKQFVYTIKTGNEDGRFTIDANGFIKLQQPLDYEKKTDYRLEIEAVDSTNQ
DMVSDPNAIVTVTVIEQNDNRPKFAKSYHRVNIEESTAIGTTVLKLSATDADAGVNEGLIRYVSLH
NTTVFNIDRTTGEITLATKLDYEVKKSYHLIAVVKDQGNPVLSARTLIDIFVNDVNDNPPVFVISSY
NNIVSEKAPIGQSIVTVSARDDDTGVNGQLKYKIRETQSMFRIDEDTGIIILNGDLNVENTNKLLYA
LTIDVVDSGVPPLQASNPATVSVQVSDVNDKQPVFALDRYQFEINENIEGNQIFGDSVTVRANDD
DLTGTNNQLTYRLVNTTMFDLVVTNQNVFIKTALGVSFDYETKKSYSFEIEAMDSAANKLYGRSLV
TVTVKDFNDNPPIITVPNIIVNISDATLIGSHIATIMAEDKDSGENGILIYTVTAGDSTNRFTMESDT
GSIILKNPVDSYTKDIYVLNVLVQDNGIPRRSATGTVTINVIPVDLTLLKFNTTCPFAISIPENQINSN
FLGLKALDAGEYVSKQLRYTINSDENDVNQIFALDSIKGEFKQLKEANYEERSRYDMTICATNERG
SKVCCHITIFITDINEPPSFIGLNNNNIYEVSISETTPIGTIIYRVLSKDQDLPGVLNGQTSYSLGQQNV
FIIDQMGQIRVNKTLDAETQLNYVFQVIVTDKNPTNPLQSAATIRVNILDYNDERPIIQNIPDITIDE
NSDNQVVRNIVVTDRDISNIGSNIELSIEPSNILTINNRQLRTVGSALDYEKVQRHTLYLTAKDKSFP
YLSSTTSFTVTINNLNDNKPKFPVNNYNLTVSEKTTVGTLILTLFAEDDDLEEFGEIANYEIVSGNQ
EDRFSIDSNGRLTLKKQLNWRNVKSYNLNIIARDKGLPSEQSQIPATVVINVADYSEEQPNFLQNL
YVKFIPETTPIDTLVLTVEATSGEPVYGPFKYTIVPKLPDTNNFQIFSNGEIKTINQFDYENQRNFTFD
VQVEDEKNGKTNRASVFIHITDVNDNIPQFTDLTRSNTQYNITEFTLPGSMITCLSATDRDHGKNA
EIRFRGIGGDGNGVFNVNLDGKVLLSQQIFYDKKNKYNLIVEAFDLGSPEQKAQLTLTIFLHKSYT
SVITTDVDINEDVETQTRVTRVSSIDPFTRSSNNFTYYLVGKSTTFFEINAQTGEIFTKATLDRETRDR
HVLTIQSRGPTTNGVGELVVNVKDINDNPPIFDPQVYQVTVSESANIGLSIVSVTATDKDIGVNGE
VTYSIEDGDQTIFSINPSTGSISLAGKLDFESKQSHTLTIKAVDRGNPPQDSVNKATVIVVVQDFND
NCPVFENSTYSLTIKEPGELGKEYLKLGITDNDNGRNQLFTCSIDEPLAEKYFEMINDCRLRLIKEL
DYEEFTSFRFVVKATDQGDVSCTTTTNMIVNVGDRADVIPFFSPLTYNISIPESTTVGTIVVSLKVGS
TFKGLLYSLFHKNTQDDLIKFGINKDTGEIFLKESVDREIQDKYEFTADVFDGSNNAVQRASVTINII
DVNDNAPFFNQPTCYQRDLTESTAIGTFVVDVSAADNDKGRNQQVTYSLLPSPSCTSFSVDETSG
RITTSQILDHEQQKLQHCLIKAKDNGTNSLSGYSCLTVNVVNVNDNKPAFIVNRFDVSVLETVPA
GTLVASLVARDVDNQGLITYSISNGNLNNQFTIDSSGKLRTRFPLDYENTKSYNLTIQASDNNGKFS
SPDDALVLITVLEVNDNRPQFLQSYYKVSIPEDTATGASILNVTAIDLDHGELTKSLFYAALENTTQ
FTVNANTGVISVAENLDYEIEDHYELVVIVQDRGTPHLSNRALVDIQITDVNDNAPMFVTTVYNR
ELSEKALIGAPVVKLNAMDADKSDKMTFTIDSVVQGAQGFFSINGDTIQLDKELDYEVPEQRTFV
LSVSVKDSRDRDGANKANVFINVRDINDREPIFDLNMYEVTVSENNVISNPIIEVTARDSDSTPEN
NNLLYSLVDGEDAASFSLLNNGQSAIITSKISFDYEKKKSYRFLVRASDKVRENFTAFSTVFVTVQD
TNDEAPVPEKDLIMVNISESTRVLSTIAIVFANDKDQGINGQLAFQIINHSEDKFEINNKGVIRLKST
LDFFQQNFYNFTVLVTDKGNPVRTSKPVTVLVYVYRTEIPGIIFEKNHYIFNINEKEIVSLVGTVNAI
DEGTYVSRQIVYSIVSGQNDIKDKFRAVNNNIIQDKEVNYKDQSEYNFVMKAINERGSIAFTHITIN
IMDVNEKPYFVGLNNKGRYEFSMSEIISVPSYIYAVRAKDDDFGLNGEVDYEIISNFRFGNFSIDQY
GRIQVLKDPDFEFTRSYEFQVRAKDRGTPSLESNLVSVVITVLDYNDNHPIFNIQEPLEISIKELVNN

TDVGTVTATDKDGSGLNRIVKYVAIYPSDHLTVNRDNGQIRVIEPFDYEIAHKHEFVVVAQDQGT
PYLNAYKVIKVEVLNENDEFPEFTVIEYNITISELTPIGSPVLFVHAQDKDRGPSGQVTNYKIKYVPP
SGGSGAGSPDKTFEITSDGQVVLKEKLEYKFQKYYQYRVTCTDNGSPPKNSINEAIIHLNIADASLE
QPIFSVNPAVVEVYENITLNSNIVKVEVDSIEPIKGPFEYSIIASEDSQPFMIDNSTGQITNKESFDYEI
KRKYVFLAEARDKHNNRTQRAMVVIFILDINDNIPTIETIAPQTFVQNISDTTYPGSVVAKLYSRDA
DSGENAMVDFTITGGDGENVFAINPNGYVTLIGPLDVRVKTQYTINVKVSDRGIPTLSNNRALTF
NIKKSKFIQNTVRFDRHHYKSNCIEENQINLNIIQLNATNSNPVAGSTIIYSLDGTLEERQPFIVNSQ
TGEISLSQTLDYEIKQEYDFVAVAVNNDGSRDVAVVTVCIKDLNDNSPIFEPQSYVVNVSESESVGY
LVYKTTVMDADKTDFNTTKSYDIIQGNVNDKFIIDSMGHIYIKSKIDYEALQVKHFDLRIRVNEGN
LVGDMVLRVNILDWNDVHPSFSTLNIYNFTIPEDKPVGEIIEILTANDGDATSPNNEVTYKLLTTED
DFILRRNDKGHGSLIVNKQLDYSKQNRYEYVALAEDKGYLNLLGTAIVIINIQDVNNHPPEFDDSE
YNTTVSEHAQIGTRVLQIHAHDKDTGLGEQIFFIYGDPTETFTISSQGFIILNKQLDYETQRDYIFDV
GARDGGNLVSLNNVTVHIKVSEVNDNEPIFDLSTYRNSYAEDTPLGTTLFTVRATDKDFIGNSIIYSI
SGANDDTSYVSVDQTTGDITLQRRMDYESKNYITFTVQAIDSNNRNKLKGSTHVIIDVTDVNDN
NPKFVFGSDLVYNVTVVENINVGTLVVVVDSQDADSGLNGQTRYHIVSGNNENKFFINELNGQI
NLINKLDALQTNKYVLTVMAKDLGNPSRSTNVTVTINVLRYFESTPMFLPSYAEVIYPENLALGSSI
YELNVTKTLPGSPMKLSFHSSSKFPEFTLQGNKIILSKEFDYEIKNRYELIIIATDEENDKAFAQVIVR
VQNLNDNIPYFTQNAYFGVVSELAFVGHSVLTITAKDDDKDFTELFYSIATQVPDNAFKLQDNIV
VVNGKLIRNKVYEFEVEAFDNTFTSKRATVSITVTPENLPSFVNATYIVTLQENYPISTNFLNISAGT
FSGITYVITSMKAKDMFNVDANGQISLTKSLDYEKQRQHVFPVTASVTSPITQVRVASVIVNVLNE
NDNAPVFVNGPYERNITEGHLVGTMVIRVDGHDDDSDKLTYSIVSGDDGKFSINSEGVISLISTLNF
DLKNKYVLNVRVSDGKFVTDTIVTINITPIVKFPPMFNQTLYLLSVKENTLVGTSILQVSTVNVIPPS
SFSINEQSGRDFFTIDANGVIRTNNALDYEAKRQHIFTLKVQDLRSMGHATVVVNIVNDDDTCPIL
TPATQTVQITEPVAPGTIVAAVQAKDIDSKSLNFTITGGNGLFRIDQHGGIKTINEVDISTSTEYELTV
TVDDGTCTKSVQVDVNVNPLAPCPDCNIYPFTQTIYEFSINENTVPASPIITVTTGQSLQRNFSIEDP
AALEYVTINRLTGEISVIKAFDYEATKHLSFNVMAKTQTTKNRNPFYVITSVSRVRITVRDLNDNCP
SIESPMYYEVSHSSPIVQDTVVTRILVEDPDTLKDHTYSISDNNFKIDSMGHIRTVQNIESFTAQFYN
LTVTVSDGQCSKTSNVGVNLAVNPTPMTYQFTSNGVYSKSVREDHNLQDVILSVALNQNYPRVFS
IVESDANDQFVIDSNSGALRLKTSLNYELKKRYDFTVMADIHGGVYKSYASVHIIVLDVNDECPIF
PVIGYNQRVSGTPVIGTIVEIIEANDADNATLEYSIVSGNEDGHFSLDNSGVVRVVKPFASSFSKTYG
LSVRATDNKCSADVPLTVQVSTCAYGEDYQFEESEYVVYYLDENRNLGIVSNIKLKSTRAAFLTLSQ
NTHFTIDQNGDFRLIQTLNYETKKEYHLTLTANFTQDNVVTRIPIKVFVRDINDNCPAFSEELYAVQ
HTEPLREGLLIAKLSVIDDDTVQNFTYNIVSGDKRFSINENGEVVMNARLVTDTTLLSTTYKLEVSV
NDGKCAKNTKVDVFVYKTVVNSFLFPQPFYKFDISENQAIPFTIDRFVNVGGHEAVYGFLVSTDFF
QIDPKTAQLQLVKKLDFEVEKTHYVLISATYDDGTQTYTVVAIHVGDENDNAPQIFAHPPITRLVQ
NTPHNSIVTKIEVEDPDTSKEFTFKITNGNPLQNNDPDKALFRIESNGMIKTTRCISEDEIGNYVLTI
SVDDQVNTPTIFSNMQVQLKTTDDRSYVTNCGLGCPANDGPFSRYQFEQSLFIVNVTETAANPTV
LKRLTRPFVEKTTYTIGAGADGKFSIDQNGTLSLTGSLDYETKPVYYFIVSAVIPSQVVGRFITSKTAY
LVMVNDVDDSKPVIEGNGGTLAVSETSEIGQIVGTVRVKDSDSSSPLTYNVVNGSQYFNIDSAGNI
VIKDTLNAAGSPYTVTYTVSDGINPPVNGEFVVSISETNNNVPKFTQNIYEVSLSEDTATDTIAVTIL
ANDGDTNDKIAYSFLQSEGPFVINRDSGEIKLIGPLDYEAQKSYNMVVQATDSGGFANIAFVQIAV
GDVNDQVPVITPQTTPTQIHESTLVNSVVYRVIASDGDGNERLTYALANSNDFFTINSATGEITIKE
NLAKSNVSSHTLSIVVTDSSGKTSTQDVALNIRRSGTSQGCVKITTSQGQSSSPVINLPENTASGTTV
FKLVAGDASVFSNMRFTLNREANGVFAVTEQGELQLVAPLDYETKQFYSIIITAEDVTRGVSSQYNL
FVRISDVNEFTPVLETIAPVAVSELANIGDVIAQAKANDQDGDSQVTYSLGEFGNNDNKFTIDAAT
GNIILNKRLGSGAPHVVEVCASDGSIKKCQNVTFIVADTNDNPPSFQDLTGVGSIDLTNTSSYVVY
KPIVTDSDQTGTLSFSLKDPAPSQFSINSTTGEVKANKDIAKGTYTFGVCASDGRYTSCQSVTVVAS
DRSQSITPSFNQDQVGAIVDISASSGKQIIQVMASTLDKAKIEYELKNEADRNIFEINKDSGLITLKN
RTALTLGTIYTIPVKASRADDPLNFDEINIVISVGNQQLDSGSFIEQVLDTQHLVNFSLSTFDISKVSY
YIILGQQVGGDSKDDEKLPVSWYYATFGDESYRHRFYYIAKLDGSKFRNTGGSRKRRSIQDKDSKS
FTYLVGEENGCSKNTSMAAICNGPLKTGTEYRYKLRAYYDDGELRGIPVDQKPTEVKRTQAPQA
GKSIDEYREQDSYVITFWIIGLVIFGLLFWFSVLVIAILLCYKRRYEVTQAYGYSVQTRDKIDGPPTEY

SNTVYRFQNPSYPDNDTVKMKKVDASESRTKTEVQTTSMVVLDDYEQEQPQPDIPVQDYDAIEY
ETRSAQIKFHEESPEWESMDIQLRIDPTGNQDPVITRKSDSGSKDSDSDASKKYENTEYIEEKEDFV
NEKEDSLTRL*
>TRINITY_DN161_c0_g1_i2.p1 type:5prime_partial len:399 gc:universal
TRINITY_DN161_c0_g1_i2:1683-487(-)
WNQEPLTCTYLYSVNSQLLKYKMADDDVAALVVDNGSGMCKAGFAGDDAPRAVFPSIVGRPRH
QGVMVGMGQKDSYVGDEAQSKRGILTLKYPIEHGIVTNWDDMEKIWHHTFYNELRVAPEEHPV
LLTEAPLNPKANREKMTQIMFETFNSPAMYVAIQAVLSLYASGRTTGIVLDSGDGVSHTVPIYEGY
ALPHAIIRLDLAGRDLTDYLMKILTERGYSFTTTAEREIVRDIKEKLCYVALDFEQEMQTAASSSSLE
KSYELPDGQVITIGNERFRCPETLFQPSFIGMESAGVHETTYNSIMKCDVDIRKDLYANTVLSGGTT
MFPGIADRMQKEISALAPPTMKIKIIAPPERKYSVWIGGSILASLSTFQQMWISKQEYDESGPSIVHR
KCF*
>TRINITY_DN982_c0_g1_i1.p1 type:5prime_partial len:6412 gc:universal
TRINITY_DN982_c0_g1_i1:19377-142(-)
KLDFETKQSHTLTIKAVDRGNPSQDSLNKATVIVVVQDFNDNCPLFSESIYNASVSESTAIGTTVLR
VSVTDKDVGNVNNEITCSIDEPLAKGFFSVNKINNNQFSVVLIKNLDYETHRGYRFVIKAKDNGE
VPCQLTTNMVVEVQDEPDVITNFKPFNYVISIPESTLIGTKLLELKVDSESSSITYTLQAKNQPGDLD
KFMLNGQSGELVLKGTVDFEKKSEYEFTATLNDGVNNGGNTANIVVNILDINDNPPVFSQSTCYI
RRMPENQQVNTTVTSVLATDSDQGINSNISYSLIKDSSCNNFEINEITGAISLTSSLNYEVRKEHYCII
KATDNGSPVLSSYSCLKVIVENVNDNSPVFLPKELEITVLESKPVNSFVIRVFATDKDEGNSITYSIV
GGNTQNAFAITPLTGHIILNQKLDYEIVKNYILEIQATDDGTPRRSSNLNAKVMVKVLEVNDNKP
KFNQQRYLRTIPEDTNIGSTIISVTATDDDAGLLTSKIQYIALENTNDFLIQPDTGDIVVSQNLDYER
QRTYQLVAVCSDSGTPPFTGRTLVDITITDVNDNAPILNPTLYNVLVSEKAPLGTSLIQVHAKDKDS
GINGGLKYQIVSGNDLNKFVINNSTGVITLQETLDREKYSSLYNLTVSVSDQATPSLNGNNAFVQI
RVLDVNDKQPVFVKSEYIFEISENQPLNSPIGLVQANDDDSTSPNNIIQYTIVPSKDSINFTMINNQI
LSNSVFDYEEQKSYEFEVQARDQSTGVNNEQLVGRTLVKVLIRDTNDNDPMIENQNIALNISDVT
LVGTLLFDIQATDKDNGENARLTYTISSGDTSTFRINENDGRFYLSKKLDFYNKNEYSLIVIVNDNG
SPQRNSVANVQINVYPIDQTLISFEPQCPIAFTIQENSLSSPMNIKAIDTGSYVSKTMRYMLKSNKSD
VSSMFQIVETNGVIIQNKAANYEERSRYDMTVCARNERGSEGCCPVTIYILDQNEQPFFDTDNDN
LDNGKYLVRVSETTPIGSVMFSVNTGDEDFGSLPNGQKTYSIKAKPGEEMLFLVDSYGRIITNTTLD
REKTPQYDFSVVVTDKGTPPRQAEAEVIVTVIDYNDQRPVFEIMSVRPVNENANNEVLGRVVAVD
IDSTSINNGIKYSIQPAGFLNISNTGTLTSVGSALDYEKNQKVTLYVTATDTGSPYLSATTSLQLDIINI
NDNKPYFLQNNYNLTVSEKTTVGTLVLTLFAEDDDLGEFGEIANYEIVSGNQEDRFSIDSNGRLTL
KNPLNWRNVKSYNLNIIARDKGLPSEQSQIPATVVINVADYSEEQPNFLQNLYVKFIPETTPIDTLV
LTVEATSGEPVYGPFKYTIVPKLPDTNNFQIFSNGEIKTINQFDYENQRNFTFDVQVEDEKNGKTN
RASVFIHITDVNDNIPQFTDLTRSNTQYNITEFTLPGSMITCLSATDRDHGKNAEIRFRGIGGDGNG
VFNVNLDGKVLLSQQIFYDKKNKYNLIVEAFDLGSPEQKAQLTLTIFLHKSYTSVITTDVDINEDVE
TQTRVTRVSSIDPFTRSSNNFTYYLVGKSTTFFEINAQTGEIFTKATLDRETRDRHVLTIQSRGPTTN
GVGELVVNVKDINDNPPIFDPQVYQVTVSESANIGLSIVSVTATDKDIGVNGEVTYSIEDGDQTIFSI
NPSTGSISLAGKLDFESKQSHTLTIKAVDRGNPPQDSVNKATVIVVVQDFNDNCPVFENSTYSLTIK
EPGELGKEYLKLGITDNDNGRNQLFTCSIDEPLAEKYFEMINDCRLRLIKELDYEEFTSFRFVVKAT
DQGDVSCTTTTNMIVNVGDRADVIPFFSPLTYNISIPESTTVGTIVVSLKVGSTFKGLLYSLFHKNTQ
DDLIKFGINKDTGEIFLKESVDREIQDKYEFTADVFDGSNNAVQRASVTINIIDVNDNAPFFNQPTC
YQRDLTESTAIGTFVVDVSAADNDKGRNQQVTYSLLPSPSCTSFSVDETSGRITTSQILDHEQQKLQ
HCLIKAKDNGTNSLSGYSCLTVNVVNVNDNKPAFIVNRFDVSVLETVPAGTLVASLVARDVDNQ
GLITYSISNGNLNNQFTIDSSGKLRTRFPLDYENTKSYNLTIQASDNNGKFSSPDDALVLITVLEVN
DNRPQFLQSYYKVSIPEDTATGASILNVTAIDLDHGELTKSLFYAALENTTQFTVNANTGVISVAEN
LDYEIEDHYELVVIVQDRGTPHLSNRALVDIQITDVNDNAPMFVTTVYNRELSEKALIGAPVVKL
NAMDADKSDKMTFTIDSVVQGAQGFFSINGDTIQLDKELDYEVPEQRTFVLSVSVKDSRDRDGA
NKANVFINVRDINDREPIFDLNMYEVTVSENNVISNPIIEVTARDSDSTPENNNLLYSLVDGEDAA
SFSLLNNGQSAIITSKISFDYEKKKSYRFLVRASDKVRENFTAFSTVFVTVQDTNDEAPVPEKDLIM
VNISESTRVLSTIAIVFANDKDQGINGQLAFQIINHSEDKFEINNKGVIRLKSTLDFFQQNFYNFTVL

VTDKGNPVRTSKPVTVLVYVYRTEIPGIIFEKNHYIFNINEKEIVSLVGTVNAIDEGTYVSRQIVYSIV
SGQNDIKDKFRAVNNNIIQDKEVNYKDQSEYNFVMKAINERGSIAFTHITINIMDVNEKPYFVGL
NNKGRYEFSMSEIISVPSYIYAVRAKDDDFGLNGEVDYEIISNFRFGNFSIDQYGRIQVLKDPDFEFT
RSYEFQVRAKDRGTPSLESNLVSVVITVLDYNDNHPIFNIQEPLEISIKELVNNTDVGTVTATDKDG
SGLNRIVKYVAIYPSDHLTVNRDNGQIRVIEPFDYEIAHKHEFVVVAQDQGTPYLNAYKVIKVEVL
NENDEFPEFTVIEYNITISELTPIGSPVLFVHAQDKDRGPSGQVTNYKIKYVPPSGGSGAGSPDKTFE
ITSDGQVVLKEKLEYKFQKYYQYRVTCTDNGSPPKNSINEAIIHLNIADASLEQPIFSVNPAVVEVY
ENITLNSNIVKVEVDSIEPIKGPFEYSIIASEDSQPFMIDNSTGQITNKESFDYEIKRKYVFLAEARDK
HNNRTQRAMVVIFILDINDNIPTIETIAPQTFVQNISDTTYPGSVVAKLYSRDADSGENAMVDFTIT
GGDGENVFAINPNGYVTLIGPLDVRVKTQYTINVKVSDRGIPTLSNNRALTFNIKKSKFIQNTVRF
DRHHYKSNCIEENQINLNIIQLNATNSNPVAGSTIIYSLDGTLEERQPFIVNSQTGEISLSQTLDYEIK
QEYDFVAVAVNNDGSRDVAVVTVCIKDLNDNSPIFEPQSYVVNVSESESVGYLVYKTTVMDADKT
DFNTTKSYDIIQGNVNDKFIIDSMGHIYIKSKIDYEALQVKHFDLRIRVNEGNLVGDMVLRVNILD
WNDVHPSFSTLNIYNFTIPEDKPVGEIIEILTANDGDATSPNNEVTYKLLTTEDDFILRRNDKGHGS
LIVNKQLDYSKQNRYEYVALAEDKGYLNLLGTAIVIINIQDVNNHPPEFDDSEYNTTVSEHAQIGT
RVLQIHAHDKDTGLGEQIFFIYGDPTETFTISSQGFIILNKQLDYETQRDYIFDVGARDGGNLVSLN
NVTVHIKVSEVNDNEPIFDLSTYRNSYAEDTPLGTTLFTVRATDKDFIGNSIIYSISGANDDTSYVSV
DQTTGDITLQRRMDYESKNYITFTVQAIDSNNRNKLKGSTHVIIDVTDVNDNNPKFVFGSDLVYN
VTVVENINVGTLVVVVDSQDADSGLNGQTRYHIVSGNNENKFFINELNGQINLINKLDALQTNK
YVLTVMAKDLGNPSRSTNVTVTINVLRYFESTPMFLPSYAEVIYPENLALGSSIYELNVTKTLPGSP
MKLSFHSSSKFPEFTLQGNKIILSKEFDYEIKNRYELIIIATDEENDKAFAQVIVRVQNLNDNIPYFTQ
NAYFGVVSELAFVGHSVLTITAKDDDKDFTELFYSIATQVPDNAFKLQDNIVVVNGKLIRNKVYEF
EVEAFDNTFTSKRATVSITVTPENLPSFVNATYIVTLQENYPISTNFLNISAGTFSGITYVITSMKAKD
MFNVDANGQISLTKSLDYEKQRQHVFPVTASVTSPITQVRVASVIVNVLNENDNAPVFVNGPYER
NITEGHLVGTMVIRVDGHDDDSDKLTYSIVSGDDGKFSINSEGVISLISTLNFDLKNKYVLNVRVSD
GKFVTDTIVTINITPIVKFPPMFNQTLYLLSVKENTLVGTSILQVSTVNVIPPSSFSINEQSGRDFFTID
ANGVIRTNNALDYEAKRQHIFTLKVQDLRSMGHATVVVNIVNDDDTCPILTPATQTVQITEPVAP
GTIVAAVQAKDIDSKSLNFTITGGNGLFRIDQHGGIKTINEVDISTSTEYELTVTVDDGTCTKSVQV
DVNVNPLAPCPDCNIYPFTQTIYEFSINENTVPASPIITVTTGQSLQRNFSIEDPAALEYVTINRLTGE
ISVIKAFDYEATKHLSFNVMAKTQTTKNRNPFYVITSVSRVRITVRDLNDNCPSIESPMYYEVSHSS
PIVQDTVVTRILVEDPDTLKDHTYSISDNNFKIDSMGHIRTVQNIESFTAQFYNLTVTVSDGQCSKT
SNVGVNLAVNPTPMTYQFTSNGVYSKSVREDHNLQDVILSVALNQNYPRVFSIVESDANDQFVID
SNSGALRLKTSLNYELKKRYDFTVMADIHGGVYKSYASVHIIVLDVNDECPIFPVIGYNQRVSGTP
VIGTIVEIIEANDADNATLEYSIVSGNEDGHFSLDNSGVVRVVKPFASSFSKTYGLSVRATDNKCSA
DVPLTVQVSTCAYGEDYQFEESEYVYYLDENRNLGIVSNIKLKSTRAAFLTLSQNTHFTIDQNGDF
RLIQTLNYETKKEYHLTLTANFTQDNVVTRIPIKVFVRDINDNCPAFSEELYAVQHTEPLREGLLIA
KLSVIDDDTVQNFTYNIVSGDKRFSINENGEVVMNARLVTDTTLLSTTYKLEVSVNDGKCAKNTK
VDVFVYKTVVNSFLFPQPFYKFDISENQAIPFTIDRFVNVGGHEAVYGFLVSTDFFQIDPKTAQLQL
VKKLDFEVEKTHYVLISATYDDGTQTYTVVAIHVGDENDNAPQIFAHPPITRLVQNTPHNSIVTKI
EVEDPDTSKEFTFKITNGNPLQNNDPDKALFRIESNGMIKTTRCISEDEIGNYVLTISVDDQVNTPTI
FSNMQVQLKTTDDRSYVTNCGLGCPANDGPFSRYQFEQSLFIVNVTETAANPTVLKRLTRPFVEK
TTYTIGAGADGKFSIDQNGTLSLTGSLDYETKPVYYFIVSAVIPSQVVGRFITSKTAYLVMVNDVDD
SKPVIEGNGGTLAVSETSEIGQIVGTVRVKDSDSSSPLTYNVVNGSQYFNIDSAGNIVIKDTLNAAG
SPYTVTYTVSDGINPPVNGEFVVSISETNNNVPKFTQNIYEVSLSEDTATDTIAVTILANDGDTNDKI
AYSFLQSEGPFVINRDSGEIKLIGPLDYEAQKSYNMVVQATDSGGFANIAFVQIAVGDVNDQVPVI
TPQTTPTQIHESTLVNSVVYRVIASDGDGNERLTYALANSNDFFTINSATGEITIKENLAKSNVSSHT
LSIVVTDSSGKTSTQDVALNIRRSGTSQGCVKITTSQGQSSSPVINLPENTASGTTVFKLVAGDASVF
SNMRFTLNREANGVFAVTEQGELQLVAPLDYETKQFYSIIITAEDVTRGVSSQYNLFVRISDVNEFT
PVLETIAPVAVSELANIGDVIAQAKANDQDGDSQVTYSLGEFGNNDNKFTIDAATGNIILNKRLG
SGAPHVVEVCASDGSIKKCQNVTFIVADTNDNPPSFQDLTGVGSIDLTNTSSYVVVYKPIVTDSDQT
GTLSFSLKDPAPSQFSINSTTGEVKANKDIAKGTYTFGVCASDGRYTSCQSVTVVASDRSQSITPSFN
QDQVGAIVDISASSGKQIIQVMASTLDKAKIEYELKNEADRNIFEINKDSGLITLKNRTALTLGTIYT

IPVKASRADDPLNFDEINIVISVGNQQLDSGSFIEQVLDTQHLVNFSLSTFDISKVSYYIILGQQVGG
DSKDDEKLPVSWYYATFGDESYRHRFYYIAKLDGSKFRNTGGSRKRRSIQDKDSKSFTYLVGEENG
CSKNTSMAAICNGPLKTGTEYRYKLRAYYDDGELRGIPVDQKPTEVKRTQAPQAGKSIDEYREQD
SYVITFWIIGLVIFGLLFWFSVLVIAILLCYKRRYEVTQAYGYSVQTRDKIDGPPTEYSNTVYRFQNPS
YPDNDTVKMKKVDASESRTKTEVQTTSMVVLDDYEQEQPQPDIPVQDYDAIEYETRSAQIKFHEE
SPEWESMDIQLRIDPTGNQDPVITRKSDSGSKDSDSDASKKYENTEYIEEKEDFVNEKEDSLTRL*
>TRINITY_DN1280_c0_g1_i1.p1 type:complete len:426 gc:universal TRINITY_DN1280_c0_g1_i1:195-1472(+)
MGFETCGPNEAMVISGCGQERPLLISGGRKFVWEFFQKVQRISLNVMTLNVESQRVYTVQGVPIS
VTGIAQVKVQGQNEEMLHAACQQFLGKHENEIRNIALETLEGHQRAIMGTMTVEEIYQDRKKFS
KSVFEVASSDLVNMGISVISYTLKDIRDDEGYLKALGQRRIAEVQCEARIGQAMAKMQSGIKEAQ
AEEVRIKAEYENKTEVARSQRDFQLNKSAYDIEVNTKKATSELAFALQSAITNQKIKEEDMQIKVIE
RAQAINVQLQEIQRREKELEAQIRKPAEAERYKIEKIAEANRMKVVLEAEAEAESIRMKAEAEAE
VIQKKATAEAEQMSKKADAWRDYQEAAMVDMLLDTLPKVAAEIAAPLSKVKKVTMISSGSSDV
GAAKITGELLDIVIRMPQAMEQLTGVDITKTFRREK*
>TRINITY_DN329_c0_g1_i2.p1 type:5prime_partial len:320 gc:universal TRINITY_DN329_c0_g1_i2:1-960(+)
FNNSTHSPSLPTHVYLYCCRIKILILSFDPSTIEILKMAPPKFDDLGKEAKDLINKSFHFGVIKLEGKT
KAKNGVEFTTEGTHNTDTGTVNGSLETKFKYADYGLTFSEKCNTDNVISTKITIDNKLAKGLKVD
FDTTFAPVTGKKTAKVKSAYQHNNIHSTCDVDFDFAGPTIHGSAVFGYNGWHAGYQASYDTANS
KLIANNASLTYKEGDLVIHSGITDAVKYSGSVHHKVNSNLTAAALIQWASGTNNTSLTVAGKYAV
DNETFVKAKIDNNLRLGLSYAQKLRPGVELTMSSLVNAKSLEQGGHKLGLSLNFEA*
>TRINITY_DN428_c0_g1_i1.p1 type:complete len:1949 gc:universal TRINITY_DN428_c0_g1_i1:96-5942(+)
MADDADLKFLMVDRRQVSDPVAHAEWAAKKLVWVPSEELGFVAASVKEEKGDKIIAQIEGGKR
VTFHKDDIQRMNPPKFDKAEDMADLTCLNEASVLHNIKDRYFSDLIYTYSGLFCVVVNPYKRLPI
YTDKVINLYKGKKRHEVPPHVFSIADTAFRSMLQDRDNQSILCTGESGAGKTENTKKVIQYLTAIA
GRANQQKADEKSQGKLEVQLLQANPILEAFGNAKTVKNDNSSRFGKFIRINFDNSGFICGANIES
YLLEKARLVRQAPDERIFHIFYQLLLGASPEVKKQFLLLDTKSYNFLSNGHVQLPNMDDRQEFKV
TLEGMRDMGITQEELDPIFKVVSAVMLFGNMEFKQERKSDQATLPDNTIAQKICHLLGIQVTDFT
KSLLKPKVKVGREFTQKSQTKAQCEFAIEAITKALYERMFKWLVGRINKSLNRGKRDGASFIGILDI
AGFEIFQLNSFEQLCINYTNEKLQQLFNHTMFILEQEEYQKEGIDWKFIDFGLDLQPTIDLIDKPMG
VLSLLDEECWFPKATDKSYVEKLHKQQTKHPKYLKPDFRSNSDFCVIHYAGRVDYSAAQWLTKN
MDPLNDNVVSLLAHSKEAFIASIWKDTENVVGMSVTEGMDTTFGASRTRKGMFRTVSQLYKEQL
SGLMTTLHCTKPNFVRCIIPNYEKRAGKIDNHLVLDQLRCNGVLEGIRICRQGFPNRILFQEFRQR
YEILCPGIVPKGFMDGRKAAKQMIDNLEMDPNLYRIGQSKIFFRAGVLAHLEEERDIKLTEIIIQFQ
AFCRGNIARRNYRKRIQQLSAIRVIQRNGRSWLKLRNWQWWRLFTKVKPLLNVSRQEDELRSKE
EEFKKLQDKSDKLESLQKEQQQANEKLTEEKNTLAEQLQAEIELCQEAEEARNRLLQRKTELEDL
IQDFEMKLGEEEEKAVKYAEDKKKLQQGIQDLEESLEEETQRQKLQLEKVQVEAKLKEMEDERQ
VAEDNNARLTRDKKQLEDRVAEFGQKLAQEEDRAKQLSKAKAKQEAFIAELEDQIAKGDKSTHE
LHKEKRKAEQDLSDLRAQLAQALQRIAELEDDLARREKELAEAIKRGDEEMAKRTTVEREKREL
QALLDELREDMESEKSAKDKVERQKRDLEQDLQELRSELEVTTDATVVQKEFQKKREEELMDLK
KQIEEEAKEHDQQMASLRTKHNGLVEDLNNQVDTFKRGKAALEKNKTALENENAEIAADLQRI
SQLKQEGDRKAKNLEQQLNEANSVRAVQDEKVSSLESEKAKYQKECDSLSTQIEEVETKAASLDR
AKQAAEAQLEDTQDQLAEETRQKLSYQTKLREASDEANRLQEQLEEEEDEKKAVQKVLSNLQV
QLDQAKKEIELKIQDFEQAEAARQKQKRENEELRGEVEKLTGDVSRLDKAKKKLQGDLDDVTVS
MERERNSANQMSSKQRKFDQLLAEEKANSQALGIERDNLEKLARQNETKALSLQNANEELEDR
LAEAERQRKQLQMELGDLVESKDDVGKSVHDLEKSKRLLEQQLAEQKTQVEEIEDELQACEDAK
LRLEVNMQAQKAQFDRELQAKEDAIEEGRRGLVKQLRELEAELEDERKLRQSASAAKRQLEGDV
KELEGQLDQANRIKEDGLKQLKKYQNQLKDVQRDLEDSRQSKDDLAAQVKDNEKKLKGLEAEF
LQMQEDLSAAERARRAIESERDELLEELNNNSALKNAVAEERKRYEAQLAALEDELEEERTQTEL
MQDKVKRSNLQLEQMNAELQEERSAAQSLDNTRMSLERQNKELQSKLADLESSLRSRQKNTVAT

LEAKINNLEIQLDQEAKERQQQNKALRRNEKRCKDLQMQIEEERSHTESYKQQLEKMNARVKN
MKRQLDESEEEVTRLNTAKRKLQRDFDENQESLESSQREANQLRQRLKGGASLSSSRSSGRGRRA
KDEDDDDDDANE*
>TRINITY_DN3034_c0_g1_i1.p1 type:complete len:1941 gc:universal TRINITY_DN3034_c0_g1_i1:6289-467(-)
MAEEEDPMKYLQVDPAQALKDQSKTFDAKKWVWVPVIGEDGYAAAQVIKVDGDNVTVETNK
GHEMEVKKEKCDQMNPPKYEKCSDMASLTYLNEASVLYNLKARYTTGLIYTYSGLFCVAVNPYR
RLPIYSDQVVKLYRGKRRAEMPPHIFAIVDTAYQDMLTDHDNQSMLITGESGAGKTENTKKVIQYI
AKVAGVEKKKSDDGPVKRDLDEQVVEANPLLEAFGNAKTTRNNNSSRFGKFIRCHFSQTGKLAG
ADIESYLLEKNRVIHQASAERNYHIFYQLLYATTDEELQKLCLVTRRSEEFEFLKKGVPSVDAIDDN
EEFGYTTNAIEVLGFDEEEKQSMFKICAAILNFGNMKFKQKPRDEQAEVADPADGERVAYLLGLPI
GEFHKSLTKPKVKVGTEFVNKGQNVSQVLYSVAALSKALYERMFWWIVSRVNKALDTKERRSYFI
GVLDIAGFEIFEYNSFEQLCINLTNEKLQQFFNHHMFVLEQEEYKKEGIHWEFIDFGMDLEETIDLI
EKPMGVFAILEEECIVPKASDQTYLQKLHKQHAGKNGSYEKPTPKQTKSGGGDFILHHYAGSVGY
SVANWLEKNKDPINAHTAELFSKATVGLVSGLFADYNPDVTGGRRKGSAFQTVSFRHKEQLKNL
MTTLMSTSPHFVRCIIPNEKKTPGLVEGTLILHQLRCNGVLEGIRICRKGFPSRMLFQDFRQRYQIL
AASAIPSGFIDGKVAAEKLIDSLQLDQSEYRVGTTKVFFRAGIIGELEDMRDERLAKIITQFQAYCK
GHLARIEYKKMCEKRIGLAVIQRNVRKFLFLRNWQWWKLYCKVQPLLSIARAEEEMKEKEEELK
KALEKAKEDEEQRKKMEEQLTENIAEKEKLFADLQAETDRLINLEDKLMQIQQQKDRLEANLNE
TLEKLDGEEHSVSVLQEKMHDAEDRIDSLEAEASELKSNCSRLEVERANRDKQIDALNEDINKQD
ESIAKLGKEKKGLEDNIQELNETLQASEDKCSSLTKAKKKVEDALKESENSLSKEKDSKARLDKEK
RRVEGELKETNDKLSEAENELKNTQDTVYKREKAIKELEEARDNQDNQIKQLQKKIQELLQRIEE
LEEELEAERKQKQKSELSRKELESQIEELQDQLETAGGATHAQVEVSKKREQECSRLRKEIEDINA
ANDEALASTKHKFNAQLSEAQEELESVKKAKSKSDKERSQLAGDLEDVQGQLDQVKKQRASSD
KNNRMLEEQLAELRGKVSSLETDMGDSESRNLKFQNESATLLKQLEEAEHKLGLANKQNKQLQ
ASLGESKNVADDEARGKHDMQNKLQNALAQIEELQEQLEDEQTAKMDLQNKYSRANADAQQ
WKNKYDMEGASRVEELEDAKRKLAARVQEMEEALAAAESKAASMEKVKNRMNEEVEDLLLDL
EKAQAQAANLEKKQKKVDQQINEWKLKCDEVQEELEKSQRDNRSYSTELLKMRTANEDTVEKY
ESLKKENRALAAELQSVSEQLSEGGKNSLEVEKLKRKLGLENEELEVALEEAELALEQEEAKFLKI
QLELTQLKQSTDRRSAEKDEELETLRKNHARQLEALQNTIDAEQKGKAEAQKLRKKLEGDLADL
ENRLEGSDRNSADYLKTIKKLQAQIKELQAMIDDESRGRDDMRDTASRSEKRAMDLAVQLDEAR
VALEQSDRLRKLAENEKLENAERVAELQTMYNSIANAKRKAEGDYHSLQEEIEELENEAKMAEE
KANRAMNECARLSSELNGAQENASNAEKSKSLMARQITELQQQLEEAESMGGKSLKNQIRKLE
QKVMEVEGELDNEARKSTESIKIARKADKRVKELQFQLEDEHKAVERAQEVSDKLNQKMKKMR
LQLEESEQQSSSWQSKFKKAVVDLEEAEERCESAEQALQKARQRARSGASGMTTRGVSRARSRVR
SPQNDD*
>TRINITY_DN262_c0_g1_i1.p1 type:complete len:1033 gc:universal TRINITY_DN262_c0_g1_i1:111-3209(+)
MADPSSLETKEKDGSYRVATKSGGKDKGKKSKKAAKKVNMDDLKKELEMTEHSCELETLVSQLE
TSLTQGLTKSIAARNLERDGPNALTPPKQTPEWVKFCKQLFGGFAMLLWVGAILCFFAYIIRSIREE
EPGMDELYLGIVLTVVVIITGCFSYYQESKSSKIMESFKKMVPQEAVVMRDGEKLNLNATECVVGD
LVFVKFGDRIPADLRIIESKGFKVDNSSLTGESEPQSRNSECSSDNPLETRNLAFFSTNAVEGTATGIV
VKTGDNTVMGRIANLASGLGSGKTPIALEIEHFIHIVTGVAVFLGVSFLIISLAMNYHWLESIIFLIGI
IVANVPEGLLATVTVCLTLTAKKMASKNCLVKHLEAVETLGSTSVICSDKTGTLTQNRMTVAHM
WFDNMVVEADTTEDQSGQAHDKGSTTWKALAKIGALCNRAEFRPNQEEEPILKRECNGDASET
AILKFVELSVGSVEDIRSKNKKIVEIPFNSTNKYQVSIHEQEDPDDPRYLLVMKGAPERILDRCSTIL
HEGVEKPLDEEWKENFNKAYVDLGGLGERVLGFCHYYLPADDFPKGFAFETEDPQNFPLEKLCF
VGLMSMIDPPRAAVPDAVSKCRHAGIKVIMVTGDHPITAKAIAKGVGIISEGNETVEDIAQRLNIP
KEEVNERDANAAVVHGSHLKDLPQEKLDDILKYHQEIVFARTSPQQKLIIVEGCQRMGAIVAVTG
DGVNDSPALKKADIGVAMGIAGSDVSKQAADMILLDDNFASIVTGVEEGRLIFDNLKKSIAYTLTS
NIPEISPFLMFILLAIPLPLGTVTILCIDLGTDLVPAISLAYEKAETDIMKRKPRDPVRDKLVNERLISL
AYGQIGMIQATAGFFTYFVIMAENGFLPGYLIGLRNDWDDKKNNAVMDSYGSEWTYNQRKNLE

LTCHTAFFTTIVVVQWADVIISKTRRLSLFQQGMTNWMLNFGIFFETALAAFLQYTPGLNTGLRLR
PMNFPWWFVGVPFSLLIFVYDELRRFLLRRNPGGWVEQETYY*
>TRINITY_DN1189_c0_g1_i1.p1 type:5prime_partial len:449 gc:universal
TRINITY_DN1189_c0_g1_i1:1554-208(-)
VSAKSQVIAKMKFFLFSIAAILLVNSALADNAVQIPKTCEDVLAMETCDRLRAVAKKFHEKVGTV
NQAVTDAFKHHKTKAVEVLTYVKEYLVDKAVNFKCEDVLPADQCHNLLEFAKRLNVKASEASIA
LREAIADGSEQAKQLYQRAVSFLVNDVPNLKCEDFFKKDDTCDKIRNFATKVNAKAKDVTAAVK
KAIVENLENAKDGLKKAKQFLKAKISCDQVLNEKMCNTIQSIGDKFSVSMKKIDAALREAYANG
VRKWSELYEAAIKYIMDKWSIFGDDEPQRERREIQPENVKKAIKNLLDSLKVKSKKVRAAIMKLIE
SGKVKFGNLRKTVKQLLASLKGDKKSEDAVTAVYKRSVVDTLKETLQKLKEQIKDLAGKAKEQA
KAKIAELKELIKGALGSLDQDQSDELDQDFEMISDVLEAAADESFDEIANNALENHLGQYY*
>TRINITY_DN36_c0_g1_i19.p1 type:complete len:223 gc:universal TRINITY_DN36_c0_g1_i19:826-158(-)
MIKLAIIVTVIAACVFESSAARCYVGYSAKGLPLAIGVRDVECGKGFDYCTTIKYRLGGIEVTQGNC
ARKYGQAMCQFMRNPHKAMTDCEETICQGDSCNKDVYDAEQAERKRVGPYKNTLKCTTGLIT
KFPAIPFYTADVPNAIDECFPGTTECVTVTYDYLFKFETQIATKDNVFKLPCVQKMCNRHNIDNC
NKWCDILSIHQSMENCVAKCCKGDNCNA*
>TRINITY_DN11435_c0_g1_i1.p1 type:complete len:430 gc:universal
TRINITY_DN11435_c0_g1_i1:1616-327(-)
MGNIYTVGPNEALVVSGGCVGQRVKRTIVGGWAFAWACVSDVQSISLEVMTLNPRCEKVETAKG
VAVTVTGVAQVKVIKEDDLLKTACEQFLGKDPHEIEDILLQTLEGHLRAILGTLSVEAIYKDRDQF
AQLVREVASPDVGRMGIEILSFTIKDIVDEVSYLDSLGKTQTAEVKKNADVGVAEANRDAGIREAE
CDTQRQDVRFKADTTIADAARAYEMEKAEFDKEVQTKNAEAKLAYELQANKEQQRIRNEEIQIE
VIERRKLIEVEEKEIQRREQELQATVKLPAEAESYKVQTIAEANKSAKVYLAQAEAEKIKMIGSAQ
AAAIEAIGKAEAERMRQKAAAYKQYGDAALMSLILEAMPKIAAEVAAPLRKTEEIVLINDDNGN
KMSSEVTKLLGQLPPSVKALTGVDLSKALGKIPGAQVTA*
>TRINITY_DN72625_c0_g1_i1.p1 type:complete len:1964 gc:universal
TRINITY_DN72625_c0_g1_i1:173-6064(+)
MKLAFNIFTISLLIGNTIAQNNDVVQVCKSQADVVAVLDSSGSVGVQDYEELKKFVGFVANLLTIN
ENGNRLGVVTYSETAEVDFNLNEIYDSDQYGREALTRLSVRPREFKTNHYAGLKVGSDLLFNKAA
GARDNAAKFLIFTTDGRNNFDETNNRNRASQLATQLKADGVNIYVIKAEVMSDNNGRLDSRLYS
TDPNKFYRFINRFGEITNENFLDEVGLEFCELVQLPDNQQATVREDRNIGTLVTTIRAPGLNNPTFE
LIPQDNLPFIVDPLTGEIKTIGLLDFEQVQQYVFQVRVRATNPDVEKIITVTINVLDVNDNDPIPETA
NYRMSVAETTPLGQRILTVKANDPDGGTNGNVEYFLISGGDGKFEVDQATGEIRLIGQLNKRIKD
TYLLTLEARDMGIPTRKAPITVAIDVTSLNNFPPQFNEMSFVRTIPENFGAGTVINAAATDRDGDT
MVYSLIGENIPFSIDQNGLITVSGNIDFETIPNNVFQFQVQAEANGDLVTQDVIITITDINDNVPVYS
MTTYSGSINENSAAGTVVNFDNVIIATDADKTNANNMITYNIIDGNDGDRFQVTKNERGEAVISL
KSTPLDQETQGQYRLTLQAIDNGVDPRSLEMNSLLIIDVIDQNDNDPIPEESVYSIAVAETSPVGQSI
LTVRANDADSGMNGKVEYFLISGGDGKFQVDQATGQITLIGMLNKRVKESYTLNIEARDMGVPT
RNAPITVNIDVTSLNSFLPEFAGAGDAFERTIPENFGSDTVIKAAATDGDGDMVFYSLVGDNLPFA
IDQNGMITISGSIDFETIPNNPIRFQVKAEANGDMTLKDVVITITDVNDVTPEFTQQVYAGKIEENS
QTETVVRFDQPVIATDGDKSPANNVVTYRIVDGNVDNIFKIVKNAQGEAVISVNNAGLDREILNQ
YTLTIEAKDNGVDPGSLSSTTQVIIDIEDVNDIPPLINTSENKAVGEADFTATISEDSPIGTSVVKVEA
TDPDSNNDDLIFTIANDVPVPFTIDPKTGLITVSGPLDADDQDEFEIAVTVSDGVNNSTAIVKVNIA
DLNDNKPIFTTDTRDITIPLDTPNGQEIIKFTGSDADKTSPNNQFEFSLVDGDGKPFTITPDGSLIIN
GPLMPGDYMLTVKIMDKGTNPGPLSNVIIYNVKVVDTNDPPTFQNNEQTVTISENTADGTDVITF
AATDPNGDAITYEIVNPGSVPFEIDPITGVIRKMGLIDFESPVRSYTLDVKATDAKGKSTMGRVFVQ
ITDFNDNTPTFGPGNTDNFILTEGVYSNQLVSSFTATDLDATSPNNVFEYTLVGAPSMFLLTTNPNT
GAADLTVNGDIDAEQNMSFNFIIRATDKGNPALSMDYPVTITVGDINDNAPVFAANTPESIDLME
GDFNNQIVATFTANDDDTTAPNNLVDFELVGAPDFFKLIVNPETGTADLVIDGNVDIDGLDPTINF
IVRAKDGGNPALVKEFPLTVNIADINDNAPVFTLEAYNQVINENTPIGTKVVEVRATDSDSGPNG
DITYRIVDGDGTFGIDPKTGDITVLKNTDFESGVRSYEFTVEASDNGNPKQSATAKVKIDIRNLND

NDPIFTSIPITGNINENRPAGSVVQNIQPVNGILNQNNVKILREPPPGAQETEGVGFDAPFEIDPVT
GEVKSTRPFDFEEEKVFTMMVQAADGDNNPMLTTMTVMVNNLNDNDPIMKKANFEITLNQDT
PVGTFVLQVQATDIDQENQTKITYSIINIDGGNMNDFTINPDTGVITLNTDLSMNTVDIYNITIQAE
DQEIQGVIRRSTALAEIKILRPAEAEPDISTLCTNSCDIPEERLKCLDYSKFEITRKFNGTCYVDKLKA
AIADVKNNTMEIMNLNASFLECRNKVQNEITNPNTQFPVNGLFDLHRKCTVAIECKLEQLAFNL
MRKTTELTNNLKAEEEGFSMYDMSLSAFFQHISKMVSMSKSSRVD*
>TRINITY_DN15274_c0_g1_i1.p1 type:complete len:1822 gc:universal
TRINITY_DN15274_c0_g1_i1:87-5552(+)
MECIRRSIIRLLLILGFYTLYISSQEECKRGGCFPATGDLLVGREKQLLANSTCGLNGPSKYCILSFLD
KTDKCFYCDSRRQVDERPDGYKNSHLPKYMVTREPSKRLQGWWQSVNGRQDVTIQLDLEAEFIF
THLIMTFKSFRPRAMYIERSSNYGNDWSDYRYFAYNCRKSFPNIPRGPQRKIDDVICEERYSKVEPS
TGGEVVMKVLDPIIQKKEDPYSLKVLNLLKLTNLRVHFKELHTFGDDILGDSPKVNQKYYYALYE
MVVRGSCSCYGHAEQCIPTKGTPANVNMVHGQCNCTHNTKGRNCEMCKDGYNDEPWRPAYQ
DQLNVCRKCNCNGHTDKCHFDPAVYKATGNTSGGVCDDCNHNTAGRQCELCKPLYYRDPYKN
IFDEDVCKPCDCDPSGSFGGGECETISDPNNSSALTVAGRCICKPNAQGLRCDSCKDGYWNLLES
NPDGCQSCNCDDKGTVELNKCDQQTGTCRCKRFVIGLTCSKCLEGFWNLGNTPEGCEPCNCDV
GGSLNAKCDDITGMCMCKPNIIGRKCDQVAPGSYFGNLDQVISEAENSLVRSDSSSVEIVREILNPT
DSRSFTGDGYLRVSDGTLVNFTNIRVPSTGFQTFVMRYSRPTPGSNNNVMVRITKTDGTILGGNGC
VQTNEREIILNGVLNDDNFLELGDMFCLEKDTDYSVQLVVSNRNSPNDGGVNRGVTNLDSIVM
MPNIDNLAIFQGREGMRLKSQYDLNNCRQYYLEPNGAANAPEICKKIQFSISAEINNGALKCNC
DPTGTNSSGSSDILTCQKSGGQCPCKPGVVGRRCEKCAPGYFGFDSNGCTACACNDTGSEKLFCN
GITGQCPCKNRIKGRQCNSCPSNHFGFPNCIACQCNEHSSTCDPISGECTNCKDNTAGPSCNLCA
EGYYGDATAGTEKDCQRCQCPGGSSLNQFSNTCHLKPDGKVVCNNCTEGYTGDQCDKCEDGY
YGNPLVPNGSCNKCDCNGNIDPFADGKCNAENGKCFNCINNSDGDHCENCKEGFFGNAKNQS
CRACVCNKLGTNSATSDSCDRETGQCECLPNVEGVECDRCKSGYYNIASGNGCVSCNCDKGGSL
SSHCNQLDGQCNCLNGYGGLDCTQCEDFYFGDPKQNTCQACGCNPVGSITLQCNRTTGVCMC
KDNVVGEKCDMCAPQTAGVMPQCEECHVCNDQWEDIVNDIEKNITTILTLKGSSPLNVSEIRAY
TNDIADLKNRLDKIEEDFGKQMVDPNKINRLRDKVYRFSYNLTGINRDADFYMEMVNETITRDE
KANDILDGLELRLATLQTNTEDFKNNVTKIVESNIGGALNSTRESLEKSRKAQESVNDSDLKIKES
KKMRKKIKKEIQNGDPEFEVMHNRSSEFVNNLADRENYLEGELEKLNRLLCGSDKRCGGCTKGG
CDTCGVSGENYTCDDGIKMMSMMASDKALEAKEAIDQKLENASELLENVNRAKQHVIQANISA
NMAEDKAEEVNKIGQAVLKNITTLIKDIQDFLATDFNHPNASKDLAMQTIALNISLTPDEVVELG
RKIRDAVKSLTGVDKILNESRNDYDTAIGLKEQALAAKERAIEVMNQTMAIIETLSQAEELQNSTL
AGVNVARADIEDVKDLLKKLKEKLDAADNDLQMADNSTASAEDKRNAILDQNLENREKLDLSE
KKIDEAEELGDKVQGSVDKMNEKYNATKAKLNEKEDMIEKVRARIKALADKTIKLYENADRKLR
RINKMKDSYIINEERAKDLYYEIRRLEIKEFFINTKLEGTAKCYSTCNPIIPESFCRDVYKNIAVKETN
AYAELEAEKASTTSLLI*
>TRINITY_DN160_c0_g1_i1.p1 type:complete len:1145 gc:universal
TRINITY_DN160_c0_g1_i1:4810-1376(-)
MRLLAIIPLLVMLDIAKSDKLWECNFEENDCWAASSTRNHERNQLTLHWNRWKMRTPTGATAS
PVTGPMEAFEGKHYLYYEATESMDKLKMADGASKGVVTFKDINLMGRTQFILSFYYQVKGNDG
DFYDFDLMVSNTENTHYDLSMKSIDGKDWKHFKHIMKVEGAGVTSIDLKFTARSYGAFADYAID
NIVIEDYPDAGPCKDNRCLANEYCTITSSTQTVCVAKCTQQACGENAECILDSLAINKCKCRQGY
LPRVSNYGDKLNCYRPDASCDIEYNSLGQCRISTSIPIMYGHPSLWHGRYSPVSGNGYYVFKYDNT
ITLDAVSSQKLSFFYLVDNPNMINSLTVEYGSRKFEVPINREKTNIWTLYEVLILASTNQGVQIKASF
YAEGGVIGIDNIQFTQQNEVVVEKIYKESMMAATFEDGSVPTDLKVAMLFVTRYGKIDFGCLPGIE
LETRNGKLYCKDGVVQRQSACSQDSDCIGSNARCVLRNCVCNTNSYQISIGTYGEKATSCAVYPC
EINSPRCHSTAICSNIKTPGQNFYNDYTCKCGRSLNNVETIINPFIGGDKSTQCLAATPSDSYVCND
NNFKPSRTTEYCLKGNQATVEYSLRATNFGNYALFKHCDDPDDEYRSSFNDPGSFWRPIDLVAAA
NENLAVSVTYKTSLSNKFIKCNFEQARFGSKLTLAVNVNSNSRVLTFTNTQTSVNKQNPVYQGQV
FQSDNPTPGAEMSVTFTVTGNQAVDQNVDAILLDNVVCKGFDDVASVNTPCGNNACASNQYC
NPLLSNSEKECLCPTGQKDQNCPDVVPSGSVVSADGPKCIGHHEKYMNGACVCDDGFEKTGDT

CTVKINCGCNDGLCLSGDKCSCPLNLLKVNQACVRTNKDCAKDCVSTLGNGGYCDVTTGMCD
CLTGFVKSGRDDGSYRCDKHAVVQKINDPKGICSATCGTGFVNEIEVCVGATGTGFECMTNIVQG
TLGVCSLPACPATSSQCSIQKECACNNVRVPIGSDGKLRLDFDHEAKLNMIFRFLSKHNQKDLEFK
FREARCDNTKLDSIARTIEDDLKQLEFDTELQAAITTMKSILDCSPSGNDHNAAYWDVFDMLIELS
SYRPHAVSDLQLKKELVMHEQSRCGMRSNLHKVVDGYAFWNKK*
>TRINITY_DN332_c0_g1_i1.p1 type:complete len:246 gc:universal TRINITY_DN332_c0_g1_i1:940-203(-)
MGALTYIFCLFLISSSFAKEEESCRTYAGGQVYPDERKSGVEHALHWSKAQISKPAPHWEGKAVV
NGEFKDLKLTDYKGKYLVFFFYPLDFTFVCPTEIIAFSDRVEEFKKINTEVIAVSVDSVFTHLAWTNT
PRKEGGLGKMKIPLLADLTKQISKDYGVLLEDAGHTLRGLFIIDDKGVLRQITMNDLPVGRSVDE
TLRLVQAFQYTDKHGEVCPAGWKPGSDTIIPDPKEKLNYFNKQKEEI*
>TRINITY_DN1377_c0_g1_i1.p1 type:complete len:878 gc:universal TRINITY_DN1377_c0_g1_i1:68-2701(+)
MTGRARGRSRGRGRAAQDASQRPGGAQGGPPPQAAAPQQGGGGRGRTRGQPAPQQVAPQQR
PAAQTAVPPTARMAQMSVKDQKPATEERQPRRRRLDPAPDVLVTRPEGRTKEGTGGTPVNIVSNF
FKVIKMPNFKGLYQYNVSFDPEINSSKLKNALLYQMADVLGQTKCFDGMTLFLPKRLPDQITTRS
VTTHQGNAIVIKIMFTNVIPEISPQVLQMINIIFRRQLRCIKMELIGRNYFNSSRKIEVKAHKMEVW
PGFSTSILQYEKDVMLCADVQHKVVRQQSVLSMMYELHQKTREKNRFYELAAKKLIGETVITRYN
KKNYKIDDIEWNKNPNSTFDKNGTQITFKEYFEKTYDAKVGDDQQPLLVSLPKAKNRKSGIVGPI
LLLPEFCLISGLSDEIRSNFSIMKDLAQHTRINPAERAKNLEQFVQDLQTNEESKKELEQWNLKFES
KLLQMTGRAYGSEKMFQREDQFSYKIQDADWSRETRGKKLIHTVNMTDWLLVYYERDEPIATDF
KQTLVKVCGPMGMNVAEPQLCRLKDDTARSYFRALSEQIRPTTQIVVCIVPNNRKDRYDSIKKLC
CVEKPIPSQVIVSRTLSKKQMLMSVCTKIGIQLNCKLGGEIWAVDIPISKNPKDPPSLMVVGFDVYH
DSASKGKSIGGFIASTNRHLTKYYSRITQQQSHQEIADQLRVCMTGALKKFNELNGTMPSRIIVYR
DGVGDGQLRQVFDHELPQFKAAFNAISQGYDPKFTMIVVKKRISTRLFLSERGMKTNPLPGTVVD
TVVTRPEWYDFFLVSQSVRQGTVSPTHYNIIADTSGWKPDIFQRLTYKLCHLYYNWPGTIRVPAPC
QYAHKLAFLVGQSIHAEPDVQLADRLYYL*
>TRINITY_DN3986_c0_g1_i1.p1 type:complete len:446 gc:universal TRINITY_DN3986_c0_g1_i1:252-1589(+)
MREIVHLQAGQCGNQIGAKFWEVISDEHGIDPTGTYHGDSDLQLERINVYYNEATGGKYVPRAV
LVDLEPGTMDSVRSGPFGQIFRPDNFVFGQSGAGNNWAKGHYTEGAELVDSVLDVVRKEAESCD
CLQGFQLTHSLGGGTGSGMGTLLISKIREEYPDRIMNTFSVVPSPKVSDTVVEPYNATLSVHQLVE
NTDETFCIDNEALYDICFRTLKLTTPTYGDLNHLVSATMSGVTTCLRFPGQLNADLRKLAVNMVP
FPRLHFFMPGFAPLTSRGSQQYRALTVPELTQQMFDAKNMMAACDPRHGRYLTVAAMFRGRMS
MKEVDEQMLNVQNKNSSYFVEWIPNNVKTAVCDIPPRGLKMSATFIGNSTAIQELFKRISEQFTA
MFRRKAFLHWYTGEGMDEMEFTEAESNMNDLVSEYQQYQDATAEDEGEFDEEEEEEDA*
>TRINITY_DN168534_c0_g1_i1.p1 type:complete len:226 gc:universal TRINITY_DN168534_c0_g1_i1:774-97(-)
MIRSAIIAIVIASHLYESSAARCYVGYSAVGLPLAIGVRDVDCQKGMDFCTTVKSTIGGITVTQGNC
ARKYGQATCQFMQNSHKAMTDCKETLCRGDYCNSALIEAEQKERKRVGPYKNTLKCTTELKTR
FPLIPFATADIPIGIDECFPGTTRCVTVEYDYIYKFKTKIATRHNHFVLPVIQKMCNRPNIDDCNKW
CTIMSIHQFMHSSCRAKCCTGDNCNAKD*
>TRINITY_DN36_c0_g1_i20.p1 type:complete len:223 gc:universal TRINITY_DN36_c0_g1_i20:1172-504(-)
MIKLAIIVTVIAACVFESSAARCYVGYSAKGLPLAIGVRDVECGKGFDYCTTIKYRLGGIEVTQGNC
ARKYGQAMCQFMRNPHKAMTDCEETICQGDSCNKDVYDAEQAERKRVGPYKNTLKCTTGLIT
KFPAIPFYTADVPNAIDECFPGTTECVTVTYDYLFKFESQIATRHNVFKLPCIQKMCNRHNIDDCK
KWCSILSIHQTMTNCEAKCCKGDNCNA*
>TRINITY_DN1369_c0_g1_i1.p1 type:3prime_partial len:1577 gc:universal TRINITY_DN1369_c0_g1_i1:93-4820(+)
MRIQLTIFVTFSIILVSGQNSPPGICSTWDGSHYQTFDSYSFNYHGTCSYALALDCRPGSDDFAVHV
ENDEKCDNHGGTDCTRAVSLYVNDIEYKLGSNNTVLVDGKSNIVLPYFNEGVRVSKHAEYYFIDA

LEGKVVVKYDGRSSIYVQLDSSYQAGVCGLCGNFNGNKKDDLKLPGDKGLTKSLKEFGKSWEKP
RIGQVCKEVTEEHFDQCTNKSKMLVYYAEKECEILRTSSEFKKCHKIVDPTPFYEKCREDLCLCGS
GDKYCKCDVLEQYTRKCAHYGTVLEWRQQNLCYKNCTGNKVFSECGPACSSVCSAIGSKSVCK
DACIDGCHCPPHTSLSGDKCVPDDECPCEHNGDMYDHGDVINMKCKSCKCNGGKWKCNPLP
CDGEASVYGNGHFVSYDGKTYNYPGKCLYLYTYLSDRSFAVYLDRTSCDSLACHMNVMILFRGD
RIKLSASNSTLALEVNRNVKSFPYISSTGYRMYRFSTVFVRFEATNGIKVDWDTYSRLHVLMPHSL
KGKVFGLSGNFDFKTKNDFLTPRKDLAHSAIEFGNSWMMYSSRLNCSALAPDYRMTSCEANHQ
VAKYAESKCSILMSSLFRECHKVVDPFIYYENCKDDTCECNGDSKCYCSVIAAYSKACSSAGKPTE
GWRKYTPCEEKCSNGMVFTECGNSCLKTCDDQTDPNPFCEKECVEGCLCPVGQVLDKITEQCV
DIKHCGCTYGNRTISNGGTVAKDCNTCKCTKGVMVCTKKECRDKKAICSDGKEWSSCMSPCPK
TCQTKDDICPETGLDCIEGCQCKKGYIESANGTCVELSKCPCYHNGKEYQETQTISKDCNTCKCT
AGEWSCTEKLCDATCSVFGDPHYKTFDGKIFDFQGKCGHVMLTDKCANNDNATENIRISVKSST
CGSQGVTCTREITLNYHDTVVHMKRGARKIDIVSNKRQDHEKDQVKVYRHAGIYVHVLTPMGV
SILWDNATRLYITAPPSLSGKICGLCGNFDGSETNDFVTYQGDTAGNAVSFGDSWRTENNCPLTKE
IEDTCHNRKHRLDPSKRECAVIKSDVFKKCHTLVDPVPYYDRCVFDACGCDSIGDCDCLCAAIGA
YAKECIDEGVYINWRKGHNICEKEMCCPTNMTFVPKGTGCPKTCKYPNGLHHCPTKYVDGCQC
PEGLVQNNDANGDVTCVRPEECKVCKLDGKIYNEGEVVLYKCKECVCRDGKMTCSGNYCPTTL
PPTTTTESTTTPSTTPETTIPSTTTTESTTTPTTEPPTTTTTASTTTASTTTESTTTEATTTTKPTTTTTEST
TTESTTTQSTTTPSTTPSTTMKPIDTCVYSNGTHNVTLERGDVIQVNECTKKTCVKDEVTGLHEIKV
TTTQCENSTCDTTCEASKSNRAILVKTPGECCPQCKKPEDLCDNPNEVPCNSTYCIPKSWFCDGN
KDCSNGRDEECCPNYCKVEGEIKKPGEVWTNKEKCLTCKCEGYYTKCRRMECPELDCPEERQVK
SEGECCPHCKPQTEPPTTTTEMTTTTTESTTTPATTPSTTSKETIIIITEEPKSTTASPQEKCDCKVLQPV
GVSDGRIPDNQMTANSV
>TRINITY_DN140702_c0_g1_i1.p1 type:complete len:2036 gc:universal TRINITY_DN140702_c0_g1_i1:94-6201(+)
MATSFKVSEIKCTELLNLEKKGAIDPLVEVEYLGLSKETENKDNDLNPSFGEVFDFDLSGKALGPE
DFLKITVKDYEKFGRNRVVGKADVYLQELLSNPSSTLSATLNDKNDRPTTGKITFKVDYIGPAGGG
AGGATGGGGDDRQAAERELEGDEDEGDPNAMPDGTPIPEGPAGDAMRAEKRKMKKNKKKRR
EHNLPDKPTDFQIRIKIIEGRQLPGGNINPTVRVHLGEETKQTRVKHSSNKPFYDETFFFNFNERPN
ELFDKLVTFEVHNSKNVLSEAMIGAFQFDVGTIYFEDSSHAIIRKWLLLTAPEDNDKKDGEETKGA
SLQPGGPPAGYLKITAIVLGPGDELPEKAKGSGKSEGGEEDIESNLLRPAGVTLRPATFLVKIYHAE
DIPQMDSAAFDGVKKLFSSGSAQKDHVDPYATFSFAGKTQKTEIKYGNNHPDFNQELRVAFKFPS
MCERLKLQMFDWDRIGNDDCIGTSFISLTAISGNGDEGFLPCFGPCYVNFYGSTREFSDLPDEHDN
LNKGRGEGCGYRGRALVEIDTRFGDHTAKPVQDIMAQELVRVQAYMRRRKYKLFASFLSANMIS
EADGLVEFEVSIGNYGNKLDPDTEPQSSSTPPTNPVYDGCAYYFLPWQESKPCCVVESHWEDVIFR
LEPINCLLKIIDKMEKNIKSVELGLKAKAQPAEVAALLIALLDQFISDVSMDLPKFAATDNGVNEL
DLKIQEIRSDELLNLKEEASTLREQATDVQEAMDTIDGYLKRMRDLCVEPQNSMPDVVIWMIAGS
KRVAYHRIPAYDIFYSENPLARGRKCASLQNFILKYPGKKGHDMEDHPEIPASVSVILWLGLEKHQ
AAWTDTEQMEGEYSVYAETYENQGKPPMFDWTAKIIKRPGFSDSEGKIRLNKEYYEPPEGWKWD
DPPDGDWFIDPEMSSNFDADAGHKKFMQDCFENESRVPAGNWGKSTPHYTNVQGDEIDHRDE
TSLPEGWSWTDDWKVDVNRACDEEGWEYCIEATGGGWGAVFRSYHFCRRRRWVRNRQLEADP
QKVMKQKKEEAKQGEGWEYAPVFGMKYHVKEHKFDFVRRRRWHRKMVCVKPGAVPMFKIPD
DDDKGKFHQIMPRIFLAYDEPHKWQFRAYLFQARDILGLDSEGTSDAYAKVCFQDQSQITDVIEET
LCPEFNQTLLFEDMQLYGSPSVFAENPPFVSIELFDKDKVGKDEFLGRVLAQPIVRLNGLKPPATRL
MWYDIHHGERTCGELLAAFELFLDEGADLPLPPPWLEKEKKFQVPSGIRPILVKTGVEILNWGVR
NMKKYQLASCTSPSVTYEIGREIITTDVIKNTKKNPNFLKNIFYKEVYLPKDPLYMPPIVIKVRDHR
TFGRKPVVGRHVIKSIDFVQPPKADPVVELPPADLVGEPEEKKDVTLDIEEKKKKKEIPVDDNLDW
WSKYFASADEIEKCGNYLKKGHKTIQIYDSELEEIKDFNGFSDFIVSYPLERGKEDDDEDDNVVGE
LKGLVKVYPLPQDPKDQTKIRYFTPKLKEKSKIVKCIVRVYVIRARDLQPKDPNGKADPFIVVQLG
KKKISTKDNYIPNEINPFFGSFFEMKADVPLDKDLKIKVMDYDFMSGDDLIGETMIDLENRFLSNC
RATCGLSPAYYTSGAYQWRDAKSPKEILDQVCRTKPQWLSNEQVVVNGQLVKIEDFEESPPTDPHI
GPVEQRLALHVLNNEDIIGHRLVPEHVESRSLKNPLQPSMDQGQLEIFVDIFPIANGVPGPPLDIAT

RKPKEYVLRVIIWNTAEVPPSETSITGEEMSDLYLKGWMEGQDDEKQKTDVHYRSMDGEGMFN
WRFVFPFKYMPEERVMVVEKKEHFWSLDTTKQTLPPRMNLQVWDNDLFSANDFIGNLELDLSK
LPRPKKAAKKATLAMLDTEGDDVMDLFQAKIAKGWWPMLSENEDGTRTMQGKVEMTLEIVTD
LEHEAKPAGQGQEEPNMNPKLDPPNRPETSFLWITSPWKTLKHIIWKNYKWYIIGFFLFIILILFIGL
VLYSVPGSIVHKIFGFQ*
>TRINITY_DN3991_c0_g1_i1.p1 type:3prime_partial len:219 gc:universal TRINITY_DN3991_c0_g1_i1:84-737(+)
MTSWILNFLVLSIIALPCSSISNCKHKLEPVGCFKDRGSARAMPEQILNERDTTSKVYSKQLMSWK
NFHEWLPGFICRCAEVAARKGYSVIGIQFWGECWSGPNAESTYNRHGQVDSCINQNLKKNRAK
LSQCTAASGKHFTNYVYRLAPACHTQYKPVGCFQDNLQKPRPMPYYVSTDRETRLAIFSGKLLN
WKNWNTHMPDMICRCAAVAKSKGHQ
>TRINITY_DN138_c0_g1_i1.p1 type:complete len:401 gc:universal TRINITY_DN138_c0_g1_i1:157-1359(+)
MAARFLFITVILIVGNLIGDVCPERCERNWIKIGCFHDRIFPNRPYPFELVNHRDPINNHWDGNLI
NWRKYPDSIEKLACICANKTRTKGYQYFGLQFYGECWSGPSYNFHRDGPSTKCIGNDFKPCDDSS
KTECVGRAFTNYIYKLKDENPKVDGKWSEWEKWSPCSATCGYGERYRERFCSNPEPENGGQTCE
GEAREYEECNHGECQTTCDKEIDFGIALDYSSSVRQVNWNRTLDFVVELSKGFNISPEKVHFGVLS
FSHRTRFDFGLSDKTYWNSESFEKKIKSLEYRWGYTWTDKALKKVKQLIFCEKCGLRKDSLKYLLV
FTDGRSNNVKTDLKTVSDSIKDMGVNVISVGAGHTINHRELEEIATDDNHVFYVGGYAYLGDNM
NAIRRLTCG*
>TRINITY_DN118_c1_g1_i3.p1 type:internal len:200 gc:universal TRINITY_DN118_c1_g1_i3:599-3(-)
KVLFLCGFILLSGLAFAYEIEEIVEDAPKPEPEHVPEPEPEPVKEPEPEPEHKPEPEPEQCSTVKHQK
CSKADKVCSPEDSHCTFTQICTPFFVKKCCKVVSVEKKCVVVRNCIPKFSYEKVCEDVKRCPPRKR
CKNVCRQVDLGEHKVKSICEQECEVVEHPEPCKLVHTCFSKWVSKKDCTPRETCSDIEKRECREV
>TRINITY_DN1616_c0_g1_i3.p1 type:complete len:3855 gc:universal TRINITY_DN1616_c0_g1_i3:218-11782(+)
MIIFLAFLAVAVGQEVLLPPYFDIAKGKNITATATCGEDYVAPKGSPGELFCTLADATGKFGIKGLK
CSHCVPGDYGDGDGKNARDHHIRNANDGSNRWWQSPPLSRGLQYRKINITMALGQSFHVAYVI
IKAANSPRPGVWALERSNDNGRTYTPWYYFAPTSSECKQYFGVDSYLPIVKDDTVICSTEYSKIPPF
KNGEIHISLINGRPGASNFSQSKTLQEWTTATNVRLRLLDIKTLLTDLLPLSLEDPTVTRRYYYSIKDI
TIGGRCMCNGFADECTPHTNIVGKYECNCQYNTEGPRCSRCKKGYVQKEWRQRTSTDKFECEKC
NCHGHSDECEYNEEINKRKLSLDVNGNYQGGGVCKNCQHNTLGINCEKCKPFFYQPANVPIDS
PNVCQPCNCDTQFSTGLCAEETGQCECKSNYVGRYCDQCNKGFHMFPVCKPCECDVNGSEDRE
CIPDQINGCKCKKGYAGRYCDECLPGYFGFPDCQPCACLTRYGAVNDNCERDNGQCSCKVGFK
GRRCEECAAGHYQFPFCKECGCDPEKTLDTVCDSGTGVCQCKSNFAGPKCESCSPGFYAHPSCFA
CGCSTAGSTSSVCDQNSGLCSCRPNYSGFKCDRCAEGYYGFPNCILCDCNPEGSRRVGICDRQDG
QCQCKPTYSGQKCDKCLPGFYGFPDCKRCNCNPAGTKPGPGGRPGDCSTSNNGQCVCKDNVQ
GLECNECKPGFFNLTESNRKGCSTCGCNGAGTNNGVKSCSVEGTCKCKPLVKQEDKCTECRDGY
YGLKRTNVFGCLGCNCEVGGSSSSVCSKDNGQCPCKDNIVGKRCESVAANSFYPDLCHIKAEME
EGKTTDGDVVRYDYQKEIFPDFSWKGFVAVNDQQPSVVVSINVPKTSEYQVKFRYALNQIQPVTA
TVTAFKGDSKIVGSINFASTQKGSGFVASLATVQDNLVQEMYFPLTEGQWNIKLEAPHSNLVVDY
AVLFPKEYAQGGALKKKVQNACDVMTDEKFCNLYRYIDVADKKDIIVLEAENPVKMRQQKPPVL
FNDTAVLSKLDFQAMVVLSPSNSRQLNHLFNVAKPGKYVVMLQYYHDSDTAKYLDVYVRSQIAV
QNGRALLQSCRYTFGCRQAVLNRGDNGVFVFNIKSTKNHLLTLLSRYAVVAVDSVMLVPYESWH
LDYSTPTLYCVTKDSKCTNMNYDVPAESRLVQANPLEQDLSEKPRYLIDRSVEVRYVKPGVNIINIR
DVKAGNYLLVAHYYQPNFPSFESRAAVYSGRTSGLRARFDFKFCPNLNGCRSPIRRPDSNSNLYVF
EEGEARIRFDIPNGLDAWIEYILMIPEGTNVSSLYTPRVLDYSDDFKKECSIDLNVCTSSNLSSFCKDS
IFTITTDFNNGSFPCKCNMDGSKSSSCQEFGGKCPCKDNVIGRECSRCKVGYYGFPNCKPCNCSN
CDEETGVCTCPRGVIGENCDQCAPGTFGFDPIIGCQKCNCNEIGTGSANAECNVTSGQCPCKEN
YSSRKCSACKPGYFNYPACTKCDCLDAGISAEKCDVLTGKCICQNNVIGLKCNECADGYFKTNDI
KGKQLCAKCWCNGRSTKSCTEAELFRATPITASKGWTVSVDTLNVIENGNGRFTISDVNTLPEPV
YWVAPNQFLGNRGLSYGSHFKYNFQYSVKQPSQNTNGDVVTDAVIKGDGYTIKSMRSVIEKEND

GYSASILMKETEWMLEDGSTIDRELFLAILSDIDTLQILATHGKNTISSTIYDISLETAGENGTDRVYG
IEKCFCPKEYADLSCSKCSKGYTRQKDGSCGKCQCLGKSNICDPQNGKCINCSGNTMGDRCEKC
KPGFYGDKCKACECPFSNPENSFANTCAINVNTSSLVCQCNAGYKGVRCDQCSDGYYGNPLQG
VTCKKCSCNGNINASFTGNCDNTTGDCLKCLYNSDGNQCQECRDGYYGDAVVEKNCTDCQCSI
CGTTTAPCNKATGQCQCKKNVEGSKCNQCKPNTWNFDACNGCEDCNCAEASETQQCDLKTG
QCKCPPTVFGRRCGFCKLGYYNYSSEGCQKCQCPDGKPCDVNTGKCCTGVLNEDCKLCPVGHII
EDGECVECDRCVQRTLQRVNTLIHNVTILEKHIIPAGGLNYGALETLGNRFSNLSNKGDEHISNL
DTLKKAIGFGEYRNHSGNMDYVRMYLVRDFKSCSFIVNIKTPPTSSAPATTMVTNTPGPDPIRDHL
SNKKISDSQCPPSLARVIDEGEKNIKLVEKKVVVLEKDAYESTENATATKKRAEDLLAKLKDFMVD
FKDVLSFANSTLKGNVVINIDGNRSLAEAEEMLKEIKDRNFNSTINRSEREKSAAINATIIAEQLMK
QASELANKAYKFNDTFKDFVKALRNLKMESELALLDANNATNSINLAKAVDFQSIIDNAQKLHS
QNNVTVTQAMEILQNAKSAYENAKDVFNKFHNQTGPLSDLMEELRNYVRKLEFDLQTTSPIVDD
SVKHAKRLRDSADRLKELLQDTKKFADAALRAAKVYQNIVDAINDALDAARMANETAHEADK
KVQQAQQDAVESSITSKVVDTEANDLTEEVKKTLKVDISDMKMRLKELRELRKRIQGRIDNVKED
VKSFAKEKEVGEKAMNISTQADGVMKVANKGNEASQDTNEKVEKDGEKVETLKVDISDINKAIK
DLASNVTLVEDTIPKIRSNIDEVNVKHEQIEEFIKTNVTNNIGYLKYLISNAHDILDQMQLSMKFNS
TSEVVLDAPKSVADPTINNKISLTFKINQGNGFLFIIGDFSGNSSGFMSLHVQNHTVVFDYKLNTG
DVQSLTNPKFIETDVNYDVMVTRNFATAVLNVQKSGNASTLMTAYQNRSNISPVLSLGQDTNVY
VGYHPSTPNQRPNFDGCINQFRFDEELMGLWHWKSAKNVGGCVQDFRSIEGQYSFDGDGFVEF
DLVSPELSGFRKIELRFATLEENGLIFLGTDPPTSLPSDIMFDIPLSNQIIAFNALEIKDGYLVSTLDLG
NGVQRLVHTNAGKVNDGKPHLFEMKVLISSLLIKFKLDKKKITEIAISPDTDIRLRLKEAFIGGLPQ
RAWLPNSNISTKGFTGAIGDAKYRGGEFKLVKRFNNIRKGFILPKGYSASFPEEKSYLRLDACNLK
QNSLFGFSFQTFEKNGIIFHSEEGSGSSRSSIEIILIDGQVKVDVSVGSQKRTLQTSVARGFSDGLWHT
LSLYFEETLQEADIVLLYLAVDKAYITGTVPKVDLKGSMYFGSKPSGTESGFNGNIRQVYCNKNEIN
LASGDVFEGGVVGSLKLLPPLKLADFTVVPTTLPPLTTSITTSESTTTETVMTTSTTSISGTTLEPPKET
TTNMAKTTENPTVKITSSEPIIETTMESTTVAKTTSAITMEESTTTPTSITTSTLETTISTLEPGSIEGSTT
TAEPITTTAEAITTTATEPITTTEPITITQPITTVEVTTEPATTQESTTTPEPITTLETTTTPEPSTTPEPTTP
EELTTTAKPTTTIRVTTEPPKCQSNLEVYDEAYGFGMNEPSFIEFGVDGRKMLKSNQFEFLFYTAEN
DGMMISSEPSNLYDYEYVYMENGAISYAFNAGAGRVRVTSTKKYNDGKWHEVKISRSKESGTLTI
DGELIGTDSKSGKTAVNGIKKIVVGGSSVPYRNIPPKSQVGFKGGMRGFKLFNEALKPLKEIGITEA
YDELPIPEGVSFGPDGGYVALNFKSEYVVRTEYKLALDVRSVSSSGYIFFIGNYGADYLSLYIESDRK
VVAECNNGGGRFSVSIDVPTICNGEFHSILLEKKYKTLDLSVEGVQKSFTTTKGSTSADTGSNPPAF
GGFNGVGSRPKNLETFTGCITAVRIKDKAVDLTAPDVTIFGQVSNGCPSNIR*
>TRINITY_DN250_c0_g1_i2.p1 type:3prime_partial len:322 gc:universal TRINITY_DN250_c0_g1_i2:131-1093(+)
MRECISVHVGQAGCQIGNACWELYCLEHGIQPDGQMPSDKTIGGGDDSFNTFFSETGAGKHVPR
AVFVDLEPTVVDEIRTGTYRQLFHPEQLITGKEDAANNYARGHYTVGKELVDQVLDRIRKLADQC
TGLQGFLIFHSFGGGTGSGFTSLLMERLSVDYGKKSKLEFAIYPAPQVSTAVVEPYNSVLTTHTTLE
HSDCAFMVDNEAIYDICRRNLDIERPTYTNLNRLMGQIVSSITASLRFDGALNVDLTEFQTNLVPY
PRIHFPLATYSPVISAEKAYHEQLTVAEITNACFEPANQMVKCDPRHGKYMACCLLYRG
>TRINITY_DN240_c0_g1_i4.p1 type:5prime_partial len:397 gc:universal TRINITY_DN240_c0_g1_i4:1327-137(-)
LKAAAAFSGVSPFALVQPRARVPSRSRVAMVRRSPLPVLGLAGAACFFARSPCFIPGGPLRRQPWA
AAAGSAAVLGATPALADPIGDAAKKLADASYPFAKEVDWNSGIYLQAPGKLAPLEALKAIDKMI
VLGSQADPKLLKAAADAHHKAISSASGPTGMTSKADWEAVNAALGRVFASVPESSVMDVYNAV
AKITDPGVPAYLKSKVNGADAEKAYAAFLEFKDVVKANQVSSAAAAPVPAAGGDKIGAAAQAL
ADQSYPFLKSVDWTSDLYLKPLPGVSAAKALKAVDKAIVMGSAMDGNLLKAAASAHHQAIGSI
DAAGVTSAADYASVTAALGRAIASAPTSKVMDVYNAFAGIVEPSVPNTLFSKVDPLAANAAASA
FYEFKDVVKAAQR*
>TRINITY_DN6390_c0_g1_i1.p1 type:3prime_partial len:1941 gc:universal TRINITY_DN6390_c0_g1_i1:5820-1(-)

MVIAPKCKFSIFSYLVFGILSYNWNELDASTNIELVDGTGVLLQRDVDNVLNDAVVQKEYQQNIK
HKPVDKRCLGEPFYQGLCKEKLEDAIQQNITEGGCHNLLVKIQKDECSNGPKTLGGKGYCNFICS
QEICNSIYDIDKCVSKRLVHHFNKDKNECEEIMQADCGSESFTSSNQCQKRCIGNSDDKRETKVY
CDQFCLNGGKCIGEVYCQCPPAYHTATCGLSYCNPSCGVGGKCVKTGEGKYGCQCFPGYKGVK
CDKEDSKNMPVVKGLCLSAIKSVLTSPNTSFHDKPEEEKNYCLKYLFDLESNCDKQRPFVNYTEE
QCNHFCASDICDNIYSNDCGSIEERYGYVSSTGRCSKFLYNGCLGNTNNFKDKAACDTKYSPING
LMTSVALLRPLNHTCYIRGKTNFITFDGKVYDLLSGCSHYLVHDSDTTSDFNVILHNDPKVNYNE
KSKRKLEITADGKNVLLNQKVSSDRFFVQVDGRSVPLPYNDVPKIRQAGRYILVLTGDGVSIAFDG
YEDISITVNDYYRTGDGGDHRLNGLCGNLDGNSHNDWLDFTGELRENVRDFAKVYQTDRSTCK
MDNLSLKSLVYNPLHNDQEVFERAERHCDVINTDAFKPCHSVVSPEQYKVLCMQDYIKCNDDK
RDDCVCSSLSFYSRVCHKYGKDIKWRRANLCPITCQRNMKYYECGSACPKTCDNLITPTKCQTDC
VDGCHCPPDLYYDKSTENCVPRNLCSCYRDGIAYKHGSVRSFHCEECTCNGGSWECNKKDCEAT
CDIAGDPHIKTFDGLNYQMYGKCSYVLVDHCHYGNTGIKEFEVIVENKNCNDDALYTSCVRHLS
VNILQTNTRVSLDTAKDNNGTVLPVAFVNGKEGKEFRSKDYTITSVGGRNIIFQYKVNQLKVSWT
GHNAYVTVGHQFENQTCGLCGTYNFNKQDDFHTRSDTVEKSVTAFTQSWVYQSSDKVQDPSKC
HTAKWETSEKPCQIYTNKAQYAKTQCSIIIKPDGPFEDCHSIVPPGEYYSMCQDDACKCDDCYCH
IVAAYAKRCTDLNIRVGNWRDETLSCKQTCQAPKVLKQCNAIDSLDDDLSESLKCPSTCYELSQN
DTSICQSRGCIEGCYCPEGYFENEQNECVKQDQCSCYHDGKVYRHDEIRKDRCNECVCKGGRFE
CTDFDCVAHCASKGLVYSDCGKTCSNFNFNPQDSKCQAGCFCPAGKVLHENGTCVNPQTECQC
KEGDQFFDAGEVSPSDCSKVCNGDRSWKNLPGKDAKPQYDCSAFGQMHYRTLDGRMFKFSGG
LCPYVLLTDCRQDSATGFCDLTKANVNIHVRNLRCVNSYEAYMCKEVTLDLRLPNGEMAEFVLM
QNKIKMKTGLVTKVFDKGSYPQPKTAVVDGVEIFKSGLYLVVRAYHLNITVKYDRQNRVYVVPGR
EYKDAGKLAGLCGNYNGLDDDDFRRPDNSNAENELQFAKSWQDSNSACPDPSESDTCSSNKER
LPWAQKGCNVMKTGNFTACHKTVSPEPFIESCQQETCLCKSGGDCACFCSAVAAYVQECNRHG
VPIYWRRDGLCKLDCCDPCGHSYGYYTDNHVPNTCGVKDKVCKENDRCCKGAVVEGCGCPPG
TYDDGIKCQNETTTNVIPCSPSSSIVPSTSISVPSTIIEPVSSSSPSPMTSSVESQSPSPSSSVVSPTPSSSII
QSMSSSEIESISTTSSSFVSSIESSVQSSTSIVFPVVSSNSGESSVSSSMLPSTSWVGIHSVSSSMGESSVST
NVLPTDYLRTTDVFPLSSSEITYPSSSSSLEPHTPSSSILQESSSSVSKPVGISSVIEPEISSIRYSSMSSQN
VMSSSVSSSVSKPVGISSVIEPEISSIRYPSMSSGSVLESSVSTSFVLPSETVLSTSVFSTNTESPSSSMTLS
TSSSGFVFPSVTSTFQTSSKVLSSLPRSGPAENTVPLSTMFTKTV
>TRINITY_DN2113_c0_g1_i1.p1 type:complete len:1704 gc:universal
TRINITY_DN2113_c0_g1_i1:5257-146(-)
MAQLLPIRFQEHLQLQNVGINGANIGFSTLTMESDKFICVREKVGEQSQVVIVDMADANNPTRR
PISADSAIMNPASKVIALKAAKTLQIFNIEMKSKMKAHNLTEDVIFWKWISVNTIGLVTETAVFHW
SMEGDSSPVKMFDRHQSLAGCQIINYKVDHSGQWLLLIGISAQQNRVVGAMQLYSVERKVSQPIE
GHAAVFSQFKVDGNPQTSNIFCFAVRGAAASKLHIIEVGTPPTGNQPFPKKAENIFFPPEADKDFP
VAMQASQKYGVVYLVTKYGYIHLYDIESAQCIYSNRISGDTIFVTASYEPTEGIIGVNRKGQVLSVSV
DEEKMVPFLIQVKQNGELARRLAERCNLPGADNLFLQAFNDKFASGNYQEAAKIAAKAPKGILR
TQQTIQRFQQLPSQQGQTSPLLQYFGILLDQGQLNKYESVELCRPVLMQNRKQLMEKWLKEDKL
DCSEELGDLAKQFDNTLALSVYLRANVPNKVILCFAETGQFQKIVLYAKKVGFEPDYVSLLRNVM
RVNPEAGANFSGMLIQDDEPLADLNQIVDVFMEFNLIPQCTSFLLDALKNNRPAEGPLQTRLLEM
NLLSAPQVADAILGNQMVTHYDRAHVAQLCEKAGLLQRALEHYSDIYDIKRAIVHTHLLNPEW
LVNYFGTLSVEDSIECLKAMLSHNIRQNLQICVQIASKYHEQLTTSSLIEIFESFKSFEGLFYFLGSIVN
FSQEEEVHFKYIQAACKTGQMKEVERICRESNNYEPERVKNFLKEAKLTDQLPLIIVCDRHDFVH
DLVLYLYKNNLQKYIEIYVQKVNPARLSIVVGGLLDVDCSEDVIKSLILSVKGQFSTDDLVAEVEKR
NRLKLLLPWLESRIHEGSEEPATHNALAKIYIDANNNPERFLRENQFYDSAVVGKYCEKRDPHLA
CVAYERGQCDQDLINVCNENSLFKSLARYLVRRRDAELWGTVLVEDNKYRRQLIDQVVQTALSET
QDPEDVSVTVKAFMTADLPNELIELLEKIVLENSVFSDHRNLQNLLILTAIKADRTRVMEYITRLD
NYDAPDIAGIAIVSELFEEAFAIFKKFEVNTSAIQVLIENIQNLDRSYEFAERCNDPAVWSILGKAQL
DQGLIKEAIDSYIKADDPSAYMEVVEASKNCGNFEDLVKFLQMARKKAKETFIDTELIFAFAHTGR
LADLEEFISSPNHANISQVGERCFEQKMFEAAKILFNNISNFARLASTLVHLGEYQNAVDGARKA
NSTRTWKEVCFACVDGQEFRLAQMCGLNIVVHADELDDLIRYYESRGYFEELISLMEAALGLERA

HMGMFTELSILYSKYKPTKMKEHLELFWSRVNIPKVLRACEQSHLWSELVFLYDKYEEYDNAVVT
MMKHPTVAWKESLFKDVITKVANIELYYKALQFYLDNKPLLINDLLVVLTPRMDHTRGVSFFQRA
CYLSLVKPYLRSVQNHNNKAINEALNSLLIDEEDYNGLRASIDAFDNFDNIALAQLLEKHELIEFR
RIGAYLYKGNNRWKQSVELCKKDHLYKDAMQFACESRDKAVAEDLLSWFLSIDKTDCFAATLYM
CYDLLEPDVVLELAWRNNMLDLAMPFMIQFMKDYYTKVDKLAESNAKRDEDKENAPPEAPIM
GVEQLMITAGPSSYVPQPNMYGMPPQGMAPQGMPPQGMPPQGMPPQGMPPQGMPPQQGFPQ
QGFYGGAM*
>TRINITY_DN116_c0_g1_i1.p1 type:complete len:297 gc:universal TRINITY_DN116_c0_g1_i1:72-962(+)
MGFVKAIKNKQYFKRFQVKFKRRREGKTDYYARKRLVTQDKNKYNTPKYRLIVRFTNKDIVCQIA
YAKIAGDVIVCAAYSHELPRYGVKVGLTNYAAAYCTGLLVARRLLHKLNLHETYSGQTEVDGDEY
NVESVDDGPAAFRCYLDIGLNRTTTGARVFGALKGAVDGGLEIPHSVSRFPGFDSESDDFNAEVH
RNHIFGKNVGDYMRALQDEDEDAFKRQFSKYIKEGVKPDNIEGMYKKCHAAIRADPAHKPSGK
EKFKGKRYGRKKMSFAQKMDSVRQKKEAFKRKMEQDED*
>TRINITY_DN22_c0_g1_i1.p1 type:5prime_partial len:353 gc:universal TRINITY_DN22_c0_g1_i1:3-1061(+)
ISNYYFHFQYQTAIFEYHTVFSLVKIINMKFAVALLVVCYFYSTEACKDVLHPSICADVKSIGNCGV
YKDLCMKTCGNCGGVLPPTRRPIFPTRGPGPGPKTPPPIVPTGECGVSSFAGGRVIAGTTAREGAW
PWQILLKSNGRAGCGGSLISANWVLTAAHCMNGGSYTVVLGEHDRNRRSGNEQEIRVSSAITHP
QWNRRTFEYDYALLRLSRPAQLNRYVKPVCLPSGDPQPGTSCYITGWGKTSSQGQMNGLLQQAP
MRVIEQNTCHKKNYAAIRVAVRPSMICGGGSGRISGCQGDSGGPFVCNEGGRFVLHGDVSHGSR
YCRSSETYTVFGRVNYVRRWIKSTANV*
>TRINITY_DN776_c0_g1_i1.p1 type:5prime_partial len:201 gc:universal TRINITY_DN776_c0_g1_i1:692-90(-)
RAGKTPHTASMVKNVCSKTYVTPRRPFEKARLDQELKLIGEYGLRNKREVWRVKYTLAKIRKRA
RDLLTLPEKDNRRLFEGNALLRRLVRIGVLDEGRMKLDYVLGLKVDDFLERRLQTQVFKLGLAKS
IHHARVLIRQRHIRVRKQLVNVPSFIVRLDSQKHIDFSTNSPYGGGRPGRVKRKNMKKGTGGGDG
DEEDED*
>TRINITY_DN1091_c0_g1_i1.p1 type:complete len:212 gc:universal TRINITY_DN1091_c0_g1_i1:200-835(+)
MKLILICLFAVLVYSIHCDQPSDPVWPNLFQQSFREKFYYPVIGTHYTNGTYYYDYKNLRYRLDRD
NGRYDRYCGLNGIRAFQNTPCTHLVLDGTRWLVYPEKKECCECCHAANGCGVLKPTWLTGAEF
VSKSNGIYKWNQKGLQDNFYSERVTDRIMTEIDQVPNDTQYYDPDSFKTKIENIEDVFKLPSYCKR
GTKCSFFSACRAVGFF*
>TRINITY_DN624_c0_g1_i1.p1 type:complete len:884 gc:universal TRINITY_DN624_c0_g1_i1:218-2869(+)
MANQEEEEHFGYYYGEEEDRGLLDPAWEKQQKKTFTAWCNSHLRKVNVQIEELNVDFCDGLKL
MLLLEVISGERLPKPERGRLRFHLISNVNKALDFIASKGVKLVGIGAEEIVDGNLKMTLGMIWTIIL
RFAIQDITVDEFSAKEGLLLWCQRKTAPYKNVNVQNFHMSFKDGLAFCALIHRHRPDLIDYDKL
AKDDPMYNLNYAFDVAEKELDIPKMLDAEDMVNAVKPDERAVMTYVSCYYHAFSQSQQAETA
AKRILKALNVNQGNEKAIEDYENLASDLLDWLNNKIPTLDDRTTDSSLDTMQNKLNEFRDYKGT
EKPPKVDDKSKLENLFNTLQTKLRLSNRPLYMPAEGKMINDIDKRWKDLEQSEKDFQEWILNELR
KLERLDHLAMKFKHKCDIHEAWSTGQDDYLRSDDITDASLASLLASRKRHEAFESDLAAQQDRV
EQIVAIAQELNTLGYSKIDEINTRCKGICDGWDTLGELTTQRSEKLAKAIEIAERLDNLWLEFAKKA
SPFNNWMDGAKEDLMDMFNVHTIEEVNDLKDAQLKFKANMPAAQEEFNTIMGLQNAISSMSS
QPNPYTNTKAEDLQEKWQEVLELIPKRDSLLESETQKQQNNERLRVQFAQMANQVGAWIKQRM
GRMTDIGMNAQGTLERQLEELENLNAEINGFKQNVLDLDASARNVQEAMIFENPHTPYTMEVI
RIQFEQLLTGVTRNINEVENQILMRDSIGITEEQVKELRTSFNHFDKNSNGRLDYKEFKQCLVSLG
QLGDVEDKGEHTFNRIIAMVDPNETGYVSFQAFLDYMTREMADTDTADQVMESFRILAGDKPYI
TADELRRELPSEQAEYCIARMSAYNGPDGVPGALDYRSFSTALYGESDL*
>TRINITY_DN4595_c0_g1_i4.p1 type:5prime_partial len:1639 gc:universal TRINITY_DN4595_c0_g1_i4:5857-941(-)

QCGVNTGTQVVYRRCNAALCKYQGNDYTRNQEVNIRTQACTVACDYTPWQNTQSCTKTCDGG
VLVQTRTERAFNKIETRNGNTPCNTQACAQELGPKQLRTCNVGQCGVNTGAQVVYRRCNAAL
CKYQGNDYTRNQEVNIRTQACTVACDYTPWQDTQSCTKTCGGGVLVQSRTERAFNKVETRNGN
TPCNTQVCSSELGPEQLRTCNVGQCGVNTGTQVVYRVCNAALCRYQGSDYTRNQEVNIRTQAC
TVACDYTPWQDTQSCTKSCDGGVLVQTRIERAFNRVETRDGTTPCNTRSCASDFSAERLRSCLNV
NQCGVNTGTQTVYIVCNGNFCRYQGREYNINEEVNVRQQNCTIACDYTAWEDTTICSKICDGGV
LEQRRLERAFNRLERRNGTTPCNTRACDSGFSAVLSRNCVNVNQCGVSNGTQLLYRVCNGEPCIL
QERSYKINDVVVVGSRACSVPCNYTEWRDTTSCSKTCGGGFFFETRTEQAFQKTESRLGKTLCNE
NSCVKDNFGNWTEWTACSKTCGMGMRYRSRECLKGFECIGLIEETETCKLGCRTETCPMGRPYP
GDCNFEDDCGKSWNQVNSIQRNAFFIVTSKDLAHQGLLPNMETTFNTKDCGRTMKLNSSVAGD
VNYLWTKRMMITDINTDVDDDVAAIDNYFKEENEQCVEFNFAVMANSLDKIGDMQVYLYYLEY
PNASLIYSKKGNTGLKWELVKRSIRVDTNKAFVILFQVNQGQSNVQYGDIGIDGFKVNPGNCQLK
GWSHWGEWKCSATCGFGTQTRMRHCLDKVNRTACVGMDRENKPCPMMKECPMNRDGNWG
QWTEFSKCSKTCDTGTYFRTRKCDSPSAKYNGSLCRVSQTKFNLPVVNDFEIDFKPCSVQQCNNT
FSQWTTWSSCSGTCGPGQRTRTRTCESGKRCSGIFEEIETCYMGCRLEKCPKFTAEIDCNFEDRNKS
DCQWKYYTVSGTSSWSFKTQPKNRLLSGPSGDSTIGCGYFLYYPSQTLSSAYAWGPNIYPTMDSYC
VKFKYSMVTSVNSSMGNLTVIKHFLNRPRKADTPLFFHSGVSQKQWNPMAIDITTDIQRSPFVIMF
KGQSDPNNAGLSDIAIDDYMLMAGKCNKSYSEWSEWSTCSRTCGVGQRTRTRVCLDDDIKVCK
MGDVATEGEACVKPLCAVPVDGNWGQWSAYSQCNARCNGTQKRERFCNNPAPSNGGRMCLL
GDTIFQLRNNYHAQIRPCSIDCGDNDTFSSYKRINDTCHFDCITGRVLQERECEQDTCFDGNTRFS
KGEKQYTQRACSIKEYCETSEYKCREFRSVCSTNTPSSCLDPQNRAVKSCECQVERGCSSMTNPGQ
TVKHKEFDVFYRPCRYVGVCFTDYETDAFSPYEKVNDTCHLSCSNGKVFEERECRQDNCIDGSNT
LTRGAKQYRETQCTTKEYCETSQYSCREISGPCSSNPPSSCQDPANKSLKVCTCQVTTGCASMLTA
GQAVSYLAEETFSRPCKYTGACNANGNTDKTFSPYERVNDTCHISCADGKVKQMRECKKDACV
DGGQNFPRGFKQFKDSSCTPNEFCETAQYSCRELSSACTSNPPSSCLVSTNKAVKMCDCNDPNGC
DSMITPGKKVNQGEKEFLQRPCRYTGVCFTDAETGT*
>TRINITY_DN477_c0_g1_i7.p1 type:3prime_partial len:1130 gc:universal TRINITY_DN477_c0_g1_i7:3387-1(-)
MMGSIKVFVGLVLLSFTAIEIAQAQSGTVLSNKQFAFDFVVDGSTSIQDEQWNLLTDFVSRLAVKM
STLNNAKLSALQFSHTLNRILQCNQYANVEEARNTIKNMVQENGATGTLAAMLTSADNLMNVC
SAGDPNRQNVMIVIVDGNQNLEQKSHSEIVQDIKVNVKDKNILVLTLGVGPQVNRNEFGDYASD
SGKGFFVDDFTLLTDELVQRMYDELIRTYGDGPDYLTAWVNGPCSATCNPFPMANRPTLRQTRQ
CQEPNGGNCGGASLEQIINCNENIACAGETYLTAWVDSSACSLTCNPFPVSNQPTKRQTRECRQP
SGSCNGASLTQTINCNENVQCPGETYLTAWIDSSCSATCDTGSNNPVFTQTRQCRQTVGNCDGA
PLQQTRSCNVGVSCTAPTFLTAWSNATPCSATCNPFPMAVRPTLTQVRQCNEPNGGNCGGASLQ
QTVQCNLDVACAGETYLTNWVDLTACSATCDDASNRPVKQQERQCQQPGGNCDNLPLQQTVN
CNIGVSCTVSSSACSSKEISFTFVIDTSASVDNIGFFVLKDFMKKYMSQSTLGTDPRTSVAIVSYGAG
DAKVEVECDTITTQQSFNTTVDNLSQLFGIADDNSFNQALSTAFDASGRCARSSTSLNIIIMLSDGD
PPALENILTRDRIQSSSNYYFMFVAIQASDGAQSTFASLANQGRGLRNASSSFTDLYNFNTDLYYLE
TLSKACDVGGQWTEWSTSACSTTCGPGVVTRTRECRNRITGKLQQDCGPGPAQETQACNLQDC
QGTLTQWTSWSCPATCNINNDVNNPPLALRTRQCLGSTGQGNCNNADKTENEPCVRNVPCPG
AWTTWSVFSACSETCRSQSAFPTRRRTRQCIGGTLNFQCPPQIVNGISYREIWQEDCINVPNCPAK
FTQWTTWSCPVTCNVNNDRNNPPTATRTRTCDDSGTINGCANANILENQPCVQDVQCPGAWS
TWSAYGACSETCQSGNRSPTYTRTRTCLGGTLGFQCPPFITQIGGFQIFGTQTSNCNSEVKCPSKLT
QWTTWSCPVTCNNRNDVNNPPQARRTRRCDTSTGSGICESPIVEEFENCVANVPCPGRLTEFTYD
NCAHTCNVNNDVNNPPQRVGTRRCLDGTLG
>TRINITY_DN286_c0_g1_i2.p1 type:3prime_partial len:106 gc:universal TRINITY_DN286_c0_g1_i2:40-354(+)
MIKVAIITIAIVACVLESTAAVRCYVGYSAADLPLAIGVRNIECGEEFNHCTTVKYRIGGITVTQGN
CAKRYGQAMCQFMRNPHKAMTECEESLCQGDNCNTAVN
>TRINITY_DN64_c0_g1_i1.p1 type:complete len:468 gc:universal TRINITY_DN64_c0_g1_i1:1555-152(-)

MPPKEAEKIHINIVVIGHVDSGKSTSTGHLIYKCGGIDKRAIEKFEKEAQEMGKGSFKYAWVLDKL
KAERERGITIDIALWKFKTGKYYVTIIDAPGHRDFIKNMITGTSQADCAVLIVASSTGEFEAGISKNG
QTREHALLAYTLGVKQLIVGVNKIDNTEPPYSEKRFDEIVKEVKTYVKKVGYDPKTVAFVPISGWH
GDNMLEASKNMGWYKGWTTERKETKTTTGKTLLEALDNILPPKRPTDRPLRLPLQDVYKIGGIG
TVPVGRVETGIIKPGMVVTFAPSNITTEVKSVEMHHEALLEAVPGDNVGFNIKNVSVKDIKRGNV
ASDSKNDPAKEAKSFYAQVIILNHPGEIHSGYQPVLDCHTAHVACKFSEIKQKIDRRSGKVLEEMP
KMVKSGDAAMIDLTPSKPMCVEAFSEYAPLGRFAVRDMRQTVAVGVIKSVTKSDGSGGKMTKSA
QKATGKKK*
>TRINITY_DN121501_c0_g1_i1.p1 type:3prime_partial len:267 gc:universal TRINITY_DN121501_c0_g1_i1:36-833(+)
MEEQKKPKLVDAVLSSLSTSKGWPQNVELYTVLEEQELLTDKAKCYSIEALLRIHELDAKYVKNRR
NNAFYMPPIKGSLPFLRIKNEIICEKDIVEFLKKCSYVLSNDLTAEEMLFHKGIFSIIEHRLIPLELYYL
WYSEANQKDTMNKFGHNHPEPLKKILCVQRYYEIKEYLKAEGVLDKTESQLQKDLKNIFDFFAK
LLEKKKFLVGDDMTEADVLLYGHIQAILESRLPNDILLKTLESYPKLTNFCLNFSQVCLGKQAML
W
>TRINITY_DN804_c0_g1_i1.p1 type:complete len:240 gc:universal TRINITY_DN804_c0_g1_i1:125-844(+)
MAPNTAAETFVKVDPGTKKGINGILMGPPGSGKGTQSQKLVDRYCVCHLATGDLLRAVVSSNSD
LGNEIKGVMDAGKLVSDELVVKLIDSNLEKPECKNGFLLDGFPRTVPQAEKLDELLEKRGTPLDQ
VIEFHIDEETLVKRITGRLFHMASGRSYHEVFNPPKKEMTDDITGEPLTRRSDDNEATLRRRLEAYR
KSTAPLVDFYQKRNLHSRINADRPMNAVFATVAAIFDGCRDKK*
>TRINITY_DN477_c0_g1_i9.p1 type:complete len:306 gc:universal TRINITY_DN477_c0_g1_i9:1840-923(-)
MTRQCLGATLNENCGNAALVQFEVCNFQVPCPGLLTNWGPYTQCSATCQKDANSMPTQSRQR
FCTGGSFGGNCGGVQLLQTIGCNQLIICPVYGYWLQWSAFGECSKTCDQGTHVRYRNCTKSIPA
NAGNNCTGPAQEVKPCQVKPCPVTCPLTKKCDCRNMNYLAAAPGISQTVTNDVMGVAAFLDY
WTLDILKQICEGCRMTQLDQSKATVDSQILRTQAVLDRLNIEKRVYRDMITCNENPYITDDLFKVY
NDIWDRIVTTDGVMIELKASKMRFDTAITACRSYDWYHDLFGRFFQIKKSS*
>TRINITY_DN5633_c0_g1_i1.p1 type:complete len:848 gc:universal TRINITY_DN5633_c0_g1_i1:2881-338(-)
MRRSNFLTIGIIAFFLIFSYVSADETSDEEVEPPKVDDDVGKSRDASKTDDEVVQREEEAINIDGLSV
AEMKQMRESAEKHAFQAEVSRMMKLIINSLYKNKEIFLRELISNSSDALDKIRFLSLTDTEALAAT
DELSVKIKADKENNVLHITDTGIGMTKDDLIKQLGTIAKSGTSEFFQKIQDAGTTDQASDLIGQFG
VGFYSAFLVADRVVVTSKNNDDKQHIWESDSVEFSISEDPRGDTLLRGTQISLYLKEEASDFLEPNT
LKDLIKKYSQFINFNIYLWSSKTEEVEEPIEEEPEKKEDEKKDDDKKEDADDKKDEEKKDDDEDD
AKVEEDKEEEKPKTKKVEKTVWDWELMNSSKPIWLRKPKEVTDDEYNEFYKAISKESDGPLTKT
HFVAEGEVTFKSVLFVPKNSPSDMFQDYGKRRESIKLYVRRVFITDDFQDMMPKYLSFIKGIVDSD
DLPLNVSREQLQQHKLLKVIKKKLVRKALDMIKKIPDEDYIEKFWKEFSTNIKLGVMEDHSNRTR
LAKLLRFRSSNDPEKLTSLTDYVERMKEKQETIFFMAGASLDEVKNSPFVERLLKKGYEVLYLIEPV
DEYCIQSLPEFDGKKFQNVAKEGLKIGNEAKEKARVDELEKEYEPLLKWLKEDALKDLIEKAAVSS
RLTESPCALVANSYGWSGNMERIMNSQAYAKSKDAGANFYATQKKLLEINVYHPLIQELQKRVKT
DKEDQTAKDLARVLFETATMRSGYTVKDSSDFAGRIERMLRLSMGVDVDAKVELPEEDDEEKEEE
KADDEGEEVNADGEEEESKEDIPEIKVDSKTVEPVAEHPTNEDAVENEAPAEGKTEL*
>TRINITY_DN2636_c0_g1_i1.p1 type:complete len:314 gc:universal TRINITY_DN2636_c0_g1_i1:986-45(-)
MSAIAVVVIVIAAVLLHFSVHKIEEGHVAVYYRGGALLSSVSGPGYHLMAPMLTKYKSVQTTLQT
DEVKNVPCGTSGGVVIYFDRIEVVNLLSVDKVHDIVKNFTADYDKALIFNKIHHELNQFCSVKSL
QEVYIDLFDQIDENLKRALQQDLNEMAPGLKILAVRVTKPKIPEQIRKNYELMEAEKTKLLIRIQH
QKVVEKEAETERKLAVIQAEKQSQVAGIQYEQKIMEKESMKRMSEIEDLTHLAKRKAMADAEFY
THQKQAESNKMKLTPEYLELMRIEAISKNNKVYYGSNIPNIFLNEGGLSQKTK*
>TRINITY_DN3545_c0_g1_i2.p1 type:5prime_partial len:236 gc:universal TRINITY_DN3545_c0_g1_i2:3-710(+)

SGPGRSSCRPILSVIKISKINNIDSGKVEGGYTMWSVYGSCSKSCGGGAQKRVRSCTNPAPANGGK
HCDGVSFETRECNINQCPVDGGYTMWSVYGSCSKSCGGGAQKRVRSCTNPAPANGGKPCDGVS
FETRECNINQCPVCPEIVGTYDISSRHHYGGAKYGTVVFKKLSNSKTVEAYYTINDGRRGVLTCKIE
SAQPDQCSGHGYYGKYFTYYKNRCQIIWSNDVIYTKQNC*
>TRINITY_DN1306_c0_g1_i1.p1 type:5prime_partial len:441 gc:universal TRINITY_DN1306_c0_g1_i1:1765-443(-)
QSSIFYNINFLELKNYFRNHIKKYIFEIGILTTMYFILLFFSSHLLLVVNAQEVLTLSTIFEKSRAAVQS
RKAVNVINVLRDYYFTDKKENSIWFHAQKEAKEHFNVELNKDDLSSIESGIRKLSNSLEECVEYSR
NKRRALCFRDLIDRLEEKQRNALWNYAPDIDREEKGKWGTLFLATLSMNTLISATISANLKYASSP
KAYLWKKNLFEFFEHWYSLLYGYEVDVIRWRVDQISAVDICEEKEHFWKRFTLACETRWKRNIDD
EVDNMAEIENIKAHLPNNEFKSEKKSMLKEKRSIANSPTFEMLSDEFKKTKQKMKALIDEEDNGR
DHPDNEPTLNALSNVIGGITSFEKIYKGKVFDFVKKKSIFEKEVLVKPADRRGDTLLSLVKEGMSKR
GVYEKKIKQELNYFNNGTSIVVRGIMAVHMNDLKKGFMQHL*
>TRINITY_DN602_c0_g1_i3.p1 type:complete len:851 gc:universal TRINITY_DN602_c0_g1_i3:3046-494(-)
MIICRLHGLSLGIILIFSILYTSDARSRHAYGHKDASSPQTGKKRAESDAGAGTEAATGANTGAATA
SDSSSESQTFTNTKVNTRIATSSNARSGANAAAKVKTYAHANTETKVDTQQQKPTPPVPPEALTT
PPPGVTPPGPPGPPGSENNNTSSGSECKEKETKPPIIKVYCPNCPCPCPPPCPCKCPCIKVAPPPPAP
PPCICLPPCPPPCPPPPPPCPPPCPEPLPAPAPCLPPCAVPPCPCNIAPITLDPSLPCPMDCPLPPCAC
PFMKKDNITTTKTKRSVIRKRNLNIKRSHRVGTKRVNAGKSPNLAYAQPDSFEKAAVAADNKKCS
EIGVEILKKGGSAADAVVSTHCCVEIVNSHSTGLGGGGFMVYYDKKSGKSRAYDFRETLPKKYTD
TQNKTQGETILVPGVLRGLEAVWKDFGKLPWEELWKPCIKYAKEGFYVHEALAAGIVKKADYIM
ANLGLRELFAPDGELLQLGSLFKRPKLARTYEQIAKHGADLFYRGEMAKQIVNEIKEAGGVMQL
EDLSSYQVNIRETLKSNINGLKLLTSPAPSSGSLMALALKIMESFKWKPEDLLNNPSLVYHQIVEAM
KFAYAPTSYLGDPRFTNGTDKVEQYMVSPQTAKEMFKRIDNKSHPVEYYKPWSELFSAEKTGTSH
MSLLDQEGSAVSLTSSINAYFGSKLRSREFGFIYNNELADFSEFWPTVYNLTSDKKAPSKRPMSKVM
PGIFLKGKTVQGVWGAAGGFFIPSALTMSIANWLFFKDNLKVAITRPRLHCQLFPPTVVYEPTFPP
SLIPKLQEFGHKYVTNSTYDVSGQLNAIMGVVQAIVRLPNGSYSAEADYRKGGRPAGF*
>TRINITY_DN730_c0_g2_i3.p1 type:complete len:235 gc:universal TRINITY_DN730_c0_g2_i3:1019-315(-)
MPKKDRTELNRELEAFAVQIPSANSGFRMQLGETFIQNVKDGKIKGFEYKVRDPYRNDLFHDTSR
IDLYTDYELIMDNLYVKNRSSYKVEVEDEKGLLAFKEKLLSKPFMCHAKLHLMLKEDLTQKLSND
FFVTVFPENPDVKAAIEKGFQHALDCVKNKKHYKVWFTPEIWFWYKDFVTNATKTQFEFGDLRY
SKIELNDKKGFFSSSKDLNCPIKPSIIELVTTEAIMPDGY*
>TRINITY_DN1199_c0_g1_i2.p1 type:complete len:296 gc:universal TRINITY_DN1199_c0_g1_i2:164-1051(+)
MDGLNDAATKAKTIYGGNLLFSAAMTAVAHPMTYVKVLIQIGHEPLPPQQVTTLFGKKVMRLP
NFFQYAGHIKKIDGWFGLYRGLGPRVVHNICSSAITNSVTNKIKGNSRDEARPNDKSVEGFLRETG
NLAIAKTAGIVVSYPFHLISIRMMVQFVGREGHYRNIFTSVQEIYREEGILGFFSGLIPHLVGELFAL
WMFKTLNFLVQNYLISEEYSQVAEIKSYSQGVSQYIASMVTYPFQLVSNIMSVNDSRLVAGNPPLM
PIYDSWTDCWIELGKKGLRNRGSTLFRRNVLT*
>TRINITY_DN15584_c0_g1_i1.p1 type:complete len:275 gc:universal TRINITY_DN15584_c0_g1_i1:874-50(-)
MADSGSAGADRGGRGGFGRGGRGGRGRGRGRGRGRGKPEDKEWTPVTKLGRLVKDLKIKTLE
QIYLYSLPIKEFEIIDFFIGPSLKDEVLKIMPVQKQTRAGQRTRFKAFVAIGDYGGHVGLGVKCSKEV
ATAIRGAIIQAKLSIIPVRRGYWGNKIGKPHTVPCKVTGKCGSVTVRLIPAPRGTGIVAAGVSKKLL
QMAGIDDCYTSACGTTATVGNFAMATYMAIARTYSYLTPDMWAENQFQKTPYQEFTDYLAKNH
NVGGKPVAPATATA*
>TRINITY_DN3078_c0_g1_i1.p1 type:complete len:1109 gc:universal TRINITY_DN3078_c0_g1_i1:3628-302(-)
MKMRIYLFLILFATVYCRVVQKGPNDIPEETKNEIKNQEPKIKFEPEKDKKYFLAKDTTCAHDVDK
HCKNIRRENNFAVFVCLQDVIHKDEDDVAEECHETLWHYKYNLTRDPNFDQAAFKVCENVINEI

QECNDSELLKSPGFVTNCLIENRHAVSEAKCKSFLQKMSAIVFEDYRLIKGFYDSCVEDVKTLKCG
TVAKPTDEEYQHDQTEVISCLEEQLDLVSEKCQAQLVKVATMQADDYHLDRPLFYACQEARERF
CPEVKSGDGRIYKCLVEHKTNEMMLEKCRKKIEIREKLTQKDIKVNELLWKSCKADFTKYNCPKD
GISEHGAASSLLLCLQGKVAEGLPVDQSCQAEMRTFAIELFENYKISPVIVSKCDVEINQFCHHLIG
KTDDGDMMDCLMTQAATNNESVSKKCYDAVSDVIKETGAGNNYKIDRALYLACEPVITTLCQG
KEDAMILGCLMDNAHNSKMPEKCAAQVFHLQYFLSRDFRLDASLYKDCKKDAEEICSANDFNA
ESESKVPDNFVIACLYRNALDRKVDDRKVSQTCAEHVFRVMHQRATNIHLMPELERPCMADLG
KHCIDKGDKEGEEIDCLQTNYEKLSAKCQNAIKVFTIQEAKDFDLDKHLVDKCAPMVQQFCQRE
LEDGNGEKVLPCLVDHKNDIDMDHQCANAVEHWQLIEMKDFEFSPALTEMCLLDIRKHCQKSK
AKIEALHCLSENIVKDASSVTEKCRAQLKNELIEQSENIKLNPELYEACTKDIELFCRDVVPSGGQL
EECLKRNHKKLTDPKCKSLLFKEEKKESFDPELDYRLMHVCKPIIKRYCTQYHQPVDIMECLRQV
THDRDMSRECRAIITERQIEQAESFELDPELSKFCQDDVNRYCKEEIKHAEKGDDDGAIFGCLVNA
MIDNKPIKNDCEMYIRRREAEAAQELDLNPQFLLQCRDEITKICPNMDEDEIIECMKMNIPQIQSK
GCVQEVKKLIVEGIEDVHVDPHLQEQCARDIRTFCQSVPSKSGNVVLCLIDVHKAKNLHLQPNC
ENFLTKRLSLYNAAKVDYAVFDSISAVMNAVSESPNRNYVYMTLLLVLTILFVGGMLFGRYTKKIRS
EIKNR*
>TRINITY_DN19237_c0_g1_i1.p1 type:complete len:209 gc:universal
TRINITY_DN19237_c0_g1_i1:128-754(+)
MMSEDEQMRMELQRMQRRADEMTDESLESTRRMLRMAEETQDTGIKTLVALDEQGEKLNRIEE
NLDEINDGMKEAERNLTGLEKFCGLCVCGCKKRKDFEGTEEYKKAYNHKSDGNKNGRSGNKA
GGSNDVAKSGYIQRITNDDREDEMDENLGEVAGIVNNLKSMALDMGNEIETQNKQIDRITDKTN
ANEIRIDHANKRAENILRS*
>TRINITY_DN3036_c0_g1_i3.p1 type:internal len:548 gc:universal
TRINITY_DN3036_c0_g1_i3:1643-3(-)
AFAGAARASPARQGSVARRALDQSSRYADLSLTEEDLIRNGKHVLVAYIMKPKAGYDYLATAAHF
AAESSTGTNVNVCTTDDFTKSVDALVYYIDPEEEEMKIAYPNLLFDRNIIDGRAMMCSFLTLTIGN
NQGMGDVEYGKIYDFFLPPEYLRLFDGPSCNVIDMWRILGRGTSNGGLVVGTIIKPKLGLQPKPFG
EACYAFWQGGDFIKNDEPQGNQVFCQMNECIPEVVKAMRAAVKETGVMKLFSANITADDPVE
MISRGKYVLSQFGPLSENCAFLVDGYVAGGTAITVARRNFPKQFLHYHRAGHGAVTSPQTQRGYT
AFVHTKISRVIGASGIHVGTMSFGKMEGDASDKNIAYMLQGDVADGPYYQQDWQGMKETTPIIS
GGMNALRLPAFFENLGHSNVILTAGGGAFGHKDGPKQGATSCRQGEEAWKLWKAGTYGDITLS
DGVIEYAKTHEEIKGAFLTFQKDADQIYPGWKEQLGYTGESSVQAASFDWKRKAGAAAFAGAAR
SPLAKQSSVARRALDQSSRYADLSLTEEDLIR
>TRINITY_DN22492_c0_g1_i1.p1 type:complete len:1026 gc:universal
TRINITY_DN22492_c0_g1_i1:171-3248(+)
MLQKTLIYTLLGCWITLLSHAAIVRKSGGDDFSLKVGKPQKPIGQPKLKDAKKEVKEEPTSDVIPQ
QDENNDTTDRQKHKEQVSPDAVESGMPPRYPLKAMSAEVSAHDNEIGGCRDHPRASRVCKNC
GNRNFAAEHESYTYSYCRKSCRHCDPELSSFHTFWRLRAASQFDPTWMVKEIEFYPKPTEYDNLA
TDPQRAYASSSYPGFKPSYAFDGNNKTSWYPSGWGLHDNNDDWIGFEFPVAVRVHAIRMVLDE
MHRTAAPQRIFVEASDKKDGPFTTKWKISNPTYEVDNVYKLLQCPVLWRKFESDEGSWCFRLIAK
FETWEVARNECTVEGGDLATIATEEEKRFLTDELNLGGFAWIGLNHKDQAYYWQDRANSSYKN
WKAGWSYSEVQKQNLNCVATDQDGFWYNFHCTNKYYFVCKKKLEPQAAPREDLILKAWPYEP
EIPPMIFLYPNITVPVINTKNLEPGVFPDENGKVPEEEEEETTADDLFYEQTVEHLKEKGLKPSEEIS
NDVDKEETDYTQQLNKIALKTQSRDDFRNETESDKLKFNSTEAVKQAKDLEEQKSVNLDQNFN
QYNSTDGVKQQSQPVVAAPSDGPSGNSGDNNTLPRIVNGKPVESPSVNPNNVGKPNTPVASPES
PENTDFAQPGDTTFTTPTPTVQSGDPGNAEKSNPQKATNDQQAGGQQPNQESGSNANQSQVQ
QQSQQQQPQQQQQQQAQNNNNNQNGQQNASNQQNNNNNNNNQNNNNNQQQNNQQQ
NNQNNQQQNNQNNQQQNNQNNQQQNGNNNGGNNNDLPNGLPPVMGGPAPQKIPEPPAP
KEGTKDNDSEKSVGGQADVMPDDAPSNDNDGGDETTTGNEENNDESNNNNNNNNNNMNNV
NNNNDNNQSRTLDKPLPNQGKNSSETVDKATNVGGTNKQENNDFMKLQKAKPGIRTHKIGHK
KSAGLKKTHMKNVVSPLKKVKTEKKINKQVKTVKKIKAVKVKHHKKALKKEVVIHKSEDDEEE
GSGSGDEGDQEDVDDILESITNGLW*

>TRINITY_DN5500_c0_g1_i2.p1 type:3prime_partial len:1183 gc:universal TRINITY_DN5500_c0_g1_i2:3546-1(-)
MEIKNFLILLSLLSLTSQLKAGLVFNETGVDQPVDLFFVIDGSGSVGYELFDGQLQFVTELTDQLGG
VSPFNVHVAIAVITTSPYQAIGLTQYQVNSEFKQAVSEIFYPRGATRIDKALLAVERELLPTSRAGVP
KVLVILTDGIQARWPGVYNTHVIIERLFNAGVSPVVVGIGSDYRDLDRLNKLAIKENHHLNEPIEK
LFLTTDDMEVGSPALVQKAASTVKKILSNVARFGYEVRSDFAFLLDGSTYAEDQLKLMEGLASYFQ
INRELSRFAVASSQGGGRTYIEFNEAQLSRDFKAMLMKLNVDSESIDIGIAAKYAYEEIFRIEEGERYS
TPRTLVIFSGNAEAKDSTLFGKYYSLLKKANVNLVMIAVGGGKGRSNLRNFPVDYYLPDFNDEVS
FTEECNIIAREMHNYIIRFASDTQRSKKCLHDGDLFFSIDVSGSAGAYYQENLNIIEKIIASFEISNED
SRVGFAIDRDGNTQVTPLDKHNSLESLMRDFDSLPYYPRGRSDMVAKLKAAYNQFLTSSRIHFQDI
LFIFTDGYLNRKSESESKELIDKLKAFGVEVFVVSLGYDVNFPFLMTLSSDKGHFFHVNQLPKDSDS
FVNSIINKVCDYTETFQDKYEIPINVALVLDGSHCSEKFHEAQKTFSNVIIDGLDNSPLAKNYLFSYS
SGVQQLDSNSSLAINFGSDGNRLDLALNEVVKLFQSISSNEVNMAIFVTNAGLESEFNATMSQAR
KLRSLGVITMVICIGDNFNYDVIRTLASSRQLIYMIPSPSQLTDTKFLRHIISAAFEMAESMPPPRLEA
GKSWYPAFFGNWVDSNSLEGEVPDAPLKAYHFGPTVLWSKPIQQKNYIIESTIIPAGSESGRFGTCG
HIFKHKVGATKKEKYISKSWGYFLWSNSFLYITTDGTETSPRIEFDYSSRDKLNLKTIVDGSKVKIYI
DGVFIRSIELEGTEADSQSHMFVGVWCHRLSAVTVEDFKVSALSNPCTVNNGGCSQICSADPYEF
KCSCIPGYRLVEDESTCVAESTCPELITTTVITTTLSSSPPTTNQSKFISTTTKPDELIDTTATIISTRESDI
TTTLILSTKSTEKPIEKIEKTTTSAIGDILEQTTVAPIKTSTVFVPTTKEEVILTTKEMAETTLTVPTTASP
VEETTTVAPVSTAAKTEATTQAVITTLAATTK
>TRINITY_DN1121_c0_g1_i1.p1 type:complete len:252 gc:universal TRINITY_DN1121_c0_g1_i1:1155-400(-)
MAEREQLIQQAKLAEQAERYEDMATHMKGVTKMADQPLNTEDRNLLSVAYKNIVGAKRSSWR
VISSIEQKATDSKKQYAEAYRIVIEKELSDICNEVLHLLDDFLVKSASATYYEDPNLTTAESAVFYLK
MKGDYYRYLAEVASESEKQSVVDKASNAYKEASNIGIKSLAATNPIRLGLALNFSVFHYEICSEPSK
ACTLAKDAFDLAIAELDSLPEESYKDSTLIMQLLRDNLTLWTADQADGNEPDAE*
>TRINITY_DN278_c0_g1_i1.p1 type:complete len:470 gc:universal TRINITY_DN278_c0_g1_i1:3091-1682(-)
MAPSFNKTNGHMEENGGMIHANGKSSEKPINLGEQTYSYMTFKKMGLIMITLPLVFLLLVGLPSY
LYPVLVLLFFIPVFAGYIGLNNVIYCLYERHNQVSKIDNYIEFLDEDLKKKFTGRFIPIRDLYELYAEG
KLNFKGDVLQCLERRNEYVSYKLQLWHLKFFLSKLLPELLHARSQDKEQVCDHYDRGNDFYNA
YLGPLMVYTSGIRYSESDTLEDMQMNKLKAVCEKVQLKEGDRHLDIGCGWGTLVNYAAENYNS
QSEGVTLAKEQVCWAEEKAKSKEIMDRVNYLCMDYRDIPKKKYDKITCLEMSEHVGIKRYHHFL
KDVRNMLEDDGIFFLQIAGLRRAYQWEDFLWGFFMDTYIFPGADASLPLQFPIAELEAAGFEVES
VKTVGIHYSDTIKYWYHNWVKSDSHDKMIQKYGRRMVRVWDIFLAWSTIIARQGNSTCYQIVAH
KNFNHYPRRRFIQPKN*
>TRINITY_DN1653_c0_g1_i1.p1 type:complete len:1122 gc:universal TRINITY_DN1653_c0_g1_i1:180-3545(+)
MMRRSIKGSEDNWVEMQNDTFKNWVNANLKSRGLEVDNFETDFEDGVRLINLFEAVSKKSIGK
YAKQPKFANQKLENVSLVLRAMESDGVKLVSIDSDHVVNGNSKMILGLIWQLILRYQISGSDDKSF
KIALKKLLLIWLQHVIPEQGIRNLTTNWNNGIALCNLINVLKPGIFPEYNNLDKRTPVENLAKGM
QLAESELDIPQIISPENMASPNVDELSMMTYLSYFTQDGGIGESWTVDLVNFWNSSLSVTNFNTD
WNDGMRLGRLVETFLPGAMPIMESTDNLDNCRIAMDVAHEKLGISKIITAEEMSNPQIPELAVMS
YIVQFTKVKRVERASDKFSASGPGLVEPKIGIVNEFYVRSDAGYPLDGLQVTIIGPNNNYIPIEIDDS
DPQLRNYSYSCEEQGQYHINIIYEGEDIPRSPIIVTSQQDLNTIRVYGERLEVSHAYEETEFTVDVGSI
NSYVTATIDGPEGSQPTPQTINNNDGTYTVRYIPSTVGTHRIYLAVNNQQLPGGPIETNITYPIVSEA
ELIETVAFSAKYEEDKHLYAELYERIGISVDTSQSGDGRITSKTIGPDEKEIYSEVDAEGDGSYTVSFTP
EVIGEYHTNVFWSGKPIHGSPFVVWVSDPNKVFARGKCLYETRVGVESSFEVATNGAGPGHITGY
AETNGVEYPINVRKREECLHDISFTANQVGIYYVYINYNNVPIKDSPFQIKVGNPSLIEIEKHEEAIM
ESNQPQSFIVNVLKDAGFGDVLCVVRGPAGEDVQCTIKDVNDSLKEIMFLPNYPGTYEVFVYYAG
GLVERSPFIFAISEPKLAIYPENIRVSGNGLSRAKLNELARFQVDTTNAGPGHLTVQIEDEKDDVEA
GIRDNNNGTYDVTYTPKRSGKYRVIIKWSDQHITDSPFEIIVDNRAEEVLVEGYGIKEGTAGQPVLF

TLDGRNAGPGQLSCRCRAPSGKMTYVLISDNKDGTYTVDLNATEPGLHTVEVEWDNQAIPGSPF
LVRIMQAPDVKKVRASGPGLRSGTLDSFNGMFQVDSKGAGPGTLKVRIHGPKGAFKVEMSRENP
KDRIINVRYNPTEPGTYTANIYWSDEHVQGSPFEIFVGQNDQEVDRWDRNKEELSKSQVNVY*
>TRINITY_DN1936_c0_g2_i1.p1 type:3prime_partial len:1894 gc:universal TRINITY_DN1936_c0_g2_i1:5680-2(-)
MLSPAVTKVFIVLSVLGGLICQSCDFEVANACQWTQYAKDKFDWTRNFGNTGTGGTGPKTDHTS
EVKILKANTTGGNSNGAPCVFPFKFRKRTYTKCVRSMQRGKSWCATVPDMNKKRQWGYCMPT
GYYMYIEASSKRGNPRNKGDNAVLLSPVISKGPKCLQFWYHMYGPHIDTLNVYKKVSNNMTKL
WSLTGTQGNRWISANVTFNGGSQFQMAIEAIRGVEYKGDIAIDDITVDDGSCNADNGQKGNVTS
YGKCTFEKDKCGFKDGNTSTLTWTRHKGKTPSKGTGPLVDHTFGTGLAYYMYVEASNNPMQSA
RLYTTYTNLDSTGSCLSFWYHMFGPRTGELKVYINPPEIGKEIPSFSVGGEQGNLWIRGEVTVNTTT
ALVVFEGTTKYSYLGDLAIDDVSMERGKCEDPDSCSFQKDLCAWYNLPKSSGLDDFDWIVGAGV
TPSRFTGPTSDHNGDIAGKYLFMESSSPVKMGDKTWLSSELFNSTGKGKCFVFFYHMYGRDVGSL
NIYVNTSSGVELVWTQSRSKGKQWQNGQVDVERPGTYKVLVEGVVGKGYLGDIAIDDFKFKPGK
CQIVPSNASPKKPQTTMRPTTKRPTQPPTPILDCDFEFGVCSDWRQDYKNDTDNWKVGKGTTST
AGTGPSFDHTKGTIYGKYIYLEASYGRLNETARLISTSIPATGDTGGKCLTFWYHMYGPHINRLNIY
VMIGGHTGKPIWSRVGTQGNKWNKGQVTLVGRQPFQAVIEAVKGAKYQGDIAVDDVSLSSKKC
PQPGATTCTFENSQCGYIDDTSSDVQFQWTWQSQATSSLNTGPKFDHTLQSVSGFYMYTEASNA
KQGDKARLISPVNPGTSGSCLKFWYHMYGAGMGTLSVRLLVNNKLQSVPLWSETGNKGNEWK
VATTTIKTKDAYQVVFQGVIGTTYASDMAIDDVKLSDGECQSPGFCNFEDGLCTYANSKTDDFDW
LVGSSNTPSSFTGPKVDHTFGNASGSYLFLEVSKPIKAGDVAVLESQQFNAVGGKTRCLNFWYHM
YGSDIGTLQVIYKVFSGSQQERVLWKLTGQQQITEKDPWKYARVPVDMDADHMVMFKGIAGSG
YAGDIAIDDIQFTTLACRVEPSIANPTPTTPKPPTTFPKTAPPTAAPTPFSCNFEKDFCSLTSDSTAVF
NWTRSQGYTASQNTGPSTDHTYGRGFVKTYGPGSIRHAAGMCIHISMGGHTKPKDETALVLYNG
CGQDRTEFKYDNGLIKSTQYNMCFKLKGASTSAGTKVVISDTCTNKWKMLANGAFQDAKTNLC
LAPSGKLAYPQNNSPLIVSKDCKAKGVIFTFSPTMGFYALIEASAPQKPNDTARLISPVLNKPKCLS
FWYHMYGANINQLNVYTKSPNGVYGNPVWSKFGSHGDKWRPAHVYMNPKSSSYAYAIEGVRG
NGYEGDIAIDDVSVSDKCPAPVECTFEETNPVACGWTNLKTDDFDWSIGQGATSSQNTGPSNDH
TYNDQRGKYLYIETSSNLKAGAKALIQSPVYRGNGRQQGQCFQFWYHMYGASIGRLSLHLDQVG
STTKNRAWIQESDQGNRWSIGQITIKSQAAFQLTFEGIRGKSFTGDIALDDFRMFDGPCNNIATCD
FENEKCTWKNSYHKDDFDWIIGQGTTLSLNTGPVTDHSTNSGDGHYIFIDASRKKQGAKARLYSE
IIPRGFFTFIPYCIYFWYHMSGPDIGTLNLIAKHGKGKALEKVIWSLSGNQGSKWLQGTAPVTSSVD
FHLVFEAIVGDGYKGDITLDDIVYKLGLCSISPKAADPKLISTTSAPTPIPTTAVPAMYDCNFEKNSL
CQWKQDLTDQFNWTLVQGQTPTLGSGPSVDHTTGNKTGYYLFIETSNPRKAGDKARILSPSMDS
DKDHGHPHCLKFWFHMYGPHV
>TRINITY_DN146208_c0_g1_i1.p1 type:5prime_partial len:284 gc:universal TRINITY_DN146208_c0_g1_i1:876-25(-)
TDSHIRTTSAFIPCAKPSKMPPKTKKIKVKKVAAAPFQTSKPAPKKSVNVLIERRPRNFGIGADVQP
KRDLTRFVRWPKYVRLQRQKRILYQRLKVPPSINQFTQTLDQQTATQLFKLLHKYRPETKAEKKQ
RLTQLAETKAAGKQESSRKKPVTIKYGINHITNLVESKRAQLVVIAHDVDPIEIVVWLPALCRKMG
VPYCIVKGKARLGKLVHKKTATAVCLTAVRQEDRHSLEKVSDAVRTNYNERFDEIRRHWGGGIM
GGKSQAKVAKAERAKAKELRV*
>TRINITY_DN165536_c0_g1_i1.p1 type:complete len:249 gc:universal TRINITY_DN165536_c0_g1_i1:851-105(-)
MSEQEISNLVEQAKLAEQAERYDDMVKYMKELTEKSTEGLSDEQRNLLSVAFKNVVGARRSAWR
IIHSLEEKKGNEGGNTELISSYRKEIEKELKERCNEVLSLIDKYLLPSAQKEGQKGTEGKVFYEKMK
GDYYRYLVEVTTGEDRANLSAQSKEAYDKATETATGLATTHPIKLGLALNFSVFHYEIMNDGKKA
CELAKKAFDDAIAELDSLKEDSYKDSTLIMQLLRDNLTLWTSENDNEENDAQ*
>TRINITY_DN13856_c0_g1_i2.p2 type:5prime_partial len:292 gc:universal TRINITY_DN13856_c0_g1_i2:3-878(+)
GAGRQAWSPSVSRQHGAAGGGAWAGAPESAATLTSSLAVGLSAGVVVAGLSSRRARTARRAVE
NKSQNGPFGELATVEPRTLELTEKVKAVIKQKGSLDRESYREIFLAPKPDAVFAGGLVGSEYHGWG

TYQWDPAGLATRYPEHLPWYREAELKHCRVAMLAWLGLIVPDFVRIPLDDFQDPTLTLENAHNK
LIYGLGSGPMWWLLLFCGIIESLRFKQLGLDFGKLTLENAGDLDFGKGFLPKTKEGIVQMKIKELK
NGRLAMLAVSGALTQSMAYGVYTFPWTLPSPN*
>TRINITY_DN3347_c0_g1_i1.p1 type:5prime_partial len:924 gc:universal TRINITY_DN3347_c0_g1_i1:2-2773(+)
YGDGSDDDMDSIDEDEDEDEDDDDDEYSDDDDMSWKVRRASAKCLSAILGSRMDLLSEFYINVA
PKLIARFKEREENVKSDIFQAYISLLRQTKPVAMNDALEDKPDSPLSMLKQQVPLVVKALHKQLM
EKNIKTRQGCFNLFTELVGVLPSGLTEHIDKIMPGIHFCITDRNSTSNMKIDTLSFLGCILQHIKAEV
FYPHIHQFLPAVVECIHDPFYKITSEALTVAQNTIKVMRPTGTENYFDFKPYINDLYQSILHRLRATD
IDQEVKEKSITCMGQLLRNMGDNLSKQLPECLPIFLDRLRNEITRLTTVKSLTTIASSPLHIDLSILLL
DGIPLMATFLRKNHRGLKLHTLTCLDLLMQNYGNVMSTKHFEAVLDELSNLISETDLHVSQLSLN
LLTTMISKQQLLLKKVSSTVLSHIVILTRSPLLQGHALNSMVSFLTTLVRYSSSGLGYTDLLQMLTEV
IYGSTTENIHKSAYHSIARCASALTIACPSEAEAVVTKFIKDIKNSGTNDSIRLFSLLTIGEIGAHLDLS
SKAELKNVIVNSFSADSEDIKTAASFSLGHITIGNMDAYLPFILKEIESNAKRQYLLLHSLKEIIVCQS
TDPSSVKSLEQYVPSIWSLLLTHCENKEEGTRNVVAECLGKLTMVEPQKLLPELKSYLSSDLYSAQ
MRSTVLTAFKFTITDQPQSIDILLKDHLQAFLQPITDNDLNVRRVALTTFNSAAHNKPGLIRDLLTK
ILPHLYNETKVKKELIREVEMGPFKHTVDDGLDIRKAAFECMYTLLDSCLDRLDIFEFLNHVENGL
KDHYDIKMLTYLMLVRLSKLSPNAVFQHLERIIDPLRTTIQAKVKANSVKQEYEKQDELKRSALRA
VCALQKIPDAESHPILSDFVSQIRSNSDTKVMFESVQRDPSSTSLDKMDTAS*
>TRINITY_DN1194_c0_g1_i1.p1 type:complete len:555 gc:universal TRINITY_DN1194_c0_g1_i1:101-1765(+)
MAARLGGGMMQPQIILLKEGTDSSQGIPQLISNINACEVVAEAIRTTLGPRGMDKLIVDKNGKSTI
SNDGATIVNLLDIVHPAARTLVDIAKSQDAEVGDGTTSVILLAVEFLHQIKSLVEENVHPQILIKGY
RKATKMALEKIKEINVQIKKDDQEEMRKLLQRCAETALSSKLVAGHKEFFGKMVCEAVMHLDEL
LPLNMIGIKKVKGGALEESQLITGVAFKKTFSYAGFEMQPKKYKDPKIALLNVELELKAEKENAEV
RVDNVEEYQKIVNAEWNILYEKLEKIVASGAKVVLSKLPIGDVATQYFADRDVFCAGRVVEEDLL
RTMKACGGSIQTSIHNLVDNVLGTCEDFEEVQIGCERYNLFQGCPRSKTVTLILRGGAEQFIEETER
SLHDAIMIVRRALKNDAVVAGGGAIEMELSKHLREHSRSIAGKEQLIIAAFAKALEIIPRQLCDNA
GFDSTTVLNKLRQKHAQGGKWFGVDILKEDIADNFEAFVWEPAIVRINALTAASEAACLVLSIDE
TVQNPKSDMSGDDNPITGKRGRMPQMGR*
>TRINITY_DN1386_c0_g1_i1.p1 type:complete len:596 gc:universal TRINITY_DN1386_c0_g1_i1:2419-632(-)
MEYIEVKSFIVALVFVFSILGTAQSDLVINNVERTLDLSTQTAKYTTKIKFHNPGKKVEKSFLLALEK
DQEKHLSYLEILDKQDDNSLEYAAENVEGQSGRFFKVSFSKPLKENDDVTLHIETVFTHVLTPFPA
KIGQTEKQLVLYKGNTVFYSPYEIKEQSTTVKLASSAIESYSRLKPSRSSENTITYGPYKNSKPYRLHE
MKIHFENNSPFLVVNEMTRWIEISHWGNIAVEETYHMTHEGALLKGHFSRYDYQRSPTHSVIKSF
KTVLPAAARDVYYRDEIGNISTSNMLVNDDSVEIELRPRFPLFGGWQTRYMLGYNVPAYQYLYNK
GDNYILKMRLIDHVFDEFVIDDLNVKIILPEGSTNIEVVKPYELEEGKRDIHKTYLDTSGRTVIVLSK
KNVVENHIQDIQVHYTFKKLQLLQEPVICVAVFYILFLSVIILVRLDFSITKDAAKESRMKVASLLEE
LLSSCGRRGSLYYSYDSAIDKLKQNRNQNDFAVSLKKLNQDYNTLTQSINELCSLLIKEDPEIGDKV
NDLQKKENERKVIVDQLVTLATRVVTNKIGRQQYIESEKNSMAKREKLGEDIDNVLATL*
>TRINITY_DN102244_c0_g1_i1.p1 type:internal len:304 gc:universal TRINITY_DN102244_c0_g1_i1:1-909(+)
LTFIESLRLRLYEILVDKMDLVFACQFFLLVLCNLVYSEEVCPTRINDEDKFETKKRFDALRSKLGDK
KLEVRFRHTSKKRENDLYDYTITLCGENNSKAVTQFDVTKSTILSAGQHDSSSVVYGGVDWLMIE
YLNGDKYTTAHHCHGKPRKTLLTIRCEEDNEKPKLLAIEEARYDTDGAGRHPNTPCYYLFEYNH
PAACSSSKVRRFHSENKLSGGAIFLIIVLSITAAYLLFGFIYKRFIVGAKGWEQIPNFSFWRKVGNAS
ADGCDFVCRREERSTTYKGMADALDIETSDDDRDDGLL
>TRINITY_DN22594_c0_g1_i1.p2 type:internal len:268 gc:universal TRINITY_DN22594_c0_g1_i1:801-1(-)
DFQDPTLNLETAHNKLIYGLGSGPMWWLLLFCGIIESLRFRQLGLDFGKLTLENAGDLDFGKGFL
PKTEEGIVQMKIKELKNGRLAMLAVSGALTQSMAYGVYTFPWTLPAPNAVQSRTALRAVEFKGQS

GPFGALDVAEPKTLELTDRVKAVIKSKGSLDRESYREIFLQPKAETVFAGGLVGSEYHGWGTYQW
DPAGLATRYPEHLPWYREAELKHCRVAMLAWLGLIVPDFVRIPLDDFQDPSLDLENAHNKLIYGL
GSGPMWW
>TRINITY_DN941_c0_g1_i9.p1 type:complete len:3186 gc:universal TRINITY_DN941_c0_g1_i9:86-9643(+)
MADPAKIVDPIDDPTASDAEWKKIQQNTFTRWCNEHLKSSNIYIYNLETDLCDGLKLIALLQVLS
HKKIVRYNKKPTFRPQKIENIAIALKFIEKEQIKLVNIDASDILKGNLKLILGLIWTLILKYQISMSMV
ADDDDDPDQKTTPKQALMGWVKSKLPPELKCGNFNKDWNDGIALASLVDSTAPGLFPEWEDL
DPANKLDNAKEAMKRAEEWLGVPQVLTPEEITNPNVDELSVMTYVSYFPESKLKPGAPLKPKMN
NASKCSASGPGLQEHGVVVNKPAEFTVFTSGSSRGKLNVQVFGPGRKAIDCMVQDNHDNTYSC
QYTAPKTGVYDVHIRVGGHHIPKSPFRVNVASDLDTSKVTARGPGLEPHGLIVGRPASFEVIATDA
GSGELVIDVIDPKGNKKTELKVEARPDNVYFCTYMPVQVGRYVIVIKYGGKEVAKSPYHVSVTPA
ADRVKIYGPGLEPGLKTGKPASFTIDCSEAGEGGLDVKIVGPGKGDIKYEVKDNGDGTYTCTYKP
TRPGQYEITVKFDGGVVPKCPVKVSVGSTADPSKITAWGPGLERGVAGRPVEFYVNCKGAPVDNL
TVGVEGPGEAKVDMKTNSDGTVTVTYYPTKPGKYIIHVMYDEMDIKESPYSATISPRGDVTKVYA
EGPGLEAGNMAGKPAEFTVFTKGAGVKGNVQVTCVDENGKPCEVTVKDNGDGTFKVTYYPKTI
GKYVITISFMDEQIPKSPINVTIIPFSDPTKVKATGPGLQGGKTNKLAKFKIDASKAGEGGIGLTIEG
PNESEIKCDDNGDGTCNVEYMPTESGDYKINVLFADQHIPGSPFTAVIGDDFDASKVKADGPGLK
PGNKAGKPCDIAIDARKAGKAPLVVEVTDEVGNPVKVDLNEEEPGLFASSYHPEKEGPHTINITY
GGKHIPRSPFKVTVKPSSDASKVKVFGPGIKPTGVKSLEPTYFEVDAGEAGEGNVDIKIEPTAKGM
PPVKDIDVKPGKTPNSYIVTYIPPKDGEYNIVVTFAGVNVPKSPFKISVTPGSDASKCKASGPGLEE
PIVGKENVFQVETPPEAGLATLTCTIDTPSGRKLKADCKKNKDGNGYDVTWTPKEVGRHKVTVK
YGDKEVPESPFYVDVLGKPDPTKVKVYGPGLENAFVEKPAEFTIETRDAGPGNLGLTIEGPEEAKI
DCKDNGDGTCSVTYIPTKEGPHDINVLFDDQHVPGSPFKVAATEPIDASKVKCKGKGIGKSTPAS
YPAEFVVDATKAGKAPLEVDVVGPGDKKYPCDIVDNNDGTFNCAYVPEKKGRYEVTPKYGGTP
VPGAPFKVSGEPTGDASKVKCSGPGLDRPHIGHAAPFEVDTTKAGDGKLNVNAKGPDGKELPV
KMVEEEEGIFACEFVPEELGPHELEVLFGGQPVKGSTFKADATPAPELDKVRTPALEEILEASPCVD
QQFEAPVDCSEVPDNKGAKLSGKITTPAGKREKVDVTDNFDGTFALNYTPTEVGKYVLDVGYDG
IPIPGSPFEFEVTEGGPDKVKVFGKGIKKGVVAVPSRFTVDTKNAGPGDLSVAVEGPSKVDIECVDN
GDGTCEVTYVPVEPGEYDVIVNFDNQQVPGSPFKPQIVQDEPAIEVGRQSDLCLTPEGISAKDIPSL
KGVLQAPSGKEKPVDITEGPNDSFIASFVPDEPGKNLLHVKKRNKPIDGSPVPVMVREKPVIGEES
GIPMDLADCDLPKDLPKLNAVLTRPNGKEGPCGVEPGPDDTVAISFVPEEPGKHIIKVYKDGKQIE
DSPFVVMVEGSPDQYPKVGHPCDKDFYQPEPCRRGDISKMKGTLRRPSGKEEEVDVKLGPDGTIA
VSFVPTEAGEHLVSVKKDGRHVPNSPFSIMVIDEPTVPTVGSECSANFDIPELDLPKDLQYLKGTLT
RPNGVEEPLELSVGADNSLAINFVPKEPGKHLVSIKKRGKHVKDSPFEIMVEEGKPTDAPTGGPVV
GHDCGVNLDIPELSLPRDLKNLDAELTRPTGKKEPLKCDMGPEETLAVNFTPTEPGKHLIDVKKR
GRPVKGSPFEVMVGSTPVSEDQIPVSEEIMAQSAPDVESGLPGGLDSLDSGKDKPVTVGSPCDVNL
DIDGVDLPKDLDKLKAELTRPSGKKEPVKCEMADNGGLSLNFVPEETGKHLLEVKKNNRPVKGS
PFEIMVEESASPESKPTAGQKCDLDLDIPDIDLPRDLKDLKAQLTRPNGKQEPIECTCTPENTLGLE
FTPDEPGKHLIDVTKRGRPVKGSPFEVMVESPESKPTVGNNCGLDFDIPDIDLPKDLKVLKAELTR
PTGKKEPIECTATPENTLGLEFTPSEPGKHVIDVTKRGKPVQGSPFEVVVGEEEPASKVPTVGSECD
VNLEIPGLNLPRDFKYLDGTLKRPNKTSDEPLELTMNENGTIGVNFVPEAPGRHEISIKKHGKHVS
GSPFDVMVESAPSDQPEVGSPTTLGLDMVIPKEDLRLLKGTLVRPNGQEEPIEITLDDNGHPNVTF
TPQEVGNHVINIKKRGQHVPGSPINVMVGPAKKPTTCDVGFEIPGIKLPDDLKYLKGSVVSPSGTE
EPVELQAGQNNTIVVSFLPKEAGIHYIHIKKNGKPVGDSPYQVNVKPEDIAAGQPDASKVKCTGR
GLRHGLPGVKADFLVDTKDAGYGGLGLSVEGPSKVEINCVDNEDGTCAVDFTPAEAGQYLINVT
FADTHVPGSPFKCKVGGPGVEDDIVPMTDDGVECASSDMLAFPSSKARSPQDFVIKFSGSGDLKA
TVTRPSGVEDEAEVVETGPDTYTVRFVPRETGEHLVNVKSRRRHIPGSPFKVLVEAPAGGAASCKA
HGPGLEGGVAGQPCRFTVITRDAGPGGLAVAVEGPAKAEIQCHDNGDGSCDITWYPVESGEYML
HIRFADEAIPGSPFKAFVLPADEGKAMPMESFKDQVLRVGQQASFAVQMKGKKGKITASVQSPSG
VQIDCSAVELEEGNYAVRFVPRELGDHLVSVFLDGHHIPGSPFTVKVGGLDSDPSKVTAYGPGLKG
GVASQPAEFTVNALNAGSGALALSIDGPAKVKMHCVEQDDGTYKVIYNPTVPGVYEISIRFGGQH

IPGSAYKVRIVGSGDDAINIFGDASKCNASGPGLNQATIGQPATFKVDASNAGRGSIMVGVEGPVI
PAKEIIVKHTGNNVYSINYILEQSGDHILRVLWADNHIPGSPFHVNV*
>TRINITY_DN79_c0_g1_i1.p1 type:5prime_partial len:478 gc:universal TRINITY_DN79_c0_g1_i1:2132-699(-)
VVSSESNVLVEFYAPWCGHCQSLAPEYAKAAKQLKESKSDIKLAKVDATVETKLAEKYEVQGFPTI
KFFKKGNALDYGGGRTDKEIVAWLNKKTGPPAKLLDNAADVKAFADERDVAILGFFNDKESSEL
AKAFLAVAEAVDDLEFAIAAPDASGDFAVTEDKIIVAKKFDDPQVVYSGEANIADIKKFVRAESIA
LVTEFTDEAAPKIFGGDIKNHLLLFISKKAENFAELKNTFQEAAKGFKGTVLFVYIDTDVEDNGRIL
EFFGLKAADAPTLRLIQLEGDMTKYVPETKDLDKDSVTSFVQNVIDGKIKPFLMSADVPEDWDK
NPVKVLVGKNFAEVALSKDKDVLVEFYAPWCGHCKQLAPIWDKLGEKYKDSENIVIAKMDSTAN
EVEEVKVSSFPTIKYFPKEGGVVDYNGGRTLDDFVKYLESGGKDVSGGADETGEDEDADEEEEGM
EEGMEEGDLKPEEPEVAKDEL*
>TRINITY_DN66856_c0_g1_i1.p1 type:internal len:443 gc:universal TRINITY_DN66856_c0_g1_i1:1327-2(-)
WSIGQGKTASTKTGPKNDHTYNDQKGKYLFMPTSNKKLRNGAKAQIQSPIYQVQPNGNSVKCF
QFWYHMYGSSIGSLNLYMKSQWLKRKLWTKVSYQGNLWWIGQVTVTSQTPYQLMFEGVRGSSY
TGDIALDDFRMLEGACNNLATCDFENDKCTWQNVLRSDDFDWLIGQGTTWTKWTGPLTDHST
DTGDGHYMFIDSSRKTKGAVARLYSERVQRSIFSFFPFCLSFWYHMQGPNIGTLNILTKTGRGKAN
EQLVWSLSGNQANSWKYATVSLKSRKGFIMVIEAIAGGGHKGDIAIDDIVFGRRFCAISPPEAKPT
VATTPGPSPPPTTPMPTVYDCNFEKKDICQWQQDVSDQFNWTLRQGATPTRGSGPTADHTTRNK
TGSYIFIETSNPRKAGDKARIISPPMDSDKYHGHPHCLQFWYHMFGSHIDTLNVYKKV
>TRINITY_DN118520_c0_g1_i1.p1 type:complete len:581 gc:universal TRINITY_DN118520_c0_g1_i1:1895-153(-)
MYRLLSVIRKTTQTAKLRQGLSACISTTTHYCSPKEIKTGTDARALMLKGVDQLADTVAVTLGPK
GKNVMIEQSFGGPKITKDGVTVAKAIELPDKYMNIGAKLVQDVANNTNEEAGDGTTTATVLARA
IARSGFEAVSKGANPNEVRKGVMAAVEVVTQGLKDLARPVTTTEEIAQVASISANNDAAIGKLIS
DAMEKVGKDGVITVKDGKTVYDELEVIEGMKFDRGYISPYFINTAKGQKVEYQNVLVLICQSKIS
NVQDIVPVLELANQHKRPLLIIAEDVDGEALTALVINRLKIGLQVVAVKAPGFGDNRKNTLKDVA
ISTGAEVFGDDLNLKLDEVQLSDLGQIGELVVTKDDCLMMKGRGNTDLVNQRANHIHDEIKATN
SDYEREKFQERLAKLVGGVAVIKVGGSSEVEVNEKKDRVTDALNATRAAAAEGIVPGGGTALIRC
AKLLDTFQVPTEDQKWGVDIIRAALFKPCSTIVANTGIESAVVVEKVSSSKDPEYGYDAASGEFAN
LIKAGIIDPVKVVRIALSDAAGVASLLTTAEAVITEIPKEDPSMPPMGGGMGGMGGMGGMM*
>TRINITY_DN31399_c0_g1_i2.p1 type:complete len:819 gc:universal TRINITY_DN31399_c0_g1_i2:85-2541(+)
MAVNVSHVYTCPSDYLCSKAEQGTLLIPLINESEWGRGFRIFLYTLALLWCFMGIAIAADLFMCAI
EIITSKTKKIKVATEGDDKMGYEEIEIRVWNDTVANLTLMALGSSAPEILLSIIEIVGNNFESGELGPG
TIVGSAAFNLLVITACCVMAIPGGESKRINAFNVFLTTSFFAVFAYIWMFIVLTVSSKGILEVWEAAV
TLLLFGILVILAYIADKDLCVSVFTRKRRKKDIELDNFDLLNENVLKKHHPDLIEYMKEITANPSISE
QEAAKLIAIKIEASKRKNYGYYRVGALRSIGGGRKVVPAVTPKLQGIIDGGSTLNVNAETNVNDSV
PDNKATIEFTATAVSVMENNKVVQLGVERSGNMSSQVSVNFETIDGTAEAGSDYIAAKDTLVFEID
EIRKSIEIVIIDDYEWEPDETFFVKLHVPEGANDRVQIGHHSICEVTIINDDEPGTFSFNKPSNIVKES
CGQAELEVERVNGADGKVEVVWKTKDQSALHGKDFIGGEGTLVFEHGETLKSISIEIIDDHMFEK
DETFMVELLEVKNEGAKFGRLKRTVVTIVSDDEYQHMFDRVVNMAHMNLHQFQLGSSTWGQQ
FREAMNVNGGDLESASFMDYVMHFITFFWKILFALVPPCTWYGGWLSFFIILGMIGCLTAVVGDIA
TIFGCLVGLRKDVTAITFVALGTSLPDLFASRNAAMNEKYADASIGNVTGSNGVNVFLGLGIPWLI
ASIYHEAKGKVFKTPTGSLATSVVTYSVCAIACILLLIIRRFVPFFGGGELGGPKGPMYASGIFMISL
WFIYVLISALVSYGHIAAL*
>TRINITY_DN73067_c0_g1_i1.p1 type:complete len:831 gc:universal TRINITY_DN73067_c0_g1_i1:3714-1222(-)
MHWFVILLLFGVIFVTVSNYEIDVLSTFELSEDIAGISPLDNKYQAYQFAADGKELLLDDSIKTEIVR
GVTNFKDFFIAVNIRHDKQKPSSLLSLQTKTTGKQKFTLWINAEEEKIGIRSHSKESKKGKKNLTFR
KVPLNTGQWHRIVLNFKKIDTDEPVVDLYVDCKFHDSKTFPFSIGHALIEDALISELRLGQLKHLP

EKDPIRFIGAMQDFRLVSNRDVHWYNRPQHCYNMMIEVKGMKAYHEPVSTISHDVDNMAAAM
KNMQKQLKLQLDEIRDIKNWLQQCTACKTAAENVLGGEIPKACSQNRCNENANCIESNGGSAF
RCECKAGYAGNGEKCGLDSDSDGVPDNKLDCLGKECSQDNCPGFPNAGQEDADEDGSGDACD
IDDDNDGVLDGDDNCQFLSNRDQKDSDADNRGDACDNCPFESNYNQLDSNNDGLGDACTRD
ADGDGIRDKSDNCPRVYNRDQIDRDSDGIGDACDNCIDIYNPIQKDVDNDNLGDACDSNVDID
NDGIQDNKDNCLYIANANQQDIDNDGIGDACDPDDDNDNIIDENDNCRLIYNPDQSDSDGDG
QGDACEDDADKDETVDLKDACPENPEIQNTNFTNYKHIMLDPEGTSQVDPEWEILNKGAEIKQ
WRNSDPGIAIGDKVFGGVDYSGTFYVHDDKDDDFAGFVFSFQSNSKFYALMWKKDKQTYWFET
PYKVTGHAGIQLKVVTSSTGPGEHMRNALWSSISIPGQTKIIWSDPSKEGWKPKTSYRWTLMHRP
SEGLVRITIKEGSQVRADTGYLIDKTYKGGKLGVLVFSQKEIVFSALQYRCTEQIPADYNPNSPN*
>TRINITY_DN374_c0_g1_i1.p1 type:5prime_partial len:290 gc:universal TRINITY_DN374_c0_g1_i1:1-870(+)
FHSGVNIDWNNFKKYLKDALCRCEKAAAAKGKKYFSFRYFGICHGLDSLPKESSEYCFHHTFEQ
CMNNHNECVGADEAEYIYKVGVQIPTNTKPALKVQRKVFCEHRGSKTVTCGAGETIKVTKAYYG
RTSSSHCGYRGFYNLNCKGSTSYSKIKSACDGKQTCRLNPHNNVFGDPCKYTVKYIDIEYVCKPP
VKKAIFCEHRGTKSITCNEGEKIEVVHGYYGRQSSAHCGFRNFYNTRCKASSSVGKIKNKCDGHR
SCNLLPANSVYGDPCVRTVKYIEVDYRCIKN*
>TRINITY_DN113_c3_g1_i1.p1 type:3prime_partial len:1162 gc:universal TRINITY_DN113_c3_g1_i1:3485-3(-)
MEIVSRSRIAIFVLLGLLISVAESQFDPECPEIDPDVECSPPESTVNECLINNDCDVDKPLCCSNGCG
LKCTARERRSITPDGFTPEDEKLCRVPVDLGFIIDVSGSIGQTNFDKIKKFIEKIVDAFHIADDGTHV
GAVFYSDNASLAFDFNHLQGDQLNEMNVVREIEKIQVTDGQTRIDLGLELASRELFNPAAGMRI
HKPKIALVLTDGKQTRGELAPDAKDLHQASRLLKQTGVQVYSLGIGLDYDIGELLDIASDDASVF
RSQDFDELVSIVDILIEQTCKGCTRAVDVHFALDASANVGEENFVFMKDFVKAIGYNFIISETGAH
MSGSVFGDVGTLVFDKSQATEINEFENEVENMPYLGDATGNMDKVLKMISNEVFNFDGFTRQSS
PKVIVLLTSSNCDTCQESLEDLARPLKAEGIHIVAIPIGDPLFDEMQVLASHPIDQYAIPRATHQELL
NAEFIQKVSEMVCAGKPGICAEPPVKGECAEIINECSVDMRCPEDQKCCIKNCVASCVLPLAACIT
PIEIAFAFETTKYGIADFNNVKNFAINLLDHFKVGPSKAHVSLSTFSEDVTSLTKFIDDFDMESIKQSI
KDLKHDGGEESNLGNFLEIINTEVFSVRGGIRQQTPRVLVIFANGDINDEQAVKAQEEALHLKAD
GKDVKIIIVNIGSSTKTLDVLNEVATEGSFYEFFGSAELDSTAIETEISEKICSTKVRRSKCQRRIRSSCA
VKDDLCDSDFDCPGHQECFSDGCRLRCQTCEFRCKDKVDLLFVMDTSARIGRDNFERGKKNIKK
MLEWFTIDSNNTNVGVITYSDTANVAMPLPSRGNNNPAQSLIDVQDRLDSIPYSGGSVSHLDNAL
QLAAEKVFPDNGPNLRRDSKKAVVVITDSLDDSASQLELASWPLRRQRVQTEDGDASNIRILAFN
VGQELDLDLWGNIVSPPVEENVFYAADFDDMYNGIRQLAEDSCKYAAAITPPPIIKQKGEPGPPG
PPGIPGTNGTDGRNGTDGRRGPKGERGDTGLKGFKGEVGLVGPPGGGAEEQKGPDPLASAVGIV
NVTDLPGGGRIVFDLREGPVGQQGLTGIIGPKGPIGDTGEVGIKGRNGTTGPGGFFGLPGPKGQP
GTDGAKGLRGDPGLRGSAGRKGPTGAPGLTGPQGPFGYK
>TRINITY_DN3190_c1_g1_i1.p1 type:complete len:315 gc:universal TRINITY_DN3190_c1_g1_i1:68-1012(+)
MATVVDYPDFDAQADAETLRKAMKGLGTDEAAITFILGSRSNKQRQEIKTQFKQMFGKDLIDDL
KSELGGHYEDVVVALMKKPDEYDASELHNAMSGLGTHENVLIEILCSRSNAEIKAIKETYKKKYK
ADLEKDLQGETSGSFKRLLTSLVQGARLEDEPADHASAQADAHALFKAGESQWGTDESRFNAIL
ASKSFAQLEMIFHEYQLISNKTLEKAIKSEFSGDIQNGLLAIGKCAQSRPAFFAERLHKSMRGLGTT
DSTLIRVMVSRCEVDMVQIKQAFERNYGKTLNSYIADDCSGDYKKILLKLCGPE*
>TRINITY_DN40728_c1_g1_i1.p2 type:5prime_partial len:124 gc:universal TRINITY_DN40728_c1_g1_i1:1-372(+)
KTLTGKTITLDVEASDTIDNVKAKIQDKEGIPPDQQRLIFAGKQLEDGRTLSDYNIQKESTLHLVLR
LRGGVIEPSLAVFARKYNCDKMICRKCYARLPARAVNCRKKKCGHTNMLRPKKKLK*
>TRINITY_DN21847_c0_g1_i1.p1 type:internal len:384 gc:universal TRINITY_DN21847_c0_g1_i1:1150-2(-)
LSADGDVTAFRRRRSVELKHGRISMLATMGYITPEITGKFPGYLSNSMSLKFADIPNGLAAISKVPA
LGWAQVVAYCGFCELSAGSSDWKTGTPGDYGFKVLTSSDPAEKEKKLAAEVANGRLAMMAIIG

MFFQDGLTGSAWGDWALYDASPLRAFENELGVQEPLGFWDPLSLSADGDVTAFRRRRSVELKHG
RISMLATMGYITPEITGKFPGYLSNSMSLKFADIPNGLAAISKVPALGWAQVVAYCGFCELSAGSSD
WKTGTPGDYGFKVLTSSDPAEKEKKLAAEVANGRLAMMAIIGMFFQDGLTGSAWGDWALYDAS
PLRAFESELGVQAPLGFWDPMGYVTDGDVEAFKQYREAEVKHGRVSMLACIGYIVPEYF
>TRINITY_DN123301_c0_g1_i1.p1 type:complete len:272 gc:universal TRINITY_DN123301_c0_g1_i1:90-905(+)
MAQSASYQKMDVKKEEYHAVSGASYGEEKIPQAITSQPTAVSGVESYVLDLDASQVATWTEQDVI
NNFVERVGLGYANELFMANKINGKSLMLLREDHLKEMGIQAIGDRVFLLDMIQMLKKKKKEMQ
TTAALWSGETPADGCEYSDSCGHCIRRYLCPCCISRTYWKVTGQGVFYRKVPPCRQYFGTVSTDY
MDYRFFKDLELKEEHQCCCFCPRYRLELYVDDKDHNASAKSDEANPSFPIPHELLHPEAPKVEKII
RNAWNEARLVSE*
>TRINITY_DN12632_c1_g1_i1.p1 type:complete len:504 gc:universal TRINITY_DN12632_c1_g1_i1:165-1676(+)
MGNCLRKPKKQRQKEEKKLDATSLEPSLVEPNPQPPNSFPAPAPLGNSNGVQGATVFVALYDYD
ARINEDLSFRKGERLQIINTADGDWWYAKSLQTGREGYIPSTYVAPEKSYEAEDWYFGDIKRAEAE
KRLMMRSLPTGTFLVRKAESAPGNFSLSVRDGDSVKHYRIRKLDSGGYFITARAPFSSLHELVQHY
SRDADGLVCALNIPCPNNDKPLTMGVAKDVWEIPRESLRLTKKLGAGQFGEVWAGVWNNTTQV
AVKTLKEGTMSPAGFLEEASVMKNLRHKHLVQLYAICSDCEPIYIVTEFMCHGSLLDYLSKGEGQ
NLQLPTLIDMSAQVAAGMAFLEAQGYIHRDLAARNILVGENYVCKVADFGLARLIEDTEYTAHE
GAKFPIKWTAPEAALYNKFTIKSDVWSFGILMTEIVAKGRIPYPGMTNAETIHQVDRGYRMPQPP
GCPEPLYQILLNCWNKDPEARPTFEYLQLTLEDYFVSTEPGYRDLNQTDS*
>TRINITY_DN1080_c0_g2_i1.p1 type:5prime_partial len:8617 gc:universal TRINITY_DN1080_c0_g2_i1:26447-597(-)
VVIRRVVRRPVPIVTRRRVYRKVILDLDGTEKGVEEKVEESLPGMQGLDEDPITAPVVAEEQESVPD
MFGDLTEQSIVSRRSDFPKEGELDGVVDIADGKIGDTDENENETNFEIAFKREVYRLAVLDKKSIPV
ERRIIRKVIKLPNGEQKVLSEEDVLIPLEEEGVPISNTEKCKIVSTRIVTKRIMQYPDGKEDVLDETIES
ETERTQPLTSPEQAYVLVVDKPDGVGSIIEKPVEIAPLEYSYSEFDEDKVDSDIVEKGKTGKIVLRRQ
TVSTSKELHRSHLNKDTEKSVIVDLITYPSEQTEDSVIVTDEQDLSKSPEIIKDEKNERGIVFRRVVRR
PLPVTTKRTVYRTIVLNDEGITTEFEASQEEPPVEKKNVLQKIISSVKPKTDGKDVPEVDDSKPEEVE
RHLIDRVVKHKDGKEELIDQYELINSLEMPAVNVEPITKRVTDSKGRVIKTQVIRRVYITVKTIVVRK
VVEKADGTEIELGSSYEESPDDSTEETPYPERSVIHRVLPQDTPRVVEESDDISPLETSYKKGQEKEFI
SKNVLDDGTIENTVYKKDEITKKKLVTRKTIEYSKGKKAKKEEPIEEIHEHPVEFGSDVKFVESTEEI
KNEKGEIVKRVIHKEKPVHTRRMIYKKLTYTPEEMNKIKLEQMGEEKVLPKESKDDKKPGKQTTA
KPLAKPSTKAKPSTKSKSVSSVKPESKESGKPSKISIEKPSKKLSTKVTTKPSTESDGKREAPPSPTKST
TKPSTDSSGKHEAPPSSTKSTTKPSTESGGKREAPPSPTKSSATKPVMKTSGVKLSKPSNEKRIVKRP
KIPGDSTKSESKSKENDPAKKNRADEKDGAEADEIISKSKNVLDVKSVPTEMNVDIDTTDKRQSE
KAAEILKEKNQKHEADEIMQPVDIEEVQSSPLDLEKPMIFDDVVKSVDMEDVQSSPFDLEKAKISD
SAFKPVDMEDVQSSPIDSEKPITSDDIVEPIDIEDVQSMPFDFVKPMTSDDVVEPINVENTQSLPYDT
KGVKGREDVFLDQGEGLSDSNDENDFIEPGKVSLMRRQSSLRKRNRPYDEKKEDIRRNSLPLQEL
FIKLGRHDSGLSAIWESLLSDSKEEYISYDDYMTWLNFLEKEYSSSKFIPGKHDLLTKEHDYNKSLK
VDLRKRKAIVDHINKMKPVLQDLAVGQDKELISQELNELTTRFDKVYVTCEHRLKEINNMVPLSK
EHEDHYVPVSTVNEIVEDELKVKPRTGVDSKKIQAEIDRVKKLKDKLDNCKPAVRQLRKSLNSVQ
KHQTDIIDKEQIEEEAVNAVSKHDDLCNNVGQYNDTLERQLEKAKKFFGECDMIKKWAPVVEA
QLYNNETDSKNPEDLKKELKQLDMLKNEIDKKRPLIMAIDENGQWLMQNLYVTDKAGRRKSTA
SEVQLSLAEITSIVHHLSEQLNGRRDKINALLADTMSFEDSATKFEKWLIVIEEKVSSQKTLVLKSNE
LKDEILRYKPLLDEIVEHKPMIQSLVHSGEKIMSSANDKDKEKYMNKIALYSDRYDKVLKTATLKA
DKLDQVLPLVREHETNTEIVENIMSLAQPVLTSIEPVGVDSEKGNDQLKKVKNVVDLLDHNEPAV
NTTKRCVVQLTRILEEDLVKSVKETTTNNAIKYEDLRAKLKEGDANLAKQYIECERFTEIFYSIESKE
EYIYVLLKEIKSCSKQPERIDDGLLKSKEISIVIEEVLEQCSVVEEIIIIIQGYCKGHPVVLKTVGEKSSVI
SSKKIMMGKVSKEVQDEQNKMISLQQANKELDQKVIDINCWLPQIEGEVVKQTPVSANYDILKV
QYEKHQPLYQDVVQKESNIKHVLLLAGNILKDKKPGEEKDKLSKSIEDIERRWNDVSEKVNKRQ
NQIQRVMPLASVYKNTSTSFCQWLSNGEKQLKDLTSLSGNLEDIAVRKRELQDLAKDIESEKPNQ

LKVNDTARKHLDVCKNEAVEEPISEKDQVQQEVDHIDARWKKLTINVKEETDRLSQIEDEAVKM
CDKERVLEDLFIEVEKTLTEQKPVNLIPEKSKEELEKIKTLIVKVDEKEKDLTVFNNCSDKLQNLLV
QYDGNKDNVEKVKPVINDKFRKIRSHLTDRHADIDDIYTNVKEFDTDVKSSHTFLHDVTKECED
VFNKKAIPKDENELKSSVDTIDKLIDRLTKKKSDLQKDYEIGDKLIEHSSRDPLVLHDVQSRLQKIQ
DPINELIDKLNDRKSSIVNESRLHQALTGKLDDLDKHLPNLENKITNLKPVSAKLTVARGQQEEFK
PVYDETMAFKPLYEAIEKEADELKSSKSKRNDEEDEMLEKKLPDVLSQWNEQFEKVEKYNNQLK
DVLPTSANYHFTFLKFSPWLENAEKQLKEIKNDSFKTEPSSNLSSDIDKFRRDIGTNEPLLKDYNTS
AGNLFDRCKEVDVTTDVPYLEDEVKHTNDRWNRLKVDFSEMTENSNKLKNALQAYEKASEPVE
ECMIAITENLNNETPSDLDLEGLEFYVDERNADLESMQEKEPALQKVQSKCNEISDLLTANGGDP
TDIRDKAKDMNARWNDTKSKLTGKRDKSEAQLKQLEQFLNATDLLDNWLNVTTTTVTNMGPA
SATSEGVKKQIDQIDAIQEEISKQNVKLLKAEELGDWLCDENKDNPQFCANVNNRLNKIKNPLD
ELRSILSARKSRLHETLVATQDFRVAFDDFSTEIDKLVSRQNKQKPLSVHHQTLKSQDDEQKATEKE
IEALKPTFEKLIENGKTIIKETEKSNERDSLEENIEQLQKKFTGISETSDKRRKSIDELFPRSEKYFTKE
DDVSDWLDKQEKVLEELDIVPVSVADVEKLENDIMKLEEEVNKEEPEYKDCLKSFTDLSEHANNE
KITKDLPETKELDEKMNTRWENLKKKKNDKKLFASKLKTLISSYSDFCEGLKNNLAQFEDAIDKK
PSHGTDSKKCNDELERIDELIRKLDTHEPEMNNVEKLNSELTELIRKEKGDSNTVELTTKNLNGRF
QKVRDTLNERKKSLEKVSRVLLQFNGIVDDVSKWSDQADEQYVKLGRVSKDPEEAKRQIHKIDLL
LEETIRQKSAVTSAEENGKWLTENCLDDKEMRANVRSVLSNVYTPINTIEAKLNDRKAKLENAVL
CGQEFDKSFNDISERVTSLEFQVDRKKPINVDWTYLVKEQQEQKLLMKEIATLKPLYNQLITTAQK
TLNSLEPGDEKEATQQRIDSIVIIWEKISVAIQHRDEVIDQLENVSQSHHDQHRLFKEWIVDPEDSI
KNLEEVPSTRDEFFKFKKSVKEFIVLIENHLPNHSSLNDSFEKIKSETEKFPDDEFVGLNSLEEDIHK
TNDRWEKLTLNMKKYRARVEKLQSPIVKYSDGYDDAGKILEDIEKALEYQPSFGADEQLGKDEL
DRIEELIKASHVASEKVTVISEDSKHLEIVIDENDGDNTNVKESTKILVKRLKDIQEQCSVRKVDVE
KKIKVVCQFHKTSEELQKFYMTITQKLVSIRSAKPTPEQVKEQLNIVNQLEEDLQKHRPQLQALKE
TANWLVEKNADDRVASSDIKNQFSIIEDPFNELADKLNEKQNLLQASLLQTQEFQDTYNNVLDN
LAEYEIKANKSEPLSVKYDKLWKQEEDFKAFETDVLQMEPMFDQLIKSAKKVKDGTDEKEEKEDI
ENKTKDIERRRKNILEKLKERRDSFEKLLPVALEEDKIHKDFKPFVENTEEQYDQIKQAPKTLQEC
KNQLESLNSLKSKVDEGKPKHKTFNTNYNKEKETANTIADIADASTLEDEVNDLNKRWEALSGN
IDTKKDQVNKLHFHFTQYQESTESALHDIKKLEDYLEQAKRFGLDSSEGQKILSNVDDMLKTLEK
TERKVDEVNRSGKDLIGELESCKSDPWEVTTQMNDVNRKLDMLKVNLKECQEKVVDEVQIVNQ
FLKDVKDCESSLTDVNNKQESFEPLSTDPTKIKEQIAAVDELLNGLEKMSPRLDQVGRQGDLICKN
NPDDFSVSTDVNFRISKIKEPTDRCILRLQERKNKLQNLLVGSQEVQDTLDDFNDKLTNFEKTYNK
MKPVSAKFIVVKKQEIITEQVLDDVSQLQPSFKLLEKTAESVLKDAEPKEQVQLTEKINSLKTRWET
TVEDLKKRRKLISKVTPLAQDYYDRDQGFSNWVGDVEKKLIPRTDDFVVDQENVSKELDRLKAIK
KDIDVHKPDYDAILSIHKPLVGTAEDDINDIDEEVKRTTERWENLNVSIDKQANEFVELNNNMN
QVDEQIVPLEEFMNQCEVTLEELEPVGVDKDKAKDQLNTLNVCLTELDNKEKDIESIEKLANDLQ
EKPGLNPDKLKDTVSSLKVRHGTIKHSFTEKKEKLSEYIVYIEEYYVIIEEITEYIIVTKKNPAFTEPISS
EPSILKTQLRNIEAIQDEFISKKPKLERSNELLIIIVQECKHDFTLCNEVKQSFDEVKKPMDEIWTKIE
VRQNRLQDSLLKSQKFQESFDDFLDNIGQLEERYNNGKQVSAKYDVVRTQKKDHDQVHNDIM
QLEPVFEQIMTEANNLIETADPEEKVKLVEKVKGTTDRYTQIKENSISRMKRLSDEIVLTQKYSDET
QSFKSWLTEIEIKIENLEPLPCYGNSVSKQFIYVKSLVEDIQEKQSKLEDIEKIKKEVVENAEIDQSSV
EDDCNQIKVSYEKLSVQINERSTKLTELTVHTQEYTVHLKPIDQLLDKVEHCVVCTLPLYGIDMQK
ISKEQEDVNVLVQQLTDILPTLKDYNDVSRVIADKADKSSPEYDAFKKETDNLNERYKRLRDQLH
DKHDKLHDYANKAAKYQELNEDFDNWMEELGKTPGVMQPIGTEPEVVKRQLNEVEAVLDDLN
KKKLLPQSIEENGHWITEANPQDKNLKSNIDDKVEKNQNSYDKFSTKVEIRYNRLQNALLKSQEF
QTTFTDFTNVLEDIKSRQDKQEPLDARYEELTKLKVENELLQQDIAQKEPVYEKILQNGENLLEAS
EPGEERNALEKRLDEVKSEWNGVKDKCCKRKDDIERLHPLSKDHDCNCVTFTVYLRDAEKRKE
NLKPISSDKNIALLQLKETEELLNDVRNHESLYNGYHQTNEPIYVTCKELNITKGLPELKDEIDDID
GRWKNLNGDLEDRVSNCNDILKRLGECSEKMRPAESLVDEIKDLMKAPTRFGDDLKKGKEAKD
QIKKLKESLNNNKPEIDKLRSEIPELLRQGGIVAEPIEEKFSALLENVDECGMNLDDLDKKVDDEL
DKAAKFQDACDKLNKWLPEANKSTCLTDLSSSDPDEIMRQIEELKALQEDIVNHKPELMDVQNL
AKDMLDANKSDPKVCENIVDKLNNVAKPFDDLVNKLEERQKKLDTIKKVIDNYNHEKQPLEEFI

TATEATVDNLPPFGLDPDEGEKQVSELEDLVKTMDKRKPELRAVNRAGQSLIRQLDSPDEIEKALD
QLSDRYYDVFDKSKGKLNETKEKVNKVKIFIKVIEVLEIWITEVVVIVVKIGKTSSTEPETVKKLLDQ
VQEIRVDILNHKPQLKEAEELKDWINDILKDDENTKNMVNERFDKVKIPMDETSNKLDELEKRL
QDSFSSSKQFVDDIDDIEKLVSKLERVVEEEKPISADKDTCTRQQNDHKYIVEETLLQEPKYENVLK
QGRELLDHTDPGEEKDKLNEKLQKIAERWDQTKKNVVDRKNTLDEVVDHATNIDNDKKDIIPW
LNDAEKLSESLDCISVDPERLGKQKDCIKKLLEDMESKRPLVEHMKEHRTPLIEKAGVDQSPVNEL
VDDVCKRFDNLAKKLKDADEKADQLADGMGSFNSQKKPIEELLEKSKNLVGKQSPLGSDEADK
DIDDMKSLLDELLKHRPDVDKLKKSGDDLIAIEDKPDNVTAIKQVTDGLEKDYDDLLEKLQEKID
EAEKGKKDFEDFNKALKDANAKINGVEDKSKNLEPIGATPEKVKEQLIELENLQAELQAANNRV
AEAESLADKIRSNSNNDPSVIKKIDDELNAEKKRLKELEDLLNDREENLKDQLQQCGALQDQMD
DFNRRLQNLDGRCENQAKKPMSMNPVNMAATKNNVDGISKDLDDEKPVFEELMKNAQKLLD
NTQSPAEKEALEAKINAMKKLFDDASKKIDEEKNREEDLLGKTKTVDDKMNDLEKFLDDQEKK
LRSLEPVAIDKLSQQQKEINSLKNDTKSQQPALEEIGKLIKELEPLCEEDVPGVKEKLAKLKAHFDE
VEKLGEDRQKELTDVQNGLDNFKNISDPLKSKLSKMEALLPSLNMYGADKDKIDEINEELAKLQ
KQIEDADKKLEDMNSITENLVNEHPTADSKALRDEKDELKKRLDKFKEDFNKKSEGVNDILGDW
DALSNDIKAAVGTVKNCSEGIDKSKPTRLAVDEIVGQIENVEALEANLNESKPLFDDIQKRGRKLR
DKGIGADLFGDLLPKIDGQVREIKKDLPEKAKELGELKDTLNDFNDKLKDADQGMTAFEEKLES
QKPVGGDKDTIDKQMKDLKELSSELDKLHGKVSKLNDVRSDIKNKYPEADSSAMDEPLNKLND
RTNNANQKLSDRQGKLDDALVQCGQFTDAVDSLLGWLNETKELVDNQKPISAADENVLKAQM
QEQKLLQRMFADREASVKNLQKTGEELLKTTEDEDKKKEIEKDLEKVKELWESLKDQTDDRKTK
LDEALVASEKFNKNYNNVMQSLDDLDKEINSDKNIPTSDPQKIEEQLDNMKKLSDKIKELEPIIQA
VSGDETELEPYCTPDDLALLQNKLDKMNEKFDKLNEDCQEKEDNLSTAKDLSEEFANKEKELNE
WMDNIGKELKDADGGKLKDLQKDITDHKPDIDRLKELGKDLKKLIKPEDYSKLKKSIEDAEEKY
KNLKGDVDENARSFFMAEGKVQEFDKKLEELAKWTSDKTNQYRTIDPVGVQPDRIKEQMNEQK
QISNELSSKEPLFKELYDLSEDLMMGCNEEEKAEMKEKIENLKKNKHQLDKTSTERDEQLVDALL
LAQQFSDVYNDVASRLSNTENLLEALDKEKGRGVEMQKEKLQNVEDNIGQLLPLMGTLKKTGS
DLIKLSGPGQDSESVQKKIDECNARWDALKTNSEEKGISVGVAAQQSEAVWNDVEDLIEKCQLV
KDDFKNQRPIPVHEEPITEEIGKLEEKEMLLKDLEDPVTSALGSIDEILKDDPKSPASTALKDRQRK
LGNLWKFISTSAKDRRNDLDSSKVAAEKFWPGIEKLNDVLIDVQTKLEDQNDPGMGIEEIEGLLK
ETDMLHNELNSNEDVITELVDATPKLVEKASPEEKTEVHHQLANVRDQWDNLEKSLQKRKGDL
ENVKALATEFDSEKDHVEKWLSEAEAFLKGEDTKPSDVNELKTRLRAMRKFHREITKHQNEITKL
EQKGESLAEKVNDEDSKKINEIVEDITKRWDNLLDQSYEKQHQLEDALLDQGHFSFAIEEILVWIN
QTRDSLKTDDKIPKHKKLIEVEQSKLKIVTNDVEAHKPSVENCRKAAEKLISENSSEKAELEVKLG
QLNEGWDEIQELLKNREGKLQDAFEEALKFQSSARELTIWLSEAKAFLKTKRPYGGKLETVQKQV
DKHQEFIRVVEEREVTYLFIVETFEYLIQSSDVSSSRELQKSLNEISSAWTVVSKLSQDTTDKLNRSLG
DAQFLQDKFNEIEIWIVRIEGTVSMFKPASTVYETIEVQIIEMEEIYIEFAEKRNGMREIRKTTDRILE
HCSPEEGRILNEEIEELTQKWKELSTSLKDRKKTLDENLDQSKIFFEGSEQLMTFLDEIEKKINEDET
IGKDAASVKAQMRIHRDLQNQIGKRQTKLNVVVKLGKTLLEKSPEEDYQIIQAKIEDLQARWEV
VCAISVERQHKLEEALLFHGMFQDAVHALLEWLHAVEPSLKTETAIMGDPDTVKLLMDNHKSFE
RELTKRQKNYNSIVNRGETMIVEGKVDNIEEISQQLEKLKNDWEAVSQMTITKHERLQSAYTLAK
EFQVGCRTCVNNLGELENKLKKQGPISDDVAGVRKQIEEFIVFEELLEIEEVKVNGCLKKGAVILRF
CHPSSLQTIRHQVAVIKKRWNDVSGWARQRKARLDEILIEMIEEEQIVQILIEWIAKQEVLLEDREK
IPLPDSYEVLNQLLDEHKMMQNEAENKNPDYSKITKRAKRKPLTDKQRKLMTPRHGTQPQRDFS
NPMVGVLSKRWQSLWLTLMNRHRRIQDKLDAIRIQKASAEFSWADWRDRYNTWLRDSKSRVLD
IWRKKDSDKDNKLSKEQFVGAIFESAFTTEKWEVELVYDQHKRGVLITYQDFMDALKDRKRRPD
KPQTEAEQIDDIIKSEVSRCCCAKSFEMFQVAERKYRFGETQKLRLVRILRSTVMVRVGGGWETLG
EFLDKNDPCRAEGRTNYELRENFIMPEHASQGMQAFKTSRPGISREGSATGTPKFSRERSGGIQRS
GGMKREKSCGMHQHDRTDGGSNLTTPTFQRRKSDLEREAKGLPSYKGKDYSGVKGSGYGRTYN
KRTTKTTSTTPDHSSTQSTPTSSTRIAREKSGDVYSRLYGSTPKTTSATPRSSTPSTTSTGIPKSAGSVS
RGGATPRSSTSTSKTPTKRTSSSSSTTATPKLSVTSPDEVTTPIARSSSNLRKAPSQGSIAKKGTESSER
KTMTKTKSTPKSAEPKKRTSTAKK*

>TRINITY_DN121866_c0_g1_i1.p1 type:5prime_partial len:460 gc:universal TRINITY_DN121866_c0_g1_i1:2-1381(+)
LYIINSCEPPSIFFSCHLHFYTLVEETMSTTTAPTTTGTSAATKRLYVGNLGSTVTTQDLKQLFGLETT
PYLRDTCFVELAVCEKTGKSKNFAFVSAPGHVFSELIKLNGIEFYGRQLVIEEAKTKPEEEGEKKDN
KEKKKTPNNKRKGGSNNNRNNNRRYGNKPKSKFNLPTLEPDQVFHLVDCGVNLTNPKFSQHT
DYVIARALSAGVQKMVITGLKLNGCKAAVVMAKTRPNILYAAVGIHPHFVKDDWSDKALDQLE
EQVKQPECVAVGECGLDYKRDYSPRDQQELAFKKQVQLGVRYQKALLVHERDSHDGIIEVLKEF
EASLPPVIIHCFTGTVEQIKTYIERGYYIGVTGYICKDKYGKTLRDAIKDGTLPLNRIIVQTNAPYMT
PNTPRDEIDPVSQTLLDHCYADNEPCTLSIIVRCIAKCLSQEPRHVADVCTETAIKVFRFQKSESE*
>TRINITY_DN1058_c0_g1_i1.p1 type:complete len:2639 gc:universal TRINITY_DN1058_c0_g1_i1:134-8050(+)
MVKYIIWILLLSIFKEAYGGDWAQWQNWSACQDSSGAPIGCGRGNQHRSRTCADNNCGAGAIIE
VRPCLDEICPTFNCQNSSEIAGMCKFDYSNSCVGDDNCHHCICKSEGFKVSGDQCYNYCVEVGC
DEEIEGVSQLKRYCGDQAISCTGTVLNRTCLCNPATSYPSKDGQYCYEATCKTSQEFMTNCGAEN
SCQGTNTSFVCECNGFGYGYEVVANVQNRKKCKLLSCEDGDLNPLEICGQGATACTGNVHNLT
CACNVTAGYIPSEDKKSCIPMPCNTDPAVGVQCLPRNTCEGSKIDWRCICSAYGFRNDPTDPKKCI
EAKCDDPNIDILGLCGTGVTSCTGFLSKRRCVCNTTENYIPTLDGSHCMPAPCNANENATKDCG
GENSCIGSFDSYMCVCNGYGFQKNATINKCEEVPCSSLPTAEVQRICGFGALSCDGTIRNPVCNC
NNDLQYYASEDMKSCIEASCSSKADYDSHCGNENSCVGNITHFECICETEGYKPDPSNKKKCILES
CELNEVDVLNVCGKGAISCKGNVRDLECVCDASKGYVQSPDTNKCEPSSCYGNATVSEQCGEEN
HCLGDLSDWICVCDAYGYKVNATNSMTCDKVMCGDTGADAVTVCGKETTSCSGHIAKPVCTC
NETAQYLPSPDSTRCVKASCKENDAARKACVPGNSCSGSYDEFICICDQFSYHQDPADNRKCIRE
KCEDLAADYDYEKICGVGGISCTGYVNQPVCVCNSTESYHPSNDRTSCVKASCSTSDAVRTQCGL
ENSCFGDIESFMCDCKAHGYKLEPINKTSCVLATCEDGDFSYHFQCGKGAVGCSGNINTLSCLCD
SAKGYVPSADMKRCVPNSCKDNPDVYIKCGMENTCLGSYADWFCVCSGAGYEANPADPKSCQL
MECSKDDAIHVCGYGAIDCKGPVLAPTCSCNYSDHYIASEDGTSCVPITCRKNDTARAQCGEENT
CKGHTDNFKCICDAYGFKADNVDHQKCVERQCTDGDADIIRICGHGGIRCTGPIRSAVCECNSTE
YYYPNADRSRCILASCKANETIMKSCGEFNSCEGNMESHICVCNGPGYKHHPSDDKQCVKETCE
MGEIDPIYLCGKGAVKCNGTYDIYTCECDNAMGYVQAPDGNSCKPFDCHANATMHKQCGDG
NRCIGSLTDWICVCDVAWYKPDPSNLMRCHEKTCKEADLELICGTGATGCSGSATNPTCECDSA
NYFVSSTDRRSCIKASCKTNITAVQQCGDGNSCYGTPDNYVCNCDAYGYRLDDNNNQACVEKK
CDDVGANYVEICGLGGIGCKGTVKNPVCECDQTKHYVASPDSTSCVLSNCRGNKTVMAACGNE
NKCFGSASSFICVCDGPGYKRNPSNLQECLPETCEAGEIKPHMVCGRGATACKGTMYHFECECN
NTLGFIRSPGHESCIPVNDCYANATMHEQCGEGNGCQGSVADWICKCDAPGYKPHKTNFQMC
HQRMCSDADIDLVEVCGVGVESCVGPIANPTCNCDTSKHYVLSADSTSCIPGSCKTNETIMAQCG
EGNSCSGTSENFVCICNGYGYRPHETDKQTCVLKKCSHSDVNAVDVCGQGALSCIGTMRDPICQ
CNNDMFFIPSDDSRSCVLASCSANSDVAVACGKVNGCFGSKSSWICDCSSPGTMLNPNNIRECLH
ETCESGKLDPIIVCGKGALGCNGTMSNVTCECDNSMGYVQSPNGMSCVASDCKTNSTMHELCG
DGNSCVGSLTDWMCVCDAPGYHPHPSDPMRCLQKMCSHANINPVDVCGIGATKCVGSITEPRC
TCDTAAYYVPSPDGTYCIPGSCQTNKTALEQCGNGNTCTGTADNFVCICNAPGYKLDDSNNKRC
IEISCNDADVDLVEICGQGSIGCIGTARQPVCQCDHSKHFVPSLESTSCQLASCRANDTIMDRCRM
GNTCFGSMTSHICVCEAKGFKVNPSDPTECVQETCEDKDIDALSACGKGATDCMGTLMNLTCQ
CDGASYVESPDKTSCLPVGCQQNVDVKKACGEGNTCEGSYDDWVCLCTNPEYIPHATDEKVCS
KKKCTDNPIGSCGIGATSCSGNVNIHKCTCNLTAGYMPSKDEQSCIPIDCKMNPKMNCGFGNTC
RGSFFSYVCECNTLGTMRNISNPKQCIEVDCSTDAVSICGVGATGCAVTPTNDVNCTCGEFYHPA
HDYHSCVLDDCKFNKLVPDACGVENECTGSISSWMCVCKGPKYIQDIKSTQLCAPGPCRIEQCGS
GKASGSFQYYAESCSGTYFEPTCNCKEGFEKDSYGGCIVKSCSRNNQSSVDCNDGAISCEGTYFKY
KCECRAPTHILNPANKRSCIPARCNPQLCGRGLVNGDYMDFASECSGSYTDFTCKCKYGLYGSGT
SKCSLEKRTCALSNDCHPNANCIDYDGHYFKCECKDGYSGDGYQCHRNGGLTPWSVVQPCDKT
CGTGYAQVMRTCDDPKPDTLGKKCNVPSPDVREVPCHQFACTACSVKKTCECENIRPYFVHEYV
EPDESLKYFTNLTAEDVEKALSAIEKWKLSVDEKEFCIKHTLPEIDGLISDINENLRSVDQTKLYTIT

ARDMMRNKLDCNVDLEKKDKYFRVYEAIVKRHFILKQVVSDLQAKEKLMVTYRSSCTKDSKVG
WLSSIFS*
>TRINITY_DN89_c0_g1_i1.p1 type:complete len:376 gc:universal TRINITY_DN89_c0_g1_i1:117-1244(+)
MKTFKIFLVACLLLASAYADLMSEMEALEKELEARELADQKEDSSSEKALKDMSTEELMDELKKE
VGKAKKSAVETHREKFDDLTKRLALEEQVEEELKRADAAAKAQASDDESDKDFDNLIKNVIEDEI
KKNSNVPAYAKGTNDATEEDTATFEELKERVKKDYYGDELVPFDQCKDQMPNCNIYKKYCEVYK
KNLIKRCPKTCNFCAKPVTTPAPAVAVHVCRDKLPKRTCKMAKKMCDSENSVQRTRVQKLCAET
CMVCRAPSPPRCENTEYGCCWDKETIKTDRKGTACPACQDEFKYICKTFKDDCVRVTHNSGLFM
YRNCKTTCGVCNNNYCEDDPRFAKSCHFWKKYLKQCEKDTAMKGLCKKTCGHC*
>TRINITY_DN8903_c0_g1_i1.p1 type:5prime_partial len:447 gc:universal TRINITY_DN8903_c0_g1_i1:1546-206(-)
DMATKACGLENMCVGSYKSWRCICQAPRYMQDPDNTQSCIHAPCRIEDCGSGYAPGSFQYYAES
CNGTFFNPICNCQEEYEKDMLGGCVPKSCRENPIAVAECGENGYCKGTYFKYECGCQGPKYVQN
PRNTRQCIEAPCNVQNCGHGFENELYVSFADSCIGKHADYTCQCKEGYTGTGTSRCIPDGKECGK
SYCHANATCAPLGKGEFDCTCKPGYSGDGLYCFLDGGWSDFVNTRECNASCGTHGYAEVTRYC
NNPVAERFGKQCGAKSPEQLEVPCNRFDCLGCTVKKDCRCTSVVKPLIVEGYNEPEETLKFFTDL
HPDDVKKAKDTLLKWKAFSNENLCRKYNKTTLHAKFFGVRDAAKEVNKMKSSYGSARDSMRI
KLDCNINIAKKTQYFGVYEMIVKRLFIMKQVVKDLHIIEDYVLKHMENCDDSPVGMIRNTFGK*
>TRINITY_DN2472_c0_g1_i1.p1 type:complete len:344 gc:universal TRINITY_DN2472_c0_g1_i1:311-1342(+)
MSSGEIESLRQEAENLRNQIKDAQKVAADTSFSSVAQNVDGINRIQLRTRRTLRGHLAKIYAMHW
ASDSRSLVSASQDGKLIVWDSYTTNKVHAIPLRSSWVMTCAYAQSGNYVACGGLDNICSIYSLKTR
EGNVRVSRELPGHTGYLSCCRFLDDSQILTSSGDTTCCLWDIETGQQLTQFVGHTGDVMSLSLCN
DSGRTFVSGACDASAKVWDIRDGMCRQTFIGHDSDINAVSYFPSGLSFGTGSDDATCRLFDIRSDQ
ELMCYSHDNIICGITSVAFSKSGRLLLAGYDDFNCNIWDTIKGDRSGVLAGHDNRVSCLGVTDDG
MAVGTGSWDSFLRIWN*
>TRINITY_DN468_c1_g1_i1.p1 type:complete len:191 gc:universal TRINITY_DN468_c1_g1_i1:676-104(-)
MKTFYTILLLAIVQTNAFFFPKVDTVKDLDLASYTGRWYEVYSSLIQRATFQRNSYCTTADYSLKPS
GAIKVINSGRLKSATGEENTIEGEAKVVDAKEPGALNVKFSTGPQSDKANYLVVKLGPVVDGKYD
YTVITTDYKALVWVLARNPETFKQKYEKDVLAYLKKNGYNWFINKPRPTYQGKDCIYI*
>TRINITY_DN10724_c0_g1_i1.p2 type:3prime_partial len:148 gc:universal TRINITY_DN10724_c0_g1_i1:442-2(-)
MLAFVGMVVQQWWQIPGLPQQPDWVKALDECYASPVAVWGIVQISVFAAVMEGHFFPEYAWE
GIMKREPGDFGFDPLKFSKRPGFDLKKKQLIELKNGRLAMIGVASMASNHVLPGSVPFLTGAYAV
RPEARAPGAPAGFCGSTGPR
>TRINITY_DN3016_c0_g1_i5.p1 type:complete len:406 gc:universal TRINITY_DN3016_c0_g1_i5:2713-1496(-)
MAPPNLKKMIQVKIKPRQDSYSDQFSRILMVKMLMLTAAFTGITWMKDKVKCIVPKNHDADGG
FVSKSCWINGFYVFKGLKEKDTSYYYGIPKDVLSDGYDKYGALCSTKNQQGRLNQGCTPLEKTFF
LQYQWFPLVIAAIAFLYYLPYLMFRIVNTDLQEIKDIIKGRNANDVDAKAIVKRFFVRKNNSTSRL
KARVLLNVVVKVLYVVVNVLGMIFVDSSTNSEFMQYGANWSSWTALKNEDMHDYTNREHEKA
GHRLLPSFGLCQVYTTAQDIKTKVANDHTYICELSQHVIYQYILIVMWYMFVFGIIISIIGLIQHMVL
KTMAILSMNAEGDSVRKIYSDLTVREKEYLDFIRRKNLPVFTKVLDEISQLKGVSRSYATSESDRSSF
GATAPEKENMMME*
>TRINITY_DN79126_c0_g1_i1.p1 type:complete len:115 gc:universal TRINITY_DN79126_c0_g1_i1:397-53(-)
MVARLVLRRRLSYNTKSNRRRISKTPGGKLVYLYTKKAGATPKCGDCKCKLRGLKAVRPKKLAKL
SKTKKTVTRCYGGSRCGGCVRERIVRAFLIEEQKLVVRVLRAQQVKKSS*
>TRINITY_DN691_c0_g1_i2.p1 type:complete len:1673 gc:universal TRINITY_DN691_c0_g1_i2:5503-485(-)

MMNLFRLIVLGVICFSYEGDAQQCGGSCSPCGNLCVGMKGDRGTAGLPGFRGPPGLPGFPGPEG
PIGQKGEKGPAGLNGMEGSQGARGKVGPPGLPGTPGRHGRPGNEGREGEAGLPGCNGTKGDR
GPEGPRGRDGIEGPQGLQGNVGPRGEPGDSTATKLKGQKGEPGSAGQQGEKGGRGMEGMKGD
TGKPGRNGRAGERGPKGEPGTSNVTIIGEKGSKGSVGPPGLPGRQCQVTGDKASNIAQGGQKGNQ
GSKGEEGAKGEKGDAGLAGPQGEEGDIGPQGDRGDDGPQGPPGPEGEQGRSGIQGNKGQKGE
VGNNGKDGNDGQTGLTGQRGRQGTPGQKGEPGKDKKGLRGPEGAKGERGNDGEKGSDGPIGP
IGPRGPKGELGPLGMKGFKGSIGSAGPKGENGNPGDPGIKGRRGELGIKGDSGVKGAPGNPGISV
TGPDGMDGDTGPRGQSGGKGAVGFPGKPGPPGVVTIDGEEVLPGEPGPQGLKGFRGVQGDAGS
KGSRGEKGNACESSKCPAGPKGEKGLSGQDGLQGGDGEEGEKGEKGDKGDIGSRGDTGSRGEP
GPQGESGEKGEKGIQGPQGNSGKDIIVQGERGDKGTKGNAGTPGSIGFPGERGSPGESGDRGLSG
VNGLRGETGDRGARGVAGKPGVNGQKGQKGEIGSGARGFKGDKGEVGPDGPAGLRGPGGPVG
PKGDPFPTAVNGKTGEPGEQGAAGPKGAQGPVGPEGPRGFPGDIGPVGEPGDGEKGERGDDGA
RGERGEKGEPGQDGKDGDPGPKGEPGQEGKEGPPGMFKGNKGEKGATGLPGVDGLPGNSAPK
GEAGKDGQDGDPGPAGEKGDPGEAGSPGTEGPIGEDGPVGDKGEPGESRAGPKGNEGRAGAK
GENGRDGRAGRPGIGRPGDKGELGQKGEAVIGAKGSKGEQGLEGPPGPIGKPGVKGDAGVPGK
DGEAGGRGKDGERGLPGDKGDTGDIGFNGSPGAPGDKGEPGYSGQKGAKGMSGPRGPPGSTSN
VVGPKGNRGLPGMKGEPGQQGEKGLIGPQGSKGESGFGSEKGIKGSRGNPGAVGPSGEKGERGD
RGKDGLQGIAGPRGEEGNPGEKGSRGDQGPQGREGPSGELGPEGQKGDPGFAGEKGEKGDRGG
DGQKGDAGEPLRGEPGVAGPPGPTGPQGERGPKGEEGNMGPKGSQGRDGLTGEDGPKGSKGEP
GLDGDVGEKGEAGFQGLQGEKGNQGPQGLEGSTGMDGQKGQIGEKGVEGPPGPPCEPVNSVR
GETGEEGPKGDRGPKGQKGKPGPQSQAATPGEKGSKGEVGQPGPQGIKGEPGREGDFGPKGSPG
AQGEKGDQGQKGEPGQEGPEGLKGEPTRAFGPKGQKGETGLEGLRGPVGPKGEDGQTGPQGP
DGPKGNEGEKGDFGINGLNGAAGPSGPKGAIGLKGDSGPPGPTGAQGPRGSGGSPDFGFYYVK
HSQTTIVPSCPAGMKTLWTGYSFLYAQGNERAYGQDLGQPGSCLKRFSTMPYLFCDLQNQCTVAS
RNDYSFWLSTDEPANMMKPAAGPEVEPYISRCAVCEAPANILAVHSQSVIVPDCPNGWENIWAG
YSFLMYNSAGADGSGQLLASPGSCLEEFRANPFIECHGRGTCWYYGPTLSFWLSTIDDSNQFQIPK
SETLKAGNLKTRVSRCSVCMKTVRNGNFP*
>TRINITY_DN79_c1_g1_i1.p1 type:5prime_partial len:450 gc:universal TRINITY_DN79_c1_g1_i1:3268-1919(-)
EFYAPWCGHCKRLAPEYETAATKLKKNDPPVPLAKVDCVGEGKETCGKHGVSGYPTLKIFRGGQ
VSQDYDGPRDGAGIVNYMKKNSGPSSVEIKDNEHLEKKLNEAEDPLVVGYFKEENDLYKRFMKA
ADKQRASFAFAHSLNGDVNSKQGHENVIVLHRPNHMHSKHEDPKEFLSNENANTNQIIDFIKEK
QNGLVGQLTAANQESFTSPLLVTVYYKVDWKRNPKGSRYWRNRVLRVAKKFVGAATFAIAAKSE
FARQIGDWGFDVDNDDVYAIGTNAKKQVFKMEDKFSVNNLEKFVNEFKDGLLKPYIKSEPLPEN
NDGPVKVVVGENFDKIVNDPTKDVLIEFYAPWCGHCKSLEPKYTELGEKLTNNKDIVIAKMDAT
ANDVPHPYEVQGFPTIFWAPMGSKDNPKKYEGGREVSNFVDYIKREATNPVEIESKKKKKKKEL
*
>TRINITY_DN1174_c2_g1_i1.p1 type:complete len:323 gc:universal TRINITY_DN1174_c2_g1_i1:48-1016(+)
MQTMRTTIAVLFCLAGYCYSAEWGYVAGNPNELEPKDWKNGFPKCAGKSQSPINIETRSTYLCH
LPPILLGKSWGTLLKSADLSLANVGSTIKIDVKSSIPITTTKEGTIWKLAQMHLHWGRDSNTGSEH
TINGKYYPAEIHFVHFNSRYATIGEAVAQPDGLLVLGFFFKVDEKAVVPSIYQKLFGALDKIKFKGQ
KMKLDLSGRALGDMIPPKGTDRYYSYAGSLTTPTCDESVNWNVMSEDNAIVVTEKEMMELRKLS
AGKMNQKFKPQSGNFRPIQPRNNRLVKRNFRTAKTAFFDVIQTYAPMVFNYFNGVPMRFWN*
>TRINITY_DN2217_c0_g1_i2.p1 type:complete len:254 gc:universal TRINITY_DN2217_c0_g1_i2:884-123(-)
MNSVDGNTDNAQKECLELCQRNAKATGCEVIWNRHNRGCYIHTQEVARGNNVDRHFCWVFS
KCTLNNVPPAVCPIEKGYCVKGNGHDQNSGVIKMNSVDGNTENAQKECLELCQRNPKATGCEV
IWNQHNRGCYIHTQEVARGNNVDRHFCWVFSKCTLNNVPPAVCPIEKGYCVKGNGHDQNSGV
IKMNSVDGNTENAQKECLELCQRNPKATGCEVIWDRHNRGCYIHTQEIARGNNVARHFCWVFS
KCNL*

>TRINITY_DN571_c0_g1_i2.p1 type:complete len:186 gc:universal TRINITY_DN571_c0_g1_i2:192-749(+)
MREYKLVVLGSGGVGKSALTVQFVQGIFVEKYDPTIEDSYRKQVEVGSQQAMLEILDTAGTEQFT
AMRDLYMKNGQGFVLVYSITAQSTFNDLMDLRDQILRVKDTEDVPMVLVGNKCDLEDERVVGK
DQGQSLAKQFGNCTFLETSAKMKINVNEIFHDLVRQINQRQPENTKSERKKKGCTIL*
>TRINITY_DN902_c1_g1_i1.p1 type:3prime_partial len:153 gc:universal TRINITY_DN902_c1_g1_i1:146-601(+)
MIRIAIIAIAIAACVYESQAVRCHVGYSAADLPLAMSVRDVECGQGFDHCVTLKYVLDGMKITQG
NCATKFGQATCQFMRNSHAAMTDCSEAICSGDHCNSAVKEAEKIEMKRVGAYENTLKCTTGLQ
SDFPNIPFASPNVPYAVDECFPGT
>TRINITY_DN2885_c0_g1_i2.p1 type:complete len:114 gc:universal TRINITY_DN2885_c0_g1_i2:31-372(+)
MAQPVRLYSKGIFVGYQRGLRNQHEQTSLIKIEGVNTKADTSFYLGKRVAFVYRVSKKTVAKGDK
KPSRIRVVWGKVTRPHGASGVVRAKFRHNLPAKAIGGSVKVMLYPSRI*
>TRINITY_DN141214_c0_g1_i1.p1 type:internal len:179 gc:universal TRINITY_DN141214_c0_g1_i1:2-535(+)
KFIQSLYNVTIPDTVQENYKIVTVSANDADSASNSNNVIRYEINSSSDTFVVNVVTGSVTLKPNINL
KGTNIKEYRIEIKAFNPDLPNSFDLAVVEIYVTKTQASLTATAIVSSYTTLTLPENQPAGAITNPITGR
NSFVIIYDSNTNPSGVIFSVDQTGRVFLNKPLDFERENQIRF
>TRINITY_DN923_c0_g1_i1.p1 type:complete len:442 gc:universal TRINITY_DN923_c0_g1_i1:53-1378(+)
MAEKYDLTYKVGAYLDRHMVFPLLEFLSSKEIYDDTELLRGRLELLQGTNMLDFVKDEYQRLHQT
DEVPDELANKRQEVIRQMRELEAATESITAAFEDPDFQKEASSARDGAQLFKYLNETFGIRSENLD
TLYQYAKFQYECGNYNGASQYLYFYRVLVHDDDKNASAAIWGKLACEILTQNWDPALEEINRLK
EVIDGDHFSHPLQLLQQRTWLIHWSLFVFFNHAKGRDLLIDMFLYSPNYLNAIQTSCPHVLRYLTT
AVITNKRRKQILKDLVRVIQQESYTYRDPITEFLECLYVNFDFDGAQKKLLECETVLLNDFFLVACL
EDFIENARLFIFETFCRIHQCISIQMLAEKLNMTPEEAERWIVNLIRNARLDAKIDSKQGHVVMGT
QAAAVHQQVIERTRSIMFKTQAITTNIDKKLASLQKQPYTEPFGLAW*
>TRINITY_DN902_c1_g2_i3.p1 type:internal len:178 gc:universal TRINITY_DN902_c1_g2_i3:2-532(+)
AVFKCIKLNYIDHIIKMIKIAIIAIVITALVHESTAVRCNVGYSTADLPMAMSVRQVECGKGLTHCN
TLSYKLDGMSVTQGNCATSGGQASCLFMKNSLQSMTECSEAICNGDHCNKAVKDAENAERARV
GPYKNTLKCTTGLQTDFPDIPFASPNVPVGVDECFPGTKECVTMTYD
>TRINITY_DN1566_c0_g1_i1.p1 type:complete len:4626 gc:universal TRINITY_DN1566_c0_g1_i1:42-13919(+)
MDTEEVPGEPPPTVLAATEDVINYVKNITCFIFDESQPCLPLDKILKEEQTKDYFRKFISDVQTKTIL
LYRINQLTDDVDGDHDQGVSQPSYGIKLNVQYVPHKESSIAFIKRGMLLESDKKINAQLHVLILD
NSSPFDTLHSYISNAVSPFFKSYVKTSGKSEREGDKMVPSVEKKIAELEMGLLHLQQNIDIPEISLQI
HPVVQSIIQKAEIERRKISPDDYAEKAEDSSFLNALQNGVNRWIREIQKVTKLERDPSSGNALQEIS
FWLNLEKALHRIEEKQQSPEVKLTLDILKYAKRFHAIVSFTNDTGLKQGLQTVASYNPLMKDFPLE
QLLSATELERIKDALIAIFSHLRKIKLSQYPIQRCLRLIEAISKDLMSQMLKVLGTRKFMLMNYELFE
KVMTSAFNVLNCWDDEYERLQQILRDMAKKKKDDSLKTMWRVNPSHKKLQSRLNNLRTFRKQ
HEQLRAVILRVLRPSKVEAKSSNEENAASSGSIEISDDNAVKEVDLAYENFKEIDPLDVSKEGTEAW
ETALKRYGERIDRVETRITSTLRDQLGTAKNANEMFRIFSRFNALFVRPRIRGAIREYQKHLIGLVKE
DIEALHDKFKVQYSHSKASKMSRVRDLPPVSGSIIWAKEIERQLHAYLRRVEDVLGKDWENHIEG
QGLKADGDSFRQKLNTQEIFDDWSQQVQKKPLLVVGRIFSIETLKTKGSNKATRKLKVNFTKEVIT
LAKEVRNLKNLSFRVPLAIVRQANDANQYYPLAISLIESVRTYEVTNDKVFEKESIIPLVAGIRKDIQ
SLLAEGMHLEWQSYKLMPYVMRLAEAVTGYQEKVDELLTSIQKIDIEVQSLATCGFKHEAFQEILI
RIQKLVDELNLHSYSNLNSWVKTLDEQVEAKLAHRLQAGIKEWTRVLRTGNENVDDKDLAIHSP
GGLPDIKPLRVEFFIRNQVIEMHPPFEKCIEHLYNELAVYCSTITSLTKIQSQRYQVGLDRDNAEETY
HNLLTKLPDPNGPNPITVYGEMESMLEDCRKYCEGWTGFQALWDAEMSNLAPRMKTLEDWMT
ILNQIKMSRSHFDTTETMKEYGPLIVDFNAIQGKVNLKYDSLQKAIVQNFGGKLGDEVNDFFTNV
TKARTDLEQQSLDVATTAEAVTFITYVQGLKRKMKTWEQNVEVFREGQKVLERQRYQFPSNWLY

SDNVDGEWSAFNDILKRKDGAIQTQVAILQQKIVAEDQAVEERTFNLLADWDKGKPVSGNTKPD
SAIQTLQIFEGRLTRLKEDRDNLQKAKEALELIEPGTVPPSDERVQVAIEELSDFKSVWGELEKIWA
QVDDLKEQPWVSIQPRKLRQSLDSIANGMKILPAHMRQYAAFDYVQKIIKGYLKANMLVVELKS
EALKERHWKQLMRKLSVKWNLSDLTLGQIWDCNLQKNEVVIRDVINIAQGEMALEEFLKQVRE
SWQAYELELINYQNRCKLIRGWDELFDKVREHINSVSAMKLSPYYKVFEEDSKQWEDKLNRILNL
FDVWIDVQRRWVYLDGIFSGSGDIQHLLPIETQRFGSISTEFLTLMRKVAKAPLVLDVLNIQGVQRS
LERLADLLGKIQKALGEYLERERASFPRFYFVGDEDLLEIIGNSKNVGRLQKHFKKMFAGVHTIIL
NEDSTLVTGICSKEGEEVIFNKAVSLIEFPKINEWLTLVEEQMRLTLATLLTLSAKEIKSFTGDSIDPK
LYMEWVDKYQSQLVVLSAQIVWSENCEAALQTCYNGDMSSLEKVLSIVEATLTVLADSVLQEQP
QVRRIKLEHLITELVHQRDVTRTLVRSKVQGTKSFAWLSQMRFYFDPKQRDVLQQLSIHMANAKF
NYGFEYLGIQEKLVQTPLTDRCYLTLTQAIERRMGGSPFGPAGTGKTESVKALGNQLGRFVLVFNC
DEKFDFQAMGRIFVGLCQVGAWGCFDEFNRLEERMLSAVSQQIQTIQVALKEGPKEIGKPIQIELI
GKDVKVSEDMAIFITMNPTYAGRSNLPDNLKKLFRSIAMTKPDMNLIAEVMLYSQGFRTAEVLAG
KVVPFFKLCFEQLSAQSHYDFGLRALKSVLVSSGNVKRDKILSIKQKMQAQGVVADVGKITETLN
EQEILIQSVMETMVPKLIAEDIPLLHSLLADVFPGIKYVNAEMTALRNELKKVCNEMHLVYGENL
ESGAHWIEKVLQVYQISCISHGLMVVGPSGSGKSTAWRTLLKALGKLEGNEGVCHVIDPKAISKD
QLYGVMDPNTREWTDGLFTHILRKIVDNVRGELSKRQWIVFDGDVDPEWVENLNSVLDNNKLL
TLPNGERLSIPPNVRIIFEVQDLKYATLATVSRCGMVWFSDDVVSPEMIIENYIKKLRNVLLEESEED
IRSKQSKSVTDSKDELVSPTLQVQRDFSDILTPHLASNGLVLKTLEYSYSKDHIMDFTRMRALNSLF
SLLNQGVRNILQYNQQHHDFPMSQDTIEAYTPKYLVHSLLWCMSGDGKLISRLDISNYIRNITTIPL
PSPVTSLIIDFEVNMQGDWVPWSNKVPQIEIETHKVASPDVVVPTLDTVRHESLLYTWLADHKPL
VLCGPPGSGKTMTLFAALRALPDMEVVGLNFSSATTPELLLKTFDHYCEYRRTPGGLVLSPVQIGK
WLILFCDEINLPDIDAYGTQRVISFLRQLVEHNGFYRPSDLQWVSLERIQFVGACNPPTDPGRKPLS
HRFLRHLPVIYVDYPGPSSLTQIYGTFNRAMLRLVPSLRSFSQPLTDAMVEFYTKSQERFTQEMQP
HYIYSPREMTRWVRGVLEAIRPLDNLSPDGLVRLWAHEALRLFHDRLITDEERRWTNTNIDLVAQ
KHFPNVDLISLKRPILYSNWLSKDYLPVDREELRDFTRARLKVFYEEELDVPLVLFDEVLDHVLRID
RIFRQPQGHLLLIGVSGSGKTTLSRFVAWMNGLSVVQVKVHRKYTGDDFDEDLRIVLRRSGCRNE
KIAFIMDESNFLDSSFLERMNTLLANGEVPGLFEGDEYTTLMTQCKEGSQRAGLMLDSGEELYKW
FTQQVMNNLHVVLTMNPSAEGLKSRAATSPALFNRCVVNWFGDWSTGALYQVGQSFTNKIDLE
RSTYKAPDYLPVVFENLPMPPTHRDVIVNTMVFVHQTMHKANEQVTARGGRTVAITPRSYLDFIS
QYMKLFNEKRSDLEEQQLHLNVGLQKIRDTVTQVEELQKSLSMKSQELESKNELANKKLKQMLQ
GQQEAEKKRITSKQIQDDLKKQTEEINKKKSQVLGELDQVEPALLDAKAAVKSIRKENLDELRRL
GNPPTAIKLALESICLLLGTPSVEWKEIRVLVSKTTFIPSIVNFKTDDISIETRKQMMKYLNHSDYNF
EKMNKASVACGPLVKWAIAQVSYAEMLQKVDPLRRELQKNEDEMMSNQSKLDSVGEMISELEK
SIAKYKEEYALLISEAQSIKADLAAVEAKVSRSTALLRSLSSERQRWENTSDGFQNQMDTIAGDVLL
SAAFLSYGGYFDQQMRSRLWGTWSEQMEQSKIKFRSDLARTEYLSTADERLHWQSNSLPADDLC
TENAVMLSRYIRYPLIIDPSGQATEFIMKEYSGKKITKTSFLDDAFRKNLESSLRFGNPLLVQDVESY
DPILNTVLNKEVRKTGGRVLITLGDQDIDLSPAFTIFLSTRDPTVEFPPDVCSRVTFVNFTVTRSSLQ
SQCLNQVLRAERPEVDTKRSDLLKLQGEFHLRLRHLEKELLQALNDSKGSILNDDKVITTLETLKT
EAADIMKKVEETDTVMSEVEAVSEQYRTLAAACSSIFFTLEEANLIMFLYQYSLKFFLEIFQSILFNN
PLLKDKRDPLVRLGILIESLFQVVYNRVARGMVHNDRVSFATLLARIRLKGKSENSYDLEFDHLLK
GKEIVVQVSDVKLTGLSSDQKMALLRLSKLNPFKELSRYIMDNSMGFQEWINSSNATLNVPRCW
NIKDSLTSSGEVLYECLVVQAFRPDNLHAMLRKYVLAVMGEKFLHTAEQELDMVNIVENEIKAET
PLLLCSAPGYDVSGRVDDLVAITNKQITSIAIGSAEGFSEAEKAINASVKSGRWVLLKNVHLATQW
LISLEKKLHTLQPHPNFRLFMTMEINPKVPVNLLRAARIFVYEPPPGLKANLLRTFSTIPASRMSEA
PGERSRLYFLLAWFNAIVQERLRYAPLGWSKIYEFSESDLRVGYDMLDIWIANVSQGRANVSPDKL
PWDAFRKLIGDSIYGGRIDNDFDQRLLQSFIDRLFTAKSFDSDFSLVTSVDGKQSENIVIPDGMRRE
QFVQWCEKLPDYQSPSWLGLPNNAEKVILTTLGERTCVKLLKMSVNTEGSELAYSADSHLKESEV
TGDIRPLWMKSLQQSVRNWLNILPKRIDPIKRTKENIKDPMFRFFEREVNLGVKLLNDILHDLNEI
LEVCEGNRKQTNDLRMLVDTLVKGVIPRSWNRYATPKGLTVIQWIVDLGERMKQLQNIARGTNA
GGSKELKSFKVWLGGLFIPEAYLTATRQYVAQSNQWSLEELYLNVTIADELNPQLKLDDCSFVVIG

LKLQGATCRNNQLSPVDTITTDLMDICLCWTKIDPRKPPTLGANDVTLPVYLNQTRCELLFTVDM
STTGAVPPRSFYERGVGFVVSALTG*
>TRINITY_DN8101_c0_g1_i2.p2 type:complete len:820 gc:universal TRINITY_DN8101_c0_g1_i2:2597-138(-)
MDGKTGASLKLTILNDRGRVWTMVAGGGASVVYADTVADYGWGHELANYGEYSGAPSTEETFV
YAKTLLSLMMKYKHPEGKFLIIGGGIANFTDVAATFTGLIQALQHFAAEIKEHKIQIWIRRAGPNY
LEGLRKVKAASDKLGLGLKVYGPETHITAVIPMALGLTTPLPEPDLSQACGPPRRAAIAGAGSKPS
TQKAAPAPSAHTIVTATPETTSIVYGLQNRAVQGMLDFDFMCKRKKPSVEAMVFPFSGNHYVKFY
WGTEEVLMPVYTTTKEAVQKHPNTSVFINFASFRSVHETSLEAMQYPNVKTLAIIAEGVPEQQTR
QLIQTAEAKGVGMIGPATVGGIKPGCIRIGNTGGMLDNIVMSRLYRAGSVAYVSKSGGMSNELNN
IVCRNSNGVYEGVAIGGDRYPGSRFLDHFLRYQDDAGAKMLLLLGEVGGTDEYDLIEAVKSGRIT
KPIVAWCVGTCASCFATEVQFGHAGAQARGDMETAAAKNKAMREAGFHVPDSFDKLPELIGQV
YTQLVQEGVVVETPEAETPQVPMDYTWAKKLGMVRKPANFISSIADDRGEELKYCGVTISEVFAQ
ELGVGGVLSLLWFRRQLPPACTKFIEMILMVTADHGPAVSGAHNTIVTARAGKDLVSALCSGLLTI
GPRFGGALDDAAKMFAEAQDSGVSAKDFIEKMKKTNQLIMGIGHRIKSLANPDQRVVLIKDYAK
KHFKNTPVLDFALEVEQLTTRKRANLILNVDGCIAVCFVDMIRSCGAFSREECDDLMAFGCLNGL
FVLGRSIGFIGHYLDQLRLKQPLYRHPWDDITYIQELAAQG*
>TRINITY_DN30329_c0_g1_i1.p1 type:5prime_partial len:213 gc:universal TRINITY_DN30329_c0_g1_i1:1-639(+)
EPIVNRVLVKRMKVMEAINDVSPAMMLDFFKTTLATNFLKFPFFEAINAFCAALPIAPSVRGLFTG
FVFTTATLPVTNYRYRKSMQLEVNWANIYEAYFPTVIRDIVYGICRNYCTIWTLALNPQWTAVSPQ
VLFIVVILGCLGSAPFNEWRGYLLQSKGQELTFKEFFKPSNFVRSTSLGAIKQGLALAVGYWCAPP
ATRLVHGLFAGLRG*
>TRINITY_DN153369_c0_g1_i1.p2 type:5prime_partial len:115 gc:universal TRINITY_DN153369_c0_g1_i1:2-346(+)
GTSTSGHMSRRWPASKCSWGWHWAAPRYFACGGVAGAPLCPRCTAASGKRCRKPPSWWHGVP
GGRWMRKSFTAYTPRLPSSYGAGTCITSARTSSNPSPPVTRCPTQNLRAPPL*
>TRINITY_DN7504_c0_g1_i1.p1 type:complete len:414 gc:universal TRINITY_DN7504_c0_g1_i1:87-1328(+)
MAKIPGGIVVEMDGDEMTRVIWDMIKTKLIFPFVDLDIKSYDLSIQNRDNTDDKVTVDAAEAML
KYNVGIKCATITPDEKRVEEFNLKKMWKSPNGTIRNILGGTVFREAIICKNIPKLVPGWTKPIVIGR
HAHADQYKATDFLVPEAGTLEMKFTPKGQTEPTISYVVVNNFAGAGVAMGMYNTDKSIRDFAHSS
FQCALEKNYPLYLSTKNTILKKYDGRFKDIFQEIYDNEYKGKYEAKGIWYEHRLIDDMVAYALKVE
GGFVWACKNYDGDVQSDTVAQGYGSLGMMTSVLMCPDGKTIEAEAAHGTVTRHYRLHQKGQ
ETSTNPVASIFAWTRGLAHRGKLDGNERLMNFAKLLEEVCVATIENGSMTKDLAICIKGMKNVTR
ADYLNTTEFLQKIADNLTTKLNLSHL*
>TRINITY_DN163801_c0_g1_i1.p1 type:complete len:298 gc:universal TRINITY_DN163801_c0_g1_i1:55-948(+)
MANPLKDIISRFSKGVPAGSSTGLGLLIGAGLAAYGIKESIYTVDGGHRAIIFSRIGGIQPGVYSEGL
HFRIPWFQYPIIYDIRARPRRIMSPTGSKDLQMVNIGLRVLARPLSSTLPHMYQRLGLDFDERVLPSI
CNEVLKSVVAQFNASQLITMRQEVSLMIRRDLTERAKDFDIILDDVSITDLSFSAQYTAAVESKQVA
QQEAQRAAFLVERAVQERQQKIVASEGEAKAATLLGEAMNENPGYLKLRRIRAAQKVARVIAQS
QNKVYLNANNLLLNIKDTDDIDDGLLSKKR*
>TRINITY_DN360_c0_g1_i1.p1 type:5prime_partial len:476 gc:universal TRINITY_DN360_c0_g1_i1:2237-810(-)
FQELLNCKNSKQQTIRLPANRENLTMALPYSSAEDFYQSVVKRTPNEVEFHQAVKEVAFALWDFL
VKNPEYAKEGVLQRLTEPERTIMFRVPWVDDTGNIQVNRGYRCEFNSAIGPYKGGLRFHPTVNLG
ILKFLGFEQIFKNSLTSLPMGGGKGGSDFDPKGKSDAEVMRFCQSFMTELSRHIGPDTDVPAGDIG
VGGREIGYMFGQYKRLRNEFTGVLTGKGYNWGGSLIRPEATGYGCVYFTKEMLETKGHSFKDKIV
VISGSGNVSLFACEKCTELGAKVVTVSDSSGYVYDKDGIDKEKLAFLMDLKFTRRGRVIEYTQKYP
SATFHAGERPWGVKCDIALPSATQNEISGEEAATLANNGVLAVAEGANMPSTPEAIEVFLKHKVL

YGPGKAANAGGVAVSGLEMSQNSTRFQWSREEVDSKLHTIMVNIHKTCQKYGTDESGFTNYVIG
ANVGGFVKIADAMIAQGIV*
>TRINITY_DN761_c0_g1_i1.p1 type:complete len:830 gc:universal TRINITY_DN761_c0_g1_i1:129-2618(+)
MTSMFRSEEMTLCQLFLQSESAYACVSELGEIGVVEFRDLNPDVNAFQRKFVNEVRRCDEMERKL
RFMQKEIDKADIAIVDTGENPDAPHPREMIDLEAQFEQLENEMKDSNSNYEALQKSLLELLELRH
ILKKTQSFFEEEGYAETQARLQADHEPGVTGGGQLGFVAGVINRERIPTFERLLWRACRGNVFFK
QAEIDTALYDPGTGDLTHKCVFIVFFQGEQLKIRVKKICEGFRATLYPCPETPNERREMAIGVKTRIE
DLETVISQTQDHRHRLLQTIAKNIRVWLVKVKKIKAIYHTMNMFNLDVTQKCLIAECWAPVADL
DSIQAGLRRGTERSGAQVSSIVNRMSTKQDPPTFNRTNKFTKGFQAIIDAYGVASYQEVNPALYTVI
TFPFLFSVMFGDCGHGAIMALFALWLIMNERKIANWKAGGEMFDTVFQGRYIVFLMGLFSIYSGL
LYNDIFSKSLNIFGSAWVPTLNRTKYDYQPENYKEWSDGDRMLNPLDAPRETPYYFGIDPIWQLTS
NKLTFTNSLKMKLSVIFGVTHMIFGVILSLFNHLHFKQYVNIFCEFIPQLLFISCIFGYLVILIFYKWIA
FSIHSPSQPSILLGLLAMFLNFAKKKTDADRLYNGQDFLEPFLVVVAVICVPWMLLIKPFYLRWQH
NKKKQHGTNFARLHEDTAPLQDNEVNHHDDSGISSKNEEEEEFDFGEVFVHQCIHTIEYCLGCIS
NTASYLRLWALSLAHAQLSEVIWTMVFKIGLSMDGYVGSVAIFAVFAAWAILSVAILLIMEGLSAFL
HTLRLHWVEFNSKFYVGAGSKFVPFSFKDIMNDEAF*
>TRINITY_DN684_c0_g1_i1.p1 type:5prime_partial len:675 gc:universal TRINITY_DN684_c0_g1_i1:2-2026(+)
LFINGNITRMAWLLLLLAAFASITSADNVNIEKRFLKCDNILAVKVCDRFRNVGATLKLKASEFEA
LVKDAIDKGKDVKQYVENKLEEKLLSKSCEELAGESFCKNLDVFGIYLQKKKEELKPYVVKAVLA
GKQKAQEIAIQVREYVKNDLMTKKCVDILPAEHCKRIEDIGAAFKLKANEIKRVTIENVLKGLSK
ARDVASATLQSMKRSAEKFECEELLNPDVCVSLRKYAAKFRIGMPKMMRAVRSAIVMGYKVGKG
FLTVVYKIADAFIDCEHFLSVENCRKIKDTASKVGAKLTEIRDAIKSAVTKGITEAEKIYSKTLKFLK
DKFGKQKRSLIDPAGLEAKLKKYRELLKKHITNADKYLKEALKMLMKKFKVADQAIRTAVEKAIV
QGKLSYEEIKSYIEELMKKNENGQVISKRDTSGGIRDLLKKALSKATGITAVIIRKMLEYPMEKLQS
MKRYVQGIVDSVLGTETKRDVDSQLVSKSYALTGIRDLLKKALSKATGITAVIIRKLLEYPMEKLQSI
KSTVQGIIDSVLGTGDTKRDVENEMMDEAADELVKTKINDMTNDLNQQINDMAEDIVEDTAALI
DEVVRKEKRSATGESDISDYIMKILKKTLGDAYDKMQDKIAKNMADVKEALKKAGTNMLDRAE
AVLKKIDEMLKKITEEKQS*
>TRINITY_DN77_c1_g1_i1.p1 type:3prime_partial len:1965 gc:universal TRINITY_DN77_c1_g1_i1:2-5893(+)
MGVQVYSLGIGSNYDIGELLDIASDDASVFRSQDVDELVSIVASITEQTCKGCTRAVDIHFALDASQ
NVGRENFQVIKNFIKAIGFKFIISETGSHMSASIFGEYGTLIFSMDQATQQEDFINEVDNVPYIGDFN
GNIDKALDMVSEDIFTFDGFSRQGVPKVLVVLTASNCDSCEKSVEEAVKPLKAQGVHVVAIPIGSN
ANMEEMDKMASYPTQDYVIPRASYEELLNGVFIQKVSEMVCSGRPGVCEEPPIEGECTEIINECTL
DVNCPANRKCCIKDCKASCEVPAAACITPIDLAFAFETTGFIEENFDEVKSFASSLLDHFRVEPAKT
HISISTYGESVNAKTAFYESYDKSALKQFIKDLEPDGGRQANIGDFLKTANDEIFNITNGIRQSSPR
MLVLFTNGVYSNDQIAKAQEESLKLKAEGQDVNILVINIGSIVEGNSVLRDVVSTPDFRNLHNVT
DLRRLVNFGFENKIAEAICASKVRKTRCKPRTPQACSVRDDLCQTDFDCPGYQECYSDGCRLRCQ
TCENRCGDKVDLLFMIDTSSRIGPEGFKNAKENIKKMLEWFTIEANNTNVAAITYSDNANYAMQ
LPAAGSNDPPQTLFDVQGRLDSIPYSGGSKSNLDKALKMAAAQVFYQNSLRLRKDSKKAIVIVTD
GVDDSTSRLEIASWPLRKQRVVVDGNDYAAIRLLAVTVGNNYNLDTLEKVVSPPIDKNIFLAADF
DDMYNGIRQLAEDSCKYTRQDVEPPVIAVKGDPGPKGEKGRQGNPGTNGTDGMDGIEGPKGER
GDDGPKGEKGDTGIAGPPGGDQGDLKGPDPMAEAVGIIEGTAVKGTRGVTYDLRDGPIGPPGPIG
GVGKTGLPGDSGEQGQKGRGGPPGLRGVFGGQGPKGNPGEDASPGLPGEPGPKGISGKKGPTGS
PGMPGPQGPFGYKGEQGIIGQPGDPGDGGKKGPKGYAGEKGDQGDIGDAGFPGPDGKVGPIGP
PGQRGLRGPEGSSGPIGKTGDIGNKGLPGPRGSRGLKGQSGNAGPRGGKGRTGDVGLVGEKGTQ
GSIGEAGQEGQPGVPGKEGPPGYPGPIGTPGPMGSRGDIGPQGEEGPKGSLGLKGNRGEAGRRG
LTGEKGETGDIGELGPKGAQGQQGKQGKTGKIGPRGQQGPLGFQGLQGQTGDPGQNGRQGPK
GNLGPTGEKGADGPKGSLGSAGLTGPKGLVGEPGNVGRPGLPGRDGKNGPQGANGKDGDPGA
SGPQGLQGSPGSMGTPGSTGDPGSPGDRGATGAVGDKGTRGDSGVRGEPGAFGQQGPPGLRGE

RGEKGPQGTNGVQGSPGPRGTPGLEGLDGIVGRKGTAGRPGNPGQSGEQGAVGSIGVPGPQGIS
GTPGERGDGGSDGGPGEQGPPGLQGSSGSPGRDGMPGRPGQQGTTGGKGKTGLRGPKGANGQ
NGVQGARGPDGLPGAPGLVGNRGTPGPNGIPGLDGNPGPAGKQGKQGKMGTPGMTGPAGLR
GPVGSPGLRGQKGPTGDRGDSGETGPIGVAGDIGPRGFTGELGLRGAIGEMGQIGETGPQGMPGS
QGTPGAAGLTGPAGPSGLPGKDGNKGPIGDPGNAGNPGRDGGPGSPGKDGTPGEPGTPGSQGK
QGLKGPFGPQGAPGEQGQRGLTGRQGSGGKDGPKGFPGNDGSPGISGKRGPRGKDGTPGSPGPI
GKDGRPGKPGINGTDGDPGNNGPKGDPGERGDSGDSGSLGASGDKGKTGSEGPAGFVGPRGSN
GKAGVPGKRGKRGDQGRDGPRGNSGPDGIDGSKGFKGNMGRFGIQGPPGDAGLKGALGPDGT
VGHTGPQGNTGDPGPDGPEGPSGSKGPAGNAGQIGNAGYPGSPGDGGIPGTAGLPGPPGPPGPP
GDMSGAFSGNYWDFVNAGGLQKGPRYYGGRSKRSLDQQDNSIEKVFKSNYETFKNFDTVWNQI
VDDLVIQKTLGSSKNPALTCGDLFKTNPITKSGDYWID
>TRINITY_DN34481_c0_g1_i1.p1 type:complete len:802 gc:universal TRINITY_DN34481_c0_g1_i1:2487-82(-)
MTTPHMSPQEEQEKLLEEALQIVKNQAFQMKRCLDKAKIMDALKHASNMLGELRTSLLSPKSYY
ELYMGVSDELRHLETFLLDEFDKGRKVNDLFELVQYAGNIVPRLYLLITVGIVYIKVGDVPKKDIIK
DLVEMCRGVQHPLRGLFLRNYLLQSTRGQLPDSETEESEGTIKDSIDFILLNFAEMNKLWVRMQH
QGHSKYKEKREREERQELRILVGTNLVRLSQLEGIDVETYKKDVLPGILEQCICCRDPIAQEYLMECII
QVFPDEFHLQTLNAFLKACAELHQDVNVKNIIIALIDRLAMFAHRGDGPGIPNDIRLFEIFSQQVA
HVIQSRSDMPTEDVVALQVSLINLALKCYPDRVDYVDKVLEYTEEIFSKLNLAYIDKNNAVSKELM
RLLRIPVDIYNNVLTILKLKHFAPLFDYFDYATRKEMAMYVINNALDNETLIPSQEEVDSVLQLAA
PLIMDQDDQPTEPEDPEDFAEEQGLMGRFVQLLYSDNPDQQYLILTTARKHFGNGGEQRIKLTLP
PLVFAAFRLAFIYRDAQEQDDKWDKKCQKIFQFCHQTICALSKAEYADLSLRLFLQGALAADQQ
KFTNAETVAYEFMSQAFAIYEDEISDSKAQLAAITLIIGTFEKTSCFTEENHEPLRTQCALAASKLLK
KPDQCRGVSVCSHLFWSGRVNEGESEEKDAASNECRDGKRVMECLKKSLRIANQCMDPTVQVQ
LFVEILNRYLYYFERGNEAVNSTILNQLIDKIREDLPSLEVNDEMEQINKHFQNTLSHIQSKKDSAD
ENEAAIYKDFIL*
>TRINITY_DN95248_c0_g1_i1.p1 type:complete len:124 gc:universal TRINITY_DN95248_c0_g1_i1:197-568(+)
MAPKTKKVSKKKSTLNFNIDCTLPVEDGIMKVDDLKKFLQEKIKVDDKLNNLDQITLEVAKTKVT
LVSERPFSKRYLKYLTKKYLKKNNLRDWLRVVASSHTDYELRYFQINNDEEEDEEDNE*
>TRINITY_DN121236_c0_g1_i1.p1 type:internal len:568 gc:universal TRINITY_DN121236_c0_g1_i1:1701-1(-)
TCIMCAHDEYSPEHSSECIKRKSCTPSDYHQYWEPCKDDKTRKSFQWNQPKICNEYTGEKLPNSG
DFIECPPCNPGQYINGSECQFCPKGFYSNGKEACQRCPPETAPAPGLFYKFWNRIPKRPMHLFCL
ARPMSGCRTLVGWVVHDSYLATGLGHSDEAQLIMYIKIDRFIKKNSLFTMEFEISDCIGLTCRTKV
QTIDKHHSVQSIGVYEGNQPRTTIRYTRYNDVGTEYSILFEKKYDQDAQTYHYQSAQLKIYSIEITD
VENGGTRQCEACTTRGSGDCVTCEPGHYLSPNSQNCWKCPRGTYMPEHLQDAVGLQSCIQCPIG
STSEPGSTKCHSDCMLAFGEGVNRKYFNLTALAGPQFVRGPLLFTSKGYKFFHGFSISFCGHPYKE
SLCVDNITSTQGFHASICRSTFIPEEHKLLSTQSIIIGDTLQKINFISEESNANHTEQKQKRDIHRNEI
QYHFHAERITDVCNNGTSGVLRLICDPVASYSSKKPNIEFGNDECTSETCDGCNYAFTLKSPYACA
KCTKDDFEAITTSCNNGEKHIKYVWKEPRACVGGMEKPE
>TRINITY_DN3271_c0_g1_i1.p1 type:complete len:399 gc:universal TRINITY_DN3271_c0_g1_i1:218-1414(+)
MTRIIWHKIKSELIMPYLDLDCKYYDLGLPYRDETNDQVTVDAAEAILKYNVGIKCATITPDEERV
EEFKLKYMWRSPNGTIRNILGGTVFREPILCDNIPRLVPGWTSPIVIGRHAHGDQYKAQDLVAPGP
GKFEMSFTPLEGSEKVTVNVFEFKENGGCMMGMYNTDDSIKDFAHSCFNYAIGKKWPLYMSTKN
TILKKYDGRFKDIFEEIFQDQYKSQFDALGIWYEHRLIDDMVAQCLKSSGAFVWACKNYDGDVQS
DTLAQGFGSLGLMTSVLLCPDGKTIESEAAHGTVTRHYREHQKGNATSTNPIASIYAWTRGLAHR
AKLDGNPELEKFSNSLELACVKAVEEGNMTKDLAGCIYGLKNVKREHYLLTNEYLDAVRDKLLQ
LWTPPSQV*
>TRINITY_DN1907_c0_g1_i2.p1 type:complete len:210 gc:universal TRINITY_DN1907_c0_g1_i2:53-682(+)

MDMDTTFPLVISLLFSILCCSFAVELTFELPDNARECFHEEIHNATKCTLEFQVVTGGHYDVDVSLK
DPHGKVLYDEKKKQYDSHTFEATVPGEYVFCFSNEFSSFTHKTVYFDFQSGDEPPLAPGMGKHHT
ALTLLESSAVTIHESLKVIIDYQTHHRLREGTSRDMAEYLNERVQYWSIGQSVIVVAMSIIQVYILRN
FFSEKRDRL*
>TRINITY_DN850_c0_g1_i1.p1 type:complete len:374 gc:universal TRINITY_DN850_c0_g1_i1:1261-140(-)
MAARPLVTVFNDQGEVSGQVTLPAVFKAPIRSDVVNFVHSNMAKNSRQPYAVHKHAGHQTSAE
SWGTGRAVARIPRVRGGGTHRSGQGAFGNMCRGGRMFAPTKTWRRWHRPINQNQRRYAVCSA
LAASSLPALVMARGHRVDGIPEVPLVISNAIESLTKTKAAVQVLKKVNAYDDIEKCKDSRKIRAGK
GKLRNRRYTMRRGPLVVYNEDHGIKQAFRNLPGVELISVDRLNLLKLAPGGHLGRFCIWSQSAFE
KLDSIYGTWRKASTEKSNYNLPRPKMTMPDLARLINSDEIQKVVRPAKPRARRASIKKNPLKNTQ
VMIRLNPHAKSTKRAAILAQERRKAAKDNVLDKKRGIKPAKETKAKKAKGKK*
>TRINITY_DN165_c0_g1_i1.p1 type:complete len:598 gc:universal TRINITY_DN165_c0_g1_i1:147-1940(+)
MTFHTGFSYCICLAFIFTIVLGSESQSEASQAVEYVKPSLDNLDYIAEPFHSLEEFQSRWVLSSAKKD
GVEENISKYDGKWAIVEPKENPLIGDKGLVFSSEARHGAVSGKLKTPFNFEDKPFIVQYEVRFQNK
HECGGAYIKLLADAPELSLDTFNDKTEYAIMFGPDKCGGDSKLHFIFQHKNPLNGKMEEKHAKK
PSGDFSNIFDDQKTHLVRLIVKPDNTFEIYVDKRRVNEGSLLHNFEPPVNPPKEIDDPNDQKPDD
WDEREKIPDPDAVKPDDWDEDAPKQIEDPNAKKPEGWLDDEPELIPDPQTERPADWDDEEDGE
WEAPQIDNPKCKEIGCGEWKPAVIDNPNYKGKWTPSLIANPKYKGIWSPKKIANPEFFEDNNPFA
MKSIVAVGFELWSMQADILFDNLLVADDLSLADQWTLQTWDLKHTQEVNKEPGVVGGLWNNI
QEATQERPWLWVIFVVLVLLPFVLIYFFCFGGKEDEYAIRKKTDEPTQDDDLVENSEDEPVEDKKES
DPEATEESVDSKKEDEESGKENEEITDEKTKSEVSPNDDNKVDTEKKLSEDSEDSKKEEDESPSPKT
RASKRKTRKE*
>TRINITY_DN96161_c0_g1_i1.p1 type:complete len:453 gc:universal TRINITY_DN96161_c0_g1_i1:1593-235(-)
MRECISIHVGQAGVQIGNNCWELYCLEHGIQPDGQMPSDKTVGGGDDSFNTFFSETDSGKHVPR
AVFVDLEPTVIDQIRIGPYRSLFHPEQMINGKEDAANNYARGHYTIGKIHIDNVIDRVRKLTESCTG
LQGFFIFHSFGGGTGSGLSSLLMERLSVDYGKKSKLEFAIYPAPHISTAVVEPYNSILTTHTTLEHSDC
AFLVDNEAIYDICRKKLDIERPTYFNLNRLISQIVSSITASLRFDGSLNVDLTEFQTNLVPYPRIHFPM
ATYSPILCAEKANHEQITVADITNACFDPRNQLVKCDPTKGKYMSCCLLYRGDVVPKDVNVAIQA
LKSKRTVQFVDWCPTGFKVGINYQPPTVVPGGDLAKVSRAVCMLSNTTAIGEAWARLDHKFDL
MYAKRAFVHWYVGEGMEEGEFSEAREDMAALEKDYEEVGQDSNADEDGDDDGDDF*
>TRINITY_DN1339_c6_g1_i1.p1 type:internal len:570 gc:universal TRINITY_DN1339_c6_g1_i1:1-1707(+)
GMFFQDGLTGSAWGDWALYVDSPLRFFGGGEASTYKTFDPATELGVQEPVGFWDPLGFASDGDE
ATFKRRRSVEIKHGRVCMAASIGYIVPEYYKFPGFLSPSEGLQFADVPNGLAALSKVPALGWVQIIF
FAGIVEGAFGVTDDYRNGTPGDYGWKVITSDDPAEKEKKLSAELANGRLAMVAIIGMFFQDGLT
GSAWGDWALYTASPLRAFESELGVQAPVGFWDPAGYTNDGSMENFKRRRATEIKHGRVSMLAC
MGYMTPEITGKLPGYLSPSMKVQFADIPNGLGALSKVPGVGWAQIFAYCAYCELSQDQSAASPGG
AGDFGFKAITSSDPATLTRKLNAEIANGRLAMMAIIGMFFQDGLTGSAWGDWALYVDSPLRFFGG
GEASTYKTFDPATELGVQEPVGFWDPLGFASDGDEATFKRRRSVEIKHGRVCMAASIGYIVPEYYK
FPGFLSPSEGLQFADVPNGLAALSKVPALGWVQIIFFAGIVEGAFGVTDDYRNGTPGDYGWKVITS
DDPAEKEKKLSAELANGRLAMVAIIGMFFQDGLTGSAWGDWALYTASPL
>TRINITY_DN1374_c0_g1_i1.p1 type:complete len:548 gc:universal TRINITY_DN1374_c0_g1_i1:1751-108(-)
MSMVLTIIHIAIIALVAQLEVEGKDFYIRTDSDVGECKYSGYIPGGKQINISPIKSTTASLETNILANIA
GFGGNAGTDAIQFPKISSTIENIAPNLGPALALFTVVLSAALNSELLTTTQEGMEDITLTGLFKLIED
VNDKFTDMENYVDQETLNVIKGRINTEYIHMFTLYSNCITEVSLQRAVECQEDANQYIRSGLPKF
MLFTDELKKFTMSSPPTADQIKKMELTFLPFREYATLSLMQLQVLKDTYRDNQNSLKNGPTIYREL
LGKLSSDAQKRIDYAQNMFKWIDLTYDEFAKNRCKDTKTSWSRCTNEGGWGSYTVCFGYTKCQF
DMTISSKKYCTTRCTVRADGQKIYTKKTYPDSFSNQDTGKKFTAEEIDLKASAYLSQKSKDVKNYY

ESEVLSSIPVWQEIKNKADAELMALNDEPNINYNFEGRQFKRTHTFNPIQRKYMPVGDFEFFEEN
DEKNSDLENRKLNYRYEENYRRSEYPILKDAEYGEFNDRFENDLSLREKKIKDGRFSPRFKERYQR
AFDTMRYFDDLNRARRF*
>TRINITY_DN495_c0_g1_i5.p1 type:3prime_partial len:407 gc:universal TRINITY_DN495_c0_g1_i5:152-1369(+)
MDFHWNTVIAGLIIVQVYSLAEYGTLNGKDSYRNAYPWLANDRNNNELGREDARSIYRSSDEID
AEYPQSDDEVDTNDIPKSYTSRDELDAEFAQRTYKFADDLDIENAQKSYASSDELDRDDEQRTNK
NTDDLDAEDAEREETINDSLDAENAQRRYTSIDELDGEDTQRVISKTITLPRLGAGRIYKSRFIHGP
YIRPKFVRPHKGIRLPHPNRIIIPGSWKRPAHGIFALKGKKAIFCEHKGIKRISCSNDKVIKVRSALY
GRTAGNVCSISCSKDEEIKVKNALYGRTAGDVCGRSRVTNCRALNSFAIVQKKCNGHNSCDLNPS
NSVFGDPCVGTVKYLNIKYFCVKRITPTTTPNGKTAIFCEHKGIKSISCSKDEEIKVKNALYGRTAG
DVCGRSRVTN
>TRINITY_DN135129_c0_g1_i1.p1 type:internal len:155 gc:universal TRINITY_DN135129_c0_g1_i1:1-462(+)
SSFSYMSTSPSRSMDISASSDEALTPGGTLIIRDNVDNRTSFYERLQEKDSFIAETSSALNTPQNSPRP
STTTPLPRTPQQDRTPPKRSPLVRMKSDSSDIEKRAKFSKDNVSSTFRPVSMLNLSVFASNRPKRLH
TGSESELSEHVPLSTHRR
>TRINITY_DN1143_c0_g1_i1.p1 type:complete len:629 gc:universal TRINITY_DN1143_c0_g1_i1:2539-653(-)
MLRSRTIITNTLPKLKSINRLEPSFSCISNARCFSVSAANKDASESFAMGIFTGQANVSQIFPYPDVLT
DDQREFLSSLVDPVEKFMSEQNNAFNNDALEEIESETLEGLKEMGAFGLQVPAELDGVGLTNTQY
ARLVEVVGRYDLGVGIVLGAHQSIGFKGILLVGNEEQKKKYLPSLATGEKFAAFCLTEPSSGSDAA
SIRTNAKLSDDGKHWILNGGKLWISNGGIADVFTVFAQTPVLDEKSGETKNKVTAFIVERGFGGIS
SGPPEKKMGIKCSNTAEVHFEDVKIPVENILGGVGQGFKVAMHILNNGRFGMAAALSGTQKGLI
AQAIDHAASRTQFGQKINNFGVIQEKIARMAVRHYVTESMAYVICGMMDKGFKDFQLEAAISKI
YASEAAWEVADETIQVLGGMGYMKDCGAERVMRDLRIFRIFEGTNDILRLFVSLTGLQGAGKELR
KLQKSLSNPLSNMGTLLEQGLLRSKRAAGISSGPDLSSNVHPDLRKEADLASKGIGKFGSTSEALL
VKYKKSIIDQQMVCSRLSDSAIDLFGMVCVLSRATKSLNNNVPNAVHEALIAKTFCSEAYKRINTN
LESCKNEHNMATDKLKAQITSDITNHMGVVTTHPLGF*
>TRINITY_DN5229_c0_g1_i1.p1 type:complete len:728 gc:universal TRINITY_DN5229_c0_g1_i1:2363-180(-)
MFKSLTISISVLTFILLFQEGSSSPVVAANSTNNAANATILIEEDESNKRQETEPKSFSDDIEDVIKIKD
SMENAGLIRDNTGAVNKAKTSRIAGKLREKARSIDSDSADSATEGVSGAGEGLLTLVEGIKGGNW
VDIVTGGLDMISSVLGMLGPMSVIASSVLSLFSMAFGLFGGATGGEESTEDMMTRVIEKALLEAKA
EELRSEVNGIKRLLQSFSSSIANFRETNSLTQEQALTLYDQGFRGVAQFGVLEHYIEKWCAVGDPN
TYDTPVKKGELNDMVSRCLDMVNLYCDLSVFRQIMLADMASLIAEVGIEGTPEVFMHLLRKEQK
SDKKLLKFLMDPANNRDKRFVITKYFTSLTKYTTITAYLKKLSTLDLSDVETKKSARAEISKDEKFFD
KKFTLVCTDIELRGNCFQFSELGAKRDASLKGWKKKVVSLYVSDGVEVQGFHRPDLQGSLFGPYY
GPAVYGKVSGSKDWESMNILASETSALDLLRVCENENMDTEGVCEAIETKNYPNLGAMDKRGT
AMYYVSTIRKKAKCRYSKCDVWNTGKWWCERSKRRDWEECLIGRYWSERIKSMTIPKGYKIYGY
SETDYGGVEYGPLYGPSTVLKVHPSAAWKSLKMVKTTQSTTKMVQMCSEEGLKGFCDYFIANSG
QTDLSNVHGVNWDSDKRIKSMMVPGSITVYTYSEKSYGGMEMGPYDGPVTITKVDGADGEEQIK
SFKIMRRY*
>TRINITY_DN167_c0_g2_i1.p1 type:complete len:658 gc:universal TRINITY_DN167_c0_g2_i1:165-2138(+)
MSKGRAIGIDLGTTYSCVGVFQHGKVEIIANDQGNRTTPSYVAFTDTERLIGDAAKNQVAMNPV
NTVFDAKRLIGRKFNDPTVVKDKEHWPFDVVEEDGRPKIRVQYKNEKKTFLPEEISSMVLTKMKD
TAESYLGGKVSDVVITVPAYFNDSQRQATKDAGVIAGLNVLRIINEPTAAAIAYGLDKKVSEERNV
LIFDLGGGTFDVSILTIEDGIFEVKSTAGDTHLGGEDFDNRMVDWFVKEFKKKNGGKDLSTNKRS
LRRLRTACERAKRTLSASKQASIEIDSLFEGLDHYSSITRAKFEDLCADLFQSTMKPVTKAIADANY
SKEQIHEVVLVGGSTRIPKIQALLKDHFSGKELNKSINPDEAVAYGAAVQAAILSGDKDEAVADLL
LLDVAPLSLGIETAGGVMTTIIKRNSTVPTKQQQKFTTYADNQPGVLIQVFEGERAMTKDNNLLG

KFELSGIPPAPRGVPQIQVTFDIDANGILNVSAVDTSTGKENKITITNDKGRLSKEEIEKMVQEAEK
YRNEDDALKEKVQAKNNLESYAYNMKSTIDDEKVKDKISDDDKKTIQDKCTEIIGWLENNPTAS
KEEIDEKQKEIEKVCAPIVTKLYQGAGGMPGGMPGGMPGGMPGGMPGGMPGDGAPSGGSGGP
TIEEVD*
>TRINITY_DN11643_c0_g1_i1.p1 type:complete len:187 gc:universal TRINITY_DN11643_c0_g1_i1:1748-1188(-)
MPAKGKLTQEEEVLLQDFSRTVSKKSQALFYGNALMISVLPLWLFWKVHLMDPMSYAALFICGTL
SAAYLVAFAYKNTKFALKHKVAQKREVAITREVNTELDSRPETKKLSRKEKDDRILWKKNNVADT
EATTFSIFYTNTLYLFIIIIASFYVLRSFSPAVNFTLSTGMAAGTVALLSTGTQSS*
>TRINITY_DN333_c0_g1_i1.p1 type:5prime_partial len:384 gc:universal TRINITY_DN333_c0_g1_i1:3-1154(+)
IFVKQIILSYRSRALITAKMDMRQLSPFIFTVIGFLLFITQECNAQRNNFCSHNGCRGIPLQTVPYLA
RRVSLKASRKGQYPDNIMRIATWNISGIPVDRAVVNRRRRRPTGLCLSILKMGLTLFSLQGVDSEQ
LKGLQQCLRSHVFLPNTLIGDGNLIGFRRQRRLVKHAAGQFYLSETPFERSKLRGASDYATCTWA
KLSFANVVGKRNETVETAYSKLLGLLKHYSNQRRGSSRRVTRLRKIYRYFTFYTVNVAKVDEENKA
IAKEQMKICLSHLYNDIMERRRYPIIVTGRLNWESSFTTSNNVRTKNENFNLKNGMDDADMTYY
TFSNIDKERNTKELKDYVTYNGFDSLLTAVLVNRNSERKKRNINRPVVSFLYHS*
>TRINITY_DN26249_c0_g1_i1.p1 type:5prime_partial len:477 gc:universal TRINITY_DN26249_c0_g1_i1:1733-303(-)
QMKMAASNMAGHVVRNNLIFLLSFVIIIVAEEGRHARFEYKFSFKGPHLANKQGTVPFWNHYGS
AIPSDDQVRITPSMRDQRGGLWTKTKSSSEFWEVEIFFRITGRGRVGGDGLAMWFTEEAGIQGPVF
GSSDHWKGMGLFFDSFDNDGEQNNPYVMVMLNDGTKQYDHFKDGNSQQLGGCMRDFRNRP
HPIRAKIRYYNNILTVFYHSGMSERSDDYELCMRAENVVLPKEGFFGITAATGGLADDHDVIKFLT
HSLTPPLTDEQKQKILSEEQRKKYEEQYGEYTQKLETKKEEFQKEHPDKKAPEEKFESVYDRDVRL
IYENQNTIHHVVRALHEKVGELTNQLNNLHVTINNNNQLAQQQQPKGDGVTRQEVNTIINLQN
QVHNAIQELKQQSGGGGGDNTKTVQMEKTLQTVLEQVGHLVSKNNDRRPSCPTVSCVTPSILILF
LVAQVLLVAGYNFYKEHQNKAAKKFY*
>TRINITY_DN172571_c0_g1_i1.p1 type:complete len:104 gc:universal TRINITY_DN172571_c0_g1_i1:30-341(+)
MSGRGKGGKGLGKGGAKRHRKILRDNIQGITKPAIRRLARRGGVKRISGLIYEETRGVLKVFLENV
IRDAVTYTEHAKRKTVTAMDVVYALKRQGRTLYGFGG*
>TRINITY_DN140696_c0_g1_i1.p1 type:complete len:260 gc:universal TRINITY_DN140696_c0_g1_i1:43-822(+)
MPRGPKKHLKRLNAPKHWMLDKLSGNFAPRPSSGPHKLRESLPLVIFLRNRLKYALNYTEVMKI
MKQRLIKVDGKVRTDICYPAGFMDVITIEKTGEFFRLLYDVKGRFVCHRISSDESLYKLGKVKRVAF
GKNSVPYLVTHDARTINYPDPSVNVNDTVVIDIKTGKINDFIKFDTGNTVMVIGGRNTGRVGIITH
RERHASSYDIVHIKDAAGNQFATRLSNIFLVGKGNKAMVTLPRGKGVRLTIAEERDRRLASK*
>TRINITY_DN8707_c0_g1_i2.p1 type:complete len:564 gc:universal TRINITY_DN8707_c0_g1_i2:2751-1060(-)
MSGFVRQSKYRHVFGSPARKDGCYEGFRVTNCAFDGNFIAANKHFIAVCVEVGGGGAFIIVPVGS
TGRLDVNIPKVSAHKEYILDLQWNPYNDFMLASCSEDGTIKIWEIPEKGLFMNIGDEKALLSLEYH
EKRCTQVLWHPSASNVLMSVSQEPKVCVWNLDEGSVEVEILEHPELIYNATWSQKGDKIGTTCK
DKKIRIFDAREGTLLVQGMGHEGAKPSRVIFTFQDKYLFTTGFSKMSERQYACWNAETIEQLGLEE
LDTANGCLVPTYDADSQLVFLNAKGDSNIRYYELVDGDPYYFYLSTYQSSAPQRGFGFIPKRCINV
NANEISKGYKLHGTKTGYIEPVSFTVPRKSETFQQDLYPDAIGDEAALEPDDWFSGKNAEPKRIA
MSTFFKGKTKPTGAAGGGLKKGGLKGLKAKKEAKTAAKSAAKSEPAKPAPVKTESKDKVDAAP
PAAAAVEQKSSSTAVAAPAAAAPVVVSGVDEKVVKELQNEIKSLKDGDKKLQKEIISLKDKLKDY
DTLRSDIKLLCDAVKKNDERITALESLVAEETDEEETGEGGDE*
>TRINITY_DN875_c0_g1_i2.p1 type:complete len:606 gc:universal TRINITY_DN875_c0_g1_i2:2136-319(-)
MLRLVLFAISASVVYGSCNPLKLGCFIHSGSDRNLLHSDNSEDIYTDFYSGTNIEWDKFSSYLQRAA
CRCDQAAANAGYRFFSLRDFGECHGIGKGASSFDALKLKKSMYCIAPNLGQCDISEHQHCTGAE

EGEFIYETKAQPAEKVIDGGYSSWSAWSVCSKECDQGLQERQRTCTNPEPKGKDAKDCSKLGKPS
ETRSCKIKDCPPSGETVIFCEHRGVKRIACENDQVVEILAANYGRTSTKHCVFGRTKVTNCRAAH
SMSIVKSQCDGFNYCDLNPHNDVFGNPCGFYTKYLEVKFRCVARKTSSRRAVLCEHRGSPPLSCP
NPDEVLSINKASYGRLSTHYCQYGHFKQTNCRALHSDALIKKTCNGLNSCSLNPSNTVYGNPCG
NVNKYISVEYTCKKTNAASRQAVFCEHQGGKTLKCANPDEVLSINKANYGRLSTDHCQFGHFK
QTNCRAEQSDALIKLACNGLNECSLNPSNDVYGNPCGNVRKYISVDYTCKKANAASLRAVFCE
HQGVKTLKCANPSQVLSITKAIYGRLSTDYCQLGLFKQTNCRAANSDGMIKKQCNGKNSCQLNP
SNAVYGDPCKYVRKYISVEYKCINK*
>TRINITY_DN540_c0_g1_i1.p1 type:complete len:480 gc:universal TRINITY_DN540_c0_g1_i1:1503-64(-)
MATDKLEGLIARLEKVTGKLESVSVRGGSGGGAAVEDDPAWYDEYIAIHEGQWESFLKATKENG
DNDMVTLEQKLRAAFNAHKDLLTIAARNGKPSPKDLPELVKPLSAAIGEVQSFREKSRTSKKFNH
LSAVSEGVAFLGWVVSPSKPGQHVIDMKDSAEFYTNKILKEFKETDKVQVEWARGLRKMLMDFA
DHLKHCHPVHLLWNPDKPAATASTKASGGSGGPPPPPPPAAAPPPPPPVAAPSSAPSGGSEDSRA
ALFSALNQGSGVTAGLKKVSDDMKTHKNPALRNAQPVPGKTSSLNPRPYAPPQLKKFNAPVAEK
PPVFELQMKKWIIEHQKNNQSLVIDETNDKQTIYMYKCNNSVLQVKGKVNSITLDGCTKCGVVF
EDLISTCEFVNCQSIKAQANGKVPTISIDKTDGTQIYLNDKSLDTQIITAKSSEMNVCITKPDGDLN
EFPVPEQYKTVWNGTKFVTTIMDLNL*
>TRINITY_DN3773_c0_g1_i1.p1 type:complete len:504 gc:universal TRINITY_DN3773_c0_g1_i1:168-1679(+)
MAPVVGDLMDLSDIDASKQHNQAVVRDYISQPRLIYKTVSGVNGPLVILDQVKFPKFAEIVTLTLP
DGTRRSGQVLEVSGSKAVVQVFEGTSGIDAKHTTCEFTGDILRTPISSDMLGRVFNGSGKPIDNGP
PILAEDFLDIMGQPINPWSRIYPEEMIQTGISAIDTMNSIARGQKIPIFSAAGLPHNEIAAQICRQAG
LVKHSKGTMDYSDDNFAIVFAAMGVNRETARFFEQDFKENGSMDNVCLFLNLANDPTIERIITPR
LALTAAEYFAYQCEKHVLVILTDMSSYAEALREVSAAREEVPGRRGFPGYMYTDLATIYERAGRVE
GRNGSITQIPILTMPNDDITHPIPDLTGYITEGQIYVDRQLQNRQIHPPINVLPSLSRLMKSAIGEGM
TRKDHSDVSNQLYANYAIGKDVQAMKAVVGEEALTPDDLLYLEFLQKFEKEFISQGAYENRTIFES
LDIGWKLLRIFPKEMLKRIPAKTLAEFYPRDGRKEETD*
>TRINITY_DN32522_c0_g1_i1.p1 type:complete len:235 gc:universal TRINITY_DN32522_c0_g1_i1:147-851(+)
MAAWLDSYRETEQLGHDIMELINERNTLKRSASNTSKVQSSIRIKMQQYSSGLQRLKDLLLRDSSS
YNVTQQELQRRQNQLDVLVSKEQQLNQAFKPTQSNYDRSNLFGEKYGDDLNGRYGGASASNGL
DDDLTPQNFRQQQEQILKEQDKGLDSLHEVIQRQKMMGKAIGNELEYQNDLIDDITKGVSRVDN
KVNATERKVKKVHRKTGSCAMIVVIILLLIAILVVAVFPTN*
>TRINITY_DN6920_c0_g1_i3.p1 type:5prime_partial len:411 gc:universal TRINITY_DN6920_c0_g1_i3:2124-892(-)
CKRHQGKMSHRKFSAPRHGSLGFLPRKRCKRHQGKIKSFPKDDAAQPCHLTGFIGFKAGMTHIV
REVDRVGSKTHKKEVVEAVTIVETPPVVVVGIVGYVETPRGLRQLTTVWAEHLNEECLRRFYKNW
YKSQRKAFTKASKKWSDDDGKKSIERDLEKMKKYCTVIRVLVHTQQKLIKLKQKRAHIMELQIN
GGTVADKVDWAKKSLESKLSVSTVFGQDEMIDVIGITKGKGFRGVTFRWGTKKLPRKTHKGLRK
VACIGAWHPARVGFSIPRAGQCGYHHRVERNKKIYRIGRGIHTKDNKVIKNNAATDYDITNKTIT
PMGGFPHYGEVNEDFIMLKGCVPGPKKRVLTLRKSLLAQTSRKALEKITLKFIDTSSKFGHGRFQH
DQEKKNFMGLLKKDKERQAVEA*
>TRINITY_DN97809_c0_g1_i1.p1 type:internal len:184 gc:universal TRINITY_DN97809_c0_g1_i1:2-550(+)
LGPDQVDVAGAGRDPRHLPRPLGRRLRVLDRHRGRVRQRHKAEADQDPKLETARLDVRRPGVQ
VRRVRHPRPSSRDQCHRDCHAPGRVEGRAQVDRGRRGLGRRRVEHEVHGAAQPAARGQEKGR
LRAQQILAVAAGQEEAVDVGARNRQGPPSALAAVSRAPHAQFARLAQRDRRVARAKGW
>TRINITY_DN909_c0_g1_i1.p1 type:5prime_partial len:313 gc:universal TRINITY_DN909_c0_g1_i1:1-939(+)
RRVLFRSLTFIESLRLSRLVNKKNKTFMEMSEKGGIYNDNEREMNFTGNSDYESGDLGFCGWFLTV
LSFIIAVCTFPLSLFFCIKIVQEYERAVIFRVGRLLSGGARGPGLFFILPCIDTYLKVDKRVISFDVPPQE

ILTRDSVTVSVDAVTYFKIQNPVASVCNVEDAQRSTKLLAQTTLRNELGTKNLSELLLEREAISRNI
SNILDQATDPWGVKVERVEVKDVRLPPMLQRAMAAEAESSREARAKVIAAEGEMNAARALKEA
SDVISESPQALQLRYLQTLTTISAEKNSTIIFPLPINMLQSMMNK*
>TRINITY_DN413_c0_g1_i4.p1 type:3prime_partial len:463 gc:universal TRINITY_DN413_c0_g1_i4:1388-3(-)
MVSLSVTCLSLLLFVTVNAEESDHCGYDEKSWYKAMGWCYHSGSRLLLGDFNGDKRTDFFCHD
SNGKNWVAVSNSDGSFGSTTWQDSKERWCASPNILLTGDFNGDGVTDILCHGTATGKKWIKYFN
KYGVPSGKSFYRAMSWCNHKGSQLYIGDFNGDKRDDLLCHDTSGRYWTSYAGTDESKVFHHTS
WYKALEWCRHATSKLYLGDFNGDKKTDVLCTDGKGYKWIMYAKTSGNLFTGTDWHTNLDWC
HESSTVFLRIGDVNGDGKDDLVCHSTAGNSWVAYTQPKGGFGNFCHLAKWCAAGKESFFLGYF
NQDSRADILCHDTKYGKKWIRYAECSFAEESDHCGYDGKSWHKAMRWCYHSGSRLLLGDFNG
DKRTDFLCHDSRGINWVAVSNSDGSFGSTTWHDSKERWCTSPNILLTGDFNGDGVTDMLCHDY
KTGKKWIKYFNKHGVPSGKSFYR
>TRINITY_DN2776_c0_g1_i6.p1 type:complete len:374 gc:universal TRINITY_DN2776_c0_g1_i6:1221-100(-)
MLNTLIRTGNANVFKLSSNSQKICSCTYMSGVGVRRSLPHNTIIKFVPQQESWIVERFGKFHKVLS
PGLNILLPIVDEIKYVQSLKEIASEVPQQSAITKDNVTLHLDGVLYFRVIDPYQASYGVEAPQFAITQ
LAQTTMRSELGKIALDDVFRERDTLNQNIVEAINSAATVWGIRCLRYEIRDIQLPAKVRESMQMQ
VEAERKKRAAVLESEGQREAQINRATGEANAILAKAKARAEAIDLISAALTQQSGNQAAGLSIAE
QYIQAFSNLAKESNTVVLPAYTGDVSSMVAQAMAVYGSISKNLPNDNNNDNDPNNSGHGGSIN
SRKQSSVENETSTLGFSGYDELSKFALKSEDEFSVTTSPTRKRDFV*
>TRINITY_DN2963_c0_g1_i1.p1 type:complete len:116 gc:universal TRINITY_DN2963_c0_g1_i1:1072-725(-)
MSATGNLVTVLRKLYDEYNDKTPSRLKIVDGFLLYVMLTGIFQFLYCCLVGTFPFNSFLSGFISCVGT
FILGVCLRVQTNPRNRTEFKGISPERAFADFIFANVVLHLVVMNFIG*
>TRINITY_DN201860_c0_g1_i1.p1 type:internal len:221 gc:universal TRINITY_DN201860_c0_g1_i1:1-660(+)
LTAITKVVCSTTSGSATGSKDGSLSSGDDYISRDLSDKLSIITFDTNETAQYCEVQIIDDSFYEEEEQFT
VKITPYFGGEVDEERADVNIVILEDKNDEPKIYLKSTLYEIDESDGVVKVAVQRTGSDLSFKSYVIIST
KQTEPVSAKANVDYYPVVKTLEFGIDEKEKLVDIGIIDDKGRPRIEGEESFEVLLGLAKNGELLDGQ
SKGVVIINDEESDK
>TRINITY_DN5554_c0_g1_i1.p1 type:complete len:316 gc:universal TRINITY_DN5554_c0_g1_i1:223-1170(+)
MRIIIALLFCVVGYCYSADWGYEAGNPNVLEPKDWKQHFPKCGGRKQSPINIATRSAILSRLPKIIL
GEGWGTLLESADLSLANNGAKVSIKSSTPITTKQGGTIWNLAQFHLHWGKDSHSGSEHTINGMS
FPAEIHFVHFNSKYPTIGDAVPQPDGLMVLGFFIKVQENAVVPSILQKFFGSLDQIKFEDQKVKLDF
GGRALGDMLPPRGTSKYFNYPGSLTTPTCDESVNWNVMSTENAIVITEKQMTQLRSLSAGKKNE
AFKPQSGNFRPIQPRNGRTIRRNFRTATTKFRSVLRRYFPRIFSYFRVTPRFE*
>TRINITY_DN2077_c0_g1_i1.p3 type:5prime_partial len:238 gc:universal TRINITY_DN2077_c0_g1_i1:2-715(+)
LAQAVSIVGPRKPGRPAAHRSSGSRHVARVGLRPPHHGLLAGGQVVPGGVRPRGGQEEPEHHV
DRIAGERLRRGRNAEKGPRQAHGQGLYHALVQHHAQHRLRHDGHAPRRAGARLPRARDRLEV
PGPERLRDPGALSRIEARQHRTGVHAARLHAPVWRQHHAYEPGRRGGAISLPDRPRRDVQGFQ
GLRGRFEGKRGDQRFGKGHQEEGGLHRGRDDPAGHRLSPAGVGHGLQVR
>TRINITY_DN128406_c0_g1_i1.p1 type:5prime_partial len:150 gc:universal TRINITY_DN128406_c0_g1_i1:474-25(-)
FGSTRKMSQTQSVQVFGRKKTATAVAHCKRGNGLLKINGCPLHLVEPSTLRYKVQEPILLLGKERF
EGVDIRVRVKGGGVVSRIYAIRQAIAKSLVAYYQKYVDEVSKKEIRDILVQYDRSLLVADPRRSEPKK
FGGPGARARYQKSYR*
>TRINITY_DN4781_c0_g1_i3.p3 type:complete len:183 gc:universal TRINITY_DN4781_c0_g1_i3:2965-2417(-)

MCTEPKAPSSCANDEFQCTKTKQCIKKHMLNLTAYTKVGSKMTKLWTKSGTYGPRWRHATVDIS
SASTFKVVIEGTASSGYMGDIALDDLFLNNGACPPSGECSFEDPKLCGWTNMNGDNFDWTRDN
AGTSSSGTGPRFDHTYGTAQGFYVYIEASNPRSKGHKAWLKSGEYKPTLGRCLSV*
>TRINITY_DN942_c0_g1_i5.p1 type:complete len:5116 gc:universal TRINITY_DN942_c0_g1_i5:15664-317(-)
MKMKLALLVLFTGTTLAAPFWVLDDNLPRAEIKEVGCSGKRFQNGKSHVYEYEGVTYNTIEGST
DKKTGIKLTATATITPMSECKFMMQLKSVELMSKSMNQDTFTTNAESAKLKDELEKLMVGFTMK
GNQIVTIHASENEPTHVLNIKRSILSSLQLDESTEKETDINGICDVEIVTKSGKIVKSKTLSECTERAQ
NEIGFQSTSIKSATTLKPLDSTSKCEYVMEGDVIKSVVCEEKHIFKPFSAGYKTPSGAMTTVKQSLSF
KEYTVAAISAVPSDFTVETNLLFDHSTEEPTATSTINDQVEKVMLRLVDPAQKSDLSAKENSAQEFK
NLVTLFRRMDEKRMEATWTKYFDCIKSGVCTDANTRDLYRQYLLDGISHCGTSTCIAMVRNVIV
NGQITGERMNMFLRSIPFVTKTTMKMIGHVLEICEKNPSRQAYLTLGTLLVRHCKQNPEDCSENL
RNPVTMAEEHLVKKLGQECEGQDDHERVEELLRVLKAIGNAQRPARVQEKLMKCAEKSKHSNI
TTSIFDAFRGFSCDALKQGELLRVFEDINNEDETRIHAFITIMKCPTVEILEKVTMMLEKELSNQVA
SYMWSYLTNLMESSDPRHQVARDILTKMTAQKPLREVNLPVHQFSKAFEKSLHSEFLKSGLTTEG
HIIFNKDKYIPKMGMLNMKANMMGNSLDMLETKIGLDGIEPILETLVGPNGMLPKDLNIDLGSY
GKFNSEFLRKMLEKKNNKRKLFNDNLTKMHLKANKQFEEPCAHLSVKIMGQEVSVLSYDDILW
MIDQIDNMNAVQLLTNIAKGGHKTFSKSLMFLEMSHNVPTGMGLPLKLKLTGSAVGTVEFNGKF
DIRNLFWGTPAVNIKGHMKPSTVVELSGQMGVEGHYVSTGMFVKSSMFTSQIVKGAIEYKHGQIL
KINMDVPEEPLAAVKFSSSPFLFMNEESELVEGTSRFVNPEHCTKSALLGYGLCASMRLPIAFREYE
APYYPLSGPAHFAVRAIRGDEKLTTWQFLVELNKQSGTTFGNIEFSTPGAAYERKLNGKVSMKQE
GKMNMFTLSTEGYDRKASVELNFNKETYSTAIITKNNIFTTKDMTTKLAIFNEEIDSGRQYGIQAL
ATMDWYTFDHITKFVRKPNVYMFQTSTTYYPKKVVMGTIVYDTKENKLSTRIQANQFQQAYEFS
GKFSDDGQEKGVTVTGKCVTSGKSITLYGGIQNTKQTKQILFTADVLGYSAKQYIRYENNNDVHT
VSQFLSVNGKIISLNGELSSFTEMKRLKFVADIIGRKADTTFLLTNNKGEKSLSVDMNAMQKHAN
FKTGCYQAKDSNECKFNFKIGTKEGEILMKQIMRDGNVITTLETLFDKYVAGFRNVYSNKNANA
KSLCSSIFYGTEEKEHEPVNVCLKYNAASDEKKILAITFDIVDRNQQGEINMELTKQTEFVKMQTE
VVYNKNSLAKAMQSMKYSKDLNNEIKMEVNVGKYNAGFRWFTLKTTNGANVGIEGYAMDKKI
QFVNKYVKDVVSGTTESKVTSELYINDKLVPVTTETIFLNGKGIAGPKFQVNIGKHSVSYATLVTYG
KGVYGIDLEFLVAKESNMLFKLFNGAAASVTPKKMDFQYKFGFGINGKQYTYGGDMSYNNLGN
DEKMEHSVKLGLQYSINRRSSMTFVLSNDKTKAALNMEFEYIPTKSVSHSIEYTKNDRILQVSVEFL
PKMFYTFKSQLVKNEGYSLASSVTLNWKNYNRVLKMINSMVNNDKKFELSTVFGKDLTFTFTFG
KENQKSMTLSASGFKRSLKVIGTFVKPNVQLQVYKNGEKIMDVTTSFDGITNKFQFALTNGKDTY
LKMMKSYNEFVLASGKETYLKMKKKYNEFVVTSGKETYLKMMKTYNEFKNQYKFQINGKIWTK
VQEWYLKMTKMTKDNKITKFLAPFLDLTVKLDRETNDMKLVLSNGKQTFVKIVKSYNEAKNQH
DFQIFGMSKEWVDLMAKYNKRNKVLKVSFSFFGKELAMFTLNMKEKDTVSLTTGSDYWKKNIV
LTGSLDRSQKSIAGNFQYGKTKVQLKLRADWNNMILENAFTFNDNVAKIGLLREQNSMVFRVKL
SPTLMAQVVMEVKNDNIIKMTIQRSEGDKLIEETSMQFKLSKDLYNLAFKWNPETFKAVIRAVQN
MIQNGYYKDIKPFITNVFVKGQRMSRKALTTTVMTFTEKVKEAKTKLIDLITNFEASVDKINFVAIR
EQIGQLSLKTIQKLKELVKVTLKELMTKLENAKENIPELLKQYKQIRNKIEKISKDTAEVLPKFYKET
LEAMDKQYNKLNGYWKDVSKVVMSNFELVSKNLTDSTKPVVEKAIYLISNFKLRGKAVKEIVSIA
RELVEKYIQILIEQGKIIMANGKIQLENMKLKSMEHYKNLKLKGMEQYNNLKLKGMELYQKGKM
PYLDETVEGIIKISVEKYMDLKKKMTDFDFEKYIQSVRDQIMEKKINGKTIVEHLNTAKEEMKNLP
PALKENYVKLIELITKLTKALNTNLKEWKIFAKPLTNYMKLVALSTKKHFGPILTKSMDKILPLITGI
ELPDFKMMALEQWKQLENFMLPLIKPVGPLYENIKTQIRNIKVSGYPIGSFVDFNIEIAKTFIQTSLK
TTKQKLDENVKMVTEYANLIKKMTPEEIMNQITLKSTELTKEYKEKVLKVYEERQERLKVLVEKLK
ELYNEFKQQYERITAVPVQDHIDDMMFKSSKFVKGNLEEISKVVKQITEIEISKPTWDAWKQADV
MGHLDKYGINQKMIDFIKAVKEVDMKRIAQKTTRLIQDMISTIYSKAFVRFVQVYGKLEKVHDYL
RSIPKKEYEEWVKELKEFSLENKERVIEFMGKMYKQTQKKSLNLYTQSKFLYAIMHKIVLKNMEKI
NINLVKPSQELYSDIKYRSEQVYGDLEQPTIDVKNHYQKIINDFVEEQYTIVKGAVLRHYKSLEEYK
EIMYTKAQEKMEEVKKLIELKYNELLAKYGDMTYEEMYDNAYKYGKSQYKVVRKTVKRGYKKL
REQMEKMEERLNALYQLYSTKYSDLVNRIKTEIIPQTQEQYQKYRLIVINLIKKYQKVLMEKMAEL

KTRGMTIYNDLKTRGMAIYNDLKTKGMTIYTDNKDKTIKALFREVKQNAIKNINTVYVLVKNFG
DEKLKQVRTIIVTYYNKAKGVMVDVIIPEVKMELVSIINQTLRSSKVMTEEIMKAYTPHMVLAKNF
ANKGAILIIERGTVAMKASEKAIREFMDKAPAMMKDIVKKIKDQEYYNKIVNHEVVKKYIAKTQE
MIENLKTKVEQLKQDPTVVKYQKMFSDKVTELKERAKIVIDKLNQRIEELKNHPMTLKYQKIVTE
KMTELKKKAQLLLEDVKAKIEELRNNPSVLKYQNMMNEKIAELKAKVKMLVKELKKRIEDLRK
HPKTIEYQNMIKQKIVDLKKQISMYKQKIENLYQHPFVTKSMVTMKRIKRSALFTADKINVNLQP
KIKEASEMAQKRIEKVQEQAMSAVQFFKNEPEEAFWTGVSNLEKSGKDLYEQIKRLFNKELIKKQ
FDVLTTDAKMQWKIFLNDYFDDWSKEASRKMYEQVVTLVKNMNMKIIDMKMNMQKLPEVYK
PIIEDYKNQIMENYKNIVEYSLQNMIQGKVYLSNQWVELNNQWKACPYRLIMENPVWSEIGDEI
MQHEAMTAVKYVSNLGNLKFQAMKTILMKEYEIKKQLLNQKITELQVSLKKTVEDLRSKGLKNY
QLAKEKAEQMVKNIKEIAIKMDKFLEEKTIGEIVEITLEKYQMLITKVEFLRTQVVGLSQWYKEKA
TVIYNEKRKRAEKIFEEQKEKVLAVYQKYKAQVMPIYQKYEDKVKQYYNKYSSIIIEQYKMLLKKS
AQKYIQLRTKYEELDLKNKLNKLQKDAIDMYQNKKESALKLWEESGVPTKLVELKQMTIRKTIEA
LKQLPGQTKEQLTAVFDKYLNELKVQYKKYYSAGYTKLIKEYNDLKAKFEAKFELNRNLLIKLAK
PYTTPVVVAYKWVENEVAETALFVYRYHKLGERYNNGRELIKSEIERLTPILKNNFKKYNAEIRKY
VKDQADKYSAQALEMSKDLKQKAIVLSKDLSKKVVASLKTYSLMTGDKILRSIHSSMKCIDNVDF
GKVQKQISKKAQDFYREFTKYVIIDPSKGEILINIPHGDMVPSFTHQFEQIKLRARRSVQVLKENGK
VLMKKINNRMAELKVKIEKVRNKVMKKVMDNTVEVRRDLLSSYNINKRIFDRVYVKAQIIYKKS
LRKATNQYKVLKTKSQDYIRKATAQAKAYYEEYMTKAKDYIRKNTELAKVYYQKYMTIMKERYL
TVNKIIMDLYNTPPAELYKQLKESKASLKVIELIMHLYNTPTAELYKQLKASPTYLRIDRIIMDIYNT
PLSQRLEQLKTSSAYLKVNKMVMDIYNTQYMDIYNQRLEQIKNSPRYIKVKGMIIRLYNTPAKELL
SKLQYRGQKLYKIWFNKVKVMINKAIEKNAPLIKARLNAIKKELRPYTESLKTMYKKIKLGYSVKD
SFYPMYKQMKFATKFYRGEAMRQARKIKAQICKEDPKLCKAIKSSAKVHSFIINKYATRVNKVLNI
ALDKYQEGVAKTHRAYRHALNMIARPKSTYAAVAMVFKDHYVTFDGKSYEFDIPSSKCQYVLAH
DFQNADFTILKTQDAIKINNKDMKVVIFKNGKTMTVFPDKKISNGLPVQAGESKCIRQHNEIICN
FVEDKLRVVVDLENDITTVSISAWHHGKLQGLLGTNNRESFDDMKMSNGDLAKDQLQFTNSYE
MTGSPKCQLNKVAFKKLQCGKVTQKCKKLFENPKSELAGFFNIINAEPFIKACERDLVSCEKSKEA
KHCSSVNAYLSMIKSRGYPVELHSECMKEGERMTGDKWQQKQSNKEFDIVVLVSQRKTVNKKPI
MKVLQKLQSSFKSNSIDTRYALIGFGGKGVNEEAHVHALGKGASVFSNMNDLKRAITNTMVFNG
DEESNDAYHAIVEASLQRFRPGAHKIFVMFNSEPHTSHKYGATLKEAIFTLEKESHASLFVFDEINF
KSLNKKGLTIGQTNSKIYTTKNLKGLRLSGMELPKSEFKDMVESTNGGMFTNELMNKQNELATV
LFDAITDTVTQNQRQCLDCEKHTTWYGSTRQLCHPVAC*
>TRINITY_DN464_c0_g1_i4.p1 type:complete len:717 gc:universal TRINITY_DN464_c0_g1_i4:2167-17(-)
MLLHFGKSLHYLKFGTLFWRLKLVAIPRDTSNIINNIFLESKSQRYIRMSANILNEYSQNVGKMEDE
ISGLTSQLNALKRKLNGEIEDEDCSDQLKELRSKNQKLHYQKNIIQRALKKEQEKADSKMLNVQ
AALNVLFESAIRSAYPQIPNPPVALTIAHNPKFGDYQCNSAMAISKMLQKLTGEKIGPHVVAKTIV
ENIPSNSFIKKVDVAGPGFINLHLEKEFLKGVLEKVIKEKVYPPGIGNKKKVVIDFSSPNIAKEMHV
GHLRSTIIGESLSRLLEFVGHDVLRLNHVGDWGTQFGMLIAHLEDKYPDYKTVSPPIGDLLLFYKA
SKKRFDDEEDFKKRAYQRVVDLQGGNPDVRKAWQMICEVSRNEFQKIYDRLDVTLIERGESFYN
DLMSKVVENMKKSGICQKSDDGRWVAFAPNMKVPLIILKSDGGYTYDTSDMAALQQRLQEEHG
DWLIYVVDAGQGDHFKQIFGVGEAAGWYNSSITRVDHVAFGVVLGEDKKKFKTRSGDTVRLIDL
LDEGLKRSMEKLLEKGRDKELTKEELLAAQESVAYGCIKYADLSHNRVMDYVFSFDKMLDDKGN
TAVYLLYAYTRVRSIQRNAKKGRSEIEEYMKSNSLHFDHEKEWKLAKLLCRYPEIVVRCLDDLTLH
LLCEYLYELSSTLTEFYDACYCIEKNRETGEIISVDMNRIALLESVARIFEAGFHILGIKPISRM*
>TRINITY_DN36471_c0_g1_i1.p1 type:internal len:286 gc:universal TRINITY_DN36471_c0_g1_i1:2-856(+)
GNKWLVDCRVSKNTINIIKINRKSYCYLPEILDRIMTSITEQPCYTLINYPMENDTVSESKLKEDLEK
GDIPVKTEALKKVMYQLLNGEKMPSMLMTIIRFILPLQDHTIKKLLLIFWEIVPKTGQDGKLLHEM
ILVCDAYRKDLEHPNEFIRGSTLRFLCKLKEAELLEPLMPAIRLCLEHRHSYVRRNAVFAIYTIFRNF
DFLIPDAPELVYEFLEKEQDASCKRNAFMMLIHVDQERALDYLSSCIDQVHSFNEILQLVIVELIYK
VCIANPQERARFIKCI

>TRINITY_DN67_c0_g1_i2.p1 type:5prime_partial len:474 gc:universal TRINITY_DN67_c0_g1_i2:1488-67(-)
VHREAPKKMKLLIAVLFALCAIACGENYMRVCYYTNWAQYRTRMEFFPEDLDANLCTHVVYAF
AKIGRGHKLTMYEWNDDKMYPRMMAMKKQNPNLKVLLAVGGWNHENGNGQGKFSKMVNS
NSNRRAFIDSSVALLRKWGFDGLDLDWEYPAGRPSGEKFPGDKQKFTQLCSELMSAFERDAGER
QTTRLMLTAAVAAGKGTIDAGYEVAKIGKILDILNLMAYDLHGAWDPVTGHHTAMATDGVDLP
NRVEFTIPYSVDYWIKKGFPAKKIALGMGTYGRAFRLKDKSQHGLGAPVTSGWPQPPKGQFTRE
AGFLAYYEICKMGLTIVKDNAVSAPYGYKGDIWVGFDDKESLIHKVNTQIKAKGLGGAMFWCLD
LDDFKGSQCGEGKYPLMGAVAKALGGYTPPPPPTPGPKPKSTRAPPGEKTDRPVKTEGPSGGVCR
AIGSWTGNANMNAWCVSNCARGNCPASVCKC*
>TRINITY_DN73587_c0_g1_i1.p1 type:complete len:533 gc:universal TRINITY_DN73587_c0_g1_i1:94-1692(+)
MLRQAVRRLSRGTNKRSLLRKSSERLLPACVNACQRFVSTDVTNNQAAAAAKAADKSPAAAVA
VGQIVAVIGAVVDVQFDAGLPPILNALEVQGHGSRLVLEVAQHLGENVVRTIAMDGTEGLVRGQ
RIVDTGNPISIPVGPETLGRIMNVIGEPIDERGPIETKSKAPIHTEAPEFVEMNVNQEILETGIKVVDL
LAPYAKGGKIGLFGGAGVGKTVLIMELINNIAKSHGGYSVFAGVGERTREGNDLYHEFIETGVINL
KDDSSKVALVYGQMNEPPGARARVALTGLTIAEYFRDVEGQDVLLFIDNIFRFTQAGSEVSALLGR
IPSAVGYQPTLATDMGSMQERITTTRKGSITSVQAIYVPADDLTDPAPATTFAHLDANTVLSRGIAE
LGIYPAVDPLDSNSRILDPNVVGEEHYQIARNVQKILQDHKSLQDIIAILGMDELSEEDKLTVARAR
KIQRFLSQPFQVAEIFTGHMGKLVELKDSINGFKQILSGELDHLPEVAFYMVGDISEVESKATRLAE
EAS*
>TRINITY_DN247_c0_g1_i1.p1 type:complete len:373 gc:universal TRINITY_DN247_c0_g1_i1:38-1156(+)
MKLVLVIFVILVYTWNVSAGGKKCISNKDFMTPYPTNFWTEHYPKFVNNRPTIGIVAMEVLGQKM
LVNVPWSWNKDYFGSSFVKLVESAGGRAVPIKEDITEDDLDKLMRKLNGVVLPGGDADVGDSGY
ERISKQAIAFSKKMAKKNVTFPVLGICRGAQMMMMAEAGKDFLEVTDSLNLSIPLDFTSEQKESR
LFGNAPQGLIKSLSEDAITFNAHAMGIPTLNFYNNSGLQKAFRAISTNYDRNGTKFISTFEGRDAPI
YGLQWHPEKSLFVFNPVLAVDHSIKGVMASQYIANFFISEARKNNHTFRDRVEEEEHLLMSYYPS
YVGNITETPYEQIYLFDFVKPFQNPKYTPGKVNKNDDDNSGDDDDE*
>TRINITY_DN751_c0_g1_i1.p1 type:complete len:227 gc:universal TRINITY_DN751_c0_g1_i1:731-51(-)
MAMKIVLCTLAALMCCAYAQDGITAITDIINPENINTALDAAEKLKNLIHTTSESKIQSREKTQGFE
FLNMKANAQYTRGMKKHHLMRMMNRYLVPFSRSSADMKEVVDTLKEFIEDDVEEHQEQFFGL
MTQGGGGTTEAIFVYYKLRDDGKYNFKRMTFRGTFRLAADLVITHSSKSGFFSSESKDVINWLPRR
GVTAQNVQELLDIIVPKLATVMSEFAPKDSI*
>TRINITY_DN1505_c0_g1_i1.p1 type:complete len:154 gc:universal TRINITY_DN1505_c0_g1_i1:98-559(+)
MSLVIPEKFQHILRVMNTNIDGKTKIMYAMTSIKGVGRRFSNICLKKADIDMTKRAGELSDEEVER
LVTIMQNPRQYKIPDYFLNRKKDIKDGKFSQVLANGLDNKLREDLERLKKIRAHRGLRHYWGLR
VRGQHTKTTGRKGRTVGVSKKKN*
>TRINITY_DN925_c0_g1_i1.p1 type:complete len:261 gc:universal TRINITY_DN925_c0_g1_i1:826-44(-)
MGRVIRGQRKGAGSIFKAHVKKRKGAAKLRSLDFAERNGYVKGVVKDIIHDPGRGAPLAIVAFR
DPYKFKLRKELFIATEGMHTGQFIYCGKKATLQIGNILPVGTMPEGTIICNLEEKSGDRGKLARAS
GDYATVVSHNRDSKVTRVKLPSGVKKAIPSFNRASVGIVAGGGRIDKPLLKAGRAYHKYKVKRNC
WPKVRGVAMNPVEHPHGGGNHQHIGFPSTVRRDTSAGRKVGLIAARRTGRIRGTAKVKDVGGD
KE*
>TRINITY_DN97534_c0_g1_i1.p1 type:complete len:563 gc:universal TRINITY_DN97534_c0_g1_i1:138-1826(+)
MSATPAMDIRSPRYGYQSPNVRTRVQEKEELSDLNDRLATYIRRMQYLESQNNKLTAEITTSKESV
SREVQNVKALYESELADARRMVDELSKEKASLTLENDKLNGIIDDLRSKLEKETNGRSKAENDLR
HVERRLHEKEALSMSLSKERKALDEKCKDLESQLQDALDQLDQEKAKLEEEILRRVDMENRYQT

LTEDKKFCDEIHEKELIDLRNVTDTFKFQMQTDGAPSDPDDILREKLEELREMFEDEAENAKQEL
RDAYGSKNDSLQKELDRSNTNRLRIIEKYDGVKTELDKLKADYGLLDAKNKQLQKQIPELENLRE
MDKDEFARQIQDYIDKNKDLEEELSNLRDDYQKLLEIKIALDIEIAAYRKLLEGEEERLHLSPSSQP
RSQSTGGKRGTKRRRPEEEQVTPAEPAVNSTAVGNVEIIECDNSGKFVKLVNKSETEDEALGGFFV
QRVVNGREDKAIEYKFTPKYVLKAGQSVTLWSFSSGVKHKSPTDLVLKNNEDWTVGQSMTTSLI
NQEAEVMAKHSMVKSPNSDRDLKRARRRRGGQDRESCFIM*
>TRINITY_DN4377_c0_g1_i1.p1 type:5prime_partial len:604 gc:universal
TRINITY_DN4377_c0_g1_i1:3-1814(+)
SFEEFQSFEALLCQTDAQYRILFEMFDLTGSGTVTFEEFKRTLECQSHAKKYPFDFDSEFVKNHFGQ
DKKYEVSYHEFTQIIKDLNKEYISHLFKQKAKSNEGDSITLLQFGEIIKVGFRHKLTPYIYENMMAA
VTAQTGRRQVSYTYCVAFYDLLSNMEQIAEIFKEATDNRKFSELTKEELLQASFHHPQVTPMQIEIL
FDLCTAHNPSKGKISLIDTVKLIPKINPLTVFSKIHEVQQYDPSYAEEEQNDQSVLMQVAESSYRFLL
GSIAGASGAAAVYPIDLVKTRMQNQRSVLATEVVYKNSWDCFRKVLSNEGVRGLYRGLIPQLVGV
SPEKAIKLTTNDFVRGKFTDKDGEIRLVGEILSGGCGGAAQVMFTNPIEIVKIRMQVAGEAGQRPS
AINLVKELGFFGLYKGARACLLRDVPFSAIYFPCYAHFKSSLADKNGYISPLSLLCAAAAAGVPAA
YLVTPADVIKTRLQVKARRGQQTYRGVIDCTRKIYVEEGGSAFFKGGVARIFRSSPQFGVTLLVYEL
LQRVFYVDFGGGYTKTQISPVNPDISLNPDHIGGLRLAVSSFSGIETKFGLLLPKFNTAPKTIPKVQP
NT*
>TRINITY_DN5263_c0_g1_i1.p1 type:complete len:243 gc:universal
TRINITY_DN5263_c0_g1_i1:827-99(-)
MATPISKKRKFVADGVFKAELNEFLKRELAEDGYSGVEVRVTPLRTEIIILATRTQNVLGEKGRRIR
ELTSVVQKRFNFPEGSVELYAEKVSFRGLCAIAQCESLRYKLLAGAPVRKACYGVVRFIMESGAKG
CEVVVSGKLRGQRAKAMKFTDGFMIHSGFPIQEYIDMAVRHVYLRQGVLGIKVKIMLPWDPTGK
TGPKKPLPDHVSIVEPKEEVLPTAPSSEQKGGKPDIQAPAPTPVP*
>TRINITY_DN5587_c0_g1_i1.p1 type:complete len:809 gc:universal
TRINITY_DN5587_c0_g1_i1:196-2622(+)
MAQNKDDLSTEILKKKSKPNRLMVEEAVTDDNSVVTMSADKMEELQLFRGDTVLLKGKRKKET
VCIVLSNDEATSNDKIGMNRVVRQNLRVRLGDIVSVQPCPDVKYGKRVHVLPIDDTVEGLTGSLF
DVYLKPYFLEAYRPVCKGDLFQIRGGMRSVDFKVVETDPSPYCIVAPDTVIHCEGEPIKREDEEDA
LNSIGYDDVGGCRKQLAQIKEMVELPLRHPQLFKALGIKPPRGILLYGPPGTGKTLIARAVANETG
SFFFLINGPEIMSKLAGESESNLRKAFEEAEKNSPAIIFIDEIDAIAPKREKTHGEVERRIVSQLLTLM
DGLKQRSHVVIMAATNRPNSIDPALRRFGRFDREIDIGIPDATGRLEIIRIHTKNMKLDDEVDLEQI
AAETHGYVGSDMASLCSEAALQQIREKMDIIDMEEETIDAEVLDSLAVSMDNFRFAMGATNPSA
LRETVVEVPTVTWGDIGGLENVKRELQELVQYPVEHPEKFLKFGMTPSKGVLFYGPPGCGKTLLA
KAIANECQANFISIKGPELLTMWFGESEANVRDVFDKARMAAPCVLFFDELDSIAKSRGGSAGDG
GGASDRVINQILTEMDGMGAKKNVFIIGATNRPDIIDSAILRPGRLDQLIYIPLPDEPSRISILKANLR
KTPISKEVDLGYLAKVTHGFSGADLTEICQRACKLAIRESIEKDIQREKQREAQGQNDADMEMDD
EDLVPEIRRDHFEESMRFARRSVSDNDIRKYEMFAQTLQQSRGLGTNFRFPGSSNQQSSSGTSQPAS
EQPSIYGNDDDDDDLYN*
>TRINITY_DN166344_c0_g1_i1.p1 type:complete len:360 gc:universal
TRINITY_DN166344_c0_g1_i1:93-1172(+)
MQKPKKYDWKDSNMALFGSDTEKKVKKESAESEPAWQGAGADVGVQVWRINKFKVEHWDKE
QYGEFYNGDSYIVLNTYKEPDTDELKYDVHFWIGKYSTQDEYGTAAYKTVELDTFLDDKPIQHRE
VQGHESSKFKTYFPTLTLLKGGADTGFKRVLPEAYEPRLFHVKKQNRTQITCTQISLKRGNLKSED
VFIIDLGDKIYQYNGSSCSADEKYKATQEVARLKSVRGKARAETIEERTVSDEHPALMVLKDGSSK
EKSTVEGEKKMLRVSDADGSIGMDEVASGDDISKDKLTSDDVYIINTGSHVYCWIGSGASIDERKN
GLSYASNYLNKTETPWLPISVVGQGKEDSQFQKAF*
>TRINITY_DN1272_c0_g1_i1.p1 type:complete len:262 gc:universal
TRINITY_DN1272_c0_g1_i1:957-172(-)
MRCQLFLLVACMAIATCSAYDWRNKVQNMLKYAKVNIFDRAAYILGDKRQHQLGKPWFCHKL
DCPAFQVVGNYTNKKDGFEERCYPETAWVMTSKEVDANENDIETRDMFMKLFKYIQGENEKQE
KIEMTVPVFTYSQLKKDDPSKRYISMHFFVPPSQEKNIPKPTRDDVTIMDVKADCYYVRSFGGYV

MSFGSKIMNEEKKIHAALEKNNLDFEARKTMAAGYDSPFRLFNRHNEVWVAKRKTMDSGCCTPRKSTNVMY*
>TRINITY_DN72959_c0_g1_i1.p1 type:complete len:216 gc:universal TRINITY_DN72959_c0_g1_i1:54-701(+)
MGRRPARCYRYCKNKPYPKSRFCRGVPDSKIRIFDLGKKKTGVDDFPKCVNLVSDEYEQLSSEALEAARICANKYMTKTAGKDAFHIRIRVHPFHIVRINKMLSCAGADRLQTGMRGAFGKPQGTVARVHIGQPLISVRCKDQHEATAIEALRRAKFKFPGRQKIFTSKKWGFTRWDREEYLERRQDGSIKPDGTNVQYYPCKGPLKDWKWRESR*
>TRINITY_DN12390_c0_g1_i1.p1 type:complete len:268 gc:universal TRINITY_DN12390_c0_g1_i1:826-23(-)
MLASQVSIALIVFYLGCGNAYRPLSFYETNGPVQEILEDFEFRDDFEDGFLDRDAGVSMAEACVHAHNVMRQRHQDTPNLVWDEGLAKAAQAYAEKLAAKDDGLHHDIKNNKKEGWGENLYFKDAARVGTCADAVISWYNEIDDYDFNLGMSRNHKAIGHFTQLVWKTTTKVGVGVATHKGQTDNDIKTYIVAKYKPPGNWFHGYHDYVKPLIIPIKHRPTSVEELDRSVCTIDAFPSRFCRGFRREGRCGEFIVKDACYRTCHSCN*
>TRINITY_DN535_c0_g1_i1.p1 type:5prime_partial len:162 gc:universal TRINITY_DN535_c0_g1_i1:712-227(-)
GKYLVFFFYPLDFTFVCPTEIIAFSDRVEEFRALGCDVLACSVDSHFSHLAWTQQSRKKGGLGQMKIPILSDLTKSISRDYGVLLENDGIALRGLFIIDDKGILRQITVNDLPVGRSVDETLRLIQAFKFTDEYGEVCPAGWKPGADTINTGKAHEYFEKQ*
>TRINITY_DN98420_c0_g1_i1.p1 type:5prime_partial len:286 gc:universal TRINITY_DN98420_c0_g1_i1:1007-150(-)
LHKEARSTVINKMASRLLNSLGKVGVGLAVVGGVVNNALYNVDGGHRAVIFDRFRGVLPDVSGEGTHFLIPWVQRAIIYDVRSKPRNVPVITGSKDLQNVNITLRILFRPVASELPKIYMSLGEDYDERVLPSITTEVLKAIVARFDASELITQRDNVSLNVQEALVERCKAFGLILDDISLTHLTFGKEFTEAVELKQVAQQEAERARFLVERAEQQKQAAIITAEGDAVGAQLLSDAFKKAGNGLIELRKIEASEEIAQNLSGSRNVVYLPDGQGMLMNLPAP*
>TRINITY_DN14830_c0_g1_i1.p1 type:3prime_partial len:834 gc:universal TRINITY_DN14830_c0_g1_i1:113-2611(+)
MNKMASAALSRSFAQLYLVWLLFQSVVGQSKVNVSPKAVSKKAKEPVYIDCSVVPKSTGIRVTWKKDNKAIPYYSKETSQKDAPYVFAENNTLYIASLKRRHTGRYECEAFDQSGNTRTVGEATIEIKYIQKLAPIDPVRSYVGSDAEIVCLQPLANPFAQPSWQFNGVDIVQSDRVDPTGWTLRIKNVSLTDEGVYTCVATNDANVRTENATFTPVVSGPVEATPEDDVITEGEDAEFTCVSKANPPLEVTWKRVGKTCEDGVCNDEVTVENSDDGRINVENGVLSVTNAIPSDKGKYKCEASLGSSFNEAFVSLNVVELLKEDPQMHQRFQSVDIREENGTIFCQFRGDGRYDIIWSRVNSTKPLRKGMNQFNNTLKITDITFEDGGTYRCTADGIKGTATADVTLNVYESPVFSIHPNNVSQNEGKPAWIDCKGEGTPAPRVSFHRNKQKESLNASHFQQLPNGTLYIAELKKEDAGTFYCWLTQKAKIEYSTFYIRVKDAEPISPGSSSPMGRTVGIAVGCAGVYILLVIGLMIYCRARRARLLKKGLITEADDCDAKDPLVNGEDIPLPTNKSLDYLQVPKGEITEICELGHGKMGRVYKGLVTNERYPNGVLSAVKEFNGNNEQQNEEFNLEVEMFLQLNHDNVVKLIGIQNNTSPHYLIIEHSEIGNLKDHLVNSPDMTDAQRVNMSLDIANGMEYLISLRYIHRNLSTRNCIAMNNDNIKVTFLSLCEDVNKDDYYLLNNILVPLRWLSPEAIANEVYSEKSDVWSFGVTLWEIFSNGQQPYSKFDDEQVRDGICKDLRLTVPDKCPSTVADLMKKCWIAETTER
>TRINITY_DN81944_c0_g1_i1.p1 type:5prime_partial len:222 gc:universal TRINITY_DN81944_c0_g1_i1:3-668(+)
VHQVIARYSINMLPQILSTFVILTVSTALADKPWFCHDLDCPSYKVAGAFTEKKDGFEVRCYPKTVWAMTQAEFSAKSSSYSTGSLFMKLFKYISGNNKEGKKVAMTAPVFTYSDLKKGDNSTRVIKMHFFVPPAQESTIPAPSGDDIEIVTVPADCYYVHSFGGFVFSMGRKIRRETGNLVKSLEKAGIKSYDLSKSMYAGYDSPFRLFGRHNEVWLAKM*
>TRINITY_DN828_c0_g1_i2.p1 type:complete len:216 gc:universal TRINITY_DN828_c0_g1_i2:1021-374(-)
MANSMIVSLFVLAACASISQAIKCHVGLSSKELTFEIGVRQVECGGKFTRCATTTFIADGKKAYRGYCASTYGPYLCILMKNSAKTMTECDTQICSGDLCNKQVEEASQREKSGKGVKCYTGIEMNLPPLD

IATGDVPASVDQCEDNTDTCMSVSFLHEVKDPVQDRVFVRVPTVMRLCKPSYSTCENHCERLSK
HITIGDCKATCCKGDMCNPF*
>TRINITY_DN1559_c0_g1_i1.p1 type:complete len:311 gc:universal TRINITY_DN1559_c0_g1_i1:221-1153(+)
MAEKKATFKLGFAENFALSGAAAVISKTAAAPIERVKLLVQNQDEMIKQGKLSEPYKGVIDCTTRT
FKTEGLAPFWRGNLANCIRYFPTQALNFAFKDKIKAAFKVSKSDGYALGFGKNIASGGCAGALSL
CFVYSLDFARTKLANDTKSSKKGGSREYNGLVDVYRKTLKSDGVAGLYRGFVLSCVGIFVYRGCYF
GFYDTLKPVLLGADASLFMSFILGYGVTVTAGLISYPIDTIRRRMMMTSGQAVKYKGSIDCATQILK
NEGAMSLMKGAGANILRGVAGAGVLAGFDKFKEVYIAMRKRQMGLE*
>TRINITY_DN10890_c0_g1_i1.p1 type:5prime_partial len:963 gc:universal TRINITY_DN10890_c0_g1_i1:3568-680(-)
NEFFAGFQISPKILDLSEKDKPRKIILENTVPIVCQDGTENCRVLVELAQTNTDNFMDYCTISFKPGP
AGQTNEVEVVAKRDFIDDGDQTMFLKLHIPDHIDPVDWNCYKNITDIKIKTTDVKTSRCLSTGDP
HITTFDGHYYNHYLVGDYTLVESKARKFKIHVRTWKCGSVSCNCGVAVQEGDDVIVVDMCRDR
VPRARFASTVEPQKGTTIERNNNGKSFLLSFPSGAFVRITCKRNYADFQIQLPSDDFQNTVGLCGTF
DRNRNNDLTSKSGQVFNSRRGRNGPNGFTESWKLSSRDSLFYYRGGPKKCLATRPKSYCTCKETC
CGKRTVNCDYEGYSQRPKYKQGFRGWKSLTFPGAEHCGRRKRRSLDDSSVVLPDDGDTAIYDYK
PAPVIPNEGRFPTPKGKSESQARNTCTRKIKESTIGKACLKIITGFDVQDYIDQCVIDVRVLDDFIVP
VESAVTNMASNCEEETLRDVKKYWKKDAEGKDAPPQDIGDSACLNDCSDNGVCRNGTCICND
GFITDDCSMTKGQQPVLLGIPNDGLCDIRRQDCVRTRVTGDYFIDSSTLQCKATEAKVSDTTPPEK
FGSGVIEQGQLLSFREIGCLLPKVPLVINGNPDYSRGKPTGAFYISVSNDGNNFSKEDTTYPDSKSET
VMVVYDSKCMDCTKEGTCSWKKNSCKMNEYCFANGDPKPDDWCQVCDPDRSRTEFVKRLDN
QPPVFNGGSTQYIFKGQQWKYELPAEDPEKQPLMYSFIGENHGMKISSAGILTWTPTELKVYSFKV
EIEDPCGLKASQEFKVDVRNCSCEGQNGGRCEWLKSNTPEDHKCVCPDGCKGISCTDPIDGRICR
VKVSPQSGDDDSSSNGAVIAVIIILLIILIVIAVVGFLIIRYLKKENKDVNMKNIKTTIADAGKNAKD
RVADAGRNAKDRVIPNKNAKDDINANDKMRSSIRINLRPQSGTSAK*
>TRINITY_DN4439_c0_g1_i1.p1 type:complete len:941 gc:universal TRINITY_DN4439_c0_g1_i1:77-2899(+)
MTDSKYFTTTKKGEIYELKAELNQHKMDRKKEAVKKVIAIMTVGKDVSALFPDVINCMQTDNLE
LKKLVYLYLMNYAKSQPDMAIMAVNTFARDCDDPNPLIRALAVRTMGCIRVDKITEYLCEPLRKC
LKDEDPYVRKTAAVCVAKLHDINSTLVEDQGFLELLRDLLSDSNPMVVANAVASLHEIAESSPNAT
QYFEMNSATINKLLTALNECTEWGQIFILDSLANYSPRDEKEAQSISERVTPRLSHANAAVVLSAV
KVLMKLMEMMPTGGQLVQNLCKKLSPPLVTLLSAEAEVQYVALRNINLIVQKRPDILKNEIKAFF
VKYNDPIYVKLEKLDIMIRLASQQNIGQVLAELKEYATEVDVDFVRKSVRAIGRCAIKVEQSAEKC
VTTLLDLIQNKVNYVVQEAIVVLKDIFRRYPNKYESIISTLCENLDSLDEPEARASMIWIIGEYAERI
DNADELLTTFLDGFSDENTQVQLQLLTAIVKLFLKRPTDTQELVQQVLSLATQDSDNPDLRDRGYI
YWRLLSTDPAAAKEVVLAEKPLISEETDLIEPSLLDELICYISTLASVYHRPPSSFVAGKTPARRAVLP
RRTGSDTNENEEVSSISEETPTPTLSQVAGMPPISSQPATDTLIGDLLMDMGPAPTPPQPSVFDAHR
QQQAPSADLLAGDLSMLNTPVTPQQQPMGMGGGLNDLFSLQGALPGSGYCPPKQVWLAASKG
KGLEIAGTFSRRQGQIFMDLTFTNRAMQQMGGLAIQFNKNSFGVAPAVPVPNTPLPPNQSAEVSL
LLNIAGPVQKMQPINNLQVAVKNNIDVFYFSCLIPPHVLFVEAGKMDRKQFLDVWKEIPQVNEV
QNTITTKGFNSDGIQNVLEANNMFTIAKRTVEGQDMLYLSLRFSNGIDVLCELKIIPGNPVVQLSL
KTKAMDVIPMVQEVIQLILQS*
>TRINITY_DN119684_c0_g1_i1.p1 type:complete len:662 gc:universal TRINITY_DN119684_c0_g1_i1:2182-197(-)
MKLEIILFTLLVALTVHCSEKKDDSKKDVGTVIGIDLGTTYSCVGVFKNGRVEIIPNDQGNRITPSYV
AFTEEGERLIGDAAKNQLTSNPENTVFDAKRLIGREWGDKAVVHDMKFFPFKVVEKGKKPHVQ
VNVKENEKKTFAAEEISAMVLVKMKEIAEAYLGKKVTHAVVTVPAYFNDAQRQATKDAGVIAGL
NVMRIINEPTAAAIAYGLDKKEGEKNILVFDLGGGTFDVSLLTIDNGVFEVVATNGDTHLGGEDF
DQRVMDHFIKLYKKKKSKDIRKDNRAVQKLRREVEKAKRALSTQHQARIEVESLFDGEDFSETLT
RAKFEELNSDLFKSTMKPVQKVIDDSGLKKKEIHEIVLVGGSTRIPKVQQLVKDFFGGKEPSRGINP
DEAVAYGAAVQGGVLGGEEDTGEIVLLDVNPLTMGIETVGGVMTKLIGRNTVVPTKKSQIFSTAA

DNQQTVTIQVFEGERPMTKDNHLLGKFDLNGIPPAPRGVPQIEVTFEIDVNGILRVSAEDKGTGK
KEKITITNDQNRLSPEDIERMVNDAEKFADEDKKVKEKVDAKNEFESYAYSLKNQISEKDKLGGK
LSEEDKGVLEKAVDEAISWMDSHQDASVDELKEAKKKLEDVATPIVSKIYQQQGGAPPPPGGEG
GEEKEDKEEL*
>TRINITY_DN794_c0_g1_i2.p1 type:complete len:250 gc:universal TRINITY_DN794_c0_g1_i2:72-821(+)
MGLTGCGAVTKWILVIFNSIFFILGIGVLGVGIWLKLEREDYEDISSYDFVTPSNIAIAIGVIVMVVSF
VGCVGAATENSCFLLLHFLLLFLIFVLQIIACVLAYVNRGKVEDGLESDFRKSIKEKYGEPEYEAVT
KVIDKFQNEFKCCGFSSYKDWRMSKYNKRTASIPKSCCIESQKESKLCGSPNPSGLLVYEKKGCYN
EVLDFLKENLLVVGAFALAMAIVQVFGLIFAIYLYILVVRANKGTFA*
>TRINITY_DN598_c1_g1_i2.p1 type:5prime_partial len:742 gc:universal TRINITY_DN598_c1_g1_i2:2-2227(+)
RWSGCSKSCGVGVKTKSRECDNPRPSGGGRYCTGERTATEACKIQDCGVDGKFSHWSTWTACSK
TCGGGAQKRARQCNSPPPSGDGKDCAGAREEFKGCSNDFCKGPTGVAKAQEIIERFLENFHKKT
YPDREPSGKFLKAIFAIQKAVAEEFISVDPSVRGKTLEQRLKDLEKFLKVATEGGSIDFARTLLGKGIF
TFMKKKIQEGLPIDDIDKAAEGFLEKLRGDVGEKKMNEILSSQGDATLMFTIDTTGSMKDEINAA
KAIAKQIISATRDYEVDYILSPFNDPRTGPVTHRSSSNRASFVTDIDRLRATGGGDCPELSFTGMLN
ALNQGPQYGSPMFVFTDASAKDDSASNIDSLKAAADLTDATINFFTNLNGCKGAKSVESYKEIAS
YTGGQVFPLKNEAELKKFTNYVKTSLQQDTVITSGGTLNSKSYKRSLYSMQAHDFMVDDGIIMIS
VAISVQKYASQVTILDPNGRRVNPSTTLSLSLVYQISHPPPGQWKINFPSSVGKYGYTVQAVSSSAIA
FAHNFIYQQSVRKNSPPIPLANPLKGVKNMMVVRLGGTKNFDLNSIKIDLVDTSGNVLQKGIGTD
RLDSDEVRHATIDPPSGPFKVRLTGKTKSGYNFERVSQGISRPVSAAMRTNFAGYDFTAKLGKGPY
AAQMLFINRGAHENFYFYAKANYGSIPNYKQTKRVTSQGFVTFTYKAPRQYNLKGKSDRITVTVK
RQSTGEIVSHHTSILLVV*
>TRINITY_DN22219_c0_g1_i1.p1 type:5prime_partial len:427 gc:universal TRINITY_DN22219_c0_g1_i1:2-1282(+)
YISKDYCKGKKNPSVCTLHIHNEFSQIKHFQHQTPVTSENAPGIILAHGQAGDALTGAGNVWFSR
NGGYNWTRIAKGSHHYAIGDNGNLIVLVPKFNSTGKTLIYSLNQGRNWYHNEFDLFDENITIHG
LAAEPHSKERSFSLWGHRLVKGKKLWHTFTVNFRKLITKECQEEDMKTWYPHINNGKDGCFLGE
RTIYKVPHKDSLCYLGRSYKPEKKVEKCSCTKDDLECDYGYERNRAGNCHRNEDIPLIEICKNGEE
MQEAFSKGYRRVPGDNCIAQSSNDEVVRFIDEHKKCVPNKEDIFGDFNRERAEVIREEEEEKEKK
MDETQKATKTKKSKKKGPNWPVIILSIILMCIIIGGAYWYWRKNYRPNNVHHVMYNASSFDNVG
MDNNDGDVDADDLFDPRPKSLREYHDETDAEDEQLLLP*
>TRINITY_DN244_c0_g1_i1.p1 type:complete len:3019 gc:universal TRINITY_DN244_c0_g1_i1:9748-692(-)
MDARGQRLLYCLLIFLSLATLLVSQQVEKCKRPVDLVFALDTSGSVKDSGFRLIQKFAKDILKTFDI
GVADSHVGVLTFSEQAEPQIKLTETFDKAELMQMIDSLVYQGYRTATDDALRVAKTDFFSTAGGA
RQGVPHVLVLLNDGKCTVCIEPVKEAAQKLKDMGVEIFTIGVTENIKKQELIDIASQPTAYHFFEL
DDFDQLVDVVGDIQTRSCEVKKGKCPIPSPTEQCSGNEINECTSDENCEGELKCCSDGCSRICKYP
LLNCFTPIDIAFALDSSSSIQPETFQEMKKFSNDIVDSFAVSQKKTRFAALVYSDRAEIGFNFVRFDD
ASSIKKAIQNLPKLDSKERIDYALELAKSDIFSLQGKVRTRRPMVLFVFYDGTVSSGMKDLSLVVEP
LKNYGVKIVSMGVGPETNYYQMRKISSNNSDVFRATNFKELLPEIYNIAKRACTEKPGRCDASLN
PLPKEACKNVKILNQCENDFDCFGDAKCCRKGCNNVCVIRGPVCPRKLDIALAVHQNMDESEF
AATKFYLQSVVDQFNVGPSQTHFSLLSFGQEPKVIFDFNSDQSSNEIVRRKIDNFQYQYGIGRISDV
LALSCDAVFCAAGGTRDDVPKMLVIVTSRTSPSSPSKLKEKLQTLRNRGVNILMIGVGDVDTSLLQ
AITSDDPLVEDRVLLSKRFDGIFQYTKDISDYACGMAPALPPPDNIKGDAKGECGAPKSCYKGPKG
SRGRPGTLGQQGESGDLGLVGPKGIQGRRGDKGPDGQMGSKGKRGPDGLIGLPGFPGYNGIPGV
PGIEGRRGRDGDCGLPGSSGEAGQAGLDGLSGPPGRAGPVGEKGEKGLPAPENPNIRIEPGDRGD
TGSVGLPGFKGIKGEEGFKGPSGDSGVDGIKGVDGEAGEMGDIGTYIYQKGPEGDRGARGPPGD
GDTQGATAGLGVQGEKGEKGMRGEAGDGSIFPGIKGRRGDQGPKGEEGSTGPAGPTGQRGPEG
EEGPKGIKGEIGDRGKDGTPGQPGQEGDLGEKGFDGLKGQDGDDAEGELGDSGGKGLKGENGL
AGSPGSIGTPGPPGSKGEPGDIQIGLSGDPGAIGPRGPRGDNGISGQPGMRGDSGPKGAKGNQGP

RGDRGRNANMLAGKDGMTGPPGPKGFTGPVGDPGFPGPPGPPSVVECVEGACPKGVPGAMGQ
IGKTGEPGPPGPPGPRGSKGVAGGPCPICPDGIQGDSGDIGQPGMPGKEGSRGRKGNIGDAGSKG
LQGRNGDDGDQGPVGKKGFIGDIGPVGENGVDALGDSVAPDGEKGSTGDTGQIGSRGPVGNDG
PKGFKGTIGERGRRGLQGEKGDMGLTGETGEDGSRGLEGAKGDLGDGLRGAKGDKGNIGQEGN
QGVVGDVGLQGPKGEQGAPPTEDELKNVKATKGSKGEIGSTGDAGEIGDIGPAGPRGPKGFDGA
AGPKGPSGVKGDRGNKGRAGDGEPGFQGFPGRPGKRGPPGDPGKPGKRGFSEPGDAGDRGDP
GKSGKKGADAPPPIGCFKFNEPTDIGFILDSSSSVDDDNFLRQLQFVKNIVRQYPVGPDQTQVGIIK
YGEKAVTGINFYDYVTEEGLFNAIDLLEHTKESESRLDLALKLARDELFTESVGARGFDAKKALVIL
GDGFISGGGTPSKTLMNNAMQYAEDLRGAGVSIFALAVGPEKNKALFHEFVSEKEYYLDIESYSQL
DNELGALQLSLQTGCISVGVPGRDGLPGYTGPKGQKGDDGPKGFSPQGPRGIKGIKGVRGTIGIG
VKGLPGDPGLQGPQGEKGRDGEDGSADPGPKGRPGDKGSRGDQIKGASGEDGDIGPIGPPGFDG
RKLCDNVLAVDLLFVLDTSSGVGVDNFNKMLKFIESIINVFDVDNQLTRVSVMTYDSDARLVIRFS
DYNDPDKLDAAIDNIPYANGDETRIDKALLLASSNAFDTSNGAREGVNKAVILMTDGQQSFAVD
QVALKDAVKPLVDANVLMYAIGIGSEISPVDLKTIAAERVLIADDFDGLSLKIEDQIVLLGRVGCQG
PPGKDGKPGANGEDGFPGLIGAKGQKGDRGEAGTEGLKGNKGSQGEAFPGLNRKGEKGDPGS
AGPLGLPGCEIKNQNEVIDLGFAIDASGSLYEEGFNREKEFIKTIVDRVGRVSEQGIRIGVIVYSDTAS
IEIRFDDHFNNEDLKNDIDNLPFFAKNTRMDLGLEEAKKMFGEQFGGRGSSKKVLILLADGQQTY
VPGVTEPSEVAKELAAAGVDIFAIGIGQEIDRTELESLISKPEHIFLASDISKLIDELTPEVTSALECEG
GPRGPRGRAGTDGPPGSKGLLGPIGDKGFPGEPGSAGFGPKGDDGENGTAGTIGDRGEKGFRGP
AGVPGKRGAVGPKGFPGPNGEKGDRGDEGFIGFEGARGLEGPKGQRGDSSDNGVAGVKGSTGD
NGFKGPKGDPGKLPPGNRIEDFKGETGGSGLEGDTGPKGKPGRALFEPGPQGLTGPKGEKGDQG
FFGMSGPIGNKGFQGLQGFRGDDGAAGPVGEDGFPGMKGLPSSSGDQGDKGKNGRKGESGEV
GQPGRVGEDGDQGEKGYAGFVGMPGKKGDQGEPGVQGPQQIRNEILDNIRPGPKGYPGSTGPA
GDEGEDGEVGEPGETVGAQGMNGDFGERGSQGPKGEQGDQGLQGMIGVIGDKGVKGRVGTG
GNPGTVGLRGLTGAPGEVIFGSEKGNKGDLGIIGLRGRAGQKGKAGAIGPRGRRGRNGAKGERG
PPGFPGLSGIEGDSGNEGDKGPRGDPGSVGNRGIKGVPGESGIAEGNYIVIHSQTHIIPDCPRDFKK
LWHGYSLMYVVGNGHSHSQDLGDAGSCSQKFSSKIFLFCEIIKVCKHASRNDFTYWLAGDVDHP
MMPVSNAAVEPFISRCAVCEAHSSHITVHSQDTYAPECPPNWSSLWSGYSFLMVAGAGKTGAGQ
SLSSSGSCLESFRPKPFVECQGARGTCHFFSDKHSFWLVTIDPNKQFEVMKPQTLIKDKNHDLTSR
VSRCRVCIRKTGTKNNTVEPNIATRIRRSIASWIL*
>TRINITY_DN1614_c0_g1_i1.p1 type:complete len:218 gc:universal TRINITY_DN1614_c0_g1_i1:56-709(+)
MHKTKICIWLVVRYRSNPFRGYNRRPDLKSAGAKMTKYSTEPENPSKSCKARGSYLRVHFKNTHE
TAQAIKKMHIRKAYRYLKDVVDMKQIIPFRRFNGGVGRKAQAKAHGCSQGRWPKKSAQFLLQL
LKNAESNADLKGLDVDSLVIDHIQVNRAPYMRRRTYRAHGRINPYMSSPCHIEMILAEKEQVVPR
AEEEVEVKKVSKKKLAREKLKSRE*
>TRINITY_DN74124_c0_g1_i1.p1 type:complete len:183 gc:universal TRINITY_DN74124_c0_g1_i1:100-648(+)
MLLYTFACLIVSTLALELETTAKLLASKTVENNVLVENIDITIKYTIFNVGTSSASNVKLVDATFPTT
DFETVRGQLSVEWQNIPVNGNVTHIVVVKPLKTGYFNFTSAYLSYVPRDGEIQNAYTSFPGEGGIM
SELDYARKHSPHLLEWSVFALMCMPSLIIPFALWYRSHSKYTMKTKKN*
>TRINITY_DN1243_c0_g1_i1.p1 type:complete len:929 gc:universal TRINITY_DN1243_c0_g1_i1:2903-117(-)
MATDKEVKQTPSDDTKNKEKDEKDKKADKKKKEKEEAEMTEEDKQLLEELNMLVERLKETDTT
LYKPALESLRSQIRASTSSMTSVPKPLKFLRAHFDTLKDLHSKWPEDDNKHFLADIISVLGMTRPEE
RDCLKYRFLGSKESLESWGHEYVRHLAEEIRLEYTEKSGGEVDHLIKLANDIIPYDMKHNAEIDAC
DLAMEIERLDLLEKCVDKSTFQRVCLYLASCVKYVPEPEDSELLKCAMNIFLKYDCYPEALNLAIQ
LNDLDKIKEIFKDCTGSEDKKLIQKQLAYVLGRLQIFLEIEDEIEESDELVEIISNSHLNNSFLALGRE
LDIMEPKIPEDIYKSHLDNRPFSSSSVSMDSARQNLASSFVNGFVNTAFGKDKLLTEDGNKWLYK
NKEHGMMSTAASLGLLLLWDVDGGLTQIDKYLYSNEDFIKAGALLACGIVNAGVRNECDPALAL
LSDYVEHNTNAMQIGSILGLGLAYAGSNREDVISLILPVLADSKSSMEVVGVASFACGLIAIATCNS
EVTSVILQTMMERSAIELKEHHAKYLALGLGLTFLGKQEAVDATVETLKVVQGPLSHWSSVMVDI

CAYAGTGNVLKIQNLLHICSEHYEDKETESDKDKKEKKDDKKKDDKTSDKDEKKEKEKSSESNES
TENEKKDGSHQGIAVVGIAAIAMGEDIGIEMALRAFNHLLQYGEPAIRRAVPLALALLSASNPRLT
VIDILSKFSHDHDAEVAHNSIFAMGVVGAGSNNARLANMLRQLALYHHKDANNLFIVRLAQGL
VHLGKGTLTVSPYQYDRTYMSPVAVGGLLGTLTAFFDVKQTILGKSHYLLYSLVAAMQPRMLVTL
DTNLKPLPCNVRVGQAVDVVGQAGKPKSITGFQTHTTPVLLAHGERAELATDEFIAMAPVLEGF
VICKKNPNYDPGE*
>TRINITY_DN11097_c0_g1_i1.p1 type:complete len:730 gc:universal
TRINITY_DN11097_c0_g1_i1:2348-159(-)
MVRPQGVTQEHSSKPSTSERNSFVFSALSKSGCFETLPSIYDSIIIQRSHWLHTEGFSMRTSSTGSQQ
TSRRAFYEELQAFSSTIPFYESLSWALLSLSGCLNPVSTPNIREEILADLEGRSVDESREVLLVFATLFD
VYKSYQNSIVVEISAILPGGILRCISLGQTEGFNSYNTVGLINYQPIVYPVGRNVLGRLFNVTGSCID
LYNEVTMDMPFVRSVSTEETHTPQKTTLIKTGANADTSSDSDCVKANALFKECTSHINVKETSLD
HLISLYCGCYMGMTSAPIAPISRLPIEAESSRAPEHMVQKLQECMGTYSGSADPSTPNQIGSKKQPF
WLSKEALESGVSPIHSTPLPISKLRVDLSLFETGIKVVDLLTPYKKGGKIGLFGGAGVGKTVVIMEFI
RNLAIEHKGISIFCGVGERTREGNDLYSEMAEAGIICKDYTTMKPSDPSSNGGPTGSIDQHSVTFTR
SSVTLVYGQMNESPGCRMRVAHAGLTVAEFFRDVFKQDVLVFVDNVFRFLQAGSELSTLLGRMPS
AVGYQPTLATEMGAFQERIIAALWGSITSIQAIYVPADDLTDPAPVTIFTHLDAVSSLSRELAAKGIY
PAVDPFNSTSRMLDPQCLTSEHFRVALEVQQILQRYRELQDVIAILGIEELSEEDLKTVERARKIERF
LSQPFSVAEVFTKIKGIFVPLEATIRSFAMVLSGALDDVPEGNFYMKGGLDTLKL*
>TRINITY_DN1534_c2_g1_i1.p1 type:complete len:547 gc:universal
TRINITY_DN1534_c2_g1_i1:1914-274(-)
MHRVNSSFVKVAERTSKLCGNLGIKNSKIPSSLIARSSSTDTTDGVGESEPGFLEMVSIFTENAHSIT
LDKLLSAKPAPGKRAEDTGVREKRIKGILDIIKPTNTALAVNFPLRRDNGDYELITGYRAQHSHHR
LPCKGGIRYAPDVNLDEVKALAMLMTFKCAVVDVPFGGAKGGICIKKSDYSENEVEKITRRFTVE
LARKGFIGPGIDVPAPDMGTGEKEMSWMADTYAMTLGYGDMNSSGCVTGKPISQGGIHGRMSA
TGRGVVYHGIDNFANNEEYMNLIGLKPGLKGKTFIVQGFGNVGLHSCRYLHRYGARCIGVIEHDG
SIMNPNGIDPKDLEDYKLDNGTIVGFPGATDTTEDLMVAECDILVPAANELQITAKNAPHIKAKII
AEGANGPTTPQADKILQQRKRLVIPDLYINAGGVTVSYFEWLKNLNHVSYGRLTWKYEESSNYNL
LSSVQESLEKHFGGNIPINPSSTFLQSIQGASEKDIVHSGLAYTMERSANRIMKTAKDYDLGLDVRA
AAYVVALEKIFNTYSEAGLTFT*
>TRINITY_DN27859_c0_g1_i1.p1 type:complete len:361 gc:universal
TRINITY_DN27859_c0_g1_i1:77-1159(+)
MKLELFYHILFFGVILTNVNSKKNRKKDNVEFNGRDNFLYSKRQHKFKALPQRSVKAVVNSNDY
NNFKNSYLSNILRVRVPGTVGHHEVKRFLISKLQEYKWNVSLDEFEEKTPLGKTKFTNIMATLNP
NADRRLALVAHYDSKLVTPKDGRYFLAATDSAVPCAMLLDFAKILSEKYEKNKEALKDVSPMIILL
DGEEAFVEWTKTDSIYGSRHLADLLSKTKHHNPKLSEFSTTALDSLDAFVLFDLIGTSTTQFIDIYPA
TSPLYQRLQQIEMSLRKSNLIASPRQYFKGQPYSPVYIEDDHVPFLEKGVPILHLISVPFPKEWHTLD
DNEKCLDETTISNLLIIFKEFLLEYFHFE*
>TRINITY_DN147947_c0_g1_i1.p1 type:internal len:168 gc:universal
TRINITY_DN147947_c0_g1_i1:501-1(-)
VGKSALTIQFIQSHFVPDYDPTIEDSYRKQCVIDDRVAHLDILDTAGQEEFSAMREQYMRTGEGFL
LVFSVTDRSSFDEIHKFHTQILRVKDKDEFPMILVGNKSDLENERTVSTAEAQELGRKLSVPYLESS
AKQRINVDAAFHDLVRAIRRSNNVEALPIKNKKK
>TRINITY_DN5643_c0_g1_i1.p1 type:complete len:932 gc:universal
TRINITY_DN5643_c0_g1_i1:2894-99(-)
MAFNQWRKFNFFERELLQSPLSDHIKNLQITACTSGRGRLLFGDSDGDLHILNRDLQFKTIKIYMK
SVTHLFQLKEHDILVTVGLEKSGVQHFIKVWKIDKVDGDITLKCLRVSIVPTKSDAIAVSCIAVHEN
LSHIAVGFVNGTVVTMKGDITKMRNVEHRIHEDRYPVTGLGYKQVGENTFLFVTTTETVMSFNIS
AKNYMTLLDHFGSNNHCSAVCDLSEENHFIIGRDDALYLYLPDVRTKCLPFEGTKEMVCWFQSY
LVVVSKGGKQLPRTAGTGGVQSQLISLSVYDIENKFIAFSAPFPTIAGILGEWGSLHIITSEGKIFQLT
EKDTNTKLQTLFRKNMYETAISLAKSQNYSDGLIPIFRTYGDHLYAKGDHDGAIQQYIQTIGHLEP
SYVIRKFLDAQRIPNLTNYLQALHEKGAANTDHTTLLLNCYTKSKDINKLNDFITSDKDFDVETAI

KVCRRAGYFDHALSLAEKYEQHESYIKVQLQDLKNYKKALNYMKLLPFEEAENNLKLYGKTLIN
HATEETTELLKQLCTDYKPASGKKEEVPNVTTGIVSVGDVIMSPIPARKIKRAYPDDFIHIFVHQKP
KLKEFLEHMVKMRNDCSNVAHNTLLELYLHDASAAFSEKNIEKGSDEESKAYNLLTDIEARYDLD
HAMVLTQMHNFRRGMLYLYEKSKSYRQILRCHMEHNEYDEIIEDCQKYGAQDPGLWSQALSYFA
KNEKDCKKHIIVVLNHIDKYNLLSPLMVIETLAHNSNVTLGVVKDYIIRRLQHENEQIAKDERMI
RKYREDTEKMRVQMEELKTSAKIFQNSKCMICSRPLELPAVHFLCGDSFHQSCFESYADGENECPV
CAPENRKFMDILRAQEHNKDLHEQFNHQLERSTDGFSVVADYFGRGCFKKVSVFTDTKTQQPIL
PSEAETERRTLFN*
>TRINITY_DN2028_c0_g1_i1.p1 type:complete len:274 gc:universal TRINITY_DN2028_c0_g1_i1:1608-787(-)
MGQSDGDDLTFGQKISREWRDFREFLYNSETGKVMGRSGTSWAKIGGFYLLFYGFLAGFFTALLFI
FYTTIDKPGIGGPKLIQYIENKPGLTRLDRSSKLQSYSASNNDRISSYKTVIDEFLDGYNTSDHSGAL
CGAAGTPKSDKPCIFDTTLLGECAPSKNTTYGLREKKPCIFIKINKVFGWTPADDGTGYLTLSCTG
HATVMPQGFPLTAFPYNGHKVYQSPIVAVKIDATKGKKNVDCTLKGKNIEVSNNFNPSRAFGKIR
IEGISATD*
>TRINITY_DN37295_c0_g1_i1.p1 type:5prime_partial len:195 gc:universal TRINITY_DN37295_c0_g1_i1:857-273(-)
LHSLDLIYRDLKPENLLIDEKGYLKVTDFGFGKRVKGRTWTLCGTPEYLAPEIILSKGYNKAVDW
WALGVLIYEMAAGYPPFFADQPIQIYEKIVSGKMRCPSHFSQDLKDVLKSLLQVDLTKRFGNLKN
GVADIKGHRWFSSTDWIAIYQRKIDAPFIPRCKGPGDPSNFDDYEEEALRISSTEKCAKEFADF*
>TRINITY_DN1378_c0_g1_i1.p1 type:complete len:443 gc:universal TRINITY_DN1378_c0_g1_i1:2151-823(-)
MLRFAVLVVVASIGVSALYDSSDDVVELTQSNFNNHVIGSDDIWLVEFYAPWCGHCRNLAPAWK
QAATALKGIVKVGAVDMDQHQSVGGPYNVRGFPTIKVFGANKMSPSDYNGARSAQAIVDEAM
KVVQRVVSDRLGGRSSGGGGRRSGGSSGGSGNKDDVIELTDSNFEKEVLNTKDMVLVEFFAPWC
GHCQRLAPEWAKAATELKGKVKLAALDATVHGQTASRFQVQGYPTIKFFPAGDKDFSSAQDYQ
GGRTASDIVAWALAKHAENVDPPEILEITSNSVLTENCNEKPLCVISFLPDILDSMAAGRNGYISTL
KELGDKYKSKMWGWLWTAPGLHSNLEQALGVGGFGYPAMAVVNIRKKVYVLLRGSFGIDGIDE
LLKSIAVGRGRTEKLTQGLPTLKDVEAWDGKDGQLPEEEDIDLSDVELDDLDDEPKQEL*
>TRINITY_DN6783_c0_g1_i1.p1 type:complete len:314 gc:universal TRINITY_DN6783_c0_g1_i1:1018-77(-)
MVREDKSTWKANYFTKLEGLLDEFPRCFLVSADNVGSKQMQQIRVALRGKAEVLMGKNTMMR
KAMRGQMQKHPSLEKLLPHIVGNVGFVFTNEDLTEIRDILLSNKKEAPARAGAISPLDVFVPAGN
TGQGPEKTSFFQALAIPTKITKGTIEILGDVHLIKKDEKVGASESALLNMLNIRPFHYGLVCRTVYD
NGSVFNPSILDIKEEDLITKFCEGVANVAAVSLAVGYPTAASVPHSMINGFKNIAAVCLEADIDIPQI
ATIKEYLADPSKFAAAVAPAAGDSGAAEVKEEKKEESEEESDDDMGFGLFD*
>TRINITY_DN61707_c0_g1_i1.p1 type:3prime_partial len:417 gc:universal TRINITY_DN61707_c0_g1_i1:1250-3(-)
MNVIAAIIRIAILALVFQHEAEGKVYYIRTDADSGRCTYQGYIPGGKEINISPIKAPTAQLETNVLA
NIAGFGGKAGTGVMHLSKFTETIGKIAPKLGPALGVFGVAFGVVADFVKPSPADILAATNEAIGKL
TEEVNDRFTDMKSYVDLELMNVVKERINVEYKHMFTMFSNCIKDVSLERVLECQEDANQYITSGS
GKFMLFNMDDYSDSSPPTANEIKKMELTFLPFREYVSLNLMQLQVLTDTYKDLQNSVKDGPLIYR
KHLSELSEEAAKLIVYARNMFKWIDKTYDEFSRQRCKETKTYWSRCTTEGSWSGNTACYATAQCQ
FDITIPDNKKCKTKCTVRVDGKKIYDRYDFKNKNYGYEEAGQLFAGGKLDKKASSYLNQQAADV
KNYWQAEVLSSIPVWEEMKKRADD
>TRINITY_DN164351_c0_g1_i1.p1 type:complete len:212 gc:universal TRINITY_DN164351_c0_g1_i1:694-59(-)
MEDWGEPGEQEAVGPTSIEVQDIKLFGKWNPDEVQVSDISLTDYIAVKEKHAVYLPHTAARYAVK
RFRKAQCPIVERLTNSLMMHGRNNGKKLMTVRIVRHAFEIIHLLTGGENPLQVLCDAIINSGPRE
DSTRIGRAGTVRRQAVDVAPLRRVNQAIWLLCTGAREASFRNIKSIAECLADELINASKGSSNSYAI
KKKDELERVAKSNR*

>TRINITY_DN773_c0_g1_i1.p1 type:complete len:440 gc:universal TRINITY_DN773_c0_g1_i1:1608-289(-)
MVSPPGPQDNKPPHKVADMSLADLGRRYIEMSENEMPGLMSMRKQYGEKKPLKGARVAGCLH
MTIQTAVLIETLTELGAEVQWSSCNIFSTQDHAAAAIAKTGVPVYAWKGETDEEYLWCIEQTLIFP
GGEPLNMILDDGGDLTNLVHEKYPELMKDIRGVSEETTTGVHNLYKMFKEGRLKCPAINVNDSV
TKSKFDNLYGCRESLVDGIKRATDVMIAGKICVVAGYGDVGKGCAEALRAFGARVMITEIDPINA
LQAAMAGYEVTTMDEACSKARMFVTSTGCSGIIEPHHFQQMPEDAIVCNIGHFDCEIDAKWLN
DNCERETVKPQVDRYTLPNGRHIILLAEGRLVNLGCAHGHPSFVMSNSFTNQVLAQIELWQNHK
NYEIGVVMLPKRLDEEVARLHLDHLSVKLTKLSKSQADYLNLPADGPFKPDHYRY*
>TRINITY_DN58867_c0_g1_i2.p1 type:complete len:354 gc:universal TRINITY_DN58867_c0_g1_i2:80-1141(+)
MVSLYGDMMFNIENGYLEGLVRGFRSGILNRTDYLNLIQCETLEDLKLHLQSTDYGNFLSNEAGP
LTVSVIDEKLREKLVIEFDHLRIQAVEPLSQFMDYITYSYMIDNIILLITGTLHQRSISELIPKCHPLGR
FPEMASINIASTPAELYNAVLVDTPLAPFFVDCISEQDLDEMNIEIIRNSLYKSYLESFSRFCDSLGGT
NSEVMKTILQFEADRRAIMITINSFGTELNKDDRSKLYPKCGKLYPDGLARLARAEDYDQVRQVA
DCYAEYQPLFEGTGTNPGDKTLEDKFFEYEVKLNVNSFMQQFHFGVFYAYVKLKEQECRNIVWIA
ECISQRNKSKIDNYINIFE*
>TRINITY_DN598_c1_g1_i1.p1 type:5prime_partial len:745 gc:universal TRINITY_DN598_c1_g1_i1:2-2236(+)
RWSGCSKSCGVGVKTKSRECDNPRPSGGGRYCTGERTATEACKIQDCGVDGKFSHWSTWTACSK
TCGGGAQKRARQCNSPPPSGDGKDCAGAREEFKGCSNDFCKGPTGVAKAQEIIERFLENFHKKT
YPDREPSGKFLKAIFAIQKAVAEEFISVDPYVRSKTLEQRLKDLENFLKVATEGGSIDFARTLLGKGV
FTFMKQKIKDGLPIDDIDKAAEGFLEKLRGDLGDKKMDEILSSQGDATLMFTIDTTGSMKQEINA
AKAIAKQIISATRDYEVDYILSPFSDPGTGPVTHRSASERASFVSDIDRLRARGGGDCPELSFGGML
NALNNDPQYGSPMFVFTDATAKDDTDANVASLKAAADATDATINFFTNLAGCNGRRDRKRGIE
SYKEIASYTGGQVFPLKNEAELIKFTSYVKTSLQQDTVITSGGTLNSKSKRSLYSMQARHFMVDDGI
SKLVISVGVQQLASQVALVDPNGKKVYPSTTLALSLIYQVSHPSPGQWKLDFPTSVGKYSYNVQAV
SSSAIAFAHNFIYQQSVRKNSPPIPLANPLKGVKNMMVVRLGGTKNFDLNSIKIDLVDTSGNVLQ
KGIGTDRLDSDEVRHATIDPPSGPFKVRLTGKTKSGYNFERVSQGISRPVSAAMRTNFAGYDFTAK
LGKGPYAAQMLFINRGAHENFYFYAKANYGSIPNYKQTKRVTSQGFVTFTYKAPRQYNLKGKSD
RITVTVKRQSTGEIVSHHTSILLVV*
>TRINITY_DN6008_c0_g1_i1.p1 type:complete len:426 gc:universal TRINITY_DN6008_c0_g1_i1:1620-343(-)
MSLEDTQLRKRKGVSEKHYTLDGFTNLQENEKKSSPLMSITKVILKMMIATFVLLGIYIAITPSPIDP
GVLELDTPLPRFEGVLAPNDKLKQARRIGEGKLLAPESLAMDKEGNIYSGLGDGRIVKITKDEEIIN
ITRTGEPHPECGKRKLGSKCGRPLGIHFDKDQKNLIICDAFFGLLKLNIATKKLSTLVGANVINEKP
LKFTNHISVSSKNVVYFSDSSSRWDLENVALLVLEGNGDGRLMSYDMESGKLQILLEGIYFANGVS
LSPDEDFVLVCEMSRNRIIRYYLKGENKGNYDIFADNLPGSPDNIRRSPNGGYWLALPSIRKAPFSP
PDLIAPYPSIRNVIAKLLSPQTLEYLLGSMRYGMIIKLDENGKVVESYHDKDGSKMYTISEVYEDSV
NKVLYLGSYKNPFMGKLQLSDSS*
>TRINITY_DN1761_c0_g1_i1.p1 type:5prime_partial len:251 gc:universal TRINITY_DN1761_c0_g1_i1:843-91(-)
IRWHKHLSVCCKMRTLLLSAILCFTSFQLSYGQLAVGAGVAAGVMGAVLNPSNWITALDAAGRI
KEMIKVTKDSRIAIREKNEGFEFLTMKANAQTSRGIKKHHLLKILRKKLIPFASRSPDMKLMVQTL
VEFIEDDVEEHQEQFFGLMSQGAGGSTQAMFVYYKIREDGKYNFKRMTFYGSFRVAADLVITRSS
RKGFFSSESKDLISYLPRRGMTAKHIKNLLTIIMPNLTKIMNEFVPQDDKPFTFE*
>TRINITY_DN26425_c0_g1_i1.p1 type:internal len:414 gc:universal TRINITY_DN26425_c0_g1_i1:3-1241(+)
LSKSVKEDHNRFSACDFNANDVVISQSEIIDMWDKVKNKEISQEELIETLKKRSKSKDNCGSPQST
PGASRRNLAKVDSLEGSKKIKDERSSSLRKAASATFYTPGKEDIKQDGINEELNISDDNKSVESELS
PSPKTSPETSKSGFFRGIRNSLRKKGRLQDESPTRTEPPVNLLKKDSSKLRSNKDSLSESRSNSLSGSL
SEEKEDGHDIDSIKYETSDNEETFSEVINKLTKKPAPPPPPRDPRSITKELNDKTIELGKPIIPPRKHK

QMSPVTPKNIEDIDKEMQQLLFGLSDELKELSESQGVIKPSFKKPLLPPKGSKLKINSDNVATTKNK
ENNNIKVATLNKNKIVDKSAQDTQTVDSKISDSQESMKQVFKRLPLKDLVSKKLKKEEIEINEVPY
TNAEGLWNI
>TRINITY_DN3052_c0_g1_i1.p1 type:complete len:362 gc:universal TRINITY_DN3052_c0_g1_i1:45-1130(+)
MAHLSQEQRNELKKNAESIVAPGKGILAADESTGTVGKRFAGINVENNEENRRLYRQLLFTADKS
ISNYLGGIILFHETLYHKDDNGKPLVELIKELGINPGIKVDKGIVPLHGTNNEGTTQGLDGLAERC
KQYKQDGCTFAKFRCVIKINTPLGVPTDIAIQENAQVLARYANICQANGLVPIVEPEILIDGDHNL
ATSQRVTERVLACVYKTLVDHHVYLEGTLLKPNMVTPGQSCPEKASAEEVGVATVKALRRTVPA
AVPGITFLSGGQSEVEATKHLDAMNKTDLARPWALSFSYGRALQASVLKAWGGKKENVAEAQK
VLLHRAHLNSLASLGKYEGENASGATGDSLFVANHQY*
>TRINITY_DN5397_c0_g1_i1.p1 type:internal len:161 gc:universal TRINITY_DN5397_c0_g1_i1:2-481(+)
CSVVCDYSPWQDTQSCTKTCDGGVLIQSRTERSFNKVESREGSTPCNTQACGSGLSEERFRKCNA
NQCGTNTGLQEVYKVCNAANCRFQGIDYTRNQEINIRTQACTTACDYTPWQDTTACSKSCNGG
VLVQSRTERTFNKIETRDGTTPCNTQACTQEL
>TRINITY_DN5153_c0_g1_i1.p1 type:complete len:862 gc:universal TRINITY_DN5153_c0_g1_i1:61-2646(+)
MSEKKEFHRLSRSVRPIHYNITLKPDLENFTFAGNEDIDLEIKKETKHIKVNSLDIKVSSAVLTVDSQ
DIEPSDIRYKEDEETVTFKFSNPIPVGNAKLSLKFTGELNDKMKGFYRSKYTSASGEEKHMAVTQFE
PTDARRAFPCWDEPAVKATFDITMIAPKDRVVLSNMDVKSEKDHAEDDSFKEVAFNTTPIMSTYL
VAFVIGEFDFIEETLENGVLARVYTPLGKTEQGTFALKITSKTLPFYEEYFGIKYPLPKIDLIAIPDFAA
GAMENWGLVTYRETALLVDEKESSASSKQWVAIVVGHELAHQWFGNLTTMEWWTHLWLNEGF
ASWIEYLCVDYCCPEYDIWTQFVTNDFTRALELDALSNSHPIEVKVGHPSEVDEIFDAISYSKGSCV
IRMLHDFVGVGDFKRGLNSYLTTYKYANGETEDLWYHLEKASGKPVANVMATWTKQMGYPVLS
VSAEYVDTQCILKISQKKFTADGSADPNDQRWAIPINIMTNDNPKFSTLMTDPEMVLFMENEPES
NYIKLNPGQIGFYRTCYSSDLITKLVPVINSLSPVDRLGLESDLFALSIAGVSKTVDFLNILKGYKDE
MNYTVWTDIDSNISSLGVILQNTDNFELFKEYVIKLYKPISEALGWEPKDDDDHLTALLRNLILRRL
GNYGDADTLAEGQRKFKAHVDGTELVNADIRSAVYSMVSKHGDEETVDQFINLYRNTTHSEEKT
RILTYIGASYKSEVIEKSLKFSISDDVRSQDTVFALCSSTGSLNGRRLTWNFVKENWDDLFKRYQGG
FLLSRLISRTISNFATVEEANDVKTFFEEHKAPAAERCIKQGIESIQLNNAWLTRDGESINQWLFQE
N*

Band 9
>TRINITY_DN982_c0_g1_i3.p1 type:complete len:9852 gc:universal TRINITY_DN982_c0_g1_i3:29697-142(-)
MSIKLCTFFVFFLIQCINSFPEIGLHLSLDRSYSFQGNGWKTVNGWEIKPRANGPYEIDAIKNISPEY
FPVPITGVYYITVSLPVEGASNGEFKSAIIVDGKNEEGRIGLMSSFQKTAFKNGASTLTFAGFVRLFA
DEKLTIEIYSDDINANYVVLESATFVFHFIGVTGSVPAYLAHTAQDQEYASDSIIKPWVTLGDSRVYE
SLSGFIKNEGVFTTICNGVHKMAVNIMIKANAGTYTLKALFQDEESATATVTINQPDSIATLNIFQN
FKWSEGMSITLQIHSSNGANFKVLKSSSWSMSFIGETTANFQEFSVFLMADQFLQPSQFPQRINIDL
NGVFQFLQSNPDQYNKGQLVSGADTKFKDNLDFIVDSTDLFYVSANIIIEGEAEDLEITIAVSQIDID
VLPEDGLSGRLTKISNSRRSISLSGIIFIREKQYLSFFVRSRDSSKSFTVSKDSFISVINMRYTVSSVSARQ
NENIDVTAGSWYDISGRFAEQKNGLYSFGKDFNINNGVFRTSHSGIHSVHANIQFSLDSDAFGDN
DDNKIVLRVVIDGQEDKISLMSIRKNPEKTATLKLFGSIYLRAGVTISFQVKSLMPNTVQYKILQRSS
ISISYIGPQWAVNSFHAVANQDLNYDGKPDDTRPDIPSPLNFWKTEAADEFISPFEVLESFDGTRYT
SVDDGLYVVSSLLILKNVDCDRSALFKLDFVTTGGSRASDKSNGFADSHFFIADSKGTEFTLSVTTA
MRLEVGFKVGLKLTVGSTSCRYTISKESSFSIAQWSKSSNTIEENRLKYIGVLARLSAPTEVSSPNTW
SNLGSNTMDINPGDKSRSSFRPGIFGGDDLAPITRTGKDFNGDVSDFTIKDPGIFFISGMITVTTTEN
ANEEFAATVQIDSEDIESGLYAAVINQGSTEMSLTFGGTIYLKSGARVRVVVKGGRTFNIAAGSSFSL
LKLQPDYKTPGIIVSIDEKITQAITRYEHKDLRADIHRTLTIGNLGFVESTGVYTIQVDGVYMVDYT
VILEATNPNAVFGAEILSDNTVVATGTSTFGNTKTIAITELVYFSKGAIVKAVLKSNTNGETLNNLQ

VDGGLTVYFLEPKEHVKDGHRSKQTSPQALPVRSFEKVTGFAAQSPDFAGGADSVDYTVPETGVY
LVAFTTRVNRVINGFKSRVLVNDNEFLNGNYQIVSAQNAPATLPLSIAMELTVNDVLSFQVMADG
SGMSLDGSTRSILKIDEVNTIRNVTEGLSAYKLRDQKVNGQQETIVEEWDTSVTEGTFLAKNVSFT
VDHSIVVVAAGVFQISVSVVVKNDRPSQSKFELRVLQGDDVKSHGLHSKVEIGGGREQTLTIGGSL
LLQKFDSLRIVVRDIADPLASYTIKAGSTVSVIIIDDNNGPIACEDEGLNMRRLPERIRKPTRESHTF
QCNVVSSLKAEKYTWFKDNVQLKEENQKNLVLNDLKLSDSGVYQCRVRFLELTGATNNATLSVY
DPYPKITSPKFIANVLENQDPPLTIEMFSLSAFKLSPEDEKASITTQIIAGDDQSFFTLSGPSKQTVTLS
TTKKLDYETQNEYNLTIKATNLEETALTTKGPYETTINYIVRVIDVNDHPPVFSQSIYSRSVPEDYPI
DGSIAKVSANDQDAGANVIYTLVGQVSGDLFKIDNTGLIQLRQNLDREIRDDHTIFVQASDGLFT
TVAEVVVTVTDVNDNFPNFMDTGFDISVSEATLTQSVILKVIATDPDLGTNSQLRYTIKSGNNENK
FSLNLLDGNLTLISKLDYEGTKIYNLEIDVQDSASPPKKSQNPLTVVIRVQDANDNNPTFTQSLYNT
RIAENTAVGTSVLKVSATDRDGEGINNPLTFSITDTDANEFFQVVSSGATAGEIRVRKSLNYDIRKD
YRFSVVAKDNNLGEPRYRTALVVINIDDTNNLRPTFEAGSASISISEGTSIGTSVHQVVAIDKDTVG
KLDYRIISGADGKFAINKETGDINLISAVDFESNKKTYTLEIQVNDGVRDALNPATVVINIQDFND
NQPEFIQKYYIFTALESISDSQAINVGNPVIARDADSTTNSQVDYSIQYDINNPSVPNEGDALFRIDS
NGLIFKKNGVILDFENVRYHTFTVVAIDRGTPKTLLGKAFVTINIQDVNDEAPSWSPAIYSVTVKES
AGINRDLVFLSTTDKDTSGAVTYSIVSGDSANQFQIFDNVIKIVKPLDFEKTETHTLKVSAQDDNG
NRAQLDATVTITVEEVNDNRPLVTNNNANSIQVSVPEDIPVNSLIVKLNGRDDDKGAPGQASNQ
FTFVSLDKTTDFTIDEVTGEIKNTVNLDYERQKSYSLVATIRDKGTPYLESKFLVDIIVENKNDERPIF
DPAIYTIKLSEKAPIDTVVAMLQATDLDTPKSQLRFSSVTTLDSHFSLDPITGIITLKTSFDKEVNGKD
GIFVISCTVNDGTSNSAQNAIVRVEVVDVNDNQPVFPTSEYFIQVNEGSSIGTILQNIAATDKDNT
NTHNQFTYTLEGKEAVDFRLNGNQLQTNKIFDYENKKSYSFDVLATNNLSPDKLVGKATVVVSIL
DQNDNNPVLSPSGVINLNISEGTIAPANIITIFATDKDSTINAEVQFTLTGDPTNTFTINQNTGEIML
AKSVDSFAKNEYTLSVTATDKGSPPRSSTATTLNISIKPVFVPGIEFENCPQIISFNENAAPNTFTKTL
TVKDRGSFNQVQFTVVSGQAGIQNLITVSQNGVITNGQRRADFEARKRHDFLVQAENERGAKAY
CHVTANINDVNEGPIFDSANYVFDVLEVIKINTVVFSVRATDPDFSSTTNGQLTYSLAGQEASQFSI
VPNNGQIKLAQPLDYEIKKQYQFNVIATDGGSLQTTSSVIVNVEDWNDNRPIFDQPQKLAINVPE
NSKPDNVFTVSATDKDSTTNGEIEYVGIYPTGIISIDKDTGTAKIIGDLNYEKRQKYDFVIVAKDKG
VPYLSNTREVSLSVLNLADEVPYFDTAAYTISISEKTSINTAVFRVMAKDNDLENFGKISSYEIISGNQ
LNRFRIDSNGNIIVQQSVVLENNNPNQYILTITAFDTQRTQSLNNTQVTINIIDLSDKQPFFIQNSYI
VEVQENNEVSKSLLSLEAQSNEPTFNPTYSIVQDPKNTDSQFFQIINGNTIVATTVFDYEKKRFYSFE
VQVIDTNNGKTNKALVVVRITDENDNKPFEVSGNLHFNITEHTLLGSTIGSLNGKDNDAGINKEF
EFSILEGNDDDKFMLLPTGQILLKNFVSVKEKDFYKIEVKITDKGTPSLFNIFTVRVTINSSQALVAS
NNPTIPEDKNTGEEITRVTAIDPNTGSSNGFIYSLVGTLSNSQFTIDAKTGAISLLASVDFETDRTHT
AVVKAEGPTSTGVTEVIVRVTDINDNKPIFDPTNFNLNINENFVVGQEIVLVRATDKDSSLNDNNV
IIYRIDSVNPSSGNDLFSVNPSTGRILLLKALDRENGDTSYVLTIAASNRGVSNKPDNTMTVTINIA
DVNDNFPIFEKSLYQGSVSEDANTGTSILTVRASDKDILLKNRNIRYSMTQPMAVQKFQIDPISGVV
SLKQEVDFETKQRYDFSVIASDTGDVAYSRTANVEITILDVPDVEPKFDPQRYEITIAESTLVSTSILK
LQVIDSVKNLNFNIKQGNSNSDFRLVASSGELFVNKQLDRERISRYQMTIEVSDSVNKAAIDAIVIV
NIADVNDNQPLFLQSGCYQANRAEGIETEQVVVNVQATDRDEGSNKEIVYSFSDACSVLDIDAN
TGAITTKQKFDYESARSHICTIKAEDKGTPKLSGFSCIILTVTDSNDLKPTFEVITSNARVLENIPVGS
VITRVLATDKDTLSSNKQFVYTIKTGNEDGRFTIDANGFIKLQQPLDYEKKTDYRLEIEAVDSTNQ
DMVSDPNAIVTVTVIEQNDNRPKFAKSYHRVNIEESTAIGTTVLKLSATDADAGVNEGLIRYVSLH
NTTVFNIDRTTGEITLATKLDYEVKKSYHLIAVVKDQGNPVLSARTLIDIFVNDVNDNPPVFVISSY
NNIVSEKAPIGQSIVTVSARDDDTGVNGQLKYKIRETQSMFRIDEDTGIIILNGDLNVENTNKLLYA
LTIDVVDSGVPPLQASNPATVSVQVSDVNDKQPVFALDRYQFEINENIEGNQIFGDSVTVRANDD
DLTGTNNQLTYRLVNTTMFDLVVTNQNVFIKTALGVSFDYETKKSYSFEIEAMDSAANKLYGRSLV
TVTVKDFNDNPPIITVPNIIVNISDATLIGSHIATIMAEDKDSGENGILIYTVTAGDSTNRFTMESDT
GSIILKNPVDSYTKDIYVLNVLVQDNGIPRRSATGTVTINVIPVDLTLLKFNTTCPFAISIPENQINSN
FLGLKALDAGEYVSKQLRYTINSDENDVNQIFALDSIKGEFKQLKEANYEERSRYDMTICATNERG
SKVCCHITIFITDINEPPSFIGLNNNNIYEVSISETTPIGTIIYRVLSKDQDLPGVLNGQTSYSLGQQNV
FIIDQMGQIRVNKTLDAETQLNYVFQVIVTDKNPTNPLQSAATIRVNILDYNDERPIIQNIPDITIDE

NSDNQVVRNIVVTDRDISNIGSNIELSIEPSNILTINNRQLRTVGSALDYEKVQRHTLYLTAKDKSFP
YLSSTTSFTVTINNLNDNKPKFPVNNYNLTVSEKTTVGTLILTLFAEDDDLEEFGEIANYEIVSGNQ
EDRFSIDSNGRLTLKKQLNWRNVKSYNLNIIARDKGLPSEQSQIPATVVINVADYSEEQPNFLQNL
YVKFIPETTPIDTLVLTVEATSGEPVYGPFKYTIVPKLPDTNNFQIFSNGEIKTINQFDYENQRNFTFD
VQVEDEKNGKTNRASVFIHITDVNDNIPQFTDLTRSNTQYNITEFTLPGSMITCLSATDRDHGKNA
EIRFRGIGGDGNGVFNVNLDGKVLLSQQIFYDKKNKYNLIVEAFDLGSPEQKAQLTLTIFLHKSYT
SVITTDVDINEDVETQTRVTRVSSIDPFTRSSNNFTYYLVGKSTTFFEINAQTGEIFTKATLDRETRDR
HVLTIQSRGPTTNGVGELVVNVKDINDNPPIFDPQVYQVTVSESANIGLSIVSVTATDKDIGVNGE
VTYSIEDGDQTIFSINPSTGSISLAGKLDFESKQSHTLTIKAVDRGNPPQDSVNKATVIVVVQDFND
NCPVFENSTYSLTIKEPGELGKEYLKLGITDNDNGRNQLFTCSIDEPLAEKYFEMINDCRLRLIKEL
DYEEFTSFRFVVKATDQGDVSCTTTTNMIVNVGDRADVIPFFSPLTYNISIPESTTVGTIVVSLKVGS
TFKGLLYSLFHKNTQDDLIKFGINKDTGEIFLKESVDREIQDKYEFTADVFDGSNNAVQRASVTINII
DVNDNAPFFNQPTCYQRDLTESTAIGTFVVDVSAADNDKGRNQQVTYSLLPSPSCTSFSVDETSG
RITTSQILDHEQQKLQHCLIKAKDNGTNSLSGYSCLTVNVVNVNDNKPAFIVNRFDVSVLETVPA
GTLVASLVARDVDNQGLITYSISNGNLNNQFTIDSSGKLRTRFPLDYENTKSYNLTIQASDNNGKFS
SPDDALVLITVLEVNDNRPQFLQSYYKVSIPEDTATGASILNVTAIDLDHGELTKSLFYAALENTTQ
FTVNANTGVISVAENLDYEIEDHYELVVIVQDRGTPHLSNRALVDIQITDVNDNAPMFVTTVYNR
ELSEKALIGAPVVKLNAMDADKSDKMTFTIDSVVQGAQGFFSINGDTIQLDKELDYEVPEQRTFV
LSVSVKDSRDRDGANKANVFINVRDINDREPIFDLNMYEVTVSENNVISNPIIEVTARDSDSTPEN
NNLLYSLVDGEDAASFSLLNNGQSAIITSKISFDYEKKKSYRFLVRASDKVRENFTAFSTVFVTVQD
TNDEAPVPEKDLIMVNISESTRVLSTIAIVFANDKDQGINGQLAFQIINHSEDKFEINNKGVIRLKST
LDFFQQNFYNFTVLVTDKGNPVRTSKPVTVLVYVYRTEIPGIIFEKNHYIFNINEKEIVSLVGTVNAI
DEGTYVSRQIVYSIVSGQNDIKDKFRAVNNNIIQDKEVNYKDQSEYNFVMKAINERGSIAFTHITIN
IMDVNEKPYFVGLNNKGRYEFSMSEIISVPSYIYAVRAKDDDFGLNGEVDYEIISNFRFGNFSIDQY
GRIQVLKDPDFEFTRSYEFQVRAKDRGTPSLESNLVSVVITVLDYNDNHPIFNIQEPLEISIKELVNN
TDVGTVTATDKDGSGLNRIVKYVAIYPSDHLTVNRDNGQIRVIEPFDYEIAHKHEFVVVAQDQGT
PYLNAYKVIKVEVLNENDEFPEFTVIEYNITISELTPIGSPVLFVHAQDKDRGPSGQVTNYKIKYVPP
SGGSGAGSPDKTFEITSDGQVVLKEKLEYKFQKYYQYRVTCTDNGSPPKNSINEAIIHLNIADASLE
QPIFSVNPAVVEVYENITLNSNIVKVEVDSIEPIKGPFEYSIIASEDSQPFMIDNSTGQITNKESFDYEI
KRKYVFLAEARDKHNNRTQRAMVVIFILDINDNIPTIETIAPQTFVQNISDTTYPGSVVAKLYSRDA
DSGENAMVDFTITGGDGENVFAINPNGYVTLIGPLDVRVKTQYTINVKVSDRGIPTLSNNRALTF
NIKKSKFIQNTVRFDRHHYKSNCIEENQINLNIIQLNATNSNPVAGSTIIYSLDGTLEERQPFIVNSQ
TGEISLSQTLDYEIKQEYDFVAVAVNNDGSRDVAVVTVCIKDLNDNSPIFEPQSYVVNVSESESVGY
LVYKTTVMDADKTDFNTTKSYDIIQGNVNDKFIIDSMGHIYIKSKIDYEALQVKHFDLRIRVNEGN
LVGDMVLRVNILDWNDVHPSFSTLNIYNFTIPEDKPVGEIIEILTANDGDATSPNNEVTYKLLTTED
DFILRRNDKGHGSLIVNKQLDYSKQNRYEYVALAEDKGYLNLLGTAIVIINIQDVNNHPPEFDDSE
YNTTVSEHAQIGTRVLQIHAHDKDTGLGEQIFFIYGDPTETFTISSQGFIILNKQLDYETQRDYIFDV
GARDGGNLVSLNNVTVHIKVSEVNDNEPIFDLSTYRNSYAEDTPLGTTLFTVRATDKDFIGNSIIYSI
SGANDDTSYVSVDQTTGDITLQRRMDYESKNYITFTVQAIDSNNRNKLKGSTHVIIDVTDVNDN
NPKFVFGSDLVYNVTVVENINVGTLVVVVDSQDADSGLNGQTRYHIVSGNNENKFFINELNGQI
NLINKLDALQTNKYVLTVMAKDLGNPSRSTNVTVTINVLRYFESTPMFLPSYAEVIYPENLALGSSI
YELNVTKTLPGSPMKLSFHSSSKFPEFTLQGNKIILSKEFDYEIKNRYELIIIATDEENDKAFAQVIVR
VQNLNDNIPYFTQNAYFGVVSELAFVGHSVLTITAKDDDKDFTELFYSIATQVPDNAFKLQDNIV
VVNGKLIRNKVYEFEVEAFDNTFTSKRATVSITVTPENLPSFVNATYIVTLQENYPISTNFLNISAGT
FSGITYVITSMKAKDMFNVDANGQISLTKSLDYEKQRQHVFPVTASVTSPITQVRVASVIVNVLNE
NDNAPVFVNGPYERNITEGHLVGTMVIRVDGHDDDSDKLTYSIVSGDDGKFSINSEGVISLISTLNF
DLKNKYVLNVRVSDGKFVTDTIVTINITPIVKFPPMFNQTLYLLSVKENTLVGTSILQVSTVNVIPPS
SFSINEQSGRDFFTIDANGVIRTNNALDYEAKRQHIFTLKVQDLRSMGHATVVVNIVNDDDTCPIL
TPATQTVQITEPVAPGTIVAAVQAKDIDSKSLNFTITGGNGLFRIDQHGGIKTINEVDISTSTEYELTV
TVDDGTCTKSVQVDVNVNPLAPCPDCNIYPFTQTIYEFSINENTVPASPIITVTTGQSLQRNFSIEDP
AALEYVTINRLTGEISVIKAFDYEATKHLSFNVMAKTQTTKNRNPFYVITSVSRVRITVRDLNDNCP
SIESPMYYEVSHSSPIVQDTVVTRILVEDPDTLKDHTYSISDNNFKIDSMGHIRTVQNIESFTAQFYN

LTVTVSDGQCSKTSNVGVNLAVNPTPMTYQFTSNGVYSKSVREDHNLQDVILSVALNQNYPRVFS
IVESDANDQFVIDSNSGALRLKTSLNYELKKRYDFTVMADIHGGVYKSYASVHIIVLDVNDECPIF
PVIGYNQRVSGTPVIGTIVEIIEANDADNATLEYSIVSGNEDGHFSLDNSGVVRVVKPFASSFSKTYG
LSVRATDNKCSADVPLTVQVSTCAYGEDYQFEESEYVVYYLDENRNLGIVSNIKLKSTRAAFLTLSQ
NTHFTIDQNGDFRLIQTLNYETKKEYHLTLTANFTQDNVVTRIPIKVFVRDINDNCPAFSEELYAVQ
HTEPLREGLLIAKLSVIDDDTVQNFTYNIVSGDKRFSINENGEVVMNARLVTDTTLLSTTYKLEVSV
NDGKCAKNTKVDVFVYKTVVNSFLFPQPFYKFDISENQAIPFTIDRFVNVGGHEAVYGFLVSTDFF
QIDPKTAQLQLVKKLDFEVEKTHYVLISATYDDGTQTYTVVAIHVGDENDNAPQIFAHPPITRLVQ
NTPHNSIVTKIEVEDPDTSKEFTFKITNGNPLQNNDPDKALFRIESNGMIKTTRCISEDEIGNYVLTI
SVDDQVNTPTIFSNMQVQLKTTDDRSYVTNCGLGCPANDGPFSRYQFEQSLFIVNVTETAANPTV
LKRLTRPFVEKTTYTIGAGADGKFSIDQNGTLSLTGSLDYETKPVYYFIVSAVIPSQVVGRFITSKTAY
LVMVNDVDDSKPVIEGNGGTLAVSETSEIGQIVGTVRVKDSDSSSPLTYNVVNGSQYFNIDSAGNI
VIKDTLNAAGSPYTVTYTVSDGINPPVNGEFVVSISETNNNVPKFTQNIYEVSLSEDTATDTIAVTIL
ANDGDTNDKIAYSFLQSEGPFVINRDSGEIKLIGPLDYEAQKSYNMVVQATDSGGFANIAFVQIAV
GDVNDQVPVITPQTTPTQIHESTLVNSVVYRVIASDGDGNERLTYALANSNDFFTINSATGEITIKE
NLAKSNVSSHTLSIVVTDSSGKTSTQDVALNIRRSGTSQGCVKITTSQGQSSSPVINLPENTASGTTV
FKLVAGDASVFSNMRFTLNREANGVFAVTEQGELQLVAPLDYETKQFYSIIITAEDVTRGVSSQYNL
FVRISDVNEFTPVLETIAPVAVSELANIGDVIAQAKANDQDGDSQVTYSLGEFGNNDNKFTIDAAT
GNIILNKRLGSGAPHVVEVCASDGSIKKCQNVTFIVADTNDNPPSFQDLTGVGSIDLTNTSSYVVY
KPIVTDSDQTGTLSFSLKDPAPSQFSINSTTGEVKANKDIAKGTYTFGVCASDGRYTSCQSVTVVAS
DRSQSITPSFNQDQVGAIVDISASSGKQIIQVMASTLDKAKIEYELKNEADRNIFEINKDSGLITLKN
RTALTLGTIYTIPVKASRADDPLNFDEINIVISVGNQQLDSGSFIEQVLDTQHLVNFSLSTFDISKVSY
YIILGQQVGGDSKDDEKLPVSWYYATFGDESYRHRFYYIAKLDGSKFRNTGGSRKRRSIQDKDSKS
FTYLVGEENGCSKNTSMAAICNGPLKTGTEYRYKLRAYYDDGELRGIPVDQKPTEVKRTQAPQA
GKSIDEYREQDSYVITFWIIGLVIFGLLFWFSVLVIAILLCYKRRYEVTQAYGYSVQTRDKIDGPPTEY
SNTVYRFQNPSYPDNDTVKMKKVDASESRTKTEVQTTSMVVLDDYEQEQPQPDIPVQDYDAIEY
ETRSAQIKFHEESPEWESMDIQLRIDPTGNQDPVITRKSDSGSKDSDSDASKKYENTEYIEEKEDFV
NEKEDSLTRL*
>TRINITY_DN982_c0_g1_i1.p1 type:5prime_partial len:6412 gc:universal TRINITY_DN982_c0_g1_i1:19377-142(-)
KLDFETKQSHTLTIKAVDRGNPSQDSLNKATVIVVVQDFNDNCPLFSESIYNASVSESTAIGTTVLR
VSVTDKDVGNVNNEITCSIDEPLAKGFFSVNKINNNQFSVVLIKNLDYETHRGYRFVIKAKDNGE
VPCQLTTNMVVEVQDEPDVITNFKPFNYVISIPESTLIGTKLLELKVDSESSSITYTLQAKNQPGDLD
KFMLNGQSGELVLKGTVDFEKKSEYEFTATLNDGVNNGGNTANIVVNILDINDNPPVFSQSTCYI
RRMPENQQVNTTVTSVLATDSDQGINSNISYSLIKDSSCNNFEINEITGAISLTSSLNYEVRKEHYCII
KATDNGSPVLSSYSCLKVIVENVNDNSPVFLPKELEITVLESKPVNSFVIRVFATDKDEGNSITYSIV
GGNTQNAFAITPLTGHIILNQKLDYEIVKNYILEIQATDDGTPRRSSNLNAKVMVKVLEVNDNKP
KFNQQRYLRTIPEDTNIGSTIISVTATDDDAGLLTSKIQYIALENTNDFLIQPDTGDIVVSQNLDYER
QRTYQLVAVCSDSGTPPFTGRTLVDITITDVNDNAPILNPTLYNVLVSEKAPLGTSLIQVHAKDKDS
GINGGLKYQIVSGNDLNKFVINNSTGVITLQETLDREKYSSLYNLTVSVSDQATPSLNGNNAFVQI
RVLDVNDKQPVFVKSEYIFEISENQPLNSPIGLVQANDDDSTSPNNIIQYTIVPSKDSINFTMINNQI
LSNSVFDYEEQKSYEFEVQARDQSTGVNNEQLVGRTLVKVLIRDTNDNDPMIENQNIALNISDVT
LVGTLLFDIQATDKDNGENARLTYTISSGDTSTFRINENDGRFYLSKKLDFYNKNEYSLIVIVNDNG
SPQRNSVANVQINVYPIDQTLISFEPQCPIAFTIQENSLSSPMNIKAIDTGSYVSKTMRYMLKSNKSD
VSSMFQIVETNGVIIQNKAANYEERSRYDMTVCARNERGSEGCCPVTIYILDQNEQPFFDTDNDN
LDNGKYLVRVSETTPIGSVMFSVNTGDEDFGSLPNGQKTYSIKAKPGEEMLFLVDSYGRIITNTTLD
REKTPQYDFSVVVTDKGTPPRQAEAEVIVTVIDYNDQRPVFEIMSVRPVNENANNEVLGRVVAVD
IDSTSINNGIKYSIQPAGFLNISNTGTLTSVGSALDYEKNQKVTLYVTATDTGSPYLSATTSLQLDIINI
NDNKPYFLQNNYNLTVSEKTTVGTLVLTLFAEDDDLGEFGEIANYEIVSGNQEDRFSIDSNGRLTL
KNPLNWRNVKSYNLNIIARDKGLPSEQSQIPATVVINVADYSEEQPNFLQNLYVKFIPETTPIDTLV
LTVEATSGEPVYGPFKYTIVPKLPDTNNFQIFSNGEIKTINQFDYENQRNFTFDVQVEDEKNGKTN
RASVFIHITDVNDNIPQFTDLTRSNTQYNITEFTLPGSMITCLSATDRDHGKNAEIRFRGIGGDGNG

VFNVNLDGKVLLSQQIFYDKKNKYNLIVEAFDLGSPEQKAQLTLTIFLHKSYTSVITTDVDINEDVE
TQTRVTRVSSIDPFTRSSNNFTYYLVGKSTTFFEINAQTGEIFTKATLDRETRDRHVLTIQSRGPTTN
GVGELVVNVKDINDNPPIFDPQVYQVTVSESANIGLSIVSVTATDKDIGVNGEVTYSIEDGDQTIFSI
NPSTGSISLAGKLDFESKQSHTLTIKAVDRGNPPQDSVNKATVIVVVQDFNDNCPVFENSTYSLTIK
EPGELGKEYLKLGITDNDNGRNQLFTCSIDEPLAEKYFEMINDCRLRLIKELDYEEFTSFRFVVKAT
DQGDVSCTTTTNMIVNVGDRADVIPFFSPLTYNISIPESTTVGTIVVSLKVGSTFKGLLYSLFHKNTQ
DDLIKFGINKDTGEIFLKESVDREIQDKYEFTADVFDGSNNAVQRASVTINIIDVNDNAPFFNQPTC
YQRDLTESTAIGTFVVDVSAADNDKGRNQQVTYSLLPSPSCTSFSVDETSGRITTSQILDHEQQKLQ
HCLIKAKDNGTNSLSGYSCLTVNVVNVNDNKPAFIVNRFDVSVLETVPAGTLVASLVARDVDNQ
GLITYSISNGNLNNQFTIDSSGKLRTRFPLDYENTKSYNLTIQASDNNGKFSSPDDALVLITVLEVN
DNRPQFLQSYYKVSIPEDTATGASILNVTAIDLDHGELTKSLFYAALENTTQFTVNANTGVISVAEN
LDYEIEDHYELVVIVQDRGTPHLSNRALVDIQITDVNDNAPMFVTTVYNRELSEKALIGAPVVKL
NAMDADKSDKMTFTIDSVVQGAQGFFSINGDTIQLDKELDYEVPEQRTFVLSVSVKDSRDRDGA
NKANVFINVRDINDREPIFDLNMYEVTVSENNVISNPIIEVTARDSDSTPENNNLLYSLVDGEDAA
SFSLLNNGQSAIITSKISFDYEKKKSYRFLVRASDKVRENFTAFSTVFVTVQDTNDEAPVPEKDLIM
VNISESTRVLSTIAIVFANDKDQGINGQLAFQIINHSEDKFEINNKGVIRLKSTLDFFQQNFYNFTVL
VTDKGNPVRTSKPVTVLVYVYRTEIPGIIFEKNHYIFNINEKEIVSLVGTVNAIDEGTYVSRQIVYSIV
SGQNDIKDKFRAVNNNIIQDKEVNYKDQSEYNFVMKAINERGSIAFTHITINIMDVNEKPYFVGL
NNKGRYEFSMSEIISVPSYIYAVRAKDDDFGLNGEVDYEIISNFRFGNFSIDQYGRIQVLKDPDFEFT
RSYEFQVRAKDRGTPSLESNLVSVVITVLDYNDNHPIFNIQEPLEISIKELVNNTDVGTVTATDKDG
SGLNRIVKYVAIYPSDHLTVNRDNGQIRVIEPFDYEIAHKHEFVVVAQDQGTPYLNAYKVIKVEVL
NENDEFPEFTVIEYNITISELTPIGSPVLFVHAQDKDRGPSGQVTNYKIKYVPPSGGSGAGSPDKTFE
ITSDGQVVLKEKLEYKFQKYYQYRVTCTDNGSPPKNSINEAIIHLNIADASLEQPIFSVNPAVVEVY
ENITLNSNIVKVEVDSIEPIKGPFEYSIIASEDSQPFMIDNSTGQITNKESFDYEIKRKYVFLAEARDK
HNNRTQRAMVVIFILDINDNIPTIETIAPQTFVQNISDTTYPGSVVAKLYSRDADSGENAMVDFTIT
GGDGENVFAINPNGYVTLIGPLDVRVKTQYTINVKVSDRGIPTLSNNRALTFNIKKSKFIQNTVRF
DRHHYKSNCIEENQINLNIIQLNATNSNPVAGSTIIYSLDGTLEERQPFIVNSQTGEISLSQTLDYEIK
QEYDFVAVAVNNDGSRDVAVVTVCIKDLNDNSPIFEPQSYVVNVSESESVGYLVYKTTVMDADKT
DFNTTKSYDIIQGNVNDKFIIDSMGHIYIKSKIDYEALQVKHFDLRIRVNEGNLVGDMVLRVNILD
WNDVHPSFSTLNIYNFTIPEDKPVGEIIEILTANDGDATSPNNEVTYKLLTTEDDFILRRNDKGHGS
LIVNKQLDYSKQNRYEYVALAEDKGYLNLLGTAIVIINIQDVNNHPPEFDDSEYNTTVSEHAQIGT
RVLQIHAHDKDTGLGEQIFFIYGDPTETFTISSQGFIILNKQLDYETQRDYIFDVGARDGGNLVSLN
NVTVHIKVSEVNDNEPIFDLSTYRNSYAEDTPLGTTLFTVRATDKDFIGNSIIYSISGANDDTSYVSV
DQTTGDITLQRRMDYESKNYITFTVQAIDSNNRNKLKGSTHVIIDVTDVNDNNPKFVFGSDLVYN
VTVVENINVGTLVVVVDSQDADSGLNGQTRYHIVSGNNENKFFINELNGQINLINKLDALQTNK
YVLTVMAKDLGNPSRSTNVTVTINVLRYFESTPMFLPSYAEVIYPENLALGSSIYELNVTKTLPGSP
MKLSFHSSSKFPEFTLQGNKIILSKEFDYEIKNRYELIIIATDEENDKAFAQVIVRVQNLNDNIPYFTQ
NAYFGVVSELAFVGHSVLTITAKDDDKDFTELFYSIATQVPDNAFKLQDNIVVVNGKLIRNKVYEF
EVEAFDNTFTSKRATVSITVTPENLPSFVNATYIVTLQENYPISTNFLNISAGTFSGITYVITSMKAKD
MFNVDANGQISLTKSLDYEKQRQHVFPVTASVTSPITQVRVASVIVNVLNENDNAPVFVNGPYER
NITEGHLVGTMVIRVDGHDDDSDKLTYSIVSGDDGKFSINSEGVISLISTLNFDLKNKYVLNVRVSD
GKFVTDTIVTINITPIVKFPPMFNQTLYLLSVKENTLVGTSILQVSTVNVIPPSSFSINEQSGRDFFTID
ANGVIRTNNALDYEAKRQHIFTLKVQDLRSMGHATVVVNIVNDDDTCPILTPATQTVQITEPVAP
GTIVAAVQAKDIDSKSLNFTITGGNGLFRIDQHGGIKTINEVDISTSTEYELTVTVDDGTCTKSVQV
DVNVNPLAPCPDCNIYPFTQTIYEFSINENTVPASPIITVTTGQSLQRNFSIEDPAALEYVTINRLTGE
ISVIKAFDYEATKHLSFNVMAKTQTTKNRNPFYVITSVSRVRITVRDLNDNCPSIESPMYYEVSHSS
PIVQDTVVTRILVEDPDTLKDHTYSISDNNFKIDSMGHIRTVQNIESFTAQFYNLTVTVSDGQCSKT
SNVGVNLAVNPTPMTYQFTSNGVYSKSVREDHNLQDVILSVALNQNYPRVFSIVESDANDQFVID
SNSGALRLKTSLNYELKKRYDFTVMADIHGGVYKSYASVHIIVLDVNDECPIFPVIGYNQRVSGTP
VIGTIVEIIEANDADNATLEYSIVSGNEDGHFSLDNSGVVRVVKPFASSFSKTYGLSVRATDNKCSA
DVPLTVQVSTCAYGEDYQFEESEYVYYLDENRNLGIVSNIKLKSTRAAFLTLSQNTHFTIDQNGDF
RLIQTLNYETKKEYHLTLTANFTQDNVVTRIPIKVFVRDINDNCPAFSEELYAVQHTEPLREGLLIA

KLSVIDDDTVQNFTYNIVSGDKRFSINENGEVVMNARLVTDTTLLSTTYKLEVSVNDGKCAKNTK
VDVFVYKTVVNSFLFPQPFYKFDISENQAIPFTIDRFVNVGGHEAVYGFLVSTDFFQIDPKTAQLQL
VKKLDFEVEKTHYVLISATYDDGTQTYTVVAIHVGDENDNAPQIFAHPPITRLVQNTPHNSIVTKI
EVEDPDTSKEFTFKITNGNPLQNNDPDKALFRIESNGMIKTTRCISEDEIGNYVLTISVDDQVNTPTI
FSNMQVQLKTTDDRSYVTNCGLGCPANDGPFSRYQFEQSLFIVNVTETAANPTVLKRLTRPFVEK
TTYTIGAGADGKFSIDQNGTLSLTGSLDYETKPVYYFIVSAVIPSQVVGRFITSKTAYLVMVNDVDD
SKPVIEGNGGTLAVSETSEIGQIVGTVRVKDSDSSSPLTYNVVNGSQYFNIDSAGNIVIKDTLNAAG
SPYTVTYTVSDGINPPVNGEFVVSISETNNNVPKFTQNIYEVSLSEDTATDTIAVTILANDGDTNDKI
AYSFLQSEGPFVINRDSGEIKLIGPLDYEAQKSYNMVVQATDSGGFANIAFVQIAVGDVNDQVPVI
TPQTTPTQIHESTLVNSVVYRVIASDGDGNERLTYALANSNDFFTINSATGEITIKENLAKSNVSSHT
LSIVVTDSSGKTSTQDVALNIRRSGTSQGCVKITTSQGQSSSPVINLPENTASGTTVFKLVAGDASVF
SNMRFTLNREANGVFAVTEQGELQLVAPLDYETKQFYSIIITAEDVTRGVSSQYNLFVRISDVNEFT
PVLETIAPVAVSELANIGDVIAQAKANDQDGDSQVTYSLGEFGNNDNKFTIDAATGNIILNKRLG
SGAPHVVEVCASDGSIKKCQNVTFIVADTNDNPPSFQDLTGVGSIDLTNTSSYVVVYKPIVTDSDQT
GTLSFSLKDPAPSQFSINSTTGEVKANKDIAKGTYTFGVCASDGRYTSCQSVTVVASDRSQSITPSFN
QDQVGAIVDISASSGKQIIQVMASTLDKAKIEYELKNEADRNIFEINKDSGLITLKNRTALTLGTIYT
IPVKASRADDPLNFDEINIVISVGNQQLDSGSFIEQVLDTQHLVNFSLSTFDISKVSYYIILGQQVGG
DSKDDEKLPVSWYYATFGDESYRHRFYYIAKLDGSKFRNTGGSRKRRSIQDKDSKSFTYLVGEENG
CSKNTSMAAICNGPLKTGTEYRYKLRAYYDDGELRGIPVDQKPTEVKRTQAPQAGKSIDEYREQD
SYVITFWIIGLVIFGLLFWFSVLVIAILLCYKRRYEVTQAYGYSVQTRDKIDGPPTEYSNTVYRFQNPS
YPDNDTVKMKKVDASESRTKTEVQTTSMVVLDDYEQEQPQPDIPVQDYDAIEYETRSAQIKFHEE
SPEWESMDIQLRIDPTGNQDPVITRKSDSGSKDSDSDASKKYENTEYIEEKEDFVNEKEDSLTRL*
>TRINITY_DN11435_c0_g1_i1.p1 type:complete len:430 gc:universal TRINITY_DN11435_c0_g1_i1:1616-327(-)
MGNIYTVGPNEALVVSGGCVGQRVKRTIVGGWAFAWACVSDVQSISLEVMTLNPRCEKVETAKG
VAVTVTGVAQVKVIKEDDLLKTACEQFLGKDPHEIEDILLQTLEGHLRAILGTLSVEAIYKDRDQF
AQLVREVASPDVGRMGIEILSFTIKDIVDEVSYLDSLGKTQTAEVKKNADVGVAEANRDAGIREAE
CDTQRQDVRFKADTTIADAARAYEMEKAEFDKEVQTKNAEAKLAYELQANKEQQRIRNEEIQIE
VIERRKLIEVEEKEIQRREQELQATVKLPAEAESYKVQTIAEANKSAKVYLAQAEAEKIKMIGSAQ
AAAIEAIGKAEAERMRQKAAYKQYGDAALMSLILEAMPKIAAEVAAPLRKTEEIVLINDDNGN
KMSSEVTKLLGQLPPSVKALTGVDLSKALGKIPGAQVTA*
>TRINITY_DN160_c0_g1_i1.p1 type:complete len:1145 gc:universal TRINITY_DN160_c0_g1_i1:4810-1376(-)
MRLLAIIPLLVMLDIAKSDKLWECNFEENDCWAASSTRNHERNQLTLHWNRWKMRTPTGATAS
PVTGPMEAFEGKHYLYYEATESMDKLKMADGASKGVVTFKDINLMGRTQFILSFYYQVKGNDG
DFYDFDLMVSNTENTHYDLSMKSIDGKDWKHFKHIMKVEGAGVTSIDLKFTARSYGAFADYAID
NIVIEDYPDAGPCKDNRCLANEYCTITSSTQTVCVAKCTQQACGENAECILDSLAINKCKCRQGY
LPRVSNYGDKLNCYRPDASCDIEYNSLGQCRISTSIPIMYGHPSLWHGRYSPVSGNGYYVFKYDNT
ITLDAVSSQKLSFFYLVDNPNMINSLTVEYGSRKFEVPINREKTNIWTLYEVLILASTNQGVQIKASF
YAEGGVIGIDNIQFTQQNEVVVEKIYKESMMAATFEDGSVPTDLKVAMLFVTRYGKIDFGCLPGIE
LETRNGKLYCKDGVVQRQSACSQDSDCIGSNARCVLRNCVCNTNSYQISIGTYGEKATSCAVYPC
EINSPRCHSTAICSNIKTPGQNFYNDYTCKCGRSLNNVETIINPFIGGDKSTQCLAATPSDSYVCND
NNFKPSRTTEYCLKGNQATVEYSLRATNFGNYALFKHCDDPDDEYRSSFNDPGSFWRPIDLVAAA
NENLAVSVTYKTSLSNKFIKCNFEQARFGSKLTLAVNVNSNSRVLTFTNTQTSVNKQNPVYQGQV
FQSDNPTPGAEMSVTFTVTGNQAVDQNVDAILLDNVVCKGFDDVASVNTPCGNNACASNQYC
NPLLSNSEKECLCPTGQKDQNCPDVVPSGSVVSADGPKCIGHHEKYMNGACVCDDGFEKTGDT
CTVKINCGCNDGLCLSGDKCSCPLNLLKVNQACVRTNKDCAKDCVSTLGNGGYCDVTTGMCD
CLTGFVKSGRDDGSYRCDKHAVVQKINDPKGICSATCGTGFVNEIEVCVGATGTGFECMTNIVQG
TLGVCSLPACPATSSQCSIQKECACNNVRVPIGSDGKLRLDFDHEAKLNMIFRFLSKHNQKDLEFK
FREARCDNTKLDSIARTIEDDLKQLEFDTELQAAITTMKSILDCSPSGNDHNAAYWDVFDMLIELS
SYRPHAVSDLQLKKELVMHEQSRCGMRSNLHKVVDGYAFWNKK*

>TRINITY_DN240_c0_g1_i4.p1 type:5prime_partial len:397 gc:universal TRINITY_DN240_c0_g1_i4:1327-137(-)
LKAAAAFSGVSPFALVQPRARVPSRSRVAMVRRSPLPVLGLAGAACFFARSPCFIPGGPLRRQPWA
AAAGSAAVLGATPALADPIGDAAKKLADASYPFAKEVDWNSGIYLQAPGKLAPLEALKAIDKMI
VLGSQADPKLLKAAADAHHKAISSASGPTGMTSKADWEAVNAALGRVFASVPESSVMDVYNAV
AKITDPGVPAYLKSKVNGADAEKAYAAFLEFKDVVKANQVSSAAAAPVPAAGGDKIGAAAQAL
ADQSYPFLKSVDWTSDLYLKPLPGVSAAKALKAVDKAIVMGSAMDGNLLKAAASAHHQAIGSI
DAAGVTSAADYASVTAALGRAIASAPTSKVMDVYNAFAGIVEPSVPNTLFSKVDPLAANAAASA
FYEFKDVVKAAQR*
>TRINITY_DN240_c0_g1_i4.p1 type:5prime_partial len:397 gc:universal TRINITY_DN240_c0_g1_i4:1327-137(-)
LKAAAAFSGVSPFALVQPRARVPSRSRVAMVRRSPLPVLGLAGAACFFARSPCFIPGGPLRRQPWA
AAAGSAAVLGATPALADPIGDAAKKLADASYPFAKEVDWNSGIYLQAPGKLAPLEALKAIDKMI
VLGSQADPKLLKAAADAHHKAISSASGPTGMTSKADWEAVNAALGRVFASVPESSVMDVYNAV
AKITDPGVPAYLKSKVNGADAEKAYAAFLEFKDVVKANQVSSAAAAPVPAAGGDKIGAAAQAL
ADQSYPFLKSVDWTSDLYLKPLPGVSAAKALKAVDKAIVMGSAMDGNLLKAAASAHHQAIGSI
DAAGVTSAADYASVTAALGRAIASAPTSKVMDVYNAFAGIVEPSVPNTLFSKVDPLAANAAASA
FYEFKDVVKAAQR*
>TRINITY_DN776_c0_g1_i1.p1 type:5prime_partial len:201 gc:universal TRINITY_DN776_c0_g1_i1:692-90(-)
RAGKTPHTASMVKNVCSKTYVTPRRPFEKARLDQELKLIGEYGLRNKREVWRVKYTLAKIRKRA
RDLLTLPEKDNRRLFEGNALLRRLVRIGVLDEGRMKLDYVLGLKVDDFLERRLQTQVFKLGLAKS
IHHARVLIRQRHIRVRKQLVNVPSFIVRLDSQKHIDFSTNSPYGGGRPGRVKRKNMKKGTGGGDG
DEEDED*
>TRINITY_DN161_c0_g1_i2.p1 type:5prime_partial len:399 gc:universal TRINITY_DN161_c0_g1_i2:1683-487(-)
WNQEPLTCTYLYSVNSQLLKYKMADDDVAALVVDNGSGMCKAGFAGDDAPRAVFPSIVGRPRH
QGVMVGMGQKDSYVGDEAQSKRGILTLKYPIEHGIVTNWDDMEKIWHHTFYNELRVAPEEHPV
LLTEAPLNPKANREKMTQIMFETFNSPAMYVAIQAVLSLYASGRTTGIVLDSGDGVSHTVPIYEGY
ALPHAIIRLDLAGRDLTDYLMKILTERGYSFTTTAEREIVRDIKEKLCYVALDFEQEMQTAASSSSLE
KSYELPDGQVITIGNERFRCPETLFQPSFIGMESAGVHETTYNSIMKCDVDIRKDLYANTVLSGGTT
MFPGIADRMQKEISALAPPTMKIKIIAPPERKYSVWIGGSILASLSTFQQMWISKQEYDESGPSIVHR
KCF*
>TRINITY_DN329_c0_g1_i2.p1 type:5prime_partial len:320 gc:universal TRINITY_DN329_c0_g1_i2:1-960(+)
FNNSTHSPSLPTHVYLYCCRIKILILSFDPSTIEILKMAPPKFDDLGKEAKDLINKSFHFGVIKLEGKT
KAKNGVEFTTEGTHNTDTGTVNGSLETKFKYADYGLTFSEKCNTDNVISTKITIDNKLAKGLKVD
FDTTFAPVTGKKTAKVKSAYQHNNIHSTCDVDFDFAGPTIHGSAVFGYNGWHAGYQASYDTANS
KLIANNASLTYKEGDLVIHSGITDAVKYSGSVHHKVNSNLTAAALIQWASGTNNTSLTVAGKYAV
DNETFVKAKIDNNLRLGLSYAQKLRPGVELTMSSLVNAKSLEQGGHKLGLSLNFEA*
>TRINITY_DN428_c0_g1_i1.p1 type:complete len:1949 gc:universal TRINITY_DN428_c0_g1_i1:96-5942(+)
MADDADLKFLMVDRRQVSDPVAHAEWAAKKLVWVPSEELGFVAASVKEEKGDKIIAQIEGGKR
VTFHKDDIQRMNPPKFDKAEDMADLTCLNEASVLHNIKDRYFSDLIYTYSGLFCVVVNPYKRLPI
YTDKVINLYKGKKRHEVPPHVFSIADTAFRSMLQDRDNQSILCTGESGAGKTENTKKVIQYLTAIA
GRANQQKADEKSQGKLEVQLLQANPILEAFGNAKTVKNDNSSRFGKFIRINFDNSGFICGANIES
YLLEKARLVRQAPDERIFHIFYQLLLGASPEVKKQFLLLDTKSYNFLSNGHVQLPNMDDRQEFKV
TLEGMRDMGITQEELDPIFKVVSAVMLFGNMEFKQERKSDQATLPDNTIAQKICHLLGIQVTDFT
KSLLKPKVKVGREFTQKSQTKAQCEFAIEAITKALYERMFKWLVGRINKSLNRGKRDGASFIGILDI
AGFEIFQLNSFEQLCINYTNEKLQQLFNHTMFILEQEEYQKEGIDWKFIDFGLDLQPTIDLIDKPMG
VLSLLDEECWFPKATDKSYVEKLHKQQTKHPKYLKPDFRSNSDFCVIHYAGRVDYSAAQWLTKN
MDPLNDNVVSLLAHSKEAFIASIWKDTENVVGMSVTEGMDTTFGASRTRKGMFRTVSQLYKEQL

SGLMTTLHCTKPNFVRCIIPNYEKRAGKIDNHLVLDQLRCNGVLEGIRICRQGFPNRILFQEFRQR
YEILCPGIVPKGFMDGRKAAKQMIDNLEMDPNLYRIGQSKIFFRAGVLAHLEEERDIKLTEIIIQFQ
AFCRGNIARRNYRKRIQQLSAIRVIQRNGRSWLKLRNWQWWRLFTKVKPLLNVSRQEDELRSKE
EEFKKLQDKSDKLESLQKEQQQANEKLTEEKNTLAEQLQAEIELCQEAEEARNRLLQRKTELEDL
IQDFEMKLGEEEEKAVKYAEDKKKLQQGIQDLEESLEEETQRQKLQLEKVQVEAKLKEMEDERQ
VAEDNNARLTRDKKQLEDRVAEFGQKLAQEEDRAKQLSKAKAKQEAFIAELEDQIAKGDKSTHE
LHKEKRKAEQDLSDLRAQLAQALQRIAELEDDLARREKELAEAIKRGDEEMAKRTTVEREKREL
QALLDELREDMESEKSAKDKVERQKRDLEQDLQELRSELEVTTDATVVQKEFQKKREEELMDLK
KQIEEEAKEHDQQMASLRTKHNGLVEDLNNQVDTFKRGKAALEKNKTALENENAEIAADLQRI
SQLKQEGDRKAKNLEQQLNEANSVRAVQDEKVSSLESEKAKYQKECDSLSTQIEEVETKAASLDR
AKQAAEAQLEDTQDQLAEETRQKLSYQTKLREASDEANRLQEQLEEEEDEKKAVQKVLSNLQV
QLDQAKKEIELKIQDFEQAEAARQKQKRENEELRGEVEKLTGDVSRLDKAKKKLQGDLDDVTVS
MERERNSANQMSSKQRKFDQLLAEEKANSQALGIERDNLEKLARQNETKALSLQNANEELEDR
LAEAERQRKQLQMELGDLVESKDDVGKSVHDLEKSKRLLEQQLAEQKTQVEEIEDELQACEDAK
LRLEVNMQAQKAQFDRELQAKEDAIEEGRRGLVKQLRELEAELEDERKLRQSASAAKRQLEGDV
KELEGQLDQANRIKEDGLKQLKKYQNQLKDVQRDLEDSRQSKDDLAAQVKDNEKKLKGLEAEF
LQMQEDLSAAERARRAIESERDELLEELNNNSALKNAVAEERKRYEAQLAALEDELEEERTQTEL
MQDKVKRSNLQLEQMNAELQEERSAAQSLDNTRMSLERQNKELQSKLADLESSLRSRQKNTVAT
LEAKINNLEIQLDQEAKERQQQNKALRRNEKRCKDLQMQIEEERSHTESYKQQLEKMNARVKN
MKRQLDESEEEVTRLNTAKRKLQRDFDENQESLESSQREANQLRQRLKGGASLSSSRSSGRGRRA
KDEDDDDDDANE*
>TRINITY_DN2217_c0_g1_i3.p1 type:complete len:566 gc:universal TRINITY_DN2217_c0_g1_i3:1820-123(-)
MLRLVLIAISAAVAYGCSPLKHGCYAHSKSDAHLLHSDNSEDTHADIFSGTVIDWNNFGPYLQKA
ACRCNKAAAKAGYPFFSLRDFGECHGVGKGSTSFSALNLKKSVYCFGTDLGICDSSQHKHCAGA
EEGEFIYETKPEELTLLSEGKPSKQSSTYLGNEKTYGSYLAFDGELSQKVKRTCCHTKKSAAPWIQV
DLLQPSTIYSIKLANVLENYRNRLNNVKIYVSNNENFDGMKQLCASFVHMKTIVQTFTCQTPLTG
RYVRLVNNNNFLHICEMQVYGVQNSESITCPIEKGFCVKGNGHDQNSGVIKMNSVDGNTDNAQ
KECLELCQRNAKATGCEVIWNRHNRGCYIHTQEVARGNNVDRHFCWVFSKCTLNNVPPAVCPI
EKGYCVKGNGHDQNSGVIKMNSVDGNTENAQKECLELCQRNPKATGCEVIWNQHNRGCYIHT
QEVARGNNVDRHFCWVFSKCTLNNVPPAVCPIEKGYCVKGNGHDQNSGVIKMNSVDGNTENA
QKECLELCQRNPKATGCEVIWDRHNRGCYIHTQEIARGNNVARHFCWVFSKCNL*
>TRINITY_DN118_c1_g1_i3.p1 type:internal len:200 gc:universal TRINITY_DN118_c1_g1_i3:599-3(-)
KVLFLCGFILLSGLAFAYEIEEIVEDAPKPEPEHVPEPEPEPVKEPEPEPEHKPEPEPEQCSTVKHQK
CSKADKVCSPEDSHCTFTQICTPFFVKKCCKVVSVEKKCVVVRNCIPKFSYEKVCEDVKRCPPRKR
CKNVCRQVDLGEHKVKSICEQECEVVEHPEPCKLVHTCFSKWVSKKDCTPRETCSDIEKRECREV
>TRINITY_DN535_c0_g1_i1.p1 type:5prime_partial len:162 gc:universal TRINITY_DN535_c0_g1_i1:712-227(-)
GKYLVFFFYPLDFTFVCPTEIIAFSDRVEEFRALGCDVLACSVDSHFSHLAWTQQSRKKGGLGQMK
IPILSDLTKSISRDYGVLLENDGIALRGLFIIDDKGILRQITVNDLPVGRSVDETLRLIQAFKFTDEYG
EVCPAGWKPGADTINTGKAHEYFEKQ*
>TRINITY_DN72625_c0_g1_i1.p1 type:complete len:1964 gc:universal TRINITY_DN72625_c0_g1_i1:173-6064(+)
MKLAFNIFTISLLIGNTIAQNNDVVQVCKSQADVVAVLDSSGSVGVQDYEELKKFVGFVANLLTIN
ENGNRLGVVTYSETAEVDFNLNEIYDSDQYGREALTRLSVRPREFKTNHYAGLKVGSDLLFNKAA
GARDNAAKFLIFTTDGRNNFDETNNRNRASQLATQLKADGVNIYVIKAEVMSDNNGRLDSRLYS
TDPNKFYRFINRFGEITNENFLDEVGLEFCELVQLPDNQQATVREDRNIGTLVTTIRAPGLNNPTFE
LIPQDNLPFIVDPLTGEIKTIGLLDFEQVQQYVFQVRVRATNPDVEKIITVTINVLDVNDNDPIPETA
NYRMSVAETTPLGQRILTVKANDPDGGTNGNVEYFLISGGDGKFEVDQATGEIRLIGQLNKRIKD
TYLLTLEARDMGIPTRKAPITVAIDVTSLNNFPPQFNEMSFVRTIPENFGAGTVINAAATDRDGDT
MVYSLIGENIPFSIDQNGLITVSGNIDFETIPNNVFQFQVQAEANGDLVTQDVIITITDINDNVPVYS
MTTYSGSINENSAAGTVVNFDNVIIATDADKTNANNMITYNIIDGNDGDRFQVTKNERGEAVISL

KSTPLDQETQGQYRLTLQAIDNGVDPRSLEMNSLLIIDVIDQNDNDPIPEESVYSIAVAETSPVGQSI
LTVRANDADSGMNGKVEYFLISGGDGKFQVDQATGQITLIGMLNKRVKESYTLNIEARDMGVPT
RNAPITVNIDVTSLNSFLPEFAGAGDAFERTIPENFGSDTVIKAAATDGDGDMVFYSLVGDNLPFA
IDQNGMITISGSIDFETIPNNPIRFQVKAEANGDMTLKDVVITITDVNDVTPEFTQQVYAGKIEENS
QTETVVRFDQPVIATDGDKSPANNVVTYRIVDGNVDNIFKIVKNAQGEAVISVNNAGLDREILNQ
YTLTIEAKDNGVDPGSLSSTTQVIIDIEDVNDIPPLINTSENKAVGEADFTATISEDSPIGTSVVKVEA
TDPDSNNDDLIFTIANDVPVPFTIDPKTGLITVSGPLDADDQDEFEIAVTVSDGVNNSTAIVKVNIA
DLNDNKPIFTTDTRDITIPLDTPNGQEIIKFTGSDADKTSPNNQFEFSLVDGDGKPFTITPDGSLIIN
GPLMPGDYMLTVKIMDKGTNPGPLSNVIIYNVKVVDTNDPPTFQNNEQTVTISENTADGTDVITF
AATDPNGDAITYEIVNPGSVPFEIDPITGVIRKMGLIDFESPVRSYTLDVKATDAKGKSTMGRVFVQ
ITDFNDNTPTFGPGNTDNFILTEGVYSNQLVSSFTATDLDATSPNNVFEYTLVGAPSMFLLTTNPNT
GAADLTVNGDIDAEQNMSFNFIIRATDKGNPALSMDYPVTITVGDINDNAPVFAANTPESIDLME
GDFNNQIVATFTANDDDTTAPNNLVDFELVGAPDFFKLIVNPETGTADLVIDGNVDIDGLDPTINF
IVRAKDGGNPALVKEFPLTVNIADINDNAPVFTLEAYNQVINENTPIGTKVVEVRATDSDSGPNG
DITYRIVDGDGTFGIDPKTGDITVLKNTDFESGVRSYEFTVEASDNGNPKQSATAKVKIDIRNLND
NDPIFTSIPITGNINENRPAGSVVQNIQPVNGILNQNNVKILREPPPGAQETEGVGFDAPFEIDPVT
GEVKSTRPFDFEEEKVFTMMVQAADGDNNPMLTTMTVMVNNLNDNDPIMKKANFEITLNQDT
PVGTFVLQVQATDIDQENQTKITYSIINIDGGNMNDFTINPDTGVITLNTDLSMNTVDIYNITIQAE
DQEIQGVIRRSTALAEIKILRPAEAEPDISTLCTNSCDIPEERLKCLDYSKFEITRKFNGTCYVDKLKA
AIADVKNNTMEIMNLNASFLECRNKVQNEITNPNTQFPVNGLFDLHRKCTVAIECKLEQLAFNL
MRKTTELTNNLKAEEEGFSMYDMSLSAFFQHISKMVSMSKSSRVD*
>TRINITY_DN15274_c0_g1_i1.p1 type:complete len:1822 gc:universal TRINITY_DN15274_c0_g1_i1:87-5552(+)
MECIRRSIIRLLLILGFYTLYISSQEECKRGGCFPATGDLLVGREKQLLANSTCGLNGPSKYCILSFLD
KTDKCFYCDSRRQVDERPDGYKNSHLPKYMVTREPSKRLQGWWQSVNGRQDVTIQLDLEAEFIF
THLIMTFKSFRPRAMYIERSSNYGNDWSDYRYFAYNCRKSFPNIPRGPQRKIDDVICEERYSKVEPS
TGGEVVMKVLDPIIQKKEDPYSLKVLNLLKLTNLRVHFKELHTFGDDILGDSPKVNQKYYYALYE
MVVRGSCSCYGHAEQCIPTKGTPANVNMVHGQCNCTHNTKGRNCEMCKDGYNDEPWRPAYQ
DQLNVCRKCNCNGHTDKCHFDPAVYKATGNTSGGVCDDCNHNTAGRQCELCKPLYYRDPYKN
IFDEDVCKPCDCDPSGSFGGGECETISDPNNSSALTVAGRCICKPNAQGLRCDSCKDGYWNLLES
NPDGCQSCNCDDKGTVELNKCDQQTGTCRCKRFVIGLTCSKCLEGFWNLGNTPEGCEPCNCDV
GGSLNAKCDDITGMCMCKPNIIGRKCDQVAPGSYFGNLDQVISEAENSLVRSDSSSVEIVREILNPT
DSRSFTGDGYLRVSDGTLVNFTNIRVPSTGFQTFVMRYSRPTPGSNNNVMVRITKTDGTILGGNGC
VQTNEREIILNGVLNDDNFLELGDMFCLEKDTDYSVQLVVSNRNSPNDGGVNRGVTNLDSIVM
MPNIDNLAIFQGREGMRLKSQYDLNNCRQYYLEPNGAANAPEICKKIQFSISAEINNGALKCNC
DPTGTNSSGSSDILTCQKSGGQCPCKPGVVGRRCEKCAPGYFGFDSNGCTACACNDTGSEKLFCN
GITGQCPCKNRIKGRQCNSCPSNHFGFPNCIACQCNEHSSTCDPISGECTNCKDNTAGPSCNLCA
EGYYGDATAGTEKDCQRCQCPGGSSLNQFSNTCHLKPDGKVVCNNCTEGYTGDQCDKCEDGY
YGNPLVPNGSCNKCDCNGNIDPFADGKCNAENGKCFNCINNSDGDHCENCKEGFFGNAKNQS
CRACVCNKLGTNSATSDSCDRETGQCECLPNVEGVECDRCKSGYYNIASGNGCVSCNCDKGGSL
SSHCNQLDGQCNCLNGYGGLDCTQCEDFYFGDPKQNTCQACGCNPVGSITLQCNRTTGVCMC
KDNVVGEKCDMCAPQTAGVMPQCEECHVCNDQWEDIVNDIEKNITTILTLKGSSPLNVSEIRAY
TNDIADLKNRLDKIEEDFGKQMVDPNKINRLRDKVYRFSYNLTGINRDADFYMEMVNETITRDE
KANDILDGLELRLATLQTNTEDFKNNVTKIVESNIGGALNSTRESLEKSRKAQESVNDSDLKIKES
KKMRKKIKKEIQNGDPEFEVMHNRSSEFVNNLADRENYLEGELEKLNRLLCGSDKRCGGCTKGG
CDTCGVSGENYTCDDGIKMMSMMASDKALEAKEAIDQKLENASELLENVNRAKQHVIQANISA
NMAEDKAEEVNKIGQAVLKNITTLIKDIQDFLATDFNHPNASKDLAMQTIALNISLTPDEVVELG
RKIRDAVKSLTGVDKILNESRNDYDTAIGLKEQALAAKERAIEVMNQTMAIIETLSQAEELQNSTL
AGVNVARADIEDVKDLLKKLKEKLDAADNDLQMADNSTASAEDKRNAILDQNLENREKLDLSE
KKIDEAEELGDKVQGSVDKMNEKYNATKAKLNEKEDMIEKVRARIKALADKTIKLYENADRKLR
RINKMKDSYIINEERAKDLYYEIRRLEIKEFFINTKLEGTAKCYSTCNPIIPESFCRDVYKNIAVKETN
AYAELEAEKASTTSLLI*

>TRINITY_DN138_c0_g1_i1.p1 type:complete len:401 gc:universal TRINITY_DN138_c0_g1_i1:157-1359(+)
MAARFLFITVILIVGNLIGDVCPERCERNWIKIGCFHDRIFPNRPYPFELVNHRDPINNHWDGNLI
NWRKYPDSIEKLACICANKTRTKGYQYFGLQFYGECWSGPSYNFHRDGPSTKCIGNDFKPCDDSS
KTECVGRAFTNYIYKLKDENPKVDGKWSEWEKWSPCSATCGYGERYRERFCSNPEPENGGQTCE
GEAREYEECNHGECQTTCDKEIDFGIALDYSSSVRQVNWNRTLDFVVELSKGFNISPEKVHFGVLS
FSHRTRFDFGLSDKTYWNSESFEKKIKSLEYRWGYTWTDKALKKVKQLIFCEKCGLRKDSLKYLLV
FTDGRSNNVKTDLKTVSDSIKDMGVNVISVGAGHTINHRELEEIATDDNHVFYVGGYAYLGDNM
NAIRRLTCG*
>TRINITY_DN332_c0_g1_i1.p1 type:complete len:246 gc:universal TRINITY_DN332_c0_g1_i1:940-203(-)
MGALTYIFCLFLISSSFAKEEESCRTYAGGQVYPDERKSGVEHALHWSKAQISKPAPHWEGKAVV
NGEFKDLKLTDYKGKYLVFFFYPLDFTFVCPTEIIAFSDRVEEFKKINTEVIAVSVDSVFTHLAWTNT
PRKEGGLGKMKIPLLADLTKQISKDYGVLLEDAGHTLRGLFIIDDKGVLRQITMNDLPVGRSVDE
TLRLVQAFQYTDKHGEVCPAGWKPGSDTIIPDPKEKLNYFNKQKEEI*
>TRINITY_DN602_c0_g1_i3.p1 type:complete len:851 gc:universal TRINITY_DN602_c0_g1_i3:3046-494(-)
MIICRLHGLSLGIILIFSILYTSDARSRHAYGHKDASSPQTGKKRAESDAGAGTEAATGANTGAATA
SDSSSESQTFTNTKVNTRIATSSNARSGANAAAKVKTYAHANTETKVDTQQQKPTPPVPPEALTT
PPPGVTPPGPPGPPGSENNNTSSGSECKEKETKPPIIKVYCPNCPCPCPPPCPCKCPCIKVAPPPPAP
PPCICLPPCPPPCPPPPPPCPPPCPEPLPAPAPCLPPCAVPPCPCNIAPITLDPSLPCPMDCPLPPCAC
PFMKKDNITTTKTKRSVIRKRNLNIKRSHRVGTKRVNAGKSPNLAYAQPDSFEKAAVAADNKKCS
EIGVEILKKGGSAADAVVSTHCCVEIVNSHSTGLGGGGFMVYYDKKSGKSRAYDFRETLPKKYTD
TQNKTQGETILVPGVLRGLEAVWKDFGKLPWEELWKPCIKYAKEGFYVHEALAAGIVKKADYIM
ANLGLRELFAPDGELLQLGSLFKRPKLARTYEQIAKHGADLFYRGEMAKQIVNEIKEAGGVMQL
EDLSSYQVNIRETLKSNINGLKLLTSPAPSSGSLMALALKIMESFKWKPEDLLNNPSLVYHQIVEAM
KFAYAPTSYLGDPRFTNGTDKVEQYMVSPQTAKEMFKRIDNKSHPVEYYKPWSELFSAEKTGTSH
MSLLDQEGSAVSLTSSINAYFGSKLRSREFGFIYNNELADFSEFWPTVYNLTSDKKAPSKRPMSKVM
PGIFLKGKTVQGVWGAAGGFFIPSALTMSIANWLFFKDNLKVAITRPRLHCQLFPPTVVYEPTFPP
SLIPKLQEFGHKYVTNSTYDVSGQLNAIMGVVQAIVRLPNGSYSAEADYRKGGRPAGF*
>TRINITY_DN761_c0_g1_i1.p1 type:complete len:830 gc:universal TRINITY_DN761_c0_g1_i1:129-2618(+)
MTSMFRSEEMTLCQLFLQSESAYACVSELGEIGVVEFRDLNPDVNAFQRKFVNEVRRCDEMERKL
RFMQKEIDKADIAIVDTGENPDAPHPREMIDLEAQFEQLENEMKDSNSNYEALQKSLLELLELRH
ILKKTQSFFEEEGYAETQARLQADHEPGVTGGGQLGFVAGVINRERIPTFERLLWRACRGNVFFK
QAEIDTALYDPGTGDLTHKCVFIVFFQGEQLKIRVKKICEGFRATLYPCPETPNERREMAIGVKTRIE
DLETVISQTQDHRHRLLQTIAKNIRVWLVKVKKIKAIYHTMNMFNLDVTQKCLIAECWAPVADL
DSIQAGLRRGTERSGAQVSSIVNRMSTKQDPPTFNRTNKFTKGFQAIIDAYGVASYQEVNPALYTVI
TFPFLFSVMFGDCGHGAIMALFALWLIMNERKIANWKAGGEMFDTVFQGRYIVFLMGLFSIYSGL
LYNDIFSKSLNIFGSAWVPTLNRTKYDYQPENYKEWSDGDRMLNPLDAPRETPYYFGIDPIWQLTS
NKLTFTNSLKMKLSVIFGVTHMIFGVILSLFNHLHFKQYVNIFCEFIPQLLFISCIFGYLVILIFYKWIA
FSIHSPSQPSILLGLLAMFLNFAKKKTDADRLYNGQDFLEPFLVVVAVICVPWMLLIKPFYLRWQH
NKKKQHGTNFARLHEDTAPLQDNEVNHHDDSGISSKNEEEEEFDFGEVFVHQCIHTIEYCLGCIS
NTASYLRLWALSLAHAQLSEVIWTMVFKIGLSMDGYVGSVAIFAVFAAWAILSVAILLIMEGLSAFL
HTLRLHWVEFNSKFYVGAGSKFVPFSFKDIMNDEAF*
>TRINITY_DN164271_c0_g1_i1.p1 type:complete len:163 gc:universal TRINITY_DN164271_c0_g1_i1:1172-684(-)
MATGIKPDPQCTDLYNKLKMTGGKAKPYEYLILHIDDKLNKVVPDVYPDLNSTLEMDEYKDVTG
QPASYKRLKDYLLAKASAYAFYHFEWSEKDGERSEYVMIAWCNDNKANITQKMKFSGTKDEVK
KVLKVGKVIQCNDADDIDYKTIIKDLSKNKAIFTK*
>TRINITY_DN22492_c0_g1_i1.p1 type:complete len:1026 gc:universal TRINITY_DN22492_c0_g1_i1:171-3248(+)

MLQKTLIYTLLGCWITLLSHAAIVRKSGGDDFSLKVGKPQKPIGQPKLKDAKKEVKEEPTSDVIPQ
QDENNDTTDRQKHKEQVSPDAVESGMPPRYPLKAMSAEVSAHDNEIGGCRDHPRASRVCKNC
GNRNFAAEHESYTYSYCRKSCRHCDPELSSFHTFWRLRAASQFDPTWMVKEIEFYPKPTEYDNLA
TDPQRAYASSSYPGFKPSYAFDGNNKTSWYPSGWGLHDNNDDWIGFEFPVAVRVHAIRMVLDE
MHRTAAPQRIFVEASDKKDGPFTTKWKISNPTYEVDNVYKLLQCPVLWRKFESDEGSWCFRLIAK
FETWEVARNECTVEGGDLATIATEEEKRFLTDELNLGGFAWIGLNHKDQAYYWQDRANSSYKN
WKAGWSYSEVQKQNLNCVATDQDGFWYNFHCTNKYYFVCKKKLEPQAAPREDLILKAWPYEP
EIPPMIFLYPNITVPVINTKNLEPGVFPDENGKVPEEEEEETTADDLFYEQTVEHLKEKGLKPSEEIS
NDVDKEETDYTQQLNKIALKTQSRDDFRNETESDKLKFNSTEAVKQAKDLEEQKSVNLDQNFN
QYNSTDGVKQQSQPVVAAPSDGPSGNSGDNNTLPRIVNGKPVESPSVNPNNVGKPNTPVASPES
PENTDFAQPGDTTFTTPTPTVQSGDPGNAEKSNPQKATNDQQAGGQQPNQESGSNANQSQVQ
QQSQQQQPQQQQQQAQNNNNNQNGQQNASNQQNNNNNNNNQNNNNNQQQNNQQQ
NNQNNQQQNNQNNQQQNNQNNQQQNGNNNGGNNNDLPNGLPPVMGGPAPQKIPEPPAP
KEGTKDNDSEKSVGGQADVMPDDAPSNDNDGGDETTTGNEENNDESNNNNNNNNNNMNNV
NNNNDNNQSRTLDKPLPNQGKNSSETVDKATNVGGTNKQENNDFMKLQKAKPGIRTHKIGHK
KSAGLKKTHMKNVVSPLKKVKTEKKINKQVKTVKKIKAVKVKHHKKALKKEVVIHKSEDDEEE
GSGSGDEGDQEDVDDILESITNGLW*
>TRINITY_DN4595_c0_g1_i4.p1 type:5prime_partial len:1639 gc:universal TRINITY_DN4595_c0_g1_i4:5857-941(-)
QCGVNTGTQVVYRRCNAALCKYQGNDYTRNQEVNIRTQACTVACDYTPWQNTQSCTKTCDGG
VLVQTRTERAFNKIETRNGNTPCNTQACAQELGPKQLRTCNVGQCGVNTGAQVVYRRCNAAL
CKYQGNDYTRNQEVNIRTQACTVACDYTPWQDTQSCTKTCGGGVLVQSRTERAFNKVETRNGN
TPCNTQVCSSELGPEQLRTCNVGQCGVNTGTQVVYRVCNAALCRYQGSDYTRNQEVNIRTQAC
TVACDYTPWQDTQSCTKSCDGGVLVQTRIERAFNRVETRDGTTPCNTRSCASDFSAERLRSCLNV
NQCGVNTGTQTVYIVCNGNFCRYQGREYNINEEVNVRQQNCTIACDYTAWEDTTICSKICDGGV
LEQRRLERAFNRLERRNGTTPCNTRACDSGFSAVLSRNCVNVNQCGVSNGTQLLYRVCNGEPCIL
QERSYKINDVVVVGSRACSVPCNYTEWRDTTSCSKTCGGGFFFETRTEQAFQKTESRLGKTLCNE
NSCVKDNFGNWTEWTACSKTCGMGMRYRSRECLKGFECIGLIEETETCKLGCRTETCPMGRPYP
GDCNFEDDCGKSWNQVNSIQRNAFFIVTSKDLAHQGLLPNMETTFNTKDCGRTMKLNSSVAGD
VNYLWTKRMMITDINTDVDDDVAAIDNYFKEENEQCVEFNFAVMANSLDKIGDMQVYLYYLEY
PNASLIYSKKGNTGLKWELVKRSIRVDTNKAFVILFQVNQGQSNVQYGDIGIDGFKVNPGNCQLK
GWSHWGEWKCSATCGFGTQTRMRHCLDKVNRTACVGMDRENKPCPMMKECPMNRDGNWG
QWTEFSKCSKTCDTGTYFRTRKCDSPSAKYNGSLCRVSQTKFNLPVVNDFEIDFKPCSVQQCNNT
FSQWTTWSSCSGTCGPGQRTRTRTCESGKRCSGIFEEIETCYMGCRLEKCPKFTAEIDCNFEDRNKS
DCQWKYYTVSGTSSWSFKTQPKNRLLSGPSGDSTIGCGYFLYYPSQTLSSAYAWGPNIYPTMDSYC
VKFKYSMVTSVNSSMGNLTVIKHFLNRPRKADTPLFFHSGVSQKQWNPMAIDITTDIQRSPFVIMF
KGQSDPNNAGLSDIAIDDYMLMAGKCNKSYSEWSEWSTCSRTCGVGQRTRTRVCLDDDIKVCK
MGDVATEGEACVKPLCAVPVDGNWGQWSAYSQCNARCNGTQKRERFCNNPAPSNGGRMCLL
GDTIFQLRNNYHAQIRPCSIDCGDNDTFSSYKRINDTCHFDCITGRVLQERECEQDTCFDGNTRFS
KGEKQYTQRACSIKEYCETSEYKCREFRSVCSTNTPSSCLDPQNRAVKSCECQVERGCSSMTNPGQ
TVKHKEFDVFYRPCRYVGVCFTDYETDAFSPYEKVNDTCHLSCSNGKVFEERECRQDNCIDGSNT
LTRGAKQYRETQCTTKEYCETSQYSCREISGPCSSNPPSSCQDPANKSLKVCTCQVTTGCASMLTA
GQAVSYLAEETFSRPCKYTGACNANGNTDKTFSPYERVNDTCHISCADGKVKQMRECKKDACV
DGGQNFPRGFKQFKDSSCTPNEFCETAQYSCRELSSACTSNPPSSCLVSTNKAVKMCDCNDPNGC
DSMITPGKKVNQGEKEFLQRPCRYTGVCFTDAETGT*
>TRINITY_DN477_c0_g1_i7.p1 type:3prime_partial len:1130 gc:universal TRINITY_DN477_c0_g1_i7:3387-1(-)
MMGSIKVFVGLVLLSFTAIEIAQAQSGTVLSNKQFAFDFVVDGSTSIQDEQWNLLTDFVSRLAVKM
STLNNAKLSALQFSHTLNRILQCNQYANVEEARNTIKNMVQENGATGTLAAMLTSADNLMNVC
SAGDPNRQNVMIVIVDGNQNLEQKSHSEIVQDIKVNVKDKNILVLTLGVGPQVNRNEFGDYASD
SGKGFFVDDFTLLTDELVQRMYDELIRTYGDGPDYLTAWVNGPCSATCNPFPMANRPTLRQTRQ
CQEPNGGNCGGASLEQIINCNENIACAGETYLTAWVDSSACSLTCNPFPVSNQPTKRQTRECRQP

SGSCNGASLTQTINCNENVQCPGETYLTAWIDSSCSATCDTGSNNPVFTQTRQCRQTVGNCDGA
PLQQTRSCNVGVSCTAPTFLTAWSNATPCSATCNPFPMAVRPTLTQVRQCNEPNGGNCGGASLQ
QTVQCNLDVACAGETYLTNWVDLTACSATCDDASNRPVKQQERQCQQPGGNCDNLPLQQTVN
CNIGVSCTVSSSACSSKEISFTFVIDTSASVDNIGFFVLKDFMKKYMSQSTLGTDPRTSVAIVSYGAG
DAKVEVECDTITTQQSFNTTVDNLSQLFGIADDNSFNQALSTAFDASGRCARSSTSLNIIIMLSDGD
PPALENILTRDRIQSSSNYYFMFVAIQASDGAQSTFASLANQGRGLRNASSSFTDLYNFNTDLYYLE
TLSKACDVGGQWTEWSTSACSTTCGPGVVTRTRECRNRITGKLQQDCGPGPAQETQACNLQDC
QGTLTQWTSWSCPATCNINNDVNNPPLALRTRQCLGSTGQGNCNNADKTENEPCVRNVPCPG
AWTTWSVFSACSETCRSQSAFPTRRRTRQCIGGTLNFQCPPQIVNGISYREIWQEDCINVPNCPAK
FTQWTTWSCPVTCNVNNDRNNPPTATRTRTCDDSGTINGCANANILENQPCVQDVQCPGAWS
TWSAYGACSETCQSGNRSPTYTRTRTCLGGTLGFQCPPFITQIGGFQIFGTQTSNCNSEVKCPSKLT
QWTTWSCPVTCNNRNDVNNPPQARRTRRCDTSTGSGICESPIVEEFENCVANVPCPGRLTEFTYD
NCAHTCNVNNDVNNPPQRVGTRRCLDGTLG
>TRINITY_DN3991_c0_g1_i1.p1 type:3prime_partial len:219 gc:universal TRINITY_DN3991_c0_g1_i1:84-737(+)
MTSWILNFLVLSIIALPCSSISNCKHKLEPVGCFKDRGSARAMPEQILNERDTTSKVYSKQLMSWK
NFHEWLPGFICRCAEVAARKGYSVIGIQFWGECWSGPNAESTYNRHGQVDSCINQNLKKNRAK
LSQCTAASGKHFTNYVYRLAPACHTQYKPVGCFQDNLQKPRPMPYYVSTDRETRLAIFSGKLLN
WKNWNTHMPDMICRCAAVAKSKGHQ
>TRINITY_DN1386_c0_g1_i1.p1 type:complete len:596 gc:universal TRINITY_DN1386_c0_g1_i1:2419-632(-)
MEYIEVKSFIVALVFVFSILGTAQSDLVINNVERTLDLSTQTAKYTTKIKFHNPGKKVEKSFLLALEK
DQEKHLSYLEILDKQDDNSLEYAAENVEGQSGRFFKVSFSKPLKENDDVTLHIETVFTHVLTPFPA
KIGQTEKQLVLYKGNTVFYSPYEIKEQSTTVKLASSAIESYSRLKPSRSSENTITYGPYKNSKPYRLHE
MKIHFENNSPFLVVNEMTRWIEISHWGNIAVEETYHMTHEGALLKGHFSRYDYQRSPTHSVIKSF
KTVLPAAARDVYYRDEIGNISTSNMLVNDDSVEIELRPRFPLFGGWQTRYMLGYNVPAYQYLYNK
GDNYILKMRLIDHVFDEFVIDDLNVKIILPEGSTNIEVVKPYELEEGKRDIHKTYLDTSGRTVIVLSK
KNVVENHIQDIQVHYTFKKLQLLQEPVICVAVFYILFLSVIILVRLDFSITKDAAKESRMKVASLLEE
LLSSCGRRGSLYYSYDSAIDKLKQNRNQNDFAVSLKKLNQDYNTLTQSINELCSLLIKEDPEIGDKV
NDLQKKENERKVIVDQLVTLATRVVTNKIGRQQYIESEKNSMAKREKLGEDIDNVLATL*
>TRINITY_DN941_c0_g1_i9.p1 type:complete len:3186 gc:universal TRINITY_DN941_c0_g1_i9:86-9643(+)
MADPAKIVDPIDDPTASDAEWKKIQQNTFTRWCNEHLKSSNIYIYNLETDLCDGLKLIALLQVLS
HKKIVRYNKKPTFRPQKIENIAIALKFIEKEQIKLVNIDASDILKGNLKLILGLIWTLILKYQISMSMV
ADDDDDPDQKTTPKQALMGWVKSKLPPELKCGNFNKDWNDGIALASLVDSTAPGLFPEWEDL
DPANKLDNAKEAMKRAEEWLGVPQVLTPEEITNPNVDELSVMTYVSYFPESKLKPGAPLKPKMN
NASKCSASGPGLQEHGVVVNKPAEFTVFTSGSSRGKLNVQVFGPGRKAIDCMVQDNHDNTYSC
QYTAPKTGVYDVHIRVGGHHIPKSPFRVNVASDLDTSKVTARGPGLEPHGLIVGRPASFEVIATDA
GSGELVIDVIDPKGNKKTELKVEARPDNVYFCTYMPVQVGRYVIVIKYGGKEVAKSPYHVSVTPA
ADRVKIYGPGLEPGLKTGKPASFTIDCSEAGEGGLDVKIVGPGKGDIKYEVKDNGDGTYTCTYKP
TRPGQYEITVKFDGGVVPKCPVKVSVGSTADPSKITAWGPGLERGVAGRPVEFYVNCKGAPVDNL
TVGVEGPGEAKVDMKTNSDGTVTVTYYPTKPGKYIIHVMYDEMDIKESPYSATISPRGDVTKVYA
EGPGLEAGNMAGKPAEFTVFTKGAGVKGNVQVTCVDENGKPCEVTVKDNGDGTFKVTYYPKTI
GKYVITISFMDEQIPKSPINVTIIPFSDPTKVKATGPGLQGGKTNKLAKFKIDASKAGEGGIGLTIEG
PNESEIKCDDNGDGTCNVEYMPTESGDYKINVLFADQHIPGSPFTAVIGDDFDASKVKADGPGLK
PGNKAGKPCDIAIDARKAGKAPLVVEVTDEVGNPVKVDLNEEEPGLFASSYHPEKEGPHTINITY
GGKHIPRSPFKVTVKPSSDASKVKVFGPGIKPTGVKSLEPTYFEVDAGEAGEGNVDIKIEPTAKGM
PPVKDIDVKPGKTPNSYIVTYIPPKDGEYNIVVTFAGVNVPKSPFKISVTPGSDASKCKASGPGLEE
PIVGKENVFQVETPPEAGLATLTCTIDTPSGRKLKADCKKNKDGNGYDVTWTPKEVGRHKVTVK
YGDKEVPESPFYVDVLGKPDPTKVKVYGPGLENAFVEKPAEFTIETRDAGPGNLGLTIEGPEEAKI
DCKDNGDGTCSVTYIPTKEGPHDINVLFDDQHVPGSPFKVAATEPIDASKVKCKGKGIGKSTPAS
YPAEFVVDATKAGKAPLEVDVVGPGDKKYPCDIVDNNDGTFNCAYVPEKKGRYEVTPKYGGTP

VPGAPFKVSGEPTGDASKVKCSGPGLDRPHIGHAAPFEVDTTKAGDGKLNVNAKGPDGKELPV
KMVEEEEGIFACEFVPEELGPHELEVLFGGQPVKGSTFKADATPAPELDKVRTPALEEILEASPCVD
QQFEAPVDCSEVPDNKGAKLSGKITTPAGKREKVDVTDNFDGTFALNYTPTEVGKYVLDVGYDG
IPIPGSPFEFEVTEGGPDKVKVFGKGIKKGVVAVPSRFTVDTKNAGPGDLSVAVEGPSKVDIECVDN
GDGTCEVTYVPVEPGEYDVIVNFDNQQVPGSPFKPQIVQDEPAIEVGRQSDLCLTPEGISAKDIPSL
KGVLQAPSGKEKPVDITEGPNDSFIASFVPDEPGKNLLHVKKRNKPIDGSPVPVMVREKPVIGEES
GIPMDLADCDLPKDLPKLNAVLTRPNGKEGPCGVEPGPDDTVAISFVPEEPGKHIIKVYKDGKQIE
DSPFVVMVEGSPDQYPKVGHPCDKDFYQPEPCRRGDISKMKGTLRRPSGKEEEVDVKLGPDGTIA
VSFVPTEAGEHLVSVKKDGRHVPNSPFSIMVIDEPTVPTVGSECSANFDIPELDLPKDLQYLKGTLT
RPNGVEEPLELSVGADNSLAINFVPKEPGKHLVSIKKRGKHVKDSPFEIMVEEGKPTDAPTGGPVV
GHDCGVNLDIPELSLPRDLKNLDAELTRPTGKKEPLKCDMGPEETLAVNFTPTEPGKHLIDVKKR
GRPVKGSPFEVMVGSTPVSEDQIPVSEEIMAQSAPDVESGLPGGLDSLDSGKDKPVTVGSPCDVNL
DIDGVDLPKDLDKLKAELTRPSGKKEPVKCEMADNGGLSLNFVPEETGKHLLEVKKNNRPVKGS
PFEIMVEESASPESKPTAGQKCDLDLDIPDIDLPRDLKDLKAQLTRPNGKQEPIECTCTPENTLGLE
FTPDEPGKHLIDVTKRGRPVKGSPFEVMVESPESKPTVGNNCGLDFDIPDIDLPKDLKVLKAELTR
PTGKKEPIECTATPENTLGLEFTPSEPGKHVIDVTKRGKPVQGSPFEVVVGEEEPASKVPTVGSECD
VNLEIPGLNLPRDFKYLDGTLKRPNKTSDEPLELTMNENGTIGVNFVPEAPGRHEISIKKHGKHVS
GSPFDVMVESAPSDQPEVGSPTTLGLDMVIPKEDLRLLKGTLVRPNGQEEPIEITLDDNGHPNVTF
TPQEVGNHVINIKKRGQHVPGSPINVMVGPAKKPTTCDVGFEIPGIKLPDDLKYLKGSVVSPSGTE
EPVELQAGQNNTIVVSFLPKEAGIHYIHIKKNGKPVGDSPYQVNVKPEDIAAGQPDASKVKCTGR
GLRHGLPGVKADFLVDTKDAGYGGLGLSVEGPSKVEINCVDNEDGTCAVDFTPAEAGQYLINVT
FADTHVPGSPFKCKVGGPGVEDDIVPMTDDGVECASSDMLAFPSSKARSPQDFVIKFSGSGDLKA
TVTRPSGVEDEAEVVETGPDTYTVRFVPRETGEHLVNVKSRRRHIPGSPFKVLVEAPAGGAASCKA
HGPGLEGGVAGQPCRFTVITRDAGPGGLAVAVEGPAKAEIQCHDNGDGSCDITWYPVESGEYML
HIRFADEAIPGSPFKAFVLPADEGKAMPMESFKDQVLRVGQQASFAVQMKGKKGKITASVQSPSG
VQIDCSAVELEEGNYAVRFVPRELGDHLVSVFLDGHHIPGSPFTVKVGGLDSDPSKVTAYGPGLKG
GVASQPAEFTVNALNAGSGALALSIDGPAKVKMHCVEQDDGTYKVIYNPTVPGVYEISIRFGGQH
IPGSAYKVRIVGSGDDAINIFGDASKCNASGPGLNQATIGQPATFKVDASNAGRGSIMVGVEGPVI
PAKEIIVKHTGNNVYSINYILEQSGDHILRVLWADNHIPGSPFHVNV*
>TRINITY_DN3986_c0_g1_i1.p1 type:complete len:446 gc:universal TRINITY_DN3986_c0_g1_i1:252-1589(+)
MREIVHLQAGQCGNQIGAKFWEVISDEHGIDPTGTYHGDSDLQLERINVYYNEATGGKYVPRAV
LVDLEPGTMDSVRSGPFGQIFRPDNFVFGQSGAGNNWAKGHYTEGAELVDSVLDVVRKEAESCD
CLQGFQLTHSLGGGTGSGMGTLLISKIREEYPDRIMNTFSVVPSPKVSDTVVEPYNATLSVHQLVE
NTDETFCIDNEALYDICFRTLKLTTPTYGDLNHLVSATMSGVTTCLRFPGQLNADLRKLAVNMVP
FPRLHFFMPGFAPLTSRGSQQYRALTVPELTQQMFDAKNMMAACDPRHGRYLTVAAMFRGRMS
MKEVDEQMLNVQNKNSSYFVEWIPNNVKTAVCDIPPRGLKMSATFIGNSTAIQELFKRISEQFTA
MFRRKAFLHWYTGEGMDEMEFTEAESNMNDLVSEYQQYQDATAEDEGEFDEEEEEEDA*
>TRINITY_DN64_c0_g1_i1.p1 type:complete len:468 gc:universal TRINITY_DN64_c0_g1_i1:1555-152(-)
MPPKEAEKIHINIVVIGHVDSGKSTSTGHLIYKCGGIDKRAIEKFEKEAQEMGKGSFKYAWVLDKL
KAERERGITIDIALWKFKTGKYYVTIIDAPGHRDFIKNMITGTSQADCAVLIVASSTGEFEAGISKNG
QTREHALLAYTLGVKQLIVGVNKIDNTEPPYSEKRFDEIVKEVKTYVKKVGYDPKTVAFVPISGWH
GDNMLEASKNMGWYKGWTTERKETKTTTGKTLLEALDNILPPKRPTDRPLRLPLQDVYKIGGIG
TVPVGRVETGIIKPGMVVTFAPSNITTEVKSVEMHHEALLEAVPGDNVGFNIKNVSVKDIKRGNV
ASDSKNDPAKEAKSFYAQVIILNHPGEIHSGYQPVLDCHTAHVACKFSEIKQKIDRRSGKVLEEMP
KMVKSGDAAMIDLTPSKPMCVEAFSEYAPLGRFAVRDMRQTVAVGVIKSVTKSDGSGGKMTKSA
QKATGKKK*
>TRINITY_DN1172_c0_g2_i2.p1 type:internal len:264 gc:universal TRINITY_DN1172_c0_g2_i2:790-2(-)
GDYGFKVLTSSDPAEKEKKLAAELANGRLAMVAIIGMFFQDGLTGSAWGDWALYTASPLRFFGG
DSGPSYKAFDPATELGVQEPLGFWDPLGFTSDNDEAAFKRRRSVELKHGRVCMAASIGYIVPNYY

TFPGFLSPSAGVKFADVPNGLAALGKVPALGWVQIIFFAGIVEGAFGFDDYKSGAPGDYGWKVLT
SDDPAEKDKKLAAELANGRLAMVATIGMFFQDGLTGSAWGDWALYTASPLRAEPVSTAGVGAA
TKGGTE
>TRINITY_DN3190_c1_g1_i1.p1 type:complete len:315 gc:universal TRINITY_DN3190_c1_g1_i1:68-1012(+)
MATVVDYPDFDAQADAETLRKAMKGLGTDEAAITFILGSRSNKQRQEIKTQFKQMFGKDLIDDL
KSELGGHYEDVVVALMKKPDEYDASELHNAMSGLGTHENVLIEILCSRSNAEIKAIKETYKKKYK
ADLEKDLQGETSGSFKRLLTSLVQGARLEDEPADHASAQADAHALFKAGESQWGTDESRFNAIL
ASKSFAQLEMIFHEYQLISNKTLEKAIKSEFSGDIQNGLLAIGKCAQSRPAFFAERLHKSMRGLGTT
DSTLIRVMVSRCEVDMVQIKQAFERNYGKTLNSYIADDCSGDYKKILLKLCGPE*
>TRINITY_DN36_c0_g1_i16.p1 type:complete len:224 gc:universal TRINITY_DN36_c0_g1_i16:829-158(-)
MMIKTTIVAVVLAACVLQSFAGRCYVGYSAKGLPLAIGVRDVECGKGYDYCTTIKYRLGGIEVTQ
GNCARKYGQAMCQFMRNPHKAMTDCEETICQGDSCNKDVYDAEQAERKRVGPYKNTLKCTT
GLITKFPAIPFYTADVPNAIDECFPGTTECVTVTYDYLFKFETQIATKDNVFKLPCVQKMCNRHNI
DNCNKWCDILSIHQSMENCVAKCCKGDNCNA*
>TRINITY_DN1369_c0_g1_i1.p1 type:3prime_partial len:1577 gc:universal TRINITY_DN1369_c0_g1_i1:93-4820(+)
MRIQLTIFVTFSIILVSGQNSPPGICSTWDGSHYQTFDSYSFNYHGTCSYALALDCRPGSDDFAVHV
ENDEKCDNHGGTDCTRAVSLYVNDIEYKLGSNNTVLVDGKSNIVLPYFNEGVRVSKHAEYYFIDA
LEGKVVVKYDGRSSIYVQLDSSYQAGVCGLCGNFNGNKKDDLKLPGDKGLTKSLKEFGKSWEKP
RIGQVCKEVTEEHFDQCTNKSKMLVYYAEKECEILRTSSEFKKCHKIVDPTPFYEKCREDLCLCGS
GDKYCKCDVLEQYTRKCAHYGTVLEWRQQNLCYKNCTGNKVFSECGPACSSVCSAIGSKSVCK
DACIDGCHCPPHTSLSGDKCVPDDECPCEHNGDMYDHGDVINMKCKSCKCNGGKWKCNPLP
CDGEASVYGNGHFVSYDGKTYNYPGKCLYLYTYLSDRSFAVYLDRTSCDSLACHMNVMILFRGD
RIKLSASNSTLALEVNRNVKSFPYISSTGYRMYRFSTVFVRFEATNGIKVDWDTYSRLHVLMPHSL
KGKVFGLSGNFDFKTKNDFLTPRKDLAHSAIEFGNSWMMYSSRLNCSALAPDYRMTSCEANHQ
VAKYAESKCSILMSSLFRECHKVVDPFIYYENCKDDTCECNGDSKCYCSVIAAYSKACSSAGKPTE
GWRKYTPCEEKCSNGMVFTECGNSCLKTCDDQTDPNPFCEKECVEGCLCPVGQVLDKITEQCV
DIKHCGCTYGNRTISNGGTVAKDCNTCKCTKGVMVCTKKECRDKKAICSDGKEWSSCMSPCPK
TCQTKDDICPETGLDCIEGCQCKKGYIESANGTCVELSKCPCYHNGKEYQETQTISKDCNTCKCT
AGEWSCTEKLCDATCSVFGDPHYKTFDGKIFDFQGKCGHVMLTDKCANNDNATENIRISVKSST
CGSQGVTCTREITLNYHDTVVHMKRGARKIDIVSNKRQDHEKDQVKVYRHAGIYVHVLTPMGV
SILWDNATRLYITAPPSLSGKICGLCGNFDGSETNDFVTYQGDTAGNAVSFGDSWRTENNCPLTKE
IEDTCHNRKHRLDPSKRECAVIKSDVFKKCHTLVDPVPYYDRCVFDACGCDSIGDCDCLCAAIGA
YAKECIDEGVYINWRKGHNICEKEMCCPTNMTFVPKGTGCPKTCKYPNGLHHCPTKYVDGCQC
PEGLVQNNDANGDVTCVRPEECKVCKLDGKIYNEGEVVLYKCKECVCRDGKMTCSGNYCPTTL
PPTTTTESTTTPSTTPETTIPSTTTTESTTTPTTEPPTTTTTASTTTASTTTESTTTEATTTTKPTTTTTEST
TTESTTTQSTTTPSTTPSTTMKPIDTCVYSNGTHNVTLERGDVIQVNECTKKTCVKDEVTGLHEIKV
TTTQCENSTCDTTCEASKSNRAILVKTPGECCPQCKKPEDLCDNPNEVPCNSTYCIPKSWFCDGN
KDCSNGRDEECCPNYCKVEGEIKKPGEVWTNKEKCLTCKCEGYYTKCRRMECPELDCPEERQVK
SEGECCPHCKPQTEPPTTTTEMTTTTTESTTTPATTPSTTSKETIIIITEEPKSTTASPQEKCDCKVLQPV
GVSDGRIPDNQMTANSV
>TRINITY_DN2636_c0_g1_i1.p1 type:complete len:314 gc:universal TRINITY_DN2636_c0_g1_i1:986-45(-)
MSAIAVVVIVIAAVLLHFSVHKIEEGHVAVYYRGGALLSSVSGPGYHLMAPMLTKYKSVQTTLQT
DEVKNVPCGTSGGVVIYFDRIEVVNLLSVDKVHDIVKNFTADYDKALIFNKIHHELNQFCSVKSL
QEVYIDLFDQIDENLKRALQQDLNEMAPGLKILAVRVTKPKIPEQIRKNYELMEAEKTKLLIRIQH
QKVVEKEAETERKLAVIQAEKQSQVAGIQYEQKIMEKESMKRMSEIEDLTHLAKRKAMADAEFY
THQKQAESNKMKLTPEYLELMRIEAISKNNKVYYGSNIPNIFLNEGGLSQKTK*
>TRINITY_DN3545_c0_g1_i2.p1 type:5prime_partial len:236 gc:universal TRINITY_DN3545_c0_g1_i2:3-710(+)

SGPGRSSCRPILSVIKISKINNIDSGKVEGGYTMWSVYGSCSKSCGGGAQKRVRSCTNPAPANGGK
HCDGVSFETRECNINQCPVDGGYTMWSVYGSCSKSCGGGAQKRVRSCTNPAPANGGKPCDGVS
FETRECNINQCPVCPEIVGTYDISSRHHYGGAKYGTVVFKKLSNSKTVEAYYTINDGRRGVLTCKIE
SAQPDQCSGHGYYGKYFTYYKNRCQIIWSNDVIYTKQNC*
>TRINITY_DN817_c0_g1_i10.p1 type:internal len:186 gc:universal TRINITY_DN817_c0_g1_i10:556-2(-)
VGLSSKELTLEISVRKVECPGFATKCATTKFVLDGTTSYRGYCASDYGPYLCTYMESSFKTMTDCKS
TLCSGHYCNTAVERASKRSTINGKGIKCYTGMQMFLPQLPIGIATGTVPTEVDQCEDQTSQCMSVI
FTHLVTDPIRKIKVKIPTQMRLCQPGYSTCENHCRRLAQHITIENCEASCCK
>TRINITY_DN165469_c0_g1_i1.p1 type:complete len:2668 gc:universal TRINITY_DN165469_c0_g1_i1:41-8044(+)
MVEEDLNRSKELDIPKTLAYCFHNVTTVSLNRKQDIFRLLTQCIESQGCQDGHLKAVCKIISLTVM
RYRDSKSRQLVLKFVQKFLDIHELQAVKCLLTEIHILAKQLIKCGASLFSASCGLALLSWCSSISKKK
VQEASILKILYEVEAMCYSVICTCDSFRVKRLLKRAYLYLQDGKDAEQLENCLGVIISNSNCQLESIC
LVGFIFQYLTTKKEIQRVDEVKKRSLIDIIIKNYIQTKVKADYHYLKHAVPFLKYITHDDFKEILPAIE
KGLLRNPETVLQGVGYLLEGLSIDSSNYIEELSKCIAVHLISKNASLQADAVFAVSVIAKQCSDFSAI
NHLLKQLIKVLNESEGKLVFPEQKNAVLQAIKSVDCTSVTGSDHKENLATTLHDGLFPFILLEGHE
GVLTQALNVMKTWCKYFKNNLPKTFVDNIKKGLSLKVSTTVIQAHYLLVVSEALKGNTVSQASG
LLETLLQVIEKVKGQSHLINNVFLCISSSMVLLKMFLSDDVQVNAAKEDSFWNVILDPVKKNLVSE
KFLLSHQKEYFYHLSQCMNTMFLNYSSRFSQKLLEPWYRAFNVLMVYGDWKTKKSVKILLSKMF
DSGNYDTDEFQSRLFADLDSLLDQEMSKSSKIEDTEDILVVNHVVDAYLFLCIWNKKDINEDEVLA
VLRKCLIYVHHDTIYQHDSSLFSKIGSVLKKDVAKEFVEKYKDDLLKYFINLESLSKADNHSLKTLL
SLYPEALLDTLVDEIIKYFTKPDLINVTSQEYEIMKTPDGVAYDQSVFESAKAANQKSGNLKRESKA
YSYKDQKAEMELRKELEKKKGKSDKIELNKKQKEQLEVILTQEKAIRDKLKSLEQGVMKHASILSL
LLDMDSDLLCYHYLDLMNLLLCLCQSHIAAPHVIPIYTKLSEKLFEGDLKHLGQLVANVILRLKKP
VCELPMGWSVENLPEQAKRALNTLHTFVNERNVYLMMTYTRKKTFSVLVFSYCLPLLKSILKDQ
GALVKGSEEFRMKALQLIHIHAKLRSPMDMGSEKNDPELLPRKEMLQILSDVIVSCEGHNKEAKV
QQQATLAFDEVCKCASGRLGCAVATEEEINILLSNILSQCSILRRVSLKGLLEFVDILPTENVVIMTQ
LTRSVLIGRFDIVDENATLAKRLWDEADICIEPSMIQHLVENVTHPLDDVRLAASLALEFSLNERKE
VDIIPQLLDIYFEKLKIPPPIIDNLGRVVSQQFVDSWEARQGVGLALEKIAETVGEDKLGDLFSFFVP
KALGDRNESVRKQMLNASLQYVNLYGQKNMDMLLGVFQDFLDSAPDSKELDTIRQSVIILTGSIA
KHFDKTDPQIKSIFSKLMAALSTPSQSVQQAVANCLPPLCGAMKSEAPELFKNLLEQLLESESYGE
RRGAAFGLGGLVKGLGILSLKQQNVIPTLNDAIQNKKIWKHREGALFAFETLCTMLGRLFEPYVV
HLLPHLLLCFGDGNQYVREAADDTAKAVMKNLSNHGVKLVLPSLLKALEEDAWRTKTGSAELL
GAMSFCAPKQLSSCLPSIVPRLTEILADSHLKVQKAGQQALRQIGGVVRNPEIQEICPIILDALSDP
NKHTVACLQALLSTSFVHFIDAPSLALIMPTLEKALGQRPTETKKMAAQILGNMYALTDPKDLQP
YLQAVVPGLKKSLLDPSPEVRAVSARALGAIVKGMGEVDDLMPWLLETLTSEASSVDRSGAAQG
LSEVLFALGKDKLDKLMPDVIATSMKTELSPNIRDGYLMLYVYLPATFEDQFLDYIGLIIPAVLKGL
ADESEFVRDTSLKAGQRMINMYADSAIELLLPQLEAGLFDDHWRIRLSSVQLLGDLLFKLSGVTG
KQTTVSEEDDNFGTAYSSQVILETLGFDRRNRLFAGLYMGRSDVALGVRQAALHVWKVIVQNTA
KTLREILPVLFELLLGCLASQSYDKRQVAARALGDLVRKLGERILPEIIPILEAGLDHEDGDKRQGV
CIGLSEIMDSTSREMISMFEDSLINTIRKALVDELPNVREAAASSFENLHNTIGHRALEGVLPTVFQ
KLDDPQLSNYALDGLKQVMTVKSKSVLPFLVPKLTTPPVNTKALAILSSVAGEALIKHIEKILPAMI
QAIQVSQGTGLDGDSEFESTRTLILSVYDEVGVRTIVDELTTSSKSQMPGIRKVAASLLMVFCKETR
GDHVPYIQSLFRTIIQLMNDVDQDVVASAWGALDALVKRLDPSEQLQHLSSLRQAVRFIKDDIRN
EQLPGFCILKKGITPVLSIFREGILNGPQEIKEQAANGLGEIIKLTSPEALRPSVVHITGPLIRILGDRF
NSSVKVAILDTLGLLLQKVGALLKPFLPQLQTTFIKSLNDSALGVRQKAAWTLGLLTVLHTRVDSL
FTELKNGIKNNEDSAICETILGALRSITLKAGSKMSEGIRNTLYETLVELISSNEDGIRLKAAAALGA
MCQILPQDQIEQLLNDNLLDINQMLDWSICHGRAVALSYALYHAPSQVFDAVDEDDIVEAVVKY
STYDRVPICTYGVHSLGHLLMFSKDNADVAPQIVVSSIKKTLLNSSVDIRLASLSTIKHVSRNTPEV
MTFERIRYLIPEFISLLRDRNTSVRSSAEITLAFTLQMDKNDILFDECVNTISEASHFLEEFKKNTFSK
LNDSLATDKDDYFFAT*

>TRINITY_DN1616_c0_g1_i2.p1 type:3prime_partial len:3398 gc:universal TRINITY_DN1616_c0_g1_i2:218-10408(+)
MIIFLAFLAVAVGQEVLLPPYFDIAKGKNITATATCGEDYVAPKGSPGELFCTLADATGKFGIKGLK
CSHCVPGDYGDGDGKNARDHHIRNANDGSNRWWQSPPLSRGLQYRKINITMALGQSFHVAYVI
IKAANSPRPGVWALERSNDNGRTYTPWYYFAPTSSECKQYFGVDSYLPIVKDDTVICSTEYSKIPPF
KNGEIHISLINGRPGASNFSQSKTLQEWTTATNVRLRLLDIKTLLTDLLPLSLEDPTVTRRYYYSIKDI
TIGGRCMCNGFADECTPHTNIVGKYECNCQYNTEGPRCSRCKKGYVQKEWRQRTSTDKFECEKC
NCHGHSDECEYNEEINKRKLSLDVNGNYQGGGVCKNCQHNTLGINCEKCKPFFYQPANVPIDS
PNVCQPCNCDTQFSTGLCAEETGQCECKSNYVGRYCDQCNKGFHMFPVCKPCECDVNGSEDRE
CIPDQINGCKCKKGYAGRYCDECLPGYFGFPDCQPCACLTRYGAVNDNCERDNGQCSCKVGFK
GRRCEECAAGHYQFPFCKECGCDPEKTLDTVCDSGTGVCQCKSNFAGPKCESCSPGFYAHPSCFA
CGCSTAGSTSSVCDQNSGLCSCRPNYSGFKCDRCAEGYYGFPNCILCDCNPEGSRRVGICDRQDG
QCQCKPTYSGQKCDKCLPGFYGFPDCKRCNCNPAGTKPGPGGRPGDCSTSNNGQCVCKDNVQ
GLECNECKPGFFNLTESNRKGCSTCGCNGAGTNNGVKSCSVEGTCKCKPLVKQEDKCTECRDGY
YGLKRTNVFGCLGCNCEVGGSSSSVCSKDNGQCPCKDNIVGKRCESVAANSFYPDLCHIKAEME
EGKTTDGDVVRYDYQKEIFPDFSWKGFVAVNDQQPSVVVSINVPKTSEYQVKFRYALNQIQPVTA
TVTAFKGDSKIVGSINFASTQKGSGFVASLATVQDNLVQEMYFPLTEGQWNIKLEAPHSNLVVDY
AVLFPKEYAQGGALKKKVQNACDVMTDEKFCNLYRYIDVADKKDIIVLEAENPVKMRQQKPPVL
FNDTAVLSKLDFQAMVVLSPSNSRQLNHLFNVAKPGKYVVMLQYYHDSDTAKYLDVYVRSQIAV
QNGRALLQSCRYTFGCRQAVLNRGDNGVFVFNIKSTKNHLLTLLSRYAVVAVDSVMLVPYESWH
LDYSTPTLYCVTKDSKCTNMNYDVPAESRLVQANPLEQDLSEKPRYLIDRSVEVRYVKPGVNIINIR
DVKAGNYLLVAHYYQPNFPSFESRAAVYSGRTSGLRARFDFKFCPNLNGCRSPIRRPDSNSNLYVF
EEGEARIRFDIPNGLDAWIEYILMIPEGTNVSSLYTPRVLDYSDDFKKECSIDLNVCTSSNLSSFCKDS
IFTITTDFNNGSFPCKCNMDGSKSSSCQEFGGKCPCKDNVIGRECSRCKVGYYGFPNCKPCNCSN
CDEETGVCTCPRGVIGENCDQCAPGTFGFDPIIGCQKCNCNEIGTGSANAECNVTSGQCPCKEN
YSSRKCSACKPGYFNYPACTKCDCLDAGISAEKCDVLTGKCICQNNVIGLKCNECADGYFKTNDI
KGKQLCAKCWCNGRSTKSCTEAELFRATPITASKGWTVSVDTLNVIENGNGRFTISDVNTLPEPV
YWVAPNQFLGNRGLSYGSHFKYNFQYSVKQPSQNTNGDVVTDAVIKGDGYTIKSMRSVIEKEND
GYSASILMKETEWMLEDGSTIDRELFLAILSDIDTLQILATHGKNTISSTIYDISLETAGENGTDRVYG
IEKCFCPKEYADLSCSKCSKGYTRQKDGSCGKCQCLGKSNICDPQNGKCINCSGNTMGDRCEKC
KPGFYGDKCKACECPFSNPENSFANTCAINVNTSSLVCQCNAGYKGVRCDQCSDGYYGNPLQG
VTCKKCSCNGNINASFTGNCDNTTGDCLKCLYNSDGNQCQECRDGYYGDAVVEKNCTDCQCSI
CGTTTAPCNKATGQCQCKKNVEGSKCNQCKPNTWNFDACNGCEDCNCAEASETQQCDLKTG
QCKCPPTVFGRRCGFCKLGYYNYSSEGCQKCQCPDGKPCDVNTGKCCTGVLNEDCKLCPVGHII
EDGECVECDRCVQRTLQRVNTLIHNVTILEKHIIPAGGLNYGALETLGNRFSNLSNKGDEHISNL
DTLKKAIGFGEYRNHSGNMDYVRMYLVRDFKSCSFIVNIKTPPTSSAPATTMVTNTPGPDPIRDHL
SNKKISDSQCPPSLARVIDEGEKNIKLVEKKVVVLEKDAYESTENATATKKRAEDLLAKLKDFMVD
FKDVLSFANSTLKGNVVINIDGNRSLAEAEEMLKEIKDRNFNSTINRSEREKSAAINATIIAEQLMK
QASELANKAYKFNDTFKDFVKALRNLKMESELALLDANNATNSINLAKAVDFQSIIDNAQKLHS
QNNVTVTQAMEILQNAKSAYENAKDVFNKFHNQTGPLSDLMEELRNYVRKLEFDLQTTSPIVDD
SVKHAKRLRDSADRLKELLQDTKKFADAALRAAKVYQNIVDAINDALDAARMANETAHEADK
KVQQAQQDAVESSITSKVVDTEANDLTEEVKKTLKVDISDMKMRLKELRELRKRIQGRIDNVKED
VKSFAKEKEVGEKAMNISTQADGVMKVANKGNEASQDTNEKVEKDGEKVETLKVDISDINKAIK
DLASNVTLVEDTIPKIRSNIDEVNVKHEQIEEFIKTNVTNNIGYLKYLISNAHDILDQMQLSMKFNS
TSEVVLDAPKSVADPTINNKISLTFKINQGNGFLFIIGDFSGNSSGFMSLHVQNHTVVFDYKLNTG
DVQSLTNPKFIETDVNYDVMVTRNFATAVLNVQKSGNASTLMTAYQNRSNISPVLSLGQDTNVY
VGYHPSTPNQRPNFDGCINQFRFDEELMGLWHWKSAKNVGGCVQDFRSIEGQYSFDGDGFVEF
DLVSPELSGFRKIELRFATLEENGLIFLGTDPPTSLPSDIMFDIPLSNQIIAFNALEIKDGYLVSTLDLG
NGVQRLVHTNAGKVNDGKPHLFEMKVLISSLLIKFKLDKKKITEIAISPDTDIRLRLKEAFIGGLPQ
RAWLPNSNISTKGFTGAIGDAKYRGGEFKLVKRFNNIRKGFILPKGYSASFPEEKSYLRLDACNLK
QNSLFGFSFQTFEKNGIIFHSEEGSGSSRSSIEIILIDGQVKVDVSVGSQKRTLQTSVARGFSDGLWHT
LSLYFEETLQEADIVLLYLAVDKAYITGTVPKVDLKGSMYFGSKPSGTESGFNGNIRQVYCNKNEIN

LASGDVFEGGVVGSLKLLPPLKLADFTVVPTTLPPLTTSITTSESTTTETVMTTSTTSISGTTLEPPKET
TTNMAKTTENPTVKITSSEPIIETTMESTTVAKTTSAITMEESTTTPTSITTSTLETTISTLEPGSIEGSTT
TAE
>TRINITY_DN577_c0_g1_i1.p1 type:5prime_partial len:269 gc:universal TRINITY_DN577_c0_g1_i1:1086-280(-)
PCQNCADMAVGKNKRLSKGKKGSKKKIVDPFTKKDWYDVKSPSNFQIRNIGKTVVSRTVGTKIA
SDGLKGRVFEIAQADLNKDEVAFRKFKLICEDVQGRCVLTNFHGMTMTTDKLRSLVKKWQTCIE
AHVDVKTTDGYLLRLFAIGFTKKRQNSVKKTAYAKTTQVRAIRKKMVEIITREVSTKDLKDVVNK
LLPDSMGSDIEKASQGIYPLHDCYIRKVKCLKKPKFDVHKLMEMHESSGTTTTDATGGVVVERPD
NFEPPVLDSV*
>TRINITY_DN15584_c0_g1_i1.p1 type:complete len:275 gc:universal TRINITY_DN15584_c0_g1_i1:874-50(-)
MADSGSAGADRGGRGGFGRGGRGGRGRGRGRGRGRGKPEDKEWTPVTKLGRLVKDLKIKTLE
QIYLYSLPIKEFEIIDFFIGPSLKDEVLKIMPVQKQTRAGQRTRFKAFVAIGDYGGHVGLGVKCSKEV
ATAIRGAIIQAKLSIIPVRRGYWGNKIGKPHTVPCKVTGKCGSVTVRLIPAPRGTGIVAAGVSKKLL
QMAGIDDCYTSACGTTATVGNFAMATYMAIARTYSYLTPDMWAENQFQKTPYQEFTDYLAKNH
NVGGKPVAPATATA*
>TRINITY_DN3034_c0_g1_i1.p1 type:complete len:1941 gc:universal TRINITY_DN3034_c0_g1_i1:6289-467(-)
MAEEEDPMKYLQVDPAQALKDQSKTFDAKKWVWVPVIGEDGYAAAQVIKVDGDNVTVETNK
GHEMEVKKEKCDQMNPPKYEKCSDMASLTYLNEASVLYNLKARYTTGLIYTYSGLFCVAVNPYR
RLPIYSDQVVKLYRGKRRAEMPPHIFAIVDTAYQDMLTDHDNQSMLITGESGAGKTENTKKVIQYI
AKVAGVEKKKSDDGPVKRDLDEQVVEANPLLEAFGNAKTTRNNNSSRFGKFIRCHFSQTGKLAG
ADIESYLLEKNRVIHQASAERNYHIFYQLLYATTDEELQKLCLVTRRSEEFEFLKKGVPSVDAIDDN
EEFGYTTNAIEVLGFDEEEKQSMFKICAAILNFGNMKFKQKPRDEQAEVADPADGERVAYLLGLPI
GEFHKSLTKPKVKVGTEFVNKGQNVSQVLYSVAALSKALYERMFWWIVSRVNKALDTKERRSYFI
GVLDIAGFEIFEYNSFEQLCINLTNEKLQQFFNHHMFVLEQEEYKKEGIHWEFIDFGMDLEETIDLI
EKPMGVFAILEEECIVPKASDQTYLQKLHKQHAGKNGSYEKPTPKQTKSGGGDFILHHYAGSVGY
SVANWLEKNKDPINAHTAELFSKATVGLVSGLFADYNPDVTGGRRKGSAFQTVSFRHKEQLKNL
MTTLMSTSPHFVRCIIPNEKKTPGLVEGTLILHQLRCNGVLEGIRICRKGFPSRMLFQDFRQRYQIL
AASAIPSGFIDGKVAAEKLIDSLQLDQSEYRVGTTKVFFRAGIIGELEDMRDERLAKIITQFQAYCK
GHLARIEYKKMCEKRIGLAVIQRNVRKFLFLRNWQWWKLYCKVQPLLSIARAEEEMKEKEEELK
KALEKAKEDEEQRKKMEEQLTENIAEKEKLFADLQAETDRLINLEDKLMQIQQQKDRLEANLNE
TLEKLDGEEHSVSVLQEKMHDAEDRIDSLEAEASELKSNCSRLEVERANRDKQIDALNEDINKQD
ESIAKLGKEKKGLEDNIQELNETLQASEDKCSSLTKAKKKVEDALKESENSLSKEKDSKARLDKEK
RRVEGELKETNDKLSEAENELKNTQDTVYKREKAIKELEEARDNQDNQIKQLQKKIQELLQRIEE
LEEELEAERKQKQKSELSRKELESQIEELQDQLETAGGATHAQVEVSKKREQECSRLRKEIEDINA
ANDEALASTKHKFNAQLSEAQEELESVKKAKSKSDKERSQLAGDLEDVQGQLDQVKKQRASSD
KNNRMLEEQLAELRGKVSSLETDMGDSESRNLKFQNESATLLKQLEEAEHKLGLANKQNKQLQ
ASLGESKNVADDEARGKHDMQNKLQNALAQIEELQEQLEDEQTAKMDLQNKYSRANADAQQ
WKNKYDMEGASRVEELEDAKRKLAARVQEMEEALAAAESKAASMEKVKNRMNEEVEDLLLDL
EKAQAQAANLEKKQKKVDQQINEWKLKCDEVQEELEKSQRDNRSYSTELLKMRTANEDTVEKY
ESLKKENRALAAELQSVSEQLSEGGKNSLEVEKLKRKLGLENEELEVALEEAELALEQEEAKFLKI
QLELTQLKQSTDRRSAEKDEELETLRKNHARQLEALQNTIDAEQKGKAEAQKLRKKLEGDLADL
ENRLEGSDRNSADYLKTIKKLQAQIKELQAMIDDESRGRDDMRDTASRSEKRAMDLAVQLDEAR
VALEQSDRLRKLAENEKLENAERVAELQTMYNSIANAKRKAEGDYHSLQEEIEELENEAKMAEE
KANRAMNECARLSSELNGAQENASNAEKSKSLMARQITELQQQLEEAESMGGKSLKNQIRKLE
QKVMEVEGELDNEARKSTESIKIARKADKRVKELQFQLEDEHKAVERAQEVSDKLNQKMKKMR
LQLEESEQQSSSWQSKFKKAVVDLEEAEERCESAEQALQKARQRARSGASGMTTRGVSRARSRVR
SPQNDD*
>TRINITY_DN1339_c0_g1_i2.p1 type:internal len:384 gc:universal TRINITY_DN1339_c0_g1_i2:1151-3(-)

IIGMFFQDGLTGSAWGDWALYTASPLRFFGGDSAPAYKSFDPATALGVQEPVGFWDPLGFSSDND
EATFNRRRSVELKHGRVCMAASIGYIVPYYYKFPGFLSPSEGVQFADVPNGLAALGKVPALGWVQ
IIFFAGIMEGAFGVTDDYKNGTPGDYGWKVITSDDPAEKEKKLAAELANGRLAMVAVIGMFFQD
GLTGSAWGDWALYTASPLRAFENELGVQDPVGFWDPVGFTSDGDVTAFRRRRTVELKHGRISML
ATMGYITPELTGKFPGYISNSLSLKFADIPTGLSAIAKVPALGWAQVVAYCGFCELSAGSDDWKTG
TPGDYGFKVLTSDDPAEKEKKLAAEVANGRLAMVAIIGMFFQDGLTGSAWGDWALYTAS
>TRINITY_DN96_c0_g1_i2.p1 type:5prime_partial len:189 gc:universal
TRINITY_DN96_c0_g1_i2:1253-687(-)
LASLGICYGADLMVTKQVYFDVSIGGQNAGKITIGLFGEVVPKTVQNFYELVTHSKGFGFKGSKF
HRVIKAFMMQGGDFTNGDGTGGKSIYGERFPDENFKLSHYGPGWLSMANAGQDTNGSQFFITF
TKTPWLDGRHVVFGKVVNGMDVVKKVEQVQTDSRDKPKKDVTIEDCGVVKVEKPFAVKKQ*
>TRINITY_DN3036_c0_g1_i3.p1 type:internal len:548 gc:universal
TRINITY_DN3036_c0_g1_i3:1643-3(-)
AFAGAARASPARQGSVARRALDQSSRYADLSLTEEDLIRNGKHVLVAYIMKPKAGYDYLATAAHF
AAESSTGTNVNVCTTDDFTKSVDALVYYIDPEEEEMKIAYPNLLFDRNIIDGRAMMCSFLTLTIGN
NQGMGDVEYGKIYDFFLPPEYLRLFDGPSCNVIDMWRILGRGTSNGGLVVGTIIKPKLGLQPKPFG
EACYAFWQGGDFIKNDEPQGNQVFCQMNECIPEVVKAMRAAVKETGVMKLFSANITADDPVE
MISRGKYVLSQFGPLSENCAFLVDGYVAGGTAITVARRNFPKQFLHYHRAGHGAVTSPQTQRGYT
AFVHTKISRVIGASGIHVGTMSFGKMEGDASDKNIAYMLQGDVADGPYYQQDWQGMKETTPIIS
GGMNALRLPAFFENLGHSNVILTAGGGAFGHKDGPKQGATSCRQGEEAWKLWKAGTYGDITLS
DGVIEYAKTHEEIKGAFLTFQKDADQIYPGWKEQLGYTGESSVQAASFDWKRKAGAAAFAGAAR
SPLAKQSSVARRALDQSSRYADLSLTEEDLIR
>TRINITY_DN6390_c0_g1_i1.p1 type:3prime_partial len:1941 gc:universal
TRINITY_DN6390_c0_g1_i1:5820-1(-)
MVIAPKCKFSIFSYLVFGILSYNWNELDASTNIELVDGTGVLLQRDVDNVLNDAVVQKEYQQNIK
HKPVDKRCLGEPFYQGLCKEKLEDAIQQNITEGGCHNLLVKIQKDECSNGPKTLGGKGYCNFICS
QEICNSIYDIDKCVSKRLVHHFNKDKNECEEIMQADCGSESFTSSNQCQKRCIGNSDDKRETKVY
CDQFCLNGGKCIGEVYCQCPPAYHTATCGLSYCNPSCGVGGKCVKTGEGKYGCQCFPGYKGVK
CDKEDSKNMPVVKGLCLSAIKSVLTSPNTSFHDKPEEEKNYCLKYLFDLESNCDKQRPFVNYTEE
QCNHFCASDICDNIYSNDCGSIEERYGYVSSTGRCSKFLYNGCLGNTNNFKDKAACDTKYSPING
LMTSVALLRPLNHTCYIRGKTNFITFDGKVYDLLSGCSHYLVHDSDTTSDFNVILHNDPKVNYNE
KSKRKLEITADGKNVLLNQKVSSDRFFVQVDGRSVPLPYNDVPKIRQAGRYILVLTGDGVSIAFDG
YEDISITVNDYYRTGDGGDHRLNGLCGNLDGNSHNDWLDFTGELRENVRDFAKVYQTDRSTCK
MDNLSLKSLVYNPLHNDQEVFERAERHCDVINTDAFKPCHSVVSPEQYKVLCMQDYIKCNDDK
RDDCVCSSLSFYSRVCHKYGKDIKWRRANLCPITCQRNMKYYECGSACPKTCDNLITPTKCQTDC
VDGCHCPPDLYYDKSTENCVPRNLCSCYRDGIAYKHGSVRSFHCEECTCNGGSWECNKKDCEAT
CDIAGDPHIKTFDGLNYQMYGKCSYVLVDHCHYGNTGIKEFEVIVENKNCNDDALYTSCVRHLS
VNILQTNTRVSLDTAKDNNGTVLPVAFVNGKEGKEFRSKDYTITSVGGRNIIFQYKVNQLKVSWT
GHNAYVTVGHQFENQTCGLCGTYNFNKQDDFHTRSDTVEKSVTAFTQSWVYQSSDKVQDPSKC
HTAKWETSEKPCQIYTNKAQYAKTQCSIIIKPDGPFEDCHSIVPPGEYYSMCQDDACKCDDCYCH
IVAAYAKRCTDLNIRVGNWRDETLSCKQTCQAPKVLKQCNAIDSLDDDLSESLKCPSTCYELSQN
DTSICQSRGCIEGCYCPEGYFENEQNECVKQDQCSCYHDGKVYRHDEIRKDRCNECVCKGGRFE
CTDFDCVAHCASKGLVYSDCGKTCSNFNFNPQDSKCQAGCFCPAGKVLHENGTCVNPQTECQC
KEGDQFFDAGEVSPSDCSKVCNGDRSWKNLPGKDAKPQYDCSAFGQMHYRTLDGRMFKFSGG
LCPYVLLTDCRQDSATGFCDLTKANVNIHVRNLRCVNSYEAYMCKEVTLDLRLPNGEMAEFVLM
QNKIKMKTGLVTKVFDKGSYPQPKTAVVDGVEIFKSGLYLVVRAYHLNITVKYDRQNRVYVVPGR
EYKDAGKLAGLCGNYNGLDDDDFRRPDNSNAENELQFAKSWQDSNSACPDPSESDTCSSNKER
LPWAQKGCNVMKTGNFTACHKTVSPEPFIESCQQETCLCKSGGDCACFCSAVAAYVQECNRHG
VPIYWRRDGLCKLDCCDPCGHSYGYYTDNHVPNTCGVKDKVCKENDRCCKGAVVEGCGCPPG
TYDDGIKCQNETTTNVIPCSPSSSIVPSTSISVPSTIIEPVSSSSPSPMTSSVESQSPSPSSSVVSPTPSSSII
QSMSSSEIESISTTSSSFVSSIESSVQSSTSIVFPVVSSNSGESSVSSSMLPSTSWVGIHSVSSSMGESSVST
NVLPTDYLRTTDVFPLSSSEITYPSSSSSLEPHTPSSSILQESSSSVSKPVGISSVIEPEISSIRYSSMSSQN

VMSSSVSSSVSKPVGISSVIEPEISSIRYPSMSSGSVLESSVSTSFVLPSETVLSTSVFSTNTESPSSSMTLS
TSSSGFVFPSVTSTFQTSSKVLSSLPRSGPAENTVPLSTMFTKTV
>TRINITY_DN6920_c0_g1_i3.p1 type:5prime_partial len:411 gc:universal TRINITY_DN6920_c0_g1_i3:2124-892(-)
CKRHQGKMSHRKFSAPRHGSLGFLPRKRCKRHQGKIKSFPKDDAAQPCHLTGFIGFKAGMTHIV
REVDRVGSKTHKKEVVEAVTIVETPPVVVVGIVGYVETPRGLRQLTTVWAEHLNEECLRRFYKNW
YKSQRKAFTKASKKWSDDDGKKSIERDLEKMKKYCTVIRVLVHTQQKLIKLKQKRAHIMELQIN
GGTVADKVDWAKKSLESKLSVSTVFGQDEMIDVIGITKGKGFRGVTFRWGTKKLPRKTHKGLRK
VACIGAWHPARVGFSIPRAGQCGYHHRVERNKKIYRIGRGIHTKDNKVIKNNAATDYDITNKTIT
PMGGFPHYGEVNEDFIMLKGCVPGPKKRVLTLRKSLLAQTSRKALEKITLKFIDTSSKFGHGRFQH
DQEKKNFMGLLKKDKERQAVEA*
>TRINITY_DN12_c0_g2_i1.p1 type:complete len:203 gc:universal TRINITY_DN12_c0_g2_i1:239-847(+)
MANRPKGFGMTAELANKRAAKFDAELAAEAMGWVKDVLEASGNDEAVNLAREIGSIDGPTDV
KGPLKNGVILCHLINSIQPGSVKRINQSSMAFKQMENIGNFLTGCEGIGCSKQDLFQTVDLYEGN
NIPQVINGLFALGRKARKTGYDGPRLGPDEASENKREFTEDQLRAGEGVIGLQAGSNKGASQAG
QNFGKTRAIID*
>TRINITY_DN278_c0_g1_i1.p1 type:complete len:470 gc:universal TRINITY_DN278_c0_g1_i1:3091-1682(-)
MAPSFNKTNGHMEENGGMIHANGKSSEKPINLGEQTYSYMTFKKMGLIMITLPLVFLLLVGLPSY
LYPVLVLLFFIPVFAGYIGLNNVIYCLYERHNQVSKIDNYIEFLDEDLKKKFTGRFIPIRDLYELYAEG
KLNFKGDVLQCLERRNEYVSYKLQLWHLKFFLSKLLPELLHARSQDKEQVCDHYDRGNDFYNA
YLGPLMVYTSGIRYSESDTLEDMQMNKLKAVCEKVQLKEGDRHLDIGCGWGTLVNYAAENYNS
QSEGVTLAKEQVCWAEEKAKSKEIMDRVNYLCMDYRDIPKKKYDKITCLEMSEHVGIKRYHHFL
KDVRNMLEDDGIFFLQIAGLRRAYQWEDFLWGFFMDTYIFPGADASLPLQFPIAELEAAGFEVES
VKTVGIHYSDTIKYWYHNWVKSDSHDKMIQKYGRRMVRVWDIFLAWSTIIARQGNSTCYQIVAH
KNFNHYPRRRFIQPKN*
>TRINITY_DN624_c0_g1_i1.p1 type:complete len:884 gc:universal TRINITY_DN624_c0_g1_i1:218-2869(+)
MANQEEEEHFGYYYGEEEDRGLLDPAWEKQQKKTFTAWCNSHLRKVNVQIEELNVDFCDGLKL
MLLLEVISGERLPKPERGRLRFHLISNVNKALDFIASKGVKLVGIGAEEIVDGNLKMTLGMIWTIIL
RFAIQDITVDEFSAKEGLLLWCQRKTAPYKNVNVQNFHMSFKDGLAFCALIHRHRPDLIDYDKL
AKDDPMYNLNYAFDVAEKELDIPKMLDAEDMVNAVKPDERAVMTYVSCYYHAFSQSQQAETA
AKRILKALNVNQGNEKAIEDYENLASDLLDWLNNKIPTLDDRTTDSSLDTMQNKLNEFRDYKGT
EKPPKVDDKSKLENLFNTLQTKLRLSNRPLYMPAEGKMINDIDKRWKDLEQSEKDFQEWILNELR
KLERLDHLAMKFKHKCDIHEAWSTGQDDYLRSDDITDASLASLLASRKRHEAFESDLAAQQDRV
EQIVAIAQELNTLGYSKIDEINTRCKGICDGWDTLGELTTQRSEKLAKAIEIAERLDNLWLEFAKKA
SPFNNWMDGAKEDLMDMFNVHTIEEVNDLKDAQLKFKANMPAAQEEFNTIMGLQNAISSMSS
QPNPYTNTKAEDLQEKWQEVLELIPKRDSLLESETQKQQNNERLRVQFAQMANQVGAWIKQRM
GRMTDIGMNAQGTLERQLEELENLNAEINGFKQNVLDLDASARNVQEAMIFENPHTPYTMEVI
RIQFEQLLTGVTRNINEVENQILMRDSIGITEEQVKELRTSFNHFDKNSNGRLDYKEFKQCLVSLG
QLGDVEDKGEHTFNRIIAMVDPNETGYVSFQAFLDYMTREMADTDTADQVMESFRILAGDKPYI
TADELRRELPSEQAEYCIARMSAYNGPDGVPGALDYRSFSTALYGESDL*
>TRINITY_DN4329_c0_g1_i1.p1 type:5prime_partial len:735 gc:universal TRINITY_DN4329_c0_g1_i1:2240-36(-)
LTFIESLRLSNNMKEKLDYVVLCFFSWMVVGSCVDRGNFKTCKDSSFCRRNRAIQAGGSPFALLS
DTLVFGSDSITGDIYNTNTNVSFKLTIDTLVKETARIRITELSPIRPRYIVQESLVREPQHIKYEKVKSD
SDGFKVKFGSSLMQVTFKPLKVDFFDAEGVLLMSLNNKGLLNFEHYRVKNSSPQDSVEKTNESEK
GEDADQNSENEGKENTEEEKPENTNTVREDINEEGMWEETFKDHTDSKPYGPSSVGFDVSFIGFE
HVYGIPQHADSLSLKQTSGGEPYRLYNLDVFEYELNNPMALYGSIPFMTAHSAYKTVGIFFNNAA
EQWIDISSSKSVLSSLISFFKSGDTPEVDTHWMAESGIIDLFVMLGPKPNDVLNQYSTITGTQTLPP
MFATAYHQCRWNYNDEDDVKNVDAGFDEHDIPYDVIWLDIEHTDGKKYFTWDALKFPDSKRMI

DHIASKGRKMVTIVDPHIKRVGGYRIHSEAESKQFYVKKKDGADYEGWCWPGSSSWIDYTNPDA
RNWWASKFFLDQYQGSSLDLFTWNDMNEPSVFNGPEVTMHKDAIHYGDWEHRDVHNIYGML
QHMSTFEGHLLRSDGKARPFILSRSFFAGSQRAGAHWTGDNTADWGHLKMSIPMLLSLNVAGFP
FVGADVGGFFGNPDSEMLVRWYQMATFFPFFRAHAHIDTRRREPWLFGEENTQLIQQAIRKRYM
ILPYIYTLMRNSFDTGMAVMR*
>TRINITY_DN453_c0_g1_i1.p1 type:complete len:249 gc:universal TRINITY_DN453_c0_g1_i1:944-198(-)
MDIDQLKYEIIELGKQIETVHVAIAVVVLTTILLWIAIRSRRSGDTVLLLGLCDSGKTLLFSLLTAGKS
AATQTSIKENKSRLTMGKKSWAIVDLPGHERLRAKYLYNHKDSARGIVLMIDSVEFPREIRDVAEL
MYDLLANRTIKKNKVSILVACNKQDLTTAKACNVIKSQLEKEINNLRKTRSAALLGIDDNTSSKN
AFIGKKGKDFEFAHVKPIKIEFCECCLKDENGESKTDYNGIEEWMKKL*
>TRINITY_DN1377_c0_g1_i1.p1 type:complete len:878 gc:universal TRINITY_DN1377_c0_g1_i1:68-2701(+)
MTGRARGRSRGRGRAAQDASQRPGGAQGGPPPQAAAPQQGGGGRGRTRGQPAPQQVAPQQR
PAAQTAVPPTARMAQMSVKDQKPATEERQPRRRRLDPAPDVLVTRPEGRTKEGTGGTPVNIVSNF
FKVIKMPNFKGLYQYNVSFDPEINSSKLKNALLYQMADVLGQTKCFDGMTLFLPKRLPDQITTRS
VTTHQGNAIVIKIMFTNVIPEISPQVLQMINIIFRRQLRCIKMELIGRNYFNSSRKIEVKAHKMEVW
PGFSTSILQYEKDVMLCADVQHKVVRQQSVLSMMYELHQKTREKNRFYELAAKKLIGETVITRYN
KKNYKIDDIEWNKNPNSTFDKNGTQITFKEYFEKTYDAKVGDDQQPLLVSLPKAKNRKSGIVGPI
LLLPEFCLISGLSDEIRSNFSIMKDLAQHTRINPAERAKNLEQFVQDLQTNEESKKELEQWNLKFES
KLLQMTGRAYGSEKMFQREDQFSYKIQDADWSRETRGKKLIHTVNMTDWLLVYYERDEPIATDF
KQTLVKVCGPMGMNVAEPQLCRLKDDTARSYFRALSEQIRPTTQIVVCIVPNNRKDRYDSIKKLC
CVEKPIPSQVIVSRTLSKKQMLMSVCTKIGIQLNCKLGGEIWAVDIPISKNPKDPPSLMVVGFDVYH
DSASKGKSIGGFIASTNRHLTKYYSRITQQQSHQEIADQLRVCMTGALKKFNELNGTMPSRIIVYR
DGVGDGQLRQVFDHELPQFKAAFNAISQGYDPKFTMIVVKKRISTRLFLSERGMKTNPLPGTVVD
TVVTRPEWYDFFLVSQSVRQGTVSPTHYNIIADTSGWKPDIFQRLTYKLCHLYYNWPGTIRVPAPC
QYAHKLAFLVGQSIHAEPDVQLADRLYYL*
>TRINITY_DN286_c0_g1_i2.p1 type:3prime_partial len:106 gc:universal TRINITY_DN286_c0_g1_i2:40-354(+)
MIKVAIITIAIVACVLESTAAVRCYVGYSAADLPLAIGVRNIECGEEFNHCTTVKYRIGGITVTQGN
CAKRYGQAMCQFMRNPHKAMTECEESLCQGDNCNTAVN
>TRINITY_DN4439_c0_g1_i1.p1 type:complete len:941 gc:universal TRINITY_DN4439_c0_g1_i1:77-2899(+)
MTDSKYFTTTKKGEIYELKAELNQHKMDRKKEAVKKVIAIMTVGKDVSALFPDVINCMQTDNLE
LKKLVYLYLMNYAKSQPDMAIMAVNTFARDCDDPNPLIRALAVRTMGCIRVDKITEYLCEPLRKC
LKDEDPYVRKTAAVCVAKLHDINSTLVEDQGFLELLRDLLSDSNPMVVANAVASLHEIAESSPNAT
QYFEMNSATINKLLTALNECTEWGQIFILDSLANYSPRDEKEAQSISERVTPRLSHANAAVVLSAV
KVLMKLMEMMPTGGQLVQNLCKKLSPPLVTLLSAEAEVQYVALRNINLIVQKRPDILKNEIKAFF
VKYNDPIYVKLEKLDIMIRLASQQNIGQVLAELKEYATEVDVDFVRKSVRAIGRCAIKVEQSAEKC
VTTLLDLIQNKVNYVVQEAIVVLKDIFRRYPNKYESIISTLCENLDSLDEPEARASMIWIIGEYAERI
DNADELLTTFLDGFSDENTQVQLQLLTAIVKLFLKRPTDTQELVQQVLSLATQDSDNPDLRDRGYI
YWRLLSTDPAAAKEVVLAEKPLISEETDLIEPSLLDELICYISTLASVYHRPPSSFVAGKTPARRAVLP
RRTGSDTNENEEVSSISEETPTPTLSQVAGMPPISSQPATDTLIGDLLMDMGPAPTPPQPSVFDAHR
QQQAPSADLLAGDLSMLNTPVTPQQQPMGMGGGLNDLFSLQGALPGSGYCPPKQVWLAASKG
KGLEIAGTFSRRQGQIFMDLTFTNRAMQQMGGLAIQFNKNSFGVAPAVPVPNTPLPPNQSAEVSL
LLNIAGPVQKMQPINNLQVAVKNNIDVFYFSCLIPPHVLFVEAGKMDRKQFLDVWKEIPQVNEV
QNTITTKGFNSDGIQNVLEANNMFTIAKRTVEGQDMLYLSLRFSNGIDVLCELKIIPGNPVVQLSL
KTKAMDVIPMVQEVIQLILQS*
>TRINITY_DN2805_c0_g1_i1.p1 type:complete len:189 gc:universal TRINITY_DN2805_c0_g1_i1:623-57(-)

MGFEKVVIIDGKGHLMGRLASVIAKSLLQGQRIVVVRCEGINISGNFYRNKLKVLDYLRKRMNTK
PSRGPYHFRAPSKCLFKVVRGMIPRKTKRGMEALNRLKVFDGIPPPYDRMKRMVVPSALRVLKLK
PGRKYCSIGRLSHETGWKYKALIESLEEKRKAKAELYYKARKQTEAVRQKAIQQVAAS*
>TRINITY_DN5793_c0_g1_i1.p1 type:complete len:213 gc:universal TRINITY_DN5793_c0_g1_i1:170-808(+)
MSYAYLFKYIIIGDTGVGKSCLLLQFTDKRFQPVHDLTIGVEFGARMITIDGKQIKLQIWDTAGQES
FRSITRSYYRGAAGALLVYDITRRDTFNHLTTWLEDARQHSSSNMVIMLIGNKSDLEARREVRKEE
GEAFAREHGLIFMETSAKTAANVEEAFIGTAKEIYQKIQEGVFDINNEANGIKLGPQHSASSPSPM
GGGGGNQQGGGCC*
>TRINITY_DN326_c0_g1_i1.p1 type:complete len:177 gc:universal TRINITY_DN326_c0_g1_i1:729-199(-)
MTSNVRQNYHAETEAAINKQINMELYASYVYLNMAYHFDRSDLALEGYHNYFKNQSDEEREHA
QKLMKYQNVRGGTILLKDVAAPPDMSVNSNDHIASLEAALELERKVNDSLLKLHALGDSHNDA
HLCDFLESEFLDEQVQSINEIAKLITNAKRCGDGLGVFQFDKLTMQEMKE*
>TRINITY_DN1614_c0_g1_i1.p1 type:complete len:218 gc:universal TRINITY_DN1614_c0_g1_i1:56-709(+)
MHKTKICIWLVVRYRSNPFRGYNRRPDLKSAGAKMTKYSTEPENPSKSCKARGSYLRVHFKNTHE
TAQAIKKMHIRKAYRYLKDVVDMKQIIPFRRFNGGVGRKAQAKAHGCSQGRWPKKSAQFLLQL
LKNAESNADLKGLDVDSLVIDHIQVNRAPYMRRRTYRAHGRINPYMSSPCHIEMILAEKEQVVPR
AEEEVEVKKVSKKKLAREKLKSRE*
>TRINITY_DN74124_c0_g1_i1.p1 type:complete len:183 gc:universal TRINITY_DN74124_c0_g1_i1:100-648(+)
MLLYTFACLIVSTLALELETTAKLLASKTVENNVLVENIDITIKYTIFNVGTSSASNVKLVDATFPTT
DFETVRGQLSVEWQNIPVNGNVTHIVVVKPLKTGYFNFTSAYLSYVPRDGEIQNAYTSFPGEGGIM
SELDYARKHSPHLLEWSVFALMCMPSLIIPFALWYRSHSKYTMKTKKN*
>TRINITY_DN147947_c0_g1_i1.p1 type:internal len:168 gc:universal TRINITY_DN147947_c0_g1_i1:501-1(-)
VGKSALTIQFIQSHFVPDYDPTIEDSYRKQCVIDDRVAHLDILDTAGQEEFSAMREQYMRTGEGFL
LVFSVTDRSSFDEIHKFHTQILRVKDKDEFPMILVGNKSDLENERTVSTAEAQELGRKLSVPYLESS
AKQRINVDAAFHDLVRAIRRSNNVEALPIKNKKK
>TRINITY_DN73067_c0_g1_i1.p1 type:complete len:831 gc:universal TRINITY_DN73067_c0_g1_i1:3714-1222(-)
MHWFVILLLFGVIFVTVSNYEIDVLSTFELSEDIAGISPLDNKYQAYQFAADGKELLLDDSIKTEIVR
GVTNFKDFFIAVNIRHDKQKPSSLLSLQTKTTGKQKFTLWINAEEEKIGIRSHSKESKKGKKNLTFR
KVPLNTGQWHRIVLNFKKIDTDEPVVDLYVDCKFHDSKTFPFSIGHALIEDALISELRLGQLKHLP
EKDPIRFIGAMQDFRLVSNRDVHWYNRPQHCYNMMIEVKGMKAYHEPVSTISHDVDNMAAAM
KNMQKQLKLQLDEIRDIKNWLQQCTACKTAAENVLGGEIPKACSQNRCNENANCIESNGGSAF
RCECKAGYAGNGEKCGLDSDSDGVPDNKLDCLGKECSQDNCPGFPNAGQEDADEDGSGDACD
IDDDNDGVLDGDDNCQFLSNRDQKDSDADNRGDACDNCPFESNYNQLDSNNDGLGDACTRD
ADGDGIRDKSDNCPRVYNRDQIDRDSDGIGDACDNCIDIYNPIQKDVDNDNLGDACDSNVDID
NDGIQDNKDNCLYIANANQQDIDNDGIGDACDPDDDNDNIIDENDNCRLIYNPDQSDSDGDG
QGDACEDDADKDETVDLKDACPENPEIQNTNFTNYKHIMLDPEGTSQVDPEWEILNKGAEIKQ
WRNSDPGIAIGDKVFGGVDYSGTFYVHDDKDDDFAGFVFSFQSNSKFYALMWKKDKQTYWFET
PYKVTGHAGIQLKVVTSSTGPGEHMRNALWSSISIPGQTKIIWSDPSKEGWKPKTSYRWTLMHRP
SEGLVRITIKEGSQVRADTGYLIDKTYKGGKLGVLVFSQKEIVFSALQYRCTEQIPADYNPNSPN*
>TRINITY_DN319_c2_g1_i3.p1 type:complete len:2588 gc:universal TRINITY_DN319_c2_g1_i3:165-7928(+)
MVLLKGQLWILGFIFLTSTKYVMGDVPECSNNATVAVHCRFDEGNTCVDDPHPMHYVCVCNNL
GYMNDTDSVEGYSICKERDCSNSNETPTDLLKRICGVNTKTCTGKILQRECNCNTDSYRSKNGQY
CFFAPCQASPTIQQNCGPGNQCYGNAPNYICDCSARGYKRSAADNRHCELKSDEELTPIEEYCGK
GSLYANGTINNITCTCDTDKGFFPSATGETCISPSCYLNPQMQEKCGPENTCLGGKDDYVCLCSNP
NYKRNATDLQHCIRRTCADTSPAIDFKSICGEGATGCSGYVDKPLCSCDVSNHYVPAADGRSCIK

ATCKGNKKALEQCGQANSCTGSFDNFLCVCDANGYYLNTTNNTCVERECTDPDLVITDICGVGS
LSCNGKAGSPECICDKDKFYYASEDLRSCVKANCLSSETISKKCGKENSCYGSMESYVCNCNAPG
YLLNTTDNQGCYLGMCEDNGLEYDKVCGEGAISCNGTIKNLECNCGMNYVQSPDKKSCIRADC
RANQTMHEQCGEDNDCVGSLEDWMCICSTYGYKPDPMDMMKCKKVMCEEEVVNNVTVCGL
GTTGCKGHILKPECTCDIAADYVPNEDASRCIKGSCENNKMAMKDCGIANSCTGSYNNYVCVC
NGFGYKKLDDTHCVEQMCSDPDIDIRNVCGEGAHYCNGTVNQRVCSCNETEYFYPSADSKSCV
KATCAVSPMVMEQCGVENSCQGSQESFICNCNALGYLLNTTTNQHCYLGTCENGDFDLMQAC
GDGALYCNGTIKNLECQCDESKGYVQAPDKMSCIPNDCRGNLTMYRQCGQLNKCVGSIDDWR
CICDSPYYVAHSDIMKCKLLHCSDSEVDHVEVCGKGTIGCSGSTLHPTCHCNTTENYFASKDGMR
CVAGSCKTNQTAVKKCGNANSCAGSHSEWTCICDGIGYKHDPADPQNCLLKTCNDADIDIVATC
GVGAISCEGYQLMPKCECNDRDYYIPSDDRTSCELASCRANRTAMRACGEMNDCRGSFSSHICL
CHDPAYKSDPSDSRVCLEKTCEAGDFELVRVCGKGATGCSGSMKNIMCECDNAKGYVASADHT
QCIPSDCNNNVTMHLQCGEGNGCVGSLTDWYCVCSAPGYEPHASDHMKCHLKTCADPGVDH
VQICGMGATSCMGPVINPVCYCNESMYYIPSMDRTSCIKASCMSNKTAIAQCGSENSCSGSPDNF
VCLCQAYGYRQDTNNNQACIPSQCEDAGIDFVEICGQGGVGCSGSVLNPVCMCDHMKHFVPSP
DQNSCVLSTCRGNETAMTSCGEQNKCFGSSSSYICVCDAVGYKPHPTNLQECVLQTCEANELNP
VAVCGKGALSCKGTIQSLMCECDNSKGYVAAPGYKSCIPADCRANMTMHAQCGTGNRCRGSM
EDWSCVCDGIGSKPHPTDHMSCIAIDCSDPNVDIKSVCGYGATGCMGLARDPICHCDTSKYYVQ
SEDGRTCVPATCHTNKTAQMQCGPDNSCIGPPNDFICECTGFGYRHHETDKQKCVPIQCNETAL
FDIERVCGKGATGCRGNIRQPICDCDHAMYFHPSEDRRTCIEASCRRNSSIRADCGVENTCFGSTR
SWICDCNSYGSILNPSDVKKCMLATCEAGQLDPVAVCGKGALSCKGSMRDVQCTCNNELGYVH
SPDGKKCLPSDCSGNVTMHHQCGDANKCMGSLTDWVCVCDAPGYKPHAKDKMKCHQISCAD
NNVDPVDVCGIGATSCKGPITNPLCQCDTDNYYITSSDGASCIPATCKTNKTAIHQCGEANSCEG
PADNFICVCNAYGYKLDDNDHQRCVEISCDDPNAHESICGTGSTGCRGTARDPICECDHTQHFV
ASSDLTSCLLASCRANDTVMNHCGDGNTCFGSLTSHICVCEAFGYKLNPNNHKHCLEETCEDD
MLNPVAVCGKGAVSCSGPLKSVKCECDNNAGFFRSPGGMKCESTSCRQNPASQMMCGSQNQCY
GSYDDWYCDCSAPGYMANQTNAAECLKVMCDDMAVDVCGLGATGCTGHTLNPICTCDSSMG
YMMAKDKHSCVPMDCSMNPASSQCGYGNTCHGSYFSYVCVCSTAGTVMNKTNPMECINVACP
ADAVHVCGEGATNCSVTSTNNVECSCEEGYHPADDLHSCVLDDCRFNPKALKACGLLNKCVGS
YKSWKCNCHYPEFTQDPMDHQTCVVGPCMVEQCGKGNAPGSFQHYAVNCSGLYDDPICNCQP
EYTNDAKGGCIPKICSENPVAVQACGSGYCEGTYFKYTCKCDEPAFVQTPDKMQCVPAKCNIQQ
CGHGFDRGLYIDFAESCSGTYADPMCQCKTGFSGTNTSKCGPAIDGCKLDKCDPLATCQTLDVD
GNVKCTCKEGYSGSGMMGECYLDGGFSAYEGTKECSEVCGVNGVAEVTRFCNNPTADPKGKPC
NGTSPETIQVPCNRFACSSCTVRKTCGCGGLKPIFVEFYHEPNSTLEYFTDLKADDVQTALGFLTK
WKELSQQDLCLMYSKIELTVFVTLMGDSETQVSTMKSSLVSARDIMRKKLDCNINLGKKAQYYEA
YETIIKRHLAISNILKDLTIIKNSITKYMNNCDDSEIGMIRDVIGMM*
>TRINITY_DN164351_c0_g1_i1.p1 type:complete len:212 gc:universal TRINITY_DN164351_c0_g1_i1:694-59(-)
MEDWGEPGEQEAVGPTSIEVQDIKLFGKWNPDEVQVSDISLTDYIAVKEKHAVYLPHTAARYAVK
RFRKAQCPIVERLTNSLMMHGRNNGKKLMTVRIVRHAFEIIHLLTGGENPLQVLCDAIINSGPRE
DSTRIGRAGTVRRQAVDVAPLRRVNQAIWLLCTGAREASFRNIKSIAECLADELINASKGSSNSYAI
KKKDELERVAKSNR*
>TRINITY_DN477_c0_g1_i9.p1 type:complete len:306 gc:universal TRINITY_DN477_c0_g1_i9:1840-923(-)
MTRQCLGATLNENCGNAALVQFEVCNFQVPCPGLLTNWGPYTQCSATCQKDANSMPTQSRQR
FCTGGSFGGNCGGVQLLQTIGCNQLIICPVYGYWLQWSAFGECSKTCDQGTHVRYRNCTKSIPA
NAGNNCTGPAQEVKPCQVKPCPVTCPLTKKCDCRNMNYLAAAPGISQTVTNDVMGVAAFLDY
WTLDILKQICEGCRMTQLDQSKATVDSQILRTQAVLDRLNIEKRVYRDMITCNENPYITDDLFKVY
NDIWDRIVTTDGVMIELKASKMRFDTAITACRSYDWYHDLFGRFFQIKKSS*
>TRINITY_DN168534_c0_g1_i1.p1 type:complete len:226 gc:universal TRINITY_DN168534_c0_g1_i1:774-97(-)

MIRSAIIAIVIASHLYESSAARCYVGYSAVGLPLAIGVRDVDCQKGMDFCTTVKSTIGGITVTQGNC
ARKYGQATCQFMQNSHKAMTDCKETLCRGDYCNSALIEAEQKERKRVGPYKNTLKCTTELKTR
FPLIPFATADIPIGIDECFPGTTRCVTVEYDYIYKFKTKIATRHNHFVLPVIQKMCNRPNIDDCNKW
CTIMSIHQFMHSSCRAKCCTGDNCNAKD*
>TRINITY_DN345_c0_g1_i1.p1 type:complete len:150 gc:universal TRINITY_DN345_c0_g1_i1:760-311(-)
MADQLTEEQIAEFKEAFSLFDKDGDGTITTKELGTVMRSLGQNPTEAELQDMINEVDADGNGTI
DFPEFLTMMARKMKDTDTEEEIKEAFRVFDKDGNGFISAAELRHVMTNLGEKLTDEEVDEMIRE
ADIDGDGQVNYEEFVKMMMAK*
>TRINITY_DN5633_c0_g1_i1.p1 type:complete len:848 gc:universal TRINITY_DN5633_c0_g1_i1:2881-338(-)
MRRSNFLTIGIIAFFLIFSYVSADETSDEEVEPPKVDDDVGKSRDASKTDDEVVQREEEAINIDGLSV
AEMKQMRESAEKHAFQAEVSRMMKLIINSLYKNKEIFLRELISNSSDALDKIRFLSLTDTEALAAT
DELSVKIKADKENNVLHITDTGIGMTKDDLIKQLGTIAKSGTSEFFQKIQDAGTTDQASDLIGQFG
VGFYSAFLVADRVVVTSKNNDDKQHIWESDSVEFSISEDPRGDTLLRGTQISLYLKEEASDFLEPNT
LKDLIKKYSQFINFNIYLWSSKTEEVEEPIEEEPEKKEDEKKDDDKKEDADDKKDEEKKDDDEDD
AKVEEDKEEEKPKTKKVEKTVWDWELMNSSKPIWLRKPKEVTDDEYNEFYKAISKESDGPLTKT
HFVAEGEVTFKSVLFVPKNSPSDMFQDYGKRRESIKLYVRRVFITDDFQDMMPKYLSFIKGIVDSD
DLPLNVSREQLQQHKLLKVIKKKLVRKALDMIKKIPDEDYIEKFWKEFSTNIKLGVMEDHSNRTR
LAKLLRFRSSNDPEKLTSLTDYVERMKEKQETIFFMAGASLDEVKNSPFVERLLKKGYEVLYLIEPV
DEYCIQSLPEFDGKKFQNVAKEGLKIGNEAKEKARVDELEKEYEPLLKWLKEDALKDLIEKAAVSS
RLTESPCALVANSYGWSGNMERIMNSQAYAKSKDAGANFYATQKKLLEINVYHPLIQELQKRVKT
DKEDQTAKDLARVLFETATMRSGYTVKDSSDFAGRIERMLRLSMGVDVDAKVELPEEDDEEKEEE
KADDEGEEVNADGEEEESKEDIPEIKVDSKTVEPVAEHPTNEDAVENEAPAEGKTEL*
>TRINITY_DN773_c0_g1_i1.p1 type:complete len:440 gc:universal TRINITY_DN773_c0_g1_i1:1608-289(-)
MVSPPGPQDNKPPHKVADMSLADLGRRYIEMSENEMPGLMSMRKQYGEKKPLKGARVAGCLH
MTIQTAVLIETLTELGAEVQWSSCNIFSTQDHAAAAIAKTGVPVYAWKGETDEEYLWCIEQTLIFP
GGEPLNMILDDGGDLTNLVHEKYPELMKDIRGVSEETTTGVHNLYKMFKEGRLKCPAINVNDSV
TKSKFDNLYGCRESLVDGIKRATDVMIAGKICVVAGYGDVGKGCAEALRAFGARVMITEIDPINA
LQAAMAGYEVTTMDEACSKARMFVTSTGCSGIIEPHHFQQMPEDAIVCNIGHFDCEIDAKWLN
DNCERETVKPQVDRYTLPNGRHIILLAEGRLVNLGCAHGHPSFVMSNSFTNQVLAQIELWQNHK
NYEIGVVMLPKRLDEEVARLHLDHLSVKLTKLSKSQADYLNLPADGPFKPDHYRY*
>TRINITY_DN374_c0_g1_i1.p1 type:5prime_partial len:290 gc:universal TRINITY_DN374_c0_g1_i1:1-870(+)
FHSGVNIDWNNFKKYLKDALCRCEKAAAAKGKKYFSFRYFGICHGLDSLPKESSEYCFHHTFEQ
CMNNHNECVGADEAEYIYKVGVQIPTNTKPALKVQRKVFCEHRGSKTVTCGAGETIKVTKAYYG
RTSSSHCGYRGFYNLNCKGSTSYSKIKSACDGKQTCRLNPHNNVFGDPCKYTVKYIDIEYVCKPP
VKKAIFCEHRGTKSITCNEGEKIEVVHGYYGRQSSAHCGFRNFYNTRCKASSSVGKIKNKCDGHR
SCNLLPANSVYGDPCVRTVKYIEVDYRCIKN*
>TRINITY_DN2904_c0_g1_i1.p1 type:complete len:221 gc:universal TRINITY_DN2904_c0_g1_i1:1339-677(-)
MSLAKIFGSGKKKEESPNAQDAIQKLRQTEEMLTKKSDFLEKKIQQELVIAKQNSSKNKRIAINAL
KKKKRYEKQLNQIDGTLSTIELQRETLEGASTNTEVLKTMGYAAKALKNAHQQLDVDDVHDM
MDEISEQTEISKEITDAISTSFGVADDIDEDELNAELEELEQEGLDEQMLNIESTSLPSVPETELPTPS
RAQPSAQKVQPDDDMAELEAWAS*
>TRINITY_DN40728_c1_g1_i1.p2 type:5prime_partial len:124 gc:universal TRINITY_DN40728_c1_g1_i1:1-372(+)
KTLTGKTITLDVEASDTIDNVKAKIQDKEGIPPDQQRLIFAGKQLEDGRTLSDYNIQKESTLHLVLR
LRGGVIEPSLAVFARKYNCDKMICRKCYARLPARAVNCRKKKCGHTNMLRPKKKLK*
>TRINITY_DN1819_c0_g1_i5.p1 type:complete len:432 gc:universal TRINITY_DN1819_c0_g1_i5:79-1374(+)

MILQLLLILSIWNVAEGLDNGLALTPPMGWLQWERFRCITDCKKYPKQCISDSLFKRTADRLVSDG
YRDVGYEYVNIDDCWAELQRTKKGEMVANHERFSQGIKALADYIHSQDLKMGIYTDFGTKTCG
GYPGSIFYMKEDTMSFADWGIDMIKVDGCYVCGDLYADGYKAFGFHMNQTARPMLYSCSWPAY
LNDKGKPYKEIAEHCNIWRNWADIQDSWDSVLSIINYNGEHQDEIVPYVGPGNFNDIDMLLIGD
FGLSQEQCKTQFALWAILAAPLFISADLDTMQDWQKEILLNKEIIDVNQDVLGKMGKRYYNENN
QQTWMRPLSNGEMAVAMMSTRTDIPVQMGFTLQQLSLNGIYNVRDLYTHEDKGNITQGYEAEV
NPTGVVMLKLTPVKSEEVEPAVIHSGYENAEVNEIKEKIEEMYEYIIY*
>TRINITY_DN1058_c0_g1_i1.p1 type:complete len:2639 gc:universal TRINITY_DN1058_c0_g1_i1:134-8050(+)
MVKYIIWILLLSIFKEAYGGDWAQWQNWSACQDSSGAPIGCGRGNQHRSRTCADNNCGAGAIIE
VRPCLDEICPTFNCQNSSEIAGMCKFDYSNSCVGDDNCHHCICKSEGFKVSGDQCYNYCVEVGC
DEEIEGVSQLKRYCGDQAISCTGTVLNRTCLCNPATSYPSKDGQYCYEATCKTSQEFMTNCGAEN
SCQGTNTSFVCECNGFGYGYEVVANVQNRKKCKLLSCEDGDLNPLEICGQGATACTGNVHNLT
CACNVTAGYIPSEDKKSCIPMPCNTDPAVGVQCLPRNTCEGSKIDWRCICSAYGFRNDPTDPKKCI
EAKCDDPNIDILGLCGTGVTSCTGFLSKRRCVCNTTENYIPTLDGSHCMPAPCNANENATKDCG
GENSCIGSFDSYMCVCNGYGFQKNATINKCEEVPCSSLPTAEVQRICGFGALSCDGTIRNPVCNC
NNDLQYYASEDMKSCIEASCSSKADYDSHCGNENSCVGNITHFECICETEGYKPDPSNKKKCILES
CELNEVDVLNVCGKGAISCKGNVRDLECVCDASKGYVQSPDTNKCEPSSCYGNATVSEQCGEEN
HCLGDLSDWICVCDAYGYKVNATNSMTCDKVMCGDTGADAVTVCGKETTSCSGHIAKPVCTC
NETAQYLPSPDSTRCVKASCKENDAARKACVPGNSCSGSYDEFICICDQFSYHQDPADNRKCIRE
KCEDLAADYDYEKICGVGGISCTGYVNQPVCVCNSTESYHPSNDRTSCVKASCSTSDAVRTQCGL
ENSCFGDIESFMCDCKAHGYKLEPINKTSCVLATCEDGDFSYHFQCGKGAVGCSGNINTLSCLCD
SAKGYVPSADMKRCVPNSCKDNPDVYIKCGMENTCLGSYADWFCVCSGAGYEANPADPKSCQL
MECSKDDAIHVCGYGAIDCKGPVLAPTCSCNYSDHYIASEDGTSCVPITCRKNDTARAQCGEENT
CKGHTDNFKCICDAYGFKADNVDHQKCVERQCTDGDADIIRICGHGGIRCTGPIRSAVCECNSTE
YYYPNADRSRCILASCKANETIMKSCGEFNSCEGNMESHICVCNGPGYKHHPSDDKQCVKETCE
MGEIDPIYLCGKGAVKCNGTYDIYTCECDNAMGYVQAPDGNSCKPFDCHANATMHKQCGDG
NRCIGSLTDWICVCDVAWYKPDPSNLMRCHEKTCKEADLELICGTGATGCSGSATNPTCECDSA
NYFVSSTDRRSCIKASCKTNITAVQQCGDGNSCYGTPDNYVCNCDAYGYRLDDNNNQACVEKK
CDDVGANYVEICGLGGIGCKGTVKNPVCECDQTKHYVASPDSTSCVLSNCRGNKTVMAACGNE
NKCFGSASSFICVCDGPGYKRNPSNLQECLPETCEAGEIKPHMVCGRGATACKGTMYHFECECN
NTLGFIRSPGHESCIPVNDCYANATMHEQCGEGNGCQGSVADWICKCDAPGYKPHKTNFQMC
HQRMCSDADIDLVEVCGVGVESCVGPIANPTCNCDTSKHYVLSADSTSCIPGSCKTNETIMAQCG
EGNSCSGTSENFVCICNGYGYRPHETDKQTCVLKKCSHSDVNAVDVCGQGALSCIGTMRDPICQ
CNNDMFFIPSDDSRSCVLASCSANSDVAVACGKVNGCFGSKSSWICDCSSPGTMLNPNNIRECLH
ETCESGKLDPIIVCGKGALGCNGTMSNVTCECDNSMGYVQSPNGMSCVASDCKTNSTMHELCG
DGNSCVGSLTDWMCVCDAPGYHPHPSDPMRCLQKMCSHANINPVDVCGIGATKCVGSITEPRC
TCDTAAYYVPSPDGTYCIPGSCQTNKTALEQCGNGNTCTGTADNFVCICNAPGYKLDDSNNKRC
IEISCNDADVDLVEICGQGSIGCIGTARQPVCQCDHSKHFVPSLESTSCQLASCRANDTIMDRCRM
GNTCFGSMTSHICVCEAKGFKVNPSDPTECVQETCEDKDIDALSACGKGATDCMGTLMNLTCQ
CDGASYVESPDKTSCLPVGCQQNVDVKKACGEGNTCEGSYDDWVCLCTNPEYIPHATDEKVCS
KKKCTDNPIGSCGIGATSCSGNVNIHKCTCNLTAGYMPSKDEQSCIPIDCKMNPKMNCGFGNTC
RGSFFSYVCECNTLGTMRNISNPKQCIEVDCSTDAVSICGVGATGCAVTPTNDVNCTCGEFYHPA
HDYHSCVLDDCKFNKLVPDACGVENECTGSISSWMCVCKGPKYIQDIKSTQLCAPGPCRIEQCGS
GKASGSFQYYAESCSGTYFEPTCNCKEGFEKDSYGGCIVKSCSRNNQSSVDCNDGAISCEGTYFKY
KCECRAPTHILNPANKRSCIPARCNPQLCGRGLVNGDYMDFASECSGSYTDFTCKCKYGLYGSGT
SKCSLEKRTCALSNDCHPNANCIDYDGHYFKCECKDGYSGDGYQCHRNGGLTPWSVVQPCDKT
CGTGYAQVMRTCDDPKPDTLGKKCNVPSPDVREVPCHQFACTACSVKKTCECENIRPYFVHEYV
EPDESLKYFTNLTAEDVEKALSAIEKWKLSVDEKEFCIKHTLPEIDGLISDINENLRSVDQTKLYTIT
ARDMMRNKLDCNVDLEKKDKYFRVYEAIVKRHFILKQVVSDLQAKEKLMVTYRSSCTKDSKVG
WLSSIFS*

>TRINITY_DN1339_c0_g2_i3.p1 type:internal len:185 gc:universal TRINITY_DN1339_c0_g2_i3:553-2(-)
NSRLAMVAILGMFFQDGLTGSAWGDWANYVDSPLRAFENELGVQEPVGFWDPAGFTADGNVE
NFARRRQTELKHGRVSMLATMGYITPELTGKLPGYLSPSAGLKFADVPNGLAAISKVPAAGWAQI
VAYCAFCELSQDQSAGSPAAAGDFGWKLLTSSDQAELTKKLSAEVANGRLAMMAIIG
>TRINITY_DN1676_c0_g1_i12.p1 type:complete len:1631 gc:universal TRINITY_DN1676_c0_g1_i12:206-5098(+)
MLIITRTAKLLLLSLTYIYLTIAQDLTNATLNNGFEAGRAIKCKTDDGMVQRCMPVFENIAFGRDII
ASNTCGQTRQTYCVQTGTSGAVKICEVCDASDPDKAHPADLMTDLQFDDQPSWWQSETILDNK
SPVYLTLNLNKKYNVAFIRLRFHSVRPHSFAIYKKSTYDSNEQWLPYQFYSRTCQETYNVPNQQSV
SYSNQKLALCTDEASGMIPLSGGNVAFLTLQYRPGKRDFDNNPGLQDWVTVTALRFELDRVNTF
GDEVFGDPKVLRSYYFAISDITVGGRCKCNGHGSSCNNNNNEGTFQCVCEHNTAGKDCEKCLPI
YNDRPWRKASGRSANPCKACDCNGLADQCEFDQQLWVDSRYRSGGRCVNCKNNTDGINCEYC
KEHHFRSNVKEACKPCRCNPLGSRSLSCTTKGECACHPGVTGLKCDKCKEGYYGFSSSGCKACN
CDRDGSIGVNCDFTGQCNCKKKTVGKRCDQCAPLTFDLDRSNPKGCKECFCFGHGVSCDAIEGI
GVKEIFTTFEADFDGWKLQDESGASFDSNMEWDFRGQYISVKPPRNKQLYLVAPQRYLGNKLSSY
GKLFTFAYGIFLDPNDPLVSAGRDIIFEGNGLQAVYPINSQNNILPEARFYQFKYRLMEPVGMSTFD
FQKILVNLTAVKIRTAYMNERTGAIDNIKLYSTKFISINSPEQVTWQEICECEEGYTGDHCEKCDSGY
TRSGGDTSPFGECKKCFCNNHGNFCNPDTGICQCLHNTTGDSCDQCAPGFYGNPTAGTPEDCK
PCPCLYGNECILIGNSVKCTDCPEGHIGDLCELCDDGYFGDPNGTIGEPSRCQKCDCNGNIDPNAI
ANCDSLTGSCLKCINNTKNGPENKCELCADGFYGNSTIDPKGNCSACSCYEKGTMKPPFHDPKL
QLPCDTNGKCECLQNVIGDKCDMCPPGHWNVASGKGCELCNCNATGSKGSDCNLQTGQCEC
QDGVGGRKCDQCAPRFYGFSKDGCIACNCHPDGSKHQNCTQDGVCECLPGVLGIKCDQCPEN
KYNLSVGCIACPRCFDLIQVAVNKLRNKLKTFNITGNLTTGSIDIKDPEFEKLFNELKRNISELEQK
MKDLLEGDMAILEFYEVLVNQSSTLIEMIPGIQKMIKKSEGAAETGSVTISKAEEITEEIQNLLGQAE
KMLEAETGRIFKNVTSSSEDVSGLSKRMSEIAEEARKHADRQEQESKDIEFNTDMAYNNSKAAYE
QAKKARDTEEAISYLLDQMALGHDEALALSKNSTEIIAKANSTSIAALNDSDHLMMKAKEPLPN
LDIEAMKEKAKNIDAEGKATEFNATDLHEKNKDKITQLQNDDKEARELMERGTQLHNDSNALY
KIAKNASDEAKQAQEDGLKVAKDAQDMLKTLMEFENNIAKSKANATEALKLIGLIRRLISKANK
TTENASSNAIMASINATAALRLASAANKTTQDAMNEIESSRREVEKLLNETMDFMMKEVAQTKD
DIDDIEKSLEESEKLAKSNSMKLGDALNDSQAATDKASMVQNELLKSLDQIEDLMNEISNLDEIDF
ETLEDAEKRFEEAKDTIENKLKNEIEIMESKLSDQKKLLQQHELDLTPLKEQIMKNEELFKYLPKSC
FREKAKPEDIDK*
>TRINITY_DN8903_c0_g1_i1.p1 type:5prime_partial len:447 gc:universal TRINITY_DN8903_c0_g1_i1:1546-206(-)
DMATKACGLENMCVGSYKSWRCICQAPRYMQDPDNTQSCIHAPCRIEDCGSGYAPGSFQYYAES
CNGTFFNPICNCQEEYEKDMLGGCVPKSCRENPIAVAECGENGYCKGTYFKYECGCQGPKYVQN
PRNTRQCIEAPCNVQNCGHGFENELYVSFADSCIGKHADYTCQCKEGYTGTGTSRCIPDGKECGK
SYCHANATCAPLGKGEFDCTCKPGYSGDGLYCFLDGGWSDFVNTRECNASCGTHGYAEVTRYC
NNPVAERFGKQCGAKSPEQLEVPCNRFDCLGCTVKKDCRCTSVVKPLIVEGYNEPEETLKFFTDL
HPDDVKKAKDTLLKWKAFSNENLCRKYNKTTLHAKFFGVRDAAKEVNKMKSSYGSARDSMRI
KLDCNINIAKKTQYFGVYEMIVKRLFIMKQVVKDLHIIEDYVLKHMENCDDSPVGMIRNTFGK*
>TRINITY_DN2472_c0_g1_i1.p1 type:complete len:344 gc:universal TRINITY_DN2472_c0_g1_i1:311-1342(+)
MSSGEIESLRQEAENLRNQIKDAQKVAADTSFSSVAQNVDGINRIQLRTRRTLRGHLAKIYAMHW
ASDSRSLVSASQDGKLIVWDSYTTNKVHAIPLRSSWVMTCAYAQSGNYVACGGLDNICSIYSLKTR
EGNVRVSRELPGHTGYLSCCRFLDDSQILTSSGDTTCCLWDIETGQQLTQFVGHTGDVMSLSLCN
DSGRTFVSGACDASAKVWDIRDGMCRQTFIGHDSDINAVSYFPSGLSFGTGSDDATCRLFDIRSDQ
ELMCYSHDNIICGITSVAFSKSGRLLLAGYDDFNCNIWDTIKGDRSGVLAGHDNRVSCLGVTDDG
MAVGTGSWDSFLRIWN*
>TRINITY_DN123121_c0_g1_i1.p1 type:internal len:127 gc:universal TRINITY_DN123121_c0_g1_i1:3-380(+)

LNQKLCAQQVIHLEITDANDNFPVFSQSRLSLNVSEYTEVGQVIATVRANDLDERSQGNLLFSILK
DEDFFGILSNGDNGVIFLKQSLDAERRLTHQIEVTVSDREGKSSLQNMEIIVFVEDENDN
>TRINITY_DN17095_c0_g1_i1.p1 type:internal len:607 gc:universal TRINITY_DN17095_c0_g1_i1:1820-3(-)
CVEHAHCKRNEFHCIGTMNCILKSSLCNSVKDCQDGSDEGPKCTFSSCNDTCSYKCQKQPSGVG
ACICEPGKILADDGHSCIDENECDQVPSPCSQHCNNTEGSFMCSCTYGYILQDDKRGCKLNVSKE
EELLFVDVKEIKRYYMRSKLLQPVFTNLSRGVAFDFDSASGKLFYTDVTQNVIYSSYLDDFAKVVH
GEKRILDRGLAIPDGIAVDWICKVIYFAESAANRIDVIEFNGKNRASLISANLTTPRGLALDPRVGY
LFITDWGGKPKILRSQMDGSNLKVLITEKVGWPNGITIDYYDKTVYWVDARYDYIDAIDYNGNN
RRNIVKGKDLIPHPFSLSILDDDIYVTDWVLNGVLKISKLPGTKKVEQLVYNLSRPMDIHVISEKRQ
PYTDNPCDTRKKGNRCDQLCVIKSATESSCLCQARYILGSDGRTCEPANEFLLFARSWEVRGISLN
ASYLNDVMTPVLGLNSAVGTDFDALSEYVYFSDVKTDKIGRTFIGNDKGGKIEWIVEGNLDNPDG
LAVDWVGRNLYWTDSRISRQSEIGVSKLDGKFRKSLIQEPLGKPRGIVVYPEKGYLFYTDWMRPAK
LGRIDMDGSHLMILVN
>TRINITY_DN468_c1_g1_i1.p1 type:complete len:191 gc:universal TRINITY_DN468_c1_g1_i1:676-104(-)
MKTFYTILLLAIVQTNAFFFPKVDTVKDLDLASYTGRWYEVYSSLIQRATFQRNSYCTTADYSLKPS
GAIKVINSGRLKSATGEENTIEGEAKVVDAKEPGALNVKFSTGPQSDKANYLVVKLGPVVDGKYD
YTVITTDYKALVWVLARNPETFKQKYEKDVLAYLKKNGYNWFINKPRPTYQGKDCIYI*
>TRINITY_DN10724_c0_g1_i1.p2 type:3prime_partial len:148 gc:universal TRINITY_DN10724_c0_g1_i1:442-2(-)
MLAFVGMVVQQWWQIPGLPQQPDWVKALDECYASPVAVWGIVQISVFAAVMEGHFFPEYAWE
GIMKREPGDFGFDPLKFSKRPGFDLKKKQLIELKNGRLAMIGVASMASNHVLPGSVPFLTGAYAV
RPEARAPGAPAGFCGSTGPR
>TRINITY_DN186979_c0_g1_i1.p1 type:internal len:357 gc:universal TRINITY_DN186979_c0_g1_i1:1-1068(+)
LGSGVTTVLIDIIDVNDFTPILSVSSSNVNIKENGLIGTIIAVFNVRDDDTFTKNNNEITYNLRTGATS
FNILKVSETTAYVVLSRQLDAEQSVSTNLELFAVDSNGGKSNSQIVTLNIEDVNDFNPQFVLTNYIF
DVQETNNIPINVGLVKAMDNDATSVNNQIIYSLLGSIDTQVLINETSGLITLNQAIDYESEKEVVFT
VVATDNSFPPISRRQASANVLIRIQNADDNKPVFKSATTIVVSESTSIGTTVYIASAVDADESDVTTF
SESTQGPFRVERDGKVILQSLLDYETKEFYTLSILALSNGQPGTVLNVQVDVQNENDERPRFINTPY
QVTIAENNALSNIINVSA
>TRINITY_DN1797_c0_g1_i2.p1 type:3prime_partial len:998 gc:universal TRINITY_DN1797_c0_g1_i2:2993-3(-)
MSKIKLNIVYILLLVLWISIVHSQSQDDRCPAIDNEQECPPPDSLNNECLENSDCAGNQICCGDGC
ELKCVVAADELCKVPIDLGFILDSSGSIGSKFDNVKIFVEKIIDAFVIGEDFTHIGIVEYSENASVVLA
FNTYQGAELTKANILTELRKMSLIGGHTRIDLALEVASRDLFSAKGGVRPGKPKIALVLTDGKQTR
GKLAPDAKDLHIASQPLKDSEIHVYSLGIGDDYDIGELLDIASDDTSVFRSQDFDELLNIVSQITEET
CKGCTRAIDIHFALDTSINVGRENFEYMKNFVEAIGYRFVISETSSHMSASIFGEEPMLMFLMNEAT
EQNMFLEEVKQLQYIESANGNMDRLLRMVYDDIFTLEGFTRQSVPKVLVMLTASNCDSCDERVEE
AVKPLKEQGVHVVTIPIGNNVNMDEINAMASYPSSEYVVPRQTYVGLMNDFFIQKVSDMICSGRP
GVCAEPPVEGSCDTIIQECTTDVKCEGNRKCCIKNCKASCELPVSACITPVDLAFAFETTRNGEAD
FENAQKFAANLLDHFKIDRSTTHVSVSTFSETIGTSTFFNEEYDKSNIQQFIKDLGSDSGDQSNLGD
FLEFASRKIFSVQAGLRQSTPRVLVIFTNGIVSAEQSLKAQQEAQKLKAEGLDVSILVVNIGGNIGSV
DLFESIISNGRIFSYVESSELSKFEAETDLAEAICATKVRVRRCPPKRTPDTCNVKDDLCSSDFDCPG
YQDCVSDGCRLRCQTCEYRCEDKLDLLFMIDSSYRIGAEGFQKAKENVNKMLEWFTIAPDNTHV
GVITYSDTANIAMSLPPLGTNNPPQSLPDVQGRINAIPYNGGSKSYLDEALKLAAAQVFVENSDRF
RKGSKKAVVIITDGKDDSTSRLDLASWPLRKQRVVVGKEDYSAIHILAVAIGDELNTNALENVVTP
TLEENFFFAANYKDMYNEIRKLAKSSCEYAGAFTAPPDIYMKGEPGPPGPPGPKGDPGPDGPDGT
R
>TRINITY_DN79126_c0_g1_i1.p1 type:complete len:115 gc:universal TRINITY_DN79126_c0_g1_i1:397-53(-)

MVARLVLRRRLSYNTKSNRRRISKTPGGKLVYLYTKKAGATPKCGDCKCKLRGLKAVRPKKLAKL
SKTKKTVTRCYGGSRCGGCVRERIVRAFLIEEQKLVVRVLRAQQVKKSS*
>TRINITY_DN691_c0_g1_i2.p1 type:complete len:1673 gc:universal TRINITY_DN691_c0_g1_i2:5503-485(-)
MMNLFRLIVLGVICFSYEGDAQQCGGSCSPCGNLCVGMKGDRGTAGLPGFRGPPGLPGFPGPEG
PIGQKGEKGPAGLNGMEGSQGARGKVGPPGLPGTPGRHGRPGNEGREGEAGLPGCNGTKGDR
GPEGPRGRDGIEGPQGLQGNVGPRGEPGDSTATKLKGQKGEPGSAGQQGEKGGRGMEGMKGD
TGKPGRNGRAGERGPKGEPGTSNVTIIGEKGSKGSVGPPGLPGRQCQVTGDKASNIAQGQKGNQ
GSKGEEGAKGEKGDAGLAGPQGEEGDIGPQGDRGDDGPQGPPGPEGEQGRSGIQGNKGQKGE
VGNNGKDGNDGQTGLTGQRGRQGTPGQKGEPGKDKKGLRGPEGAKGERGNDGEKGSDGPIGP
IGPRGPKGELGPLGMKGFKGSIGSAGPKGENGNPGDPGIKGRRGELGIKGDSGVKGAPGNPGISV
TGPDGMDGDTGPRGQSGGKGAVGFPGKPGPPGVVTIDGEEVLPGEPGPQGLKGFRGVQGDAGS
KGSRGEKGNACESSKCPAGPKGEKGLSGQDGLQGGDGEEGEKGEKGDKGDIGSRGDTGSRGEP
GPQGESGEKGEKGIQGPQGNSGKDIIVQGERGDKGTKGNAGTPGSIGFPGERGSPGESGDRGLSG
VNGLRGETGDRGARGVAGKPGVNGQKGQKGEIGSGARGFKGDKGEVGPDGPAGLRGPGGPVG
PKGDPFPTAVNGKTGEPGEQGAAGPKGAQGPVGPEGPRGFPGDIGPVGEPGDGEKGERGDDGA
RGERGEKGEPGQDGKDGDPGPKGEPGQEGKEGPPGMFKGNKGEKGATGLPGVDGLPGNSAPK
GEAGKDGQDGDPGPAGEKGDPGEAGSPGTEGPIGEDGPVGDKGEPGESRAGPKGNEGRAGAK
GENGRDGRAGRPGIGRPGDKGELGQKGEAVIGAKGSKGEQGLEGPPGPIGKPGVKGDAGVPGK
DGEAGGRGKDGERGLPGDKGDTGDIGFNGSPGAPGDKGEPGYSGQKGAKGMSGPRGPPGSTSN
VVGPKGNRGLPGMKGEPGQQGEKGLIGPQGSKGESGFGSEKGIKGSRGNPGAVGPSGEKGERGD
RGKDGLQGIAGPRGEEGNPGEKGSRGDQGPQGREGPSGELGPEGQKGDPGFAGEKGEKGDRGG
DGQKGDAGEPLRGEPGVAGPPGPTGPQGERGPKGEEGNMGPKGSQGRDGLTGEDGPKGSKGEP
GLDGDVGEKGEAGFQGLQGEKGNQGPQGLEGSTGMDGQKGQIGEKGVEGPPGPPCEPVNSVR
GETGEEGPKGDRGPKGQKGKPGPQSQAATPGEKGSKGEVGQPGPQGIKGEPGREGDFGPKGSPG
AQGEKGDQGQKGEPGQEGPEGLKGEPTRAFGPKGQKGETGLEGLRGPVGPKGEDGQTGPQGP
DGPKGNEGEKGDFGINGLNGAAGPSGPKGAIGLKGDSGPPGPTGAQGPRGSGGSPDFGFYYVK
HSQTTIVPSCPAGMKTLWTGYSFLYAQGNERAYGQDLGQPGSCLKRFSTMPYLFCDLQNQCTVAS
RNDYSFWLSTDEPANMMKPAAGPEVEPYISRCAVCEAPANILAVHSQSVIVPDCPNGWENIWAG
YSFLMYNSAGADGSGQLLASPGSCLEEFRANPFIECHGRGTCWYYGPTLSFWLSTIDDSNQFQIPK
SETLKAGNLKTRVSRCSVCMKTVRNGNFP*
>TRINITY_DN6044_c0_g2_i1.p1 type:5prime_partial len:661 gc:universal TRINITY_DN6044_c0_g2_i1:2126-144(-)
YMTPSNSPNDKQYGKVEGSGPSVTFVFQNVLPGKYKVFPVRKNWCWSPLNKEIAVEESNIGDIEL
THSGYHLKCKISHNITLNFTLEGRNEVVGSFELKQGSNQFCVKAAGTYSLTPKSCYQFEKLTYTYK
TSDPKSLTLSVAFYKVIIQIISSKKVSDLQVKVRTSTSEDILTPTYVESKDQNYGFLYEIVHWAKLAQQ
LIVTPISKEVLFLPKSSSVTVRENCPGGQVQFNGKEGIFVEGEIVPPFSNVFVIIKTESNEYEEGKVME
VMTDENGKYRVGPLHGGTVYSVDAYKEGYFITPFDKKKGSFVAQKLGHITVKVVDENNEPMSSV
LLSLSGGQYRNNNLTQMNGAFTFSNLGPGQYFLRPMKKEYNFEPSSKMIDVSEGEDVIFHIKGTR
VAFSVSGFVSTLSGIPEKSIAIEAVGMHKCSEYQESTITDNNGNYRLRGLMPGCAYNIRMMRSKEN
EQVERLAPANHVFQIQKEDISNVNFIVFMKPTKFHLSVQVETEYDYLPNLKVVLYAEDNPDSPIHT
LTMGAIKYIKFPPLPPKTYVVKLHSNLSPSTHETKSTSATIEASQDLSKKHVKLKFDVKKRRVDMEP
TQSVIALPFAVALVFLAYNYDAVLVFLLRAKAFMQSLNQQEEDENESNQENEGLSLNKKKKKL*
>TRINITY_DN10563_c0_g1_i1.p1 type:internal len:307 gc:universal TRINITY_DN10563_c0_g1_i1:1-918(+)
GMFFQDGLTGSAWGDWGLYVDSPLRAFESELGVQAPVGFWDPIGFTKDGSAENFSRRRQTELKH
GRISMLATMGYITPELTGKWSGYISPSMGLRFEDIPNGLGALSKVPALGWAQIIGYMAFCEISQDQS
AGSKGAAGDFGFKVLTASDPEALKRKLNAEIANGRLAMMAIIGMFFQDGLTGSAWGDWGLYVD
SPLRAFESELGVQAPVGFWDPIGFTKDGSAENFSRRRQTELKHGRISMLATMGYITPELTGKWSGY
ISPSMGLRFEDIPNGLGALSKVPALGWAQIIGYMAFCEISQDQPAG

>TRINITY_DN95387_c0_g1_i1.p1 type:complete len:169 gc:universal TRINITY_DN95387_c0_g1_i1:96-602(+)
MAATLKPYLNAVRATLEAAMCMENFSSQVVERHVKPEVEVQSSKELLLTPVCIARNEKEKVLIEG
SVNSVRVSIGVKQADEIEKILCHKFMRFMMLRAENFFVLRRKPIKGYDISFLITNIHTEQMYKKKLV
DFVIQFMEEIDKEISEMKLSLNARARISAEEFLKNF*
>TRINITY_DN194146_c0_g1_i1.p1 type:complete len:190 gc:universal TRINITY_DN194146_c0_g1_i1:165-734(+)
MKTVLASQTVTIPEDVSVSVNGRTIVVKGPRGTLKRNFNHLNLVLTRVNKTTIRVDVWFALRKHL
ACLRTICSHIENMMKGVRFGFLYKMRSVYAHFPINIIVNDGGKVVEVRNYLGENYVRVVKMREG
VICKPSGNKDEIIVEGNDIELVSNSAALINQCVKAKNKDIRKFLDGIYVSEKTTIVPMTE*
>TRINITY_DN6397_c0_g2_i3.p1 type:internal len:130 gc:universal TRINITY_DN6397_c0_g2_i3:387-1(-)
LTSSDQAELTKKLSAEVANGRLAMMAIIGMFFQDGLTGSAWGDWSLYTASPLRAFENELGVQDP
VGFWDPAGFTADGNVENFRRRRQTELKHGRVSMLATMGYITPELTGKLPGYLSPSAGLKFADVP
N
>TRINITY_DN16548_c0_g1_i4.p1 type:internal len:171 gc:universal TRINITY_DN16548_c0_g1_i4:2-511(+)
LYTDSPLRAFEGELGVQAPVGFWDPLGLAADGDSVAFRRRRVTEIKHGRISMLACMGYITPELVG
KFPGSLSPSQGIEFADVPNGLAAISKIPSYGWFQIIAYCLYCEASALNFGYFNEAFDQKAPGDYGW
RPPLLATDDAALKTKRLNAELANGRLAMVAIIGMFFQDG
>TRINITY_DN571_c0_g1_i2.p1 type:complete len:186 gc:universal TRINITY_DN571_c0_g1_i2:192-749(+)
MREYKLVVLGSGGVGKSALTVQFVQGIFVEKYDPTIEDSYRKQVEVGSQQAMLEILDTAGTEQFT
AMRDLYMKNGQGFVLVYSITAQSTFNDLMDLRDQILRVKDTEDVPMVLVGNKCDLEDERVVGK
DQGQSLAKQFGNCTFLETSAKMKINVNEIFHDLVRQINQRQPENTKSERKKKGCTIL*
>TRINITY_DN902_c1_g1_i1.p1 type:3prime_partial len:153 gc:universal TRINITY_DN902_c1_g1_i1:146-601(+)
MIRIAIIAIAIAACVYESQAVRCHVGYSAADLPLAMSVRDVECGQGFDHCVTLKYVLDGMKITQG
NCATKFGQATCQFMRNSHAAMTDCSEAICSGDHCNSAVKEAEKIEMKRVGAYENTLKCTTGLQ
SDFPNIPFASPNVPYAVDECFPGT
>TRINITY_DN2885_c0_g1_i2.p1 type:complete len:114 gc:universal TRINITY_DN2885_c0_g1_i2:31-372(+)
MAQPVRLYSKGIFVGYQRGLRNQHEQTSLIKIEGVNTKADTSFYLGKRVAFVYRVSKKTVAKGDK
KPSRIRVVWGKVTRPHGASGVVRAKFRHNLPAKAIGGSVKVMLYPSRI*
>TRINITY_DN141214_c0_g1_i1.p1 type:internal len:179 gc:universal TRINITY_DN141214_c0_g1_i1:2-535(+)
KFIQSLYNVTIPDTVQENYKIVTVSANDADSASNSNNVIRYEINSSSDTFVVNVVTGSVTLKPNINL
KGTNIKEYRIEIKAFNPDLPNSFDLAVVEIYVTKTQASLTATAIVSSYTTLTLPENQPAGAITNPITGR
NSFVIIYDSNTNPSGVIFSVDQTGRVFLNKPLDFERENQIRF
>TRINITY_DN6132_c0_g1_i1.p1 type:complete len:161 gc:universal TRINITY_DN6132_c0_g1_i1:95-577(+)
MTNPHGKRRGTRYLFSKQFRKRGVEHLSTYLKTYRVGDIVDVKGNGAIQKGMPYKYYHGKTGR
VFNVTRRAVGVVVNKKLGNRIIPKKINVRVEHIKHSKCRDDFLRRVKENEIKKKEGKAKGIKVDC
RRQPVQPRGAHFVSSKNNAPSLVQPIPYEFII*
>TRINITY_DN2487_c1_g1_i3.p1 type:internal len:317 gc:universal TRINITY_DN2487_c1_g1_i3:1-948(+)
EINNGRLAMVAIIGMWFQNGLTGSAWGDWALYTDSPLRAAAPDFSKEIGACPPLGFWDPLGFSV
YKDPELAAAQFRRRRLVELQHGRVSMIACIGYIVPEYFRWPGFISPSEKLAFEDIPNGLGAAAKVPI
LGWGQMIGFAGAIEISNLSSPSSKTPGDYAGYGAFGLPFGKGIDDPAKREKSLLAEINNGRLAMVA
IIGMWFQNGLTGSAWGDWALYTDSPLRAAAPDFSKEIGACPPLGFWDPLGFSVYKDPELAAAQF
RRRRLVELQHGRVSMIACIGYIVPEYFRWPGFISPSEGLAFEDIPNGLGAAAKVP

>TRINITY_DN923_c0_g1_i1.p1 type:complete len:442 gc:universal TRINITY_DN923_c0_g1_i1:53-1378(+)
MAEKYDLTYKVGAYLDRHMVFPLLEFLSSKEIYDDTELLRGRLELLQGTNMLDFVKDEYQRLHQT
DEVPDELANKRQEVIRQMRELEAATESITAAFEDPDFQKEASSARDGAQLFKYLNETFGIRSENLD
TLYQYAKFQYECGNYNGASQYLYFYRVLVHDDDKNASAAIWGKLACEILTQNWDPALEEINRLK
EVIDGDHFSHPLQLLQQRTWLIHWSLFVFFNHAKGRDLLIDMFLYSPNYLNAIQTSCPHVLRYLTT
AVITNKRRKQILKDLVRVIQQESYTYRDPITEFLECLYVNFDFDGAQKKLLECETVLLNDFFLVACL
EDFIENARLFIFETFCRIHQCISIQMLAEKLNMTPEEAERWIVNLIRNARLDAKIDSKQGHVVMGT
QAAAVHQQVIERTRSIMFKTQAITTNIDKKLASLQKQPYTEPFGLAW*
>TRINITY_DN6294_c0_g3_i2.p1 type:complete len:134 gc:universal TRINITY_DN6294_c0_g3_i2:817-416(-)
MGYKRFVEAGRVALINKGKYDGKLVVIADIVDQKRLLCENPVKSVPRHVVRTQDVNLTDLKVNI
PCGARGGKIRKEYEKEEVDSKWEKTVWAQKIKARHVKRTLSDFDRFKSKIQKQRRNRKVKAVLK
SMKKN*
>TRINITY_DN20278_c0_g1_i1.p2 type:internal len:154 gc:universal TRINITY_DN20278_c0_g1_i1:460-2(-)
ISMLATMGYITPEVTGKFPGYLSPSMDLKFADIPTGLAAVSKVPALGWVQMVAYAAYCELSGPRTR
AANVEDYADITPGDYGFKVLTSSDPDELTKKLNAEIANSRLAMVAILGMFFQDGLTGQAWGDWS
LYTDSPLRAFETELGVQAPLGYW
>TRINITY_DN902_c1_g2_i3.p1 type:internal len:178 gc:universal TRINITY_DN902_c1_g2_i3:2-532(+)
AVFKCIKLNYIDHIIKMIKIAIIAIVITALVHESTAVRCNVGYSTADLPMAMSVRQVECGKGLTHCN
TLSYKLDGMSVTQGNCATSGGQASCLFMKNSLQSMTECSEAICNGDHCNKAVKDAENAERARV
GPYKNTLKCTTGLQTDFPDIPFASPNVPVGVDECFPGTKECVTMTYD
>TRINITY_DN5188_c0_g1_i8.p1 type:complete len:520 gc:universal TRINITY_DN5188_c0_g1_i8:154-1713(+)
MELSNIVIVSTILFLELVLAVPDQPPFTAQIVESLVKKEIAKKMPAAVSSDHKKKSMAGVHLKEATD
AINKLVDESIVDRVQEAEKEVSQMLLRSIERHIQDAGKVLLKQDENTCKDKIDSCDRLKEAGVCES
SKDAMVTNCPATCGFCGMLDNMKAAKAEGKKEEKEENETKPMQEQPQKLRADDKKEEELAEK
IEQEMEHVNNAPIVNKDKKPKKEKLNRNHLKKHLTPKLKTSIPEEVRLLHQNMIDETFDHANLP
LAVKTEPVEFEIEGSKAHILPKNDRQEEEESDNEQNEEVENEEKTKKQRQLEEEEKYKADKLKKQ
KEEKLKQEKLRQEQLKEERKKEEKLKIEKLRRAKKIKEERERIVKEAREKEAKERKKVEEKRLKEA
EDKRKAELAKKKKLAKKVKEQQSIMQTLSGNDTGSSGNGETETETIEQTNLQQQTNDQIQQVQL
TPSTEYAYGEPVVRYIQYSQPYEKPTKGVNQDATKRNTDKKVNTNRKLKDEIEVMVDKAFGELLL
*
>TRINITY_DN250_c0_g1_i6.p1 type:complete len:452 gc:universal TRINITY_DN250_c0_g1_i6:131-1486(+)
MRECISVHVGQAGCQIGNACWELYCLEHGIQPDGQMPSDKTIGGGDDSFNTFFSETGAGKHVPR
AVFVDLEPTVVDEIRTGTYRQLFHPEQLITGKEDAANNYARGHYTVGKELVDQALDRIRKLADQ
CTGLQGFLIFHSFGGGTGSGFTSLLMERLSVDYGKKSKLEFSIYPAPQVSTAVVEPYNAVLTTHTTLE
HSDCAFMVDNEAIYDICRRNLDIERPTYTNLNRLIGQIVSSITASLRFDGALNVDLTEFQTNLVPYP
RIHFPLATYSPVISAEKAYHEQLTVSEITNACFEPANQMVKCDPRHGKYMACCLLYRGDVVPKDV
NAAIATIKTKRTIQFVDWCPTGFKVGINYQPPTVVPGGDLAKVQRAVCMLSNTTAVAEAWARLD
HKFDLMYAKRAFVHWYVGEGMEEGEFSEAREDMAALEKDYEEVGVDSVDAEDEEEGDEY*
>TRINITY_DN1566_c0_g1_i1.p1 type:complete len:4626 gc:universal TRINITY_DN1566_c0_g1_i1:42-13919(+)
MDTEEVPGEPPPTVLAATEDVINYVKNITCFIFDESQPCLPLDKILKEEQTKDYFRKFISDVQTKTIL
LYRINQLTDDVDGDHDQGVSQPSYGIKLNVQYVPHKESSIAFIKRGMLLESDKKINAQLHVLILD
NSSPFDTLHSYISNAVSPFFKSYVKTSGKSEREGDKMVPSVEKKIAELEMGLLHLQQNIDIPEISLQI
HPVVQSIIQKAEIERRKISPDDYAEKAEDSSFLNALQNGVNRWIREIQKVTKLERDPSSGNALQEIS
FWLNLEKALHRIEEKQQSPEVKLTLDILKYAKRFHAIVSFTNDTGLKQGLQTVASYNPLMKDFPLE
QLLSATELERIKDALIAIFSHLRKIKLSQYPIQRCLRLIEAISKDLMSQMLKVLGTRKFMLMNYELFE
KVMTSAFNVLNCWDDEYERLQQILRDMAKKKKDDSLKTMWRVNPSHKKLQSRLNNLRTFRKQ

HEQLRAVILRVLRPSKVEAKSSNEENAASSGSIEISDDNAVKEVDLAYENFKEIDPLDVSKEGTEAW
ETALKRYGERIDRVETRITSTLRDQLGTAKNANEMFRIFSRFNALFVRPRIRGAIREYQKHLIGLVKE
DIEALHDKFKVQYSHSKASKMSRVRDLPPVSGSIIWAKEIERQLHAYLRRVEDVLGKDWENHIEG
QGLKADGDSFRQKLNTQEIFDDWSQQVQKKPLLVVGRIFSIETLKTKGSNKATRKLKVNFTKEVIT
LAKEVRNLKNLSFRVPLAIVRQANDANQYYPLAISLIESVRTYEVTNDKVFEKESIIPLVAGIRKDIQ
SLLAEGMHLEWQSYKLMPYVMRLAEAVTGYQEKVDELLTSIQKIDIEVQSLATCGFKHEAFQEILI
RIQKLVDELNLHSYSNLNSWVKTLDEQVEAKLAHRLQAGIKEWTRVLRTGNENVDDKDLAIHSP
GGLPDIKPLRVEFFIRNQVIEMHPPFEKCIEHLYNELAVYCSTITSLTKIQSQRYQVGLDRDNAEETY
HNLLTKLPDPNGPNPITVYGEMESMLEDCRKYCEGWTGFQALWDAEMSNLAPRMKTLEDWMT
ILNQIKMSRSHFDTTETMKEYGPLIVDFNAIQGKVNLKYDSLQKAIVQNFGGKLGDEVNDFFTNV
TKARTDLEQQSLDVATTAEAVTFITYVQGLKRKMKTWEQNVEVFREGQKVLERQRYQFPSNWLY
SDNVDGEWSAFNDILKRKDGAIQTQVAILQQKIVAEDQAVEERTFNLLADWDKGKPVSGNTKPD
SAIQTLQIFEGRLTRLKEDRDNLQKAKEALELIEPGTVPPSDERVQVAIEELSDFKSVWGELEKIWA
QVDDLKEQPWVSIQPRKLRQSLDSIANGMKILPAHMRQYAAFDYVQKIIKGYLKANMLVVELKS
EALKERHWKQLMRKLSVKWNLSDLTLGQIWDCNLQKNEVVIRDVINIAQGEMALEEFLKQVRE
SWQAYELELINYQNRCKLIRGWDELFDKVREHINSVSAMKLSPYYKVFEEDSKQWEDKLNRILNL
FDVWIDVQRRWVYLDGIFSGSGDIQHLLPIETQRFGSISTEFLTLMRKVAKAPLVLDVLNIQGVQRS
LERLADLLGKIQKALGEYLERERASFPRFYFVGDEDLLEIIGNSKNVGRLQKHFKKMFAGVHTIIL
NEDSTLVTGICSKEGEEVIFNKAVSLIEFPKINEWLTLVEEQMRLTLATLLTLSAKEIKSFTGDSIDPK
LYMEWVDKYQSQLVVLSAQIVWSENCEAALQTCYNGDMSSLEKVLSIVEATLTVLADSVLQEQP
QVRRIKLEHLITELVHQRDVTRTLVRSKVQGTKSFAWLSQMRFYFDPKQRDVLQQLSIHMANAKF
NYGFEYLGIQEKLVQTPLTDRCYLTLTQAIERRMGGSPFGPAGTGKTESVKALGNQLGRFVLVFNC
DEKFDFQAMGRIFVGLCQVGAWGCFDEFNRLEERMLSAVSQQIQTIQVALKEGPKEIGKPIQIELI
GKDVKVSEDMAIFITMNPTYAGRSNLPDNLKKLFRSIAMTKPDMNLIAEVMLYSQGFRTAEVLAG
KVVPFFKLCFEQLSAQSHYDFGLRALKSVLVSSGNVKRDKILSIKQKMQAQGVVADVGKITETLN
EQEILIQSVMETMVPKLIAEDIPLLHSLLADVFPGIKYVNAEMTALRNELKKVCNEMHLVYGENL
ESGAHWIEKVLQVYQISCISHGLMVVGPSGSGKSTAWRTLLKALGKLEGNEGVCHVIDPKAISKD
QLYGVMDPNTREWTDGLFTHILRKIVDNVRGELSKRQWIVFDGDVDPEWVENLNSVLDNNKLL
TLPNGERLSIPPNVRIIFEVQDLKYATLATVSRCGMVWFSDDVVSPEMIIENYIKKLRNVLLEESEED
IRSKQSKSVTDSKDELVSPTLQVQRDFSDILTPHLASNGLVLKTLEYSYSKDHIMDFTRMRALNSLF
SLLNQGVRNILQYNQQHHDFPMSQDTIEAYTPKYLVHSLLWCMSGDGKLISRLDISNYIRNITTIPL
PSPVTSLIIDFEVNMQGDWVPWSNKVPQIEIETHKVASPDVVVPTLDTVRHESLLYTWLADHKPL
VLCGPPGSGKTMTLFAALRALPDMEVVGLNFSSATTPELLLKTFDHYCEYRRTPGGLVLSPVQIGK
WLILFCDEINLPDIDAYGTQRVISFLRQLVEHNGFYRPSDLQWVSLERIQFVGACNPPTDPGRKPLS
HRFLRHLPVIYVDYPGPSSLTQIYGTFNRAMLRLVPSLRSFSQPLTDAMVEFYTKSQERFTQEMQP
HYIYSPREMTRWVRGVLEAIRPLDNLSPDGLVRLWAHEALRLFHDRLITDEERRWTNTNIDLVAQ
KHFPNVDLISLKRPILYSNWLSKDYLPVDREELRDFTRARLKVFYEEELDVPLVLFDEVLDHVLRID
RIFRQPQGHLLLIGVSGSGKTTLSRFVAWMNGLSVVQVKVHRKYTGDDFDEDLRIVLRRSGCRNE
KIAFIMDESNFLDSSFLERMNTLLANGEVPGLFEGDEYTTLMTQCKEGSQRAGLMLDSGEELYKW
FTQQVMNNLHVVLTMNPSAEGLKSRAATSPALFNRCVVNWFGDWSTGALYQVGQSFTNKIDLE
RSTYKAPDYLPVVFENLPMPPTHRDVIVNTMVFVHQTMHKANEQVTARGGRTVAITPRSYLDFIS
QYMKLFNEKRSDLEEQQLHLNVGLQKIRDTVTQVEELQKSLSMKSQELESKNELANKKLKQMLQ
GQQEAEKKRITSKQIQDDLKKQTEEINKKKSQVLGELDQVEPALLDAKAAVKSIRKENLDELRRL
GNPPTAIKLALESICLLLGTPSVEWKEIRVLVSKTTFIPSIVNFKTDDISIETRKQMMKYLNHSDYNF
EKMNKASVACGPLVKWAIAQVSYAEMLQKVDPLRRELQKNEDEMMSNQSKLDSVGEMISELEK
SIAKYKEEYALLISEAQSIKADLAAVEAKVSRSTALLRSLSSERQRWENTSDGFQNQMDTIAGDVLL
SAAFLSYGGYFDQQMRSRLWGTWSEQMEQSKIKFRSDLARTEYLSTADERLHWQSNSLPADDLC
TENAVMLSRYIRYPLIIDPSGQATEFIMKEYSGKKITKTSFLDDAFRKNLESSLRFGNPLLVQDVESY
DPILNTVLNKEVRKTGGRVLITLGDQDIDLSPAFTIFLSTRDPTVEFPPDVCSRVTFVNFTVTRSSLQ
SQCLNQVLRAERPEVDTKRSDLLKLQGEFHLRLRHLEKELLQALNDSKGSILNDDKVITTLETLKT
EAADIMKKVEETDTVMSEVEAVSEQYRTLAAACSSIFFTLEEANLIMFLYQYSLKFFLEIFQSILFNN
PLLKDKRDPLVRLGILIESLFQVVYNRVARGMVHNDRVSFATLLARIRLKGKSENSYDLEFDHLLK

GKEIVVQVSDVKLTGLSSDQKMALLRLSKLNPFKELSRYIMDNSMGFQEWINSSNATLNVPRCW
NIKDSLTSSGEVLYECLVVQAFRPDNLHAMLRKYVLAVMGEKFLHTAEQELDMVNIVENEIKAET
PLLLCSAPGYDVSGRVDDLVAITNKQITSIAIGSAEGFSEAEKAINASVKSGRWVLLKNVHLATQW
LISLEKKLHTLQPHPNFRLFMTMEINPKVPVNLLRAARIFVYEPPPGLKANLLRTFSTIPASRMSEA
PGERSRLYFLLAWFNAIVQERLRYAPLGWSKIYEFSESDLRVGYDMLDIWIANVSQGRANVSPDKL
PWDAFRKLIGDSIYGGRIDNDFDQRLLQSFIDRLFTAKSFDSDFSLVTSVDGKQSENIVIPDGMRRE
QFVQWCEKLPDYQSPSWLGLPNNAEKVILTTLGERTCVKLLKMSVNTEGSELAYSADSHLKESEV
TGDIRPLWMKSLQQSVRNWLNILPKRIDPIKRTKENIKDPMFRFFEREVNLGVKLLNDILHDLNEI
LEVCEGNRKQTNDLRMLVDTLVKGVIPRSWNRYATPKGLTVIQWIVDLGERMKQLQNIARGTNA
GGSKELKSFKVWLGGLFIPEAYLTATRQYVAQSNQWSLEELYLNVTIADELNPQLKLDDCSFVVIG
LKLQGATCRNNQLSPVDTITTDLMDICLCWTKIDPRKPPTLGANDVTLPVYLNQTRCELLFTVDM
STTGAVPPRSFYERGVGFVVSALTG*
>TRINITY_DN30329_c0_g1_i1.p1 type:5prime_partial len:213 gc:universal TRINITY_DN30329_c0_g1_i1:1-639(+)
EPIVNRVLVKRMKVMEAINDVSPAMMLDFFKTTLATNFLKFPFFEAINAFCAALPIAPSVRGLFTG
FVFTTATLPVTNYRYRKSMQLEVNWANIYEAYFPTVIRDIVYGICRNYCTIWTLALNPQWTAVSPQ
VLFIVVILGCLGSAPFNEWRGYLLQSKGQELTFKEFFKPSNFVRSTSLGAIKQGLALAVGYWCAPP
ATRLVHGLFAGLRG*
>TRINITY_DN7504_c0_g1_i1.p1 type:complete len:414 gc:universal TRINITY_DN7504_c0_g1_i1:87-1328(+)
MAKIPGGIVVEMDGDEMTRVIWDMIKTKLIFPFVDLDIKSYDLSIQNRDNTDDKVTVDAAEAML
KYNVGIKCATITPDEKRVEEFNLKKMWKSPNGTIRNILGGTVFREAIICKNIPKLVPGWTKPIVIGR
HAHADQYKATDFLVPEAGTLEMKFTPKGQTEPTISYVVVNNFAGAGVAMGMYNTDKSIRDFAHSS
FQCALEKNYPLYLSTKNTILKKYDGRFKDIFQEIYDNEYKGKYEAKGIWYEHRLIDDMVAYALKVE
GGFVWACKNYDGDVQSDTVAQGYGSLGMMTSVLMCPDGKTIEAEAAHGTVTRHYRLHQKGQ
ETSTNPVASIFAWTRGLAHRGKLDGNERLMNFAKLLEEVCVATIENGSMTKDLAICIKGMKNVTR
ADYLNTTEFLQKIADNLTTKLNLSHL*
>TRINITY_DN163801_c0_g1_i1.p1 type:complete len:298 gc:universal TRINITY_DN163801_c0_g1_i1:55-948(+)
MANPLKDIISRFSKGVPAGSSTGLGLLIGAGLAAYGIKESIYTVDGGHRAIIFSRIGGIQPGVYSEGL
HFRIPWFQYPIIYDIRARPRRIMSPTGSKDLQMVNIGLRVLARPLSSTLPHMYQRLGLDFDERVLPSI
CNEVLKSVVAQFNASQLITMRQEVSLMIRRDLTERAKDFDIILDDVSITDLSFSAQYTAAVESKQVA
QQEAQRAAFLVERAVQERQQKIVASEGEAKAATLLGEAMNENPGYLKLRRIRAAQKVARVIAQS
QNKVYLNANNLLLNIKDTDDIDDGLLSKKR*
>TRINITY_DN18025_c0_g1_i1.p1 type:5prime_partial len:225 gc:universal TRINITY_DN18025_c0_g1_i1:1248-574(-)
PCHNTAERCRVTTKSEMATMEAAYLATFLSLILILRCNSLTFTLSAGNTKCLREEVHKDVLVTGEY
KLSEAPIQTHLTVIDTNGHVLYKKEDAQKGKFAFTSDDYDMFEICFRSLGNHGQGIDREIFLDVKT
GFEAKNYDDLAKTEKLKPMELELKKLEDLSEAIVNDFAYMRGREEQMRDTNESTSDRVLYFSIFS
MLCLIGLAMWQIFYLRRFFIAKKLIEN*
>TRINITY_DN198705_c0_g1_i1.p1 type:complete len:190 gc:universal TRINITY_DN198705_c0_g1_i1:80-649(+)
MADEYYKRFTDRIIIDFERRGCSPCLQGGIDGDLKDNIPKELSDVGITPAKWHELLTEFEEIDATDG
TSIWCQMVGALIPTCGFQMIFACCFPPAVKHSCSCLPGCYGKWTVRMTDWLEKVNEFLNHYGM
HMKFTTYKPYTKAPRSVNFKKRVRWQDPRKYQMTFIEIALTREESQSYKKNIGVMEFRL*
>TRINITY_DN199_c0_g1_i1.p1 type:complete len:229 gc:universal TRINITY_DN199_c0_g1_i1:207-893(+)
MVQGSMTIVKYLMFFFNFIFWLSGLALIVIGAVIRDKYGDYFSYADSRFTNVAVFLIVVGVIVFIIGFL
GCCGAIKENYCMVTTFAVILGIIFILEIVAGAIGFAYKGQVEEKADKALVRASKNYNNDDEPGAKK
LLDWAQQRFECCEVASATGNGTCADKSPKSCHPDNDCNKTKYSQKCKEGFVEFMKDNLVTVGA
VALAIAFIQILGIVFACCLMKAIKGEYEVV*

>TRINITY_DN77_c1_g1_i1.p1 type:3prime_partial len:1965 gc:universal TRINITY_DN77_c1_g1_i1:2-5893(+)
MGVQVYSLGIGSNYDIGELLDIASDDASVFRSQDVDELVSIVASITEQTCKGCTRAVDIHFALDASQ
NVGRENFQVIKNFIKAIGFKFIISETGSHMSASIFGEYGTLIFSMDQATQQEDFINEVDNVPYIGDFN
GNIDKALDMVSEDIFTFDGFSRQGVPKVLVVLTASNCDSCEKSVEEAVKPLKAQGVHVVAIPIGSN
ANMEEMDKMASYPTQDYVIPRASYEELLNGVFIQKVSEMVCSGRPGVCEEPPIEGECTEIINECTL
DVNCPANRKCCIKDCKASCEVPAAACITPIDLAFAFETTGFIEENFDEVKSFASSLLDHFRVEPAKT
HISISTYGESVNAKTAFYESYDKSALKQFIKDLEPDGGRQANIGDFLKTANDEIFNITNGIRQSSPR
MLVLFTNGVYSNDQIAKAQEESLKLKAEGQDVNILVINIGSIVEGNSVLRDVVSTPDFRNLHNVT
DLRRLVNFGFENKIAEAICASKVRKTRCKPRTPQACSVRDDLCQTDFDCPGYQECYSDGCRLRCQ
TCENRCGDKVDLLFMIDTSSRIGPEGFKNAKENIKKMLEWFTIEANNTNVAAITYSDNANYAMQ
LPAAGSNDPPQTLFDVQGRLDSIPYSGGSKSNLDKALKMAAAQVFYQNSLRLRKDSKKAIVIVTD
GVDDSTSRLEIASWPLRKQRVVVDGNDYAAIRLLAVTVGNNYNLDTLEKVVSPPIDKNIFLAADF
DDMYNGIRQLAEDSCKYTRQDVEPPVIAVKGDPGPKGEKGRQGNPGTNGTDGMDGIEGPKGER
GDDGPKGEKGDTGIAGPPGGDQGDLKGPDPMAEAVGIIEGTAVKGTRGVTYDLRDGPIGPPGPIG
GVGKTGLPGDSGEQGQKGRGGPPGLRGVFGGQGPKGNPGEDASPGLPGEPGPKGISGKKGPTGS
PGMPGPQGPFGYKGEQGIIGQPGDPGDGGKKGPKGYAGEKGDQGDIGDAGFPGPDGKVGPIGP
PGQRGLRGPEGSSGPIGKTGDIGNKGLPGPRGSRGLKGQSGNAGPRGGKGRTGDVGLVGEKGTQ
GSIGEAGQEGQPGVPGKEGPPGYPGPIGTPGPMGSRGDIGPQGEEGPKGSLGLKGNRGEAGRRG
LTGEKGETGDIGELGPKGAQGQQGKQGKTGKIGPRGQQGPLGFQGLQGQTGDPGQNGRQGPK
GNLGPTGEKGADGPKGSLGSAGLTGPKGLVGEPGNVGRPGLPGRDGKNGPQGANGKDGDPGA
SGPQGLQGSPGSMGTPGSTGDPGSPGDRGATGAVGDKGTRGDSGVRGEPGAFGQQGPPGLRGE
RGEKGPQGTNGVQGSPGPRGTPGLEGLDGIVGRKGTAGRPGNPGQSGEQGAVGSIGVPGPQGIS
GTPGERGDGGSDGGPGEQGPPGLQGSSGSPGRDGMPGRPGQQGTTGGKGKTGLRGPKGANGQ
NGVQGARGPDGLPGAPGLVGNRGTPGPNGIPGLDGNPGPAGKQGKQGKMGTPGMTGPAGLR
GPVGSPGLRGQKGPTGDRGDSGETGPIGVAGDIGPRGFTGELGLRGAIGEMGQIGETGPQGMPGS
QGTPGAAGLTGPAGPSGLPGKDGNKGPIGDPGNAGNPGRDGGPGSPGKDGTPGEPGTPGSQGK
QGLKGPFGPQGAPGEQGQRGLTGRQGSGGKDGPKGFPGNDGSPGISGKRGPRGKDGTPGSPGPI
GKDGRPGKPGINGTDGDPGNNGPKGDPGERGDSGDSGSLGASGDKGKTGSEGPAGFVGPRGSN
GKAGVPGKRGKRGDQGRDGPRGNSGPDGIDGSKGFKGNMGRFGIQGPPGDAGLKGALGPDGT
VGHTGPQGNTGDPGPDGPEGPSGSKGPAGNAGQIGNAGYPGSPGDGGIPGTAGLPGPPGPPGPP
GDMSGAFSGNYWDFVNAGGLQKGPRYYGGRSKRSLDQQDNSIEKVFKSNYETFKNFDTVWNQI
VDDLVIQKTLGSSKNPALTCGDLFKTNPITKSGDYWID
>TRINITY_DN1222_c0_g1_i2.p1 type:5prime_partial len:213 gc:universal TRINITY_DN1222_c0_g1_i2:2-640(+)
FKNMVPYSLFLLQQGCSSGRLIKVGMFNKIVKPGGEQPDELEKSIGEALLELEVNSDLKTQLRELYI
AGAREVDIGDKRKCIIIFVPVPLLRQFQKIQVRLVRELEKKFSGKHVVVVAQRKILPKPSRKTRNQK
QMRPRSRTLTAVHDSILEDLCFPSEIVGKSIRVKMDTSRMIKVVLEKAQQTNVEHKLDTFTSVYKK
LTGKDTHFSFEI*
>TRINITY_DN3078_c0_g1_i1.p1 type:complete len:1109 gc:universal TRINITY_DN3078_c0_g1_i1:3628-302(-)
MKMRIYLFLILFATVYCRVVQKGPNDIPEETKNEIKNQEPKIKFEPEKDKKYFLAKDTTCAHDVDK
HCKNIRRENNFAVFVCLQDVIHKDEDDVAEECHETLWHYKYNLTRDPNFDQAAFKVCENVINEI
QECNDSELLKSPGFVTNCLIENRHAVSEAKCKSFLQKMSAIVFEDYRLIKGFYDSCVEDVKTLKCG
TVAKPTDEEYQHDQTEVISCLEEQLDLVSEKCQAQLVKVATMQADDYHLDRPLFYACQEARERF
CPEVKSGDGRIYKCLVEHKTNEMMLEKCRKKIEIREKLTQKDIKVNELLWKSCKADFTKYNCPKD
GISEHGAASSLLLCLQGKVAEGLPVDQSCQAEMRTFAIELFENYKISPVIVSKCDVEINQFCHHLIG
KTDDGDMMDCLMTQAATNNESVSKKCYDAVSDVIKETGAGNNYKIDRALYLACEPVITTLCQG
KEDAMILGCLMDNAHNSKMPEKCAAQVFHLQYFLSRDFRLDASLYKDCKKDAEEICSANDFNA
ESESKVPDNFVIACLYRNALDRKVDDRKVSQTCAEHVFRVMHQRATNIHLMPELERPCMADLG
KHCIDKGDKEGEEIDCLQTNYEKLSAKCQNAIKVFTIQEAKDFDLDKHLVDKCAPMVQQFCQRE
LEDGNGEKVLPCLVDHKNDIDMDHQCANAVEHWQLIEMKDFEFSPALTEMCLLDIRKHCQKSK

AKIEALHCLSENIVKDASSVTEKCRAQLKNELIEQSENIKLNPELYEACTKDIELFCRDVVPSGGQL
EECLKRNHKKLTDPKCKSLLFKEEKKESFDPELDYRLMHVCKPIIKRYCTQYHQPVDIMECLRQV
THDRDMSRECRAIITERQIEQAESFELDPELSKFCQDDVNRYCKEEIKHAEKGDDDGAIFGCLVNA
MIDNKPIKNDCEMYIRRREAEAAQELDLNPQFLLQCRDEITKICPNMDEDEIIECMKMNIPQIQSK
GCVQEVKKLIVEGIEDVHVDPHLQEQCARDIRTFCQSVPSKSGNVVLCLIDVHKAKNLHLQPNC
ENFLTKRLSLYNAAKVDYAVFDSISAVMNAVSESPNRNYVYMTLLLVLTILFVGGMLFGRYTKKIRS
EIKNR*
>TRINITY_DN6388_c0_g1_i2.p2 type:5prime_partial len:143 gc:universal TRINITY_DN6388_c0_g1_i2:1-429(+)
QWWKDTCYPVWFKYVKGPYERYQYEHAVAELRGYGLMFDDQHNDKDPIVERAIELLPHDLAV
GRHRRLMRAFEMNTKKTHLPVEEQNYDPMVPYLAPFMEEAKFQMQEEQELLNFHPWDRRLYS
GPTTGFGETSPYTCFSCW*
>TRINITY_DN95248_c0_g1_i1.p1 type:complete len:124 gc:universal TRINITY_DN95248_c0_g1_i1:197-568(+)
MAPKTKKVSKKKSTLNFNIDCTLPVEDGIMKVDDLKKFLQEKIKVDDKLNNLDQITLEVAKTKVT
LVSERPFSKRYLKYLTKKYLKKNNLRDWLRVVASSHTDYELRYFQINNDEEEDEEDNE*
>TRINITY_DN4762_c0_g1_i1.p1 type:3prime_partial len:542 gc:universal TRINITY_DN4762_c0_g1_i1:171-1793(+)
MSLTSPEDIENWFKGTQSYDHGDIDTALKQFREVTQNSRILFNTGCCHLTLNDIKTASENYKRAVE
NDQHLAVGYFMLGLTAFLEKDFENAKVAFERSLVLLRGNRCVDYKQLGLKYQLYSCEILTNQAV
MSYHLNDFDKARELLLEAVGCKAEKRHEKIFEMLVSLQAGAEISPFTLPKNQIFRPPKSYVQNLKT
KDYLGKATVVTTSNEDDIYACFSGIRDQVMAAKDQKMAAKANGQTKVENDKEKKPVTRALSHS
IMLKELRKPERKKTLKKTIVKRREPTIKLGKILEAPDVENVPLITPKPKKPRPKSPCPPRKSSSVDSYV
GDKDRNSSASSPPPRLSPPNSVFSSQASLAITDSDESPYESINENLDNSHNESSFTSISSNKNMERLR
QNRSREDRFSMPNLDPNQEKRLNITLLKPQKKQPALPDRAMSLDEVGLYHNAQYSNTLPIPGHIE
NSLKHLSLQKPKKKPPSPLLEESQEDHDNRRRHSNIDVVRKSRTSSLPLNNLKEEKLQTSSKSKDFP
KKKVSLDDKFLYW
>TRINITY_DN801_c0_g1_i4.p1 type:5prime_partial len:182 gc:universal TRINITY_DN801_c0_g1_i4:1-546(+)
SSLAQGAPRPPPNDPLPRPARPRRARLGLPSPMADQLTEEQIAEFKEAFSLFDKDGDGTITTKELGT
VMRSLGQNPTEAELQDMINEVDADGNGTIDFPEFLSLMARKMKDTDTEEELIEAFKVFDRDGNG
FISAAELRHVMTNLGEKLTDEEVDEMIREADVDGDGQINYEEFVKMMMAK*
>TRINITY_DN3271_c0_g1_i1.p1 type:complete len:399 gc:universal TRINITY_DN3271_c0_g1_i1:218-1414(+)
MTRIIWHKIKSELIMPYLDLDCKYYDLGLPYRDETNDQVTVDAAEAILKYNVGIKCATITPDEERV
EEFKLKYMWRSPNGTIRNILGGTVFREPILCDNIPRLVPGWTSPIVIGRHAHGDQYKAQDLVAPGP
GKFEMSFTPLEGSEKVTVNVFEFKENGGCMMGMYNTDDSIKDFAHSCFNYAIGKKWPLYMSTKN
TILKKYDGRFKDIFEEIFQDQYKSQFDALGIWYEHRLIDDMVAQCLKSSGAFVWACKNYDGDVQS
DTLAQGFGSLGLMTSVLLCPDGKTIESEAAHGTVTRHYREHQKGNATSTNPIASIYAWTRGLAHR
AKLDGNPELEKFSNSLELACVKAVEEGNMTKDLAGCIYGLKNVKREHYLLTNEYLDAVRDKLLQ
LWTPPSQV*
>TRINITY_DN651_c0_g1_i1.p1 type:complete len:408 gc:universal TRINITY_DN651_c0_g1_i1:122-1345(+)
MSGHQGGVKRSNDGGNASGGGGPKKMRGDNVTFRFLLLSKHAGGIIGKGGQTIKRLRTEYSAT
VNVPDSPTQERVLSIGASQESAIKVLCECLPLFHEPPYSVGGNHQNQGKYEINFLVHSSQVGGIIGR
GGFKIKELREATNANIKVFQECLPNSTERVVAISGNEDEIVNLLHKILEIMQEAPIKGNIALYDPSQE
AWNGPNDNFGGPPMNMGMGGGNRGGMRGGMRGGRGGGAGVGGNQGGRGGFQGGFQGG
QGGPGGPGGPGGPPGNFGGPNNNRGGFNGGPMGGNGNFNDDYRNNQNFYNQGPGNQNQG
AAPGGGTGNDFSIGDSQTTQVTIPNDLAGAIIGKGGERIRNIRQRSSADIKISDPIEGKSDRIITITGT
QEQIQYSQFLMQQSVRQYSGKPMK*
>TRINITY_DN33065_c0_g2_i1.p1 type:3prime_partial len:804 gc:universal TRINITY_DN33065_c0_g2_i1:2409-1(-)

MWQFVWLAVAITSQINGNVHHKHTKPRHQNLIDDIVPNDDFDLDYSHNIFIPFDAYDNYEKFLE
YAERSDLAKRGGVTSLPAIQSLPGDGSHTYAAPGVNCQVQLLNKKVEGTDKRVIERTSCAEGWTL
KNALGFFFAGVGHDLATKSAGLVHMNQLRFKMLIFEAEGYTFVADTDNEVYSNLQSKLSLKNAE
CRLTISNSYTFENSEIVLTGNHEVIEKITNAKKTLEVTFEKGKDTSSFDVKMKVPSMPLETFATIFLES
ETEFITADMGDTSYEINIDPIKAVIIESTSNSVTGVFDGSDGKFQLTGKFLSNDQNAFMGQATDIYLT
VNRKSLGSRTTYNLMLGFAKEVKVMDIIPKYVGKTEIQDEFDDNFLKTTIEKWHLLFTSKRIIVAD
GAEYKKTFANIFDADEYQEFKQGVGIKATVDLQKTLTACSSGSTVPDSLKSVKIKGLLGRRGLSMV
FPPGTSVPLIPLIKCKLRQIQDDLGFELPISLKGYEQKSKFKLEEIAYSSDSGAFKMSLLYENTITIYKD
VLEITNPQLTIEREGKDEPWQFTIAADVSIFQVDASVMISYSKTGYTVEVSSGNVPLTNLLRAFSFSD
KPAITFEIHDFKMQIEVKDKEEEEENEEEGEDEGNKNQLAIKRQDNKEEKKRDLKFSIAGKPVFN
GFDKVSIEALVWTSQTPAGVAVAVIMENIRLDEVLEKILDVPEVPMAWFSDMTLTMVISNVDAEG
CDNPAQSEDDCEEADEEEEEEEEGEGEEEEGEEGEKTQEGEEGEKGEEGGGGGPGGGPGGGGRG
PGGGRPRRG
>TRINITY_DN1319_c0_g1_i2.p1 type:complete len:134 gc:universal TRINITY_DN1319_c0_g1_i2:86-487(+)
MVVPSAKRKIVKKRTKKFIRHQSERYKTVKQNWRKPKGIDNRVRRRFKGQYLMPSIGYGSNQKT
RHLMPDGFKKFVVHNVKELELLMMLKGTHAAEIAHCVSRRKRIEIVERANQLAVKVTNATARV
RSEENE*
>TRINITY_DN3180_c0_g1_i1.p1 type:complete len:1889 gc:universal TRINITY_DN3180_c0_g1_i1:5857-191(-)
MGYLRVTWICVITLSTIVLSEFIFDDDEFGEKRTFGDPDSLVKRSSSGSTKKHVSTFTRTQRFSGGGST
CNYRVIKRSGRQYVVFRSSRHTRVANGCTKIHVQSFLNNHFGGKNLLTTFPNLNGDNFEVVQMM
YSLETRVAFMEAISKTSMAFFGGGNLEFGKSGDVPLTLKLQWTEDHDSSAWDPSDIQVELETTKV
VLSAASFHFKAKKSYHNPDHFMIEKFDTQGVGNFRVSAYTSAIAGLDNTAFGADFSQDKYGALT
WNDFRGYGFFDPAENFQLVLHPSTYVRNSRLFNNEETSQFIILSNPVSDDTTYVTLMAQFGQSTNI
KDTLNGIMTSVNADIGIIKRINSRAFCLTVSSGEYDFLFTPPYTKDRNLMNPCFPLEYQDARDNQN
DLSKQKKKMTADINIHVTINFKENVGSCNDDSSIDTSSLPPKMTINGNENWKGFSWSFPEQAKGP
MMAIMKCMWQNSAAFQGLPYSLPFGDSEVVSISDFLDSPTEKTIGLKITPPEQWRTIPGIDLTFNYL
EFTASYNIPDQTWELSGTADMRIDKFEVFLKLEYSRTTVEVEDDDDDDSSNEDDGEGPDKKAKKK
SDKKKKQSKNSKSSKSESKLMAKLRKRKSMDALLKSLPDTFQDDDFNNNHHVFYDNLIIPRSLPV
KNNQSANVSLNKDAGTNIVPAAVKNSSLATAATTSKRTTEVEKQWTFTILMPTLLVKDIYTIFSPQ
STLPAEYPKVFNNGIKNLQISWAWGNEFEVRFRGQLVIMGWDKILLETVFWKQMPSRSLKFGIGIY
FQKIDVDAILKHIFQDESISVPWLSNLDCKLVIGNSEGFKDVSKKKKKIKKIKKAKSNKKVSKKQR
KTSKSKSKTSAALTRSTRGMEEYIYADLAEKRQADDDDDGDDDDAEPDVKFTYGSLADNALIAG
AGFEMTWYRPTEEEKCLGNTLCKFIYEKVQSNTQFVLAGQVDTGGITISAEVKDAGIVLATDLKL
ENAKLEIDGRAGGGIKLSCQMIYTNSEIATNPLTFEGAIEIGFAGELTFRFTMGGTIDSAFGYNRIAL
GNILLGATIDFKAASFTIEFGVTVLMGLLNEDPITIRGYFGLSPTNWIDSWVYVKASKLTIGSLGKAF
DFSVPDTFKDEGFPEGFQFSLSRKVVNIESLGIEIPSGLYLVGKFKFKFFELSADCTIDPANKAFDVKL
EMNKQNWIGGMITISSYTDENKGPMFHIKVDENTPFYANIDGKLTVLGLSQEILVRIDENSGALIH
LRGTPFPFIFDAALDINAALDPNTKSFNGGTFKITVSSSEFIPTLARKIRDWLEGLKSEGTQAKQDG
MTAITKGEEDSETITGKKSLWDSALDTWKSNLQRRESELAAKQRAITGALQACIDECELVCVPFFD
WEEDCFTIFGWSAPCPWPNSCAKKVPNYFCYAGCALDQLGSAFLTWVESTLTKIGLALQSTAVFL
TDMVEGIAAALTKLGEYIVAAKDWLINQFQAALNWVADLLTKQIVLRKLVLEATVQPPFKYCFG
YDIDITLFTWHITDGGHVCLDTLIRDLIYSLFGYNQDSKKALGMKDQIEEAKSKTLSIKEAAEELEE
SAETTQAEVDMQTAKTFEEELKRKRTMPTKEMFKFIDEKSKNTKYSKAHQKIMDTPHGMPAYQI
HNNHMLENLEKRSPWVVVRSFKDGNRNLEPKRIADHEIVKRSLSFGDGSKPCSRLRRSLATYDTI
VNVLIRSARNLENMEKNHFAHKEFHGHGIRKLDKRIAELELKNMTDEQREDAYFWYDKANEGY
EDFHNKVRRNIDYHDNYALPLLHNQLNYILKKSGKEGGFLKRVEDMHSEALHDFKRSIKNEEDR
ADGQELLSKIKKSMLAIVTDENTPLSRYTRSINGMKNDITRMNKMVLAC*
>TRINITY_DN1995_c0_g1_i1.p1 type:internal len:514 gc:universal TRINITY_DN1995_c0_g1_i1:1539-1(-)

LTFIESLRLRTSKKRMGKEFKHQKNEKLEEDNEDISKTKGRLTNDTISYYKRVDSVLTENNFESDEL
YEAFIRNVFAQLKEDSVQVLRHTLTSKVIEKIIPLLSKSQFIELLEFVKEDVELLCCDRFASHVMETIV
ITYNGISDSTSDENFIKQFVSFCKVIRELIDHLIRDVFGCHVVSKLLQVLSGVKVSDRITKSKASINSK
KKAQQKYQIKEKELSAKEVTIINVADSLKKQYSKHVRVLLNVDNFGELLCHRNASPVLQVLLITTS
HINKKKYSVLSNQILQRARLIENRSLMEEENEDTIHKLPVVLTDAVGSHLIEVLLQTADDGLYRLLS
SQCFKNIIVSLCIQPVANFLAQTFLRLLEEKEQALFYVDKLFMYMEDILAVGNMGCISRLAELISRL
DLIDKQKKFIKVISNALHIPDQKLYKTDQKSNVLVTLIITMTTFDMFYKKESESENKTDIKVETDTN
KEIQNSDIENLCIKKNSINYHGACILKNCFKFKKCKTIVQS
>TRINITY_DN123_c0_g1_i1.p1 type:5prime_partial len:1231 gc:universal TRINITY_DN123_c0_g1_i1:2-3694(+)
RGGFRPFGTGLMGSPMLGPYAFGPARSGALGGFGIAPLVRPPTRGGAAGANRGSRSNSNRGGKK
TTPVKNNKSTPEAKKDNNINAKNDKKEDKKDEKKDDKKDEKKDDKKDDKKGGKTPTGKEIKV
EPKDDMKNIKKNFFKDPKVEPKNEKKPDTKAVTVKPEIKKESGPQNVTLDKFNSELHLSIDADGL
SAGNMKGEGFQYMWGGVRGTHGVKKGKVCYEVYLLENIKVHCPGENNPHVLRVGWSVDNSD
YQLGEDKLSWGYGGTAKSSYDSKFLDYGKKYTVGDTITCYIDFESNPKAIFFMLNGEYLGVAFRFG
NELGDKAVFPHITSKNMRFRVNFGKEKPKHHVTNGFRMMQDIPVDQLVLPSPSPKTPKEAEIIM
MVGLPACGKSFWCEKYAKENPDKKYYILGTNNIIERMKVSGLTRKRNYHGRWEMLIKRASLVLN
KLFTVAKNNPRNYVLDQTNVYFTARRRKMESFAGYKKIAAVIINKPDVLASRTEKQQKIEGKMVP
EGAVMEMKFNFTLPEKGPSFNEVWYLEETLPKAQELAANYRREGSNFKTKSKRKPESSPSQPESSP
SSEKKPKLDPKIISRGFDKPFSKPNTNSPQQSTFKAPSPAQNNANKTPNQDRFSKNEKSTSKVETNS
DNSRSKAGNFNKGHDGNKSNERPFFSNTPDRGNASNRGNDSNRGNSSDRGNAPNKGNSSDRG
NAPNKGNFADKGNAPNKGNSSDRGNAPNKGNASDSANKNSKNEKPKEESKKTPKRAIRGTLD
QLNSIINSEARKTENNSGNIRDSAQSGKNEKMGSLMDIDRTPQRESDSYQNLQSRNRDALSNQG
GKNKESQSAGLMDIRNVPQGNQDYRGKAGILDSSRNTSQDYRGKPGILDSSRNTPQDYRGKPGIL
DSSRNTARRNEYESPVGRGNNSYSDSPARGVSTPRGRASFNDSPARGARGGRGQSDISRSGSLAKS
QGSGGNRGGRGRGRSKHLGDVNKYDPVGNMGGYYSALKALPSVKDLIGNRSSFSSYSTRIDEPSR
FDQLYSSYEPYSFGRTQNANDSRSLNYSFGQTDSYSSLERDGGYRDSQNSKDFLGSYQQRKDSSYS
DNRGSQGGQRGYQDSSYNADGLDAKDQYQNDDYQESYSSQKDKSSYDQNTQSSRSYNQETSYE
ESSDYGRSRNQEQDEYARSGTYERGDTSYGTDDANRSSRRYEDDLQESEPMDGNYSTQDYGRSSQ
SSQRARIW*
>TRINITY_DN495_c0_g1_i5.p1 type:3prime_partial len:407 gc:universal TRINITY_DN495_c0_g1_i5:152-1369(+)
MDFHWNTVIAGLIIVQVYSLAEYGTLNGKDSYRNAYPWLANDRNNNELGREDARSIYRSSDEID
AEYPQSDDEVDTNDIPKSYTSRDELDAEFAQRTYKFADDLDIENAQKSYASSDELDRDDEQRTNK
NTDDLDAEDAEREETINDSLDAENAQRRYTSIDELDGEDTQRVISKTITLPRLGAGRIYKSRFIHGP
YIRPKFVRPHKGIRLPHPNRIIIPGSWKRPAHGIFALKGKKAIFCEHKGIKRISCSNDKVIKVRSALY
GRTAGNVCSISCSKDEEIKVKNALYGRTAGDVCGRSRVTNCRALNSFAIVQKKCNGHNSCDLNPS
NSVFGDPCVGTVKYLNIKYFCVKRITPTTTPNGKTAIFCEHKGIKSISCSKDEEIKVKNALYGRTAG
DVCGRSRVTN
>TRINITY_DN135129_c0_g1_i1.p1 type:internal len:155 gc:universal TRINITY_DN135129_c0_g1_i1:1-462(+)
SSFSYMSTSPSRSMDISASSDEALTPGGTLIIRDNVDNRTSFYERLQEKDSFIAETSSALNTPQNSPRP
STTTPLPRTPQQDRTPPKRSPLVRMKSDSSDIEKRAKFSKDNVSSTFRPVSMLNLSVFASNRPKRLH
TGSESELSEHVPLSTHRR
>TRINITY_DN34_c0_g1_i1.p1 type:complete len:149 gc:universal TRINITY_DN34_c0_g1_i1:477-31(-)
MDAAQAAEIKKKRTFRKFTFRGVDLDQLLDLSSEQLMELVHARARRRFSRGLKMKPLALMKKLR
KAKKEAPPMEKPAVVKTHLRNMIVLPDMIGSIVGVHNGKVFNQVEIKPEMIGHYLGEFSMTYKP
VRHGRPGIGATHSSRFIPLK*
>TRINITY_DN56792_c0_g1_i3.p1 type:5prime_partial len:1003 gc:universal TRINITY_DN56792_c0_g1_i3:3453-445(-)
DNELKVIECNLRVSRSFPFISKTLDHDFVAMATKVIAGLKVERVLNIMNNCNKIGCKVPQFSFSRL
AGADFQLGVEMASTGEVACFGENRYEAYLKALISTGFTIPKKSILLTVGSYKAKNEMLGSVRLLEQ

MGFELYASMGTGDFYQEHGVKIQVIDWPFEENGIEGQEQSIQEYLSRNKFDLVINIPMRQTSSKRN
PHFITHGYRTRRLAIDHAVSLITNIKCAKLFCEALYRTIGQIKVKTHIDCVSSGNIIRLPGLIDVHVH
MREPGAEYKEDFQSGTAAALAGGITMVLCMPNTRPSITDTLSFKQAQKAAQKGARCDYALYAG
ASNKNAKHIKQIASSCAGLKMYLNDTFSTLKMDNLSDWMNHFENWPKHMPIVCHAESQTTAA
IILVAELFNRPVHIAHVARKEEIMVIRAAKLRGIQVTCEVSPQHLFMTVDDIGGECEGKYRVKPPIV
TAEDQKALWDNIDIIDCFATDHAPHHVDEKNSENPPPGYPGLETMLPLLFTAVHDGRLTVDDIIL
RLHTNPRRIFGLPEQHDTYVEVDMDEEWVIPDAMTYTKSKWTPFAGRKVKGKILRVVLRGELAVI
ENEVLAKPGMGEDVRTWEPTHCMPPVSTTGLVSSKDAVDPTRSAYLGVTAPSVPVEDIHKTIESPS
SPARKLFGTQKDNEGMHNILETIGPVSVAPSISQPSPLFAQTHLFHGQHILTVESLSKEQLYALFNL
AGYMRQEVRKGKSFDVLKGRVLALLFYEASTRTSCSFTTAMQRLGGSTIEIKPSDSSMKKGESLED
TVQTMSCYSDVLAIRHPKKGAVQNAAEHCKRPVINAGDGTGEHPTQALLDIFTIREEIGTVNGITI
TMVGDLKHGRTVHSLARLLTMYRVNLQYVSPKSLKMPESVKEYVKQRGIPQVEISTLEDALTNTD
VLYVTRIQKERFDNEEEYNKVHGSYIVTPKLLTKAKEKMVVMHPLPRVNEISTEVDSDPRAAYFRQ
VECGMYIRMALLYMLVAR*
>TRINITY_DN1143_c0_g1_i1.p1 type:complete len:629 gc:universal TRINITY_DN1143_c0_g1_i1:2539-653(-)
MLRSRTIITNTLPKLKSINRLEPSFSCISNARCFSVSAANKDASESFAMGIFTGQANVSQIFPYPDVLT
DDQREFLSSLVDPVEKFMSEQNNAFNNDALEEIESETLEGLKEMGAFGLQVPAELDGVGLTNTQY
ARLVEVVGRYDLGVGIVLGAHQSIGFKGILLVGNEEQKKKYLPSLATGEKFAAFCLTEPSSGSDAA
SIRTNAKLSDDGKHWILNGGKLWISNGGIADVFTVFAQTPVLDEKSGETKNKVTAFIVERGFGGIS
SGPPEKKMGIKCSNTAEVHFEDVKIPVENILGGVGQGFKVAMHILNNGRFGMAAALSGTQKGLI
AQAIDHAASRTQFGQKINNFGVIQEKIARMAVRHYVTESMAYVICGMMDKGFKDFQLEAAISKI
YASEAAWEVADETIQVLGGMGYMKDCGAERVMRDLRIFRIFEGTNDILRLFVSLTGLQGAGKELR
KLQKSLSNPLSNMGTLLEQGLLRSKRAAGISSGPDLSSNVHPDLRKEADLASKGIGKFGSTSEALL
VKYKKSIIDQQMVCSRLSDSAIDLFGMVCVLSRATKSLNNNVPNAVHEALIAKTFCSEAYKRINTN
LESCKNEHNMATDKLKAQITSDITNHMGVVTTHPLGF*
>TRINITY_DN1339_c1_g1_i1.p2 type:internal len:183 gc:universal TRINITY_DN1339_c1_g1_i1:546-1(-)
ENELGVQAPLGFWDPLGLSSDGDVYSFKRRRSVEVKHGRICMLATMGYITPEVAGKFGGYISPSSK
LAFQDIPNGLAAIGKVPGVGWLQIFAYCGWCELTYDFDEEVNTEPGNVGWKPPLLATSDPEVRK
RRLNAELANGRLAMMAIIGMFFQDGLTGSAWGDWANYTDSPLRAFENELGVQ
>TRINITY_DN11643_c0_g1_i1.p1 type:complete len:187 gc:universal TRINITY_DN11643_c0_g1_i1:1748-1188(-)
MPAKGKLTQEEEVLLQDFSRTVSKKSQALFYGNALMISVLPLWLFWKVHLMDPMSYAALFICGTL
SAAYLVAFAYKNTKFALKHKVAQKREVAITREVNTELDSRPETKKLSRKEKDDRILWKKNNVADT
EATTFSIFYTNTLYLFIIIIASFYVLRSFSPAVNFTLSTGMAAGTVALLSTGTQSS*
>TRINITY_DN19618_c0_g1_i1.p1 type:internal len:223 gc:universal TRINITY_DN19618_c0_g1_i1:3-668(+)
AEGRTRKLNAELANGRLAMVAIMGMLFQDGTMGTAGPEMWGFSPAFEKELGVQAPFGFWDPA
GLTKDGDIDAFKRRRNTEVKHGRVSMIACLGYIVPEFAKFPGYLSPSQGIAFADVPNGLAALSKVP
GAGVAQWVLFCGLIEKSLLVYDPSRAAGDFANGGVLGVPNGSTMPEGEGKTRKLNAEIANGRL
AMVAIIGMFYQDGLTGSAWGDWSLYVDSPLR
>TRINITY_DN140702_c0_g1_i1.p1 type:complete len:2036 gc:universal TRINITY_DN140702_c0_g1_i1:94-6201(+)
MATSFKVSEIKCTELLNLEKKGAIDPLVEVEYLGLSKETENKDNDLNPSFGEVFDFDLSGKALGPE
DFLKITVKDYEKFGRNRVVGKADVYLQELLSNPSSTLSATLNDKNDRPTTGKITFKVDYIGPAGGG
AGGATGGGGDDRQAAERELEGDEDEGDPNAMPDGTPIPEGPAGDAMRAEKRKMKKNKKKRR
EHNLPDKPTDFQIRIKIIEGRQLPGGNINPTVRVHLGEETKQTRVKHSSNKPFYDETFFFNFNERPN
ELFDKLVTFEVHNSKNVLSEAMIGAFQFDVGTIYFEDSSHAIIRKWLLLTAPEDNDKKDGEETKGA
SLQPGGPPAGYLKITAIVLGPGDELPEKAKGSGKSEGGEEDIESNLLRPAGVTLRPATFLVKIYHAE
DIPQMDSAAFDGVKKLFSSGSAQKDHVDPYATFSFAGKTQKTEIKYGNNHPDFNQELRVAFKFPS
MCERLKLQMFDWDRIGNDDCIGTSFISLTAISGNGDEGFLPCFGPCYVNFYGSTREFSDLPDEHDN

LNKGRGEGCGYRGRALVEIDTRFGDHTAKPVQDIMAQELVRVQAYMRRRKYKLFASFLSANMIS
EADGLVEFEVSIGNYGNKLDPDTEPQSSSTPPTNPVYDGCAYYFLPWQESKPCCVVESHWEDVIFR
LEPINCLLKIIDKMEKNIKSVELGLKAKAQPAEVAALLIALLDQFISDVSMDLPKFAATDNGVNEL
DLKIQEIRSDELLNLKEEASTLREQATDVQEAMDTIDGYLKRMRDLCVEPQNSMPDVVIWMIAGS
KRVAYHRIPAYDIFYSENPLARGRKCASLQNFILKYPGKKGHDMEDHPEIPASVSVILWLGLEKHQ
AAWTDTEQMEGEYSVYAETYENQGKPPMFDWTAKIIKRPGFSDSEGKIRLNKEYYEPPEGWKWD
DPPDGDWFIDPEMSSNFDADAGHKKFMQDCFENESRVPAGNWGKSTPHYTNVQGDEIDHRDE
TSLPEGWSWTDDWKVDVNRACDEEGWEYCIEATGGGWGAVFRSYHFCRRRRWVRNRQLEADP
QKVMKQKKEEAKQGEGWEYAPVFGMKYHVKEHKFDFVRRRRWHRKMVCVKPGAVPMFKIPD
DDDKGKFHQIMPRIFLAYDEPHKWQFRAYLFQARDILGLDSEGTSDAYAKVCFQDQSQITDVIEET
LCPEFNQTLLFEDMQLYGSPSVFAENPPFVSIELFDKDKVGKDEFLGRVLAQPIVRLNGLKPPATRL
MWYDIHHGERTCGELLAAFELFLDEGADLPLPPPWLEKEKKFQVPSGIRPILVKTGVEILNWGVR
NMKKYQLASCTSPSVTYEIGREIITTDVIKNTKKNPNFLKNIFYKEVYLPKDPLYMPPIVIKVRDHR
TFGRKPVVGRHVIKSIDFVQPPKADPVVELPPADLVGEPEEKKDVTLDIEEKKKKKEIPVDDNLDW
WSKYFASADEIEKCGNYLKKGHKTIQIYDSELEEIKDFNGFSDFIVSYPLERGKEDDDEDDNVVGE
LKGLVKVYPLPQDPKDQTKIRYFTPKLKEKSKIVKCIVRVYVIRARDLQPKDPNGKADPFIVVQLG
KKKISTKDNYIPNEINPFFGSFFEMKADVPLDKDLKIKVMDYDFMSGDDLIGETMIDLENRFLSNC
RATCGLSPAYYTSGAYQWRDAKSPKEILDQVCRTKPQWLSNEQVVVNGQLVKIEDFEESPPTDPHI
GPVEQRLALHVLNNEDIIGHRLVPEHVESRSLKNPLQPSMDQGQLEIFVDIFPIANGVPGPPLDIAT
RKPKEYVLRVIIWNTAEVPPSETSITGEEMSDLYLKGWMEGQDDEKQKTDVHYRSMDGEGMFN
WRFVFPFKYMPEERVMVVEKKEHFWSLDTTKQTLPPRMNLQVWDNDLFSANDFIGNLELDLSK
LPRPKKAAKKATLAMLDTEGDDVMDLFQAKIAKGWWPMLSENEDGTRTMQGKVEMTLEIVTD
LEHEAKPAGQGQEEPNMNPKLDPPNRPETSFLWITSPWKTLKHIIWKNYKWYIIGFFLFIILILFIGL
VLYSVPGSIVHKIFGFQ*

>TRINITY_DN57_c0_g1_i1.p1 type:complete len:712 gc:universal TRINITY_DN57_c0_g1_i1:140-2275(+)

MGHDGSTLPSFLPKMSFERQTTLLKLLILSIAAILSFSTRLFSVLRFESVIHEFDPYFNYRTTRFLAEEG
FYEFHNWFDDRAWYPLGRIIGGTIYPGLMITSAVLYHFMHFFHVTIEVRNVCVFLAPLFSSLTALVT
YQLTKELKDAGAGLIAASMIAIVPGYISRSVAGSYDNEGIAIFCMLLTYYLWIKSVKTGSLFWSTFCS
IAYFYMVSSWGGYVFLINLIPMHVFALILTGRFSHRIYVAYSTVYCIGTILSMQISFVGFQPVQTSEH
MAALGIFGLCQIHAFVDYLRYRLTPSYFEILFKSLVIISATIAGIIGAILTVTGKVAPWTGRFYSLLDPS
YAKNNIPIIASVSEHQPTTWSSFYFDLQMLVFMFPVGLYYCFAKLTDANIFIIIYGVTSIYFAGVMVR
LMLVLAPVACILAGIGISSVLTTYMRNIELSNDKPKPIKKAKQADPSYPIKSEVARCMVALITFFLITY
TFHCTWVTSEAYSSPSIVLSAKQSDGSRVIFDDFREAYYWLRQNTPEDAKVMSWWDYGYQITAM
ANRTILVDNNTWNNTHISRVGQAMASPEDKAYDIMRELDVDYVLVIFGGLTGYASDDINKFLWM
VRIGGSTDTGRHIKEHDYYTPAGEFRIDKDGSPVLLNCMMYKMCFYRFGSVYTEQGQPSGYDRVR
NAEIGNKEFELDVLEEAYTTEHWLVRIYKVKDLDNRGQ*

>TRINITY_DN371_c1_g1_i1.p1 type:complete len:173 gc:universal TRINITY_DN371_c1_g1_i1:157-675(+)

MASAVRQNYHEECEAAINKQINMELYASYVYLNMAYYFDVSDVALEGLFKYFKDQSDEEREHAQ
KLMKYQNLRGGQIVLKEIKAPEPVDFNGKAHLTALENAFQLEKKVNESLLNLHGVASKNNDPH
LCDFLESEFLDEQVKSLSEINRLITKAKRCGDGLGVFQFDKLALE*

>TRINITY_DN26249_c0_g1_i1.p1 type:5prime_partial len:477 gc:universal TRINITY_DN26249_c0_g1_i1:1733-303(-)

QMKMAASNMAGHVVRNNLIFLLSFVIIIVAEEGRHARFEYKFSFKGPHLANKQGTVPFWNHYGS
AIPSDDQVRITPSMRDQRGGLWTKTKSSSEFWEVEIFFRITGRGRVGGDGLAMWFTEEAGIQGPVF
GSSDHWKGMGLFFDSFDNDGEQNNPYVMVMLNDGTKQYDHFKDGNSQQLGGCMRDFRNRP
HPIRAKIRYYNNILTVFYHSGMSERSDDYELCMRAENVVLPKEGFFGITAATGGLADDHDVIKFLT
HSLTPPLTDEQKQKILSEEQRKKYEEQYGEYTQKLETKKEEFQKEHPDKKAPEEKFESVYDRDVRL
IYENQNTIHHVVRALHEKVGELTNQLNNLHVTINNNNQLAQQQQPKGDGVTRQEVNTIINLQN
QVHNAIQELKQQSGGGGGDNTKTVQMEKTLQTVLEQVGHLVSKNNDRRPSCPTVSCVTPSILILF
LVAQVLLVAGYNFYKEHQNKAAKKFY*

>TRINITY_DN172571_c0_g1_i1.p1 type:complete len:104 gc:universal TRINITY_DN172571_c0_g1_i1:30-341(+)
MSGRGKGGKGLGKGGAKRHRKILRDNIQGITKPAIRRLARRGGVKRISGLIYEETRGVLKVFLENV
IRDAVTYTEHAKRKTVTAMDVVYALKRQGRTLYGFGG*
>TRINITY_DN8707_c0_g1_i2.p1 type:complete len:564 gc:universal TRINITY_DN8707_c0_g1_i2:2751-1060(-)
MSGFVRQSKYRHVFGSPARKDGCYEGFRVTNCAFDGNFIAANKHFIAVCVEVGGGGAFIIVPVGS
TGRLDVNIPKVSAHKEYILDLQWNPYNDFMLASCSEDGTIKIWEIPEKGLFMNIGDEKALLSLEYH
EKRCTQVLWHPSASNVLMSVSQEPKVCVWNLDEGSVEVEILEHPELIYNATWSQKGDKIGTTCK
DKKIRIFDAREGTLLVQGMGHEGAKPSRVIFTFQDKYLFTTGFSKMSERQYACWNAETIEQLGLEE
LDTANGCLVPTYDADSQLVFLNAKGDSNIRYYELVDGDPYYFYLSTYQSSAPQRGFGFIPKRCINV
NANEISKGYKLHGTKTGYIEPVSFTVPRKSETFQQDLYPDAIGDEAALEPDDWFSGKNAEPKRIA
MSTFFKGKTKPTGAAGGGLKKGGLKGLKAKKEAKTAAKSAAKSEPAKPAPVKTESKDKVDAAP
PAAAAVEQKSSSTAVAAPAAAAPVVVSGVDEKVVKELQNEIKSLKDGDKKLQKEIISLKDKLKDY
DTLRSDIKLLCDAVKKNDERITALESLVAEETDEEETGEGGDE*
>TRINITY_DN2113_c0_g1_i1.p1 type:complete len:1704 gc:universal TRINITY_DN2113_c0_g1_i1:5257-146(-)
MAQLLPIRFQEHLQLQNVGINGANIGFSTLTMESDKFICVREKVGEQSQVVIVDMADANNPTRR
PISADSAIMNPASKVIALKAAKTLQIFNIEMKSKMKAHNLTEDVIFWKWISVNTIGLVTETAVFHW
SMEGDSSPVKMFDRHQSLAGCQIINYKVDHSGQWLLLIGISAQQNRVVGAMQLYSVERKVSQPIE
GHAAVFSQFKVDGNPQTSNIFCFAVRGAAASKLHIIEVGTPPTGNQPFPKKAENIFFPPEADKDFP
VAMQASQKYGVVYLVTKYGYIHLYDIESAQCIYSNRISGDTIFVTASYEPTEGIIGVNRKGQVLSVSV
DEEKMVPFLIQVKQNGELARRLAERCNLPGADNLFLQAFNDKFASGNYQEAAKIAAKAPKGILR
TQQTIQRFQQLPSQQGQTSPLLQYFGILLDQGQLNKYESVELCRPVLMQNRKQLMEKWLKEDKL
DCSEELGDLAKQFDNTLALSVYLRANVPNKVILCFAETGQFQKIVLYAKKVGFEPDYVSLLRNVM
RVNPEAGANFSGMLIQDDEPLADLNQIVDVFMEFNLIPQCTSFLLDALKNNRPAEGPLQTRLLEM
NLLSAPQVADAILGNQMVTHYDRAHVAQLCEKAGLLQRALEHYSDIYDIKRAIVHTHLLNPEW
LVNYFGTLSVEDSIECLKAMLSHNIRQNLQICVQIASKYHEQLTTSSLIEIFESFKSFEGLFYFLGSIVN
FSQEEEVHFKYIQAACKTGQMKEVERICRESNNYEPERVKNFLKEAKLTDQLPLIIVCDRHDFVH
DLVLYLYKNNLQKYIEIYVQKVNPARLSIVVGGLLDVDCSEDVIKSLILSVKGQFSTDDLVAEVEKR
NRLKLLLPWLESRIHEGSEEPATHNALAKIYIDANNNPERFLRENQFYDSAVVGKYCEKRDPHLA
CVAYERGQCDQDLINVCNENSLFKSLARYLVRRRDAELWGTVLVEDNKYRRQLIDQVVQTALSET
QDPEDVSVTVKAFMTADLPNELIELLEKIVLENSVFSDHRNLQNLLILTAIKADRTRVMEYITRLD
NYDAPDIAGIAIVSELFEEAFAIFKKFEVNTSAIQVLIENIQNLDRSYEFAERCNDPAVWSILGKAQL
DQGLIKEAIDSYIKADDPSAYMEVVEASKNCGNFEDLVKFLQMARKKAKETFIDTELIFAFAHTGR
LADLEEFISSPNHANISQVGERCFEQKMFEAAKILFNNISNFARLASTLVHLGEYQNAVDGARKA
NSTRTWKEVCFACVDGQEFRLAQMCGLNIVVHADELDDLIRYYESRGYFEELISLMEAALGLERA
HMGMFTELSILYSKYKPTKMKEHLELFWSRVNIPKVLRACEQSHLWSELVFLYDKYEEYDNAVVT
MMKHPTVAWKESLFKDVITKVANIELYYKALQFYLDNKPLLINDLLVVLTPRMDHTRGVSFFQRA
CYLSLVKPYLRSVQNHNNKAINEALNSLLIDEEDYNGLRASIDAFDNFDNIALAQLLEKHELIEFR
RIGAYLYKGNNRWKQSVELCKKDHLYKDAMQFACESRDKAVAEDLLSWFLSIDKTDCFAATLYM
CYDLLEPDVVLELAWRNNMLDLAMPFMIQFMKDYYTKVDKLAESNAKRDEDKENAPPEAPIM
GVEQLMITAGPSSYVPQPNMYGMPPQGMAPQGMPPQGMPPQGMPPQGMPPQGMPPQQGFPQ
QGFYGGAM*
>TRINITY_DN32763_c0_g1_i1.p1 type:internal len:104 gc:universal TRINITY_DN32763_c0_g1_i1:2-310(+)
GQERPGHVGRDADPGRLPVVVRRGWARRGPVRRQRGARGPGPQRIGLAQRRGRVTFGRFADRE
SLARPRGRVAQHAVVARWAGRALRQPQWAGGGRHLGADRG
>TRINITY_DN1130_c0_g1_i1.p1 type:complete len:317 gc:universal TRINITY_DN1130_c0_g1_i1:1199-249(-)
MDSSQRILVSFFCLIGLAYSASWNYDEGAATGPDAWQNSYPKCSGKSQSPIDIITADVTYDELPTIQ
FGSNWDMLLDNSSLELSNNGHTAVVNVKHPTQITTTQGGKTYILAQFHLHWGTTDDTGSEHKI

DGKFYPAEVHFVHYNSKYADLGTAVPKADGLMVLGYFIKVDNSALTSSFLHLIMEGLDDVVYKD
EKTSLPANGRSLYDLFHPFSTTKYFHYSGSLTTPTCDESVAWNVMHPENGLTITSTMMAKLRAMS
QNKRGEAMKVQSGNYRPVLALNGRSVRRNFDKSSASSIGSLLSITIPMLAIAIKLSS*
>TRINITY_DN116_c0_g1_i1.p1 type:complete len:297 gc:universal TRINITY_DN116_c0_g1_i1:72-962(+)
MGFVKAIKNKQYFKRFQVKFKRRREGKTDYYARKRLVTQDKNKYNTPKYRLIVRFTNKDIVCQIA
YAKIAGDVIVCAAYSHELPRYGVKVGLTNYAAAYCTGLLVARRLLHKLNLHETYSGQTEVDGDEY
NVESVDDGPAAFRCYLDIGLNRTTTGARVFGALKGAVDGGLEIPHSVSRFPGFDSESDDFNAEVH
RNHIFGKNVGDYMRALQDEDEDAFKRQFSKYIKEGVKPDNIEGMYKKCHAAIRADPAHKPSGK
EKFKGKRYGRKKMSFAQKMDSVRQKKEAFKRKMEQDED*
>TRINITY_DN4525_c0_g2_i1.p1 type:internal len:111 gc:universal TRINITY_DN4525_c0_g2_i1:1-330(+)
RLAMMAIIGMFFQDGLTGSAWGDWSLYTDSPLRAYESEPGVQAPVGFWDPLGLSTSGNEFDYKR
RREVELKHGRVCMFATIGYIVPEYTRLPGFLSPSYGVKFDDVPNGL
>TRINITY_DN97809_c0_g1_i1.p1 type:internal len:184 gc:universal TRINITY_DN97809_c0_g1_i1:2-550(+)
LGPDQVDVAGAGRDPRHLPRPLGRRLRVLDRHRGRVRQRHKAEADQDPKLETARLDVRRPGVQ
VRRVRHPRPSSRDQCHRDCHAPGRVEGRAQVDRGRRGLGRRRVEHEVHGAAQPAARGQEKGR
LRAQQILAVAAGQEEAVDVGARNRQGPPSALAAVSRAPHAQFARLAQRDRRVARAKGW
>TRINITY_DN41662_c0_g1_i2.p1 type:complete len:289 gc:universal TRINITY_DN41662_c0_g1_i2:168-1034(+)
MNNNYNNKQYPQIEDGNYSISTENDEKKFSKCTWVLTILSYVVVICLFPFSLLFCLKIVQEFERAVI
FRLGRLLSGDARGPGIFFVLPCIDTYMKVDLRVVSFDVPPQEILTRDSVTVAVDAVVYFRISNPIWSV
VNVEDAQRSTKLLAQTTLRNELGTKNLSDLLLERESISQSIQRILDVATDPWGMKVERVEVKDVRL
PQQLQRAMAAEAEASREARAKVIAAEGEMNAALALKQASEVIVESPQALQLRYLQTLQTISAEK
NSTIIFPLPIDILNAGLRDRKTQ*
>TRINITY_DN413_c0_g1_i4.p1 type:3prime_partial len:463 gc:universal TRINITY_DN413_c0_g1_i4:1388-3(-)
MVSLSVTCLSLLLFVTVNAEESDHCGYDEKSWYKAMGWCYHSGSRLLLGDFNGDKRTDFFCHD
SNGKNWVAVSNSDGSFGSTTWQDSKERWCASPNILLTGDFNGDGVTDILCHGTATGKKWIKYFN
KYGVPSGKSFYRAMSWCNHKGSQLYIGDFNGDKRDDLLCHDTSGRYWTSYAGTDESKVFHHTS
WYKALEWCRHATSKLYLGDFNGDKKTDVLCTDGKGYKWIMYAKTSGNLFTGTDWHTNLDWC
HESSTVFLRIGDVNGDGKDDLVCHSTAGNSWVAYTQPKGGFGNFCHLAKWCAAGKESFFLGYF
NQDSRADILCHDTKYGKKWIRYAECSFAEESDHCGYDGKSWHKAMRWCYHSGSRLLLGDFNG
DKRTDFLCHDSRGINWVAVSNSDGSFGSTTWHDSKERWCTSPNILLTGDFNGDGVTDMLCHDY
KTGKKWIKYFNKHGVPSGKSFYR
>TRINITY_DN2963_c0_g1_i1.p1 type:complete len:116 gc:universal TRINITY_DN2963_c0_g1_i1:1072-725(-)
MSATGNLVTVLRKLYDEYNDKTPSRLKIVDGFLLYVMLTGIFQFLYCCLVGTFPFNSFLSGFISCVGT
FILGVCLRVQTNPRNRTEFKGISPERAFADFIFANVVLHLVVMNFIG*
>TRINITY_DN6_c0_g1_i1.p1 type:complete len:196 gc:universal TRINITY_DN6_c0_g1_i1:843-256(-)
MIFTVFLIGLQFVGIYNHAIPHLHPGFHPFHHYQPQGLLNQMDKEFTFSCPNGKAISFIGSVHDNR
KEDRQWTVYCANDVMEPRGTSTWTYYVNDFDGEMDFACADGNIVTGIASFHKNKYEDRRYKF
RCTYMPGTIRKGCSWSKYSTYDAMFSEHIEPGRYLTGMRSSHDNNKEDRIFQIETCRFDHYYEQQF
H*
>TRINITY_DN33328_c0_g1_i1.p1 type:3prime_partial len:1422 gc:universal TRINITY_DN33328_c0_g1_i1:4264-2(-)
MLLKYFFLFLCLLLLNDIVYGKRRGGKKPARRPPPARRPPPSRKPVRRPPSRKPSPPSRAPSPPSPPSS
PPNRKPSSNKPANQASNDQGGPGKPASNAQGGGKAQELPDLNAILRAFAGKQTDSGSTQYEYG
GCVLDIGKDKCKIKFIPKKQSCHNKLALGKLFDKFVPKGKIKTLFKKYAPVDVFQVTRFEVDKCQ
GDLDFETEAKEAFNVINDKLKVNSGKLSVSFDYKKFMAAPSFDKLGIDIDGEVQLFGKKVPLTLK
KKRDSGEGIFSIKTASIKASDFAKLFSTKSVANAQSPPQTKMFTDAVIQNPSISGSRDDDGGFELVVK

GQVTGIKGIGAISLFVIIQKSSDSPVSVAIVADIKKIQPTVLLSSLTGKNLNSIPIIKDITANLAVEFANN
DMMMLKDPELNKLLGKYIANGKTIASGTKIKVELPIKEILTKVKTTASLSHVPSTIYMQMFLNGGK
VCFKFPDNIAMDLANVLVALLPKVPDLLFSKVLKTPPKIVIEKFDVNVATKYVDMVLEAPEEIVLG
SDLFKIKNAKFELKHEENKPWDFNIEATKQIGKTMIDINVKSEAKNYIFKATIDSLTTKELATTFGS
KATADGILTKMSFLNFGIRTLTVTAEFGDKFTLNIKGNPVLFNWEGAFFEGFIVGSGLNKKMAFGI
VVKNIRIDDIVFKLFKKRILQTTWMSELTATFLISNTDPDDNIDFTLPELKEHVKLKQGVFFKSELTI
PTDCRAEKICEVLRDKMKPGTKLYLEGEITLNGFKLTAGYKGHGLNFATDLTLTSAKLVLTVGTST
EFYLEGEVQMKNPNVKFIGKLGVGVTGIVELSLIMEGMWRTPFGLSYIAFGNLQLAVAIQPGVPVP
KLEFTAEMWVGKIGNNQEIKTKTAVGINPSNPLDMYFYGEINKLTLAEVAKAFEFKSTLPKVVQET
GFPEGLAAGYTTNPKGKTISHLKVNLKTGFFFRGAINILGYKLECEILVQIPKAIKVSVDMGPIKIAK
GLIKLAKGAKDLDNGPSLYVAISSNEVAVKIQAYAELLGLGAEVNIDVSDKGMKFFVYGNIFNLIE
ANVTVNAPYGNGDLAKSEFSVSSCISAGISDIQNTAFEEVDEGAKESQKIQKVADDEVKDASSWY
NSAMDKLLSYEKKLEEKTAIINRKSGKLEKLKEYLDASCVDHCPDVCQGGPVMRKRKGRFFNKK
PGWDDCKEMVKSIVCLGKCKGKKAYYQGKAAKKVVTMEIIEGSVKIAKKLIVIAKGFAERSKGL
MDLTQKAANKVVSLTKAGKDALGALAKYTKENLINIHLLCFDTTLAKAEGACFGVNVDLTVLG
NKKLQYEGEVCFNADFFKNMAKQVADKVFPGIISIKDKIMQAKNLFSDSDSKKKELEEYEKQSAQ
EEKNLEGQLERQNEDDDDDDDEEFNGKYKASFDP
>TRINITY_DN201860_c0_g1_i1.p1 type:internal len:221 gc:universal TRINITY_DN201860_c0_g1_i1:1-660(+)
LTAITKVVCSTTSGSATGSKDGSLSSGDDYISRDLSDKLSIITFDTNETAQYCEVQIIDDSFYEEEEQFT
VKITPYFGGEVDEERADVNIVILEDKNDEPKIYLKSTLYEIDESDGVVKVAVQRTGSDLSFKSYVIIST
KQTEPVSAKANVDYYPVVKTLEFGIDEKEKLVDIGIIDDKGRPRIEGEESFEVLLGLAKNGELLDGQ
SKGVVIINDEESDK
>TRINITY_DN76040_c0_g1_i1.p1 type:5prime_partial len:383 gc:universal TRINITY_DN76040_c0_g1_i1:1-1149(+)
RTSSKAGHRFAEITKMASEQETDAEMIKECEDYVKKHNIQVLLKDAIVQLCINKPDNPYQFLRDH
FEKLENEHGGKTGKDDHVSATEPPPQATTRRRRGAVSAAVISEEDAASYVKKVIPKDYKTTAALS
KAIEKNILFSHLDESERSDIFDAMFMVKHSEGEVIIQQGDEGDNFYITDSGEVDVYVNDNLVVSISP
GGSFGELALIYGTPRAATVKAKTDVKLWAIDRVTYRRILMGSTMRKRKMYETFLEKVSILESLDKW
ERLTVCDALEPCNFNDGEEIVVQGEPGNDFYIISEGQAAVLQRRSPNEDPIEVSRLNVSDYFGEIAL
VLNRPRAATVVARGTLKCVKLDRQRFERLLGPCGDILRRNIQQYNSFVSLVV*
>TRINITY_DN10706_c0_g1_i1.p1 type:complete len:213 gc:universal TRINITY_DN10706_c0_g1_i1:122-760(+)
MNIFRLIGDLSHLLAILLLLWKIWKTRSCAGVSGKSQCLFALVFITRYLDLFTSFVSLYNTVMKCIFL
AATISTCYLILVKFKATYDSNHDSFRAEFLVVPVAGLSVLVNHELSVMEVLWTFSIYLESVAILPQLF
MISKTGEAESITSHYLFSLGSYRGLYLFNWVYRYYFDGFFDLIAIVAGCVQTVLYCDFFYLYITKVLK
GKKLSLPA*
>TRINITY_DN129732_c0_g1_i1.p2 type:internal len:112 gc:universal TRINITY_DN129732_c0_g1_i1:2-334(+)
WDPAGYTNDGSVENFKRRRATEIKHGRVSMLACMGYMTPEITGKFPGYLSPSLKLQFSDIPNGLG
ALSKVPGVGWAQIFAYCAYCELSQDQSAASPGGAGDFGFKAITSSD
>TRINITY_DN464_c0_g1_i4.p1 type:complete len:717 gc:universal TRINITY_DN464_c0_g1_i4:2167-17(-)
MLLHFGKSLHYLKFGTLFWRLKLVAIPRDTSNIINNIFLESKSQRYIRMSANILNEYSQNVGKMEDE
ISGLTSQLNALKRKLNGEIEDEDCSDQLKELRSKNQKLHYQKNIIQRALKKEQEKADSKMLNVQ
AALNVLFESAIRSAYPQIPNPPVALTIAHNPKFGDYQCNSAMAISKMLQKLTGEKIGPHVVAKTIV
ENIPSNSFIKKVDVAGPGFINLHLEKEFLKGVLEKVIKEKVYPPGIGNKKKVVIDFSSPNIAKEMHV
GHLRSTIIGESLSRLLEFVGHDVLRLNHVGDWGTQFGMLIAHLEDKYPDYKTVSPPIGDLLLFYKA
SKKRFDDEEDFKKRAYQRVVDLQGGNPDVRKAWQMICEVSRNEFQKIYDRLDVTLIERGESFYN
DLMSKVVENMKKSGICQKSDDGRWVAFAPNMKVPLIILKSDGGYTYDTSDMAALQQRLQEEHG
DWLIYVVDAGQGDHFKQIFGVGEAAGWYNSSITRVDHVAFGVVLGEDKKKFKTRSGDTVRLIDL
LDEGLKRSMEKLLEKGRDKELTKEELLAAQESVAYGCIKYADLSHNRVMDYVFSFDKMLDDKGN

TAVYLLYAYTRVRSIQRNAKKGRSEIEEYMKSNSLHFDHEKEWKLAKLLCRYPEIVVRCLDDLTLH
LLCEYLYELSSTLTEFYDACYCIEKNRETGEIISVDMNRIALLESVARIFEAGFHILGIKPISRM*
>TRINITY_DN1091_c0_g1_i1.p1 type:complete len:212 gc:universal TRINITY_DN1091_c0_g1_i1:200-835(+)
MKLILICLFAVLVYSIHCDQPSDPVWPNLFQQSFREKFYYPVIGTHYTNGTYYYDYKNLRYRLDRD
NGRYDRYCGLNGIRAFQNTPCTHLVLDGTRWLVYPEKKECCECCHAANGCGVLKPTWLTGAEF
VSKSNGIYKWNQKGLQDNFYSERVTDRIMTEIDQVPNDTQYYDPDSFKTKIENIEDVFKLPSYCKR
GTKCSFFSACRAVGFF*
>TRINITY_DN1653_c0_g1_i1.p1 type:complete len:1122 gc:universal TRINITY_DN1653_c0_g1_i1:180-3545(+)
MMRRSIKGSEDNWVEMQNDTFKNWVNANLKSRGLEVDNFETDFEDGVRLINLFEAVSKKSIGK
YAKQPKFANQKLENVSLVLRAMESDGVKLVSIDSDHVVNGNSKMILGLIWQLILRYQISGSDDKSF
KIALKKLLLIWLQHVIPEQGIRNLTTNWNNGIALCNLINVLKPGIFPEYNNLDKRTPVENLAKGM
QLAESELDIPQIISPENMASPNVDELSMMTYLSYFTQDGGIGESWTVDLVNFWNSSLSVTNFNTD
WNDGMRLGRLVETFLPGAMPIMESTDNLDNCRIAMDVAHEKLGISKIITAEEMSNPQIPELAVMS
YIVQFTKVKRVERASDKFSASGPGLVEPKIGIVNEFYVRSDAGYPLDGLQVTIIGPNNNYIPIEIDDS
DPQLRNYSYSCEEQGQYHINIIYEGEDIPRSPIIVTSQQDLNTIRVYGERLEVSHAYEETEFTVDVGSI
NSYVTATIDGPEGSQPTPQTINNNDGTYTVRYIPSTVGTHRIYLAVNNQQLPGGPIETNITYPIVSEA
ELIETVAFSAKYEEDKHLYAELYERIGISVDTSQSGDGRITSKTIGPDEKEIYSEVDAEGDGSYTVSFTP
EVIGEYHTNVFWSGKPIHGSPFVVWVSDPNKVFARGKCLYETRVGVESSFEVATNGAGPGHITGY
AETNGVEYPINVRKREECLHDISFTANQVGIYYVYINYNNVPIKDSPFQIKVGNPSLIEIEKHEEAIM
ESNQPQSFIVNVLKDAGFGDVLCVVRGPAGEDVQCTIKDVNDSLKEIMFLPNYPGTYEVFVYYAG
GLVERSPFIFAISEPKLAIYPENIRVSGNGLSRAKLNELARFQVDTTNAGPGHLTVQIEDEKDDVEA
GIRDNNNGTYDVTYTPKRSGKYRVIIKWSDQHITDSPFEIIVDNRAEEVLVEGYGIKEGTAGQPVLF
TLDGRNAGPGQLSCRCRAPSGKMTYVLISDNKDGTYTVDLNATEPGLHTVEVEWDNQAIPGSPF
LVRIMQAPDVKKVRASGPGLRSGTLDSFNGMFQVDSKGAGPGTLKVRIHGPKGAFKVEMSRENP
KDRIINVRYNPTEPGTYTANIYWSDEHVQGSPFEIFVGQNDQEVDRWDRNKEELSKSQVNVY*
>TRINITY_DN2230_c0_g1_i1.p1 type:complete len:249 gc:universal TRINITY_DN2230_c0_g1_i1:796-50(-)
MKLNVAYPANGTQKLLEIEDEIKLRSFYEKRMAQELSVDFLGDEWKGYVVRITGGNDKQGFPMK
QGVLTPGRARLLLSKGHSCYRPRRTGERKRKSVRGCIVDGNLSVLNLVVIKKGDNEIPGLTDRVIP
RRLGPKRVGKIRKLFNLSKEDDVRQYIIRRPIATPEGKPDPKKPKTKAPKIQRLVTPQRLQRKRHVK
ALKRERANKAKLEAATYAKLLAKRHKEAKEKRHQEHMKRRRLSSLRESKSQ*
>TRINITY_DN109115_c0_g1_i1.p1 type:internal len:125 gc:universal TRINITY_DN109115_c0_g1_i1:3-374(+)
NEFLTRELAEDGYSGVEVRVTPIRTEIIILATRTQNVLGEKGRRIRELTSVVQKRFGFPEGTVELYAEK
VATRGLCAIAQCESLRYKLIGGLAVRRACYGVLRFIMESGAKGCEVVVSGKLRGQ
>TRINITY_DN247_c0_g1_i1.p1 type:complete len:373 gc:universal TRINITY_DN247_c0_g1_i1:38-1156(+)
MKLVLVIFVILVYTWNVSAGGKKCISNKDFMTPYPTNFWTEHYPKFVNNRPTIGIVAMEVLGQKM
LVNVPWSWNKDYFGSSFVKLVESAGGRAVPIKEDITEDDLDKLMRKLNGVVLPGGDADVGDSGY
ERISKQAIAFSKKMAKKNVTFPVLGICRGAQMMMMAEAGKDFLEVTDSLNLSIPLDFTSEQKESR
LFGNAPQGLIKSLSEDAITFNAHAMGIPTLNFYNNSGLQKAFRAISTNYDRNGTKFISTFEGRDAPI
YGLQWHPEKSLFVFNPVLAVDHSIKGVMASQYIANFFISEARKNNHTFRDRVEEEEHLLMSYYPS
YVGNITETPYEQIYLFDFVKPFQNPKYTPGKVNKNDDDNSGDDDDE*
>TRINITY_DN751_c0_g1_i1.p1 type:complete len:227 gc:universal TRINITY_DN751_c0_g1_i1:731-51(-)
MAMKIVLCTLAALMCCAYAQDGITAITDIINPENINTALDAAEKLKNLIHTTSESKIQSREKTQGFE
FLNMKANAQYTRGMKKHHLMRMMNRYLVPFSRSSADMKEVVDTLKEFIEDDVEEHQEQFFGL
MTQGGGGTTEAIFVYYKLRDDGKYNFKRMTFRGTFRLAADLVITHSSKSGFFSSESKDVINWLPRR
GVTAQNVQELLDIIVPKLATVMSEFAPKDSI*

>TRINITY_DN3153_c0_g1_i3.p1 type:complete len:196 gc:universal TRINITY_DN3153_c0_g1_i3:154-741(+)
MANDKSIREKVDEFLHQKNYFTDGLVFIEQKTGINRLYLLVGCVVFLALYLVVGYASGFIVTLLGFL
YPAYASVKAIESSAKDDDTQWLTYWVVYTAFSLLEYFSDIFLSWFPLYFLFKCSFLAWCMAPFSWN
GSVLIYRRLIKPFVLKHESKVDEYLHKGEELAKEYYGKAKKDVTKAVVEGMTADSDESKKDE*
>TRINITY_DN1505_c0_g1_i1.p1 type:complete len:154 gc:universal TRINITY_DN1505_c0_g1_i1:98-559(+)
MSLVIPEKFQHILRVMNTNIDGKTKIMYAMTSIKGVGRRFSNICLKKADIDMTKRAGELSDEEVER
LVTIMQNPRQYKIPDYFLNRKKDIKDGKFSQVLANGLDNKLREDLERLKKIRAHRGLRHYWGLR
VRGQHTKTTGRKGRTVGVSKKKN*
>TRINITY_DN1189_c0_g1_i1.p1 type:5prime_partial len:449 gc:universal TRINITY_DN1189_c0_g1_i1:1554-208(-)
VSAKSQVIAKMKFFLFSIAAILLVNSALADNAVQIPKTCEDVLAMETCDRLRAVAKKFHEKVGTV
NQAVTDAFKHHKTKAVEVLTYVKEYLVDKAVNFKCEDVLPADQCHNLLEFAKRLNVKASEASIA
LREAIADGSEQAKQLYQRAVSFLVNDVPNLKCEDFFKKDDTCDKIRNFATKVNAKAKDVTAAVK
KAIVENLENAKDGLKKAKQFLKAKISCDQVLNEKMCNTIQSIGDKFSVSMKKIDAALREAYANG
VRKWSELYEAAIKYIMDKWSIFGDDEPQRERREIQPENVKKAIKNLLDSLKVKSKKVRAAIMKLIE
SGKVKFGNLRKTVKQLLASLKGDKKSEDAVTAVYKRSVVDTLKETLQKLKEQIKDLAGKAKEQA
KAKIAELKELIKGALGSLDQDQSDELDQDFEMISDVLEAAADESFDEIANNALENHLGQYY*
>TRINITY_DN925_c0_g1_i1.p1 type:complete len:261 gc:universal TRINITY_DN925_c0_g1_i1:826-44(-)
MGRVIRGQRKGAGSIFKAHVKKRKGAAKLRSLDFAERNGYVKGVVKDIIHDPGRGAPLAIVAFR
DPYKFKLRKELFIATEGMHTGQFIYCGKKATLQIGNILPVGTMPEGTIICNLEEKSGDRGKLARAS
GDYATVVSHNRDSKVTRVKLPSGVKKAIPSFNRASVGIVAGGGRIDKPLLKAGRAYHKYKVKRNC
WPKVRGVAMNPVEHPHGGGNHQHIGFPSTVRRDTSAGRKVGLIAARRTGRIRGTAKVKDVGGD
KE*
>TRINITY_DN146208_c0_g1_i1.p1 type:5prime_partial len:284 gc:universal TRINITY_DN146208_c0_g1_i1:876-25(-)
TDSHIRTTSAFIPCAKPSKMPPKTKKIKVKKVAAAPFQTSKPAPKKSVNVLIERRPRNFGIGADVQP
KRDLTRFVRWPKYVRLQRQKRILYQRLKVPPSINQFTQTLDQQTATQLFKLLHKYRPETKAEKKQ
RLTQLAETKAAGKQESSRKKPVTIKYGINHITNLVESKRAQLVVIAHDVDPIEIVVWLPALCRKMG
VPYCIVKGKARLGKLVHKKTATAVCLTAVRQEDRHSLEKVSDAVRTNYNERFDEIRRHWGGGIM
GGKSQAKVAKAERAKAKELRV*
>TRINITY_DN165536_c0_g1_i1.p1 type:complete len:249 gc:universal TRINITY_DN165536_c0_g1_i1:851-105(-)
MSEQEISNLVEQAKLAEQAERYDDMVKYMKELTEKSTEGLSDEQRNLLSVAFKNVVGARRSAWR
IIHSLEEKKGNEGGNTELISSYRKEIEKELKERCNEVLSLIDKYLLPSAQKEGQKGTEGKVFYEKMK
GDYYRYLVEVTTGEDRANLSAQSKEAYDKATETATGLATTHPIKLGLALNFSVFHYEIMNDGKKA
CELAKKAFDDAIAELDSLKEDSYKDSTLIMQLLRDNLTLWTSENDNEENDAQ*
>TRINITY_DN13856_c0_g1_i2.p2 type:5prime_partial len:292 gc:universal TRINITY_DN13856_c0_g1_i2:3-878(+)
GAGRQAWSPSVSRQHGAAGGGAWAGAPESAATLTSSLAVGLSAGVVVAGLSSRRARTARRAVE
NKSQNGPFGELATVEPRTLELTEKVKAVIKQKGSLDRESYREIFLAPKPDAVFAGGLVGSEYHGWG
TYQWDPAGLATRYPEHLPWYREAELKHCRVAMLAWLGLIVPDFVRIPLDDFQDPTLTLENAHNK
LIYGLGSGPMWWLLLFCGIIESLRFKQLGLDFGKLTLENAGDLDFGKGFLPKTKEGIVQMKIKELK
NGRLAMLAVSGALTQSMAYGVYTFPWTLPSPN*
>TRINITY_DN21847_c1_g1_i1.p1 type:internal len:133 gc:universal TRINITY_DN21847_c1_g1_i1:397-2(-)
DTKKRKLSAELANGRLAMMAIIGMFFQDGLTGAAWGDWELYVDSPLRAFENELGVQDPVGFW
DPAGFTADGSVENFKRRRQTELKHGRVSMLATMGYITPEITGKLPGYLSPSAGLKFADIPNGLAAI
SKVP

>TRINITY_DN166344_c0_g1_i1.p1 type:complete len:360 gc:universal TRINITY_DN166344_c0_g1_i1:93-1172(+)
MQKPKKYDWKDSNMALFGSDTEKKVKKESAESEPAWQGAGADVGVQVWRINKFKVEHWDKE
QYGEFYNGDSYIVLNTYKEPDTDELKYDVHFWIGKYSTQDEYGTAAYKTVELDTFLDDKPIQHRE
VQGHESSKFKTYFPTLTLLKGGADTGFKRVLPEAYEPRLFHVKKQNRTQITCTQISLKRGNLKSED
VFIIDLGDKIYQYNGSSCSADEKYKATQEVARLKSVRGKARAETIEERTVSDEHPALMVLKDGSSK
EKSTVEGEKKMLRVSDADGSIGMDEVASGDDISKDKLTSDDVYIINTGSHVYCWIGSGASIDERKN
GLSYASNYLNKTETPWLPISVVGQGKEDSQFQKAF*
>TRINITY_DN484_c0_g1_i1.p1 type:complete len:554 gc:universal TRINITY_DN484_c0_g1_i1:164-1825(+)
MASMILNGERTTGNDVREQNVMAAASIANIVKSSLGPVGLDKMLVDDIGDVTITNDGATILKLLE
VEHPAAKILCELADLQDQEVGDGTTSVVIIAAELLKNAGNLIKYKLHPTSIITGYRLACREACKYIQ
ENLAVKTADLEREHVINCAKTSMSSKLVGVDSDFFSTMIVDAAMSIKRTGNKGETKIPIKSINILKA
HGGNMKESISVPGYALNCTIACEGMPKKIENAKIALLDFSLQKAKMHLGVQVLIEDPEKLEAVRQ
REMDITKERIEKVLASGANVILLSGGIDDLCMKYFVEAGAMAVRRIKKVDLKRIARATGGTVCLSL
ANLEGEESFEASYLGHADEVAQERICDDELILIRHPKTKAAASIILRGANDFMVDEMERSIHDALC
VVKRVLESKSIVPGGGAVEAALSIYLENFATSLASREQLAIAEFANSLLVIPKTLTVNAAKDSADLVA
KLRAYHNASQTQSDKAHLKWVGLDLINGVIRDNKKAGVLEPTMSKIKCLKFATEAAITILRIDDL
VKIEPEKKDDPNSYQNAIAGGRMG*
>TRINITY_DN82805_c0_g1_i1.p1 type:internal len:381 gc:universal TRINITY_DN82805_c0_g1_i1:2-1141(+)
FILANVRFPKETRYQRACMDKIIADVSNVNSEYEKKLNVLVMGDFHFDRLSGADAKGELYKIITN
LQIDGENYHSPITYLEKDPITTVYGRRFDNILSNINSKGNIKTIGPDFPLEPESVILPITLQRLYNNYIT
ALYTTDSDESDKIYECIKAFQMAGKKELEGDMCKNTWVPSSYISDHLPIQFKVKMSLPGKTNPVE
VKLASWNIQGDTFKERLLDPSVRRGFLNALKQYHVAVFHEFPPHDRIIDDQELGSSDIVRLKQGTI
TTEFGIYVSENVKDGETDMYVLRKKFKDEPNGVEDVVNEVTPEDSKNLITTVGLFAPKIVVDTVKI
RPCLYTVKTSKTQTSGVDFSRIINAIDKDVQERKCDFKVMLASLNLEE
>TRINITY_DN131028_c0_g1_i1.p2 type:5prime_partial len:104 gc:universal TRINITY_DN131028_c0_g1_i1:2-313(+)
FDKYHPGYFGKVGMRNFHFIKHGKVIPTINVERLWSLVSEQTRTYYKEHTDKAPIIDVLKAGYMK
VTGKGQLPEQAVIVKARYFTKEAETKIKEAGGVCVLCA*
>TRINITY_DN1272_c0_g1_i1.p1 type:complete len:262 gc:universal TRINITY_DN1272_c0_g1_i1:957-172(-)
MRCQLFLLVACMAIATCSAYDWRNKVQNMLKYAKVNIFDRAAYILGDKRQHQLGKPWFCHKL
DCPAFQVVGNYTNKKDGFEERCYPETAWVMTSKEVDANENDIETRDMFMKLFKYIQGENEKQE
KIEMTVPVFTYSQLKKDDPSKRYISMHFFVPPSQEKNIPKPTRDDVTIMDVKADCYYVRSFGGYV
MSFGSKIMNEEKKIHAALEKNNLDFEARKTMAAGYDSPFRLFNRHNEVWVAKRKTMDSGCCTP
RKSTNVMY*
>TRINITY_DN95381_c0_g1_i1.p1 type:complete len:192 gc:universal TRINITY_DN95381_c0_g1_i1:70-645(+)
MGKQNSKLKPEVINDLREQTEFSENELQEWYKGFLKDCPDGTLTVEEFKKIYGNFFPYGDAAKFA
EHVFRTFDANADGRIDFREFICALSVTSRGTLEQKLKWAFSMYDLDANGYISRDEMLEIVCAIYK
MVGNVVKMPEDESTPEKRVDKIFSQMDKNVDGKLSLAEFIEGAKSDPSIVRLLQCDPHAPK*
>TRINITY_DN59667_c0_g1_i1.p1 type:3prime_partial len:225 gc:universal TRINITY_DN59667_c0_g1_i1:673-2(-)
MGKAKKGQKIDESKFEGKGTDFKCKLIGFVNVPSARGDDICQEAITKLKHLALKLNKEKKEHKQ
KITVNITVKGIRLIDEPTERIEHAHPINRISYICHDNDDKRTFGYIVGTKEGYKLFAIRTMKEAEYITK
TLKDLFQVVFERHQAQKAASDTSEIKGQEQVEEKDDEEKFEVGEEQNEEPTESPLLDWEHGINEQ
IQAMQNDAMSTSGFPDPFGDAQFDSA
>TRINITY_DN72959_c0_g1_i1.p1 type:complete len:216 gc:universal TRINITY_DN72959_c0_g1_i1:54-701(+)

MGRRPARCYRYCKNKPYPKSRFCRGVPDSKIRIFDLGKKKTGVDDFPKCVNLVSDEYEQLSSEALE
AARICANKYMTKTAGKDAFHIRIRVHPFHIVRINKMLSCAGADRLQTGMRGAFGKPQGTVARV
HIGQPLISVRCKDQHEATAIEALRRAKFKFPGRQKIFTSKKWGFTRWDREEYLERRQDGSIKPDGT
NVQYYPCKGPLKDWKWRESR*
>TRINITY_DN4815_c0_g1_i2.p1 type:complete len:638 gc:universal TRINITY_DN4815_c0_g1_i2:79-1992(+)
MVSSLLLVLICTVVYTAGISAFFQCDFEKVDICGFKSSSSNYTDTGEAPNWRRHTGTTPSAKDQNN
AHVTGPLEAQSGDYYMYFEASNLPSGASSTGTLTSRLFDATQFGVTDIVFHVSSKGESLSLLVLDFI
DYTTNDIFDEKVVFPRNKAAWQKYTFNLDEKIFKRKFYMQFIGYVREVNGKKDNGDWAIDNLRI
DGIAIVEKCENVKCNVNTNCKDTSNGPNCECLPGYSTKAGTSGKNTECIETCQAFTCNLNAECS
NTENGPDCKCESGYKAINGTSGKTTRCEETCQAFTCNLNAECSNTENGPDCKCESGYKAINGTS
GKTTRCEDDTCSNFQCSNNTNCENTVSGPNCTCKEGYRPVNGTRGRDTICEDINECAIKNICGGL
KVCRNVPGSHHCDCPPNYTFFAAENKCYRNGAFSQWRNVGSCSKTCGAGDQKQTRSCSNPSRE
APHGRDCPNEPTERAVACNSGPCLAGCPRNKTCTCENVRQYKTGEEPKKDFSSFNGLSITDGETS
RRYVHTYNREFIEKTIKDVNCNNNQLKSVYKNITDIIGILEGINKKYSSTKMEMQDIIDCNANIDIE
KNLWDVYDVLAEHSICNYQVQKDLEMKADRIQAEITRCNNRGWYSTILDAII*
>TRINITY_DN22594_c0_g1_i1.p2 type:internal len:268 gc:universal TRINITY_DN22594_c0_g1_i1:801-1(-)
DFQDPTLNLETAHNKLIYGLGSGPMWWLLLFCGIIESLRFRQLGLDFGKLTLENAGDLDFGKGFL
PKTEEGIVQMKIKELKNGRLAMLAVSGALTQSMAYGVYTFPWTLPAPNAVQSRTALRAVEFKGQS
GPFGALDVAEPKTLELTDRVKAVIKSKGSLDRESYREIFLQPKAETVFAGGLVGSEYHGWGTYQW
DPAGLATRYPEHLPWYREAELKHCRVAMLAWLGLIVPDFVRIPLDDFQDPSLDLENAHNKLIYGL
GSGPMWW
>TRINITY_DN101992_c0_g1_i1.p1 type:internal len:139 gc:universal TRINITY_DN101992_c0_g1_i1:414-1(-)
AERYDHHSGIVESMDSNKISDNLKELTRLPHPPGRSRNKELGDMLADFWQNEGMDKIEKFKYNI
YLSYPEKPSTILLFGGAKTEKYTIEAEPPVDESEKGGTPVVPFNAFSANDIVKAELVFVNHCRESDF
DEVKKAG
>TRINITY_DN15599_c0_g1_i2.p1 type:internal len:206 gc:universal TRINITY_DN15599_c0_g1_i2:1-615(+)
KALTSSDPAERTKKLQSELANGRLAMIAIIAMFVQNGITGTTGPEMWRGGGAFENELGVQAPVG
FYDPVGFTLDGDVQSFRRRRSVELKHGRISMLACMGYLTPEIVGKWPGYISPSLGLKFTDIPSGLDA
IGKVPALGVAQIVAYFGFVEFAAGFDDYKPGGTPGDYGWKALTSSDPAERTKKLQAELANGRLA
MIAIIAMFVQ
>TRINITY_DN98420_c0_g1_i1.p1 type:5prime_partial len:286 gc:universal TRINITY_DN98420_c0_g1_i1:1007-150(-)
LHKEARSTVINKMASRLLNSLGKVGVGLAVVGGVVNNALYNVDGGHRAVIFDRFRGVLPDVSG
EGTHFLIPWVQRAIIYDVRSKPRNVPVITGSKDLQNVNITLRILFRPVASELPKIYMSLGEDYDERVL
PSITTEVLKAIVARFDASELITQRDNVSLNVQEALVERCKAFGLILDDISLTHLTFGKEFTEAVELKQ
VAQQEAERARFLVERAEQQKQAAIITAEGDAVGAQLLSDAFKKAGNGLIELRKIEASEEIAQNLSG
SRNVVYLPDGQGMLMNLPAP*
>TRINITY_DN35672_c0_g1_i1.p1 type:3prime_partial len:668 gc:universal TRINITY_DN35672_c0_g1_i1:2001-1(-)
MLKFYLSLILTWTIATAINSQNINRPFQNVYRQYNSNQNNQQKQTQVRNGHYPSNRNSNFRSRN
SKYYSMPTTNAGPNRCQGGQVCCPGWSLNPSTRMCTIPTCRNPCGYGFCRSPNLCQCNNGRFG
SRCGGQGGGHIGCQQRCMNGGQCQGDQCNCRQGYVGLSCEQPICAQPCLNNGRCIGPNRCAC
PYGYTGQRCELDHRIGPCFTRIEDNMCRGQLVGVKCSRDSCCATVGAAWGVPCSRCPSKRECKL
GFFKDPRTGKCEDMNECRGIPNICAMGTCINTIGSYRCECGQGRVYNAEKFECSAVETFKGCFSD
HTCRAGTQLSNRLGKSKCCCGKPGRSFGLCDKCPVKGEEAYRSLGCSDDVGNIGEGGDGKDGV
GDGKEVKPEVNECKLNPGLCEFGKCVNINGGDFKCECNDGFEPTDDGKRCAAKQTFDKCYIDH
SCNGKSVEGVSQKSCCCGKPERAFGFCKKCPVKGSDAYRTLVCSDDQGNIGEGGDGKGGIGDDD
NDDHDDDGGGDGRDDKKEVDECALNNGLCEFGKCVNIDGGDFKCECNDGYEPINDGKRCAA

KQTFDRCYKDHSCSVKYSNDGISKKSCCCGRPENAFGICKKCPVKGSDTYRELGCSDDQGNIGEG GDGKGGIGDDNDGDDDDDDDDDDDDDDDDDDDEN
>TRINITY_DN79_c0_g1_i1.p1 type:5prime_partial len:478 gc:universal TRINITY_DN79_c0_g1_i1:2132-699(-)
VVSSESNVLVEFYAPWCGHCQSLAPEYAKAAKQLKESKSDIKLAKVDATVETKLAEKYEVQGFPTI KFFKKGNALDYGGGRTDKEIVAWLNKKTGPPAKLLDNAADVKAFADERDVAILGFFNDKESSEL AKAFLAVAEAVDDLEFAIAAPDASGDFAVTEDKIIVAKKFDDPQVVYSGEANIADIKKFVRAESIA LVTEFTDEAAPKIFGGDIKNHLLLFISKKAENFAELKNTFQEAAKGFKGTVLFVYIDTDVEDNGRIL EFFGLKAADAPTLRLIQLEGDMTKYVPETKDLDKDSVTSFVQNVIDGKIKPFLMSADVPEDWDK NPVKVLVGKNFAEVALSKDKDVLVEFYAPWCGHCKQLAPIWDKLGEKYKDSENIVIAKMDSTAN EVEEVKVSSFPTIKYFPKEGGVVDYNGGRTLDDFVKYLESGGKDVSGGADETGEDEDADEEEEGM EEGMEEGDLKPEEPEVAKDEL*
>TRINITY_DN1559_c0_g1_i1.p1 type:complete len:311 gc:universal TRINITY_DN1559_c0_g1_i1:221-1153(+)
MAEKKATFKLGFAENFALSGAAAVISKTAAAPIERVKLLVQNQDEMIKQGKLSEPYKGVIDCTTRT FKTEGLAPFWRGNLANCIRYFPTQALNFAFKDKIKAAFKVSKSDGYALGFGKNIASGGCAGALSL CFVYSLDFARTKLANDTKSSKKGGSREYNGLVDVYRKTLKSDGVAGLYRGFVLSCVGIFVYRGCYF GFYDTLKPVLLGADASLFMSFILGYGVTVTAGLISYPIDTIRRRMMMTSGQAVKYKGSIDCATQILK NEGAMSLMKGAGANILRGVAGAGVLAGFDKFKEVYIAMRKRQMGLE*
>TRINITY_DN10890_c0_g1_i1.p1 type:5prime_partial len:963 gc:universal TRINITY_DN10890_c0_g1_i1:3568-680(-)
NEFFAGFQISPKILDLSEKDKPRKIILENTVPIVCQDGTENCRVLVELAQTNTDNFMDYCTISFKPGP AGQTNEVEVVAKRDFIDDGDQTMFLKLHIPDHIDPVDWNCYKNITDIKIKTTDVKTSRCLSTGDP HITTFDGHYYNHYLVGDYTLVESKARKFKIHVRTWKCGSVSCNCGVAVQEGDDVIVVDMCRDR VPRARFASTVEPQKGTTIERNNNGKSFLLSFPSGAFVRITCKRNYADFQIQLPSDDFQNTVGLCGTF DRNRNNDLTSKSGQVFNSRRGRNGPNGFTESWKLSSRDSLFYYRGGPKKCLATRPKSYCTCKETC CGKRTVNCDYEGYSQRPKYKQGFRGWKSLTFPGAEHCGRRKRRSLDDSSVVLPDDGDTAIYDYK PAPVIPNEGRFPTPKGKSESQARNTCTRKIKESTIGKACLKIITGFDVQDYIDQCVIDVRVLDDFIVP VESAVTNMASNCEEETLRDVKKYWKKDAEGKDAPPQDIGDSACLNDCSDNGVCRNGTCICND GFITDDCSMTKGQQPVLLGIPNDGLCDIRRQDCVRTRVTGDYFIDSSTLQCKATEAKVSDTTPPEK FGSGVIEQGQLLSFREIGCLLPKVPLVINGNPDYSRGKPTGAFYISVSNDGNNFSKEDTTYPDSKSET VMVVYDSKCMDCTKEGTCSWKKNSCKMNEYCFANGDPKPDDWCQVCDPDRSRTEFVKRLDN QPPVFNGGSTQYIFKGQQWKYELPAEDPEKQPLMYSFIGENHGMKISSAGILTWTPTELKVYSFKV EIEDPCGLKASQEFKVDVRNCSCEGQNGGRCEWLKSNTPEDHKCVCPDGCKGISCTDPIDGRICR VKVSPQSGDDDSSSNGAVIAVIIILLIILIVIAVVGFLIIRYLKKENKDVNMKNIKTTIADAGKNAKD RVADAGRNAKDRVIPNKNAKDDINANDKMRSSIRINLRPQSGTSAK*
>TRINITY_DN1183_c0_g1_i1.p1 type:complete len:193 gc:universal TRINITY_DN1183_c0_g1_i1:54-632(+)
MAAIRKKLVIVGDGACGKTCLLIVFSKDQFPEVYVPTVFENYVADIEVDGKQVELALWDTAGQED YDRLRPLSYPDTDVILMCFSIDSPDSLENIPEKWTPEVKHFCPNVPIILVGNKKDLRGDENVKWELS KMKQEPVKIDQGREMTDKINAFAYLECSAKSKEGVREVFETATRAALQTKKKKGKKCLIL*
>TRINITY_DN12304_c0_g1_i1.p1 type:complete len:195 gc:universal TRINITY_DN12304_c0_g1_i1:1971-1387(-)
MSSSSEPTKFKMVVVGAGGVGKSALTIQLIQNHFVEDYDPTIEDSYIKQVVVDGAICILDILDTAGQ EEYSAMREHYMRTGEGFLCVFAVTSLKSFQEIDNFRTQALRVKDAETVPMVLVGNKVDLPKRDVS TKHGNEKAQSFGIPYVETSAKTKQGVEEAFFTLVREIIADRRRRESQMKNDMKNKKFKCTIL*
>TRINITY_DN119684_c0_g1_i1.p1 type:complete len:662 gc:universal TRINITY_DN119684_c0_g1_i1:2182-197(-)
MKLEIILFTLLVALTVHCSEKKDDSKKDVGTVIGIDLGTTYSCVGVFKNGRVEIIPNDQGNRITPSYV AFTEEGERLIGDAAKNQLTSNPENTVFDAKRLIGREWGDKAVVHDMKFFPFKVVEKGKKPHVQ VNVKENEKKTFAAEEISAMVLVKMKEIAEAYLGKKVTHAVVTVPAYFNDAQRQATKDAGVIAGL NVMRIINEPTAAAIAYGLDKKEGEKNILVFDLGGGTFDVSLLTIDNGVFEVVATNGDTHLGGEDF

DQRVMDHFIKLYKKKKSKDIRKDNRAVQKLRREVEKAKRALSTQHQARIEVESLFDGEDFSETLT
RAKFEELNSDLFKSTMKPVQKVIDDSGLKKKEIHEIVLVGGSTRIPKVQQLVKDFFGGKEPSRGINP
DEAVAYGAAVQGGVLGGEEDTGEIVLLDVNPLTMGIETVGGVMTKLIGRNTVVPTKKSQIFSTAA
DNQQTVTIQVFEGERPMTKDNHLLGKFDLNGIPPAPRGVPQIEVTFEIDVNGILRVSAEDKGTGK
KEKITITNDQNRLSPEDIERMVNDAEKFADEDKKVKEKVDAKNEFESYAYSLKNQISEKDKLGGK
LSEEDKGVLEKAVDEAISWMDSHQDASVDELKEAKKKLEDVATPIVSKIYQQQGGAPPPPGGEG
GEEKEDKEEL*
>TRINITY_DN118520_c0_g1_i1.p1 type:complete len:581 gc:universal TRINITY_DN118520_c0_g1_i1:1895-153(-)
MYRLLSVIRKTTQTAKLRQGLSACISTTTHYCSPKEIKTGTDARALMLKGVDQLADTVAVTLGPK
GKNVMIEQSFGGPKITKDGVTVAKAIELPDKYMNIGAKLVQDVANNTNEEAGDGTTTATVLARA
IARSGFEAVSKGANPNEVRKGVMAAVEVVTQGLKDLARPVTTTEEIAQVASISANNDAAIGKLIS
DAMEKVGKDGVITVKDGKTVYDELEVIEGMKFDRGYISPYFINTAKGQKVEYQNVLVLICQSKIS
NVQDIVPVLELANQHKRPLLIIAEDVDGEALTALVINRLKIGLQVVAVKAPGFGDNRKNTLKDVA
ISTGAEVFGDDLNLKLDEVQLSDLGQIGELVVTKDDCLMMKGRGNTDLVNQRANHIHDEIKATN
SDYEREKFQERLAKLVGGVAVIKVGGSSEVEVNEKKDRVTDALNATRAAAAEGIVPGGGTALIRC
AKLLDTFQVPTEDQKWGVDIIRAALFKPCSTIVANTGIESAVVVEKVSSSKDPEYGYDAASGEFAN
LIKAGIIDPVKVVRIALSDAAGVASLLTTAEAVITEIPKEDPSMPPMGGGMGGMGGMGGMM*
>TRINITY_DN252_c0_g1_i1.p1 type:complete len:182 gc:universal TRINITY_DN252_c0_g1_i1:1409-864(-)
MASTLVILLICFVGGTFAQGCPRNKTCRCDNVIKYTTGNQPNKDLSSFPGLSLSDGPTARSYVQTY
TKMYIDDKIKEAKCDNNKLRAVDMDLNPIVMSLKTMNTNYVDKKTEMQDIIDCNPNLDIESDL
WDVYDVLADHCVCISQIHKDLKMKMEMVEAEMKRCDERGWYRSMLESFSIIS*
>TRINITY_DN598_c1_g1_i2.p1 type:5prime_partial len:742 gc:universal TRINITY_DN598_c1_g1_i2:2-2227(+)
RWSGCSKSCGVGVKTKSRECDNPRPSGGGRYCTGERTATEACKIQDCGVDGKFSHWSTWTACSK
TCGGGAQKRARQCNSPPPSGDGKDCAGAREEFKGCSNDFCKGPTGVAKAQEIIERFLENFHKKT
YPDREPSGKFLKAIFAIQKAVAEEFISVDPSVRGKTLEQRLKDLEKFLKVATEGGSIDFARTLLGKGIF
TFMKKKIQEGLPIDDIDKAAEGFLEKLRGDVGEKKMNEILSSQGDATLMFTIDTTGSMKDEINAA
KAIAKQIISATRDYEVDYILSPFNDPRTGPVTHRSSSNRASFVTDIDRLRATGGGDCPELSFTGMLN
ALNQGPQYGSPMFVFTDASAKDDSASNIDSLKAAADLTDATINFFTNLNGCKGAKSVESYKEIAS
YTGGQVFPLKNEAELKKFTNYVKTSLQQDTVITSGGTLNSKSYKRSLYSMQAHDFMVDDGIIMIS
VAISVQKYASQVTILDPNGRRVNPSTTLSLSLVYQISHPPPGQWKINFPSSVGKYGYTVQAVSSSAIA
FAHNFIYQQSVRKNSPPIPLANPLKGVKNMMVVRLGGTKNFDLNSIKIDLVDTSGNVLQKGIGTD
RLDSDEVRHATIDPPSGPFKVRLTGKTKSGYNFERVSQGISRPVSAAMRTNFAGYDFTAKLGKGPY
AAQMLFINRGAHENFYFYAKANYGSIPNYKQTKRVTSQGFVTFTYKAPRQYNLKGKSDRITVTVK
RQSTGEIVSHHTSILLVV*
>TRINITY_DN22219_c0_g1_i1.p1 type:5prime_partial len:427 gc:universal TRINITY_DN22219_c0_g1_i1:2-1282(+)
YISKDYCKGKKNPSVCTLHIHNEFSQIKHFQHQTPVTSENAPGIILAHGQAGDALTGAGNVWFSR
NGGYNWTRIAKGSHHYAIGDNGNLIVLVPKFNSTGKTLIYSLNQGRNWYHNEFDLFDENITIHG
LAAEPHSKERSFSLWGHRLVKGKKLWHTFTVNFRKLITKECQEEDMKTWYPHINNGKDGCFLGE
RTIYKVPHKDSLCYLGRSYKPEKKVEKCSCTKDDLECDYGYERNRAGNCHRNEDIPLIEICKNGEE
MQEAFSKGYRRVPGDNCIAQSSNDEVVRFIDEHKKCVPNKEDIFGDFNRERAEVIREEEEEKEKK
MDETQKATKTKKSKKKGPNWPVIILSIILMCIIIGGAYWYWRKNYRPNNVHHVMYNASSFDNVG
MDNNDGDVDADDLFDPRPKSLREYHDETDAEDEQLLLP*
>TRINITY_DN15248_c0_g1_i1.p2 type:3prime_partial len:250 gc:universal TRINITY_DN15248_c0_g1_i1:748-2(-)
MARVWAFALPAAVALGTRLAAAAPLNFTLLSAPVKLRYGEVYNRMQVPMPLPPDVVSRFADGD
KVMAVSGFEVDMVRIAADGTESRVPLYDHYVHHYVLRIGRRPSMESLFDAAAKDRASAQMLTG
CHAMTGADLHAFDARLGGQAAVFGSGAGAEYRGTPNSLEAPFRRLLHRPEGWAPVLHIINTNR
PVAGQTYSPLAECPCTAQRRINASAGTIDGQVSRPPIHCSPEFAATGNPSCQLATYTGGL

>TRINITY_DN244_c0_g1_i1.p1 type:complete len:3019 gc:universal TRINITY_DN244_c0_g1_i1:9748-692(-)
MDARGQRLLYCLLIFLSLATLLVSQQVEKCKRPVDLVFALDTSGSVKDSGFRLIQKFAKDILKTFDI
GVADSHVGVLTFSEQAEPQIKLTETFDKAELMQMIDSLVYQGYRTATDDALRVAKTDFFSTAGGA
RQGVPHVLVLLNDGKCTVCIEPVKEAAQKLKDMGVEIFTIGVTENIKKQELIDIASQPTAYHFFEL
DDFDQLVDVVGDIQTRSCEVKKGKCPIPSPTEQCSGNEINECTSDENCEGELKCCSDGCSRICKYP
LLNCFTPIDIAFALDSSSSIQPETFQEMKKFSNDIVDSFAVSQKKTRFAALVYSDRAEIGFNFVRFDD
ASSIKKAIQNLPKLDSKERIDYALELAKSDIFSLQGKVRTRRPMVLFVFYDGTVSSGMKDLSLVVEP
LKNYGVKIVSMGVGPETNYYQMRKISSNNSDVFRATNFKELLPEIYNIAKRACTEKPGRCDASLN
PLPKEACKNVKILNQCENDFDCFGDAKCCRKGCNNVCVIRGPVCPRKLDIALAVHQNMDESEF
AATKFYLQSVVDQFNVGPSQTHFSLLSFGQEPKVIFDFNSDQSSNEIVRRKIDNFQYQYGIGRISDV
LALSCDAVFCAAGGTRDDVPKMLVIVTSRTSPSSPSKLKEKLQTLRNRGVNILMIGVGDVDTSLLQ
AITSDDPLVEDRVLLSKRFDGIFQYTKDISDYACGMAPALPPPDNIKGDAKGECGAPKSCYKGPKG
SRGRPGTLGQQGESGDLGLVGPKGIQGRRGDKGPDGQMGSKGKRGPDGLIGLPGFPGYNGIPGV
PGIEGRRGRDGDCGLPGSSGEAGQAGLDGLSGPPGRAGPVGEKGEKGLPAPENPNIRIEPGDRGD
TGSVGLPGFKGIKGEEGFKGPSGDSGVDGIKGVDGEAGEMGDIGTYIYQKGPEGDRGARGPPGD
GDTQGATAGLGVQGEKGEKGMRGEAGDGSIFPGIKGRRGDQGPKGEEGSTGPAGPTGQRGPEG
EEGPKGIKGEIGDRGKDGTPGQPGQEGDLGEKGFDGLKGQDGDDAEGELGDSGGKGLKGENGL
AGSPGSIGTPGPPGSKGEPGDIQIGLSGDPGAIGPRGPRGDNGISGQPGMRGDSGPKGAKGNQGP
RGDRGRNANMLAGKDGMTGPPGPKGFTGPVGDPGFPGPPGPPSVVECVEGACPKGVPGAMGQ
IGKTGEPGPPGPPGPRGSKGVAGGPCPICPDGIQGDSGDIGQPGMPGKEGSRGRKGNIGDAGSKG
LQGRNGDDGDQGPVGKKGFIGDIGPVGENGVDALGDSVAPDGEKGSTGDTGQIGSRGPVGNDG
PKGFKGTIGERGRRGLQGEKGDMGLTGETGEDGSRGLEGAKGDLGDGLRGAKGDKGNIGQEGN
QGVVGDVGLQGPKGEQGAPPTEDELKNVKATKGSKGEIGSTGDAGEIGDIGPAGPRGPKGFDGA
AGPKGPSGVKGDRGNKGRAGDGEPGFQGFPGRPGKRGPPGDPGKPGKRGFSEPGDAGDRGDP
GKSGKKGADAPPPIGCFKFNEPTDIGFILDSSSSVDDDNFLRQLQFVKNIVRQYPVGPDQTQVGIIK
YGEKAVTGINFYDYVTEEGLFNAIDLLEHTKESESRLDLALKLARDELFTESVGARGFDAKKALVIL
GDGFISGGGTPSKTLMNNAMQYAEDLRGAGVSIFALAVGPEKNKALFHEFVSEKEYYLDIESYSQL
DNELGALQLSLQTGCISVGVPGRDGLPGYTGPKGQKGDDGPKGFSPQGPRGIKGIKGVRGTIGIG
VKGLPGDPGLQGPQGEKGRDGEDGSADPGPKGRPGDKGSRGDQIKGASGEDGDIGPIGPPGFDG
RKLCDNVLAVDLLFVLDTSSGVGVDNFNKMLKFIESIINVFDVDNQLTRVSVMTYDSDARLVIRFS
DYNDPDKLDAAIDNIPYANGDETRIDKALLLASSNAFDTSNGAREGVNKAVILMTDGQQSFAVD
QVALKDAVKPLVDANVLMYAIGIGSEISPVDLKTIAAERVLIADDFDGLSLKIEDQIVLLGRVGCQG
PPGKDGKPGANGEDGFPGLIGAKGQKGDRGEAGTEGLKGNKGSQGEAFPGLNRKGEKGDPGS
AGPLGLPGCEIKNQNEVIDLGFAIDASGSLYEEGFNREKEFIKTIVDRVGRVSEQGIRIGVIVYSDTAS
IEIRFDDHFNNEDLKNDIDNLPFFAKNTRMDLGLEEAKKMFGEQFGGRGSSKKVLILLADGQQTY
VPGVTEPSEVAKELAAAGVDIFAIGIGQEIDRTELESLISKPEHIFLASDISKLIDELTPEVTSALECEG
GPRGPRGRAGTDGPPGSKGLLGPIGDKGFPGEPGSAGFGPKGDDGENGTAGTIGDRGEKGFRGP
AGVPGKRGAVGPKGFPGPNGEKGDRGDEGFIGFEGARGLEGPKGQRGDSSDNGVAGVKGSTGD
NGFKGPKGDPGKLPPGNRIEDFKGETGGSGLEGDTGPKGKPGRALFEPGPQGLTGPKGEKGDQG
FFGMSGPIGNKGFQGLQGFRGDDGAAGPVGEDGFPGMKGLPSSSGDQGDKGKNGRKGESGEV
GQPGRVGEDGDQGEKGYAGFVGMPGKKGDQGEPGVQGPQQIRNEILDNIRPGPKGYPGSTGPA
GDEGEDGEVGEPGETVGAQGMNGDFGERGSQGPKGEQGDQGLQGMIGVIGDKGVKGRVGTG
GNPGTVGLRGLTGAPGEVIFGSEKGNKGDLGIIGLRGRAGQKGKAGAIGPRGRRGRNGAKGERG
PPGFPGLSGIEGDSGNEGDKGPRGDPGSVGNRGIKGVPGESGIAEGNYIVIHSQTHIIPDCPRDFKK
LWHGYSLMYVVGNGHSHSQDLGDAGSCSQKFSSKIFLFCEIIKVCKHASRNDFTYWLAGDVDHP
MMPVSNAAVEPFISRCAVCEAHSSHITVHSQDTYAPECPPNWSSLWSGYSFLMVAGAGKTGAGQ
SLSSSGSCLESFRPKPFVECQGARGTCHFFSDKHSFWLVTIDPNKQFEVMKPQTLIKDKNHDLTSR
VSRCRVCIRKTGTKNNTVEPNIATRIRRSIASWIL*
>TRINITY_DN785_c0_g1_i1.p1 type:complete len:289 gc:universal TRINITY_DN785_c0_g1_i1:1062-196(-)

MIIESKMLSSIMLKMIFVVVFLCGITLSEGIEKCDLQLEPIGCFKDDVWKRTMPEQILNERDPLNKV
YNNRMINWKKFDTYLPGFICRCAEAAFKKGYRVIGMQFWGECWSGPEAHLHFDSLGAADTCSN
QKFEREYDNITQCVSAIGMDSTNYVYRIAPTVCDTHYEPVGCFKDEQKVPRPMPHYISTDRDYSL
KIFSGKTIDWRNWNTYSPEMICRCATLAKSKGHALFSIQFWGECWSGPETNGYMRDGVTTDCRG
KDFEMCPCNGYHCVGKAYTNYVYRLGHHH*
>TRINITY_DN1534_c2_g1_i1.p1 type:complete len:547 gc:universal
TRINITY_DN1534_c2_g1_i1:1914-274(-)
MHRVNSSFVKVAERTSKLCGNLGIKNSKIPSSLIARSSSTDTTDGVGESEPGFLEMVSIFTENAHSIT
LDKLLSAKPAPGKRAEDTGVREKRIKGILDIIKPTNTALAVNFPLRRDNGDYELITGYRAQHSHHR
LPCKGGIRYAPDVNLDEVKALAMLMTFKCAVVDVPFGGAKGGICIKKSDYSENEVEKITRRFTVE
LARKGFIGPGIDVPAPDMGTGEKEMSWMADTYAMTLGYGDMNSSGCVTGKPISQGGIHGRMSA
TGRGVYHGIDNFANNEEYMNLIGLKPGLKGKTFIVQGFGNVGLHSCRYLHRYGARCIGVIEHDG
SIMNPNGIDPKDLEDYKLDNGTIVGFPGATDTTEDLMVAECDILVPAANELQITAKNAPHIKAKII
AEGANGPTTPQADKILQQRKRLVIPDLYINAGGVTVSYFEWLKNLNHVSYGRLTWKYEESSNYNL
LSSVQESLEKHFGGNIPINPSSTFLQSIQGASEKDIVHSGLAYTMERSANRIMKTAKDYDLGLDVRA
AAYVVALEKIFNTYSEAGLTFT*
>TRINITY_DN3619_c0_g1_i1.p1 type:5prime_partial len:1653 gc:universal
TRINITY_DN3619_c0_g1_i1:5108-150(-)
ISSRKKSKMGKWDDFVTVAIFVFTANYVVCGEIDKRSSLNTIDFNRYQNALVSGGCSIRRRGQTFN
LNNQYCSMKVVVNGIKDIFTSRPNAKIPNIKDATRNFIDPDIFKACQSIRFTENTASTVKILISEVTN
GESSSPLLPWISIMEVTVDVEITVDLTSQKEATVGLVFSETTFPAELTGNHLPSTPGLQIRITKENAYD
DINYSILQLATLEMSLEDFLRGIGVKTEYMLDDLDFRVRGPINGVVAEGLWTVRINGDNFFNQISD
KSKKFWSGSNAFLFCQYNKEDEKGPKCSLNSEQISGTDWWNLVTTVDMPNKFLGGGSFDDSPIS
VLQSSKVEIDVAFSTEVKQKLLPFSERSFSVLYGSRRNRQENQAYVSTGTCLRYLRESFQYTYSDGNS
YGTTLIPYIFNVCLEQNSVTGTFLPAFSLPLDMLIAIVMHQPMRDALKTKIDNYGILVKSMKYIGSQ
TCATFTSLTEKHHEFIKDQFPPENTVVTVCGDDNMATITAEGREKVLDAETSVDAKLMSEYYTDD
PAATVISTGWFLTGSVKIFDIEYVLEKTGQHFMGEGELEWFPSFKKFALTDTVFKAPSSSQLYLTLEG
TTHINHNKFKFELVSRSENTNDEIPASLALCLRKEGGTSDEIFSIFYKEKYKELPYSFEKTVISVQKDV
NFYTNHQASGTGIIFRCQNVEIKHKEEGVRITGSHAGSVRAMLETLVFGCDASGEGSKKSSSCQVI
KNGFGGGDTLEGNWYGRVAMRDLRLSALINKPFIVADGIEIKSGLQHVLIQNTNDEVQEMLRNQ
ISGQIDLGSHTTTASIIFNDDTAVFELHEYTSETSFNLYGVGGISQNRIFKSKTAMTNTRWSNLEIQG
NIILGSAPGFEIDIENDFEGAVRQFMYGDTTKNRFYACAPTKKEFTMSLVLQAFGAYDGTSLFSTTI
LKEVCVAYHNDENDWDIKFQGFKAKQGLRLYGTITSLFGYEQLKANVKLDGDVEVNIELPVLKF
GARTKVNERTGQTETRYAFTFSKMKSLEDYGPTFTAIFNNRNSFGQGKLTGHIKLPMYEGQVTLTF
NSYGHINQDIQLVNSESGDSKIKPGKINLSGTLLLGNVLTMGASRFTFSADLLLAAQANLAESCTK
EFTEINKRSALALTEIGNQYTSVLSNITSMETQIRLVNGNRLKRSIQELETQMLTEIRKVKKQNQEH
CKPLKLSGFKLEKSCGKRNSLQNATCLKFIDDAFDATDTTCLEESKNNRKGAHQNALKKNNIIRA
SSLSLREKRTCYDLFDQINNHYKAARDFLLTAKSTDLPNKINSLQSLGTYFSLRVAKYRTVNPMGL
SSIPNDVCIDFELEYNTGTPHTETKKLCALDSMFDDLGKRIVTEYIRGKEDERNEVYNEAITKAAH
EIYMANLIKKKNEDDYELCKRVQRARKREKVFVPEFFRSGYKNQAFRTNAENTESMMKYYAPW
NVMKRNNPMTIMSNSKPNRYLRMNHVEGSGPCSDARNKIESYGTMVDYMKTWVDNYQKGLA
KFKKINENVVRGLKKRNMTQHQGFAEFTKETESQLNQLNHFAPSYMKNELNALINPKQQDQLE
NFLYQTSTDTSNLIKKSFPQNDQTFSNLSNMLIQQFSGNKTLDEMAPSVKEMEKMMTALNKRTLV
CHDEL*
>TRINITY_DN2028_c0_g1_i1.p1 type:complete len:274 gc:universal
TRINITY_DN2028_c0_g1_i1:1608-787(-)
MGQSDGDDLTFGQKISREWRDFREFLYNSETGKVMGRSGTSWAKIGGFYLLFYGFLAGFFTALLFI
FYTTIDKPGIGGPKLIQYIENKPGLTRLDRSSKLQSYSASNNDRISSYKTVIDEFLDGYNTSDHSGAL
CGAAGTPKSDKPCIFDTTLLGECAPSKNTTYGLREKKPCIFIKINKVFGWTPADDGTGYLTLSCTG
HATVMPQGFPLTAFPYNGHKVYQSPIVAVKIDATKGKKNVDCTLKGKNIEVSNNFNPSRAFGKIR
IEGISATD*

>TRINITY_DN757_c0_g1_i1.p1 type:3prime_partial len:218 gc:universal TRINITY_DN757_c0_g1_i1:653-3(-)
MNAALVAIVFLLLSTVYGKTTPTPSTDKATTPTPKSPVCPKQGKDFYVQIKVLSLGEYVPEMSRVES
QSFQDIREKIYGCELKTYEDYDFYEDVIILDIQNSTAGFIVAFAIRYTKEGDGHLNRLSQAVASGKLC
DMEVSPKFFECKELDPSILYPMANCPAPCGGDLNCWPTCDATCCGTQEQTIYIPAAAPPPPPPPP
PPPPPPPPPAPQMVM
>TRINITY_DN578_c0_g1_i1.p1 type:complete len:429 gc:universal TRINITY_DN578_c0_g1_i1:379-1665(+)
MDMKALFLSLALVVVAWNTAYGSSNHKLVAYWGQNAVYNLKRDKQYWEKDLSYFCDNSKYDV
IVLAFMHVFFDRRQKDALPGLNFAFHCETSMSADLPFLLRCPQIEAGIKRCQANGKKVILSLGGA
TGGYGFANDGEAKTFAHTVHNLVLAGNKMQNIRPFGSAVLDGIDLDIEGGSNVGYATFVRELRA
LQGSNHYLIAAAPQCPFPDHFLGPSPGTAFGEVADLFDEIYIQFYNNYCHTGDAKQFYPNFQQWI
NFSKKSNGPKIFIGMPSHERAAFGQGYWRTPAEVAAIYENVKNEPGFGGFMLWDASFDDVNRFN
GRPYSDSIAALFKNGGGPRPTYRPNTMRPNPVTRPTPKTRRPNTQRPVTQGPVGPVSCSAGADGL
IPHQDCSKFIECSHGRATVKSCPPGLKFSSAKKYCDWPRNVQC*
>TRINITY_DN10907_c0_g1_i1.p1 type:complete len:206 gc:universal TRINITY_DN10907_c0_g1_i1:1010-393(-)
MAKKAYDYLFKLLLIGDSGVGKTCLLFRFSDDAFNTTFISTIGIDFKIKTVEVDGKKIKLQIWDTAG
QERFHTITTSYYRGAMGIMLVYDITQEKTFDNIAKWLRNIDEHASEGVERMILGNKCDMEEKRM
VSKEKGEGIAREHSIKFFETSAKENICVEEAFMQLTQDILRKHQAGATHEQSPSGVVIEKDSHNRS
KGGMNCCN*
>TRINITY_DN1280_c0_g1_i1.p1 type:complete len:426 gc:universal TRINITY_DN1280_c0_g1_i1:195-1472(+)
MGFETCGPNEAMVISGCGQERPLLISGGRKFVWEFFQKVQRISLNVMTLNVESQRVYTVQGVPIS
VTGIAQVKVQGQNEEMLHAACQQFLGKHENEIRNIALETLEGHQRAIMGTMTVEEIYQDRKKFS
KSVFEVASSDLVNMGISVISYTLKDIRDDEGYLKALGQRRIAEVQCEARIGQAMAKMQSGIKEAQ
AEEVRIKAEYENKTEVARSQRDFQLNKSAYDIEVNTKKATSELAFALQSAITNQKIKEEDMQIKVIE
RAQAINVQLQEIQRREKELEAQIRKPAEAERYKIEKIAEANRMKVVLEAEAEAESIRMKAEAEAE
VIQKKATAEAEQMSKKADAWRDYQEAAMVDMLLDTLPKVAAEIAAPLSKVKKVTMISSGSSDV
GAAKITGELLDIVIRMPQAMEQLTGVDITKTFRREK*
>TRINITY_DN598_c1_g1_i1.p1 type:5prime_partial len:745 gc:universal TRINITY_DN598_c1_g1_i1:2-2236(+)
RWSGCSKSCGVGVKTKSRECDNPRPSGGGRYCTGERTATEACKIQDCGVDGKFSHWSTWTACSK
TCGGGAQKRARQCNSPPPSGDGKDCAGAREEFKGCSNDFCKGPTGVAKAQEIIERFLENFHKKT
YPDREPSGKFLKAIFAIQKAVAEEFISVDPYVRSKTLEQRLKDLENFLKVATEGGSIDFARTLLGKGV
FTFMKQKIKDGLPIDDIDKAAEGFLEKLRGDLGDKKMDEILSSQGDATLMFTIDTTGSMKQEINA
AKAIAKQIISATRDYEVDYILSPFSDPGTGPVTHRSASERASFVSDIDRLRARGGGDCPELSFGGML
NALNNDPQYGSPMFVFTDATAKDDTDANVASLKAAADATDATINFFTNLAGCNGRRDRKRGIE
SYKEIASYTGGQVFPLKNEAELIKFTSYVKTSLQQDTVITSGGTLNSKSKRSLYSMQARHFMVDDGI
SKLVISVGVQQLASQVALVDPNGKKVYPSTTLALSLIYQVSHPSPGQWKLDFPTSVGKYSYNVQAV
SSSAIAFAHNFIYQQSVRKNSPPIPLANPLKGVKNMMVVRLGGTKNFDLNSIKIDLVDTSGNVLQ
KGIGTDRLDSDEVRHATIDPPSGPFKVRLTGKTKSGYNFERVSQGISRPVSAAMRTNFAGYDFTAK
LGKGPYAAQMLFINRGAHENFYFYAKANYGSIPNYKQTKRVTSQGFVTFTYKAPRQYNLKGKSD
RITVTVKRQSTGEIVSHHTSILLVV*
>TRINITY_DN164469_c0_g1_i1.p1 type:complete len:184 gc:universal TRINITY_DN164469_c0_g1_i1:45-596(+)
MDGLFDISSWFDEVKRMNKRQLYYQVLNFGMIVSSALMIWKCLIVVTGSESPIVVVLSGSMEPAFF
RGDLLFLTNDNTKPIRAGEIVVFRIEGREIPIVHRVIKVHEKDSGYVKFLTKGDNNNVDDRGLYVP
GQMWLEKKDVIGRANGFLPYIGIVTILMNDYPKIKYFILAALGFFVLIRKE*
>TRINITY_DN614_c0_g1_i1.p1 type:complete len:183 gc:universal TRINITY_DN614_c0_g1_i1:194-742(+)

MGQMFGKLFSGLFGKKEMRILMVGLDAAGKTTILYKLKLGEIVTTIPTIGFNVETVEYKNISFTVW
DVGGQDKIRPLWRHYFQNTQGLIFVVDSNDKERVTEAKGELAKMLGEDELREAALLVFANKQD
LPNAMNAADLTKQLALHDLKSRSWYIQATCATSGDGLYEGLDWLSTELKKKKN*
>TRINITY_DN1613_c0_g2_i1.p1 type:3prime_partial len:1062 gc:universal TRINITY_DN1613_c0_g2_i1:70-3252(+)
MWLRIYFALAAFALTANAYDFKTCAKTYKKIGCYEDKNHDMVMLENYRDTRRDDFHQLSPVYW
KKFNQTMHSLACECIKIAQSKGMDYIQIGFYGECKGGKDFASDIITRKEIPNDQCVNTKFKKCVTA
SDLDCVGKAHTGYIYLVEGGRDRNERKVDGGYSYWSEWTECSSSCGRGIQERERQCDSPLPKGG
GKDCSEIGEPFEIKDCNLRECPVDGGFTDWSQYEKCSATCGGGFQKSTRSCTNPAPKFGGQDCVG
DVEKTRDCGTKPCPVDGGWSLWDDYGECSKECGGGVQVSKRSCTNPSPAHGGKECVGVDERSR
ECNTFACPVDGGFGKWSEYDTCTATCGGGTQTSTRQCDSPSPAHGGQDCAGESERVKECGMKP
CPVDGGFSAWSDYSACPVTCGGATQTSTRTCTNPTPAHGGEKCVGKTTREKSCANNPCPVDGGF
TAWSEYGSCSEECGGGIQESKRSCTNPAPEHGGKDCVGDTERTKQCNTQACPIDGEFTVWSEFTK
CTVSCGGGVQESRRSCTNPAPEHGGEECFGPVMRTRACNTKKCPINGGFSKWGEYSVCSEECGG
GTQSRTRTCTNPKPQYGGEPCSGDSLEERDCNVQPCPVNGKYSSWSAYSKCSASCGGGSQKSTRT
CSNPAPAHGGAQCQGDAERTRECGTKPCPVDGGLTKWSEFGACTASCGGGEQTATRSCTNPSPA
HGGKGCLGELEKVRECGTAPCPVDGGFTDWTQYGECTKSCGGGSQTSTRTCTNPSPAHGGQEC
DGSTMKTRTCNSDPCPVDGGFSKWSEFDKCSTTCGGGYQTKTRSCNNPAPAHGGKSCSGETEMT
QSCNSNPCPVDGGFTAWSKYSICSATCGNGIATSTRSCTNPAPANGGDDCVGLKIKSKKCFVKHC
PVNGGLTDWSEYDTCTEKCGGGTQTSTRSCTNPRPAHGGDDCDGELERTRSCGMDPCPVDGGL
TKWSDYDQCSKPCGGGYQTSTRTCTNPLPAHGGKECTGETDRTRECNTHHCPVDGGLSQWSSY
GKCSDECGGGIQERRRSCTNPKPMYGGKPCTGLFMET
>TRINITY_DN932_c0_g1_i1.p1 type:complete len:475 gc:universal TRINITY_DN932_c0_g1_i1:1940-516(-)
MGFKFLELLKPFISVLPEVSKPERKIQFREKVLWTAITLFIFLVCCQIPLFGIMSSDSADPFYWLRVIM
ASNRGTLMELGISPIVTSGLIMQLLAGAKILEVGDTPRDRALFNGAQKLFGMVITVGQAIVYVMT
GMYGEPSELGAGVCLLIIIQLFCAGLIVLLLDELLQKGYGLGSGISLFIATNICETIVWKAFSPATVNT
GRGTEFEGAIIALFHLLATRQDKVRALREAFYRQNLPNLMNLLATVFVFAIVIYFQGFRVDLPIKSA
RYRGQYSSYPIKLFYTSNIPIILQSALVSNVYIISQMLSAKFAGNFLVNLLGVWNEQGGPSRSYPIGG
LCYYLSPPESLGQIGQDPVHALLYIVFMLGSCAFFSKTWIDVSGSSAKDVAKQLKEQQMVMKGHR
EKSMIHELNRYIPTAAAFGGLCIGALSVLADFMGAIGSGTGILLAVTIIYQYFELFVKEQSEQGGMS
TLLF*
>TRINITY_DN76073_c0_g1_i1.p1 type:complete len:107 gc:universal TRINITY_DN76073_c0_g1_i1:794-1114(+)
MNTNVENDYDQCFIGSQLKDECHKKNYLRTTGTILLQDLLEKDRSLLQWRCEVKTSDSDLEKRIC
YHHEKIYLTRYVGLQKYCCDPCNMHGNRFVTSKWKDFSQYF*
>TRINITY_DN11097_c0_g2_i1.p1 type:internal len:222 gc:universal TRINITY_DN11097_c0_g2_i1:665-3(-)
GEAKVCESSRLGDSKHLEQPSTLEFDANENHVRGSDLMTTGSRIQKSEPHWLSKEALQSGVSPIHS
TPLPVSKLRVDLSLFETGIKVVDLLTPYKKGGKIGLFGGAGVGKTVVIMEFIRNLAIEHKGISIFCGV
GERTREGNDLYSEMAEAGIIQVDGGYCYDLDSKDKQFVKDFTFTSSSVTLVYGQMNESPGCRMRV
AHAGLTVAEFFRDVFKQDVLVL
>TRINITY_DN113_c3_g1_i1.p1 type:3prime_partial len:1162 gc:universal TRINITY_DN113_c3_g1_i1:3485-3(-)
MEIVSRSRIAIFVLLGLLISVAESQFDPECPEIDPDVECSPPESTVNECLINNDCDVDKPLCCSNGCG
LKCTARERRSITPDGFTPEDEKLCRVPVDLGFIIDVSGSIGQTNFDKIKKFIEKIVDAFHIADDGTHV
GAVFYSDNASLAFDFNHLQGDQLNEMNVVREIEKIQVTDGQTRIDLGLELASRELFNPAAGMRI
HKPKIALVLTDGKQTRGELAPDAKDLHQASRLLKQTGVQVYSLGIGLDYDIGELLDIASDDASVF
RSQDFDELVSIVDILIEQTCKGCTRAVDVHFALDASANVGEENFVFMKDFVKAIGYNFIISETGAH
MSGSVFGDVGTLVFDKSQATEINEFENEVENMPYLGDATGNMDKVLKMISNEVFNFDGFTRQSS
PKVIVLLTSSNCDTCQESLEDLARPLKAEGIHIVAIPIGDPLFDEMQVLASHPIDQYAIPRATHQELL
NAEFIQKVSEMVCAGKPGICAEPPVKGECAEIINECSVDMRCPEDQKCCIKNCVASCVLPLAACIT

PIEIAFAFETTKYGIADFNNVKNFAINLLDHFKVGPSKAHVSLSTFSEDVTSLTKFIDDFDMESIKQSI
KDLKHDGGEESNLGNFLEIINTEVFSVRGGIRQQTPRVLVIFANGDINDEQAVKAQEEALHLKAD
GKDVKIIIVNIGSSTKTLDVLNEVATEGSFYEFFGSAELDSTAIETEISEKICSTKVRRSKCQRRIRSSCA
VKDDLCDSDFDCPGHQECFSDGCRLRCQTCEFRCKDKVDLLFVMDTSARIGRDNFERGKKNIKK
MLEWFTIDSNNTNVGVITYSDTANVAMPLPSRGNNNPAQSLIDVQDRLDSIPYSGGSVSHLDNAL
QLAAEKVFPDNGPNLRRDSKKAVVVITDSLDDSASQLELASWPLRRQRVQTEDGDASNIRILAFN
VGQELDLDLWGNIVSPPVEENVFYAADFDDMYNGIRQLAEDSCKYAAAITPPPIIKQKGEPGPPG
PPGIPGTNGTDGRNGTDGRRGPKGERGDTGLKGFKGEVGLVGPPGGGAEEQKGPDPLASAVGIV
NVTDLPGGGRIVFDLREGPVGQQGLTGIIGPKGPIGDTGEVGIKGRNGTTGPGGFFGLPGPKGQP
GTDGAKGLRGDPGLRGSAGRKGPTGAPGLTGPQGPFGYK
>TRINITY_DN3052_c0_g1_i1.p1 type:complete len:362 gc:universal TRINITY_DN3052_c0_g1_i1:45-1130(+)
MAHLSQEQRNELKKNAESIVAPGKGILAADESTGTVGKRFAGINVENNEENRRLYRQLLFTADKS
ISNYLGGIILFHETLYHKDDNGKPLVELIKELGINPGIKVDKGIVPLHGTNNEGTTQGLDGLAERC
KQYKQDGCTFAKFRCVIKINTPLGVPTDIAIQENAQVLARYANICQANGLVPIVEPEILIDGDHNL
ATSQRVTERVLACVYKTLVDHHVYLEGTLLKPNMVTPGQSCPEKASAEEVGVATVKALRRTVPA
AVPGITFLSGGQSEVEATKHLDAMNKTDLARPWALSFSYGRALQASVLKAWGGKKENVAEAQK
VLLHRAHLNSLASLGKYEGENASGATGDSLFVANHQY*
>TRINITY_DN10385_c0_g1_i1.p1 type:5prime_partial len:968 gc:universal TRINITY_DN10385_c0_g1_i1:2-2905(+)
TATPKGGSEPEGNAFKKPSSDNPNESPLPIDGSEKKPAQKPLEPVESKNPLEKPSFSNTPESANKPK
EEEAKNPLETPEEAKSPLSLPEEETKKPLDQPEIGKNPLEEPLNEGKNPLEQAPNPQEQPKEGKNP
LEQPPEQEGANPMEKPKSSNTPSENPLGVENPSEEGKNPLDKPNDNAPEKQSDDKFPLQQTGEK
NPLAKPTTEKNPSLEGKNPLEQPNDNAPGKQPDEKNPLQQAEGEKKPLAKPATEKNPLQEGKN
PLEQPQEGKNPLEPPQGGAKPQELPNNVPNTPLGKQPEDQTPLKKPNEKPLEKPQEGKNPLDQP
EEGKNPLEQPMGEKNAAGGKPGDENPLNKPEDAKKPPKIPQEAKNPFGKPSEEPSEEAKNPLEK
LEEGKNPLENPQPQETPSNKPEDNNSPSTPGEADQSQSKPLGNTTAPGSPNGAEAPKKPTGIPEK
VSGTEEGEGPSSPAPAMSTSLKNGLAAHNALRGIHGSKRMKPNEDLEAKSREIAASIADAGGKM
ENKKRQDIADFGENVNVNCDTSSKTVEEEATLKWWSQDFCDTGPSTISDKRMGAGTQVIWDAT
DEIGMATSAFMKDGKLCYVVVALYTPKGNDRKTYDQNVFQGDFDFETMCDDKNNTLQTAKEL
DKDKNQEATEPQTPSSSESTPAGSEPNQTTGGGAAQKPGGGAVQKPGGGAAQKPGGGAGQKPG
GGADQKPGGEGSGKPGGGEEKKPEKGQPEAAGGKGLDQKKFAQEGLEAHNKLRKIHGSPPMA
LDDQMNKEASEYAAKIAKLGELKHSTSAERNDDGENLAYVCGSLANGYKGEQPTASWYNEVCE
YNYPFSNEVVGSTLHFTQVVWKQSVKLGMGYAKGTKYGMTCYYAVGRYRIAGNMMGNFQENV
QKGSFSKATTCSEFWKMLNVTPDKRSKVRTHVGHALMNKSKKHKTPGSTANIQTMKGTLKKKG
YIQQKRAKSKKA*
>TRINITY_DN1070_c2_g1_i1.p1 type:complete len:209 gc:universal TRINITY_DN1070_c2_g1_i1:1431-805(-)
MSGSEFGNPLRKFKLVFLGEQSVGKTSLITRFMYDSFDNTYQATIGIDFLSKTMYLEDRTVRLQLW
DTAGQERFRSLIPSYIRDSSVAVVVYDITNINSFQQTNKWIDDVRAERGNDVIIMLVGNKTDLAEK
RQVATESGEAKSKELNVMFIETSAKAGHNVKQLFRKVASALPGMENTPDKKEDLIEVQLKDTPLD
QNQQETAGCAC*
>TRINITY_DN5397_c0_g1_i1.p1 type:internal len:161 gc:universal TRINITY_DN5397_c0_g1_i1:2-481(+)
CSVVCDYSPWQDTQSCTKTCDGGVLIQSRTERSFNKVESREGSTPCNTQACGSGLSEERFRKCNA
NQCGTNTGLQEVYKVCNAANCRFQGIDYTRNQEINIRTQACTTACDYTPWQDTTACSKSCNGG
VLVQSRTERTFNKIETRDGTTPCNTQACTQEL
>TRINITY_DN11662_c0_g1_i1.p1 type:complete len:398 gc:universal TRINITY_DN11662_c0_g1_i1:75-1268(+)
MALSRPVYIIAAKRTAFGSFGGSFKGLTATQLGQKAAAAAIVDAKLDPSLINSVIFGNVAQSSSDA
AYVARHIGLGNDIPFASTALTVNRLCGSGFQAVVNGAQEVVLGEADIVLAGGTENMSQCPFAIRN
ARWGINLGETREIEDTLWSALTDSFIQTPMGITAENLAEQYGITRLECDEYALASQQRWAAANDN

GLFKAEIAPIEVKQKRKMVPVDKDEHPRAGITIEALNKLPPVFKKNGTVSAGNASGICDGAAALV
LASEEACKQHNLAPLAKVVSYGISGVEPSIMGIGPCPAIKQALSRADMSLSDMAFIEVNEAFAAQY
LAVEKELGLDRSITNACGGAIALGHPLATSGARILTHLVHTLKRTKENHAIGSACIGGGQGIAVLL
ENVM*

Band 10
>TRINITY_DN982_c0_g1_i3.p1 type:complete len:9852 gc:universal TRINITY_DN982_c0_g1_i3:29697-142(-)
MSIKLCTFFVFFLIQCINSFPEIGLHLSLDRSYSFQGNGWKTVNGWEIKPRANGPYEIDAIKNISPEY
FPVPITGVYYITVSLPVEGASNGEFKSAIIVDGKNEEGRIGLMSSFQKTAFKNGASTLTFAGFVRLFA
DEKLTIEIYSDDINANYVVLESATFVFHFIGVTGSVPAYLAHTAQDQEYASDSIIKPWVTLGDSRVYE
SLSGFIKNEGVFTTICNGVHKMAVNIMIKANAGTYTLKALFQDEESATATVTINQPDSIATLNIFQN
FKWSEGMSITLQIHSSNGANFKVLKSSSWSMSFIGETTANFQEFSVFLMADQFLQPSQFPQRINIDL
NGVFQFLQSNPDQYNKGQLVSGADTKFKDNLDFIVDSTDLFYVSANIIIEGEAEDLEITIAVSQIDID
VLPEDGLSGRLTKISNSRRSISLSGIIFIREKQYLSFFVRSRDSSKSFTVSKDSFISVINMRYTVSSVSARQ
NENIDVTAGSWYDISGRFAEQKNGLYSFGKDFNINNGVFRTSHSGIHSVHANIQFSLDSDAFGDN
DDNKIVLRVVIDGQEDKISLMSIRKNPEKTATLKLFGSIYLRAGVTISFQVKSLMPNTVQYKILQRSS
ISISYIGPQWAVNSFHAVANQDLNYDGKPDDTRPDIPSPLNFWKTEAADEFISPFEVLESFDGTRYT
SVDDGLYVVSSLLILKNVDCDRSALFKLDFVTTGGSRASDKSNGFADSHFFIADSKGTEFTLSVTTA
MRLEVGFKVGLKLTVGSTSCRYTISKESSFSIAQWSKSSNTIEENRLKYIGVLARLSAPTEVSSPNTW
SNLGSNTMDINPGDKSRSSFRPGIFGGDDLAPITRTGKDFNGDVSDFTIKDPGIFFISGMITVTTTEN
ANEEFAATVQIDSEDIESGLYAAVINQGSTEMSLTFGGTIYLKSGARVRVVVKGGRTFNIAAGSSFSL
LKLQPDYKTPGIIVSIDEKITQAITRYEHKDLRADIHRTLTIGNLGFVESTGVYTIQVDGVYMVDYT
VILEATNPNAVFGAEILSDNTVVATGTSTFGNTKTIAITELVYFSKGAIVKAVLKSNTNGETLNNLQ
VDGGLTVYFLEPKEHVKDGHRSKQTSPQALPVRSFEKVTGFAAQSPDFAGGADSVDYTVPETGVY
LVAFTTRVNRVINGFKSRVLVNDNEFLNGNYQIVSAQNAPATLPLSIAMELTVNDVLSFQVMADG
SGMSLDGSTRSILKIDEVNTIRNVTEGLSAYKLRDQKVNGQQETIVEEWDTSVTEGTFLAKNVSFT
VDHSIVVVAAGVFQISVSVVVKNDRPSQSKFELRVLQGDDVKSHGLHSKVEIGGGREQTLTIGGSL
LLQKFDSLRIVVRDIADPLASYTIKAGSTVSVIIIDDNNGPIACEDEGLNMRRLPERIRKPTRESHTF
QCNVVSSLKAEKYTWFKDNVQLKEENQKNLVLNDLKLSDSGVYQCRVRFLELTGATNNATLSVY
DPYPKITSPKFIANVLENQDPPLTIEMFSLSAFKLSPEDEKASITTQIIAGDDQSFFTLSGPSKQTVTLS
TTKKLDYETQNEYNLTIKATNLEETALTTKGPYETTINYIVRVIDVNDHPPVFSQSIYSRSVPEDYPI
DGSIAKVSANDQDAGANVIYTLVGQVSGDLFKIDNTGLIQLRQNLDREIRDDHTIFVQASDGLFT
TVAEVVVTVTDVNDNFPNFMDTGFDISVSEATLTQSVILKVIATDPDLGTNSQLRYTIKSGNNENK
FSLNLLDGNLTLISKLDYEGTKIYNLEIDVQDSASPPKKSQNPLTVVIRVQDANDNNPTFTQSLYNT
RIAENTAVGTSVLKVSATDRDGEGINNPLTFSITDTDANEFFQVVSSGATAGEIRVRKSLNYDIRKD
YRFSVVAKDNNLGEPRYRTALVVINIDDTNNLRPTFEAGSASISISEGTSIGTSVHQVVAIDKDTVG
KLDYRIISGADGKFAINKETGDINLISAVDFESNKKTYTLEIQVNDGVRDALNPATVVINIQDFND
NQPEFIQKYYIFTALESISDSQAINVGNPVIARDADSTTNSQVDYSIQYDINNPSVPNEGDALFRIDS
NGLIFKKNGVILDFENVRYHTFTVVAIDRGTPKTLLGKAFVTINIQDVNDEAPSWSPAIYSVTVKES
AGINRDLVFLSTTDKDTSGAVTYSIVSGDSANQFQIFDNVIKIVKPLDFEKTETHTLKVSAQDDNG
NRAQLDATVTITVEEVNDNRPLVTNNNANSIQVSVPEDIPVNSLIVKLNGRDDDKGAPGQASNQ
FTFVSLDKTTDFTIDEVTGEIKNTVNLDYERQKSYSLVATIRDKGTPYLESKFLVDIIVENKNDERPIF
DPAIYTIKLSEKAPIDTVVAMLQATDLDTPKSQLRFSSVTTLDSHFSLDPITGIITLKTSFDKEVNGKD
GIFVISCTVNDGTSNSAQNAIVRVEVVDVNDNQPVFPTSEYFIQVNEGSSIGTILQNIAATDKDNT
NTHNQFTYTLEGKEAVDFRLNGNQLQTNKIFDYENKKSYSFDVLATNNLSPDKLVGKATVVVSIL
DQNDNNPVLSPSGVINLNISEGTIAPANIITIFATDKDSTINAEVQFTLTGDPTNTFTINQNTGEIML
AKSVDSFAKNEYTLSVTATDKGSPPRSSTATTLNISIKPVFVPGIEFENCPQIISFNENAAPNTFTKTL
TVKDRGSFNQVQFTVVSGQAGIQNLITVSQNGVITNGQRRADFEARKRHDFLVQAENERGAKAY
CHVTANINDVNEGPIFDSANYVFDVLEVIKINTVVFSVRATDPDFSSTTNGQLTYSLAGQEASQFSI
VPNNGQIKLAQPLDYEIKKQYQFNVIATDGGSLQTTSSVIVNVEDWNDNRPIFDQPQKLAINVPE
NSKPDNVFTVSATDKDSTTNGEIEYVGIYPTGIISIDKDTGTAKIIGDLNYEKRQKYDFVIVAKDKG

VPYLSNTREVSLSVLNLADEVPYFDTAAYTISISEKTSINTAVFRVMAKDNDLENFGKISSYEIISGNQ
LNRFRIDSNGNIIVQQSVVLENNNPNQYILTITAFDTQRTQSLNNTQVTINIIDLSDKQPFFIQNSYI
VEVQENNEVSKSLLSLEAQSNEPTFNPTYSIVQDPKNTDSQFFQIINGNTIVATTVFDYEKKRFYSFE
VQVIDTNNGKTNKALVVVRITDENDNKPFEVSGNLHFNITEHTLLGSTIGSLNGKDNDAGINKEF
EFSILEGNDDDKFMLLPTGQILLKNFVSVKEKDFYKIEVKITDKGTPSLFNIFTVRVTINSSQALVAS
NNPTIPEDKNTGEEITRVTAIDPNTGSSNGFIYSLVGTLSNSQFTIDAKTGAISLLASVDFETDRTHT
AVVKAEGPTSTGVTEVIVRVTDINDNKPIFDPTNFNLNINENFVVGQEIVLVRATDKDSSLNDNNV
IIYRIDSVNPSSGNDLFSVNPSTGRILLLKALDRENGDTSYVLTIAASNRGVSNKPDNTMTVTINIA
DVNDNFPIFEKSLYQGSVSEDANTGTSILTVRASDKDILLKNRNIRYSMTQPMAVQKFQIDPISGVV
SLKQEVDFETKQRYDFSVIASDTGDVAYSRTANVEITILDVPDVEPKFDPQRYEITIAESTLVSTSILK
LQVIDSVKNLNFNIKQGNSNSDFRLVASSGELFVNKQLDRERISRYQMTIEVSDSVNKAAIDAIVIV
NIADVNDNQPLFLQSGCYQANRAEGIETEQVVVNVQATDRDEGSNKEIVYSFSDACSVLDIDAN
TGAITTKQKFDYESARSHICTIKAEDKGTPKLSGFSCIILTVTDSNDLKPTFEVITSNARVLENIPVGS
VITRVLATDKDTLSSNKQFVYTIKTGNEDGRFTIDANGFIKLQQPLDYEKKTDYRLEIEAVDSTNQ
DMVSDPNAIVTVTVIEQNDNRPKFAKSYHRVNIEESTAIGTTVLKLSATDADAGVNEGLIRYVSLH
NTTVFNIDRTTGEITLATKLDYEVKKSYHLIAVVKDQGNPVLSARTLIDIFVNDVNDNPPVFVISSY
NNIVSEKAPIGQSIVTVSARDDDTGVNGQLKYKIRETQSMFRIDEDTGIIILNGDLNVENTNKLLYA
LTIDVVDSGVPPLQASNPATVSVQVSDVNDKQPVFALDRYQFEINENIEGNQIFGDSVTVRANDD
DLTGTNNQLTYRLVNTTMFDLVVTNQNVFIKTALGVSFDYETKKSYSFEIEAMDSAANKLYGRSLV
TVTVKDFNDNPPIITVPNIIVNISDATLIGSHIATIMAEDKDSGENGILIYTVTAGDSTNRFTMESDT
GSIILKNPVDSYTKDIYVLNVLVQDNGIPRRSATGTVTINVIPVDLTLLKFNTTCPFAISIPENQINSN
FLGLKALDAGEYVSKQLRYTINSDENDVNQIFALDSIKGEFKQLKEANYEERSRYDMTICATNERG
SKVCCHITIFITDINEPPSFIGLNNNNIYEVSISETTPIGTIIYRVLSKDQDLPGVLNGQTSYSLGQQNV
FIIDQMGQIRVNKTLDAETQLNYVFQVIVTDKNPTNPLQSAATIRVNILDYNDERPIIQNIPDITIDE
NSDNQVVRNIVVTDRDISNIGSNIELSIEPSNILTINNRQLRTVGSALDYEKVQRHTLYLTAKDKSFP
YLSSTTSFTVTINNLNDNKPKFPVNNYNLTVSEKTTVGTLILTLFAEDDDLEEFGEIANYEIVSGNQ
EDRFSIDSNGRLTLKKQLNWRNVKSYNLNIIARDKGLPSEQSQIPATVVINVADYSEEQPNFLQNL
YVKFIPETTPIDTLVLTVEATSGEPVYGPFKYTIVPKLPDTNNFQIFSNGEIKTINQFDYENQRNFTFD
VQVEDEKNGKTNRASVFIHITDVNDNIPQFTDLTRSNTQYNITEFTLPGSMITCLSATDRDHGKNA
EIRFRGIGGDGNGVFNVNLDGKVLLSQQIFYDKKNKYNLIVEAFDLGSPEQKAQLTLTIFLHKSYT
SVITTDVDINEDVETQTRVTRVSSIDPFTRSSNNFTYYLVGKSTTFFEINAQTGEIFTKATLDRETRDR
HVLTIQSRGPTTNGVGELVVNVKDINDNPPIFDPQVYQVTVSESANIGLSIVSVTATDKDIGVNGE
VTYSIEDGDQTIFSINPSTGSISLAGKLDFESKQSHTLTIKAVDRGNPPQDSVNKATVIVVVQDFND
NCPVFENSTYSLTIKEPGELGKEYLKLGITDNDNGRNQLFTCSIDEPLAEKYFEMINDCRLRLIKEL
DYEEFTSFRFVVKATDQGDVSCTTTTNMIVNVGDRADVIPFFSPLTYNISIPESTTVGTIVVSLKVGS
TFKGLLYSLFHKNTQDDLIKFGINKDTGEIFLKESVDREIQDKYEFTADVFDGSNNAVQRASVTINII
DVNDNAPFFNQPTCYQRDLTESTAIGTFVVDVSAADNDKGRNQQVTYSLLPSPSCTSFSVDETSG
RITTSQILDHEQQKLQHCLIKAKDNGTNSLSGYSCLTVNVVNVNDNKPAFIVNRFDVSVLETVPA
GTLVASLVARDVDNQGLITYSISNGNLNNQFTIDSSGKLRTRFPLDYENTKSYNLTIQASDNNGKFS
SPDDALVLITVLEVNDNRPQFLQSYYKVSIPEDTATGASILNVTAIDLDHGELTKSLFYAALENTTQ
FTVNANTGVISVAENLDYEIEDHYELVVIVQDRGTPHLSNRALVDIQITDVNDNAPMFVTTVYNR
ELSEKALIGAPVVKLNAMDADKSDKMTFTIDSVVQGAQGFFSINGDTIQLDKELDYEVPEQRTFV
LSVSVKDSRDRDGANKANVFINVRDINDREPIFDLNMYEVTVSENNVISNPIIEVTARDSDSTPEN
NNLLYSLVDGEDAASFSLLNNGQSAIITSKISFDYEKKKSYRFLVRASDKVRENFTAFSTVFVTVQD
TNDEAPVPEKDLIMVNISESTRVLSTIAIVFANDKDQGINGQLAFQIINHSEDKFEINNKGVIRLKST
LDFFQQNFYNFTVLVTDKGNPVRTSKPVTVLVYVYRTEIPGIIFEKNHYIFNINEKEIVSLVGTVNAI
DEGTYVSRQIVYSIVSGQNDIKDKFRAVNNNIIQDKEVNYKDQSEYNFVMKAINERGSIAFTHITIN
IMDVNEKPYFVGLNNKGRYEFSMSEIISVPSYIYAVRAKDDDFGLNGEVDYEIISNFRFGNFSIDQY
GRIQVLKDPDFEFTRSYEFQVRAKDRGTPSLESNLVSVVITVLDYNDNHPIFNIQEPLEISIKELVNN
TDVGTVTATDKDGSGLNRIVKYVAIYPSDHLTVNRDNGQIRVIEPFDYEIAHKHEFVVVAQDQGT
PYLNAYKVIKVEVLNENDEFPEFTVIEYNITISELTPIGSPVLFVHAQDKDRGPSGQVTNYKIKYVPP
SGGSGAGSPDKTFEITSDGQVVLKEKLEYKFQKYYQYRVTCTDNGSPPKNSINEAIIHLNIADASLE

QPIFSVNPAVVEVYENITLNSNIVKVEVDSIEPIKGPFEYSIIASEDSQPFMIDNSTGQITNKESFDYEI
KRKYVFLAEARDKHNNRTQRAMVVIFILDINDNIPTIETIAPQTFVQNISDTTYPGSVVAKLYSRDA
DSGENAMVDFTITGGDGENVFAINPNGYVTLIGPLDVRVKTQYTINVKVSDRGIPTLSNNRALTF
NIKKSKFIQNTVRFDRHHYKSNCIEENQINLNIIQLNATNSNPVAGSTIIYSLDGTLEERQPFIVNSQ
TGEISLSQTLDYEIKQEYDFVAVAVNNDGSRDVAVVTVCIKDLNDNSPIFEPQSYVVNVSESESVGY
LVYKTTVMDADKTDFNTTKSYDIIQGNVNDKFIIDSMGHIYIKSKIDYEALQVKHFDLRIRVNEGN
LVGDMVLRVNILDWNDVHPSFSTLNIYNFTIPEDKPVGEIIEILTANDGDATSPNNEVTYKLLTTED
DFILRRNDKGHGSLIVNKQLDYSKQNRYEYVALAEDKGYLNLLGTAIVIINIQDVNNHPPEFDDSE
YNTTVSEHAQIGTRVLQIHAHDKDTGLGEQIFFIYGDPTETFTISSQGFIILNKQLDYETQRDYIFDV
GARDGGNLVSLNNVTVHIKVSEVNDNEPIFDLSTYRNSYAEDTPLGTTLFTVRATDKDFIGNSIIYSI
SGANDDTSYVSVDQTTGDITLQRRMDYESKNYITFTVQAIDSNNRNKLKGSTHVIIDVTDVNDN
NPKFVFGSDLVYNVTVVENINVGTLVVVVDSQDADSGLNGQTRYHIVSGNNENKFFINELNGQI
NLINKLDALQTNKYVLTVMAKDLGNPSRSTNVTVTINVLRYFESTPMFLPSYAEVIYPENLALGSSI
YELNVTKTLPGSPMKLSFHSSSKFPEFTLQGNKIILSKEFDYEIKNRYELIIIATDEENDKAFAQVIVR
VQNLNDNIPYFTQNAYFGVVSELAFVGHSVLTITAKDDDKDFTELFYSIATQVPDNAFKLQDNIV
VVNGKLIRNKVYEFEVEAFDNTFTSKRATVSITVTPENLPSFVNATYIVTLQENYPISTNFLNISAGT
FSGITYVITSMKAKDMFNVDANGQISLTKSLDYEKQRQHVFPVTASVTSPITQVRVASVIVNVLNE
NDNAPVFVNGPYERNITEGHLVGTMVIRVDGHDDDSDKLTYSIVSGDDGKFSINSEGVISLISTLNF
DLKNKYVLNVRVSDGKFVTDTIVTINITPIVKFPPMFNQTLYLLSVKENTLVGTSILQVSTVNVIPPS
SFSINEQSGRDFFTIDANGVIRTNNALDYEAKRQHIFTLKVQDLRSMGHATVVVNIVNDDDTCPIL
TPATQTVQITEPVAPGTIVAAVQAKDIDSKSLNFTITGGNGLFRIDQHGGIKTINEVDISTSTEYELTV
TVDDGTCTKSVQVDVNVNPLAPCPDCNIYPFTQTIYEFSINENTVPASPIITVTTGQSLQRNFSIEDP
AALEYVTINRLTGEISVIKAFDYEATKHLSFNVMAKTQTTKNRNPFYVITSVSRVRITVRDLNDNCP
SIESPMYYEVSHSSPIVQDTVVTRILVEDPDTLKDHTYSISDNNFKIDSMGHIRTVQNIESFTAQFYN
LTVTVSDGQCSKTSNVGVNLAVNPTPMTYQFTSNGVYSKSVREDHNLQDVILSVALNQNYPRVFS
IVESDANDQFVIDSNSGALRLKTSLNYELKKRYDFTVMADIHGGVYKSYASVHIIVLDVNDECPIF
PVIGYNQRVSGTPVIGTIVEIIEANDADNATLEYSIVSGNEDGHFSLDNSGVVRVVKPFASSFSKTYG
LSVRATDNKCSADVPLTVQVSTCAYGEDYQFEESEYVVYYLDENRNLGIVSNIKLKSTRAAFLTLSQ
NTHFTIDQNGDFRLIQTLNYETKKEYHLTLTANFTQDNVVTRIPIKVFVRDINDNCPAFSEELYAVQ
HTEPLREGLLIAKLSVIDDDTVQNFTYNIVSGDKRFSINENGEVVMNARLVTDTTLLSTTYKLEVSV
NDGKCAKNTKVDVFVYKTVVNSFLFPQPFYKFDISENQAIPFTIDRFVNVGGHEAVYGFLVSTDFF
QIDPKTAQLQLVKKLDFEVEKTHYVLISATYDDGTQTYTVVAIHVGDENDNAPQIFAHPPITRLVQ
NTPHNSIVTKIEVEDPDTSKEFTFKITNGNPLQNNDPDKALFRIESNGMIKTTRCISEDEIGNYVLTI
SVDDQVNTPTIFSNMQVQLKTTDDRSYVTNCGLGCPANDGPFSRYQFEQSLFIVNVTETAANPTV
LKRLTRPFVEKTTYTIGAGADGKFSIDQNGTLSLTGSLDYETKPVYYFIVSAVIPSQVVGRFITSKTAY
LVMVNDVDDSKPVIEGNGGTLAVSETSEIGQIVGTVRVKDSDSSSPLTYNVVNGSQYFNIDSAGNI
VIKDTLNAAGSPYTVTYTVSDGINPPVNGEFVVSISETNNNVPKFTQNIYEVSLSEDTATDTIAVTIL
ANDGDTNDKIAYSFLQSEGPFVINRDSGEIKLIGPLDYEAQKSYNMVVQATDSGGFANIAFVQIAV
GDVNDQVPVITPQTTPTQIHESTLVNSVVYRVIASDGDGNERLTYALANSNDFFTINSATGEITIKE
NLAKSNVSSHTLSIVVTDSSGKTSTQDVALNIRRSGTSQGCVKITTSQGQSSSPVINLPENTASGTTV
FKLVAGDASVFSNMRFTLNREANGVFAVTEQGELQLVAPLDYETKQFYSIIITAEDVTRGVSSQYNL
FVRISDVNEFTPVLETIAPVAVSELANIGDVIAQAKANDQDGDSQVTYSLGEFGNNDNKFTIDAAT
GNIILNKRLGSGAPHVVEVCASDGSIKKCQNVTFIVADTNDNPPSFQDLTGVGSIDLTNTSSYVVY
KPIVTDSDQTGTLSFSLKDPAPSQFSINSTTGEVKANKDIAKGTYTFGVCASDGRYTSCQSVTVVAS
DRSQSITPSFNQDQVGAIVDISASSGKQIIQVMASTLDKAKIEYELKNEADRNIFEINKDSGLITLKN
RTALTLGTIYTIPVKASRADDPLNFDEINIVISVGNQQLDSGSFIEQVLDTQHLVNFSLSTFDISKVSY
YIILGQQVGGDSKDDEKLPVSWYYATFGDESYRHRFYYIAKLDGSKFRNTGGSRKRRSIQDKDSKS
FTYLVGEENGCSKNTSMAAICNGPLKTGTEYRYKLRAYYDDGELRGIPVDQKPTEVKRTQAPQA
GKSIDEYREQDSYVITFWIIGLVIFGLLFWFSVLVIAILLCYKRRYEVTQAYGYSVQTRDKIDGPPTEY
SNTVYRFQNPSYPDNDTVKMKKVDASESRTKTEVQTTSMVVLDDYEQEQPQPDIPVQDYDAIEY
ETRSAQIKFHEESPEWESMDIQLRIDPTGNQDPVITRKSDSGSKDSDSDASKKYENTEYIEEKEDFV
NEKEDSLTRL*

>TRINITY_DN161_c0_g1_i2.p1 type:5prime_partial len:399 gc:universal TRINITY_DN161_c0_g1_i2:1683-487(-)
WNQEPLTCTYLYSVNSQLLKYKMADDDVAALVVDNGSGMCKAGFAGDDAPRAVFPSIVGRPRH
QGVMVGMGQKDSYVGDEAQSKRGILTLKYPIEHGIVTNWDDMEKIWHHTFYNELRVAPEEHPV
LLTEAPLNPKANREKMTQIMFETFNSPAMYVAIQAVLSLYASGRTTGIVLDSGDGVSHTVPIYEGY
ALPHAIIRLDLAGRDLTDYLMKILTERGYSFTTTAEREIVRDIKEKLCYVALDFEQEMQTAASSSSLE
KSYELPDGQVITIGNERFRCPETLFQPSFIGMESAGVHETTYNSIMKCDVDIRKDLYANTVLSGGTT
MFPGIADRMQKEISALAPPTMKIKIIAPPERKYSVWIGGSILASLSTFQQMWISKQEYDESGPSIVHR
KCF*
>TRINITY_DN329_c0_g1_i2.p1 type:5prime_partial len:320 gc:universal TRINITY_DN329_c0_g1_i2:1-960(+)
FNNSTHSPSLPTHVYLYCCRIKILILSFDPSTIEILKMAPPKFDDLGKEAKDLINKSFHFGVIKLEGKT
KAKNGVEFTTEGTHNTDTGTVNGSLETKFKYADYGLTFSEKCNTDNVISTKITIDNKLAKGLKVD
FDTTFAPVTGKKTAKVKSAYQHNNIHSTCDVDFDFAGPTIHGSAVFGYNGWHAGYQASYDTANS
KLIANNASLTYKEGDLVIHSGITDAVKYSGSVHHKVNSNLTAAALIQWASGTNNTSLTVAGKYAV
DNETFVKAKIDNNLRLGLSYAQKLRPGVELTMSSLVNAKSLEQGGHKLGLSLNFEA*
>TRINITY_DN3986_c0_g1_i1.p1 type:complete len:446 gc:universal TRINITY_DN3986_c0_g1_i1:252-1589(+)
MREIVHLQAGQCGNQIGAKFWEVISDEHGIDPTGTYHGDSDLQLERINVYYNEATGGKYVPRAV
LVDLEPGTMDSVRSGPFGQIFRPDNFVFGQSGAGNNWAKGHYTEGAELVDSVLDVVRKEAESCD
CLQGFQLTHSLGGGTGSGMGTLLISKIREEYPDRIMNTFSVVPSPKVSDTVVEPYNATLSVHQLVE
NTDETFCIDNEALYDICFRTLKLTTPTYGDLNHLVSATMSGVTTCLRFPGQLNADLRKLAVNMVP
FPRLHFFMPGFAPLTSRGSQQYRALTVPELTQQMFDAKNMMAACDPRHGRYLTVAAMFRGRMS
MKEVDEQMLNVQNKNSSYFVEWIPNNVKTAVCDIPPRGLKMSATFIGNSTAIQELFKRISEQFTA
MFRRKAFLHWYTGEGMDEMEFTEAESNMNDLVSEYQQYQDATAEDEGEFDEEEEEEDA*
>TRINITY_DN1280_c0_g1_i1.p1 type:complete len:426 gc:universal TRINITY_DN1280_c0_g1_i1:195-1472(+)
MGFETCGPNEAMVISGCGQERPLLISGGRKFVWEFFQKVQRISLNVMTLNVESQRVYTVQGVPIS
VTGIAQVKVQGQNEEMLHAACQQFLGKHENEIRNIALETLEGHQRAIMGTMTVEEIYQDRKKFS
KSVFEVASSDLVNMGISVISYTLKDIRDDEGYLKALGQRRIAEVQCEARIGQAMAKMQSGIKEAQ
AEEVRIKAEYENKTEVARSQRDFQLNKSAYDIEVNTKKATSELAFALQSAITNQKIKEEDMQIKVIE
RAQAINVQLQEIQRREKELEAQIRKPAEAERYKIEKIAEANRMKVVLEAEAEAESIRMKAEAEAE
VIQKKATAEAEQMSKKADAWRDYQEAAMVDMLLDTLPKVAAEIAAPLSKVKKVTMISSGSSDV
GAAKITGELLDIVIRMPQAMEQLTGVDITKTFRREK*
>TRINITY_DN160_c0_g1_i1.p1 type:complete len:1145 gc:universal TRINITY_DN160_c0_g1_i1:4810-1376(-)
MRLLAIIPLLVMLDIAKSDKLWECNFEENDCWAASSTRNHERNQLTLHWNRWKMRTPTGATAS
PVTGPMEAFEGKHYLYYEATESMDKLKMADGASKGVVTFKDINLMGRTQFILSFYYQVKGNDG
DFYDFDLMVSNTENTHYDLSMKSIDGKDWKHFKHIMKVEGAGVTSIDLKFTARSYGAFADYAID
NIVIEDYPDAGPCKDNRCLANEYCTITSSTQTVCVAKCTQQACGENAECILDSLAINKCKCRQGY
LPRVSNYGDKLNCYRPDASCDIEYNSLGQCRISTSIPIMYGHPSLWHGRYSPVSGNGYYVFKYDNT
ITLDAVSSQKLSFFYLVDNPNMINSLTVEYGSRKFEVPINREKTNIWTLYEVLILASTNQGVQIKASF
YAEGGVIGIDNIQFTQQNEVVVEKIYKESMMAATFEDGSVPTDLKVAMLFVTRYGKIDFGCLPGIE
LETRNGKLYCKDGVVQRQSACSQDSDCIGSNARCVLRNCVCNTNSYQISIGTYGEKATSCAVYPC
EINSPRCHSTAICSNIKTPGQNFYNDYTCKCGRSLNNVETIINPFIGGDKSTQCLAATPSDSYVCND
NNFKPSRTTEYCLKGNQATVEYSLRATNFGNYALFKHCDDPDDEYRSSFNDPGSFWRPIDLVAAA
NENLAVSVTYKTSLSNKFIKCNFEQARFGSKLTLAVNVNSNSRVLTFTNTQTSVNKQNPVYQGQV
FQSDNPTPGAEMSVTFTVTGNQAVDQNVDAILLDNVVCKGFDDVASVNTPCGNNACASNQYC
NPLLSNSEKECLCPTGQKDQNCPDVVPSGSVVSADGPKCIGHHEKYMNGACVCDDGFEKTGDT
CTVKINCGCNDGLCLSGDKCSCPLNLLKVNQACVRTNKDCAKDCVSTLGNGGYCDVTTGMCD
CLTGFVKSGRDDGSYRCDKHAVVQKINDPKGICSATCGTGFVNEIEVCVGATGTGFECMTNIVQG
TLGVCSLPACPATSSQCSIQKECACNNVRVPIGSDGKLRLDFDHEAKLNMIFRFLSKHNQKDLEFK

FREARCDNTKLDSIARTIEDDLKQLEFDTELQAAITTMKSILDCSPSGNDHNAAYWDVFDMLIELS
SYRPHAVSDLQLKKELVMHEQSRCGMRSNLHKVVDGYAFWNKK*
>TRINITY_DN776_c0_g1_i1.p1 type:5prime_partial len:201 gc:universal TRINITY_DN776_c0_g1_i1:692-90(-)
RAGKTPHTASMVKNVCSKTYVTPRRPFEKARLDQELKLIGEYGLRNKREVWRVKYTLAKIRKRA
RDLLTLPEKDNRRLFEGNALLRRLVRIGVLDEGRMKLDYVLGLKVDDFLERRLQTQVFKLGLAKS
IHHARVLIRQRHIRVRKQLVNVPSFIVRLDSQKHIDFSTNSPYGGGRPGRVKRKNMKKGTGGGDG
DEEDED*
>TRINITY_DN36_c0_g1_i19.p1 type:complete len:223 gc:universal TRINITY_DN36_c0_g1_i19:826-158(-)
MIKLAIIVTVIAACVFESSAARCYVGYSAKGLPLAIGVRDVECGKGFDYCTTIKYRLGGIEVTQGNC
ARKYGQAMCQFMRNPHKAMTDCEETICQGDSCNKDVYDAEQAERKRVGPYKNTLKCTTGLIT
KFPAIPFYTADVPNAIDECFPGTTECVTVTYDYLFKFETQIATKDNVFKLPCVQKMCNRHNIDNC
NKWCDILSIHQSMENCVAKCCKGDNCNA*
>TRINITY_DN428_c0_g1_i1.p1 type:complete len:1949 gc:universal TRINITY_DN428_c0_g1_i1:96-5942(+)
MADDADLKFLMVDRRQVSDPVAHAEWAAKKLVWVPSEELGFVAASVKEEKGDKIIAQIEGGKR
VTFHKDDIQRMNPPKFDKAEDMADLTCLNEASVLHNIKDRYFSDLIYTYSGLFCVVVNPYKRLPI
YTDKVINLYKGKKRHEVPPHVFSIADTAFRSMLQDRDNQSILCTGESGAGKTENTKKVIQYLTAIA
GRANQQKADEKSQGKLEVQLLQANPILEAFGNAKTVKNDNSSRFGKFIRINFDNSGFICGANIES
YLLEKARLVRQAPDERIFHIFYQLLLGASPEVKKQFLLLDTKSYNFLSNGHVQLPNMDDRQEFKV
TLEGMRDMGITQEELDPIFKVVSAVMLFGNMEFKQERKSDQATLPDNTIAQKICHLLGIQVTDFT
KSLLKPKVKVGREFTQKSQTKAQCEFAIEAITKALYERMFKWLVGRINKSLNRGKRDGASFIGILDI
AGFEIFQLNSFEQLCINYTNEKLQQLFNHTMFILEQEEYQKEGIDWKFIDFGLDLQPTIDLIDKPMG
VLSLLDEECWFPKATDKSYVEKLHKQQTKHPKYLKPDFRSNSDFCVIHYAGRVDYSAAQWLTKN
MDPLNDNVVSLLAHSKEAFIASIWKDTENVVGMSVTEGMDTTFGASRTRKGMFRTVSQLYKEQL
SGLMTTLHCTKPNFVRCIIPNYEKRAGKIDNHLVLDQLRCNGVLEGIRICRQGFPNRILFQEFRQR
YEILCPGIVPKGFMDGRKAAKQMIDNLEMDPNLYRIGQSKIFFRAGVLAHLEEERDIKLTEIIIQFQ
AFCRGNIARRNYRKRIQQLSAIRVIQRNGRSWLKLRNWQWWRLFTKVKPLLNVSRQEDELRSKE
EEFKKLQDKSDKLESLQKEQQQANEKLTEEKNTLAEQLQAEIELCQEAEEARNRLLQRKTELEDL
IQDFEMKLGEEEEKAVKYAEDKKKLQQGIQDLEESLEEEETQRQKLQLEKVQVEAKLKEMEDERQ
VAEDNNARLTRDKKQLEDRVAEFGQKLAQEEDRAKQLSKAKAKQEAFIAELEDQIAKGDKSTHE
LHKEKRKAEQDLSDLRAQLAQALQRIAELEDDLARREKELAEAIKRGDEEMAKRTTVEREKREL
QALLDELREDMESEKSAKDKVERQKRDLEQDLQELRSELEVTTDATVVQKEFQKKREEELMDLK
KQIEEEAKEHDQQMASLRTKHNGLVEDLNNQVDTFKRGKAALEKNKTALENENAEIAADLQRI
SQLKQEGDRKAKNLEQQLNEANSVRAVQDEKVSSLESEKAKYQKECDSLSTQIEEVETKAASLDR
AKQAAEAQLEDTQDQLAEETRQKLSYQTKLREASDEANRLQEQLEEEEDEKKAVQKVLSNLQV
QLDQAKKEIELKIQDFEQAEAARQKQKRENEELRGEVEKLTGDVSRLDKAKKKLQGDLDDVTVS
MERERNSANQMSSKQRKFDQLLAEEKANSQALGIERDNLEKLARQNETKALSLQNANEELEDR
LAEAERQRKQLQMELGDLVESKDDVGKSVHDLEKSKRLLEQQLAEQKTQVEEIEDELQACEDAK
LRLEVNMQAQKAQFDRELQAKEDAIEEGRRGLVKQLRELEAELEDERKLRQSASAAKRQLEGDV
KELEGQLDQANRIKEDGLKQLKKYQNQLKDVQRDLEDSRQSKDDLAAQVKDNEKKLKGLEAEF
LQMQEDLSAAERARRAIESERDELLEELNNNSALKNAVAEERKRYEAQLAALEDELEEERTQTEL
MQDKVKRSNLQLEQMNAELQEERSAAQSLDNTRMSLERQNKELQSKLADLESSLRSRQKNTVAT
LEAKINNLEIQLDQEAKERQQQNKALRRNEKRCKDLQMQIEEERSHTESYKQQLEKMNARVKN
MKRQLDESEEEVTRLNTAKRKLQRDFDENQESLESSQREANQLRQRLKGGASLSSSRSSGRGRRA
KDEDDDDDDANE*
>TRINITY_DN11435_c0_g1_i1.p1 type:complete len:430 gc:universal TRINITY_DN11435_c0_g1_i1:1616-327(-)
MGNIYTVGPNEALVVSGGCVGQRVKRTIVGGWAFAWACVSDVQSISLEVMTLNPRCEKVETAKG
VAVTVTGVAQVKVIKEDDLLKTACEQFLGKDPHEIEDILLQTLEGHLRAILGTLSVEAIYKDRDQF
AQLVREVASPDVGRMGIEILSFTIKDIVDEVSYLDSLGKTQTAEVKKNADVGVAEANRDAGIREAE

CDTQRQDVRFKADTTIADAARAYEMEKAEFDKEVQTKNAEAKLAYELQANKEQQRIRNEEIQIE
VIERRKLIEVEEKEIQRREQELQATVKLPAEAESYKVQTIAEANKSAKVYLAQAEAEKIKMIGSAQ
AAAIEAIGKAEAERMRQKAAAYKQYGDAALMSLILEAMPKIAAEVAAPLRKTEEIVLINDDNGN
KMSSEVTKLLGQLPPSVKALTGVDLSKALGKIPGAQVTA*
>TRINITY_DN80458_c0_g1_i1.p1 type:5prime_partial len:157 gc:universal
TRINITY_DN80458_c0_g1_i1:519-49(-)
SKSAKMGRMHANGKGISGSALPYRRSVPTWLKLTEEDVKDQIYKLAKKGLTPSQIGVILRDSHGV
AQVRYVTGNKILRILKAKGLAPSLPEDLHCLIKKAVAVRKHLERNRKDRDSKFRLILIEARIHRLAR
YYKVKRVLPPNWKYESSTASALVA*
>TRINITY_DN2217_c0_g1_i2.p1 type:complete len:254 gc:universal
TRINITY_DN2217_c0_g1_i2:884-123(-)
MNSVDGNTDNAQKECLELCQRNAKATGCEVIWNRHNRGCYIHTQEVARGNNVDRHFCWVFS
KCTLNNVPPAVCPIEKGYCVKGNGHDQNSGVIKMNSVDGNTENAQKECLELCQRNPKATGCEV
IWNQHNRGCYIHTQEVARGNNVDRHFCWVFSKCTLNNVPPAVCPIEKGYCVKGNGHDQNSGV
IKMNSVDGNTENAQKECLELCQRNPKATGCEVIWDRHNRGCYIHTQEIARGNNVARHFCWVFS
KCNL*
>TRINITY_DN3036_c0_g1_i3.p1 type:internal len:548 gc:universal
TRINITY_DN3036_c0_g1_i3:1643-3(-)
AFAGAARASPARQGSVARRALDQSSRYADLSLTEEDLIRNGKHVLVAYIMKPKAGYDYLATAAHF
AAESSTGTNVNVCTTDDFTKSVDALVYYIDPEEEEMKIAYPNLLFDRNIIDGRAMMCSFLTLTIGN
NQGMGDVEYGKIYDFFLPPEYLRLFDGPSCNVIDMWRILGRGTSNGGLVVGTIIKPKLGLQPKPFG
EACYAFWQGGDFIKNDEPQGNQVFCQMNECIPEVVKAMRAAVKETGVMKLFSANITADDPVE
MISRGKYVLSQFGPLSENCAFLVDGYVAGGTAITVARRNFPKQFLHYHRAGHGAVTSPQTQRGYT
AFVHTKISRVIGASGIHVGTMSFGKMEGDASDKNIAYMLQGDVADGPYYQQDWQGMKETTPIIS
GGMNALRLPAFFENLGHSNVILTAGGGAFGHKDGPKQGATSCRQGEEAWKLWKAGTYGDITLS
DGVIEYAKTHEEIKGAFLTFQKDADQIYPGWKEQLGYTGESSVQAASFDWKRKAGAAAFAGAAR
SPLAKQSSVARRALDQSSRYADLSLTEEDLIR
>TRINITY_DN3991_c0_g1_i1.p1 type:3prime_partial len:219 gc:universal
TRINITY_DN3991_c0_g1_i1:84-737(+)
MTSWILNFLVLSIIALPCSSISNCKHKLEPVGCFKDRGSARAMPEQILNERDTTSKVYSKQLMSWK
NFHEWLPGFICRCAEVAARKGYSVIGIQFWGECWSGPNAESTYNRHGQVDSCINQNLKKNRAK
LSQCTAASGKHFTNYVYRLAPACHTQYKPVGCFQDNLQKPRPMPYYVSTDRETRLAIFSGKLLN
WKNWNTHMPDMICRCAAVAKSKGHQ
>TRINITY_DN286_c0_g1_i2.p1 type:3prime_partial len:106 gc:universal
TRINITY_DN286_c0_g1_i2:40-354(+)
MIKVAIITIAIVACVLESTAAVRCYVGYSAADLPLAIGVRNIECGEEFNHCTTVKYRIGGITVTQGN
CAKRYGQAMCQFMRNPHKAMTECEESLCQGDNCNTAVN
>TRINITY_DN1616_c0_g1_i2.p1 type:3prime_partial len:3398 gc:universal
TRINITY_DN1616_c0_g1_i2:218-10408(+)
MIIFLAFLAVAVGQEVLLPPYFDIAKGKNITATATCGEDYVAPKGSPGELFCTLADATGKFGIKGLK
CSHCVPGDYGDGDGKNARDHHIRNANDGSNRWWQSPPLSRGLQYRKINITMALGQSFHVAYVI
IKAANSPRPGVWALERSNDNGRTYTPWYYFAPTSSECKQYFGVDSYLPIVKDDTVICSTEYSKIPPF
KNGEIHISLINGRPGASNFSQSKTLQEWTTATNVRLRLLDIKTLLTDLLPLSLEDPTVTRRYYYSIKDI
TIGGRCMCNGFADECTPHTNIVGKYECNCQYNTEGPRCSRCKKGYVQKEWRQRTSTDKFECEKC
NCHGHSDECEYNEEINKRKLSLDVNGNYQGGGVCKNCQHNTLGINCEKCKPFFYQPANVPIDS
PNVCQPCNCDTQFSTGLCAEETGQCECKSNYVGRYCDQCNKGFHMFPVCKPCECDVNGSEDRE
CIPDQINGCKCKKGYAGRYCDECLPGYFGFPDCQPCACLTRYGAVNDNCERDNGQCSCKVGFK
GRRCEECAAGHYQFPFCKECGCDPEKTLDTVCDSGTGVCQCKSNFAGPKCESCSPGFYAHPSCFA
CGCSTAGSTSSVCDQNSGLCSCRPNYSGFKCDRCAEGYYGFPNCILCDCNPEGSRRVGICDRQDG
QCQCKPTYSGQKCDKCLPGFYGFPDCKRCNCNPAGTKPGPGGRPGDCSTSNNGQCVCKDNVQ
GLECNECKPGFFNLTESNRKGCSTCGCNGAGTNNGVKSCSVEGTCKCKPLVKQEDKCTECRDGY
YGLKRTNVFGCLGCNCEVGGSSSSVCSKDNGQCPCKDNIVGKRCESVAANSFYPDLCHIKAEME

EGKTTDGDVVRYDYQKEIFPDFSWKGFVAVNDQQPSVVVSINVPKTSEYQVKFRYALNQIQPVTA
TVTAFKGDSKIVGSINFASTQKGSGFVASLATVQDNLVQEMYFPLTEGQWNIKLEAPHSNLVVDY
AVLFPKEYAQGGALKKKVQNACDVMTDEKFCNLYRYIDVADKKDIIVLEAENPVKMRQQKPPVL
FNDTAVLSKLDFQAMVVLSPSNSRQLNHLFNVAKPGKYVVMLQYYHDSDTAKYLDVYVRSQIAV
QNGRALLQSCRYTFGCRQAVLNRGDNGVFVFNIKSTKNHLLTLLSRYAVVAVDSVMLVPYESWH
LDYSTPTLYCVTKDSKCTNMNYDVPAESRLVQANPLEQDLSEKPRYLIDRSVEVRYVKPGVNIINIR
DVKAGNYLLVAHYYQPNFPSFESRAAVYSGRTSGLRARFDFKFCPNLNGCRSPIRRPDSNSNLYVF
EEGEARIRFDIPNGLDAWIEYILMIPEGTNVSSLYTPRVLDYSDDFKKECSIDLNVCTSSNLSSFCKDS
IFTITTDFNNGSFPCKCNMDGSKSSSCQEFGGKCPCKDNVIGRECSRCKVGYYGFPNCKPCNCSN
CDEETGVCTCPRGVIGENCDQCAPGTFGFDPIIGCQKCNCNEIGTGSANAECNVTSGQCPCKEN
YSSRKCSACKPGYFNYPACTKCDCLDAGISAEKCDVLTGKCICQNNVIGLKCNECADGYFKTNDI
KGKQLCAKCWCNGRSTKSCTEAELFRATPITASKGWTVSVDTLNVIENGNGRFTISDVNTLPEPV
YWVAPNQFLGNRGLSYGSHFKYNFQYSVKQPSQNTNGDVVTDAVIKGDGYTIKSMRSVIEKEND
GYSASILMKETEWMLEDGSTIDRELFLAILSDIDTLQILATHGKNTISSTIYDISLETAGENGTDRVYG
IEKCFCPKEYADLSCSKCSKGYTRQKDGSCGKCQCLGKSNICDPQNGKCINCSGNTMGDRCEKC
KPGFYGDKCKACECPFSNPENSFANTCAINVNTSSLVCQCNAGYKGVRCDQCSDGYYGNPLQG
VTCKKCSCNGNINASFTGNCDNTTGDCLKCLYNSDGNQCQECRDGYYGDAVVEKNCTDCQCSI
CGTTTAPCNKATGQCQCKKNVEGSKCNQCKPNTWNFDACNGCEDCNCAEASETQQCDLKTG
QCKCPPTVFGRRCGFCKLGYYNYSSEGCQKCQCPDGKPCDVNTGKCCTGVLNEDCKLCPVGHII
EDGECVECDRCVQRTLQRVNTLIHNVTILEKHIIPAGGLNYGALETLGNRFSNLSNKGDEHISNL
DTLKKAIGFGEYRNHSGNMDYVRMYLVRDFKSCSFIVNIKTPPTSSAPATTMVTNTPGPDPIRDHL
SNKKISDSQCPPSLARVIDEGEKNIKLVEKKVVVLEKDAYESTENATATKKRAEDLLAKLKDFMVD
FKDVLSFANSTLKGNVVINIDGNRSLAEAEEMLKEIKDRNFNSTINRSEREKSAAINATIIAEQLMK
QASELANKAYKFNDTFKDFVKALRNLKMESELALLDANNATNSINLAKAVDFQSIIDNAQKLHS
QNNVTVTQAMEILQNAKSAYENAKDVFNKFHNQTGPLSDLMEELRNYVRKLEFDLQTTSPIVDD
SVKHAKRLRDSADRLKELLQDTKKFADAALRAAKVYQNIVDAINDALDAARMANETAHEADK
KVQQAQQDAVESSITSKVVDTEANDLTEEVKKTLKVDISDMKMRLKELRELRKRIQGRIDNVKED
VKSFAKEKEVGEKAMNISTQADGVMKVANKGNEASQDTNEKVEKDGEKVETLKVDISDINKAIK
DLASNVTLVEDTIPKIRSNIDEVNVKHEQIEEFIKTNVTNNIGYLKYLISNAHDILDQMQLSMKFNS
TSEVVLDAPKSVADPTINNKISLTFKINQGNGFLFIIGDFSGNSSGFMSLHVQNHTVVFDYKLNTG
DVQSLTNPKFIETDVNYDVMVTRNFATAVLNVQKSGNASTLMTAYQNRSNISPVLSLGQDTNVY
VGYHPSTPNQRPNFDGCINQFRFDEELMGLWHWKSAKNVGGCVQDFRSIEGQYSFDGDGFVEF
DLVSPELSGFRKIELRFATLEENGLIFLGTDPPTSLPSDIMFDIPLSNQIIAFNALEIKDGYLVSTLDLG
NGVQRLVHTNAGKVNDGKPHLFEMKVLISSLLIKFKLDKKKITEIAISPDTDIRLRLKEAFIGGLPQ
RAWLPNSNISTKGFTGAIGDAKYRGGEFKLVKRFNNIRKGFILPKGYSASFPEEKSYLRLDACNLK
QNSLFGFSFQTFEKNGIIFHSEEGSGSSRSSIEIILIDGQVKVDVSVGSQKRTLQTSVARGFSDGLWHT
LSLYFEETLQEADIVLLYLAVDKAYITGTVPKVDLKGSMYFGSKPSGTESGFNGNIRQVYCNKNEIN
LASGDVFEGGVVGSLKLLPPLKLADFTVVPTTLPPLTTSITTSESTTTETVMTTSTTSISGTTLEPPKET
TTNMAKTTENPTVKITSSEPIIETTMESTTVAKTTSAITMEESTTTPTSITTSTLETTISTLEPGSIEGSTT
TAE
>TRINITY_DN118_c1_g1_i3.p1 type:internal len:200 gc:universal TRINITY_DN118_c1_g1_i3:599-3(-)
KVLFLCGFILLSGLAFAYEIEEIVEDAPKPEPEHVPEPEPEPVKEPEPEPEHKPEPEPEQCSTVKHQK
CSKADKVCSPEDSHCTFTQICTPFFVKKCCKVVSVEKKCVVVRNCIPKFSYEKVCEDVKRCPPRKR
CKNVCRQVDLGEHKVKSICEQECEVVEHPEPCKLVHTCFSKWVSKKDCTPRETCSDIEKRECREV
>TRINITY_DN15584_c0_g1_i1.p1 type:complete len:275 gc:universal TRINITY_DN15584_c0_g1_i1:874-50(-)
MADSGSAGADRGGRGGFGRGGRGGRGRGRGRGRGRGKPEDKEWTPVTKLGRLVKDLKIKTLE
QIYLYSLPIKEFEIIDFFIGPSLKDEVLKIMPVQKQTRAGQRTRFKAFVAIGDYGGHVGLGVKCSKEV
ATAIRGAIIQAKLSIIPVRRGYWGNKIGKPHTVPCKVTGKCGSVTVRLIPAPRGTGIVAAGVSKKLL
QMAGIDDCYTSACGTTATVGNFAMATYMAIARTYSYLTPDMWAENQFQKTPYQEFTDYLAKNH
NVGGKPVAPATATA*

>TRINITY_DN3034_c0_g1_i1.p1 type:complete len:1941 gc:universal TRINITY_DN3034_c0_g1_i1:6289-467(-)
MAEEEDPMKYLQVDPAQALKDQSKTFDAKKWVWVPVIGEDGYAAAQVIKVDGDNVTVETNK
GHEMEVKKEKCDQMNPPKYEKCSDMASLTYLNEASVLYNLKARYTTGLIYTYSGLFCVAVNPYR
RLPIYSDQVVKLYRGKRRAEMPPHIFAIVDTAYQDMLTDHDNQSMLITGESGAGKTENTKKVIQYI
AKVAGVEKKKSDDGPVKRDLDEQVVEANPLLEAFGNAKTTRNNNSSRFGKFIRCHFSQTGKLAG
ADIESYLLEKNRVIHQASAERNYHIFYQLLYATTDEELQKLCLVTRRSEEFEFLKKGVPSVDAIDDN
EEFGYTTNAIEVLGFDEEEKQSMFKICAAILNFGNMKFKQKPRDEQAEVADPADGERVAYLLGLPI
GEFHKSLTKPKVKVGTEFVNKGQNVSQVLYSVAALSKALYERMFWWIVSRVNKALDTKERRSYFI
GVLDIAGFEIFEYNSFEQLCINLTNEKLQQFFNHHMFVLEQEEYKKEGIHWEFIDFGMDLEETIDLI
EKPMGVFAILEEECIVPKASDQTYLQKLHKQHAGKNGSYEKPTPKQTKSGGGDFILHHYAGSVGY
SVANWLEKNKDPINAHTAELFSKATVGLVSGLFADYNPDVTGGRRKGSAFQTVSFRHKEQLKNL
MTTLMSTSPHFVRCIIPNEKKTPGLVEGTLILHQLRCNGVLEGIRICRKGFPSRMLFQDFRQRYQIL
AASAIPSGFIDGKVAAEKLIDSLQLDQSEYRVGTTKVFFRAGIIGELEDMRDERLAKIITQFQAYCK
GHLARIEYKKMCEKRIGLAVIQRNVRKFLFLRNWQWWKLYCKVQPLLSIARAEEEMKEKEEELK
KALEKAKEDEEQRKKMEEQLTENIAEKEKLFADLQAETDRLINLEDKLMQIQQQKDRLEANLNE
TLEKLDGEEHSVSVLQEKMHDAEDRIDSLEAEASELKSNCSRLEVERANRDKQIDALNEDINKQD
ESIAKLGKEKKGLEDNIQELNETLQASEDKCSSLTKAKKKVEDALKESENSLSKEKDSKARLDKEK
RRVEGELKETNDKLSEAENELKNTQDTVYKREKAIKELEEARDNQDNQIKQLQKKIQELLQRIEE
LEEELEAERKQKQKSELSRKELESQIEELQDQLETAGGATHAQVEVSKKREQECSRLRKEIEDINA
ANDEALASTKHKFNAQLSEAQEELESVKKAKSKSDKERSQLAGDLEDVQGQLDQVKKQRASSD
KNNRMLEEQLAELRGKVSSLETDMGDSESRNLKFQNESATLLKQLEEAEHKLGLANKQNKQLQ
ASLGESKNVADDEARGKHDMQNKLQNALAQIEELQEQLEDEQTAKMDLQNKYSRANADAQQ
WKNKYDMEGASRVEELEDAKRKLAARVQEMEEALAAAESKAASMEKVKNRMNEEVEDLLLDL
EKAQAQAANLEKKQKKVDQQINEWKLKCDEVQEELEKSQRDNRSYSTELLKMRTANEDTVEKY
ESLKKENRALAAELQSVSEQLSEGGKNSLEVEKLKRKLGLENEELEVALEEAELALEQEEAKFLKI
QLELTQLKQSTDRRSAEKDEELETLRKNHARQLEALQNTIDAEQKGKAEAQKLRKKLEGDLADL
ENRLEGSDRNSADYLKTIKKLQAQIKELQAMIDDESRGRDDMRDTASRSEKRAMDLAVQLDEAR
VALEQSDRLRKLAENEKLENAERVAELQTMYNSIANAKRKAEGDYHSLQEEIEELENEAKMAEE
KANRAMNECARLSSELNGAQENASNAEKSKSLMARQITELQQQLEEAESMGGKSLKNQIRKLE
QKVMEVEGELDNEARKSTESIKIARKADKRVKELQFQLEDEHKAVERAQEVSDKLNQKMKKMR
LQLEESEQQSSSWQSKFKKAVVDLEEAEERCESAEQALQKARQRARSGASGMTTRGVSRARSRVR
SPQNDD*
>TRINITY_DN761_c0_g1_i1.p1 type:complete len:830 gc:universal TRINITY_DN761_c0_g1_i1:129-2618(+)
MTSMFRSEEMTLCQLFLQSESAYACVSELGEIGVVEFRDLNPDVNAFQRKFVNEVRRCDEMERKL
RFMQKEIDKADIAIVDTGENPDAPHPREMIDLEAQFEQLENEMKDSNSNYEALQKSLLELLELRH
ILKKTQSFFEEEGYAETQARLQADHEPGVTGGGQLGFVAGVINRERIPTFERLLWRACRGNVFFK
QAEIDTALYDPGTGDLTHKCVFIVFFQGEQLKIRVKKICEGFRATLYPCPETPNERREMAIGVKTRIE
DLETVISQTQDHRHRLLQTIAKNIRVWLVKVKKIKAIYHTMNMFNLDVTQKCLIAECWAPVADL
DSIQAGLRRGTERSGAQVSSIVNRMSTKQDPPTFNRTNKFTKGFQAIIDAYGVASYQEVNPALYTVI
TFPFLFSVMFGDCGHGAIMALFALWLIMNERKIANWKAGGEMFDTVFQGRYIVFLMGLFSIYSGL
LYNDIFSKSLNIFGSAWVPTLNRTKYDYQPENYKEWSDGDRMLNPLDAPRETPYYFGIDPIWQLTS
NKLTFTNSLKMKLSVIFGVTHMIFGVILSLFNHLHFKQYVNIFCEFIPQLLFISCIFGYLVILIFYKWIA
FSIHSPSQPSILLGLLAMFLNFAKKKTDADRLYNGQDFLEPFLVVVAVICVPWMLLIKPFYLRWQH
NKKKQHGTNFARLHEDTAPLQDNEVNHHDDSGISSKNEEEEEFDFGEVFVHQCIHTIEYCLGCIS
NTASYLRLWALSLAHAQLSEVIWTMVFKIGLSMDGYVGSVAIFAVFAAWAILSVAILLIMEGLSAFL
HTLRLHWVEFNSKFYVGAGSKFVPFSFKDIMNDEAF*
>TRINITY_DN77_c1_g1_i1.p1 type:3prime_partial len:1965 gc:universal TRINITY_DN77_c1_g1_i1:2-5893(+)
MGVQVYSLGIGSNYDIGELLDIASDDASVFRSQDVDELVSIVASITEQTCKGCTRAVDIHFALDASQ
NVGRENFQVIKNFIKAIGFKFIISETGSHMSASIFGEYGTLIFSMDQATQQEDFINEVDNVPYIGDFN

GNIDKALDMVSEDIFTFDGFSRQGVPKVLVVLTASNCDSCEKSVEEAVKPLKAQGVHVVAIPIGSN
ANMEEMDKMASYPTQDYVIPRASYEELLNGVFIQKVSEMVCSGRPGVCEEPPIEGECTEIINECTL
DVNCPANRKCCIKDCKASCEVPAAACITPIDLAFAFETTGFIEENFDEVKSFASSLLDHFRVEPAKT
HISISTYGESVNAKTAFYESYDKSALKQFIKDLEPDGGRQANIGDFLKTANDEIFNITNGIRQSSPR
MLVLFTNGVYSNDQIAKAQEESLKLKAEGQDVNILVINIGSIVEGNSVLRDVVSTPDFRNLHNVT
DLRRLVNFGFENKIAEAICASKVRKTRCKPRTPQACSVRDDLCQTDFDCPGYQECYSDGCRLRCQ
TCENRCGDKVDLLFMIDTSSRIGPEGFKNAKENIKKMLEWFTIEANNTNVAAITYSDNANYAMQ
LPAAGSNDPPQTLFDVQGRLDSIPYSGGSKSNLDKALKMAAAQVFYQNSLRLRKDSKKAIVIVTD
GVDDSTSRLEIASWPLRKQRVVVDGNDYAAIRLLAVTVGNNYNLDTLEKVVSPPIDKNIFLAADF
DDMYNGIRQLAEDSCKYTRQDVEPPVIAVKGDPGPKGEKGRQGNPGTNGTDGMDGIEGPKGER
GDDGPKGEKGDTGIAGPPGGDQGDLKGPDPMAEAVGIIEGTAVKGTRGVTYDLRDGPIGPPGPIG
GVGKTGLPGDSGEQGQKGRGGPPGLRGVFGGQGPKGNPGEDASPGLPGEPGPKGISGKKGPTGS
PGMPGPQGPFGYKGEQGIIGQPGDPGDGGKKGPKGYAGEKGDQGDIGDAGFPGPDGKVGPIGP
PGQRGLRGPEGSSGPIGKTGDIGNKGLPGPRGSRGLKGQSGNAGPRGGKGRTGDVGLVGEKGTQ
GSIGEAGQEGQPGVPGKEGPPGYPGPIGTPGPMGSRGDIGPQGEEGPKGSLGLKGNRGEAGRRG
LTGEKGETGDIGELGPKGAQGQQGKQGKTGKIGPRGQQGPLGFQGLQGQTGDPGQNGRQGPK
GNLGPTGEKGADGPKGSLGSAGLTGPKGLVGEPGNVGRPGLPGRDGKNGPQGANGKDGDPGA
SGPQGLQGSPGSMGTPGSTGDPGSPGDRGATGAVGDKGTRGDSGVRGEPGAFGQQGPPGLRGE
RGEKGPQGTNGVQGSPGPRGTPGLEGLDGIVGRKGTAGRPGNPGQSGEQGAVGSIGVPGPQGIS
GTPGERGDGGSDGGPGEQGPPGLQGSSGSPGRDGMPGRPGQQGTTGGKGKTGLRGPKGANGQ
NGVQGARGPDGLPGAPGLVGNRGTPGPNGIPGLDGNPGPAGKQGKQGKMGTPGMTGPAGLR
GPVGSPGLRGQKGPTGDRGDSGETGPIGVAGDIGPRGFTGELGLRGAIGEMGQIGETGPQGMPGS
QGTPGAAGLTGPAGPSGLPGKDGNKGPIGDPGNAGNPGRDGGPGSPGKDGTPGEPGTPGSQGK
QGLKGPFGPQGAPGEQGQRGLTGRQGSGGKDGPKGFPGNDGSPGISGKRGPRGKDGTPGSPGPI
GKDGRPGKPGINGTDGDPGNNGPKGDPGERGDSGDSGSLGASGDKGKTGSEGPAGFVGPRGSN
GKAGVPGKRGKRGDQGRDGPRGNSGPDGIDGSKGFKGNMGRFGIQGPPGDAGLKGALGPDGT
VGHTGPQGNTGDPGPDGPEGPSGSKGPAGNAGQIGNAGYPGSPGDGGIPGTAGLPGPPGPPGPP
GDMSGAFSGNYWDFVNAGGLQKGPRYYGGRSKRSLDQQDNSIEKVFKSNYETFKNFDTVWNQI
VDDLVIQKTLGSSKNPALTCGDLFKTNPITKSGDYWID
>TRINITY_DN95248_c0_g1_i1.p1 type:complete len:124 gc:universal TRINITY_DN95248_c0_g1_i1:197-568(+)
MAPKTKKVSKKKSTLNFNIDCTLPVEDGIMKVDDLKKFLQEKIKVDDKLNNLDQITLEVAKTKVT
LVSERPFSKRYLKYLTKKYLKKNNLRDWLRVVASSHTDYELRYFQINNDEEEDEEDNE*
>TRINITY_DN240_c0_g1_i4.p1 type:5prime_partial len:397 gc:universal TRINITY_DN240_c0_g1_i4:1327-137(-)
LKAAAAFSGVSPFALVQPRARVPSRSRVAMVRRSPLPVLGLAGAACFFARSPCFIPGGPLRRQPWA
AAAGSAAVLGATPALADPIGDAAKKLADASYPFAKEVDWNSGIYLQAPGKLAPLEALKAIDKMI
VLGSQADPKLLKAAADAHHKAISSASGPTGMTSKADWEAVNAALGRVFASVPESSVMDVYNAV
AKITDPGVPAYLKSKVNGADAEKAYAAFLEFKDVVKANQVSSAAAAPVPAAGGDKIGAAAQAL
ADQSYPFLKSVDWTSDLYLKPLPGVSAAKALKAVDKAIVMGSAMDGNLLKAAASAHHQAIGSI
DAAGVTSAADYASVTAALGRAIASAPTSKVMDVYNAFAGIVEPSVPNTLFSKVDPLAANAAASA
FYEFKDVVKAAQR*
>TRINITY_DN1112_c0_g1_i1.p1 type:complete len:200 gc:universal TRINITY_DN1112_c0_g1_i1:740-141(-)
MSTLKLQKRLAASVLKCGKKKIWLDPNEASEIANANSRQNVRKLIKDGLIIKKPQVVHSRARTRA
NLEAKRKGRHKGFGKRHGTANARMPEKVIWMNRMRVLRRLLRKYRESKKIDLHLYHELYMKA
KGNVFKNKRVLMEYIHKKKAEKKRSKELSDQAEARRNKVKAARTRREERQAQKRAEILQAYAK
EDTAATPAS*
>TRINITY_DN116_c0_g1_i1.p1 type:complete len:297 gc:universal TRINITY_DN116_c0_g1_i1:72-962(+)
MGFVKAIKNKQYFKRFQVKFKRRREGKTDYYARKRLVTQDKNKYNTPKYRLIVRFTNKDIVCQIA
YAKIAGDVIVCAAYSHELPRYGVKVGLTNYAAAYCTGLLVARRLLHKLNLHETYSGQTEVDGDEY

NVESVDDGPAAFRCYLDIGLNRTTTGARVFGALKGAVDGGLEIPHSVSRFPGFDSESDDFNAEVH
RNHIFGKNVGDYMRALQDEDEDAFKRQFSKYIKEGVKPDNIEGMYKKCHAAIRADPAHKPSGK
EKFKGKRYGRKKMSFAQKMDSVRQKKEAFKRKMEQDED*
>TRINITY_DN262_c0_g1_i1.p1 type:complete len:1033 gc:universal TRINITY_DN262_c0_g1_i1:111-3209(+)
MADPSSLETKEKDGSYRVATKSGGKDKGKKSKKAAKKVNMDDLKKELEMTEHSCELETLVSQLE
TSLTQGLTKSIAARNLERDGPNALTPPKQTPEWVKFCKQLFGGFAMLLWVGAILCFFAYIIRSIREE
EPGMDELYLGIVLTVVVIITGCFSYYQESKSSKIMESFKKMVPQEAVVMRDGEKLNLNATECVVGD
LVFVKFGDRIPADLRIIESKGFKVDNSSLTGESEPQSRNSECSSDNPLETRNLAFFSTNAVEGTATGIV
VKTGDNTVMGRIANLASGLGSGKTPIALEIEHFIHIVTGVAVFLGVSFLIISLAMNYHWLESIIFLIGI
IVANVPEGLLATVTVCLTLTAKKMASKNCLVKHLEAVETLGSTSVICSDKTGTLTQNRMTVAHM
WFDNMVVEADTTEDQSGQAHDKGSTTWKALAKIGALCNRAEFRPNQEEEPILKRECNGDASET
AILKFVELSVGSVEDIRSKNKKIVEIPFNSTNKYQVSIHEQEDPDDPRYLLVMKGAPERILDRCSTIL
HEGVEKPLDEEWKENFNKAYVDLGGLGERVLGFCHYYLPADDFPKGFAFETEDPQNFPLEKLCF
VGLMSMIDPPRAAVPDAVSKCRHAGIKVIMVTGDHPITAKAIAKGVGIISEGNETVEDIAQRLNIP
KEEVNERDANAAVVHGSHLKDLPQEKLDDILKYHQEIVFARTSPQQKLIIVEGCQRMGAIVAVTG
DGVNDSPALKKADIGVAMGIAGSDVSKQAADMILLDDNFASIVTGVEEGRLIFDNLKKSIAYTLTS
NIPEISPFLMFILLAIPLPLGTVTILCIDLGTDLVPAISLAYEKAETDIMKRKPRDPVRDKLVNERLISL
AYGQIGMIQATAGFFTYFVIMAENGFLPGYLIGLRNDWDDKKNNAVMDSYGSEWTYNQRKNLE
LTCHTAFFTTIVVVQWADVIISKTRRLSLFQQGMTNWMLNFGIFFETALAAFLQYTPGLNTGLRLR
PMNFPWWFVGVPFSLLIFVYDELRRFLLRRNPGGWVEQETYY*
>TRINITY_DN477_c0_g1_i7.p1 type:3prime_partial len:1130 gc:universal TRINITY_DN477_c0_g1_i7:3387-1(-)
MMGSIKVFVGLVLLSFTAIEIAQAQSGTVLSNKQFAFDFVVDGSTSIQDEQWNLLTDFVSRLAVKM
STLNNAKLSALQFSHTLNRILQCNQYANVEEARNTIKNMVQENGATGTLAAMLTSADNLMNVC
SAGDPNRQNVMIVIVDGNQNLEQKSHSEIVQDIKVNVKDKNILVLTLGVGPQVNRNEFGDYASD
SGKGFFVDDFTLLTDELVQRMYDELIRTYGDGPDYLTAWVNGPCSATCNPFPMANRPTLRQTRQ
CQEPNGGNCGGASLEQIINCNENIACAGETYLTAWVDSSACSLTCNPFPVSNQPTKRQTRECRQP
SGSCNGASLTQTINCNENVQCPGETYLTAWIDSSCSATCDTGSNNPVFTQTRQCRQTVGNCDGA
PLQQTRSCNVGVSCTAPTFLTAWSNATPCSATCNPFPMAVRPTLTQVRQCNEPNGGNCGGASLQ
QTVQCNLDVACAGETYLTNWVDLTACSATCDDASNRPVKQQERQCQQPGGNCDNLPLQQTVN
CNIGVSCTVSSSACSSKEISFTFVIDTSASVDNIGFFVLKDFMKKYMSQSTLGTDPRTSVAIVSYGAG
DAKVEVECDTITTQQSFNTTVDNLSQLFGIADDNSFNQALSTAFDASGRCARSSTSLNIIIMLSDGD
PPALENILTRDRIQSSSNYYFMFVAIQASDGAQSTFASLANQGRGLRNASSSFTDLYNFNTDLYYLE
TLSKACDVGGQWTEWSTSACSTTCGPGVVTRTRECRNRITGKLQQDCGPGPAQETQACNLQDC
QGTLTQWTSWSCPATCNINNDVNNPPLALRTRQCLGSTGQGNCNNADKTENEPCVRNVPCPG
AWTTWSVFSACSETCRSQSAFPTRRRTRQCIGGTLNFQCPPQIVNGISYREIWQEDCINVPNCPAK
FTQWTTWSCPVTCNVNNDRNNPPTATRTRTCDDSGTINGCANANILENQPCVQDVQCPGAWS
TWSAYGACSETCQSGNRSPTYTRTRTCLGGTLGFQCPPFITQIGGFQIFGTQTSNCNSEVKCPSKLT
QWTTWSCPVTCNNRNDVNNPPQARRTRRCDTSTGSGICESPIVEEFENCVANVPCPGRLTEFTYD
NCAHTCNVNNDVNNPPQRVGTRRCLDGTLG
>TRINITY_DN1386_c0_g1_i1.p1 type:complete len:596 gc:universal TRINITY_DN1386_c0_g1_i1:2419-632(-)
MEYIEVKSFIVALVFVFSILGTAQSDLVINNVERTLDLSTQTAKYTTKIKFHNPGKKVEKSFLLALEK
DQEKHLSYLEILDKQDDNSLEYAAENVEGQSGRFFKVSFSKPLKENDDVTLHIETVFTHVLTPFPA
KIGQTEKQLVLYKGNTVFYSPYEIKEQSTTVKLASSAIESYSRLKPSRSSENTITYGPYKNSKPYRLHE
MKIHFENNSPFLVVNEMTRWIEISHWGNIAVEETYHMTHEGALLKGHFSRYDYQRSPTHSVIKSF
KTVLPAAARDVYYRDEIGNISTSNMLVNDDSVEIELRPRFPLFGGWQTRYMLGYNVPAYQYLYNK
GDNYILKMRLIDHVFDEFVIDDLNVKIILPEGSTNIEVVKPYELEEGKRDIHKTYLDTSGRTVIVLSK
KNVVENHIQDIQVHYTFKKLQLLQEPVICVAVFYILFLSVIILVRLDFSITKDAAKESRMKVASLLEE
LLSSCGRRGSLYYSYDSAIDKLKQNRNQNDFAVSLKKLNQDYNTLTQSINELCSLLIKEDPEIGDKV
NDLQKKENERKVIVDQLVTLATRVVTNKIGRQQYIESEKNSMAKREKLGEDIDNVLATL*

>TRINITY_DN250_c0_g1_i5.p1 type:complete len:452 gc:universal TRINITY_DN250_c0_g1_i5:131-1486(+)
MRECISVHVGQAGCQIGNACWELYCLEHGIQPDGQMPSDKTIGGGDDSFNTFFSETGAGKHVPR
AVFVDLEPTVVDEIRTGTYRQLFHPEQLITGKEDAANNYARGHYTVGKELVDQVLDRIRKLADQC
TGLQGFLIFHSFGGGTGSGFTSLLMERLSVDYGKKSKLEFAIYPAPQVSTAVVEPYNSVLTTHTTLE
HSDCAFMVDNEAIYDICRRNLDIERPTYTNLNRLIGQIVSSITASLRFDGALNVDLTEFQTNLVPYP
RIHFPLATYSPVISAEKAYHEQLTVSEITNACFEPANQMVKCDPRHGKYMACCLLYRGDVVPKDV
NAAIATIKTKRTIQFVDWCPTGFKVGINYQPPTVVPGGDLAKVQRAVCMLSNTTAIAEAWARLD
HKFDLMYAKRAFVHWYVGEGMEEGEFSEAREDMAALEKDYEEVGVDSVDAEDEEEGDEY*
>TRINITY_DN5633_c0_g1_i1.p1 type:complete len:848 gc:universal TRINITY_DN5633_c0_g1_i1:2881-338(-)
MRRSNFLTIGIIAFFLIFSYVSADETSDEEVEPPKVDDDVGKSRDASKTDDEVVQREEEAINIDGLSV
AEMKQMRESAEKHAFQAEVSRMMKLIINSLYKNKEIFLRELISNSSDALDKIRFLSLTDTEALAAT
DELSVKIKADKENNVLHITDTGIGMTKDDLIKQLGTIAKSGTSEFFQKIQDAGTTDQASDLIGQFG
VGFYSAFLVADRVVVTSKNNDDKQHIWESDSVEFSISEDPRGDTLLRGTQISLYLKEEASDFLEPNT
LKDLIKKYSQFINFNIYLWSSKTEEVEEPIEEEPEKKEDEKKDDDKKEDADDKKDEEKKDDDEDD
AKVEEDKEEEKPKTKKVEKTVWDWELMNSSKPIWLRKPKEVTDDEYNEFYKAISKESDGPLTKT
HFVAEGEVTFKSVLFVPKNSPSDMFQDYGKRRESIKLYVRRVFITDDFQDMMPKYLSFIKGIVDSD
DLPLNVSREQLQQHKLLKVIKKKLVRKALDMIKKIPDEDYIEKFWKEFSTNIKLGVMEDHSNRTR
LAKLLRFRSSNDPEKLTSLTDYVERMKEKQETIFFMAGASLDEVKNSPFVERLLKKGYEVLYLIEPV
DEYCIQSLPEFDGKKFQNVAKEGLKIGNEAKEKARVDELEKEYEPLLKWLKEDALKDLIEKAAVSS
RLTESPCALVANSYGWSGNMERIMNSQAYAKSKDAGANFYATQKKLLEINVYHPLIQELQKRVKT
DKEDQTAKDLARVLFETATMRSGYTVKDSSDFAGRIERMLRLSMGVDVDAKVELPEEDDEEKEEE
KADDEGEEVNADGEEEESKEDIPEIKVDSKTVEPVAEHPTNEDAVENEAPAEGKTEL*
>TRINITY_DN1613_c0_g2_i1.p1 type:3prime_partial len:1062 gc:universal TRINITY_DN1613_c0_g2_i1:70-3252(+)
MWLRIYFALAAFALTANAYDFKTCAKTYKKIGCYEDKNHDMVMLENYRDTRRDDFHQLSPVYYW
KKFNQTMHSLACECIKIAQSKGMDYIQIGFYGECKGGKDFASDIITRKEIPNDQCVNTKFKKCVTA
SDLDCVGKAHTGYIYLVEGGRDRNERKVDGGYSYWSEWTECSSSCGRGIQERERQCDSPLPKGG
GKDCSEIGEPFEIKDCNLRECPVDGGFTDWSQYEKCSATCGGGFQKSTRSCTNPAPKFGGQDCVG
DVEKTRDCGTKPCPVDGGWSLWDDYGECSKECGGGVQVSKRSCTNPSPAHGGKECVGVDERSR
ECNTFACPVDGGFGKWSEYDTCTATCGGGTQTSTRQCDSPSPAHGGQDCAGESERVKECGMKP
CPVDGGFSAWSDYSACPVTCGGATQTSTRTCTNPTPAHGGEKCVGKTTREKSCANNPCPVDGGF
TAWSEYGSCSEECGGGIQESKRSCTNPAPEHGGKDCVGDTERTKQCNTQACPIDGEFTVWSEFTK
CTVSCGGGVQESRRSCTNPAPEHGGEECFGPVMRTRACNTKKCPINGGFSKWGEYSVCSEECGG
GTQSRTRTCTNPKPQYGGEPCSGDSLEERDCNVQPCPVNGKYSSWSAYSKCSASCGGGSQKSTRT
CSNPAPAHGGAQCQGDAERTRECGTKPCPVDGGLTKWSEFGACTASCGGGEQTATRSCTNPSPA
HGGKGCLGELEKVRECGTAPCPVDGGFTDWTQYGECTKSCGGGSQTSTRTCTNPSPAHGGQEC
DGSTMKTRTCNSDPCPVDGGFSKWSEFDKCSTTCGGGYQTKTRSCNNPAPAHGGKSCSGETEMT
QSCNSNPCPVDGGFTAWSKYSICSATCGNGIATSTRSCTNPAPANGGDDCVGLKIKSKKCFVKHC
PVNGGLTDWSEYDTCTEKCGGGTQTSTRSCTNPRPAHGGDDCDGELERTRSCGMDPCPVDGGL
TKWSDYDQCSKPCGGGYQTSTRTCTNPLPAHGGKECTGETDRTRECNTHHCPVDGGLSQWSSY
GKCSDECGGGIQERRRSCTNPKPMYGGKPCTGLFMET
>TRINITY_DN1369_c0_g1_i1.p1 type:3prime_partial len:1577 gc:universal TRINITY_DN1369_c0_g1_i1:93-4820(+)
MRIQLTIFVTFSIILVSGQNSPPGICSTWDGSHYQTFDSYSFNYHGTCSYALALDCRPGSDDFAVHV
ENDEKCDNHGGTDCTRAVSLYVNDIEYKLGSNNTVLVDGKSNIVLPYFNEGVRVSKHAEYYFIDA
LEGKVVVKYDGRSSIYVQLDSSYQAGVCGLCGNFNGNKKDDLKLPGDKGLTKSLKEFGKSWEKP
RIGQVCKEVTEEHFDQCTNKSKMLVYYAEKECEILRTSSEFKKCHKIVDPTPFYEKCREDLCLCGS
GDKYCKCDVLEQYTRKCAHYGTVLEWRQQNLCYKNCTGNKVFSECGPACSSVCSAIGSKSVCK
DACIDGCHCPPHTSLSGDKCVPDDECPCEHNGDMYDHGDVINMKCKSCKCNGGKWKCNPLP
CDGEASVYGNGHFVSYDGKTYNYPGKCLYLYTYLSDRSFAVYLDRTSCDSLACHMNVMILFRGD

RIKLSASNSTLALEVNRNVKSFPYISSTGYRMYRFSTVFVRFEATNGIKVDWDTYSRLHVLMPHSL
KGKVFGLSGNFDFKTKNDFLTPRKDLAHSAIEFGNSWMMYSSRLNCSALAPDYRMTSCEANHQ
VAKYAESKCSILMSSLFRECHKVVDPFIYYENCKDDTCECNGDSKCYCSVIAAYSKACSSAGKPTE
GWRKYTPCEEKCSNGMVFTECGNSCLKTCDDQTDPNPFCEKECVEGCLCPVGQVLDKITEQCV
DIKHCGCTYGNRTISNGGTVAKDCNTCKCTKGVMVCTKKECRDKKAICSDGKEWSSCMSPCPK
TCQTKDDICPETGLDCIEGCQCKKGYIESANGTCVELSKCPCYHNGKEYQETQTISKDCNTCKCT
AGEWSCTEKLCDATCSVFGDPHYKTFDGKIFDFQGKCGHVMLTDKCANNDNATENIRISVKSST
CGSQGVTCTREITLNYHDTVVHMKRGARKIDIVSNKRQDHEKDQVKVYRHAGIYVHVLTPMGV
SILWDNATRLYITAPPSLSGKICGLCGNFDGSETNDFVTYQGDTAGNAVSFGDSWRTENNCPLTKE
IEDTCHNRKHRLDPSKRECAVIKSDVFKKCHTLVDPVPYYDRCVFDACGCDSIGDCDCLCAAIGA
YAKECIDEGVYINWRKGHNICEKEMCCPTNMTFVPKGTGCPKTCKYPNGLHHCPTKYVDGCQC
PEGLVQNNDANGDVTCVRPEECKVCKLDGKIYNEGEVVLYKCKECVCRDGKMTCSGNYCPTTL
PPTTTTESTTTPSTTPETTIPSTTTTESTTTPTTEPPTTTTTASTTTASTTTESTTTEATTTTKPTTTTTEST
TTESTTTQSTTTPSTTPSTTMKPIDTCVYSNGTHNVTLERGDVIQVNECTKKTCVKDEVTGLHEIKV
TTTQCENSTCDTTCEASKSNRAILVKTPGECCPQCKKPEDLCDNPNEVPCNSTYCIPKSWFCDGN
KDCSNGRDEECCPNYCKVEGEIKKPGEVWTNKEKCLTCKCEGYYTKCRRMECPELDCPEERQVK
SEGECCPHCKPQTEPPTTTTEMTTTTTESTTTPATTPSTTSKETIIIITEEPKSTTASPQEKCDCKVLQPV
GVSDGRIPDNQMTANSV
>TRINITY_DN3545_c0_g1_i2.p1 type:5prime_partial len:236 gc:universal TRINITY_DN3545_c0_g1_i2:3-710(+)
SGPGRSSCRPILSVIKISKINNIDSGKVEGGYTMWSVYGSCSKSCGGGAQKRVRSCTNPAPANGGK
HCDGVSFETRECNINQCPVDGGYTMWSVYGSCSKSCGGGAQKRVRSCTNPAPANGGKPCDGVS
FETRECNINQCPVCPEIVGTYDISSRHHYGGAKYGTVVFKKLSNSKTVEAYYTINDGRRGVLTCKIE
SAQPDQCSGHGYYGKYFTYYKNRCQIIWSNDVIYTKQNC*
>TRINITY_DN332_c0_g1_i1.p1 type:complete len:246 gc:universal TRINITY_DN332_c0_g1_i1:940-203(-)
MGALTYIFCLFLISSSFAKEEESCRTYAGGQVYPDERKSGVEHALHWSKAQISKPAPHWEGKAVV
NGEFKDLKLTDYKGKYLVFFFYPLDFTFVCPTEIIAFSDRVEEFKKINTEVIAVSVDSVFTHLAWTNT
PRKEGGLGKMKIPLLADLTKQISKDYGVLLEDAGHTLRGLFIIDDKGVLRQITMNDLPVGRSVDE
TLRLVQAFQYTDKHGEVCPAGWKPGSDTIIPDPKEKLNYFNKQKEEI*
>TRINITY_DN577_c0_g1_i1.p1 type:5prime_partial len:269 gc:universal TRINITY_DN577_c0_g1_i1:1086-280(-)
PCQNCADMAVGKNKRLSKGKKGSKKKIVDPFTKKDWYDVKSPSNFQIRNIGKTVVSRTVGTKIA
SDGLKGRVFEIAQADLNKDEVAFRKFKLICEDVQGRCVLTNFHGMTMTTDKLRSLVKKWQTCIE
AHVDVKTTDGYLLRLFAIGFTKKRQNSVKKTAYAKTTQVRAIRKKMVEIITREVSTKDLKDVVNK
LLPDSMGSDIEKASQGIYPLHDCYIRKVKCLKKPKFDVHKLMEMHESSGTTTTDATGGVVVERPD
NFEPPVLDSV*
>TRINITY_DN673_c0_g1_i1.p1 type:internal len:100 gc:universal TRINITY_DN673_c0_g1_i1:1-297(+)
AKSHTLQVYEFNCKLDIYKFIRMSSPDKPSYMAFFGAMGATSAMVFSSLGAAYGTAKSGTGIAAM
SVMRPELIMKSIIPVVMAGIIAIYGLVVAVLIAN
>TRINITY_DN194146_c0_g1_i1.p1 type:complete len:190 gc:universal TRINITY_DN194146_c0_g1_i1:165-734(+)
MKTVLASQTVTIPEDVSVSVNGRTIVVKGPRGTLKRNFNHLNLVLTRVNKTTIRVDVWFALRKHL
ACLRTICSHIENMMKGVRFGFLYKMRSVYAHFPINIIVNDGGKVVEVRNYLGENYVRVVKMREG
VICKPSGNKDEIIVEGNDIELVSNSAALINQCVKAKNKDIRKFLDGIYVSEKTTIVPMTE*
>TRINITY_DN2885_c0_g1_i2.p1 type:complete len:114 gc:universal TRINITY_DN2885_c0_g1_i2:31-372(+)
MAQPVRLYSKGIFVGYQRGLRNQHEQTSLIKIEGVNTKADTSFYLGKRVAFVYRVSKKTVAKGDK
KPSRIRVVWGKVTRPHGASGVVRAKFRHNLPAKAIGGSVKVMLYPSRI*
>TRINITY_DN2126_c0_g1_i1.p1 type:complete len:114 gc:universal TRINITY_DN2126_c0_g1_i1:55-396(+)

MAPVKKKTAESINSRLALVMKSGKYTLGYRSTLKTLRQGKAKLVIISSNTPQLRKSEIEYYAMLAKT
GVHHYTGNNIELGTACGKYFRVSVLSITDPGDSDIIRSMPSEQETK*
>TRINITY_DN7504_c0_g1_i1.p1 type:complete len:414 gc:universal TRINITY_DN7504_c0_g1_i1:87-1328(+)
MAKIPGGIVVEMDGDEMTRVIWDMIKTKLIFPFVDLDIKSYDLSIQNRDNTDDKVTVDAAEAML
KYNVGIKCATITPDEKRVEEFNLKKMWKSPNGTIRNILGGTVFREAIICKNIPKLVPGWTKPIVIGR
HAHADQYKATDFLVPEAGTLEMKFTPKGQTEPTISYVVVNNFAGAGVAMGMYNTDKSIRDFAHSS
FQCALEKNYPLYLSTKNTILKKYDGRFKDIFQEIYDNEYKGKYEAKGIWYEHRLIDDMVAYALKVE
GGFVWACKNYDGDVQSDTVAQGYGSLGMMTSVLMCPDGKTIEAEAAHGTVTRHYRLHQKGQ
ETSTNPVASIFAWTRGLAHRGKLDGNERLMNFAKLLEEVCVATIENGSMTKDLAICIKGMKNVTR
ADYLNTTEFLQKIADNLTTKLNLSHL*
>TRINITY_DN141338_c2_g1_i1.p1 type:complete len:720 gc:universal TRINITY_DN141338_c2_g1_i1:2850-691(-)
MSEADETETFAFQAEIAQLMSLIINTFYSNKEIFLRELISNSSDALDKIRYQSLTDSGVLESNKDMKIE
IIANKDEKTLTLFDTGIGMTKADLVNNLGTIAKSGTKAFMEALTAGADISMIGQFGVGFYSAYLVA
DKVEVVTKNNDDEQYIWCSSAGGSFTVKRDTINEPIGRGTKLVLYMKEDQLDFLDEKKLKDIIKK
HSQFIGYPIYLETQKTRDKEVSDDEADEEESTDKKEDEENEDKEDDGPKIEDVGDEEKEKEKKKKT
IKENYTEMEQINKTKPLWTRNPDDISTEEYAEFYKSLTNDWEEHLAVKHFSVEGQLEFRAILFVPK
RAPFDLFENKKQKNSIKLYVRRVFIMDNCEELMPEWLNFVKGVVDSEDLPLNISRETLQQSKILKV
IRKNLVKKCLELFAEISEDKDNYKKFYEQFSKNVKLGIHEDTQNRNKVAELLRYSSSASGDELTSFK
DYVSRMKENQKDIYYITGESKEIVAASAFVERVKKKGFEVLYLVDPIDEYAVQQLKEYDGKKLVCV
TKEGLELPEDEDEKKRQEELKAQFEDLCKVIKDILEKKVEKVVVSNRLVDSPCCIVTSTYGWSAN
MERIMKAQALRDTSTMGYMAAKKHLEINPEHQIMIALKKKIEADKNDKSIKDLIILLYETSLLSSG
FSLEDPQNHSSRIHRMIKLGLGVDDDGDVEEIETDDLPPLEGEGDDDEDKARMEEVD*
>TRINITY_DN3078_c0_g1_i1.p1 type:complete len:1109 gc:universal TRINITY_DN3078_c0_g1_i1:3628-302(-)
MKMRIYLFLILFATVYCRVVQKGPNDIPEETKNEIKNQEPKIKFEPEKDKKYFLAKDTTCAHDVDK
HCKNIRRENNFAVFVCLQDVIHKDEDDVAEECHETLWHYKYNLTRDPNFDQAAFKVCENVINEI
QECNDSELLKSPGFVTNCLIENRHAVSEAKCKSFLQKMSAIVFEDYRLIKGFYDSCVEDVKTLKCG
TVAKPTDEEYQHDQTEVISCLEEQLDLVSEKCQAQLVKVATMQADDYHLDRPLFYACQEARERF
CPEVKSGDGRIYKCLVEHKTNEMMLEKCRKKIEIREKLTQKDIKVNELLWKSCKADFTKYNCPKD
GISEHGAASSLLLCLQGKVAEGLPVDQSCQAEMRTFAIELFENYKISPVIVSKCDVEINQFCHHLIG
KTDDGDMMDCLMTQAATNNESVSKKCYDAVSDVIKETGAGNNYKIDRALYLACEPVITTLCQG
KEDAMILGCLMDNAHNSKMPEKCAAQVFHLQYFLSRDFRLDASLYKDCKKDAEEICSANDFNA
ESESKVPDNFVIACLYRNALDRKVDDRKVSQTCAEHVFRVMHQRATNIHLMPELERPCMADLG
KHCIDKGDKEGEEIDCLQTNYEKLSAKCQNAIKVFTIQEAKDFDLDKHLVDKCAPMVQQFCQRE
LEDGNGEKVLPCLVDHKNDIDMDHQCANAVEHWQLIEMKDFEFSPALTEMCLLDIRKHCQKSK
AKIEALHCLSENIVKDASSVTEKCRAQLKNELIEQSENIKLNPELYEACTKDIELFCRDVVPSGGQL
EECLKRNHKKLTDPKCKSLLFKEEKKESFDPELDYRLMHVCKPIIKRYCTQYHQPVDIMECLRQV
THDRDMSRECRAIITERQIEQAESFELDPELSKFCQDDVNRYCKEEIKHAEKGDDDGAIFGCLVNA
MIDNKPIKNDCEMYIRRREAEAAQELDLNPQFLLQCRDEITKICPNMDEDEIIECMKMNIPQIQSK
GCVQEVKKLIVEGIEDVHVDPHLQEQCARDIRTFCQSVPSKSGNVVLCLIDVHKAKNLHLQPNC
ENFLTKRLSLYNAAKVDYAVFDSISAVMNAVSESPNRNYVYMTLLLVLTILFVGGMLFGRYTKKIRS
EIKNR*
>TRINITY_DN1989_c0_g1_i1.p1 type:complete len:546 gc:universal TRINITY_DN1989_c0_g1_i1:150-1787(+)
MRGNNRGSGGQRKSFDGNRTPNNNSNRGNYGGNKRNWSPSPYKKPQETPPKVSKDEPKHLTP
PVASMSKPTQKNTPDVIITKEVDIPKAQPDFMRHNDPVGKVPMNMKNDMAPPVMQGNPNNG
QGNNELENTKKEEKAPVNRVKYTGREGEKKFSGRCRLFIANMNNGTTEEDLRELFGQFGETGEV
FVNKEKGFGFIRFDFRHNAEIAKSTLDKTEFKGRNIQVRFATHASALEIHGLDRFASNEFIEKAMSI
FGKVERAVVVCDDRGRSKGYAIVEYEWKKTAQKVLDRFKGEMFVLGRLPKPVFAKPLSQVDEEE
GVREEDLERLQGIEGEREFPPRFISPSSFEYTWAKKWRDLYIEEEDKKARLDQELRDSRYKLELEME

SALQEQEALKMREGLIIELRSRQEELRRLEEGLMRRQEITARITRDQDDRKQEFNRSRDESQYPME
GMADTNQSDYNIQQQTEFDTHQGNFDVNVRQDLMHQNANIPISGVAGFPQHVVHRMPHGIIQ
AGGKPLPPPPAIPGIHLSNVYPYIDEALPFDE*
>TRINITY_DN3339_c0_g1_i1.p1 type:complete len:131 gc:universal TRINITY_DN3339_c0_g1_i1:31-423(+)
MVRVNVLNDALNSICNAEKRGKRQVLLRPSSKVTVKFLTVMMKHGYIGEFEVIDDHRSGKIVVN
LTGRMNKCGVISPRFDVKLKDIEKWASYLMPSRQFGYIVMTTSAGIMDHEEARRKNTGGKILGYF
Y*
>TRINITY_DN15847_c0_g1_i1.p1 type:5prime_partial len:166 gc:universal TRINITY_DN15847_c0_g1_i1:3-500(+)
NRQGATQYKMSEQTERAFQKQPTIFQNKKRVVGKESKGKEQRFSKSVGLGFKTPREAIEGTYIDK
KCPFTGNVSIRGRILSGIVQSMKMKRTIIIRRDYLHFIKKYQRYEKRHKNLAAHMSPCFRDVQLGD
LVTVGQCRPLSKTVRFNVLKVNKSTSAKKSFEKF*
>TRINITY_DN7659_c0_g1_i2.p1 type:complete len:359 gc:universal TRINITY_DN7659_c0_g1_i2:82-1158(+)
MASAATDNRKVALITGVTGQDGSYLAEFLLDKGYEVHGIIRRSSSFNTGRIQHLYKDRTAHLEGN
LHLHYGDLTDSSCLLHVVSKAQPTEIYNLGAQSHVKVSFDLAEYTADVDAMGTLRLLEAVKICGL
MHKVKFYQASTSELYGKVHEIPQTETTPFHPRSPYGAAKLYAYWIVQNYRESYNLFGCNGILFNH
ESPRRGETFVTRKITRAVAKIHLGLQEELVLGNIDSVRDWGHARDYVEAMWLMLQQEQPETYVIA
TGETHTVREFVEKSFGLIDVKIRWEGTGENEIGYDAASNKVRVRISSKYYRPAEVEYLHGNPAKAE
KELGWKRKVSFDELVKEMIDSDIALMKKDPEA*
>TRINITY_DN172571_c0_g1_i1.p1 type:complete len:104 gc:universal TRINITY_DN172571_c0_g1_i1:30-341(+)
MSGRGKGGKGLGKGGAKRHRKILRDNIQGITKPAIRRLARRGGVKRISGLIYEETRGVLKVFLENV
IRDAVTYTEHAKRKTVTAMDVVYALKRQGRTLYGFGG*
>TRINITY_DN22077_c0_g1_i1.p3 type:5prime_partial len:151 gc:universal TRINITY_DN22077_c0_g1_i1:520-68(-)
SVCGTAVVQLGDPPPPPCAPLALPRLSLRAMSSAAASSSKASKGDKFSMGRESDKAPIGKGGATR
TKKVLRESIQGITKPAIRRLARRGGVKRISGLIYEETRGVLKSFLENVLRDSITYTEHARRKTVTALDI
VYALKRQGRTIYGFGV*
>TRINITY_DN6390_c0_g1_i1.p1 type:3prime_partial len:1941 gc:universal TRINITY_DN6390_c0_g1_i1:5820-1(-)
MVIAPKCKFSIFSYLVFGILSYNWNELDASTNIELVDGTGVLLQRDVDNVLNDAVVQKEYQQNIK
HKPVDKRCLGEPFYQGLCKEKLEDAIQQNITEGGCHNLLVKIQKDECSNGPKTLGGKGYCNFICS
QEICNSIYDIDKCVSKRLVHHFNKDKNECEEIMQADCGSESFTSSNQCQKRCIGNSDDKRETKVY
CDQFCLNGGKCIGEVYCQCPPAYHTATCGLSYCNPSCGVGGKCVKTGEGKYGCQCFPGYKGVK
CDKEDSKNMPVVKGLCLSAIKSVLTSPNTSFHDKPEEEKNYCLKYLFDLESNCDKQRPFVNYTEE
QCNHFCASDICDNIYSNDCGSIEERYGYVSSTGRCSKFLYNGCLGNTNNFKDKAACDTKYSPING
LMTSVALLRPLNHTCYIRGKTNFITFDGKVYDLLSGCSHYLVHDSDTTSDFNVILHNDPKVNYNE
KSKRKLEITADGKNVLLNQKVSSDRFFVQVDGRSVPLPYNDVPKIRQAGRYILVLTGDGVSIAFDG
YEDISITVNDYYRTGDGGDHRLNGLCGNLDGNSHNDWLDFTGELRENVRDFAKVYQTDRSTCK
MDNLSLKSLVYNPLHNDQEVFERAERHCDVINTDAFKPCHSVVSPEQYKVLCMQDYIKCNDDK
RDDCVCSSLSFYSRVCHKYGKDIKWRRANLCPITCQRNMKYYECGSACPKTCDNLITPTKCQTDC
VDGCHCPPDLYYDKSTENCVPRNLCSCYRDGIAYKHGSVRSFHCEECTCNGGSWECNKKDCEAT
CDIAGDPHIKTFDGLNYQMYGKCSYVLVDHCHYGNTGIKEFEVIVENKNCNDDALYTSCVRHLS
VNILQTNTRVSLDTAKDNNGTVLPVAFVNGKEGKEFRSKDYTITSVGGRNIIFQYKVNQLKVSWT
GHNAYVTVGHQFENQTCGLCGTYNFNKQDDFHTRSDTVEKSVTAFTQSWVYQSSDKVQDPSKC
HTAKWETSEKPCQIYTNKAQYAKTQCSIIIKPDGPFEDCHSIVPPGEYYSMCQDDACKCDDCYCH
IVAAYAKRCTDLNIRVGNWRDETLSCKQTCQAPKVLKQCNAIDSLDDDLSESLKCPSTCYELSQN
DTSICQSRGCIEGCYCPEGYFENEQNECVKQDQCSCYHDGKVYRHDEIRKDRCNECVCKGGRFE
CTDFDCVAHCASKGLVYSDCGKTCSNFNFNPQDSKCQAGCFCPAGKVLHENGTCVNPQTECQC
KEGDQFFDAGEVSPSDCSKVCNGDRSWKNLPGKDAKPQYDCSAFGQMHYRTLDGRMFKFSGG

LCPYVLLTDCRQDSATGFCDLTKANVNIHVRNLRCVNSYEAYMCKEVTLDLRLPNGEMAEFVLM
QNKIKMKTGLVTKVFDKGSYPQPKTAVVDGVEIFKSGLYLVVRAYHLNITVKYDRQNRVYVVPGR
EYKDAGKLAGLCGNYNGLDDDDFRRPDNSNAENELQFAKSWQDSNSACPDPSESDTCSSNKER
LPWAQKGCNVMKTGNFTACHKTVSPEPFIESCQQETCLCKSGGDCACFCSAVAAYVQECNRHG
VPIYWRRDGLCKLDCCDPCGHSYGYYTDNHVPNTCGVKDKVCKENDRCCKGAVVEGCGCPPG
TYDDGIKCQNETTTNVIPCSPSSSIVPSTSISVPSTIIEPVSSSSPSPMTSSVESQSPSPSSSVVSPTPSSSII
QSMSSSEIESISTTSSSFVSSIESSVQSSTSIVFPVVSSNSGESSVSSSMLPSTSWVGIHSVSSSMGESSVST
NVLPTDYLRTTDVFPLSSSEITYPSSSSSLEPHTPSSSILQESSSSVSKPVGISSVIEPEISSIRYSSMSSQN
VMSSSVSSSVSKPVGISSVIEPEISSIRYPSMSSGSVLESSVSTSFVLPSETVLSTSVFSTNTESPSSSMTLS
TSSSGFVFPSVTSTFQTSSKVLSSLPRSGPAENTVPLSTMFTKTV
>TRINITY_DN162_c0_g1_i1.p1 type:5prime_partial len:206 gc:universal
TRINITY_DN162_c0_g1_i1:654-37(-)
LSTNCGPSPASFFKIIKMGIDLDHRNDKKKHRKEPVSQDPYLRLLVKLYRFLARRTNAKFNEVILK
RLFMSKTNRPPVSLARVTKRMGKPGCEGKICVIVGTVTDDNRLLEVPKLKICALRFTEGARTRILK
AGGEILTFDQLALKAPTGQNTLLMQGPRNSREANRHFGKAPGVPHSSTKPFVRAKGRKFEKARG
RRASRGYKV*
>TRINITY_DN22492_c0_g1_i1.p1 type:complete len:1026 gc:universal
TRINITY_DN22492_c0_g1_i1:171-3248(+)
MLQKTLIYTLLGCWITLLSHAAIVRKSGGDDFSLKVGKPQKPIGQPKLKDAKKEVKEEPTSDVIPQ
QDENNDTTDRQKHKEQVSPDAVESGMPPRYPLKAMSAEVSAHDNEIGGCRDHPRASRVCKNC
GNRNFAAEHESYTYSYCRKSCRHCDPELSSFHTFWRLRAASQFDPTWMVKEIEFYPKPTEYDNLA
TDPQRAYASSSYPGFKPSYAFDGNNKTSWYPSGWGLHDNNDDWIGFEFPVAVRVHAIRMVLDE
MHRTAAPQRIFVEASDKKDGPFTTKWKISNPTYEVDNVYKLLQCPVLWRKFESDEGSWCFRLIAK
FETWEVARNECTVEGGDLATIATEEEKRFLTDELNLGGFAWIGLNHKDQAYYWQDRANSSYKN
WKAGWSYSEVQKQNLNCVATDQDGFWYNFHCTNKYYFVCKKKLEPQAAPREDLILKAWPYEP
EIPPMIFLYPNITVPVINTKNLEPGVFPDENGKVPEEEEEETTADDLFYEQTVEHLKEKGLKPSEEIS
NDVDKEETDYTQQLNKIALKTQSRDDFRNETESDKLKFNSTEAVKQAKDLEEQKSVNLDQNFN
QYNSTDGVKQQSQPVVAAPSDGPSGNSGDNNTLPRIVNGKPVESPSVNPNNVGKPNTPVASPES
PENTDFAQPGDTTFTTPTPTVQSGDPGNAEKSNPQKATNDQQAGGQQPNQESGSNANQSQVQ
QQSQQQQPQQQQQQQAQNNNNNQNGQQNASNQQNNNNNNNNNQNNNNNQQQNNQQQ
NNQNNQQQNNQNNQQQNNQNNQQQNGNNNGGNNNDLPNGLPPVMGGPAPQKIPEPPAP
KEGTKDNDSEKSVGGQADVMPDDAPSNDNDGGDETTTGNEENNDESNNNNNNNNNNMNNV
NNNNDNNQSRTLDKPLPNQGKNSSETVDKATNVGGTNKQENNDFMKLQKAKPGIRTHKIGHK
KSAGLKKTHMKNVVSPLKKVKTEKKINKQVKTVKKIKAVKVKHHKKALKKEVVIHKSEDDEEE
GSGSGDEGDQEDVDDILESITNGLW*
>TRINITY_DN2113_c0_g1_i1.p1 type:complete len:1704 gc:universal
TRINITY_DN2113_c0_g1_i1:5257-146(-)
MAQLLPIRFQEHLQLQNVGINGANIGFSTLTMESDKFICVREKVGEQSQVVIVDMADANNPTRR
PISADSAIMNPASKVIALKAAKTLQIFNIEMKSKMKAHNLTEDVIFWKWISVNTIGLVTETAVFHW
SMEGDSSPVKMFDRHQSLAGCQIINYKVDHSGQWLLLIGISAQQNRVVGAMQLYSVERKVSQPIE
GHAAVFSQFKVDGNPQTSNIFCFAVRGAAASKLHIIEVGTPPTGNQPFPKKAENIFFPPEADKDFP
VAMQASQKYGVVYLVTKYGYIHLYDIESAQCIYSNRISGDTIFVTASYEPTEGIIGVNRKGQVLSVSV
DEEKMVPFLIQVKQNGELARRLAERCNLPGADNLFLQAFNDKFASGNYQEAAKIAAKAPKGILR
TQQTIQRFQQLPSQQGQTSPLLQYFGILLDQGQLNKYESVELCRPVLMQNRKQLMEKWLKEDKL
DCSEELGDLAKQFDNTLALSVYLRANVPNKVILCFAETGQFQKIVLYAKKVGFEPDYVSLLRNVM
RVNPEAGANFSGMLIQDDEPLADLNQIVDVFMEFNLIPQCTSFLLDALKNNRPAEGPLQTRLLEM
NLLSAPQVADAILGNQMVTHYDRAHVAQLCEKAGLLQRALEHYSDIYDIKRAIVHTHLLNPEW
LVNYFGTLSVEDSIECLKAMLSHNIRQNLQICVQIASKYHEQLTTSSLIEIFESFKSFEGLFYFLGSIVN
FSQEEEVHFKYIQAACKTGQMKEVERICRESNNYEPERVKNFLKEAKLTDQLPLIIVCDRHDFVH
DLVLYLYKNNLQKYIEIYVQKVNPARLSIVVGGLLDVDCSEDVIKSLILSVKGQFSTDDLVAEVEKR
NRLKLLLPWLESRIHEGSEEPATHNALAKIYIDANNNPERFLRENQFYDSAVVGKYCEKRDPHLA
CVAYERGQCDQDLINVCNENSLFKSLARYLVRRRDAELWGTVLVEDNKYRRQLIDQVVQTALSET

QDPEDVSVTVKAFMTADLPNELIELLEKIVLENSVFSDHRNLQNLLILTAIKADRTRVMEYITRLD
NYDAPDIAGIAIVSELFEEAFAIFKKFEVNTSAIQVLIENIQNLDRSYEFAERCNDPAVWSILGKAQL
DQGLIKEAIDSYIKADDPSAYMEVVEASKNCGNFEDLVKFLQMARKKAKETFIDTELIFAFAHTGR
LADLEEFISSPNHANISQVGERCFEQKMFEAAKILFNNISNFARLASTLVHLGEYQNAVDGARKA
NSTRTWKEVCFACVDGQEFRLAQMCGLNIVVHADELDDLIRYYESRGYFEELISLMEAALGLERA
HMGMFTELSILYSKYKPTKMKEHLELFWSRVNIPKVLRACEQSHLWSELVFLYDKYEEYDNAVVT
MMKHPTVAWKESLFKDVITKVANIELYYKALQFYLDNKPLLINDLLVVLTPRMDHTRGVSFFQRA
CYLSLVKPYLRSVQNHNNKAINEALNSLLIDEEDYNGLRASIDAFDNFDNIALAQLLEKHELIEFR
RIGAYLYKGNNRWKQSVELCKKDHLYKDAMQFACESRDKAVAEDLLSWFLSIDKTDCFAATLYM
CYDLLEPDVVLELAWRNNMLDLAMPFMIQFMKDYYTKVDKLAESNAKRDEDKENAPPEAPIM
GVEQLMITAGPSSYVPQPNMYGMPPQGMAPQGMPPQGMPPQGMPPQGMPPQGMPPQQGFPQ
QGFYGGAM*
>TRINITY_DN909_c0_g1_i1.p1 type:5prime_partial len:313 gc:universal TRINITY_DN909_c0_g1_i1:1-939(+)
RRVLFRSLTFIESLRLSRLVNKKNKTFMEMSEKGGIYNDNEREMNFTGNSDYESGDLGFCGWFLTV
LSFIIAVCTFPLSLFFCIKIVQEYERAVIFRVGRLLSGGARGPGLFFILPCIDTYLKVDKRVISFDVPPQE
ILTRDSVTVSVDAVTYFKIQNPVASVCNVEDAQRSTKLLAQTTLRNELGTKNLSELLLEREAISRNI
SNILDQATDPWGVKVERVEVKDVRLPPMLQRAMAAEAESSREARAKVIAAEGEMNAARALKEA
SDVISESPQALQLRYLQTLTTISAEKNSTIIFPLPINMLQSMMNK*
>TRINITY_DN2963_c0_g1_i1.p1 type:complete len:116 gc:universal TRINITY_DN2963_c0_g1_i1:1072-725(-)
MSATGNLVTVLRKLYDEYNDKTPSRLKIVDGFLLYVMLTGIFQFLYCCLVGTFPFNSFLSGFISCVGT
FILGVCLRVQTNPRNRTEFKGISPERAFADFIFANVVLHLVVMNFIG*
>TRINITY_DN464_c0_g1_i4.p1 type:complete len:717 gc:universal TRINITY_DN464_c0_g1_i4:2167-17(-)
MLLHFGKSLHYLKFGTLFWRLKLVAIPRDTSNIINNIFLESKSQRYIRMSANILNEYSQNVGKMEDE
ISGLTSQLNALKRKLNGEIEDEDCSDQLKELRSKNQKLHYQKNIIQRALKKEQEKADSKMLNVQ
AALNVLFESAIRSAYPQIPNPPVALTIAHNPKFGDYQCNSAMAISKMLQKLTGEKIGPHVVAKTIV
ENIPSNSFIKKVDVAGPGFINLHLEKEFLKGVLEKVIKEKVYPPGIGNKKKVVIDFSSPNIAKEMHV
GHLRSTIIGESLSRLLEFVGHDVLRLNHVGDWGTQFGMLIAHLEDKYPDYKTVSPPIGDLLLFYKA
SKKRFDDEEDFKKRAYQRVVDLQGGNPDVRKAWQMICEVSRNEFQKIYDRLDVTLIERGESFYN
DLMSKVVENMKKSGICQKSDDGRWVAFAPNMKVPLIILKSDGGYTYDTSDMAALQQRLQEEHG
DWLIYVVDAGQGDHFKQIFGVGEAAGWYNSSITRVDHVAFGVVLGEDKKKFKTRSGDTVRLIDL
LDEGLKRSMEKLLEKGRDKELTKEELLAAQESVAYGCIKYADLSHNRVMDYVFSFDKMLDDKGN
TAVYLLYAYTRVRSIQRNAKKGRSEIEEYMKSNSLHFDHEKEWKLAKLLCRYPEIVVRCLDDLTLH
LLCEYLYELSSTLTEFYDACYCIEKNRETGEIISVDMNRIALLESVARIFEAGFHILGIKPISRM*
>TRINITY_DN3202_c2_g1_i1.p1 type:5prime_partial len:263 gc:universal TRINITY_DN3202_c2_g1_i1:949-161(-)
WSQGNRGCYIHTQEVARGNNARRHMCWVFSKCSKNPDVNLKSCPIESGLCVKENGLDQNSGV
RKLHHYDVTTPYAEADCFKRCSTYRYGEITGCEVIWNQGNRGCYLHSQPISRGNGIARHMCWVF
SKCTTSKVPATTAPKALSMTDTYFGKTELDPIIGVKRIGCYRMHRHRFRKKVNMKGSMDPLSFIDR
VKQCRDIVNNKEAEFEIFGVSRYGSQCDFYLPAEVKNEKPKPDSEDCHFDTMGGDTAYYVYKLCK
DEKQC*
>TRINITY_DN2230_c0_g1_i1.p1 type:complete len:249 gc:universal TRINITY_DN2230_c0_g1_i1:796-50(-)
MKLNVAYPANGTQKLLEIEDEIKLRSFYEKRMAQELSVDFLGDEWKGYVVRITGGNDKQGFPMK
QGVLTPGRARLLLSKGHSCYRPRRTGERKRKSVRGCIVDGNLSVLNLVVIKKGDNEIPGLTDRVIP
RRLGPKRVGKIRKLFNLSKEDDVRQYIIRRPIATPEGKPDPKKPKTKAPKIQRLVTPQRLQRKRHVK
ALKRERANKAKLEAATYAKLLAKRHKEAKEKRHQEHMKRRRLSSLRESKSQ*
>TRINITY_DN197343_c0_g1_i1.p1 type:complete len:124 gc:universal TRINITY_DN197343_c0_g1_i1:399-28(-)

MAKLKAHELRGKRKEELCHQLTELKTELQQLHVAKVTGGAASKLSKIKIVRKSIARVLTVISHNQ
RENIKKFYKTRKYKPLDVRSKKTRAMRRALTTHEASRKTLRQIKKDKHFAIRKYAVKE*
>TRINITY_DN1505_c0_g1_i1.p1 type:complete len:154 gc:universal TRINITY_DN1505_c0_g1_i1:98-559(+)
MSLVIPEKFQHILRVMNTNIDGKTKIMYAMTSIKGVGRRFSNICLKKADIDMTKRAGELSDEEVER
LVTIMQNPRQYKIPDYFLNRKKDIKDGKFSQVLANGLDNKLREDLERLKKIRAHRGLRHYWGLR
VRGQHTKTTGRKGRTVGVSKKKN*
>TRINITY_DN4329_c0_g1_i1.p1 type:5prime_partial len:735 gc:universal TRINITY_DN4329_c0_g1_i1:2240-36(-)
LTFIESLRLSNNMKEKLDYVVLCFFSWMVVGSCVDRGNFKTCKDSSFCRRNRAIQAGGSPFALLS
DTLVFGSDSITGDIYNTNTNVSFKLTIDTLVKETARIRITELSPIRPRYIVQESLVREPQHIKYEKVKSD
SDGFKVKFGSSLMQVTFKPLKVDFFDAEGVLLMSLNNKGLLNFEHYRVKNSSPQDSVEKTNESEK
GEDADQNSENEGKENTEEEKPENTNTVREDINEEGMWEETFKDHTDSKPYGPSSVGFDVSFIGFE
HVYGIPQHADSLSLKQTSGGEPYRLYNLDVFEYELNNPMALYGSIPFMTAHSAYKTVGIFFNNAA
EQWIDISSSKSVLSSLISFFKSGDTPEVDTHWMAESGIIDLFVMLGPKPNDVLNQYSTITGTQTLPP
MFATAYHQCRWNYNDEDDVKNVDAGFDEHDIPYDVIWLDIEHTDGKKYFTWDALKFPDSKRMI
DHIASKGRKMVTIVDPHIKRVGGYRIHSEAESKQFYVKKKDGADYEGWCWPGSSSWIDYTNPDA
RNWWASKFFLDQYQGSSLDLFTWNDMNEPSVFNGPEVTMHKDAIHYGDWEHRDVHNIYGML
QHMSTFEGHLLRSDGKARPFILSRSFFAGSQRAGAHWTGDNTADWGHLKMSIPMLLSLNVAGFP
FVGADVGGFFGNPDSEMLVRWYQMATFFPFFRAHAHIDTRRREPWLFGEENTQLIQQAIRKRYM
ILPYIYTLMRNSFDTGMAVMR*
>TRINITY_DN1194_c0_g1_i1.p1 type:complete len:555 gc:universal TRINITY_DN1194_c0_g1_i1:101-1765(+)
MAARLGGGMMQPQIILLKEGTDSSQGIPQLISNINACEVVAEAIRTTLGPRGMDKLIVDKNGKSTI
SNDGATIVNLLDIVHPAARTLVDIAKSQDAEVGDGTTSVILLAVEFLHQIKSLVEENVHPQILIKGY
RKATKMALEKIKEINVQIKKDDQEEMRKLLQRCAETALSSKLVAGHKEFFGKMVCEAVMHLDEL
LPLNMIGIKKVKGGALEESQLITGVAFKKTFSYAGFEMQPKKYKDPKIALLNVELELKAEKENAEV
RVDNVEEYQKIVNAEWNILYEKLEKIVASGAKVVLSKLPIGDVATQYFADRDVFCAGRVVEEDLL
RTMKACGGSIQTSIHNLVDNVLGTCEDFEEVQIGCERYNLFQGCPRSKTVTLILRGGAEQFIEETER
SLHDAIMIVRRALKNDAVVAGGGAIEMELSKHLREHSRSIAGKEQLIIAAFAKALEIIPRQLCDNA
GFDSTTVLNKLRQKHAQGGKWFGVDILKEDIADNFEAFVWEPAIVRINALTAASEAACLVLSIDE
TVQNPKSDMSGDDNPITGKRGRMPQMGR*
>TRINITY_DN1377_c0_g1_i1.p1 type:complete len:878 gc:universal TRINITY_DN1377_c0_g1_i1:68-2701(+)
MTGRARGRSRGRGRAAQDASQRPGGAQGGPPPQAAAPQQGGGGRGRTRGQPAPQQVAPQQR
PAAQTAVPPTARMAQMSVKDQKPATEERQPRRRRLDPAPDVLVTRPEGRTKEGTGGTPVNIVSNF
FKVIKMPNFKGLYQYNVSFDPEINSSKLKNALLYQMADVLGQTKCFDGMTLFLPKRLPDQITTRS
VTTHQGNAIVIKIMFTNVIPEISPQVLQMINIIFRRQLRCIKMELIGRNYFNSSRKIEVKAHKMEVW
PGFSTSILQYEKDVMLCADVQHKVVRQQSVLSMMYELHQKTREKNRFYELAAKKLIGETVITRYN
KKNYKIDDIEWNKNPNSTFDKNGTQITFKEYFEKTYDAKVGDDQQPLLVSLPKAKNRKSGIVGPI
LLLPEFCLISGLSDEIRSNFSIMKDLAQHTRINPAERAKNLEQFVQDLQTNEESKKELEQWNLKFES
KLLQMTGRAYGSEKMFQREDQFSYKIQDADWSRETRGKKLIHTVNMTDWLLVYYERDEPIATDF
KQTLVKVCGPMGMNVAEPQLCRLKDDTARSYFRALSEQIRPTTQIVVCIVPNNRKDRYDSIKKLC
CVEKPIPSQVIVSRTLSKKQMLMSVCTKIGIQLNCKLGGEIWAVDIPISKNPKDPPSLMVVGFDVYH
DSASKGKSIGGFIASTNRHLTKYYSRITQQQSHQEIADQLRVCMTGALKKFNELNGTMPSRIIVYR
DGVGDGQLRQVFDHELPQFKAAFNAISQGYDPKFTMIVVKKRISTRLFLSERGMKTNPLPGTVVD
TVVTRPEWYDFFLVSQSVRQGTVSPTHYNIIADTSGWKPDIFQRLTYKLCHLYYNWPGTIRVPAPC
QYAHKLAFLVGQSIHAEPDVQLADRLYYL*
>TRINITY_DN2637_c0_g1_i9.p2 type:5prime_partial len:340 gc:universal TRINITY_DN2637_c0_g1_i9:3-1022(+)
GYCARDALVACRAQLSDAGVPSAEETDAHIEELKAAAQRLVAGAAAAVVFATTAAGDAFAYPIF
AQQNYKEPRDATGKIACANCHLASKLIEAKMPSEVLPDTIFKATIEIPAKYEKRYQPIADGSKAKM

NVGAIAIMPEGWKLAPRDRLPKALKKEMKGLAWAPYSKEQPNILVAGPVPGERYETMVLPILAP DPATNPNVVFDKASIFFGGNRGRGQVYPEGNQSNNNQFFSAATGTVLAVEGTKVSVTLADGTVA TQEVLPGATIVVKAGDAVQKDQPITTNPNVGGFGQAEKEVVLQDMSRVYAYCALSVSIFLSQLSF VLKKKQFEKVQLAEGF*
>TRINITY_DN72959_c0_g1_i1.p1 type:complete len:216 gc:universal TRINITY_DN72959_c0_g1_i1:54-701(+)
MGRRPARCYRYCKNKPYPKSRFCRGVPDSKIRIFDLGKKKTGVDDFPKCVNLVSDEYEQLSSEALE AARICANKYMTKTAGKDAFHIRIRVHPFHIVRINKMLSCAGADRLQTGMRGAFGKPQGTVARV HIGQPLISVRCKDQHEATAIEALRRAKFKFPGRQKIFTSKKWGFTRWDREEYLERRQDGSIKPDGT NVQYYPCKGPLKDWKWRESR*
>TRINITY_DN535_c0_g1_i1.p1 type:5prime_partial len:162 gc:universal TRINITY_DN535_c0_g1_i1:712-227(-)
GKYLVFFFYPLDFTFVCPTEIIAFSDRVEEFRALGCDVLACSVDSHFSHLAWTQQSRKKGGLGQMK IPILSDLTKSISRDYGVLLENDGIALRGLFIIDDKGILRQITVNDLPVGRSVDETLRLIQAFKFTDEYG EVCPAGWKPGADTINTGKAHEYFEKQ*
>TRINITY_DN167384_c0_g1_i1.p1 type:complete len:316 gc:universal TRINITY_DN167384_c0_g1_i1:1004-57(-)
MDQLTLGGTLKGHNGWVTQIATTPQDPNQILSASRDKTLILWELTRDDQMNYGVPKRSLTGHN HFVSDVVMSSDGQFALSGSWDKTLRLWDLSAGITTRQFVGHTKDVLSVAFSADNRQIVSGSRDNT IKLWNTLGQCKYTIQDESHSEWVSCVRFSPNTQNPIIVSCGWDKMVKVWNLTNCKLKTNHFGH AGYLNTVTVSPDGSLCASGGKDGKAMLWDLNEGKHLYTLDGGNIINSLCFSPNRYWLCAATGPS IKIWDLESKSIVDELKPEIISISTKAKAADCISLAWSADGQTLFAGYTDNLIRVWQVTRM*
>TRINITY_DN72625_c0_g1_i1.p1 type:complete len:1964 gc:universal TRINITY_DN72625_c0_g1_i1:173-6064(+)
MKLAFNIFTISLLIGNTIAQNNDVVQVCKSQADVVAVLDSSGSVGVQDYEELKKFVGFVANLLTIN ENGNRLGVVTYSETAEVDFNLNEIYDSDQYGREALTRLSVRPREFKTNHYAGLKVGSDLLFNKAA GARDNAAKFLIFTTDGRNNFDETNNRNRASQLATQLKADGVNIYVIKAEVMSDNNGRLDSRLYS TDPNKFYRFINRFGEITNENFLDEVGLEFCELVQLPDNQQATVREDRNIGTLVTTIRAPGLNNPTFE LIPQDNLPFIVDPLTGEIKTIGLLDFEQVQQYVFQVRVRATNPDVEKIITVTINVLDVNDNDPIPETA NYRMSVAETTPLGQRILTVKANDPDGGTNGNVEYFLISGGDGKFEVDQATGEIRLIGQLNKRIKD TYLLTLEARDMGIPTRKAPITVAIDVTSLNNFPPQFNEMSFVRTIPENFGAGTVINAAATDRDGDT MVYSLIGENIPFSIDQNGLITVSGNIDFETIPNNVFQFQVQAEANGDLVTQDVIITITDINDNVPVYS MTTYSGSINENSAAGTVVNFDNVIIATDADKTNANNMITYNIIDGNDGDRFQVTKNERGEAVISL KSTPLDQETQGQYRLTLQAIDNGVDPRSLEMNSLLIIDVIDQNDNDPIPEESVYSIAVAETSPVGQSI LTVRANDADSGMNGKVEYFLISGGDGKFQVDQATGQITLIGMLNKRVKESYTLNIEARDMGVPT RNAPITVNIDVTSLNSFLPEFAGAGDAFERTIPENFGSDTVIKAAATDGDGDMVFYSLVGDNLPFA IDQNGMITISGSIDFETIPNNPIRFQVKAEANGDMTLKDVVITITDVNDVTPEFTQQVYAGKIEENS QTETVVRFDQPVIATDGDKSPANNVVTYRIVDGNVDNIFKIVKNAQGEAVISVNNAGLDREILNQ YTLTIEAKDNGVDPGSLSSTTQVIIDIEDVNDIPPLINTSENKAVGEADFTATISEDSPIGTSVVKVEA TDPDSNNDDLIFTIANDVPVPFTIDPKTGLITVSGPLDADDQDEFEIAVTVSDGVNNSTAIVKVNIA DLNDNKPIFTTDTRDITIPLDTPNGQEIIKFTGSDADKTSPNNQFEFSLVDGDGKPFTITPDGSLIIN GPLMPGDYMLTVKIMDKGTNPGPLSNVIIYNVKVVDTNDPPTFQNNEQTVTISENTADGTDVITF AATDPNGDAITYEIVNPGSVPFEIDPITGVIRKMGLIDFESPVRSYTLDVKATDAKGKSTMGRVFVQ ITDFNDNTPTFGPGNTDNFILTEGVYSNQLVSSFTATDLDATSPNNVFEYTLVGAPSMFLLTTNPNT GAADLTVNGDIDAEQNMSFNFIIRATDKGNPALSMDYPVTITVGDINDNAPVFAANTPESIDLME GDFNNQIVATFTANDDDTTAPNNLVDFELVGAPDFFKLIVNPETGTADLVIDGNVDIDGLDPTINF IVRAKDGGNPALVKEFPLTVNIADINDNAPVFTLEAYNQVINENTPIGTKVVEVRATDSDSGPNG DITYRIVDGDGTFGIDPKTGDITVLKNTDFESGVRSYEFTVEASDNGNPKQSATAKVKIDIRNLND NDPIFTSIPITGNINENRPAGSVVQNIQPVNGILNQNNVKILREPPPGAQETEGVGFDAPFEIDPVT GEVKSTRPFDFEEEKVFTMMVQAADGDNNPMLTTMTVMVNNLNDNDPIMKKANFEITLNQDT PVGTFVLQVQATDIDQENQTKITYSIINIDGGNMNDFTINPDTGVITLNTDLSMNTVDIYNITIQAE DQEIQGVIRRSTALAEIKILRPAEAEPDISTLCTNSCDIPEERLKCLDYSKFEITRKFNGTCYVDKLKA

AIADVKNNTMEIMNLNASFLECRNKVQNEITNPNTQFPVNGLFDLHRKCTVAIECKLEQLAFNL
MRKTTELTNNLKAEEEGFSMYDMSLSAFFQHISKMVSMSKSSRVD*
>TRINITY_DN1559_c0_g1_i1.p1 type:complete len:311 gc:universal TRINITY_DN1559_c0_g1_i1:221-1153(+)
MAEKKATFKLGFAENFALSGAAAVISKTAAAPIERVKLLVQNQDEMIKQGKLSEPYKGVIDCTTRT
FKTEGLAPFWRGNLANCIRYFPTQALNFAFKDKIKAAFKVSKSDGYALGFGKNIASGGCAGALSL
CFVYSLDFARTKLANDTKSSKKGGSREYNGLVDVYRKTLKSDGVAGLYRGFVLSCVGIFVYRGCYF
GFYDTLKPVLLGADASLFMSFILGYGVTVTAGLISYPIDTIRRRMMMTSGQAVKYKGSIDCATQILK
NEGAMSLMKGAGANILRGVAGAGVLAGFDKFKEVYIAMRKRQMGLE*
>TRINITY_DN64_c0_g1_i1.p1 type:complete len:468 gc:universal TRINITY_DN64_c0_g1_i1:1555-152(-)
MPPKEAEKIHINIVVIGHVDSGKSTSTGHLIYKCGGIDKRAIEKFEKEAQEMGKGSFKYAWVLDKL
KAERERGITIDIALWKFKTGKYYVTIIDAPGHRDFIKNMITGTSQADCAVLIVASSTGEFEAGISKNG
QTREHALLAYTLGVKQLIVGVNKIDNTEPPYSEKRFDEIVKEVKTYVKKVGYDPKTVAFVPISGWH
GDNMLEASKNMGWYKGWTTERKETKTTTGKTLLEALDNILPPKRPTDRPLRLPLQDVYKIGGIG
TVPVGRVETGIIKPGMVVTFAPSNITTEVKSVEMHHEALLEAVPGDNVGFNIKNVSVKDIKRGNV
ASDSKNDPAKEAKSFYAQVIILNHPGEIHSGYQPVLDCHTAHVACKFSEIKQKIDRRSGKVLEEMP
KMVKSGDAAMIDLTPSKPMCVEAFSEYAPLGRFAVRDMRQTVAVGVIKSVTKSDGSGGKMTKSA
QKATGKKK*
>TRINITY_DN119684_c0_g1_i1.p1 type:complete len:662 gc:universal TRINITY_DN119684_c0_g1_i1:2182-197(-)
MKLEIILFTLLVALTVHCSEKKDDSKKDVGTVIGIDLGTTYSCVGVFKNGRVEIIPNDQGNRITPSYV
AFTEEGERLIGDAAKNQLTSNPENTVFDAKRLIGREWGDKAVVHDMKFFPFKVVEKGKKPHVQ
VNVKENEKKTFAAEEISAMVLVKMKEIAEAYLGKKVTHAVVTVPAYFNDAQRQATKDAGVIAGL
NVMRIINEPTAAAIAYGLDKKEGEKNILVFDLGGGTFDVSLLTIDNGVFEVVATNGDTHLGGEDF
DQRVMDHFIKLYKKKKSKDIRKDNRAVQKLRREVEKAKRALSTQHQARIEVESLFDGEDFSETLT
RAKFEELNSDLFKSTMKPVQKVIDDSGLKKKEIHEIVLVGGSTRIPKVQQLVKDFFGGKEPSRGINP
DEAVAYGAAVQGGVLGGEEDTGEIVLLDVNPLTMGIETVGGVMTKLIGRNTVVPTKKSQIFSTAA
DNQQTVTIQVFEGERPMTKDNHLLGKFDLNGIPPAPRGVPQIEVTFEIDVNGILRVSAEDKGTGK
KEKITITNDQNRLSPEDIERMVNDAEKFADEDKKVKEKVDAKNEFESYAYSLKNQISEKDKLGGK
LSEEDKGVLEKAVDEAISWMDSHQDASVDELKEAKKKLEDVATPIVSKIYQQQGGAPPPPGGEG
GEEKEDKEEL*
>TRINITY_DN15274_c0_g1_i1.p1 type:complete len:1822 gc:universal TRINITY_DN15274_c0_g1_i1:87-5552(+)
MECIRRSIIRLLLILGFYTLYISSQEECKRGGCFPATGDLLVGREKQLLANSTCGLNGPSKYCILSFLD
KTDKCFYCDSRRQVDERPDGYKNSHLPKYMVTREPSKRLQGWWQSVNGRQDVTIQLDLEAEFIF
THLIMTFKSFRPRAMYIERSSNYGNDWSDYRYFAYNCRKSFPNIPRGPQRKIDDVICEERYSKVEPS
TGGEVVMKVLDPIIQKKEDPYSLKVLNLLKLTNLRVHFKELHTFGDDILGDSPKVNQKYYYALYE
MVVRGSCSCYGHAEQCIPTKGTPANVNMVHGQCNCTHNTKGRNCEMCKDGYNDEPWRPAYQ
DQLNVCRKCNCNGHTDKCHFDPAVYKATGNTSGGVCDDCNHNTAGRQCELCKPLYYRDPYKN
IFDEDVCKPCDCDPSGSFGGGECETISDPNNSSALTVAGRCICKPNAQGLRCDSCKDGYWNLLES
NPDGCQSCNCDDKGTVELNKCDQQTGTCRCKRFVIGLTCSKCLEGFWNLGNTPEGCEPCNCDV
GGSLNAKCDDITGMCMCKPNIIGRKCDQVAPGSYFGNLDQVISEAENSLVRSDSSSVEIVREILNPT
DSRSFTGDGYLRVSDGTLVNFTNIRVPSTGFQTFVMRYSRPTPGSNNNVMVRITKTDGTILGGNGC
VQTNEREIILNGVLNDDNFLELGDMFCLEKDTDYSVQLVVSNRNSPNDGGVNRGVTNLDSIVM
MPNIDNLAIFQGREGMRLKSQYDLNNCRQYYLEPNGAANAPEICKKIQFSISAEINNGALKCNC
DPTGTNSSGSSDILTCQKSGGQCPCKPGVVGRRCEKCAPGYFGFDSNGCTACACNDTGSEKLFCN
GITGQCPCKNRIKGRQCNSCPSNHFGFPNCIACQCNEHSSTCDPISGECTNCKDNTAGPSCNLCA
EGYYGDATAGTEKDCQRCQCPGGSSLNQFSNTCHLKPDGKVVCNNCTEGYTGDQCDKCEDGY
YGNPLVPNGSCNKCDCNGNIDPFADGKCNAENGKCFNCINNSDGDHCENCKEGFFGNAKNQS
CRACVCNKLGTNSATSDSCDRETGQCECLPNVEGVECDRCKSGYYNIASGNGCVSCNCDKGGSL
SSHCNQLDGQCNCLNGYGGLDCTQCEDFYFGDPKQNTCQACGCNPVGSITLQCNRTTGVCMC

KDNVVGEKCDMCAPQTAGVMPQCEECHVCNDQWEDIVNDIEKNITTILTLKGSSPLNVSEIRAY
TNDIADLKNRLDKIEEDFGKQMVDPNKINRLRDKVYRFSYNLTGINRDADFYMEMVNETITRDE
KANDILDGLELRLATLQTNTEDFKNNVTKIVESNIGGALNSTRESLEKSRKAQESVNDSDLKIKES
KKMRKKIKKEIQNGDPEFEVMHNRSSEFVNNLADRENYLEGELEKLNRLLCGSDKRCGGCTKGG
CDTCGVSGENYTCDDGIKMMSMMASDKALEAKEAIDQKLENASELLENVNRAKQHVIQANISA
NMAEDKAEEVNKIGQAVLKNITTLIKDIQDFLATDFNHPNASKDLAMQTIALNISLTPDEVVELG
RKIRDAVKSLTGVDKILNESRNDYDTAIGLKEQALAAKERAIEVMNQTMAIIETLSQAEELQNSTL
AGVNVARADIEDVKDLLKKLKEKLDAADNDLQMADNSTASAEDKRNAILDQNLENREKLDLSE
KKIDEAEELGDKVQGSVDKMNEKYNATKAKLNEKEDMIEKVRARIKALADKTIKLYENADRKLR
RINKMKDSYIINEERAKDLYYEIRRLEIKEFFINTKLEGTAKCYSTCNPIIPESFCRDVYKNIAVKETN
AYAELEAEKASTTSLLI*
>TRINITY_DN118520_c0_g1_i1.p1 type:complete len:581 gc:universal TRINITY_DN118520_c0_g1_i1:1895-153(-)
MYRLLSVIRKTTQTAKLRQGLSACISTTTHYCSPKEIKTGTDARALMLKGVDQLADTVAVTLGPK
GKNVMIEQSFGGPKITKDGVTVAKAIELPDKYMNIGAKLVQDVANNTNEEAGDGTTTATVLARA
IARSGFEAVSKGANPNEVRKGVMAAVEVVTQGLKDLARPVTTTEEIAQVASISANNDAAIGKLIS
DAMEKVGKDGVITVKDGKTVYDELEVIEGMKFDRGYISPYFINTAKGQKVEYQNVLVLICQSKIS
NVQDIVPVLELANQHKRPLLIIAEDVDGEALTALVINRLKIGLQVVAVKAPGFGDNRKNTLKDVA
ISTGAEVFGDDLNLKLDEVQLSDLGQIGELVVTKDDCLMMKGRGNTDLVNQRANHIHDEIKATN
SDYEREKFQERLAKLVGGVAVIKVGGSSEVEVNEKKDRVTDALNATRAAAAEGIVPGGGTALIRC
AKLLDTFQVPTEDQKWGVDIIRAALFKPCSTIVANTGIESAVVVEKVSSSKDPEYGYDAASGEFAN
LIKAGIIDPVKVVRIALSDAAGVASLLTTAEAVITEIPKEDPSMPPMGGGMGGMGGMGGMM*
>TRINITY_DN899_c0_g1_i1.p1 type:complete len:375 gc:universal TRINITY_DN899_c0_g1_i1:70-1194(+)
MVFRALLALSFCVASRGFVLPYIGWKLPSMQIPSLDMSMQFPSIFGAEDPLGQAMESNTEAAMDR
EFNMDDNMPSYKYAFKMPEVNTTSNDTKTSRKVKHKDGYKIITTETKFPGGQSVMQTMEKEGN
DTGKSFSMSNLEESIGESDLSKMNQFIPTETKGKCKCGKDEFCDPLFGKCHKKLPEGATCAMKKQ
CKAGLMCVWGKCNKGKAGDPGTFCKKKEECKGEACCVKKPGQYHAICTPLGAETAICGNKME
GFLNIFQVKQVKETCNPCKKGLKCATTGDSTYKRCLPESFVEEPKLEDKKGKDDDKKDDKSDGK
DDGSSDEDSSDDDDDEKSKLSTNAAGKIPIKLDPPNKSAESKKKKPKSKREKNAE*
>TRINITY_DN1614_c0_g1_i1.p1 type:complete len:218 gc:universal TRINITY_DN1614_c0_g1_i1:56-709(+)
MHKTKICIWLVVRYRSNPFRGYNRRPDLKSAGAKMTKYSTEPENPSKSCKARGSYLRVHFKNTHE
TAQAIKKMHIRKAYRYLKDVVDMKQIIPFRRFNGGVGRKAQAKAHGCSQGRWPKKSAQFLLQL
LKNAESNADLKGLDVDSLVIDHIQVNRAPYMRRRTYRAHGRINPYMSSPCHIEMILAEKEQVVPR
AEEEVEVKKVSKKKLAREKLKSRE*
>TRINITY_DN11097_c0_g1_i1.p1 type:complete len:730 gc:universal TRINITY_DN11097_c0_g1_i1:2348-159(-)
MVRPQGVTQEHSSKPSTSERNSFVFSALSKSGCFETLPSIYDSIIIQRSHWLHTEGFSMRTSSTGSQQ
TSRRAFYEELQAFSSTIPFYESLSWALLSLSGCLNPVSTPNIREEILADLEGRSVDESREVLLVFATLFD
VYKSYQNSIVVEISAILPGGILRCISLGQTEGFNSYNTVGLINYQPIVYPVGRNVLGRLFNVTGSCID
LYNEVTMDMPFVRSVSTEETHTPQKTTLIKTGANADTSSDSDCVKANALFKECTSHINVKETSLD
HLISLYCGCYMGMTSAPIAPISRLPIEAESSRAPEHMVQKLQECMGTYSGSADPSTPNQIGSKKQPF
WLSKEALESGVSPIHSTPLPISKLRVDLSLFETGIKVVDLLTPYKKGGKIGLFGGAGVGKTVVIMEFI
RNLAIEHKGISIFCGVGERTREGNDLYSEMAEAGIICKDYTTMKPSDPSSNGGPTGSIDQHSVTFTR
SSVTLVYGQMNESPGCRMRVAHAGLTVAEFFRDVFKQDVLVFVDNVFRFLQAGSELSTLLGRMPS
AVGYQPTLATEMGAFQERIIAALWGSITSIQAIYVPADDLTDPAPVTIFTHLDAVSSLSRELAAKGIY
PAVDPFNSTSRMLDPQCLTSEHFRVALEVQQILQRYRELQDVIAILGIEELSEEDLKTVERARKIERF
LSQPFSVAEVFTKIKGIFVPLEATIRSFAMVLSGALDDVPEGNFYMKGGLDTLKL*
>TRINITY_DN73067_c0_g1_i1.p1 type:complete len:831 gc:universal TRINITY_DN73067_c0_g1_i1:3714-1222(-)

MHWFVILLLFGVIFVTVSNYEIDVLSTFELSEDIAGISPLDNKYQAYQFAADGKELLLDDSIKTEIVR
GVTNFKDFFIAVNIRHDKQKPSSLLSLQTKTTGKQKFTLWINAEEEKIGIRSHSKESKKGKKNLTFR
KVPLNTGQWHRIVLNFKKIDTDEPVVDLYVDCKFHDSKTFPFSIGHALIEDALISELRLGQLKHLP
EKDPIRFIGAMQDFRLVSNRDVHWYNRPQHCYNMMIEVKGMKAYHEPVSTISHDVDNMAAAM
KNMQKQLKLQLDEIRDIKNWLQQCTACKTAAENVLGGEIPKACSQNRCNENANCIESNGGSAF
RCECKAGYAGNGEKCGLDSDSDGVPDNKLDCLGKECSQDNCPGFPNAGQEDADEDGSGDACD
IDDDNDGVLDGDDNCQFLSNRDQKDSDADNRGDACDNCPFESNYNQLDSNNDGLGDACTRD
ADGDGIRDKSDNCPRVYNRDQIDRDSDGIGDACDNCIDIYNPIQKDVDNDNLGDACDSNVDID
NDGIQDNKDNCLYIANANQQDIDNDGIGDACDPDDDNDNIIDENDNCRLIYNPDQSDSDGDG
QGDACEDDADKDETVDLKDACPENPEIQNTNFTNYKHIMLDPEGTSQVDPEWEILNKGAEIKQ
WRNSDPGIAIGDKVFGGVDYSGTFYVHDDKDDDFAGFVFSFQSNSKFYALMWKKDKQTYWFET
PYKVTGHAGIQLKVVTSSTGPGEHMRNALWSSISIPGQTKIIWSDPSKEGWKPKTSYRWTLMHRP
SEGLVRITIKEGSQVRADTGYLIDKTYKGGKLGVLVFSQKEIVFSALQYRCTEQIPADYNPNSPN*
>TRINITY_DN477_c0_g1_i9.p1 type:complete len:306 gc:universal TRINITY_DN477_c0_g1_i9:1840-923(-)
MTRQCLGATLNENCGNAALVQFEVCNFQVPCPGLLTNWGPYTQCSATCQKDANSMPTQSRQR
FCTGGSFGGNCGGVQLLQTIGCNQLIICPVYGYWLQWSAFGECSKTCDQGTHVRYRNCTKSIPA
NAGNNCTGPAQEVKPCQVKPCPVTCPLTKKCDCRNMNYLAAAPGISQTVTNDVMGVAAFLDY
WTLDILKQICEGCRMTQLDQSKATVDSQILRTQAVLDRLNIEKRVYRDMITCNENPYITDDLFKVY
NDIWDRIVTTDGVMIELKASKMRFDTAITACRSYDWYHDLFGRFFQIKKSS*
>TRINITY_DN168534_c0_g1_i1.p1 type:complete len:226 gc:universal TRINITY_DN168534_c0_g1_i1:774-97(-)
MIRSAIIAIVIASHLYESSAARCYVGYSAVGLPLAIGVRDVDCQKGMDFCTTVKSTIGGITVTQGNC
ARKYGQATCQFMQNSHKAMTDCKETLCRGDYCNSALIEAEQKERKRVGPYKNTLKCTTELKTR
FPLIPFATADIPIGIDECFPGTTRCVTVEYDYIYKFKTKIATRHNHFVLPVIQKMCNRPNIDDCNKW
CTIMSIHQFMHSSCRAKCCTGDNCNAKD*
>TRINITY_DN15599_c0_g1_i1.p1 type:internal len:206 gc:universal TRINITY_DN15599_c0_g1_i1:1-615(+)
KALTSSDPAERTKKLQAELANGRLAMIAIIAMFVQNGITGTTGPEMWRGGGAFENELGVQAPVG
FYDPVGFTLDGDVQSFRRRRSVELKHGRISMLACMGYLTPEIVGKWPGYISPSLGVKFADIPSGLD
AIGKVPALGVAQIVAYFGFVEFAAGFDDYKPGGTPGDYGWKALTSSDPAERTKKLQAELANGRL
AMIAIIAMFVQ
>TRINITY_DN58867_c0_g1_i2.p1 type:complete len:354 gc:universal TRINITY_DN58867_c0_g1_i2:80-1141(+)
MVSLYGDMMFNIENGYLEGLVRGFRSGILNRTDYLNLIQCETLEDLKLHLQSTDYGNFLSNEAGP
LTVSVIDEKLREKLVIEFDHLRIQAVEPLSQFMDYITYSYMIDNIILLITGTLHQRSISELIPKCHPLGR
FPEMASINIASTPAELYNAVLVDTPLAPFFVDCISEQDLDEMNIEIIRNSLYKSYLESFSRFCDSLGGT
NSEVMKTILQFEADRRAIMITINSFGTELNKDDRSKLYPKCGKLYPDGLARLARAEDYDQVRQVA
DCYAEYQPLFEGTGTNPGDKTLEDKFFEYEVKLNVNSFMQQFHFGVFYAYVKLKEQECRNIVWIA
ECISQRNKSKIDNYINIFE*
>TRINITY_DN1172_c0_g2_i2.p1 type:internal len:264 gc:universal TRINITY_DN1172_c0_g2_i2:790-2(-)
GDYGFKVLTSSDPAEKEKKLAAELANGRLAMVAIIGMFFQDGLTGSAWGDWALYTASPLRFFGG
DSGPSYKAFDPATELGVQEPLGFWDPLGFTSDNDEAAFKRRRSVELKHGRVCMAASIGYIVPNYY
TFPGFLSPSAGVKFADVPNGLAALGKVPALGWVQIIFFAGIVEGAFGFDDYKSGAPGDYGWKVLT
SDDPAEKDKKLAAELANGRLAMVATIGMFFQDGLTGSAWGDWALYTASPLRAEPVSTAGVGAA
TKGGTE
>TRINITY_DN21847_c0_g1_i1.p1 type:internal len:384 gc:universal TRINITY_DN21847_c0_g1_i1:1150-2(-)
LSADGDVTAFRRRRSVELKHGRISMLATMGYITPEITGKFPGYLSNSMSLKFADIPNGLAAISKVPA
LGWAQVVAYCGFCELSAGSSDWKTGTPGDYGFKVLTSSDPAEKEKKLAAEVANGRLAMMAIIG
MFFQDGLTGSAWGDWALYDASPLRAFENELGVQEPLGFWDPLSLSADGDVTAFRRRRSVELKHG

RISMLATMGYITPEITGKFPGYLSNSMSLKFADIPNGLAAISKVPALGWAQVVAYCGFCELSAGSSD
WKTGTPGDYGFKVLTSSDPAEKEKKLAAEVANGRLAMMAIIGMFFQDGLTGSAWGDWALYDAS
PLRAFESELGVQAPLGFWDPMGYVTDGDVEAFKQYREAEVKHGRVSMLACIGYIVPEYF
>TRINITY_DN4497_c0_g1_i1.p1 type:internal len:160 gc:universal TRINITY_DN4497_c0_g1_i1:1-477(+)
NELGVQEPVGFWDPVGFTNDGDVTSFKRRRTVELKHGRISMLATMGYITPELTGKFPGYISNSLAL
KFDDIPSGLAAISKVPALGWAQVVAYCGFCELSAGSEDWKSGTPGDYGFKVLTSSDPAEKEKKLA
AEIANGRLAMVAIIGMFFQDGLTGSAWG
>TRINITY_DN478_c0_g1_i2.p1 type:complete len:152 gc:universal TRINITY_DN478_c0_g1_i2:99-554(+)
MADERTFIMVKPDGVARGLVAKIIKRFEQRGYKLVAMKFMQASEDLLHQHYGALKDLPFFAGLIK
YSSSGPVLAMVWEGRGVVLQGRAMLGETKPSESKPGSIRGDYSIDVGRNIVHGSDSVETAQKEIKL
WFKDEELVAWENPLDHAIHE*
>TRINITY_DN40728_c1_g1_i1.p2 type:5prime_partial len:124 gc:universal TRINITY_DN40728_c1_g1_i1:1-372(+)
KTLTGKTITLDVEASDTIDNVKAKIQDKEGIPPDQQRLIFAGKQLEDGRTLSDYNIQKESTLHLVLR
LRGGVIEPSLAVFARKYNCDKMICRKCYARLPARAVNCRKKKCGHTNMLRPKKKLK*
>TRINITY_DN195631_c0_g1_i1.p2 type:internal len:145 gc:universal TRINITY_DN195631_c0_g1_i1:2-433(+)
EEWRCMYRLDLPREDADALLIEHWVEPEAADGLGIVLHTLGQVRNSTEDDWLDVELHLVANEL
MILGAGGGARGRELTNALQEVAAPRDGGTMQIFIKTLTGKTVTLDVSASDTIDAIKAKIQDKEGIP
PDQQRLIFAGKQLED
>TRINITY_DN8227_c0_g1_i1.p1 type:internal len:135 gc:universal TRINITY_DN8227_c0_g1_i1:404-3(-)
GPGRAGGRWKPPGGGGAAARHGAAMQIFVKTLTGKTITLDVEASDTIDNVKAKIQDKEGIPPDQ
QRLIFAGKQLEDGRTLSDYNIQKESTLHLVLRLRGGVIEPSLAVLARKYNCDKMICRKCYARLPAR
AVNC
>TRINITY_DN152_c0_g1_i15.p1 type:internal len:141 gc:universal TRINITY_DN152_c0_g1_i15:2-421(+)
FGSRNIGPSHHCSHAGVAQHHQFDRRPIAAMQIFVKTLTGKTITLDVEASDTIDNVKAKIQDKEGI
PPDQQRLIFAGKQLEDGRTLSDYNIQKESTLHLVLRLRGGMQIFVKTLTGKTITLDVEASDTIDNV
KAKIQDKE
>TRINITY_DN585_c0_g1_i10.p1 type:3prime_partial len:236 gc:universal TRINITY_DN585_c0_g1_i10:705-1(-)
MQIFVKTLTGKTITLEVEPSDTIENVKSKIQDKEGIPPDQQRLIFAGKQLEDGRTLSDYNIQKESTLH
LVLRLRGGMQIFVKTLTGKTITLEVEPSDTIKIQDKEGIPPDQQRLIFAGKQLEDGRTLSDYNIQKES
TLHLVLRLRGGMQIENVKSKIQDKEGIPPDQQRLIFAGKQLEDGRTLSDYNIQKESTLHLVLRLRG
GMQIFVKTLTGKTITLEVEPSDTIENVKSKIQD
>TRINITY_DN554_c0_g1_i3.p2 type:complete len:268 gc:universal TRINITY_DN554_c0_g1_i3:1464-661(-)
MQIFVKTLTGKTITLEVEPSDTIENVKSKIQDKEGIPPDQQRLIFAGKQLEDGRTLSDYNIQKESTLH
LVLRLRGGMQIFVKTLTGKTITLEVEPSDTIENVKSKIQDKEGIPPDQQRLIFAGKQLEDGRTLSDY
NIQKESTLHLVLRLRGGMQIFVKTLTGKTITLEVEPSDTIENVLEVEPSDTIENVKSKIQDKEGIPPD
QQRLIFAGKQLEDGRTLSDYNIQKESTLHLVLRLRGGMQIFVTITRRDANFCQNLNWKNNNTRS*
>TRINITY_DN1465_c1_g1_i1.p1 type:internal len:102 gc:universal TRINITY_DN1465_c1_g1_i1:2-304(+)
QRAAMQIFVKTLTGKTITLDVEASDTIDNVKAKIQDKEGIPPDQQRLIFAGKQLEDGRTLSDYNIQ
KESTLHLVLRLRGGVIEPSLAVLARKYNCDKMICR
>TRINITY_DN585_c0_g1_i2.p1 type:internal len:184 gc:universal TRINITY_DN585_c0_g1_i2:549-1(-)
KIQDKEGIPPDQQRLIFAGKQLEDGRTLSDYNIQKESTLHLVLRLRGGMQIFVKTLTGKTITLEVEPS
DTIENVKSKIQDKEGIPPDQQRLIFAGKQLEDGRTLSDYNIQKESTLHLVLRLRGGMQIFVKTLTGK
TITLEVEPSDTILRGGMQIFVKTLTGKTITLEVEPSDTIENVKSKIQD

>TRINITY_DN173718_c0_g1_i1.p1 type:3prime_partial len:112 gc:universal TRINITY_DN173718_c0_g1_i1:37-369(+)
MQIFVKTLTGKTITLEVEPSDSIENVKTKIQDKEGIPPDQQRLIFAGKQLEDGRTLSDYNIQKESTLH
LVLRLRGGAKKRKKKNYTTPKKIKHKRKKVKLAVLKYYKVDEN
>TRINITY_DN1819_c0_g1_i5.p1 type:complete len:432 gc:universal TRINITY_DN1819_c0_g1_i5:79-1374(+)
MILQLLLILSIWNVAEGLDNGLALTPPMGWLQWERFRCITDCKKYPKQCISDSLFKRTADRLVSDG
YRDVGYEYVNIDDCWAELQRTKKGEMVANHERFSQGIKALADYIHSQDLKMGIYTDFGTKTCG
GYPGSIFYMKEDTMSFADWGIDMIKVDGCYVCGDLYADGYKAFGFHMNQTARPMLYSCSWPAY
LNDKGKPYKEIAEHCNIWRNWADIQDSWDSVLSIINYNGEHQDEIVPYVGPGNFNDIDMLLIGD
FGLSQEQCKTQFALWAILAAPLFISADLDTMQDWQKEILLNKEIIDVNQDVLGKMGKRYYNENN
QQTWMRPLSNGEMAVAMMSTRTDIPVQMGFTLQQLSLNGIYNVRDLYTHEDKGNITQGYEAEV
NPTGVVMLKLTPVKSEEVEPAVIHSGYENAEVNEIKEKIEEMYEYIIY*
>TRINITY_DN2636_c0_g1_i1.p1 type:complete len:314 gc:universal TRINITY_DN2636_c0_g1_i1:986-45(-)
MSAIAVVVIVIAAVLLHFSVHKIEEGHVAVYYRGGALLSSVSGPGYHLMAPMLTKYKSVQTTLQT
DEVKNVPCGTSGGVVIYFDRIEVVNLLSVDKVHDIVKNFTADYDKALIFNKIHHELNQFCSVKSL
QEVYIDLFDQIDENLKRALQQDLNEMAPGLKILAVRVTKPKIPEQIRKNYELMEAEKTKLLIRIQH
QKVVEKEAETERKLAVIQAEKQSQVAGIQYEQKIMEKESMKRMSEIEDLTHLAKRKAMADAEFY
THQKQAESNKMKLTPEYLELMRIEAISKNNKVYYGSNIPNIFLNEGGLSQKTK*
>TRINITY_DN5107_c0_g1_i6.p1 type:internal len:138 gc:universal TRINITY_DN5107_c0_g1_i6:1-411(+)
FWLKRLPQPQRSSLPVRVRITMGKKSYQNYGKTFRKPKRPFEKERLDSEMKIIGEYGLKNKREVW
RVNYALAKIRTAARTLLTLDEKSEQRMFQGEALLRRMVRLGLLLESEKKLDYVLGLTTAKIMERRL
QTKVFK
>TRINITY_DN3589_c0_g1_i1.p1 type:3prime_partial len:548 gc:universal TRINITY_DN3589_c0_g1_i1:63-1703(+)
MDVTKVETKPFEGQKPGTSGLRKPVMTFFQENYTENFIQSVFNAVGPQLYGRTLIVGGDGRFFMS
EAIKVIVQMAAANKVGKLIIGLNGILSTPAVSCLIRKRKAIGGIILTASHNPGGKDGDFGIKYNTSN
GGPAPETITNDIYNETTKITEYELCKDVTVDLSKSGYSNFDVNGKKFYVEVIDSVKDYLGMMKRVF
DFKLLKEFLSQEDFKFVASGMNGVTGPYLKRIFCVELGAPETSLMNAEPLEDFGGEHPDPNMTYA
ASFVDKMRTGEYNIGAAFDGDGDRNMILGHKGFFVNPSDSVAVIAAHHSIIPYFKKTGLKGLARS
MPTSGALDRVAEKLEIKLYEVPTGWKFFGNLMDSGKISICGEESFGTGSYHVREKDGIWAFLAWLS
ILASTKKSVEQILEDHWATYGRNFFTRYDYENVEDESGNKMMTKLRELICTNELKGQQLGAYVVD
TLDEFKYVDPVDESVTEKQGVRLLFTDGSRIVFRLSGTGSQGTTIRLYIESYVTDAIKIKENTQSALK
PLIDIALKISNLPSLTGREK
>TRINITY_DN10026_c0_g1_i1.p1 type:3prime_partial len:105 gc:universal TRINITY_DN10026_c0_g1_i1:284-595(+)
MVYFFTVSRKEDVSEFFDIKEEVGRGATSVVKIVVQKGSQQKYAMKQIKKNIDKKIIRTEIGILLRLK
HPNIIRLKEIFESENELFLILELVTGGELFDRIVAR
>TRINITY_DN384_c0_g1_i2.p1 type:complete len:314 gc:universal TRINITY_DN384_c0_g1_i2:1018-77(-)
MRIAVALFLVFYLYPTEACKDYLNPTVCRIGVKQKGDCEYYKRLCMKTCGGCGGVIRPTQGPGPG
PVIPAGDCGVSSFQGGRIISGTTAREGAWPWQILLKANGRAGCGGSLISANWVLTAAHCITGGSYS
VVLGEHDRRRRSGHEQEIRVSRAIVHPRWNKRSFEYDYALLRLSQPARMNQYVKPVCLPTGEPRP
GQSCYITGWGKTSSRGQMHYILQQAPMQVIDSNTCNRKNYRAIRIAIRPSMVCGGGSGRISGCQG
DSGGPFVCNEGGKFVLHGDVSHGSRECSSSKTYTVFGRVNHVRSWIRQTANV*
>TRINITY_DN22_c0_g1_i1.p1 type:5prime_partial len:353 gc:universal TRINITY_DN22_c0_g1_i1:3-1061(+)
ISNYYFHFQYQTAIFEYHTVFSLVKIINMKFAVALLVVCYFYSTEACKDVLHPSICADVKSIGNCGV
YKDLCMKTCGNCGGVLPPTRRPIFPTRGPGPGPKTPPPIVPTGECGVSSFAGGRVIAGTTAREGAW
PWQILLKSNGRAGCGGSLISANWVLTAAHCMNGGSYTVVLGEHDRNRRSGNEQEIRVSSAITHP

QWNRRTFEYDYALLRLSRPAQLNRYVKPVCLPSGDPQPGTSCYITGWGKTSSQGQMNGLLQQAP
MRVIEQNTCHKKNYAAIRVAVRPSMICGGGSGRISGCQGDSGGPFVCNEGGRFVLHGDVSHGSR
YCRSSETYTVFGRVNYVRRWIKSTANV*
>TRINITY_DN12632_c1_g1_i1.p1 type:complete len:504 gc:universal
TRINITY_DN12632_c1_g1_i1:165-1676(+)
MGNCLRKPKKQRQKEEKKLDATSLEPSLVEPNPQPPNSFPAPAPLGNSNGVQGATVFVALYDYD
ARINEDLSFRKGERLQIINTADGDWWYAKSLQTGREGYIPSTYVAPEKSYEAEDWYFGDIKRAEAE
KRLMMRSLPTGTFLVRKAESAPGNFSLSVRDGDSVKHYRIRKLDSGGYFITARAPFSSLHELVQHY
SRDADGLVCALNIPCPNNDKPLTMGVAKDVWEIPRESLRLTKKLGAGQFGEVWAGVWNNTTQV
AVKTLKEGTMSPAGFLEEASVMKNLRHKHLVQLYAICSDCEPIYIVTEFMCHGSLLDYLSKGEGQ
NLQLPTLIDMSAQVAAGMAFLEAQGYIHRDLAARNILVGENYVCKVADFGLARLIEDTEYTAHE
GAKFPIKWTAPEAALYNKFTIKSDVWSFGILMTEIVAKGRIPYPGMTNAETIHQVDRGYRMPQPP
GCPEPLYQILLNCWNKDPEARPTFEYLQLTLEDYFVSTEPGYRDLNQTDS*
>TRINITY_DN1080_c0_g2_i1.p1 type:5prime_partial len:8617 gc:universal
TRINITY_DN1080_c0_g2_i1:26447-597(-)
VVIRRVVRRPVPIVTRRRVYRKVILDLDGTEKGVEEKVEESLPGMQGLDEDPITAPVVAEEQESVPD
MFGDLTEQSIVSRRSDFPKEGELDGVVDIADGKIGDTDENENETNFEIAFKREVYRLAVLDKKSIPV
ERRIIRKVIKLPNGEQKVLSEEDVLIPLEEEGVPISNTEKCKIVSTRIVTKRIMQYPDGKEDVLDETIES
ETERTQPLTSPEQAYVLVVDKPDGVGSIIEKPVEIAPLEYSYSEFDEDKVDSDIVEKGKTGKIVLRRQ
TVSTSKELHRSHLNKDTEKSVIVDLITYPSEQTEDSVIVTDEQDLSKSPEIIKDEKNERGIVFRRVVRR
PLPVTTKRTVYRTIVLNDEGITTEFEASQEEPPVEKKNVLQKIISSVKPKTDGKDVPEVDDSKPEEVE
RHLIDRVVKHKDGKEELIDQYELINSLEMPAVNVEPITKRVTDSKGRVIKTQVIRRVYITVKTIVVRK
VVEKADGTEIELGSSYEESPDDSTEETPYPERSVIHRVLPQDTPRVVEESDDISPLETSYKKGQEKEFI
SKNVLDDGTIENTVYKKDEITKKKLVTRKTIEYSKGKKAKKEEPIEEIHEHPVEFGSDVKFVESTEEI
KNEKGEIVKRVIHKEKPVHTRRMIYKKLTYTPEEMNKIKLEQMGEEKVLPKESKDDKKPGKQTTA
KPLAKPSTKAKPSTKSKSVSSVKPESKESGKPSKISIEKPSKKLSTKVTTKPSTESDGKREAPPSPTKST
TKPSTDSSGKHEAPPSSTKSTTKPSTESGGKREAPPSPTKSSATKPVMKTSGVKLSKPSNEKRIVKRP
KIPGDSTKSESKSKENDPAKKNRADEKDGAEADEIISKSKNVLDVKSVPTEMNVDIDTTDKRQSE
KAAEILKEKNQKHEADEIMQPVDIEEVQSSPLDLEKPMIFDDVVKSVDMEDVQSSPFDLEKAKISD
SAFKPVDMEDVQSSPIDSEKPITSDDIVEPIDIEDVQSMPFDFVKPMTSDDVVEPINVENTQSLPYDT
KGVKGREDVFLDQGEGLSDSNDENDFIEPGKVSLMRRQSSLRKRNRPYDEKKEDIRRNSLPLQEL
FIKLGRHDSGLSAIWESLLSDSKEEYISYDDYMTWLNFLEKEYSSSKFIPGKHDLLTKEHDYNKSLK
VDLRKRKAIVDHINKMKPVLQDLAVGQDKELISQELNELTTRFDKVYVTCEHRLKEINNMVPLSK
EHEDHYVPVSTVNEIVEDELKVKPRTGVDSKKIQAEIDRVKKLKDKLDNCKPAVRQLRKSLNSVQ
KHQTDIIDKEQIEEEAVNAVSKHDDLCNNVGQYNDTLERQLEKAKKFFGECDMIKKWAPVVEA
QLYNNETDSKNPEDLKKELKQLDMLKNEIDKKRPLIMAIDENGQWLMQNLYVTDKAGRRKSTA
SEVQLSLAEITSIVHHLSEQLNGRRDKINALLADTMSFEDSATKFEKWLIVIEEKVSSQKTLVLKSNE
LKDEILRYKPLLDEIVEHKPMIQSLVHSGEKIMSSANDKDKEKYMNKIALYSDRYDKVLKTATLKA
DKLDQVLPLVREHETNTEIVENIMSLAQPVLTSIEPVGVDSEKGNDQLKKVKNVVDLLDHNEPAV
NTTKRCVVQLTRILEEDLVKSVKETTTNNAIKYEDLRAKLKEGDANLAKQYIECERFTEIFYSIESKE
EYIYVLLKEIKSCSKQPERIDDGLLKSKEISIVIEEVLEQCSVVEEIIIIIQGYCKGHPVVLKTVGEKSSVI
SSKKIMMGKVSKEVQDEQNKMISLQQANKELDQKVIDINCWLPQIEGEVVKQTPVSANYDILKV
QYEKHQPLYQDVVQKESNIKHVLLLAGNILKDKKPGEEKDKLSKSIEDIERRWNDVSEKVNKRQ
NQIQRVMPLASVYKNTSTSFCQWLSNGEKQLKDLTSLSGNLEDIAVRKRELQDLAKDIESEKPNQ
LKVNDTARKHLDVCKNEAVEEPISEKDQVQQEVDHIDARWKKLTINVKEETDRLSQIEDEAVKM
CDKERVLEDLFIEVEKTLTEQKPVNLIPEKSKEELEKIKTLIVKVDEKEKDLTVFNNCSDKLQNLLV
QYDGNKDNVEKVKPVINDKFRKIRSHLTDRHADIDDIYTNVKEFDTDVKSSHTFLHDVTKECED
VFNKKAIPKDENELKSSVDTIDKLIDRLTKKKSDLQKDYEIGDKLIEHSSRDPLVLHDVQSRLQKIQ
DPINELIDKLNDRKSSIVNESRLHQALTGKLDDLDKHLPNLENKITNLKPVSAKLTVARGQQEEFK
PVYDETMAFKPLYEAIEKEADELKSSKSKRNDEEDEMLEKKLPDVLSQWNEQFEKVEKYNNQLK
DVLPTSANYHFTFLKFSPWLENAEKQLKEIKNDSFKTEPSSNLSSDIDKFRRDIGTNEPLLKDYNTS
AGNLFDRCKEVDVTTDVPYLEDEVKHTNDRWNRLKVDFSEMTENSNKLKNALQAYEKASEPVE

ECMIAITENLNNETPSDLDLEGLEFYVDERNADLESMQEKEPALQKVQSKCNEISDLLTANGGDP
TDIRDKAKDMNARWNDTKSKLTGKRDKSEAQLKQLEQFLNATDLLDNWLNVTTTTVTNMGPA
SATSEGVKKQIDQIDAIQEEISKQNVKLLKAEELGDWLCDENKDNPQFCANVNNRLNKIKNPLD
ELRSILSARKSRLHETLVATQDFRVAFDDFSTEIDKLVSRQNKQKPLSVHHQTLKSQDDEQKATEKE
IEALKPTFEKLIENGKTIIKETEKSNERDSLEENIEQLQKKFTGISETSDKRRKSIDELFPRSEKYFTKE
DDVSDWLDKQEKVLEELDIVPVSVADVEKLENDIMKLEEEVNKEEPEYKDCLKSFTDLSEHANNE
KITKDLPETKELDEKMNTRWENLKKKKNDKKLFASKLKTLISSYSDFCEGLKNNLAQFEDAIDKK
PSHGTDSKKCNDELERIDELIRKLDTHEPEMNNVEKLNSELTELIRKEKGDSNTVELTTKNLNGRF
QKVRDTLNERKKSLEKVSRVLLQFNGIVDDVSKWSDQADEQYVKLGRVSKDPEEAKRQIHKIDLL
LEETIRQKSAVTSAEENGKWLTENCLDDKEMRANVRSVLSNVYTPINTIEAKLNDRKAKLENAVL
CGQEFDKSFNDISERVTSLEFQVDRKKPINVDWTYLVKEQQEQKLLMKEIATLKPLYNQLITTAQK
TLNSLEPGDEKEATQQRIDSIVIIWEKISVAIQHRDEVIDQLENVSQSHHDQHRLFKEWIVDPEDSI
KNLEEVPSTRDEFFKFKKSVKEFIVLIENHLPNHSSLNDSFEKIKSETEKFPDDEFVGLNSLEEDIHK
TNDRWEKLTLNMKKYRARVEKLQSPIVKYSDGYDDAGKILEDIEKALEYQPSFGADEQLGKDEL
DRIEELIKASHVASEKVTVISEDSKHLEIVIDENDGDNTNVKESTKILVKRLKDIQEQCSVRKVDVE
KKIKVVCQFHKTSEELQKFYMTITQKLVSIRSAKPTPEQVKEQLNIVNQLEEDLQKHRPQLQALKE
TANWLVEKNADDRVASSDIKNQFSIIEDPFNELADKLNEKQNLLQASLLQTQEFQDTYNNVLDN
LAEYEIKANKSEPLSVKYDKLWKQEEDFKAFETDVLQMEPMFDQLIKSAKKVKDGTDEKEEKEDI
ENKTKDIERRRKNILEKLKERRDSFEKLLPVALEEDKIHKDFKPFVENTEEQYDQIKQAPKTLQEC
KNQLESLNSLKSKVDEGKPKHKTFNTNYNKEKETANTIADIADASTLEDEVNDLNKRWEALSGN
IDTKKDQVNKLHFHFTQYQESTESALHDIKKLEDYLEQAKRFGLDSSEGQKILSNVDDMLKTLEK
TERKVDEVNRSGKDLIGELESCKSDPWEVTTQMNDVNRKLDMLKVNLKECQEKVVDEVQIVNQ
FLKDVKDCESSLTDVNNKQESFEPLSTDPTKIKEQIAAVDELLNGLEKMSPRLDQVGRQGDLICKN
NPDDFSVSTDVNFRISKIKEPTDRCILRLQERKNKLQNLLVGSQEVQDTLDDFNDKLTNFEKTYNK
MKPVSAKFIVVKKQEIITEQVLDDVSQLQPSFKLLEKTAESVLKDAEPKEQVQLTEKINSLKTRWET
TVEDLKKRRKLISKVTPLAQDYYDRDQGFSNWVGDVEKKLIPRTDDFVVDQENVSKELDRLKAIK
KDIDVHKPDYDAILSIHKPLVGTAEDDINDIDEEVKRTTERWENLNVSIDKQANEFVELNNNMN
QVDEQIVPLEEFMNQCEVTLEELEPVGVDKDKAKDQLNTLNVCLTELDNKEKDIESIEKLANDLQ
EKPGLNPDKLKDTVSSLKVRHGTIKHSFTEKKEKLSEYIVYIEEYYVIIEEITEYIIVTKKNPAFTEPISS
EPSILKTQLRNIEAIQDEFISKKPKLERSNELLIIIVQECKHDFTLCNEVKQSFDEVKKPMDEIWTKIE
VRQNRLQDSLLKSQKFQESFDDFLDNIGQLEERYNNGKQVSAKYDVVRTQKKDHDQVHNDIM
QLEPVFEQIMTEANNLIETADPEEKVKLVEKVKGTTDRYTQIKENSISRMKRLSDEIVLTQKYSDET
QSFKSWLTEIEIKIENLEPLPCYGNSVSKQFIYVKSLVEDIQEKQSKLEDIEKIKKEVVENAEIDQSSV
EDDCNQIKVSYEKLSVQINERSTKLTELTVHTQEYTVHLKPIDQLLDKVEHCVVCTLPLYGIDMQK
ISKEQEDVNVLVQQLTDILPTLKDYNDVSRVIADKADKSSPEYDAFKKETDNLNERYKRLRDQLH
DKHDKLHDYANKAAKYQELNEDFDNWMEELGKTPGVMQPIGTEPEVVKRQLNEVEAVLDDLN
KKKLLPQSIEENGHWITEANPQDKNLKSNIDDKVEKNQNSYDKFSTKVEIRYNRLQNALLKSQEF
QTTFTDFTNVLEDIKSRQDKQEPLDARYEELTKLKVENELLQQDIAQKEPVYEKILQNGENLLEAS
EPGEERNALEKRLDEVKSEWNGVKDKCCKRKDDIERLHPLSKDHDCNCVTFTVYLRDAEKRKE
NLKPISSDKNIALLQLKETEELLNDVRNHESLYNGYHQTNEPIYVTCKELNITKGLPELKDEIDDID
GRWKNLNGDLEDRVSNCNDILKRLGECSEKMRPAESLVDEIKDLMKAPTRFGDDLKKGKEAKD
QIKKLKESLNNNKPEIDKLRSEIPELLRQGGIVAEPIEEKFSALLENVDECGMNLDDLDKKVDDEL
DKAAKFQDACDKLNKWLPEANKSTCLTDLSSSDPDEIMRQIEELKALQEDIVNHKPELMDVQNL
AKDMLDANKSDPKVCENIVDKLNNVAKPFDDLVNKLEERQKKLDTIKKVIDNYNHEKQPLEEFI
TATEATVDNLPPFGLDPDEGEKQVSELEDLVKTMDKRKPELRAVNRAGQSLIRQLDSPDEIEKALD
QLSDRYYDVFDKSKGKLNETKEKVNKVKIFIKVIEVLEIWITEVVVIVVKIGKTSSTEPETVKKLLDQ
VQEIRVDILNHKPQLKEAEELKDWINDILKDDENTKNMVNERFDKVKIPMDETSNKLDELEKRL
QDSFSSSKQFVDDIDDIEKLVSKLERVVEEEKPISADKDTCTRQQNDHKYIVEETLLQEPKYENVLK
QGRELLDHTDPGEEKDKLNEKLQKIAERWDQTKKNVVDRKNTLDEVVDHATNIDNDKKDIIPW
LNDAEKLSESLDCISVDPERLGKQKDCIKKLLEDMESKRPLVEHMKEHRTPLIEKAGVDQSPVNEL
VDDVCKRFDNLAKKLKDADEKADQLADGMGSFNSQKKPIEELLEKSKNLVGKQSPLGSDEADK
DIDDMKSLLDELLKHRPDVDKLKKSGDDLIAIEDKPDNVTAIKQVTDGLEKDYDDLLEKLQEKID

EAEKGKKDFEDFNKALKDANAKINGVEDKSKNLEPIGATPEKVKEQLIELENLQAELQAANNRV
AEAESLADKIRSNSNNDPSVIKKIDDELNAEKKRLKELEDLLNDREENLKDQLQQCGALQDQMD
DFNRRLQNLDGRCENQAKKPMSMNPVNMAATKNNVDGISKDLDDEKPVFEELMKNAQKLLD
NTQSPAEKEALEAKINAMKKLFDDASKKIDEEKNREEDLLGKTKTVDDKMNDLEKFLDDQEKK
LRSLEPVAIDKLSQQQKEINSLKNDTKSQQPALEEIGKLIKELEPLCEEDVPGVKEKLAKLKAHFDE
VEKLGEDRQKELTDVQNGLDNFKNISDPLKSKLSKMEALLPSLNMYGADKDKIDEINEELAKLQ
KQIEDADKKLEDMNSITENLVNEHPTADSKALRDEKDELKKRLDKFKEDFNKKSEGVNDILGDW
DALSNDIKAAVGTVKNCSEGIDKSKPTRLAVDEIVGQIENVEALEANLNESKPLFDDIQKRGRKLR
DKGIGADLFGDLLPKIDGQVREIKKDLPEKAKELGELKDTLNDFNDKLKDADQGMTAFEEKLES
QKPVGGDKDTIDKQMKDLKELSSELDKLHGKVSKLNDVRSDIKNKYPEADSSAMDEPLNKLND
RTNNANQKLSDRQGKLDDALVQCGQFTDAVDSLLGWLNETKELVDNQKPISAADENVLKAQM
QEQKLLQRMFADREASVKNLQKTGEELLKTTEDEDKKKEIEKDLEKVKELWESLKDQTDDRKTK
LDEALVASEKFNKNYNNVMQSLDDLDKEINSDKNIPTSDPQKIEEQLDNMKKLSDKIKELEPIIQA
VSGDETELEPYCTPDDLALLQNKLDKMNEKFDKLNEDCQEKEDNLSTAKDLSEEFANKEKELNE
WMDNIGKELKDADGGKLKDLQKDITDHKPDIDRLKELGKDLKKLIKPEDYSKLKKSIEDAEEKY
KNLKGDVDENARSFFMAEGKVQEFDKKLEELAKWTSDKTNQYRTIDPVGVQPDRIKEQMNEQK
QISNELSSKEPLFKELYDLSEDLMMGCNEEEKAEMKEKIENLKKNKHQLDKTSTERDEQLVDALL
LAQQFSDVYNDVASRLSNTENLLEALDKEKGRGVEMQKEKLQNVEDNIGQLLPLMGTLKKTGS
DLIKLSGPGQDSESVQKKIDECNARWDALKTNSEEKGISVGVAAQQSEAVWNDVEDLIEKCQLV
KDDFKNQRPIPVHEEPITEEIGKLEEKEMLLKDLEDPVTSALGSIDEILKDDPKSPASTALKDRQRK
LGNLWKFISTSAKDRRNDLDSSKVAAEKFWPGIEKLNDVLIDVQTKLEDQNDPGMGIEEIEGLLK
ETDMLHNELNSNEDVITELVDATPKLVEKASPEEKTEVHHQLANVRDQWDNLEKSLQKRKGDL
ENVKALATEFDSEKDHVEKWLSEAEAFLKGEDTKPSDVNELKTRLRAMRKFHREITKHQNEITKL
EQKGESLAEKVNDEDSKKINEIVEDITKRWDNLLDQSYEKQHQLEDALLDQGHFSFAIEEILVWIN
QTRDSLKTDDKIPKHKKLIEVEQSKLKIVTNDVEAHKPSVENCRKAAEKLISENSSEKAELEVKLG
QLNEGWDEIQELLKNREGKLQDAFEEALKFQSSARELTIWLSEAKAFLKTKRPYGGKLETVQKQV
DKHQEFIRVVEEREVTYLFIVETFEYLIQSSDVSSSRELQKSLNEISSAWTVVSKLSQDTTDKLNRSLG
DAQFLQDKFNEIEIWIVRIEGTVSMFKPASTVYETIEVQIIEMEEIYIEFAEKRNGMREIRKTTDRILE
HCSPEEGRILNEEIEELTQKWKELSTSLKDRKKTLDENLDQSKIFFEGSEQLMTFLDEIEKKINEDET
IGKDAASVKAQMRIHRDLQNQIGKRQTKLNVVVKLGKTLLEKSPEEDYQIIQAKIEDLQARWEV
VCAISVERQHKLEEALLFHGMFQDAVHALLEWLHAVEPSLKTETAIMGDPDTVKLLMDNHKSFE
RELTKRQKNYNSIVNRGETMIVEGKVDNIEEISQQLEKLKNDWEAVSQMTITKHERLQSAYTLAK
EFQVGCRTCVNNLGELENKLKKQGPISDDVAGVRKQIEEFIVFEELLEIEEVKVNGCLKKGAVILRF
CHPSSLQTIRHQVAVIKKRWNDVSGWARQRKARLDEILIEMIEEEQIVQILIEWIAKQEVLLEDREK
IPLPDSYEVLNQLLDEHKMMQNEAENKNPDYSKITKRAKRKPLTDKQRKLMTPRHGTQPQRDFS
NPMVGVLSKRWQSLWLTLMNRHRRIQDKLDAIRIQKASAEFSWADWRDRYNTWLRDSKSRVLD
IWRKKDSDKDNKLSKEQFVGAIFESAFTTEKWEVELVYDQHKRGVLITYQDFMDALKDRKRRPD
KPQTEAEQIDDIIKSEVSRCCCAKSFEMFQVAERKYRFGETQKLRLVRILRSTVMVRVGGGWETLG
EFLDKNDPCRAEGRTNYELRENFIMPEHASQGMQAFKTSRPGISREGSATGTPKFSRERSGGIQRS
GGMKREKSCGMHQHDRTDGGSNLTTPTFQRRKSDLEREAKGLPSYKGKDYSGVKGSGYGRTYN
KRTTKTTSTTPDHSSTQSTPTSSTRIAREKSGDVYSRLYGSTPKTTSATPRSSTPSTTSTGIPKSAGSVS
RGGATPRSSTSTSKTPTKRTSSSSSTTATPKLSVTSPDEVTTPIARSSSNLRKAPSQGSIAKKGTESSER
KTMTKTKSTPKSAEPKKRTSTAKK*
>TRINITY_DN817_c0_g1_i4.p1 type:internal len:176 gc:universal TRINITY_DN817_c0_g1_i4:526-2(-)
TLEISVRKVECPGFATKCATTKFVLDGTTSYRGYCASDYGPYLCTYMESSVKTMTDCKSTLCSGHY
CNIEVERASRKLTINGKGIKCYTGVQMFLPQLPIATGTVPTEVDQCEDQTSQCMSVIFTHLVTDPIR
KIKVKIPTQMRLCQPGYSTCENHCRRLAQHITIENCEASCCK
>TRINITY_DN121866_c0_g1_i1.p1 type:5prime_partial len:460 gc:universal TRINITY_DN121866_c0_g1_i1:2-1381(+)
LYIINSCEPPSIFFSCHLHFYTLVEETMSTTTAPTTTGTSAATKRLYVGNLGSTVTTQDLKQLFGLETT
PYLRDTCFVELAVCEKTGKSKNFAFVSAPGHVFSELIKLNGIEFYGRQLVIEEAKTKPEEEGEKKDN
KEKKKTPNNKRKGGSNNNRNNNRRYGNKPKSKFNLPTLEPDQVFHLVDCGVNLTNPKFSQHT

DYVIARALSAGVQKMVITGLKLNGCKAAVVMAKTRPNILYAAVGIHPHFVKDDWSDKALDQLE
EQVKQPECVAVGECGLDYKRDYSPRDQQELAFKKQVQLGVRYQKALLVHERDSHDGIIEVLKEF
EASLPPVIIHCFTGTVEQIKTYIERGYYIGVTGYICKDKYGKTLRDAIKDGTLPLNRIIVQTNAPYMT
PNTPRDEIDPVSQTLLDHCYADNEPCTLSIIVRCIAKCLSQEPRHVADVCTETAIKVFRFQKSESE*
>TRINITY_DN1058_c0_g1_i1.p1 type:complete len:2639 gc:universal TRINITY_DN1058_c0_g1_i1:134-8050(+)
MVKYIIWILLLSIFKEAYGGDWAQWQNWSACQDSSGAPIGCGRGNQHRSRTCADNNCGAGAIIE
VRPCLDEICPTFNCQNSSEIAGMCKFDYSNSCVGDDNCHHCICKSEGFKVSGDQCYNYCVEVGC
DEEIEGVSQLKRYCGDQAISCTGTVLNRTCLCNPATSYPSKDGQYCYEATCKTSQEFMTNCGAEN
SCQGTNTSFVCECNGFGYGYEVVANVQNRKKCKLLSCEDGDLNPLEICGQGATACTGNVHNLT
CACNVTAGYIPSEDKKSCIPMPCNTDPAVGVQCLPRNTCEGSKIDWRCICSAYGFRNDPTDPKKCI
EAKCDDPNIDILGLCGTGVTSCTGFLSKRRCVCNTTENYIPTLDGSHCMPAPCNANENATKDCG
GENSCIGSFDSYMCVCNGYGFQKNATINKCEEVPCSSLPTAEVQRICGFGALSCDGTIRNPVCNC
NNDLQYYASEDMKSCIEASCSSKADYDSHCGNENSCVGNITHFECICETEGYKPDPSNKKKCILES
CELNEVDVLNVCGKGAISCKGNVRDLECVCDASKGYVQSPDTNKCEPSSCYGNATVSEQCGEEN
HCLGDLSDWICVCDAYGYKVNATNSMTCDKVMCGDTGADAVTVCGKETTSCSGHIAKPVCTC
NETAQYLPSPDSTRCVKASCKENDAARKACVPGNSCSGSYDEFICICDQFSYHQDPADNRKCIRE
KCEDLAADYDYEKICGVGGISCTGYVNQPVCVCNSTESYHPSNDRTSCVKASCSTSDAVRTQCGL
ENSCFGDIESFMCDCKAHGYKLEPINKTSCVLATCEDGDFSYHFQCGKGAVGCSGNINTLSCLCD
SAKGYVPSADMKRCVPNSCKDNPDVYIKCGMENTCLGSYADWFCVCSGAGYEANPADPKSCQL
MECSKDDAIHVCGYGAIDCKGPVLAPTCSCNYSDHYIASEDGTSCVPITCRKNDTARAQCGEENT
CKGHTDNFKCICDAYGFKADNVDHQKCVERQCTDGDADIIRICGHGGIRCTGPIRSAVCECNSTE
YYYPNADRSRCILASCKANETIMKSCGEFNSCEGNMESHICVCNGPGYKHHPSDDKQCVKETCE
MGEIDPIYLCGKGAVKCNGTYDIYTCECDNAMGYVQAPDGNSCKPFDCHANATMHKQCGDG
NRCIGSLTDWICVCDVAWYKPDPSNLMRCHEKTCKEADLELICGTGATGCSGSATNPTCECDSA
NYFVSSTDRRSCIKASCKTNITAVQQCGDGNSCYGTPDNYVCNCDAYGYRLDDNNNQACVEKK
CDDVGANYVEICGLGGIGCKGTVKNPVCECDQTKHYVASPDSTSCVLSNCRGNKTVMAACGNE
NKCFGSASSFICVCDGPGYKRNPSNLQECLPETCEAGEIKPHMVCGRGATACKGTMYHFECECN
NTLGFIRSPGHESCIPVNDCYANATMHEQCGEGNGCQGSVADWICKCDAPGYKPHKTNFQMC
HQRMCSDADIDLVEVCGVGVESCVGPIANPTCNCDTSKHYVLSADSTSCIPGSCKTNETIMAQCG
EGNSCSGTSENFVCICNGYGYRPHETDKQTCVLKKCSHSDVNAVDVCGQGALSCIGTMRDPICQ
CNNDMFFIPSDDSRSCVLASCSANSDVAVACGKVNGCFGSKSSWICDCSSPGTMLNPNNIRECLH
ETCESGKLDPIIVCGKGALGCNGTMSNVTCECDNSMGYVQSPNGMSCVASDCKTNSTMHELCG
DGNSCVGSLTDWMCVCDAPGYHPHPSDPMRCLQKMCSHANINPVDVCGIGATKCVGSITEPRC
TCDTAAYYVPSPDGTYCIPGSCQTNKTALEQCGNGNTCTGTADNFVCICNAPGYKLDDSNNKRC
IEISCNDADVDLVEICGQGSIGCIGTARQPVCQCDHSKHFVPSLESTSCQLASCRANDTIMDRCRM
GNTCFGSMTSHICVCEAKGFKVNPSDPTECVQETCEDKDIDALSACGKGATDCMGTLMNLTCQ
CDGASYVESPDKTSCLPVGCQQNVDVKKACGEGNTCEGSYDDWVCLCTNPEYIPHATDEKVCS
KKKCTDNPIGSCGIGATSCSGNVNIHKCTCNLTAGYMPSKDEQSCIPIDCKMNPKMNCGFGNTC
RGSFFSYVCECNTLGTMRNISNPKQCIEVDCSTDAVSICGVGATGCAVTPTNDVNCTCGEFYHPA
HDYHSCVLDDCKFNKLVPDACGVENECTGSISSWMCVCKGPKYIQDIKSTQLCAPGPCRIEQCGS
GKASGSFQYYAESCSGTYFEPTCNCKEGFEKDSYGGCIVKSCSRNNQSSVDCNDGAISCEGTYFKY
KCECRAPTHILNPANKRSCIPARCNPQLCGRGLVNGDYMDFASECSGSYTDFTCKCKYGLYGSGT
SKCSLEKRTCALSNDCHPNANCIDYDGHYFKCECKDGYSGDGYQCHRNGGLTPWSVVQPCDKT
CGTGYAQVMRTCDDPKPDTLGKKCNVPSPDVREVPCHQFACTACSVKKTCECENIRPYFVHEYV
EPDESLKYFTNLTAEDVEKALSAIEKWKLSVDEKEFCIKHTLPEIDGLISDINENLRSVDQTKLYTIT
ARDMMRNKLDCNVDLEKKDKYFRVYEAIVKRHFILKQVVSDLQAKEKLMVTYRSSCTKDSKVG
WLSSIFS*
>TRINITY_DN1339_c0_g2_i3.p1 type:internal len:185 gc:universal TRINITY_DN1339_c0_g2_i3:553-2(-)

NSRLAMVAILGMFFQDGLTGSAWGDWANYVDSPLRAFENELGVQEPVGFWDPAGFTADGNVE
NFARRRQTELKHGRVSMLATMGYITPELTGKLPGYLSPSAGLKFADVPNGLAAISKVPAAGWAQI
VAYCAFCELSQDQSAGSPAAAGDFGWKLLTSSDQAELTKKLSAEVANGRLAMMAIIG
>TRINITY_DN21998_c0_g1_i1.p1 type:5prime_partial len:507 gc:universal TRINITY_DN21998_c0_g1_i1:1561-41(-)
AFLKYLRDKMSKREFFYLFNIAVIGVAGIVFLAVVVLTYAGYIAPWSGRFYSLYDTGYAKIHIPIIASV
SEHQPTTWVSFFFDLHVLICVFPYGLWLCVKNINSERVFIVLYAVSAVYFAGVMVRLMLTLTPVVCI
LAAIGFSRTLDRYLNDQDEEQEKEPEQQEAEPKTNEGDNKKKKKESSKKENKLEAGMQDSTSMG
SHLKMLVVIVILMLLMLFTVHCTWVTSNAYSSPSIVLATSNYDGSRNILDDFREAYYWLSQNTPDD
ARIMSWWDYGYQIAGMANRTTLVDNNTWNNSHIALVGKAMSSNESAAYEIMRSLDVDYVLVIF
GGVIGYSGDDINKFLWMVRIAEGEHPKDIKESDYFTPQGEFRVDRGGSPTLLNCLMYKLCYYRFG
DLKLDYRAPSGYDRTRNTEIGNKNIKLTHLEEAYTTEHWLVRIYKVKPLETRTKSSMKQRNTSMK
KNKKKYYSKKTRSNKAGQIKDKLTIKKGTSAKSTKKTKKGKSKTKKY*
>TRINITY_DN998_c0_g1_i5.p2 type:5prime_partial len:178 gc:universal TRINITY_DN998_c0_g1_i5:2-535(+)
LAQGSLRSALPCLLSALDSEAMSVQFQSQNFQHILRMCNTNVDGRQKVMYALTRIRGIGRRISNI
VCKKCDIDLNKRAGELTTDEVNKIVAVISNPLQFKIPAWMLNRQRDTKDGKTSQKYANFLDQAL
REDLERMKRVRLHRGLRHYWGIRVRGQHTKTTGRHRAFADAGGPPKKK*
>TRINITY_DN2142_c0_g1_i1.p2 type:5prime_partial len:246 gc:universal TRINITY_DN2142_c0_g1_i1:3-740(+)
WLKPFWLRVPFGRLRDELVASRLPRPMLVLRARQLALQRLADGGRRGVATSAARLGGHPPYMH
MPPAPPPEFEPDGTPKKYPLHQPIWDHGYDWEHWALMPAGVFHTVYYGDFAQDYPPYWAWLQ
KFPMWVVTPFLTLWAITTMVVFQNAGSIGIKPKRYTIEWMQATKERERQENTNPVTRYLDRRRTE
RGGTWIAQYNLPWHPYWIWMTQSHDPDYPEYYLPKPGSPHLHYTDSLTTPFVKDE*
>TRINITY_DN143799_c0_g1_i1.p1 type:complete len:178 gc:universal TRINITY_DN143799_c0_g1_i1:576-43(-)
MKCLGQLKEYKIVGRRIPTEKNANPPLYRMRIFAPDVVSAKSRFWYFMKKLKKLKKSVGEIVQCS
EIFDKSPTTVKNFGIWIRYNSRSGTHNMYREYRDVITTGAVTQCYVDMAARHRARASSIQILKVEV
VPSSKCRRPHVKAMHNAKIRFPLPHRVMRTQFRSIFQAKRPHTHLL*
>TRINITY_DN433_c0_g2_i1.p1 type:complete len:144 gc:universal TRINITY_DN433_c0_g2_i1:1496-1065(-)
MAQQTTVPRSFKLLEELEEGQKGGDGFVSWGLEKDDDMNLEFWTGMILGPPRTPFENRMYNLK
IRCTDRYPLEPPIISFVTRISLSGVDANGKIDRRACNVLKGWQTKDSIKAVLTDLRRMMTLKDNLR
LSQPPENSQYGPSR*
>TRINITY_DN2472_c0_g1_i1.p1 type:complete len:344 gc:universal TRINITY_DN2472_c0_g1_i1:311-1342(+)
MSSGEIESLRQEAENLRNQIKDAQKVAADTSFSSVAQNVDGINRIQLRTRRTLRGHLAKIYAMHW
ASDSRSLVSASQDGKLIVWDSYTTNKVHAIPLRSSWVMTCAYAQSGNYVACGGLDNICSIYSLKTR
EGNVRVSRELPGHTGYLSCCRFLDDSQILTSSGDTTCCLWDIETGQQLTQFVGHTGDVMSLSLCN
DSGRTFVSGACDASAKVWDIRDGMCRQTFIGHDSDINAVSYFPSGLSFGTGSDDATCRLFDIRSDQ
ELMCYSHDNIICGITSVAFSKSGRLLLAGYDDFNCNIWDTIKGDRSGVLAGHDNRVSCLGVTDDG
MAVGTGSWDSFLRIWN*
>TRINITY_DN45599_c0_g1_i1.p1 type:internal len:642 gc:universal TRINITY_DN45599_c0_g1_i1:1923-1(-)
LFQCKIKEATKISEVINNARDRKQSQMLNGNSNGQIEGNYLNQPVVIDKGYQKPETRTQMNLQQ
RHAVARAVEAFQTNNGPVNDAVDGQPSSPRPNGITPLQDAKITRDVDILNKCIEEIENFVVWLKR
INEARKELQSKSKKKKNKKSENILVLQSQPPPEEQIIDIYRKFKHALNLLGKLRQHISEPSAPELIHY
LFIPLNIIVKVTGIERARSVKQPLLSQTCVDQLNSCLDSREYYFWQNLGPNWTLTKQNDNFKDEHI
PPFFPTFADGWVPPEVTISEKQEAAAAAVARMQSSQDMVDSSKMNGHQMTQPQLNTQNSFHER
QSSIESNRSSNSGDLRTQRRLVGIVVYDYLAHNNKELSIRAGEKVYILDNSRRWWLVRNDHGEQG
YVPSTVLEIAAPETPDRNAERITNASLISSYSPSPTHMDKVDANRSSKSSIIYDANQNYVNDNSAVV
NGSPNNRQYNMNPNNQQTVTPVVISVQLPDGGRVETNSDGTRIVATPASVVNQTHNRTSSNGA

AYPQRSVTPVRESPTSYAAVNKQSITRQTSNGTSTYQQTNGYDDVSNRRQSNEPNTYQKQVVAPRI
SQTSQAQISYQRTTPPVQQSANRIVTQSNSPQYGAMPPPPPPPPPPPPPVAQKG
>TRINITY_DN1787_c0_g2_i1.p1 type:complete len:197 gc:universal TRINITY_DN1787_c0_g2_i1:144-734(+)
MWCVTMWRIKILAFTSIFLQLSIAEKVLKPIPPSLAKRNDIQPRMLAPGYYSQECNAHKACPEKKY
CHLFLCVHCLKENVACTQNGQCCEGQCTYGRCKAGVSEGQPGTFCDRHEDCAGEGKAACCVR
EPAINPHISICKPPLAENMVCGPINFFRNVYVGAQVQKACGPCKQNLICKQVGLFGIHEICMKED
DKK*
>TRINITY_DN468_c1_g1_i1.p1 type:complete len:191 gc:universal TRINITY_DN468_c1_g1_i1:676-104(-)
MKTFYTILLLAIVQTNAFFFPKVDTVKDLDLASYTGRWYEVYSSLIQRATFQRNSYCTTADYSLKPS
GAIKVINSGRLKSATGEENTIEGEAKVVDAKEPGALNVKFSTGPQSDKANYLVVKLGPVVDGKYD
YTVITTDYKALVWVLARNPETFKQKYEKDVLAYLKKNGYNWFINKPRPTYQGKDCIYI*
>TRINITY_DN4911_c0_g1_i1.p1 type:complete len:271 gc:universal TRINITY_DN4911_c0_g1_i1:896-84(-)
MFSKLLLIVFISYFKSYRLEDPFSCFDGPLGGFYCSNDRTGYIDCSNPVSVPGNVSVIQPCPSGTFCS
CFINTKCQVEESNICQALKKPPLPKSFDITFNLTTNMISPITPQDGSTSYYIQRVALNGDLKKFYLRL
KNLETNEEFFEHIGKEGSLFVKYEGSYPSSCSKKVLESFPPSFINPDDFQLTSKEGSMETFEYKVGSR
NMSNPFLVTKSVLFDMDGEHAVPLIYEHIFDSSRIYAEVLHFHAEEKQADFFKVPEICNQEDIISK*
>TRINITY_DN7314_c0_g2_i1.p1 type:5prime_partial len:142 gc:universal TRINITY_DN7314_c0_g2_i1:541-116(-)
AEATEAPVKYVESAADKRLFEQVYVQYAQEYLKGPLYWDPNKLQGIDPQFPGNPITVDGKLTSNT
IGNLKTFSSNELAFFSFLFFGIGLYGNYMFNYGDPQFEKVIRDGGYLNASYIVEAIFLPISFFFHIACYI
QRKNGK*
>TRINITY_DN79126_c0_g1_i1.p1 type:complete len:115 gc:universal TRINITY_DN79126_c0_g1_i1:397-53(-)
MVARLVLRRRLSYNTKSNRRRISKTPGGKLVYLYTKKAGATPKCGDCKCKLRGLKAVRPKKLAKL
SKTKKTVTRCYGGSRCGGCVRERIVRAFLIEEQKLVVRVLRAQQVKKSS*
>TRINITY_DN602_c0_g1_i3.p1 type:complete len:851 gc:universal TRINITY_DN602_c0_g1_i3:3046-494(-)
MIICRLHGLSLGIILIFSILYTSDARSRHAYGHKDASSPQTGKKRAESDAGAGTEAATGANTGAATA
SDSSSESQTFTNTKVNTRIATSSNARSGANAAAKVKTYAHANTETKVDTQQQKPTPPVPPEALTT
PPPGVTPPGPPGPPGSENNNTSSGSECKEKETKPPIIKVYCPNCPCPCPPPCPCKCPCIKVAPPPPAP
PPCICLPPCPPPCPPPPPPCPPPCPEPLPAPAPCLPPCAVPPCPCNIAPITLDPSLPCPMDCPLPPCAC
PFMKKDNITTTKTKRSVIRKRNLNIKRSHRVGTKRVNAGKSPNLAYAQPDSFEKAAVAADNKKCS
EIGVEILKKGGSAADAVVSTHCCVEIVNSHSTGLGGGGFMVYYDKKSGKSRAYDFRETLPKKYTD
TQNKTQGETILVPGVLRGLEAVWKDFGKLPWEELWKPCIKYAKEGFYVHEALAAGIVKKADYIM
ANLGLRELFAPDGELLQLGSLFKRPKLARTYEQIAKHGADLFYRGEMAKQIVNEIKEAGGVMQL
EDLSSYQVNIRETLKSNINGLKLLTSPAPSSGSLMALALKIMESFKWKPEDLLNNPSLVYHQIVEAM
KFAYAPTSYLGDPRFTNGTDKVEQYMVSPQTAKEMFKRIDNKSHPVEYYKPWSELFSAEKTGTSH
MSLLDQEGSAVSLTSSINAYFGSKLRSREFGFIYNNELADFSEFWPTVYNLTSDKKAPSKRPMSKVM
PGIFLKGKTVQGVWGAAGGFFIPSALTMSIANWLFFKDNLKVAITRPRLHCQLFPPTVVYEPTFPP
SLIPKLQEFGHKYVTNSTYDVSGQLNAIMGVVQAIVRLPNGSYSAEADYRKGGRPAGF*
>TRINITY_DN691_c0_g1_i2.p1 type:complete len:1673 gc:universal TRINITY_DN691_c0_g1_i2:5503-485(-)
MMNLFRLIVLGVICFSYEGDAQQCGGSCSPCGNLCVGMKGDRGTAGLPGFRGPPGLPGFPGPEG
PIGQKGEKGPAGLNGMEGSQGARGKVGPPGLPGTPGRHGRPGNEGREGEAGLPGCNGTKGDR
GPEGPRGRDGIEGPQGLQGNVGPRGEPGDSTATKLKGQKGEPGSAGQQGEKGGRGMEGMKGD
TGKPGRNGRAGERGPKGEPGTSNVTIIGEKGSKGSVGPPGLPGRQCQVTGDKASNIAQGQKGNQ
GSKGEEGAKGEKGDAGLAGPQGEEGDIGPQGDRGDDGPQGPPGPEGEQGRSGIQGNKGQKGE
VGNNGKDGNDGQTGLTGQRGRQGTPGQKGEPGKDKKGLRGPEGAKGERGNDGEKGSDGPIGP
IGPRGPKGELGPLGMKGFKGSIGSAGPKGENGNPGDPGIKGRRGELGIKGDSGVKGAPGNPGISV

TGPDGMDGDTGPRGQSGGKGAVGFPGKPGPPGVVTIDGEEVLPGEPGPQGLKGFRGVQGDAGS
KGSRGEKGNACESSKCPAGPKGEKGLSGQDGLQGGDGEEGEKGEKGDKGDIGSRGDTGSRGEP
GPQGESGEKGEKGIQGPQGNSGKDIIVQGERGDKGTKGNAGTPGSIGFPGERGSPGESGDRGLSG
VNGLRGETGDRGARGVAGKPGVNGQKGQKGEIGSGARGFKGDKGEVGPDGPAGLRGPGGPVG
PKGDPFPTAVNGKTGEPGEQGAAGPKGAQGPVGPEGPRGFPGDIGPVGEPGDGEKGERGDDGA
RGERGEKGEPGQDGKDGDPGPKGEPGQEGKEGPPGMFKGNKGEKGATGLPGVDGLPGNSAPK
GEAGKDGQDGDPGPAGEKGDPGEAGSPGTEGPIGEDGPVGDKGEPGESRAGPKGNEGRAGAK
GENGRDGRAGRPGIGRPGDKGELGQKGEAVIGAKGSKGEQGLEGPPGPIGKPGVKGDAGVPGK
DGEAGGRGKDGERGLPGDKGDTGDIGFNGSPGAPGDKGEPGYSGQKGAKGMSGPRGPPGSTSN
VVGPKGNRGLPGMKGEPGQQGEKGLIGPQGSKGESGFGSEKGIKGSRGNPGAVGPSGEKGERGD
RGKDGLQGIAGPRGEEGNPGEKGSRGDQGPQGREGPSGELGPEGQKGDPGFAGEKGEKGDRGG
DGQKGDAGEPLRGEPGVAGPPGPTGPQGERGPKGEEGNMGPKGSQGRDGLTGEDGPKGSKGEP
GLDGDVGEKGEAGFQGLQGEKGNQGPQGLEGSTGMDGQKGQIGEKGVEGPPGPPCEPVNSVR
GETGEEGPKGDRGPKGQKGKPGPQSQAATPGEKGSKGEVGQPGPQGIKGEPGREGDFGPKGSPG
AQGEKGDQGQKGEPGQEGPEGLKGEPTRAFGPKGQKGETGLEGLRGPVGPKGEDGQTGPQGP
DGPKGNEGEKGDFGINGLNGAAGPSGPKGAIGLKGDSGPPGPTGAQGPRGSGGSPDFGFYYVK
HSQTTIVPSCPAGMKTLWTGYSFLYAQGNERAYGQDLGQPGSCLKRFSTMPYLFCDLQNQCTVAS
RNDYSFWLSTDEPANMMKPAAGPEVEPYISRCAVCEAPANILAVHSQSVIVPDCPNGWENIWAG
YSFLMYNSAGADGSGQLLASPGSCLEEFRANPFIECHGRGTCWYYGPTLSFWLSTIDDSNQFQIPK
SETLKAGNLKTRVSRCSVCMKTVRNGNFP*

>TRINITY_DN2772_c0_g1_i3.p1 type:complete len:142 gc:universal TRINITY_DN2772_c0_g1_i3:476-51(-)
MSDAEGDDVPVQQEVAAGGPMDINTALQEILKLSMINDGLARGLRESVKALDKREALLCVLAN
NCDEQMYVKLIEALCAEHEIKLIKVDDNKKLGEWAGLCKIDKEGKARKIVGCSCVVIKDWGKET
QAHEVLKDYFQSKS*

>TRINITY_DN34765_c0_g1_i1.p1 type:internal len:204 gc:universal TRINITY_DN34765_c0_g1_i1:1-609(+)
IPDKLLLYDYLGTNPSKGGLFRSGAQIKGDGVVDSWTGHSNFESKALALYVRRMPAFFETFPVILV
DRGGTVRGDIPFRRAESRYSIEQVGISVYFTGGVLDGSSIEKSSVVKAFARKSQFGEIFSFDKKVAAS
DGVFRTTSRGWYSFSHLGLSLCFLFGHLWHSGRAFFRDVWVGLLTSSSAVESIEYGRNEKLGDNTS
KTS

>TRINITY_DN14344_c0_g1_i1.p1 type:complete len:512 gc:universal TRINITY_DN14344_c0_g1_i1:191-1726(+)
MATNSSYVRLPWFRVHIVVLNDVGRLLSVHLMHSALVCGWSSSMTLFELIVVDPSDPVFNPVWR
QGAYVLPFAARLGVTSSVYLWSLGATTLNASSLWFFESVSISHLILSGLLALSAFWHWAYWDLDLF
VQSSSGRIVLDLVKVFGIHLLLASIASFGFGGLHLSGVFGPGMWTSDYCGLLGSARSVKPVYSLISLS
PFSYGAIPANHIVTGIFGILVSLWQVSSRPGPALFNLVLMSSLESVLSSSIAAVLSVSLISSAVFWYGSV
TSPIELFGPTRYQWDNGVFSLEAERRVKNNEINSWSQIPDKLLLYDYLGTNPSKGGLFRSGAQIKG
DGVVDSWTGHSNFESKSLALYVRRMPAFFETFPVILVDRGGTVRGDIPFRRAESRYSIEQVGISVYFS
GGVLDGSSIEKASVVKAFARKSQFGEIFSFDKKVAASDGVFRTTSRGWYSFSHLGLSLCFLFGHLW
HSGRAFFRDVWVGLLTSSATVESIEYGRNEKLGDNTSKTSSFL*

>TRINITY_DN2626_c0_g1_i7.p2 type:5prime_partial len:361 gc:universal TRINITY_DN2626_c0_g1_i7:1099-17(-)
GVGDLRDGRDGPIVRLLARRVDAAVVAVAHGVDARVALVVGAPNFALARLDGKRHVHQDPTA
AARPLLAQGLRGHEVVRGLHADANVLLVWAVRQADAPVELRVLGVVLQVVKLLHALPLHVPG
AVGRRLRRQRAQALRQRVDDRQLQAGEEVLQLRDPFHADEASAHDQDRGALPVQVADGLVLL
QDVPAAPLQEALVQVRPGALRPRGLVDRWEPQRLAPLVEDAEVAARGDDAIVKIDDLILRREHRL
DRGRPIRPVQLLHLAPDELAAHAPLDDRVQRERQGVEVLRLDVRAEHARRVLEELLGVHDGDE
VMVLEVPRDAQTREAAPDDQDAALGGRHGSRPRARGRLPARDQEWT*

>TRINITY_DN190525_c0_g1_i1.p2 type:internal len:218 gc:universal TRINITY_DN190525_c0_g1_i1:3-653(+)

NHPARPEPWGPAALHAARPGARRRADGDLAVPPRALRHLLPQAAVQGTRADGGEIQFAQVLR
APGRGRRPQRVVRVGHRRRPLGLGGHRPQRPLGTRRHGQHARCFLRGGAGRLEADAARDDPG
LHRRARQGGREGHPGGGQGGAGPGEVHPASRGHRPAHRAGDAQEGGLAGAHRRREQAAGLR
LLGLLRRARCPGGGPAVRRGAPHPGEPPGLSG
>TRINITY_DN10563_c0_g1_i1.p1 type:internal len:307 gc:universal TRINITY_DN10563_c0_g1_i1:1-918(+)
GMFFQDGLTGSAWGDWGLYVDSPLRAFESELGVQAPVGFWDPIGFTKDGSAENFSRRRQTELKH
GRISMLATMGYITPELTGKWSGYISPSMGLRFEDIPNGLGALSKVPALGWAQIIGYMAFCEISQDQS
AGSKGAAGDFGFKVLTASDPEALKRKLNAEIANGRLAMMAIIGMFFQDGLTGSAWGDWGLYVD
SPLRAFESELGVQAPVGFWDPIGFTKDGSAENFSRRRQTELKHGRISMLATMGYITPELTGKWSGY
ISPSMGLRFEDIPNGLGALSKVPALGWAQIIGYMAFCEISQDQPAG
>TRINITY_DN95387_c0_g1_i1.p1 type:complete len:169 gc:universal TRINITY_DN95387_c0_g1_i1:96-602(+)
MAATLKPYLNAVRATLEAAMCMENFSSQVVERHVKPEVEVQSSKELLLTPVCIARNEKEKVLIEG
SVNSVRVSIGVKQADEIEKILCHKFMRFMMLRAENFFVLRRKPIKGYDISFLITNIHTEQMYKKKLV
DFVIQFMEEIDKEISEMKLSLNARARISAEEFLKNF*
>TRINITY_DN30378_c0_g1_i2.p1 type:complete len:403 gc:universal TRINITY_DN30378_c0_g1_i2:1370-162(-)
MDVKFILLWLSSVTMLTCEDYQGYDPTGGVSAENKIKIDDLFKMDDCGLVSDDGDTIHWNYKGT
LLTGKVFDQGKFTATLGQHHVIKGVDVGMRGMCVGDKRMMTIHSDWAYGDRGTEGIPPKSTLR
FEVELTGVDRPGVGSETIDDILKKTGGVSMSQKIKVEDIFKVDPEQCKMQSQEHDTIHWHYKGTF
LDGKVFDEGTFTAKIGHHQVITGVDIGMRGLCVGDERKMIIHPAWGYGERGVQGVIPPNSVLVFV
VVLKSIERPDGTKDVHDDDTEEKYNMGGVSKKNKLKIDITHAVKDCQIRSKEDDTIQWHYKGTF
LDGSEFHTGHFKATLGHGHVIEGVDRGMRGMCVGDKRRITIHPDWAYGEHGRPGSIPPNSVLIFD
VEMTNIQRLDPRKGEL*
>TRINITY_DN6397_c0_g2_i3.p1 type:internal len:130 gc:universal TRINITY_DN6397_c0_g2_i3:387-1(-)
LTSSDQAELTKKLSAEVANGRLAMMAIIGMFFQDGLTGSAWGDWSLYTASPLRAFENELGVQDP
VGFWDPAGFTADGNVENFRRRRQTELKHGRVSMLATMGYITPELTGKLPGYLSPSAGLKFADVP
N
>TRINITY_DN16548_c0_g1_i4.p1 type:internal len:171 gc:universal TRINITY_DN16548_c0_g1_i4:2-511(+)
LYTDSPLRAFEGELGVQAPVGFWDPLGLAADGDSVAFRRRRVTEIKHGRISMLACMGYITPELVG
KFPGSLSPSQGIEFADVPNGLAAISKIPSYGWFQIIAYCLYCEASALNFGYFNEAFDQKAPGDYGW
RPPLLATDDAALKTKRLNAELANGRLAMVAIIGMFFQDG
>TRINITY_DN6132_c0_g1_i1.p1 type:complete len:161 gc:universal TRINITY_DN6132_c0_g1_i1:95-577(+)
MTNPHGKRRGTRYLFSKQFRKRGVEHLSTYLKTYRVGDIVDVKGNGAIQKGMPYKYYHGKTGR
VFNVTRRAVGVVVNKKLGNRIIPKKINVRVEHIKHSKCRDDFLRRVKENEIKKKEGKAKGIKVDC
RRQPVQPRGAHFVSSKNNAPSLVQPIPYEFII*
>TRINITY_DN17516_c0_g1_i1.p1 type:complete len:335 gc:universal TRINITY_DN17516_c0_g1_i1:1523-519(-)
MNQLLIAVLLVLSVSIVFHECAKTRKSKESVAAQLEQKIQSLVELNHKRPVIRMNGEKFRQYVKSS
PRNYSIIVMLTALAPQRQCGICKEVNHEYQIIASSWKYSSGFFSNRLFFAMVDFDEGSDIFQSLKLN
SAPVIMHFPSKGRPKRVDTYDMARQGFTAEQIGKWVNERCDVSIRVVRPPNYMMAITIGALLVGI
GGLLYIKRKSIEVIYNKDYWAILAVTIAFIMISGQMWNHIRSPPYAHRNPQTGDIAYIHGSSDGQLI
AETYIVLVLYACICVGFIFLNEKSSEQKDIKKARICTVVGWGMVVLFFSLLLSVFRSKYHGYPYSFMI
K*
>TRINITY_DN6294_c0_g3_i2.p1 type:complete len:134 gc:universal TRINITY_DN6294_c0_g3_i2:817-416(-)

MGYKRFVEAGRVALINKGKYDGKLVVIADIVDQKRLLCENPVKSVPRHVVRTQDVNLTDLKVNI
PCGARGGKIRKEYEKEEVDSKWEKTVWAQKIKARHVKRTLSDFDRFKSKIQKQRRNRKVKAVLK
SMKKN*
>TRINITY_DN3649_c0_g1_i1.p1 type:complete len:246 gc:universal TRINITY_DN3649_c0_g1_i1:2360-1623(-)
MVSQLLEQNLTLGFFLKGLNGPPGPPGPPGPPGEAGPPGLPGSPGAPGHVGEDGAPGPQGPPGA
QGMPGSPGYPGEKGDPGPVGLPGAAGLPGAPGLPGPQGIAGESGPEPVNPDIIITCEGEKNWLQC
KQYELIKVNKVFWGREDHVTCQKVPAGLTADRLCESDPENAKKKIEGQCKNEQGCEVVASNIFF
DDNTCGNVYKYLKVWYECIPDEANSVDILREGGRRRRKRKRSAKEKRSYRDS*
>TRINITY_DN902_c1_g2_i3.p1 type:internal len:178 gc:universal TRINITY_DN902_c1_g2_i3:2-532(+)
AVFKCIKLNYIDHIIKMIKIAIIAIVITALVHESTAVRCNVGYSTADLPMAMSVRQVECGKGLTHCN
TLSYKLDGMSVTQGNCATSGGQASCLFMKNSLQSMTECSEAICNGDHCNKAVKDAENAERARV
GPYKNTLKCTTGLQTDFPDIPFASPNVPVGVDECFPGTKECVTMTYD
>TRINITY_DN4166_c0_g3_i2.p1 type:complete len:490 gc:universal TRINITY_DN4166_c0_g3_i2:1876-407(-)
MAASIFIAVYIFMWITYIWETYLSYRQHKIYVVTKEIPNELKDTLDKETFEKSRLYQIDKSRFSFYASI
YSQIESSLILLLDGIPYLWAVSAVVNQYFGYDSTHEIKQSITFTVLGTIFSTITGLPWSLYSSFVIEEKHG
FNKQTIGFFFTDTLKKLLVSMAIMLPVTAALLWIIKWGGEFFFLYAWLFALLVSLFLIAIYHDYIAPL
FDRFIPLPDGNLRTNIENLAKSIQFPLSKIFVVEGSKRSSHSNAYFFGFYKKKVIVLFDTLLKVSPFEE
EKKKEEEVEVIKQEEGGEKETAEVEKVSGDDNDKIELLDKATENEINNVIEGDKKKKEDKGCSDE
EILAILGHELGHWKLNHVLKNLVISQVNLLFSFFVFGLLMNYDTLYESFGFYGERPVLIGLLIIFQFIF
SPYNELFGFLMTALSRKFEFEADNFGKSLGYSVQLQAALIKLHKENLGFPVTDELYSAYHYSHPPL
LERLRALKTKQD*
>TRINITY_DN1566_c0_g1_i1.p1 type:complete len:4626 gc:universal TRINITY_DN1566_c0_g1_i1:42-13919(+)
MDTEEVPGEPPPTVLAATEDVINYVKNITCFIFDESQPCLPLDKILKEEQTKDYFRKFISDVQTKTIL
LYRINQLTDDVDGDHDQGVSQPSYGIKLNVQYVPHKESSIAFIKRGMLLESDKKINAQLHVLILD
NSSPFDTLHSYISNAVSPFFKSYVKTSGKSEREGDKMVPSVEKKIAELEMGLLHLQQNIDIPEISLQI
HPVVQSIIQKAEIERRKISPDDYAEKAEDSSFLNALQNGVNRWIREIQKVTKLERDPSSGNALQEIS
FWLNLEKALHRIEEKQQSPEVKLTLDILKYAKRFHAIVSFTNDTGLKQGLQTVASYNPLMKDFPLE
QLLSATELERIKDALIAIFSHLRKIKLSQYPIQRCLRLIEAISKDLMSQMLKVLGTRKFMLMNYELFE
KVMTSAFNVLNCWDDEYERLQQILRDMAKKKKDDSLKTMWRVNPSHKKLQSRLNNLRTFRKQ
HEQLRAVILRVLRPSKVEAKSSNEENAASSGSIEISDDNAVKEVDLAYENFKEIDPLDVSKEGTEAW
ETALKRYGERIDRVETRITSTLRDQLGTAKNANEMFRIFSRFNALFVRPRIRGAIREYQKHLIGLVKE
DIEALHDKFKVQYSHSKASKMSRVRDLPPVSGSIIWAKEIERQLHAYLRRVEDVLGKDWENHIEG
QGLKADGDSFRQKLNTQEIFDDWSQQVQKKPLLVVGRIFSIETLKTKGSNKATRKLKVNFTKEVIT
LAKEVRNLKNLSFRVPLAIVRQANDANQYYPLAISLIESVRTYEVTNDKVFEKESIIPLVAGIRKDIQ
SLLAEGMHLEWQSYKLMPYVMRLAEAVTGYQEKVDELLTSIQKIDIEVQSLATCGFKHEAFQEILI
RIQKLVDELNLHSYSNLNSWVKTLDEQVEAKLAHRLQAGIKEWTRVLRTGNENVDDKDLAIHSP
GGLPDIKPLRVEFFIRNQVIEMHPPFEKCIEHLYNELAVYCSTITSLTKIQSQRYQVGLDRDNAEETY
HNLLTKLPDPNGPNPITVYGEMESMLEDCRKYCEGWTGFQALWDAEMSNLAPRMKTLEDWMT
ILNQIKMSRSHFDTTETMKEYGPLIVDFNAIQGKVNLKYDSLQKAIVQNFGGKLGDEVNDFFTNV
TKARTDLEQQSLDVATTAEAVTFITYVQGLKRKMKTWEQNVEVFREGQKVLERQRYQFPSNWLY
SDNVDGEWSAFNDILKRKDGAIQTQVAILQQKIVAEDQAVEERTFNLLADWDKGKPVSGNTKPD
SAIQTLQIFEGRLTRLKEDRDNLQKAKEALELIEPGTVPPSDERVQVAIEELSDFKSVWGELEKIWA
QVDDLKEQPWVSIQPRKLRQSLDSIANGMKILPAHMRQYAAFDYVQKIIKGYLKANMLVVELKS
EALKERHWKQLMRKLSVKWNLSDLTLGQIWDCNLQKNEVVIRDVINIAQGEMALEEFLKQVRE
SWQAYELELINYQNRCKLIRGWDELFDKVREHINSVSAMKLSPYYKVFEEDSKQWEDKLNRILNL
FDVWIDVQRRWVYLDGIFSGSGDIQHLLPIETQRFGSISTEFLTLMRKVAKAPLVLDVLNIQGVQRS
LERLADLLGKIQKALGEYLERERASFPRFYFVGDEDLLEIIGNSKNVGRLQKHFKKMFAGVHTIIL
NEDSTLVTGICSKEGEEVIFNKAVSLIEFPKINEWLTLVEEQMRLTLATLLTLSAKEIKSFTGDSIDPK
LYMEWVDKYQSQLVVLSAQIVWSENCEAALQTCYNGDMSSLEKVLSIVEATLTVLADSVLQEQP

QVRRIKLEHLITELVHQRDVTRTLVRSKVQGTKSFAWLSQMRFYFDPKQRDVLQQLSIHMANAKF
NYGFEYLGIQEKLVQTPLTDRCYLTLTQAIERRMGGSPFGPAGTGKTESVKALGNQLGRFVLVFNC
DEKFDFQAMGRIFVGLCQVGAWGCFDEFNRLEERMLSAVSQQIQTIQVALKEGPKEIGKPIQIELI
GKDVKVSEDMAIFITMNPTYAGRSNLPDNLKKLFRSIAMTKPDMNLIAEVMLYSQGFRTAEVLAG
KVVPFFKLCFEQLSAQSHYDFGLRALKSVLVSSGNVKRDKILSIKQKMQAQGVVADVGKITETLN
EQEILIQSVMETMVPKLIAEDIPLLHSLLADVFPGIKYVNAEMTALRNELKKVCNEMHLVYGENL
ESGAHWIEKVLQVYQISCISHGLMVVGPSGSGKSTAWRTLLKALGKLEGNEGVCHVIDPKAISKD
QLYGVMDPNTREWTDGLFTHILRKIVDNVRGELSKRQWIVFDGDVDPEWVENLNSVLDNNKLL
TLPNGERLSIPPNVRIIFEVQDLKYATLATVSRCGMVWFSDDVVSPEMIIENYIKKLRNVLLEESEED
IRSKQSKSVTDSKDELVSPTLQVQRDFSDILTPHLASNGLVLKTLEYSYSKDHIMDFTRMRALNSLF
SLLNQGVRNILQYNQQHHDFPMSQDTIEAYTPKYLVHSLLWCMSGDGKLISRLDISNYIRNITTIPL
PSPVTSLIIDFEVNMQGDWVPWSNKVPQIEIETHKVASPDVVVPTLDTVRHESLLYTWLADHKPL
VLCGPPGSGKTMTLFAALRALPDMEVVGLNFSSATTPELLLKTFDHYCEYRRTPGGLVLSPVQIGK
WLILFCDEINLPDIDAYGTQRVISFLRQLVEHNGFYRPSDLQWVSLERIQFVGACNPPTDPGRKPLS
HRFLRHLPVIYVDYPGPSSLTQIYGTFNRAMLRLVPSLRSFSQPLTDAMVEFYTKSQERFTQEMQP
HYIYSPREMTRWVRGVLEAIRPLDNLSPDGLVRLWAHEALRLFHDRLITDEERRWTNTNIDLVAQ
KHFPNVDLISLKRPILYSNWLSKDYLPVDREELRDFTRARLKVFYEEELDVPLVLFDEVLDHVLRID
RIFRQPQGHLLLIGVSGSGKTTLSRFVAWMNGLSVVQVKVHRKYTGDDFDEDLRIVLRRSGCRNE
KIAFIMDESNFLDSSFLERMNTLLANGEVPGLFEGDEYTTLMTQCKEGSQRAGLMLDSGEELYKW
FTQQVMNNLHVVLTMNPSAEGLKSRAATSPALFNRCVVNWFGDWSTGALYQVGQSFTNKIDLE
RSTYKAPDYLPVVFENLPMPPTHRDVIVNTMVFVHQTMHKANEQVTARGGRTVAITPRSYLDFIS
QYMKLFNEKRSDLEEQQLHLNVGLQKIRDTVTQVEELQKSLSMKSQELESKNELANKKLKQMLQ
GQQEAEKKRITSKQIQDDLKKQTEEINKKKSQVLGELDQVEPALLDAKAAVKSIRKENLDELRRL
GNPPTAIKLALESICLLLGTPSVEWKEIRVLVSKTTFIPSIVNFKTDDISIETRKQMMKYLNHSDYNF
EKMNKASVACGPLVKWAIAQVSYAEMLQKVDPLRRELQKNEDEMMSNQSKLDSVGEMISELEK
SIAKYKEEYALLISEAQSIKADLAAVEAKVSRSTALLRSLSSERQRWENTSDGFQNQMDTIAGDVLL
SAAFLSYGGYFDQQMRSRLWGTWSEQMEQSKIKFRSDLARTEYLSTADERLHWQSNSLPADDLC
TENAVMLSRYIRYPLIIDPSGQATEFIMKEYSGKKITKTSFLDDAFRKNLESSLRFGNPLLVQDVESY
DPILNTVLNKEVRKTGGRVLITLGDQDIDLSPAFTIFLSTRDPTVEFPPDVCSRVTFVNFTVTRSSLQ
SQCLNQVLRAERPEVDTKRSDLLKLQGEFHLRLRHLEKELLQALNDSKGSILNDDKVITTLETLKT
EAADIMKKVEETDTVMSEVEAVSEQYRTLAAACSSIFFTLEEANLIMFLYQYSLKFFLEIFQSILFNN
PLLKDKRDPLVRLGILIESLFQVVYNRVARGMVHNDRVSFATLLARIRLKGKSENSYDLEFDHLLK
GKEIVVQVSDVKLTGLSSDQKMALLRLSKLNPFKELSRYIMDNSMGFQEWINSSNATLNVPRCW
NIKDSLTSSGEVLYECLVVQAFRPDNLHAMLRKYVLAVMGEKFLHTAEQELDMVNIVENEIKAET
PLLLCSAPGYDVSGRVDDLVAITNKQITSIAIGSAEGFSEAEKAINASVKSGRWVLLKNVHLATQW
LISLEKKLHTLQPHPNFRLFMTMEINPKVPVNLLRAARIFVYEPPPGLKANLLRTFSTIPASRMSEA
PGERSRLYFLLAWFNAIVQERLRYAPLGWSKIYEFSESDLRVGYDMLDIWIANVSQGRANVSPDKL
PWDAFRKLIGDSIYGGRIDNDFDQRLLQSFIDRLFTAKSFDSDFSLVTSVDGKQSENIVIPDGMRRE
QFVQWCEKLPDYQSPSWLGLPNNAEKVILTTLGERTCVKLLKMSVNTEGSELAYSADSHLKESEV
TGDIRPLWMKSLQQSVRNWLNILPKRIDPIKRTKENIKDPMFRFFEREVNLGVKLLNDILHDLNEI
LEVCEGNRKQTNDLRMLVDTLVKGVIPRSWNRYATPKGLTVIQWIVDLGERMKQLQNIARGTNA
GGSKELKSFKVWLGGLFIPEAYLTATRQYVAQSNQWSLEELYLNVTIADELNPQLKLDDCSFVVIG
LKLQGATCRNNQLSPVDTITTDLMDICLCWTKIDPRKPPTLGANDVTLPVYLNQTRCELLFTVDM
STTGAVPPRSFYERGVGFVVSALTG*
>TRINITY_DN1631_c0_g1_i1.p1 type:complete len:124 gc:universal TRINITY_DN1631_c0_g1_i1:62-433(+)
MAEAPKPKPAPKSRYGPSFDDIERRDPAQWHAAHNAWVLERLVEQETVKIYRERMDECYWKEQ
ENFPQNCRKHILKYWKAWNDWKKKDWGQTEEGSVYRFRVPVEEYYREVEELYKNKDKGNE*
>TRINITY_DN5131_c1_g2_i1.p1 type:complete len:164 gc:universal TRINITY_DN5131_c1_g2_i1:518-1009(+)

MGVDYGMLTRVTVALIGFALSVYALQVEVMASRNKNYKAYCDINEHISCSKVFTSPYGRGFGIIGN
ILGEKSPLNLPNPVYGIVFYVLVIFLTLNRKNYFAASLSVILSFASCLMSFYLATILYKLKDTCVVCIST
YVVNFLLMYLNFRHLNRFNVEVKPKEE*
>TRINITY_DN95606_c0_g1_i1.p1 type:complete len:538 gc:universal TRINITY_DN95606_c0_g1_i1:82-1695(+)
MVLYTLFEHAAGYAAFKVNSNEEIGALLPEVQEAVLDLERFGRIVKLVAFSPFKSAANALENINSIS
EGVVHEDLKVFLESNITCGKKKSKVALGISDAKLGAAIVESCNLQCEYGATVAEILRGIRFHFHKLI
QGLTAETSGKAQLGLGHSYSRAKVKFNVHRVDNMIIQTIALVDQLDKDINTFAMRIREWYSYHFP
ELIKVVSDNYMYAKVAQYIKSRKNLTEDKLEGLEEIVMDEGKAQAIYDAARASMGMDISPIDLINI
ESFAARVIGLVEYRRKLHDYLQSKMSQVAPNLQALIGDLVGARLIAHAGSLTNLAKYPASTVQILG
AEKALFRAIKTRGNTPKYGLIFHSSFIGRAGAKNKGRISRYLANKCSIASRIDCFTDNPSSLFGKKL
HEQVEDRLKFYETGEAPMKNADAMQEVLDEIADIETTATSEKKKKKKKKKKQQENGDDNQDSN
NNDADENIKIGKKRKLEDVAQEVVENGEEPKSKKKKKKKKLDDTVNGEEDNEIDTTIEELIPKKK
KKKKKHSENEE*
>TRINITY_DN30329_c0_g1_i1.p1 type:5prime_partial len:213 gc:universal TRINITY_DN30329_c0_g1_i1:1-639(+)
EPIVNRVLVKRMKVMEAINDVSPAMMLDFFKTTLATNFLKFPFFEAINAFCAALPIAPSVRGLFTG
FVFTTATLPVTNYRYRKSMQLEVNWANIYEAYFPTVIRDIVYGICRNYCTIWTLALNPQWTAVSPQ
VLFIVVILGCLGSAPFNEWRGYLLQSKGQELTFKEFFKPSNFVRSTSLGAIKQGLALAVGYWCAPP
ATRLVHGLFAGLRG*
>TRINITY_DN7808_c4_g1_i3.p2 type:3prime_partial len:280 gc:universal TRINITY_DN7808_c4_g1_i3:837-1(-)
MWVPGAALARRAFEGELGVTMPFRYWDPLQLAKDGDAEAFRRRRAAEIKNGRVAMIACIGYIV
PEYFRFPGELSPSASLKFADVPNGLAALSKVPSEGWAQIGMFCAFLELFPFRQPQDRMPGDLPGA
GKLGLPVFLGGVESKPCDADGSARSLNAEINNGRLAMVAIVGMVTQNGFLGTTGPGMWVPGA
ALARRAFEGELGVTMPFRYWDPLQLAKDGDAEAFRRRRAAEIKNGRVAMIACIGYIVPEYFRFPG
ELSPSAGLKFTDVPNGVAALSKVP
>TRINITY_DN163801_c0_g1_i1.p1 type:complete len:298 gc:universal TRINITY_DN163801_c0_g1_i1:55-948(+)
MANPLKDIISRFSKGVPAGSSTGLGLLIGAGLAAYGIKESIYTVDGGHRAIIFSRIGGIQPGVYSEGL
HFRIPWFQYPIIYDIRARPRRIMSPTGSKDLQMVNIGLRVLARPLSSTLPHMYQRLGLDFDERVLPSI
CNEVLKSVVAQFNASQLITMRQEVSLMIRRDLTERAKDFDIILDDVSITDLSFSAQYTAAVESKQVA
QQEAQRAAFLVERAVQERQQKIVASEGEAKAATLLGEAMNENPGYLKLRRIRAAQKVARVIAQS
QNKVYLNANNLLLNIKDTDDIDDGLLSKKR*
>TRINITY_DN1739_c0_g1_i1.p1 type:complete len:400 gc:universal TRINITY_DN1739_c0_g1_i1:86-1285(+)
MAITDNIKDILSVKIKPRHDTYSDQYSRIFMVKIMMMSALLVGLNWYSDKIKCIIPDSISADGGFVG
AACWINGFYVYEKIRYHVNEVGYFGMPREIDHDGMFPDGQTCNSKDTLSSDPSMECQPMEKTFF
LQYQYMTFVMAAFALLYYAPYAIFRWINVDLVSLKGSIQDNSAEKIIGNYFNHRVNSPNKMRMRV
MGNIVVKILYIVVNIAAFLLLDSILNGGFTTYGTLWTQWAKLDNDLAYDYMGIRHSPKPANALLP
TFGFCEVYMSAKDIKTTVNNKFKFICEISQHILYQYVFIVVWFAMVFGIVLSIVGLLMQLIEHALTIT
CFLKQGDQARRMYSILTLRECEYLEYIRRKNIPLYNDVIKKLKEQFQDGFHKPRDHYNNDGLPKY
QDTQA*
>TRINITY_DN309_c0_g1_i1.p1 type:complete len:454 gc:universal TRINITY_DN309_c0_g1_i1:65-1426(+)
MSNCEHKTQKITIQKKTVKVTSQREEDEYVKARKEGKKYILPWKTPDGLQPPDGFTNLKYIFTPDS
SNVPSSAEKIDEEPLMQKMKPDPSRIENPPETGIRATWLGHCSVLFQLDNVNVLVNPNFNDRGIK
YYFPGDNKRYRTCVYKVDDLPRIDVIFITNTHYDYLDLSSVRALNNKYGEMLLWYVPIGVGEWM
TKAGCHNVVEMNWWREDEVDFIDHTKIDDDEETTTTTFNISCTPSQSFHSRTFDEDNAALWCSW
VIKSPRYKLFISGATGYCDIFKVIGRKYGPFHMAALPIGGYHPKDKFGYGNVTPEEAVQIHQDVLA
MCSLAVGWGTFTLSNEYYLEPPRRLNEELSKQKLSEMQFFLLKHGESRLIEIKEADKDDDEPPQEN
GVPPVVPPVDDTIEGEVCGEEKKDDDDSDVLQQLVDNFTMDEEKKSEEEEEKSEEKEEEKEE*

>TRINITY_DN3452_c0_g1_i1.p1 type:complete len:466 gc:universal TRINITY_DN3452_c0_g1_i1:139-1536(+)
MDVACFRFRMILSIISFTLLTFDLPAVNSEALHLTEENYQDTLDKNKLVLVNFYADWCMFSQQLAP IFHQTADIIHEEYSNVKLGRVDCEEQQKIALDNMVSKYPTIKIFRNGKALRKEYRGQRSVDAFAQF IKEHLKPVVQDITTSPNDMKINENRNTIVGYLESKDSPGYQSFAKIAEDLGETTDFFALLGDASHK ERAVGESVVFKPKKSYGESESTYSGSVSNLDRLKAWATEKGNPLVREITFENGEELTEEGLPFLLLFY KPGDTTSIQRFKNGVMRELLPERPRINFLIADGTTFSHPLHHLGKSLDDLPLICIDSFRHMYLFKKF DEVDVPGKLIAFINDLHSGKLHQDFHNPSPDDQAAQGHHHGGQAQIDNNAQQPPQEQQPDQ KQPDQKGEDTIQNTDQPPSGSGNKGSGKKPASGSGSGQSPPETVFHKLAPSENRYTLLHWRDYS GNYRDEL*
>TRINITY_DN47849_c0_g1_i1.p1 type:complete len:689 gc:universal TRINITY_DN47849_c0_g1_i1:176-2242(+)
MCGIFAYLNYGVPRSRRYILETLVNGLKRLEYRGYDSAGVAFDSEETLKNHECGISIVKTKGKVQSL NDEIFDNDARAFEAELSYHLGISHTRWATHGEPSPLNAHPHRSSDDNEFVVVHNGIITNYKDIKL LLAKKGYHTESETDTEVIVKLLKYMYDRDQESDNEKRLNFPELVELVIHQLEGAFALVFKSTHFPN ECVATRRGSPLLIGVKSNKPIVVDQIPVMCTENGSPGDRTPNGTSIFDSGASAIKANFCVADDAAM EYYFASDASAIIEHTKRVIFLEDGDIAWSKEGNLNIARMNNSTPAIRDVQTLQLEIQQIMKGSYSTF MQKEIMEQPESVFNTMRGRVNFEKRKVLLGGLVSHMDYIKRCRRLIFIACGTSYHSAVATRQLLEE LTELPVMVELASDFLDRQTPIFRDDVCFFISQSGETADTLMALRYCKSRGALLVGITNTVGSSISRET MCGVHINAGPEIGVASTKAYTSQFLSLVMFACMMAEDRISKQQRVDDIIGALEVFPEKVEEVLKLD KKIEELAAHFYKQKSLLVMGRGYQFATCLEGALKVKEITYMHSEGILAGELKHGPLALVDEDMPV LIIALRDKTYDKCMNALQQVLARKGQPIIICSKGDEETKKLGNFCIEIPEVPDCLAGILTVIPLQLLS FHLAVKRGFDVDFPRNLAKSVTVE*
>TRINITY_DN15151_c0_g1_i3.p1 type:internal len:560 gc:universal TRINITY_DN15151_c0_g1_i3:1-1677(+)
LDGAERPRILAAMLSTLRRAPRLAARAPLAAPAAARAFAAAKDIRHGSEARALMLEGCNRLAD AVAVTMGPKGRNVVIEQPFGAPKVTKDGVTVAKSIDLKAAPMINVGAQLVKQVASKTNDVAGD GTTTATVLARAIFREGCKAVAAGMNPMDIKRGMDAAAKVVLEDLAAQATTIESPTSIRNVATIAA NGDAQIGEMITDAFEKVGKDGTITVQDGKTLEHELEVVEGMKLDRGFISPYFITNVKAQKVELEN PLVLLYEKKISSVQAILPVLEQVAKVQKPLLLIAEDIDGEALAMLIVNKLRGGLKVAAVKAPGFGD NRKEILKDLAVLTGAELLSDELGDKLEDLQITQLGTVGSATITKDDTVLLDGSGNKSAIDERCEAIR DAIELSTSEYEKDKLKERLAKLSGGVAVIKVGGASEVEVNEVKDRLNDALCATKAALEEGIVPGG GTALLYSAQKLEALTLDSLDQQVGVDAIKAALSQPCVQIAQNAGEEGAVVVRALLQEGNAARGF NAATGQYVDMVEAGIIDPTKVVRTALADAVSVASLMTTTEA
>TRINITY_DN801_c0_g1_i4.p1 type:5prime_partial len:182 gc:universal TRINITY_DN801_c0_g1_i4:1-546(+)
SSLAQGAPRPPPNDPLPRPARPRRARLGLPSPMADQLTEEQIAEFKEAFSLFDKDGDGTITTKELGT VMRSLGQNPTEAELQDMINEVDADGNGTIDFPEFLSLMARKMKDTDTEEELIEAFKVFDRDGNG FISAAELRHVMTNLGEKLTDEEVDEMIREADVDGDGQINYEEFVKMMMAK*
>TRINITY_DN30386_c0_g1_i1.p2 type:internal len:165 gc:universal TRINITY_DN30386_c0_g1_i1:3-494(+)
ACRQRAAARPAARRLARFVSEGAGAMADQLIEEQIAEFKETFDFFDKDNDQRLSIKEFGCMMNS LGQNHTEDDLHKMVAEVETDFEMRINFPEWLSLMAKKMKDTDTEEELIEAFKVFDRDGNGFISA AELRHVMTNLGEKLTDEEVDEMIREADVDGDGQINY
>TRINITY_DN3271_c0_g1_i1.p1 type:complete len:399 gc:universal TRINITY_DN3271_c0_g1_i1:218-1414(+)
MTRIIWHKIKSELIMPYLDLDCKYYDLGLPYRDETNDQVTVDAAEAILKYNVGIKCATITPDEERV EEFKLKYMWRSPNGTIRNILGGTVFREPILCDNIPRLVPGWTSPIVIGRHAHGDQYKAQDLVAPGP GKFEMSFTPLEGSEKVTVNVFEFKENGGCMMGMYNTDDSIKDFAHSCFNYAIGKKWPLYMSTKN TILKKYDGRFKDIFEEIFQDQYKSQFDALGIWYEHRLIDDMVAQCLKSSGAFVWACKNYDGDVQS DTLAQGFGSLGLMTSVLLCPDGKTIESEAAHGTVTRHYREHQKGNATSTNPIASIYAWTRGLAHR

AKLDGNPELEKFSNSLELACVKAVEEGNMTKDLAGCIYGLKNVKREHYLLTNEYLDAVRDKLLQLWTPPSQV*
>TRINITY_DN123073_c0_g1_i1.p1 type:internal len:179 gc:universal TRINITY_DN123073_c0_g1_i1:2-535(+)
EGETYPRPLPGQEPLPPRVFLYRGFAPSTVQVARGPCLKVDLSVRMIQGQTALVKLTTLRDLLIAKHKNEGLPPPTKEEMLDHLKFQMAGRTCMTKHNHIHYRIKEVCIDMSPTSTFPFEEGEISYLEYFQRRHGITLKRNQPMLHCPFRGKSELYLPAEVALLTGVDEDWRSDREFA
>TRINITY_DN33065_c0_g2_i1.p1 type:3prime_partial len:804 gc:universal TRINITY_DN33065_c0_g2_i1:2409-1(-)
MWQFVWLAVAITSQINGNVHHKHTKPRHQNLIDDIVPNDDFDLDYSHNIFIPFDAYDNYEKFLEYAERSDLAKRGGVTSLPAIQSLPGDGSHTYAAPGVNCQVQLLNKKVEGTDKRVIERTSCAEGWTLKNALGFFFAGVGHDLATKSAGLVHMNQLRFKMLIFEAEGYTFVADTDNEVYSNLQSKLSLKNAECRLTISNSYTFENSEIVLTGNHEVIEKITNAKKTLEVTFEKGKDTSSFDVKMKVPSMPLETFATIFLESETEFITADMGDTSYEINIDPIKAVIIESTSNSVTGVFDGSDGKFQLTGKFLSNDQNAFMGQATDIYLTVNRKSLGSRTTYNLMLGFAKEVKVMDIIPKYVGKTEIQDEFDDNFLKTTIEKWHLLFTSKRIIVADGAEYKKTFANIFDADEYQEFKQGVGIKATVDLQKTLTACSSGSTVPDSLKSVKIKGLLGRRGLSMVFPPGTSVPLIPLIKCKLRQIQDDLGFELPISLKGYEQKSKFKLEEIAYSSDSGAFKMSLLYENTITIYKDVLEITNPQLTIEREGKDEPWQFTIAADVSIFQVDASVMISYSKTGYTVEVSSGNVPLTNLLRAFSFSDKPAITFEIHDFKMQIEVKDKEEEEENEEEGEDEGNKNQLAIKRQDNKEEKKRDLKFSIAGKPVFNGFDKVSIEALVWTSQTPAGVAVAVIMENIRLDEVLEKILDVPEVPMAWFSDMTLTMVISNVDAEGCDNPAQSEDDCEEADEEEEEEEEGEGEEEEGEEGEKTQEGEEGEKGEEGGGGGPGGGPGGGGRGPGGGRPRRG
>TRINITY_DN1319_c0_g1_i2.p1 type:complete len:134 gc:universal TRINITY_DN1319_c0_g1_i2:86-487(+)
MVVPSAKRKIVKKRTKKFIRHQSERYKTVKQNWRKPKGIDNRVRRRFKGQYLMPSIGYGSNQKTRHLMPDGFKKFVVHNVKELELLMMLKGTHAAEIAHCVSRRKRIEIVERANQLAVKVTNATARVRSEENE*
>TRINITY_DN3180_c0_g1_i1.p1 type:complete len:1889 gc:universal TRINITY_DN3180_c0_g1_i1:5857-191(-)
MGYLRVTWICVITLSTIVLSEFIFDDDEFGEKRTFGDPDSLVKRSSSGSTKKHVSTFTRTQRFSGGGSTCNYRVIKRSGRQYVVFRSSRHTRVANGCTKIHVQSFLNNHFGGKNLLTTFPNLNGDNFEVVQMMYSLETRVAFMEAISKTSMAFFGGGNLEFGKSGDVPLTLKLQWTEDHDSSAWDPSDIQVELETTKVVLSAASFHFKAKKSYHNPDHFMIEKFDTQGVGNFRVSAYTSAIAGLDNTAFGADFSQDKYGALTWNDFRGYGFFDPAENFQLVLHPSTYVRNSRLFNNEETSQFIILSNPVSDDTTYVTLMAQFGQSTNIKDTLNGIMTSVNADIGIIKRINSRAFCLTVSSGEYDFLFTPPYTKDRNLMNPCFPLEYQDARDNQNDLSKQKKKMTADINIHVTINFKENVGSCNDDSSIDTSSLPPKMTINGNENWKGFSWSFPEQAKGPMMAIMKCMWQNSAAFQGLPYSLPFGDSEVVSISDFLDSPTEKTIGLKITPPEQWRTIPGIDLTFNYLEFTASYNIPDQTWELSGTADMRIDKFEVFLKLEYSRTTVEVEDDDDDDSSNEDDGEGPDKKAKKKSDKKKKQSKNSKSSKSESKLMAKLRKRKSMDALLKSLPDTFQDDDFNNNHHVFYDNLIIPRSLPVKNNQSANVSLNKDAGTNIVPAAVKNSSLATAATTSKRTTEVEKQWTFTILMPTLLVKDIYTIFSPQSTLPAEYPKVFNNGIKNLQISWAWGNEFEVRFRGQLVIMGWDKILLETVFWKQMPSRSLKFGIGIYFQKIDVDAILKHIFQDESISVPWLSNLDCKLVIGNSEGFKDVSKKKKKIKKIKKAKSNKKVSKKQRKTSKSKSKTSAALTRSTRGMEEYIYADLAEKRQADDDDDGDDDDAEPDVKFTYGSLADNALIAGAGFEMTWYRPTEEEKCLGNTLCKFIYEKVQSNTQFVLAGQVDTGGITISAEVKDAGIVLATDLKLENAKLEIDGRAGGGIKLSCQMIYTNSEIATNPLTFEGAIEIGFAGELTFRFTMGGTIDSAFGYNRIALGNILLGATIDFKAASFTIEFGVTVLMGLLNEDPITIRGYFGLSPTNWIDSWVYVKASKLTIGSLGKAFDFSVPDTFKDEGFPEGFQFSLSRKVVNIESLGIEIPSGLYLVGKFKFKFFELSADCTIDPANKAFDVKLEMNKQNWIGGMITISSYTDENKGPMFHIKVDENTPFYANIDGKLTVLGLSQEILVRIDENSGALIHLRGTPFPFIFDAALDINAALDPNTKSFNGGTFKITVSSSEFIPTLARKIRDWLEGLKSEGTQAKQDGMTAITKGEEDSETITGKKSLWDSALDTWKSNLQRRESELAAKQRAITGALQACIDECELVCVPFFDWEEDCFTIFGWSAPCPWPNSCAKKVPNYFCYAGCALDQLGSAFLTWVESTLTKIGLALQSTAVFLTDMVEGIAAALTKLGEYIVAAKDWLINQFQAALNWVADLLTKQIVLRKLVLEATVQPPFKYCFG

YDIDITLFTWHITDGGHVCLDTLIRDLIYSLFGYNQDSKKALGMKDQIEEAKSKTLSIKEAAEELEE
SAETTQAEVDMQTAKTFEEELKRKRTMPTKEMFKFIDEKSKNTKYSKAHQKIMDTPHGMPAYQI
HNNHMLENLEKRSPWVVVRSFKDGNRNLEPKRIADHEIVKRSLSFGDGSKPCSRLRRSLATYDTI
VNVLIRSARNLENMEKNHFAHKEFHGHGIRKLDKRIAELELKNMTDEQREDAYFWYDKANEGY
EDFHNKVRRNIDYHDNYALPLLHNQLNYILKKSGKEGGFLKRVEDMHSEALHDFKRSIKNEEDR
ADGQELLSKIKKSMLAIVTDENTPLSRYTRSINGMKNDITRMNKMVLAC
>TRINITY_DN1339_c6_g1_i1.p1 type:internal len:570 gc:universal TRINITY_DN1339_c6_g1_i1:1-1707(+)
GMFFQDGLTGSAWGDWALYVDSPLRFFGGGEASTYKTFDPATELGVQEPVGFWDPLGFASDGDE
ATFKRRRSVEIKHGRVCMAASIGYIVPEYYKFPGFLSPSEGLQFADVPNGLAALSKVPALGWVQIIF
FAGIVEGAFGVTDDYRNGTPGDYGWKVITSDDPAEKEKKLSAELANGRLAMVAIIGMFFQDGLT
GSAWGDWALYTASPLRAFESELGVQAPVGFWDPAGYTNDGSMENFKRRRATEIKHGRVSMLAC
MGYMTPEITGKLPGYLSPSMKVQFADIPNGLGALSKVPGVGWAQIFAYCAYCELSQDQSAASPGG
AGDFGFKAITSSDPATLTRKLNAEIANGRLAMMAIIGMFFQDGLTGSAWGDWALYVDSPLRFFGG
GEASTYKTFDPATELGVQEPVGFWDPLGFASDGDEATFKRRRSVEIKHGRVCMAASIGYIVPEYYK
FPGFLSPSEGLQFADVPNGLAALSKVPALGWVQIIFFAGIVEGAFGVTDDYRNGTPGDYGWKVITS
DDPAEKEKKLSAELANGRLAMVAIIGMFFQDGLTGSAWGDWALYTASPL
>TRINITY_DN124769_c0_g1_i1.p1 type:internal len:113 gc:universal TRINITY_DN124769_c0_g1_i1:1-336(+)
NRRRSVELKHGRVCMAATIGYIVPEYYKFPGLLSPSEGLKFADVPNGLAALGKVPALGWVQIIFFA
GIVEGAFGVTDDYRNGTPGDYGWKVITSDDPAEKEKKLSAELANGR
>TRINITY_DN24414_c1_g1_i2.p2 type:internal len:146 gc:universal TRINITY_DN24414_c1_g1_i2:435-1(-)
VTSFKRRRTVELKHGRISMLATMGYITPEITGKFPGYISNSLGLKFDDIPNGLAAISKVPALGWAQV
VAYCGFCELSAGSEDWKSGTPGDYGFKVLTSDDPAEKEKKLAAEIANGRLAMVAIIGMFFQDGLT
GSAWGDWALYTAS
>TRINITY_DN1995_c0_g1_i1.p1 type:internal len:514 gc:universal TRINITY_DN1995_c0_g1_i1:1539-1(-)
LTFIESLRLRTSKKRMGKEFKHQKNEKLEEDNEDISKTKGRLTNDTISYYKRVDSVLTENNFESDEL
YEAFIRNVFAQLKEDSVQVLRHTLTSKVIEKIIPLLSKSQFIELLEFVKEDVELLCCDRFASHVMETIV
ITYNGISDSTSDENFIKQFVSFCKVIRELIDHLIRDVFGCHVVSKLLQVLSGVKVSDRITKSKASINSK
KKAQQKYQIKEKELSAKEVTIINVADSLKKQYSKHVRVLLNVDNFGELLCHRNASPVLQVLLITTS
HINKKKYSVLSNQILQRARLIENRSLMEEENEDTIHKLPVVLTDAVGSHLIEVLLQTADDGLYRLLS
SQCFKNIIVSLCIQPVANFLAQTFLRLLEEKEQALFYVDKLFMYMEDILAVGNMGCISRLAELISRL
DLIDKQKKFIKVISNALHIPDQKLYKTDQKSNVLVTLIITMTTFDMFYKKESESENKTDIKVETDTN
KEIQNSDIENLCIKKNSINYHGACILKNCFKFKKCKTIVQS
>TRINITY_DN123_c0_g1_i1.p1 type:5prime_partial len:1231 gc:universal TRINITY_DN123_c0_g1_i1:2-3694(+)
RGGFRPFGTGLMGSPMLGPYAFGPARSGALGGFGIAPLVRPPTRGGAAGANRGSRSNSNRGGKK
TTPVKNNKSTPEAKKDNNINAKNDKKEDKKDEKKDDKKDEKKDDKKDDKKGGKTPTGKEIKV
EPKDDMKNIKKNFFKDPKVEPKNEKKPDTKAVTVKPEIKKESGPQNVTLDKFNSELHLSIDADGL
SAGNMKGEGFQYMWGGVRGTHGVKKGKVCYEVYLLENIKVHCPGENNPHVLRVGWSVDNSD
YQLGEDKLSWGYGGTAKSSYDSKFLDYGKKYTVGDTITCYIDFESNPKAIFFMLNGEYLGVAFRFG
NELGDKAVFPHITSKNMRFRVNFGKEKPKHHVTNGFRMMQDIPVDQLVLPSPSPKTPKEAEIIM
MVGLPACGKSFWCEKYAKENPDKKYYILGTNNIIERMKVSGLTRKRNYHGRWEMLIKRASLVLN
KLFTVAKNNPRNYVLDQTNVYFTARRRKMESFAGYKKIAAVIINKPDVLASRTEKQQKIEGKMVP
EGAVMEMKFNFTLPEKGPSFNEVWYLEETLPKAQELAANYRREGSNFKTKSKRKPESSPSQPESSP
SSEKKPKLDPKIISRGFDKPFSKPNTNSPQQSTFKAPSPAQNNANKTPNQDRFSKNEKSTSKVETNS
DNSRSKAGNFNKGHDGNKSNERPFFSNTPDRGNASNRGNDSNRGNSSDRGNAPNKGNSSDRG
NAPNKGNFADKGNAPNKGNSSDRGNAPNKGNASDSANKNSKNEKPKEESKKTPKRAIRGTLD
QLNSIINSEARKTENNSGNIRDSAQSGKNEKMGSLMDIDRTPQRESDSYQNLQSRNRDALSNQG
GKNKESQSAGLMDIRNVPQGNQDYRGKAGILDSSRNTSQDYRGKPGILDSSRNTPQDYRGKPGIL

DSSRNTARRNEYESPVGRGNNSYSDSPARGVSTPRGRASFNDSPARGARGGRGQSDISRSGSLAKS
QGSGGNRGGRGRGRSKHLGDVNKYDPVGNMGGYYSALKALPSVKDLIGNRSSFSSYSTRIDEPSR
FDQLYSSYEPYSFGRTQNANDSRSLNYSFGQTDSYSSLERDGGYRDSQNSKDFLGSYQQRKDSSYS
DNRGSQGGQRGYQDSSYNADGLDAKDQYQNDDYQESYSSQKDKSSYDQNTQSSRSYNQETSYE
ESSDYGRSRNQEQDEYARSGTYERGDTSYGTDDANRSSRRYEDDLQESEPMDGNYSTQDYGRSSQ
SSQRARIW*
>TRINITY_DN495_c0_g1_i5.p1 type:3prime_partial len:407 gc:universal
TRINITY_DN495_c0_g1_i5:152-1369(+)
MDFHWNTVIAGLIIVQVYSLAEYGTLNGKDSYRNAYPWLANDRNNNELGREDARSIYRSSDEID
AEYPQSDDEVDTNDIPKSYTSRDELDAEFAQRTYKFADDLDIENAQKSYASSDELDRDDEQRTNK
NTDDLDAEDAEREETINDSLDAENAQRRYTSIDELDGEDTQRVISKTITLPRLGAGRIYKSRFIHGP
YIRPKFVRPHKGIRLPHPNRIIIPGSWKRPAHGIFALKGKKAIFCEHKGIKRISCSNDKVIKVRSALY
GRTAGNVCSISCSKDEEIKVKNALYGRTAGDVCGRSRVTNCRALNSFAIVQKKCNGHNSCDLNPS
NSVFGDPCVGTVKYLNIKYFCVKRITPTTTPNGKTAIFCEHKGIKSISCSKDEEIKVKNALYGRTAG
DVCGRSRVTN
>TRINITY_DN495_c0_g1_i6.p1 type:3prime_partial len:382 gc:universal
TRINITY_DN495_c0_g1_i6:152-1294(+)
MDFHWNTVIAGLIIVQVYSLAEYGTLNGKDSYRNAYPWLANDRNNNELGREDARSIYRSSDEID
AEYPQSDDEVDTNDIPKSYTSRDELDAEFAQRTYKFADDLDIENAQKSYASSDELDRDDEQRTNK
NTDDLDAEDAEREETINDSLDAENAQRRYTSIDELDGEDTQRVISKTITLPRLGAGRIYKSRFIHGP
YIRPKFVRPHKGIRLPHPNRIIIPGSWKRPAHGIFALKGKKAIFCEHKGIKSISCSKDEEIKVKNALYG
RTAGDVCGRSRVTNCRALNSFAIVQKKCNGHNSCDLNPSNSVFGDPCVGTVKYLNIKYFCVKRIT
PTTTPNGKTAIFCEHKGIKSISCSKDEEIKVKNALYGRTAGDVCGRSRVTN
>TRINITY_DN135129_c0_g1_i1.p1 type:internal len:155 gc:universal
TRINITY_DN135129_c0_g1_i1:1-462(+)
SSFSYMSTSPSRSMDISASSDEALTPGGTLIIRDNVDNRTSFYERLQEKDSFIAETSSALNTPQNSPRP
STTTPLPRTPQQDRTPPKRSPLVRMKSDSSDIEKRAKFSKDNVSSTFRPVSMLNLSVFASNRPKRLH
TGSESELSEHVPLSTHRR
>TRINITY_DN34_c0_g1_i1.p1 type:complete len:149 gc:universal TRINITY_DN34_c0_g1_i1:477-31(-)
MDAAQAAEIKKKRTFRKFTFRGVDLDQLLDLSSEQLMELVHARARRRFSRGLKMKPLALMKKLR
KAKKEAPPMEKPAVVKTHLRNMIVLPDMIGSIVGVHNGKVFNQVEIKPEMIGHYLGEFSMTYKP
VRHGRPGIGATHSSRFIPLK*
>TRINITY_DN56792_c0_g1_i3.p1 type:5prime_partial len:1003 gc:universal
TRINITY_DN56792_c0_g1_i3:3453-445(-)
DNELKVIECNLRVSRSFPFISKTLDHDFVAMATKVIAGLKVERVLNIMNNCNKIGCKVPQFSFSRL
AGADFQLGVEMASTGEVACFGENRYEAYLKALISTGFTIPKKSILLTVGSYKAKNEMLGSVRLLEQ
MGFELYASMGTGDFYQEHGVKIQVIDWPFEENGIEGQEQSIQEYLSRNKFDLVINIPMRQTSSKRN
PHFITHGYRTRRLAIDHAVSLITNIKCAKLFCEALYRTIGQIKVKTHIDCVSSGNIIRLPGLIDVHVH
MREPGAEYKEDFQSGTAAALAGGITMVLCMPNTRPSITDTLSFKQAQKAAQKGARCDYALYAG
ASNKNAKHIKQIASSCAGLKMYLNDTFSTLKMDNLSDWMNHFENWPKHMPIVCHAESQTTAA
IILVAELFNRPVHIAHVARKEEIMVIRAAKLRGIQVTCEVSPQHLFMTVDDIGGECEGKYRVKPPIV
TAEDQKALWDNIDIIDCFATDHAPHHVDEKNSENPPPGYPGLETMLPLLFTAVHDGRLTVDDIIL
RLHTNPRRIFGLPEQHDTYVEVDMDEEWVIPDAMTYTKSKWTPFAGRKVKGKILRVVLRGELAVI
ENEVLAKPGMGEDVRTWEPTHCMPPVSTTGLVSSKDAVDPTRSAYLGVTAPSVPVEDIHKTIESPS
SPARKLFGTQKDNEGMHNILETIGPVSVAPSISQPSPLFAQTHLFHGQHILTVESLSKEQLYALFNL
AGYMRQEVRKGKSFDVLKGRVLALLFYEASTRTSCSFTTAMQRLGGSTIEIKPSDSSMKKGESLED
TVQTMSCYSDVLAIRHPKKGAVQNAAEHCKRPVINAGDGTGEHPTQALLDIFTIREEIGTVNGITI
TMVGDLKHGRTVHSLARLLTMYRVNLQYVSPKSLKMPESVKEYVKQRGIPQVEISTLEDALTNTD
VLYVTRIQKERFDNEEEYNKVHGSYIVTPKLLTKAKEKMVVMHPLPRVNEISTEVDSDPRAAYFRQ
VECGMYIRMALLYMLVAR*
>TRINITY_DN167719_c0_g1_i1.p1 type:internal len:144 gc:universal
TRINITY_DN167719_c0_g1_i1:431-3(-)

SRRAMSAPYKAMDDGGSRMNVPAEVGALVLRLTVCVTVVHHGLQKLQNPEGFAQNMVAKWF
PFLPAPLFWTYAAAGMEIVAPILLGLGVFARLAALGLLVTMVFANAFHFLFTGSEGYPLGVPASGA
YAFEPSLLCGAIFAYF
>TRINITY_DN164271_c0_g1_i1.p1 type:complete len:163 gc:universal
TRINITY_DN164271_c0_g1_i1:1172-684(-)
MATGIKPDPQCTDLYNKLKMTGGKAKPYEYLILHIDDKLNKVVPDVYPDLNSTLEMDEYKDVTG
QPASYKRLKDYLLAKASAYAFYHFEWSEKDGERSEYVMIAWCNDNKANITQKMKFSGTKDEVK
KVLKVGKVIQCNDADDIDYKTIIKDLSKNKAIFTK*
>TRINITY_DN19237_c0_g1_i1.p1 type:complete len:209 gc:universal
TRINITY_DN19237_c0_g1_i1:128-754(+)
MMSEDEQMRMELQRMQRRADEMTDESLESTRRMLRMAEETQDTGIKTLVALDEQGEKLNRIEE
NLDEINDGMKEAERNLTGLEKFCGLCVCGCKKRKDFEGTEEYKKAYNHKSDGNKNGRSGNKA
GGSNDVAKSGYIQRITNDDREDEMDENLGEVAGIVNNLKSMALDMGNEIETQNKQIDRITDKTN
ANEIRIDHANKRAENILRS*
>TRINITY_DN11643_c0_g1_i1.p1 type:complete len:187 gc:universal
TRINITY_DN11643_c0_g1_i1:1748-1188(-)
MPAKGKLTQEEEVLLQDFSRTVSKKSQALFYGNALMISVLPLWLFWKVHLMDPMSYAALFICGTL
SAAYLVAFAYKNTKFALKHKVAQKREVAITREVNTELDSRPETKKLSRKEKDDRILWKKNNVADT
EATTFSIFYTNTLYLFIIIIASFYVLRSFSPAVNFTLSTGMAAGTVALLSTGTQSS*
>TRINITY_DN140702_c0_g1_i1.p1 type:complete len:2036 gc:universal
TRINITY_DN140702_c0_g1_i1:94-6201(+)
MATSFKVSEIKCTELLNLEKKGAIDPLVEVEYLGLSKETENKDNDLNPSFGEVFDFDLSGKALGPE
DFLKITVKDYEKFGRNRVVGKADVYLQELLSNPSSTLSATLNDKNDRPTTGKITFKVDYIGPAGGG
AGGATGGGGDDRQAAERELEGDEDEGDPNAMPDGTPIPEGPAGDAMRAEKRKMKKNKKKRR
EHNLPDKPTDFQIRIKIIEGRQLPGGNINPTVRVHLGEETKQTRVKHSSNKPFYDETFFFNFNERPN
ELFDKLVTFEVHNSKNVLSEAMIGAFQFDVGTIYFEDSSHAIIRKWLLLTAPEDNDKKDGEETKGA
SLQPGGPPAGYLKITAIVLGPGDELPEKAKGSGKSEGGEEDIESNLLRPAGVTLRPATFLVKIYHAE
DIPQMDSAAFDGVKKLFSSGSAQKDHVDPYATFSFAGKTQKTEIKYGNNHPDFNQELRVAFKFPS
MCERLKLQMFDWDRIGNDDCIGTSFISLTAISGNGDEGFLPCFGPCYVNFYGSTREFSDLPDEHDN
LNKGRGEGCGYRGRALVEIDTRFGDHTAKPVQDIMAQELVRVQAYMRRRKYKLFASFLSANMIS
EADGLVEFEVSIGNYGNKLDPDTEPQSSSTPPTNPVYDGCAYYFLPWQESKPCCVVESHWEDVIFR
LEPINCLLKIIDKMEKNIKSVELGLKAKAQPAEVAALLIALLDQFISDVSMDLPKFAATDNGVNEL
DLKIQEIRSDELLNLKEEASTLREQATDVQEAMDTIDGYLKRMRDLCVEPQNSMPDVVIWMIAGS
KRVAYHRIPAYDIFYSENPLARGRKCASLQNFILKYPGKKGHDMEDHPEIPASVSVILWLGLEKHQ
AAWTDTEQMEGEYSVYAETYENQGKPPMFDWTAKIIKRPGFSDSEGKIRLNKEYYEPPEGWKWD
DPPDGDWFIDPEMSSNFDADAGHKKFMQDCFENESRVPAGNWGKSTPHYTNVQGDEIDHRDE
TSLPEGWSWTDDWKVDVNRACDEEGWEYCIEATGGGWGAVFRSYHFCRRRRWVRNRQLEADP
QKVMKQKKEEAKQGEGWEYAPVFGMKYHVKEHKFDFVRRRRWHRKMVCVKPGAVPMFKIPD
DDDKGKFHQIMPRIFLAYDEPHKWQFRAYLFQARDILGLDSEGTSDAYAKVCFQDQSQITDVIEET
LCPEFNQTLLFEDMQLYGSPSVFAENPPFVSIELFDKDKVGKDEFLGRVLAQPIVRLNGLKPPATRL
MWYDIHHGERTCGELLAAFELFLDEGADLPLPPPWLEKEKKFQVPSGIRPILVKTGVEILNWGVR
NMKKYQLASCTSPSVTYEIGREIITTDVIKNTKKNPNFLKNIFYKEVYLPKDPLYMPPIVIKVRDHR
TFGRKPVVGRHVIKSIDFVQPPKADPVVELPPADLVGEPEEKKDVTLDIEEKKKKKEIPVDDNLDW
WSKYFASADEIEKCGNYLKKGHKTIQIYDSELEEIKDFNGFSDFIVSYPLERGKEDDDEDDNVVGE
LKGLVKVYPLPQDPKDQTKIRYFTPKLKEKSKIVKCIVRVYVIRARDLQPKDPNGKADPFIVVQLG
KKKISTKDNYIPNEINPFFGSFFEMKADVPLDKDLKIKVMDYDFMSGDDLIGETMIDLENRFLSNC
RATCGLSPAYYTSGAYQWRDAKSPKEILDQVCRTKPQWLSNEQVVVNGQLVKIEDFEESPPTDPHI
GPVEQRLALHVLNNEDIIGHRLVPEHVESRSLKNPLQPSMDQGQLEIFVDIFPIANGVPGPPLDIAT
RKPKEYVLRVIIWNTAEVPPSETSITGEEMSDLYLKGWMEGQDDEKQKTDVHYRSMDGEGMFN
WRFVFPFKYMPEERVMVVEKKEHFWSLDTTKQTLPPRMNLQVWDNDLFSANDFIGNLELDLSK
LPRPKKAAKKATLAMLDTEGDDVMDLFQAKIAKGWWPMLSENEDGTRTMQGKVEMTLEIVTD

LEHEAKPAGQGQEEPNMNPKLDPPNRPETSFLWITSPWKTLKHIIWKNYKWYIIGFFLFIILILFIGL
VLYSVPGSIVHKIFGFQ*
>TRINITY_DN96_c0_g1_i2.p1 type:5prime_partial len:189 gc:universal TRINITY_DN96_c0_g1_i2:1253-687(-)
LASLGICYGADLMVTKQVYFDVSIGGQNAGKITIGLFGEVVPKTVQNFYELVTHSKGFGFKGSKF
HRVIKAFMMQGGDFTNGDGTGGKSIYGERFPDENFKLSHYGPGWLSMANAGQDTNGSQFFITF
TKTPWLDGRHVVFGKVVNGMDVVKKVEQVQTDSRDKPKKDVTIEDCGVVKVEKPFAVKKQ*
>TRINITY_DN26249_c0_g1_i1.p1 type:5prime_partial len:477 gc:universal TRINITY_DN26249_c0_g1_i1:1733-303(-)
QMKMAASNMAGHVVRNNLIFLLSFVIIIVAEEGRHARFEYKFSFKGPHLANKQGTVPFWNHYGS
AIPSDDQVRITPSMRDQRGGLWTKTKSSSEFWEVEIFFRITGRGRVGGDGLAMWFTEEAGIQGPVF
GSSDHWKGMGLFFDSFDNDGEQNNPYVMVMLNDGTKQYDHFKDGNSQQLGGCMRDFRNRP
HPIRAKIRYYNNILTVFYHSGMSERSDDYELCMRAENVVLPKEGFFGITAATGGLADDHDVIKFLT
HSLTPPLTDEQKQKILSEEQRKKYEEQYGEYTQKLETKKEEFQKEHPDKKAPEEKFESVYDRDVRL
IYENQNTIHHVVRALHEKVGELTNQLNNLHVTINNNNQLAQQQQPKGDGVTRQEVNTIINLQN
QVHNAIQELKQQSGGGGGDNTKTVQMEKTLQTVLEQVGHLVSKNNDRRPSCPTVSCVTPSILILF
LVAQVLLVAGYNFYKEHQNKAAKKFY*
>TRINITY_DN140696_c0_g1_i1.p1 type:complete len:260 gc:universal TRINITY_DN140696_c0_g1_i1:43-822(+)
MPRGPKKHLKRLNAPKHWMLDKLSGNFAPRPSSGPHKLRESLPLVIFLRNRLKYALNYTEVMKI
MKQRLIKVDGKVRTDICYPAGFMDVITIEKTGEFFRLLYDVKGRFVCHRISSDESLYKLGKVKRVAF
GKNSVPYLVTHDARTINYPDPSVNVNDTVVIDIKTGKINDFIKFDTGNTVMVIGGRNTGRVGIITH
RERHASSYDIVHIKDAAGNQFATRLSNIFLVGKGNKAMVTLPRGKGVRLTIAEERDRRLASK*
>TRINITY_DN8707_c0_g1_i2.p1 type:complete len:564 gc:universal TRINITY_DN8707_c0_g1_i2:2751-1060(-)
MSGFVRQSKYRHVFGSPARKDGCYEGFRVTNCAFDGNFIAANKHFIAVCVEVGGGGAFIIVPVGS
TGRLDVNIPKVSAHKEYILDLQWNPYNDFMLASCSEDGTIKIWEIPEKGLFMNIGDEKALLSLEYH
EKRCTQVLWHPSASNVLMSVSQEPKVCVWNLDEGSVEVEILEHPELIYNATWSQKGDKIGTTCK
DKKIRIFDAREGTLLVQGMGHEGAKPSRVIFTFQDKYLFTTGFSKMSERQYACWNAETIEQLGLEE
LDTANGCLVPTYDADSQLVFLNAKGDSNIRYYELVDGDPYYFYLSTYQSSAPQRGFGFIPKRCINV
NANEISKGYKLHGTKTGYIEPVSFTVPRKSETFQQDLYPDAIGDEAALEPDDWFSGKNAEPKRIA
MSTFFKGKTKPTGAAGGGLKKGGLKGLKAKKEAKTAAKSAAKSEPAKPAPVKTESKDKVDAAP
PAAAAVEQKSSSTAVAAPAAAAPVVVSGVDEKVVKELQNEIKSLKDGDKKLQKEIISLKDKLKDY
DTLRSDIKLLCDAVKKNDERITALESLVAEETDEEETGEGGDE*
>TRINITY_DN41831_c0_g1_i1.p3 type:internal len:115 gc:universal TRINITY_DN41831_c0_g1_i1:342-1(-)
WGSWSSVRTRGEDKDARTQRSSKTPPQYRSSCLKNGKLRVCWMQRLTASTVLMAGIGKLTMESP
ASLTMSMTRSGGASPIDLRANSPVRPMSNARARSLTWAWRRACLPNKQAR
>TRINITY_DN2490_c0_g1_i1.p1 type:complete len:1044 gc:universal TRINITY_DN2490_c0_g1_i1:354-3485(+)
MDVNKKVNQAYIDDDDDITINFNPKSGDPNPQAQRFKISKLPSQSDSNNSMEDIEEADDVDGLE
KDNVQNTVSKHVQLTIPAEHYHNDHNDTRRASHTHTQTIGYMTHDAVPMSVFYRNQASMENA
ANGDMRPTLDQLHKGQGLDEARKRHWDALNEEDEQQDAERGTSNTKTKLGWIKGVLFPTLLN
IWGVILFLRMPWIVGQAGIGLSSVIVLLATVVTTVTALSMSAVCTNGEVKGGGAYFLISRSLGPQFG
GSIGIIFSVANAVAVALYLVGFGETVQALMARSDTYMVDATNDIRIIGLIALILIFVIAMIGLEWVIHT
QTFLLLLLVAAILCVAVGCVYPAPQESREKALAWGLPGYKSKSFADNFVPDYRDGEEFFGVFAIFFP
AATGIMAGSNLSGDLKDPQKAIPLGTLLAIGMTSTSYVALSWILGSFVMRDASGIIPVINVTVNGTT
MNSHTCEIGSCEYGSVNDLQIIEKISLWGPLVLIGIFAATLSSALASLVSAPKVFQAVCKDKIFPHIEL
FGKEYGKNGEPRNGYLLTLVISACFIAIGELNVIAPIISNFFLMAYALINYSVFAASIAKSPGWRPSFK
YYNKWCALVGFFLCIVIMFMINWWAALITIIVVAALYKYVDVRKPDINWGSSGQAHTYLKALRFT
RRLEDTDEHVKNFRLQVLCLTGQPSLRPNLVHFCSHITKHLGLLVCGEVNVTDKILSPSGNEGKW
LKKQKIKAFHQIIHSATLRNGAQSMLQSVGLGKLKPNTLIMGYLNRWQSIEVAKVDEYVHIIHDA

FHLRYGVGILRLQDGFDITDGVVDDQIENGVVENHEPDWYTEDWDENQKDTRGSDGDGSDQA
KEERQETFKPPDIVSAFPKKRKGYIDVWWLYDDGGLTILAPHLLALSSHWRGCKLRIFTPASDKKI
KANQIRMANLLKKFRIDFSSVVEFRGINKHPKEDSINQFKQLRRGEHLPQDGLLDKKTLRQIRLGE
LLHEHSSEAKLIVLTLPIPKEGMVTSLMYLSWLEVLSADLPPVLMLRGNQTSVLTFYS*
>TRINITY_DN39385_c0_g1_i1.p1 type:5prime_partial len:154 gc:universal TRINITY_DN39385_c0_g1_i1:2-463(+)
SNNITIKLSDNVTKINRSDGKEVAKIFLISDTHVYLLNSKTLQLIHQIEISKINKVTLTPYKDGIIVLH
VNQGSDKSLQKGDFILNSSNVIENVTRLAMLYESISQKNLDIDFSSNLCINTKGKNIDLVVQENDD
NNQLVFRKKGNQIMVSVQ*
>TRINITY_DN367_c0_g2_i1.p1 type:complete len:199 gc:universal TRINITY_DN367_c0_g2_i1:67-663(+)
MNILYLSGLLLLCGLAFCDEEDIEVEDFGQSCTKVPTRKCEHVKRCSRVDKHHPEVCKVVEECKH
VLVEKCVPKPKPRFETKCEVVDTVRKCETVYKCIPKYFYVKECKSRQVCKHIPFFKKVCKDFCTKK
LVKHHYGHPVFKKVCHPRCYTAPFPKSECKDEPFCFQKWQSKKNCGNFKHCWDQDIRKCRKVL
VSHH*
>TRINITY_DN1121_c0_g1_i1.p1 type:complete len:252 gc:universal TRINITY_DN1121_c0_g1_i1:1155-400(-)
MAEREQLIQQAKLAEQAERYEDMATHMKGVTKMADQPLNTEDRNLLSVAYKNIVGAKRSSWR
VISSIEQKATDSKKQYAEAYRIVIEKELSDICNEVLHLLDDFLVKSASATYYEDPNLTTAESAVFYLK
MKGDYYRYLAEVASESEKQSVVDKASNAYKEASNIGIKSLAATNPIRLGLALNFSVFHYEICSEPSK
ACTLAKDAFDLAIAELDSLPEESYKDSTLIMQLLRDNLTLWTADQADGNEPDAE*
>TRINITY_DN3773_c0_g1_i1.p1 type:complete len:504 gc:universal TRINITY_DN3773_c0_g1_i1:168-1679(+)
MAPVVGDLMDLSDIDASKQHNQAVVRDYISQPRLIYKTVSGVNGPLVILDQVKFPKFAEIVTLTLP
DGTRRSGQVLEVSGSKAVVQVFEGTSGIDAKHTTCEFTGDILRTPISSDMLGRVFNGSGKPIDNGP
PILAEDFLDIMGQPINPWSRIYPEEMIQTGISAIDTMNSIARGQKIPIFSAAGLPHNEIAAQICRQAG
LVKHSKGTMDYSDDNFAIVFAAMGVNRETARFFEQDFKENGSMDNVCLFLNLANDPTIERIITPR
LALTAAEYFAYQCEKHVLVILTDMSSYAEALREVSAAREEVPGRRGFPGYMYTDLATIYERAGRVE
GRNGSITQIPILTMPNDDITHPIPDLTGYITEGQIYVDRQLQNRQIHPPINVLPSLSRLMKSAIGEGM
TRKDHSDVSNQLYANYAIGKDVQAMKAVVGEEALTPDDLLYLEFLQKFEKEFISQGAYENRTIFES
LDIGWKLLRIFPKEMLKRIPAKTLAEFYPRDGRKEETD*
>TRINITY_DN32522_c0_g1_i1.p1 type:complete len:235 gc:universal TRINITY_DN32522_c0_g1_i1:147-851(+)
MAAWLDSYRETEQLGHDIMELINERNTLKRSASNTSKVQSSIRIKMQQYSSGLQRLKDLLLRDSSS
YNVTQQELQRRQNQLDVLVSKEQQLNQAFKPTQSNYDRSNLFGEKYGDDLNGRYGGASASNGL
DDDLTPQNFRQQQEQILKEQDKGLDSLHEVIQRQKMMGKAIGNELEYQNDLIDDITKGVSRVDN
KVNATERKVKKVHRKTGSCAMIVVIILLLIAILVVAVFPTN*
>TRINITY_DN18187_c0_g1_i4.p3 type:5prime_partial len:112 gc:universal TRINITY_DN18187_c0_g1_i4:361-26(-)
SRKAMPVQGSIKKPHRYRPGTVALREIRKYQKSTELLIRKLPFQRLIRELAQDFKTDLKFQSQAVLA
LQEAAEAYLVGLFEDTNLCAIHAKRVTIMPKDMQLARRLRGERL*
>TRINITY_DN34191_c0_g1_i1.p1 type:internal len:238 gc:universal TRINITY_DN34191_c0_g1_i1:1-711(+)
VRQRSAPAPGGGPPFATMAGRFDPSKLLQAFASRGAPLAAGGAVLFGGAYAVQNAVYTVDAGH
VAIKYHRMSGIGSSTYREGVHFMLPWFERPIVFDVRARPHTMTSLTGSRDLQMVNISLRALCKPDP
SRLPDIYRNLGPDYDEKVLPSIINEVLKSVVAQYNAAQLIMQRELVSRMIRQRLVQRANDFSILLDD
VAITHLSFSSEYEKAVESKQVAQQQAERARYLVLKAQEEKK
>TRINITY_DN12_c0_g2_i1.p1 type:complete len:203 gc:universal TRINITY_DN12_c0_g2_i1:239-847(+)
MANRPKGFGMTAELANKRAAKFDAELAAEAMGWVKDVLEASGNDEAVNLAREIGSIDGPTDV
KGPLKNGVILCHLINSIQPGSVKRINQSSMAFKQMENIGNFLTGCEGIGCSKQDLFQTVDLYEGN

NIPQVINGLFALGRKARKTGYDGPRLGPDEASENKREFTEDQLRAGEGVIGLQAGSNKGASQAG
QNFGKTRAIID*
>TRINITY_DN2301_c0_g1_i1.p1 type:5prime_partial len:452 gc:universal TRINITY_DN2301_c0_g1_i1:2886-1531(-)
TCVCRNHIAKMEGKSTCQQLLFVITIILSCISALFGKTLVLLDNANIKETHSIFFENLKDKGFELVFK
AADDPSLTLVKHGEYLYKNLIVFSPTVEEFGGSVDVPAITTFVDNGGNVLVAGSSNVGNPIRELGSE
CGIEYDEEKTSVIDHHNFDQSDDGTHTTILTDAKNLIKSNVIIPSKVDSPIMFNGVGMTADTDNPL
VLEILSAESTAYSYYPDMKITEYPLAVGKNTLLIGGQQARNNARIVFCGSLDFFSDQYFMSGAQNA
AQSGSTYFIQSGNMKVAVDVALWTFRHNGVLRVSQVNHHLLGEVDPPPAYTIEQEVVYSIKIEEFS
NGQWVPFQAKDVQLEFFRIDPFVRTYLKSSTDGVYNVNFKLPDVYGVFKFKVDYDRVGYTHLFSS
TQVSVRPKQHTQYERFIVSAYPYYASAFSMMAGICIFSVVFLHHKETSKVKDE*
>TRINITY_DN97809_c0_g1_i1.p1 type:internal len:184 gc:universal TRINITY_DN97809_c0_g1_i1:2-550(+)
LGPDQVDVAGAGRDPRHLPRPLGRRLRVLDRHRGRVRQRHKAEADQDPKLETARLDVRRPGVQ
VRRVRHPRPSSRDQCHRDCHAPGRVEGRAQVDRGRRGLGRRRVEHEVHGAAQPAARGQEKGR
LRAQQILAVAAGQEEAVDVGARNRQGPPSALAAVSRAPHAQFARLAQRDRRVARAKGW
>TRINITY_DN278_c0_g1_i1.p1 type:complete len:470 gc:universal TRINITY_DN278_c0_g1_i1:3091-1682(-)
MAPSFNKTNGHMEENGGMIHANGKSSEKPINLGEQTYSYMTFKKMGLIMITLPLVFLLLVGLPSY
LYPVLVLLFFIPVFAGYIGLNNVIYCLYERHNQVSKIDNYIEFLDEDLKKKFTGRFIPIRDLYELYAEG
KLNFKGDVLQCLERRNEYVSYKLQLWHLKFFLSKLLPELLHARSQDKEQVCDHYDRGNDFYNA
YLGPLMVYTSGIRYSESDTLEDMQMNKLKAVCEKVQLKEGDRHLDIGCGWGTLVNYAAENYNS
QSEGVTLAKEQVCWAEEKAKSKEIMDRVNYLCMDYRDIPKKKYDKITCLEMSEHVGIKRYHHFL
KDVRNMLEDDGIFFLQIAGLRRAYQWEDFLWGFFMDTYIFPGADASLPLQFPIAELEAAGFEVES
VKTVGIHYSDTIKYWYHNWVKSDSHDKMIQKYGRRMVRVWDIFLAWSTIIARQGNSTCYQIVAH
KNFNHYPRRRFIQPKN*
>TRINITY_DN413_c0_g1_i4.p1 type:3prime_partial len:463 gc:universal TRINITY_DN413_c0_g1_i4:1388-3(-)
MVSLSVTCLSLLLFVTVNAEESDHCGYDEKSWYKAMGWCYHSGSRLLLGDFNGDKRTDFFCHD
SNGKNWVAVSNSDGSFGSTTWQDSKERWCASPNILLTGDFNGDGVTDILCHGTATGKKWIKYFN
KYGVPSGKSFYRAMSWCNHKGSQLYIGDFNGDKRDDLLCHDTSGRYWTSYAGTDESKVFHHTS
WYKALEWCRHATSKLYLGDFNGDKKTDVLCTDGKGYKWIMYAKTSGNLFTGTDWHTNLDWC
HESSTVFLRIGDVNGDGKDDLVCHSTAGNSWVAYTQPKGGFGNFCHLAKWCAAGKESFFLGYF
NQDSRADILCHDTKYGKKWIRYAECSFAEESDHCGYDGKSWHKAMRWCYHSGSRLLLGDFNG
DKRTDFLCHDSRGINWVAVSNSDGSFGSTTWHDSKERWCTSPNILLTGDFNGDGVTDMLCHDY
KTGKKWIKYFNKHGVPSGKSFYR
>TRINITY_DN26892_c0_g1_i1.p1 type:complete len:233 gc:universal TRINITY_DN26892_c0_g1_i1:108-806(+)
MHMEERNMSSEKFEEYEDEFQTYFQEFKSIVHNKLPKLTAEPRKKEVRNAEKIYEDADFQLTQMT
DEAQKAPGVHKSRLMTKTRGYRADLNKVKRELVKISTAVIMSNSGAREDLLESSRPERQMNIQQ
QSRMKEGLDYLERGSQSVARSQRIAAETEEVGVQIIGDLDDQKETLHRVKDRLKDTEADLGKSRRI
LNSMAVRLATNKLLLAVIIFVELLILAGVTYLKFFKK*
>TRINITY_DN6_c0_g1_i1.p1 type:complete len:196 gc:universal TRINITY_DN6_c0_g1_i1:843-256(-)
MIFTVFLIGLQFVGIYNHAIPHLHPGFHPFHHYQPQGLLNQMDKEFTFSCPNGKAISFIGSVHDNR
KEDRQWTVYCANDVMEPRGTSTWTYYVNDFDGEMDFACADGNIVTGIASFHKNKYEDRRYKF
RCTYMPGTIRKGCSWSKYSTYDAMFSEHIEPGRYLTGMRSSHDNNKEDRIFQIETCRFDHYYEQQF
H*
>TRINITY_DN76040_c0_g1_i1.p1 type:5prime_partial len:383 gc:universal TRINITY_DN76040_c0_g1_i1:1-1149(+)
RTSSKAGHRFAEITKMASEQETDAEMIKECEDYVKKHNIQVLLKDAIVQLCINKPDNPYQFLRDH
FEKLENEHGGKTGKDDHVSATEPPPQATTRRRRGAVSAAVISEEDAASYVKKVIPKDYKTTAALS
KAIEKNILFSHLDESERSDIFDAMFMVKHSEGEVIIQQGDEGDNFYITDSGEVDVYVNDNLVVSISP

GGSFGELALIYGTPRAATVKAKTDVKLWAIDRVTYRRILMGSTMRKRKMYETFLEKVSILESLDKW
ERLTVCDALEPCNFNDGEEIVVQGEPGNDFYIISEGQAAVLQRRSPNEDPIEVSRLNVSDYFGEIAL
VLNRPRAATVVARGTLKCVKLDRQRFERLLGPCGDILRRNIQQYNSFVSLVV*
>TRINITY_DN15016_c0_g1_i2.p1 type:complete len:566 gc:universal TRINITY_DN15016_c0_g1_i2:94-1791(+)
MGQVLPCLPSPSAGSRSKLPLNMDNIIMLTDSYKVSHYVQYPEGTDEVYSYYESRGFDSSCVAGHP
TVCFFGLQYFIKRYLEGPVVTMEKIQAAKAFFDLHFVGTGAKFNLDGWMHILKAHGGCLPIEIKA
VAEGTEVPTSTVLFTLKSTDPKCYWLTNYLETLLVQVWYPMTVATNCMYQRRMLLEYLKETGSPE
GIDFKLVDFGFRGVSSVESAAVGDLAHLVSFCSTDTVAGMVAAQSFYGAKMPGASIPASEHSTITS
WGREHEVDAMRNMLSKYPTGMVACVSDSFNIYDACDKKWGQILKEQICSRDGILVVRPDSGEPK
EIVPELLEKLAAAFKAGTCGPSPGTKKNEKGYVVLDPHVRLIQGDGVNTQTVLEILHEIKNRGFS
ADNVTFGSGGALLQRMDRDTQKIAFKCCQISVKGKKSDVYKDPIHDKGKKSKMGEMFLIRDPSM
PKKSPEVRGAMDPNRFAGECYDLVTVCHYEGGTKRAVWASDAMGKAGRADPESIGRSRAPTAD
GLAKDKAADLLVPVFRNGVCLKEYTLKEVRALAAKSWPGDVGCNTLQ*
>TRINITY_DN1397_c0_g1_i2.p1 type:5prime_partial len:107 gc:universal TRINITY_DN1397_c0_g1_i2:2-322(+)
NTLKCTTGLQTKWPAIPFATADVPSAVDECFPGTKECVTVKYDYIFQFETKIATPKNEFRLPVVQK
MCNRHNIDKCNKWCTILSIHQRMENCDADCCKGDNCNANA*
>TRINITY_DN128406_c0_g1_i1.p1 type:5prime_partial len:150 gc:universal TRINITY_DN128406_c0_g1_i1:474-25(-)
FGSTRKMSQTQSVQVFGRKKTATAVAHCKRGNGLLKINGCPLHLVEPSTLRYKVQEPILLLGKERF
EGVDIRVRVKGGGVVSRIYAIRQAIAKSLVAYYQKYVDEVSKKEIRDILVQYDRSLLVADPRRSEPKK
FGGPGARARYQKSYR*
>TRINITY_DN1315_c0_g1_i1.p1 type:complete len:371 gc:universal TRINITY_DN1315_c0_g1_i1:246-1358(+)
MKILLAVLCTICAVYGKSLPQGPEEDNGEDMSATDIIFNVNQYAGKTDESISTFGAGSDSNPDLFE
GDIVMTNRLKSQINQMNHSNGNDKFDAIRNGAWPKGVVPYTFASNFARTYRSAVMSAIKEYNA
KTCIRWKPYTAALARAAGGYVQFFHSGGCYSMIGRQGGLQKISLGTGCGTKGVAIHEMMHALG
FFHEQSRRDRDSYIRINWNNINPSTRYNFEKYRSGQASTLGEPYDKSSIMHYGNYAFSINRQKTIVSI
SNYNEVLGQRNGLSVIDARQLNKYYKCSNTGTGVVKPPVVTNNLTNKYKFCVDLSKWCGDSFV
KANCKKTCGCKDIKTQKECSYWSNQGFCRHTWVTFMKVNCPKTCNMC*
>TRINITY_DN33483_c0_g1_i1.p1 type:complete len:344 gc:universal TRINITY_DN33483_c0_g1_i1:56-1087(+)
MMTMSEEKGTTTVTLTNPKDIIEKVVVHPLVLLSVVDHFNRMSKIGNQKRVAGVLLGSIRNKVLD
VANSFAVPFDEDDKDASVWFLDHDYLENMAAMFKKVNAKERIVGWYHTGPKLHANDIRINEVI
RRFNSNSVLVVIDAKPKELGLPTEAYIAIEEVHDDGTPTSKTFAHIPSEIGAEEAEEVGVEHLLRDIR
NITVGTLSQQITNQLSSLKGLHVHLQEIQTYLEQVISGKLPMNHTIMYHLQGIFNLLPNLNIEEFIK
SITVKTNDQMVVIYLASVIRSVIAMHNLINNKLQNKELEKEKEEGKTKKDKDAKDDKSSSKDEKE
KKDEKKDSKKDEKKT*
>TRINITY_DN1091_c0_g1_i1.p1 type:complete len:212 gc:universal TRINITY_DN1091_c0_g1_i1:200-835(+)
MKLILICLFAVLVYSIHCDQPSDPVWPNLFQQSFREKFYYPVIGTHYTNGTYYYDYKNLRYRLDRD
NGRYDRYCGLNGIRAFQNTPCTHLVLDGTRWLVYPEKKECCECCHAANGCGVLKPTWLTGAEF
VSKSNGIYKWNQKGLQDNFYSERVTDRIMTEIDQVPNDTQYYDPDSFKTKIENIEDVFKLPSYCKR
GTKCSFFSACRAVGFF*
>TRINITY_DN26679_c0_g1_i1.p3 type:5prime_partial len:108 gc:universal TRINITY_DN26679_c0_g1_i1:1-324(+)
TKSLNRRGWRLAAPAFRARKPTTRLKLPAPGCACAALKPAHLDQATAVGAHEDQDRHQDGPH
QLVVRVHLHATLPLRKLSGQRLLLREGHVAPLARPHQLDLHLRRQ*
>TRINITY_DN36471_c0_g1_i1.p1 type:internal len:286 gc:universal TRINITY_DN36471_c0_g1_i1:2-856(+)

GNKWLVDCRVSKNTINIIKINRKSYCYLPEILDRIMTSITEQPCYTLINYPMENDTVSESKLKEDLEK
GDIPVKTEALKKVMYQLLNGEKMPSMLMTIIRFILPLQDHTIKKLLLIFWEIVPKTGQDGKLLHEM
ILVCDAYRKDLEHPNEFIRGSTLRFLCKLKEAELLEPLMPAIRLCLEHRHSYVRRNAVFAIYTIFRNF
DFLIPDAPELVYEFLEKEQDASCKRNAFMMLIHVDQERALDYLSSCIDQVHSFNEILQLVIVELIYK
VCIANPQERARFIKCI
>TRINITY_DN1479_c0_g1_i1.p1 type:5prime_partial len:107 gc:universal TRINITY_DN1479_c0_g1_i1:397-77(-)
GPYKNTLKCTTGLQTDFPNIPFASPNVPYAVDECFPGTKECVTLEYDFIYKFHSTIAQKNVFKLPFV
QKMCNRPYMPCNAWCTFFYARQATESCKAKCCKGDNCNA*
>TRINITY_DN73587_c0_g1_i1.p1 type:complete len:533 gc:universal TRINITY_DN73587_c0_g1_i1:94-1692(+)
MLRQAVRRLSRGTNKRSLLRKSSERLLPACVNACQRFVSTDVTNNQAAAAAKAADKSPAAAVA
VGQIVAVIGAVVDVQFDAGLPPILNALEVQGHGSRLVLEVAQHLGENVVRTIAMDGTEGLVRGQ
RIVDTGNPISIPVGPETLGRIMNVIGEPIDERGPIETKSKAPIHTEAPEFVEMNVNQEILETGIKVVDL
LAPYAKGGKIGLFGGAGVGKTVLIMELINNIAKSHGGYSVFAGVGERTREGNDLYHEFIETGVINL
KDDSSKVALVYGQMNEPPGARARVALTGLTIAEYFRDVEGQDVLLFIDNIFRFTQAGSEVSALLGR
IPSAVGYQPTLATDMGSMQERITTTRKGSITSVQAIYVPADDLTDPAPATTFAHLDANTVLSRGIAE
LGIYPAVDPLDSNSRILDPNVVGEEHYQIARNVQKILQDHKSLQDIIAILGMDELSEEDKLTVARAR
KIQRFLSQPFQVAEIFTGHMGKLVELKDSINGFKQILSGELDHLPEVAFYMVGDISEVESKATRLAE
EAS*
>TRINITY_DN493_c0_g1_i1.p1 type:complete len:473 gc:universal TRINITY_DN493_c0_g1_i1:2681-1263(-)
MKCMKFISLMLITFTSILVAQDTGFIYYDDSSSSDIDSLTEPYGYQSVMDNREKSYEKPDMRYNFDK
PSASDIDSLNTPQYGYEPVMDDRERSYKRPDMRYNSDEPSARDIDSLNIEPYDYESLMDYRDTPSE
RPGMRFDFDESITGDIDSIDTPLDDYQSLMDHKRKPYARKRRSIRHRRFIGQRRWYGRRKFIRKNQ
RQRNGFRKINSQRKRVCSKGSNCRHKLEPMGCFKDIKSERAMTEEILNERDTASKVFNNRLINWR
QYHEWLPGFICRCAQAASERGYSVIGIQFWGECWSGPNSESTYNKHGQSDSCVNKNLQENRAN
LTQCAQAAGKDSTNYVYRIAPACNTKYEPVGCFQDDQKEPRPIPHYISTDRDSSLDIFSGKLLNW
KQWNTHMPDMICRCAALAKSKGHKMFAIQFWGECWSGPLSNDYKRNGASGECRGKDFKECSC
NSYYCVGKTHTNYVYRLS*
>TRINITY_DN751_c0_g1_i1.p1 type:complete len:227 gc:universal TRINITY_DN751_c0_g1_i1:731-51(-)
MAMKIVLCTLAALMCCAYAQDGITAITDIINPENINTALDAAEKLKNLIHTTSESKIQSREKTQGFE
FLNMKANAQYTRGMKKHHLMRMMNRYLVPFSRSSADMKEVVDTLKEFIEDDVEEHQEQFFGL
MTQGGGGTTEAIFVYYKLRDDGKYNFKRMTFRGTFRLAADLVITHSSKSGFFSSESKDVINWLPRR
GVTAQNVQELLDIIVPKLATVMSEFAPKDSI*
>TRINITY_DN165222_c0_g1_i1.p2 type:5prime_partial len:133 gc:universal TRINITY_DN165222_c0_g1_i1:1-399(+)
FRHDLGGARRLFLHVGRGGRPPDRFLLDRAGAPAGGGPRGGRLDGDVLLRRGERIPHIFVLLHA
VHDLRDHIRLLLRWRILHRAVRRNHADLLHPVDEEQGLRRARGRPGDARRGHHRRPSVDHRGV
LGLPS*
>TRINITY_DN925_c0_g1_i1.p1 type:complete len:261 gc:universal TRINITY_DN925_c0_g1_i1:826-44(-)
MGRVIRGQRKGAGSIFKAHVKKRKGAAKLRSLDFAERNGYVKGVVKDIIHDPGRGAPLAIVAFR
DPYKFKLRKELFIATEGMHTGQFIYCGKKATLQIGNILPVGTMPEGTIICNLEEKSGDRGKLARAS
GDYATVVSHNRDSKVTRVKLPSGVKKAIPSFNRASVGIVAGGGRIDKPLLKAGRAYHKYKVKRNC
WPKVRGVAMNPVEHPHGGGNHQHIGFPSTVRRDTSAGRKVGLIAARRTGRIRGTAKVKDVGGD
KE*
>TRINITY_DN624_c0_g1_i1.p1 type:complete len:884 gc:universal TRINITY_DN624_c0_g1_i1:218-2869(+)
MANQEEEEHFGYYYGEEEDRGLLDPAWEKQQKKTFTAWCNSHLRKVNVQIEELNVDFCDGLKL
MLLLEVISGERLPKPERGRLRFHLISNVNKALDFIASKGVKLVGIGAEEIVDGNLKMTLGMIWTIIL

RFAIQDITVDEFSAKEGLLLWCQRKTAPYKNVNVQNFHMSFKDGLAFCALIHRHRPDLIDYDKL
AKDDPMYNLNYAFDVAEKELDIPKMLDAEDMVNAVKPDERAVMTYVSCYYHAFSQSQQAETA
AKRILKALNVNQGNEKAIEDYENLASDLLDWLNNKIPTLDDRTTDSSLDTMQNKLNEFRDYKGT
EKPPKVDDKSKLENLFNTLQTKLRLSNRPLYMPAEGKMINDIDKRWKDLEQSEKDFQEWILNELR
KLERLDHLAMKFKHKCDIHEAWSTGQDDYLRSDDITDASLASLLASRKRHEAFESDLAAQQDRV
EQIVAIAQELNTLGYSKIDEINTRCKGICDGWDTLGELTTQRSEKLAKAIEIAERLDNLWLEFAKKA
SPFNNWMDGAKEDLMDMFNVHTIEEVNDLKDAQLKFKANMPAAQEEFNTIMGLQNAISSMSS
QPNPYTNTKAEDLQEKWQEVLELIPKRDSLLESETQKQQNNERLRVQFAQMANQVGAWIKQRM
GRMTDIGMNAQGTLERQLEELENLNAEINGFKQNVLDLDASARNVQEAMIFENPHTPYTMEVI
RIQFEQLLTGVTRNINEVENQILMRDSIGITEEQVKELRTSFNHFDKNSNGRLDYKEFKQCLVSLG
QLGDVEDKGEHTFNRIIAMVDPNETGYVSFQAFLDYMTREMADTDTADQVMESFRILAGDKPYI
TADELRRELPSEQAEYCIARMSAYNGPDGVPGALDYRSFSTALYGESDL*
>TRINITY_DN146208_c0_g1_i1.p1 type:5prime_partial len:284 gc:universal TRINITY_DN146208_c0_g1_i1:876-25(-)
TDSHIRTTSAFIPCAKPSKMPPKTKKIKVKKVAAAPFQTSKPAPKKSVNVLIERRPRNFGIGADVQP
KRDLTRFVRWPKYVRLQRQKRILYQRLKVPPSINQFTQTLDQQTATQLFKLLHKYRPETKAEKKQ
RLTQLAETKAAGKQESSRKKPVTIKYGINHITNLVESKRAQLVVIAHDVDPIEIVVWLPALCRKMG
VPYCIVKGKARLGKLVHKKTATAVCLTAVRQEDRHSLEKVSDAVRTNYNERFDEIRRHWGGGIM
GGKSQAKVAKAERAKAKELRV*
>TRINITY_DN120478_c0_g1_i1.p1 type:complete len:544 gc:universal TRINITY_DN120478_c0_g1_i1:2674-1043(-)
MMRAPVMVLNSNTKRESGKKVQISNIEAAKTVADIIRTCLGPKAMLKMLMDPMGGIVMTNDG
NAILREIQVQHPAAKSMLEISRTQDEEVGDGTTSVVILAGEVLSVASQFIEDKIHPTLIIAAYRKALD
DMLDVLKEQISIPVDVDNDEEMINILKSCLGTKFVNQWSKLACNMALKASRTVSTEASGRREIDI
KRYAKVEKVPGGSLEESCVLDGIMLNKDILHPRMKRYIENPRILLLDCNLEFKKGESQTNIELSGET
DFNRILQLEEEFIKKMCDAIIKHNPTLVFTEKGVSDLAQHYLVKAGIAVIRRIKKTDNNRIARAVGA
TIVNRPEEIREEDLGLKCGLFEVKKIGDEYFSFLTKCEDPKACTILLRGASKDILNEVERNLQDAMH
VARNVMVDPRLVPGGGAAEMALSCALEEKAKSITGVVQWPYKAIAKAVEVIPKTLIQNCGVNAI
RTLTALRAKHAEGGNSSWGINGETGEIANMKDLGIWDCYSVKAQTLKTAVETAMLLLRIDDIVSG
SKKSKGDEGATSGGMPTM*
>TRINITY_DN165536_c0_g1_i1.p1 type:complete len:249 gc:universal TRINITY_DN165536_c0_g1_i1:851-105(-)
MSEQEISNLVEQAKLAEQAERYDDMVKYMKELTEKSTEGLSDEQRNLLSVAFKNVVGARRSAWR
IIHSLEEKKGNEGGNTELISSYRKEIEKELKERCNEVLSLIDKYLLPSAQKEGQKGTEGKVFYEKMK
GDYYRYLVEVTTGEDRANLSAQSKEAYDKATETATGLATTHPIKLGLALNFSVFHYEIMNDGKKA
CELAKKAFDDAIAELDSLKEDSYKDSTLIMQLLRDNLTLWTSENDNEENDAQ*
>TRINITY_DN13856_c0_g1_i2.p2 type:5prime_partial len:292 gc:universal TRINITY_DN13856_c0_g1_i2:3-878(+)
GAGRQAWSPSVSRQHGAAGGGAWAGAPESAATLTSSLAVGLSAGVVVAGLSSRRARTARRAVE
NKSQNGPFGELATVEPRTLELTEKVKAVIKQKGSLDRESYREIFLAPKPDAVFAGGLVGSEYHGWG
TYQWDPAGLATRYPEHLPWYREAELKHCRVAMLAWLGLIVPDFVRIPLDDFQDPTLTLENAHNK
LIYGLGSGPMWWLLLFCGIIESLRFKQLGLDFGKLTLENAGDLDFGKGFLPKTKEGIVQMKIKELK
NGRLAMLAVSGALTQSMAYGVYTFPWTLPSPN*
>TRINITY_DN140996_c0_g1_i1.p1 type:internal len:109 gc:universal TRINITY_DN140996_c0_g1_i1:326-3(-)
VPHQPEQCVTVKHQSCSKPDRVCLPHDHHCTFSQLCRPFNVRKCCTVVSVERRCQFVNRCVPKY
SYTRVCRHVQQCPPKCRNVCRQVDLGEHQVKSICEQKCDDSAHR
>TRINITY_DN200_c0_g1_i2.p1 type:complete len:551 gc:universal TRINITY_DN200_c0_g1_i2:123-1775(+)
MITARLARSLAKNAPKNLLKQYQGAGAVAAIIASRHVQTSKAASATAEVSAILEERILKTSSQVQL
EEAGKVLSVGDGIARVYGLKNVQAEEMVEFSSGLKGMALNLEPDNVGVVVFGNDKLIKEGDVV
KRTGAIVDVPVGEGLLGRVVDALGNPIDGLGPIPADNRARVGVKAPGIIPRTSVKEPMLTGIKAVD

SLVPIGRGQRELIIGDRQTGKTAIAIDTIINQKRFNDAQDEKKKLYCIYVAIGQKRSTVAQLVKRLGD
ADALKYTIIVSATASDAAPLQYLAPYSGCAMGEYFRDNGKHALIIYDDLSKQAVAYRQMSLLLRR
PPGREAYPGDVFYLHSRLLERAAKMSDDFGGGSLTALPVIETQAGDVSAYIPTNVISITDGQIFLESE
LFYKGIRPAINVGLSVSRVGSAAQTKAMKQVAGSMKLELAQYREVAAFAQFGSDLDAATQALLN
RGVRLTELLKQGQYVPMDIGEQVAAIYAGVRGHLDKIDPSKVVKFEEEFVKHVKTAHQDLLDLIR
KEGQISEATDAKLKEIVVAFMQSFE*
>TRINITY_DN21847_c1_g1_i1.p1 type:internal len:133 gc:universal TRINITY_DN21847_c1_g1_i1:397-2(-)
DTKKRKLSAELANGRLAMMAIIGMFFQDGLTGAAWGDWELYVDSPLRAFENELGVQDPVGFW
DPAGFTADGSVENFKRRRQTELKHGRVSMLATMGYITPEITGKLPGYLSPSAGLKFADIPNGLAAI
SKVP
>TRINITY_DN7808_c2_g1_i2.p1 type:3prime_partial len:277 gc:universal TRINITY_DN7808_c2_g1_i2:830-3(-)
MWFGPAALARRAGVVEFVDGKARFVVDEAAKAAAAEAAMAGVFRVDNELGVQAPLGFWDP
AGFCDGCTELEFKRFRSCELKHGRLSMLATIGYIQPFYGKLGGFLSPSAGLKFADMPLGFAAASKV
PSGGWIQILLLAALCEFGLGIEEWKERAGGDYGKGFLGMFGPVTDPEKKRRSLNAEIANGRLAM
VAITGMFVQEGVFGTTDMWFGPAALARRAGVVEFVDGKARFVVDEAAKAAAAEAAMAGVFR
VDNELGVQAPLGFWDPAGFCDGCTE
>TRINITY_DN2487_c0_g1_i4.p1 type:internal len:370 gc:universal TRINITY_DN2487_c0_g1_i4:2-1108(+)
LTGSAWGDWSLYTESPLRAFESELGVQAPVGFWDPLGFCKDADQIKFKRRRQVELKHGRVCMLA
CLGYITPEYFRLPGYLSTSLGLQFNEVPNGLAAIGKVPVAGGLQMFFFAGAMERGLLQQDPSRDP
GDFANIGNLGVPYSEDIPDPEARKRSLNAELANGRLAMVAIMGMMFQDGTFGTTGPEMWLPGS
AFENELGVQAPFGFWDPLGLSKDGDADDFYRRRCTEIKHGRISMIACIGYITPEYFKFPGFCSPTLG
VEFTYIPNGLAALKEIPAPGIAQWFLFCGFCEVYAARQDPKDPPGKLSGNDEGFEQTTYGNLGLY
KGSSIADPDKKKRGLNSEIANGRLAMMAIIGMFFQDGLTGSAWGD
>TRINITY_DN142212_c0_g1_i1.p1 type:internal len:178 gc:universal TRINITY_DN142212_c0_g1_i1:3-533(+)
GLAKDGDSTAFARRRATELKHGRVSMLACVGYIVPEYVKFPGYLAPKSGIMFEEVPNGLTALAKV
PGAGVAQIIAFAGAMELNFLTADPGRAPGDFKNAGRLGIPFGGGIEDAEKRKRSLNAEIANGRLA
MMAIIGMFFQDGLTGSAWGDWLLYDDSPLRSFAPDPGYNTLPDWSKP
>TRINITY_DN7671_c0_g1_i1.p1 type:complete len:123 gc:universal TRINITY_DN7671_c0_g1_i1:425-57(-)
MTSKRRNGGRNKNGRGHVRYVRCTNCARCVPKDKAIKKFVIRNIVEAAAVRDLSEASVYDGYA
LPKLYAKLSYCVSCAIHSKVVRNRSREDRKIRTPPPRFGMRREDNKRPGQPGAPGAQRT*
>TRINITY_DN1468_c0_g1_i1.p1 type:complete len:245 gc:universal TRINITY_DN1468_c0_g1_i1:766-32(-)
MADSNKLPGVPETLLKRRKTLEKVKAARAAAQLKTKKHLKEKRKVIFKRAEQYVKEYRSKERDE
IRLRRMAKKAGNFYVPAEAKLAFVIRIRGINQVSPKVKKALQLLRLRQIHNGVFVKLNKSTLNML
RLVEPYIAWGYPNLKSVRELIYKRGFAKVNHQRIPITDNQIIENVLGEQDIICVEDLIHEIFTVGSNF
KKANNFLWPFKLSAPTGGMRLKTNHYVEGGDHGNREDFINNLIRQMN*
>TRINITY_DN5587_c0_g1_i1.p1 type:complete len:809 gc:universal TRINITY_DN5587_c0_g1_i1:196-2622(+)
MAQNKDDLSTEILKKKSKPNRLMVEEAVTDDNSVVTMSADKMEELQLFRGDTVLLKGKRKKET
VCIVLSNDEATSNDKIGMNRVVRQNLRVRLGDIVSVQPCPDVKYGKRVHVLPIDDTVEGLTGSLF
DVYLKPYFLEAYRPVCKGDLFQIRGGMRSVDFKVVETDPSPYCIVAPDTVIHCEGEPIKREDEEDA
LNSIGYDDVGGCRKQLAQIKEMVELPLRHPQLFKALGIKPPRGILLYGPPGTGKTLIARAVANETG
SFFFLINGPEIMSKLAGESESNLRKAFEEAEKNSPAIIFIDEIDAIAPKREKTHGEVERRIVSQLLTLM
DGLKQRSHVVIMAATNRPNSIDPALRRFGRFDREIDIGIPDATGRLEIIRIHTKNMKLDDEVDLEQI
AAETHGYVGSDMASLCSEAALQQIREKMDIIDMEEETIDAEVLDSLAVSMDNFRFAMGATNPSA
LRETVVEVPTVTWGDIGGLENVKRELQELVQYPVEHPEKFLKFGMTPSKGVLFYGPPGCGKTLLA
KAIANECQANFISIKGPELLTMWFGESEANVRDVFDKARMAAPCVLFFDELDSIAKSRGGSAGDG

GGASDRVINQILTEMDGMGAKKNVFIIGATNRPDIIDSAILRPGRLDQLIYIPLPDEPSRISILKANLR
KTPISKEVDLGYLAKVTHGFSGADLTEICQRACKLAIRESIEKDIQREKQREAQGQNDADMEMDD
EDLVPEIRRDHFEESMRFARRSVSDNDIRKYEMFAQTLQQSRGLGTNFRFPGSSNQQSSSGTSQPAS
EQPSIYGNDDDDDDLYN*
>TRINITY_DN4595_c0_g1_i4.p1 type:5prime_partial len:1639 gc:universal TRINITY_DN4595_c0_g1_i4:5857-941(-)
QCGVNTGTQVVYRRCNAALCKYQGNDYTRNQEVNIRTQACTVACDYTPWQNTQSCTKTCDGG
VLVQTRTERAFNKIETRNGNTPCNTQACAQELGPKQLRTCNVGQCGVNTGAQVVYRRCNAAL
CKYQGNDYTRNQEVNIRTQACTVACDYTPWQDTQSCTKTCGGGVLVQSRTERAFNKVETRNGN
TPCNTQVCSSELGPEQLRTCNVGQCGVNTGTQVVYRVCNAALCRYQGSDYTRNQEVNIRTQAC
TVACDYTPWQDTQSCTKSCDGGVLVQTRIERAFNRVETRDGTTPCNTRSCASDFSAERLRSCLNV
NQCGVNTGTQTVYIVCNGNFCRYQGREYNINEEVNVRQQNCTIACDYTAWEDTTICSKICDGGV
LEQRRLERAFNRLERRNGTTPCNTRACDSGFSAVLSRNCVNVNQCGVSNGTQLLYRVCNGEPCIL
QERSYKINDVVVVGSRACSVPCNYTEWRDTTSCSKTCGGGFFFETRTEQAFQKTESRLGKTLCNE
NSCVKDNFGNWTEWTACSKTCGMGMRYRSRECLKGFECIGLIEETETCKLGCRTETCPMGRPYP
GDCNFEDDCGKSWNQVNSIQRNAFFIVTSKDLAHQGLLPNMETTFNTKDCGRTMKLNSSVAGD
VNYLWTKRMMITDINTDVDDDVAAIDNYFKEENEQCVEFNFAVMANSLDKIGDMQVYLYYLEY
PNASLIYSKKGNTGLKWELVKRSIRVDTNKAFVILFQVNQGQSNVQYGDIGIDGFKVNPGNCQLK
GWSHWGEWKCSATCGFGTQTRMRHCLDKVNRTACVGMDRENKPCPMMKECPMNRDGNWG
QWTEFSKCSKTCDTGTYFRTRKCDSPSAKYNGSLCRVSQTKFNLPVVNDFEIDFKPCSVQQCNNT
FSQWTTWSSCSGTCGPGQRTRTRTCESGKRCSGIFEEIETCYMGCRLEKCPKFTAEIDCNFEDRNKS
DCQWKYYTVSGTSSWSFKTQPKNRLLSGPSGDSTIGCGYFLYYPSQTLSSAYAWGPNIYPTMDSYC
VKFKYSMVTSVNSSMGNLTVIKHFLNRPRKADTPLFFHSGVSQKQWNPMAIDITTDIQRSPFVIMF
KGQSDPNNAGLSDIAIDDYMLMAGKCNKSYSEWSEWSTCSRTCGVGQRTRTRVCLDDDIKVCK
MGDVATEGEACVKPLCAVPVDGNWGQWSAYSQCNARCNGTQKRERFCNNPAPSNGGRMCLL
GDTIFQLRNNYHAQIRPCSIDCGDNDTFSSYKRINDTCHFDCITGRVLQERECEQDTCFDGNTRFS
KGEKQYTQRACSIKEYCETSEYKCREFRSVCSTNTPSSCLDPQNRAVKSCECQVERGCSSMTNPGQ
TVKHKEFDVFYRPCRYVGVCFTDYETDAFSPYEKVNDTCHLSCSNGKVFEERECRQDNCIDGSNT
LTRGAKQYRETQCTTKEYCETSQYSCREISGPCSSNPPSSCQDPANKSLKVCTCQVTTGCASMLTA
GQAVSYLAEETFSRPCKYTGACNANGNTDKTFSPYERVNDTCHISCADGKVKQMRECKKDACV
DGGQNFPRGFKQFKDSSCTPNEFCETAQYSCRELSSACTSNPPSSCLVSTNKAVKMCDCNDPNGC
DSMITPGKKVNQGEKEFLQRPCRYTGVCFTDAETGT*
>TRINITY_DN166344_c0_g1_i1.p1 type:complete len:360 gc:universal TRINITY_DN166344_c0_g1_i1:93-1172(+)
MQKPKKYDWKDSNMALFGSDTEKKVKKESAESEPAWQGAGADVGVQVWRINKFKVEHWDKE
QYGEFYNGDSYIVLNTYKEPDTDELKYDVHFWIGKYSTQDEYGTAAYKTVELDTFLDDKPIQHRE
VQGHESSKFKTYFPTLTLLKGGADTGFKRVLPEAYEPRLFHVKKQNRTQITCTQISLKRGNLKSED
VFIIDLGDKIYQYNGSSCSADEKYKATQEVARLKSVRGKARAETIEERTVSDEHPALMVLKDGSSK
EKSTVEGEKKMLRVSDADGSIGMDEVASGDDISKDKLTSDDVYIINTGSHVYCWIGSGASIDERKN
GLSYASNYLNKTETPWLPISVVGQGKEDSQFQKAF*
>TRINITY_DN484_c0_g1_i1.p1 type:complete len:554 gc:universal TRINITY_DN484_c0_g1_i1:164-1825(+)
MASMILNGERTTGNDVREQNVMAAASIANIVKSSLGPVGLDKMLVDDIGDVTITNDGATILKLLE
VEHPAAKILCELADLQDQEVGDGTTSVVIIAAELLKNAGNLIKYKLHPTSIITGYRLACREACKYIQ
ENLAVKTADLEREHVINCAKTSMSSKLVGVDSDFFSTMIVDAAMSIKRTGNKGETKIPIKSINILKA
HGGNMKESISVPGYALNCTIACEGMPKKIENAKIALLDFSLQKAKMHLGVQVLIEDPEKLEAVRQ
REMDITKERIEKVLASGANVILLSGGIDDLCMKYFVEAGAMAVRRIKKVDLKRIARATGGTVCLSL
ANLEGEESFEASYLGHADEVAQERICDDELILIRHPKTKAAASIILRGANDFMVDEMERSIHDALC
VVKRVLESKSIVPGGGAVEAALSIYLENFATSLASREQLAIAEFANSLLVIPKTLTVNAAKDSADLVA
KLRAYHNASQTQSDKAHLKWVGLDLINGVIRDNKKAGVLEPTMSKIKCLKFATEAAITILRIDDL
VKIEPEKKDDPNSYQNAIAGGRMG*

>TRINITY_DN119650_c0_g1_i1.p1 type:complete len:211 gc:universal TRINITY_DN119650_c0_g1_i1:883-251(-)
MKHNNILSNGHFHKHWQNYVKTWFNQPGRKKRRRVARQKKALAIAPRPVAGSLRPVVRCPTF
KYNTKVRSGRGFTLEELKTAGIPVKSAKTIGISVDHRRKNRSTESLQANVQRLKEYNSKLIIFPRKQ
SAPKKGDSDASELSVAAQLQGPVLPIMQSKPSTKARKITDDERKISVFRSMRIARANARLVGVRAK
RVREAEADAQMKAKK*
>TRINITY_DN7808_c1_g3_i1.p1 type:internal len:212 gc:universal TRINITY_DN7808_c1_g3_i1:635-3(-)
ESELGVQAPVGFWDPAGLARDCDVDTFTRRRTTELKHGRVAMYATLGYLVPEYFRFPGYLSPSEG
LKFADVPNGLAALSKVPLNGWFQIVLFCGCYEFPTYNSNRAPGDLGWKAITSSDPEVLKRKLNCE
LANGRLAMVAIMGMLFQNGTVGTTGPEMWFPAGAFEGELGVQAPFGYWDPAGFTKDGDAEM
FKRRREIEVKHGRVSMIACL
>TRINITY_DN131028_c0_g1_i1.p2 type:5prime_partial len:104 gc:universal TRINITY_DN131028_c0_g1_i1:2-313(+)
FDKYHPGYFGKVGMRNFHFIKHGKVIPTINVERLWSLVSEQTRTYYKEHTDKAPIIDVLKAGYMK
VTGKGQLPEQAVIVKARYFTKEAETKIKEAGGVCVLCA*
>TRINITY_DN1272_c0_g1_i1.p1 type:complete len:262 gc:universal TRINITY_DN1272_c0_g1_i1:957-172(-)
MRCQLFLLVACMAIATCSAYDWRNKVQNMLKYAKVNIFDRAAYILGDKRQHQLGKPWFCHKL
DCPAFQVVGNYTNKKDGFEERCYPETAWVMTSKEVDANENDIETRDMFMKLFKYIQGENEKQE
KIEMTVPVFTYSQLKKDDPSKRYISMHFFVPPSQEKNIPKPTRDDVTIMDVKADCYYVRSFGGYV
MSFGSKIMNEEKKIHAALEKNNLDFEARKTMAAGYDSPFRLFNRHNEVWVAKRKTMDSGCCTP
RKSTNVMY*
>TRINITY_DN884_c0_g1_i1.p1 type:complete len:542 gc:universal TRINITY_DN884_c0_g1_i1:1783-158(-)
MLRVLHQTCLSISRSTTPISGTETKLFFSSSSFRMVLDLELFRPDKGGCPEKIRENQSKRFKDVKKVD
NVIDCDNKWRKLRFQADNWNKLKNLCSKTIGMKMKKKEPVGDNDAIPEELSKTESLQGLNPQ
QLESFTVNQIKKIRTLIDEHMQNCEAERLECEKVRHQNLFEIGNWLHESVVVSNDEDENEIVRTV
GDCTTKKQYSHIDLLYMIDGVEYERGATVAGNRGYFLKGPGVLLQQGLINLATQTLIKNGFTPLYT
PFFMKKDVMQEVAQLSQFDDELYKVIGKGSEKEDDTSFEEKYLIATSEQPIAAFHRGEWMDTNEL
PKKYCGISTCFRQEVGSHGRDTRGIFRVHQFEKVEQFVFCSPHENESWKMLDLMCNNAEEFYKSL
NIPYRIVGIVSGALNHAASKKLDLEAWFPGSGAFRELCSCSNCLDYQARRLLIRYGQTKKMNDKA
EFCHMLNATMCASTRVICCILENNQTEEGIIVPELLRPFMGNIELIKFVKSAPIQEQKESKGKGKKK
GKEQNIATKLENMDVNGK*
>TRINITY_DN453_c0_g1_i1.p1 type:complete len:249 gc:universal TRINITY_DN453_c0_g1_i1:944-198(-)
MDIDQLKYEIIELGKQIETVHVAIAVVVLTTILLWIAIRSRRSGDTVLLLGLCDSGKTLLFSLLTAGKS
AATQTSIKENKSRLTMGKKSWAIVDLPGHERLRAKYLYNHKDSARGIVLMIDSVEFPREIRDVAEL
MYDLLANRTIKKNKVSILVACNKQDLTTAKACNVIKSQLEKEINNLRKTRSAALLGIDDNTSSKN
AFIGKKGKDFEFAHVKPIKIEFCECCLKDENGESKTDYNGIEEWMKKL*
>TRINITY_DN59667_c0_g1_i1.p1 type:3prime_partial len:225 gc:universal TRINITY_DN59667_c0_g1_i1:673-2(-)
MGKAKKGQKIDESKFEGKGTDFKCKLIGFVNVPSARGDDICQEAITKLKHLALKLNKEKKEHKQ
KITVNITVKGIRLIDEPTERIEHAHPINRISYICHDNDDKRTFGYIVGTKEGYKLFAIRTMKEAEYITK
TLKDLFQVVFERHQAQKAASDTSEIKGQEQVEEKDDEEKFEVGEEQNEEPTESPLLDWEHGINEQ
IQAMQNDAMSTSGFPDPFGDAQFDSA
>TRINITY_DN12390_c0_g1_i1.p1 type:complete len:268 gc:universal TRINITY_DN12390_c0_g1_i1:826-23(-)
MLASQVSIALIVFYLGCGNAYRPLSFYETNGPVQEILEDFEFRDDFEDGFLDRDAGVSMAEACVH
AHNVMRQRHQDTPNLVWDEGLAKAAQAYAEKLAAKDDGLHHDIKNNKKEGWGENLYFKDA
ARVGTCADAVISWYNEIDDYDFNLGMSRNHKAIGHFTQLVWKTTTKVGVGVATHKGQTDNDIK

TYIVAKYKPPGNWFHGYHDYVKPLIIPIKHRPTSVEELDRSVCTIDAFPSRFCRGFRREGRCGEFIVK
DACYRTCHSCN*
>TRINITY_DN4815_c0_g1_i2.p1 type:complete len:638 gc:universal TRINITY_DN4815_c0_g1_i2:79-1992(+)
MVSSLLLVLICTVVYTAGISAFFQCDFEKVDICGFKSSSSNYTDTGEAPNWRRHTGTTPSAKDQNN
AHVTGPLEAQSGDYYMYFEASNLPSGASSTGTLTSRLFDATQFGVTDIVFHVSSKGESLSLLVLDFI
DYTTNDIFDEKVVFPRNKAAWQKYTFNLDEKIFKRKFYMQFIGYVREVNGKKDNGDWAIDNLRI
DGIAIVEKCENVKCNVNTNCKDTSNGPNCECLPGYSTKAGTSGKNTECIETCQAFTCNLNAECS
NTENGPDCKCESGYKAINGTSGKTTRCEETCQAFTCNLNAECSNTENGPDCKCESGYKAINGTS
GKTTRCEDDTCSNFQCSNNTNCENTVSGPNCTCKEGYRPVNGTRGRDTICEDINECAIKNICGGL
KVCRNVPGSHHCDCPPNYTFFAAENKCYRNGAFSQWRNVGSCSKTCGAGDQKQTRSCSNPSRE
APHGRDCPNEPTERAVACNSGPCLAGCPRNKTCTCENVRQYKTGEEPKKDFSSFNGLSITDGETS
RRYVHTYNREFIEKTIKDVNCNNNQLKSVYKNITDIIGILEGINKKYSSTKMEMQDIIDCNANIDIE
KNLWDVYDVLAEHSICNYQVQKDLEMKADRIQAEITRCNNRGWYSTILDAII*
>TRINITY_DN22594_c0_g1_i1.p2 type:internal len:268 gc:universal TRINITY_DN22594_c0_g1_i1:801-1(-)
DFQDPTLNLETAHNKLIYGLGSGPMWWLLLFCGIIESLRFRQLGLDFGKLTLENAGDLDFGKGFL
PKTEEGIVQMKIKELKNGRLAMLAVSGALTQSMAYGVYTFPWTLPAPNAVQSRTALRAVEFKGQS
GPFGALDVAEPKTLELTDRVKAVIKSKGSLDRESYREIFLQPKAETVFAGGLVGSEYHGWGTYQW
DPAGLATRYPEHLPWYREAELKHCRVAMLAWLGLIVPDFVRIPLDDFQDPSLDLENAHNKLIYGL
GSGPMWW
>TRINITY_DN6701_c0_g2_i1.p1 type:internal len:169 gc:universal TRINITY_DN6701_c0_g2_i1:2-505(+)
ADSPLRAFEGELGVQAPVGFWDPLKLSADGDAEAFKRRRSTELKHGRVSMYACLGYIVPEYFRFP
GYLSPSEGLRFEDVPNGLAAFSKVPVNGWLQIVLFAGFYEFPTYNSDRAPGDLGWKAITASEPEAL
KRKLNAELANGRLAMVAIMGMLFQNGTVGSTGPEMWF
>TRINITY_DN647_c0_g1_i1.p1 type:complete len:344 gc:universal TRINITY_DN647_c0_g1_i1:1110-79(-)
MNPIFALRPSSKLIREGIKKFSTSNQKNVKVAVLGASGGIGQPLALLLKHSPMISHLSLYDLAPFTPG
VGKDLSHIETTSEVTTHLGPDNLDECLKGCSLVLIPAGLPRKPGMTRDDLFNTNASIAMNLADAC
ARNCPNAVVGIITNPVNSTVPIAAEVYKKHGCYNSNKLFGVTTLDVVRANTFVAEKKGLDITKTN
VPVIGGHSGVTILPLLSQVLPKTTFTNEELETLTHRIQNAGTEVVEAKAGAGSATLSMAYAGARFG
FSILEAMNGKKGVVECAYMASEETDASHFATPILLGPEGAERNLGMGELSDFEKEKLKEVLPELQ
NNIAKGEEFTAKNL*
>TRINITY_DN187147_c0_g1_i1.p1 type:complete len:1223 gc:universal TRINITY_DN187147_c0_g1_i1:3708-40(-)
MLTKFETKSARVKGLTFHSKRPWVLASLHNGVIQLWDYRMCTLLERFDEHDGPVRGIDFHENQP
LFVSGGDDYKIKVWNYKQKKCIFTLLGHLDYIRTSFFHKEHPWIISCSDDQTIRIWNWQSRNCISV
LTGHNHYVMCAQFHAVEDYIVSASLDQTVRVWDISGLRKKYCAPGAKPKEDITAPKTPGQMDLF
GHSDAIVKHVLEGHDRGVNWVTFHPTMPLIVSAADDRQVKLWRMNDSKAWEVDTCRGHYNN
VSSVIFHPRLELILSNSEDKSIRVWDMSKRTGVQTFRREHDRFWILGAHPTLNLFAAGHDSGMIVF
KLQKERPAYAVHQNTLYYIKERYLRMYELGTSKDIPIMQLRKSSGRSTPLKLIFNPAERCILITYNND
TSAGESQVYELHTLPKDIRSTDPEQSEGRRAAGTAAVWVARNRFAVLDKQQNVVIKNLKNEVTK
KVQTPACDMLFYAGTGSLLLRDAEQVMLFDVQQKRVMATVKIAKAKNVIWSSDMSYAAILAKH
SIAICNRKMENLCTVHESVKVKSGGWDDSGVFVYTTSNHIKYTLTNGDAGIIRTLDLPIYITRVKGS
NVYCLDRECKTKVLNIDPTEFKFKLALVNHRYDEVLYMVRNAKLVGQSIISYLQKKGYPEVALHF
VKDEKTRFGLALECGNLEIALEAAKTLDDKACWEKLSEMALLQGNHQIVEMCYQRTKNFDRLS
FLYLTTGNSDKLRKMLKISEIRKDVSGHLHNALYCGDVEERIKVLKQVGQGSLAYLTAATHGLVEE
CEEIKATFDLNPEKLPPINPNAKLLRPAVPILKNEGNWPLLSVSKTLFEGAAAMKDAGTSNMAAA
VDMDDEGEGWGDSDLVLEEDAGFVDPEEKLDIDEEGGGWDVDDDDLELPPDLDVGPVSSGGED
YYVPPTRGNSQAQVWCTNSQIPVDHLVAGSFDSAARLLNEQLGVVSFEPYKQLFMLAYARGRVA
APGLPSTPPLFYYPHRNWKEAGAKAGLPAVAIKLNDLVQRLQTAYQLTTGGKFADAVERLHQILL

SVTLLSVESRQDVTEAQQLLGICREYILGLQMELNRKELPKQNAQQQKRICEMAAYFTHCNLQPV
HLVLTLRTALNLAFKIKNYKLASSFARRLLELGPKPEVSQQTRKILLACEKNPTNAVQFDYDELNP
FSICAVSYTPIYRGKPEEKCPLCQASYKPEYKGTICKICRVAEVGRPGSGLKISMGR*
>TRINITY_DN148467_c0_g1_i1.p1 type:complete len:218 gc:universal TRINITY_DN148467_c0_g1_i1:105-758(+)
MGNIITLMYVDDDPVIKGLMLASGPLMVAGGLLLPVNLENATVATFLNMTEYGCFSIWFGVQTW
MSFVAGITLIKILPRPKFSQVQKALFGTYYKAGYKIGSIMLGIHLLRYRNVSWQNDILYRGCAMFG
TMLLSASGMLLKPQLDAVVSEMRAVEAKYGQSDVIGVVTSEELLKNPEYQVLRKNFAKGHILAM
VSNFTMLGVSGYLMYTMAKRINF*
>TRINITY_DN101992_c0_g1_i1.p1 type:internal len:139 gc:universal TRINITY_DN101992_c0_g1_i1:414-1(-)
AERYDHHSGIVESMDSNKISDNLKELTRLPHPPGRSRNKELGDMLADFWQNEGMDKIEKFKYNI
YLSYPEKPSTILLFGGAKTEKYTIEAEPPVDESEKGGTPVVPFNAFSANDIVKAELVFVNHCRESDF
DEVKKAG
>TRINITY_DN98420_c0_g1_i1.p1 type:5prime_partial len:286 gc:universal TRINITY_DN98420_c0_g1_i1:1007-150(-)
LHKEARSTVINKMASRLLNSLGKVGVGLAVVGGVVNNALYNVDGGHRAVIFDRFRGVLPDVSG
EGTHFLIPWVQRAIIYDVRSKPRNVPVITGSKDLQNVNITLRILFRPVASELPKIYMSLGEDYDERVL
PSITTEVLKAIVARFDASELITQRDNVSLNVQEALVERCKAFGLILDDISLTHLTFGKEFTEAVELKQ
VAQQEAERARFLVERAEQQKQAAIITAEGDAVGAQLLSDAFKKAGNGLIELRKIEASEEIAQNLSG
SRNVVYLPDGQGMLMNLPAP*
>TRINITY_DN478_c2_g1_i1.p1 type:complete len:145 gc:universal TRINITY_DN478_c2_g1_i1:3-437(+)
MIKPDGVVRGLVADIIKRFEQRGYKLVAMKMMWPDEDLLNVHYGDLKTKPFFPGLIKHITSGPVV
AMVWEGTDVVKQGRQMLGETDPKKSKPGSIRGDYSIDMGRNIIHGSDSVESANDEIELWFKKGE
LMDYQLPLHSQVFEK*
>TRINITY_DN187527_c0_g1_i1.p1 type:complete len:102 gc:universal TRINITY_DN187527_c0_g1_i1:392-87(-)
MSGERTPITSDKRLQQTQKQVDQVVDIMRQNMDKVLERDTKLSELDNRADALQLGASQFETSA
GKLKRKMWWQNCKMWIILIAVLLVFIIIIVVWVVLENQ*
>TRINITY_DN79_c0_g1_i1.p1 type:5prime_partial len:478 gc:universal TRINITY_DN79_c0_g1_i1:2132-699(-)
VVSSESNVLVEFYAPWCGHCQSLAPEYAKAAKQLKESKSDIKLAKVDATVETKLAEKYEVQGFPTI
KFFKKGNALDYGGGRTDKEIVAWLNKKTGPPAKLLDNAADVKAFADERDVAILGFFNDKESSEL
AKAFLAVAEAVDDLEFAIAAPDASGDFAVTEDKIIVAKKFDDPQVVYSGEANIADIKKFVRAESIA
LVTEFTDEAAPKIFGGDIKNHLLLFISKKAENFAELKNTFQEAAKGFKGTVLFVYIDTDVEDNGRIL
EFFGLKAADAPTLRLIQLEGDMTKYVPETKDLDKDSVTSFVQNVIDGKIKPFLMSADVPEDWDK
NPVKVLVGKNFAEVALSKDKDVLVEFYAPWCGHCKQLAPIWDKLGEKYKDSENIVIAKMDSTAN
EVEEVKVSSFPTIKYFPKEGGVVDYNGGRTLDDFVKYLESGGKDVSGGADETGEDEDADEEEEGM
EEGMEEGDLKPEEPEVAKDEL*
>TRINITY_DN7808_c1_g2_i1.p1 type:internal len:237 gc:universal TRINITY_DN7808_c1_g2_i1:710-3(-)
VSLAAFENELGVQAPVGFWDPAGLSKDSDAETFKRRRTTELKHGRVAMYATLGYLVPEYFRFPGY
LSPSEGLRFEDVPNGLAALSKVPLNGWLQIVLFCGFYEFPTYNSNRAPGDLGWKAITSSDPDALK
RKLNCELANGRLAMVAIMGMLFQNGTFGTTGPEMWFPTGAFEGELGVQAPFGYWDPAGFTKD
GDAEMFRRRREIEVKHGRVSMIACLGYIVPEYFKWPGYLSPSLP
>TRINITY_DN36276_c0_g1_i1.p1 type:complete len:449 gc:universal TRINITY_DN36276_c0_g1_i1:104-1450(+)
MKFSQCCQLSQKTSFISMMVLTSTFFLVEIIVGYLTNSMALVADSFHMLSDVVSLIVGFVALKYSKG
GQQKGRYTFGWARAEVLGALVNAVFLVALCFSILVEAMKRMVIPEDIENPRLVLLVGTIGLVVNVI
GLFLFHQHGHSHGGSSHGHSHGGSNHSHTISPNITPREPALPKKDTSGTVAKVEVPLLDEHDLEM
DQDSSDGEGKENSPGASASQLNIRGVYLHVLGDALGSVIVMISALIIMYQHGVWTLYVDPGMSII

MVIIILKTSIPLLKESSMILMQTVPTHIKIQEIQDKLVQRIPGVISVHEFHIWQLAGNRIIASAHVRCQ
TLADYMAIANKLKEFFHDEGIHSTTIQPEFVEDENSTIVKECDYVIDKNDCILECKKDCVERTCCG
ALTNNHDNSTDSGMDIPPISPPLPQPNESEVPLEVVTTDPVSRLKEDSAA*
>TRINITY_DN165969_c0_g1_i1.p1 type:internal len:128 gc:universal
TRINITY_DN165969_c0_g1_i1:2-382(+)
RILGNGGSAVHRGRDGPPAARQVRREQDVLVHRHVLLRLGAGQRVGLGQPHRGGQSDQIHGRR
VCEQGPGRCTHARGAESTNLHRGGGLPHPVLRQVALAVPALHVAATPRDGRPAGAARGPRVRE
P
>TRINITY_DN1428_c0_g1_i1.p1 type:5prime_partial len:165 gc:universal
TRINITY_DN1428_c0_g1_i1:1-495(+)
HLSLNEQASSGLFVNLKMSDALATITIHKEYGYVVLTAFSSFVMLMYKAIKVGKARKLYNVPYPIM
YASDDNDKAKIFNCVQRAHQNTLEVYAPTMISLLLGGIQHPVLSSVAGMVWILGRIAYAQGYATG
DPSKRMRGTFGYLGLFTMYGCTISFALHLLKVV*
>TRINITY_DN2805_c0_g1_i1.p1 type:complete len:189 gc:universal
TRINITY_DN2805_c0_g1_i1:623-57(-)
MGFEKVVIIDGKGHLMGRLASVIAKSLLQGQRIVVVRCEGINISGNFYRNKLKVLDYLRKRMNTK
PSRGPYHFRAPSKCLFKVVRGMIPRKTKRGMEALNRLKVFDGIPPPYDRMKRMVVPSALRVLKLK
PGRKYCSIGRLSHETGWKYKALIESLEEKRKAKAELYYKARKQTEAVRQKAIQQVAAS*
>TRINITY_DN8008_c0_g1_i2.p1 type:5prime_partial len:1298 gc:universal
TRINITY_DN8008_c0_g1_i2:4205-312(-)
IMSPKERTCPLTQYLFTPIYTGSCKISDAEQRANDRQVKSAVQMAGSAIGTVVGTIFGGGIGGAIGG
FIGGAIGSLIGGIIGLFSSCDKSYEVVTRSWVEERKCILLEPDVRSITRPYSECFDAKIKANTGFKVPY
TCNCKVNDCIVKSPSPDCLLQNQQCERKRKDFLERTKTVPPTFSKLYKSLKQCQDSVGASILKVQS
AKKTVQFYEKEHTRIKNMLLALEEENKFSKQALSDATSILQDDLCVSNAFMNNKNINELIDIKNV
QFDTLLPLVNNIRINAHVKVKGNDVLVPFVHELSNDNKVSLNAFGRKIIMNTICKKIRKRRSLDL
NEDEKNDLAFVPWINNVNGTATVADLSCVSLDKTLEFIRHTVKTLNKKSSYANDLLMQLQYSTN
KLNPSLYTAFTAYSGITTEKNDIIKANEALLMSANNELNKIQDQVSVSNVLDIWGNEAEIVTGFNN
VSVCLNYKDCIISAIDTLKNLPTMFRNSREAYMKEVMELEKELTKIYEEKNLIGLSAKAQSVERIVS
RIKYMSFHCSKRPIVTLKEVKDMDVEKGKNITLRCIVRSARAIKYYWQFNDDILDEENSHSLTIPVS
NETSGIYRCIAKNLVGNGTSNQIRITTYTKPSFIEEPKDQIYILPSHPQSNLVLTCNVTNNYDTTIAW
YYRSFTIGKRPNIINGQTSILLDLDGTKQENSGYYFCQAFNKHGRIKSREARVNILNSNIARLEVNIS
LDILQTDEISSNSFKKISGDMLSEIKDLGRTLTTTVIKSKTKTRVTFNLKVKPITDNVKSLSESFNISSR
MRQNLGSAAAVLLGKLSENSFKLPINDGEFAKVDNDTMTYGMKLNICQEGYKLHKNGFLCVQC
PFGTAGKDCKKCPLNTYADAPGLGICKKCETSKITEQIGSTSKSQCIGTTERKVDIIFAVDVSKRFLE
QINGLQKIRELLSKFIKTYSGTKYNTRFALLTYYKYVDVYLSLTEYYHSDDILGLLEILGEGSRMPDT
KLALVEIETMFDEESSSDRKKVAVFITNGGTSDSYYLMELFKYKRQAITLQNKGVHIISVGLGTVDH
RGLRALASYPKLENAIFPSSNSVAVQKMIYSIGNELKDNCYTPAVDFGIIYHPNHKESLVQITKEQF
LRNANWLSKLLLPIYRMFKQQSYYFMYKHSVPSNSFGSFSETGFQERIVSYLQQQTSGFDIYKKIST
AINVDMTEIYRSFSKVKVLIIITEEPLDEAVLKQYEISVHGEKIDIYNFVKDASSIEKFEGPFSDKPRA
HRLVYGIDEVNFLVNAINCNRTKICG*
>TRINITY_DN2068_c0_g1_i1.p1 type:complete len:204 gc:universal
TRINITY_DN2068_c0_g1_i1:160-771(+)
MNPEYDYLFKLLLIGDSGVGKSCLLLRFADDTYTESYISTIGVDFKIRTIELDGKTIKLQIWDTAGQE
RFRTITSSYYRGAHGIIIVYDVTDQDTFNNVKQWLQEIDRYACENVNKLLVGNKCDLTSKKVVDY
TTAKEFADSLGVPFLETSAKNATNVEQAFMTMAAEIKNRMGPPQTTADNSSKKVPLTSSSPVKQG
GGGCC*
>TRINITY_DN87064_c0_g1_i1.p1 type:internal len:179 gc:universal
TRINITY_DN87064_c0_g1_i1:536-3(-)
PAPRARGRPSRRRRPPPPRRRPAGKMGIDIEAGGRVWNKPRVTKSENPYIRLLIKLYKFMARRTDS
KFNKVILKRLNMTRKCKPPLSLQKLAKHMDGKDGKIAVVVGAVTDDKRLYEVPKLTVCALRFTE
AARASIVKAGGECLTFDKLALRSPLGKGTVLLRGPVKAREAERYFGKA

>TRINITY_DN1084_c0_g1_i1.p1 type:complete len:301 gc:universal TRINITY_DN1084_c0_g1_i1:1131-229(-)
MGRPGFGGGGGRGGFGGGRGGRGGGGGRGGGRGGGGGRGGGRGGTPRGRGGRGGGRGGMR
GGKKTIIEPHRHEGVFIARGKEDALVTKNLVAGNTVYGEKKITVEGDETKTEYRVWNPFRSKLAA
AILGGVESIHMKPGSKVLYLGAASGTTVSHVSDLVGPDGLVYAVEFSHRSGRDLLNVAKQRTNIIPI
IEDARHPHKYRMLVGMVDTIFADVAQPDQARIVAINAHHFLKNSGHFVISIKANCIDSTAAPAAV
FAGEVKKLQSEKLKPQEQLTLEPYERDHAVVVGTYRPPPKKKD*
>TRINITY_DN1339_c0_g2_i5.p1 type:internal len:362 gc:universal TRINITY_DN1339_c0_g2_i5:1085-3(-)
NSRLAMVAILGMFFQDGLTGSAWGDWANYVDSPLRAFENELGVQEPVGFWDPAGFTADGNVE
NFARRRQTELKHGRVSMLATMGYITPELTGKLPGYLSPSAGLKFADVPNGLAAISKVPAAGWAQI
VAYCAFCELSQDQSAGSPAAAGDFGWKLLTSSDQGELTKKLSAEVANGRLAMMAIIGMFFQDGL
TGSAWGDWALYTASPLRAFENELGVQEPVGFWDPAGFTADGNVENFRRRRQTELKHGRVSMLA
TMGYITPELTGKLPGFLSPSAGLKFADIPNGLGAISKVPAAGWAQIVAYCAFCELSQDQSAGSPAA
AGDFGWKLLTSSDQGELTKKLSAEVANGRLAMMAIIGMFFQ
>TRINITY_DN598_c1_g1_i2.p1 type:5prime_partial len:742 gc:universal TRINITY_DN598_c1_g1_i2:2-2227(+)
RWSGCSKSCGVGVKTKSRECDNPRPSGGGRYCTGERTATEACKIQDCGVDGKFSHWSTWTACSK
TCGGGAQKRARQCNSPPPSGDGKDCAGAREEFKGCSNDFCKGPTGVAKAQEIIERFLENFHKKT
YPDREPSGKFLKAIFAIQKAVAEEFISVDPSVRGKTLEQRLKDLEKFLKVATEGGSIDFARTLLGKGIF
TFMKKKIQEGLPIDDIDKAAEGFLEKLRGDVGEKKMNEILSSQGDATLMFTIDTTGSMKDEINAA
KAIAKQIISATRDYEVDYILSPFNDPRTGPVTHRSSSNRASFVTDIDRLRATGGGDCPELSFTGMLN
ALNQGPQYGSPMFVFTDASAKDDSASNIDSLKAAADLTDATINFFTNLNGCKGAKSVESYKEIAS
YTGGQVFPLKNEAELKKFTNYVKTSLQQDTVITSGGTLNSKSYKRSLYSMQAHDFMVDDGIIMIS
VAISVQKYASQVTILDPNGRRVNPSTTLSLSLVYQISHPPPGQWKINFPSSVGKYGYTVQAVSSSAIA
FAHNFIYQQSVRKNSPPIPLANPLKGVKNMMVVRLGGTKNFDLNSIKIDLVDTSGNVLQKGIGTD
RLDSDEVRHATIDPPSGPFKVRLTGKTKSGYNFERVSQGISRPVSAAMRTNFAGYDFTAKLGKGPY
AAQMLFINRGAHENFYFYAKANYGSIPNYKQTKRVTSQGFVTFTYKAPRQYNLKGKSDRITVTVK
RQSTGEIVSHHTSILLVV*
>TRINITY_DN22219_c0_g1_i1.p1 type:5prime_partial len:427 gc:universal TRINITY_DN22219_c0_g1_i1:2-1282(+)
YISKDYCKGKKNPSVCTLHIHNEFSQIKHFQHQTPVTSENAPGIILAHGQAGDALTGAGNVWFSR
NGGYNWTRIAKGSHHYAIGDNGNLIVLVPKFNSTGKTLIYSLNQGRNWYHNEFDLFDENITIHG
LAAEPHSKERSFSLWGHRLVKGKKLWHTFTVNFRKLITKECQEEDMKTWYPHINNGKDGCFLGE
RTIYKVPHKDSLCYLGRSYKPEKKVEKCSCTKDDLECDYGYERNRAGNCHRNEDIPLIEICKNGEE
MQEAFSKGYRRVPGDNCIAQSSNDEVVRFIDEHKKCVPNKEDIFGDFNRERAEVIREEEEEKEKK
MDETQKATKTKKSKKKGPNWPVIILSIILMCIIIGGAYWYWRKNYRPNNVHHVMYNASSFDNVG
MDNNDGDVDADDLFDPRPKSLREYHDETDAEDEQLLLP*
>TRINITY_DN5001_c0_g1_i1.p1 type:complete len:1575 gc:universal TRINITY_DN5001_c0_g1_i1:40-4764(+)
MHAGLTSVFLTKRFISHFVLQKQIRSTFPVIQTFYRGFTSNMIEVNADGKNPASVVLLAVEHLKKH
EIQVTVKDNNKTFIKVSNTIVEGENDVSRFLARKYPEVGLYGKSGIESSQIDSWLDFSCLYIKGSQDL
LPSLKELNRAIGQADYLVGPKLTVADLSVWSALHGNSKWKNLLDSGKAGEPVSKWFKTCMQQG
TFKVPTESSPNKIKSKDKGSNPKNSKSNMEEGGKFIDLPGAKIGEVVVRFPPEASGYLHIGHAKAA
LLNQYYKDHYKGKLIMRFDDTNPAKENSHYEKIILEDIKLLEMTPDIYSRTSDYFEEMFVYAEQLIK
QGDAYIDDTPADLMKTLREQRLPSPKRESSIECNLKMFDEMKKGTPEGLKCCLRAKIDYKSDNGC
LRDPVFYRCRDEEHVQHGNKYKAYPTYDFACPIVDSVEGVTHAMRTTEYLDRDVQYAWVIEKLG
IRKVDIYSYSRLNLINTVLSKRKLTYLVDEGFVEGWDDPRFPTVRGVLRRGMTIEGLREFIAAQGSS
KSNVHMEWDKIWAFNKKVIDPIAPRYNALLKEEVVPVTVHGVEESTAQYPQHPQNPGSEKKTVY
YSNKVFVEGADANLMKEGDLVTFINWGNLRIAKVNKSDDKVTSLEANLELDNKDFKKTLKVTW
LAEHSEAPFIPTKCGFFENIIEKADMTREDDFKQHIRKDTKKVSDMMGDQCLANLKKGDIIQLQR
RGYFICDEPYQPPSRHSCKESPCILFHIPDGSTAGMMNQGKSKNTAGGKTSSSTKSSDNKNKGSSK

KQNKEKSPPADTGTIVEHTAVQQELFDSIVVQGDKIRNLKAQKATKDEITAEVATLKELKSKYKTS
TGKEFVNPTAATAAAPSKQPSKQPSKQQAASLSTSATVNHTPEQQALFDKIVEQGNKIRTLKAEK
ASKDQITSEVNVLKDLKSQYKSSTGSEFSNPSANNQSPTKKSKEKMAPVPVATPEAVTVESPEVEA
VKQKIVAQGDKIRELKSKKIEKAELQPEIDNLLKFKAEYKELTGSEYKPPGGGGNQKKGKEKKQN
EPKKAPEKKVTQQEAADGSAKKITRLGLEAKKEESLSDWYQQVITKAELIEYYDVSGCYILRPMAY
GIWERIKAFFDTRIKSIGVENCYFPMFVSQAALEKEKEHIADFAPEVAWVTKSGQSELAEPIAIRPTS
ETVMYPAYAKWIQSHRDLPLRLNQWCNIVRWEFKHPQPFLRTREFLWQEGHTAFATREEACEEV
HIILDWYEKVYTDLLAIPVVKGRKTEKEKFAGGDFTTTVEAFIAASGRAIQGATSHHLGQNFSKMF
DIVIEDPNNPDSKDKIHVHQNSWGLTTRTIGVMVMVHGDNKGLVIPPNVSNVQVVIVPVGVTAN
TSDDQRKLIKDTSLSYRDLLLDSGVRVKVDDRENYSPGWKFNHWELKGVPIRIELGPRDIEKNEYV
AVRRDTGEKISMKRESASTDLSNLLKTIQEDMYKSACKQLNDNLTVVKSWEEFLSMLDQNKLIQA
PFCGAIPCEEKIKKDSARDDGTADAGAPSMGAKSLCIPFNQPGKVDGLKCVCPCCSKDAVSYTLF
GRSY*
>TRINITY_DN244_c0_g1_i1.p1 type:complete len:3019 gc:universal
TRINITY_DN244_c0_g1_i1:9748-692(-)
MDARGQRLLYCLLIFLSLATLLVSQQVEKCKRPVDLVFALDTSGSVKDSGFRLIQKFAKDILKTFDI
GVADSHVGVLTFSEQAEPQIKLTETFDKAELMQMIDSLVYQGYRTATDDALRVAKTDFFSTAGGA
RQGVPHVLVLLNDGKCTVCIEPVKEAAQKLKDMGVEIFTIGVTENIKKQELIDIASQPTAYHFFEL
DDFDQLVDVVGDIQTRSCEVKKGKCPIPSPTEQCSGNEINECTSDENCEGELKCCSDGCSRICKYP
LLNCFTPIDIAFALDSSSSIQPETFQEMKKFSNDIVDSFAVSQKKTRFAALVYSDRAEIGFNFVRFDD
ASSIKKAIQNLPKLDSKERIDYALELAKSDIFSLQGKVRTRRPMVLFVFYDGTVSSGMKDLSLVVEP
LKNYGVKIVSMGVGPETNYYQMRKISSNNSDVFRATNFKELLPEIYNIAKRACTEKPGRCDASLN
PLPKEACKNVKILNQCENDFDCFGDAKCCRKGCNNVCVIRGPVCPRKLDIALAVHQNMDESEF
AATKFYLQSVVDQFNVGPSQTHFSLLSFGQEPKVIFDFNSDQSSNEIVRRKIDNFQYQYGIGRISDV
LALSCDAVFCAAGGTRDDVPKMLVIVTSRTSPSSPSKLKEKLQTLRNRGVNILMIGVGDVDTSLLQ
AITSDDPLVEDRVLLSKRFDGIFQYTKDISDYACGMAPALPPPDNIKGDAKGECGAPKSCYKGPKG
SRGRPGTLGQQGESGDLGLVGPKGIQGRRGDKGPDGQMGSKGKRGPDGLIGLPGFPGYNGIPGV
PGIEGRRGRDGDCGLPGSSGEAGQAGLDGLSGPPGRAGPVGEKGEKGLPAPENPNIRIEPGDRGD
TGSVGLPGFKGIKGEEGFKGPSGDSGVDGIKGVDGEAGEMGDIGTYIYQKGPEGDRGARGPPGD
GDTQGATAGLGVQGEKGEKGMRGEAGDGSIFPGIKGRRGDQGPKGEEGSTGPAGPTGQRGPEG
EEGPKGIKGEIGDRGKDGTPGQPGQEGDLGEKGFDGLKGQDGDDAEGELGDSGGKGLKGENGL
AGSPGSIGTPGPPGSKGEPGDIQIGLSGDPGAIGPRGPRGDNGISGQPGMRGDSGPKGAKGNQGP
RGDRGRNANMLAGKDGMTGPPGPKGFTGPVGDPGFPGPPGPPSVVECVEGACPKGVPGAMGQ
IGKTGEPGPPGPPGPRGSKGVAGGPCPICPDGIQGDSGDIGQPGMPGKEGSRGRKGNIGDAGSKG
LQGRNGDDGDQGPVGKKGFIGDIGPVGENGVDALGDSVAPDGEKGSTGDTGQIGSRGPVGNDG
PKGFKGTIGERGRRGLQGEKGDMGLTGETGEDGSRGLEGAKGDLGDGLRGAKGDKGNIGQEGN
QGVVGDVGLQGPKGEQGAPPTEDELKNVKATKGSKGEIGSTGDAGEIGDIGPAGPRGPKGFDGA
AGPKGPSGVKGDRGNKGRAGDGEPGFQGFPGRPGKRGPPGDPGKPGKRGFSEPGDAGDRGDP
GKSGKKGADAPPPIGCFKFNEPTDIGFILDSSSSVDDDNFLRQLQFVKNIVRQYPVGPDQTQVGIIK
YGEKAVTGINFYDYVTEEGLFNAIDLLEHTKESESRLDLALKLARDELFTESVGARGFDAKKALVIL
GDGFISGGGTPSKTLMNNAMQYAEDLRGAGVSIFALAVGPEKNKALFHEFVSEKEYYLDIESYSQL
DNELGALQLSLQTGCISVGVPGRDGLPGYTGPKGQKGDDGPKGFSPQGPRGIKGIKGVRGTIGIG
VKGLPGDPGLQGPQGEKGRDGEDGSADPGPKGRPGDKGSRGDQIKGASGEDGDIGPIGPPGFDG
RKLCDNVLAVDLLFVLDTSSGVGVDNFNKMLKFIESIINVFDVDNQLTRVSVMTYDSDARLVIRFS
DYNDPDKLDAAIDNIPYANGDETRIDKALLLASSNAFDTSNGAREGVNKAVILMTDGQQSFAVD
QVALKDAVKPLVDANVLMYAIGIGSEISPVDLKTIAAERVLIADDFDGLSLKIEDQIVLLGRVGCQG
PPGKDGKPGANGEDGFPGLIGAKGQKGDRGEAGTEGLKGNKGSQGEAFPGLNRKGEKGDPGS
AGPLGLPGCEIKNQNEVIDLGFAIDASGSLYEEGFNREKEFIKTIVDRVGRVSEQGIRIGVIVYSDTAS
IEIRFDDHFNNEDLKNDIDNLPFFAKNTRMDLGLEEAKKMFGEQFGGRGSSKKVLILLADGQQTY
VPGVTEPSEVAKELAAAGVDIFAIGIGQEIDRTELESLISKPEHIFLASDISKLIDELTPEVTSALECEG
GPRGPRGRAGTDGPPGSKGLLGPIGDKGFPGEPGSAGFGPKGDDGENGTAGTIGDRGEKGFRGP
AGVPGKRGAVGPKGFPGPNGEKGDRGDEGFIGFEGARGLEGPKGQRGDSSDNGVAGVKGSTGD

NGFKGPKGDPGKLPPGNRIEDFKGETGGSGLEGDTGPKGKPGRALFEPGPQGLTGPKGEKGDQG
FFGMSGPIGNKGFQGLQGFRGDDGAAGPVGEDGFPGMKGLPSSSGDQGDKGKNGRKGESGEV
GQPGRVGEDGDQGEKGYAGFVGMPGKKGDQGEPGVQGPQQIRNEILDNIRPGPKGYPGSTGPA
GDEGEDGEVGEPGETVGAQGMNGDFGERGSQGPKGEQGDQGLQGMIGVIGDKGVKGRVGTG
GNPGTVGLRGLTGAPGEVIFGSEKGNKGDLGIIGLRGRAGQKGKAGAIGPRGRRGRNGAKGERG
PPGFPGLSGIEGDSGNEGDKGPRGDPGSVGNRGIKGVPGESGIAEGNYIVIHSQTHIIPDCPRDFKK
LWHGYSLMYVVGNGHSHSQDLGDAGSCSQKFSSKIFLFCEIIKVCKHASRNDFTYWLAGDVDHP
MMPVSNAAVEPFISRCAVCEAHSSHITVHSQDTYAPECPPNWSSLWSGYSFLMVAGAGKTGAGQ
SLSSSGSCLESFRPKPFVECQGARGTCHFFSDKHSFWLVTIDPNKQFEVMKPQTLIKDKNHDLTSR
VSRCRVCIRKTGTKNNTVEPNIATRIRRSIASWIL*
>TRINITY_DN785_c0_g1_i1.p1 type:complete len:289 gc:universal TRINITY_DN785_c0_g1_i1:1062-196(-)
MIIESKMLSSIMLKMIFVVVFLCGITLSEGIEKCDLQLEPIGCFKDDVWKRTMPEQILNERDPLNKV
YNNRMINWKKFDTYLPGFICRCAEAAFKKGYRVIGMQFWGECWSGPEAHLHFDSLGAADTCSN
QKFEREYDNITQCVSAIGMDSTNYVYRIAPTVCDTHYEPVGCFKDEQKVPRPMPHYISTDRDYSL
KIFSGKTIDWRNWNTYSPEMICRCATLAKSKGHALFSIQFWGECWSGPETNGYMRDGVTTDCRG
KDFEMCPCNGYHCVGKAYTNYVYRLGHHH*
>TRINITY_DN1534_c2_g1_i1.p1 type:complete len:547 gc:universal TRINITY_DN1534_c2_g1_i1:1914-274(-)
MHRVNSSFVKVAERTSKLCGNLGIKNSKIPSSLIARSSSTDTTDGVGESEPGFLEMVSIFTENAHSIT
LDKLLSAKPAPGKRAEDTGVREKRIKGILDIIKPTNTALAVNFPLRRDNGDYELITGYRAQHSHHR
LPCKGGIRYAPDVNLDEVKALAMLMTFKCAVVDVPFGGAKGGICIKKSDYSENEVEKITRRFTVE
LARKGFIGPGIDVPAPDMGTGEKEMSWMADTYAMTLGYGDMNSSGCVTGKPISQGGIHGRMSA
TGRGVYHGIDNFANNEEYMNLIGLKPGLKGKTFIVQGFGNVGLHSCRYLHRYGARCIGVIEHDG
SIMNPNGIDPKDLEDYKLDNGTIVGFPGATDTTEDLMVAECDILVPAANELQITAKNAPHIKAKII
AEGANGPTTPQADKILQQRKRLVIPDLYINAGGVTVSYFEWLKNLNHVSYGRLTWKYEESSNYNL
LSSVQESLEKHFGGNIPINPSSTFLQSIQGASEKDIVHSGLAYTMERSANRIMKTAKDYDLGLDVRA
AAYVVALEKIFNTYSEAGLTFT*
>TRINITY_DN4781_c0_g1_i1.p1 type:5prime_partial len:2696 gc:universal TRINITY_DN4781_c0_g1_i1:10504-2417(-)
QCIKKHMLCDMDRNCCDGSDEAAKLCKGYKTLDFETGLQQWIQFRNDDFDFDIQSGKTSSSGT
GPQTDHTLEEEHGKYIFIESSLRNENETANLGSPTLAATTSSLYASGDCSLRFWYHMYGSTIGNLTL
FLRRDNKDTLLWAKSKPEGNQWRPALVTVNSWYKPFQLIFVGTVGKSYFGDIAIDDLMMTDEKC
PSPGDCDFEDFDKCTYTQDLVEDDFDWIRGHGGTGSWRTGPSLDNTKKDNSGHYYFIEASAPRT
QGDRARLVSELFDPTSSTRGRCLQFYRHMYGTAVGTLNVYVKTGPGNSSDAESLVWTNAGNQG
NSWIQGQVPIYSSKPFRLVFEAVRGNGYAGDIAIDDINFNNNFAPCSFLPSNAAPPTLPPTTITPEDS
NCTFDKGLCRWKQMKSNDQFDWTLVSGSTGSVGTGPLGDHTSGKGSYIYIEASYPRKENDSAILQ
SPPIRPSLGSVCFKFAYHMHGAHVNALYIYKNVKGKRTMVWKRVGTQGNQWRNGQVNIRGVS
GIFNIEMKGIRGVDYQGDISLDDFVVTNGQCPTPSHCDFENNQCGWFNIGRGDDFNWKRSNGG
TPSLGTGPGTDHTTGVALGSYMFIETSYPRKYRDVANLYSPYYPGSKTGYCFNFWYHMYGSHIGSL
NLYQDTVKSTPIWNKTDNQGNMWKHGRVTVNSRAKFRLIFVGTVGKSFLGDIALDDVWVDDFP
CPAPGACDFESKSFCTWSQVPNKFNTSNYDDFDWELGNGPTPSFQTGPAVDHTKGTSVGTYAYV
EVSGLIKRGQKAQLMSEKFPPTNGKCMSFWYHMYGSGVGDLNIYLKYLGVKKSHLVKSISGQQG
NKWIQGEVGLHSQKDFNVIIEAVKGSSYAGDISLDDISILDGNCVGLCSSVQPVARVACGPQSVTS
ASCVVSYGCCYDNSVPNVPACFQHPSSCKAVPIIARQQCGYYSISKYFCARRGCCFDDSGAGGPP
CYHSLSKPTDFPTTRAPPTTQPPSKFDCNFEKNQCQYTNLRNDQFNWARHKGDTASFGTGPKFD
HTLRNQNGHYMFVETSFNKENDTANLGTPTIDFPTGGDDVCVRFWYHMYGQHVGAFKVYHKL
TTRVPTKPIWERIGSMVNKWVYAQLIVPRRPAFQIVFQGVRGKGYQGDIAVDDVGFVNGPCPPSL
QCDYQNGDLCGWAQDADDEFDWSVGNGKTMSTQTGPKADHTYETDFGKYLFIEGSSPRQPGD
KARIESFSHRPTKGMCFEFFYHMKGTGIGTLNIWIRRGKTMDSKPVWTLSGEQGDEWYRAKVTLV
EDTENWTVIMEGIRGADFNSDIAIDDVIFHQGDCAPQGDCDFENDDVSACGYENWPRNKVDWL
IYSGGTPSYNTGPMVDHTLGTKQGHYLYMETSSPAKKGDFGYFMSEKFRVNPNYKWCVDFYYH

MFGDGVGSFMVKMMYQTKYSRGRYLLQTLFKKEGNHDDIWQHGQVNITSSYDFQIVFQGVVGG
GITHRGDIAIDDITVKKGLCPAPKAPPKPNPCAIKCKDGTCVPSNKVCDFYPDCAQTSDNTDDTD
EKGCDGCDFEKGTCNWNDTSKGLFDWNRQNGGTSSYNTGPSVDHTKKNALGYYVYVEASKGQ
SSDLATLSSPVLNQASSTCKMTFWYHMYGTSIGTLNVYLVNGYRLTLHWTLSGNQGNLWKQGTV
YVNRQLKPYSVRFVAERSFSYRGDIAIDDIKFEGCELPRPAGFCNSRYQHRCAVTRACISYTRLCDY
TDDCGDGTDELNCRWTQYPYRCNFEYDLCYWRNLKDDDFDFTRQKGQTDSANTGPLFDHTTG
TSSGYYIFVETSWPRKTGDKARIGSGYLGPITRTYCILRFYYHMYGDHIGALSVKLRTCIGCPETQI
WRRNTTVGNYWVRHAMYLRSDKPFQVIFEAEKGSDYQGDLALDDISFDGCTPYQGTFPTVAPST
KPPPTTSPCPSGKFYCAKDAKCIKGSMKCDYIKDCSDGQDEQHCGPCDFEKDMCGYQDVSIGTL
EFKRTQPINDGSKNGPSVDHTTNSTKGYFAMAQKGSGNKGSPVSFLLSAQLSQTAPSCKVQLFYH
AKAGANYVRVSHRQPGSNGKTVISQVQSLSIRNMDKWQNDTVGVGQRPFGWQVLIGLLSEYAA
IDDIKFIDCFYPRPTGVCNRMYTQCDNGVCLDPEKMCDFSNDCGDNSDEKPSVCKNFKERCDFE
TDTCNWYQDQSDNFEWKRIKGGTPSGSTGTGTDHTTGTSQGYYMYIEASNPRKPNDTARLLSPV
FKPHGNKKCAMRFHYHMFGIHVDSLSVKMKFSQDDNSTMKTVWTKRGNQGNQWNTAKVYLL
SKVNFQVVMEARRGISFLGDIAIDDVSFSPDCLPDFKATLNPNPITKAPPPGCKAGQFSCGDGQCI
DSDFFCNFKQDCKNNADEIRCPSSCDFEGGKSCNWFNAKFGDIMDWTVHQGKTPSNGTGPPFD
HTKGTKDGWFVYMEATQRKSSNFPNAHFVSPMFYQGAYDCKFSFWYNMYHYQISIRNDARLNL
YYRKNGKETRIWSNARSTGNKWVQQIVQLPRCPQDFRLVFEGYHYWATKTDAAIDDLKFMCTEP
KAPSSCANDEFQCTKTKQCIKKHMLCDMDRNCCDGSDEAAKLCKGYKTLDFETGLQQWIQFRN
DDFDFDIQSGKTSSSGTGPQTDHTLEEEHGKYIFIESSLRNENETANLGSPTLAATTSSLYASGDCSL
RL*
>TRINITY_DN2028_c0_g1_i1.p1 type:complete len:274 gc:universal TRINITY_DN2028_c0_g1_i1:1608-787(-)
MGQSDGDDLTFGQKISREWRDFREFLYNSETGKVMGRSGTSWAKIGGFYLLFYGFLAGFFTALLFI
FYTTIDKPGIGGPKLIQYIENKPGLTRLDRSSKLQSYSASNNDRISSYKTVIDEFLDGYNTSDHSGAL
CGAAGTPKSDKPCIFDTTLLGECAPSKNTTYGLREKKPCIFIKINKVFGWTPADDGTGYLTLSCTG
HATVMPQGFPLTAFPYNGHKVYQSPIVAVKIDATKGKKNVDCTLKGKNIEVSNNFNPSRAFGKIR
IEGISATD*
>TRINITY_DN436_c0_g1_i1.p1 type:complete len:116 gc:universal TRINITY_DN436_c0_g1_i1:74-421(+)
MAVSRAAGSASTFQKFANAFRSWYIYAAGYRQLGLRADDLHMEENPDVSAAIKRLPEEDQYLRIF
RIKRALDLSLKHNILPKEEWTKPEEDQYYLTPIIEAVKAERKEREQWDRM*
>TRINITY_DN1378_c0_g1_i1.p1 type:complete len:443 gc:universal TRINITY_DN1378_c0_g1_i1:2151-823(-)
MLRFAVLVVVASIGVSALYDSSDDVVELTQSNFNNHVIGSDDIWLVEFYAPWCGHCRNLAPAWK
QAATALKGIVKVGAVDMDQHQSVGGPYNVRGFPTIKVFGANKMSPSDYNGARSAQAIVDEAM
KVVQRVVSDRLGGRSSGGGGRRSGGSSGGSGNKDDVIELTDSNFEKEVLNTKDMVLVEFFAPWC
GHCQRLAPEWAKAATELKGKVKLAALDATVHGQTASRFQVQGYPTIKFFPAGDKDFSSAQDYQ
GGRTASDIVAWALAKHAENVDPPEILEITSNSVLTENCNEKPLCVISFLPDILDSMAAGRNGYISTL
KELGDKYKSKMWGWLWTAPGLHSNLEQALGVGGFGYPAMAVVNIRKKVYVLLRGSFGIDGIDE
LLKSIAVGRGRTEKLTQGLPTLKDVEAWDGKDGQLPEEEDIDLSDVELDDLDDEPKQEL*
>TRINITY_DN6783_c0_g1_i1.p1 type:complete len:314 gc:universal TRINITY_DN6783_c0_g1_i1:1018-77(-)
MVREDKSTWKANYFTKLEGLLDEFPRCFLVSADNVGSKQMQQIRVALRGKAEVLMGKNTMMR
KAMRGQMQKHPSLEKLLPHIVGNVGFVFTNEDLTEIRDILLSNKKEAPARAGAISPLDVFVPAGN
TGQGPEKTSFFQALAIPTKITKGTIEILGDVHLIKKDEKVGASESALLNMLNIRPFHYGLVCRTVYD
NGSVFNPSILDIKEEDLITKFCEGVANVAAVSLAVGYPTAASVPHSMINGFKNIAAVCLEADIDIPQI
ATIKEYLADPSKFAAAVAPAAGDSGAAEVKEEKKEESEEESDDDMGFGLFD*
>TRINITY_DN98968_c0_g1_i1.p1 type:3prime_partial len:424 gc:universal TRINITY_DN98968_c0_g1_i1:1269-1(-)
MEYKNILTSCIFLISVITNTQQTSYVNYFNDQSDLLRSFNIPVKPERSFNTGLPASYMKYARTNDFY
HLYARLFNENQDGKQMAFLTKPISFPSNIETVCVYFEYWMHNLGNTQLYIVGDTTDSTQMQNIN

VNNLVNTENRKERLVQTANEPNSCWREVNSCFNVKGKTKFTLHIQLVPPSTGIVSTNGAIALDSVI
VTTDKRIESTACTRRVGRRIKRDVSFTNLVSNPAAIPGMNLIFHNYDASKTTQDFECGDDLGLCGN
KYLGWTLTNQCSSSCGNGFIIESRTYRGQAIIAQYRQNFQQACNRGSCSTGPPQPPGNNLGPEQL
ATCSVDQCGVNTGTQVVFRVCNAALCQYQGTDYSLNQQVTIRVQSCTVACDYTAWQDTSACSK
TCDGGVLVQRRTERAFNKVETRNGNTPCNTQA
>TRINITY_DN319_c2_g1_i3.p1 type:complete len:2588 gc:universal TRINITY_DN319_c2_g1_i3:165-7928(+)
MVLLKGQLWILGFIFLTSTKYVMGDVPECSNNATVAVHCRFDEGNTCVDDPHPMHYVCVCNNL
GYMNDTDSVEGYSICKERDCSNSNETPTDLLKRICGVNTKTCTGKILQRECNCNTDSYRSKNGQY
CFFAPCQASPTIQQNCGPGNQCYGNAPNYICDCSARGYKRSAADNRHCELKSDEELTPIEEYCGK
GSLYANGTINNITCTCDTDKGFFPSATGETCISPSCYLNPQMQEKCGPENTCLGGKDDYVCLCSNP
NYKRNATDLQHCIRRTCADTSPAIDFKSICGEGATGCSGYVDKPLCSCDVSNHYVPAADGRSCIK
ATCKGNKKALEQCGQANSCTGSFDNFLCVCDANGYYLNTTNNTCVERECTDPDLVITDICGVGS
LSCNGKAGSPECICDKDKFYYASEDLRSCVKANCLSSETISKKCGKENSCYGSMESYVCNCNAPG
YLLNTTDNQGCYLGMCEDNGLEYDKVCGEGAISCNGTIKNLECNCGMNYVQSPDKKSCIRADC
RANQTMHEQCGEDNDCVGSLEDWMCICSTYGYKPDPMDMMKCKKVMCEEEVVNNVTVCGL
GTTGCKGHILKPECTCDIAADYVPNEDASRCIKGSCENNKMAMKDCGIANSCTGSYNNYVCVC
NGFGYKKLDDTHCVEQMCSDPDIDIRNVCGEGAHYCNGTVNQRVCSCNETEYFYPSADSKSCV
KATCAVSPMVMEQCGVENSCQGSQESFICNCNALGYLLNTTTNQHCYLGTCENGDFDLMQAC
GDGALYCNGTIKNLECQCDESKGYVQAPDKMSCIPNDCRGNLTMYRQCGQLNKCVGSIDDWR
CICDSPYYVAHSDIMKCKLLHCSDSEVDHVEVCGKGTIGCSGSTLHPTCHCNTTENYFASKDGMR
CVAGSCKTNQTAVKKCGNANSCAGSHSEWTCICDGIGYKHDPADPQNCLLKTCNDADIDIVATC
GVGAISCEGYQLMPKCECNDRDYYIPSDDRTSCELASCRANRTAMRACGEMNDCRGSFSSHICL
CHDPAYKSDPSDSRVCLEKTCEAGDFELVRVCGKGATGCSGSMKNIMCECDNAKGYVASADHT
QCIPSDCNNNVTMHLQCGEGNGCVGSLTDWYCVCSAPGYEPHASDHMKCHLKTCADPGVDH
VQICGMGATSCMGPVINPVCYCNESMYYIPSMDRTSCIKASCMSNKTAIAQCGSENSCSGSPDNF
VCLCQAYGYRQDTNNNQACIPSQCEDAGIDFVEICGQGGVGCSGSVLNPVCMCDHMKHFVPSP
DQNSCVLSTCRGNETAMTSCGEQNKCFGSSSSYICVCDAVGYKPHPTNLQECVLQTCEANELNP
VAVCGKGALSCKGTIQSLMCECDNSKGYVAAPGYKSCIPADCRANMTMHAQCGTGNRCRGSM
EDWSCVCDGIGSKPHPTDHMSCIAIDCSDPNVDIKSVCGYGATGCMGLARDPICHCDTSKYYVQ
SEDGRTCVPATCHTNKTAQMQCGPDNSCIGPPNDFICECTGFGYRHHETDKQKCVPIQCNETAL
FDIERVCGKGATGCRGNIRQPICDCDHAMYFHPSEDRRTCIEASCRRNSSIRADCGVENTCFGSTR
SWICDCNSYGSILNPSDVKKCMLATCEAGQLDPVAVCGKGALSCKGSMRDVQCTCNNELGYVH
SPDGKKCLPSDCSGNVTMHHQCGDANKCMGSLTDWVCVCDAPGYKPHAKDKMKCHQISCAD
NNVDPVDVCGIGATSCKGPITNPLCQCDTDNYYITSSDGASCIPATCKTNKTAIHQCGEANSCEG
PADNFICVCNAYGYKLDDNDHQRCVEISCDDPNAHESICGTGSTGCRGTARDPICECDHTQHFV
ASSDLTSCLLASCRANDTVMNHCGDGNTCFGSLTSHICVCEAFGYKLNPNNHKHCLEETCEDD
MLNPVAVCGKGAVSCSGPLKSVKCECDNNAGFFRSPGGMKCESTSCRQNPASQMMCGSQNQCY
GSYDDWYCDCSAPGYMANQTNAAECLKVMCDDMAVDVCGLGATGCTGHTLNPICTCDSSMG
YMMAKDKHSCVPMDCSMNPASSQCGYGNTCHGSYFSYVCVCSTAGTVMNKTNPMECINVACP
ADAVHVCGEGATNCSVTSTNNVECSCEEGYHPADDLHSCVLDDCRFNPKALKACGLLNKCVGS
YKSWKCNCHYPEFTQDPMDHQTCVVGPCMVEQCGKGNAPGSFQHYAVNCSGLYDDPICNCQP
EYTNDAKGGCIPKICSENPVAVQACGSGYCEGTYFKYTCKCDEPAFVQTPDKMQCVPAKCNIQQ
CGHGFDRGLYIDFAESCSGTYADPMCQCKTGFSGTNTSKCGPAIDGCKLDKCDPLATCQTLDVD
GNVKCTCKEGYSGSGMMGECYLDGGFSAYEGTKECSEVCGVNGVAEVTRFCNNPTADPKGKPC
NGTSPETIQVPCNRFACSSCTVRKTCGCGGLKPIFVEFYHEPNSTLEYFTDLKADDVQTALGFLTK
WKELSQQDLCLMYSKIELTVFVTLMGDSETQVSTMKSSLVSARDIMRKKLDCNINLGKKAQYYEA
YETIIKRHLAISNILKDLTIIKNSITKYMNNCDDSEIGMIRDVIGMM*
>TRINITY_DN164351_c0_g1_i1.p1 type:complete len:212 gc:universal TRINITY_DN164351_c0_g1_i1:694-59(-)
MEDWGEPGEQEAVGPTSIEVQDIKLFGKWNPDEVQVSDISLTDYIAVKEKHAVYLPHTAARYAVK
RFRKAQCPIVERLTNSLMMHGRNNGKKLMTVRIVRHAFEIIHLLTGGENPLQVLCDAIINSGPRE

DSTRIGIRAGTVRRQAVDVAPLRRVNQAIWLLCTGAREASFRNIKSIAECLADELINASKGSSNSYAI
KKKDELERVAKSNR*
>TRINITY_DN578_c0_g1_i1.p1 type:complete len:429 gc:universal TRINITY_DN578_c0_g1_i1:379-1665(+)
MDMKALFLSLALVVVAWNTAYGSSNHKLVAYWGQNAVYNLKRDKQYWEKDLSYFCDNSKYDV
IVLAFMHVFFDRRQKDALPGLNFAFHCETSMSADLPFLLRCPQIEAGIKRCQANGKKVILSLGGA
TGGYGFANDGEAKTFAHTVHNLVLAGNKMQNIRPFGSAVLDGIDLDIEGGSNVGYATFVRELRA
LQGSNHYLIAAAPQCPFPDHFLGPSPGTAFGEVADLFDEIYIQFYNNYCHTGDAKQFYPNFQQWI
NFSKKSNGPKIFIGMPSHERAAFGQGYWRTPAEVAAIYENVKNEPGFGGFMLWDASFDDVNRFN
GRPYSDSIAALFKNGGGPRPTYRPNTMRPNPVTRPTPKTRRPNTQRPVTQGPVGPVSCSAGADGL
IPHQDCSKFIECSHGRATVKSCPPGLKFSSAKKYCDWPRNVQC*
>TRINITY_DN10907_c0_g1_i1.p1 type:complete len:206 gc:universal TRINITY_DN10907_c0_g1_i1:1010-393(-)
MAKKAYDYLFKLLLIGDSGVGKTCLLFRFSDDAFNTTFISTIGIDFKIKTVEVDGKKIKLQIWDTAG
QERFHTITTSYYRGAMGIMLVYDITQEKTFDNIAKWLRNIDEHASEGVERMILGNKCDMEEKRM
VSKEKGEGIAREHSIKFFETSAKENICVEEAFMQLTQDILRKHQAGATHEQSPSGVVIEKDSHNRS
KGGMNCCN*
>TRINITY_DN138_c0_g1_i1.p1 type:complete len:401 gc:universal TRINITY_DN138_c0_g1_i1:157-1359(+)
MAARFLFITVILIVGNLIGDVCPERCERNWIKIGCFHDRIFPNRPYPFELVNHRDPINNHWDGNLI
NWRKYPDSIEKLACICANKTRTKGYQYFGLQFYGECWSGPSYNFHRDGPSTKCIGNDFKPCDDSS
KTECVGRAFTNYIYKLKDENPKVDGKWSEWEKWSPCSATCGYGERYRERFCSNPEPENGGQTCE
GEAREYEECNHGECQTTCDKEIDFGIALDYSSSVRQVNWNRTLDFVVELSKGFNISPEKVHFGVLS
FSHRTRFDFGLSDKTYWNSESFEKKIKSLEYRWGYTWTDKALKKVKQLIFCEKCGLRKDSLKYLLV
FTDGRSNNVKTDLKTVSDSIKDMGVNVISVGAGHTINHRELEEIATDDNHVFYVGGYAYLGDNM
NAIRRLTCG*
>TRINITY_DN1320_c0_g2_i1.p1 type:complete len:260 gc:universal TRINITY_DN1320_c0_g2_i1:97-876(+)
MRCLSMIFAILLVYLFDQVSSRACRDYVNYQCRKYLCHVPKYHRFVNMYCKATCGLCPVIHGLQ
MSTMSTPTTTATTTTTTTTTTKTTTTTTTTTTTTTTTTTTTTTTTTTTAATIVLLSGGKTISGTPEDAS
GPAVRCTDGDVTTLCITTQAIDAYFQVDLGSQKKVSRMEIKNYFHYENQLELKFLVSNDVTFATST
ICDTWDVRSVNEGDVQDKKRDCNSNVSSQTFRYARVQTVNNANEPEHLRVGEFEVYGVA*
>TRINITY_DN1114_c0_g1_i1.p1 type:complete len:118 gc:universal TRINITY_DN1114_c0_g1_i1:462-815(+)
MGKKKDDGTIPAGDSTKGAKVFKQRCSQCHTVEAGGKHKTGPNLYGLIGRKTGQQPGYSYTDA
NKAKGITWARDTLWVYLENPAKYIPGTKMVFAGIKKKGERADLIAYLEESTTKK*
>TRINITY_DN773_c0_g1_i1.p1 type:complete len:440 gc:universal TRINITY_DN773_c0_g1_i1:1608-289(-)
MVSPPGPQDNKPPHKVADMSLADLGRRYIEMSENEMPGLMSMRKQYGEKKPLKGARVAGCLH
MTIQTAVLIETLTELGAEVQWSSCNIFSTQDHAAAAIAKTGVPVYAWKGETDEEYLWCIEQTLIFP
GGEPLNMILDDGGDLTNLVHEKYPELMKDIRGVSEETTTGVHNLYKMFKEGRLKCPAINVNDSV
TKSKFDNLYGCRESLVDGIKRATDVMIAGKICVVAGYGDVGKGCAEALRAFGARVMITEIDPINA
LQAAMAGYEVTTMDEACSKARMFVTSTGCSGIIEPHHFQQMPEDAIVCNIGHFDCEIDAKWLN
DNCERETVKPQVDRYTLPNGRHIILLAEGRLVNLGCAHGHPSFVMSNSFTNQVLAQIELWQNHK
NYEIGVVMLPKRLDEEVARLHLDHLSVKLTKLSKSQADYLNLPADGPFKPDHYRY*
>TRINITY_DN164128_c0_g1_i1.p1 type:complete len:166 gc:universal TRINITY_DN164128_c0_g1_i1:117-614(+)
MGPKFDPTAITIVYLRVVGGEVGATSSLAPKIGPLGLSPKKVGDDIAKSTGDWKGLKVTVQLTIQN
RQAKISVVPSAASLIIKALKEPPRDRKKVKHVQHNGDISFETVLDIARQMRERSMARKLEGTVKEI
LGTAQSVGCTIDGENPHDMIDMVNSGEKEVPES*
>TRINITY_DN51048_c0_g1_i1.p1 type:3prime_partial len:761 gc:universal TRINITY_DN51048_c0_g1_i1:2282-3(-)

MEKSVDSWILVDYASKCRSVSSPSNNSSREIKVGKDTGRVKVFVRIKPFTNEKNIKYESLPYEISEEK
LQVRILSDERGHTEKTYQFDHVFGPDRCNDHVCSIMTPPLVEAAMNGFNGTLMAYGQTGSGKTY
SLMSANGITQNMIDQCFRRIHDDGKHDYKVTMSYLQIYQEKLYDLLTNSNDKTDLTIRENPKTGV
YVENLTEYVVRSPHEVIQFLEVGKKKLVFGETRMNRTSSRSHSVFQMTIERTLQDGKDVQTITRKR
NSNHTQINKDPNSLDLNSFGKDVIIRGKIHICDLAGSERIKKTGATGDRLSEAQHINSSLLELGNVI
QALADEKTMHIPFRNSVLTRLLQESLGGNCKTSLIICTSPSNRDLHETKCTLNFGSRAMKVKNTAY
VNMEVDFKKLSDELMKQLQRKEDELFQVRQKYQDDIDRECDKVRNVLEKANKEKLLSFELEREV
MEEKMICMNAELCELKNELNNRRNRIKGLEDSILSMDDILKHERDTGYQMLKTENHIKSFMSSS
NMIENIVALTVILEHCILETESNYTEELLLENFLYIQQSVVELEKYKTKNMDTTEELLEKLKKRLEKL
SNQVAEKNNNSNQYDDSDIVWLRYNLFLGNVINNLLQSLKVFIMNKKKNDMKAVMERKTEKTL
DLDDDVSDRSDNRQETVKAKYQTYGETWNNAEDTIVKLPILLEKKQLDNGTQYDISDFSPNRRL
VDTTVEEIEKIITEIQSENITDRRDIKRSGEIFSGFSTTLG
>TRINITY_DN23911_c0_g1_i1.p1 type:3prime_partial len:203 gc:universal TRINITY_DN23911_c0_g1_i1:606-1(-)
MVTNDAESLIRKSESFDQHYDVLHELGRGRFAVVKKCVCKKTGKQFAAKIIKKSRTSSQGRSGRE
QLLLEIDILNNSNHPKLVRLYDVFETRSEMQLILELAAGGDLHRHCIEADVTRTEKEICYLIRQITEA
VRYLHDQSIVHLDLKPDNILLAKEGEVFPEIRLIDFGLSRRLDVPYSQFDIVGTAEYVAPEVLAFEPL
D
>TRINITY_DN598_c1_g1_i1.p1 type:5prime_partial len:745 gc:universal TRINITY_DN598_c1_g1_i1:2-2236(+)
RWSGCSKSCGVGVKTKSRECDNPRPSGGGRYCTGERTATEACKIQDCGVDGKFSHWSTWTACSK
TCGGGAQKRARQCNSPPPSGDGKDCAGAREEFKGCSNDFCKGPTGVAKAQEIIERFLENFHKKT
YPDREPSGKFLKAIFAIQKAVAEEFISVDPYVRSKTLEQRLKDLENFLKVATEGGSIDFARTLLGKGV
FTFMKQKIKDGLPIDDIDKAAEGFLEKLRGDLGDKKMDEILSSQGDATLMFTIDTTGSMKQEINA
AKAIAKQIISATRDYEVDYILSPFSDPGTGPVTHRSASERASFVSDIDRLRARGGGDCPELSFGGML
NALNNDPQYGSPMFVFTDATAKDDTDANVASLKAAADATDATINFFTNLAGCNGRRDRKRGIE
SYKEIASYTGGQVFPLKNEAELIKFTSYVKTSLQQDTVITSGGTLNSKSKRSLYSMQARHFMVDDGI
SKLVISVGVQQLASQVALVDPNGKKVYPSTTLALSLIYQVSHPSPGQWKLDFPTSVGKYSYNVQAV
SSSAIAFAHNFIYQQSVRKNSPPIPLANPLKGVKNMMVVRLGGTKNFDLNSIKIDLVDTSGNVLQ
KGIGTDRLDSDEVRHATIDPPSGPFKVRLTGKTKSGYNFERVSQGISRPVSAAMRTNFAGYDFTAK
LGKGPYAAQMLFINRGAHENFYFYAKANYGSIPNYKQTKRVTSQGFVTFTYKAPRQYNLKGKSD
RITVTVKRQSTGEIVSHHTSILLVV*
>TRINITY_DN6008_c0_g1_i1.p1 type:complete len:426 gc:universal TRINITY_DN6008_c0_g1_i1:1620-343(-)
MSLEDTQLRKRKGVSEKHYTLDGFTNLQENEKKSSPLMSITKVILKMMIATFVLLGIYIAITPSPIDP
GVLELDTPLPRFEGVLAPNDKLKQARRIGEGKLLAPESLAMDKEGNIYSGLGDGRIVKITKDEEIIN
ITRTGEPHPECGKRKLGSKCGRPLGIHFDKDQKNLIICDAFFGLLKLNIATKKLSTLVGANVINEKP
LKFTNHISVSSKNVVYFSDSSSRWDLENVALLVLEGNGDGRLMSYDMESGKLQILLEGIYFANGVS
LSPDEDFVLVCEMSRNRIIRYYLKGENKGNYDIFADNLPGSPDNIRRSPNGGYWLALPSIRKAPFSP
PDLIAPYPSIRNVIAKLLSPQTLEYLLGSMRYGMIIKLDENGKVVESYHDKDGSKMYTISEVYEDSV
NKVLYLGSYKNPFMGKLQLSDSS*
>TRINITY_DN614_c0_g1_i1.p1 type:complete len:183 gc:universal TRINITY_DN614_c0_g1_i1:194-742(+)
MGQMFGKLFSGLFGKKEMRILMVGLDAAGKTTILYKLKLGEIVTTIPTIGFNVETVEYKNISFTVW
DVGGQDKIRPLWRHYFQNTQGLIFVVDSNDKERVTEAKGELAKMLGEDELREAALLVFANKQD
LPNAMNAADLTKQLALHDLKSRSWYIQATCATSGDGLYEGLDWLSTELKKKKN*
>TRINITY_DN7573_c0_g1_i4.p2 type:internal len:151 gc:universal TRINITY_DN7573_c0_g1_i4:3-452(+)
SAPPQPLRAPSAAAMAPKDTVEATTTLGPQVRDGEIVFGVAHIYASFNDTFVHVTDLSGRETIVRV
TGGMKVKADRDESSPYAAMLAAQDVAARCKELGIAALHIKLRATGGTKTRTPGPGAQSALRAL
ARSGMKIGRIEDVTPIPTDCT

>TRINITY_DN76073_c0_g1_i1.p1 type:complete len:107 gc:universal TRINITY_DN76073_c0_g1_i1:794-1114(+)
MNTNVENDYDQCFIGSQLKDECHKKNYLRTTGTILLQDLLEKDRSLLQWRCEVKTSDSDLEKRIC
YHHEKIYLTRYVGLQKYCCDPCNMHGNRFVTSKWKDFSQYF*
>TRINITY_DN113_c3_g1_i1.p1 type:3prime_partial len:1162 gc:universal TRINITY_DN113_c3_g1_i1:3485-3(-)
MEIVSRSRIAIFVLLGLLISVAESQFDPECPEIDPDVECSPPESTVNECLINNDCDVDKPLCCSNGCG
LKCTARERRSITPDGFTPEDEKLCRVPVDLGFIIDVSGSIGQTNFDKIKKFIEKIVDAFHIADDGTHV
GAVFYSDNASLAFDFNHLQGDQLNEMNVVREIEKIQVTDGQTRIDLGLELASRELFNPAAGMRI
HKPKIALVLTDGKQTRGELAPDAKDLHQASRLLKQTGVQVYSLGIGLDYDIGELLDIASDDASVF
RSQDFDELVSIVDILIEQTCKGCTRAVDVHFALDASANVGEENFVFMKDFVKAIGYNFIISETGAH
MSGSVFGDVGTLVFDKSQATEINEFENEVENMPYLGDATGNMDKVLKMISNEVFNFDGFTRQSS
PKVIVLLTSSNCDTCQESLEDLARPLKAEGIHIVAIPIGDPLFDEMQVLASHPIDQYAIPRATHQELL
NAEFIQKVSEMVCAGKPGICAEPPVKGECAEIINECSVDMRCPEDQKCCIKNCVASCVLPLAACIT
PIEIAFAFETTKYGIADFNNVKNFAINLLDHFKVGPSKAHVSLSTFSEDVTSLTKFIDDFDMESIKQSI
KDLKHDGGEESNLGNFLEIINTEVFSVRGGIRQQTPRVLVIFANGDINDEQAVKAQEEALHLKAD
GKDVKIIIVNIGSSTKTLDVLNEVATEGSFYEFFGSAELDSTAIETEISEKICSTKVRRSKCQRRIRSSCA
VKDDLCDSDFDCPGHQECFSDGCRLRCQTCEFRCKDKVDLLFVMDTSARIGRDNFERGKKNIKK
MLEWFTIDSNNTNVGVITYSDTANVAMPLPSRGNNNPAQSLIDVQDRLDSIPYSGGSVSHLDNAL
QLAAEKVFPDNGPNLRRDSKKAVVVITDSLDDSASQLELASWPLRRQRVQTEDGDASNIRILAFN
VGQELDLDLWGNIVSPPVEENVFYAADFDDMYNGIRQLAEDSCKYAAAITPPPIIKQKGEPGPPG
PPGIPGTNGTDGRNGTDGRRGPKGERGDTGLKGFKGEVGLVGPPGGGAEEQKGPDPLASAVGIV
NVTDLPGGGRIVFDLREGPVGQQGLTGIIGPKGPIGDTGEVGIKGRNGTTGPGGFFGLPGPKGQP
GTDGAKGLRGDPGLRGSAGRKGPTGAPGLTGPQGPFGYK
>TRINITY_DN2904_c0_g1_i1.p1 type:complete len:221 gc:universal TRINITY_DN2904_c0_g1_i1:1339-677(-)
MSLAKIFGSGKKKEESPNAQDAIQKLRQTEEMLTKKSDFLEKKIQQELVIAKQNSSKNKRIAINAL
KKKKRYEKQLNQIDGTLSTIELQRETLEGASTNTEVLKTMGYAAKALKNAHQQLDVDDVHDM
MDEISEQTEISKEITDAISTSFGVADDIDEDELNAELEELEQEGLDEQMLNIESTSLPSVPETELPTPS
RAQPSAQKVQPDDDMAELEAWAS*
>TRINITY_DN11662_c0_g1_i1.p1 type:complete len:398 gc:universal TRINITY_DN11662_c0_g1_i1:75-1268(+)
MALSRPVYIIAAKRTAFGSFGGSFKGLTATQLGQKAAAAAIVDAKLDPSLINSVIFGNVAQSSSDA
AYVARHIGLGNDIPFASTALTVNRLCGSGFQAVVNGAQEVVLGEADIVLAGGTENMSQCPFAIRN
ARWGINLGETREIEDTLWSALTDSFIQTPMGITAENLAEQYGITRLECDEYALASQQRWAAANDN
GLFKAEIAPIEVKQKRKMVPVDKDEHPRAGITIEALNKLPPVFKKNGTVSAGNASGICDGAAALV
LASEEACKQHNLAPLAKVVSYGISGVEPSIMGIGPCPAIKQALSRADMSLSDMAFIEVNEAFAAQY
LAVEKELGLDRSITNACGGAIALGHPLATSGARILTHLVHTLKRTKENHAIGSACIGGGQGIAVLL
ENVM*
>TRINITY_DN1065_c0_g1_i1.p1 type:complete len:373 gc:universal TRINITY_DN1065_c0_g1_i1:53-1171(+)
MSGRGKVKAQRISLSKRAGTIFPVGRIRRLMKGCTHKFRVGIGAPIYQAAVMEYLAAEILELAGN
AARDNKRRRITPRHILLAIANDEELNKLLRNVTIAQGGVMPHIQPELLRRKEGNKFSLPSNMKAK
ALKAQNAGVQKAKAKPAPVKKAAPAKKAAPAKAESKQAPKKAPQTNASPKKKSDQPWTVLSE
KTLFLGQKFLVVQGNVADIKCDAVVHPTNASFSTTGEVGKALIAAGGDDLKKAISELHKSHGDL
QVATALLGDAPNLKAKHVVHVHSPVWGTGNPEQDLENVVKSALTLADEKNVQTIAFPAIGSGA
GFKKNVAAQLMVRSMSNYFVDVSPSSIKNIYIFVTDAESIGFYTLELAKVDSS*

Band 11
>TRINITY_DN161_c0_g1_i2.p1 type:5prime_partial len:399 gc:universal TRINITY_DN161_c0_g1_i2:1683-487(-)

WNQEPLTCTYLYSVNSQLLKYKMADDDVAALVVDNGSGMCKAGFAGDDAPRAVFPSIVGRPRH
QGVMVGMGQKDSYVGDEAQSKRGILTLKYPIEHGIVTNWDDMEKIWHHTFYNELRVAPEEHPV
LLTEAPLNPKANREKMTQIMFETFNSPAMYVAIQAVLSLYASGRTTGIVLDSGDGVSHTVPIYEGY
ALPHAIIRLDLAGRDLTDYLMKILTERGYSFTTTAEREIVRDIKEKLCYVALDFEQEMQTAASSSLE
KSYELPDGQVITIGNERFRCPETLFQPSFIGMESAGVHETTYNSIMKCDVDIRKDLYANTVLSGGTT
MFPGIADRMQKEISALAPPTMKIKIIAPPERKYSVWIGGSILASLSTFQQMWISKQEYDESGPSIVHR
KCF*

>TRINITY_DN2477_c0_g1_i10.p1 type:5prime_partial len:344 gc:universal TRINITY_DN2477_c0_g1_i10:1215-184(-)
SIVGRPKMPGIMVGMDQKDSYVGDEAQSKRGVLTLKYPIEHGIVTNWDDMEKIWHHTFYNELR
VAPEEHPVLLTEAPLNPKANRERMTQIMFETFNVPAMYVAIQAVLSLYASGRTTGVVMDSGDGVS
HTVPIYEGYALPHAILRLDLAGRDLTEYMMKILTERGYSFTTTAEREIVRDVKEKLSYIALDFDTEM
KAATESSDKEKTYELPDGNIITVGSERFRCPEVLFQPSFVGKEASGIHDTTFQSIMKCDVDIRKDLYA
NVVLSGGTTMFAGIGERMTKELTALAPSTMKIKVVAPPERKYSVWIGGSILSSLSTFQQMWISKGE
YDESGPTIVHRKCF*

>TRINITY_DN195934_c0_g1_i1.p2 type:internal len:127 gc:universal TRINITY_DN195934_c0_g1_i1:3-380(+)
GVMVGMGQKDSYVGDEAQSKRGILTLKYPIEHGIVTNWDDMEKIWHHTFYNELRVAPEEHPVL
LTEAPLNPKANREKMTQIMFETFNSPAMYVAIQAVLSLYASGRTTGIVLDSGDGVSHTVPIYK

>TRINITY_DN2941_c0_g1_i4.p1 type:internal len:116 gc:universal TRINITY_DN2941_c0_g1_i4:2-346(+)
LKYPIEHGIVTNWDDMEKIWHHTFYNELRVAPEEHPVLLTEAPLNPKANRERMTQIMFETFNVP
AMYVAIQAVLSLYASGRTTGVVMDSGDGVSHTVPIYEGYALPHAILRLDLA

>TRINITY_DN1280_c0_g1_i1.p1 type:complete len:426 gc:universal TRINITY_DN1280_c0_g1_i1:195-1472(+)
MGFETCGPNEAMVISGCGQERPLLISGGRKFVWEFFQKVQRISLNVMTLNVESQRVYTVQGVPIS
VTGIAQVKVQGQNEEMLHAACQQFLGKHENEIRNIALETLEGHQRAIMGTMTVEEIYQDRKKFS
KSVFEVASSDLVNMGISVISYTLKDIRDDEGYLKALGQRRIAEVQCEARIGQAMAKMQSGIKEAQ
AEEVRIKAEYENKTEVARSQRDFQLNKSAYDIEVNTKKATSELAFALQSAITNQKIKEEDMQIKVIE
RAQAINVQLQEIQRREKELEAQIRKPAEAERYKIEKIAEANRMKVVLEAEAEAESIRMKAEAEAE
VIQKKATAEAEQMSKKADAWRDYQEAAMVDMLLDTLPKVAAEIAAPLSKVKKVTMISSGSSDV
GAAKITGELLDIVIRMPQAMEQLTGVDITKTFRREK*

>TRINITY_DN11435_c0_g1_i1.p1 type:complete len:430 gc:universal TRINITY_DN11435_c0_g1_i1:1616-327(-)
MGNIYTVGPNEALVVSGGCVGQRVKRTIVGGWAFAWACVSDVQSISLEVMTLNPRCEKVETAKG
VAVTVTGVAQVKVIKEDDLLKTACEQFLGKDPHEIEDILLQTLEGHLRAILGTLSVEAIYKDRDQF
AQLVREVASPDVGRMGIEILSFTIKDIVDEVSYLDSLGKTQTAEVKKNADVGVAEANRDAGIREAE
CDTQRQDVRFKADTTIADAARAYEMEKAEFDKEVQTKNAEAKLAYELQANKEQQRIRNEEIQIE
VIERRKLIEVEEKEIQRREQELQATVKLPAEAESYKVQTIAEANKSAKVYLAQAEAEKIKMIGSAQ
AAAIEAIGKAEAERMRQKAAYKQYGDAALMSLILEAMPKIAAEVAAPLRKTEEIVLINDDNGN
KMSSEVTKLLGQLPPSVKALTGVDLSKALGKIPGAQVTA*

>TRINITY_DN22077_c0_g1_i1.p3 type:5prime_partial len:151 gc:universal TRINITY_DN22077_c0_g1_i1:520-68(-)
SVCGTAVVQLGDPPPPPCAPLALPRLSLRAMSSAAASSSKASKGDKFSMGRESDKAPIGKGGATR
TKKVLRESIQGITKPAIRRLARRGGVKRISGLIYEETRGVLKSFLENVLRDSITYTEHARRKTVTALDI
VYALKRQGRTIYGFGV*

>TRINITY_DN329_c0_g1_i2.p1 type:5prime_partial len:320 gc:universal TRINITY_DN329_c0_g1_i2:1-960(+)
FNNSTHSPSLPTHVYLYCCRIKILILSFDPSTIEILKMAPPKFDDLGKEAKDLINKSFHFGVIKLEGKT
KAKNGVEFTTEGTHNTDTGTVNGSLETKFKYADYGLTFSEKCNTDNVISTKITIDNKLAKGLKVD
FDTTFAPVTGKKTAKVKSAYQHNNIHSTCDVDFDFAGPTIHGSAVFGYNGWHAGYQASYDTANS

KLIANNASLTYKEGDLVIHSGITDAVKYSGSVHHKVNSNLTAAALIQWASGTNNTSLTVAGKYAV
DNETFVKAKIDNNLRLGLSYAQKLRPGVELTMSSLVNAKSLEQGGHKLGLSLNFEA*
>TRINITY_DN3986_c0_g1_i1.p1 type:complete len:446 gc:universal
TRINITY_DN3986_c0_g1_i1:252-1589(+)
MREIVHLQAGQCGNQIGAKFWEVISDEHGIDPTGTYHGDSDLQLERINVYYNEATGGKYVPRAV
LVDLEPGTMDSVRSGPFGQIFRPDNFVFGQSGAGNNWAKGHYTEGAELVDSVLDVVRKEAESCD
CLQGFQLTHSLGGGTGSGMGTLLISKIREEYPDRIMNTFSVVPSPKVSDTVVEPYNATLSVHQLVE
NTDETFCIDNEALYDICFRTLKLTTPTYGDLNHLVSATMSGVTTCLRFPGQLNADLRKLAVNMVP
FPRLHFFMPGFAPLTSRGSQQYRALTVPELTQQMFDAKNMMAACDPRHGRYLTVAAMFRGRMS
MKEVDEQMLNVQNKNSSYFVEWIPNNVKTAVCDIPPRGLKMSATFIGNSTAIQELFKRISEQFTA
MFRRKAFLHWYTGEGMDEMEFTEAESNMNDLVSEYQQYQDATAEDEGEFDEEEEEEDA*
>TRINITY_DN982_c0_g1_i3.p1 type:complete len:9852 gc:universal
TRINITY_DN982_c0_g1_i3:29697-142(-)
MSIKLCTFFVFFLIQCINSFPEIGLHLSLDRSYSFQGNGWKTVNGWEIKPRANGPYEIDAIKNISPEY
FPVPITGVYYITVSLPVEGASNGEFKSAIIVDGKNEEGRIGLMSSFQKTAFKNGASTLTFAGFVRLFA
DEKLTIEIYSDDINANYVVLESATFVFHFIGVTGSVPAYLAHTAQDQEYASDSIIKPWVTLGDSRVYE
SLSGFIKNEGVFTTICNGVHKMAVNIMIKANAGTYTLKALFQDEESATATVTINQPDSIATLNIFQN
FKWSEGMSITLQIHSSNGANFKVLKSSSWSMSFIGETTANFQEFSVFLMADQFLQPSQFPQRINIDL
NGVFQFLQSNPDQYNKGQLVSGADTKFKDNLDFIVDSTDLFYVSANIIIEGEAEDLEITIAVSQIDID
VLPEDGLSGRLTKISNSRRSISLSGIIFIREKQYLSFFVRSRDSSKSFTVSKDSFISVINMRYTVSSVSARQ
NENIDVTAGSWYDISGRFAEQKNGLYSFGKDFNINNGVFRTSHSGIHSVHANIQFSLDSDAFGDN
DDNKIVLRVVIDGQEDKISLMSIRKNPEKTATLKLFGSIYLRAGVTISFQVKSLMPNTVQYKILQRSS
ISISYIGPQWAVNSFHAVANQDLNYDGKPDDTRPDIPSPLNFWKTEAADEFISPFEVLESFDGTRYT
SVDDGLYVVSSLLILKNVDCDRSALFKLDFVTTGGSRASDKSNGFADSHFFIADSKGTEFTLSVTTA
MRLEVGFKVGLKLTVGSTSCRYTISKESSFSIAQWSKSSNTIEENRLKYIGVLARLSAPTEVSSPNTW
SNLGSNTMDINPGDKSRSSFRPGIFGGDDLAPITRTGKDFNGDVSDFTIKDPGIFFISGMITVTTTEN
ANEEFAATVQIDSEDIESGLYAAVINQGSTEMSLTFGGTIYLKSGARVRVVVKGGRTFNIAAGSSFSL
LKLQPDYKTPGIIVSIDEKITQAITRYEHKDLRADIHRTLTIGNLGFVESTGVYTIQVDGVYMVDYT
VILEATNPNAVFGAEILSDNTVVATGTSTFGNTKTIAITELVYFSKGAIVKAVLKSNTNGETLNNLQ
VDGGLTVYFLEPKEHVKDGHRSKQTSPQALPVRSFEKVTGFAAQSPDFAGGADSVDYTVPETGVY
LVAFTTRVNRVINGFKSRVLVNDNEFLNGNYQIVSAQNAPATLPLSIAMELTVNDVLSFQVMADG
SGMSLDGSTRSILKIDEVNTIRNVTEGLSAYKLRDQKVNGQQETIVEEWDTSVTEGTFLAKNVSFT
VDHSIVVVAAGVFQISVSVVVKNDRPSQSKFELRVLQGDDVKSHGLHSKVEIGGGREQTLTIGGSL
LLQKFDSLRIVVRDIADPLASYTIKAGSTVSVIIIDDNNGPIACEDEGLNMRRLPERIRKPTRESHTF
QCNVVSSLKAEKYTWFKDNVQLKEENQKNLVLNDLKLSDSGVYQCRVRFLELTGATNNATLSVY
DPYPKITSPKFIANVLENQDPPLTIEMFSLSAFKLSPEDEKASITTQIIAGDDQSFFTLSGPSKQTVTLS
TTKKLDYETQNEYNLTIKATNLEETALTTKGPYETTINYIVRVIDVNDHPPVFSQSIYSRSVPEDYPI
DGSIAKVSANDQDAGANVIYTLVGQVSGDLFKIDNTGLIQLRQNLDREIRDDHTIFVQASDGLFT
TVAEVVVTVTDVNDNFPNFMDTGFDISVSEATLTQSVILKVIATDPDLGTNSQLRYTIKSGNNENK
FSLNLLDGNLTLISKLDYEGTKIYNLEIDVQDSASPPKKSQNPLTVVIRVQDANDNNPTFTQSLYNT
RIAENTAVGTSVLKVSATDRDGEGINNPLTFSITDTDANEFFQVVSSGATAGEIRVRKSLNYDIRKD
YRFSVVAKDNNLGEPRYRTALVVINIDDTNNLRPTFEAGSASISISEGTSIGTSVHQVVAIDKDTVG
KLDYRIISGADGKFAINKETGDINLISAVDFESNKKTYTLEIQVNDGVRDALNPATVVINIQDFND
NQPEFIQKYYIFTALESISDSQAINVGNPVIARDADSTTNSQVDYSIQYDINNPSVPNEGDALFRIDS
NGLIFKKNGVILDFENVRYHTFTVVAIDRGTPKTLLGKAFVTINIQDVNDEAPSWSPAIYSVTVKES
AGINRDLVFLSTTDKDTSGAVTYSIVSGDSANQFQIFDNVIKIVKPLDFEKTETHTLKVSAQDDNG
NRAQLDATVTITVEEVNDNRPLVTNNNANSIQVSVPEDIPVNSLIVKLNGRDDDKGAPGQASNQ
FTFVSLDKTTDFTIDEVTGEIKNTVNLDYERQKSYSLVATIRDKGTPYLESKFLVDIIVENKNDERPIF
DPAIYTIKLSEKAPIDTVVAMLQATDLDTPKSQLRFSSVTTLDSHFSLDPITGIITLKTSFDKEVNGKD
GIFVISCTVNDGTSNSAQNAIVRVEVVDVNDNQPVFPTSEYFIQVNEGSSIGTILQNIAATDKDNT
NTHNQFTYTLEGKEAVDFRLNGNQLQTNKIFDYENKKSYSFDVLATNNLSPDKLVGKATVVVSIL
DQNDNNPVLSPSGVINLNISEGTIAPANIITIFATDKDSTINAEVQFTLTGDPTNTFTINQNTGEIML

AKSVDSFAKNEYTLSVTATDKGSPPRSSTATTLNISIKPVFVPGIEFENCPQIISFNENAAPNTFTKTL
TVKDRGSFNQVQFTVVSGQAGIQNLITVSQNGVITNGQRRADFEARKRHDFLVQAENERGAKAY
CHVTANINDVNEGPIFDSANYVFDVLEVIKINTVVFSVRATDPDFSSTTNGQLTYSLAGQEASQFSI
VPNNGQIKLAQPLDYEIKKQYQFNVIATDGGSLQTTSSVIVNVEDWNDNRPIFDQPQKLAINVPE
NSKPDNVFTVSATDKDSTTNGEIEYVGIYPTGIISIDKDTGTAKIIGDLNYEKRQKYDFVIVAKDKG
VPYLSNTREVSLSVLNLADEVPYFDTAAYTISISEKTSINTAVFRVMAKDNDLENFGKISSYEIISGNQ
LNRFRIDSNGNIIVQQSVVLENNNPNQYILTITAFDTQRTQSLNNTQVTINIIDLSDKQPFFIQNSYI
VEVQENNEVSKSLLSLEAQSNEPTFNPTYSIVQDPKNTDSQFFQIINGNTIVATTVFDYEKKRFYSFE
VQVIDTNNGKTNKALVVVRITDENDNKPFEVSGNLHFNITEHTLLGSTIGSLNGKDNDAGINKEF
EFSILEGNDDDKFMLLPTGQILLKNFVSVKEKDFYKIEVKITDKGTPSLFNIFTVRVTINSSQALVAS
NNPTIPEDKNTGEEITRVTAIDPNTGSSNGFIYSLVGTLSNSQFTIDAKTGAISLLASVDFETDRTHT
AVVKAEGPTSTGVTEVIVRVTDINDNKPIFDPTNFNLNINENFVVGQEIVLVRATDKDSSLNDNNV
IIYRIDSVNPSSGNDLFSVNPSTGRILLLKALDRENGDTSYVLTIAASNRGVSNKPDNTMTVTINIA
DVNDNFPIFEKSLYQGSVSEDANTGTSILTVRASDKDILLKNRNIRYSMTQPMAVQKFQIDPISGVV
SLKQEVDFETKQRYDFSVIASDTGDVAYSRTANVEITILDVPDVEPKFDPQRYEITIAESTLVSTSILK
LQVIDSVKNLNFNIKQGNSNSDFRLVASSGELFVNKQLDRERISRYQMTIEVSDSVNKAAIDAIVIV
NIADVNDNQPLFLQSGCYQANRAEGIETEQVVVNVQATDRDEGSNKEIVYSFSDACSVLDIDAN
TGAITTKQKFDYESARSHICTIKAEDKGTPKLSGFSCIILTVTDSNDLKPTFEVITSNARVLENIPVGS
VITRVLATDKDTLSSNKQFVYTIKTGNEDGRFTIDANGFIKLQQPLDYEKKTDYRLEIEAVDSTNQ
DMVSDPNAIVTVTVIEQNDNRPKFAKSYHRVNIEESTAIGTTVLKLSATDADAGVNEGLIRYVSLH
NTTVFNIDRTTGEITLATKLDYEVKKSYHLIAVVKDQGNPVLSARTLIDIFVNDVNDNPPVFVISSY
NNIVSEKAPIGQSIVTVSARDDDTGVNGQLKYKIRETQSMFRIDEDTGIIILNGDLNVENTNKLLYA
LTIDVVDSGVPPLQASNPATVSVQVSDVNDKQPVFALDRYQFEINENIEGNQIFGDSVTVRANDD
DLTGTNNQLTYRLVNTTMFDLVVTNQNVFIKTALGVSFDYETKKSYSFEIEAMDSAANKLYGRSLV
TVTVKDFNDNPPIITVPNIIVNISDATLIGSHIATIMAEDKDSGENGILIYTVTAGDSTNRFTMESDT
GSIILKNPVDSYTKDIYVLNVLVQDNGIPRRSATGTVTINVIPVDLTLLKFNTTCPFAISIPENQINSN
FLGLKALDAGEYVSKQLRYTINSDENDVNQIFALDSIKGEFKQLKEANYEERSRYDMTICATNERG
SKVCCHITIFITDINEPPSFIGLNNNNIYEVSISETTPIGTIIYRVLSKDQDLPGVLNGQTSYSLGQQNV
FIIDQMGQIRVNKTLDAETQLNYVFQVIVTDKNPTNPLQSAATIRVNILDYNDERPIIQNIPDITIDE
NSDNQVVRNIVVTDRDISNIGSNIELSIEPSNILTINNRQLRTVGSALDYEKVQRHTLYLTAKDKSFP
YLSSTTSFTVTINNLNDNKPKFPVNNYNLTVSEKTTVGTLILTLFAEDDDLEEFGEIANYEIVSGNQ
EDRFSIDSNGRLTLKKQLNWRNVKSYNLNIIARDKGLPSEQSQIPATVVINVADYSEEQPNFLQNL
YVKFIPETTPIDTLVLTVEATSGEPVYGPFKYTIVPKLPDTNNFQIFSNGEIKTINQFDYENQRNFTFD
VQVEDEKNGKTNRASVFIHITDVNDNIPQFTDLTRSNTQYNITEFTLPGSMITCLSATDRDHGKNA
EIRFRGIGGDGNGVFNVNLDGKVLLSQQIFYDKKNKYNLIVEAFDLGSPEQKAQLTLTIFLHKSYT
SVITTDVDINEDVETQTRVTRVSSIDPFTRSSNNFTYYLVGKSTTFFEINAQTGEIFTKATLDRETRDR
HVLTIQSRGPTTNGVGELVVNVKDINDNPPIFDPQVYQVTVSESANIGLSIVSVTATDKDIGVNGE
VTYSIEDGDQTIFSINPSTGSISLAGKLDFESKQSHTLTIKAVDRGNPPQDSVNKATVIVVVQDFND
NCPVFENSTYSLTIKEPGELGKEYLKLGITDNDNGRNQLFTCSIDEPLAEKYFEMINDCRLRLIKEL
DYEEFTSFRFVVKATDQGDVSCTTTTNMIVNVGDRADVIPFFSPLTYNISIPESTTVGTIVVSLKVGS
TFKGLLYSLFHKNTQDDLIKFGINKDTGEIFLKESVDREIQDKYEFTADVFDGSNNAVQRASVTINII
DVNDNAPFFNQPTCYQRDLTESTAIGTFVVDVSAADNDKGRNQQVTYSLLPSPSCTSFSVDETSG
RITTSQILDHEQQKLQHCLIKAKDNGTNSLSGYSCLTVNVVNVNDNKPAFIVNRFDVSVLETVPA
GTLVASLVARDVDNQGLITYSISNGNLNNQFTIDSSGKLRTRFPLDYENTKSYNLTIQASDNNGKFS
SPDDALVLITVLEVNDNRPQFLQSYYKVSIPEDTATGASILNVTAIDLDHGELTKSLFYAALENTTQ
FTVNANTGVISVAENLDYEIEDHYELVVIVQDRGTPHLSNRALVDIQITDVNDNAPMFVTTVYNR
ELSEKALIGAPVVKLNAMDADKSDKMTFTIDSVVQGAQGFFSINGDTIQLDKELDYEVPEQRTFV
LSVSVKDSRDRDGANKANVFINVRDINDREPIFDLNMYEVTVSENNVISNPIIEVTARDSDSTPEN
NNLLYSLVDGEDAASFSLLNNGQSAIITSKISFDYEKKKSYRFLVRASDKVRENFTAFSTVFVTVQD
TNDEAPVPEKDLIMVNISESTRVLSTIAIVFANDKDQGINGQLAFQIINHSEDKFEINNKGVIRLKST
LDFFQQNFYNFTVLVTDKGNPVRTSKPVTVLVYVYRTEIPGIIFEKNHYIFNINEKEIVSLVGTVNAI
DEGTYVSRQIVYSIVSGQNDIKDKFRAVNNNIIQDKEVNYKDQSEYNFVMKAINERGSIAFTHITIN

IMDVNEKPYFVGLNNKGRYEFSMSEIISVPSYIYAVRAKDDDFGLNGEVDYEIISNFRFGNFSIDQY
GRIQVLKDPDFEFTRSYEFQVRAKDRGTPSLESNLVSVVITVLDYNDNHPIFNIQEPLEISIKELVNN
TDVGTVTATDKDGSGLNRIVKYVAIYPSDHLTVNRDNGQIRVIEPFDYEIAHKHEFVVVAQDQGT
PYLNAYKVIKVEVLNENDEFPEFTVIEYNITISELTPIGSPVLFVHAQDKDRGPSGQVTNYKIKYVPP
SGGSGAGSPDKTFEITSDGQVVLKEKLEYKFQKYYQYRVTCTDNGSPPKNSINEAIIHLNIADASLE
QPIFSVNPAVVEVYENITLNSNIVKVEVDSIEPIKGPFEYSIIASEDSQPFMIDNSTGQITNKESFDYEI
KRKYVFLAEARDKHNNRTQRAMVVIFILDINDNIPTIETIAPQTFVQNISDTTYPGSVVAKLYSRDA
DSGENAMVDFTITGGDGENVFAINPNGYVTLIGPLDVRVKTQYTINVKVSDRGIPTLSNNRALTF
NIKKSKFIQNTVRFDRHHYKSNCIEENQINLNIIQLNATNSNPVAGSTIIYSLDGTLEERQPFIVNSQ
TGEISLSQTLDYEIKQEYDFVAVAVNNDGSRDVAVVTVCIKDLNDNSPIFEPQSYVVNVSESESVGY
LVYKTTVMDADKTDFNTTKSYDIIQGNVNDKFIIDSMGHIYIKSKIDYEALQVKHFDLRIRVNEGN
LVGDMVLRVNILDWNDVHPSFSTLNIYNFTIPEDKPVGEIIEILTANDGDATSPNNEVTYKLLTTED
DFILRRNDKGHGSLIVNKQLDYSKQNRYEYVALAEDKGYLNLLGTAIVIINIQDVNNHPPEFDDSE
YNTTVSEHAQIGTRVLQIHAHDKDTGLGEQIFFIYGDPTETFTISSQGFIILNKQLDYETQRDYIFDV
GARDGGNLVSLNNVTVHIKVSEVNDNEPIFDLSTYRNSYAEDTPLGTTLFTVRATDKDFIGNSIIYSI
SGANDDTSYVSVDQTTGDITLQRRMDYESKNYITFTVQAIDSNNRNKLKGSTHVIIDVTDVNDN
NPKFVFGSDLVYNVTVVENINVGTLVVVVDSQDADSGLNGQTRYHIVSGNNENKFFINELNGQI
NLINKLDALQTNKYVLTVMAKDLGNPSRSTNVTVTINVLRYFESTPMFLPSYAEVIYPENLALGSSI
YELNVTKTLPGSPMKLSFHSSSKFPEFTLQGNKIILSKEFDYEIKNRYELIIIATDEENDKAFAQVIVR
VQNLNDNIPYFTQNAYFGVVSELAFVGHSVLTITAKDDDKDFTELFYSIATQVPDNAFKLQDNIV
VVNGKLIRNKVYEFEVEAFDNTFTSKRATVSITVTPENLPSFVNATYIVTLQENYPISTNFLNISAGT
FSGITYVITSMKAKDMFNVDANGQISLTKSLDYEKQRQHVFPVTASVTSPITQVRVASVIVNVLNE
NDNAPVFVNGPYERNITEGHLVGTMVIRVDGHDDDSDKLTYSIVSGDDGKFSINSEGVISLISTLNF
DLKNKYVLNVRVSDGKFVTDTIVTINITPIVKFPPMFNQTLYLLSVKENTLVGTSILQVSTVNVIPPS
SFSINEQSGRDFFTIDANGVIRTNNALDYEAKRQHIFTLKVQDLRSMGHATVVVNIVNDDDTCPIL
TPATQTVQITEPVAPGTIVAAVQAKDIDSKSLNFTITGGNGLFRIDQHGGIKTINEVDISTSTEYELTV
TVDDGTCTKSVQVDVNVNPLAPCPDCNIYPFTQTIYEFSINENTVPASPIITVTTGQSLQRNFSIEDP
AALEYVTINRLTGEISVIKAFDYEATKHLSFNVMAKTQTTKNRNPFYVITSVSRVRITVRDLNDNCP
SIESPMYYEVSHSSPIVQDTVVTRILVEDPDTLKDHTYSISDNNFKIDSMGHIRTVQNIESFTAQFYN
LTVTVSDGQCSKTSNVGVNLAVNPTPMTYQFTSNGVYSKSVREDHNLQDVILSVALNQNYPRVFS
IVESDANDQFVIDSNSGALRLKTSLNYELKKRYDFTVMADIHGGVYKSYASVHIIVLDVNDECPIF
PVIGYNQRVSGTPVIGTIVEIIEANDADNATLEYSIVSGNEDGHFSLDNSGVVRVVKPFASSFSKTYG
LSVRATDNKCSADVPLTVQVSTCAYGEDYQFEESEYVVYYLDENRNLGIVSNIKLKSTRAAFLTLSQ
NTHFTIDQNGDFRLIQTLNYETKKEYHLTLTANFTQDNVVTRIPIKVFVRDINDNCPAFSEELYAVQ
HTEPLREGLLIAKLSVIDDDTVQNFTYNIVSGDKRFSINENGEVVMNARLVTDTTLLSTTYKLEVSV
NDGKCAKNTKVDVFVYKTVVNSFLFPQPFYKFDISENQAIPFTIDRFVNVGGHEAVYGFLVSTDFF
QIDPKTAQLQLVKKLDFEVEKTHYVLISATYDDGTQTYTVVAIHVGDENDNAPQIFAHPPITRLVQ
NTPHNSIVTKIEVEDPDTSKEFTFKITNGNPLQNNDPDKALFRIESNGMIKTTRCISEDEIGNYVLTI
SVDDQVNTPTIFSNMQVQLKTTDDRSYVTNCGLGCPANDGPFSRYQFEQSLFIVNVTETAANPTV
LKRLTRPFVEKTTYTIGAGADGKFSIDQNGTLSLTGSLDYETKPVYYFIVSAVIPSQVVGRFITSKTAY
LVMVNDVDDSKPVIEGNGGTLAVSETSEIGQIVGTVRVKDSDSSSPLTYNVVNGSQYFNIDSAGNI
VIKDTLNAAGSPYTVTYTVSDGINPPVNGEFVVSISETNNNVPKFTQNIYEVSLSEDTATDTIAVTIL
ANDGDTNDKIAYSFLQSEGPFVINRDSGEIKLIGPLDYEAQKSYNMVVQATDSGGFANIAFVQIAV
GDVNDQVPVITPQTTPTQIHESTLVNSVVYRVIASDGDGNERLTYALANSNDFFTINSATGEITIKE
NLAKSNVSSHTLSIVVTDSSGKTSTQDVALNIRRSGTSQGCVKITTSQGQSSSPVINLPENTASGTTV
FKLVAGDASVFSNMRFTLNREANGVFAVTEQGELQLVAPLDYETKQFYSIIITAEDVTRGVSSQYNL
FVRISDVNEFTPVLETIAPVAVSELANIGDVIAQAKANDQDGDSQVTYSLGEFGNNDNKFTIDAAT
GNIILNKRLGSGAPHVVEVCASDGSIKKCQNVTFIVADTNDNPPSFQDLTGVGSIDLTNTSSYVVY
KPIVTDSDQTGTLSFSLKDPAPSQFSINSTTGEVKANKDIAKGTYTFGVCASDGRYTSCQSVTVVAS
DRSQSITPSFNQDQVGAIVDISASSGKQIIQVMASTLDKAKIEYELKNEADRNIFEINKDSGLITLKN
RTALTLGTIYTIPVKASRADDPLNFDEINIVISVGNQQLDSGSFIEQVLDTQHLVNFSLSTFDISKVSY
YIILGQQVGGDSKDDEKLPVSWYYATFGDESYRHRFYYIAKLDGSKFRNTGGSRKRRSIQDKDSKS

FTYLVGEENGCSKNTSMAAICNGPLKTGTEYRYKLRAYYDDGELRGIPVDQKPTEVKRTQAPQA
GKSIDEYREQDSYVITFWIIGLVIFGLLFWFSVLVIAILLCYKRRYEVTQAYGYSVQTRDKIDGPPTEY
SNTVYRFQNPSYPDNDTVKMKKVDASESRTKTEVQTTSMVVLDDYEQEQPQPDIPVQDYDAIEY
ETRSAQIKFHEESPEWESMDIQLRIDPTGNQDPVITRKSDSGSKDSDSDASKKYENTEYIEEKEDFV
NEKEDSLTRL
>TRINITY_DN95248_c0_g1_i1.p1 type:complete len:124 gc:universal TRINITY_DN95248_c0_g1_i1:197-568(+)
MAPKTKKVSKKKSTLNFNIDCTLPVEDGIMKVDDLKKFLQEKIKVDDKLNNLDQITLEVAKTKVT
LVSERPFSKRYLKYLTKKYLKKNNLRDWLRVVASSHTDYELRYFQINNDEEEDEEDNE*
>TRINITY_DN250_c0_g1_i5.p1 type:complete len:452 gc:universal TRINITY_DN250_c0_g1_i5:131-1486(+)
MRECISVHVGQAGCQIGNACWELYCLEHGIQPDGQMPSDKTIGGGDDSFNTFFSETGAGKHVPR
AVFVDLEPTVVDEIRTGTYRQLFHPEQLITGKEDAANNYARGHYTVGKELVDQVLDRIRKLADQC
TGLQGFLIFHSFGGGTGSGFTSLLMERLSVDYGKKSKLEFAIYPAPQVSTAVVEPYNSVLTTHTTLE
HSDCAFMVDNEAIYDICRRNLDIERPTYTNLNRLIGQIVSSITASLRFDGALNVDLTEFQTNLVPYP
RIHFPLATYSPVISAEKAYHEQLTVSEITNACFEPANQMVKCDPRHGKYMACCLLYRGDVVPKDV
NAAIATIKTKRTIQFVDWCPTGFKVGINYQPPTVVPGGDLAKVQRAVCMLSNTTAIAEAWARLD
HKFDLMYAKRAFVHWYVGEGMEEGEFSEAREDMAALEKDYEEVGVDSVDAEDEEEGDEY*
>TRINITY_DN118_c1_g1_i3.p1 type:internal len:200 gc:universal TRINITY_DN118_c1_g1_i3:599-3(-)
KVLFLCGFILLSGLAFAYEIEEIVEDAPKPEPEHVPEPEPEPVKEPEPEPEHKPEPEPEQCSTVKHQK
CSKADKVCSPEDSHCTFTQICTPFFVKKCCKVVSVEKKCVVVRNCIPKFSYEKVCEDVKRCPPRKR
CKNVCRQVDLGEHKVKSICEQECEVVEHPEPCKLVHTCFSKWVSKKDCTPRETCSDIEKRECREV
>TRINITY_DN15584_c0_g1_i1.p1 type:complete len:275 gc:universal TRINITY_DN15584_c0_g1_i1:874-50(-)
MADSGSAGADRGGRGGFGRGGRGGRGRGRGRGRGRGKPEDKEWTPVTKLGRLVKDLKIKTLE
QIYLYSLPIKEFEIIDFFIGPSLKDEVLKIMPVQKQTRAGQRTRFKAFVAIGDYGGHVGLGVKCSKEV
ATAIRGAIIQAKLSIIPVRRGYWGNKIGKPHTVPCKVTGKCGSVTVRLIPAPRGTGIVAAGVSKKLL
QMAGIDDCYTSACGTTATVGNFAMATYMAIARTYSYLTPDMWAENQFQKTPYQEFTDYLAKNH
NVGGKPVAPATATA*
>TRINITY_DN2126_c0_g1_i1.p1 type:complete len:114 gc:universal TRINITY_DN2126_c0_g1_i1:55-396(+)
MAPVKKKTAESINSRLALVMKSGKYTLGYRSTLKTLRQGKAKLVIISSNTPQLRKSEIEYYAMLAKT
GVHHYTGNNIELGTACGKYFRVSVLSITDPGDSDIIRSMPSEQETK*
>TRINITY_DN1566_c0_g1_i1.p1 type:complete len:4626 gc:universal TRINITY_DN1566_c0_g1_i1:42-13919(+)
MDTEEVPGEPPPTVLAATEDVINYVKNITCFIFDESQPCLPLDKILKEEQTKDYFRKFISDVQTKTIL
LYRINQLTDDVDGDHDQGVSQPSYGIKLNVQYVPHKESSIAFIKRGMLLESDKKINAQLHVLILD
NSSPFDTLHSYISNAVSPFFKSYVKTSGKSEREGDKMVPSVEKKIAELEMGLLHLQQNIDIPEISLQI
HPVVQSIIQKAEIERRKISPDDYAEKAEDSSFLNALQNGVNRWIREIQKVTKLERDPSSGNALQEIS
FWLNLEKALHRIEEKQQSPEVKLTLDILKYAKRFHAIVSFTNDTGLKQGLQTVASYNPLMKDFPLE
QLLSATELERIKDALIAIFSHLRKIKLSQYPIQRCLRLIEAISKDLMSQMLKVLGTRKFMLMNYELFE
KVMTSAFNVLNCWDDEYERLQQILRDMAKKKKDDSLKTMWRVNPSHKKLQSRLNNLRTFRKQ
HEQLRAVILRVLRPSKVEAKSSNEENAASSGSIEISDDNAVKEVDLAYENFKEIDPLDVSKEGTEAW
ETALKRYGERIDRVETRITSTLRDQLGTAKNANEMFRIFSRFNALFVRPRIRGAIREYQKHLIGLVKE
DIEALHDKFKVQYSHSKASKMSRVRDLPPVSGSIIWAKEIERQLHAYLRRVEDVLGKDWENHIEG
QGLKADGDSFRQKLNTQEIFDDWSQQVQKKPLLVVGRIFSIETLKTKGSNKATRKLKVNFTKEVIT
LAKEVRNLKNLSFRVPLAIVRQANDANQYYPLAISLIESVRTYEVTNDKVFEKESIIPLVAGIRKDIQ
SLLAEGMHLEWQSYKLMPYVMRLAEAVTGYQEKVDELLTSIQKIDIEVQSLATCGFKHEAFQEILI
RIQKLVDELNLHSYSNLNSWVKTLDEQVEAKLAHRLQAGIKEWTRVLRTGNENVDDKDLAIHSP
GGLPDIKPLRVEFFIRNQVIEMHPPFEKCIEHLYNELAVYCSTITSLTKIQSQRYQVGLDRDNAEETY
HNLLTKLPDPNGPNPITVYGEMESMLEDCRKYCEGWTGFQALWDAEMSNLAPRMKTLEDWMT
ILNQIKMSRSHFDTTETMKEYGPLIVDFNAIQGKVNLKYDSLQKAIVQNFGGKLGDEVNDFFTNV

TKARTDLEQQSLDVATTAEAVTFITYVQGLKRKMKTWEQNVEVFREGQKVLERQRYQFPSNWLY
SDNVDGEWSAFNDILKRKDGAIQTQVAILQQKIVAEDQAVEERTFNLLADWDKGKPVSGNTKPD
SAIQTLQIFEGRLTRLKEDRDNLQKAKEALELIEPGTVPPSDERVQVAIEELSDFKSVWGELEKIWA
QVDDLKEQPWVSIQPRKLRQSLDSIANGMKILPAHMRQYAAFDYVQKIIKGYLKANMLVVELKS
EALKERHWKQLMRKLSVKWNLSDLTLGQIWDCNLQKNEVVIRDVINIAQGEMALEEFLKQVRE
SWQAYELELINYQNRCKLIRGWDELFDKVREHINSVSAMKLSPYYKVFEEDSKQWEDKLNRILNL
FDVWIDVQRRWVYLDGIFSGSGDIQHLLPIETQRFGSISTEFLTLMRKVAKAPLVLDVLNIQGVQRS
LERLADLLGKIQKALGEYLERERASFPRFYFVGDEDLLEIIGNSKNVGRLQKHFKKMFAGVHTIIL
NEDSTLVTGICSKEGEEVIFNKAVSLIEFPKINEWLTLVEEQMRLTLATLLTLSAKEIKSFTGDSIDPK
LYMEWVDKYQSQLVVLSAQIVWSENCEAALQTCYNGDMSSLEKVLSIVEATLTVLADSVLQEQP
QVRRIKLEHLITELVHQRDVTRTLVRSKVQGTKSFAWLSQMRFYFDPKQRDVLQQLSIHMANAKF
NYGFEYLGIQEKLVQTPLTDRCYLTLTQAIERRMGGSPFGPAGTGKTESVKALGNQLGRFVLVFNC
DEKFDFQAMGRIFVGLCQVGAWGCFDEFNRLEERMLSAVSQQIQTIQVALKEGPKEIGKPIQIELI
GKDVKVSEDMAIFITMNPTYAGRSNLPDNLKKLFRSIAMTKPDMNLIAEVMLYSQGFRTAEVLAG
KVVPFFKLCFEQLSAQSHYDFGLRALKSVLVSSGNVKRDKILSIKQKMQAQGVVADVGKITETLN
EQEILIQSVMETMVPKLIAEDIPLLHSLLADVFPGIKYVNAEMTALRNELKKVCNEMHLVYGENL
ESGAHWIEKVLQVYQISCISHGLMVVGPSGSGKSTAWRTLLKALGKLEGNEGVCHVIDPKAISKD
QLYGVMDPNTREWTDGLFTHILRKIVDNVRGELSKRQWIVFDGDVDPEWVENLNSVLDNNKLL
TLPNGERLSIPPNVRIIFEVQDLKYATLATVSRCGMVWFSDDVVSPEMIIENYIKKLRNVLLEESEED
IRSKQSKSVTDSKDELVSPTLQVQRDFSDILTPHLASNGLVLKTLEYSYSKDHIMDFTRMRALNSLF
SLLNQGVRNILQYNQQHHDFPMSQDTIEAYTPKYLVHSLLWCMSGDGKLISRLDISNYIRNITTIPL
PSPVTSLIIDFEVNMQGDWVPWSNKVPQIEIETHKVASPDVVVPTLDTVRHESLLYTWLADHKPL
VLCGPPGSGKTMTLFAALRALPDMEVVGLNFSSATTPELLLKTFDHYCEYRRTPGGLVLSPVQIGK
WLILFCDEINLPDIDAYGTQRVISFLRQLVEHNGFYRPSDLQWVSLERIQFVGACNPPTDPGRKPLS
HRFLRHLPVIYVDYPGPSSLTQIYGTFNRAMLRLVPSLRSFSQPLTDAMVEFYTKSQERFTQEMQP
HYIYSPREMTRWVRGVLEAIRPLDNLSPDGLVRLWAHEALRLFHDRLITDEERRWTNTNIDLVAQ
KHFPNVDLISLKRPILYSNWLSKDYLPVDREELRDFTRARLKVFYEEELDVPLVLFDEVLDHVLRID
RIFRQPQGHLLLIGVSGSGKTTLSRFVAWMNGLSVVQVKVHRKYTGDDFDEDLRIVLRRSGCRNE
KIAFIMDESNFLDSSFLERMNTLLANGEVPGLFEGDEYTTLMTQCKEGSQRAGLMLDSGEELYKW
FTQQVMNNLHVVLTMNPSAEGLKSRAATSPALFNRCVVNWFGDWSTGALYQVGQSFTNKIDLE
RSTYKAPDYLPVVFENLPMPPTHRDVIVNTMVFVHQTMHKANEQVTARGGRTVAITPRSYLDFIS
QYMKLFNEKRSDLEEQQLHLNVGLQKIRDTVTQVEELQKSLSMKSQELESKNELANKKLKQMLQ
GQQEAEKKRITSKQIQDDLKKQTEEINKKKSQVLGELDQVEPALLDAKAAVKSIRKENLDELRRL
GNPPTAIKLALESICLLLGTPSVEWKEIRVLVSKTTFIPSIVNFKTDDISIETRKQMMKYLNHSDYNF
EKMNKASVACGPLVKWAIAQVSYAEMLQKVDPLRRELQKNEDEMMSNQSKLDSVGEMISELEK
SIAKYKEEYALLISEAQSIKADLAAVEAKVSRSTALLRSLSSERQRWENTSDGFQNQMDTIAGDVLL
SAAFLSYGGYFDQQMRSRLWGTWSEQMEQSKIKFRSDLARTEYLSTADERLHWQSNSLPADDLC
TENAVMLSRYIRYPLIIDPSGQATEFIMKEYSGKKITKTSFLDDAFRKNLESSLRFGNPLLVQDVESY
DPILNTVLNKEVRKTGGRVLITLGDQDIDLSPAFTIFLSTRDPTVEFPPDVCSRVTFVNFTVTRSSLQ
SQCLNQVLRAERPEVDTKRSDLLKLQGEFHLRLRHLEKELLQALNDSKGSILNDDKVITTLETLKT
EAADIMKKVEETDTVMSEVEAVSEQYRTLAAACSSIFFTLEEANLIMFLYQYSLKFFLEIFQSILFNN
PLLKDKRDPLVRLGILIESLFQVVYNRVARGMVHNDRVSFATLLARIRLKGKSENSYDLEFDHLLK
GKEIVVQVSDVKLTGLSSDQKMALLRLSKLNPFKELSRYIMDNSMGFQEWINSSNATLNVPRCW
NIKDSLTSSGEVLYECLVVQAFRPDNLHAMLRKYVLAVMGEKFLHTAEQELDMVNIVENEIKAET
PLLLCSAPGYDVSGRVDDLVAITNKQITSIAIGSAEGFSEAEKAINASVKSGRWVLLKNVHLATQW
LISLEKKLHTLQPHPNFRLFMTMEINPKVPVNLLRAARIFVYEPPPGLKANLLRTFSTIPASRMSEA
PGERSRLYFLLAWFNAIVQERLRYAPLGWSKIYEFSESDLRVGYDMLDIWIANVSQGRANVSPDKL
PWDAFRKLIGDSIYGGRIDNDFDQRLLQSFIDRLFTAKSFDSDFSLVTSVDGKQSENIVIPDGMRRE
QFVQWCEKLPDYQSPSWLGLPNNAEKVILTTLGERTCVKLLKMSVNTEGSELAYSADSHLKESEV
TGDIRPLWMKSLQQSVRNWLNILPKRIDPIKRTKENIKDPMFRFFEREVNLGVKLLNDILHDLNEI
LEVCEGNRKQTNDLRMLVDTLVKGVIPRSWNRYATPKGLTVIQWIVDLGERMKQLQNIARGTNA
GGSKELKSFKVWLGGLFIPEAYLTATRQYVAQSNQWSLEELYLNVTIADELNPQLKLDDCSFVVIG

LKLQGATCRNNQLSPVDTITTDLMDICLCWTKIDPRKPPTLGANDVTLPVYLNQTRCELLFTVDM
STTGAVPPRSFYERGVGFVVSALTG*
>TRINITY_DN189323_c0_g1_i1.p1 type:5prime_partial len:156 gc:universal TRINITY_DN189323_c0_g1_i1:2-469(+)
KREVWRVKYTLAKIRKAARELLTLEEKDAKRLFEGNALLRRLVRIGVLDENRMKLDYVLGLRIED
FLERRLQTQVFKLGLAKSIHHARVLIRQRHIRVRKQLVNVPSFIVRLDSQKHIDFALNSPYGGGRP
GRVKRKNLKKKQGGGSGGQEEDED*
>TRINITY_DN7659_c0_g1_i2.p1 type:complete len:359 gc:universal TRINITY_DN7659_c0_g1_i2:82-1158(+)
MASAATDNRKVALITGVTGQDGSYLAEFLLDKGYEVHGIIRRSSSFNTGRIQHLYKDRTAHLEGN
LHLHYGDLTDSSCLLHVVSKAQPTEIYNLGAQSHVKVSFDLAEYTADVDAMGTLRLLEAVKICGL
MHKVKFYQASTSELYGKVHEIPQTETTPFHPRSPYGAAKLYAYWIVQNYRESYNLFGCNGILFNH
ESPRRGETFVTRKITRAVAKIHLGLQEELVLGNIDSVRDWGHARDYVEAMWLMLQQEQPETYVIA
TGETHTVREFVEKSFGLIDVKIRWEGTGENEIGYDAASNKVRVRISSKYYRPAEVEYLHGNPAKAE
KELGWKRKVSFDELVKEMIDSDIALMKKDPEA*
>TRINITY_DN3036_c0_g1_i3.p1 type:internal len:548 gc:universal TRINITY_DN3036_c0_g1_i3:1643-3(-)
AFAGAARASPARQGSVARRALDQSSRYADLSLTEEDLIRNGKHVLVAYIMKPKAGYDYLATAAHF
AAESSTGTNVNVCTTDDFTKSVDALVYYIDPEEEEMKIAYPNLLFDRNIIDGRAMMCSFLTLTIGN
NQGMGDVEYGKIYDFFLPPEYLRLFDGPSCNVIDMWRILGRGTSNGGLVVGTIIKPKLGLQPKPFG
EACYAFWQGGDFIKNDEPQGNQVFCQMNECIPEVVKAMRAAVKETGVMKLFSANITADDPVE
MISRGKYVLSQFGPLSENCAFLVDGYVAGGTAITVARRNFPKQFLHYHRAGHGAVTSPQTQRGYT
AFVHTKISRVIGASGIHVGTMSFGKMEGDASDKNIAYMLQGDVADGPYYQQDWQGMKETTPIIS
GGMNALRLPAFFENLGHSNVILTAGGGAFGHKDGPKQGATSCRQGEEAWKLWKAGTYGDITLS
DGVIEYAKTHEEIKGAFLTFQKDADQIYPGWKEQLGYTGESSVQAASFDWKRKAGAAAFAGAAR
SPLAKQSSVARRALDQSSRYADLSLTEEDLIR
>TRINITY_DN6920_c0_g1_i3.p1 type:5prime_partial len:411 gc:universal TRINITY_DN6920_c0_g1_i3:2124-892(-)
CKRHQGKMSHRKFSAPRHGSLGFLPRKRCKRHQGKIKSFPKDDAAQPCHLTGFIGFKAGMTHIV
REVDRVGSKTHKKEVVEAVTIVETPPVVVVGIVGYVETPRGLRQLTTVWAEHLNEECLRRFYKNW
YKSQRKAFTKASKKWSDDDGKKSIERDLEKMKKYCTVIRVLVHTQQKLIKLKQKRAHIMELQIN
GGTVADKVDWAKKSLESKLSVSTVFGQDEMIDVIGITKGKGFRGVTFRWGTKKLPRKTHKGLRK
VACIGAWHPARVGFSIPRAGQCGYHHRVERNKKIYRIGRGIHTKDNKVIKNNAATDYDITNKTIT
PMGGFPHYGEVNEDFIMLKGCVPGPKKRVLTLRKSLLAQTSRKALEKITLKFIDTSSKFGHGRFQH
DQEKKNFMGLLKKDKERQAVEA*
>TRINITY_DN262_c0_g1_i1.p1 type:complete len:1033 gc:universal TRINITY_DN262_c0_g1_i1:111-3209(+)
MADPSSLETKEKDGSYRVATKSGGKDKGKKSKKAAKKVNMDDLKKELEMTEHSCELETLVSQLE
TSLTQGLTKSIAARNLERDGPNALTPPKQTPEWVKFCKQLFGGFAMLLWVGAILCFFAYIIRSIREE
EPGMDELYLGIVLTVVVIITGCFSYYQESKSSKIMESFKKMVPQEAVVMRDGEKLNLNATECVVGD
LVFVKFGDRIPADLRIIESKGFKVDNSSLTGESEPQSRNSECSSDNPLETRNLAFFSTNAVEGTATGIV
VKTGDNTVMGRIANLASGLGSGKTPIALEIEHFIHIVTGVAVFLGVSFLIISLAMNYHWLESIIFLIGI
IVANVPEGLLATVTVCLTLTAKKMASKNCLVKHLEAVETLGSTSVICSDKTGTLTQNRMTVAHM
WFDNMVVEADTTEDQSGQAHDKGSTTWKALAKIGALCNRAEFRPNQEEEPILKRECNGDASET
AILKFVELSVGSVEDIRSKNKKIVEIPFNSTNKYQVSIHEQEDPDDPRYLLVMKGAPERILDRCSTIL
HEGVEKPLDEEWKENFNKAYVDLGGLGERVLGFCHYYLPADDFPKGFAFETEDPQNFPLEKLCF
VGLMSMIDPPRAAVPDAVSKCRHAGIKVIMVTGDHPITAKAIAKGVGIISEGNETVEDIAQRLNIP
KEEVNERDANAAVVHGSHLKDLPQEKLDDILKYHQEIVFARTSPQQKLIIVEGCQRMGAIVAVTG
DGVNDSPALKKADIGVAMGIAGSDVSKQAADMILLDDNFASIVTGVEEGRLIFDNLKKSIAYTLTS
NIPEISPFLMFILLAIPLPLGTVTILCIDLGTDLVPAISLAYEKAETDIMKRKPRDPVRDKLVNERLISL
AYGQIGMIQATAGFFTYFVIMAENGFLPGYLIGLRNDWDDKKNNAVMDSYGSEWTYNQRKNLE

LTCHTAFFTTIVVVQWADVIISKTRRLSLFQQGMTNWMLNFGIFFETALAAFLQYTPGLNTGLRLR
PMNFPWWFVGVPFSLLIFVYDELRRFLLRRNPGGWVEQETYY*
>TRINITY_DN2963_c0_g1_i1.p1 type:complete len:116 gc:universal TRINITY_DN2963_c0_g1_i1:1072-725(-)
MSATGNLVTVLRKLYDEYNDKTPSRLKIVDGFLLYVMLTGIFQFLYCCLVGTFPFNSFLSGFISCVGT
FILGVCLRVQTNPRNRTEFKGISPERAFADFIFANVVLHLVVMNFIG*
>TRINITY_DN36_c0_g1_i19.p1 type:complete len:223 gc:universal TRINITY_DN36_c0_g1_i19:826-158(-)
MIKLAIIVTVIAACVFESSAARCYVGYSAKGLPLAIGVRDVECGKGFDYCTTIKYRLGGIEVTQGNC
ARKYGQAMCQFMRNPHKAMTDCEETICQGDSCNKDVYDAEQAERKRVGPYKNTLKCTTGLIT
KFPAIPFYTADVPNAIDECFPGTTECVTVTYDYLFKFETQIATKDNVFKLPCVQKMCNRHNIDNC
NKWCDILSIHQSMENCVAKCCKGDNCNA*
>TRINITY_DN3036_c0_g1_i3.p2 type:3prime_partial len:342 gc:universal TRINITY_DN3036_c0_g1_i3:621-1643(+)
MDPAGADDPRDLRVYESRVSALRLGARHGAVPGTVVMQELLREVPPRHSDRRAAGDVAVHEEG
AILRQRTKLRQHILPAGNHLDRVVRGDVRRKQLHHASLLDGGPHRLHHFWDAFVHLTEDLIAL
RLIVLDEVSTLPERVARLPERLRLQAELRLDDGADHQAAIGRAAAQDAPHVDDVARRAVEQAQV
LRRQEEVVDLAILDVAHALVVADRQRQEAAHHGAAINDVAVEQQVRVRDLHLLFLRVDVVHQR
VDAFGEVIRRADIHVRARGRLGREVRRGGEIVVPGLRFHDVRNEDMLPVPDEVLLRQGQIGVAA
GLIQSAAGHRALPRRRRASRARKS
>TRINITY_DN1386_c0_g1_i1.p1 type:complete len:596 gc:universal TRINITY_DN1386_c0_g1_i1:2419-632(-)
MEYIEVKSFIVALVFVFSILGTAQSDLVINNVERTLDLSTQTAKYTTKIKFHNPGKKVEKSFLLALEK
DQEKHLSYLEILDKQDDNSLEYAAENVEGQSGRFFKVSFSKPLKENDDVTLHIETVFTHVLTPFPA
KIGQTEKQLVLYKGNTVFYSPYEIKEQSTTVKLASSAIESYSRLKPSRSSENTITYGPYKNSKPYRLHE
MKIHFENNSPFLVVNEMTRWIEISHWGNIAVEETYHMTHEGALLKGHFSRYDYQRSPTHSVIKSF
KTVLPAAARDVYYRDEIGNISTSNMLVNDDSVEIELRPRFPLFGGWQTRYMLGYNVPAYQYLYNK
GDNYILKMRLIDHVFDEFVIDDLNVKIILPEGSTNIEVVKPYELEEGKRDIHKTYLDTSGRTVIVLSK
KNVVENHIQDIQVHYTFKKLQLLQEPVICVAVFYILFLSVIILVRLDFSITKDAAKESRMKVASLLEE
LLSSCGRRGSLYYSYDSAIDKLKQNRNQNDFAVSLKKLNQDYNTLTQSINELCSLLIKEDPEIGDKV
NDLQKKENERKVIVDQLVTLATRVVTNKIGRQQYIESEKNSMAKREKLGEDIDNVLATL
>TRINITY_DN286_c0_g1_i2.p1 type:3prime_partial len:106 gc:universal TRINITY_DN286_c0_g1_i2:40-354(+)
MIKVAIITIAIVACVLESTAAVRCYVGYSAADLPLAIGVRNIECGEEFNHCTTVKYRIGGITVTQGN
CAKRYGQAMCQFMRNPHKAMTECEESLCQGDNCNTAVN
>TRINITY_DN982_c0_g1_i1.p1 type:5prime_partial len:6412 gc:universal TRINITY_DN982_c0_g1_i1:19377-142(-)
KLDFETKQSHTLTIKAVDRGNPSQDSLNKATVIVVVQDFNDNCPLFSESIYNASVSESTAIGTTVLR
VSVTDKDVGNVNNEITCSIDEPLAKGFFSVNKINNNQFSVVLIKNLDYETHRGYRFVIKAKDNGE
VPCQLTTNMVVEVQDEPDVITNFKPFNYVISIPESTLIGTKLLELKVDSESSSITYTLQAKNQPGDLD
KFMLNGQSGELVLKGTVDFEKKSEYEFTATLNDGVNNGGNTANIVVNILDINDNPPVFSQSTCYI
RRMPENQQVNTTVTSVLATDSDQGINSNISYSLIKDSSCNNFEINEITGAISLTSSLNYEVRKEHYCII
KATDNGSPVLSSYSCLKVIVENVNDNSPVFLPKELEITVLESKPVNSFVIRVFATDKDEGNSITYSIV
GGNTQNAFAITPLTGHIILNQKLDYEIVKNYILEIQATDDGTPRRSSNLNAKVMVKVLEVNDNKP
KFNQQRYLRTIPEDTNIGSTIISVTATDDDAGLLTSKIQYIALENTNDFLIQPDTGDIVVSQNLDYER
QRTYQLVAVCSDSGTPPFTGRTLVDITITDVNDNAPILNPTLYNVLVSEKAPLGTSLIQVHAKDKDS
GINGGLKYQIVSGNDLNKFVINNSTGVITLQETLDREKYSSLYNLTVSVSDQATPSLNGNNAFVQI
RVLDVNDKQPVFVKSEYIFEISENQPLNSPIGLVQANDDDSTSPNNIIQYTIVPSKDSINFTMINNQI
LSNSVFDYEEQKSYEFEVQARDQSTGVNNEQLVGRTLVKVLIRDTNDNDPMIENQNIALNISDVT
LVGTLLFDIQATDKDNGENARLTYTISSGDTSTFRINENDGRFYLSKKLDFYNKNEYSLIVIVNDNG
SPQRNSVANVQINVYPIDQTLISFEPQCPIAFTIQENSLSSPMNIKAIDTGSYVSKTMRYMLKSNKSD
VSSMFQIVETNGVIIQNKAANYEERSRYDMTVCARNERGSEGCCPVTIYILDQNEQPFFDTDNDN

LDNGKYLVRVSETTPIGSVMFSVNTGDEDFGSLPNGQKTYSIKAKPGEEMLFLVDSYGRIITNTTLD
REKTPQYDFSVVVTDKGTPPRQAEAEVIVTVIDYNDQRPVFEIMSVRPVNENANNEVLGRVVAVD
IDSTSINNGIKYSIQPAGFLNISNTGTLTSVGSALDYEKNQKVTLYVTATDTGSPYLSATTSLQLDIINI
NDNKPYFLQNNYNLTVSEKTTVGTLVLTLFAEDDDLGEFGEIANYEIVSGNQEDRFSIDSNGRLTL
KNPLNWRNVKSYNLNIIARDKGLPSEQSQIPATVVINVADYSEEQPNFLQNLYVKFIPETTPIDTLV
LTVEATSGEPVYGPFKYTIVPKLPDTNNFQIFSNGEIKTINQFDYENQRNFTFDVQVEDEKNGKTN
RASVFIHITDVNDNIPQFTDLTRSNTQYNITEFTLPGSMITCLSATDRDHGKNAEIRFRGIGGDGNG
VFNVNLDGKVLLSQQIFYDKKNKYNLIVEAFDLGSPEQKAQLTLTIFLHKSYTSVITTDVDINEDVE
TQTRVTRVSSIDPFTRSSNNFTYYLVGKSTTFFEINAQTGEIFTKATLDRETRDRHVLTIQSRGPTTN
GVGELVVNVKDINDNPPIFDPQVYQVTVSESANIGLSIVSVTATDKDIGVNGEVTYSIEDGDQTIFSI
NPSTGSISLAGKLDFESKQSHTLTIKAVDRGNPPQDSVNKATVIVVVQDFNDNCPVFENSTYSLTIK
EPGELGKEYLKLGITDNDNGRNQLFTCSIDEPLAEKYFEMINDCRLRLIKELDYEEFTSFRFVVKAT
DQGDVSCTTTTNMIVNVGDRADVIPFFSPLTYNISIPESTTVGTIVVSLKVGSTFKGLLYSLFHKNTQ
DDLIKFGINKDTGEIFLKESVDREIQDKYEFTADVFDGSNNAVQRASVTINIIDVNDNAPFFNQPTC
YQRDLTESTAIGTFVVDVSAADNDKGRNQQVTYSLLPSPSCTSFSVDETSGRITTSQILDHEQQKLQ
HCLIKAKDNGTNSLSGYSCLTVNVVNVNDNKPAFIVNRFDVSVLETVPAGTLVASLVARDVDNQ
GLITYSISNGNLNNQFTIDSSGKLRTRFPLDYENTKSYNLTIQASDNNGKFSSPDDALVLITVLEVN
DNRPQFLQSYYKVSIPEDTATGASILNVTAIDLDHGELTKSLFYAALENTTQFTVNANTGVISVAEN
LDYEIEDHYELVVIVQDRGTPHLSNRALVDIQITDVNDNAPMFVTTVYNRELSEKALIGAPVVKL
NAMDADKSDKMTFTIDSVVQGAQGFFSINGDTIQLDKELDYEVPEQRTFVLSVSVKDSRDRDGA
NKANVFINVRDINDREPIFDLNMYEVTVSENNVISNPIIEVTARDSDSTPENNNLLYSLVDGEDAA
SFSLLNNGQSAIITSKISFDYEKKKSYRFLVRASDKVRENFTAFSTVFVTVQDTNDEAPVPEKDLIM
VNISESTRVLSTIAIVFANDKDQGINGQLAFQIINHSEDKFEINNKGVIRLKSTLDFFQQNFYNFTVL
VTDKGNPVRTSKPVTVLVYVYRTEIPGIIFEKNHYIFNINEKEIVSLVGTVNAIDEGTYVSRQIVYSIV
SGQNDIKDKFRAVNNNIIQDKEVNYKDQSEYNFVMKAINERGSIAFTHITINIMDVNEKPYFVGL
NNKGRYEFSMSEIISVPSYIYAVRAKDDDFGLNGEVDYEIISNFRFGNFSIDQYGRIQVLKDPDFEFT
RSYEFQVRAKDRGTPSLESNLVSVVITVLDYNDNHPIFNIQEPLEISIKELVNNTDVGTVTATDKDG
SGLNRIVKYVAIYPSDHLTVNRDNGQIRVIEPFDYEIAHKHEFVVVAQDQGTPYLNAYKVIKVEVL
NENDEFPEFTVIEYNITISELTPIGSPVLFVHAQDKDRGPSGQVTNYKIKYVPPSGGSGAGSPDKTFE
ITSDGQVVLKEKLEYKFQKYYQYRVTCTDNGSPPKNSINEAIIHLNIADASLEQPIFSVNPAVVEVY
ENITLNSNIVKVEVDSIEPIKGPFEYSIIASEDSQPFMIDNSTGQITNKESFDYEIKRKYVFLAEARDK
HNNRTQRAMVVIFILDINDNIPTIETIAPQTFVQNISDTTYPGSVVAKLYSRDADSGENAMVDFTIT
GGDGENVFAINPNGYVTLIGPLDVRVKTQYTINVKVSDRGIPTLSNNRALTFNIKKSKFIQNTVRF
DRHHYKSNCIEENQINLNIIQLNATNSNPVAGSTIIYSLDGTLEERQPFIVNSQTGEISLSQTLDYEIK
QEYDFVAVAVNNDGSRDVAVVTVCIKDLNDNSPIFEPQSYVVNVSESESVGYLVYKTTVMDADKT
DFNTTKSYDIIQGNVNDKFIIDSMGHIYIKSKIDYEALQVKHFDLRIRVNEGNLVGDMVLRVNILD
WNDVHPSFSTLNIYNFTIPEDKPVGEIIEILTANDGDATSPNNEVTYKLLTTEDDFILRRNDKGHGS
LIVNKQLDYSKQNRYEYVALAEDKGYLNLLGTAIVIINIQDVNNHPPEFDDSEYNTTVSEHAQIGT
RVLQIHAHDKDTGLGEQIFFIYGDPTETFTISSQGFIILNKQLDYETQRDYIFDVGARDGGNLVSLN
NVTVHIKVSEVNDNEPIFDLSTYRNSYAEDTPLGTTLFTVRATDKDFIGNSIIYSISGANDDTSYVSV
DQTTGDITLQRRMDYESKNYITFTVQAIDSNNRNKLKGSTHVIIDVTDVNDNNPKFVFGSDLVYN
VTVVENINVGTLVVVVDSQDADSGLNGQTRYHIVSGNNENKFFINELNGQINLINKLDALQTNK
YVLTVMAKDLGNPSRSTNVTVTINVLRYFESTPMFLPSYAEVIYPENLALGSSIYELNVTKTLPGSP
MKLSFHSSSKFPEFTLQGNKIILSKEFDYEIKNRYELIIIATDEENDKAFAQVIVRVQNLNDNIPYFTQ
NAYFGVVSELAFVGHSVLTITAKDDDKDFTELFYSIATQVPDNAFKLQDNIVVVNGKLIRNKVYEF
EVEAFDNTFTSKRATVSITVTPENLPSFVNATYIVTLQENYPISTNFLNISAGTFSGITYVITSMKAKD
MFNVDANGQISLTKSLDYEKQRQHVFPVTASVTSPITQVRVASVIVNVLNENDNAPVFVNGPYER
NITEGHLVGTMVIRVDGHDDDSDKLTYSIVSGDDGKFSINSEGVISLISTLNFDLKNKYVLNVRVSD
GKFVTDTIVTINITPIVKFPPMFNQTLYLLSVKENTLVGTSILQVSTVNVIPPSSFSINEQSGRDFFTID
ANGVIRTNNALDYEAKRQHIFTLKVQDLRSMGHATVVVNIVNDDDTCPILTPATQTVQITEPVAP
GTIVAAVQAKDIDSKSLNFTITGGNGLFRIDQHGGIKTINEVDISTSTEYELTVTVDDGTCTKSVQV
DVNVNPLAPCPDCNIYPFTQTIYEFSINENTVPASPIITVTTGQSLQRNFSIEDPAALEYVTINRLTGE

ISVIKAFDYEATKHLSFNVMAKTQTTKNRNPFYVITSVSRVRITVRDLNDNCPSIESPMYYEVSHSS
PIVQDTVVTRILVEDPDTLKDHTYSISDNNFKIDSMGHIRTVQNIESFTAQFYNLTVTVSDGQCSKT
SNVGVNLAVNPTPMTYQFTSNGVYSKSVREDHNLQDVILSVALNQNYPRVFSIVESDANDQFVID
SNSGALRLKTSLNYELKKRYDFTVMADIHGGVYKSYASVHIIVLDVNDECPIFPVIGYNQRVSGTP
VIGTIVEIIEANDADNATLEYSIVSGNEDGHFSLDNSGVVRVVKPFASSFSKTYGLSVRATDNKCSA
DVPLTVQVSTCAYGEDYQFEESEYVVYLDENRNLGIVSNIKLKSTRAAFLTLSQNTHFTIDQNGDF
RLIQTLNYETKKEYHLTLTANFTQDNVVTRIPIKVFVRDINDNCPAFSEELYAVQHTEPLREGLLIA
KLSVIDDDTVQNFTYNIVSGDKRFSINENGEVVMNARLVTDTTLLSTTYKLEVSVNDGKCAKNTK
VDVFVYKTVVNSFLFPQPFYKFDISENQAIPFTIDRFVNVGGHEAVYGFLVSTDFFQIDPKTAQLQL
VKKLDFEVEKTHYVLISATYDDGTQTYTVVAIHVGDENDNAPQIFAHPPITRLVQNTPHNSIVTKI
EVEDPDTSKEFTFKITNGNPLQNNDPDKALFRIESNGMIKTTRCISEDEIGNYVLTISVDDQVNTPTI
FSNMQVQLKTTDDRSYVTNCGLGCPANDGPFSRYQFEQSLFIVNVTETAANPTVLKRLTRPFVEK
TTYTIGAGADGKFSIDQNGTLSLTGSLDYETKPVYYFIVSAVIPSQVVGRFITSKTAYLVMVNDVDD
SKPVIEGNGGTLAVSETSEIGQIVGTVRVKDSDSSSPLTYNVVNGSQYFNIDSAGNIVIKDTLNAAG
SPYTVTYTVSDGINPPVNGEFVVSISETNNNVPKFTQNIYEVSLSEDTATDTIAVTILANDGDTNDKI
AYSFLQSEGPFVINRDSGEIKLIGPLDYEAQKSYNMVVQATDSGGFANIAFVQIAVGDVNDQVPVI
TPQTTPTQIHESTLVNSVVYRVIASDGDGNERLTYALANSNDFFTINSATGEITIKENLAKSNVSSHT
LSIVVTDSSGKTSTQDVALNIRRSGTSQGCVKITTSQGQSSSPVINLPENTASGTTVFKLVAGDASVF
SNMRFTLNREANGVFAVTEQGELQLVAPLDYETKQFYSIIITAEDVTRGVSSQYNLFVRISDVNEFT
PVLETIAPVAVSELANIGDVIAQAKANDQDGDSQVTYSLGEFGNNDNKFTIDAATGNIILNKRLG
SGAPHVVEVCASDGSIKKCQNVTFIVADTNDNPPSFQDLTGVGSIDLTNTSSYVVVYKPIVTDSDQT
GTLSFSLKDPAPSQFSINSTTGEVKANKDIAKGTYTFGVCASDGRYTSCQSVTVVASDRSQSITPSFN
QDQVGAIVDISASSGKQIIQVMASTLDKAKIEYELKNEADRNIFEINKDSGLITLKNRTALTLGTIYT
IPVKASRADDPLNFDEINIVISVGNQQLDSGSFIEQVLDTQHLVNFSLSTFDISKVSYYIILGQQVGG
DSKDDEKLPVSWYYATFGDESYRHRFYYIAKLDGSKFRNTGGSRKRRSIQDKDSKSFTYLVGEENG
CSKNTSMAAICNGPLKTGTEYRYKLRAYYDDGELRGIPVDQKPTEVKRTQAPQAGKSIDEYREQD
SYVITFWIIGLVIFGLLFWFSVLVIAILLCYKRRYEVTQAYGYSVQTRDKIDGPPTEYSNTVYRFQNPS
YPDNDTVKMKKVDASESRTKTEVQTTSMVVLDDYEQEQPQPDIPVQDYDAIEYETRSAQIKFHEE
SPEWESMDIQLRIDPTGNQDPVITRKSDSGSKDSDSDASKKYENTEYIEEKEDFVNEKEDSLTRL
>TRINITY_DN64_c0_g1_i1.p1 type:complete len:468 gc:universal TRINITY_DN64_c0_g1_i1:1555-152(-)
MPPKEAEKIHINIVVIGHVDSGKSTSTGHLIYKCGGIDKRAIEKFEKEAQEMGKGSFKYAWVLDKL
KAERERGITIDIALWKFKTGKYYVTIIDAPGHRDFIKNMITGTSQADCAVLIVASSTGEFEAGISKNG
QTREHALLAYTLGVKQLIVGVNKIDNTEPPYSEKRFDEIVKEVKTYVKKVGYDPKTVAFVPISGWH
GDNMLEASKNMGWYKGWTTERKETKTTTGKTLLEALDNILPPKRPTDRPLRLPLQDVYKIGGIG
TVPVGRVETGIIKPGMVVTFAPSNITTEVKSVEMHHEALLEAVPGDNVGFNIKNVSVKDIKRGNV
ASDSKNDPAKEAKSFYAQVIILNHPGEIHSGYQPVLDCHTAHVACKFSEIKQKIDRRSGKVLEEMP
KMVKSGDAAMIDLTPSKPMCVEAFSEYAPLGRFAVRDMRQTVAVGVIKSVTKSDGSGGKMTKSA
QKATGKKK*
>TRINITY_DN1614_c0_g1_i1.p1 type:complete len:218 gc:universal TRINITY_DN1614_c0_g1_i1:56-709(+)
MHKTKICIWLVVRYRSNPFRGYNRRPDLKSAGAKMTKYSTEPENPSKSCKARGSYLRVHFKNTHE
TAQAIKKMHIRKAYRYLKDVVDMKQIIPFRRFNGGVGRKAQAKAHGCSQGRWPKKSAQFLLQL
LKNAESNADLKGLDVDSLVIDHIQVNRAPYMRRRTYRAHGRINPYMSSPCHIEMILAEKEQVVPR
AEEEVEVKKVSKKKLAREKLKSRE*
>TRINITY_DN477_c0_g1_i9.p1 type:complete len:306 gc:universal TRINITY_DN477_c0_g1_i9:1840-923(-)
MTRQCLGATLNENCGNAALVQFEVCNFQVPCPGLLTNWGPYTQCSATCQKDANSMPTQSRQR
FCTGGSFGGNCGGVQLLQTIGCNQLIICPVYGYWLQWSAFGECSKTCDQGTHVRYRNCTKSIPA
NAGNNCTGPAQEVKPCQVKPCPVTCPLTKKCDCRNMNYLAAAPGISQTVTNDVMGVAAFLDY
WTLDILKQICEGCRMTQLDQSKATVDSQILRTQAVLDRLNIEKRVYRDMITCNENPYITDDLFKVY
NDIWDRIVTTDGVMIELKASKMRFDTAITACRSYDWYHDLFGRFFQIKKSS*

>TRINITY_DN5633_c0_g1_i1.p1 type:complete len:848 gc:universal TRINITY_DN5633_c0_g1_i1:2881-338(-)
MRRSNFLTIGIIAFFLIFSYVSADETSDEEVEPPKVDDDVGKSRDASKTDDEVVQREEEAINIDGLSV
AEMKQMRESAEKHAFQAEVSRMMKLIINSLYKNKEIFLRELISNSSDALDKIRFLSLTDTEALAAT
DELSVKIKADKENNVLHITDTGIGMTKDDLIKQLGTIAKSGTSEFFQKIQDAGTTDQASDLIGQFG
VGFYSAFLVADRVVVTSKNNDDKQHIWESDSVEFSISEDPRGDTLLRGTQISLYLKEEASDFLEPNT
LKDLIKKYSQFINFNIYLWSSKTEEVEEPIEEEPEKKEDEKKDDDKKEDADDKKDEEKKDDDEDD
AKVEEDKEEEKPKTKKVEKTVWDWELMNSSKPIWLRKPKEVTDDEYNEFYKAISKESDGPLTKT
HFVAEGEVTFKSVLFVPKNSPSDMFQDYGKRRESIKLYVRRVFITDDFQDMMPKYLSFIKGIVDSD
DLPLNVSREQLQQHKLLKVIKKKLVRKALDMIKKIPDEDYIEKFWKEFSTNIKLGVMEDHSNRTR
LAKLLRFRSSNDPEKLTSLTDYVERMKEKQETIFFMAGASLDEVKNSPFVERLLKKGYEVLYLIEPV
DEYCIQSLPEFDGKKFQNVAKEGLKIGNEAKEKARVDELEKEYEPLLKWLKEDALKDLIEKAAVSS
RLTESPCALVANSYGWSGNMERIMNSQAYAKSKDAGANFYATQKKLLEINVYHPLIQELQKRVKT
DKEDQTAKDLARVLFETATMRSGYTVKDSSDFAGRIERMLRLSMGVDVDAKVELPEEDDEEKEEE
KADDEGEEVNADGEEEESKEDIPEIKVDSKTVEPVAEHPTNEDAVENEAPAEGKTEL*
>TRINITY_DN2472_c0_g1_i1.p1 type:complete len:344 gc:universal TRINITY_DN2472_c0_g1_i1:311-1342(+)
MSSGEIESLRQEAENLRNQIKDAQKVAADTSFSSVAQNVDGINRIQLRTRRTLRGHLAKIYAMHW
ASDSRSLVSASQDGKLIVWDSYTTNKVHAIPLRSSWVMTCAYAQSGNYVACGGLDNICSIYSLKTR
EGNVRVSRELPGHTGYLSCCRFLDDSQILTSSGDTTCCLWDIETGQQLTQFVGHTGDVMSLSLCN
DSGRTFVSGACDASAKVWDIRDGMCRQTFIGHDSDINAVSYFPSGLSFGTGSDDATCRLFDIRSDQ
ELMCYSHDNIICGITSVAFSKSGRLLLAGYDDFNCNIWDTIKGDRSGVLAGHDNRVSCLGVTDDG
MAVGTGSWDSFLRIWN*
>TRINITY_DN332_c0_g1_i1.p1 type:complete len:246 gc:universal TRINITY_DN332_c0_g1_i1:940-203(-)
MGALTYIFCLFLISSSFAKEEESCRTYAGGQVYPDERKSGVEHALHWSKAQISKPAPHWEGKAVV
NGEFKDLKLTDYKGKYLVFFFYPLDFTFVCPTEIIAFSDRVEEFKKINTEVIAVSVDSVFTHLAWTNT
PRKEGGLGKMKIPLLADLTKQISKDYGVLLEDAGHTLRGLFIIDDKGVLRQITMNDLPVGRSVDE
TLRLVQAFQYTDKHGEVCPAGWKPGSDTIIPDPKEKLNYFNKQKEEI*
>TRINITY_DN1616_c0_g1_i3.p1 type:complete len:3855 gc:universal TRINITY_DN1616_c0_g1_i3:218-11782(+)
MIIFLAFLAVAVGQEVLLPPYFDIAKGKNITATATCGEDYVAPKGSPGELFCTLADATGKFGIKGLK
CSHCVPGDYGDGDGKNARDHHIRNANDGSNRWWQSPPLSRGLQYRKINITMALGQSFHVAYVI
IKAANSPRPGVWALERSNDNGRTYTPWYYFAPTSSECKQYFGVDSYLPIVKDDTVICSTEYSKIPPF
KNGEIHISLINGRPGASNFSQSKTLQEWTTATNVRLRLLDIKTLLTDLLPLSLEDPTVTRRYYYSIKDI
TIGGRCMCNGFADECTPHTNIVGKYECNCQYNTEGPRCSRCKKGYVQKEWRQRTSTDKFECEKC
NCHGHSDECEYNEEINKRKLSLDVNGNYQGGGVCKNCQHNTLGINCEKCKPFFYQPANVPIDS
PNVCQPCNCDTQFSTGLCAEETGQCECKSNYVGRYCDQCNKGFHMFPVCKPCECDVNGSEDRE
CIPDQINGCKCKKGYAGRYCDECLPGYFGFPDCQPCACLTRYGAVNDNCERDNGQCSCKVGFK
GRRCEECAAGHYQFPFCKECGCDPEKTLDTVCDSGTGVCQCKSNFAGPKCESCSPGFYAHPSCFA
CGCSTAGSTSSVCDQNSGLCSCRPNYSGFKCDRCAEGYYGFPNCILCDCNPEGSRRVGICDRQDG
QCQCKPTYSGQKCDKCLPGFYGFPDCKRCNCNPAGTKPGPGGRPGDCSTSNNGQCVCKDNVQ
GLECNECKPGFFNLTESNRKGCSTCGCNGAGTNNGVKSCSVEGTCKCKPLVKQEDKCTECRDGY
YGLKRTNVFGCLGCNCEVGGSSSSVCSKDNGQCPCKDNIVGKRCESVAANSFYPDLCHIKAEME
EGKTTDGDVVRYDYQKEIFPDFSWKGFVAVNDQQPSVVVSINVPKTSEYQVKFRYALNQIQPVTA
TVTAFKGDSKIVGSINFASTQKGSGFVASLATVQDNLVQEMYFPLTEGQWNIKLEAPHSNLVVDY
AVLFPKEYAQGGALKKKVQNACDVMTDEKFCNLYRYIDVADKKDIIVLEAENPVKMRQQKPPVL
FNDTAVLSKLDFQAMVVLSPSNSRQLNHLFNVAKPGKYVVMLQYYHDSDTAKYLDVYVRSQIAV
QNGRALLQSCRYTFGCRQAVLNRGDNGVFVFNIKSTKNHLLTLLSRYAVVAVDSVMLVPYESWH
LDYSTPTLYCVTKDSKCTNMNYDVPAESRLVQANPLEQDLSEKPRYLIDRSVEVRYVKPGVNIINIR
DVKAGNYLLVAHYYQPNFPSFESRAAVYSGRTSGLRARFDFKFCPNLNGCRSPIRRPDSNSNLYVF
EEGEARIRFDIPNGLDAWIEYILMIPEGTNVSSLYTPRVLDYSDDFKKECSIDLNVCTSSNLSSFCKDS

IFTITTDFNNGSFPCKCNMDGSKSSSCQEFGGKCPCKDNVIGRECSRCKVGYYGFPNCKPCNCSN
CDEETGVCTCPRGVIGENCDQCAPGTFGFDPIIGCQKCNCNEIGTGSANAECNVTSGQCPCKEN
YSSRKCSACKPGYFNYPACTKCDCLDAGISAEKCDVLTGKCICQNNVIGLKCNECADGYFKTNDI
KGKQLCAKCWCNGRSTKSCTEAELFRATPITASKGWTVSVDTLNVIENGNGRFTISDVNTLPEPV
YWVAPNQFLGNRGLSYGSHFKYNFQYSVKQPSQNTNGDVVTDAVIKGDGYTIKSMRSVIEKEND
GYSASILMKETEWMLEDGSTIDRELFLAILSDIDTLQILATHGKNTISSTIYDISLETAGENGTDRVYG
IEKCFCPKEYADLSCSKCSKGYTRQKDGSCGKCQCLGKSNICDPQNGKCINCSGNTMGDRCEKC
KPGFYGDKCKACECPFSNPENSFANTCAINVNTSSLVCQCNAGYKGVRCDQCSDGYYGNPLQG
VTCKKCSCNGNINASFTGNCDNTTGDCLKCLYNSDGNQCQECRDGYYGDAVVEKNCTDCQCSI
CGTTTAPCNKATGQCQCKKNVEGSKCNQCKPNTWNFDACNGCEDCNCAEASETQQCDLKTG
QCKCPPTVFGRRCGFCKLGYYNYSSEGCQKCQCPDGKPCDVNTGKCCTGVLNEDCKLCPVGHII
EDGECVECDRCVQRTLQRVNTLIHNVTILEKHIIPAGGLNYGALETLGNRFSNLSNKGDEHISNL
DTLKKAIGFGEYRNHSGNMDYVRMYLVRDFKSCSFIVNIKTPPTSSAPATTMVTNTPGPDPIRDHL
SNKKISDSQCPPSLARVIDEGEKNIKLVEKKVVVLEKDAYESTENATATKKRAEDLLAKLKDFMVD
FKDVLSFANSTLKGNVVINIDGNRSLAEAEEMLKEIKDRNFNSTINRSEREKSAAINATIIAEQLMK
QASELANKAYKFNDTFKDFVKALRNLKMESELALLDANNATNSINLAKAVDFQSIIDNAQKLHS
QNNVTVTQAMEILQNAKSAYENAKDVFNKFHNQTGPLSDLMEELRNYVRKLEFDLQTTSPIVDD
SVKHAKRLRDSADRLKELLQDTKKFADAALRAAKVYQNIVDAINDALDAARMANETAHEADK
KVQQAQQDAVESSITSKVVDTEANDLTEEVKKTLKVDISDMKMRLKELRELRKRIQGRIDNVKED
VKSFAKEKEVGEKAMNISTQADGVMKVANKGNEASQDTNEKVEKDGEKVETLKVDISDINKAIK
DLASNVTLVEDTIPKIRSNIDEVNVKHEQIEEFIKTNVTNNIGYLKYLISNAHDILDQMQLSMKFNS
TSEVVLDAPKSVADPTINNKISLTFKINQGNGFLFIIGDFSGNSSGFMSLHVQNHTVVFDYKLNTG
DVQSLTNPKFIETDVNYDVMVTRNFATAVLNVQKSGNASTLMTAYQNRSNISPVLSLGQDTNVY
VGYHPSTPNQRPNFDGCINQFRFDEELMGLWHWKSAKNVGGCVQDFRSIEGQYSFDGDGFVEF
DLVSPELSGFRKIELRFATLEENGLIFLGTDPPTSLPSDIMFDIPLSNQIIAFNALEIKDGYLVSTLDLG
NGVQRLVHTNAGKVNDGKPHLFEMKVLISSLLIKFKLDKKKITEIAISPDTDIRLRLKEAFIGGLPQ
RAWLPNSNISTKGFTGAIGDAKYRGGEFKLVKRFNNIRKGFILPKGYSASFPEEKSYLRLDACNLK
QNSLFGFSFQTFEKNGIIFHSEEGSGSSRSSIEIILIDGQVKVDVSVGSQKRTLQTSVARGFSDGLWHT
LSLYFEETLQEADIVLLYLAVDKAYITGTVPKVDLKGSMYFGSKPSGTESGFNGNIRQVYCNKNEIN
LASGDVFEGGVVGSLKLLPPLKLADFTVVPTTLPPLTTSITTSESTTTETVMTTSTTSISGTTLEPPKET
TTNMAKTTENPTVKITSSEPIIETTMESTTVAKTTSAITMEESTTTPTSITTSTLETTISTLEPGSIEGSTT
TAEPITTTAEAITTTATEPITTTEPITITQPITTVEVTTEPATTQESTTTPEPITTLETTTTPEPSTTPEPTTP
EELTTTAKPTTTIRVTTEPPKCQSNLEVYDEAYGFGMNEPSFIEFGVDGRKMLKSNQFEFLFYTAEN
DGMMISSEPSNLYDYEYVYMENGAISYAFNAGAGRVRVTSTKKYNDGKWHEVKISRSKESGTLTI
DGELIGTDSKSGKTAVNGIKKIVVGGSSVPYRNIPPKSQVGFKGGMRGFKLFNEALKPLKEIGITEA
YDELPIPEGVSFGPDGGYVALNFKSEYVVRTEYKLALDVRSVSSSGYIFFIGNYGADYLSLYIESDRK
VVAECNNGGGRFSVSIDVPTICNGEFHSILLEKKYKTLDLSVEGVQKSFTTTKGSTSADTGSNPPAF
GGFNGVGSRPKNLETFTGCITAVRIKDKAVDLTAPDVTIFGQVSNGCPSNIR*
>TRINITY_DN3078_c0_g1_i1.p1 type:complete len:1109 gc:universal
TRINITY_DN3078_c0_g1_i1:3628-302(-)
MKMRIYLFLILFATVYCRVVQKGPNDIPEETKNEIKNQEPKIKFEPEKDKKYFLAKDTTCAHDVDK
HCKNIRRENNFAVFVCLQDVIHKDEDDVAEECHETLWHYKYNLTRDPNFDQAAFKVCENVINEI
QECNDSELLKSPGFVTNCLIENRHAVSEAKCKSFLQKMSAIVFEDYRLIKGFYDSCVEDVKTLKCG
TVAKPTDEEYQHDQTEVISCLEEQLDLVSEKCQAQLVKVATMQADDYHLDRPLFYACQEARERF
CPEVKSGDGRIYKCLVEHKTNEMMLEKCRKKIEIREKLTQKDIKVNELLWKSCKADFTKYNCPKD
GISEHGAASSLLLCLQGKVAEGLPVDQSCQAEMRTFAIELFENYKISPVIVSKCDVEINQFCHHLIG
KTDDGDMMDCLMTQAATNNESVSKKCYDAVSDVIKETGAGNNYKIDRALYLACEPVITTLCQG
KEDAMILGCLMDNAHNSKMPEKCAAQVFHLQYFLSRDFRLDASLYKDCKKDAEEICSANDFNA
ESESKVPDNFVIACLYRNALDRKVDDRKVSQTCAEHVFRVMHQRATNIHLMPELERPCMADLG
KHCIDKGDKEGEEIDCLQTNYEKLSAKCQNAIKVFTIQEAKDFDLDKHLVDKCAPMVQQFCQRE
LEDGNGEKVLPCLVDHKNDIDMDHQCANAVEHWQLIEMKDFEFSPALTEMCLLDIRKHCQKSK
AKIEALHCLSENIVKDASSVTEKCRAQLKNELIEQSENIKLNPELYEACTKDIELFCRDVVPSGGQL

EECLKRNHKKLTDPKCKSLLFKEEKKESFDPELDYRLMHVCKPIIKRYCTQYHQPVDIMECLRQV
THDRDMSRECRAIITERQIEQAESFELDPELSKFCQDDVNRYCKEEIKHAEKGDDDGAIFGCLVNA
MIDNKPIKNDCEMYIRRREAEAAQELDLNPQFLLQCRDEITKICPNMDEDEIIECMKMNIPQIQSK
GCVQEVKKLIVEGIEDVHVDPHLQEQCARDIRTFCQSVPSKSGNVVLCLIDVHKAKNLHLQPNC
ENFLTKRLSLYNAAKVDYAVFDSISAVMNAVSESPNRNYVYMTLLLVLTILFVGGMLFGRYTKKIRS
EIKNR*
TRINITY_DN3339_c0_g1_i1.p1 type:complete len:131 gc:universal TRINITY_DN3339_c0_g1_i1:31-423(+)
MVRVNVLNDALNSICNAEKRGKRQVLLRPSSKVTVKFLTVMMKHGYIGEFEVIDDHRSGKIVVN
LTGRMNKCGVISPRFDVKLKDIEKWASYLMPSRQFGYIVMTTSAGIMDHEEARRKNTGGKILGYF
Y*
>TRINITY_DN1071_c0_g1_i3.p1 type:internal len:375 gc:universal TRINITY_DN1071_c0_g1_i3:1124-3(-)
DPKVAMRVEFYVCPPCGVTTPAITTTEQTTMPTTTTTESTTTPTTTPATTTESTTTTLPTTTTTTPTTT
QSTTSKETIIITEEPKPTTGLPQKKTTTTPRTTTESPTTTEATTTTPATTTEATTTTQATTTTTKATTTTP
ATTTTKATTTTKATTTTPATTTTKATTTTKATTTTPATTTTKATTTTEATTTPTTKPCECKVLQPVGV
SDGRIPDNQMTANSVLVNVSGPTFTGASQARLNNKPSKDGVGAWEPAQQEEIYLEVHFNKIEDV
REIRIQGHPTDKKFTKNFFLSTSQDGVNFEFTDEDLYEGNKDGNTIKSTKVSFKTKAVQIHPFNDVS
VGDPKVAMRVEFYVCPPCGVTTPAITTTEQTTMPT
>TRINITY_DN1112_c0_g1_i1.p1 type:complete len:200 gc:universal TRINITY_DN1112_c0_g1_i1:740-141(-)
MSTLKLQKRLAASVLKCGKKKIWLDPNEASEIANANSRQNVRKLIKDGLIIKKPQVVHSRARTRA
NLEAKRKGRHKGFGKRHGTANARMPEKVIWMNRMRVLRRLLRKYRESKKIDLHLYHELYMKA
KGNVFKNKRVLMEYIHKKKAEKKRSKELSDQAEARRNKVKAARTRREERQAQKRAEILQAYAK
EDTAATPAS*
>TRINITY_DN2113_c0_g1_i1.p1 type:complete len:1704 gc:universal TRINITY_DN2113_c0_g1_i1:5257-146(-)
MAQLLPIRFQEHLQLQNVGINGANIGFSTLTMESDKFICVREKVGEQSQVVIVDMADANNPTRR
PISADSAIMNPASKVIALKAAKTLQIFNIEMKSKMKAHNLTEDVIFWKWISVNTIGLVTETAVFHW
SMEGDSSPVKMFDRHQSLAGCQIINYKVDHSGQWLLLIGISAQQNRVVGAMQLYSVERKVSQPIE
GHAAVFSQFKVDGNPQTSNIFCFAVRGAAASKLHIIEVGTPPTGNQPFPKKAENIFFPPEADKDFP
VAMQASQKYGVVYLVTKYGYIHLYDIESAQCIYSNRISGDTIFVTASYEPTEGIIGVNRKGQVLSVSV
DEEKMVPFLIQVKQNGELARRLAERCNLPGADNLFLQAFNDKFASGNYQEAAKIAAKAPKGILR
TQQTIQRFQQLPSQQGQTSPLLQYFGILLDQGQLNKYESVELCRPVLMQNRKQLMEKWLKEDKL
DCSEELGDLAKQFDNTLALSVYLRANVPNKVILCFAETGQFQKIVLYAKKVGFEPDYVSLLRNVM
RVNPEAGANFSGMLIQDDEPLADLNQIVDVFMEFNLIPQCTSFLLDALKNNRPAEGPLQTRLLEM
NLLSAPQVADAILGNQMVTHYDRAHVAQLCEKAGLLQRALEHYSDIYDIKRAIVHTHLLNPEW
LVNYFGTLSVEDSIECLKAMLSHNIRQNLQICVQIASKYHEQLTTSSLIEIFESFKSFEGLFYFLGSIVN
FSQEEEVHFKYIQAACKTGQMKEVERICRESNNYEPERVKNFLKEAKLTDQLPLIIVCDRHDFVH
DLVLYLYKNNLQKYIEIYVQKVNPARLSIVVGGLLDVDCSEDVIKSLILSVKGQFSTDDLVAEVEKR
NRLKLLLPWLESRIHEGSEEPATHNALAKIYIDANNNPERFLRENQFYDSAVVGKYCEKRDPHLA
CVAYERGQCDQDLINVCNENSLFKSLARYLVRRRDAELWGTVLVEDNKYRRQLIDQVVQTALSET
QDPEDVSVTVKAFMTADLPNELIELLEKIVLENSVFSDHRNLQNLLILTAIKADRTRVMEYITRLD
NYDAPDIAGIAIVSELFEEAFAIFKKFEVNTSAIQVLIENIQNLDRSYEFAERCNDPAVWSILGKAQL
DQGLIKEAIDSYIKADDPSAYMEVVEASKNCGNFEDLVKFLQMARKKAKETFIDTELIFAFAHTGR
LADLEEFISSPNHANISQVGERCFEQKMFEAAKILFNNISNFARLASTLVHLGEYQNAVDGARKA
NSTRTWKEVCFACVDGQEFRLAQMCGLNIVVHADELDDLIRYYESRGYFEELISLMEAALGLERA
HMGMFTELSILYSKYKPTKMKEHLELFWSRVNIPKVLRACEQSHLWSELVFLYDKYEEYDNAVVT
MMKHPTVAWKESLFKDVITKVANIELYYKALQFYLDNKPLLINDLLVVLTPRMDHTRGVSFFQRA
CYLSLVKPYLRSVQNHNNKAINEALNSLLIDEEDYNGLRASIDAFDNFDNIALAQLLEKHELIEFR
RIGAYLYKGNNRWKQSVELCKKDHLYKDAMQFACESRDKAVAEDLLSWFLSIDKTDCFAATLYM
CYDLLEPDVVLELAWRNNMLDLAMPFMIQFMKDYYTKVDKLAESNAKRDEDKENAPPEAPIM

GVEQLMITAGPSSYVPQPNMYGMPPQGMAPQGMPPQGMPPQGMPPQGMPPQGMPPQQGFPQ
QGFYGGAM*
>TRINITY_DN197343_c0_g1_i1.p1 type:complete len:124 gc:universal TRINITY_DN197343_c0_g1_i1:399-28(-)
MAKLKAHELRGKRKEELCHQLTELKTELQQLHVAKVTGGAASKLSKIKIVRKSIARVLTVISHNQ
RENIKKFYKTRKYKPLDVRSKKTRAMRRALTTHEASRKTLRQIKKDKHFAIRKYAVKE*
>TRINITY_DN9509_c0_g1_i10.p1 type:5prime_partial len:424 gc:universal TRINITY_DN9509_c0_g1_i10:1493-222(-)
VRVGDRRRKAARAAAVVRHAKPHVNLGTVGHVDHGKTTLSAAISLVCAQFSETQDTKQKTYEEI
DNAPEERARGITINASHIEYETSNRHYCHVDCPGHADYVKNMITGACQMDGGVLVISSPDGPMA
QTREHILLSKQVGVPRLVCFMNKVDVMDDEELLELVELETREMLTQYGFPGDDTPFIQGSALQAL
EAMKANPGIKKGDDKWCDKILELMETVDEYVELPERDTDKPFLLAVEDVFSISGRGTVATGRVEQ
GIVKKGDDVEILGRGKKPQKSVVTGIRMFNTDLPEGPAGYTVGVLCRGIERSSVLRGQVICAPGAT
STHTKFKANIYLSKKDEGGRSNPIMPGYMPVFYFRTCDVTGQIKTMASSEGNEVQMAMPGDDVT
IQCELISGTPIETGMRFAMREGGRTIGQGLITETM*
>TRINITY_DN477_c0_g1_i7.p1 type:3prime_partial len:1130 gc:universal TRINITY_DN477_c0_g1_i7:3387-1(-)
MMGSIKVFVGLVLLSFTAIEIAQAQSGTVLSNKQFAFDFVVDGSTSIQDEQWNLLTDFVSRLAVKM
STLNNAKLSALQFSHTLNRILQCNQYANVEEARNTIKNMVQENGATGTLAAMLTSADNLMNVC
SAGDPNRQNVMIVIVDGNQNLEQKSHSEIVQDIKVNVKDKNILVLTLGVGPQVNRNEFGDYASD
SGKGFFVDDFTLLTDELVQRMYDELIRTYGDGPDYLTAWVNGPCSATCNPFPMANRPTLRQTRQ
CQEPNGGNCGGASLEQIINCNENIACAGETYLTAWVDSSACSLTCNPFPVSNQPTKRQTRECRQP
SGSCNGASLTQTINCNENVQCPGETYLTAWIDSSCSATCDTGSNNPVFTQTRQCRQTVGNCDGA
PLQQTRSCNVGVSCTAPTFLTAWSNATPCSATCNPFPMAVRPTLTQVRQCNEPNGGNCGGASLQ
QTVQCNLDVACAGETYLTNWVDLTACSATCDDASNRPVKQQERQCQQPGGNCDNLPLQQTVN
CNIGVSCTVSSSACSSKEISFTFVIDTSASVDNIGFFVLKDFMKKYMSQSTLGTDPRTSVAIVSYGAG
DAKVEVECDTITTQQSFNTTVDNLSQLFGIADDNSFNQALSTAFDASGRCARSSTSLNIIIMLSDGD
PPALENILTRDRIQSSSNYYFMFVAIQASDGAQSTFASLANQGRGLRNASSSFTDLYNFNTDLYYLE
TLSKACDVGGQWTEWSTSACSTTCGPGVVTRTRECRNRITGKLQQDCGPGPAQETQACNLQDC
QGTLTQWTSWSCPATCNINNDVNNPPLALRTRQCLGSTGQGNCNNADKTENEPCVRNVPCPG
AWTTWSVFSACSETCRSQSAFPTRRRTRQCIGGTLNFQCPPQIVNGISYREIWQEDCINVPNCPAK
FTQWTTWSCPVTCNVNNDRNNPPTATRTRTCDDSGTINGCANANILENQPCVQDVQCPGAWS
TWSAYGACSETCQSGNRSPTYTRTRTCLGGTLGFQCPPFITQIGGFQIFGTQTSNCNSEVKCPSKLT
QWTTWSCPVTCNNRNDVNNPPQARRTRRCDTSTGSGICESPIVEEFENCVANVPCPGRLTEFTYD
NCAHTCNVNNDVNNPPQRVGTRRCLDGTLG
>TRINITY_DN484_c0_g1_i1.p1 type:complete len:554 gc:universal TRINITY_DN484_c0_g1_i1:164-1825(+)
MASMILNGERTTGNDVREQNVMAAASIANIVKSSLGPVGLDKMLVDDIGDVTITNDGATILKLLE
VEHPAAKILCELADLQDQEVGDGTTSVVIIAAELLKNAGNLIKYKLHPTSIITGYRLACREACKYIQ
ENLAVKTADLEREHVINCAKTSMSSKLVGVDSDFFSTMIVDAAMSIKRTGNKGETKIPIKSINILKA
HGGNMKESISVPGYALNCTIACEGMPKKIENAKIALLDFSLQKAKMHLGVQVLIEDPEKLEAVRQ
REMDITKERIEKVLASGANVILLSGGIDDLCMKYFVEAGAMAVRRIKKVDLKRIARATGGTVCLSL
ANLEGEESFEASYLGHADEVAQERICDDELILIRHPKTKAAASIILRGANDFMVDEMERSIHDALC
VVKRVLESKSIVPGGGAVEAALSIYLENFATSLASREQLAIAEFANSLLVIPKTLTVNAAKDSADLVA
KLRAYHNASQTQSDKAHLKWVGLDLINGVIRDNKKAGVLEPTMSKIKCLKFATEAAITILRIDDL
VKIEPEKKDDPNSYQNAIAGGRMG*
>TRINITY_DN3991_c0_g1_i1.p1 type:3prime_partial len:219 gc:universal TRINITY_DN3991_c0_g1_i1:84-737(+)
MTSWILNFLVLSIIALPCSSISNCKHKLEPVGCFKDRGSARAMPEQILNERDTTSKVYSKQLMSWK
NFHEWLPGFICRCAEVAARKGYSVIGIQFWGECWSGPNAESTYNRHGQVDSCINQNLKKNRAK
LSQCTAASGKHFTNYVYRLAPACHTQYKPVGCFQDNLQKPRPMPYYVSTDRETRLAIFSGKLLN
WKNWNTHMPDMICRCAAVAKSKGHQ

>TRINITY_DN1377_c0_g1_i1.p1 type:complete len:878 gc:universal TRINITY_DN1377_c0_g1_i1:68-2701(+)
MTGRARGRSRGRGRAAQDASQRPGGAQGGPPPQAAAPQQGGGGRGRTRGQPAPQQVAPQQR
PAAQTAVPPTARMAQMSVKDQKPATEERQPRRRRLDPAPDVLVTRPEGRTKEGTGGTPVNIVSNF
FKVIKMPNFKGLYQYNVSFDPEINSSKLKNALLYQMADVLGQTKCFDGMTLFLPKRLPDQITTRS
VTTHQGNAIVIKIMFTNVIPEISPQVLQMINIIFRRQLRCIKMELIGRNYFNSSRKIEVKAHKMEVW
PGFSTSILQYEKDVMLCADVQHKVVRQQSVLSMMYELHQKTREKNRFYELAAKKLIGETVITRYN
KKNYKIDDIEWNKNPNSTFDKNGTQITFKEYFEKTYDAKVGDDQQPLLVSLPKAKNRKSGIVGPI
LLLPEFCLISGLSDEIRSNFSIMKDLAQHTRINPAERAKNLEQFVQDLQTNEESKKELEQWNLKFES
KLLQMTGRAYGSEKMFQREDQFSYKIQDADWSRETRGKKLIHTVNMTDWLLVYYERDEPIATDF
KQTLVKVCGPMGMNVAEPQLCRLKDDTARSYFRALSEQIRPTTQIVVCIVPNNRKDRYDSIKKLC
CVEKPIPSQVIVSRTLSKKQMLMSVCTKIGIQLNCKLGGEIWAVDIPISKNPKDPPSLMVVGFDVYH
DSASKGKSIGGFIASTNRHLTKYYSRITQQQSHQEIADQLRVCMTGALKKFNELNGTMPSRIIVYR
DGVGDGQLRQVFDHELPQFKAAFNAISQGYDPKFTMIVVKKRISTRLFLSERGMKTNPLPGTVVD
TVVTRPEWYDFFLVSQSVRQGTVSPTHYNIIADTSGWKPDIFQRLTYKLCHLYYNWPGTIRVPAPC
QYAHKLAFLVGQSIHAEPDVQLADRLYYL*
>TRINITY_DN428_c0_g1_i1.p1 type:complete len:1949 gc:universal TRINITY_DN428_c0_g1_i1:96-5942(+)
MADDADLKFLMVDRRQVSDPVAHAEWAAKKLVWVPSEELGFVAASVKEEKGDKIIAQIEGGKR
VTFHKDDIQRMNPPKFDKAEDMADLTCLNEASVLHNIKDRYFSDLIYTYSGLFCVVVNPYKRLPI
YTDKVINLYKGKKRHEVPPHVFSIADTAFRSMLQDRDNQSILCTGESGAGKTENTKKVIQYLTAIA
GRANQQKADEKSQGKLEVQLLQANPILEAFGNAKTVKNDNSSRFGKFIRINFDNSGFICGANIES
YLLEKARLVRQAPDERIFHIFYQLLLGASPEVKKQFLLLDTKSYNFLSNGHVQLPNMDDRQEFKV
TLEGMRDMGITQEELDPIFKVVSAVMLFGNMEFKQERKSDQATLPDNTIAQKICHLLGIQVTDFT
KSLLKPKVKVGREFTQKSQTKAQCEFAIEAITKALYERMFKWLVGRINKSLNRGKRDGASFIGILDI
AGFEIFQLNSFEQLCINYTNEKLQQLFNHTMFILEQEEYQKEGIDWKFIDFGLDLQPTIDLIDKPMG
VLSLLDEECWFPKATDKSYVEKLHKQQTKHPKYLKPDFRSNSDFCVIHYAGRVDYSAAQWLTKN
MDPLNDNVVSLLAHSKEAFIASIWKDTENVVGMSVTEGMDTTFGASRTRKGMFRTVSQLYKEQL
SGLMTTLHCTKPNFVRCIIPNYEKRAGKIDNHLVLDQLRCNGVLEGIRICRQGFPNRILFQEFRQR
YEILCPGIVPKGFMDGRKAAKQMIDNLEMDPNLYRIGQSKIFFRAGVLAHLEEERDIKLTEIIIQFQ
AFCRGNIARRNYRKRIQQLSAIRVIQRNGRSWLKLRNWQWWRLFTKVKPLLNVSRQEDELRSKE
EEFKKLQDKSDKLESLQKEQQQANEKLTEEKNTLAEQLQAEIELCQEAEEARNRLLQRKTELEDL
IQDFEMKLGEEEEKAVKYAEDKKKLQQGIQDLEESLEEEETQRQKLQLEKVQVEAKLKEMEDERQ
VAEDNNARLTRDKKQLEDRVAEFGQKLAQEEDRAKQLSKAKAKQEAFIAELEDQIAKGDKSTHE
LHKEKRKAEQDLSDLRAQLAQALQRIAELEDDLARREKELAEAIKRGDEEMAKRTTVEREKREL
QALLDELREDMESEKSAKDKVERQKRDLEQDLQELRSELEVTTDATVVQKEFQKKREEELMDLK
KQIEEEAKEHDQQMASLRTKHNGLVEDLNNQVDTFKRGKAALEKNKTALENENAEIAADLQRI
SQLKQEGDRKAKNLEQQLNEANSVRAVQDEKVSSLESEKAKYQKECDSLSTQIEEVETKAASLDR
AKQAAEAQLEDTQDQLAEETRQKLSYQTKLREASDEANRLQEQLEEEEDEKKAVQKVLSNLQV
QLDQAKKEIELKIQDFEQAEAARQKQKRENEELRGEVEKLTGDVSRLDKAKKKLQGDLDDVTVS
MERERNSANQMSSKQRKFDQLLAEEKANSQALGIERDNLEKLARQNETKALSLQNANEELEDR
LAEAERQRKQLQMELGDLVESKDDVGKSVHDLEKSKRLLEQQLAEQKTQVEEIEDELQACEDAK
LRLEVNMQAQKAQFDRELQAKEDAIEEGRRGLVKQLRELEAELEDERKLRQSASAAKRQLEGDV
KELEGQLDQANRIKEDGLKQLKKYQNQLKDVQRDLEDSRQSKDDLAAQVKDNEKKLKGLEAEF
LQMQEDLSAAERARRAIESERDELLEELNNNSALKNAVAEERKRYEAQLAALEDELEEERTQTEL
MQDKVKRSNLQLEQMNAELQEERSAAQSLDNTRMSLERQNKELQSKLADLESSLRSRQKNTVAT
LEAKINNLEIQLDQEAKERQQQNKALRRNEKRCKDLQMQIEEERSHTESYKQQLEKMNARVKN
MKRQLDESEEEVTRLNTAKRKLQRDFDENQESLESSQREANQLRQRLKGGASLSSSRSSGRGRRA
KDEDDDDDDANE*
>TRINITY_DN72959_c0_g1_i1.p1 type:complete len:216 gc:universal TRINITY_DN72959_c0_g1_i1:54-701(+)

MGRRPARCYRYCKNKPYPKSRFCRGVPDSKIRIFDLGKKKTGVDDFPKCVNLVSDEYEQLSSEALE
AARICANKYMTKTAGKDAFHIRIRVHPFHIVRINKMLSCAGADRLQTGMRGAFGKPQGTVARV
HIGQPLISVRCKDQHEATAIEALRRAKFKFPGRQKIFTSKKWGFTRWDREEYLERRQDGSIKPDGT
NVQYYPCKGPLKDWKWRESR*
>TRINITY_DN72753_c0_g1_i1.p1 type:complete len:137 gc:universal
TRINITY_DN72753_c0_g1_i1:95-505(+)
MARTKQTARKSTGGKAPRKQLATKAARKSAPSTGGVKKPHRYRPGTVALREIRRYQKSTELLIRK
LPFQRLVREIAQDFKTDLRFQSAAIGALQEAAEAYLVGLFEDTNLCAIHAKRVTIMPKDIQLARRIR
GERA*
>TRINITY_DN535_c0_g1_i1.p1 type:5prime_partial len:162 gc:universal
TRINITY_DN535_c0_g1_i1:712-227(-)
GKYLVFFFYPLDFTFVCPTEIIAFSDRVEEFRALGCDVLACSVDSHFSHLAWTQQSRKKGGLGQMK
IPILSDLTKSISRDYGVLLENDGIALRGLFIIDDKGILRQITVNDLPVGRSVDETLRLIQAFKFTDEYG
EVCPAGWKPGADTINTGKAHEYFEKQ*
>TRINITY_DN79_c0_g1_i1.p1 type:5prime_partial len:478 gc:universal
TRINITY_DN79_c0_g1_i1:2132-699(-)
VVSSESNVLVEFYAPWCGHCQSLAPEYAKAAKQLKESKSDIKLAKVDATVETKLAEKYEVQGFPTI
KFFKKGNALDYGGGRTDKEIVAWLNKKTGPPAKLLDNAADVKAFADERDVAILGFFNDKESSEL
AKAFLAVAEAVDDLEFAIAAPDASGDFAVTEDKIIVAKKFDDPQVVYSGEANIADIKKFVRAESIA
LVTEFTDEAAPKIFGGDIKNHLLLFISKKAENFAELKNTFQEAAKGFKGTVLFVYIDTDVEDNGRIL
EFFGLKAADAPTLRLIQLEGDMTKYVPETKDLDKDSVTSFVQNVIDGKIKPFLMSADVPEDWDK
NPVKVLVGKNFAEVALSKDKDVLVEFYAPWCGHCKQLAPIWDKLGEKYKDSENIVIAKMDSTAN
EVEEVKVSSFPTIKYFPKEGGVVDYNGGRTLDDFVKYLESGGKDVSGGADETGEDEDADEEEEGM
EEGMEEGDLKPEEPEVAKDEL*
>TRINITY_DN119684_c0_g1_i1.p1 type:complete len:662 gc:universal
TRINITY_DN119684_c0_g1_i1:2182-197(-)
MKLEIILFTLLVALTVHCSEKKDDSKKDVGTVIGIDLGTTYSCVGVFKNGRVEIIPNDQGNRITPSYV
AFTEEGERLIGDAAKNQLTSNPENTVFDAKRLIGREWGDKAVVHDMKFFPFKVVEKGKKPHVQ
VNVKENEKKTFAAEEISAMVLVKMKEIAEAYLGKKVTHAVVTVPAYFNDAQRQATKDAGVIAGL
NVMRIINEPTAAAIAYGLDKKEGEKNILVFDLGGGTFDVSLLTIDNGVFEVVATNGDTHLGGEDF
DQRVMDHFIKLYKKKKSKDIRKDNRAVQKLRREVEKAKRALSTQHQARIEVESLFDGEDFSETLT
RAKFEELNSDLFKSTMKPVQKVIDDSGLKKKEIHEIVLVGGSTRIPKVQQLVKDFFGGKEPSRGINP
DEAVAYGAAVQGGVLGGEEDTGEIVLLDVNPLTMGIETVGGVMTKLIGRNTVVPTKKSQIFSTAA
DNQQTVTIQVFEGERPMTKDNHLLGKFDLNGIPPAPRGVPQIEVTFEIDVNGILRVSAEDKGTGK
KEKITITNDQNRLSPEDIERMVNDAEKFADEDKKVKEKVDAKNEFESYAYSLKNQISEKDKLGGK
LSEEDKGVLEKAVDEAISWMDSHQDASVDELKEAKKKLEDVATPIVSKIYQQQGGAPPPPGGEG
GEEKEDKEEL*
>TRINITY_DN195948_c0_g1_i1.p1 type:5prime_partial len:170 gc:universal
TRINITY_DN195948_c0_g1_i1:602-93(-)
NLLRWSRMRLPFRIDLAKNRSRCKMPGGATVKDVDPQYFVTAFAAHLKKSGKLKVPEFVEIVKTS
KGAELGPTDPDWYYIRAAAVARHVYLRPNVGVGAVRKIYGRAKRNGTKPNHFCLGSASVARKV
MQSLEGVKIVSKDVNGGRTITSTGRRDMDRIAGQVLSKEEN*
>TRINITY_DN1080_c0_g1_i3.p1 type:internal len:425 gc:universal
TRINITY_DN1080_c0_g1_i3:1274-3(-)
EVPVEDEDPSQSIDVTRRQVHRQYMPDPERQGKKQPKPVERLYVTRVIRKPDGTEQLVDESETVYP
LEVTPEEQAPEAIDEEKDSRGVVIRRVVRRPVMVTTKRTVIRRAEVLPSGEEKEIEKSVEEAPKTEQ
EPQKTRRVVSRVVKEPSGQERVVQEPSYVSPLESSTTEESEPEVVEKRLRGQVIRTVKRRSITTSVKR
VIRRVVEGTDGKPSDGPVEDTLEEPVEPTKYSLVRRTVTKSDGTTETVEEPHYEMPVESEPTVEEEK
DRRGVVIRRVVRRPVPIVTRRRVYRKVILALDGTEKGVEEKVEEPTPGMQGLEEEPIAAPMVVDEQ
ESVPDMFGDSGDQTSVLRKTVTIRKRIVRKIIVLPDGSRKEVEEEVPVEDEDPSQSIDVTRRQVHRQ
YMPDPERQGKKQPKPVERLYVTR

>TRINITY_DN2028_c0_g1_i1.p1 type:complete len:274 gc:universal TRINITY_DN2028_c0_g1_i1:1608-787(-)
MGQSDGDDLTFGQKISREWRDFREFLYNSETGKVMGRSGTSWAKIGGFYLLFYGFLAGFFTALLFI
FYTTIDKPGIGGPKLIQYIENKPGLTRLDRSSKLQSYSASNNDRISSYKTVIDEFLDGYNTSDHSGAL
CGAAGTPKSDKPCIFDTTLLGECAPSKNTTYGLREKKPCIFIKINKVFGWTPADDGTGYLTLSCTG
HATVMPQGFPLTAFPYNGHKVYQSPIVAVKIDATKGKKNVDCTLKGKNIEVSNNFNPSRAFGKIR
IEGISATD*
>TRINITY_DN319_c2_g1_i3.p1 type:complete len:2588 gc:universal TRINITY_DN319_c2_g1_i3:165-7928(+)
MVLLKGQLWILGFIFLTSTKYVMGDVPECSNNATVAVHCRFDEGNTCVDDPHPMHYVCVCNNL
GYMNDTDSVEGYSICKERDCSNSNETPTDLLKRICGVNTKTCTGKILQRECNCNTDSYRSKNGQY
CFFAPCQASPTIQQNCGPGNQCYGNAPNYICDCSARGYKRSAADNRHCELKSDEELTPIEEYCGK
GSLYANGTINNITCTCDTDKGFFPSATGETCISPSCYLNPQMQEKCGPENTCLGGKDDYVCLCSNP
NYKRNATDLQHCIRRTCADTSPAIDFKSICGEGATGCSGYVDKPLCSCDVSNHYVPAADGRSCIK
ATCKGNKKALEQCGQANSCTGSFDNFLCVCDANGYYLNTTNNTCVERECTDPDLVITDICGVGS
LSCNGKAGSPECICDKDKFYYASEDLRSCVKANCLSSETISKKCGKENSCYGSMESYVCNCNAPG
YLLNTTDNQGCYLGMCEDNGLEYDKVCGEGAISCNGTIKNLECNCGMNYVQSPDKKSCIRADC
RANQTMHEQCGEDNDCVGSLEDWMCICSTYGYKPDPMDMMKCKKVMCEEEVVNNVTVCGL
GTTGCKGHILKPECTCDIAADYVPNEDASRCIKGSCENNKMAMKDCGIANSCTGSYNNYVCVC
NGFGYKKLDDTHCVEQMCSDPDIDIRNVCGEGAHYCNGTVNQRVCSCNETEYFYPSADSKSCV
KATCAVSPMVMEQCGVENSCQGSQESFICNCNALGYLLNTTTNQHCYLGTCENGDFDLMQAC
GDGALYCNGTIKNLECQCDESKGYVQAPDKMSCIPNDCRGNLTMYRQCGQLNKCVGSIDDWR
CICDSPYYVAHSDIMKCKLLHCSDSEVDHVEVCGKGTIGCSGSTLHPTCHCNTTENYFASKDGMR
CVAGSCKTNQTAVKKCGNANSCAGSHSEWTCICDGIGYKHDPADPQNCLLKTCNDADIDIVATC
GVGAISCEGYQLMPKCECNDRDYYIPSDDRTSCELASCRANRTAMRACGEMNDCRGSFSSHICL
CHDPAYKSDPSDSRVCLEKTCEAGDFELVRVCGKGATGCSGSMKNIMCECDNAKGYVASADHT
QCIPSDCNNNVTMHLQCGEGNGCVGSLTDWYCVCSAPGYEPHASDHMKCHLKTCADPGVDH
VQICGMGATSCMGPVINPVCYCNESMYYIPSMDRTSCIKASCMSNKTAIAQCGSENSCSGSPDNF
VCLCQAYGYRQDTNNNQACIPSQCEDAGIDFVEICGQGGVGCSGSVLNPVCMCDHMKHFVPSP
DQNSCVLSTCRGNETAMTSCGEQNKCFGSSSSYICVCDAVGYKPHPTNLQECVLQTCEANELNP
VAVCGKGALSCKGTIQSLMCECDNSKGYVAAPGYKSCIPADCRANMTMHAQCGTGNRCRGSM
EDWSCVCDGIGSKPHPTDHMSCIAIDCSDPNVDIKSVCGYGATGCMGLARDPICHCDTSKYYVQ
SEDGRTCVPATCHTNKTAQMQCGPDNSCIGPPNDFICECTGFGYRHHETDKQKCVPIQCNETAL
FDIERVCGKGATGCRGNIRQPICDCDHAMYFHPSEDRRTCIEASCRRNSSIRADCGVENTCFGSTR
SWICDCNSYGSILNPSDVKKCMLATCEAGQLDPVAVCGKGALSCKGSMRDVQCTCNNELGYVH
SPDGKKCLPSDCSGNVTMHHQCGDANKCMGSLTDWVCVCDAPGYKPHAKDKMKCHQISCAD
NNVDPVDVCGIGATSCKGPITNPLCQCDTDNYYITSSDGASCIPATCKTNKTAIHQCGEANSCEG
PADNFICVCNAYGYKLDDNDHQRCVEISCDDPNAHESICGTGSTGCRGTARDPICECDHTQHFV
ASSDLTSCLLASCRANDTVMNHCGDGNTCFGSLTSHICVCEAFGYKLNPNNHKHCLEETCEDD
MLNPVAVCGKGAVSCSGPLKSVKCECDNNAGFFRSPGGMKCESTSCRQNPASQMMCGSQNQCY
GSYDDWYCDCSAPGYMANQTNAAECLKVMCDDMAVDVCGLGATGCTGHTLNPICTCDSSMG
YMMAKDKHSCVPMDCSMNPASSQCGYGNTCHGSYFSYVCVCSTAGTVMNKTNPMECINVACP
ADAVHVCGEGATNCSVTSTNNVECSCEEGYHPADDLHSCVLDDCRFNPKALKACGLLNKCVGS
YKSWKCNCHYPEFTQDPMDHQTCVVGPCMVEQCGKGNAPGSFQHYAVNCSGLYDDPICNCQP
EYTNDAKGGCIPKICSENPVAVQACGSGYCEGTYFKYTCKCDEPAFVQTPDKMQCVPAKCNIQQ
CGHGFDRGLYIDFAESCSGTYADPMCQCKTGFSGTNTSKCGPAIDGCKLDKCDPLATCQTLDVD
GNVKCTCKEGYSGSGMMGECYLDGGFSAYEGTKECSEVCGVNGVAEVTRFCNNPTADPKGKPC
NGTSPETIQVPCNRFACSSCTVRKTCGCGGLKPIFVEFYHEPNSTLEYFTDLKADDVQTALGFLTK
WKELSQQDLCLMYSKIELTVFVTLMGDSETQVSTMKSSLVSARDIMRKKLDCNINLGKKAQYYEA
YETIIKRHLAISNILKDLTIIKNSITKYMNNCDDSEIGMIRDVIGMM*
>TRINITY_DN168534_c0_g1_i1.p1 type:complete len:226 gc:universal TRINITY_DN168534_c0_g1_i1:774-97(-)

MIRSAIIAIVIASHLYESSAARCYVGYSAVGLPLAIGVRDVDCQKGMDFCTTVKSTIGGITVTQGNC
ARKYGQATCQFMQNSHKAMTDCKETLCRGDYCNSALIEAEQKERKRVGPYKNTLKCTTELKTR
FPLIPFATADIPIGIDECFPGTTRCVTVEYDYIYKFKTKIATRHNHFVLPVIQKMCNRPNIDDCNKW
CTIMSIHQFMHSSCRAKCCTGDNCNAKD*
>TRINITY_DN15599_c0_g1_i2.p1 type:internal len:206 gc:universal TRINITY_DN15599_c0_g1_i2:1-615(+)
KALTSSDPAERTKKLQSELANGRLAMIAIIAMFVQNGITGTTGPEMWRGGGAFENELGVQAPVG
FYDPVGFTLDGDVQSFRRRRSVELKHGRISMLACMGYLTPEIVGKWPGYISPSLGLKFTDIPSGLDA
IGKVPALGVAQIVAYFGFVEFAAGFDDYKPGGTPGDYGWKALTSSDPAERTKKLQAELANGRLA
MIAIIAMFVQ
>TRINITY_DN773_c0_g1_i1.p1 type:complete len:440 gc:universal TRINITY_DN773_c0_g1_i1:1608-289(-)
MVSPPGPQDNKPPHKVADMSLADLGRRYIEMSENEMPGLMSMRKQYGEKKPLKGARVAGCLH
MTIQTAVLIETLTELGAEVQWSSCNIFSTQDHAAAAIAKTGVPVYAWKGETDEEYLWCIEQTLIFP
GGEPLNMILDDGGDLTNLVHEKYPELMKDIRGVSEETTTGVHNLYKMFKEGRLKCPAINVNDSV
TKSKFDNLYGCRESLVDGIKRATDVMIAGKICVVAGYGDVGKGCAEALRAFGARVMITEIDPINA
LQAAMAGYEVTTMDEACSKARMFVTSTGCSGIIEPHHFQQMPEDAIVCNIGHFDCEIDAKWLN
DNCERETVKPQVDRYTLPNGRHIILLAEGRLVNLGCAHGHPSFVMSNSFTNQVLAQIELWQNHK
NYEIGVVMLPKRLDEEVARLHLDHLSVKLTKLSKSQADYLNLPADGPFKPDHYRY*
>TRINITY_DN1172_c0_g2_i2.p1 type:internal len:264 gc:universal TRINITY_DN1172_c0_g2_i2:790-2(-)
GDYGFKVLTSSDPAEKEKKLAAELANGRLAMVAIIGMFFQDGLTGSAWGDWALYTASPLRFFGG
DSGPSYKAFDPATELGVQEPLGFWDPLGFTSDNDEAAFKRRRSVELKHGRVCMAASIGYIVPNYY
TFPGFLSPSAGVKFADVPNGLAALGKVPALGWVQIIFFAGIVEGAFGFDDYKSGAPGDYGWKVLT
SDDPAEKDKKLAAELANGRLAMVATIGMFFQDGLTGSAWGDWALYTASPLRAEPVSTAGVGAA
TKGGTE
>TRINITY_DN40728_c1_g1_i1.p2 type:5prime_partial len:124 gc:universal TRINITY_DN40728_c1_g1_i1:1-372(+)
KTLTGKTITLDVEASDTIDNVKAKIQDKEGIPPDQQRLIFAGKQLEDGRTLSDYNIQKESTLHLVLR
LRGGVIEPSLAVFARKYNCDKMICRKCYARLPARAVNCRKKKCGHTNMLRPKKKLK*
>TRINITY_DN265_c0_g1_i9.p1 type:complete len:136 gc:universal TRINITY_DN265_c0_g1_i9:136-543(+)
MGRVRTKTVKKAARVIIEKYYTRLTHDFHTNKRMCEEIAIIPSKKLKNKIAGFITHLMKRIELGPVR
GISIKLQEEERERRDNYVPEISALEQDPLEVDPDTKEMLKIIDFANIPNLQVMQPITASGLGTRIPGN
*
>TRINITY_DN2636_c0_g1_i1.p1 type:complete len:314 gc:universal TRINITY_DN2636_c0_g1_i1:986-45(-)
MSAIAVVVIVIAAVLLHFSVHKIEEGHVAVYYRGGALLSSVSGPGYHLMAPMLTKYKSVQTTLQT
DEVKNVPCGTSGGVVIYFDRIEVVNLLSVDKVHDIVKNFTADYDKALIFNKIHHELNQFCSVKSL
QEVYIDLFDQIDENLKRALQQDLNEMAPGLKILAVRVTKPKIPEQIRKNYELMEAEKTKLLIRIQH
QKVVEKEAETERKLAVIQAEKQSQVAGIQYEQKIMEKESMKRMSEIEDLTHLAKRKAMADAEFY
THQKQAESNKMKLTPEYLELMRIEAISKNNKVYYGSNIPNIFLNEGGLSQKTK*
>TRINITY_DN3545_c0_g1_i2.p1 type:5prime_partial len:236 gc:universal TRINITY_DN3545_c0_g1_i2:3-710(+)
SGPGRSSCRPILSVIKISKINNIDSGKVEGGYTMWSVYGSCSKSCGGGAQKRVRSCTNPAPANGGK
HCDGVSFETRECNINQCPVDGGYTMWSVYGSCSKSCGGGAQKRVRSCTNPAPANGGKPCDGVS
FETRECNINQCPVCPEIVGTYDISSRHHYGGAKYGTVVFKKLSNSKTVEAYYTINDGRRGVLTCKIE
SAQPDQCSGHGYYGKYFTYYKNRCQIIWSNDVIYTKQNC*
>TRINITY_DN384_c0_g1_i2.p1 type:complete len:314 gc:universal TRINITY_DN384_c0_g1_i2:1018-77(-)
MRIAVALFLVFYLYPTEACKDYLNPTVCRIGVKQKGDCEYYKRLCMKTCGGCGGVIRPTQGPGPG
PVIPAGDCGVSSFQGGRIISGTTAREGAWPWQILLKANGRAGCGGSLISANWVLTAAHCITGGSYS

VVLGEHDRRRRSGHEQEIRVSRAIVHPRWNKRSFEYDYALLRLSQPARMNQYVKPVCLPTGEPRP
GQSCYITGWGKTSSRGQMHYILQQAPMQVIDSNTCNRKNYRAIRIAIRPSMVCGGGSGRISGCQG
DSGGPFVCNEGGKFVLHGDVSHGSRECSSSKTYTVFGRVNHVRSWIRQTANV*
>TRINITY_DN817_c0_g1_i4.p1 type:internal len:176 gc:universal TRINITY_DN817_c0_g1_i4:526-2(-)
TLEISVRKVECPGFATKCATTKFVLDGTTSYRGYCASDYGPYLCTYMESSVKTMTDCKSTLCSGHY
CNIEVERASRKLTINGKGIKCYTGVQMFLPQLPIATGTVPTEVDQCEDQTSQCMSVIFTHLVTDPIR
KIKVKIPTQMRLCQPGYSTCENHCRRLAQHITIENCEASCCK
>TRINITY_DN121866_c0_g1_i1.p1 type:5prime_partial len:460 gc:universal TRINITY_DN121866_c0_g1_i1:2-1381(+)
LYIINSCEPPSIFFSCHLHFYTLVEETMSTTTAPTTTGTSAATKRLYVGNLGSTVTTQDLKQLFGLETT
PYLRDTCFVELAVCEKTGKSKNFAFVSAPGHVFSELIKLNGIEFYGRQLVIEEAKTKPEEEGEKKDN
KEKKKTPNNKRKGGSNNNRNNNRRYGNKPKSKFNLPTLEPDQVFHLVDCGVNLTNPKFSQHT
DYVIARALSAGVQKMVITGLKLNGCKAAVVMAKTRPNILYAAVGIHPHFVKDDWSDKALDQLE
EQVKQPECVAVGECGLDYKRDYSPRDQQELAFKKQVQLGVRYQKALLVHERDSHDGIIEVLKEF
EASLPPVIIHCFTGTVEQIKTYIERGYYIGVTGYICKDKYGKTLRDAIKDGTLPLNRIIVQTNAPYMT
PNTPRDEIDPVSQTLLDHCYADNEPCTLSIIVRCIAKCLSQEPRHVADVCTETAIKVFRFQKSESE*
>TRINITY_DN1339_c0_g2_i3.p1 type:internal len:185 gc:universal TRINITY_DN1339_c0_g2_i3:553-2(-)
NSRLAMVAILGMFFQDGLTGSAWGDWANYVDSPLRAFENELGVQEPVGFWDPAGFTADGNVE
NFARRRQTELKHGRVSMLATMGYITPELTGKLPGYLSPSAGLKFADVPNGLAAISKVPAAGWAQI
VAYCAFCELSQDQSAGSPAAAGDFGWKLLTSSDQAELTKKLSAEVANGRLAMMAIIG
>TRINITY_DN89_c0_g1_i1.p1 type:complete len:376 gc:universal TRINITY_DN89_c0_g1_i1:117-1244(+)
MKTFKIFLVACLLLASAYADLMSEMEALEKELEARELADQKEDSSSEKALKDMSTEELMDELKKE
VGKAKKSAVETHREKFDDLTKRLALEEQVEEELKRADAAAKAQASDDESDKDFDNLIKNVIEDEI
KKNSNVPAYAKGTNDATEEDTATFEELKERVKKDYYGDELVPFDQCKDQMPNCNIYKKYCEVYK
KNLIKRCPKTCNFCAKPVTTPAPAVAVHVCRDKLPKRTCKMAKKMCDSENSVQRTRVQKLCAET
CMVCRAPSPPRCENTEYGCCWDKETIKTDRKGTACPACQDEFKYICKTFKDDCVRVTHNSGLFM
YRNCKTTCGVCNNNYCEDDPRFAKSCHFWKKYLKQCEKDTAMKGLCKKTCGHC*
>TRINITY_DN165469_c0_g1_i1.p1 type:complete len:2668 gc:universal TRINITY_DN165469_c0_g1_i1:41-8044(+)
MVEEDLNRSKELDIPKTLAYCFHNVTTVSLNRKQDIFRLLTQCIESQGCQDGHLKAVCKIISLTVM
RYRDSKSRQLVLKFVQKFLDIHELQAVKCLLTEIHILAKQLIKCGASLFSASCGLALLSWCSSISKKK
VQEASILKILYEVEAMCYSVICTCDSFRVKRLLKRAYLYLQDGKDAEQLENCLGVIISNSNCQLESIC
LVGFIFQYLTTKKEIQRVDEVKKRSLIDIIIKNYIQTKVKADYHYLKHAVPFLKYITHDDFKEILPAIE
KGLLRNPETVLQGVGYLLEGLSIDSSNYIEELSKCIAVHLISKNASLQADAVFAVSVIAKQCSDFSAI
NHLLKQLIKVLNESEGKLVFPEQKNAVLQAIKSVDCTSVTGSDHKENLATTLHDGLFPFILLEGHE
GVLTQALNVMKTWCKYFKNNLPKTFVDNIKKGLSLKVSTTVIQAHYLLVVSEALKGNTVSQASG
LLETLLQVIEKVKGQSHLINNVFLCISSSMVLLKMFLSDDVQVNAAKEDSFWNVILDPVKKNLVSE
KFLLSHQKEYFYHLSQCMNTMFLNYSSRFSQKLLEPWYRAFNVLMVYGDWKTKKSVKILLSKMF
DSGNYDTDEFQSRLFADLDSLLDQEMSKSSKIEDTEDILVVNHVVDAYLFLCIWNKKDINEDEVLA
VLRKCLIYVHHDTIYQHDSSLFSKIGSVLKKDVAKEFVEKYKDDLLKYFINLESLSKADNHSLKTLL
SLYPEALLDTLVDEIIKYFTKPDLINVTSQEYEIMKTPDGVAYDQSVFESAKAANQKSGNLKRESKA
YSYKDQKAEMELRKELEKKKGKSDKIELNKKQKEQLEVILTQEKAIRDKLKSLEQGVMKHASILSL
LLDMDSDLLCYHYLDLMNLLLCLCQSHIAAPHVIPIYTKLSEKLFEGDLKHLGQLVANVILRLKKP
VCELPMGWSVENLPEQAKRALNTLHTFVNERNVYLMMTYTRKKTFSVLVFSYCLPLLKSILKDQ
GALVKGSEEFRMKALQLIHIHAKLRSPMDMGSEKNDPELLPRKEMLQILSDVIVSCEGHNKEAKV
QQQATLAFDEVCKCASGRLGCAVATEEEINILLSNILSQCSILRRVSLKGLLEFVDILPTENVVIMTQ
LTRSVLIGRFDIVDENATLAKRLWDEADICIEPSMIQHLVENVTHPLDDVRLAASLALEFSLNERKE
VDIIPQLLDIYFEKLKIPPPIIDNLGRVVSQQFVDSWEARQGVGLALEKIAETVGEDKLGDLFSFFVP
KALGDRNESVRKQMLNASLQYVNLYGQKNMDMLLGVFQDFLDSAPDSKELDTIRQSVIILTGSIA
KHFDKTDPQIKSIFSKLMAALSTPSQSVQQAVANCLPPLCGAMKSEAPELFKNLLEQLLESESYGE

RRGAAFGLGGLVKGLGILSLKQQNVIPTLNDAIQNKKIWKHREGALFAFETLCTMLGRLFEPYVV
HLLPHLLLCFGDGNQYVREAADDTAKAVMKNLSNHGVKLVLPSLLKALEEDAWRTKTGSAELL
GAMSFCAPKQLSSCLPSIVPRLTEILADSHLKVQKAGQQALRQIGGVVRNPEIQEICPIILDALSDP
NKHTVACLQALLSTSFVHFIDAPSLALIMPTLEKALGQRPTETKKMAAQILGNMYALTDPKDLQP
YLQAVVPGLKKSLLDPSPEVRAVSARALGAIVKGMGEVDDLMPWLLETLTSEASSVDRSGAAQG
LSEVLFALGKDKLDKLMPDVIATSMKTELSPNIRDGYLMLYVYLPATFEDQFLDYIGLIIPAVLKGL
ADESEFVRDTSLKAGQRMINMYADSAIELLLPQLEAGLFDDHWRIRLSSVQLLGDLLFKLSGVTG
KQTTVSEEDDNFGTAYSSQVILETLGFDRRNRLFAGLYMGRSDVALGVRQAALHVWKVIVQNTA
KTLREILPVLFELLLGCLASQSYDKRQVAARALGDLVRKLGERILPEIIPILEAGLDHEDGDKRQGV
CIGLSEIMDSTSREMISMFEDSLINTIRKALVDELPNVREAAASSFENLHNTIGHRALEGVLPTVFQ
KLDDPQLSNYALDGLKQVMTVKSKSVLPFLVPKLTTPPVNTKALAILSSVAGEALIKHIEKILPAMI
QAIQVSQGTGLDGDSEFESTRTLILSVYDEVGVRTIVDELTTSSKSQMPGIRKVAASLLMVFCKETR
GDHVPYIQSLFRTIIQLMNDVDQDVVASAWGALDALVKRLDPSEQLQHLSSLRQAVRFIKDDIRN
EQLPGFCILKKGITPVLSIFREGILNGPQEIKEQAANGLGEIIKLTSPEALRPSVVHITGPLIRILGDRF
NSSVKVAILDTLGLLLQKVGALLKPFLPQLQTTFIKSLNDSALGVRQKAAWTLGLLTVLHTRVDSL
FTELKNGIKNNEDSAICETILGALRSITLKAGSKMSEGIRNTLYETLVELISSNEDGIRLKAAAALGA
MCQILPQDQIEQLLNDNLLDINQMLDWSICHGRAVALSYALYHAPSQVFDAVDEDDIVEAVVKY
STYDRVPICTYGVHSLGHLLMFSKDNADVAPQIVVSSIKKTLLNSSVDIRLASLSTIKHVSRNTPEV
MTFERIRYLIPEFISLLRDRNTSVRSSAEITLAFTLQMDKNDILFDECVNTISEASHFLEEFKKNTFSK
LNDSLATDKDDYFFAT*
>TRINITY_DN8903_c0_g1_i1.p1 type:5prime_partial len:447 gc:universal TRINITY_DN8903_c0_g1_i1:1546-206(-)
DMATKACGLENMCVGSYKSWRCICQAPRYMQDPDNTQSCIHAPCRIEDCGSGYAPGSFQYYAES
CNGTFFNPICNCQEEYEKDMLGGCVPKSCRENPIAVAECGENGYCKGTYFKYECGCQGPKYVQN
PRNTRQCIEAPCNVQNCGHGFENELYVSFADSCIGKHADYTCQCKEGYTGTGTSRCIPDGKECGK
SYCHANATCAPLGKGEFDCTCKPGYSGDGLYCFLDGGWSDFVNTRECNASCGTHGYAEVTRYC
NNPVAERFGKQCGAKSPEQLEVPCNRFDCLGCTVKKDCRCTSVVKPLIVEGYNEPEETLKFFTDL
HPDDVKKAKDTLLKWKAFSNENLCRKYNKTTLHAKFFGVRDAAKEVNKMKSSYGSARDSMRI
KLDCNINIAKKTQYFGVYEMIVKRLFIMKQVVKDLHIIEDYVLKHMENCDDSPVGMIRNTFGK*
>TRINITY_DN106952_c0_g1_i1.p1 type:3prime_partial len:135 gc:universal TRINITY_DN106952_c0_g1_i1:18-419(+)
MSDAAAPKGGKHAPKVAKKGEKRAGKKGGKIGGGSEKKRKRKRKETYAIYIYNVLRQVHPDVG
VSSKAMSIMNSFVNDIFERIASEASRLSLQNKKSTISSREIQTAVRLLLPGELAKHAVSEGTKAVTKY
TSS
>TRINITY_DN433_c0_g2_i1.p1 type:complete len:144 gc:universal TRINITY_DN433_c0_g2_i1:1496-1065(-)
MAQQTTVPRSFKLLEELEEGQKGGDGFVSWGLEKDDDMNLEFWTGMILGPPRTPFENRMYNLK
IRCTDRYPLEPPIISFVTRISLSGVDANGKIDRRACNVLKGWQTKDSIKAVLTDLRRMMTLKDNLR
LSQPPENSQYGPSR*
>TRINITY_DN5276_c0_g1_i12.p2 type:internal len:228 gc:universal TRINITY_DN5276_c0_g1_i12:683-3(-)
AQVFLYAANDSGWFDASGNTDFDGMLMLSAMKKEERAPGDEGWRPPLLATDDAELKKKRLNA
EIANCRLATMAISGMFFQDGLTGSAWGDWSLYTASPLRAFESELGVQAPVGFWDPLGLSKDGIM
DNFKRRRATDVKHGRISMLATMGYSTPEFFKWPGFLSPGNSLKFADIPNGLGAISKVPLAGWAQI
FLYAGFVENSANDFGWFDGSGNTDFDGKLMSSAMKK
>TRINITY_DN4101_c0_g1_i3.p2 type:5prime_partial len:133 gc:universal TRINITY_DN4101_c0_g1_i3:3-401(+)
QAILAQGLPLPPGGGPPAACRAPRGGVMSGIGAFAQLAHKFVTWPQGSTQGFCNVIKIGTFCRT
VVWPCLPPVLMYQYIRGVDEDAYTLEVLHYKAGSKDPKAFFDSSRIGLSGHWRLQQDLETIRAAV
NAE*
>TRINITY_DN468_c1_g1_i1.p1 type:complete len:191 gc:universal TRINITY_DN468_c1_g1_i1:676-104(-)

MKTFYTILLLAIVQTNAFFFPKVDTVKDLDLASYTGRWYEVYSSLIQRATFQRNSYCTTADYSLKPS
GAIKVINSGRLKSATGEENTIEGEAKVVDAKEPGALNVKFSTGPQSDKANYLVVKLGPVVDGKYD
YTVITTDYKALVWVLARNPETFKQKYEKDVLAYLKKNGYNWFINKPRPTYQGKDCIYI*
>TRINITY_DN167010_c0_g1_i1.p1 type:5prime_partial len:506 gc:universal TRINITY_DN167010_c0_g1_i1:1755-238(-)
SQQNKLLGCYRYLHYNYNYKQNMAGVTSGPKFFDIYELKEELGKGAFSIVKRCVHKENRMEYA
AKIFNTRKLSTRDLQKLEREARICRMLKHPNIVRLHASIPEEGMHYLVFDLVTGGELFEDIVAREYY
SESDASHCIQQILESVLHCHQLHVVHRDLKPENLLLASKEKGAAVKLADFGLAVEIENDKYTWH
GFAGTPGYLSPEVLRKEPYGKPVDVWACGVILYILLVGYPPFWDEDQQRLYGQIKAAAYDYPSPE
WDTVTKEAKELIDCMLTVDPKRRITATEALKHPWIAERHTCASTFHRQETINGLKRFNARRKLKG
AILTTVFARRISGTSGGLFASAAKEKLINDKARKPSEDEVMLPTEEEDAMKATREQEIIKLTQKLIA
GITMWDFETYTKLCDDRMTCFEPEAKGNLVEGLDFHKYYFDNMKQHSLPINTTILSPHVILLSEES
ACISYIRLTQLMDCNGRNSTFQHEETRIWQRKGSQWRCVHLHRSSNST*
>TRINITY_DN160_c0_g1_i1.p1 type:complete len:1145 gc:universal TRINITY_DN160_c0_g1_i1:4810-1376(-)
MRLLAIIPLLVMLDIAKSDKLWECNFEENDCWAASSTRNHERNQLTLHWNRWKMRTPTGATAS
PVTGPMEAFEGKHYLYYEATESMDKLKMADGASKGVVTFKDINLMGRTQFILSFYYQVKGNDG
DFYDFDLMVSNTENTHYDLSMKSIDGKDWKHFKHIMKVEGAGVTSIDLKFTARSYGAFADYAID
NIVIEDYPDAGPCKDNRCLANEYCTITSSTQTVCVAKCTQQACGENAECILDSLAINKCKCRQGY
LPRVSNYGDKLNCYRPDASCDIEYNSLGQCRISTSIPIMYGHPSLWHGRYSPVSGNGYYVFKYDNT
ITLDAVSSQKLSFFYLVDNPNMINSLTVEYGSRKFEVPINREKTNIWTLYEVLILASTNQGVQIKASF
YAEGGVIGIDNIQFTQQNEVVVEKIYKESMMAATFEDGSVPTDLKVAMLFVTRYGKIDFGCLPGIE
LETRNGKLYCKDGVVQRQSACSQDSDCIGSNARCVLRNCVCNTNSYQISIGTYGEKATSCAVYPC
EINSPRCHSTAICSNIKTPGQNFYNDYTCKCGRSLNNVETIINPFIGGDKSTQCLAATPSDSYVCND
NNFKPSRTTEYCLKGNQATVEYSLRATNFGNYALFKHCDDPDDEYRSSFNDPGSFWRPIDLVAAA
NENLAVSVTYKTSLSNKFIKCNFEQARFGSKLTLAVNVNSNSRVLTFTNTQTSVNKQNPVYQGQV
FQSDNPTPGAEMSVTFTVTGNQAVDQNVDAILLDNVVCKGFDDVASVNTPCGNNACASNQYC
NPLLSNSEKECLCPTGQKDQNCPDVVPSGSVVSADGPKCIGHHEKYMNGACVCDDGFEKTGDT
CTVKINCGCNDGLCLSGDKCSCPLNLLKVNQACVRTNKDCAKDCVSTLGNGGYCDVTTGMCD
CLTGFVKSGRDDGSYRCDKHAVVQKINDPKGICSATCGTGFVNEIEVCVGATGTGFECMTNIVQG
TLGVCSLPACPATSSQCSIQKECACNNVRVPIGSDGKLRLDFDHEAKLNMIFRFLSKHNQKDLEFK
FREARCDNTKLDSIARTIEDDLKQLEFDTELQAAITTMKSILDCSPSGNDHNAAYWDVFDMLIELS
SYRPHAVSDLQLKKELVMHEQSRCGMRSNLHKVVDGYAFWNKK*
>TRINITY_DN1797_c0_g1_i2.p1 type:3prime_partial len:998 gc:universal TRINITY_DN1797_c0_g1_i2:2993-3(-)
MSKIKLNIVYILLLVLWISIVHSQSQDDRCPAIDNEQECPPPDSLNNECLENSDCAGNQICCGDGC
ELKCVVAADELCKVPIDLGFILDSSGSIGSKFDNVKIFVEKIIDAFVIGEDFTHIGIVEYSENASVVLA
FNTYQGAELTKANILTELRKMSLIGGHTRIDLALEVASRDLFSAKGGVRPGKPKIALVLTDGKQTR
GKLAPDAKDLHIASQPLKDSEIHVYSLGIGDDYDIGELLDIASDDTSVFRSQDFDELLNIVSQITEET
CKGCTRAIDIHFALDTSINVGRENFEYMKNFVEAIGYRFVISETSSHMSASIFGEEPMLMFLMNEAT
EQNMFLEEVKQLQYIESANGNMDRLLRMVYDDIFTLEGFTRQSVPKVLVMLTASNCDSCDERVEE
AVKPLKEQGVHVVTIPIGNNVNMDEINAMASYPSSEYVVPRQTYVGLMNDFFIQKVSDMICSGRP
GVCAEPPVEGSCDTIIQECTTDVKCEGNRKCCIKNCKASCELPVSACITPVDLAFAFETTRNGEAD
FENAQKFAANLLDHFKIDRSTTHVSVSTFSETIGTSTFFNEEYDKSNIQQFIKDLGSDSGDQSNLGD
FLEFASRKIFSVQAGLRQSTPRVLVIFTNGIVSAEQSLKAQQEAQKLKAEGLDVSILVVNIGGNIGSV
DLFESIISNGRIFSYVESSELSKFEAETDLAEAICATKVRVRRCPPKRTPDTCNVKDDLCSSDFDCPG
YQDCVSDGCRLRCQTCEYRCEDKLDLLFMIDSSYRIGAEGFQKAKENVNKMLEWFTIAPDNTHV
GVITYSDTANIAMSLPPLGTNNPPQSLPDVQGRINAIPYNGGSKSYLDEALKLAAAQVFVENSDRF
RKGSKKAVVIITDGKDDSTSRLDLASWPLRKQRVVVGKEDYSAIHILAVAIGDELNTNALENVVTP
TLEENFFFAANYKDMYNEIRKLAKSSCEYAGAFTAPPDIYMKGEPGPPGPPGPKGDPGPDGPDGT
R

>TRINITY_DN7314_c0_g2_i1.p1 type:5prime_partial len:142 gc:universal TRINITY_DN7314_c0_g2_i1:541-116(-)
AEATEAPVKYVESAADKRLFEQVYVQYAQEYLKGPLYWDPNKLQGIDPQFPGNPITVDGKLTSNT
IGNLKTFSSNELAFFSFLFFGIGLYGNYMFNYGDPQFEKVIRDGGYLNASYIVEAIFLPISFFFHIACYI
QRKNGK*
>TRINITY_DN79126_c0_g1_i1.p1 type:complete len:115 gc:universal TRINITY_DN79126_c0_g1_i1:397-53(-)
MVARLVLRRRLSYNTKSNRRRISKTPGGKLVYLYTKKAGATPKCGDCKCKLRGLKAVRPKKLAKL
SKTKKTVTRCYGGSRCGGCVRERIVRAFLIEEQKLVVRVLRAQQVKKSS*
>TRINITY_DN691_c0_g1_i2.p1 type:complete len:1673 gc:universal TRINITY_DN691_c0_g1_i2:5503-485(-)
MMNLFRLIVLGVICFSYEGDAQQCGGSCSPCGNLCVGMKGDRGTAGLPGFRGPPGLPGFPGPEG
PIGQKGEKGPAGLNGMEGSQGARGKVGPPGLPGTPGRHGRPGNEGREGEAGLPGCNGTKGDR
GPEGPRGRDGIEGPQGLQGNVGPRGEPGDSTATKLKGQKGEPGSAGQQGEKGGRGMEGMKGD
TGKPGRNGRAGERGPKGEPGTSNVTIIGEKGSKGSVGPPGLPGRQCQVTGDKASNIAQGQKGNQ
GSKGEEGAKGEKGDAGLAGPQGEEGDIGPQGDRGDDGPQGPPGPEGEQGRSGIQGNKGQKGE
VGNNGKDGNDGQTGLTGQRGRQGTPGQKGEPGKDKKGLRGPEGAKGERGNDGEKGSDGPIGP
IGPRGPKGELGPLGMKGFKGSIGSAGPKGENGNPGDPGIKGRRGELGIKGDSGVKGAPGNPGISV
TGPDGMDGDTGPRGQSGGKGAVGFPGKPGPPGVVTIDGEEVLPGEPGPQGLKGFRGVQGDAGS
KGSRGEKGNACESSKCPAGPKGEKGLSGQDGLQGGDGEEGEKGEKGDKGDIGSRGDTGSRGEP
GPQGESGEKGEKGIQGPQGNSGKDIIVQGERGDKGTKGNAGTPGSIGFPGERGSPGESGDRGLSG
VNGLRGETGDRGARGVAGKPGVNGQKGQKGEIGSGARGFKGDKGEVGPDGPAGLRGPGGPVG
PKGDPFPTAVNGKTGEPGEQGAAGPKGAQGPVGPEGPRGFPGDIGPVGEPGDGEKGERGDDGA
RGERGEKGEPGQDGKDGDPGPKGEPGQEGKEGPPGMFKGNKGEKGATGLPGVDGLPGNSAPK
GEAGKDGQDGDPGPAGEKGDPGEAGSPGTEGPIGEDGPVGDKGEPGESRAGPKGNEGRAGAK
GENGRDGRAGRPGIGRPGDKGELGQKGEAVIGAKGSKGEQGLEGPPGPIGKPGVKGDAGVPGK
DGEAGGRGKDGERGLPGDKGDTGDIGFNGSPGAPGDKGEPGYSGQKGAKGMSGPRGPPGSTSN
VVGPKGNRGLPGMKGEPGQQGEKGLIGPQGSKGESGFGSEKGIKGSRGNPGAVGPSGEKGERGD
RGKDGLQGIAGPRGEEGNPGEKGSRGDQGPQGREGPSGELGPEGQKGDPGFAGEKGEKGDRGG
DGQKGDAGEPLRGEPGVAGPPGPTGPQGERGPKGEEGNMGPKGSQGRDGLTGEDGPKGSKGEP
GLDGDVGEKGEAGFQGLQGEKGNQGPQGLEGSTGMDGQKGQIGEKGVEGPPGPPCEPVNSVR
GETGEEGPKGDRGPKGQKGKPGPQSQAATPGEKGSKGEVGQPGPQGIKGEPGREGDFGPKGSPG
AQGEKGDQGQKGEPGQEGPEGLKGEPTRAFGPKGQKGETGLEGLRGPVGPKGEDGQTGPQGP
DGPKGNEGEKGDFGINGLNGAAGPSGPKGAIGLKGDSGPPGPTGAQGPRGSGGSPDFGFYYVK
HSQTTIVPSCPAGMKTLWTGYSFLYAQGNERAYGQDLGQPGSCLKRFSTMPYLFCDLQNQCTVAS
RNDYSFWLSTDEPANMMKPAAGPEVEPYISRCAVCEAPANILAVHSQSVIVPDCPNGWENIWAG
YSFLMYNSAGADGSGQLLASPGSCLEEFRANPFIECHGRGTCWYYGPTLSFWLSTIDDSNQFQIPK
SETLKAGNLKTRVSRCSVCMKTVRNGNFP*
>TRINITY_DN187823_c0_g1_i1.p1 type:complete len:611 gc:universal TRINITY_DN187823_c0_g1_i1:1943-111(-)
MWRRIFNFRVSQLDLIKSPEFRYHYLFILPKSLCTMGDNEKISKNELKRRLKLEQKAKDKAEKQAA
TTAIAQEKKAVKSTLNVLVPDEENLDPNQYYKIRQQTVANLKSSGDNPYPHKFEVSISLTDFITKYS
SIEAGKWQDDRVSVAGRIHAKRLSGQKLIFYDLRGEGVKLQVMADQRSSEQDFAQIHGKLRRGDI
VGIKGKPGKTKKGELSIQPSHVELLSPCLHMLPHLHFGLKDKETRFRQRYLDLILNEKVRQKFYTR
SRIINYVRRYLDQMGFLEIETPMMNMIPGGATAKPFVTHHNDLNMDLYLRVAPELYHKMLVVGG
IDRVYEIGRQFRNEGIDMTHNPEFTTCEFYMAYADYNDLIDLTEKMISGMVKLLFGKYKVTYHPD
RNEEGDGEPLEVDFTPPFRRMSMLEELQKVLNISFPNFDLHSEEARKYFDDLCAQKEVDCSSPRTV
SRLLDKLVGEFLESQCVNPTFIMDHPQIMSPLAKWHRSKQGLTERFELFVCCKEICNAYTELNDPL
VQRQMFSNQAKDKAAGDDESMFYDEGYCTALEYGLPPTAGWGMGIDRLCMFLTDSNNIKEVLF
FPAMKPEEKVREVPLSPTKVNP*
>TRINITY_DN2772_c0_g1_i3.p1 type:complete len:142 gc:universal TRINITY_DN2772_c0_g1_i3:476-51(-)

MSDAEGDDVPVQQEVAAGGPMDINTALQEILKLSMINDGLARGLRESVKALDKREALLCVLAN
NCDEQMYVKLIEALCAEHEIKLIKVDDNKKLGEWAGLCKIDKEGKARKIVGCSCVVIKDWGKET
QAHEVLKDYFQSKS*
>TRINITY_DN276_c0_g1_i1.p1 type:complete len:151 gc:universal TRINITY_DN276_c0_g1_i1:653-201(-)
MSTEYSESEMMEYRDTFSLFDKQGDGKVECSEIGEMLRALSLNPTEAEIKKIIAEIDPTGQKRVTFE
EFIPLYHTQSKKKVTGNFESFAEGFRIFDRDSNGMVNVGEVRHLLTSLGEKLSADDVNILVQGME
DSNGMVNYEEFVRGVMNG*
>TRINITY_DN2217_c0_g1_i2.p1 type:complete len:254 gc:universal TRINITY_DN2217_c0_g1_i2:884-123(-)
MNSVDGNTDNAQKECLELCQRNAKATGCEVIWNRHNRGCYIHTQEVARGNNVDRHFCWVFS
KCTLNNVPPAVCPIEKGYCVKGNGHDQNSGVIKMNSVDGNTENAQKECLELCQRNPKATGCEV
IWNQHNRGCYIHTQEVARGNNVDRHFCWVFSKCTLNNVPPAVCPIEKGYCVKGNGHDQNSGV
IKMNSVDGNTENAQKECLELCQRNPKATGCEVIWDRHNRGCYIHTQEIARGNNVARHFCWVFS
KCNL*
>TRINITY_DN6397_c0_g2_i3.p1 type:internal len:130 gc:universal TRINITY_DN6397_c0_g2_i3:387-1(-)
LTSSDQAELTKKLSAEVANGRLAMMAIIGMFFQDGLTGSAWGDWSLYTASPLRAFENELGVQDP
VGFWDPAGFTADGNVENFRRRRQTELKHGRVSMLATMGYITPELTGKLPGYLSPSAGLKFADVP
N
>TRINITY_DN16548_c0_g1_i4.p1 type:internal len:171 gc:universal TRINITY_DN16548_c0_g1_i4:2-511(+)
LYTDSPLRAFEGELGVQAPVGFWDPLGLAADGDSVAFRRRRVTEIKHGRISMLACMGYITPELVG
KFPGSLSPSQGIEFADVPNGLAAISKIPSYGWFQIIAYCLYCEASALNFGYFNEAFDQKAPGDYGW
RPPLLATDDAALKTKRLNAELANGRLAMVAIIGMFFQDG
>TRINITY_DN571_c0_g1_i2.p1 type:complete len:186 gc:universal TRINITY_DN571_c0_g1_i2:192-749(+)
MREYKLVVLGSGGVGKSALTVQFVQGIFVEKYDPTIEDSYRKQVEVGSQQAMLEILDTAGTEQFT
AMRDLYMKNGQGFVLVYSITAQSTFNDLMDLRDQILRVKDTEDVPMVLVGNKCDLEDERVVGK
DQGQSLAKQFGNCTFLETSAKMKINVNEIFHDLVRQINQRQPENTKSERKKKGCTIL*
>TRINITY_DN902_c1_g1_i1.p1 type:3prime_partial len:153 gc:universal TRINITY_DN902_c1_g1_i1:146-601(+)
MIRIAIIAIAIAACVYESQAVRCHVGYSAADLPLAMSVRDVECGQGFDHCVTLKYVLDGMKITQG
NCATKFGQATCQFMRNSHAAMTDCSEAICSGDHCNSAVKEAEKIEMKRVGAYENTLKCTTGLQ
SDFPNIPFASPNVPYAVDECFPGT
>TRINITY_DN2885_c0_g1_i2.p1 type:complete len:114 gc:universal TRINITY_DN2885_c0_g1_i2:31-372(+)
MAQPVRLYSKGIFVGYQRGLRNQHEQTSLIKIEGVNTKADTSFYLGKRVAFVYRVSKKTVAKGDK
KPSRIRVVWGKVTRPHGASGVVRAKFRHNLPAKAIGGSVKVMLYPSRI*
>TRINITY_DN6294_c0_g3_i2.p1 type:complete len:134 gc:universal TRINITY_DN6294_c0_g3_i2:817-416(-)
MGYKRFVEAGRVALINKGKYDGKLVVIADIVDQKRLLCENPVKSVPRHVVRTQDVNLTDLKVNI
PCGARGGKIRKEYEKEEVDSKWEKTVWAQKIKARHVKRTLSDFDRFKSKIQKQRRNRKVKAVLK
SMKKN*
>TRINITY_DN902_c1_g2_i3.p1 type:internal len:178 gc:universal TRINITY_DN902_c1_g2_i3:2-532(+)
AVFKCIKLNYIDHIIKMIKIAIIAIVITALVHESTAVRCNVGYSTADLPMAMSVRQVECGKGLTHCN
TLSYKLDGMSVTQGNCATSGGQASCLFMKNSLQSMTECSEAICNGDHCNKAVKDAENAERARV
GPYKNTLKCTTGLQTDFPDIPFASPNVPVGVDECFPGTKECVTMTYD
>TRINITY_DN3870_c0_g1_i1.p1 type:3prime_partial len:990 gc:universal TRINITY_DN3870_c0_g1_i1:2969-3(-)
MPQYFQKPENALKRANEFIDIGKKEAALDVLYDVIKSKKHRNWQKIHEPILRAYLTLCVDLKRSV
NAKEGLYQYKLICQQVNIVSLEEVISFFLQLAEKHADEAKNQSIDKVVVEDLDQVQTPEGILLSSV

SAEDAQDRSDRVLLTPWVKFLWEAYRNVLELLRNNNRVERLYQETSQSAFRFCLKYNRRAEFRKL
CTLIKNHLDQVLKYQGQPTAINLNNPESLQMHLETRLFQLENAITMELWQEAFKAIEGIHYLMQ
MSKKPPKPHMMANYYEKVALVFFKADNKLFHAAALLKLFVLIKEQKKTVTHGELEKIGNKVLL
AVLSIPSPPGRSSMEEYLSYDTVSYDKERRLTTLLGLIGIPSRKSLIGDLKSQILPHVSSDLQELFSLLET
KFQPLTMGKRVNEILESVKENDTLVQYIQSLQRITMVKTLKQVSQIYETITFPRLLRILPFTDVHQLE
RSIVSCVKEEDLQISINHQTHSLSFGSTLQVALKEEVPDGPYIQAMPSEILRSQLCSLSEGITEIVRVLE
PEKIQNGILTQRKTLIDQYMRQERKEHVQLLNRKSVIEARKERIESMKNAQERQEQSQLHKQREQ
LKKAEEQRLEAEREKREKERKIQEMKDIQKQQVWDRVESLRGTDIGKRAFKNLSAKEIDEMDPD
DILQRQFEQIDRDKREQTLKLRTQEKRVDHLARAMRMEEIPLREKFIQEKYDLDKEQWNKDEEER
IKNAEKEHHIALKTKDRLIRMLDDRNIFLDKLFGNRNAEYQARLEEFEENLNKIKQERLEARRRK
RMSQRRQEFIMARREEKRKEREEQLRREKEREEQRRKEEEEQRLREQEEKQKKLEELAQKQRERE
LEIEQRQRQRAEESENLRDRGDGPRDRIDGPPSRDSRPTGSVAGGKYVPPSRRSVNPSDGGGDGG
GWRRGDDTPKRDGDRFRRDDDRFRRDDDRPRQEDDRFRRDDDRFRRDDDRPRQEDDRFRRDD
DRFRRD
>TRINITY_DN1631_c0_g1_i1.p1 type:complete len:124 gc:universal TRINITY_DN1631_c0_g1_i1:62-433(+)
MAEAPKPKPAPKSRYGPSFDDIERRDPAQWHAAHNAWVLERLVEQETVKIYRERMDECYWKEQ
ENFPQNCRKHILKYWKAWNDWKKKDWGQTEEGSVYRFRVPVEEYYREVEELYKNKDKGNE*
>TRINITY_DN8101_c0_g1_i2.p2 type:complete len:820 gc:universal TRINITY_DN8101_c0_g1_i2:2597-138(-)
MDGKTGASLKLTILNDRGRVWTMVAGGGASVVYADTVADYGWGHELANYGEYSGAPSTEETFV
YAKTLLSLMMKYKHPEGKFLIIGGGIANFTDVAATFTGLIQALQHFAAEIKEHKIQIWIRRAGPNY
LEGLRKVKAASDKLGLGLKVYGPETHITAVIPMALGLTTPLPEPDLSQACGPPRRAAIAGAGSKPS
TQKAAPAPSAHTIVTATPETTSIVYGLQNRAVQGMLDFDFMCKRKKPSVEAMVFPFSGNHYVKFY
WGTEEVLMPVYTTTKEAVQKHPNTSVFINFASFRSVHETSLEAMQYPNVKTLAIIAEGVPEQQTR
QLIQTAEAKGVGMIGPATVGGIKPGCIRIGNTGGMLDNIVMSRLYRAGSVAYVSKSGGMSNELNN
IVCRNSNGVYEGVAIGGDRYPGSRFLDHFLRYQDDAGAKMLLLLGEVGGTDEYDLIEAVKSGRIT
KPIVAWCVGTCASCFATEVQFGHAGAQARGDMETAAAKNKAMREAGFHVPDSFDKLPELIGQV
YTQLVQEGVVVETPEAETPQVPMDYTWAKKLGMVRKPANFISSIADDRGEELKYCGVTISEVFAQ
ELGVGGVLSLLWFRRQLPPACTKFIEMILMVTADHGPAVSGAHNTIVTARAGKDLVSALCSGLLTI
GPRFGGALDDAAKMFAEAQDSGVSAKDFIEKMKKTNQLIMGIGHRIKSLANPDQRVVLIKDYAK
KHFKNTPVLDFALEVEQLTTRKRANLILNVDGCIAVCFVDMIRSCGAFSREECDDLMAFGCLNGL
FVLGRSIGFIGHYLDQLRLKQPLYRHPWDDITYIQELAAQG*
>TRINITY_DN3034_c0_g1_i1.p1 type:complete len:1941 gc:universal TRINITY_DN3034_c0_g1_i1:6289-467(-)
MAEEEDPMKYLQVDPAQALKDQSKTFDAKKWVWVPVIGEDGYAAAQVIKVDGDNVTVETNK
GHEMEVKKEKCDQMNPPKYEKCSDMASLTYLNEASVLYNLKARYTTGLIYTYSGLFCVAVNPYR
RLPIYSDQVVKLYRGKRRAEMPPHIFAIVDTAYQDMLTDHDNQSMLITGESGAGKTENTKKVIQYI
AKVAGVEKKKSDDGPVKRDLDEQVVEANPLLEAFGNAKTTRNNNSSRFGKFIRCHFSQTGKLAG
ADIESYLLEKNRVIHQASAERNYHIFYQLLYATTDEELQKLCLVTRRSEEFEFLKKGVPSVDAIDDN
EEFGYTTNAIEVLGFDEEEKQSMFKICAAILNFGNMKFKQKPRDEQAEVADPADGERVAYLLGLPI
GEFHKSLTKPKVKVGTEFVNKGQNVSQVLYSVAALSKALYERMFWWIVSRVNKALDTKERRSYFI
GVLDIAGFEIFEYNSFEQLCINLTNEKLQQFFNHHMFVLEQEEYKKEGIHWEFIDFGMDLEETIDLI
EKPMGVFAILEEECIVPKASDQTYLQKLHKQHAGKNGSYEKPTPKQTKSGGGDFILHHYAGSVGY
SVANWLEKNKDPINAHTAELFSKATVGLVSGLFADYNPDVTGGRRKGSAFQTVSFRHKEQLKNL
MTTLMSTSPHFVRCIIPNEKKTPGLVEGTLILHQLRCNGVLEGIRICRKGFPSRMLFQDFRQRYQIL
AASAIPSGFIDGKVAAEKLIDSLQLDQSEYRVGTTKVFFRAGIIGELEDMRDERLAKIITQFQAYCK
GHLARIEYKKMCEKRIGLAVIQRNVRKFLFLRNWQWWKLYCKVQPLLSIARAEEEMKEKEEELK
KALEKAKEDEEQRKKMEEQLTENIAEKEKLFADLQAETDRLINLEDKLMQIQQQKDRLEANLNE
TLEKLDGEEHSVSVLQEKMHDAEDRIDSLEAEASELKSNCSRLEVERANRDKQIDALNEDINKQD
ESIAKLGKEKKGLEDNIQELNETLQASEDKCSSLTKAKKKVEDALKESENSLSKEKDSKARLDKEK
RRVEGELKETNDKLSEAENELKNTQDTVYKREKAIKELEEARDNQDNQIKQLQKKIQELLQRIEE

LEEELEAERKQKQKSELSRKELESQIEELQDQLETAGGATHAQVEVSKKREQECSRLRKEIEDINA
ANDEALASTKHKFNAQLSEAQEELESVKKAKSKSDKERSQLAGDLEDVQGQLDQVKKQRASSD
KNNRMLEEQLAELRGKVSSLETDMGDSESRNLKFQNESATLLKQLEEAEHKLGLANKQNKQLQ
ASLGESKNVADDEARGKHDMQNKLQNALAQIEELQEQLEDEQTAKMDLQNKYSRANADAQQ
WKNKYDMEGASRVEELEDAKRKLAARVQEMEEALAAAESKAASMEKVKNRMNEEVEDLLLDL
EKAQAQAANLEKKQKKVDQQINEWKLKCDEVQEELEKSQRDNRSYSTELLKMRTANEDTVEKY
ESLKKENRALAAELQSVSEQLSEGGKNSLEVEKLKRKLGLENEELEVALEEAELALEQEEAKFLKI
QLELTQLKQSTDRRSAEKDEELETLRKNHARQLEALQNTIDAEQKGKAEAQKLRKKLEGDLADL
ENRLEGSDRNSADYLKTIKKLQAQIKELQAMIDDESRGRDDMRDTASRSEKRAMDLAVQLDEAR
VALEQSDRLRKLAENEKLENAERVAELQTMYNSIANAKRKAEGDYHSLQEEIEELENEAKMAEE
KANRAMNECARLSSELNGAQENASNAEKSKSLMARQITELQQQLEEAESMGGKSLKNQIRKLE
QKVMEVEGELDNEARKSTESIKIARKADKRVKELQFQLEDEHKAVERAQEVSDKLNQKMKKMR
LQLEESEQQSSSWQSKFKKAVVDLEEAEERCESAEQALQKARQRARSGASGMTTRGVSRARSRVR
SPQNDD*
>TRINITY_DN7504_c0_g1_i1.p1 type:complete len:414 gc:universal TRINITY_DN7504_c0_g1_i1:87-1328(+)
MAKIPGGIVVEMDGDEMTRVIWDMIKTKLIFPFVDLDIKSYDLSIQNRDNTDDKVTVDAAEAML
KYNVGIKCATITPDEKRVEEFNLKKMWKSPNGTIRNILGGTVFREAIICKNIPKLVPGWTKPIVIGR
HAHADQYKATDFLVPEAGTLEMKFTPKGQTEPTISYVVNNFAGAGVAMGMYNTDKSIRDFAHSS
FQCALEKNYPLYLSTKNTILKKYDGRFKDIFQEIYDNEYKGKYEAKGIWYEHRLIDDMVAYALKVE
GGFVWACKNYDGDVQSDTVAQGYGSLGMMTSVLMCPDGKTIEAEAAHGTVTRHYRLHQKGQ
ETSTNPVASIFAWTRGLAHRGKLDGNERLMNFAKLLEEVCVATIENGSMTKDLAICIKGMKNVTR
ADYLNTTEFLQKIADNLTTKLNLSHL*
>TRINITY_DN163801_c0_g1_i1.p1 type:complete len:298 gc:universal TRINITY_DN163801_c0_g1_i1:55-948(+)
MANPLKDIISRFSKGVPAGSSTGLGLLIGAGLAAYGIKESIYTVDGGHRAIIFSRIGGIQPGVYSEGL
HFRIPWFQYPIIYDIRARPRRIMSPTGSKDLQMVNIGLRVLARPLSSTLPHMYQRLGLDFDERVLPSI
CNEVLKSVVAQFNASQLITMRQEVSLMIRRDLTERAKDFDIILDDVSITDLSFSAQYTAAVESKQVA
QQEAQRAAFLVERAVQERQQKIVASEGEAKAATLLGEAMNENPGYLKLRRIRAAQKVARVIAQS
QNKVYLNANNLLLNIKDTDDIDDGLLSKKR*
>TRINITY_DN761_c0_g1_i1.p1 type:complete len:830 gc:universal TRINITY_DN761_c0_g1_i1:129-2618(+)
MTSMFRSEEMTLCQLFLQSESAYACVSELGEIGVVEFRDLNPDVNAFQRKFVNEVRRCDEMERKL
RFMQKEIDKADIAIVDTGENPDAPHPREMIDLEAQFEQLENEMKDSNSNYEALQKSLLELLELRH
ILKKTQSFFEEEGYAETQARLQADHEPGVTGGGQLGFVAGVINRERIPTFERLLWRACRGNVFFK
QAEIDTALYDPGTGDLTHKCVFIVFFQGEQLKIRVKKICEGFRATLYPCPETPNERREMAIGVKTRIE
DLETVISQTQDHRHRLLQTIAKNIRVWLVKVKKIKAIYHTMNMFNLDVTQKCLIAECWAPVADL
DSIQAGLRRGTERSGAQVSSIVNRMSTKQDPPTFNRTNKFTKGFQAIIDAYGVASYQEVNPALYTVI
TFPFLFSVMFGDCGHGAIMALFALWLIMNERKIANWKAGGEMFDTVFQGRYIVFLMGLFSIYSGL
LYNDIFSKSLNIFGSAWVPTLNRTKYDYQPENYKEWSDGDRMLNPLDAPRETPYYFGIDPIWQLTS
NKLTFTNSLKMKLSVIFGVTHMIFGVILSLFNHLHFKQYVNIFCEFIPQLLFISCIFGYLVILIFYKWIA
FSIHSPSQPSILLGLLAMFLNFAKKKTDADRLYNGQDFLEPFLVVVAVICVPWMLLIKPFYLRWQH
NKKKQHGTNFARLHEDTAPLQDNEVNHHDDSGISSKNEEEEEFDFGEVFVHQCIHTIEYCLGCIS
NTASYLRLWALSLAHAQLSEVIWTMVFKIGLSMDGYVGSVAIFAVFAAWAILSVAILLIMEGLSAFL
HTLRLHWVEFNSKFYVGAGSKFVPFSFKDIMNDEAF*
>TRINITY_DN309_c0_g1_i1.p1 type:complete len:454 gc:universal TRINITY_DN309_c0_g1_i1:65-1426(+)
MSNCEHKTQKITIQKKTVKVTSQREEDEYVKARKEGKKYILPWKTPDGLQPPDGFTNLKYIFTPDS
SNVPSSAEKIDEEPLMQKMKPDPSRIENPPETGIRATWLGHCSVLFQLDNVNVLVNPNFNDRGIK
YYFPGDNKRYRTCVYKVDDLPRIDVIFITNTHYDYLDLSSVRALNNKYGEMLLWYVPIGVGEWM
TKAGCHNVVEMNWWREDEVDFIDHTKIDDDEETTTTTFNISCTPSQSFHSRTFDEDNAALWCSW
VIKSPRYKLFISGATGYCDIFKVIGRKYGPFHMAALPIGGYHPKDKFGYGNVTPEEAVQIHQDVLA

MCSLAVGWGTFTLSNEYYLEPPRRLNEELSKQKLSEMQFFLLKHGESRLIEIKEADKDDDEPPQEN
GVPPVVPPVDDTIEGEVCGEEKKDDDDSDVLQQLVDNFTMDEEKKSEEEEEKSEEKEEEKEE*
>TRINITY_DN77_c1_g1_i1.p1 type:3prime_partial len:1965 gc:universal TRINITY_DN77_c1_g1_i1:2-5893(+)
MGVQVYSLGIGSNYDIGELLDIASDDASVFRSQDVDELVSIVASITEQTCKGCTRAVDIHFALDASQ
NVGRENFQVIKNFIKAIGFKFIISETGSHMSASIFGEYGTLIFSMDQATQQEDFINEVDNVPYIGDFN
GNIDKALDMVSEDIFTFDGFSRQGVPKVLVVLTASNCDSCEKSVEEAVKPLKAQGVHVVAIPIGSN
ANMEEMDKMASYPTQDYVIPRASYEELLNGVFIQKVSEMVCSGRPGVCEEPPIEGECTEIINECTL
DVNCPANRKCCIKDCKASCEVPAACITPIDLAFAFETTGFIEENFDEVKSFASSLLDHFRVEPAKT
HISISTYGESVNAKTAFYESYDKSALKQFIKDLEPDGGRQANIGDFLKTANDEIFNITNGIRQSSPR
MLVLFTNGVYSNDQIAKAQEESLKLKAEGQDVNILVINIGSIVEGNSVLRDVVSTPDFRNLHNVT
DLRRLVNFGFENKIAEAICASKVRKTRCKPRTPQACSVRDDLCQTDFDCPGYQECYSDGCRLRCQ
TCENRCGDKVDLLFMIDTSSRIGPEGFKNAKENIKKMLEWFTIEANNTNVAAITYSDNANYAMQ
LPAAGSNDPPQTLFDVQGRLDSIPYSGGSKSNLDKALKMAAAQVFYQNSLRLRKDSKKAIVIVTD
GVDDSTSRLEIASWPLRKQRVVVDGNDYAAIRLLAVTVGNNYNLDTLEKVVSPPIDKNIFLAADF
DDMYNGIRQLAEDSCKYTRQDVEPPVIAVKGDPGPKGEKGRQGNPGTNGTDGMDGIEGPKGER
GDDGPKGEKGDTGIAGPPGGDQGDLKGPDPMAEAVGIIEGTAVKGTRGVTYDLRDGPIGPPGPIG
GVGKTGLPGDSGEQGQKGRGGPPGLRGVFGGQGPKGNPGEDASPGLPGEPGPKGISGKKGPTGS
PGMPGPQGPFGYKGEQGIIGQPGDPGDGGKKGPKGYAGEKGDQGDIGDAGFPGPDGKVGPIGP
PGQRGLRGPEGSSGPIGKTGDIGNKGLPGPRGSRGLKGQSGNAGPRGGKGRTGDVGLVGEKGTQ
GSIGEAGQEGQPGVPGKEGPPGYPGPIGTPGPMGSRGDIGPQGEEGPKGSLGLKGNRGEAGRRG
LTGEKGETGDIGELGPKGAQGQQGKQGKTGKIGPRGQQGPLGFQGLQGQTGDPGQNGRQGPK
GNLGPTGEKGADGPKGSLGSAGLTGPKGLVGEPGNVGRPGLPGRDGKNGPQGANGKDGDPGA
SGPQGLQGSPGSMGTPGSTGDPGSPGDRGATGAVGDKGTRGDSGVRGEPGAFGQQGPPGLRGE
RGEKGPQGTNGVQGSPGPRGTPGLEGLDGIVGRKGTAGRPGNPGQSGEQGAVGSIGVPGPQGIS
GTPGERGDGGSDGGPGEQGPPGLQGSSGSPGRDGMPGRPGQQGTTGGKGKTGLRGPKGANGQ
NGVQGARGPDGLPGAPGLVGNRGTPGPNGIPGLDGNPGPAGKQGKQGKMGTPGMTGPAGLR
GPVGSPGLRGQKGPTGDRGDSGETGPIGVAGDIGPRGFTGELGLRGAIGEMGQIGETGPQGMPGS
QGTPGAAGLTGPAGPSGLPGKDGNKGPIGDPGNAGNPGRDGGPGSPGKDGTPGEPGTPGSQGK
QGLKGPFGPQGAPGEQGQRGLTGRQGSGGKDGPKGFPGNDGSPGISGKRGPRGKDGTPGSPGPI
GKDGRPGKPGINGTDGDPGNNGPKGDPGERGDSGDSGSLGASGDKGKTGSEGPAGFVGPRGSN
GKAGVPGKRGKRGDQGRDGPRGNSGPDGIDGSKGFKGNMGRFGIQGPPGDAGLKGALGPDGT
VGHTGPQGNTGDPGPDGPEGPSGSKGPAGNAGQIGNAGYPGSPGDGGIPGTAGLPGPPGPPGPP
GDMSGAFSGNYWDFVNAGGLQKGPRYYGGRSKRSLDQQDNSIEKVFKSNYETFKNFDTVWNQI
VDDLVIQKTLGSSKNPALTCGDLFKTNPITKSGDYWID
>TRINITY_DN30365_c0_g1_i3.p1 type:internal len:239 gc:universal TRINITY_DN30365_c0_g1_i3:1-714(+)
LTFIESLRLRYKKLQKQLEFLEVQEEYIKDEQKNLKKEYLHAQEEVKRIQSVPLVIGQFLEAIDQNT
GIVGSTTGSNYYVRILSTIDRELLKPNASVALHKHSNALVDILPPEADSSIAMLQADEKPEVSYVDI
GGMDIQKQEMREAVELPLTHFDLYKQIGIDPPRGVLMYGPPGCGKTMLAKAVAHHTTASFIRVV
GSEFVQKYLGEGPRMVKDVFRLAKENAPAIPSRHLLVQSQ
>TRINITY_DN1789_c0_g1_i1.p1 type:complete len:393 gc:universal TRINITY_DN1789_c0_g1_i1:1193-15(-)
MTIDTSNRDKALSDYSKKLIEHREVEARLKQVREQLKELTNDYDKSENNLKALQSVGQIVGEVLK
QLTEEKYIVKATNGPRYVVGIRRGVDKTKLRQGTRVALDMTTLTVMRMLPREVDPLVYNMSHED
PGNISYAQIGGLSEQIRELREVIELPLINPELFQRVGITPPKGCLLYGPPGTGKTLLAKAVASQLDCN
FLKVVSSAIVDKYIGESARLIREMFAYARDHQPCIIFMDEIDAIGARRFSEGTSADREIQRTLMELLN
QMDGFDVLGQVKMIMATNRPDTLDPALLRPGRLDRKIHIDLPNEQGRMDILKIHAAPITKHGEI
DYEAVVKLSDSFNGADLRNVCTEAGMFAIRSERDYVIQEDFMKAVRKVSDNKKLETKLDYKPV*
>TRINITY_DN47849_c0_g1_i1.p1 type:complete len:689 gc:universal TRINITY_DN47849_c0_g1_i1:176-2242(+)

MCGIFAYLNYGVPRSRRYILETLVNGLKRLEYRGYDSAGVAFDSEETLKNHECGISIVKTKGKVQSL
NDEIFDNDARAFEAELSYHLGISHTRWATHGEPSPLNAHPHRSSDDNEFVVVHNGIITNYKDIKL
LLAKKGYHTESETDTEVIVKLLKYMYDRDQESDNEKRLNFPELVELVIHQLEGAFALVFKSTHFPN
ECVATRRGSPLLIGVKSNKPIVVDQIPVMCTENGSPGDRTPNGTSIFDSGASAIKANFCVADDAAM
EYYFASDASAIIEHTKRVIFLEDGDIAWSKEGNLNIARMNNSTPAIRDVQTLQLEIQQIMKGSYSTF
MQKEIMEQPESVFNTMRGRVNFEKRKVLLGGLVSHMDYIKRCRRLIFIACGTSYHSAVATRQLLEE
LTELPVMVELASDFLDRQTPIFRDDVCFFISQSGETADTLMALRYCKSRGALLVGITNTVGSSISRET
MCGVHINAGPEIGVASTKAYTSQFLSLVMFACMMAEDRISKQQRVDDIIGALEVFPEKVEEVLKLD
KKIEELAAHFYKQKSLLVMGRGYQFATCLEGALKVKEITYMHSEGILAGELKHGPLALVDEDMPV
LIIALRDKTYDKCMNALQQVLARKGQPIIICSKGDEETKKLGNFCIEIPEVPDCLAGILTVIPLQLLS
FHLAVKRGFDVDFPRNLAKSVTVE*
>TRINITY_DN1989_c0_g1_i1.p1 type:complete len:546 gc:universal TRINITY_DN1989_c0_g1_i1:150-1787(+)
MRGNNRGSGGQRKSFDGNRTPNNNSNRGNYGGNKRNWSPSPYKKPQETPPKVSKDEPKHLTP
PVASMSKPTQKNTPDVIITKEVDIPKAQPDFMRHNDPVGKVPMNMKNDMAPPVMQGNPNNG
QGNNELENTKKEEKAPVNRVKYTGREGEKKFSGRCRLFIANMNNGTTEEDLRELFGQFGETGEV
FVNKEKGFGFIRFDFRHNAEIAKSTLDKTEFKGRNIQVRFATHASALEIHGLDRFASNEFIEKAMSI
FGKVERAVVVCDDRGRSKGYAIVEYEWKKTAQKVLDRFKGEMFVLGRLPKPVFAKPLSQVDEEE
GVREEDLERLQGIEGEREFPPRFISPSSFEYTWAKKWRDLYIEEEDKKARLDQELRDSRYKLELEME
SALQEQEALKMREGLIIELRSRQEELRRLEEGLMRRQEITARITRDQDDRKQEFNRSRDESQYPME
GMADTNQSDYNIQQQTEFDTHQGNFDVNVRQDLMHQNANIPISGVAGFPQHVVHRMPHGIIQ
AGGKPLPPPPAIPGIHLSNVYPYIDEALPFDE*
>TRINITY_DN3271_c0_g1_i1.p1 type:complete len:399 gc:universal TRINITY_DN3271_c0_g1_i1:218-1414(+)
MTRIIWHKIKSELIMPYLDLDCKYYDLGLPYRDETNDQVTVDAAEAILKYNVGIKCATITPDEERV
EEFKLKYMWRSPNGTIRNILGGTVFREPILCDNIPRLVPGWTSPIVIGRHAHGDQYKAQDLVAPGP
GKFEMSFTPLEGSEKVTVNVFEFKENGGCMMGMYNTDDSIKDFAHSCFNYAIGKKWPLYMSTKN
TILKKYDGRFKDIFEEIFQDQYKSQFDALGIWYEHRLIDDMVAQCLKSSGGAFVWACKNYDGDVQS
DTLAQGFGSLGLMTSVLLCPDGKTIESEAAHGTVTRHYREHQKGNATSTNPIASIYAWTRGLAHR
AKLDGNPELEKFSNSLELACVKAVEEGNMTKDLAGCIYGLKNVKREHYLLTNEYLDAVRDKLLQ
LWTPPSQV*
>TRINITY_DN1907_c0_g1_i2.p1 type:complete len:210 gc:universal TRINITY_DN1907_c0_g1_i2:53-682(+)
MDMDTTFPLVISLLFSILCCSFAVELTFELPDNARECFHEEIHNATKCTLEFQVVTGGHYDVDVSLK
DPHGKVLYDEKKKQYDSHTFEATVPGEYVFCFSNEFSSFTHKTVYFDFQSGDEPPLAPGMGKHHT
ALTLLESSAVTIHESLKVIIDYQTHHRLREGTSRDMAEYLNERVQYWSIGQSVIVVAMSIIQVYILRN
FFSEKRDRL*
>TRINITY_DN651_c0_g1_i1.p1 type:complete len:408 gc:universal TRINITY_DN651_c0_g1_i1:122-1345(+)
MSGHQGGVKRSNDGGNASGGGGPKKMRGDNVTFRFLLLSKHAGGIIGKGGQTIKRLRTEYSAT
VNVPDSPTQERVLSIGASQESAIKVLCECLPLFHEPPYSVGGNHQNQGKYEINFLVHSSQVGGIIGR
GGFKIKELREATNANIKVFQECLPNSTERVVAISGNEDEIVNLLHKILEIMQEAPIKGNIALYDPSQE
AWNGPNDNFGGPPMNMGMGGGNRGGMRGGMRGGRGGGAGVGGNQGGRGGFQGGFQGG
QGGPGGPGGPGGPPGNFGGPNNNRGGFNGGPMGGNGNFNDDYRNNQNFYNQGPGNQNQG
AAPGGGTGNDFSIGDSQTTQVTIPNDLAGAIIGKGGERIRNIRQRSSADIKISDPIEGKSDRIITITGT
QEQIQYSQFLMQQSVRQYSGKPMK*
>TRINITY_DN165_c0_g1_i1.p1 type:complete len:598 gc:universal TRINITY_DN165_c0_g1_i1:147-1940(+)
MTFHTGFSYCICLAFIFTIVLGSESQSEASQAVEYVKPSLDNLDYIAEPFHSLEEFQSRWVLSSAKKD
GVEENISKYDGKWAIVEPKENPLIGDKGLVFSSEARHGAVSGKLKTPFNFEDKPFIVQYEVRFQNK
HECGGAYIKLLADAPELSLDTFNDKTEYAIMFGPDKCGGDSKLHFIFQHKNPLNGKMEEKHAKK
PSGDFSNIFDDQKTHLVRLIVKPDNTFEIYVDKRRVNEGSLLHNFEPPVNPPKEIDDPNDQKPDD

WDEREKIPDPDAVKPDDWDEDAPKQIEDPNAKKPEGWLDDEPELIPDPQTERPADWDDEEDGE
WEAPQIDNPKCKEIGCGEWKPAVIDNPNYKGKWTPSLIANPKYKGIWSPKKIANPEFFEDNNPFA
MKSIVAVGFELWSMQADILFDNLLVADDLSLADQWTLQTWDLKHTQEVNKEPGVVGGLWNNI
QEATQERPWLWVIFVVLVLLPFVLIYFFCFGGKEDEYAIRKKTDEPTQDDDLVENSEDEPVEDKKES
DPEATEESVDSKKEDEESGKENEEITDEKTKSEVSPNDDNKVDTEKKLSEDSEDSKKEEDESPSPKT
RASKRKTRKE*
>TRINITY_DN1319_c0_g1_i2.p1 type:complete len:134 gc:universal TRINITY_DN1319_c0_g1_i2:86-487(+)
MVVPSAKRKIVKKRTKKFIRHQSERYKTVKQNWRKPKGIDNRVRRRFKGQYLMPSIGYGSNQKT
RHLMPDGFKKFVVHNVKELELLMMLKGTHAAEIAHCVSRRKRIEIVERANQLAVKVTNATARV
RSEENE*
>TRINITY_DN1339_c6_g1_i1.p1 type:internal len:570 gc:universal TRINITY_DN1339_c6_g1_i1:1-1707(+)
GMFFQDGLTGSAWGDWALYVDSPLRFFGGGEASTYKTFDPATELGVQEPVGFWDPLGFASDGDE
ATFKRRRSVEIKHGRVCMAASIGYIVPEYYKFPGFLSPSEGLQFADVPNGLAALSKVPALGWVQIIF
FAGIVEGAFGVTDDYRNGTPGDYGWKVITSDDPAEKEKKLSAELANGRLAMVAIIGMFFQDGLT
GSAWGDWALYTASPLRAFESELGVQAPVGFWDPAGYTNDGSMENFKRRRATEIKHGRVSMLAC
MGYMTPEITGKLPGYLSPSMKVQFADIPNGLGALSKVPGVGWAQIFAYCAYCELSQDQSAASPGG
AGDFGFKAITSSDPATLTRKLNAEIANGRLAMMAIIGMFFQDGLTGSAWGDWALYVDSPLRFFGG
GEASTYKTFDPATELGVQEPVGFWDPLGFASDGDEATFKRRRSVEIKHGRVCMAASIGYIVPEYYK
FPGFLSPSEGLQFADVPNGLAALSKVPALGWVQIIFFAGIVEGAFGVTDDYRNGTPGDYGWKVITS
DDPAEKEKKLSAELANGRLAMVAIIGMFFQDGLTGSAWGDWALYTASPL
>TRINITY_DN123_c0_g1_i1.p1 type:5prime_partial len:1231 gc:universal TRINITY_DN123_c0_g1_i1:2-3694(+)
RGGFRPFGTGLMGSPMLGPYAFGPARSGALGGFGIAPLVRPPTRGGAAGANRGSRSNSNRGGKK
TTPVKNNKSTPEAKKDNNINAKNDKKEDKKDEKKDDKKDEKKDDKKDDKKGGKTPTGKEIKV
EPKDDMKNIKKNFFKDPKVEPKNEKKPDTKAVTVKPEIKKESGPQNVTLDKFNSELHLSIDADGL
SAGNMKGEGFQYMWGGVRGTHGVKKGKVCYEVYLLENIKVHCPGENNPHVLRVGWSVDNSD
YQLGEDKLSWGYGGTAKSSYDSKFLDYGKKYTVGDTITCYIDFESNPKAIFFMLNGEYLGVAFRFG
NELGDKAVFPHITSKNMRFRVNFGKEKPKHHVTNGFRMMQDIPVDQLVLPSPSPKTPKEAEIIM
MVGLPACGKSFWCEKYAKENPDKKYYILGTNNIIERMKVSGLTRKRNYHGRWEMLIKRASLVLN
KLFTVAKNNPRNYVLDQTNVYFTARRRKMESFAGYKKIAAVIINKPDVLASRTEKQQKIEGKMVP
EGAVMEMKFNFTLPEKGPSFNEVWYLEETLPKAQELAANYRREGSNFKTKSKRKPESSPSQPESSP
SSEKKPKLDPKIISRGFDKPFSKPNTNSPQQSTFKAPSPAQNNANKTPNQDRFSKNEKSTSKVETNS
DNSRSKAGNFNKGHDGNKSNERPFFSNTPDRGNASNRGNDSNRGNSSDRGNAPNKGNSSDRG
NAPNKGNFADKGNAPNKGNSSDRGNAPNKGNASDSANKNSKNEKPKEESKKTPKRAIRGTLD
QLNSIINSEARKTENNSGNIRDSAQSGKNEKMGSLMDIDRTPQRESDSYQNLQSRNRDALSNQG
GKNKESQSAGLMDIRNVPQGNQDYRGKAGILDSSRNTSQDYRGKPGILDSSRNTPQDYRGKPGIL
DSSRNTARRNEYESPVGRGNNSYSDSPARGVSTPRGRASFNDSPARGARGGRGQSDISRSGSLAKS
QGSGGNRGGRGRGRSKHLGDVNKYDPVGNMGGYYSALKALPSVKDLIGNRSSFSSYSTRIDEPSR
FDQLYSSYEPYSFGRTQNANDSRSLNYSFGQTDSYSSLERDGGYRDSQNSKDFLGSYQQRKDSSYS
DNRGSQGGQRGYQDSSYNADGLDAKDQYQNDDYQESYSSQKDKSSYDQNTQSSRSYNQETSYE
ESSDYGRSRNQEQDEYARSGTYERGDTSYGTDDANRSSRRYEDDLQESEPMDGNYSTQDYGRSSQ
SSQRARIW*
>TRINITY_DN15847_c0_g1_i1.p1 type:5prime_partial len:166 gc:universal TRINITY_DN15847_c0_g1_i1:3-500(+)
NRQGATQYKMSEQTERAFQKQPTIFQNKKRVVGKESKGKEQRFSKSVGLGFKTPREAIEGTYIDK
KCPFTGNVSIRGRILSGIVQSMKMKRTIIIRRDYLHFIKKYQRYEKRHKNLAAHMSPCFRDVQLGD
LVTVGQCRPLSKTVRFNVLKVNKSTSAKKSFEKF*
>TRINITY_DN135129_c0_g1_i1.p1 type:internal len:155 gc:universal TRINITY_DN135129_c0_g1_i1:1-462(+)

SSFSYMSTSPSRSMDISASSDEALTPGGTLIIRDNVDNRTSFYERLQEKDSFIAETSSALNTPQNSPRP
STTTPLPRTPQQDRTPPKRSPLVRMKSDSSDIEKRAKFSKDNVSSTFRPVSMLNLSVFASNRPKRLH
TGSESELSEHVPLSTHRR
>TRINITY_DN240_c0_g1_i4.p1 type:5prime_partial len:397 gc:universal
TRINITY_DN240_c0_g1_i4:1327-137(-)
LKAAAAFSGVSPFALVQPRARVPSRSRVAMVRRSPLPVLGLAGAACFFARSPCFIPGGPLRRQPWA
AAAGSAAVLGATPALADPIGDAAKKLADASYPFAKEVDWNSGIYLQAPGKLAPLEALKAIDKMI
VLGSQADPKLLKAAADAHHKAISSASGPTGMTSKADWEAVNAALGRVFASVPESSVMDVYNAV
AKITDPGVPAYLKSKVNGADAEKAYAAFLEFKDVVKANQVSSAAAAPVPAAGGDKIGAAAQAL
ADQSYPFLKSVDWTSDLYLKPLPGVSAAKALKAVDKAIVMGSAMDGNLLKAAASAHHQAIGSI
DAAGVTSAADYASVTAALGRAIASAPTSKVMDVYNAFAGIVEPSVPNTLFSKVDPLAANAAASA
FYEFKDVVKAAQR*
>TRINITY_DN240_c0_g1_i4.p1 type:5prime_partial len:397 gc:universal
TRINITY_DN240_c0_g1_i4:1327-137(-)
LKAAAAFSGVSPFALVQPRARVPSRSRVAMVRRSPLPVLGLAGAACFFARSPCFIPGGPLRRQPWA
AAAGSAAVLGATPALADPIGDAAKKLADASYPFAKEVDWNSGIYLQAPGKLAPLEALKAIDKMI
VLGSQADPKLLKAAADAHHKAISSASGPTGMTSKADWEAVNAALGRVFASVPESSVMDVYNAV
AKITDPGVPAYLKSKVNGADAEKAYAAFLEFKDVVKANQVSSAAAAPVPAAGGDKIGAAAQAL
ADQSYPFLKSVDWTSDLYLKPLPGVSAAKALKAVDKAIVMGSAMDGNLLKAAASAHHQAIGSI
DAAGVTSAADYASVTAALGRAIASAPTSKVMDVYNAFAGIVEPSVPNTLFSKVDPLAANAAASA
FYEFKDVVKAAQR*
>TRINITY_DN11643_c0_g1_i1.p1 type:complete len:187 gc:universal
TRINITY_DN11643_c0_g1_i1:1748-1188(-)
MPAKGKLTQEEEVLLQDFSRTVSKKSQALFYGNALMISVLPLWLFWKVHLMDPMSYAALFICGTL
SAAYLVAFAYKNTKFALKHKVAQKREVAITREVNTELDSRPETKKLSRKEKDDRILWKKNNVADT
EATTFSIFYTNTLYLFIIIIASFYVLRSFSPAVNFTLSTGMAAGTVALLSTGTQSS*
>TRINITY_DN333_c0_g1_i1.p1 type:5prime_partial len:384 gc:universal
TRINITY_DN333_c0_g1_i1:3-1154(+)
IFVKQIILSYRSRALITAKMDMRQLSPFIFTVIGFLLFITQECNAQRNNFCSHNGCRGIPLQTVPYLA
RRVSLKASRKGQYPDNIMRIATWNISGIPVDRAVVNRRRRRPTGLCLSILKMGLTLFSLQGVDSEQ
LKGLQQCLRSHVFLPNTLIGDGNLIGFRRQRRLVKHAAGQFYLSETPFERSKLRGASDYATCTWA
KLSFANVVGKRNETVETAYSKLLGLLKHYSNQRRGSSRRVTRLRKIYRYFTFYTVNVAKVDEENKA
IAKEQMKICLSHLYNDIMERRRYPIIVTGRLNWESSFTTSNNVRTKNENFNLKNGMDDADMTYY
TFSNIDKERNTKELKDYVTYNGFDSLLTAVLVNRNSERKKRNINRPVVSFLYHS*
>TRINITY_DN1744_c0_g1_i2.p1 type:3prime_partial len:141 gc:universal
TRINITY_DN1744_c0_g1_i2:422-3(-)
MPLSQDDWIKALSHISDADKLQATIKGSFYGSLGIGLLAMVCSVILGPFGLLIGGISGSIIAYRYMKG
SYKPLSSVLTSLNPEQRALFFKEIENIRSQITVQDYLELILLLQGHGGLLMKKELLKSTFEFVKKNFN
MDVP
>TRINITY_DN6390_c0_g1_i1.p1 type:3prime_partial len:1941 gc:universal
TRINITY_DN6390_c0_g1_i1:5820-1(-)
MVIAPKCKFSIFSYLVFGILSYNWNELDASTNIELVDGTGVLLQRDVDNVLNDAVVQKEYQQNIK
HKPVDKRCLGEPFYQGLCKEKLEDAIQQNITEGGCHNLLVKIQKDECSNGPKTLGGKGYCNFICS
QEICNSIYDIDKCVSKRLVHHFNKDKNECEEIMQADCGSESFTSSNQCQKRCIGNSDDKRETKVY
CDQFCLNGGKCIGEVYCQCPPAYHTATCGLSYCNPSCGVGGKCVKTGEGKYGCQCFPGYKGVK
CDKEDSKNMPVVKGLCLSAIKSVLTSPNTSFHDKPEEEKNYCLKYLFDLESNCDKQRPFVNYTEE
QCNHFCASDICDNIYSNDCGSIEERYGYVSSTGRCSKFLYNGCLGNTNNFKDKAACDTKYSPING
LMTSVALLRPLNHTCYIRGKTNFITFDGKVYDLLSGCSHYLVHDSDTTSDFNVILHNDPKVNYNE
KSKRKLEITADGKNVLLNQKVSSDRFFVQVDGRSVPLPYNDVPKIRQAGRYILVLTGDGVSIAFDG
YEDISITVNDYYRTGDGGDHRLNGLCGNLDGNSHNDWLDFTGELRENVRDFAKVYQTDRSTCK
MDNLSLKSLVYNPLHNDQEVFERAERHCDVINTDAFKPCHSVVSPEQYKVLCMQDYIKCNDDK
RDDCVCSSLSFYSRVCHKYGKDIKWRRANLCPITCQRNMKYYECGSACPKTCDNLITPTKCQTDC

VDGCHCPPDLYYDKSTENCVPRNLCSCYRDGIAYKHGSVRSFHCEECTCNGGSWECNKKDCEAT
CDIAGDPHIKTFDGLNYQMYGKCSYVLVDHCHYGNTGIKEFEVIVENKNCNDDALYTSCVRHLS
VNILQTNTRVSLDTAKDNNGTVLPVAFVNGKEGKEFRSKDYTITSVGGRNIIFQYKVNQLKVSWT
GHNAYVTVGHQFENQTCGLCGTYNFNKQDDFHTRSDTVEKSVTAFTQSWVYQSSDKVQDPSKC
HTAKWETSEKPCQIYTNKAQYAKTQCSIIIKPDGPFEDCHSIVPPGEYYSMCQDDACKCDDCYCH
IVAAYAKRCTDLNIRVGNWRDETLSCKQTCQAPKVLKQCNAIDSLDDDLSESLKCPSTCYELSQN
DTSICQSRGCIEGCYCPEGYFENEQNECVKQDQCSCYHDGKVYRHDEIRKDRCNECVCKGGRFE
CTDFDCVAHCASKGLVYSDCGKTCSNFNFNPQDSKCQAGCFCPAGKVLHENGTCVNPQTECQC
KEGDQFFDAGEVSPSDCSKVCNGDRSWKNLPGKDAKPQYDCSAFGQMHYRTLDGRMFKFSGG
LCPYVLLTDCRQDSATGFCDLTKANVNIHVRNLRCVNSYEAYMCKEVTLDLRLPNGEMAEFVLM
QNKIKMKTGLVTKVFDKGSYPQPKTAVVDGVEIFKSGLYLVVRAYHLNITVKYDRQNRVYVVPGR
EYKDAGKLAGLCGNYNGLDDDDFRRPDNSNAENELQFAKSWQDSNSACPDPSESDTCSSNKER
LPWAQKGCNVMKTGNFTACHKTVSPEPFIESCQQETCLCKSGGDCACFCSAVAAYVQECNRHG
VPIYWRRDGLCKLDCCDPCGHSYGYYTDNHVPNTCGVKDKVCKENDRCCKGAVVEGCGCPPG
TYDDGIKCQNETTTNVIPCSPSSSIVPSTSISVPSTIIEPVSSSSPSPMTSSVESQSPSPSSSVVSPTPSSSII
QSMSSSEIESISTTSSSFVSSIESSVQSSTSIVFPVVSSNSGESSVSSSMLPSTSWVGIHSVSSSMGESSVST
NVLPTDYLRTTDVFPLSSSEITYPSSSSSLEPHTPSSSILQESSSSVSKPVGISSVIEPEISSIRYSSMSSQN
VMSSSVSSSVSKPVGISSVIEPEISSIRYPSMSSGSVLESSVSTSFVLPSETVLSTSVFSTNTESPSSSMTLS
TSSSGFVFPSVTSTFQTSSKVLSSLPRSGPAENTVPLSTMFTKTV
>TRINITY_DN8707_c0_g1_i2.p1 type:complete len:564 gc:universal TRINITY_DN8707_c0_g1_i2:2751-1060(-)
MSGFVRQSKYRHVFGSPARKDGCYEGFRVTNCAFDGNFIAANKHFIAVCVEVGGGGAFIIVPVGS
TGRLDVNIPKVSAHKEYILDLQWNPYNDFMLASCSEDGTIKIWEIPEKGLFMNIGDEKALLSLEYH
EKRCTQVLWHPSASNVLMSVSQEPKVCVWNLDEGSVEVEILEHPELIYNATWSQKGDKIGTTCK
DKKIRIFDAREGTLLVQGMGHEGAKPSRVIFTFQDKYLFTTGFSKMSERQYACWNAETIEQLGLEE
LDTANGCLVPTYDADSQLVFLNAKGDSNIRYYELVDGDPYYFYLSTYQSSAPQRGFGFIPKRCINV
NANEISKGYKLHGTKTGYIEPVSFTVPRKSETFQQDLYPDAIGDEAALEPDDWFSGKNAEPKRIA
MSTFFKGKTKPTGAAGGGLKKGGLKGLKAKKEAKTAAKSAAKSEPAKPAPVKTESKDKVDAAP
PAAAAVEQKSSSTAVAAPAAAAPVVVSGVDEKVVKELQNEIKSLKDGDKKLQKEIISLKDKLKDY
DTLRSDIKLLCDAVKKNDERITALESLVAEETDEEETGEGGDE*
>TRINITY_DN22492_c0_g1_i1.p1 type:complete len:1026 gc:universal TRINITY_DN22492_c0_g1_i1:171-3248(+)
MLQKTLIYTLLGCWITLLSHAAIVRKSGGDDFSLKVGKPQKPIGQPKLKDAKKEVKEEPTSDVIPQ
QDENNDTTDRQKHKEQVSPDAVESGMPPRYPLKAMSAEVSAHDNEIGGCRDHPRASRVCKNC
GNRNFAAEHESYTYSYCRKSCRHCDPELSSFHTFWRLRAASQFDPTWMVKEIEFYPKPTEYDNLA
TDPQRAYASSSYPGFKPSYAFDGNNKTSWYPSGWGLHDNNDDWIGFEFPVAVRVHAIRMVLDE
MHRTAAPQRIFVEASDKKDGPFTTKWKISNPTYEVDNVYKLLQCPVLWRKFESDEGSWCFRLIAK
FETWEVARNECTVEGGDLATIATEEEKRFLTDELNLGGFAWIGLNHKDQAYYWQDRANSSYKN
WKAGWSYSEVQKQNLNCVATDQDGFWYNFHCTNKYYFVCKKKLEPQAAPREDLILKAWPYEP
EIPPMIFLYPNITVPVINTKNLEPGVFPDENGKVPEEEEEETTADDLFYEQTVEHLKEKGLKPSEEIS
NDVDKEETDYTQQLNKIALKTQSRDDFRNETESDKLKFNSTEAVKQAKDLEEQKSVNLDQNFN
QYNSTDGVKQQSQPVVAAPSDGPSGNSGDNNTLPRIVNGKPVESPSVNPNNVGKPNTPVASPES
PENTDFAQPGDTTFTTPTPTVQSGDPGNAEKSNPQKATNDQQAGGQQPNQESGSNANQSQVQ
QQSQQQQPQQQQQQQAQNNNNNQNGQQNASNQQNNNNNNNNQNNNNNQQQNNQQQ
NNQNNQQQNNQNNQQQNNQNNQQQNGNNNGGNNNDLPNGLPPVMGGPAPQKIPEPPAP
KEGTKDNDSEKSVGGQADVMPDDAPSNDNDGGDETTTGNEENNDESNNNNNNNNNNMNNV
NNNNDNNQSRTLDKPLPNQGKNSSETVDKATNVGGTNKQENNDFMKLQKAKPGIRTHKIGHK
KSAGLKKTHMKNVVSPLKKVKTEKKINKQVKTVKKIKAVKVKHHKKALKKEVVIHKSEDDEEE
GSGSGDEGDQEDVDDILESITNGLW*
>TRINITY_DN956_c0_g1_i2.p1 type:complete len:267 gc:universal TRINITY_DN956_c0_g1_i2:93-893(+)

MSRYKLFIGHLSPDVRTKDLERFFKDHGFSKTIQEVVVKAGYGFVIFDDRRDADDAIYELNGKELL
GTRIQVEYAKPTGRFDDGRGGGGGGGGGGGGGRGGGGGYDRGSRYDDRGRGSAQGGGYSSKY
GRPYNTEYRLIIENLSTRCNWQDIKDYFRQAGEVTFAKCHREKMGEGVVEFASYKDMKNAIRKL
DDTDLFGKRIKLTYRGGGSRSRSRSRSRSPRRRSRSPSGSPPPRRYSRSASRSPRKSLSRSPRRSPSRSRS
RSR*
>TRINITY_DN3773_c0_g1_i1.p1 type:complete len:504 gc:universal TRINITY_DN3773_c0_g1_i1:168-1679(+)
MAPVVGDLMDLSDIDASKQHNQAVVRDYISQPRLIYKTVSGVNGPLVILDQVKFPKFAEIVTLTLP
DGTRRSGQVLEVSGSKAVVQVFEGTSGIDAKHTTCEFTGDILRTPISSDMLGRVFNGSGKPIDNGP
PILAEDFLDIMGQPINPWSRIYPEEMIQTGISAIDTMNSIARGQKIPIFSAAGLPHNEIAAQICRQAG
LVKHSKGTMDYSDDNFAIVFAAMGVNRETARFFEQDFKENGSMDNVCLFLNLANDPTIERIITPR
LALTAAEYFAYQCEKHVLVILTDMSSYAEALREVSAAREEVPGRRGFPGYMYTDLATIYERAGRVE
GRNGSITQIPILTMPNDDITHPIPDLTGYITEGQIYVDRQLQNRQIHPPINVLPSLSRLMKSAIGEGM
TRKDHSDVSNQLYANYAIGKDVQAMKAVVGEEALTPDDLLYLEFLQKFEKEFISQGAYENRTIFES
LDIGWKLLRIFPKEMLKRIPAKTLAEFYPRDGRKEETD*
>TRINITY_DN32522_c0_g1_i1.p1 type:complete len:235 gc:universal TRINITY_DN32522_c0_g1_i1:147-851(+)
MAAWLDSYRETEQLGHDIMELINERNTLKRSASNTSKVQSSIRIKMQQYSSGLQRLKDLLLRDSSS
YNVTQQELQRRQNQLDVLVSKEQQLNQAFKPTQSNYDRSNLFGEKYGDDLNGRYGGASASNGL
DDDLTPQNFRQQQEQILKEQDKGLDSLHEVIQRQKMMGKAIGNELEYQNDLIDDITKGVSRVDN
KVNATERKVKKVHRKTGSCAMIVVIILLLIAILVVAVFPTN*
>TRINITY_DN1130_c0_g1_i1.p1 type:complete len:317 gc:universal TRINITY_DN1130_c0_g1_i1:1199-249(-)
MDSSQRILVSFFCLIGLAYSASWNYDEGAATGPDAWQNSYPKCSGKSQSPIDIITADVTYDELPTIQ
FGSNWDMLLDNSSLELSNNGHTAVVNVKHPTQITTTQGGKTYILAQFHLHWGTTDDTGSEHKI
DGKFYPAEVHFVHYNSKYADLGTAVPKADGLMVLGYFIKVDNSALTSSFLHLIMEGLDDVVYKD
EKTSLPANGRSLYDLFHPFSTTKYFHYSGSLTTPTCDESVAWNVMHPENGLTITSTMMAKLRAMS
QNKRGEAMKVQSGNYRPVLALNGRSVRRNFDKSSASSIGSLLSITIPMLAIAIKLSS*
>TRINITY_DN116_c0_g1_i1.p1 type:complete len:297 gc:universal TRINITY_DN116_c0_g1_i1:72-962(+)
MGFVKAIKNKQYFKRFQVKFKRRREGKTDYYARKRLVTQDKNKYNTPKYRLIVRFTNKDIVCQIA
YAKIAGDVIVCAAYSHELPRYGVKVGLTNYAAAYCTGLLVARRLLHKLNLHETYSGQTEVDGDEY
NVESVDDGPAAFRCYLDIGLNRTTTGARVFGALKGAVDGGLEIPHSVSRFPGFDSESDDFNAEVH
RNHIFGKNVGDYMRALQDEDEDAFKRQFSKYIKEGVKPDNIEGMYKKCHAAIRADPAHKPSGK
EKFKGKRYGRKKMSFAQKMDSVRQKKEAFKRKMEQDED*
>TRINITY_DN34191_c0_g1_i1.p1 type:internal len:238 gc:universal TRINITY_DN34191_c0_g1_i1:1-711(+)
VRQRSAPAPGGGPPFATMAGRFDPSKLLQAFASRGAPLAAGGAVLFGGAYAVQNAVYTVDAGH
VAIKYHRMSGIGSSTYREGVHFMLPWFERPIVFDVRARPHTMTSLTGSRDLQMVNISLRALCKPDP
SRLPDIYRNLGPDYDEKVLPSIINEVLKSVVAQYNAAQLIMQRELVSRMIRQRLVQRANDFSILLDD
VAITHLSFSSEYEKAVESKQVAQQQAERARYLVLKAQEEKK
>TRINITY_DN2301_c0_g1_i1.p1 type:5prime_partial len:452 gc:universal TRINITY_DN2301_c0_g1_i1:2886-1531(-)
TCVCRNHIAKMEGKSTCQQLLFVITIILSCISALFGKTLVLLDNANIKETHSIFFENLKDKGFELVFK
AADDPSLTLVKHGEYLYKNLIVFSPTVEEFGGSVDVPAITTFVDNGGNVLVAGSSNVGNPIRELGSE
CGIEYDEEKTSVIDHHNFDQSDDGTHTTILTDAKNLIKSNVIIPSKVDSPIMFNGVGMTADTDNPL
VLEILSAESTAYSYYPDMKITEYPLAVGKNTLLIGGQQARNNARIVFCGSLDFFSDQYFMSGAQNA
AQSGSTYFIQSGNMKVAVDVALWTFRHNGVLRVSQVNHHLLGEVDPPPAYTIEQEVVYSIKIEEFS
NGQWVPFQAKDVQLEFFRIDPFVRTYLKSSTDGVYNVNFKLPDVYGVFKFKVDYDRVGYTHLFSS
TQVSVRPKQHTQYERFIVSAYPYYASAFSMMAGICIFSVVFLHHKETSKVKDE*
>TRINITY_DN97809_c0_g1_i1.p1 type:internal len:184 gc:universal TRINITY_DN97809_c0_g1_i1:2-550(+)

LGPDQVDVAGAGRDPRHLPRPLGRRLRVLDRHRGRVRQRHKAEADQDPKLETARLDVRRPGVQ
VRRVRHPRPSSRDQCHRDCHAPGRVEGRAQVDRGRRGLGRRRVEHEVHGAAQPAARGQEKGR
LRAQQILAVAAGQEEAVDVGARNRQGPPSALAAVSRAPHAQFARLAQRDRRVARAKGW
>TRINITY_DN413_c0_g1_i4.p1 type:3prime_partial len:463 gc:universal TRINITY_DN413_c0_g1_i4:1388-3(-)
MVSLSVTCLSLLLFVTVNAEESDHCGYDEKSWYKAMGWCYHSGSRLLLGDFNGDKRTDFFCHD
SNGKNWVAVSNSDGSFGSTTWQDSKERWCASPNILLTGDFNGDGVTDILCHGTATGKKWIKYFN
KYGVPSGKSFYRAMSWCNHKGSQLYIGDFNGDKRDDLLCHDTSGRYWTSYAGTDESKVFHHTS
WYKALEWCRHATSKLYLGDFNGDKKTDVLCTDGKGYKWIMYAKTSGNLFTGTDWHTNLDWC
HESSTVFLRIGDVNGDGKDDLVCHSTAGNSWVAYTQPKGGFGNFCHLAKWCAAGKESFFLGYF
NQDSRADILCHDTKYGKKWIRYAECSFAEESDHCGYDGKSWHKAMRWCYHSGSRLLLGDFNG
DKRTDFLCHDSRGINWVAVSNSDGSFGSTTWHDSKERWCTSPNILLTGDFNGDGVTDMLCHDY
KTGKKWIKYFNKHGVPSGKSFYR
>TRINITY_DN55_c0_g1_i1.p1 type:5prime_partial len:104 gc:universal TRINITY_DN55_c0_g1_i1:3-314(+)
TGLHTDFPDIPFASPNVPFAVDECFPGTKECVTMEYEYLYRFESAIATPNNEFKLPFVQKMCNRPYI
PCNAWCTLFSVRQTIGDCKATCCEGDNCNAGGRDEL*
>TRINITY_DN104063_c0_g1_i1.p1 type:internal len:181 gc:universal TRINITY_DN104063_c0_g1_i1:542-3(-)
PLPLLLRLGVDLDRVLGVAAVRRVVRAGVLAVTHEVGEYLVRREALGREPRLHGLGDRRAGLAL
HRDLVAPPREEACQRVPRLVVAPVRRIHRPRVLPGAQQEPLKRGDGVGVQVAGRRDVLLHSLRG
GFGGVQELLQGHGGLRAPEGRVGAQVLLLDGKALARQPRLERFDRQRLERAV
>TRINITY_DN26679_c0_g1_i1.p3 type:5prime_partial len:108 gc:universal TRINITY_DN26679_c0_g1_i1:1-324(+)
TKSLNRRGWRLAAPAFRARKPTTRLKLPAPGCACAALKPAHLDQATAVGAHEDQDRHQDGPH
QLVVRVHLHATLPLRKLSGQRLLLREGHVAPLARPHQLDLHLRRQ*
>TRINITY_DN2230_c0_g1_i1.p1 type:complete len:249 gc:universal TRINITY_DN2230_c0_g1_i1:796-50(-)
MKLNVAYPANGTQKLLEIEDEIKLRSFYEKRMAQELSVDFLGDEWKGYVVRITGGNDKQGFPMK
QGVLTPGRARLLLSKGHSCYRPRRTGERKRKSVRGCIVDGNLSVLNLVVIKKGDNEIPGLTDRVIP
RRLGPKRVGKIRKLFNLSKEDDVRQYIIRRPIATPEGKPDPKKPKTKAPKIQRLVTPQRLQRKRHVK
ALKRERANKAKLEAATYAKLLAKRHKEAKEKRHQEHMKRRRLSSLRESKSQ*
>TRINITY_DN109115_c0_g1_i1.p1 type:internal len:125 gc:universal TRINITY_DN109115_c0_g1_i1:3-374(+)
NEFLTRELAEDGYSGVEVRVTPIRTEIIILATRTQNVLGEKGRRIRELTSVVQKRFGFPEGTVELYAEK
VATRGLCAIAQCESLRYKLIGGLAVRRACYGVLRFIMESGAKGCEVVVSGKLRGQ
>TRINITY_DN73587_c0_g1_i1.p1 type:complete len:533 gc:universal TRINITY_DN73587_c0_g1_i1:94-1692(+)
MLRQAVRRLSRGTNKRSLLRKSSERLLPACVNACQRFVSTDVTNNQAAAAAKAADKSPAAAVA
VGQIVAVIGAVVDVQFDAGLPPILNALEVQGHGSRLVLEVAQHLGENVVRTIAMDGTEGLVRGQ
RIVDTGNPISIPVGPETLGRIMNVIGEPIDERGPIETKSKAPIHTEAPEFVEMNVNQEILETGIKVVDL
LAPYAKGGKIGLFGGAGVGKTVLIMELINNIAKSHGGYSVFAGVGERTREGNDLYHEFIETGVINL
KDDSSKVALVYGQMNEPPGARARVALTGLTIAEYFRDVEGQDVLLFIDNIFRFTQAGSEVSALLGR
IPSAVGYQPTLATDMGSMQERITTTRKGSITSVQAIYVPADDLTDPAPATTFAHLDANTVLSRGIAE
LGIYPAVDPLDSNSRILDPNVVGEEHYQIARNVQKILQDHKSLQDIIAILGMDELSEEDKLTVARAR
KIQRFLSQPFQVAEIFTGHMGKLVELKDSINGFKQILSGELDHLPEVAFYMVGDISEVESKATRLAE
EAS*
>TRINITY_DN751_c0_g1_i1.p1 type:complete len:227 gc:universal TRINITY_DN751_c0_g1_i1:731-51(-)
MAMKIVLCTLAALMCCAYAQDGITAITDIINPENINTALDAAEKLKNLIHTTSESKIQSREKTQGFE
FLNMKANAQYTRGMKKHHLMRMMNRYLVPFSRSSADMKEVVDTLKEFIEDDVEEHQEQFFGL

MTQGGGGTTEAIFVYYKLRDDGKYNFKRMTFRGTFRLAADLVITHSSKSGFFSSESKDVINWLPRR
GVTAQNVQELLDIIVPKLATVMSEFAPKDSI*
>TRINITY_DN1505_c0_g1_i1.p1 type:complete len:154 gc:universal TRINITY_DN1505_c0_g1_i1:98-559(+)
MSLVIPEKFQHILRVMNTNIDGKTKIMYAMTSIKGVGRRFSNICLKKADIDMTKRAGELSDEEVER
LVTIMQNPRQYKIPDYFLNRKKDIKDGKFSQVLANGLDNKLREDLERLKKIRAHRGLRHYWGLR
VRGQHTKTTGRKGRTVGVSKKKN*
>TRINITY_DN925_c0_g1_i1.p1 type:complete len:261 gc:universal TRINITY_DN925_c0_g1_i1:826-44(-)
MGRVIRGQRKGAGSIFKAHVKKRKGAAKLRSLDFAERNGYVKGVVKDIIHDPGRGAPLAIVAFR
DPYKFKLRKELFIATEGMHTGQFIYCGKKATLQIGNILPVGTMPEGTIICNLEEKSGDRGKLARAS
GDYATVVSHNRDSKVTRVKLPSGVKKAIPSFNRASVGIVAGGGRIDKPLLKAGRAYHKYKVKRNC
WPKVRGVAMNPVEHPHGGGNHQHIGFPSTVRRDTSAGRKVGLIAARRTGRIRGTAKVKDVGGD
KE*
>TRINITY_DN165536_c0_g1_i1.p1 type:complete len:249 gc:universal TRINITY_DN165536_c0_g1_i1:851-105(-)
MSEQEISNLVEQAKLAEQAERYDDMVKYMKELTEKSTEGLSDEQRNLLSVAFKNVVGARRSAWR
IIHSLEEKKGNEGGNTELISSYRKEIEKELKERCNEVLSLIDKYLLPSAQKEGQKGTEGKVFYEKMK
GDYYRYLVEVTTGEDRANLSAQSKEAYDKATETATGLATTHPIKLGLALNFSVFHYEIMNDGKKA
CELAKKAFDDAIAELDSLKEDSYKDSTLIMQLLRDNLTLWTSENDNEENDAQ*
>TRINITY_DN1283_c0_g1_i1.p1 type:complete len:542 gc:universal TRINITY_DN1283_c0_g1_i1:81-1706(+)
MPRWNKKQRAPEVFDSIADGLQKVYKEKLLPLEETYKFHDFHSPKLENSDFFGKPMVLLVGQYS
TGKTTFLKYLIEEEFPGMRVGPEPTTDCFTVLMNGNHSRVVPGNAVVVDTQKRFKSLSAFGNAF
MNRFCCSELPNPVLETMTFVDSPGILSGEKQDIDRGYDFTGILEWFAERVDRIILLFDAHKLDISDE
FRRCIEALHGFDDKIRIVLNKADMISTQQLMRIYGALMWSLGKVIQTPEVTRVYIGSFWDQPLTID
DNRKFFEMEETDLFDDLQSLPQNAALRKLNDLIKRARLAKVHAYIISILNEEMPSVFAKDRKKAEL
IKNLGDIFKRVQMEHKISPGDFPNLERMQEYLKISDFSKFSTLKEGLIKNVDELLGHDIPRLIGMLP
QETDDNMEAQKVKGGVFQNTNGDFNPFAAGAAEGFQLGSETDKWAVEMYGKLEEYNRLFESL
NPVDGRITGKAAKDEMVKSKLAVNTLGKIWKLSDVDKDGHLDKEEFALAMYLIHVKKNSADKI
PEKLFPHLIPPSKKNLIIPNGH*
>TRINITY_DN200_c0_g1_i2.p1 type:complete len:551 gc:universal TRINITY_DN200_c0_g1_i2:123-1775(+)
MITARLARSLAKNAPKNLLKQYQGAGAVAAIIASRHVQTSKAASATAEVSAILEERILKTSSQVQL
EEAGKVLSVGDGIARVYGLKNVQAEEMVEFSSGLKGMALNLEPDNVGVVVFGNDKLIKEGDVV
KRTGAIVDVPVGEGLLGRVVDALGNPIDGLGPIPADNRARVGVKAPGIIPRTSVKEPMLTGIKAVD
SLVPIGRGQRELIIGDRQTGKTAIAIDTIINQKRFNDAQDEKKKLYCIYVAIGQKRSTVAQLVKRLGD
ADALKYTIIVSATASDAAPLQYLAPYSGCAMGEYFRDNGKHALIIYDDLSKQAVAYRQMSLLLRR
PPGREAYPGDVFYLHSRLLERAAKMSDDFGGGSLTALPVIETQAGDVSAYIPTNVISITDGQIFLESE
LFYKGIRPAINVGLSVSRVGSAAQTKAMKQVAGSMKLELAQYREVAAFAQFGSDLDAATQALLN
RGVRLTELLKQGQYVPMDIGEQVAAIYAGVRGHLDKIDPSKVVKFEEEFVKHVKTAHQDLLDLIR
KEGQISEATDAKLKEIVVAFMQSFE*
>TRINITY_DN7671_c0_g1_i1.p1 type:complete len:123 gc:universal TRINITY_DN7671_c0_g1_i1:425-57(-)
MTSKRRNGGRNKNGRGHVRYVRCTNCARCVPKDKAIKKFVIRNIVEAAAVRDLSEASVYDGYA
LPKLYAKLSYCVSCAIHSKVVRNRSREDRKIRTPPPRFGMRREDNKRPGQPGAPGAQRT*
>TRINITY_DN1468_c0_g1_i1.p1 type:complete len:245 gc:universal TRINITY_DN1468_c0_g1_i1:766-32(-)
MADSNKLPGVPETLLKRRKTLEKVKAARAAAQLKTKKHLKEKRKVIFKRAEQYVKEYRSKERDE
IRLRRMAKKAGNFYVPAEAKLAFVIRIRGINQVSPKVKKALQLLRLRQIHNGVFVKLNKSTLNML
RLVEPYIAWGYPNLKSVRELIYKRGFAKVNHQRIPITDNQIIENVLGEQDIICVEDLIHEIFTVGSNF
KKANNFLWPFKLSAPTGGMRLKTNHYVEGGDHGNREDFINNLIRQMN*

>TRINITY_DN4595_c0_g1_i4.p1 type:5prime_partial len:1639 gc:universal TRINITY_DN4595_c0_g1_i4:5857-941(-)
QCGVNTGTQVVYRRCNAALCKYQGNDYTRNQEVNIRTQACTVACDYTPWQNTQSCTKTCDGG
VLVQTRTERAFNKIETRNGNTPCNTQACAQELGPKQLRTCNVGQCGVNTGAQVVYRRCNAAL
CKYQGNDYTRNQEVNIRTQACTVACDYTPWQDTQSCTKTCGGGVLVQSRTERAFNKVETRNGN
TPCNTQVCSSELGPEQLRTCNVGQCGVNTGTQVVYRVCNAALCRYQGSDYTRNQEVNIRTQAC
TVACDYTPWQDTQSCTKSCDGGVLVQTRIERAFNRVETRDGTTPCNTRSCASDFSAERLRSCLNV
NQCGVNTGTQTVYIVCNGNFCRYQGREYNINEEVNVRQQNCTIACDYTAWEDTTICSKICDGGV
LEQRRLERAFNRLERRNGTTPCNTRACDSGFSAVLSRNCVNVNQCGVSNGTQLLYRVCNGEPCIL
QERSYKINDVVVVGSRACSVPCNYTEWRDTTSCSKTCGGGFFFETRTEQAFQKTESRLGKTLCNE
NSCVKDNFGNWTEWTACSKTCGMGMRYRSRECLKGFECIGLIEETETCKLGCRTETCPMGRPYP
GDCNFEDDCGKSWNQVNSIQRNAFFIVTSKDLAHQGLLPNMETTFNTKDCGRTMKLNSSVAGD
VNYLWTKRMMITDINTDVDDDVAAIDNYFKEENEQCVEFNFAVMANSLDKIGDMQVYLYYLEY
PNASLIYSKKGNTGLKWELVKRSIRVDTNKAFVILFQVNQGQSNVQYGDIGIDGFKVNPGNCQLK
GWSHWGEWKCSATCGFGTQTRMRHCLDKVNRTACVGMDRENKPCPMMKECPMNRDGNWG
QWTEFSKCSKTCDTGTYFRTRKCDSPSAKYNGSLCRVSQTKFNLPVVNDFEIDFKPCSVQQCNNT
FSQWTTWSSCSGTCGPGQRTRTRTCESGKRCSGIFEEIETCYMGCRLEKCPKFTAEIDCNFEDRNKS
DCQWKYYTVSGTSSWSFKTQPKNRLLSGPSGDSTIGCGYFLYYPSQTLSSAYAWGPNIYPTMDSYC
VKFKYSMVTSVNSSMGNLTVIKHFLNRPRKADTPLFFHSGVSQKQWNPMAIDITTDIQRSPFVIMF
KGQSDPNNAGLSDIAIDDYMLMAGKCNKSYSEWSEWSTCSRTCGVGQRTRTRVCLDDDIKVCK
MGDVATEGEACVKPLCAVPVDGNWGQWSAYSQCNARCNGTQKRERFCNNPAPSNGGRMCLL
GDTIFQLRNNYHAQIRPCSIDCGDNDTFSSYKRINDTCHFDCITGRVLQERECEQDTCFDGNTRFS
KGEKQYTQRACSIKEYCETSEYKCREFRSVCSTNTPSSCLDPQNRAVKSCECQVERGCSSMTNPGQ
TVKHKEFDVFYRPCRYVGVCFTDYETDAFSPYEKVNDTCHLSCSNGKVFEERECRQDNCIDGSNT
LTRGAKQYRETQCTTKEYCETSQYSCREISGPCSSNPPSSCQDPANKSLKVCTCQVTTGCASMLTA
GQAVSYLAEETFSRPCKYTGACNANGNTDKTFSPYERVNDTCHISCADGKVKQMRECKKDACV
DGGQNFPRGFKQFKDSSCTPNEFCETAQYSCRELSSACTSNPPSSCLVSTNKAVKMCDCNDPNGC
DSMITPGKKVNQGEKEFLQRPCRYTGVCFTDAETGT*
>TRINITY_DN12629_c0_g1_i1.p1 type:complete len:727 gc:universal TRINITY_DN12629_c0_g1_i1:250-2430(+)
MSDRKRRLDVDGSSSQFKKTREEDMRKQVLSSSFPPSNHYVMTSFKETPPKINPFTQKPYSEKFQTI
LKKRLLLPVWEYRDEFIKLTTQNQCLVLVGETGSGKTTQIPQWCLDLVLLGRRKHVCCTQPRRVA
AMSVAQRVADEMDCVLGQEVGYSIRFEDCSSSRTVMKYMTDGMLLREAMTDPLLDRYGVILLDE
AHERTLATDILMGLIKEVIKQRPDLKVIVMSATLDAGKFQNYFDNAPLMTIPGRTHPVEIFYTPEP
ERDYLEAAIRTVVQIHLCEESQGDILLFLTGQEEIDEACKRIKREVDNLGPEVGEMKVIPLYSTLPP
NLQQRIFEAAPPAKANGAIGRKVVVSTNIAETSLTIDGVVFVIDPGFSKQKVYNPRIRVESLLVSAIS
KASAQQRAGRAGRTRPGKCFRLYTEKAYKSEMQSNTYPEILRSNLGTVVLQLKKLGIDDLVHFDF
MDPPAPETLMRALELLNYLGALDDDGDLTELGSMMAEFPLDPQLAKMVIASTDYNCSNEALSIV
AMLSVQQVFMRPNEQKKAADEAKMKFAHIDGDHLTLLNVYHAFKQNHEEAQWCYDNFLNVR
SLKSADNVRQQLARIMDRFNLKRTSTDFNSREYYVNIRRSLVTGFFMQIAHLERTGHYLTVKDNQ
VVQLHPSTCLDHKPEWVLYNEFVLTTKNYIRTCTDVKAEWLIRTAAQYYDMNNFPNCEAKKVLE
RVIERLKRGK*
>TRINITY_DN131028_c0_g1_i1.p2 type:5prime_partial len:104 gc:universal TRINITY_DN131028_c0_g1_i1:2-313(+)
FDKYHPGYFGKVGMRNFHFIKHGKVIPTINVERLWSLVSEQTRTYYKEHTDKAPIIDVLKAGYMK
VTGKGQLPEQAVIVKARYFTKEAETKIKEAGGVCVLCA*
>TRINITY_DN1272_c0_g1_i1.p1 type:complete len:262 gc:universal TRINITY_DN1272_c0_g1_i1:957-172(-)
MRCQLFLLVACMAIATCSAYDWRNKVQNMLKYAKVNIFDRAAYILGDKRQHQLGKPWFCHKL
DCPAFQVVGNYTNKKDGFEERCYPETAWVMTSKEVDANENDIETRDMFMKLFKYIQGENEKQE
KIEMTVPVFTYSQLKKDDPSKRYISMHFFVPPSQEKNIPKPTRDDVTIMDVKADCYYVRSFGGYV

MSFGSKIMNEEKKIHAALEKNNLDFEARKTMAAGYDSPFRLFNRHNEVWVAKRKTMDSGCCTP
RKSTNVMY*
>TRINITY_DN453_c0_g1_i1.p1 type:complete len:249 gc:universal TRINITY_DN453_c0_g1_i1:944-198(-)
MDIDQLKYEIIELGKQIETVHVAIAVVVLTTILLWIAIRSRRSGDTVLLLGLCDSGKTLLFSLLTAGKS
AATQTSIKENKSRLTMGKKSWAIVDLPGHERLRAKYLYNHKDSARGIVLMIDSVEFPREIRDVAEL
MYDLLANRTIKKNKVSILVACNKQDLTTAKACNVIKSQLEKEINNLRKTRSAALLGIDDNTSSKN
AFIGKKGKDFEFAHVKPIKIEFCECCLKDENGESKTDYNGIEEWMKKL*
>TRINITY_DN59667_c0_g1_i1.p1 type:3prime_partial len:225 gc:universal TRINITY_DN59667_c0_g1_i1:673-2(-)
MGKAKKGQKIDESKFEGKGTDFKCKLIGFVNVPSARGDDICQEAITKLKHLALKLNKEKKEHKQ
KITVNITVKGIRLIDEPTERIEHAHPINRISYICHDNDDKRTFGYIVGTKEGYKLFAIRTMKEAEYITK
TLKDLFQVVFERHQAQKAASDTSEIKGQEQVEEKDDEEKFEVGEEQNEEPTESPLLDWEHGINEQ
IQAMQNDAMSTSGFPDPFGDAQFDSA
>TRINITY_DN16120_c0_g1_i2.p1 type:5prime_partial len:232 gc:universal TRINITY_DN16120_c0_g1_i2:734-39(-)
GFFLKGLNGPPGPPGPPGPSGEPGPPGLPGAPGAPGHVGEDGAPGPMGPPGPPGPGGPPGFPGE
KGDPGVAGLPGSAGLPGAPGLPGPQGPPGIAGPEPITPNFTVICEGEKGWVQCKQYELIKITKAF
WGRDDYETCPKVPAGLTAGRLCETNTANTEAKVNGQCKNEQGCEVVASNIFFDDNSCGNVYKY
LKIGYECIPDESNAVDVLREGGRRKRKRATKDKRSFRDS*
>TRINITY_DN22594_c0_g1_i1.p2 type:internal len:268 gc:universal TRINITY_DN22594_c0_g1_i1:801-1(-)
DFQDPTLNLETAHNKLIYGLGSGPMWWLLLFCGIIESLRFRQLGLDFGKLTLENAGDLDFGKGFL
PKTEEGIVQMKIKELKNGRLAMLAVSGALTQSMAYGVYTFPWTLPAPNAVQSRTALRAVEFKGQS
GPFGALDVAEPKTLELTDRVKAVIKSKGSLDRESYREIFLQPKAETVFAGGLVGSEYHGWGTYQW
DPAGLATRYPEHLPWYREAELKHCRVAMLAWLGLIVPDFVRIPLDDFQDPSLDLENAHNKLIYGL
GSGPMWW
>TRINITY_DN98420_c0_g1_i1.p1 type:5prime_partial len:286 gc:universal TRINITY_DN98420_c0_g1_i1:1007-150(-)
LHKEARSTVINKMASRLLNSLGKVGVGLAVVGGVVNNALYNVDGGHRAVIFDRFRGVLPDVSG
EGTHFLIPWVQRAIIYDVRSKPRNVPVITGSKDLQNVNITLRILFRPVASELPKIYMSLGEDYDERVL
PSITTEVLKAIVARFDASELITQRDNVSLNVQEALVERCKAFGLILDDISLTHLTFGKEFTEAVELKQ
VAQQEAERARFLVERAEQQKQAAIITAEGDAVGAQLLSDAFKKAGNGLIELRKIEASEEIAQNLSG
SRNVVYLPDGQGMLMNLPAP*
>TRINITY_DN1559_c0_g1_i1.p1 type:complete len:311 gc:universal TRINITY_DN1559_c0_g1_i1:221-1153(+)
MAEKKATFKLGFAENFALSGAAAVISKTAAAPIERVKLLVQNQDEMIKQGKLSEPYKGVIDCTTRT
FKTEGLAPFWRGNLANCIRYFPTQALNFAFKDKIKAAFKVSKSDGYALGFGKNIASGGCAGALSL
CFVYSLDFARTKLANDTKSSKKGGSREYNGLVDVYRKTLKSDGVAGLYRGFVLSCVGIFVYRGCYF
GFYDTLKPVLLGADASLFMSFILGYGVTVTAGLISYPIDTIRRRMMMTSGQAVKYKGSIDCATQILK
NEGAMSLMKGAGANILRGVAGAGVLAGFDKFKEVYIAMRKRQMGLE*
>TRINITY_DN1583_c0_g1_i1.p1 type:complete len:314 gc:universal TRINITY_DN1583_c0_g1_i1:1007-66(-)
MSEGIEALQLKEDDVTKFLASGTHLGAQNVDSHCESYVFKRKADGIHIINLRKTWEKLILAARIIA
GIENPADVCVISSRPYGTRAVLKFAQATGATPIAGRFTPGTFTNQIQKAFREPRLLVTTDPQFDHQA
ITEASYVNIPVIALCNTDSPLKYVDCAIPCNNKGIQSIGTMWWMLAREVLYLRGIISRKTPWEVMP
DLYFYREPEDIEKEEQAAALAAAKPEETYQPGEWTQPPQTGDQQPGDWTEPGPIATTTDWGAEP
AVFGSTSKDWAAEPMPASNDEWGGKAADWESKEVATTNADWAAPTTEDWS*
>TRINITY_DN1428_c0_g1_i1.p1 type:5prime_partial len:165 gc:universal TRINITY_DN1428_c0_g1_i1:1-495(+)

HLSLNEQASSGLFVNLKMSDALATITIHKEYGYVVLTAFSSFVMLMYKAIKVGKARKLYNVPYPIM
YASDDNDKAKIFNCVQRAHQNTLEVYAPTMISLLLGGIQHPVLSSVAGMVWILGRIAYAQGYATG
DPSKRMRGTFGYLGLFTMYGCTISFALHLLKVV*
>TRINITY_DN2805_c0_g1_i1.p1 type:complete len:189 gc:universal TRINITY_DN2805_c0_g1_i1:623-57(-)
MGFEKVVIIDGKGHLMGRLASVIAKSLLQGQRIVVVRCEGINISGNFYRNKLKVLDYLRKRMNTK
PSRGPYHFRAPSKCLFKVVRGMIPRKTKRGMEALNRLKVFDGIPPPYDRMKRMVVPSALRVLKLK
PGRKYCSIGRLSHETGWKYKALIESLEEKRKAKAELYYKARKQTEAVRQKAIQQVAAS*
>TRINITY_DN20323_c0_g1_i1.p1 type:5prime_partial len:338 gc:universal TRINITY_DN20323_c0_g1_i1:3-1016(+)
GFWKERLPEIMAAVSVTRRAVASLQRTLQRTISYEATRPSLMIGKDTKVICQGFTGKQGTFHSEQAI
AYGTNVVGGVSPKKAGTLHLGQPVFASVKEAKDATSADATVIYVPPPGAAAAIIEAVEAEVPLIV
CITEGIPQQDMVKVKHVLERQDKSRLIGPNCPGIIKPGECKIGIMPGHIHQPGTVGIVSRSGTLTYE
AVHQTTKVGLGQTLCVGIGGDPFNGTNFIDCLDCFLKDPSTKAIIMIGEIGGAAEERAAEFLIKNN
SGENRKPVVGFIAGLTAPPGRRMGHAGAIISGGKGGAEDKIKALEDAGVVVTRSPAQMGSTLRQI
YDKHYMV*
>TRINITY_DN95987_c0_g1_i1.p1 type:complete len:113 gc:universal TRINITY_DN95987_c0_g1_i1:390-52(-)
MSEGSPQFPGCEGPESMFVKLISSDGFEFIVKREHALTSGTIKAMLSCPGQFEENDTNEVLFREIPSH
VLEKVCSYFCYKVKYTNSSEEIPEFPIQPDIALELLMAANFLDC*
>TRINITY_DN118520_c0_g1_i1.p1 type:complete len:581 gc:universal TRINITY_DN118520_c0_g1_i1:1895-153(-)
MYRLLSVIRKTTQTAKLRQGLSACISTTTHYCSPKEIKTGTDARALMLKGVDQLADTVAVTLGPK
GKNVMIEQSFGGPKITKDGVTVAKAIELPDKYMNIGAKLVQDVANNTNEEAGDGTTTATVLARA
IARSGFEAVSKGANPNEVRKGVMAAVEVVTQGLKDLARPVTTTEEIAQVASISANNDAAIGKLIS
DAMEKVGKDGVITVKDGKTVYDELEVIEGMKFDRGYISPYFINTAKGQKVEYQNVLVLICQSKIS
NVQDIVPVLELANQHKRPLLIIAEDVDGEALTALVINRLKIGLQVVAVKAPGFGDNRKNTLKDVA
ISTGAEVFGDDLNLKLDEVQLSDLGQIGELVVTKDDCLMMKGRGNTDLVNQRANHIHDEIKATN
SDYEREKFQERLAKLVGGVAVIKVGGSSEVEVNEKKDRVTDALNATRAAAAEGIVPGGGTALIRC
AKLLDTFQVPTEDQKWGVDIIRAALFKPCSTIVANTGIESAVVVEKVSSSKDPEYGYDAASGEFAN
LIKAGIIDPVKVVRIALSDAAGVASLLTTAEAVITEIPKEDPSMPPMGGGMGGMGGMGGMM*
>TRINITY_DN7333_c0_g1_i1.p1 type:complete len:192 gc:universal TRINITY_DN7333_c0_g1_i1:47-622(+)
MQTIKCVVVGDGAVGKTCLLISYTTNKFPSEYVPTVFDNYAVTVMIGGEPYTLGLFDTAGQEDYD
RLRPLSYPQTDVFLVCFSVVAPASFENVKEKWVPEITHHCPKTPFLLVGTQVDLRDDAAIVEKLAK
TKQKPISYEQGEKLARELKAVKYVECSALTQKGLKNVFDEAILAALEPPEQIKKRKCTII*
>TRINITY_DN1084_c0_g1_i1.p1 type:complete len:301 gc:universal TRINITY_DN1084_c0_g1_i1:1131-229(-)
MGRPGFGGGGGRGGFGGGRGGRGGGGGRGGGRGGGGGRGGGRGGTPRGRGGRGGGRGGMR
GGKKTIIEPHRHEGVFIARGKEDALVTKNLVAGNTVYGEKKITVEGDETKTEYRVWNPFRSKLAA
AILGGVESIHMKPGSKVLYLGAASGTTVSHVSDLVGPDGLVYAVEFSHRSGRDLLNVAKQRTNIIPI
IEDARHPHKYRMLVGMVDTIFADVAQPDQARIVAINAHHFLKNSGHFVISIKANCIDSTAAPAAV
FAGEVKKLQSEKLKPQEQLTLEPYERDHAVVVGTYRPPPKKKD*
>TRINITY_DN10497_c0_g1_i1.p1 type:complete len:206 gc:universal TRINITY_DN10497_c0_g1_i1:84-701(+)
MEDDQRMSNFGDGNKINMNIPERIVLSAGYDGDDNKDTNRIFQDSSSVFDETPIVNPDQVMGG
MLTPPSVLTIDHSSDIFKVASPNWHTEGDGADQDESVSSLQKYTRLKEEEAENNEDDYSRSSYMSP
QRPITIAGEENHSNSMLPLDDPRAMQKYFISMQRRVTILENELDYQHRVSNVWKYLLLTLTVVNP
FIINYFFGRRR*
>TRINITY_DN36_c0_g1_i12.p1 type:complete len:222 gc:universal TRINITY_DN36_c0_g1_i12:931-266(-)

MFKLSVIALIIATCVFESTAIRCYVGYSAPDLPLAISVRDVECGKQFDHCTTIKYTLGGQAVIQGNC
ARKYGQAMCQFMRNPHKTMTDCSETLCEGDHCNTAVHESEQAERKKVGPFANTLQCYTGLLTE
FPDIPFASADVPLGVDACFPGSEECVTMTYNYIYKFETQVATKDNIFKLPVIQKMCNRPKVNCDH
WCGMLSKNQAMENCKTTCCKGNYCNA*
>TRINITY_DN10940_c0_g1_i1.p1 type:complete len:135 gc:universal
TRINITY_DN10940_c0_g1_i1:1786-1382(-)
MSWDGYIDNLLGHCKDNADKAAIIGIDGSKWTSDASPKAIKVSPAEAKNIGDVFKNKAFQTFQG
SGVIVEGQKYTYLRVLDDKLVFAKKKGEGSLSLQCTNTAVVLAHTIEGKSQGETNKGVAVIAEYLE
SLNM*
>TRINITY_DN1339_c0_g2_i5.p1 type:internal len:362 gc:universal
TRINITY_DN1339_c0_g2_i5:1085-3(-)
NSRLAMVAILGMFFQDGLTGSAWGDWANYVDSPLRAFENELGVQEPVGFWDPAGFTADGNVE
NFARRRQTELKHGRVSMLATMGYITPELTGKLPGYLSPSAGLKFADVPNGLAAISKVPAAGWAQI
VAYCAFCELSQDQSAGSPAAAGDFGWKLLTSSDQGELTKKLSAEVANGRLAMMAIIGMFFQDGL
TGSAWGDWALYTASPLRAFENELGVQEPVGFWDPAGFTADGNVENFRRRRQTELKHGRVSMLA
TMGYITPELTGKLPGFLSPSAGLKFADIPNGLGAISKVPAAGWAQIVAYCAFCELSQDQSAGSPAA
AGDFGWKLLTSSDQGELTKKLSAEVANGRLAMMAIIGMFFQ
>TRINITY_DN118208_c0_g1_i1.p1 type:complete len:422 gc:universal
TRINITY_DN118208_c0_g1_i1:121-1386(+)
MVSGTLYTYNGSHQAYKILITAGYSGAEVKLADFEFGKTNTSDAFLQKFPLGRVPAFEDANGHHL
SETNAITRYLSNDQLKGGSNAIDQALVQQYIDFADHEIQPSACAWTYPTLGFKQYNKQDTEKAM
THIKKCMDLLNNLLLGRTFLVGERVTLADISLCCNLLSLYVQVLDPKFREAYGNVNRWFLTCINQ
PAFKKVLGEIKLCEKMAAFDNKKYQELHPKQQKKATKESKPKEQPKKEEKVAKPEAEEPPPKPK
KIDYFADVPKSDFVLDDWKKMYSNNEVDVSTPWLWNNFDNNAWSFWYCEYQYNEECTVDFLS
MNLIGGFYQRIEGLRKHAFANMLLVKSDKEEHFCLRGVWMIRGKKLAFSLNQDWNVDAEQYTF
RPLDPVGNAEDKKLVNNYLEWEGDAFTEYKVRDGHTFK*
>TRINITY_DN598_c1_g1_i2.p1 type:5prime_partial len:742 gc:universal
TRINITY_DN598_c1_g1_i2:2-2227(+)
RWSGCSKSCGVGVKTKSRECDNPRPSGGGRYCTGERTATEACKIQDCGVDGKFSHWSTWTACSK
TCGGGAQKRARQCNSPPPSGDGKDCAGAREEFKGCSNDFCKGPTGVAKAQEIIERFLENFHKKT
YPDREPSGKFLKAIFAIQKAVAEEFISVDPSVRGKTLEQRLKDLEKFLKVATEGGSIDFARTLLGKGIF
TFMKKKIQEGLPIDDIDKAAEGFLEKLRGDVGEKKMNEILSSQGDATLMFTIDTTGSMKDEINAA
KAIAKQIISATRDYEVDYILSPFNDPRTGPVTHRSSSNRASFVTDIDRLRATGGGDCPELSFTGMLN
ALNQGPQYGSPMFVFTDASAKDDSASNIDSLKAAADLTDATINFFTNLNGCKGAKSVESYKEIAS
YTGGQVFPLKNEAELKKFTNYVKTSLQQDTVITSGGTLNSKSYKRSLYSMQAHDFMVDDGIIMIS
VAISVQKYASQVTILDPNGRRVNPSTTLSLSLVYQISHPPPGQWKINFPSSVGKYGYTVQAVSSSAIA
FAHNFIYQQSVRKNSPPIPLANPLKGVKNMMVVRLGGTKNFDLNSIKIDLVDTSGNVLQKGIGTD
RLDSDEVRHATIDPPSGPFKVRLTGKTKSGYNFERVSQGISRPVSAAMRTNFAGYDFTAKLGKGPY
AAQMLFINRGAHENFYFYAKANYGSIPNYKQTKRVTSQGFVTFTYKAPRQYNLKGKSDRITVTVK
RQSTGEIVSHHTSILLVV*
>TRINITY_DN123_c1_g1_i1.p1 type:3prime_partial len:1051 gc:universal
TRINITY_DN123_c1_g1_i1:49-3198(+)
MSFQRGGFRPFGRGGMMGNSMGGPPFGPRRGGLGGPRFGLNPIVRPPAHGDFRGGYMGGPRGP
RPPFSKDTKYHSPAASRSNVFQKAPPLLETKKDNRVNNEKDDKKEEKKDDKQEEKKDSKTEEKK
DDNKVEKKDDKKDDKKDDKKDDKKDDKRDGKSSDGKDIKEEPIEKSDPKKDEKKDEKKNEVN
KDSKVGSKSDPKPVKEEKPDIKPLIANPEVTTEFATANVTLDRLNSELHLTIESDGFTAGNMQGDG
FQFMWAGVRGTHGVSKGKVCYEVHLLENIKVNCPDEKSPHVLRVGWSADNTDFQLGEDKLSW
GYGGTAKSSYDSKFLDYGKRYTVGDVVTCYIDLDARPRAIFFMLNGEYLGVAYRFGSELGDKAVF
PHVTCKNMRFRVNFGKEKPKHNVTNGFRMMSDVPADQLELPSPSPKSPKEAEIIMMVGLPACGK
SFWCEKYAKENKDKKYYILGTNNIIDRMKVSGLLRKRNYHGRWEELIKRASLVLNKLFTITKSNPR
NYILDQTNVYFTARRRKMEAFAGYKKVAAVMINKPEVLADRTEKQQKVEGKIVPEAAVMEMKY
NFTLPEKGPSFDEVWYLEETMPKAQELVASYRREGSNFKNQAKRKPDGQINPENSPANEKKPKL

DSKPISRGFDKPLGKPDNDLSKQSSTKDSNMKKTDQDRFSKSERPMLKDETHGDSTSSSSGNFIRR
PDGDINKDRGSFGNTSDRGKSFDSGNREERNDRQFPPREDRNTPMKDGRDSLDQRSPFQPSDAL
GMTKDHDDFRLPTQFGKPEGRSSLMDIDRLPRRDSDSFQSPQFRGREELSNRGGGRGRGTPPFRG
GFNDFRNSPQGSQDFRSGPGMRDTPRNTRWDAPGSRGPSEFTNTPGRGGFTPGGRGGPNDFTDS
PGRGGFSPSGRGHSNFNDPSARGSFTAGIGRGGSMLRSQDSAKEREDQLTEDLEELIPPKMRKTRF
SDAPPINKYDPVGNSRVGFNAPEINSSSGNIRSFPPPAGNQGLKDTPSRFDGPPRFDRTNPQNEPP
RFDRTNPQNEPPRFDRTNPQ
>TRINITY_DN60705_c0_g1_i1.p1 type:5prime_partial len:169 gc:universal
TRINITY_DN60705_c0_g1_i1:1-507(+)
SITHPNDVKSTTLLCPHGIKNKCEEEFAERARKEKKLWLMPESLEEYREMMKVACHKELHIPELYL
KGAFQIYVLKQEVYGKVAVALREEENLHLLERNLEKLTKPVLLVWGKEDKILDVACVDVIKSKLP
APPTVHLLDNCGHTFVIERARKTANLMSEFINTIELN*
>TRINITY_DN11097_c0_g1_i1.p1 type:complete len:730 gc:universal
TRINITY_DN11097_c0_g1_i1:2348-159(-)
MVRPQGVTQEHSSKPSTSERNSFVFSALSKSGCFETLPSIYDSIIIQRSHWLHTEGFSMRTSSTGSQQ
TSRRAFYEELQAFSSTIPFYESLSWALLSLSGCLNPVSTPNIREEILADLEGRSVDESREVLLVFATLFD
VYKSYQNSIVVEISAILPGGILRCISLGQTEGFNSYNTVGLINYQPIVYPVGRNVLGRLFNVTGSCID
LYNEVTMDMPFVRSVSTEETHTPQKTTLIKTGANADTSSDSDCVKANALFKECTSHINVKETSLD
HLISLYCGCYMGMTSAPIAPISRLPIEAESSRAPEHMVQKLQECMGTYSGSADPSTPNQIGSKKQPF
WLSKEALESGVSPIHSTPLPISKLRVDLSLFETGIKVVDLLTPYKKGGKIGLFGGAGVGKTVVIMEFI
RNLAIEHKGISIFCGVGERTREGNDLYSEMAEAGIICKDYTTMKPSDPSSNGGPTGSIDQHSVTFTR
SSVTLVYGQMNESPGCRMRVAHAGLTVAEFFRDVFKQDVLVFVDNVFRFLQAGSELSTLLGRMPS
AVGYQPTLATEMGAFQERIIAALWGSITSIQAIYVPADDLTDPAPVTIFTHLDAVSSLSRELAAKGIY
PAVDPFNSTSRMLDPQCLTSEHFRVALEVQQILQRYRELQDVIAILGIEELSEEDLKTVERARKIERF
LSQPFSVAEVFTKIKGIFVPLEATIRSFAMVLSGALDDVPEGNFYMKGGLDTLKL*
>TRINITY_DN1534_c2_g1_i1.p1 type:complete len:547 gc:universal
TRINITY_DN1534_c2_g1_i1:1914-274(-)
MHRVNSSFVKVAERTSKLCGNLGIKNSKIPSSLIARSSSTDTTDGVGESEPGFLEMVSIFTENAHSIT
LDKLLSAKPAPGKRAEDTGVREKRIKGILDIIKPTNTALAVNFPLRRDNGDYELITGYRAQHSHHR
LPCKGGIRYAPDVNLDEVKALAMLMTFKCAVVDVPFGGAKGGICIKKSDYSENEVEKITRRFTVE
LARKGFIGPGIDVPAPDMGTGEKEMSWMADTYAMTLGYGDMNSSGCVTGKPISQGGIHGRMSA
TGRGVYHGIDNFANNEEYMNLIGLKPGLKGKTFIVQGFGNVGLHSCRYLHRYGARCIGVIEHDG
SIMNPNGIDPKDLEDYKLDNGTIVGFPGATDTTEDLMVAECDILVPAANELQITAKNAPHIKAKII
AEGANGPTTPQADKILQQRKRLVIPDLYINAGGVTVSYFEWLKNLNHVSYGRLTWKYEESSNYNL
LSSVQESLEKHFGGNIPINPSSTFLQSIQGASEKDIVHSGLAYTMERSANRIMKTAKDYDLGLDVRA
AAYVVALEKIFNTYSEAGLTFT*
>TRINITY_DN1534_c2_g1_i1.p1 type:complete len:547 gc:universal
TRINITY_DN1534_c2_g1_i1:1914-274(-)
MHRVNSSFVKVAERTSKLCGNLGIKNSKIPSSLIARSSSTDTTDGVGESEPGFLEMVSIFTENAHSIT
LDKLLSAKPAPGKRAEDTGVREKRIKGILDIIKPTNTALAVNFPLRRDNGDYELITGYRAQHSHHR
LPCKGGIRYAPDVNLDEVKALAMLMTFKCAVVDVPFGGAKGGICIKKSDYSENEVEKITRRFTVE
LARKGFIGPGIDVPAPDMGTGEKEMSWMADTYAMTLGYGDMNSSGCVTGKPISQGGIHGRMSA
TGRGVYHGIDNFANNEEYMNLIGLKPGLKGKTFIVQGFGNVGLHSCRYLHRYGARCIGVIEHDG
SIMNPNGIDPKDLEDYKLDNGTIVGFPGATDTTEDLMVAECDILVPAANELQITAKNAPHIKAKII
AEGANGPTTPQADKILQQRKRLVIPDLYINAGGVTVSYFEWLKNLNHVSYGRLTWKYEESSNYNL
LSSVQESLEKHFGGNIPINPSSTFLQSIQGASEKDIVHSGLAYTMERSANRIMKTAKDYDLGLDVRA
AAYVVALEKIFNTYSEAGLTFT*
>TRINITY_DN147947_c0_g1_i1.p1 type:internal len:168 gc:universal
TRINITY_DN147947_c0_g1_i1:501-1(-)
VGKSALTIQFIQSHFVPDYDPTIEDSYRKQCVIDDRVAHLDILDTAGQEEFSAMREQYMRTGEGFL
LVFSVTDRSSFDEIHKFHTQILRVKDKDEFPMILVGNKSDLENERTVSTAEAQELGRKLSVPYLESS
AKQRINVDAAFHDLVRAIRRSNNVEALPIKNKKK

>TRINITY_DN436_c0_g1_i1.p1 type:complete len:116 gc:universal TRINITY_DN436_c0_g1_i1:74-421(+)
MAVSRAAGSASTFQKFANAFRSWYIYAAGYRQLGLRADDLHMEENPDVSAAIKRLPEEDQYLRIF
RIKRALDLSLKHNILPKEEWTKPEEDQYYLTPIIEAVKAERKEREQWDRM*
>TRINITY_DN7112_c0_g1_i2.p1 type:complete len:224 gc:universal TRINITY_DN7112_c0_g1_i2:79-750(+)
MAPMQVGYWTIRGLAQPIRVLLGYTNTEFEDVLYDCGEAPSYDRSCWMNVKESMGLAFPNLPY
LIDGDHKITQSNAIMRYIARKHNLEGETEEERSRVDVMENQSMDFRNGFVRLCYGSTPSDIEQKS
KAYDQSVSVLLGRFEKYIGDRKFFAGDKLTFVDFLMYELLQHHLVYKSSILDDFPTLQAFCKRFEE
VPEIAAFMKSDKCFKGPINNKMALIGSKV*
>TRINITY_DN1378_c0_g1_i1.p1 type:complete len:443 gc:universal TRINITY_DN1378_c0_g1_i1:2151-823(-)
MLRFAVLVVVASIGVSALYDSSDDVVELTQSNFNNHVIGSDDIWLVEFYAPWCGHCRNLAPAWK
QAATALKGIVKVGAVDMDQHQSVGGPYNVRGFPTIKVFGANKMSPSDYNGARSAQAIVDEAM
KVVQRVVSDRLGGRSSGGGGRRSGGSSGGSGNKDDVIELTDSNFEKEVLNTKDMVLVEFFAPWC
GHCQRLAPEWAKAATELKGKVKLAALDATVHGQTASRFQVQGYPTIKFFPAGDKDFSSAQDYQ
GGRTASDIVAWALAKHAENVDPPEILEITSNSVLTENCNEKPLCVISFLPDILDSMAAGRNGYISTL
KELGDKYKSKMWGWLWTAPGLHSNLEQALGVGGFGYPAMAVVNIRKKVYVLLRGSFGIDGIDE
LLKSIAVGRGRTEKLTQGLPTLKDVEAWDGKDGQLPEEEDIDLSDVELDDLDDEPKQEL*
>TRINITY_DN98968_c0_g1_i1.p1 type:3prime_partial len:424 gc:universal TRINITY_DN98968_c0_g1_i1:1269-1(-)
MEYKNILTSCIFLISVITNTQQTSYVNYFNDQSDLLRSFNIPVKPERSFNTGLPASYMKYARTNDFY
HLYARLFNENQDGKQMAFLTKPISFPSNIETVCVYFEYWMHNLGNTQLYIVGDTTDSTQMQNIN
VNNLVNTENRKERLVQTANEPNSCWREVNSCFNVKGKTKFTLHIQLVPPSTGIVSTNGAIALDSVI
VTTDKRIESTACTRRVGRRIKRDVSFTNLVSNPAAIPGMNLIFHNYDASKTTQDFECGDDLGLCGN
KYLGWTLTNQCSSSCGNGFIIESRTYRGQAIIAQYRQNFQQACNRGSCSTGPPQPPGNNLGPEQL
ATCSVDQCGVNTGTQVVFRVCNAALCQYQGTDYSLNQQVTIRVQSCTVACDYTAWQDTSACSK
TCDGGVLVQRRTERAFNKVETRNGNTPCNTQA
>TRINITY_DN164351_c0_g1_i1.p1 type:complete len:212 gc:universal TRINITY_DN164351_c0_g1_i1:694-59(-)
MEDWGEPGEQEAVGPTSIEVQDIKLFGKWNPDEVQVSDISLTDYIAVKEKHAVYLPHTAARYAVK
RFRKAQCPIVERLTNSLMMHGRNNGKKLMTVRIVRHAFEIIHLLTGGENPLQVLCDAIINSGPRE
DSTRIGRAGTVRRQAVDVAPLRRVNQAIWLLCTGAREASFRNIKSIAECLADELINASKGSSNSYAI
KKKDELERVAKSNR*
>TRINITY_DN10907_c0_g1_i1.p1 type:complete len:206 gc:universal TRINITY_DN10907_c0_g1_i1:1010-393(-)
MAKKAYDYLFKLLLIGDSGVGKTCLLFRFSDDAFNTTFISTIGIDFKIKTVEVDGKKIKLQIWDTAG
QERFHTITTSYYRGAMGIMLVYDITQEKTFDNIAKWLRNIDEHASEGVERMILGNKCDMEEKRM
VSKEKGEGIAREHSIKFFETSAKENICVEEAFMQLTQDILRKHQAGATHEQSPSGVVIEKDSHNRS
KGGMNCCN*
>TRINITY_DN138_c0_g1_i1.p1 type:complete len:401 gc:universal TRINITY_DN138_c0_g1_i1:157-1359(+)
MAARFLFITVILIVGNLIGDVCPERCERNWIKIGCFHDRIFPNRPYPFELVNHRDPINNHWDGNLI
NWRKYPDSIEKLACICANKTRTKGYQYFGLQFYGECWSGPSYNFHRDGPSTKCIGNDFKPCDDSS
KTECVGRAFTNYIYKLKDENPKVDGKWSEWEKWSPCSATCGYGERYRERFCSNPEPENGGQTCE
GEAREYEECNHGECQTTCDKEIDFGIALDYSSSVRQVNWNRTLDFVVELSKGFNISPEKVHFGVLS
FSHRTRFDFGLSDKTYWNSESFEKKIKSLEYRWGYTWTDKALKKVKQLIFCEKCGLRKDSLKYLLV
FTDGRSNNVKTDLKTVSDSIKDMGVNVISVGAGHTINHRELEEIATDDNHVFYVGGYAYLGDNM
NAIRRLTCG*
>TRINITY_DN1320_c0_g2_i1.p1 type:complete len:260 gc:universal TRINITY_DN1320_c0_g2_i1:97-876(+)

MRCLSMIFAILLVYLFDQVSSRACRDYVNYQCRKYLCHVPKYHRFVNMYCKATCGLCPVIHGLQ
MSTMSTPTTTATTTTTTTTTKTTTTTTTTTTTTTTTTTTTTTTTTTTAATIVLLSGGKTISGTPEDAS
GPAVRCTDGDVTTLCITTQAIDAYFQVDLGSQKKVSRMEIKNYFHYENQLELKFLVSNDVTFATST
ICDTWDVRSVNEGDVQDKKRDCNSNVSSQTFRYARVQTVNNANEPEHLRVGEFEVYGVA*
>TRINITY_DN164128_c0_g1_i1.p1 type:complete len:166 gc:universal TRINITY_DN164128_c0_g1_i1:117-614(+)
MGPKFDPTAITIVYLRVVGGEVGATSSLAPKIGPLGLSPKKVGDDIAKSTGDWKGLKVTVQLTIQN
RQAKISVVPSAASLIIKALKEPPRDRKKVKHVQHNGDISFETVLDIARQMRERSMARKLEGTVKEI
LGTAQSVGCTIDGENPHDMIDMVNSGEKEVPES*
>TRINITY_DN164753_c0_g1_i1.p1 type:complete len:143 gc:universal TRINITY_DN164753_c0_g1_i1:491-63(-)
MAQSSVQVFGRKKTATAVAFCKRGNGMIKVNGRPLDLVQPVTLRMKVQEPVLLLGKERFAGIDI
RVRVKGGGQVAQLYAIRQAISKAIVAYYQKFVDEASKKEIKDLLVQYDRSLLVADPRRCEAKKFGG
PAARARFQKSYR*
>TRINITY_DN58867_c0_g1_i2.p1 type:complete len:354 gc:universal TRINITY_DN58867_c0_g1_i2:80-1141(+)
MVSLYGDMMFNIENGYLEGLVRGFRSGILNRTDYLNLIQCETLEDLKLHLQSTDYGNFLSNEAGP
LTVSVIDEKLREKLVIEFDHLRIQAVEPLSQFMDYITYSYMIDNIILLITGTLHQRSISELIPKCHPLGR
FPEMASINIASTPAELYNAVLVDTPLAPFFVDCISEQDLDEMNIEIIRNSLYKSYLESFSRFCDSLGGT
NSEVMKTILQFEADRRAIMITINSFGTELNKDDRSKLYPKCGKLYPDGLARLARAEDYDQVRQVA
DCYAEYQPLFEGTGTNPGDKTLEDKFFEYEVKLNVNSFMQQFHFGVFYAYVKLKEQECRNIVWIA
ECISQRNKSKIDNYINIFE*
>TRINITY_DN598_c1_g1_i1.p1 type:5prime_partial len:745 gc:universal TRINITY_DN598_c1_g1_i1:2-2236(+)
RWSGCSKSCGVGVKTKSRECDNPRPSGGGRYCTGERTATEACKIQDCGVDGKFSHWSTWTACSK
TCGGGAQKRARQCNSPPPSGDGKDCAGAREEFKGCSNDFCKGPTGVAKAQEIIERFLENFHKKT
YPDREPSGKFLKAIFAIQKAVAEEFISVDPYVRSKTLEQRLKDLENFLKVATEGGSIDFARTLLGKGV
FTFMKQKIKDGLPIDDIDKAAEGFLEKLRGDLGDKKMDEILSSQGDATLMFTIDTTGSMKQEINA
AKAIAKQIISATRDYEVDYILSPFSDPGTGPVTHRSASERASFVSDIDRLRARGGGDCPELSFGGML
NALNNDPQYGSPMFVFTDATAKDDTDANVASLKAAADATDATINFFTNLAGCNGRRDRKRGIE
SYKEIASYTGGQVFPLKNEAELIKFTSYVKTSLQQDTVITSGGTLNSKSKRSLYSMQARHFMVDDGI
SKLVISVGVQQLASQVALVDPNGKKVYPSTTLALSLIYQVSHPSPGQWKLDFPTSVGKYSYNVQAV
SSSAIAFAHNFIYQQSVRKNSPPIPLANPLKGVKNMMVVRLGGTKNFDLNSIKIDLVDTSGNVLQ
KGIGTDRLDSDEVRHATIDPPSGPFKVRLTGKTKSGYNFERVSQGISRPVSAAMRTNFAGYDFTAK
LGKGPYAAQMLFINRGAHENFYFYAKANYGSIPNYKQTKRVTSQGFVTFTYKAPRQYNLKGKSD
RITVTVKRQSTGEIVSHHTSILLVV*
>TRINITY_DN614_c0_g1_i1.p1 type:complete len:183 gc:universal TRINITY_DN614_c0_g1_i1:194-742(+)
MGQMFGKLFSGLFGKKEMRILMVGLDAAGKTTILYKLKLGEIVTTIPTIGFNVETVEYKNISFTVW
DVGGQDKIRPLWRHYFQNTQGLIFVVDSNDKERVTEAKGELAKMLGEDELREAALLVFANKQD
LPNAMNAADLTKQLALHDLKSRSWYIQATCATSGDGLYEGLDWLSTELKKKKN*
>TRINITY_DN4_c0_g1_i1.p1 type:5prime_partial len:586 gc:universal TRINITY_DN4_c0_g1_i1:2-1759(+)
SMAEIVSVLFFHTMRYKVSDPRDASSDRFVLSKGHSAPILYAAWSLAGLFPESDLMNLRKIDCDLE
GHPTPRLSFVDVATGSLGQGLSCACGMAYTGKYFDKSSYRVFAVLGDGESAEGSVIEAANFASFYK
LDNLVAIVDINRLGQSDPTSLGHELDVYQRRWESSGWNTIVIDGHDVEALCKAFFTAEEPSAKPT
VILAKTFKGRGIPGIEDQMNWHGKPLGAKTDAAIEAIESCIVNKEINGEKVNSRMVVDDAAPTD
MSPITLSEKPNYSPDVKVATRNAYGTALAKLGRTSNKVIALDGDTKNSTFAITFQKEFPDRFVECFI
AEQNMVGVGIGCATRNRTVAFASAFACFLSRAYDQIRMGAISQTQLKMAGSHVGVSIGEDGPSQ
MALEDLAMFRAIPNCTVFYPSDPVSCERAVELAANLRNMCYIRTSRPATVPVYSNDEVFEPGKGK
VIRQSENDTCLLVGAGITLHECVKAHETLKAEGINVRVFDPFTLKPFDNDGLIANAKACHGNVV
TVEDHFPQGGLGETVCGAVDMETGIRVKRLAVNGVPRSGPSSVLLDMFGISASHVVNAVKTAFN*

>TRINITY_DN932_c0_g1_i1.p1 type:complete len:475 gc:universal TRINITY_DN932_c0_g1_i1:1940-516(-)
MGFKFLELLKPFISVLPEVSKPERKIQFREKVLWTAITLFIFLVCCQIPLFGIMSSDSADPFYWLRVIM
ASNRGTLMELGISPIVTSGLIMQLLAGAKILEVGDTPRDRALFNGAQKLFGMVITVGQAIVYVMT
GMYGEPSELGAGVCLLIIIQLFCAGLIVLLLDELLQKGYGLGSGISLFIATNICETIVWKAFSPATVNT
GRGTEFEGAIIALFHLLATRQDKVRALREAFYRQNLPNLMNLLATVFVFAIVIYFQGFRVDLPIKSA
RYRGQYSSYPIKLFYTSNIPIILQSALVSNVYIISQMLSAKFAGNFLVNLLGVWNEQGGPSRSYPIGG
LCYYLSPPESLGQIGQDPVHALLYIVFMLGSCAFFSKTWIDVSGSSAKDVAKQLKEQQMVMKGHR
EKSMIHELNRYIPTAAAFGGLCIGALSVLADFMGAIGSGTGILLAVTIIYQYFELFVKEQSEQGGMS
TLLF*
>TRINITY_DN11545_c0_g1_i1.p1 type:5prime_partial len:452 gc:universal TRINITY_DN11545_c0_g1_i1:1-1356(+)
LTFIESLRLSRFMYSIRSKLKEIHRLKSIRKMASLSSVGDNLKDVTLNGQNEVEENQVVNILHVEAK
DNKGIDYDKLIKQFGSNKVTDAQIEKIEKLTGKKAHHFLKRGIFFSQRDLDFILSQVDKGKPFFLYT
GRGPSSDSMHLGHLIPFMFTKYLQDAFDVPLVIQITDDEKFLMREHLSLEETYRLAYENVKDIIAC
GFDKDKTFIFSDIDYVGNSSAFYRNILKVQRCVTFNQVKGIFGFTDGDKIGKISFPAIQAAPSFSSSFP
QIFGDKSNIPCLIPCAIDQDPYFRMTRDVAPRLNLHKPALLHSTFFPALQGAKTKMSSSDANSSIFL
TDTAKQIKTKINKYAFSGGRDTIEEHREMGGDITVDISYQYLTFFLEDDDRLQQIKEDYASGKMLT
GELKKELIAVLQPLVAAQIERRKLVTDDLVKEFMTPRPLNFKGVSKSMN*
>TRINITY_DN3052_c0_g1_i1.p1 type:complete len:362 gc:universal TRINITY_DN3052_c0_g1_i1:45-1130(+)
MAHLSQEQRNELKKNAESIVAPGKGILAADESTGTVGKRFAGINVENNEENRRLYRQLLFTADKS
ISNYLGGIILFHETLYHKDDNGKPLVELIKELGINPGIKVDKGIVPLHGTNNEGTTQGLDGLAERC
KQYKQDGCTFAKFRCVIKINTPLGVPTDIAIQENAQVLARYANICQANGLVPIVEPEILIDGDHNL
ATSQRVTERVLACVYKTLVDHHVYLEGTLLKPNMVTPGQSCPEKASAEEVGVATVKALRRTVPA
AVPGITFLSGGQSEVEATKHLDAMNKTDLARPWALSFSYGRALQASVLKAWGGKKENVAEAQK
VLLHRAHLNSLASLGKYEGENASGATGDSLFVANHQY*
>TRINITY_DN11662_c0_g1_i1.p1 type:complete len:398 gc:universal TRINITY_DN11662_c0_g1_i1:75-1268(+)
MALSRPVYIIAAKRTAFGSFGGSFKGLTATQLGQKAAAAAIVDAKLDPSLINSVIFGNVAQSSSDA
AYVARHIGLGNDIPFASTALTVNRLCGSGFQAVVNGAQEVVLGEADIVLAGGTENMSQCPFAIRN
ARWGINLGETREIEDTLWSALTDSFIQTPMGITAENLAEQYGITRLECDEYALASQQRWAAANDN
GLFKAEIAPIEVKQKRKMVPVDKDEHPRAGITIEALNKLPPVFKKNGTVSAGNASGICDGAAALV
LASEEACKQHNLAPLAKVVSYGISGVEPSIMGIGPCPAIKQALSRADMSLSDMAFIEVNEAFAAQY
LAVEKELGLDRSITNACGGAIALGHPLATSGARILTHLVHTLKRTKENHAIGSACIGGGQGIAVLL
ENVM*
>TRINITY_DN1065_c0_g1_i1.p1 type:complete len:373 gc:universal TRINITY_DN1065_c0_g1_i1:53-1171(+)
MSGRGKVKAQRISLSKRAGTIFPVGRIRRLMKGCTHKFRVGIGAPIYQAAVMEYLAAEILELAGN
AARDNKRRRITPRHILLAIANDEELNKLLRNVTIAQGGVMPHIQPELLRRKEGNKFSLPSNMKAK
ALKAQNAGVQKAKAKPAPVKKAAPAKKAAPAKAESKQAPKKAPQTNASPKKKSDQPWTVLSE
KTLFLGQKFLVVQGNVADIKCDAVVHPTNASFSTTGEVGKALIAAGGDDLKKAISELHKSHGDL
QVATALLGDAPNLKAKHVVHVHSPVWGTGNPEQDLENVVKSALTLADEKNVQTIAFPAIGSGA
GFKKNVAAQLMVRSMSNYFVDVSPSSIKNIYIFVTDAESIGFYTLELAKVDSS*
>TRINITY_DN10995_c0_g1_i2.p3 type:5prime_partial len:152 gc:universal TRINITY_DN10995_c0_g1_i2:3-458(+)
FWLKPRRPKAAGSPTLRDRGRPERVGACSLDRQGMASRSRALLSAVRASRVRRSGGFGSPRDHEP
AWIENWNKDHRAGKDSLDWFFRAFNSTGIYEIFLKSSPRFYGFIIGAGVLGGYYWSRMWDHIWN
HINQGKLYAHNPYVYPPPEDDE*
>TRINITY_DN5153_c0_g1_i1.p1 type:complete len:862 gc:universal TRINITY_DN5153_c0_g1_i1:61-2646(+)

MSEKKEFHRLSRSVRPIHYNITLKPDLENFTFAGNEDIDLEIKKETKHIKVNSLDIKVSSAVLTVDSQ
DIEPSDIRYKEDEETVTFKFSNPIPVGNAKLSLKFTGELNDKMKGFYRSKYTSASGEEKHMAVTQFE
PTDARRAFPCWDEPAVKATFDITMIAPKDRVVLSNMDVKSEKDHAEDDSFKEVAFNTTPIMSTYL
VAFVIGEFDFIEETLENGVLARVYTPLGKTEQGTFALKITSKTLPFYEEYFGIKYPLPKIDLIAIPDFAA
GAMENWGLVTYRETALLVDEKESSASSKQWVAIVVGHELAHQWFGNLTTMEWWTHLWLNEGF
ASWIEYLCVDYCCPEYDIWTQFVTNDFTRALELDALSNSHPIEVKVGHPSEVDEIFDAISYSKGSCV
IRMLHDFVGVGDFKRGLNSYLTTYKYANGETEDLWYHLEKASGKPVANVMATWTKQMGYPVLS
VSAEYVDTQCILKISQKKFTADGSADPNDQRWAIPINIMTNDNPKFSTLMTDPEMVLFMENEPES
NYIKLNPGQIGFYRTCYSSDLITKLVPVINSLSPVDRLGLESDLFALSIAGVSKTVDFLNILKGYKDE
MNYTVWTDIDSNISSLGVILQNTDNFELFKEYVIKLYKPISEALGWEPKDDDDHLTALLRNLILRRL
GNYGDADTLAEGQRKFKAHVDGTELVNADIRSAVYSMVSKHGDEETVDQFINLYRNTTHSEEKT
RILTYIGASYKSEVIEKSLKFSISDDVRSQDTVFALCSSTGSLNGRRLTWNFVKENWDDLFKRYQGG
FLLSRLISRTISNFATVEEANDVKTFFEEHKAPAAERCIKQGIESIQLNNAWLTRDGESINQWLFQE
N*
