# Supplementary material for: Proteomic Analysis and Biochemical Characterization of the Nematocyst Extract of the Hydrozoan Velella velella
Source: Mar Drugs. 2024 Oct 12;22(10):468. doi: 10.3390/md22100468 (PMC11509761; doi:10.3390/md22100468)
Supplement: Supplementary file 1 [file marinedrugs-22-00468-s001.zip › Supplementary File S1.zip!/Sample_1_Vellela/db.peptides.html]

peptide list


Peptide List

  

| Peptide | -10lgP | Mass | Length | ppm | m/z | RT | 1/k0 Start | 1/k0 End | Precursor ID | Area Sample 3 | #Feature | #Feature Sample 3 | Accession | PTM | AScore | Found By |
| --- | --- | --- | --- | --- | --- | --- | --- | --- | --- | --- | --- | --- | --- | --- | --- | --- |
| DKIFPHIELFGK | 36.06 | 1442.7921 | 12 | 4.76 | 481.9375 | 18.63 | 0.8696 | 0.8769 | 60334 | 4.52e2 | 1 | 1 | TRINITY\_DN2490\_c0\_g1\_i1.p1 |  |  | DB Search |
| AEFRPNQEEEPILKR | 35.37 | 1854.9587 | 15 | 5.35 | 619.3266 | 13.84 | 0.8753 | 0.8826 | 35617 | 4.88e3 | 1 | 1 | TRINITY\_DN262\_c0\_g1\_i1.p1 |  |  | DB Search |
| RMSEIAEEARK | 34.53 | 1318.6663 | 11 | 6.48 | 440.563 | 10.97 | 0.7587 | 0.7661 | 21230 | 4.84e2 | 1 | 1 | TRINITY\_DN1676\_c0\_g1\_i6.p1:TRINITY\_DN1676\_c0\_g1\_i12.p1 |  |  | DB Search |
| KTC(+57.02)VKDEVTGLHEIK | 33.64 | 1755.9189 | 15 | 5.41 | 586.3134 | 12.81 | 0.8846 | 0.8919 | 30711 | 0 | 0 | 0 | TRINITY\_DN1369\_c0\_g1\_i1.p1 | Carbamidomethylation |  | DB Search |
| KSEGAAETGSVTISK | 33.52 | 1463.7467 | 15 | 4.25 | 732.8795 | 11.40 | 0.9892 | 0.9965 | 23419 | 7.43e2 | 1 | 1 | TRINITY\_DN1676\_c0\_g1\_i6.p1:TRINITY\_DN1676\_c0\_g1\_i12.p1 |  |  | DB Search |
| LC(+57.02)FVGLM(+15.99)SM(+15.99)IDPPR | 33.39 | 1666.7881 | 14 | 2.76 | 834.3988 | 18.80 | 1.0733 | 1.0805 | 61317 | 6.39e2 | 1 | 1 | TRINITY\_DN262\_c0\_g1\_i1.p1 | Carbamidomethylation, Oxidation (M), Oxidation (M) | M7:Oxidation (M):1000 M9:Oxidation (M):1000 | DB Search |
| FELDRVNTFGDEVFGDPK | 33.39 | 2083.9851 | 18 | 9.37 | 695.6714 | 19.27 | 0.9133 | 0.9206 | 64506 | 4.68e2 | 1 | 1 | TRINITY\_DN1676\_c0\_g1\_i6.p1:TRINITY\_DN1676\_c0\_g1\_i12.p1 |  |  | DB Search |
| GVGIISEGNETVEDIAQR | 33.02 | 1885.9381 | 18 | 3.38 | 943.9741 | 17.88 | 1.1414 | 1.1486 | 55578 | 6.3e2 | 1 | 1 | TRINITY\_DN262\_c0\_g1\_i1.p1 |  |  | DB Search |
| AYGQDLGQPGSC(+57.02)LKR | 32.5 | 1648.7991 | 15 | 5.96 | 825.407 | 13.47 | 1.0733 | 1.0805 | 33986 | 7.47e2 | 1 | 1 | TRINITY\_DN691\_c0\_g1\_i2.p1:TRINITY\_DN691\_c0\_g1\_i1.p1 | Carbamidomethylation |  | DB Search |
| AKQEAFIAELEDQIAK | 32.28 | 1802.9414 | 16 | 9.12 | 601.9897 | 19.97 | 0.8793 | 0.8866 | 69132 | 0 | 0 | 0 | TRINITY\_DN428\_c0\_g1\_i1.p1 |  |  | DB Search |
| YLIEQEIDHFSQFNR | 32.17 | 1937.9271 | 15 | 6.69 | 646.9836 | 19.66 | 0.8854 | 0.8927 | 67188 | 0 | 0 | 0 | TRINITY\_DN4927\_c0\_g1\_i9.p1:TRINITY\_DN4927\_c0\_g1\_i10.p1 |  |  | DB Search |
| GKDGETGEQGPQGQEGPTGPR | 32.03 | 2080.9409 | 21 | 5.04 | 694.6537 | 10.74 | 0.8498 | 0.8571 | 19974 | 6.04e3 | 2 | 2 | TRINITY\_DN113\_c0\_g1\_i1.p1 |  |  | DB Search |
| IHINIVVIGHVDSGK | 32.02 | 1599.9097 | 15 | 5.66 | 534.3104 | 16.64 | 0.8412 | 0.8485 | 48792 | 1.31e2 | 1 | 1 | TRINITY\_DN64\_c0\_g1\_i1.p1 |  |  | DB Search |
| ADC(+57.02)GVENTC(+57.02)FGSTR | 31.98 | 1572.6296 | 14 | 3.17 | 787.3201 | 13.66 | 1.0206 | 1.0279 | 34821 | 4.97e2 | 1 | 1 | TRINITY\_DN319\_c2\_g1\_i3.p1:TRINITY\_DN319\_c2\_g1\_i1.p1:TRINITY\_DN319\_c2\_g1\_i2.p1 | Carbamidomethylation, Carbamidomethylation |  | DB Search |
| S(+42.01)LVIPEKFQHILR | 31.98 | 1620.9352 | 13 | 4.73 | 541.3184 | 19.70 | 0.8388 | 0.8461 | 67303 | 3.39e2 | 1 | 1 | TRINITY\_DN1505\_c0\_g1\_i1.p1 | Acetylation (Protein N-term) | S1:Acetylation (Protein N-term):1000 | DB Search |
| FDFHSWIGLKK | 31.98 | 1376.7241 | 11 | 3.29 | 459.9142 | 18.36 | 0.7437 | 0.751 | 58604 | 4.29e2 | 1 | 1 | TRINITY\_DN12627\_c0\_g1\_i1.p1 |  |  | DB Search |
| YPNGLHHC(+57.02)PTK | 31.97 | 1322.619 | 11 | 4.7 | 441.8798 | 10.39 | 0.7677 | 0.775 | 18227 | 6.71e2 | 1 | 1 | TRINITY\_DN1369\_c0\_g1\_i1.p1 | Carbamidomethylation |  | DB Search |
| GLQGQQGEQGSTGVQGSVGGR | 31.96 | 1985.9514 | 21 | 15.33 | 993.9925 | 12.02 | 1.1526 | 1.1598 | 26652 | 4.41e3 | 1 | 1 | TRINITY\_DN1030\_c0\_g1\_i1.p1:TRINITY\_DN1030\_c0\_g1\_i2.p1 |  |  | DB Search |
| RYGQAMC(+57.02)QFMR | 31.95 | 1446.6318 | 11 | -3.39 | 724.3166 | 17.38 | 0.9808 | 0.988 | 52561 | 3.82e2 | 1 | 1 | TRINITY\_DN286\_c0\_g1\_i4.p1:TRINITY\_DN286\_c0\_g1\_i2.p1 | Carbamidomethylation |  | DB Search |
| FFGSLDQIKFEDQK | 31.94 | 1700.8409 | 14 | 6.15 | 567.9545 | 18.24 | 0.814 | 0.8214 | 57825 | 3.3e2 | 1 | 1 | TRINITY\_DN5554\_c0\_g1\_i1.p1 |  |  | DB Search |
| SYYFAISDITVGGR | 31.92 | 1547.762 | 14 | 8.82 | 774.8906 | 19.95 | 1.0452 | 1.0524 | 68928 | 3.71e2 | 1 | 1 | TRINITY\_DN1676\_c0\_g1\_i6.p1:TRINITY\_DN1676\_c0\_g1\_i12.p1 |  |  | DB Search |
| YAAYVGC(+57.02)EFQSVDFSSVAK | 31.89 | 2126.9619 | 19 | 7.79 | 1064.4904 | 18.86 | 1.2324 | 1.2396 | 61864 | 0 | 0 | 0 | TRINITY\_DN17714\_c0\_g1\_i1.p1 | Carbamidomethylation |  | DB Search |
| IC(+57.02)HLLGIQVTDFTK | 31.85 | 1643.8705 | 14 | 3.68 | 548.963 | 18.57 | 0.8161 | 0.8234 | 59993 | 2.47e2 | 1 | 1 | TRINITY\_DN428\_c0\_g1\_i1.p1 | Carbamidomethylation |  | DB Search |
| KALVILGDGFISGGGTPSK | 31.85 | 1816.0094 | 19 | 6.44 | 909.0126 | 18.13 | 1.1734 | 1.1806 | 57036 | 4.06e2 | 1 | 1 | TRINITY\_DN244\_c0\_g1\_i1.p1 |  |  | DB Search |
| GVTSITLELQDLQR | 31.84 | 1571.8518 | 14 | 2.39 | 786.9305 | 19.40 | 1.0576 | 1.0648 | 65430 | 3.99e2 | 1 | 1 | TRINITY\_DN51907\_c0\_g1\_i1.p1 |  |  | DB Search |
| GKLTQEEEVLLQDFSR | 31.76 | 1890.9686 | 16 | 2.44 | 631.3281 | 19.70 | 0.9048 | 0.9121 | 67409 | 0 | 0 | 0 | TRINITY\_DN11643\_c0\_g1\_i1.p1 |  |  | DB Search |
| IVIEKFDVNVATK | 31.76 | 1474.8395 | 13 | -5.09 | 738.419 | 16.54 | 1.0419 | 1.0492 | 48332 | 0 | 0 | 0 | TRINITY\_DN33328\_c0\_g1\_i1.p1 |  |  | DB Search |
| GQAGPAGQDGVDGAVGAR | 31.48 | 1581.7495 | 18 | 2.61 | 791.8795 | 12.51 | 0.9997 | 1.0069 | 29162 | 3.92e2 | 1 | 1 | TRINITY\_DN1030\_c0\_g1\_i1.p1:TRINITY\_DN1030\_c0\_g1\_i2.p1 |  |  | DB Search |
| DPIC(+57.02)HC(+57.02)DTSK | 31.43 | 1231.4961 | 10 | 6.8 | 411.5064 | 9.93 | 0.7331 | 0.7404 | 15608 | 1e3 | 1 | 1 | TRINITY\_DN319\_c2\_g1\_i3.p1:TRINITY\_DN319\_c2\_g1\_i1.p1:TRINITY\_DN319\_c2\_g1\_i2.p1 | Carbamidomethylation, Carbamidomethylation |  | DB Search |
| GSFSSHIC(+57.02)LC(+57.02)HDPAYK | 31.43 | 1877.8188 | 16 | 3.63 | 626.9456 | 14.39 | 0.8814 | 0.8887 | 38439 | 1.55e3 | 1 | 1 | TRINITY\_DN319\_c2\_g1\_i3.p1:TRINITY\_DN319\_c2\_g1\_i1.p1:TRINITY\_DN319\_c2\_g1\_i2.p1 | Carbamidomethylation, Carbamidomethylation |  | DB Search |
| M(+42.01)(+15.99)DAIFGERPISFK | 31.43 | 1567.7704 | 13 | 6.82 | 784.8933 | 19.02 | 1.062 | 1.0693 | 62921 | 3.12e3 | 1 | 1 | TRINITY\_DN2971\_c0\_g1\_i1.p1 | Acetylation (Protein N-term), Oxidation (M) | M1:Acetylation (Protein N-term):1000 M1:Oxidation (M):1000 | DB Search |
| KPGEVWTNKEK | 31.43 | 1314.6931 | 11 | 3.3 | 439.2372 | 10.87 | 0.7551 | 0.7624 | 20789 | 0 | 0 | 0 | TRINITY\_DN1369\_c0\_g1\_i1.p1 |  |  | DB Search |
| LALHVLNNEDIIGHR | 31.43 | 1712.9321 | 15 | 3.56 | 571.9834 | 16.44 | 0.8538 | 0.8611 | 47846 | 7.52e2 | 1 | 1 | TRINITY\_DN140702\_c0\_g1\_i1.p1 |  |  | DB Search |
| SAVNHNIIFEGR | 31.43 | 1355.6946 | 12 | -4.41 | 678.8477 | 14.70 | 0.9675 | 0.9747 | 39863 | 0 | 0 | 0 | TRINITY\_DN1936\_c0\_g1\_i3.p1 |  |  | DB Search |
| TPGEC(+57.02)C(+57.02)PQC(+57.02)K | 31.43 | 1235.4733 | 10 | 6.61 | 618.7444 | 9.98 | 0.8915 | 0.8988 | 15843 | 1.95e3 | 1 | 1 | TRINITY\_DN1369\_c0\_g1\_i1.p1 | Carbamidomethylation, Carbamidomethylation, Carbamidomethylation |  | DB Search |
| ALVILGDGFISGGGTPSK | 31.42 | 1687.9144 | 18 | 9.81 | 844.9679 | 19.77 | 1.1682 | 1.1754 | 67505 | 1.82e4 | 1 | 1 | TRINITY\_DN244\_c0\_g1\_i1.p1 |  |  | DB Search |
| AVQIHPFNDVSVGDPK | 31.42 | 1721.8737 | 16 | 5.27 | 861.9437 | 15.88 | 1.1142 | 1.1214 | 45122 | 5.31e3 | 2 | 2 | TRINITY\_DN1071\_c0\_g1\_i2.p1:TRINITY\_DN1071\_c0\_g1\_i3.p1:TRINITY\_DN1071\_c0\_g1\_i4.p1:TRINITY\_DN1071\_c0\_g1\_i1.p1 |  |  | DB Search |
| LLAVTVGNNYNLDTLEK | 31.42 | 1875.9941 | 17 | 6.79 | 939.0053 | 18.68 | 1.1462 | 1.1534 | 60547 | 3.02e3 | 1 | 1 | TRINITY\_DN77\_c1\_g1\_i1.p1 |  |  | DB Search |
| AFLIEEQKLVVR | 31.42 | 1443.8448 | 12 | 4.96 | 722.9291 | 17.47 | 1.0443 | 1.0516 | 53047 | 7.81e2 | 1 | 1 | TRINITY\_DN79126\_c0\_g1\_i1.p1 |  |  | DB Search |
| AVFPSIVGRPR | 31.42 | 1197.6982 | 11 | 5.9 | 599.8564 | 15.80 | 0.9533 | 0.9606 | 44974 | 8.41e3 | 1 | 1 | TRINITY\_DN161\_c0\_g1\_i1.p1:TRINITY\_DN161\_c0\_g1\_i3.p1:TRINITY\_DN161\_c0\_g1\_i2.p1 |  |  | DB Search |
| KVIVDQLVTLATR | 31.42 | 1454.8821 | 13 | 5.37 | 728.448 | 18.39 | 1.0246 | 1.0319 | 58671 | 8.62e2 | 1 | 1 | TRINITY\_DN1386\_c0\_g1\_i1.p1 |  |  | DB Search |
| KAQYYEAYETIIKR | 31.42 | 1774.9254 | 14 | 2.25 | 592.647 | 15.86 | 0.8575 | 0.8648 | 45137 | 7.72e2 | 1 | 1 | TRINITY\_DN319\_c2\_g1\_i3.p1:TRINITY\_DN319\_c2\_g1\_i1.p1 |  |  | DB Search |
| HLAEVC(+57.02)HER | 31.42 | 1149.5349 | 9 | 14.7 | 384.189 | 9.74 | 0.731 | 0.7384 | 14869 | 0 | 0 | 0 | TRINITY\_DN4364\_c0\_g1\_i1.p1 | Carbamidomethylation |  | DB Search |
| ALLLASSNAFDTSNGAR | 31.42 | 1706.8588 | 17 | -0.8 | 854.431 | 17.58 | 1.129 | 1.1362 | 53833 | 3.18e3 | 2 | 2 | TRINITY\_DN244\_c0\_g1\_i1.p1 |  |  | DB Search |
| LLQQHELDLTPLK | 31.42 | 1546.8718 | 13 | -0.58 | 774.4383 | 16.85 | 1.0552 | 1.0624 | 49653 | 2.38e2 | 1 | 1 | TRINITY\_DN1676\_c0\_g1\_i6.p1:TRINITY\_DN1676\_c0\_g1\_i12.p1 |  |  | DB Search |
| AIHPTIIDASK | 31.42 | 1164.6503 | 11 | 5.47 | 583.3322 | 13.51 | 0.9602 | 0.9675 | 34115 | 5.55e2 | 1 | 1 | TRINITY\_DN17721\_c0\_g1\_i2.p1:TRINITY\_DN17721\_c0\_g1\_i1.p1 |  |  | DB Search |
| AENDIGDWFR | 31.42 | 1221.5414 | 10 | 1.83 | 611.7756 | 19.30 | 0.8862 | 0.8935 | 64616 | 2.38e3 | 1 | 1 | TRINITY\_DN121418\_c0\_g1\_i1.p1 |  |  | DB Search |
| GVAGAGVLAGFDKFK | 31.42 | 1435.7823 | 15 | 7.26 | 479.6021 | 18.38 | 0.8469 | 0.8542 | 58601 | 6.91e3 | 2 | 2 | TRINITY\_DN1559\_c0\_g1\_i1.p1 |  |  | DB Search |
| KVNMPISIIALNHR | 31.42 | 1604.9185 | 14 | 5.12 | 535.9797 | 17.25 | 0.8453 | 0.8526 | 51799 | 5.03e2 | 1 | 1 | TRINITY\_DN2433\_c0\_g1\_i2.p1:TRINITY\_DN2433\_c0\_g1\_i1.p1 |  |  | DB Search |
| GSDEVEHNVK | 31.42 | 1112.5098 | 10 | 10.01 | 557.2645 | 9.66 | 0.8923 | 0.8996 | 14398 | 0 | 0 | 0 | TRINITY\_DN5854\_c0\_g1\_i1.p1 |  |  | DB Search |
| ADIEDVKDLLK | 31.42 | 1257.6816 | 11 | 7.3 | 420.2351 | 17.68 | 0.7571 | 0.7644 | 54439 | 1.59e4 | 2 | 2 | TRINITY\_DN15274\_c0\_g1\_i1.p1 |  |  | DB Search |
| ALFHEFVSEK | 31.41 | 1205.608 | 10 | 4.03 | 603.8102 | 15.95 | 0.9247 | 0.9319 | 45506 | 5.22e2 | 1 | 1 | TRINITY\_DN244\_c0\_g1\_i1.p1 |  |  | DB Search |
| ITQLQNDDKEAR | 31.41 | 1429.7161 | 12 | 7.95 | 715.8669 | 10.85 | 1.0255 | 1.0327 | 20687 | 0 | 0 | 0 | TRINITY\_DN1676\_c0\_g1\_i6.p1:TRINITY\_DN1676\_c0\_g1\_i12.p1 |  |  | DB Search |
| IPITC(+57.02)YYPTSGK | 31.41 | 1398.6853 | 12 | 7.57 | 700.3512 | 15.79 | 0.9763 | 0.9836 | 44811 | 2.56e3 | 1 | 1 | TRINITY\_DN581\_c1\_g1\_i1.p1 | Carbamidomethylation |  | DB Search |
| LVHGDVFRPPR | 31.41 | 1291.7148 | 11 | 7.13 | 646.8656 | 13.43 | 0.9699 | 0.9771 | 33689 | 4.13e2 | 1 | 1 | TRINITY\_DN1285\_c0\_g1\_i1.p1:TRINITY\_DN11595\_c0\_g1\_i2.p1:TRINITY\_DN11595\_c0\_g1\_i1.p1 |  |  | DB Search |
| TC(+57.02)VKDEVTGLHEIK | 31.41 | 1627.824 | 14 | 1.81 | 543.6131 | 13.77 | 0.853 | 0.8603 | 35449 | 1.2e3 | 1 | 1 | TRINITY\_DN1369\_c0\_g1\_i1.p1 | Carbamidomethylation |  | DB Search |
| A(+42.01)NPLKDIISR | 31.4 | 1167.6611 | 10 | 6.58 | 584.8383 | 18.69 | 0.9166 | 0.9238 | 60626 | 6.71e2 | 1 | 1 | TRINITY\_DN163801\_c0\_g1\_i1.p1 | Acetylation (Protein N-term) | A1:Acetylation (Protein N-term):1000 | DB Search |
| S(+42.01)ATGNLVTVLRK | 31.4 | 1299.751 | 12 | 10.13 | 650.8856 | 17.20 | 0.9949 | 1.0021 | 51417 | 5.92e3 | 1 | 1 | TRINITY\_DN2963\_c0\_g1\_i1.p1 | Acetylation (Protein N-term) | S1:Acetylation (Protein N-term):1000 | DB Search |
| ADIEDVKDLLKK | 31.4 | 1385.7765 | 12 | 5.27 | 693.8952 | 16.35 | 0.9908 | 0.9981 | 47347 | 1.16e3 | 1 | 1 | TRINITY\_DN15274\_c0\_g1\_i1.p1 |  |  | DB Search |
| APTHILNPANKR | 31.4 | 1330.7469 | 12 | 6.7 | 444.59 | 11.12 | 0.7445 | 0.7518 | 21628 | 4.55e3 | 2 | 2 | TRINITY\_DN1058\_c0\_g1\_i4.p1:TRINITY\_DN1058\_c0\_g1\_i1.p1:TRINITY\_DN1058\_c0\_g1\_i3.p1:TRINITY\_DN1058\_c0\_g1\_i2.p2 |  |  | DB Search |
| TLSIDLLPFEKPR | 31.38 | 1527.8661 | 13 | 9.86 | 510.298 | 19.94 | 0.8218 | 0.8291 | 68839 | 3e2 | 1 | 1 | TRINITY\_DN31906\_c0\_g1\_i1.p1 |  |  | DB Search |
| SGDPLTIGNFIK | 31.38 | 1260.6714 | 12 | -7.98 | 631.3343 | 19.73 | 0.8996 | 0.9069 | 67627 | 0 | 0 | 0 | TRINITY\_DN2577\_c1\_g1\_i1.p1:TRINITY\_DN2577\_c1\_g1\_i2.p1 |  |  | DB Search |
| SGDPLTLGNFIK | 31.38 | 1260.6714 | 12 | -7.98 | 631.3343 | 19.73 | 0.8996 | 0.9069 | 67627 | 0 | 0 | 0 | TRINITY\_DN3795\_c0\_g1\_i1.p1 |  |  | DB Search |
| AFVAIGDYGGHVGLGVK | 31.38 | 1658.8779 | 17 | 7.13 | 830.4474 | 17.72 | 1.163 | 1.1702 | 54593 | 5.16e2 | 1 | 1 | TRINITY\_DN15584\_c0\_g1\_i1.p1 |  |  | DB Search |
| DANSM(+15.99)PTQSR | 31.37 | 1121.4772 | 10 | -0.52 | 561.7423 | 9.05 | 0.836 | 0.8433 | 11164 | 1.28e2 | 1 | 1 | TRINITY\_DN477\_c0\_g1\_i9.p1 | Oxidation (M) | M5:Oxidation (M):1000 | DB Search |
| ESTLHLVLR | 31.37 | 1066.6135 | 9 | -2.66 | 534.3095 | 15.64 | 0.8785 | 0.8858 | 44242 | 0 | 0 | 0 | TRINITY\_DN554\_c0\_g1\_i3.p2:TRINITY\_DN585\_c0\_g1\_i3.p1:TRINITY\_DN554\_c0\_g1\_i4.p1:TRINITY\_DN1580\_c0\_g1\_i3.p1:TRINITY\_DN40728\_c1\_g1\_i1.p2:TRINITY\_DN152\_c0\_g1\_i2.p1:TRINITY\_DN585\_c0\_g1\_i5.p1:TRINITY\_DN585\_c0\_g2\_i1.p1:TRINITY\_DN585\_c0\_g1\_i4.p1:TRINITY\_DN152\_c0\_g1\_i15.p1:TRINITY\_DN103165\_c0\_g1\_i1.p1:TRINITY\_DN152\_c0\_g1\_i10.p1:TRINITY\_DN585\_c0\_g1\_i8.p1:TRINITY\_DN101\_c0\_g1\_i1.p1:TRINITY\_DN10545\_c0\_g1\_i1.p3:TRINITY\_DN173718\_c0\_g1\_i1.p1:TRINITY\_DN1465\_c1\_g1\_i1.p1:TRINITY\_DN40728\_c0\_g1\_i1.p1:TRINITY\_DN152\_c0\_g1\_i9.p1:TRINITY\_DN554\_c0\_g1\_i2.p1:TRINITY\_DN110760\_c0\_g1\_i1.p1:TRINITY\_DN585\_c0\_g1\_i10.p1:TRINITY\_DN8227\_c0\_g1\_i1.p1:TRINITY\_DN554\_c0\_g1\_i5.p1:TRINITY\_DN585\_c0\_g1\_i2.p1 |  |  | DB Search |
| DGEKVETLKVDISDINK | 31.36 | 1901.9945 | 17 | 4.01 | 635.0043 | 15.97 | 0.8976 | 0.9048 | 45629 | 4.35e2 | 1 | 1 | TRINITY\_DN1616\_c0\_g1\_i3.p1:TRINITY\_DN1616\_c0\_g1\_i6.p1:TRINITY\_DN1616\_c0\_g1\_i2.p1:TRINITY\_DN1616\_c0\_g1\_i7.p1 |  |  | DB Search |
| AGAIAEEVISSIR | 31.36 | 1314.7142 | 13 | 6.18 | 658.3647 | 19.95 | 0.9755 | 0.9828 | 68980 | 5.91e2 | 1 | 1 | TRINITY\_DN7102\_c0\_g1\_i1.p1 |  |  | DB Search |
| RIVLLATDALPK | 31.32 | 1308.8129 | 12 | 4.44 | 655.4128 | 17.23 | 1.0126 | 1.0198 | 51672 | 1.04e3 | 1 | 1 | TRINITY\_DN11377\_c0\_g1\_i1.p1 |  |  | DB Search |
| AITDNIKDILSVK | 31.32 | 1428.8187 | 13 | 12.14 | 715.4212 | 19.28 | 1.0504 | 1.0576 | 64605 | 0 | 0 | 0 | TRINITY\_DN1739\_c0\_g1\_i1.p1 |  |  | DB Search |
| DEKANDILDGLELR | 31.31 | 1599.8104 | 14 | 10.48 | 534.2799 | 19.01 | 0.8063 | 0.8136 | 62845 | 3.83e2 | 1 | 1 | TRINITY\_DN15274\_c0\_g1\_i1.p1 |  |  | DB Search |
| GLYISAEFAIPEDC(+57.02)KK | 31.26 | 1839.9076 | 16 | 9.12 | 920.9642 | 18.33 | 1.2017 | 1.2089 | 58227 | 4.93e2 | 1 | 1 | TRINITY\_DN8155\_c0\_g1\_i1.p1 | Carbamidomethylation |  | DB Search |
| ANDILDGLELR | 31.21 | 1227.6459 | 11 | -0.51 | 614.8264 | 19.49 | 0.9267 | 0.9339 | 66003 | 0 | 0 | 0 | TRINITY\_DN15274\_c0\_g1\_i1.p1 |  |  | DB Search |
| IGVIVYSDTASIEIR | 31.21 | 1634.8879 | 15 | 6.98 | 818.4522 | 18.66 | 1.1041 | 1.1114 | 60549 | 2.62e2 | 1 | 1 | TRINITY\_DN244\_c0\_g1\_i1.p1 |  |  | DB Search |
| AVAEEFISVDPYVR | 31.16 | 1593.8038 | 14 | 9.9 | 797.9125 | 19.05 | 1.0588 | 1.066 | 63171 | 0 | 0 | 0 | TRINITY\_DN598\_c1\_g1\_i1.p1 |  |  | DB Search |
| EVNTELDSRPETK | 31.11 | 1516.7369 | 13 | -3.14 | 759.369 | 11.76 | 1.0226 | 1.0299 | 25293 | 0 | 0 | 0 | TRINITY\_DN11643\_c0\_g1\_i1.p1 |  |  | DB Search |
| AKDLYYEIR | 31.08 | 1169.608 | 9 | 5.22 | 585.811 | 14.60 | 0.9158 | 0.923 | 39418 | 0 | 0 | 0 | TRINITY\_DN15274\_c0\_g1\_i1.p1 |  |  | DB Search |
| K(+42.01)LAAELANGR | 31.07 | 1083.6036 | 10 | 6.78 | 542.8096 | 15.40 | 0.8644 | 0.8716 | 43065 | 1.23e3 | 1 | 1 | TRINITY\_DN1172\_c0\_g2\_i2.p1 | Acetylation (Protein N-term) | K1:Acetylation (Protein N-term):1000 | DB Search |
| DLPQEKLDDILK | 31.07 | 1425.7715 | 12 | 0.29 | 713.8891 | 17.79 | 1.0355 | 1.0427 | 55100 | 0 | 0 | 0 | TRINITY\_DN262\_c0\_g1\_i1.p1 |  |  | DB Search |
| TQAVLDRLNIEK | 31.03 | 1398.7831 | 12 | 4.65 | 700.398 | 15.43 | 1.0017 | 1.009 | 43214 | 1.51e3 | 1 | 1 | TRINITY\_DN477\_c0\_g1\_i9.p1 |  |  | DB Search |
| N(+42.01)VLLWHEPR | 31 | 1204.6353 | 9 | 6.46 | 603.3253 | 19.76 | 0.9461 | 0.9533 | 67733 | 1.94e3 | 1 | 1 | TRINITY\_DN15109\_c0\_g2\_i1.p1 | Acetylation (Protein N-term) | N1:Acetylation (Protein N-term):1000 | DB Search |
| VYESLSGFIKNEGVFTTIC(+57.02)NGVHK | 30.89 | 2698.3425 | 24 | 2.53 | 900.4518 | 19.61 | 1.1218 | 1.129 | 66836 | 0 | 0 | 0 | TRINITY\_DN982\_c0\_g1\_i3.p1 | Carbamidomethylation |  | DB Search |
| VLILLADGQQTYVPGVTEPSEVAK | 30.81 | 2526.3582 | 24 | 5.22 | 843.1262 | 19.61 | 1.1382 | 1.1454 | 66707 | 1.73e3 | 1 | 1 | TRINITY\_DN244\_c0\_g1\_i1.p1 |  |  | DB Search |
| AAGDLGIYENGC(+57.02)YDQLLK | 30.76 | 1998.9357 | 18 | 1.98 | 1000.4713 | 18.85 | 1.1742 | 1.1813 | 61754 | 0 | 0 | 0 | TRINITY\_DN661\_c0\_g1\_i10.p1 | Carbamidomethylation |  | DB Search |
| SPFVIMFK | 30.75 | 967.5201 | 8 | 3.16 | 484.7661 | 19.85 | 0.8104 | 0.8177 | 68318 | 2.17e3 | 1 | 1 | TRINITY\_DN4595\_c0\_g1\_i10.p1:TRINITY\_DN4595\_c0\_g1\_i4.p1 |  |  | DB Search |
| SAIIVDGKNEEGR | 30.72 | 1386.7103 | 13 | 5.71 | 694.3624 | 11.88 | 0.9969 | 1.0041 | 25934 | 0 | 0 | 0 | TRINITY\_DN982\_c0\_g1\_i3.p1 |  |  | DB Search |
| AAAPDFSK | 30.68 | 805.397 | 8 | -4.73 | 403.7015 | 14.81 | 0.7363 | 0.7437 | 40420 | 0 | 0 | 0 | TRINITY\_DN2487\_c1\_g1\_i3.p1 |  |  | DB Search |
| DANAAVVHGSHLK | 30.67 | 1317.679 | 13 | 7.18 | 659.8477 | 10.89 | 1.0142 | 1.0214 | 20842 | 6.5e2 | 1 | 1 | TRINITY\_DN262\_c0\_g1\_i1.p1 |  |  | DB Search |
| YPGYHEHAFPFER | 30.35 | 1648.7422 | 13 | 5.76 | 550.588 | 15.54 | 0.8481 | 0.8554 | 43744 | 4.4e2 | 1 | 1 | TRINITY\_DN11726\_c0\_g1\_i1.p1 |  |  | DB Search |
| TRIEDLETVISQTQDHR | 30.31 | 2040.0236 | 17 | 3.9 | 681.0139 | 17.48 | 0.9594 | 0.9666 | 53079 | 1.31e3 | 1 | 1 | TRINITY\_DN761\_c0\_g1\_i3.p1:TRINITY\_DN761\_c0\_g1\_i1.p1:TRINITY\_DN761\_c0\_g1\_i2.p1 |  |  | DB Search |
| DLHIIEDYVLK | 30.28 | 1356.7289 | 11 | 3.24 | 679.37 | 19.65 | 0.992 | 0.9993 | 67106 | 3.64e2 | 1 | 1 | TRINITY\_DN8903\_c0\_g1\_i1.p1 |  |  | DB Search |
| RVDHQGGGVR | 30.22 | 1079.5583 | 10 | -9.32 | 360.8546 | 10.47 | 0.7298 | 0.7371 | 18621 | 5.44e3 | 1 | 1 | TRINITY\_DN191687\_c0\_g1\_i1.p1 |  |  | DB Search |
| LIDELTPEVTSALEC(+57.02)EGGPR | 30.13 | 2185.0571 | 20 | 7.57 | 1093.5378 | 19.37 | 1.2177 | 1.2248 | 65094 | 7.91e2 | 1 | 1 | TRINITY\_DN244\_c0\_g1\_i1.p1 | Carbamidomethylation |  | DB Search |
| VLIVISDGNEDFKEK | 30.08 | 1704.8933 | 15 | 3.65 | 853.4521 | 16.81 | 1.1694 | 1.1766 | 49463 | 2.58e2 | 1 | 1 | TRINITY\_DN195348\_c0\_g1\_i1.p1 |  |  | DB Search |
| AITKPFTNVIIQLDKK | 30.07 | 1828.0822 | 16 | 4.04 | 610.3669 | 17.67 | 0.9004 | 0.9077 | 54223 | 6.21e2 | 1 | 1 | TRINITY\_DN1785\_c0\_g1\_i1.p1 |  |  | DB Search |
| GDVIQVNEC(+57.02)TKK | 30.07 | 1389.6921 | 12 | 5.72 | 695.8533 | 12.08 | 1.0094 | 1.0166 | 26957 | 1.81e3 | 1 | 1 | TRINITY\_DN1369\_c0\_g1\_i1.p1 | Carbamidomethylation |  | DB Search |
| VIM(+15.99)VTGDHPITAK | 30.07 | 1396.7384 | 13 | 4.97 | 699.3759 | 13.23 | 1.0182 | 1.0255 | 32647 | 4.44e2 | 1 | 1 | TRINITY\_DN262\_c0\_g1\_i1.p1 | Oxidation (M) | M3:Oxidation (M):1000 | DB Search |
| TITLEVEPSDTIENVK | 30.07 | 1786.92 | 16 | 6.27 | 894.4677 | 17.53 | 1.1438 | 1.151 | 53352 | 6.91e2 | 1 | 1 | TRINITY\_DN554\_c0\_g1\_i3.p2:TRINITY\_DN585\_c0\_g1\_i8.p1:TRINITY\_DN101\_c0\_g1\_i1.p1:TRINITY\_DN585\_c0\_g1\_i4.p1:TRINITY\_DN585\_c0\_g1\_i3.p1:TRINITY\_DN554\_c0\_g1\_i4.p1:TRINITY\_DN554\_c0\_g1\_i2.p1:TRINITY\_DN585\_c0\_g1\_i10.p1:TRINITY\_DN585\_c0\_g1\_i5.p1:TRINITY\_DN554\_c0\_g1\_i5.p1:TRINITY\_DN585\_c0\_g1\_i2.p1:TRINITY\_DN585\_c0\_g2\_i1.p1 |  |  | DB Search |
| GSVASLTC(+57.02)EC(+57.02)NNHK | 30.07 | 1575.677 | 14 | 7.87 | 788.8474 | 11.94 | 1.0596 | 1.0668 | 26202 | 4.23e2 | 1 | 1 | TRINITY\_DN1058\_c3\_g1\_i1.p1 | Carbamidomethylation, Carbamidomethylation |  | DB Search |
| ALSLQNANEELEDRLAEAER | 30.07 | 2270.1138 | 20 | 8.88 | 757.7142 | 18.86 | 0.982 | 0.9892 | 61796 | 4.24e2 | 1 | 1 | TRINITY\_DN428\_c0\_g1\_i1.p1 |  |  | DB Search |
| total 100 peptides |  |  |  |  |  |  |  |  |  |  |  |  |  |  |  |  |
| --- | --- | --- | --- | --- | --- | --- | --- | --- | --- | --- | --- | --- | --- | --- | --- | --- |
