# Supplementary material for: Proteomic Analysis and Biochemical Characterization of the Nematocyst Extract of the Hydrozoan Velella velella
Source: Mar Drugs. 2024 Oct 12;22(10):468. doi: 10.3390/md22100468 (PMC11509761; doi:10.3390/md22100468)
Supplement: Supplementary file 1 [file marinedrugs-22-00468-s001.zip › Supplementary File S1.zip!/Sample_1_Vellela/db.proteins.html]

proteins


Protein List

  

| Protein Group | Protein ID | Accession | -10lgP | Coverage (%) | Coverage (%) Sample 3 | Area Sample 3 | #Peptides | #Unique | #Spec Sample 3 | PTM | Avg. Mass | Description |
| --- | --- | --- | --- | --- | --- | --- | --- | --- | --- | --- | --- | --- |
| 1 | 1 | TRINITY\_DN244\_c0\_g1\_i1.p1 | 81.57 | 3 | 3 | 2.51e4 | 7 | 7 | 14 | Carbamidomethylation | 313594.0938 | type:complete len:3019 gc:universal TRINITY\_DN244\_c0\_g1\_i1:9748-692(-) |
| 2 | 2 | TRINITY\_DN1369\_c0\_g1\_i1.p1 | 79.25 | 4 | 4 | 5.63e3 | 6 | 6 | 7 | Carbamidomethylation | 174229.4219 | type:3prime\_partial len:1577 gc:universal TRINITY\_DN1369\_c0\_g1\_i1:93-4820(+) |
| 3 | 3 | TRINITY\_DN1676\_c0\_g1\_i12.p1 | 81.92 | 5 | 5 | 2.3e3 | 6 | 6 | 6 |  | 180975.5156 | type:complete len:1631 gc:universal TRINITY\_DN1676\_c0\_g1\_i12:206-5098(+) |
| 3 | 4 | TRINITY\_DN1676\_c0\_g1\_i6.p1 | 81.92 | 5 | 5 | 2.3e3 | 6 | 6 | 6 |  | 180975.5156 | type:complete len:1631 gc:universal TRINITY\_DN1676\_c0\_g1\_i6:206-5098(+) |
| 4 | 5 | TRINITY\_DN262\_c0\_g1\_i1.p1 | 81.99 | 8 | 8 | 7.24e3 | 6 | 6 | 8 | Carbamidomethylation, Oxidation (M) | 114520.6406 | type:complete len:1033 gc:universal TRINITY\_DN262\_c0\_g1\_i1:111-3209(+) |
| 5 | 6 | TRINITY\_DN15274\_c0\_g1\_i1.p1 | 71.58 | 2 | 2 | 1.75e4 | 5 | 5 | 9 |  | 201897.7344 | type:complete len:1822 gc:universal TRINITY\_DN15274\_c0\_g1\_i1:87-5552(+) |
| 6 | 7 | TRINITY\_DN319\_c2\_g1\_i3.p1 | 66.03 | 2 | 2 | 3.82e3 | 4 | 4 | 9 | Carbamidomethylation | 279087.1875 | type:complete len:2588 gc:universal TRINITY\_DN319\_c2\_g1\_i3:165-7928(+) |
| 6 | 8 | TRINITY\_DN319\_c2\_g1\_i1.p1 | 66.03 | 2 | 2 | 3.82e3 | 4 | 4 | 9 | Carbamidomethylation | 279168.25 | type:complete len:2588 gc:universal TRINITY\_DN319\_c2\_g1\_i1:165-7928(+) |
| 6 | 9 | TRINITY\_DN319\_c2\_g1\_i2.p1 | 58.17 | 2 | 2 | 3.05e3 | 3 | 3 | 8 | Carbamidomethylation | 215796.4062 | type:3prime\_partial len:2004 gc:universal TRINITY\_DN319\_c2\_g1\_i2:165-6173(+) |
| 7 | 10 | TRINITY\_DN428\_c0\_g1\_i1.p1 | 58.23 | 3 | 3 | 6.71e2 | 3 | 3 | 3 | Carbamidomethylation | 225824.1719 | type:complete len:1949 gc:universal TRINITY\_DN428\_c0\_g1\_i1:96-5942(+) |
| 8 | 11 | TRINITY\_DN585\_c0\_g1\_i10.p1 | 46.41 | 11 | 11 | 6.91e2 | 2 | 2 | 2 |  | 26563.5332 | type:3prime\_partial len:236 gc:universal TRINITY\_DN585\_c0\_g1\_i10:705-1(-) |
| 8 | 12 | TRINITY\_DN585\_c0\_g1\_i5.p1 | 46.41 | 10 | 10 | 6.91e2 | 2 | 2 | 2 |  | 28098.1602 | type:internal len:250 gc:universal TRINITY\_DN585\_c0\_g1\_i5:747-1(-) |
| 8 | 13 | TRINITY\_DN585\_c0\_g2\_i1.p1 | 46.41 | 16 | 16 | 6.91e2 | 2 | 2 | 2 |  | 17391.9824 | type:5prime\_partial len:152 gc:universal TRINITY\_DN585\_c0\_g2\_i1:598-143(-) |
| 8 | 14 | TRINITY\_DN554\_c0\_g1\_i2.p1 | 46.41 | 9 | 9 | 6.91e2 | 2 | 2 | 2 |  | 29982.375 | type:internal len:266 gc:universal TRINITY\_DN554\_c0\_g1\_i2:797-3(-) |
| 8 | 15 | TRINITY\_DN554\_c0\_g1\_i3.p2 | 46.41 | 9 | 9 | 6.91e2 | 2 | 2 | 2 |  | 30419.4961 | type:complete len:268 gc:universal TRINITY\_DN554\_c0\_g1\_i3:1464-661(-) |
| 8 | 16 | TRINITY\_DN554\_c0\_g1\_i4.p1 | 46.41 | 8 | 8 | 6.91e2 | 2 | 2 | 2 |  | 35966.3984 | type:3prime\_partial len:319 gc:universal TRINITY\_DN554\_c0\_g1\_i4:956-3(-) |
| 8 | 17 | TRINITY\_DN585\_c0\_g1\_i8.p1 | 46.41 | 7 | 7 | 6.91e2 | 2 | 2 | 2 |  | 37858.4844 | type:3prime\_partial len:337 gc:universal TRINITY\_DN585\_c0\_g1\_i8:1008-1(-) |
| 8 | 18 | TRINITY\_DN585\_c0\_g1\_i2.p1 | 46.41 | 14 | 14 | 6.91e2 | 2 | 2 | 2 |  | 20558.6738 | type:internal len:184 gc:universal TRINITY\_DN585\_c0\_g1\_i2:549-1(-) |
| 8 | 19 | TRINITY\_DN585\_c0\_g1\_i3.p1 | 46.41 | 16 | 16 | 6.91e2 | 2 | 2 | 2 |  | 17628.2207 | type:internal len:157 gc:universal TRINITY\_DN585\_c0\_g1\_i3:468-1(-) |
| 8 | 20 | TRINITY\_DN101\_c0\_g1\_i1.p1 | 46.41 | 19 | 19 | 6.91e2 | 2 | 2 | 2 |  | 14847.2393 | type:complete len:129 gc:universal TRINITY\_DN101\_c0\_g1\_i1:416-30(-) |
| 8 | 21 | TRINITY\_DN585\_c0\_g1\_i4.p1 | 46.41 | 11 | 11 | 6.91e2 | 2 | 2 | 2 |  | 26348.0742 | type:complete len:232 gc:universal TRINITY\_DN585\_c0\_g1\_i4:847-152(-) |
| 8 | 22 | TRINITY\_DN554\_c0\_g1\_i5.p1 | 46.41 | 7 | 7 | 6.91e2 | 2 | 2 | 2 |  | 42081.4141 | type:3prime\_partial len:373 gc:universal TRINITY\_DN554\_c0\_g1\_i5:1118-3(-) |
| 8 | 23 | TRINITY\_DN40728\_c1\_g1\_i1.p2 | 31.37 | 7 | 7 | 0 | 1 | 1 | 1 |  | 14073.3066 | type:5prime\_partial len:124 gc:universal TRINITY\_DN40728\_c1\_g1\_i1:1-372(+) |
| 8 | 24 | TRINITY\_DN152\_c0\_g1\_i15.p1 | 31.37 | 6 | 6 | 0 | 1 | 1 | 1 |  | 15560.71 | type:internal len:141 gc:universal TRINITY\_DN152\_c0\_g1\_i15:2-421(+) |
| 8 | 25 | TRINITY\_DN152\_c0\_g1\_i2.p1 | 31.37 | 9 | 9 | 0 | 1 | 1 | 1 |  | 11754.375 | type:internal len:105 gc:universal TRINITY\_DN152\_c0\_g1\_i2:1-312(+) |
| 8 | 26 | TRINITY\_DN152\_c0\_g1\_i10.p1 | 31.37 | 5 | 5 | 0 | 1 | 1 | 1 |  | 20063.498 | type:internal len:180 gc:universal TRINITY\_DN152\_c0\_g1\_i10:1-537(+) |
| 8 | 27 | TRINITY\_DN173718\_c0\_g1\_i1.p1 | 31.37 | 8 | 8 | 0 | 1 | 1 | 1 |  | 12865.0928 | type:3prime\_partial len:112 gc:universal TRINITY\_DN173718\_c0\_g1\_i1:37-369(+) |
| 8 | 28 | TRINITY\_DN110760\_c0\_g1\_i1.p1 | 31.37 | 9 | 9 | 0 | 1 | 1 | 1 |  | 11456.2695 | type:internal len:104 gc:universal TRINITY\_DN110760\_c0\_g1\_i1:2-310(+) |
| 8 | 29 | TRINITY\_DN1580\_c0\_g1\_i3.p1 | 31.37 | 5 | 5 | 0 | 1 | 1 | 1 |  | 19665.5898 | type:5prime\_partial len:179 gc:universal TRINITY\_DN1580\_c0\_g1\_i3:1-537(+) |
| 8 | 30 | TRINITY\_DN40728\_c0\_g1\_i1.p1 | 31.37 | 9 | 9 | 0 | 1 | 1 | 1 |  | 11225.8301 | type:internal len:105 gc:universal TRINITY\_DN40728\_c0\_g1\_i1:1-312(+) |
| 8 | 31 | TRINITY\_DN1465\_c1\_g1\_i1.p1 | 31.37 | 9 | 9 | 0 | 1 | 1 | 1 |  | 11342.1572 | type:internal len:102 gc:universal TRINITY\_DN1465\_c1\_g1\_i1:2-304(+) |
| 8 | 32 | TRINITY\_DN152\_c0\_g1\_i9.p1 | 31.37 | 6 | 6 | 0 | 1 | 1 | 1 |  | 17213.1973 | type:5prime\_partial len:152 gc:universal TRINITY\_DN152\_c0\_g1\_i9:1-456(+) |
| 8 | 33 | TRINITY\_DN10545\_c0\_g1\_i1.p3 | 31.37 | 6 | 6 | 0 | 1 | 1 | 1 |  | 17872.502 | type:5prime\_partial len:162 gc:universal TRINITY\_DN10545\_c0\_g1\_i1:3-488(+) |
| 8 | 34 | TRINITY\_DN103165\_c0\_g1\_i1.p1 | 31.37 | 4 | 4 | 0 | 1 | 1 | 1 |  | 26936.2734 | type:internal len:242 gc:universal TRINITY\_DN103165\_c0\_g1\_i1:723-1(-) |
| 8 | 35 | TRINITY\_DN8227\_c0\_g1\_i1.p1 | 31.37 | 7 | 7 | 0 | 1 | 1 | 1 |  | 14513.8135 | type:internal len:135 gc:universal TRINITY\_DN8227\_c0\_g1\_i1:404-3(-) |
| 9 | 36 | TRINITY\_DN1030\_c0\_g1\_i2.p1 | 47.71 | 3 | 3 | 4.8e3 | 2 | 2 | 3 |  | 140221.3281 | type:complete len:1426 gc:universal TRINITY\_DN1030\_c0\_g1\_i2:4483-206(-) |
| 9 | 37 | TRINITY\_DN1030\_c0\_g1\_i1.p1 | 47.71 | 3 | 3 | 4.8e3 | 2 | 2 | 3 |  | 141535.875 | type:complete len:1439 gc:universal TRINITY\_DN1030\_c0\_g1\_i1:4522-206(-) |
| 10 | 38 | TRINITY\_DN982\_c0\_g1\_i3.p1 | 46.25 | 0 | 0 | 0 | 2 | 2 | 2 | Carbamidomethylation | 1090854.75 | type:complete len:9852 gc:universal TRINITY\_DN982\_c0\_g1\_i3:29697-142(-) |
| 11 | 39 | TRINITY\_DN11643\_c0\_g1\_i1.p1 | 47.31 | 16 | 16 | 0 | 2 | 2 | 3 |  | 21061.2832 | type:complete len:187 gc:universal TRINITY\_DN11643\_c0\_g1\_i1:1748-1188(-) |
| 12 | 40 | TRINITY\_DN477\_c0\_g1\_i9.p1 | 46.89 | 7 | 7 | 1.64e3 | 2 | 2 | 2 | Oxidation (M) | 34222.9453 | type:complete len:306 gc:universal TRINITY\_DN477\_c0\_g1\_i9:1840-923(-) |
| 13 | 41 | TRINITY\_DN3795\_c0\_g1\_i1.p1 | 31.38 | 1 | 1 | 0 | 1 | 1 | 1 |  | 97717.9844 | type:5prime\_partial len:867 gc:universal TRINITY\_DN3795\_c0\_g1\_i1:2719-119(-) |
| 13 | 42 | TRINITY\_DN2577\_c1\_g1\_i1.p1 | 31.38 | 1 | 1 | 0 | 1 | 1 | 1 |  | 105277.2734 | type:complete len:937 gc:universal TRINITY\_DN2577\_c1\_g1\_i1:3082-272(-) |
| 13 | 43 | TRINITY\_DN2577\_c1\_g1\_i2.p1 | 31.38 | 1 | 1 | 0 | 1 | 1 | 1 |  | 121869.7266 | type:5prime\_partial len:1087 gc:universal TRINITY\_DN2577\_c1\_g1\_i2:3532-272(-) |
| 14 | 44 | TRINITY\_DN286\_c0\_g1\_i4.p1 | 31.95 | 5 | 5 | 3.82e2 | 1 | 1 | 1 | Carbamidomethylation | 25084.5293 | type:complete len:224 gc:universal TRINITY\_DN286\_c0\_g1\_i4:40-711(+) |
| 14 | 45 | TRINITY\_DN286\_c0\_g1\_i2.p1 | 31.95 | 10 | 10 | 3.82e2 | 1 | 1 | 1 | Carbamidomethylation | 11368.1738 | type:3prime\_partial len:106 gc:universal TRINITY\_DN286\_c0\_g1\_i2:40-354(+) |
| 15 | 46 | TRINITY\_DN1505\_c0\_g1\_i1.p1 | 31.98 | 8 | 8 | 3.39e2 | 1 | 1 | 1 | Acetylation (Protein N-term) | 17930.752 | type:complete len:154 gc:universal TRINITY\_DN1505\_c0\_g1\_i1:98-559(+) |
| 16 | 47 | TRINITY\_DN1058\_c3\_g1\_i1.p1 | 30.07 | 3 | 3 | 4.23e2 | 1 | 1 | 1 | Carbamidomethylation | 51455.6484 | type:internal len:485 gc:universal TRINITY\_DN1058\_c3\_g1\_i1:1452-1(-) |
| 17 | 48 | TRINITY\_DN2971\_c0\_g1\_i1.p1 | 31.43 | 5 | 5 | 3.12e3 | 1 | 1 | 2 | Acetylation (Protein N-term), Oxidation (M) | 26549.3965 | type:complete len:239 gc:universal TRINITY\_DN2971\_c0\_g1\_i1:125-841(+) |
| 18 | 49 | TRINITY\_DN8155\_c0\_g1\_i1.p1 | 31.26 | 6 | 6 | 4.93e2 | 1 | 1 | 1 | Carbamidomethylation | 27519.8945 | type:internal len:249 gc:universal TRINITY\_DN8155\_c0\_g1\_i1:744-1(-) |
| 19 | 50 | TRINITY\_DN161\_c0\_g1\_i2.p1 | 31.42 | 3 | 3 | 8.41e3 | 1 | 1 | 1 |  | 44577.6484 | type:5prime\_partial len:399 gc:universal TRINITY\_DN161\_c0\_g1\_i2:1683-487(-) |
| 19 | 51 | TRINITY\_DN161\_c0\_g1\_i3.p1 | 31.42 | 3 | 3 | 8.41e3 | 1 | 1 | 1 |  | 44592.6602 | type:5prime\_partial len:399 gc:universal TRINITY\_DN161\_c0\_g1\_i3:1450-254(-) |
| 19 | 52 | TRINITY\_DN161\_c0\_g1\_i1.p1 | 31.42 | 3 | 3 | 8.41e3 | 1 | 1 | 1 |  | 41918.5938 | type:complete len:377 gc:universal TRINITY\_DN161\_c0\_g1\_i1:1384-254(-) |
| 20 | 53 | TRINITY\_DN1058\_c0\_g1\_i1.p1 | 31.4 | 0 | 0 | 4.55e3 | 1 | 1 | 3 |  | 284466.2812 | type:complete len:2639 gc:universal TRINITY\_DN1058\_c0\_g1\_i1:134-8050(+) |
| 20 | 54 | TRINITY\_DN1058\_c0\_g1\_i3.p1 | 31.4 | 0 | 0 | 4.55e3 | 1 | 1 | 3 |  | 277633.5312 | type:complete len:2581 gc:universal TRINITY\_DN1058\_c0\_g1\_i3:134-7876(+) |
| 20 | 55 | TRINITY\_DN1058\_c0\_g1\_i4.p1 | 31.4 | 2 | 2 | 4.55e3 | 1 | 1 | 3 |  | 79375.5703 | type:complete len:723 gc:universal TRINITY\_DN1058\_c0\_g1\_i4:366-2534(+) |
| 20 | 56 | TRINITY\_DN1058\_c0\_g1\_i2.p2 | 31.4 | 1 | 1 | 4.55e3 | 1 | 1 | 3 |  | 140284.0938 | type:complete len:1300 gc:universal TRINITY\_DN1058\_c0\_g1\_i2:4090-7989(+) |
| 21 | 57 | TRINITY\_DN691\_c0\_g1\_i1.p1 | 32.5 | 1 | 1 | 7.47e2 | 1 | 1 | 2 | Carbamidomethylation | 169920.1562 | type:complete len:1726 gc:universal TRINITY\_DN691\_c0\_g1\_i1:5662-485(-) |
| 21 | 58 | TRINITY\_DN691\_c0\_g1\_i2.p1 | 32.5 | 1 | 1 | 7.47e2 | 1 | 1 | 2 | Carbamidomethylation | 164625.2188 | type:complete len:1673 gc:universal TRINITY\_DN691\_c0\_g1\_i2:5503-485(-) |
| 22 | 59 | TRINITY\_DN1616\_c0\_g1\_i2.p1 | 31.36 | 1 | 1 | 4.35e2 | 1 | 1 | 1 |  | 374396 | type:3prime\_partial len:3398 gc:universal TRINITY\_DN1616\_c0\_g1\_i2:218-10408(+) |
| 22 | 60 | TRINITY\_DN1616\_c0\_g1\_i3.p1 | 31.36 | 0 | 0 | 4.35e2 | 1 | 1 | 1 |  | 423947.2812 | type:complete len:3855 gc:universal TRINITY\_DN1616\_c0\_g1\_i3:218-11782(+) |
| 22 | 61 | TRINITY\_DN1616\_c0\_g1\_i7.p1 | 31.36 | 0 | 0 | 4.35e2 | 1 | 1 | 1 |  | 423875.2188 | type:complete len:3855 gc:universal TRINITY\_DN1616\_c0\_g1\_i7:218-11782(+) |
| 22 | 62 | TRINITY\_DN1616\_c0\_g1\_i6.p1 | 31.36 | 0 | 0 | 4.35e2 | 1 | 1 | 1 |  | 423911.25 | type:complete len:3855 gc:universal TRINITY\_DN1616\_c0\_g1\_i6:218-11782(+) |
| 23 | 63 | TRINITY\_DN8903\_c0\_g1\_i1.p1 | 30.28 | 2 | 2 | 3.64e2 | 1 | 1 | 1 |  | 50029.5586 | type:5prime\_partial len:447 gc:universal TRINITY\_DN8903\_c0\_g1\_i1:1546-206(-) |
| 24 | 64 | TRINITY\_DN11377\_c0\_g1\_i1.p1 | 31.32 | 1 | 1 | 1.04e3 | 1 | 1 | 2 |  | 89751.1094 | type:complete len:804 gc:universal TRINITY\_DN11377\_c0\_g1\_i1:3179-768(-) |
| 25 | 65 | TRINITY\_DN1071\_c0\_g1\_i3.p1 | 31.42 | 4 | 4 | 5.31e3 | 1 | 1 | 5 |  | 39803.1602 | type:internal len:375 gc:universal TRINITY\_DN1071\_c0\_g1\_i3:1124-3(-) |
| 25 | 66 | TRINITY\_DN1071\_c0\_g1\_i2.p1 | 31.42 | 5 | 5 | 5.31e3 | 1 | 1 | 5 |  | 36243.2383 | type:internal len:339 gc:universal TRINITY\_DN1071\_c0\_g1\_i2:1016-3(-) |
| 25 | 67 | TRINITY\_DN1071\_c0\_g1\_i1.p1 | 31.42 | 2 | 2 | 5.31e3 | 1 | 1 | 5 |  | 71848.4062 | type:internal len:660 gc:universal TRINITY\_DN1071\_c0\_g1\_i1:1977-1(-) |
| 25 | 68 | TRINITY\_DN1071\_c0\_g1\_i4.p1 | 31.42 | 2 | 2 | 5.31e3 | 1 | 1 | 5 |  | 75408.3359 | type:internal len:696 gc:universal TRINITY\_DN1071\_c0\_g1\_i4:2085-1(-) |
| 26 | 69 | TRINITY\_DN51907\_c0\_g1\_i1.p1 | 31.84 | 3 | 3 | 3.99e2 | 1 | 1 | 1 |  | 47638.75 | type:3prime\_partial len:421 gc:universal TRINITY\_DN51907\_c0\_g1\_i1:1262-3(-) |
| 27 | 70 | TRINITY\_DN4364\_c0\_g1\_i1.p1 | 31.42 | 2 | 2 | 0 | 1 | 1 | 1 | Carbamidomethylation | 61209.3516 | type:complete len:545 gc:universal TRINITY\_DN4364\_c0\_g1\_i1:1962-328(-) |
| 28 | 71 | TRINITY\_DN11595\_c0\_g1\_i1.p1 | 31.41 | 2 | 2 | 4.13e2 | 1 | 1 | 1 |  | 58738.1367 | type:complete len:510 gc:universal TRINITY\_DN11595\_c0\_g1\_i1:1768-239(-) |
| 28 | 72 | TRINITY\_DN11595\_c0\_g1\_i2.p1 | 31.41 | 2 | 2 | 4.13e2 | 1 | 1 | 1 |  | 70926.875 | type:complete len:615 gc:universal TRINITY\_DN11595\_c0\_g1\_i2:1952-108(-) |
| 28 | 73 | TRINITY\_DN1285\_c0\_g1\_i1.p1 | 31.41 | 2 | 2 | 4.13e2 | 1 | 1 | 1 |  | 74668.875 | type:complete len:658 gc:universal TRINITY\_DN1285\_c0\_g1\_i1:2316-343(-) |
| 29 | 74 | TRINITY\_DN661\_c0\_g1\_i10.p1 | 30.76 | 7 | 7 | 0 | 1 | 1 | 2 | Carbamidomethylation | 28014.7773 | type:complete len:253 gc:universal TRINITY\_DN661\_c0\_g1\_i10:1712-2470(+) |
| 30 | 75 | TRINITY\_DN79126\_c0\_g1\_i1.p1 | 31.42 | 10 | 10 | 7.81e2 | 1 | 1 | 1 |  | 13015.4727 | type:complete len:115 gc:universal TRINITY\_DN79126\_c0\_g1\_i1:397-53(-) |
| 31 | 76 | TRINITY\_DN4595\_c0\_g1\_i4.p1 | 30.75 | 0 | 0 | 2.17e3 | 1 | 1 | 1 |  | 183684.4844 | type:5prime\_partial len:1639 gc:universal TRINITY\_DN4595\_c0\_g1\_i4:5857-941(-) |
| 31 | 77 | TRINITY\_DN4595\_c0\_g1\_i10.p1 | 30.75 | 1 | 1 | 2.17e3 | 1 | 1 | 1 |  | 133760.0625 | type:complete len:1188 gc:universal TRINITY\_DN4595\_c0\_g1\_i10:4405-842(-) |
| 32 | 78 | TRINITY\_DN140702\_c0\_g1\_i1.p1 | 31.43 | 1 | 1 | 7.52e2 | 1 | 1 | 1 |  | 231919.1562 | type:complete len:2036 gc:universal TRINITY\_DN140702\_c0\_g1\_i1:94-6201(+) |
| 33 | 79 | TRINITY\_DN121418\_c0\_g1\_i1.p1 | 31.42 | 4 | 4 | 2.38e3 | 1 | 1 | 1 |  | 29750.6836 | type:complete len:259 gc:universal TRINITY\_DN121418\_c0\_g1\_i1:1061-285(-) |
| 34 | 80 | TRINITY\_DN1936\_c0\_g1\_i3.p1 | 31.43 | 1 | 1 | 0 | 1 | 1 | 1 |  | 119975.3906 | type:internal len:1070 gc:universal TRINITY\_DN1936\_c0\_g1\_i3:3208-2(-) |
| 35 | 81 | TRINITY\_DN1386\_c0\_g1\_i1.p1 | 31.42 | 2 | 2 | 8.62e2 | 1 | 1 | 1 |  | 68692.1953 | type:complete len:596 gc:universal TRINITY\_DN1386\_c0\_g1\_i1:2419-632(-) |
| 36 | 82 | TRINITY\_DN2490\_c0\_g1\_i1.p1 | 36.06 | 1 | 1 | 4.52e2 | 1 | 1 | 1 |  | 116032.3672 | type:complete len:1044 gc:universal TRINITY\_DN2490\_c0\_g1\_i1:354-3485(+) |
| 37 | 83 | TRINITY\_DN17721\_c0\_g1\_i2.p1 | 31.42 | 3 | 3 | 5.55e2 | 1 | 1 | 1 |  | 39372.3242 | type:5prime\_partial len:364 gc:universal TRINITY\_DN17721\_c0\_g1\_i2:2-1093(+) |
| 37 | 84 | TRINITY\_DN17721\_c0\_g1\_i1.p1 | 31.42 | 3 | 3 | 5.55e2 | 1 | 1 | 1 |  | 39368.3164 | type:5prime\_partial len:364 gc:universal TRINITY\_DN17721\_c0\_g1\_i1:2-1093(+) |
| 38 | 85 | TRINITY\_DN15584\_c0\_g1\_i1.p1 | 31.38 | 6 | 6 | 5.16e2 | 1 | 1 | 1 |  | 29534.9648 | type:complete len:275 gc:universal TRINITY\_DN15584\_c0\_g1\_i1:874-50(-) |
| 39 | 86 | TRINITY\_DN1785\_c0\_g1\_i1.p1 | 30.07 | 3 | 3 | 6.21e2 | 1 | 1 | 1 |  | 67156.3125 | type:complete len:615 gc:universal TRINITY\_DN1785\_c0\_g1\_i1:2316-472(-) |
| 40 | 87 | TRINITY\_DN2487\_c1\_g1\_i3.p1 | 30.68 | 3 | 3 | 0 | 1 | 1 | 1 |  | 34312.3867 | type:internal len:317 gc:universal TRINITY\_DN2487\_c1\_g1\_i3:1-948(+) |
| 41 | 88 | TRINITY\_DN2433\_c0\_g1\_i1.p1 | 31.42 | 5 | 5 | 5.03e2 | 1 | 1 | 1 |  | 31350.3457 | type:5prime\_partial len:267 gc:universal TRINITY\_DN2433\_c0\_g1\_i1:1-801(+) |
| 41 | 89 | TRINITY\_DN2433\_c0\_g1\_i2.p1 | 31.42 | 10 | 10 | 5.03e2 | 1 | 1 | 1 |  | 17063.7168 | type:5prime\_partial len:147 gc:universal TRINITY\_DN2433\_c0\_g1\_i2:1-441(+) |
| 42 | 90 | TRINITY\_DN17714\_c0\_g1\_i1.p1 | 31.89 | 3 | 3 | 0 | 1 | 1 | 1 | Carbamidomethylation | 72261.3516 | type:complete len:644 gc:universal TRINITY\_DN17714\_c0\_g1\_i1:102-2033(+) |
| 43 | 91 | TRINITY\_DN12627\_c0\_g1\_i1.p1 | 31.98 | 1 | 1 | 4.29e2 | 1 | 1 | 1 |  | 120592.0703 | type:5prime\_partial len:1061 gc:universal TRINITY\_DN12627\_c0\_g1\_i1:3456-274(-) |
| 44 | 92 | TRINITY\_DN4927\_c0\_g1\_i10.p1 | 32.17 | 1 | 1 | 0 | 1 | 1 | 1 |  | 198368.5156 | type:3prime\_partial len:1754 gc:universal TRINITY\_DN4927\_c0\_g1\_i10:5260-2(-) |
| 44 | 93 | TRINITY\_DN4927\_c0\_g1\_i9.p1 | 32.17 | 1 | 1 | 0 | 1 | 1 | 1 |  | 198368.5156 | type:3prime\_partial len:1754 gc:universal TRINITY\_DN4927\_c0\_g1\_i9:5260-2(-) |
| 45 | 94 | TRINITY\_DN598\_c1\_g1\_i1.p1 | 31.16 | 2 | 2 | 0 | 1 | 1 | 1 |  | 81322.8672 | type:5prime\_partial len:745 gc:universal TRINITY\_DN598\_c1\_g1\_i1:2-2236(+) |
| 46 | 95 | TRINITY\_DN2963\_c0\_g1\_i1.p1 | 31.4 | 10 | 10 | 5.92e3 | 1 | 1 | 2 | Acetylation (Protein N-term) | 13082.1787 | type:complete len:116 gc:universal TRINITY\_DN2963\_c0\_g1\_i1:1072-725(-) |
| 47 | 96 | TRINITY\_DN1172\_c0\_g2\_i2.p1 | 31.07 | 4 | 4 | 1.23e3 | 1 | 1 | 1 | Acetylation (Protein N-term) | 27813.4355 | type:internal len:264 gc:universal TRINITY\_DN1172\_c0\_g2\_i2:790-2(-) |
| 48 | 97 | TRINITY\_DN1559\_c0\_g1\_i1.p1 | 31.42 | 5 | 5 | 6.91e3 | 1 | 1 | 2 |  | 33672.0977 | type:complete len:311 gc:universal TRINITY\_DN1559\_c0\_g1\_i1:221-1153(+) |
| 49 | 98 | TRINITY\_DN33328\_c0\_g1\_i1.p1 | 31.76 | 1 | 1 | 0 | 1 | 1 | 1 |  | 156121.4062 | type:3prime\_partial len:1422 gc:universal TRINITY\_DN33328\_c0\_g1\_i1:4264-2(-) |
| 50 | 99 | TRINITY\_DN191687\_c0\_g1\_i1.p1 | 30.22 | 4 | 4 | 5.44e3 | 1 | 1 | 1 |  | 25234.6445 | type:internal len:241 gc:universal TRINITY\_DN191687\_c0\_g1\_i1:1-720(+) |
| 51 | 100 | TRINITY\_DN5554\_c0\_g1\_i1.p1 | 31.94 | 4 | 4 | 3.3e2 | 1 | 1 | 1 |  | 35530.5234 | type:complete len:316 gc:universal TRINITY\_DN5554\_c0\_g1\_i1:223-1170(+) |
| 52 | 101 | TRINITY\_DN195348\_c0\_g1\_i1.p1 | 30.08 | 5 | 5 | 2.58e2 | 1 | 1 | 1 |  | 34993.6055 | type:internal len:311 gc:universal TRINITY\_DN195348\_c0\_g1\_i1:1-930(+) |
| 53 | 102 | TRINITY\_DN113\_c0\_g1\_i1.p1 | 32.03 | 1 | 1 | 6.04e3 | 1 | 1 | 2 |  | 139235.5938 | type:complete len:1417 gc:universal TRINITY\_DN113\_c0\_g1\_i1:4413-163(-) |
| 54 | 103 | TRINITY\_DN761\_c0\_g1\_i2.p1 | 30.31 | 2 | 2 | 1.31e3 | 1 | 1 | 1 |  | 95330.7812 | type:complete len:830 gc:universal TRINITY\_DN761\_c0\_g1\_i2:87-2576(+) |
| 54 | 104 | TRINITY\_DN761\_c0\_g1\_i1.p1 | 30.31 | 2 | 2 | 1.31e3 | 1 | 1 | 1 |  | 95330.7812 | type:complete len:830 gc:universal TRINITY\_DN761\_c0\_g1\_i1:129-2618(+) |
| 54 | 105 | TRINITY\_DN761\_c0\_g1\_i3.p1 | 30.31 | 2 | 2 | 1.31e3 | 1 | 1 | 1 |  | 96968.6562 | type:complete len:845 gc:universal TRINITY\_DN761\_c0\_g1\_i3:129-2663(+) |
| 55 | 106 | TRINITY\_DN163801\_c0\_g1\_i1.p1 | 31.4 | 3 | 3 | 6.71e2 | 1 | 1 | 1 | Acetylation (Protein N-term) | 33076.9062 | type:complete len:298 gc:universal TRINITY\_DN163801\_c0\_g1\_i1:55-948(+) |
| 56 | 107 | TRINITY\_DN1739\_c0\_g1\_i1.p1 | 31.32 | 3 | 3 | 0 | 1 | 1 | 1 |  | 46350.6953 | type:complete len:400 gc:universal TRINITY\_DN1739\_c0\_g1\_i1:86-1285(+) |
| 57 | 108 | TRINITY\_DN581\_c1\_g1\_i1.p1 | 31.41 | 2 | 2 | 2.56e3 | 1 | 1 | 1 | Carbamidomethylation | 53141.4414 | type:5prime\_partial len:481 gc:universal TRINITY\_DN581\_c1\_g1\_i1:2139-697(-) |
| 58 | 109 | TRINITY\_DN11726\_c0\_g1\_i1.p1 | 30.35 | 1 | 1 | 4.4e2 | 1 | 1 | 1 |  | 101973.8359 | type:3prime\_partial len:877 gc:universal TRINITY\_DN11726\_c0\_g1\_i1:2630-3(-) |
| 59 | 110 | TRINITY\_DN77\_c1\_g1\_i1.p1 | 31.42 | 1 | 1 | 3.02e3 | 1 | 1 | 2 |  | 198350.2344 | type:3prime\_partial len:1965 gc:universal TRINITY\_DN77\_c1\_g1\_i1:2-5893(+) |
| 60 | 111 | TRINITY\_DN31906\_c0\_g1\_i1.p1 | 31.38 | 2 | 2 | 3e2 | 1 | 1 | 1 |  | 66488.1875 | type:5prime\_partial len:595 gc:universal TRINITY\_DN31906\_c0\_g1\_i1:1953-169(-) |
| 61 | 112 | TRINITY\_DN5854\_c0\_g1\_i1.p1 | 31.42 | 2 | 2 | 0 | 1 | 1 | 1 |  | 49734.1484 | type:complete len:464 gc:universal TRINITY\_DN5854\_c0\_g1\_i1:1435-44(-) |
| 62 | 113 | TRINITY\_DN64\_c0\_g1\_i1.p1 | 32.02 | 3 | 3 | 1.31e2 | 1 | 1 | 1 |  | 51450.1914 | type:complete len:468 gc:universal TRINITY\_DN64\_c0\_g1\_i1:1555-152(-) |
| 63 | 114 | TRINITY\_DN15109\_c0\_g2\_i1.p1 | 31 | 4 | 4 | 1.94e3 | 1 | 1 | 1 | Acetylation (Protein N-term) | 23973.3203 | type:3prime\_partial len:209 gc:universal TRINITY\_DN15109\_c0\_g2\_i1:624-1(-) |
| 64 | 115 | TRINITY\_DN7102\_c0\_g1\_i1.p1 | 31.36 | 1 | 1 | 5.91e2 | 1 | 1 | 1 |  | 139040.8594 | type:5prime\_partial len:1263 gc:universal TRINITY\_DN7102\_c0\_g1\_i1:3-3791(+) |
| total 115 proteins |  |  |  |  |  |  |  |  |  |  |  |  |
| --- | --- | --- | --- | --- | --- | --- | --- | --- | --- | --- | --- | --- |

  

TRINITY\_DN2487\_c1\_g1\_i3.p1

back to list

  

| Protein Coverage
| Supporting Peptides
| Best Unique PSM
|

Protein Coverage:

Supporting Peptides:

| Peptide | Uniq | -10lgP | Mass | Length | ppm | m/z | z | RT | 1/k0 Start | 1/k0 End | Precursor ID | Area Sample 3 | #Feature | #Feature Sample 3 | Start | End | PTM | AScore | Found By |
| --- | --- | --- | --- | --- | --- | --- | --- | --- | --- | --- | --- | --- | --- | --- | --- | --- | --- | --- | --- |
| R.AAAPDFSK.E | Y | 30.68 | 805.397 | 8 | -4.73 | 403.7015 | 2 | 14.81 | 0.7363 | 0.7437 | 40420 | 0 | 0 | 0 | 39 | 46 |  |  | DB Search |
| total 1 peptides |  |  |  |  |  |  |  |  |  |  |  |  |  |  |  |  |  |  |  |
| --- | --- | --- | --- | --- | --- | --- | --- | --- | --- | --- | --- | --- | --- | --- | --- | --- | --- | --- | --- |

Best Unique PSM (Scan Sample\_1\_A1\_1\_3639.d:40420, m/z=403.7015, z=2, RT=14.81, ppm=-10.51):


TRINITY\_DN33328\_c0\_g1\_i1.p1

back to list

  

| Protein Coverage
| Supporting Peptides
| Best Unique PSM
|

Protein Coverage:

Supporting Peptides:

| Peptide | Uniq | -10lgP | Mass | Length | ppm | m/z | z | RT | 1/k0 Start | 1/k0 End | Precursor ID | Area Sample 3 | #Feature | #Feature Sample 3 | Start | End | PTM | AScore | Found By |
| --- | --- | --- | --- | --- | --- | --- | --- | --- | --- | --- | --- | --- | --- | --- | --- | --- | --- | --- | --- |
| K.IVIEKFDVNVATK.Y | Y | 31.76 | 1474.8395 | 13 | -5.09 | 738.419 | 2 | 16.54 | 1.0419 | 1.0492 | 48332 | 0 | 0 | 0 | 506 | 518 |  |  | DB Search |
| total 1 peptides |  |  |  |  |  |  |  |  |  |  |  |  |  |  |  |  |  |  |  |
| --- | --- | --- | --- | --- | --- | --- | --- | --- | --- | --- | --- | --- | --- | --- | --- | --- | --- | --- | --- |

Best Unique PSM (Scan Sample\_1\_A1\_1\_3639.d:48332, m/z=738.419, z=2, RT=16.54, ppm=-10.87):


TRINITY\_DN1559\_c0\_g1\_i1.p1

back to list

  

| Protein Coverage
| Supporting Peptides
| Best Unique PSM
|

Protein Coverage:

Supporting Peptides:

| Peptide | Uniq | -10lgP | Mass | Length | ppm | m/z | z | RT | 1/k0 Start | 1/k0 End | Precursor ID | Area Sample 3 | #Feature | #Feature Sample 3 | Start | End | PTM | AScore | Found By |
| --- | --- | --- | --- | --- | --- | --- | --- | --- | --- | --- | --- | --- | --- | --- | --- | --- | --- | --- | --- |
| R.GVAGAGVLAGFDKFK.E | Y | 31.42 | 1435.7823 | 15 | 7.26 | 479.6021 | 3 | 18.38 | 0.8469 | 0.8542 | 58601 | 6.91e3 | 2 | 2 | 282 | 296 |  |  | DB Search |
| total 1 peptides |  |  |  |  |  |  |  |  |  |  |  |  |  |  |  |  |  |  |  |
| --- | --- | --- | --- | --- | --- | --- | --- | --- | --- | --- | --- | --- | --- | --- | --- | --- | --- | --- | --- |

Best Unique PSM (Scan Sample\_1\_A1\_1\_3639.d:58601, m/z=479.6021, z=3, RT=18.38, ppm=1.48):


TRINITY\_DN477\_c0\_g1\_i9.p1

back to list

  

| Protein Coverage
| Supporting Peptides
| Best Unique PSM
|

Protein Coverage:

Supporting Peptides:

| Peptide | Uniq | -10lgP | Mass | Length | ppm | m/z | z | RT | 1/k0 Start | 1/k0 End | Precursor ID | Area Sample 3 | #Feature | #Feature Sample 3 | Start | End | PTM | AScore | Found By |
| --- | --- | --- | --- | --- | --- | --- | --- | --- | --- | --- | --- | --- | --- | --- | --- | --- | --- | --- | --- |
| K.DANSM(+15.99)PTQSR.Q | Y | 31.37 | 1121.4771 | 10 | -0.52 | 561.7423 | 2 | 9.05 | 0.836 | 0.8433 | 11164 | 1.28e2 | 1 | 1 | 51 | 60 | Oxidation (M) | M5:Oxidation (M):1000 | DB Search |
| R.TQAVLDRLNIEK.R | Y | 31.03 | 1398.783 | 12 | 4.65 | 700.398 | 2 | 15.43 | 1.0017 | 1.009 | 43214 | 1.51e3 | 1 | 1 | 220 | 231 |  |  | DB Search |
| total 2 peptides |  |  |  |  |  |  |  |  |  |  |  |  |  |  |  |  |  |  |  |
| --- | --- | --- | --- | --- | --- | --- | --- | --- | --- | --- | --- | --- | --- | --- | --- | --- | --- | --- | --- |

Best Unique PSM (Scan Sample\_1\_A1\_1\_3639.d:11164, m/z=561.2378, z=2, RT=9.06, ppm=-6.3):


TRINITY\_DN319\_c2\_g1\_i2.p1

back to list

  

| Protein Coverage
| Supporting Peptides
| Best Unique PSM
|

Protein Coverage:

Supporting Peptides:

| Peptide | Uniq | -10lgP | Mass | Length | ppm | m/z | z | RT | 1/k0 Start | 1/k0 End | Precursor ID | Area Sample 3 | #Feature | #Feature Sample 3 | Start | End | PTM | AScore | Found By |
| --- | --- | --- | --- | --- | --- | --- | --- | --- | --- | --- | --- | --- | --- | --- | --- | --- | --- | --- | --- |
| R.ADC(+57.02)GVENTC(+57.02)FGSTR.S | Y | 31.98 | 1572.6297 | 14 | 3.17 | 787.3201 | 2 | 13.66 | 1.0206 | 1.0279 | 34821 | 4.97e2 | 1 | 1 | 1578 | 1591 | Carbamidomethylation, Carbamidomethylation |  | DB Search |
| R.DPIC(+57.02)HC(+57.02)DTSK.Y | Y | 31.43 | 1231.4961 | 10 | 6.8 | 411.5064 | 3 | 9.93 | 0.7331 | 0.7404 | 15608 | 1e3 | 1 | 1 | 1448 | 1457 | Carbamidomethylation, Carbamidomethylation |  | DB Search |
| R.GSFSSHIC(+57.02)LC(+57.02)HDPAYK.S | Y | 31.43 | 1877.8189 | 16 | 3.63 | 626.9456 | 3 | 14.39 | 0.8814 | 0.8887 | 38439 | 1.55e3 | 1 | 1 | 1011 | 1026 | Carbamidomethylation, Carbamidomethylation |  | DB Search |
| total 3 peptides |  |  |  |  |  |  |  |  |  |  |  |  |  |  |  |  |  |  |  |
| --- | --- | --- | --- | --- | --- | --- | --- | --- | --- | --- | --- | --- | --- | --- | --- | --- | --- | --- | --- |

Best Unique PSM (Scan Sample\_1\_A1\_1\_3639.d:34821, m/z=787.3201, z=2, RT=13.66, ppm=-2.6):


TRINITY\_DN161\_c0\_g1\_i3.p1

back to list

  

| Protein Coverage
| Supporting Peptides
| Best Unique PSM
|

Protein Coverage:

Supporting Peptides:

| Peptide | Uniq | -10lgP | Mass | Length | ppm | m/z | z | RT | 1/k0 Start | 1/k0 End | Precursor ID | Area Sample 3 | #Feature | #Feature Sample 3 | Start | End | PTM | AScore | Found By |
| --- | --- | --- | --- | --- | --- | --- | --- | --- | --- | --- | --- | --- | --- | --- | --- | --- | --- | --- | --- |
| R.AVFPSIVGRPR.H | Y | 31.42 | 1197.6982 | 11 | 5.9 | 599.8564 | 2 | 15.80 | 0.9533 | 0.9606 | 44974 | 8.41e3 | 1 | 1 | 52 | 62 |  |  | DB Search |
| total 1 peptides |  |  |  |  |  |  |  |  |  |  |  |  |  |  |  |  |  |  |  |
| --- | --- | --- | --- | --- | --- | --- | --- | --- | --- | --- | --- | --- | --- | --- | --- | --- | --- | --- | --- |

Best Unique PSM (Scan Sample\_1\_A1\_1\_3639.d:44974, m/z=599.8564, z=2, RT=15.80, ppm=0.13):


TRINITY\_DN1785\_c0\_g1\_i1.p1

back to list

  

| Protein Coverage
| Supporting Peptides
| Best Unique PSM
|

Protein Coverage:

Supporting Peptides:

| Peptide | Uniq | -10lgP | Mass | Length | ppm | m/z | z | RT | 1/k0 Start | 1/k0 End | Precursor ID | Area Sample 3 | #Feature | #Feature Sample 3 | Start | End | PTM | AScore | Found By |
| --- | --- | --- | --- | --- | --- | --- | --- | --- | --- | --- | --- | --- | --- | --- | --- | --- | --- | --- | --- |
| K.AITKPFTNVIIQLDKK.I | Y | 30.07 | 1828.0822 | 16 | 4.04 | 610.3669 | 3 | 17.67 | 0.9004 | 0.9077 | 54223 | 6.21e2 | 1 | 1 | 253 | 268 |  |  | DB Search |
| total 1 peptides |  |  |  |  |  |  |  |  |  |  |  |  |  |  |  |  |  |  |  |
| --- | --- | --- | --- | --- | --- | --- | --- | --- | --- | --- | --- | --- | --- | --- | --- | --- | --- | --- | --- |

Best Unique PSM (Scan Sample\_1\_A1\_1\_3639.d:54223, m/z=610.3669, z=3, RT=17.67, ppm=-1.74):


TRINITY\_DN101\_c0\_g1\_i1.p1

back to list

  

| Protein Coverage
| Supporting Peptides
| Best Unique PSM
|

Protein Coverage:

Supporting Peptides:

| Peptide | Uniq | -10lgP | Mass | Length | ppm | m/z | z | RT | 1/k0 Start | 1/k0 End | Precursor ID | Area Sample 3 | #Feature | #Feature Sample 3 | Start | End | PTM | AScore | Found By |
| --- | --- | --- | --- | --- | --- | --- | --- | --- | --- | --- | --- | --- | --- | --- | --- | --- | --- | --- | --- |
| K.ESTLHLVLR.L | Y | 31.37 | 1066.6135 | 9 | -2.66 | 534.3095 | 2 | 15.64 | 0.8785 | 0.8858 | 44242 | 0 | 0 | 0 | 64 | 72 |  |  | DB Search |
| K.TITLEVEPSDTIENVK.A | Y | 30.07 | 1786.92 | 16 | 6.27 | 894.4677 | 2 | 17.53 | 1.1438 | 1.151 | 53352 | 6.91e2 | 1 | 1 | 12 | 27 |  |  | DB Search |
| total 2 peptides |  |  |  |  |  |  |  |  |  |  |  |  |  |  |  |  |  |  |  |
| --- | --- | --- | --- | --- | --- | --- | --- | --- | --- | --- | --- | --- | --- | --- | --- | --- | --- | --- | --- |

Best Unique PSM (Scan Sample\_1\_A1\_1\_3639.d:44242, m/z=534.3095, z=2, RT=15.64, ppm=-8.44):


TRINITY\_DN2577\_c1\_g1\_i2.p1

back to list

  

| Protein Coverage
| Supporting Peptides
| Best Unique PSM
|

Protein Coverage:

Supporting Peptides:

| Peptide | Uniq | -10lgP | Mass | Length | ppm | m/z | z | RT | 1/k0 Start | 1/k0 End | Precursor ID | Area Sample 3 | #Feature | #Feature Sample 3 | Start | End | PTM | AScore | Found By |
| --- | --- | --- | --- | --- | --- | --- | --- | --- | --- | --- | --- | --- | --- | --- | --- | --- | --- | --- | --- |
| R.SGDPLTIGNFIK.N | Y | 31.38 | 1260.6714 | 12 | -7.98 | 631.3343 | 2 | 19.73 | 0.8996 | 0.9069 | 67627 | 0 | 0 | 0 | 322 | 333 |  |  | DB Search |
| total 1 peptides |  |  |  |  |  |  |  |  |  |  |  |  |  |  |  |  |  |  |  |
| --- | --- | --- | --- | --- | --- | --- | --- | --- | --- | --- | --- | --- | --- | --- | --- | --- | --- | --- | --- |

Best Unique PSM (Scan Sample\_1\_A1\_1\_3639.d:67627, m/z=631.3343, z=2, RT=19.73, ppm=-13.76):


TRINITY\_DN10545\_c0\_g1\_i1.p3

back to list

  

| Protein Coverage
| Supporting Peptides
| Best Unique PSM
|

Protein Coverage:

Supporting Peptides:

| Peptide | Uniq | -10lgP | Mass | Length | ppm | m/z | z | RT | 1/k0 Start | 1/k0 End | Precursor ID | Area Sample 3 | #Feature | #Feature Sample 3 | Start | End | PTM | AScore | Found By |
| --- | --- | --- | --- | --- | --- | --- | --- | --- | --- | --- | --- | --- | --- | --- | --- | --- | --- | --- | --- |
| K.ESTLHLVLR.L | Y | 31.37 | 1066.6135 | 9 | -2.66 | 534.3095 | 2 | 15.64 | 0.8785 | 0.8858 | 44242 | 0 | 0 | 0 | 16 | 24 |  |  | DB Search |
| total 1 peptides |  |  |  |  |  |  |  |  |  |  |  |  |  |  |  |  |  |  |  |
| --- | --- | --- | --- | --- | --- | --- | --- | --- | --- | --- | --- | --- | --- | --- | --- | --- | --- | --- | --- |

Best Unique PSM (Scan Sample\_1\_A1\_1\_3639.d:44242, m/z=534.3095, z=2, RT=15.64, ppm=-8.44):


TRINITY\_DN2963\_c0\_g1\_i1.p1

back to list

  

| Protein Coverage
| Supporting Peptides
| Best Unique PSM
|

Protein Coverage:

Supporting Peptides:

| Peptide | Uniq | -10lgP | Mass | Length | ppm | m/z | z | RT | 1/k0 Start | 1/k0 End | Precursor ID | Area Sample 3 | #Feature | #Feature Sample 3 | Start | End | PTM | AScore | Found By |
| --- | --- | --- | --- | --- | --- | --- | --- | --- | --- | --- | --- | --- | --- | --- | --- | --- | --- | --- | --- |
| M.S(+42.01)ATGNLVTVLRK.L | Y | 31.4 | 1299.751 | 12 | 10.13 | 650.8856 | 2 | 17.20 | 0.9949 | 1.0021 | 51417 | 5.92e3 | 1 | 1 | 2 | 13 | Acetylation (Protein N-term) | S1:Acetylation (Protein N-term):1000 | DB Search |
| total 1 peptides |  |  |  |  |  |  |  |  |  |  |  |  |  |  |  |  |  |  |  |
| --- | --- | --- | --- | --- | --- | --- | --- | --- | --- | --- | --- | --- | --- | --- | --- | --- | --- | --- | --- |

Best Unique PSM (Scan Sample\_1\_A1\_1\_3639.d:51417, m/z=650.8856, z=2, RT=17.20, ppm=4.36):


TRINITY\_DN1071\_c0\_g1\_i1.p1

back to list

  

| Protein Coverage
| Supporting Peptides
| Best Unique PSM
|

Protein Coverage:

Supporting Peptides:

| Peptide | Uniq | -10lgP | Mass | Length | ppm | m/z | z | RT | 1/k0 Start | 1/k0 End | Precursor ID | Area Sample 3 | #Feature | #Feature Sample 3 | Start | End | PTM | AScore | Found By |
| --- | --- | --- | --- | --- | --- | --- | --- | --- | --- | --- | --- | --- | --- | --- | --- | --- | --- | --- | --- |
| K.AVQIHPFNDVSVGDPK.V | Y | 31.42 | 1721.8736 | 16 | 5.27 | 861.9437 | 2 | 15.88 | 1.1142 | 1.1214 | 45122 | 5.31e3 | 2 | 2 | 293 | 308 |  |  | DB Search |
| total 1 peptides |  |  |  |  |  |  |  |  |  |  |  |  |  |  |  |  |  |  |  |
| --- | --- | --- | --- | --- | --- | --- | --- | --- | --- | --- | --- | --- | --- | --- | --- | --- | --- | --- | --- |

Best Unique PSM (Scan Sample\_1\_A1\_1\_3639.d:45122, m/z=861.9437, z=2, RT=15.88, ppm=-0.5):


TRINITY\_DN1676\_c0\_g1\_i6.p1

back to list

  

| Protein Coverage
| Supporting Peptides
| Best Unique PSM
|

Protein Coverage:

Supporting Peptides:

| Peptide | Uniq | -10lgP | Mass | Length | ppm | m/z | z | RT | 1/k0 Start | 1/k0 End | Precursor ID | Area Sample 3 | #Feature | #Feature Sample 3 | Start | End | PTM | AScore | Found By |
| --- | --- | --- | --- | --- | --- | --- | --- | --- | --- | --- | --- | --- | --- | --- | --- | --- | --- | --- | --- |
| K.RMSEIAEEARK.H | Y | 34.53 | 1318.6663 | 11 | 6.48 | 440.563 | 3 | 10.97 | 0.7587 | 0.7661 | 21230 | 4.84e2 | 1 | 1 | 1192 | 1202 |  |  | DB Search |
| K.KSEGAAETGSVTISK.A | Y | 33.52 | 1463.7467 | 15 | 4.25 | 732.8795 | 2 | 11.40 | 0.9892 | 0.9965 | 23419 | 7.43e2 | 1 | 1 | 1135 | 1149 |  |  | DB Search |
| R.FELDRVNTFGDEVFGDPK.V | Y | 33.39 | 2083.985 | 18 | 9.37 | 695.6714 | 3 | 19.27 | 0.9133 | 0.9206 | 64506 | 4.68e2 | 1 | 1 | 252 | 269 |  |  | DB Search |
| R.SYYFAISDITVGGR.C | Y | 31.92 | 1547.762 | 14 | 8.82 | 774.8906 | 2 | 19.95 | 1.0452 | 1.0524 | 68928 | 3.71e2 | 1 | 1 | 273 | 286 |  |  | DB Search |
| K.LLQQHELDLTPLK.E | Y | 31.42 | 1546.8719 | 13 | -0.58 | 774.4383 | 2 | 16.85 | 1.0552 | 1.0624 | 49653 | 2.38e2 | 1 | 1 | 1589 | 1601 |  |  | DB Search |
| K.ITQLQNDDKEAR.E | Y | 31.41 | 1429.7161 | 12 | 7.95 | 715.8669 | 2 | 10.85 | 1.0255 | 1.0327 | 20687 | 0 | 0 | 0 | 1329 | 1340 |  |  | DB Search |
| total 6 peptides |  |  |  |  |  |  |  |  |  |  |  |  |  |  |  |  |  |  |  |
| --- | --- | --- | --- | --- | --- | --- | --- | --- | --- | --- | --- | --- | --- | --- | --- | --- | --- | --- | --- |

Best Unique PSM (Scan Sample\_1\_A1\_1\_3639.d:21230, m/z=440.563, z=3, RT=10.97, ppm=0.7):


TRINITY\_DN319\_c2\_g1\_i1.p1

back to list

  

| Protein Coverage
| Supporting Peptides
| Best Unique PSM
|

Protein Coverage:

Supporting Peptides:

| Peptide | Uniq | -10lgP | Mass | Length | ppm | m/z | z | RT | 1/k0 Start | 1/k0 End | Precursor ID | Area Sample 3 | #Feature | #Feature Sample 3 | Start | End | PTM | AScore | Found By |
| --- | --- | --- | --- | --- | --- | --- | --- | --- | --- | --- | --- | --- | --- | --- | --- | --- | --- | --- | --- |
| R.ADC(+57.02)GVENTC(+57.02)FGSTR.S | Y | 31.98 | 1572.6297 | 14 | 3.17 | 787.3201 | 2 | 13.66 | 1.0206 | 1.0279 | 34821 | 4.97e2 | 1 | 1 | 1578 | 1591 | Carbamidomethylation, Carbamidomethylation |  | DB Search |
| R.DPIC(+57.02)HC(+57.02)DTSK.Y | Y | 31.43 | 1231.4961 | 10 | 6.8 | 411.5064 | 3 | 9.93 | 0.7331 | 0.7404 | 15608 | 1e3 | 1 | 1 | 1448 | 1457 | Carbamidomethylation, Carbamidomethylation |  | DB Search |
| R.GSFSSHIC(+57.02)LC(+57.02)HDPAYK.S | Y | 31.43 | 1877.8189 | 16 | 3.63 | 626.9456 | 3 | 14.39 | 0.8814 | 0.8887 | 38439 | 1.55e3 | 1 | 1 | 1011 | 1026 | Carbamidomethylation, Carbamidomethylation |  | DB Search |
| K.KAQYYEAYETIIKR.H | Y | 31.42 | 1774.9254 | 14 | 2.25 | 592.647 | 3 | 15.86 | 0.8575 | 0.8648 | 45137 | 7.72e2 | 1 | 1 | 2534 | 2547 |  |  | DB Search |
| total 4 peptides |  |  |  |  |  |  |  |  |  |  |  |  |  |  |  |  |  |  |  |
| --- | --- | --- | --- | --- | --- | --- | --- | --- | --- | --- | --- | --- | --- | --- | --- | --- | --- | --- | --- |

Best Unique PSM (Scan Sample\_1\_A1\_1\_3639.d:34821, m/z=787.3201, z=2, RT=13.66, ppm=-2.6):


TRINITY\_DN761\_c0\_g1\_i2.p1

back to list

  

| Protein Coverage
| Supporting Peptides
| Best Unique PSM
|

Protein Coverage:

Supporting Peptides:

| Peptide | Uniq | -10lgP | Mass | Length | ppm | m/z | z | RT | 1/k0 Start | 1/k0 End | Precursor ID | Area Sample 3 | #Feature | #Feature Sample 3 | Start | End | PTM | AScore | Found By |
| --- | --- | --- | --- | --- | --- | --- | --- | --- | --- | --- | --- | --- | --- | --- | --- | --- | --- | --- | --- |
| K.TRIEDLETVISQTQDHR.H | Y | 30.31 | 2040.0236 | 17 | 3.9 | 681.0139 | 3 | 17.48 | 0.9594 | 0.9666 | 53079 | 1.31e3 | 1 | 1 | 260 | 276 |  |  | DB Search |
| total 1 peptides |  |  |  |  |  |  |  |  |  |  |  |  |  |  |  |  |  |  |  |
| --- | --- | --- | --- | --- | --- | --- | --- | --- | --- | --- | --- | --- | --- | --- | --- | --- | --- | --- | --- |

Best Unique PSM (Scan Sample\_1\_A1\_1\_3639.d:53079, m/z=681.0139, z=3, RT=17.48, ppm=-1.88):


TRINITY\_DN2433\_c0\_g1\_i1.p1

back to list

  

| Protein Coverage
| Supporting Peptides
| Best Unique PSM
|

Protein Coverage:

Supporting Peptides:

| Peptide | Uniq | -10lgP | Mass | Length | ppm | m/z | z | RT | 1/k0 Start | 1/k0 End | Precursor ID | Area Sample 3 | #Feature | #Feature Sample 3 | Start | End | PTM | AScore | Found By |
| --- | --- | --- | --- | --- | --- | --- | --- | --- | --- | --- | --- | --- | --- | --- | --- | --- | --- | --- | --- |
| R.KVNMPISIIALNHR.K | Y | 31.42 | 1604.9184 | 14 | 5.12 | 535.9797 | 3 | 17.25 | 0.8453 | 0.8526 | 51799 | 5.03e2 | 1 | 1 | 131 | 144 |  |  | DB Search |
| total 1 peptides |  |  |  |  |  |  |  |  |  |  |  |  |  |  |  |  |  |  |  |
| --- | --- | --- | --- | --- | --- | --- | --- | --- | --- | --- | --- | --- | --- | --- | --- | --- | --- | --- | --- |

Best Unique PSM (Scan Sample\_1\_A1\_1\_3639.d:51799, m/z=535.9797, z=3, RT=17.25, ppm=-0.66):


TRINITY\_DN152\_c0\_g1\_i9.p1

back to list

  

| Protein Coverage
| Supporting Peptides
| Best Unique PSM
|

Protein Coverage:

Supporting Peptides:

| Peptide | Uniq | -10lgP | Mass | Length | ppm | m/z | z | RT | 1/k0 Start | 1/k0 End | Precursor ID | Area Sample 3 | #Feature | #Feature Sample 3 | Start | End | PTM | AScore | Found By |
| --- | --- | --- | --- | --- | --- | --- | --- | --- | --- | --- | --- | --- | --- | --- | --- | --- | --- | --- | --- |
| K.ESTLHLVLR.L | Y | 31.37 | 1066.6135 | 9 | -2.66 | 534.3095 | 2 | 15.64 | 0.8785 | 0.8858 | 44242 | 0 | 0 | 0 | 24 | 32 |  |  | DB Search |
| total 1 peptides |  |  |  |  |  |  |  |  |  |  |  |  |  |  |  |  |  |  |  |
| --- | --- | --- | --- | --- | --- | --- | --- | --- | --- | --- | --- | --- | --- | --- | --- | --- | --- | --- | --- |

Best Unique PSM (Scan Sample\_1\_A1\_1\_3639.d:44242, m/z=534.3095, z=2, RT=15.64, ppm=-8.44):


TRINITY\_DN12627\_c0\_g1\_i1.p1

back to list

  

| Protein Coverage
| Supporting Peptides
| Best Unique PSM
|

Protein Coverage:

Supporting Peptides:

| Peptide | Uniq | -10lgP | Mass | Length | ppm | m/z | z | RT | 1/k0 Start | 1/k0 End | Precursor ID | Area Sample 3 | #Feature | #Feature Sample 3 | Start | End | PTM | AScore | Found By |
| --- | --- | --- | --- | --- | --- | --- | --- | --- | --- | --- | --- | --- | --- | --- | --- | --- | --- | --- | --- |
| K.FDFHSWIGLKK.I | Y | 31.98 | 1376.7241 | 11 | 3.29 | 459.9142 | 3 | 18.36 | 0.7437 | 0.751 | 58604 | 4.29e2 | 1 | 1 | 193 | 203 |  |  | DB Search |
| total 1 peptides |  |  |  |  |  |  |  |  |  |  |  |  |  |  |  |  |  |  |  |
| --- | --- | --- | --- | --- | --- | --- | --- | --- | --- | --- | --- | --- | --- | --- | --- | --- | --- | --- | --- |

Best Unique PSM (Scan Sample\_1\_A1\_1\_3639.d:58604, m/z=459.9142, z=3, RT=18.36, ppm=-2.49):


TRINITY\_DN1172\_c0\_g2\_i2.p1

back to list

  

| Protein Coverage
| Supporting Peptides
| Best Unique PSM
|

Protein Coverage:

Supporting Peptides:

| Peptide | Uniq | -10lgP | Mass | Length | ppm | m/z | z | RT | 1/k0 Start | 1/k0 End | Precursor ID | Area Sample 3 | #Feature | #Feature Sample 3 | Start | End | PTM | AScore | Found By |
| --- | --- | --- | --- | --- | --- | --- | --- | --- | --- | --- | --- | --- | --- | --- | --- | --- | --- | --- | --- |
| K.K(+42.01)LAAELANGR.L | Y | 31.07 | 1083.6036 | 10 | 6.78 | 542.8096 | 2 | 15.40 | 0.8644 | 0.8716 | 43065 | 1.23e3 | 1 | 1 | 19 | 28 | Acetylation (Protein N-term) | K1:Acetylation (Protein N-term):1000 | DB Search |
| total 1 peptides |  |  |  |  |  |  |  |  |  |  |  |  |  |  |  |  |  |  |  |
| --- | --- | --- | --- | --- | --- | --- | --- | --- | --- | --- | --- | --- | --- | --- | --- | --- | --- | --- | --- |

Best Unique PSM (Scan Sample\_1\_A1\_1\_3639.d:43065, m/z=542.8096, z=2, RT=15.40, ppm=1):


TRINITY\_DN4595\_c0\_g1\_i4.p1

back to list

  

| Protein Coverage
| Supporting Peptides
| Best Unique PSM
|

Protein Coverage:

Supporting Peptides:

| Peptide | Uniq | -10lgP | Mass | Length | ppm | m/z | z | RT | 1/k0 Start | 1/k0 End | Precursor ID | Area Sample 3 | #Feature | #Feature Sample 3 | Start | End | PTM | AScore | Found By |
| --- | --- | --- | --- | --- | --- | --- | --- | --- | --- | --- | --- | --- | --- | --- | --- | --- | --- | --- | --- |
| R.SPFVIMFK.G | Y | 30.75 | 967.5201 | 8 | 3.16 | 484.7661 | 2 | 19.85 | 0.8104 | 0.8177 | 68318 | 2.17e3 | 1 | 1 | 1081 | 1088 |  |  | DB Search |
| total 1 peptides |  |  |  |  |  |  |  |  |  |  |  |  |  |  |  |  |  |  |  |
| --- | --- | --- | --- | --- | --- | --- | --- | --- | --- | --- | --- | --- | --- | --- | --- | --- | --- | --- | --- |

Best Unique PSM (Scan Sample\_1\_A1\_1\_3639.d:68318, m/z=484.7661, z=2, RT=19.85, ppm=-2.62):


TRINITY\_DN15584\_c0\_g1\_i1.p1

back to list

  

| Protein Coverage
| Supporting Peptides
| Best Unique PSM
|

Protein Coverage:

Supporting Peptides:

| Peptide | Uniq | -10lgP | Mass | Length | ppm | m/z | z | RT | 1/k0 Start | 1/k0 End | Precursor ID | Area Sample 3 | #Feature | #Feature Sample 3 | Start | End | PTM | AScore | Found By |
| --- | --- | --- | --- | --- | --- | --- | --- | --- | --- | --- | --- | --- | --- | --- | --- | --- | --- | --- | --- |
| K.AFVAIGDYGGHVGLGVK.C | Y | 31.38 | 1658.878 | 17 | 7.13 | 830.4474 | 2 | 17.72 | 1.163 | 1.1702 | 54593 | 5.16e2 | 1 | 1 | 109 | 125 |  |  | DB Search |
| total 1 peptides |  |  |  |  |  |  |  |  |  |  |  |  |  |  |  |  |  |  |  |
| --- | --- | --- | --- | --- | --- | --- | --- | --- | --- | --- | --- | --- | --- | --- | --- | --- | --- | --- | --- |

Best Unique PSM (Scan Sample\_1\_A1\_1\_3639.d:54593, m/z=830.4474, z=2, RT=17.72, ppm=1.35):


TRINITY\_DN598\_c1\_g1\_i1.p1

back to list

  

| Protein Coverage
| Supporting Peptides
| Best Unique PSM
|

Protein Coverage:

Supporting Peptides:

| Peptide | Uniq | -10lgP | Mass | Length | ppm | m/z | z | RT | 1/k0 Start | 1/k0 End | Precursor ID | Area Sample 3 | #Feature | #Feature Sample 3 | Start | End | PTM | AScore | Found By |
| --- | --- | --- | --- | --- | --- | --- | --- | --- | --- | --- | --- | --- | --- | --- | --- | --- | --- | --- | --- |
| K.AVAEEFISVDPYVR.S | Y | 31.16 | 1593.8038 | 14 | 9.9 | 797.9125 | 2 | 19.05 | 1.0588 | 1.066 | 63171 | 0 | 0 | 0 | 148 | 161 |  |  | DB Search |
| total 1 peptides |  |  |  |  |  |  |  |  |  |  |  |  |  |  |  |  |  |  |  |
| --- | --- | --- | --- | --- | --- | --- | --- | --- | --- | --- | --- | --- | --- | --- | --- | --- | --- | --- | --- |

Best Unique PSM (Scan Sample\_1\_A1\_1\_3639.d:63171, m/z=797.9125, z=2, RT=19.05, ppm=4.12):


TRINITY\_DN585\_c0\_g1\_i2.p1

back to list

  

| Protein Coverage
| Supporting Peptides
| Best Unique PSM
|

Protein Coverage:

Supporting Peptides:

| Peptide | Uniq | -10lgP | Mass | Length | ppm | m/z | z | RT | 1/k0 Start | 1/k0 End | Precursor ID | Area Sample 3 | #Feature | #Feature Sample 3 | Start | End | PTM | AScore | Found By |
| --- | --- | --- | --- | --- | --- | --- | --- | --- | --- | --- | --- | --- | --- | --- | --- | --- | --- | --- | --- |
| K.ESTLHLVLR.L | Y | 31.37 | 1066.6135 | 9 | -2.66 | 534.3095 | 2 | 15.64 | 0.8785 | 0.8858 | 44242 | 0 | 0 | 0 | 36 | 44 |  |  | DB Search |
| K.TITLEVEPSDTIENVK.S | Y | 30.07 | 1786.92 | 16 | 6.27 | 894.4677 | 2 | 17.53 | 1.1438 | 1.151 | 53352 | 6.91e2 | 1 | 1 | 60 | 75 |  |  | DB Search |
| total 2 peptides |  |  |  |  |  |  |  |  |  |  |  |  |  |  |  |  |  |  |  |
| --- | --- | --- | --- | --- | --- | --- | --- | --- | --- | --- | --- | --- | --- | --- | --- | --- | --- | --- | --- |

Best Unique PSM (Scan Sample\_1\_A1\_1\_3639.d:44242, m/z=534.3095, z=2, RT=15.64, ppm=-8.44):


TRINITY\_DN4927\_c0\_g1\_i10.p1

back to list

  

| Protein Coverage
| Supporting Peptides
| Best Unique PSM
|

Protein Coverage:

Supporting Peptides:

| Peptide | Uniq | -10lgP | Mass | Length | ppm | m/z | z | RT | 1/k0 Start | 1/k0 End | Precursor ID | Area Sample 3 | #Feature | #Feature Sample 3 | Start | End | PTM | AScore | Found By |
| --- | --- | --- | --- | --- | --- | --- | --- | --- | --- | --- | --- | --- | --- | --- | --- | --- | --- | --- | --- |
| K.YLIEQEIDHFSQFNR.H | Y | 32.17 | 1937.9271 | 15 | 6.69 | 646.9836 | 3 | 19.66 | 0.8854 | 0.8927 | 67188 | 0 | 0 | 0 | 1039 | 1053 |  |  | DB Search |
| total 1 peptides |  |  |  |  |  |  |  |  |  |  |  |  |  |  |  |  |  |  |  |
| --- | --- | --- | --- | --- | --- | --- | --- | --- | --- | --- | --- | --- | --- | --- | --- | --- | --- | --- | --- |

Best Unique PSM (Scan Sample\_1\_A1\_1\_3639.d:67188, m/z=646.9836, z=3, RT=19.66, ppm=0.91):


TRINITY\_DN113\_c0\_g1\_i1.p1

back to list

  

| Protein Coverage
| Supporting Peptides
| Best Unique PSM
|

Protein Coverage:

Supporting Peptides:

| Peptide | Uniq | -10lgP | Mass | Length | ppm | m/z | z | RT | 1/k0 Start | 1/k0 End | Precursor ID | Area Sample 3 | #Feature | #Feature Sample 3 | Start | End | PTM | AScore | Found By |
| --- | --- | --- | --- | --- | --- | --- | --- | --- | --- | --- | --- | --- | --- | --- | --- | --- | --- | --- | --- |
| R.GKDGETGEQGPQGQEGPTGPR.G | Y | 32.03 | 2080.9409 | 21 | 5.04 | 694.6537 | 3 | 10.74 | 0.8498 | 0.8571 | 19974 | 6.04e3 | 2 | 2 | 693 | 713 |  |  | DB Search |
| total 1 peptides |  |  |  |  |  |  |  |  |  |  |  |  |  |  |  |  |  |  |  |
| --- | --- | --- | --- | --- | --- | --- | --- | --- | --- | --- | --- | --- | --- | --- | --- | --- | --- | --- | --- |

Best Unique PSM (Scan Sample\_1\_A1\_1\_3639.d:19974, m/z=694.6537, z=3, RT=10.74, ppm=-0.73):


TRINITY\_DN4927\_c0\_g1\_i9.p1

back to list

  

| Protein Coverage
| Supporting Peptides
| Best Unique PSM
|

Protein Coverage:

Supporting Peptides:

| Peptide | Uniq | -10lgP | Mass | Length | ppm | m/z | z | RT | 1/k0 Start | 1/k0 End | Precursor ID | Area Sample 3 | #Feature | #Feature Sample 3 | Start | End | PTM | AScore | Found By |
| --- | --- | --- | --- | --- | --- | --- | --- | --- | --- | --- | --- | --- | --- | --- | --- | --- | --- | --- | --- |
| K.YLIEQEIDHFSQFNR.H | Y | 32.17 | 1937.9271 | 15 | 6.69 | 646.9836 | 3 | 19.66 | 0.8854 | 0.8927 | 67188 | 0 | 0 | 0 | 1039 | 1053 |  |  | DB Search |
| total 1 peptides |  |  |  |  |  |  |  |  |  |  |  |  |  |  |  |  |  |  |  |
| --- | --- | --- | --- | --- | --- | --- | --- | --- | --- | --- | --- | --- | --- | --- | --- | --- | --- | --- | --- |

Best Unique PSM (Scan Sample\_1\_A1\_1\_3639.d:67188, m/z=646.9836, z=3, RT=19.66, ppm=0.91):


TRINITY\_DN1616\_c0\_g1\_i6.p1

back to list

  

| Protein Coverage
| Supporting Peptides
| Best Unique PSM
|

Protein Coverage:

Supporting Peptides:

| Peptide | Uniq | -10lgP | Mass | Length | ppm | m/z | z | RT | 1/k0 Start | 1/k0 End | Precursor ID | Area Sample 3 | #Feature | #Feature Sample 3 | Start | End | PTM | AScore | Found By |
| --- | --- | --- | --- | --- | --- | --- | --- | --- | --- | --- | --- | --- | --- | --- | --- | --- | --- | --- | --- |
| K.DGEKVETLKVDISDINK.A | Y | 31.36 | 1901.9945 | 17 | 4.01 | 635.0043 | 3 | 15.97 | 0.8976 | 0.9048 | 45629 | 4.35e2 | 1 | 1 | 2634 | 2650 |  |  | DB Search |
| total 1 peptides |  |  |  |  |  |  |  |  |  |  |  |  |  |  |  |  |  |  |  |
| --- | --- | --- | --- | --- | --- | --- | --- | --- | --- | --- | --- | --- | --- | --- | --- | --- | --- | --- | --- |

Best Unique PSM (Scan Sample\_1\_A1\_1\_3639.d:45629, m/z=635.0043, z=3, RT=15.97, ppm=-1.77):


TRINITY\_DN1739\_c0\_g1\_i1.p1

back to list

  

| Protein Coverage
| Supporting Peptides
| Best Unique PSM
|

Protein Coverage:

Supporting Peptides:

| Peptide | Uniq | -10lgP | Mass | Length | ppm | m/z | z | RT | 1/k0 Start | 1/k0 End | Precursor ID | Area Sample 3 | #Feature | #Feature Sample 3 | Start | End | PTM | AScore | Found By |
| --- | --- | --- | --- | --- | --- | --- | --- | --- | --- | --- | --- | --- | --- | --- | --- | --- | --- | --- | --- |
| M.AITDNIKDILSVK.I | Y | 31.32 | 1428.8188 | 13 | 12.14 | 715.4212 | 2 | 19.28 | 1.0504 | 1.0576 | 64605 | 0 | 0 | 0 | 2 | 14 |  |  | DB Search |
| total 1 peptides |  |  |  |  |  |  |  |  |  |  |  |  |  |  |  |  |  |  |  |
| --- | --- | --- | --- | --- | --- | --- | --- | --- | --- | --- | --- | --- | --- | --- | --- | --- | --- | --- | --- |

Best Unique PSM (Scan Sample\_1\_A1\_1\_3639.d:64605, m/z=715.4212, z=2, RT=19.28, ppm=6.37):


TRINITY\_DN1058\_c0\_g1\_i3.p1

back to list

  

| Protein Coverage
| Supporting Peptides
| Best Unique PSM
|

Protein Coverage:

Supporting Peptides:

| Peptide | Uniq | -10lgP | Mass | Length | ppm | m/z | z | RT | 1/k0 Start | 1/k0 End | Precursor ID | Area Sample 3 | #Feature | #Feature Sample 3 | Start | End | PTM | AScore | Found By |
| --- | --- | --- | --- | --- | --- | --- | --- | --- | --- | --- | --- | --- | --- | --- | --- | --- | --- | --- | --- |
| R.APTHILNPANKR.S | Y | 31.4 | 1330.7469 | 12 | 6.7 | 444.59 | 3 | 11.12 | 0.7445 | 0.7518 | 21628 | 4.55e3 | 2 | 2 | 2314 | 2325 |  |  | DB Search |
| total 1 peptides |  |  |  |  |  |  |  |  |  |  |  |  |  |  |  |  |  |  |  |
| --- | --- | --- | --- | --- | --- | --- | --- | --- | --- | --- | --- | --- | --- | --- | --- | --- | --- | --- | --- |

Best Unique PSM (Scan Sample\_1\_A1\_1\_3639.d:21628, m/z=444.59, z=3, RT=11.12, ppm=0.92):


TRINITY\_DN191687\_c0\_g1\_i1.p1

back to list

  

| Protein Coverage
| Supporting Peptides
| Best Unique PSM
|

Protein Coverage:

Supporting Peptides:

| Peptide | Uniq | -10lgP | Mass | Length | ppm | m/z | z | RT | 1/k0 Start | 1/k0 End | Precursor ID | Area Sample 3 | #Feature | #Feature Sample 3 | Start | End | PTM | AScore | Found By |
| --- | --- | --- | --- | --- | --- | --- | --- | --- | --- | --- | --- | --- | --- | --- | --- | --- | --- | --- | --- |
| R.RVDHQGGGVR.G | Y | 30.22 | 1079.5584 | 10 | -9.32 | 360.8546 | 3 | 10.47 | 0.7298 | 0.7371 | 18621 | 5.44e3 | 1 | 1 | 98 | 107 |  |  | DB Search |
| total 1 peptides |  |  |  |  |  |  |  |  |  |  |  |  |  |  |  |  |  |  |  |
| --- | --- | --- | --- | --- | --- | --- | --- | --- | --- | --- | --- | --- | --- | --- | --- | --- | --- | --- | --- |

Best Unique PSM (Scan Sample\_1\_A1\_1\_3639.d:18621, m/z=360.8546, z=3, RT=10.47, ppm=-15.1):


TRINITY\_DN2433\_c0\_g1\_i2.p1

back to list

  

| Protein Coverage
| Supporting Peptides
| Best Unique PSM
|

Protein Coverage:

Supporting Peptides:

| Peptide | Uniq | -10lgP | Mass | Length | ppm | m/z | z | RT | 1/k0 Start | 1/k0 End | Precursor ID | Area Sample 3 | #Feature | #Feature Sample 3 | Start | End | PTM | AScore | Found By |
| --- | --- | --- | --- | --- | --- | --- | --- | --- | --- | --- | --- | --- | --- | --- | --- | --- | --- | --- | --- |
| R.KVNMPISIIALNHR.K | Y | 31.42 | 1604.9184 | 14 | 5.12 | 535.9797 | 3 | 17.25 | 0.8453 | 0.8526 | 51799 | 5.03e2 | 1 | 1 | 11 | 24 |  |  | DB Search |
| total 1 peptides |  |  |  |  |  |  |  |  |  |  |  |  |  |  |  |  |  |  |  |
| --- | --- | --- | --- | --- | --- | --- | --- | --- | --- | --- | --- | --- | --- | --- | --- | --- | --- | --- | --- |

Best Unique PSM (Scan Sample\_1\_A1\_1\_3639.d:51799, m/z=535.9797, z=3, RT=17.25, ppm=-0.66):


TRINITY\_DN585\_c0\_g2\_i1.p1

back to list

  

| Protein Coverage
| Supporting Peptides
| Best Unique PSM
|

Protein Coverage:

Supporting Peptides:

| Peptide | Uniq | -10lgP | Mass | Length | ppm | m/z | z | RT | 1/k0 Start | 1/k0 End | Precursor ID | Area Sample 3 | #Feature | #Feature Sample 3 | Start | End | PTM | AScore | Found By |
| --- | --- | --- | --- | --- | --- | --- | --- | --- | --- | --- | --- | --- | --- | --- | --- | --- | --- | --- | --- |
| K.ESTLHLVLR.L | Y | 31.37 | 1066.6135 | 9 | -2.66 | 534.3095 | 2 | 15.64 | 0.8785 | 0.8858 | 44242 | 0 | 0 | 0 | 57 | 65 |  |  | DB Search |
| K.TITLEVEPSDTIENVK.S | Y | 30.07 | 1786.92 | 16 | 6.27 | 894.4677 | 2 | 17.53 | 1.1438 | 1.151 | 53352 | 6.91e2 | 1 | 1 | 5 | 20 |  |  | DB Search |
| total 2 peptides |  |  |  |  |  |  |  |  |  |  |  |  |  |  |  |  |  |  |  |
| --- | --- | --- | --- | --- | --- | --- | --- | --- | --- | --- | --- | --- | --- | --- | --- | --- | --- | --- | --- |

Best Unique PSM (Scan Sample\_1\_A1\_1\_3639.d:44242, m/z=534.3095, z=2, RT=15.64, ppm=-8.44):


TRINITY\_DN31906\_c0\_g1\_i1.p1

back to list

  

| Protein Coverage
| Supporting Peptides
| Best Unique PSM
|

Protein Coverage:

Supporting Peptides:

| Peptide | Uniq | -10lgP | Mass | Length | ppm | m/z | z | RT | 1/k0 Start | 1/k0 End | Precursor ID | Area Sample 3 | #Feature | #Feature Sample 3 | Start | End | PTM | AScore | Found By |
| --- | --- | --- | --- | --- | --- | --- | --- | --- | --- | --- | --- | --- | --- | --- | --- | --- | --- | --- | --- |
| K.TLSIDLLPFEKPR.Y | Y | 31.38 | 1527.866 | 13 | 9.86 | 510.298 | 3 | 19.94 | 0.8218 | 0.8291 | 68839 | 3e2 | 1 | 1 | 369 | 381 |  |  | DB Search |
| total 1 peptides |  |  |  |  |  |  |  |  |  |  |  |  |  |  |  |  |  |  |  |
| --- | --- | --- | --- | --- | --- | --- | --- | --- | --- | --- | --- | --- | --- | --- | --- | --- | --- | --- | --- |

Best Unique PSM (Scan Sample\_1\_A1\_1\_3639.d:68839, m/z=510.298, z=3, RT=19.94, ppm=4.08):


TRINITY\_DN581\_c1\_g1\_i1.p1

back to list

  

| Protein Coverage
| Supporting Peptides
| Best Unique PSM
|

Protein Coverage:

Supporting Peptides:

| Peptide | Uniq | -10lgP | Mass | Length | ppm | m/z | z | RT | 1/k0 Start | 1/k0 End | Precursor ID | Area Sample 3 | #Feature | #Feature Sample 3 | Start | End | PTM | AScore | Found By |
| --- | --- | --- | --- | --- | --- | --- | --- | --- | --- | --- | --- | --- | --- | --- | --- | --- | --- | --- | --- |
| R.IPITC(+57.02)YYPTSGK.A | Y | 31.41 | 1398.6853 | 12 | 7.57 | 700.3512 | 2 | 15.79 | 0.9763 | 0.9836 | 44811 | 2.56e3 | 1 | 1 | 222 | 233 | Carbamidomethylation |  | DB Search |
| total 1 peptides |  |  |  |  |  |  |  |  |  |  |  |  |  |  |  |  |  |  |  |
| --- | --- | --- | --- | --- | --- | --- | --- | --- | --- | --- | --- | --- | --- | --- | --- | --- | --- | --- | --- |

Best Unique PSM (Scan Sample\_1\_A1\_1\_3639.d:44811, m/z=700.3512, z=2, RT=15.79, ppm=1.8):


TRINITY\_DN5854\_c0\_g1\_i1.p1

back to list

  

| Protein Coverage
| Supporting Peptides
| Best Unique PSM
|

Protein Coverage:

Supporting Peptides:

| Peptide | Uniq | -10lgP | Mass | Length | ppm | m/z | z | RT | 1/k0 Start | 1/k0 End | Precursor ID | Area Sample 3 | #Feature | #Feature Sample 3 | Start | End | PTM | AScore | Found By |
| --- | --- | --- | --- | --- | --- | --- | --- | --- | --- | --- | --- | --- | --- | --- | --- | --- | --- | --- | --- |
| R.GSDEVEHNVK.D | Y | 31.42 | 1112.5098 | 10 | 10.01 | 557.2645 | 2 | 9.66 | 0.8923 | 0.8996 | 14398 | 0 | 0 | 0 | 407 | 416 |  |  | DB Search |
| total 1 peptides |  |  |  |  |  |  |  |  |  |  |  |  |  |  |  |  |  |  |  |
| --- | --- | --- | --- | --- | --- | --- | --- | --- | --- | --- | --- | --- | --- | --- | --- | --- | --- | --- | --- |

Best Unique PSM (Scan Sample\_1\_A1\_1\_3639.d:14398, m/z=557.2645, z=2, RT=9.66, ppm=4.24):


TRINITY\_DN195348\_c0\_g1\_i1.p1

back to list

  

| Protein Coverage
| Supporting Peptides
| Best Unique PSM
|

Protein Coverage:

Supporting Peptides:

| Peptide | Uniq | -10lgP | Mass | Length | ppm | m/z | z | RT | 1/k0 Start | 1/k0 End | Precursor ID | Area Sample 3 | #Feature | #Feature Sample 3 | Start | End | PTM | AScore | Found By |
| --- | --- | --- | --- | --- | --- | --- | --- | --- | --- | --- | --- | --- | --- | --- | --- | --- | --- | --- | --- |
| K.VLIVISDGNEDFKEK.Q | Y | 30.08 | 1704.8934 | 15 | 3.65 | 853.4521 | 2 | 16.81 | 1.1694 | 1.1766 | 49463 | 2.58e2 | 1 | 1 | 132 | 146 |  |  | DB Search |
| total 1 peptides |  |  |  |  |  |  |  |  |  |  |  |  |  |  |  |  |  |  |  |
| --- | --- | --- | --- | --- | --- | --- | --- | --- | --- | --- | --- | --- | --- | --- | --- | --- | --- | --- | --- |

Best Unique PSM (Scan Sample\_1\_A1\_1\_3639.d:49463, m/z=853.4521, z=2, RT=16.81, ppm=-2.13):


TRINITY\_DN1285\_c0\_g1\_i1.p1

back to list

  

| Protein Coverage
| Supporting Peptides
| Best Unique PSM
|

Protein Coverage:

Supporting Peptides:

| Peptide | Uniq | -10lgP | Mass | Length | ppm | m/z | z | RT | 1/k0 Start | 1/k0 End | Precursor ID | Area Sample 3 | #Feature | #Feature Sample 3 | Start | End | PTM | AScore | Found By |
| --- | --- | --- | --- | --- | --- | --- | --- | --- | --- | --- | --- | --- | --- | --- | --- | --- | --- | --- | --- |
| K.LVHGDVFRPPR.A | Y | 31.41 | 1291.7149 | 11 | 7.13 | 646.8656 | 2 | 13.43 | 0.9699 | 0.9771 | 33689 | 4.13e2 | 1 | 1 | 344 | 354 |  |  | DB Search |
| total 1 peptides |  |  |  |  |  |  |  |  |  |  |  |  |  |  |  |  |  |  |  |
| --- | --- | --- | --- | --- | --- | --- | --- | --- | --- | --- | --- | --- | --- | --- | --- | --- | --- | --- | --- |

Best Unique PSM (Scan Sample\_1\_A1\_1\_3639.d:33689, m/z=646.8656, z=2, RT=13.43, ppm=1.36):


TRINITY\_DN585\_c0\_g1\_i4.p1

back to list

  

| Protein Coverage
| Supporting Peptides
| Best Unique PSM
|

Protein Coverage:

Supporting Peptides:

| Peptide | Uniq | -10lgP | Mass | Length | ppm | m/z | z | RT | 1/k0 Start | 1/k0 End | Precursor ID | Area Sample 3 | #Feature | #Feature Sample 3 | Start | End | PTM | AScore | Found By |
| --- | --- | --- | --- | --- | --- | --- | --- | --- | --- | --- | --- | --- | --- | --- | --- | --- | --- | --- | --- |
| K.ESTLHLVLR.L | Y | 31.37 | 1066.6135 | 9 | -2.66 | 534.3095 | 2 | 15.64 | 0.8785 | 0.8858 | 44242 | 0 | 0 | 0 | 64 | 72 |  |  | DB Search |
| K.TITLEVEPSDTIENVK.S | Y | 30.07 | 1786.92 | 16 | 6.27 | 894.4677 | 2 | 17.53 | 1.1438 | 1.151 | 53352 | 6.91e2 | 1 | 1 | 12 | 27 |  |  | DB Search |
| total 2 peptides |  |  |  |  |  |  |  |  |  |  |  |  |  |  |  |  |  |  |  |
| --- | --- | --- | --- | --- | --- | --- | --- | --- | --- | --- | --- | --- | --- | --- | --- | --- | --- | --- | --- |

Best Unique PSM (Scan Sample\_1\_A1\_1\_3639.d:44242, m/z=534.3095, z=2, RT=15.64, ppm=-8.44):


TRINITY\_DN1071\_c0\_g1\_i3.p1

back to list

  

| Protein Coverage
| Supporting Peptides
| Best Unique PSM
|

Protein Coverage:

Supporting Peptides:

| Peptide | Uniq | -10lgP | Mass | Length | ppm | m/z | z | RT | 1/k0 Start | 1/k0 End | Precursor ID | Area Sample 3 | #Feature | #Feature Sample 3 | Start | End | PTM | AScore | Found By |
| --- | --- | --- | --- | --- | --- | --- | --- | --- | --- | --- | --- | --- | --- | --- | --- | --- | --- | --- | --- |
| K.AVQIHPFNDVSVGDPK.V | Y | 31.42 | 1721.8736 | 16 | 5.27 | 861.9437 | 2 | 15.88 | 1.1142 | 1.1214 | 45122 | 5.31e3 | 2 | 2 | 329 | 344 |  |  | DB Search |
| total 1 peptides |  |  |  |  |  |  |  |  |  |  |  |  |  |  |  |  |  |  |  |
| --- | --- | --- | --- | --- | --- | --- | --- | --- | --- | --- | --- | --- | --- | --- | --- | --- | --- | --- | --- |

Best Unique PSM (Scan Sample\_1\_A1\_1\_3639.d:45122, m/z=861.9437, z=2, RT=15.88, ppm=-0.5):


TRINITY\_DN761\_c0\_g1\_i1.p1

back to list

  

| Protein Coverage
| Supporting Peptides
| Best Unique PSM
|

Protein Coverage:

Supporting Peptides:

| Peptide | Uniq | -10lgP | Mass | Length | ppm | m/z | z | RT | 1/k0 Start | 1/k0 End | Precursor ID | Area Sample 3 | #Feature | #Feature Sample 3 | Start | End | PTM | AScore | Found By |
| --- | --- | --- | --- | --- | --- | --- | --- | --- | --- | --- | --- | --- | --- | --- | --- | --- | --- | --- | --- |
| K.TRIEDLETVISQTQDHR.H | Y | 30.31 | 2040.0236 | 17 | 3.9 | 681.0139 | 3 | 17.48 | 0.9594 | 0.9666 | 53079 | 1.31e3 | 1 | 1 | 260 | 276 |  |  | DB Search |
| total 1 peptides |  |  |  |  |  |  |  |  |  |  |  |  |  |  |  |  |  |  |  |
| --- | --- | --- | --- | --- | --- | --- | --- | --- | --- | --- | --- | --- | --- | --- | --- | --- | --- | --- | --- |

Best Unique PSM (Scan Sample\_1\_A1\_1\_3639.d:53079, m/z=681.0139, z=3, RT=17.48, ppm=-1.88):


TRINITY\_DN761\_c0\_g1\_i3.p1

back to list

  

| Protein Coverage
| Supporting Peptides
| Best Unique PSM
|

Protein Coverage:

Supporting Peptides:

| Peptide | Uniq | -10lgP | Mass | Length | ppm | m/z | z | RT | 1/k0 Start | 1/k0 End | Precursor ID | Area Sample 3 | #Feature | #Feature Sample 3 | Start | End | PTM | AScore | Found By |
| --- | --- | --- | --- | --- | --- | --- | --- | --- | --- | --- | --- | --- | --- | --- | --- | --- | --- | --- | --- |
| K.TRIEDLETVISQTQDHR.H | Y | 30.31 | 2040.0236 | 17 | 3.9 | 681.0139 | 3 | 17.48 | 0.9594 | 0.9666 | 53079 | 1.31e3 | 1 | 1 | 275 | 291 |  |  | DB Search |
| total 1 peptides |  |  |  |  |  |  |  |  |  |  |  |  |  |  |  |  |  |  |  |
| --- | --- | --- | --- | --- | --- | --- | --- | --- | --- | --- | --- | --- | --- | --- | --- | --- | --- | --- | --- |

Best Unique PSM (Scan Sample\_1\_A1\_1\_3639.d:53079, m/z=681.0139, z=3, RT=17.48, ppm=-1.88):


TRINITY\_DN5554\_c0\_g1\_i1.p1

back to list

  

| Protein Coverage
| Supporting Peptides
| Best Unique PSM
|

Protein Coverage:

Supporting Peptides:

| Peptide | Uniq | -10lgP | Mass | Length | ppm | m/z | z | RT | 1/k0 Start | 1/k0 End | Precursor ID | Area Sample 3 | #Feature | #Feature Sample 3 | Start | End | PTM | AScore | Found By |
| --- | --- | --- | --- | --- | --- | --- | --- | --- | --- | --- | --- | --- | --- | --- | --- | --- | --- | --- | --- |
| K.FFGSLDQIKFEDQK.V | Y | 31.94 | 1700.8409 | 14 | 6.15 | 567.9545 | 3 | 18.24 | 0.814 | 0.8214 | 57825 | 3.3e2 | 1 | 1 | 180 | 193 |  |  | DB Search |
| total 1 peptides |  |  |  |  |  |  |  |  |  |  |  |  |  |  |  |  |  |  |  |
| --- | --- | --- | --- | --- | --- | --- | --- | --- | --- | --- | --- | --- | --- | --- | --- | --- | --- | --- | --- |

Best Unique PSM (Scan Sample\_1\_A1\_1\_3639.d:57825, m/z=567.9545, z=3, RT=18.24, ppm=0.37):


TRINITY\_DN103165\_c0\_g1\_i1.p1

back to list

  

| Protein Coverage
| Supporting Peptides
| Best Unique PSM
|

Protein Coverage:

Supporting Peptides:

| Peptide | Uniq | -10lgP | Mass | Length | ppm | m/z | z | RT | 1/k0 Start | 1/k0 End | Precursor ID | Area Sample 3 | #Feature | #Feature Sample 3 | Start | End | PTM | AScore | Found By |
| --- | --- | --- | --- | --- | --- | --- | --- | --- | --- | --- | --- | --- | --- | --- | --- | --- | --- | --- | --- |
| K.ESTLHLVLR.L | Y | 31.37 | 1066.6135 | 9 | -2.66 | 534.3095 | 2 | 15.64 | 0.8785 | 0.8858 | 44242 | 0 | 0 | 0 | 16 | 24 |  |  | DB Search |
| total 1 peptides |  |  |  |  |  |  |  |  |  |  |  |  |  |  |  |  |  |  |  |
| --- | --- | --- | --- | --- | --- | --- | --- | --- | --- | --- | --- | --- | --- | --- | --- | --- | --- | --- | --- |

Best Unique PSM (Scan Sample\_1\_A1\_1\_3639.d:44242, m/z=534.3095, z=2, RT=15.64, ppm=-8.44):


TRINITY\_DN17721\_c0\_g1\_i1.p1

back to list

  

| Protein Coverage
| Supporting Peptides
| Best Unique PSM
|

Protein Coverage:

Supporting Peptides:

| Peptide | Uniq | -10lgP | Mass | Length | ppm | m/z | z | RT | 1/k0 Start | 1/k0 End | Precursor ID | Area Sample 3 | #Feature | #Feature Sample 3 | Start | End | PTM | AScore | Found By |
| --- | --- | --- | --- | --- | --- | --- | --- | --- | --- | --- | --- | --- | --- | --- | --- | --- | --- | --- | --- |
| R.AIHPTIIDASK.S | Y | 31.42 | 1164.6502 | 11 | 5.47 | 583.3322 | 2 | 13.51 | 0.9602 | 0.9675 | 34115 | 5.55e2 | 1 | 1 | 56 | 66 |  |  | DB Search |
| total 1 peptides |  |  |  |  |  |  |  |  |  |  |  |  |  |  |  |  |  |  |  |
| --- | --- | --- | --- | --- | --- | --- | --- | --- | --- | --- | --- | --- | --- | --- | --- | --- | --- | --- | --- |

Best Unique PSM (Scan Sample\_1\_A1\_1\_3639.d:34115, m/z=583.3322, z=2, RT=13.51, ppm=-0.31):


TRINITY\_DN11726\_c0\_g1\_i1.p1

back to list

  

| Protein Coverage
| Supporting Peptides
| Best Unique PSM
|

Protein Coverage:

Supporting Peptides:

| Peptide | Uniq | -10lgP | Mass | Length | ppm | m/z | z | RT | 1/k0 Start | 1/k0 End | Precursor ID | Area Sample 3 | #Feature | #Feature Sample 3 | Start | End | PTM | AScore | Found By |
| --- | --- | --- | --- | --- | --- | --- | --- | --- | --- | --- | --- | --- | --- | --- | --- | --- | --- | --- | --- |
| R.YPGYHEHAFPFER.S | Y | 30.35 | 1648.7422 | 13 | 5.76 | 550.588 | 3 | 15.54 | 0.8481 | 0.8554 | 43744 | 4.4e2 | 1 | 1 | 790 | 802 |  |  | DB Search |
| total 1 peptides |  |  |  |  |  |  |  |  |  |  |  |  |  |  |  |  |  |  |  |
| --- | --- | --- | --- | --- | --- | --- | --- | --- | --- | --- | --- | --- | --- | --- | --- | --- | --- | --- | --- |

Best Unique PSM (Scan Sample\_1\_A1\_1\_3639.d:43744, m/z=550.2775, z=2, RT=15.55, ppm=-0.02):


TRINITY\_DN1030\_c0\_g1\_i1.p1

back to list

  

| Protein Coverage
| Supporting Peptides
| Best Unique PSM
|

Protein Coverage:

Supporting Peptides:

| Peptide | Uniq | -10lgP | Mass | Length | ppm | m/z | z | RT | 1/k0 Start | 1/k0 End | Precursor ID | Area Sample 3 | #Feature | #Feature Sample 3 | Start | End | PTM | AScore | Found By |
| --- | --- | --- | --- | --- | --- | --- | --- | --- | --- | --- | --- | --- | --- | --- | --- | --- | --- | --- | --- |
| R.GLQGQQGEQGSTGVQGSVGGR.G | Y | 31.96 | 1985.9515 | 21 | 15.33 | 993.9925 | 2 | 12.02 | 1.1526 | 1.1598 | 26652 | 4.41e3 | 1 | 1 | 361 | 381 |  |  | DB Search |
| R.GQAGPAGQDGVDGAVGAR.G | Y | 31.48 | 1581.7495 | 18 | 2.61 | 791.8795 | 2 | 12.51 | 0.9997 | 1.0069 | 29162 | 3.92e2 | 1 | 1 | 316 | 333 |  |  | DB Search |
| total 2 peptides |  |  |  |  |  |  |  |  |  |  |  |  |  |  |  |  |  |  |  |
| --- | --- | --- | --- | --- | --- | --- | --- | --- | --- | --- | --- | --- | --- | --- | --- | --- | --- | --- | --- |

Best Unique PSM (Scan Sample\_1\_A1\_1\_3639.d:26652, m/z=993.9925, z=2, RT=12.02, ppm=9.55):


TRINITY\_DN15109\_c0\_g2\_i1.p1

back to list

  

| Protein Coverage
| Supporting Peptides
| Best Unique PSM
|

Protein Coverage:

Supporting Peptides:

| Peptide | Uniq | -10lgP | Mass | Length | ppm | m/z | z | RT | 1/k0 Start | 1/k0 End | Precursor ID | Area Sample 3 | #Feature | #Feature Sample 3 | Start | End | PTM | AScore | Found By |
| --- | --- | --- | --- | --- | --- | --- | --- | --- | --- | --- | --- | --- | --- | --- | --- | --- | --- | --- | --- |
| R.N(+42.01)VLLWHEPR.I | Y | 31 | 1204.6353 | 9 | 6.46 | 603.3253 | 2 | 19.76 | 0.9461 | 0.9533 | 67733 | 1.94e3 | 1 | 1 | 7 | 15 | Acetylation (Protein N-term) | N1:Acetylation (Protein N-term):1000 | DB Search |
| total 1 peptides |  |  |  |  |  |  |  |  |  |  |  |  |  |  |  |  |  |  |  |
| --- | --- | --- | --- | --- | --- | --- | --- | --- | --- | --- | --- | --- | --- | --- | --- | --- | --- | --- | --- |

Best Unique PSM (Scan Sample\_1\_A1\_1\_3639.d:67733, m/z=603.3253, z=2, RT=19.76, ppm=0.68):


TRINITY\_DN262\_c0\_g1\_i1.p1

back to list

  

| Protein Coverage
| Supporting Peptides
| Best Unique PSM
|

Protein Coverage:

Supporting Peptides:

| Peptide | Uniq | -10lgP | Mass | Length | ppm | m/z | z | RT | 1/k0 Start | 1/k0 End | Precursor ID | Area Sample 3 | #Feature | #Feature Sample 3 | Start | End | PTM | AScore | Found By |
| --- | --- | --- | --- | --- | --- | --- | --- | --- | --- | --- | --- | --- | --- | --- | --- | --- | --- | --- | --- |
| R.AEFRPNQEEEPILKR.E | Y | 35.37 | 1854.9588 | 15 | 5.35 | 619.3266 | 3 | 13.84 | 0.8753 | 0.8826 | 35617 | 4.88e3 | 1 | 1 | 439 | 453 |  |  | DB Search |
| K.LC(+57.02)FVGLM(+15.99)SM(+15.99)IDPPR.A | Y | 33.39 | 1666.7881 | 14 | 2.76 | 834.3988 | 2 | 18.80 | 1.0733 | 1.0805 | 61317 | 6.39e2 | 1 | 1 | 592 | 605 | Carbamidomethylation, Oxidation (M), Oxidation (M) | M7:Oxidation (M):1000 M9:Oxidation (M):1000 | DB Search |
| K.GVGIISEGNETVEDIAQR.L | Y | 33.02 | 1885.9381 | 18 | 3.38 | 943.9741 | 2 | 17.88 | 1.1414 | 1.1486 | 55578 | 6.3e2 | 1 | 1 | 639 | 656 |  |  | DB Search |
| K.DLPQEKLDDILK.Y | Y | 31.07 | 1425.7715 | 12 | 0.29 | 713.8891 | 2 | 17.79 | 1.0355 | 1.0427 | 55100 | 0 | 0 | 0 | 681 | 692 |  |  | DB Search |
| R.DANAAVVHGSHLK.D | Y | 30.67 | 1317.6789 | 13 | 7.18 | 659.8477 | 2 | 10.89 | 1.0142 | 1.0214 | 20842 | 6.5e2 | 1 | 1 | 668 | 680 |  |  | DB Search |
| K.VIM(+15.99)VTGDHPITAK.A | Y | 30.07 | 1396.7384 | 13 | 4.97 | 699.3759 | 2 | 13.23 | 1.0182 | 1.0255 | 32647 | 4.44e2 | 1 | 1 | 622 | 634 | Oxidation (M) | M3:Oxidation (M):1000 | DB Search |
| total 6 peptides |  |  |  |  |  |  |  |  |  |  |  |  |  |  |  |  |  |  |  |
| --- | --- | --- | --- | --- | --- | --- | --- | --- | --- | --- | --- | --- | --- | --- | --- | --- | --- | --- | --- |

Best Unique PSM (Scan Sample\_1\_A1\_1\_3639.d:35617, m/z=619.3266, z=3, RT=13.84, ppm=-0.43):


TRINITY\_DN163801\_c0\_g1\_i1.p1

back to list

  

| Protein Coverage
| Supporting Peptides
| Best Unique PSM
|

Protein Coverage:

Supporting Peptides:

| Peptide | Uniq | -10lgP | Mass | Length | ppm | m/z | z | RT | 1/k0 Start | 1/k0 End | Precursor ID | Area Sample 3 | #Feature | #Feature Sample 3 | Start | End | PTM | AScore | Found By |
| --- | --- | --- | --- | --- | --- | --- | --- | --- | --- | --- | --- | --- | --- | --- | --- | --- | --- | --- | --- |
| M.A(+42.01)NPLKDIISR.F | Y | 31.4 | 1167.6611 | 10 | 6.58 | 584.8383 | 2 | 18.69 | 0.9166 | 0.9238 | 60626 | 6.71e2 | 1 | 1 | 2 | 11 | Acetylation (Protein N-term) | A1:Acetylation (Protein N-term):1000 | DB Search |
| total 1 peptides |  |  |  |  |  |  |  |  |  |  |  |  |  |  |  |  |  |  |  |
| --- | --- | --- | --- | --- | --- | --- | --- | --- | --- | --- | --- | --- | --- | --- | --- | --- | --- | --- | --- |

Best Unique PSM (Scan Sample\_1\_A1\_1\_3639.d:60626, m/z=584.8383, z=2, RT=18.69, ppm=0.8):


TRINITY\_DN1071\_c0\_g1\_i4.p1

back to list

  

| Protein Coverage
| Supporting Peptides
| Best Unique PSM
|

Protein Coverage:

Supporting Peptides:

| Peptide | Uniq | -10lgP | Mass | Length | ppm | m/z | z | RT | 1/k0 Start | 1/k0 End | Precursor ID | Area Sample 3 | #Feature | #Feature Sample 3 | Start | End | PTM | AScore | Found By |
| --- | --- | --- | --- | --- | --- | --- | --- | --- | --- | --- | --- | --- | --- | --- | --- | --- | --- | --- | --- |
| K.AVQIHPFNDVSVGDPK.V | Y | 31.42 | 1721.8736 | 16 | 5.27 | 861.9437 | 2 | 15.88 | 1.1142 | 1.1214 | 45122 | 5.31e3 | 2 | 2 | 329 | 344 |  |  | DB Search |
| total 1 peptides |  |  |  |  |  |  |  |  |  |  |  |  |  |  |  |  |  |  |  |
| --- | --- | --- | --- | --- | --- | --- | --- | --- | --- | --- | --- | --- | --- | --- | --- | --- | --- | --- | --- |

Best Unique PSM (Scan Sample\_1\_A1\_1\_3639.d:45122, m/z=861.9437, z=2, RT=15.88, ppm=-0.5):


TRINITY\_DN1058\_c0\_g1\_i2.p2

back to list

  

| Protein Coverage
| Supporting Peptides
| Best Unique PSM
|

Protein Coverage:

Supporting Peptides:

| Peptide | Uniq | -10lgP | Mass | Length | ppm | m/z | z | RT | 1/k0 Start | 1/k0 End | Precursor ID | Area Sample 3 | #Feature | #Feature Sample 3 | Start | End | PTM | AScore | Found By |
| --- | --- | --- | --- | --- | --- | --- | --- | --- | --- | --- | --- | --- | --- | --- | --- | --- | --- | --- | --- |
| R.APTHILNPANKR.S | Y | 31.4 | 1330.7469 | 12 | 6.7 | 444.59 | 3 | 11.12 | 0.7445 | 0.7518 | 21628 | 4.55e3 | 2 | 2 | 975 | 986 |  |  | DB Search |
| total 1 peptides |  |  |  |  |  |  |  |  |  |  |  |  |  |  |  |  |  |  |  |
| --- | --- | --- | --- | --- | --- | --- | --- | --- | --- | --- | --- | --- | --- | --- | --- | --- | --- | --- | --- |

Best Unique PSM (Scan Sample\_1\_A1\_1\_3639.d:21628, m/z=444.59, z=3, RT=11.12, ppm=0.92):


TRINITY\_DN77\_c1\_g1\_i1.p1

back to list

  

| Protein Coverage
| Supporting Peptides
| Best Unique PSM
|

Protein Coverage:

Supporting Peptides:

| Peptide | Uniq | -10lgP | Mass | Length | ppm | m/z | z | RT | 1/k0 Start | 1/k0 End | Precursor ID | Area Sample 3 | #Feature | #Feature Sample 3 | Start | End | PTM | AScore | Found By |
| --- | --- | --- | --- | --- | --- | --- | --- | --- | --- | --- | --- | --- | --- | --- | --- | --- | --- | --- | --- |
| R.LLAVTVGNNYNLDTLEK.V | Y | 31.42 | 1875.9942 | 17 | 6.79 | 939.0053 | 2 | 18.68 | 1.1462 | 1.1534 | 60547 | 3.02e3 | 1 | 1 | 693 | 709 |  |  | DB Search |
| total 1 peptides |  |  |  |  |  |  |  |  |  |  |  |  |  |  |  |  |  |  |  |
| --- | --- | --- | --- | --- | --- | --- | --- | --- | --- | --- | --- | --- | --- | --- | --- | --- | --- | --- | --- |

Best Unique PSM (Scan Sample\_1\_A1\_1\_3639.d:60547, m/z=939.0053, z=2, RT=18.68, ppm=1.02):


TRINITY\_DN1505\_c0\_g1\_i1.p1

back to list

  

| Protein Coverage
| Supporting Peptides
| Best Unique PSM
|

Protein Coverage:

Supporting Peptides:

| Peptide | Uniq | -10lgP | Mass | Length | ppm | m/z | z | RT | 1/k0 Start | 1/k0 End | Precursor ID | Area Sample 3 | #Feature | #Feature Sample 3 | Start | End | PTM | AScore | Found By |
| --- | --- | --- | --- | --- | --- | --- | --- | --- | --- | --- | --- | --- | --- | --- | --- | --- | --- | --- | --- |
| M.S(+42.01)LVIPEKFQHILR.V | Y | 31.98 | 1620.9351 | 13 | 4.73 | 541.3184 | 3 | 19.70 | 0.8388 | 0.8461 | 67303 | 3.39e2 | 1 | 1 | 2 | 14 | Acetylation (Protein N-term) | S1:Acetylation (Protein N-term):1000 | DB Search |
| total 1 peptides |  |  |  |  |  |  |  |  |  |  |  |  |  |  |  |  |  |  |  |
| --- | --- | --- | --- | --- | --- | --- | --- | --- | --- | --- | --- | --- | --- | --- | --- | --- | --- | --- | --- |

Best Unique PSM (Scan Sample\_1\_A1\_1\_3639.d:67303, m/z=541.3184, z=3, RT=19.70, ppm=-1.04):


TRINITY\_DN161\_c0\_g1\_i1.p1

back to list

  

| Protein Coverage
| Supporting Peptides
| Best Unique PSM
|

Protein Coverage:

Supporting Peptides:

| Peptide | Uniq | -10lgP | Mass | Length | ppm | m/z | z | RT | 1/k0 Start | 1/k0 End | Precursor ID | Area Sample 3 | #Feature | #Feature Sample 3 | Start | End | PTM | AScore | Found By |
| --- | --- | --- | --- | --- | --- | --- | --- | --- | --- | --- | --- | --- | --- | --- | --- | --- | --- | --- | --- |
| R.AVFPSIVGRPR.H | Y | 31.42 | 1197.6982 | 11 | 5.9 | 599.8564 | 2 | 15.80 | 0.9533 | 0.9606 | 44974 | 8.41e3 | 1 | 1 | 30 | 40 |  |  | DB Search |
| total 1 peptides |  |  |  |  |  |  |  |  |  |  |  |  |  |  |  |  |  |  |  |
| --- | --- | --- | --- | --- | --- | --- | --- | --- | --- | --- | --- | --- | --- | --- | --- | --- | --- | --- | --- |

Best Unique PSM (Scan Sample\_1\_A1\_1\_3639.d:44974, m/z=599.8564, z=2, RT=15.80, ppm=0.13):


TRINITY\_DN64\_c0\_g1\_i1.p1

back to list

  

| Protein Coverage
| Supporting Peptides
| Best Unique PSM
|

Protein Coverage:

Supporting Peptides:

| Peptide | Uniq | -10lgP | Mass | Length | ppm | m/z | z | RT | 1/k0 Start | 1/k0 End | Precursor ID | Area Sample 3 | #Feature | #Feature Sample 3 | Start | End | PTM | AScore | Found By |
| --- | --- | --- | --- | --- | --- | --- | --- | --- | --- | --- | --- | --- | --- | --- | --- | --- | --- | --- | --- |
| K.IHINIVVIGHVDSGK.S | Y | 32.02 | 1599.9096 | 15 | 5.66 | 534.3104 | 3 | 16.64 | 0.8412 | 0.8485 | 48792 | 1.31e2 | 1 | 1 | 9 | 23 |  |  | DB Search |
| total 1 peptides |  |  |  |  |  |  |  |  |  |  |  |  |  |  |  |  |  |  |  |
| --- | --- | --- | --- | --- | --- | --- | --- | --- | --- | --- | --- | --- | --- | --- | --- | --- | --- | --- | --- |

Best Unique PSM (Scan Sample\_1\_A1\_1\_3639.d:48792, m/z=534.3104, z=3, RT=16.64, ppm=-0.11):


TRINITY\_DN7102\_c0\_g1\_i1.p1

back to list

  

| Protein Coverage
| Supporting Peptides
| Best Unique PSM
|

Protein Coverage:

Supporting Peptides:

| Peptide | Uniq | -10lgP | Mass | Length | ppm | m/z | z | RT | 1/k0 Start | 1/k0 End | Precursor ID | Area Sample 3 | #Feature | #Feature Sample 3 | Start | End | PTM | AScore | Found By |
| --- | --- | --- | --- | --- | --- | --- | --- | --- | --- | --- | --- | --- | --- | --- | --- | --- | --- | --- | --- |
| K.AGAIAEEVISSIR.T | Y | 31.36 | 1314.7143 | 13 | 6.18 | 658.3647 | 2 | 19.95 | 0.9755 | 0.9828 | 68980 | 5.91e2 | 1 | 1 | 212 | 224 |  |  | DB Search |
| total 1 peptides |  |  |  |  |  |  |  |  |  |  |  |  |  |  |  |  |  |  |  |
| --- | --- | --- | --- | --- | --- | --- | --- | --- | --- | --- | --- | --- | --- | --- | --- | --- | --- | --- | --- |

Best Unique PSM (Scan Sample\_1\_A1\_1\_3639.d:68980, m/z=658.3647, z=2, RT=19.95, ppm=0.4):


TRINITY\_DN554\_c0\_g1\_i5.p1

back to list

  

| Protein Coverage
| Supporting Peptides
| Best Unique PSM
|

Protein Coverage:

Supporting Peptides:

| Peptide | Uniq | -10lgP | Mass | Length | ppm | m/z | z | RT | 1/k0 Start | 1/k0 End | Precursor ID | Area Sample 3 | #Feature | #Feature Sample 3 | Start | End | PTM | AScore | Found By |
| --- | --- | --- | --- | --- | --- | --- | --- | --- | --- | --- | --- | --- | --- | --- | --- | --- | --- | --- | --- |
| K.ESTLHLVLR.L | Y | 31.37 | 1066.6135 | 9 | -2.66 | 534.3095 | 2 | 15.64 | 0.8785 | 0.8858 | 44242 | 0 | 0 | 0 | 64 | 72 |  |  | DB Search |
| K.TITLEVEPSDTIENVK.S | Y | 30.07 | 1786.92 | 16 | 6.27 | 894.4677 | 2 | 17.53 | 1.1438 | 1.151 | 53352 | 6.91e2 | 1 | 1 | 12 | 27 |  |  | DB Search |
| total 2 peptides |  |  |  |  |  |  |  |  |  |  |  |  |  |  |  |  |  |  |  |
| --- | --- | --- | --- | --- | --- | --- | --- | --- | --- | --- | --- | --- | --- | --- | --- | --- | --- | --- | --- |

Best Unique PSM (Scan Sample\_1\_A1\_1\_3639.d:44242, m/z=534.3095, z=2, RT=15.64, ppm=-8.44):


TRINITY\_DN8227\_c0\_g1\_i1.p1

back to list

  

| Protein Coverage
| Supporting Peptides
| Best Unique PSM
|

Protein Coverage:

Supporting Peptides:

| Peptide | Uniq | -10lgP | Mass | Length | ppm | m/z | z | RT | 1/k0 Start | 1/k0 End | Precursor ID | Area Sample 3 | #Feature | #Feature Sample 3 | Start | End | PTM | AScore | Found By |
| --- | --- | --- | --- | --- | --- | --- | --- | --- | --- | --- | --- | --- | --- | --- | --- | --- | --- | --- | --- |
| K.ESTLHLVLR.L | Y | 31.37 | 1066.6135 | 9 | -2.66 | 534.3095 | 2 | 15.64 | 0.8785 | 0.8858 | 44242 | 0 | 0 | 0 | 88 | 96 |  |  | DB Search |
| total 1 peptides |  |  |  |  |  |  |  |  |  |  |  |  |  |  |  |  |  |  |  |
| --- | --- | --- | --- | --- | --- | --- | --- | --- | --- | --- | --- | --- | --- | --- | --- | --- | --- | --- | --- |

Best Unique PSM (Scan Sample\_1\_A1\_1\_3639.d:44242, m/z=534.3095, z=2, RT=15.64, ppm=-8.44):


TRINITY\_DN1616\_c0\_g1\_i7.p1

back to list

  

| Protein Coverage
| Supporting Peptides
| Best Unique PSM
|

Protein Coverage:

Supporting Peptides:

| Peptide | Uniq | -10lgP | Mass | Length | ppm | m/z | z | RT | 1/k0 Start | 1/k0 End | Precursor ID | Area Sample 3 | #Feature | #Feature Sample 3 | Start | End | PTM | AScore | Found By |
| --- | --- | --- | --- | --- | --- | --- | --- | --- | --- | --- | --- | --- | --- | --- | --- | --- | --- | --- | --- |
| K.DGEKVETLKVDISDINK.A | Y | 31.36 | 1901.9945 | 17 | 4.01 | 635.0043 | 3 | 15.97 | 0.8976 | 0.9048 | 45629 | 4.35e2 | 1 | 1 | 2634 | 2650 |  |  | DB Search |
| total 1 peptides |  |  |  |  |  |  |  |  |  |  |  |  |  |  |  |  |  |  |  |
| --- | --- | --- | --- | --- | --- | --- | --- | --- | --- | --- | --- | --- | --- | --- | --- | --- | --- | --- | --- |

Best Unique PSM (Scan Sample\_1\_A1\_1\_3639.d:45629, m/z=635.0043, z=3, RT=15.97, ppm=-1.77):


TRINITY\_DN15274\_c0\_g1\_i1.p1

back to list

  

| Protein Coverage
| Supporting Peptides
| Best Unique PSM
|

Protein Coverage:

Supporting Peptides:

| Peptide | Uniq | -10lgP | Mass | Length | ppm | m/z | z | RT | 1/k0 Start | 1/k0 End | Precursor ID | Area Sample 3 | #Feature | #Feature Sample 3 | Start | End | PTM | AScore | Found By |
| --- | --- | --- | --- | --- | --- | --- | --- | --- | --- | --- | --- | --- | --- | --- | --- | --- | --- | --- | --- |
| R.ADIEDVKDLLK.K | Y | 31.42 | 1257.6816 | 11 | 7.3 | 420.2351 | 3 | 17.68 | 0.7571 | 0.7644 | 54439 | 1.59e4 | 2 | 2 | 1616 | 1626 |  |  | DB Search |
| R.ADIEDVKDLLKK.L | Y | 31.4 | 1385.7765 | 12 | 5.27 | 693.8952 | 2 | 16.35 | 0.9908 | 0.9981 | 47347 | 1.16e3 | 1 | 1 | 1616 | 1627 |  |  | DB Search |
| R.DEKANDILDGLELR.L | Y | 31.31 | 1599.8104 | 14 | 10.48 | 534.2799 | 3 | 19.01 | 0.8063 | 0.8136 | 62845 | 3.83e2 | 1 | 1 | 1283 | 1296 |  |  | DB Search |
| K.ANDILDGLELR.L | Y | 31.21 | 1227.6459 | 11 | -0.51 | 614.8264 | 2 | 19.49 | 0.9267 | 0.9339 | 66003 | 0 | 0 | 0 | 1286 | 1296 |  |  | DB Search |
| R.AKDLYYEIR.R | Y | 31.08 | 1169.608 | 9 | 5.22 | 585.811 | 2 | 14.60 | 0.9158 | 0.923 | 39418 | 0 | 0 | 0 | 1751 | 1759 |  |  | DB Search |
| total 5 peptides |  |  |  |  |  |  |  |  |  |  |  |  |  |  |  |  |  |  |  |
| --- | --- | --- | --- | --- | --- | --- | --- | --- | --- | --- | --- | --- | --- | --- | --- | --- | --- | --- | --- |

Best Unique PSM (Scan Sample\_1\_A1\_1\_3639.d:54439, m/z=420.2351, z=3, RT=17.68, ppm=1.52):


TRINITY\_DN4364\_c0\_g1\_i1.p1

back to list

  

| Protein Coverage
| Supporting Peptides
| Best Unique PSM
|

Protein Coverage:

Supporting Peptides:

| Peptide | Uniq | -10lgP | Mass | Length | ppm | m/z | z | RT | 1/k0 Start | 1/k0 End | Precursor ID | Area Sample 3 | #Feature | #Feature Sample 3 | Start | End | PTM | AScore | Found By |
| --- | --- | --- | --- | --- | --- | --- | --- | --- | --- | --- | --- | --- | --- | --- | --- | --- | --- | --- | --- |
| K.HLAEVC(+57.02)HER.Y | Y | 31.42 | 1149.5349 | 9 | 14.7 | 384.189 | 3 | 9.74 | 0.731 | 0.7384 | 14869 | 0 | 0 | 0 | 108 | 116 | Carbamidomethylation |  | DB Search |
| total 1 peptides |  |  |  |  |  |  |  |  |  |  |  |  |  |  |  |  |  |  |  |
| --- | --- | --- | --- | --- | --- | --- | --- | --- | --- | --- | --- | --- | --- | --- | --- | --- | --- | --- | --- |

Best Unique PSM (Scan Sample\_1\_A1\_1\_3639.d:14869, m/z=384.189, z=3, RT=9.74, ppm=8.92):


TRINITY\_DN1058\_c0\_g1\_i1.p1

back to list

  

| Protein Coverage
| Supporting Peptides
| Best Unique PSM
|

Protein Coverage:

Supporting Peptides:

| Peptide | Uniq | -10lgP | Mass | Length | ppm | m/z | z | RT | 1/k0 Start | 1/k0 End | Precursor ID | Area Sample 3 | #Feature | #Feature Sample 3 | Start | End | PTM | AScore | Found By |
| --- | --- | --- | --- | --- | --- | --- | --- | --- | --- | --- | --- | --- | --- | --- | --- | --- | --- | --- | --- |
| R.APTHILNPANKR.S | Y | 31.4 | 1330.7469 | 12 | 6.7 | 444.59 | 3 | 11.12 | 0.7445 | 0.7518 | 21628 | 4.55e3 | 2 | 2 | 2314 | 2325 |  |  | DB Search |
| total 1 peptides |  |  |  |  |  |  |  |  |  |  |  |  |  |  |  |  |  |  |  |
| --- | --- | --- | --- | --- | --- | --- | --- | --- | --- | --- | --- | --- | --- | --- | --- | --- | --- | --- | --- |

Best Unique PSM (Scan Sample\_1\_A1\_1\_3639.d:21628, m/z=444.59, z=3, RT=11.12, ppm=0.92):


TRINITY\_DN1369\_c0\_g1\_i1.p1

back to list

  

| Protein Coverage
| Supporting Peptides
| Best Unique PSM
|

Protein Coverage:

Supporting Peptides:

| Peptide | Uniq | -10lgP | Mass | Length | ppm | m/z | z | RT | 1/k0 Start | 1/k0 End | Precursor ID | Area Sample 3 | #Feature | #Feature Sample 3 | Start | End | PTM | AScore | Found By |
| --- | --- | --- | --- | --- | --- | --- | --- | --- | --- | --- | --- | --- | --- | --- | --- | --- | --- | --- | --- |
| K.KTC(+57.02)VKDEVTGLHEIK.V | Y | 33.64 | 1755.9189 | 15 | 5.41 | 586.3134 | 3 | 12.81 | 0.8846 | 0.8919 | 30711 | 0 | 0 | 0 | 1345 | 1359 | Carbamidomethylation |  | DB Search |
| K.YPNGLHHC(+57.02)PTK.Y | Y | 31.97 | 1322.619 | 11 | 4.7 | 441.8798 | 3 | 10.39 | 0.7677 | 0.775 | 18227 | 6.71e2 | 1 | 1 | 1140 | 1150 | Carbamidomethylation |  | DB Search |
| K.KPGEVWTNKEK.C | Y | 31.43 | 1314.6932 | 11 | 3.3 | 439.2372 | 3 | 10.87 | 0.7551 | 0.7624 | 20789 | 0 | 0 | 0 | 1449 | 1459 |  |  | DB Search |
| K.TPGEC(+57.02)C(+57.02)PQC(+57.02)K.K | Y | 31.43 | 1235.4733 | 10 | 6.61 | 618.7444 | 2 | 9.98 | 0.8915 | 0.8988 | 15843 | 1.95e3 | 1 | 1 | 1387 | 1396 | Carbamidomethylation, Carbamidomethylation, Carbamidomethylation |  | DB Search |
| K.TC(+57.02)VKDEVTGLHEIK.V | Y | 31.41 | 1627.8239 | 14 | 1.81 | 543.6131 | 3 | 13.77 | 0.853 | 0.8603 | 35449 | 1.2e3 | 1 | 1 | 1346 | 1359 | Carbamidomethylation |  | DB Search |
| R.GDVIQVNEC(+57.02)TKK.T | Y | 30.07 | 1389.6922 | 12 | 5.72 | 695.8533 | 2 | 12.08 | 1.0094 | 1.0166 | 26957 | 1.81e3 | 1 | 1 | 1334 | 1345 | Carbamidomethylation |  | DB Search |
| total 6 peptides |  |  |  |  |  |  |  |  |  |  |  |  |  |  |  |  |  |  |  |
| --- | --- | --- | --- | --- | --- | --- | --- | --- | --- | --- | --- | --- | --- | --- | --- | --- | --- | --- | --- |

Best Unique PSM (Scan Sample\_1\_A1\_1\_3639.d:30711, m/z=586.3134, z=3, RT=12.81, ppm=-0.37):


TRINITY\_DN1676\_c0\_g1\_i12.p1

back to list

  

| Protein Coverage
| Supporting Peptides
| Best Unique PSM
|

Protein Coverage:

Supporting Peptides:

| Peptide | Uniq | -10lgP | Mass | Length | ppm | m/z | z | RT | 1/k0 Start | 1/k0 End | Precursor ID | Area Sample 3 | #Feature | #Feature Sample 3 | Start | End | PTM | AScore | Found By |
| --- | --- | --- | --- | --- | --- | --- | --- | --- | --- | --- | --- | --- | --- | --- | --- | --- | --- | --- | --- |
| K.RMSEIAEEARK.H | Y | 34.53 | 1318.6663 | 11 | 6.48 | 440.563 | 3 | 10.97 | 0.7587 | 0.7661 | 21230 | 4.84e2 | 1 | 1 | 1192 | 1202 |  |  | DB Search |
| K.KSEGAAETGSVTISK.A | Y | 33.52 | 1463.7467 | 15 | 4.25 | 732.8795 | 2 | 11.40 | 0.9892 | 0.9965 | 23419 | 7.43e2 | 1 | 1 | 1135 | 1149 |  |  | DB Search |
| R.FELDRVNTFGDEVFGDPK.V | Y | 33.39 | 2083.985 | 18 | 9.37 | 695.6714 | 3 | 19.27 | 0.9133 | 0.9206 | 64506 | 4.68e2 | 1 | 1 | 252 | 269 |  |  | DB Search |
| R.SYYFAISDITVGGR.C | Y | 31.92 | 1547.762 | 14 | 8.82 | 774.8906 | 2 | 19.95 | 1.0452 | 1.0524 | 68928 | 3.71e2 | 1 | 1 | 273 | 286 |  |  | DB Search |
| K.LLQQHELDLTPLK.E | Y | 31.42 | 1546.8719 | 13 | -0.58 | 774.4383 | 2 | 16.85 | 1.0552 | 1.0624 | 49653 | 2.38e2 | 1 | 1 | 1589 | 1601 |  |  | DB Search |
| K.ITQLQNDDKEAR.E | Y | 31.41 | 1429.7161 | 12 | 7.95 | 715.8669 | 2 | 10.85 | 1.0255 | 1.0327 | 20687 | 0 | 0 | 0 | 1329 | 1340 |  |  | DB Search |
| total 6 peptides |  |  |  |  |  |  |  |  |  |  |  |  |  |  |  |  |  |  |  |
| --- | --- | --- | --- | --- | --- | --- | --- | --- | --- | --- | --- | --- | --- | --- | --- | --- | --- | --- | --- |

Best Unique PSM (Scan Sample\_1\_A1\_1\_3639.d:21230, m/z=440.563, z=3, RT=10.97, ppm=0.7):


TRINITY\_DN40728\_c1\_g1\_i1.p2

back to list

  

| Protein Coverage
| Supporting Peptides
| Best Unique PSM
|

Protein Coverage:

Supporting Peptides:

| Peptide | Uniq | -10lgP | Mass | Length | ppm | m/z | z | RT | 1/k0 Start | 1/k0 End | Precursor ID | Area Sample 3 | #Feature | #Feature Sample 3 | Start | End | PTM | AScore | Found By |
| --- | --- | --- | --- | --- | --- | --- | --- | --- | --- | --- | --- | --- | --- | --- | --- | --- | --- | --- | --- |
| K.ESTLHLVLR.L | Y | 31.37 | 1066.6135 | 9 | -2.66 | 534.3095 | 2 | 15.64 | 0.8785 | 0.8858 | 44242 | 0 | 0 | 0 | 59 | 67 |  |  | DB Search |
| total 1 peptides |  |  |  |  |  |  |  |  |  |  |  |  |  |  |  |  |  |  |  |
| --- | --- | --- | --- | --- | --- | --- | --- | --- | --- | --- | --- | --- | --- | --- | --- | --- | --- | --- | --- |

Best Unique PSM (Scan Sample\_1\_A1\_1\_3639.d:44242, m/z=534.3095, z=2, RT=15.64, ppm=-8.44):


TRINITY\_DN585\_c0\_g1\_i5.p1

back to list

  

| Protein Coverage
| Supporting Peptides
| Best Unique PSM
|

Protein Coverage:

Supporting Peptides:

| Peptide | Uniq | -10lgP | Mass | Length | ppm | m/z | z | RT | 1/k0 Start | 1/k0 End | Precursor ID | Area Sample 3 | #Feature | #Feature Sample 3 | Start | End | PTM | AScore | Found By |
| --- | --- | --- | --- | --- | --- | --- | --- | --- | --- | --- | --- | --- | --- | --- | --- | --- | --- | --- | --- |
| K.ESTLHLVLR.L | Y | 31.37 | 1066.6135 | 9 | -2.66 | 534.3095 | 2 | 15.64 | 0.8785 | 0.8858 | 44242 | 0 | 0 | 0 | 36 | 44 |  |  | DB Search |
| K.TITLEVEPSDTIENVK.S | Y | 30.07 | 1786.92 | 16 | 6.27 | 894.4677 | 2 | 17.53 | 1.1438 | 1.151 | 53352 | 6.91e2 | 1 | 1 | 60 | 75 |  |  | DB Search |
| total 2 peptides |  |  |  |  |  |  |  |  |  |  |  |  |  |  |  |  |  |  |  |
| --- | --- | --- | --- | --- | --- | --- | --- | --- | --- | --- | --- | --- | --- | --- | --- | --- | --- | --- | --- |

Best Unique PSM (Scan Sample\_1\_A1\_1\_3639.d:44242, m/z=534.3095, z=2, RT=15.64, ppm=-8.44):


TRINITY\_DN152\_c0\_g1\_i2.p1

back to list

  

| Protein Coverage
| Supporting Peptides
| Best Unique PSM
|

Protein Coverage:

Supporting Peptides:

| Peptide | Uniq | -10lgP | Mass | Length | ppm | m/z | z | RT | 1/k0 Start | 1/k0 End | Precursor ID | Area Sample 3 | #Feature | #Feature Sample 3 | Start | End | PTM | AScore | Found By |
| --- | --- | --- | --- | --- | --- | --- | --- | --- | --- | --- | --- | --- | --- | --- | --- | --- | --- | --- | --- |
| K.ESTLHLVLR.L | Y | 31.37 | 1066.6135 | 9 | -2.66 | 534.3095 | 2 | 15.64 | 0.8785 | 0.8858 | 44242 | 0 | 0 | 0 | 24 | 32 |  |  | DB Search |
| total 1 peptides |  |  |  |  |  |  |  |  |  |  |  |  |  |  |  |  |  |  |  |
| --- | --- | --- | --- | --- | --- | --- | --- | --- | --- | --- | --- | --- | --- | --- | --- | --- | --- | --- | --- |

Best Unique PSM (Scan Sample\_1\_A1\_1\_3639.d:44242, m/z=534.3095, z=2, RT=15.64, ppm=-8.44):


TRINITY\_DN8155\_c0\_g1\_i1.p1

back to list

  

| Protein Coverage
| Supporting Peptides
| Best Unique PSM
|

Protein Coverage:

Supporting Peptides:

| Peptide | Uniq | -10lgP | Mass | Length | ppm | m/z | z | RT | 1/k0 Start | 1/k0 End | Precursor ID | Area Sample 3 | #Feature | #Feature Sample 3 | Start | End | PTM | AScore | Found By |
| --- | --- | --- | --- | --- | --- | --- | --- | --- | --- | --- | --- | --- | --- | --- | --- | --- | --- | --- | --- |
| R.GLYISAEFAIPEDC(+57.02)KK.D | Y | 31.26 | 1839.9076 | 16 | 9.12 | 920.9642 | 2 | 18.33 | 1.2017 | 1.2089 | 58227 | 4.93e2 | 1 | 1 | 123 | 138 | Carbamidomethylation |  | DB Search |
| total 1 peptides |  |  |  |  |  |  |  |  |  |  |  |  |  |  |  |  |  |  |  |
| --- | --- | --- | --- | --- | --- | --- | --- | --- | --- | --- | --- | --- | --- | --- | --- | --- | --- | --- | --- |

Best Unique PSM (Scan Sample\_1\_A1\_1\_3639.d:58227, m/z=920.9642, z=2, RT=18.33, ppm=3.34):


TRINITY\_DN2971\_c0\_g1\_i1.p1

back to list

  

| Protein Coverage
| Supporting Peptides
| Best Unique PSM
|

Protein Coverage:

Supporting Peptides:

| Peptide | Uniq | -10lgP | Mass | Length | ppm | m/z | z | RT | 1/k0 Start | 1/k0 End | Precursor ID | Area Sample 3 | #Feature | #Feature Sample 3 | Start | End | PTM | AScore | Found By |
| --- | --- | --- | --- | --- | --- | --- | --- | --- | --- | --- | --- | --- | --- | --- | --- | --- | --- | --- | --- |
| M(+42.01)(+15.99)DAIFGERPISFK.A | Y | 31.43 | 1567.7704 | 13 | 6.82 | 784.8933 | 2 | 19.02 | 1.062 | 1.0693 | 62921 | 3.12e3 | 1 | 1 | 1 | 13 | Acetylation (Protein N-term), Oxidation (M) | M1:Acetylation (Protein N-term):1000 M1:Oxidation (M):1000 | DB Search |
| total 1 peptides |  |  |  |  |  |  |  |  |  |  |  |  |  |  |  |  |  |  |  |
| --- | --- | --- | --- | --- | --- | --- | --- | --- | --- | --- | --- | --- | --- | --- | --- | --- | --- | --- | --- |

Best Unique PSM (Scan Sample\_1\_A1\_1\_3639.d:62921, m/z=784.8933, z=2, RT=19.02, ppm=1.05):


TRINITY\_DN8903\_c0\_g1\_i1.p1

back to list

  

| Protein Coverage
| Supporting Peptides
| Best Unique PSM
|

Protein Coverage:

Supporting Peptides:

| Peptide | Uniq | -10lgP | Mass | Length | ppm | m/z | z | RT | 1/k0 Start | 1/k0 End | Precursor ID | Area Sample 3 | #Feature | #Feature Sample 3 | Start | End | PTM | AScore | Found By |
| --- | --- | --- | --- | --- | --- | --- | --- | --- | --- | --- | --- | --- | --- | --- | --- | --- | --- | --- | --- |
| K.DLHIIEDYVLK.H | Y | 30.28 | 1356.7289 | 11 | 3.24 | 679.37 | 2 | 19.65 | 0.992 | 0.9993 | 67106 | 3.64e2 | 1 | 1 | 417 | 427 |  |  | DB Search |
| total 1 peptides |  |  |  |  |  |  |  |  |  |  |  |  |  |  |  |  |  |  |  |
| --- | --- | --- | --- | --- | --- | --- | --- | --- | --- | --- | --- | --- | --- | --- | --- | --- | --- | --- | --- |

Best Unique PSM (Scan Sample\_1\_A1\_1\_3639.d:67106, m/z=679.37, z=2, RT=19.65, ppm=-2.54):


TRINITY\_DN244\_c0\_g1\_i1.p1

back to list

  

| Protein Coverage
| Supporting Peptides
| Best Unique PSM
|

Protein Coverage:

Supporting Peptides:

| Peptide | Uniq | -10lgP | Mass | Length | ppm | m/z | z | RT | 1/k0 Start | 1/k0 End | Precursor ID | Area Sample 3 | #Feature | #Feature Sample 3 | Start | End | PTM | AScore | Found By |
| --- | --- | --- | --- | --- | --- | --- | --- | --- | --- | --- | --- | --- | --- | --- | --- | --- | --- | --- | --- |
| K.KALVILGDGFISGGGTPSK.T | Y | 31.85 | 1816.0094 | 19 | 6.44 | 909.0126 | 2 | 18.13 | 1.1734 | 1.1806 | 57036 | 4.06e2 | 1 | 1 | 1618 | 1636 |  |  | DB Search |
| K.ALVILGDGFISGGGTPSK.T | Y | 31.42 | 1687.9144 | 18 | 9.81 | 844.9679 | 2 | 19.77 | 1.1682 | 1.1754 | 67505 | 1.82e4 | 1 | 1 | 1619 | 1636 |  |  | DB Search |
| K.ALLLASSNAFDTSNGAR.E | Y | 31.42 | 1706.8587 | 17 | -0.8 | 854.431 | 2 | 17.58 | 1.129 | 1.1362 | 53833 | 3.18e3 | 2 | 2 | 1912 | 1928 |  |  | DB Search |
| K.ALFHEFVSEK.E | Y | 31.41 | 1205.608 | 10 | 4.03 | 603.8102 | 2 | 15.95 | 0.9247 | 0.9319 | 45506 | 5.22e2 | 1 | 1 | 1668 | 1677 |  |  | DB Search |
| R.IGVIVYSDTASIEIR.F | Y | 31.21 | 1634.8879 | 15 | 6.98 | 818.4522 | 2 | 18.66 | 1.1041 | 1.1114 | 60549 | 2.62e2 | 1 | 1 | 2136 | 2150 |  |  | DB Search |
| K.VLILLADGQQTYVPGVTEPSEVAK.E | Y | 30.81 | 2526.3581 | 24 | 5.22 | 843.1262 | 3 | 19.61 | 1.1382 | 1.1454 | 66707 | 1.73e3 | 1 | 1 | 2200 | 2223 |  |  | DB Search |
| K.LIDELTPEVTSALEC(+57.02)EGGPR.G | Y | 30.13 | 2185.0572 | 20 | 7.57 | 1093.5378 | 2 | 19.37 | 1.2177 | 1.2248 | 65094 | 7.91e2 | 1 | 1 | 2265 | 2284 | Carbamidomethylation |  | DB Search |
| total 7 peptides |  |  |  |  |  |  |  |  |  |  |  |  |  |  |  |  |  |  |  |
| --- | --- | --- | --- | --- | --- | --- | --- | --- | --- | --- | --- | --- | --- | --- | --- | --- | --- | --- | --- |

Best Unique PSM (Scan Sample\_1\_A1\_1\_3639.d:57036, m/z=909.0126, z=2, RT=18.13, ppm=0.66):


TRINITY\_DN3795\_c0\_g1\_i1.p1

back to list

  

| Protein Coverage
| Supporting Peptides
| Best Unique PSM
|

Protein Coverage:

Supporting Peptides:

| Peptide | Uniq | -10lgP | Mass | Length | ppm | m/z | z | RT | 1/k0 Start | 1/k0 End | Precursor ID | Area Sample 3 | #Feature | #Feature Sample 3 | Start | End | PTM | AScore | Found By |
| --- | --- | --- | --- | --- | --- | --- | --- | --- | --- | --- | --- | --- | --- | --- | --- | --- | --- | --- | --- |
| R.SGDPLTLGNFIK.N | Y | 31.38 | 1260.6714 | 12 | -7.98 | 631.3343 | 2 | 19.73 | 0.8996 | 0.9069 | 67627 | 0 | 0 | 0 | 100 | 111 |  |  | DB Search |
| total 1 peptides |  |  |  |  |  |  |  |  |  |  |  |  |  |  |  |  |  |  |  |
| --- | --- | --- | --- | --- | --- | --- | --- | --- | --- | --- | --- | --- | --- | --- | --- | --- | --- | --- | --- |

Best Unique PSM (Scan Sample\_1\_A1\_1\_3639.d:67627, m/z=631.3343, z=2, RT=19.73, ppm=-13.76):


TRINITY\_DN152\_c0\_g1\_i15.p1

back to list

  

| Protein Coverage
| Supporting Peptides
| Best Unique PSM
|

Protein Coverage:

Supporting Peptides:

| Peptide | Uniq | -10lgP | Mass | Length | ppm | m/z | z | RT | 1/k0 Start | 1/k0 End | Precursor ID | Area Sample 3 | #Feature | #Feature Sample 3 | Start | End | PTM | AScore | Found By |
| --- | --- | --- | --- | --- | --- | --- | --- | --- | --- | --- | --- | --- | --- | --- | --- | --- | --- | --- | --- |
| K.ESTLHLVLR.L | Y | 31.37 | 1066.6135 | 9 | -2.66 | 534.3095 | 2 | 15.64 | 0.8785 | 0.8858 | 44242 | 0 | 0 | 0 | 94 | 102 |  |  | DB Search |
| total 1 peptides |  |  |  |  |  |  |  |  |  |  |  |  |  |  |  |  |  |  |  |
| --- | --- | --- | --- | --- | --- | --- | --- | --- | --- | --- | --- | --- | --- | --- | --- | --- | --- | --- | --- |

Best Unique PSM (Scan Sample\_1\_A1\_1\_3639.d:44242, m/z=534.3095, z=2, RT=15.64, ppm=-8.44):


TRINITY\_DN1058\_c3\_g1\_i1.p1

back to list

  

| Protein Coverage
| Supporting Peptides
| Best Unique PSM
|

Protein Coverage:

Supporting Peptides:

| Peptide | Uniq | -10lgP | Mass | Length | ppm | m/z | z | RT | 1/k0 Start | 1/k0 End | Precursor ID | Area Sample 3 | #Feature | #Feature Sample 3 | Start | End | PTM | AScore | Found By |
| --- | --- | --- | --- | --- | --- | --- | --- | --- | --- | --- | --- | --- | --- | --- | --- | --- | --- | --- | --- |
| K.GSVASLTC(+57.02)EC(+57.02)NNHK.G | Y | 30.07 | 1575.677 | 14 | 7.87 | 788.8474 | 2 | 11.94 | 1.0596 | 1.0668 | 26202 | 4.23e2 | 1 | 1 | 243 | 256 | Carbamidomethylation, Carbamidomethylation |  | DB Search |
| total 1 peptides |  |  |  |  |  |  |  |  |  |  |  |  |  |  |  |  |  |  |  |
| --- | --- | --- | --- | --- | --- | --- | --- | --- | --- | --- | --- | --- | --- | --- | --- | --- | --- | --- | --- |

Best Unique PSM (Scan Sample\_1\_A1\_1\_3639.d:26202, m/z=788.8474, z=2, RT=11.94, ppm=2.1):


TRINITY\_DN152\_c0\_g1\_i10.p1

back to list

  

| Protein Coverage
| Supporting Peptides
| Best Unique PSM
|

Protein Coverage:

Supporting Peptides:

| Peptide | Uniq | -10lgP | Mass | Length | ppm | m/z | z | RT | 1/k0 Start | 1/k0 End | Precursor ID | Area Sample 3 | #Feature | #Feature Sample 3 | Start | End | PTM | AScore | Found By |
| --- | --- | --- | --- | --- | --- | --- | --- | --- | --- | --- | --- | --- | --- | --- | --- | --- | --- | --- | --- |
| K.ESTLHLVLR.L | Y | 31.37 | 1066.6135 | 9 | -2.66 | 534.3095 | 2 | 15.64 | 0.8785 | 0.8858 | 44242 | 0 | 0 | 0 | 52 | 60 |  |  | DB Search |
| total 1 peptides |  |  |  |  |  |  |  |  |  |  |  |  |  |  |  |  |  |  |  |
| --- | --- | --- | --- | --- | --- | --- | --- | --- | --- | --- | --- | --- | --- | --- | --- | --- | --- | --- | --- |

Best Unique PSM (Scan Sample\_1\_A1\_1\_3639.d:44242, m/z=534.3095, z=2, RT=15.64, ppm=-8.44):


TRINITY\_DN161\_c0\_g1\_i2.p1

back to list

  

| Protein Coverage
| Supporting Peptides
| Best Unique PSM
|

Protein Coverage:

Supporting Peptides:

| Peptide | Uniq | -10lgP | Mass | Length | ppm | m/z | z | RT | 1/k0 Start | 1/k0 End | Precursor ID | Area Sample 3 | #Feature | #Feature Sample 3 | Start | End | PTM | AScore | Found By |
| --- | --- | --- | --- | --- | --- | --- | --- | --- | --- | --- | --- | --- | --- | --- | --- | --- | --- | --- | --- |
| R.AVFPSIVGRPR.H | Y | 31.42 | 1197.6982 | 11 | 5.9 | 599.8564 | 2 | 15.80 | 0.9533 | 0.9606 | 44974 | 8.41e3 | 1 | 1 | 52 | 62 |  |  | DB Search |
| total 1 peptides |  |  |  |  |  |  |  |  |  |  |  |  |  |  |  |  |  |  |  |
| --- | --- | --- | --- | --- | --- | --- | --- | --- | --- | --- | --- | --- | --- | --- | --- | --- | --- | --- | --- |

Best Unique PSM (Scan Sample\_1\_A1\_1\_3639.d:44974, m/z=599.8564, z=2, RT=15.80, ppm=0.13):


TRINITY\_DN661\_c0\_g1\_i10.p1

back to list

  

| Protein Coverage
| Supporting Peptides
| Best Unique PSM
|

Protein Coverage:

Supporting Peptides:

| Peptide | Uniq | -10lgP | Mass | Length | ppm | m/z | z | RT | 1/k0 Start | 1/k0 End | Precursor ID | Area Sample 3 | #Feature | #Feature Sample 3 | Start | End | PTM | AScore | Found By |
| --- | --- | --- | --- | --- | --- | --- | --- | --- | --- | --- | --- | --- | --- | --- | --- | --- | --- | --- | --- |
| K.AAGDLGIYENGC(+57.02)YDQLLK.E | Y | 30.76 | 1998.9357 | 18 | 1.98 | 1000.4713 | 2 | 18.85 | 1.1742 | 1.1813 | 61754 | 0 | 0 | 0 | 192 | 209 | Carbamidomethylation |  | DB Search |
| total 1 peptides |  |  |  |  |  |  |  |  |  |  |  |  |  |  |  |  |  |  |  |
| --- | --- | --- | --- | --- | --- | --- | --- | --- | --- | --- | --- | --- | --- | --- | --- | --- | --- | --- | --- |

Best Unique PSM (Scan Sample\_1\_A1\_1\_3639.d:61754, m/z=1000.4713, z=2, RT=18.85, ppm=-3.79):


TRINITY\_DN121418\_c0\_g1\_i1.p1

back to list

  

| Protein Coverage
| Supporting Peptides
| Best Unique PSM
|

Protein Coverage:

Supporting Peptides:

| Peptide | Uniq | -10lgP | Mass | Length | ppm | m/z | z | RT | 1/k0 Start | 1/k0 End | Precursor ID | Area Sample 3 | #Feature | #Feature Sample 3 | Start | End | PTM | AScore | Found By |
| --- | --- | --- | --- | --- | --- | --- | --- | --- | --- | --- | --- | --- | --- | --- | --- | --- | --- | --- | --- |
| M.AENDIGDWFR.S | Y | 31.42 | 1221.5414 | 10 | 1.83 | 611.7756 | 2 | 19.30 | 0.8862 | 0.8935 | 64616 | 2.38e3 | 1 | 1 | 2 | 11 |  |  | DB Search |
| total 1 peptides |  |  |  |  |  |  |  |  |  |  |  |  |  |  |  |  |  |  |  |
| --- | --- | --- | --- | --- | --- | --- | --- | --- | --- | --- | --- | --- | --- | --- | --- | --- | --- | --- | --- |

Best Unique PSM (Scan Sample\_1\_A1\_1\_3639.d:64616, m/z=611.7756, z=2, RT=19.30, ppm=-3.95):


TRINITY\_DN1058\_c0\_g1\_i4.p1

back to list

  

| Protein Coverage
| Supporting Peptides
| Best Unique PSM
|

Protein Coverage:

Supporting Peptides:

| Peptide | Uniq | -10lgP | Mass | Length | ppm | m/z | z | RT | 1/k0 Start | 1/k0 End | Precursor ID | Area Sample 3 | #Feature | #Feature Sample 3 | Start | End | PTM | AScore | Found By |
| --- | --- | --- | --- | --- | --- | --- | --- | --- | --- | --- | --- | --- | --- | --- | --- | --- | --- | --- | --- |
| R.APTHILNPANKR.S | Y | 31.4 | 1330.7469 | 12 | 6.7 | 444.59 | 3 | 11.12 | 0.7445 | 0.7518 | 21628 | 4.55e3 | 2 | 2 | 398 | 409 |  |  | DB Search |
| total 1 peptides |  |  |  |  |  |  |  |  |  |  |  |  |  |  |  |  |  |  |  |
| --- | --- | --- | --- | --- | --- | --- | --- | --- | --- | --- | --- | --- | --- | --- | --- | --- | --- | --- | --- |

Best Unique PSM (Scan Sample\_1\_A1\_1\_3639.d:21628, m/z=444.59, z=3, RT=11.12, ppm=0.92):


TRINITY\_DN1030\_c0\_g1\_i2.p1

back to list

  

| Protein Coverage
| Supporting Peptides
| Best Unique PSM
|

Protein Coverage:

Supporting Peptides:

| Peptide | Uniq | -10lgP | Mass | Length | ppm | m/z | z | RT | 1/k0 Start | 1/k0 End | Precursor ID | Area Sample 3 | #Feature | #Feature Sample 3 | Start | End | PTM | AScore | Found By |
| --- | --- | --- | --- | --- | --- | --- | --- | --- | --- | --- | --- | --- | --- | --- | --- | --- | --- | --- | --- |
| R.GLQGQQGEQGSTGVQGSVGGR.G | Y | 31.96 | 1985.9515 | 21 | 15.33 | 993.9925 | 2 | 12.02 | 1.1526 | 1.1598 | 26652 | 4.41e3 | 1 | 1 | 348 | 368 |  |  | DB Search |
| R.GQAGPAGQDGVDGAVGAR.G | Y | 31.48 | 1581.7495 | 18 | 2.61 | 791.8795 | 2 | 12.51 | 0.9997 | 1.0069 | 29162 | 3.92e2 | 1 | 1 | 303 | 320 |  |  | DB Search |
| total 2 peptides |  |  |  |  |  |  |  |  |  |  |  |  |  |  |  |  |  |  |  |
| --- | --- | --- | --- | --- | --- | --- | --- | --- | --- | --- | --- | --- | --- | --- | --- | --- | --- | --- | --- |

Best Unique PSM (Scan Sample\_1\_A1\_1\_3639.d:26652, m/z=993.9925, z=2, RT=12.02, ppm=9.55):


TRINITY\_DN173718\_c0\_g1\_i1.p1

back to list

  

| Protein Coverage
| Supporting Peptides
| Best Unique PSM
|

Protein Coverage:

Supporting Peptides:

| Peptide | Uniq | -10lgP | Mass | Length | ppm | m/z | z | RT | 1/k0 Start | 1/k0 End | Precursor ID | Area Sample 3 | #Feature | #Feature Sample 3 | Start | End | PTM | AScore | Found By |
| --- | --- | --- | --- | --- | --- | --- | --- | --- | --- | --- | --- | --- | --- | --- | --- | --- | --- | --- | --- |
| K.ESTLHLVLR.L | Y | 31.37 | 1066.6135 | 9 | -2.66 | 534.3095 | 2 | 15.64 | 0.8785 | 0.8858 | 44242 | 0 | 0 | 0 | 64 | 72 |  |  | DB Search |
| total 1 peptides |  |  |  |  |  |  |  |  |  |  |  |  |  |  |  |  |  |  |  |
| --- | --- | --- | --- | --- | --- | --- | --- | --- | --- | --- | --- | --- | --- | --- | --- | --- | --- | --- | --- |

Best Unique PSM (Scan Sample\_1\_A1\_1\_3639.d:44242, m/z=534.3095, z=2, RT=15.64, ppm=-8.44):


TRINITY\_DN11595\_c0\_g1\_i1.p1

back to list

  

| Protein Coverage
| Supporting Peptides
| Best Unique PSM
|

Protein Coverage:

Supporting Peptides:

| Peptide | Uniq | -10lgP | Mass | Length | ppm | m/z | z | RT | 1/k0 Start | 1/k0 End | Precursor ID | Area Sample 3 | #Feature | #Feature Sample 3 | Start | End | PTM | AScore | Found By |
| --- | --- | --- | --- | --- | --- | --- | --- | --- | --- | --- | --- | --- | --- | --- | --- | --- | --- | --- | --- |
| K.LVHGDVFRPPR.Y | Y | 31.41 | 1291.7149 | 11 | 7.13 | 646.8656 | 2 | 13.43 | 0.9699 | 0.9771 | 33689 | 4.13e2 | 1 | 1 | 301 | 311 |  |  | DB Search |
| total 1 peptides |  |  |  |  |  |  |  |  |  |  |  |  |  |  |  |  |  |  |  |
| --- | --- | --- | --- | --- | --- | --- | --- | --- | --- | --- | --- | --- | --- | --- | --- | --- | --- | --- | --- |

Best Unique PSM (Scan Sample\_1\_A1\_1\_3639.d:33689, m/z=646.8656, z=2, RT=13.43, ppm=1.36):


TRINITY\_DN11377\_c0\_g1\_i1.p1

back to list

  

| Protein Coverage
| Supporting Peptides
| Best Unique PSM
|

Protein Coverage:

Supporting Peptides:

| Peptide | Uniq | -10lgP | Mass | Length | ppm | m/z | z | RT | 1/k0 Start | 1/k0 End | Precursor ID | Area Sample 3 | #Feature | #Feature Sample 3 | Start | End | PTM | AScore | Found By |
| --- | --- | --- | --- | --- | --- | --- | --- | --- | --- | --- | --- | --- | --- | --- | --- | --- | --- | --- | --- |
| R.RIVLLATDALPK.I | Y | 31.32 | 1308.8129 | 12 | 4.44 | 655.4128 | 2 | 17.23 | 1.0126 | 1.0198 | 51672 | 1.04e3 | 1 | 1 | 265 | 276 |  |  | DB Search |
| total 1 peptides |  |  |  |  |  |  |  |  |  |  |  |  |  |  |  |  |  |  |  |
| --- | --- | --- | --- | --- | --- | --- | --- | --- | --- | --- | --- | --- | --- | --- | --- | --- | --- | --- | --- |

Best Unique PSM (Scan Sample\_1\_A1\_1\_3639.d:51672, m/z=655.4128, z=2, RT=17.23, ppm=-1.33):


TRINITY\_DN110760\_c0\_g1\_i1.p1

back to list

  

| Protein Coverage
| Supporting Peptides
| Best Unique PSM
|

Protein Coverage:

Supporting Peptides:

| Peptide | Uniq | -10lgP | Mass | Length | ppm | m/z | z | RT | 1/k0 Start | 1/k0 End | Precursor ID | Area Sample 3 | #Feature | #Feature Sample 3 | Start | End | PTM | AScore | Found By |
| --- | --- | --- | --- | --- | --- | --- | --- | --- | --- | --- | --- | --- | --- | --- | --- | --- | --- | --- | --- |
| K.ESTLHLVLR.L | Y | 31.37 | 1066.6135 | 9 | -2.66 | 534.3095 | 2 | 15.64 | 0.8785 | 0.8858 | 44242 | 0 | 0 | 0 | 18 | 26 |  |  | DB Search |
| total 1 peptides |  |  |  |  |  |  |  |  |  |  |  |  |  |  |  |  |  |  |  |
| --- | --- | --- | --- | --- | --- | --- | --- | --- | --- | --- | --- | --- | --- | --- | --- | --- | --- | --- | --- |

Best Unique PSM (Scan Sample\_1\_A1\_1\_3639.d:44242, m/z=534.3095, z=2, RT=15.64, ppm=-8.44):


TRINITY\_DN286\_c0\_g1\_i4.p1

back to list

  

| Protein Coverage
| Supporting Peptides
| Best Unique PSM
|

Protein Coverage:

Supporting Peptides:

| Peptide | Uniq | -10lgP | Mass | Length | ppm | m/z | z | RT | 1/k0 Start | 1/k0 End | Precursor ID | Area Sample 3 | #Feature | #Feature Sample 3 | Start | End | PTM | AScore | Found By |
| --- | --- | --- | --- | --- | --- | --- | --- | --- | --- | --- | --- | --- | --- | --- | --- | --- | --- | --- | --- |
| K.RYGQAMC(+57.02)QFMR.N | Y | 31.95 | 1446.6319 | 11 | -3.39 | 724.3166 | 2 | 17.38 | 0.9808 | 0.988 | 52561 | 3.82e2 | 1 | 1 | 71 | 81 | Carbamidomethylation |  | DB Search |
| total 1 peptides |  |  |  |  |  |  |  |  |  |  |  |  |  |  |  |  |  |  |  |
| --- | --- | --- | --- | --- | --- | --- | --- | --- | --- | --- | --- | --- | --- | --- | --- | --- | --- | --- | --- |

Best Unique PSM (Scan Sample\_1\_A1\_1\_3639.d:52561, m/z=724.3166, z=2, RT=17.38, ppm=-9.16):


TRINITY\_DN585\_c0\_g1\_i10.p1

back to list

  

| Protein Coverage
| Supporting Peptides
| Best Unique PSM
|

Protein Coverage:

Supporting Peptides:

| Peptide | Uniq | -10lgP | Mass | Length | ppm | m/z | z | RT | 1/k0 Start | 1/k0 End | Precursor ID | Area Sample 3 | #Feature | #Feature Sample 3 | Start | End | PTM | AScore | Found By |
| --- | --- | --- | --- | --- | --- | --- | --- | --- | --- | --- | --- | --- | --- | --- | --- | --- | --- | --- | --- |
| K.ESTLHLVLR.L | Y | 31.37 | 1066.6135 | 9 | -2.66 | 534.3095 | 2 | 15.64 | 0.8785 | 0.8858 | 44242 | 0 | 0 | 0 | 64 | 72 |  |  | DB Search |
| K.TITLEVEPSDTIENVK.S | Y | 30.07 | 1786.92 | 16 | 6.27 | 894.4677 | 2 | 17.53 | 1.1438 | 1.151 | 53352 | 6.91e2 | 1 | 1 | 12 | 27 |  |  | DB Search |
| total 2 peptides |  |  |  |  |  |  |  |  |  |  |  |  |  |  |  |  |  |  |  |
| --- | --- | --- | --- | --- | --- | --- | --- | --- | --- | --- | --- | --- | --- | --- | --- | --- | --- | --- | --- |

Best Unique PSM (Scan Sample\_1\_A1\_1\_3639.d:44242, m/z=534.3095, z=2, RT=15.64, ppm=-8.44):


TRINITY\_DN51907\_c0\_g1\_i1.p1

back to list

  

| Protein Coverage
| Supporting Peptides
| Best Unique PSM
|

Protein Coverage:

Supporting Peptides:

| Peptide | Uniq | -10lgP | Mass | Length | ppm | m/z | z | RT | 1/k0 Start | 1/k0 End | Precursor ID | Area Sample 3 | #Feature | #Feature Sample 3 | Start | End | PTM | AScore | Found By |
| --- | --- | --- | --- | --- | --- | --- | --- | --- | --- | --- | --- | --- | --- | --- | --- | --- | --- | --- | --- |
| R.GVTSITLELQDLQR.N | Y | 31.84 | 1571.8519 | 14 | 2.39 | 786.9305 | 2 | 19.40 | 1.0576 | 1.0648 | 65430 | 3.99e2 | 1 | 1 | 5 | 18 |  |  | DB Search |
| total 1 peptides |  |  |  |  |  |  |  |  |  |  |  |  |  |  |  |  |  |  |  |
| --- | --- | --- | --- | --- | --- | --- | --- | --- | --- | --- | --- | --- | --- | --- | --- | --- | --- | --- | --- |

Best Unique PSM (Scan Sample\_1\_A1\_1\_3639.d:65430, m/z=786.9305, z=2, RT=19.40, ppm=-3.39):


TRINITY\_DN1936\_c0\_g1\_i3.p1

back to list

  

| Protein Coverage
| Supporting Peptides
| Best Unique PSM
|

Protein Coverage:

Supporting Peptides:

| Peptide | Uniq | -10lgP | Mass | Length | ppm | m/z | z | RT | 1/k0 Start | 1/k0 End | Precursor ID | Area Sample 3 | #Feature | #Feature Sample 3 | Start | End | PTM | AScore | Found By |
| --- | --- | --- | --- | --- | --- | --- | --- | --- | --- | --- | --- | --- | --- | --- | --- | --- | --- | --- | --- |
| K.SAVNHNIIFEGR.T | Y | 31.43 | 1355.6946 | 12 | -4.41 | 678.8477 | 2 | 14.70 | 0.9675 | 0.9747 | 39863 | 0 | 0 | 0 | 59 | 70 |  |  | DB Search |
| total 1 peptides |  |  |  |  |  |  |  |  |  |  |  |  |  |  |  |  |  |  |  |
| --- | --- | --- | --- | --- | --- | --- | --- | --- | --- | --- | --- | --- | --- | --- | --- | --- | --- | --- | --- |

Best Unique PSM (Scan Sample\_1\_A1\_1\_3639.d:39863, m/z=678.8477, z=2, RT=14.70, ppm=-10.19):


TRINITY\_DN554\_c0\_g1\_i3.p2

back to list

  

| Protein Coverage
| Supporting Peptides
| Best Unique PSM
|

Protein Coverage:

Supporting Peptides:

| Peptide | Uniq | -10lgP | Mass | Length | ppm | m/z | z | RT | 1/k0 Start | 1/k0 End | Precursor ID | Area Sample 3 | #Feature | #Feature Sample 3 | Start | End | PTM | AScore | Found By |
| --- | --- | --- | --- | --- | --- | --- | --- | --- | --- | --- | --- | --- | --- | --- | --- | --- | --- | --- | --- |
| K.ESTLHLVLR.L | Y | 31.37 | 1066.6135 | 9 | -2.66 | 534.3095 | 2 | 15.64 | 0.8785 | 0.8858 | 44242 | 0 | 0 | 0 | 64 | 72 |  |  | DB Search |
| K.TITLEVEPSDTIENVK.S | Y | 30.07 | 1786.92 | 16 | 6.27 | 894.4677 | 2 | 17.53 | 1.1438 | 1.151 | 53352 | 6.91e2 | 1 | 1 | 12 | 27 |  |  | DB Search |
| total 2 peptides |  |  |  |  |  |  |  |  |  |  |  |  |  |  |  |  |  |  |  |
| --- | --- | --- | --- | --- | --- | --- | --- | --- | --- | --- | --- | --- | --- | --- | --- | --- | --- | --- | --- |

Best Unique PSM (Scan Sample\_1\_A1\_1\_3639.d:44242, m/z=534.3095, z=2, RT=15.64, ppm=-8.44):


TRINITY\_DN4595\_c0\_g1\_i10.p1

back to list

  

| Protein Coverage
| Supporting Peptides
| Best Unique PSM
|

Protein Coverage:

Supporting Peptides:

| Peptide | Uniq | -10lgP | Mass | Length | ppm | m/z | z | RT | 1/k0 Start | 1/k0 End | Precursor ID | Area Sample 3 | #Feature | #Feature Sample 3 | Start | End | PTM | AScore | Found By |
| --- | --- | --- | --- | --- | --- | --- | --- | --- | --- | --- | --- | --- | --- | --- | --- | --- | --- | --- | --- |
| R.SPFVIMFK.G | Y | 30.75 | 967.5201 | 8 | 3.16 | 484.7661 | 2 | 19.85 | 0.8104 | 0.8177 | 68318 | 2.17e3 | 1 | 1 | 630 | 637 |  |  | DB Search |
| total 1 peptides |  |  |  |  |  |  |  |  |  |  |  |  |  |  |  |  |  |  |  |
| --- | --- | --- | --- | --- | --- | --- | --- | --- | --- | --- | --- | --- | --- | --- | --- | --- | --- | --- | --- |

Best Unique PSM (Scan Sample\_1\_A1\_1\_3639.d:68318, m/z=484.7661, z=2, RT=19.85, ppm=-2.62):


TRINITY\_DN585\_c0\_g1\_i3.p1

back to list

  

| Protein Coverage
| Supporting Peptides
| Best Unique PSM
|

Protein Coverage:

Supporting Peptides:

| Peptide | Uniq | -10lgP | Mass | Length | ppm | m/z | z | RT | 1/k0 Start | 1/k0 End | Precursor ID | Area Sample 3 | #Feature | #Feature Sample 3 | Start | End | PTM | AScore | Found By |
| --- | --- | --- | --- | --- | --- | --- | --- | --- | --- | --- | --- | --- | --- | --- | --- | --- | --- | --- | --- |
| K.ESTLHLVLR.L | Y | 31.37 | 1066.6135 | 9 | -2.66 | 534.3095 | 2 | 15.64 | 0.8785 | 0.8858 | 44242 | 0 | 0 | 0 | 36 | 44 |  |  | DB Search |
| K.TITLEVEPSDTIENVK.S | Y | 30.07 | 1786.92 | 16 | 6.27 | 894.4677 | 2 | 17.53 | 1.1438 | 1.151 | 53352 | 6.91e2 | 1 | 1 | 60 | 75 |  |  | DB Search |
| total 2 peptides |  |  |  |  |  |  |  |  |  |  |  |  |  |  |  |  |  |  |  |
| --- | --- | --- | --- | --- | --- | --- | --- | --- | --- | --- | --- | --- | --- | --- | --- | --- | --- | --- | --- |

Best Unique PSM (Scan Sample\_1\_A1\_1\_3639.d:44242, m/z=534.3095, z=2, RT=15.64, ppm=-8.44):


TRINITY\_DN2577\_c1\_g1\_i1.p1

back to list

  

| Protein Coverage
| Supporting Peptides
| Best Unique PSM
|

Protein Coverage:

Supporting Peptides:

| Peptide | Uniq | -10lgP | Mass | Length | ppm | m/z | z | RT | 1/k0 Start | 1/k0 End | Precursor ID | Area Sample 3 | #Feature | #Feature Sample 3 | Start | End | PTM | AScore | Found By |
| --- | --- | --- | --- | --- | --- | --- | --- | --- | --- | --- | --- | --- | --- | --- | --- | --- | --- | --- | --- |
| R.SGDPLTIGNFIK.N | Y | 31.38 | 1260.6714 | 12 | -7.98 | 631.3343 | 2 | 19.73 | 0.8996 | 0.9069 | 67627 | 0 | 0 | 0 | 172 | 183 |  |  | DB Search |
| total 1 peptides |  |  |  |  |  |  |  |  |  |  |  |  |  |  |  |  |  |  |  |
| --- | --- | --- | --- | --- | --- | --- | --- | --- | --- | --- | --- | --- | --- | --- | --- | --- | --- | --- | --- |

Best Unique PSM (Scan Sample\_1\_A1\_1\_3639.d:67627, m/z=631.3343, z=2, RT=19.73, ppm=-13.76):


TRINITY\_DN554\_c0\_g1\_i4.p1

back to list

  

| Protein Coverage
| Supporting Peptides
| Best Unique PSM
|

Protein Coverage:

Supporting Peptides:

| Peptide | Uniq | -10lgP | Mass | Length | ppm | m/z | z | RT | 1/k0 Start | 1/k0 End | Precursor ID | Area Sample 3 | #Feature | #Feature Sample 3 | Start | End | PTM | AScore | Found By |
| --- | --- | --- | --- | --- | --- | --- | --- | --- | --- | --- | --- | --- | --- | --- | --- | --- | --- | --- | --- |
| K.ESTLHLVLR.L | Y | 31.37 | 1066.6135 | 9 | -2.66 | 534.3095 | 2 | 15.64 | 0.8785 | 0.8858 | 44242 | 0 | 0 | 0 | 64 | 72 |  |  | DB Search |
| K.TITLEVEPSDTIENVK.S | Y | 30.07 | 1786.92 | 16 | 6.27 | 894.4677 | 2 | 17.53 | 1.1438 | 1.151 | 53352 | 6.91e2 | 1 | 1 | 12 | 27 |  |  | DB Search |
| total 2 peptides |  |  |  |  |  |  |  |  |  |  |  |  |  |  |  |  |  |  |  |
| --- | --- | --- | --- | --- | --- | --- | --- | --- | --- | --- | --- | --- | --- | --- | --- | --- | --- | --- | --- |

Best Unique PSM (Scan Sample\_1\_A1\_1\_3639.d:44242, m/z=534.3095, z=2, RT=15.64, ppm=-8.44):


TRINITY\_DN17721\_c0\_g1\_i2.p1

back to list

  

| Protein Coverage
| Supporting Peptides
| Best Unique PSM
|

Protein Coverage:

Supporting Peptides:

| Peptide | Uniq | -10lgP | Mass | Length | ppm | m/z | z | RT | 1/k0 Start | 1/k0 End | Precursor ID | Area Sample 3 | #Feature | #Feature Sample 3 | Start | End | PTM | AScore | Found By |
| --- | --- | --- | --- | --- | --- | --- | --- | --- | --- | --- | --- | --- | --- | --- | --- | --- | --- | --- | --- |
| R.AIHPTIIDASK.S | Y | 31.42 | 1164.6502 | 11 | 5.47 | 583.3322 | 2 | 13.51 | 0.9602 | 0.9675 | 34115 | 5.55e2 | 1 | 1 | 56 | 66 |  |  | DB Search |
| total 1 peptides |  |  |  |  |  |  |  |  |  |  |  |  |  |  |  |  |  |  |  |
| --- | --- | --- | --- | --- | --- | --- | --- | --- | --- | --- | --- | --- | --- | --- | --- | --- | --- | --- | --- |

Best Unique PSM (Scan Sample\_1\_A1\_1\_3639.d:34115, m/z=583.3322, z=2, RT=13.51, ppm=-0.31):


TRINITY\_DN982\_c0\_g1\_i3.p1

back to list

  

| Protein Coverage
| Supporting Peptides
| Best Unique PSM
|

Protein Coverage:

Supporting Peptides:

| Peptide | Uniq | -10lgP | Mass | Length | ppm | m/z | z | RT | 1/k0 Start | 1/k0 End | Precursor ID | Area Sample 3 | #Feature | #Feature Sample 3 | Start | End | PTM | AScore | Found By |
| --- | --- | --- | --- | --- | --- | --- | --- | --- | --- | --- | --- | --- | --- | --- | --- | --- | --- | --- | --- |
| R.VYESLSGFIKNEGVFTTIC(+57.02)NGVHK.M | Y | 30.89 | 2698.3424 | 24 | 2.53 | 900.4518 | 3 | 19.61 | 1.1218 | 1.129 | 66836 | 0 | 0 | 0 | 202 | 225 | Carbamidomethylation |  | DB Search |
| K.SAIIVDGKNEEGR.I | Y | 30.72 | 1386.7103 | 13 | 5.71 | 694.3624 | 2 | 11.88 | 0.9969 | 1.0041 | 25934 | 0 | 0 | 0 | 95 | 107 |  |  | DB Search |
| total 2 peptides |  |  |  |  |  |  |  |  |  |  |  |  |  |  |  |  |  |  |  |
| --- | --- | --- | --- | --- | --- | --- | --- | --- | --- | --- | --- | --- | --- | --- | --- | --- | --- | --- | --- |

Best Unique PSM (Scan Sample\_1\_A1\_1\_3639.d:66836, m/z=898.9257, z=2, RT=19.62, ppm=-3.24):


TRINITY\_DN1580\_c0\_g1\_i3.p1

back to list

  

| Protein Coverage
| Supporting Peptides
| Best Unique PSM
|

Protein Coverage:

Supporting Peptides:

| Peptide | Uniq | -10lgP | Mass | Length | ppm | m/z | z | RT | 1/k0 Start | 1/k0 End | Precursor ID | Area Sample 3 | #Feature | #Feature Sample 3 | Start | End | PTM | AScore | Found By |
| --- | --- | --- | --- | --- | --- | --- | --- | --- | --- | --- | --- | --- | --- | --- | --- | --- | --- | --- | --- |
| K.ESTLHLVLR.L | Y | 31.37 | 1066.6135 | 9 | -2.66 | 534.3095 | 2 | 15.64 | 0.8785 | 0.8858 | 44242 | 0 | 0 | 0 | 23 | 31 |  |  | DB Search |
| total 1 peptides |  |  |  |  |  |  |  |  |  |  |  |  |  |  |  |  |  |  |  |
| --- | --- | --- | --- | --- | --- | --- | --- | --- | --- | --- | --- | --- | --- | --- | --- | --- | --- | --- | --- |

Best Unique PSM (Scan Sample\_1\_A1\_1\_3639.d:44242, m/z=534.3095, z=2, RT=15.64, ppm=-8.44):


TRINITY\_DN1616\_c0\_g1\_i2.p1

back to list

  

| Protein Coverage
| Supporting Peptides
| Best Unique PSM
|

Protein Coverage:

Supporting Peptides:

| Peptide | Uniq | -10lgP | Mass | Length | ppm | m/z | z | RT | 1/k0 Start | 1/k0 End | Precursor ID | Area Sample 3 | #Feature | #Feature Sample 3 | Start | End | PTM | AScore | Found By |
| --- | --- | --- | --- | --- | --- | --- | --- | --- | --- | --- | --- | --- | --- | --- | --- | --- | --- | --- | --- |
| K.DGEKVETLKVDISDINK.A | Y | 31.36 | 1901.9945 | 17 | 4.01 | 635.0043 | 3 | 15.97 | 0.8976 | 0.9048 | 45629 | 4.35e2 | 1 | 1 | 2634 | 2650 |  |  | DB Search |
| total 1 peptides |  |  |  |  |  |  |  |  |  |  |  |  |  |  |  |  |  |  |  |
| --- | --- | --- | --- | --- | --- | --- | --- | --- | --- | --- | --- | --- | --- | --- | --- | --- | --- | --- | --- |

Best Unique PSM (Scan Sample\_1\_A1\_1\_3639.d:45629, m/z=635.0043, z=3, RT=15.97, ppm=-1.77):


TRINITY\_DN11643\_c0\_g1\_i1.p1

back to list

  

| Protein Coverage
| Supporting Peptides
| Best Unique PSM
|

Protein Coverage:

Supporting Peptides:

| Peptide | Uniq | -10lgP | Mass | Length | ppm | m/z | z | RT | 1/k0 Start | 1/k0 End | Precursor ID | Area Sample 3 | #Feature | #Feature Sample 3 | Start | End | PTM | AScore | Found By |
| --- | --- | --- | --- | --- | --- | --- | --- | --- | --- | --- | --- | --- | --- | --- | --- | --- | --- | --- | --- |
| K.GKLTQEEEVLLQDFSR.T | Y | 31.76 | 1890.9687 | 16 | 2.44 | 631.3281 | 3 | 19.70 | 0.9048 | 0.9121 | 67409 | 0 | 0 | 0 | 5 | 20 |  |  | DB Search |
| R.EVNTELDSRPETK.K | Y | 31.11 | 1516.7369 | 13 | -3.14 | 759.369 | 2 | 11.76 | 1.0226 | 1.0299 | 25293 | 0 | 0 | 0 | 97 | 109 |  |  | DB Search |
| total 2 peptides |  |  |  |  |  |  |  |  |  |  |  |  |  |  |  |  |  |  |  |
| --- | --- | --- | --- | --- | --- | --- | --- | --- | --- | --- | --- | --- | --- | --- | --- | --- | --- | --- | --- |

Best Unique PSM (Scan Sample\_1\_A1\_1\_3639.d:67409, m/z=631.3281, z=3, RT=19.70, ppm=-3.33):


TRINITY\_DN140702\_c0\_g1\_i1.p1

back to list

  

| Protein Coverage
| Supporting Peptides
| Best Unique PSM
|

Protein Coverage:

Supporting Peptides:

| Peptide | Uniq | -10lgP | Mass | Length | ppm | m/z | z | RT | 1/k0 Start | 1/k0 End | Precursor ID | Area Sample 3 | #Feature | #Feature Sample 3 | Start | End | PTM | AScore | Found By |
| --- | --- | --- | --- | --- | --- | --- | --- | --- | --- | --- | --- | --- | --- | --- | --- | --- | --- | --- | --- |
| R.LALHVLNNEDIIGHR.L | Y | 31.43 | 1712.9322 | 15 | 3.56 | 571.9834 | 3 | 16.44 | 0.8538 | 0.8611 | 47846 | 7.52e2 | 1 | 1 | 1702 | 1716 |  |  | DB Search |
| total 1 peptides |  |  |  |  |  |  |  |  |  |  |  |  |  |  |  |  |  |  |  |
| --- | --- | --- | --- | --- | --- | --- | --- | --- | --- | --- | --- | --- | --- | --- | --- | --- | --- | --- | --- |

Best Unique PSM (Scan Sample\_1\_A1\_1\_3639.d:47846, m/z=571.9834, z=3, RT=16.44, ppm=-2.21):


TRINITY\_DN79126\_c0\_g1\_i1.p1

back to list

  

| Protein Coverage
| Supporting Peptides
| Best Unique PSM
|

Protein Coverage:

Supporting Peptides:

| Peptide | Uniq | -10lgP | Mass | Length | ppm | m/z | z | RT | 1/k0 Start | 1/k0 End | Precursor ID | Area Sample 3 | #Feature | #Feature Sample 3 | Start | End | PTM | AScore | Found By |
| --- | --- | --- | --- | --- | --- | --- | --- | --- | --- | --- | --- | --- | --- | --- | --- | --- | --- | --- | --- |
| R.AFLIEEQKLVVR.V | Y | 31.42 | 1443.8449 | 12 | 4.96 | 722.9291 | 2 | 17.47 | 1.0443 | 1.0516 | 53047 | 7.81e2 | 1 | 1 | 92 | 103 |  |  | DB Search |
| total 1 peptides |  |  |  |  |  |  |  |  |  |  |  |  |  |  |  |  |  |  |  |
| --- | --- | --- | --- | --- | --- | --- | --- | --- | --- | --- | --- | --- | --- | --- | --- | --- | --- | --- | --- |

Best Unique PSM (Scan Sample\_1\_A1\_1\_3639.d:53047, m/z=722.9291, z=2, RT=17.47, ppm=-0.82):


TRINITY\_DN1071\_c0\_g1\_i2.p1

back to list

  

| Protein Coverage
| Supporting Peptides
| Best Unique PSM
|

Protein Coverage:

Supporting Peptides:

| Peptide | Uniq | -10lgP | Mass | Length | ppm | m/z | z | RT | 1/k0 Start | 1/k0 End | Precursor ID | Area Sample 3 | #Feature | #Feature Sample 3 | Start | End | PTM | AScore | Found By |
| --- | --- | --- | --- | --- | --- | --- | --- | --- | --- | --- | --- | --- | --- | --- | --- | --- | --- | --- | --- |
| K.AVQIHPFNDVSVGDPK.V | Y | 31.42 | 1721.8736 | 16 | 5.27 | 861.9437 | 2 | 15.88 | 1.1142 | 1.1214 | 45122 | 5.31e3 | 2 | 2 | 293 | 308 |  |  | DB Search |
| total 1 peptides |  |  |  |  |  |  |  |  |  |  |  |  |  |  |  |  |  |  |  |
| --- | --- | --- | --- | --- | --- | --- | --- | --- | --- | --- | --- | --- | --- | --- | --- | --- | --- | --- | --- |

Best Unique PSM (Scan Sample\_1\_A1\_1\_3639.d:45122, m/z=861.9437, z=2, RT=15.88, ppm=-0.5):


TRINITY\_DN428\_c0\_g1\_i1.p1

back to list

  

| Protein Coverage
| Supporting Peptides
| Best Unique PSM
|

Protein Coverage:

Supporting Peptides:

| Peptide | Uniq | -10lgP | Mass | Length | ppm | m/z | z | RT | 1/k0 Start | 1/k0 End | Precursor ID | Area Sample 3 | #Feature | #Feature Sample 3 | Start | End | PTM | AScore | Found By |
| --- | --- | --- | --- | --- | --- | --- | --- | --- | --- | --- | --- | --- | --- | --- | --- | --- | --- | --- | --- |
| K.AKQEAFIAELEDQIAK.G | Y | 32.28 | 1802.9414 | 16 | 9.12 | 601.9897 | 3 | 19.97 | 0.8793 | 0.8866 | 69132 | 0 | 0 | 0 | 1020 | 1035 |  |  | DB Search |
| K.IC(+57.02)HLLGIQVTDFTK.S | Y | 31.85 | 1643.8705 | 14 | 3.68 | 548.963 | 3 | 18.57 | 0.8161 | 0.8234 | 59993 | 2.47e2 | 1 | 1 | 377 | 390 | Carbamidomethylation |  | DB Search |
| K.ALSLQNANEELEDRLAEAER.Q | Y | 30.07 | 2270.1138 | 20 | 8.88 | 757.7142 | 3 | 18.86 | 0.982 | 0.9892 | 61796 | 4.24e2 | 1 | 1 | 1475 | 1494 |  |  | DB Search |
| total 3 peptides |  |  |  |  |  |  |  |  |  |  |  |  |  |  |  |  |  |  |  |
| --- | --- | --- | --- | --- | --- | --- | --- | --- | --- | --- | --- | --- | --- | --- | --- | --- | --- | --- | --- |

Best Unique PSM (Scan Sample\_1\_A1\_1\_3639.d:69132, m/z=601.9897, z=3, RT=19.97, ppm=3.35):


TRINITY\_DN585\_c0\_g1\_i8.p1

back to list

  

| Protein Coverage
| Supporting Peptides
| Best Unique PSM
|

Protein Coverage:

Supporting Peptides:

| Peptide | Uniq | -10lgP | Mass | Length | ppm | m/z | z | RT | 1/k0 Start | 1/k0 End | Precursor ID | Area Sample 3 | #Feature | #Feature Sample 3 | Start | End | PTM | AScore | Found By |
| --- | --- | --- | --- | --- | --- | --- | --- | --- | --- | --- | --- | --- | --- | --- | --- | --- | --- | --- | --- |
| K.ESTLHLVLR.L | Y | 31.37 | 1066.6135 | 9 | -2.66 | 534.3095 | 2 | 15.64 | 0.8785 | 0.8858 | 44242 | 0 | 0 | 0 | 64 | 72 |  |  | DB Search |
| K.TITLEVEPSDTIENVK.S | Y | 30.07 | 1786.92 | 16 | 6.27 | 894.4677 | 2 | 17.53 | 1.1438 | 1.151 | 53352 | 6.91e2 | 1 | 1 | 12 | 27 |  |  | DB Search |
| total 2 peptides |  |  |  |  |  |  |  |  |  |  |  |  |  |  |  |  |  |  |  |
| --- | --- | --- | --- | --- | --- | --- | --- | --- | --- | --- | --- | --- | --- | --- | --- | --- | --- | --- | --- |

Best Unique PSM (Scan Sample\_1\_A1\_1\_3639.d:44242, m/z=534.3095, z=2, RT=15.64, ppm=-8.44):


TRINITY\_DN1616\_c0\_g1\_i3.p1

back to list

  

| Protein Coverage
| Supporting Peptides
| Best Unique PSM
|

Protein Coverage:

Supporting Peptides:

| Peptide | Uniq | -10lgP | Mass | Length | ppm | m/z | z | RT | 1/k0 Start | 1/k0 End | Precursor ID | Area Sample 3 | #Feature | #Feature Sample 3 | Start | End | PTM | AScore | Found By |
| --- | --- | --- | --- | --- | --- | --- | --- | --- | --- | --- | --- | --- | --- | --- | --- | --- | --- | --- | --- |
| K.DGEKVETLKVDISDINK.A | Y | 31.36 | 1901.9945 | 17 | 4.01 | 635.0043 | 3 | 15.97 | 0.8976 | 0.9048 | 45629 | 4.35e2 | 1 | 1 | 2634 | 2650 |  |  | DB Search |
| total 1 peptides |  |  |  |  |  |  |  |  |  |  |  |  |  |  |  |  |  |  |  |
| --- | --- | --- | --- | --- | --- | --- | --- | --- | --- | --- | --- | --- | --- | --- | --- | --- | --- | --- | --- |

Best Unique PSM (Scan Sample\_1\_A1\_1\_3639.d:45629, m/z=635.0043, z=3, RT=15.97, ppm=-1.77):


TRINITY\_DN1465\_c1\_g1\_i1.p1

back to list

  

| Protein Coverage
| Supporting Peptides
| Best Unique PSM
|

Protein Coverage:

Supporting Peptides:

| Peptide | Uniq | -10lgP | Mass | Length | ppm | m/z | z | RT | 1/k0 Start | 1/k0 End | Precursor ID | Area Sample 3 | #Feature | #Feature Sample 3 | Start | End | PTM | AScore | Found By |
| --- | --- | --- | --- | --- | --- | --- | --- | --- | --- | --- | --- | --- | --- | --- | --- | --- | --- | --- | --- |
| K.ESTLHLVLR.L | Y | 31.37 | 1066.6135 | 9 | -2.66 | 534.3095 | 2 | 15.64 | 0.8785 | 0.8858 | 44242 | 0 | 0 | 0 | 68 | 76 |  |  | DB Search |
| total 1 peptides |  |  |  |  |  |  |  |  |  |  |  |  |  |  |  |  |  |  |  |
| --- | --- | --- | --- | --- | --- | --- | --- | --- | --- | --- | --- | --- | --- | --- | --- | --- | --- | --- | --- |

Best Unique PSM (Scan Sample\_1\_A1\_1\_3639.d:44242, m/z=534.3095, z=2, RT=15.64, ppm=-8.44):


TRINITY\_DN17714\_c0\_g1\_i1.p1

back to list

  

| Protein Coverage
| Supporting Peptides
| Best Unique PSM
|

Protein Coverage:

Supporting Peptides:

| Peptide | Uniq | -10lgP | Mass | Length | ppm | m/z | z | RT | 1/k0 Start | 1/k0 End | Precursor ID | Area Sample 3 | #Feature | #Feature Sample 3 | Start | End | PTM | AScore | Found By |
| --- | --- | --- | --- | --- | --- | --- | --- | --- | --- | --- | --- | --- | --- | --- | --- | --- | --- | --- | --- |
| K.YAAYVGC(+57.02)EFQSVDFSSVAK.G | Y | 31.89 | 2126.9619 | 19 | 7.79 | 1064.4904 | 2 | 18.86 | 1.2324 | 1.2396 | 61864 | 0 | 0 | 0 | 537 | 555 | Carbamidomethylation |  | DB Search |
| total 1 peptides |  |  |  |  |  |  |  |  |  |  |  |  |  |  |  |  |  |  |  |
| --- | --- | --- | --- | --- | --- | --- | --- | --- | --- | --- | --- | --- | --- | --- | --- | --- | --- | --- | --- |

Best Unique PSM (Scan Sample\_1\_A1\_1\_3639.d:61864, m/z=1064.4904, z=2, RT=18.86, ppm=2.02):


TRINITY\_DN286\_c0\_g1\_i2.p1

back to list

  

| Protein Coverage
| Supporting Peptides
| Best Unique PSM
|

Protein Coverage:

Supporting Peptides:

| Peptide | Uniq | -10lgP | Mass | Length | ppm | m/z | z | RT | 1/k0 Start | 1/k0 End | Precursor ID | Area Sample 3 | #Feature | #Feature Sample 3 | Start | End | PTM | AScore | Found By |
| --- | --- | --- | --- | --- | --- | --- | --- | --- | --- | --- | --- | --- | --- | --- | --- | --- | --- | --- | --- |
| K.RYGQAMC(+57.02)QFMR.N | Y | 31.95 | 1446.6319 | 11 | -3.39 | 724.3166 | 2 | 17.38 | 0.9808 | 0.988 | 52561 | 3.82e2 | 1 | 1 | 71 | 81 | Carbamidomethylation |  | DB Search |
| total 1 peptides |  |  |  |  |  |  |  |  |  |  |  |  |  |  |  |  |  |  |  |
| --- | --- | --- | --- | --- | --- | --- | --- | --- | --- | --- | --- | --- | --- | --- | --- | --- | --- | --- | --- |

Best Unique PSM (Scan Sample\_1\_A1\_1\_3639.d:52561, m/z=724.3166, z=2, RT=17.38, ppm=-9.16):


TRINITY\_DN40728\_c0\_g1\_i1.p1

back to list

  

| Protein Coverage
| Supporting Peptides
| Best Unique PSM
|

Protein Coverage:

Supporting Peptides:

| Peptide | Uniq | -10lgP | Mass | Length | ppm | m/z | z | RT | 1/k0 Start | 1/k0 End | Precursor ID | Area Sample 3 | #Feature | #Feature Sample 3 | Start | End | PTM | AScore | Found By |
| --- | --- | --- | --- | --- | --- | --- | --- | --- | --- | --- | --- | --- | --- | --- | --- | --- | --- | --- | --- |
| K.ESTLHLVLR.L | Y | 31.37 | 1066.6135 | 9 | -2.66 | 534.3095 | 2 | 15.64 | 0.8785 | 0.8858 | 44242 | 0 | 0 | 0 | 87 | 95 |  |  | DB Search |
| total 1 peptides |  |  |  |  |  |  |  |  |  |  |  |  |  |  |  |  |  |  |  |
| --- | --- | --- | --- | --- | --- | --- | --- | --- | --- | --- | --- | --- | --- | --- | --- | --- | --- | --- | --- |

Best Unique PSM (Scan Sample\_1\_A1\_1\_3639.d:44242, m/z=534.3095, z=2, RT=15.64, ppm=-8.44):


TRINITY\_DN319\_c2\_g1\_i3.p1

back to list

  

| Protein Coverage
| Supporting Peptides
| Best Unique PSM
|

Protein Coverage:

Supporting Peptides:

| Peptide | Uniq | -10lgP | Mass | Length | ppm | m/z | z | RT | 1/k0 Start | 1/k0 End | Precursor ID | Area Sample 3 | #Feature | #Feature Sample 3 | Start | End | PTM | AScore | Found By |
| --- | --- | --- | --- | --- | --- | --- | --- | --- | --- | --- | --- | --- | --- | --- | --- | --- | --- | --- | --- |
| R.ADC(+57.02)GVENTC(+57.02)FGSTR.S | Y | 31.98 | 1572.6297 | 14 | 3.17 | 787.3201 | 2 | 13.66 | 1.0206 | 1.0279 | 34821 | 4.97e2 | 1 | 1 | 1578 | 1591 | Carbamidomethylation, Carbamidomethylation |  | DB Search |
| R.DPIC(+57.02)HC(+57.02)DTSK.Y | Y | 31.43 | 1231.4961 | 10 | 6.8 | 411.5064 | 3 | 9.93 | 0.7331 | 0.7404 | 15608 | 1e3 | 1 | 1 | 1448 | 1457 | Carbamidomethylation, Carbamidomethylation |  | DB Search |
| R.GSFSSHIC(+57.02)LC(+57.02)HDPAYK.S | Y | 31.43 | 1877.8189 | 16 | 3.63 | 626.9456 | 3 | 14.39 | 0.8814 | 0.8887 | 38439 | 1.55e3 | 1 | 1 | 1011 | 1026 | Carbamidomethylation, Carbamidomethylation |  | DB Search |
| K.KAQYYEAYETIIKR.H | Y | 31.42 | 1774.9254 | 14 | 2.25 | 592.647 | 3 | 15.86 | 0.8575 | 0.8648 | 45137 | 7.72e2 | 1 | 1 | 2534 | 2547 |  |  | DB Search |
| total 4 peptides |  |  |  |  |  |  |  |  |  |  |  |  |  |  |  |  |  |  |  |
| --- | --- | --- | --- | --- | --- | --- | --- | --- | --- | --- | --- | --- | --- | --- | --- | --- | --- | --- | --- |

Best Unique PSM (Scan Sample\_1\_A1\_1\_3639.d:34821, m/z=787.3201, z=2, RT=13.66, ppm=-2.6):


TRINITY\_DN691\_c0\_g1\_i1.p1

back to list

  

| Protein Coverage
| Supporting Peptides
| Best Unique PSM
|

Protein Coverage:

Supporting Peptides:

| Peptide | Uniq | -10lgP | Mass | Length | ppm | m/z | z | RT | 1/k0 Start | 1/k0 End | Precursor ID | Area Sample 3 | #Feature | #Feature Sample 3 | Start | End | PTM | AScore | Found By |
| --- | --- | --- | --- | --- | --- | --- | --- | --- | --- | --- | --- | --- | --- | --- | --- | --- | --- | --- | --- |
| R.AYGQDLGQPGSC(+57.02)LKR.F | Y | 32.5 | 1648.7991 | 15 | 5.96 | 825.407 | 2 | 13.47 | 1.0733 | 1.0805 | 33986 | 7.47e2 | 1 | 1 | 1533 | 1547 | Carbamidomethylation |  | DB Search |
| total 1 peptides |  |  |  |  |  |  |  |  |  |  |  |  |  |  |  |  |  |  |  |
| --- | --- | --- | --- | --- | --- | --- | --- | --- | --- | --- | --- | --- | --- | --- | --- | --- | --- | --- | --- |

Best Unique PSM (Scan Sample\_1\_A1\_1\_3639.d:33986, m/z=825.407, z=2, RT=13.47, ppm=0.18):


TRINITY\_DN1386\_c0\_g1\_i1.p1

back to list

  

| Protein Coverage
| Supporting Peptides
| Best Unique PSM
|

Protein Coverage:

Supporting Peptides:

| Peptide | Uniq | -10lgP | Mass | Length | ppm | m/z | z | RT | 1/k0 Start | 1/k0 End | Precursor ID | Area Sample 3 | #Feature | #Feature Sample 3 | Start | End | PTM | AScore | Found By |
| --- | --- | --- | --- | --- | --- | --- | --- | --- | --- | --- | --- | --- | --- | --- | --- | --- | --- | --- | --- |
| R.KVIVDQLVTLATR.V | Y | 31.42 | 1454.882 | 13 | 5.37 | 728.448 | 2 | 18.39 | 1.0246 | 1.0319 | 58671 | 8.62e2 | 1 | 1 | 547 | 559 |  |  | DB Search |
| total 1 peptides |  |  |  |  |  |  |  |  |  |  |  |  |  |  |  |  |  |  |  |
| --- | --- | --- | --- | --- | --- | --- | --- | --- | --- | --- | --- | --- | --- | --- | --- | --- | --- | --- | --- |

Best Unique PSM (Scan Sample\_1\_A1\_1\_3639.d:58671, m/z=728.448, z=2, RT=18.39, ppm=-0.4):


TRINITY\_DN691\_c0\_g1\_i2.p1

back to list

  

| Protein Coverage
| Supporting Peptides
| Best Unique PSM
|

Protein Coverage:

Supporting Peptides:

| Peptide | Uniq | -10lgP | Mass | Length | ppm | m/z | z | RT | 1/k0 Start | 1/k0 End | Precursor ID | Area Sample 3 | #Feature | #Feature Sample 3 | Start | End | PTM | AScore | Found By |
| --- | --- | --- | --- | --- | --- | --- | --- | --- | --- | --- | --- | --- | --- | --- | --- | --- | --- | --- | --- |
| R.AYGQDLGQPGSC(+57.02)LKR.F | Y | 32.5 | 1648.7991 | 15 | 5.96 | 825.407 | 2 | 13.47 | 1.0733 | 1.0805 | 33986 | 7.47e2 | 1 | 1 | 1480 | 1494 | Carbamidomethylation |  | DB Search |
| total 1 peptides |  |  |  |  |  |  |  |  |  |  |  |  |  |  |  |  |  |  |  |
| --- | --- | --- | --- | --- | --- | --- | --- | --- | --- | --- | --- | --- | --- | --- | --- | --- | --- | --- | --- |

Best Unique PSM (Scan Sample\_1\_A1\_1\_3639.d:33986, m/z=825.407, z=2, RT=13.47, ppm=0.18):


TRINITY\_DN2490\_c0\_g1\_i1.p1

back to list

  

| Protein Coverage
| Supporting Peptides
| Best Unique PSM
|

Protein Coverage:

Supporting Peptides:

| Peptide | Uniq | -10lgP | Mass | Length | ppm | m/z | z | RT | 1/k0 Start | 1/k0 End | Precursor ID | Area Sample 3 | #Feature | #Feature Sample 3 | Start | End | PTM | AScore | Found By |
| --- | --- | --- | --- | --- | --- | --- | --- | --- | --- | --- | --- | --- | --- | --- | --- | --- | --- | --- | --- |
| K.DKIFPHIELFGK.E | Y | 36.06 | 1442.7921 | 12 | 4.76 | 481.9375 | 3 | 18.63 | 0.8696 | 0.8769 | 60334 | 4.52e2 | 1 | 1 | 519 | 530 |  |  | DB Search |
| total 1 peptides |  |  |  |  |  |  |  |  |  |  |  |  |  |  |  |  |  |  |  |
| --- | --- | --- | --- | --- | --- | --- | --- | --- | --- | --- | --- | --- | --- | --- | --- | --- | --- | --- | --- |

Best Unique PSM (Scan Sample\_1\_A1\_1\_3639.d:60334, m/z=481.9375, z=3, RT=18.63, ppm=-1.02):


TRINITY\_DN554\_c0\_g1\_i2.p1

back to list

  

| Protein Coverage
| Supporting Peptides
| Best Unique PSM
|

Protein Coverage:

Supporting Peptides:

| Peptide | Uniq | -10lgP | Mass | Length | ppm | m/z | z | RT | 1/k0 Start | 1/k0 End | Precursor ID | Area Sample 3 | #Feature | #Feature Sample 3 | Start | End | PTM | AScore | Found By |
| --- | --- | --- | --- | --- | --- | --- | --- | --- | --- | --- | --- | --- | --- | --- | --- | --- | --- | --- | --- |
| K.ESTLHLVLR.L | Y | 31.37 | 1066.6135 | 9 | -2.66 | 534.3095 | 2 | 15.64 | 0.8785 | 0.8858 | 44242 | 0 | 0 | 0 | 29 | 37 |  |  | DB Search |
| K.TITLEVEPSDTIENVK.S | Y | 30.07 | 1786.92 | 16 | 6.27 | 894.4677 | 2 | 17.53 | 1.1438 | 1.151 | 53352 | 6.91e2 | 1 | 1 | 53 | 68 |  |  | DB Search |
| total 2 peptides |  |  |  |  |  |  |  |  |  |  |  |  |  |  |  |  |  |  |  |
| --- | --- | --- | --- | --- | --- | --- | --- | --- | --- | --- | --- | --- | --- | --- | --- | --- | --- | --- | --- |

Best Unique PSM (Scan Sample\_1\_A1\_1\_3639.d:44242, m/z=534.3095, z=2, RT=15.64, ppm=-8.44):


TRINITY\_DN11595\_c0\_g1\_i2.p1

back to list

  

| Protein Coverage
| Supporting Peptides
| Best Unique PSM
|

Protein Coverage:

Supporting Peptides:

| Peptide | Uniq | -10lgP | Mass | Length | ppm | m/z | z | RT | 1/k0 Start | 1/k0 End | Precursor ID | Area Sample 3 | #Feature | #Feature Sample 3 | Start | End | PTM | AScore | Found By |
| --- | --- | --- | --- | --- | --- | --- | --- | --- | --- | --- | --- | --- | --- | --- | --- | --- | --- | --- | --- |
| K.LVHGDVFRPPR.Y | Y | 31.41 | 1291.7149 | 11 | 7.13 | 646.8656 | 2 | 13.43 | 0.9699 | 0.9771 | 33689 | 4.13e2 | 1 | 1 | 301 | 311 |  |  | DB Search |
| total 1 peptides |  |  |  |  |  |  |  |  |  |  |  |  |  |  |  |  |  |  |  |
| --- | --- | --- | --- | --- | --- | --- | --- | --- | --- | --- | --- | --- | --- | --- | --- | --- | --- | --- | --- |

Best Unique PSM (Scan Sample\_1\_A1\_1\_3639.d:33689, m/z=646.8656, z=2, RT=13.43, ppm=1.36):


---

Prepared with PEAKS ™ (bioinfor.com)
