# Supplementary material for: Proteomic Analysis and Biochemical Characterization of the Nematocyst Extract of the Hydrozoan Velella velella
Source: Mar Drugs. 2024 Oct 12;22(10):468. doi: 10.3390/md22100468 (PMC11509761; doi:10.3390/md22100468)
Supplement: Supplementary file 1 [file marinedrugs-22-00468-s001.zip › Supplementary File S1.zip!/Sample_10_Vellela/db.peptides.html]

peptide list


Peptide List

  

| Peptide | -10lgP | Mass | Length | ppm | m/z | RT | 1/k0 Start | 1/k0 End | Precursor ID | Area Sample 1 | #Feature | #Feature Sample 1 | Accession | PTM | AScore | Found By |
| --- | --- | --- | --- | --- | --- | --- | --- | --- | --- | --- | --- | --- | --- | --- | --- | --- |
| LTHLEEAYTTEHWLVR | 66.93 | 1997.0006 | 16 | 0.43 | 666.6714 | 17.14 | 0.9574 | 0.9646 | 53465 | 3.26e2 | 1 | 1 | TRINITY\_DN21998\_c0\_g1\_i1.p1 |  |  | DB Search |
| GFDYC(+57.02)TTIKYR | 66.92 | 1422.6602 | 11 | -0.86 | 712.3336 | 15.08 | 1.023 | 1.0303 | 42693 | 0 | 0 | 0 | TRINITY\_DN36\_c0\_g1\_i13.p1:TRINITY\_DN36\_c0\_g1\_i19.p1:TRINITY\_DN36\_c0\_g1\_i20.p1:TRINITY\_DN36\_c0\_g1\_i5.p1 | Carbamidomethylation |  | DB Search |
| FSQHTDYVIAR | 66.91 | 1335.6571 | 11 | 1.81 | 668.834 | 13.45 | 0.9763 | 0.9836 | 33690 | 0 | 0 | 0 | TRINITY\_DN121866\_c0\_g1\_i1.p1 |  |  | DB Search |
| AIELLPHDLAVGR | 66.91 | 1402.7932 | 13 | -9.52 | 702.394 | 17.77 | 1.0275 | 1.0347 | 57279 | 0 | 0 | 0 | TRINITY\_DN4954\_c0\_g1\_i2.p2:TRINITY\_DN6388\_c0\_g1\_i1.p2:TRINITY\_DN6388\_c0\_g1\_i2.p2 |  |  | DB Search |
| GLKVDFDTTFAPVTGKK | 66.86 | 1822.9828 | 17 | 1.5 | 912.496 | 17.12 | 1.1802 | 1.1873 | 53325 | 2.2e3 | 2 | 2 | TRINITY\_DN329\_c0\_g1\_i1.p1:TRINITY\_DN329\_c0\_g1\_i2.p1 |  |  | DB Search |
| AYAAFLEFKDVVK | 66.86 | 1499.8024 | 13 | 1.14 | 750.9059 | 19.02 | 1.0945 | 1.1017 | 64841 | 9.59e3 | 1 | 1 | TRINITY\_DN240\_c0\_g1\_i6.p1:TRINITY\_DN240\_c0\_g1\_i1.p1:TRINITY\_DN240\_c0\_g1\_i4.p1 |  |  | DB Search |
| FSYEKVC(+57.02)EDVKR | 66.86 | 1558.745 | 12 | -4.31 | 520.5844 | 13.47 | 0.7945 | 0.8019 | 33820 | 0 | 0 | 0 | TRINITY\_DN118\_c1\_g1\_i3.p1 | Carbamidomethylation |  | DB Search |
| S(+42.01)LVIPEKFQHILR | 66.85 | 1620.9352 | 13 | 0.67 | 811.4717 | 19.55 | 1.1226 | 1.1298 | 68036 | 3.14e4 | 2 | 2 | TRINITY\_DN1505\_c0\_g1\_i1.p1 | Acetylation (Protein N-term) | S1:Acetylation (Protein N-term):1000 | DB Search |
| VSILESLDKWER | 66.83 | 1473.7827 | 12 | -1.01 | 492.2655 | 19.59 | 0.7868 | 0.7941 | 68471 | 0 | 0 | 0 | TRINITY\_DN76040\_c0\_g1\_i1.p1 |  |  | DB Search |
| TIEAEAAHGTVTR | 66.82 | 1354.6841 | 13 | 1.88 | 678.3475 | 11.81 | 1.013 | 1.0202 | 24117 | 1.54e3 | 1 | 1 | TRINITY\_DN7504\_c0\_g1\_i1.p1 |  |  | DB Search |
| ILQC(+57.02)NQYANVEEAR | 66.82 | 1706.8046 | 14 | -0.34 | 854.4055 | 14.19 | 1.0693 | 1.0765 | 37690 | 1.33e3 | 1 | 1 | TRINITY\_DN477\_c0\_g1\_i8.p1:TRINITY\_DN477\_c0\_g1\_i7.p1 | Carbamidomethylation |  | DB Search |
| AIGHFTQLVWK | 66.81 | 1298.7135 | 11 | 0.43 | 650.3614 | 17.56 | 0.9755 | 0.9828 | 56062 | 0 | 0 | 0 | TRINITY\_DN12390\_c0\_g1\_i1.p1 |  |  | DB Search |
| AFLIEEQKLVVR | 66.8 | 1443.8448 | 12 | 1.31 | 722.9274 | 17.34 | 1.0496 | 1.0568 | 54531 | 8.16e3 | 1 | 1 | TRINITY\_DN79126\_c0\_g1\_i1.p1 |  |  | DB Search |
| AMTDC(+57.02)KETLC(+57.02)R | 66.8 | 1383.5945 | 11 | 1.03 | 692.8021 | 11.63 | 0.9888 | 0.9961 | 23189 | 1.32e3 | 1 | 1 | TRINITY\_DN168534\_c0\_g1\_i1.p1 | Carbamidomethylation, Carbamidomethylation |  | DB Search |
| IC(+57.02)HLLGIQVTDFTK | 66.8 | 1643.8705 | 14 | 2.77 | 822.9411 | 18.45 | 1.0837 | 1.0909 | 61351 | 7.71e2 | 1 | 1 | TRINITY\_DN428\_c0\_g1\_i1.p1 | Carbamidomethylation |  | DB Search |
| NFNHLNLVLTR | 66.8 | 1339.7361 | 11 | 6.41 | 670.8766 | 16.83 | 0.9783 | 0.9856 | 51799 | 1.21e3 | 1 | 1 | TRINITY\_DN194146\_c0\_g1\_i1.p1 |  |  | DB Search |
| SFHFGVIKLEGK | 66.8 | 1360.7502 | 12 | 0.14 | 681.3795 | 16.99 | 1.0275 | 1.0347 | 52570 | 1.81e3 | 1 | 1 | TRINITY\_DN329\_c0\_g1\_i1.p1:TRINITY\_DN329\_c0\_g1\_i2.p1 |  |  | DB Search |
| SLVEENVHPQILIK | 66.8 | 1617.9089 | 14 | 4.08 | 809.9614 | 16.31 | 1.1013 | 1.1086 | 49172 | 0 | 0 | 0 | TRINITY\_DN1194\_c0\_g1\_i1.p1 |  |  | DB Search |
| LIYENQNTIHHVVR | 66.79 | 1734.9165 | 14 | -1.94 | 868.46 | 13.18 | 1.1274 | 1.1346 | 31991 | 5.25e2 | 1 | 1 | TRINITY\_DN26249\_c0\_g1\_i1.p1 |  |  | DB Search |
| VAHAGLTVAEFFR | 66.78 | 1416.7513 | 13 | 2.84 | 709.3818 | 18.86 | 1.0267 | 1.0339 | 63839 | 8.41e2 | 1 | 1 | TRINITY\_DN11097\_c0\_g1\_i1.p1:TRINITY\_DN11097\_c0\_g2\_i1.p1 |  |  | DB Search |
| KGLTPSQIGVILR | 66.78 | 1380.8452 | 13 | -0.23 | 691.4266 | 17.08 | 0.9981 | 1.0053 | 53138 | 4.79e3 | 1 | 1 | TRINITY\_DN80458\_c0\_g1\_i1.p1 |  |  | DB Search |
| LVIGNSEGFKDVSK | 66.78 | 1491.7932 | 14 | 0.35 | 746.9008 | 14.67 | 1.0809 | 1.0881 | 40349 | 0 | 0 | 0 | TRINITY\_DN3180\_c0\_g1\_i1.p1 |  |  | DB Search |
| DANAAVVHGSHLK | 66.78 | 1317.679 | 13 | -0.21 | 659.8436 | 10.89 | 1.0154 | 1.0226 | 18821 | 6.63e2 | 1 | 1 | TRINITY\_DN262\_c0\_g1\_i1.p1 |  |  | DB Search |
| LTVC(+57.02)VTVVHHGLQK | 66.78 | 1589.8711 | 14 | 7.51 | 795.9453 | 13.99 | 1.1158 | 1.123 | 36644 | 0 | 0 | 0 | TRINITY\_DN167719\_c0\_g1\_i1.p1 | Carbamidomethylation |  | DB Search |
| GLKVDFDTTFAPVTGK | 66.78 | 1694.8879 | 16 | 3.14 | 848.4501 | 18.13 | 1.0933 | 1.1005 | 59417 | 1.18e3 | 1 | 1 | TRINITY\_DN329\_c0\_g1\_i1.p1:TRINITY\_DN329\_c0\_g1\_i2.p1 |  |  | DB Search |
| DMGVNVISVGAGHTINHR | 66.78 | 1875.9374 | 18 | 3.53 | 938.975 | 15.24 | 1.2443 | 1.2515 | 43380 | 3.44e2 | 1 | 1 | TRINITY\_DN138\_c0\_g1\_i1.p1 |  |  | DB Search |
| M(+15.99)SATFIGNSTAIQELFKR | 66.78 | 2029.0303 | 18 | 0.01 | 677.3477 | 19.94 | 0.9348 | 0.942 | 70482 | 1.29e3 | 1 | 1 | TRINITY\_DN3986\_c0\_g1\_i1.p1 | Oxidation (M) | M1:Oxidation (M):1000 | DB Search |
| LVQNTPHNSIVTK | 66.78 | 1449.7939 | 13 | 2.36 | 725.9027 | 11.65 | 1.0628 | 1.0701 | 23219 | 0 | 0 | 0 | TRINITY\_DN982\_c0\_g1\_i3.p1:TRINITY\_DN982\_c0\_g1\_i1.p1 |  |  | DB Search |
| LTEEDVKDQIYK | 66.77 | 1479.7456 | 12 | 4.19 | 740.8799 | 14.60 | 1.0226 | 1.0299 | 39982 | 0 | 0 | 0 | TRINITY\_DN80458\_c0\_g1\_i1.p1 |  |  | DB Search |
| KIDLHLYHELYM(+15.99)K | 66.77 | 1717.8861 | 13 | -3.92 | 859.9431 | 15.60 | 1.1414 | 1.1486 | 45433 | 4.78e2 | 1 | 1 | TRINITY\_DN1112\_c0\_g1\_i1.p1 | Oxidation (M) | M12:Oxidation (M):1000 | DB Search |
| VYLAQAEAEKIK | 66.77 | 1361.7554 | 12 | 0.13 | 681.882 | 14.27 | 1.0347 | 1.0419 | 38198 | 3.91e2 | 1 | 1 | TRINITY\_DN11435\_c0\_g1\_i1.p1 |  |  | DB Search |
| IVAINAHHFLK | 66.77 | 1261.7295 | 11 | 1.77 | 631.8703 | 14.46 | 1.0162 | 1.0234 | 39273 | 0 | 0 | 0 | TRINITY\_DN1084\_c0\_g1\_i1.p1 |  |  | DB Search |
| SGNVVLC(+57.02)LIDVHK | 66.77 | 1452.7759 | 13 | -0.46 | 727.3916 | 18.50 | 1.0391 | 1.0464 | 61767 | 0 | 0 | 0 | TRINITY\_DN3078\_c0\_g1\_i1.p1 | Carbamidomethylation |  | DB Search |
| AEYENKTEVAR | 66.77 | 1308.631 | 11 | 4.86 | 655.323 | 10.97 | 0.9783 | 0.9856 | 19355 | 0 | 0 | 0 | TRINITY\_DN1280\_c0\_g1\_i1.p1 |  |  | DB Search |
| AVC(+57.02)M(+15.99)LSNTTAIAEAWAR | 66.77 | 1879.8921 | 17 | -0.29 | 940.9488 | 18.63 | 1.1418 | 1.149 | 62475 | 6.54e2 | 1 | 1 | TRINITY\_DN687\_c0\_g2\_i1.p1:TRINITY\_DN250\_c0\_g1\_i5.p1:TRINITY\_DN687\_c0\_g1\_i1.p1:TRINITY\_DN250\_c0\_g1\_i1.p1:TRINITY\_DN250\_c0\_g1\_i3.p1 | Carbamidomethylation, Oxidation (M) | M4:Oxidation (M):1000 | DB Search |
| LVFEGYHYWATK | 66.76 | 1512.7401 | 12 | 1.97 | 757.3754 | 17.66 | 1.0439 | 1.0512 | 56486 | 7.22e2 | 1 | 1 | TRINITY\_DN4781\_c0\_g1\_i1.p1 |  |  | DB Search |
| MRFDTAITAC(+57.02)R | 66.76 | 1340.6329 | 11 | 3.22 | 671.3229 | 14.89 | 0.9675 | 0.9747 | 41498 | 1.49e3 | 1 | 1 | TRINITY\_DN477\_c0\_g1\_i9.p1 | Carbamidomethylation |  | DB Search |
| VLTASDPEALKR | 66.76 | 1298.7194 | 12 | 2.49 | 650.3657 | 12.93 | 0.9912 | 0.9985 | 30634 | 7.44e2 | 1 | 1 | TRINITY\_DN10563\_c0\_g1\_i1.p1 |  |  | DB Search |
| LIEDTEYTAHEGAK | 66.76 | 1575.7416 | 14 | -1.83 | 788.8731 | 12.66 | 1.0789 | 1.0861 | 29096 | 0 | 0 | 0 | TRINITY\_DN12632\_c1\_g1\_i1.p1 |  |  | DB Search |
| A(+42.01)EAPKPKPAPK | 66.75 | 1174.671 | 11 | 10.9 | 392.5668 | 9.90 | 0.7665 | 0.7738 | 13029 | 1.04e4 | 2 | 2 | TRINITY\_DN1631\_c0\_g1\_i1.p1 | Acetylation (Protein N-term) | A1:Acetylation (Protein N-term):1000 | DB Search |
| LC(+57.02)ESDPENAKK | 66.75 | 1289.5922 | 11 | 2.03 | 645.8018 | 10.10 | 0.9489 | 0.9562 | 14132 | 7.73e2 | 1 | 1 | TRINITY\_DN3649\_c0\_g1\_i2.p1:TRINITY\_DN3649\_c0\_g1\_i1.p1 | Carbamidomethylation |  | DB Search |
| AYC(+57.02)DINEHISC(+57.02)SK | 66.75 | 1595.6708 | 13 | 2.31 | 798.8409 | 12.79 | 1.0564 | 1.0636 | 29794 | 7.14e2 | 1 | 1 | TRINITY\_DN5131\_c1\_g2\_i1.p1 | Carbamidomethylation, Carbamidomethylation |  | DB Search |
| ASGDYATVVSHNR | 66.75 | 1375.6481 | 13 | -1.79 | 688.827 | 12.07 | 0.9957 | 1.0029 | 25649 | 0 | 0 | 0 | TRINITY\_DN925\_c0\_g1\_i1.p1 |  |  | DB Search |
| RYGQAMC(+57.02)QFMR | 66.75 | 1446.6318 | 11 | -6.11 | 724.3156 | 17.23 | 0.9868 | 0.9941 | 53709 | 8.19e3 | 1 | 1 | TRINITY\_DN286\_c0\_g1\_i4.p1:TRINITY\_DN286\_c0\_g1\_i2.p1 | Carbamidomethylation |  | DB Search |
| AVFPSIVGRPR | 66.75 | 1197.6982 | 11 | -0.27 | 599.8535 | 15.64 | 0.9307 | 0.938 | 45498 | 3.34e4 | 1 | 1 | TRINITY\_DN161\_c0\_g1\_i1.p1:TRINITY\_DN161\_c0\_g1\_i3.p1:TRINITY\_DN161\_c0\_g1\_i2.p1 |  |  | DB Search |
| YIQGENEKQEK | 66.74 | 1364.6572 | 11 | -0.74 | 455.8906 | 9.56 | 0.7697 | 0.777 | 11206 | 9.07e2 | 1 | 1 | TRINITY\_DN1272\_c0\_g1\_i1.p1 |  |  | DB Search |
| YC(+57.02)ILSFLDKTDK | 66.74 | 1501.7487 | 12 | -2.59 | 501.5866 | 19.64 | 0.8002 | 0.8075 | 68673 | 3.17e2 | 1 | 1 | TRINITY\_DN15274\_c0\_g1\_i1.p1 | Carbamidomethylation |  | DB Search |
| IAAVIINKPDVLASR | 66.74 | 1578.9457 | 15 | -2.48 | 790.4746 | 15.97 | 1.1013 | 1.1086 | 47200 | 1.29e3 | 1 | 1 | TRINITY\_DN123\_c0\_g1\_i1.p1 |  |  | DB Search |
| DSYVGDEAQSKR | 66.73 | 1353.6161 | 12 | 2.52 | 677.814 | 10.94 | 1.0069 | 1.0142 | 19045 | 5.51e3 | 1 | 1 | TRINITY\_DN2477\_c0\_g1\_i5.p1:TRINITY\_DN2477\_c0\_g1\_i6.p1:TRINITY\_DN195934\_c0\_g1\_i1.p2:TRINITY\_DN161\_c0\_g1\_i1.p1:TRINITY\_DN2477\_c0\_g1\_i9.p1:TRINITY\_DN2477\_c0\_g1\_i10.p1:TRINITY\_DN2477\_c0\_g1\_i2.p1:TRINITY\_DN161\_c0\_g1\_i3.p1:TRINITY\_DN161\_c0\_g1\_i2.p1 |  |  | DB Search |
| DLQGETSGSFKR | 66.73 | 1323.6418 | 12 | -3.84 | 662.8227 | 12.59 | 0.9553 | 0.9626 | 28698 | 0 | 0 | 0 | TRINITY\_DN3190\_c1\_g1\_i1.p1 |  |  | DB Search |
| ITTSQILDHEQQK | 66.73 | 1539.7893 | 13 | 2.99 | 770.9008 | 13.83 | 1.0945 | 1.1017 | 35680 | 3.16e2 | 1 | 1 | TRINITY\_DN982\_c0\_g1\_i3.p1:TRINITY\_DN982\_c0\_g1\_i1.p1 |  |  | DB Search |
| LVQANPLEQDLSEKPR | 66.73 | 1835.9741 | 16 | 3.23 | 918.9932 | 15.63 | 1.1837 | 1.1909 | 45493 | 3.81e2 | 1 | 1 | TRINITY\_DN1616\_c0\_g1\_i3.p1:TRINITY\_DN1616\_c0\_g1\_i6.p1:TRINITY\_DN1616\_c0\_g1\_i2.p1:TRINITY\_DN1616\_c0\_g1\_i7.p1 |  |  | DB Search |
| TPGLVEGTLILHQLR | 66.72 | 1645.9515 | 15 | -1.96 | 549.6542 | 18.79 | 0.8635 | 0.8708 | 63448 | 6.54e2 | 1 | 1 | TRINITY\_DN3034\_c0\_g1\_i1.p1 |  |  | DB Search |
| TVQNFYELVTHSK | 66.72 | 1564.7886 | 13 | 1.3 | 783.399 | 18.24 | 1.0913 | 1.0985 | 60147 | 1.44e3 | 2 | 2 | TRINITY\_DN96\_c0\_g1\_i2.p1:TRINITY\_DN96\_c0\_g1\_i1.p1 |  |  | DB Search |
| VLTVISHNQR | 66.72 | 1165.6567 | 10 | 2.9 | 583.8347 | 12.24 | 0.9315 | 0.9388 | 26669 | 5.13e2 | 1 | 1 | TRINITY\_DN197343\_c0\_g1\_i1.p1 |  |  | DB Search |
| LVIISSNTPQLRK | 66.71 | 1467.8773 | 13 | 0.59 | 734.9431 | 14.91 | 1.058 | 1.0652 | 41605 | 1.53e3 | 1 | 1 | TRINITY\_DN2126\_c0\_g1\_i1.p1 |  |  | DB Search |
| LALHVLNNEDIIGHR | 66.71 | 1712.9321 | 15 | 1.25 | 857.4706 | 16.30 | 1.1346 | 1.1418 | 49035 | 4.05e2 | 1 | 1 | TRINITY\_DN140702\_c0\_g1\_i1.p1 |  |  | DB Search |
| TLQLIHQIEISK | 66.71 | 1421.8242 | 12 | 0.03 | 711.9162 | 17.16 | 1.0367 | 1.0439 | 53670 | 0 | 0 | 0 | TRINITY\_DN39385\_c0\_g1\_i2.p1:TRINITY\_DN39385\_c0\_g1\_i1.p1 |  |  | DB Search |
| VVTSSTGPGEHMR | 66.7 | 1356.6456 | 13 | -0.17 | 679.3269 | 10.84 | 0.9844 | 0.9916 | 18517 | 3.16e3 | 1 | 1 | TRINITY\_DN73067\_c0\_g1\_i1.p1 |  |  | DB Search |
| VITSDDPAEKEK | 66.7 | 1330.6616 | 12 | 2.85 | 666.337 | 10.71 | 0.9836 | 0.9908 | 17367 | 7.2e3 | 1 | 1 | TRINITY\_DN1339\_c6\_g1\_i2.p1:TRINITY\_DN1339\_c6\_g1\_i1.p1:TRINITY\_DN6701\_c0\_g1\_i1.p1:TRINITY\_DN6701\_c0\_g1\_i2.p2:TRINITY\_DN1339\_c0\_g1\_i2.p1:TRINITY\_DN124769\_c0\_g1\_i1.p1:TRINITY\_DN1172\_c0\_g1\_i5.p1:TRINITY\_DN1172\_c0\_g1\_i3.p1:TRINITY\_DN1339\_c0\_g1\_i1.p1 |  |  | DB Search |
| VLTSDDPAEKEK | 66.7 | 1330.6616 | 12 | 2.85 | 666.337 | 10.71 | 0.9836 | 0.9908 | 17367 | 7.2e3 | 1 | 1 | TRINITY\_DN119673\_c0\_g1\_i1.p1:TRINITY\_DN24414\_c1\_g1\_i2.p2 |  |  | DB Search |
| EIRDILVQYDR | 66.7 | 1418.7517 | 11 | -3.73 | 710.3773 | 19.70 | 1.0323 | 1.0395 | 68136 | 1.75e4 | 1 | 1 | TRINITY\_DN128406\_c0\_g1\_i1.p1 |  |  | DB Search |
| G(+42.01)C(+57.02)YIHTQEVAR | 66.7 | 1374.635 | 11 | -1.48 | 688.3207 | 13.98 | 0.9916 | 0.9989 | 36588 | 6.57e2 | 1 | 1 | TRINITY\_DN3202\_c2\_g1\_i1.p1 | Acetylation (Protein N-term), Carbamidomethylation | G1:Acetylation (Protein N-term):1000 | DB Search |
| LIEALC(+57.02)AEHEIK | 66.7 | 1424.7333 | 12 | 0.68 | 713.3712 | 15.09 | 1.0709 | 1.0781 | 42629 | 1.49e3 | 1 | 1 | TRINITY\_DN2772\_c0\_g1\_i3.p1:TRINITY\_DN2772\_c0\_g1\_i2.p1:TRINITY\_DN2772\_c0\_g1\_i1.p1 | Carbamidomethylation |  | DB Search |
| ADLIAYLEESTTKK | 66.7 | 1580.8297 | 14 | 1.07 | 791.4194 | 19.58 | 1.0985 | 1.1057 | 68341 | 3e2 | 1 | 1 | TRINITY\_DN1114\_c0\_g1\_i2.p1:TRINITY\_DN1114\_c0\_g1\_i1.p1 |  |  | DB Search |
| YGINHITNLVESKR | 66.7 | 1642.879 | 14 | -0.91 | 548.6307 | 14.97 | 0.8226 | 0.8299 | 42030 | 3.39e2 | 1 | 1 | TRINITY\_DN146208\_c0\_g1\_i1.p1 |  |  | DB Search |
| VLTSSDPAEKEK | 66.69 | 1302.6666 | 12 | 3.87 | 652.3402 | 10.57 | 0.9977 | 1.0049 | 16696 | 7.52e3 | 1 | 1 | TRINITY\_DN4497\_c0\_g2\_i1.p1:TRINITY\_DN1172\_c0\_g2\_i2.p1:TRINITY\_DN21847\_c0\_g1\_i1.p1:TRINITY\_DN4497\_c0\_g1\_i1.p1 |  |  | DB Search |
| VTRPHGASGVVR | 66.69 | 1234.6895 | 12 | 2.23 | 618.3506 | 10.05 | 0.9319 | 0.9392 | 13758 | 2.92e4 | 3 | 3 | TRINITY\_DN2885\_c0\_g1\_i2.p1:TRINITY\_DN2885\_c0\_g1\_i5.p1:TRINITY\_DN2885\_c0\_g1\_i4.p1 |  |  | DB Search |
| AKLSQC(+57.02)TAASGK | 66.69 | 1220.6183 | 12 | 2.46 | 611.3152 | 9.68 | 0.9533 | 0.9606 | 11765 | 7.57e3 | 1 | 1 | TRINITY\_DN3991\_c0\_g1\_i1.p1 | Carbamidomethylation |  | DB Search |
| AGVLAHLEEER | 66.69 | 1222.6306 | 11 | -5.26 | 612.3166 | 14.81 | 0.9541 | 0.9614 | 41119 | 0 | 0 | 0 | TRINITY\_DN428\_c0\_g1\_i1.p1 |  |  | DB Search |
| SYELPDGQVITIGNER | 66.69 | 1789.8846 | 16 | -3.48 | 895.9424 | 18.53 | 0.9795 | 0.9868 | 61913 | 1.88e3 | 2 | 2 | TRINITY\_DN161\_c0\_g1\_i1.p1:TRINITY\_DN161\_c0\_g1\_i4.p1:TRINITY\_DN161\_c0\_g1\_i3.p1:TRINITY\_DN161\_c0\_g1\_i2.p1 |  |  | DB Search |
| DNIQGITKPAIR | 66.68 | 1324.7462 | 12 | 3.7 | 663.3799 | 13.75 | 0.9791 | 0.9864 | 35280 | 0 | 0 | 0 | TRINITY\_DN172571\_c0\_g1\_i1.p1 |  |  | DB Search |
| AFFDLHFVGTGAK | 66.68 | 1408.7139 | 13 | -4.47 | 705.3579 | 19.23 | 1.0371 | 1.0443 | 66248 | 0 | 0 | 0 | TRINITY\_DN15016\_c0\_g1\_i3.p1:TRINITY\_DN15016\_c0\_g1\_i2.p1 |  |  | DB Search |
| IRNEEIQIEVIERR | 66.68 | 1795.9904 | 14 | 1.69 | 599.6691 | 15.10 | 0.8798 | 0.887 | 42640 | 8.61e2 | 1 | 1 | TRINITY\_DN11435\_c0\_g1\_i1.p1 |  |  | DB Search |
| APTHILNPANKR | 66.68 | 1330.7469 | 12 | 2.53 | 444.5887 | 11.11 | 0.7526 | 0.7599 | 20079 | 1.09e3 | 2 | 2 | TRINITY\_DN1058\_c0\_g1\_i4.p1:TRINITY\_DN1058\_c0\_g1\_i1.p1:TRINITY\_DN1058\_c0\_g1\_i3.p1:TRINITY\_DN1058\_c0\_g1\_i2.p2 |  |  | DB Search |
| AEFRPNQEEEPILKR | 66.68 | 1854.9587 | 15 | 1.2 | 619.3248 | 13.73 | 0.8745 | 0.8818 | 35104 | 1.03e3 | 1 | 1 | TRINITY\_DN262\_c0\_g1\_i1.p1 |  |  | DB Search |
| IAYAQGYATGDPSKR | 66.68 | 1596.7896 | 15 | 6.44 | 799.4036 | 12.71 | 1.1069 | 1.1142 | 29416 | 2.71e2 | 1 | 1 | TRINITY\_DN1428\_c0\_g1\_i1.p1 |  |  | DB Search |
| S(+42.01)ATGNLVTVLRK | 66.68 | 1299.751 | 12 | 3.4 | 650.8821 | 17.06 | 0.9977 | 1.0049 | 52945 | 3.07e3 | 1 | 1 | TRINITY\_DN2963\_c0\_g1\_i1.p1 | Acetylation (Protein N-term) | S1:Acetylation (Protein N-term):1000 | DB Search |
| LLEFVGHDVLR | 66.68 | 1296.719 | 11 | 5.9 | 649.3677 | 17.75 | 1.0033 | 1.0106 | 57204 | 0 | 0 | 0 | TRINITY\_DN464\_c0\_g1\_i4.p1 |  |  | DB Search |
| IC(+57.02)VIVGTVTDDNRLLEVPK | 66.68 | 2140.1562 | 19 | 0.02 | 714.3895 | 18.48 | 0.9473 | 0.9545 | 61525 | 1.93e3 | 1 | 1 | TRINITY\_DN162\_c0\_g1\_i1.p1 | Carbamidomethylation |  | DB Search |
| YC(+57.02)HLFLC(+57.02)VHC(+57.02)LK | 66.68 | 1648.7676 | 12 | -5.26 | 825.3831 | 16.15 | 1.1098 | 1.117 | 48282 | 9.73e2 | 2 | 2 | TRINITY\_DN1787\_c0\_g2\_i1.p1:TRINITY\_DN1787\_c0\_g2\_i2.p1 | Carbamidomethylation, Carbamidomethylation, Carbamidomethylation |  | DB Search |
| IPIFSAAGLPHNEIAAQIC(+57.02)R | 66.68 | 2177.1416 | 20 | 1.41 | 726.7189 | 18.83 | 1.0098 | 1.017 | 63727 | 5.71e2 | 1 | 1 | TRINITY\_DN3773\_c0\_g1\_i1.p1 | Carbamidomethylation |  | DB Search |
| FVLHGDVSHGSR | 66.68 | 1309.6527 | 12 | 4.95 | 655.8339 | 12.00 | 0.9892 | 0.9965 | 25296 | 0 | 0 | 0 | TRINITY\_DN22\_c0\_g1\_i1.p1:TRINITY\_DN384\_c0\_g1\_i2.p1:TRINITY\_DN384\_c0\_g1\_i1.p1 |  |  | DB Search |
| RYGQAM(+15.99)C(+57.02)QFM(+15.99)R | 66.67 | 1478.6217 | 11 | -8.74 | 740.3083 | 12.90 | 0.9759 | 0.9832 | 30310 | 4.97e3 | 1 | 1 | TRINITY\_DN286\_c0\_g1\_i4.p1:TRINITY\_DN286\_c0\_g1\_i2.p1 | Oxidation (M), Carbamidomethylation, Oxidation (M) | M6:Oxidation (M):1000 M10:Oxidation (M):1000 | DB Search |
| LAIDHAVSLITNIK | 66.67 | 1506.877 | 14 | 0.6 | 754.4428 | 19.10 | 1.0761 | 1.0833 | 65382 | 4.12e2 | 1 | 1 | TRINITY\_DN56792\_c0\_g1\_i1.p1:TRINITY\_DN56792\_c0\_g1\_i3.p1 |  |  | DB Search |
| GLDVDSLVIDHIQVNR | 66.67 | 1791.9479 | 16 | -2.06 | 896.9753 | 19.01 | 1.1794 | 1.1865 | 64835 | 3.83e3 | 1 | 1 | TRINITY\_DN1614\_c0\_g1\_i1.p1 |  |  | DB Search |
| LSYC(+57.02)VSC(+57.02)AIHSK | 66.67 | 1423.6588 | 12 | 2.43 | 712.8352 | 14.01 | 1.0315 | 1.0387 | 36741 | 1.87e3 | 1 | 1 | TRINITY\_DN7671\_c0\_g1\_i1.p1 | Carbamidomethylation, Carbamidomethylation |  | DB Search |
| IAVVVGAVTDDKR | 66.67 | 1341.7616 | 13 | 6.04 | 671.8891 | 14.15 | 1.0323 | 1.0395 | 37500 | 1.94e2 | 1 | 1 | TRINITY\_DN87064\_c0\_g1\_i1.p1 |  |  | DB Search |
| KAQYYEAYETIIKR | 66.67 | 1774.9254 | 14 | 2.41 | 592.6478 | 15.69 | 0.8627 | 0.87 | 45929 | 0 | 0 | 0 | TRINITY\_DN319\_c2\_g1\_i3.p1:TRINITY\_DN319\_c2\_g1\_i1.p1 |  |  | DB Search |
| K(+42.01)LSAELANGR | 66.66 | 1099.5985 | 10 | 2.66 | 550.8055 | 14.21 | 0.8712 | 0.8785 | 37894 | 8.99e2 | 1 | 1 | TRINITY\_DN21847\_c1\_g1\_i1.p1:TRINITY\_DN8165\_c0\_g2\_i2.p2:TRINITY\_DN8165\_c0\_g2\_i1.p2 | Acetylation (Protein N-term) | K1:Acetylation (Protein N-term):1000 | DB Search |
| DSHGVAQVR | 66.66 | 967.4835 | 9 | 8.7 | 484.7511 | 10.11 | 0.8193 | 0.8266 | 14162 | 8.11e2 | 1 | 1 | TRINITY\_DN80458\_c0\_g1\_i1.p1 |  |  | DB Search |
| RLFEGNALLR | 66.66 | 1187.6775 | 10 | 1.42 | 594.8442 | 16.07 | 0.9384 | 0.9457 | 47820 | 1.65e3 | 1 | 1 | TRINITY\_DN189323\_c0\_g1\_i1.p1:TRINITY\_DN776\_c0\_g1\_i1.p1 |  |  | DB Search |
| ETC(+57.02)SDIEKR | 66.66 | 1136.5132 | 9 | 3.94 | 569.2635 | 10.07 | 0.8931 | 0.9004 | 13965 | 4.9e3 | 1 | 1 | TRINITY\_DN118\_c1\_g1\_i3.p1:TRINITY\_DN118\_c1\_g1\_i5.p1 | Carbamidomethylation |  | DB Search |
| AITDNIKDILSVK | 66.66 | 1428.8187 | 13 | -1.1 | 715.4127 | 19.15 | 1.0512 | 1.0584 | 65748 | 5.17e2 | 1 | 1 | TRINITY\_DN1739\_c0\_g1\_i1.p1 |  |  | DB Search |
| NM(+15.99)M(+15.99)AAC(+57.02)DPR | 66.66 | 1096.41 | 9 | 0.96 | 549.2103 | 9.51 | 0.8591 | 0.8664 | 10859 | 2.33e3 | 1 | 1 | TRINITY\_DN1910\_c0\_g1\_i1.p1:TRINITY\_DN1910\_c0\_g1\_i5.p1:TRINITY\_DN3986\_c0\_g1\_i1.p1 | Oxidation (M), Oxidation (M), Carbamidomethylation | M2:Oxidation (M):1000 M3:Oxidation (M):1000 | DB Search |
| EVNTELDSRPETK | 66.66 | 1516.7369 | 13 | -0.4 | 759.372 | 11.74 | 1.0222 | 1.0295 | 23643 | 2.54e3 | 2 | 2 | TRINITY\_DN11643\_c0\_g1\_i1.p1 |  |  | DB Search |
| AGHGAVTSPQTQR | 66.66 | 1308.6534 | 13 | 4.48 | 655.334 | 9.70 | 0.9109 | 0.9182 | 11470 | 2.1e4 | 2 | 2 | TRINITY\_DN3036\_c0\_g1\_i13.p1:TRINITY\_DN3036\_c0\_g1\_i19.p1:TRINITY\_DN3036\_c0\_g1\_i11.p1:TRINITY\_DN3036\_c0\_g1\_i3.p1:TRINITY\_DN3036\_c0\_g1\_i16.p1:TRINITY\_DN3036\_c0\_g1\_i2.p2:TRINITY\_DN3036\_c0\_g1\_i8.p1 |  |  | DB Search |
| IVGIVSGALNHAASK | 66.65 | 1435.8147 | 15 | 2.8 | 718.9134 | 15.20 | 1.0556 | 1.0628 | 43196 | 2.91e2 | 1 | 1 | TRINITY\_DN884\_c0\_g1\_i1.p1 |  |  | DB Search |
| AM(+15.99)AAEAESSR | 66.65 | 1037.4447 | 10 | -0.52 | 519.7271 | 9.04 | 0.8149 | 0.8222 | 9454 | 2.47e2 | 1 | 1 | TRINITY\_DN909\_c0\_g1\_i1.p1 | Oxidation (M) | M2:Oxidation (M):1000 | DB Search |
| IGKPHTVPC(+57.02)K | 66.65 | 1135.6172 | 10 | 2.23 | 568.8146 | 9.75 | 0.8947 | 0.902 | 12052 | 1.76e4 | 2 | 2 | TRINITY\_DN15584\_c0\_g1\_i1.p1 | Carbamidomethylation |  | DB Search |
| ELVM(+15.99)HEQSR | 66.65 | 1143.5343 | 9 | 4.42 | 572.7744 | 9.76 | 0.8895 | 0.8968 | 12266 | 8.53e2 | 1 | 1 | TRINITY\_DN160\_c0\_g1\_i2.p1:TRINITY\_DN160\_c0\_g1\_i1.p1 | Oxidation (M) | M4:Oxidation (M):1000 | DB Search |
| IEHAHPINR | 66.65 | 1085.573 | 9 | 6.4 | 362.8656 | 9.48 | 0.7363 | 0.7437 | 10908 | 0 | 0 | 0 | TRINITY\_DN59667\_c0\_g1\_i1.p1 |  |  | DB Search |
| ATGC(+57.02)EVIWNQHNR | 66.64 | 1583.7263 | 13 | 0.95 | 792.8676 | 13.84 | 1.0785 | 1.0857 | 35783 | 1.6e3 | 1 | 1 | TRINITY\_DN2217\_c0\_g1\_i3.p1:TRINITY\_DN2217\_c0\_g1\_i2.p1 | Carbamidomethylation |  | DB Search |
| LIAFINDLHSGK | 66.64 | 1326.7295 | 12 | 1.4 | 664.37 | 17.48 | 1.0238 | 1.0311 | 55497 | 7.23e2 | 1 | 1 | TRINITY\_DN3452\_c0\_g1\_i1.p1 |  |  | DB Search |
| LLTSSDQGELTKK | 66.63 | 1418.7616 | 13 | 2.72 | 710.3868 | 12.36 | 1.0383 | 1.0456 | 27367 | 0 | 0 | 0 | TRINITY\_DN1339\_c0\_g2\_i5.p1 |  |  | DB Search |
| AVVVC(+57.02)DDRGR | 66.63 | 1145.5612 | 10 | 1.85 | 573.7863 | 10.61 | 0.883 | 0.8903 | 17052 | 2.15e3 | 1 | 1 | TRINITY\_DN1989\_c0\_g1\_i1.p1 | Carbamidomethylation |  | DB Search |
| S(+42.01)SGEIESLRQEAENLR | 66.62 | 1858.9021 | 16 | 0.69 | 930.4548 | 18.69 | 1.1973 | 1.2045 | 62988 | 1.64e3 | 1 | 1 | TRINITY\_DN2472\_c0\_g1\_i1.p1 | Acetylation (Protein N-term) | S1:Acetylation (Protein N-term):1000 | DB Search |
| GVHIISVGLGTVDHR | 66.61 | 1558.8579 | 15 | -17.11 | 520.6154 | 17.30 | 0.8116 | 0.8189 | 54443 | 0 | 0 | 0 | TRINITY\_DN8008\_c0\_g1\_i2.p1:TRINITY\_DN8008\_c0\_g1\_i3.p1 |  |  | DB Search |
| VVASSHTDYELR | 66.61 | 1375.6732 | 12 | 3.98 | 688.8435 | 12.52 | 1.0086 | 1.0158 | 28165 | 3.7e3 | 1 | 1 | TRINITY\_DN95248\_c0\_g1\_i1.p1 |  |  | DB Search |
| LLLGDFNGDKR | 66.61 | 1246.667 | 11 | 0.98 | 624.3386 | 16.00 | 0.9848 | 0.992 | 47446 | 9.37e2 | 1 | 1 | TRINITY\_DN413\_c0\_g1\_i1.p1:TRINITY\_DN413\_c0\_g1\_i4.p1:TRINITY\_DN413\_c0\_g1\_i2.p1:TRINITY\_DN413\_c0\_g1\_i3.p1 |  |  | DB Search |
| RLPEEDQYLR | 66.61 | 1317.6677 | 10 | -4.11 | 659.8354 | 14.09 | 0.9735 | 0.9808 | 36893 | 1.37e3 | 1 | 1 | TRINITY\_DN436\_c0\_g1\_i1.p1 |  |  | DB Search |
| FNSNSVLVVIDAKPK | 66.6 | 1629.9089 | 15 | 6.93 | 815.9637 | 17.38 | 1.1674 | 1.1746 | 54871 | 2.33e2 | 1 | 1 | TRINITY\_DN33483\_c0\_g1\_i1.p1 |  |  | DB Search |
| DYGVLLEDAGHTLR | 66.59 | 1557.7787 | 14 | -2.26 | 779.8914 | 18.22 | 1.1286 | 1.1358 | 59935 | 3.98e2 | 1 | 1 | TRINITY\_DN332\_c0\_g1\_i1.p1 |  |  | DB Search |
| GLFIIDDKGVLR | 66.59 | 1344.7765 | 12 | 0.84 | 673.3931 | 18.67 | 1.0017 | 1.009 | 62753 | 5.91e2 | 1 | 1 | TRINITY\_DN332\_c0\_g1\_i1.p1 |  |  | DB Search |
| TILQFEADRR | 66.59 | 1247.6622 | 10 | 3.81 | 624.838 | 15.07 | 0.9505 | 0.9578 | 42491 | 5.6e2 | 1 | 1 | TRINITY\_DN58867\_c0\_g1\_i2.p1 |  |  | DB Search |
| GTSNGGLVVGTIIKPK | 66.59 | 1539.8984 | 16 | 0.22 | 770.9532 | 15.68 | 1.1262 | 1.1334 | 45813 | 3.23e3 | 1 | 1 | TRINITY\_DN3036\_c0\_g1\_i3.p1:TRINITY\_DN3036\_c0\_g1\_i2.p2:TRINITY\_DN3036\_c0\_g1\_i8.p1 |  |  | DB Search |
| LSQC(+57.02)TAASGK | 66.58 | 1021.4862 | 10 | -1.41 | 511.7474 | 9.65 | 0.8356 | 0.8429 | 11604 | 2.54e4 | 1 | 1 | TRINITY\_DN3991\_c0\_g1\_i1.p1 | Carbamidomethylation |  | DB Search |
| FATHASALEIHGLDR | 66.57 | 1636.8322 | 15 | 3.46 | 819.4225 | 14.84 | 1.1378 | 1.145 | 41195 | 5.09e2 | 1 | 1 | TRINITY\_DN1989\_c0\_g1\_i1.p1 |  |  | DB Search |
| A(+42.01)ATLKPYLNAVR | 66.57 | 1357.7717 | 12 | -0.04 | 679.8901 | 18.20 | 1.0452 | 1.0524 | 59968 | 1.22e3 | 1 | 1 | TRINITY\_DN95387\_c0\_g1\_i1.p1 | Acetylation (Protein N-term) | A1:Acetylation (Protein N-term):1000 | DB Search |
| GC(+57.02)YIHTQEIAR | 66.57 | 1346.6401 | 11 | -0.12 | 674.3242 | 12.88 | 1.0158 | 1.023 | 30410 | 6.22e2 | 1 | 1 | TRINITY\_DN2217\_c0\_g1\_i3.p1:TRINITY\_DN38397\_c0\_g1\_i1.p1:TRINITY\_DN2217\_c0\_g1\_i2.p1 | Carbamidomethylation |  | DB Search |
| AFSFSDKPAITFEIHDFK | 66.57 | 2099.0364 | 18 | -0.42 | 700.6826 | 18.87 | 0.98 | 0.9872 | 64039 | 1.14e2 | 1 | 1 | TRINITY\_DN33065\_c0\_g2\_i1.p1 |  |  | DB Search |
| YRPGTVALR | 66.57 | 1031.5876 | 9 | 0.86 | 516.7992 | 12.50 | 0.8554 | 0.8627 | 28119 | 7.47e2 | 1 | 1 | TRINITY\_DN18187\_c0\_g1\_i4.p3:TRINITY\_DN72753\_c0\_g1\_i1.p1:TRINITY\_DN18187\_c0\_g1\_i6.p2:TRINITY\_DN5023\_c0\_g1\_i3.p2:TRINITY\_DN5023\_c0\_g1\_i2.p2:TRINITY\_DN18187\_c0\_g1\_i3.p4:TRINITY\_DN18187\_c0\_g1\_i5.p1 |  |  | DB Search |
| VSQTC(+57.02)AEHVFR | 66.57 | 1332.6245 | 11 | 0.3 | 667.3167 | 11.88 | 0.9832 | 0.9904 | 24552 | 0 | 0 | 0 | TRINITY\_DN3078\_c0\_g1\_i1.p1 | Carbamidomethylation |  | DB Search |
| LDFDHEAKLNMIFR | 66.57 | 1747.8716 | 14 | 2.05 | 583.6297 | 18.33 | 0.8558 | 0.8631 | 60685 | 0 | 0 | 0 | TRINITY\_DN160\_c0\_g1\_i2.p1:TRINITY\_DN160\_c0\_g1\_i1.p1 |  |  | DB Search |
| GLTHC(+57.02)NTLSYK | 66.56 | 1292.6183 | 11 | 2.85 | 647.3154 | 12.41 | 0.9699 | 0.9771 | 27654 | 0 | 0 | 0 | TRINITY\_DN902\_c1\_g2\_i4.p1:TRINITY\_DN902\_c1\_g2\_i1.p1:TRINITY\_DN902\_c1\_g2\_i3.p1 | Carbamidomethylation |  | DB Search |
| IKLEHLITELVHQR | 66.56 | 1728.0046 | 14 | -0.31 | 577.006 | 18.36 | 0.8676 | 0.8749 | 60768 | 2.25e2 | 1 | 1 | TRINITY\_DN1566\_c0\_g1\_i1.p1 |  |  | DB Search |
| ALQDEDEDAFKR | 66.56 | 1435.658 | 12 | 0.52 | 718.8334 | 13.14 | 0.9937 | 1.0009 | 31899 | 0 | 0 | 0 | TRINITY\_DN116\_c0\_g1\_i1.p1 |  |  | DB Search |
| VVTSSTGPGEHM(+15.99)R | 66.55 | 1372.6405 | 13 | 4.36 | 458.554 | 9.70 | 0.7212 | 0.7286 | 11985 | 1.74e3 | 2 | 2 | TRINITY\_DN73067\_c0\_g1\_i1.p1 | Oxidation (M) | M12:Oxidation (M):1000 | DB Search |
| AITASEPEALKR | 66.55 | 1284.7037 | 12 | -4.07 | 643.3536 | 12.85 | 0.9727 | 0.98 | 30111 | 2.02e3 | 1 | 1 | TRINITY\_DN6701\_c0\_g2\_i1.p1 |  |  | DB Search |
| ASFVTDIDRLR | 66.55 | 1291.6885 | 11 | -3.8 | 646.8461 | 17.02 | 0.988 | 0.9953 | 52753 | 1.2e3 | 1 | 1 | TRINITY\_DN598\_c1\_g1\_i2.p1 |  |  | DB Search |
| AEAEAEVIQKK | 66.54 | 1214.6506 | 11 | 5.58 | 608.3333 | 11.13 | 0.9545 | 0.9618 | 20225 | 0 | 0 | 0 | TRINITY\_DN1280\_c0\_g1\_i1.p1 |  |  | DB Search |
| AFVAIGDYGGHVGLGVK | 66.54 | 1658.8779 | 17 | 1.87 | 830.4441 | 17.61 | 1.1638 | 1.171 | 55921 | 1.18e4 | 1 | 1 | TRINITY\_DN15584\_c0\_g1\_i1.p1 |  |  | DB Search |
| ASFVSDIDRLR | 66.54 | 1277.6727 | 11 | 1.77 | 639.8419 | 16.87 | 0.9828 | 0.99 | 51974 | 1.96e3 | 1 | 1 | TRINITY\_DN598\_c1\_g1\_i1.p1 |  |  | DB Search |
| TIESEAAHGTVTR | 66.53 | 1370.679 | 13 | 2.05 | 686.3451 | 11.89 | 1.0194 | 1.0267 | 24550 | 7.11e2 | 1 | 1 | TRINITY\_DN3271\_c0\_g1\_i1.p1:TRINITY\_DN3271\_c0\_g1\_i2.p1 |  |  | DB Search |
| TFAHTVHNLVLAGNK | 66.52 | 1620.8735 | 15 | -4.74 | 811.4366 | 14.93 | 1.1166 | 1.1238 | 41769 | 0 | 0 | 0 | TRINITY\_DN578\_c0\_g1\_i1.p1 |  |  | DB Search |
| VLLSQQIFYDKK | 66.52 | 1480.829 | 12 | -1.68 | 741.4172 | 16.70 | 1.06 | 1.0672 | 51062 | 6.76e2 | 1 | 1 | TRINITY\_DN982\_c0\_g1\_i3.p1:TRINITY\_DN982\_c0\_g1\_i1.p1:TRINITY\_DN982\_c0\_g1\_i2.p1 |  |  | DB Search |
| AIGGIILTASHNPGGK | 66.51 | 1504.8362 | 16 | 0.62 | 753.4224 | 15.47 | 1.0961 | 1.1033 | 44709 | 6.94e2 | 1 | 1 | TRINITY\_DN3589\_c0\_g1\_i1.p1 |  |  | DB Search |
| A(+42.01)NPLKDIISR | 66.51 | 1167.6611 | 10 | 0.91 | 584.8358 | 18.57 | 0.919 | 0.9263 | 61990 | 1.77e3 | 1 | 1 | TRINITY\_DN163801\_c0\_g1\_i1.p1 | Acetylation (Protein N-term) | A1:Acetylation (Protein N-term):1000 | DB Search |
| ATETATGLATTHPIK | 66.51 | 1510.7991 | 15 | 0.02 | 756.4034 | 12.89 | 1.1053 | 1.1126 | 30404 | 8.59e2 | 1 | 1 | TRINITY\_DN165536\_c0\_g1\_i1.p1 |  |  | DB Search |
| IGSIMLGIHLLR | 66.5 | 1321.7904 | 12 | -0.58 | 661.8991 | 19.69 | 1.0375 | 1.0448 | 69001 | 4.62e2 | 1 | 1 | TRINITY\_DN148467\_c0\_g1\_i1.p1 |  |  | DB Search |
| VVEKEAETER | 66.5 | 1188.5986 | 10 | 7.91 | 397.2082 | 9.54 | 0.729 | 0.7363 | 11130 | 2.07e3 | 2 | 2 | TRINITY\_DN2636\_c0\_g1\_i2.p1:TRINITY\_DN2636\_c0\_g1\_i1.p1 |  |  | DB Search |
| LGGIEVTQGNC(+57.02)AR | 66.48 | 1373.6721 | 13 | 5.18 | 687.8438 | 13.73 | 0.963 | 0.9703 | 35192 | 0 | 0 | 0 | TRINITY\_DN36\_c0\_g1\_i9.p1:TRINITY\_DN36\_c0\_g1\_i14.p1:TRINITY\_DN36\_c0\_g1\_i13.p1:TRINITY\_DN36\_c0\_g1\_i2.p1:TRINITY\_DN36\_c0\_g1\_i3.p1:TRINITY\_DN36\_c0\_g1\_i16.p1:TRINITY\_DN36\_c0\_g1\_i17.p1:TRINITY\_DN36\_c0\_g1\_i4.p1:TRINITY\_DN36\_c0\_g1\_i11.p1:TRINITY\_DN36\_c0\_g1\_i1.p1:TRINITY\_DN36\_c0\_g1\_i8.p1:TRINITY\_DN36\_c0\_g1\_i19.p1:TRINITY\_DN36\_c0\_g1\_i20.p1:TRINITY\_DN36\_c0\_g1\_i5.p1 | Carbamidomethylation |  | DB Search |
| VTLVSERPFSK | 66.47 | 1261.703 | 11 | -6.46 | 631.8519 | 15.24 | 0.9614 | 0.9687 | 43493 | 0 | 0 | 0 | TRINITY\_DN95248\_c0\_g1\_i1.p1 |  |  | DB Search |
| K(+42.01)LSAEVANGR | 66.46 | 1085.5829 | 10 | 2.21 | 543.7975 | 12.83 | 0.8595 | 0.8668 | 30189 | 3.02e3 | 1 | 1 | TRINITY\_DN6397\_c0\_g2\_i4.p1:TRINITY\_DN6397\_c0\_g2\_i3.p1 | Acetylation (Protein N-term) | K1:Acetylation (Protein N-term):1000 | DB Search |
| GLFIIDDKGILR | 66.46 | 1358.7921 | 12 | 2.12 | 680.4017 | 19.27 | 1.0122 | 1.0194 | 66460 | 6.42e3 | 1 | 1 | TRINITY\_DN535\_c0\_g1\_i2.p1:TRINITY\_DN535\_c0\_g1\_i1.p1 |  |  | DB Search |
| HGYIGEFEVIDDHR | 66.45 | 1685.7798 | 14 | 0.12 | 562.9314 | 16.03 | 0.8716 | 0.8789 | 47611 | 9.53e2 | 2 | 2 | TRINITY\_DN3339\_c0\_g1\_i1.p1 |  |  | DB Search |
| YVVMLQYYHDSDTAK | 66.45 | 1831.8451 | 15 | 2.49 | 916.928 | 16.54 | 1.1798 | 1.1869 | 50323 | 0 | 0 | 0 | TRINITY\_DN1616\_c0\_g1\_i3.p1:TRINITY\_DN1616\_c0\_g1\_i6.p1:TRINITY\_DN1616\_c0\_g1\_i2.p1:TRINITY\_DN1616\_c0\_g1\_i7.p1 |  |  | DB Search |
| RLPDQITTR | 66.44 | 1098.6145 | 9 | 1.99 | 550.3132 | 12.54 | 0.8741 | 0.8814 | 28273 | 1.51e3 | 1 | 1 | TRINITY\_DN1377\_c0\_g1\_i1.p1 |  |  | DB Search |
| NYFHYENQLELK | 66.44 | 1596.7572 | 12 | 8.12 | 799.3888 | 16.35 | 1.0861 | 1.0933 | 49354 | 0 | 0 | 0 | TRINITY\_DN1320\_c0\_g2\_i1.p1 |  |  | DB Search |
| LAADLVITHSSK | 66.4 | 1253.6979 | 12 | 1.42 | 627.8543 | 14.21 | 0.9965 | 1.0037 | 37795 | 1.72e3 | 1 | 1 | TRINITY\_DN751\_c0\_g1\_i1.p1:TRINITY\_DN7214\_c0\_g1\_i1.p1 |  |  | DB Search |
| LVVIADIVDQKR | 66.4 | 1367.8136 | 12 | 1.88 | 684.9123 | 17.50 | 1.0415 | 1.0488 | 55496 | 4.87e3 | 1 | 1 | TRINITY\_DN6294\_c0\_g3\_i1.p1:TRINITY\_DN6294\_c0\_g3\_i2.p1 |  |  | DB Search |
| FTNKDIVC(+57.02)QIAYAK | 66.39 | 1669.8497 | 14 | -7.67 | 835.922 | 15.97 | 1.1214 | 1.1286 | 47367 | 0 | 0 | 0 | TRINITY\_DN116\_c0\_g1\_i1.p1 | Carbamidomethylation |  | DB Search |
| IHINIVVIGHVDSGK | 66.38 | 1599.9097 | 15 | 1.53 | 800.9597 | 16.52 | 1.0993 | 1.1065 | 50056 | 1.41e3 | 2 | 2 | TRINITY\_DN64\_c0\_g1\_i1.p1 |  |  | DB Search |
| ALLSLEYHEKR | 66.36 | 1357.7354 | 11 | 4.28 | 679.8748 | 14.42 | 1.0279 | 1.0351 | 38931 | 3.27e3 | 1 | 1 | TRINITY\_DN8707\_c0\_g1\_i2.p1 |  |  | DB Search |
| TRIEDLETVISQTQDHR | 66.35 | 2040.0236 | 17 | 3.15 | 681.0142 | 17.32 | 0.9622 | 0.9695 | 54563 | 0 | 0 | 0 | TRINITY\_DN761\_c0\_g1\_i3.p1:TRINITY\_DN761\_c0\_g1\_i1.p1:TRINITY\_DN761\_c0\_g1\_i2.p1 |  |  | DB Search |
| DKNILVLTLGVGPQVNR | 66.34 | 1835.0629 | 17 | -1.89 | 918.5328 | 19.38 | 1.1326 | 1.1398 | 66901 | 2.29e3 | 2 | 2 | TRINITY\_DN477\_c0\_g1\_i8.p1:TRINITY\_DN477\_c0\_g1\_i7.p1 |  |  | DB Search |
| KSAQFLLQLLK | 66.34 | 1287.7914 | 11 | 0.84 | 644.9006 | 19.70 | 0.9783 | 0.9856 | 69083 | 8.25e2 | 1 | 1 | TRINITY\_DN1614\_c0\_g1\_i1.p1 |  |  | DB Search |
| VIGASGIHVGTMSFGK | 66.34 | 1559.813 | 16 | -0.2 | 780.9101 | 16.89 | 1.1037 | 1.111 | 52086 | 2.86e2 | 1 | 1 | TRINITY\_DN3036\_c0\_g1\_i6.p1:TRINITY\_DN3036\_c0\_g1\_i13.p1:TRINITY\_DN3036\_c0\_g1\_i19.p1:TRINITY\_DN3036\_c0\_g1\_i11.p1:TRINITY\_DN3036\_c0\_g1\_i3.p1:TRINITY\_DN3036\_c0\_g1\_i4.p1:TRINITY\_DN3036\_c0\_g1\_i16.p1:TRINITY\_DN3036\_c0\_g1\_i2.p2:TRINITY\_DN3036\_c0\_g1\_i8.p1 |  |  | DB Search |
| TQITC(+57.02)TQISLKR | 66.31 | 1447.7817 | 12 | 1.05 | 724.8956 | 13.79 | 1.0255 | 1.0327 | 35470 | 1.17e3 | 1 | 1 | TRINITY\_DN166344\_c0\_g1\_i1.p1 | Carbamidomethylation |  | DB Search |
| M(+15.99)KLDYVLGLKVDDFLER | 66.31 | 2069.0867 | 17 | 5.54 | 690.7036 | 19.97 | 0.9731 | 0.9804 | 70746 | 0 | 0 | 0 | TRINITY\_DN776\_c0\_g1\_i1.p1 | Oxidation (M) | M1:Oxidation (M):1000 | DB Search |
| LLTSSDQAELTKK | 66.26 | 1432.7773 | 13 | 0.59 | 717.3931 | 12.73 | 1.0504 | 1.0576 | 29502 | 1.34e3 | 1 | 1 | TRINITY\_DN1339\_c0\_g2\_i1.p2:TRINITY\_DN1339\_c0\_g2\_i2.p1:TRINITY\_DN1339\_c0\_g2\_i3.p1:TRINITY\_DN1339\_c0\_g2\_i4.p2 |  |  | DB Search |
| KVIVDQLVTLATR | 66.24 | 1454.8821 | 13 | -0.52 | 728.4446 | 18.26 | 1.0271 | 1.0343 | 60277 | 0 | 0 | 0 | TRINITY\_DN1386\_c0\_g1\_i1.p1 |  |  | DB Search |
| GLAPSLPEDLHC(+57.02)LIK | 66.23 | 1661.881 | 15 | 1.43 | 831.9453 | 19.09 | 1.1406 | 1.1478 | 65296 | 1.58e3 | 1 | 1 | TRINITY\_DN80458\_c0\_g1\_i1.p1 | Carbamidomethylation |  | DB Search |
| LFEGNALLRR | 66.2 | 1187.6775 | 10 | 0.49 | 594.8436 | 15.56 | 0.9675 | 0.9747 | 45173 | 2.25e3 | 1 | 1 | TRINITY\_DN189323\_c0\_g1\_i1.p1:TRINITY\_DN776\_c0\_g1\_i1.p1 |  |  | DB Search |
| IYAEVLHFHAEEK | 66.19 | 1584.7936 | 13 | 2.08 | 793.4022 | 15.23 | 1.1366 | 1.1438 | 43384 | 3.89e2 | 1 | 1 | TRINITY\_DN4911\_c0\_g1\_i1.p1 |  |  | DB Search |
| AKLVIISSNTPQLR | 66.17 | 1538.9144 | 14 | 3.07 | 770.4634 | 15.57 | 1.0809 | 1.0881 | 45325 | 0 | 0 | 0 | TRINITY\_DN2126\_c0\_g1\_i1.p1 |  |  | DB Search |
| LIEVEEKEIQR | 66.16 | 1384.7561 | 11 | -8.44 | 693.3764 | 13.80 | 1.0146 | 1.0218 | 35646 | 0 | 0 | 0 | TRINITY\_DN11435\_c0\_g1\_i1.p1 |  |  | DB Search |
| SFLLALEKDQEK | 66 | 1419.7609 | 12 | 0.75 | 710.8851 | 17.37 | 1.0733 | 1.0805 | 54745 | 7.32e3 | 1 | 1 | TRINITY\_DN1386\_c0\_g1\_i1.p1 |  |  | DB Search |
| AITSSDPDALKR | 65.96 | 1272.6674 | 12 | 2.56 | 637.3397 | 11.98 | 0.9558 | 0.963 | 25174 | 2.08e3 | 1 | 1 | TRINITY\_DN7808\_c1\_g2\_i1.p1 |  |  | DB Search |
| LLEIEDEIKLR | 65.81 | 1369.7816 | 11 | 1.07 | 685.8958 | 18.00 | 1.0468 | 1.054 | 58561 | 1.84e3 | 1 | 1 | TRINITY\_DN2230\_c0\_g1\_i1.p1 |  |  | DB Search |
| TQAVLDRLNIEKR | 65.76 | 1554.8842 | 13 | -5.27 | 519.2969 | 14.53 | 0.8023 | 0.8096 | 39606 | 8.83e2 | 1 | 1 | TRINITY\_DN477\_c0\_g1\_i9.p1 |  |  | DB Search |
| GTHAAEIAHC(+57.02)VSR | 65.75 | 1407.6677 | 13 | 1.54 | 704.8391 | 11.03 | 1.0283 | 1.0355 | 19632 | 6.69e2 | 1 | 1 | TRINITY\_DN1319\_c0\_g1\_i2.p1:TRINITY\_DN1319\_c0\_g1\_i1.p1 | Carbamidomethylation |  | DB Search |
| IPGVISVHEFHIWQLAGNR | 65.56 | 2172.1592 | 19 | -17.22 | 725.0446 | 19.24 | 0.98 | 0.9872 | 66361 | 0 | 0 | 0 | TRINITY\_DN36276\_c0\_g1\_i1.p1 |  |  | DB Search |
| KKEESPNAQDAIQK | 65.45 | 1584.8107 | 14 | -1.03 | 529.2746 | 10.32 | 0.7909 | 0.7982 | 15344 | 4.71e2 | 1 | 1 | TRINITY\_DN2904\_c0\_g1\_i1.p1 |  |  | DB Search |
| VSILVAC(+57.02)NKQDLTTAK | 65.21 | 1759.9502 | 16 | -1.35 | 880.9772 | 14.68 | 1.1829 | 1.1901 | 40439 | 0 | 0 | 0 | TRINITY\_DN453\_c0\_g1\_i1.p1:TRINITY\_DN453\_c0\_g1\_i2.p1 | Carbamidomethylation |  | DB Search |
| KEDVSEFFDIKEEVGR | 65.16 | 1925.937 | 16 | 2.76 | 642.9852 | 17.77 | 0.9105 | 0.9178 | 57244 | 2.14e2 | 1 | 1 | TRINITY\_DN10026\_c0\_g1\_i1.p1 |  |  | DB Search |
| LPIYTDKVINLYK | 65.11 | 1578.9021 | 13 | -1.17 | 790.4539 | 17.72 | 1.0989 | 1.1061 | 56954 | 4.39e2 | 1 | 1 | TRINITY\_DN428\_c0\_g1\_i1.p1 |  |  | DB Search |
| IQDKEGIPPDQQR | 65.08 | 1522.7739 | 13 | -0.66 | 762.3903 | 11.01 | 1.0339 | 1.0411 | 19352 | 9.49e2 | 1 | 1 | TRINITY\_DN554\_c0\_g1\_i3.p2:TRINITY\_DN585\_c0\_g1\_i3.p1:TRINITY\_DN173718\_c0\_g1\_i1.p1:TRINITY\_DN1465\_c1\_g1\_i1.p1:TRINITY\_DN40728\_c1\_g1\_i1.p2:TRINITY\_DN152\_c0\_g1\_i2.p1:TRINITY\_DN585\_c0\_g1\_i5.p1:TRINITY\_DN152\_c0\_g1\_i14.p2:TRINITY\_DN40728\_c0\_g1\_i1.p1:TRINITY\_DN152\_c0\_g1\_i9.p1:TRINITY\_DN585\_c0\_g1\_i4.p1:TRINITY\_DN152\_c0\_g1\_i15.p1:TRINITY\_DN195631\_c0\_g1\_i1.p2:TRINITY\_DN585\_c0\_g1\_i10.p1:TRINITY\_DN554\_c0\_g1\_i5.p1:TRINITY\_DN585\_c0\_g1\_i2.p1:TRINITY\_DN8227\_c0\_g1\_i1.p1:TRINITY\_DN152\_c0\_g1\_i10.p1 |  |  | DB Search |
| K(+42.01)LAAELANGR | 64.95 | 1083.6036 | 10 | -0.25 | 542.8065 | 15.17 | 0.8648 | 0.8721 | 42282 | 4.9e3 | 1 | 1 | TRINITY\_DN1172\_c0\_g2\_i2.p1 | Acetylation (Protein N-term) | K1:Acetylation (Protein N-term):1000 | DB Search |
| SVTTHQGNAIVIK | 64.94 | 1366.7568 | 13 | 0.36 | 684.3829 | 12.49 | 0.9945 | 1.0017 | 28112 | 2.8e2 | 1 | 1 | TRINITY\_DN1377\_c0\_g1\_i1.p1 |  |  | DB Search |
| VIGASGIHVGTM(+15.99)SFGK | 64.9 | 1575.8079 | 16 | 0.52 | 788.9081 | 15.11 | 1.1262 | 1.1334 | 42748 | 6.68e2 | 1 | 1 | TRINITY\_DN3036\_c0\_g1\_i6.p1:TRINITY\_DN3036\_c0\_g1\_i13.p1:TRINITY\_DN3036\_c0\_g1\_i19.p1:TRINITY\_DN3036\_c0\_g1\_i11.p1:TRINITY\_DN3036\_c0\_g1\_i3.p1:TRINITY\_DN3036\_c0\_g1\_i4.p1:TRINITY\_DN3036\_c0\_g1\_i16.p1:TRINITY\_DN3036\_c0\_g1\_i2.p2:TRINITY\_DN3036\_c0\_g1\_i8.p1 | Oxidation (M) | M12:Oxidation (M):1000 | DB Search |
| A(+42.01)EREQLIQQAK | 64.88 | 1354.7205 | 11 | 2.32 | 678.366 | 13.60 | 0.9775 | 0.9848 | 34443 | 1.59e3 | 1 | 1 | TRINITY\_DN1121\_c0\_g1\_i1.p1 | Acetylation (Protein N-term) | A1:Acetylation (Protein N-term):1000 | DB Search |
| VGINYQPPTVVPGGDLAK | 64.86 | 1823.9781 | 18 | -3.39 | 912.9891 | 17.23 | 1.1198 | 1.127 | 54086 | 0 | 0 | 0 | TRINITY\_DN687\_c0\_g2\_i1.p1:TRINITY\_DN250\_c0\_g1\_i5.p1:TRINITY\_DN687\_c0\_g1\_i1.p1:TRINITY\_DN250\_c0\_g1\_i1.p1:TRINITY\_DN250\_c0\_g1\_i6.p1:TRINITY\_DN250\_c0\_g1\_i4.p1:TRINITY\_DN96161\_c0\_g1\_i1.p1:TRINITY\_DN250\_c0\_g1\_i3.p1 |  |  | DB Search |
| VDFDTTFAPVTGKK | 64.84 | 1524.7823 | 14 | 4.3 | 763.3983 | 18.74 | 1.048 | 1.0552 | 63303 | 9.89e2 | 2 | 2 | TRINITY\_DN329\_c0\_g1\_i1.p1:TRINITY\_DN329\_c0\_g1\_i2.p1 |  |  | DB Search |
| AGVLAHLEEERDIK | 64.78 | 1578.8365 | 14 | 1.02 | 790.4228 | 14.83 | 1.1053 | 1.1126 | 41168 | 6.95e2 | 1 | 1 | TRINITY\_DN428\_c0\_g1\_i1.p1 |  |  | DB Search |
| YYVTIIDAPGHRDFIK | 64.76 | 1906.9941 | 16 | -3.62 | 954.4966 | 16.71 | 1.2077 | 1.2149 | 51222 | 0 | 0 | 0 | TRINITY\_DN64\_c0\_g1\_i1.p1 |  |  | DB Search |
| TAIAIDTIINQKR | 64.76 | 1455.8409 | 13 | -0.36 | 728.9242 | 16.58 | 1.0588 | 1.066 | 50506 | 4.46e2 | 1 | 1 | TRINITY\_DN200\_c0\_g1\_i1.p1:TRINITY\_DN200\_c0\_g1\_i5.p1:TRINITY\_DN200\_c0\_g1\_i2.p1:TRINITY\_DN200\_c0\_g1\_i3.p1 |  |  | DB Search |
| NIDEHASEGVER | 64.74 | 1354.6113 | 12 | 4.41 | 678.3129 | 11.03 | 0.9683 | 0.9755 | 19561 | 6.21e2 | 1 | 1 | TRINITY\_DN10907\_c0\_g1\_i1.p1 |  |  | DB Search |
| LRLDFDHEAKLNMIFR | 64.71 | 2017.0568 | 16 | -0.61 | 673.3561 | 18.47 | 0.9449 | 0.9521 | 61552 | 3.77e3 | 2 | 2 | TRINITY\_DN160\_c0\_g1\_i2.p1:TRINITY\_DN160\_c0\_g1\_i1.p1 |  |  | DB Search |
| NSSYFVEWIPNNVK | 64.67 | 1695.8257 | 14 | -4.28 | 848.9127 | 19.92 | 1.0889 | 1.0961 | 70457 | 0 | 0 | 0 | TRINITY\_DN1910\_c0\_g1\_i1.p1:TRINITY\_DN3986\_c0\_g1\_i2.p1:TRINITY\_DN3986\_c0\_g1\_i1.p1 |  |  | DB Search |
| RAGELSDEEVER | 64.61 | 1388.6532 | 12 | 3.19 | 695.3329 | 11.78 | 0.9707 | 0.9779 | 23916 | 1.24e3 | 1 | 1 | TRINITY\_DN1505\_c0\_g1\_i1.p1 |  |  | DB Search |
| VTSQREEDEYVK | 64.6 | 1481.6998 | 12 | 3.77 | 741.8566 | 11.19 | 1.05 | 1.0572 | 20488 | 4.16e2 | 1 | 1 | TRINITY\_DN309\_c0\_g1\_i1.p1 |  |  | DB Search |
| YQYEHAVAELR | 64.48 | 1377.6677 | 11 | 2.2 | 689.8395 | 14.37 | 1.0102 | 1.0174 | 38774 | 1.67e3 | 1 | 1 | TRINITY\_DN4954\_c0\_g1\_i2.p2:TRINITY\_DN6388\_c0\_g1\_i1.p2:TRINITY\_DN6388\_c0\_g1\_i2.p2 |  |  | DB Search |
| LRLDFDHEAKLNM(+15.99)IFR | 64.35 | 2033.0516 | 16 | 4.9 | 509.2704 | 17.76 | 0.8558 | 0.8631 | 57084 | 1.58e3 | 1 | 1 | TRINITY\_DN160\_c0\_g1\_i2.p1:TRINITY\_DN160\_c0\_g1\_i1.p1 | Oxidation (M) | M13:Oxidation (M):1000 | DB Search |
| HILLAIANDEELNKLLR | 64.29 | 1974.1261 | 17 | -1.92 | 659.0451 | 19.51 | 0.9852 | 0.9924 | 67939 | 3.02e2 | 1 | 1 | TRINITY\_DN1065\_c0\_g1\_i1.p1 |  |  | DB Search |
| ATLGHGHVIEGVDR | 64.13 | 1459.7532 | 14 | -3.35 | 730.8781 | 12.12 | 1.011 | 1.0182 | 25970 | 0 | 0 | 0 | TRINITY\_DN30378\_c0\_g1\_i1.p1:TRINITY\_DN30378\_c0\_g1\_i2.p1 |  |  | DB Search |
| WC(+57.02)DILSIHQSMENC(+57.02)VAK | 63.98 | 2089.9382 | 17 | -2.35 | 697.6486 | 17.89 | 0.8777 | 0.885 | 58058 | 0 | 0 | 0 | TRINITY\_DN36\_c0\_g1\_i3.p1:TRINITY\_DN36\_c0\_g1\_i16.p1:TRINITY\_DN36\_c0\_g1\_i14.p1:TRINITY\_DN36\_c0\_g1\_i17.p1:TRINITY\_DN36\_c0\_g1\_i13.p1:TRINITY\_DN36\_c0\_g1\_i8.p1:TRINITY\_DN36\_c0\_g1\_i19.p1 | Carbamidomethylation, Carbamidomethylation |  | DB Search |
| KGELMDYQLPLHSQVFEK | 63.95 | 2161.0876 | 18 | 0.45 | 721.3669 | 17.73 | 0.9896 | 0.9969 | 57002 | 5.77e2 | 1 | 1 | TRINITY\_DN478\_c2\_g1\_i1.p1 |  |  | DB Search |
| LYDEYNDKTPSR | 63.94 | 1499.6892 | 12 | 17.25 | 750.8615 | 11.79 | 1.0098 | 1.017 | 24101 | 0 | 0 | 0 | TRINITY\_DN2963\_c0\_g1\_i1.p1 |  |  | DB Search |
| EGFYVHEALAAGIVK | 63.7 | 1602.8406 | 15 | -2.05 | 802.4223 | 17.52 | 1.0889 | 1.0961 | 55709 | 2.51e2 | 1 | 1 | TRINITY\_DN602\_c0\_g1\_i4.p1:TRINITY\_DN602\_c0\_g1\_i3.p1:TRINITY\_DN602\_c0\_g1\_i1.p1 |  |  | DB Search |
| TNRPPVSLAR | 63.65 | 1109.6305 | 10 | 2.36 | 555.8214 | 12.15 | 0.883 | 0.8903 | 26078 | 3.42e3 | 1 | 1 | TRINITY\_DN162\_c0\_g1\_i1.p1 |  |  | DB Search |
| AVIFDRFR | 63.61 | 1022.5661 | 8 | -0.98 | 512.2875 | 15.89 | 0.8684 | 0.8757 | 46912 | 1.42e3 | 1 | 1 | TRINITY\_DN98420\_c0\_g1\_i1.p1 |  |  | DB Search |
| VRVNVLNDALNSIC(+57.02)NAEKR | 63.58 | 2184.1433 | 19 | -8.4 | 729.0457 | 18.74 | 0.9828 | 0.99 | 63282 | 0 | 0 | 0 | TRINITY\_DN3339\_c0\_g1\_i1.p1 | Carbamidomethylation |  | DB Search |
| KLDYVLGLTTAK | 63.57 | 1320.7653 | 12 | -2.37 | 661.3854 | 16.69 | 0.9908 | 0.9981 | 51148 | 2.13e2 | 1 | 1 | TRINITY\_DN5107\_c0\_g1\_i11.p1:TRINITY\_DN5107\_c0\_g1\_i5.p2:TRINITY\_DN5107\_c0\_g2\_i1.p1:TRINITY\_DN5107\_c0\_g1\_i2.p1:TRINITY\_DN5107\_c0\_g1\_i7.p2:TRINITY\_DN5107\_c0\_g1\_i9.p1:TRINITY\_DN5107\_c0\_g1\_i8.p2:TRINITY\_DN5107\_c0\_g1\_i3.p1:TRINITY\_DN5107\_c0\_g1\_i6.p1:TRINITY\_DN5107\_c0\_g1\_i10.p1:TRINITY\_DN5107\_c0\_g1\_i1.p2 |  |  | DB Search |
| HLSVNILQTNTR | 63.57 | 1394.7629 | 12 | -0.64 | 698.3852 | 15.23 | 1.0057 | 1.013 | 43441 | 4.4e2 | 1 | 1 | TRINITY\_DN6390\_c0\_g1\_i1.p1 |  |  | DB Search |
| GGKDFASDIITRK | 63.55 | 1406.7517 | 13 | 2.63 | 704.3818 | 14.09 | 1.0009 | 1.0081 | 37275 | 1.13e3 | 1 | 1 | TRINITY\_DN1613\_c0\_g2\_i1.p1 |  |  | DB Search |
| AYHNASQTQSDKAHLK | 63.47 | 1797.8757 | 16 | 0.02 | 450.4742 | 9.43 | 0.7412 | 0.7485 | 10705 | 0 | 0 | 0 | TRINITY\_DN484\_c0\_g1\_i1.p1 |  |  | DB Search |
| AFQNTPC(+57.02)THLVLDGTR | 63.4 | 1828.889 | 16 | -0.04 | 915.4476 | 15.25 | 1.1762 | 1.1833 | 43382 | 3.21e2 | 1 | 1 | TRINITY\_DN1091\_c0\_g1\_i1.p1 | Carbamidomethylation |  | DB Search |
| TVLASQTVTIPEDVSVSVNGR | 63.4 | 2171.1433 | 21 | 6.66 | 1086.5813 | 17.80 | 1.2272 | 1.2344 | 57498 | 0 | 0 | 0 | TRINITY\_DN194146\_c0\_g1\_i1.p1 |  |  | DB Search |
| A(+42.01)TVVDYPDFDAQADAETLRK | 63.34 | 2266.0752 | 20 | -6.61 | 1134.0323 | 18.33 | 1.2869 | 1.2941 | 60630 | 0 | 0 | 0 | TRINITY\_DN3190\_c1\_g1\_i1.p1 | Acetylation (Protein N-term) | A1:Acetylation (Protein N-term):1000 | DB Search |
| DLSLVVEPLKNYGVK | 63.33 | 1672.9399 | 15 | 2.59 | 837.4756 | 19.05 | 1.1346 | 1.1418 | 65061 | 1.67e2 | 1 | 1 | TRINITY\_DN244\_c0\_g1\_i1.p1 |  |  | DB Search |
| A(+42.01)LSRPVYIIAAK | 63.33 | 1342.7972 | 12 | -0.52 | 672.4025 | 17.88 | 1.0528 | 1.06 | 57936 | 2.31e3 | 1 | 1 | TRINITY\_DN11662\_c0\_g1\_i1.p1 | Acetylation (Protein N-term) | A1:Acetylation (Protein N-term):1000 | DB Search |
| FAVLDKQQNVVIK | 63.28 | 1500.8663 | 13 | 4.2 | 751.4402 | 15.20 | 1.0777 | 1.0849 | 43259 | 2.65e2 | 1 | 1 | TRINITY\_DN187147\_c0\_g1\_i1.p1 |  |  | DB Search |
| DLEHPNEFIR | 63.27 | 1268.6149 | 10 | -1.34 | 635.311 | 14.83 | 0.9198 | 0.9271 | 41210 | 1.17e2 | 1 | 1 | TRINITY\_DN36471\_c0\_g1\_i1.p1 |  |  | DB Search |
| K(+42.01)LQAELANGR | 63.27 | 1140.6251 | 10 | -2.89 | 571.3156 | 14.26 | 0.8919 | 0.8992 | 37952 | 3.72e3 | 1 | 1 | TRINITY\_DN15599\_c0\_g1\_i1.p1 | Acetylation (Protein N-term) | K1:Acetylation (Protein N-term):1000 | DB Search |
| VFEIAQADLNKDEVAFR | 63.21 | 1964.0002 | 17 | -4.68 | 982.9984 | 18.05 | 1.2479 | 1.2551 | 59001 | 0 | 0 | 0 | TRINITY\_DN577\_c0\_g1\_i1.p1 |  |  | DB Search |
| A(+42.01)SAATDNRK | 63.17 | 974.4781 | 9 | -3.25 | 488.2426 | 9.45 | 0.8205 | 0.8279 | 10765 | 0 | 0 | 0 | TRINITY\_DN7659\_c0\_g1\_i2.p1:TRINITY\_DN7659\_c0\_g1\_i1.p1 | Acetylation (Protein N-term) | A1:Acetylation (Protein N-term):1000 | DB Search |
| DVQLGDLVTVGQC(+57.02)RPLSK | 63.15 | 1984.0411 | 18 | 4.67 | 993.028 | 17.98 | 1.2726 | 1.2797 | 58548 | 8.93e2 | 1 | 1 | TRINITY\_DN15847\_c0\_g1\_i1.p1:TRINITY\_DN15847\_c0\_g1\_i2.p1 | Carbamidomethylation |  | DB Search |
| IDLHLYHELYM(+15.99)K | 63.13 | 1589.7911 | 12 | -2.23 | 795.8975 | 15.63 | 1.0953 | 1.1025 | 45494 | 5.53e2 | 1 | 1 | TRINITY\_DN1112\_c0\_g1\_i1.p1 | Oxidation (M) | M11:Oxidation (M):1000 | DB Search |
| SISC(+57.02)SKDEEIK | 63.06 | 1294.6074 | 11 | 1.99 | 648.3094 | 10.84 | 0.9755 | 0.9828 | 18539 | 0 | 0 | 0 | TRINITY\_DN495\_c0\_g1\_i5.p1:TRINITY\_DN495\_c0\_g1\_i6.p1 | Carbamidomethylation |  | DB Search |
| RYGQAMC(+57.02)QFM(+15.99)R | 63.05 | 1462.6268 | 11 | -11.86 | 732.3087 | 14.93 | 0.9779 | 0.9852 | 41834 | 0 | 0 | 0 | TRINITY\_DN286\_c0\_g1\_i4.p1:TRINITY\_DN286\_c0\_g1\_i2.p1 | Carbamidomethylation, Oxidation (M) | M10:Oxidation (M):25.11 | DB Search |
| YVESAADKR | 63 | 1037.5142 | 9 | 0.83 | 519.7625 | 9.57 | 0.8538 | 0.8611 | 11283 | 0 | 0 | 0 | TRINITY\_DN7314\_c0\_g2\_i1.p1 |  |  | DB Search |
| IVNAEWNILYEKLEK | 62.98 | 1860.9985 | 15 | -4.36 | 931.4983 | 19.47 | 1.1881 | 1.1953 | 67707 | 0 | 0 | 0 | TRINITY\_DN1194\_c0\_g1\_i1.p1 |  |  | DB Search |
| HGQVDSC(+57.02)INQNLKK | 62.95 | 1639.8101 | 14 | 1.44 | 547.6089 | 11.98 | 0.8315 | 0.8388 | 25203 | 5.94e2 | 1 | 1 | TRINITY\_DN3991\_c0\_g1\_i1.p1 | Carbamidomethylation |  | DB Search |
| FDKYHPGYFGK | 62.93 | 1357.6455 | 11 | 1.88 | 679.8282 | 18.01 | 0.988 | 0.9953 | 58678 | 4.03e3 | 1 | 1 | TRINITY\_DN131028\_c0\_g1\_i1.p2 |  |  | DB Search |
| KSQFGEIFSFDKK | 62.93 | 1559.7983 | 13 | -6.3 | 520.9344 | 17.14 | 0.7888 | 0.7962 | 53558 | 6.09e2 | 1 | 1 | TRINITY\_DN34765\_c0\_g1\_i1.p1:TRINITY\_DN14344\_c0\_g1\_i1.p1 |  |  | DB Search |
| RLLTSLVQGAR | 62.93 | 1212.7302 | 11 | 1.05 | 607.3703 | 15.15 | 0.9775 | 0.9848 | 43011 | 0 | 0 | 0 | TRINITY\_DN3190\_c1\_g1\_i1.p1 |  |  | DB Search |
| KFVWEFFQK | 62.91 | 1257.6545 | 9 | -1.78 | 629.8306 | 19.87 | 0.9449 | 0.9521 | 70107 | 0 | 0 | 0 | TRINITY\_DN1280\_c0\_g1\_i1.p1 |  |  | DB Search |
| LDLAGRDLTDYLM(+15.99)K | 62.87 | 1638.8286 | 14 | 4.29 | 820.4214 | 18.15 | 1.143 | 1.1502 | 59617 | 0 | 0 | 0 | TRINITY\_DN161\_c0\_g1\_i1.p1:TRINITY\_DN161\_c0\_g1\_i4.p1:TRINITY\_DN161\_c0\_g1\_i3.p1:TRINITY\_DN161\_c0\_g1\_i2.p1 | Oxidation (M) | M13:Oxidation (M):1000 | DB Search |
| AHVSLSTFSEDVTSLTK | 62.84 | 1820.9155 | 17 | 4.58 | 911.4651 | 18.78 | 1.141 | 1.1482 | 63505 | 0 | 0 | 0 | TRINITY\_DN113\_c3\_g1\_i1.p1 |  |  | DB Search |
| ARPHTM(+15.99)TSLTGSR | 62.83 | 1429.7096 | 13 | 4.71 | 477.5772 | 9.97 | 0.849 | 0.8562 | 13362 | 0 | 0 | 0 | TRINITY\_DN34191\_c0\_g1\_i1.p1 | Oxidation (M) | M6:Oxidation (M):1000 | DB Search |
| AIELPDKYMNIGAK | 62.82 | 1561.8174 | 14 | -1.23 | 781.9115 | 17.04 | 1.0733 | 1.0805 | 52963 | 0 | 0 | 0 | TRINITY\_DN118520\_c0\_g1\_i1.p1 |  |  | DB Search |
| VC(+57.02)EDVKR | 62.75 | 904.4437 | 7 | 2.55 | 453.2282 | 10.47 | 0.7953 | 0.8027 | 16197 | 2.08e3 | 2 | 2 | TRINITY\_DN118\_c1\_g1\_i3.p1:TRINITY\_DN118\_c1\_g1\_i5.p1 | Carbamidomethylation |  | DB Search |
| AAASAHHQAIGSIDAAGVTSAADYASVTAALGR | 62.75 | 3080.5273 | 33 | 0.95 | 771.1364 | 18.15 | 1.0636 | 1.0709 | 59524 | 1.07e3 | 1 | 1 | TRINITY\_DN240\_c0\_g1\_i6.p1:TRINITY\_DN240\_c0\_g1\_i1.p1:TRINITY\_DN240\_c0\_g1\_i4.p1 |  |  | DB Search |
| VVIIDGKGHLM(+15.99)GR | 62.74 | 1409.7812 | 13 | 0.05 | 705.8948 | 12.97 | 1.023 | 1.0303 | 30926 | 1.45e3 | 1 | 1 | TRINITY\_DN2805\_c0\_g1\_i1.p1 | Oxidation (M) | M11:Oxidation (M):1000 | DB Search |
| LGLPVFLGGVESKPC(+57.02)DADGSAR | 62.56 | 2244.1208 | 22 | 1.07 | 749.045 | 19.31 | 0.9271 | 0.9344 | 66694 | 2.92e2 | 1 | 1 | TRINITY\_DN7808\_c4\_g1\_i3.p2 | Carbamidomethylation |  | DB Search |
| SGTGIAAM(+15.99)SVM(+15.99)RPELIMK | 62.55 | 1922.9628 | 18 | 4.55 | 962.4887 | 15.49 | 1.273 | 1.2801 | 44794 | 1.46e2 | 1 | 1 | TRINITY\_DN673\_c0\_g1\_i1.p1 | Oxidation (M), Oxidation (M) | M8:Oxidation (M):50.04 M11:Oxidation (M):62.39 | DB Search |
| VGYTHLFSSTQVSVRPK | 62.53 | 1905.0109 | 17 | 8.5 | 636.0134 | 15.20 | 0.9392 | 0.9465 | 43249 | 8.92e1 | 1 | 1 | TRINITY\_DN2301\_c0\_g1\_i1.p1 |  |  | DB Search |
| AYGQDLGQPGSC(+57.02)LKR | 62.49 | 1648.7991 | 15 | -3.4 | 825.4003 | 13.37 | 1.0725 | 1.0797 | 33176 | 9e2 | 1 | 1 | TRINITY\_DN691\_c0\_g1\_i2.p1:TRINITY\_DN691\_c0\_g1\_i1.p1 | Carbamidomethylation |  | DB Search |
| KGDNEIPGLTDRVIPR | 62.47 | 1778.9639 | 16 | -2.38 | 593.9911 | 15.59 | 0.8972 | 0.9044 | 45402 | 0 | 0 | 0 | TRINITY\_DN2230\_c0\_g1\_i1.p1 |  |  | DB Search |
| IKVDDKLNNLDQITLEVAK | 62.46 | 2168.2051 | 19 | -0.11 | 723.739 | 17.81 | 0.9105 | 0.9178 | 57433 | 1.3e3 | 1 | 1 | TRINITY\_DN95248\_c0\_g1\_i1.p1 |  |  | DB Search |
| GVAGAGVLAGFDKFK | 62.44 | 1435.7823 | 15 | 3.16 | 718.8975 | 18.24 | 1.0869 | 1.0941 | 60071 | 4.33e3 | 1 | 1 | TRINITY\_DN1559\_c0\_g1\_i1.p1 |  |  | DB Search |
| LIQYIENKPGLTR | 62.38 | 1543.8722 | 13 | 0.76 | 772.9405 | 14.75 | 1.0773 | 1.0845 | 40651 | 3.68e3 | 1 | 1 | TRINITY\_DN2028\_c0\_g1\_i1.p1 |  |  | DB Search |
| ISGLIYEETRGVLK | 62.35 | 1576.8824 | 14 | 2.48 | 789.4469 | 17.34 | 1.0929 | 1.1001 | 54665 | 0 | 0 | 0 | TRINITY\_DN172571\_c0\_g1\_i1.p1:TRINITY\_DN22077\_c0\_g1\_i1.p3 |  |  | DB Search |
| RLVGIVVYDYLAHNNK | 62.34 | 1873.021 | 16 | 4.78 | 625.3478 | 18.53 | 0.9344 | 0.9416 | 62014 | 0 | 0 | 0 | TRINITY\_DN45599\_c0\_g1\_i1.p1 |  |  | DB Search |
| GFTLEELKTAGIPVK | 62.3 | 1601.9028 | 15 | -1.3 | 801.954 | 18.20 | 1.1138 | 1.121 | 59964 | 0 | 0 | 0 | TRINITY\_DN119650\_c0\_g1\_i1.p1 |  |  | DB Search |
| AFHQIIHSATLR | 62.3 | 1392.7626 | 12 | -5.44 | 697.3817 | 13.45 | 1.009 | 1.0162 | 33729 | 0 | 0 | 0 | TRINITY\_DN2490\_c0\_g1\_i1.p1 |  |  | DB Search |
| IFEAGFHILGIKPISR | 62.22 | 1797.03 | 16 | -0.24 | 600.0145 | 19.00 | 0.9117 | 0.919 | 64761 | 4.9e2 | 1 | 1 | TRINITY\_DN464\_c0\_g1\_i4.p1 |  |  | DB Search |
| GAFGKPQGTVAR | 62.21 | 1187.6411 | 12 | -0.01 | 594.8251 | 11.39 | 0.9234 | 0.9307 | 21663 | 1.25e3 | 1 | 1 | TRINITY\_DN72959\_c0\_g1\_i1.p1 |  |  | DB Search |
| NIVHGSDSVETAQK | 62.14 | 1483.7267 | 14 | -1.27 | 742.8663 | 11.13 | 0.9993 | 1.0065 | 20026 | 1.18e3 | 1 | 1 | TRINITY\_DN478\_c0\_g1\_i2.p1 |  |  | DB Search |
| LLAVTVGNNYNLDTLEK | 62.12 | 1875.9941 | 17 | 1.09 | 939.0012 | 18.57 | 1.1462 | 1.1534 | 62222 | 0 | 0 | 0 | TRINITY\_DN77\_c1\_g1\_i1.p1 |  |  | DB Search |
| VLATDKDTLSSNK | 62.11 | 1390.7303 | 13 | 2.98 | 696.3714 | 11.50 | 1.0154 | 1.0226 | 22335 | 9.57e2 | 1 | 1 | TRINITY\_DN982\_c0\_g1\_i3.p1 |  |  | DB Search |
| SGTGIAAM(+15.99)SVMRPELIM(+15.99)K | 62.1 | 1922.9628 | 18 | -7.32 | 962.4773 | 15.70 | 1.2364 | 1.2436 | 45909 | 1.55e2 | 1 | 1 | TRINITY\_DN673\_c0\_g1\_i1.p1 | Oxidation (M), Oxidation (M) | M8:Oxidation (M):22.45 M17:Oxidation (M):22.09 | DB Search |
| ALGC(+57.02)DVLAC(+57.02)SVDSHFSHLAWTQQSR | 62.1 | 2844.3071 | 25 | 4.64 | 712.0842 | 18.79 | 0.9222 | 0.9295 | 63472 | 1.49e3 | 1 | 1 | TRINITY\_DN535\_c0\_g1\_i2.p1:TRINITY\_DN535\_c0\_g1\_i1.p1 | Carbamidomethylation, Carbamidomethylation |  | DB Search |
| AVDIHFALDASQNVGR | 62.09 | 1711.8641 | 16 | 0.55 | 856.936 | 17.40 | 1.0989 | 1.1061 | 54900 | 6.01e2 | 1 | 1 | TRINITY\_DN77\_c1\_g1\_i1.p1 |  |  | DB Search |
| LAALDATVHGQTASR | 62.09 | 1509.7899 | 15 | 0.19 | 755.899 | 13.42 | 1.0721 | 1.0793 | 33378 | 4.46e2 | 1 | 1 | TRINITY\_DN1378\_c0\_g1\_i1.p1 |  |  | DB Search |
| AESIALVTEFTDEAAPK | 62.08 | 1790.8938 | 17 | -2.25 | 896.4481 | 19.81 | 1.1198 | 1.127 | 69737 | 2.36e2 | 1 | 1 | TRINITY\_DN79\_c0\_g1\_i1.p1 |  |  | DB Search |
| FPGQLNADIRK | 62.07 | 1257.683 | 11 | -5.49 | 629.8425 | 14.27 | 0.9428 | 0.9501 | 38203 | 0 | 0 | 0 | TRINITY\_DN5365\_c0\_g1\_i3.p1:TRINITY\_DN5365\_c0\_g1\_i2.p1 |  |  | DB Search |
| FPGQLNADLRK | 62.07 | 1257.683 | 11 | -5.49 | 629.8425 | 14.27 | 0.9428 | 0.9501 | 38203 | 0 | 0 | 0 | TRINITY\_DN1910\_c0\_g1\_i1.p1:TRINITY\_DN1910\_c0\_g1\_i5.p1:TRINITY\_DN3986\_c0\_g1\_i2.p1:TRINITY\_DN3986\_c0\_g1\_i1.p1 |  |  | DB Search |
| ILQLEEEFIKK | 62.05 | 1388.7915 | 11 | -3.77 | 695.3973 | 17.67 | 1.019 | 1.0263 | 56718 | 2.93e2 | 1 | 1 | TRINITY\_DN120478\_c0\_g1\_i1.p1 |  |  | DB Search |
| IAGDVIVC(+57.02)AAYSHELPR | 62.03 | 1869.9407 | 17 | 2.59 | 935.9758 | 17.14 | 1.2001 | 1.2073 | 53423 | 2.89e2 | 1 | 1 | TRINITY\_DN116\_c0\_g1\_i1.p1 | Carbamidomethylation |  | DB Search |
| SAIIVDGKNEEGR | 62.03 | 1386.7103 | 13 | -2.52 | 694.3575 | 11.84 | 0.9953 | 1.0025 | 24410 | 0 | 0 | 0 | TRINITY\_DN982\_c0\_g1\_i3.p1 |  |  | DB Search |
| DQHEATAIEALRR | 62.02 | 1508.7695 | 13 | 4.8 | 503.9306 | 13.82 | 0.8027 | 0.81 | 35722 | 0 | 0 | 0 | TRINITY\_DN72959\_c0\_g1\_i1.p1 |  |  | DB Search |
| SFASSLLDHFRVEPAK | 62.01 | 1802.9315 | 16 | 3.47 | 601.9838 | 19.81 | 0.9129 | 0.9202 | 69718 | 3.84e2 | 1 | 1 | TRINITY\_DN77\_c1\_g1\_i1.p1 |  |  | DB Search |
| AITSSDPEVLKR | 62.01 | 1314.7144 | 12 | -5.42 | 658.3579 | 12.95 | 0.9844 | 0.9916 | 30804 | 0 | 0 | 0 | TRINITY\_DN7808\_c1\_g3\_i2.p2:TRINITY\_DN7808\_c1\_g3\_i1.p1:TRINITY\_DN7808\_c1\_g3\_i3.p1 |  |  | DB Search |
| IDNYIEFLDEDLKK | 62.01 | 1753.8774 | 14 | 3.11 | 585.6323 | 19.83 | 0.8453 | 0.8526 | 69916 | 0 | 0 | 0 | TRINITY\_DN278\_c0\_g1\_i1.p1 |  |  | DB Search |
| LC(+57.02)QPGYSTC(+57.02)ENHC(+57.02)R | 62.01 | 1780.708 | 14 | 5.39 | 891.3621 | 11.41 | 1.0893 | 1.0965 | 21785 | 1.84e2 | 1 | 1 | TRINITY\_DN817\_c0\_g1\_i4.p1:TRINITY\_DN817\_c0\_g1\_i7.p1:TRINITY\_DN817\_c0\_g1\_i1.p1:TRINITY\_DN817\_c0\_g1\_i3.p1:TRINITY\_DN817\_c0\_g1\_i8.p1:TRINITY\_DN817\_c0\_g1\_i6.p1:TRINITY\_DN817\_c0\_g1\_i2.p1:TRINITY\_DN817\_c0\_g1\_i10.p1:TRINITY\_DN817\_c0\_g1\_i5.p1:TRINITY\_DN817\_c0\_g1\_i9.p1 | Carbamidomethylation, Carbamidomethylation, Carbamidomethylation |  | DB Search |
| SDSSDIEKR | 62 | 1035.4833 | 9 | -18.84 | 518.7368 | 11.06 | 0.8558 | 0.8631 | 19937 | 8.59e3 | 1 | 1 | TRINITY\_DN135129\_c0\_g1\_i1.p1 |  |  | DB Search |
| APGDYGWRPPLLATDDAALK | 61.97 | 2126.0796 | 20 | -2.01 | 709.6959 | 18.98 | 0.9081 | 0.9154 | 64807 | 3.62e3 | 1 | 1 | TRINITY\_DN16548\_c0\_g1\_i4.p1:TRINITY\_DN16548\_c0\_g1\_i1.p1:TRINITY\_DN16548\_c0\_g1\_i3.p1 |  |  | DB Search |
| LYSTDPNKFYR | 61.95 | 1402.6881 | 11 | -1.52 | 702.3471 | 14.18 | 1.0303 | 1.0375 | 37720 | 6.29e2 | 1 | 1 | TRINITY\_DN72625\_c0\_g1\_i1.p1 |  |  | DB Search |
| ELISNSSDALDKIR | 61.95 | 1559.8154 | 14 | -1.81 | 780.9101 | 15.46 | 1.0753 | 1.0825 | 44710 | 0 | 0 | 0 | TRINITY\_DN141338\_c2\_g1\_i1.p1:TRINITY\_DN5633\_c0\_g1\_i1.p1 |  |  | DB Search |
| EVNHEYQIIASSWK | 61.95 | 1702.8314 | 14 | -0.63 | 852.4186 | 17.08 | 1.1258 | 1.133 | 53181 | 0 | 0 | 0 | TRINITY\_DN17516\_c0\_g1\_i3.p1:TRINITY\_DN17516\_c0\_g1\_i1.p1 |  |  | DB Search |
| S(+42.01)GERTPITSDKR | 61.94 | 1387.7056 | 12 | -0.02 | 463.5737 | 10.89 | 0.7514 | 0.7587 | 18882 | 0 | 0 | 0 | TRINITY\_DN187527\_c0\_g1\_i1.p1 | Acetylation (Protein N-term) | S1:Acetylation (Protein N-term):1000 | DB Search |
| AVAAGM(+15.99)NPM(+15.99)DIKR | 61.93 | 1404.6853 | 13 | -5.88 | 703.3427 | 11.20 | 1.0122 | 1.0194 | 20513 | 4.12e2 | 1 | 1 | TRINITY\_DN15151\_c0\_g1\_i2.p1:TRINITY\_DN15151\_c0\_g1\_i3.p1:TRINITY\_DN15151\_c0\_g1\_i1.p1 | Oxidation (M), Oxidation (M) | M6:Oxidation (M):1000 M9:Oxidation (M):1000 | DB Search |
| EIDKADIAIVDTGENPDAPHPR | 61.91 | 2372.1606 | 22 | 3.12 | 791.7264 | 14.88 | 1.0415 | 1.0488 | 41517 | 0 | 0 | 0 | TRINITY\_DN761\_c0\_g1\_i3.p1:TRINITY\_DN761\_c0\_g1\_i1.p1:TRINITY\_DN761\_c0\_g1\_i2.p1 |  |  | DB Search |
| ATIEIPAKYEK | 61.89 | 1261.6918 | 11 | 0.22 | 631.8505 | 14.08 | 0.982 | 0.9892 | 37168 | 0 | 0 | 0 | TRINITY\_DN2637\_c0\_g1\_i6.p1:TRINITY\_DN2637\_c0\_g1\_i14.p2:TRINITY\_DN2637\_c0\_g1\_i5.p2:TRINITY\_DN2637\_c0\_g1\_i8.p2:TRINITY\_DN2637\_c0\_g1\_i9.p2:TRINITY\_DN2637\_c0\_g1\_i13.p1:TRINITY\_DN2637\_c0\_g1\_i7.p2:TRINITY\_DN2637\_c0\_g1\_i2.p2:TRINITY\_DN2637\_c0\_g1\_i4.p1 |  |  | DB Search |
| IPLDDFQDPTLTLENAHNK | 61.84 | 2180.075 | 19 | 2.14 | 727.6972 | 18.19 | 0.9896 | 0.9969 | 59846 | 3.57e2 | 1 | 1 | TRINITY\_DN13856\_c0\_g1\_i1.p2:TRINITY\_DN13856\_c0\_g1\_i2.p2 |  |  | DB Search |
| LIQTLNYETKK | 61.82 | 1349.7555 | 11 | 0.35 | 675.8822 | 13.59 | 1.0098 | 1.017 | 34441 | 0 | 0 | 0 | TRINITY\_DN982\_c0\_g1\_i3.p1:TRINITY\_DN982\_c0\_g1\_i1.p1 |  |  | DB Search |
| VKELQFQLEDEHK | 61.8 | 1641.8362 | 13 | -15.13 | 548.2753 | 14.58 | 0.8124 | 0.8197 | 39893 | 0 | 0 | 0 | TRINITY\_DN3034\_c0\_g1\_i1.p1 |  |  | DB Search |
| KLLEINVYHPLIQELQK | 61.8 | 2077.1936 | 17 | 2.34 | 693.4036 | 18.48 | 0.9521 | 0.9594 | 61635 | 0 | 0 | 0 | TRINITY\_DN5633\_c0\_g1\_i1.p1 |  |  | DB Search |
| YANFLDQALREDLER | 61.79 | 1851.9115 | 15 | -3.63 | 618.3061 | 19.80 | 0.8741 | 0.8814 | 69772 | 0 | 0 | 0 | TRINITY\_DN53760\_c0\_g1\_i1.p1:TRINITY\_DN998\_c0\_g1\_i2.p2:TRINITY\_DN998\_c0\_g1\_i6.p2:TRINITY\_DN998\_c0\_g1\_i4.p2:TRINITY\_DN4928\_c0\_g1\_i1.p1:TRINITY\_DN998\_c0\_g1\_i5.p2:TRINITY\_DN998\_c0\_g1\_i3.p2 |  |  | DB Search |
| GAHGIIIVYDVTDQDTFNNVK | 61.79 | 2318.1543 | 21 | -3.18 | 773.7194 | 18.44 | 0.9198 | 0.9271 | 61374 | 2.77e2 | 1 | 1 | TRINITY\_DN2068\_c0\_g1\_i1.p1 |  |  | DB Search |
| LAYELQANKEQQR | 61.78 | 1589.8162 | 13 | -0.05 | 795.9117 | 12.90 | 1.0508 | 1.058 | 30507 | 0 | 0 | 0 | TRINITY\_DN11435\_c0\_g1\_i1.p1 |  |  | DB Search |
| IGAAAQALADQSYPFLK | 61.76 | 1762.9253 | 17 | 1.13 | 882.467 | 19.07 | 1.161 | 1.1682 | 65059 | 6.63e2 | 1 | 1 | TRINITY\_DN240\_c0\_g1\_i6.p1:TRINITY\_DN240\_c0\_g1\_i1.p1:TRINITY\_DN240\_c0\_g1\_i4.p1 |  |  | DB Search |
| IAAETEEVGVQIIGDLDDQKETLHR | 61.7 | 2778.4036 | 25 | 2.04 | 695.6064 | 19.02 | 0.9392 | 0.9465 | 64964 | 0 | 0 | 0 | TRINITY\_DN26892\_c0\_g1\_i1.p1 |  |  | DB Search |
| GVGIISEGNETVEDIAQR | 61.69 | 1885.9381 | 18 | 0.15 | 943.9722 | 17.77 | 1.1438 | 1.151 | 57272 | 0 | 0 | 0 | TRINITY\_DN262\_c0\_g1\_i1.p1 |  |  | DB Search |
| LADLEEFISSPNHANISQVGER | 61.67 | 2425.1873 | 22 | 1.36 | 809.4005 | 18.74 | 0.9263 | 0.9335 | 63253 | 4.69e2 | 1 | 1 | TRINITY\_DN2113\_c0\_g1\_i1.p1:TRINITY\_DN2113\_c0\_g1\_i2.p1 |  |  | DB Search |
| HC(+57.02)DPELSSFHTFWR | 61.65 | 1817.7943 | 14 | -6 | 606.9324 | 17.68 | 0.8887 | 0.8959 | 56846 | 2.37e2 | 1 | 1 | TRINITY\_DN22492\_c0\_g1\_i1.p1:TRINITY\_DN22492\_c0\_g1\_i2.p1 | Carbamidomethylation |  | DB Search |
| ISNVQDIVPVLELANQHK | 61.64 | 2016.1003 | 18 | -1.98 | 673.0364 | 19.58 | 0.9501 | 0.9574 | 68461 | 5.02e2 | 1 | 1 | TRINITY\_DN118520\_c0\_g1\_i1.p1 |  |  | DB Search |
| FETQIATK | 61.61 | 936.4916 | 8 | 0.97 | 937.4956 | 13.84 | 1.2037 | 1.2109 | 35727 | 1.28e3 | 1 | 1 | TRINITY\_DN649\_c0\_g1\_i2.p1:TRINITY\_DN36\_c0\_g1\_i3.p1:TRINITY\_DN36\_c0\_g1\_i16.p1:TRINITY\_DN36\_c0\_g1\_i14.p1:TRINITY\_DN36\_c0\_g1\_i17.p1:TRINITY\_DN649\_c0\_g1\_i5.p1:TRINITY\_DN36\_c0\_g1\_i13.p1:TRINITY\_DN36\_c0\_g1\_i8.p1:TRINITY\_DN649\_c0\_g1\_i1.p1:TRINITY\_DN36\_c0\_g1\_i19.p1 |  |  | DB Search |
| LGPDQVDVAGAGR | 61.56 | 1253.6364 | 13 | 3 | 627.8245 | 13.56 | 0.9311 | 0.9384 | 34257 | 0 | 0 | 0 | TRINITY\_DN97809\_c0\_g1\_i1.p1 |  |  | DB Search |
| KQLVNVPSFIVR | 61.54 | 1398.8347 | 12 | 1.44 | 700.4225 | 17.76 | 1.0065 | 1.0138 | 57229 | 4.4e2 | 1 | 1 | TRINITY\_DN189323\_c0\_g1\_i1.p1:TRINITY\_DN776\_c0\_g1\_i1.p1 |  |  | DB Search |
| GC(+57.02)YIHTQEVAR | 61.54 | 1332.6245 | 11 | -1.99 | 667.3152 | 12.14 | 1.0086 | 1.0158 | 26094 | 0 | 0 | 0 | TRINITY\_DN2217\_c0\_g1\_i3.p1:TRINITY\_DN3202\_c2\_g1\_i1.p1:TRINITY\_DN2217\_c0\_g1\_i2.p1 | Carbamidomethylation |  | DB Search |
| IKAEYENKTEVAR | 61.53 | 1549.8099 | 13 | -4.14 | 775.9056 | 10.97 | 1.0556 | 1.0628 | 19328 | 0 | 0 | 0 | TRINITY\_DN1280\_c0\_g1\_i1.p1 |  |  | DB Search |
| FAIQDITVDEFSAK | 61.52 | 1582.7878 | 14 | -2.01 | 792.3961 | 19.66 | 1.0443 | 1.0516 | 68879 | 0 | 0 | 0 | TRINITY\_DN624\_c0\_g1\_i1.p1 |  |  | DB Search |
| A(+42.01)TGIKPDPQC(+57.02)TDLYNK | 61.48 | 1861.8879 | 16 | -0.79 | 931.9464 | 14.86 | 1.1614 | 1.1686 | 41244 | 3.84e2 | 1 | 1 | TRINITY\_DN164271\_c0\_g1\_i1.p1 | Acetylation (Protein N-term), Carbamidomethylation | A1:Acetylation (Protein N-term):1000 | DB Search |
| DYLHFIK | 61.47 | 934.4912 | 7 | -4.37 | 468.2487 | 18.67 | 0.8242 | 0.8315 | 62776 | 3.12e2 | 1 | 1 | TRINITY\_DN15847\_c0\_g1\_i1.p1:TRINITY\_DN15847\_c0\_g1\_i2.p1 |  |  | DB Search |
| GVSEETTTGVHNLYK | 61.46 | 1633.7948 | 15 | -0.34 | 817.9007 | 13.56 | 1.0717 | 1.0789 | 34190 | 6.1e2 | 1 | 1 | TRINITY\_DN773\_c0\_g1\_i1.p1 |  |  | DB Search |
| VDNKVNATER | 61.28 | 1144.5836 | 10 | -1.08 | 573.2959 | 9.55 | 0.8866 | 0.8939 | 11254 | 0 | 0 | 0 | TRINITY\_DN32522\_c0\_g1\_i1.p1 |  |  | DB Search |
| AM(+15.99)TDC(+57.02)KETLC(+57.02)R | 61.26 | 1399.5894 | 11 | -4.02 | 700.796 | 10.58 | 1.0013 | 1.0086 | 16952 | 0 | 0 | 0 | TRINITY\_DN168534\_c0\_g1\_i1.p1 | Oxidation (M), Carbamidomethylation, Carbamidomethylation | M2:Oxidation (M):1000 | DB Search |
| FGFSILEAMNGKK | 61.26 | 1440.7435 | 13 | 2.19 | 481.254 | 19.76 | 0.7819 | 0.7892 | 69523 | 0 | 0 | 0 | TRINITY\_DN647\_c0\_g1\_i1.p1 |  |  | DB Search |
| TLVALDEQGEKLNR | 61.23 | 1584.847 | 14 | -0.56 | 793.4268 | 14.58 | 1.0985 | 1.1057 | 39877 | 0 | 0 | 0 | TRINITY\_DN19237\_c0\_g1\_i1.p1 |  |  | DB Search |
| HQGVM(+15.99)VGM(+15.99)GQK | 61.11 | 1202.5536 | 11 | 7.61 | 602.2859 | 10.70 | 0.9089 | 0.9162 | 17721 | 0 | 0 | 0 | TRINITY\_DN161\_c0\_g1\_i1.p1:TRINITY\_DN161\_c0\_g1\_i3.p1:TRINITY\_DN161\_c0\_g1\_i2.p1 | Oxidation (M), Oxidation (M) | M5:Oxidation (M):1000 M8:Oxidation (M):1000 | DB Search |
| LLEINVYHPLIQELQKR | 61.04 | 2105.1997 | 17 | 3.51 | 702.7398 | 18.83 | 0.9884 | 0.9957 | 63817 | 0 | 0 | 0 | TRINITY\_DN5633\_c0\_g1\_i1.p1 |  |  | DB Search |
| SPTHSVIK | 60.92 | 867.4814 | 8 | 0.09 | 434.7461 | 9.91 | 0.7876 | 0.7949 | 13010 | 3.62e2 | 1 | 1 | TRINITY\_DN1386\_c0\_g1\_i1.p1 |  |  | DB Search |
| KVAC(+57.02)IGAWHPAR | 60.86 | 1364.7135 | 12 | 9.11 | 683.3672 | 13.20 | 0.9711 | 0.9783 | 32100 | 9.16e2 | 1 | 1 | TRINITY\_DN6920\_c0\_g1\_i1.p1:TRINITY\_DN6920\_c0\_g1\_i3.p1:TRINITY\_DN6920\_c0\_g1\_i4.p1:TRINITY\_DN6920\_c0\_g1\_i2.p1 | Carbamidomethylation |  | DB Search |
| LRPGVELTM(+15.99)SSLVNAK | 60.81 | 1729.9396 | 16 | -1.76 | 865.9717 | 15.17 | 1.1358 | 1.143 | 42935 | 1.5e3 | 1 | 1 | TRINITY\_DN329\_c0\_g1\_i1.p1:TRINITY\_DN329\_c0\_g1\_i2.p1 | Oxidation (M) | M9:Oxidation (M):1000 | DB Search |
| EGDLVIHSGITDAVK | 60.79 | 1552.8097 | 15 | -9.48 | 777.4012 | 15.72 | 1.0668 | 1.0741 | 46131 | 0 | 0 | 0 | TRINITY\_DN329\_c0\_g1\_i1.p1:TRINITY\_DN329\_c0\_g1\_i2.p1 |  |  | DB Search |
| VFEIAQADLNKDEVAFRK | 60.64 | 2092.0952 | 18 | -6.42 | 698.3647 | 16.92 | 0.9505 | 0.9578 | 52375 | 0 | 0 | 0 | TRINITY\_DN577\_c0\_g1\_i1.p1 |  |  | DB Search |
| LGIHEDTQNR | 60.32 | 1181.5789 | 10 | -1.02 | 591.7935 | 11.11 | 0.8943 | 0.9016 | 20072 | 6.52e2 | 1 | 1 | TRINITY\_DN141338\_c2\_g1\_i1.p1 |  |  | DB Search |
| LSTLVGANVINEKPLK | 60.22 | 1694.993 | 16 | 7.58 | 848.5064 | 16.33 | 1.1817 | 1.1889 | 49261 | 0 | 0 | 0 | TRINITY\_DN6008\_c0\_g1\_i1.p1 |  |  | DB Search |
| YVAIYPSDHLTVNR | 60.16 | 1646.8417 | 14 | -9.73 | 824.4164 | 15.50 | 1.1098 | 1.117 | 44890 | 0 | 0 | 0 | TRINITY\_DN982\_c0\_g1\_i3.p1:TRINITY\_DN982\_c0\_g1\_i1.p1 |  |  | DB Search |
| EIVHLQAGQC(+57.02)GNQIGAK | 59.9 | 1821.9155 | 17 | -14.62 | 911.9476 | 13.73 | 1.113 | 1.1202 | 35183 | 0 | 0 | 0 | TRINITY\_DN1910\_c0\_g1\_i1.p1:TRINITY\_DN1910\_c0\_g1\_i5.p1:TRINITY\_DN3986\_c0\_g1\_i1.p1 | Carbamidomethylation |  | DB Search |
| ELVHIQAGQC(+57.02)GNQIGAK | 59.9 | 1821.9155 | 17 | -14.62 | 911.9476 | 13.73 | 1.113 | 1.1202 | 35183 | 0 | 0 | 0 | TRINITY\_DN2031\_c1\_g1\_i4.p1 | Carbamidomethylation |  | DB Search |
| LGPVVDGKYDYTVITTDYK | 59.89 | 2146.0833 | 19 | 3.69 | 1074.0481 | 17.48 | 1.242 | 1.2491 | 55518 | 0 | 0 | 0 | TRINITY\_DN468\_c1\_g1\_i1.p1 |  |  | DB Search |
| ALADYIHSQDLK | 59.54 | 1372.6986 | 12 | 0.98 | 687.3542 | 15.62 | 1.0061 | 1.0134 | 45560 | 3.85e2 | 1 | 1 | TRINITY\_DN1819\_c0\_g1\_i5.p1:TRINITY\_DN1819\_c0\_g1\_i7.p1 |  |  | DB Search |
| AIQGATSHHLGQNFSK | 59.41 | 1694.8489 | 16 | 4.36 | 848.4316 | 11.89 | 1.1406 | 1.1478 | 24619 | 0 | 0 | 0 | TRINITY\_DN5001\_c0\_g1\_i1.p1 |  |  | DB Search |
| YLHDQSIVHLDLKPDNILLAK | 59.04 | 2444.3428 | 21 | 13 | 612.0981 | 18.20 | 0.9061 | 0.9133 | 59977 | 0 | 0 | 0 | TRINITY\_DN23911\_c0\_g1\_i1.p1 |  |  | DB Search |
| LVLEVAQHLGENVVR | 58.94 | 1674.9417 | 15 | 9.79 | 838.4825 | 17.91 | 1.1414 | 1.1486 | 58140 | 0 | 0 | 0 | TRINITY\_DN73587\_c0\_g1\_i1.p1 |  |  | DB Search |
| VLLGGLVSHM(+15.99)DYIKR | 58.58 | 1715.9392 | 15 | -1.08 | 858.9721 | 16.10 | 1.1897 | 1.1969 | 47995 | 1.27e2 | 1 | 1 | TRINITY\_DN47849\_c0\_g1\_i1.p1 | Oxidation (M) | M10:Oxidation (M):1000 | DB Search |
| YVKPGVNIINIR | 58.56 | 1384.8191 | 12 | 3.46 | 693.4161 | 15.95 | 1.0025 | 1.0098 | 47296 | 0 | 0 | 0 | TRINITY\_DN1616\_c0\_g1\_i3.p1:TRINITY\_DN1616\_c0\_g1\_i6.p1:TRINITY\_DN1616\_c0\_g1\_i2.p1:TRINITY\_DN1616\_c0\_g1\_i7.p1 |  |  | DB Search |
| GQFSTDDLVAEVEKR | 58.01 | 1692.8318 | 15 | 1.27 | 847.4205 | 17.68 | 1.1374 | 1.1446 | 56739 | 3.43e2 | 1 | 1 | TRINITY\_DN2113\_c0\_g1\_i1.p1:TRINITY\_DN2113\_c0\_g1\_i2.p1 |  |  | DB Search |
| IREEYPDR | 56.96 | 1076.525 | 8 | 1.05 | 539.2679 | 10.96 | 0.8644 | 0.8716 | 19132 | 3.24e3 | 1 | 1 | TRINITY\_DN1910\_c0\_g1\_i5.p1:TRINITY\_DN3986\_c0\_g1\_i1.p1 |  |  | DB Search |
| NIALETLEGHQR | 56.79 | 1379.7157 | 12 | 3.41 | 690.8644 | 15.22 | 1.0178 | 1.025 | 43440 | 5.81e2 | 1 | 1 | TRINITY\_DN1280\_c0\_g1\_i1.p1 |  |  | DB Search |
| EITLNYHDTVVHMK | 55.14 | 1698.84 | 14 | -0.31 | 850.4232 | 15.27 | 1.1126 | 1.1198 | 43604 | 5.8e2 | 1 | 1 | TRINITY\_DN1369\_c0\_g1\_i1.p1 |  |  | DB Search |
| VGDIVDVKGNGAIQK | 52.97 | 1511.8307 | 15 | -2.72 | 756.9172 | 13.61 | 1.0721 | 1.0793 | 34539 | 0 | 0 | 0 | TRINITY\_DN6132\_c0\_g1\_i1.p1 |  |  | DB Search |
| VTEAKGELAK | 52.31 | 1044.5815 | 10 | -5.06 | 523.293 | 9.99 | 0.8947 | 0.902 | 13499 | 0 | 0 | 0 | TRINITY\_DN614\_c0\_g1\_i1.p1 |  |  | DB Search |
| RSLNAEIANGR | 51.82 | 1199.6371 | 11 | 2.77 | 600.8248 | 11.99 | 0.9259 | 0.9331 | 25198 | 6.38e2 | 1 | 1 | TRINITY\_DN7808\_c2\_g1\_i1.p1:TRINITY\_DN142212\_c0\_g1\_i1.p1:TRINITY\_DN7808\_c2\_g1\_i2.p1:TRINITY\_DN7808\_c2\_g1\_i3.p1 |  |  | DB Search |
| RSLNAELANGR | 51.82 | 1199.6371 | 11 | 2.77 | 600.8248 | 11.99 | 0.9259 | 0.9331 | 25198 | 6.38e2 | 1 | 1 | TRINITY\_DN2487\_c0\_g1\_i4.p1 |  |  | DB Search |
| MTSC(+57.02)EANHQVAK | 50.44 | 1374.6021 | 12 | -2.69 | 688.3033 | 9.67 | 0.9767 | 0.984 | 11763 | 0 | 0 | 0 | TRINITY\_DN1369\_c0\_g1\_i1.p1 | Carbamidomethylation |  | DB Search |
| SSC(+57.02)RPILSVIK | 49.81 | 1258.7067 | 11 | -8.86 | 630.3522 | 16.81 | 0.9691 | 0.9763 | 51630 | 2.1e3 | 1 | 1 | TRINITY\_DN158421\_c0\_g1\_i1.p1:TRINITY\_DN3545\_c0\_g1\_i2.p1:TRINITY\_DN3545\_c0\_g1\_i1.p1 | Carbamidomethylation |  | DB Search |
| LRQIHNGVFVK | 49.48 | 1309.7618 | 11 | 7.63 | 655.8903 | 12.45 | 0.9727 | 0.98 | 27858 | 0 | 0 | 0 | TRINITY\_DN1468\_c0\_g1\_i1.p1 |  |  | DB Search |
| DKTLILWELTR | 49.3 | 1386.7871 | 11 | 3.36 | 694.4 | 19.94 | 0.9945 | 1.0017 | 70462 | 1.74e2 | 1 | 1 | TRINITY\_DN167384\_c0\_g1\_i1.p1 |  |  | DB Search |
| M(+42.01)(+15.99)DAAQAAEIKK | 49.25 | 1232.6071 | 11 | -4.25 | 617.3054 | 12.14 | 0.9477 | 0.9549 | 26098 | 0 | 0 | 0 | TRINITY\_DN34\_c0\_g1\_i1.p1 | Acetylation (Protein N-term), Oxidation (M) | M1:Acetylation (Protein N-term):1000 M1:Oxidation (M):1000 | DB Search |
| LAGESESNLRK | 48.88 | 1202.6255 | 11 | 5.59 | 602.3207 | 10.70 | 0.9291 | 0.9364 | 17678 | 0 | 0 | 0 | TRINITY\_DN5587\_c0\_g1\_i1.p1 |  |  | DB Search |
| SKELSDQAEAR | 48.59 | 1232.5996 | 11 | 0.56 | 617.3047 | 10.16 | 0.9372 | 0.9444 | 14479 | 1.07e3 | 1 | 1 | TRINITY\_DN1112\_c0\_g1\_i1.p1 |  |  | DB Search |
| TIKDVNC(+57.02)NNNQLK | 47.49 | 1559.7726 | 13 | 8.47 | 780.8967 | 11.21 | 1.05 | 1.0572 | 20684 | 1.74e2 | 1 | 1 | TRINITY\_DN4815\_c0\_g1\_i2.p1:TRINITY\_DN4815\_c0\_g1\_i1.p1 | Carbamidomethylation |  | DB Search |
| LVPGWTKPIVIGR | 46.21 | 1434.8711 | 13 | 2.88 | 718.4417 | 17.27 | 1.0411 | 1.0484 | 54264 | 0 | 0 | 0 | TRINITY\_DN7504\_c0\_g1\_i1.p1 |  |  | DB Search |
| GTIEILGDVHLIK | 45.38 | 1406.8132 | 13 | -2.67 | 704.4089 | 18.96 | 1.0616 | 1.0688 | 64620 | 5.41e2 | 1 | 1 | TRINITY\_DN6783\_c0\_g1\_i1.p1 |  |  | DB Search |
| AFVHWYVGEGM(+15.99)EEGEFSEAR | 44.9 | 2345.0059 | 20 | -0.18 | 782.6722 | 17.81 | 0.9251 | 0.9323 | 57560 | 6.2e2 | 1 | 1 | TRINITY\_DN471\_c0\_g1\_i3.p1:TRINITY\_DN250\_c0\_g1\_i5.p1:TRINITY\_DN471\_c0\_g1\_i11.p1:TRINITY\_DN250\_c0\_g1\_i6.p1:TRINITY\_DN250\_c0\_g1\_i4.p1:TRINITY\_DN471\_c0\_g1\_i10.p1:TRINITY\_DN96161\_c0\_g1\_i1.p1:TRINITY\_DN471\_c0\_g1\_i2.p1:TRINITY\_DN471\_c0\_g1\_i4.p1 | Oxidation (M) | M11:Oxidation (M):1000 | DB Search |
| LEPVGC(+57.02)FKDR | 43.43 | 1219.6019 | 10 | 10.59 | 610.812 | 12.97 | 0.9352 | 0.9424 | 30908 | 0 | 0 | 0 | TRINITY\_DN3991\_c0\_g1\_i1.p1 | Carbamidomethylation |  | DB Search |
| RQAVDVAPLRR | 43.13 | 1279.7473 | 11 | 9.42 | 427.5918 | 12.00 | 0.7648 | 0.7722 | 25287 | 0 | 0 | 0 | TRINITY\_DN164351\_c0\_g1\_i1.p1 |  |  | DB Search |
| KKLEPQAAPR | 43.1 | 1136.6665 | 10 | 2.05 | 379.8952 | 9.66 | 0.7604 | 0.7677 | 11777 | 3.9e3 | 2 | 2 | TRINITY\_DN22492\_c0\_g1\_i1.p1:TRINITY\_DN22492\_c0\_g1\_i2.p1 |  |  | DB Search |
| ITELSPIRPR | 43.05 | 1180.6927 | 10 | -1.76 | 591.35 | 13.86 | 0.9255 | 0.9327 | 35968 | 5.64e2 | 1 | 1 | TRINITY\_DN4329\_c0\_g1\_i1.p1 |  |  | DB Search |
| RIQQLSAIR | 42.81 | 1083.6512 | 9 | 5.14 | 542.8333 | 13.13 | 0.9146 | 0.9218 | 31805 | 1.14e3 | 1 | 1 | TRINITY\_DN428\_c0\_g1\_i1.p1 |  |  | DB Search |
| LRLDFDHEAK | 42.61 | 1242.6356 | 10 | 8.37 | 415.2208 | 14.18 | 0.7282 | 0.7355 | 37619 | 6.56e2 | 1 | 1 | TRINITY\_DN160\_c0\_g1\_i2.p1:TRINITY\_DN160\_c0\_g1\_i1.p1 |  |  | DB Search |
| RIAEVQC(+57.02)EAR | 42.53 | 1230.6139 | 10 | 0.71 | 616.3119 | 10.93 | 0.9214 | 0.9287 | 19029 | 9.5e2 | 1 | 1 | TRINITY\_DN1280\_c0\_g1\_i1.p1 | Carbamidomethylation |  | DB Search |
| KLQAELANGR | 41.93 | 1098.6145 | 10 | 1.23 | 550.3127 | 11.49 | 0.8878 | 0.8951 | 21900 | 9.13e3 | 1 | 1 | TRINITY\_DN15599\_c0\_g1\_i1.p1:TRINITY\_DN15599\_c0\_g1\_i2.p1 |  |  | DB Search |
| HFC(+57.02)WVFSK | 41.91 | 1109.5116 | 8 | -7.68 | 555.7563 | 19.76 | 0.8712 | 0.8785 | 69479 | 2.04e2 | 1 | 1 | TRINITY\_DN2217\_c0\_g1\_i3.p1:TRINITY\_DN2217\_c0\_g1\_i2.p1 | Carbamidomethylation |  | DB Search |
| TSDSDLEKR | 41.64 | 1049.4989 | 9 | -18.21 | 525.7448 | 11.26 | 0.8611 | 0.8684 | 20698 | 3.58e3 | 1 | 1 | TRINITY\_DN76073\_c0\_g1\_i1.p1 |  |  | DB Search |
| ALKEPPRDR | 41.49 | 1080.604 | 9 | 9.05 | 361.2102 | 9.43 | 0.7314 | 0.7388 | 10516 | 1.16e4 | 1 | 1 | TRINITY\_DN164128\_c0\_g1\_i1.p1 |  |  | DB Search |
| VTRPHGASGVVRAK | 41.36 | 1433.8215 | 14 | 4.92 | 359.4628 | 9.64 | 0.7151 | 0.7224 | 11644 | 0 | 0 | 0 | TRINITY\_DN2885\_c0\_g1\_i2.p1:TRINITY\_DN2885\_c0\_g1\_i5.p1:TRINITY\_DN2885\_c0\_g1\_i4.p1 |  |  | DB Search |
| FRQLGLDFGK | 41.31 | 1179.64 | 10 | -5.77 | 590.8212 | 19.79 | 0.9214 | 0.9287 | 69618 | 2.9e3 | 1 | 1 | TRINITY\_DN22594\_c0\_g1\_i1.p2 |  |  | DB Search |
| EC(+57.02)VTLEYDFIYK | 41.22 | 1578.7275 | 12 | -0.88 | 790.3668 | 19.93 | 1.0552 | 1.0624 | 70514 | 5.43e2 | 1 | 1 | TRINITY\_DN1479\_c0\_g1\_i1.p1 | Carbamidomethylation |  | DB Search |
| AEVIREEEEEKEK | 40.71 | 1616.7893 | 13 | 5.8 | 539.9377 | 10.70 | 0.7897 | 0.797 | 17726 | 8.74e2 | 1 | 1 | TRINITY\_DN22219\_c0\_g1\_i1.p1 |  |  | DB Search |
| IRITELSPIRPR | 40.64 | 1449.8779 | 12 | 0.33 | 484.2979 | 15.22 | 0.8238 | 0.8311 | 43346 | 6.45e2 | 1 | 1 | TRINITY\_DN4329\_c0\_g1\_i1.p1 |  |  | DB Search |
| KFDTYLPGFIC(+57.02)R | 40.39 | 1515.7544 | 12 | -0.6 | 758.8806 | 18.56 | 1.0339 | 1.0411 | 62069 | 5.32e2 | 1 | 1 | TRINITY\_DN785\_c0\_g1\_i1.p1 | Carbamidomethylation |  | DB Search |
| AHTGYIYLVEGGRDR | 40.28 | 1705.8536 | 15 | 3.15 | 853.9329 | 14.97 | 1.1478 | 1.155 | 41960 | 6.86e2 | 1 | 1 | TRINITY\_DN1613\_c0\_g2\_i1.p1 |  |  | DB Search |
| SC(+57.02)TNPAPANGGK | 39.93 | 1172.5244 | 12 | 0 | 587.2668 | 9.43 | 0.8404 | 0.8477 | 10729 | 0 | 0 | 0 | TRINITY\_DN495\_c0\_g1\_i2.p1:TRINITY\_DN3545\_c0\_g1\_i2.p1:TRINITY\_DN3545\_c0\_g1\_i1.p1:TRINITY\_DN495\_c0\_g1\_i3.p1 | Carbamidomethylation |  | DB Search |
| ATNPDVEK | 39.91 | 872.424 | 8 | 4.9 | 437.2194 | 9.68 | 0.799 | 0.8063 | 11736 | 1.23e3 | 1 | 1 | TRINITY\_DN72625\_c0\_g1\_i1.p1 |  |  | DB Search |
| DFASDIITR | 39.77 | 1036.5189 | 9 | -0.03 | 1037.5215 | 16.56 | 1.2841 | 1.2913 | 50406 | 3.96e2 | 1 | 1 | TRINITY\_DN1613\_c0\_g2\_i1.p1 |  |  | DB Search |
| KIDDVIC(+57.02)EER | 39.44 | 1275.6129 | 10 | -9.66 | 638.8047 | 13.45 | 0.9255 | 0.9327 | 33693 | 0 | 0 | 0 | TRINITY\_DN15274\_c0\_g1\_i1.p1 | Carbamidomethylation |  | DB Search |
| LSPEDIER | 39.28 | 957.4767 | 8 | 13.25 | 479.7498 | 14.37 | 0.827 | 0.8343 | 38738 | 1.06e4 | 1 | 1 | TRINITY\_DN119684\_c0\_g1\_i1.p1 |  |  | DB Search |
| FVNEVRR | 39.18 | 918.5035 | 7 | -2.89 | 460.2556 | 12.63 | 0.8096 | 0.8169 | 28832 | 9.86e2 | 1 | 1 | TRINITY\_DN761\_c0\_g1\_i3.p1:TRINITY\_DN761\_c0\_g1\_i1.p1:TRINITY\_DN761\_c0\_g1\_i2.p1 |  |  | DB Search |
| AISFIGSVHDNRK | 39.17 | 1442.7629 | 13 | -1.14 | 722.3847 | 14.03 | 1.0415 | 1.0488 | 36874 | 0 | 0 | 0 | TRINITY\_DN6\_c0\_g1\_i1.p1 |  |  | DB Search |
| VFDRDGNGFISAAELR | 39.11 | 1765.8748 | 16 | -1.5 | 883.9393 | 19.71 | 1.1234 | 1.1306 | 69271 | 0 | 0 | 0 | TRINITY\_DN801\_c0\_g1\_i10.p1:TRINITY\_DN801\_c0\_g1\_i4.p1:TRINITY\_DN801\_c0\_g1\_i7.p1:TRINITY\_DN801\_c0\_g1\_i1.p1:TRINITY\_DN801\_c0\_g1\_i9.p2:TRINITY\_DN30386\_c0\_g1\_i1.p2 |  |  | DB Search |
| KEELC(+57.02)HQLTELKTELQQLHVAK | 38.46 | 2674.4111 | 22 | -0.85 | 535.8867 | 18.43 | 0.8437 | 0.851 | 61365 | 0 | 0 | 0 | TRINITY\_DN197343\_c0\_g1\_i1.p1 | Carbamidomethylation |  | DB Search |
| LSIIPVRR | 38.41 | 952.6182 | 8 | 1.59 | 477.315 | 14.73 | 0.8453 | 0.8526 | 40459 | 4.64e3 | 1 | 1 | TRINITY\_DN15584\_c0\_g1\_i1.p1 |  |  | DB Search |
| ERQENTNPVTR | 37.82 | 1342.6589 | 11 | 1.48 | 672.3347 | 9.98 | 0.9558 | 0.963 | 13356 | 6.23e2 | 1 | 1 | TRINITY\_DN2142\_c0\_g1\_i2.p2:TRINITY\_DN2142\_c0\_g1\_i1.p2 |  |  | DB Search |
| DLLTLPEKDNRR | 36.23 | 1468.7998 | 12 | 2.6 | 735.4058 | 14.56 | 1.0283 | 1.0355 | 39735 | 1e3 | 1 | 1 | TRINITY\_DN776\_c0\_g1\_i1.p1 |  |  | DB Search |
| AQPTEIYNLGAQSHVK | 35.72 | 1754.8951 | 16 | 0.32 | 878.4512 | 14.67 | 1.1722 | 1.1794 | 40344 | 0 | 0 | 0 | TRINITY\_DN7659\_c0\_g1\_i2.p1:TRINITY\_DN7659\_c0\_g1\_i1.p1 |  |  | DB Search |
| IESTAC(+57.02)TR | 34.99 | 936.4335 | 8 | 6.23 | 469.2248 | 9.63 | 0.812 | 0.8193 | 11550 | 1.4e3 | 1 | 1 | TRINITY\_DN98968\_c0\_g1\_i1.p1 | Carbamidomethylation |  | DB Search |
| LLAQTTLRNELGTK | 34.86 | 1556.8885 | 14 | -1.74 | 779.4467 | 15.17 | 1.1102 | 1.1174 | 43095 | 4.27e2 | 1 | 1 | TRINITY\_DN5818\_c1\_g1\_i2.p1:TRINITY\_DN41662\_c0\_g1\_i2.p1:TRINITY\_DN5818\_c1\_g1\_i1.p1:TRINITY\_DN909\_c0\_g1\_i1.p1 |  |  | DB Search |
| EC(+57.02)DKVR | 32.35 | 805.3752 | 6 | 4.12 | 806.3822 | 10.47 | 1.1074 | 1.1146 | 16279 | 8.92e3 | 1 | 1 | TRINITY\_DN51048\_c0\_g1\_i1.p1 | Carbamidomethylation |  | DB Search |
| LTIAEERDR | 31.8 | 1101.5778 | 9 | -3 | 551.7921 | 11.69 | 0.9004 | 0.9077 | 23456 | 0 | 0 | 0 | TRINITY\_DN140696\_c0\_g1\_i1.p1 |  |  | DB Search |
| VIGLVEYRR | 31.43 | 1103.6451 | 9 | 4.18 | 552.8297 | 14.49 | 0.9097 | 0.917 | 39428 | 0 | 0 | 0 | TRINITY\_DN95606\_c0\_g1\_i1.p1 |  |  | DB Search |
| AIM(+15.99)ITINSFGTELNKDDR | 31.12 | 2053.0149 | 18 | 3.55 | 1027.5138 | 18.19 | 1.2495 | 1.2567 | 59830 | 0 | 0 | 0 | TRINITY\_DN58867\_c0\_g1\_i2.p1 | Oxidation (M) | M3:Oxidation (M):1000 | DB Search |
| SQSAFPTRR | 31.06 | 1048.5414 | 9 | 5.06 | 525.2783 | 10.99 | 0.8433 | 0.8506 | 19385 | 6.25e2 | 1 | 1 | TRINITY\_DN477\_c0\_g1\_i8.p1:TRINITY\_DN477\_c0\_g1\_i7.p1 |  |  | DB Search |
| LGGIEVTQGNC(+57.02)ARK | 31.04 | 1501.7671 | 14 | -2.67 | 751.8854 | 12.64 | 1.0624 | 1.0697 | 29000 | 0 | 0 | 0 | TRINITY\_DN36\_c0\_g1\_i9.p1:TRINITY\_DN36\_c0\_g1\_i14.p1:TRINITY\_DN36\_c0\_g1\_i13.p1:TRINITY\_DN36\_c0\_g1\_i2.p1:TRINITY\_DN36\_c0\_g1\_i3.p1:TRINITY\_DN36\_c0\_g1\_i16.p1:TRINITY\_DN36\_c0\_g1\_i17.p1:TRINITY\_DN36\_c0\_g1\_i4.p1:TRINITY\_DN36\_c0\_g1\_i11.p1:TRINITY\_DN36\_c0\_g1\_i1.p1:TRINITY\_DN36\_c0\_g1\_i8.p1:TRINITY\_DN36\_c0\_g1\_i19.p1:TRINITY\_DN36\_c0\_g1\_i20.p1:TRINITY\_DN36\_c0\_g1\_i5.p1 | Carbamidomethylation |  | DB Search |
| LGPDEASENKR | 29.91 | 1214.5891 | 11 | -1.14 | 608.2984 | 10.44 | 0.9166 | 0.9238 | 16086 | 0 | 0 | 0 | TRINITY\_DN12\_c0\_g1\_i1.p1:TRINITY\_DN12\_c0\_g2\_i1.p1 |  |  | DB Search |
| GVAIHEMMHALGFFHEQSR | 29.88 | 2196.0356 | 19 | -11.88 | 550.0072 | 18.95 | 0.8793 | 0.8866 | 64544 | 0 | 0 | 0 | TRINITY\_DN1315\_c0\_g1\_i1.p1 |  |  | DB Search |
| RYGQAM(+15.99)C(+57.02)QFMR | 29.61 | 1462.6268 | 11 | -5.85 | 732.3131 | 14.49 | 0.9751 | 0.9824 | 39230 | 2.67e3 | 1 | 1 | TRINITY\_DN286\_c0\_g1\_i4.p1:TRINITY\_DN286\_c0\_g1\_i2.p1 | Oxidation (M), Carbamidomethylation | M6:Oxidation (M):34.3 | DB Search |
| GKKPHVQVNVK | 29.58 | 1232.7354 | 11 | 0.24 | 411.9173 | 9.77 | 0.7913 | 0.7986 | 12271 | 6.19e2 | 1 | 1 | TRINITY\_DN119684\_c0\_g1\_i1.p1 |  |  | DB Search |
| KLPEGATC(+57.02)AM(+15.99)KK | 29.28 | 1348.6843 | 12 | 0.14 | 450.5667 | 9.88 | 0.7974 | 0.8047 | 12910 | 0 | 0 | 0 | TRINITY\_DN899\_c0\_g1\_i1.p1 | Carbamidomethylation, Oxidation (M) | M10:Oxidation (M):1000 | DB Search |
| SLGYSVQLQAALIK | 29.2 | 1489.8503 | 14 | -3.67 | 745.9264 | 18.69 | 1.0516 | 1.0588 | 62924 | 0 | 0 | 0 | TRINITY\_DN4166\_c0\_g3\_i1.p1:TRINITY\_DN4166\_c0\_g3\_i2.p1 |  |  | DB Search |
| LDLAGRDLTDYLMK | 27.85 | 1622.8337 | 14 | -0.39 | 541.9492 | 19.26 | 0.7974 | 0.8047 | 66371 | 6.87e2 | 1 | 1 | TRINITY\_DN161\_c0\_g1\_i1.p1:TRINITY\_DN161\_c0\_g1\_i4.p1:TRINITY\_DN161\_c0\_g1\_i3.p1:TRINITY\_DN161\_c0\_g1\_i2.p1 |  |  | DB Search |
| TPPQYR | 27.5 | 760.3868 | 6 | 0.77 | 381.1992 | 11.81 | 0.7106 | 0.718 | 24175 | 4.83e2 | 1 | 1 | TRINITY\_DN41831\_c0\_g1\_i1.p3 |  |  | DB Search |
| C(+57.02)EHVKR | 27.34 | 827.4072 | 6 | 5.54 | 414.7113 | 10.41 | 0.7599 | 0.7673 | 15886 | 1.02e4 | 1 | 1 | TRINITY\_DN367\_c0\_g2\_i1.p1 | Carbamidomethylation |  | DB Search |
| L(+42.01)AAPAFR | 26.73 | 786.4388 | 7 | 17.1 | 394.2316 | 14.97 | 0.7506 | 0.7579 | 41907 | 2.53e3 | 1 | 1 | TRINITY\_DN26679\_c0\_g1\_i1.p3 | Acetylation (Protein N-term) | L1:Acetylation (Protein N-term):1000 | DB Search |
| LRVDLSLFETGIK | 25.55 | 1489.8503 | 13 | 2.4 | 745.9309 | 19.94 | 1.0407 | 1.048 | 70562 | 4.53e2 | 1 | 1 | TRINITY\_DN11097\_c0\_g1\_i1.p1:TRINITY\_DN11097\_c0\_g2\_i1.p1 |  |  | DB Search |
| FSPNTQNPIIVSC(+57.02)GWDK | 25.22 | 1961.9305 | 17 | 3.93 | 981.972 | 17.89 | 1.1638 | 1.171 | 58040 | 0 | 0 | 0 | TRINITY\_DN167384\_c0\_g1\_i1.p1 | Carbamidomethylation |  | DB Search |
| HGSLGFLPR | 25.17 | 982.5348 | 9 | -2.89 | 492.2711 | 17.17 | 0.8408 | 0.8481 | 53699 | 1.07e3 | 1 | 1 | TRINITY\_DN2799\_c0\_g1\_i3.p1:TRINITY\_DN2799\_c0\_g1\_i1.p1:TRINITY\_DN6920\_c0\_g1\_i3.p1:TRINITY\_DN6920\_c0\_g1\_i2.p1:TRINITY\_DN5906\_c3\_g1\_i1.p2:TRINITY\_DN2799\_c0\_g1\_i4.p2:TRINITY\_DN2799\_c0\_g1\_i2.p1 |  |  | DB Search |
| LIEVEEKEIQRR | 24.84 | 1540.8573 | 12 | -0.66 | 514.6237 | 13.09 | 0.7836 | 0.7909 | 31641 | 0 | 0 | 0 | TRINITY\_DN11435\_c0\_g1\_i1.p1 |  |  | DB Search |
| GFAPSTVQVAR | 24.23 | 1131.6036 | 11 | 9.77 | 566.8121 | 14.99 | 0.8955 | 0.9028 | 41874 | 6.64e3 | 1 | 1 | TRINITY\_DN123073\_c0\_g1\_i1.p1 |  |  | DB Search |
| AELVFVNHC(+57.02)R | 23.34 | 1243.6132 | 10 | -1.51 | 622.8101 | 14.29 | 0.9537 | 0.961 | 38337 | 0 | 0 | 0 | TRINITY\_DN101992\_c0\_g1\_i1.p1 | Carbamidomethylation |  | DB Search |
| LSEPYKGVIDC(+57.02)TTR | 23.08 | 1637.8082 | 14 | -1.74 | 819.9063 | 14.48 | 1.0592 | 1.0664 | 39224 | 3.32e2 | 1 | 1 | TRINITY\_DN1559\_c0\_g1\_i1.p1 | Carbamidomethylation |  | DB Search |
| AFNRVETR | 22.7 | 991.5199 | 8 | -1.27 | 496.7644 | 10.70 | 0.8465 | 0.8538 | 17683 | 0 | 0 | 0 | TRINITY\_DN4595\_c0\_g1\_i7.p1:TRINITY\_DN4595\_c0\_g1\_i4.p1:TRINITY\_DN4595\_c0\_g1\_i10.p2 |  |  | DB Search |
| AVLTDLRR | 22.55 | 942.561 | 8 | -1.62 | 472.2849 | 13.15 | 0.84 | 0.8473 | 31971 | 4.27e2 | 1 | 1 | TRINITY\_DN433\_c0\_g2\_i1.p1 |  |  | DB Search |
| LPEGATC(+57.02)AM(+15.99)KK | 22.51 | 1220.5894 | 11 | -2.49 | 611.2977 | 11.46 | 0.9586 | 0.9658 | 22172 | 0 | 0 | 0 | TRINITY\_DN899\_c0\_g1\_i1.p1 | Carbamidomethylation, Oxidation (M) | M9:Oxidation (M):1000 | DB Search |
| ELIDHLIR | 22.02 | 1007.5764 | 8 | -0.07 | 504.7932 | 19.91 | 0.8652 | 0.8725 | 70328 | 3.38e3 | 1 | 1 | TRINITY\_DN1995\_c0\_g1\_i1.p1 |  |  | DB Search |
| ADGGEIQFAQVLR | 21.23 | 1402.7205 | 13 | 7.88 | 468.5823 | 14.99 | 0.7656 | 0.773 | 42194 | 2e3 | 1 | 1 | TRINITY\_DN190525\_c0\_g1\_i1.p2 |  |  | DB Search |
| RC(+57.02)QFVNR | 20.95 | 978.4818 | 7 | -6.21 | 490.2429 | 11.89 | 0.8079 | 0.8153 | 24685 | 0 | 0 | 0 | TRINITY\_DN140996\_c0\_g1\_i1.p1 | Carbamidomethylation |  | DB Search |
| SVGEIVQC(+57.02)SEIFDKSPTTVK | 20.8 | 2223.1094 | 20 | 0.96 | 1112.558 | 17.35 | 1.2893 | 1.2964 | 54732 | 0 | 0 | 0 | TRINITY\_DN143799\_c0\_g1\_i1.p1 | Carbamidomethylation |  | DB Search |
| VSVTLADGTVATQEVLPGATIVVK | 20.47 | 2367.3259 | 24 | 4.39 | 1184.6702 | 19.66 | 1.2833 | 1.2905 | 68866 | 0 | 0 | 0 | TRINITY\_DN2637\_c0\_g1\_i6.p1:TRINITY\_DN2637\_c0\_g1\_i9.p2 |  |  | DB Search |
| GQELTFKEFFKPSNFVR | 20.36 | 2073.0684 | 17 | -5.6 | 692.0231 | 19.23 | 0.9311 | 0.9384 | 66186 | 6.45e2 | 1 | 1 | TRINITY\_DN30329\_c0\_g1\_i1.p1:TRINITY\_DN30329\_c0\_g2\_i1.p2 |  |  | DB Search |
| AGM(+15.99)THIVR | 19.81 | 899.4647 | 8 | 2.72 | 450.7388 | 9.73 | 0.8226 | 0.8299 | 12038 | 6.74e2 | 1 | 1 | TRINITY\_DN32727\_c0\_g1\_i2.p1:TRINITY\_DN2799\_c0\_g1\_i3.p1:TRINITY\_DN2799\_c0\_g1\_i1.p1:TRINITY\_DN6920\_c0\_g1\_i3.p1:TRINITY\_DN6920\_c0\_g1\_i2.p1:TRINITY\_DN5906\_c3\_g1\_i1.p2:TRINITY\_DN2799\_c0\_g1\_i4.p2:TRINITY\_DN2799\_c0\_g1\_i2.p1 | Oxidation (M) | M3:Oxidation (M):1000 | DB Search |
| RC(+57.02)TDLNIR | 19.02 | 1046.5291 | 8 | 7.46 | 524.2734 | 11.91 | 0.8664 | 0.8737 | 24737 | 0 | 0 | 0 | TRINITY\_DN6390\_c0\_g1\_i1.p1 | Carbamidomethylation |  | DB Search |
| TRTPGPGAQSALR | 18.84 | 1310.7054 | 13 | -4.39 | 656.3542 | 12.39 | 0.9469 | 0.9541 | 27541 | 3.36e2 | 1 | 1 | TRINITY\_DN7573\_c0\_g1\_i4.p2 |  |  | DB Search |
| D(+42.01)GPIVR | 18.66 | 697.3759 | 6 | -4.48 | 349.6921 | 11.01 | 0.7587 | 0.7661 | 19714 | 2e2 | 1 | 1 | TRINITY\_DN2626\_c0\_g1\_i1.p1:TRINITY\_DN2626\_c0\_g1\_i7.p2 | Acetylation (Protein N-term) | D1:Acetylation (Protein N-term):1000 | DB Search |
| AMLGETKPSESKPGSIR | 18.29 | 1786.9247 | 17 | 1.94 | 894.4673 | 12.10 | 1.1394 | 1.1466 | 25825 | 3.88e2 | 1 | 1 | TRINITY\_DN478\_c0\_g1\_i2.p1 |  |  | DB Search |
| AGAPAGGGPR | 17.62 | 809.4144 | 10 | -12.48 | 810.4079 | 11.05 | 1.1298 | 1.137 | 19747 | 5.12e3 | 1 | 1 | TRINITY\_DN165222\_c0\_g1\_i1.p2 |  |  | DB Search |
| RFTVELAR | 17.27 | 990.561 | 8 | 5.33 | 496.2882 | 14.42 | 0.8656 | 0.8729 | 39040 | 0 | 0 | 0 | TRINITY\_DN1534\_c2\_g1\_i1.p1 |  |  | DB Search |
| NEFRLPVVQK | 17.17 | 1228.6927 | 10 | -5.88 | 615.3473 | 17.44 | 0.9327 | 0.94 | 55344 | 4.76e2 | 1 | 1 | TRINITY\_DN1397\_c0\_g1\_i3.p1:TRINITY\_DN1397\_c0\_g1\_i2.p1:TRINITY\_DN1397\_c0\_g1\_i1.p1 |  |  | DB Search |
| RPVPIVTR | 17.05 | 936.5869 | 8 | 4.27 | 469.3006 | 11.83 | 0.8071 | 0.8144 | 24513 | 2.28e2 | 1 | 1 | TRINITY\_DN1080\_c0\_g1\_i2.p1:TRINITY\_DN1080\_c0\_g2\_i1.p1:TRINITY\_DN1080\_c0\_g1\_i3.p1 |  |  | DB Search |
| LQQFFNHHM(+15.99)FVLEQEEYKK | 16.72 | 2510.2053 | 19 | 1.98 | 628.557 | 15.85 | 0.8721 | 0.8793 | 46749 | 0 | 0 | 0 | TRINITY\_DN3034\_c0\_g1\_i1.p1 | Oxidation (M) | M9:Oxidation (M):1000 | DB Search |
| DGPPAAR | 16.47 | 682.3398 | 7 | 1.31 | 683.3449 | 9.82 | 1.056 | 1.0632 | 12476 | 4.59e3 | 1 | 1 | TRINITY\_DN165969\_c0\_g1\_i1.p1 |  |  | DB Search |
| EC(+57.02)SC(+57.02)NSYYC(+57.02)VGK | 16.2 | 1525.5636 | 12 | -6.8 | 763.7805 | 11.79 | 1.0045 | 1.0118 | 24015 | 0 | 0 | 0 | TRINITY\_DN493\_c0\_g1\_i1.p1 | Carbamidomethylation, Carbamidomethylation, Carbamidomethylation |  | DB Search |
| total 417 peptides |  |  |  |  |  |  |  |  |  |  |  |  |  |  |  |  |
| --- | --- | --- | --- | --- | --- | --- | --- | --- | --- | --- | --- | --- | --- | --- | --- | --- |
