# Supplementary material for: Proteomic Analysis and Biochemical Characterization of the Nematocyst Extract of the Hydrozoan Velella velella
Source: Mar Drugs. 2024 Oct 12;22(10):468. doi: 10.3390/md22100468 (PMC11509761; doi:10.3390/md22100468)
Supplement: Supplementary file 1 [file marinedrugs-22-00468-s001.zip › Supplementary File S1.zip!/Sample_10_Vellela/db.proteins.html]

proteins


Protein List

  

| Protein Group | Protein ID | Accession | -10lgP | Coverage (%) | Coverage (%) Sample 1 | Area Sample 1 | #Peptides | #Unique | #Spec Sample 1 | PTM | Avg. Mass | Description |
| --- | --- | --- | --- | --- | --- | --- | --- | --- | --- | --- | --- | --- |
| 1 | 1 | TRINITY\_DN982\_c0\_g1\_i3.p1 | 169.14 | 1 | 1 | 1.95e3 | 7 | 7 | 8 |  | 1090854.75 | type:complete len:9852 gc:universal TRINITY\_DN982\_c0\_g1\_i3:29697-142(-) |
| 1 | 2 | TRINITY\_DN982\_c0\_g1\_i1.p1 | 149.8 | 1 | 1 | 9.92e2 | 5 | 5 | 5 |  | 712566.0625 | type:5prime\_partial len:6412 gc:universal TRINITY\_DN982\_c0\_g1\_i1:19377-142(-) |
| 1 | 3 | TRINITY\_DN982\_c0\_g1\_i2.p1 | 66.52 | 1 | 1 | 6.76e2 | 1 | 1 | 1 |  | 190367.1406 | type:internal len:1718 gc:universal TRINITY\_DN982\_c0\_g1\_i2:5151-1(-) |
| 2 | 4 | TRINITY\_DN161\_c0\_g1\_i2.p1 | 150.28 | 16 | 16 | 4.15e4 | 5 | 5 | 10 | Oxidation (M) | 44577.6484 | type:5prime\_partial len:399 gc:universal TRINITY\_DN161\_c0\_g1\_i2:1683-487(-) |
| 2 | 5 | TRINITY\_DN161\_c0\_g1\_i3.p1 | 150.28 | 16 | 16 | 4.15e4 | 5 | 5 | 10 | Oxidation (M) | 44592.6602 | type:5prime\_partial len:399 gc:universal TRINITY\_DN161\_c0\_g1\_i3:1450-254(-) |
| 2 | 6 | TRINITY\_DN161\_c0\_g1\_i1.p1 | 150.28 | 17 | 17 | 4.15e4 | 5 | 5 | 10 | Oxidation (M) | 41918.5938 | type:complete len:377 gc:universal TRINITY\_DN161\_c0\_g1\_i1:1384-254(-) |
| 2 | 7 | TRINITY\_DN161\_c0\_g1\_i4.p1 | 98.12 | 13 | 13 | 2.57e3 | 2 | 2 | 4 | Oxidation (M) | 25887.2988 | type:5prime\_partial len:232 gc:universal TRINITY\_DN161\_c0\_g1\_i4:949-254(-) |
| 2 | 8 | TRINITY\_DN2477\_c0\_g1\_i10.p1 | 66.73 | 3 | 3 | 5.51e3 | 1 | 1 | 1 |  | 38566.9688 | type:5prime\_partial len:344 gc:universal TRINITY\_DN2477\_c0\_g1\_i10:1215-184(-) |
| 2 | 9 | TRINITY\_DN195934\_c0\_g1\_i1.p2 | 66.73 | 10 | 10 | 5.51e3 | 1 | 1 | 1 |  | 14094.0938 | type:internal len:127 gc:universal TRINITY\_DN195934\_c0\_g1\_i1:3-380(+) |
| 2 | 10 | TRINITY\_DN2477\_c0\_g1\_i5.p1 | 66.73 | 4 | 4 | 5.51e3 | 1 | 1 | 1 |  | 37416.5195 | type:complete len:333 gc:universal TRINITY\_DN2477\_c0\_g1\_i5:1182-184(-) |
| 2 | 11 | TRINITY\_DN2477\_c0\_g1\_i2.p1 | 66.73 | 3 | 3 | 5.51e3 | 1 | 1 | 1 |  | 38566.9688 | type:5prime\_partial len:344 gc:universal TRINITY\_DN2477\_c0\_g1\_i2:1215-184(-) |
| 2 | 12 | TRINITY\_DN2477\_c0\_g1\_i6.p1 | 66.73 | 3 | 3 | 5.51e3 | 1 | 1 | 1 |  | 41878.6875 | type:complete len:377 gc:universal TRINITY\_DN2477\_c0\_g1\_i6:1314-184(-) |
| 2 | 13 | TRINITY\_DN2477\_c0\_g1\_i9.p1 | 66.73 | 3 | 3 | 5.51e3 | 1 | 1 | 1 |  | 41878.6875 | type:complete len:377 gc:universal TRINITY\_DN2477\_c0\_g1\_i9:1314-184(-) |
| 3 | 14 | TRINITY\_DN329\_c0\_g1\_i2.p1 | 161.02 | 19 | 19 | 7.67e3 | 6 | 6 | 13 | Oxidation (M) | 34761.9609 | type:5prime\_partial len:320 gc:universal TRINITY\_DN329\_c0\_g1\_i2:1-960(+) |
| 3 | 15 | TRINITY\_DN329\_c0\_g1\_i1.p1 | 161.02 | 21 | 21 | 7.67e3 | 6 | 6 | 13 | Oxidation (M) | 30514.9648 | type:complete len:283 gc:universal TRINITY\_DN329\_c0\_g1\_i1:158-1006(+) |
| 4 | 16 | TRINITY\_DN3986\_c0\_g1\_i1.p1 | 158.65 | 17 | 17 | 6.86e3 | 6 | 6 | 6 | Carbamidomethylation, Oxidation (M) | 50011.8867 | type:complete len:446 gc:universal TRINITY\_DN3986\_c0\_g1\_i1:252-1589(+) |
| 4 | 17 | TRINITY\_DN1910\_c0\_g1\_i1.p1 | 134.66 | 11 | 11 | 2.33e3 | 4 | 4 | 4 | Carbamidomethylation, Oxidation (M) | 50135.0117 | type:complete len:447 gc:universal TRINITY\_DN1910\_c0\_g1\_i1:2183-843(-) |
| 4 | 18 | TRINITY\_DN1910\_c0\_g1\_i5.p1 | 131.9 | 10 | 10 | 5.57e3 | 4 | 4 | 4 | Carbamidomethylation, Oxidation (M) | 50120.9062 | type:complete len:447 gc:universal TRINITY\_DN1910\_c0\_g1\_i5:2031-691(-) |
| 4 | 19 | TRINITY\_DN3986\_c0\_g1\_i2.p1 | 95.7 | 6 | 6 | 0 | 2 | 2 | 2 |  | 50284.332 | type:complete len:446 gc:universal TRINITY\_DN3986\_c0\_g1\_i2:47-1384(+) |
| 4 | 20 | TRINITY\_DN5365\_c0\_g1\_i2.p1 | 62.07 | 2 | 2 | 0 | 1 | 1 | 1 |  | 51878.4453 | type:complete len:458 gc:universal TRINITY\_DN5365\_c0\_g1\_i2:1448-75(-) |
| 4 | 21 | TRINITY\_DN5365\_c0\_g1\_i3.p1 | 62.07 | 2 | 2 | 0 | 1 | 1 | 1 |  | 51878.4453 | type:complete len:458 gc:universal TRINITY\_DN5365\_c0\_g1\_i3:1448-75(-) |
| 4 | 22 | TRINITY\_DN2031\_c1\_g1\_i4.p1 | 59.9 | 14 | 14 | 0 | 1 | 1 | 1 | Carbamidomethylation | 13658.1758 | type:internal len:124 gc:universal TRINITY\_DN2031\_c1\_g1\_i4:369-1(-) |
| 5 | 23 | TRINITY\_DN1280\_c0\_g1\_i1.p1 | 154.84 | 13 | 13 | 1.53e3 | 6 | 6 | 8 | Carbamidomethylation | 47818.6641 | type:complete len:426 gc:universal TRINITY\_DN1280\_c0\_g1\_i1:195-1472(+) |
| 6 | 24 | TRINITY\_DN160\_c0\_g1\_i1.p1 | 132.15 | 2 | 2 | 6.86e3 | 4 | 4 | 9 | Oxidation (M) | 126611.5703 | type:complete len:1145 gc:universal TRINITY\_DN160\_c0\_g1\_i1:4810-1376(-) |
| 6 | 25 | TRINITY\_DN160\_c0\_g1\_i2.p1 | 132.15 | 2 | 2 | 6.86e3 | 4 | 4 | 9 | Oxidation (M) | 126611.5703 | type:complete len:1145 gc:universal TRINITY\_DN160\_c0\_g1\_i2:4810-1376(-) |
| 7 | 26 | TRINITY\_DN776\_c0\_g1\_i1.p1 | 144.51 | 25 | 25 | 5.34e3 | 5 | 5 | 6 | Oxidation (M) | 23300.8359 | type:5prime\_partial len:201 gc:universal TRINITY\_DN776\_c0\_g1\_i1:692-90(-) |
| 7 | 27 | TRINITY\_DN189323\_c0\_g1\_i1.p1 | 120.27 | 15 | 15 | 4.34e3 | 3 | 3 | 4 |  | 18178.9902 | type:5prime\_partial len:156 gc:universal TRINITY\_DN189323\_c0\_g1\_i1:2-469(+) |
| 8 | 28 | TRINITY\_DN36\_c0\_g1\_i19.p1 | 143.1 | 22 | 22 | 1.28e3 | 5 | 5 | 9 | Carbamidomethylation | 24821.4141 | type:complete len:223 gc:universal TRINITY\_DN36\_c0\_g1\_i19:826-158(-) |
| 8 | 29 | TRINITY\_DN36\_c0\_g1\_i13.p1 | 143.1 | 22 | 22 | 1.28e3 | 5 | 5 | 9 | Carbamidomethylation | 24821.4141 | type:complete len:223 gc:universal TRINITY\_DN36\_c0\_g1\_i13:816-148(-) |
| 8 | 30 | TRINITY\_DN36\_c0\_g1\_i16.p1 | 126.77 | 17 | 17 | 1.28e3 | 4 | 4 | 8 | Carbamidomethylation | 24953.5938 | type:complete len:224 gc:universal TRINITY\_DN36\_c0\_g1\_i16:829-158(-) |
| 8 | 31 | TRINITY\_DN36\_c0\_g1\_i3.p1 | 126.77 | 17 | 17 | 1.28e3 | 4 | 4 | 8 | Carbamidomethylation | 24953.5938 | type:complete len:224 gc:universal TRINITY\_DN36\_c0\_g1\_i3:829-158(-) |
| 8 | 32 | TRINITY\_DN36\_c0\_g1\_i14.p1 | 126.77 | 17 | 17 | 1.28e3 | 4 | 4 | 8 | Carbamidomethylation | 24953.5938 | type:complete len:224 gc:universal TRINITY\_DN36\_c0\_g1\_i14:819-148(-) |
| 8 | 33 | TRINITY\_DN36\_c0\_g1\_i8.p1 | 126.77 | 17 | 17 | 1.28e3 | 4 | 4 | 8 | Carbamidomethylation | 24953.5938 | type:complete len:224 gc:universal TRINITY\_DN36\_c0\_g1\_i8:819-148(-) |
| 8 | 34 | TRINITY\_DN36\_c0\_g1\_i17.p1 | 126.77 | 17 | 17 | 1.28e3 | 4 | 4 | 8 | Carbamidomethylation | 24953.5938 | type:complete len:224 gc:universal TRINITY\_DN36\_c0\_g1\_i17:819-148(-) |
| 8 | 35 | TRINITY\_DN36\_c0\_g1\_i5.p1 | 110.51 | 11 | 11 | 0 | 3 | 3 | 3 | Carbamidomethylation | 24548.0977 | type:complete len:222 gc:universal TRINITY\_DN36\_c0\_g1\_i5:931-266(-) |
| 8 | 36 | TRINITY\_DN36\_c0\_g1\_i20.p1 | 110.51 | 11 | 11 | 0 | 3 | 3 | 3 | Carbamidomethylation | 24874.5234 | type:complete len:223 gc:universal TRINITY\_DN36\_c0\_g1\_i20:1172-504(-) |
| 8 | 37 | TRINITY\_DN36\_c0\_g1\_i9.p1 | 82 | 6 | 6 | 0 | 2 | 2 | 2 | Carbamidomethylation | 24680.2773 | type:complete len:223 gc:universal TRINITY\_DN36\_c0\_g1\_i9:934-266(-) |
| 8 | 38 | TRINITY\_DN36\_c0\_g1\_i1.p1 | 82 | 6 | 6 | 0 | 2 | 2 | 2 | Carbamidomethylation | 25006.7051 | type:complete len:224 gc:universal TRINITY\_DN36\_c0\_g1\_i1:1175-504(-) |
| 8 | 39 | TRINITY\_DN36\_c0\_g1\_i11.p1 | 82 | 6 | 6 | 0 | 2 | 2 | 2 | Carbamidomethylation | 25006.7051 | type:complete len:224 gc:universal TRINITY\_DN36\_c0\_g1\_i11:1175-504(-) |
| 8 | 40 | TRINITY\_DN36\_c0\_g1\_i4.p1 | 82 | 6 | 6 | 0 | 2 | 2 | 2 | Carbamidomethylation | 24680.2773 | type:complete len:223 gc:universal TRINITY\_DN36\_c0\_g1\_i4:932-264(-) |
| 8 | 41 | TRINITY\_DN36\_c0\_g1\_i2.p1 | 82 | 6 | 6 | 0 | 2 | 2 | 2 | Carbamidomethylation | 24680.2773 | type:complete len:223 gc:universal TRINITY\_DN36\_c0\_g1\_i2:934-266(-) |
| 8 | 42 | TRINITY\_DN649\_c0\_g1\_i5.p1 | 61.61 | 7 | 7 | 1.28e3 | 1 | 1 | 5 |  | 13746.6875 | type:internal len:124 gc:universal TRINITY\_DN649\_c0\_g1\_i5:371-3(-) |
| 8 | 43 | TRINITY\_DN649\_c0\_g1\_i2.p1 | 61.61 | 6 | 6 | 1.28e3 | 1 | 1 | 5 |  | 13819.7393 | type:internal len:125 gc:universal TRINITY\_DN649\_c0\_g1\_i2:374-3(-) |
| 8 | 44 | TRINITY\_DN649\_c0\_g1\_i1.p1 | 61.61 | 7 | 7 | 1.28e3 | 1 | 1 | 5 |  | 13690.5801 | type:internal len:124 gc:universal TRINITY\_DN649\_c0\_g1\_i1:371-3(-) |
| 9 | 45 | TRINITY\_DN428\_c0\_g1\_i1.p1 | 146.61 | 3 | 3 | 3.04e3 | 5 | 5 | 7 | Carbamidomethylation | 225824.1719 | type:complete len:1949 gc:universal TRINITY\_DN428\_c0\_g1\_i1:96-5942(+) |
| 10 | 46 | TRINITY\_DN11435\_c0\_g1\_i1.p1 | 142.58 | 12 | 12 | 1.25e3 | 5 | 5 | 5 |  | 47019.5312 | type:complete len:430 gc:universal TRINITY\_DN11435\_c0\_g1\_i1:1616-327(-) |
| 11 | 47 | TRINITY\_DN80458\_c0\_g1\_i1.p1 | 138.95 | 31 | 31 | 7.19e3 | 4 | 4 | 8 | Carbamidomethylation | 17768.7109 | type:5prime\_partial len:157 gc:universal TRINITY\_DN80458\_c0\_g1\_i1:519-49(-) |
| 12 | 48 | TRINITY\_DN2217\_c0\_g1\_i2.p1 | 130.92 | 17 | 17 | 2.43e3 | 4 | 3 | 4 | Carbamidomethylation | 28618.9531 | type:complete len:254 gc:universal TRINITY\_DN2217\_c0\_g1\_i2:884-123(-) |
| 12 | 49 | TRINITY\_DN2217\_c0\_g1\_i3.p1 | 130.92 | 8 | 8 | 2.43e3 | 4 | 3 | 4 | Carbamidomethylation | 63077.0508 | type:complete len:566 gc:universal TRINITY\_DN2217\_c0\_g1\_i3:1820-123(-) |
| 12 | 50 | TRINITY\_DN38397\_c0\_g1\_i1.p1 | 66.57 | 3 | 3 | 6.22e2 | 1 | 1 | 1 | Carbamidomethylation | 40487.2539 | type:internal len:364 gc:universal TRINITY\_DN38397\_c0\_g1\_i1:3-1091(+) |
| 13 | 51 | TRINITY\_DN3036\_c0\_g1\_i3.p1 | 122.06 | 8 | 8 | 2.52e4 | 3 | 3 | 14 | Oxidation (M) | 59630.4023 | type:internal len:548 gc:universal TRINITY\_DN3036\_c0\_g1\_i3:1643-3(-) |
| 13 | 52 | TRINITY\_DN3036\_c0\_g1\_i8.p1 | 122.06 | 8 | 8 | 2.52e4 | 3 | 3 | 14 | Oxidation (M) | 59630.4023 | type:internal len:548 gc:universal TRINITY\_DN3036\_c0\_g1\_i8:1643-3(-) |
| 13 | 53 | TRINITY\_DN3036\_c0\_g1\_i2.p2 | 122.06 | 8 | 8 | 2.52e4 | 3 | 3 | 14 | Oxidation (M) | 59586.3086 | type:internal len:548 gc:universal TRINITY\_DN3036\_c0\_g1\_i2:1643-3(-) |
| 13 | 54 | TRINITY\_DN3036\_c0\_g1\_i11.p1 | 99.82 | 8 | 8 | 2.2e4 | 2 | 2 | 12 | Oxidation (M) | 40331.5547 | type:internal len:374 gc:universal TRINITY\_DN3036\_c0\_g1\_i11:1121-3(-) |
| 13 | 55 | TRINITY\_DN3036\_c0\_g1\_i16.p1 | 99.82 | 8 | 8 | 2.2e4 | 2 | 2 | 12 | Oxidation (M) | 40331.5547 | type:internal len:374 gc:universal TRINITY\_DN3036\_c0\_g1\_i16:1121-3(-) |
| 13 | 56 | TRINITY\_DN3036\_c0\_g1\_i19.p1 | 99.82 | 8 | 8 | 2.2e4 | 2 | 2 | 12 | Oxidation (M) | 40287.4609 | type:internal len:374 gc:universal TRINITY\_DN3036\_c0\_g1\_i19:1121-3(-) |
| 13 | 57 | TRINITY\_DN3036\_c0\_g1\_i13.p1 | 99.82 | 8 | 8 | 2.2e4 | 2 | 2 | 12 | Oxidation (M) | 40287.4609 | type:internal len:374 gc:universal TRINITY\_DN3036\_c0\_g1\_i13:1121-3(-) |
| 13 | 58 | TRINITY\_DN3036\_c0\_g1\_i4.p1 | 66.34 | 7 | 7 | 9.54e2 | 1 | 1 | 3 | Oxidation (M) | 25489.3926 | type:internal len:238 gc:universal TRINITY\_DN3036\_c0\_g1\_i4:713-3(-) |
| 13 | 59 | TRINITY\_DN3036\_c0\_g1\_i6.p1 | 66.34 | 7 | 7 | 9.54e2 | 1 | 1 | 3 | Oxidation (M) | 25533.4863 | type:internal len:238 gc:universal TRINITY\_DN3036\_c0\_g1\_i6:713-3(-) |
| 14 | 60 | TRINITY\_DN3991\_c0\_g1\_i1.p1 | 131.82 | 17 | 17 | 3.36e4 | 4 | 4 | 5 | Carbamidomethylation | 24846.6543 | type:3prime\_partial len:219 gc:universal TRINITY\_DN3991\_c0\_g1\_i1:84-737(+) |
| 15 | 61 | TRINITY\_DN286\_c0\_g1\_i2.p1 | 66.75 | 10 | 10 | 1.58e4 | 1 | 1 | 7 | Oxidation (M), Carbamidomethylation | 11368.1738 | type:3prime\_partial len:106 gc:universal TRINITY\_DN286\_c0\_g1\_i2:40-354(+) |
| 15 | 62 | TRINITY\_DN286\_c0\_g1\_i4.p1 | 66.75 | 5 | 5 | 1.58e4 | 1 | 1 | 7 | Oxidation (M), Carbamidomethylation | 25084.5293 | type:complete len:224 gc:universal TRINITY\_DN286\_c0\_g1\_i4:40-711(+) |
| 16 | 63 | TRINITY\_DN1616\_c0\_g1\_i2.p1 | 119.47 | 1 | 1 | 3.81e2 | 3 | 3 | 5 |  | 374396 | type:3prime\_partial len:3398 gc:universal TRINITY\_DN1616\_c0\_g1\_i2:218-10408(+) |
| 16 | 64 | TRINITY\_DN1616\_c0\_g1\_i3.p1 | 119.47 | 1 | 1 | 3.81e2 | 3 | 3 | 5 |  | 423947.2812 | type:complete len:3855 gc:universal TRINITY\_DN1616\_c0\_g1\_i3:218-11782(+) |
| 16 | 65 | TRINITY\_DN1616\_c0\_g1\_i7.p1 | 119.47 | 1 | 1 | 3.81e2 | 3 | 3 | 5 |  | 423875.2188 | type:complete len:3855 gc:universal TRINITY\_DN1616\_c0\_g1\_i7:218-11782(+) |
| 16 | 66 | TRINITY\_DN1616\_c0\_g1\_i6.p1 | 119.47 | 1 | 1 | 3.81e2 | 3 | 3 | 5 |  | 423911.25 | type:complete len:3855 gc:universal TRINITY\_DN1616\_c0\_g1\_i6:218-11782(+) |
| 17 | 67 | TRINITY\_DN118\_c1\_g1\_i3.p1 | 121.1 | 11 | 11 | 6.98e3 | 3 | 3 | 5 | Carbamidomethylation | 22860.4883 | type:internal len:200 gc:universal TRINITY\_DN118\_c1\_g1\_i3:599-3(-) |
| 17 | 68 | TRINITY\_DN118\_c1\_g1\_i5.p1 | 98.04 | 8 | 8 | 6.98e3 | 2 | 2 | 3 | Carbamidomethylation | 23277.6309 | type:5prime\_partial len:203 gc:universal TRINITY\_DN118\_c1\_g1\_i5:620-12(-) |
| 18 | 69 | TRINITY\_DN15584\_c0\_g1\_i1.p1 | 112.72 | 13 | 13 | 3.4e4 | 3 | 3 | 6 | Carbamidomethylation | 29534.9648 | type:complete len:275 gc:universal TRINITY\_DN15584\_c0\_g1\_i1:874-50(-) |
| 19 | 70 | TRINITY\_DN3034\_c0\_g1\_i1.p1 | 103.19 | 2 | 2 | 6.54e2 | 3 | 3 | 5 | Oxidation (M) | 222266.9844 | type:complete len:1941 gc:universal TRINITY\_DN3034\_c0\_g1\_i1:6289-467(-) |
| 20 | 71 | TRINITY\_DN761\_c0\_g1\_i1.p1 | 110.36 | 6 | 6 | 9.86e2 | 3 | 3 | 4 |  | 95330.7812 | type:complete len:830 gc:universal TRINITY\_DN761\_c0\_g1\_i1:129-2618(+) |
| 20 | 72 | TRINITY\_DN761\_c0\_g1\_i2.p1 | 110.36 | 6 | 6 | 9.86e2 | 3 | 3 | 4 |  | 95330.7812 | type:complete len:830 gc:universal TRINITY\_DN761\_c0\_g1\_i2:87-2576(+) |
| 20 | 73 | TRINITY\_DN761\_c0\_g1\_i3.p1 | 110.36 | 5 | 5 | 9.86e2 | 3 | 3 | 4 |  | 96968.6562 | type:complete len:845 gc:universal TRINITY\_DN761\_c0\_g1\_i3:129-2663(+) |
| 21 | 74 | TRINITY\_DN77\_c1\_g1\_i1.p1 | 113.83 | 2 | 2 | 9.86e2 | 3 | 3 | 3 |  | 198350.2344 | type:3prime\_partial len:1965 gc:universal TRINITY\_DN77\_c1\_g1\_i1:2-5893(+) |
| 22 | 75 | TRINITY\_DN95248\_c0\_g1\_i1.p1 | 120.67 | 34 | 34 | 5e3 | 3 | 3 | 4 |  | 14722.6113 | type:complete len:124 gc:universal TRINITY\_DN95248\_c0\_g1\_i1:197-568(+) |
| 23 | 76 | TRINITY\_DN240\_c0\_g1\_i4.p1 | 118.82 | 16 | 16 | 1.13e4 | 3 | 3 | 4 |  | 40419.2031 | type:5prime\_partial len:397 gc:universal TRINITY\_DN240\_c0\_g1\_i4:1327-137(-) |
| 23 | 77 | TRINITY\_DN240\_c0\_g1\_i6.p1 | 118.82 | 16 | 16 | 1.13e4 | 3 | 3 | 4 |  | 40418.2188 | type:5prime\_partial len:397 gc:universal TRINITY\_DN240\_c0\_g1\_i6:1494-304(-) |
| 23 | 78 | TRINITY\_DN240\_c0\_g1\_i1.p1 | 118.82 | 16 | 16 | 1.13e4 | 3 | 3 | 4 |  | 40418.2188 | type:5prime\_partial len:397 gc:universal TRINITY\_DN240\_c0\_g1\_i1:1518-328(-) |
| 24 | 79 | TRINITY\_DN1112\_c0\_g1\_i1.p1 | 114.53 | 12 | 12 | 2.1e3 | 3 | 3 | 5 | Oxidation (M) | 23407.2832 | type:complete len:200 gc:universal TRINITY\_DN1112\_c0\_g1\_i1:740-141(-) |
| 25 | 80 | TRINITY\_DN116\_c0\_g1\_i1.p1 | 120.43 | 14 | 14 | 2.89e2 | 3 | 3 | 4 | Carbamidomethylation | 34095.6328 | type:complete len:297 gc:universal TRINITY\_DN116\_c0\_g1\_i1:72-962(+) |
| 26 | 81 | TRINITY\_DN6920\_c0\_g1\_i3.p1 | 80.06 | 7 | 7 | 2.66e3 | 3 | 3 | 3 | Oxidation (M), Carbamidomethylation | 46932.582 | type:5prime\_partial len:411 gc:universal TRINITY\_DN6920\_c0\_g1\_i3:2124-892(-) |
| 26 | 82 | TRINITY\_DN6920\_c0\_g1\_i2.p1 | 80.06 | 7 | 7 | 2.66e3 | 3 | 3 | 3 | Oxidation (M), Carbamidomethylation | 46932.582 | type:5prime\_partial len:411 gc:universal TRINITY\_DN6920\_c0\_g1\_i2:2147-915(-) |
| 26 | 83 | TRINITY\_DN6920\_c0\_g1\_i4.p1 | 60.86 | 4 | 4 | 9.16e2 | 1 | 1 | 1 | Carbamidomethylation | 32195.3809 | type:5prime\_partial len:282 gc:universal TRINITY\_DN6920\_c0\_g1\_i4:1321-476(-) |
| 26 | 84 | TRINITY\_DN6920\_c0\_g1\_i1.p1 | 60.86 | 4 | 4 | 9.16e2 | 1 | 1 | 1 | Carbamidomethylation | 32882.168 | type:complete len:287 gc:universal TRINITY\_DN6920\_c0\_g1\_i1:1775-915(-) |
| 26 | 85 | TRINITY\_DN2799\_c0\_g1\_i3.p1 | 35.08 | 15 | 15 | 1.74e3 | 2 | 2 | 2 | Oxidation (M) | 12365.4863 | type:internal len:112 gc:universal TRINITY\_DN2799\_c0\_g1\_i3:334-2(-) |
| 26 | 86 | TRINITY\_DN5906\_c3\_g1\_i1.p2 | 35.08 | 13 | 13 | 1.74e3 | 2 | 2 | 2 | Oxidation (M) | 15031.4814 | type:internal len:137 gc:universal TRINITY\_DN5906\_c3\_g1\_i1:3-410(+) |
| 26 | 87 | TRINITY\_DN2799\_c0\_g1\_i2.p1 | 35.08 | 15 | 15 | 1.74e3 | 2 | 2 | 2 | Oxidation (M) | 12149.2383 | type:internal len:111 gc:universal TRINITY\_DN2799\_c0\_g1\_i2:331-2(-) |
| 26 | 88 | TRINITY\_DN2799\_c0\_g1\_i4.p2 | 35.08 | 15 | 15 | 1.74e3 | 2 | 2 | 2 | Oxidation (M) | 12149.2383 | type:internal len:111 gc:universal TRINITY\_DN2799\_c0\_g1\_i4:331-2(-) |
| 26 | 89 | TRINITY\_DN2799\_c0\_g1\_i1.p1 | 35.08 | 15 | 15 | 1.74e3 | 2 | 2 | 2 | Oxidation (M) | 12875.0586 | type:internal len:117 gc:universal TRINITY\_DN2799\_c0\_g1\_i1:349-2(-) |
| 26 | 90 | TRINITY\_DN32727\_c0\_g1\_i2.p1 | 19.81 | 5 | 5 | 6.74e2 | 1 | 1 | 1 | Oxidation (M) | 17342.1152 | type:internal len:154 gc:universal TRINITY\_DN32727\_c0\_g1\_i2:460-2(-) |
| 27 | 91 | TRINITY\_DN262\_c0\_g1\_i1.p1 | 120.68 | 4 | 4 | 1.69e3 | 3 | 3 | 3 |  | 114520.6406 | type:complete len:1033 gc:universal TRINITY\_DN262\_c0\_g1\_i1:111-3209(+) |
| 28 | 92 | TRINITY\_DN477\_c0\_g1\_i7.p1 | 110.34 | 4 | 4 | 4.24e3 | 3 | 3 | 11 | Carbamidomethylation | 122478.2578 | type:3prime\_partial len:1130 gc:universal TRINITY\_DN477\_c0\_g1\_i7:3387-1(-) |
| 28 | 93 | TRINITY\_DN477\_c0\_g1\_i8.p1 | 110.34 | 4 | 4 | 4.24e3 | 3 | 3 | 11 | Carbamidomethylation | 107165.2031 | type:complete len:985 gc:universal TRINITY\_DN477\_c0\_g1\_i8:3548-594(-) |
| 29 | 94 | TRINITY\_DN1386\_c0\_g1\_i1.p1 | 119.55 | 6 | 6 | 7.68e3 | 3 | 3 | 4 |  | 68692.1953 | type:complete len:596 gc:universal TRINITY\_DN1386\_c0\_g1\_i1:2419-632(-) |
| 30 | 95 | TRINITY\_DN250\_c0\_g1\_i5.p1 | 114.16 | 12 | 12 | 1.27e3 | 3 | 3 | 5 | Oxidation (M), Carbamidomethylation | 50305.4453 | type:complete len:452 gc:universal TRINITY\_DN250\_c0\_g1\_i5:131-1486(+) |
| 30 | 96 | TRINITY\_DN687\_c0\_g2\_i1.p1 | 99.2 | 8 | 8 | 6.54e2 | 2 | 2 | 4 | Carbamidomethylation, Oxidation (M) | 50237.3242 | type:complete len:453 gc:universal TRINITY\_DN687\_c0\_g2\_i1:90-1448(+) |
| 30 | 97 | TRINITY\_DN687\_c0\_g1\_i1.p1 | 99.2 | 8 | 8 | 6.54e2 | 2 | 2 | 4 | Carbamidomethylation, Oxidation (M) | 50621.6914 | type:complete len:453 gc:universal TRINITY\_DN687\_c0\_g1\_i1:95-1453(+) |
| 30 | 98 | TRINITY\_DN250\_c0\_g1\_i3.p1 | 99.2 | 9 | 9 | 6.54e2 | 2 | 2 | 4 | Carbamidomethylation, Oxidation (M) | 44456.6445 | type:3prime\_partial len:402 gc:universal TRINITY\_DN250\_c0\_g1\_i3:131-1333(+) |
| 30 | 99 | TRINITY\_DN250\_c0\_g1\_i1.p1 | 99.2 | 9 | 9 | 6.54e2 | 2 | 2 | 4 | Carbamidomethylation, Oxidation (M) | 44454.6094 | type:3prime\_partial len:402 gc:universal TRINITY\_DN250\_c0\_g1\_i1:131-1333(+) |
| 30 | 100 | TRINITY\_DN250\_c0\_g1\_i6.p1 | 87.31 | 8 | 8 | 6.2e2 | 2 | 2 | 2 | Oxidation (M) | 50263.3672 | type:complete len:452 gc:universal TRINITY\_DN250\_c0\_g1\_i6:131-1486(+) |
| 30 | 101 | TRINITY\_DN96161\_c0\_g1\_i1.p1 | 87.31 | 8 | 8 | 6.2e2 | 2 | 2 | 2 | Oxidation (M) | 50498.6562 | type:complete len:453 gc:universal TRINITY\_DN96161\_c0\_g1\_i1:1593-235(-) |
| 30 | 102 | TRINITY\_DN250\_c0\_g1\_i4.p1 | 87.31 | 8 | 8 | 6.2e2 | 2 | 2 | 2 | Oxidation (M) | 50305.4453 | type:complete len:452 gc:universal TRINITY\_DN250\_c0\_g1\_i4:131-1486(+) |
| 30 | 103 | TRINITY\_DN471\_c0\_g1\_i3.p1 | 44.9 | 6 | 6 | 6.2e2 | 1 | 1 | 1 | Oxidation (M) | 36097.9961 | type:5prime\_partial len:324 gc:universal TRINITY\_DN471\_c0\_g1\_i3:1-972(+) |
| 30 | 104 | TRINITY\_DN471\_c0\_g1\_i2.p1 | 44.9 | 4 | 4 | 6.2e2 | 1 | 1 | 1 | Oxidation (M) | 54220.6289 | type:5prime\_partial len:492 gc:universal TRINITY\_DN471\_c0\_g1\_i2:3-1478(+) |
| 30 | 105 | TRINITY\_DN471\_c0\_g1\_i11.p1 | 44.9 | 4 | 4 | 6.2e2 | 1 | 1 | 1 | Oxidation (M) | 54220.6289 | type:5prime\_partial len:492 gc:universal TRINITY\_DN471\_c0\_g1\_i11:3-1478(+) |
| 30 | 106 | TRINITY\_DN471\_c0\_g1\_i10.p1 | 44.9 | 4 | 4 | 6.2e2 | 1 | 1 | 1 | Oxidation (M) | 54220.6289 | type:5prime\_partial len:492 gc:universal TRINITY\_DN471\_c0\_g1\_i10:3-1478(+) |
| 30 | 107 | TRINITY\_DN471\_c0\_g1\_i4.p1 | 44.9 | 4 | 4 | 6.2e2 | 1 | 1 | 1 | Oxidation (M) | 54220.6289 | type:5prime\_partial len:492 gc:universal TRINITY\_DN471\_c0\_g1\_i4:3-1478(+) |
| 31 | 108 | TRINITY\_DN5633\_c0\_g1\_i1.p1 | 113.2 | 4 | 4 | 0 | 3 | 2 | 3 |  | 97310.9922 | type:complete len:848 gc:universal TRINITY\_DN5633\_c0\_g1\_i1:2881-338(-) |
| 32 | 109 | TRINITY\_DN1613\_c0\_g2\_i1.p1 | 96.95 | 3 | 3 | 2.21e3 | 3 | 3 | 4 |  | 112924.5938 | type:3prime\_partial len:1062 gc:universal TRINITY\_DN1613\_c0\_g2\_i1:70-3252(+) |
| 33 | 110 | TRINITY\_DN3190\_c1\_g1\_i1.p1 | 119.37 | 13 | 13 | 0 | 3 | 3 | 3 | Acetylation (Protein N-term) | 35061.5508 | type:complete len:315 gc:universal TRINITY\_DN3190\_c1\_g1\_i1:68-1012(+) |
| 34 | 111 | TRINITY\_DN1369\_c0\_g1\_i1.p1 | 80.37 | 2 | 2 | 5.8e2 | 2 | 2 | 2 | Carbamidomethylation | 174229.4219 | type:3prime\_partial len:1577 gc:universal TRINITY\_DN1369\_c0\_g1\_i1:93-4820(+) |
| 35 | 112 | TRINITY\_DN3545\_c0\_g1\_i2.p1 | 69.77 | 10 | 10 | 2.1e3 | 2 | 2 | 3 | Carbamidomethylation | 25563.4785 | type:5prime\_partial len:236 gc:universal TRINITY\_DN3545\_c0\_g1\_i2:3-710(+) |
| 35 | 113 | TRINITY\_DN3545\_c0\_g1\_i1.p1 | 69.77 | 8 | 8 | 2.1e3 | 2 | 2 | 3 | Carbamidomethylation | 31576.2656 | type:5prime\_partial len:293 gc:universal TRINITY\_DN3545\_c0\_g1\_i1:3-881(+) |
| 35 | 114 | TRINITY\_DN158421\_c0\_g1\_i1.p1 | 49.81 | 10 | 10 | 2.1e3 | 1 | 1 | 2 | Carbamidomethylation | 12814.7959 | type:internal len:115 gc:universal TRINITY\_DN158421\_c0\_g1\_i1:1-342(+) |
| 35 | 115 | TRINITY\_DN495\_c0\_g1\_i2.p1 | 39.93 | 2 | 2 | 0 | 1 | 1 | 1 | Carbamidomethylation | 72975.0312 | type:complete len:655 gc:universal TRINITY\_DN495\_c0\_g1\_i2:152-2116(+) |
| 35 | 116 | TRINITY\_DN495\_c0\_g1\_i3.p1 | 39.93 | 2 | 2 | 0 | 1 | 1 | 1 | Carbamidomethylation | 73673.9297 | type:complete len:659 gc:universal TRINITY\_DN495\_c0\_g1\_i3:152-2128(+) |
| 36 | 117 | TRINITY\_DN332\_c0\_g1\_i1.p1 | 99.89 | 11 | 11 | 9.9e2 | 2 | 2 | 2 |  | 27839.6836 | type:complete len:246 gc:universal TRINITY\_DN332\_c0\_g1\_i1:940-203(-) |
| 37 | 118 | TRINITY\_DN577\_c0\_g1\_i1.p1 | 93.53 | 7 | 7 | 0 | 2 | 2 | 2 |  | 30281.0371 | type:5prime\_partial len:269 gc:universal TRINITY\_DN577\_c0\_g1\_i1:1086-280(-) |
| 38 | 119 | TRINITY\_DN673\_c0\_g1\_i1.p1 | 62.55 | 18 | 18 | 3e2 | 1 | 1 | 2 | Oxidation (M) | 10504.5596 | type:internal len:100 gc:universal TRINITY\_DN673\_c0\_g1\_i1:1-297(+) |
| 39 | 120 | TRINITY\_DN194146\_c0\_g1\_i1.p1 | 98.5 | 17 | 17 | 1.21e3 | 2 | 2 | 3 |  | 21439.9336 | type:complete len:190 gc:universal TRINITY\_DN194146\_c0\_g1\_i1:165-734(+) |
| 40 | 121 | TRINITY\_DN2885\_c0\_g1\_i2.p1 | 87.37 | 12 | 12 | 2.92e4 | 2 | 2 | 6 |  | 12758.7783 | type:complete len:114 gc:universal TRINITY\_DN2885\_c0\_g1\_i2:31-372(+) |
| 40 | 122 | TRINITY\_DN2885\_c0\_g1\_i5.p1 | 87.37 | 13 | 13 | 2.92e4 | 2 | 2 | 6 |  | 12383.5439 | type:3prime\_partial len:112 gc:universal TRINITY\_DN2885\_c0\_g1\_i5:31-363(+) |
| 40 | 123 | TRINITY\_DN2885\_c0\_g1\_i4.p1 | 87.37 | 13 | 13 | 2.92e4 | 2 | 2 | 6 |  | 12245.125 | type:5prime\_partial len:109 gc:universal TRINITY\_DN2885\_c0\_g1\_i4:1-327(+) |
| 41 | 124 | TRINITY\_DN2126\_c0\_g1\_i1.p1 | 99.8 | 13 | 13 | 1.53e3 | 2 | 2 | 3 |  | 12631.3887 | type:complete len:114 gc:universal TRINITY\_DN2126\_c0\_g1\_i1:55-396(+) |
| 42 | 125 | TRINITY\_DN7504\_c0\_g1\_i1.p1 | 89.93 | 6 | 6 | 1.54e3 | 2 | 2 | 2 |  | 46519.1953 | type:complete len:414 gc:universal TRINITY\_DN7504\_c0\_g1\_i1:87-1328(+) |
| 43 | 126 | TRINITY\_DN141338\_c2\_g1\_i1.p1 | 92.11 | 3 | 3 | 6.52e2 | 2 | 1 | 2 |  | 82716.2266 | type:complete len:720 gc:universal TRINITY\_DN141338\_c2\_g1\_i1:2850-691(-) |
| 44 | 127 | TRINITY\_DN3078\_c0\_g1\_i1.p1 | 100.05 | 2 | 2 | 0 | 2 | 2 | 2 | Carbamidomethylation | 127689.1797 | type:complete len:1109 gc:universal TRINITY\_DN3078\_c0\_g1\_i1:3628-302(-) |
| 45 | 128 | TRINITY\_DN1989\_c0\_g1\_i1.p1 | 99.92 | 5 | 5 | 2.65e3 | 2 | 2 | 2 | Carbamidomethylation | 62731.1836 | type:complete len:546 gc:universal TRINITY\_DN1989\_c0\_g1\_i1:150-1787(+) |
| 46 | 129 | TRINITY\_DN6388\_c0\_g1\_i2.p2 | 99.15 | 17 | 17 | 1.67e3 | 2 | 2 | 2 |  | 17280.2852 | type:5prime\_partial len:143 gc:universal TRINITY\_DN6388\_c0\_g1\_i2:1-429(+) |
| 46 | 130 | TRINITY\_DN6388\_c0\_g1\_i1.p2 | 99.15 | 16 | 16 | 1.67e3 | 2 | 2 | 2 |  | 17821.9297 | type:5prime\_partial len:148 gc:universal TRINITY\_DN6388\_c0\_g1\_i1:1-444(+) |
| 46 | 131 | TRINITY\_DN4954\_c0\_g1\_i2.p2 | 99.15 | 12 | 12 | 1.67e3 | 2 | 2 | 2 |  | 23731.8477 | type:5prime\_partial len:200 gc:universal TRINITY\_DN4954\_c0\_g1\_i2:1-600(+) |
| 47 | 132 | TRINITY\_DN3339\_c0\_g1\_i1.p1 | 98.24 | 25 | 25 | 9.53e2 | 2 | 2 | 4 | Carbamidomethylation | 14934.2627 | type:complete len:131 gc:universal TRINITY\_DN3339\_c0\_g1\_i1:31-423(+) |
| 48 | 133 | TRINITY\_DN15847\_c0\_g1\_i1.p1 | 93.88 | 15 | 15 | 1.2e3 | 2 | 2 | 4 | Carbamidomethylation | 19271.252 | type:5prime\_partial len:166 gc:universal TRINITY\_DN15847\_c0\_g1\_i1:3-500(+) |
| 48 | 134 | TRINITY\_DN15847\_c0\_g1\_i2.p1 | 93.88 | 15 | 15 | 1.2e3 | 2 | 2 | 4 | Carbamidomethylation | 19271.252 | type:5prime\_partial len:166 gc:universal TRINITY\_DN15847\_c0\_g1\_i2:3-500(+) |
| 49 | 135 | TRINITY\_DN7659\_c0\_g1\_i2.p1 | 81.03 | 7 | 7 | 0 | 2 | 2 | 3 | Acetylation (Protein N-term) | 40699.8555 | type:complete len:359 gc:universal TRINITY\_DN7659\_c0\_g1\_i2:82-1158(+) |
| 49 | 136 | TRINITY\_DN7659\_c0\_g1\_i1.p1 | 81.03 | 7 | 7 | 0 | 2 | 2 | 3 | Acetylation (Protein N-term) | 40699.8555 | type:complete len:359 gc:universal TRINITY\_DN7659\_c0\_g1\_i1:69-1145(+) |
| 50 | 137 | TRINITY\_DN172571\_c0\_g1\_i1.p1 | 97.86 | 25 | 25 | 0 | 2 | 2 | 3 |  | 11500.2529 | type:complete len:104 gc:universal TRINITY\_DN172571\_c0\_g1\_i1:30-341(+) |
| 50 | 138 | TRINITY\_DN22077\_c0\_g1\_i1.p3 | 62.35 | 9 | 9 | 0 | 1 | 1 | 1 |  | 16229.6445 | type:5prime\_partial len:151 gc:universal TRINITY\_DN22077\_c0\_g1\_i1:520-68(-) |
| 51 | 139 | TRINITY\_DN6390\_c0\_g1\_i1.p1 | 73.08 | 1 | 1 | 4.4e2 | 2 | 2 | 3 | Carbamidomethylation | 214480.2344 | type:3prime\_partial len:1941 gc:universal TRINITY\_DN6390\_c0\_g1\_i1:5820-1(-) |
| 52 | 140 | TRINITY\_DN162\_c0\_g1\_i1.p1 | 98.5 | 14 | 14 | 5.36e3 | 2 | 2 | 4 | Carbamidomethylation | 23228.1055 | type:5prime\_partial len:206 gc:universal TRINITY\_DN162\_c0\_g1\_i1:654-37(-) |
| 53 | 141 | TRINITY\_DN22492\_c0\_g1\_i1.p1 | 83.2 | 2 | 2 | 4.14e3 | 2 | 2 | 5 | Carbamidomethylation | 115296.5156 | type:complete len:1026 gc:universal TRINITY\_DN22492\_c0\_g1\_i1:171-3248(+) |
| 53 | 142 | TRINITY\_DN22492\_c0\_g1\_i2.p1 | 83.2 | 2 | 2 | 4.14e3 | 2 | 2 | 5 | Carbamidomethylation | 115355.5859 | type:complete len:1026 gc:universal TRINITY\_DN22492\_c0\_g1\_i2:39-3116(+) |
| 54 | 143 | TRINITY\_DN2113\_c0\_g1\_i1.p1 | 90.67 | 2 | 2 | 8.12e2 | 2 | 2 | 2 |  | 193799.0156 | type:complete len:1704 gc:universal TRINITY\_DN2113\_c0\_g1\_i1:5257-146(-) |
| 54 | 144 | TRINITY\_DN2113\_c0\_g1\_i2.p1 | 90.67 | 2 | 2 | 8.12e2 | 2 | 2 | 2 |  | 193799.0156 | type:complete len:1704 gc:universal TRINITY\_DN2113\_c0\_g1\_i2:5257-146(-) |
| 55 | 145 | TRINITY\_DN909\_c0\_g1\_i1.p1 | 84.08 | 8 | 8 | 6.75e2 | 2 | 2 | 2 | Oxidation (M) | 35211.4883 | type:5prime\_partial len:313 gc:universal TRINITY\_DN909\_c0\_g1\_i1:1-939(+) |
| 55 | 146 | TRINITY\_DN41662\_c0\_g1\_i2.p1 | 34.86 | 5 | 5 | 4.27e2 | 1 | 1 | 1 |  | 32617.3926 | type:complete len:289 gc:universal TRINITY\_DN41662\_c0\_g1\_i2:168-1034(+) |
| 55 | 147 | TRINITY\_DN5818\_c1\_g1\_i1.p1 | 34.86 | 5 | 5 | 4.27e2 | 1 | 1 | 1 |  | 31405.1387 | type:complete len:282 gc:universal TRINITY\_DN5818\_c1\_g1\_i1:49-894(+) |
| 55 | 148 | TRINITY\_DN5818\_c1\_g1\_i2.p1 | 34.86 | 5 | 5 | 4.27e2 | 1 | 1 | 1 |  | 32704.6465 | type:complete len:293 gc:universal TRINITY\_DN5818\_c1\_g1\_i2:178-1056(+) |
| 56 | 149 | TRINITY\_DN2963\_c0\_g1\_i1.p1 | 98.65 | 21 | 21 | 3.07e3 | 2 | 2 | 3 | Acetylation (Protein N-term) | 13082.1787 | type:complete len:116 gc:universal TRINITY\_DN2963\_c0\_g1\_i1:1072-725(-) |
| 57 | 150 | TRINITY\_DN464\_c0\_g1\_i4.p1 | 97.79 | 4 | 4 | 4.9e2 | 2 | 2 | 2 |  | 82057.25 | type:complete len:717 gc:universal TRINITY\_DN464\_c0\_g1\_i4:2167-17(-) |
| 58 | 151 | TRINITY\_DN3202\_c2\_g1\_i1.p1 | 66.7 | 4 | 4 | 6.57e2 | 1 | 1 | 2 | Acetylation (Protein N-term), Carbamidomethylation | 30131.0488 | type:5prime\_partial len:263 gc:universal TRINITY\_DN3202\_c2\_g1\_i1:949-161(-) |
| 59 | 152 | TRINITY\_DN2230\_c0\_g1\_i1.p1 | 97.04 | 11 | 11 | 1.84e3 | 2 | 2 | 2 |  | 28784.541 | type:complete len:249 gc:universal TRINITY\_DN2230\_c0\_g1\_i1:796-50(-) |
| 60 | 153 | TRINITY\_DN197343\_c0\_g1\_i1.p1 | 85.95 | 26 | 26 | 5.13e2 | 2 | 2 | 2 | Carbamidomethylation | 14546.0635 | type:complete len:124 gc:universal TRINITY\_DN197343\_c0\_g1\_i1:399-28(-) |
| 61 | 154 | TRINITY\_DN1505\_c0\_g1\_i1.p1 | 99.16 | 16 | 16 | 3.26e4 | 2 | 2 | 10 | Acetylation (Protein N-term) | 17930.752 | type:complete len:154 gc:universal TRINITY\_DN1505\_c0\_g1\_i1:98-559(+) |
| 62 | 155 | TRINITY\_DN4329\_c0\_g1\_i1.p1 | 63.36 | 2 | 2 | 1.21e3 | 2 | 2 | 2 |  | 84233.3359 | type:5prime\_partial len:735 gc:universal TRINITY\_DN4329\_c0\_g1\_i1:2240-36(-) |
| 63 | 156 | TRINITY\_DN1194\_c0\_g1\_i1.p1 | 98.29 | 5 | 5 | 0 | 2 | 2 | 2 |  | 61081.3672 | type:complete len:555 gc:universal TRINITY\_DN1194\_c0\_g1\_i1:101-1765(+) |
| 64 | 157 | TRINITY\_DN1377\_c0\_g1\_i1.p1 | 98.91 | 3 | 3 | 1.79e3 | 2 | 2 | 3 |  | 99936.9219 | type:complete len:878 gc:universal TRINITY\_DN1377\_c0\_g1\_i1:68-2701(+) |
| 65 | 158 | TRINITY\_DN2637\_c0\_g1\_i9.p2 | 72.13 | 10 | 10 | 0 | 2 | 2 | 2 |  | 36257.3242 | type:5prime\_partial len:340 gc:universal TRINITY\_DN2637\_c0\_g1\_i9:3-1022(+) |
| 65 | 159 | TRINITY\_DN2637\_c0\_g1\_i6.p1 | 72.13 | 11 | 11 | 0 | 2 | 2 | 2 |  | 34214.9922 | type:5prime\_partial len:320 gc:universal TRINITY\_DN2637\_c0\_g1\_i6:1-960(+) |
| 65 | 160 | TRINITY\_DN2637\_c0\_g1\_i7.p2 | 61.89 | 5 | 5 | 0 | 1 | 1 | 1 |  | 24919.5234 | type:internal len:233 gc:universal TRINITY\_DN2637\_c0\_g1\_i7:3-698(+) |
| 65 | 161 | TRINITY\_DN2637\_c0\_g1\_i4.p1 | 61.89 | 4 | 4 | 0 | 1 | 1 | 1 |  | 29205.3301 | type:internal len:277 gc:universal TRINITY\_DN2637\_c0\_g1\_i4:1-828(+) |
| 65 | 162 | TRINITY\_DN2637\_c0\_g1\_i8.p2 | 61.89 | 4 | 4 | 0 | 1 | 1 | 1 |  | 31853.3555 | type:internal len:302 gc:universal TRINITY\_DN2637\_c0\_g1\_i8:3-905(+) |
| 65 | 163 | TRINITY\_DN2637\_c0\_g1\_i2.p2 | 61.89 | 4 | 4 | 0 | 1 | 1 | 1 |  | 31274.6855 | type:internal len:297 gc:universal TRINITY\_DN2637\_c0\_g1\_i2:3-890(+) |
| 65 | 164 | TRINITY\_DN2637\_c0\_g1\_i14.p2 | 61.89 | 3 | 3 | 0 | 1 | 1 | 1 |  | 34349.1445 | type:5prime\_partial len:320 gc:universal TRINITY\_DN2637\_c0\_g1\_i14:1-960(+) |
| 65 | 165 | TRINITY\_DN2637\_c0\_g1\_i5.p2 | 61.89 | 4 | 4 | 0 | 1 | 1 | 1 |  | 28455.5449 | type:internal len:270 gc:universal TRINITY\_DN2637\_c0\_g1\_i5:3-809(+) |
| 65 | 166 | TRINITY\_DN2637\_c0\_g1\_i13.p1 | 61.89 | 4 | 4 | 0 | 1 | 1 | 1 |  | 31247.6602 | type:internal len:297 gc:universal TRINITY\_DN2637\_c0\_g1\_i13:3-890(+) |
| 66 | 167 | TRINITY\_DN72959\_c0\_g1\_i1.p1 | 93.22 | 12 | 12 | 1.25e3 | 2 | 2 | 3 |  | 25064.7695 | type:complete len:216 gc:universal TRINITY\_DN72959\_c0\_g1\_i1:54-701(+) |
| 67 | 168 | TRINITY\_DN535\_c0\_g1\_i1.p1 | 97.51 | 23 | 23 | 7.91e3 | 2 | 2 | 3 | Carbamidomethylation | 18221.5957 | type:5prime\_partial len:162 gc:universal TRINITY\_DN535\_c0\_g1\_i1:712-227(-) |
| 67 | 169 | TRINITY\_DN535\_c0\_g1\_i2.p1 | 97.51 | 23 | 23 | 7.91e3 | 2 | 2 | 3 | Carbamidomethylation | 18221.5957 | type:5prime\_partial len:162 gc:universal TRINITY\_DN535\_c0\_g1\_i2:699-214(-) |
| 68 | 170 | TRINITY\_DN167384\_c0\_g1\_i1.p1 | 61.91 | 9 | 9 | 1.74e2 | 2 | 2 | 2 | Carbamidomethylation | 35025.4102 | type:complete len:316 gc:universal TRINITY\_DN167384\_c0\_g1\_i1:1004-57(-) |
| 69 | 171 | TRINITY\_DN72625\_c0\_g1\_i1.p1 | 81.91 | 1 | 1 | 1.86e3 | 2 | 2 | 2 |  | 214200.3906 | type:complete len:1964 gc:universal TRINITY\_DN72625\_c0\_g1\_i1:173-6064(+) |
| 70 | 172 | TRINITY\_DN1559\_c0\_g1\_i1.p1 | 73.98 | 9 | 9 | 4.66e3 | 2 | 2 | 2 | Carbamidomethylation | 33672.0977 | type:complete len:311 gc:universal TRINITY\_DN1559\_c0\_g1\_i1:221-1153(+) |
| 71 | 173 | TRINITY\_DN64\_c0\_g1\_i1.p1 | 98.76 | 7 | 7 | 1.41e3 | 2 | 2 | 4 |  | 51450.1914 | type:complete len:468 gc:universal TRINITY\_DN64\_c0\_g1\_i1:1555-152(-) |
| 72 | 174 | TRINITY\_DN119684\_c0\_g1\_i1.p1 | 54.07 | 3 | 3 | 1.12e4 | 2 | 2 | 2 |  | 72994.7266 | type:complete len:662 gc:universal TRINITY\_DN119684\_c0\_g1\_i1:2182-197(-) |
| 73 | 175 | TRINITY\_DN15274\_c0\_g1\_i1.p1 | 86.45 | 1 | 1 | 3.17e2 | 2 | 2 | 2 | Carbamidomethylation | 201897.7344 | type:complete len:1822 gc:universal TRINITY\_DN15274\_c0\_g1\_i1:87-5552(+) |
| 74 | 176 | TRINITY\_DN118520\_c0\_g1\_i1.p1 | 93.64 | 6 | 6 | 5.02e2 | 2 | 2 | 3 |  | 61587.4766 | type:complete len:581 gc:universal TRINITY\_DN118520\_c0\_g1\_i1:1895-153(-) |
| 75 | 177 | TRINITY\_DN899\_c0\_g1\_i1.p1 | 40.53 | 3 | 3 | 0 | 2 | 2 | 2 | Carbamidomethylation, Oxidation (M) | 41364.9766 | type:complete len:375 gc:universal TRINITY\_DN899\_c0\_g1\_i1:70-1194(+) |
| 76 | 178 | TRINITY\_DN1614\_c0\_g1\_i1.p1 | 99.84 | 12 | 12 | 4.66e3 | 2 | 2 | 3 |  | 25367.3418 | type:complete len:218 gc:universal TRINITY\_DN1614\_c0\_g1\_i1:56-709(+) |
| 77 | 179 | TRINITY\_DN11097\_c0\_g1\_i1.p1 | 79.56 | 4 | 4 | 1.29e3 | 2 | 2 | 3 |  | 80111.1172 | type:complete len:730 gc:universal TRINITY\_DN11097\_c0\_g1\_i1:2348-159(-) |
| 77 | 180 | TRINITY\_DN11097\_c0\_g2\_i1.p1 | 79.56 | 12 | 12 | 1.29e3 | 2 | 2 | 3 |  | 24192.5488 | type:internal len:222 gc:universal TRINITY\_DN11097\_c0\_g2\_i1:665-3(-) |
| 78 | 181 | TRINITY\_DN73067\_c0\_g1\_i1.p1 | 66.7 | 2 | 2 | 4.9e3 | 1 | 1 | 5 | Oxidation (M) | 93020.375 | type:complete len:831 gc:universal TRINITY\_DN73067\_c0\_g1\_i1:3714-1222(-) |
| 79 | 182 | TRINITY\_DN477\_c0\_g1\_i9.p1 | 99.64 | 8 | 8 | 2.37e3 | 2 | 2 | 3 | Carbamidomethylation | 34222.9453 | type:complete len:306 gc:universal TRINITY\_DN477\_c0\_g1\_i9:1840-923(-) |
| 80 | 183 | TRINITY\_DN168534\_c0\_g1\_i1.p1 | 66.8 | 5 | 5 | 1.32e3 | 1 | 1 | 2 | Oxidation (M), Carbamidomethylation | 25265.0449 | type:complete len:226 gc:universal TRINITY\_DN168534\_c0\_g1\_i1:774-97(-) |
| 81 | 184 | TRINITY\_DN15599\_c0\_g1\_i1.p1 | 63.27 | 5 | 5 | 1.28e4 | 1 | 1 | 3 | Acetylation (Protein N-term) | 21907.2578 | type:internal len:206 gc:universal TRINITY\_DN15599\_c0\_g1\_i1:1-615(+) |
| 81 | 185 | TRINITY\_DN15599\_c0\_g1\_i2.p1 | 41.93 | 5 | 5 | 9.13e3 | 1 | 1 | 1 |  | 21967.3105 | type:internal len:206 gc:universal TRINITY\_DN15599\_c0\_g1\_i2:1-615(+) |
| 82 | 186 | TRINITY\_DN58867\_c0\_g1\_i2.p1 | 82.15 | 8 | 8 | 5.6e2 | 2 | 2 | 2 | Oxidation (M) | 40822.2812 | type:complete len:354 gc:universal TRINITY\_DN58867\_c0\_g1\_i2:80-1141(+) |
| 83 | 187 | TRINITY\_DN1172\_c0\_g2\_i2.p1 | 99.17 | 8 | 8 | 1.24e4 | 2 | 2 | 7 | Acetylation (Protein N-term) | 27813.4355 | type:internal len:264 gc:universal TRINITY\_DN1172\_c0\_g2\_i2:790-2(-) |
| 83 | 188 | TRINITY\_DN21847\_c0\_g1\_i1.p1 | 66.69 | 3 | 3 | 7.52e3 | 1 | 1 | 3 |  | 41400.2812 | type:internal len:384 gc:universal TRINITY\_DN21847\_c0\_g1\_i1:1150-2(-) |
| 83 | 189 | TRINITY\_DN4497\_c0\_g1\_i1.p1 | 66.69 | 8 | 8 | 7.52e3 | 1 | 1 | 3 |  | 17016.3789 | type:internal len:160 gc:universal TRINITY\_DN4497\_c0\_g1\_i1:1-477(+) |
| 83 | 190 | TRINITY\_DN4497\_c0\_g2\_i1.p1 | 66.69 | 7 | 7 | 7.52e3 | 1 | 1 | 3 |  | 19644.3613 | type:internal len:183 gc:universal TRINITY\_DN4497\_c0\_g2\_i1:2-547(+) |
| 84 | 191 | TRINITY\_DN478\_c0\_g1\_i2.p1 | 71.28 | 20 | 20 | 1.57e3 | 2 | 2 | 2 |  | 17033.3613 | type:complete len:152 gc:universal TRINITY\_DN478\_c0\_g1\_i2:99-554(+) |
| 85 | 192 | TRINITY\_DN40728\_c1\_g1\_i1.p2 | 65.08 | 10 | 10 | 9.49e2 | 1 | 1 | 1 |  | 14073.3066 | type:5prime\_partial len:124 gc:universal TRINITY\_DN40728\_c1\_g1\_i1:1-372(+) |
| 85 | 193 | TRINITY\_DN40728\_c0\_g1\_i1.p1 | 65.08 | 13 | 13 | 9.49e2 | 1 | 1 | 1 |  | 11225.8301 | type:internal len:105 gc:universal TRINITY\_DN40728\_c0\_g1\_i1:1-312(+) |
| 85 | 194 | TRINITY\_DN195631\_c0\_g1\_i1.p2 | 65.08 | 9 | 9 | 9.49e2 | 1 | 1 | 1 |  | 15844.8984 | type:internal len:145 gc:universal TRINITY\_DN195631\_c0\_g1\_i1:2-433(+) |
| 85 | 195 | TRINITY\_DN8227\_c0\_g1\_i1.p1 | 65.08 | 10 | 10 | 9.49e2 | 1 | 1 | 1 |  | 14513.8135 | type:internal len:135 gc:universal TRINITY\_DN8227\_c0\_g1\_i1:404-3(-) |
| 85 | 196 | TRINITY\_DN152\_c0\_g1\_i15.p1 | 65.08 | 9 | 9 | 9.49e2 | 1 | 1 | 1 |  | 15560.71 | type:internal len:141 gc:universal TRINITY\_DN152\_c0\_g1\_i15:2-421(+) |
| 85 | 197 | TRINITY\_DN585\_c0\_g1\_i10.p1 | 65.08 | 6 | 6 | 9.49e2 | 1 | 1 | 1 |  | 26563.5332 | type:3prime\_partial len:236 gc:universal TRINITY\_DN585\_c0\_g1\_i10:705-1(-) |
| 85 | 198 | TRINITY\_DN152\_c0\_g1\_i2.p1 | 65.08 | 13 | 13 | 9.49e2 | 1 | 1 | 1 |  | 11754.375 | type:internal len:105 gc:universal TRINITY\_DN152\_c0\_g1\_i2:1-312(+) |
| 85 | 199 | TRINITY\_DN152\_c0\_g1\_i10.p1 | 65.08 | 7 | 7 | 9.49e2 | 1 | 1 | 1 |  | 20063.498 | type:internal len:180 gc:universal TRINITY\_DN152\_c0\_g1\_i10:1-537(+) |
| 85 | 200 | TRINITY\_DN152\_c0\_g1\_i14.p2 | 65.08 | 11 | 11 | 9.49e2 | 1 | 1 | 1 |  | 12547.4834 | type:5prime\_partial len:115 gc:universal TRINITY\_DN152\_c0\_g1\_i14:3-347(+) |
| 85 | 201 | TRINITY\_DN554\_c0\_g1\_i3.p2 | 65.08 | 5 | 5 | 9.49e2 | 1 | 1 | 1 |  | 30419.4961 | type:complete len:268 gc:universal TRINITY\_DN554\_c0\_g1\_i3:1464-661(-) |
| 85 | 202 | TRINITY\_DN1465\_c1\_g1\_i1.p1 | 65.08 | 13 | 13 | 9.49e2 | 1 | 1 | 1 |  | 11342.1572 | type:internal len:102 gc:universal TRINITY\_DN1465\_c1\_g1\_i1:2-304(+) |
| 85 | 203 | TRINITY\_DN152\_c0\_g1\_i9.p1 | 65.08 | 9 | 9 | 9.49e2 | 1 | 1 | 1 |  | 17213.1973 | type:5prime\_partial len:152 gc:universal TRINITY\_DN152\_c0\_g1\_i9:1-456(+) |
| 85 | 204 | TRINITY\_DN585\_c0\_g1\_i2.p1 | 65.08 | 7 | 7 | 9.49e2 | 1 | 1 | 1 |  | 20558.6738 | type:internal len:184 gc:universal TRINITY\_DN585\_c0\_g1\_i2:549-1(-) |
| 85 | 205 | TRINITY\_DN585\_c0\_g1\_i5.p1 | 65.08 | 5 | 5 | 9.49e2 | 1 | 1 | 1 |  | 28098.1602 | type:internal len:250 gc:universal TRINITY\_DN585\_c0\_g1\_i5:747-1(-) |
| 85 | 206 | TRINITY\_DN173718\_c0\_g1\_i1.p1 | 65.08 | 12 | 12 | 9.49e2 | 1 | 1 | 1 |  | 12865.0928 | type:3prime\_partial len:112 gc:universal TRINITY\_DN173718\_c0\_g1\_i1:37-369(+) |
| 85 | 207 | TRINITY\_DN585\_c0\_g1\_i3.p1 | 65.08 | 8 | 8 | 9.49e2 | 1 | 1 | 1 |  | 17628.2207 | type:internal len:157 gc:universal TRINITY\_DN585\_c0\_g1\_i3:468-1(-) |
| 85 | 208 | TRINITY\_DN585\_c0\_g1\_i4.p1 | 65.08 | 6 | 6 | 9.49e2 | 1 | 1 | 1 |  | 26348.0742 | type:complete len:232 gc:universal TRINITY\_DN585\_c0\_g1\_i4:847-152(-) |
| 85 | 209 | TRINITY\_DN554\_c0\_g1\_i5.p1 | 65.08 | 3 | 3 | 9.49e2 | 1 | 1 | 1 |  | 42081.4141 | type:3prime\_partial len:373 gc:universal TRINITY\_DN554\_c0\_g1\_i5:1118-3(-) |
| 86 | 210 | TRINITY\_DN1819\_c0\_g1\_i5.p1 | 59.54 | 3 | 3 | 3.85e2 | 1 | 1 | 1 |  | 49581.1055 | type:complete len:432 gc:universal TRINITY\_DN1819\_c0\_g1\_i5:79-1374(+) |
| 86 | 211 | TRINITY\_DN1819\_c0\_g1\_i7.p1 | 59.54 | 3 | 3 | 3.85e2 | 1 | 1 | 1 |  | 49581.1055 | type:complete len:432 gc:universal TRINITY\_DN1819\_c0\_g1\_i7:67-1362(+) |
| 87 | 212 | TRINITY\_DN2636\_c0\_g1\_i1.p1 | 66.5 | 3 | 3 | 2.07e3 | 1 | 1 | 2 |  | 35741.2305 | type:complete len:314 gc:universal TRINITY\_DN2636\_c0\_g1\_i1:986-45(-) |
| 87 | 213 | TRINITY\_DN2636\_c0\_g1\_i2.p1 | 66.5 | 3 | 3 | 2.07e3 | 1 | 1 | 2 |  | 35741.2305 | type:complete len:314 gc:universal TRINITY\_DN2636\_c0\_g1\_i2:986-45(-) |
| 88 | 214 | TRINITY\_DN5107\_c0\_g1\_i6.p1 | 63.57 | 9 | 9 | 2.13e2 | 1 | 1 | 1 |  | 16361.4258 | type:internal len:138 gc:universal TRINITY\_DN5107\_c0\_g1\_i6:1-411(+) |
| 88 | 215 | TRINITY\_DN5107\_c0\_g1\_i5.p2 | 63.57 | 7 | 7 | 2.13e2 | 1 | 1 | 1 |  | 18530.3633 | type:5prime\_partial len:163 gc:universal TRINITY\_DN5107\_c0\_g1\_i5:2-490(+) |
| 88 | 216 | TRINITY\_DN5107\_c0\_g1\_i8.p2 | 63.57 | 6 | 6 | 2.13e2 | 1 | 1 | 1 |  | 22145.5762 | type:5prime\_partial len:192 gc:universal TRINITY\_DN5107\_c0\_g1\_i8:2-577(+) |
| 88 | 217 | TRINITY\_DN5107\_c0\_g1\_i9.p1 | 63.57 | 12 | 12 | 2.13e2 | 1 | 1 | 1 |  | 12326.5479 | type:internal len:105 gc:universal TRINITY\_DN5107\_c0\_g1\_i9:2-313(+) |
| 88 | 218 | TRINITY\_DN5107\_c0\_g1\_i1.p2 | 63.57 | 7 | 7 | 2.13e2 | 1 | 1 | 1 |  | 18530.3633 | type:5prime\_partial len:163 gc:universal TRINITY\_DN5107\_c0\_g1\_i1:2-490(+) |
| 88 | 219 | TRINITY\_DN5107\_c0\_g2\_i1.p1 | 63.57 | 11 | 11 | 2.13e2 | 1 | 1 | 1 |  | 12484.9023 | type:internal len:111 gc:universal TRINITY\_DN5107\_c0\_g2\_i1:1-330(+) |
| 88 | 220 | TRINITY\_DN5107\_c0\_g1\_i11.p1 | 63.57 | 10 | 10 | 2.13e2 | 1 | 1 | 1 |  | 13801.3359 | type:3prime\_partial len:117 gc:universal TRINITY\_DN5107\_c0\_g1\_i11:61-408(+) |
| 88 | 221 | TRINITY\_DN5107\_c0\_g1\_i3.p1 | 63.57 | 10 | 10 | 2.13e2 | 1 | 1 | 1 |  | 13801.3359 | type:3prime\_partial len:117 gc:universal TRINITY\_DN5107\_c0\_g1\_i3:61-408(+) |
| 88 | 222 | TRINITY\_DN5107\_c0\_g1\_i10.p1 | 63.57 | 6 | 6 | 2.13e2 | 1 | 1 | 1 |  | 24893.9121 | type:5prime\_partial len:215 gc:universal TRINITY\_DN5107\_c0\_g1\_i10:1-645(+) |
| 88 | 223 | TRINITY\_DN5107\_c0\_g1\_i7.p2 | 63.57 | 5 | 5 | 2.13e2 | 1 | 1 | 1 |  | 25575.6934 | type:5prime\_partial len:221 gc:universal TRINITY\_DN5107\_c0\_g1\_i7:1-663(+) |
| 88 | 224 | TRINITY\_DN5107\_c0\_g1\_i2.p1 | 63.57 | 7 | 7 | 2.13e2 | 1 | 1 | 1 |  | 18514.3633 | type:5prime\_partial len:163 gc:universal TRINITY\_DN5107\_c0\_g1\_i2:2-490(+) |
| 89 | 225 | TRINITY\_DN3589\_c0\_g1\_i1.p1 | 66.51 | 3 | 3 | 6.94e2 | 1 | 1 | 1 |  | 60561.1484 | type:3prime\_partial len:548 gc:universal TRINITY\_DN3589\_c0\_g1\_i1:63-1703(+) |
| 90 | 226 | TRINITY\_DN10026\_c0\_g1\_i1.p1 | 65.16 | 15 | 15 | 2.14e2 | 1 | 1 | 1 |  | 12114.2949 | type:3prime\_partial len:105 gc:universal TRINITY\_DN10026\_c0\_g1\_i1:284-595(+) |
| 91 | 227 | TRINITY\_DN384\_c0\_g1\_i2.p1 | 66.68 | 4 | 4 | 0 | 1 | 1 | 1 |  | 34316.0703 | type:complete len:314 gc:universal TRINITY\_DN384\_c0\_g1\_i2:1018-77(-) |
| 91 | 228 | TRINITY\_DN22\_c0\_g1\_i1.p1 | 66.68 | 3 | 3 | 0 | 1 | 1 | 1 |  | 38528.8086 | type:5prime\_partial len:353 gc:universal TRINITY\_DN22\_c0\_g1\_i1:3-1061(+) |
| 91 | 229 | TRINITY\_DN384\_c0\_g1\_i1.p1 | 66.68 | 4 | 4 | 0 | 1 | 1 | 1 |  | 34317.9414 | type:complete len:313 gc:universal TRINITY\_DN384\_c0\_g1\_i1:1015-77(-) |
| 92 | 230 | TRINITY\_DN12632\_c1\_g1\_i1.p1 | 66.76 | 3 | 3 | 0 | 1 | 1 | 1 |  | 56568.082 | type:complete len:504 gc:universal TRINITY\_DN12632\_c1\_g1\_i1:165-1676(+) |
| 93 | 231 | TRINITY\_DN1080\_c0\_g2\_i1.p1 | 17.05 | 0 | 0 | 2.28e2 | 1 | 1 | 1 |  | 990519.0625 | type:5prime\_partial len:8617 gc:universal TRINITY\_DN1080\_c0\_g2\_i1:26447-597(-) |
| 93 | 232 | TRINITY\_DN1080\_c0\_g1\_i3.p1 | 17.05 | 2 | 2 | 2.28e2 | 1 | 1 | 1 |  | 48680.9648 | type:internal len:425 gc:universal TRINITY\_DN1080\_c0\_g1\_i3:1274-3(-) |
| 93 | 233 | TRINITY\_DN1080\_c0\_g1\_i2.p1 | 17.05 | 2 | 2 | 2.28e2 | 1 | 1 | 1 |  | 49162.0703 | type:5prime\_partial len:431 gc:universal TRINITY\_DN1080\_c0\_g1\_i2:1300-8(-) |
| 94 | 234 | TRINITY\_DN817\_c0\_g1\_i4.p1 | 62.01 | 8 | 8 | 1.84e2 | 1 | 1 | 1 | Carbamidomethylation | 19497.5664 | type:internal len:176 gc:universal TRINITY\_DN817\_c0\_g1\_i4:526-2(-) |
| 94 | 235 | TRINITY\_DN817\_c0\_g1\_i10.p1 | 62.01 | 8 | 8 | 1.84e2 | 1 | 1 | 1 | Carbamidomethylation | 20465.6582 | type:internal len:186 gc:universal TRINITY\_DN817\_c0\_g1\_i10:556-2(-) |
| 94 | 236 | TRINITY\_DN817\_c0\_g1\_i5.p1 | 62.01 | 8 | 8 | 1.84e2 | 1 | 1 | 1 | Carbamidomethylation | 19443.4023 | type:internal len:176 gc:universal TRINITY\_DN817\_c0\_g1\_i5:526-2(-) |
| 94 | 237 | TRINITY\_DN817\_c0\_g1\_i2.p1 | 62.01 | 8 | 8 | 1.84e2 | 1 | 1 | 1 | Carbamidomethylation | 19573.5801 | type:internal len:176 gc:universal TRINITY\_DN817\_c0\_g1\_i2:526-2(-) |
| 94 | 238 | TRINITY\_DN817\_c0\_g1\_i3.p1 | 62.01 | 7 | 7 | 1.84e2 | 1 | 1 | 1 | Carbamidomethylation | 23798.4922 | type:complete len:215 gc:universal TRINITY\_DN817\_c0\_g1\_i3:848-204(-) |
| 94 | 239 | TRINITY\_DN817\_c0\_g1\_i9.p1 | 62.01 | 7 | 7 | 1.84e2 | 1 | 1 | 1 | Carbamidomethylation | 21303.3516 | type:5prime\_partial len:191 gc:universal TRINITY\_DN817\_c0\_g1\_i9:776-204(-) |
| 94 | 240 | TRINITY\_DN817\_c0\_g1\_i7.p1 | 62.01 | 12 | 12 | 1.84e2 | 1 | 1 | 1 | Carbamidomethylation | 13259.333 | type:internal len:120 gc:universal TRINITY\_DN817\_c0\_g1\_i7:358-2(-) |
| 94 | 241 | TRINITY\_DN817\_c0\_g1\_i6.p1 | 62.01 | 8 | 8 | 1.84e2 | 1 | 1 | 1 | Carbamidomethylation | 19716.6973 | type:internal len:178 gc:universal TRINITY\_DN817\_c0\_g1\_i6:532-2(-) |
| 94 | 242 | TRINITY\_DN817\_c0\_g1\_i1.p1 | 62.01 | 6 | 6 | 1.84e2 | 1 | 1 | 1 | Carbamidomethylation | 24026.7148 | type:complete len:217 gc:universal TRINITY\_DN817\_c0\_g1\_i1:854-204(-) |
| 94 | 243 | TRINITY\_DN817\_c0\_g1\_i8.p1 | 62.01 | 12 | 12 | 1.84e2 | 1 | 1 | 1 | Carbamidomethylation | 13423.584 | type:internal len:122 gc:universal TRINITY\_DN817\_c0\_g1\_i8:364-2(-) |
| 95 | 244 | TRINITY\_DN121866\_c0\_g1\_i1.p1 | 66.91 | 2 | 2 | 0 | 1 | 1 | 2 |  | 51719.8477 | type:5prime\_partial len:460 gc:universal TRINITY\_DN121866\_c0\_g1\_i1:2-1381(+) |
| 96 | 245 | TRINITY\_DN1058\_c0\_g1\_i1.p1 | 66.68 | 0 | 0 | 1.09e3 | 1 | 1 | 2 |  | 284466.2812 | type:complete len:2639 gc:universal TRINITY\_DN1058\_c0\_g1\_i1:134-8050(+) |
| 96 | 246 | TRINITY\_DN1058\_c0\_g1\_i4.p1 | 66.68 | 2 | 2 | 1.09e3 | 1 | 1 | 2 |  | 79375.5703 | type:complete len:723 gc:universal TRINITY\_DN1058\_c0\_g1\_i4:366-2534(+) |
| 96 | 247 | TRINITY\_DN1058\_c0\_g1\_i3.p1 | 66.68 | 0 | 0 | 1.09e3 | 1 | 1 | 2 |  | 277633.5312 | type:complete len:2581 gc:universal TRINITY\_DN1058\_c0\_g1\_i3:134-7876(+) |
| 96 | 248 | TRINITY\_DN1058\_c0\_g1\_i2.p2 | 66.68 | 1 | 1 | 1.09e3 | 1 | 1 | 2 |  | 140284.0938 | type:complete len:1300 gc:universal TRINITY\_DN1058\_c0\_g1\_i2:4090-7989(+) |
| 97 | 249 | TRINITY\_DN1339\_c0\_g2\_i3.p1 | 66.26 | 7 | 7 | 1.34e3 | 1 | 1 | 1 |  | 19523.1797 | type:internal len:185 gc:universal TRINITY\_DN1339\_c0\_g2\_i3:553-2(-) |
| 97 | 250 | TRINITY\_DN1339\_c0\_g2\_i2.p1 | 66.26 | 3 | 3 | 1.34e3 | 1 | 1 | 1 |  | 42663.3828 | type:internal len:398 gc:universal TRINITY\_DN1339\_c0\_g2\_i2:1193-3(-) |
| 97 | 251 | TRINITY\_DN1339\_c0\_g2\_i4.p2 | 66.26 | 6 | 6 | 1.34e3 | 1 | 1 | 1 |  | 22988.9766 | type:internal len:216 gc:universal TRINITY\_DN1339\_c0\_g2\_i4:646-2(-) |
| 97 | 252 | TRINITY\_DN1339\_c0\_g2\_i1.p2 | 66.26 | 3 | 3 | 1.34e3 | 1 | 1 | 1 |  | 43129.793 | type:internal len:402 gc:universal TRINITY\_DN1339\_c0\_g2\_i1:1203-1(-) |
| 98 | 253 | TRINITY\_DN21998\_c0\_g1\_i1.p1 | 66.93 | 3 | 3 | 3.26e2 | 1 | 1 | 1 |  | 58159.8398 | type:5prime\_partial len:507 gc:universal TRINITY\_DN21998\_c0\_g1\_i1:1561-41(-) |
| 99 | 254 | TRINITY\_DN998\_c0\_g1\_i5.p2 | 61.79 | 8 | 8 | 0 | 1 | 1 | 1 |  | 20375.3574 | type:5prime\_partial len:178 gc:universal TRINITY\_DN998\_c0\_g1\_i5:2-535(+) |
| 99 | 255 | TRINITY\_DN998\_c0\_g1\_i2.p2 | 61.79 | 10 | 10 | 0 | 1 | 1 | 1 |  | 16490.1328 | type:internal len:145 gc:universal TRINITY\_DN998\_c0\_g1\_i2:2-433(+) |
| 99 | 256 | TRINITY\_DN998\_c0\_g1\_i3.p2 | 61.79 | 8 | 8 | 0 | 1 | 1 | 1 |  | 20612.5449 | type:5prime\_partial len:179 gc:universal TRINITY\_DN998\_c0\_g1\_i3:3-539(+) |
| 99 | 257 | TRINITY\_DN53760\_c0\_g1\_i1.p1 | 61.79 | 14 | 14 | 0 | 1 | 1 | 1 |  | 12426.4873 | type:internal len:107 gc:universal TRINITY\_DN53760\_c0\_g1\_i1:2-319(+) |
| 99 | 258 | TRINITY\_DN4928\_c0\_g1\_i1.p1 | 61.79 | 15 | 15 | 0 | 1 | 1 | 1 |  | 11917.8975 | type:internal len:102 gc:universal TRINITY\_DN4928\_c0\_g1\_i1:303-1(-) |
| 99 | 259 | TRINITY\_DN998\_c0\_g1\_i6.p2 | 61.79 | 8 | 8 | 0 | 1 | 1 | 1 |  | 20538.3555 | type:5prime\_partial len:181 gc:universal TRINITY\_DN998\_c0\_g1\_i6:1-543(+) |
| 99 | 260 | TRINITY\_DN998\_c0\_g1\_i4.p2 | 61.79 | 9 | 9 | 0 | 1 | 1 | 1 |  | 18680.3047 | type:5prime\_partial len:161 gc:universal TRINITY\_DN998\_c0\_g1\_i4:2-484(+) |
| 100 | 261 | TRINITY\_DN2142\_c0\_g1\_i1.p2 | 37.82 | 4 | 4 | 6.23e2 | 1 | 1 | 1 |  | 29103.0977 | type:5prime\_partial len:246 gc:universal TRINITY\_DN2142\_c0\_g1\_i1:3-740(+) |
| 100 | 262 | TRINITY\_DN2142\_c0\_g1\_i2.p2 | 37.82 | 4 | 4 | 6.23e2 | 1 | 1 | 1 |  | 29103.0977 | type:5prime\_partial len:246 gc:universal TRINITY\_DN2142\_c0\_g1\_i2:3-740(+) |
| 101 | 263 | TRINITY\_DN143799\_c0\_g1\_i1.p1 | 20.8 | 11 | 11 | 0 | 1 | 1 | 1 | Carbamidomethylation | 20897.4434 | type:complete len:178 gc:universal TRINITY\_DN143799\_c0\_g1\_i1:576-43(-) |
| 102 | 264 | TRINITY\_DN433\_c0\_g2\_i1.p1 | 22.55 | 6 | 6 | 4.27e2 | 1 | 1 | 1 |  | 16483.6387 | type:complete len:144 gc:universal TRINITY\_DN433\_c0\_g2\_i1:1496-1065(-) |
| 103 | 265 | TRINITY\_DN2472\_c0\_g1\_i1.p1 | 66.62 | 5 | 5 | 1.64e3 | 1 | 1 | 2 | Acetylation (Protein N-term) | 37676.8203 | type:complete len:344 gc:universal TRINITY\_DN2472\_c0\_g1\_i1:311-1342(+) |
| 104 | 266 | TRINITY\_DN45599\_c0\_g1\_i1.p1 | 62.34 | 2 | 2 | 0 | 1 | 1 | 1 |  | 71803.0391 | type:internal len:642 gc:universal TRINITY\_DN45599\_c0\_g1\_i1:1923-1(-) |
| 105 | 267 | TRINITY\_DN1787\_c0\_g2\_i1.p1 | 66.68 | 6 | 6 | 9.73e2 | 1 | 1 | 3 | Carbamidomethylation | 21908.5 | type:complete len:197 gc:universal TRINITY\_DN1787\_c0\_g2\_i1:144-734(+) |
| 105 | 268 | TRINITY\_DN1787\_c0\_g2\_i2.p1 | 66.68 | 6 | 6 | 9.73e2 | 1 | 1 | 3 | Carbamidomethylation | 21908.5 | type:complete len:197 gc:universal TRINITY\_DN1787\_c0\_g2\_i2:144-734(+) |
| 106 | 269 | TRINITY\_DN468\_c1\_g1\_i1.p1 | 59.89 | 10 | 10 | 0 | 1 | 1 | 1 |  | 21670.5645 | type:complete len:191 gc:universal TRINITY\_DN468\_c1\_g1\_i1:676-104(-) |
| 107 | 270 | TRINITY\_DN4911\_c0\_g1\_i1.p1 | 66.19 | 5 | 5 | 3.89e2 | 1 | 1 | 1 |  | 30774.7383 | type:complete len:271 gc:universal TRINITY\_DN4911\_c0\_g1\_i1:896-84(-) |
| 108 | 271 | TRINITY\_DN7314\_c0\_g2\_i1.p1 | 63 | 6 | 6 | 0 | 1 | 1 | 1 |  | 16177.1182 | type:5prime\_partial len:142 gc:universal TRINITY\_DN7314\_c0\_g2\_i1:541-116(-) |
| 109 | 272 | TRINITY\_DN79126\_c0\_g1\_i1.p1 | 66.8 | 10 | 10 | 8.16e3 | 1 | 1 | 2 |  | 13015.4727 | type:complete len:115 gc:universal TRINITY\_DN79126\_c0\_g1\_i1:397-53(-) |
| 110 | 273 | TRINITY\_DN602\_c0\_g1\_i3.p1 | 63.7 | 2 | 2 | 2.51e2 | 1 | 1 | 1 |  | 92279.0078 | type:complete len:851 gc:universal TRINITY\_DN602\_c0\_g1\_i3:3046-494(-) |
| 110 | 274 | TRINITY\_DN602\_c0\_g1\_i4.p1 | 63.7 | 3 | 3 | 2.51e2 | 1 | 1 | 1 |  | 61175.9766 | type:5prime\_partial len:552 gc:universal TRINITY\_DN602\_c0\_g1\_i4:3498-1843(-) |
| 110 | 275 | TRINITY\_DN602\_c0\_g1\_i1.p1 | 63.7 | 3 | 3 | 2.51e2 | 1 | 1 | 1 |  | 65701.2422 | type:complete len:593 gc:universal TRINITY\_DN602\_c0\_g1\_i1:3622-1844(-) |
| 111 | 276 | TRINITY\_DN691\_c0\_g1\_i2.p1 | 62.49 | 1 | 1 | 9e2 | 1 | 1 | 1 | Carbamidomethylation | 164625.2188 | type:complete len:1673 gc:universal TRINITY\_DN691\_c0\_g1\_i2:5503-485(-) |
| 111 | 277 | TRINITY\_DN691\_c0\_g1\_i1.p1 | 62.49 | 1 | 1 | 9e2 | 1 | 1 | 1 | Carbamidomethylation | 169920.1562 | type:complete len:1726 gc:universal TRINITY\_DN691\_c0\_g1\_i1:5662-485(-) |
| 112 | 278 | TRINITY\_DN2772\_c0\_g1\_i3.p1 | 66.7 | 8 | 8 | 1.49e3 | 1 | 1 | 1 | Carbamidomethylation | 15694.8809 | type:complete len:142 gc:universal TRINITY\_DN2772\_c0\_g1\_i3:476-51(-) |
| 112 | 279 | TRINITY\_DN2772\_c0\_g1\_i2.p1 | 66.7 | 8 | 8 | 1.49e3 | 1 | 1 | 1 | Carbamidomethylation | 15694.8809 | type:complete len:142 gc:universal TRINITY\_DN2772\_c0\_g1\_i2:476-51(-) |
| 112 | 280 | TRINITY\_DN2772\_c0\_g1\_i1.p1 | 66.7 | 8 | 8 | 1.49e3 | 1 | 1 | 1 | Carbamidomethylation | 15694.8809 | type:complete len:142 gc:universal TRINITY\_DN2772\_c0\_g1\_i1:476-51(-) |
| 113 | 281 | TRINITY\_DN34765\_c0\_g1\_i1.p1 | 62.93 | 6 | 6 | 6.09e2 | 1 | 1 | 1 |  | 22438.3496 | type:internal len:204 gc:universal TRINITY\_DN34765\_c0\_g1\_i1:1-609(+) |
| 113 | 282 | TRINITY\_DN14344\_c0\_g1\_i1.p1 | 62.93 | 3 | 3 | 6.09e2 | 1 | 1 | 1 |  | 56232.1914 | type:complete len:512 gc:universal TRINITY\_DN14344\_c0\_g1\_i1:191-1726(+) |
| 114 | 283 | TRINITY\_DN2626\_c0\_g1\_i7.p2 | 18.66 | 2 | 2 | 2e2 | 1 | 1 | 1 | Acetylation (Protein N-term) | 39690.0312 | type:5prime\_partial len:361 gc:universal TRINITY\_DN2626\_c0\_g1\_i7:1099-17(-) |
| 114 | 284 | TRINITY\_DN2626\_c0\_g1\_i1.p1 | 18.66 | 2 | 2 | 2e2 | 1 | 1 | 1 | Acetylation (Protein N-term) | 39417.0977 | type:internal len:365 gc:universal TRINITY\_DN2626\_c0\_g1\_i1:1093-2(-) |
| 115 | 285 | TRINITY\_DN190525\_c0\_g1\_i1.p2 | 21.23 | 6 | 6 | 2e3 | 1 | 1 | 2 |  | 22824.7207 | type:internal len:218 gc:universal TRINITY\_DN190525\_c0\_g1\_i1:3-653(+) |
| 116 | 286 | TRINITY\_DN10563\_c0\_g1\_i1.p1 | 66.76 | 4 | 4 | 7.44e2 | 1 | 1 | 1 |  | 33159.7109 | type:internal len:307 gc:universal TRINITY\_DN10563\_c0\_g1\_i1:1-918(+) |
| 117 | 287 | TRINITY\_DN95387\_c0\_g1\_i1.p1 | 66.57 | 7 | 7 | 1.22e3 | 1 | 1 | 2 | Acetylation (Protein N-term) | 19610.8633 | type:complete len:169 gc:universal TRINITY\_DN95387\_c0\_g1\_i1:96-602(+) |
| 118 | 288 | TRINITY\_DN30378\_c0\_g1\_i2.p1 | 64.13 | 3 | 3 | 0 | 1 | 1 | 1 |  | 44977.9023 | type:complete len:403 gc:universal TRINITY\_DN30378\_c0\_g1\_i2:1370-162(-) |
| 118 | 289 | TRINITY\_DN30378\_c0\_g1\_i1.p1 | 64.13 | 5 | 5 | 0 | 1 | 1 | 1 |  | 30261.1582 | type:complete len:272 gc:universal TRINITY\_DN30378\_c0\_g1\_i1:977-162(-) |
| 119 | 290 | TRINITY\_DN6397\_c0\_g2\_i3.p1 | 66.46 | 8 | 8 | 3.02e3 | 1 | 1 | 4 | Acetylation (Protein N-term) | 14030.8945 | type:internal len:130 gc:universal TRINITY\_DN6397\_c0\_g2\_i3:387-1(-) |
| 119 | 291 | TRINITY\_DN6397\_c0\_g2\_i4.p1 | 66.46 | 9 | 9 | 3.02e3 | 1 | 1 | 4 | Acetylation (Protein N-term) | 12470.125 | type:internal len:115 gc:universal TRINITY\_DN6397\_c0\_g2\_i4:343-2(-) |
| 120 | 292 | TRINITY\_DN16548\_c0\_g1\_i4.p1 | 61.97 | 12 | 12 | 3.62e3 | 1 | 1 | 3 |  | 18505.2012 | type:internal len:171 gc:universal TRINITY\_DN16548\_c0\_g1\_i4:2-511(+) |
| 120 | 293 | TRINITY\_DN16548\_c0\_g1\_i3.p1 | 61.97 | 12 | 12 | 3.62e3 | 1 | 1 | 3 |  | 18505.2012 | type:internal len:171 gc:universal TRINITY\_DN16548\_c0\_g1\_i3:2-511(+) |
| 120 | 294 | TRINITY\_DN16548\_c0\_g1\_i1.p1 | 61.97 | 17 | 17 | 3.62e3 | 1 | 1 | 3 |  | 12774.7002 | type:internal len:119 gc:universal TRINITY\_DN16548\_c0\_g1\_i1:1-354(+) |
| 121 | 295 | TRINITY\_DN6132\_c0\_g1\_i1.p1 | 52.97 | 9 | 9 | 0 | 1 | 1 | 1 |  | 18615.5566 | type:complete len:161 gc:universal TRINITY\_DN6132\_c0\_g1\_i1:95-577(+) |
| 122 | 296 | TRINITY\_DN17516\_c0\_g1\_i1.p1 | 61.95 | 4 | 4 | 0 | 1 | 1 | 1 |  | 38198.582 | type:complete len:335 gc:universal TRINITY\_DN17516\_c0\_g1\_i1:1523-519(-) |
| 122 | 297 | TRINITY\_DN17516\_c0\_g1\_i3.p1 | 61.95 | 4 | 4 | 0 | 1 | 1 | 1 |  | 38198.582 | type:complete len:335 gc:universal TRINITY\_DN17516\_c0\_g1\_i3:1648-644(-) |
| 123 | 298 | TRINITY\_DN6294\_c0\_g3\_i2.p1 | 66.4 | 9 | 9 | 4.87e3 | 1 | 1 | 1 |  | 15623.1504 | type:complete len:134 gc:universal TRINITY\_DN6294\_c0\_g3\_i2:817-416(-) |
| 123 | 299 | TRINITY\_DN6294\_c0\_g3\_i1.p1 | 66.4 | 9 | 9 | 4.87e3 | 1 | 1 | 1 |  | 15623.1504 | type:complete len:134 gc:universal TRINITY\_DN6294\_c0\_g3\_i1:706-305(-) |
| 124 | 300 | TRINITY\_DN3649\_c0\_g1\_i1.p1 | 66.75 | 4 | 4 | 7.73e2 | 1 | 1 | 1 | Carbamidomethylation | 25900.9395 | type:complete len:246 gc:universal TRINITY\_DN3649\_c0\_g1\_i1:2360-1623(-) |
| 124 | 301 | TRINITY\_DN3649\_c0\_g1\_i2.p1 | 66.75 | 4 | 4 | 7.73e2 | 1 | 1 | 1 | Carbamidomethylation | 31445.3438 | type:complete len:293 gc:universal TRINITY\_DN3649\_c0\_g1\_i2:2501-1623(-) |
| 125 | 302 | TRINITY\_DN902\_c1\_g2\_i3.p1 | 66.56 | 6 | 6 | 0 | 1 | 1 | 1 | Carbamidomethylation | 19070.0254 | type:internal len:178 gc:universal TRINITY\_DN902\_c1\_g2\_i3:2-532(+) |
| 125 | 303 | TRINITY\_DN902\_c1\_g2\_i1.p1 | 66.56 | 6 | 6 | 0 | 1 | 1 | 1 | Carbamidomethylation | 18964.9824 | type:internal len:178 gc:universal TRINITY\_DN902\_c1\_g2\_i1:2-532(+) |
| 125 | 304 | TRINITY\_DN902\_c1\_g2\_i4.p1 | 66.56 | 6 | 6 | 0 | 1 | 1 | 1 | Carbamidomethylation | 19994.1074 | type:internal len:182 gc:universal TRINITY\_DN902\_c1\_g2\_i4:2-544(+) |
| 126 | 305 | TRINITY\_DN4166\_c0\_g3\_i2.p1 | 29.2 | 3 | 3 | 0 | 1 | 1 | 1 |  | 56732.3477 | type:complete len:490 gc:universal TRINITY\_DN4166\_c0\_g3\_i2:1876-407(-) |
| 126 | 306 | TRINITY\_DN4166\_c0\_g3\_i1.p1 | 29.2 | 3 | 3 | 0 | 1 | 1 | 1 |  | 56865.4062 | type:complete len:491 gc:universal TRINITY\_DN4166\_c0\_g3\_i1:1879-407(-) |
| 127 | 307 | TRINITY\_DN1566\_c0\_g1\_i1.p1 | 66.56 | 0 | 0 | 2.25e2 | 1 | 1 | 1 |  | 529444 | type:complete len:4626 gc:universal TRINITY\_DN1566\_c0\_g1\_i1:42-13919(+) |
| 128 | 308 | TRINITY\_DN1631\_c0\_g1\_i1.p1 | 66.75 | 9 | 9 | 1.04e4 | 1 | 1 | 4 | Acetylation (Protein N-term) | 15272.8457 | type:complete len:124 gc:universal TRINITY\_DN1631\_c0\_g1\_i1:62-433(+) |
| 129 | 309 | TRINITY\_DN5131\_c1\_g2\_i1.p1 | 66.75 | 8 | 8 | 7.14e2 | 1 | 1 | 1 | Carbamidomethylation | 18551.6797 | type:complete len:164 gc:universal TRINITY\_DN5131\_c1\_g2\_i1:518-1009(+) |
| 130 | 310 | TRINITY\_DN95606\_c0\_g1\_i1.p1 | 31.43 | 2 | 2 | 0 | 1 | 1 | 1 |  | 60167.6328 | type:complete len:538 gc:universal TRINITY\_DN95606\_c0\_g1\_i1:82-1695(+) |
| 131 | 311 | TRINITY\_DN30329\_c0\_g1\_i1.p1 | 20.36 | 8 | 8 | 6.45e2 | 1 | 1 | 1 |  | 23962.9473 | type:5prime\_partial len:213 gc:universal TRINITY\_DN30329\_c0\_g1\_i1:1-639(+) |
| 131 | 312 | TRINITY\_DN30329\_c0\_g2\_i1.p2 | 20.36 | 7 | 7 | 6.45e2 | 1 | 1 | 1 |  | 26093.5586 | type:5prime\_partial len:237 gc:universal TRINITY\_DN30329\_c0\_g2\_i1:1-711(+) |
| 132 | 313 | TRINITY\_DN7808\_c4\_g1\_i3.p2 | 62.56 | 8 | 8 | 2.92e2 | 1 | 1 | 1 | Carbamidomethylation | 30146.9023 | type:3prime\_partial len:280 gc:universal TRINITY\_DN7808\_c4\_g1\_i3:837-1(-) |
| 133 | 314 | TRINITY\_DN163801\_c0\_g1\_i1.p1 | 66.51 | 3 | 3 | 1.77e3 | 1 | 1 | 1 | Acetylation (Protein N-term) | 33076.9062 | type:complete len:298 gc:universal TRINITY\_DN163801\_c0\_g1\_i1:55-948(+) |
| 134 | 315 | TRINITY\_DN1739\_c0\_g1\_i1.p1 | 66.66 | 3 | 3 | 5.17e2 | 1 | 1 | 1 |  | 46350.6953 | type:complete len:400 gc:universal TRINITY\_DN1739\_c0\_g1\_i1:86-1285(+) |
| 135 | 316 | TRINITY\_DN309\_c0\_g1\_i1.p1 | 64.6 | 3 | 3 | 4.16e2 | 1 | 1 | 1 |  | 52371.3906 | type:complete len:454 gc:universal TRINITY\_DN309\_c0\_g1\_i1:65-1426(+) |
| 136 | 317 | TRINITY\_DN3452\_c0\_g1\_i1.p1 | 66.64 | 3 | 3 | 7.23e2 | 1 | 1 | 1 |  | 52685.7344 | type:complete len:466 gc:universal TRINITY\_DN3452\_c0\_g1\_i1:139-1536(+) |
| 137 | 318 | TRINITY\_DN47849\_c0\_g1\_i1.p1 | 58.58 | 2 | 2 | 1.27e2 | 1 | 1 | 1 | Oxidation (M) | 77056.3125 | type:complete len:689 gc:universal TRINITY\_DN47849\_c0\_g1\_i1:176-2242(+) |
| 138 | 319 | TRINITY\_DN15151\_c0\_g1\_i3.p1 | 61.93 | 2 | 2 | 4.12e2 | 1 | 1 | 1 | Oxidation (M) | 58451.293 | type:internal len:560 gc:universal TRINITY\_DN15151\_c0\_g1\_i3:1-1677(+) |
| 138 | 320 | TRINITY\_DN15151\_c0\_g1\_i1.p1 | 61.93 | 2 | 2 | 4.12e2 | 1 | 1 | 1 | Oxidation (M) | 62118.2383 | type:5prime\_partial len:592 gc:universal TRINITY\_DN15151\_c0\_g1\_i1:1-1776(+) |
| 138 | 321 | TRINITY\_DN15151\_c0\_g1\_i2.p1 | 61.93 | 2 | 2 | 4.12e2 | 1 | 1 | 1 | Oxidation (M) | 58324.2109 | type:internal len:560 gc:universal TRINITY\_DN15151\_c0\_g1\_i2:1-1677(+) |
| 139 | 322 | TRINITY\_DN801\_c0\_g1\_i4.p1 | 39.11 | 9 | 9 | 0 | 1 | 1 | 1 |  | 20287.4453 | type:5prime\_partial len:182 gc:universal TRINITY\_DN801\_c0\_g1\_i4:1-546(+) |
| 139 | 323 | TRINITY\_DN801\_c0\_g1\_i1.p1 | 39.11 | 9 | 9 | 0 | 1 | 1 | 1 |  | 19577.4629 | type:5prime\_partial len:177 gc:universal TRINITY\_DN801\_c0\_g1\_i1:3-533(+) |
| 139 | 324 | TRINITY\_DN801\_c0\_g1\_i10.p1 | 39.11 | 11 | 11 | 0 | 1 | 1 | 1 |  | 16657.5469 | type:internal len:153 gc:universal TRINITY\_DN801\_c0\_g1\_i10:3-458(+) |
| 139 | 325 | TRINITY\_DN801\_c0\_g1\_i9.p2 | 39.11 | 11 | 11 | 0 | 1 | 1 | 1 |  | 16657.5469 | type:internal len:153 gc:universal TRINITY\_DN801\_c0\_g1\_i9:3-458(+) |
| 139 | 326 | TRINITY\_DN801\_c0\_g1\_i7.p1 | 39.11 | 10 | 10 | 0 | 1 | 1 | 1 |  | 17665.748 | type:internal len:160 gc:universal TRINITY\_DN801\_c0\_g1\_i7:1-477(+) |
| 139 | 327 | TRINITY\_DN30386\_c0\_g1\_i1.p2 | 39.11 | 10 | 10 | 0 | 1 | 1 | 1 |  | 18794.9727 | type:internal len:165 gc:universal TRINITY\_DN30386\_c0\_g1\_i1:3-494(+) |
| 140 | 328 | TRINITY\_DN3271\_c0\_g1\_i1.p1 | 66.53 | 3 | 3 | 7.11e2 | 1 | 1 | 1 |  | 45246.3555 | type:complete len:399 gc:universal TRINITY\_DN3271\_c0\_g1\_i1:218-1414(+) |
| 140 | 329 | TRINITY\_DN3271\_c0\_g1\_i2.p1 | 66.53 | 3 | 3 | 7.11e2 | 1 | 1 | 1 |  | 53020.332 | type:5prime\_partial len:468 gc:universal TRINITY\_DN3271\_c0\_g1\_i2:2-1405(+) |
| 141 | 330 | TRINITY\_DN123073\_c0\_g1\_i1.p1 | 24.23 | 6 | 6 | 6.64e3 | 1 | 1 | 5 |  | 20490.7109 | type:internal len:179 gc:universal TRINITY\_DN123073\_c0\_g1\_i1:2-535(+) |
| 142 | 331 | TRINITY\_DN33065\_c0\_g2\_i1.p1 | 66.57 | 2 | 2 | 1.14e2 | 1 | 1 | 1 |  | 89122.9766 | type:3prime\_partial len:804 gc:universal TRINITY\_DN33065\_c0\_g2\_i1:2409-1(-) |
| 143 | 332 | TRINITY\_DN1319\_c0\_g1\_i2.p1 | 65.75 | 10 | 10 | 6.69e2 | 1 | 1 | 1 | Carbamidomethylation | 15785.3457 | type:complete len:134 gc:universal TRINITY\_DN1319\_c0\_g1\_i2:86-487(+) |
| 143 | 333 | TRINITY\_DN1319\_c0\_g1\_i1.p1 | 65.75 | 10 | 10 | 6.69e2 | 1 | 1 | 1 | Carbamidomethylation | 15785.3457 | type:complete len:134 gc:universal TRINITY\_DN1319\_c0\_g1\_i1:362-763(+) |
| 144 | 334 | TRINITY\_DN3180\_c0\_g1\_i1.p1 | 66.78 | 1 | 1 | 0 | 1 | 1 | 1 |  | 212833.2969 | type:complete len:1889 gc:universal TRINITY\_DN3180\_c0\_g1\_i1:5857-191(-) |
| 145 | 335 | TRINITY\_DN1339\_c6\_g1\_i1.p1 | 66.7 | 2 | 2 | 7.2e3 | 1 | 1 | 2 |  | 61274.3594 | type:internal len:570 gc:universal TRINITY\_DN1339\_c6\_g1\_i1:1-1707(+) |
| 145 | 336 | TRINITY\_DN1339\_c0\_g1\_i2.p1 | 66.7 | 3 | 3 | 7.2e3 | 1 | 1 | 2 |  | 41222.6992 | type:internal len:384 gc:universal TRINITY\_DN1339\_c0\_g1\_i2:1151-3(-) |
| 145 | 337 | TRINITY\_DN124769\_c0\_g1\_i1.p1 | 66.7 | 11 | 11 | 7.2e3 | 1 | 1 | 2 |  | 12144.8828 | type:internal len:113 gc:universal TRINITY\_DN124769\_c0\_g1\_i1:1-336(+) |
| 145 | 338 | TRINITY\_DN24414\_c1\_g1\_i2.p2 | 66.7 | 8 | 8 | 7.2e3 | 1 | 1 | 2 |  | 15590.8623 | type:internal len:146 gc:universal TRINITY\_DN24414\_c1\_g1\_i2:435-1(-) |
| 145 | 339 | TRINITY\_DN1172\_c0\_g1\_i3.p1 | 66.7 | 7 | 7 | 7.2e3 | 1 | 1 | 2 |  | 19709.3613 | type:internal len:182 gc:universal TRINITY\_DN1172\_c0\_g1\_i3:545-3(-) |
| 145 | 340 | TRINITY\_DN119673\_c0\_g1\_i1.p1 | 66.7 | 9 | 9 | 7.2e3 | 1 | 1 | 2 |  | 13988.7559 | type:internal len:129 gc:universal TRINITY\_DN119673\_c0\_g1\_i1:2-385(+) |
| 145 | 341 | TRINITY\_DN1339\_c0\_g1\_i1.p1 | 66.7 | 5 | 5 | 7.2e3 | 1 | 1 | 2 |  | 24722.7773 | type:internal len:229 gc:universal TRINITY\_DN1339\_c0\_g1\_i1:684-1(-) |
| 145 | 342 | TRINITY\_DN6701\_c0\_g1\_i1.p1 | 66.7 | 6 | 6 | 7.2e3 | 1 | 1 | 2 |  | 21246.0547 | type:internal len:196 gc:universal TRINITY\_DN6701\_c0\_g1\_i1:1-585(+) |
| 145 | 343 | TRINITY\_DN1172\_c0\_g1\_i5.p1 | 66.7 | 5 | 5 | 7.2e3 | 1 | 1 | 2 |  | 24525.6855 | type:internal len:226 gc:universal TRINITY\_DN1172\_c0\_g1\_i5:677-3(-) |
| 145 | 344 | TRINITY\_DN6701\_c0\_g1\_i2.p2 | 66.7 | 8 | 8 | 7.2e3 | 1 | 1 | 2 |  | 16512.8535 | type:internal len:152 gc:universal TRINITY\_DN6701\_c0\_g1\_i2:3-455(+) |
| 145 | 345 | TRINITY\_DN1339\_c6\_g1\_i2.p1 | 66.7 | 3 | 3 | 7.2e3 | 1 | 1 | 2 |  | 43821.8359 | type:internal len:408 gc:universal TRINITY\_DN1339\_c6\_g1\_i2:3-1223(+) |
| 146 | 346 | TRINITY\_DN1995\_c0\_g1\_i1.p1 | 22.02 | 2 | 2 | 3.38e3 | 1 | 1 | 1 |  | 59145.6719 | type:internal len:514 gc:universal TRINITY\_DN1995\_c0\_g1\_i1:1539-1(-) |
| 147 | 347 | TRINITY\_DN123\_c0\_g1\_i1.p1 | 66.74 | 1 | 1 | 1.29e3 | 1 | 1 | 1 |  | 136822.0312 | type:5prime\_partial len:1231 gc:universal TRINITY\_DN123\_c0\_g1\_i1:2-3694(+) |
| 148 | 348 | TRINITY\_DN495\_c0\_g1\_i5.p1 | 63.06 | 3 | 3 | 0 | 1 | 1 | 1 | Carbamidomethylation | 45678.0781 | type:3prime\_partial len:407 gc:universal TRINITY\_DN495\_c0\_g1\_i5:152-1369(+) |
| 148 | 349 | TRINITY\_DN495\_c0\_g1\_i6.p1 | 63.06 | 3 | 3 | 0 | 1 | 1 | 1 | Carbamidomethylation | 42984.9375 | type:3prime\_partial len:382 gc:universal TRINITY\_DN495\_c0\_g1\_i6:152-1294(+) |
| 149 | 350 | TRINITY\_DN135129\_c0\_g1\_i1.p1 | 62 | 6 | 6 | 8.59e3 | 1 | 1 | 3 |  | 17174.043 | type:internal len:155 gc:universal TRINITY\_DN135129\_c0\_g1\_i1:1-462(+) |
| 150 | 351 | TRINITY\_DN34\_c0\_g1\_i1.p1 | 49.25 | 7 | 7 | 0 | 1 | 1 | 1 | Acetylation (Protein N-term), Oxidation (M) | 16992.0059 | type:complete len:149 gc:universal TRINITY\_DN34\_c0\_g1\_i1:477-31(-) |
| 151 | 352 | TRINITY\_DN56792\_c0\_g1\_i3.p1 | 66.67 | 1 | 1 | 4.12e2 | 1 | 1 | 1 |  | 112051.7656 | type:5prime\_partial len:1003 gc:universal TRINITY\_DN56792\_c0\_g1\_i3:3453-445(-) |
| 151 | 353 | TRINITY\_DN56792\_c0\_g1\_i1.p1 | 66.67 | 1 | 1 | 4.12e2 | 1 | 1 | 1 |  | 111385.9531 | type:5prime\_partial len:997 gc:universal TRINITY\_DN56792\_c0\_g1\_i1:3435-445(-) |
| 152 | 354 | TRINITY\_DN167719\_c0\_g1\_i1.p1 | 66.78 | 10 | 10 | 0 | 1 | 1 | 1 | Carbamidomethylation | 15286.0078 | type:internal len:144 gc:universal TRINITY\_DN167719\_c0\_g1\_i1:431-3(-) |
| 153 | 355 | TRINITY\_DN164271\_c0\_g1\_i1.p1 | 61.48 | 10 | 10 | 3.84e2 | 1 | 1 | 1 | Acetylation (Protein N-term), Carbamidomethylation | 18729.2617 | type:complete len:163 gc:universal TRINITY\_DN164271\_c0\_g1\_i1:1172-684(-) |
| 154 | 356 | TRINITY\_DN19237\_c0\_g1\_i1.p1 | 61.23 | 7 | 7 | 0 | 1 | 1 | 1 |  | 23866.248 | type:complete len:209 gc:universal TRINITY\_DN19237\_c0\_g1\_i1:128-754(+) |
| 155 | 357 | TRINITY\_DN11643\_c0\_g1\_i1.p1 | 66.66 | 7 | 7 | 2.54e3 | 1 | 1 | 2 |  | 21061.2832 | type:complete len:187 gc:universal TRINITY\_DN11643\_c0\_g1\_i1:1748-1188(-) |
| 156 | 358 | TRINITY\_DN140702\_c0\_g1\_i1.p1 | 66.71 | 1 | 1 | 4.05e2 | 1 | 1 | 3 |  | 231919.1562 | type:complete len:2036 gc:universal TRINITY\_DN140702\_c0\_g1\_i1:94-6201(+) |
| 157 | 359 | TRINITY\_DN96\_c0\_g1\_i2.p1 | 66.72 | 7 | 7 | 1.44e3 | 1 | 1 | 3 |  | 20764.5137 | type:5prime\_partial len:189 gc:universal TRINITY\_DN96\_c0\_g1\_i2:1253-687(-) |
| 157 | 360 | TRINITY\_DN96\_c0\_g1\_i1.p1 | 66.72 | 7 | 7 | 1.44e3 | 1 | 1 | 3 |  | 20737.4844 | type:5prime\_partial len:189 gc:universal TRINITY\_DN96\_c0\_g1\_i1:1253-687(-) |
| 158 | 361 | TRINITY\_DN26249\_c0\_g1\_i1.p1 | 66.79 | 3 | 3 | 5.25e2 | 1 | 1 | 2 |  | 54490.1055 | type:5prime\_partial len:477 gc:universal TRINITY\_DN26249\_c0\_g1\_i1:1733-303(-) |
| 159 | 362 | TRINITY\_DN140696\_c0\_g1\_i1.p1 | 31.8 | 3 | 3 | 0 | 1 | 1 | 1 |  | 29465.1973 | type:complete len:260 gc:universal TRINITY\_DN140696\_c0\_g1\_i1:43-822(+) |
| 160 | 363 | TRINITY\_DN8707\_c0\_g1\_i2.p1 | 66.36 | 2 | 2 | 3.27e3 | 1 | 1 | 1 |  | 61942.9023 | type:complete len:564 gc:universal TRINITY\_DN8707\_c0\_g1\_i2:2751-1060(-) |
| 161 | 364 | TRINITY\_DN41831\_c0\_g1\_i1.p3 | 27.5 | 5 | 5 | 4.83e2 | 1 | 1 | 1 |  | 12698.6445 | type:internal len:115 gc:universal TRINITY\_DN41831\_c0\_g1\_i1:342-1(-) |
| 162 | 365 | TRINITY\_DN2490\_c0\_g1\_i1.p1 | 62.3 | 1 | 1 | 0 | 1 | 1 | 1 |  | 116032.3672 | type:complete len:1044 gc:universal TRINITY\_DN2490\_c0\_g1\_i1:354-3485(+) |
| 163 | 366 | TRINITY\_DN39385\_c0\_g1\_i1.p1 | 66.71 | 8 | 8 | 0 | 1 | 1 | 1 |  | 17334.5918 | type:5prime\_partial len:154 gc:universal TRINITY\_DN39385\_c0\_g1\_i1:2-463(+) |
| 163 | 367 | TRINITY\_DN39385\_c0\_g1\_i2.p1 | 66.71 | 10 | 10 | 0 | 1 | 1 | 1 |  | 13149.8369 | type:5prime\_partial len:117 gc:universal TRINITY\_DN39385\_c0\_g1\_i2:2-352(+) |
| 164 | 368 | TRINITY\_DN367\_c0\_g2\_i1.p1 | 27.34 | 3 | 3 | 1.02e4 | 1 | 1 | 1 | Carbamidomethylation | 23551.5996 | type:complete len:199 gc:universal TRINITY\_DN367\_c0\_g2\_i1:67-663(+) |
| 165 | 369 | TRINITY\_DN1121\_c0\_g1\_i1.p1 | 64.88 | 4 | 4 | 1.59e3 | 1 | 1 | 1 | Acetylation (Protein N-term) | 28282.5449 | type:complete len:252 gc:universal TRINITY\_DN1121\_c0\_g1\_i1:1155-400(-) |
| 166 | 370 | TRINITY\_DN3773\_c0\_g1\_i1.p1 | 66.68 | 4 | 4 | 5.71e2 | 1 | 1 | 2 | Carbamidomethylation | 56129.7773 | type:complete len:504 gc:universal TRINITY\_DN3773\_c0\_g1\_i1:168-1679(+) |
| 167 | 371 | TRINITY\_DN32522\_c0\_g1\_i1.p1 | 61.28 | 4 | 4 | 0 | 1 | 1 | 2 |  | 26779.0059 | type:complete len:235 gc:universal TRINITY\_DN32522\_c0\_g1\_i1:147-851(+) |
| 168 | 372 | TRINITY\_DN18187\_c0\_g1\_i4.p3 | 66.57 | 8 | 8 | 7.47e2 | 1 | 1 | 1 |  | 13023.127 | type:5prime\_partial len:112 gc:universal TRINITY\_DN18187\_c0\_g1\_i4:361-26(-) |
| 168 | 373 | TRINITY\_DN18187\_c0\_g1\_i6.p2 | 66.57 | 5 | 5 | 7.47e2 | 1 | 1 | 1 |  | 19395.627 | type:5prime\_partial len:178 gc:universal TRINITY\_DN18187\_c0\_g1\_i6:559-26(-) |
| 168 | 374 | TRINITY\_DN5023\_c0\_g1\_i2.p2 | 66.57 | 8 | 8 | 7.47e2 | 1 | 1 | 1 |  | 12713.9521 | type:internal len:117 gc:universal TRINITY\_DN5023\_c0\_g1\_i2:348-1(-) |
| 168 | 375 | TRINITY\_DN72753\_c0\_g1\_i1.p1 | 66.57 | 7 | 7 | 7.47e2 | 1 | 1 | 1 |  | 15430.793 | type:complete len:137 gc:universal TRINITY\_DN72753\_c0\_g1\_i1:95-505(+) |
| 168 | 376 | TRINITY\_DN18187\_c0\_g1\_i3.p4 | 66.57 | 6 | 6 | 7.47e2 | 1 | 1 | 1 |  | 15989.5195 | type:5prime\_partial len:143 gc:universal TRINITY\_DN18187\_c0\_g1\_i3:454-26(-) |
| 168 | 377 | TRINITY\_DN5023\_c0\_g1\_i3.p2 | 66.57 | 9 | 9 | 7.47e2 | 1 | 1 | 1 |  | 11215.2041 | type:internal len:105 gc:universal TRINITY\_DN5023\_c0\_g1\_i3:313-2(-) |
| 168 | 378 | TRINITY\_DN18187\_c0\_g1\_i5.p1 | 66.57 | 5 | 5 | 7.47e2 | 1 | 1 | 1 |  | 19887.0645 | type:5prime\_partial len:182 gc:universal TRINITY\_DN18187\_c0\_g1\_i5:664-119(-) |
| 169 | 379 | TRINITY\_DN34191\_c0\_g1\_i1.p1 | 62.83 | 5 | 5 | 0 | 1 | 1 | 1 | Oxidation (M) | 26288.2148 | type:internal len:238 gc:universal TRINITY\_DN34191\_c0\_g1\_i1:1-711(+) |
| 170 | 380 | TRINITY\_DN12\_c0\_g2\_i1.p1 | 29.91 | 5 | 5 | 0 | 1 | 1 | 1 |  | 21647.1582 | type:complete len:203 gc:universal TRINITY\_DN12\_c0\_g2\_i1:239-847(+) |
| 170 | 381 | TRINITY\_DN12\_c0\_g1\_i1.p1 | 29.91 | 5 | 5 | 0 | 1 | 1 | 1 |  | 21891.4863 | type:complete len:203 gc:universal TRINITY\_DN12\_c0\_g1\_i1:141-749(+) |
| 171 | 382 | TRINITY\_DN2301\_c0\_g1\_i1.p1 | 62.53 | 4 | 4 | 8.92e1 | 1 | 1 | 1 |  | 50428.9648 | type:5prime\_partial len:452 gc:universal TRINITY\_DN2301\_c0\_g1\_i1:2886-1531(-) |
| 172 | 383 | TRINITY\_DN97809\_c0\_g1\_i1.p1 | 61.56 | 7 | 7 | 0 | 1 | 1 | 2 |  | 20461.1211 | type:internal len:184 gc:universal TRINITY\_DN97809\_c0\_g1\_i1:2-550(+) |
| 173 | 384 | TRINITY\_DN278\_c0\_g1\_i1.p1 | 62.01 | 3 | 3 | 0 | 1 | 1 | 1 |  | 55197.2969 | type:complete len:470 gc:universal TRINITY\_DN278\_c0\_g1\_i1:3091-1682(-) |
| 174 | 385 | TRINITY\_DN413\_c0\_g1\_i4.p1 | 66.61 | 2 | 2 | 9.37e2 | 1 | 1 | 1 |  | 52354.1758 | type:3prime\_partial len:463 gc:universal TRINITY\_DN413\_c0\_g1\_i4:1388-3(-) |
| 174 | 386 | TRINITY\_DN413\_c0\_g1\_i1.p1 | 66.61 | 3 | 3 | 9.37e2 | 1 | 1 | 1 |  | 40351.7656 | type:complete len:355 gc:universal TRINITY\_DN413\_c0\_g1\_i1:1352-288(-) |
| 174 | 387 | TRINITY\_DN413\_c0\_g1\_i2.p1 | 66.61 | 8 | 8 | 9.37e2 | 1 | 1 | 1 |  | 15739.6523 | type:3prime\_partial len:140 gc:universal TRINITY\_DN413\_c0\_g1\_i2:419-3(-) |
| 174 | 388 | TRINITY\_DN413\_c0\_g1\_i3.p1 | 66.61 | 3 | 3 | 9.37e2 | 1 | 1 | 1 |  | 39501.6133 | type:complete len:350 gc:universal TRINITY\_DN413\_c0\_g1\_i3:1286-237(-) |
| 175 | 389 | TRINITY\_DN26892\_c0\_g1\_i1.p1 | 61.7 | 11 | 11 | 0 | 1 | 1 | 1 |  | 27019.6895 | type:complete len:233 gc:universal TRINITY\_DN26892\_c0\_g1\_i1:108-806(+) |
| 176 | 390 | TRINITY\_DN6\_c0\_g1\_i1.p1 | 39.17 | 7 | 7 | 0 | 1 | 1 | 1 |  | 23154.6738 | type:complete len:196 gc:universal TRINITY\_DN6\_c0\_g1\_i1:843-256(-) |
| 177 | 391 | TRINITY\_DN76040\_c0\_g1\_i1.p1 | 66.83 | 3 | 3 | 0 | 1 | 1 | 1 |  | 43150.668 | type:5prime\_partial len:383 gc:universal TRINITY\_DN76040\_c0\_g1\_i1:1-1149(+) |
| 178 | 392 | TRINITY\_DN15016\_c0\_g1\_i2.p1 | 66.68 | 2 | 2 | 0 | 1 | 1 | 1 |  | 62089.082 | type:complete len:566 gc:universal TRINITY\_DN15016\_c0\_g1\_i2:94-1791(+) |
| 178 | 393 | TRINITY\_DN15016\_c0\_g1\_i3.p1 | 66.68 | 2 | 2 | 0 | 1 | 1 | 1 |  | 62084.0977 | type:complete len:566 gc:universal TRINITY\_DN15016\_c0\_g1\_i3:94-1791(+) |
| 179 | 394 | TRINITY\_DN1397\_c0\_g1\_i2.p1 | 17.17 | 9 | 9 | 4.76e2 | 1 | 1 | 1 |  | 12114.6553 | type:5prime\_partial len:107 gc:universal TRINITY\_DN1397\_c0\_g1\_i2:2-322(+) |
| 179 | 395 | TRINITY\_DN1397\_c0\_g1\_i3.p1 | 17.17 | 9 | 9 | 4.76e2 | 1 | 1 | 1 |  | 12502.1777 | type:5prime\_partial len:110 gc:universal TRINITY\_DN1397\_c0\_g1\_i3:1-330(+) |
| 179 | 396 | TRINITY\_DN1397\_c0\_g1\_i1.p1 | 17.17 | 9 | 9 | 4.76e2 | 1 | 1 | 1 |  | 12085.7002 | type:5prime\_partial len:106 gc:universal TRINITY\_DN1397\_c0\_g1\_i1:2-319(+) |
| 180 | 397 | TRINITY\_DN128406\_c0\_g1\_i1.p1 | 66.7 | 7 | 7 | 1.75e4 | 1 | 1 | 3 |  | 16958.5273 | type:5prime\_partial len:150 gc:universal TRINITY\_DN128406\_c0\_g1\_i1:474-25(-) |
| 181 | 398 | TRINITY\_DN1315\_c0\_g1\_i1.p1 | 29.88 | 5 | 5 | 0 | 1 | 1 | 1 |  | 41477.8164 | type:complete len:371 gc:universal TRINITY\_DN1315\_c0\_g1\_i1:246-1358(+) |
| 182 | 399 | TRINITY\_DN33483\_c0\_g1\_i1.p1 | 66.6 | 4 | 4 | 2.33e2 | 1 | 1 | 1 |  | 38962.4062 | type:complete len:344 gc:universal TRINITY\_DN33483\_c0\_g1\_i1:56-1087(+) |
| 183 | 400 | TRINITY\_DN1091\_c0\_g1\_i1.p1 | 63.4 | 8 | 8 | 3.21e2 | 1 | 1 | 2 | Carbamidomethylation | 24868.0293 | type:complete len:212 gc:universal TRINITY\_DN1091\_c0\_g1\_i1:200-835(+) |
| 184 | 401 | TRINITY\_DN26679\_c0\_g1\_i1.p3 | 26.73 | 6 | 6 | 2.53e3 | 1 | 1 | 2 | Acetylation (Protein N-term) | 12077.7803 | type:5prime\_partial len:108 gc:universal TRINITY\_DN26679\_c0\_g1\_i1:1-324(+) |
| 185 | 402 | TRINITY\_DN36471\_c0\_g1\_i1.p1 | 63.27 | 4 | 4 | 1.17e2 | 1 | 1 | 1 |  | 33492.4688 | type:internal len:286 gc:universal TRINITY\_DN36471\_c0\_g1\_i1:2-856(+) |
| 186 | 403 | TRINITY\_DN1479\_c0\_g1\_i1.p1 | 41.22 | 11 | 11 | 5.43e2 | 1 | 1 | 1 | Carbamidomethylation | 12183.8027 | type:5prime\_partial len:107 gc:universal TRINITY\_DN1479\_c0\_g1\_i1:397-77(-) |
| 187 | 404 | TRINITY\_DN73587\_c0\_g1\_i1.p1 | 58.94 | 3 | 3 | 0 | 1 | 1 | 1 |  | 57376.2227 | type:complete len:533 gc:universal TRINITY\_DN73587\_c0\_g1\_i1:94-1692(+) |
| 188 | 405 | TRINITY\_DN493\_c0\_g1\_i1.p1 | 16.2 | 3 | 3 | 0 | 1 | 1 | 1 | Carbamidomethylation | 55371.7461 | type:complete len:473 gc:universal TRINITY\_DN493\_c0\_g1\_i1:2681-1263(-) |
| 189 | 406 | TRINITY\_DN751\_c0\_g1\_i1.p1 | 66.4 | 5 | 5 | 1.72e3 | 1 | 1 | 1 |  | 25720.373 | type:complete len:227 gc:universal TRINITY\_DN751\_c0\_g1\_i1:731-51(-) |
| 189 | 407 | TRINITY\_DN7214\_c0\_g1\_i1.p1 | 66.4 | 5 | 5 | 1.72e3 | 1 | 1 | 1 |  | 25800.4062 | type:complete len:228 gc:universal TRINITY\_DN7214\_c0\_g1\_i1:39-722(+) |
| 190 | 408 | TRINITY\_DN165222\_c0\_g1\_i1.p2 | 17.62 | 8 | 8 | 5.12e3 | 1 | 1 | 1 |  | 15107.1035 | type:5prime\_partial len:133 gc:universal TRINITY\_DN165222\_c0\_g1\_i1:1-399(+) |
| 191 | 409 | TRINITY\_DN925\_c0\_g1\_i1.p1 | 66.75 | 5 | 5 | 0 | 1 | 1 | 2 |  | 28336.7676 | type:complete len:261 gc:universal TRINITY\_DN925\_c0\_g1\_i1:826-44(-) |
| 192 | 410 | TRINITY\_DN624\_c0\_g1\_i1.p1 | 61.52 | 2 | 2 | 0 | 1 | 1 | 1 |  | 101743.5391 | type:complete len:884 gc:universal TRINITY\_DN624\_c0\_g1\_i1:218-2869(+) |
| 193 | 411 | TRINITY\_DN146208\_c0\_g1\_i1.p1 | 66.7 | 5 | 5 | 3.39e2 | 1 | 1 | 1 |  | 32340.8027 | type:5prime\_partial len:284 gc:universal TRINITY\_DN146208\_c0\_g1\_i1:876-25(-) |
| 194 | 412 | TRINITY\_DN120478\_c0\_g1\_i1.p1 | 62.05 | 2 | 2 | 2.93e2 | 1 | 1 | 1 |  | 59788.2227 | type:complete len:544 gc:universal TRINITY\_DN120478\_c0\_g1\_i1:2674-1043(-) |
| 195 | 413 | TRINITY\_DN165536\_c0\_g1\_i1.p1 | 66.51 | 6 | 6 | 8.59e2 | 1 | 1 | 1 |  | 28339.4531 | type:complete len:249 gc:universal TRINITY\_DN165536\_c0\_g1\_i1:851-105(-) |
| 196 | 414 | TRINITY\_DN13856\_c0\_g1\_i2.p2 | 61.84 | 7 | 7 | 3.57e2 | 1 | 1 | 1 |  | 31731.2012 | type:5prime\_partial len:292 gc:universal TRINITY\_DN13856\_c0\_g1\_i2:3-878(+) |
| 196 | 415 | TRINITY\_DN13856\_c0\_g1\_i1.p2 | 61.84 | 7 | 7 | 3.57e2 | 1 | 1 | 1 |  | 31646.0527 | type:5prime\_partial len:292 gc:universal TRINITY\_DN13856\_c0\_g1\_i1:3-878(+) |
| 197 | 416 | TRINITY\_DN140996\_c0\_g1\_i1.p1 | 20.95 | 6 | 6 | 0 | 1 | 1 | 1 | Carbamidomethylation | 12619.5664 | type:internal len:109 gc:universal TRINITY\_DN140996\_c0\_g1\_i1:326-3(-) |
| 198 | 417 | TRINITY\_DN200\_c0\_g1\_i2.p1 | 64.76 | 2 | 2 | 4.46e2 | 1 | 1 | 1 |  | 59337.1367 | type:complete len:551 gc:universal TRINITY\_DN200\_c0\_g1\_i2:123-1775(+) |
| 198 | 418 | TRINITY\_DN200\_c0\_g1\_i3.p1 | 64.76 | 2 | 2 | 4.46e2 | 1 | 1 | 1 |  | 59435.2422 | type:complete len:551 gc:universal TRINITY\_DN200\_c0\_g1\_i3:110-1762(+) |
| 198 | 419 | TRINITY\_DN200\_c0\_g1\_i5.p1 | 64.76 | 2 | 2 | 4.46e2 | 1 | 1 | 1 |  | 59339.1094 | type:complete len:551 gc:universal TRINITY\_DN200\_c0\_g1\_i5:110-1762(+) |
| 198 | 420 | TRINITY\_DN200\_c0\_g1\_i1.p1 | 64.76 | 2 | 2 | 4.46e2 | 1 | 1 | 1 |  | 59435.2422 | type:complete len:551 gc:universal TRINITY\_DN200\_c0\_g1\_i1:168-1820(+) |
| 199 | 421 | TRINITY\_DN21847\_c1\_g1\_i1.p1 | 66.66 | 8 | 8 | 8.99e2 | 1 | 1 | 2 | Acetylation (Protein N-term) | 14363.4707 | type:internal len:133 gc:universal TRINITY\_DN21847\_c1\_g1\_i1:397-2(-) |
| 199 | 422 | TRINITY\_DN8165\_c0\_g2\_i2.p2 | 66.66 | 5 | 5 | 8.99e2 | 1 | 1 | 2 | Acetylation (Protein N-term) | 21784.791 | type:internal len:204 gc:universal TRINITY\_DN8165\_c0\_g2\_i2:3-611(+) |
| 199 | 423 | TRINITY\_DN8165\_c0\_g2\_i1.p2 | 66.66 | 10 | 10 | 8.99e2 | 1 | 1 | 2 | Acetylation (Protein N-term) | 10817.4883 | type:internal len:102 gc:universal TRINITY\_DN8165\_c0\_g2\_i1:3-305(+) |
| 200 | 424 | TRINITY\_DN7808\_c2\_g1\_i2.p1 | 51.82 | 4 | 4 | 6.38e2 | 1 | 1 | 1 |  | 29272.7227 | type:3prime\_partial len:277 gc:universal TRINITY\_DN7808\_c2\_g1\_i2:830-3(-) |
| 200 | 425 | TRINITY\_DN2487\_c0\_g1\_i4.p1 | 51.82 | 3 | 3 | 6.38e2 | 1 | 1 | 1 |  | 40387.0664 | type:internal len:370 gc:universal TRINITY\_DN2487\_c0\_g1\_i4:2-1108(+) |
| 200 | 426 | TRINITY\_DN142212\_c0\_g1\_i1.p1 | 51.82 | 6 | 6 | 6.38e2 | 1 | 1 | 1 |  | 18882.6484 | type:internal len:178 gc:universal TRINITY\_DN142212\_c0\_g1\_i1:3-533(+) |
| 200 | 427 | TRINITY\_DN7808\_c2\_g1\_i3.p1 | 51.82 | 4 | 4 | 6.38e2 | 1 | 1 | 1 |  | 31066.7188 | type:internal len:294 gc:universal TRINITY\_DN7808\_c2\_g1\_i3:881-3(-) |
| 200 | 428 | TRINITY\_DN7808\_c2\_g1\_i1.p1 | 51.82 | 6 | 6 | 6.38e2 | 1 | 1 | 1 |  | 19420.2109 | type:5prime\_partial len:180 gc:universal TRINITY\_DN7808\_c2\_g1\_i1:568-29(-) |
| 201 | 429 | TRINITY\_DN7671\_c0\_g1\_i1.p1 | 66.67 | 10 | 10 | 1.87e3 | 1 | 1 | 1 | Carbamidomethylation | 13919.8574 | type:complete len:123 gc:universal TRINITY\_DN7671\_c0\_g1\_i1:425-57(-) |
| 202 | 430 | TRINITY\_DN1468\_c0\_g1\_i1.p1 | 49.48 | 4 | 4 | 0 | 1 | 1 | 1 |  | 28461.1699 | type:complete len:245 gc:universal TRINITY\_DN1468\_c0\_g1\_i1:766-32(-) |
| 203 | 431 | TRINITY\_DN5587\_c0\_g1\_i1.p1 | 48.88 | 1 | 1 | 0 | 1 | 1 | 1 |  | 89981.1016 | type:complete len:809 gc:universal TRINITY\_DN5587\_c0\_g1\_i1:196-2622(+) |
| 204 | 432 | TRINITY\_DN4595\_c0\_g1\_i4.p1 | 22.7 | 0 | 0 | 0 | 1 | 1 | 1 |  | 183684.4844 | type:5prime\_partial len:1639 gc:universal TRINITY\_DN4595\_c0\_g1\_i4:5857-941(-) |
| 204 | 433 | TRINITY\_DN4595\_c0\_g1\_i7.p1 | 22.7 | 2 | 2 | 0 | 1 | 1 | 1 |  | 38387.582 | type:internal len:348 gc:universal TRINITY\_DN4595\_c0\_g1\_i7:1042-2(-) |
| 204 | 434 | TRINITY\_DN4595\_c0\_g1\_i10.p2 | 22.7 | 2 | 2 | 0 | 1 | 1 | 1 |  | 39822.0156 | type:5prime\_partial len:358 gc:universal TRINITY\_DN4595\_c0\_g1\_i10:5369-4296(-) |
| 205 | 435 | TRINITY\_DN166344\_c0\_g1\_i1.p1 | 66.31 | 3 | 3 | 1.17e3 | 1 | 1 | 1 | Carbamidomethylation | 40794.2773 | type:complete len:360 gc:universal TRINITY\_DN166344\_c0\_g1\_i1:93-1172(+) |
| 206 | 436 | TRINITY\_DN484\_c0\_g1\_i1.p1 | 63.47 | 3 | 3 | 0 | 1 | 1 | 1 |  | 59940.2617 | type:complete len:554 gc:universal TRINITY\_DN484\_c0\_g1\_i1:164-1825(+) |
| 207 | 437 | TRINITY\_DN119650\_c0\_g1\_i1.p1 | 62.3 | 7 | 7 | 0 | 1 | 1 | 1 |  | 23946.6621 | type:complete len:211 gc:universal TRINITY\_DN119650\_c0\_g1\_i1:883-251(-) |
| 208 | 438 | TRINITY\_DN7808\_c1\_g3\_i1.p1 | 62.01 | 6 | 6 | 0 | 1 | 1 | 1 |  | 23305.7031 | type:internal len:212 gc:universal TRINITY\_DN7808\_c1\_g3\_i1:635-3(-) |
| 208 | 439 | TRINITY\_DN7808\_c1\_g3\_i2.p2 | 62.01 | 7 | 7 | 0 | 1 | 1 | 1 |  | 18163.7988 | type:internal len:166 gc:universal TRINITY\_DN7808\_c1\_g3\_i2:495-1(-) |
| 208 | 440 | TRINITY\_DN7808\_c1\_g3\_i3.p1 | 62.01 | 6 | 6 | 0 | 1 | 1 | 1 |  | 23224.3848 | type:internal len:210 gc:universal TRINITY\_DN7808\_c1\_g3\_i3:628-2(-) |
| 209 | 441 | TRINITY\_DN131028\_c0\_g1\_i1.p2 | 62.93 | 11 | 11 | 4.03e3 | 1 | 1 | 2 |  | 11838.5801 | type:5prime\_partial len:104 gc:universal TRINITY\_DN131028\_c0\_g1\_i1:2-313(+) |
| 210 | 442 | TRINITY\_DN1272\_c0\_g1\_i1.p1 | 66.74 | 4 | 4 | 9.07e2 | 1 | 1 | 2 |  | 30690.1172 | type:complete len:262 gc:universal TRINITY\_DN1272\_c0\_g1\_i1:957-172(-) |
| 211 | 443 | TRINITY\_DN884\_c0\_g1\_i1.p1 | 66.65 | 3 | 3 | 2.91e2 | 1 | 1 | 1 |  | 62089.875 | type:complete len:542 gc:universal TRINITY\_DN884\_c0\_g1\_i1:1783-158(-) |
| 212 | 444 | TRINITY\_DN453\_c0\_g1\_i1.p1 | 65.21 | 6 | 6 | 0 | 1 | 1 | 1 | Carbamidomethylation | 27993.4082 | type:complete len:249 gc:universal TRINITY\_DN453\_c0\_g1\_i1:944-198(-) |
| 212 | 445 | TRINITY\_DN453\_c0\_g1\_i2.p1 | 65.21 | 6 | 6 | 0 | 1 | 1 | 1 | Carbamidomethylation | 27993.4082 | type:complete len:249 gc:universal TRINITY\_DN453\_c0\_g1\_i2:944-198(-) |
| 213 | 446 | TRINITY\_DN59667\_c0\_g1\_i1.p1 | 66.65 | 4 | 4 | 0 | 1 | 1 | 2 |  | 25518.6816 | type:3prime\_partial len:225 gc:universal TRINITY\_DN59667\_c0\_g1\_i1:673-2(-) |
| 214 | 447 | TRINITY\_DN12390\_c0\_g1\_i1.p1 | 66.81 | 4 | 4 | 0 | 1 | 1 | 1 |  | 30457.0371 | type:complete len:268 gc:universal TRINITY\_DN12390\_c0\_g1\_i1:826-23(-) |
| 215 | 448 | TRINITY\_DN4815\_c0\_g1\_i2.p1 | 47.49 | 2 | 2 | 1.74e2 | 1 | 1 | 1 | Carbamidomethylation | 70695.4688 | type:complete len:638 gc:universal TRINITY\_DN4815\_c0\_g1\_i2:79-1992(+) |
| 215 | 449 | TRINITY\_DN4815\_c0\_g1\_i1.p1 | 47.49 | 2 | 2 | 1.74e2 | 1 | 1 | 1 | Carbamidomethylation | 66133.5156 | type:complete len:595 gc:universal TRINITY\_DN4815\_c0\_g1\_i1:79-1863(+) |
| 216 | 450 | TRINITY\_DN22594\_c0\_g1\_i1.p2 | 41.31 | 4 | 4 | 2.9e3 | 1 | 1 | 1 |  | 29863.3594 | type:internal len:268 gc:universal TRINITY\_DN22594\_c0\_g1\_i1:801-1(-) |
| 217 | 451 | TRINITY\_DN6701\_c0\_g2\_i1.p1 | 66.55 | 7 | 7 | 2.02e3 | 1 | 1 | 1 |  | 18503.1309 | type:internal len:169 gc:universal TRINITY\_DN6701\_c0\_g2\_i1:2-505(+) |
| 218 | 452 | TRINITY\_DN647\_c0\_g1\_i1.p1 | 61.26 | 4 | 4 | 0 | 1 | 1 | 1 |  | 36573.8047 | type:complete len:344 gc:universal TRINITY\_DN647\_c0\_g1\_i1:1110-79(-) |
| 219 | 453 | TRINITY\_DN187147\_c0\_g1\_i1.p1 | 63.28 | 1 | 1 | 2.65e2 | 1 | 1 | 1 |  | 138182.3125 | type:complete len:1223 gc:universal TRINITY\_DN187147\_c0\_g1\_i1:3708-40(-) |
| 220 | 454 | TRINITY\_DN148467\_c0\_g1\_i1.p1 | 66.5 | 6 | 6 | 4.62e2 | 1 | 1 | 1 |  | 24229.5137 | type:complete len:218 gc:universal TRINITY\_DN148467\_c0\_g1\_i1:105-758(+) |
| 221 | 455 | TRINITY\_DN101992\_c0\_g1\_i1.p1 | 23.34 | 7 | 7 | 0 | 1 | 1 | 1 | Carbamidomethylation | 15607.4678 | type:internal len:139 gc:universal TRINITY\_DN101992\_c0\_g1\_i1:414-1(-) |
| 222 | 456 | TRINITY\_DN98420\_c0\_g1\_i1.p1 | 63.61 | 3 | 3 | 1.42e3 | 1 | 1 | 1 |  | 31374.8145 | type:5prime\_partial len:286 gc:universal TRINITY\_DN98420\_c0\_g1\_i1:1007-150(-) |
| 223 | 457 | TRINITY\_DN478\_c2\_g1\_i1.p1 | 63.95 | 12 | 12 | 5.77e2 | 1 | 1 | 2 |  | 16466.8184 | type:complete len:145 gc:universal TRINITY\_DN478\_c2\_g1\_i1:3-437(+) |
| 224 | 458 | TRINITY\_DN187527\_c0\_g1\_i1.p1 | 61.94 | 12 | 12 | 0 | 1 | 1 | 2 | Acetylation (Protein N-term) | 11905.7393 | type:complete len:102 gc:universal TRINITY\_DN187527\_c0\_g1\_i1:392-87(-) |
| 225 | 459 | TRINITY\_DN79\_c0\_g1\_i1.p1 | 62.08 | 4 | 4 | 2.36e2 | 1 | 1 | 1 |  | 52590.9805 | type:5prime\_partial len:478 gc:universal TRINITY\_DN79\_c0\_g1\_i1:2132-699(-) |
| 226 | 460 | TRINITY\_DN7808\_c1\_g2\_i1.p1 | 65.96 | 5 | 5 | 2.08e3 | 1 | 1 | 1 |  | 26262.0195 | type:internal len:237 gc:universal TRINITY\_DN7808\_c1\_g2\_i1:710-3(-) |
| 227 | 461 | TRINITY\_DN36276\_c0\_g1\_i1.p1 | 65.56 | 4 | 4 | 0 | 1 | 1 | 1 |  | 49209.5391 | type:complete len:449 gc:universal TRINITY\_DN36276\_c0\_g1\_i1:104-1450(+) |
| 228 | 462 | TRINITY\_DN165969\_c0\_g1\_i1.p1 | 16.47 | 6 | 6 | 4.59e3 | 1 | 1 | 1 |  | 13455.2549 | type:internal len:128 gc:universal TRINITY\_DN165969\_c0\_g1\_i1:2-382(+) |
| 229 | 463 | TRINITY\_DN1428\_c0\_g1\_i1.p1 | 66.68 | 9 | 9 | 2.71e2 | 1 | 1 | 1 |  | 18259.2207 | type:5prime\_partial len:165 gc:universal TRINITY\_DN1428\_c0\_g1\_i1:1-495(+) |
| 230 | 464 | TRINITY\_DN2805\_c0\_g1\_i1.p1 | 62.74 | 7 | 7 | 1.45e3 | 1 | 1 | 1 | Oxidation (M) | 21692.6699 | type:complete len:189 gc:universal TRINITY\_DN2805\_c0\_g1\_i1:623-57(-) |
| 231 | 465 | TRINITY\_DN8008\_c0\_g1\_i2.p1 | 66.61 | 1 | 1 | 0 | 1 | 1 | 2 |  | 146342.75 | type:5prime\_partial len:1298 gc:universal TRINITY\_DN8008\_c0\_g1\_i2:4205-312(-) |
| 231 | 466 | TRINITY\_DN8008\_c0\_g1\_i3.p1 | 66.61 | 1 | 1 | 0 | 1 | 1 | 2 |  | 146351.7188 | type:5prime\_partial len:1298 gc:universal TRINITY\_DN8008\_c0\_g1\_i3:4205-312(-) |
| 232 | 467 | TRINITY\_DN2068\_c0\_g1\_i1.p1 | 61.79 | 10 | 10 | 2.77e2 | 1 | 1 | 1 |  | 22662.4531 | type:complete len:204 gc:universal TRINITY\_DN2068\_c0\_g1\_i1:160-771(+) |
| 233 | 468 | TRINITY\_DN87064\_c0\_g1\_i1.p1 | 66.67 | 7 | 7 | 1.94e2 | 1 | 1 | 1 |  | 19998.8125 | type:internal len:179 gc:universal TRINITY\_DN87064\_c0\_g1\_i1:536-3(-) |
| 234 | 469 | TRINITY\_DN1084\_c0\_g1\_i1.p1 | 66.77 | 4 | 4 | 0 | 1 | 1 | 1 |  | 31571.6562 | type:complete len:301 gc:universal TRINITY\_DN1084\_c0\_g1\_i1:1131-229(-) |
| 235 | 470 | TRINITY\_DN1339\_c0\_g2\_i5.p1 | 66.63 | 4 | 4 | 0 | 1 | 1 | 1 |  | 38453.7148 | type:internal len:362 gc:universal TRINITY\_DN1339\_c0\_g2\_i5:1085-3(-) |
| 236 | 471 | TRINITY\_DN598\_c1\_g1\_i2.p1 | 66.55 | 1 | 1 | 1.2e3 | 1 | 1 | 1 |  | 81043.625 | type:5prime\_partial len:742 gc:universal TRINITY\_DN598\_c1\_g1\_i2:2-2227(+) |
| 237 | 472 | TRINITY\_DN22219\_c0\_g1\_i1.p1 | 40.71 | 3 | 3 | 8.74e2 | 1 | 1 | 1 |  | 49350.1836 | type:5prime\_partial len:427 gc:universal TRINITY\_DN22219\_c0\_g1\_i1:2-1282(+) |
| 238 | 473 | TRINITY\_DN5001\_c0\_g1\_i1.p1 | 59.41 | 1 | 1 | 0 | 1 | 1 | 1 |  | 177237.6562 | type:complete len:1575 gc:universal TRINITY\_DN5001\_c0\_g1\_i1:40-4764(+) |
| 239 | 474 | TRINITY\_DN244\_c0\_g1\_i1.p1 | 63.33 | 0 | 0 | 1.67e2 | 1 | 1 | 1 |  | 313594.0938 | type:complete len:3019 gc:universal TRINITY\_DN244\_c0\_g1\_i1:9748-692(-) |
| 240 | 475 | TRINITY\_DN785\_c0\_g1\_i1.p1 | 40.39 | 4 | 4 | 5.32e2 | 1 | 1 | 1 | Carbamidomethylation | 33410.082 | type:complete len:289 gc:universal TRINITY\_DN785\_c0\_g1\_i1:1062-196(-) |
| 241 | 476 | TRINITY\_DN1534\_c2\_g1\_i1.p1 | 17.27 | 1 | 1 | 0 | 1 | 1 | 1 |  | 59603.6055 | type:complete len:547 gc:universal TRINITY\_DN1534\_c2\_g1\_i1:1914-274(-) |
| 242 | 477 | TRINITY\_DN4781\_c0\_g1\_i1.p1 | 66.76 | 0 | 0 | 7.22e2 | 1 | 1 | 1 |  | 302042.125 | type:5prime\_partial len:2696 gc:universal TRINITY\_DN4781\_c0\_g1\_i1:10504-2417(-) |
| 243 | 478 | TRINITY\_DN2028\_c0\_g1\_i1.p1 | 62.38 | 5 | 5 | 3.68e3 | 1 | 1 | 1 |  | 30106.9453 | type:complete len:274 gc:universal TRINITY\_DN2028\_c0\_g1\_i1:1608-787(-) |
| 244 | 479 | TRINITY\_DN436\_c0\_g1\_i1.p1 | 66.61 | 9 | 9 | 1.37e3 | 1 | 1 | 1 |  | 13711.3193 | type:complete len:116 gc:universal TRINITY\_DN436\_c0\_g1\_i1:74-421(+) |
| 245 | 480 | TRINITY\_DN1378\_c0\_g1\_i1.p1 | 62.09 | 3 | 3 | 4.46e2 | 1 | 1 | 1 |  | 47978.9219 | type:complete len:443 gc:universal TRINITY\_DN1378\_c0\_g1\_i1:2151-823(-) |
| 246 | 481 | TRINITY\_DN6783\_c0\_g1\_i1.p1 | 45.38 | 4 | 4 | 5.41e2 | 1 | 1 | 1 |  | 34142.9297 | type:complete len:314 gc:universal TRINITY\_DN6783\_c0\_g1\_i1:1018-77(-) |
| 247 | 482 | TRINITY\_DN98968\_c0\_g1\_i1.p1 | 34.99 | 2 | 2 | 1.4e3 | 1 | 1 | 2 | Carbamidomethylation | 47268.0352 | type:3prime\_partial len:424 gc:universal TRINITY\_DN98968\_c0\_g1\_i1:1269-1(-) |
| 248 | 483 | TRINITY\_DN319\_c2\_g1\_i3.p1 | 66.67 | 1 | 1 | 0 | 1 | 1 | 2 |  | 279087.1875 | type:complete len:2588 gc:universal TRINITY\_DN319\_c2\_g1\_i3:165-7928(+) |
| 248 | 484 | TRINITY\_DN319\_c2\_g1\_i1.p1 | 66.67 | 1 | 1 | 0 | 1 | 1 | 2 |  | 279168.25 | type:complete len:2588 gc:universal TRINITY\_DN319\_c2\_g1\_i1:165-7928(+) |
| 249 | 485 | TRINITY\_DN164351\_c0\_g1\_i1.p1 | 43.13 | 5 | 5 | 0 | 1 | 1 | 1 |  | 23723.9492 | type:complete len:212 gc:universal TRINITY\_DN164351\_c0\_g1\_i1:694-59(-) |
| 250 | 486 | TRINITY\_DN578\_c0\_g1\_i1.p1 | 66.52 | 3 | 3 | 0 | 1 | 1 | 1 |  | 47588.7344 | type:complete len:429 gc:universal TRINITY\_DN578\_c0\_g1\_i1:379-1665(+) |
| 251 | 487 | TRINITY\_DN10907\_c0\_g1\_i1.p1 | 64.74 | 6 | 6 | 6.21e2 | 1 | 1 | 1 |  | 23412.5352 | type:complete len:206 gc:universal TRINITY\_DN10907\_c0\_g1\_i1:1010-393(-) |
| 252 | 488 | TRINITY\_DN138\_c0\_g1\_i1.p1 | 66.78 | 4 | 4 | 3.44e2 | 1 | 1 | 1 |  | 46324.8945 | type:complete len:401 gc:universal TRINITY\_DN138\_c0\_g1\_i1:157-1359(+) |
| 253 | 489 | TRINITY\_DN1320\_c0\_g2\_i1.p1 | 66.44 | 5 | 5 | 0 | 1 | 1 | 1 |  | 28787.1875 | type:complete len:260 gc:universal TRINITY\_DN1320\_c0\_g2\_i1:97-876(+) |
| 254 | 490 | TRINITY\_DN1114\_c0\_g1\_i1.p1 | 66.7 | 12 | 12 | 3e2 | 1 | 1 | 1 |  | 12915.5664 | type:complete len:118 gc:universal TRINITY\_DN1114\_c0\_g1\_i1:462-815(+) |
| 254 | 491 | TRINITY\_DN1114\_c0\_g1\_i2.p1 | 66.7 | 12 | 12 | 3e2 | 1 | 1 | 1 |  | 12915.5664 | type:complete len:118 gc:universal TRINITY\_DN1114\_c0\_g1\_i2:165-518(+) |
| 255 | 492 | TRINITY\_DN773\_c0\_g1\_i1.p1 | 61.46 | 3 | 3 | 6.1e2 | 1 | 1 | 1 |  | 48865.75 | type:complete len:440 gc:universal TRINITY\_DN773\_c0\_g1\_i1:1608-289(-) |
| 256 | 493 | TRINITY\_DN164128\_c0\_g1\_i1.p1 | 41.49 | 5 | 5 | 1.16e4 | 1 | 1 | 1 |  | 17885.4395 | type:complete len:166 gc:universal TRINITY\_DN164128\_c0\_g1\_i1:117-614(+) |
| 257 | 494 | TRINITY\_DN51048\_c0\_g1\_i1.p1 | 32.35 | 1 | 1 | 8.92e3 | 1 | 1 | 1 | Carbamidomethylation | 87809.5703 | type:3prime\_partial len:761 gc:universal TRINITY\_DN51048\_c0\_g1\_i1:2282-3(-) |
| 258 | 495 | TRINITY\_DN23911\_c0\_g1\_i1.p1 | 59.04 | 10 | 10 | 0 | 1 | 1 | 1 |  | 23109.5176 | type:3prime\_partial len:203 gc:universal TRINITY\_DN23911\_c0\_g1\_i1:606-1(-) |
| 259 | 496 | TRINITY\_DN598\_c1\_g1\_i1.p1 | 66.54 | 1 | 1 | 1.96e3 | 1 | 1 | 1 |  | 81322.8672 | type:5prime\_partial len:745 gc:universal TRINITY\_DN598\_c1\_g1\_i1:2-2236(+) |
| 260 | 497 | TRINITY\_DN6008\_c0\_g1\_i1.p1 | 60.22 | 4 | 4 | 0 | 1 | 1 | 1 |  | 47488.5547 | type:complete len:426 gc:universal TRINITY\_DN6008\_c0\_g1\_i1:1620-343(-) |
| 261 | 498 | TRINITY\_DN614\_c0\_g1\_i1.p1 | 52.31 | 5 | 5 | 0 | 1 | 1 | 1 |  | 20573.5293 | type:complete len:183 gc:universal TRINITY\_DN614\_c0\_g1\_i1:194-742(+) |
| 262 | 499 | TRINITY\_DN7573\_c0\_g1\_i4.p2 | 18.84 | 9 | 9 | 3.36e2 | 1 | 1 | 1 |  | 15652.9033 | type:internal len:151 gc:universal TRINITY\_DN7573\_c0\_g1\_i4:3-452(+) |
| 263 | 500 | TRINITY\_DN76073\_c0\_g1\_i1.p1 | 41.64 | 8 | 8 | 3.58e3 | 1 | 1 | 2 |  | 12905.3965 | type:complete len:107 gc:universal TRINITY\_DN76073\_c0\_g1\_i1:794-1114(+) |
| 264 | 501 | TRINITY\_DN113\_c3\_g1\_i1.p1 | 62.84 | 1 | 1 | 0 | 1 | 1 | 1 |  | 125422.4922 | type:3prime\_partial len:1162 gc:universal TRINITY\_DN113\_c3\_g1\_i1:3485-3(-) |
| 265 | 502 | TRINITY\_DN2904\_c0\_g1\_i1.p1 | 65.45 | 6 | 6 | 4.71e2 | 1 | 1 | 1 |  | 24728.3496 | type:complete len:221 gc:universal TRINITY\_DN2904\_c0\_g1\_i1:1339-677(-) |
| 266 | 503 | TRINITY\_DN11662\_c0\_g1\_i1.p1 | 63.33 | 3 | 3 | 2.31e3 | 1 | 1 | 1 | Acetylation (Protein N-term) | 41619.3711 | type:complete len:398 gc:universal TRINITY\_DN11662\_c0\_g1\_i1:75-1268(+) |
| 267 | 504 | TRINITY\_DN1065\_c0\_g1\_i1.p1 | 64.29 | 5 | 5 | 3.02e2 | 1 | 1 | 1 |  | 40043.2539 | type:complete len:373 gc:universal TRINITY\_DN1065\_c0\_g1\_i1:53-1171(+) |
| total 504 proteins |  |  |  |  |  |  |  |  |  |  |  |  |
| --- | --- | --- | --- | --- | --- | --- | --- | --- | --- | --- | --- | --- |

  

TRINITY\_DN11435\_c0\_g1\_i1.p1

back to list

  

| Protein Coverage
| Supporting Peptides
| Best Unique PSM
|

Protein Coverage:

Supporting Peptides:

| Peptide | Uniq | -10lgP | Mass | Length | ppm | m/z | z | RT | 1/k0 Start | 1/k0 End | Precursor ID | Area Sample 1 | #Feature | #Feature Sample 1 | Start | End | PTM | AScore | Found By |
| --- | --- | --- | --- | --- | --- | --- | --- | --- | --- | --- | --- | --- | --- | --- | --- | --- | --- | --- | --- |
| K.VYLAQAEAEKIK.M | Y | 66.77 | 1361.7554 | 12 | 0.13 | 681.882 | 2 | 14.27 | 1.0347 | 1.0419 | 38198 | 3.91e2 | 1 | 1 | 309 | 320 |  |  | DB Search |
| R.IRNEEIQIEVIERR.K | Y | 66.68 | 1795.9904 | 14 | 1.69 | 599.6691 | 3 | 15.10 | 0.8798 | 0.887 | 42640 | 8.61e2 | 1 | 1 | 252 | 265 |  |  | DB Search |
| K.LIEVEEKEIQR.R | Y | 66.16 | 1384.7562 | 11 | -8.44 | 693.3764 | 2 | 13.80 | 1.0146 | 1.0218 | 35646 | 0 | 0 | 0 | 267 | 277 |  |  | DB Search |
| K.LAYELQANKEQQR.I | Y | 61.78 | 1589.8161 | 13 | -0.05 | 795.9117 | 2 | 12.90 | 1.0508 | 1.058 | 30507 | 0 | 0 | 0 | 239 | 251 |  |  | DB Search |
| K.LIEVEEKEIQRR.E | Y | 24.84 | 1540.8573 | 12 | -0.66 | 514.6237 | 3 | 13.09 | 0.7836 | 0.7909 | 31641 | 0 | 0 | 0 | 267 | 278 |  |  | DB Search |
| total 5 peptides |  |  |  |  |  |  |  |  |  |  |  |  |  |  |  |  |  |  |  |
| --- | --- | --- | --- | --- | --- | --- | --- | --- | --- | --- | --- | --- | --- | --- | --- | --- | --- | --- | --- |

Best Unique PSM (Scan Sample\_10\_A1\_1\_3647.d:38198, m/z=681.882, z=2, RT=14.27, ppm=-4.36):


TRINITY\_DN5107\_c0\_g2\_i1.p1

back to list

  

| Protein Coverage
| Supporting Peptides
| Best Unique PSM
|

Protein Coverage:

Supporting Peptides:

| Peptide | Uniq | -10lgP | Mass | Length | ppm | m/z | z | RT | 1/k0 Start | 1/k0 End | Precursor ID | Area Sample 1 | #Feature | #Feature Sample 1 | Start | End | PTM | AScore | Found By |
| --- | --- | --- | --- | --- | --- | --- | --- | --- | --- | --- | --- | --- | --- | --- | --- | --- | --- | --- | --- |
| K.KLDYVLGLTTAK.I | Y | 63.57 | 1320.7653 | 12 | -2.37 | 661.3854 | 2 | 16.69 | 0.9908 | 0.9981 | 51148 | 2.13e2 | 1 | 1 | 18 | 29 |  |  | DB Search |
| total 1 peptides |  |  |  |  |  |  |  |  |  |  |  |  |  |  |  |  |  |  |  |
| --- | --- | --- | --- | --- | --- | --- | --- | --- | --- | --- | --- | --- | --- | --- | --- | --- | --- | --- | --- |

Best Unique PSM (Scan Sample\_10\_A1\_1\_3647.d:51148, m/z=661.3854, z=2, RT=16.69, ppm=-6.87):


TRINITY\_DN72753\_c0\_g1\_i1.p1

back to list

  

| Protein Coverage
| Supporting Peptides
| Best Unique PSM
|

Protein Coverage:

Supporting Peptides:

| Peptide | Uniq | -10lgP | Mass | Length | ppm | m/z | z | RT | 1/k0 Start | 1/k0 End | Precursor ID | Area Sample 1 | #Feature | #Feature Sample 1 | Start | End | PTM | AScore | Found By |
| --- | --- | --- | --- | --- | --- | --- | --- | --- | --- | --- | --- | --- | --- | --- | --- | --- | --- | --- | --- |
| R.YRPGTVALR.E | Y | 66.57 | 1031.5876 | 9 | 0.86 | 516.7992 | 2 | 12.50 | 0.8554 | 0.8627 | 28119 | 7.47e2 | 1 | 1 | 42 | 50 |  |  | DB Search |
| total 1 peptides |  |  |  |  |  |  |  |  |  |  |  |  |  |  |  |  |  |  |  |
| --- | --- | --- | --- | --- | --- | --- | --- | --- | --- | --- | --- | --- | --- | --- | --- | --- | --- | --- | --- |

Best Unique PSM (Scan Sample\_10\_A1\_1\_3647.d:28119, m/z=516.7992, z=2, RT=12.50, ppm=-3.64):


TRINITY\_DN5818\_c1\_g1\_i2.p1

back to list

  

| Protein Coverage
| Supporting Peptides
| Best Unique PSM
|

Protein Coverage:

Supporting Peptides:

| Peptide | Uniq | -10lgP | Mass | Length | ppm | m/z | z | RT | 1/k0 Start | 1/k0 End | Precursor ID | Area Sample 1 | #Feature | #Feature Sample 1 | Start | End | PTM | AScore | Found By |
| --- | --- | --- | --- | --- | --- | --- | --- | --- | --- | --- | --- | --- | --- | --- | --- | --- | --- | --- | --- |
| K.LLAQTTLRNELGTK.N | Y | 34.86 | 1556.8886 | 14 | -1.74 | 779.4467 | 2 | 15.17 | 1.1102 | 1.1174 | 43095 | 4.27e2 | 1 | 1 | 155 | 168 |  |  | DB Search |
| total 1 peptides |  |  |  |  |  |  |  |  |  |  |  |  |  |  |  |  |  |  |  |
| --- | --- | --- | --- | --- | --- | --- | --- | --- | --- | --- | --- | --- | --- | --- | --- | --- | --- | --- | --- |

Best Unique PSM (Scan Sample\_10\_A1\_1\_3647.d:43095, m/z=779.4467, z=2, RT=15.17, ppm=-6.23):


TRINITY\_DN647\_c0\_g1\_i1.p1

back to list

  

| Protein Coverage
| Supporting Peptides
| Best Unique PSM
|

Protein Coverage:

Supporting Peptides:

| Peptide | Uniq | -10lgP | Mass | Length | ppm | m/z | z | RT | 1/k0 Start | 1/k0 End | Precursor ID | Area Sample 1 | #Feature | #Feature Sample 1 | Start | End | PTM | AScore | Found By |
| --- | --- | --- | --- | --- | --- | --- | --- | --- | --- | --- | --- | --- | --- | --- | --- | --- | --- | --- | --- |
| R.FGFSILEAMNGKK.G | Y | 61.26 | 1440.7435 | 13 | 2.19 | 481.254 | 3 | 19.76 | 0.7819 | 0.7892 | 69523 | 0 | 0 | 0 | 263 | 275 |  |  | DB Search |
| total 1 peptides |  |  |  |  |  |  |  |  |  |  |  |  |  |  |  |  |  |  |  |
| --- | --- | --- | --- | --- | --- | --- | --- | --- | --- | --- | --- | --- | --- | --- | --- | --- | --- | --- | --- |

Best Unique PSM (Scan Sample\_10\_A1\_1\_3647.d:69523, m/z=481.254, z=3, RT=19.76, ppm=-2.31):


TRINITY\_DN72959\_c0\_g1\_i1.p1

back to list

  

| Protein Coverage
| Supporting Peptides
| Best Unique PSM
|

Protein Coverage:

Supporting Peptides:

| Peptide | Uniq | -10lgP | Mass | Length | ppm | m/z | z | RT | 1/k0 Start | 1/k0 End | Precursor ID | Area Sample 1 | #Feature | #Feature Sample 1 | Start | End | PTM | AScore | Found By |
| --- | --- | --- | --- | --- | --- | --- | --- | --- | --- | --- | --- | --- | --- | --- | --- | --- | --- | --- | --- |
| R.GAFGKPQGTVAR.V | Y | 62.21 | 1187.6411 | 12 | -0.01 | 594.8251 | 2 | 11.39 | 0.9234 | 0.9307 | 21663 | 1.25e3 | 1 | 1 | 117 | 128 |  |  | DB Search |
| K.DQHEATAIEALRR.A | Y | 62.02 | 1508.7695 | 13 | 4.8 | 503.9306 | 3 | 13.82 | 0.8027 | 0.81 | 35722 | 0 | 0 | 0 | 142 | 154 |  |  | DB Search |
| total 2 peptides |  |  |  |  |  |  |  |  |  |  |  |  |  |  |  |  |  |  |  |
| --- | --- | --- | --- | --- | --- | --- | --- | --- | --- | --- | --- | --- | --- | --- | --- | --- | --- | --- | --- |

Best Unique PSM (Scan Sample\_10\_A1\_1\_3647.d:21663, m/z=594.8251, z=2, RT=11.39, ppm=-4.51):


TRINITY\_DN164128\_c0\_g1\_i1.p1

back to list

  

| Protein Coverage
| Supporting Peptides
| Best Unique PSM
|

Protein Coverage:

Supporting Peptides:

| Peptide | Uniq | -10lgP | Mass | Length | ppm | m/z | z | RT | 1/k0 Start | 1/k0 End | Precursor ID | Area Sample 1 | #Feature | #Feature Sample 1 | Start | End | PTM | AScore | Found By |
| --- | --- | --- | --- | --- | --- | --- | --- | --- | --- | --- | --- | --- | --- | --- | --- | --- | --- | --- | --- |
| K.ALKEPPRDR.K | Y | 41.49 | 1080.604 | 9 | 9.05 | 361.2102 | 3 | 9.43 | 0.7314 | 0.7388 | 10516 | 1.16e4 | 1 | 1 | 84 | 92 |  |  | DB Search |
| total 1 peptides |  |  |  |  |  |  |  |  |  |  |  |  |  |  |  |  |  |  |  |
| --- | --- | --- | --- | --- | --- | --- | --- | --- | --- | --- | --- | --- | --- | --- | --- | --- | --- | --- | --- |

Best Unique PSM (Scan Sample\_10\_A1\_1\_3647.d:10516, m/z=361.2102, z=3, RT=9.43, ppm=4.55):


TRINITY\_DN101992\_c0\_g1\_i1.p1

back to list

  

| Protein Coverage
| Supporting Peptides
| Best Unique PSM
|

Protein Coverage:

Supporting Peptides:

| Peptide | Uniq | -10lgP | Mass | Length | ppm | m/z | z | RT | 1/k0 Start | 1/k0 End | Precursor ID | Area Sample 1 | #Feature | #Feature Sample 1 | Start | End | PTM | AScore | Found By |
| --- | --- | --- | --- | --- | --- | --- | --- | --- | --- | --- | --- | --- | --- | --- | --- | --- | --- | --- | --- |
| K.AELVFVNHC(+57.02)R.E | Y | 23.34 | 1243.6132 | 10 | -1.51 | 622.8101 | 2 | 14.29 | 0.9537 | 0.961 | 38337 | 0 | 0 | 0 | 118 | 127 | Carbamidomethylation |  | DB Search |
| total 1 peptides |  |  |  |  |  |  |  |  |  |  |  |  |  |  |  |  |  |  |  |
| --- | --- | --- | --- | --- | --- | --- | --- | --- | --- | --- | --- | --- | --- | --- | --- | --- | --- | --- | --- |

Best Unique PSM (Scan Sample\_10\_A1\_1\_3647.d:38337, m/z=622.8101, z=2, RT=14.29, ppm=-6):


TRINITY\_DN453\_c0\_g1\_i1.p1

back to list

  

| Protein Coverage
| Supporting Peptides
| Best Unique PSM
|

Protein Coverage:

Supporting Peptides:

| Peptide | Uniq | -10lgP | Mass | Length | ppm | m/z | z | RT | 1/k0 Start | 1/k0 End | Precursor ID | Area Sample 1 | #Feature | #Feature Sample 1 | Start | End | PTM | AScore | Found By |
| --- | --- | --- | --- | --- | --- | --- | --- | --- | --- | --- | --- | --- | --- | --- | --- | --- | --- | --- | --- |
| K.VSILVAC(+57.02)NKQDLTTAK.A | Y | 65.21 | 1759.9502 | 16 | -1.35 | 880.9772 | 2 | 14.68 | 1.1829 | 1.1901 | 40439 | 0 | 0 | 0 | 150 | 165 | Carbamidomethylation |  | DB Search |
| total 1 peptides |  |  |  |  |  |  |  |  |  |  |  |  |  |  |  |  |  |  |  |
| --- | --- | --- | --- | --- | --- | --- | --- | --- | --- | --- | --- | --- | --- | --- | --- | --- | --- | --- | --- |

Best Unique PSM (Scan Sample\_10\_A1\_1\_3647.d:40439, m/z=880.9772, z=2, RT=14.68, ppm=-5.84):


TRINITY\_DN1377\_c0\_g1\_i1.p1

back to list

  

| Protein Coverage
| Supporting Peptides
| Best Unique PSM
|

Protein Coverage:

Supporting Peptides:

| Peptide | Uniq | -10lgP | Mass | Length | ppm | m/z | z | RT | 1/k0 Start | 1/k0 End | Precursor ID | Area Sample 1 | #Feature | #Feature Sample 1 | Start | End | PTM | AScore | Found By |
| --- | --- | --- | --- | --- | --- | --- | --- | --- | --- | --- | --- | --- | --- | --- | --- | --- | --- | --- | --- |
| K.RLPDQITTR.S | Y | 66.44 | 1098.6145 | 9 | 1.99 | 550.3132 | 2 | 12.54 | 0.8741 | 0.8814 | 28273 | 1.51e3 | 1 | 1 | 182 | 190 |  |  | DB Search |
| R.SVTTHQGNAIVIK.I | Y | 64.94 | 1366.7568 | 13 | 0.36 | 684.3829 | 2 | 12.49 | 0.9945 | 1.0017 | 28112 | 2.8e2 | 1 | 1 | 191 | 203 |  |  | DB Search |
| total 2 peptides |  |  |  |  |  |  |  |  |  |  |  |  |  |  |  |  |  |  |  |
| --- | --- | --- | --- | --- | --- | --- | --- | --- | --- | --- | --- | --- | --- | --- | --- | --- | --- | --- | --- |

Best Unique PSM (Scan Sample\_10\_A1\_1\_3647.d:28273, m/z=550.3132, z=2, RT=12.54, ppm=-2.5):


TRINITY\_DN59667\_c0\_g1\_i1.p1

back to list

  

| Protein Coverage
| Supporting Peptides
| Best Unique PSM
|

Protein Coverage:

Supporting Peptides:

| Peptide | Uniq | -10lgP | Mass | Length | ppm | m/z | z | RT | 1/k0 Start | 1/k0 End | Precursor ID | Area Sample 1 | #Feature | #Feature Sample 1 | Start | End | PTM | AScore | Found By |
| --- | --- | --- | --- | --- | --- | --- | --- | --- | --- | --- | --- | --- | --- | --- | --- | --- | --- | --- | --- |
| R.IEHAHPINR.I | Y | 66.65 | 1085.573 | 9 | 6.4 | 362.8656 | 3 | 9.48 | 0.7363 | 0.7437 | 10908 | 0 | 0 | 0 | 85 | 93 |  |  | DB Search |
| total 1 peptides |  |  |  |  |  |  |  |  |  |  |  |  |  |  |  |  |  |  |  |
| --- | --- | --- | --- | --- | --- | --- | --- | --- | --- | --- | --- | --- | --- | --- | --- | --- | --- | --- | --- |

Best Unique PSM (Scan Sample\_10\_A1\_1\_3647.d:10908, m/z=362.8656, z=3, RT=9.48, ppm=1.9):


TRINITY\_DN6701\_c0\_g2\_i1.p1

back to list

  

| Protein Coverage
| Supporting Peptides
| Best Unique PSM
|

Protein Coverage:

Supporting Peptides:

| Peptide | Uniq | -10lgP | Mass | Length | ppm | m/z | z | RT | 1/k0 Start | 1/k0 End | Precursor ID | Area Sample 1 | #Feature | #Feature Sample 1 | Start | End | PTM | AScore | Found By |
| --- | --- | --- | --- | --- | --- | --- | --- | --- | --- | --- | --- | --- | --- | --- | --- | --- | --- | --- | --- |
| K.AITASEPEALKR.K | Y | 66.55 | 1284.7037 | 12 | -4.07 | 643.3536 | 2 | 12.85 | 0.9727 | 0.98 | 30111 | 2.02e3 | 1 | 1 | 122 | 133 |  |  | DB Search |
| total 1 peptides |  |  |  |  |  |  |  |  |  |  |  |  |  |  |  |  |  |  |  |
| --- | --- | --- | --- | --- | --- | --- | --- | --- | --- | --- | --- | --- | --- | --- | --- | --- | --- | --- | --- |

Best Unique PSM (Scan Sample\_10\_A1\_1\_3647.d:30111, m/z=643.3536, z=2, RT=12.85, ppm=-8.57):


TRINITY\_DN1172\_c0\_g1\_i3.p1

back to list

  

| Protein Coverage
| Supporting Peptides
| Best Unique PSM
|

Protein Coverage:

Supporting Peptides:

| Peptide | Uniq | -10lgP | Mass | Length | ppm | m/z | z | RT | 1/k0 Start | 1/k0 End | Precursor ID | Area Sample 1 | #Feature | #Feature Sample 1 | Start | End | PTM | AScore | Found By |
| --- | --- | --- | --- | --- | --- | --- | --- | --- | --- | --- | --- | --- | --- | --- | --- | --- | --- | --- | --- |
| K.VITSDDPAEKEK.K | Y | 66.7 | 1330.6616 | 12 | 2.85 | 666.337 | 2 | 10.71 | 0.9836 | 0.9908 | 17367 | 7.2e3 | 1 | 1 | 123 | 134 |  |  | DB Search |
| total 1 peptides |  |  |  |  |  |  |  |  |  |  |  |  |  |  |  |  |  |  |  |
| --- | --- | --- | --- | --- | --- | --- | --- | --- | --- | --- | --- | --- | --- | --- | --- | --- | --- | --- | --- |

Best Unique PSM (Scan Sample\_10\_A1\_1\_3647.d:17367, m/z=666.337, z=2, RT=10.71, ppm=-1.65):


TRINITY\_DN2637\_c0\_g1\_i9.p2

back to list

  

| Protein Coverage
| Supporting Peptides
| Best Unique PSM
|

Protein Coverage:

Supporting Peptides:

| Peptide | Uniq | -10lgP | Mass | Length | ppm | m/z | z | RT | 1/k0 Start | 1/k0 End | Precursor ID | Area Sample 1 | #Feature | #Feature Sample 1 | Start | End | PTM | AScore | Found By |
| --- | --- | --- | --- | --- | --- | --- | --- | --- | --- | --- | --- | --- | --- | --- | --- | --- | --- | --- | --- |
| K.ATIEIPAKYEK.R | Y | 61.89 | 1261.6918 | 11 | 0.22 | 631.8505 | 2 | 14.08 | 0.982 | 0.9892 | 37168 | 0 | 0 | 0 | 107 | 117 |  |  | DB Search |
| K.VSVTLADGTVATQEVLPGATIVVK.A | Y | 20.47 | 2367.326 | 24 | 4.39 | 1184.6702 | 2 | 19.66 | 1.2833 | 1.2905 | 68866 | 0 | 0 | 0 | 248 | 271 |  |  | DB Search |
| total 2 peptides |  |  |  |  |  |  |  |  |  |  |  |  |  |  |  |  |  |  |  |
| --- | --- | --- | --- | --- | --- | --- | --- | --- | --- | --- | --- | --- | --- | --- | --- | --- | --- | --- | --- |

Best Unique PSM (Scan Sample\_10\_A1\_1\_3647.d:37168, m/z=631.8505, z=2, RT=14.08, ppm=-4.27):


TRINITY\_DN22594\_c0\_g1\_i1.p2

back to list

  

| Protein Coverage
| Supporting Peptides
| Best Unique PSM
|

Protein Coverage:

Supporting Peptides:

| Peptide | Uniq | -10lgP | Mass | Length | ppm | m/z | z | RT | 1/k0 Start | 1/k0 End | Precursor ID | Area Sample 1 | #Feature | #Feature Sample 1 | Start | End | PTM | AScore | Found By |
| --- | --- | --- | --- | --- | --- | --- | --- | --- | --- | --- | --- | --- | --- | --- | --- | --- | --- | --- | --- |
| R.FRQLGLDFGK.L | Y | 41.31 | 1179.64 | 10 | -5.77 | 590.8212 | 2 | 19.79 | 0.9214 | 0.9287 | 69618 | 2.9e3 | 1 | 1 | 40 | 49 |  |  | DB Search |
| total 1 peptides |  |  |  |  |  |  |  |  |  |  |  |  |  |  |  |  |  |  |  |
| --- | --- | --- | --- | --- | --- | --- | --- | --- | --- | --- | --- | --- | --- | --- | --- | --- | --- | --- | --- |

Best Unique PSM (Scan Sample\_10\_A1\_1\_3647.d:69618, m/z=590.8212, z=2, RT=19.79, ppm=-10.26):


TRINITY\_DN167384\_c0\_g1\_i1.p1

back to list

  

| Protein Coverage
| Supporting Peptides
| Best Unique PSM
|

Protein Coverage:

Supporting Peptides:

| Peptide | Uniq | -10lgP | Mass | Length | ppm | m/z | z | RT | 1/k0 Start | 1/k0 End | Precursor ID | Area Sample 1 | #Feature | #Feature Sample 1 | Start | End | PTM | AScore | Found By |
| --- | --- | --- | --- | --- | --- | --- | --- | --- | --- | --- | --- | --- | --- | --- | --- | --- | --- | --- | --- |
| R.DKTLILWELTR.D | Y | 49.3 | 1386.7871 | 11 | 3.36 | 694.4 | 2 | 19.94 | 0.9945 | 1.0017 | 70462 | 1.74e2 | 1 | 1 | 36 | 46 |  |  | DB Search |
| R.FSPNTQNPIIVSC(+57.02)GWDK.M | Y | 25.22 | 1961.9305 | 17 | 3.93 | 981.972 | 2 | 17.89 | 1.1638 | 1.171 | 58040 | 0 | 0 | 0 | 156 | 172 | Carbamidomethylation |  | DB Search |
| total 2 peptides |  |  |  |  |  |  |  |  |  |  |  |  |  |  |  |  |  |  |  |
| --- | --- | --- | --- | --- | --- | --- | --- | --- | --- | --- | --- | --- | --- | --- | --- | --- | --- | --- | --- |

Best Unique PSM (Scan Sample\_10\_A1\_1\_3647.d:70462, m/z=694.4, z=2, RT=19.94, ppm=-1.13):


TRINITY\_DN982\_c0\_g1\_i2.p1

back to list

  

| Protein Coverage
| Supporting Peptides
| Best Unique PSM
|

Protein Coverage:

Supporting Peptides:

| Peptide | Uniq | -10lgP | Mass | Length | ppm | m/z | z | RT | 1/k0 Start | 1/k0 End | Precursor ID | Area Sample 1 | #Feature | #Feature Sample 1 | Start | End | PTM | AScore | Found By |
| --- | --- | --- | --- | --- | --- | --- | --- | --- | --- | --- | --- | --- | --- | --- | --- | --- | --- | --- | --- |
| K.VLLSQQIFYDKK.N | Y | 66.52 | 1480.8289 | 12 | -1.68 | 741.4172 | 2 | 16.70 | 1.06 | 1.0672 | 51062 | 6.76e2 | 1 | 1 | 1479 | 1490 |  |  | DB Search |
| total 1 peptides |  |  |  |  |  |  |  |  |  |  |  |  |  |  |  |  |  |  |  |
| --- | --- | --- | --- | --- | --- | --- | --- | --- | --- | --- | --- | --- | --- | --- | --- | --- | --- | --- | --- |

Best Unique PSM (Scan Sample\_10\_A1\_1\_3647.d:51062, m/z=741.4172, z=2, RT=16.70, ppm=-6.17):


TRINITY\_DN413\_c0\_g1\_i3.p1

back to list

  

| Protein Coverage
| Supporting Peptides
| Best Unique PSM
|

Protein Coverage:

Supporting Peptides:

| Peptide | Uniq | -10lgP | Mass | Length | ppm | m/z | z | RT | 1/k0 Start | 1/k0 End | Precursor ID | Area Sample 1 | #Feature | #Feature Sample 1 | Start | End | PTM | AScore | Found By |
| --- | --- | --- | --- | --- | --- | --- | --- | --- | --- | --- | --- | --- | --- | --- | --- | --- | --- | --- | --- |
| R.LLLGDFNGDKR.T | Y | 66.61 | 1246.6669 | 11 | 0.98 | 624.3386 | 2 | 16.00 | 0.9848 | 0.992 | 47446 | 9.37e2 | 1 | 1 | 46 | 56 |  |  | DB Search |
| total 1 peptides |  |  |  |  |  |  |  |  |  |  |  |  |  |  |  |  |  |  |  |
| --- | --- | --- | --- | --- | --- | --- | --- | --- | --- | --- | --- | --- | --- | --- | --- | --- | --- | --- | --- |

Best Unique PSM (Scan Sample\_10\_A1\_1\_3647.d:47446, m/z=624.3386, z=2, RT=16.00, ppm=-3.51):


TRINITY\_DN478\_c2\_g1\_i1.p1

back to list

  

| Protein Coverage
| Supporting Peptides
| Best Unique PSM
|

Protein Coverage:

Supporting Peptides:

| Peptide | Uniq | -10lgP | Mass | Length | ppm | m/z | z | RT | 1/k0 Start | 1/k0 End | Precursor ID | Area Sample 1 | #Feature | #Feature Sample 1 | Start | End | PTM | AScore | Found By |
| --- | --- | --- | --- | --- | --- | --- | --- | --- | --- | --- | --- | --- | --- | --- | --- | --- | --- | --- | --- |
| K.KGELMDYQLPLHSQVFEK.X | Y | 63.95 | 2161.0877 | 18 | 0.45 | 721.3669 | 3 | 17.73 | 0.9896 | 0.9969 | 57002 | 5.77e2 | 1 | 1 | 127 | 144 |  |  | DB Search |
| total 1 peptides |  |  |  |  |  |  |  |  |  |  |  |  |  |  |  |  |  |  |  |
| --- | --- | --- | --- | --- | --- | --- | --- | --- | --- | --- | --- | --- | --- | --- | --- | --- | --- | --- | --- |

Best Unique PSM (Scan Sample\_10\_A1\_1\_3647.d:57002, m/z=721.3669, z=3, RT=17.73, ppm=-4.04):


TRINITY\_DN2487\_c0\_g1\_i4.p1

back to list

  

| Protein Coverage
| Supporting Peptides
| Best Unique PSM
|

Protein Coverage:

Supporting Peptides:

| Peptide | Uniq | -10lgP | Mass | Length | ppm | m/z | z | RT | 1/k0 Start | 1/k0 End | Precursor ID | Area Sample 1 | #Feature | #Feature Sample 1 | Start | End | PTM | AScore | Found By |
| --- | --- | --- | --- | --- | --- | --- | --- | --- | --- | --- | --- | --- | --- | --- | --- | --- | --- | --- | --- |
| K.RSLNAELANGR.L | Y | 51.82 | 1199.6371 | 11 | 2.77 | 600.8248 | 2 | 11.99 | 0.9259 | 0.9331 | 25198 | 6.38e2 | 1 | 1 | 154 | 164 |  |  | DB Search |
| total 1 peptides |  |  |  |  |  |  |  |  |  |  |  |  |  |  |  |  |  |  |  |
| --- | --- | --- | --- | --- | --- | --- | --- | --- | --- | --- | --- | --- | --- | --- | --- | --- | --- | --- | --- |

Best Unique PSM (Scan Sample\_10\_A1\_1\_3647.d:25198, m/z=600.8248, z=2, RT=11.99, ppm=-1.73):


TRINITY\_DN776\_c0\_g1\_i1.p1

back to list

  

| Protein Coverage
| Supporting Peptides
| Best Unique PSM
|

Protein Coverage:

Supporting Peptides:

| Peptide | Uniq | -10lgP | Mass | Length | ppm | m/z | z | RT | 1/k0 Start | 1/k0 End | Precursor ID | Area Sample 1 | #Feature | #Feature Sample 1 | Start | End | PTM | AScore | Found By |
| --- | --- | --- | --- | --- | --- | --- | --- | --- | --- | --- | --- | --- | --- | --- | --- | --- | --- | --- | --- |
| R.RLFEGNALLR.R | Y | 66.66 | 1187.6775 | 10 | 1.42 | 594.8442 | 2 | 16.07 | 0.9384 | 0.9457 | 47820 | 1.65e3 | 1 | 1 | 77 | 86 |  |  | DB Search |
| R.M(+15.99)KLDYVLGLKVDDFLER.R | Y | 66.31 | 2069.0867 | 17 | 5.54 | 690.7036 | 3 | 19.97 | 0.9731 | 0.9804 | 70746 | 0 | 0 | 0 | 99 | 115 | Oxidation (M) | M1:Oxidation (M):1000 | DB Search |
| R.LFEGNALLRR.L | Y | 66.2 | 1187.6775 | 10 | 0.49 | 594.8436 | 2 | 15.56 | 0.9675 | 0.9747 | 45173 | 2.25e3 | 1 | 1 | 78 | 87 |  |  | DB Search |
| R.KQLVNVPSFIVR.L | Y | 61.54 | 1398.8347 | 12 | 1.44 | 700.4225 | 2 | 17.76 | 1.0065 | 1.0138 | 57229 | 4.4e2 | 1 | 1 | 146 | 157 |  |  | DB Search |
| R.DLLTLPEKDNRR.L | Y | 36.23 | 1468.7998 | 12 | 2.6 | 735.4058 | 2 | 14.56 | 1.0283 | 1.0355 | 39735 | 1e3 | 1 | 1 | 66 | 77 |  |  | DB Search |
| total 5 peptides |  |  |  |  |  |  |  |  |  |  |  |  |  |  |  |  |  |  |  |
| --- | --- | --- | --- | --- | --- | --- | --- | --- | --- | --- | --- | --- | --- | --- | --- | --- | --- | --- | --- |

Best Unique PSM (Scan Sample\_10\_A1\_1\_3647.d:47820, m/z=594.8442, z=2, RT=16.07, ppm=-3.08):


TRINITY\_DN5818\_c1\_g1\_i1.p1

back to list

  

| Protein Coverage
| Supporting Peptides
| Best Unique PSM
|

Protein Coverage:

Supporting Peptides:

| Peptide | Uniq | -10lgP | Mass | Length | ppm | m/z | z | RT | 1/k0 Start | 1/k0 End | Precursor ID | Area Sample 1 | #Feature | #Feature Sample 1 | Start | End | PTM | AScore | Found By |
| --- | --- | --- | --- | --- | --- | --- | --- | --- | --- | --- | --- | --- | --- | --- | --- | --- | --- | --- | --- |
| K.LLAQTTLRNELGTK.N | Y | 34.86 | 1556.8886 | 14 | -1.74 | 779.4467 | 2 | 15.17 | 1.1102 | 1.1174 | 43095 | 4.27e2 | 1 | 1 | 144 | 157 |  |  | DB Search |
| total 1 peptides |  |  |  |  |  |  |  |  |  |  |  |  |  |  |  |  |  |  |  |
| --- | --- | --- | --- | --- | --- | --- | --- | --- | --- | --- | --- | --- | --- | --- | --- | --- | --- | --- | --- |

Best Unique PSM (Scan Sample\_10\_A1\_1\_3647.d:43095, m/z=779.4467, z=2, RT=15.17, ppm=-6.23):


TRINITY\_DN3986\_c0\_g1\_i2.p1

back to list

  

| Protein Coverage
| Supporting Peptides
| Best Unique PSM
|

Protein Coverage:

Supporting Peptides:

| Peptide | Uniq | -10lgP | Mass | Length | ppm | m/z | z | RT | 1/k0 Start | 1/k0 End | Precursor ID | Area Sample 1 | #Feature | #Feature Sample 1 | Start | End | PTM | AScore | Found By |
| --- | --- | --- | --- | --- | --- | --- | --- | --- | --- | --- | --- | --- | --- | --- | --- | --- | --- | --- | --- |
| K.NSSYFVEWIPNNVK.T | Y | 64.67 | 1695.8256 | 14 | -4.28 | 848.9127 | 2 | 19.92 | 1.0889 | 1.0961 | 70457 | 0 | 0 | 0 | 337 | 350 |  |  | DB Search |
| R.FPGQLNADLRK.L | Y | 62.07 | 1257.6829 | 11 | -5.49 | 629.8425 | 2 | 14.27 | 0.9428 | 0.9501 | 38203 | 0 | 0 | 0 | 242 | 252 |  |  | DB Search |
| total 2 peptides |  |  |  |  |  |  |  |  |  |  |  |  |  |  |  |  |  |  |  |
| --- | --- | --- | --- | --- | --- | --- | --- | --- | --- | --- | --- | --- | --- | --- | --- | --- | --- | --- | --- |

Best Unique PSM (Scan Sample\_10\_A1\_1\_3647.d:70457, m/z=848.9127, z=2, RT=19.92, ppm=-8.78):


TRINITY\_DN319\_c2\_g1\_i1.p1

back to list

  

| Protein Coverage
| Supporting Peptides
| Best Unique PSM
|

Protein Coverage:

Supporting Peptides:

| Peptide | Uniq | -10lgP | Mass | Length | ppm | m/z | z | RT | 1/k0 Start | 1/k0 End | Precursor ID | Area Sample 1 | #Feature | #Feature Sample 1 | Start | End | PTM | AScore | Found By |
| --- | --- | --- | --- | --- | --- | --- | --- | --- | --- | --- | --- | --- | --- | --- | --- | --- | --- | --- | --- |
| K.KAQYYEAYETIIKR.H | Y | 66.67 | 1774.9254 | 14 | 2.41 | 592.6478 | 3 | 15.69 | 0.8627 | 0.87 | 45929 | 0 | 0 | 0 | 2534 | 2547 |  |  | DB Search |
| total 1 peptides |  |  |  |  |  |  |  |  |  |  |  |  |  |  |  |  |  |  |  |
| --- | --- | --- | --- | --- | --- | --- | --- | --- | --- | --- | --- | --- | --- | --- | --- | --- | --- | --- | --- |

Best Unique PSM (Scan Sample\_10\_A1\_1\_3647.d:45929, m/z=592.6478, z=3, RT=15.69, ppm=-2.09):


TRINITY\_DN11097\_c0\_g2\_i1.p1

back to list

  

| Protein Coverage
| Supporting Peptides
| Best Unique PSM
|

Protein Coverage:

Supporting Peptides:

| Peptide | Uniq | -10lgP | Mass | Length | ppm | m/z | z | RT | 1/k0 Start | 1/k0 End | Precursor ID | Area Sample 1 | #Feature | #Feature Sample 1 | Start | End | PTM | AScore | Found By |
| --- | --- | --- | --- | --- | --- | --- | --- | --- | --- | --- | --- | --- | --- | --- | --- | --- | --- | --- | --- |
| R.VAHAGLTVAEFFR.D | Y | 66.78 | 1416.7513 | 13 | 2.84 | 709.3818 | 2 | 18.86 | 1.0267 | 1.0339 | 63839 | 8.41e2 | 1 | 1 | 199 | 211 |  |  | DB Search |
| K.LRVDLSLFETGIK.V | Y | 25.55 | 1489.8504 | 13 | 2.4 | 745.9309 | 2 | 19.94 | 1.0407 | 1.048 | 70562 | 4.53e2 | 1 | 1 | 74 | 86 |  |  | DB Search |
| total 2 peptides |  |  |  |  |  |  |  |  |  |  |  |  |  |  |  |  |  |  |  |
| --- | --- | --- | --- | --- | --- | --- | --- | --- | --- | --- | --- | --- | --- | --- | --- | --- | --- | --- | --- |

Best Unique PSM (Scan Sample\_10\_A1\_1\_3647.d:63839, m/z=709.3818, z=2, RT=18.86, ppm=-1.65):


TRINITY\_DN484\_c0\_g1\_i1.p1

back to list

  

| Protein Coverage
| Supporting Peptides
| Best Unique PSM
|

Protein Coverage:

Supporting Peptides:

| Peptide | Uniq | -10lgP | Mass | Length | ppm | m/z | z | RT | 1/k0 Start | 1/k0 End | Precursor ID | Area Sample 1 | #Feature | #Feature Sample 1 | Start | End | PTM | AScore | Found By |
| --- | --- | --- | --- | --- | --- | --- | --- | --- | --- | --- | --- | --- | --- | --- | --- | --- | --- | --- | --- |
| R.AYHNASQTQSDKAHLK.W | Y | 63.47 | 1797.8758 | 16 | 0.02 | 450.4742 | 4 | 9.43 | 0.7412 | 0.7485 | 10705 | 0 | 0 | 0 | 468 | 483 |  |  | DB Search |
| total 1 peptides |  |  |  |  |  |  |  |  |  |  |  |  |  |  |  |  |  |  |  |
| --- | --- | --- | --- | --- | --- | --- | --- | --- | --- | --- | --- | --- | --- | --- | --- | --- | --- | --- | --- |

Best Unique PSM (Scan Sample\_10\_A1\_1\_3647.d:10705, m/z=450.4742, z=4, RT=9.43, ppm=-4.47):


TRINITY\_DN5107\_c0\_g1\_i11.p1

back to list

  

| Protein Coverage
| Supporting Peptides
| Best Unique PSM
|

Protein Coverage:

Supporting Peptides:

| Peptide | Uniq | -10lgP | Mass | Length | ppm | m/z | z | RT | 1/k0 Start | 1/k0 End | Precursor ID | Area Sample 1 | #Feature | #Feature Sample 1 | Start | End | PTM | AScore | Found By |
| --- | --- | --- | --- | --- | --- | --- | --- | --- | --- | --- | --- | --- | --- | --- | --- | --- | --- | --- | --- |
| K.KLDYVLGLTTAK.I | Y | 63.57 | 1320.7653 | 12 | -2.37 | 661.3854 | 2 | 16.69 | 0.9908 | 0.9981 | 51148 | 2.13e2 | 1 | 1 | 93 | 104 |  |  | DB Search |
| total 1 peptides |  |  |  |  |  |  |  |  |  |  |  |  |  |  |  |  |  |  |  |
| --- | --- | --- | --- | --- | --- | --- | --- | --- | --- | --- | --- | --- | --- | --- | --- | --- | --- | --- | --- |

Best Unique PSM (Scan Sample\_10\_A1\_1\_3647.d:51148, m/z=661.3854, z=2, RT=16.69, ppm=-6.87):


TRINITY\_DN649\_c0\_g1\_i2.p1

back to list

  

| Protein Coverage
| Supporting Peptides
| Best Unique PSM
|

Protein Coverage:

Supporting Peptides:

| Peptide | Uniq | -10lgP | Mass | Length | ppm | m/z | z | RT | 1/k0 Start | 1/k0 End | Precursor ID | Area Sample 1 | #Feature | #Feature Sample 1 | Start | End | PTM | AScore | Found By |
| --- | --- | --- | --- | --- | --- | --- | --- | --- | --- | --- | --- | --- | --- | --- | --- | --- | --- | --- | --- |
| K.FETQIATK.N | Y | 61.61 | 936.4916 | 8 | 0.97 | 937.4956 | 1 | 13.84 | 1.2037 | 1.2109 | 35727 | 1.28e3 | 1 | 1 | 103 | 110 |  |  | DB Search |
| total 1 peptides |  |  |  |  |  |  |  |  |  |  |  |  |  |  |  |  |  |  |  |
| --- | --- | --- | --- | --- | --- | --- | --- | --- | --- | --- | --- | --- | --- | --- | --- | --- | --- | --- | --- |

Best Unique PSM (Scan Sample\_10\_A1\_1\_3647.d:35727, m/z=937.4956, z=1, RT=13.84, ppm=-3.52):


TRINITY\_DN413\_c0\_g1\_i4.p1

back to list

  

| Protein Coverage
| Supporting Peptides
| Best Unique PSM
|

Protein Coverage:

Supporting Peptides:

| Peptide | Uniq | -10lgP | Mass | Length | ppm | m/z | z | RT | 1/k0 Start | 1/k0 End | Precursor ID | Area Sample 1 | #Feature | #Feature Sample 1 | Start | End | PTM | AScore | Found By |
| --- | --- | --- | --- | --- | --- | --- | --- | --- | --- | --- | --- | --- | --- | --- | --- | --- | --- | --- | --- |
| R.LLLGDFNGDKR.T | Y | 66.61 | 1246.6669 | 11 | 0.98 | 624.3386 | 2 | 16.00 | 0.9848 | 0.992 | 47446 | 9.37e2 | 1 | 1 | 46 | 56 |  |  | DB Search |
| total 1 peptides |  |  |  |  |  |  |  |  |  |  |  |  |  |  |  |  |  |  |  |
| --- | --- | --- | --- | --- | --- | --- | --- | --- | --- | --- | --- | --- | --- | --- | --- | --- | --- | --- | --- |

Best Unique PSM (Scan Sample\_10\_A1\_1\_3647.d:47446, m/z=624.3386, z=2, RT=16.00, ppm=-3.51):


TRINITY\_DN152\_c0\_g1\_i9.p1

back to list

  

| Protein Coverage
| Supporting Peptides
| Best Unique PSM
|

Protein Coverage:

Supporting Peptides:

| Peptide | Uniq | -10lgP | Mass | Length | ppm | m/z | z | RT | 1/k0 Start | 1/k0 End | Precursor ID | Area Sample 1 | #Feature | #Feature Sample 1 | Start | End | PTM | AScore | Found By |
| --- | --- | --- | --- | --- | --- | --- | --- | --- | --- | --- | --- | --- | --- | --- | --- | --- | --- | --- | --- |
| K.IQDKEGIPPDQQR.L | Y | 65.08 | 1522.7739 | 13 | -0.66 | 762.3903 | 2 | 11.01 | 1.0339 | 1.0411 | 19352 | 9.49e2 | 1 | 1 | 66 | 78 |  |  | DB Search |
| total 1 peptides |  |  |  |  |  |  |  |  |  |  |  |  |  |  |  |  |  |  |  |
| --- | --- | --- | --- | --- | --- | --- | --- | --- | --- | --- | --- | --- | --- | --- | --- | --- | --- | --- | --- |

Best Unique PSM (Scan Sample\_10\_A1\_1\_3647.d:19352, m/z=762.3903, z=2, RT=11.01, ppm=-5.16):


TRINITY\_DN1114\_c0\_g1\_i2.p1

back to list

  

| Protein Coverage
| Supporting Peptides
| Best Unique PSM
|

Protein Coverage:

Supporting Peptides:

| Peptide | Uniq | -10lgP | Mass | Length | ppm | m/z | z | RT | 1/k0 Start | 1/k0 End | Precursor ID | Area Sample 1 | #Feature | #Feature Sample 1 | Start | End | PTM | AScore | Found By |
| --- | --- | --- | --- | --- | --- | --- | --- | --- | --- | --- | --- | --- | --- | --- | --- | --- | --- | --- | --- |
| R.ADLIAYLEESTTKK.X | Y | 66.7 | 1580.8297 | 14 | 1.07 | 791.4194 | 2 | 19.58 | 1.0985 | 1.1057 | 68341 | 3e2 | 1 | 1 | 104 | 117 |  |  | DB Search |
| total 1 peptides |  |  |  |  |  |  |  |  |  |  |  |  |  |  |  |  |  |  |  |
| --- | --- | --- | --- | --- | --- | --- | --- | --- | --- | --- | --- | --- | --- | --- | --- | --- | --- | --- | --- |

Best Unique PSM (Scan Sample\_10\_A1\_1\_3647.d:68341, m/z=791.4194, z=2, RT=19.58, ppm=-3.42):


TRINITY\_DN278\_c0\_g1\_i1.p1

back to list

  

| Protein Coverage
| Supporting Peptides
| Best Unique PSM
|

Protein Coverage:

Supporting Peptides:

| Peptide | Uniq | -10lgP | Mass | Length | ppm | m/z | z | RT | 1/k0 Start | 1/k0 End | Precursor ID | Area Sample 1 | #Feature | #Feature Sample 1 | Start | End | PTM | AScore | Found By |
| --- | --- | --- | --- | --- | --- | --- | --- | --- | --- | --- | --- | --- | --- | --- | --- | --- | --- | --- | --- |
| K.IDNYIEFLDEDLKK.K | Y | 62.01 | 1753.8774 | 14 | 3.11 | 585.6323 | 3 | 19.83 | 0.8453 | 0.8526 | 69916 | 0 | 0 | 0 | 102 | 115 |  |  | DB Search |
| total 1 peptides |  |  |  |  |  |  |  |  |  |  |  |  |  |  |  |  |  |  |  |
| --- | --- | --- | --- | --- | --- | --- | --- | --- | --- | --- | --- | --- | --- | --- | --- | --- | --- | --- | --- |

Best Unique PSM (Scan Sample\_10\_A1\_1\_3647.d:69916, m/z=585.6323, z=3, RT=19.83, ppm=-1.38):


TRINITY\_DN36276\_c0\_g1\_i1.p1

back to list

  

| Protein Coverage
| Supporting Peptides
| Best Unique PSM
|

Protein Coverage:

Supporting Peptides:

| Peptide | Uniq | -10lgP | Mass | Length | ppm | m/z | z | RT | 1/k0 Start | 1/k0 End | Precursor ID | Area Sample 1 | #Feature | #Feature Sample 1 | Start | End | PTM | AScore | Found By |
| --- | --- | --- | --- | --- | --- | --- | --- | --- | --- | --- | --- | --- | --- | --- | --- | --- | --- | --- | --- |
| R.IPGVISVHEFHIWQLAGNR.I | Y | 65.56 | 2172.1592 | 19 | -17.22 | 725.0446 | 3 | 19.24 | 0.98 | 0.9872 | 66361 | 0 | 0 | 0 | 304 | 322 |  |  | DB Search |
| total 1 peptides |  |  |  |  |  |  |  |  |  |  |  |  |  |  |  |  |  |  |  |
| --- | --- | --- | --- | --- | --- | --- | --- | --- | --- | --- | --- | --- | --- | --- | --- | --- | --- | --- | --- |

Best Unique PSM (Scan Sample\_10\_A1\_1\_3647.d:66361, m/z=725.0446, z=3, RT=19.24, ppm=-21.71):


TRINITY\_DN1172\_c0\_g2\_i2.p1

back to list

  

| Protein Coverage
| Supporting Peptides
| Best Unique PSM
|

Protein Coverage:

Supporting Peptides:

| Peptide | Uniq | -10lgP | Mass | Length | ppm | m/z | z | RT | 1/k0 Start | 1/k0 End | Precursor ID | Area Sample 1 | #Feature | #Feature Sample 1 | Start | End | PTM | AScore | Found By |
| --- | --- | --- | --- | --- | --- | --- | --- | --- | --- | --- | --- | --- | --- | --- | --- | --- | --- | --- | --- |
| K.VLTSSDPAEKEK.K | Y | 66.69 | 1302.6667 | 12 | 3.87 | 652.3402 | 2 | 10.57 | 0.9977 | 1.0049 | 16696 | 7.52e3 | 1 | 1 | 7 | 18 |  |  | DB Search |
| K.K(+42.01)LAAELANGR.L | Y | 64.95 | 1083.6036 | 10 | -0.25 | 542.8065 | 2 | 15.17 | 0.8648 | 0.8721 | 42282 | 4.9e3 | 1 | 1 | 19 | 28 | Acetylation (Protein N-term) | K1:Acetylation (Protein N-term):1000 | DB Search |
| total 2 peptides |  |  |  |  |  |  |  |  |  |  |  |  |  |  |  |  |  |  |  |
| --- | --- | --- | --- | --- | --- | --- | --- | --- | --- | --- | --- | --- | --- | --- | --- | --- | --- | --- | --- |

Best Unique PSM (Scan Sample\_10\_A1\_1\_3647.d:16696, m/z=652.3402, z=2, RT=10.57, ppm=-0.62):


TRINITY\_DN4595\_c0\_g1\_i4.p1

back to list

  

| Protein Coverage
| Supporting Peptides
| Best Unique PSM
|

Protein Coverage:

Supporting Peptides:

| Peptide | Uniq | -10lgP | Mass | Length | ppm | m/z | z | RT | 1/k0 Start | 1/k0 End | Precursor ID | Area Sample 1 | #Feature | #Feature Sample 1 | Start | End | PTM | AScore | Found By |
| --- | --- | --- | --- | --- | --- | --- | --- | --- | --- | --- | --- | --- | --- | --- | --- | --- | --- | --- | --- |
| R.AFNRVETR.D | Y | 22.7 | 991.5199 | 8 | -1.27 | 496.7644 | 2 | 10.70 | 0.8465 | 0.8538 | 17683 | 0 | 0 | 0 | 282 | 289 |  |  | DB Search |
| total 1 peptides |  |  |  |  |  |  |  |  |  |  |  |  |  |  |  |  |  |  |  |
| --- | --- | --- | --- | --- | --- | --- | --- | --- | --- | --- | --- | --- | --- | --- | --- | --- | --- | --- | --- |

Best Unique PSM (Scan Sample\_10\_A1\_1\_3647.d:17683, m/z=496.7644, z=2, RT=10.70, ppm=-5.76):


TRINITY\_DN15599\_c0\_g1\_i2.p1

back to list

  

| Protein Coverage
| Supporting Peptides
| Best Unique PSM
|

Protein Coverage:

Supporting Peptides:

| Peptide | Uniq | -10lgP | Mass | Length | ppm | m/z | z | RT | 1/k0 Start | 1/k0 End | Precursor ID | Area Sample 1 | #Feature | #Feature Sample 1 | Start | End | PTM | AScore | Found By |
| --- | --- | --- | --- | --- | --- | --- | --- | --- | --- | --- | --- | --- | --- | --- | --- | --- | --- | --- | --- |
| K.KLQAELANGR.L | Y | 41.93 | 1098.6145 | 10 | 1.23 | 550.3127 | 2 | 11.49 | 0.8878 | 0.8951 | 21900 | 9.13e3 | 1 | 1 | 184 | 193 |  |  | DB Search |
| total 1 peptides |  |  |  |  |  |  |  |  |  |  |  |  |  |  |  |  |  |  |  |
| --- | --- | --- | --- | --- | --- | --- | --- | --- | --- | --- | --- | --- | --- | --- | --- | --- | --- | --- | --- |

Best Unique PSM (Scan Sample\_10\_A1\_1\_3647.d:21900, m/z=550.3127, z=2, RT=11.49, ppm=-3.27):


TRINITY\_DN72625\_c0\_g1\_i1.p1

back to list

  

| Protein Coverage
| Supporting Peptides
| Best Unique PSM
|

Protein Coverage:

Supporting Peptides:

| Peptide | Uniq | -10lgP | Mass | Length | ppm | m/z | z | RT | 1/k0 Start | 1/k0 End | Precursor ID | Area Sample 1 | #Feature | #Feature Sample 1 | Start | End | PTM | AScore | Found By |
| --- | --- | --- | --- | --- | --- | --- | --- | --- | --- | --- | --- | --- | --- | --- | --- | --- | --- | --- | --- |
| R.LYSTDPNKFYR.F | Y | 61.95 | 1402.6881 | 11 | -1.52 | 702.3471 | 2 | 14.18 | 1.0303 | 1.0375 | 37720 | 6.29e2 | 1 | 1 | 193 | 203 |  |  | DB Search |
| R.ATNPDVEK.I | Y | 39.91 | 872.4239 | 8 | 4.9 | 437.2194 | 2 | 9.68 | 0.799 | 0.8063 | 11736 | 1.23e3 | 1 | 1 | 302 | 309 |  |  | DB Search |
| total 2 peptides |  |  |  |  |  |  |  |  |  |  |  |  |  |  |  |  |  |  |  |
| --- | --- | --- | --- | --- | --- | --- | --- | --- | --- | --- | --- | --- | --- | --- | --- | --- | --- | --- | --- |

Best Unique PSM (Scan Sample\_10\_A1\_1\_3647.d:37720, m/z=702.3471, z=2, RT=14.18, ppm=-6.01):


TRINITY\_DN98420\_c0\_g1\_i1.p1

back to list

  

| Protein Coverage
| Supporting Peptides
| Best Unique PSM
|

Protein Coverage:

Supporting Peptides:

| Peptide | Uniq | -10lgP | Mass | Length | ppm | m/z | z | RT | 1/k0 Start | 1/k0 End | Precursor ID | Area Sample 1 | #Feature | #Feature Sample 1 | Start | End | PTM | AScore | Found By |
| --- | --- | --- | --- | --- | --- | --- | --- | --- | --- | --- | --- | --- | --- | --- | --- | --- | --- | --- | --- |
| R.AVIFDRFR.G | Y | 63.61 | 1022.5661 | 8 | -0.98 | 512.2875 | 2 | 15.89 | 0.8684 | 0.8757 | 46912 | 1.42e3 | 1 | 1 | 48 | 55 |  |  | DB Search |
| total 1 peptides |  |  |  |  |  |  |  |  |  |  |  |  |  |  |  |  |  |  |  |
| --- | --- | --- | --- | --- | --- | --- | --- | --- | --- | --- | --- | --- | --- | --- | --- | --- | --- | --- | --- |

Best Unique PSM (Scan Sample\_10\_A1\_1\_3647.d:46912, m/z=512.2875, z=2, RT=15.89, ppm=-5.47):


TRINITY\_DN79\_c0\_g1\_i1.p1

back to list

  

| Protein Coverage
| Supporting Peptides
| Best Unique PSM
|

Protein Coverage:

Supporting Peptides:

| Peptide | Uniq | -10lgP | Mass | Length | ppm | m/z | z | RT | 1/k0 Start | 1/k0 End | Precursor ID | Area Sample 1 | #Feature | #Feature Sample 1 | Start | End | PTM | AScore | Found By |
| --- | --- | --- | --- | --- | --- | --- | --- | --- | --- | --- | --- | --- | --- | --- | --- | --- | --- | --- | --- |
| R.AESIALVTEFTDEAAPK.I | Y | 62.08 | 1790.8938 | 17 | -2.25 | 896.4481 | 2 | 19.81 | 1.1198 | 1.127 | 69737 | 2.36e2 | 1 | 1 | 192 | 208 |  |  | DB Search |
| total 1 peptides |  |  |  |  |  |  |  |  |  |  |  |  |  |  |  |  |  |  |  |
| --- | --- | --- | --- | --- | --- | --- | --- | --- | --- | --- | --- | --- | --- | --- | --- | --- | --- | --- | --- |

Best Unique PSM (Scan Sample\_10\_A1\_1\_3647.d:69737, m/z=896.4481, z=2, RT=19.81, ppm=-6.74):


TRINITY\_DN585\_c0\_g1\_i2.p1

back to list

  

| Protein Coverage
| Supporting Peptides
| Best Unique PSM
|

Protein Coverage:

Supporting Peptides:

| Peptide | Uniq | -10lgP | Mass | Length | ppm | m/z | z | RT | 1/k0 Start | 1/k0 End | Precursor ID | Area Sample 1 | #Feature | #Feature Sample 1 | Start | End | PTM | AScore | Found By |
| --- | --- | --- | --- | --- | --- | --- | --- | --- | --- | --- | --- | --- | --- | --- | --- | --- | --- | --- | --- |
| K.IQDKEGIPPDQQR.L | Y | 65.08 | 1522.7739 | 13 | -0.66 | 762.3903 | 2 | 11.01 | 1.0339 | 1.0411 | 19352 | 9.49e2 | 1 | 1 | 2 | 14 |  |  | DB Search |
| total 1 peptides |  |  |  |  |  |  |  |  |  |  |  |  |  |  |  |  |  |  |  |
| --- | --- | --- | --- | --- | --- | --- | --- | --- | --- | --- | --- | --- | --- | --- | --- | --- | --- | --- | --- |

Best Unique PSM (Scan Sample\_10\_A1\_1\_3647.d:19352, m/z=762.3903, z=2, RT=11.01, ppm=-5.16):


TRINITY\_DN36\_c0\_g1\_i8.p1

back to list

  

| Protein Coverage
| Supporting Peptides
| Best Unique PSM
|

Protein Coverage:

Supporting Peptides:

| Peptide | Uniq | -10lgP | Mass | Length | ppm | m/z | z | RT | 1/k0 Start | 1/k0 End | Precursor ID | Area Sample 1 | #Feature | #Feature Sample 1 | Start | End | PTM | AScore | Found By |
| --- | --- | --- | --- | --- | --- | --- | --- | --- | --- | --- | --- | --- | --- | --- | --- | --- | --- | --- | --- |
| R.LGGIEVTQGNC(+57.02)AR.K | Y | 66.48 | 1373.6721 | 13 | 5.18 | 687.8438 | 2 | 13.73 | 0.963 | 0.9703 | 35192 | 0 | 0 | 0 | 58 | 70 | Carbamidomethylation |  | DB Search |
| K.WC(+57.02)DILSIHQSMENC(+57.02)VAK.C | Y | 63.98 | 2089.9383 | 17 | -2.35 | 697.6486 | 3 | 17.89 | 0.8777 | 0.885 | 58058 | 0 | 0 | 0 | 198 | 214 | Carbamidomethylation, Carbamidomethylation |  | DB Search |
| K.FETQIATK.D | Y | 61.61 | 936.4916 | 8 | 0.97 | 937.4956 | 1 | 13.84 | 1.2037 | 1.2109 | 35727 | 1.28e3 | 1 | 1 | 167 | 174 |  |  | DB Search |
| R.LGGIEVTQGNC(+57.02)ARK.Y | Y | 31.04 | 1501.7671 | 14 | -2.67 | 751.8854 | 2 | 12.64 | 1.0624 | 1.0697 | 29000 | 0 | 0 | 0 | 58 | 71 | Carbamidomethylation |  | DB Search |
| total 4 peptides |  |  |  |  |  |  |  |  |  |  |  |  |  |  |  |  |  |  |  |
| --- | --- | --- | --- | --- | --- | --- | --- | --- | --- | --- | --- | --- | --- | --- | --- | --- | --- | --- | --- |

Best Unique PSM (Scan Sample\_10\_A1\_1\_3647.d:35192, m/z=687.8438, z=2, RT=13.73, ppm=0.69):


TRINITY\_DN3190\_c1\_g1\_i1.p1

back to list

  

| Protein Coverage
| Supporting Peptides
| Best Unique PSM
|

Protein Coverage:

Supporting Peptides:

| Peptide | Uniq | -10lgP | Mass | Length | ppm | m/z | z | RT | 1/k0 Start | 1/k0 End | Precursor ID | Area Sample 1 | #Feature | #Feature Sample 1 | Start | End | PTM | AScore | Found By |
| --- | --- | --- | --- | --- | --- | --- | --- | --- | --- | --- | --- | --- | --- | --- | --- | --- | --- | --- | --- |
| K.DLQGETSGSFKR.L | Y | 66.73 | 1323.6419 | 12 | -3.84 | 662.8227 | 2 | 12.59 | 0.9553 | 0.9626 | 28698 | 0 | 0 | 0 | 135 | 146 |  |  | DB Search |
| M.A(+42.01)TVVDYPDFDAQADAETLRK.A | Y | 63.34 | 2266.0753 | 20 | -6.61 | 1134.0323 | 2 | 18.33 | 1.2869 | 1.2941 | 60630 | 0 | 0 | 0 | 2 | 21 | Acetylation (Protein N-term) | A1:Acetylation (Protein N-term):1000 | DB Search |
| K.RLLTSLVQGAR.L | Y | 62.93 | 1212.7302 | 11 | 1.05 | 607.3703 | 2 | 15.15 | 0.9775 | 0.9848 | 43011 | 0 | 0 | 0 | 146 | 156 |  |  | DB Search |
| total 3 peptides |  |  |  |  |  |  |  |  |  |  |  |  |  |  |  |  |  |  |  |
| --- | --- | --- | --- | --- | --- | --- | --- | --- | --- | --- | --- | --- | --- | --- | --- | --- | --- | --- | --- |

Best Unique PSM (Scan Sample\_10\_A1\_1\_3647.d:28698, m/z=662.8227, z=2, RT=12.59, ppm=-8.34):


TRINITY\_DN1616\_c0\_g1\_i6.p1

back to list

  

| Protein Coverage
| Supporting Peptides
| Best Unique PSM
|

Protein Coverage:

Supporting Peptides:

| Peptide | Uniq | -10lgP | Mass | Length | ppm | m/z | z | RT | 1/k0 Start | 1/k0 End | Precursor ID | Area Sample 1 | #Feature | #Feature Sample 1 | Start | End | PTM | AScore | Found By |
| --- | --- | --- | --- | --- | --- | --- | --- | --- | --- | --- | --- | --- | --- | --- | --- | --- | --- | --- | --- |
| R.LVQANPLEQDLSEKPR.Y | Y | 66.73 | 1835.9741 | 16 | 3.23 | 918.9932 | 2 | 15.63 | 1.1837 | 1.1909 | 45493 | 3.81e2 | 1 | 1 | 1191 | 1206 |  |  | DB Search |
| K.YVVMLQYYHDSDTAK.Y | Y | 66.45 | 1831.8451 | 15 | 2.49 | 916.928 | 2 | 16.54 | 1.1798 | 1.1869 | 50323 | 0 | 0 | 0 | 1071 | 1085 |  |  | DB Search |
| R.YVKPGVNIINIR.D | Y | 58.56 | 1384.819 | 12 | 3.46 | 693.4161 | 2 | 15.95 | 1.0025 | 1.0098 | 47296 | 0 | 0 | 0 | 1217 | 1228 |  |  | DB Search |
| total 3 peptides |  |  |  |  |  |  |  |  |  |  |  |  |  |  |  |  |  |  |  |
| --- | --- | --- | --- | --- | --- | --- | --- | --- | --- | --- | --- | --- | --- | --- | --- | --- | --- | --- | --- |

Best Unique PSM (Scan Sample\_10\_A1\_1\_3647.d:45493, m/z=918.9932, z=2, RT=15.63, ppm=-1.26):


TRINITY\_DN3202\_c2\_g1\_i1.p1

back to list

  

| Protein Coverage
| Supporting Peptides
| Best Unique PSM
|

Protein Coverage:

Supporting Peptides:

| Peptide | Uniq | -10lgP | Mass | Length | ppm | m/z | z | RT | 1/k0 Start | 1/k0 End | Precursor ID | Area Sample 1 | #Feature | #Feature Sample 1 | Start | End | PTM | AScore | Found By |
| --- | --- | --- | --- | --- | --- | --- | --- | --- | --- | --- | --- | --- | --- | --- | --- | --- | --- | --- | --- |
| R.G(+42.01)C(+57.02)YIHTQEVAR.G | Y | 66.7 | 1374.635 | 11 | -1.48 | 688.3207 | 2 | 13.98 | 0.9916 | 0.9989 | 36588 | 6.57e2 | 1 | 1 | 7 | 17 | Acetylation (Protein N-term), Carbamidomethylation | G1:Acetylation (Protein N-term):1000 | DB Search |
| R.GC(+57.02)YIHTQEVAR.G | N | 61.54 | 1332.6245 | 11 | -1.99 | 667.3152 | 2 | 12.14 | 1.0086 | 1.0158 | 26094 | 0 | 0 | 0 | 7 | 17 | Carbamidomethylation |  | DB Search |
| total 2 peptides |  |  |  |  |  |  |  |  |  |  |  |  |  |  |  |  |  |  |  |
| --- | --- | --- | --- | --- | --- | --- | --- | --- | --- | --- | --- | --- | --- | --- | --- | --- | --- | --- | --- |

Best Unique PSM (Scan Sample\_10\_A1\_1\_3647.d:36588, m/z=688.3207, z=2, RT=13.98, ppm=-5.98):


TRINITY\_DN2805\_c0\_g1\_i1.p1

back to list

  

| Protein Coverage
| Supporting Peptides
| Best Unique PSM
|

Protein Coverage:

Supporting Peptides:

| Peptide | Uniq | -10lgP | Mass | Length | ppm | m/z | z | RT | 1/k0 Start | 1/k0 End | Precursor ID | Area Sample 1 | #Feature | #Feature Sample 1 | Start | End | PTM | AScore | Found By |
| --- | --- | --- | --- | --- | --- | --- | --- | --- | --- | --- | --- | --- | --- | --- | --- | --- | --- | --- | --- |
| K.VVIIDGKGHLM(+15.99)GR.L | Y | 62.74 | 1409.7813 | 13 | 0.05 | 705.8948 | 2 | 12.97 | 1.023 | 1.0303 | 30926 | 1.45e3 | 1 | 1 | 6 | 18 | Oxidation (M) | M11:Oxidation (M):1000 | DB Search |
| total 1 peptides |  |  |  |  |  |  |  |  |  |  |  |  |  |  |  |  |  |  |  |
| --- | --- | --- | --- | --- | --- | --- | --- | --- | --- | --- | --- | --- | --- | --- | --- | --- | --- | --- | --- |

Best Unique PSM (Scan Sample\_10\_A1\_1\_3647.d:30926, m/z=705.8948, z=2, RT=12.97, ppm=-4.44):


TRINITY\_DN96\_c0\_g1\_i1.p1

back to list

  

| Protein Coverage
| Supporting Peptides
| Best Unique PSM
|

Protein Coverage:

Supporting Peptides:

| Peptide | Uniq | -10lgP | Mass | Length | ppm | m/z | z | RT | 1/k0 Start | 1/k0 End | Precursor ID | Area Sample 1 | #Feature | #Feature Sample 1 | Start | End | PTM | AScore | Found By |
| --- | --- | --- | --- | --- | --- | --- | --- | --- | --- | --- | --- | --- | --- | --- | --- | --- | --- | --- | --- |
| K.TVQNFYELVTHSK.G | Y | 66.72 | 1564.7885 | 13 | 1.3 | 783.399 | 2 | 18.24 | 1.0913 | 1.0985 | 60147 | 1.44e3 | 2 | 2 | 44 | 56 |  |  | DB Search |
| total 1 peptides |  |  |  |  |  |  |  |  |  |  |  |  |  |  |  |  |  |  |  |
| --- | --- | --- | --- | --- | --- | --- | --- | --- | --- | --- | --- | --- | --- | --- | --- | --- | --- | --- | --- |

Best Unique PSM (Scan Sample\_10\_A1\_1\_3647.d:60147, m/z=783.399, z=2, RT=18.24, ppm=-3.19):


TRINITY\_DN36471\_c0\_g1\_i1.p1

back to list

  

| Protein Coverage
| Supporting Peptides
| Best Unique PSM
|

Protein Coverage:

Supporting Peptides:

| Peptide | Uniq | -10lgP | Mass | Length | ppm | m/z | z | RT | 1/k0 Start | 1/k0 End | Precursor ID | Area Sample 1 | #Feature | #Feature Sample 1 | Start | End | PTM | AScore | Found By |
| --- | --- | --- | --- | --- | --- | --- | --- | --- | --- | --- | --- | --- | --- | --- | --- | --- | --- | --- | --- |
| K.DLEHPNEFIR.G | Y | 63.27 | 1268.6149 | 10 | -1.34 | 635.311 | 2 | 14.83 | 0.9198 | 0.9271 | 41210 | 1.17e2 | 1 | 1 | 144 | 153 |  |  | DB Search |
| total 1 peptides |  |  |  |  |  |  |  |  |  |  |  |  |  |  |  |  |  |  |  |
| --- | --- | --- | --- | --- | --- | --- | --- | --- | --- | --- | --- | --- | --- | --- | --- | --- | --- | --- | --- |

Best Unique PSM (Scan Sample\_10\_A1\_1\_3647.d:41210, m/z=635.311, z=2, RT=14.83, ppm=-5.83):


TRINITY\_DN165969\_c0\_g1\_i1.p1

back to list

  

| Protein Coverage
| Supporting Peptides
| Best Unique PSM
|

Protein Coverage:

Supporting Peptides:

| Peptide | Uniq | -10lgP | Mass | Length | ppm | m/z | z | RT | 1/k0 Start | 1/k0 End | Precursor ID | Area Sample 1 | #Feature | #Feature Sample 1 | Start | End | PTM | AScore | Found By |
| --- | --- | --- | --- | --- | --- | --- | --- | --- | --- | --- | --- | --- | --- | --- | --- | --- | --- | --- | --- |
| R.DGPPAAR.Q | Y | 16.47 | 682.3398 | 7 | 1.31 | 683.3449 | 1 | 9.82 | 1.056 | 1.0632 | 12476 | 4.59e3 | 1 | 1 | 15 | 21 |  |  | DB Search |
| total 1 peptides |  |  |  |  |  |  |  |  |  |  |  |  |  |  |  |  |  |  |  |
| --- | --- | --- | --- | --- | --- | --- | --- | --- | --- | --- | --- | --- | --- | --- | --- | --- | --- | --- | --- |

Best Unique PSM (Scan Sample\_10\_A1\_1\_3647.d:12476, m/z=683.3449, z=1, RT=9.82, ppm=-3.19):


TRINITY\_DN38397\_c0\_g1\_i1.p1

back to list

  

| Protein Coverage
| Supporting Peptides
| Best Unique PSM
|

Protein Coverage:

Supporting Peptides:

| Peptide | Uniq | -10lgP | Mass | Length | ppm | m/z | z | RT | 1/k0 Start | 1/k0 End | Precursor ID | Area Sample 1 | #Feature | #Feature Sample 1 | Start | End | PTM | AScore | Found By |
| --- | --- | --- | --- | --- | --- | --- | --- | --- | --- | --- | --- | --- | --- | --- | --- | --- | --- | --- | --- |
| R.GC(+57.02)YIHTQEIAR.G | Y | 66.57 | 1346.6401 | 11 | -0.12 | 674.3242 | 2 | 12.88 | 1.0158 | 1.023 | 30410 | 6.22e2 | 1 | 1 | 202 | 212 | Carbamidomethylation |  | DB Search |
| total 1 peptides |  |  |  |  |  |  |  |  |  |  |  |  |  |  |  |  |  |  |  |
| --- | --- | --- | --- | --- | --- | --- | --- | --- | --- | --- | --- | --- | --- | --- | --- | --- | --- | --- | --- |

Best Unique PSM (Scan Sample\_10\_A1\_1\_3647.d:30410, m/z=674.3242, z=2, RT=12.88, ppm=-4.62):


TRINITY\_DN761\_c0\_g1\_i3.p1

back to list

  

| Protein Coverage
| Supporting Peptides
| Best Unique PSM
|

Protein Coverage:

Supporting Peptides:

| Peptide | Uniq | -10lgP | Mass | Length | ppm | m/z | z | RT | 1/k0 Start | 1/k0 End | Precursor ID | Area Sample 1 | #Feature | #Feature Sample 1 | Start | End | PTM | AScore | Found By |
| --- | --- | --- | --- | --- | --- | --- | --- | --- | --- | --- | --- | --- | --- | --- | --- | --- | --- | --- | --- |
| K.TRIEDLETVISQTQDHR.H | Y | 66.35 | 2040.0236 | 17 | 3.15 | 681.0142 | 3 | 17.32 | 0.9622 | 0.9695 | 54563 | 0 | 0 | 0 | 275 | 291 |  |  | DB Search |
| K.EIDKADIAIVDTGENPDAPHPR.E | Y | 61.91 | 2372.1607 | 22 | 3.12 | 791.7264 | 3 | 14.88 | 1.0415 | 1.0488 | 41517 | 0 | 0 | 0 | 71 | 92 |  |  | DB Search |
| K.FVNEVRR.C | Y | 39.18 | 918.5035 | 7 | -2.89 | 460.2556 | 2 | 12.63 | 0.8096 | 0.8169 | 28832 | 9.86e2 | 1 | 1 | 51 | 57 |  |  | DB Search |
| total 3 peptides |  |  |  |  |  |  |  |  |  |  |  |  |  |  |  |  |  |  |  |
| --- | --- | --- | --- | --- | --- | --- | --- | --- | --- | --- | --- | --- | --- | --- | --- | --- | --- | --- | --- |

Best Unique PSM (Scan Sample\_10\_A1\_1\_3647.d:54563, m/z=681.0142, z=3, RT=17.32, ppm=-1.34):


TRINITY\_DN2230\_c0\_g1\_i1.p1

back to list

  

| Protein Coverage
| Supporting Peptides
| Best Unique PSM
|

Protein Coverage:

Supporting Peptides:

| Peptide | Uniq | -10lgP | Mass | Length | ppm | m/z | z | RT | 1/k0 Start | 1/k0 End | Precursor ID | Area Sample 1 | #Feature | #Feature Sample 1 | Start | End | PTM | AScore | Found By |
| --- | --- | --- | --- | --- | --- | --- | --- | --- | --- | --- | --- | --- | --- | --- | --- | --- | --- | --- | --- |
| K.LLEIEDEIKLR.S | Y | 65.81 | 1369.7816 | 11 | 1.07 | 685.8958 | 2 | 18.00 | 1.0468 | 1.054 | 58561 | 1.84e3 | 1 | 1 | 15 | 25 |  |  | DB Search |
| K.KGDNEIPGLTDRVIPR.R | Y | 62.47 | 1778.9638 | 16 | -2.38 | 593.9911 | 3 | 15.59 | 0.8972 | 0.9044 | 45402 | 0 | 0 | 0 | 116 | 131 |  |  | DB Search |
| total 2 peptides |  |  |  |  |  |  |  |  |  |  |  |  |  |  |  |  |  |  |  |
| --- | --- | --- | --- | --- | --- | --- | --- | --- | --- | --- | --- | --- | --- | --- | --- | --- | --- | --- | --- |

Best Unique PSM (Scan Sample\_10\_A1\_1\_3647.d:58561, m/z=685.8958, z=2, RT=18.00, ppm=-3.42):


TRINITY\_DN982\_c0\_g1\_i1.p1

back to list

  

| Protein Coverage
| Supporting Peptides
| Best Unique PSM
|

Protein Coverage:

Supporting Peptides:

| Peptide | Uniq | -10lgP | Mass | Length | ppm | m/z | z | RT | 1/k0 Start | 1/k0 End | Precursor ID | Area Sample 1 | #Feature | #Feature Sample 1 | Start | End | PTM | AScore | Found By |
| --- | --- | --- | --- | --- | --- | --- | --- | --- | --- | --- | --- | --- | --- | --- | --- | --- | --- | --- | --- |
| R.LVQNTPHNSIVTK.I | Y | 66.78 | 1449.794 | 13 | 2.36 | 725.9027 | 2 | 11.65 | 1.0628 | 1.0701 | 23219 | 0 | 0 | 0 | 5129 | 5141 |  |  | DB Search |
| R.ITTSQILDHEQQK.L | Y | 66.73 | 1539.7893 | 13 | 2.99 | 770.9008 | 2 | 13.83 | 1.0945 | 1.1017 | 35680 | 3.16e2 | 1 | 1 | 1994 | 2006 |  |  | DB Search |
| K.VLLSQQIFYDKK.N | Y | 66.52 | 1480.8289 | 12 | -1.68 | 741.4172 | 2 | 16.70 | 1.06 | 1.0672 | 51062 | 6.76e2 | 1 | 1 | 1479 | 1490 |  |  | DB Search |
| R.LIQTLNYETKK.E | Y | 61.82 | 1349.7554 | 11 | 0.35 | 675.8822 | 2 | 13.59 | 1.0098 | 1.017 | 34441 | 0 | 0 | 0 | 4879 | 4889 |  |  | DB Search |
| K.YVAIYPSDHLTVNR.D | Y | 60.16 | 1646.8416 | 14 | -9.73 | 824.4164 | 2 | 15.50 | 1.1098 | 1.117 | 44890 | 0 | 0 | 0 | 2814 | 2827 |  |  | DB Search |
| total 5 peptides |  |  |  |  |  |  |  |  |  |  |  |  |  |  |  |  |  |  |  |
| --- | --- | --- | --- | --- | --- | --- | --- | --- | --- | --- | --- | --- | --- | --- | --- | --- | --- | --- | --- |

Best Unique PSM (Scan Sample\_10\_A1\_1\_3647.d:23219, m/z=725.9027, z=2, RT=11.65, ppm=-2.13):


TRINITY\_DN15016\_c0\_g1\_i2.p1

back to list

  

| Protein Coverage
| Supporting Peptides
| Best Unique PSM
|

Protein Coverage:

Supporting Peptides:

| Peptide | Uniq | -10lgP | Mass | Length | ppm | m/z | z | RT | 1/k0 Start | 1/k0 End | Precursor ID | Area Sample 1 | #Feature | #Feature Sample 1 | Start | End | PTM | AScore | Found By |
| --- | --- | --- | --- | --- | --- | --- | --- | --- | --- | --- | --- | --- | --- | --- | --- | --- | --- | --- | --- |
| K.AFFDLHFVGTGAK.F | Y | 66.68 | 1408.7139 | 13 | -4.47 | 705.3579 | 2 | 19.23 | 1.0371 | 1.0443 | 66248 | 0 | 0 | 0 | 96 | 108 |  |  | DB Search |
| total 1 peptides |  |  |  |  |  |  |  |  |  |  |  |  |  |  |  |  |  |  |  |
| --- | --- | --- | --- | --- | --- | --- | --- | --- | --- | --- | --- | --- | --- | --- | --- | --- | --- | --- | --- |

Best Unique PSM (Scan Sample\_10\_A1\_1\_3647.d:66248, m/z=705.3579, z=2, RT=19.23, ppm=-8.96):


TRINITY\_DN6701\_c0\_g1\_i2.p2

back to list

  

| Protein Coverage
| Supporting Peptides
| Best Unique PSM
|

Protein Coverage:

Supporting Peptides:

| Peptide | Uniq | -10lgP | Mass | Length | ppm | m/z | z | RT | 1/k0 Start | 1/k0 End | Precursor ID | Area Sample 1 | #Feature | #Feature Sample 1 | Start | End | PTM | AScore | Found By |
| --- | --- | --- | --- | --- | --- | --- | --- | --- | --- | --- | --- | --- | --- | --- | --- | --- | --- | --- | --- |
| K.VITSDDPAEKEK.K | Y | 66.7 | 1330.6616 | 12 | 2.85 | 666.337 | 2 | 10.71 | 0.9836 | 0.9908 | 17367 | 7.2e3 | 1 | 1 | 114 | 125 |  |  | DB Search |
| total 1 peptides |  |  |  |  |  |  |  |  |  |  |  |  |  |  |  |  |  |  |  |
| --- | --- | --- | --- | --- | --- | --- | --- | --- | --- | --- | --- | --- | --- | --- | --- | --- | --- | --- | --- |

Best Unique PSM (Scan Sample\_10\_A1\_1\_3647.d:17367, m/z=666.337, z=2, RT=10.71, ppm=-1.65):


TRINITY\_DN4595\_c0\_g1\_i7.p1

back to list

  

| Protein Coverage
| Supporting Peptides
| Best Unique PSM
|

Protein Coverage:

Supporting Peptides:

| Peptide | Uniq | -10lgP | Mass | Length | ppm | m/z | z | RT | 1/k0 Start | 1/k0 End | Precursor ID | Area Sample 1 | #Feature | #Feature Sample 1 | Start | End | PTM | AScore | Found By |
| --- | --- | --- | --- | --- | --- | --- | --- | --- | --- | --- | --- | --- | --- | --- | --- | --- | --- | --- | --- |
| R.AFNRVETR.D | Y | 22.7 | 991.5199 | 8 | -1.27 | 496.7644 | 2 | 10.70 | 0.8465 | 0.8538 | 17683 | 0 | 0 | 0 | 282 | 289 |  |  | DB Search |
| total 1 peptides |  |  |  |  |  |  |  |  |  |  |  |  |  |  |  |  |  |  |  |
| --- | --- | --- | --- | --- | --- | --- | --- | --- | --- | --- | --- | --- | --- | --- | --- | --- | --- | --- | --- |

Best Unique PSM (Scan Sample\_10\_A1\_1\_3647.d:17683, m/z=496.7644, z=2, RT=10.70, ppm=-5.76):


TRINITY\_DN8008\_c0\_g1\_i3.p1

back to list

  

| Protein Coverage
| Supporting Peptides
| Best Unique PSM
|

Protein Coverage:

Supporting Peptides:

| Peptide | Uniq | -10lgP | Mass | Length | ppm | m/z | z | RT | 1/k0 Start | 1/k0 End | Precursor ID | Area Sample 1 | #Feature | #Feature Sample 1 | Start | End | PTM | AScore | Found By |
| --- | --- | --- | --- | --- | --- | --- | --- | --- | --- | --- | --- | --- | --- | --- | --- | --- | --- | --- | --- |
| K.GVHIISVGLGTVDHR.G | Y | 66.61 | 1558.8579 | 15 | -17.11 | 520.6154 | 3 | 17.30 | 0.8116 | 0.8189 | 54443 | 0 | 0 | 0 | 1055 | 1069 |  |  | DB Search |
| total 1 peptides |  |  |  |  |  |  |  |  |  |  |  |  |  |  |  |  |  |  |  |
| --- | --- | --- | --- | --- | --- | --- | --- | --- | --- | --- | --- | --- | --- | --- | --- | --- | --- | --- | --- |

Best Unique PSM (Scan Sample\_10\_A1\_1\_3647.d:54443, m/z=520.6154, z=3, RT=17.30, ppm=-21.61):


TRINITY\_DN6294\_c0\_g3\_i1.p1

back to list

  

| Protein Coverage
| Supporting Peptides
| Best Unique PSM
|

Protein Coverage:

Supporting Peptides:

| Peptide | Uniq | -10lgP | Mass | Length | ppm | m/z | z | RT | 1/k0 Start | 1/k0 End | Precursor ID | Area Sample 1 | #Feature | #Feature Sample 1 | Start | End | PTM | AScore | Found By |
| --- | --- | --- | --- | --- | --- | --- | --- | --- | --- | --- | --- | --- | --- | --- | --- | --- | --- | --- | --- |
| K.LVVIADIVDQKR.L | Y | 66.4 | 1367.8136 | 12 | 1.88 | 684.9123 | 2 | 17.50 | 1.0415 | 1.0488 | 55496 | 4.87e3 | 1 | 1 | 24 | 35 |  |  | DB Search |
| total 1 peptides |  |  |  |  |  |  |  |  |  |  |  |  |  |  |  |  |  |  |  |
| --- | --- | --- | --- | --- | --- | --- | --- | --- | --- | --- | --- | --- | --- | --- | --- | --- | --- | --- | --- |

Best Unique PSM (Scan Sample\_10\_A1\_1\_3647.d:55496, m/z=684.9123, z=2, RT=17.50, ppm=-2.61):


TRINITY\_DN2301\_c0\_g1\_i1.p1

back to list

  

| Protein Coverage
| Supporting Peptides
| Best Unique PSM
|

Protein Coverage:

Supporting Peptides:

| Peptide | Uniq | -10lgP | Mass | Length | ppm | m/z | z | RT | 1/k0 Start | 1/k0 End | Precursor ID | Area Sample 1 | #Feature | #Feature Sample 1 | Start | End | PTM | AScore | Found By |
| --- | --- | --- | --- | --- | --- | --- | --- | --- | --- | --- | --- | --- | --- | --- | --- | --- | --- | --- | --- |
| R.VGYTHLFSSTQVSVRPK.Q | Y | 62.53 | 1905.0108 | 17 | 8.5 | 636.0134 | 3 | 15.20 | 0.9392 | 0.9465 | 43249 | 8.92e1 | 1 | 1 | 390 | 406 |  |  | DB Search |
| total 1 peptides |  |  |  |  |  |  |  |  |  |  |  |  |  |  |  |  |  |  |  |
| --- | --- | --- | --- | --- | --- | --- | --- | --- | --- | --- | --- | --- | --- | --- | --- | --- | --- | --- | --- |

Best Unique PSM (Scan Sample\_10\_A1\_1\_3647.d:43249, m/z=636.0134, z=3, RT=15.20, ppm=4.01):


TRINITY\_DN113\_c3\_g1\_i1.p1

back to list

  

| Protein Coverage
| Supporting Peptides
| Best Unique PSM
|

Protein Coverage:

Supporting Peptides:

| Peptide | Uniq | -10lgP | Mass | Length | ppm | m/z | z | RT | 1/k0 Start | 1/k0 End | Precursor ID | Area Sample 1 | #Feature | #Feature Sample 1 | Start | End | PTM | AScore | Found By |
| --- | --- | --- | --- | --- | --- | --- | --- | --- | --- | --- | --- | --- | --- | --- | --- | --- | --- | --- | --- |
| K.AHVSLSTFSEDVTSLTK.F | Y | 62.84 | 1820.9156 | 17 | 4.58 | 911.4651 | 2 | 18.78 | 1.141 | 1.1482 | 63505 | 0 | 0 | 0 | 568 | 584 |  |  | DB Search |
| total 1 peptides |  |  |  |  |  |  |  |  |  |  |  |  |  |  |  |  |  |  |  |
| --- | --- | --- | --- | --- | --- | --- | --- | --- | --- | --- | --- | --- | --- | --- | --- | --- | --- | --- | --- |

Best Unique PSM (Scan Sample\_10\_A1\_1\_3647.d:63505, m/z=911.4651, z=2, RT=18.78, ppm=0.09):


TRINITY\_DN413\_c0\_g1\_i2.p1

back to list

  

| Protein Coverage
| Supporting Peptides
| Best Unique PSM
|

Protein Coverage:

Supporting Peptides:

| Peptide | Uniq | -10lgP | Mass | Length | ppm | m/z | z | RT | 1/k0 Start | 1/k0 End | Precursor ID | Area Sample 1 | #Feature | #Feature Sample 1 | Start | End | PTM | AScore | Found By |
| --- | --- | --- | --- | --- | --- | --- | --- | --- | --- | --- | --- | --- | --- | --- | --- | --- | --- | --- | --- |
| R.LLLGDFNGDKR.T | Y | 66.61 | 1246.6669 | 11 | 0.98 | 624.3386 | 2 | 16.00 | 0.9848 | 0.992 | 47446 | 9.37e2 | 1 | 1 | 46 | 56 |  |  | DB Search |
| total 1 peptides |  |  |  |  |  |  |  |  |  |  |  |  |  |  |  |  |  |  |  |
| --- | --- | --- | --- | --- | --- | --- | --- | --- | --- | --- | --- | --- | --- | --- | --- | --- | --- | --- | --- |

Best Unique PSM (Scan Sample\_10\_A1\_1\_3647.d:47446, m/z=624.3386, z=2, RT=16.00, ppm=-3.51):


TRINITY\_DN471\_c0\_g1\_i10.p1

back to list

  

| Protein Coverage
| Supporting Peptides
| Best Unique PSM
|

Protein Coverage:

Supporting Peptides:

| Peptide | Uniq | -10lgP | Mass | Length | ppm | m/z | z | RT | 1/k0 Start | 1/k0 End | Precursor ID | Area Sample 1 | #Feature | #Feature Sample 1 | Start | End | PTM | AScore | Found By |
| --- | --- | --- | --- | --- | --- | --- | --- | --- | --- | --- | --- | --- | --- | --- | --- | --- | --- | --- | --- |
| R.AFVHWYVGEGM(+15.99)EEGEFSEAR.E | Y | 44.9 | 2345.0058 | 20 | -0.18 | 782.6722 | 3 | 17.81 | 0.9251 | 0.9323 | 57560 | 6.2e2 | 1 | 1 | 441 | 460 | Oxidation (M) | M11:Oxidation (M):1000 | DB Search |
| total 1 peptides |  |  |  |  |  |  |  |  |  |  |  |  |  |  |  |  |  |  |  |
| --- | --- | --- | --- | --- | --- | --- | --- | --- | --- | --- | --- | --- | --- | --- | --- | --- | --- | --- | --- |

Best Unique PSM (Scan Sample\_10\_A1\_1\_3647.d:57560, m/z=782.6722, z=3, RT=17.81, ppm=-4.67):


TRINITY\_DN649\_c0\_g1\_i1.p1

back to list

  

| Protein Coverage
| Supporting Peptides
| Best Unique PSM
|

Protein Coverage:

Supporting Peptides:

| Peptide | Uniq | -10lgP | Mass | Length | ppm | m/z | z | RT | 1/k0 Start | 1/k0 End | Precursor ID | Area Sample 1 | #Feature | #Feature Sample 1 | Start | End | PTM | AScore | Found By |
| --- | --- | --- | --- | --- | --- | --- | --- | --- | --- | --- | --- | --- | --- | --- | --- | --- | --- | --- | --- |
| K.FETQIATK.N | Y | 61.61 | 936.4916 | 8 | 0.97 | 937.4956 | 1 | 13.84 | 1.2037 | 1.2109 | 35727 | 1.28e3 | 1 | 1 | 102 | 109 |  |  | DB Search |
| total 1 peptides |  |  |  |  |  |  |  |  |  |  |  |  |  |  |  |  |  |  |  |
| --- | --- | --- | --- | --- | --- | --- | --- | --- | --- | --- | --- | --- | --- | --- | --- | --- | --- | --- | --- |

Best Unique PSM (Scan Sample\_10\_A1\_1\_3647.d:35727, m/z=937.4956, z=1, RT=13.84, ppm=-3.52):


TRINITY\_DN1058\_c0\_g1\_i2.p2

back to list

  

| Protein Coverage
| Supporting Peptides
| Best Unique PSM
|

Protein Coverage:

Supporting Peptides:

| Peptide | Uniq | -10lgP | Mass | Length | ppm | m/z | z | RT | 1/k0 Start | 1/k0 End | Precursor ID | Area Sample 1 | #Feature | #Feature Sample 1 | Start | End | PTM | AScore | Found By |
| --- | --- | --- | --- | --- | --- | --- | --- | --- | --- | --- | --- | --- | --- | --- | --- | --- | --- | --- | --- |
| R.APTHILNPANKR.S | Y | 66.68 | 1330.7469 | 12 | 2.53 | 444.5887 | 3 | 11.11 | 0.7526 | 0.7599 | 20079 | 1.09e3 | 2 | 2 | 975 | 986 |  |  | DB Search |
| total 1 peptides |  |  |  |  |  |  |  |  |  |  |  |  |  |  |  |  |  |  |  |
| --- | --- | --- | --- | --- | --- | --- | --- | --- | --- | --- | --- | --- | --- | --- | --- | --- | --- | --- | --- |

Best Unique PSM (Scan Sample\_10\_A1\_1\_3647.d:20079, m/z=444.5887, z=3, RT=11.11, ppm=-1.97):


TRINITY\_DN6701\_c0\_g1\_i1.p1

back to list

  

| Protein Coverage
| Supporting Peptides
| Best Unique PSM
|

Protein Coverage:

Supporting Peptides:

| Peptide | Uniq | -10lgP | Mass | Length | ppm | m/z | z | RT | 1/k0 Start | 1/k0 End | Precursor ID | Area Sample 1 | #Feature | #Feature Sample 1 | Start | End | PTM | AScore | Found By |
| --- | --- | --- | --- | --- | --- | --- | --- | --- | --- | --- | --- | --- | --- | --- | --- | --- | --- | --- | --- |
| K.VITSDDPAEKEK.K | Y | 66.7 | 1330.6616 | 12 | 2.85 | 666.337 | 2 | 10.71 | 0.9836 | 0.9908 | 17367 | 7.2e3 | 1 | 1 | 158 | 169 |  |  | DB Search |
| total 1 peptides |  |  |  |  |  |  |  |  |  |  |  |  |  |  |  |  |  |  |  |
| --- | --- | --- | --- | --- | --- | --- | --- | --- | --- | --- | --- | --- | --- | --- | --- | --- | --- | --- | --- |

Best Unique PSM (Scan Sample\_10\_A1\_1\_3647.d:17367, m/z=666.337, z=2, RT=10.71, ppm=-1.65):


TRINITY\_DN119673\_c0\_g1\_i1.p1

back to list

  

| Protein Coverage
| Supporting Peptides
| Best Unique PSM
|

Protein Coverage:

Supporting Peptides:

| Peptide | Uniq | -10lgP | Mass | Length | ppm | m/z | z | RT | 1/k0 Start | 1/k0 End | Precursor ID | Area Sample 1 | #Feature | #Feature Sample 1 | Start | End | PTM | AScore | Found By |
| --- | --- | --- | --- | --- | --- | --- | --- | --- | --- | --- | --- | --- | --- | --- | --- | --- | --- | --- | --- |
| K.VLTSDDPAEKEK | Y | 66.7 | 1330.6616 | 12 | 2.85 | 666.337 | 2 | 10.71 | 0.9836 | 0.9908 | 17367 | 7.2e3 | 1 | 1 | 117 | 128 |  |  | DB Search |
| total 1 peptides |  |  |  |  |  |  |  |  |  |  |  |  |  |  |  |  |  |  |  |
| --- | --- | --- | --- | --- | --- | --- | --- | --- | --- | --- | --- | --- | --- | --- | --- | --- | --- | --- | --- |

Best Unique PSM (Scan Sample\_10\_A1\_1\_3647.d:17367, m/z=666.337, z=2, RT=10.71, ppm=-1.65):


TRINITY\_DN1505\_c0\_g1\_i1.p1

back to list

  

| Protein Coverage
| Supporting Peptides
| Best Unique PSM
|

Protein Coverage:

Supporting Peptides:

| Peptide | Uniq | -10lgP | Mass | Length | ppm | m/z | z | RT | 1/k0 Start | 1/k0 End | Precursor ID | Area Sample 1 | #Feature | #Feature Sample 1 | Start | End | PTM | AScore | Found By |
| --- | --- | --- | --- | --- | --- | --- | --- | --- | --- | --- | --- | --- | --- | --- | --- | --- | --- | --- | --- |
| M.S(+42.01)LVIPEKFQHILR.V | Y | 66.85 | 1620.9351 | 13 | 0.67 | 811.4717 | 2 | 19.55 | 1.1226 | 1.1298 | 68036 | 3.14e4 | 2 | 2 | 2 | 14 | Acetylation (Protein N-term) | S1:Acetylation (Protein N-term):1000 | DB Search |
| K.RAGELSDEEVER.L | Y | 64.61 | 1388.6531 | 12 | 3.19 | 695.3329 | 2 | 11.78 | 0.9707 | 0.9779 | 23916 | 1.24e3 | 1 | 1 | 55 | 66 |  |  | DB Search |
| total 2 peptides |  |  |  |  |  |  |  |  |  |  |  |  |  |  |  |  |  |  |  |
| --- | --- | --- | --- | --- | --- | --- | --- | --- | --- | --- | --- | --- | --- | --- | --- | --- | --- | --- | --- |

Best Unique PSM (Scan Sample\_10\_A1\_1\_3647.d:68036, m/z=811.4717, z=2, RT=19.55, ppm=-3.82):


TRINITY\_DN36\_c0\_g1\_i11.p1

back to list

  

| Protein Coverage
| Supporting Peptides
| Best Unique PSM
|

Protein Coverage:

Supporting Peptides:

| Peptide | Uniq | -10lgP | Mass | Length | ppm | m/z | z | RT | 1/k0 Start | 1/k0 End | Precursor ID | Area Sample 1 | #Feature | #Feature Sample 1 | Start | End | PTM | AScore | Found By |
| --- | --- | --- | --- | --- | --- | --- | --- | --- | --- | --- | --- | --- | --- | --- | --- | --- | --- | --- | --- |
| R.LGGIEVTQGNC(+57.02)AR.K | Y | 66.48 | 1373.6721 | 13 | 5.18 | 687.8438 | 2 | 13.73 | 0.963 | 0.9703 | 35192 | 0 | 0 | 0 | 58 | 70 | Carbamidomethylation |  | DB Search |
| R.LGGIEVTQGNC(+57.02)ARK.Y | Y | 31.04 | 1501.7671 | 14 | -2.67 | 751.8854 | 2 | 12.64 | 1.0624 | 1.0697 | 29000 | 0 | 0 | 0 | 58 | 71 | Carbamidomethylation |  | DB Search |
| total 2 peptides |  |  |  |  |  |  |  |  |  |  |  |  |  |  |  |  |  |  |  |
| --- | --- | --- | --- | --- | --- | --- | --- | --- | --- | --- | --- | --- | --- | --- | --- | --- | --- | --- | --- |

Best Unique PSM (Scan Sample\_10\_A1\_1\_3647.d:35192, m/z=687.8438, z=2, RT=13.73, ppm=0.69):


TRINITY\_DN187147\_c0\_g1\_i1.p1

back to list

  

| Protein Coverage
| Supporting Peptides
| Best Unique PSM
|

Protein Coverage:

Supporting Peptides:

| Peptide | Uniq | -10lgP | Mass | Length | ppm | m/z | z | RT | 1/k0 Start | 1/k0 End | Precursor ID | Area Sample 1 | #Feature | #Feature Sample 1 | Start | End | PTM | AScore | Found By |
| --- | --- | --- | --- | --- | --- | --- | --- | --- | --- | --- | --- | --- | --- | --- | --- | --- | --- | --- | --- |
| R.FAVLDKQQNVVIK.N | Y | 63.28 | 1500.8664 | 13 | 4.2 | 751.4402 | 2 | 15.20 | 1.0777 | 1.0849 | 43259 | 2.65e2 | 1 | 1 | 433 | 445 |  |  | DB Search |
| total 1 peptides |  |  |  |  |  |  |  |  |  |  |  |  |  |  |  |  |  |  |  |
| --- | --- | --- | --- | --- | --- | --- | --- | --- | --- | --- | --- | --- | --- | --- | --- | --- | --- | --- | --- |

Best Unique PSM (Scan Sample\_10\_A1\_1\_3647.d:43259, m/z=751.4402, z=2, RT=15.20, ppm=-0.29):


TRINITY\_DN6397\_c0\_g2\_i4.p1

back to list

  

| Protein Coverage
| Supporting Peptides
| Best Unique PSM
|

Protein Coverage:

Supporting Peptides:

| Peptide | Uniq | -10lgP | Mass | Length | ppm | m/z | z | RT | 1/k0 Start | 1/k0 End | Precursor ID | Area Sample 1 | #Feature | #Feature Sample 1 | Start | End | PTM | AScore | Found By |
| --- | --- | --- | --- | --- | --- | --- | --- | --- | --- | --- | --- | --- | --- | --- | --- | --- | --- | --- | --- |
| K.K(+42.01)LSAEVANGR.L | Y | 66.46 | 1085.5829 | 10 | 2.21 | 543.7975 | 2 | 12.83 | 0.8595 | 0.8668 | 30189 | 3.02e3 | 1 | 1 | 12 | 21 | Acetylation (Protein N-term) | K1:Acetylation (Protein N-term):1000 | DB Search |
| total 1 peptides |  |  |  |  |  |  |  |  |  |  |  |  |  |  |  |  |  |  |  |
| --- | --- | --- | --- | --- | --- | --- | --- | --- | --- | --- | --- | --- | --- | --- | --- | --- | --- | --- | --- |

Best Unique PSM (Scan Sample\_10\_A1\_1\_3647.d:30189, m/z=543.7975, z=2, RT=12.83, ppm=-2.28):


TRINITY\_DN33483\_c0\_g1\_i1.p1

back to list

  

| Protein Coverage
| Supporting Peptides
| Best Unique PSM
|

Protein Coverage:

Supporting Peptides:

| Peptide | Uniq | -10lgP | Mass | Length | ppm | m/z | z | RT | 1/k0 Start | 1/k0 End | Precursor ID | Area Sample 1 | #Feature | #Feature Sample 1 | Start | End | PTM | AScore | Found By |
| --- | --- | --- | --- | --- | --- | --- | --- | --- | --- | --- | --- | --- | --- | --- | --- | --- | --- | --- | --- |
| R.FNSNSVLVVIDAKPK.E | Y | 66.6 | 1629.909 | 15 | 6.93 | 815.9637 | 2 | 17.38 | 1.1674 | 1.1746 | 54871 | 2.33e2 | 1 | 1 | 131 | 145 |  |  | DB Search |
| total 1 peptides |  |  |  |  |  |  |  |  |  |  |  |  |  |  |  |  |  |  |  |
| --- | --- | --- | --- | --- | --- | --- | --- | --- | --- | --- | --- | --- | --- | --- | --- | --- | --- | --- | --- |

Best Unique PSM (Scan Sample\_10\_A1\_1\_3647.d:54871, m/z=815.9637, z=2, RT=17.38, ppm=2.44):


TRINITY\_DN1080\_c0\_g1\_i2.p1

back to list

  

| Protein Coverage
| Supporting Peptides
| Best Unique PSM
|

Protein Coverage:

Supporting Peptides:

| Peptide | Uniq | -10lgP | Mass | Length | ppm | m/z | z | RT | 1/k0 Start | 1/k0 End | Precursor ID | Area Sample 1 | #Feature | #Feature Sample 1 | Start | End | PTM | AScore | Found By |
| --- | --- | --- | --- | --- | --- | --- | --- | --- | --- | --- | --- | --- | --- | --- | --- | --- | --- | --- | --- |
| R.RPVPIVTR.R | Y | 17.05 | 936.5869 | 8 | 4.27 | 469.3006 | 2 | 11.83 | 0.8071 | 0.8144 | 24513 | 2.28e2 | 1 | 1 | 281 | 288 |  |  | DB Search |
| total 1 peptides |  |  |  |  |  |  |  |  |  |  |  |  |  |  |  |  |  |  |  |
| --- | --- | --- | --- | --- | --- | --- | --- | --- | --- | --- | --- | --- | --- | --- | --- | --- | --- | --- | --- |

Best Unique PSM (Scan Sample\_10\_A1\_1\_3647.d:24513, m/z=469.3006, z=2, RT=11.83, ppm=-0.23):


TRINITY\_DN148467\_c0\_g1\_i1.p1

back to list

  

| Protein Coverage
| Supporting Peptides
| Best Unique PSM
|

Protein Coverage:

Supporting Peptides:

| Peptide | Uniq | -10lgP | Mass | Length | ppm | m/z | z | RT | 1/k0 Start | 1/k0 End | Precursor ID | Area Sample 1 | #Feature | #Feature Sample 1 | Start | End | PTM | AScore | Found By |
| --- | --- | --- | --- | --- | --- | --- | --- | --- | --- | --- | --- | --- | --- | --- | --- | --- | --- | --- | --- |
| K.IGSIMLGIHLLR.Y | Y | 66.5 | 1321.7904 | 12 | -0.58 | 661.8991 | 2 | 19.69 | 1.0375 | 1.0448 | 69001 | 4.62e2 | 1 | 1 | 100 | 111 |  |  | DB Search |
| total 1 peptides |  |  |  |  |  |  |  |  |  |  |  |  |  |  |  |  |  |  |  |
| --- | --- | --- | --- | --- | --- | --- | --- | --- | --- | --- | --- | --- | --- | --- | --- | --- | --- | --- | --- |

Best Unique PSM (Scan Sample\_10\_A1\_1\_3647.d:69001, m/z=661.8991, z=2, RT=19.69, ppm=-5.08):


TRINITY\_DN384\_c0\_g1\_i1.p1

back to list

  

| Protein Coverage
| Supporting Peptides
| Best Unique PSM
|

Protein Coverage:

Supporting Peptides:

| Peptide | Uniq | -10lgP | Mass | Length | ppm | m/z | z | RT | 1/k0 Start | 1/k0 End | Precursor ID | Area Sample 1 | #Feature | #Feature Sample 1 | Start | End | PTM | AScore | Found By |
| --- | --- | --- | --- | --- | --- | --- | --- | --- | --- | --- | --- | --- | --- | --- | --- | --- | --- | --- | --- |
| K.FVLHGDVSHGSR.E | Y | 66.68 | 1309.6527 | 12 | 4.95 | 655.8339 | 2 | 12.00 | 0.9892 | 0.9965 | 25296 | 0 | 0 | 0 | 274 | 285 |  |  | DB Search |
| total 1 peptides |  |  |  |  |  |  |  |  |  |  |  |  |  |  |  |  |  |  |  |
| --- | --- | --- | --- | --- | --- | --- | --- | --- | --- | --- | --- | --- | --- | --- | --- | --- | --- | --- | --- |

Best Unique PSM (Scan Sample\_10\_A1\_1\_3647.d:25296, m/z=655.8339, z=2, RT=12.00, ppm=0.46):


TRINITY\_DN36\_c0\_g1\_i19.p1

back to list

  

| Protein Coverage
| Supporting Peptides
| Best Unique PSM
|

Protein Coverage:

Supporting Peptides:

| Peptide | Uniq | -10lgP | Mass | Length | ppm | m/z | z | RT | 1/k0 Start | 1/k0 End | Precursor ID | Area Sample 1 | #Feature | #Feature Sample 1 | Start | End | PTM | AScore | Found By |
| --- | --- | --- | --- | --- | --- | --- | --- | --- | --- | --- | --- | --- | --- | --- | --- | --- | --- | --- | --- |
| K.GFDYC(+57.02)TTIKYR.L | Y | 66.92 | 1422.6602 | 11 | -0.86 | 712.3336 | 2 | 15.08 | 1.023 | 1.0303 | 42693 | 0 | 0 | 0 | 46 | 56 | Carbamidomethylation |  | DB Search |
| R.LGGIEVTQGNC(+57.02)AR.K | Y | 66.48 | 1373.6721 | 13 | 5.18 | 687.8438 | 2 | 13.73 | 0.963 | 0.9703 | 35192 | 0 | 0 | 0 | 57 | 69 | Carbamidomethylation |  | DB Search |
| K.WC(+57.02)DILSIHQSMENC(+57.02)VAK.C | Y | 63.98 | 2089.9383 | 17 | -2.35 | 697.6486 | 3 | 17.89 | 0.8777 | 0.885 | 58058 | 0 | 0 | 0 | 197 | 213 | Carbamidomethylation, Carbamidomethylation |  | DB Search |
| K.FETQIATK.D | Y | 61.61 | 936.4916 | 8 | 0.97 | 937.4956 | 1 | 13.84 | 1.2037 | 1.2109 | 35727 | 1.28e3 | 1 | 1 | 166 | 173 |  |  | DB Search |
| R.LGGIEVTQGNC(+57.02)ARK.Y | Y | 31.04 | 1501.7671 | 14 | -2.67 | 751.8854 | 2 | 12.64 | 1.0624 | 1.0697 | 29000 | 0 | 0 | 0 | 57 | 70 | Carbamidomethylation |  | DB Search |
| total 5 peptides |  |  |  |  |  |  |  |  |  |  |  |  |  |  |  |  |  |  |  |
| --- | --- | --- | --- | --- | --- | --- | --- | --- | --- | --- | --- | --- | --- | --- | --- | --- | --- | --- | --- |

Best Unique PSM (Scan Sample\_10\_A1\_1\_3647.d:42693, m/z=712.3336, z=2, RT=15.08, ppm=-5.35):


TRINITY\_DN4329\_c0\_g1\_i1.p1

back to list

  

| Protein Coverage
| Supporting Peptides
| Best Unique PSM
|

Protein Coverage:

Supporting Peptides:

| Peptide | Uniq | -10lgP | Mass | Length | ppm | m/z | z | RT | 1/k0 Start | 1/k0 End | Precursor ID | Area Sample 1 | #Feature | #Feature Sample 1 | Start | End | PTM | AScore | Found By |
| --- | --- | --- | --- | --- | --- | --- | --- | --- | --- | --- | --- | --- | --- | --- | --- | --- | --- | --- | --- |
| R.ITELSPIRPR.Y | Y | 43.05 | 1180.6928 | 10 | -1.76 | 591.35 | 2 | 13.86 | 0.9255 | 0.9327 | 35968 | 5.64e2 | 1 | 1 | 104 | 113 |  |  | DB Search |
| R.IRITELSPIRPR.Y | Y | 40.64 | 1449.8779 | 12 | 0.33 | 484.2979 | 3 | 15.22 | 0.8238 | 0.8311 | 43346 | 6.45e2 | 1 | 1 | 102 | 113 |  |  | DB Search |
| total 2 peptides |  |  |  |  |  |  |  |  |  |  |  |  |  |  |  |  |  |  |  |
| --- | --- | --- | --- | --- | --- | --- | --- | --- | --- | --- | --- | --- | --- | --- | --- | --- | --- | --- | --- |

Best Unique PSM (Scan Sample\_10\_A1\_1\_3647.d:35968, m/z=591.35, z=2, RT=13.86, ppm=-6.25):


TRINITY\_DN36\_c0\_g1\_i5.p1

back to list

  

| Protein Coverage
| Supporting Peptides
| Best Unique PSM
|

Protein Coverage:

Supporting Peptides:

| Peptide | Uniq | -10lgP | Mass | Length | ppm | m/z | z | RT | 1/k0 Start | 1/k0 End | Precursor ID | Area Sample 1 | #Feature | #Feature Sample 1 | Start | End | PTM | AScore | Found By |
| --- | --- | --- | --- | --- | --- | --- | --- | --- | --- | --- | --- | --- | --- | --- | --- | --- | --- | --- | --- |
| K.GFDYC(+57.02)TTIKYR.L | Y | 66.92 | 1422.6602 | 11 | -0.86 | 712.3336 | 2 | 15.08 | 1.023 | 1.0303 | 42693 | 0 | 0 | 0 | 46 | 56 | Carbamidomethylation |  | DB Search |
| R.LGGIEVTQGNC(+57.02)AR.K | Y | 66.48 | 1373.6721 | 13 | 5.18 | 687.8438 | 2 | 13.73 | 0.963 | 0.9703 | 35192 | 0 | 0 | 0 | 57 | 69 | Carbamidomethylation |  | DB Search |
| R.LGGIEVTQGNC(+57.02)ARK.Y | Y | 31.04 | 1501.7671 | 14 | -2.67 | 751.8854 | 2 | 12.64 | 1.0624 | 1.0697 | 29000 | 0 | 0 | 0 | 57 | 70 | Carbamidomethylation |  | DB Search |
| total 3 peptides |  |  |  |  |  |  |  |  |  |  |  |  |  |  |  |  |  |  |  |
| --- | --- | --- | --- | --- | --- | --- | --- | --- | --- | --- | --- | --- | --- | --- | --- | --- | --- | --- | --- |

Best Unique PSM (Scan Sample\_10\_A1\_1\_3647.d:42693, m/z=712.3336, z=2, RT=15.08, ppm=-5.35):


TRINITY\_DN7214\_c0\_g1\_i1.p1

back to list

  

| Protein Coverage
| Supporting Peptides
| Best Unique PSM
|

Protein Coverage:

Supporting Peptides:

| Peptide | Uniq | -10lgP | Mass | Length | ppm | m/z | z | RT | 1/k0 Start | 1/k0 End | Precursor ID | Area Sample 1 | #Feature | #Feature Sample 1 | Start | End | PTM | AScore | Found By |
| --- | --- | --- | --- | --- | --- | --- | --- | --- | --- | --- | --- | --- | --- | --- | --- | --- | --- | --- | --- |
| R.LAADLVITHSSK.K | Y | 66.4 | 1253.6979 | 12 | 1.42 | 627.8543 | 2 | 14.21 | 0.9965 | 1.0037 | 37795 | 1.72e3 | 1 | 1 | 167 | 178 |  |  | DB Search |
| total 1 peptides |  |  |  |  |  |  |  |  |  |  |  |  |  |  |  |  |  |  |  |
| --- | --- | --- | --- | --- | --- | --- | --- | --- | --- | --- | --- | --- | --- | --- | --- | --- | --- | --- | --- |

Best Unique PSM (Scan Sample\_10\_A1\_1\_3647.d:37795, m/z=627.8543, z=2, RT=14.21, ppm=-3.07):


TRINITY\_DN817\_c0\_g1\_i2.p1

back to list

  

| Protein Coverage
| Supporting Peptides
| Best Unique PSM
|

Protein Coverage:

Supporting Peptides:

| Peptide | Uniq | -10lgP | Mass | Length | ppm | m/z | z | RT | 1/k0 Start | 1/k0 End | Precursor ID | Area Sample 1 | #Feature | #Feature Sample 1 | Start | End | PTM | AScore | Found By |
| --- | --- | --- | --- | --- | --- | --- | --- | --- | --- | --- | --- | --- | --- | --- | --- | --- | --- | --- | --- |
| R.LC(+57.02)QPGYSTC(+57.02)ENHC(+57.02)R.R | Y | 62.01 | 1780.708 | 14 | 5.39 | 891.3621 | 2 | 11.41 | 1.0893 | 1.0965 | 21785 | 1.84e2 | 1 | 1 | 145 | 158 | Carbamidomethylation, Carbamidomethylation, Carbamidomethylation |  | DB Search |
| total 1 peptides |  |  |  |  |  |  |  |  |  |  |  |  |  |  |  |  |  |  |  |
| --- | --- | --- | --- | --- | --- | --- | --- | --- | --- | --- | --- | --- | --- | --- | --- | --- | --- | --- | --- |

Best Unique PSM (Scan Sample\_10\_A1\_1\_3647.d:21785, m/z=891.3621, z=2, RT=11.41, ppm=0.9):


TRINITY\_DN925\_c0\_g1\_i1.p1

back to list

  

| Protein Coverage
| Supporting Peptides
| Best Unique PSM
|

Protein Coverage:

Supporting Peptides:

| Peptide | Uniq | -10lgP | Mass | Length | ppm | m/z | z | RT | 1/k0 Start | 1/k0 End | Precursor ID | Area Sample 1 | #Feature | #Feature Sample 1 | Start | End | PTM | AScore | Found By |
| --- | --- | --- | --- | --- | --- | --- | --- | --- | --- | --- | --- | --- | --- | --- | --- | --- | --- | --- | --- |
| R.ASGDYATVVSHNR.D | Y | 66.75 | 1375.648 | 13 | -1.79 | 688.827 | 2 | 12.07 | 0.9957 | 1.0029 | 25649 | 0 | 0 | 0 | 129 | 141 |  |  | DB Search |
| total 1 peptides |  |  |  |  |  |  |  |  |  |  |  |  |  |  |  |  |  |  |  |
| --- | --- | --- | --- | --- | --- | --- | --- | --- | --- | --- | --- | --- | --- | --- | --- | --- | --- | --- | --- |

Best Unique PSM (Scan Sample\_10\_A1\_1\_3647.d:25649, m/z=687.8253, z=2, RT=12.10, ppm=-6.28):


TRINITY\_DN152\_c0\_g1\_i2.p1

back to list

  

| Protein Coverage
| Supporting Peptides
| Best Unique PSM
|

Protein Coverage:

Supporting Peptides:

| Peptide | Uniq | -10lgP | Mass | Length | ppm | m/z | z | RT | 1/k0 Start | 1/k0 End | Precursor ID | Area Sample 1 | #Feature | #Feature Sample 1 | Start | End | PTM | AScore | Found By |
| --- | --- | --- | --- | --- | --- | --- | --- | --- | --- | --- | --- | --- | --- | --- | --- | --- | --- | --- | --- |
| K.IQDKEGIPPDQQR.L | Y | 65.08 | 1522.7739 | 13 | -0.66 | 762.3903 | 2 | 11.01 | 1.0339 | 1.0411 | 19352 | 9.49e2 | 1 | 1 | 66 | 78 |  |  | DB Search |
| total 1 peptides |  |  |  |  |  |  |  |  |  |  |  |  |  |  |  |  |  |  |  |
| --- | --- | --- | --- | --- | --- | --- | --- | --- | --- | --- | --- | --- | --- | --- | --- | --- | --- | --- | --- |

Best Unique PSM (Scan Sample\_10\_A1\_1\_3647.d:19352, m/z=762.3903, z=2, RT=11.01, ppm=-5.16):


TRINITY\_DN5906\_c3\_g1\_i1.p2

back to list

  

| Protein Coverage
| Supporting Peptides
| Best Unique PSM
|

Protein Coverage:

Supporting Peptides:

| Peptide | Uniq | -10lgP | Mass | Length | ppm | m/z | z | RT | 1/k0 Start | 1/k0 End | Precursor ID | Area Sample 1 | #Feature | #Feature Sample 1 | Start | End | PTM | AScore | Found By |
| --- | --- | --- | --- | --- | --- | --- | --- | --- | --- | --- | --- | --- | --- | --- | --- | --- | --- | --- | --- |
| R.HGSLGFLPR.K | Y | 25.17 | 982.5348 | 9 | -2.89 | 492.2711 | 2 | 17.17 | 0.8408 | 0.8481 | 53699 | 1.07e3 | 1 | 1 | 36 | 44 |  |  | DB Search |
| K.AGM(+15.99)THIVR.D | Y | 19.81 | 899.4647 | 8 | 2.72 | 450.7388 | 2 | 9.73 | 0.8226 | 0.8299 | 12038 | 6.74e2 | 1 | 1 | 76 | 83 | Oxidation (M) | M3:Oxidation (M):1000 | DB Search |
| total 2 peptides |  |  |  |  |  |  |  |  |  |  |  |  |  |  |  |  |  |  |  |
| --- | --- | --- | --- | --- | --- | --- | --- | --- | --- | --- | --- | --- | --- | --- | --- | --- | --- | --- | --- |

Best Unique PSM (Scan Sample\_10\_A1\_1\_3647.d:53699, m/z=492.2711, z=2, RT=17.17, ppm=-7.39):


TRINITY\_DN4595\_c0\_g1\_i10.p2

back to list

  

| Protein Coverage
| Supporting Peptides
| Best Unique PSM
|

Protein Coverage:

Supporting Peptides:

| Peptide | Uniq | -10lgP | Mass | Length | ppm | m/z | z | RT | 1/k0 Start | 1/k0 End | Precursor ID | Area Sample 1 | #Feature | #Feature Sample 1 | Start | End | PTM | AScore | Found By |
| --- | --- | --- | --- | --- | --- | --- | --- | --- | --- | --- | --- | --- | --- | --- | --- | --- | --- | --- | --- |
| R.AFNRVETR.D | Y | 22.7 | 991.5199 | 8 | -1.27 | 496.7644 | 2 | 10.70 | 0.8465 | 0.8538 | 17683 | 0 | 0 | 0 | 152 | 159 |  |  | DB Search |
| total 1 peptides |  |  |  |  |  |  |  |  |  |  |  |  |  |  |  |  |  |  |  |
| --- | --- | --- | --- | --- | --- | --- | --- | --- | --- | --- | --- | --- | --- | --- | --- | --- | --- | --- | --- |

Best Unique PSM (Scan Sample\_10\_A1\_1\_3647.d:17683, m/z=496.7644, z=2, RT=10.70, ppm=-5.76):


TRINITY\_DN33065\_c0\_g2\_i1.p1

back to list

  

| Protein Coverage
| Supporting Peptides
| Best Unique PSM
|

Protein Coverage:

Supporting Peptides:

| Peptide | Uniq | -10lgP | Mass | Length | ppm | m/z | z | RT | 1/k0 Start | 1/k0 End | Precursor ID | Area Sample 1 | #Feature | #Feature Sample 1 | Start | End | PTM | AScore | Found By |
| --- | --- | --- | --- | --- | --- | --- | --- | --- | --- | --- | --- | --- | --- | --- | --- | --- | --- | --- | --- |
| R.AFSFSDKPAITFEIHDFK.M | Y | 66.57 | 2099.0363 | 18 | -0.42 | 700.6826 | 3 | 18.87 | 0.98 | 0.9872 | 64039 | 1.14e2 | 1 | 1 | 595 | 612 |  |  | DB Search |
| total 1 peptides |  |  |  |  |  |  |  |  |  |  |  |  |  |  |  |  |  |  |  |
| --- | --- | --- | --- | --- | --- | --- | --- | --- | --- | --- | --- | --- | --- | --- | --- | --- | --- | --- | --- |

Best Unique PSM (Scan Sample\_10\_A1\_1\_3647.d:64039, m/z=700.6826, z=3, RT=18.87, ppm=-4.91):


TRINITY\_DN165222\_c0\_g1\_i1.p2

back to list

  

| Protein Coverage
| Supporting Peptides
| Best Unique PSM
|

Protein Coverage:

Supporting Peptides:

| Peptide | Uniq | -10lgP | Mass | Length | ppm | m/z | z | RT | 1/k0 Start | 1/k0 End | Precursor ID | Area Sample 1 | #Feature | #Feature Sample 1 | Start | End | PTM | AScore | Found By |
| --- | --- | --- | --- | --- | --- | --- | --- | --- | --- | --- | --- | --- | --- | --- | --- | --- | --- | --- | --- |
| R.AGAPAGGGPR.G | Y | 17.62 | 809.4144 | 10 | -12.48 | 810.4079 | 1 | 11.05 | 1.1298 | 1.137 | 19747 | 5.12e3 | 1 | 1 | 30 | 39 |  |  | DB Search |
| total 1 peptides |  |  |  |  |  |  |  |  |  |  |  |  |  |  |  |  |  |  |  |
| --- | --- | --- | --- | --- | --- | --- | --- | --- | --- | --- | --- | --- | --- | --- | --- | --- | --- | --- | --- |

Best Unique PSM (Scan Sample\_10\_A1\_1\_3647.d:19747, m/z=810.4079, z=1, RT=11.05, ppm=-16.97):


TRINITY\_DN36\_c0\_g1\_i17.p1

back to list

  

| Protein Coverage
| Supporting Peptides
| Best Unique PSM
|

Protein Coverage:

Supporting Peptides:

| Peptide | Uniq | -10lgP | Mass | Length | ppm | m/z | z | RT | 1/k0 Start | 1/k0 End | Precursor ID | Area Sample 1 | #Feature | #Feature Sample 1 | Start | End | PTM | AScore | Found By |
| --- | --- | --- | --- | --- | --- | --- | --- | --- | --- | --- | --- | --- | --- | --- | --- | --- | --- | --- | --- |
| R.LGGIEVTQGNC(+57.02)AR.K | Y | 66.48 | 1373.6721 | 13 | 5.18 | 687.8438 | 2 | 13.73 | 0.963 | 0.9703 | 35192 | 0 | 0 | 0 | 58 | 70 | Carbamidomethylation |  | DB Search |
| K.WC(+57.02)DILSIHQSMENC(+57.02)VAK.C | Y | 63.98 | 2089.9383 | 17 | -2.35 | 697.6486 | 3 | 17.89 | 0.8777 | 0.885 | 58058 | 0 | 0 | 0 | 198 | 214 | Carbamidomethylation, Carbamidomethylation |  | DB Search |
| K.FETQIATK.D | Y | 61.61 | 936.4916 | 8 | 0.97 | 937.4956 | 1 | 13.84 | 1.2037 | 1.2109 | 35727 | 1.28e3 | 1 | 1 | 167 | 174 |  |  | DB Search |
| R.LGGIEVTQGNC(+57.02)ARK.Y | Y | 31.04 | 1501.7671 | 14 | -2.67 | 751.8854 | 2 | 12.64 | 1.0624 | 1.0697 | 29000 | 0 | 0 | 0 | 58 | 71 | Carbamidomethylation |  | DB Search |
| total 4 peptides |  |  |  |  |  |  |  |  |  |  |  |  |  |  |  |  |  |  |  |
| --- | --- | --- | --- | --- | --- | --- | --- | --- | --- | --- | --- | --- | --- | --- | --- | --- | --- | --- | --- |

Best Unique PSM (Scan Sample\_10\_A1\_1\_3647.d:35192, m/z=687.8438, z=2, RT=13.73, ppm=0.69):


TRINITY\_DN5001\_c0\_g1\_i1.p1

back to list

  

| Protein Coverage
| Supporting Peptides
| Best Unique PSM
|

Protein Coverage:

Supporting Peptides:

| Peptide | Uniq | -10lgP | Mass | Length | ppm | m/z | z | RT | 1/k0 Start | 1/k0 End | Precursor ID | Area Sample 1 | #Feature | #Feature Sample 1 | Start | End | PTM | AScore | Found By |
| --- | --- | --- | --- | --- | --- | --- | --- | --- | --- | --- | --- | --- | --- | --- | --- | --- | --- | --- | --- |
| R.AIQGATSHHLGQNFSK.M | Y | 59.41 | 1694.8488 | 16 | 4.36 | 848.4316 | 2 | 11.89 | 1.1406 | 1.1478 | 24619 | 0 | 0 | 0 | 1293 | 1308 |  |  | DB Search |
| total 1 peptides |  |  |  |  |  |  |  |  |  |  |  |  |  |  |  |  |  |  |  |
| --- | --- | --- | --- | --- | --- | --- | --- | --- | --- | --- | --- | --- | --- | --- | --- | --- | --- | --- | --- |

Best Unique PSM (Scan Sample\_10\_A1\_1\_3647.d:24619, m/z=848.4316, z=2, RT=11.89, ppm=-0.14):


TRINITY\_DN3991\_c0\_g1\_i1.p1

back to list

  

| Protein Coverage
| Supporting Peptides
| Best Unique PSM
|

Protein Coverage:

Supporting Peptides:

| Peptide | Uniq | -10lgP | Mass | Length | ppm | m/z | z | RT | 1/k0 Start | 1/k0 End | Precursor ID | Area Sample 1 | #Feature | #Feature Sample 1 | Start | End | PTM | AScore | Found By |
| --- | --- | --- | --- | --- | --- | --- | --- | --- | --- | --- | --- | --- | --- | --- | --- | --- | --- | --- | --- |
| R.AKLSQC(+57.02)TAASGK.H | Y | 66.69 | 1220.6183 | 12 | 2.46 | 611.3152 | 2 | 9.68 | 0.9533 | 0.9606 | 11765 | 7.57e3 | 1 | 1 | 128 | 139 | Carbamidomethylation |  | DB Search |
| K.LSQC(+57.02)TAASGK.H | Y | 66.58 | 1021.4862 | 10 | -1.41 | 511.7474 | 2 | 9.65 | 0.8356 | 0.8429 | 11604 | 2.54e4 | 1 | 1 | 130 | 139 | Carbamidomethylation |  | DB Search |
| R.HGQVDSC(+57.02)INQNLKK.N | Y | 62.95 | 1639.81 | 14 | 1.44 | 547.6089 | 3 | 11.98 | 0.8315 | 0.8388 | 25203 | 5.94e2 | 1 | 1 | 112 | 125 | Carbamidomethylation |  | DB Search |
| K.LEPVGC(+57.02)FKDR.G | Y | 43.43 | 1219.6019 | 10 | 10.59 | 610.812 | 2 | 12.97 | 0.9352 | 0.9424 | 30908 | 0 | 0 | 0 | 28 | 37 | Carbamidomethylation |  | DB Search |
| total 4 peptides |  |  |  |  |  |  |  |  |  |  |  |  |  |  |  |  |  |  |  |
| --- | --- | --- | --- | --- | --- | --- | --- | --- | --- | --- | --- | --- | --- | --- | --- | --- | --- | --- | --- |

Best Unique PSM (Scan Sample\_10\_A1\_1\_3647.d:11765, m/z=611.3152, z=2, RT=9.68, ppm=-2.04):


TRINITY\_DN120478\_c0\_g1\_i1.p1

back to list

  

| Protein Coverage
| Supporting Peptides
| Best Unique PSM
|

Protein Coverage:

Supporting Peptides:

| Peptide | Uniq | -10lgP | Mass | Length | ppm | m/z | z | RT | 1/k0 Start | 1/k0 End | Precursor ID | Area Sample 1 | #Feature | #Feature Sample 1 | Start | End | PTM | AScore | Found By |
| --- | --- | --- | --- | --- | --- | --- | --- | --- | --- | --- | --- | --- | --- | --- | --- | --- | --- | --- | --- |
| R.ILQLEEEFIKK.M | Y | 62.05 | 1388.7915 | 11 | -3.77 | 695.3973 | 2 | 17.67 | 1.019 | 1.0263 | 56718 | 2.93e2 | 1 | 1 | 267 | 277 |  |  | DB Search |
| total 1 peptides |  |  |  |  |  |  |  |  |  |  |  |  |  |  |  |  |  |  |  |
| --- | --- | --- | --- | --- | --- | --- | --- | --- | --- | --- | --- | --- | --- | --- | --- | --- | --- | --- | --- |

Best Unique PSM (Scan Sample\_10\_A1\_1\_3647.d:56718, m/z=696.3618, z=2, RT=17.72, ppm=-8.26):


TRINITY\_DN152\_c0\_g1\_i15.p1

back to list

  

| Protein Coverage
| Supporting Peptides
| Best Unique PSM
|

Protein Coverage:

Supporting Peptides:

| Peptide | Uniq | -10lgP | Mass | Length | ppm | m/z | z | RT | 1/k0 Start | 1/k0 End | Precursor ID | Area Sample 1 | #Feature | #Feature Sample 1 | Start | End | PTM | AScore | Found By |
| --- | --- | --- | --- | --- | --- | --- | --- | --- | --- | --- | --- | --- | --- | --- | --- | --- | --- | --- | --- |
| K.IQDKEGIPPDQQR.L | Y | 65.08 | 1522.7739 | 13 | -0.66 | 762.3903 | 2 | 11.01 | 1.0339 | 1.0411 | 19352 | 9.49e2 | 1 | 1 | 60 | 72 |  |  | DB Search |
| total 1 peptides |  |  |  |  |  |  |  |  |  |  |  |  |  |  |  |  |  |  |  |
| --- | --- | --- | --- | --- | --- | --- | --- | --- | --- | --- | --- | --- | --- | --- | --- | --- | --- | --- | --- |

Best Unique PSM (Scan Sample\_10\_A1\_1\_3647.d:19352, m/z=762.3903, z=2, RT=11.01, ppm=-5.16):


TRINITY\_DN2477\_c0\_g1\_i2.p1

back to list

  

| Protein Coverage
| Supporting Peptides
| Best Unique PSM
|

Protein Coverage:

Supporting Peptides:

| Peptide | Uniq | -10lgP | Mass | Length | ppm | m/z | z | RT | 1/k0 Start | 1/k0 End | Precursor ID | Area Sample 1 | #Feature | #Feature Sample 1 | Start | End | PTM | AScore | Found By |
| --- | --- | --- | --- | --- | --- | --- | --- | --- | --- | --- | --- | --- | --- | --- | --- | --- | --- | --- | --- |
| K.DSYVGDEAQSKR.G | Y | 66.73 | 1353.616 | 12 | 2.52 | 677.814 | 2 | 10.94 | 1.0069 | 1.0142 | 19045 | 5.51e3 | 1 | 1 | 19 | 30 |  |  | DB Search |
| total 1 peptides |  |  |  |  |  |  |  |  |  |  |  |  |  |  |  |  |  |  |  |
| --- | --- | --- | --- | --- | --- | --- | --- | --- | --- | --- | --- | --- | --- | --- | --- | --- | --- | --- | --- |

Best Unique PSM (Scan Sample\_10\_A1\_1\_3647.d:19045, m/z=677.814, z=2, RT=10.94, ppm=-1.97):


TRINITY\_DN2885\_c0\_g1\_i4.p1

back to list

  

| Protein Coverage
| Supporting Peptides
| Best Unique PSM
|

Protein Coverage:

Supporting Peptides:

| Peptide | Uniq | -10lgP | Mass | Length | ppm | m/z | z | RT | 1/k0 Start | 1/k0 End | Precursor ID | Area Sample 1 | #Feature | #Feature Sample 1 | Start | End | PTM | AScore | Found By |
| --- | --- | --- | --- | --- | --- | --- | --- | --- | --- | --- | --- | --- | --- | --- | --- | --- | --- | --- | --- |
| K.VTRPHGASGVVR.A | Y | 66.69 | 1234.6894 | 12 | 2.23 | 618.3506 | 2 | 10.05 | 0.9319 | 0.9392 | 13758 | 2.92e4 | 3 | 3 | 72 | 83 |  |  | DB Search |
| K.VTRPHGASGVVRAK.F | Y | 41.36 | 1433.8215 | 14 | 4.92 | 359.4628 | 4 | 9.64 | 0.7151 | 0.7224 | 11644 | 0 | 0 | 0 | 72 | 85 |  |  | DB Search |
| total 2 peptides |  |  |  |  |  |  |  |  |  |  |  |  |  |  |  |  |  |  |  |
| --- | --- | --- | --- | --- | --- | --- | --- | --- | --- | --- | --- | --- | --- | --- | --- | --- | --- | --- | --- |

Best Unique PSM (Scan Sample\_10\_A1\_1\_3647.d:13758, m/z=618.3506, z=2, RT=10.05, ppm=-2.27):


TRINITY\_DN152\_c0\_g1\_i10.p1

back to list

  

| Protein Coverage
| Supporting Peptides
| Best Unique PSM
|

Protein Coverage:

Supporting Peptides:

| Peptide | Uniq | -10lgP | Mass | Length | ppm | m/z | z | RT | 1/k0 Start | 1/k0 End | Precursor ID | Area Sample 1 | #Feature | #Feature Sample 1 | Start | End | PTM | AScore | Found By |
| --- | --- | --- | --- | --- | --- | --- | --- | --- | --- | --- | --- | --- | --- | --- | --- | --- | --- | --- | --- |
| K.IQDKEGIPPDQQR.L | Y | 65.08 | 1522.7739 | 13 | -0.66 | 762.3903 | 2 | 11.01 | 1.0339 | 1.0411 | 19352 | 9.49e2 | 1 | 1 | 141 | 153 |  |  | DB Search |
| total 1 peptides |  |  |  |  |  |  |  |  |  |  |  |  |  |  |  |  |  |  |  |
| --- | --- | --- | --- | --- | --- | --- | --- | --- | --- | --- | --- | --- | --- | --- | --- | --- | --- | --- | --- |

Best Unique PSM (Scan Sample\_10\_A1\_1\_3647.d:19352, m/z=762.3903, z=2, RT=11.01, ppm=-5.16):


TRINITY\_DN1428\_c0\_g1\_i1.p1

back to list

  

| Protein Coverage
| Supporting Peptides
| Best Unique PSM
|

Protein Coverage:

Supporting Peptides:

| Peptide | Uniq | -10lgP | Mass | Length | ppm | m/z | z | RT | 1/k0 Start | 1/k0 End | Precursor ID | Area Sample 1 | #Feature | #Feature Sample 1 | Start | End | PTM | AScore | Found By |
| --- | --- | --- | --- | --- | --- | --- | --- | --- | --- | --- | --- | --- | --- | --- | --- | --- | --- | --- | --- |
| R.IAYAQGYATGDPSKR.M | Y | 66.68 | 1596.7896 | 15 | 6.44 | 799.4036 | 2 | 12.71 | 1.1069 | 1.1142 | 29416 | 2.71e2 | 1 | 1 | 122 | 136 |  |  | DB Search |
| total 1 peptides |  |  |  |  |  |  |  |  |  |  |  |  |  |  |  |  |  |  |  |
| --- | --- | --- | --- | --- | --- | --- | --- | --- | --- | --- | --- | --- | --- | --- | --- | --- | --- | --- | --- |

Best Unique PSM (Scan Sample\_10\_A1\_1\_3647.d:29416, m/z=799.4036, z=2, RT=12.71, ppm=1.95):


TRINITY\_DN30378\_c0\_g1\_i1.p1

back to list

  

| Protein Coverage
| Supporting Peptides
| Best Unique PSM
|

Protein Coverage:

Supporting Peptides:

| Peptide | Uniq | -10lgP | Mass | Length | ppm | m/z | z | RT | 1/k0 Start | 1/k0 End | Precursor ID | Area Sample 1 | #Feature | #Feature Sample 1 | Start | End | PTM | AScore | Found By |
| --- | --- | --- | --- | --- | --- | --- | --- | --- | --- | --- | --- | --- | --- | --- | --- | --- | --- | --- | --- |
| K.ATLGHGHVIEGVDR.G | Y | 64.13 | 1459.7531 | 14 | -3.35 | 730.8781 | 2 | 12.12 | 1.011 | 1.0182 | 25970 | 0 | 0 | 0 | 203 | 216 |  |  | DB Search |
| total 1 peptides |  |  |  |  |  |  |  |  |  |  |  |  |  |  |  |  |  |  |  |
| --- | --- | --- | --- | --- | --- | --- | --- | --- | --- | --- | --- | --- | --- | --- | --- | --- | --- | --- | --- |

Best Unique PSM (Scan Sample\_10\_A1\_1\_3647.d:25970, m/z=730.8781, z=2, RT=12.12, ppm=-7.85):


TRINITY\_DN4166\_c0\_g3\_i1.p1

back to list

  

| Protein Coverage
| Supporting Peptides
| Best Unique PSM
|

Protein Coverage:

Supporting Peptides:

| Peptide | Uniq | -10lgP | Mass | Length | ppm | m/z | z | RT | 1/k0 Start | 1/k0 End | Precursor ID | Area Sample 1 | #Feature | #Feature Sample 1 | Start | End | PTM | AScore | Found By |
| --- | --- | --- | --- | --- | --- | --- | --- | --- | --- | --- | --- | --- | --- | --- | --- | --- | --- | --- | --- |
| K.SLGYSVQLQAALIK.L | Y | 29.2 | 1489.8504 | 14 | -3.67 | 745.9264 | 2 | 18.69 | 1.0516 | 1.0588 | 62924 | 0 | 0 | 0 | 440 | 453 |  |  | DB Search |
| total 1 peptides |  |  |  |  |  |  |  |  |  |  |  |  |  |  |  |  |  |  |  |
| --- | --- | --- | --- | --- | --- | --- | --- | --- | --- | --- | --- | --- | --- | --- | --- | --- | --- | --- | --- |

Best Unique PSM (Scan Sample\_10\_A1\_1\_3647.d:62924, m/z=745.9264, z=2, RT=18.69, ppm=-8.17):


TRINITY\_DN189323\_c0\_g1\_i1.p1

back to list

  

| Protein Coverage
| Supporting Peptides
| Best Unique PSM
|

Protein Coverage:

Supporting Peptides:

| Peptide | Uniq | -10lgP | Mass | Length | ppm | m/z | z | RT | 1/k0 Start | 1/k0 End | Precursor ID | Area Sample 1 | #Feature | #Feature Sample 1 | Start | End | PTM | AScore | Found By |
| --- | --- | --- | --- | --- | --- | --- | --- | --- | --- | --- | --- | --- | --- | --- | --- | --- | --- | --- | --- |
| K.RLFEGNALLR.R | Y | 66.66 | 1187.6775 | 10 | 1.42 | 594.8442 | 2 | 16.07 | 0.9384 | 0.9457 | 47820 | 1.65e3 | 1 | 1 | 31 | 40 |  |  | DB Search |
| R.LFEGNALLRR.L | Y | 66.2 | 1187.6775 | 10 | 0.49 | 594.8436 | 2 | 15.56 | 0.9675 | 0.9747 | 45173 | 2.25e3 | 1 | 1 | 32 | 41 |  |  | DB Search |
| R.KQLVNVPSFIVR.L | Y | 61.54 | 1398.8347 | 12 | 1.44 | 700.4225 | 2 | 17.76 | 1.0065 | 1.0138 | 57229 | 4.4e2 | 1 | 1 | 100 | 111 |  |  | DB Search |
| total 3 peptides |  |  |  |  |  |  |  |  |  |  |  |  |  |  |  |  |  |  |  |
| --- | --- | --- | --- | --- | --- | --- | --- | --- | --- | --- | --- | --- | --- | --- | --- | --- | --- | --- | --- |

Best Unique PSM (Scan Sample\_10\_A1\_1\_3647.d:47820, m/z=594.8442, z=2, RT=16.07, ppm=-3.08):


TRINITY\_DN200\_c0\_g1\_i2.p1

back to list

  

| Protein Coverage
| Supporting Peptides
| Best Unique PSM
|

Protein Coverage:

Supporting Peptides:

| Peptide | Uniq | -10lgP | Mass | Length | ppm | m/z | z | RT | 1/k0 Start | 1/k0 End | Precursor ID | Area Sample 1 | #Feature | #Feature Sample 1 | Start | End | PTM | AScore | Found By |
| --- | --- | --- | --- | --- | --- | --- | --- | --- | --- | --- | --- | --- | --- | --- | --- | --- | --- | --- | --- |
| K.TAIAIDTIINQKR.F | Y | 64.76 | 1455.8409 | 13 | -0.36 | 728.9242 | 2 | 16.58 | 1.0588 | 1.066 | 50506 | 4.46e2 | 1 | 1 | 217 | 229 |  |  | DB Search |
| total 1 peptides |  |  |  |  |  |  |  |  |  |  |  |  |  |  |  |  |  |  |  |
| --- | --- | --- | --- | --- | --- | --- | --- | --- | --- | --- | --- | --- | --- | --- | --- | --- | --- | --- | --- |

Best Unique PSM (Scan Sample\_10\_A1\_1\_3647.d:50506, m/z=728.9242, z=2, RT=16.58, ppm=-4.85):


TRINITY\_DN1172\_c0\_g1\_i5.p1

back to list

  

| Protein Coverage
| Supporting Peptides
| Best Unique PSM
|

Protein Coverage:

Supporting Peptides:

| Peptide | Uniq | -10lgP | Mass | Length | ppm | m/z | z | RT | 1/k0 Start | 1/k0 End | Precursor ID | Area Sample 1 | #Feature | #Feature Sample 1 | Start | End | PTM | AScore | Found By |
| --- | --- | --- | --- | --- | --- | --- | --- | --- | --- | --- | --- | --- | --- | --- | --- | --- | --- | --- | --- |
| K.VITSDDPAEKEK.K | Y | 66.7 | 1330.6616 | 12 | 2.85 | 666.337 | 2 | 10.71 | 0.9836 | 0.9908 | 17367 | 7.2e3 | 1 | 1 | 123 | 134 |  |  | DB Search |
| total 1 peptides |  |  |  |  |  |  |  |  |  |  |  |  |  |  |  |  |  |  |  |
| --- | --- | --- | --- | --- | --- | --- | --- | --- | --- | --- | --- | --- | --- | --- | --- | --- | --- | --- | --- |

Best Unique PSM (Scan Sample\_10\_A1\_1\_3647.d:17367, m/z=666.337, z=2, RT=10.71, ppm=-1.65):


TRINITY\_DN2637\_c0\_g1\_i2.p2

back to list

  

| Protein Coverage
| Supporting Peptides
| Best Unique PSM
|

Protein Coverage:

Supporting Peptides:

| Peptide | Uniq | -10lgP | Mass | Length | ppm | m/z | z | RT | 1/k0 Start | 1/k0 End | Precursor ID | Area Sample 1 | #Feature | #Feature Sample 1 | Start | End | PTM | AScore | Found By |
| --- | --- | --- | --- | --- | --- | --- | --- | --- | --- | --- | --- | --- | --- | --- | --- | --- | --- | --- | --- |
| K.ATIEIPAKYEK.R | Y | 61.89 | 1261.6918 | 11 | 0.22 | 631.8505 | 2 | 14.08 | 0.982 | 0.9892 | 37168 | 0 | 0 | 0 | 107 | 117 |  |  | DB Search |
| total 1 peptides |  |  |  |  |  |  |  |  |  |  |  |  |  |  |  |  |  |  |  |
| --- | --- | --- | --- | --- | --- | --- | --- | --- | --- | --- | --- | --- | --- | --- | --- | --- | --- | --- | --- |

Best Unique PSM (Scan Sample\_10\_A1\_1\_3647.d:37168, m/z=631.8505, z=2, RT=14.08, ppm=-4.27):


TRINITY\_DN817\_c0\_g1\_i9.p1

back to list

  

| Protein Coverage
| Supporting Peptides
| Best Unique PSM
|

Protein Coverage:

Supporting Peptides:

| Peptide | Uniq | -10lgP | Mass | Length | ppm | m/z | z | RT | 1/k0 Start | 1/k0 End | Precursor ID | Area Sample 1 | #Feature | #Feature Sample 1 | Start | End | PTM | AScore | Found By |
| --- | --- | --- | --- | --- | --- | --- | --- | --- | --- | --- | --- | --- | --- | --- | --- | --- | --- | --- | --- |
| R.LC(+57.02)QPGYSTC(+57.02)ENHC(+57.02)R.K | Y | 62.01 | 1780.708 | 14 | 5.39 | 891.3621 | 2 | 11.41 | 1.0893 | 1.0965 | 21785 | 1.84e2 | 1 | 1 | 153 | 166 | Carbamidomethylation, Carbamidomethylation, Carbamidomethylation |  | DB Search |
| total 1 peptides |  |  |  |  |  |  |  |  |  |  |  |  |  |  |  |  |  |  |  |
| --- | --- | --- | --- | --- | --- | --- | --- | --- | --- | --- | --- | --- | --- | --- | --- | --- | --- | --- | --- |

Best Unique PSM (Scan Sample\_10\_A1\_1\_3647.d:21785, m/z=891.3621, z=2, RT=11.41, ppm=0.9):


TRINITY\_DN56792\_c0\_g1\_i3.p1

back to list

  

| Protein Coverage
| Supporting Peptides
| Best Unique PSM
|

Protein Coverage:

Supporting Peptides:

| Peptide | Uniq | -10lgP | Mass | Length | ppm | m/z | z | RT | 1/k0 Start | 1/k0 End | Precursor ID | Area Sample 1 | #Feature | #Feature Sample 1 | Start | End | PTM | AScore | Found By |
| --- | --- | --- | --- | --- | --- | --- | --- | --- | --- | --- | --- | --- | --- | --- | --- | --- | --- | --- | --- |
| R.LAIDHAVSLITNIK.C | Y | 66.67 | 1506.8769 | 14 | 0.6 | 754.4428 | 2 | 19.10 | 1.0761 | 1.0833 | 65382 | 4.12e2 | 1 | 1 | 211 | 224 |  |  | DB Search |
| total 1 peptides |  |  |  |  |  |  |  |  |  |  |  |  |  |  |  |  |  |  |  |
| --- | --- | --- | --- | --- | --- | --- | --- | --- | --- | --- | --- | --- | --- | --- | --- | --- | --- | --- | --- |

Best Unique PSM (Scan Sample\_10\_A1\_1\_3647.d:65382, m/z=754.4428, z=2, RT=19.10, ppm=-3.9):


TRINITY\_DN152\_c0\_g1\_i14.p2

back to list

  

| Protein Coverage
| Supporting Peptides
| Best Unique PSM
|

Protein Coverage:

Supporting Peptides:

| Peptide | Uniq | -10lgP | Mass | Length | ppm | m/z | z | RT | 1/k0 Start | 1/k0 End | Precursor ID | Area Sample 1 | #Feature | #Feature Sample 1 | Start | End | PTM | AScore | Found By |
| --- | --- | --- | --- | --- | --- | --- | --- | --- | --- | --- | --- | --- | --- | --- | --- | --- | --- | --- | --- |
| K.IQDKEGIPPDQQR.S | Y | 65.08 | 1522.7739 | 13 | -0.66 | 762.3903 | 2 | 11.01 | 1.0339 | 1.0411 | 19352 | 9.49e2 | 1 | 1 | 40 | 52 |  |  | DB Search |
| total 1 peptides |  |  |  |  |  |  |  |  |  |  |  |  |  |  |  |  |  |  |  |
| --- | --- | --- | --- | --- | --- | --- | --- | --- | --- | --- | --- | --- | --- | --- | --- | --- | --- | --- | --- |

Best Unique PSM (Scan Sample\_10\_A1\_1\_3647.d:19352, m/z=762.3903, z=2, RT=11.01, ppm=-5.16):


TRINITY\_DN1339\_c6\_g1\_i1.p1

back to list

  

| Protein Coverage
| Supporting Peptides
| Best Unique PSM
|

Protein Coverage:

Supporting Peptides:

| Peptide | Uniq | -10lgP | Mass | Length | ppm | m/z | z | RT | 1/k0 Start | 1/k0 End | Precursor ID | Area Sample 1 | #Feature | #Feature Sample 1 | Start | End | PTM | AScore | Found By |
| --- | --- | --- | --- | --- | --- | --- | --- | --- | --- | --- | --- | --- | --- | --- | --- | --- | --- | --- | --- |
| K.VITSDDPAEKEK.K | Y | 66.7 | 1330.6616 | 12 | 2.85 | 666.337 | 2 | 10.71 | 0.9836 | 0.9908 | 17367 | 7.2e3 | 1 | 1 | 158 | 169 |  |  | DB Search |
| total 1 peptides |  |  |  |  |  |  |  |  |  |  |  |  |  |  |  |  |  |  |  |
| --- | --- | --- | --- | --- | --- | --- | --- | --- | --- | --- | --- | --- | --- | --- | --- | --- | --- | --- | --- |

Best Unique PSM (Scan Sample\_10\_A1\_1\_3647.d:17367, m/z=666.337, z=2, RT=10.71, ppm=-1.65):


TRINITY\_DN801\_c0\_g1\_i10.p1

back to list

  

| Protein Coverage
| Supporting Peptides
| Best Unique PSM
|

Protein Coverage:

Supporting Peptides:

| Peptide | Uniq | -10lgP | Mass | Length | ppm | m/z | z | RT | 1/k0 Start | 1/k0 End | Precursor ID | Area Sample 1 | #Feature | #Feature Sample 1 | Start | End | PTM | AScore | Found By |
| --- | --- | --- | --- | --- | --- | --- | --- | --- | --- | --- | --- | --- | --- | --- | --- | --- | --- | --- | --- |
| K.VFDRDGNGFISAAELR.H | Y | 39.11 | 1765.8747 | 16 | -1.5 | 883.9393 | 2 | 19.71 | 1.1234 | 1.1306 | 69271 | 0 | 0 | 0 | 117 | 132 |  |  | DB Search |
| total 1 peptides |  |  |  |  |  |  |  |  |  |  |  |  |  |  |  |  |  |  |  |
| --- | --- | --- | --- | --- | --- | --- | --- | --- | --- | --- | --- | --- | --- | --- | --- | --- | --- | --- | --- |

Best Unique PSM (Scan Sample\_10\_A1\_1\_3647.d:69271, m/z=883.9393, z=2, RT=19.71, ppm=-6):


TRINITY\_DN598\_c1\_g1\_i2.p1

back to list

  

| Protein Coverage
| Supporting Peptides
| Best Unique PSM
|

Protein Coverage:

Supporting Peptides:

| Peptide | Uniq | -10lgP | Mass | Length | ppm | m/z | z | RT | 1/k0 Start | 1/k0 End | Precursor ID | Area Sample 1 | #Feature | #Feature Sample 1 | Start | End | PTM | AScore | Found By |
| --- | --- | --- | --- | --- | --- | --- | --- | --- | --- | --- | --- | --- | --- | --- | --- | --- | --- | --- | --- |
| R.ASFVTDIDRLR.A | Y | 66.55 | 1291.6884 | 11 | -3.8 | 646.8461 | 2 | 17.02 | 0.988 | 0.9953 | 52753 | 1.2e3 | 1 | 1 | 302 | 312 |  |  | DB Search |
| total 1 peptides |  |  |  |  |  |  |  |  |  |  |  |  |  |  |  |  |  |  |  |
| --- | --- | --- | --- | --- | --- | --- | --- | --- | --- | --- | --- | --- | --- | --- | --- | --- | --- | --- | --- |

Best Unique PSM (Scan Sample\_10\_A1\_1\_3647.d:52753, m/z=646.8461, z=2, RT=17.02, ppm=-8.29):


TRINITY\_DN21847\_c1\_g1\_i1.p1

back to list

  

| Protein Coverage
| Supporting Peptides
| Best Unique PSM
|

Protein Coverage:

Supporting Peptides:

| Peptide | Uniq | -10lgP | Mass | Length | ppm | m/z | z | RT | 1/k0 Start | 1/k0 End | Precursor ID | Area Sample 1 | #Feature | #Feature Sample 1 | Start | End | PTM | AScore | Found By |
| --- | --- | --- | --- | --- | --- | --- | --- | --- | --- | --- | --- | --- | --- | --- | --- | --- | --- | --- | --- |
| R.K(+42.01)LSAELANGR.L | Y | 66.66 | 1099.5985 | 10 | 2.66 | 550.8055 | 2 | 14.21 | 0.8712 | 0.8785 | 37894 | 8.99e2 | 1 | 1 | 6 | 15 | Acetylation (Protein N-term) | K1:Acetylation (Protein N-term):1000 | DB Search |
| total 1 peptides |  |  |  |  |  |  |  |  |  |  |  |  |  |  |  |  |  |  |  |
| --- | --- | --- | --- | --- | --- | --- | --- | --- | --- | --- | --- | --- | --- | --- | --- | --- | --- | --- | --- |

Best Unique PSM (Scan Sample\_10\_A1\_1\_3647.d:37894, m/z=550.8055, z=2, RT=14.21, ppm=-1.83):


TRINITY\_DN15847\_c0\_g1\_i1.p1

back to list

  

| Protein Coverage
| Supporting Peptides
| Best Unique PSM
|

Protein Coverage:

Supporting Peptides:

| Peptide | Uniq | -10lgP | Mass | Length | ppm | m/z | z | RT | 1/k0 Start | 1/k0 End | Precursor ID | Area Sample 1 | #Feature | #Feature Sample 1 | Start | End | PTM | AScore | Found By |
| --- | --- | --- | --- | --- | --- | --- | --- | --- | --- | --- | --- | --- | --- | --- | --- | --- | --- | --- | --- |
| R.DVQLGDLVTVGQC(+57.02)RPLSK.T | Y | 63.15 | 1984.0411 | 18 | 4.67 | 993.028 | 2 | 17.98 | 1.2726 | 1.2797 | 58548 | 8.93e2 | 1 | 1 | 126 | 143 | Carbamidomethylation |  | DB Search |
| R.DYLHFIK.K | Y | 61.47 | 934.4912 | 7 | -4.37 | 468.2487 | 2 | 18.67 | 0.8242 | 0.8315 | 62776 | 3.12e2 | 1 | 1 | 98 | 104 |  |  | DB Search |
| total 2 peptides |  |  |  |  |  |  |  |  |  |  |  |  |  |  |  |  |  |  |  |
| --- | --- | --- | --- | --- | --- | --- | --- | --- | --- | --- | --- | --- | --- | --- | --- | --- | --- | --- | --- |

Best Unique PSM (Scan Sample\_10\_A1\_1\_3647.d:58548, m/z=993.028, z=2, RT=17.98, ppm=0.18):


TRINITY\_DN1819\_c0\_g1\_i7.p1

back to list

  

| Protein Coverage
| Supporting Peptides
| Best Unique PSM
|

Protein Coverage:

Supporting Peptides:

| Peptide | Uniq | -10lgP | Mass | Length | ppm | m/z | z | RT | 1/k0 Start | 1/k0 End | Precursor ID | Area Sample 1 | #Feature | #Feature Sample 1 | Start | End | PTM | AScore | Found By |
| --- | --- | --- | --- | --- | --- | --- | --- | --- | --- | --- | --- | --- | --- | --- | --- | --- | --- | --- | --- |
| K.ALADYIHSQDLK.M | Y | 59.54 | 1372.6986 | 12 | 0.98 | 687.3542 | 2 | 15.62 | 1.0061 | 1.0134 | 45560 | 3.85e2 | 1 | 1 | 105 | 116 |  |  | DB Search |
| total 1 peptides |  |  |  |  |  |  |  |  |  |  |  |  |  |  |  |  |  |  |  |
| --- | --- | --- | --- | --- | --- | --- | --- | --- | --- | --- | --- | --- | --- | --- | --- | --- | --- | --- | --- |

Best Unique PSM (Scan Sample\_10\_A1\_1\_3647.d:45560, m/z=687.3542, z=2, RT=15.62, ppm=-3.52):


TRINITY\_DN7808\_c1\_g3\_i2.p2

back to list

  

| Protein Coverage
| Supporting Peptides
| Best Unique PSM
|

Protein Coverage:

Supporting Peptides:

| Peptide | Uniq | -10lgP | Mass | Length | ppm | m/z | z | RT | 1/k0 Start | 1/k0 End | Precursor ID | Area Sample 1 | #Feature | #Feature Sample 1 | Start | End | PTM | AScore | Found By |
| --- | --- | --- | --- | --- | --- | --- | --- | --- | --- | --- | --- | --- | --- | --- | --- | --- | --- | --- | --- |
| K.AITSSDPEVLKR.K | Y | 62.01 | 1314.7143 | 12 | -5.42 | 658.3579 | 2 | 12.95 | 0.9844 | 0.9916 | 30804 | 0 | 0 | 0 | 114 | 125 |  |  | DB Search |
| total 1 peptides |  |  |  |  |  |  |  |  |  |  |  |  |  |  |  |  |  |  |  |
| --- | --- | --- | --- | --- | --- | --- | --- | --- | --- | --- | --- | --- | --- | --- | --- | --- | --- | --- | --- |

Best Unique PSM (Scan Sample\_10\_A1\_1\_3647.d:30804, m/z=658.3579, z=2, RT=12.95, ppm=-9.91):


TRINITY\_DN554\_c0\_g1\_i3.p2

back to list

  

| Protein Coverage
| Supporting Peptides
| Best Unique PSM
|

Protein Coverage:

Supporting Peptides:

| Peptide | Uniq | -10lgP | Mass | Length | ppm | m/z | z | RT | 1/k0 Start | 1/k0 End | Precursor ID | Area Sample 1 | #Feature | #Feature Sample 1 | Start | End | PTM | AScore | Found By |
| --- | --- | --- | --- | --- | --- | --- | --- | --- | --- | --- | --- | --- | --- | --- | --- | --- | --- | --- | --- |
| K.IQDKEGIPPDQQR.L | Y | 65.08 | 1522.7739 | 13 | -0.66 | 762.3903 | 2 | 11.01 | 1.0339 | 1.0411 | 19352 | 9.49e2 | 1 | 1 | 30 | 42 |  |  | DB Search |
| total 1 peptides |  |  |  |  |  |  |  |  |  |  |  |  |  |  |  |  |  |  |  |
| --- | --- | --- | --- | --- | --- | --- | --- | --- | --- | --- | --- | --- | --- | --- | --- | --- | --- | --- | --- |

Best Unique PSM (Scan Sample\_10\_A1\_1\_3647.d:19352, m/z=762.3903, z=2, RT=11.01, ppm=-5.16):


TRINITY\_DN3036\_c0\_g1\_i16.p1

back to list

  

| Protein Coverage
| Supporting Peptides
| Best Unique PSM
|

Protein Coverage:

Supporting Peptides:

| Peptide | Uniq | -10lgP | Mass | Length | ppm | m/z | z | RT | 1/k0 Start | 1/k0 End | Precursor ID | Area Sample 1 | #Feature | #Feature Sample 1 | Start | End | PTM | AScore | Found By |
| --- | --- | --- | --- | --- | --- | --- | --- | --- | --- | --- | --- | --- | --- | --- | --- | --- | --- | --- | --- |
| R.AGHGAVTSPQTQR.G | Y | 66.66 | 1308.6534 | 13 | 4.48 | 655.334 | 2 | 9.70 | 0.9109 | 0.9182 | 11470 | 2.1e4 | 2 | 2 | 135 | 147 |  |  | DB Search |
| R.VIGASGIHVGTMSFGK.M | Y | 66.34 | 1559.813 | 16 | -0.2 | 780.9101 | 2 | 16.89 | 1.1037 | 1.111 | 52086 | 2.86e2 | 1 | 1 | 160 | 175 |  |  | DB Search |
| R.VIGASGIHVGTM(+15.99)SFGK.M | Y | 64.9 | 1575.8079 | 16 | 0.52 | 788.9081 | 2 | 15.11 | 1.1262 | 1.1334 | 42748 | 6.68e2 | 1 | 1 | 160 | 175 | Oxidation (M) | M12:Oxidation (M):1000 | DB Search |
| total 3 peptides |  |  |  |  |  |  |  |  |  |  |  |  |  |  |  |  |  |  |  |
| --- | --- | --- | --- | --- | --- | --- | --- | --- | --- | --- | --- | --- | --- | --- | --- | --- | --- | --- | --- |

Best Unique PSM (Scan Sample\_10\_A1\_1\_3647.d:11470, m/z=655.334, z=2, RT=9.70, ppm=-0.02):


TRINITY\_DN22492\_c0\_g1\_i2.p1

back to list

  

| Protein Coverage
| Supporting Peptides
| Best Unique PSM
|

Protein Coverage:

Supporting Peptides:

| Peptide | Uniq | -10lgP | Mass | Length | ppm | m/z | z | RT | 1/k0 Start | 1/k0 End | Precursor ID | Area Sample 1 | #Feature | #Feature Sample 1 | Start | End | PTM | AScore | Found By |
| --- | --- | --- | --- | --- | --- | --- | --- | --- | --- | --- | --- | --- | --- | --- | --- | --- | --- | --- | --- |
| R.HC(+57.02)DPELSSFHTFWR.L | Y | 61.65 | 1817.7944 | 14 | -6 | 606.9324 | 3 | 17.68 | 0.8887 | 0.8959 | 56846 | 2.37e2 | 1 | 1 | 151 | 164 | Carbamidomethylation |  | DB Search |
| K.KKLEPQAAPR.E | Y | 43.1 | 1136.6666 | 10 | 2.05 | 379.8952 | 3 | 9.66 | 0.7604 | 0.7677 | 11777 | 3.9e3 | 2 | 2 | 425 | 434 |  |  | DB Search |
| total 2 peptides |  |  |  |  |  |  |  |  |  |  |  |  |  |  |  |  |  |  |  |
| --- | --- | --- | --- | --- | --- | --- | --- | --- | --- | --- | --- | --- | --- | --- | --- | --- | --- | --- | --- |

Best Unique PSM (Scan Sample\_10\_A1\_1\_3647.d:56846, m/z=606.9324, z=3, RT=17.68, ppm=-10.49):


TRINITY\_DN240\_c0\_g1\_i6.p1

back to list

  

| Protein Coverage
| Supporting Peptides
| Best Unique PSM
|

Protein Coverage:

Supporting Peptides:

| Peptide | Uniq | -10lgP | Mass | Length | ppm | m/z | z | RT | 1/k0 Start | 1/k0 End | Precursor ID | Area Sample 1 | #Feature | #Feature Sample 1 | Start | End | PTM | AScore | Found By |
| --- | --- | --- | --- | --- | --- | --- | --- | --- | --- | --- | --- | --- | --- | --- | --- | --- | --- | --- | --- |
| K.AYAAFLEFKDVVK.A | Y | 66.86 | 1499.8024 | 13 | 1.14 | 750.9059 | 2 | 19.02 | 1.0945 | 1.1017 | 64841 | 9.59e3 | 1 | 1 | 217 | 229 |  |  | DB Search |
| K.AAASAHHQAIGSIDAAGVTSAADYASVTAALGR.A | Y | 62.75 | 3080.5275 | 33 | 0.95 | 771.1364 | 4 | 18.15 | 1.0636 | 1.0709 | 59524 | 1.07e3 | 1 | 1 | 307 | 339 |  |  | DB Search |
| K.IGAAAQALADQSYPFLK.S | Y | 61.76 | 1762.9253 | 17 | 1.13 | 882.467 | 2 | 19.07 | 1.161 | 1.1682 | 65059 | 6.63e2 | 1 | 1 | 249 | 265 |  |  | DB Search |
| total 3 peptides |  |  |  |  |  |  |  |  |  |  |  |  |  |  |  |  |  |  |  |
| --- | --- | --- | --- | --- | --- | --- | --- | --- | --- | --- | --- | --- | --- | --- | --- | --- | --- | --- | --- |

Best Unique PSM (Scan Sample\_10\_A1\_1\_3647.d:64841, m/z=750.9059, z=2, RT=19.02, ppm=-3.35):


TRINITY\_DN5107\_c0\_g1\_i1.p2

back to list

  

| Protein Coverage
| Supporting Peptides
| Best Unique PSM
|

Protein Coverage:

Supporting Peptides:

| Peptide | Uniq | -10lgP | Mass | Length | ppm | m/z | z | RT | 1/k0 Start | 1/k0 End | Precursor ID | Area Sample 1 | #Feature | #Feature Sample 1 | Start | End | PTM | AScore | Found By |
| --- | --- | --- | --- | --- | --- | --- | --- | --- | --- | --- | --- | --- | --- | --- | --- | --- | --- | --- | --- |
| K.KLDYVLGLTTAK.I | Y | 63.57 | 1320.7653 | 12 | -2.37 | 661.3854 | 2 | 16.69 | 0.9908 | 0.9981 | 51148 | 2.13e2 | 1 | 1 | 62 | 73 |  |  | DB Search |
| total 1 peptides |  |  |  |  |  |  |  |  |  |  |  |  |  |  |  |  |  |  |  |
| --- | --- | --- | --- | --- | --- | --- | --- | --- | --- | --- | --- | --- | --- | --- | --- | --- | --- | --- | --- |

Best Unique PSM (Scan Sample\_10\_A1\_1\_3647.d:51148, m/z=661.3854, z=2, RT=16.69, ppm=-6.87):


TRINITY\_DN119650\_c0\_g1\_i1.p1

back to list

  

| Protein Coverage
| Supporting Peptides
| Best Unique PSM
|

Protein Coverage:

Supporting Peptides:

| Peptide | Uniq | -10lgP | Mass | Length | ppm | m/z | z | RT | 1/k0 Start | 1/k0 End | Precursor ID | Area Sample 1 | #Feature | #Feature Sample 1 | Start | End | PTM | AScore | Found By |
| --- | --- | --- | --- | --- | --- | --- | --- | --- | --- | --- | --- | --- | --- | --- | --- | --- | --- | --- | --- |
| R.GFTLEELKTAGIPVK.S | Y | 62.3 | 1601.9028 | 15 | -1.3 | 801.954 | 2 | 18.20 | 1.1138 | 1.121 | 59964 | 0 | 0 | 0 | 73 | 87 |  |  | DB Search |
| total 1 peptides |  |  |  |  |  |  |  |  |  |  |  |  |  |  |  |  |  |  |  |
| --- | --- | --- | --- | --- | --- | --- | --- | --- | --- | --- | --- | --- | --- | --- | --- | --- | --- | --- | --- |

Best Unique PSM (Scan Sample\_10\_A1\_1\_3647.d:59964, m/z=801.954, z=2, RT=18.20, ppm=-5.79):


TRINITY\_DN250\_c0\_g1\_i5.p1

back to list

  

| Protein Coverage
| Supporting Peptides
| Best Unique PSM
|

Protein Coverage:

Supporting Peptides:

| Peptide | Uniq | -10lgP | Mass | Length | ppm | m/z | z | RT | 1/k0 Start | 1/k0 End | Precursor ID | Area Sample 1 | #Feature | #Feature Sample 1 | Start | End | PTM | AScore | Found By |
| --- | --- | --- | --- | --- | --- | --- | --- | --- | --- | --- | --- | --- | --- | --- | --- | --- | --- | --- | --- |
| R.AVC(+57.02)M(+15.99)LSNTTAIAEAWAR.L | Y | 66.77 | 1879.892 | 17 | -0.29 | 940.9488 | 2 | 18.63 | 1.1418 | 1.149 | 62475 | 6.54e2 | 1 | 1 | 374 | 390 | Carbamidomethylation, Oxidation (M) | M4:Oxidation (M):1000 | DB Search |
| K.VGINYQPPTVVPGGDLAK.V | Y | 64.86 | 1823.9781 | 18 | -3.39 | 912.9891 | 2 | 17.23 | 1.1198 | 1.127 | 54086 | 0 | 0 | 0 | 353 | 370 |  |  | DB Search |
| R.AFVHWYVGEGM(+15.99)EEGEFSEAR.E | Y | 44.9 | 2345.0058 | 20 | -0.18 | 782.6722 | 3 | 17.81 | 0.9251 | 0.9323 | 57560 | 6.2e2 | 1 | 1 | 403 | 422 | Oxidation (M) | M11:Oxidation (M):1000 | DB Search |
| total 3 peptides |  |  |  |  |  |  |  |  |  |  |  |  |  |  |  |  |  |  |  |
| --- | --- | --- | --- | --- | --- | --- | --- | --- | --- | --- | --- | --- | --- | --- | --- | --- | --- | --- | --- |

Best Unique PSM (Scan Sample\_10\_A1\_1\_3647.d:62475, m/z=940.9488, z=2, RT=18.63, ppm=-4.79):


TRINITY\_DN3036\_c0\_g1\_i11.p1

back to list

  

| Protein Coverage
| Supporting Peptides
| Best Unique PSM
|

Protein Coverage:

Supporting Peptides:

| Peptide | Uniq | -10lgP | Mass | Length | ppm | m/z | z | RT | 1/k0 Start | 1/k0 End | Precursor ID | Area Sample 1 | #Feature | #Feature Sample 1 | Start | End | PTM | AScore | Found By |
| --- | --- | --- | --- | --- | --- | --- | --- | --- | --- | --- | --- | --- | --- | --- | --- | --- | --- | --- | --- |
| R.AGHGAVTSPQTQR.G | Y | 66.66 | 1308.6534 | 13 | 4.48 | 655.334 | 2 | 9.70 | 0.9109 | 0.9182 | 11470 | 2.1e4 | 2 | 2 | 135 | 147 |  |  | DB Search |
| R.VIGASGIHVGTMSFGK.M | Y | 66.34 | 1559.813 | 16 | -0.2 | 780.9101 | 2 | 16.89 | 1.1037 | 1.111 | 52086 | 2.86e2 | 1 | 1 | 160 | 175 |  |  | DB Search |
| R.VIGASGIHVGTM(+15.99)SFGK.M | Y | 64.9 | 1575.8079 | 16 | 0.52 | 788.9081 | 2 | 15.11 | 1.1262 | 1.1334 | 42748 | 6.68e2 | 1 | 1 | 160 | 175 | Oxidation (M) | M12:Oxidation (M):1000 | DB Search |
| total 3 peptides |  |  |  |  |  |  |  |  |  |  |  |  |  |  |  |  |  |  |  |
| --- | --- | --- | --- | --- | --- | --- | --- | --- | --- | --- | --- | --- | --- | --- | --- | --- | --- | --- | --- |

Best Unique PSM (Scan Sample\_10\_A1\_1\_3647.d:11470, m/z=655.334, z=2, RT=9.70, ppm=-0.02):


TRINITY\_DN8165\_c0\_g2\_i2.p2

back to list

  

| Protein Coverage
| Supporting Peptides
| Best Unique PSM
|

Protein Coverage:

Supporting Peptides:

| Peptide | Uniq | -10lgP | Mass | Length | ppm | m/z | z | RT | 1/k0 Start | 1/k0 End | Precursor ID | Area Sample 1 | #Feature | #Feature Sample 1 | Start | End | PTM | AScore | Found By |
| --- | --- | --- | --- | --- | --- | --- | --- | --- | --- | --- | --- | --- | --- | --- | --- | --- | --- | --- | --- |
| K.K(+42.01)LSAELANGR.L | Y | 66.66 | 1099.5985 | 10 | 2.66 | 550.8055 | 2 | 14.21 | 0.8712 | 0.8785 | 37894 | 8.99e2 | 1 | 1 | 4 | 13 | Acetylation (Protein N-term) | K1:Acetylation (Protein N-term):1000 | DB Search |
| total 1 peptides |  |  |  |  |  |  |  |  |  |  |  |  |  |  |  |  |  |  |  |
| --- | --- | --- | --- | --- | --- | --- | --- | --- | --- | --- | --- | --- | --- | --- | --- | --- | --- | --- | --- |

Best Unique PSM (Scan Sample\_10\_A1\_1\_3647.d:37894, m/z=550.8055, z=2, RT=14.21, ppm=-1.83):


TRINITY\_DN3036\_c0\_g1\_i2.p2

back to list

  

| Protein Coverage
| Supporting Peptides
| Best Unique PSM
|

Protein Coverage:

Supporting Peptides:

| Peptide | Uniq | -10lgP | Mass | Length | ppm | m/z | z | RT | 1/k0 Start | 1/k0 End | Precursor ID | Area Sample 1 | #Feature | #Feature Sample 1 | Start | End | PTM | AScore | Found By |
| --- | --- | --- | --- | --- | --- | --- | --- | --- | --- | --- | --- | --- | --- | --- | --- | --- | --- | --- | --- |
| R.AGHGAVTSPQTQR.G | Y | 66.66 | 1308.6534 | 13 | 4.48 | 655.334 | 2 | 9.70 | 0.9109 | 0.9182 | 11470 | 2.1e4 | 2 | 2 | 309 | 321 |  |  | DB Search |
| R.GTSNGGLVVGTIIKPK.L | Y | 66.59 | 1539.8984 | 16 | 0.22 | 770.9532 | 2 | 15.68 | 1.1262 | 1.1334 | 45813 | 3.23e3 | 1 | 1 | 173 | 188 |  |  | DB Search |
| R.VIGASGIHVGTMSFGK.M | Y | 66.34 | 1559.813 | 16 | -0.2 | 780.9101 | 2 | 16.89 | 1.1037 | 1.111 | 52086 | 2.86e2 | 1 | 1 | 334 | 349 |  |  | DB Search |
| R.VIGASGIHVGTM(+15.99)SFGK.M | Y | 64.9 | 1575.8079 | 16 | 0.52 | 788.9081 | 2 | 15.11 | 1.1262 | 1.1334 | 42748 | 6.68e2 | 1 | 1 | 334 | 349 | Oxidation (M) | M12:Oxidation (M):1000 | DB Search |
| total 4 peptides |  |  |  |  |  |  |  |  |  |  |  |  |  |  |  |  |  |  |  |
| --- | --- | --- | --- | --- | --- | --- | --- | --- | --- | --- | --- | --- | --- | --- | --- | --- | --- | --- | --- |

Best Unique PSM (Scan Sample\_10\_A1\_1\_3647.d:11470, m/z=655.334, z=2, RT=9.70, ppm=-0.02):


TRINITY\_DN477\_c0\_g1\_i7.p1

back to list

  

| Protein Coverage
| Supporting Peptides
| Best Unique PSM
|

Protein Coverage:

Supporting Peptides:

| Peptide | Uniq | -10lgP | Mass | Length | ppm | m/z | z | RT | 1/k0 Start | 1/k0 End | Precursor ID | Area Sample 1 | #Feature | #Feature Sample 1 | Start | End | PTM | AScore | Found By |
| --- | --- | --- | --- | --- | --- | --- | --- | --- | --- | --- | --- | --- | --- | --- | --- | --- | --- | --- | --- |
| R.ILQC(+57.02)NQYANVEEAR.N | Y | 66.82 | 1706.8046 | 14 | -0.34 | 854.4055 | 2 | 14.19 | 1.0693 | 1.0765 | 37690 | 1.33e3 | 1 | 1 | 86 | 99 | Carbamidomethylation |  | DB Search |
| K.DKNILVLTLGVGPQVNR.N | Y | 66.34 | 1835.0628 | 17 | -1.89 | 918.5328 | 2 | 19.38 | 1.1326 | 1.1398 | 66901 | 2.29e3 | 2 | 2 | 168 | 184 |  |  | DB Search |
| R.SQSAFPTRR.R | Y | 31.06 | 1048.5414 | 9 | 5.06 | 525.2783 | 2 | 10.99 | 0.8433 | 0.8506 | 19385 | 6.25e2 | 1 | 1 | 856 | 864 |  |  | DB Search |
| total 3 peptides |  |  |  |  |  |  |  |  |  |  |  |  |  |  |  |  |  |  |  |
| --- | --- | --- | --- | --- | --- | --- | --- | --- | --- | --- | --- | --- | --- | --- | --- | --- | --- | --- | --- |

Best Unique PSM (Scan Sample\_10\_A1\_1\_3647.d:37690, m/z=854.4055, z=2, RT=14.19, ppm=-4.83):


TRINITY\_DN140702\_c0\_g1\_i1.p1

back to list

  

| Protein Coverage
| Supporting Peptides
| Best Unique PSM
|

Protein Coverage:

Supporting Peptides:

| Peptide | Uniq | -10lgP | Mass | Length | ppm | m/z | z | RT | 1/k0 Start | 1/k0 End | Precursor ID | Area Sample 1 | #Feature | #Feature Sample 1 | Start | End | PTM | AScore | Found By |
| --- | --- | --- | --- | --- | --- | --- | --- | --- | --- | --- | --- | --- | --- | --- | --- | --- | --- | --- | --- |
| R.LALHVLNNEDIIGHR.L | Y | 66.71 | 1712.9322 | 15 | 1.25 | 857.4706 | 2 | 16.30 | 1.1346 | 1.1418 | 49035 | 4.05e2 | 1 | 1 | 1702 | 1716 |  |  | DB Search |
| total 1 peptides |  |  |  |  |  |  |  |  |  |  |  |  |  |  |  |  |  |  |  |
| --- | --- | --- | --- | --- | --- | --- | --- | --- | --- | --- | --- | --- | --- | --- | --- | --- | --- | --- | --- |

Best Unique PSM (Scan Sample\_10\_A1\_1\_3647.d:49035, m/z=857.4706, z=2, RT=16.30, ppm=-3.24):


TRINITY\_DN1194\_c0\_g1\_i1.p1

back to list

  

| Protein Coverage
| Supporting Peptides
| Best Unique PSM
|

Protein Coverage:

Supporting Peptides:

| Peptide | Uniq | -10lgP | Mass | Length | ppm | m/z | z | RT | 1/k0 Start | 1/k0 End | Precursor ID | Area Sample 1 | #Feature | #Feature Sample 1 | Start | End | PTM | AScore | Found By |
| --- | --- | --- | --- | --- | --- | --- | --- | --- | --- | --- | --- | --- | --- | --- | --- | --- | --- | --- | --- |
| K.SLVEENVHPQILIK.G | Y | 66.8 | 1617.909 | 14 | 4.08 | 809.9614 | 2 | 16.31 | 1.1013 | 1.1086 | 49172 | 0 | 0 | 0 | 118 | 131 |  |  | DB Search |
| K.IVNAEWNILYEKLEK.I | Y | 62.98 | 1860.9985 | 15 | -4.36 | 931.4983 | 2 | 19.47 | 1.1881 | 1.1953 | 67707 | 0 | 0 | 0 | 274 | 288 |  |  | DB Search |
| total 2 peptides |  |  |  |  |  |  |  |  |  |  |  |  |  |  |  |  |  |  |  |
| --- | --- | --- | --- | --- | --- | --- | --- | --- | --- | --- | --- | --- | --- | --- | --- | --- | --- | --- | --- |

Best Unique PSM (Scan Sample\_10\_A1\_1\_3647.d:49172, m/z=809.9614, z=2, RT=16.31, ppm=-0.41):


TRINITY\_DN602\_c0\_g1\_i4.p1

back to list

  

| Protein Coverage
| Supporting Peptides
| Best Unique PSM
|

Protein Coverage:

Supporting Peptides:

| Peptide | Uniq | -10lgP | Mass | Length | ppm | m/z | z | RT | 1/k0 Start | 1/k0 End | Precursor ID | Area Sample 1 | #Feature | #Feature Sample 1 | Start | End | PTM | AScore | Found By |
| --- | --- | --- | --- | --- | --- | --- | --- | --- | --- | --- | --- | --- | --- | --- | --- | --- | --- | --- | --- |
| K.EGFYVHEALAAGIVK.K | Y | 63.7 | 1602.8405 | 15 | -2.05 | 802.4223 | 2 | 17.52 | 1.0889 | 1.0961 | 55709 | 2.51e2 | 1 | 1 | 144 | 158 |  |  | DB Search |
| total 1 peptides |  |  |  |  |  |  |  |  |  |  |  |  |  |  |  |  |  |  |  |
| --- | --- | --- | --- | --- | --- | --- | --- | --- | --- | --- | --- | --- | --- | --- | --- | --- | --- | --- | --- |

Best Unique PSM (Scan Sample\_10\_A1\_1\_3647.d:55709, m/z=802.4223, z=2, RT=17.52, ppm=-6.54):


TRINITY\_DN691\_c0\_g1\_i1.p1

back to list

  

| Protein Coverage
| Supporting Peptides
| Best Unique PSM
|

Protein Coverage:

Supporting Peptides:

| Peptide | Uniq | -10lgP | Mass | Length | ppm | m/z | z | RT | 1/k0 Start | 1/k0 End | Precursor ID | Area Sample 1 | #Feature | #Feature Sample 1 | Start | End | PTM | AScore | Found By |
| --- | --- | --- | --- | --- | --- | --- | --- | --- | --- | --- | --- | --- | --- | --- | --- | --- | --- | --- | --- |
| R.AYGQDLGQPGSC(+57.02)LKR.F | Y | 62.49 | 1648.7991 | 15 | -3.4 | 825.4003 | 2 | 13.37 | 1.0725 | 1.0797 | 33176 | 9e2 | 1 | 1 | 1533 | 1547 | Carbamidomethylation |  | DB Search |
| total 1 peptides |  |  |  |  |  |  |  |  |  |  |  |  |  |  |  |  |  |  |  |
| --- | --- | --- | --- | --- | --- | --- | --- | --- | --- | --- | --- | --- | --- | --- | --- | --- | --- | --- | --- |

Best Unique PSM (Scan Sample\_10\_A1\_1\_3647.d:33176, m/z=824.3564, z=2, RT=13.40, ppm=-7.89):


TRINITY\_DN7659\_c0\_g1\_i1.p1

back to list

  

| Protein Coverage
| Supporting Peptides
| Best Unique PSM
|

Protein Coverage:

Supporting Peptides:

| Peptide | Uniq | -10lgP | Mass | Length | ppm | m/z | z | RT | 1/k0 Start | 1/k0 End | Precursor ID | Area Sample 1 | #Feature | #Feature Sample 1 | Start | End | PTM | AScore | Found By |
| --- | --- | --- | --- | --- | --- | --- | --- | --- | --- | --- | --- | --- | --- | --- | --- | --- | --- | --- | --- |
| M.A(+42.01)SAATDNRK.V | Y | 63.17 | 974.4781 | 9 | -3.25 | 488.2426 | 2 | 9.45 | 0.8205 | 0.8279 | 10765 | 0 | 0 | 0 | 2 | 10 | Acetylation (Protein N-term) | A1:Acetylation (Protein N-term):1000 | DB Search |
| K.AQPTEIYNLGAQSHVK.V | Y | 35.72 | 1754.8951 | 16 | 0.32 | 878.4512 | 2 | 14.67 | 1.1722 | 1.1794 | 40344 | 0 | 0 | 0 | 86 | 101 |  |  | DB Search |
| total 2 peptides |  |  |  |  |  |  |  |  |  |  |  |  |  |  |  |  |  |  |  |
| --- | --- | --- | --- | --- | --- | --- | --- | --- | --- | --- | --- | --- | --- | --- | --- | --- | --- | --- | --- |

Best Unique PSM (Scan Sample\_10\_A1\_1\_3647.d:10765, m/z=488.2426, z=2, RT=9.45, ppm=-7.75):


TRINITY\_DN1465\_c1\_g1\_i1.p1

back to list

  

| Protein Coverage
| Supporting Peptides
| Best Unique PSM
|

Protein Coverage:

Supporting Peptides:

| Peptide | Uniq | -10lgP | Mass | Length | ppm | m/z | z | RT | 1/k0 Start | 1/k0 End | Precursor ID | Area Sample 1 | #Feature | #Feature Sample 1 | Start | End | PTM | AScore | Found By |
| --- | --- | --- | --- | --- | --- | --- | --- | --- | --- | --- | --- | --- | --- | --- | --- | --- | --- | --- | --- |
| K.IQDKEGIPPDQQR.L | Y | 65.08 | 1522.7739 | 13 | -0.66 | 762.3903 | 2 | 11.01 | 1.0339 | 1.0411 | 19352 | 9.49e2 | 1 | 1 | 34 | 46 |  |  | DB Search |
| total 1 peptides |  |  |  |  |  |  |  |  |  |  |  |  |  |  |  |  |  |  |  |
| --- | --- | --- | --- | --- | --- | --- | --- | --- | --- | --- | --- | --- | --- | --- | --- | --- | --- | --- | --- |

Best Unique PSM (Scan Sample\_10\_A1\_1\_3647.d:19352, m/z=762.3903, z=2, RT=11.01, ppm=-5.16):


TRINITY\_DN286\_c0\_g1\_i2.p1

back to list

  

| Protein Coverage
| Supporting Peptides
| Best Unique PSM
|

Protein Coverage:

Supporting Peptides:

| Peptide | Uniq | -10lgP | Mass | Length | ppm | m/z | z | RT | 1/k0 Start | 1/k0 End | Precursor ID | Area Sample 1 | #Feature | #Feature Sample 1 | Start | End | PTM | AScore | Found By |
| --- | --- | --- | --- | --- | --- | --- | --- | --- | --- | --- | --- | --- | --- | --- | --- | --- | --- | --- | --- |
| K.RYGQAMC(+57.02)QFMR.N | Y | 66.75 | 1446.6319 | 11 | -6.11 | 724.3156 | 2 | 17.23 | 0.9868 | 0.9941 | 53709 | 8.19e3 | 1 | 1 | 71 | 81 | Carbamidomethylation |  | DB Search |
| K.RYGQAM(+15.99)C(+57.02)QFM(+15.99)R.N | Y | 66.67 | 1478.6217 | 11 | -8.74 | 740.3083 | 2 | 12.90 | 0.9759 | 0.9832 | 30310 | 4.97e3 | 1 | 1 | 71 | 81 | Oxidation (M), Carbamidomethylation, Oxidation (M) | M6:Oxidation (M):1000 M10:Oxidation (M):1000 | DB Search |
| K.RYGQAMC(+57.02)QFM(+15.99)R.N | Y | 63.05 | 1462.6268 | 11 | -11.86 | 732.3087 | 2 | 14.93 | 0.9779 | 0.9852 | 41834 | 0 | 0 | 0 | 71 | 81 | Carbamidomethylation, Oxidation (M) | M10:Oxidation (M):25.11 | DB Search |
| K.RYGQAM(+15.99)C(+57.02)QFMR.N | Y | 29.61 | 1462.6268 | 11 | -5.85 | 732.3131 | 2 | 14.49 | 0.9751 | 0.9824 | 39230 | 2.67e3 | 1 | 1 | 71 | 81 | Oxidation (M), Carbamidomethylation | M6:Oxidation (M):34.3 | DB Search |
| total 4 peptides |  |  |  |  |  |  |  |  |  |  |  |  |  |  |  |  |  |  |  |
| --- | --- | --- | --- | --- | --- | --- | --- | --- | --- | --- | --- | --- | --- | --- | --- | --- | --- | --- | --- |

Best Unique PSM (Scan Sample\_10\_A1\_1\_3647.d:53709, m/z=724.3156, z=2, RT=17.23, ppm=-10.6):


TRINITY\_DN172571\_c0\_g1\_i1.p1

back to list

  

| Protein Coverage
| Supporting Peptides
| Best Unique PSM
|

Protein Coverage:

Supporting Peptides:

| Peptide | Uniq | -10lgP | Mass | Length | ppm | m/z | z | RT | 1/k0 Start | 1/k0 End | Precursor ID | Area Sample 1 | #Feature | #Feature Sample 1 | Start | End | PTM | AScore | Found By |
| --- | --- | --- | --- | --- | --- | --- | --- | --- | --- | --- | --- | --- | --- | --- | --- | --- | --- | --- | --- |
| R.DNIQGITKPAIR.R | Y | 66.68 | 1324.7463 | 12 | 3.7 | 663.3799 | 2 | 13.75 | 0.9791 | 0.9864 | 35280 | 0 | 0 | 0 | 25 | 36 |  |  | DB Search |
| R.ISGLIYEETRGVLK.V | Y | 62.35 | 1576.8824 | 14 | 2.48 | 789.4469 | 2 | 17.34 | 1.0929 | 1.1001 | 54665 | 0 | 0 | 0 | 47 | 60 |  |  | DB Search |
| total 2 peptides |  |  |  |  |  |  |  |  |  |  |  |  |  |  |  |  |  |  |  |
| --- | --- | --- | --- | --- | --- | --- | --- | --- | --- | --- | --- | --- | --- | --- | --- | --- | --- | --- | --- |

Best Unique PSM (Scan Sample\_10\_A1\_1\_3647.d:35280, m/z=663.3799, z=2, RT=13.75, ppm=-0.8):


TRINITY\_DN2490\_c0\_g1\_i1.p1

back to list

  

| Protein Coverage
| Supporting Peptides
| Best Unique PSM
|

Protein Coverage:

Supporting Peptides:

| Peptide | Uniq | -10lgP | Mass | Length | ppm | m/z | z | RT | 1/k0 Start | 1/k0 End | Precursor ID | Area Sample 1 | #Feature | #Feature Sample 1 | Start | End | PTM | AScore | Found By |
| --- | --- | --- | --- | --- | --- | --- | --- | --- | --- | --- | --- | --- | --- | --- | --- | --- | --- | --- | --- |
| K.AFHQIIHSATLR.N | Y | 62.3 | 1392.7626 | 12 | -5.44 | 697.3817 | 2 | 13.45 | 1.009 | 1.0162 | 33729 | 0 | 0 | 0 | 734 | 745 |  |  | DB Search |
| total 1 peptides |  |  |  |  |  |  |  |  |  |  |  |  |  |  |  |  |  |  |  |
| --- | --- | --- | --- | --- | --- | --- | --- | --- | --- | --- | --- | --- | --- | --- | --- | --- | --- | --- | --- |

Best Unique PSM (Scan Sample\_10\_A1\_1\_3647.d:33729, m/z=697.3817, z=2, RT=13.45, ppm=-9.93):


TRINITY\_DN6390\_c0\_g1\_i1.p1

back to list

  

| Protein Coverage
| Supporting Peptides
| Best Unique PSM
|

Protein Coverage:

Supporting Peptides:

| Peptide | Uniq | -10lgP | Mass | Length | ppm | m/z | z | RT | 1/k0 Start | 1/k0 End | Precursor ID | Area Sample 1 | #Feature | #Feature Sample 1 | Start | End | PTM | AScore | Found By |
| --- | --- | --- | --- | --- | --- | --- | --- | --- | --- | --- | --- | --- | --- | --- | --- | --- | --- | --- | --- |
| R.HLSVNILQTNTR.V | Y | 63.57 | 1394.763 | 12 | -0.64 | 698.3852 | 2 | 15.23 | 1.0057 | 1.013 | 43441 | 4.4e2 | 1 | 1 | 836 | 847 |  |  | DB Search |
| K.RC(+57.02)TDLNIR.V | Y | 19.02 | 1046.5291 | 8 | 7.46 | 524.2734 | 2 | 11.91 | 0.8664 | 0.8737 | 24737 | 0 | 0 | 0 | 1039 | 1046 | Carbamidomethylation |  | DB Search |
| total 2 peptides |  |  |  |  |  |  |  |  |  |  |  |  |  |  |  |  |  |  |  |
| --- | --- | --- | --- | --- | --- | --- | --- | --- | --- | --- | --- | --- | --- | --- | --- | --- | --- | --- | --- |

Best Unique PSM (Scan Sample\_10\_A1\_1\_3647.d:43441, m/z=698.3852, z=2, RT=15.23, ppm=-5.14):


TRINITY\_DN24414\_c1\_g1\_i2.p2

back to list

  

| Protein Coverage
| Supporting Peptides
| Best Unique PSM
|

Protein Coverage:

Supporting Peptides:

| Peptide | Uniq | -10lgP | Mass | Length | ppm | m/z | z | RT | 1/k0 Start | 1/k0 End | Precursor ID | Area Sample 1 | #Feature | #Feature Sample 1 | Start | End | PTM | AScore | Found By |
| --- | --- | --- | --- | --- | --- | --- | --- | --- | --- | --- | --- | --- | --- | --- | --- | --- | --- | --- | --- |
| K.VLTSDDPAEKEK.K | Y | 66.7 | 1330.6616 | 12 | 2.85 | 666.337 | 2 | 10.71 | 0.9836 | 0.9908 | 17367 | 7.2e3 | 1 | 1 | 95 | 106 |  |  | DB Search |
| total 1 peptides |  |  |  |  |  |  |  |  |  |  |  |  |  |  |  |  |  |  |  |
| --- | --- | --- | --- | --- | --- | --- | --- | --- | --- | --- | --- | --- | --- | --- | --- | --- | --- | --- | --- |

Best Unique PSM (Scan Sample\_10\_A1\_1\_3647.d:17367, m/z=666.337, z=2, RT=10.71, ppm=-1.65):


TRINITY\_DN140696\_c0\_g1\_i1.p1

back to list

  

| Protein Coverage
| Supporting Peptides
| Best Unique PSM
|

Protein Coverage:

Supporting Peptides:

| Peptide | Uniq | -10lgP | Mass | Length | ppm | m/z | z | RT | 1/k0 Start | 1/k0 End | Precursor ID | Area Sample 1 | #Feature | #Feature Sample 1 | Start | End | PTM | AScore | Found By |
| --- | --- | --- | --- | --- | --- | --- | --- | --- | --- | --- | --- | --- | --- | --- | --- | --- | --- | --- | --- |
| R.LTIAEERDR.R | Y | 31.8 | 1101.5778 | 9 | -3 | 551.7921 | 2 | 11.69 | 0.9004 | 0.9077 | 23456 | 0 | 0 | 0 | 246 | 254 |  |  | DB Search |
| total 1 peptides |  |  |  |  |  |  |  |  |  |  |  |  |  |  |  |  |  |  |  |
| --- | --- | --- | --- | --- | --- | --- | --- | --- | --- | --- | --- | --- | --- | --- | --- | --- | --- | --- | --- |

Best Unique PSM (Scan Sample\_10\_A1\_1\_3647.d:23456, m/z=551.7921, z=2, RT=11.69, ppm=-7.49):


TRINITY\_DN39385\_c0\_g1\_i1.p1

back to list

  

| Protein Coverage
| Supporting Peptides
| Best Unique PSM
|

Protein Coverage:

Supporting Peptides:

| Peptide | Uniq | -10lgP | Mass | Length | ppm | m/z | z | RT | 1/k0 Start | 1/k0 End | Precursor ID | Area Sample 1 | #Feature | #Feature Sample 1 | Start | End | PTM | AScore | Found By |
| --- | --- | --- | --- | --- | --- | --- | --- | --- | --- | --- | --- | --- | --- | --- | --- | --- | --- | --- | --- |
| K.TLQLIHQIEISK.I | Y | 66.71 | 1421.8242 | 12 | 0.03 | 711.9162 | 2 | 17.16 | 1.0367 | 1.0439 | 53670 | 0 | 0 | 0 | 41 | 52 |  |  | DB Search |
| total 1 peptides |  |  |  |  |  |  |  |  |  |  |  |  |  |  |  |  |  |  |  |
| --- | --- | --- | --- | --- | --- | --- | --- | --- | --- | --- | --- | --- | --- | --- | --- | --- | --- | --- | --- |

Best Unique PSM (Scan Sample\_10\_A1\_1\_3647.d:53670, m/z=711.9162, z=2, RT=17.16, ppm=-4.46):


TRINITY\_DN240\_c0\_g1\_i1.p1

back to list

  

| Protein Coverage
| Supporting Peptides
| Best Unique PSM
|

Protein Coverage:

Supporting Peptides:

| Peptide | Uniq | -10lgP | Mass | Length | ppm | m/z | z | RT | 1/k0 Start | 1/k0 End | Precursor ID | Area Sample 1 | #Feature | #Feature Sample 1 | Start | End | PTM | AScore | Found By |
| --- | --- | --- | --- | --- | --- | --- | --- | --- | --- | --- | --- | --- | --- | --- | --- | --- | --- | --- | --- |
| K.AYAAFLEFKDVVK.A | Y | 66.86 | 1499.8024 | 13 | 1.14 | 750.9059 | 2 | 19.02 | 1.0945 | 1.1017 | 64841 | 9.59e3 | 1 | 1 | 217 | 229 |  |  | DB Search |
| K.AAASAHHQAIGSIDAAGVTSAADYASVTAALGR.A | Y | 62.75 | 3080.5275 | 33 | 0.95 | 771.1364 | 4 | 18.15 | 1.0636 | 1.0709 | 59524 | 1.07e3 | 1 | 1 | 307 | 339 |  |  | DB Search |
| K.IGAAAQALADQSYPFLK.S | Y | 61.76 | 1762.9253 | 17 | 1.13 | 882.467 | 2 | 19.07 | 1.161 | 1.1682 | 65059 | 6.63e2 | 1 | 1 | 249 | 265 |  |  | DB Search |
| total 3 peptides |  |  |  |  |  |  |  |  |  |  |  |  |  |  |  |  |  |  |  |
| --- | --- | --- | --- | --- | --- | --- | --- | --- | --- | --- | --- | --- | --- | --- | --- | --- | --- | --- | --- |

Best Unique PSM (Scan Sample\_10\_A1\_1\_3647.d:64841, m/z=750.9059, z=2, RT=19.02, ppm=-3.35):


TRINITY\_DN884\_c0\_g1\_i1.p1

back to list

  

| Protein Coverage
| Supporting Peptides
| Best Unique PSM
|

Protein Coverage:

Supporting Peptides:

| Peptide | Uniq | -10lgP | Mass | Length | ppm | m/z | z | RT | 1/k0 Start | 1/k0 End | Precursor ID | Area Sample 1 | #Feature | #Feature Sample 1 | Start | End | PTM | AScore | Found By |
| --- | --- | --- | --- | --- | --- | --- | --- | --- | --- | --- | --- | --- | --- | --- | --- | --- | --- | --- | --- |
| R.IVGIVSGALNHAASK.K | Y | 66.65 | 1435.8147 | 15 | 2.8 | 718.9134 | 2 | 15.20 | 1.0556 | 1.0628 | 43196 | 2.91e2 | 1 | 1 | 397 | 411 |  |  | DB Search |
| total 1 peptides |  |  |  |  |  |  |  |  |  |  |  |  |  |  |  |  |  |  |  |
| --- | --- | --- | --- | --- | --- | --- | --- | --- | --- | --- | --- | --- | --- | --- | --- | --- | --- | --- | --- |

Best Unique PSM (Scan Sample\_10\_A1\_1\_3647.d:43196, m/z=718.9134, z=2, RT=15.20, ppm=-1.7):


TRINITY\_DN26249\_c0\_g1\_i1.p1

back to list

  

| Protein Coverage
| Supporting Peptides
| Best Unique PSM
|

Protein Coverage:

Supporting Peptides:

| Peptide | Uniq | -10lgP | Mass | Length | ppm | m/z | z | RT | 1/k0 Start | 1/k0 End | Precursor ID | Area Sample 1 | #Feature | #Feature Sample 1 | Start | End | PTM | AScore | Found By |
| --- | --- | --- | --- | --- | --- | --- | --- | --- | --- | --- | --- | --- | --- | --- | --- | --- | --- | --- | --- |
| R.LIYENQNTIHHVVR.A | Y | 66.79 | 1734.9165 | 14 | -1.94 | 868.46 | 2 | 13.18 | 1.1274 | 1.1346 | 31991 | 5.25e2 | 1 | 1 | 322 | 335 |  |  | DB Search |
| total 1 peptides |  |  |  |  |  |  |  |  |  |  |  |  |  |  |  |  |  |  |  |
| --- | --- | --- | --- | --- | --- | --- | --- | --- | --- | --- | --- | --- | --- | --- | --- | --- | --- | --- | --- |

Best Unique PSM (Scan Sample\_10\_A1\_1\_3647.d:31991, m/z=868.46, z=2, RT=13.18, ppm=-6.43):


TRINITY\_DN3036\_c0\_g1\_i6.p1

back to list

  

| Protein Coverage
| Supporting Peptides
| Best Unique PSM
|

Protein Coverage:

Supporting Peptides:

| Peptide | Uniq | -10lgP | Mass | Length | ppm | m/z | z | RT | 1/k0 Start | 1/k0 End | Precursor ID | Area Sample 1 | #Feature | #Feature Sample 1 | Start | End | PTM | AScore | Found By |
| --- | --- | --- | --- | --- | --- | --- | --- | --- | --- | --- | --- | --- | --- | --- | --- | --- | --- | --- | --- |
| R.VIGASGIHVGTMSFGK.M | Y | 66.34 | 1559.813 | 16 | -0.2 | 780.9101 | 2 | 16.89 | 1.1037 | 1.111 | 52086 | 2.86e2 | 1 | 1 | 24 | 39 |  |  | DB Search |
| R.VIGASGIHVGTM(+15.99)SFGK.M | Y | 64.9 | 1575.8079 | 16 | 0.52 | 788.9081 | 2 | 15.11 | 1.1262 | 1.1334 | 42748 | 6.68e2 | 1 | 1 | 24 | 39 | Oxidation (M) | M12:Oxidation (M):1000 | DB Search |
| total 2 peptides |  |  |  |  |  |  |  |  |  |  |  |  |  |  |  |  |  |  |  |
| --- | --- | --- | --- | --- | --- | --- | --- | --- | --- | --- | --- | --- | --- | --- | --- | --- | --- | --- | --- |

Best Unique PSM (Scan Sample\_10\_A1\_1\_3647.d:52086, m/z=780.9101, z=2, RT=16.89, ppm=-4.69):


TRINITY\_DN187527\_c0\_g1\_i1.p1

back to list

  

| Protein Coverage
| Supporting Peptides
| Best Unique PSM
|

Protein Coverage:

Supporting Peptides:

| Peptide | Uniq | -10lgP | Mass | Length | ppm | m/z | z | RT | 1/k0 Start | 1/k0 End | Precursor ID | Area Sample 1 | #Feature | #Feature Sample 1 | Start | End | PTM | AScore | Found By |
| --- | --- | --- | --- | --- | --- | --- | --- | --- | --- | --- | --- | --- | --- | --- | --- | --- | --- | --- | --- |
| M.S(+42.01)GERTPITSDKR.L | Y | 61.94 | 1387.7055 | 12 | -0.02 | 463.5737 | 3 | 10.89 | 0.7514 | 0.7587 | 18882 | 0 | 0 | 0 | 2 | 13 | Acetylation (Protein N-term) | S1:Acetylation (Protein N-term):1000 | DB Search |
| total 1 peptides |  |  |  |  |  |  |  |  |  |  |  |  |  |  |  |  |  |  |  |
| --- | --- | --- | --- | --- | --- | --- | --- | --- | --- | --- | --- | --- | --- | --- | --- | --- | --- | --- | --- |

Best Unique PSM (Scan Sample\_10\_A1\_1\_3647.d:18882, m/z=463.5737, z=3, RT=10.89, ppm=-4.51):


TRINITY\_DN142212\_c0\_g1\_i1.p1

back to list

  

| Protein Coverage
| Supporting Peptides
| Best Unique PSM
|

Protein Coverage:

Supporting Peptides:

| Peptide | Uniq | -10lgP | Mass | Length | ppm | m/z | z | RT | 1/k0 Start | 1/k0 End | Precursor ID | Area Sample 1 | #Feature | #Feature Sample 1 | Start | End | PTM | AScore | Found By |
| --- | --- | --- | --- | --- | --- | --- | --- | --- | --- | --- | --- | --- | --- | --- | --- | --- | --- | --- | --- |
| K.RSLNAEIANGR.L | Y | 51.82 | 1199.6371 | 11 | 2.77 | 600.8248 | 2 | 11.99 | 0.9259 | 0.9331 | 25198 | 6.38e2 | 1 | 1 | 118 | 128 |  |  | DB Search |
| total 1 peptides |  |  |  |  |  |  |  |  |  |  |  |  |  |  |  |  |  |  |  |
| --- | --- | --- | --- | --- | --- | --- | --- | --- | --- | --- | --- | --- | --- | --- | --- | --- | --- | --- | --- |

Best Unique PSM (Scan Sample\_10\_A1\_1\_3647.d:25198, m/z=600.8248, z=2, RT=11.99, ppm=-1.73):


TRINITY\_DN168534\_c0\_g1\_i1.p1

back to list

  

| Protein Coverage
| Supporting Peptides
| Best Unique PSM
|

Protein Coverage:

Supporting Peptides:

| Peptide | Uniq | -10lgP | Mass | Length | ppm | m/z | z | RT | 1/k0 Start | 1/k0 End | Precursor ID | Area Sample 1 | #Feature | #Feature Sample 1 | Start | End | PTM | AScore | Found By |
| --- | --- | --- | --- | --- | --- | --- | --- | --- | --- | --- | --- | --- | --- | --- | --- | --- | --- | --- | --- |
| K.AMTDC(+57.02)KETLC(+57.02)R.G | Y | 66.8 | 1383.5945 | 11 | 1.03 | 692.8021 | 2 | 11.63 | 0.9888 | 0.9961 | 23189 | 1.32e3 | 1 | 1 | 85 | 95 | Carbamidomethylation, Carbamidomethylation |  | DB Search |
| K.AM(+15.99)TDC(+57.02)KETLC(+57.02)R.G | Y | 61.26 | 1399.5894 | 11 | -4.02 | 700.796 | 2 | 10.58 | 1.0013 | 1.0086 | 16952 | 0 | 0 | 0 | 85 | 95 | Oxidation (M), Carbamidomethylation, Carbamidomethylation | M2:Oxidation (M):1000 | DB Search |
| total 2 peptides |  |  |  |  |  |  |  |  |  |  |  |  |  |  |  |  |  |  |  |
| --- | --- | --- | --- | --- | --- | --- | --- | --- | --- | --- | --- | --- | --- | --- | --- | --- | --- | --- | --- |

Best Unique PSM (Scan Sample\_10\_A1\_1\_3647.d:23189, m/z=692.8021, z=2, RT=11.63, ppm=-3.46):


TRINITY\_DN10907\_c0\_g1\_i1.p1

back to list

  

| Protein Coverage
| Supporting Peptides
| Best Unique PSM
|

Protein Coverage:

Supporting Peptides:

| Peptide | Uniq | -10lgP | Mass | Length | ppm | m/z | z | RT | 1/k0 Start | 1/k0 End | Precursor ID | Area Sample 1 | #Feature | #Feature Sample 1 | Start | End | PTM | AScore | Found By |
| --- | --- | --- | --- | --- | --- | --- | --- | --- | --- | --- | --- | --- | --- | --- | --- | --- | --- | --- | --- |
| R.NIDEHASEGVER.M | Y | 64.74 | 1354.6113 | 12 | 4.41 | 678.3129 | 2 | 11.03 | 0.9683 | 0.9755 | 19561 | 6.21e2 | 1 | 1 | 106 | 117 |  |  | DB Search |
| total 1 peptides |  |  |  |  |  |  |  |  |  |  |  |  |  |  |  |  |  |  |  |
| --- | --- | --- | --- | --- | --- | --- | --- | --- | --- | --- | --- | --- | --- | --- | --- | --- | --- | --- | --- |

Best Unique PSM (Scan Sample\_10\_A1\_1\_3647.d:19561, m/z=678.3129, z=2, RT=11.03, ppm=-0.08):


TRINITY\_DN161\_c0\_g1\_i3.p1

back to list

  

| Protein Coverage
| Supporting Peptides
| Best Unique PSM
|

Protein Coverage:

Supporting Peptides:

| Peptide | Uniq | -10lgP | Mass | Length | ppm | m/z | z | RT | 1/k0 Start | 1/k0 End | Precursor ID | Area Sample 1 | #Feature | #Feature Sample 1 | Start | End | PTM | AScore | Found By |
| --- | --- | --- | --- | --- | --- | --- | --- | --- | --- | --- | --- | --- | --- | --- | --- | --- | --- | --- | --- |
| R.AVFPSIVGRPR.H | Y | 66.75 | 1197.6982 | 11 | -0.27 | 599.8535 | 2 | 15.64 | 0.9307 | 0.938 | 45498 | 3.34e4 | 1 | 1 | 52 | 62 |  |  | DB Search |
| K.DSYVGDEAQSKR.G | Y | 66.73 | 1353.616 | 12 | 2.52 | 677.814 | 2 | 10.94 | 1.0069 | 1.0142 | 19045 | 5.51e3 | 1 | 1 | 74 | 85 |  |  | DB Search |
| K.SYELPDGQVITIGNER.F | Y | 66.69 | 1789.8846 | 16 | -3.48 | 895.9424 | 2 | 18.53 | 0.9795 | 0.9868 | 61913 | 1.88e3 | 2 | 2 | 262 | 277 |  |  | DB Search |
| R.LDLAGRDLTDYLM(+15.99)K.I | Y | 62.87 | 1638.8287 | 14 | 4.29 | 820.4214 | 2 | 18.15 | 1.143 | 1.1502 | 59617 | 0 | 0 | 0 | 201 | 214 | Oxidation (M) | M13:Oxidation (M):1000 | DB Search |
| R.HQGVM(+15.99)VGM(+15.99)GQK.D | Y | 61.11 | 1202.5536 | 11 | 7.61 | 602.2859 | 2 | 10.70 | 0.9089 | 0.9162 | 17721 | 0 | 0 | 0 | 63 | 73 | Oxidation (M), Oxidation (M) | M5:Oxidation (M):1000 M8:Oxidation (M):1000 | DB Search |
| R.LDLAGRDLTDYLMK.I | Y | 27.85 | 1622.8338 | 14 | -0.39 | 541.9492 | 3 | 19.26 | 0.7974 | 0.8047 | 66371 | 6.87e2 | 1 | 1 | 201 | 214 |  |  | DB Search |
| total 6 peptides |  |  |  |  |  |  |  |  |  |  |  |  |  |  |  |  |  |  |  |
| --- | --- | --- | --- | --- | --- | --- | --- | --- | --- | --- | --- | --- | --- | --- | --- | --- | --- | --- | --- |

Best Unique PSM (Scan Sample\_10\_A1\_1\_3647.d:45498, m/z=599.8535, z=2, RT=15.64, ppm=-4.77):


TRINITY\_DN138\_c0\_g1\_i1.p1

back to list

  

| Protein Coverage
| Supporting Peptides
| Best Unique PSM
|

Protein Coverage:

Supporting Peptides:

| Peptide | Uniq | -10lgP | Mass | Length | ppm | m/z | z | RT | 1/k0 Start | 1/k0 End | Precursor ID | Area Sample 1 | #Feature | #Feature Sample 1 | Start | End | PTM | AScore | Found By |
| --- | --- | --- | --- | --- | --- | --- | --- | --- | --- | --- | --- | --- | --- | --- | --- | --- | --- | --- | --- |
| K.DMGVNVISVGAGHTINHR.E | Y | 66.78 | 1875.9373 | 18 | 3.53 | 938.975 | 2 | 15.24 | 1.2443 | 1.2515 | 43380 | 3.44e2 | 1 | 1 | 349 | 366 |  |  | DB Search |
| total 1 peptides |  |  |  |  |  |  |  |  |  |  |  |  |  |  |  |  |  |  |  |
| --- | --- | --- | --- | --- | --- | --- | --- | --- | --- | --- | --- | --- | --- | --- | --- | --- | --- | --- | --- |

Best Unique PSM (Scan Sample\_10\_A1\_1\_3647.d:43380, m/z=938.975, z=2, RT=15.24, ppm=-0.96):


TRINITY\_DN471\_c0\_g1\_i4.p1

back to list

  

| Protein Coverage
| Supporting Peptides
| Best Unique PSM
|

Protein Coverage:

Supporting Peptides:

| Peptide | Uniq | -10lgP | Mass | Length | ppm | m/z | z | RT | 1/k0 Start | 1/k0 End | Precursor ID | Area Sample 1 | #Feature | #Feature Sample 1 | Start | End | PTM | AScore | Found By |
| --- | --- | --- | --- | --- | --- | --- | --- | --- | --- | --- | --- | --- | --- | --- | --- | --- | --- | --- | --- |
| R.AFVHWYVGEGM(+15.99)EEGEFSEAR.E | Y | 44.9 | 2345.0058 | 20 | -0.18 | 782.6722 | 3 | 17.81 | 0.9251 | 0.9323 | 57560 | 6.2e2 | 1 | 1 | 441 | 460 | Oxidation (M) | M11:Oxidation (M):1000 | DB Search |
| total 1 peptides |  |  |  |  |  |  |  |  |  |  |  |  |  |  |  |  |  |  |  |
| --- | --- | --- | --- | --- | --- | --- | --- | --- | --- | --- | --- | --- | --- | --- | --- | --- | --- | --- | --- |

Best Unique PSM (Scan Sample\_10\_A1\_1\_3647.d:57560, m/z=782.6722, z=3, RT=17.81, ppm=-4.67):


TRINITY\_DN1397\_c0\_g1\_i3.p1

back to list

  

| Protein Coverage
| Supporting Peptides
| Best Unique PSM
|

Protein Coverage:

Supporting Peptides:

| Peptide | Uniq | -10lgP | Mass | Length | ppm | m/z | z | RT | 1/k0 Start | 1/k0 End | Precursor ID | Area Sample 1 | #Feature | #Feature Sample 1 | Start | End | PTM | AScore | Found By |
| --- | --- | --- | --- | --- | --- | --- | --- | --- | --- | --- | --- | --- | --- | --- | --- | --- | --- | --- | --- |
| K.NEFRLPVVQK.M | Y | 17.17 | 1228.6928 | 10 | -5.88 | 615.3473 | 2 | 17.44 | 0.9327 | 0.94 | 55344 | 4.76e2 | 1 | 1 | 62 | 71 |  |  | DB Search |
| total 1 peptides |  |  |  |  |  |  |  |  |  |  |  |  |  |  |  |  |  |  |  |
| --- | --- | --- | --- | --- | --- | --- | --- | --- | --- | --- | --- | --- | --- | --- | --- | --- | --- | --- | --- |

Best Unique PSM (Scan Sample\_10\_A1\_1\_3647.d:55344, m/z=615.3473, z=2, RT=17.44, ppm=-10.38):


TRINITY\_DN36\_c0\_g1\_i4.p1

back to list

  

| Protein Coverage
| Supporting Peptides
| Best Unique PSM
|

Protein Coverage:

Supporting Peptides:

| Peptide | Uniq | -10lgP | Mass | Length | ppm | m/z | z | RT | 1/k0 Start | 1/k0 End | Precursor ID | Area Sample 1 | #Feature | #Feature Sample 1 | Start | End | PTM | AScore | Found By |
| --- | --- | --- | --- | --- | --- | --- | --- | --- | --- | --- | --- | --- | --- | --- | --- | --- | --- | --- | --- |
| R.LGGIEVTQGNC(+57.02)AR.K | Y | 66.48 | 1373.6721 | 13 | 5.18 | 687.8438 | 2 | 13.73 | 0.963 | 0.9703 | 35192 | 0 | 0 | 0 | 58 | 70 | Carbamidomethylation |  | DB Search |
| R.LGGIEVTQGNC(+57.02)ARK.Y | Y | 31.04 | 1501.7671 | 14 | -2.67 | 751.8854 | 2 | 12.64 | 1.0624 | 1.0697 | 29000 | 0 | 0 | 0 | 58 | 71 | Carbamidomethylation |  | DB Search |
| total 2 peptides |  |  |  |  |  |  |  |  |  |  |  |  |  |  |  |  |  |  |  |
| --- | --- | --- | --- | --- | --- | --- | --- | --- | --- | --- | --- | --- | --- | --- | --- | --- | --- | --- | --- |

Best Unique PSM (Scan Sample\_10\_A1\_1\_3647.d:35192, m/z=687.8438, z=2, RT=13.73, ppm=0.69):


TRINITY\_DN3036\_c0\_g1\_i19.p1

back to list

  

| Protein Coverage
| Supporting Peptides
| Best Unique PSM
|

Protein Coverage:

Supporting Peptides:

| Peptide | Uniq | -10lgP | Mass | Length | ppm | m/z | z | RT | 1/k0 Start | 1/k0 End | Precursor ID | Area Sample 1 | #Feature | #Feature Sample 1 | Start | End | PTM | AScore | Found By |
| --- | --- | --- | --- | --- | --- | --- | --- | --- | --- | --- | --- | --- | --- | --- | --- | --- | --- | --- | --- |
| R.AGHGAVTSPQTQR.G | Y | 66.66 | 1308.6534 | 13 | 4.48 | 655.334 | 2 | 9.70 | 0.9109 | 0.9182 | 11470 | 2.1e4 | 2 | 2 | 135 | 147 |  |  | DB Search |
| R.VIGASGIHVGTMSFGK.M | Y | 66.34 | 1559.813 | 16 | -0.2 | 780.9101 | 2 | 16.89 | 1.1037 | 1.111 | 52086 | 2.86e2 | 1 | 1 | 160 | 175 |  |  | DB Search |
| R.VIGASGIHVGTM(+15.99)SFGK.M | Y | 64.9 | 1575.8079 | 16 | 0.52 | 788.9081 | 2 | 15.11 | 1.1262 | 1.1334 | 42748 | 6.68e2 | 1 | 1 | 160 | 175 | Oxidation (M) | M12:Oxidation (M):1000 | DB Search |
| total 3 peptides |  |  |  |  |  |  |  |  |  |  |  |  |  |  |  |  |  |  |  |
| --- | --- | --- | --- | --- | --- | --- | --- | --- | --- | --- | --- | --- | --- | --- | --- | --- | --- | --- | --- |

Best Unique PSM (Scan Sample\_10\_A1\_1\_3647.d:11470, m/z=655.334, z=2, RT=9.70, ppm=-0.02):


TRINITY\_DN477\_c0\_g1\_i9.p1

back to list

  

| Protein Coverage
| Supporting Peptides
| Best Unique PSM
|

Protein Coverage:

Supporting Peptides:

| Peptide | Uniq | -10lgP | Mass | Length | ppm | m/z | z | RT | 1/k0 Start | 1/k0 End | Precursor ID | Area Sample 1 | #Feature | #Feature Sample 1 | Start | End | PTM | AScore | Found By |
| --- | --- | --- | --- | --- | --- | --- | --- | --- | --- | --- | --- | --- | --- | --- | --- | --- | --- | --- | --- |
| K.MRFDTAITAC(+57.02)R.S | Y | 66.76 | 1340.6329 | 11 | 3.22 | 671.3229 | 2 | 14.89 | 0.9675 | 0.9747 | 41498 | 1.49e3 | 1 | 1 | 276 | 286 | Carbamidomethylation |  | DB Search |
| R.TQAVLDRLNIEKR.V | Y | 65.76 | 1554.8842 | 13 | -5.27 | 519.2969 | 3 | 14.53 | 0.8023 | 0.8096 | 39606 | 8.83e2 | 1 | 1 | 220 | 232 |  |  | DB Search |
| total 2 peptides |  |  |  |  |  |  |  |  |  |  |  |  |  |  |  |  |  |  |  |
| --- | --- | --- | --- | --- | --- | --- | --- | --- | --- | --- | --- | --- | --- | --- | --- | --- | --- | --- | --- |

Best Unique PSM (Scan Sample\_10\_A1\_1\_3647.d:41498, m/z=671.3229, z=2, RT=14.89, ppm=-1.28):


TRINITY\_DN3271\_c0\_g1\_i2.p1

back to list

  

| Protein Coverage
| Supporting Peptides
| Best Unique PSM
|

Protein Coverage:

Supporting Peptides:

| Peptide | Uniq | -10lgP | Mass | Length | ppm | m/z | z | RT | 1/k0 Start | 1/k0 End | Precursor ID | Area Sample 1 | #Feature | #Feature Sample 1 | Start | End | PTM | AScore | Found By |
| --- | --- | --- | --- | --- | --- | --- | --- | --- | --- | --- | --- | --- | --- | --- | --- | --- | --- | --- | --- |
| K.TIESEAAHGTVTR.H | Y | 66.53 | 1370.679 | 13 | 2.05 | 686.3451 | 2 | 11.89 | 1.0194 | 1.0267 | 24550 | 7.11e2 | 1 | 1 | 354 | 366 |  |  | DB Search |
| total 1 peptides |  |  |  |  |  |  |  |  |  |  |  |  |  |  |  |  |  |  |  |
| --- | --- | --- | --- | --- | --- | --- | --- | --- | --- | --- | --- | --- | --- | --- | --- | --- | --- | --- | --- |

Best Unique PSM (Scan Sample\_10\_A1\_1\_3647.d:24550, m/z=686.3451, z=2, RT=11.89, ppm=-2.44):


TRINITY\_DN250\_c0\_g1\_i1.p1

back to list

  

| Protein Coverage
| Supporting Peptides
| Best Unique PSM
|

Protein Coverage:

Supporting Peptides:

| Peptide | Uniq | -10lgP | Mass | Length | ppm | m/z | z | RT | 1/k0 Start | 1/k0 End | Precursor ID | Area Sample 1 | #Feature | #Feature Sample 1 | Start | End | PTM | AScore | Found By |
| --- | --- | --- | --- | --- | --- | --- | --- | --- | --- | --- | --- | --- | --- | --- | --- | --- | --- | --- | --- |
| R.AVC(+57.02)M(+15.99)LSNTTAIAEAWAR.L | Y | 66.77 | 1879.892 | 17 | -0.29 | 940.9488 | 2 | 18.63 | 1.1418 | 1.149 | 62475 | 6.54e2 | 1 | 1 | 374 | 390 | Carbamidomethylation, Oxidation (M) | M4:Oxidation (M):1000 | DB Search |
| K.VGINYQPPTVVPGGDLAK.V | Y | 64.86 | 1823.9781 | 18 | -3.39 | 912.9891 | 2 | 17.23 | 1.1198 | 1.127 | 54086 | 0 | 0 | 0 | 353 | 370 |  |  | DB Search |
| total 2 peptides |  |  |  |  |  |  |  |  |  |  |  |  |  |  |  |  |  |  |  |
| --- | --- | --- | --- | --- | --- | --- | --- | --- | --- | --- | --- | --- | --- | --- | --- | --- | --- | --- | --- |

Best Unique PSM (Scan Sample\_10\_A1\_1\_3647.d:62475, m/z=940.9488, z=2, RT=18.63, ppm=-4.79):


TRINITY\_DN6920\_c0\_g1\_i2.p1

back to list

  

| Protein Coverage
| Supporting Peptides
| Best Unique PSM
|

Protein Coverage:

Supporting Peptides:

| Peptide | Uniq | -10lgP | Mass | Length | ppm | m/z | z | RT | 1/k0 Start | 1/k0 End | Precursor ID | Area Sample 1 | #Feature | #Feature Sample 1 | Start | End | PTM | AScore | Found By |
| --- | --- | --- | --- | --- | --- | --- | --- | --- | --- | --- | --- | --- | --- | --- | --- | --- | --- | --- | --- |
| R.KVAC(+57.02)IGAWHPAR.V | Y | 60.86 | 1364.7135 | 12 | 9.11 | 683.3672 | 2 | 13.20 | 0.9711 | 0.9783 | 32100 | 9.16e2 | 1 | 1 | 257 | 268 | Carbamidomethylation |  | DB Search |
| R.HGSLGFLPR.K | Y | 25.17 | 982.5348 | 9 | -2.89 | 492.2711 | 2 | 17.17 | 0.8408 | 0.8481 | 53699 | 1.07e3 | 1 | 1 | 18 | 26 |  |  | DB Search |
| K.AGM(+15.99)THIVR.E | Y | 19.81 | 899.4647 | 8 | 2.72 | 450.7388 | 2 | 9.73 | 0.8226 | 0.8299 | 12038 | 6.74e2 | 1 | 1 | 58 | 65 | Oxidation (M) | M3:Oxidation (M):1000 | DB Search |
| total 3 peptides |  |  |  |  |  |  |  |  |  |  |  |  |  |  |  |  |  |  |  |
| --- | --- | --- | --- | --- | --- | --- | --- | --- | --- | --- | --- | --- | --- | --- | --- | --- | --- | --- | --- |

Best Unique PSM (Scan Sample\_10\_A1\_1\_3647.d:32100, m/z=683.3672, z=2, RT=13.20, ppm=4.62):


TRINITY\_DN2799\_c0\_g1\_i2.p1

back to list

  

| Protein Coverage
| Supporting Peptides
| Best Unique PSM
|

Protein Coverage:

Supporting Peptides:

| Peptide | Uniq | -10lgP | Mass | Length | ppm | m/z | z | RT | 1/k0 Start | 1/k0 End | Precursor ID | Area Sample 1 | #Feature | #Feature Sample 1 | Start | End | PTM | AScore | Found By |
| --- | --- | --- | --- | --- | --- | --- | --- | --- | --- | --- | --- | --- | --- | --- | --- | --- | --- | --- | --- |
| R.HGSLGFLPR.K | Y | 25.17 | 982.5348 | 9 | -2.89 | 492.2711 | 2 | 17.17 | 0.8408 | 0.8481 | 53699 | 1.07e3 | 1 | 1 | 28 | 36 |  |  | DB Search |
| K.AGM(+15.99)THIVR.D | Y | 19.81 | 899.4647 | 8 | 2.72 | 450.7388 | 2 | 9.73 | 0.8226 | 0.8299 | 12038 | 6.74e2 | 1 | 1 | 68 | 75 | Oxidation (M) | M3:Oxidation (M):1000 | DB Search |
| total 2 peptides |  |  |  |  |  |  |  |  |  |  |  |  |  |  |  |  |  |  |  |
| --- | --- | --- | --- | --- | --- | --- | --- | --- | --- | --- | --- | --- | --- | --- | --- | --- | --- | --- | --- |

Best Unique PSM (Scan Sample\_10\_A1\_1\_3647.d:53699, m/z=492.2711, z=2, RT=17.17, ppm=-7.39):


TRINITY\_DN2799\_c0\_g1\_i4.p2

back to list

  

| Protein Coverage
| Supporting Peptides
| Best Unique PSM
|

Protein Coverage:

Supporting Peptides:

| Peptide | Uniq | -10lgP | Mass | Length | ppm | m/z | z | RT | 1/k0 Start | 1/k0 End | Precursor ID | Area Sample 1 | #Feature | #Feature Sample 1 | Start | End | PTM | AScore | Found By |
| --- | --- | --- | --- | --- | --- | --- | --- | --- | --- | --- | --- | --- | --- | --- | --- | --- | --- | --- | --- |
| R.HGSLGFLPR.K | Y | 25.17 | 982.5348 | 9 | -2.89 | 492.2711 | 2 | 17.17 | 0.8408 | 0.8481 | 53699 | 1.07e3 | 1 | 1 | 28 | 36 |  |  | DB Search |
| K.AGM(+15.99)THIVR.D | Y | 19.81 | 899.4647 | 8 | 2.72 | 450.7388 | 2 | 9.73 | 0.8226 | 0.8299 | 12038 | 6.74e2 | 1 | 1 | 68 | 75 | Oxidation (M) | M3:Oxidation (M):1000 | DB Search |
| total 2 peptides |  |  |  |  |  |  |  |  |  |  |  |  |  |  |  |  |  |  |  |
| --- | --- | --- | --- | --- | --- | --- | --- | --- | --- | --- | --- | --- | --- | --- | --- | --- | --- | --- | --- |

Best Unique PSM (Scan Sample\_10\_A1\_1\_3647.d:53699, m/z=492.2711, z=2, RT=17.17, ppm=-7.39):


TRINITY\_DN51048\_c0\_g1\_i1.p1

back to list

  

| Protein Coverage
| Supporting Peptides
| Best Unique PSM
|

Protein Coverage:

Supporting Peptides:

| Peptide | Uniq | -10lgP | Mass | Length | ppm | m/z | z | RT | 1/k0 Start | 1/k0 End | Precursor ID | Area Sample 1 | #Feature | #Feature Sample 1 | Start | End | PTM | AScore | Found By |
| --- | --- | --- | --- | --- | --- | --- | --- | --- | --- | --- | --- | --- | --- | --- | --- | --- | --- | --- | --- |
| R.EC(+57.02)DKVR.N | Y | 32.35 | 805.3752 | 6 | 4.12 | 806.3822 | 1 | 10.47 | 1.1074 | 1.1146 | 16279 | 8.92e3 | 1 | 1 | 435 | 440 | Carbamidomethylation |  | DB Search |
| total 1 peptides |  |  |  |  |  |  |  |  |  |  |  |  |  |  |  |  |  |  |  |
| --- | --- | --- | --- | --- | --- | --- | --- | --- | --- | --- | --- | --- | --- | --- | --- | --- | --- | --- | --- |

Best Unique PSM (Scan Sample\_10\_A1\_1\_3647.d:16279, m/z=806.3822, z=1, RT=10.47, ppm=-0.37):


TRINITY\_DN1280\_c0\_g1\_i1.p1

back to list

  

| Protein Coverage
| Supporting Peptides
| Best Unique PSM
|

Protein Coverage:

Supporting Peptides:

| Peptide | Uniq | -10lgP | Mass | Length | ppm | m/z | z | RT | 1/k0 Start | 1/k0 End | Precursor ID | Area Sample 1 | #Feature | #Feature Sample 1 | Start | End | PTM | AScore | Found By |
| --- | --- | --- | --- | --- | --- | --- | --- | --- | --- | --- | --- | --- | --- | --- | --- | --- | --- | --- | --- |
| K.AEYENKTEVAR.S | Y | 66.77 | 1308.631 | 11 | 4.86 | 655.323 | 2 | 10.97 | 0.9783 | 0.9856 | 19355 | 0 | 0 | 0 | 202 | 212 |  |  | DB Search |
| K.AEAEAEVIQKK.A | Y | 66.54 | 1214.6506 | 11 | 5.58 | 608.3333 | 2 | 11.13 | 0.9545 | 0.9618 | 20225 | 0 | 0 | 0 | 321 | 331 |  |  | DB Search |
| R.KFVWEFFQK.V | Y | 62.91 | 1257.6546 | 9 | -1.78 | 629.8306 | 2 | 19.87 | 0.9449 | 0.9521 | 70107 | 0 | 0 | 0 | 30 | 38 |  |  | DB Search |
| R.IKAEYENKTEVAR.S | Y | 61.53 | 1549.81 | 13 | -4.14 | 775.9056 | 2 | 10.97 | 1.0556 | 1.0628 | 19328 | 0 | 0 | 0 | 200 | 212 |  |  | DB Search |
| R.NIALETLEGHQR.A | Y | 56.79 | 1379.7157 | 12 | 3.41 | 690.8644 | 2 | 15.22 | 1.0178 | 1.025 | 43440 | 5.81e2 | 1 | 1 | 99 | 110 |  |  | DB Search |
| R.RIAEVQC(+57.02)EAR.I | Y | 42.53 | 1230.6139 | 10 | 0.71 | 616.3119 | 2 | 10.93 | 0.9214 | 0.9287 | 19029 | 9.5e2 | 1 | 1 | 169 | 178 | Carbamidomethylation |  | DB Search |
| total 6 peptides |  |  |  |  |  |  |  |  |  |  |  |  |  |  |  |  |  |  |  |
| --- | --- | --- | --- | --- | --- | --- | --- | --- | --- | --- | --- | --- | --- | --- | --- | --- | --- | --- | --- |

Best Unique PSM (Scan Sample\_10\_A1\_1\_3647.d:19355, m/z=655.323, z=2, RT=10.97, ppm=0.36):


TRINITY\_DN2799\_c0\_g1\_i3.p1

back to list

  

| Protein Coverage
| Supporting Peptides
| Best Unique PSM
|

Protein Coverage:

Supporting Peptides:

| Peptide | Uniq | -10lgP | Mass | Length | ppm | m/z | z | RT | 1/k0 Start | 1/k0 End | Precursor ID | Area Sample 1 | #Feature | #Feature Sample 1 | Start | End | PTM | AScore | Found By |
| --- | --- | --- | --- | --- | --- | --- | --- | --- | --- | --- | --- | --- | --- | --- | --- | --- | --- | --- | --- |
| R.HGSLGFLPR.K | Y | 25.17 | 982.5348 | 9 | -2.89 | 492.2711 | 2 | 17.17 | 0.8408 | 0.8481 | 53699 | 1.07e3 | 1 | 1 | 29 | 37 |  |  | DB Search |
| K.AGM(+15.99)THIVR.D | Y | 19.81 | 899.4647 | 8 | 2.72 | 450.7388 | 2 | 9.73 | 0.8226 | 0.8299 | 12038 | 6.74e2 | 1 | 1 | 69 | 76 | Oxidation (M) | M3:Oxidation (M):1000 | DB Search |
| total 2 peptides |  |  |  |  |  |  |  |  |  |  |  |  |  |  |  |  |  |  |  |
| --- | --- | --- | --- | --- | --- | --- | --- | --- | --- | --- | --- | --- | --- | --- | --- | --- | --- | --- | --- |

Best Unique PSM (Scan Sample\_10\_A1\_1\_3647.d:53699, m/z=492.2711, z=2, RT=17.17, ppm=-7.39):


TRINITY\_DN535\_c0\_g1\_i2.p1

back to list

  

| Protein Coverage
| Supporting Peptides
| Best Unique PSM
|

Protein Coverage:

Supporting Peptides:

| Peptide | Uniq | -10lgP | Mass | Length | ppm | m/z | z | RT | 1/k0 Start | 1/k0 End | Precursor ID | Area Sample 1 | #Feature | #Feature Sample 1 | Start | End | PTM | AScore | Found By |
| --- | --- | --- | --- | --- | --- | --- | --- | --- | --- | --- | --- | --- | --- | --- | --- | --- | --- | --- | --- |
| R.GLFIIDDKGILR.Q | Y | 66.46 | 1358.7921 | 12 | 2.12 | 680.4017 | 2 | 19.27 | 1.0122 | 1.0194 | 66460 | 6.42e3 | 1 | 1 | 94 | 105 |  |  | DB Search |
| R.ALGC(+57.02)DVLAC(+57.02)SVDSHFSHLAWTQQSR.K | Y | 62.1 | 2844.3071 | 25 | 4.64 | 712.0842 | 4 | 18.79 | 0.9222 | 0.9295 | 63472 | 1.49e3 | 1 | 1 | 33 | 57 | Carbamidomethylation, Carbamidomethylation |  | DB Search |
| total 2 peptides |  |  |  |  |  |  |  |  |  |  |  |  |  |  |  |  |  |  |  |
| --- | --- | --- | --- | --- | --- | --- | --- | --- | --- | --- | --- | --- | --- | --- | --- | --- | --- | --- | --- |

Best Unique PSM (Scan Sample\_10\_A1\_1\_3647.d:66460, m/z=680.4017, z=2, RT=19.27, ppm=-2.37):


TRINITY\_DN578\_c0\_g1\_i1.p1

back to list

  

| Protein Coverage
| Supporting Peptides
| Best Unique PSM
|

Protein Coverage:

Supporting Peptides:

| Peptide | Uniq | -10lgP | Mass | Length | ppm | m/z | z | RT | 1/k0 Start | 1/k0 End | Precursor ID | Area Sample 1 | #Feature | #Feature Sample 1 | Start | End | PTM | AScore | Found By |
| --- | --- | --- | --- | --- | --- | --- | --- | --- | --- | --- | --- | --- | --- | --- | --- | --- | --- | --- | --- |
| K.TFAHTVHNLVLAGNK.M | Y | 66.52 | 1620.8736 | 15 | -4.74 | 811.4366 | 2 | 14.93 | 1.1166 | 1.1238 | 41769 | 0 | 0 | 0 | 141 | 155 |  |  | DB Search |
| total 1 peptides |  |  |  |  |  |  |  |  |  |  |  |  |  |  |  |  |  |  |  |
| --- | --- | --- | --- | --- | --- | --- | --- | --- | --- | --- | --- | --- | --- | --- | --- | --- | --- | --- | --- |

Best Unique PSM (Scan Sample\_10\_A1\_1\_3647.d:41769, m/z=811.4366, z=2, RT=14.93, ppm=-9.24):


TRINITY\_DN15599\_c0\_g1\_i1.p1

back to list

  

| Protein Coverage
| Supporting Peptides
| Best Unique PSM
|

Protein Coverage:

Supporting Peptides:

| Peptide | Uniq | -10lgP | Mass | Length | ppm | m/z | z | RT | 1/k0 Start | 1/k0 End | Precursor ID | Area Sample 1 | #Feature | #Feature Sample 1 | Start | End | PTM | AScore | Found By |
| --- | --- | --- | --- | --- | --- | --- | --- | --- | --- | --- | --- | --- | --- | --- | --- | --- | --- | --- | --- |
| K.K(+42.01)LQAELANGR.L | Y | 63.27 | 1140.6251 | 10 | -2.89 | 571.3156 | 2 | 14.26 | 0.8919 | 0.8992 | 37952 | 3.72e3 | 1 | 1 | 14 | 23 | Acetylation (Protein N-term) | K1:Acetylation (Protein N-term):1000 | DB Search |
| K.KLQAELANGR.L | Y | 41.93 | 1098.6145 | 10 | 1.23 | 550.3127 | 2 | 11.49 | 0.8878 | 0.8951 | 21900 | 9.13e3 | 1 | 1 | 14 | 23 |  |  | DB Search |
| total 2 peptides |  |  |  |  |  |  |  |  |  |  |  |  |  |  |  |  |  |  |  |
| --- | --- | --- | --- | --- | --- | --- | --- | --- | --- | --- | --- | --- | --- | --- | --- | --- | --- | --- | --- |

Best Unique PSM (Scan Sample\_10\_A1\_1\_3647.d:37952, m/z=571.3156, z=2, RT=14.26, ppm=-7.38):


TRINITY\_DN4166\_c0\_g3\_i2.p1

back to list

  

| Protein Coverage
| Supporting Peptides
| Best Unique PSM
|

Protein Coverage:

Supporting Peptides:

| Peptide | Uniq | -10lgP | Mass | Length | ppm | m/z | z | RT | 1/k0 Start | 1/k0 End | Precursor ID | Area Sample 1 | #Feature | #Feature Sample 1 | Start | End | PTM | AScore | Found By |
| --- | --- | --- | --- | --- | --- | --- | --- | --- | --- | --- | --- | --- | --- | --- | --- | --- | --- | --- | --- |
| K.SLGYSVQLQAALIK.L | Y | 29.2 | 1489.8504 | 14 | -3.67 | 745.9264 | 2 | 18.69 | 1.0516 | 1.0588 | 62924 | 0 | 0 | 0 | 439 | 452 |  |  | DB Search |
| total 1 peptides |  |  |  |  |  |  |  |  |  |  |  |  |  |  |  |  |  |  |  |
| --- | --- | --- | --- | --- | --- | --- | --- | --- | --- | --- | --- | --- | --- | --- | --- | --- | --- | --- | --- |

Best Unique PSM (Scan Sample\_10\_A1\_1\_3647.d:62924, m/z=745.9264, z=2, RT=18.69, ppm=-8.17):


TRINITY\_DN2477\_c0\_g1\_i9.p1

back to list

  

| Protein Coverage
| Supporting Peptides
| Best Unique PSM
|

Protein Coverage:

Supporting Peptides:

| Peptide | Uniq | -10lgP | Mass | Length | ppm | m/z | z | RT | 1/k0 Start | 1/k0 End | Precursor ID | Area Sample 1 | #Feature | #Feature Sample 1 | Start | End | PTM | AScore | Found By |
| --- | --- | --- | --- | --- | --- | --- | --- | --- | --- | --- | --- | --- | --- | --- | --- | --- | --- | --- | --- |
| K.DSYVGDEAQSKR.G | Y | 66.73 | 1353.616 | 12 | 2.52 | 677.814 | 2 | 10.94 | 1.0069 | 1.0142 | 19045 | 5.51e3 | 1 | 1 | 52 | 63 |  |  | DB Search |
| total 1 peptides |  |  |  |  |  |  |  |  |  |  |  |  |  |  |  |  |  |  |  |
| --- | --- | --- | --- | --- | --- | --- | --- | --- | --- | --- | --- | --- | --- | --- | --- | --- | --- | --- | --- |

Best Unique PSM (Scan Sample\_10\_A1\_1\_3647.d:19045, m/z=677.814, z=2, RT=10.94, ppm=-1.97):


TRINITY\_DN1114\_c0\_g1\_i1.p1

back to list

  

| Protein Coverage
| Supporting Peptides
| Best Unique PSM
|

Protein Coverage:

Supporting Peptides:

| Peptide | Uniq | -10lgP | Mass | Length | ppm | m/z | z | RT | 1/k0 Start | 1/k0 End | Precursor ID | Area Sample 1 | #Feature | #Feature Sample 1 | Start | End | PTM | AScore | Found By |
| --- | --- | --- | --- | --- | --- | --- | --- | --- | --- | --- | --- | --- | --- | --- | --- | --- | --- | --- | --- |
| R.ADLIAYLEESTTKK.X | Y | 66.7 | 1580.8297 | 14 | 1.07 | 791.4194 | 2 | 19.58 | 1.0985 | 1.1057 | 68341 | 3e2 | 1 | 1 | 104 | 117 |  |  | DB Search |
| total 1 peptides |  |  |  |  |  |  |  |  |  |  |  |  |  |  |  |  |  |  |  |
| --- | --- | --- | --- | --- | --- | --- | --- | --- | --- | --- | --- | --- | --- | --- | --- | --- | --- | --- | --- |

Best Unique PSM (Scan Sample\_10\_A1\_1\_3647.d:68341, m/z=791.4194, z=2, RT=19.58, ppm=-3.42):


TRINITY\_DN250\_c0\_g1\_i4.p1

back to list

  

| Protein Coverage
| Supporting Peptides
| Best Unique PSM
|

Protein Coverage:

Supporting Peptides:

| Peptide | Uniq | -10lgP | Mass | Length | ppm | m/z | z | RT | 1/k0 Start | 1/k0 End | Precursor ID | Area Sample 1 | #Feature | #Feature Sample 1 | Start | End | PTM | AScore | Found By |
| --- | --- | --- | --- | --- | --- | --- | --- | --- | --- | --- | --- | --- | --- | --- | --- | --- | --- | --- | --- |
| K.VGINYQPPTVVPGGDLAK.V | Y | 64.86 | 1823.9781 | 18 | -3.39 | 912.9891 | 2 | 17.23 | 1.1198 | 1.127 | 54086 | 0 | 0 | 0 | 353 | 370 |  |  | DB Search |
| R.AFVHWYVGEGM(+15.99)EEGEFSEAR.E | Y | 44.9 | 2345.0058 | 20 | -0.18 | 782.6722 | 3 | 17.81 | 0.9251 | 0.9323 | 57560 | 6.2e2 | 1 | 1 | 403 | 422 | Oxidation (M) | M11:Oxidation (M):1000 | DB Search |
| total 2 peptides |  |  |  |  |  |  |  |  |  |  |  |  |  |  |  |  |  |  |  |
| --- | --- | --- | --- | --- | --- | --- | --- | --- | --- | --- | --- | --- | --- | --- | --- | --- | --- | --- | --- |

Best Unique PSM (Scan Sample\_10\_A1\_1\_3647.d:54086, m/z=912.9891, z=2, RT=17.23, ppm=-7.88):


TRINITY\_DN998\_c0\_g1\_i3.p2

back to list

  

| Protein Coverage
| Supporting Peptides
| Best Unique PSM
|

Protein Coverage:

Supporting Peptides:

| Peptide | Uniq | -10lgP | Mass | Length | ppm | m/z | z | RT | 1/k0 Start | 1/k0 End | Precursor ID | Area Sample 1 | #Feature | #Feature Sample 1 | Start | End | PTM | AScore | Found By |
| --- | --- | --- | --- | --- | --- | --- | --- | --- | --- | --- | --- | --- | --- | --- | --- | --- | --- | --- | --- |
| K.YANFLDQALREDLER.M | Y | 61.79 | 1851.9115 | 15 | -3.63 | 618.3061 | 3 | 19.80 | 0.8741 | 0.8814 | 69772 | 0 | 0 | 0 | 122 | 136 |  |  | DB Search |
| total 1 peptides |  |  |  |  |  |  |  |  |  |  |  |  |  |  |  |  |  |  |  |
| --- | --- | --- | --- | --- | --- | --- | --- | --- | --- | --- | --- | --- | --- | --- | --- | --- | --- | --- | --- |

Best Unique PSM (Scan Sample\_10\_A1\_1\_3647.d:69772, m/z=618.3061, z=3, RT=19.80, ppm=-8.12):


TRINITY\_DN1080\_c0\_g1\_i3.p1

back to list

  

| Protein Coverage
| Supporting Peptides
| Best Unique PSM
|

Protein Coverage:

Supporting Peptides:

| Peptide | Uniq | -10lgP | Mass | Length | ppm | m/z | z | RT | 1/k0 Start | 1/k0 End | Precursor ID | Area Sample 1 | #Feature | #Feature Sample 1 | Start | End | PTM | AScore | Found By |
| --- | --- | --- | --- | --- | --- | --- | --- | --- | --- | --- | --- | --- | --- | --- | --- | --- | --- | --- | --- |
| R.RPVPIVTR.R | Y | 17.05 | 936.5869 | 8 | 4.27 | 469.3006 | 2 | 11.83 | 0.8071 | 0.8144 | 24513 | 2.28e2 | 1 | 1 | 281 | 288 |  |  | DB Search |
| total 1 peptides |  |  |  |  |  |  |  |  |  |  |  |  |  |  |  |  |  |  |  |
| --- | --- | --- | --- | --- | --- | --- | --- | --- | --- | --- | --- | --- | --- | --- | --- | --- | --- | --- | --- |

Best Unique PSM (Scan Sample\_10\_A1\_1\_3647.d:24513, m/z=469.3006, z=2, RT=11.83, ppm=-0.23):


TRINITY\_DN1320\_c0\_g2\_i1.p1

back to list

  

| Protein Coverage
| Supporting Peptides
| Best Unique PSM
|

Protein Coverage:

Supporting Peptides:

| Peptide | Uniq | -10lgP | Mass | Length | ppm | m/z | z | RT | 1/k0 Start | 1/k0 End | Precursor ID | Area Sample 1 | #Feature | #Feature Sample 1 | Start | End | PTM | AScore | Found By |
| --- | --- | --- | --- | --- | --- | --- | --- | --- | --- | --- | --- | --- | --- | --- | --- | --- | --- | --- | --- |
| K.NYFHYENQLELK.F | Y | 66.44 | 1596.7572 | 12 | 8.12 | 799.3888 | 2 | 16.35 | 1.0861 | 1.0933 | 49354 | 0 | 0 | 0 | 176 | 187 |  |  | DB Search |
| total 1 peptides |  |  |  |  |  |  |  |  |  |  |  |  |  |  |  |  |  |  |  |
| --- | --- | --- | --- | --- | --- | --- | --- | --- | --- | --- | --- | --- | --- | --- | --- | --- | --- | --- | --- |

Best Unique PSM (Scan Sample\_10\_A1\_1\_3647.d:49354, m/z=799.3888, z=2, RT=16.35, ppm=3.63):


TRINITY\_DN902\_c1\_g2\_i3.p1

back to list

  

| Protein Coverage
| Supporting Peptides
| Best Unique PSM
|

Protein Coverage:

Supporting Peptides:

| Peptide | Uniq | -10lgP | Mass | Length | ppm | m/z | z | RT | 1/k0 Start | 1/k0 End | Precursor ID | Area Sample 1 | #Feature | #Feature Sample 1 | Start | End | PTM | AScore | Found By |
| --- | --- | --- | --- | --- | --- | --- | --- | --- | --- | --- | --- | --- | --- | --- | --- | --- | --- | --- | --- |
| K.GLTHC(+57.02)NTLSYK.L | Y | 66.56 | 1292.6183 | 11 | 2.85 | 647.3154 | 2 | 12.41 | 0.9699 | 0.9771 | 27654 | 0 | 0 | 0 | 62 | 72 | Carbamidomethylation |  | DB Search |
| total 1 peptides |  |  |  |  |  |  |  |  |  |  |  |  |  |  |  |  |  |  |  |
| --- | --- | --- | --- | --- | --- | --- | --- | --- | --- | --- | --- | --- | --- | --- | --- | --- | --- | --- | --- |

Best Unique PSM (Scan Sample\_10\_A1\_1\_3647.d:27654, m/z=647.3154, z=2, RT=12.41, ppm=-1.65):


TRINITY\_DN998\_c0\_g1\_i4.p2

back to list

  

| Protein Coverage
| Supporting Peptides
| Best Unique PSM
|

Protein Coverage:

Supporting Peptides:

| Peptide | Uniq | -10lgP | Mass | Length | ppm | m/z | z | RT | 1/k0 Start | 1/k0 End | Precursor ID | Area Sample 1 | #Feature | #Feature Sample 1 | Start | End | PTM | AScore | Found By |
| --- | --- | --- | --- | --- | --- | --- | --- | --- | --- | --- | --- | --- | --- | --- | --- | --- | --- | --- | --- |
| K.YANFLDQALREDLER.M | Y | 61.79 | 1851.9115 | 15 | -3.63 | 618.3061 | 3 | 19.80 | 0.8741 | 0.8814 | 69772 | 0 | 0 | 0 | 104 | 118 |  |  | DB Search |
| total 1 peptides |  |  |  |  |  |  |  |  |  |  |  |  |  |  |  |  |  |  |  |
| --- | --- | --- | --- | --- | --- | --- | --- | --- | --- | --- | --- | --- | --- | --- | --- | --- | --- | --- | --- |

Best Unique PSM (Scan Sample\_10\_A1\_1\_3647.d:69772, m/z=618.3061, z=3, RT=19.80, ppm=-8.12):


TRINITY\_DN15584\_c0\_g1\_i1.p1

back to list

  

| Protein Coverage
| Supporting Peptides
| Best Unique PSM
|

Protein Coverage:

Supporting Peptides:

| Peptide | Uniq | -10lgP | Mass | Length | ppm | m/z | z | RT | 1/k0 Start | 1/k0 End | Precursor ID | Area Sample 1 | #Feature | #Feature Sample 1 | Start | End | PTM | AScore | Found By |
| --- | --- | --- | --- | --- | --- | --- | --- | --- | --- | --- | --- | --- | --- | --- | --- | --- | --- | --- | --- |
| K.IGKPHTVPC(+57.02)K.V | Y | 66.65 | 1135.6172 | 10 | 2.23 | 568.8146 | 2 | 9.75 | 0.8947 | 0.902 | 12052 | 1.76e4 | 2 | 2 | 157 | 166 | Carbamidomethylation |  | DB Search |
| K.AFVAIGDYGGHVGLGVK.C | Y | 66.54 | 1658.878 | 17 | 1.87 | 830.4441 | 2 | 17.61 | 1.1638 | 1.171 | 55921 | 1.18e4 | 1 | 1 | 109 | 125 |  |  | DB Search |
| K.LSIIPVRR.G | Y | 38.41 | 952.6182 | 8 | 1.59 | 477.315 | 2 | 14.73 | 0.8453 | 0.8526 | 40459 | 4.64e3 | 1 | 1 | 143 | 150 |  |  | DB Search |
| total 3 peptides |  |  |  |  |  |  |  |  |  |  |  |  |  |  |  |  |  |  |  |
| --- | --- | --- | --- | --- | --- | --- | --- | --- | --- | --- | --- | --- | --- | --- | --- | --- | --- | --- | --- |

Best Unique PSM (Scan Sample\_10\_A1\_1\_3647.d:12052, m/z=568.8146, z=2, RT=9.75, ppm=-2.27):


TRINITY\_DN598\_c1\_g1\_i1.p1

back to list

  

| Protein Coverage
| Supporting Peptides
| Best Unique PSM
|

Protein Coverage:

Supporting Peptides:

| Peptide | Uniq | -10lgP | Mass | Length | ppm | m/z | z | RT | 1/k0 Start | 1/k0 End | Precursor ID | Area Sample 1 | #Feature | #Feature Sample 1 | Start | End | PTM | AScore | Found By |
| --- | --- | --- | --- | --- | --- | --- | --- | --- | --- | --- | --- | --- | --- | --- | --- | --- | --- | --- | --- |
| R.ASFVSDIDRLR.A | Y | 66.54 | 1277.6728 | 11 | 1.77 | 639.8419 | 2 | 16.87 | 0.9828 | 0.99 | 51974 | 1.96e3 | 1 | 1 | 302 | 312 |  |  | DB Search |
| total 1 peptides |  |  |  |  |  |  |  |  |  |  |  |  |  |  |  |  |  |  |  |
| --- | --- | --- | --- | --- | --- | --- | --- | --- | --- | --- | --- | --- | --- | --- | --- | --- | --- | --- | --- |

Best Unique PSM (Scan Sample\_10\_A1\_1\_3647.d:51974, m/z=639.8419, z=2, RT=16.87, ppm=-2.73):


TRINITY\_DN30378\_c0\_g1\_i2.p1

back to list

  

| Protein Coverage
| Supporting Peptides
| Best Unique PSM
|

Protein Coverage:

Supporting Peptides:

| Peptide | Uniq | -10lgP | Mass | Length | ppm | m/z | z | RT | 1/k0 Start | 1/k0 End | Precursor ID | Area Sample 1 | #Feature | #Feature Sample 1 | Start | End | PTM | AScore | Found By |
| --- | --- | --- | --- | --- | --- | --- | --- | --- | --- | --- | --- | --- | --- | --- | --- | --- | --- | --- | --- |
| K.ATLGHGHVIEGVDR.G | Y | 64.13 | 1459.7531 | 14 | -3.35 | 730.8781 | 2 | 12.12 | 1.011 | 1.0182 | 25970 | 0 | 0 | 0 | 334 | 347 |  |  | DB Search |
| total 1 peptides |  |  |  |  |  |  |  |  |  |  |  |  |  |  |  |  |  |  |  |
| --- | --- | --- | --- | --- | --- | --- | --- | --- | --- | --- | --- | --- | --- | --- | --- | --- | --- | --- | --- |

Best Unique PSM (Scan Sample\_10\_A1\_1\_3647.d:25970, m/z=730.8781, z=2, RT=12.12, ppm=-7.85):


TRINITY\_DN250\_c0\_g1\_i3.p1

back to list

  

| Protein Coverage
| Supporting Peptides
| Best Unique PSM
|

Protein Coverage:

Supporting Peptides:

| Peptide | Uniq | -10lgP | Mass | Length | ppm | m/z | z | RT | 1/k0 Start | 1/k0 End | Precursor ID | Area Sample 1 | #Feature | #Feature Sample 1 | Start | End | PTM | AScore | Found By |
| --- | --- | --- | --- | --- | --- | --- | --- | --- | --- | --- | --- | --- | --- | --- | --- | --- | --- | --- | --- |
| R.AVC(+57.02)M(+15.99)LSNTTAIAEAWAR.L | Y | 66.77 | 1879.892 | 17 | -0.29 | 940.9488 | 2 | 18.63 | 1.1418 | 1.149 | 62475 | 6.54e2 | 1 | 1 | 374 | 390 | Carbamidomethylation, Oxidation (M) | M4:Oxidation (M):1000 | DB Search |
| K.VGINYQPPTVVPGGDLAK.V | Y | 64.86 | 1823.9781 | 18 | -3.39 | 912.9891 | 2 | 17.23 | 1.1198 | 1.127 | 54086 | 0 | 0 | 0 | 353 | 370 |  |  | DB Search |
| total 2 peptides |  |  |  |  |  |  |  |  |  |  |  |  |  |  |  |  |  |  |  |
| --- | --- | --- | --- | --- | --- | --- | --- | --- | --- | --- | --- | --- | --- | --- | --- | --- | --- | --- | --- |

Best Unique PSM (Scan Sample\_10\_A1\_1\_3647.d:62475, m/z=940.9488, z=2, RT=18.63, ppm=-4.79):


TRINITY\_DN158421\_c0\_g1\_i1.p1

back to list

  

| Protein Coverage
| Supporting Peptides
| Best Unique PSM
|

Protein Coverage:

Supporting Peptides:

| Peptide | Uniq | -10lgP | Mass | Length | ppm | m/z | z | RT | 1/k0 Start | 1/k0 End | Precursor ID | Area Sample 1 | #Feature | #Feature Sample 1 | Start | End | PTM | AScore | Found By |
| --- | --- | --- | --- | --- | --- | --- | --- | --- | --- | --- | --- | --- | --- | --- | --- | --- | --- | --- | --- |
| R.SSC(+57.02)RPILSVIK.I | Y | 49.81 | 1258.7067 | 11 | -8.86 | 630.3522 | 2 | 16.81 | 0.9691 | 0.9763 | 51630 | 2.1e3 | 1 | 1 | 90 | 100 | Carbamidomethylation |  | DB Search |
| total 1 peptides |  |  |  |  |  |  |  |  |  |  |  |  |  |  |  |  |  |  |  |
| --- | --- | --- | --- | --- | --- | --- | --- | --- | --- | --- | --- | --- | --- | --- | --- | --- | --- | --- | --- |

Best Unique PSM (Scan Sample\_10\_A1\_1\_3647.d:51630, m/z=630.3522, z=2, RT=16.81, ppm=-13.35):


TRINITY\_DN2068\_c0\_g1\_i1.p1

back to list

  

| Protein Coverage
| Supporting Peptides
| Best Unique PSM
|

Protein Coverage:

Supporting Peptides:

| Peptide | Uniq | -10lgP | Mass | Length | ppm | m/z | z | RT | 1/k0 Start | 1/k0 End | Precursor ID | Area Sample 1 | #Feature | #Feature Sample 1 | Start | End | PTM | AScore | Found By |
| --- | --- | --- | --- | --- | --- | --- | --- | --- | --- | --- | --- | --- | --- | --- | --- | --- | --- | --- | --- |
| R.GAHGIIIVYDVTDQDTFNNVK.Q | Y | 61.79 | 2318.1542 | 21 | -3.18 | 773.7194 | 3 | 18.44 | 0.9198 | 0.9271 | 61374 | 2.77e2 | 1 | 1 | 80 | 100 |  |  | DB Search |
| total 1 peptides |  |  |  |  |  |  |  |  |  |  |  |  |  |  |  |  |  |  |  |
| --- | --- | --- | --- | --- | --- | --- | --- | --- | --- | --- | --- | --- | --- | --- | --- | --- | --- | --- | --- |

Best Unique PSM (Scan Sample\_10\_A1\_1\_3647.d:61374, m/z=773.7194, z=3, RT=18.44, ppm=-7.68):


TRINITY\_DN7573\_c0\_g1\_i4.p2

back to list

  

| Protein Coverage
| Supporting Peptides
| Best Unique PSM
|

Protein Coverage:

Supporting Peptides:

| Peptide | Uniq | -10lgP | Mass | Length | ppm | m/z | z | RT | 1/k0 Start | 1/k0 End | Precursor ID | Area Sample 1 | #Feature | #Feature Sample 1 | Start | End | PTM | AScore | Found By |
| --- | --- | --- | --- | --- | --- | --- | --- | --- | --- | --- | --- | --- | --- | --- | --- | --- | --- | --- | --- |
| K.TRTPGPGAQSALR.A | Y | 18.84 | 1310.7055 | 13 | -4.39 | 656.3542 | 2 | 12.39 | 0.9469 | 0.9541 | 27541 | 3.36e2 | 1 | 1 | 115 | 127 |  |  | DB Search |
| total 1 peptides |  |  |  |  |  |  |  |  |  |  |  |  |  |  |  |  |  |  |  |
| --- | --- | --- | --- | --- | --- | --- | --- | --- | --- | --- | --- | --- | --- | --- | --- | --- | --- | --- | --- |

Best Unique PSM (Scan Sample\_10\_A1\_1\_3647.d:27541, m/z=655.8232, z=2, RT=12.39, ppm=-8.89):


TRINITY\_DN3036\_c0\_g1\_i13.p1

back to list

  

| Protein Coverage
| Supporting Peptides
| Best Unique PSM
|

Protein Coverage:

Supporting Peptides:

| Peptide | Uniq | -10lgP | Mass | Length | ppm | m/z | z | RT | 1/k0 Start | 1/k0 End | Precursor ID | Area Sample 1 | #Feature | #Feature Sample 1 | Start | End | PTM | AScore | Found By |
| --- | --- | --- | --- | --- | --- | --- | --- | --- | --- | --- | --- | --- | --- | --- | --- | --- | --- | --- | --- |
| R.AGHGAVTSPQTQR.G | Y | 66.66 | 1308.6534 | 13 | 4.48 | 655.334 | 2 | 9.70 | 0.9109 | 0.9182 | 11470 | 2.1e4 | 2 | 2 | 135 | 147 |  |  | DB Search |
| R.VIGASGIHVGTMSFGK.M | Y | 66.34 | 1559.813 | 16 | -0.2 | 780.9101 | 2 | 16.89 | 1.1037 | 1.111 | 52086 | 2.86e2 | 1 | 1 | 160 | 175 |  |  | DB Search |
| R.VIGASGIHVGTM(+15.99)SFGK.M | Y | 64.9 | 1575.8079 | 16 | 0.52 | 788.9081 | 2 | 15.11 | 1.1262 | 1.1334 | 42748 | 6.68e2 | 1 | 1 | 160 | 175 | Oxidation (M) | M12:Oxidation (M):1000 | DB Search |
| total 3 peptides |  |  |  |  |  |  |  |  |  |  |  |  |  |  |  |  |  |  |  |
| --- | --- | --- | --- | --- | --- | --- | --- | --- | --- | --- | --- | --- | --- | --- | --- | --- | --- | --- | --- |

Best Unique PSM (Scan Sample\_10\_A1\_1\_3647.d:11470, m/z=655.334, z=2, RT=9.70, ppm=-0.02):


TRINITY\_DN3986\_c0\_g1\_i1.p1

back to list

  

| Protein Coverage
| Supporting Peptides
| Best Unique PSM
|

Protein Coverage:

Supporting Peptides:

| Peptide | Uniq | -10lgP | Mass | Length | ppm | m/z | z | RT | 1/k0 Start | 1/k0 End | Precursor ID | Area Sample 1 | #Feature | #Feature Sample 1 | Start | End | PTM | AScore | Found By |
| --- | --- | --- | --- | --- | --- | --- | --- | --- | --- | --- | --- | --- | --- | --- | --- | --- | --- | --- | --- |
| K.M(+15.99)SATFIGNSTAIQELFKR.I | Y | 66.78 | 2029.0302 | 18 | 0.01 | 677.3477 | 3 | 19.94 | 0.9348 | 0.942 | 70482 | 1.29e3 | 1 | 1 | 363 | 380 | Oxidation (M) | M1:Oxidation (M):1000 | DB Search |
| K.NM(+15.99)M(+15.99)AAC(+57.02)DPR.H | Y | 66.66 | 1096.41 | 9 | 0.96 | 549.2103 | 2 | 9.51 | 0.8591 | 0.8664 | 10859 | 2.33e3 | 1 | 1 | 298 | 306 | Oxidation (M), Oxidation (M), Carbamidomethylation | M2:Oxidation (M):1000 M3:Oxidation (M):1000 | DB Search |
| K.NSSYFVEWIPNNVK.T | Y | 64.67 | 1695.8256 | 14 | -4.28 | 848.9127 | 2 | 19.92 | 1.0889 | 1.0961 | 70457 | 0 | 0 | 0 | 337 | 350 |  |  | DB Search |
| R.FPGQLNADLRK.L | Y | 62.07 | 1257.6829 | 11 | -5.49 | 629.8425 | 2 | 14.27 | 0.9428 | 0.9501 | 38203 | 0 | 0 | 0 | 242 | 252 |  |  | DB Search |
| R.EIVHLQAGQC(+57.02)GNQIGAK.F | Y | 59.9 | 1821.9155 | 17 | -14.62 | 911.9476 | 2 | 13.73 | 1.113 | 1.1202 | 35183 | 0 | 0 | 0 | 3 | 19 | Carbamidomethylation |  | DB Search |
| K.IREEYPDR.I | Y | 56.96 | 1076.525 | 8 | 1.05 | 539.2679 | 2 | 10.96 | 0.8644 | 0.8716 | 19132 | 3.24e3 | 1 | 1 | 155 | 162 |  |  | DB Search |
| total 6 peptides |  |  |  |  |  |  |  |  |  |  |  |  |  |  |  |  |  |  |  |
| --- | --- | --- | --- | --- | --- | --- | --- | --- | --- | --- | --- | --- | --- | --- | --- | --- | --- | --- | --- |

Best Unique PSM (Scan Sample\_10\_A1\_1\_3647.d:70482, m/z=677.3477, z=3, RT=19.94, ppm=-4.48):


TRINITY\_DN1058\_c0\_g1\_i3.p1

back to list

  

| Protein Coverage
| Supporting Peptides
| Best Unique PSM
|

Protein Coverage:

Supporting Peptides:

| Peptide | Uniq | -10lgP | Mass | Length | ppm | m/z | z | RT | 1/k0 Start | 1/k0 End | Precursor ID | Area Sample 1 | #Feature | #Feature Sample 1 | Start | End | PTM | AScore | Found By |
| --- | --- | --- | --- | --- | --- | --- | --- | --- | --- | --- | --- | --- | --- | --- | --- | --- | --- | --- | --- |
| R.APTHILNPANKR.S | Y | 66.68 | 1330.7469 | 12 | 2.53 | 444.5887 | 3 | 11.11 | 0.7526 | 0.7599 | 20079 | 1.09e3 | 2 | 2 | 2314 | 2325 |  |  | DB Search |
| total 1 peptides |  |  |  |  |  |  |  |  |  |  |  |  |  |  |  |  |  |  |  |
| --- | --- | --- | --- | --- | --- | --- | --- | --- | --- | --- | --- | --- | --- | --- | --- | --- | --- | --- | --- |

Best Unique PSM (Scan Sample\_10\_A1\_1\_3647.d:20079, m/z=444.5887, z=3, RT=11.11, ppm=-1.97):


TRINITY\_DN2637\_c0\_g1\_i13.p1

back to list

  

| Protein Coverage
| Supporting Peptides
| Best Unique PSM
|

Protein Coverage:

Supporting Peptides:

| Peptide | Uniq | -10lgP | Mass | Length | ppm | m/z | z | RT | 1/k0 Start | 1/k0 End | Precursor ID | Area Sample 1 | #Feature | #Feature Sample 1 | Start | End | PTM | AScore | Found By |
| --- | --- | --- | --- | --- | --- | --- | --- | --- | --- | --- | --- | --- | --- | --- | --- | --- | --- | --- | --- |
| K.ATIEIPAKYEK.R | Y | 61.89 | 1261.6918 | 11 | 0.22 | 631.8505 | 2 | 14.08 | 0.982 | 0.9892 | 37168 | 0 | 0 | 0 | 107 | 117 |  |  | DB Search |
| total 1 peptides |  |  |  |  |  |  |  |  |  |  |  |  |  |  |  |  |  |  |  |
| --- | --- | --- | --- | --- | --- | --- | --- | --- | --- | --- | --- | --- | --- | --- | --- | --- | --- | --- | --- |

Best Unique PSM (Scan Sample\_10\_A1\_1\_3647.d:37168, m/z=631.8505, z=2, RT=14.08, ppm=-4.27):


TRINITY\_DN7808\_c2\_g1\_i1.p1

back to list

  

| Protein Coverage
| Supporting Peptides
| Best Unique PSM
|

Protein Coverage:

Supporting Peptides:

| Peptide | Uniq | -10lgP | Mass | Length | ppm | m/z | z | RT | 1/k0 Start | 1/k0 End | Precursor ID | Area Sample 1 | #Feature | #Feature Sample 1 | Start | End | PTM | AScore | Found By |
| --- | --- | --- | --- | --- | --- | --- | --- | --- | --- | --- | --- | --- | --- | --- | --- | --- | --- | --- | --- |
| R.RSLNAEIANGR.L | Y | 51.82 | 1199.6371 | 11 | 2.77 | 600.8248 | 2 | 11.99 | 0.9259 | 0.9331 | 25198 | 6.38e2 | 1 | 1 | 132 | 142 |  |  | DB Search |
| total 1 peptides |  |  |  |  |  |  |  |  |  |  |  |  |  |  |  |  |  |  |  |
| --- | --- | --- | --- | --- | --- | --- | --- | --- | --- | --- | --- | --- | --- | --- | --- | --- | --- | --- | --- |

Best Unique PSM (Scan Sample\_10\_A1\_1\_3647.d:25198, m/z=600.8248, z=2, RT=11.99, ppm=-1.73):


TRINITY\_DN817\_c0\_g1\_i1.p1

back to list

  

| Protein Coverage
| Supporting Peptides
| Best Unique PSM
|

Protein Coverage:

Supporting Peptides:

| Peptide | Uniq | -10lgP | Mass | Length | ppm | m/z | z | RT | 1/k0 Start | 1/k0 End | Precursor ID | Area Sample 1 | #Feature | #Feature Sample 1 | Start | End | PTM | AScore | Found By |
| --- | --- | --- | --- | --- | --- | --- | --- | --- | --- | --- | --- | --- | --- | --- | --- | --- | --- | --- | --- |
| R.LC(+57.02)QPGYSTC(+57.02)ENHC(+57.02)R.K | Y | 62.01 | 1780.708 | 14 | 5.39 | 891.3621 | 2 | 11.41 | 1.0893 | 1.0965 | 21785 | 1.84e2 | 1 | 1 | 179 | 192 | Carbamidomethylation, Carbamidomethylation, Carbamidomethylation |  | DB Search |
| total 1 peptides |  |  |  |  |  |  |  |  |  |  |  |  |  |  |  |  |  |  |  |
| --- | --- | --- | --- | --- | --- | --- | --- | --- | --- | --- | --- | --- | --- | --- | --- | --- | --- | --- | --- |

Best Unique PSM (Scan Sample\_10\_A1\_1\_3647.d:21785, m/z=891.3621, z=2, RT=11.41, ppm=0.9):


TRINITY\_DN761\_c0\_g1\_i1.p1

back to list

  

| Protein Coverage
| Supporting Peptides
| Best Unique PSM
|

Protein Coverage:

Supporting Peptides:

| Peptide | Uniq | -10lgP | Mass | Length | ppm | m/z | z | RT | 1/k0 Start | 1/k0 End | Precursor ID | Area Sample 1 | #Feature | #Feature Sample 1 | Start | End | PTM | AScore | Found By |
| --- | --- | --- | --- | --- | --- | --- | --- | --- | --- | --- | --- | --- | --- | --- | --- | --- | --- | --- | --- |
| K.TRIEDLETVISQTQDHR.H | Y | 66.35 | 2040.0236 | 17 | 3.15 | 681.0142 | 3 | 17.32 | 0.9622 | 0.9695 | 54563 | 0 | 0 | 0 | 260 | 276 |  |  | DB Search |
| K.EIDKADIAIVDTGENPDAPHPR.E | Y | 61.91 | 2372.1607 | 22 | 3.12 | 791.7264 | 3 | 14.88 | 1.0415 | 1.0488 | 41517 | 0 | 0 | 0 | 71 | 92 |  |  | DB Search |
| K.FVNEVRR.C | Y | 39.18 | 918.5035 | 7 | -2.89 | 460.2556 | 2 | 12.63 | 0.8096 | 0.8169 | 28832 | 9.86e2 | 1 | 1 | 51 | 57 |  |  | DB Search |
| total 3 peptides |  |  |  |  |  |  |  |  |  |  |  |  |  |  |  |  |  |  |  |
| --- | --- | --- | --- | --- | --- | --- | --- | --- | --- | --- | --- | --- | --- | --- | --- | --- | --- | --- | --- |

Best Unique PSM (Scan Sample\_10\_A1\_1\_3647.d:54563, m/z=681.0142, z=3, RT=17.32, ppm=-1.34):


TRINITY\_DN1613\_c0\_g2\_i1.p1

back to list

  

| Protein Coverage
| Supporting Peptides
| Best Unique PSM
|

Protein Coverage:

Supporting Peptides:

| Peptide | Uniq | -10lgP | Mass | Length | ppm | m/z | z | RT | 1/k0 Start | 1/k0 End | Precursor ID | Area Sample 1 | #Feature | #Feature Sample 1 | Start | End | PTM | AScore | Found By |
| --- | --- | --- | --- | --- | --- | --- | --- | --- | --- | --- | --- | --- | --- | --- | --- | --- | --- | --- | --- |
| K.GGKDFASDIITRK.E | Y | 63.55 | 1406.7517 | 13 | 2.63 | 704.3818 | 2 | 14.09 | 1.0009 | 1.0081 | 37275 | 1.13e3 | 1 | 1 | 99 | 111 |  |  | DB Search |
| K.AHTGYIYLVEGGRDR.N | Y | 40.28 | 1705.8536 | 15 | 3.15 | 853.9329 | 2 | 14.97 | 1.1478 | 1.155 | 41960 | 6.86e2 | 1 | 1 | 138 | 152 |  |  | DB Search |
| K.DFASDIITR.K | Y | 39.77 | 1036.5189 | 9 | -0.03 | 1037.5215 | 1 | 16.56 | 1.2841 | 1.2913 | 50406 | 3.96e2 | 1 | 1 | 102 | 110 |  |  | DB Search |
| total 3 peptides |  |  |  |  |  |  |  |  |  |  |  |  |  |  |  |  |  |  |  |
| --- | --- | --- | --- | --- | --- | --- | --- | --- | --- | --- | --- | --- | --- | --- | --- | --- | --- | --- | --- |

Best Unique PSM (Scan Sample\_10\_A1\_1\_3647.d:37275, m/z=704.3818, z=2, RT=14.09, ppm=-1.86):


TRINITY\_DN18187\_c0\_g1\_i5.p1

back to list

  

| Protein Coverage
| Supporting Peptides
| Best Unique PSM
|

Protein Coverage:

Supporting Peptides:

| Peptide | Uniq | -10lgP | Mass | Length | ppm | m/z | z | RT | 1/k0 Start | 1/k0 End | Precursor ID | Area Sample 1 | #Feature | #Feature Sample 1 | Start | End | PTM | AScore | Found By |
| --- | --- | --- | --- | --- | --- | --- | --- | --- | --- | --- | --- | --- | --- | --- | --- | --- | --- | --- | --- |
| R.YRPGTVALR.E | Y | 66.57 | 1031.5876 | 9 | 0.86 | 516.7992 | 2 | 12.50 | 0.8554 | 0.8627 | 28119 | 7.47e2 | 1 | 1 | 87 | 95 |  |  | DB Search |
| total 1 peptides |  |  |  |  |  |  |  |  |  |  |  |  |  |  |  |  |  |  |  |
| --- | --- | --- | --- | --- | --- | --- | --- | --- | --- | --- | --- | --- | --- | --- | --- | --- | --- | --- | --- |

Best Unique PSM (Scan Sample\_10\_A1\_1\_3647.d:28119, m/z=516.7992, z=2, RT=12.50, ppm=-3.64):


TRINITY\_DN36\_c0\_g1\_i20.p1

back to list

  

| Protein Coverage
| Supporting Peptides
| Best Unique PSM
|

Protein Coverage:

Supporting Peptides:

| Peptide | Uniq | -10lgP | Mass | Length | ppm | m/z | z | RT | 1/k0 Start | 1/k0 End | Precursor ID | Area Sample 1 | #Feature | #Feature Sample 1 | Start | End | PTM | AScore | Found By |
| --- | --- | --- | --- | --- | --- | --- | --- | --- | --- | --- | --- | --- | --- | --- | --- | --- | --- | --- | --- |
| K.GFDYC(+57.02)TTIKYR.L | Y | 66.92 | 1422.6602 | 11 | -0.86 | 712.3336 | 2 | 15.08 | 1.023 | 1.0303 | 42693 | 0 | 0 | 0 | 46 | 56 | Carbamidomethylation |  | DB Search |
| R.LGGIEVTQGNC(+57.02)AR.K | Y | 66.48 | 1373.6721 | 13 | 5.18 | 687.8438 | 2 | 13.73 | 0.963 | 0.9703 | 35192 | 0 | 0 | 0 | 57 | 69 | Carbamidomethylation |  | DB Search |
| R.LGGIEVTQGNC(+57.02)ARK.Y | Y | 31.04 | 1501.7671 | 14 | -2.67 | 751.8854 | 2 | 12.64 | 1.0624 | 1.0697 | 29000 | 0 | 0 | 0 | 57 | 70 | Carbamidomethylation |  | DB Search |
| total 3 peptides |  |  |  |  |  |  |  |  |  |  |  |  |  |  |  |  |  |  |  |
| --- | --- | --- | --- | --- | --- | --- | --- | --- | --- | --- | --- | --- | --- | --- | --- | --- | --- | --- | --- |

Best Unique PSM (Scan Sample\_10\_A1\_1\_3647.d:42693, m/z=712.3336, z=2, RT=15.08, ppm=-5.35):


TRINITY\_DN5107\_c0\_g1\_i2.p1

back to list

  

| Protein Coverage
| Supporting Peptides
| Best Unique PSM
|

Protein Coverage:

Supporting Peptides:

| Peptide | Uniq | -10lgP | Mass | Length | ppm | m/z | z | RT | 1/k0 Start | 1/k0 End | Precursor ID | Area Sample 1 | #Feature | #Feature Sample 1 | Start | End | PTM | AScore | Found By |
| --- | --- | --- | --- | --- | --- | --- | --- | --- | --- | --- | --- | --- | --- | --- | --- | --- | --- | --- | --- |
| K.KLDYVLGLTTAK.I | Y | 63.57 | 1320.7653 | 12 | -2.37 | 661.3854 | 2 | 16.69 | 0.9908 | 0.9981 | 51148 | 2.13e2 | 1 | 1 | 62 | 73 |  |  | DB Search |
| total 1 peptides |  |  |  |  |  |  |  |  |  |  |  |  |  |  |  |  |  |  |  |
| --- | --- | --- | --- | --- | --- | --- | --- | --- | --- | --- | --- | --- | --- | --- | --- | --- | --- | --- | --- |

Best Unique PSM (Scan Sample\_10\_A1\_1\_3647.d:51148, m/z=661.3854, z=2, RT=16.69, ppm=-6.87):


TRINITY\_DN15151\_c0\_g1\_i2.p1

back to list

  

| Protein Coverage
| Supporting Peptides
| Best Unique PSM
|

Protein Coverage:

Supporting Peptides:

| Peptide | Uniq | -10lgP | Mass | Length | ppm | m/z | z | RT | 1/k0 Start | 1/k0 End | Precursor ID | Area Sample 1 | #Feature | #Feature Sample 1 | Start | End | PTM | AScore | Found By |
| --- | --- | --- | --- | --- | --- | --- | --- | --- | --- | --- | --- | --- | --- | --- | --- | --- | --- | --- | --- |
| K.AVAAGM(+15.99)NPM(+15.99)DIKR.G | Y | 61.93 | 1404.6853 | 13 | -5.88 | 703.3427 | 2 | 11.20 | 1.0122 | 1.0194 | 20513 | 4.12e2 | 1 | 1 | 145 | 157 | Oxidation (M), Oxidation (M) | M6:Oxidation (M):1000 M9:Oxidation (M):1000 | DB Search |
| total 1 peptides |  |  |  |  |  |  |  |  |  |  |  |  |  |  |  |  |  |  |  |
| --- | --- | --- | --- | --- | --- | --- | --- | --- | --- | --- | --- | --- | --- | --- | --- | --- | --- | --- | --- |

Best Unique PSM (Scan Sample\_10\_A1\_1\_3647.d:20513, m/z=703.3427, z=2, RT=11.20, ppm=-10.38):


TRINITY\_DN3036\_c0\_g1\_i4.p1

back to list

  

| Protein Coverage
| Supporting Peptides
| Best Unique PSM
|

Protein Coverage:

Supporting Peptides:

| Peptide | Uniq | -10lgP | Mass | Length | ppm | m/z | z | RT | 1/k0 Start | 1/k0 End | Precursor ID | Area Sample 1 | #Feature | #Feature Sample 1 | Start | End | PTM | AScore | Found By |
| --- | --- | --- | --- | --- | --- | --- | --- | --- | --- | --- | --- | --- | --- | --- | --- | --- | --- | --- | --- |
| R.VIGASGIHVGTMSFGK.M | Y | 66.34 | 1559.813 | 16 | -0.2 | 780.9101 | 2 | 16.89 | 1.1037 | 1.111 | 52086 | 2.86e2 | 1 | 1 | 24 | 39 |  |  | DB Search |
| R.VIGASGIHVGTM(+15.99)SFGK.M | Y | 64.9 | 1575.8079 | 16 | 0.52 | 788.9081 | 2 | 15.11 | 1.1262 | 1.1334 | 42748 | 6.68e2 | 1 | 1 | 24 | 39 | Oxidation (M) | M12:Oxidation (M):1000 | DB Search |
| total 2 peptides |  |  |  |  |  |  |  |  |  |  |  |  |  |  |  |  |  |  |  |
| --- | --- | --- | --- | --- | --- | --- | --- | --- | --- | --- | --- | --- | --- | --- | --- | --- | --- | --- | --- |

Best Unique PSM (Scan Sample\_10\_A1\_1\_3647.d:52086, m/z=780.9101, z=2, RT=16.89, ppm=-4.69):


TRINITY\_DN11662\_c0\_g1\_i1.p1

back to list

  

| Protein Coverage
| Supporting Peptides
| Best Unique PSM
|

Protein Coverage:

Supporting Peptides:

| Peptide | Uniq | -10lgP | Mass | Length | ppm | m/z | z | RT | 1/k0 Start | 1/k0 End | Precursor ID | Area Sample 1 | #Feature | #Feature Sample 1 | Start | End | PTM | AScore | Found By |
| --- | --- | --- | --- | --- | --- | --- | --- | --- | --- | --- | --- | --- | --- | --- | --- | --- | --- | --- | --- |
| M.A(+42.01)LSRPVYIIAAK.R | Y | 63.33 | 1342.7972 | 12 | -0.52 | 672.4025 | 2 | 17.88 | 1.0528 | 1.06 | 57936 | 2.31e3 | 1 | 1 | 2 | 13 | Acetylation (Protein N-term) | A1:Acetylation (Protein N-term):1000 | DB Search |
| total 1 peptides |  |  |  |  |  |  |  |  |  |  |  |  |  |  |  |  |  |  |  |
| --- | --- | --- | --- | --- | --- | --- | --- | --- | --- | --- | --- | --- | --- | --- | --- | --- | --- | --- | --- |

Best Unique PSM (Scan Sample\_10\_A1\_1\_3647.d:57936, m/z=672.4025, z=2, RT=17.88, ppm=-5.01):


TRINITY\_DN5633\_c0\_g1\_i1.p1

back to list

  

| Protein Coverage
| Supporting Peptides
| Best Unique PSM
|

Protein Coverage:

Supporting Peptides:

| Peptide | Uniq | -10lgP | Mass | Length | ppm | m/z | z | RT | 1/k0 Start | 1/k0 End | Precursor ID | Area Sample 1 | #Feature | #Feature Sample 1 | Start | End | PTM | AScore | Found By |
| --- | --- | --- | --- | --- | --- | --- | --- | --- | --- | --- | --- | --- | --- | --- | --- | --- | --- | --- | --- |
| R.ELISNSSDALDKIR.F | N | 61.95 | 1559.8155 | 14 | -1.81 | 780.9101 | 2 | 15.46 | 1.0753 | 1.0825 | 44710 | 0 | 0 | 0 | 108 | 121 |  |  | DB Search |
| K.KLLEINVYHPLIQELQK.R | Y | 61.8 | 2077.1935 | 17 | 2.34 | 693.4036 | 3 | 18.48 | 0.9521 | 0.9594 | 61635 | 0 | 0 | 0 | 705 | 721 |  |  | DB Search |
| K.LLEINVYHPLIQELQKR.V | Y | 61.04 | 2105.1997 | 17 | 3.51 | 702.7398 | 3 | 18.83 | 0.9884 | 0.9957 | 63817 | 0 | 0 | 0 | 706 | 722 |  |  | DB Search |
| total 3 peptides |  |  |  |  |  |  |  |  |  |  |  |  |  |  |  |  |  |  |  |
| --- | --- | --- | --- | --- | --- | --- | --- | --- | --- | --- | --- | --- | --- | --- | --- | --- | --- | --- | --- |

Best Unique PSM (Scan Sample\_10\_A1\_1\_3647.d:61635, m/z=693.4036, z=3, RT=18.48, ppm=-2.15):


TRINITY\_DN2477\_c0\_g1\_i10.p1

back to list

  

| Protein Coverage
| Supporting Peptides
| Best Unique PSM
|

Protein Coverage:

Supporting Peptides:

| Peptide | Uniq | -10lgP | Mass | Length | ppm | m/z | z | RT | 1/k0 Start | 1/k0 End | Precursor ID | Area Sample 1 | #Feature | #Feature Sample 1 | Start | End | PTM | AScore | Found By |
| --- | --- | --- | --- | --- | --- | --- | --- | --- | --- | --- | --- | --- | --- | --- | --- | --- | --- | --- | --- |
| K.DSYVGDEAQSKR.G | Y | 66.73 | 1353.616 | 12 | 2.52 | 677.814 | 2 | 10.94 | 1.0069 | 1.0142 | 19045 | 5.51e3 | 1 | 1 | 19 | 30 |  |  | DB Search |
| total 1 peptides |  |  |  |  |  |  |  |  |  |  |  |  |  |  |  |  |  |  |  |
| --- | --- | --- | --- | --- | --- | --- | --- | --- | --- | --- | --- | --- | --- | --- | --- | --- | --- | --- | --- |

Best Unique PSM (Scan Sample\_10\_A1\_1\_3647.d:19045, m/z=677.814, z=2, RT=10.94, ppm=-1.97):


TRINITY\_DN309\_c0\_g1\_i1.p1

back to list

  

| Protein Coverage
| Supporting Peptides
| Best Unique PSM
|

Protein Coverage:

Supporting Peptides:

| Peptide | Uniq | -10lgP | Mass | Length | ppm | m/z | z | RT | 1/k0 Start | 1/k0 End | Precursor ID | Area Sample 1 | #Feature | #Feature Sample 1 | Start | End | PTM | AScore | Found By |
| --- | --- | --- | --- | --- | --- | --- | --- | --- | --- | --- | --- | --- | --- | --- | --- | --- | --- | --- | --- |
| K.VTSQREEDEYVK.A | Y | 64.6 | 1481.6998 | 12 | 3.77 | 741.8566 | 2 | 11.19 | 1.05 | 1.0572 | 20488 | 4.16e2 | 1 | 1 | 20 | 31 |  |  | DB Search |
| total 1 peptides |  |  |  |  |  |  |  |  |  |  |  |  |  |  |  |  |  |  |  |
| --- | --- | --- | --- | --- | --- | --- | --- | --- | --- | --- | --- | --- | --- | --- | --- | --- | --- | --- | --- |

Best Unique PSM (Scan Sample\_10\_A1\_1\_3647.d:20488, m/z=741.8566, z=2, RT=11.19, ppm=-0.72):


TRINITY\_DN161\_c0\_g1\_i1.p1

back to list

  

| Protein Coverage
| Supporting Peptides
| Best Unique PSM
|

Protein Coverage:

Supporting Peptides:

| Peptide | Uniq | -10lgP | Mass | Length | ppm | m/z | z | RT | 1/k0 Start | 1/k0 End | Precursor ID | Area Sample 1 | #Feature | #Feature Sample 1 | Start | End | PTM | AScore | Found By |
| --- | --- | --- | --- | --- | --- | --- | --- | --- | --- | --- | --- | --- | --- | --- | --- | --- | --- | --- | --- |
| R.AVFPSIVGRPR.H | Y | 66.75 | 1197.6982 | 11 | -0.27 | 599.8535 | 2 | 15.64 | 0.9307 | 0.938 | 45498 | 3.34e4 | 1 | 1 | 30 | 40 |  |  | DB Search |
| K.DSYVGDEAQSKR.G | Y | 66.73 | 1353.616 | 12 | 2.52 | 677.814 | 2 | 10.94 | 1.0069 | 1.0142 | 19045 | 5.51e3 | 1 | 1 | 52 | 63 |  |  | DB Search |
| K.SYELPDGQVITIGNER.F | Y | 66.69 | 1789.8846 | 16 | -3.48 | 895.9424 | 2 | 18.53 | 0.9795 | 0.9868 | 61913 | 1.88e3 | 2 | 2 | 240 | 255 |  |  | DB Search |
| R.LDLAGRDLTDYLM(+15.99)K.I | Y | 62.87 | 1638.8287 | 14 | 4.29 | 820.4214 | 2 | 18.15 | 1.143 | 1.1502 | 59617 | 0 | 0 | 0 | 179 | 192 | Oxidation (M) | M13:Oxidation (M):1000 | DB Search |
| R.HQGVM(+15.99)VGM(+15.99)GQK.D | Y | 61.11 | 1202.5536 | 11 | 7.61 | 602.2859 | 2 | 10.70 | 0.9089 | 0.9162 | 17721 | 0 | 0 | 0 | 41 | 51 | Oxidation (M), Oxidation (M) | M5:Oxidation (M):1000 M8:Oxidation (M):1000 | DB Search |
| R.LDLAGRDLTDYLMK.I | Y | 27.85 | 1622.8338 | 14 | -0.39 | 541.9492 | 3 | 19.26 | 0.7974 | 0.8047 | 66371 | 6.87e2 | 1 | 1 | 179 | 192 |  |  | DB Search |
| total 6 peptides |  |  |  |  |  |  |  |  |  |  |  |  |  |  |  |  |  |  |  |
| --- | --- | --- | --- | --- | --- | --- | --- | --- | --- | --- | --- | --- | --- | --- | --- | --- | --- | --- | --- |

Best Unique PSM (Scan Sample\_10\_A1\_1\_3647.d:45498, m/z=599.8535, z=2, RT=15.64, ppm=-4.77):


TRINITY\_DN3078\_c0\_g1\_i1.p1

back to list

  

| Protein Coverage
| Supporting Peptides
| Best Unique PSM
|

Protein Coverage:

Supporting Peptides:

| Peptide | Uniq | -10lgP | Mass | Length | ppm | m/z | z | RT | 1/k0 Start | 1/k0 End | Precursor ID | Area Sample 1 | #Feature | #Feature Sample 1 | Start | End | PTM | AScore | Found By |
| --- | --- | --- | --- | --- | --- | --- | --- | --- | --- | --- | --- | --- | --- | --- | --- | --- | --- | --- | --- |
| K.SGNVVLC(+57.02)LIDVHK.A | Y | 66.77 | 1452.7759 | 13 | -0.46 | 727.3916 | 2 | 18.50 | 1.0391 | 1.0464 | 61767 | 0 | 0 | 0 | 1014 | 1026 | Carbamidomethylation |  | DB Search |
| K.VSQTC(+57.02)AEHVFR.V | Y | 66.57 | 1332.6245 | 11 | 0.3 | 667.3167 | 2 | 11.88 | 0.9832 | 0.9904 | 24552 | 0 | 0 | 0 | 547 | 557 | Carbamidomethylation |  | DB Search |
| total 2 peptides |  |  |  |  |  |  |  |  |  |  |  |  |  |  |  |  |  |  |  |
| --- | --- | --- | --- | --- | --- | --- | --- | --- | --- | --- | --- | --- | --- | --- | --- | --- | --- | --- | --- |

Best Unique PSM (Scan Sample\_10\_A1\_1\_3647.d:61767, m/z=727.3916, z=2, RT=18.50, ppm=-4.96):


TRINITY\_DN478\_c0\_g1\_i2.p1

back to list

  

| Protein Coverage
| Supporting Peptides
| Best Unique PSM
|

Protein Coverage:

Supporting Peptides:

| Peptide | Uniq | -10lgP | Mass | Length | ppm | m/z | z | RT | 1/k0 Start | 1/k0 End | Precursor ID | Area Sample 1 | #Feature | #Feature Sample 1 | Start | End | PTM | AScore | Found By |
| --- | --- | --- | --- | --- | --- | --- | --- | --- | --- | --- | --- | --- | --- | --- | --- | --- | --- | --- | --- |
| R.NIVHGSDSVETAQK.E | Y | 62.14 | 1483.7267 | 14 | -1.27 | 742.8663 | 2 | 11.13 | 0.9993 | 1.0065 | 20026 | 1.18e3 | 1 | 1 | 114 | 127 |  |  | DB Search |
| R.AMLGETKPSESKPGSIR.G | Y | 18.29 | 1786.9247 | 17 | 1.94 | 894.4673 | 2 | 12.10 | 1.1394 | 1.1466 | 25825 | 3.88e2 | 1 | 1 | 88 | 104 |  |  | DB Search |
| total 2 peptides |  |  |  |  |  |  |  |  |  |  |  |  |  |  |  |  |  |  |  |
| --- | --- | --- | --- | --- | --- | --- | --- | --- | --- | --- | --- | --- | --- | --- | --- | --- | --- | --- | --- |

Best Unique PSM (Scan Sample\_10\_A1\_1\_3647.d:20026, m/z=742.8663, z=2, RT=11.13, ppm=-5.76):


TRINITY\_DN118520\_c0\_g1\_i1.p1

back to list

  

| Protein Coverage
| Supporting Peptides
| Best Unique PSM
|

Protein Coverage:

Supporting Peptides:

| Peptide | Uniq | -10lgP | Mass | Length | ppm | m/z | z | RT | 1/k0 Start | 1/k0 End | Precursor ID | Area Sample 1 | #Feature | #Feature Sample 1 | Start | End | PTM | AScore | Found By |
| --- | --- | --- | --- | --- | --- | --- | --- | --- | --- | --- | --- | --- | --- | --- | --- | --- | --- | --- | --- |
| K.AIELPDKYMNIGAK.L | Y | 62.82 | 1561.8174 | 14 | -1.23 | 781.9115 | 2 | 17.04 | 1.0733 | 1.0805 | 52963 | 0 | 0 | 0 | 90 | 103 |  |  | DB Search |
| K.ISNVQDIVPVLELANQHK.R | Y | 61.64 | 2016.1003 | 18 | -1.98 | 673.0364 | 3 | 19.58 | 0.9501 | 0.9574 | 68461 | 5.02e2 | 1 | 1 | 258 | 275 |  |  | DB Search |
| total 2 peptides |  |  |  |  |  |  |  |  |  |  |  |  |  |  |  |  |  |  |  |
| --- | --- | --- | --- | --- | --- | --- | --- | --- | --- | --- | --- | --- | --- | --- | --- | --- | --- | --- | --- |

Best Unique PSM (Scan Sample\_10\_A1\_1\_3647.d:52963, m/z=781.9115, z=2, RT=17.04, ppm=-5.72):


TRINITY\_DN7808\_c4\_g1\_i3.p2

back to list

  

| Protein Coverage
| Supporting Peptides
| Best Unique PSM
|

Protein Coverage:

Supporting Peptides:

| Peptide | Uniq | -10lgP | Mass | Length | ppm | m/z | z | RT | 1/k0 Start | 1/k0 End | Precursor ID | Area Sample 1 | #Feature | #Feature Sample 1 | Start | End | PTM | AScore | Found By |
| --- | --- | --- | --- | --- | --- | --- | --- | --- | --- | --- | --- | --- | --- | --- | --- | --- | --- | --- | --- |
| K.LGLPVFLGGVESKPC(+57.02)DADGSAR.S | Y | 62.56 | 2244.1208 | 22 | 1.07 | 749.045 | 3 | 19.31 | 0.9271 | 0.9344 | 66694 | 2.92e2 | 1 | 1 | 131 | 152 | Carbamidomethylation |  | DB Search |
| total 1 peptides |  |  |  |  |  |  |  |  |  |  |  |  |  |  |  |  |  |  |  |
| --- | --- | --- | --- | --- | --- | --- | --- | --- | --- | --- | --- | --- | --- | --- | --- | --- | --- | --- | --- |

Best Unique PSM (Scan Sample\_10\_A1\_1\_3647.d:66694, m/z=748.05, z=3, RT=19.36, ppm=-3.42):


TRINITY\_DN554\_c0\_g1\_i5.p1

back to list

  

| Protein Coverage
| Supporting Peptides
| Best Unique PSM
|

Protein Coverage:

Supporting Peptides:

| Peptide | Uniq | -10lgP | Mass | Length | ppm | m/z | z | RT | 1/k0 Start | 1/k0 End | Precursor ID | Area Sample 1 | #Feature | #Feature Sample 1 | Start | End | PTM | AScore | Found By |
| --- | --- | --- | --- | --- | --- | --- | --- | --- | --- | --- | --- | --- | --- | --- | --- | --- | --- | --- | --- |
| K.IQDKEGIPPDQQR.L | Y | 65.08 | 1522.7739 | 13 | -0.66 | 762.3903 | 2 | 11.01 | 1.0339 | 1.0411 | 19352 | 9.49e2 | 1 | 1 | 30 | 42 |  |  | DB Search |
| total 1 peptides |  |  |  |  |  |  |  |  |  |  |  |  |  |  |  |  |  |  |  |
| --- | --- | --- | --- | --- | --- | --- | --- | --- | --- | --- | --- | --- | --- | --- | --- | --- | --- | --- | --- |

Best Unique PSM (Scan Sample\_10\_A1\_1\_3647.d:19352, m/z=762.3903, z=2, RT=11.01, ppm=-5.16):


TRINITY\_DN3589\_c0\_g1\_i1.p1

back to list

  

| Protein Coverage
| Supporting Peptides
| Best Unique PSM
|

Protein Coverage:

Supporting Peptides:

| Peptide | Uniq | -10lgP | Mass | Length | ppm | m/z | z | RT | 1/k0 Start | 1/k0 End | Precursor ID | Area Sample 1 | #Feature | #Feature Sample 1 | Start | End | PTM | AScore | Found By |
| --- | --- | --- | --- | --- | --- | --- | --- | --- | --- | --- | --- | --- | --- | --- | --- | --- | --- | --- | --- |
| K.AIGGIILTASHNPGGK.D | Y | 66.51 | 1504.8361 | 16 | 0.62 | 753.4224 | 2 | 15.47 | 1.0961 | 1.1033 | 44709 | 6.94e2 | 1 | 1 | 105 | 120 |  |  | DB Search |
| total 1 peptides |  |  |  |  |  |  |  |  |  |  |  |  |  |  |  |  |  |  |  |
| --- | --- | --- | --- | --- | --- | --- | --- | --- | --- | --- | --- | --- | --- | --- | --- | --- | --- | --- | --- |

Best Unique PSM (Scan Sample\_10\_A1\_1\_3647.d:44709, m/z=753.4224, z=2, RT=15.47, ppm=-3.88):


TRINITY\_DN21847\_c0\_g1\_i1.p1

back to list

  

| Protein Coverage
| Supporting Peptides
| Best Unique PSM
|

Protein Coverage:

Supporting Peptides:

| Peptide | Uniq | -10lgP | Mass | Length | ppm | m/z | z | RT | 1/k0 Start | 1/k0 End | Precursor ID | Area Sample 1 | #Feature | #Feature Sample 1 | Start | End | PTM | AScore | Found By |
| --- | --- | --- | --- | --- | --- | --- | --- | --- | --- | --- | --- | --- | --- | --- | --- | --- | --- | --- | --- |
| K.VLTSSDPAEKEK.K | Y | 66.69 | 1302.6667 | 12 | 3.87 | 652.3402 | 2 | 10.57 | 0.9977 | 1.0049 | 16696 | 7.52e3 | 1 | 1 | 101 | 112 |  |  | DB Search |
| total 1 peptides |  |  |  |  |  |  |  |  |  |  |  |  |  |  |  |  |  |  |  |
| --- | --- | --- | --- | --- | --- | --- | --- | --- | --- | --- | --- | --- | --- | --- | --- | --- | --- | --- | --- |

Best Unique PSM (Scan Sample\_10\_A1\_1\_3647.d:16696, m/z=652.3402, z=2, RT=10.57, ppm=-0.62):


TRINITY\_DN1080\_c0\_g2\_i1.p1

back to list

  

| Protein Coverage
| Supporting Peptides
| Best Unique PSM
|

Protein Coverage:

Supporting Peptides:

| Peptide | Uniq | -10lgP | Mass | Length | ppm | m/z | z | RT | 1/k0 Start | 1/k0 End | Precursor ID | Area Sample 1 | #Feature | #Feature Sample 1 | Start | End | PTM | AScore | Found By |
| --- | --- | --- | --- | --- | --- | --- | --- | --- | --- | --- | --- | --- | --- | --- | --- | --- | --- | --- | --- |
| R.RPVPIVTR.R | Y | 17.05 | 936.5869 | 8 | 4.27 | 469.3006 | 2 | 11.83 | 0.8071 | 0.8144 | 24513 | 2.28e2 | 1 | 1 | 9 | 16 |  |  | DB Search |
| total 1 peptides |  |  |  |  |  |  |  |  |  |  |  |  |  |  |  |  |  |  |  |
| --- | --- | --- | --- | --- | --- | --- | --- | --- | --- | --- | --- | --- | --- | --- | --- | --- | --- | --- | --- |

Best Unique PSM (Scan Sample\_10\_A1\_1\_3647.d:24513, m/z=469.3006, z=2, RT=11.83, ppm=-0.23):


TRINITY\_DN22077\_c0\_g1\_i1.p3

back to list

  

| Protein Coverage
| Supporting Peptides
| Best Unique PSM
|

Protein Coverage:

Supporting Peptides:

| Peptide | Uniq | -10lgP | Mass | Length | ppm | m/z | z | RT | 1/k0 Start | 1/k0 End | Precursor ID | Area Sample 1 | #Feature | #Feature Sample 1 | Start | End | PTM | AScore | Found By |
| --- | --- | --- | --- | --- | --- | --- | --- | --- | --- | --- | --- | --- | --- | --- | --- | --- | --- | --- | --- |
| R.ISGLIYEETRGVLK.S | Y | 62.35 | 1576.8824 | 14 | 2.48 | 789.4469 | 2 | 17.34 | 1.0929 | 1.1001 | 54665 | 0 | 0 | 0 | 94 | 107 |  |  | DB Search |
| total 1 peptides |  |  |  |  |  |  |  |  |  |  |  |  |  |  |  |  |  |  |  |
| --- | --- | --- | --- | --- | --- | --- | --- | --- | --- | --- | --- | --- | --- | --- | --- | --- | --- | --- | --- |

Best Unique PSM (Scan Sample\_10\_A1\_1\_3647.d:54665, m/z=789.4469, z=2, RT=17.34, ppm=-2.01):


TRINITY\_DN15151\_c0\_g1\_i1.p1

back to list

  

| Protein Coverage
| Supporting Peptides
| Best Unique PSM
|

Protein Coverage:

Supporting Peptides:

| Peptide | Uniq | -10lgP | Mass | Length | ppm | m/z | z | RT | 1/k0 Start | 1/k0 End | Precursor ID | Area Sample 1 | #Feature | #Feature Sample 1 | Start | End | PTM | AScore | Found By |
| --- | --- | --- | --- | --- | --- | --- | --- | --- | --- | --- | --- | --- | --- | --- | --- | --- | --- | --- | --- |
| K.AVAAGM(+15.99)NPM(+15.99)DIKR.G | Y | 61.93 | 1404.6853 | 13 | -5.88 | 703.3427 | 2 | 11.20 | 1.0122 | 1.0194 | 20513 | 4.12e2 | 1 | 1 | 145 | 157 | Oxidation (M), Oxidation (M) | M6:Oxidation (M):1000 M9:Oxidation (M):1000 | DB Search |
| total 1 peptides |  |  |  |  |  |  |  |  |  |  |  |  |  |  |  |  |  |  |  |
| --- | --- | --- | --- | --- | --- | --- | --- | --- | --- | --- | --- | --- | --- | --- | --- | --- | --- | --- | --- |

Best Unique PSM (Scan Sample\_10\_A1\_1\_3647.d:20513, m/z=703.3427, z=2, RT=11.20, ppm=-10.38):


TRINITY\_DN165536\_c0\_g1\_i1.p1

back to list

  

| Protein Coverage
| Supporting Peptides
| Best Unique PSM
|

Protein Coverage:

Supporting Peptides:

| Peptide | Uniq | -10lgP | Mass | Length | ppm | m/z | z | RT | 1/k0 Start | 1/k0 End | Precursor ID | Area Sample 1 | #Feature | #Feature Sample 1 | Start | End | PTM | AScore | Found By |
| --- | --- | --- | --- | --- | --- | --- | --- | --- | --- | --- | --- | --- | --- | --- | --- | --- | --- | --- | --- |
| K.ATETATGLATTHPIK.L | Y | 66.51 | 1510.7991 | 15 | 0.02 | 756.4034 | 2 | 12.89 | 1.1053 | 1.1126 | 30404 | 8.59e2 | 1 | 1 | 161 | 175 |  |  | DB Search |
| total 1 peptides |  |  |  |  |  |  |  |  |  |  |  |  |  |  |  |  |  |  |  |
| --- | --- | --- | --- | --- | --- | --- | --- | --- | --- | --- | --- | --- | --- | --- | --- | --- | --- | --- | --- |

Best Unique PSM (Scan Sample\_10\_A1\_1\_3647.d:30404, m/z=756.4034, z=2, RT=12.89, ppm=-4.47):


TRINITY\_DN7808\_c1\_g3\_i3.p1

back to list

  

| Protein Coverage
| Supporting Peptides
| Best Unique PSM
|

Protein Coverage:

Supporting Peptides:

| Peptide | Uniq | -10lgP | Mass | Length | ppm | m/z | z | RT | 1/k0 Start | 1/k0 End | Precursor ID | Area Sample 1 | #Feature | #Feature Sample 1 | Start | End | PTM | AScore | Found By |
| --- | --- | --- | --- | --- | --- | --- | --- | --- | --- | --- | --- | --- | --- | --- | --- | --- | --- | --- | --- |
| K.AITSSDPEVLKR.K | Y | 62.01 | 1314.7143 | 12 | -5.42 | 658.3579 | 2 | 12.95 | 0.9844 | 0.9916 | 30804 | 0 | 0 | 0 | 114 | 125 |  |  | DB Search |
| total 1 peptides |  |  |  |  |  |  |  |  |  |  |  |  |  |  |  |  |  |  |  |
| --- | --- | --- | --- | --- | --- | --- | --- | --- | --- | --- | --- | --- | --- | --- | --- | --- | --- | --- | --- |

Best Unique PSM (Scan Sample\_10\_A1\_1\_3647.d:30804, m/z=658.3579, z=2, RT=12.95, ppm=-9.91):


TRINITY\_DN56792\_c0\_g1\_i1.p1

back to list

  

| Protein Coverage
| Supporting Peptides
| Best Unique PSM
|

Protein Coverage:

Supporting Peptides:

| Peptide | Uniq | -10lgP | Mass | Length | ppm | m/z | z | RT | 1/k0 Start | 1/k0 End | Precursor ID | Area Sample 1 | #Feature | #Feature Sample 1 | Start | End | PTM | AScore | Found By |
| --- | --- | --- | --- | --- | --- | --- | --- | --- | --- | --- | --- | --- | --- | --- | --- | --- | --- | --- | --- |
| R.LAIDHAVSLITNIK.C | Y | 66.67 | 1506.8769 | 14 | 0.6 | 754.4428 | 2 | 19.10 | 1.0761 | 1.0833 | 65382 | 4.12e2 | 1 | 1 | 211 | 224 |  |  | DB Search |
| total 1 peptides |  |  |  |  |  |  |  |  |  |  |  |  |  |  |  |  |  |  |  |
| --- | --- | --- | --- | --- | --- | --- | --- | --- | --- | --- | --- | --- | --- | --- | --- | --- | --- | --- | --- |

Best Unique PSM (Scan Sample\_10\_A1\_1\_3647.d:65382, m/z=754.4428, z=2, RT=19.10, ppm=-3.9):


TRINITY\_DN1339\_c0\_g2\_i1.p2

back to list

  

| Protein Coverage
| Supporting Peptides
| Best Unique PSM
|

Protein Coverage:

Supporting Peptides:

| Peptide | Uniq | -10lgP | Mass | Length | ppm | m/z | z | RT | 1/k0 Start | 1/k0 End | Precursor ID | Area Sample 1 | #Feature | #Feature Sample 1 | Start | End | PTM | AScore | Found By |
| --- | --- | --- | --- | --- | --- | --- | --- | --- | --- | --- | --- | --- | --- | --- | --- | --- | --- | --- | --- |
| K.LLTSSDQAELTKK.L | Y | 66.26 | 1432.7773 | 13 | 0.59 | 717.3931 | 2 | 12.73 | 1.0504 | 1.0576 | 29502 | 1.34e3 | 1 | 1 | 155 | 167 |  |  | DB Search |
| total 1 peptides |  |  |  |  |  |  |  |  |  |  |  |  |  |  |  |  |  |  |  |
| --- | --- | --- | --- | --- | --- | --- | --- | --- | --- | --- | --- | --- | --- | --- | --- | --- | --- | --- | --- |

Best Unique PSM (Scan Sample\_10\_A1\_1\_3647.d:29502, m/z=717.3931, z=2, RT=12.73, ppm=-3.91):


TRINITY\_DN244\_c0\_g1\_i1.p1

back to list

  

| Protein Coverage
| Supporting Peptides
| Best Unique PSM
|

Protein Coverage:

Supporting Peptides:

| Peptide | Uniq | -10lgP | Mass | Length | ppm | m/z | z | RT | 1/k0 Start | 1/k0 End | Precursor ID | Area Sample 1 | #Feature | #Feature Sample 1 | Start | End | PTM | AScore | Found By |
| --- | --- | --- | --- | --- | --- | --- | --- | --- | --- | --- | --- | --- | --- | --- | --- | --- | --- | --- | --- |
| K.DLSLVVEPLKNYGVK.I | Y | 63.33 | 1672.9399 | 15 | 2.59 | 837.4756 | 2 | 19.05 | 1.1346 | 1.1418 | 65061 | 1.67e2 | 1 | 1 | 392 | 406 |  |  | DB Search |
| total 1 peptides |  |  |  |  |  |  |  |  |  |  |  |  |  |  |  |  |  |  |  |
| --- | --- | --- | --- | --- | --- | --- | --- | --- | --- | --- | --- | --- | --- | --- | --- | --- | --- | --- | --- |

Best Unique PSM (Scan Sample\_10\_A1\_1\_3647.d:65061, m/z=837.4756, z=2, RT=19.05, ppm=-1.9):


TRINITY\_DN477\_c0\_g1\_i8.p1

back to list

  

| Protein Coverage
| Supporting Peptides
| Best Unique PSM
|

Protein Coverage:

Supporting Peptides:

| Peptide | Uniq | -10lgP | Mass | Length | ppm | m/z | z | RT | 1/k0 Start | 1/k0 End | Precursor ID | Area Sample 1 | #Feature | #Feature Sample 1 | Start | End | PTM | AScore | Found By |
| --- | --- | --- | --- | --- | --- | --- | --- | --- | --- | --- | --- | --- | --- | --- | --- | --- | --- | --- | --- |
| R.ILQC(+57.02)NQYANVEEAR.N | Y | 66.82 | 1706.8046 | 14 | -0.34 | 854.4055 | 2 | 14.19 | 1.0693 | 1.0765 | 37690 | 1.33e3 | 1 | 1 | 86 | 99 | Carbamidomethylation |  | DB Search |
| K.DKNILVLTLGVGPQVNR.N | Y | 66.34 | 1835.0628 | 17 | -1.89 | 918.5328 | 2 | 19.38 | 1.1326 | 1.1398 | 66901 | 2.29e3 | 2 | 2 | 168 | 184 |  |  | DB Search |
| R.SQSAFPTRR.R | Y | 31.06 | 1048.5414 | 9 | 5.06 | 525.2783 | 2 | 10.99 | 0.8433 | 0.8506 | 19385 | 6.25e2 | 1 | 1 | 856 | 864 |  |  | DB Search |
| total 3 peptides |  |  |  |  |  |  |  |  |  |  |  |  |  |  |  |  |  |  |  |
| --- | --- | --- | --- | --- | --- | --- | --- | --- | --- | --- | --- | --- | --- | --- | --- | --- | --- | --- | --- |

Best Unique PSM (Scan Sample\_10\_A1\_1\_3647.d:37690, m/z=854.4055, z=2, RT=14.19, ppm=-4.83):


TRINITY\_DN1397\_c0\_g1\_i1.p1

back to list

  

| Protein Coverage
| Supporting Peptides
| Best Unique PSM
|

Protein Coverage:

Supporting Peptides:

| Peptide | Uniq | -10lgP | Mass | Length | ppm | m/z | z | RT | 1/k0 Start | 1/k0 End | Precursor ID | Area Sample 1 | #Feature | #Feature Sample 1 | Start | End | PTM | AScore | Found By |
| --- | --- | --- | --- | --- | --- | --- | --- | --- | --- | --- | --- | --- | --- | --- | --- | --- | --- | --- | --- |
| K.NEFRLPVVQK.M | Y | 17.17 | 1228.6928 | 10 | -5.88 | 615.3473 | 2 | 17.44 | 0.9327 | 0.94 | 55344 | 4.76e2 | 1 | 1 | 58 | 67 |  |  | DB Search |
| total 1 peptides |  |  |  |  |  |  |  |  |  |  |  |  |  |  |  |  |  |  |  |
| --- | --- | --- | --- | --- | --- | --- | --- | --- | --- | --- | --- | --- | --- | --- | --- | --- | --- | --- | --- |

Best Unique PSM (Scan Sample\_10\_A1\_1\_3647.d:55344, m/z=615.3473, z=2, RT=17.44, ppm=-10.38):


TRINITY\_DN6920\_c0\_g1\_i4.p1

back to list

  

| Protein Coverage
| Supporting Peptides
| Best Unique PSM
|

Protein Coverage:

Supporting Peptides:

| Peptide | Uniq | -10lgP | Mass | Length | ppm | m/z | z | RT | 1/k0 Start | 1/k0 End | Precursor ID | Area Sample 1 | #Feature | #Feature Sample 1 | Start | End | PTM | AScore | Found By |
| --- | --- | --- | --- | --- | --- | --- | --- | --- | --- | --- | --- | --- | --- | --- | --- | --- | --- | --- | --- |
| R.KVAC(+57.02)IGAWHPAR.V | Y | 60.86 | 1364.7135 | 12 | 9.11 | 683.3672 | 2 | 13.20 | 0.9711 | 0.9783 | 32100 | 9.16e2 | 1 | 1 | 128 | 139 | Carbamidomethylation |  | DB Search |
| total 1 peptides |  |  |  |  |  |  |  |  |  |  |  |  |  |  |  |  |  |  |  |
| --- | --- | --- | --- | --- | --- | --- | --- | --- | --- | --- | --- | --- | --- | --- | --- | --- | --- | --- | --- |

Best Unique PSM (Scan Sample\_10\_A1\_1\_3647.d:32100, m/z=683.3672, z=2, RT=13.20, ppm=4.62):


TRINITY\_DN5107\_c0\_g1\_i3.p1

back to list

  

| Protein Coverage
| Supporting Peptides
| Best Unique PSM
|

Protein Coverage:

Supporting Peptides:

| Peptide | Uniq | -10lgP | Mass | Length | ppm | m/z | z | RT | 1/k0 Start | 1/k0 End | Precursor ID | Area Sample 1 | #Feature | #Feature Sample 1 | Start | End | PTM | AScore | Found By |
| --- | --- | --- | --- | --- | --- | --- | --- | --- | --- | --- | --- | --- | --- | --- | --- | --- | --- | --- | --- |
| K.KLDYVLGLTTAK.I | Y | 63.57 | 1320.7653 | 12 | -2.37 | 661.3854 | 2 | 16.69 | 0.9908 | 0.9981 | 51148 | 2.13e2 | 1 | 1 | 93 | 104 |  |  | DB Search |
| total 1 peptides |  |  |  |  |  |  |  |  |  |  |  |  |  |  |  |  |  |  |  |
| --- | --- | --- | --- | --- | --- | --- | --- | --- | --- | --- | --- | --- | --- | --- | --- | --- | --- | --- | --- |

Best Unique PSM (Scan Sample\_10\_A1\_1\_3647.d:51148, m/z=661.3854, z=2, RT=16.69, ppm=-6.87):


TRINITY\_DN200\_c0\_g1\_i1.p1

back to list

  

| Protein Coverage
| Supporting Peptides
| Best Unique PSM
|

Protein Coverage:

Supporting Peptides:

| Peptide | Uniq | -10lgP | Mass | Length | ppm | m/z | z | RT | 1/k0 Start | 1/k0 End | Precursor ID | Area Sample 1 | #Feature | #Feature Sample 1 | Start | End | PTM | AScore | Found By |
| --- | --- | --- | --- | --- | --- | --- | --- | --- | --- | --- | --- | --- | --- | --- | --- | --- | --- | --- | --- |
| K.TAIAIDTIINQKR.F | Y | 64.76 | 1455.8409 | 13 | -0.36 | 728.9242 | 2 | 16.58 | 1.0588 | 1.066 | 50506 | 4.46e2 | 1 | 1 | 217 | 229 |  |  | DB Search |
| total 1 peptides |  |  |  |  |  |  |  |  |  |  |  |  |  |  |  |  |  |  |  |
| --- | --- | --- | --- | --- | --- | --- | --- | --- | --- | --- | --- | --- | --- | --- | --- | --- | --- | --- | --- |

Best Unique PSM (Scan Sample\_10\_A1\_1\_3647.d:50506, m/z=728.9242, z=2, RT=16.58, ppm=-4.85):


TRINITY\_DN16548\_c0\_g1\_i1.p1

back to list

  

| Protein Coverage
| Supporting Peptides
| Best Unique PSM
|

Protein Coverage:

Supporting Peptides:

| Peptide | Uniq | -10lgP | Mass | Length | ppm | m/z | z | RT | 1/k0 Start | 1/k0 End | Precursor ID | Area Sample 1 | #Feature | #Feature Sample 1 | Start | End | PTM | AScore | Found By |
| --- | --- | --- | --- | --- | --- | --- | --- | --- | --- | --- | --- | --- | --- | --- | --- | --- | --- | --- | --- |
| K.APGDYGWRPPLLATDDAALK.T | Y | 61.97 | 2126.0796 | 20 | -2.01 | 709.6959 | 3 | 18.98 | 0.9081 | 0.9154 | 64807 | 3.62e3 | 1 | 1 | 73 | 92 |  |  | DB Search |
| total 1 peptides |  |  |  |  |  |  |  |  |  |  |  |  |  |  |  |  |  |  |  |
| --- | --- | --- | --- | --- | --- | --- | --- | --- | --- | --- | --- | --- | --- | --- | --- | --- | --- | --- | --- |

Best Unique PSM (Scan Sample\_10\_A1\_1\_3647.d:64807, m/z=709.6959, z=3, RT=18.98, ppm=-6.51):


TRINITY\_DN433\_c0\_g2\_i1.p1

back to list

  

| Protein Coverage
| Supporting Peptides
| Best Unique PSM
|

Protein Coverage:

Supporting Peptides:

| Peptide | Uniq | -10lgP | Mass | Length | ppm | m/z | z | RT | 1/k0 Start | 1/k0 End | Precursor ID | Area Sample 1 | #Feature | #Feature Sample 1 | Start | End | PTM | AScore | Found By |
| --- | --- | --- | --- | --- | --- | --- | --- | --- | --- | --- | --- | --- | --- | --- | --- | --- | --- | --- | --- |
| K.AVLTDLRR.M | Y | 22.55 | 942.561 | 8 | -1.62 | 472.2849 | 2 | 13.15 | 0.84 | 0.8473 | 31971 | 4.27e2 | 1 | 1 | 113 | 120 |  |  | DB Search |
| total 1 peptides |  |  |  |  |  |  |  |  |  |  |  |  |  |  |  |  |  |  |  |
| --- | --- | --- | --- | --- | --- | --- | --- | --- | --- | --- | --- | --- | --- | --- | --- | --- | --- | --- | --- |

Best Unique PSM (Scan Sample\_10\_A1\_1\_3647.d:31971, m/z=472.2849, z=2, RT=13.15, ppm=-6.12):


TRINITY\_DN7808\_c2\_g1\_i2.p1

back to list

  

| Protein Coverage
| Supporting Peptides
| Best Unique PSM
|

Protein Coverage:

Supporting Peptides:

| Peptide | Uniq | -10lgP | Mass | Length | ppm | m/z | z | RT | 1/k0 Start | 1/k0 End | Precursor ID | Area Sample 1 | #Feature | #Feature Sample 1 | Start | End | PTM | AScore | Found By |
| --- | --- | --- | --- | --- | --- | --- | --- | --- | --- | --- | --- | --- | --- | --- | --- | --- | --- | --- | --- |
| R.RSLNAEIANGR.L | Y | 51.82 | 1199.6371 | 11 | 2.77 | 600.8248 | 2 | 11.99 | 0.9259 | 0.9331 | 25198 | 6.38e2 | 1 | 1 | 177 | 187 |  |  | DB Search |
| total 1 peptides |  |  |  |  |  |  |  |  |  |  |  |  |  |  |  |  |  |  |  |
| --- | --- | --- | --- | --- | --- | --- | --- | --- | --- | --- | --- | --- | --- | --- | --- | --- | --- | --- | --- |

Best Unique PSM (Scan Sample\_10\_A1\_1\_3647.d:25198, m/z=600.8248, z=2, RT=11.99, ppm=-1.73):


TRINITY\_DN4497\_c0\_g2\_i1.p1

back to list

  

| Protein Coverage
| Supporting Peptides
| Best Unique PSM
|

Protein Coverage:

Supporting Peptides:

| Peptide | Uniq | -10lgP | Mass | Length | ppm | m/z | z | RT | 1/k0 Start | 1/k0 End | Precursor ID | Area Sample 1 | #Feature | #Feature Sample 1 | Start | End | PTM | AScore | Found By |
| --- | --- | --- | --- | --- | --- | --- | --- | --- | --- | --- | --- | --- | --- | --- | --- | --- | --- | --- | --- |
| K.VLTSSDPAEKEK.K | Y | 66.69 | 1302.6667 | 12 | 3.87 | 652.3402 | 2 | 10.57 | 0.9977 | 1.0049 | 16696 | 7.52e3 | 1 | 1 | 126 | 137 |  |  | DB Search |
| total 1 peptides |  |  |  |  |  |  |  |  |  |  |  |  |  |  |  |  |  |  |  |
| --- | --- | --- | --- | --- | --- | --- | --- | --- | --- | --- | --- | --- | --- | --- | --- | --- | --- | --- | --- |

Best Unique PSM (Scan Sample\_10\_A1\_1\_3647.d:16696, m/z=652.3402, z=2, RT=10.57, ppm=-0.62):


TRINITY\_DN998\_c0\_g1\_i6.p2

back to list

  

| Protein Coverage
| Supporting Peptides
| Best Unique PSM
|

Protein Coverage:

Supporting Peptides:

| Peptide | Uniq | -10lgP | Mass | Length | ppm | m/z | z | RT | 1/k0 Start | 1/k0 End | Precursor ID | Area Sample 1 | #Feature | #Feature Sample 1 | Start | End | PTM | AScore | Found By |
| --- | --- | --- | --- | --- | --- | --- | --- | --- | --- | --- | --- | --- | --- | --- | --- | --- | --- | --- | --- |
| K.YANFLDQALREDLER.M | Y | 61.79 | 1851.9115 | 15 | -3.63 | 618.3061 | 3 | 19.80 | 0.8741 | 0.8814 | 69772 | 0 | 0 | 0 | 124 | 138 |  |  | DB Search |
| total 1 peptides |  |  |  |  |  |  |  |  |  |  |  |  |  |  |  |  |  |  |  |
| --- | --- | --- | --- | --- | --- | --- | --- | --- | --- | --- | --- | --- | --- | --- | --- | --- | --- | --- | --- |

Best Unique PSM (Scan Sample\_10\_A1\_1\_3647.d:69772, m/z=618.3061, z=3, RT=19.80, ppm=-8.12):


TRINITY\_DN817\_c0\_g1\_i8.p1

back to list

  

| Protein Coverage
| Supporting Peptides
| Best Unique PSM
|

Protein Coverage:

Supporting Peptides:

| Peptide | Uniq | -10lgP | Mass | Length | ppm | m/z | z | RT | 1/k0 Start | 1/k0 End | Precursor ID | Area Sample 1 | #Feature | #Feature Sample 1 | Start | End | PTM | AScore | Found By |
| --- | --- | --- | --- | --- | --- | --- | --- | --- | --- | --- | --- | --- | --- | --- | --- | --- | --- | --- | --- |
| R.LC(+57.02)QPGYSTC(+57.02)ENHC(+57.02)R.R | Y | 62.01 | 1780.708 | 14 | 5.39 | 891.3621 | 2 | 11.41 | 1.0893 | 1.0965 | 21785 | 1.84e2 | 1 | 1 | 91 | 104 | Carbamidomethylation, Carbamidomethylation, Carbamidomethylation |  | DB Search |
| total 1 peptides |  |  |  |  |  |  |  |  |  |  |  |  |  |  |  |  |  |  |  |
| --- | --- | --- | --- | --- | --- | --- | --- | --- | --- | --- | --- | --- | --- | --- | --- | --- | --- | --- | --- |

Best Unique PSM (Scan Sample\_10\_A1\_1\_3647.d:21785, m/z=891.3621, z=2, RT=11.41, ppm=0.9):


TRINITY\_DN173718\_c0\_g1\_i1.p1

back to list

  

| Protein Coverage
| Supporting Peptides
| Best Unique PSM
|

Protein Coverage:

Supporting Peptides:

| Peptide | Uniq | -10lgP | Mass | Length | ppm | m/z | z | RT | 1/k0 Start | 1/k0 End | Precursor ID | Area Sample 1 | #Feature | #Feature Sample 1 | Start | End | PTM | AScore | Found By |
| --- | --- | --- | --- | --- | --- | --- | --- | --- | --- | --- | --- | --- | --- | --- | --- | --- | --- | --- | --- |
| K.IQDKEGIPPDQQR.L | Y | 65.08 | 1522.7739 | 13 | -0.66 | 762.3903 | 2 | 11.01 | 1.0339 | 1.0411 | 19352 | 9.49e2 | 1 | 1 | 30 | 42 |  |  | DB Search |
| total 1 peptides |  |  |  |  |  |  |  |  |  |  |  |  |  |  |  |  |  |  |  |
| --- | --- | --- | --- | --- | --- | --- | --- | --- | --- | --- | --- | --- | --- | --- | --- | --- | --- | --- | --- |

Best Unique PSM (Scan Sample\_10\_A1\_1\_3647.d:19352, m/z=762.3903, z=2, RT=11.01, ppm=-5.16):


TRINITY\_DN1339\_c0\_g2\_i5.p1

back to list

  

| Protein Coverage
| Supporting Peptides
| Best Unique PSM
|

Protein Coverage:

Supporting Peptides:

| Peptide | Uniq | -10lgP | Mass | Length | ppm | m/z | z | RT | 1/k0 Start | 1/k0 End | Precursor ID | Area Sample 1 | #Feature | #Feature Sample 1 | Start | End | PTM | AScore | Found By |
| --- | --- | --- | --- | --- | --- | --- | --- | --- | --- | --- | --- | --- | --- | --- | --- | --- | --- | --- | --- |
| K.LLTSSDQGELTKK.L | Y | 66.63 | 1418.7616 | 13 | 2.72 | 710.3868 | 2 | 12.36 | 1.0383 | 1.0456 | 27367 | 0 | 0 | 0 | 155 | 167 |  |  | DB Search |
| total 1 peptides |  |  |  |  |  |  |  |  |  |  |  |  |  |  |  |  |  |  |  |
| --- | --- | --- | --- | --- | --- | --- | --- | --- | --- | --- | --- | --- | --- | --- | --- | --- | --- | --- | --- |

Best Unique PSM (Scan Sample\_10\_A1\_1\_3647.d:27367, m/z=710.3868, z=2, RT=12.36, ppm=-1.78):


TRINITY\_DN4815\_c0\_g1\_i1.p1

back to list

  

| Protein Coverage
| Supporting Peptides
| Best Unique PSM
|

Protein Coverage:

Supporting Peptides:

| Peptide | Uniq | -10lgP | Mass | Length | ppm | m/z | z | RT | 1/k0 Start | 1/k0 End | Precursor ID | Area Sample 1 | #Feature | #Feature Sample 1 | Start | End | PTM | AScore | Found By |
| --- | --- | --- | --- | --- | --- | --- | --- | --- | --- | --- | --- | --- | --- | --- | --- | --- | --- | --- | --- |
| K.TIKDVNC(+57.02)NNNQLK.S | Y | 47.49 | 1559.7726 | 13 | 8.47 | 780.8967 | 2 | 11.21 | 1.05 | 1.0572 | 20684 | 1.74e2 | 1 | 1 | 490 | 502 | Carbamidomethylation |  | DB Search |
| total 1 peptides |  |  |  |  |  |  |  |  |  |  |  |  |  |  |  |  |  |  |  |
| --- | --- | --- | --- | --- | --- | --- | --- | --- | --- | --- | --- | --- | --- | --- | --- | --- | --- | --- | --- |

Best Unique PSM (Scan Sample\_10\_A1\_1\_3647.d:20684, m/z=780.8967, z=2, RT=11.21, ppm=3.97):


TRINITY\_DN899\_c0\_g1\_i1.p1

back to list

  

| Protein Coverage
| Supporting Peptides
| Best Unique PSM
|

Protein Coverage:

Supporting Peptides:

| Peptide | Uniq | -10lgP | Mass | Length | ppm | m/z | z | RT | 1/k0 Start | 1/k0 End | Precursor ID | Area Sample 1 | #Feature | #Feature Sample 1 | Start | End | PTM | AScore | Found By |
| --- | --- | --- | --- | --- | --- | --- | --- | --- | --- | --- | --- | --- | --- | --- | --- | --- | --- | --- | --- |
| K.KLPEGATC(+57.02)AM(+15.99)KK.Q | Y | 29.28 | 1348.6843 | 12 | 0.14 | 450.5667 | 3 | 9.88 | 0.7974 | 0.8047 | 12910 | 0 | 0 | 0 | 181 | 192 | Carbamidomethylation, Oxidation (M) | M10:Oxidation (M):1000 | DB Search |
| K.LPEGATC(+57.02)AM(+15.99)KK.Q | Y | 22.51 | 1220.5893 | 11 | -2.49 | 611.2977 | 2 | 11.46 | 0.9586 | 0.9658 | 22172 | 0 | 0 | 0 | 182 | 192 | Carbamidomethylation, Oxidation (M) | M9:Oxidation (M):1000 | DB Search |
| total 2 peptides |  |  |  |  |  |  |  |  |  |  |  |  |  |  |  |  |  |  |  |
| --- | --- | --- | --- | --- | --- | --- | --- | --- | --- | --- | --- | --- | --- | --- | --- | --- | --- | --- | --- |

Best Unique PSM (Scan Sample\_10\_A1\_1\_3647.d:12910, m/z=450.5667, z=3, RT=9.88, ppm=-4.35):


TRINITY\_DN1339\_c6\_g1\_i2.p1

back to list

  

| Protein Coverage
| Supporting Peptides
| Best Unique PSM
|

Protein Coverage:

Supporting Peptides:

| Peptide | Uniq | -10lgP | Mass | Length | ppm | m/z | z | RT | 1/k0 Start | 1/k0 End | Precursor ID | Area Sample 1 | #Feature | #Feature Sample 1 | Start | End | PTM | AScore | Found By |
| --- | --- | --- | --- | --- | --- | --- | --- | --- | --- | --- | --- | --- | --- | --- | --- | --- | --- | --- | --- |
| K.VITSDDPAEKEK.K | Y | 66.7 | 1330.6616 | 12 | 2.85 | 666.337 | 2 | 10.71 | 0.9836 | 0.9908 | 17367 | 7.2e3 | 1 | 1 | 355 | 366 |  |  | DB Search |
| total 1 peptides |  |  |  |  |  |  |  |  |  |  |  |  |  |  |  |  |  |  |  |
| --- | --- | --- | --- | --- | --- | --- | --- | --- | --- | --- | --- | --- | --- | --- | --- | --- | --- | --- | --- |

Best Unique PSM (Scan Sample\_10\_A1\_1\_3647.d:17367, m/z=666.337, z=2, RT=10.71, ppm=-1.65):


TRINITY\_DN2217\_c0\_g1\_i3.p1

back to list

  

| Protein Coverage
| Supporting Peptides
| Best Unique PSM
|

Protein Coverage:

Supporting Peptides:

| Peptide | Uniq | -10lgP | Mass | Length | ppm | m/z | z | RT | 1/k0 Start | 1/k0 End | Precursor ID | Area Sample 1 | #Feature | #Feature Sample 1 | Start | End | PTM | AScore | Found By |
| --- | --- | --- | --- | --- | --- | --- | --- | --- | --- | --- | --- | --- | --- | --- | --- | --- | --- | --- | --- |
| K.ATGC(+57.02)EVIWNQHNR.G | Y | 66.64 | 1583.7263 | 13 | 0.95 | 792.8676 | 2 | 13.84 | 1.0785 | 1.0857 | 35783 | 1.6e3 | 1 | 1 | 431 | 443 | Carbamidomethylation |  | DB Search |
| R.GC(+57.02)YIHTQEIAR.G | Y | 66.57 | 1346.6401 | 11 | -0.12 | 674.3242 | 2 | 12.88 | 1.0158 | 1.023 | 30410 | 6.22e2 | 1 | 1 | 538 | 548 | Carbamidomethylation |  | DB Search |
| R.GC(+57.02)YIHTQEVAR.G | N | 61.54 | 1332.6245 | 11 | -1.99 | 667.3152 | 2 | 12.14 | 1.0086 | 1.0158 | 26094 | 0 | 0 | 0 | 350 | 360 | Carbamidomethylation |  | DB Search |
| R.HFC(+57.02)WVFSK.C | Y | 41.91 | 1109.5117 | 8 | -7.68 | 555.7563 | 2 | 19.76 | 0.8712 | 0.8785 | 69479 | 2.04e2 | 1 | 1 | 367 | 374 | Carbamidomethylation |  | DB Search |
| total 4 peptides |  |  |  |  |  |  |  |  |  |  |  |  |  |  |  |  |  |  |  |
| --- | --- | --- | --- | --- | --- | --- | --- | --- | --- | --- | --- | --- | --- | --- | --- | --- | --- | --- | --- |

Best Unique PSM (Scan Sample\_10\_A1\_1\_3647.d:35783, m/z=792.8676, z=2, RT=13.84, ppm=-3.55):


TRINITY\_DN7808\_c2\_g1\_i3.p1

back to list

  

| Protein Coverage
| Supporting Peptides
| Best Unique PSM
|

Protein Coverage:

Supporting Peptides:

| Peptide | Uniq | -10lgP | Mass | Length | ppm | m/z | z | RT | 1/k0 Start | 1/k0 End | Precursor ID | Area Sample 1 | #Feature | #Feature Sample 1 | Start | End | PTM | AScore | Found By |
| --- | --- | --- | --- | --- | --- | --- | --- | --- | --- | --- | --- | --- | --- | --- | --- | --- | --- | --- | --- |
| R.RSLNAEIANGR.L | Y | 51.82 | 1199.6371 | 11 | 2.77 | 600.8248 | 2 | 11.99 | 0.9259 | 0.9331 | 25198 | 6.38e2 | 1 | 1 | 194 | 204 |  |  | DB Search |
| total 1 peptides |  |  |  |  |  |  |  |  |  |  |  |  |  |  |  |  |  |  |  |
| --- | --- | --- | --- | --- | --- | --- | --- | --- | --- | --- | --- | --- | --- | --- | --- | --- | --- | --- | --- |

Best Unique PSM (Scan Sample\_10\_A1\_1\_3647.d:25198, m/z=600.8248, z=2, RT=11.99, ppm=-1.73):


TRINITY\_DN5023\_c0\_g1\_i3.p2

back to list

  

| Protein Coverage
| Supporting Peptides
| Best Unique PSM
|

Protein Coverage:

Supporting Peptides:

| Peptide | Uniq | -10lgP | Mass | Length | ppm | m/z | z | RT | 1/k0 Start | 1/k0 End | Precursor ID | Area Sample 1 | #Feature | #Feature Sample 1 | Start | End | PTM | AScore | Found By |
| --- | --- | --- | --- | --- | --- | --- | --- | --- | --- | --- | --- | --- | --- | --- | --- | --- | --- | --- | --- |
| R.YRPGTVALR.E | Y | 66.57 | 1031.5876 | 9 | 0.86 | 516.7992 | 2 | 12.50 | 0.8554 | 0.8627 | 28119 | 7.47e2 | 1 | 1 | 78 | 86 |  |  | DB Search |
| total 1 peptides |  |  |  |  |  |  |  |  |  |  |  |  |  |  |  |  |  |  |  |
| --- | --- | --- | --- | --- | --- | --- | --- | --- | --- | --- | --- | --- | --- | --- | --- | --- | --- | --- | --- |

Best Unique PSM (Scan Sample\_10\_A1\_1\_3647.d:28119, m/z=516.7992, z=2, RT=12.50, ppm=-3.64):


TRINITY\_DN11097\_c0\_g1\_i1.p1

back to list

  

| Protein Coverage
| Supporting Peptides
| Best Unique PSM
|

Protein Coverage:

Supporting Peptides:

| Peptide | Uniq | -10lgP | Mass | Length | ppm | m/z | z | RT | 1/k0 Start | 1/k0 End | Precursor ID | Area Sample 1 | #Feature | #Feature Sample 1 | Start | End | PTM | AScore | Found By |
| --- | --- | --- | --- | --- | --- | --- | --- | --- | --- | --- | --- | --- | --- | --- | --- | --- | --- | --- | --- |
| R.VAHAGLTVAEFFR.D | Y | 66.78 | 1416.7513 | 13 | 2.84 | 709.3818 | 2 | 18.86 | 1.0267 | 1.0339 | 63839 | 8.41e2 | 1 | 1 | 493 | 505 |  |  | DB Search |
| K.LRVDLSLFETGIK.V | Y | 25.55 | 1489.8504 | 13 | 2.4 | 745.9309 | 2 | 19.94 | 1.0407 | 1.048 | 70562 | 4.53e2 | 1 | 1 | 362 | 374 |  |  | DB Search |
| total 2 peptides |  |  |  |  |  |  |  |  |  |  |  |  |  |  |  |  |  |  |  |
| --- | --- | --- | --- | --- | --- | --- | --- | --- | --- | --- | --- | --- | --- | --- | --- | --- | --- | --- | --- |

Best Unique PSM (Scan Sample\_10\_A1\_1\_3647.d:63839, m/z=709.3818, z=2, RT=18.86, ppm=-1.65):


TRINITY\_DN140996\_c0\_g1\_i1.p1

back to list

  

| Protein Coverage
| Supporting Peptides
| Best Unique PSM
|

Protein Coverage:

Supporting Peptides:

| Peptide | Uniq | -10lgP | Mass | Length | ppm | m/z | z | RT | 1/k0 Start | 1/k0 End | Precursor ID | Area Sample 1 | #Feature | #Feature Sample 1 | Start | End | PTM | AScore | Found By |
| --- | --- | --- | --- | --- | --- | --- | --- | --- | --- | --- | --- | --- | --- | --- | --- | --- | --- | --- | --- |
| R.RC(+57.02)QFVNR.C | Y | 20.95 | 978.4818 | 7 | -6.21 | 490.2429 | 2 | 11.89 | 0.8079 | 0.8153 | 24685 | 0 | 0 | 0 | 53 | 59 | Carbamidomethylation |  | DB Search |
| total 1 peptides |  |  |  |  |  |  |  |  |  |  |  |  |  |  |  |  |  |  |  |
| --- | --- | --- | --- | --- | --- | --- | --- | --- | --- | --- | --- | --- | --- | --- | --- | --- | --- | --- | --- |

Best Unique PSM (Scan Sample\_10\_A1\_1\_3647.d:24685, m/z=490.2429, z=2, RT=11.89, ppm=-10.71):


TRINITY\_DN7808\_c1\_g3\_i1.p1

back to list

  

| Protein Coverage
| Supporting Peptides
| Best Unique PSM
|

Protein Coverage:

Supporting Peptides:

| Peptide | Uniq | -10lgP | Mass | Length | ppm | m/z | z | RT | 1/k0 Start | 1/k0 End | Precursor ID | Area Sample 1 | #Feature | #Feature Sample 1 | Start | End | PTM | AScore | Found By |
| --- | --- | --- | --- | --- | --- | --- | --- | --- | --- | --- | --- | --- | --- | --- | --- | --- | --- | --- | --- |
| K.AITSSDPEVLKR.K | Y | 62.01 | 1314.7143 | 12 | -5.42 | 658.3579 | 2 | 12.95 | 0.9844 | 0.9916 | 30804 | 0 | 0 | 0 | 114 | 125 |  |  | DB Search |
| total 1 peptides |  |  |  |  |  |  |  |  |  |  |  |  |  |  |  |  |  |  |  |
| --- | --- | --- | --- | --- | --- | --- | --- | --- | --- | --- | --- | --- | --- | --- | --- | --- | --- | --- | --- |

Best Unique PSM (Scan Sample\_10\_A1\_1\_3647.d:30804, m/z=658.3579, z=2, RT=12.95, ppm=-9.91):


TRINITY\_DN39385\_c0\_g1\_i2.p1

back to list

  

| Protein Coverage
| Supporting Peptides
| Best Unique PSM
|

Protein Coverage:

Supporting Peptides:

| Peptide | Uniq | -10lgP | Mass | Length | ppm | m/z | z | RT | 1/k0 Start | 1/k0 End | Precursor ID | Area Sample 1 | #Feature | #Feature Sample 1 | Start | End | PTM | AScore | Found By |
| --- | --- | --- | --- | --- | --- | --- | --- | --- | --- | --- | --- | --- | --- | --- | --- | --- | --- | --- | --- |
| K.TLQLIHQIEISK.I | Y | 66.71 | 1421.8242 | 12 | 0.03 | 711.9162 | 2 | 17.16 | 1.0367 | 1.0439 | 53670 | 0 | 0 | 0 | 41 | 52 |  |  | DB Search |
| total 1 peptides |  |  |  |  |  |  |  |  |  |  |  |  |  |  |  |  |  |  |  |
| --- | --- | --- | --- | --- | --- | --- | --- | --- | --- | --- | --- | --- | --- | --- | --- | --- | --- | --- | --- |

Best Unique PSM (Scan Sample\_10\_A1\_1\_3647.d:53670, m/z=711.9162, z=2, RT=17.16, ppm=-4.46):


TRINITY\_DN2772\_c0\_g1\_i3.p1

back to list

  

| Protein Coverage
| Supporting Peptides
| Best Unique PSM
|

Protein Coverage:

Supporting Peptides:

| Peptide | Uniq | -10lgP | Mass | Length | ppm | m/z | z | RT | 1/k0 Start | 1/k0 End | Precursor ID | Area Sample 1 | #Feature | #Feature Sample 1 | Start | End | PTM | AScore | Found By |
| --- | --- | --- | --- | --- | --- | --- | --- | --- | --- | --- | --- | --- | --- | --- | --- | --- | --- | --- | --- |
| K.LIEALC(+57.02)AEHEIK.L | Y | 66.7 | 1424.7333 | 12 | 0.68 | 713.3712 | 2 | 15.09 | 1.0709 | 1.0781 | 42629 | 1.49e3 | 1 | 1 | 73 | 84 | Carbamidomethylation |  | DB Search |
| total 1 peptides |  |  |  |  |  |  |  |  |  |  |  |  |  |  |  |  |  |  |  |
| --- | --- | --- | --- | --- | --- | --- | --- | --- | --- | --- | --- | --- | --- | --- | --- | --- | --- | --- | --- |

Best Unique PSM (Scan Sample\_10\_A1\_1\_3647.d:42629, m/z=713.3712, z=2, RT=15.09, ppm=-3.81):


TRINITY\_DN585\_c0\_g1\_i3.p1

back to list

  

| Protein Coverage
| Supporting Peptides
| Best Unique PSM
|

Protein Coverage:

Supporting Peptides:

| Peptide | Uniq | -10lgP | Mass | Length | ppm | m/z | z | RT | 1/k0 Start | 1/k0 End | Precursor ID | Area Sample 1 | #Feature | #Feature Sample 1 | Start | End | PTM | AScore | Found By |
| --- | --- | --- | --- | --- | --- | --- | --- | --- | --- | --- | --- | --- | --- | --- | --- | --- | --- | --- | --- |
| K.IQDKEGIPPDQQR.L | Y | 65.08 | 1522.7739 | 13 | -0.66 | 762.3903 | 2 | 11.01 | 1.0339 | 1.0411 | 19352 | 9.49e2 | 1 | 1 | 2 | 14 |  |  | DB Search |
| total 1 peptides |  |  |  |  |  |  |  |  |  |  |  |  |  |  |  |  |  |  |  |
| --- | --- | --- | --- | --- | --- | --- | --- | --- | --- | --- | --- | --- | --- | --- | --- | --- | --- | --- | --- |

Best Unique PSM (Scan Sample\_10\_A1\_1\_3647.d:19352, m/z=762.3903, z=2, RT=11.01, ppm=-5.16):


TRINITY\_DN5587\_c0\_g1\_i1.p1

back to list

  

| Protein Coverage
| Supporting Peptides
| Best Unique PSM
|

Protein Coverage:

Supporting Peptides:

| Peptide | Uniq | -10lgP | Mass | Length | ppm | m/z | z | RT | 1/k0 Start | 1/k0 End | Precursor ID | Area Sample 1 | #Feature | #Feature Sample 1 | Start | End | PTM | AScore | Found By |
| --- | --- | --- | --- | --- | --- | --- | --- | --- | --- | --- | --- | --- | --- | --- | --- | --- | --- | --- | --- |
| K.LAGESESNLRK.A | Y | 48.88 | 1202.6255 | 11 | 5.59 | 602.3207 | 2 | 10.70 | 0.9291 | 0.9364 | 17678 | 0 | 0 | 0 | 275 | 285 |  |  | DB Search |
| total 1 peptides |  |  |  |  |  |  |  |  |  |  |  |  |  |  |  |  |  |  |  |
| --- | --- | --- | --- | --- | --- | --- | --- | --- | --- | --- | --- | --- | --- | --- | --- | --- | --- | --- | --- |

Best Unique PSM (Scan Sample\_10\_A1\_1\_3647.d:17678, m/z=602.3207, z=2, RT=10.70, ppm=1.1):


TRINITY\_DN2031\_c1\_g1\_i4.p1

back to list

  

| Protein Coverage
| Supporting Peptides
| Best Unique PSM
|

Protein Coverage:

Supporting Peptides:

| Peptide | Uniq | -10lgP | Mass | Length | ppm | m/z | z | RT | 1/k0 Start | 1/k0 End | Precursor ID | Area Sample 1 | #Feature | #Feature Sample 1 | Start | End | PTM | AScore | Found By |
| --- | --- | --- | --- | --- | --- | --- | --- | --- | --- | --- | --- | --- | --- | --- | --- | --- | --- | --- | --- |
| R.ELVHIQAGQC(+57.02)GNQIGAK.F | Y | 59.9 | 1821.9155 | 17 | -14.62 | 911.9476 | 2 | 13.73 | 1.113 | 1.1202 | 35183 | 0 | 0 | 0 | 23 | 39 | Carbamidomethylation |  | DB Search |
| total 1 peptides |  |  |  |  |  |  |  |  |  |  |  |  |  |  |  |  |  |  |  |
| --- | --- | --- | --- | --- | --- | --- | --- | --- | --- | --- | --- | --- | --- | --- | --- | --- | --- | --- | --- |

Best Unique PSM (Scan Sample\_10\_A1\_1\_3647.d:35183, m/z=911.9476, z=2, RT=13.73, ppm=-19.11):


TRINITY\_DN73067\_c0\_g1\_i1.p1

back to list

  

| Protein Coverage
| Supporting Peptides
| Best Unique PSM
|

Protein Coverage:

Supporting Peptides:

| Peptide | Uniq | -10lgP | Mass | Length | ppm | m/z | z | RT | 1/k0 Start | 1/k0 End | Precursor ID | Area Sample 1 | #Feature | #Feature Sample 1 | Start | End | PTM | AScore | Found By |
| --- | --- | --- | --- | --- | --- | --- | --- | --- | --- | --- | --- | --- | --- | --- | --- | --- | --- | --- | --- |
| K.VVTSSTGPGEHMR.N | Y | 66.7 | 1356.6456 | 13 | -0.17 | 679.3269 | 2 | 10.84 | 0.9844 | 0.9916 | 18517 | 3.16e3 | 1 | 1 | 714 | 726 |  |  | DB Search |
| K.VVTSSTGPGEHM(+15.99)R.N | Y | 66.55 | 1372.6405 | 13 | 4.36 | 458.554 | 3 | 9.70 | 0.7212 | 0.7286 | 11985 | 1.74e3 | 2 | 2 | 714 | 726 | Oxidation (M) | M12:Oxidation (M):1000 | DB Search |
| total 2 peptides |  |  |  |  |  |  |  |  |  |  |  |  |  |  |  |  |  |  |  |
| --- | --- | --- | --- | --- | --- | --- | --- | --- | --- | --- | --- | --- | --- | --- | --- | --- | --- | --- | --- |

Best Unique PSM (Scan Sample\_10\_A1\_1\_3647.d:18517, m/z=679.3269, z=2, RT=10.84, ppm=-4.66):


TRINITY\_DN2028\_c0\_g1\_i1.p1

back to list

  

| Protein Coverage
| Supporting Peptides
| Best Unique PSM
|

Protein Coverage:

Supporting Peptides:

| Peptide | Uniq | -10lgP | Mass | Length | ppm | m/z | z | RT | 1/k0 Start | 1/k0 End | Precursor ID | Area Sample 1 | #Feature | #Feature Sample 1 | Start | End | PTM | AScore | Found By |
| --- | --- | --- | --- | --- | --- | --- | --- | --- | --- | --- | --- | --- | --- | --- | --- | --- | --- | --- | --- |
| K.LIQYIENKPGLTR.L | Y | 62.38 | 1543.8722 | 13 | 0.76 | 772.9405 | 2 | 14.75 | 1.0773 | 1.0845 | 40651 | 3.68e3 | 1 | 1 | 81 | 93 |  |  | DB Search |
| total 1 peptides |  |  |  |  |  |  |  |  |  |  |  |  |  |  |  |  |  |  |  |
| --- | --- | --- | --- | --- | --- | --- | --- | --- | --- | --- | --- | --- | --- | --- | --- | --- | --- | --- | --- |

Best Unique PSM (Scan Sample\_10\_A1\_1\_3647.d:40651, m/z=772.9405, z=2, RT=14.75, ppm=-3.74):


TRINITY\_DN6920\_c0\_g1\_i1.p1

back to list

  

| Protein Coverage
| Supporting Peptides
| Best Unique PSM
|

Protein Coverage:

Supporting Peptides:

| Peptide | Uniq | -10lgP | Mass | Length | ppm | m/z | z | RT | 1/k0 Start | 1/k0 End | Precursor ID | Area Sample 1 | #Feature | #Feature Sample 1 | Start | End | PTM | AScore | Found By |
| --- | --- | --- | --- | --- | --- | --- | --- | --- | --- | --- | --- | --- | --- | --- | --- | --- | --- | --- | --- |
| R.KVAC(+57.02)IGAWHPAR.V | Y | 60.86 | 1364.7135 | 12 | 9.11 | 683.3672 | 2 | 13.20 | 0.9711 | 0.9783 | 32100 | 9.16e2 | 1 | 1 | 133 | 144 | Carbamidomethylation |  | DB Search |
| total 1 peptides |  |  |  |  |  |  |  |  |  |  |  |  |  |  |  |  |  |  |  |
| --- | --- | --- | --- | --- | --- | --- | --- | --- | --- | --- | --- | --- | --- | --- | --- | --- | --- | --- | --- |

Best Unique PSM (Scan Sample\_10\_A1\_1\_3647.d:32100, m/z=683.3672, z=2, RT=13.20, ppm=4.62):


TRINITY\_DN23911\_c0\_g1\_i1.p1

back to list

  

| Protein Coverage
| Supporting Peptides
| Best Unique PSM
|

Protein Coverage:

Supporting Peptides:

| Peptide | Uniq | -10lgP | Mass | Length | ppm | m/z | z | RT | 1/k0 Start | 1/k0 End | Precursor ID | Area Sample 1 | #Feature | #Feature Sample 1 | Start | End | PTM | AScore | Found By |
| --- | --- | --- | --- | --- | --- | --- | --- | --- | --- | --- | --- | --- | --- | --- | --- | --- | --- | --- | --- |
| R.YLHDQSIVHLDLKPDNILLAK.E | Y | 59.04 | 2444.3427 | 21 | 13 | 612.0981 | 4 | 18.20 | 0.9061 | 0.9133 | 59977 | 0 | 0 | 0 | 136 | 156 |  |  | DB Search |
| total 1 peptides |  |  |  |  |  |  |  |  |  |  |  |  |  |  |  |  |  |  |  |
| --- | --- | --- | --- | --- | --- | --- | --- | --- | --- | --- | --- | --- | --- | --- | --- | --- | --- | --- | --- |

Best Unique PSM (Scan Sample\_10\_A1\_1\_3647.d:59977, m/z=612.0981, z=4, RT=18.20, ppm=8.5):


TRINITY\_DN6008\_c0\_g1\_i1.p1

back to list

  

| Protein Coverage
| Supporting Peptides
| Best Unique PSM
|

Protein Coverage:

Supporting Peptides:

| Peptide | Uniq | -10lgP | Mass | Length | ppm | m/z | z | RT | 1/k0 Start | 1/k0 End | Precursor ID | Area Sample 1 | #Feature | #Feature Sample 1 | Start | End | PTM | AScore | Found By |
| --- | --- | --- | --- | --- | --- | --- | --- | --- | --- | --- | --- | --- | --- | --- | --- | --- | --- | --- | --- |
| K.LSTLVGANVINEKPLK.F | Y | 60.22 | 1694.993 | 16 | 7.58 | 848.5064 | 2 | 16.33 | 1.1817 | 1.1889 | 49261 | 0 | 0 | 0 | 189 | 204 |  |  | DB Search |
| total 1 peptides |  |  |  |  |  |  |  |  |  |  |  |  |  |  |  |  |  |  |  |
| --- | --- | --- | --- | --- | --- | --- | --- | --- | --- | --- | --- | --- | --- | --- | --- | --- | --- | --- | --- |

Best Unique PSM (Scan Sample\_10\_A1\_1\_3647.d:49261, m/z=848.5064, z=2, RT=16.33, ppm=3.09):


TRINITY\_DN1616\_c0\_g1\_i2.p1

back to list

  

| Protein Coverage
| Supporting Peptides
| Best Unique PSM
|

Protein Coverage:

Supporting Peptides:

| Peptide | Uniq | -10lgP | Mass | Length | ppm | m/z | z | RT | 1/k0 Start | 1/k0 End | Precursor ID | Area Sample 1 | #Feature | #Feature Sample 1 | Start | End | PTM | AScore | Found By |
| --- | --- | --- | --- | --- | --- | --- | --- | --- | --- | --- | --- | --- | --- | --- | --- | --- | --- | --- | --- |
| R.LVQANPLEQDLSEKPR.Y | Y | 66.73 | 1835.9741 | 16 | 3.23 | 918.9932 | 2 | 15.63 | 1.1837 | 1.1909 | 45493 | 3.81e2 | 1 | 1 | 1191 | 1206 |  |  | DB Search |
| K.YVVMLQYYHDSDTAK.Y | Y | 66.45 | 1831.8451 | 15 | 2.49 | 916.928 | 2 | 16.54 | 1.1798 | 1.1869 | 50323 | 0 | 0 | 0 | 1071 | 1085 |  |  | DB Search |
| R.YVKPGVNIINIR.D | Y | 58.56 | 1384.819 | 12 | 3.46 | 693.4161 | 2 | 15.95 | 1.0025 | 1.0098 | 47296 | 0 | 0 | 0 | 1217 | 1228 |  |  | DB Search |
| total 3 peptides |  |  |  |  |  |  |  |  |  |  |  |  |  |  |  |  |  |  |  |
| --- | --- | --- | --- | --- | --- | --- | --- | --- | --- | --- | --- | --- | --- | --- | --- | --- | --- | --- | --- |

Best Unique PSM (Scan Sample\_10\_A1\_1\_3647.d:45493, m/z=918.9932, z=2, RT=15.63, ppm=-1.26):


TRINITY\_DN22219\_c0\_g1\_i1.p1

back to list

  

| Protein Coverage
| Supporting Peptides
| Best Unique PSM
|

Protein Coverage:

Supporting Peptides:

| Peptide | Uniq | -10lgP | Mass | Length | ppm | m/z | z | RT | 1/k0 Start | 1/k0 End | Precursor ID | Area Sample 1 | #Feature | #Feature Sample 1 | Start | End | PTM | AScore | Found By |
| --- | --- | --- | --- | --- | --- | --- | --- | --- | --- | --- | --- | --- | --- | --- | --- | --- | --- | --- | --- |
| R.AEVIREEEEEKEK.K | Y | 40.71 | 1616.7893 | 13 | 5.8 | 539.9377 | 3 | 10.70 | 0.7897 | 0.797 | 17726 | 8.74e2 | 1 | 1 | 311 | 323 |  |  | DB Search |
| total 1 peptides |  |  |  |  |  |  |  |  |  |  |  |  |  |  |  |  |  |  |  |
| --- | --- | --- | --- | --- | --- | --- | --- | --- | --- | --- | --- | --- | --- | --- | --- | --- | --- | --- | --- |

Best Unique PSM (Scan Sample\_10\_A1\_1\_3647.d:17726, m/z=539.9377, z=3, RT=10.70, ppm=1.3):


TRINITY\_DN15847\_c0\_g1\_i2.p1

back to list

  

| Protein Coverage
| Supporting Peptides
| Best Unique PSM
|

Protein Coverage:

Supporting Peptides:

| Peptide | Uniq | -10lgP | Mass | Length | ppm | m/z | z | RT | 1/k0 Start | 1/k0 End | Precursor ID | Area Sample 1 | #Feature | #Feature Sample 1 | Start | End | PTM | AScore | Found By |
| --- | --- | --- | --- | --- | --- | --- | --- | --- | --- | --- | --- | --- | --- | --- | --- | --- | --- | --- | --- |
| R.DVQLGDLVTVGQC(+57.02)RPLSK.T | Y | 63.15 | 1984.0411 | 18 | 4.67 | 993.028 | 2 | 17.98 | 1.2726 | 1.2797 | 58548 | 8.93e2 | 1 | 1 | 126 | 143 | Carbamidomethylation |  | DB Search |
| R.DYLHFIK.K | Y | 61.47 | 934.4912 | 7 | -4.37 | 468.2487 | 2 | 18.67 | 0.8242 | 0.8315 | 62776 | 3.12e2 | 1 | 1 | 98 | 104 |  |  | DB Search |
| total 2 peptides |  |  |  |  |  |  |  |  |  |  |  |  |  |  |  |  |  |  |  |
| --- | --- | --- | --- | --- | --- | --- | --- | --- | --- | --- | --- | --- | --- | --- | --- | --- | --- | --- | --- |

Best Unique PSM (Scan Sample\_10\_A1\_1\_3647.d:58548, m/z=993.028, z=2, RT=17.98, ppm=0.18):


TRINITY\_DN471\_c0\_g1\_i3.p1

back to list

  

| Protein Coverage
| Supporting Peptides
| Best Unique PSM
|

Protein Coverage:

Supporting Peptides:

| Peptide | Uniq | -10lgP | Mass | Length | ppm | m/z | z | RT | 1/k0 Start | 1/k0 End | Precursor ID | Area Sample 1 | #Feature | #Feature Sample 1 | Start | End | PTM | AScore | Found By |
| --- | --- | --- | --- | --- | --- | --- | --- | --- | --- | --- | --- | --- | --- | --- | --- | --- | --- | --- | --- |
| R.AFVHWYVGEGM(+15.99)EEGEFSEAR.E | Y | 44.9 | 2345.0058 | 20 | -0.18 | 782.6722 | 3 | 17.81 | 0.9251 | 0.9323 | 57560 | 6.2e2 | 1 | 1 | 273 | 292 | Oxidation (M) | M11:Oxidation (M):1000 | DB Search |
| total 1 peptides |  |  |  |  |  |  |  |  |  |  |  |  |  |  |  |  |  |  |  |
| --- | --- | --- | --- | --- | --- | --- | --- | --- | --- | --- | --- | --- | --- | --- | --- | --- | --- | --- | --- |

Best Unique PSM (Scan Sample\_10\_A1\_1\_3647.d:57560, m/z=782.6722, z=3, RT=17.81, ppm=-4.67):


TRINITY\_DN12632\_c1\_g1\_i1.p1

back to list

  

| Protein Coverage
| Supporting Peptides
| Best Unique PSM
|

Protein Coverage:

Supporting Peptides:

| Peptide | Uniq | -10lgP | Mass | Length | ppm | m/z | z | RT | 1/k0 Start | 1/k0 End | Precursor ID | Area Sample 1 | #Feature | #Feature Sample 1 | Start | End | PTM | AScore | Found By |
| --- | --- | --- | --- | --- | --- | --- | --- | --- | --- | --- | --- | --- | --- | --- | --- | --- | --- | --- | --- |
| R.LIEDTEYTAHEGAK.F | Y | 66.76 | 1575.7416 | 14 | -1.83 | 788.8731 | 2 | 12.66 | 1.0789 | 1.0861 | 29096 | 0 | 0 | 0 | 378 | 391 |  |  | DB Search |
| total 1 peptides |  |  |  |  |  |  |  |  |  |  |  |  |  |  |  |  |  |  |  |
| --- | --- | --- | --- | --- | --- | --- | --- | --- | --- | --- | --- | --- | --- | --- | --- | --- | --- | --- | --- |

Best Unique PSM (Scan Sample\_10\_A1\_1\_3647.d:29096, m/z=788.8731, z=2, RT=12.66, ppm=-6.32):


TRINITY\_DN58867\_c0\_g1\_i2.p1

back to list

  

| Protein Coverage
| Supporting Peptides
| Best Unique PSM
|

Protein Coverage:

Supporting Peptides:

| Peptide | Uniq | -10lgP | Mass | Length | ppm | m/z | z | RT | 1/k0 Start | 1/k0 End | Precursor ID | Area Sample 1 | #Feature | #Feature Sample 1 | Start | End | PTM | AScore | Found By |
| --- | --- | --- | --- | --- | --- | --- | --- | --- | --- | --- | --- | --- | --- | --- | --- | --- | --- | --- | --- |
| K.TILQFEADRR.A | Y | 66.59 | 1247.6622 | 10 | 3.81 | 624.838 | 2 | 15.07 | 0.9505 | 0.9578 | 42491 | 5.6e2 | 1 | 1 | 211 | 220 |  |  | DB Search |
| R.AIM(+15.99)ITINSFGTELNKDDR.S | Y | 31.12 | 2053.015 | 18 | 3.55 | 1027.5138 | 2 | 18.19 | 1.2495 | 1.2567 | 59830 | 0 | 0 | 0 | 221 | 238 | Oxidation (M) | M3:Oxidation (M):1000 | DB Search |
| total 2 peptides |  |  |  |  |  |  |  |  |  |  |  |  |  |  |  |  |  |  |  |
| --- | --- | --- | --- | --- | --- | --- | --- | --- | --- | --- | --- | --- | --- | --- | --- | --- | --- | --- | --- |

Best Unique PSM (Scan Sample\_10\_A1\_1\_3647.d:42491, m/z=624.838, z=2, RT=15.07, ppm=-0.69):


TRINITY\_DN36\_c0\_g1\_i2.p1

back to list

  

| Protein Coverage
| Supporting Peptides
| Best Unique PSM
|

Protein Coverage:

Supporting Peptides:

| Peptide | Uniq | -10lgP | Mass | Length | ppm | m/z | z | RT | 1/k0 Start | 1/k0 End | Precursor ID | Area Sample 1 | #Feature | #Feature Sample 1 | Start | End | PTM | AScore | Found By |
| --- | --- | --- | --- | --- | --- | --- | --- | --- | --- | --- | --- | --- | --- | --- | --- | --- | --- | --- | --- |
| R.LGGIEVTQGNC(+57.02)AR.K | Y | 66.48 | 1373.6721 | 13 | 5.18 | 687.8438 | 2 | 13.73 | 0.963 | 0.9703 | 35192 | 0 | 0 | 0 | 58 | 70 | Carbamidomethylation |  | DB Search |
| R.LGGIEVTQGNC(+57.02)ARK.Y | Y | 31.04 | 1501.7671 | 14 | -2.67 | 751.8854 | 2 | 12.64 | 1.0624 | 1.0697 | 29000 | 0 | 0 | 0 | 58 | 71 | Carbamidomethylation |  | DB Search |
| total 2 peptides |  |  |  |  |  |  |  |  |  |  |  |  |  |  |  |  |  |  |  |
| --- | --- | --- | --- | --- | --- | --- | --- | --- | --- | --- | --- | --- | --- | --- | --- | --- | --- | --- | --- |

Best Unique PSM (Scan Sample\_10\_A1\_1\_3647.d:35192, m/z=687.8438, z=2, RT=13.73, ppm=0.69):


TRINITY\_DN535\_c0\_g1\_i1.p1

back to list

  

| Protein Coverage
| Supporting Peptides
| Best Unique PSM
|

Protein Coverage:

Supporting Peptides:

| Peptide | Uniq | -10lgP | Mass | Length | ppm | m/z | z | RT | 1/k0 Start | 1/k0 End | Precursor ID | Area Sample 1 | #Feature | #Feature Sample 1 | Start | End | PTM | AScore | Found By |
| --- | --- | --- | --- | --- | --- | --- | --- | --- | --- | --- | --- | --- | --- | --- | --- | --- | --- | --- | --- |
| R.GLFIIDDKGILR.Q | Y | 66.46 | 1358.7921 | 12 | 2.12 | 680.4017 | 2 | 19.27 | 1.0122 | 1.0194 | 66460 | 6.42e3 | 1 | 1 | 94 | 105 |  |  | DB Search |
| R.ALGC(+57.02)DVLAC(+57.02)SVDSHFSHLAWTQQSR.K | Y | 62.1 | 2844.3071 | 25 | 4.64 | 712.0842 | 4 | 18.79 | 0.9222 | 0.9295 | 63472 | 1.49e3 | 1 | 1 | 33 | 57 | Carbamidomethylation, Carbamidomethylation |  | DB Search |
| total 2 peptides |  |  |  |  |  |  |  |  |  |  |  |  |  |  |  |  |  |  |  |
| --- | --- | --- | --- | --- | --- | --- | --- | --- | --- | --- | --- | --- | --- | --- | --- | --- | --- | --- | --- |

Best Unique PSM (Scan Sample\_10\_A1\_1\_3647.d:66460, m/z=680.4017, z=2, RT=19.27, ppm=-2.37):


TRINITY\_DN12390\_c0\_g1\_i1.p1

back to list

  

| Protein Coverage
| Supporting Peptides
| Best Unique PSM
|

Protein Coverage:

Supporting Peptides:

| Peptide | Uniq | -10lgP | Mass | Length | ppm | m/z | z | RT | 1/k0 Start | 1/k0 End | Precursor ID | Area Sample 1 | #Feature | #Feature Sample 1 | Start | End | PTM | AScore | Found By |
| --- | --- | --- | --- | --- | --- | --- | --- | --- | --- | --- | --- | --- | --- | --- | --- | --- | --- | --- | --- |
| K.AIGHFTQLVWK.T | Y | 66.81 | 1298.7135 | 11 | 0.43 | 650.3614 | 2 | 17.56 | 0.9755 | 0.9828 | 56062 | 0 | 0 | 0 | 157 | 167 |  |  | DB Search |
| total 1 peptides |  |  |  |  |  |  |  |  |  |  |  |  |  |  |  |  |  |  |  |
| --- | --- | --- | --- | --- | --- | --- | --- | --- | --- | --- | --- | --- | --- | --- | --- | --- | --- | --- | --- |

Best Unique PSM (Scan Sample\_10\_A1\_1\_3647.d:56062, m/z=649.3444, z=2, RT=17.57, ppm=-4.07):


TRINITY\_DN2217\_c0\_g1\_i2.p1

back to list

  

| Protein Coverage
| Supporting Peptides
| Best Unique PSM
|

Protein Coverage:

Supporting Peptides:

| Peptide | Uniq | -10lgP | Mass | Length | ppm | m/z | z | RT | 1/k0 Start | 1/k0 End | Precursor ID | Area Sample 1 | #Feature | #Feature Sample 1 | Start | End | PTM | AScore | Found By |
| --- | --- | --- | --- | --- | --- | --- | --- | --- | --- | --- | --- | --- | --- | --- | --- | --- | --- | --- | --- |
| K.ATGC(+57.02)EVIWNQHNR.G | Y | 66.64 | 1583.7263 | 13 | 0.95 | 792.8676 | 2 | 13.84 | 1.0785 | 1.0857 | 35783 | 1.6e3 | 1 | 1 | 119 | 131 | Carbamidomethylation |  | DB Search |
| R.GC(+57.02)YIHTQEIAR.G | Y | 66.57 | 1346.6401 | 11 | -0.12 | 674.3242 | 2 | 12.88 | 1.0158 | 1.023 | 30410 | 6.22e2 | 1 | 1 | 226 | 236 | Carbamidomethylation |  | DB Search |
| R.GC(+57.02)YIHTQEVAR.G | N | 61.54 | 1332.6245 | 11 | -1.99 | 667.3152 | 2 | 12.14 | 1.0086 | 1.0158 | 26094 | 0 | 0 | 0 | 38 | 48 | Carbamidomethylation |  | DB Search |
| R.HFC(+57.02)WVFSK.C | Y | 41.91 | 1109.5117 | 8 | -7.68 | 555.7563 | 2 | 19.76 | 0.8712 | 0.8785 | 69479 | 2.04e2 | 1 | 1 | 55 | 62 | Carbamidomethylation |  | DB Search |
| total 4 peptides |  |  |  |  |  |  |  |  |  |  |  |  |  |  |  |  |  |  |  |
| --- | --- | --- | --- | --- | --- | --- | --- | --- | --- | --- | --- | --- | --- | --- | --- | --- | --- | --- | --- |

Best Unique PSM (Scan Sample\_10\_A1\_1\_3647.d:35783, m/z=792.8676, z=2, RT=13.84, ppm=-3.55):


TRINITY\_DN2637\_c0\_g1\_i14.p2

back to list

  

| Protein Coverage
| Supporting Peptides
| Best Unique PSM
|

Protein Coverage:

Supporting Peptides:

| Peptide | Uniq | -10lgP | Mass | Length | ppm | m/z | z | RT | 1/k0 Start | 1/k0 End | Precursor ID | Area Sample 1 | #Feature | #Feature Sample 1 | Start | End | PTM | AScore | Found By |
| --- | --- | --- | --- | --- | --- | --- | --- | --- | --- | --- | --- | --- | --- | --- | --- | --- | --- | --- | --- |
| K.ATIEIPAKYEK.R | Y | 61.89 | 1261.6918 | 11 | 0.22 | 631.8505 | 2 | 14.08 | 0.982 | 0.9892 | 37168 | 0 | 0 | 0 | 87 | 97 |  |  | DB Search |
| total 1 peptides |  |  |  |  |  |  |  |  |  |  |  |  |  |  |  |  |  |  |  |
| --- | --- | --- | --- | --- | --- | --- | --- | --- | --- | --- | --- | --- | --- | --- | --- | --- | --- | --- | --- |

Best Unique PSM (Scan Sample\_10\_A1\_1\_3647.d:37168, m/z=631.8505, z=2, RT=14.08, ppm=-4.27):


TRINITY\_DN80458\_c0\_g1\_i1.p1

back to list

  

| Protein Coverage
| Supporting Peptides
| Best Unique PSM
|

Protein Coverage:

Supporting Peptides:

| Peptide | Uniq | -10lgP | Mass | Length | ppm | m/z | z | RT | 1/k0 Start | 1/k0 End | Precursor ID | Area Sample 1 | #Feature | #Feature Sample 1 | Start | End | PTM | AScore | Found By |
| --- | --- | --- | --- | --- | --- | --- | --- | --- | --- | --- | --- | --- | --- | --- | --- | --- | --- | --- | --- |
| K.KGLTPSQIGVILR.D | Y | 66.78 | 1380.8452 | 13 | -0.23 | 691.4266 | 2 | 17.08 | 0.9981 | 1.0053 | 53138 | 4.79e3 | 1 | 1 | 48 | 60 |  |  | DB Search |
| K.LTEEDVKDQIYK.L | Y | 66.77 | 1479.7457 | 12 | 4.19 | 740.8799 | 2 | 14.60 | 1.0226 | 1.0299 | 39982 | 0 | 0 | 0 | 33 | 44 |  |  | DB Search |
| R.DSHGVAQVR.Y | Y | 66.66 | 967.4835 | 9 | 8.7 | 484.7511 | 2 | 10.11 | 0.8193 | 0.8266 | 14162 | 8.11e2 | 1 | 1 | 61 | 69 |  |  | DB Search |
| K.GLAPSLPEDLHC(+57.02)LIK.K | Y | 66.23 | 1661.881 | 15 | 1.43 | 831.9453 | 2 | 19.09 | 1.1406 | 1.1478 | 65296 | 1.58e3 | 1 | 1 | 84 | 98 | Carbamidomethylation |  | DB Search |
| total 4 peptides |  |  |  |  |  |  |  |  |  |  |  |  |  |  |  |  |  |  |  |
| --- | --- | --- | --- | --- | --- | --- | --- | --- | --- | --- | --- | --- | --- | --- | --- | --- | --- | --- | --- |

Best Unique PSM (Scan Sample\_10\_A1\_1\_3647.d:53138, m/z=691.4266, z=2, RT=17.08, ppm=-4.73):


TRINITY\_DN200\_c0\_g1\_i5.p1

back to list

  

| Protein Coverage
| Supporting Peptides
| Best Unique PSM
|

Protein Coverage:

Supporting Peptides:

| Peptide | Uniq | -10lgP | Mass | Length | ppm | m/z | z | RT | 1/k0 Start | 1/k0 End | Precursor ID | Area Sample 1 | #Feature | #Feature Sample 1 | Start | End | PTM | AScore | Found By |
| --- | --- | --- | --- | --- | --- | --- | --- | --- | --- | --- | --- | --- | --- | --- | --- | --- | --- | --- | --- |
| K.TAIAIDTIINQKR.F | Y | 64.76 | 1455.8409 | 13 | -0.36 | 728.9242 | 2 | 16.58 | 1.0588 | 1.066 | 50506 | 4.46e2 | 1 | 1 | 217 | 229 |  |  | DB Search |
| total 1 peptides |  |  |  |  |  |  |  |  |  |  |  |  |  |  |  |  |  |  |  |
| --- | --- | --- | --- | --- | --- | --- | --- | --- | --- | --- | --- | --- | --- | --- | --- | --- | --- | --- | --- |

Best Unique PSM (Scan Sample\_10\_A1\_1\_3647.d:50506, m/z=728.9242, z=2, RT=16.58, ppm=-4.85):


TRINITY\_DN98968\_c0\_g1\_i1.p1

back to list

  

| Protein Coverage
| Supporting Peptides
| Best Unique PSM
|

Protein Coverage:

Supporting Peptides:

| Peptide | Uniq | -10lgP | Mass | Length | ppm | m/z | z | RT | 1/k0 Start | 1/k0 End | Precursor ID | Area Sample 1 | #Feature | #Feature Sample 1 | Start | End | PTM | AScore | Found By |
| --- | --- | --- | --- | --- | --- | --- | --- | --- | --- | --- | --- | --- | --- | --- | --- | --- | --- | --- | --- |
| R.IESTAC(+57.02)TR.R | Y | 34.99 | 936.4335 | 8 | 6.23 | 469.2248 | 2 | 9.63 | 0.812 | 0.8193 | 11550 | 1.4e3 | 1 | 1 | 204 | 211 | Carbamidomethylation |  | DB Search |
| total 1 peptides |  |  |  |  |  |  |  |  |  |  |  |  |  |  |  |  |  |  |  |
| --- | --- | --- | --- | --- | --- | --- | --- | --- | --- | --- | --- | --- | --- | --- | --- | --- | --- | --- | --- |

Best Unique PSM (Scan Sample\_10\_A1\_1\_3647.d:11550, m/z=469.2248, z=2, RT=9.63, ppm=1.73):


TRINITY\_DN817\_c0\_g1\_i7.p1

back to list

  

| Protein Coverage
| Supporting Peptides
| Best Unique PSM
|

Protein Coverage:

Supporting Peptides:

| Peptide | Uniq | -10lgP | Mass | Length | ppm | m/z | z | RT | 1/k0 Start | 1/k0 End | Precursor ID | Area Sample 1 | #Feature | #Feature Sample 1 | Start | End | PTM | AScore | Found By |
| --- | --- | --- | --- | --- | --- | --- | --- | --- | --- | --- | --- | --- | --- | --- | --- | --- | --- | --- | --- |
| R.LC(+57.02)QPGYSTC(+57.02)ENHC(+57.02)R.R | Y | 62.01 | 1780.708 | 14 | 5.39 | 891.3621 | 2 | 11.41 | 1.0893 | 1.0965 | 21785 | 1.84e2 | 1 | 1 | 89 | 102 | Carbamidomethylation, Carbamidomethylation, Carbamidomethylation |  | DB Search |
| total 1 peptides |  |  |  |  |  |  |  |  |  |  |  |  |  |  |  |  |  |  |  |
| --- | --- | --- | --- | --- | --- | --- | --- | --- | --- | --- | --- | --- | --- | --- | --- | --- | --- | --- | --- |

Best Unique PSM (Scan Sample\_10\_A1\_1\_3647.d:21785, m/z=891.3621, z=2, RT=11.41, ppm=0.9):


TRINITY\_DN8165\_c0\_g2\_i1.p2

back to list

  

| Protein Coverage
| Supporting Peptides
| Best Unique PSM
|

Protein Coverage:

Supporting Peptides:

| Peptide | Uniq | -10lgP | Mass | Length | ppm | m/z | z | RT | 1/k0 Start | 1/k0 End | Precursor ID | Area Sample 1 | #Feature | #Feature Sample 1 | Start | End | PTM | AScore | Found By |
| --- | --- | --- | --- | --- | --- | --- | --- | --- | --- | --- | --- | --- | --- | --- | --- | --- | --- | --- | --- |
| K.K(+42.01)LSAELANGR.L | Y | 66.66 | 1099.5985 | 10 | 2.66 | 550.8055 | 2 | 14.21 | 0.8712 | 0.8785 | 37894 | 8.99e2 | 1 | 1 | 4 | 13 | Acetylation (Protein N-term) | K1:Acetylation (Protein N-term):1000 | DB Search |
| total 1 peptides |  |  |  |  |  |  |  |  |  |  |  |  |  |  |  |  |  |  |  |
| --- | --- | --- | --- | --- | --- | --- | --- | --- | --- | --- | --- | --- | --- | --- | --- | --- | --- | --- | --- |

Best Unique PSM (Scan Sample\_10\_A1\_1\_3647.d:37894, m/z=550.8055, z=2, RT=14.21, ppm=-1.83):


TRINITY\_DN5107\_c0\_g1\_i10.p1

back to list

  

| Protein Coverage
| Supporting Peptides
| Best Unique PSM
|

Protein Coverage:

Supporting Peptides:

| Peptide | Uniq | -10lgP | Mass | Length | ppm | m/z | z | RT | 1/k0 Start | 1/k0 End | Precursor ID | Area Sample 1 | #Feature | #Feature Sample 1 | Start | End | PTM | AScore | Found By |
| --- | --- | --- | --- | --- | --- | --- | --- | --- | --- | --- | --- | --- | --- | --- | --- | --- | --- | --- | --- |
| K.KLDYVLGLTTAK.I | Y | 63.57 | 1320.7653 | 12 | -2.37 | 661.3854 | 2 | 16.69 | 0.9908 | 0.9981 | 51148 | 2.13e2 | 1 | 1 | 114 | 125 |  |  | DB Search |
| total 1 peptides |  |  |  |  |  |  |  |  |  |  |  |  |  |  |  |  |  |  |  |
| --- | --- | --- | --- | --- | --- | --- | --- | --- | --- | --- | --- | --- | --- | --- | --- | --- | --- | --- | --- |

Best Unique PSM (Scan Sample\_10\_A1\_1\_3647.d:51148, m/z=661.3854, z=2, RT=16.69, ppm=-6.87):


TRINITY\_DN1631\_c0\_g1\_i1.p1

back to list

  

| Protein Coverage
| Supporting Peptides
| Best Unique PSM
|

Protein Coverage:

Supporting Peptides:

| Peptide | Uniq | -10lgP | Mass | Length | ppm | m/z | z | RT | 1/k0 Start | 1/k0 End | Precursor ID | Area Sample 1 | #Feature | #Feature Sample 1 | Start | End | PTM | AScore | Found By |
| --- | --- | --- | --- | --- | --- | --- | --- | --- | --- | --- | --- | --- | --- | --- | --- | --- | --- | --- | --- |
| M.A(+42.01)EAPKPKPAPK.S | Y | 66.75 | 1174.671 | 11 | 10.9 | 392.5668 | 3 | 9.90 | 0.7665 | 0.7738 | 13029 | 1.04e4 | 2 | 2 | 2 | 12 | Acetylation (Protein N-term) | A1:Acetylation (Protein N-term):1000 | DB Search |
| total 1 peptides |  |  |  |  |  |  |  |  |  |  |  |  |  |  |  |  |  |  |  |
| --- | --- | --- | --- | --- | --- | --- | --- | --- | --- | --- | --- | --- | --- | --- | --- | --- | --- | --- | --- |

Best Unique PSM (Scan Sample\_10\_A1\_1\_3647.d:13029, m/z=392.5668, z=3, RT=9.90, ppm=6.41):


TRINITY\_DN13856\_c0\_g1\_i1.p2

back to list

  

| Protein Coverage
| Supporting Peptides
| Best Unique PSM
|

Protein Coverage:

Supporting Peptides:

| Peptide | Uniq | -10lgP | Mass | Length | ppm | m/z | z | RT | 1/k0 Start | 1/k0 End | Precursor ID | Area Sample 1 | #Feature | #Feature Sample 1 | Start | End | PTM | AScore | Found By |
| --- | --- | --- | --- | --- | --- | --- | --- | --- | --- | --- | --- | --- | --- | --- | --- | --- | --- | --- | --- |
| R.IPLDDFQDPTLTLENAHNK.L | Y | 61.84 | 2180.0749 | 19 | 2.14 | 727.6972 | 3 | 18.19 | 0.9896 | 0.9969 | 59846 | 3.57e2 | 1 | 1 | 175 | 193 |  |  | DB Search |
| total 1 peptides |  |  |  |  |  |  |  |  |  |  |  |  |  |  |  |  |  |  |  |
| --- | --- | --- | --- | --- | --- | --- | --- | --- | --- | --- | --- | --- | --- | --- | --- | --- | --- | --- | --- |

Best Unique PSM (Scan Sample\_10\_A1\_1\_3647.d:59846, m/z=727.6972, z=3, RT=18.19, ppm=-2.35):


TRINITY\_DN902\_c1\_g2\_i4.p1

back to list

  

| Protein Coverage
| Supporting Peptides
| Best Unique PSM
|

Protein Coverage:

Supporting Peptides:

| Peptide | Uniq | -10lgP | Mass | Length | ppm | m/z | z | RT | 1/k0 Start | 1/k0 End | Precursor ID | Area Sample 1 | #Feature | #Feature Sample 1 | Start | End | PTM | AScore | Found By |
| --- | --- | --- | --- | --- | --- | --- | --- | --- | --- | --- | --- | --- | --- | --- | --- | --- | --- | --- | --- |
| K.GLTHC(+57.02)NTLSYK.L | Y | 66.56 | 1292.6183 | 11 | 2.85 | 647.3154 | 2 | 12.41 | 0.9699 | 0.9771 | 27654 | 0 | 0 | 0 | 62 | 72 | Carbamidomethylation |  | DB Search |
| total 1 peptides |  |  |  |  |  |  |  |  |  |  |  |  |  |  |  |  |  |  |  |
| --- | --- | --- | --- | --- | --- | --- | --- | --- | --- | --- | --- | --- | --- | --- | --- | --- | --- | --- | --- |

Best Unique PSM (Scan Sample\_10\_A1\_1\_3647.d:27654, m/z=647.3154, z=2, RT=12.41, ppm=-1.65):


TRINITY\_DN5107\_c0\_g1\_i9.p1

back to list

  

| Protein Coverage
| Supporting Peptides
| Best Unique PSM
|

Protein Coverage:

Supporting Peptides:

| Peptide | Uniq | -10lgP | Mass | Length | ppm | m/z | z | RT | 1/k0 Start | 1/k0 End | Precursor ID | Area Sample 1 | #Feature | #Feature Sample 1 | Start | End | PTM | AScore | Found By |
| --- | --- | --- | --- | --- | --- | --- | --- | --- | --- | --- | --- | --- | --- | --- | --- | --- | --- | --- | --- |
| K.KLDYVLGLTTAK.I | Y | 63.57 | 1320.7653 | 12 | -2.37 | 661.3854 | 2 | 16.69 | 0.9908 | 0.9981 | 51148 | 2.13e2 | 1 | 1 | 91 | 102 |  |  | DB Search |
| total 1 peptides |  |  |  |  |  |  |  |  |  |  |  |  |  |  |  |  |  |  |  |
| --- | --- | --- | --- | --- | --- | --- | --- | --- | --- | --- | --- | --- | --- | --- | --- | --- | --- | --- | --- |

Best Unique PSM (Scan Sample\_10\_A1\_1\_3647.d:51148, m/z=661.3854, z=2, RT=16.69, ppm=-6.87):


TRINITY\_DN773\_c0\_g1\_i1.p1

back to list

  

| Protein Coverage
| Supporting Peptides
| Best Unique PSM
|

Protein Coverage:

Supporting Peptides:

| Peptide | Uniq | -10lgP | Mass | Length | ppm | m/z | z | RT | 1/k0 Start | 1/k0 End | Precursor ID | Area Sample 1 | #Feature | #Feature Sample 1 | Start | End | PTM | AScore | Found By |
| --- | --- | --- | --- | --- | --- | --- | --- | --- | --- | --- | --- | --- | --- | --- | --- | --- | --- | --- | --- |
| R.GVSEETTTGVHNLYK.M | Y | 61.46 | 1633.7947 | 15 | -0.34 | 817.9007 | 2 | 13.56 | 1.0717 | 1.0789 | 34190 | 6.1e2 | 1 | 1 | 160 | 174 |  |  | DB Search |
| total 1 peptides |  |  |  |  |  |  |  |  |  |  |  |  |  |  |  |  |  |  |  |
| --- | --- | --- | --- | --- | --- | --- | --- | --- | --- | --- | --- | --- | --- | --- | --- | --- | --- | --- | --- |

Best Unique PSM (Scan Sample\_10\_A1\_1\_3647.d:34190, m/z=817.9007, z=2, RT=13.56, ppm=-4.83):


TRINITY\_DN2637\_c0\_g1\_i8.p2

back to list

  

| Protein Coverage
| Supporting Peptides
| Best Unique PSM
|

Protein Coverage:

Supporting Peptides:

| Peptide | Uniq | -10lgP | Mass | Length | ppm | m/z | z | RT | 1/k0 Start | 1/k0 End | Precursor ID | Area Sample 1 | #Feature | #Feature Sample 1 | Start | End | PTM | AScore | Found By |
| --- | --- | --- | --- | --- | --- | --- | --- | --- | --- | --- | --- | --- | --- | --- | --- | --- | --- | --- | --- |
| K.ATIEIPAKYEK.R | Y | 61.89 | 1261.6918 | 11 | 0.22 | 631.8505 | 2 | 14.08 | 0.982 | 0.9892 | 37168 | 0 | 0 | 0 | 107 | 117 |  |  | DB Search |
| total 1 peptides |  |  |  |  |  |  |  |  |  |  |  |  |  |  |  |  |  |  |  |
| --- | --- | --- | --- | --- | --- | --- | --- | --- | --- | --- | --- | --- | --- | --- | --- | --- | --- | --- | --- |

Best Unique PSM (Scan Sample\_10\_A1\_1\_3647.d:37168, m/z=631.8505, z=2, RT=14.08, ppm=-4.27):


TRINITY\_DN164351\_c0\_g1\_i1.p1

back to list

  

| Protein Coverage
| Supporting Peptides
| Best Unique PSM
|

Protein Coverage:

Supporting Peptides:

| Peptide | Uniq | -10lgP | Mass | Length | ppm | m/z | z | RT | 1/k0 Start | 1/k0 End | Precursor ID | Area Sample 1 | #Feature | #Feature Sample 1 | Start | End | PTM | AScore | Found By |
| --- | --- | --- | --- | --- | --- | --- | --- | --- | --- | --- | --- | --- | --- | --- | --- | --- | --- | --- | --- |
| R.RQAVDVAPLRR.V | Y | 43.13 | 1279.7473 | 11 | 9.42 | 427.5918 | 3 | 12.00 | 0.7648 | 0.7722 | 25287 | 0 | 0 | 0 | 143 | 153 |  |  | DB Search |
| total 1 peptides |  |  |  |  |  |  |  |  |  |  |  |  |  |  |  |  |  |  |  |
| --- | --- | --- | --- | --- | --- | --- | --- | --- | --- | --- | --- | --- | --- | --- | --- | --- | --- | --- | --- |

Best Unique PSM (Scan Sample\_10\_A1\_1\_3647.d:25287, m/z=427.5918, z=3, RT=12.00, ppm=4.92):


TRINITY\_DN97809\_c0\_g1\_i1.p1

back to list

  

| Protein Coverage
| Supporting Peptides
| Best Unique PSM
|

Protein Coverage:

Supporting Peptides:

| Peptide | Uniq | -10lgP | Mass | Length | ppm | m/z | z | RT | 1/k0 Start | 1/k0 End | Precursor ID | Area Sample 1 | #Feature | #Feature Sample 1 | Start | End | PTM | AScore | Found By |
| --- | --- | --- | --- | --- | --- | --- | --- | --- | --- | --- | --- | --- | --- | --- | --- | --- | --- | --- | --- |
| LGPDQVDVAGAGR.D | Y | 61.56 | 1253.6364 | 13 | 3 | 627.8245 | 2 | 13.56 | 0.9311 | 0.9384 | 34257 | 0 | 0 | 0 | 1 | 13 |  |  | DB Search |
| total 1 peptides |  |  |  |  |  |  |  |  |  |  |  |  |  |  |  |  |  |  |  |
| --- | --- | --- | --- | --- | --- | --- | --- | --- | --- | --- | --- | --- | --- | --- | --- | --- | --- | --- | --- |

Best Unique PSM (Scan Sample\_10\_A1\_1\_3647.d:34257, m/z=627.8245, z=2, RT=13.56, ppm=-1.49):


TRINITY\_DN1339\_c0\_g2\_i2.p1

back to list

  

| Protein Coverage
| Supporting Peptides
| Best Unique PSM
|

Protein Coverage:

Supporting Peptides:

| Peptide | Uniq | -10lgP | Mass | Length | ppm | m/z | z | RT | 1/k0 Start | 1/k0 End | Precursor ID | Area Sample 1 | #Feature | #Feature Sample 1 | Start | End | PTM | AScore | Found By |
| --- | --- | --- | --- | --- | --- | --- | --- | --- | --- | --- | --- | --- | --- | --- | --- | --- | --- | --- | --- |
| K.LLTSSDQAELTKK.L | Y | 66.26 | 1432.7773 | 13 | 0.59 | 717.3931 | 2 | 12.73 | 1.0504 | 1.0576 | 29502 | 1.34e3 | 1 | 1 | 155 | 167 |  |  | DB Search |
| total 1 peptides |  |  |  |  |  |  |  |  |  |  |  |  |  |  |  |  |  |  |  |
| --- | --- | --- | --- | --- | --- | --- | --- | --- | --- | --- | --- | --- | --- | --- | --- | --- | --- | --- | --- |

Best Unique PSM (Scan Sample\_10\_A1\_1\_3647.d:29502, m/z=717.3931, z=2, RT=12.73, ppm=-3.91):


TRINITY\_DN5107\_c0\_g1\_i7.p2

back to list

  

| Protein Coverage
| Supporting Peptides
| Best Unique PSM
|

Protein Coverage:

Supporting Peptides:

| Peptide | Uniq | -10lgP | Mass | Length | ppm | m/z | z | RT | 1/k0 Start | 1/k0 End | Precursor ID | Area Sample 1 | #Feature | #Feature Sample 1 | Start | End | PTM | AScore | Found By |
| --- | --- | --- | --- | --- | --- | --- | --- | --- | --- | --- | --- | --- | --- | --- | --- | --- | --- | --- | --- |
| K.KLDYVLGLTTAK.I | Y | 63.57 | 1320.7653 | 12 | -2.37 | 661.3854 | 2 | 16.69 | 0.9908 | 0.9981 | 51148 | 2.13e2 | 1 | 1 | 120 | 131 |  |  | DB Search |
| total 1 peptides |  |  |  |  |  |  |  |  |  |  |  |  |  |  |  |  |  |  |  |
| --- | --- | --- | --- | --- | --- | --- | --- | --- | --- | --- | --- | --- | --- | --- | --- | --- | --- | --- | --- |

Best Unique PSM (Scan Sample\_10\_A1\_1\_3647.d:51148, m/z=661.3854, z=2, RT=16.69, ppm=-6.87):


TRINITY\_DN34191\_c0\_g1\_i1.p1

back to list

  

| Protein Coverage
| Supporting Peptides
| Best Unique PSM
|

Protein Coverage:

Supporting Peptides:

| Peptide | Uniq | -10lgP | Mass | Length | ppm | m/z | z | RT | 1/k0 Start | 1/k0 End | Precursor ID | Area Sample 1 | #Feature | #Feature Sample 1 | Start | End | PTM | AScore | Found By |
| --- | --- | --- | --- | --- | --- | --- | --- | --- | --- | --- | --- | --- | --- | --- | --- | --- | --- | --- | --- |
| R.ARPHTM(+15.99)TSLTGSR.D | Y | 62.83 | 1429.7096 | 13 | 4.71 | 477.5772 | 3 | 9.97 | 0.849 | 0.8562 | 13362 | 0 | 0 | 0 | 100 | 112 | Oxidation (M) | M6:Oxidation (M):1000 | DB Search |
| total 1 peptides |  |  |  |  |  |  |  |  |  |  |  |  |  |  |  |  |  |  |  |
| --- | --- | --- | --- | --- | --- | --- | --- | --- | --- | --- | --- | --- | --- | --- | --- | --- | --- | --- | --- |

Best Unique PSM (Scan Sample\_10\_A1\_1\_3647.d:13362, m/z=477.5772, z=3, RT=9.97, ppm=0.22):


TRINITY\_DN2772\_c0\_g1\_i2.p1

back to list

  

| Protein Coverage
| Supporting Peptides
| Best Unique PSM
|

Protein Coverage:

Supporting Peptides:

| Peptide | Uniq | -10lgP | Mass | Length | ppm | m/z | z | RT | 1/k0 Start | 1/k0 End | Precursor ID | Area Sample 1 | #Feature | #Feature Sample 1 | Start | End | PTM | AScore | Found By |
| --- | --- | --- | --- | --- | --- | --- | --- | --- | --- | --- | --- | --- | --- | --- | --- | --- | --- | --- | --- |
| K.LIEALC(+57.02)AEHEIK.L | Y | 66.7 | 1424.7333 | 12 | 0.68 | 713.3712 | 2 | 15.09 | 1.0709 | 1.0781 | 42629 | 1.49e3 | 1 | 1 | 73 | 84 | Carbamidomethylation |  | DB Search |
| total 1 peptides |  |  |  |  |  |  |  |  |  |  |  |  |  |  |  |  |  |  |  |
| --- | --- | --- | --- | --- | --- | --- | --- | --- | --- | --- | --- | --- | --- | --- | --- | --- | --- | --- | --- |

Best Unique PSM (Scan Sample\_10\_A1\_1\_3647.d:42629, m/z=713.3712, z=2, RT=15.09, ppm=-3.81):


TRINITY\_DN413\_c0\_g1\_i1.p1

back to list

  

| Protein Coverage
| Supporting Peptides
| Best Unique PSM
|

Protein Coverage:

Supporting Peptides:

| Peptide | Uniq | -10lgP | Mass | Length | ppm | m/z | z | RT | 1/k0 Start | 1/k0 End | Precursor ID | Area Sample 1 | #Feature | #Feature Sample 1 | Start | End | PTM | AScore | Found By |
| --- | --- | --- | --- | --- | --- | --- | --- | --- | --- | --- | --- | --- | --- | --- | --- | --- | --- | --- | --- |
| R.LLLGDFNGDKR.T | Y | 66.61 | 1246.6669 | 11 | 0.98 | 624.3386 | 2 | 16.00 | 0.9848 | 0.992 | 47446 | 9.37e2 | 1 | 1 | 46 | 56 |  |  | DB Search |
| total 1 peptides |  |  |  |  |  |  |  |  |  |  |  |  |  |  |  |  |  |  |  |
| --- | --- | --- | --- | --- | --- | --- | --- | --- | --- | --- | --- | --- | --- | --- | --- | --- | --- | --- | --- |

Best Unique PSM (Scan Sample\_10\_A1\_1\_3647.d:47446, m/z=624.3386, z=2, RT=16.00, ppm=-3.51):


TRINITY\_DN817\_c0\_g1\_i6.p1

back to list

  

| Protein Coverage
| Supporting Peptides
| Best Unique PSM
|

Protein Coverage:

Supporting Peptides:

| Peptide | Uniq | -10lgP | Mass | Length | ppm | m/z | z | RT | 1/k0 Start | 1/k0 End | Precursor ID | Area Sample 1 | #Feature | #Feature Sample 1 | Start | End | PTM | AScore | Found By |
| --- | --- | --- | --- | --- | --- | --- | --- | --- | --- | --- | --- | --- | --- | --- | --- | --- | --- | --- | --- |
| R.LC(+57.02)QPGYSTC(+57.02)ENHC(+57.02)R.R | Y | 62.01 | 1780.708 | 14 | 5.39 | 891.3621 | 2 | 11.41 | 1.0893 | 1.0965 | 21785 | 1.84e2 | 1 | 1 | 147 | 160 | Carbamidomethylation, Carbamidomethylation, Carbamidomethylation |  | DB Search |
| total 1 peptides |  |  |  |  |  |  |  |  |  |  |  |  |  |  |  |  |  |  |  |
| --- | --- | --- | --- | --- | --- | --- | --- | --- | --- | --- | --- | --- | --- | --- | --- | --- | --- | --- | --- |

Best Unique PSM (Scan Sample\_10\_A1\_1\_3647.d:21785, m/z=891.3621, z=2, RT=11.41, ppm=0.9):


TRINITY\_DN614\_c0\_g1\_i1.p1

back to list

  

| Protein Coverage
| Supporting Peptides
| Best Unique PSM
|

Protein Coverage:

Supporting Peptides:

| Peptide | Uniq | -10lgP | Mass | Length | ppm | m/z | z | RT | 1/k0 Start | 1/k0 End | Precursor ID | Area Sample 1 | #Feature | #Feature Sample 1 | Start | End | PTM | AScore | Found By |
| --- | --- | --- | --- | --- | --- | --- | --- | --- | --- | --- | --- | --- | --- | --- | --- | --- | --- | --- | --- |
| R.VTEAKGELAK.M | Y | 52.31 | 1044.5815 | 10 | -5.06 | 523.293 | 2 | 9.99 | 0.8947 | 0.902 | 13499 | 0 | 0 | 0 | 100 | 109 |  |  | DB Search |
| total 1 peptides |  |  |  |  |  |  |  |  |  |  |  |  |  |  |  |  |  |  |  |
| --- | --- | --- | --- | --- | --- | --- | --- | --- | --- | --- | --- | --- | --- | --- | --- | --- | --- | --- | --- |

Best Unique PSM (Scan Sample\_10\_A1\_1\_3647.d:13499, m/z=523.293, z=2, RT=9.99, ppm=-9.55):


TRINITY\_DN3649\_c0\_g1\_i1.p1

back to list

  

| Protein Coverage
| Supporting Peptides
| Best Unique PSM
|

Protein Coverage:

Supporting Peptides:

| Peptide | Uniq | -10lgP | Mass | Length | ppm | m/z | z | RT | 1/k0 Start | 1/k0 End | Precursor ID | Area Sample 1 | #Feature | #Feature Sample 1 | Start | End | PTM | AScore | Found By |
| --- | --- | --- | --- | --- | --- | --- | --- | --- | --- | --- | --- | --- | --- | --- | --- | --- | --- | --- | --- |
| R.LC(+57.02)ESDPENAKK.K | Y | 66.75 | 1289.5921 | 11 | 2.03 | 645.8018 | 2 | 10.10 | 0.9489 | 0.9562 | 14132 | 7.73e2 | 1 | 1 | 162 | 172 | Carbamidomethylation |  | DB Search |
| total 1 peptides |  |  |  |  |  |  |  |  |  |  |  |  |  |  |  |  |  |  |  |
| --- | --- | --- | --- | --- | --- | --- | --- | --- | --- | --- | --- | --- | --- | --- | --- | --- | --- | --- | --- |

Best Unique PSM (Scan Sample\_10\_A1\_1\_3647.d:14132, m/z=645.8018, z=2, RT=10.10, ppm=-2.47):


TRINITY\_DN2637\_c0\_g1\_i7.p2

back to list

  

| Protein Coverage
| Supporting Peptides
| Best Unique PSM
|

Protein Coverage:

Supporting Peptides:

| Peptide | Uniq | -10lgP | Mass | Length | ppm | m/z | z | RT | 1/k0 Start | 1/k0 End | Precursor ID | Area Sample 1 | #Feature | #Feature Sample 1 | Start | End | PTM | AScore | Found By |
| --- | --- | --- | --- | --- | --- | --- | --- | --- | --- | --- | --- | --- | --- | --- | --- | --- | --- | --- | --- |
| K.ATIEIPAKYEK.R | Y | 61.89 | 1261.6918 | 11 | 0.22 | 631.8505 | 2 | 14.08 | 0.982 | 0.9892 | 37168 | 0 | 0 | 0 | 107 | 117 |  |  | DB Search |
| total 1 peptides |  |  |  |  |  |  |  |  |  |  |  |  |  |  |  |  |  |  |  |
| --- | --- | --- | --- | --- | --- | --- | --- | --- | --- | --- | --- | --- | --- | --- | --- | --- | --- | --- | --- |

Best Unique PSM (Scan Sample\_10\_A1\_1\_3647.d:37168, m/z=631.8505, z=2, RT=14.08, ppm=-4.27):


TRINITY\_DN817\_c0\_g1\_i5.p1

back to list

  

| Protein Coverage
| Supporting Peptides
| Best Unique PSM
|

Protein Coverage:

Supporting Peptides:

| Peptide | Uniq | -10lgP | Mass | Length | ppm | m/z | z | RT | 1/k0 Start | 1/k0 End | Precursor ID | Area Sample 1 | #Feature | #Feature Sample 1 | Start | End | PTM | AScore | Found By |
| --- | --- | --- | --- | --- | --- | --- | --- | --- | --- | --- | --- | --- | --- | --- | --- | --- | --- | --- | --- |
| R.LC(+57.02)QPGYSTC(+57.02)ENHC(+57.02)R.R | Y | 62.01 | 1780.708 | 14 | 5.39 | 891.3621 | 2 | 11.41 | 1.0893 | 1.0965 | 21785 | 1.84e2 | 1 | 1 | 145 | 158 | Carbamidomethylation, Carbamidomethylation, Carbamidomethylation |  | DB Search |
| total 1 peptides |  |  |  |  |  |  |  |  |  |  |  |  |  |  |  |  |  |  |  |
| --- | --- | --- | --- | --- | --- | --- | --- | --- | --- | --- | --- | --- | --- | --- | --- | --- | --- | --- | --- |

Best Unique PSM (Scan Sample\_10\_A1\_1\_3647.d:21785, m/z=891.3621, z=2, RT=11.41, ppm=0.9):


TRINITY\_DN495\_c0\_g1\_i3.p1

back to list

  

| Protein Coverage
| Supporting Peptides
| Best Unique PSM
|

Protein Coverage:

Supporting Peptides:

| Peptide | Uniq | -10lgP | Mass | Length | ppm | m/z | z | RT | 1/k0 Start | 1/k0 End | Precursor ID | Area Sample 1 | #Feature | #Feature Sample 1 | Start | End | PTM | AScore | Found By |
| --- | --- | --- | --- | --- | --- | --- | --- | --- | --- | --- | --- | --- | --- | --- | --- | --- | --- | --- | --- |
| R.SC(+57.02)TNPAPANGGK.A | Y | 39.93 | 1172.5244 | 12 | 0 | 587.2668 | 2 | 9.43 | 0.8404 | 0.8477 | 10729 | 0 | 0 | 0 | 503 | 514 | Carbamidomethylation |  | DB Search |
| total 1 peptides |  |  |  |  |  |  |  |  |  |  |  |  |  |  |  |  |  |  |  |
| --- | --- | --- | --- | --- | --- | --- | --- | --- | --- | --- | --- | --- | --- | --- | --- | --- | --- | --- | --- |

Best Unique PSM (Scan Sample\_10\_A1\_1\_3647.d:10729, m/z=587.2668, z=2, RT=9.43, ppm=-4.49):


TRINITY\_DN7504\_c0\_g1\_i1.p1

back to list

  

| Protein Coverage
| Supporting Peptides
| Best Unique PSM
|

Protein Coverage:

Supporting Peptides:

| Peptide | Uniq | -10lgP | Mass | Length | ppm | m/z | z | RT | 1/k0 Start | 1/k0 End | Precursor ID | Area Sample 1 | #Feature | #Feature Sample 1 | Start | End | PTM | AScore | Found By |
| --- | --- | --- | --- | --- | --- | --- | --- | --- | --- | --- | --- | --- | --- | --- | --- | --- | --- | --- | --- |
| K.TIEAEAAHGTVTR.H | Y | 66.82 | 1354.6841 | 13 | 1.88 | 678.3475 | 2 | 11.81 | 1.013 | 1.0202 | 24117 | 1.54e3 | 1 | 1 | 301 | 313 |  |  | DB Search |
| K.LVPGWTKPIVIGR.H | Y | 46.21 | 1434.8711 | 13 | 2.88 | 718.4417 | 2 | 17.27 | 1.0411 | 1.0484 | 54264 | 0 | 0 | 0 | 119 | 131 |  |  | DB Search |
| total 2 peptides |  |  |  |  |  |  |  |  |  |  |  |  |  |  |  |  |  |  |  |
| --- | --- | --- | --- | --- | --- | --- | --- | --- | --- | --- | --- | --- | --- | --- | --- | --- | --- | --- | --- |

Best Unique PSM (Scan Sample\_10\_A1\_1\_3647.d:24117, m/z=678.3475, z=2, RT=11.81, ppm=-2.61):


TRINITY\_DN6\_c0\_g1\_i1.p1

back to list

  

| Protein Coverage
| Supporting Peptides
| Best Unique PSM
|

Protein Coverage:

Supporting Peptides:

| Peptide | Uniq | -10lgP | Mass | Length | ppm | m/z | z | RT | 1/k0 Start | 1/k0 End | Precursor ID | Area Sample 1 | #Feature | #Feature Sample 1 | Start | End | PTM | AScore | Found By |
| --- | --- | --- | --- | --- | --- | --- | --- | --- | --- | --- | --- | --- | --- | --- | --- | --- | --- | --- | --- |
| K.AISFIGSVHDNRK.E | Y | 39.17 | 1442.763 | 13 | -1.14 | 722.3847 | 2 | 14.03 | 1.0415 | 1.0488 | 36874 | 0 | 0 | 0 | 55 | 67 |  |  | DB Search |
| total 1 peptides |  |  |  |  |  |  |  |  |  |  |  |  |  |  |  |  |  |  |  |
| --- | --- | --- | --- | --- | --- | --- | --- | --- | --- | --- | --- | --- | --- | --- | --- | --- | --- | --- | --- |

Best Unique PSM (Scan Sample\_10\_A1\_1\_3647.d:36874, m/z=722.3847, z=2, RT=14.03, ppm=-5.63):


TRINITY\_DN1339\_c0\_g2\_i4.p2

back to list

  

| Protein Coverage
| Supporting Peptides
| Best Unique PSM
|

Protein Coverage:

Supporting Peptides:

| Peptide | Uniq | -10lgP | Mass | Length | ppm | m/z | z | RT | 1/k0 Start | 1/k0 End | Precursor ID | Area Sample 1 | #Feature | #Feature Sample 1 | Start | End | PTM | AScore | Found By |
| --- | --- | --- | --- | --- | --- | --- | --- | --- | --- | --- | --- | --- | --- | --- | --- | --- | --- | --- | --- |
| K.LLTSSDQAELTKK.L | Y | 66.26 | 1432.7773 | 13 | 0.59 | 717.3931 | 2 | 12.73 | 1.0504 | 1.0576 | 29502 | 1.34e3 | 1 | 1 | 155 | 167 |  |  | DB Search |
| total 1 peptides |  |  |  |  |  |  |  |  |  |  |  |  |  |  |  |  |  |  |  |
| --- | --- | --- | --- | --- | --- | --- | --- | --- | --- | --- | --- | --- | --- | --- | --- | --- | --- | --- | --- |

Best Unique PSM (Scan Sample\_10\_A1\_1\_3647.d:29502, m/z=717.3931, z=2, RT=12.73, ppm=-3.91):


TRINITY\_DN95606\_c0\_g1\_i1.p1

back to list

  

| Protein Coverage
| Supporting Peptides
| Best Unique PSM
|

Protein Coverage:

Supporting Peptides:

| Peptide | Uniq | -10lgP | Mass | Length | ppm | m/z | z | RT | 1/k0 Start | 1/k0 End | Precursor ID | Area Sample 1 | #Feature | #Feature Sample 1 | Start | End | PTM | AScore | Found By |
| --- | --- | --- | --- | --- | --- | --- | --- | --- | --- | --- | --- | --- | --- | --- | --- | --- | --- | --- | --- |
| R.VIGLVEYRR.K | Y | 31.43 | 1103.6451 | 9 | 4.18 | 552.8297 | 2 | 14.49 | 0.9097 | 0.917 | 39428 | 0 | 0 | 0 | 272 | 280 |  |  | DB Search |
| total 1 peptides |  |  |  |  |  |  |  |  |  |  |  |  |  |  |  |  |  |  |  |
| --- | --- | --- | --- | --- | --- | --- | --- | --- | --- | --- | --- | --- | --- | --- | --- | --- | --- | --- | --- |

Best Unique PSM (Scan Sample\_10\_A1\_1\_3647.d:39428, m/z=552.8297, z=2, RT=14.49, ppm=-0.32):


TRINITY\_DN76073\_c0\_g1\_i1.p1

back to list

  

| Protein Coverage
| Supporting Peptides
| Best Unique PSM
|

Protein Coverage:

Supporting Peptides:

| Peptide | Uniq | -10lgP | Mass | Length | ppm | m/z | z | RT | 1/k0 Start | 1/k0 End | Precursor ID | Area Sample 1 | #Feature | #Feature Sample 1 | Start | End | PTM | AScore | Found By |
| --- | --- | --- | --- | --- | --- | --- | --- | --- | --- | --- | --- | --- | --- | --- | --- | --- | --- | --- | --- |
| K.TSDSDLEKR.I | Y | 41.64 | 1049.4989 | 9 | -18.21 | 525.7448 | 2 | 11.26 | 0.8611 | 0.8684 | 20698 | 3.58e3 | 1 | 1 | 55 | 63 |  |  | DB Search |
| total 1 peptides |  |  |  |  |  |  |  |  |  |  |  |  |  |  |  |  |  |  |  |
| --- | --- | --- | --- | --- | --- | --- | --- | --- | --- | --- | --- | --- | --- | --- | --- | --- | --- | --- | --- |

Best Unique PSM (Scan Sample\_10\_A1\_1\_3647.d:20698, m/z=525.7448, z=2, RT=11.26, ppm=-22.7):


TRINITY\_DN128406\_c0\_g1\_i1.p1

back to list

  

| Protein Coverage
| Supporting Peptides
| Best Unique PSM
|

Protein Coverage:

Supporting Peptides:

| Peptide | Uniq | -10lgP | Mass | Length | ppm | m/z | z | RT | 1/k0 Start | 1/k0 End | Precursor ID | Area Sample 1 | #Feature | #Feature Sample 1 | Start | End | PTM | AScore | Found By |
| --- | --- | --- | --- | --- | --- | --- | --- | --- | --- | --- | --- | --- | --- | --- | --- | --- | --- | --- | --- |
| K.EIRDILVQYDR.S | Y | 66.7 | 1418.7517 | 11 | -3.73 | 710.3773 | 2 | 19.70 | 1.0323 | 1.0395 | 68136 | 1.75e4 | 1 | 1 | 110 | 120 |  |  | DB Search |
| total 1 peptides |  |  |  |  |  |  |  |  |  |  |  |  |  |  |  |  |  |  |  |
| --- | --- | --- | --- | --- | --- | --- | --- | --- | --- | --- | --- | --- | --- | --- | --- | --- | --- | --- | --- |

Best Unique PSM (Scan Sample\_10\_A1\_1\_3647.d:68136, m/z=710.3773, z=2, RT=19.70, ppm=-8.22):


TRINITY\_DN801\_c0\_g1\_i7.p1

back to list

  

| Protein Coverage
| Supporting Peptides
| Best Unique PSM
|

Protein Coverage:

Supporting Peptides:

| Peptide | Uniq | -10lgP | Mass | Length | ppm | m/z | z | RT | 1/k0 Start | 1/k0 End | Precursor ID | Area Sample 1 | #Feature | #Feature Sample 1 | Start | End | PTM | AScore | Found By |
| --- | --- | --- | --- | --- | --- | --- | --- | --- | --- | --- | --- | --- | --- | --- | --- | --- | --- | --- | --- |
| K.VFDRDGNGFISAAELR.H | Y | 39.11 | 1765.8747 | 16 | -1.5 | 883.9393 | 2 | 19.71 | 1.1234 | 1.1306 | 69271 | 0 | 0 | 0 | 124 | 139 |  |  | DB Search |
| total 1 peptides |  |  |  |  |  |  |  |  |  |  |  |  |  |  |  |  |  |  |  |
| --- | --- | --- | --- | --- | --- | --- | --- | --- | --- | --- | --- | --- | --- | --- | --- | --- | --- | --- | --- |

Best Unique PSM (Scan Sample\_10\_A1\_1\_3647.d:69271, m/z=883.9393, z=2, RT=19.71, ppm=-6):


TRINITY\_DN4911\_c0\_g1\_i1.p1

back to list

  

| Protein Coverage
| Supporting Peptides
| Best Unique PSM
|

Protein Coverage:

Supporting Peptides:

| Peptide | Uniq | -10lgP | Mass | Length | ppm | m/z | z | RT | 1/k0 Start | 1/k0 End | Precursor ID | Area Sample 1 | #Feature | #Feature Sample 1 | Start | End | PTM | AScore | Found By |
| --- | --- | --- | --- | --- | --- | --- | --- | --- | --- | --- | --- | --- | --- | --- | --- | --- | --- | --- | --- |
| R.IYAEVLHFHAEEK.Q | Y | 66.19 | 1584.7936 | 13 | 2.08 | 793.4022 | 2 | 15.23 | 1.1366 | 1.1438 | 43384 | 3.89e2 | 1 | 1 | 239 | 251 |  |  | DB Search |
| total 1 peptides |  |  |  |  |  |  |  |  |  |  |  |  |  |  |  |  |  |  |  |
| --- | --- | --- | --- | --- | --- | --- | --- | --- | --- | --- | --- | --- | --- | --- | --- | --- | --- | --- | --- |

Best Unique PSM (Scan Sample\_10\_A1\_1\_3647.d:43384, m/z=793.4022, z=2, RT=15.23, ppm=-2.42):


TRINITY\_DN801\_c0\_g1\_i1.p1

back to list

  

| Protein Coverage
| Supporting Peptides
| Best Unique PSM
|

Protein Coverage:

Supporting Peptides:

| Peptide | Uniq | -10lgP | Mass | Length | ppm | m/z | z | RT | 1/k0 Start | 1/k0 End | Precursor ID | Area Sample 1 | #Feature | #Feature Sample 1 | Start | End | PTM | AScore | Found By |
| --- | --- | --- | --- | --- | --- | --- | --- | --- | --- | --- | --- | --- | --- | --- | --- | --- | --- | --- | --- |
| K.VFDRDGNGFISAAELR.H | Y | 39.11 | 1765.8747 | 16 | -1.5 | 883.9393 | 2 | 19.71 | 1.1234 | 1.1306 | 69271 | 0 | 0 | 0 | 119 | 134 |  |  | DB Search |
| total 1 peptides |  |  |  |  |  |  |  |  |  |  |  |  |  |  |  |  |  |  |  |
| --- | --- | --- | --- | --- | --- | --- | --- | --- | --- | --- | --- | --- | --- | --- | --- | --- | --- | --- | --- |

Best Unique PSM (Scan Sample\_10\_A1\_1\_3647.d:69271, m/z=883.9393, z=2, RT=19.71, ppm=-6):


TRINITY\_DN1739\_c0\_g1\_i1.p1

back to list

  

| Protein Coverage
| Supporting Peptides
| Best Unique PSM
|

Protein Coverage:

Supporting Peptides:

| Peptide | Uniq | -10lgP | Mass | Length | ppm | m/z | z | RT | 1/k0 Start | 1/k0 End | Precursor ID | Area Sample 1 | #Feature | #Feature Sample 1 | Start | End | PTM | AScore | Found By |
| --- | --- | --- | --- | --- | --- | --- | --- | --- | --- | --- | --- | --- | --- | --- | --- | --- | --- | --- | --- |
| M.AITDNIKDILSVK.I | Y | 66.66 | 1428.8188 | 13 | -1.1 | 715.4127 | 2 | 19.15 | 1.0512 | 1.0584 | 65748 | 5.17e2 | 1 | 1 | 2 | 14 |  |  | DB Search |
| total 1 peptides |  |  |  |  |  |  |  |  |  |  |  |  |  |  |  |  |  |  |  |
| --- | --- | --- | --- | --- | --- | --- | --- | --- | --- | --- | --- | --- | --- | --- | --- | --- | --- | --- | --- |

Best Unique PSM (Scan Sample\_10\_A1\_1\_3647.d:65748, m/z=715.4127, z=2, RT=19.15, ppm=-5.59):


TRINITY\_DN1339\_c0\_g1\_i1.p1

back to list

  

| Protein Coverage
| Supporting Peptides
| Best Unique PSM
|

Protein Coverage:

Supporting Peptides:

| Peptide | Uniq | -10lgP | Mass | Length | ppm | m/z | z | RT | 1/k0 Start | 1/k0 End | Precursor ID | Area Sample 1 | #Feature | #Feature Sample 1 | Start | End | PTM | AScore | Found By |
| --- | --- | --- | --- | --- | --- | --- | --- | --- | --- | --- | --- | --- | --- | --- | --- | --- | --- | --- | --- |
| K.VITSDDPAEKEK.K | Y | 66.7 | 1330.6616 | 12 | 2.85 | 666.337 | 2 | 10.71 | 0.9836 | 0.9908 | 17367 | 7.2e3 | 1 | 1 | 160 | 171 |  |  | DB Search |
| total 1 peptides |  |  |  |  |  |  |  |  |  |  |  |  |  |  |  |  |  |  |  |
| --- | --- | --- | --- | --- | --- | --- | --- | --- | --- | --- | --- | --- | --- | --- | --- | --- | --- | --- | --- |

Best Unique PSM (Scan Sample\_10\_A1\_1\_3647.d:17367, m/z=666.337, z=2, RT=10.71, ppm=-1.65):


TRINITY\_DN468\_c1\_g1\_i1.p1

back to list

  

| Protein Coverage
| Supporting Peptides
| Best Unique PSM
|

Protein Coverage:

Supporting Peptides:

| Peptide | Uniq | -10lgP | Mass | Length | ppm | m/z | z | RT | 1/k0 Start | 1/k0 End | Precursor ID | Area Sample 1 | #Feature | #Feature Sample 1 | Start | End | PTM | AScore | Found By |
| --- | --- | --- | --- | --- | --- | --- | --- | --- | --- | --- | --- | --- | --- | --- | --- | --- | --- | --- | --- |
| K.LGPVVDGKYDYTVITTDYK.A | Y | 59.89 | 2146.0834 | 19 | 3.69 | 1074.0481 | 2 | 17.48 | 1.242 | 1.2491 | 55518 | 0 | 0 | 0 | 123 | 141 |  |  | DB Search |
| total 1 peptides |  |  |  |  |  |  |  |  |  |  |  |  |  |  |  |  |  |  |  |
| --- | --- | --- | --- | --- | --- | --- | --- | --- | --- | --- | --- | --- | --- | --- | --- | --- | --- | --- | --- |

Best Unique PSM (Scan Sample\_10\_A1\_1\_3647.d:55518, m/z=1074.0481, z=2, RT=17.48, ppm=-0.8):


TRINITY\_DN143799\_c0\_g1\_i1.p1

back to list

  

| Protein Coverage
| Supporting Peptides
| Best Unique PSM
|

Protein Coverage:

Supporting Peptides:

| Peptide | Uniq | -10lgP | Mass | Length | ppm | m/z | z | RT | 1/k0 Start | 1/k0 End | Precursor ID | Area Sample 1 | #Feature | #Feature Sample 1 | Start | End | PTM | AScore | Found By |
| --- | --- | --- | --- | --- | --- | --- | --- | --- | --- | --- | --- | --- | --- | --- | --- | --- | --- | --- | --- |
| K.SVGEIVQC(+57.02)SEIFDKSPTTVK.N | Y | 20.8 | 2223.1093 | 20 | 0.96 | 1112.558 | 2 | 17.35 | 1.2893 | 1.2964 | 54732 | 0 | 0 | 0 | 57 | 76 | Carbamidomethylation |  | DB Search |
| total 1 peptides |  |  |  |  |  |  |  |  |  |  |  |  |  |  |  |  |  |  |  |
| --- | --- | --- | --- | --- | --- | --- | --- | --- | --- | --- | --- | --- | --- | --- | --- | --- | --- | --- | --- |

Best Unique PSM (Scan Sample\_10\_A1\_1\_3647.d:54732, m/z=1112.558, z=2, RT=17.35, ppm=-3.53):


TRINITY\_DN585\_c0\_g1\_i4.p1

back to list

  

| Protein Coverage
| Supporting Peptides
| Best Unique PSM
|

Protein Coverage:

Supporting Peptides:

| Peptide | Uniq | -10lgP | Mass | Length | ppm | m/z | z | RT | 1/k0 Start | 1/k0 End | Precursor ID | Area Sample 1 | #Feature | #Feature Sample 1 | Start | End | PTM | AScore | Found By |
| --- | --- | --- | --- | --- | --- | --- | --- | --- | --- | --- | --- | --- | --- | --- | --- | --- | --- | --- | --- |
| K.IQDKEGIPPDQQR.L | Y | 65.08 | 1522.7739 | 13 | -0.66 | 762.3903 | 2 | 11.01 | 1.0339 | 1.0411 | 19352 | 9.49e2 | 1 | 1 | 30 | 42 |  |  | DB Search |
| total 1 peptides |  |  |  |  |  |  |  |  |  |  |  |  |  |  |  |  |  |  |  |
| --- | --- | --- | --- | --- | --- | --- | --- | --- | --- | --- | --- | --- | --- | --- | --- | --- | --- | --- | --- |

Best Unique PSM (Scan Sample\_10\_A1\_1\_3647.d:19352, m/z=762.3903, z=2, RT=11.01, ppm=-5.16):


TRINITY\_DN5131\_c1\_g2\_i1.p1

back to list

  

| Protein Coverage
| Supporting Peptides
| Best Unique PSM
|

Protein Coverage:

Supporting Peptides:

| Peptide | Uniq | -10lgP | Mass | Length | ppm | m/z | z | RT | 1/k0 Start | 1/k0 End | Precursor ID | Area Sample 1 | #Feature | #Feature Sample 1 | Start | End | PTM | AScore | Found By |
| --- | --- | --- | --- | --- | --- | --- | --- | --- | --- | --- | --- | --- | --- | --- | --- | --- | --- | --- | --- |
| K.AYC(+57.02)DINEHISC(+57.02)SK.V | Y | 66.75 | 1595.6708 | 13 | 2.31 | 798.8409 | 2 | 12.79 | 1.0564 | 1.0636 | 29794 | 7.14e2 | 1 | 1 | 39 | 51 | Carbamidomethylation, Carbamidomethylation |  | DB Search |
| total 1 peptides |  |  |  |  |  |  |  |  |  |  |  |  |  |  |  |  |  |  |  |
| --- | --- | --- | --- | --- | --- | --- | --- | --- | --- | --- | --- | --- | --- | --- | --- | --- | --- | --- | --- |

Best Unique PSM (Scan Sample\_10\_A1\_1\_3647.d:29794, m/z=798.8409, z=2, RT=12.79, ppm=-2.18):


TRINITY\_DN3034\_c0\_g1\_i1.p1

back to list

  

| Protein Coverage
| Supporting Peptides
| Best Unique PSM
|

Protein Coverage:

Supporting Peptides:

| Peptide | Uniq | -10lgP | Mass | Length | ppm | m/z | z | RT | 1/k0 Start | 1/k0 End | Precursor ID | Area Sample 1 | #Feature | #Feature Sample 1 | Start | End | PTM | AScore | Found By |
| --- | --- | --- | --- | --- | --- | --- | --- | --- | --- | --- | --- | --- | --- | --- | --- | --- | --- | --- | --- |
| K.TPGLVEGTLILHQLR.C | Y | 66.72 | 1645.9515 | 15 | -1.96 | 549.6542 | 3 | 18.79 | 0.8635 | 0.8708 | 63448 | 6.54e2 | 1 | 1 | 671 | 685 |  |  | DB Search |
| R.VKELQFQLEDEHK.A | Y | 61.8 | 1641.8362 | 13 | -15.13 | 548.2753 | 3 | 14.58 | 0.8124 | 0.8197 | 39893 | 0 | 0 | 0 | 1837 | 1849 |  |  | DB Search |
| K.LQQFFNHHM(+15.99)FVLEQEEYKK.E | Y | 16.72 | 2510.2052 | 19 | 1.98 | 628.557 | 4 | 15.85 | 0.8721 | 0.8793 | 46749 | 0 | 0 | 0 | 481 | 499 | Oxidation (M) | M9:Oxidation (M):1000 | DB Search |
| total 3 peptides |  |  |  |  |  |  |  |  |  |  |  |  |  |  |  |  |  |  |  |
| --- | --- | --- | --- | --- | --- | --- | --- | --- | --- | --- | --- | --- | --- | --- | --- | --- | --- | --- | --- |

Best Unique PSM (Scan Sample\_10\_A1\_1\_3647.d:63448, m/z=549.6542, z=3, RT=18.79, ppm=-6.45):


TRINITY\_DN2636\_c0\_g1\_i2.p1

back to list

  

| Protein Coverage
| Supporting Peptides
| Best Unique PSM
|

Protein Coverage:

Supporting Peptides:

| Peptide | Uniq | -10lgP | Mass | Length | ppm | m/z | z | RT | 1/k0 Start | 1/k0 End | Precursor ID | Area Sample 1 | #Feature | #Feature Sample 1 | Start | End | PTM | AScore | Found By |
| --- | --- | --- | --- | --- | --- | --- | --- | --- | --- | --- | --- | --- | --- | --- | --- | --- | --- | --- | --- |
| K.VVEKEAETER.K | Y | 66.5 | 1188.5986 | 10 | 7.91 | 397.2082 | 3 | 9.54 | 0.729 | 0.7363 | 11130 | 2.07e3 | 2 | 2 | 199 | 208 |  |  | DB Search |
| total 1 peptides |  |  |  |  |  |  |  |  |  |  |  |  |  |  |  |  |  |  |  |
| --- | --- | --- | --- | --- | --- | --- | --- | --- | --- | --- | --- | --- | --- | --- | --- | --- | --- | --- | --- |

Best Unique PSM (Scan Sample\_10\_A1\_1\_3647.d:11130, m/z=397.2082, z=3, RT=9.54, ppm=3.41):


TRINITY\_DN817\_c0\_g1\_i4.p1

back to list

  

| Protein Coverage
| Supporting Peptides
| Best Unique PSM
|

Protein Coverage:

Supporting Peptides:

| Peptide | Uniq | -10lgP | Mass | Length | ppm | m/z | z | RT | 1/k0 Start | 1/k0 End | Precursor ID | Area Sample 1 | #Feature | #Feature Sample 1 | Start | End | PTM | AScore | Found By |
| --- | --- | --- | --- | --- | --- | --- | --- | --- | --- | --- | --- | --- | --- | --- | --- | --- | --- | --- | --- |
| R.LC(+57.02)QPGYSTC(+57.02)ENHC(+57.02)R.R | Y | 62.01 | 1780.708 | 14 | 5.39 | 891.3621 | 2 | 11.41 | 1.0893 | 1.0965 | 21785 | 1.84e2 | 1 | 1 | 145 | 158 | Carbamidomethylation, Carbamidomethylation, Carbamidomethylation |  | DB Search |
| total 1 peptides |  |  |  |  |  |  |  |  |  |  |  |  |  |  |  |  |  |  |  |
| --- | --- | --- | --- | --- | --- | --- | --- | --- | --- | --- | --- | --- | --- | --- | --- | --- | --- | --- | --- |

Best Unique PSM (Scan Sample\_10\_A1\_1\_3647.d:21785, m/z=891.3621, z=2, RT=11.41, ppm=0.9):


TRINITY\_DN2799\_c0\_g1\_i1.p1

back to list

  

| Protein Coverage
| Supporting Peptides
| Best Unique PSM
|

Protein Coverage:

Supporting Peptides:

| Peptide | Uniq | -10lgP | Mass | Length | ppm | m/z | z | RT | 1/k0 Start | 1/k0 End | Precursor ID | Area Sample 1 | #Feature | #Feature Sample 1 | Start | End | PTM | AScore | Found By |
| --- | --- | --- | --- | --- | --- | --- | --- | --- | --- | --- | --- | --- | --- | --- | --- | --- | --- | --- | --- |
| R.HGSLGFLPR.K | Y | 25.17 | 982.5348 | 9 | -2.89 | 492.2711 | 2 | 17.17 | 0.8408 | 0.8481 | 53699 | 1.07e3 | 1 | 1 | 34 | 42 |  |  | DB Search |
| K.AGM(+15.99)THIVR.D | Y | 19.81 | 899.4647 | 8 | 2.72 | 450.7388 | 2 | 9.73 | 0.8226 | 0.8299 | 12038 | 6.74e2 | 1 | 1 | 74 | 81 | Oxidation (M) | M3:Oxidation (M):1000 | DB Search |
| total 2 peptides |  |  |  |  |  |  |  |  |  |  |  |  |  |  |  |  |  |  |  |
| --- | --- | --- | --- | --- | --- | --- | --- | --- | --- | --- | --- | --- | --- | --- | --- | --- | --- | --- | --- |

Best Unique PSM (Scan Sample\_10\_A1\_1\_3647.d:53699, m/z=492.2711, z=2, RT=17.17, ppm=-7.39):


TRINITY\_DN1065\_c0\_g1\_i1.p1

back to list

  

| Protein Coverage
| Supporting Peptides
| Best Unique PSM
|

Protein Coverage:

Supporting Peptides:

| Peptide | Uniq | -10lgP | Mass | Length | ppm | m/z | z | RT | 1/k0 Start | 1/k0 End | Precursor ID | Area Sample 1 | #Feature | #Feature Sample 1 | Start | End | PTM | AScore | Found By |
| --- | --- | --- | --- | --- | --- | --- | --- | --- | --- | --- | --- | --- | --- | --- | --- | --- | --- | --- | --- |
| R.HILLAIANDEELNKLLR.N | Y | 64.29 | 1974.1262 | 17 | -1.92 | 659.0451 | 3 | 19.51 | 0.9852 | 0.9924 | 67939 | 3.02e2 | 1 | 1 | 79 | 95 |  |  | DB Search |
| total 1 peptides |  |  |  |  |  |  |  |  |  |  |  |  |  |  |  |  |  |  |  |
| --- | --- | --- | --- | --- | --- | --- | --- | --- | --- | --- | --- | --- | --- | --- | --- | --- | --- | --- | --- |

Best Unique PSM (Scan Sample\_10\_A1\_1\_3647.d:67939, m/z=659.0451, z=3, RT=19.51, ppm=-6.42):


TRINITY\_DN163801\_c0\_g1\_i1.p1

back to list

  

| Protein Coverage
| Supporting Peptides
| Best Unique PSM
|

Protein Coverage:

Supporting Peptides:

| Peptide | Uniq | -10lgP | Mass | Length | ppm | m/z | z | RT | 1/k0 Start | 1/k0 End | Precursor ID | Area Sample 1 | #Feature | #Feature Sample 1 | Start | End | PTM | AScore | Found By |
| --- | --- | --- | --- | --- | --- | --- | --- | --- | --- | --- | --- | --- | --- | --- | --- | --- | --- | --- | --- |
| M.A(+42.01)NPLKDIISR.F | Y | 66.51 | 1167.6611 | 10 | 0.91 | 584.8358 | 2 | 18.57 | 0.919 | 0.9263 | 61990 | 1.77e3 | 1 | 1 | 2 | 11 | Acetylation (Protein N-term) | A1:Acetylation (Protein N-term):1000 | DB Search |
| total 1 peptides |  |  |  |  |  |  |  |  |  |  |  |  |  |  |  |  |  |  |  |
| --- | --- | --- | --- | --- | --- | --- | --- | --- | --- | --- | --- | --- | --- | --- | --- | --- | --- | --- | --- |

Best Unique PSM (Scan Sample\_10\_A1\_1\_3647.d:61990, m/z=584.8358, z=2, RT=18.57, ppm=-3.59):


TRINITY\_DN95248\_c0\_g1\_i1.p1

back to list

  

| Protein Coverage
| Supporting Peptides
| Best Unique PSM
|

Protein Coverage:

Supporting Peptides:

| Peptide | Uniq | -10lgP | Mass | Length | ppm | m/z | z | RT | 1/k0 Start | 1/k0 End | Precursor ID | Area Sample 1 | #Feature | #Feature Sample 1 | Start | End | PTM | AScore | Found By |
| --- | --- | --- | --- | --- | --- | --- | --- | --- | --- | --- | --- | --- | --- | --- | --- | --- | --- | --- | --- |
| R.VVASSHTDYELR.Y | Y | 66.61 | 1375.6732 | 12 | 3.98 | 688.8435 | 2 | 12.52 | 1.0086 | 1.0158 | 28165 | 3.7e3 | 1 | 1 | 96 | 107 |  |  | DB Search |
| K.VTLVSERPFSK.R | Y | 66.47 | 1261.703 | 11 | -6.46 | 631.8519 | 2 | 15.24 | 0.9614 | 0.9687 | 43493 | 0 | 0 | 0 | 64 | 74 |  |  | DB Search |
| K.IKVDDKLNNLDQITLEVAK.T | Y | 62.46 | 2168.2052 | 19 | -0.11 | 723.739 | 3 | 17.81 | 0.9105 | 0.9178 | 57433 | 1.3e3 | 1 | 1 | 43 | 61 |  |  | DB Search |
| total 3 peptides |  |  |  |  |  |  |  |  |  |  |  |  |  |  |  |  |  |  |  |
| --- | --- | --- | --- | --- | --- | --- | --- | --- | --- | --- | --- | --- | --- | --- | --- | --- | --- | --- | --- |

Best Unique PSM (Scan Sample\_10\_A1\_1\_3647.d:28165, m/z=688.8435, z=2, RT=12.52, ppm=-0.52):


TRINITY\_DN1091\_c0\_g1\_i1.p1

back to list

  

| Protein Coverage
| Supporting Peptides
| Best Unique PSM
|

Protein Coverage:

Supporting Peptides:

| Peptide | Uniq | -10lgP | Mass | Length | ppm | m/z | z | RT | 1/k0 Start | 1/k0 End | Precursor ID | Area Sample 1 | #Feature | #Feature Sample 1 | Start | End | PTM | AScore | Found By |
| --- | --- | --- | --- | --- | --- | --- | --- | --- | --- | --- | --- | --- | --- | --- | --- | --- | --- | --- | --- |
| R.AFQNTPC(+57.02)THLVLDGTR.W | Y | 63.4 | 1828.889 | 16 | -0.04 | 915.4476 | 2 | 15.25 | 1.1762 | 1.1833 | 43382 | 3.21e2 | 1 | 1 | 81 | 96 | Carbamidomethylation |  | DB Search |
| total 1 peptides |  |  |  |  |  |  |  |  |  |  |  |  |  |  |  |  |  |  |  |
| --- | --- | --- | --- | --- | --- | --- | --- | --- | --- | --- | --- | --- | --- | --- | --- | --- | --- | --- | --- |

Best Unique PSM (Scan Sample\_10\_A1\_1\_3647.d:43382, m/z=915.4476, z=2, RT=15.25, ppm=-4.53):


TRINITY\_DN3452\_c0\_g1\_i1.p1

back to list

  

| Protein Coverage
| Supporting Peptides
| Best Unique PSM
|

Protein Coverage:

Supporting Peptides:

| Peptide | Uniq | -10lgP | Mass | Length | ppm | m/z | z | RT | 1/k0 Start | 1/k0 End | Precursor ID | Area Sample 1 | #Feature | #Feature Sample 1 | Start | End | PTM | AScore | Found By |
| --- | --- | --- | --- | --- | --- | --- | --- | --- | --- | --- | --- | --- | --- | --- | --- | --- | --- | --- | --- |
| K.LIAFINDLHSGK.L | Y | 66.64 | 1326.7295 | 12 | 1.4 | 664.37 | 2 | 17.48 | 1.0238 | 1.0311 | 55497 | 7.23e2 | 1 | 1 | 342 | 353 |  |  | DB Search |
| total 1 peptides |  |  |  |  |  |  |  |  |  |  |  |  |  |  |  |  |  |  |  |
| --- | --- | --- | --- | --- | --- | --- | --- | --- | --- | --- | --- | --- | --- | --- | --- | --- | --- | --- | --- |

Best Unique PSM (Scan Sample\_10\_A1\_1\_3647.d:55497, m/z=664.37, z=2, RT=17.48, ppm=-3.1):


TRINITY\_DN4954\_c0\_g1\_i2.p2

back to list

  

| Protein Coverage
| Supporting Peptides
| Best Unique PSM
|

Protein Coverage:

Supporting Peptides:

| Peptide | Uniq | -10lgP | Mass | Length | ppm | m/z | z | RT | 1/k0 Start | 1/k0 End | Precursor ID | Area Sample 1 | #Feature | #Feature Sample 1 | Start | End | PTM | AScore | Found By |
| --- | --- | --- | --- | --- | --- | --- | --- | --- | --- | --- | --- | --- | --- | --- | --- | --- | --- | --- | --- |
| R.AIELLPHDLAVGR.H | Y | 66.91 | 1402.7932 | 13 | -9.52 | 702.394 | 2 | 17.77 | 1.0275 | 1.0347 | 57279 | 0 | 0 | 0 | 109 | 121 |  |  | DB Search |
| R.YQYEHAVAELR.G | Y | 64.48 | 1377.6677 | 11 | 2.2 | 689.8395 | 2 | 14.37 | 1.0102 | 1.0174 | 38774 | 1.67e3 | 1 | 1 | 79 | 89 |  |  | DB Search |
| total 2 peptides |  |  |  |  |  |  |  |  |  |  |  |  |  |  |  |  |  |  |  |
| --- | --- | --- | --- | --- | --- | --- | --- | --- | --- | --- | --- | --- | --- | --- | --- | --- | --- | --- | --- |

Best Unique PSM (Scan Sample\_10\_A1\_1\_3647.d:57279, m/z=702.394, z=2, RT=17.77, ppm=-14.02):


TRINITY\_DN2904\_c0\_g1\_i1.p1

back to list

  

| Protein Coverage
| Supporting Peptides
| Best Unique PSM
|

Protein Coverage:

Supporting Peptides:

| Peptide | Uniq | -10lgP | Mass | Length | ppm | m/z | z | RT | 1/k0 Start | 1/k0 End | Precursor ID | Area Sample 1 | #Feature | #Feature Sample 1 | Start | End | PTM | AScore | Found By |
| --- | --- | --- | --- | --- | --- | --- | --- | --- | --- | --- | --- | --- | --- | --- | --- | --- | --- | --- | --- |
| K.KKEESPNAQDAIQK.L | Y | 65.45 | 1584.8107 | 14 | -1.03 | 529.2746 | 3 | 10.32 | 0.7909 | 0.7982 | 15344 | 4.71e2 | 1 | 1 | 12 | 25 |  |  | DB Search |
| total 1 peptides |  |  |  |  |  |  |  |  |  |  |  |  |  |  |  |  |  |  |  |
| --- | --- | --- | --- | --- | --- | --- | --- | --- | --- | --- | --- | --- | --- | --- | --- | --- | --- | --- | --- |

Best Unique PSM (Scan Sample\_10\_A1\_1\_3647.d:15344, m/z=529.2746, z=3, RT=10.32, ppm=-5.52):


TRINITY\_DN32727\_c0\_g1\_i2.p1

back to list

  

| Protein Coverage
| Supporting Peptides
| Best Unique PSM
|

Protein Coverage:

Supporting Peptides:

| Peptide | Uniq | -10lgP | Mass | Length | ppm | m/z | z | RT | 1/k0 Start | 1/k0 End | Precursor ID | Area Sample 1 | #Feature | #Feature Sample 1 | Start | End | PTM | AScore | Found By |
| --- | --- | --- | --- | --- | --- | --- | --- | --- | --- | --- | --- | --- | --- | --- | --- | --- | --- | --- | --- |
| K.AGM(+15.99)THIVR.D | Y | 19.81 | 899.4647 | 8 | 2.72 | 450.7388 | 2 | 9.73 | 0.8226 | 0.8299 | 12038 | 6.74e2 | 1 | 1 | 40 | 47 | Oxidation (M) | M3:Oxidation (M):1000 | DB Search |
| total 1 peptides |  |  |  |  |  |  |  |  |  |  |  |  |  |  |  |  |  |  |  |
| --- | --- | --- | --- | --- | --- | --- | --- | --- | --- | --- | --- | --- | --- | --- | --- | --- | --- | --- | --- |

Best Unique PSM (Scan Sample\_10\_A1\_1\_3647.d:12038, m/z=450.7388, z=2, RT=9.73, ppm=-1.77):


TRINITY\_DN77\_c1\_g1\_i1.p1

back to list

  

| Protein Coverage
| Supporting Peptides
| Best Unique PSM
|

Protein Coverage:

Supporting Peptides:

| Peptide | Uniq | -10lgP | Mass | Length | ppm | m/z | z | RT | 1/k0 Start | 1/k0 End | Precursor ID | Area Sample 1 | #Feature | #Feature Sample 1 | Start | End | PTM | AScore | Found By |
| --- | --- | --- | --- | --- | --- | --- | --- | --- | --- | --- | --- | --- | --- | --- | --- | --- | --- | --- | --- |
| R.LLAVTVGNNYNLDTLEK.V | Y | 62.12 | 1875.9942 | 17 | 1.09 | 939.0012 | 2 | 18.57 | 1.1462 | 1.1534 | 62222 | 0 | 0 | 0 | 693 | 709 |  |  | DB Search |
| R.AVDIHFALDASQNVGR.E | Y | 62.09 | 1711.8641 | 16 | 0.55 | 856.936 | 2 | 17.40 | 1.0989 | 1.1061 | 54900 | 6.01e2 | 1 | 1 | 56 | 71 |  |  | DB Search |
| K.SFASSLLDHFRVEPAK.T | Y | 62.01 | 1802.9315 | 16 | 3.47 | 601.9838 | 3 | 19.81 | 0.9129 | 0.9202 | 69718 | 3.84e2 | 1 | 1 | 318 | 333 |  |  | DB Search |
| total 3 peptides |  |  |  |  |  |  |  |  |  |  |  |  |  |  |  |  |  |  |  |
| --- | --- | --- | --- | --- | --- | --- | --- | --- | --- | --- | --- | --- | --- | --- | --- | --- | --- | --- | --- |

Best Unique PSM (Scan Sample\_10\_A1\_1\_3647.d:62222, m/z=939.4923, z=1, RT=18.61, ppm=-3.41):


TRINITY\_DN17516\_c0\_g1\_i3.p1

back to list

  

| Protein Coverage
| Supporting Peptides
| Best Unique PSM
|

Protein Coverage:

Supporting Peptides:

| Peptide | Uniq | -10lgP | Mass | Length | ppm | m/z | z | RT | 1/k0 Start | 1/k0 End | Precursor ID | Area Sample 1 | #Feature | #Feature Sample 1 | Start | End | PTM | AScore | Found By |
| --- | --- | --- | --- | --- | --- | --- | --- | --- | --- | --- | --- | --- | --- | --- | --- | --- | --- | --- | --- |
| K.EVNHEYQIIASSWK.Y | Y | 61.95 | 1702.8315 | 14 | -0.63 | 852.4186 | 2 | 17.08 | 1.1258 | 1.133 | 53181 | 0 | 0 | 0 | 90 | 103 |  |  | DB Search |
| total 1 peptides |  |  |  |  |  |  |  |  |  |  |  |  |  |  |  |  |  |  |  |
| --- | --- | --- | --- | --- | --- | --- | --- | --- | --- | --- | --- | --- | --- | --- | --- | --- | --- | --- | --- |

Best Unique PSM (Scan Sample\_10\_A1\_1\_3647.d:53181, m/z=852.4186, z=2, RT=17.08, ppm=-5.13):


TRINITY\_DN15151\_c0\_g1\_i3.p1

back to list

  

| Protein Coverage
| Supporting Peptides
| Best Unique PSM
|

Protein Coverage:

Supporting Peptides:

| Peptide | Uniq | -10lgP | Mass | Length | ppm | m/z | z | RT | 1/k0 Start | 1/k0 End | Precursor ID | Area Sample 1 | #Feature | #Feature Sample 1 | Start | End | PTM | AScore | Found By |
| --- | --- | --- | --- | --- | --- | --- | --- | --- | --- | --- | --- | --- | --- | --- | --- | --- | --- | --- | --- |
| K.AVAAGM(+15.99)NPM(+15.99)DIKR.G | Y | 61.93 | 1404.6853 | 13 | -5.88 | 703.3427 | 2 | 11.20 | 1.0122 | 1.0194 | 20513 | 4.12e2 | 1 | 1 | 145 | 157 | Oxidation (M), Oxidation (M) | M6:Oxidation (M):1000 M9:Oxidation (M):1000 | DB Search |
| total 1 peptides |  |  |  |  |  |  |  |  |  |  |  |  |  |  |  |  |  |  |  |
| --- | --- | --- | --- | --- | --- | --- | --- | --- | --- | --- | --- | --- | --- | --- | --- | --- | --- | --- | --- |

Best Unique PSM (Scan Sample\_10\_A1\_1\_3647.d:20513, m/z=703.3427, z=2, RT=11.20, ppm=-10.38):


TRINITY\_DN5023\_c0\_g1\_i2.p2

back to list

  

| Protein Coverage
| Supporting Peptides
| Best Unique PSM
|

Protein Coverage:

Supporting Peptides:

| Peptide | Uniq | -10lgP | Mass | Length | ppm | m/z | z | RT | 1/k0 Start | 1/k0 End | Precursor ID | Area Sample 1 | #Feature | #Feature Sample 1 | Start | End | PTM | AScore | Found By |
| --- | --- | --- | --- | --- | --- | --- | --- | --- | --- | --- | --- | --- | --- | --- | --- | --- | --- | --- | --- |
| R.YRPGTVALR.E | Y | 66.57 | 1031.5876 | 9 | 0.86 | 516.7992 | 2 | 12.50 | 0.8554 | 0.8627 | 28119 | 7.47e2 | 1 | 1 | 78 | 86 |  |  | DB Search |
| total 1 peptides |  |  |  |  |  |  |  |  |  |  |  |  |  |  |  |  |  |  |  |
| --- | --- | --- | --- | --- | --- | --- | --- | --- | --- | --- | --- | --- | --- | --- | --- | --- | --- | --- | --- |

Best Unique PSM (Scan Sample\_10\_A1\_1\_3647.d:28119, m/z=516.7992, z=2, RT=12.50, ppm=-3.64):


TRINITY\_DN1989\_c0\_g1\_i1.p1

back to list

  

| Protein Coverage
| Supporting Peptides
| Best Unique PSM
|

Protein Coverage:

Supporting Peptides:

| Peptide | Uniq | -10lgP | Mass | Length | ppm | m/z | z | RT | 1/k0 Start | 1/k0 End | Precursor ID | Area Sample 1 | #Feature | #Feature Sample 1 | Start | End | PTM | AScore | Found By |
| --- | --- | --- | --- | --- | --- | --- | --- | --- | --- | --- | --- | --- | --- | --- | --- | --- | --- | --- | --- |
| R.AVVVC(+57.02)DDRGR.S | Y | 66.63 | 1145.5611 | 10 | 1.85 | 573.7863 | 2 | 10.61 | 0.883 | 0.8903 | 17052 | 2.15e3 | 1 | 1 | 261 | 270 | Carbamidomethylation |  | DB Search |
| R.FATHASALEIHGLDR.F | Y | 66.57 | 1636.8321 | 15 | 3.46 | 819.4225 | 2 | 14.84 | 1.1378 | 1.145 | 41195 | 5.09e2 | 1 | 1 | 227 | 241 |  |  | DB Search |
| total 2 peptides |  |  |  |  |  |  |  |  |  |  |  |  |  |  |  |  |  |  |  |
| --- | --- | --- | --- | --- | --- | --- | --- | --- | --- | --- | --- | --- | --- | --- | --- | --- | --- | --- | --- |

Best Unique PSM (Scan Sample\_10\_A1\_1\_3647.d:17052, m/z=573.7863, z=2, RT=10.61, ppm=-2.65):


TRINITY\_DN2885\_c0\_g1\_i5.p1

back to list

  

| Protein Coverage
| Supporting Peptides
| Best Unique PSM
|

Protein Coverage:

Supporting Peptides:

| Peptide | Uniq | -10lgP | Mass | Length | ppm | m/z | z | RT | 1/k0 Start | 1/k0 End | Precursor ID | Area Sample 1 | #Feature | #Feature Sample 1 | Start | End | PTM | AScore | Found By |
| --- | --- | --- | --- | --- | --- | --- | --- | --- | --- | --- | --- | --- | --- | --- | --- | --- | --- | --- | --- |
| K.VTRPHGASGVVR.A | Y | 66.69 | 1234.6894 | 12 | 2.23 | 618.3506 | 2 | 10.05 | 0.9319 | 0.9392 | 13758 | 2.92e4 | 3 | 3 | 77 | 88 |  |  | DB Search |
| K.VTRPHGASGVVRAK.F | Y | 41.36 | 1433.8215 | 14 | 4.92 | 359.4628 | 4 | 9.64 | 0.7151 | 0.7224 | 11644 | 0 | 0 | 0 | 77 | 90 |  |  | DB Search |
| total 2 peptides |  |  |  |  |  |  |  |  |  |  |  |  |  |  |  |  |  |  |  |
| --- | --- | --- | --- | --- | --- | --- | --- | --- | --- | --- | --- | --- | --- | --- | --- | --- | --- | --- | --- |

Best Unique PSM (Scan Sample\_10\_A1\_1\_3647.d:13758, m/z=618.3506, z=2, RT=10.05, ppm=-2.27):


TRINITY\_DN30386\_c0\_g1\_i1.p2

back to list

  

| Protein Coverage
| Supporting Peptides
| Best Unique PSM
|

Protein Coverage:

Supporting Peptides:

| Peptide | Uniq | -10lgP | Mass | Length | ppm | m/z | z | RT | 1/k0 Start | 1/k0 End | Precursor ID | Area Sample 1 | #Feature | #Feature Sample 1 | Start | End | PTM | AScore | Found By |
| --- | --- | --- | --- | --- | --- | --- | --- | --- | --- | --- | --- | --- | --- | --- | --- | --- | --- | --- | --- |
| K.VFDRDGNGFISAAELR.H | Y | 39.11 | 1765.8747 | 16 | -1.5 | 883.9393 | 2 | 19.71 | 1.1234 | 1.1306 | 69271 | 0 | 0 | 0 | 117 | 132 |  |  | DB Search |
| total 1 peptides |  |  |  |  |  |  |  |  |  |  |  |  |  |  |  |  |  |  |  |
| --- | --- | --- | --- | --- | --- | --- | --- | --- | --- | --- | --- | --- | --- | --- | --- | --- | --- | --- | --- |

Best Unique PSM (Scan Sample\_10\_A1\_1\_3647.d:69271, m/z=883.9393, z=2, RT=19.71, ppm=-6):


TRINITY\_DN8227\_c0\_g1\_i1.p1

back to list

  

| Protein Coverage
| Supporting Peptides
| Best Unique PSM
|

Protein Coverage:

Supporting Peptides:

| Peptide | Uniq | -10lgP | Mass | Length | ppm | m/z | z | RT | 1/k0 Start | 1/k0 End | Precursor ID | Area Sample 1 | #Feature | #Feature Sample 1 | Start | End | PTM | AScore | Found By |
| --- | --- | --- | --- | --- | --- | --- | --- | --- | --- | --- | --- | --- | --- | --- | --- | --- | --- | --- | --- |
| K.IQDKEGIPPDQQR.L | Y | 65.08 | 1522.7739 | 13 | -0.66 | 762.3903 | 2 | 11.01 | 1.0339 | 1.0411 | 19352 | 9.49e2 | 1 | 1 | 54 | 66 |  |  | DB Search |
| total 1 peptides |  |  |  |  |  |  |  |  |  |  |  |  |  |  |  |  |  |  |  |
| --- | --- | --- | --- | --- | --- | --- | --- | --- | --- | --- | --- | --- | --- | --- | --- | --- | --- | --- | --- |

Best Unique PSM (Scan Sample\_10\_A1\_1\_3647.d:19352, m/z=762.3903, z=2, RT=11.01, ppm=-5.16):


TRINITY\_DN197343\_c0\_g1\_i1.p1

back to list

  

| Protein Coverage
| Supporting Peptides
| Best Unique PSM
|

Protein Coverage:

Supporting Peptides:

| Peptide | Uniq | -10lgP | Mass | Length | ppm | m/z | z | RT | 1/k0 Start | 1/k0 End | Precursor ID | Area Sample 1 | #Feature | #Feature Sample 1 | Start | End | PTM | AScore | Found By |
| --- | --- | --- | --- | --- | --- | --- | --- | --- | --- | --- | --- | --- | --- | --- | --- | --- | --- | --- | --- |
| R.VLTVISHNQR.E | Y | 66.72 | 1165.6567 | 10 | 2.9 | 583.8347 | 2 | 12.24 | 0.9315 | 0.9388 | 26669 | 5.13e2 | 1 | 1 | 57 | 66 |  |  | DB Search |
| R.KEELC(+57.02)HQLTELKTELQQLHVAK.V | Y | 38.46 | 2674.4112 | 22 | -0.85 | 535.8867 | 5 | 18.43 | 0.8437 | 0.851 | 61365 | 0 | 0 | 0 | 14 | 35 | Carbamidomethylation |  | DB Search |
| total 2 peptides |  |  |  |  |  |  |  |  |  |  |  |  |  |  |  |  |  |  |  |
| --- | --- | --- | --- | --- | --- | --- | --- | --- | --- | --- | --- | --- | --- | --- | --- | --- | --- | --- | --- |

Best Unique PSM (Scan Sample\_10\_A1\_1\_3647.d:26669, m/z=583.8347, z=2, RT=12.24, ppm=-1.59):


TRINITY\_DN1616\_c0\_g1\_i7.p1

back to list

  

| Protein Coverage
| Supporting Peptides
| Best Unique PSM
|

Protein Coverage:

Supporting Peptides:

| Peptide | Uniq | -10lgP | Mass | Length | ppm | m/z | z | RT | 1/k0 Start | 1/k0 End | Precursor ID | Area Sample 1 | #Feature | #Feature Sample 1 | Start | End | PTM | AScore | Found By |
| --- | --- | --- | --- | --- | --- | --- | --- | --- | --- | --- | --- | --- | --- | --- | --- | --- | --- | --- | --- |
| R.LVQANPLEQDLSEKPR.Y | Y | 66.73 | 1835.9741 | 16 | 3.23 | 918.9932 | 2 | 15.63 | 1.1837 | 1.1909 | 45493 | 3.81e2 | 1 | 1 | 1191 | 1206 |  |  | DB Search |
| K.YVVMLQYYHDSDTAK.Y | Y | 66.45 | 1831.8451 | 15 | 2.49 | 916.928 | 2 | 16.54 | 1.1798 | 1.1869 | 50323 | 0 | 0 | 0 | 1071 | 1085 |  |  | DB Search |
| R.YVKPGVNIINIR.D | Y | 58.56 | 1384.819 | 12 | 3.46 | 693.4161 | 2 | 15.95 | 1.0025 | 1.0098 | 47296 | 0 | 0 | 0 | 1217 | 1228 |  |  | DB Search |
| total 3 peptides |  |  |  |  |  |  |  |  |  |  |  |  |  |  |  |  |  |  |  |
| --- | --- | --- | --- | --- | --- | --- | --- | --- | --- | --- | --- | --- | --- | --- | --- | --- | --- | --- | --- |

Best Unique PSM (Scan Sample\_10\_A1\_1\_3647.d:45493, m/z=918.9932, z=2, RT=15.63, ppm=-1.26):


TRINITY\_DN36\_c0\_g1\_i9.p1

back to list

  

| Protein Coverage
| Supporting Peptides
| Best Unique PSM
|

Protein Coverage:

Supporting Peptides:

| Peptide | Uniq | -10lgP | Mass | Length | ppm | m/z | z | RT | 1/k0 Start | 1/k0 End | Precursor ID | Area Sample 1 | #Feature | #Feature Sample 1 | Start | End | PTM | AScore | Found By |
| --- | --- | --- | --- | --- | --- | --- | --- | --- | --- | --- | --- | --- | --- | --- | --- | --- | --- | --- | --- |
| R.LGGIEVTQGNC(+57.02)AR.K | Y | 66.48 | 1373.6721 | 13 | 5.18 | 687.8438 | 2 | 13.73 | 0.963 | 0.9703 | 35192 | 0 | 0 | 0 | 58 | 70 | Carbamidomethylation |  | DB Search |
| R.LGGIEVTQGNC(+57.02)ARK.Y | Y | 31.04 | 1501.7671 | 14 | -2.67 | 751.8854 | 2 | 12.64 | 1.0624 | 1.0697 | 29000 | 0 | 0 | 0 | 58 | 71 | Carbamidomethylation |  | DB Search |
| total 2 peptides |  |  |  |  |  |  |  |  |  |  |  |  |  |  |  |  |  |  |  |
| --- | --- | --- | --- | --- | --- | --- | --- | --- | --- | --- | --- | --- | --- | --- | --- | --- | --- | --- | --- |

Best Unique PSM (Scan Sample\_10\_A1\_1\_3647.d:35192, m/z=687.8438, z=2, RT=13.73, ppm=0.69):


TRINITY\_DN123073\_c0\_g1\_i1.p1

back to list

  

| Protein Coverage
| Supporting Peptides
| Best Unique PSM
|

Protein Coverage:

Supporting Peptides:

| Peptide | Uniq | -10lgP | Mass | Length | ppm | m/z | z | RT | 1/k0 Start | 1/k0 End | Precursor ID | Area Sample 1 | #Feature | #Feature Sample 1 | Start | End | PTM | AScore | Found By |
| --- | --- | --- | --- | --- | --- | --- | --- | --- | --- | --- | --- | --- | --- | --- | --- | --- | --- | --- | --- |
| R.GFAPSTVQVAR.G | Y | 24.23 | 1131.6036 | 11 | 9.77 | 566.8121 | 2 | 14.99 | 0.8955 | 0.9028 | 41874 | 6.64e3 | 1 | 1 | 24 | 34 |  |  | DB Search |
| total 1 peptides |  |  |  |  |  |  |  |  |  |  |  |  |  |  |  |  |  |  |  |
| --- | --- | --- | --- | --- | --- | --- | --- | --- | --- | --- | --- | --- | --- | --- | --- | --- | --- | --- | --- |

Best Unique PSM (Scan Sample\_10\_A1\_1\_3647.d:41874, m/z=566.8121, z=2, RT=14.99, ppm=5.27):


TRINITY\_DN3545\_c0\_g1\_i2.p1

back to list

  

| Protein Coverage
| Supporting Peptides
| Best Unique PSM
|

Protein Coverage:

Supporting Peptides:

| Peptide | Uniq | -10lgP | Mass | Length | ppm | m/z | z | RT | 1/k0 Start | 1/k0 End | Precursor ID | Area Sample 1 | #Feature | #Feature Sample 1 | Start | End | PTM | AScore | Found By |
| --- | --- | --- | --- | --- | --- | --- | --- | --- | --- | --- | --- | --- | --- | --- | --- | --- | --- | --- | --- |
| R.SSC(+57.02)RPILSVIK.I | Y | 49.81 | 1258.7067 | 11 | -8.86 | 630.3522 | 2 | 16.81 | 0.9691 | 0.9763 | 51630 | 2.1e3 | 1 | 1 | 6 | 16 | Carbamidomethylation |  | DB Search |
| R.SC(+57.02)TNPAPANGGK.H | Y | 39.93 | 1172.5244 | 12 | 0 | 587.2668 | 2 | 9.43 | 0.8404 | 0.8477 | 10729 | 0 | 0 | 0 | 55 | 66 | Carbamidomethylation |  | DB Search |
| total 2 peptides |  |  |  |  |  |  |  |  |  |  |  |  |  |  |  |  |  |  |  |
| --- | --- | --- | --- | --- | --- | --- | --- | --- | --- | --- | --- | --- | --- | --- | --- | --- | --- | --- | --- |

Best Unique PSM (Scan Sample\_10\_A1\_1\_3647.d:51630, m/z=630.3522, z=2, RT=16.81, ppm=-13.35):


TRINITY\_DN118\_c1\_g1\_i3.p1

back to list

  

| Protein Coverage
| Supporting Peptides
| Best Unique PSM
|

Protein Coverage:

Supporting Peptides:

| Peptide | Uniq | -10lgP | Mass | Length | ppm | m/z | z | RT | 1/k0 Start | 1/k0 End | Precursor ID | Area Sample 1 | #Feature | #Feature Sample 1 | Start | End | PTM | AScore | Found By |
| --- | --- | --- | --- | --- | --- | --- | --- | --- | --- | --- | --- | --- | --- | --- | --- | --- | --- | --- | --- |
| K.FSYEKVC(+57.02)EDVKR.C | Y | 66.86 | 1558.745 | 12 | -4.31 | 520.5844 | 3 | 13.47 | 0.7945 | 0.8019 | 33820 | 0 | 0 | 0 | 117 | 128 | Carbamidomethylation |  | DB Search |
| R.ETC(+57.02)SDIEKR.E | Y | 66.66 | 1136.5132 | 9 | 3.94 | 569.2635 | 2 | 10.07 | 0.8931 | 0.9004 | 13965 | 4.9e3 | 1 | 1 | 186 | 194 | Carbamidomethylation |  | DB Search |
| K.VC(+57.02)EDVKR.C | Y | 62.75 | 904.4436 | 7 | 2.55 | 453.2282 | 2 | 10.47 | 0.7953 | 0.8027 | 16197 | 2.08e3 | 2 | 2 | 122 | 128 | Carbamidomethylation |  | DB Search |
| total 3 peptides |  |  |  |  |  |  |  |  |  |  |  |  |  |  |  |  |  |  |  |
| --- | --- | --- | --- | --- | --- | --- | --- | --- | --- | --- | --- | --- | --- | --- | --- | --- | --- | --- | --- |

Best Unique PSM (Scan Sample\_10\_A1\_1\_3647.d:33820, m/z=520.5844, z=3, RT=13.47, ppm=-8.81):


TRINITY\_DN1369\_c0\_g1\_i1.p1

back to list

  

| Protein Coverage
| Supporting Peptides
| Best Unique PSM
|

Protein Coverage:

Supporting Peptides:

| Peptide | Uniq | -10lgP | Mass | Length | ppm | m/z | z | RT | 1/k0 Start | 1/k0 End | Precursor ID | Area Sample 1 | #Feature | #Feature Sample 1 | Start | End | PTM | AScore | Found By |
| --- | --- | --- | --- | --- | --- | --- | --- | --- | --- | --- | --- | --- | --- | --- | --- | --- | --- | --- | --- |
| R.EITLNYHDTVVHMK.R | Y | 55.14 | 1698.8399 | 14 | -0.31 | 850.4232 | 2 | 15.27 | 1.1126 | 1.1198 | 43604 | 5.8e2 | 1 | 1 | 911 | 924 |  |  | DB Search |
| R.MTSC(+57.02)EANHQVAK.Y | Y | 50.44 | 1374.602 | 12 | -2.69 | 688.3033 | 2 | 9.67 | 0.9767 | 0.984 | 11763 | 0 | 0 | 0 | 571 | 582 | Carbamidomethylation |  | DB Search |
| total 2 peptides |  |  |  |  |  |  |  |  |  |  |  |  |  |  |  |  |  |  |  |
| --- | --- | --- | --- | --- | --- | --- | --- | --- | --- | --- | --- | --- | --- | --- | --- | --- | --- | --- | --- |

Best Unique PSM (Scan Sample\_10\_A1\_1\_3647.d:43604, m/z=850.4232, z=2, RT=15.27, ppm=-4.8):


TRINITY\_DN18187\_c0\_g1\_i3.p4

back to list

  

| Protein Coverage
| Supporting Peptides
| Best Unique PSM
|

Protein Coverage:

Supporting Peptides:

| Peptide | Uniq | -10lgP | Mass | Length | ppm | m/z | z | RT | 1/k0 Start | 1/k0 End | Precursor ID | Area Sample 1 | #Feature | #Feature Sample 1 | Start | End | PTM | AScore | Found By |
| --- | --- | --- | --- | --- | --- | --- | --- | --- | --- | --- | --- | --- | --- | --- | --- | --- | --- | --- | --- |
| R.YRPGTVALR.E | Y | 66.57 | 1031.5876 | 9 | 0.86 | 516.7992 | 2 | 12.50 | 0.8554 | 0.8627 | 28119 | 7.47e2 | 1 | 1 | 48 | 56 |  |  | DB Search |
| total 1 peptides |  |  |  |  |  |  |  |  |  |  |  |  |  |  |  |  |  |  |  |
| --- | --- | --- | --- | --- | --- | --- | --- | --- | --- | --- | --- | --- | --- | --- | --- | --- | --- | --- | --- |

Best Unique PSM (Scan Sample\_10\_A1\_1\_3647.d:28119, m/z=516.7992, z=2, RT=12.50, ppm=-3.64):


TRINITY\_DN40728\_c1\_g1\_i1.p2

back to list

  

| Protein Coverage
| Supporting Peptides
| Best Unique PSM
|

Protein Coverage:

Supporting Peptides:

| Peptide | Uniq | -10lgP | Mass | Length | ppm | m/z | z | RT | 1/k0 Start | 1/k0 End | Precursor ID | Area Sample 1 | #Feature | #Feature Sample 1 | Start | End | PTM | AScore | Found By |
| --- | --- | --- | --- | --- | --- | --- | --- | --- | --- | --- | --- | --- | --- | --- | --- | --- | --- | --- | --- |
| K.IQDKEGIPPDQQR.L | Y | 65.08 | 1522.7739 | 13 | -0.66 | 762.3903 | 2 | 11.01 | 1.0339 | 1.0411 | 19352 | 9.49e2 | 1 | 1 | 25 | 37 |  |  | DB Search |
| total 1 peptides |  |  |  |  |  |  |  |  |  |  |  |  |  |  |  |  |  |  |  |
| --- | --- | --- | --- | --- | --- | --- | --- | --- | --- | --- | --- | --- | --- | --- | --- | --- | --- | --- | --- |

Best Unique PSM (Scan Sample\_10\_A1\_1\_3647.d:19352, m/z=762.3903, z=2, RT=11.01, ppm=-5.16):


TRINITY\_DN585\_c0\_g1\_i5.p1

back to list

  

| Protein Coverage
| Supporting Peptides
| Best Unique PSM
|

Protein Coverage:

Supporting Peptides:

| Peptide | Uniq | -10lgP | Mass | Length | ppm | m/z | z | RT | 1/k0 Start | 1/k0 End | Precursor ID | Area Sample 1 | #Feature | #Feature Sample 1 | Start | End | PTM | AScore | Found By |
| --- | --- | --- | --- | --- | --- | --- | --- | --- | --- | --- | --- | --- | --- | --- | --- | --- | --- | --- | --- |
| K.IQDKEGIPPDQQR.L | Y | 65.08 | 1522.7739 | 13 | -0.66 | 762.3903 | 2 | 11.01 | 1.0339 | 1.0411 | 19352 | 9.49e2 | 1 | 1 | 2 | 14 |  |  | DB Search |
| total 1 peptides |  |  |  |  |  |  |  |  |  |  |  |  |  |  |  |  |  |  |  |
| --- | --- | --- | --- | --- | --- | --- | --- | --- | --- | --- | --- | --- | --- | --- | --- | --- | --- | --- | --- |

Best Unique PSM (Scan Sample\_10\_A1\_1\_3647.d:19352, m/z=762.3903, z=2, RT=11.01, ppm=-5.16):


TRINITY\_DN6388\_c0\_g1\_i2.p2

back to list

  

| Protein Coverage
| Supporting Peptides
| Best Unique PSM
|

Protein Coverage:

Supporting Peptides:

| Peptide | Uniq | -10lgP | Mass | Length | ppm | m/z | z | RT | 1/k0 Start | 1/k0 End | Precursor ID | Area Sample 1 | #Feature | #Feature Sample 1 | Start | End | PTM | AScore | Found By |
| --- | --- | --- | --- | --- | --- | --- | --- | --- | --- | --- | --- | --- | --- | --- | --- | --- | --- | --- | --- |
| R.AIELLPHDLAVGR.H | Y | 66.91 | 1402.7932 | 13 | -9.52 | 702.394 | 2 | 17.77 | 1.0275 | 1.0347 | 57279 | 0 | 0 | 0 | 52 | 64 |  |  | DB Search |
| R.YQYEHAVAELR.G | Y | 64.48 | 1377.6677 | 11 | 2.2 | 689.8395 | 2 | 14.37 | 1.0102 | 1.0174 | 38774 | 1.67e3 | 1 | 1 | 22 | 32 |  |  | DB Search |
| total 2 peptides |  |  |  |  |  |  |  |  |  |  |  |  |  |  |  |  |  |  |  |
| --- | --- | --- | --- | --- | --- | --- | --- | --- | --- | --- | --- | --- | --- | --- | --- | --- | --- | --- | --- |

Best Unique PSM (Scan Sample\_10\_A1\_1\_3647.d:57279, m/z=702.394, z=2, RT=17.77, ppm=-14.02):


TRINITY\_DN141338\_c2\_g1\_i1.p1

back to list

  

| Protein Coverage
| Supporting Peptides
| Best Unique PSM
|

Protein Coverage:

Supporting Peptides:

| Peptide | Uniq | -10lgP | Mass | Length | ppm | m/z | z | RT | 1/k0 Start | 1/k0 End | Precursor ID | Area Sample 1 | #Feature | #Feature Sample 1 | Start | End | PTM | AScore | Found By |
| --- | --- | --- | --- | --- | --- | --- | --- | --- | --- | --- | --- | --- | --- | --- | --- | --- | --- | --- | --- |
| R.ELISNSSDALDKIR.Y | N | 61.95 | 1559.8155 | 14 | -1.81 | 780.9101 | 2 | 15.46 | 1.0753 | 1.0825 | 44710 | 0 | 0 | 0 | 37 | 50 |  |  | DB Search |
| K.LGIHEDTQNR.N | Y | 60.32 | 1181.5789 | 10 | -1.02 | 591.7935 | 2 | 11.11 | 0.8943 | 0.9016 | 20072 | 6.52e2 | 1 | 1 | 434 | 443 |  |  | DB Search |
| total 2 peptides |  |  |  |  |  |  |  |  |  |  |  |  |  |  |  |  |  |  |  |
| --- | --- | --- | --- | --- | --- | --- | --- | --- | --- | --- | --- | --- | --- | --- | --- | --- | --- | --- | --- |

Best Unique PSM (Scan Sample\_10\_A1\_1\_3647.d:20072, m/z=591.7935, z=2, RT=11.11, ppm=-5.52):


TRINITY\_DN2477\_c0\_g1\_i6.p1

back to list

  

| Protein Coverage
| Supporting Peptides
| Best Unique PSM
|

Protein Coverage:

Supporting Peptides:

| Peptide | Uniq | -10lgP | Mass | Length | ppm | m/z | z | RT | 1/k0 Start | 1/k0 End | Precursor ID | Area Sample 1 | #Feature | #Feature Sample 1 | Start | End | PTM | AScore | Found By |
| --- | --- | --- | --- | --- | --- | --- | --- | --- | --- | --- | --- | --- | --- | --- | --- | --- | --- | --- | --- |
| K.DSYVGDEAQSKR.G | Y | 66.73 | 1353.616 | 12 | 2.52 | 677.814 | 2 | 10.94 | 1.0069 | 1.0142 | 19045 | 5.51e3 | 1 | 1 | 52 | 63 |  |  | DB Search |
| total 1 peptides |  |  |  |  |  |  |  |  |  |  |  |  |  |  |  |  |  |  |  |
| --- | --- | --- | --- | --- | --- | --- | --- | --- | --- | --- | --- | --- | --- | --- | --- | --- | --- | --- | --- |

Best Unique PSM (Scan Sample\_10\_A1\_1\_3647.d:19045, m/z=677.814, z=2, RT=10.94, ppm=-1.97):


TRINITY\_DN87064\_c0\_g1\_i1.p1

back to list

  

| Protein Coverage
| Supporting Peptides
| Best Unique PSM
|

Protein Coverage:

Supporting Peptides:

| Peptide | Uniq | -10lgP | Mass | Length | ppm | m/z | z | RT | 1/k0 Start | 1/k0 End | Precursor ID | Area Sample 1 | #Feature | #Feature Sample 1 | Start | End | PTM | AScore | Found By |
| --- | --- | --- | --- | --- | --- | --- | --- | --- | --- | --- | --- | --- | --- | --- | --- | --- | --- | --- | --- |
| K.IAVVVGAVTDDKR.L | Y | 66.67 | 1341.7616 | 13 | 6.04 | 671.8891 | 2 | 14.15 | 1.0323 | 1.0395 | 37500 | 1.94e2 | 1 | 1 | 102 | 114 |  |  | DB Search |
| total 1 peptides |  |  |  |  |  |  |  |  |  |  |  |  |  |  |  |  |  |  |  |
| --- | --- | --- | --- | --- | --- | --- | --- | --- | --- | --- | --- | --- | --- | --- | --- | --- | --- | --- | --- |

Best Unique PSM (Scan Sample\_10\_A1\_1\_3647.d:37500, m/z=671.8891, z=2, RT=14.15, ppm=1.55):


TRINITY\_DN649\_c0\_g1\_i5.p1

back to list

  

| Protein Coverage
| Supporting Peptides
| Best Unique PSM
|

Protein Coverage:

Supporting Peptides:

| Peptide | Uniq | -10lgP | Mass | Length | ppm | m/z | z | RT | 1/k0 Start | 1/k0 End | Precursor ID | Area Sample 1 | #Feature | #Feature Sample 1 | Start | End | PTM | AScore | Found By |
| --- | --- | --- | --- | --- | --- | --- | --- | --- | --- | --- | --- | --- | --- | --- | --- | --- | --- | --- | --- |
| K.FETQIATK.N | Y | 61.61 | 936.4916 | 8 | 0.97 | 937.4956 | 1 | 13.84 | 1.2037 | 1.2109 | 35727 | 1.28e3 | 1 | 1 | 102 | 109 |  |  | DB Search |
| total 1 peptides |  |  |  |  |  |  |  |  |  |  |  |  |  |  |  |  |  |  |  |
| --- | --- | --- | --- | --- | --- | --- | --- | --- | --- | --- | --- | --- | --- | --- | --- | --- | --- | --- | --- |

Best Unique PSM (Scan Sample\_10\_A1\_1\_3647.d:35727, m/z=937.4956, z=1, RT=13.84, ppm=-3.52):


TRINITY\_DN47849\_c0\_g1\_i1.p1

back to list

  

| Protein Coverage
| Supporting Peptides
| Best Unique PSM
|

Protein Coverage:

Supporting Peptides:

| Peptide | Uniq | -10lgP | Mass | Length | ppm | m/z | z | RT | 1/k0 Start | 1/k0 End | Precursor ID | Area Sample 1 | #Feature | #Feature Sample 1 | Start | End | PTM | AScore | Found By |
| --- | --- | --- | --- | --- | --- | --- | --- | --- | --- | --- | --- | --- | --- | --- | --- | --- | --- | --- | --- |
| K.VLLGGLVSHM(+15.99)DYIKR.C | Y | 58.58 | 1715.9392 | 15 | -1.08 | 858.9721 | 2 | 16.10 | 1.1897 | 1.1969 | 47995 | 1.27e2 | 1 | 1 | 358 | 372 | Oxidation (M) | M10:Oxidation (M):1000 | DB Search |
| total 1 peptides |  |  |  |  |  |  |  |  |  |  |  |  |  |  |  |  |  |  |  |
| --- | --- | --- | --- | --- | --- | --- | --- | --- | --- | --- | --- | --- | --- | --- | --- | --- | --- | --- | --- |

Best Unique PSM (Scan Sample\_10\_A1\_1\_3647.d:47995, m/z=858.9721, z=2, RT=16.10, ppm=-5.57):


TRINITY\_DN902\_c1\_g2\_i1.p1

back to list

  

| Protein Coverage
| Supporting Peptides
| Best Unique PSM
|

Protein Coverage:

Supporting Peptides:

| Peptide | Uniq | -10lgP | Mass | Length | ppm | m/z | z | RT | 1/k0 Start | 1/k0 End | Precursor ID | Area Sample 1 | #Feature | #Feature Sample 1 | Start | End | PTM | AScore | Found By |
| --- | --- | --- | --- | --- | --- | --- | --- | --- | --- | --- | --- | --- | --- | --- | --- | --- | --- | --- | --- |
| K.GLTHC(+57.02)NTLSYK.L | Y | 66.56 | 1292.6183 | 11 | 2.85 | 647.3154 | 2 | 12.41 | 0.9699 | 0.9771 | 27654 | 0 | 0 | 0 | 62 | 72 | Carbamidomethylation |  | DB Search |
| total 1 peptides |  |  |  |  |  |  |  |  |  |  |  |  |  |  |  |  |  |  |  |
| --- | --- | --- | --- | --- | --- | --- | --- | --- | --- | --- | --- | --- | --- | --- | --- | --- | --- | --- | --- |

Best Unique PSM (Scan Sample\_10\_A1\_1\_3647.d:27654, m/z=647.3154, z=2, RT=12.41, ppm=-1.65):


TRINITY\_DN121866\_c0\_g1\_i1.p1

back to list

  

| Protein Coverage
| Supporting Peptides
| Best Unique PSM
|

Protein Coverage:

Supporting Peptides:

| Peptide | Uniq | -10lgP | Mass | Length | ppm | m/z | z | RT | 1/k0 Start | 1/k0 End | Precursor ID | Area Sample 1 | #Feature | #Feature Sample 1 | Start | End | PTM | AScore | Found By |
| --- | --- | --- | --- | --- | --- | --- | --- | --- | --- | --- | --- | --- | --- | --- | --- | --- | --- | --- | --- |
| K.FSQHTDYVIAR.A | Y | 66.91 | 1335.6571 | 11 | 1.81 | 668.834 | 2 | 13.45 | 0.9763 | 0.9836 | 33690 | 0 | 0 | 0 | 194 | 204 |  |  | DB Search |
| total 1 peptides |  |  |  |  |  |  |  |  |  |  |  |  |  |  |  |  |  |  |  |
| --- | --- | --- | --- | --- | --- | --- | --- | --- | --- | --- | --- | --- | --- | --- | --- | --- | --- | --- | --- |

Best Unique PSM (Scan Sample\_10\_A1\_1\_3647.d:33690, m/z=668.834, z=2, RT=13.45, ppm=-2.69):


TRINITY\_DN2113\_c0\_g1\_i2.p1

back to list

  

| Protein Coverage
| Supporting Peptides
| Best Unique PSM
|

Protein Coverage:

Supporting Peptides:

| Peptide | Uniq | -10lgP | Mass | Length | ppm | m/z | z | RT | 1/k0 Start | 1/k0 End | Precursor ID | Area Sample 1 | #Feature | #Feature Sample 1 | Start | End | PTM | AScore | Found By |
| --- | --- | --- | --- | --- | --- | --- | --- | --- | --- | --- | --- | --- | --- | --- | --- | --- | --- | --- | --- |
| R.LADLEEFISSPNHANISQVGER.C | Y | 61.67 | 2425.1873 | 22 | 1.36 | 809.4005 | 3 | 18.74 | 0.9263 | 0.9335 | 63253 | 4.69e2 | 1 | 1 | 1183 | 1204 |  |  | DB Search |
| K.GQFSTDDLVAEVEKR.N | Y | 58.01 | 1692.8318 | 15 | 1.27 | 847.4205 | 2 | 17.68 | 1.1374 | 1.1446 | 56739 | 3.43e2 | 1 | 1 | 838 | 852 |  |  | DB Search |
| total 2 peptides |  |  |  |  |  |  |  |  |  |  |  |  |  |  |  |  |  |  |  |
| --- | --- | --- | --- | --- | --- | --- | --- | --- | --- | --- | --- | --- | --- | --- | --- | --- | --- | --- | --- |

Best Unique PSM (Scan Sample\_10\_A1\_1\_3647.d:63253, m/z=809.4005, z=3, RT=18.74, ppm=-3.14):


TRINITY\_DN34765\_c0\_g1\_i1.p1

back to list

  

| Protein Coverage
| Supporting Peptides
| Best Unique PSM
|

Protein Coverage:

Supporting Peptides:

| Peptide | Uniq | -10lgP | Mass | Length | ppm | m/z | z | RT | 1/k0 Start | 1/k0 End | Precursor ID | Area Sample 1 | #Feature | #Feature Sample 1 | Start | End | PTM | AScore | Found By |
| --- | --- | --- | --- | --- | --- | --- | --- | --- | --- | --- | --- | --- | --- | --- | --- | --- | --- | --- | --- |
| R.KSQFGEIFSFDKK.V | Y | 62.93 | 1559.7984 | 13 | -6.3 | 520.9344 | 3 | 17.14 | 0.7888 | 0.7962 | 53558 | 6.09e2 | 1 | 1 | 118 | 130 |  |  | DB Search |
| total 1 peptides |  |  |  |  |  |  |  |  |  |  |  |  |  |  |  |  |  |  |  |
| --- | --- | --- | --- | --- | --- | --- | --- | --- | --- | --- | --- | --- | --- | --- | --- | --- | --- | --- | --- |

Best Unique PSM (Scan Sample\_10\_A1\_1\_3647.d:53558, m/z=520.9344, z=3, RT=17.14, ppm=-10.8):


TRINITY\_DN1614\_c0\_g1\_i1.p1

back to list

  

| Protein Coverage
| Supporting Peptides
| Best Unique PSM
|

Protein Coverage:

Supporting Peptides:

| Peptide | Uniq | -10lgP | Mass | Length | ppm | m/z | z | RT | 1/k0 Start | 1/k0 End | Precursor ID | Area Sample 1 | #Feature | #Feature Sample 1 | Start | End | PTM | AScore | Found By |
| --- | --- | --- | --- | --- | --- | --- | --- | --- | --- | --- | --- | --- | --- | --- | --- | --- | --- | --- | --- |
| K.GLDVDSLVIDHIQVNR.A | Y | 66.67 | 1791.9479 | 16 | -2.06 | 896.9753 | 2 | 19.01 | 1.1794 | 1.1865 | 64835 | 3.83e3 | 1 | 1 | 140 | 155 |  |  | DB Search |
| K.KSAQFLLQLLK.N | Y | 66.34 | 1287.7914 | 11 | 0.84 | 644.9006 | 2 | 19.70 | 0.9783 | 0.9856 | 69083 | 8.25e2 | 1 | 1 | 120 | 130 |  |  | DB Search |
| total 2 peptides |  |  |  |  |  |  |  |  |  |  |  |  |  |  |  |  |  |  |  |
| --- | --- | --- | --- | --- | --- | --- | --- | --- | --- | --- | --- | --- | --- | --- | --- | --- | --- | --- | --- |

Best Unique PSM (Scan Sample\_10\_A1\_1\_3647.d:64835, m/z=896.9753, z=2, RT=19.01, ppm=-6.55):


TRINITY\_DN1910\_c0\_g1\_i1.p1

back to list

  

| Protein Coverage
| Supporting Peptides
| Best Unique PSM
|

Protein Coverage:

Supporting Peptides:

| Peptide | Uniq | -10lgP | Mass | Length | ppm | m/z | z | RT | 1/k0 Start | 1/k0 End | Precursor ID | Area Sample 1 | #Feature | #Feature Sample 1 | Start | End | PTM | AScore | Found By |
| --- | --- | --- | --- | --- | --- | --- | --- | --- | --- | --- | --- | --- | --- | --- | --- | --- | --- | --- | --- |
| K.NM(+15.99)M(+15.99)AAC(+57.02)DPR.H | Y | 66.66 | 1096.41 | 9 | 0.96 | 549.2103 | 2 | 9.51 | 0.8591 | 0.8664 | 10859 | 2.33e3 | 1 | 1 | 298 | 306 | Oxidation (M), Oxidation (M), Carbamidomethylation | M2:Oxidation (M):1000 M3:Oxidation (M):1000 | DB Search |
| K.NSSYFVEWIPNNVK.T | Y | 64.67 | 1695.8256 | 14 | -4.28 | 848.9127 | 2 | 19.92 | 1.0889 | 1.0961 | 70457 | 0 | 0 | 0 | 337 | 350 |  |  | DB Search |
| R.FPGQLNADLRK.L | Y | 62.07 | 1257.6829 | 11 | -5.49 | 629.8425 | 2 | 14.27 | 0.9428 | 0.9501 | 38203 | 0 | 0 | 0 | 242 | 252 |  |  | DB Search |
| R.EIVHLQAGQC(+57.02)GNQIGAK.F | Y | 59.9 | 1821.9155 | 17 | -14.62 | 911.9476 | 2 | 13.73 | 1.113 | 1.1202 | 35183 | 0 | 0 | 0 | 3 | 19 | Carbamidomethylation |  | DB Search |
| total 4 peptides |  |  |  |  |  |  |  |  |  |  |  |  |  |  |  |  |  |  |  |
| --- | --- | --- | --- | --- | --- | --- | --- | --- | --- | --- | --- | --- | --- | --- | --- | --- | --- | --- | --- |

Best Unique PSM (Scan Sample\_10\_A1\_1\_3647.d:10859, m/z=549.2103, z=2, RT=9.51, ppm=-3.54):


TRINITY\_DN123\_c0\_g1\_i1.p1

back to list

  

| Protein Coverage
| Supporting Peptides
| Best Unique PSM
|

Protein Coverage:

Supporting Peptides:

| Peptide | Uniq | -10lgP | Mass | Length | ppm | m/z | z | RT | 1/k0 Start | 1/k0 End | Precursor ID | Area Sample 1 | #Feature | #Feature Sample 1 | Start | End | PTM | AScore | Found By |
| --- | --- | --- | --- | --- | --- | --- | --- | --- | --- | --- | --- | --- | --- | --- | --- | --- | --- | --- | --- |
| K.IAAVIINKPDVLASR.T | Y | 66.74 | 1578.9457 | 15 | -2.48 | 790.4746 | 2 | 15.97 | 1.1013 | 1.1086 | 47200 | 1.29e3 | 1 | 1 | 484 | 498 |  |  | DB Search |
| total 1 peptides |  |  |  |  |  |  |  |  |  |  |  |  |  |  |  |  |  |  |  |
| --- | --- | --- | --- | --- | --- | --- | --- | --- | --- | --- | --- | --- | --- | --- | --- | --- | --- | --- | --- |

Best Unique PSM (Scan Sample\_10\_A1\_1\_3647.d:47200, m/z=790.4746, z=2, RT=15.97, ppm=-6.97):


TRINITY\_DN1058\_c0\_g1\_i4.p1

back to list

  

| Protein Coverage
| Supporting Peptides
| Best Unique PSM
|

Protein Coverage:

Supporting Peptides:

| Peptide | Uniq | -10lgP | Mass | Length | ppm | m/z | z | RT | 1/k0 Start | 1/k0 End | Precursor ID | Area Sample 1 | #Feature | #Feature Sample 1 | Start | End | PTM | AScore | Found By |
| --- | --- | --- | --- | --- | --- | --- | --- | --- | --- | --- | --- | --- | --- | --- | --- | --- | --- | --- | --- |
| R.APTHILNPANKR.S | Y | 66.68 | 1330.7469 | 12 | 2.53 | 444.5887 | 3 | 11.11 | 0.7526 | 0.7599 | 20079 | 1.09e3 | 2 | 2 | 398 | 409 |  |  | DB Search |
| total 1 peptides |  |  |  |  |  |  |  |  |  |  |  |  |  |  |  |  |  |  |  |
| --- | --- | --- | --- | --- | --- | --- | --- | --- | --- | --- | --- | --- | --- | --- | --- | --- | --- | --- | --- |

Best Unique PSM (Scan Sample\_10\_A1\_1\_3647.d:20079, m/z=444.5887, z=3, RT=11.11, ppm=-1.97):


TRINITY\_DN5107\_c0\_g1\_i8.p2

back to list

  

| Protein Coverage
| Supporting Peptides
| Best Unique PSM
|

Protein Coverage:

Supporting Peptides:

| Peptide | Uniq | -10lgP | Mass | Length | ppm | m/z | z | RT | 1/k0 Start | 1/k0 End | Precursor ID | Area Sample 1 | #Feature | #Feature Sample 1 | Start | End | PTM | AScore | Found By |
| --- | --- | --- | --- | --- | --- | --- | --- | --- | --- | --- | --- | --- | --- | --- | --- | --- | --- | --- | --- |
| K.KLDYVLGLTTAK.I | Y | 63.57 | 1320.7653 | 12 | -2.37 | 661.3854 | 2 | 16.69 | 0.9908 | 0.9981 | 51148 | 2.13e2 | 1 | 1 | 91 | 102 |  |  | DB Search |
| total 1 peptides |  |  |  |  |  |  |  |  |  |  |  |  |  |  |  |  |  |  |  |
| --- | --- | --- | --- | --- | --- | --- | --- | --- | --- | --- | --- | --- | --- | --- | --- | --- | --- | --- | --- |

Best Unique PSM (Scan Sample\_10\_A1\_1\_3647.d:51148, m/z=661.3854, z=2, RT=16.69, ppm=-6.87):


TRINITY\_DN785\_c0\_g1\_i1.p1

back to list

  

| Protein Coverage
| Supporting Peptides
| Best Unique PSM
|

Protein Coverage:

Supporting Peptides:

| Peptide | Uniq | -10lgP | Mass | Length | ppm | m/z | z | RT | 1/k0 Start | 1/k0 End | Precursor ID | Area Sample 1 | #Feature | #Feature Sample 1 | Start | End | PTM | AScore | Found By |
| --- | --- | --- | --- | --- | --- | --- | --- | --- | --- | --- | --- | --- | --- | --- | --- | --- | --- | --- | --- |
| K.KFDTYLPGFIC(+57.02)R.C | Y | 40.39 | 1515.7544 | 12 | -0.6 | 758.8806 | 2 | 18.56 | 1.0339 | 1.0411 | 62069 | 5.32e2 | 1 | 1 | 77 | 88 | Carbamidomethylation |  | DB Search |
| total 1 peptides |  |  |  |  |  |  |  |  |  |  |  |  |  |  |  |  |  |  |  |
| --- | --- | --- | --- | --- | --- | --- | --- | --- | --- | --- | --- | --- | --- | --- | --- | --- | --- | --- | --- |

Best Unique PSM (Scan Sample\_10\_A1\_1\_3647.d:62069, m/z=758.8806, z=2, RT=18.56, ppm=-5.09):


TRINITY\_DN602\_c0\_g1\_i1.p1

back to list

  

| Protein Coverage
| Supporting Peptides
| Best Unique PSM
|

Protein Coverage:

Supporting Peptides:

| Peptide | Uniq | -10lgP | Mass | Length | ppm | m/z | z | RT | 1/k0 Start | 1/k0 End | Precursor ID | Area Sample 1 | #Feature | #Feature Sample 1 | Start | End | PTM | AScore | Found By |
| --- | --- | --- | --- | --- | --- | --- | --- | --- | --- | --- | --- | --- | --- | --- | --- | --- | --- | --- | --- |
| K.EGFYVHEALAAGIVK.K | Y | 63.7 | 1602.8405 | 15 | -2.05 | 802.4223 | 2 | 17.52 | 1.0889 | 1.0961 | 55709 | 2.51e2 | 1 | 1 | 185 | 199 |  |  | DB Search |
| total 1 peptides |  |  |  |  |  |  |  |  |  |  |  |  |  |  |  |  |  |  |  |
| --- | --- | --- | --- | --- | --- | --- | --- | --- | --- | --- | --- | --- | --- | --- | --- | --- | --- | --- | --- |

Best Unique PSM (Scan Sample\_10\_A1\_1\_3647.d:55709, m/z=802.4223, z=2, RT=17.52, ppm=-6.54):


TRINITY\_DN1819\_c0\_g1\_i5.p1

back to list

  

| Protein Coverage
| Supporting Peptides
| Best Unique PSM
|

Protein Coverage:

Supporting Peptides:

| Peptide | Uniq | -10lgP | Mass | Length | ppm | m/z | z | RT | 1/k0 Start | 1/k0 End | Precursor ID | Area Sample 1 | #Feature | #Feature Sample 1 | Start | End | PTM | AScore | Found By |
| --- | --- | --- | --- | --- | --- | --- | --- | --- | --- | --- | --- | --- | --- | --- | --- | --- | --- | --- | --- |
| K.ALADYIHSQDLK.M | Y | 59.54 | 1372.6986 | 12 | 0.98 | 687.3542 | 2 | 15.62 | 1.0061 | 1.0134 | 45560 | 3.85e2 | 1 | 1 | 105 | 116 |  |  | DB Search |
| total 1 peptides |  |  |  |  |  |  |  |  |  |  |  |  |  |  |  |  |  |  |  |
| --- | --- | --- | --- | --- | --- | --- | --- | --- | --- | --- | --- | --- | --- | --- | --- | --- | --- | --- | --- |

Best Unique PSM (Scan Sample\_10\_A1\_1\_3647.d:45560, m/z=687.3542, z=2, RT=15.62, ppm=-3.52):


TRINITY\_DN36\_c0\_g1\_i16.p1

back to list

  

| Protein Coverage
| Supporting Peptides
| Best Unique PSM
|

Protein Coverage:

Supporting Peptides:

| Peptide | Uniq | -10lgP | Mass | Length | ppm | m/z | z | RT | 1/k0 Start | 1/k0 End | Precursor ID | Area Sample 1 | #Feature | #Feature Sample 1 | Start | End | PTM | AScore | Found By |
| --- | --- | --- | --- | --- | --- | --- | --- | --- | --- | --- | --- | --- | --- | --- | --- | --- | --- | --- | --- |
| R.LGGIEVTQGNC(+57.02)AR.K | Y | 66.48 | 1373.6721 | 13 | 5.18 | 687.8438 | 2 | 13.73 | 0.963 | 0.9703 | 35192 | 0 | 0 | 0 | 58 | 70 | Carbamidomethylation |  | DB Search |
| K.WC(+57.02)DILSIHQSMENC(+57.02)VAK.C | Y | 63.98 | 2089.9383 | 17 | -2.35 | 697.6486 | 3 | 17.89 | 0.8777 | 0.885 | 58058 | 0 | 0 | 0 | 198 | 214 | Carbamidomethylation, Carbamidomethylation |  | DB Search |
| K.FETQIATK.D | Y | 61.61 | 936.4916 | 8 | 0.97 | 937.4956 | 1 | 13.84 | 1.2037 | 1.2109 | 35727 | 1.28e3 | 1 | 1 | 167 | 174 |  |  | DB Search |
| R.LGGIEVTQGNC(+57.02)ARK.Y | Y | 31.04 | 1501.7671 | 14 | -2.67 | 751.8854 | 2 | 12.64 | 1.0624 | 1.0697 | 29000 | 0 | 0 | 0 | 58 | 71 | Carbamidomethylation |  | DB Search |
| total 4 peptides |  |  |  |  |  |  |  |  |  |  |  |  |  |  |  |  |  |  |  |
| --- | --- | --- | --- | --- | --- | --- | --- | --- | --- | --- | --- | --- | --- | --- | --- | --- | --- | --- | --- |

Best Unique PSM (Scan Sample\_10\_A1\_1\_3647.d:35192, m/z=687.8438, z=2, RT=13.73, ppm=0.69):


TRINITY\_DN21998\_c0\_g1\_i1.p1

back to list

  

| Protein Coverage
| Supporting Peptides
| Best Unique PSM
|

Protein Coverage:

Supporting Peptides:

| Peptide | Uniq | -10lgP | Mass | Length | ppm | m/z | z | RT | 1/k0 Start | 1/k0 End | Precursor ID | Area Sample 1 | #Feature | #Feature Sample 1 | Start | End | PTM | AScore | Found By |
| --- | --- | --- | --- | --- | --- | --- | --- | --- | --- | --- | --- | --- | --- | --- | --- | --- | --- | --- | --- |
| K.LTHLEEAYTTEHWLVR.I | Y | 66.93 | 1997.0006 | 16 | 0.43 | 666.6714 | 3 | 17.14 | 0.9574 | 0.9646 | 53465 | 3.26e2 | 1 | 1 | 421 | 436 |  |  | DB Search |
| total 1 peptides |  |  |  |  |  |  |  |  |  |  |  |  |  |  |  |  |  |  |  |
| --- | --- | --- | --- | --- | --- | --- | --- | --- | --- | --- | --- | --- | --- | --- | --- | --- | --- | --- | --- |

Best Unique PSM (Scan Sample\_10\_A1\_1\_3647.d:53465, m/z=666.6714, z=3, RT=17.14, ppm=-4.07):


TRINITY\_DN1787\_c0\_g2\_i1.p1

back to list

  

| Protein Coverage
| Supporting Peptides
| Best Unique PSM
|

Protein Coverage:

Supporting Peptides:

| Peptide | Uniq | -10lgP | Mass | Length | ppm | m/z | z | RT | 1/k0 Start | 1/k0 End | Precursor ID | Area Sample 1 | #Feature | #Feature Sample 1 | Start | End | PTM | AScore | Found By |
| --- | --- | --- | --- | --- | --- | --- | --- | --- | --- | --- | --- | --- | --- | --- | --- | --- | --- | --- | --- |
| K.YC(+57.02)HLFLC(+57.02)VHC(+57.02)LK.E | Y | 66.68 | 1648.7676 | 12 | -5.26 | 825.3831 | 2 | 16.15 | 1.1098 | 1.117 | 48282 | 9.73e2 | 2 | 2 | 66 | 77 | Carbamidomethylation, Carbamidomethylation, Carbamidomethylation |  | DB Search |
| total 1 peptides |  |  |  |  |  |  |  |  |  |  |  |  |  |  |  |  |  |  |  |
| --- | --- | --- | --- | --- | --- | --- | --- | --- | --- | --- | --- | --- | --- | --- | --- | --- | --- | --- | --- |

Best Unique PSM (Scan Sample\_10\_A1\_1\_3647.d:48282, m/z=825.3831, z=2, RT=16.15, ppm=-9.75):


TRINITY\_DN286\_c0\_g1\_i4.p1

back to list

  

| Protein Coverage
| Supporting Peptides
| Best Unique PSM
|

Protein Coverage:

Supporting Peptides:

| Peptide | Uniq | -10lgP | Mass | Length | ppm | m/z | z | RT | 1/k0 Start | 1/k0 End | Precursor ID | Area Sample 1 | #Feature | #Feature Sample 1 | Start | End | PTM | AScore | Found By |
| --- | --- | --- | --- | --- | --- | --- | --- | --- | --- | --- | --- | --- | --- | --- | --- | --- | --- | --- | --- |
| K.RYGQAMC(+57.02)QFMR.N | Y | 66.75 | 1446.6319 | 11 | -6.11 | 724.3156 | 2 | 17.23 | 0.9868 | 0.9941 | 53709 | 8.19e3 | 1 | 1 | 71 | 81 | Carbamidomethylation |  | DB Search |
| K.RYGQAM(+15.99)C(+57.02)QFM(+15.99)R.N | Y | 66.67 | 1478.6217 | 11 | -8.74 | 740.3083 | 2 | 12.90 | 0.9759 | 0.9832 | 30310 | 4.97e3 | 1 | 1 | 71 | 81 | Oxidation (M), Carbamidomethylation, Oxidation (M) | M6:Oxidation (M):1000 M10:Oxidation (M):1000 | DB Search |
| K.RYGQAMC(+57.02)QFM(+15.99)R.N | Y | 63.05 | 1462.6268 | 11 | -11.86 | 732.3087 | 2 | 14.93 | 0.9779 | 0.9852 | 41834 | 0 | 0 | 0 | 71 | 81 | Carbamidomethylation, Oxidation (M) | M10:Oxidation (M):25.11 | DB Search |
| K.RYGQAM(+15.99)C(+57.02)QFMR.N | Y | 29.61 | 1462.6268 | 11 | -5.85 | 732.3131 | 2 | 14.49 | 0.9751 | 0.9824 | 39230 | 2.67e3 | 1 | 1 | 71 | 81 | Oxidation (M), Carbamidomethylation | M6:Oxidation (M):34.3 | DB Search |
| total 4 peptides |  |  |  |  |  |  |  |  |  |  |  |  |  |  |  |  |  |  |  |
| --- | --- | --- | --- | --- | --- | --- | --- | --- | --- | --- | --- | --- | --- | --- | --- | --- | --- | --- | --- |

Best Unique PSM (Scan Sample\_10\_A1\_1\_3647.d:53709, m/z=724.3156, z=2, RT=17.23, ppm=-10.6):


TRINITY\_DN2142\_c0\_g1\_i1.p2

back to list

  

| Protein Coverage
| Supporting Peptides
| Best Unique PSM
|

Protein Coverage:

Supporting Peptides:

| Peptide | Uniq | -10lgP | Mass | Length | ppm | m/z | z | RT | 1/k0 Start | 1/k0 End | Precursor ID | Area Sample 1 | #Feature | #Feature Sample 1 | Start | End | PTM | AScore | Found By |
| --- | --- | --- | --- | --- | --- | --- | --- | --- | --- | --- | --- | --- | --- | --- | --- | --- | --- | --- | --- |
| R.ERQENTNPVTR.Y | Y | 37.82 | 1342.6589 | 11 | 1.48 | 672.3347 | 2 | 9.98 | 0.9558 | 0.963 | 13356 | 6.23e2 | 1 | 1 | 172 | 182 |  |  | DB Search |
| total 1 peptides |  |  |  |  |  |  |  |  |  |  |  |  |  |  |  |  |  |  |  |
| --- | --- | --- | --- | --- | --- | --- | --- | --- | --- | --- | --- | --- | --- | --- | --- | --- | --- | --- | --- |

Best Unique PSM (Scan Sample\_10\_A1\_1\_3647.d:13356, m/z=672.3347, z=2, RT=9.98, ppm=-3.01):


TRINITY\_DN3180\_c0\_g1\_i1.p1

back to list

  

| Protein Coverage
| Supporting Peptides
| Best Unique PSM
|

Protein Coverage:

Supporting Peptides:

| Peptide | Uniq | -10lgP | Mass | Length | ppm | m/z | z | RT | 1/k0 Start | 1/k0 End | Precursor ID | Area Sample 1 | #Feature | #Feature Sample 1 | Start | End | PTM | AScore | Found By |
| --- | --- | --- | --- | --- | --- | --- | --- | --- | --- | --- | --- | --- | --- | --- | --- | --- | --- | --- | --- |
| K.LVIGNSEGFKDVSK.K | Y | 66.78 | 1491.7933 | 14 | 0.35 | 746.9008 | 2 | 14.67 | 1.0809 | 1.0881 | 40349 | 0 | 0 | 0 | 819 | 832 |  |  | DB Search |
| total 1 peptides |  |  |  |  |  |  |  |  |  |  |  |  |  |  |  |  |  |  |  |
| --- | --- | --- | --- | --- | --- | --- | --- | --- | --- | --- | --- | --- | --- | --- | --- | --- | --- | --- | --- |

Best Unique PSM (Scan Sample\_10\_A1\_1\_3647.d:40349, m/z=746.9008, z=2, RT=14.67, ppm=-4.15):


TRINITY\_DN495\_c0\_g1\_i5.p1

back to list

  

| Protein Coverage
| Supporting Peptides
| Best Unique PSM
|

Protein Coverage:

Supporting Peptides:

| Peptide | Uniq | -10lgP | Mass | Length | ppm | m/z | z | RT | 1/k0 Start | 1/k0 End | Precursor ID | Area Sample 1 | #Feature | #Feature Sample 1 | Start | End | PTM | AScore | Found By |
| --- | --- | --- | --- | --- | --- | --- | --- | --- | --- | --- | --- | --- | --- | --- | --- | --- | --- | --- | --- |
| C.SISC(+57.02)SKDEEIK.V | Y | 63.06 | 1294.6075 | 11 | 1.99 | 648.3094 | 2 | 10.84 | 0.9755 | 0.9828 | 18539 | 0 | 0 | 0 | 273 | 283 | Carbamidomethylation |  | DB Search |
| total 1 peptides |  |  |  |  |  |  |  |  |  |  |  |  |  |  |  |  |  |  |  |
| --- | --- | --- | --- | --- | --- | --- | --- | --- | --- | --- | --- | --- | --- | --- | --- | --- | --- | --- | --- |

Best Unique PSM (Scan Sample\_10\_A1\_1\_3647.d:18539, m/z=648.3094, z=2, RT=10.84, ppm=-2.5):


TRINITY\_DN135129\_c0\_g1\_i1.p1

back to list

  

| Protein Coverage
| Supporting Peptides
| Best Unique PSM
|

Protein Coverage:

Supporting Peptides:

| Peptide | Uniq | -10lgP | Mass | Length | ppm | m/z | z | RT | 1/k0 Start | 1/k0 End | Precursor ID | Area Sample 1 | #Feature | #Feature Sample 1 | Start | End | PTM | AScore | Found By |
| --- | --- | --- | --- | --- | --- | --- | --- | --- | --- | --- | --- | --- | --- | --- | --- | --- | --- | --- | --- |
| K.SDSSDIEKR.A | Y | 62 | 1035.4832 | 9 | -18.84 | 518.7368 | 2 | 11.06 | 0.8558 | 0.8631 | 19937 | 8.59e3 | 1 | 1 | 96 | 104 |  |  | DB Search |
| total 1 peptides |  |  |  |  |  |  |  |  |  |  |  |  |  |  |  |  |  |  |  |
| --- | --- | --- | --- | --- | --- | --- | --- | --- | --- | --- | --- | --- | --- | --- | --- | --- | --- | --- | --- |

Best Unique PSM (Scan Sample\_10\_A1\_1\_3647.d:19937, m/z=518.7368, z=2, RT=11.06, ppm=-23.34):


TRINITY\_DN45599\_c0\_g1\_i1.p1

back to list

  

| Protein Coverage
| Supporting Peptides
| Best Unique PSM
|

Protein Coverage:

Supporting Peptides:

| Peptide | Uniq | -10lgP | Mass | Length | ppm | m/z | z | RT | 1/k0 Start | 1/k0 End | Precursor ID | Area Sample 1 | #Feature | #Feature Sample 1 | Start | End | PTM | AScore | Found By |
| --- | --- | --- | --- | --- | --- | --- | --- | --- | --- | --- | --- | --- | --- | --- | --- | --- | --- | --- | --- |
| R.RLVGIVVYDYLAHNNK.E | Y | 62.34 | 1873.021 | 16 | 4.78 | 625.3478 | 3 | 18.53 | 0.9344 | 0.9416 | 62014 | 0 | 0 | 0 | 346 | 361 |  |  | DB Search |
| total 1 peptides |  |  |  |  |  |  |  |  |  |  |  |  |  |  |  |  |  |  |  |
| --- | --- | --- | --- | --- | --- | --- | --- | --- | --- | --- | --- | --- | --- | --- | --- | --- | --- | --- | --- |

Best Unique PSM (Scan Sample\_10\_A1\_1\_3647.d:62014, m/z=625.3478, z=3, RT=18.53, ppm=0.28):


TRINITY\_DN7314\_c0\_g2\_i1.p1

back to list

  

| Protein Coverage
| Supporting Peptides
| Best Unique PSM
|

Protein Coverage:

Supporting Peptides:

| Peptide | Uniq | -10lgP | Mass | Length | ppm | m/z | z | RT | 1/k0 Start | 1/k0 End | Precursor ID | Area Sample 1 | #Feature | #Feature Sample 1 | Start | End | PTM | AScore | Found By |
| --- | --- | --- | --- | --- | --- | --- | --- | --- | --- | --- | --- | --- | --- | --- | --- | --- | --- | --- | --- |
| K.YVESAADKR.L | Y | 63 | 1037.5141 | 9 | 0.83 | 519.7625 | 2 | 9.57 | 0.8538 | 0.8611 | 11283 | 0 | 0 | 0 | 10 | 18 |  |  | DB Search |
| total 1 peptides |  |  |  |  |  |  |  |  |  |  |  |  |  |  |  |  |  |  |  |
| --- | --- | --- | --- | --- | --- | --- | --- | --- | --- | --- | --- | --- | --- | --- | --- | --- | --- | --- | --- |

Best Unique PSM (Scan Sample\_10\_A1\_1\_3647.d:11283, m/z=519.7625, z=2, RT=9.57, ppm=-3.66):


TRINITY\_DN6783\_c0\_g1\_i1.p1

back to list

  

| Protein Coverage
| Supporting Peptides
| Best Unique PSM
|

Protein Coverage:

Supporting Peptides:

| Peptide | Uniq | -10lgP | Mass | Length | ppm | m/z | z | RT | 1/k0 Start | 1/k0 End | Precursor ID | Area Sample 1 | #Feature | #Feature Sample 1 | Start | End | PTM | AScore | Found By |
| --- | --- | --- | --- | --- | --- | --- | --- | --- | --- | --- | --- | --- | --- | --- | --- | --- | --- | --- | --- |
| K.GTIEILGDVHLIK.K | Y | 45.38 | 1406.8133 | 13 | -2.67 | 704.4089 | 2 | 18.96 | 1.0616 | 1.0688 | 64620 | 5.41e2 | 1 | 1 | 150 | 162 |  |  | DB Search |
| total 1 peptides |  |  |  |  |  |  |  |  |  |  |  |  |  |  |  |  |  |  |  |
| --- | --- | --- | --- | --- | --- | --- | --- | --- | --- | --- | --- | --- | --- | --- | --- | --- | --- | --- | --- |

Best Unique PSM (Scan Sample\_10\_A1\_1\_3647.d:64620, m/z=704.4089, z=2, RT=18.96, ppm=-7.16):


TRINITY\_DN982\_c0\_g1\_i3.p1

back to list

  

| Protein Coverage
| Supporting Peptides
| Best Unique PSM
|

Protein Coverage:

Supporting Peptides:

| Peptide | Uniq | -10lgP | Mass | Length | ppm | m/z | z | RT | 1/k0 Start | 1/k0 End | Precursor ID | Area Sample 1 | #Feature | #Feature Sample 1 | Start | End | PTM | AScore | Found By |
| --- | --- | --- | --- | --- | --- | --- | --- | --- | --- | --- | --- | --- | --- | --- | --- | --- | --- | --- | --- |
| R.LVQNTPHNSIVTK.I | Y | 66.78 | 1449.794 | 13 | 2.36 | 725.9027 | 2 | 11.65 | 1.0628 | 1.0701 | 23219 | 0 | 0 | 0 | 8569 | 8581 |  |  | DB Search |
| R.ITTSQILDHEQQK.L | Y | 66.73 | 1539.7893 | 13 | 2.99 | 770.9008 | 2 | 13.83 | 1.0945 | 1.1017 | 35680 | 3.16e2 | 1 | 1 | 5434 | 5446 |  |  | DB Search |
| K.VLLSQQIFYDKK.N | Y | 66.52 | 1480.8289 | 12 | -1.68 | 741.4172 | 2 | 16.70 | 1.06 | 1.0672 | 51062 | 6.76e2 | 1 | 1 | 4919 | 4930 |  |  | DB Search |
| R.VLATDKDTLSSNK.Q | Y | 62.11 | 1390.7303 | 13 | 2.98 | 696.3714 | 2 | 11.50 | 1.0154 | 1.0226 | 22335 | 9.57e2 | 1 | 1 | 3827 | 3839 |  |  | DB Search |
| K.SAIIVDGKNEEGR.I | Y | 62.03 | 1386.7103 | 13 | -2.52 | 694.3575 | 2 | 11.84 | 0.9953 | 1.0025 | 24410 | 0 | 0 | 0 | 95 | 107 |  |  | DB Search |
| R.LIQTLNYETKK.E | Y | 61.82 | 1349.7554 | 11 | 0.35 | 675.8822 | 2 | 13.59 | 1.0098 | 1.017 | 34441 | 0 | 0 | 0 | 8319 | 8329 |  |  | DB Search |
| K.YVAIYPSDHLTVNR.D | Y | 60.16 | 1646.8416 | 14 | -9.73 | 824.4164 | 2 | 15.50 | 1.1098 | 1.117 | 44890 | 0 | 0 | 0 | 6254 | 6267 |  |  | DB Search |
| total 7 peptides |  |  |  |  |  |  |  |  |  |  |  |  |  |  |  |  |  |  |  |
| --- | --- | --- | --- | --- | --- | --- | --- | --- | --- | --- | --- | --- | --- | --- | --- | --- | --- | --- | --- |

Best Unique PSM (Scan Sample\_10\_A1\_1\_3647.d:23219, m/z=725.9027, z=2, RT=11.65, ppm=-2.13):


TRINITY\_DN332\_c0\_g1\_i1.p1

back to list

  

| Protein Coverage
| Supporting Peptides
| Best Unique PSM
|

Protein Coverage:

Supporting Peptides:

| Peptide | Uniq | -10lgP | Mass | Length | ppm | m/z | z | RT | 1/k0 Start | 1/k0 End | Precursor ID | Area Sample 1 | #Feature | #Feature Sample 1 | Start | End | PTM | AScore | Found By |
| --- | --- | --- | --- | --- | --- | --- | --- | --- | --- | --- | --- | --- | --- | --- | --- | --- | --- | --- | --- |
| K.DYGVLLEDAGHTLR.G | Y | 66.59 | 1557.7787 | 14 | -2.26 | 779.8914 | 2 | 18.22 | 1.1286 | 1.1358 | 59935 | 3.98e2 | 1 | 1 | 158 | 171 |  |  | DB Search |
| R.GLFIIDDKGVLR.Q | Y | 66.59 | 1344.7765 | 12 | 0.84 | 673.3931 | 2 | 18.67 | 1.0017 | 1.009 | 62753 | 5.91e2 | 1 | 1 | 172 | 183 |  |  | DB Search |
| total 2 peptides |  |  |  |  |  |  |  |  |  |  |  |  |  |  |  |  |  |  |  |
| --- | --- | --- | --- | --- | --- | --- | --- | --- | --- | --- | --- | --- | --- | --- | --- | --- | --- | --- | --- |

Best Unique PSM (Scan Sample\_10\_A1\_1\_3647.d:59935, m/z=779.8914, z=2, RT=18.22, ppm=-6.75):


TRINITY\_DN160\_c0\_g1\_i1.p1

back to list

  

| Protein Coverage
| Supporting Peptides
| Best Unique PSM
|

Protein Coverage:

Supporting Peptides:

| Peptide | Uniq | -10lgP | Mass | Length | ppm | m/z | z | RT | 1/k0 Start | 1/k0 End | Precursor ID | Area Sample 1 | #Feature | #Feature Sample 1 | Start | End | PTM | AScore | Found By |
| --- | --- | --- | --- | --- | --- | --- | --- | --- | --- | --- | --- | --- | --- | --- | --- | --- | --- | --- | --- |
| K.ELVM(+15.99)HEQSR.C | Y | 66.65 | 1143.5342 | 9 | 4.42 | 572.7744 | 2 | 9.76 | 0.8895 | 0.8968 | 12266 | 8.53e2 | 1 | 1 | 1116 | 1124 | Oxidation (M) | M4:Oxidation (M):1000 | DB Search |
| R.LDFDHEAKLNMIFR.F | Y | 66.57 | 1747.8715 | 14 | 2.05 | 583.6297 | 3 | 18.33 | 0.8558 | 0.8631 | 60685 | 0 | 0 | 0 | 1009 | 1022 |  |  | DB Search |
| K.LRLDFDHEAKLNMIFR.F | Y | 64.71 | 2017.0567 | 16 | -0.61 | 673.3561 | 3 | 18.47 | 0.9449 | 0.9521 | 61552 | 3.77e3 | 2 | 2 | 1007 | 1022 |  |  | DB Search |
| K.LRLDFDHEAKLNM(+15.99)IFR.F | Y | 64.35 | 2033.0516 | 16 | 4.9 | 509.2704 | 4 | 17.76 | 0.8558 | 0.8631 | 57084 | 1.58e3 | 1 | 1 | 1007 | 1022 | Oxidation (M) | M13:Oxidation (M):1000 | DB Search |
| K.LRLDFDHEAK.L | Y | 42.61 | 1242.6356 | 10 | 8.37 | 415.2208 | 3 | 14.18 | 0.7282 | 0.7355 | 37619 | 6.56e2 | 1 | 1 | 1007 | 1016 |  |  | DB Search |
| total 5 peptides |  |  |  |  |  |  |  |  |  |  |  |  |  |  |  |  |  |  |  |
| --- | --- | --- | --- | --- | --- | --- | --- | --- | --- | --- | --- | --- | --- | --- | --- | --- | --- | --- | --- |

Best Unique PSM (Scan Sample\_10\_A1\_1\_3647.d:12266, m/z=572.7744, z=2, RT=9.76, ppm=-0.08):


TRINITY\_DN1534\_c2\_g1\_i1.p1

back to list

  

| Protein Coverage
| Supporting Peptides
| Best Unique PSM
|

Protein Coverage:

Supporting Peptides:

| Peptide | Uniq | -10lgP | Mass | Length | ppm | m/z | z | RT | 1/k0 Start | 1/k0 End | Precursor ID | Area Sample 1 | #Feature | #Feature Sample 1 | Start | End | PTM | AScore | Found By |
| --- | --- | --- | --- | --- | --- | --- | --- | --- | --- | --- | --- | --- | --- | --- | --- | --- | --- | --- | --- |
| R.RFTVELAR.K | Y | 17.27 | 990.561 | 8 | 5.33 | 496.2882 | 2 | 14.42 | 0.8656 | 0.8729 | 39040 | 0 | 0 | 0 | 195 | 202 |  |  | DB Search |
| total 1 peptides |  |  |  |  |  |  |  |  |  |  |  |  |  |  |  |  |  |  |  |
| --- | --- | --- | --- | --- | --- | --- | --- | --- | --- | --- | --- | --- | --- | --- | --- | --- | --- | --- | --- |

Best Unique PSM (Scan Sample\_10\_A1\_1\_3647.d:39040, m/z=496.2882, z=2, RT=14.42, ppm=0.83):


TRINITY\_DN602\_c0\_g1\_i3.p1

back to list

  

| Protein Coverage
| Supporting Peptides
| Best Unique PSM
|

Protein Coverage:

Supporting Peptides:

| Peptide | Uniq | -10lgP | Mass | Length | ppm | m/z | z | RT | 1/k0 Start | 1/k0 End | Precursor ID | Area Sample 1 | #Feature | #Feature Sample 1 | Start | End | PTM | AScore | Found By |
| --- | --- | --- | --- | --- | --- | --- | --- | --- | --- | --- | --- | --- | --- | --- | --- | --- | --- | --- | --- |
| K.EGFYVHEALAAGIVK.K | Y | 63.7 | 1602.8405 | 15 | -2.05 | 802.4223 | 2 | 17.52 | 1.0889 | 1.0961 | 55709 | 2.51e2 | 1 | 1 | 443 | 457 |  |  | DB Search |
| total 1 peptides |  |  |  |  |  |  |  |  |  |  |  |  |  |  |  |  |  |  |  |
| --- | --- | --- | --- | --- | --- | --- | --- | --- | --- | --- | --- | --- | --- | --- | --- | --- | --- | --- | --- |

Best Unique PSM (Scan Sample\_10\_A1\_1\_3647.d:55709, m/z=802.4223, z=2, RT=17.52, ppm=-6.54):


TRINITY\_DN453\_c0\_g1\_i2.p1

back to list

  

| Protein Coverage
| Supporting Peptides
| Best Unique PSM
|

Protein Coverage:

Supporting Peptides:

| Peptide | Uniq | -10lgP | Mass | Length | ppm | m/z | z | RT | 1/k0 Start | 1/k0 End | Precursor ID | Area Sample 1 | #Feature | #Feature Sample 1 | Start | End | PTM | AScore | Found By |
| --- | --- | --- | --- | --- | --- | --- | --- | --- | --- | --- | --- | --- | --- | --- | --- | --- | --- | --- | --- |
| K.VSILVAC(+57.02)NKQDLTTAK.A | Y | 65.21 | 1759.9502 | 16 | -1.35 | 880.9772 | 2 | 14.68 | 1.1829 | 1.1901 | 40439 | 0 | 0 | 0 | 150 | 165 | Carbamidomethylation |  | DB Search |
| total 1 peptides |  |  |  |  |  |  |  |  |  |  |  |  |  |  |  |  |  |  |  |
| --- | --- | --- | --- | --- | --- | --- | --- | --- | --- | --- | --- | --- | --- | --- | --- | --- | --- | --- | --- |

Best Unique PSM (Scan Sample\_10\_A1\_1\_3647.d:40439, m/z=880.9772, z=2, RT=14.68, ppm=-5.84):


TRINITY\_DN19237\_c0\_g1\_i1.p1

back to list

  

| Protein Coverage
| Supporting Peptides
| Best Unique PSM
|

Protein Coverage:

Supporting Peptides:

| Peptide | Uniq | -10lgP | Mass | Length | ppm | m/z | z | RT | 1/k0 Start | 1/k0 End | Precursor ID | Area Sample 1 | #Feature | #Feature Sample 1 | Start | End | PTM | AScore | Found By |
| --- | --- | --- | --- | --- | --- | --- | --- | --- | --- | --- | --- | --- | --- | --- | --- | --- | --- | --- | --- |
| K.TLVALDEQGEKLNR.I | Y | 61.23 | 1584.8471 | 14 | -0.56 | 793.4268 | 2 | 14.58 | 1.0985 | 1.1057 | 39877 | 0 | 0 | 0 | 47 | 60 |  |  | DB Search |
| total 1 peptides |  |  |  |  |  |  |  |  |  |  |  |  |  |  |  |  |  |  |  |
| --- | --- | --- | --- | --- | --- | --- | --- | --- | --- | --- | --- | --- | --- | --- | --- | --- | --- | --- | --- |

Best Unique PSM (Scan Sample\_10\_A1\_1\_3647.d:39877, m/z=793.4268, z=2, RT=14.58, ppm=-5.06):


TRINITY\_DN79126\_c0\_g1\_i1.p1

back to list

  

| Protein Coverage
| Supporting Peptides
| Best Unique PSM
|

Protein Coverage:

Supporting Peptides:

| Peptide | Uniq | -10lgP | Mass | Length | ppm | m/z | z | RT | 1/k0 Start | 1/k0 End | Precursor ID | Area Sample 1 | #Feature | #Feature Sample 1 | Start | End | PTM | AScore | Found By |
| --- | --- | --- | --- | --- | --- | --- | --- | --- | --- | --- | --- | --- | --- | --- | --- | --- | --- | --- | --- |
| R.AFLIEEQKLVVR.V | Y | 66.8 | 1443.8449 | 12 | 1.31 | 722.9274 | 2 | 17.34 | 1.0496 | 1.0568 | 54531 | 8.16e3 | 1 | 1 | 92 | 103 |  |  | DB Search |
| total 1 peptides |  |  |  |  |  |  |  |  |  |  |  |  |  |  |  |  |  |  |  |
| --- | --- | --- | --- | --- | --- | --- | --- | --- | --- | --- | --- | --- | --- | --- | --- | --- | --- | --- | --- |

Best Unique PSM (Scan Sample\_10\_A1\_1\_3647.d:54531, m/z=722.9274, z=2, RT=17.34, ppm=-3.19):


TRINITY\_DN10563\_c0\_g1\_i1.p1

back to list

  

| Protein Coverage
| Supporting Peptides
| Best Unique PSM
|

Protein Coverage:

Supporting Peptides:

| Peptide | Uniq | -10lgP | Mass | Length | ppm | m/z | z | RT | 1/k0 Start | 1/k0 End | Precursor ID | Area Sample 1 | #Feature | #Feature Sample 1 | Start | End | PTM | AScore | Found By |
| --- | --- | --- | --- | --- | --- | --- | --- | --- | --- | --- | --- | --- | --- | --- | --- | --- | --- | --- | --- |
| K.VLTASDPEALKR.K | Y | 66.76 | 1298.7194 | 12 | 2.49 | 650.3657 | 2 | 12.93 | 0.9912 | 0.9985 | 30634 | 7.44e2 | 1 | 1 | 145 | 156 |  |  | DB Search |
| total 1 peptides |  |  |  |  |  |  |  |  |  |  |  |  |  |  |  |  |  |  |  |
| --- | --- | --- | --- | --- | --- | --- | --- | --- | --- | --- | --- | --- | --- | --- | --- | --- | --- | --- | --- |

Best Unique PSM (Scan Sample\_10\_A1\_1\_3647.d:30634, m/z=650.3657, z=2, RT=12.93, ppm=-2.01):


TRINITY\_DN1616\_c0\_g1\_i3.p1

back to list

  

| Protein Coverage
| Supporting Peptides
| Best Unique PSM
|

Protein Coverage:

Supporting Peptides:

| Peptide | Uniq | -10lgP | Mass | Length | ppm | m/z | z | RT | 1/k0 Start | 1/k0 End | Precursor ID | Area Sample 1 | #Feature | #Feature Sample 1 | Start | End | PTM | AScore | Found By |
| --- | --- | --- | --- | --- | --- | --- | --- | --- | --- | --- | --- | --- | --- | --- | --- | --- | --- | --- | --- |
| R.LVQANPLEQDLSEKPR.Y | Y | 66.73 | 1835.9741 | 16 | 3.23 | 918.9932 | 2 | 15.63 | 1.1837 | 1.1909 | 45493 | 3.81e2 | 1 | 1 | 1191 | 1206 |  |  | DB Search |
| K.YVVMLQYYHDSDTAK.Y | Y | 66.45 | 1831.8451 | 15 | 2.49 | 916.928 | 2 | 16.54 | 1.1798 | 1.1869 | 50323 | 0 | 0 | 0 | 1071 | 1085 |  |  | DB Search |
| R.YVKPGVNIINIR.D | Y | 58.56 | 1384.819 | 12 | 3.46 | 693.4161 | 2 | 15.95 | 1.0025 | 1.0098 | 47296 | 0 | 0 | 0 | 1217 | 1228 |  |  | DB Search |
| total 3 peptides |  |  |  |  |  |  |  |  |  |  |  |  |  |  |  |  |  |  |  |
| --- | --- | --- | --- | --- | --- | --- | --- | --- | --- | --- | --- | --- | --- | --- | --- | --- | --- | --- | --- |

Best Unique PSM (Scan Sample\_10\_A1\_1\_3647.d:45493, m/z=918.9932, z=2, RT=15.63, ppm=-1.26):


TRINITY\_DN1319\_c0\_g1\_i1.p1

back to list

  

| Protein Coverage
| Supporting Peptides
| Best Unique PSM
|

Protein Coverage:

Supporting Peptides:

| Peptide | Uniq | -10lgP | Mass | Length | ppm | m/z | z | RT | 1/k0 Start | 1/k0 End | Precursor ID | Area Sample 1 | #Feature | #Feature Sample 1 | Start | End | PTM | AScore | Found By |
| --- | --- | --- | --- | --- | --- | --- | --- | --- | --- | --- | --- | --- | --- | --- | --- | --- | --- | --- | --- |
| K.GTHAAEIAHC(+57.02)VSR.R | Y | 65.75 | 1407.6677 | 13 | 1.54 | 704.8391 | 2 | 11.03 | 1.0283 | 1.0355 | 19632 | 6.69e2 | 1 | 1 | 91 | 103 | Carbamidomethylation |  | DB Search |
| total 1 peptides |  |  |  |  |  |  |  |  |  |  |  |  |  |  |  |  |  |  |  |
| --- | --- | --- | --- | --- | --- | --- | --- | --- | --- | --- | --- | --- | --- | --- | --- | --- | --- | --- | --- |

Best Unique PSM (Scan Sample\_10\_A1\_1\_3647.d:19632, m/z=704.8391, z=2, RT=11.03, ppm=-2.95):


TRINITY\_DN1378\_c0\_g1\_i1.p1

back to list

  

| Protein Coverage
| Supporting Peptides
| Best Unique PSM
|

Protein Coverage:

Supporting Peptides:

| Peptide | Uniq | -10lgP | Mass | Length | ppm | m/z | z | RT | 1/k0 Start | 1/k0 End | Precursor ID | Area Sample 1 | #Feature | #Feature Sample 1 | Start | End | PTM | AScore | Found By |
| --- | --- | --- | --- | --- | --- | --- | --- | --- | --- | --- | --- | --- | --- | --- | --- | --- | --- | --- | --- |
| K.LAALDATVHGQTASR.F | Y | 62.09 | 1509.7899 | 15 | 0.19 | 755.899 | 2 | 13.42 | 1.0721 | 1.0793 | 33378 | 4.46e2 | 1 | 1 | 213 | 227 |  |  | DB Search |
| total 1 peptides |  |  |  |  |  |  |  |  |  |  |  |  |  |  |  |  |  |  |  |
| --- | --- | --- | --- | --- | --- | --- | --- | --- | --- | --- | --- | --- | --- | --- | --- | --- | --- | --- | --- |

Best Unique PSM (Scan Sample\_10\_A1\_1\_3647.d:33378, m/z=755.899, z=2, RT=13.42, ppm=-4.3):


TRINITY\_DN95387\_c0\_g1\_i1.p1

back to list

  

| Protein Coverage
| Supporting Peptides
| Best Unique PSM
|

Protein Coverage:

Supporting Peptides:

| Peptide | Uniq | -10lgP | Mass | Length | ppm | m/z | z | RT | 1/k0 Start | 1/k0 End | Precursor ID | Area Sample 1 | #Feature | #Feature Sample 1 | Start | End | PTM | AScore | Found By |
| --- | --- | --- | --- | --- | --- | --- | --- | --- | --- | --- | --- | --- | --- | --- | --- | --- | --- | --- | --- |
| M.A(+42.01)ATLKPYLNAVR.A | Y | 66.57 | 1357.7718 | 12 | -0.04 | 679.8901 | 2 | 18.20 | 1.0452 | 1.0524 | 59968 | 1.22e3 | 1 | 1 | 2 | 13 | Acetylation (Protein N-term) | A1:Acetylation (Protein N-term):1000 | DB Search |
| total 1 peptides |  |  |  |  |  |  |  |  |  |  |  |  |  |  |  |  |  |  |  |
| --- | --- | --- | --- | --- | --- | --- | --- | --- | --- | --- | --- | --- | --- | --- | --- | --- | --- | --- | --- |

Best Unique PSM (Scan Sample\_10\_A1\_1\_3647.d:59968, m/z=679.8901, z=2, RT=18.20, ppm=-4.53):


TRINITY\_DN4781\_c0\_g1\_i1.p1

back to list

  

| Protein Coverage
| Supporting Peptides
| Best Unique PSM
|

Protein Coverage:

Supporting Peptides:

| Peptide | Uniq | -10lgP | Mass | Length | ppm | m/z | z | RT | 1/k0 Start | 1/k0 End | Precursor ID | Area Sample 1 | #Feature | #Feature Sample 1 | Start | End | PTM | AScore | Found By |
| --- | --- | --- | --- | --- | --- | --- | --- | --- | --- | --- | --- | --- | --- | --- | --- | --- | --- | --- | --- |
| R.LVFEGYHYWATK.T | Y | 66.76 | 1512.7401 | 12 | 1.97 | 757.3754 | 2 | 17.66 | 1.0439 | 1.0512 | 56486 | 7.22e2 | 1 | 1 | 2536 | 2547 |  |  | DB Search |
| total 1 peptides |  |  |  |  |  |  |  |  |  |  |  |  |  |  |  |  |  |  |  |
| --- | --- | --- | --- | --- | --- | --- | --- | --- | --- | --- | --- | --- | --- | --- | --- | --- | --- | --- | --- |

Best Unique PSM (Scan Sample\_10\_A1\_1\_3647.d:56486, m/z=757.3754, z=2, RT=17.66, ppm=-2.52):


TRINITY\_DN495\_c0\_g1\_i2.p1

back to list

  

| Protein Coverage
| Supporting Peptides
| Best Unique PSM
|

Protein Coverage:

Supporting Peptides:

| Peptide | Uniq | -10lgP | Mass | Length | ppm | m/z | z | RT | 1/k0 Start | 1/k0 End | Precursor ID | Area Sample 1 | #Feature | #Feature Sample 1 | Start | End | PTM | AScore | Found By |
| --- | --- | --- | --- | --- | --- | --- | --- | --- | --- | --- | --- | --- | --- | --- | --- | --- | --- | --- | --- |
| R.SC(+57.02)TNPAPANGGK.A | Y | 39.93 | 1172.5244 | 12 | 0 | 587.2668 | 2 | 9.43 | 0.8404 | 0.8477 | 10729 | 0 | 0 | 0 | 499 | 510 | Carbamidomethylation |  | DB Search |
| total 1 peptides |  |  |  |  |  |  |  |  |  |  |  |  |  |  |  |  |  |  |  |
| --- | --- | --- | --- | --- | --- | --- | --- | --- | --- | --- | --- | --- | --- | --- | --- | --- | --- | --- | --- |

Best Unique PSM (Scan Sample\_10\_A1\_1\_3647.d:10729, m/z=587.2668, z=2, RT=9.43, ppm=-4.49):


TRINITY\_DN319\_c2\_g1\_i3.p1

back to list

  

| Protein Coverage
| Supporting Peptides
| Best Unique PSM
|

Protein Coverage:

Supporting Peptides:

| Peptide | Uniq | -10lgP | Mass | Length | ppm | m/z | z | RT | 1/k0 Start | 1/k0 End | Precursor ID | Area Sample 1 | #Feature | #Feature Sample 1 | Start | End | PTM | AScore | Found By |
| --- | --- | --- | --- | --- | --- | --- | --- | --- | --- | --- | --- | --- | --- | --- | --- | --- | --- | --- | --- |
| K.KAQYYEAYETIIKR.H | Y | 66.67 | 1774.9254 | 14 | 2.41 | 592.6478 | 3 | 15.69 | 0.8627 | 0.87 | 45929 | 0 | 0 | 0 | 2534 | 2547 |  |  | DB Search |
| total 1 peptides |  |  |  |  |  |  |  |  |  |  |  |  |  |  |  |  |  |  |  |
| --- | --- | --- | --- | --- | --- | --- | --- | --- | --- | --- | --- | --- | --- | --- | --- | --- | --- | --- | --- |

Best Unique PSM (Scan Sample\_10\_A1\_1\_3647.d:45929, m/z=592.6478, z=3, RT=15.69, ppm=-2.09):


TRINITY\_DN691\_c0\_g1\_i2.p1

back to list

  

| Protein Coverage
| Supporting Peptides
| Best Unique PSM
|

Protein Coverage:

Supporting Peptides:

| Peptide | Uniq | -10lgP | Mass | Length | ppm | m/z | z | RT | 1/k0 Start | 1/k0 End | Precursor ID | Area Sample 1 | #Feature | #Feature Sample 1 | Start | End | PTM | AScore | Found By |
| --- | --- | --- | --- | --- | --- | --- | --- | --- | --- | --- | --- | --- | --- | --- | --- | --- | --- | --- | --- |
| R.AYGQDLGQPGSC(+57.02)LKR.F | Y | 62.49 | 1648.7991 | 15 | -3.4 | 825.4003 | 2 | 13.37 | 1.0725 | 1.0797 | 33176 | 9e2 | 1 | 1 | 1480 | 1494 | Carbamidomethylation |  | DB Search |
| total 1 peptides |  |  |  |  |  |  |  |  |  |  |  |  |  |  |  |  |  |  |  |
| --- | --- | --- | --- | --- | --- | --- | --- | --- | --- | --- | --- | --- | --- | --- | --- | --- | --- | --- | --- |

Best Unique PSM (Scan Sample\_10\_A1\_1\_3647.d:33176, m/z=824.3564, z=2, RT=13.40, ppm=-7.89):


TRINITY\_DN8707\_c0\_g1\_i2.p1

back to list

  

| Protein Coverage
| Supporting Peptides
| Best Unique PSM
|

Protein Coverage:

Supporting Peptides:

| Peptide | Uniq | -10lgP | Mass | Length | ppm | m/z | z | RT | 1/k0 Start | 1/k0 End | Precursor ID | Area Sample 1 | #Feature | #Feature Sample 1 | Start | End | PTM | AScore | Found By |
| --- | --- | --- | --- | --- | --- | --- | --- | --- | --- | --- | --- | --- | --- | --- | --- | --- | --- | --- | --- |
| K.ALLSLEYHEKR.C | Y | 66.36 | 1357.7354 | 11 | 4.28 | 679.8748 | 2 | 14.42 | 1.0279 | 1.0351 | 38931 | 3.27e3 | 1 | 1 | 124 | 134 |  |  | DB Search |
| total 1 peptides |  |  |  |  |  |  |  |  |  |  |  |  |  |  |  |  |  |  |  |
| --- | --- | --- | --- | --- | --- | --- | --- | --- | --- | --- | --- | --- | --- | --- | --- | --- | --- | --- | --- |

Best Unique PSM (Scan Sample\_10\_A1\_1\_3647.d:38931, m/z=679.8748, z=2, RT=14.42, ppm=-0.21):


TRINITY\_DN801\_c0\_g1\_i9.p2

back to list

  

| Protein Coverage
| Supporting Peptides
| Best Unique PSM
|

Protein Coverage:

Supporting Peptides:

| Peptide | Uniq | -10lgP | Mass | Length | ppm | m/z | z | RT | 1/k0 Start | 1/k0 End | Precursor ID | Area Sample 1 | #Feature | #Feature Sample 1 | Start | End | PTM | AScore | Found By |
| --- | --- | --- | --- | --- | --- | --- | --- | --- | --- | --- | --- | --- | --- | --- | --- | --- | --- | --- | --- |
| K.VFDRDGNGFISAAELR.H | Y | 39.11 | 1765.8747 | 16 | -1.5 | 883.9393 | 2 | 19.71 | 1.1234 | 1.1306 | 69271 | 0 | 0 | 0 | 117 | 132 |  |  | DB Search |
| total 1 peptides |  |  |  |  |  |  |  |  |  |  |  |  |  |  |  |  |  |  |  |
| --- | --- | --- | --- | --- | --- | --- | --- | --- | --- | --- | --- | --- | --- | --- | --- | --- | --- | --- | --- |

Best Unique PSM (Scan Sample\_10\_A1\_1\_3647.d:69271, m/z=883.9393, z=2, RT=19.71, ppm=-6):


TRINITY\_DN436\_c0\_g1\_i1.p1

back to list

  

| Protein Coverage
| Supporting Peptides
| Best Unique PSM
|

Protein Coverage:

Supporting Peptides:

| Peptide | Uniq | -10lgP | Mass | Length | ppm | m/z | z | RT | 1/k0 Start | 1/k0 End | Precursor ID | Area Sample 1 | #Feature | #Feature Sample 1 | Start | End | PTM | AScore | Found By |
| --- | --- | --- | --- | --- | --- | --- | --- | --- | --- | --- | --- | --- | --- | --- | --- | --- | --- | --- | --- |
| K.RLPEEDQYLR.I | Y | 66.61 | 1317.6677 | 10 | -4.11 | 659.8354 | 2 | 14.09 | 0.9735 | 0.9808 | 36893 | 1.37e3 | 1 | 1 | 54 | 63 |  |  | DB Search |
| total 1 peptides |  |  |  |  |  |  |  |  |  |  |  |  |  |  |  |  |  |  |  |
| --- | --- | --- | --- | --- | --- | --- | --- | --- | --- | --- | --- | --- | --- | --- | --- | --- | --- | --- | --- |

Best Unique PSM (Scan Sample\_10\_A1\_1\_3647.d:36893, m/z=659.8354, z=2, RT=14.09, ppm=-8.61):


TRINITY\_DN1084\_c0\_g1\_i1.p1

back to list

  

| Protein Coverage
| Supporting Peptides
| Best Unique PSM
|

Protein Coverage:

Supporting Peptides:

| Peptide | Uniq | -10lgP | Mass | Length | ppm | m/z | z | RT | 1/k0 Start | 1/k0 End | Precursor ID | Area Sample 1 | #Feature | #Feature Sample 1 | Start | End | PTM | AScore | Found By |
| --- | --- | --- | --- | --- | --- | --- | --- | --- | --- | --- | --- | --- | --- | --- | --- | --- | --- | --- | --- |
| R.IVAINAHHFLK.N | Y | 66.77 | 1261.7295 | 11 | 1.77 | 631.8703 | 2 | 14.46 | 1.0162 | 1.0234 | 39273 | 0 | 0 | 0 | 224 | 234 |  |  | DB Search |
| total 1 peptides |  |  |  |  |  |  |  |  |  |  |  |  |  |  |  |  |  |  |  |
| --- | --- | --- | --- | --- | --- | --- | --- | --- | --- | --- | --- | --- | --- | --- | --- | --- | --- | --- | --- |

Best Unique PSM (Scan Sample\_10\_A1\_1\_3647.d:39273, m/z=631.8703, z=2, RT=14.46, ppm=-2.73):


TRINITY\_DN577\_c0\_g1\_i1.p1

back to list

  

| Protein Coverage
| Supporting Peptides
| Best Unique PSM
|

Protein Coverage:

Supporting Peptides:

| Peptide | Uniq | -10lgP | Mass | Length | ppm | m/z | z | RT | 1/k0 Start | 1/k0 End | Precursor ID | Area Sample 1 | #Feature | #Feature Sample 1 | Start | End | PTM | AScore | Found By |
| --- | --- | --- | --- | --- | --- | --- | --- | --- | --- | --- | --- | --- | --- | --- | --- | --- | --- | --- | --- |
| R.VFEIAQADLNKDEVAFR.K | Y | 63.21 | 1964.0003 | 17 | -4.68 | 982.9984 | 2 | 18.05 | 1.2479 | 1.2551 | 59001 | 0 | 0 | 0 | 72 | 88 |  |  | DB Search |
| R.VFEIAQADLNKDEVAFRK.F | Y | 60.64 | 2092.0952 | 18 | -6.42 | 698.3647 | 3 | 16.92 | 0.9505 | 0.9578 | 52375 | 0 | 0 | 0 | 72 | 89 |  |  | DB Search |
| total 2 peptides |  |  |  |  |  |  |  |  |  |  |  |  |  |  |  |  |  |  |  |
| --- | --- | --- | --- | --- | --- | --- | --- | --- | --- | --- | --- | --- | --- | --- | --- | --- | --- | --- | --- |

Best Unique PSM (Scan Sample\_10\_A1\_1\_3647.d:59001, m/z=982.9984, z=2, RT=18.05, ppm=-9.17):


TRINITY\_DN687\_c0\_g1\_i1.p1

back to list

  

| Protein Coverage
| Supporting Peptides
| Best Unique PSM
|

Protein Coverage:

Supporting Peptides:

| Peptide | Uniq | -10lgP | Mass | Length | ppm | m/z | z | RT | 1/k0 Start | 1/k0 End | Precursor ID | Area Sample 1 | #Feature | #Feature Sample 1 | Start | End | PTM | AScore | Found By |
| --- | --- | --- | --- | --- | --- | --- | --- | --- | --- | --- | --- | --- | --- | --- | --- | --- | --- | --- | --- |
| R.AVC(+57.02)M(+15.99)LSNTTAIAEAWAR.L | Y | 66.77 | 1879.892 | 17 | -0.29 | 940.9488 | 2 | 18.63 | 1.1418 | 1.149 | 62475 | 6.54e2 | 1 | 1 | 374 | 390 | Carbamidomethylation, Oxidation (M) | M4:Oxidation (M):1000 | DB Search |
| K.VGINYQPPTVVPGGDLAK.V | Y | 64.86 | 1823.9781 | 18 | -3.39 | 912.9891 | 2 | 17.23 | 1.1198 | 1.127 | 54086 | 0 | 0 | 0 | 353 | 370 |  |  | DB Search |
| total 2 peptides |  |  |  |  |  |  |  |  |  |  |  |  |  |  |  |  |  |  |  |
| --- | --- | --- | --- | --- | --- | --- | --- | --- | --- | --- | --- | --- | --- | --- | --- | --- | --- | --- | --- |

Best Unique PSM (Scan Sample\_10\_A1\_1\_3647.d:62475, m/z=940.9488, z=2, RT=18.63, ppm=-4.79):


TRINITY\_DN367\_c0\_g2\_i1.p1

back to list

  

| Protein Coverage
| Supporting Peptides
| Best Unique PSM
|

Protein Coverage:

Supporting Peptides:

| Peptide | Uniq | -10lgP | Mass | Length | ppm | m/z | z | RT | 1/k0 Start | 1/k0 End | Precursor ID | Area Sample 1 | #Feature | #Feature Sample 1 | Start | End | PTM | AScore | Found By |
| --- | --- | --- | --- | --- | --- | --- | --- | --- | --- | --- | --- | --- | --- | --- | --- | --- | --- | --- | --- |
| K.C(+57.02)EHVKR.C | Y | 27.34 | 827.4072 | 6 | 5.54 | 414.7113 | 2 | 10.41 | 0.7599 | 0.7673 | 15886 | 1.02e4 | 1 | 1 | 40 | 45 | Carbamidomethylation |  | DB Search |
| total 1 peptides |  |  |  |  |  |  |  |  |  |  |  |  |  |  |  |  |  |  |  |
| --- | --- | --- | --- | --- | --- | --- | --- | --- | --- | --- | --- | --- | --- | --- | --- | --- | --- | --- | --- |

Best Unique PSM (Scan Sample\_10\_A1\_1\_3647.d:15886, m/z=414.7113, z=2, RT=10.41, ppm=1.04):


TRINITY\_DN6920\_c0\_g1\_i3.p1

back to list

  

| Protein Coverage
| Supporting Peptides
| Best Unique PSM
|

Protein Coverage:

Supporting Peptides:

| Peptide | Uniq | -10lgP | Mass | Length | ppm | m/z | z | RT | 1/k0 Start | 1/k0 End | Precursor ID | Area Sample 1 | #Feature | #Feature Sample 1 | Start | End | PTM | AScore | Found By |
| --- | --- | --- | --- | --- | --- | --- | --- | --- | --- | --- | --- | --- | --- | --- | --- | --- | --- | --- | --- |
| R.KVAC(+57.02)IGAWHPAR.V | Y | 60.86 | 1364.7135 | 12 | 9.11 | 683.3672 | 2 | 13.20 | 0.9711 | 0.9783 | 32100 | 9.16e2 | 1 | 1 | 257 | 268 | Carbamidomethylation |  | DB Search |
| R.HGSLGFLPR.K | Y | 25.17 | 982.5348 | 9 | -2.89 | 492.2711 | 2 | 17.17 | 0.8408 | 0.8481 | 53699 | 1.07e3 | 1 | 1 | 18 | 26 |  |  | DB Search |
| K.AGM(+15.99)THIVR.E | Y | 19.81 | 899.4647 | 8 | 2.72 | 450.7388 | 2 | 9.73 | 0.8226 | 0.8299 | 12038 | 6.74e2 | 1 | 1 | 58 | 65 | Oxidation (M) | M3:Oxidation (M):1000 | DB Search |
| total 3 peptides |  |  |  |  |  |  |  |  |  |  |  |  |  |  |  |  |  |  |  |
| --- | --- | --- | --- | --- | --- | --- | --- | --- | --- | --- | --- | --- | --- | --- | --- | --- | --- | --- | --- |

Best Unique PSM (Scan Sample\_10\_A1\_1\_3647.d:32100, m/z=683.3672, z=2, RT=13.20, ppm=4.62):


TRINITY\_DN2885\_c0\_g1\_i2.p1

back to list

  

| Protein Coverage
| Supporting Peptides
| Best Unique PSM
|

Protein Coverage:

Supporting Peptides:

| Peptide | Uniq | -10lgP | Mass | Length | ppm | m/z | z | RT | 1/k0 Start | 1/k0 End | Precursor ID | Area Sample 1 | #Feature | #Feature Sample 1 | Start | End | PTM | AScore | Found By |
| --- | --- | --- | --- | --- | --- | --- | --- | --- | --- | --- | --- | --- | --- | --- | --- | --- | --- | --- | --- |
| K.VTRPHGASGVVR.A | Y | 66.69 | 1234.6894 | 12 | 2.23 | 618.3506 | 2 | 10.05 | 0.9319 | 0.9392 | 13758 | 2.92e4 | 3 | 3 | 77 | 88 |  |  | DB Search |
| K.VTRPHGASGVVRAK.F | Y | 41.36 | 1433.8215 | 14 | 4.92 | 359.4628 | 4 | 9.64 | 0.7151 | 0.7224 | 11644 | 0 | 0 | 0 | 77 | 90 |  |  | DB Search |
| total 2 peptides |  |  |  |  |  |  |  |  |  |  |  |  |  |  |  |  |  |  |  |
| --- | --- | --- | --- | --- | --- | --- | --- | --- | --- | --- | --- | --- | --- | --- | --- | --- | --- | --- | --- |

Best Unique PSM (Scan Sample\_10\_A1\_1\_3647.d:13758, m/z=618.3506, z=2, RT=10.05, ppm=-2.27):


TRINITY\_DN6294\_c0\_g3\_i2.p1

back to list

  

| Protein Coverage
| Supporting Peptides
| Best Unique PSM
|

Protein Coverage:

Supporting Peptides:

| Peptide | Uniq | -10lgP | Mass | Length | ppm | m/z | z | RT | 1/k0 Start | 1/k0 End | Precursor ID | Area Sample 1 | #Feature | #Feature Sample 1 | Start | End | PTM | AScore | Found By |
| --- | --- | --- | --- | --- | --- | --- | --- | --- | --- | --- | --- | --- | --- | --- | --- | --- | --- | --- | --- |
| K.LVVIADIVDQKR.L | Y | 66.4 | 1367.8136 | 12 | 1.88 | 684.9123 | 2 | 17.50 | 1.0415 | 1.0488 | 55496 | 4.87e3 | 1 | 1 | 24 | 35 |  |  | DB Search |
| total 1 peptides |  |  |  |  |  |  |  |  |  |  |  |  |  |  |  |  |  |  |  |
| --- | --- | --- | --- | --- | --- | --- | --- | --- | --- | --- | --- | --- | --- | --- | --- | --- | --- | --- | --- |

Best Unique PSM (Scan Sample\_10\_A1\_1\_3647.d:55496, m/z=684.9123, z=2, RT=17.50, ppm=-2.61):


TRINITY\_DN32522\_c0\_g1\_i1.p1

back to list

  

| Protein Coverage
| Supporting Peptides
| Best Unique PSM
|

Protein Coverage:

Supporting Peptides:

| Peptide | Uniq | -10lgP | Mass | Length | ppm | m/z | z | RT | 1/k0 Start | 1/k0 End | Precursor ID | Area Sample 1 | #Feature | #Feature Sample 1 | Start | End | PTM | AScore | Found By |
| --- | --- | --- | --- | --- | --- | --- | --- | --- | --- | --- | --- | --- | --- | --- | --- | --- | --- | --- | --- |
| R.VDNKVNATER.K | Y | 61.28 | 1144.5836 | 10 | -1.08 | 573.2959 | 2 | 9.55 | 0.8866 | 0.8939 | 11254 | 0 | 0 | 0 | 191 | 200 |  |  | DB Search |
| total 1 peptides |  |  |  |  |  |  |  |  |  |  |  |  |  |  |  |  |  |  |  |
| --- | --- | --- | --- | --- | --- | --- | --- | --- | --- | --- | --- | --- | --- | --- | --- | --- | --- | --- | --- |

Best Unique PSM (Scan Sample\_10\_A1\_1\_3647.d:11254, m/z=573.2959, z=2, RT=9.55, ppm=-5.58):


TRINITY\_DN6397\_c0\_g2\_i3.p1

back to list

  

| Protein Coverage
| Supporting Peptides
| Best Unique PSM
|

Protein Coverage:

Supporting Peptides:

| Peptide | Uniq | -10lgP | Mass | Length | ppm | m/z | z | RT | 1/k0 Start | 1/k0 End | Precursor ID | Area Sample 1 | #Feature | #Feature Sample 1 | Start | End | PTM | AScore | Found By |
| --- | --- | --- | --- | --- | --- | --- | --- | --- | --- | --- | --- | --- | --- | --- | --- | --- | --- | --- | --- |
| K.K(+42.01)LSAEVANGR.L | Y | 66.46 | 1085.5829 | 10 | 2.21 | 543.7975 | 2 | 12.83 | 0.8595 | 0.8668 | 30189 | 3.02e3 | 1 | 1 | 12 | 21 | Acetylation (Protein N-term) | K1:Acetylation (Protein N-term):1000 | DB Search |
| total 1 peptides |  |  |  |  |  |  |  |  |  |  |  |  |  |  |  |  |  |  |  |
| --- | --- | --- | --- | --- | --- | --- | --- | --- | --- | --- | --- | --- | --- | --- | --- | --- | --- | --- | --- |

Best Unique PSM (Scan Sample\_10\_A1\_1\_3647.d:30189, m/z=543.7975, z=2, RT=12.83, ppm=-2.28):


TRINITY\_DN17516\_c0\_g1\_i1.p1

back to list

  

| Protein Coverage
| Supporting Peptides
| Best Unique PSM
|

Protein Coverage:

Supporting Peptides:

| Peptide | Uniq | -10lgP | Mass | Length | ppm | m/z | z | RT | 1/k0 Start | 1/k0 End | Precursor ID | Area Sample 1 | #Feature | #Feature Sample 1 | Start | End | PTM | AScore | Found By |
| --- | --- | --- | --- | --- | --- | --- | --- | --- | --- | --- | --- | --- | --- | --- | --- | --- | --- | --- | --- |
| K.EVNHEYQIIASSWK.Y | Y | 61.95 | 1702.8315 | 14 | -0.63 | 852.4186 | 2 | 17.08 | 1.1258 | 1.133 | 53181 | 0 | 0 | 0 | 90 | 103 |  |  | DB Search |
| total 1 peptides |  |  |  |  |  |  |  |  |  |  |  |  |  |  |  |  |  |  |  |
| --- | --- | --- | --- | --- | --- | --- | --- | --- | --- | --- | --- | --- | --- | --- | --- | --- | --- | --- | --- |

Best Unique PSM (Scan Sample\_10\_A1\_1\_3647.d:53181, m/z=852.4186, z=2, RT=17.08, ppm=-5.13):


TRINITY\_DN12\_c0\_g2\_i1.p1

back to list

  

| Protein Coverage
| Supporting Peptides
| Best Unique PSM
|

Protein Coverage:

Supporting Peptides:

| Peptide | Uniq | -10lgP | Mass | Length | ppm | m/z | z | RT | 1/k0 Start | 1/k0 End | Precursor ID | Area Sample 1 | #Feature | #Feature Sample 1 | Start | End | PTM | AScore | Found By |
| --- | --- | --- | --- | --- | --- | --- | --- | --- | --- | --- | --- | --- | --- | --- | --- | --- | --- | --- | --- |
| R.LGPDEASENKR.E | Y | 29.91 | 1214.5891 | 11 | -1.14 | 608.2984 | 2 | 10.44 | 0.9166 | 0.9238 | 16086 | 0 | 0 | 0 | 153 | 163 |  |  | DB Search |
| total 1 peptides |  |  |  |  |  |  |  |  |  |  |  |  |  |  |  |  |  |  |  |
| --- | --- | --- | --- | --- | --- | --- | --- | --- | --- | --- | --- | --- | --- | --- | --- | --- | --- | --- | --- |

Best Unique PSM (Scan Sample\_10\_A1\_1\_3647.d:16086, m/z=608.2984, z=2, RT=10.44, ppm=-5.64):


TRINITY\_DN2113\_c0\_g1\_i1.p1

back to list

  

| Protein Coverage
| Supporting Peptides
| Best Unique PSM
|

Protein Coverage:

Supporting Peptides:

| Peptide | Uniq | -10lgP | Mass | Length | ppm | m/z | z | RT | 1/k0 Start | 1/k0 End | Precursor ID | Area Sample 1 | #Feature | #Feature Sample 1 | Start | End | PTM | AScore | Found By |
| --- | --- | --- | --- | --- | --- | --- | --- | --- | --- | --- | --- | --- | --- | --- | --- | --- | --- | --- | --- |
| R.LADLEEFISSPNHANISQVGER.C | Y | 61.67 | 2425.1873 | 22 | 1.36 | 809.4005 | 3 | 18.74 | 0.9263 | 0.9335 | 63253 | 4.69e2 | 1 | 1 | 1183 | 1204 |  |  | DB Search |
| K.GQFSTDDLVAEVEKR.N | Y | 58.01 | 1692.8318 | 15 | 1.27 | 847.4205 | 2 | 17.68 | 1.1374 | 1.1446 | 56739 | 3.43e2 | 1 | 1 | 838 | 852 |  |  | DB Search |
| total 2 peptides |  |  |  |  |  |  |  |  |  |  |  |  |  |  |  |  |  |  |  |
| --- | --- | --- | --- | --- | --- | --- | --- | --- | --- | --- | --- | --- | --- | --- | --- | --- | --- | --- | --- |

Best Unique PSM (Scan Sample\_10\_A1\_1\_3647.d:63253, m/z=809.4005, z=3, RT=18.74, ppm=-3.14):


TRINITY\_DN16548\_c0\_g1\_i4.p1

back to list

  

| Protein Coverage
| Supporting Peptides
| Best Unique PSM
|

Protein Coverage:

Supporting Peptides:

| Peptide | Uniq | -10lgP | Mass | Length | ppm | m/z | z | RT | 1/k0 Start | 1/k0 End | Precursor ID | Area Sample 1 | #Feature | #Feature Sample 1 | Start | End | PTM | AScore | Found By |
| --- | --- | --- | --- | --- | --- | --- | --- | --- | --- | --- | --- | --- | --- | --- | --- | --- | --- | --- | --- |
| K.APGDYGWRPPLLATDDAALK.T | Y | 61.97 | 2126.0796 | 20 | -2.01 | 709.6959 | 3 | 18.98 | 0.9081 | 0.9154 | 64807 | 3.62e3 | 1 | 1 | 125 | 144 |  |  | DB Search |
| total 1 peptides |  |  |  |  |  |  |  |  |  |  |  |  |  |  |  |  |  |  |  |
| --- | --- | --- | --- | --- | --- | --- | --- | --- | --- | --- | --- | --- | --- | --- | --- | --- | --- | --- | --- |

Best Unique PSM (Scan Sample\_10\_A1\_1\_3647.d:64807, m/z=709.6959, z=3, RT=18.98, ppm=-6.51):


TRINITY\_DN5365\_c0\_g1\_i3.p1

back to list

  

| Protein Coverage
| Supporting Peptides
| Best Unique PSM
|

Protein Coverage:

Supporting Peptides:

| Peptide | Uniq | -10lgP | Mass | Length | ppm | m/z | z | RT | 1/k0 Start | 1/k0 End | Precursor ID | Area Sample 1 | #Feature | #Feature Sample 1 | Start | End | PTM | AScore | Found By |
| --- | --- | --- | --- | --- | --- | --- | --- | --- | --- | --- | --- | --- | --- | --- | --- | --- | --- | --- | --- |
| R.FPGQLNADIRK.L | Y | 62.07 | 1257.6829 | 11 | -5.49 | 629.8425 | 2 | 14.27 | 0.9428 | 0.9501 | 38203 | 0 | 0 | 0 | 244 | 254 |  |  | DB Search |
| total 1 peptides |  |  |  |  |  |  |  |  |  |  |  |  |  |  |  |  |  |  |  |
| --- | --- | --- | --- | --- | --- | --- | --- | --- | --- | --- | --- | --- | --- | --- | --- | --- | --- | --- | --- |

Best Unique PSM (Scan Sample\_10\_A1\_1\_3647.d:38203, m/z=629.8425, z=2, RT=14.27, ppm=-9.98):


TRINITY\_DN194146\_c0\_g1\_i1.p1

back to list

  

| Protein Coverage
| Supporting Peptides
| Best Unique PSM
|

Protein Coverage:

Supporting Peptides:

| Peptide | Uniq | -10lgP | Mass | Length | ppm | m/z | z | RT | 1/k0 Start | 1/k0 End | Precursor ID | Area Sample 1 | #Feature | #Feature Sample 1 | Start | End | PTM | AScore | Found By |
| --- | --- | --- | --- | --- | --- | --- | --- | --- | --- | --- | --- | --- | --- | --- | --- | --- | --- | --- | --- |
| R.NFNHLNLVLTR.V | Y | 66.8 | 1339.736 | 11 | 6.41 | 670.8766 | 2 | 16.83 | 0.9783 | 0.9856 | 51799 | 1.21e3 | 1 | 1 | 37 | 47 |  |  | DB Search |
| K.TVLASQTVTIPEDVSVSVNGR.T | Y | 63.4 | 2171.1433 | 21 | 6.66 | 1086.5813 | 2 | 17.80 | 1.2272 | 1.2344 | 57498 | 0 | 0 | 0 | 3 | 23 |  |  | DB Search |
| total 2 peptides |  |  |  |  |  |  |  |  |  |  |  |  |  |  |  |  |  |  |  |
| --- | --- | --- | --- | --- | --- | --- | --- | --- | --- | --- | --- | --- | --- | --- | --- | --- | --- | --- | --- |

Best Unique PSM (Scan Sample\_10\_A1\_1\_3647.d:51799, m/z=670.8766, z=2, RT=16.83, ppm=1.92):


TRINITY\_DN41662\_c0\_g1\_i2.p1

back to list

  

| Protein Coverage
| Supporting Peptides
| Best Unique PSM
|

Protein Coverage:

Supporting Peptides:

| Peptide | Uniq | -10lgP | Mass | Length | ppm | m/z | z | RT | 1/k0 Start | 1/k0 End | Precursor ID | Area Sample 1 | #Feature | #Feature Sample 1 | Start | End | PTM | AScore | Found By |
| --- | --- | --- | --- | --- | --- | --- | --- | --- | --- | --- | --- | --- | --- | --- | --- | --- | --- | --- | --- |
| K.LLAQTTLRNELGTK.N | Y | 34.86 | 1556.8886 | 14 | -1.74 | 779.4467 | 2 | 15.17 | 1.1102 | 1.1174 | 43095 | 4.27e2 | 1 | 1 | 147 | 160 |  |  | DB Search |
| total 1 peptides |  |  |  |  |  |  |  |  |  |  |  |  |  |  |  |  |  |  |  |
| --- | --- | --- | --- | --- | --- | --- | --- | --- | --- | --- | --- | --- | --- | --- | --- | --- | --- | --- | --- |

Best Unique PSM (Scan Sample\_10\_A1\_1\_3647.d:43095, m/z=779.4467, z=2, RT=15.17, ppm=-6.23):


TRINITY\_DN6132\_c0\_g1\_i1.p1

back to list

  

| Protein Coverage
| Supporting Peptides
| Best Unique PSM
|

Protein Coverage:

Supporting Peptides:

| Peptide | Uniq | -10lgP | Mass | Length | ppm | m/z | z | RT | 1/k0 Start | 1/k0 End | Precursor ID | Area Sample 1 | #Feature | #Feature Sample 1 | Start | End | PTM | AScore | Found By |
| --- | --- | --- | --- | --- | --- | --- | --- | --- | --- | --- | --- | --- | --- | --- | --- | --- | --- | --- | --- |
| R.VGDIVDVKGNGAIQK.G | Y | 52.97 | 1511.8307 | 15 | -2.72 | 756.9172 | 2 | 13.61 | 1.0721 | 1.0793 | 34539 | 0 | 0 | 0 | 36 | 50 |  |  | DB Search |
| total 1 peptides |  |  |  |  |  |  |  |  |  |  |  |  |  |  |  |  |  |  |  |
| --- | --- | --- | --- | --- | --- | --- | --- | --- | --- | --- | --- | --- | --- | --- | --- | --- | --- | --- | --- |

Best Unique PSM (Scan Sample\_10\_A1\_1\_3647.d:34539, m/z=756.9172, z=2, RT=13.61, ppm=-7.22):


TRINITY\_DN329\_c0\_g1\_i1.p1

back to list

  

| Protein Coverage
| Supporting Peptides
| Best Unique PSM
|

Protein Coverage:

Supporting Peptides:

| Peptide | Uniq | -10lgP | Mass | Length | ppm | m/z | z | RT | 1/k0 Start | 1/k0 End | Precursor ID | Area Sample 1 | #Feature | #Feature Sample 1 | Start | End | PTM | AScore | Found By |
| --- | --- | --- | --- | --- | --- | --- | --- | --- | --- | --- | --- | --- | --- | --- | --- | --- | --- | --- | --- |
| K.GLKVDFDTTFAPVTGKK.T | Y | 66.86 | 1822.9828 | 17 | 1.5 | 912.496 | 2 | 17.12 | 1.1802 | 1.1873 | 53325 | 2.2e3 | 2 | 2 | 93 | 109 |  |  | DB Search |
| K.SFHFGVIKLEGK.T | Y | 66.8 | 1360.7503 | 12 | 0.14 | 681.3795 | 2 | 16.99 | 1.0275 | 1.0347 | 52570 | 1.81e3 | 1 | 1 | 20 | 31 |  |  | DB Search |
| K.GLKVDFDTTFAPVTGK.K | Y | 66.78 | 1694.8879 | 16 | 3.14 | 848.4501 | 2 | 18.13 | 1.0933 | 1.1005 | 59417 | 1.18e3 | 1 | 1 | 93 | 108 |  |  | DB Search |
| K.VDFDTTFAPVTGKK.T | Y | 64.84 | 1524.7824 | 14 | 4.3 | 763.3983 | 2 | 18.74 | 1.048 | 1.0552 | 63303 | 9.89e2 | 2 | 2 | 96 | 109 |  |  | DB Search |
| K.LRPGVELTM(+15.99)SSLVNAK.S | Y | 60.81 | 1729.9396 | 16 | -1.76 | 865.9717 | 2 | 15.17 | 1.1358 | 1.143 | 42935 | 1.5e3 | 1 | 1 | 250 | 265 | Oxidation (M) | M9:Oxidation (M):1000 | DB Search |
| K.EGDLVIHSGITDAVK.Y | Y | 60.79 | 1552.8096 | 15 | -9.48 | 777.4012 | 2 | 15.72 | 1.0668 | 1.0741 | 46131 | 0 | 0 | 0 | 174 | 188 |  |  | DB Search |
| total 6 peptides |  |  |  |  |  |  |  |  |  |  |  |  |  |  |  |  |  |  |  |
| --- | --- | --- | --- | --- | --- | --- | --- | --- | --- | --- | --- | --- | --- | --- | --- | --- | --- | --- | --- |

Best Unique PSM (Scan Sample\_10\_A1\_1\_3647.d:53325, m/z=912.496, z=2, RT=17.12, ppm=-2.99):


TRINITY\_DN1559\_c0\_g1\_i1.p1

back to list

  

| Protein Coverage
| Supporting Peptides
| Best Unique PSM
|

Protein Coverage:

Supporting Peptides:

| Peptide | Uniq | -10lgP | Mass | Length | ppm | m/z | z | RT | 1/k0 Start | 1/k0 End | Precursor ID | Area Sample 1 | #Feature | #Feature Sample 1 | Start | End | PTM | AScore | Found By |
| --- | --- | --- | --- | --- | --- | --- | --- | --- | --- | --- | --- | --- | --- | --- | --- | --- | --- | --- | --- |
| R.GVAGAGVLAGFDKFK.E | Y | 62.44 | 1435.7823 | 15 | 3.16 | 718.8975 | 2 | 18.24 | 1.0869 | 1.0941 | 60071 | 4.33e3 | 1 | 1 | 282 | 296 |  |  | DB Search |
| K.LSEPYKGVIDC(+57.02)TTR.T | Y | 23.08 | 1637.8083 | 14 | -1.74 | 819.9063 | 2 | 14.48 | 1.0592 | 1.0664 | 39224 | 3.32e2 | 1 | 1 | 52 | 65 | Carbamidomethylation |  | DB Search |
| total 2 peptides |  |  |  |  |  |  |  |  |  |  |  |  |  |  |  |  |  |  |  |
| --- | --- | --- | --- | --- | --- | --- | --- | --- | --- | --- | --- | --- | --- | --- | --- | --- | --- | --- | --- |

Best Unique PSM (Scan Sample\_10\_A1\_1\_3647.d:60071, m/z=718.8975, z=2, RT=18.24, ppm=-1.34):


TRINITY\_DN18187\_c0\_g1\_i6.p2

back to list

  

| Protein Coverage
| Supporting Peptides
| Best Unique PSM
|

Protein Coverage:

Supporting Peptides:

| Peptide | Uniq | -10lgP | Mass | Length | ppm | m/z | z | RT | 1/k0 Start | 1/k0 End | Precursor ID | Area Sample 1 | #Feature | #Feature Sample 1 | Start | End | PTM | AScore | Found By |
| --- | --- | --- | --- | --- | --- | --- | --- | --- | --- | --- | --- | --- | --- | --- | --- | --- | --- | --- | --- |
| R.YRPGTVALR.E | Y | 66.57 | 1031.5876 | 9 | 0.86 | 516.7992 | 2 | 12.50 | 0.8554 | 0.8627 | 28119 | 7.47e2 | 1 | 1 | 83 | 91 |  |  | DB Search |
| total 1 peptides |  |  |  |  |  |  |  |  |  |  |  |  |  |  |  |  |  |  |  |
| --- | --- | --- | --- | --- | --- | --- | --- | --- | --- | --- | --- | --- | --- | --- | --- | --- | --- | --- | --- |

Best Unique PSM (Scan Sample\_10\_A1\_1\_3647.d:28119, m/z=516.7992, z=2, RT=12.50, ppm=-3.64):


TRINITY\_DN2963\_c0\_g1\_i1.p1

back to list

  

| Protein Coverage
| Supporting Peptides
| Best Unique PSM
|

Protein Coverage:

Supporting Peptides:

| Peptide | Uniq | -10lgP | Mass | Length | ppm | m/z | z | RT | 1/k0 Start | 1/k0 End | Precursor ID | Area Sample 1 | #Feature | #Feature Sample 1 | Start | End | PTM | AScore | Found By |
| --- | --- | --- | --- | --- | --- | --- | --- | --- | --- | --- | --- | --- | --- | --- | --- | --- | --- | --- | --- |
| M.S(+42.01)ATGNLVTVLRK.L | Y | 66.68 | 1299.751 | 12 | 3.4 | 650.8821 | 2 | 17.06 | 0.9977 | 1.0049 | 52945 | 3.07e3 | 1 | 1 | 2 | 13 | Acetylation (Protein N-term) | S1:Acetylation (Protein N-term):1000 | DB Search |
| K.LYDEYNDKTPSR.L | Y | 63.94 | 1499.6892 | 12 | 17.25 | 750.8615 | 2 | 11.79 | 1.0098 | 1.017 | 24101 | 0 | 0 | 0 | 14 | 25 |  |  | DB Search |
| total 2 peptides |  |  |  |  |  |  |  |  |  |  |  |  |  |  |  |  |  |  |  |
| --- | --- | --- | --- | --- | --- | --- | --- | --- | --- | --- | --- | --- | --- | --- | --- | --- | --- | --- | --- |

Best Unique PSM (Scan Sample\_10\_A1\_1\_3647.d:52945, m/z=650.8821, z=2, RT=17.06, ppm=-1.09):


TRINITY\_DN250\_c0\_g1\_i6.p1

back to list

  

| Protein Coverage
| Supporting Peptides
| Best Unique PSM
|

Protein Coverage:

Supporting Peptides:

| Peptide | Uniq | -10lgP | Mass | Length | ppm | m/z | z | RT | 1/k0 Start | 1/k0 End | Precursor ID | Area Sample 1 | #Feature | #Feature Sample 1 | Start | End | PTM | AScore | Found By |
| --- | --- | --- | --- | --- | --- | --- | --- | --- | --- | --- | --- | --- | --- | --- | --- | --- | --- | --- | --- |
| K.VGINYQPPTVVPGGDLAK.V | Y | 64.86 | 1823.9781 | 18 | -3.39 | 912.9891 | 2 | 17.23 | 1.1198 | 1.127 | 54086 | 0 | 0 | 0 | 353 | 370 |  |  | DB Search |
| R.AFVHWYVGEGM(+15.99)EEGEFSEAR.E | Y | 44.9 | 2345.0058 | 20 | -0.18 | 782.6722 | 3 | 17.81 | 0.9251 | 0.9323 | 57560 | 6.2e2 | 1 | 1 | 403 | 422 | Oxidation (M) | M11:Oxidation (M):1000 | DB Search |
| total 2 peptides |  |  |  |  |  |  |  |  |  |  |  |  |  |  |  |  |  |  |  |
| --- | --- | --- | --- | --- | --- | --- | --- | --- | --- | --- | --- | --- | --- | --- | --- | --- | --- | --- | --- |

Best Unique PSM (Scan Sample\_10\_A1\_1\_3647.d:54086, m/z=912.9891, z=2, RT=17.23, ppm=-7.88):


TRINITY\_DN2126\_c0\_g1\_i1.p1

back to list

  

| Protein Coverage
| Supporting Peptides
| Best Unique PSM
|

Protein Coverage:

Supporting Peptides:

| Peptide | Uniq | -10lgP | Mass | Length | ppm | m/z | z | RT | 1/k0 Start | 1/k0 End | Precursor ID | Area Sample 1 | #Feature | #Feature Sample 1 | Start | End | PTM | AScore | Found By |
| --- | --- | --- | --- | --- | --- | --- | --- | --- | --- | --- | --- | --- | --- | --- | --- | --- | --- | --- | --- |
| K.LVIISSNTPQLRK.S | Y | 66.71 | 1467.8773 | 13 | 0.59 | 734.9431 | 2 | 14.91 | 1.058 | 1.0652 | 41605 | 1.53e3 | 1 | 1 | 43 | 55 |  |  | DB Search |
| K.AKLVIISSNTPQLR.K | Y | 66.17 | 1538.9144 | 14 | 3.07 | 770.4634 | 2 | 15.57 | 1.0809 | 1.0881 | 45325 | 0 | 0 | 0 | 41 | 54 |  |  | DB Search |
| total 2 peptides |  |  |  |  |  |  |  |  |  |  |  |  |  |  |  |  |  |  |  |
| --- | --- | --- | --- | --- | --- | --- | --- | --- | --- | --- | --- | --- | --- | --- | --- | --- | --- | --- | --- |

Best Unique PSM (Scan Sample\_10\_A1\_1\_3647.d:41605, m/z=734.9431, z=2, RT=14.91, ppm=-3.91):


TRINITY\_DN3036\_c0\_g1\_i8.p1

back to list

  

| Protein Coverage
| Supporting Peptides
| Best Unique PSM
|

Protein Coverage:

Supporting Peptides:

| Peptide | Uniq | -10lgP | Mass | Length | ppm | m/z | z | RT | 1/k0 Start | 1/k0 End | Precursor ID | Area Sample 1 | #Feature | #Feature Sample 1 | Start | End | PTM | AScore | Found By |
| --- | --- | --- | --- | --- | --- | --- | --- | --- | --- | --- | --- | --- | --- | --- | --- | --- | --- | --- | --- |
| R.AGHGAVTSPQTQR.G | Y | 66.66 | 1308.6534 | 13 | 4.48 | 655.334 | 2 | 9.70 | 0.9109 | 0.9182 | 11470 | 2.1e4 | 2 | 2 | 309 | 321 |  |  | DB Search |
| R.GTSNGGLVVGTIIKPK.L | Y | 66.59 | 1539.8984 | 16 | 0.22 | 770.9532 | 2 | 15.68 | 1.1262 | 1.1334 | 45813 | 3.23e3 | 1 | 1 | 173 | 188 |  |  | DB Search |
| R.VIGASGIHVGTMSFGK.M | Y | 66.34 | 1559.813 | 16 | -0.2 | 780.9101 | 2 | 16.89 | 1.1037 | 1.111 | 52086 | 2.86e2 | 1 | 1 | 334 | 349 |  |  | DB Search |
| R.VIGASGIHVGTM(+15.99)SFGK.M | Y | 64.9 | 1575.8079 | 16 | 0.52 | 788.9081 | 2 | 15.11 | 1.1262 | 1.1334 | 42748 | 6.68e2 | 1 | 1 | 334 | 349 | Oxidation (M) | M12:Oxidation (M):1000 | DB Search |
| total 4 peptides |  |  |  |  |  |  |  |  |  |  |  |  |  |  |  |  |  |  |  |
| --- | --- | --- | --- | --- | --- | --- | --- | --- | --- | --- | --- | --- | --- | --- | --- | --- | --- | --- | --- |

Best Unique PSM (Scan Sample\_10\_A1\_1\_3647.d:11470, m/z=655.334, z=2, RT=9.70, ppm=-0.02):


TRINITY\_DN761\_c0\_g1\_i2.p1

back to list

  

| Protein Coverage
| Supporting Peptides
| Best Unique PSM
|

Protein Coverage:

Supporting Peptides:

| Peptide | Uniq | -10lgP | Mass | Length | ppm | m/z | z | RT | 1/k0 Start | 1/k0 End | Precursor ID | Area Sample 1 | #Feature | #Feature Sample 1 | Start | End | PTM | AScore | Found By |
| --- | --- | --- | --- | --- | --- | --- | --- | --- | --- | --- | --- | --- | --- | --- | --- | --- | --- | --- | --- |
| K.TRIEDLETVISQTQDHR.H | Y | 66.35 | 2040.0236 | 17 | 3.15 | 681.0142 | 3 | 17.32 | 0.9622 | 0.9695 | 54563 | 0 | 0 | 0 | 260 | 276 |  |  | DB Search |
| K.EIDKADIAIVDTGENPDAPHPR.E | Y | 61.91 | 2372.1607 | 22 | 3.12 | 791.7264 | 3 | 14.88 | 1.0415 | 1.0488 | 41517 | 0 | 0 | 0 | 71 | 92 |  |  | DB Search |
| K.FVNEVRR.C | Y | 39.18 | 918.5035 | 7 | -2.89 | 460.2556 | 2 | 12.63 | 0.8096 | 0.8169 | 28832 | 9.86e2 | 1 | 1 | 51 | 57 |  |  | DB Search |
| total 3 peptides |  |  |  |  |  |  |  |  |  |  |  |  |  |  |  |  |  |  |  |
| --- | --- | --- | --- | --- | --- | --- | --- | --- | --- | --- | --- | --- | --- | --- | --- | --- | --- | --- | --- |

Best Unique PSM (Scan Sample\_10\_A1\_1\_3647.d:54563, m/z=681.0142, z=3, RT=17.32, ppm=-1.34):


TRINITY\_DN2637\_c0\_g1\_i4.p1

back to list

  

| Protein Coverage
| Supporting Peptides
| Best Unique PSM
|

Protein Coverage:

Supporting Peptides:

| Peptide | Uniq | -10lgP | Mass | Length | ppm | m/z | z | RT | 1/k0 Start | 1/k0 End | Precursor ID | Area Sample 1 | #Feature | #Feature Sample 1 | Start | End | PTM | AScore | Found By |
| --- | --- | --- | --- | --- | --- | --- | --- | --- | --- | --- | --- | --- | --- | --- | --- | --- | --- | --- | --- |
| K.ATIEIPAKYEK.R | Y | 61.89 | 1261.6918 | 11 | 0.22 | 631.8505 | 2 | 14.08 | 0.982 | 0.9892 | 37168 | 0 | 0 | 0 | 87 | 97 |  |  | DB Search |
| total 1 peptides |  |  |  |  |  |  |  |  |  |  |  |  |  |  |  |  |  |  |  |
| --- | --- | --- | --- | --- | --- | --- | --- | --- | --- | --- | --- | --- | --- | --- | --- | --- | --- | --- | --- |

Best Unique PSM (Scan Sample\_10\_A1\_1\_3647.d:37168, m/z=631.8505, z=2, RT=14.08, ppm=-4.27):


TRINITY\_DN22\_c0\_g1\_i1.p1

back to list

  

| Protein Coverage
| Supporting Peptides
| Best Unique PSM
|

Protein Coverage:

Supporting Peptides:

| Peptide | Uniq | -10lgP | Mass | Length | ppm | m/z | z | RT | 1/k0 Start | 1/k0 End | Precursor ID | Area Sample 1 | #Feature | #Feature Sample 1 | Start | End | PTM | AScore | Found By |
| --- | --- | --- | --- | --- | --- | --- | --- | --- | --- | --- | --- | --- | --- | --- | --- | --- | --- | --- | --- |
| R.FVLHGDVSHGSR.Y | Y | 66.68 | 1309.6527 | 12 | 4.95 | 655.8339 | 2 | 12.00 | 0.9892 | 0.9965 | 25296 | 0 | 0 | 0 | 314 | 325 |  |  | DB Search |
| total 1 peptides |  |  |  |  |  |  |  |  |  |  |  |  |  |  |  |  |  |  |  |
| --- | --- | --- | --- | --- | --- | --- | --- | --- | --- | --- | --- | --- | --- | --- | --- | --- | --- | --- | --- |

Best Unique PSM (Scan Sample\_10\_A1\_1\_3647.d:25296, m/z=655.8339, z=2, RT=12.00, ppm=0.46):


TRINITY\_DN26892\_c0\_g1\_i1.p1

back to list

  

| Protein Coverage
| Supporting Peptides
| Best Unique PSM
|

Protein Coverage:

Supporting Peptides:

| Peptide | Uniq | -10lgP | Mass | Length | ppm | m/z | z | RT | 1/k0 Start | 1/k0 End | Precursor ID | Area Sample 1 | #Feature | #Feature Sample 1 | Start | End | PTM | AScore | Found By |
| --- | --- | --- | --- | --- | --- | --- | --- | --- | --- | --- | --- | --- | --- | --- | --- | --- | --- | --- | --- |
| R.IAAETEEVGVQIIGDLDDQKETLHR.V | Y | 61.7 | 2778.4035 | 25 | 2.04 | 695.6064 | 4 | 19.02 | 0.9392 | 0.9465 | 64964 | 0 | 0 | 0 | 153 | 177 |  |  | DB Search |
| total 1 peptides |  |  |  |  |  |  |  |  |  |  |  |  |  |  |  |  |  |  |  |
| --- | --- | --- | --- | --- | --- | --- | --- | --- | --- | --- | --- | --- | --- | --- | --- | --- | --- | --- | --- |

Best Unique PSM (Scan Sample\_10\_A1\_1\_3647.d:64964, m/z=695.6064, z=4, RT=19.02, ppm=-2.45):


TRINITY\_DN3773\_c0\_g1\_i1.p1

back to list

  

| Protein Coverage
| Supporting Peptides
| Best Unique PSM
|

Protein Coverage:

Supporting Peptides:

| Peptide | Uniq | -10lgP | Mass | Length | ppm | m/z | z | RT | 1/k0 Start | 1/k0 End | Precursor ID | Area Sample 1 | #Feature | #Feature Sample 1 | Start | End | PTM | AScore | Found By |
| --- | --- | --- | --- | --- | --- | --- | --- | --- | --- | --- | --- | --- | --- | --- | --- | --- | --- | --- | --- |
| K.IPIFSAAGLPHNEIAAQIC(+57.02)R.Q | Y | 66.68 | 2177.1415 | 20 | 1.41 | 726.7189 | 3 | 18.83 | 1.0098 | 1.017 | 63727 | 5.71e2 | 1 | 1 | 178 | 197 | Carbamidomethylation |  | DB Search |
| total 1 peptides |  |  |  |  |  |  |  |  |  |  |  |  |  |  |  |  |  |  |  |
| --- | --- | --- | --- | --- | --- | --- | --- | --- | --- | --- | --- | --- | --- | --- | --- | --- | --- | --- | --- |

Best Unique PSM (Scan Sample\_10\_A1\_1\_3647.d:63727, m/z=726.7189, z=3, RT=18.83, ppm=-3.08):


TRINITY\_DN909\_c0\_g1\_i1.p1

back to list

  

| Protein Coverage
| Supporting Peptides
| Best Unique PSM
|

Protein Coverage:

Supporting Peptides:

| Peptide | Uniq | -10lgP | Mass | Length | ppm | m/z | z | RT | 1/k0 Start | 1/k0 End | Precursor ID | Area Sample 1 | #Feature | #Feature Sample 1 | Start | End | PTM | AScore | Found By |
| --- | --- | --- | --- | --- | --- | --- | --- | --- | --- | --- | --- | --- | --- | --- | --- | --- | --- | --- | --- |
| R.AM(+15.99)AAEAESSR.E | Y | 66.65 | 1037.4448 | 10 | -0.52 | 519.7271 | 2 | 9.04 | 0.8149 | 0.8222 | 9454 | 2.47e2 | 1 | 1 | 236 | 245 | Oxidation (M) | M2:Oxidation (M):1000 | DB Search |
| K.LLAQTTLRNELGTK.N | Y | 34.86 | 1556.8886 | 14 | -1.74 | 779.4467 | 2 | 15.17 | 1.1102 | 1.1174 | 43095 | 4.27e2 | 1 | 1 | 175 | 188 |  |  | DB Search |
| total 2 peptides |  |  |  |  |  |  |  |  |  |  |  |  |  |  |  |  |  |  |  |
| --- | --- | --- | --- | --- | --- | --- | --- | --- | --- | --- | --- | --- | --- | --- | --- | --- | --- | --- | --- |

Best Unique PSM (Scan Sample\_10\_A1\_1\_3647.d:9454, m/z=519.7271, z=2, RT=9.04, ppm=-5.02):


TRINITY\_DN195631\_c0\_g1\_i1.p2

back to list

  

| Protein Coverage
| Supporting Peptides
| Best Unique PSM
|

Protein Coverage:

Supporting Peptides:

| Peptide | Uniq | -10lgP | Mass | Length | ppm | m/z | z | RT | 1/k0 Start | 1/k0 End | Precursor ID | Area Sample 1 | #Feature | #Feature Sample 1 | Start | End | PTM | AScore | Found By |
| --- | --- | --- | --- | --- | --- | --- | --- | --- | --- | --- | --- | --- | --- | --- | --- | --- | --- | --- | --- |
| K.IQDKEGIPPDQQR.L | Y | 65.08 | 1522.7739 | 13 | -0.66 | 762.3903 | 2 | 11.01 | 1.0339 | 1.0411 | 19352 | 9.49e2 | 1 | 1 | 122 | 134 |  |  | DB Search |
| total 1 peptides |  |  |  |  |  |  |  |  |  |  |  |  |  |  |  |  |  |  |  |
| --- | --- | --- | --- | --- | --- | --- | --- | --- | --- | --- | --- | --- | --- | --- | --- | --- | --- | --- | --- |

Best Unique PSM (Scan Sample\_10\_A1\_1\_3647.d:19352, m/z=762.3903, z=2, RT=11.01, ppm=-5.16):


TRINITY\_DN1315\_c0\_g1\_i1.p1

back to list

  

| Protein Coverage
| Supporting Peptides
| Best Unique PSM
|

Protein Coverage:

Supporting Peptides:

| Peptide | Uniq | -10lgP | Mass | Length | ppm | m/z | z | RT | 1/k0 Start | 1/k0 End | Precursor ID | Area Sample 1 | #Feature | #Feature Sample 1 | Start | End | PTM | AScore | Found By |
| --- | --- | --- | --- | --- | --- | --- | --- | --- | --- | --- | --- | --- | --- | --- | --- | --- | --- | --- | --- |
| K.GVAIHEMMHALGFFHEQSR.R | Y | 29.88 | 2196.0357 | 19 | -11.88 | 550.0072 | 4 | 18.95 | 0.8793 | 0.8866 | 64544 | 0 | 0 | 0 | 181 | 199 |  |  | DB Search |
| total 1 peptides |  |  |  |  |  |  |  |  |  |  |  |  |  |  |  |  |  |  |  |
| --- | --- | --- | --- | --- | --- | --- | --- | --- | --- | --- | --- | --- | --- | --- | --- | --- | --- | --- | --- |

Best Unique PSM (Scan Sample\_10\_A1\_1\_3647.d:64544, m/z=550.0072, z=4, RT=18.95, ppm=-16.37):


TRINITY\_DN1566\_c0\_g1\_i1.p1

back to list

  

| Protein Coverage
| Supporting Peptides
| Best Unique PSM
|

Protein Coverage:

Supporting Peptides:

| Peptide | Uniq | -10lgP | Mass | Length | ppm | m/z | z | RT | 1/k0 Start | 1/k0 End | Precursor ID | Area Sample 1 | #Feature | #Feature Sample 1 | Start | End | PTM | AScore | Found By |
| --- | --- | --- | --- | --- | --- | --- | --- | --- | --- | --- | --- | --- | --- | --- | --- | --- | --- | --- | --- |
| R.IKLEHLITELVHQR.D | Y | 66.56 | 1728.0046 | 14 | -0.31 | 577.006 | 3 | 18.36 | 0.8676 | 0.8749 | 60768 | 2.25e2 | 1 | 1 | 1781 | 1794 |  |  | DB Search |
| total 1 peptides |  |  |  |  |  |  |  |  |  |  |  |  |  |  |  |  |  |  |  |
| --- | --- | --- | --- | --- | --- | --- | --- | --- | --- | --- | --- | --- | --- | --- | --- | --- | --- | --- | --- |

Best Unique PSM (Scan Sample\_10\_A1\_1\_3647.d:60768, m/z=577.006, z=3, RT=18.36, ppm=-4.8):


TRINITY\_DN16548\_c0\_g1\_i3.p1

back to list

  

| Protein Coverage
| Supporting Peptides
| Best Unique PSM
|

Protein Coverage:

Supporting Peptides:

| Peptide | Uniq | -10lgP | Mass | Length | ppm | m/z | z | RT | 1/k0 Start | 1/k0 End | Precursor ID | Area Sample 1 | #Feature | #Feature Sample 1 | Start | End | PTM | AScore | Found By |
| --- | --- | --- | --- | --- | --- | --- | --- | --- | --- | --- | --- | --- | --- | --- | --- | --- | --- | --- | --- |
| K.APGDYGWRPPLLATDDAALK.T | Y | 61.97 | 2126.0796 | 20 | -2.01 | 709.6959 | 3 | 18.98 | 0.9081 | 0.9154 | 64807 | 3.62e3 | 1 | 1 | 125 | 144 |  |  | DB Search |
| total 1 peptides |  |  |  |  |  |  |  |  |  |  |  |  |  |  |  |  |  |  |  |
| --- | --- | --- | --- | --- | --- | --- | --- | --- | --- | --- | --- | --- | --- | --- | --- | --- | --- | --- | --- |

Best Unique PSM (Scan Sample\_10\_A1\_1\_3647.d:64807, m/z=709.6959, z=3, RT=18.98, ppm=-6.51):


TRINITY\_DN464\_c0\_g1\_i4.p1

back to list

  

| Protein Coverage
| Supporting Peptides
| Best Unique PSM
|

Protein Coverage:

Supporting Peptides:

| Peptide | Uniq | -10lgP | Mass | Length | ppm | m/z | z | RT | 1/k0 Start | 1/k0 End | Precursor ID | Area Sample 1 | #Feature | #Feature Sample 1 | Start | End | PTM | AScore | Found By |
| --- | --- | --- | --- | --- | --- | --- | --- | --- | --- | --- | --- | --- | --- | --- | --- | --- | --- | --- | --- |
| R.LLEFVGHDVLR.L | Y | 66.68 | 1296.719 | 11 | 5.9 | 649.3677 | 2 | 17.75 | 1.0033 | 1.0106 | 57204 | 0 | 0 | 0 | 279 | 289 |  |  | DB Search |
| R.IFEAGFHILGIKPISR.M | Y | 62.22 | 1797.0301 | 16 | -0.24 | 600.0145 | 3 | 19.00 | 0.9117 | 0.919 | 64761 | 4.9e2 | 1 | 1 | 700 | 715 |  |  | DB Search |
| total 2 peptides |  |  |  |  |  |  |  |  |  |  |  |  |  |  |  |  |  |  |  |
| --- | --- | --- | --- | --- | --- | --- | --- | --- | --- | --- | --- | --- | --- | --- | --- | --- | --- | --- | --- |

Best Unique PSM (Scan Sample\_10\_A1\_1\_3647.d:57204, m/z=649.3677, z=2, RT=17.75, ppm=1.4):


TRINITY\_DN1397\_c0\_g1\_i2.p1

back to list

  

| Protein Coverage
| Supporting Peptides
| Best Unique PSM
|

Protein Coverage:

Supporting Peptides:

| Peptide | Uniq | -10lgP | Mass | Length | ppm | m/z | z | RT | 1/k0 Start | 1/k0 End | Precursor ID | Area Sample 1 | #Feature | #Feature Sample 1 | Start | End | PTM | AScore | Found By |
| --- | --- | --- | --- | --- | --- | --- | --- | --- | --- | --- | --- | --- | --- | --- | --- | --- | --- | --- | --- |
| K.NEFRLPVVQK.M | Y | 17.17 | 1228.6928 | 10 | -5.88 | 615.3473 | 2 | 17.44 | 0.9327 | 0.94 | 55344 | 4.76e2 | 1 | 1 | 57 | 66 |  |  | DB Search |
| total 1 peptides |  |  |  |  |  |  |  |  |  |  |  |  |  |  |  |  |  |  |  |
| --- | --- | --- | --- | --- | --- | --- | --- | --- | --- | --- | --- | --- | --- | --- | --- | --- | --- | --- | --- |

Best Unique PSM (Scan Sample\_10\_A1\_1\_3647.d:55344, m/z=615.3473, z=2, RT=17.44, ppm=-10.38):


TRINITY\_DN2637\_c0\_g1\_i5.p2

back to list

  

| Protein Coverage
| Supporting Peptides
| Best Unique PSM
|

Protein Coverage:

Supporting Peptides:

| Peptide | Uniq | -10lgP | Mass | Length | ppm | m/z | z | RT | 1/k0 Start | 1/k0 End | Precursor ID | Area Sample 1 | #Feature | #Feature Sample 1 | Start | End | PTM | AScore | Found By |
| --- | --- | --- | --- | --- | --- | --- | --- | --- | --- | --- | --- | --- | --- | --- | --- | --- | --- | --- | --- |
| K.ATIEIPAKYEK.R | Y | 61.89 | 1261.6918 | 11 | 0.22 | 631.8505 | 2 | 14.08 | 0.982 | 0.9892 | 37168 | 0 | 0 | 0 | 107 | 117 |  |  | DB Search |
| total 1 peptides |  |  |  |  |  |  |  |  |  |  |  |  |  |  |  |  |  |  |  |
| --- | --- | --- | --- | --- | --- | --- | --- | --- | --- | --- | --- | --- | --- | --- | --- | --- | --- | --- | --- |

Best Unique PSM (Scan Sample\_10\_A1\_1\_3647.d:37168, m/z=631.8505, z=2, RT=14.08, ppm=-4.27):


TRINITY\_DN76040\_c0\_g1\_i1.p1

back to list

  

| Protein Coverage
| Supporting Peptides
| Best Unique PSM
|

Protein Coverage:

Supporting Peptides:

| Peptide | Uniq | -10lgP | Mass | Length | ppm | m/z | z | RT | 1/k0 Start | 1/k0 End | Precursor ID | Area Sample 1 | #Feature | #Feature Sample 1 | Start | End | PTM | AScore | Found By |
| --- | --- | --- | --- | --- | --- | --- | --- | --- | --- | --- | --- | --- | --- | --- | --- | --- | --- | --- | --- |
| K.VSILESLDKWER.L | Y | 66.83 | 1473.7827 | 12 | -1.01 | 492.2655 | 3 | 19.59 | 0.7868 | 0.7941 | 68471 | 0 | 0 | 0 | 254 | 265 |  |  | DB Search |
| total 1 peptides |  |  |  |  |  |  |  |  |  |  |  |  |  |  |  |  |  |  |  |
| --- | --- | --- | --- | --- | --- | --- | --- | --- | --- | --- | --- | --- | --- | --- | --- | --- | --- | --- | --- |

Best Unique PSM (Scan Sample\_10\_A1\_1\_3647.d:68471, m/z=492.2655, z=3, RT=19.59, ppm=-5.51):


TRINITY\_DN36\_c0\_g1\_i3.p1

back to list

  

| Protein Coverage
| Supporting Peptides
| Best Unique PSM
|

Protein Coverage:

Supporting Peptides:

| Peptide | Uniq | -10lgP | Mass | Length | ppm | m/z | z | RT | 1/k0 Start | 1/k0 End | Precursor ID | Area Sample 1 | #Feature | #Feature Sample 1 | Start | End | PTM | AScore | Found By |
| --- | --- | --- | --- | --- | --- | --- | --- | --- | --- | --- | --- | --- | --- | --- | --- | --- | --- | --- | --- |
| R.LGGIEVTQGNC(+57.02)AR.K | Y | 66.48 | 1373.6721 | 13 | 5.18 | 687.8438 | 2 | 13.73 | 0.963 | 0.9703 | 35192 | 0 | 0 | 0 | 58 | 70 | Carbamidomethylation |  | DB Search |
| K.WC(+57.02)DILSIHQSMENC(+57.02)VAK.C | Y | 63.98 | 2089.9383 | 17 | -2.35 | 697.6486 | 3 | 17.89 | 0.8777 | 0.885 | 58058 | 0 | 0 | 0 | 198 | 214 | Carbamidomethylation, Carbamidomethylation |  | DB Search |
| K.FETQIATK.D | Y | 61.61 | 936.4916 | 8 | 0.97 | 937.4956 | 1 | 13.84 | 1.2037 | 1.2109 | 35727 | 1.28e3 | 1 | 1 | 167 | 174 |  |  | DB Search |
| R.LGGIEVTQGNC(+57.02)ARK.Y | Y | 31.04 | 1501.7671 | 14 | -2.67 | 751.8854 | 2 | 12.64 | 1.0624 | 1.0697 | 29000 | 0 | 0 | 0 | 58 | 71 | Carbamidomethylation |  | DB Search |
| total 4 peptides |  |  |  |  |  |  |  |  |  |  |  |  |  |  |  |  |  |  |  |
| --- | --- | --- | --- | --- | --- | --- | --- | --- | --- | --- | --- | --- | --- | --- | --- | --- | --- | --- | --- |

Best Unique PSM (Scan Sample\_10\_A1\_1\_3647.d:35192, m/z=687.8438, z=2, RT=13.73, ppm=0.69):


TRINITY\_DN2637\_c0\_g1\_i6.p1

back to list

  

| Protein Coverage
| Supporting Peptides
| Best Unique PSM
|

Protein Coverage:

Supporting Peptides:

| Peptide | Uniq | -10lgP | Mass | Length | ppm | m/z | z | RT | 1/k0 Start | 1/k0 End | Precursor ID | Area Sample 1 | #Feature | #Feature Sample 1 | Start | End | PTM | AScore | Found By |
| --- | --- | --- | --- | --- | --- | --- | --- | --- | --- | --- | --- | --- | --- | --- | --- | --- | --- | --- | --- |
| K.ATIEIPAKYEK.R | Y | 61.89 | 1261.6918 | 11 | 0.22 | 631.8505 | 2 | 14.08 | 0.982 | 0.9892 | 37168 | 0 | 0 | 0 | 87 | 97 |  |  | DB Search |
| K.VSVTLADGTVATQEVLPGATIVVK.A | Y | 20.47 | 2367.326 | 24 | 4.39 | 1184.6702 | 2 | 19.66 | 1.2833 | 1.2905 | 68866 | 0 | 0 | 0 | 228 | 251 |  |  | DB Search |
| total 2 peptides |  |  |  |  |  |  |  |  |  |  |  |  |  |  |  |  |  |  |  |
| --- | --- | --- | --- | --- | --- | --- | --- | --- | --- | --- | --- | --- | --- | --- | --- | --- | --- | --- | --- |

Best Unique PSM (Scan Sample\_10\_A1\_1\_3647.d:37168, m/z=631.8505, z=2, RT=14.08, ppm=-4.27):


TRINITY\_DN195934\_c0\_g1\_i1.p2

back to list

  

| Protein Coverage
| Supporting Peptides
| Best Unique PSM
|

Protein Coverage:

Supporting Peptides:

| Peptide | Uniq | -10lgP | Mass | Length | ppm | m/z | z | RT | 1/k0 Start | 1/k0 End | Precursor ID | Area Sample 1 | #Feature | #Feature Sample 1 | Start | End | PTM | AScore | Found By |
| --- | --- | --- | --- | --- | --- | --- | --- | --- | --- | --- | --- | --- | --- | --- | --- | --- | --- | --- | --- |
| K.DSYVGDEAQSKR.G | Y | 66.73 | 1353.616 | 12 | 2.52 | 677.814 | 2 | 10.94 | 1.0069 | 1.0142 | 19045 | 5.51e3 | 1 | 1 | 10 | 21 |  |  | DB Search |
| total 1 peptides |  |  |  |  |  |  |  |  |  |  |  |  |  |  |  |  |  |  |  |
| --- | --- | --- | --- | --- | --- | --- | --- | --- | --- | --- | --- | --- | --- | --- | --- | --- | --- | --- | --- |

Best Unique PSM (Scan Sample\_10\_A1\_1\_3647.d:19045, m/z=677.814, z=2, RT=10.94, ppm=-1.97):


TRINITY\_DN493\_c0\_g1\_i1.p1

back to list

  

| Protein Coverage
| Supporting Peptides
| Best Unique PSM
|

Protein Coverage:

Supporting Peptides:

| Peptide | Uniq | -10lgP | Mass | Length | ppm | m/z | z | RT | 1/k0 Start | 1/k0 End | Precursor ID | Area Sample 1 | #Feature | #Feature Sample 1 | Start | End | PTM | AScore | Found By |
| --- | --- | --- | --- | --- | --- | --- | --- | --- | --- | --- | --- | --- | --- | --- | --- | --- | --- | --- | --- |
| K.EC(+57.02)SC(+57.02)NSYYC(+57.02)VGK.T | Y | 16.2 | 1525.5636 | 12 | -6.8 | 763.7805 | 2 | 11.79 | 1.0045 | 1.0118 | 24015 | 0 | 0 | 0 | 451 | 462 | Carbamidomethylation, Carbamidomethylation, Carbamidomethylation |  | DB Search |
| total 1 peptides |  |  |  |  |  |  |  |  |  |  |  |  |  |  |  |  |  |  |  |
| --- | --- | --- | --- | --- | --- | --- | --- | --- | --- | --- | --- | --- | --- | --- | --- | --- | --- | --- | --- |

Best Unique PSM (Scan Sample\_10\_A1\_1\_3647.d:24015, m/z=763.7805, z=2, RT=11.79, ppm=-11.3):


TRINITY\_DN26679\_c0\_g1\_i1.p3

back to list

  

| Protein Coverage
| Supporting Peptides
| Best Unique PSM
|

Protein Coverage:

Supporting Peptides:

| Peptide | Uniq | -10lgP | Mass | Length | ppm | m/z | z | RT | 1/k0 Start | 1/k0 End | Precursor ID | Area Sample 1 | #Feature | #Feature Sample 1 | Start | End | PTM | AScore | Found By |
| --- | --- | --- | --- | --- | --- | --- | --- | --- | --- | --- | --- | --- | --- | --- | --- | --- | --- | --- | --- |
| R.L(+42.01)AAPAFR.A | Y | 26.73 | 786.4388 | 7 | 17.1 | 394.2316 | 2 | 14.97 | 0.7506 | 0.7579 | 41907 | 2.53e3 | 1 | 1 | 11 | 17 | Acetylation (Protein N-term) | L1:Acetylation (Protein N-term):1000 | DB Search |
| total 1 peptides |  |  |  |  |  |  |  |  |  |  |  |  |  |  |  |  |  |  |  |
| --- | --- | --- | --- | --- | --- | --- | --- | --- | --- | --- | --- | --- | --- | --- | --- | --- | --- | --- | --- |

Best Unique PSM (Scan Sample\_10\_A1\_1\_3647.d:41907, m/z=394.2316, z=2, RT=14.97, ppm=12.6):


TRINITY\_DN30329\_c0\_g1\_i1.p1

back to list

  

| Protein Coverage
| Supporting Peptides
| Best Unique PSM
|

Protein Coverage:

Supporting Peptides:

| Peptide | Uniq | -10lgP | Mass | Length | ppm | m/z | z | RT | 1/k0 Start | 1/k0 End | Precursor ID | Area Sample 1 | #Feature | #Feature Sample 1 | Start | End | PTM | AScore | Found By |
| --- | --- | --- | --- | --- | --- | --- | --- | --- | --- | --- | --- | --- | --- | --- | --- | --- | --- | --- | --- |
| K.GQELTFKEFFKPSNFVR.S | Y | 20.36 | 2073.0683 | 17 | -5.6 | 692.0231 | 3 | 19.23 | 0.9311 | 0.9384 | 66186 | 6.45e2 | 1 | 1 | 160 | 176 |  |  | DB Search |
| total 1 peptides |  |  |  |  |  |  |  |  |  |  |  |  |  |  |  |  |  |  |  |
| --- | --- | --- | --- | --- | --- | --- | --- | --- | --- | --- | --- | --- | --- | --- | --- | --- | --- | --- | --- |

Best Unique PSM (Scan Sample\_10\_A1\_1\_3647.d:66186, m/z=692.0231, z=3, RT=19.23, ppm=-10.1):


TRINITY\_DN30329\_c0\_g2\_i1.p2

back to list

  

| Protein Coverage
| Supporting Peptides
| Best Unique PSM
|

Protein Coverage:

Supporting Peptides:

| Peptide | Uniq | -10lgP | Mass | Length | ppm | m/z | z | RT | 1/k0 Start | 1/k0 End | Precursor ID | Area Sample 1 | #Feature | #Feature Sample 1 | Start | End | PTM | AScore | Found By |
| --- | --- | --- | --- | --- | --- | --- | --- | --- | --- | --- | --- | --- | --- | --- | --- | --- | --- | --- | --- |
| K.GQELTFKEFFKPSNFVR.S | Y | 20.36 | 2073.0683 | 17 | -5.6 | 692.0231 | 3 | 19.23 | 0.9311 | 0.9384 | 66186 | 6.45e2 | 1 | 1 | 184 | 200 |  |  | DB Search |
| total 1 peptides |  |  |  |  |  |  |  |  |  |  |  |  |  |  |  |  |  |  |  |
| --- | --- | --- | --- | --- | --- | --- | --- | --- | --- | --- | --- | --- | --- | --- | --- | --- | --- | --- | --- |

Best Unique PSM (Scan Sample\_10\_A1\_1\_3647.d:66186, m/z=692.0231, z=3, RT=19.23, ppm=-10.1):


TRINITY\_DN7808\_c1\_g2\_i1.p1

back to list

  

| Protein Coverage
| Supporting Peptides
| Best Unique PSM
|

Protein Coverage:

Supporting Peptides:

| Peptide | Uniq | -10lgP | Mass | Length | ppm | m/z | z | RT | 1/k0 Start | 1/k0 End | Precursor ID | Area Sample 1 | #Feature | #Feature Sample 1 | Start | End | PTM | AScore | Found By |
| --- | --- | --- | --- | --- | --- | --- | --- | --- | --- | --- | --- | --- | --- | --- | --- | --- | --- | --- | --- |
| K.AITSSDPDALKR.K | Y | 65.96 | 1272.6673 | 12 | 2.56 | 637.3397 | 2 | 11.98 | 0.9558 | 0.963 | 25174 | 2.08e3 | 1 | 1 | 120 | 131 |  |  | DB Search |
| total 1 peptides |  |  |  |  |  |  |  |  |  |  |  |  |  |  |  |  |  |  |  |
| --- | --- | --- | --- | --- | --- | --- | --- | --- | --- | --- | --- | --- | --- | --- | --- | --- | --- | --- | --- |

Best Unique PSM (Scan Sample\_10\_A1\_1\_3647.d:25174, m/z=637.3397, z=2, RT=11.98, ppm=-1.93):


TRINITY\_DN471\_c0\_g1\_i2.p1

back to list

  

| Protein Coverage
| Supporting Peptides
| Best Unique PSM
|

Protein Coverage:

Supporting Peptides:

| Peptide | Uniq | -10lgP | Mass | Length | ppm | m/z | z | RT | 1/k0 Start | 1/k0 End | Precursor ID | Area Sample 1 | #Feature | #Feature Sample 1 | Start | End | PTM | AScore | Found By |
| --- | --- | --- | --- | --- | --- | --- | --- | --- | --- | --- | --- | --- | --- | --- | --- | --- | --- | --- | --- |
| R.AFVHWYVGEGM(+15.99)EEGEFSEAR.E | Y | 44.9 | 2345.0058 | 20 | -0.18 | 782.6722 | 3 | 17.81 | 0.9251 | 0.9323 | 57560 | 6.2e2 | 1 | 1 | 441 | 460 | Oxidation (M) | M11:Oxidation (M):1000 | DB Search |
| total 1 peptides |  |  |  |  |  |  |  |  |  |  |  |  |  |  |  |  |  |  |  |
| --- | --- | --- | --- | --- | --- | --- | --- | --- | --- | --- | --- | --- | --- | --- | --- | --- | --- | --- | --- |

Best Unique PSM (Scan Sample\_10\_A1\_1\_3647.d:57560, m/z=782.6722, z=3, RT=17.81, ppm=-4.67):


TRINITY\_DN751\_c0\_g1\_i1.p1

back to list

  

| Protein Coverage
| Supporting Peptides
| Best Unique PSM
|

Protein Coverage:

Supporting Peptides:

| Peptide | Uniq | -10lgP | Mass | Length | ppm | m/z | z | RT | 1/k0 Start | 1/k0 End | Precursor ID | Area Sample 1 | #Feature | #Feature Sample 1 | Start | End | PTM | AScore | Found By |
| --- | --- | --- | --- | --- | --- | --- | --- | --- | --- | --- | --- | --- | --- | --- | --- | --- | --- | --- | --- |
| R.LAADLVITHSSK.S | Y | 66.4 | 1253.6979 | 12 | 1.42 | 627.8543 | 2 | 14.21 | 0.9965 | 1.0037 | 37795 | 1.72e3 | 1 | 1 | 166 | 177 |  |  | DB Search |
| total 1 peptides |  |  |  |  |  |  |  |  |  |  |  |  |  |  |  |  |  |  |  |
| --- | --- | --- | --- | --- | --- | --- | --- | --- | --- | --- | --- | --- | --- | --- | --- | --- | --- | --- | --- |

Best Unique PSM (Scan Sample\_10\_A1\_1\_3647.d:37795, m/z=627.8543, z=2, RT=14.21, ppm=-3.07):


TRINITY\_DN8008\_c0\_g1\_i2.p1

back to list

  

| Protein Coverage
| Supporting Peptides
| Best Unique PSM
|

Protein Coverage:

Supporting Peptides:

| Peptide | Uniq | -10lgP | Mass | Length | ppm | m/z | z | RT | 1/k0 Start | 1/k0 End | Precursor ID | Area Sample 1 | #Feature | #Feature Sample 1 | Start | End | PTM | AScore | Found By |
| --- | --- | --- | --- | --- | --- | --- | --- | --- | --- | --- | --- | --- | --- | --- | --- | --- | --- | --- | --- |
| K.GVHIISVGLGTVDHR.G | Y | 66.61 | 1558.8579 | 15 | -17.11 | 520.6154 | 3 | 17.30 | 0.8116 | 0.8189 | 54443 | 0 | 0 | 0 | 1055 | 1069 |  |  | DB Search |
| total 1 peptides |  |  |  |  |  |  |  |  |  |  |  |  |  |  |  |  |  |  |  |
| --- | --- | --- | --- | --- | --- | --- | --- | --- | --- | --- | --- | --- | --- | --- | --- | --- | --- | --- | --- |

Best Unique PSM (Scan Sample\_10\_A1\_1\_3647.d:54443, m/z=520.6154, z=3, RT=17.30, ppm=-21.61):


TRINITY\_DN36\_c0\_g1\_i14.p1

back to list

  

| Protein Coverage
| Supporting Peptides
| Best Unique PSM
|

Protein Coverage:

Supporting Peptides:

| Peptide | Uniq | -10lgP | Mass | Length | ppm | m/z | z | RT | 1/k0 Start | 1/k0 End | Precursor ID | Area Sample 1 | #Feature | #Feature Sample 1 | Start | End | PTM | AScore | Found By |
| --- | --- | --- | --- | --- | --- | --- | --- | --- | --- | --- | --- | --- | --- | --- | --- | --- | --- | --- | --- |
| R.LGGIEVTQGNC(+57.02)AR.K | Y | 66.48 | 1373.6721 | 13 | 5.18 | 687.8438 | 2 | 13.73 | 0.963 | 0.9703 | 35192 | 0 | 0 | 0 | 58 | 70 | Carbamidomethylation |  | DB Search |
| K.WC(+57.02)DILSIHQSMENC(+57.02)VAK.C | Y | 63.98 | 2089.9383 | 17 | -2.35 | 697.6486 | 3 | 17.89 | 0.8777 | 0.885 | 58058 | 0 | 0 | 0 | 198 | 214 | Carbamidomethylation, Carbamidomethylation |  | DB Search |
| K.FETQIATK.D | Y | 61.61 | 936.4916 | 8 | 0.97 | 937.4956 | 1 | 13.84 | 1.2037 | 1.2109 | 35727 | 1.28e3 | 1 | 1 | 167 | 174 |  |  | DB Search |
| R.LGGIEVTQGNC(+57.02)ARK.Y | Y | 31.04 | 1501.7671 | 14 | -2.67 | 751.8854 | 2 | 12.64 | 1.0624 | 1.0697 | 29000 | 0 | 0 | 0 | 58 | 71 | Carbamidomethylation |  | DB Search |
| total 4 peptides |  |  |  |  |  |  |  |  |  |  |  |  |  |  |  |  |  |  |  |
| --- | --- | --- | --- | --- | --- | --- | --- | --- | --- | --- | --- | --- | --- | --- | --- | --- | --- | --- | --- |

Best Unique PSM (Scan Sample\_10\_A1\_1\_3647.d:35192, m/z=687.8438, z=2, RT=13.73, ppm=0.69):


TRINITY\_DN817\_c0\_g1\_i3.p1

back to list

  

| Protein Coverage
| Supporting Peptides
| Best Unique PSM
|

Protein Coverage:

Supporting Peptides:

| Peptide | Uniq | -10lgP | Mass | Length | ppm | m/z | z | RT | 1/k0 Start | 1/k0 End | Precursor ID | Area Sample 1 | #Feature | #Feature Sample 1 | Start | End | PTM | AScore | Found By |
| --- | --- | --- | --- | --- | --- | --- | --- | --- | --- | --- | --- | --- | --- | --- | --- | --- | --- | --- | --- |
| R.LC(+57.02)QPGYSTC(+57.02)ENHC(+57.02)R.K | Y | 62.01 | 1780.708 | 14 | 5.39 | 891.3621 | 2 | 11.41 | 1.0893 | 1.0965 | 21785 | 1.84e2 | 1 | 1 | 177 | 190 | Carbamidomethylation, Carbamidomethylation, Carbamidomethylation |  | DB Search |
| total 1 peptides |  |  |  |  |  |  |  |  |  |  |  |  |  |  |  |  |  |  |  |
| --- | --- | --- | --- | --- | --- | --- | --- | --- | --- | --- | --- | --- | --- | --- | --- | --- | --- | --- | --- |

Best Unique PSM (Scan Sample\_10\_A1\_1\_3647.d:21785, m/z=891.3621, z=2, RT=11.41, ppm=0.9):


TRINITY\_DN262\_c0\_g1\_i1.p1

back to list

  

| Protein Coverage
| Supporting Peptides
| Best Unique PSM
|

Protein Coverage:

Supporting Peptides:

| Peptide | Uniq | -10lgP | Mass | Length | ppm | m/z | z | RT | 1/k0 Start | 1/k0 End | Precursor ID | Area Sample 1 | #Feature | #Feature Sample 1 | Start | End | PTM | AScore | Found By |
| --- | --- | --- | --- | --- | --- | --- | --- | --- | --- | --- | --- | --- | --- | --- | --- | --- | --- | --- | --- |
| R.DANAAVVHGSHLK.D | Y | 66.78 | 1317.6789 | 13 | -0.21 | 659.8436 | 2 | 10.89 | 1.0154 | 1.0226 | 18821 | 6.63e2 | 1 | 1 | 668 | 680 |  |  | DB Search |
| R.AEFRPNQEEEPILKR.E | Y | 66.68 | 1854.9588 | 15 | 1.2 | 619.3248 | 3 | 13.73 | 0.8745 | 0.8818 | 35104 | 1.03e3 | 1 | 1 | 439 | 453 |  |  | DB Search |
| K.GVGIISEGNETVEDIAQR.L | Y | 61.69 | 1885.9381 | 18 | 0.15 | 943.9722 | 2 | 17.77 | 1.1438 | 1.151 | 57272 | 0 | 0 | 0 | 639 | 656 |  |  | DB Search |
| total 3 peptides |  |  |  |  |  |  |  |  |  |  |  |  |  |  |  |  |  |  |  |
| --- | --- | --- | --- | --- | --- | --- | --- | --- | --- | --- | --- | --- | --- | --- | --- | --- | --- | --- | --- |

Best Unique PSM (Scan Sample\_10\_A1\_1\_3647.d:18821, m/z=659.8436, z=2, RT=10.89, ppm=-4.71):


TRINITY\_DN119684\_c0\_g1\_i1.p1

back to list

  

| Protein Coverage
| Supporting Peptides
| Best Unique PSM
|

Protein Coverage:

Supporting Peptides:

| Peptide | Uniq | -10lgP | Mass | Length | ppm | m/z | z | RT | 1/k0 Start | 1/k0 End | Precursor ID | Area Sample 1 | #Feature | #Feature Sample 1 | Start | End | PTM | AScore | Found By |
| --- | --- | --- | --- | --- | --- | --- | --- | --- | --- | --- | --- | --- | --- | --- | --- | --- | --- | --- | --- |
| R.LSPEDIER.M | Y | 39.28 | 957.4767 | 8 | 13.25 | 479.7498 | 2 | 14.37 | 0.827 | 0.8343 | 38738 | 1.06e4 | 1 | 1 | 535 | 542 |  |  | DB Search |
| K.GKKPHVQVNVK.E | Y | 29.58 | 1232.7353 | 11 | 0.24 | 411.9173 | 3 | 9.77 | 0.7913 | 0.7986 | 12271 | 6.19e2 | 1 | 1 | 125 | 135 |  |  | DB Search |
| total 2 peptides |  |  |  |  |  |  |  |  |  |  |  |  |  |  |  |  |  |  |  |
| --- | --- | --- | --- | --- | --- | --- | --- | --- | --- | --- | --- | --- | --- | --- | --- | --- | --- | --- | --- |

Best Unique PSM (Scan Sample\_10\_A1\_1\_3647.d:38738, m/z=479.7498, z=2, RT=14.37, ppm=8.76):


TRINITY\_DN14344\_c0\_g1\_i1.p1

back to list

  

| Protein Coverage
| Supporting Peptides
| Best Unique PSM
|

Protein Coverage:

Supporting Peptides:

| Peptide | Uniq | -10lgP | Mass | Length | ppm | m/z | z | RT | 1/k0 Start | 1/k0 End | Precursor ID | Area Sample 1 | #Feature | #Feature Sample 1 | Start | End | PTM | AScore | Found By |
| --- | --- | --- | --- | --- | --- | --- | --- | --- | --- | --- | --- | --- | --- | --- | --- | --- | --- | --- | --- |
| R.KSQFGEIFSFDKK.V | Y | 62.93 | 1559.7984 | 13 | -6.3 | 520.9344 | 3 | 17.14 | 0.7888 | 0.7962 | 53558 | 6.09e2 | 1 | 1 | 423 | 435 |  |  | DB Search |
| total 1 peptides |  |  |  |  |  |  |  |  |  |  |  |  |  |  |  |  |  |  |  |
| --- | --- | --- | --- | --- | --- | --- | --- | --- | --- | --- | --- | --- | --- | --- | --- | --- | --- | --- | --- |

Best Unique PSM (Scan Sample\_10\_A1\_1\_3647.d:53558, m/z=520.9344, z=3, RT=17.14, ppm=-10.8):


TRINITY\_DN4928\_c0\_g1\_i1.p1

back to list

  

| Protein Coverage
| Supporting Peptides
| Best Unique PSM
|

Protein Coverage:

Supporting Peptides:

| Peptide | Uniq | -10lgP | Mass | Length | ppm | m/z | z | RT | 1/k0 Start | 1/k0 End | Precursor ID | Area Sample 1 | #Feature | #Feature Sample 1 | Start | End | PTM | AScore | Found By |
| --- | --- | --- | --- | --- | --- | --- | --- | --- | --- | --- | --- | --- | --- | --- | --- | --- | --- | --- | --- |
| K.YANFLDQALREDLER.M | Y | 61.79 | 1851.9115 | 15 | -3.63 | 618.3061 | 3 | 19.80 | 0.8741 | 0.8814 | 69772 | 0 | 0 | 0 | 70 | 84 |  |  | DB Search |
| total 1 peptides |  |  |  |  |  |  |  |  |  |  |  |  |  |  |  |  |  |  |  |
| --- | --- | --- | --- | --- | --- | --- | --- | --- | --- | --- | --- | --- | --- | --- | --- | --- | --- | --- | --- |

Best Unique PSM (Scan Sample\_10\_A1\_1\_3647.d:69772, m/z=618.3061, z=3, RT=19.80, ppm=-8.12):


TRINITY\_DN6388\_c0\_g1\_i1.p2

back to list

  

| Protein Coverage
| Supporting Peptides
| Best Unique PSM
|

Protein Coverage:

Supporting Peptides:

| Peptide | Uniq | -10lgP | Mass | Length | ppm | m/z | z | RT | 1/k0 Start | 1/k0 End | Precursor ID | Area Sample 1 | #Feature | #Feature Sample 1 | Start | End | PTM | AScore | Found By |
| --- | --- | --- | --- | --- | --- | --- | --- | --- | --- | --- | --- | --- | --- | --- | --- | --- | --- | --- | --- |
| R.AIELLPHDLAVGR.H | Y | 66.91 | 1402.7932 | 13 | -9.52 | 702.394 | 2 | 17.77 | 1.0275 | 1.0347 | 57279 | 0 | 0 | 0 | 57 | 69 |  |  | DB Search |
| R.YQYEHAVAELR.G | Y | 64.48 | 1377.6677 | 11 | 2.2 | 689.8395 | 2 | 14.37 | 1.0102 | 1.0174 | 38774 | 1.67e3 | 1 | 1 | 27 | 37 |  |  | DB Search |
| total 2 peptides |  |  |  |  |  |  |  |  |  |  |  |  |  |  |  |  |  |  |  |
| --- | --- | --- | --- | --- | --- | --- | --- | --- | --- | --- | --- | --- | --- | --- | --- | --- | --- | --- | --- |

Best Unique PSM (Scan Sample\_10\_A1\_1\_3647.d:57279, m/z=702.394, z=2, RT=17.77, ppm=-14.02):


TRINITY\_DN18187\_c0\_g1\_i4.p3

back to list

  

| Protein Coverage
| Supporting Peptides
| Best Unique PSM
|

Protein Coverage:

Supporting Peptides:

| Peptide | Uniq | -10lgP | Mass | Length | ppm | m/z | z | RT | 1/k0 Start | 1/k0 End | Precursor ID | Area Sample 1 | #Feature | #Feature Sample 1 | Start | End | PTM | AScore | Found By |
| --- | --- | --- | --- | --- | --- | --- | --- | --- | --- | --- | --- | --- | --- | --- | --- | --- | --- | --- | --- |
| R.YRPGTVALR.E | Y | 66.57 | 1031.5876 | 9 | 0.86 | 516.7992 | 2 | 12.50 | 0.8554 | 0.8627 | 28119 | 7.47e2 | 1 | 1 | 17 | 25 |  |  | DB Search |
| total 1 peptides |  |  |  |  |  |  |  |  |  |  |  |  |  |  |  |  |  |  |  |
| --- | --- | --- | --- | --- | --- | --- | --- | --- | --- | --- | --- | --- | --- | --- | --- | --- | --- | --- | --- |

Best Unique PSM (Scan Sample\_10\_A1\_1\_3647.d:28119, m/z=516.7992, z=2, RT=12.50, ppm=-3.64):


TRINITY\_DN1121\_c0\_g1\_i1.p1

back to list

  

| Protein Coverage
| Supporting Peptides
| Best Unique PSM
|

Protein Coverage:

Supporting Peptides:

| Peptide | Uniq | -10lgP | Mass | Length | ppm | m/z | z | RT | 1/k0 Start | 1/k0 End | Precursor ID | Area Sample 1 | #Feature | #Feature Sample 1 | Start | End | PTM | AScore | Found By |
| --- | --- | --- | --- | --- | --- | --- | --- | --- | --- | --- | --- | --- | --- | --- | --- | --- | --- | --- | --- |
| M.A(+42.01)EREQLIQQAK.L | Y | 64.88 | 1354.7205 | 11 | 2.32 | 678.366 | 2 | 13.60 | 0.9775 | 0.9848 | 34443 | 1.59e3 | 1 | 1 | 2 | 12 | Acetylation (Protein N-term) | A1:Acetylation (Protein N-term):1000 | DB Search |
| total 1 peptides |  |  |  |  |  |  |  |  |  |  |  |  |  |  |  |  |  |  |  |
| --- | --- | --- | --- | --- | --- | --- | --- | --- | --- | --- | --- | --- | --- | --- | --- | --- | --- | --- | --- |

Best Unique PSM (Scan Sample\_10\_A1\_1\_3647.d:34443, m/z=678.366, z=2, RT=13.60, ppm=-2.18):


TRINITY\_DN73587\_c0\_g1\_i1.p1

back to list

  

| Protein Coverage
| Supporting Peptides
| Best Unique PSM
|

Protein Coverage:

Supporting Peptides:

| Peptide | Uniq | -10lgP | Mass | Length | ppm | m/z | z | RT | 1/k0 Start | 1/k0 End | Precursor ID | Area Sample 1 | #Feature | #Feature Sample 1 | Start | End | PTM | AScore | Found By |
| --- | --- | --- | --- | --- | --- | --- | --- | --- | --- | --- | --- | --- | --- | --- | --- | --- | --- | --- | --- |
| R.LVLEVAQHLGENVVR.T | Y | 58.94 | 1674.9417 | 15 | 9.79 | 838.4825 | 2 | 17.91 | 1.1414 | 1.1486 | 58140 | 0 | 0 | 0 | 99 | 113 |  |  | DB Search |
| total 1 peptides |  |  |  |  |  |  |  |  |  |  |  |  |  |  |  |  |  |  |  |
| --- | --- | --- | --- | --- | --- | --- | --- | --- | --- | --- | --- | --- | --- | --- | --- | --- | --- | --- | --- |

Best Unique PSM (Scan Sample\_10\_A1\_1\_3647.d:58140, m/z=838.4825, z=2, RT=17.91, ppm=5.3):


TRINITY\_DN64\_c0\_g1\_i1.p1

back to list

  

| Protein Coverage
| Supporting Peptides
| Best Unique PSM
|

Protein Coverage:

Supporting Peptides:

| Peptide | Uniq | -10lgP | Mass | Length | ppm | m/z | z | RT | 1/k0 Start | 1/k0 End | Precursor ID | Area Sample 1 | #Feature | #Feature Sample 1 | Start | End | PTM | AScore | Found By |
| --- | --- | --- | --- | --- | --- | --- | --- | --- | --- | --- | --- | --- | --- | --- | --- | --- | --- | --- | --- |
| K.IHINIVVIGHVDSGK.S | Y | 66.38 | 1599.9096 | 15 | 1.53 | 800.9597 | 2 | 16.52 | 1.0993 | 1.1065 | 50056 | 1.41e3 | 2 | 2 | 9 | 23 |  |  | DB Search |
| K.YYVTIIDAPGHRDFIK.N | Y | 64.76 | 1906.9941 | 16 | -3.62 | 954.4966 | 2 | 16.71 | 1.2077 | 1.2149 | 51222 | 0 | 0 | 0 | 88 | 103 |  |  | DB Search |
| total 2 peptides |  |  |  |  |  |  |  |  |  |  |  |  |  |  |  |  |  |  |  |
| --- | --- | --- | --- | --- | --- | --- | --- | --- | --- | --- | --- | --- | --- | --- | --- | --- | --- | --- | --- |

Best Unique PSM (Scan Sample\_10\_A1\_1\_3647.d:50056, m/z=800.9597, z=2, RT=16.52, ppm=-2.97):


TRINITY\_DN15274\_c0\_g1\_i1.p1

back to list

  

| Protein Coverage
| Supporting Peptides
| Best Unique PSM
|

Protein Coverage:

Supporting Peptides:

| Peptide | Uniq | -10lgP | Mass | Length | ppm | m/z | z | RT | 1/k0 Start | 1/k0 End | Precursor ID | Area Sample 1 | #Feature | #Feature Sample 1 | Start | End | PTM | AScore | Found By |
| --- | --- | --- | --- | --- | --- | --- | --- | --- | --- | --- | --- | --- | --- | --- | --- | --- | --- | --- | --- |
| K.YC(+57.02)ILSFLDKTDK.C | Y | 66.74 | 1501.7486 | 12 | -2.59 | 501.5866 | 3 | 19.64 | 0.8002 | 0.8075 | 68673 | 3.17e2 | 1 | 1 | 62 | 73 | Carbamidomethylation |  | DB Search |
| R.KIDDVIC(+57.02)EER.Y | Y | 39.44 | 1275.6129 | 10 | -9.66 | 638.8047 | 2 | 13.45 | 0.9255 | 0.9327 | 33693 | 0 | 0 | 0 | 184 | 193 | Carbamidomethylation |  | DB Search |
| total 2 peptides |  |  |  |  |  |  |  |  |  |  |  |  |  |  |  |  |  |  |  |
| --- | --- | --- | --- | --- | --- | --- | --- | --- | --- | --- | --- | --- | --- | --- | --- | --- | --- | --- | --- |

Best Unique PSM (Scan Sample\_10\_A1\_1\_3647.d:68673, m/z=501.5866, z=3, RT=19.64, ppm=-7.09):


TRINITY\_DN146208\_c0\_g1\_i1.p1

back to list

  

| Protein Coverage
| Supporting Peptides
| Best Unique PSM
|

Protein Coverage:

Supporting Peptides:

| Peptide | Uniq | -10lgP | Mass | Length | ppm | m/z | z | RT | 1/k0 Start | 1/k0 End | Precursor ID | Area Sample 1 | #Feature | #Feature Sample 1 | Start | End | PTM | AScore | Found By |
| --- | --- | --- | --- | --- | --- | --- | --- | --- | --- | --- | --- | --- | --- | --- | --- | --- | --- | --- | --- |
| K.YGINHITNLVESKR.A | Y | 66.7 | 1642.8791 | 14 | -0.91 | 548.6307 | 3 | 14.97 | 0.8226 | 0.8299 | 42030 | 3.39e2 | 1 | 1 | 158 | 171 |  |  | DB Search |
| total 1 peptides |  |  |  |  |  |  |  |  |  |  |  |  |  |  |  |  |  |  |  |
| --- | --- | --- | --- | --- | --- | --- | --- | --- | --- | --- | --- | --- | --- | --- | --- | --- | --- | --- | --- |

Best Unique PSM (Scan Sample\_10\_A1\_1\_3647.d:42030, m/z=548.6307, z=3, RT=14.97, ppm=-5.4):


TRINITY\_DN3649\_c0\_g1\_i2.p1

back to list

  

| Protein Coverage
| Supporting Peptides
| Best Unique PSM
|

Protein Coverage:

Supporting Peptides:

| Peptide | Uniq | -10lgP | Mass | Length | ppm | m/z | z | RT | 1/k0 Start | 1/k0 End | Precursor ID | Area Sample 1 | #Feature | #Feature Sample 1 | Start | End | PTM | AScore | Found By |
| --- | --- | --- | --- | --- | --- | --- | --- | --- | --- | --- | --- | --- | --- | --- | --- | --- | --- | --- | --- |
| R.LC(+57.02)ESDPENAKK.K | Y | 66.75 | 1289.5921 | 11 | 2.03 | 645.8018 | 2 | 10.10 | 0.9489 | 0.9562 | 14132 | 7.73e2 | 1 | 1 | 209 | 219 | Carbamidomethylation |  | DB Search |
| total 1 peptides |  |  |  |  |  |  |  |  |  |  |  |  |  |  |  |  |  |  |  |
| --- | --- | --- | --- | --- | --- | --- | --- | --- | --- | --- | --- | --- | --- | --- | --- | --- | --- | --- | --- |

Best Unique PSM (Scan Sample\_10\_A1\_1\_3647.d:14132, m/z=645.8018, z=2, RT=10.10, ppm=-2.47):


TRINITY\_DN3271\_c0\_g1\_i1.p1

back to list

  

| Protein Coverage
| Supporting Peptides
| Best Unique PSM
|

Protein Coverage:

Supporting Peptides:

| Peptide | Uniq | -10lgP | Mass | Length | ppm | m/z | z | RT | 1/k0 Start | 1/k0 End | Precursor ID | Area Sample 1 | #Feature | #Feature Sample 1 | Start | End | PTM | AScore | Found By |
| --- | --- | --- | --- | --- | --- | --- | --- | --- | --- | --- | --- | --- | --- | --- | --- | --- | --- | --- | --- |
| K.TIESEAAHGTVTR.H | Y | 66.53 | 1370.679 | 13 | 2.05 | 686.3451 | 2 | 11.89 | 1.0194 | 1.0267 | 24550 | 7.11e2 | 1 | 1 | 285 | 297 |  |  | DB Search |
| total 1 peptides |  |  |  |  |  |  |  |  |  |  |  |  |  |  |  |  |  |  |  |
| --- | --- | --- | --- | --- | --- | --- | --- | --- | --- | --- | --- | --- | --- | --- | --- | --- | --- | --- | --- |

Best Unique PSM (Scan Sample\_10\_A1\_1\_3647.d:24550, m/z=686.3451, z=2, RT=11.89, ppm=-2.44):


TRINITY\_DN10026\_c0\_g1\_i1.p1

back to list

  

| Protein Coverage
| Supporting Peptides
| Best Unique PSM
|

Protein Coverage:

Supporting Peptides:

| Peptide | Uniq | -10lgP | Mass | Length | ppm | m/z | z | RT | 1/k0 Start | 1/k0 End | Precursor ID | Area Sample 1 | #Feature | #Feature Sample 1 | Start | End | PTM | AScore | Found By |
| --- | --- | --- | --- | --- | --- | --- | --- | --- | --- | --- | --- | --- | --- | --- | --- | --- | --- | --- | --- |
| R.KEDVSEFFDIKEEVGR.G | Y | 65.16 | 1925.937 | 16 | 2.76 | 642.9852 | 3 | 17.77 | 0.9105 | 0.9178 | 57244 | 2.14e2 | 1 | 1 | 10 | 25 |  |  | DB Search |
| total 1 peptides |  |  |  |  |  |  |  |  |  |  |  |  |  |  |  |  |  |  |  |
| --- | --- | --- | --- | --- | --- | --- | --- | --- | --- | --- | --- | --- | --- | --- | --- | --- | --- | --- | --- |

Best Unique PSM (Scan Sample\_10\_A1\_1\_3647.d:57244, m/z=642.9852, z=3, RT=17.77, ppm=-1.73):


TRINITY\_DN624\_c0\_g1\_i1.p1

back to list

  

| Protein Coverage
| Supporting Peptides
| Best Unique PSM
|

Protein Coverage:

Supporting Peptides:

| Peptide | Uniq | -10lgP | Mass | Length | ppm | m/z | z | RT | 1/k0 Start | 1/k0 End | Precursor ID | Area Sample 1 | #Feature | #Feature Sample 1 | Start | End | PTM | AScore | Found By |
| --- | --- | --- | --- | --- | --- | --- | --- | --- | --- | --- | --- | --- | --- | --- | --- | --- | --- | --- | --- |
| R.FAIQDITVDEFSAK.E | Y | 61.52 | 1582.7878 | 14 | -2.01 | 792.3961 | 2 | 19.66 | 1.0443 | 1.0516 | 68879 | 0 | 0 | 0 | 132 | 145 |  |  | DB Search |
| total 1 peptides |  |  |  |  |  |  |  |  |  |  |  |  |  |  |  |  |  |  |  |
| --- | --- | --- | --- | --- | --- | --- | --- | --- | --- | --- | --- | --- | --- | --- | --- | --- | --- | --- | --- |

Best Unique PSM (Scan Sample\_10\_A1\_1\_3647.d:68879, m/z=791.3914, z=1, RT=19.69, ppm=-6.5):


TRINITY\_DN12\_c0\_g1\_i1.p1

back to list

  

| Protein Coverage
| Supporting Peptides
| Best Unique PSM
|

Protein Coverage:

Supporting Peptides:

| Peptide | Uniq | -10lgP | Mass | Length | ppm | m/z | z | RT | 1/k0 Start | 1/k0 End | Precursor ID | Area Sample 1 | #Feature | #Feature Sample 1 | Start | End | PTM | AScore | Found By |
| --- | --- | --- | --- | --- | --- | --- | --- | --- | --- | --- | --- | --- | --- | --- | --- | --- | --- | --- | --- |
| R.LGPDEASENKR.E | Y | 29.91 | 1214.5891 | 11 | -1.14 | 608.2984 | 2 | 10.44 | 0.9166 | 0.9238 | 16086 | 0 | 0 | 0 | 153 | 163 |  |  | DB Search |
| total 1 peptides |  |  |  |  |  |  |  |  |  |  |  |  |  |  |  |  |  |  |  |
| --- | --- | --- | --- | --- | --- | --- | --- | --- | --- | --- | --- | --- | --- | --- | --- | --- | --- | --- | --- |

Best Unique PSM (Scan Sample\_10\_A1\_1\_3647.d:16086, m/z=608.2984, z=2, RT=10.44, ppm=-5.64):


TRINITY\_DN384\_c0\_g1\_i2.p1

back to list

  

| Protein Coverage
| Supporting Peptides
| Best Unique PSM
|

Protein Coverage:

Supporting Peptides:

| Peptide | Uniq | -10lgP | Mass | Length | ppm | m/z | z | RT | 1/k0 Start | 1/k0 End | Precursor ID | Area Sample 1 | #Feature | #Feature Sample 1 | Start | End | PTM | AScore | Found By |
| --- | --- | --- | --- | --- | --- | --- | --- | --- | --- | --- | --- | --- | --- | --- | --- | --- | --- | --- | --- |
| K.FVLHGDVSHGSR.E | Y | 66.68 | 1309.6527 | 12 | 4.95 | 655.8339 | 2 | 12.00 | 0.9892 | 0.9965 | 25296 | 0 | 0 | 0 | 275 | 286 |  |  | DB Search |
| total 1 peptides |  |  |  |  |  |  |  |  |  |  |  |  |  |  |  |  |  |  |  |
| --- | --- | --- | --- | --- | --- | --- | --- | --- | --- | --- | --- | --- | --- | --- | --- | --- | --- | --- | --- |

Best Unique PSM (Scan Sample\_10\_A1\_1\_3647.d:25296, m/z=655.8339, z=2, RT=12.00, ppm=0.46):


TRINITY\_DN1058\_c0\_g1\_i1.p1

back to list

  

| Protein Coverage
| Supporting Peptides
| Best Unique PSM
|

Protein Coverage:

Supporting Peptides:

| Peptide | Uniq | -10lgP | Mass | Length | ppm | m/z | z | RT | 1/k0 Start | 1/k0 End | Precursor ID | Area Sample 1 | #Feature | #Feature Sample 1 | Start | End | PTM | AScore | Found By |
| --- | --- | --- | --- | --- | --- | --- | --- | --- | --- | --- | --- | --- | --- | --- | --- | --- | --- | --- | --- |
| R.APTHILNPANKR.S | Y | 66.68 | 1330.7469 | 12 | 2.53 | 444.5887 | 3 | 11.11 | 0.7526 | 0.7599 | 20079 | 1.09e3 | 2 | 2 | 2314 | 2325 |  |  | DB Search |
| total 1 peptides |  |  |  |  |  |  |  |  |  |  |  |  |  |  |  |  |  |  |  |
| --- | --- | --- | --- | --- | --- | --- | --- | --- | --- | --- | --- | --- | --- | --- | --- | --- | --- | --- | --- |

Best Unique PSM (Scan Sample\_10\_A1\_1\_3647.d:20079, m/z=444.5887, z=3, RT=11.11, ppm=-1.97):


TRINITY\_DN1339\_c0\_g2\_i3.p1

back to list

  

| Protein Coverage
| Supporting Peptides
| Best Unique PSM
|

Protein Coverage:

Supporting Peptides:

| Peptide | Uniq | -10lgP | Mass | Length | ppm | m/z | z | RT | 1/k0 Start | 1/k0 End | Precursor ID | Area Sample 1 | #Feature | #Feature Sample 1 | Start | End | PTM | AScore | Found By |
| --- | --- | --- | --- | --- | --- | --- | --- | --- | --- | --- | --- | --- | --- | --- | --- | --- | --- | --- | --- |
| K.LLTSSDQAELTKK.L | Y | 66.26 | 1432.7773 | 13 | 0.59 | 717.3931 | 2 | 12.73 | 1.0504 | 1.0576 | 29502 | 1.34e3 | 1 | 1 | 155 | 167 |  |  | DB Search |
| total 1 peptides |  |  |  |  |  |  |  |  |  |  |  |  |  |  |  |  |  |  |  |
| --- | --- | --- | --- | --- | --- | --- | --- | --- | --- | --- | --- | --- | --- | --- | --- | --- | --- | --- | --- |

Best Unique PSM (Scan Sample\_10\_A1\_1\_3647.d:29502, m/z=717.3931, z=2, RT=12.73, ppm=-3.91):


TRINITY\_DN329\_c0\_g1\_i2.p1

back to list

  

| Protein Coverage
| Supporting Peptides
| Best Unique PSM
|

Protein Coverage:

Supporting Peptides:

| Peptide | Uniq | -10lgP | Mass | Length | ppm | m/z | z | RT | 1/k0 Start | 1/k0 End | Precursor ID | Area Sample 1 | #Feature | #Feature Sample 1 | Start | End | PTM | AScore | Found By |
| --- | --- | --- | --- | --- | --- | --- | --- | --- | --- | --- | --- | --- | --- | --- | --- | --- | --- | --- | --- |
| K.GLKVDFDTTFAPVTGKK.T | Y | 66.86 | 1822.9828 | 17 | 1.5 | 912.496 | 2 | 17.12 | 1.1802 | 1.1873 | 53325 | 2.2e3 | 2 | 2 | 130 | 146 |  |  | DB Search |
| K.SFHFGVIKLEGK.T | Y | 66.8 | 1360.7503 | 12 | 0.14 | 681.3795 | 2 | 16.99 | 1.0275 | 1.0347 | 52570 | 1.81e3 | 1 | 1 | 57 | 68 |  |  | DB Search |
| K.GLKVDFDTTFAPVTGK.K | Y | 66.78 | 1694.8879 | 16 | 3.14 | 848.4501 | 2 | 18.13 | 1.0933 | 1.1005 | 59417 | 1.18e3 | 1 | 1 | 130 | 145 |  |  | DB Search |
| K.VDFDTTFAPVTGKK.T | Y | 64.84 | 1524.7824 | 14 | 4.3 | 763.3983 | 2 | 18.74 | 1.048 | 1.0552 | 63303 | 9.89e2 | 2 | 2 | 133 | 146 |  |  | DB Search |
| K.LRPGVELTM(+15.99)SSLVNAK.S | Y | 60.81 | 1729.9396 | 16 | -1.76 | 865.9717 | 2 | 15.17 | 1.1358 | 1.143 | 42935 | 1.5e3 | 1 | 1 | 287 | 302 | Oxidation (M) | M9:Oxidation (M):1000 | DB Search |
| K.EGDLVIHSGITDAVK.Y | Y | 60.79 | 1552.8096 | 15 | -9.48 | 777.4012 | 2 | 15.72 | 1.0668 | 1.0741 | 46131 | 0 | 0 | 0 | 211 | 225 |  |  | DB Search |
| total 6 peptides |  |  |  |  |  |  |  |  |  |  |  |  |  |  |  |  |  |  |  |
| --- | --- | --- | --- | --- | --- | --- | --- | --- | --- | --- | --- | --- | --- | --- | --- | --- | --- | --- | --- |

Best Unique PSM (Scan Sample\_10\_A1\_1\_3647.d:53325, m/z=912.496, z=2, RT=17.12, ppm=-2.99):


TRINITY\_DN53760\_c0\_g1\_i1.p1

back to list

  

| Protein Coverage
| Supporting Peptides
| Best Unique PSM
|

Protein Coverage:

Supporting Peptides:

| Peptide | Uniq | -10lgP | Mass | Length | ppm | m/z | z | RT | 1/k0 Start | 1/k0 End | Precursor ID | Area Sample 1 | #Feature | #Feature Sample 1 | Start | End | PTM | AScore | Found By |
| --- | --- | --- | --- | --- | --- | --- | --- | --- | --- | --- | --- | --- | --- | --- | --- | --- | --- | --- | --- |
| K.YANFLDQALREDLER.M | Y | 61.79 | 1851.9115 | 15 | -3.63 | 618.3061 | 3 | 19.80 | 0.8741 | 0.8814 | 69772 | 0 | 0 | 0 | 78 | 92 |  |  | DB Search |
| total 1 peptides |  |  |  |  |  |  |  |  |  |  |  |  |  |  |  |  |  |  |  |
| --- | --- | --- | --- | --- | --- | --- | --- | --- | --- | --- | --- | --- | --- | --- | --- | --- | --- | --- | --- |

Best Unique PSM (Scan Sample\_10\_A1\_1\_3647.d:69772, m/z=618.3061, z=3, RT=19.80, ppm=-8.12):


TRINITY\_DN1319\_c0\_g1\_i2.p1

back to list

  

| Protein Coverage
| Supporting Peptides
| Best Unique PSM
|

Protein Coverage:

Supporting Peptides:

| Peptide | Uniq | -10lgP | Mass | Length | ppm | m/z | z | RT | 1/k0 Start | 1/k0 End | Precursor ID | Area Sample 1 | #Feature | #Feature Sample 1 | Start | End | PTM | AScore | Found By |
| --- | --- | --- | --- | --- | --- | --- | --- | --- | --- | --- | --- | --- | --- | --- | --- | --- | --- | --- | --- |
| K.GTHAAEIAHC(+57.02)VSR.R | Y | 65.75 | 1407.6677 | 13 | 1.54 | 704.8391 | 2 | 11.03 | 1.0283 | 1.0355 | 19632 | 6.69e2 | 1 | 1 | 91 | 103 | Carbamidomethylation |  | DB Search |
| total 1 peptides |  |  |  |  |  |  |  |  |  |  |  |  |  |  |  |  |  |  |  |
| --- | --- | --- | --- | --- | --- | --- | --- | --- | --- | --- | --- | --- | --- | --- | --- | --- | --- | --- | --- |

Best Unique PSM (Scan Sample\_10\_A1\_1\_3647.d:19632, m/z=704.8391, z=2, RT=11.03, ppm=-2.95):


TRINITY\_DN1995\_c0\_g1\_i1.p1

back to list

  

| Protein Coverage
| Supporting Peptides
| Best Unique PSM
|

Protein Coverage:

Supporting Peptides:

| Peptide | Uniq | -10lgP | Mass | Length | ppm | m/z | z | RT | 1/k0 Start | 1/k0 End | Precursor ID | Area Sample 1 | #Feature | #Feature Sample 1 | Start | End | PTM | AScore | Found By |
| --- | --- | --- | --- | --- | --- | --- | --- | --- | --- | --- | --- | --- | --- | --- | --- | --- | --- | --- | --- |
| R.ELIDHLIR.D | Y | 22.02 | 1007.5763 | 8 | -0.07 | 504.7932 | 2 | 19.91 | 0.8652 | 0.8725 | 70328 | 3.38e3 | 1 | 1 | 164 | 171 |  |  | DB Search |
| total 1 peptides |  |  |  |  |  |  |  |  |  |  |  |  |  |  |  |  |  |  |  |
| --- | --- | --- | --- | --- | --- | --- | --- | --- | --- | --- | --- | --- | --- | --- | --- | --- | --- | --- | --- |

Best Unique PSM (Scan Sample\_10\_A1\_1\_3647.d:70328, m/z=504.7932, z=2, RT=19.91, ppm=-4.56):


TRINITY\_DN2142\_c0\_g1\_i2.p2

back to list

  

| Protein Coverage
| Supporting Peptides
| Best Unique PSM
|

Protein Coverage:

Supporting Peptides:

| Peptide | Uniq | -10lgP | Mass | Length | ppm | m/z | z | RT | 1/k0 Start | 1/k0 End | Precursor ID | Area Sample 1 | #Feature | #Feature Sample 1 | Start | End | PTM | AScore | Found By |
| --- | --- | --- | --- | --- | --- | --- | --- | --- | --- | --- | --- | --- | --- | --- | --- | --- | --- | --- | --- |
| R.ERQENTNPVTR.Y | Y | 37.82 | 1342.6589 | 11 | 1.48 | 672.3347 | 2 | 9.98 | 0.9558 | 0.963 | 13356 | 6.23e2 | 1 | 1 | 172 | 182 |  |  | DB Search |
| total 1 peptides |  |  |  |  |  |  |  |  |  |  |  |  |  |  |  |  |  |  |  |
| --- | --- | --- | --- | --- | --- | --- | --- | --- | --- | --- | --- | --- | --- | --- | --- | --- | --- | --- | --- |

Best Unique PSM (Scan Sample\_10\_A1\_1\_3647.d:13356, m/z=672.3347, z=2, RT=9.98, ppm=-3.01):


TRINITY\_DN3545\_c0\_g1\_i1.p1

back to list

  

| Protein Coverage
| Supporting Peptides
| Best Unique PSM
|

Protein Coverage:

Supporting Peptides:

| Peptide | Uniq | -10lgP | Mass | Length | ppm | m/z | z | RT | 1/k0 Start | 1/k0 End | Precursor ID | Area Sample 1 | #Feature | #Feature Sample 1 | Start | End | PTM | AScore | Found By |
| --- | --- | --- | --- | --- | --- | --- | --- | --- | --- | --- | --- | --- | --- | --- | --- | --- | --- | --- | --- |
| R.SSC(+57.02)RPILSVIK.I | Y | 49.81 | 1258.7067 | 11 | -8.86 | 630.3522 | 2 | 16.81 | 0.9691 | 0.9763 | 51630 | 2.1e3 | 1 | 1 | 6 | 16 | Carbamidomethylation |  | DB Search |
| R.SC(+57.02)TNPAPANGGK.P | Y | 39.93 | 1172.5244 | 12 | 0 | 587.2668 | 2 | 9.43 | 0.8404 | 0.8477 | 10729 | 0 | 0 | 0 | 55 | 66 | Carbamidomethylation |  | DB Search |
| total 2 peptides |  |  |  |  |  |  |  |  |  |  |  |  |  |  |  |  |  |  |  |
| --- | --- | --- | --- | --- | --- | --- | --- | --- | --- | --- | --- | --- | --- | --- | --- | --- | --- | --- | --- |

Best Unique PSM (Scan Sample\_10\_A1\_1\_3647.d:51630, m/z=630.3522, z=2, RT=16.81, ppm=-13.35):


TRINITY\_DN3339\_c0\_g1\_i1.p1

back to list

  

| Protein Coverage
| Supporting Peptides
| Best Unique PSM
|

Protein Coverage:

Supporting Peptides:

| Peptide | Uniq | -10lgP | Mass | Length | ppm | m/z | z | RT | 1/k0 Start | 1/k0 End | Precursor ID | Area Sample 1 | #Feature | #Feature Sample 1 | Start | End | PTM | AScore | Found By |
| --- | --- | --- | --- | --- | --- | --- | --- | --- | --- | --- | --- | --- | --- | --- | --- | --- | --- | --- | --- |
| K.HGYIGEFEVIDDHR.S | Y | 66.45 | 1685.7797 | 14 | 0.12 | 562.9314 | 3 | 16.03 | 0.8716 | 0.8789 | 47611 | 9.53e2 | 2 | 2 | 44 | 57 |  |  | DB Search |
| M.VRVNVLNDALNSIC(+57.02)NAEKR.G | Y | 63.58 | 2184.1433 | 19 | -8.4 | 729.0457 | 3 | 18.74 | 0.9828 | 0.99 | 63282 | 0 | 0 | 0 | 2 | 20 | Carbamidomethylation |  | DB Search |
| total 2 peptides |  |  |  |  |  |  |  |  |  |  |  |  |  |  |  |  |  |  |  |
| --- | --- | --- | --- | --- | --- | --- | --- | --- | --- | --- | --- | --- | --- | --- | --- | --- | --- | --- | --- |

Best Unique PSM (Scan Sample\_10\_A1\_1\_3647.d:47611, m/z=562.9314, z=3, RT=16.03, ppm=-4.38):


TRINITY\_DN5107\_c0\_g1\_i6.p1

back to list

  

| Protein Coverage
| Supporting Peptides
| Best Unique PSM
|

Protein Coverage:

Supporting Peptides:

| Peptide | Uniq | -10lgP | Mass | Length | ppm | m/z | z | RT | 1/k0 Start | 1/k0 End | Precursor ID | Area Sample 1 | #Feature | #Feature Sample 1 | Start | End | PTM | AScore | Found By |
| --- | --- | --- | --- | --- | --- | --- | --- | --- | --- | --- | --- | --- | --- | --- | --- | --- | --- | --- | --- |
| K.KLDYVLGLTTAK.I | Y | 63.57 | 1320.7653 | 12 | -2.37 | 661.3854 | 2 | 16.69 | 0.9908 | 0.9981 | 51148 | 2.13e2 | 1 | 1 | 114 | 125 |  |  | DB Search |
| total 1 peptides |  |  |  |  |  |  |  |  |  |  |  |  |  |  |  |  |  |  |  |
| --- | --- | --- | --- | --- | --- | --- | --- | --- | --- | --- | --- | --- | --- | --- | --- | --- | --- | --- | --- |

Best Unique PSM (Scan Sample\_10\_A1\_1\_3647.d:51148, m/z=661.3854, z=2, RT=16.69, ppm=-6.87):


TRINITY\_DN1272\_c0\_g1\_i1.p1

back to list

  

| Protein Coverage
| Supporting Peptides
| Best Unique PSM
|

Protein Coverage:

Supporting Peptides:

| Peptide | Uniq | -10lgP | Mass | Length | ppm | m/z | z | RT | 1/k0 Start | 1/k0 End | Precursor ID | Area Sample 1 | #Feature | #Feature Sample 1 | Start | End | PTM | AScore | Found By |
| --- | --- | --- | --- | --- | --- | --- | --- | --- | --- | --- | --- | --- | --- | --- | --- | --- | --- | --- | --- |
| K.YIQGENEKQEK.I | Y | 66.74 | 1364.6572 | 11 | -0.74 | 455.8906 | 3 | 9.56 | 0.7697 | 0.777 | 11206 | 9.07e2 | 1 | 1 | 116 | 126 |  |  | DB Search |
| total 1 peptides |  |  |  |  |  |  |  |  |  |  |  |  |  |  |  |  |  |  |  |
| --- | --- | --- | --- | --- | --- | --- | --- | --- | --- | --- | --- | --- | --- | --- | --- | --- | --- | --- | --- |

Best Unique PSM (Scan Sample\_10\_A1\_1\_3647.d:11206, m/z=455.8906, z=3, RT=9.56, ppm=-5.23):


TRINITY\_DN1112\_c0\_g1\_i1.p1

back to list

  

| Protein Coverage
| Supporting Peptides
| Best Unique PSM
|

Protein Coverage:

Supporting Peptides:

| Peptide | Uniq | -10lgP | Mass | Length | ppm | m/z | z | RT | 1/k0 Start | 1/k0 End | Precursor ID | Area Sample 1 | #Feature | #Feature Sample 1 | Start | End | PTM | AScore | Found By |
| --- | --- | --- | --- | --- | --- | --- | --- | --- | --- | --- | --- | --- | --- | --- | --- | --- | --- | --- | --- |
| K.KIDLHLYHELYM(+15.99)K.A | Y | 66.77 | 1717.8861 | 13 | -3.92 | 859.9431 | 2 | 15.60 | 1.1414 | 1.1486 | 45433 | 4.78e2 | 1 | 1 | 114 | 126 | Oxidation (M) | M12:Oxidation (M):1000 | DB Search |
| K.IDLHLYHELYM(+15.99)K.A | Y | 63.13 | 1589.7912 | 12 | -2.23 | 795.8975 | 2 | 15.63 | 1.0953 | 1.1025 | 45494 | 5.53e2 | 1 | 1 | 115 | 126 | Oxidation (M) | M11:Oxidation (M):1000 | DB Search |
| R.SKELSDQAEAR.R | Y | 48.59 | 1232.5997 | 11 | 0.56 | 617.3047 | 2 | 10.16 | 0.9372 | 0.9444 | 14479 | 1.07e3 | 1 | 1 | 152 | 162 |  |  | DB Search |
| total 3 peptides |  |  |  |  |  |  |  |  |  |  |  |  |  |  |  |  |  |  |  |
| --- | --- | --- | --- | --- | --- | --- | --- | --- | --- | --- | --- | --- | --- | --- | --- | --- | --- | --- | --- |

Best Unique PSM (Scan Sample\_10\_A1\_1\_3647.d:45433, m/z=859.9431, z=2, RT=15.60, ppm=-8.41):


TRINITY\_DN161\_c0\_g1\_i2.p1

back to list

  

| Protein Coverage
| Supporting Peptides
| Best Unique PSM
|

Protein Coverage:

Supporting Peptides:

| Peptide | Uniq | -10lgP | Mass | Length | ppm | m/z | z | RT | 1/k0 Start | 1/k0 End | Precursor ID | Area Sample 1 | #Feature | #Feature Sample 1 | Start | End | PTM | AScore | Found By |
| --- | --- | --- | --- | --- | --- | --- | --- | --- | --- | --- | --- | --- | --- | --- | --- | --- | --- | --- | --- |
| R.AVFPSIVGRPR.H | Y | 66.75 | 1197.6982 | 11 | -0.27 | 599.8535 | 2 | 15.64 | 0.9307 | 0.938 | 45498 | 3.34e4 | 1 | 1 | 52 | 62 |  |  | DB Search |
| K.DSYVGDEAQSKR.G | Y | 66.73 | 1353.616 | 12 | 2.52 | 677.814 | 2 | 10.94 | 1.0069 | 1.0142 | 19045 | 5.51e3 | 1 | 1 | 74 | 85 |  |  | DB Search |
| K.SYELPDGQVITIGNER.F | Y | 66.69 | 1789.8846 | 16 | -3.48 | 895.9424 | 2 | 18.53 | 0.9795 | 0.9868 | 61913 | 1.88e3 | 2 | 2 | 262 | 277 |  |  | DB Search |
| R.LDLAGRDLTDYLM(+15.99)K.I | Y | 62.87 | 1638.8287 | 14 | 4.29 | 820.4214 | 2 | 18.15 | 1.143 | 1.1502 | 59617 | 0 | 0 | 0 | 201 | 214 | Oxidation (M) | M13:Oxidation (M):1000 | DB Search |
| R.HQGVM(+15.99)VGM(+15.99)GQK.D | Y | 61.11 | 1202.5536 | 11 | 7.61 | 602.2859 | 2 | 10.70 | 0.9089 | 0.9162 | 17721 | 0 | 0 | 0 | 63 | 73 | Oxidation (M), Oxidation (M) | M5:Oxidation (M):1000 M8:Oxidation (M):1000 | DB Search |
| R.LDLAGRDLTDYLMK.I | Y | 27.85 | 1622.8338 | 14 | -0.39 | 541.9492 | 3 | 19.26 | 0.7974 | 0.8047 | 66371 | 6.87e2 | 1 | 1 | 201 | 214 |  |  | DB Search |
| total 6 peptides |  |  |  |  |  |  |  |  |  |  |  |  |  |  |  |  |  |  |  |
| --- | --- | --- | --- | --- | --- | --- | --- | --- | --- | --- | --- | --- | --- | --- | --- | --- | --- | --- | --- |

Best Unique PSM (Scan Sample\_10\_A1\_1\_3647.d:45498, m/z=599.8535, z=2, RT=15.64, ppm=-4.77):


TRINITY\_DN2477\_c0\_g1\_i5.p1

back to list

  

| Protein Coverage
| Supporting Peptides
| Best Unique PSM
|

Protein Coverage:

Supporting Peptides:

| Peptide | Uniq | -10lgP | Mass | Length | ppm | m/z | z | RT | 1/k0 Start | 1/k0 End | Precursor ID | Area Sample 1 | #Feature | #Feature Sample 1 | Start | End | PTM | AScore | Found By |
| --- | --- | --- | --- | --- | --- | --- | --- | --- | --- | --- | --- | --- | --- | --- | --- | --- | --- | --- | --- |
| K.DSYVGDEAQSKR.G | Y | 66.73 | 1353.616 | 12 | 2.52 | 677.814 | 2 | 10.94 | 1.0069 | 1.0142 | 19045 | 5.51e3 | 1 | 1 | 8 | 19 |  |  | DB Search |
| total 1 peptides |  |  |  |  |  |  |  |  |  |  |  |  |  |  |  |  |  |  |  |
| --- | --- | --- | --- | --- | --- | --- | --- | --- | --- | --- | --- | --- | --- | --- | --- | --- | --- | --- | --- |

Best Unique PSM (Scan Sample\_10\_A1\_1\_3647.d:19045, m/z=677.814, z=2, RT=10.94, ppm=-1.97):


TRINITY\_DN2626\_c0\_g1\_i1.p1

back to list

  

| Protein Coverage
| Supporting Peptides
| Best Unique PSM
|

Protein Coverage:

Supporting Peptides:

| Peptide | Uniq | -10lgP | Mass | Length | ppm | m/z | z | RT | 1/k0 Start | 1/k0 End | Precursor ID | Area Sample 1 | #Feature | #Feature Sample 1 | Start | End | PTM | AScore | Found By |
| --- | --- | --- | --- | --- | --- | --- | --- | --- | --- | --- | --- | --- | --- | --- | --- | --- | --- | --- | --- |
| R.D(+42.01)GPIVR.L | Y | 18.66 | 697.3759 | 6 | -4.48 | 349.6921 | 2 | 11.01 | 0.7587 | 0.7661 | 19714 | 2e2 | 1 | 1 | 10 | 15 | Acetylation (Protein N-term) | D1:Acetylation (Protein N-term):1000 | DB Search |
| total 1 peptides |  |  |  |  |  |  |  |  |  |  |  |  |  |  |  |  |  |  |  |
| --- | --- | --- | --- | --- | --- | --- | --- | --- | --- | --- | --- | --- | --- | --- | --- | --- | --- | --- | --- |

Best Unique PSM (Scan Sample\_10\_A1\_1\_3647.d:19714, m/z=349.6921, z=2, RT=11.01, ppm=-8.98):


TRINITY\_DN7671\_c0\_g1\_i1.p1

back to list

  

| Protein Coverage
| Supporting Peptides
| Best Unique PSM
|

Protein Coverage:

Supporting Peptides:

| Peptide | Uniq | -10lgP | Mass | Length | ppm | m/z | z | RT | 1/k0 Start | 1/k0 End | Precursor ID | Area Sample 1 | #Feature | #Feature Sample 1 | Start | End | PTM | AScore | Found By |
| --- | --- | --- | --- | --- | --- | --- | --- | --- | --- | --- | --- | --- | --- | --- | --- | --- | --- | --- | --- |
| K.LSYC(+57.02)VSC(+57.02)AIHSK.V | Y | 66.67 | 1423.6588 | 12 | 2.43 | 712.8352 | 2 | 14.01 | 1.0315 | 1.0387 | 36741 | 1.87e3 | 1 | 1 | 71 | 82 | Carbamidomethylation, Carbamidomethylation |  | DB Search |
| total 1 peptides |  |  |  |  |  |  |  |  |  |  |  |  |  |  |  |  |  |  |  |
| --- | --- | --- | --- | --- | --- | --- | --- | --- | --- | --- | --- | --- | --- | --- | --- | --- | --- | --- | --- |

Best Unique PSM (Scan Sample\_10\_A1\_1\_3647.d:36741, m/z=712.8352, z=2, RT=14.01, ppm=-2.07):


TRINITY\_DN2472\_c0\_g1\_i1.p1

back to list

  

| Protein Coverage
| Supporting Peptides
| Best Unique PSM
|

Protein Coverage:

Supporting Peptides:

| Peptide | Uniq | -10lgP | Mass | Length | ppm | m/z | z | RT | 1/k0 Start | 1/k0 End | Precursor ID | Area Sample 1 | #Feature | #Feature Sample 1 | Start | End | PTM | AScore | Found By |
| --- | --- | --- | --- | --- | --- | --- | --- | --- | --- | --- | --- | --- | --- | --- | --- | --- | --- | --- | --- |
| M.S(+42.01)SGEIESLRQEAENLR.N | Y | 66.62 | 1858.9021 | 16 | 0.69 | 930.4548 | 2 | 18.69 | 1.1973 | 1.2045 | 62988 | 1.64e3 | 1 | 1 | 2 | 17 | Acetylation (Protein N-term) | S1:Acetylation (Protein N-term):1000 | DB Search |
| total 1 peptides |  |  |  |  |  |  |  |  |  |  |  |  |  |  |  |  |  |  |  |
| --- | --- | --- | --- | --- | --- | --- | --- | --- | --- | --- | --- | --- | --- | --- | --- | --- | --- | --- | --- |

Best Unique PSM (Scan Sample\_10\_A1\_1\_3647.d:62988, m/z=930.4548, z=2, RT=18.69, ppm=-3.8):


TRINITY\_DN7659\_c0\_g1\_i2.p1

back to list

  

| Protein Coverage
| Supporting Peptides
| Best Unique PSM
|

Protein Coverage:

Supporting Peptides:

| Peptide | Uniq | -10lgP | Mass | Length | ppm | m/z | z | RT | 1/k0 Start | 1/k0 End | Precursor ID | Area Sample 1 | #Feature | #Feature Sample 1 | Start | End | PTM | AScore | Found By |
| --- | --- | --- | --- | --- | --- | --- | --- | --- | --- | --- | --- | --- | --- | --- | --- | --- | --- | --- | --- |
| M.A(+42.01)SAATDNRK.V | Y | 63.17 | 974.4781 | 9 | -3.25 | 488.2426 | 2 | 9.45 | 0.8205 | 0.8279 | 10765 | 0 | 0 | 0 | 2 | 10 | Acetylation (Protein N-term) | A1:Acetylation (Protein N-term):1000 | DB Search |
| K.AQPTEIYNLGAQSHVK.V | Y | 35.72 | 1754.8951 | 16 | 0.32 | 878.4512 | 2 | 14.67 | 1.1722 | 1.1794 | 40344 | 0 | 0 | 0 | 86 | 101 |  |  | DB Search |
| total 2 peptides |  |  |  |  |  |  |  |  |  |  |  |  |  |  |  |  |  |  |  |
| --- | --- | --- | --- | --- | --- | --- | --- | --- | --- | --- | --- | --- | --- | --- | --- | --- | --- | --- | --- |

Best Unique PSM (Scan Sample\_10\_A1\_1\_3647.d:10765, m/z=488.2426, z=2, RT=9.45, ppm=-7.75):


TRINITY\_DN15016\_c0\_g1\_i3.p1

back to list

  

| Protein Coverage
| Supporting Peptides
| Best Unique PSM
|

Protein Coverage:

Supporting Peptides:

| Peptide | Uniq | -10lgP | Mass | Length | ppm | m/z | z | RT | 1/k0 Start | 1/k0 End | Precursor ID | Area Sample 1 | #Feature | #Feature Sample 1 | Start | End | PTM | AScore | Found By |
| --- | --- | --- | --- | --- | --- | --- | --- | --- | --- | --- | --- | --- | --- | --- | --- | --- | --- | --- | --- |
| K.AFFDLHFVGTGAK.F | Y | 66.68 | 1408.7139 | 13 | -4.47 | 705.3579 | 2 | 19.23 | 1.0371 | 1.0443 | 66248 | 0 | 0 | 0 | 96 | 108 |  |  | DB Search |
| total 1 peptides |  |  |  |  |  |  |  |  |  |  |  |  |  |  |  |  |  |  |  |
| --- | --- | --- | --- | --- | --- | --- | --- | --- | --- | --- | --- | --- | --- | --- | --- | --- | --- | --- | --- |

Best Unique PSM (Scan Sample\_10\_A1\_1\_3647.d:66248, m/z=705.3579, z=2, RT=19.23, ppm=-8.96):


TRINITY\_DN1479\_c0\_g1\_i1.p1

back to list

  

| Protein Coverage
| Supporting Peptides
| Best Unique PSM
|

Protein Coverage:

Supporting Peptides:

| Peptide | Uniq | -10lgP | Mass | Length | ppm | m/z | z | RT | 1/k0 Start | 1/k0 End | Precursor ID | Area Sample 1 | #Feature | #Feature Sample 1 | Start | End | PTM | AScore | Found By |
| --- | --- | --- | --- | --- | --- | --- | --- | --- | --- | --- | --- | --- | --- | --- | --- | --- | --- | --- | --- |
| K.EC(+57.02)VTLEYDFIYK.F | Y | 41.22 | 1578.7276 | 12 | -0.88 | 790.3668 | 2 | 19.93 | 1.0552 | 1.0624 | 70514 | 5.43e2 | 1 | 1 | 40 | 51 | Carbamidomethylation |  | DB Search |
| total 1 peptides |  |  |  |  |  |  |  |  |  |  |  |  |  |  |  |  |  |  |  |
| --- | --- | --- | --- | --- | --- | --- | --- | --- | --- | --- | --- | --- | --- | --- | --- | --- | --- | --- | --- |

Best Unique PSM (Scan Sample\_10\_A1\_1\_3647.d:70514, m/z=790.3668, z=2, RT=19.93, ppm=-5.37):


TRINITY\_DN36\_c0\_g1\_i13.p1

back to list

  

| Protein Coverage
| Supporting Peptides
| Best Unique PSM
|

Protein Coverage:

Supporting Peptides:

| Peptide | Uniq | -10lgP | Mass | Length | ppm | m/z | z | RT | 1/k0 Start | 1/k0 End | Precursor ID | Area Sample 1 | #Feature | #Feature Sample 1 | Start | End | PTM | AScore | Found By |
| --- | --- | --- | --- | --- | --- | --- | --- | --- | --- | --- | --- | --- | --- | --- | --- | --- | --- | --- | --- |
| K.GFDYC(+57.02)TTIKYR.L | Y | 66.92 | 1422.6602 | 11 | -0.86 | 712.3336 | 2 | 15.08 | 1.023 | 1.0303 | 42693 | 0 | 0 | 0 | 46 | 56 | Carbamidomethylation |  | DB Search |
| R.LGGIEVTQGNC(+57.02)AR.K | Y | 66.48 | 1373.6721 | 13 | 5.18 | 687.8438 | 2 | 13.73 | 0.963 | 0.9703 | 35192 | 0 | 0 | 0 | 57 | 69 | Carbamidomethylation |  | DB Search |
| K.WC(+57.02)DILSIHQSMENC(+57.02)VAK.C | Y | 63.98 | 2089.9383 | 17 | -2.35 | 697.6486 | 3 | 17.89 | 0.8777 | 0.885 | 58058 | 0 | 0 | 0 | 197 | 213 | Carbamidomethylation, Carbamidomethylation |  | DB Search |
| K.FETQIATK.D | Y | 61.61 | 936.4916 | 8 | 0.97 | 937.4956 | 1 | 13.84 | 1.2037 | 1.2109 | 35727 | 1.28e3 | 1 | 1 | 166 | 173 |  |  | DB Search |
| R.LGGIEVTQGNC(+57.02)ARK.Y | Y | 31.04 | 1501.7671 | 14 | -2.67 | 751.8854 | 2 | 12.64 | 1.0624 | 1.0697 | 29000 | 0 | 0 | 0 | 57 | 70 | Carbamidomethylation |  | DB Search |
| total 5 peptides |  |  |  |  |  |  |  |  |  |  |  |  |  |  |  |  |  |  |  |
| --- | --- | --- | --- | --- | --- | --- | --- | --- | --- | --- | --- | --- | --- | --- | --- | --- | --- | --- | --- |

Best Unique PSM (Scan Sample\_10\_A1\_1\_3647.d:42693, m/z=712.3336, z=2, RT=15.08, ppm=-5.35):


TRINITY\_DN2626\_c0\_g1\_i7.p2

back to list

  

| Protein Coverage
| Supporting Peptides
| Best Unique PSM
|

Protein Coverage:

Supporting Peptides:

| Peptide | Uniq | -10lgP | Mass | Length | ppm | m/z | z | RT | 1/k0 Start | 1/k0 End | Precursor ID | Area Sample 1 | #Feature | #Feature Sample 1 | Start | End | PTM | AScore | Found By |
| --- | --- | --- | --- | --- | --- | --- | --- | --- | --- | --- | --- | --- | --- | --- | --- | --- | --- | --- | --- |
| R.D(+42.01)GPIVR.L | Y | 18.66 | 697.3759 | 6 | -4.48 | 349.6921 | 2 | 11.01 | 0.7587 | 0.7661 | 19714 | 2e2 | 1 | 1 | 10 | 15 | Acetylation (Protein N-term) | D1:Acetylation (Protein N-term):1000 | DB Search |
| total 1 peptides |  |  |  |  |  |  |  |  |  |  |  |  |  |  |  |  |  |  |  |
| --- | --- | --- | --- | --- | --- | --- | --- | --- | --- | --- | --- | --- | --- | --- | --- | --- | --- | --- | --- |

Best Unique PSM (Scan Sample\_10\_A1\_1\_3647.d:19714, m/z=349.6921, z=2, RT=11.01, ppm=-8.98):


TRINITY\_DN96161\_c0\_g1\_i1.p1

back to list

  

| Protein Coverage
| Supporting Peptides
| Best Unique PSM
|

Protein Coverage:

Supporting Peptides:

| Peptide | Uniq | -10lgP | Mass | Length | ppm | m/z | z | RT | 1/k0 Start | 1/k0 End | Precursor ID | Area Sample 1 | #Feature | #Feature Sample 1 | Start | End | PTM | AScore | Found By |
| --- | --- | --- | --- | --- | --- | --- | --- | --- | --- | --- | --- | --- | --- | --- | --- | --- | --- | --- | --- |
| K.VGINYQPPTVVPGGDLAK.V | Y | 64.86 | 1823.9781 | 18 | -3.39 | 912.9891 | 2 | 17.23 | 1.1198 | 1.127 | 54086 | 0 | 0 | 0 | 353 | 370 |  |  | DB Search |
| R.AFVHWYVGEGM(+15.99)EEGEFSEAR.E | Y | 44.9 | 2345.0058 | 20 | -0.18 | 782.6722 | 3 | 17.81 | 0.9251 | 0.9323 | 57560 | 6.2e2 | 1 | 1 | 403 | 422 | Oxidation (M) | M11:Oxidation (M):1000 | DB Search |
| total 2 peptides |  |  |  |  |  |  |  |  |  |  |  |  |  |  |  |  |  |  |  |
| --- | --- | --- | --- | --- | --- | --- | --- | --- | --- | --- | --- | --- | --- | --- | --- | --- | --- | --- | --- |

Best Unique PSM (Scan Sample\_10\_A1\_1\_3647.d:54086, m/z=912.9891, z=2, RT=17.23, ppm=-7.88):


TRINITY\_DN1787\_c0\_g2\_i2.p1

back to list

  

| Protein Coverage
| Supporting Peptides
| Best Unique PSM
|

Protein Coverage:

Supporting Peptides:

| Peptide | Uniq | -10lgP | Mass | Length | ppm | m/z | z | RT | 1/k0 Start | 1/k0 End | Precursor ID | Area Sample 1 | #Feature | #Feature Sample 1 | Start | End | PTM | AScore | Found By |
| --- | --- | --- | --- | --- | --- | --- | --- | --- | --- | --- | --- | --- | --- | --- | --- | --- | --- | --- | --- |
| K.YC(+57.02)HLFLC(+57.02)VHC(+57.02)LK.E | Y | 66.68 | 1648.7676 | 12 | -5.26 | 825.3831 | 2 | 16.15 | 1.1098 | 1.117 | 48282 | 9.73e2 | 2 | 2 | 66 | 77 | Carbamidomethylation, Carbamidomethylation, Carbamidomethylation |  | DB Search |
| total 1 peptides |  |  |  |  |  |  |  |  |  |  |  |  |  |  |  |  |  |  |  |
| --- | --- | --- | --- | --- | --- | --- | --- | --- | --- | --- | --- | --- | --- | --- | --- | --- | --- | --- | --- |

Best Unique PSM (Scan Sample\_10\_A1\_1\_3647.d:48282, m/z=825.3831, z=2, RT=16.15, ppm=-9.75):


TRINITY\_DN240\_c0\_g1\_i4.p1

back to list

  

| Protein Coverage
| Supporting Peptides
| Best Unique PSM
|

Protein Coverage:

Supporting Peptides:

| Peptide | Uniq | -10lgP | Mass | Length | ppm | m/z | z | RT | 1/k0 Start | 1/k0 End | Precursor ID | Area Sample 1 | #Feature | #Feature Sample 1 | Start | End | PTM | AScore | Found By |
| --- | --- | --- | --- | --- | --- | --- | --- | --- | --- | --- | --- | --- | --- | --- | --- | --- | --- | --- | --- |
| K.AYAAFLEFKDVVK.A | Y | 66.86 | 1499.8024 | 13 | 1.14 | 750.9059 | 2 | 19.02 | 1.0945 | 1.1017 | 64841 | 9.59e3 | 1 | 1 | 217 | 229 |  |  | DB Search |
| K.AAASAHHQAIGSIDAAGVTSAADYASVTAALGR.A | Y | 62.75 | 3080.5275 | 33 | 0.95 | 771.1364 | 4 | 18.15 | 1.0636 | 1.0709 | 59524 | 1.07e3 | 1 | 1 | 307 | 339 |  |  | DB Search |
| K.IGAAAQALADQSYPFLK.S | Y | 61.76 | 1762.9253 | 17 | 1.13 | 882.467 | 2 | 19.07 | 1.161 | 1.1682 | 65059 | 6.63e2 | 1 | 1 | 249 | 265 |  |  | DB Search |
| total 3 peptides |  |  |  |  |  |  |  |  |  |  |  |  |  |  |  |  |  |  |  |
| --- | --- | --- | --- | --- | --- | --- | --- | --- | --- | --- | --- | --- | --- | --- | --- | --- | --- | --- | --- |

Best Unique PSM (Scan Sample\_10\_A1\_1\_3647.d:64841, m/z=750.9059, z=2, RT=19.02, ppm=-3.35):


TRINITY\_DN801\_c0\_g1\_i4.p1

back to list

  

| Protein Coverage
| Supporting Peptides
| Best Unique PSM
|

Protein Coverage:

Supporting Peptides:

| Peptide | Uniq | -10lgP | Mass | Length | ppm | m/z | z | RT | 1/k0 Start | 1/k0 End | Precursor ID | Area Sample 1 | #Feature | #Feature Sample 1 | Start | End | PTM | AScore | Found By |
| --- | --- | --- | --- | --- | --- | --- | --- | --- | --- | --- | --- | --- | --- | --- | --- | --- | --- | --- | --- |
| K.VFDRDGNGFISAAELR.H | Y | 39.11 | 1765.8747 | 16 | -1.5 | 883.9393 | 2 | 19.71 | 1.1234 | 1.1306 | 69271 | 0 | 0 | 0 | 124 | 139 |  |  | DB Search |
| total 1 peptides |  |  |  |  |  |  |  |  |  |  |  |  |  |  |  |  |  |  |  |
| --- | --- | --- | --- | --- | --- | --- | --- | --- | --- | --- | --- | --- | --- | --- | --- | --- | --- | --- | --- |

Best Unique PSM (Scan Sample\_10\_A1\_1\_3647.d:69271, m/z=883.9393, z=2, RT=19.71, ppm=-6):


TRINITY\_DN167719\_c0\_g1\_i1.p1

back to list

  

| Protein Coverage
| Supporting Peptides
| Best Unique PSM
|

Protein Coverage:

Supporting Peptides:

| Peptide | Uniq | -10lgP | Mass | Length | ppm | m/z | z | RT | 1/k0 Start | 1/k0 End | Precursor ID | Area Sample 1 | #Feature | #Feature Sample 1 | Start | End | PTM | AScore | Found By |
| --- | --- | --- | --- | --- | --- | --- | --- | --- | --- | --- | --- | --- | --- | --- | --- | --- | --- | --- | --- |
| R.LTVC(+57.02)VTVVHHGLQK.L | Y | 66.78 | 1589.8712 | 14 | 7.51 | 795.9453 | 2 | 13.99 | 1.1158 | 1.123 | 36644 | 0 | 0 | 0 | 32 | 45 | Carbamidomethylation |  | DB Search |
| total 1 peptides |  |  |  |  |  |  |  |  |  |  |  |  |  |  |  |  |  |  |  |
| --- | --- | --- | --- | --- | --- | --- | --- | --- | --- | --- | --- | --- | --- | --- | --- | --- | --- | --- | --- |

Best Unique PSM (Scan Sample\_10\_A1\_1\_3647.d:36644, m/z=795.9453, z=2, RT=13.99, ppm=3.02):


TRINITY\_DN13856\_c0\_g1\_i2.p2

back to list

  

| Protein Coverage
| Supporting Peptides
| Best Unique PSM
|

Protein Coverage:

Supporting Peptides:

| Peptide | Uniq | -10lgP | Mass | Length | ppm | m/z | z | RT | 1/k0 Start | 1/k0 End | Precursor ID | Area Sample 1 | #Feature | #Feature Sample 1 | Start | End | PTM | AScore | Found By |
| --- | --- | --- | --- | --- | --- | --- | --- | --- | --- | --- | --- | --- | --- | --- | --- | --- | --- | --- | --- |
| R.IPLDDFQDPTLTLENAHNK.L | Y | 61.84 | 2180.0749 | 19 | 2.14 | 727.6972 | 3 | 18.19 | 0.9896 | 0.9969 | 59846 | 3.57e2 | 1 | 1 | 175 | 193 |  |  | DB Search |
| total 1 peptides |  |  |  |  |  |  |  |  |  |  |  |  |  |  |  |  |  |  |  |
| --- | --- | --- | --- | --- | --- | --- | --- | --- | --- | --- | --- | --- | --- | --- | --- | --- | --- | --- | --- |

Best Unique PSM (Scan Sample\_10\_A1\_1\_3647.d:59846, m/z=727.6972, z=3, RT=18.19, ppm=-2.35):


TRINITY\_DN585\_c0\_g1\_i10.p1

back to list

  

| Protein Coverage
| Supporting Peptides
| Best Unique PSM
|

Protein Coverage:

Supporting Peptides:

| Peptide | Uniq | -10lgP | Mass | Length | ppm | m/z | z | RT | 1/k0 Start | 1/k0 End | Precursor ID | Area Sample 1 | #Feature | #Feature Sample 1 | Start | End | PTM | AScore | Found By |
| --- | --- | --- | --- | --- | --- | --- | --- | --- | --- | --- | --- | --- | --- | --- | --- | --- | --- | --- | --- |
| K.IQDKEGIPPDQQR.L | Y | 65.08 | 1522.7739 | 13 | -0.66 | 762.3903 | 2 | 11.01 | 1.0339 | 1.0411 | 19352 | 9.49e2 | 1 | 1 | 30 | 42 |  |  | DB Search |
| total 1 peptides |  |  |  |  |  |  |  |  |  |  |  |  |  |  |  |  |  |  |  |
| --- | --- | --- | --- | --- | --- | --- | --- | --- | --- | --- | --- | --- | --- | --- | --- | --- | --- | --- | --- |

Best Unique PSM (Scan Sample\_10\_A1\_1\_3647.d:19352, m/z=762.3903, z=2, RT=11.01, ppm=-5.16):


TRINITY\_DN817\_c0\_g1\_i10.p1

back to list

  

| Protein Coverage
| Supporting Peptides
| Best Unique PSM
|

Protein Coverage:

Supporting Peptides:

| Peptide | Uniq | -10lgP | Mass | Length | ppm | m/z | z | RT | 1/k0 Start | 1/k0 End | Precursor ID | Area Sample 1 | #Feature | #Feature Sample 1 | Start | End | PTM | AScore | Found By |
| --- | --- | --- | --- | --- | --- | --- | --- | --- | --- | --- | --- | --- | --- | --- | --- | --- | --- | --- | --- |
| R.LC(+57.02)QPGYSTC(+57.02)ENHC(+57.02)R.R | Y | 62.01 | 1780.708 | 14 | 5.39 | 891.3621 | 2 | 11.41 | 1.0893 | 1.0965 | 21785 | 1.84e2 | 1 | 1 | 155 | 168 | Carbamidomethylation, Carbamidomethylation, Carbamidomethylation |  | DB Search |
| total 1 peptides |  |  |  |  |  |  |  |  |  |  |  |  |  |  |  |  |  |  |  |
| --- | --- | --- | --- | --- | --- | --- | --- | --- | --- | --- | --- | --- | --- | --- | --- | --- | --- | --- | --- |

Best Unique PSM (Scan Sample\_10\_A1\_1\_3647.d:21785, m/z=891.3621, z=2, RT=11.41, ppm=0.9):


TRINITY\_DN34\_c0\_g1\_i1.p1

back to list

  

| Protein Coverage
| Supporting Peptides
| Best Unique PSM
|

Protein Coverage:

Supporting Peptides:

| Peptide | Uniq | -10lgP | Mass | Length | ppm | m/z | z | RT | 1/k0 Start | 1/k0 End | Precursor ID | Area Sample 1 | #Feature | #Feature Sample 1 | Start | End | PTM | AScore | Found By |
| --- | --- | --- | --- | --- | --- | --- | --- | --- | --- | --- | --- | --- | --- | --- | --- | --- | --- | --- | --- |
| M(+42.01)(+15.99)DAAQAAEIKK.K | Y | 49.25 | 1232.6071 | 11 | -4.25 | 617.3054 | 2 | 12.14 | 0.9477 | 0.9549 | 26098 | 0 | 0 | 0 | 1 | 11 | Acetylation (Protein N-term), Oxidation (M) | M1:Acetylation (Protein N-term):1000 M1:Oxidation (M):1000 | DB Search |
| total 1 peptides |  |  |  |  |  |  |  |  |  |  |  |  |  |  |  |  |  |  |  |
| --- | --- | --- | --- | --- | --- | --- | --- | --- | --- | --- | --- | --- | --- | --- | --- | --- | --- | --- | --- |

Best Unique PSM (Scan Sample\_10\_A1\_1\_3647.d:26098, m/z=617.3054, z=2, RT=12.14, ppm=-8.74):


TRINITY\_DN36\_c0\_g1\_i1.p1

back to list

  

| Protein Coverage
| Supporting Peptides
| Best Unique PSM
|

Protein Coverage:

Supporting Peptides:

| Peptide | Uniq | -10lgP | Mass | Length | ppm | m/z | z | RT | 1/k0 Start | 1/k0 End | Precursor ID | Area Sample 1 | #Feature | #Feature Sample 1 | Start | End | PTM | AScore | Found By |
| --- | --- | --- | --- | --- | --- | --- | --- | --- | --- | --- | --- | --- | --- | --- | --- | --- | --- | --- | --- |
| R.LGGIEVTQGNC(+57.02)AR.K | Y | 66.48 | 1373.6721 | 13 | 5.18 | 687.8438 | 2 | 13.73 | 0.963 | 0.9703 | 35192 | 0 | 0 | 0 | 58 | 70 | Carbamidomethylation |  | DB Search |
| R.LGGIEVTQGNC(+57.02)ARK.Y | Y | 31.04 | 1501.7671 | 14 | -2.67 | 751.8854 | 2 | 12.64 | 1.0624 | 1.0697 | 29000 | 0 | 0 | 0 | 58 | 71 | Carbamidomethylation |  | DB Search |
| total 2 peptides |  |  |  |  |  |  |  |  |  |  |  |  |  |  |  |  |  |  |  |
| --- | --- | --- | --- | --- | --- | --- | --- | --- | --- | --- | --- | --- | --- | --- | --- | --- | --- | --- | --- |

Best Unique PSM (Scan Sample\_10\_A1\_1\_3647.d:35192, m/z=687.8438, z=2, RT=13.73, ppm=0.69):


TRINITY\_DN2636\_c0\_g1\_i1.p1

back to list

  

| Protein Coverage
| Supporting Peptides
| Best Unique PSM
|

Protein Coverage:

Supporting Peptides:

| Peptide | Uniq | -10lgP | Mass | Length | ppm | m/z | z | RT | 1/k0 Start | 1/k0 End | Precursor ID | Area Sample 1 | #Feature | #Feature Sample 1 | Start | End | PTM | AScore | Found By |
| --- | --- | --- | --- | --- | --- | --- | --- | --- | --- | --- | --- | --- | --- | --- | --- | --- | --- | --- | --- |
| K.VVEKEAETER.K | Y | 66.5 | 1188.5986 | 10 | 7.91 | 397.2082 | 3 | 9.54 | 0.729 | 0.7363 | 11130 | 2.07e3 | 2 | 2 | 199 | 208 |  |  | DB Search |
| total 1 peptides |  |  |  |  |  |  |  |  |  |  |  |  |  |  |  |  |  |  |  |
| --- | --- | --- | --- | --- | --- | --- | --- | --- | --- | --- | --- | --- | --- | --- | --- | --- | --- | --- | --- |

Best Unique PSM (Scan Sample\_10\_A1\_1\_3647.d:11130, m/z=397.2082, z=3, RT=9.54, ppm=3.41):


TRINITY\_DN673\_c0\_g1\_i1.p1

back to list

  

| Protein Coverage
| Supporting Peptides
| Best Unique PSM
|

Protein Coverage:

Supporting Peptides:

| Peptide | Uniq | -10lgP | Mass | Length | ppm | m/z | z | RT | 1/k0 Start | 1/k0 End | Precursor ID | Area Sample 1 | #Feature | #Feature Sample 1 | Start | End | PTM | AScore | Found By |
| --- | --- | --- | --- | --- | --- | --- | --- | --- | --- | --- | --- | --- | --- | --- | --- | --- | --- | --- | --- |
| K.SGTGIAAM(+15.99)SVM(+15.99)RPELIMK.S | Y | 62.55 | 1922.9627 | 18 | 4.55 | 962.4887 | 2 | 15.49 | 1.273 | 1.2801 | 44794 | 1.46e2 | 1 | 1 | 58 | 75 | Oxidation (M), Oxidation (M) | M8:Oxidation (M):50.04 M11:Oxidation (M):62.39 | DB Search |
| K.SGTGIAAM(+15.99)SVMRPELIM(+15.99)K.S | Y | 62.1 | 1922.9627 | 18 | -7.32 | 962.4773 | 2 | 15.70 | 1.2364 | 1.2436 | 45909 | 1.55e2 | 1 | 1 | 58 | 75 | Oxidation (M), Oxidation (M) | M8:Oxidation (M):22.45 M17:Oxidation (M):22.09 | DB Search |
| total 2 peptides |  |  |  |  |  |  |  |  |  |  |  |  |  |  |  |  |  |  |  |
| --- | --- | --- | --- | --- | --- | --- | --- | --- | --- | --- | --- | --- | --- | --- | --- | --- | --- | --- | --- |

Best Unique PSM (Scan Sample\_10\_A1\_1\_3647.d:44794, m/z=962.4887, z=2, RT=15.49, ppm=0.06):


TRINITY\_DN1339\_c0\_g1\_i2.p1

back to list

  

| Protein Coverage
| Supporting Peptides
| Best Unique PSM
|

Protein Coverage:

Supporting Peptides:

| Peptide | Uniq | -10lgP | Mass | Length | ppm | m/z | z | RT | 1/k0 Start | 1/k0 End | Precursor ID | Area Sample 1 | #Feature | #Feature Sample 1 | Start | End | PTM | AScore | Found By |
| --- | --- | --- | --- | --- | --- | --- | --- | --- | --- | --- | --- | --- | --- | --- | --- | --- | --- | --- | --- |
| K.VITSDDPAEKEK.K | Y | 66.7 | 1330.6616 | 12 | 2.85 | 666.337 | 2 | 10.71 | 0.9836 | 0.9908 | 17367 | 7.2e3 | 1 | 1 | 160 | 171 |  |  | DB Search |
| total 1 peptides |  |  |  |  |  |  |  |  |  |  |  |  |  |  |  |  |  |  |  |
| --- | --- | --- | --- | --- | --- | --- | --- | --- | --- | --- | --- | --- | --- | --- | --- | --- | --- | --- | --- |

Best Unique PSM (Scan Sample\_10\_A1\_1\_3647.d:17367, m/z=666.337, z=2, RT=10.71, ppm=-1.65):


TRINITY\_DN471\_c0\_g1\_i11.p1

back to list

  

| Protein Coverage
| Supporting Peptides
| Best Unique PSM
|

Protein Coverage:

Supporting Peptides:

| Peptide | Uniq | -10lgP | Mass | Length | ppm | m/z | z | RT | 1/k0 Start | 1/k0 End | Precursor ID | Area Sample 1 | #Feature | #Feature Sample 1 | Start | End | PTM | AScore | Found By |
| --- | --- | --- | --- | --- | --- | --- | --- | --- | --- | --- | --- | --- | --- | --- | --- | --- | --- | --- | --- |
| R.AFVHWYVGEGM(+15.99)EEGEFSEAR.E | Y | 44.9 | 2345.0058 | 20 | -0.18 | 782.6722 | 3 | 17.81 | 0.9251 | 0.9323 | 57560 | 6.2e2 | 1 | 1 | 441 | 460 | Oxidation (M) | M11:Oxidation (M):1000 | DB Search |
| total 1 peptides |  |  |  |  |  |  |  |  |  |  |  |  |  |  |  |  |  |  |  |
| --- | --- | --- | --- | --- | --- | --- | --- | --- | --- | --- | --- | --- | --- | --- | --- | --- | --- | --- | --- |

Best Unique PSM (Scan Sample\_10\_A1\_1\_3647.d:57560, m/z=782.6722, z=3, RT=17.81, ppm=-4.67):


TRINITY\_DN160\_c0\_g1\_i2.p1

back to list

  

| Protein Coverage
| Supporting Peptides
| Best Unique PSM
|

Protein Coverage:

Supporting Peptides:

| Peptide | Uniq | -10lgP | Mass | Length | ppm | m/z | z | RT | 1/k0 Start | 1/k0 End | Precursor ID | Area Sample 1 | #Feature | #Feature Sample 1 | Start | End | PTM | AScore | Found By |
| --- | --- | --- | --- | --- | --- | --- | --- | --- | --- | --- | --- | --- | --- | --- | --- | --- | --- | --- | --- |
| K.ELVM(+15.99)HEQSR.C | Y | 66.65 | 1143.5342 | 9 | 4.42 | 572.7744 | 2 | 9.76 | 0.8895 | 0.8968 | 12266 | 8.53e2 | 1 | 1 | 1116 | 1124 | Oxidation (M) | M4:Oxidation (M):1000 | DB Search |
| R.LDFDHEAKLNMIFR.F | Y | 66.57 | 1747.8715 | 14 | 2.05 | 583.6297 | 3 | 18.33 | 0.8558 | 0.8631 | 60685 | 0 | 0 | 0 | 1009 | 1022 |  |  | DB Search |
| K.LRLDFDHEAKLNMIFR.F | Y | 64.71 | 2017.0567 | 16 | -0.61 | 673.3561 | 3 | 18.47 | 0.9449 | 0.9521 | 61552 | 3.77e3 | 2 | 2 | 1007 | 1022 |  |  | DB Search |
| K.LRLDFDHEAKLNM(+15.99)IFR.F | Y | 64.35 | 2033.0516 | 16 | 4.9 | 509.2704 | 4 | 17.76 | 0.8558 | 0.8631 | 57084 | 1.58e3 | 1 | 1 | 1007 | 1022 | Oxidation (M) | M13:Oxidation (M):1000 | DB Search |
| K.LRLDFDHEAK.L | Y | 42.61 | 1242.6356 | 10 | 8.37 | 415.2208 | 3 | 14.18 | 0.7282 | 0.7355 | 37619 | 6.56e2 | 1 | 1 | 1007 | 1016 |  |  | DB Search |
| total 5 peptides |  |  |  |  |  |  |  |  |  |  |  |  |  |  |  |  |  |  |  |
| --- | --- | --- | --- | --- | --- | --- | --- | --- | --- | --- | --- | --- | --- | --- | --- | --- | --- | --- | --- |

Best Unique PSM (Scan Sample\_10\_A1\_1\_3647.d:12266, m/z=572.7744, z=2, RT=9.76, ppm=-0.08):


TRINITY\_DN164271\_c0\_g1\_i1.p1

back to list

  

| Protein Coverage
| Supporting Peptides
| Best Unique PSM
|

Protein Coverage:

Supporting Peptides:

| Peptide | Uniq | -10lgP | Mass | Length | ppm | m/z | z | RT | 1/k0 Start | 1/k0 End | Precursor ID | Area Sample 1 | #Feature | #Feature Sample 1 | Start | End | PTM | AScore | Found By |
| --- | --- | --- | --- | --- | --- | --- | --- | --- | --- | --- | --- | --- | --- | --- | --- | --- | --- | --- | --- |
| M.A(+42.01)TGIKPDPQC(+57.02)TDLYNK.L | Y | 61.48 | 1861.888 | 16 | -0.79 | 931.9464 | 2 | 14.86 | 1.1614 | 1.1686 | 41244 | 3.84e2 | 1 | 1 | 2 | 17 | Acetylation (Protein N-term), Carbamidomethylation | A1:Acetylation (Protein N-term):1000 | DB Search |
| total 1 peptides |  |  |  |  |  |  |  |  |  |  |  |  |  |  |  |  |  |  |  |
| --- | --- | --- | --- | --- | --- | --- | --- | --- | --- | --- | --- | --- | --- | --- | --- | --- | --- | --- | --- |

Best Unique PSM (Scan Sample\_10\_A1\_1\_3647.d:41244, m/z=931.9464, z=2, RT=14.86, ppm=-5.29):


TRINITY\_DN1910\_c0\_g1\_i5.p1

back to list

  

| Protein Coverage
| Supporting Peptides
| Best Unique PSM
|

Protein Coverage:

Supporting Peptides:

| Peptide | Uniq | -10lgP | Mass | Length | ppm | m/z | z | RT | 1/k0 Start | 1/k0 End | Precursor ID | Area Sample 1 | #Feature | #Feature Sample 1 | Start | End | PTM | AScore | Found By |
| --- | --- | --- | --- | --- | --- | --- | --- | --- | --- | --- | --- | --- | --- | --- | --- | --- | --- | --- | --- |
| K.NM(+15.99)M(+15.99)AAC(+57.02)DPR.H | Y | 66.66 | 1096.41 | 9 | 0.96 | 549.2103 | 2 | 9.51 | 0.8591 | 0.8664 | 10859 | 2.33e3 | 1 | 1 | 298 | 306 | Oxidation (M), Oxidation (M), Carbamidomethylation | M2:Oxidation (M):1000 M3:Oxidation (M):1000 | DB Search |
| R.FPGQLNADLRK.L | Y | 62.07 | 1257.6829 | 11 | -5.49 | 629.8425 | 2 | 14.27 | 0.9428 | 0.9501 | 38203 | 0 | 0 | 0 | 242 | 252 |  |  | DB Search |
| R.EIVHLQAGQC(+57.02)GNQIGAK.F | Y | 59.9 | 1821.9155 | 17 | -14.62 | 911.9476 | 2 | 13.73 | 1.113 | 1.1202 | 35183 | 0 | 0 | 0 | 3 | 19 | Carbamidomethylation |  | DB Search |
| K.IREEYPDR.I | Y | 56.96 | 1076.525 | 8 | 1.05 | 539.2679 | 2 | 10.96 | 0.8644 | 0.8716 | 19132 | 3.24e3 | 1 | 1 | 155 | 162 |  |  | DB Search |
| total 4 peptides |  |  |  |  |  |  |  |  |  |  |  |  |  |  |  |  |  |  |  |
| --- | --- | --- | --- | --- | --- | --- | --- | --- | --- | --- | --- | --- | --- | --- | --- | --- | --- | --- | --- |

Best Unique PSM (Scan Sample\_10\_A1\_1\_3647.d:10859, m/z=549.2103, z=2, RT=9.51, ppm=-3.54):


TRINITY\_DN118\_c1\_g1\_i5.p1

back to list

  

| Protein Coverage
| Supporting Peptides
| Best Unique PSM
|

Protein Coverage:

Supporting Peptides:

| Peptide | Uniq | -10lgP | Mass | Length | ppm | m/z | z | RT | 1/k0 Start | 1/k0 End | Precursor ID | Area Sample 1 | #Feature | #Feature Sample 1 | Start | End | PTM | AScore | Found By |
| --- | --- | --- | --- | --- | --- | --- | --- | --- | --- | --- | --- | --- | --- | --- | --- | --- | --- | --- | --- |
| R.ETC(+57.02)SDIEKR.E | Y | 66.66 | 1136.5132 | 9 | 3.94 | 569.2635 | 2 | 10.07 | 0.8931 | 0.9004 | 13965 | 4.9e3 | 1 | 1 | 186 | 194 | Carbamidomethylation |  | DB Search |
| K.VC(+57.02)EDVKR.C | Y | 62.75 | 904.4436 | 7 | 2.55 | 453.2282 | 2 | 10.47 | 0.7953 | 0.8027 | 16197 | 2.08e3 | 2 | 2 | 122 | 128 | Carbamidomethylation |  | DB Search |
| total 2 peptides |  |  |  |  |  |  |  |  |  |  |  |  |  |  |  |  |  |  |  |
| --- | --- | --- | --- | --- | --- | --- | --- | --- | --- | --- | --- | --- | --- | --- | --- | --- | --- | --- | --- |

Best Unique PSM (Scan Sample\_10\_A1\_1\_3647.d:13965, m/z=569.2635, z=2, RT=10.07, ppm=-0.55):


TRINITY\_DN495\_c0\_g1\_i6.p1

back to list

  

| Protein Coverage
| Supporting Peptides
| Best Unique PSM
|

Protein Coverage:

Supporting Peptides:

| Peptide | Uniq | -10lgP | Mass | Length | ppm | m/z | z | RT | 1/k0 Start | 1/k0 End | Precursor ID | Area Sample 1 | #Feature | #Feature Sample 1 | Start | End | PTM | AScore | Found By |
| --- | --- | --- | --- | --- | --- | --- | --- | --- | --- | --- | --- | --- | --- | --- | --- | --- | --- | --- | --- |
| K.SISC(+57.02)SKDEEIK.V | Y | 63.06 | 1294.6075 | 11 | 1.99 | 648.3094 | 2 | 10.84 | 0.9755 | 0.9828 | 18539 | 0 | 0 | 0 | 248 | 258 | Carbamidomethylation |  | DB Search |
| total 1 peptides |  |  |  |  |  |  |  |  |  |  |  |  |  |  |  |  |  |  |  |
| --- | --- | --- | --- | --- | --- | --- | --- | --- | --- | --- | --- | --- | --- | --- | --- | --- | --- | --- | --- |

Best Unique PSM (Scan Sample\_10\_A1\_1\_3647.d:18539, m/z=648.3094, z=2, RT=10.84, ppm=-2.5):


TRINITY\_DN166344\_c0\_g1\_i1.p1

back to list

  

| Protein Coverage
| Supporting Peptides
| Best Unique PSM
|

Protein Coverage:

Supporting Peptides:

| Peptide | Uniq | -10lgP | Mass | Length | ppm | m/z | z | RT | 1/k0 Start | 1/k0 End | Precursor ID | Area Sample 1 | #Feature | #Feature Sample 1 | Start | End | PTM | AScore | Found By |
| --- | --- | --- | --- | --- | --- | --- | --- | --- | --- | --- | --- | --- | --- | --- | --- | --- | --- | --- | --- |
| R.TQITC(+57.02)TQISLKR.G | Y | 66.31 | 1447.7817 | 12 | 1.05 | 724.8956 | 2 | 13.79 | 1.0255 | 1.0327 | 35470 | 1.17e3 | 1 | 1 | 174 | 185 | Carbamidomethylation |  | DB Search |
| total 1 peptides |  |  |  |  |  |  |  |  |  |  |  |  |  |  |  |  |  |  |  |
| --- | --- | --- | --- | --- | --- | --- | --- | --- | --- | --- | --- | --- | --- | --- | --- | --- | --- | --- | --- |

Best Unique PSM (Scan Sample\_10\_A1\_1\_3647.d:35470, m/z=724.8956, z=2, RT=13.79, ppm=-3.44):


TRINITY\_DN1468\_c0\_g1\_i1.p1

back to list

  

| Protein Coverage
| Supporting Peptides
| Best Unique PSM
|

Protein Coverage:

Supporting Peptides:

| Peptide | Uniq | -10lgP | Mass | Length | ppm | m/z | z | RT | 1/k0 Start | 1/k0 End | Precursor ID | Area Sample 1 | #Feature | #Feature Sample 1 | Start | End | PTM | AScore | Found By |
| --- | --- | --- | --- | --- | --- | --- | --- | --- | --- | --- | --- | --- | --- | --- | --- | --- | --- | --- | --- |
| R.LRQIHNGVFVK.L | Y | 49.48 | 1309.7619 | 11 | 7.63 | 655.8903 | 2 | 12.45 | 0.9727 | 0.98 | 27858 | 0 | 0 | 0 | 110 | 120 |  |  | DB Search |
| total 1 peptides |  |  |  |  |  |  |  |  |  |  |  |  |  |  |  |  |  |  |  |
| --- | --- | --- | --- | --- | --- | --- | --- | --- | --- | --- | --- | --- | --- | --- | --- | --- | --- | --- | --- |

Best Unique PSM (Scan Sample\_10\_A1\_1\_3647.d:27858, m/z=655.8903, z=2, RT=12.45, ppm=3.14):


TRINITY\_DN998\_c0\_g1\_i5.p2

back to list

  

| Protein Coverage
| Supporting Peptides
| Best Unique PSM
|

Protein Coverage:

Supporting Peptides:

| Peptide | Uniq | -10lgP | Mass | Length | ppm | m/z | z | RT | 1/k0 Start | 1/k0 End | Precursor ID | Area Sample 1 | #Feature | #Feature Sample 1 | Start | End | PTM | AScore | Found By |
| --- | --- | --- | --- | --- | --- | --- | --- | --- | --- | --- | --- | --- | --- | --- | --- | --- | --- | --- | --- |
| K.YANFLDQALREDLER.M | Y | 61.79 | 1851.9115 | 15 | -3.63 | 618.3061 | 3 | 19.80 | 0.8741 | 0.8814 | 69772 | 0 | 0 | 0 | 121 | 135 |  |  | DB Search |
| total 1 peptides |  |  |  |  |  |  |  |  |  |  |  |  |  |  |  |  |  |  |  |
| --- | --- | --- | --- | --- | --- | --- | --- | --- | --- | --- | --- | --- | --- | --- | --- | --- | --- | --- | --- |

Best Unique PSM (Scan Sample\_10\_A1\_1\_3647.d:69772, m/z=618.3061, z=3, RT=19.80, ppm=-8.12):


TRINITY\_DN11643\_c0\_g1\_i1.p1

back to list

  

| Protein Coverage
| Supporting Peptides
| Best Unique PSM
|

Protein Coverage:

Supporting Peptides:

| Peptide | Uniq | -10lgP | Mass | Length | ppm | m/z | z | RT | 1/k0 Start | 1/k0 End | Precursor ID | Area Sample 1 | #Feature | #Feature Sample 1 | Start | End | PTM | AScore | Found By |
| --- | --- | --- | --- | --- | --- | --- | --- | --- | --- | --- | --- | --- | --- | --- | --- | --- | --- | --- | --- |
| R.EVNTELDSRPETK.K | Y | 66.66 | 1516.7369 | 13 | -0.4 | 759.372 | 2 | 11.74 | 1.0222 | 1.0295 | 23643 | 2.54e3 | 2 | 2 | 97 | 109 |  |  | DB Search |
| total 1 peptides |  |  |  |  |  |  |  |  |  |  |  |  |  |  |  |  |  |  |  |
| --- | --- | --- | --- | --- | --- | --- | --- | --- | --- | --- | --- | --- | --- | --- | --- | --- | --- | --- | --- |

Best Unique PSM (Scan Sample\_10\_A1\_1\_3647.d:23643, m/z=759.372, z=2, RT=11.74, ppm=-4.89):


TRINITY\_DN200\_c0\_g1\_i3.p1

back to list

  

| Protein Coverage
| Supporting Peptides
| Best Unique PSM
|

Protein Coverage:

Supporting Peptides:

| Peptide | Uniq | -10lgP | Mass | Length | ppm | m/z | z | RT | 1/k0 Start | 1/k0 End | Precursor ID | Area Sample 1 | #Feature | #Feature Sample 1 | Start | End | PTM | AScore | Found By |
| --- | --- | --- | --- | --- | --- | --- | --- | --- | --- | --- | --- | --- | --- | --- | --- | --- | --- | --- | --- |
| K.TAIAIDTIINQKR.F | Y | 64.76 | 1455.8409 | 13 | -0.36 | 728.9242 | 2 | 16.58 | 1.0588 | 1.066 | 50506 | 4.46e2 | 1 | 1 | 217 | 229 |  |  | DB Search |
| total 1 peptides |  |  |  |  |  |  |  |  |  |  |  |  |  |  |  |  |  |  |  |
| --- | --- | --- | --- | --- | --- | --- | --- | --- | --- | --- | --- | --- | --- | --- | --- | --- | --- | --- | --- |

Best Unique PSM (Scan Sample\_10\_A1\_1\_3647.d:50506, m/z=728.9242, z=2, RT=16.58, ppm=-4.85):


TRINITY\_DN4497\_c0\_g1\_i1.p1

back to list

  

| Protein Coverage
| Supporting Peptides
| Best Unique PSM
|

Protein Coverage:

Supporting Peptides:

| Peptide | Uniq | -10lgP | Mass | Length | ppm | m/z | z | RT | 1/k0 Start | 1/k0 End | Precursor ID | Area Sample 1 | #Feature | #Feature Sample 1 | Start | End | PTM | AScore | Found By |
| --- | --- | --- | --- | --- | --- | --- | --- | --- | --- | --- | --- | --- | --- | --- | --- | --- | --- | --- | --- |
| K.VLTSSDPAEKEK.K | Y | 66.69 | 1302.6667 | 12 | 3.87 | 652.3402 | 2 | 10.57 | 0.9977 | 1.0049 | 16696 | 7.52e3 | 1 | 1 | 117 | 128 |  |  | DB Search |
| total 1 peptides |  |  |  |  |  |  |  |  |  |  |  |  |  |  |  |  |  |  |  |
| --- | --- | --- | --- | --- | --- | --- | --- | --- | --- | --- | --- | --- | --- | --- | --- | --- | --- | --- | --- |

Best Unique PSM (Scan Sample\_10\_A1\_1\_3647.d:16696, m/z=652.3402, z=2, RT=10.57, ppm=-0.62):


TRINITY\_DN5365\_c0\_g1\_i2.p1

back to list

  

| Protein Coverage
| Supporting Peptides
| Best Unique PSM
|

Protein Coverage:

Supporting Peptides:

| Peptide | Uniq | -10lgP | Mass | Length | ppm | m/z | z | RT | 1/k0 Start | 1/k0 End | Precursor ID | Area Sample 1 | #Feature | #Feature Sample 1 | Start | End | PTM | AScore | Found By |
| --- | --- | --- | --- | --- | --- | --- | --- | --- | --- | --- | --- | --- | --- | --- | --- | --- | --- | --- | --- |
| R.FPGQLNADIRK.L | Y | 62.07 | 1257.6829 | 11 | -5.49 | 629.8425 | 2 | 14.27 | 0.9428 | 0.9501 | 38203 | 0 | 0 | 0 | 244 | 254 |  |  | DB Search |
| total 1 peptides |  |  |  |  |  |  |  |  |  |  |  |  |  |  |  |  |  |  |  |
| --- | --- | --- | --- | --- | --- | --- | --- | --- | --- | --- | --- | --- | --- | --- | --- | --- | --- | --- | --- |

Best Unique PSM (Scan Sample\_10\_A1\_1\_3647.d:38203, m/z=629.8425, z=2, RT=14.27, ppm=-9.98):


TRINITY\_DN5107\_c0\_g1\_i5.p2

back to list

  

| Protein Coverage
| Supporting Peptides
| Best Unique PSM
|

Protein Coverage:

Supporting Peptides:

| Peptide | Uniq | -10lgP | Mass | Length | ppm | m/z | z | RT | 1/k0 Start | 1/k0 End | Precursor ID | Area Sample 1 | #Feature | #Feature Sample 1 | Start | End | PTM | AScore | Found By |
| --- | --- | --- | --- | --- | --- | --- | --- | --- | --- | --- | --- | --- | --- | --- | --- | --- | --- | --- | --- |
| K.KLDYVLGLTTAK.I | Y | 63.57 | 1320.7653 | 12 | -2.37 | 661.3854 | 2 | 16.69 | 0.9908 | 0.9981 | 51148 | 2.13e2 | 1 | 1 | 62 | 73 |  |  | DB Search |
| total 1 peptides |  |  |  |  |  |  |  |  |  |  |  |  |  |  |  |  |  |  |  |
| --- | --- | --- | --- | --- | --- | --- | --- | --- | --- | --- | --- | --- | --- | --- | --- | --- | --- | --- | --- |

Best Unique PSM (Scan Sample\_10\_A1\_1\_3647.d:51148, m/z=661.3854, z=2, RT=16.69, ppm=-6.87):


TRINITY\_DN22492\_c0\_g1\_i1.p1

back to list

  

| Protein Coverage
| Supporting Peptides
| Best Unique PSM
|

Protein Coverage:

Supporting Peptides:

| Peptide | Uniq | -10lgP | Mass | Length | ppm | m/z | z | RT | 1/k0 Start | 1/k0 End | Precursor ID | Area Sample 1 | #Feature | #Feature Sample 1 | Start | End | PTM | AScore | Found By |
| --- | --- | --- | --- | --- | --- | --- | --- | --- | --- | --- | --- | --- | --- | --- | --- | --- | --- | --- | --- |
| R.HC(+57.02)DPELSSFHTFWR.L | Y | 61.65 | 1817.7944 | 14 | -6 | 606.9324 | 3 | 17.68 | 0.8887 | 0.8959 | 56846 | 2.37e2 | 1 | 1 | 151 | 164 | Carbamidomethylation |  | DB Search |
| K.KKLEPQAAPR.E | Y | 43.1 | 1136.6666 | 10 | 2.05 | 379.8952 | 3 | 9.66 | 0.7604 | 0.7677 | 11777 | 3.9e3 | 2 | 2 | 425 | 434 |  |  | DB Search |
| total 2 peptides |  |  |  |  |  |  |  |  |  |  |  |  |  |  |  |  |  |  |  |
| --- | --- | --- | --- | --- | --- | --- | --- | --- | --- | --- | --- | --- | --- | --- | --- | --- | --- | --- | --- |

Best Unique PSM (Scan Sample\_10\_A1\_1\_3647.d:56846, m/z=606.9324, z=3, RT=17.68, ppm=-10.49):


TRINITY\_DN428\_c0\_g1\_i1.p1

back to list

  

| Protein Coverage
| Supporting Peptides
| Best Unique PSM
|

Protein Coverage:

Supporting Peptides:

| Peptide | Uniq | -10lgP | Mass | Length | ppm | m/z | z | RT | 1/k0 Start | 1/k0 End | Precursor ID | Area Sample 1 | #Feature | #Feature Sample 1 | Start | End | PTM | AScore | Found By |
| --- | --- | --- | --- | --- | --- | --- | --- | --- | --- | --- | --- | --- | --- | --- | --- | --- | --- | --- | --- |
| K.IC(+57.02)HLLGIQVTDFTK.S | Y | 66.8 | 1643.8705 | 14 | 2.77 | 822.9411 | 2 | 18.45 | 1.0837 | 1.0909 | 61351 | 7.71e2 | 1 | 1 | 377 | 390 | Carbamidomethylation |  | DB Search |
| R.AGVLAHLEEER.D | Y | 66.69 | 1222.6306 | 11 | -5.26 | 612.3166 | 2 | 14.81 | 0.9541 | 0.9614 | 41119 | 0 | 0 | 0 | 762 | 772 |  |  | DB Search |
| R.LPIYTDKVINLYK.G | Y | 65.11 | 1578.9021 | 13 | -1.17 | 790.4539 | 2 | 17.72 | 1.0989 | 1.1061 | 56954 | 4.39e2 | 1 | 1 | 126 | 138 |  |  | DB Search |
| R.AGVLAHLEEERDIK.L | Y | 64.78 | 1578.8365 | 14 | 1.02 | 790.4228 | 2 | 14.83 | 1.1053 | 1.1126 | 41168 | 6.95e2 | 1 | 1 | 762 | 775 |  |  | DB Search |
| K.RIQQLSAIR.V | Y | 42.81 | 1083.6513 | 9 | 5.14 | 542.8333 | 2 | 13.13 | 0.9146 | 0.9218 | 31805 | 1.14e3 | 1 | 1 | 799 | 807 |  |  | DB Search |
| total 5 peptides |  |  |  |  |  |  |  |  |  |  |  |  |  |  |  |  |  |  |  |
| --- | --- | --- | --- | --- | --- | --- | --- | --- | --- | --- | --- | --- | --- | --- | --- | --- | --- | --- | --- |

Best Unique PSM (Scan Sample\_10\_A1\_1\_3647.d:61351, m/z=822.9411, z=2, RT=18.45, ppm=-1.72):


TRINITY\_DN96\_c0\_g1\_i2.p1

back to list

  

| Protein Coverage
| Supporting Peptides
| Best Unique PSM
|

Protein Coverage:

Supporting Peptides:

| Peptide | Uniq | -10lgP | Mass | Length | ppm | m/z | z | RT | 1/k0 Start | 1/k0 End | Precursor ID | Area Sample 1 | #Feature | #Feature Sample 1 | Start | End | PTM | AScore | Found By |
| --- | --- | --- | --- | --- | --- | --- | --- | --- | --- | --- | --- | --- | --- | --- | --- | --- | --- | --- | --- |
| K.TVQNFYELVTHSK.G | Y | 66.72 | 1564.7885 | 13 | 1.3 | 783.399 | 2 | 18.24 | 1.0913 | 1.0985 | 60147 | 1.44e3 | 2 | 2 | 44 | 56 |  |  | DB Search |
| total 1 peptides |  |  |  |  |  |  |  |  |  |  |  |  |  |  |  |  |  |  |  |
| --- | --- | --- | --- | --- | --- | --- | --- | --- | --- | --- | --- | --- | --- | --- | --- | --- | --- | --- | --- |

Best Unique PSM (Scan Sample\_10\_A1\_1\_3647.d:60147, m/z=783.399, z=2, RT=18.24, ppm=-3.19):


TRINITY\_DN161\_c0\_g1\_i4.p1

back to list

  

| Protein Coverage
| Supporting Peptides
| Best Unique PSM
|

Protein Coverage:

Supporting Peptides:

| Peptide | Uniq | -10lgP | Mass | Length | ppm | m/z | z | RT | 1/k0 Start | 1/k0 End | Precursor ID | Area Sample 1 | #Feature | #Feature Sample 1 | Start | End | PTM | AScore | Found By |
| --- | --- | --- | --- | --- | --- | --- | --- | --- | --- | --- | --- | --- | --- | --- | --- | --- | --- | --- | --- |
| K.SYELPDGQVITIGNER.F | Y | 66.69 | 1789.8846 | 16 | -3.48 | 895.9424 | 2 | 18.53 | 0.9795 | 0.9868 | 61913 | 1.88e3 | 2 | 2 | 95 | 110 |  |  | DB Search |
| R.LDLAGRDLTDYLM(+15.99)K.I | Y | 62.87 | 1638.8287 | 14 | 4.29 | 820.4214 | 2 | 18.15 | 1.143 | 1.1502 | 59617 | 0 | 0 | 0 | 34 | 47 | Oxidation (M) | M13:Oxidation (M):1000 | DB Search |
| R.LDLAGRDLTDYLMK.I | Y | 27.85 | 1622.8338 | 14 | -0.39 | 541.9492 | 3 | 19.26 | 0.7974 | 0.8047 | 66371 | 6.87e2 | 1 | 1 | 34 | 47 |  |  | DB Search |
| total 3 peptides |  |  |  |  |  |  |  |  |  |  |  |  |  |  |  |  |  |  |  |
| --- | --- | --- | --- | --- | --- | --- | --- | --- | --- | --- | --- | --- | --- | --- | --- | --- | --- | --- | --- |

Best Unique PSM (Scan Sample\_10\_A1\_1\_3647.d:61913, m/z=895.9424, z=2, RT=18.53, ppm=-7.97):


TRINITY\_DN162\_c0\_g1\_i1.p1

back to list

  

| Protein Coverage
| Supporting Peptides
| Best Unique PSM
|

Protein Coverage:

Supporting Peptides:

| Peptide | Uniq | -10lgP | Mass | Length | ppm | m/z | z | RT | 1/k0 Start | 1/k0 End | Precursor ID | Area Sample 1 | #Feature | #Feature Sample 1 | Start | End | PTM | AScore | Found By |
| --- | --- | --- | --- | --- | --- | --- | --- | --- | --- | --- | --- | --- | --- | --- | --- | --- | --- | --- | --- |
| K.IC(+57.02)VIVGTVTDDNRLLEVPK.L | Y | 66.68 | 2140.1561 | 19 | 0.02 | 714.3895 | 3 | 18.48 | 0.9473 | 0.9545 | 61525 | 1.93e3 | 1 | 1 | 96 | 114 | Carbamidomethylation |  | DB Search |
| K.TNRPPVSLAR.V | Y | 63.65 | 1109.6305 | 10 | 2.36 | 555.8214 | 2 | 12.15 | 0.883 | 0.8903 | 26078 | 3.42e3 | 1 | 1 | 73 | 82 |  |  | DB Search |
| total 2 peptides |  |  |  |  |  |  |  |  |  |  |  |  |  |  |  |  |  |  |  |
| --- | --- | --- | --- | --- | --- | --- | --- | --- | --- | --- | --- | --- | --- | --- | --- | --- | --- | --- | --- |

Best Unique PSM (Scan Sample\_10\_A1\_1\_3647.d:61525, m/z=714.3895, z=3, RT=18.48, ppm=-4.47):


TRINITY\_DN2772\_c0\_g1\_i1.p1

back to list

  

| Protein Coverage
| Supporting Peptides
| Best Unique PSM
|

Protein Coverage:

Supporting Peptides:

| Peptide | Uniq | -10lgP | Mass | Length | ppm | m/z | z | RT | 1/k0 Start | 1/k0 End | Precursor ID | Area Sample 1 | #Feature | #Feature Sample 1 | Start | End | PTM | AScore | Found By |
| --- | --- | --- | --- | --- | --- | --- | --- | --- | --- | --- | --- | --- | --- | --- | --- | --- | --- | --- | --- |
| K.LIEALC(+57.02)AEHEIK.L | Y | 66.7 | 1424.7333 | 12 | 0.68 | 713.3712 | 2 | 15.09 | 1.0709 | 1.0781 | 42629 | 1.49e3 | 1 | 1 | 73 | 84 | Carbamidomethylation |  | DB Search |
| total 1 peptides |  |  |  |  |  |  |  |  |  |  |  |  |  |  |  |  |  |  |  |
| --- | --- | --- | --- | --- | --- | --- | --- | --- | --- | --- | --- | --- | --- | --- | --- | --- | --- | --- | --- |

Best Unique PSM (Scan Sample\_10\_A1\_1\_3647.d:42629, m/z=713.3712, z=2, RT=15.09, ppm=-3.81):


TRINITY\_DN40728\_c0\_g1\_i1.p1

back to list

  

| Protein Coverage
| Supporting Peptides
| Best Unique PSM
|

Protein Coverage:

Supporting Peptides:

| Peptide | Uniq | -10lgP | Mass | Length | ppm | m/z | z | RT | 1/k0 Start | 1/k0 End | Precursor ID | Area Sample 1 | #Feature | #Feature Sample 1 | Start | End | PTM | AScore | Found By |
| --- | --- | --- | --- | --- | --- | --- | --- | --- | --- | --- | --- | --- | --- | --- | --- | --- | --- | --- | --- |
| K.IQDKEGIPPDQQR.L | Y | 65.08 | 1522.7739 | 13 | -0.66 | 762.3903 | 2 | 11.01 | 1.0339 | 1.0411 | 19352 | 9.49e2 | 1 | 1 | 53 | 65 |  |  | DB Search |
| total 1 peptides |  |  |  |  |  |  |  |  |  |  |  |  |  |  |  |  |  |  |  |
| --- | --- | --- | --- | --- | --- | --- | --- | --- | --- | --- | --- | --- | --- | --- | --- | --- | --- | --- | --- |

Best Unique PSM (Scan Sample\_10\_A1\_1\_3647.d:19352, m/z=762.3903, z=2, RT=11.01, ppm=-5.16):


TRINITY\_DN687\_c0\_g2\_i1.p1

back to list

  

| Protein Coverage
| Supporting Peptides
| Best Unique PSM
|

Protein Coverage:

Supporting Peptides:

| Peptide | Uniq | -10lgP | Mass | Length | ppm | m/z | z | RT | 1/k0 Start | 1/k0 End | Precursor ID | Area Sample 1 | #Feature | #Feature Sample 1 | Start | End | PTM | AScore | Found By |
| --- | --- | --- | --- | --- | --- | --- | --- | --- | --- | --- | --- | --- | --- | --- | --- | --- | --- | --- | --- |
| R.AVC(+57.02)M(+15.99)LSNTTAIAEAWAR.L | Y | 66.77 | 1879.892 | 17 | -0.29 | 940.9488 | 2 | 18.63 | 1.1418 | 1.149 | 62475 | 6.54e2 | 1 | 1 | 374 | 390 | Carbamidomethylation, Oxidation (M) | M4:Oxidation (M):1000 | DB Search |
| K.VGINYQPPTVVPGGDLAK.V | Y | 64.86 | 1823.9781 | 18 | -3.39 | 912.9891 | 2 | 17.23 | 1.1198 | 1.127 | 54086 | 0 | 0 | 0 | 353 | 370 |  |  | DB Search |
| total 2 peptides |  |  |  |  |  |  |  |  |  |  |  |  |  |  |  |  |  |  |  |
| --- | --- | --- | --- | --- | --- | --- | --- | --- | --- | --- | --- | --- | --- | --- | --- | --- | --- | --- | --- |

Best Unique PSM (Scan Sample\_10\_A1\_1\_3647.d:62475, m/z=940.9488, z=2, RT=18.63, ppm=-4.79):


TRINITY\_DN190525\_c0\_g1\_i1.p2

back to list

  

| Protein Coverage
| Supporting Peptides
| Best Unique PSM
|

Protein Coverage:

Supporting Peptides:

| Peptide | Uniq | -10lgP | Mass | Length | ppm | m/z | z | RT | 1/k0 Start | 1/k0 End | Precursor ID | Area Sample 1 | #Feature | #Feature Sample 1 | Start | End | PTM | AScore | Found By |
| --- | --- | --- | --- | --- | --- | --- | --- | --- | --- | --- | --- | --- | --- | --- | --- | --- | --- | --- | --- |
| R.ADGGEIQFAQVLR.A | Y | 21.23 | 1402.7204 | 13 | 7.88 | 468.5823 | 3 | 14.99 | 0.7656 | 0.773 | 42194 | 2e3 | 1 | 1 | 50 | 62 |  |  | DB Search |
| total 1 peptides |  |  |  |  |  |  |  |  |  |  |  |  |  |  |  |  |  |  |  |
| --- | --- | --- | --- | --- | --- | --- | --- | --- | --- | --- | --- | --- | --- | --- | --- | --- | --- | --- | --- |

Best Unique PSM (Scan Sample\_10\_A1\_1\_3647.d:42194, m/z=468.5823, z=3, RT=14.99, ppm=3.38):


TRINITY\_DN4815\_c0\_g1\_i2.p1

back to list

  

| Protein Coverage
| Supporting Peptides
| Best Unique PSM
|

Protein Coverage:

Supporting Peptides:

| Peptide | Uniq | -10lgP | Mass | Length | ppm | m/z | z | RT | 1/k0 Start | 1/k0 End | Precursor ID | Area Sample 1 | #Feature | #Feature Sample 1 | Start | End | PTM | AScore | Found By |
| --- | --- | --- | --- | --- | --- | --- | --- | --- | --- | --- | --- | --- | --- | --- | --- | --- | --- | --- | --- |
| K.TIKDVNC(+57.02)NNNQLK.S | Y | 47.49 | 1559.7726 | 13 | 8.47 | 780.8967 | 2 | 11.21 | 1.05 | 1.0572 | 20684 | 1.74e2 | 1 | 1 | 533 | 545 | Carbamidomethylation |  | DB Search |
| total 1 peptides |  |  |  |  |  |  |  |  |  |  |  |  |  |  |  |  |  |  |  |
| --- | --- | --- | --- | --- | --- | --- | --- | --- | --- | --- | --- | --- | --- | --- | --- | --- | --- | --- | --- |

Best Unique PSM (Scan Sample\_10\_A1\_1\_3647.d:20684, m/z=780.8967, z=2, RT=11.21, ppm=3.97):


TRINITY\_DN1386\_c0\_g1\_i1.p1

back to list

  

| Protein Coverage
| Supporting Peptides
| Best Unique PSM
|

Protein Coverage:

Supporting Peptides:

| Peptide | Uniq | -10lgP | Mass | Length | ppm | m/z | z | RT | 1/k0 Start | 1/k0 End | Precursor ID | Area Sample 1 | #Feature | #Feature Sample 1 | Start | End | PTM | AScore | Found By |
| --- | --- | --- | --- | --- | --- | --- | --- | --- | --- | --- | --- | --- | --- | --- | --- | --- | --- | --- | --- |
| R.KVIVDQLVTLATR.V | Y | 66.24 | 1454.882 | 13 | -0.52 | 728.4446 | 2 | 18.26 | 1.0271 | 1.0343 | 60277 | 0 | 0 | 0 | 547 | 559 |  |  | DB Search |
| K.SFLLALEKDQEK.H | Y | 66 | 1419.7609 | 12 | 0.75 | 710.8851 | 2 | 17.37 | 1.0733 | 1.0805 | 54745 | 7.32e3 | 1 | 1 | 61 | 72 |  |  | DB Search |
| R.SPTHSVIK.S | Y | 60.92 | 867.4814 | 8 | 0.09 | 434.7461 | 2 | 9.91 | 0.7876 | 0.7949 | 13010 | 3.62e2 | 1 | 1 | 259 | 266 |  |  | DB Search |
| total 3 peptides |  |  |  |  |  |  |  |  |  |  |  |  |  |  |  |  |  |  |  |
| --- | --- | --- | --- | --- | --- | --- | --- | --- | --- | --- | --- | --- | --- | --- | --- | --- | --- | --- | --- |

Best Unique PSM (Scan Sample\_10\_A1\_1\_3647.d:60277, m/z=728.4446, z=2, RT=18.26, ppm=-5.02):


TRINITY\_DN998\_c0\_g1\_i2.p2

back to list

  

| Protein Coverage
| Supporting Peptides
| Best Unique PSM
|

Protein Coverage:

Supporting Peptides:

| Peptide | Uniq | -10lgP | Mass | Length | ppm | m/z | z | RT | 1/k0 Start | 1/k0 End | Precursor ID | Area Sample 1 | #Feature | #Feature Sample 1 | Start | End | PTM | AScore | Found By |
| --- | --- | --- | --- | --- | --- | --- | --- | --- | --- | --- | --- | --- | --- | --- | --- | --- | --- | --- | --- |
| K.YANFLDQALREDLER.M | Y | 61.79 | 1851.9115 | 15 | -3.63 | 618.3061 | 3 | 19.80 | 0.8741 | 0.8814 | 69772 | 0 | 0 | 0 | 121 | 135 |  |  | DB Search |
| total 1 peptides |  |  |  |  |  |  |  |  |  |  |  |  |  |  |  |  |  |  |  |
| --- | --- | --- | --- | --- | --- | --- | --- | --- | --- | --- | --- | --- | --- | --- | --- | --- | --- | --- | --- |

Best Unique PSM (Scan Sample\_10\_A1\_1\_3647.d:69772, m/z=618.3061, z=3, RT=19.80, ppm=-8.12):


TRINITY\_DN116\_c0\_g1\_i1.p1

back to list

  

| Protein Coverage
| Supporting Peptides
| Best Unique PSM
|

Protein Coverage:

Supporting Peptides:

| Peptide | Uniq | -10lgP | Mass | Length | ppm | m/z | z | RT | 1/k0 Start | 1/k0 End | Precursor ID | Area Sample 1 | #Feature | #Feature Sample 1 | Start | End | PTM | AScore | Found By |
| --- | --- | --- | --- | --- | --- | --- | --- | --- | --- | --- | --- | --- | --- | --- | --- | --- | --- | --- | --- |
| R.ALQDEDEDAFKR.Q | Y | 66.56 | 1435.6579 | 12 | 0.52 | 718.8334 | 2 | 13.14 | 0.9937 | 1.0009 | 31899 | 0 | 0 | 0 | 210 | 221 |  |  | DB Search |
| R.FTNKDIVC(+57.02)QIAYAK.I | Y | 66.39 | 1669.8498 | 14 | -7.67 | 835.922 | 2 | 15.97 | 1.1214 | 1.1286 | 47367 | 0 | 0 | 0 | 55 | 68 | Carbamidomethylation |  | DB Search |
| K.IAGDVIVC(+57.02)AAYSHELPR.Y | Y | 62.03 | 1869.9407 | 17 | 2.59 | 935.9758 | 2 | 17.14 | 1.2001 | 1.2073 | 53423 | 2.89e2 | 1 | 1 | 69 | 85 | Carbamidomethylation |  | DB Search |
| total 3 peptides |  |  |  |  |  |  |  |  |  |  |  |  |  |  |  |  |  |  |  |
| --- | --- | --- | --- | --- | --- | --- | --- | --- | --- | --- | --- | --- | --- | --- | --- | --- | --- | --- | --- |

Best Unique PSM (Scan Sample\_10\_A1\_1\_3647.d:31899, m/z=718.8334, z=2, RT=13.14, ppm=-3.97):


TRINITY\_DN124769\_c0\_g1\_i1.p1

back to list

  

| Protein Coverage
| Supporting Peptides
| Best Unique PSM
|

Protein Coverage:

Supporting Peptides:

| Peptide | Uniq | -10lgP | Mass | Length | ppm | m/z | z | RT | 1/k0 Start | 1/k0 End | Precursor ID | Area Sample 1 | #Feature | #Feature Sample 1 | Start | End | PTM | AScore | Found By |
| --- | --- | --- | --- | --- | --- | --- | --- | --- | --- | --- | --- | --- | --- | --- | --- | --- | --- | --- | --- |
| K.VITSDDPAEKEK.K | Y | 66.7 | 1330.6616 | 12 | 2.85 | 666.337 | 2 | 10.71 | 0.9836 | 0.9908 | 17367 | 7.2e3 | 1 | 1 | 91 | 102 |  |  | DB Search |
| total 1 peptides |  |  |  |  |  |  |  |  |  |  |  |  |  |  |  |  |  |  |  |
| --- | --- | --- | --- | --- | --- | --- | --- | --- | --- | --- | --- | --- | --- | --- | --- | --- | --- | --- | --- |

Best Unique PSM (Scan Sample\_10\_A1\_1\_3647.d:17367, m/z=666.337, z=2, RT=10.71, ppm=-1.65):


TRINITY\_DN131028\_c0\_g1\_i1.p2

back to list

  

| Protein Coverage
| Supporting Peptides
| Best Unique PSM
|

Protein Coverage:

Supporting Peptides:

| Peptide | Uniq | -10lgP | Mass | Length | ppm | m/z | z | RT | 1/k0 Start | 1/k0 End | Precursor ID | Area Sample 1 | #Feature | #Feature Sample 1 | Start | End | PTM | AScore | Found By |
| --- | --- | --- | --- | --- | --- | --- | --- | --- | --- | --- | --- | --- | --- | --- | --- | --- | --- | --- | --- |
| FDKYHPGYFGK.V | Y | 62.93 | 1357.6455 | 11 | 1.88 | 679.8282 | 2 | 18.01 | 0.988 | 0.9953 | 58678 | 4.03e3 | 1 | 1 | 1 | 11 |  |  | DB Search |
| total 1 peptides |  |  |  |  |  |  |  |  |  |  |  |  |  |  |  |  |  |  |  |
| --- | --- | --- | --- | --- | --- | --- | --- | --- | --- | --- | --- | --- | --- | --- | --- | --- | --- | --- | --- |

Best Unique PSM (Scan Sample\_10\_A1\_1\_3647.d:58678, m/z=679.8282, z=2, RT=18.01, ppm=-2.61):


TRINITY\_DN3036\_c0\_g1\_i3.p1

back to list

  

| Protein Coverage
| Supporting Peptides
| Best Unique PSM
|

Protein Coverage:

Supporting Peptides:

| Peptide | Uniq | -10lgP | Mass | Length | ppm | m/z | z | RT | 1/k0 Start | 1/k0 End | Precursor ID | Area Sample 1 | #Feature | #Feature Sample 1 | Start | End | PTM | AScore | Found By |
| --- | --- | --- | --- | --- | --- | --- | --- | --- | --- | --- | --- | --- | --- | --- | --- | --- | --- | --- | --- |
| R.AGHGAVTSPQTQR.G | Y | 66.66 | 1308.6534 | 13 | 4.48 | 655.334 | 2 | 9.70 | 0.9109 | 0.9182 | 11470 | 2.1e4 | 2 | 2 | 309 | 321 |  |  | DB Search |
| R.GTSNGGLVVGTIIKPK.L | Y | 66.59 | 1539.8984 | 16 | 0.22 | 770.9532 | 2 | 15.68 | 1.1262 | 1.1334 | 45813 | 3.23e3 | 1 | 1 | 173 | 188 |  |  | DB Search |
| R.VIGASGIHVGTMSFGK.M | Y | 66.34 | 1559.813 | 16 | -0.2 | 780.9101 | 2 | 16.89 | 1.1037 | 1.111 | 52086 | 2.86e2 | 1 | 1 | 334 | 349 |  |  | DB Search |
| R.VIGASGIHVGTM(+15.99)SFGK.M | Y | 64.9 | 1575.8079 | 16 | 0.52 | 788.9081 | 2 | 15.11 | 1.1262 | 1.1334 | 42748 | 6.68e2 | 1 | 1 | 334 | 349 | Oxidation (M) | M12:Oxidation (M):1000 | DB Search |
| total 4 peptides |  |  |  |  |  |  |  |  |  |  |  |  |  |  |  |  |  |  |  |
| --- | --- | --- | --- | --- | --- | --- | --- | --- | --- | --- | --- | --- | --- | --- | --- | --- | --- | --- | --- |

Best Unique PSM (Scan Sample\_10\_A1\_1\_3647.d:11470, m/z=655.334, z=2, RT=9.70, ppm=-0.02):


TRINITY\_DN41831\_c0\_g1\_i1.p3

back to list

  

| Protein Coverage
| Supporting Peptides
| Best Unique PSM
|

Protein Coverage:

Supporting Peptides:

| Peptide | Uniq | -10lgP | Mass | Length | ppm | m/z | z | RT | 1/k0 Start | 1/k0 End | Precursor ID | Area Sample 1 | #Feature | #Feature Sample 1 | Start | End | PTM | AScore | Found By |
| --- | --- | --- | --- | --- | --- | --- | --- | --- | --- | --- | --- | --- | --- | --- | --- | --- | --- | --- | --- |
| K.TPPQYR.S | Y | 27.5 | 760.3868 | 6 | 0.77 | 381.1992 | 2 | 11.81 | 0.7106 | 0.718 | 24175 | 4.83e2 | 1 | 1 | 24 | 29 |  |  | DB Search |
| total 1 peptides |  |  |  |  |  |  |  |  |  |  |  |  |  |  |  |  |  |  |  |
| --- | --- | --- | --- | --- | --- | --- | --- | --- | --- | --- | --- | --- | --- | --- | --- | --- | --- | --- | --- |

Best Unique PSM (Scan Sample\_10\_A1\_1\_3647.d:24175, m/z=381.1992, z=2, RT=11.81, ppm=-3.72):


---

Prepared with PEAKS ™ (bioinfor.com)
