# Supplementary material for: Proteomic Analysis and Biochemical Characterization of the Nematocyst Extract of the Hydrozoan Velella velella
Source: Mar Drugs. 2024 Oct 12;22(10):468. doi: 10.3390/md22100468 (PMC11509761; doi:10.3390/md22100468)
Supplement: Supplementary file 1 [file marinedrugs-22-00468-s001.zip › Supplementary File S1.zip!/Sample_11_Vellela/db.peptides.html]

peptide list


Peptide List

  

| Peptide | -10lgP | Mass | Length | ppm | m/z | RT | 1/k0 Start | 1/k0 End | Precursor ID | Area Sample 2 | #Feature | #Feature Sample 2 | Accession | PTM | AScore | Found By |
| --- | --- | --- | --- | --- | --- | --- | --- | --- | --- | --- | --- | --- | --- | --- | --- | --- |
| DSYVGDEAQSKR | 59.21 | 1353.6161 | 12 | 5.76 | 677.8155 | 10.93 | 1.0037 | 1.011 | 18642 | 9.97e3 | 1 | 1 | TRINITY\_DN2477\_c0\_g1\_i6.p1:TRINITY\_DN2477\_c0\_g1\_i5.p1:TRINITY\_DN195934\_c0\_g1\_i1.p2:TRINITY\_DN161\_c0\_g1\_i1.p1:TRINITY\_DN2477\_c0\_g1\_i9.p1:TRINITY\_DN2477\_c0\_g1\_i10.p1:TRINITY\_DN2477\_c0\_g1\_i2.p1:TRINITY\_DN161\_c0\_g1\_i3.p1:TRINITY\_DN161\_c0\_g1\_i2.p1 |  |  | DB Search |
| GTHAAEIAHC(+57.02)VSR | 59.14 | 1407.6677 | 13 | 2.78 | 704.8392 | 11.02 | 1.0263 | 1.0335 | 19137 | 2.25e3 | 1 | 1 | TRINITY\_DN1319\_c0\_g1\_i2.p1:TRINITY\_DN1319\_c0\_g1\_i1.p1 | Carbamidomethylation |  | DB Search |
| VVASSHTDYELR | 59.14 | 1375.6732 | 12 | 4.96 | 688.8435 | 12.47 | 1.0094 | 1.0166 | 27423 | 6.35e3 | 1 | 1 | TRINITY\_DN95248\_c0\_g1\_i1.p1 |  |  | DB Search |
| AKLSQC(+57.02)TAASGK | 59.12 | 1220.6183 | 12 | 6.64 | 611.3171 | 9.69 | 0.9529 | 0.9602 | 11719 | 0 | 0 | 0 | TRINITY\_DN3991\_c0\_g1\_i1.p1 | Carbamidomethylation |  | DB Search |
| ILQC(+57.02)NQYANVEEAR | 59.1 | 1706.8046 | 14 | -0.86 | 854.4042 | 14.11 | 1.0873 | 1.0945 | 36622 | 2.02e3 | 1 | 1 | TRINITY\_DN477\_c0\_g1\_i8.p1:TRINITY\_DN477\_c0\_g1\_i7.p1 | Carbamidomethylation |  | DB Search |
| ATETATGLATTHPIK | 59 | 1510.7991 | 15 | 3.59 | 756.4054 | 12.84 | 1.1053 | 1.1126 | 29583 | 1.89e3 | 1 | 1 | TRINITY\_DN165536\_c0\_g1\_i1.p1 |  |  | DB Search |
| AGHGAVTSPQTQR | 58.95 | 1308.6534 | 13 | 4.15 | 655.3331 | 9.70 | 0.9146 | 0.9218 | 11889 | 1.61e4 | 2 | 2 | TRINITY\_DN3036\_c0\_g1\_i19.p1:TRINITY\_DN3036\_c0\_g1\_i13.p1:TRINITY\_DN3036\_c0\_g1\_i11.p1:TRINITY\_DN3036\_c0\_g1\_i3.p1:TRINITY\_DN3036\_c0\_g1\_i16.p1:TRINITY\_DN3036\_c0\_g1\_i2.p2:TRINITY\_DN3036\_c0\_g1\_i8.p1 |  |  | DB Search |
| VYLAQAEAEKIK | 58.92 | 1361.7554 | 12 | 3.89 | 681.8839 | 14.17 | 1.0355 | 1.0427 | 37026 | 0 | 0 | 0 | TRINITY\_DN11435\_c0\_g1\_i1.p1 |  |  | DB Search |
| VTRPHGASGVVR | 58.91 | 1234.6895 | 12 | 6.71 | 412.5709 | 10.04 | 0.8209 | 0.8283 | 13404 | 2.81e4 | 3 | 3 | TRINITY\_DN2885\_c0\_g1\_i2.p1:TRINITY\_DN2885\_c0\_g1\_i4.p1:TRINITY\_DN2885\_c0\_g1\_i5.p1 |  |  | DB Search |
| YGINHITNLVESKR | 58.9 | 1642.879 | 14 | 4.87 | 548.6333 | 14.82 | 0.823 | 0.8303 | 40480 | 6.96e2 | 1 | 1 | TRINITY\_DN146208\_c0\_g1\_i1.p1 |  |  | DB Search |
| LVQNTPHNSIVTK | 58.89 | 1449.7939 | 13 | 3.18 | 725.9026 | 11.65 | 1.0628 | 1.0701 | 22758 | 1.18e3 | 1 | 1 | TRINITY\_DN982\_c0\_g1\_i3.p1:TRINITY\_DN982\_c0\_g1\_i1.p1 |  |  | DB Search |
| TIESEAAHGTVTR | 58.89 | 1370.679 | 13 | 4.55 | 686.3461 | 11.86 | 1.021 | 1.0283 | 23944 | 8.27e2 | 1 | 1 | TRINITY\_DN3271\_c0\_g1\_i1.p1:TRINITY\_DN3271\_c0\_g1\_i2.p1 |  |  | DB Search |
| RYGQAMC(+57.02)QFMR | 58.88 | 1446.6318 | 11 | -6.73 | 724.3144 | 17.00 | 0.9836 | 0.9908 | 51341 | 4.96e3 | 1 | 1 | TRINITY\_DN286\_c0\_g1\_i4.p1:TRINITY\_DN286\_c0\_g1\_i2.p1 | Carbamidomethylation |  | DB Search |
| FGQATC(+57.02)QFMR | 58.88 | 1244.5431 | 10 | -15.5 | 623.2657 | 13.50 | 0.9194 | 0.9267 | 33267 | 2.65e3 | 1 | 1 | TRINITY\_DN902\_c1\_g1\_i1.p1:TRINITY\_DN902\_c1\_g1\_i2.p1 | Carbamidomethylation |  | DB Search |
| LVLEVAQHLGENVVR | 58.88 | 1674.9417 | 15 | -1.69 | 838.4721 | 17.70 | 1.1418 | 1.149 | 55180 | 1.21e3 | 1 | 1 | TRINITY\_DN73587\_c0\_g1\_i1.p1 |  |  | DB Search |
| LPIYTDKVINLYK | 58.88 | 1578.9021 | 13 | -0.18 | 790.4539 | 17.48 | 1.1009 | 1.1082 | 53935 | 7.95e2 | 1 | 1 | TRINITY\_DN428\_c0\_g1\_i1.p1 |  |  | DB Search |
| IAYAQGYATGDPSKR | 58.88 | 1596.7896 | 15 | 3.91 | 799.4008 | 12.65 | 1.1069 | 1.1142 | 28431 | 1.68e3 | 1 | 1 | TRINITY\_DN1428\_c0\_g1\_i1.p1 |  |  | DB Search |
| DNIQGITKPAIR | 58.88 | 1324.7462 | 12 | 3.85 | 663.3793 | 13.67 | 0.9747 | 0.982 | 34267 | 5.79e3 | 2 | 2 | TRINITY\_DN172571\_c0\_g1\_i1.p1 |  |  | DB Search |
| AQYYEAYETIIKR | 58.88 | 1646.8304 | 13 | 5.56 | 824.4225 | 15.51 | 1.1025 | 1.1098 | 44074 | 4.92e2 | 1 | 1 | TRINITY\_DN319\_c2\_g1\_i3.p1:TRINITY\_DN319\_c2\_g1\_i1.p1 |  |  | DB Search |
| IREEYPDR | 58.88 | 1076.525 | 8 | 2.94 | 539.2684 | 10.94 | 0.866 | 0.8733 | 18650 | 6.17e3 | 1 | 1 | TRINITY\_DN1910\_c0\_g1\_i5.p1:TRINITY\_DN3986\_c0\_g1\_i1.p1 |  |  | DB Search |
| DYGVLLEDAGHTLR | 58.88 | 1557.7787 | 14 | -3.16 | 779.8899 | 18.01 | 1.1286 | 1.1358 | 56870 | 4.46e2 | 1 | 1 | TRINITY\_DN332\_c0\_g1\_i1.p1 |  |  | DB Search |
| LVIISSNTPQLRK | 58.88 | 1467.8773 | 13 | 1.32 | 734.9429 | 14.77 | 1.058 | 1.0652 | 40172 | 1.16e3 | 1 | 1 | TRINITY\_DN2126\_c0\_g1\_i1.p1 |  |  | DB Search |
| LAALDATVHGQTASR | 58.87 | 1509.7899 | 15 | 1.9 | 755.8995 | 13.35 | 1.0713 | 1.0785 | 32461 | 7.36e2 | 1 | 1 | TRINITY\_DN1378\_c0\_g1\_i1.p1 |  |  | DB Search |
| ATGC(+57.02)EVIWNQHNR | 58.87 | 1583.7263 | 13 | 1.47 | 792.8672 | 13.74 | 1.0773 | 1.0845 | 34668 | 9.8e2 | 1 | 1 | TRINITY\_DN2217\_c0\_g1\_i3.p1:TRINITY\_DN2217\_c0\_g1\_i2.p1 | Carbamidomethylation |  | DB Search |
| LSQC(+57.02)TAASGK | 58.87 | 1021.4862 | 10 | -0.07 | 511.7476 | 9.83 | 0.838 | 0.8453 | 11562 | 6.46e4 | 1 | 1 | TRINITY\_DN3991\_c0\_g1\_i1.p1 | Carbamidomethylation |  | DB Search |
| VITSDDPAEKEK | 58.87 | 1330.6616 | 12 | 2.64 | 666.3362 | 10.69 | 0.9848 | 0.992 | 17191 | 9.01e3 | 1 | 1 | TRINITY\_DN1339\_c6\_g1\_i2.p1:TRINITY\_DN1339\_c6\_g1\_i1.p1:TRINITY\_DN1339\_c0\_g1\_i2.p1:TRINITY\_DN6701\_c0\_g1\_i1.p1:TRINITY\_DN6701\_c0\_g1\_i2.p2:TRINITY\_DN124769\_c0\_g1\_i1.p1:TRINITY\_DN1172\_c0\_g1\_i5.p1:TRINITY\_DN1172\_c0\_g1\_i3.p1:TRINITY\_DN1339\_c0\_g1\_i1.p1 |  |  | DB Search |
| VLTSDDPAEKEK | 58.87 | 1330.6616 | 12 | 2.64 | 666.3362 | 10.69 | 0.9848 | 0.992 | 17191 | 9.01e3 | 1 | 1 | TRINITY\_DN119673\_c0\_g1\_i1.p1:TRINITY\_DN24414\_c1\_g1\_i2.p2 |  |  | DB Search |
| ASFVSDIDRLR | 58.87 | 1277.6727 | 11 | -0.78 | 639.8397 | 16.62 | 0.986 | 0.9932 | 49505 | 2.05e3 | 1 | 1 | TRINITY\_DN598\_c1\_g1\_i1.p1 |  |  | DB Search |
| LDLAGRDLTDYLM(+15.99)K | 58.87 | 1638.8286 | 14 | 0.58 | 820.4176 | 17.97 | 1.1438 | 1.151 | 56587 | 5.44e3 | 1 | 1 | TRINITY\_DN161\_c0\_g1\_i1.p1:TRINITY\_DN161\_c0\_g1\_i4.p1:TRINITY\_DN161\_c0\_g1\_i3.p1:TRINITY\_DN161\_c0\_g1\_i2.p1 | Oxidation (M) | M13:Oxidation (M):1000 | DB Search |
| GLDVDSLVIDHIQVNR | 58.87 | 1791.9479 | 16 | 3.22 | 896.9792 | 18.76 | 1.1794 | 1.1865 | 60798 | 1.04e3 | 1 | 1 | TRINITY\_DN1614\_c0\_g1\_i1.p1 |  |  | DB Search |
| TIEAEAAHGTVTR | 58.87 | 1354.6841 | 13 | 3.13 | 678.3477 | 11.77 | 1.0134 | 1.0206 | 23572 | 0 | 0 | 0 | TRINITY\_DN7504\_c0\_g1\_i1.p1 |  |  | DB Search |
| AFVAIGDYGGHVGLGVK | 58.87 | 1658.8779 | 17 | 0.35 | 830.442 | 17.35 | 1.1626 | 1.1698 | 53157 | 4.11e3 | 1 | 1 | TRINITY\_DN15584\_c0\_g1\_i1.p1 |  |  | DB Search |
| GFDYC(+57.02)TTIKYR | 58.87 | 1422.6602 | 11 | 2.87 | 712.3355 | 14.94 | 1.0242 | 1.0315 | 41159 | 9.5e2 | 1 | 1 | TRINITY\_DN36\_c0\_g1\_i13.p1:TRINITY\_DN36\_c0\_g1\_i19.p1:TRINITY\_DN36\_c0\_g1\_i20.p1:TRINITY\_DN36\_c0\_g1\_i5.p1 | Carbamidomethylation |  | DB Search |
| HILLAIANDEELNKLLR | 58.87 | 1974.1261 | 17 | -3.82 | 659.0432 | 19.18 | 0.9856 | 0.9928 | 63150 | 5.97e2 | 1 | 1 | TRINITY\_DN1065\_c0\_g1\_i1.p1 |  |  | DB Search |
| ATC(+57.02)C(+57.02)EGDNC(+57.02)NAGGR | 58.87 | 1540.5453 | 14 | 5.37 | 771.2798 | 9.36 | 0.9553 | 0.9626 | 10231 | 2.8e2 | 1 | 1 | TRINITY\_DN55\_c0\_g1\_i1.p1 | Carbamidomethylation, Carbamidomethylation, Carbamidomethylation |  | DB Search |
| LLTSSDQAELTKK | 58.87 | 1432.7773 | 13 | 4.21 | 717.395 | 12.69 | 1.0496 | 1.0568 | 28711 | 7.94e2 | 1 | 1 | TRINITY\_DN1339\_c0\_g2\_i1.p2:TRINITY\_DN1339\_c0\_g2\_i2.p1:TRINITY\_DN1339\_c0\_g2\_i3.p1:TRINITY\_DN1339\_c0\_g2\_i4.p2 |  |  | DB Search |
| GTSNGGLVVGTIIKPK | 58.87 | 1539.8984 | 16 | 2.71 | 770.9543 | 15.49 | 1.1254 | 1.1326 | 43901 | 6.84e3 | 1 | 1 | TRINITY\_DN3036\_c0\_g1\_i3.p1:TRINITY\_DN3036\_c0\_g1\_i2.p2:TRINITY\_DN3036\_c0\_g1\_i8.p1 |  |  | DB Search |
| S(+42.01)LVIPEKFQHILR | 58.87 | 1620.9352 | 13 | -2.12 | 541.3149 | 19.23 | 0.8368 | 0.8441 | 63378 | 4.49e4 | 2 | 2 | TRINITY\_DN1505\_c0\_g1\_i1.p1 | Acetylation (Protein N-term) | S1:Acetylation (Protein N-term):1000 | DB Search |
| AM(+15.99)SIM(+15.99)NSFVNDIFER | 58.87 | 1804.8124 | 15 | 0.11 | 903.4086 | 19.92 | 1.0508 | 1.058 | 66700 | 2.51e4 | 4 | 4 | TRINITY\_DN106952\_c0\_g1\_i1.p1 | Oxidation (M), Oxidation (M) | M2:Oxidation (M):1000 M5:Oxidation (M):1000 | DB Search |
| ELVDQVLDRIR | 58.86 | 1354.7568 | 11 | 2.67 | 678.3838 | 18.48 | 1.009 | 1.0162 | 59338 | 1.45e3 | 1 | 1 | TRINITY\_DN250\_c0\_g1\_i5.p1:TRINITY\_DN250\_c0\_g1\_i1.p1:TRINITY\_DN250\_c0\_g1\_i4.p1:TRINITY\_DN250\_c0\_g1\_i2.p1:TRINITY\_DN250\_c0\_g1\_i3.p1 |  |  | DB Search |
| SFHFGVIKLEGK | 58.86 | 1360.7502 | 12 | -0.4 | 681.3784 | 16.70 | 1.0291 | 1.0363 | 49881 | 5.45e2 | 1 | 1 | TRINITY\_DN329\_c0\_g1\_i1.p1:TRINITY\_DN329\_c0\_g1\_i2.p1 |  |  | DB Search |
| VLATDKDTLSSNK | 58.86 | 1390.7303 | 13 | 0.63 | 696.3691 | 11.49 | 1.015 | 1.0222 | 21855 | 2.04e3 | 1 | 1 | TRINITY\_DN982\_c0\_g1\_i3.p1 |  |  | DB Search |
| AVFPSIVGRPR | 58.86 | 1197.6982 | 11 | -0.21 | 599.853 | 15.44 | 0.9586 | 0.9658 | 43471 | 2.65e4 | 1 | 1 | TRINITY\_DN161\_c0\_g1\_i1.p1:TRINITY\_DN161\_c0\_g1\_i3.p1:TRINITY\_DN161\_c0\_g1\_i2.p1 |  |  | DB Search |
| TFIVQGFGNVGLHSC(+57.02)R | 58.86 | 1790.8885 | 16 | 4.75 | 896.4509 | 17.62 | 1.2236 | 1.2308 | 54796 | 1.16e2 | 1 | 1 | TRINITY\_DN1534\_c2\_g1\_i1.p1 | Carbamidomethylation |  | DB Search |
| IFGGDIKNHLLLFISK | 58.85 | 1814.0454 | 16 | -0.66 | 605.6854 | 19.80 | 0.8611 | 0.8684 | 66221 | 1.45e3 | 1 | 1 | TRINITY\_DN79\_c0\_g1\_i1.p1 |  |  | DB Search |
| RISGLIYEETR | 58.85 | 1335.7146 | 11 | 5.98 | 668.8649 | 14.62 | 0.9695 | 0.9767 | 39433 | 6.26e3 | 1 | 1 | TRINITY\_DN172571\_c0\_g1\_i1.p1:TRINITY\_DN22077\_c0\_g1\_i1.p3 |  |  | DB Search |
| GLFIIDDKGILR | 58.85 | 1358.7921 | 12 | -0.67 | 680.3992 | 18.97 | 1.0106 | 1.0178 | 62014 | 1.63e3 | 1 | 1 | TRINITY\_DN535\_c0\_g1\_i2.p1:TRINITY\_DN535\_c0\_g1\_i1.p1 |  |  | DB Search |
| LTNSLM(+15.99)M(+15.99)HGR | 58.85 | 1190.5536 | 10 | 1.85 | 596.2819 | 10.72 | 0.9105 | 0.9178 | 17299 | 2.75e3 | 1 | 1 | TRINITY\_DN164351\_c0\_g1\_i1.p1 | Oxidation (M), Oxidation (M) | M6:Oxidation (M):1000 M7:Oxidation (M):1000 | DB Search |
| LYDEYNDKTPSR | 58.85 | 1499.6892 | 12 | 0.4 | 750.8481 | 13.35 | 1.0142 | 1.0214 | 32540 | 3.34e3 | 2 | 2 | TRINITY\_DN2963\_c0\_g1\_i1.p1 |  |  | DB Search |
| AEAEAEVIQKK | 58.85 | 1214.6506 | 11 | -1.98 | 608.3281 | 11.12 | 0.9558 | 0.963 | 19778 | 1.01e3 | 1 | 1 | TRINITY\_DN1280\_c0\_g1\_i1.p1 |  |  | DB Search |
| YYVTIIDAPGHR | 58.85 | 1403.7197 | 12 | 0.07 | 702.8633 | 15.65 | 1.0427 | 1.05 | 44823 | 2.81e2 | 1 | 1 | TRINITY\_DN64\_c0\_g1\_i1.p1 |  |  | DB Search |
| KSAQFLLQLLK | 58.85 | 1287.7914 | 11 | -0.17 | 644.8994 | 19.31 | 0.9775 | 0.9848 | 63852 | 4.01e2 | 1 | 1 | TRINITY\_DN1614\_c0\_g1\_i1.p1 |  |  | DB Search |
| TKVTLVSERPFSK | 58.85 | 1490.8457 | 13 | 0.25 | 746.4262 | 13.79 | 1.0632 | 1.0705 | 34922 | 3.55e2 | 1 | 1 | TRINITY\_DN95248\_c0\_g1\_i1.p1 |  |  | DB Search |
| VIGASGIHVGTM(+15.99)SFGK | 58.84 | 1575.8079 | 16 | 2.27 | 788.9087 | 14.96 | 1.1306 | 1.1378 | 41219 | 1.68e3 | 1 | 1 | TRINITY\_DN3036\_c0\_g1\_i6.p1:TRINITY\_DN3036\_c0\_g1\_i19.p1:TRINITY\_DN3036\_c0\_g1\_i13.p1:TRINITY\_DN3036\_c0\_g1\_i11.p1:TRINITY\_DN3036\_c0\_g1\_i3.p1:TRINITY\_DN3036\_c0\_g1\_i4.p1:TRINITY\_DN3036\_c0\_g1\_i16.p1:TRINITY\_DN3036\_c0\_g1\_i2.p2:TRINITY\_DN3036\_c0\_g1\_i8.p1 | Oxidation (M) | M12:Oxidation (M):1000 | DB Search |
| GVDEDAYTLEVLHYK | 58.84 | 1750.8413 | 15 | 4.4 | 876.427 | 17.48 | 1.1802 | 1.1873 | 54020 | 1.93e2 | 1 | 1 | TRINITY\_DN4101\_c0\_g1\_i1.p3:TRINITY\_DN4101\_c0\_g1\_i3.p2:TRINITY\_DN4101\_c0\_g1\_i4.p2 |  |  | DB Search |
| GLTHC(+57.02)NTLSYK | 58.83 | 1292.6183 | 11 | 4.02 | 647.3155 | 12.36 | 0.9695 | 0.9767 | 26924 | 0 | 0 | 0 | TRINITY\_DN902\_c1\_g2\_i4.p1:TRINITY\_DN902\_c1\_g2\_i1.p1:TRINITY\_DN902\_c1\_g2\_i3.p1 | Carbamidomethylation |  | DB Search |
| A(+42.01)NPLKDIISR | 58.83 | 1167.6611 | 10 | -5.53 | 584.8314 | 18.34 | 0.919 | 0.9263 | 58511 | 1.06e3 | 1 | 1 | TRINITY\_DN163801\_c0\_g1\_i1.p1 | Acetylation (Protein N-term) | A1:Acetylation (Protein N-term):1000 | DB Search |
| LAYELQANKEQQR | 58.82 | 1589.8162 | 13 | -0.91 | 795.9103 | 12.85 | 1.0548 | 1.062 | 29615 | 1.33e3 | 1 | 1 | TRINITY\_DN11435\_c0\_g1\_i1.p1 |  |  | DB Search |
| ASFVTDIDRLR | 58.82 | 1291.6885 | 11 | 5.03 | 646.8512 | 16.77 | 0.9888 | 0.9961 | 50096 | 2.99e3 | 1 | 1 | TRINITY\_DN598\_c1\_g1\_i2.p1 |  |  | DB Search |
| LSYC(+57.02)VSC(+57.02)AIHSK | 58.81 | 1423.6588 | 12 | 2.72 | 712.8347 | 13.91 | 1.0303 | 1.0375 | 35578 | 0 | 0 | 0 | TRINITY\_DN7671\_c0\_g1\_i1.p1 | Carbamidomethylation, Carbamidomethylation |  | DB Search |
| VVEKEAETER | 58.8 | 1188.5986 | 10 | 7.97 | 397.2078 | 9.54 | 0.7322 | 0.7396 | 11020 | 5.76e2 | 1 | 1 | TRINITY\_DN2636\_c0\_g1\_i2.p1:TRINITY\_DN2636\_c0\_g1\_i1.p1 |  |  | DB Search |
| AVIFDRFR | 58.8 | 1022.5661 | 8 | 1.91 | 512.2885 | 15.68 | 0.8704 | 0.8777 | 44863 | 2.49e3 | 1 | 1 | TRINITY\_DN98420\_c0\_g1\_i1.p1 |  |  | DB Search |
| K(+42.01)LSAEVANGR | 58.78 | 1085.5829 | 10 | 5.56 | 543.7988 | 12.85 | 0.8538 | 0.8611 | 29679 | 1.02e3 | 1 | 1 | TRINITY\_DN6397\_c0\_g2\_i4.p1:TRINITY\_DN6397\_c0\_g2\_i3.p1 | Acetylation (Protein N-term) | K1:Acetylation (Protein N-term):1000 | DB Search |
| FDKYHPGYFGK | 58.78 | 1357.6455 | 11 | -2.44 | 679.8246 | 17.74 | 0.9876 | 0.9949 | 55445 | 8.83e2 | 1 | 1 | TRINITY\_DN131028\_c0\_g1\_i1.p2 |  |  | DB Search |
| EIAQDFKTDLR | 58.76 | 1334.683 | 11 | -3.43 | 668.3428 | 14.60 | 0.9892 | 0.9965 | 39397 | 0 | 0 | 0 | TRINITY\_DN72753\_c0\_g1\_i1.p1 |  |  | DB Search |
| AVAHHTTASFIR | 58.76 | 1309.6891 | 12 | -1.72 | 655.8471 | 11.68 | 0.9703 | 0.9775 | 22954 | 1.45e3 | 1 | 1 | TRINITY\_DN30365\_c0\_g1\_i2.p1:TRINITY\_DN30365\_c0\_g1\_i3.p1 |  |  | DB Search |
| GKKPHVQVNVK | 58.75 | 1232.7354 | 11 | 5.53 | 411.9191 | 9.78 | 0.7917 | 0.799 | 12069 | 7.14e2 | 1 | 1 | TRINITY\_DN119684\_c0\_g1\_i1.p1 |  |  | DB Search |
| LTHDFHTNKR | 58.75 | 1267.6421 | 10 | 2.44 | 423.5534 | 9.64 | 0.7441 | 0.7514 | 11479 | 1.15e3 | 1 | 1 | TRINITY\_DN265\_c0\_g1\_i2.p1:TRINITY\_DN265\_c0\_g1\_i1.p1:TRINITY\_DN265\_c0\_g1\_i7.p1:TRINITY\_DN265\_c0\_g1\_i3.p1:TRINITY\_DN265\_c0\_g1\_i9.p1:TRINITY\_DN265\_c0\_g1\_i5.p1:TRINITY\_DN265\_c0\_g1\_i8.p1:TRINITY\_DN265\_c0\_g1\_i6.p1:TRINITY\_DN265\_c0\_g1\_i4.p1 |  |  | DB Search |
| LIEALC(+57.02)AEHEIK | 58.73 | 1424.7333 | 12 | 3.46 | 713.3725 | 14.93 | 1.0717 | 1.0789 | 41037 | 2.22e3 | 1 | 1 | TRINITY\_DN2772\_c0\_g1\_i3.p1:TRINITY\_DN2772\_c0\_g1\_i2.p1:TRINITY\_DN2772\_c0\_g1\_i1.p1 | Carbamidomethylation |  | DB Search |
| AYAAFLEFKDVVK | 58.73 | 1499.8024 | 13 | -0.24 | 750.9042 | 18.72 | 1.0941 | 1.1013 | 60609 | 3.07e3 | 1 | 1 | TRINITY\_DN240\_c0\_g1\_i6.p1:TRINITY\_DN240\_c0\_g1\_i1.p1:TRINITY\_DN240\_c0\_g1\_i4.p1 |  |  | DB Search |
| A(+42.01)EAPKPKPAPK | 58.73 | 1174.671 | 11 | 4.56 | 392.5639 | 9.99 | 0.7665 | 0.7738 | 13134 | 4.33e3 | 2 | 2 | TRINITY\_DN1631\_c0\_g1\_i1.p1 | Acetylation (Protein N-term) | A1:Acetylation (Protein N-term):1000 | DB Search |
| VLTSSDPAEKEK | 58.73 | 1302.6666 | 12 | -2.55 | 652.3354 | 10.56 | 0.9969 | 1.0041 | 16353 | 2.66e3 | 1 | 1 | TRINITY\_DN4497\_c0\_g2\_i1.p1:TRINITY\_DN1172\_c0\_g2\_i2.p1:TRINITY\_DN21847\_c0\_g1\_i1.p1:TRINITY\_DN4497\_c0\_g1\_i1.p1 |  |  | DB Search |
| TAIAIDTIINQKR | 58.72 | 1455.8409 | 13 | 0.37 | 728.924 | 16.37 | 1.0604 | 1.0676 | 48298 | 0 | 0 | 0 | TRINITY\_DN200\_c0\_g1\_i1.p1:TRINITY\_DN200\_c0\_g1\_i5.p1:TRINITY\_DN200\_c0\_g1\_i2.p1:TRINITY\_DN200\_c0\_g1\_i3.p1 |  |  | DB Search |
| A(+42.01)SAATDNRK | 58.7 | 974.4781 | 9 | 5.12 | 488.2462 | 9.48 | 0.827 | 0.8343 | 10772 | 6.6e2 | 1 | 1 | TRINITY\_DN7659\_c0\_g1\_i1.p1:TRINITY\_DN7659\_c0\_g1\_i2.p1 | Acetylation (Protein N-term) | A1:Acetylation (Protein N-term):1000 | DB Search |
| FATHASALEIHGLDR | 58.7 | 1636.8322 | 15 | 6.31 | 819.424 | 14.70 | 1.1366 | 1.1438 | 39824 | 2.68e2 | 1 | 1 | TRINITY\_DN1989\_c0\_g1\_i1.p1 |  |  | DB Search |
| ALLSLEYHEKR | 58.7 | 1357.7354 | 11 | 1.49 | 679.8723 | 14.29 | 1.0271 | 1.0343 | 37627 | 2.25e3 | 1 | 1 | TRINITY\_DN8707\_c0\_g1\_i2.p1 |  |  | DB Search |
| HGYIGEFEVIDDHR | 58.64 | 1685.7798 | 14 | 0.78 | 562.9312 | 17.55 | 0.8725 | 0.8798 | 54397 | 3.69e2 | 1 | 1 | TRINITY\_DN3339\_c0\_g1\_i1.p1 |  |  | DB Search |
| FSQHTDYVIAR | 58.61 | 1335.6571 | 11 | 2.7 | 668.834 | 13.36 | 0.9759 | 0.9832 | 32542 | 1.21e3 | 1 | 1 | TRINITY\_DN121866\_c0\_g1\_i1.p1 |  |  | DB Search |
| GLFIIDDKGVLR | 58.59 | 1344.7765 | 12 | 1.55 | 673.3929 | 18.40 | 1.0009 | 1.0081 | 58995 | 0 | 0 | 0 | TRINITY\_DN332\_c0\_g1\_i1.p1 |  |  | DB Search |
| YVVMLQYYHDSDTAK | 58.58 | 1831.8451 | 15 | 5.67 | 916.93 | 16.32 | 1.1813 | 1.1885 | 48049 | 0 | 0 | 0 | TRINITY\_DN1616\_c0\_g1\_i3.p1:TRINITY\_DN1616\_c0\_g1\_i6.p1:TRINITY\_DN1616\_c0\_g1\_i2.p1:TRINITY\_DN1616\_c0\_g1\_i7.p1 |  |  | DB Search |
| IGKPHTVPC(+57.02)K | 58.56 | 1135.6172 | 10 | 9.01 | 379.5477 | 9.76 | 0.7709 | 0.7783 | 11898 | 7.38e3 | 2 | 2 | TRINITY\_DN15584\_c0\_g1\_i1.p1 | Carbamidomethylation |  | DB Search |
| SAIIVDGKNEEGR | 58.54 | 1386.7103 | 13 | 3.91 | 694.3613 | 11.82 | 0.9961 | 1.0033 | 23812 | 1.85e3 | 1 | 1 | TRINITY\_DN982\_c0\_g1\_i3.p1 |  |  | DB Search |
| LDLAGRDLTDYLMK | 58.52 | 1622.8337 | 14 | 2.44 | 812.4217 | 18.99 | 1.0961 | 1.1033 | 62153 | 2.2e3 | 1 | 1 | TRINITY\_DN161\_c0\_g1\_i1.p1:TRINITY\_DN161\_c0\_g1\_i4.p1:TRINITY\_DN161\_c0\_g1\_i3.p1:TRINITY\_DN161\_c0\_g1\_i2.p1 |  |  | DB Search |
| TPGLVEGTLILHQLR | 58.52 | 1645.9515 | 15 | -1.31 | 549.6541 | 18.53 | 0.8644 | 0.8716 | 59662 | 7.05e2 | 1 | 1 | TRINITY\_DN3034\_c0\_g1\_i1.p1 |  |  | DB Search |
| S(+42.01)ATGNLVTVLRK | 58.51 | 1299.751 | 12 | 3.45 | 650.8815 | 16.84 | 0.9973 | 1.0045 | 50424 | 5.95e3 | 1 | 1 | TRINITY\_DN2963\_c0\_g1\_i1.p1 | Acetylation (Protein N-term) | S1:Acetylation (Protein N-term):1000 | DB Search |
| DLHIIEDYVLK | 58.51 | 1356.7289 | 11 | 1.5 | 679.369 | 19.23 | 0.9932 | 1.0005 | 63393 | 3.95e2 | 1 | 1 | TRINITY\_DN8903\_c0\_g1\_i1.p1 |  |  | DB Search |
| HC(+57.02)DPELSSFHTFWR | 58.51 | 1817.7943 | 14 | -2.5 | 606.9339 | 18.48 | 0.8862 | 0.8935 | 59444 | 3.29e2 | 1 | 1 | TRINITY\_DN22492\_c0\_g1\_i1.p1:TRINITY\_DN22492\_c0\_g1\_i2.p1 | Carbamidomethylation |  | DB Search |
| KLQAELANGR | 58.46 | 1098.6145 | 10 | 6.54 | 550.3151 | 11.42 | 0.8907 | 0.898 | 21463 | 3.82e3 | 1 | 1 | TRINITY\_DN15599\_c0\_g1\_i1.p1:TRINITY\_DN15599\_c0\_g1\_i2.p1 |  |  | DB Search |
| LIQYIENKPGLTR | 58.44 | 1543.8722 | 13 | 3.24 | 772.9417 | 14.62 | 1.0761 | 1.0833 | 39327 | 2e3 | 1 | 1 | TRINITY\_DN2028\_c0\_g1\_i1.p1 |  |  | DB Search |
| AEYENKTEVAR | 58.43 | 1308.631 | 11 | 6.12 | 655.3232 | 10.97 | 0.9771 | 0.9844 | 18941 | 1.06e3 | 1 | 1 | TRINITY\_DN1280\_c0\_g1\_i1.p1 |  |  | DB Search |
| ALEHYSDIYDIKR | 58.43 | 1621.8099 | 13 | -3.12 | 541.606 | 14.46 | 0.8238 | 0.8311 | 38607 | 5.17e2 | 1 | 1 | TRINITY\_DN2113\_c0\_g1\_i1.p1:TRINITY\_DN2113\_c0\_g1\_i2.p1 |  |  | DB Search |
| DNIQGITKPAIRR | 58.42 | 1480.8474 | 13 | 5.31 | 494.623 | 12.73 | 0.7852 | 0.7925 | 28785 | 1.32e3 | 1 | 1 | TRINITY\_DN172571\_c0\_g1\_i1.p1 |  |  | DB Search |
| VDNKVNATER | 58.41 | 1144.5836 | 10 | 1.18 | 573.2966 | 9.57 | 0.8862 | 0.8935 | 11155 | 1.2e3 | 1 | 1 | TRINITY\_DN32522\_c0\_g1\_i1.p1 |  |  | DB Search |
| GVAGAGVLAGFDKFK | 58.4 | 1435.7823 | 15 | 1.33 | 718.8954 | 17.98 | 1.0873 | 1.0945 | 56680 | 2.72e3 | 1 | 1 | TRINITY\_DN1559\_c0\_g1\_i1.p1 |  |  | DB Search |
| YVESAADKR | 58.39 | 1037.5142 | 9 | 8.99 | 519.7662 | 9.59 | 0.8575 | 0.8648 | 11233 | 0 | 0 | 0 | TRINITY\_DN7314\_c0\_g2\_i1.p1 |  |  | DB Search |
| KAQYYEAYETIIKR | 58.38 | 1774.9254 | 14 | -0.12 | 592.6458 | 15.51 | 0.8644 | 0.8716 | 44111 | 1.84e3 | 2 | 2 | TRINITY\_DN319\_c2\_g1\_i3.p1:TRINITY\_DN319\_c2\_g1\_i1.p1 |  |  | DB Search |
| SAATQTSIKENK | 58.38 | 1276.6622 | 12 | -2.61 | 639.3333 | 9.66 | 0.9646 | 0.9719 | 11591 | 0 | 0 | 0 | TRINITY\_DN453\_c0\_g1\_i1.p1:TRINITY\_DN453\_c0\_g1\_i2.p1 |  |  | DB Search |
| FVDEASKK | 58.37 | 922.476 | 8 | 2.26 | 462.2438 | 9.81 | 0.8128 | 0.8201 | 12343 | 2.44e3 | 1 | 1 | TRINITY\_DN164753\_c0\_g1\_i1.p1 |  |  | DB Search |
| AYDQSVSVLLGRFEK | 58.37 | 1710.894 | 15 | -2.61 | 856.4474 | 18.83 | 1.1482 | 1.1554 | 61287 | 0 | 0 | 0 | TRINITY\_DN7112\_c0\_g1\_i2.p1 |  |  | DB Search |
| EIRDILVQYDR | 58.36 | 1418.7517 | 11 | -6.7 | 710.3745 | 19.40 | 1.0339 | 1.0411 | 64335 | 5.13e3 | 1 | 1 | TRINITY\_DN128406\_c0\_g1\_i1.p1 |  |  | DB Search |
| IRNEEIQIEVIERR | 58.34 | 1795.9904 | 14 | 0.23 | 599.6676 | 14.93 | 0.881 | 0.8883 | 41049 | 1.01e3 | 1 | 1 | TRINITY\_DN11435\_c0\_g1\_i1.p1 |  |  | DB Search |
| AFLIEEQKLVVR | 58.34 | 1443.8448 | 12 | 1.95 | 722.9272 | 17.10 | 1.0492 | 1.0564 | 51842 | 4.02e3 | 1 | 1 | TRINITY\_DN79126\_c0\_g1\_i1.p1 |  |  | DB Search |
| M(+15.99)SATFIGNSTAIQELFKR | 58.33 | 2029.0303 | 18 | -2.43 | 677.3453 | 19.58 | 0.9344 | 0.9416 | 65134 | 3.85e3 | 1 | 1 | TRINITY\_DN3986\_c0\_g1\_i1.p1 | Oxidation (M) | M1:Oxidation (M):1000 | DB Search |
| IAGDVIVC(+57.02)AAYSHELPR | 58.33 | 1869.9407 | 17 | 6.24 | 935.9783 | 16.90 | 1.2033 | 1.2105 | 50851 | 1.42e2 | 1 | 1 | TRINITY\_DN116\_c0\_g1\_i1.p1 | Carbamidomethylation |  | DB Search |
| EVNTELDSRPETK | 58.3 | 1516.7369 | 13 | 4.85 | 759.3752 | 11.70 | 1.025 | 1.0323 | 23146 | 0 | 0 | 0 | TRINITY\_DN11643\_c0\_g1\_i1.p1 |  |  | DB Search |
| LGGIEVTQGNC(+57.02)ARK | 58.27 | 1501.7671 | 14 | 1.48 | 751.8878 | 12.59 | 1.0616 | 1.0688 | 28229 | 7.18e2 | 1 | 1 | TRINITY\_DN36\_c0\_g1\_i9.p1:TRINITY\_DN36\_c0\_g1\_i14.p1:TRINITY\_DN36\_c0\_g1\_i13.p1:TRINITY\_DN36\_c0\_g1\_i2.p1:TRINITY\_DN36\_c0\_g1\_i3.p1:TRINITY\_DN36\_c0\_g1\_i16.p1:TRINITY\_DN36\_c0\_g1\_i17.p1:TRINITY\_DN36\_c0\_g1\_i4.p1:TRINITY\_DN36\_c0\_g1\_i11.p1:TRINITY\_DN36\_c0\_g1\_i8.p1:TRINITY\_DN36\_c0\_g1\_i1.p1:TRINITY\_DN36\_c0\_g1\_i19.p1:TRINITY\_DN36\_c0\_g1\_i20.p1:TRINITY\_DN36\_c0\_g1\_i5.p1 | Carbamidomethylation |  | DB Search |
| IKAEYENKTEVAR | 58.27 | 1549.8099 | 13 | 1.64 | 775.9093 | 10.97 | 1.056 | 1.0632 | 18831 | 5.28e2 | 1 | 1 | TRINITY\_DN1280\_c0\_g1\_i1.p1 |  |  | DB Search |
| VTLVSERPFSK | 58.25 | 1261.703 | 11 | 2.16 | 631.8567 | 15.09 | 0.9602 | 0.9675 | 41854 | 1.01e3 | 1 | 1 | TRINITY\_DN95248\_c0\_g1\_i1.p1 |  |  | DB Search |
| RLPEEDQYLR | 58.24 | 1317.6677 | 10 | 3.82 | 659.84 | 13.95 | 0.9711 | 0.9783 | 35764 | 3.56e3 | 1 | 1 | TRINITY\_DN436\_c0\_g1\_i1.p1 |  |  | DB Search |
| GAFGKPQGTVAR | 58.24 | 1187.6411 | 12 | 4.16 | 594.827 | 11.37 | 0.9247 | 0.9319 | 21197 | 7.61e2 | 1 | 1 | TRINITY\_DN72959\_c0\_g1\_i1.p1 |  |  | DB Search |
| VLTVISHNQR | 58.24 | 1165.6567 | 10 | 5.45 | 583.8356 | 12.20 | 0.9315 | 0.9388 | 25932 | 5.23e2 | 1 | 1 | TRINITY\_DN197343\_c0\_g1\_i1.p1 |  |  | DB Search |
| AERPEVDTKR | 58.22 | 1199.6259 | 10 | 3.55 | 400.8818 | 9.63 | 0.7143 | 0.7216 | 11402 | 6.01e2 | 1 | 1 | TRINITY\_DN1566\_c0\_g1\_i1.p1 |  |  | DB Search |
| NM(+15.99)M(+15.99)AAC(+57.02)DPR | 58.21 | 1096.41 | 9 | 1.94 | 549.2103 | 9.53 | 0.8627 | 0.87 | 10857 | 1.37e3 | 1 | 1 | TRINITY\_DN1910\_c0\_g1\_i1.p1:TRINITY\_DN1910\_c0\_g1\_i5.p1:TRINITY\_DN3986\_c0\_g1\_i1.p1 | Oxidation (M), Oxidation (M), Carbamidomethylation | M2:Oxidation (M):1000 M3:Oxidation (M):1000 | DB Search |
| RVTILENELDYQHR | 58.2 | 1784.9169 | 14 | 1.12 | 595.977 | 15.12 | 0.8895 | 0.8968 | 42087 | 3.46e2 | 1 | 1 | TRINITY\_DN10497\_c0\_g1\_i1.p1 |  |  | DB Search |
| VAPEEHPVLLTEAPLNPK | 58.19 | 1953.0571 | 18 | -0.07 | 977.5304 | 16.19 | 1.2089 | 1.2161 | 47306 | 2.52e2 | 1 | 1 | TRINITY\_DN2477\_c0\_g1\_i6.p1:TRINITY\_DN2477\_c0\_g1\_i5.p1:TRINITY\_DN2477\_c0\_g1\_i8.p1:TRINITY\_DN195934\_c0\_g1\_i1.p2:TRINITY\_DN161\_c0\_g1\_i1.p1:TRINITY\_DN2477\_c0\_g1\_i9.p1:TRINITY\_DN2477\_c0\_g1\_i10.p1:TRINITY\_DN2477\_c0\_g1\_i2.p1:TRINITY\_DN161\_c0\_g1\_i3.p1:TRINITY\_DN2941\_c0\_g1\_i4.p1:TRINITY\_DN161\_c0\_g1\_i2.p1 |  |  | DB Search |
| LIEVEEKEIQR | 58.17 | 1384.7561 | 11 | 5.59 | 693.3854 | 13.72 | 1.0158 | 1.023 | 34552 | 5.04e2 | 1 | 1 | TRINITY\_DN11435\_c0\_g1\_i1.p1 |  |  | DB Search |
| ALGC(+57.02)DVLAC(+57.02)SVDSHFSHLAWTQQSR | 58.16 | 2844.3071 | 25 | -3.91 | 712.0774 | 18.53 | 0.9238 | 0.9311 | 59620 | 3.52e2 | 1 | 1 | TRINITY\_DN535\_c0\_g1\_i2.p1:TRINITY\_DN535\_c0\_g1\_i1.p1 | Carbamidomethylation, Carbamidomethylation |  | DB Search |
| LISSDGFEFIVKR | 58.16 | 1509.8191 | 13 | 0.29 | 755.9129 | 18.04 | 1.0969 | 1.1041 | 57047 | 2.11e2 | 1 | 1 | TRINITY\_DN95987\_c0\_g1\_i1.p1 |  |  | DB Search |
| AYLTLC(+57.02)VDLKR | 58.15 | 1350.7329 | 11 | -2.24 | 676.3685 | 17.00 | 1.019 | 1.0263 | 51369 | 5.99e2 | 1 | 1 | TRINITY\_DN3870\_c0\_g1\_i1.p1 | Carbamidomethylation |  | DB Search |
| KVIVDQLVTLATR | 58.15 | 1454.8821 | 13 | -1.14 | 728.4435 | 18.03 | 1.0275 | 1.0347 | 57007 | 5.01e2 | 1 | 1 | TRINITY\_DN1386\_c0\_g1\_i1.p1 |  |  | DB Search |
| GLKVDFDTTFAPVTGKK | 58.14 | 1822.9828 | 17 | 1.48 | 912.4951 | 16.88 | 1.1802 | 1.1873 | 50684 | 2.54e2 | 1 | 1 | TRINITY\_DN329\_c0\_g1\_i1.p1:TRINITY\_DN329\_c0\_g1\_i2.p1 |  |  | DB Search |
| KEELC(+57.02)HQLTELKTELQQLHVAK | 58.14 | 2674.4111 | 22 | -1.57 | 535.8857 | 18.20 | 0.8408 | 0.8481 | 57883 | 8.64e2 | 2 | 2 | TRINITY\_DN197343\_c0\_g1\_i1.p1 | Carbamidomethylation |  | DB Search |
| ASGDYATVVSHNR | 58.13 | 1375.6481 | 13 | 5.41 | 688.8312 | 12.05 | 0.9937 | 1.0009 | 25145 | 0 | 0 | 0 | TRINITY\_DN925\_c0\_g1\_i1.p1 |  |  | DB Search |
| LEGLFTVTVSHPGAR | 58.13 | 1582.8467 | 15 | -1.11 | 792.4254 | 17.01 | 1.0937 | 1.1009 | 51427 | 1.13e2 | 1 | 1 | TRINITY\_DN189999\_c0\_g1\_i1.p1 |  |  | DB Search |
| SDSSDIEKR | 58.12 | 1035.4833 | 9 | -14.56 | 518.7385 | 11.06 | 0.8567 | 0.864 | 19468 | 4.49e3 | 1 | 1 | TRINITY\_DN135129\_c0\_g1\_i1.p1 |  |  | DB Search |
| LGPVVDGKYDYTVITTDYK | 58.12 | 2146.0833 | 19 | 7.52 | 1074.0511 | 17.25 | 1.2428 | 1.2499 | 52770 | 0 | 0 | 0 | TRINITY\_DN468\_c1\_g1\_i1.p1 |  |  | DB Search |
| GYSFTTTAEREIVR | 58.1 | 1628.8158 | 14 | 2.53 | 815.4128 | 15.79 | 1.121 | 1.1282 | 45442 | 6.41e3 | 1 | 1 | TRINITY\_DN2477\_c0\_g1\_i6.p1:TRINITY\_DN2477\_c0\_g1\_i5.p1:TRINITY\_DN2477\_c0\_g1\_i8.p1:TRINITY\_DN2477\_c0\_g1\_i7.p1:TRINITY\_DN161\_c0\_g1\_i1.p1:TRINITY\_DN2477\_c0\_g1\_i9.p1:TRINITY\_DN2477\_c0\_g1\_i10.p1:TRINITY\_DN161\_c0\_g1\_i4.p1:TRINITY\_DN2477\_c0\_g1\_i2.p1:TRINITY\_DN161\_c0\_g1\_i3.p1:TRINITY\_DN161\_c0\_g1\_i2.p1 |  |  | DB Search |
| IVAINAHHFLK | 58.09 | 1261.7295 | 11 | 2.94 | 631.8704 | 14.34 | 1.0166 | 1.0238 | 37934 | 0 | 0 | 0 | TRINITY\_DN1084\_c0\_g1\_i1.p1 |  |  | DB Search |
| VIIDYQTHHR | 58.05 | 1280.6626 | 10 | -2.89 | 641.3332 | 12.03 | 0.9533 | 0.9606 | 25056 | 0 | 0 | 0 | TRINITY\_DN1907\_c0\_g1\_i1.p1:TRINITY\_DN1907\_c0\_g1\_i2.p1 |  |  | DB Search |
| ETC(+57.02)SDIEKR | 58.04 | 1136.5132 | 9 | 2.35 | 569.2621 | 10.08 | 0.8947 | 0.902 | 13694 | 6.3e3 | 1 | 1 | TRINITY\_DN118\_c1\_g1\_i3.p1:TRINITY\_DN118\_c1\_g1\_i5.p1 | Carbamidomethylation |  | DB Search |
| NIDEHASEGVER | 58.02 | 1354.6113 | 12 | 8.37 | 678.3149 | 11.02 | 0.9695 | 0.9767 | 19141 | 1.13e3 | 1 | 1 | TRINITY\_DN10907\_c0\_g1\_i1.p1 |  |  | DB Search |
| SFLLALEKDQEK | 58 | 1419.7609 | 12 | 1.3 | 710.8848 | 17.09 | 1.0733 | 1.0805 | 51721 | 4.89e3 | 1 | 1 | TRINITY\_DN1386\_c0\_g1\_i1.p1 |  |  | DB Search |
| GQVIC(+57.02)APGATSTHTK | 58 | 1526.7511 | 15 | 1.7 | 764.3799 | 11.76 | 1.0291 | 1.0363 | 23431 | 4.83e2 | 1 | 1 | TRINITY\_DN9509\_c0\_g1\_i6.p1:TRINITY\_DN9509\_c0\_g1\_i8.p1:TRINITY\_DN9509\_c0\_g1\_i10.p1 | Carbamidomethylation |  | DB Search |
| VGNWRDETLSC(+57.02)K | 57.99 | 1463.6827 | 12 | 3.82 | 732.8474 | 13.58 | 1.0114 | 1.0186 | 33747 | 3.4e2 | 1 | 1 | TRINITY\_DN6390\_c0\_g1\_i1.p1 | Carbamidomethylation |  | DB Search |
| VTEAKGELAK | 57.99 | 1044.5815 | 10 | 1.77 | 523.2961 | 10.00 | 0.8947 | 0.902 | 13309 | 9.44e2 | 1 | 1 | TRINITY\_DN614\_c0\_g1\_i1.p1 |  |  | DB Search |
| DFVKM(+15.99)LLLLM(+15.99)LSK | 57.98 | 1581.8873 | 13 | 1.83 | 528.3011 | 19.45 | 0.7742 | 0.7815 | 64632 | 0 | 0 | 0 | TRINITY\_DN126137\_c0\_g1\_i1.p1 | Oxidation (M), Oxidation (M) | M5:Oxidation (M):1000 M10:Oxidation (M):1000 | DB Search |
| ALKEPPRDR | 57.98 | 1080.604 | 9 | 10.29 | 361.2103 | 9.44 | 0.7229 | 0.7302 | 10425 | 1.55e4 | 1 | 1 | TRINITY\_DN164128\_c0\_g1\_i1.p1 |  |  | DB Search |
| TQAVLDRLNIEKR | 57.97 | 1554.8842 | 13 | -4.75 | 519.2967 | 14.41 | 0.8031 | 0.8104 | 38344 | 0 | 0 | 0 | TRINITY\_DN477\_c0\_g1\_i9.p1 |  |  | DB Search |
| FVLHGDVSHGSR | 57.97 | 1309.6527 | 12 | -8.24 | 655.8246 | 11.96 | 0.9872 | 0.9945 | 24686 | 0 | 0 | 0 | TRINITY\_DN22\_c0\_g1\_i1.p1:TRINITY\_DN384\_c0\_g1\_i2.p1:TRINITY\_DN384\_c0\_g1\_i1.p1 |  |  | DB Search |
| VVAEC(+57.02)NNGGGR | 57.96 | 1131.509 | 11 | -8.94 | 566.7537 | 9.41 | 0.8234 | 0.8307 | 10484 | 0 | 0 | 0 | TRINITY\_DN1616\_c0\_g1\_i3.p1:TRINITY\_DN1616\_c0\_g1\_i6.p1:TRINITY\_DN1616\_c0\_g1\_i7.p1 | Carbamidomethylation |  | DB Search |
| LC(+57.02)ETNTANTEAK | 57.96 | 1350.6085 | 12 | -0.88 | 676.3073 | 10.37 | 0.9537 | 0.961 | 15355 | 0 | 0 | 0 | TRINITY\_DN16120\_c0\_g1\_i2.p1:TRINITY\_DN16120\_c0\_g1\_i1.p1 | Carbamidomethylation |  | DB Search |
| FETQIATK | 57.95 | 936.4916 | 8 | 1.49 | 937.4952 | 13.74 | 1.2208 | 1.228 | 34637 | 7.44e2 | 2 | 2 | TRINITY\_DN649\_c0\_g1\_i2.p1:TRINITY\_DN36\_c0\_g1\_i3.p1:TRINITY\_DN36\_c0\_g1\_i16.p1:TRINITY\_DN36\_c0\_g1\_i14.p1:TRINITY\_DN36\_c0\_g1\_i17.p1:TRINITY\_DN649\_c0\_g1\_i5.p1:TRINITY\_DN36\_c0\_g1\_i13.p1:TRINITY\_DN36\_c0\_g1\_i8.p1:TRINITY\_DN36\_c0\_g1\_i19.p1:TRINITY\_DN649\_c0\_g1\_i1.p1 |  |  | DB Search |
| AYGQDLGQPGSC(+57.02)LKR | 57.94 | 1648.7991 | 15 | 3.8 | 825.4055 | 13.30 | 1.0721 | 1.0793 | 32177 | 9.59e2 | 1 | 1 | TRINITY\_DN691\_c0\_g1\_i2.p1:TRINITY\_DN691\_c0\_g1\_i1.p1 | Carbamidomethylation |  | DB Search |
| LAADLVITHSSK | 57.93 | 1253.6979 | 12 | -0.91 | 627.8522 | 14.11 | 0.9969 | 1.0041 | 36628 | 1.76e3 | 1 | 1 | TRINITY\_DN751\_c0\_g1\_i1.p1:TRINITY\_DN7214\_c0\_g1\_i1.p1 |  |  | DB Search |
| AM(+15.99)TDC(+57.02)KETLC(+57.02)R | 57.92 | 1399.5894 | 11 | 4.81 | 700.8015 | 10.58 | 0.9993 | 1.0065 | 16561 | 0 | 0 | 0 | TRINITY\_DN168534\_c0\_g1\_i1.p1 | Oxidation (M), Carbamidomethylation, Carbamidomethylation | M2:Oxidation (M):1000 | DB Search |
| AEFRPNQEEEPILKR | 57.91 | 1854.9587 | 15 | 1.99 | 619.3247 | 13.64 | 0.8761 | 0.8834 | 34085 | 5.81e2 | 1 | 1 | TRINITY\_DN262\_c0\_g1\_i1.p1 |  |  | DB Search |
| VTSQREEDEYVK | 57.91 | 1481.6998 | 12 | 3.51 | 741.8557 | 11.18 | 1.0488 | 1.056 | 20043 | 1.4e3 | 1 | 1 | TRINITY\_DN309\_c0\_g1\_i1.p1 |  |  | DB Search |
| KFVWEFFQK | 57.89 | 1257.6545 | 9 | -4.78 | 629.8281 | 19.49 | 0.9461 | 0.9533 | 64710 | 2.12e3 | 1 | 1 | TRINITY\_DN1280\_c0\_g1\_i1.p1 |  |  | DB Search |
| IAAVIINKPDVLASR | 57.88 | 1578.9457 | 15 | -5.91 | 790.4711 | 15.76 | 1.0989 | 1.1061 | 45377 | 0 | 0 | 0 | TRINITY\_DN123\_c0\_g1\_i1.p1 |  |  | DB Search |
| SGTLTYEAVHQTTK | 57.82 | 1534.7627 | 14 | 1.94 | 768.3859 | 12.71 | 1.0701 | 1.0773 | 28882 | 4.08e2 | 1 | 1 | TRINITY\_DN20323\_c0\_g1\_i1.p1 |  |  | DB Search |
| VLVHTQQK | 57.79 | 951.5502 | 8 | -1.07 | 476.7792 | 9.55 | 0.8352 | 0.8425 | 11014 | 5.75e2 | 1 | 1 | TRINITY\_DN6920\_c0\_g1\_i1.p1:TRINITY\_DN6920\_c0\_g1\_i3.p1:TRINITY\_DN6920\_c0\_g1\_i4.p1:TRINITY\_DN6920\_c0\_g1\_i2.p1 |  |  | DB Search |
| RIAEVQC(+57.02)EAR | 57.78 | 1230.6139 | 10 | 3.28 | 616.3129 | 10.93 | 0.923 | 0.9303 | 18587 | 2.26e3 | 1 | 1 | TRINITY\_DN1280\_c0\_g1\_i1.p1 | Carbamidomethylation |  | DB Search |
| IPIFSAAGLPHNEIAAQIC(+57.02)R | 57.77 | 2177.1416 | 20 | 1.64 | 726.7183 | 18.57 | 1.0086 | 1.0158 | 59833 | 6.81e2 | 1 | 1 | TRINITY\_DN3773\_c0\_g1\_i1.p1 | Carbamidomethylation |  | DB Search |
| AVDIHFALDASQNVGR | 57.77 | 1711.8641 | 16 | -0.25 | 856.9344 | 17.17 | 1.1033 | 1.1106 | 52342 | 2.27e2 | 1 | 1 | TRINITY\_DN77\_c1\_g1\_i1.p1 |  |  | DB Search |
| LFEGNALLRR | 57.75 | 1187.6775 | 10 | 3.63 | 594.8449 | 15.37 | 0.9683 | 0.9755 | 43382 | 1.83e3 | 1 | 1 | TRINITY\_DN776\_c0\_g1\_i1.p1:TRINITY\_DN189323\_c0\_g1\_i1.p1 |  |  | DB Search |
| SSFDEIHKFHTQILR | 57.72 | 1856.9532 | 15 | 0.93 | 619.9889 | 15.24 | 0.8708 | 0.8781 | 42768 | 0 | 0 | 0 | TRINITY\_DN147947\_c0\_g1\_i1.p1 |  |  | DB Search |
| DPVRDKLVNER | 57.67 | 1339.7208 | 11 | 2.48 | 447.5795 | 11.98 | 0.7428 | 0.7502 | 24731 | 1.17e2 | 1 | 1 | TRINITY\_DN262\_c0\_g1\_i1.p1 |  |  | DB Search |
| LVVIADIVDQKR | 57.66 | 1367.8136 | 12 | 0.99 | 684.911 | 17.26 | 1.0419 | 1.0492 | 52731 | 1.74e3 | 1 | 1 | TRINITY\_DN6294\_c0\_g3\_i1.p1:TRINITY\_DN6294\_c0\_g3\_i2.p1 |  |  | DB Search |
| A(+42.01)HLSQEQRNELKK | 57.66 | 1621.8536 | 13 | 3.86 | 541.6243 | 11.22 | 0.8071 | 0.8144 | 20267 | 8.15e2 | 1 | 1 | TRINITY\_DN3052\_c0\_g1\_i1.p1 | Acetylation (Protein N-term) | A1:Acetylation (Protein N-term):1000 | DB Search |
| TQITC(+57.02)TQISLKR | 57.65 | 1447.7817 | 12 | -4.63 | 724.8908 | 13.68 | 1.025 | 1.0323 | 34409 | 0 | 0 | 0 | TRINITY\_DN166344\_c0\_g1\_i1.p1 | Carbamidomethylation |  | DB Search |
| ISGLIYEETRGVLK | 57.65 | 1576.8824 | 14 | 4.01 | 789.4473 | 17.11 | 1.0945 | 1.1017 | 51906 | 3.57e3 | 1 | 1 | TRINITY\_DN172571\_c0\_g1\_i1.p1:TRINITY\_DN22077\_c0\_g1\_i1.p3 |  |  | DB Search |
| RPVPIVTRR | 57.63 | 1092.688 | 9 | 6 | 365.2368 | 10.84 | 0.7045 | 0.7118 | 18035 | 1.06e3 | 1 | 1 | TRINITY\_DN1080\_c0\_g1\_i2.p1:TRINITY\_DN1080\_c0\_g2\_i1.p1:TRINITY\_DN1080\_c0\_g1\_i3.p1 |  |  | DB Search |
| GITINASHIEYETSNR | 57.62 | 1803.8751 | 16 | -0.09 | 902.9398 | 14.89 | 1.1674 | 1.1746 | 40938 | 0 | 0 | 0 | TRINITY\_DN9509\_c0\_g1\_i6.p1:TRINITY\_DN9509\_c0\_g1\_i2.p1:TRINITY\_DN9509\_c0\_g1\_i10.p1 |  |  | DB Search |
| LRVDLSLFETGIK | 57.58 | 1489.8503 | 13 | -1.87 | 745.927 | 19.61 | 1.0363 | 1.0435 | 65344 | 1.84e3 | 1 | 1 | TRINITY\_DN11097\_c0\_g1\_i1.p1:TRINITY\_DN11097\_c0\_g2\_i1.p1 |  |  | DB Search |
| VC(+57.02)EDVKR | 57.57 | 904.4437 | 7 | 2.45 | 453.2277 | 9.38 | 0.7722 | 0.7795 | 10303 | 3.95e3 | 2 | 2 | TRINITY\_DN118\_c1\_g1\_i3.p1:TRINITY\_DN118\_c1\_g1\_i5.p1 | Carbamidomethylation |  | DB Search |
| K(+42.01)LAAELANGR | 57.57 | 1083.6036 | 10 | 2.65 | 542.8076 | 15.11 | 0.8668 | 0.8741 | 42049 | 7.44e2 | 1 | 1 | TRINITY\_DN1172\_c0\_g2\_i2.p1 | Acetylation (Protein N-term) | K1:Acetylation (Protein N-term):1000 | DB Search |
| VGYTHLFSSTQVSVRPK | 57.32 | 1905.0109 | 17 | 9.78 | 953.5168 | 15.02 | 1.2479 | 1.2551 | 41579 | 9.02e1 | 1 | 1 | TRINITY\_DN2301\_c0\_g1\_i1.p1 |  |  | DB Search |
| YLDLILNEKVR | 57.31 | 1374.7871 | 11 | 3.44 | 688.3994 | 17.46 | 1.0407 | 1.048 | 53939 | 3.63e2 | 1 | 1 | TRINITY\_DN187823\_c0\_g1\_i1.p1 |  |  | DB Search |
| FVWEFFQK | 57.31 | 1129.5597 | 8 | -6.33 | 1130.5536 | 19.47 | 1.2833 | 1.2905 | 64690 | 0 | 0 | 0 | TRINITY\_DN1280\_c0\_g1\_i1.p1 |  |  | DB Search |
| NIALETLEGHQR | 57.29 | 1379.7157 | 12 | -4.63 | 690.8582 | 15.05 | 1.017 | 1.0242 | 41788 | 0 | 0 | 0 | TRINITY\_DN1280\_c0\_g1\_i1.p1 |  |  | DB Search |
| ISNVQDIVPVLELANQHK | 57.22 | 2016.1003 | 18 | -0.45 | 673.0367 | 19.27 | 0.9509 | 0.9582 | 63623 | 2.54e2 | 1 | 1 | TRINITY\_DN118520\_c0\_g1\_i1.p1 |  |  | DB Search |
| SDKEEHFC(+57.02)LR | 57.1 | 1319.5928 | 10 | 6.18 | 440.8719 | 12.41 | 0.7844 | 0.7917 | 27277 | 0 | 0 | 0 | TRINITY\_DN118208\_c0\_g1\_i1.p1 | Carbamidomethylation |  | DB Search |
| AKLVIISSNTPQLRK | 57.08 | 1667.0094 | 15 | 0.8 | 834.5081 | 14.23 | 1.1362 | 1.1434 | 37393 | 0 | 0 | 0 | TRINITY\_DN2126\_c0\_g1\_i1.p1 |  |  | DB Search |
| TPFLLVGTQVDLRDDAAIVEK | 56.85 | 2299.2422 | 21 | 2.14 | 767.4188 | 19.81 | 1.0319 | 1.0391 | 66139 | 4.65e2 | 1 | 1 | TRINITY\_DN7333\_c0\_g1\_i1.p1 |  |  | DB Search |
| AHGC(+57.02)SQGRWPK | 56.78 | 1282.5989 | 11 | 7.43 | 428.5411 | 10.35 | 0.7591 | 0.7665 | 15181 | 5.1e2 | 1 | 1 | TRINITY\_DN1614\_c0\_g1\_i1.p1 | Carbamidomethylation |  | DB Search |
| KVSQTC(+57.02)AEHVFR | 56.73 | 1460.7194 | 12 | 9.26 | 487.9156 | 11.84 | 0.7982 | 0.8055 | 23975 | 0 | 0 | 0 | TRINITY\_DN3078\_c0\_g1\_i1.p1 | Carbamidomethylation |  | DB Search |
| YGINHITNLVESK | 56.67 | 1486.778 | 13 | -4.57 | 744.3888 | 15.60 | 1.0371 | 1.0443 | 44593 | 0 | 0 | 0 | TRINITY\_DN146208\_c0\_g1\_i1.p1 |  |  | DB Search |
| EKSM(+15.99)IHELNR | 56.52 | 1271.6292 | 10 | 3.88 | 636.8209 | 10.41 | 0.9202 | 0.9275 | 15609 | 4.13e2 | 1 | 1 | TRINITY\_DN932\_c0\_g1\_i1.p1 | Oxidation (M) | M4:Oxidation (M):1000 | DB Search |
| IEHAHPINR | 56.51 | 1085.573 | 9 | 12.45 | 362.8675 | 9.50 | 0.7416 | 0.7489 | 10850 | 5.9e2 | 1 | 1 | TRINITY\_DN59667\_c0\_g1\_i1.p1 |  |  | DB Search |
| VSQTC(+57.02)AEHVFR | 56.5 | 1332.6245 | 11 | -12.91 | 667.3073 | 11.84 | 0.9868 | 0.9941 | 23983 | 0 | 0 | 0 | TRINITY\_DN3078\_c0\_g1\_i1.p1 | Carbamidomethylation |  | DB Search |
| KKGEGSLSLQC(+57.02)TNTAVVLAHTIEGK | 56.21 | 2640.3904 | 25 | -9.74 | 661.0948 | 17.15 | 0.9339 | 0.9412 | 52200 | 0 | 0 | 0 | TRINITY\_DN10940\_c0\_g1\_i1.p1 | Carbamidomethylation |  | DB Search |
| SNGGLVVGTIIKPK | 56.21 | 1381.8292 | 14 | 0.75 | 691.9186 | 15.49 | 1.0528 | 1.06 | 43994 | 1.43e3 | 1 | 1 | TRINITY\_DN3036\_c0\_g1\_i19.p1:TRINITY\_DN3036\_c0\_g1\_i13.p1:TRINITY\_DN3036\_c0\_g1\_i11.p1:TRINITY\_DN3036\_c0\_g1\_i16.p1 |  |  | DB Search |
| RRYPIIVTGR | 56.14 | 1229.7356 | 10 | 4.66 | 410.9188 | 12.79 | 0.777 | 0.7844 | 29251 | 7.87e2 | 1 | 1 | TRINITY\_DN333\_c0\_g1\_i2.p1:TRINITY\_DN333\_c0\_g1\_i1.p1 |  |  | DB Search |
| RYGQAM(+15.99)C(+57.02)QFMR | 56 | 1462.6268 | 11 | -6.37 | 732.312 | 14.39 | 0.9755 | 0.9828 | 37937 | 1.17e3 | 1 | 1 | TRINITY\_DN286\_c0\_g1\_i4.p1:TRINITY\_DN286\_c0\_g1\_i2.p1 | Oxidation (M), Carbamidomethylation | M6:Oxidation (M):38.03 | DB Search |
| DVQLGDLVTVGQC(+57.02)RPLSK | 55.96 | 1984.0411 | 18 | 3.74 | 993.0261 | 17.77 | 1.2766 | 1.2837 | 55652 | 0 | 0 | 0 | TRINITY\_DN15847\_c0\_g1\_i1.p1:TRINITY\_DN15847\_c0\_g1\_i2.p1 | Carbamidomethylation |  | DB Search |
| VVAISGNEDEIVNLLHK | 55.96 | 1848.9945 | 17 | 2.17 | 925.5015 | 18.50 | 1.2109 | 1.2181 | 59519 | 0 | 0 | 0 | TRINITY\_DN651\_c0\_g1\_i2.p1:TRINITY\_DN651\_c0\_g1\_i1.p1 |  |  | DB Search |
| RSITTSVKR | 55.92 | 1046.6196 | 9 | 6.21 | 349.8807 | 9.78 | 0.7037 | 0.711 | 12172 | 0 | 0 | 0 | TRINITY\_DN1080\_c0\_g1\_i2.p1:TRINITY\_DN1080\_c0\_g1\_i3.p1 |  |  | DB Search |
| TRIEDLETVISQTQDHR | 55.76 | 2040.0236 | 17 | -2.78 | 681.0095 | 17.11 | 0.9558 | 0.963 | 52031 | 0 | 0 | 0 | TRINITY\_DN761\_c0\_g1\_i1.p1:TRINITY\_DN761\_c0\_g1\_i3.p1:TRINITY\_DN761\_c0\_g1\_i2.p1 |  |  | DB Search |
| NGEDLSKLEGLFTVTVSHPGAR | 55.71 | 2326.1917 | 22 | 0.49 | 776.4006 | 19.69 | 1.0267 | 1.0339 | 65768 | 5.58e2 | 1 | 1 | TRINITY\_DN189999\_c0\_g1\_i1.p1 |  |  | DB Search |
| GDYC(+57.02)NSALIEAEQKER | 55.62 | 1881.8527 | 16 | 4.16 | 941.9324 | 14.25 | 1.2069 | 1.2141 | 37442 | 0 | 0 | 0 | TRINITY\_DN168534\_c0\_g1\_i1.p1 | Carbamidomethylation |  | DB Search |
| APGDYGWRPPLLATDDAALK | 55.58 | 2126.0796 | 20 | 0.95 | 709.6973 | 18.70 | 0.8919 | 0.8992 | 60584 | 2.4e3 | 1 | 1 | TRINITY\_DN16548\_c0\_g1\_i4.p1 |  |  | DB Search |
| A(+42.01)LSRPVYIIAAK | 55.54 | 1342.7972 | 12 | -0.08 | 672.4022 | 17.65 | 1.0532 | 1.0604 | 54904 | 5.44e3 | 1 | 1 | TRINITY\_DN11662\_c0\_g1\_i1.p1 | Acetylation (Protein N-term) | A1:Acetylation (Protein N-term):1000 | DB Search |
| KGDNEIPGLTDRVIPR | 55.53 | 1778.9639 | 16 | 1.69 | 593.993 | 15.42 | 0.8955 | 0.9028 | 43603 | 3.5e2 | 1 | 1 | TRINITY\_DN2230\_c0\_g1\_i1.p1 |  |  | DB Search |
| RISGLIYEETRGVLK | 55.36 | 1732.9835 | 15 | 1.37 | 578.6661 | 16.41 | 0.8911 | 0.8984 | 48406 | 2.02e3 | 1 | 1 | TRINITY\_DN172571\_c0\_g1\_i1.p1:TRINITY\_DN22077\_c0\_g1\_i1.p3 |  |  | DB Search |
| ELISNSSDALDKIR | 55.36 | 1559.8154 | 14 | 3.71 | 780.9136 | 15.32 | 1.0745 | 1.0817 | 43130 | 3.07e2 | 1 | 1 | TRINITY\_DN141338\_c2\_g1\_i1.p1:TRINITY\_DN5633\_c0\_g1\_i1.p1 |  |  | DB Search |
| RYGQAM(+15.99)C(+57.02)QFM(+15.99)R | 55.34 | 1478.6217 | 11 | -6.44 | 740.3093 | 12.85 | 0.9739 | 0.9812 | 29490 | 4.93e3 | 1 | 1 | TRINITY\_DN286\_c0\_g1\_i4.p1:TRINITY\_DN286\_c0\_g1\_i2.p1 | Oxidation (M), Carbamidomethylation, Oxidation (M) | M6:Oxidation (M):1000 M10:Oxidation (M):1000 | DB Search |
| KIDLHLYHELYM(+15.99)K | 55.33 | 1717.8861 | 13 | 6.51 | 859.9512 | 15.43 | 1.1406 | 1.1478 | 43636 | 4.16e2 | 1 | 1 | TRINITY\_DN1112\_c0\_g1\_i1.p1 | Oxidation (M) | M12:Oxidation (M):1000 | DB Search |
| IKVDDKLNNLDQITLEVAK | 55.27 | 2168.2051 | 19 | -2.25 | 723.7368 | 17.58 | 0.9125 | 0.9198 | 54654 | 0 | 0 | 0 | TRINITY\_DN95248\_c0\_g1\_i1.p1 |  |  | DB Search |
| DMGVNVISVGAGHTINHR | 55.26 | 1875.9374 | 18 | 0.67 | 938.9714 | 15.08 | 1.2451 | 1.2523 | 41936 | 0 | 0 | 0 | TRINITY\_DN138\_c0\_g1\_i1.p1 |  |  | DB Search |
| IQGHPTDKKFTK | 55.25 | 1398.762 | 12 | 4.46 | 467.2608 | 9.57 | 0.7677 | 0.775 | 11206 | 0 | 0 | 0 | TRINITY\_DN1071\_c0\_g1\_i2.p1:TRINITY\_DN1071\_c0\_g1\_i3.p1:TRINITY\_DN1071\_c0\_g1\_i1.p1:TRINITY\_DN1071\_c0\_g1\_i4.p1 |  |  | DB Search |
| KAENFAELKNTFQEAAK | 55.22 | 1937.9846 | 17 | 1.66 | 646.9997 | 15.88 | 0.9194 | 0.9267 | 45985 | 0 | 0 | 0 | TRINITY\_DN79\_c0\_g1\_i1.p1 |  |  | DB Search |
| DKNILVLTLGVGPQVNR | 55.18 | 1835.0629 | 17 | -2.24 | 918.5316 | 19.07 | 1.1314 | 1.1386 | 62691 | 0 | 0 | 0 | TRINITY\_DN477\_c0\_g1\_i8.p1:TRINITY\_DN477\_c0\_g1\_i7.p1 |  |  | DB Search |
| VVGKDQGQSLAK | 55.18 | 1228.6775 | 12 | -1.12 | 615.342 | 10.28 | 0.9255 | 0.9327 | 14860 | 0 | 0 | 0 | TRINITY\_DN571\_c0\_g1\_i1.p1:TRINITY\_DN571\_c0\_g1\_i2.p1 |  |  | DB Search |
| TGVHHYTGNNIELGTAC(+57.02)GK | 55.14 | 2027.9482 | 19 | 3.09 | 1014.979 | 12.60 | 1.173 | 1.1802 | 28328 | 0 | 0 | 0 | TRINITY\_DN2126\_c0\_g1\_i1.p1 | Carbamidomethylation |  | DB Search |
| AFFTAEEPSAKPTVILAK | 55.13 | 1919.0404 | 18 | -0.05 | 960.5222 | 16.95 | 1.2228 | 1.23 | 51036 | 1.07e2 | 1 | 1 | TRINITY\_DN4\_c0\_g1\_i1.p1 |  |  | DB Search |
| HQGVM(+15.99)VGM(+15.99)GQK | 55.12 | 1202.5536 | 11 | 3.32 | 602.2828 | 9.73 | 0.9105 | 0.9178 | 11903 | 0 | 0 | 0 | TRINITY\_DN161\_c0\_g1\_i1.p1:TRINITY\_DN161\_c0\_g1\_i3.p1:TRINITY\_DN161\_c0\_g1\_i2.p1 | Oxidation (M), Oxidation (M) | M5:Oxidation (M):1000 M8:Oxidation (M):1000 | DB Search |
| GVSEETTTGVHNLYK | 55.11 | 1633.7948 | 15 | 0.86 | 817.9009 | 13.48 | 1.0684 | 1.0757 | 33215 | 5.53e2 | 1 | 1 | TRINITY\_DN773\_c0\_g1\_i1.p1 |  |  | DB Search |
| IQDKEGIPPDQQR | 55.06 | 1522.7739 | 13 | 2.88 | 762.3923 | 11.00 | 1.0331 | 1.0403 | 18584 | 2.97e3 | 2 | 2 | TRINITY\_DN554\_c0\_g1\_i3.p2:TRINITY\_DN585\_c0\_g1\_i3.p1:TRINITY\_DN173718\_c0\_g1\_i1.p1:TRINITY\_DN1465\_c1\_g1\_i1.p1:TRINITY\_DN40728\_c1\_g1\_i1.p2:TRINITY\_DN585\_c0\_g1\_i5.p1:TRINITY\_DN152\_c0\_g1\_i2.p1:TRINITY\_DN152\_c0\_g1\_i14.p2:TRINITY\_DN40728\_c0\_g1\_i1.p1:TRINITY\_DN152\_c0\_g1\_i9.p1:TRINITY\_DN585\_c0\_g1\_i4.p1:TRINITY\_DN152\_c0\_g1\_i15.p1:TRINITY\_DN195631\_c0\_g1\_i1.p2:TRINITY\_DN585\_c0\_g1\_i10.p1:TRINITY\_DN585\_c0\_g1\_i2.p1:TRINITY\_DN554\_c0\_g1\_i5.p1:TRINITY\_DN8227\_c0\_g1\_i1.p1:TRINITY\_DN152\_c0\_g1\_i10.p1 |  |  | DB Search |
| K(+42.01)LQAELANGR | 55.01 | 1140.6251 | 10 | -7.15 | 571.3126 | 14.17 | 0.8927 | 0.9 | 37018 | 0 | 0 | 0 | TRINITY\_DN15599\_c0\_g1\_i1.p1 | Acetylation (Protein N-term) | K1:Acetylation (Protein N-term):1000 | DB Search |
| KLIEVEEKEIQR | 54.86 | 1512.8511 | 12 | -6.51 | 757.4238 | 13.18 | 1.0636 | 1.0709 | 31563 | 0 | 0 | 0 | TRINITY\_DN11435\_c0\_g1\_i1.p1 |  |  | DB Search |
| TIQFVDWC(+57.02)PTGFK | 54.82 | 1597.7599 | 13 | -6.53 | 799.8776 | 19.88 | 1.0351 | 1.0423 | 66701 | 3.31e2 | 1 | 1 | TRINITY\_DN471\_c0\_g1\_i3.p1:TRINITY\_DN687\_c0\_g1\_i1.p1:TRINITY\_DN471\_c0\_g1\_i11.p1:TRINITY\_DN7467\_c0\_g1\_i2.p3:TRINITY\_DN250\_c0\_g1\_i6.p1:TRINITY\_DN250\_c0\_g1\_i4.p1:TRINITY\_DN471\_c0\_g1\_i10.p1:TRINITY\_DN471\_c0\_g1\_i4.p1:TRINITY\_DN687\_c0\_g2\_i1.p1:TRINITY\_DN250\_c0\_g1\_i5.p1:TRINITY\_DN250\_c0\_g1\_i1.p1:TRINITY\_DN471\_c0\_g1\_i5.p1:TRINITY\_DN250\_c0\_g1\_i3.p1:TRINITY\_DN471\_c0\_g1\_i2.p1 | Carbamidomethylation |  | DB Search |
| SIGFIGHYLDQLR | 54.32 | 1517.7991 | 13 | 1.06 | 759.9034 | 19.56 | 1.0849 | 1.0921 | 65092 | 4.69e2 | 1 | 1 | TRINITY\_DN8101\_c0\_g1\_i10.p2:TRINITY\_DN8101\_c0\_g1\_i9.p2:TRINITY\_DN8101\_c0\_g1\_i8.p1:TRINITY\_DN8101\_c0\_g1\_i4.p1:TRINITY\_DN8101\_c0\_g1\_i2.p2:TRINITY\_DN8101\_c0\_g1\_i5.p1:TRINITY\_DN8101\_c0\_g1\_i6.p2:TRINITY\_DN8101\_c0\_g1\_i11.p2 |  |  | DB Search |
| S(+42.01)SGEIESLRQEAENLRNQIK | 54.3 | 2342.1826 | 20 | -2.81 | 781.7283 | 19.42 | 1.0086 | 1.0158 | 64378 | 7.58e2 | 1 | 1 | TRINITY\_DN2472\_c0\_g1\_i1.p1 | Acetylation (Protein N-term) | S1:Acetylation (Protein N-term):1000 | DB Search |
| IRVESLLVSAISK | 54.29 | 1413.8555 | 13 | -0.42 | 707.9308 | 18.45 | 1.0311 | 1.0383 | 59251 | 0 | 0 | 0 | TRINITY\_DN12629\_c0\_g1\_i1.p1 |  |  | DB Search |
| S(+42.01)SGEIESLRQEAENLR | 54.25 | 1858.9021 | 16 | 3.78 | 930.4567 | 18.51 | 1.1985 | 1.2057 | 59475 | 1.24e3 | 1 | 1 | TRINITY\_DN2472\_c0\_g1\_i1.p1 | Acetylation (Protein N-term) | S1:Acetylation (Protein N-term):1000 | DB Search |
| IDLHLYHELYM(+15.99)K | 54.04 | 1589.7911 | 12 | 3.21 | 795.9011 | 15.41 | 1.0925 | 1.0997 | 43639 | 0 | 0 | 0 | TRINITY\_DN1112\_c0\_g1\_i1.p1 | Oxidation (M) | M11:Oxidation (M):1000 | DB Search |
| TPFNFEDKPFIVQYEVR | 53.86 | 2128.063 | 17 | -6.4 | 710.3531 | 19.12 | 0.9453 | 0.9525 | 62983 | 0 | 0 | 0 | TRINITY\_DN165\_c0\_g1\_i1.p1 |  |  | DB Search |
| TQAVLDRLNIEK | 53.78 | 1398.7831 | 12 | 2.78 | 700.3969 | 15.15 | 1.009 | 1.0162 | 42294 | 0 | 0 | 0 | TRINITY\_DN477\_c0\_g1\_i9.p1 |  |  | DB Search |
| TILQFEADRR | 53.68 | 1247.6622 | 10 | 2.15 | 624.8363 | 14.91 | 0.9505 | 0.9578 | 40952 | 9.36e2 | 1 | 1 | TRINITY\_DN58867\_c0\_g1\_i2.p1 |  |  | DB Search |
| RLDPSEQLQHLSSLR | 53.55 | 1777.9435 | 15 | 4.12 | 593.6543 | 16.18 | 0.9065 | 0.9137 | 47359 | 1.79e2 | 1 | 1 | TRINITY\_DN165469\_c0\_g1\_i1.p1 |  |  | DB Search |
| VTFEEFIPLYHTQSK | 53.33 | 1837.925 | 15 | -7.94 | 919.9575 | 19.63 | 1.1482 | 1.1554 | 65543 | 0 | 0 | 0 | TRINITY\_DN276\_c0\_g1\_i2.p1:TRINITY\_DN276\_c0\_g1\_i1.p1 |  |  | DB Search |
| IRELTSVVQKR | 53.19 | 1327.7936 | 11 | 12.38 | 443.6082 | 12.34 | 0.7522 | 0.7595 | 26874 | 0 | 0 | 0 | TRINITY\_DN109115\_c0\_g1\_i1.p1:TRINITY\_DN5263\_c0\_g1\_i1.p1 |  |  | DB Search |
| RLFEGNALLR | 53.09 | 1187.6775 | 10 | 1.58 | 594.8437 | 15.84 | 0.9384 | 0.9457 | 45707 | 5.33e2 | 1 | 1 | TRINITY\_DN776\_c0\_g1\_i1.p1:TRINITY\_DN189323\_c0\_g1\_i1.p1 |  |  | DB Search |
| VASDKFLTSFIK | 52.71 | 1354.7496 | 12 | 1.41 | 678.3793 | 18.05 | 1.0234 | 1.0307 | 57158 | 3.75e2 | 1 | 1 | TRINITY\_DN4523\_c0\_g1\_i2.p1:TRINITY\_DN4523\_c0\_g1\_i1.p1 |  |  | DB Search |
| YIQGENEKQEK | 52.47 | 1364.6572 | 11 | 1.28 | 683.333 | 9.57 | 0.9679 | 0.9751 | 11149 | 0 | 0 | 0 | TRINITY\_DN1272\_c0\_g1\_i1.p1 |  |  | DB Search |
| TTGIVLDSGDGVSHTVPIYEGYALPHAIIR | 52.38 | 3150.635 | 30 | -8.99 | 788.6546 | 18.79 | 1.0693 | 1.0765 | 61107 | 0 | 0 | 0 | TRINITY\_DN161\_c0\_g1\_i1.p1:TRINITY\_DN161\_c0\_g1\_i4.p1:TRINITY\_DN161\_c0\_g1\_i3.p1:TRINITY\_DN161\_c0\_g1\_i2.p1 |  |  | DB Search |
| TTC(+57.02)GVC(+57.02)NNNYC(+57.02)EDDPR | 52.33 | 1973.7302 | 16 | -1.89 | 987.8651 | 12.26 | 1.127 | 1.1342 | 26302 | 0 | 0 | 0 | TRINITY\_DN89\_c0\_g1\_i1.p1 | Carbamidomethylation, Carbamidomethylation, Carbamidomethylation |  | DB Search |
| HVYLRPNVGVGAVR | 52.14 | 1535.8684 | 14 | -2.59 | 512.9593 | 15.00 | 0.8307 | 0.838 | 41514 | 0 | 0 | 0 | TRINITY\_DN195948\_c0\_g1\_i1.p1 |  |  | DB Search |
| RYILETLVNGLKR | 52.1 | 1573.9304 | 13 | 3.66 | 525.6498 | 18.46 | 0.8384 | 0.8457 | 59376 | 0 | 0 | 0 | TRINITY\_DN47849\_c0\_g1\_i1.p1 |  |  | DB Search |
| YFVEAGAM(+15.99)AVRR | 52.04 | 1384.6921 | 12 | 2.12 | 693.351 | 13.54 | 1.0484 | 1.0556 | 33548 | 1.06e3 | 1 | 1 | TRINITY\_DN484\_c0\_g1\_i1.p1 | Oxidation (M) | M8:Oxidation (M):1000 | DB Search |
| DANAAVVHGSHLK | 51.68 | 1317.679 | 13 | 1.05 | 659.8438 | 10.89 | 1.017 | 1.0242 | 18446 | 0 | 0 | 0 | TRINITY\_DN262\_c0\_g1\_i1.p1 |  |  | DB Search |
| LLEINVYHPLIQELQK | 51.52 | 1949.0985 | 16 | 3.87 | 650.7057 | 19.28 | 0.9852 | 0.9924 | 63703 | 0 | 0 | 0 | TRINITY\_DN5633\_c0\_g1\_i1.p1 |  |  | DB Search |
| RLPDQITTR | 51.26 | 1098.6145 | 9 | -16.36 | 550.3026 | 12.47 | 0.8777 | 0.885 | 27597 | 0 | 0 | 0 | TRINITY\_DN1377\_c0\_g1\_i1.p1 |  |  | DB Search |
| ARPHTM(+15.99)TSLTGSR | 50.91 | 1429.7096 | 13 | 5.95 | 477.5774 | 9.99 | 0.8506 | 0.8579 | 13238 | 0 | 0 | 0 | TRINITY\_DN34191\_c0\_g2\_i1.p2:TRINITY\_DN34191\_c0\_g1\_i1.p1 | Oxidation (M) | M6:Oxidation (M):1000 | DB Search |
| AQPTEIYNLGAQSHVK | 50.58 | 1754.8951 | 16 | 0.53 | 878.4505 | 14.56 | 1.173 | 1.1802 | 39052 | 1.18e2 | 1 | 1 | TRINITY\_DN7659\_c0\_g1\_i1.p1:TRINITY\_DN7659\_c0\_g1\_i2.p1 |  |  | DB Search |
| LRPGVELTMSSLVNAK | 50.37 | 1713.9447 | 16 | -0.1 | 857.9749 | 17.45 | 1.145 | 1.1522 | 53751 | 1.26e3 | 1 | 1 | TRINITY\_DN329\_c0\_g1\_i1.p1:TRINITY\_DN329\_c0\_g1\_i2.p1 |  |  | DB Search |
| MRFDTAITAC(+57.02)R | 50.32 | 1340.6329 | 11 | 4.02 | 671.3228 | 14.75 | 0.9622 | 0.9695 | 40083 | 6.01e2 | 1 | 1 | TRINITY\_DN477\_c0\_g1\_i9.p1 | Carbamidomethylation |  | DB Search |
| LLTSSDQGELTKK | 50.28 | 1418.7616 | 13 | -0.77 | 710.3837 | 12.33 | 1.0391 | 1.0464 | 26741 | 3.62e2 | 1 | 1 | TRINITY\_DN1339\_c0\_g2\_i5.p1 |  |  | DB Search |
| KVGFEPDYVSLLR | 49.91 | 1521.8191 | 13 | -1.44 | 761.9116 | 18.24 | 1.0435 | 1.0508 | 58056 | 8.15e2 | 1 | 1 | TRINITY\_DN2113\_c0\_g1\_i1.p1:TRINITY\_DN2113\_c0\_g1\_i2.p1 |  |  | DB Search |
| LRLDFDHEAKLNMIFR | 49.33 | 2017.0568 | 16 | 6.27 | 505.2719 | 18.22 | 0.8627 | 0.87 | 58067 | 0 | 0 | 0 | TRINITY\_DN160\_c0\_g1\_i2.p1:TRINITY\_DN160\_c0\_g1\_i1.p1 |  |  | DB Search |
| RLDEEVAR | 48.47 | 986.5145 | 8 | -5.51 | 494.2591 | 11.06 | 0.8481 | 0.8554 | 19512 | 0 | 0 | 0 | TRINITY\_DN773\_c0\_g1\_i1.p1 |  |  | DB Search |
| ADILC(+57.02)HDTKYGK | 47.79 | 1419.6816 | 12 | 13.12 | 710.8535 | 12.07 | 1.0134 | 1.0206 | 25280 | 0 | 0 | 0 | TRINITY\_DN413\_c0\_g1\_i4.p1:TRINITY\_DN413\_c0\_g1\_i1.p1:TRINITY\_DN413\_c0\_g1\_i3.p1 | Carbamidomethylation |  | DB Search |
| SVNEGDVQDKKR | 46.88 | 1373.6898 | 12 | -2.59 | 458.9002 | 9.40 | 0.7453 | 0.7526 | 10436 | 0 | 0 | 0 | TRINITY\_DN1320\_c0\_g2\_i1.p1 |  |  | DB Search |
| RNLDIERPTYTNLNR | 46.58 | 1873.9758 | 15 | -2.43 | 625.6609 | 13.94 | 0.8858 | 0.8931 | 35770 | 1.17e3 | 1 | 1 | TRINITY\_DN1933\_c0\_g1\_i8.p2:TRINITY\_DN1933\_c0\_g1\_i3.p2:TRINITY\_DN471\_c0\_g1\_i3.p1:TRINITY\_DN471\_c0\_g1\_i7.p1:TRINITY\_DN471\_c0\_g1\_i6.p1:TRINITY\_DN471\_c0\_g1\_i9.p1:TRINITY\_DN471\_c0\_g1\_i11.p1:TRINITY\_DN1933\_c0\_g1\_i4.p1:TRINITY\_DN250\_c0\_g1\_i6.p1:TRINITY\_DN1933\_c0\_g1\_i7.p1:TRINITY\_DN250\_c0\_g1\_i4.p1:TRINITY\_DN250\_c0\_g1\_i2.p1:TRINITY\_DN471\_c0\_g1\_i10.p1:TRINITY\_DN471\_c0\_g1\_i4.p1:TRINITY\_DN687\_c0\_g2\_i1.p1:TRINITY\_DN471\_c0\_g1\_i1.p1:TRINITY\_DN250\_c0\_g1\_i5.p1:TRINITY\_DN1933\_c0\_g1\_i1.p2:TRINITY\_DN250\_c0\_g1\_i1.p1:TRINITY\_DN471\_c0\_g1\_i5.p1:TRINITY\_DN250\_c0\_g1\_i3.p1:TRINITY\_DN471\_c0\_g1\_i2.p1 |  |  | DB Search |
| KVM(+15.99)QSLEGVK | 46.14 | 1133.6115 | 10 | 3 | 567.8116 | 11.20 | 0.904 | 0.9113 | 20184 | 2.1e2 | 1 | 1 | TRINITY\_DN195948\_c0\_g1\_i1.p1 | Oxidation (M) | M3:Oxidation (M):1000 | DB Search |
| AIEKFEKEAQEM(+15.99)GK | 45.67 | 1652.808 | 14 | 0.54 | 827.4072 | 12.02 | 1.1009 | 1.1082 | 24955 | 0 | 0 | 0 | TRINITY\_DN64\_c0\_g1\_i1.p1 | Oxidation (M) | M12:Oxidation (M):1000 | DB Search |
| YRPGTVALR | 44.05 | 1031.5876 | 9 | -1.83 | 516.7973 | 12.44 | 0.8575 | 0.8648 | 27332 | 1.61e3 | 1 | 1 | TRINITY\_DN18187\_c0\_g1\_i4.p3:TRINITY\_DN72753\_c0\_g1\_i1.p1:TRINITY\_DN5023\_c0\_g1\_i3.p2:TRINITY\_DN18187\_c0\_g1\_i6.p2:TRINITY\_DN5023\_c0\_g1\_i2.p2:TRINITY\_DN18187\_c0\_g1\_i3.p4:TRINITY\_DN18187\_c0\_g1\_i5.p1 |  |  | DB Search |
| ADGIHIINLRK | 43.17 | 1248.7302 | 11 | 2.43 | 625.3705 | 13.40 | 0.9646 | 0.9719 | 32870 | 0 | 0 | 0 | TRINITY\_DN1583\_c0\_g1\_i1.p1 |  |  | DB Search |
| IKLEHLITELVHQR | 42.73 | 1728.0046 | 14 | -6.21 | 577.0021 | 18.10 | 0.8672 | 0.8745 | 57432 | 0 | 0 | 0 | TRINITY\_DN1566\_c0\_g1\_i1.p1 |  |  | DB Search |
| LRPGVELTM(+15.99)SSLVNAK | 42.38 | 1729.9396 | 16 | 3.46 | 865.9753 | 15.03 | 1.1338 | 1.141 | 41582 | 4.17e3 | 1 | 1 | TRINITY\_DN329\_c0\_g1\_i1.p1:TRINITY\_DN329\_c0\_g1\_i2.p1 | Oxidation (M) | M9:Oxidation (M):1000 | DB Search |
| YYVTIIDAPGHRDFIK | 42.12 | 1906.9941 | 16 | 6.13 | 954.5049 | 16.49 | 1.2037 | 1.2109 | 48851 | 0 | 0 | 0 | TRINITY\_DN64\_c0\_g1\_i1.p1 |  |  | DB Search |
| AGYGFVIFDDRR | 41.74 | 1414.6993 | 12 | 1.94 | 708.3544 | 18.09 | 1.0379 | 1.0452 | 57312 | 3.6e2 | 1 | 1 | TRINITY\_DN956\_c0\_g1\_i2.p1:TRINITY\_DN956\_c0\_g1\_i1.p1 |  |  | DB Search |
| RIQQLSAIR | 41.24 | 1083.6512 | 9 | 2.29 | 542.8312 | 13.06 | 0.9141 | 0.9214 | 30814 | 1.68e3 | 1 | 1 | TRINITY\_DN428\_c0\_g1\_i1.p1 |  |  | DB Search |
| LDHKFDLM(+15.99)YAKR | 40.99 | 1551.7867 | 12 | 3.09 | 776.8988 | 13.19 | 1.0881 | 1.0953 | 31591 | 1.18e2 | 1 | 1 | TRINITY\_DN687\_c0\_g2\_i1.p1:TRINITY\_DN687\_c0\_g1\_i1.p1:TRINITY\_DN250\_c0\_g1\_i5.p1:TRINITY\_DN250\_c0\_g1\_i6.p1:TRINITY\_DN250\_c0\_g1\_i4.p1:TRINITY\_DN96161\_c0\_g1\_i1.p1 | Oxidation (M) | M8:Oxidation (M):1000 | DB Search |
| FSYEKVC(+57.02)EDVKR | 40.84 | 1558.745 | 12 | 0 | 780.3755 | 13.41 | 1.0709 | 1.0781 | 32836 | 5.73e2 | 1 | 1 | TRINITY\_DN118\_c1\_g1\_i3.p1 | Carbamidomethylation |  | DB Search |
| LRQIHNGVFVK | 40.8 | 1309.7618 | 11 | 2.18 | 655.886 | 12.40 | 0.9763 | 0.9836 | 27123 | 3.3e2 | 1 | 1 | TRINITY\_DN1468\_c0\_g1\_i1.p1 |  |  | DB Search |
| FRQLGLDFGK | 40.24 | 1179.64 | 10 | -11.3 | 590.8174 | 19.48 | 0.9222 | 0.9295 | 64712 | 1.03e3 | 1 | 1 | TRINITY\_DN22594\_c0\_g1\_i1.p2 |  |  | DB Search |
| RGETFVTR | 39.93 | 964.509 | 8 | -4.2 | 483.2571 | 11.39 | 0.8201 | 0.8274 | 21342 | 0 | 0 | 0 | TRINITY\_DN7659\_c0\_g1\_i1.p1:TRINITY\_DN7659\_c0\_g1\_i2.p1 |  |  | DB Search |
| RISEQFTAM(+15.99)FR | 39.68 | 1400.687 | 11 | 1.59 | 701.3481 | 15.05 | 0.9848 | 0.992 | 41767 | 1.88e3 | 1 | 1 | TRINITY\_DN1910\_c0\_g1\_i1.p1:TRINITY\_DN3986\_c0\_g1\_i1.p1 | Oxidation (M) | M9:Oxidation (M):1000 | DB Search |
| ALFFKEIENIR | 39.5 | 1378.7609 | 11 | 6.8 | 690.3886 | 18.36 | 1.0279 | 1.0351 | 58836 | 0 | 0 | 0 | TRINITY\_DN1744\_c0\_g1\_i2.p1 |  |  | DB Search |
| DFREFLYNSETGKVM(+15.99)GR | 39.36 | 2063.9734 | 17 | -3.15 | 688.9925 | 17.26 | 0.9271 | 0.9344 | 52789 | 3.31e2 | 1 | 1 | TRINITY\_DN2028\_c0\_g1\_i1.p1 | Oxidation (M) | M15:Oxidation (M):1000 | DB Search |
| VVSSAIVDKYIGESAR | 39.14 | 1692.9047 | 16 | 3.9 | 847.4583 | 16.24 | 1.1626 | 1.1698 | 47705 | 3.83e2 | 1 | 1 | TRINITY\_DN1789\_c0\_g1\_i1.p1 |  |  | DB Search |
| LC(+57.02)QPGYSTC(+57.02)ENHC(+57.02)R | 38.48 | 1780.708 | 14 | 5.76 | 891.3615 | 11.39 | 1.0877 | 1.0949 | 21326 | 1.4e2 | 1 | 1 | TRINITY\_DN817\_c0\_g1\_i4.p1:TRINITY\_DN817\_c0\_g1\_i6.p1:TRINITY\_DN817\_c0\_g1\_i2.p1:TRINITY\_DN817\_c0\_g1\_i10.p1 | Carbamidomethylation, Carbamidomethylation, Carbamidomethylation |  | DB Search |
| REQELQATVK | 38.36 | 1200.6462 | 10 | -10.4 | 601.3209 | 11.17 | 0.9125 | 0.9198 | 19974 | 1.07e3 | 1 | 1 | TRINITY\_DN11435\_c0\_g1\_i1.p1 |  |  | DB Search |
| AGM(+15.99)THIVR | 38.19 | 899.4647 | 8 | 6.96 | 450.7403 | 9.74 | 0.8254 | 0.8327 | 11994 | 0 | 0 | 0 | TRINITY\_DN2799\_c0\_g1\_i3.p1:TRINITY\_DN3448\_c0\_g1\_i7.p2:TRINITY\_DN6920\_c0\_g1\_i3.p1:TRINITY\_DN2799\_c0\_g1\_i1.p1:TRINITY\_DN3448\_c0\_g1\_i9.p3:TRINITY\_DN3448\_c0\_g2\_i1.p1:TRINITY\_DN3448\_c0\_g1\_i5.p2:TRINITY\_DN5906\_c3\_g1\_i1.p2:TRINITY\_DN32727\_c0\_g1\_i2.p1:TRINITY\_DN3448\_c0\_g2\_i3.p2:TRINITY\_DN6920\_c0\_g1\_i2.p1:TRINITY\_DN2799\_c0\_g1\_i2.p1:TRINITY\_DN2799\_c0\_g1\_i4.p2 | Oxidation (M) | M3:Oxidation (M):1000 | DB Search |
| KLYDEYNDKTPSR | 37.19 | 1627.7842 | 13 | -2.71 | 814.8927 | 11.79 | 1.0387 | 1.046 | 23644 | 0 | 0 | 0 | TRINITY\_DN2963\_c0\_g1\_i1.p1 |  |  | DB Search |
| AYHNASQTQSDKAHLK | 36.97 | 1797.8757 | 16 | -0.62 | 450.4735 | 9.45 | 0.7428 | 0.7502 | 10634 | 0 | 0 | 0 | TRINITY\_DN484\_c0\_g1\_i1.p1 |  |  | DB Search |
| DVEC(+57.02)GKQFDHC(+57.02)TTIK | 36.83 | 1836.8135 | 15 | 4.81 | 613.278 | 12.65 | 0.8571 | 0.8644 | 28563 | 3.3e2 | 1 | 1 | TRINITY\_DN36\_c0\_g1\_i12.p1 | Carbamidomethylation, Carbamidomethylation |  | DB Search |
| AGKDSLDWFFR | 36.4 | 1340.6512 | 11 | -0.05 | 671.3292 | 19.38 | 0.963 | 0.9703 | 64246 | 0 | 0 | 0 | TRINITY\_DN10995\_c0\_g1\_i2.p3 |  |  | DB Search |
| IESTAC(+57.02)TR | 34.92 | 936.4335 | 8 | 2.84 | 469.2228 | 9.65 | 0.8136 | 0.8209 | 11495 | 1.18e3 | 1 | 1 | TRINITY\_DN98968\_c0\_g1\_i1.p1 | Carbamidomethylation |  | DB Search |
| VAVALREEENLHLLER | 34.22 | 1890.0322 | 16 | -0.18 | 631.0145 | 16.17 | 0.8927 | 0.9 | 47345 | 1.03e2 | 1 | 1 | TRINITY\_DN60705\_c0\_g1\_i1.p1 |  |  | DB Search |
| ALELDALSNSHPIEVK | 33.06 | 1734.9152 | 16 | 3.63 | 868.4633 | 16.96 | 1.1706 | 1.1778 | 51172 | 0 | 0 | 0 | TRINITY\_DN5153\_c0\_g1\_i1.p1 |  |  | DB Search |
| ITSTLRDQLGTAK | 32.95 | 1402.778 | 13 | 4.84 | 702.3958 | 13.11 | 1.0198 | 1.0271 | 31190 | 3.61e2 | 1 | 1 | TRINITY\_DN1566\_c0\_g1\_i1.p1 |  |  | DB Search |
| HGSLGFLPR | 32.83 | 982.5348 | 9 | -0.04 | 492.272 | 16.93 | 0.8408 | 0.8481 | 51108 | 4.36e2 | 1 | 1 | TRINITY\_DN2799\_c0\_g1\_i3.p1:TRINITY\_DN2799\_c0\_g1\_i1.p1:TRINITY\_DN6920\_c0\_g1\_i3.p1:TRINITY\_DN6920\_c0\_g1\_i2.p1:TRINITY\_DN5906\_c3\_g1\_i1.p2:TRINITY\_DN2799\_c0\_g1\_i4.p2:TRINITY\_DN2799\_c0\_g1\_i2.p1 |  |  | DB Search |
| AVQIHPFNDVSVGDPK | 31.99 | 1721.8737 | 16 | -1.26 | 861.9383 | 15.56 | 1.1158 | 1.123 | 44381 | 1.32e2 | 1 | 1 | TRINITY\_DN1071\_c0\_g1\_i2.p1:TRINITY\_DN1071\_c0\_g1\_i3.p1:TRINITY\_DN1071\_c0\_g1\_i1.p1:TRINITY\_DN1071\_c0\_g1\_i4.p1 |  |  | DB Search |
| AVVIITDGKDDSTSR | 31.58 | 1575.8104 | 15 | -9 | 788.9011 | 13.19 | 1.0937 | 1.1009 | 31695 | 0 | 0 | 0 | TRINITY\_DN1797\_c0\_g1\_i2.p1:TRINITY\_DN1797\_c0\_g1\_i1.p1:TRINITY\_DN1797\_c0\_g1\_i3.p1 |  |  | DB Search |
| VRVNVLNDALNSIC(+57.02)NAEKR | 31.55 | 2184.1433 | 19 | -0.13 | 729.051 | 18.48 | 0.9824 | 0.9896 | 59403 | 5.64e2 | 1 | 1 | TRINITY\_DN3339\_c0\_g1\_i1.p1 | Carbamidomethylation |  | DB Search |
| KLLEINVYHPLIQELQKR | 30.15 | 2233.2947 | 18 | -0.32 | 559.3277 | 17.67 | 0.866 | 0.8733 | 55108 | 0 | 0 | 0 | TRINITY\_DN5633\_c0\_g1\_i1.p1 |  |  | DB Search |
| VVIIDGKGHLM(+15.99)GR | 29.79 | 1409.7812 | 13 | 3.46 | 705.8965 | 12.92 | 1.023 | 1.0303 | 30102 | 0 | 0 | 0 | TRINITY\_DN2805\_c0\_g1\_i1.p1 | Oxidation (M) | M11:Oxidation (M):1000 | DB Search |
| VQSGNYRPVLALNGR | 29.19 | 1642.8903 | 15 | 5.65 | 822.4526 | 14.70 | 1.0837 | 1.0909 | 39738 | 6.08e3 | 1 | 1 | TRINITY\_DN1130\_c0\_g1\_i1.p1 |  |  | DB Search |
| SSC(+57.02)RPILSVIK | 27.61 | 1258.7067 | 11 | -7.39 | 630.3525 | 16.61 | 0.9687 | 0.9759 | 49383 | 6.05e2 | 1 | 1 | TRINITY\_DN158421\_c0\_g1\_i1.p1:TRINITY\_DN3545\_c0\_g1\_i2.p1:TRINITY\_DN3545\_c0\_g1\_i1.p1 | Carbamidomethylation |  | DB Search |
| SPTHSVIK | 27.03 | 867.4814 | 8 | -9.62 | 434.7414 | 9.92 | 0.7876 | 0.7949 | 12850 | 0 | 0 | 0 | TRINITY\_DN1386\_c0\_g1\_i1.p1 |  |  | DB Search |
| YTVGDVVTC(+57.02)YIDLDARPR | 26.69 | 2112.031 | 18 | 0.25 | 705.0139 | 19.89 | 0.94 | 0.9473 | 66734 | 0 | 0 | 0 | TRINITY\_DN123\_c1\_g1\_i1.p1 | Carbamidomethylation |  | DB Search |
| VLLSQQIFYDKK | 26.23 | 1480.829 | 12 | -0.12 | 741.4176 | 16.48 | 1.0608 | 1.068 | 48785 | 6.44e2 | 1 | 1 | TRINITY\_DN982\_c0\_g1\_i3.p1:TRINITY\_DN982\_c0\_g1\_i1.p1:TRINITY\_DN982\_c0\_g1\_i2.p1 |  |  | DB Search |
| LSIIPVRR | 26.05 | 952.6182 | 8 | 2.32 | 477.3148 | 14.61 | 0.8441 | 0.8514 | 39163 | 2.05e3 | 1 | 1 | TRINITY\_DN15584\_c0\_g1\_i1.p1 |  |  | DB Search |
| VLEVNDNKPK | 26.02 | 1154.6295 | 10 | -0.56 | 578.3185 | 10.96 | 0.9234 | 0.9307 | 18881 | 0 | 0 | 0 | TRINITY\_DN982\_c0\_g1\_i1.p1:TRINITY\_DN982\_c0\_g1\_i2.p1 |  |  | DB Search |
| ITQQQSHQEIADQLR | 25.96 | 1793.902 | 15 | 0.41 | 897.9537 | 13.04 | 1.1222 | 1.1294 | 30831 | 0 | 0 | 0 | TRINITY\_DN1377\_c0\_g1\_i1.p1 |  |  | DB Search |
| AFNRVETR | 25.84 | 991.5199 | 8 | -0.1 | 496.7645 | 10.70 | 0.8477 | 0.855 | 17302 | 3.57e3 | 1 | 1 | TRINITY\_DN4595\_c0\_g1\_i7.p1:TRINITY\_DN4595\_c0\_g1\_i4.p1:TRINITY\_DN4595\_c0\_g1\_i10.p2 |  |  | DB Search |
| LDISDEFRR | 25.79 | 1149.5778 | 9 | -5.84 | 575.7897 | 15.31 | 0.9093 | 0.9166 | 43114 | 0 | 0 | 0 | TRINITY\_DN61835\_c0\_g1\_i1.p1:TRINITY\_DN49493\_c0\_g1\_i1.p1:TRINITY\_DN1283\_c0\_g1\_i1.p1 |  |  | DB Search |
| FPGQLNADIRK | 25.64 | 1257.683 | 11 | -1.3 | 629.8445 | 14.18 | 0.9457 | 0.9529 | 37015 | 6.11e2 | 1 | 1 | TRINITY\_DN5365\_c0\_g1\_i3.p1:TRINITY\_DN5365\_c0\_g1\_i2.p1 |  |  | DB Search |
| FPGQLNADLRK | 25.64 | 1257.683 | 11 | -1.3 | 629.8445 | 14.18 | 0.9457 | 0.9529 | 37015 | 6.11e2 | 1 | 1 | TRINITY\_DN1910\_c0\_g1\_i1.p1:TRINITY\_DN1910\_c0\_g1\_i5.p1:TRINITY\_DN3986\_c0\_g1\_i1.p1:TRINITY\_DN3986\_c0\_g1\_i2.p1 |  |  | DB Search |
| DQHEATAIEALRR | 24.21 | 1508.7695 | 13 | 5.66 | 503.9305 | 13.52 | 0.8039 | 0.8112 | 33520 | 0 | 0 | 0 | TRINITY\_DN72959\_c0\_g1\_i1.p1 |  |  | DB Search |
| APGDEGWRPPLLATDDAELK | 23.14 | 2150.0642 | 20 | -9.02 | 717.685 | 18.26 | 0.917 | 0.9243 | 58289 | 0 | 0 | 0 | TRINITY\_DN5276\_c0\_g1\_i12.p2:TRINITY\_DN5276\_c0\_g1\_i14.p1 |  |  | DB Search |
| VTDAQIEKIEK | 22.24 | 1272.6925 | 11 | 12.94 | 637.3583 | 12.56 | 0.9622 | 0.9695 | 28055 | 1.37e3 | 1 | 1 | TRINITY\_DN11545\_c0\_g1\_i1.p1 |  |  | DB Search |
| V(+42.01)LGVAAVRR | 22.14 | 981.6083 | 9 | -0.62 | 491.8084 | 18.27 | 0.8652 | 0.8725 | 58336 | 1.02e3 | 1 | 1 | TRINITY\_DN104063\_c0\_g1\_i1.p1 | Acetylation (Protein N-term) | V1:Acetylation (Protein N-term):1000 | DB Search |
| L(+42.01)AAPAFR | 21.67 | 786.4388 | 7 | 12.34 | 394.2294 | 14.83 | 0.7257 | 0.7331 | 40486 | 1.26e3 | 1 | 1 | TRINITY\_DN26679\_c0\_g1\_i1.p3 | Acetylation (Protein N-term) | L1:Acetylation (Protein N-term):1000 | DB Search |
| EGDLVIHSGITDAVK | 21.65 | 1552.8097 | 15 | -3.94 | 777.4048 | 15.22 | 1.0644 | 1.0717 | 42664 | 0 | 0 | 0 | TRINITY\_DN329\_c0\_g1\_i1.p1:TRINITY\_DN329\_c0\_g1\_i2.p1 |  |  | DB Search |
| IC(+57.02)RM(+15.99)LK | 21.14 | 835.4408 | 6 | -1.07 | 418.7249 | 10.37 | 0.7884 | 0.7958 | 15283 | 1.3e3 | 1 | 1 | TRINITY\_DN167010\_c0\_g1\_i1.p1 | Carbamidomethylation, Oxidation (M) | M4:Oxidation (M):1000 | DB Search |
| LGPDQVDVAGAGR | 20.68 | 1253.6364 | 13 | 1.35 | 627.8229 | 13.51 | 0.9327 | 0.94 | 33440 | 0 | 0 | 0 | TRINITY\_DN97809\_c0\_g1\_i1.p1 |  |  | DB Search |
| RLFEGNALLRR | 20.39 | 1343.7786 | 11 | 6.92 | 448.9341 | 14.60 | 0.7538 | 0.7612 | 39347 | 0 | 0 | 0 | TRINITY\_DN776\_c0\_g1\_i1.p1:TRINITY\_DN189323\_c0\_g1\_i1.p1 |  |  | DB Search |
| LSPEDIER | 20.37 | 957.4767 | 8 | 15.64 | 479.7505 | 14.28 | 0.8262 | 0.8335 | 37640 | 4.81e3 | 1 | 1 | TRINITY\_DN119684\_c0\_g1\_i1.p1 |  |  | DB Search |
| AVLTDLRR | 19.88 | 942.561 | 8 | 2.21 | 472.2863 | 13.09 | 0.8408 | 0.8481 | 31027 | 6.5e2 | 1 | 1 | TRINITY\_DN433\_c0\_g2\_i1.p1 |  |  | DB Search |
| total 302 peptides |  |  |  |  |  |  |  |  |  |  |  |  |  |  |  |  |
| --- | --- | --- | --- | --- | --- | --- | --- | --- | --- | --- | --- | --- | --- | --- | --- | --- |
