# Supplementary material for: Proteomic Analysis and Biochemical Characterization of the Nematocyst Extract of the Hydrozoan Velella velella
Source: Mar Drugs. 2024 Oct 12;22(10):468. doi: 10.3390/md22100468 (PMC11509761; doi:10.3390/md22100468)
Supplement: Supplementary file 1 [file marinedrugs-22-00468-s001.zip › Supplementary File S1.zip!/Sample_11_Vellela/db.proteins.html]

proteins


Protein List

  

| Protein Group | Protein ID | Accession | -10lgP | Coverage (%) | Coverage (%) Sample 2 | Area Sample 2 | #Peptides | #Unique | #Spec Sample 2 | PTM | Avg. Mass | Description |
| --- | --- | --- | --- | --- | --- | --- | --- | --- | --- | --- | --- | --- |
| 1 | 1 | TRINITY\_DN161\_c0\_g1\_i2.p1 | 151.1 | 28 | 28 | 5.08e4 | 7 | 7 | 12 | Oxidation (M) | 44577.6484 | type:5prime\_partial len:399 gc:universal TRINITY\_DN161\_c0\_g1\_i2:1683-487(-) |
| 1 | 2 | TRINITY\_DN161\_c0\_g1\_i3.p1 | 151.1 | 28 | 28 | 5.08e4 | 7 | 7 | 12 | Oxidation (M) | 44592.6602 | type:5prime\_partial len:399 gc:universal TRINITY\_DN161\_c0\_g1\_i3:1450-254(-) |
| 1 | 3 | TRINITY\_DN161\_c0\_g1\_i1.p1 | 151.1 | 29 | 29 | 5.08e4 | 7 | 7 | 12 | Oxidation (M) | 41918.5938 | type:complete len:377 gc:universal TRINITY\_DN161\_c0\_g1\_i1:1384-254(-) |
| 1 | 4 | TRINITY\_DN2477\_c0\_g1\_i10.p1 | 107.67 | 13 | 13 | 1.66e4 | 3 | 3 | 4 |  | 38566.9688 | type:5prime\_partial len:344 gc:universal TRINITY\_DN2477\_c0\_g1\_i10:1215-184(-) |
| 1 | 5 | TRINITY\_DN2477\_c0\_g1\_i5.p1 | 107.67 | 13 | 13 | 1.66e4 | 3 | 3 | 4 |  | 37416.5195 | type:complete len:333 gc:universal TRINITY\_DN2477\_c0\_g1\_i5:1182-184(-) |
| 1 | 6 | TRINITY\_DN2477\_c0\_g1\_i2.p1 | 107.67 | 13 | 13 | 1.66e4 | 3 | 3 | 4 |  | 38566.9688 | type:5prime\_partial len:344 gc:universal TRINITY\_DN2477\_c0\_g1\_i2:1215-184(-) |
| 1 | 7 | TRINITY\_DN2477\_c0\_g1\_i6.p1 | 107.67 | 12 | 12 | 1.66e4 | 3 | 3 | 4 |  | 41878.6875 | type:complete len:377 gc:universal TRINITY\_DN2477\_c0\_g1\_i6:1314-184(-) |
| 1 | 8 | TRINITY\_DN2477\_c0\_g1\_i9.p1 | 107.67 | 12 | 12 | 1.66e4 | 3 | 3 | 4 |  | 41878.6875 | type:complete len:377 gc:universal TRINITY\_DN2477\_c0\_g1\_i9:1314-184(-) |
| 1 | 9 | TRINITY\_DN161\_c0\_g1\_i4.p1 | 105.38 | 25 | 25 | 1.4e4 | 3 | 3 | 6 | Oxidation (M) | 25887.2988 | type:5prime\_partial len:232 gc:universal TRINITY\_DN161\_c0\_g1\_i4:949-254(-) |
| 1 | 10 | TRINITY\_DN195934\_c0\_g1\_i1.p2 | 88.3 | 24 | 24 | 1.02e4 | 2 | 2 | 3 |  | 14094.0938 | type:internal len:127 gc:universal TRINITY\_DN195934\_c0\_g1\_i1:3-380(+) |
| 1 | 11 | TRINITY\_DN2477\_c0\_g1\_i8.p1 | 87.24 | 10 | 10 | 6.66e3 | 2 | 2 | 2 |  | 34820.5938 | type:5prime\_partial len:309 gc:universal TRINITY\_DN2477\_c0\_g1\_i8:1110-184(-) |
| 1 | 12 | TRINITY\_DN2941\_c0\_g1\_i4.p1 | 58.19 | 16 | 16 | 2.52e2 | 1 | 1 | 1 |  | 13034.9365 | type:internal len:116 gc:universal TRINITY\_DN2941\_c0\_g1\_i4:2-346(+) |
| 1 | 13 | TRINITY\_DN2477\_c0\_g1\_i7.p1 | 58.1 | 5 | 5 | 6.41e3 | 1 | 1 | 1 |  | 31152.4473 | type:5prime\_partial len:280 gc:universal TRINITY\_DN2477\_c0\_g1\_i7:1023-184(-) |
| 2 | 14 | TRINITY\_DN1280\_c0\_g1\_i1.p1 | 151.25 | 13 | 13 | 6.97e3 | 7 | 7 | 10 | Carbamidomethylation | 47818.6641 | type:complete len:426 gc:universal TRINITY\_DN1280\_c0\_g1\_i1:195-1472(+) |
| 3 | 15 | TRINITY\_DN11435\_c0\_g1\_i1.p1 | 139.68 | 14 | 14 | 3.91e3 | 6 | 6 | 7 |  | 47019.5312 | type:complete len:430 gc:universal TRINITY\_DN11435\_c0\_g1\_i1:1616-327(-) |
| 4 | 16 | TRINITY\_DN172571\_c0\_g1\_i1.p1 | 133.26 | 27 | 27 | 1.9e4 | 5 | 5 | 9 |  | 11500.2529 | type:complete len:104 gc:universal TRINITY\_DN172571\_c0\_g1\_i1:30-341(+) |
| 4 | 17 | TRINITY\_DN22077\_c0\_g1\_i1.p3 | 106.13 | 10 | 10 | 1.19e4 | 3 | 3 | 5 |  | 16229.6445 | type:5prime\_partial len:151 gc:universal TRINITY\_DN22077\_c0\_g1\_i1:520-68(-) |
| 5 | 18 | TRINITY\_DN329\_c0\_g1\_i2.p1 | 110.14 | 19 | 19 | 6.22e3 | 4 | 4 | 5 | Oxidation (M) | 34761.9609 | type:5prime\_partial len:320 gc:universal TRINITY\_DN329\_c0\_g1\_i2:1-960(+) |
| 5 | 19 | TRINITY\_DN329\_c0\_g1\_i1.p1 | 110.14 | 21 | 21 | 6.22e3 | 4 | 4 | 5 | Oxidation (M) | 30514.9648 | type:complete len:283 gc:universal TRINITY\_DN329\_c0\_g1\_i1:158-1006(+) |
| 6 | 20 | TRINITY\_DN3986\_c0\_g1\_i1.p1 | 122.5 | 13 | 13 | 1.39e4 | 5 | 5 | 6 | Oxidation (M), Carbamidomethylation | 50011.8867 | type:complete len:446 gc:universal TRINITY\_DN3986\_c0\_g1\_i1:252-1589(+) |
| 6 | 21 | TRINITY\_DN1910\_c0\_g1\_i5.p1 | 96.53 | 6 | 6 | 8.15e3 | 3 | 3 | 3 | Oxidation (M), Carbamidomethylation | 50120.9062 | type:complete len:447 gc:universal TRINITY\_DN1910\_c0\_g1\_i5:2031-691(-) |
| 6 | 22 | TRINITY\_DN1910\_c0\_g1\_i1.p1 | 86.6 | 7 | 7 | 3.86e3 | 3 | 3 | 3 | Oxidation (M), Carbamidomethylation | 50135.0117 | type:complete len:447 gc:universal TRINITY\_DN1910\_c0\_g1\_i1:2183-843(-) |
| 6 | 23 | TRINITY\_DN5365\_c0\_g1\_i2.p1 | 25.64 | 2 | 2 | 6.11e2 | 1 | 1 | 1 |  | 51878.4453 | type:complete len:458 gc:universal TRINITY\_DN5365\_c0\_g1\_i2:1448-75(-) |
| 6 | 24 | TRINITY\_DN3986\_c0\_g1\_i2.p1 | 25.64 | 2 | 2 | 6.11e2 | 1 | 1 | 1 |  | 50284.332 | type:complete len:446 gc:universal TRINITY\_DN3986\_c0\_g1\_i2:47-1384(+) |
| 6 | 25 | TRINITY\_DN5365\_c0\_g1\_i3.p1 | 25.64 | 2 | 2 | 6.11e2 | 1 | 1 | 1 |  | 51878.4453 | type:complete len:458 gc:universal TRINITY\_DN5365\_c0\_g1\_i3:1448-75(-) |
| 7 | 26 | TRINITY\_DN982\_c0\_g1\_i3.p1 | 114.39 | 1 | 1 | 3.89e3 | 4 | 2 | 5 |  | 1090854.75 | type:complete len:9852 gc:universal TRINITY\_DN982\_c0\_g1\_i3:29697-142(-) |
| 8 | 27 | TRINITY\_DN95248\_c0\_g1\_i1.p1 | 121.8 | 35 | 35 | 7.72e3 | 4 | 4 | 6 |  | 14722.6113 | type:complete len:124 gc:universal TRINITY\_DN95248\_c0\_g1\_i1:197-568(+) |
| 9 | 28 | TRINITY\_DN250\_c0\_g1\_i5.p1 | 112.05 | 11 | 11 | 3.07e3 | 4 | 4 | 4 | Oxidation (M), Carbamidomethylation | 50305.4453 | type:complete len:452 gc:universal TRINITY\_DN250\_c0\_g1\_i5:131-1486(+) |
| 9 | 29 | TRINITY\_DN250\_c0\_g1\_i4.p1 | 112.05 | 11 | 11 | 3.07e3 | 4 | 4 | 4 | Oxidation (M), Carbamidomethylation | 50305.4453 | type:complete len:452 gc:universal TRINITY\_DN250\_c0\_g1\_i4:131-1486(+) |
| 9 | 30 | TRINITY\_DN250\_c0\_g1\_i3.p1 | 101.8 | 10 | 10 | 2.96e3 | 3 | 3 | 3 | Carbamidomethylation | 44456.6445 | type:3prime\_partial len:402 gc:universal TRINITY\_DN250\_c0\_g1\_i3:131-1333(+) |
| 9 | 31 | TRINITY\_DN250\_c0\_g1\_i1.p1 | 101.8 | 10 | 10 | 2.96e3 | 3 | 3 | 3 | Carbamidomethylation | 44454.6094 | type:3prime\_partial len:402 gc:universal TRINITY\_DN250\_c0\_g1\_i1:131-1333(+) |
| 9 | 32 | TRINITY\_DN250\_c0\_g1\_i6.p1 | 91.77 | 9 | 9 | 1.62e3 | 3 | 3 | 3 | Oxidation (M), Carbamidomethylation | 50263.3672 | type:complete len:452 gc:universal TRINITY\_DN250\_c0\_g1\_i6:131-1486(+) |
| 9 | 33 | TRINITY\_DN687\_c0\_g2\_i1.p1 | 91.77 | 9 | 9 | 1.62e3 | 3 | 3 | 3 | Oxidation (M), Carbamidomethylation | 50237.3242 | type:complete len:453 gc:universal TRINITY\_DN687\_c0\_g2\_i1:90-1448(+) |
| 9 | 34 | TRINITY\_DN250\_c0\_g1\_i2.p1 | 82.15 | 8 | 8 | 2.63e3 | 2 | 2 | 2 |  | 35668.3789 | type:3prime\_partial len:322 gc:universal TRINITY\_DN250\_c0\_g1\_i2:131-1093(+) |
| 9 | 35 | TRINITY\_DN471\_c0\_g1\_i3.p1 | 78.11 | 9 | 9 | 1.5e3 | 2 | 2 | 2 | Carbamidomethylation | 36097.9961 | type:5prime\_partial len:324 gc:universal TRINITY\_DN471\_c0\_g1\_i3:1-972(+) |
| 9 | 36 | TRINITY\_DN471\_c0\_g1\_i5.p1 | 78.11 | 8 | 8 | 1.5e3 | 2 | 2 | 2 | Carbamidomethylation | 39722.6172 | type:3prime\_partial len:359 gc:universal TRINITY\_DN471\_c0\_g1\_i5:117-1190(+) |
| 9 | 37 | TRINITY\_DN471\_c0\_g1\_i2.p1 | 78.11 | 6 | 6 | 1.5e3 | 2 | 2 | 2 | Carbamidomethylation | 54220.6289 | type:5prime\_partial len:492 gc:universal TRINITY\_DN471\_c0\_g1\_i2:3-1478(+) |
| 9 | 38 | TRINITY\_DN471\_c0\_g1\_i11.p1 | 78.11 | 6 | 6 | 1.5e3 | 2 | 2 | 2 | Carbamidomethylation | 54220.6289 | type:5prime\_partial len:492 gc:universal TRINITY\_DN471\_c0\_g1\_i11:3-1478(+) |
| 9 | 39 | TRINITY\_DN471\_c0\_g1\_i10.p1 | 78.11 | 6 | 6 | 1.5e3 | 2 | 2 | 2 | Carbamidomethylation | 54220.6289 | type:5prime\_partial len:492 gc:universal TRINITY\_DN471\_c0\_g1\_i10:3-1478(+) |
| 9 | 40 | TRINITY\_DN471\_c0\_g1\_i4.p1 | 78.11 | 6 | 6 | 1.5e3 | 2 | 2 | 2 | Carbamidomethylation | 54220.6289 | type:5prime\_partial len:492 gc:universal TRINITY\_DN471\_c0\_g1\_i4:3-1478(+) |
| 9 | 41 | TRINITY\_DN687\_c0\_g1\_i1.p1 | 75.31 | 6 | 6 | 4.49e2 | 2 | 2 | 2 | Oxidation (M), Carbamidomethylation | 50621.6914 | type:complete len:453 gc:universal TRINITY\_DN687\_c0\_g1\_i1:95-1453(+) |
| 9 | 42 | TRINITY\_DN7467\_c0\_g1\_i2.p3 | 54.82 | 12 | 12 | 3.31e2 | 1 | 1 | 1 | Carbamidomethylation | 12002.1533 | type:internal len:110 gc:universal TRINITY\_DN7467\_c0\_g1\_i2:328-2(-) |
| 9 | 43 | TRINITY\_DN1933\_c0\_g1\_i4.p1 | 46.58 | 10 | 10 | 1.17e3 | 1 | 1 | 1 |  | 16888.3496 | type:internal len:153 gc:universal TRINITY\_DN1933\_c0\_g1\_i4:3-458(+) |
| 9 | 44 | TRINITY\_DN471\_c0\_g1\_i9.p1 | 46.58 | 4 | 4 | 1.17e3 | 1 | 1 | 1 |  | 37621.1953 | type:3prime\_partial len:341 gc:universal TRINITY\_DN471\_c0\_g1\_i9:117-1136(+) |
| 9 | 45 | TRINITY\_DN471\_c0\_g1\_i1.p1 | 46.58 | 14 | 14 | 1.17e3 | 1 | 1 | 1 |  | 12340.0938 | type:internal len:109 gc:universal TRINITY\_DN471\_c0\_g1\_i1:2-325(+) |
| 9 | 46 | TRINITY\_DN1933\_c0\_g1\_i1.p2 | 46.58 | 11 | 11 | 1.17e3 | 1 | 1 | 1 |  | 14806.9961 | type:internal len:134 gc:universal TRINITY\_DN1933\_c0\_g1\_i1:3-401(+) |
| 9 | 47 | TRINITY\_DN1933\_c0\_g1\_i3.p2 | 46.58 | 11 | 11 | 1.17e3 | 1 | 1 | 1 |  | 14806.9961 | type:internal len:134 gc:universal TRINITY\_DN1933\_c0\_g1\_i3:3-401(+) |
| 9 | 48 | TRINITY\_DN471\_c0\_g1\_i7.p1 | 46.58 | 7 | 7 | 1.17e3 | 1 | 1 | 1 |  | 23321.0215 | type:internal len:211 gc:universal TRINITY\_DN471\_c0\_g1\_i7:1-630(+) |
| 9 | 49 | TRINITY\_DN1933\_c0\_g1\_i8.p2 | 46.58 | 11 | 11 | 1.17e3 | 1 | 1 | 1 |  | 14806.9961 | type:internal len:134 gc:universal TRINITY\_DN1933\_c0\_g1\_i8:3-401(+) |
| 9 | 50 | TRINITY\_DN471\_c0\_g1\_i6.p1 | 46.58 | 7 | 7 | 1.17e3 | 1 | 1 | 1 |  | 23321.0215 | type:internal len:211 gc:universal TRINITY\_DN471\_c0\_g1\_i6:1-630(+) |
| 9 | 51 | TRINITY\_DN1933\_c0\_g1\_i7.p1 | 46.58 | 10 | 10 | 1.17e3 | 1 | 1 | 1 |  | 17019.541 | type:internal len:154 gc:universal TRINITY\_DN1933\_c0\_g1\_i7:3-461(+) |
| 9 | 52 | TRINITY\_DN96161\_c0\_g1\_i1.p1 | 40.99 | 3 | 3 | 1.18e2 | 1 | 1 | 1 | Oxidation (M) | 50498.6562 | type:complete len:453 gc:universal TRINITY\_DN96161\_c0\_g1\_i1:1593-235(-) |
| 10 | 53 | TRINITY\_DN118\_c1\_g1\_i3.p1 | 100.44 | 11 | 11 | 1.08e4 | 3 | 3 | 6 | Carbamidomethylation | 22860.4883 | type:internal len:200 gc:universal TRINITY\_DN118\_c1\_g1\_i3:599-3(-) |
| 10 | 54 | TRINITY\_DN118\_c1\_g1\_i5.p1 | 86.83 | 8 | 8 | 1.02e4 | 2 | 2 | 5 | Carbamidomethylation | 23277.6309 | type:5prime\_partial len:203 gc:universal TRINITY\_DN118\_c1\_g1\_i5:620-12(-) |
| 11 | 55 | TRINITY\_DN15584\_c0\_g1\_i1.p1 | 96.83 | 13 | 13 | 1.35e4 | 3 | 3 | 6 | Carbamidomethylation | 29534.9648 | type:complete len:275 gc:universal TRINITY\_DN15584\_c0\_g1\_i1:874-50(-) |
| 12 | 56 | TRINITY\_DN2126\_c0\_g1\_i1.p1 | 105.79 | 30 | 30 | 1.16e3 | 3 | 3 | 3 | Carbamidomethylation | 12631.3887 | type:complete len:114 gc:universal TRINITY\_DN2126\_c0\_g1\_i1:55-396(+) |
| 13 | 57 | TRINITY\_DN1566\_c0\_g1\_i1.p1 | 90.57 | 1 | 1 | 9.62e2 | 3 | 3 | 3 |  | 529444 | type:complete len:4626 gc:universal TRINITY\_DN1566\_c0\_g1\_i1:42-13919(+) |
| 14 | 58 | TRINITY\_DN189323\_c0\_g1\_i1.p1 | 91.09 | 7 | 7 | 2.36e3 | 3 | 3 | 3 |  | 18178.9902 | type:5prime\_partial len:156 gc:universal TRINITY\_DN189323\_c0\_g1\_i1:2-469(+) |
| 14 | 59 | TRINITY\_DN776\_c0\_g1\_i1.p1 | 91.09 | 5 | 5 | 2.36e3 | 3 | 3 | 3 |  | 23300.8359 | type:5prime\_partial len:201 gc:universal TRINITY\_DN776\_c0\_g1\_i1:692-90(-) |
| 15 | 60 | TRINITY\_DN7659\_c0\_g1\_i2.p1 | 97.3 | 9 | 9 | 7.78e2 | 3 | 3 | 4 | Acetylation (Protein N-term) | 40699.8555 | type:complete len:359 gc:universal TRINITY\_DN7659\_c0\_g1\_i2:82-1158(+) |
| 15 | 61 | TRINITY\_DN7659\_c0\_g1\_i1.p1 | 97.3 | 9 | 9 | 7.78e2 | 3 | 3 | 4 | Acetylation (Protein N-term) | 40699.8555 | type:complete len:359 gc:universal TRINITY\_DN7659\_c0\_g1\_i1:69-1145(+) |
| 16 | 62 | TRINITY\_DN3036\_c0\_g1\_i3.p1 | 107.99 | 8 | 8 | 6.84e3 | 3 | 1 | 10 | Oxidation (M) | 59630.4023 | type:internal len:548 gc:universal TRINITY\_DN3036\_c0\_g1\_i3:1643-3(-) |
| 16 | 63 | TRINITY\_DN3036\_c0\_g1\_i8.p1 | 107.99 | 8 | 8 | 6.84e3 | 3 | 1 | 10 | Oxidation (M) | 59630.4023 | type:internal len:548 gc:universal TRINITY\_DN3036\_c0\_g1\_i8:1643-3(-) |
| 16 | 64 | TRINITY\_DN3036\_c0\_g1\_i2.p2 | 107.99 | 8 | 8 | 6.84e3 | 3 | 1 | 10 | Oxidation (M) | 59586.3086 | type:internal len:548 gc:universal TRINITY\_DN3036\_c0\_g1\_i2:1643-3(-) |
| 17 | 65 | TRINITY\_DN6920\_c0\_g1\_i3.p1 | 87.83 | 6 | 6 | 1.01e3 | 3 | 3 | 3 | Oxidation (M) | 46932.582 | type:5prime\_partial len:411 gc:universal TRINITY\_DN6920\_c0\_g1\_i3:2124-892(-) |
| 17 | 66 | TRINITY\_DN6920\_c0\_g1\_i2.p1 | 87.83 | 6 | 6 | 1.01e3 | 3 | 3 | 3 | Oxidation (M) | 46932.582 | type:5prime\_partial len:411 gc:universal TRINITY\_DN6920\_c0\_g1\_i2:2147-915(-) |
| 17 | 67 | TRINITY\_DN6920\_c0\_g1\_i4.p1 | 57.79 | 3 | 3 | 5.75e2 | 1 | 1 | 1 |  | 32195.3809 | type:5prime\_partial len:282 gc:universal TRINITY\_DN6920\_c0\_g1\_i4:1321-476(-) |
| 17 | 68 | TRINITY\_DN6920\_c0\_g1\_i1.p1 | 57.79 | 3 | 3 | 5.75e2 | 1 | 1 | 1 |  | 32882.168 | type:complete len:287 gc:universal TRINITY\_DN6920\_c0\_g1\_i1:1775-915(-) |
| 17 | 69 | TRINITY\_DN2799\_c0\_g1\_i3.p1 | 54.6 | 15 | 15 | 4.36e2 | 2 | 2 | 2 | Oxidation (M) | 12365.4863 | type:internal len:112 gc:universal TRINITY\_DN2799\_c0\_g1\_i3:334-2(-) |
| 17 | 70 | TRINITY\_DN5906\_c3\_g1\_i1.p2 | 54.6 | 13 | 13 | 4.36e2 | 2 | 2 | 2 | Oxidation (M) | 15031.4814 | type:internal len:137 gc:universal TRINITY\_DN5906\_c3\_g1\_i1:3-410(+) |
| 17 | 71 | TRINITY\_DN2799\_c0\_g1\_i2.p1 | 54.6 | 15 | 15 | 4.36e2 | 2 | 2 | 2 | Oxidation (M) | 12149.2383 | type:internal len:111 gc:universal TRINITY\_DN2799\_c0\_g1\_i2:331-2(-) |
| 17 | 72 | TRINITY\_DN2799\_c0\_g1\_i4.p2 | 54.6 | 15 | 15 | 4.36e2 | 2 | 2 | 2 | Oxidation (M) | 12149.2383 | type:internal len:111 gc:universal TRINITY\_DN2799\_c0\_g1\_i4:331-2(-) |
| 17 | 73 | TRINITY\_DN2799\_c0\_g1\_i1.p1 | 54.6 | 15 | 15 | 4.36e2 | 2 | 2 | 2 | Oxidation (M) | 12875.0586 | type:internal len:117 gc:universal TRINITY\_DN2799\_c0\_g1\_i1:349-2(-) |
| 17 | 74 | TRINITY\_DN3448\_c0\_g1\_i9.p3 | 38.19 | 8 | 8 | 0 | 1 | 1 | 1 | Oxidation (M) | 11358.1816 | type:internal len:102 gc:universal TRINITY\_DN3448\_c0\_g1\_i9:303-1(-) |
| 17 | 75 | TRINITY\_DN3448\_c0\_g1\_i5.p2 | 38.19 | 8 | 8 | 0 | 1 | 1 | 1 | Oxidation (M) | 11358.1816 | type:internal len:102 gc:universal TRINITY\_DN3448\_c0\_g1\_i5:303-1(-) |
| 17 | 76 | TRINITY\_DN3448\_c0\_g2\_i3.p2 | 38.19 | 8 | 8 | 0 | 1 | 1 | 1 | Oxidation (M) | 12003.9434 | type:internal len:107 gc:universal TRINITY\_DN3448\_c0\_g2\_i3:318-1(-) |
| 17 | 77 | TRINITY\_DN3448\_c0\_g2\_i1.p1 | 38.19 | 7 | 7 | 0 | 1 | 1 | 1 | Oxidation (M) | 12264.2549 | type:internal len:109 gc:universal TRINITY\_DN3448\_c0\_g2\_i1:326-3(-) |
| 17 | 78 | TRINITY\_DN3448\_c0\_g1\_i7.p2 | 38.19 | 7 | 7 | 0 | 1 | 1 | 1 | Oxidation (M) | 12485.4922 | type:internal len:111 gc:universal TRINITY\_DN3448\_c0\_g1\_i7:330-1(-) |
| 17 | 79 | TRINITY\_DN32727\_c0\_g1\_i2.p1 | 38.19 | 5 | 5 | 0 | 1 | 1 | 1 | Oxidation (M) | 17342.1152 | type:internal len:154 gc:universal TRINITY\_DN32727\_c0\_g1\_i2:460-2(-) |
| 18 | 80 | TRINITY\_DN262\_c0\_g1\_i1.p1 | 103.98 | 4 | 4 | 6.98e2 | 3 | 3 | 3 |  | 114520.6406 | type:complete len:1033 gc:universal TRINITY\_DN262\_c0\_g1\_i1:111-3209(+) |
| 19 | 81 | TRINITY\_DN2963\_c0\_g1\_i1.p1 | 100.5 | 21 | 21 | 9.3e3 | 3 | 3 | 5 | Acetylation (Protein N-term) | 13082.1787 | type:complete len:116 gc:universal TRINITY\_DN2963\_c0\_g1\_i1:1072-725(-) |
| 20 | 82 | TRINITY\_DN36\_c0\_g1\_i19.p1 | 107.32 | 15 | 15 | 2.41e3 | 3 | 3 | 5 | Carbamidomethylation | 24821.4141 | type:complete len:223 gc:universal TRINITY\_DN36\_c0\_g1\_i19:826-158(-) |
| 20 | 83 | TRINITY\_DN36\_c0\_g1\_i13.p1 | 107.32 | 15 | 15 | 2.41e3 | 3 | 3 | 5 | Carbamidomethylation | 24821.4141 | type:complete len:223 gc:universal TRINITY\_DN36\_c0\_g1\_i13:816-148(-) |
| 20 | 84 | TRINITY\_DN36\_c0\_g1\_i5.p1 | 88.01 | 11 | 11 | 1.67e3 | 2 | 2 | 2 | Carbamidomethylation | 24548.0977 | type:complete len:222 gc:universal TRINITY\_DN36\_c0\_g1\_i5:931-266(-) |
| 20 | 85 | TRINITY\_DN36\_c0\_g1\_i20.p1 | 88.01 | 11 | 11 | 1.67e3 | 2 | 2 | 2 | Carbamidomethylation | 24874.5234 | type:complete len:223 gc:universal TRINITY\_DN36\_c0\_g1\_i20:1172-504(-) |
| 20 | 86 | TRINITY\_DN36\_c0\_g1\_i16.p1 | 87.25 | 10 | 10 | 1.46e3 | 2 | 2 | 4 | Carbamidomethylation | 24953.5938 | type:complete len:224 gc:universal TRINITY\_DN36\_c0\_g1\_i16:829-158(-) |
| 20 | 87 | TRINITY\_DN36\_c0\_g1\_i3.p1 | 87.25 | 10 | 10 | 1.46e3 | 2 | 2 | 4 | Carbamidomethylation | 24953.5938 | type:complete len:224 gc:universal TRINITY\_DN36\_c0\_g1\_i3:829-158(-) |
| 20 | 88 | TRINITY\_DN36\_c0\_g1\_i14.p1 | 87.25 | 10 | 10 | 1.46e3 | 2 | 2 | 4 | Carbamidomethylation | 24953.5938 | type:complete len:224 gc:universal TRINITY\_DN36\_c0\_g1\_i14:819-148(-) |
| 20 | 89 | TRINITY\_DN36\_c0\_g1\_i8.p1 | 87.25 | 10 | 10 | 1.46e3 | 2 | 2 | 4 | Carbamidomethylation | 24953.5938 | type:complete len:224 gc:universal TRINITY\_DN36\_c0\_g1\_i8:819-148(-) |
| 20 | 90 | TRINITY\_DN36\_c0\_g1\_i17.p1 | 87.25 | 10 | 10 | 1.46e3 | 2 | 2 | 4 | Carbamidomethylation | 24953.5938 | type:complete len:224 gc:universal TRINITY\_DN36\_c0\_g1\_i17:819-148(-) |
| 20 | 91 | TRINITY\_DN36\_c0\_g1\_i9.p1 | 58.27 | 6 | 6 | 7.18e2 | 1 | 1 | 1 | Carbamidomethylation | 24680.2773 | type:complete len:223 gc:universal TRINITY\_DN36\_c0\_g1\_i9:934-266(-) |
| 20 | 92 | TRINITY\_DN36\_c0\_g1\_i1.p1 | 58.27 | 6 | 6 | 7.18e2 | 1 | 1 | 1 | Carbamidomethylation | 25006.7051 | type:complete len:224 gc:universal TRINITY\_DN36\_c0\_g1\_i1:1175-504(-) |
| 20 | 93 | TRINITY\_DN36\_c0\_g1\_i11.p1 | 58.27 | 6 | 6 | 7.18e2 | 1 | 1 | 1 | Carbamidomethylation | 25006.7051 | type:complete len:224 gc:universal TRINITY\_DN36\_c0\_g1\_i11:1175-504(-) |
| 20 | 94 | TRINITY\_DN36\_c0\_g1\_i4.p1 | 58.27 | 6 | 6 | 7.18e2 | 1 | 1 | 1 | Carbamidomethylation | 24680.2773 | type:complete len:223 gc:universal TRINITY\_DN36\_c0\_g1\_i4:932-264(-) |
| 20 | 95 | TRINITY\_DN36\_c0\_g1\_i2.p1 | 58.27 | 6 | 6 | 7.18e2 | 1 | 1 | 1 | Carbamidomethylation | 24680.2773 | type:complete len:223 gc:universal TRINITY\_DN36\_c0\_g1\_i2:934-266(-) |
| 20 | 96 | TRINITY\_DN649\_c0\_g1\_i5.p1 | 57.95 | 7 | 7 | 7.44e2 | 1 | 1 | 3 |  | 13746.6875 | type:internal len:124 gc:universal TRINITY\_DN649\_c0\_g1\_i5:371-3(-) |
| 20 | 97 | TRINITY\_DN649\_c0\_g1\_i2.p1 | 57.95 | 6 | 6 | 7.44e2 | 1 | 1 | 3 |  | 13819.7393 | type:internal len:125 gc:universal TRINITY\_DN649\_c0\_g1\_i2:374-3(-) |
| 20 | 98 | TRINITY\_DN649\_c0\_g1\_i1.p1 | 57.95 | 7 | 7 | 7.44e2 | 1 | 1 | 3 |  | 13690.5801 | type:internal len:124 gc:universal TRINITY\_DN649\_c0\_g1\_i1:371-3(-) |
| 21 | 99 | TRINITY\_DN3036\_c0\_g1\_i11.p1 | 107.1 | 12 | 12 | 1.43e3 | 3 | 1 | 9 | Oxidation (M) | 40331.5547 | type:internal len:374 gc:universal TRINITY\_DN3036\_c0\_g1\_i11:1121-3(-) |
| 21 | 100 | TRINITY\_DN3036\_c0\_g1\_i16.p1 | 107.1 | 12 | 12 | 1.43e3 | 3 | 1 | 9 | Oxidation (M) | 40331.5547 | type:internal len:374 gc:universal TRINITY\_DN3036\_c0\_g1\_i16:1121-3(-) |
| 21 | 101 | TRINITY\_DN3036\_c0\_g1\_i19.p1 | 107.1 | 12 | 12 | 1.43e3 | 3 | 1 | 9 | Oxidation (M) | 40287.4609 | type:internal len:374 gc:universal TRINITY\_DN3036\_c0\_g1\_i19:1121-3(-) |
| 21 | 102 | TRINITY\_DN3036\_c0\_g1\_i13.p1 | 107.1 | 12 | 12 | 1.43e3 | 3 | 1 | 9 | Oxidation (M) | 40287.4609 | type:internal len:374 gc:universal TRINITY\_DN3036\_c0\_g1\_i13:1121-3(-) |
| 22 | 103 | TRINITY\_DN1386\_c0\_g1\_i1.p1 | 96.15 | 6 | 6 | 5.4e3 | 3 | 3 | 7 |  | 68692.1953 | type:complete len:596 gc:universal TRINITY\_DN1386\_c0\_g1\_i1:2419-632(-) |
| 23 | 104 | TRINITY\_DN286\_c0\_g1\_i2.p1 | 58.88 | 10 | 10 | 1.11e4 | 1 | 1 | 4 | Oxidation (M), Carbamidomethylation | 11368.1738 | type:3prime\_partial len:106 gc:universal TRINITY\_DN286\_c0\_g1\_i2:40-354(+) |
| 23 | 105 | TRINITY\_DN286\_c0\_g1\_i4.p1 | 58.88 | 5 | 5 | 1.11e4 | 1 | 1 | 4 | Oxidation (M), Carbamidomethylation | 25084.5293 | type:complete len:224 gc:universal TRINITY\_DN286\_c0\_g1\_i4:40-711(+) |
| 24 | 106 | TRINITY\_DN982\_c0\_g1\_i1.p1 | 80.68 | 1 | 1 | 0 | 3 | 1 | 3 |  | 712566.0625 | type:5prime\_partial len:6412 gc:universal TRINITY\_DN982\_c0\_g1\_i1:19377-142(-) |
| 24 | 107 | TRINITY\_DN982\_c0\_g1\_i2.p1 | 39.23 | 1 | 1 | 0 | 2 | 1 | 2 |  | 190367.1406 | type:internal len:1718 gc:universal TRINITY\_DN982\_c0\_g1\_i2:5151-1(-) |
| 25 | 108 | TRINITY\_DN64\_c0\_g1\_i1.p1 | 95.72 | 6 | 6 | 2.81e2 | 3 | 3 | 3 | Oxidation (M) | 51450.1914 | type:complete len:468 gc:universal TRINITY\_DN64\_c0\_g1\_i1:1555-152(-) |
| 26 | 109 | TRINITY\_DN1614\_c0\_g1\_i1.p1 | 107.22 | 17 | 17 | 1.95e3 | 3 | 3 | 4 | Carbamidomethylation | 25367.3418 | type:complete len:218 gc:universal TRINITY\_DN1614\_c0\_g1\_i1:56-709(+) |
| 27 | 110 | TRINITY\_DN477\_c0\_g1\_i9.p1 | 101.63 | 8 | 8 | 6.01e2 | 3 | 3 | 4 | Carbamidomethylation | 34222.9453 | type:complete len:306 gc:universal TRINITY\_DN477\_c0\_g1\_i9:1840-923(-) |
| 28 | 111 | TRINITY\_DN5633\_c0\_g1\_i1.p1 | 91.17 | 4 | 4 | 3.07e2 | 3 | 3 | 3 |  | 97310.9922 | type:complete len:848 gc:universal TRINITY\_DN5633\_c0\_g1\_i1:2881-338(-) |
| 28 | 112 | TRINITY\_DN141338\_c2\_g1\_i1.p1 | 55.36 | 2 | 2 | 3.07e2 | 1 | 1 | 1 |  | 82716.2266 | type:complete len:720 gc:universal TRINITY\_DN141338\_c2\_g1\_i1:2850-691(-) |
| 29 | 113 | TRINITY\_DN2472\_c0\_g1\_i1.p1 | 81.42 | 6 | 6 | 2e3 | 2 | 2 | 2 | Acetylation (Protein N-term) | 37676.8203 | type:complete len:344 gc:universal TRINITY\_DN2472\_c0\_g1\_i1:311-1342(+) |
| 30 | 114 | TRINITY\_DN332\_c0\_g1\_i1.p1 | 88.17 | 11 | 11 | 4.46e2 | 2 | 2 | 2 |  | 27839.6836 | type:complete len:246 gc:universal TRINITY\_DN332\_c0\_g1\_i1:940-203(-) |
| 31 | 115 | TRINITY\_DN1616\_c0\_g1\_i3.p1 | 87.56 | 1 | 1 | 0 | 2 | 2 | 2 | Carbamidomethylation | 423947.2812 | type:complete len:3855 gc:universal TRINITY\_DN1616\_c0\_g1\_i3:218-11782(+) |
| 31 | 116 | TRINITY\_DN1616\_c0\_g1\_i7.p1 | 87.56 | 1 | 1 | 0 | 2 | 2 | 2 | Carbamidomethylation | 423875.2188 | type:complete len:3855 gc:universal TRINITY\_DN1616\_c0\_g1\_i7:218-11782(+) |
| 31 | 117 | TRINITY\_DN1616\_c0\_g1\_i6.p1 | 87.56 | 1 | 1 | 0 | 2 | 2 | 2 | Carbamidomethylation | 423911.25 | type:complete len:3855 gc:universal TRINITY\_DN1616\_c0\_g1\_i6:218-11782(+) |
| 31 | 118 | TRINITY\_DN1616\_c0\_g1\_i2.p1 | 58.58 | 0 | 0 | 0 | 1 | 1 | 1 |  | 374396 | type:3prime\_partial len:3398 gc:universal TRINITY\_DN1616\_c0\_g1\_i2:218-10408(+) |
| 32 | 119 | TRINITY\_DN3078\_c0\_g1\_i1.p1 | 84.98 | 1 | 1 | 0 | 2 | 2 | 2 | Carbamidomethylation | 127689.1797 | type:complete len:1109 gc:universal TRINITY\_DN3078\_c0\_g1\_i1:3628-302(-) |
| 33 | 120 | TRINITY\_DN3339\_c0\_g1\_i1.p1 | 74.41 | 25 | 25 | 9.33e2 | 2 | 2 | 3 | Carbamidomethylation | 14934.2627 | type:complete len:131 gc:universal TRINITY\_DN3339\_c0\_g1\_i1:31-423(+) |
| 34 | 121 | TRINITY\_DN1071\_c0\_g1\_i3.p1 | 71.25 | 7 | 7 | 1.32e2 | 2 | 2 | 3 |  | 39803.1602 | type:internal len:375 gc:universal TRINITY\_DN1071\_c0\_g1\_i3:1124-3(-) |
| 34 | 122 | TRINITY\_DN1071\_c0\_g1\_i1.p1 | 71.25 | 4 | 4 | 1.32e2 | 2 | 2 | 3 |  | 71848.4062 | type:internal len:660 gc:universal TRINITY\_DN1071\_c0\_g1\_i1:1977-1(-) |
| 34 | 123 | TRINITY\_DN1071\_c0\_g1\_i4.p1 | 71.25 | 4 | 4 | 1.32e2 | 2 | 2 | 3 |  | 75408.3359 | type:internal len:696 gc:universal TRINITY\_DN1071\_c0\_g1\_i4:2085-1(-) |
| 34 | 124 | TRINITY\_DN1071\_c0\_g1\_i2.p1 | 71.25 | 8 | 8 | 1.32e2 | 2 | 2 | 3 |  | 36243.2383 | type:internal len:339 gc:universal TRINITY\_DN1071\_c0\_g1\_i2:1016-3(-) |
| 35 | 125 | TRINITY\_DN1112\_c0\_g1\_i1.p1 | 82.35 | 7 | 7 | 4.16e2 | 2 | 2 | 2 | Oxidation (M) | 23407.2832 | type:complete len:200 gc:universal TRINITY\_DN1112\_c0\_g1\_i1:740-141(-) |
| 36 | 126 | TRINITY\_DN2113\_c0\_g1\_i1.p1 | 83.39 | 2 | 2 | 1.33e3 | 2 | 2 | 2 |  | 193799.0156 | type:complete len:1704 gc:universal TRINITY\_DN2113\_c0\_g1\_i1:5257-146(-) |
| 36 | 127 | TRINITY\_DN2113\_c0\_g1\_i2.p1 | 83.39 | 2 | 2 | 1.33e3 | 2 | 2 | 2 |  | 193799.0156 | type:complete len:1704 gc:universal TRINITY\_DN2113\_c0\_g1\_i2:5257-146(-) |
| 37 | 128 | TRINITY\_DN197343\_c0\_g1\_i1.p1 | 87.3 | 26 | 26 | 1.39e3 | 2 | 2 | 3 | Carbamidomethylation | 14546.0635 | type:complete len:124 gc:universal TRINITY\_DN197343\_c0\_g1\_i1:399-28(-) |
| 38 | 129 | TRINITY\_DN146208\_c0\_g1\_i1.p1 | 87.23 | 5 | 5 | 6.96e2 | 2 | 2 | 2 |  | 32340.8027 | type:5prime\_partial len:284 gc:universal TRINITY\_DN146208\_c0\_g1\_i1:876-25(-) |
| 39 | 130 | TRINITY\_DN9509\_c0\_g1\_i10.p1 | 86.81 | 7 | 7 | 4.83e2 | 2 | 2 | 3 | Carbamidomethylation | 46377.625 | type:5prime\_partial len:424 gc:universal TRINITY\_DN9509\_c0\_g1\_i10:1493-222(-) |
| 39 | 131 | TRINITY\_DN9509\_c0\_g1\_i6.p1 | 86.81 | 7 | 7 | 4.83e2 | 2 | 2 | 3 | Carbamidomethylation | 46379.5938 | type:5prime\_partial len:424 gc:universal TRINITY\_DN9509\_c0\_g1\_i6:1493-222(-) |
| 39 | 132 | TRINITY\_DN9509\_c0\_g1\_i8.p1 | 58 | 7 | 7 | 4.83e2 | 1 | 1 | 2 | Carbamidomethylation | 23738.0078 | type:5prime\_partial len:218 gc:universal TRINITY\_DN9509\_c0\_g1\_i8:875-222(-) |
| 39 | 133 | TRINITY\_DN9509\_c0\_g1\_i2.p1 | 57.62 | 6 | 6 | 0 | 1 | 1 | 1 |  | 30688.8438 | type:internal len:279 gc:universal TRINITY\_DN9509\_c0\_g1\_i2:836-3(-) |
| 40 | 134 | TRINITY\_DN477\_c0\_g1\_i7.p1 | 86.69 | 3 | 3 | 2.02e3 | 2 | 2 | 4 | Carbamidomethylation | 122478.2578 | type:3prime\_partial len:1130 gc:universal TRINITY\_DN477\_c0\_g1\_i7:3387-1(-) |
| 40 | 135 | TRINITY\_DN477\_c0\_g1\_i8.p1 | 86.69 | 3 | 3 | 2.02e3 | 2 | 2 | 4 | Carbamidomethylation | 107165.2031 | type:complete len:985 gc:universal TRINITY\_DN477\_c0\_g1\_i8:3548-594(-) |
| 41 | 136 | TRINITY\_DN484\_c0\_g1\_i1.p1 | 70.52 | 5 | 5 | 1.06e3 | 2 | 2 | 2 | Oxidation (M) | 59940.2617 | type:complete len:554 gc:universal TRINITY\_DN484\_c0\_g1\_i1:164-1825(+) |
| 42 | 137 | TRINITY\_DN3991\_c0\_g1\_i1.p1 | 88.56 | 6 | 6 | 6.46e4 | 2 | 2 | 4 | Carbamidomethylation | 24846.6543 | type:3prime\_partial len:219 gc:universal TRINITY\_DN3991\_c0\_g1\_i1:84-737(+) |
| 43 | 138 | TRINITY\_DN1377\_c0\_g1\_i1.p1 | 64.24 | 3 | 3 | 0 | 2 | 2 | 2 |  | 99936.9219 | type:complete len:878 gc:universal TRINITY\_DN1377\_c0\_g1\_i1:68-2701(+) |
| 44 | 139 | TRINITY\_DN428\_c0\_g1\_i1.p1 | 79.5 | 1 | 1 | 2.47e3 | 2 | 2 | 3 |  | 225824.1719 | type:complete len:1949 gc:universal TRINITY\_DN428\_c0\_g1\_i1:96-5942(+) |
| 45 | 140 | TRINITY\_DN72959\_c0\_g1\_i1.p1 | 70.35 | 12 | 12 | 7.61e2 | 2 | 2 | 2 |  | 25064.7695 | type:complete len:216 gc:universal TRINITY\_DN72959\_c0\_g1\_i1:54-701(+) |
| 46 | 141 | TRINITY\_DN72753\_c0\_g1\_i1.p1 | 80.79 | 15 | 15 | 1.61e3 | 2 | 2 | 3 |  | 15430.793 | type:complete len:137 gc:universal TRINITY\_DN72753\_c0\_g1\_i1:95-505(+) |
| 46 | 142 | TRINITY\_DN18187\_c0\_g1\_i4.p3 | 44.05 | 8 | 8 | 1.61e3 | 1 | 1 | 1 |  | 13023.127 | type:5prime\_partial len:112 gc:universal TRINITY\_DN18187\_c0\_g1\_i4:361-26(-) |
| 46 | 143 | TRINITY\_DN18187\_c0\_g1\_i6.p2 | 44.05 | 5 | 5 | 1.61e3 | 1 | 1 | 1 |  | 19395.627 | type:5prime\_partial len:178 gc:universal TRINITY\_DN18187\_c0\_g1\_i6:559-26(-) |
| 46 | 144 | TRINITY\_DN5023\_c0\_g1\_i2.p2 | 44.05 | 8 | 8 | 1.61e3 | 1 | 1 | 1 |  | 12713.9521 | type:internal len:117 gc:universal TRINITY\_DN5023\_c0\_g1\_i2:348-1(-) |
| 46 | 145 | TRINITY\_DN18187\_c0\_g1\_i3.p4 | 44.05 | 6 | 6 | 1.61e3 | 1 | 1 | 1 |  | 15989.5195 | type:5prime\_partial len:143 gc:universal TRINITY\_DN18187\_c0\_g1\_i3:454-26(-) |
| 46 | 146 | TRINITY\_DN5023\_c0\_g1\_i3.p2 | 44.05 | 9 | 9 | 1.61e3 | 1 | 1 | 1 |  | 11215.2041 | type:internal len:105 gc:universal TRINITY\_DN5023\_c0\_g1\_i3:313-2(-) |
| 46 | 147 | TRINITY\_DN18187\_c0\_g1\_i5.p1 | 44.05 | 5 | 5 | 1.61e3 | 1 | 1 | 1 |  | 19887.0645 | type:5prime\_partial len:182 gc:universal TRINITY\_DN18187\_c0\_g1\_i5:664-119(-) |
| 47 | 148 | TRINITY\_DN535\_c0\_g1\_i1.p1 | 87.93 | 23 | 23 | 1.98e3 | 2 | 2 | 3 | Carbamidomethylation | 18221.5957 | type:5prime\_partial len:162 gc:universal TRINITY\_DN535\_c0\_g1\_i1:712-227(-) |
| 47 | 149 | TRINITY\_DN535\_c0\_g1\_i2.p1 | 87.93 | 23 | 23 | 1.98e3 | 2 | 2 | 3 | Carbamidomethylation | 18221.5957 | type:5prime\_partial len:162 gc:universal TRINITY\_DN535\_c0\_g1\_i2:699-214(-) |
| 48 | 150 | TRINITY\_DN79\_c0\_g1\_i1.p1 | 86.47 | 7 | 7 | 1.45e3 | 2 | 2 | 4 |  | 52590.9805 | type:5prime\_partial len:478 gc:universal TRINITY\_DN79\_c0\_g1\_i1:2132-699(-) |
| 49 | 151 | TRINITY\_DN119684\_c0\_g1\_i1.p1 | 68.93 | 3 | 3 | 5.52e3 | 2 | 2 | 2 |  | 72994.7266 | type:complete len:662 gc:universal TRINITY\_DN119684\_c0\_g1\_i1:2182-197(-) |
| 50 | 152 | TRINITY\_DN195948\_c0\_g1\_i1.p1 | 75.21 | 14 | 14 | 2.1e2 | 2 | 2 | 2 | Oxidation (M) | 18888.6914 | type:5prime\_partial len:170 gc:universal TRINITY\_DN195948\_c0\_g1\_i1:602-93(-) |
| 51 | 153 | TRINITY\_DN1080\_c0\_g1\_i3.p1 | 85.59 | 4 | 4 | 1.06e3 | 2 | 2 | 2 |  | 48680.9648 | type:internal len:425 gc:universal TRINITY\_DN1080\_c0\_g1\_i3:1274-3(-) |
| 51 | 154 | TRINITY\_DN1080\_c0\_g1\_i2.p1 | 85.59 | 4 | 4 | 1.06e3 | 2 | 2 | 2 |  | 49162.0703 | type:5prime\_partial len:431 gc:universal TRINITY\_DN1080\_c0\_g1\_i2:1300-8(-) |
| 51 | 155 | TRINITY\_DN1080\_c0\_g2\_i1.p1 | 57.63 | 0 | 0 | 1.06e3 | 1 | 1 | 1 |  | 990519.0625 | type:5prime\_partial len:8617 gc:universal TRINITY\_DN1080\_c0\_g2\_i1:26447-597(-) |
| 52 | 156 | TRINITY\_DN2028\_c0\_g1\_i1.p1 | 78.12 | 11 | 11 | 2.33e3 | 2 | 2 | 2 | Oxidation (M) | 30106.9453 | type:complete len:274 gc:universal TRINITY\_DN2028\_c0\_g1\_i1:1608-787(-) |
| 53 | 157 | TRINITY\_DN319\_c2\_g1\_i3.p1 | 88.07 | 1 | 1 | 2.33e3 | 2 | 2 | 4 |  | 279087.1875 | type:complete len:2588 gc:universal TRINITY\_DN319\_c2\_g1\_i3:165-7928(+) |
| 53 | 158 | TRINITY\_DN319\_c2\_g1\_i1.p1 | 88.07 | 1 | 1 | 2.33e3 | 2 | 2 | 4 |  | 279168.25 | type:complete len:2588 gc:universal TRINITY\_DN319\_c2\_g1\_i1:165-7928(+) |
| 54 | 159 | TRINITY\_DN168534\_c0\_g1\_i1.p1 | 85.73 | 12 | 12 | 0 | 2 | 2 | 2 | Oxidation (M), Carbamidomethylation | 25265.0449 | type:complete len:226 gc:universal TRINITY\_DN168534\_c0\_g1\_i1:774-97(-) |
| 55 | 160 | TRINITY\_DN15599\_c0\_g1\_i2.p1 | 58.46 | 5 | 5 | 3.82e3 | 1 | 1 | 1 |  | 21967.3105 | type:internal len:206 gc:universal TRINITY\_DN15599\_c0\_g1\_i2:1-615(+) |
| 55 | 161 | TRINITY\_DN15599\_c0\_g1\_i1.p1 | 58.46 | 5 | 5 | 3.82e3 | 1 | 1 | 2 | Acetylation (Protein N-term) | 21907.2578 | type:internal len:206 gc:universal TRINITY\_DN15599\_c0\_g1\_i1:1-615(+) |
| 56 | 162 | TRINITY\_DN773\_c0\_g1\_i1.p1 | 79.35 | 5 | 5 | 5.53e2 | 2 | 2 | 2 |  | 48865.75 | type:complete len:440 gc:universal TRINITY\_DN773\_c0\_g1\_i1:1608-289(-) |
| 57 | 163 | TRINITY\_DN1172\_c0\_g2\_i2.p1 | 87.51 | 8 | 8 | 3.4e3 | 2 | 2 | 3 | Acetylation (Protein N-term) | 27813.4355 | type:internal len:264 gc:universal TRINITY\_DN1172\_c0\_g2\_i2:790-2(-) |
| 57 | 164 | TRINITY\_DN21847\_c0\_g1\_i1.p1 | 58.73 | 3 | 3 | 2.66e3 | 1 | 1 | 2 |  | 41400.2812 | type:internal len:384 gc:universal TRINITY\_DN21847\_c0\_g1\_i1:1150-2(-) |
| 57 | 165 | TRINITY\_DN4497\_c0\_g1\_i1.p1 | 58.73 | 8 | 8 | 2.66e3 | 1 | 1 | 2 |  | 17016.3789 | type:internal len:160 gc:universal TRINITY\_DN4497\_c0\_g1\_i1:1-477(+) |
| 57 | 166 | TRINITY\_DN4497\_c0\_g2\_i1.p1 | 58.73 | 7 | 7 | 2.66e3 | 1 | 1 | 2 |  | 19644.3613 | type:internal len:183 gc:universal TRINITY\_DN4497\_c0\_g2\_i1:2-547(+) |
| 58 | 167 | TRINITY\_DN189999\_c0\_g1\_i1.p1 | 85.98 | 13 | 13 | 6.7e2 | 2 | 2 | 3 |  | 18662.8945 | type:complete len:170 gc:universal TRINITY\_DN189999\_c0\_g1\_i1:599-90(-) |
| 59 | 168 | TRINITY\_DN40728\_c1\_g1\_i1.p2 | 55.06 | 10 | 10 | 2.97e3 | 1 | 1 | 2 |  | 14073.3066 | type:5prime\_partial len:124 gc:universal TRINITY\_DN40728\_c1\_g1\_i1:1-372(+) |
| 59 | 169 | TRINITY\_DN40728\_c0\_g1\_i1.p1 | 55.06 | 13 | 13 | 2.97e3 | 1 | 1 | 2 |  | 11225.8301 | type:internal len:105 gc:universal TRINITY\_DN40728\_c0\_g1\_i1:1-312(+) |
| 59 | 170 | TRINITY\_DN195631\_c0\_g1\_i1.p2 | 55.06 | 9 | 9 | 2.97e3 | 1 | 1 | 2 |  | 15844.8984 | type:internal len:145 gc:universal TRINITY\_DN195631\_c0\_g1\_i1:2-433(+) |
| 59 | 171 | TRINITY\_DN8227\_c0\_g1\_i1.p1 | 55.06 | 10 | 10 | 2.97e3 | 1 | 1 | 2 |  | 14513.8135 | type:internal len:135 gc:universal TRINITY\_DN8227\_c0\_g1\_i1:404-3(-) |
| 59 | 172 | TRINITY\_DN152\_c0\_g1\_i15.p1 | 55.06 | 9 | 9 | 2.97e3 | 1 | 1 | 2 |  | 15560.71 | type:internal len:141 gc:universal TRINITY\_DN152\_c0\_g1\_i15:2-421(+) |
| 59 | 173 | TRINITY\_DN585\_c0\_g1\_i10.p1 | 55.06 | 6 | 6 | 2.97e3 | 1 | 1 | 2 |  | 26563.5332 | type:3prime\_partial len:236 gc:universal TRINITY\_DN585\_c0\_g1\_i10:705-1(-) |
| 59 | 174 | TRINITY\_DN152\_c0\_g1\_i2.p1 | 55.06 | 13 | 13 | 2.97e3 | 1 | 1 | 2 |  | 11754.375 | type:internal len:105 gc:universal TRINITY\_DN152\_c0\_g1\_i2:1-312(+) |
| 59 | 175 | TRINITY\_DN152\_c0\_g1\_i10.p1 | 55.06 | 7 | 7 | 2.97e3 | 1 | 1 | 2 |  | 20063.498 | type:internal len:180 gc:universal TRINITY\_DN152\_c0\_g1\_i10:1-537(+) |
| 59 | 176 | TRINITY\_DN152\_c0\_g1\_i14.p2 | 55.06 | 11 | 11 | 2.97e3 | 1 | 1 | 2 |  | 12547.4834 | type:5prime\_partial len:115 gc:universal TRINITY\_DN152\_c0\_g1\_i14:3-347(+) |
| 59 | 177 | TRINITY\_DN554\_c0\_g1\_i3.p2 | 55.06 | 5 | 5 | 2.97e3 | 1 | 1 | 2 |  | 30419.4961 | type:complete len:268 gc:universal TRINITY\_DN554\_c0\_g1\_i3:1464-661(-) |
| 59 | 178 | TRINITY\_DN1465\_c1\_g1\_i1.p1 | 55.06 | 13 | 13 | 2.97e3 | 1 | 1 | 2 |  | 11342.1572 | type:internal len:102 gc:universal TRINITY\_DN1465\_c1\_g1\_i1:2-304(+) |
| 59 | 179 | TRINITY\_DN152\_c0\_g1\_i9.p1 | 55.06 | 9 | 9 | 2.97e3 | 1 | 1 | 2 |  | 17213.1973 | type:5prime\_partial len:152 gc:universal TRINITY\_DN152\_c0\_g1\_i9:1-456(+) |
| 59 | 180 | TRINITY\_DN585\_c0\_g1\_i2.p1 | 55.06 | 7 | 7 | 2.97e3 | 1 | 1 | 2 |  | 20558.6738 | type:internal len:184 gc:universal TRINITY\_DN585\_c0\_g1\_i2:549-1(-) |
| 59 | 181 | TRINITY\_DN585\_c0\_g1\_i5.p1 | 55.06 | 5 | 5 | 2.97e3 | 1 | 1 | 2 |  | 28098.1602 | type:internal len:250 gc:universal TRINITY\_DN585\_c0\_g1\_i5:747-1(-) |
| 59 | 182 | TRINITY\_DN173718\_c0\_g1\_i1.p1 | 55.06 | 12 | 12 | 2.97e3 | 1 | 1 | 2 |  | 12865.0928 | type:3prime\_partial len:112 gc:universal TRINITY\_DN173718\_c0\_g1\_i1:37-369(+) |
| 59 | 183 | TRINITY\_DN585\_c0\_g1\_i3.p1 | 55.06 | 8 | 8 | 2.97e3 | 1 | 1 | 2 |  | 17628.2207 | type:internal len:157 gc:universal TRINITY\_DN585\_c0\_g1\_i3:468-1(-) |
| 59 | 184 | TRINITY\_DN585\_c0\_g1\_i4.p1 | 55.06 | 6 | 6 | 2.97e3 | 1 | 1 | 2 |  | 26348.0742 | type:complete len:232 gc:universal TRINITY\_DN585\_c0\_g1\_i4:847-152(-) |
| 59 | 185 | TRINITY\_DN554\_c0\_g1\_i5.p1 | 55.06 | 3 | 3 | 2.97e3 | 1 | 1 | 2 |  | 42081.4141 | type:3prime\_partial len:373 gc:universal TRINITY\_DN554\_c0\_g1\_i5:1118-3(-) |
| 60 | 186 | TRINITY\_DN265\_c0\_g1\_i9.p1 | 58.75 | 7 | 7 | 1.15e3 | 1 | 1 | 1 |  | 15628.0479 | type:complete len:136 gc:universal TRINITY\_DN265\_c0\_g1\_i9:136-543(+) |
| 60 | 187 | TRINITY\_DN265\_c0\_g1\_i6.p1 | 58.75 | 7 | 7 | 1.15e3 | 1 | 1 | 1 |  | 15638.0283 | type:complete len:136 gc:universal TRINITY\_DN265\_c0\_g1\_i6:269-676(+) |
| 60 | 188 | TRINITY\_DN265\_c0\_g1\_i4.p1 | 58.75 | 7 | 7 | 1.15e3 | 1 | 1 | 1 |  | 15670.1318 | type:complete len:136 gc:universal TRINITY\_DN265\_c0\_g1\_i4:269-676(+) |
| 60 | 189 | TRINITY\_DN265\_c0\_g1\_i8.p1 | 58.75 | 7 | 7 | 1.15e3 | 1 | 1 | 1 |  | 15642.1182 | type:complete len:136 gc:universal TRINITY\_DN265\_c0\_g1\_i8:107-514(+) |
| 60 | 190 | TRINITY\_DN265\_c0\_g1\_i2.p1 | 58.75 | 7 | 7 | 1.15e3 | 1 | 1 | 1 |  | 15656.0615 | type:complete len:136 gc:universal TRINITY\_DN265\_c0\_g1\_i2:107-514(+) |
| 60 | 191 | TRINITY\_DN265\_c0\_g1\_i3.p1 | 58.75 | 7 | 7 | 1.15e3 | 1 | 1 | 1 |  | 15652.0986 | type:complete len:136 gc:universal TRINITY\_DN265\_c0\_g1\_i3:136-543(+) |
| 60 | 192 | TRINITY\_DN265\_c0\_g1\_i7.p1 | 58.75 | 7 | 7 | 1.15e3 | 1 | 1 | 1 |  | 15664.1533 | type:complete len:136 gc:universal TRINITY\_DN265\_c0\_g1\_i7:136-543(+) |
| 60 | 193 | TRINITY\_DN265\_c0\_g1\_i1.p1 | 58.75 | 7 | 7 | 1.15e3 | 1 | 1 | 1 |  | 15642.1182 | type:complete len:136 gc:universal TRINITY\_DN265\_c0\_g1\_i1:261-668(+) |
| 60 | 194 | TRINITY\_DN265\_c0\_g1\_i5.p1 | 58.75 | 7 | 7 | 1.15e3 | 1 | 1 | 1 |  | 15638.0283 | type:complete len:136 gc:universal TRINITY\_DN265\_c0\_g1\_i5:136-543(+) |
| 61 | 195 | TRINITY\_DN2636\_c0\_g1\_i1.p1 | 58.8 | 3 | 3 | 5.76e2 | 1 | 1 | 3 |  | 35741.2305 | type:complete len:314 gc:universal TRINITY\_DN2636\_c0\_g1\_i1:986-45(-) |
| 61 | 196 | TRINITY\_DN2636\_c0\_g1\_i2.p1 | 58.8 | 3 | 3 | 5.76e2 | 1 | 1 | 3 |  | 35741.2305 | type:complete len:314 gc:universal TRINITY\_DN2636\_c0\_g1\_i2:986-45(-) |
| 62 | 197 | TRINITY\_DN3545\_c0\_g1\_i2.p1 | 27.61 | 5 | 5 | 6.05e2 | 1 | 1 | 1 | Carbamidomethylation | 25563.4785 | type:5prime\_partial len:236 gc:universal TRINITY\_DN3545\_c0\_g1\_i2:3-710(+) |
| 62 | 198 | TRINITY\_DN3545\_c0\_g1\_i1.p1 | 27.61 | 4 | 4 | 6.05e2 | 1 | 1 | 1 | Carbamidomethylation | 31576.2656 | type:5prime\_partial len:293 gc:universal TRINITY\_DN3545\_c0\_g1\_i1:3-881(+) |
| 62 | 199 | TRINITY\_DN158421\_c0\_g1\_i1.p1 | 27.61 | 10 | 10 | 6.05e2 | 1 | 1 | 1 | Carbamidomethylation | 12814.7959 | type:internal len:115 gc:universal TRINITY\_DN158421\_c0\_g1\_i1:1-342(+) |
| 63 | 200 | TRINITY\_DN384\_c0\_g1\_i2.p1 | 57.97 | 4 | 4 | 0 | 1 | 1 | 1 |  | 34316.0703 | type:complete len:314 gc:universal TRINITY\_DN384\_c0\_g1\_i2:1018-77(-) |
| 63 | 201 | TRINITY\_DN22\_c0\_g1\_i1.p1 | 57.97 | 3 | 3 | 0 | 1 | 1 | 1 |  | 38528.8086 | type:5prime\_partial len:353 gc:universal TRINITY\_DN22\_c0\_g1\_i1:3-1061(+) |
| 63 | 202 | TRINITY\_DN384\_c0\_g1\_i1.p1 | 57.97 | 4 | 4 | 0 | 1 | 1 | 1 |  | 34317.9414 | type:complete len:313 gc:universal TRINITY\_DN384\_c0\_g1\_i1:1015-77(-) |
| 64 | 203 | TRINITY\_DN817\_c0\_g1\_i4.p1 | 38.48 | 8 | 8 | 1.4e2 | 1 | 1 | 1 | Carbamidomethylation | 19497.5664 | type:internal len:176 gc:universal TRINITY\_DN817\_c0\_g1\_i4:526-2(-) |
| 64 | 204 | TRINITY\_DN817\_c0\_g1\_i10.p1 | 38.48 | 8 | 8 | 1.4e2 | 1 | 1 | 1 | Carbamidomethylation | 20465.6582 | type:internal len:186 gc:universal TRINITY\_DN817\_c0\_g1\_i10:556-2(-) |
| 64 | 205 | TRINITY\_DN817\_c0\_g1\_i2.p1 | 38.48 | 8 | 8 | 1.4e2 | 1 | 1 | 1 | Carbamidomethylation | 19573.5801 | type:internal len:176 gc:universal TRINITY\_DN817\_c0\_g1\_i2:526-2(-) |
| 64 | 206 | TRINITY\_DN817\_c0\_g1\_i6.p1 | 38.48 | 8 | 8 | 1.4e2 | 1 | 1 | 1 | Carbamidomethylation | 19716.6973 | type:internal len:178 gc:universal TRINITY\_DN817\_c0\_g1\_i6:532-2(-) |
| 65 | 207 | TRINITY\_DN121866\_c0\_g1\_i1.p1 | 58.61 | 2 | 2 | 1.21e3 | 1 | 1 | 1 |  | 51719.8477 | type:5prime\_partial len:460 gc:universal TRINITY\_DN121866\_c0\_g1\_i1:2-1381(+) |
| 66 | 208 | TRINITY\_DN1339\_c0\_g2\_i3.p1 | 58.87 | 7 | 7 | 7.94e2 | 1 | 1 | 1 |  | 19523.1797 | type:internal len:185 gc:universal TRINITY\_DN1339\_c0\_g2\_i3:553-2(-) |
| 66 | 209 | TRINITY\_DN1339\_c0\_g2\_i2.p1 | 58.87 | 3 | 3 | 7.94e2 | 1 | 1 | 1 |  | 42663.3828 | type:internal len:398 gc:universal TRINITY\_DN1339\_c0\_g2\_i2:1193-3(-) |
| 66 | 210 | TRINITY\_DN1339\_c0\_g2\_i4.p2 | 58.87 | 6 | 6 | 7.94e2 | 1 | 1 | 1 |  | 22988.9766 | type:internal len:216 gc:universal TRINITY\_DN1339\_c0\_g2\_i4:646-2(-) |
| 66 | 211 | TRINITY\_DN1339\_c0\_g2\_i1.p2 | 58.87 | 3 | 3 | 7.94e2 | 1 | 1 | 1 |  | 43129.793 | type:internal len:402 gc:universal TRINITY\_DN1339\_c0\_g2\_i1:1203-1(-) |
| 67 | 212 | TRINITY\_DN89\_c0\_g1\_i1.p1 | 52.33 | 4 | 4 | 0 | 1 | 1 | 3 | Carbamidomethylation | 43014.0469 | type:complete len:376 gc:universal TRINITY\_DN89\_c0\_g1\_i1:117-1244(+) |
| 68 | 213 | TRINITY\_DN165469\_c0\_g1\_i1.p1 | 53.55 | 1 | 1 | 1.79e2 | 1 | 1 | 1 |  | 298128.9688 | type:complete len:2668 gc:universal TRINITY\_DN165469\_c0\_g1\_i1:41-8044(+) |
| 69 | 214 | TRINITY\_DN8903\_c0\_g1\_i1.p1 | 58.51 | 2 | 2 | 3.95e2 | 1 | 1 | 1 |  | 50029.5586 | type:5prime\_partial len:447 gc:universal TRINITY\_DN8903\_c0\_g1\_i1:1546-206(-) |
| 70 | 215 | TRINITY\_DN106952\_c0\_g1\_i1.p1 | 58.87 | 11 | 11 | 2.51e4 | 1 | 1 | 10 | Oxidation (M) | 14528.8164 | type:3prime\_partial len:135 gc:universal TRINITY\_DN106952\_c0\_g1\_i1:18-419(+) |
| 71 | 216 | TRINITY\_DN433\_c0\_g2\_i1.p1 | 19.88 | 6 | 6 | 6.5e2 | 1 | 1 | 1 |  | 16483.6387 | type:complete len:144 gc:universal TRINITY\_DN433\_c0\_g2\_i1:1496-1065(-) |
| 72 | 217 | TRINITY\_DN5276\_c0\_g1\_i12.p2 | 23.14 | 9 | 9 | 0 | 1 | 1 | 1 |  | 24814.1113 | type:internal len:228 gc:universal TRINITY\_DN5276\_c0\_g1\_i12:683-3(-) |
| 72 | 218 | TRINITY\_DN5276\_c0\_g1\_i14.p1 | 23.14 | 7 | 7 | 0 | 1 | 1 | 1 |  | 33293.9062 | type:internal len:304 gc:universal TRINITY\_DN5276\_c0\_g1\_i14:911-3(-) |
| 73 | 219 | TRINITY\_DN4101\_c0\_g1\_i3.p2 | 58.84 | 11 | 11 | 1.93e2 | 1 | 1 | 1 |  | 14374.332 | type:5prime\_partial len:133 gc:universal TRINITY\_DN4101\_c0\_g1\_i3:3-401(+) |
| 73 | 220 | TRINITY\_DN4101\_c0\_g1\_i4.p2 | 58.84 | 11 | 11 | 1.93e2 | 1 | 1 | 1 |  | 14374.332 | type:5prime\_partial len:133 gc:universal TRINITY\_DN4101\_c0\_g1\_i4:3-401(+) |
| 73 | 221 | TRINITY\_DN4101\_c0\_g1\_i1.p3 | 58.84 | 13 | 13 | 1.93e2 | 1 | 1 | 1 |  | 12995.6113 | type:5prime\_partial len:118 gc:universal TRINITY\_DN4101\_c0\_g1\_i1:3-356(+) |
| 74 | 222 | TRINITY\_DN468\_c1\_g1\_i1.p1 | 58.12 | 10 | 10 | 0 | 1 | 1 | 2 |  | 21670.5645 | type:complete len:191 gc:universal TRINITY\_DN468\_c1\_g1\_i1:676-104(-) |
| 75 | 223 | TRINITY\_DN167010\_c0\_g1\_i1.p1 | 21.14 | 1 | 1 | 1.3e3 | 1 | 1 | 1 | Carbamidomethylation, Oxidation (M) | 58355.5547 | type:5prime\_partial len:506 gc:universal TRINITY\_DN167010\_c0\_g1\_i1:1755-238(-) |
| 76 | 224 | TRINITY\_DN160\_c0\_g1\_i1.p1 | 49.33 | 1 | 1 | 0 | 1 | 1 | 1 |  | 126611.5703 | type:complete len:1145 gc:universal TRINITY\_DN160\_c0\_g1\_i1:4810-1376(-) |
| 76 | 225 | TRINITY\_DN160\_c0\_g1\_i2.p1 | 49.33 | 1 | 1 | 0 | 1 | 1 | 1 |  | 126611.5703 | type:complete len:1145 gc:universal TRINITY\_DN160\_c0\_g1\_i2:4810-1376(-) |
| 77 | 226 | TRINITY\_DN1797\_c0\_g1\_i2.p1 | 31.58 | 2 | 2 | 0 | 1 | 1 | 1 |  | 109594.9688 | type:3prime\_partial len:998 gc:universal TRINITY\_DN1797\_c0\_g1\_i2:2993-3(-) |
| 77 | 227 | TRINITY\_DN1797\_c0\_g1\_i1.p1 | 31.58 | 1 | 1 | 0 | 1 | 1 | 1 |  | 110096.6328 | type:3prime\_partial len:1004 gc:universal TRINITY\_DN1797\_c0\_g1\_i1:3011-3(-) |
| 77 | 228 | TRINITY\_DN1797\_c0\_g1\_i3.p1 | 31.58 | 2 | 2 | 0 | 1 | 1 | 1 |  | 109462.9766 | type:3prime\_partial len:998 gc:universal TRINITY\_DN1797\_c0\_g1\_i3:2993-3(-) |
| 78 | 229 | TRINITY\_DN7314\_c0\_g2\_i1.p1 | 58.39 | 6 | 6 | 0 | 1 | 1 | 1 |  | 16177.1182 | type:5prime\_partial len:142 gc:universal TRINITY\_DN7314\_c0\_g2\_i1:541-116(-) |
| 79 | 230 | TRINITY\_DN79126\_c0\_g1\_i1.p1 | 58.34 | 10 | 10 | 4.02e3 | 1 | 1 | 1 |  | 13015.4727 | type:complete len:115 gc:universal TRINITY\_DN79126\_c0\_g1\_i1:397-53(-) |
| 80 | 231 | TRINITY\_DN691\_c0\_g1\_i2.p1 | 57.94 | 1 | 1 | 9.59e2 | 1 | 1 | 1 | Carbamidomethylation | 164625.2188 | type:complete len:1673 gc:universal TRINITY\_DN691\_c0\_g1\_i2:5503-485(-) |
| 80 | 232 | TRINITY\_DN691\_c0\_g1\_i1.p1 | 57.94 | 1 | 1 | 9.59e2 | 1 | 1 | 1 | Carbamidomethylation | 169920.1562 | type:complete len:1726 gc:universal TRINITY\_DN691\_c0\_g1\_i1:5662-485(-) |
| 81 | 233 | TRINITY\_DN187823\_c0\_g1\_i1.p1 | 57.31 | 2 | 2 | 3.63e2 | 1 | 1 | 1 |  | 70773.125 | type:complete len:611 gc:universal TRINITY\_DN187823\_c0\_g1\_i1:1943-111(-) |
| 82 | 234 | TRINITY\_DN2772\_c0\_g1\_i3.p1 | 58.73 | 8 | 8 | 2.22e3 | 1 | 1 | 1 | Carbamidomethylation | 15694.8809 | type:complete len:142 gc:universal TRINITY\_DN2772\_c0\_g1\_i3:476-51(-) |
| 82 | 235 | TRINITY\_DN2772\_c0\_g1\_i2.p1 | 58.73 | 8 | 8 | 2.22e3 | 1 | 1 | 1 | Carbamidomethylation | 15694.8809 | type:complete len:142 gc:universal TRINITY\_DN2772\_c0\_g1\_i2:476-51(-) |
| 82 | 236 | TRINITY\_DN2772\_c0\_g1\_i1.p1 | 58.73 | 8 | 8 | 2.22e3 | 1 | 1 | 1 | Carbamidomethylation | 15694.8809 | type:complete len:142 gc:universal TRINITY\_DN2772\_c0\_g1\_i1:476-51(-) |
| 83 | 237 | TRINITY\_DN276\_c0\_g1\_i1.p1 | 53.33 | 10 | 10 | 0 | 1 | 1 | 2 |  | 17071.9023 | type:complete len:151 gc:universal TRINITY\_DN276\_c0\_g1\_i1:653-201(-) |
| 83 | 238 | TRINITY\_DN276\_c0\_g1\_i2.p1 | 53.33 | 10 | 10 | 0 | 1 | 1 | 2 |  | 16557.4121 | type:complete len:147 gc:universal TRINITY\_DN276\_c0\_g1\_i2:641-201(-) |
| 84 | 239 | TRINITY\_DN2217\_c0\_g1\_i2.p1 | 58.87 | 5 | 5 | 9.8e2 | 1 | 1 | 1 | Carbamidomethylation | 28618.9531 | type:complete len:254 gc:universal TRINITY\_DN2217\_c0\_g1\_i2:884-123(-) |
| 84 | 240 | TRINITY\_DN2217\_c0\_g1\_i3.p1 | 58.87 | 2 | 2 | 9.8e2 | 1 | 1 | 1 | Carbamidomethylation | 63077.0508 | type:complete len:566 gc:universal TRINITY\_DN2217\_c0\_g1\_i3:1820-123(-) |
| 85 | 241 | TRINITY\_DN6397\_c0\_g2\_i3.p1 | 58.78 | 8 | 8 | 1.02e3 | 1 | 1 | 3 | Acetylation (Protein N-term) | 14030.8945 | type:internal len:130 gc:universal TRINITY\_DN6397\_c0\_g2\_i3:387-1(-) |
| 85 | 242 | TRINITY\_DN6397\_c0\_g2\_i4.p1 | 58.78 | 9 | 9 | 1.02e3 | 1 | 1 | 3 | Acetylation (Protein N-term) | 12470.125 | type:internal len:115 gc:universal TRINITY\_DN6397\_c0\_g2\_i4:343-2(-) |
| 86 | 243 | TRINITY\_DN16548\_c0\_g1\_i4.p1 | 55.58 | 12 | 12 | 2.4e3 | 1 | 1 | 1 |  | 18505.2012 | type:internal len:171 gc:universal TRINITY\_DN16548\_c0\_g1\_i4:2-511(+) |
| 87 | 244 | TRINITY\_DN571\_c0\_g1\_i2.p1 | 55.18 | 6 | 6 | 0 | 1 | 1 | 1 |  | 21030.8027 | type:complete len:186 gc:universal TRINITY\_DN571\_c0\_g1\_i2:192-749(+) |
| 87 | 245 | TRINITY\_DN571\_c0\_g1\_i1.p1 | 55.18 | 6 | 6 | 0 | 1 | 1 | 1 |  | 20785.6484 | type:complete len:186 gc:universal TRINITY\_DN571\_c0\_g1\_i1:192-749(+) |
| 88 | 246 | TRINITY\_DN902\_c1\_g1\_i1.p1 | 58.88 | 7 | 7 | 2.65e3 | 1 | 1 | 2 | Carbamidomethylation | 16370.7676 | type:3prime\_partial len:153 gc:universal TRINITY\_DN902\_c1\_g1\_i1:146-601(+) |
| 88 | 247 | TRINITY\_DN902\_c1\_g1\_i2.p1 | 58.88 | 7 | 7 | 2.65e3 | 1 | 1 | 2 | Carbamidomethylation | 16400.9395 | type:3prime\_partial len:153 gc:universal TRINITY\_DN902\_c1\_g1\_i2:90-545(+) |
| 89 | 248 | TRINITY\_DN2885\_c0\_g1\_i2.p1 | 58.91 | 11 | 11 | 2.81e4 | 1 | 1 | 4 |  | 12758.7783 | type:complete len:114 gc:universal TRINITY\_DN2885\_c0\_g1\_i2:31-372(+) |
| 89 | 249 | TRINITY\_DN2885\_c0\_g1\_i5.p1 | 58.91 | 11 | 11 | 2.81e4 | 1 | 1 | 4 |  | 12383.5439 | type:3prime\_partial len:112 gc:universal TRINITY\_DN2885\_c0\_g1\_i5:31-363(+) |
| 89 | 250 | TRINITY\_DN2885\_c0\_g1\_i4.p1 | 58.91 | 11 | 11 | 2.81e4 | 1 | 1 | 4 |  | 12245.125 | type:5prime\_partial len:109 gc:universal TRINITY\_DN2885\_c0\_g1\_i4:1-327(+) |
| 90 | 251 | TRINITY\_DN6294\_c0\_g3\_i2.p1 | 57.66 | 9 | 9 | 1.74e3 | 1 | 1 | 1 |  | 15623.1504 | type:complete len:134 gc:universal TRINITY\_DN6294\_c0\_g3\_i2:817-416(-) |
| 90 | 252 | TRINITY\_DN6294\_c0\_g3\_i1.p1 | 57.66 | 9 | 9 | 1.74e3 | 1 | 1 | 1 |  | 15623.1504 | type:complete len:134 gc:universal TRINITY\_DN6294\_c0\_g3\_i1:706-305(-) |
| 91 | 253 | TRINITY\_DN902\_c1\_g2\_i3.p1 | 58.83 | 6 | 6 | 0 | 1 | 1 | 3 | Carbamidomethylation | 19070.0254 | type:internal len:178 gc:universal TRINITY\_DN902\_c1\_g2\_i3:2-532(+) |
| 91 | 254 | TRINITY\_DN902\_c1\_g2\_i1.p1 | 58.83 | 6 | 6 | 0 | 1 | 1 | 3 | Carbamidomethylation | 18964.9824 | type:internal len:178 gc:universal TRINITY\_DN902\_c1\_g2\_i1:2-532(+) |
| 91 | 255 | TRINITY\_DN902\_c1\_g2\_i4.p1 | 58.83 | 6 | 6 | 0 | 1 | 1 | 3 | Carbamidomethylation | 19994.1074 | type:internal len:182 gc:universal TRINITY\_DN902\_c1\_g2\_i4:2-544(+) |
| 92 | 256 | TRINITY\_DN3870\_c0\_g1\_i1.p1 | 58.15 | 1 | 1 | 5.99e2 | 1 | 1 | 1 | Carbamidomethylation | 117885.8984 | type:3prime\_partial len:990 gc:universal TRINITY\_DN3870\_c0\_g1\_i1:2969-3(-) |
| 93 | 257 | TRINITY\_DN1631\_c0\_g1\_i1.p1 | 58.73 | 9 | 9 | 4.33e3 | 1 | 1 | 2 | Acetylation (Protein N-term) | 15272.8457 | type:complete len:124 gc:universal TRINITY\_DN1631\_c0\_g1\_i1:62-433(+) |
| 94 | 258 | TRINITY\_DN8101\_c0\_g1\_i2.p2 | 54.32 | 2 | 2 | 4.69e2 | 1 | 1 | 1 |  | 88889.1172 | type:complete len:820 gc:universal TRINITY\_DN8101\_c0\_g1\_i2:2597-138(-) |
| 94 | 259 | TRINITY\_DN8101\_c0\_g1\_i9.p2 | 54.32 | 1 | 1 | 4.69e2 | 1 | 1 | 1 |  | 121647.5547 | type:5prime\_partial len:1113 gc:universal TRINITY\_DN8101\_c0\_g1\_i9:3476-138(-) |
| 94 | 260 | TRINITY\_DN8101\_c0\_g1\_i10.p2 | 54.32 | 6 | 6 | 4.69e2 | 1 | 1 | 1 |  | 23508.5586 | type:internal len:214 gc:universal TRINITY\_DN8101\_c0\_g1\_i10:639-1(-) |
| 94 | 261 | TRINITY\_DN8101\_c0\_g1\_i5.p1 | 54.32 | 2 | 2 | 4.69e2 | 1 | 1 | 1 |  | 88960.2812 | type:complete len:820 gc:universal TRINITY\_DN8101\_c0\_g1\_i5:2531-72(-) |
| 94 | 262 | TRINITY\_DN8101\_c0\_g1\_i8.p1 | 54.32 | 1 | 1 | 4.69e2 | 1 | 1 | 1 |  | 121763.7734 | type:5prime\_partial len:1113 gc:universal TRINITY\_DN8101\_c0\_g1\_i8:3410-72(-) |
| 94 | 263 | TRINITY\_DN8101\_c0\_g1\_i11.p2 | 54.32 | 1 | 1 | 4.69e2 | 1 | 1 | 1 |  | 121692.6094 | type:5prime\_partial len:1113 gc:universal TRINITY\_DN8101\_c0\_g1\_i11:3410-72(-) |
| 94 | 264 | TRINITY\_DN8101\_c0\_g1\_i4.p1 | 54.32 | 3 | 3 | 4.69e2 | 1 | 1 | 1 |  | 55522.8164 | type:5prime\_partial len:511 gc:universal TRINITY\_DN8101\_c0\_g1\_i4:1604-72(-) |
| 94 | 265 | TRINITY\_DN8101\_c0\_g1\_i6.p2 | 54.32 | 2 | 2 | 4.69e2 | 1 | 1 | 1 |  | 88934.1797 | type:complete len:820 gc:universal TRINITY\_DN8101\_c0\_g1\_i6:2531-72(-) |
| 95 | 266 | TRINITY\_DN3034\_c0\_g1\_i1.p1 | 58.52 | 1 | 1 | 7.05e2 | 1 | 1 | 1 |  | 222266.9844 | type:complete len:1941 gc:universal TRINITY\_DN3034\_c0\_g1\_i1:6289-467(-) |
| 96 | 267 | TRINITY\_DN7504\_c0\_g1\_i1.p1 | 58.87 | 3 | 3 | 0 | 1 | 1 | 1 |  | 46519.1953 | type:complete len:414 gc:universal TRINITY\_DN7504\_c0\_g1\_i1:87-1328(+) |
| 97 | 268 | TRINITY\_DN163801\_c0\_g1\_i1.p1 | 58.83 | 3 | 3 | 1.06e3 | 1 | 1 | 1 | Acetylation (Protein N-term) | 33076.9062 | type:complete len:298 gc:universal TRINITY\_DN163801\_c0\_g1\_i1:55-948(+) |
| 98 | 269 | TRINITY\_DN761\_c0\_g1\_i1.p1 | 55.76 | 2 | 2 | 0 | 1 | 1 | 1 |  | 95330.7812 | type:complete len:830 gc:universal TRINITY\_DN761\_c0\_g1\_i1:129-2618(+) |
| 98 | 270 | TRINITY\_DN761\_c0\_g1\_i2.p1 | 55.76 | 2 | 2 | 0 | 1 | 1 | 1 |  | 95330.7812 | type:complete len:830 gc:universal TRINITY\_DN761\_c0\_g1\_i2:87-2576(+) |
| 98 | 271 | TRINITY\_DN761\_c0\_g1\_i3.p1 | 55.76 | 2 | 2 | 0 | 1 | 1 | 1 |  | 96968.6562 | type:complete len:845 gc:universal TRINITY\_DN761\_c0\_g1\_i3:129-2663(+) |
| 99 | 272 | TRINITY\_DN309\_c0\_g1\_i1.p1 | 57.91 | 3 | 3 | 1.4e3 | 1 | 1 | 1 |  | 52371.3906 | type:complete len:454 gc:universal TRINITY\_DN309\_c0\_g1\_i1:65-1426(+) |
| 100 | 273 | TRINITY\_DN77\_c1\_g1\_i1.p1 | 57.77 | 1 | 1 | 2.27e2 | 1 | 1 | 1 |  | 198350.2344 | type:3prime\_partial len:1965 gc:universal TRINITY\_DN77\_c1\_g1\_i1:2-5893(+) |
| 101 | 274 | TRINITY\_DN30365\_c0\_g1\_i3.p1 | 58.76 | 5 | 5 | 1.45e3 | 1 | 1 | 1 |  | 26810.9355 | type:internal len:239 gc:universal TRINITY\_DN30365\_c0\_g1\_i3:1-714(+) |
| 101 | 275 | TRINITY\_DN30365\_c0\_g1\_i2.p1 | 58.76 | 3 | 3 | 1.45e3 | 1 | 1 | 1 |  | 47444.8828 | type:complete len:420 gc:universal TRINITY\_DN30365\_c0\_g1\_i2:123-1382(+) |
| 102 | 276 | TRINITY\_DN1789\_c0\_g1\_i1.p1 | 39.14 | 4 | 4 | 3.83e2 | 1 | 1 | 1 |  | 44467.0234 | type:complete len:393 gc:universal TRINITY\_DN1789\_c0\_g1\_i1:1193-15(-) |
| 103 | 277 | TRINITY\_DN47849\_c0\_g1\_i1.p1 | 52.1 | 2 | 2 | 0 | 1 | 1 | 1 |  | 77056.3125 | type:complete len:689 gc:universal TRINITY\_DN47849\_c0\_g1\_i1:176-2242(+) |
| 104 | 278 | TRINITY\_DN1989\_c0\_g1\_i1.p1 | 58.7 | 3 | 3 | 2.68e2 | 1 | 1 | 1 |  | 62731.1836 | type:complete len:546 gc:universal TRINITY\_DN1989\_c0\_g1\_i1:150-1787(+) |
| 105 | 279 | TRINITY\_DN3271\_c0\_g1\_i1.p1 | 58.89 | 3 | 3 | 8.27e2 | 1 | 1 | 1 |  | 45246.3555 | type:complete len:399 gc:universal TRINITY\_DN3271\_c0\_g1\_i1:218-1414(+) |
| 105 | 280 | TRINITY\_DN3271\_c0\_g1\_i2.p1 | 58.89 | 3 | 3 | 8.27e2 | 1 | 1 | 1 |  | 53020.332 | type:5prime\_partial len:468 gc:universal TRINITY\_DN3271\_c0\_g1\_i2:2-1405(+) |
| 106 | 281 | TRINITY\_DN4523\_c0\_g1\_i1.p1 | 52.71 | 1 | 1 | 3.75e2 | 1 | 1 | 1 |  | 188936.4688 | type:complete len:1645 gc:universal TRINITY\_DN4523\_c0\_g1\_i1:5332-398(-) |
| 106 | 282 | TRINITY\_DN4523\_c0\_g1\_i2.p1 | 52.71 | 1 | 1 | 3.75e2 | 1 | 1 | 1 |  | 195964.2344 | type:5prime\_partial len:1710 gc:universal TRINITY\_DN4523\_c0\_g1\_i2:5527-398(-) |
| 107 | 283 | TRINITY\_DN1907\_c0\_g1\_i2.p1 | 58.05 | 5 | 5 | 0 | 1 | 1 | 1 |  | 24166.1172 | type:complete len:210 gc:universal TRINITY\_DN1907\_c0\_g1\_i2:53-682(+) |
| 107 | 284 | TRINITY\_DN1907\_c0\_g1\_i1.p1 | 58.05 | 5 | 5 | 0 | 1 | 1 | 1 |  | 24233.2676 | type:complete len:211 gc:universal TRINITY\_DN1907\_c0\_g1\_i1:56-688(+) |
| 108 | 285 | TRINITY\_DN651\_c0\_g1\_i1.p1 | 55.96 | 4 | 4 | 0 | 1 | 1 | 1 |  | 42527.9297 | type:complete len:408 gc:universal TRINITY\_DN651\_c0\_g1\_i1:122-1345(+) |
| 108 | 286 | TRINITY\_DN651\_c0\_g1\_i2.p1 | 55.96 | 4 | 4 | 0 | 1 | 1 | 1 |  | 45414.0391 | type:complete len:431 gc:universal TRINITY\_DN651\_c0\_g1\_i2:122-1414(+) |
| 109 | 287 | TRINITY\_DN165\_c0\_g1\_i1.p1 | 53.86 | 3 | 3 | 0 | 1 | 1 | 1 |  | 68397.5781 | type:complete len:598 gc:universal TRINITY\_DN165\_c0\_g1\_i1:147-1940(+) |
| 110 | 288 | TRINITY\_DN1319\_c0\_g1\_i2.p1 | 59.14 | 10 | 10 | 2.25e3 | 1 | 1 | 2 | Carbamidomethylation | 15785.3457 | type:complete len:134 gc:universal TRINITY\_DN1319\_c0\_g1\_i2:86-487(+) |
| 110 | 289 | TRINITY\_DN1319\_c0\_g1\_i1.p1 | 59.14 | 10 | 10 | 2.25e3 | 1 | 1 | 2 | Carbamidomethylation | 15785.3457 | type:complete len:134 gc:universal TRINITY\_DN1319\_c0\_g1\_i1:362-763(+) |
| 111 | 290 | TRINITY\_DN1339\_c6\_g1\_i1.p1 | 58.87 | 2 | 2 | 9.01e3 | 1 | 1 | 2 |  | 61274.3594 | type:internal len:570 gc:universal TRINITY\_DN1339\_c6\_g1\_i1:1-1707(+) |
| 111 | 291 | TRINITY\_DN1339\_c0\_g1\_i2.p1 | 58.87 | 3 | 3 | 9.01e3 | 1 | 1 | 2 |  | 41222.6992 | type:internal len:384 gc:universal TRINITY\_DN1339\_c0\_g1\_i2:1151-3(-) |
| 111 | 292 | TRINITY\_DN124769\_c0\_g1\_i1.p1 | 58.87 | 11 | 11 | 9.01e3 | 1 | 1 | 2 |  | 12144.8828 | type:internal len:113 gc:universal TRINITY\_DN124769\_c0\_g1\_i1:1-336(+) |
| 111 | 293 | TRINITY\_DN24414\_c1\_g1\_i2.p2 | 58.87 | 8 | 8 | 9.01e3 | 1 | 1 | 2 |  | 15590.8623 | type:internal len:146 gc:universal TRINITY\_DN24414\_c1\_g1\_i2:435-1(-) |
| 111 | 294 | TRINITY\_DN1172\_c0\_g1\_i3.p1 | 58.87 | 7 | 7 | 9.01e3 | 1 | 1 | 2 |  | 19709.3613 | type:internal len:182 gc:universal TRINITY\_DN1172\_c0\_g1\_i3:545-3(-) |
| 111 | 295 | TRINITY\_DN119673\_c0\_g1\_i1.p1 | 58.87 | 9 | 9 | 9.01e3 | 1 | 1 | 2 |  | 13988.7559 | type:internal len:129 gc:universal TRINITY\_DN119673\_c0\_g1\_i1:2-385(+) |
| 111 | 296 | TRINITY\_DN1339\_c0\_g1\_i1.p1 | 58.87 | 5 | 5 | 9.01e3 | 1 | 1 | 2 |  | 24722.7773 | type:internal len:229 gc:universal TRINITY\_DN1339\_c0\_g1\_i1:684-1(-) |
| 111 | 297 | TRINITY\_DN6701\_c0\_g1\_i1.p1 | 58.87 | 6 | 6 | 9.01e3 | 1 | 1 | 2 |  | 21246.0547 | type:internal len:196 gc:universal TRINITY\_DN6701\_c0\_g1\_i1:1-585(+) |
| 111 | 298 | TRINITY\_DN1172\_c0\_g1\_i5.p1 | 58.87 | 5 | 5 | 9.01e3 | 1 | 1 | 2 |  | 24525.6855 | type:internal len:226 gc:universal TRINITY\_DN1172\_c0\_g1\_i5:677-3(-) |
| 111 | 299 | TRINITY\_DN6701\_c0\_g1\_i2.p2 | 58.87 | 8 | 8 | 9.01e3 | 1 | 1 | 2 |  | 16512.8535 | type:internal len:152 gc:universal TRINITY\_DN6701\_c0\_g1\_i2:3-455(+) |
| 111 | 300 | TRINITY\_DN1339\_c6\_g1\_i2.p1 | 58.87 | 3 | 3 | 9.01e3 | 1 | 1 | 2 |  | 43821.8359 | type:internal len:408 gc:universal TRINITY\_DN1339\_c6\_g1\_i2:3-1223(+) |
| 112 | 301 | TRINITY\_DN123\_c0\_g1\_i1.p1 | 57.88 | 1 | 1 | 0 | 1 | 1 | 1 |  | 136822.0312 | type:5prime\_partial len:1231 gc:universal TRINITY\_DN123\_c0\_g1\_i1:2-3694(+) |
| 113 | 302 | TRINITY\_DN15847\_c0\_g1\_i1.p1 | 55.96 | 11 | 11 | 0 | 1 | 1 | 1 | Carbamidomethylation | 19271.252 | type:5prime\_partial len:166 gc:universal TRINITY\_DN15847\_c0\_g1\_i1:3-500(+) |
| 113 | 303 | TRINITY\_DN15847\_c0\_g1\_i2.p1 | 55.96 | 11 | 11 | 0 | 1 | 1 | 1 | Carbamidomethylation | 19271.252 | type:5prime\_partial len:166 gc:universal TRINITY\_DN15847\_c0\_g1\_i2:3-500(+) |
| 114 | 304 | TRINITY\_DN135129\_c0\_g1\_i1.p1 | 58.12 | 6 | 6 | 4.49e3 | 1 | 1 | 3 |  | 17174.043 | type:internal len:155 gc:universal TRINITY\_DN135129\_c0\_g1\_i1:1-462(+) |
| 115 | 305 | TRINITY\_DN240\_c0\_g1\_i4.p1 | 58.73 | 3 | 3 | 3.07e3 | 1 | 1 | 1 |  | 40419.2031 | type:5prime\_partial len:397 gc:universal TRINITY\_DN240\_c0\_g1\_i4:1327-137(-) |
| 115 | 306 | TRINITY\_DN240\_c0\_g1\_i6.p1 | 58.73 | 3 | 3 | 3.07e3 | 1 | 1 | 1 |  | 40418.2188 | type:5prime\_partial len:397 gc:universal TRINITY\_DN240\_c0\_g1\_i6:1494-304(-) |
| 115 | 307 | TRINITY\_DN240\_c0\_g1\_i1.p1 | 58.73 | 3 | 3 | 3.07e3 | 1 | 1 | 1 |  | 40418.2188 | type:5prime\_partial len:397 gc:universal TRINITY\_DN240\_c0\_g1\_i1:1518-328(-) |
| 116 | 308 | TRINITY\_DN11643\_c0\_g1\_i1.p1 | 58.3 | 7 | 7 | 0 | 1 | 1 | 1 |  | 21061.2832 | type:complete len:187 gc:universal TRINITY\_DN11643\_c0\_g1\_i1:1748-1188(-) |
| 117 | 309 | TRINITY\_DN333\_c0\_g1\_i1.p1 | 56.14 | 3 | 3 | 7.87e2 | 1 | 1 | 1 |  | 44545.1602 | type:5prime\_partial len:384 gc:universal TRINITY\_DN333\_c0\_g1\_i1:3-1154(+) |
| 117 | 310 | TRINITY\_DN333\_c0\_g1\_i2.p1 | 56.14 | 3 | 3 | 7.87e2 | 1 | 1 | 1 |  | 44545.1602 | type:5prime\_partial len:384 gc:universal TRINITY\_DN333\_c0\_g1\_i2:3-1154(+) |
| 118 | 311 | TRINITY\_DN1744\_c0\_g1\_i2.p1 | 39.5 | 8 | 8 | 0 | 1 | 1 | 1 |  | 15485.2461 | type:3prime\_partial len:141 gc:universal TRINITY\_DN1744\_c0\_g1\_i2:422-3(-) |
| 119 | 312 | TRINITY\_DN6390\_c0\_g1\_i1.p1 | 57.99 | 1 | 1 | 3.4e2 | 1 | 1 | 1 | Carbamidomethylation | 214480.2344 | type:3prime\_partial len:1941 gc:universal TRINITY\_DN6390\_c0\_g1\_i1:5820-1(-) |
| 120 | 313 | TRINITY\_DN8707\_c0\_g1\_i2.p1 | 58.7 | 2 | 2 | 2.25e3 | 1 | 1 | 1 |  | 61942.9023 | type:complete len:564 gc:universal TRINITY\_DN8707\_c0\_g1\_i2:2751-1060(-) |
| 121 | 314 | TRINITY\_DN22492\_c0\_g1\_i1.p1 | 58.51 | 1 | 1 | 3.29e2 | 1 | 1 | 1 | Carbamidomethylation | 115296.5156 | type:complete len:1026 gc:universal TRINITY\_DN22492\_c0\_g1\_i1:171-3248(+) |
| 121 | 315 | TRINITY\_DN22492\_c0\_g1\_i2.p1 | 58.51 | 1 | 1 | 3.29e2 | 1 | 1 | 1 | Carbamidomethylation | 115355.5859 | type:complete len:1026 gc:universal TRINITY\_DN22492\_c0\_g1\_i2:39-3116(+) |
| 122 | 316 | TRINITY\_DN956\_c0\_g1\_i2.p1 | 41.74 | 4 | 4 | 3.6e2 | 1 | 1 | 1 |  | 29904.0254 | type:complete len:267 gc:universal TRINITY\_DN956\_c0\_g1\_i2:93-893(+) |
| 122 | 317 | TRINITY\_DN956\_c0\_g1\_i1.p1 | 41.74 | 8 | 8 | 3.6e2 | 1 | 1 | 1 |  | 17427 | type:complete len:159 gc:universal TRINITY\_DN956\_c0\_g1\_i1:93-569(+) |
| 123 | 318 | TRINITY\_DN3773\_c0\_g1\_i1.p1 | 57.77 | 4 | 4 | 6.81e2 | 1 | 1 | 2 | Carbamidomethylation | 56129.7773 | type:complete len:504 gc:universal TRINITY\_DN3773\_c0\_g1\_i1:168-1679(+) |
| 124 | 319 | TRINITY\_DN32522\_c0\_g1\_i1.p1 | 58.41 | 4 | 4 | 1.2e3 | 1 | 1 | 2 |  | 26779.0059 | type:complete len:235 gc:universal TRINITY\_DN32522\_c0\_g1\_i1:147-851(+) |
| 125 | 320 | TRINITY\_DN1130\_c0\_g1\_i1.p1 | 29.19 | 5 | 5 | 6.08e3 | 1 | 1 | 1 |  | 34883.0859 | type:complete len:317 gc:universal TRINITY\_DN1130\_c0\_g1\_i1:1199-249(-) |
| 126 | 321 | TRINITY\_DN116\_c0\_g1\_i1.p1 | 58.33 | 6 | 6 | 1.42e2 | 1 | 1 | 2 | Carbamidomethylation | 34095.6328 | type:complete len:297 gc:universal TRINITY\_DN116\_c0\_g1\_i1:72-962(+) |
| 127 | 322 | TRINITY\_DN34191\_c0\_g1\_i1.p1 | 50.91 | 5 | 5 | 0 | 1 | 1 | 1 | Oxidation (M) | 26288.2148 | type:internal len:238 gc:universal TRINITY\_DN34191\_c0\_g1\_i1:1-711(+) |
| 127 | 323 | TRINITY\_DN34191\_c0\_g2\_i1.p2 | 50.91 | 4 | 4 | 0 | 1 | 1 | 1 | Oxidation (M) | 32547.0195 | type:5prime\_partial len:293 gc:universal TRINITY\_DN34191\_c0\_g2\_i1:2-880(+) |
| 128 | 324 | TRINITY\_DN2301\_c0\_g1\_i1.p1 | 57.32 | 4 | 4 | 9.02e1 | 1 | 1 | 1 |  | 50428.9648 | type:5prime\_partial len:452 gc:universal TRINITY\_DN2301\_c0\_g1\_i1:2886-1531(-) |
| 129 | 325 | TRINITY\_DN97809\_c0\_g1\_i1.p1 | 20.68 | 7 | 7 | 0 | 1 | 1 | 1 |  | 20461.1211 | type:internal len:184 gc:universal TRINITY\_DN97809\_c0\_g1\_i1:2-550(+) |
| 130 | 326 | TRINITY\_DN413\_c0\_g1\_i4.p1 | 47.79 | 3 | 3 | 0 | 1 | 1 | 1 | Carbamidomethylation | 52354.1758 | type:3prime\_partial len:463 gc:universal TRINITY\_DN413\_c0\_g1\_i4:1388-3(-) |
| 130 | 327 | TRINITY\_DN413\_c0\_g1\_i1.p1 | 47.79 | 3 | 3 | 0 | 1 | 1 | 1 | Carbamidomethylation | 40351.7656 | type:complete len:355 gc:universal TRINITY\_DN413\_c0\_g1\_i1:1352-288(-) |
| 130 | 328 | TRINITY\_DN413\_c0\_g1\_i3.p1 | 47.79 | 3 | 3 | 0 | 1 | 1 | 1 | Carbamidomethylation | 39501.6133 | type:complete len:350 gc:universal TRINITY\_DN413\_c0\_g1\_i3:1286-237(-) |
| 131 | 329 | TRINITY\_DN55\_c0\_g1\_i1.p1 | 58.87 | 13 | 13 | 2.8e2 | 1 | 1 | 3 | Carbamidomethylation | 11694.9785 | type:5prime\_partial len:104 gc:universal TRINITY\_DN55\_c0\_g1\_i1:3-314(+) |
| 132 | 330 | TRINITY\_DN104063\_c0\_g1\_i1.p1 | 22.14 | 5 | 5 | 1.02e3 | 1 | 1 | 1 | Acetylation (Protein N-term) | 19755.082 | type:internal len:181 gc:universal TRINITY\_DN104063\_c0\_g1\_i1:542-3(-) |
| 133 | 331 | TRINITY\_DN128406\_c0\_g1\_i1.p1 | 58.36 | 7 | 7 | 5.13e3 | 1 | 1 | 2 |  | 16958.5273 | type:5prime\_partial len:150 gc:universal TRINITY\_DN128406\_c0\_g1\_i1:474-25(-) |
| 134 | 332 | TRINITY\_DN26679\_c0\_g1\_i1.p3 | 21.67 | 6 | 6 | 1.26e3 | 1 | 1 | 1 | Acetylation (Protein N-term) | 12077.7803 | type:5prime\_partial len:108 gc:universal TRINITY\_DN26679\_c0\_g1\_i1:1-324(+) |
| 135 | 333 | TRINITY\_DN2230\_c0\_g1\_i1.p1 | 55.53 | 6 | 6 | 3.5e2 | 1 | 1 | 1 |  | 28784.541 | type:complete len:249 gc:universal TRINITY\_DN2230\_c0\_g1\_i1:796-50(-) |
| 136 | 334 | TRINITY\_DN109115\_c0\_g1\_i1.p1 | 53.19 | 9 | 9 | 0 | 1 | 1 | 1 |  | 13721.9717 | type:internal len:125 gc:universal TRINITY\_DN109115\_c0\_g1\_i1:3-374(+) |
| 136 | 335 | TRINITY\_DN5263\_c0\_g1\_i1.p1 | 53.19 | 5 | 5 | 0 | 1 | 1 | 1 |  | 26869.2422 | type:complete len:243 gc:universal TRINITY\_DN5263\_c0\_g1\_i1:827-99(-) |
| 137 | 336 | TRINITY\_DN73587\_c0\_g1\_i1.p1 | 58.88 | 3 | 3 | 1.21e3 | 1 | 1 | 1 |  | 57376.2227 | type:complete len:533 gc:universal TRINITY\_DN73587\_c0\_g1\_i1:94-1692(+) |
| 138 | 337 | TRINITY\_DN751\_c0\_g1\_i1.p1 | 57.93 | 5 | 5 | 1.76e3 | 1 | 1 | 1 |  | 25720.373 | type:complete len:227 gc:universal TRINITY\_DN751\_c0\_g1\_i1:731-51(-) |
| 138 | 338 | TRINITY\_DN7214\_c0\_g1\_i1.p1 | 57.93 | 5 | 5 | 1.76e3 | 1 | 1 | 1 |  | 25800.4062 | type:complete len:228 gc:universal TRINITY\_DN7214\_c0\_g1\_i1:39-722(+) |
| 139 | 339 | TRINITY\_DN1505\_c0\_g1\_i1.p1 | 58.87 | 8 | 8 | 4.49e4 | 1 | 1 | 8 | Acetylation (Protein N-term) | 17930.752 | type:complete len:154 gc:universal TRINITY\_DN1505\_c0\_g1\_i1:98-559(+) |
| 140 | 340 | TRINITY\_DN925\_c0\_g1\_i1.p1 | 58.13 | 5 | 5 | 0 | 1 | 1 | 1 |  | 28336.7676 | type:complete len:261 gc:universal TRINITY\_DN925\_c0\_g1\_i1:826-44(-) |
| 141 | 341 | TRINITY\_DN165536\_c0\_g1\_i1.p1 | 59 | 6 | 6 | 1.89e3 | 1 | 1 | 1 |  | 28339.4531 | type:complete len:249 gc:universal TRINITY\_DN165536\_c0\_g1\_i1:851-105(-) |
| 142 | 342 | TRINITY\_DN1283\_c0\_g1\_i1.p1 | 25.79 | 2 | 2 | 0 | 1 | 1 | 1 |  | 61937 | type:complete len:542 gc:universal TRINITY\_DN1283\_c0\_g1\_i1:81-1706(+) |
| 142 | 343 | TRINITY\_DN61835\_c0\_g1\_i1.p1 | 25.79 | 2 | 2 | 0 | 1 | 1 | 1 |  | 57843.9023 | type:3prime\_partial len:513 gc:universal TRINITY\_DN61835\_c0\_g1\_i1:1537-2(-) |
| 142 | 344 | TRINITY\_DN49493\_c0\_g1\_i1.p1 | 25.79 | 7 | 7 | 0 | 1 | 1 | 1 |  | 15437.627 | type:internal len:135 gc:universal TRINITY\_DN49493\_c0\_g1\_i1:404-3(-) |
| 143 | 345 | TRINITY\_DN200\_c0\_g1\_i2.p1 | 58.72 | 2 | 2 | 0 | 1 | 1 | 1 |  | 59337.1367 | type:complete len:551 gc:universal TRINITY\_DN200\_c0\_g1\_i2:123-1775(+) |
| 143 | 346 | TRINITY\_DN200\_c0\_g1\_i3.p1 | 58.72 | 2 | 2 | 0 | 1 | 1 | 1 |  | 59435.2422 | type:complete len:551 gc:universal TRINITY\_DN200\_c0\_g1\_i3:110-1762(+) |
| 143 | 347 | TRINITY\_DN200\_c0\_g1\_i5.p1 | 58.72 | 2 | 2 | 0 | 1 | 1 | 1 |  | 59339.1094 | type:complete len:551 gc:universal TRINITY\_DN200\_c0\_g1\_i5:110-1762(+) |
| 143 | 348 | TRINITY\_DN200\_c0\_g1\_i1.p1 | 58.72 | 2 | 2 | 0 | 1 | 1 | 1 |  | 59435.2422 | type:complete len:551 gc:universal TRINITY\_DN200\_c0\_g1\_i1:168-1820(+) |
| 144 | 349 | TRINITY\_DN7671\_c0\_g1\_i1.p1 | 58.81 | 10 | 10 | 0 | 1 | 1 | 1 | Carbamidomethylation | 13919.8574 | type:complete len:123 gc:universal TRINITY\_DN7671\_c0\_g1\_i1:425-57(-) |
| 145 | 350 | TRINITY\_DN1468\_c0\_g1\_i1.p1 | 40.8 | 4 | 4 | 3.3e2 | 1 | 1 | 1 |  | 28461.1699 | type:complete len:245 gc:universal TRINITY\_DN1468\_c0\_g1\_i1:766-32(-) |
| 146 | 351 | TRINITY\_DN4595\_c0\_g1\_i4.p1 | 25.84 | 0 | 0 | 3.57e3 | 1 | 1 | 1 |  | 183684.4844 | type:5prime\_partial len:1639 gc:universal TRINITY\_DN4595\_c0\_g1\_i4:5857-941(-) |
| 146 | 352 | TRINITY\_DN4595\_c0\_g1\_i7.p1 | 25.84 | 2 | 2 | 3.57e3 | 1 | 1 | 1 |  | 38387.582 | type:internal len:348 gc:universal TRINITY\_DN4595\_c0\_g1\_i7:1042-2(-) |
| 146 | 353 | TRINITY\_DN4595\_c0\_g1\_i10.p2 | 25.84 | 2 | 2 | 3.57e3 | 1 | 1 | 1 |  | 39822.0156 | type:5prime\_partial len:358 gc:universal TRINITY\_DN4595\_c0\_g1\_i10:5369-4296(-) |
| 147 | 354 | TRINITY\_DN166344\_c0\_g1\_i1.p1 | 57.65 | 3 | 3 | 0 | 1 | 1 | 1 | Carbamidomethylation | 40794.2773 | type:complete len:360 gc:universal TRINITY\_DN166344\_c0\_g1\_i1:93-1172(+) |
| 148 | 355 | TRINITY\_DN12629\_c0\_g1\_i1.p1 | 54.29 | 2 | 2 | 0 | 1 | 1 | 1 |  | 83116.4453 | type:complete len:727 gc:universal TRINITY\_DN12629\_c0\_g1\_i1:250-2430(+) |
| 149 | 356 | TRINITY\_DN126137\_c0\_g1\_i1.p1 | 57.98 | 5 | 5 | 0 | 1 | 1 | 1 | Oxidation (M) | 31557.7129 | type:5prime\_partial len:287 gc:universal TRINITY\_DN126137\_c0\_g1\_i1:3-863(+) |
| 150 | 357 | TRINITY\_DN131028\_c0\_g1\_i1.p2 | 58.78 | 11 | 11 | 8.83e2 | 1 | 1 | 1 |  | 11838.5801 | type:5prime\_partial len:104 gc:universal TRINITY\_DN131028\_c0\_g1\_i1:2-313(+) |
| 151 | 358 | TRINITY\_DN1272\_c0\_g1\_i1.p1 | 52.47 | 4 | 4 | 0 | 1 | 1 | 1 |  | 30690.1172 | type:complete len:262 gc:universal TRINITY\_DN1272\_c0\_g1\_i1:957-172(-) |
| 152 | 359 | TRINITY\_DN453\_c0\_g1\_i1.p1 | 58.38 | 5 | 5 | 0 | 1 | 1 | 1 |  | 27993.4082 | type:complete len:249 gc:universal TRINITY\_DN453\_c0\_g1\_i1:944-198(-) |
| 152 | 360 | TRINITY\_DN453\_c0\_g1\_i2.p1 | 58.38 | 5 | 5 | 0 | 1 | 1 | 1 |  | 27993.4082 | type:complete len:249 gc:universal TRINITY\_DN453\_c0\_g1\_i2:944-198(-) |
| 153 | 361 | TRINITY\_DN59667\_c0\_g1\_i1.p1 | 56.51 | 4 | 4 | 5.9e2 | 1 | 1 | 1 |  | 25518.6816 | type:3prime\_partial len:225 gc:universal TRINITY\_DN59667\_c0\_g1\_i1:673-2(-) |
| 154 | 362 | TRINITY\_DN16120\_c0\_g1\_i2.p1 | 57.96 | 5 | 5 | 0 | 1 | 1 | 1 | Carbamidomethylation | 23723.4336 | type:5prime\_partial len:232 gc:universal TRINITY\_DN16120\_c0\_g1\_i2:734-39(-) |
| 154 | 363 | TRINITY\_DN16120\_c0\_g1\_i1.p1 | 57.96 | 5 | 5 | 0 | 1 | 1 | 1 | Carbamidomethylation | 22724.2812 | type:5prime\_partial len:220 gc:universal TRINITY\_DN16120\_c0\_g1\_i1:698-39(-) |
| 155 | 364 | TRINITY\_DN22594\_c0\_g1\_i1.p2 | 40.24 | 4 | 4 | 1.03e3 | 1 | 1 | 1 |  | 29863.3594 | type:internal len:268 gc:universal TRINITY\_DN22594\_c0\_g1\_i1:801-1(-) |
| 156 | 365 | TRINITY\_DN98420\_c0\_g1\_i1.p1 | 58.8 | 3 | 3 | 2.49e3 | 1 | 1 | 1 |  | 31374.8145 | type:5prime\_partial len:286 gc:universal TRINITY\_DN98420\_c0\_g1\_i1:1007-150(-) |
| 157 | 366 | TRINITY\_DN1559\_c0\_g1\_i1.p1 | 58.4 | 5 | 5 | 2.72e3 | 1 | 1 | 1 |  | 33672.0977 | type:complete len:311 gc:universal TRINITY\_DN1559\_c0\_g1\_i1:221-1153(+) |
| 158 | 367 | TRINITY\_DN1583\_c0\_g1\_i1.p1 | 43.17 | 4 | 4 | 0 | 1 | 1 | 1 |  | 34724.7109 | type:complete len:314 gc:universal TRINITY\_DN1583\_c0\_g1\_i1:1007-66(-) |
| 159 | 368 | TRINITY\_DN1428\_c0\_g1\_i1.p1 | 58.88 | 9 | 9 | 1.68e3 | 1 | 1 | 2 |  | 18259.2207 | type:5prime\_partial len:165 gc:universal TRINITY\_DN1428\_c0\_g1\_i1:1-495(+) |
| 160 | 369 | TRINITY\_DN2805\_c0\_g1\_i1.p1 | 29.79 | 7 | 7 | 0 | 1 | 1 | 1 | Oxidation (M) | 21692.6699 | type:complete len:189 gc:universal TRINITY\_DN2805\_c0\_g1\_i1:623-57(-) |
| 161 | 370 | TRINITY\_DN20323\_c0\_g1\_i1.p1 | 57.82 | 4 | 4 | 4.08e2 | 1 | 1 | 1 |  | 35637.9492 | type:5prime\_partial len:338 gc:universal TRINITY\_DN20323\_c0\_g1\_i1:3-1016(+) |
| 162 | 371 | TRINITY\_DN95987\_c0\_g1\_i1.p1 | 58.16 | 12 | 12 | 2.11e2 | 1 | 1 | 1 |  | 12698.2246 | type:complete len:113 gc:universal TRINITY\_DN95987\_c0\_g1\_i1:390-52(-) |
| 163 | 372 | TRINITY\_DN118520\_c0\_g1\_i1.p1 | 57.22 | 3 | 3 | 2.54e2 | 1 | 1 | 1 |  | 61587.4766 | type:complete len:581 gc:universal TRINITY\_DN118520\_c0\_g1\_i1:1895-153(-) |
| 164 | 373 | TRINITY\_DN7333\_c0\_g1\_i1.p1 | 56.85 | 11 | 11 | 4.65e2 | 1 | 1 | 1 |  | 21384.5684 | type:complete len:192 gc:universal TRINITY\_DN7333\_c0\_g1\_i1:47-622(+) |
| 165 | 374 | TRINITY\_DN1084\_c0\_g1\_i1.p1 | 58.09 | 4 | 4 | 0 | 1 | 1 | 1 |  | 31571.6562 | type:complete len:301 gc:universal TRINITY\_DN1084\_c0\_g1\_i1:1131-229(-) |
| 166 | 375 | TRINITY\_DN10497\_c0\_g1\_i1.p1 | 58.2 | 7 | 7 | 3.46e2 | 1 | 1 | 1 |  | 23686.9492 | type:complete len:206 gc:universal TRINITY\_DN10497\_c0\_g1\_i1:84-701(+) |
| 167 | 376 | TRINITY\_DN36\_c0\_g1\_i12.p1 | 36.83 | 7 | 7 | 3.3e2 | 1 | 1 | 1 | Carbamidomethylation | 24684.2031 | type:complete len:222 gc:universal TRINITY\_DN36\_c0\_g1\_i12:931-266(-) |
| 168 | 377 | TRINITY\_DN10940\_c0\_g1\_i1.p1 | 56.21 | 19 | 19 | 0 | 1 | 1 | 1 | Carbamidomethylation | 14531.2686 | type:complete len:135 gc:universal TRINITY\_DN10940\_c0\_g1\_i1:1786-1382(-) |
| 169 | 378 | TRINITY\_DN1339\_c0\_g2\_i5.p1 | 50.28 | 4 | 4 | 3.62e2 | 1 | 1 | 1 |  | 38453.7148 | type:internal len:362 gc:universal TRINITY\_DN1339\_c0\_g2\_i5:1085-3(-) |
| 170 | 379 | TRINITY\_DN118208\_c0\_g1\_i1.p1 | 57.1 | 2 | 2 | 0 | 1 | 1 | 1 | Carbamidomethylation | 48778.1367 | type:complete len:422 gc:universal TRINITY\_DN118208\_c0\_g1\_i1:121-1386(+) |
| 171 | 380 | TRINITY\_DN598\_c1\_g1\_i2.p1 | 58.82 | 1 | 1 | 2.99e3 | 1 | 1 | 1 |  | 81043.625 | type:5prime\_partial len:742 gc:universal TRINITY\_DN598\_c1\_g1\_i2:2-2227(+) |
| 172 | 381 | TRINITY\_DN123\_c1\_g1\_i1.p1 | 26.69 | 2 | 2 | 0 | 1 | 1 | 1 | Carbamidomethylation | 117906.4141 | type:3prime\_partial len:1051 gc:universal TRINITY\_DN123\_c1\_g1\_i1:49-3198(+) |
| 173 | 382 | TRINITY\_DN60705\_c0\_g1\_i1.p1 | 34.22 | 9 | 9 | 1.03e2 | 1 | 1 | 1 |  | 19534.6289 | type:5prime\_partial len:169 gc:universal TRINITY\_DN60705\_c0\_g1\_i1:1-507(+) |
| 174 | 383 | TRINITY\_DN11097\_c0\_g1\_i1.p1 | 57.58 | 2 | 2 | 1.84e3 | 1 | 1 | 1 |  | 80111.1172 | type:complete len:730 gc:universal TRINITY\_DN11097\_c0\_g1\_i1:2348-159(-) |
| 174 | 384 | TRINITY\_DN11097\_c0\_g2\_i1.p1 | 57.58 | 6 | 6 | 1.84e3 | 1 | 1 | 1 |  | 24192.5488 | type:internal len:222 gc:universal TRINITY\_DN11097\_c0\_g2\_i1:665-3(-) |
| 175 | 385 | TRINITY\_DN1534\_c2\_g1\_i1.p1 | 58.86 | 3 | 3 | 1.16e2 | 1 | 1 | 1 | Carbamidomethylation | 59603.6055 | type:complete len:547 gc:universal TRINITY\_DN1534\_c2\_g1\_i1:1914-274(-) |
| 176 | 386 | TRINITY\_DN147947\_c0\_g1\_i1.p1 | 57.72 | 9 | 9 | 0 | 1 | 1 | 1 |  | 19144.6445 | type:internal len:168 gc:universal TRINITY\_DN147947\_c0\_g1\_i1:501-1(-) |
| 177 | 387 | TRINITY\_DN436\_c0\_g1\_i1.p1 | 58.24 | 9 | 9 | 3.56e3 | 1 | 1 | 2 |  | 13711.3193 | type:complete len:116 gc:universal TRINITY\_DN436\_c0\_g1\_i1:74-421(+) |
| 178 | 388 | TRINITY\_DN7112\_c0\_g1\_i2.p1 | 58.37 | 7 | 7 | 0 | 1 | 1 | 1 |  | 25819.3359 | type:complete len:224 gc:universal TRINITY\_DN7112\_c0\_g1\_i2:79-750(+) |
| 179 | 389 | TRINITY\_DN1378\_c0\_g1\_i1.p1 | 58.87 | 3 | 3 | 7.36e2 | 1 | 1 | 1 |  | 47978.9219 | type:complete len:443 gc:universal TRINITY\_DN1378\_c0\_g1\_i1:2151-823(-) |
| 180 | 390 | TRINITY\_DN98968\_c0\_g1\_i1.p1 | 34.92 | 2 | 2 | 1.18e3 | 1 | 1 | 1 | Carbamidomethylation | 47268.0352 | type:3prime\_partial len:424 gc:universal TRINITY\_DN98968\_c0\_g1\_i1:1269-1(-) |
| 181 | 391 | TRINITY\_DN164351\_c0\_g1\_i1.p1 | 58.85 | 5 | 5 | 2.75e3 | 1 | 1 | 3 | Oxidation (M) | 23723.9492 | type:complete len:212 gc:universal TRINITY\_DN164351\_c0\_g1\_i1:694-59(-) |
| 182 | 392 | TRINITY\_DN10907\_c0\_g1\_i1.p1 | 58.02 | 6 | 6 | 1.13e3 | 1 | 1 | 1 |  | 23412.5352 | type:complete len:206 gc:universal TRINITY\_DN10907\_c0\_g1\_i1:1010-393(-) |
| 183 | 393 | TRINITY\_DN138\_c0\_g1\_i1.p1 | 55.26 | 4 | 4 | 0 | 1 | 1 | 1 |  | 46324.8945 | type:complete len:401 gc:universal TRINITY\_DN138\_c0\_g1\_i1:157-1359(+) |
| 184 | 394 | TRINITY\_DN1320\_c0\_g2\_i1.p1 | 46.88 | 5 | 5 | 0 | 1 | 1 | 1 |  | 28787.1875 | type:complete len:260 gc:universal TRINITY\_DN1320\_c0\_g2\_i1:97-876(+) |
| 185 | 395 | TRINITY\_DN164128\_c0\_g1\_i1.p1 | 57.98 | 5 | 5 | 1.55e4 | 1 | 1 | 1 |  | 17885.4395 | type:complete len:166 gc:universal TRINITY\_DN164128\_c0\_g1\_i1:117-614(+) |
| 186 | 396 | TRINITY\_DN164753\_c0\_g1\_i1.p1 | 58.37 | 6 | 6 | 2.44e3 | 1 | 1 | 2 |  | 15990.6094 | type:complete len:143 gc:universal TRINITY\_DN164753\_c0\_g1\_i1:491-63(-) |
| 187 | 397 | TRINITY\_DN58867\_c0\_g1\_i2.p1 | 53.68 | 3 | 3 | 9.36e2 | 1 | 1 | 1 |  | 40822.2812 | type:complete len:354 gc:universal TRINITY\_DN58867\_c0\_g1\_i2:80-1141(+) |
| 188 | 398 | TRINITY\_DN598\_c1\_g1\_i1.p1 | 58.87 | 1 | 1 | 2.05e3 | 1 | 1 | 2 |  | 81322.8672 | type:5prime\_partial len:745 gc:universal TRINITY\_DN598\_c1\_g1\_i1:2-2236(+) |
| 189 | 399 | TRINITY\_DN614\_c0\_g1\_i1.p1 | 57.99 | 5 | 5 | 9.44e2 | 1 | 1 | 1 |  | 20573.5293 | type:complete len:183 gc:universal TRINITY\_DN614\_c0\_g1\_i1:194-742(+) |
| 190 | 400 | TRINITY\_DN4\_c0\_g1\_i1.p1 | 55.13 | 3 | 3 | 1.07e2 | 1 | 1 | 2 |  | 63285.6953 | type:5prime\_partial len:586 gc:universal TRINITY\_DN4\_c0\_g1\_i1:2-1759(+) |
| 191 | 401 | TRINITY\_DN932\_c0\_g1\_i1.p1 | 56.52 | 2 | 2 | 4.13e2 | 1 | 1 | 1 | Oxidation (M) | 52305.4961 | type:complete len:475 gc:universal TRINITY\_DN932\_c0\_g1\_i1:1940-516(-) |
| 192 | 402 | TRINITY\_DN11545\_c0\_g1\_i1.p1 | 22.24 | 2 | 2 | 1.37e3 | 1 | 1 | 1 |  | 51486.9375 | type:5prime\_partial len:452 gc:universal TRINITY\_DN11545\_c0\_g1\_i1:1-1356(+) |
| 193 | 403 | TRINITY\_DN3052\_c0\_g1\_i1.p1 | 57.66 | 4 | 4 | 8.15e2 | 1 | 1 | 1 | Acetylation (Protein N-term) | 39261.4453 | type:complete len:362 gc:universal TRINITY\_DN3052\_c0\_g1\_i1:45-1130(+) |
| 194 | 404 | TRINITY\_DN11662\_c0\_g1\_i1.p1 | 55.54 | 3 | 3 | 5.44e3 | 1 | 1 | 1 | Acetylation (Protein N-term) | 41619.3711 | type:complete len:398 gc:universal TRINITY\_DN11662\_c0\_g1\_i1:75-1268(+) |
| 195 | 405 | TRINITY\_DN1065\_c0\_g1\_i1.p1 | 58.87 | 5 | 5 | 5.97e2 | 1 | 1 | 1 |  | 40043.2539 | type:complete len:373 gc:universal TRINITY\_DN1065\_c0\_g1\_i1:53-1171(+) |
| 196 | 406 | TRINITY\_DN10995\_c0\_g1\_i2.p3 | 36.4 | 7 | 7 | 0 | 1 | 1 | 1 |  | 17560.5059 | type:5prime\_partial len:152 gc:universal TRINITY\_DN10995\_c0\_g1\_i2:3-458(+) |
| 197 | 407 | TRINITY\_DN5153\_c0\_g1\_i1.p1 | 33.06 | 2 | 2 | 0 | 1 | 1 | 1 |  | 98168.6016 | type:complete len:862 gc:universal TRINITY\_DN5153\_c0\_g1\_i1:61-2646(+) |
| total 407 proteins |  |  |  |  |  |  |  |  |  |  |  |  |
| --- | --- | --- | --- | --- | --- | --- | --- | --- | --- | --- | --- | --- |

  

TRINITY\_DN11435\_c0\_g1\_i1.p1

back to list

  

| Protein Coverage
| Supporting Peptides
| Best Unique PSM
|

Protein Coverage:

Supporting Peptides:

| Peptide | Uniq | -10lgP | Mass | Length | ppm | m/z | z | RT | 1/k0 Start | 1/k0 End | Precursor ID | Area Sample 2 | #Feature | #Feature Sample 2 | Start | End | PTM | AScore | Found By |
| --- | --- | --- | --- | --- | --- | --- | --- | --- | --- | --- | --- | --- | --- | --- | --- | --- | --- | --- | --- |
| K.VYLAQAEAEKIK.M | Y | 58.92 | 1361.7554 | 12 | 3.89 | 681.8839 | 2 | 14.17 | 1.0355 | 1.0427 | 37026 | 0 | 0 | 0 | 309 | 320 |  |  | DB Search |
| K.LAYELQANKEQQR.I | Y | 58.82 | 1589.8161 | 13 | -0.91 | 795.9103 | 2 | 12.85 | 1.0548 | 1.062 | 29615 | 1.33e3 | 1 | 1 | 239 | 251 |  |  | DB Search |
| R.IRNEEIQIEVIERR.K | Y | 58.34 | 1795.9904 | 14 | 0.23 | 599.6676 | 3 | 14.93 | 0.881 | 0.8883 | 41049 | 1.01e3 | 1 | 1 | 252 | 265 |  |  | DB Search |
| K.LIEVEEKEIQR.R | Y | 58.17 | 1384.7562 | 11 | 5.59 | 693.3854 | 2 | 13.72 | 1.0158 | 1.023 | 34552 | 5.04e2 | 1 | 1 | 267 | 277 |  |  | DB Search |
| R.KLIEVEEKEIQR.R | Y | 54.86 | 1512.8511 | 12 | -6.51 | 757.4238 | 2 | 13.18 | 1.0636 | 1.0709 | 31563 | 0 | 0 | 0 | 266 | 277 |  |  | DB Search |
| R.REQELQATVK.L | Y | 38.36 | 1200.6462 | 10 | -10.4 | 601.3209 | 2 | 11.17 | 0.9125 | 0.9198 | 19974 | 1.07e3 | 1 | 1 | 278 | 287 |  |  | DB Search |
| total 6 peptides |  |  |  |  |  |  |  |  |  |  |  |  |  |  |  |  |  |  |  |
| --- | --- | --- | --- | --- | --- | --- | --- | --- | --- | --- | --- | --- | --- | --- | --- | --- | --- | --- | --- |

Best Unique PSM (Scan Sample\_11\_A1\_1\_3648.d:37026, m/z=681.8839, z=2, RT=14.17, ppm=-1.58):


TRINITY\_DN453\_c0\_g1\_i1.p1

back to list

  

| Protein Coverage
| Supporting Peptides
| Best Unique PSM
|

Protein Coverage:

Supporting Peptides:

| Peptide | Uniq | -10lgP | Mass | Length | ppm | m/z | z | RT | 1/k0 Start | 1/k0 End | Precursor ID | Area Sample 2 | #Feature | #Feature Sample 2 | Start | End | PTM | AScore | Found By |
| --- | --- | --- | --- | --- | --- | --- | --- | --- | --- | --- | --- | --- | --- | --- | --- | --- | --- | --- | --- |
| K.SAATQTSIKENK.S | Y | 58.38 | 1276.6623 | 12 | -2.61 | 639.3333 | 2 | 9.66 | 0.9646 | 0.9719 | 11591 | 0 | 0 | 0 | 69 | 80 |  |  | DB Search |
| total 1 peptides |  |  |  |  |  |  |  |  |  |  |  |  |  |  |  |  |  |  |  |
| --- | --- | --- | --- | --- | --- | --- | --- | --- | --- | --- | --- | --- | --- | --- | --- | --- | --- | --- | --- |

Best Unique PSM (Scan Sample\_11\_A1\_1\_3648.d:11591, m/z=639.3333, z=2, RT=9.66, ppm=-8.09):


TRINITY\_DN72753\_c0\_g1\_i1.p1

back to list

  

| Protein Coverage
| Supporting Peptides
| Best Unique PSM
|

Protein Coverage:

Supporting Peptides:

| Peptide | Uniq | -10lgP | Mass | Length | ppm | m/z | z | RT | 1/k0 Start | 1/k0 End | Precursor ID | Area Sample 2 | #Feature | #Feature Sample 2 | Start | End | PTM | AScore | Found By |
| --- | --- | --- | --- | --- | --- | --- | --- | --- | --- | --- | --- | --- | --- | --- | --- | --- | --- | --- | --- |
| R.EIAQDFKTDLR.F | Y | 58.76 | 1334.683 | 11 | -3.43 | 668.3428 | 2 | 14.60 | 0.9892 | 0.9965 | 39397 | 0 | 0 | 0 | 74 | 84 |  |  | DB Search |
| R.YRPGTVALR.E | Y | 44.05 | 1031.5876 | 9 | -1.83 | 516.7973 | 2 | 12.44 | 0.8575 | 0.8648 | 27332 | 1.61e3 | 1 | 1 | 42 | 50 |  |  | DB Search |
| total 2 peptides |  |  |  |  |  |  |  |  |  |  |  |  |  |  |  |  |  |  |  |
| --- | --- | --- | --- | --- | --- | --- | --- | --- | --- | --- | --- | --- | --- | --- | --- | --- | --- | --- | --- |

Best Unique PSM (Scan Sample\_11\_A1\_1\_3648.d:39397, m/z=668.3428, z=2, RT=14.60, ppm=-8.9):


TRINITY\_DN72959\_c0\_g1\_i1.p1

back to list

  

| Protein Coverage
| Supporting Peptides
| Best Unique PSM
|

Protein Coverage:

Supporting Peptides:

| Peptide | Uniq | -10lgP | Mass | Length | ppm | m/z | z | RT | 1/k0 Start | 1/k0 End | Precursor ID | Area Sample 2 | #Feature | #Feature Sample 2 | Start | End | PTM | AScore | Found By |
| --- | --- | --- | --- | --- | --- | --- | --- | --- | --- | --- | --- | --- | --- | --- | --- | --- | --- | --- | --- |
| R.GAFGKPQGTVAR.V | Y | 58.24 | 1187.6411 | 12 | 4.16 | 594.827 | 2 | 11.37 | 0.9247 | 0.9319 | 21197 | 7.61e2 | 1 | 1 | 117 | 128 |  |  | DB Search |
| K.DQHEATAIEALRR.A | Y | 24.21 | 1508.7695 | 13 | 5.66 | 503.9305 | 3 | 13.52 | 0.8039 | 0.8112 | 33520 | 0 | 0 | 0 | 142 | 154 |  |  | DB Search |
| total 2 peptides |  |  |  |  |  |  |  |  |  |  |  |  |  |  |  |  |  |  |  |
| --- | --- | --- | --- | --- | --- | --- | --- | --- | --- | --- | --- | --- | --- | --- | --- | --- | --- | --- | --- |

Best Unique PSM (Scan Sample\_11\_A1\_1\_3648.d:21197, m/z=594.827, z=2, RT=11.37, ppm=-1.32):


TRINITY\_DN30365\_c0\_g1\_i2.p1

back to list

  

| Protein Coverage
| Supporting Peptides
| Best Unique PSM
|

Protein Coverage:

Supporting Peptides:

| Peptide | Uniq | -10lgP | Mass | Length | ppm | m/z | z | RT | 1/k0 Start | 1/k0 End | Precursor ID | Area Sample 2 | #Feature | #Feature Sample 2 | Start | End | PTM | AScore | Found By |
| --- | --- | --- | --- | --- | --- | --- | --- | --- | --- | --- | --- | --- | --- | --- | --- | --- | --- | --- | --- |
| K.AVAHHTTASFIR.V | Y | 58.76 | 1309.6891 | 12 | -1.72 | 655.8471 | 2 | 11.68 | 0.9703 | 0.9775 | 22954 | 1.45e3 | 1 | 1 | 219 | 230 |  |  | DB Search |
| total 1 peptides |  |  |  |  |  |  |  |  |  |  |  |  |  |  |  |  |  |  |  |
| --- | --- | --- | --- | --- | --- | --- | --- | --- | --- | --- | --- | --- | --- | --- | --- | --- | --- | --- | --- |

Best Unique PSM (Scan Sample\_11\_A1\_1\_3648.d:22954, m/z=655.8471, z=2, RT=11.68, ppm=-7.2):


TRINITY\_DN164128\_c0\_g1\_i1.p1

back to list

  

| Protein Coverage
| Supporting Peptides
| Best Unique PSM
|

Protein Coverage:

Supporting Peptides:

| Peptide | Uniq | -10lgP | Mass | Length | ppm | m/z | z | RT | 1/k0 Start | 1/k0 End | Precursor ID | Area Sample 2 | #Feature | #Feature Sample 2 | Start | End | PTM | AScore | Found By |
| --- | --- | --- | --- | --- | --- | --- | --- | --- | --- | --- | --- | --- | --- | --- | --- | --- | --- | --- | --- |
| K.ALKEPPRDR.K | Y | 57.98 | 1080.604 | 9 | 10.29 | 361.2103 | 3 | 9.44 | 0.7229 | 0.7302 | 10425 | 1.55e4 | 1 | 1 | 84 | 92 |  |  | DB Search |
| total 1 peptides |  |  |  |  |  |  |  |  |  |  |  |  |  |  |  |  |  |  |  |
| --- | --- | --- | --- | --- | --- | --- | --- | --- | --- | --- | --- | --- | --- | --- | --- | --- | --- | --- | --- |

Best Unique PSM (Scan Sample\_11\_A1\_1\_3648.d:10425, m/z=361.2103, z=3, RT=9.44, ppm=4.81):


TRINITY\_DN265\_c0\_g1\_i2.p1

back to list

  

| Protein Coverage
| Supporting Peptides
| Best Unique PSM
|

Protein Coverage:

Supporting Peptides:

| Peptide | Uniq | -10lgP | Mass | Length | ppm | m/z | z | RT | 1/k0 Start | 1/k0 End | Precursor ID | Area Sample 2 | #Feature | #Feature Sample 2 | Start | End | PTM | AScore | Found By |
| --- | --- | --- | --- | --- | --- | --- | --- | --- | --- | --- | --- | --- | --- | --- | --- | --- | --- | --- | --- |
| R.LTHDFHTNKR.M | Y | 58.75 | 1267.6421 | 10 | 2.44 | 423.5534 | 3 | 9.64 | 0.7441 | 0.7514 | 11479 | 1.15e3 | 1 | 1 | 24 | 33 |  |  | DB Search |
| total 1 peptides |  |  |  |  |  |  |  |  |  |  |  |  |  |  |  |  |  |  |  |
| --- | --- | --- | --- | --- | --- | --- | --- | --- | --- | --- | --- | --- | --- | --- | --- | --- | --- | --- | --- |

Best Unique PSM (Scan Sample\_11\_A1\_1\_3648.d:11479, m/z=423.5534, z=3, RT=9.64, ppm=-3.04):


TRINITY\_DN16120\_c0\_g1\_i2.p1

back to list

  

| Protein Coverage
| Supporting Peptides
| Best Unique PSM
|

Protein Coverage:

Supporting Peptides:

| Peptide | Uniq | -10lgP | Mass | Length | ppm | m/z | z | RT | 1/k0 Start | 1/k0 End | Precursor ID | Area Sample 2 | #Feature | #Feature Sample 2 | Start | End | PTM | AScore | Found By |
| --- | --- | --- | --- | --- | --- | --- | --- | --- | --- | --- | --- | --- | --- | --- | --- | --- | --- | --- | --- |
| R.LC(+57.02)ETNTANTEAK.V | Y | 57.96 | 1350.6085 | 12 | -0.88 | 676.3073 | 2 | 10.37 | 0.9537 | 0.961 | 15355 | 0 | 0 | 0 | 150 | 161 | Carbamidomethylation |  | DB Search |
| total 1 peptides |  |  |  |  |  |  |  |  |  |  |  |  |  |  |  |  |  |  |  |
| --- | --- | --- | --- | --- | --- | --- | --- | --- | --- | --- | --- | --- | --- | --- | --- | --- | --- | --- | --- |

Best Unique PSM (Scan Sample\_11\_A1\_1\_3648.d:15355, m/z=676.3073, z=2, RT=10.37, ppm=-6.36):


TRINITY\_DN1377\_c0\_g1\_i1.p1

back to list

  

| Protein Coverage
| Supporting Peptides
| Best Unique PSM
|

Protein Coverage:

Supporting Peptides:

| Peptide | Uniq | -10lgP | Mass | Length | ppm | m/z | z | RT | 1/k0 Start | 1/k0 End | Precursor ID | Area Sample 2 | #Feature | #Feature Sample 2 | Start | End | PTM | AScore | Found By |
| --- | --- | --- | --- | --- | --- | --- | --- | --- | --- | --- | --- | --- | --- | --- | --- | --- | --- | --- | --- |
| K.RLPDQITTR.S | Y | 51.26 | 1098.6145 | 9 | -16.36 | 550.3026 | 2 | 12.47 | 0.8777 | 0.885 | 27597 | 0 | 0 | 0 | 182 | 190 |  |  | DB Search |
| R.ITQQQSHQEIADQLR.V | Y | 25.96 | 1793.902 | 15 | 0.41 | 897.9537 | 2 | 13.04 | 1.1222 | 1.1294 | 30831 | 0 | 0 | 0 | 678 | 692 |  |  | DB Search |
| total 2 peptides |  |  |  |  |  |  |  |  |  |  |  |  |  |  |  |  |  |  |  |
| --- | --- | --- | --- | --- | --- | --- | --- | --- | --- | --- | --- | --- | --- | --- | --- | --- | --- | --- | --- |

Best Unique PSM (Scan Sample\_11\_A1\_1\_3648.d:27597, m/z=550.3026, z=2, RT=12.47, ppm=-21.83):


TRINITY\_DN59667\_c0\_g1\_i1.p1

back to list

  

| Protein Coverage
| Supporting Peptides
| Best Unique PSM
|

Protein Coverage:

Supporting Peptides:

| Peptide | Uniq | -10lgP | Mass | Length | ppm | m/z | z | RT | 1/k0 Start | 1/k0 End | Precursor ID | Area Sample 2 | #Feature | #Feature Sample 2 | Start | End | PTM | AScore | Found By |
| --- | --- | --- | --- | --- | --- | --- | --- | --- | --- | --- | --- | --- | --- | --- | --- | --- | --- | --- | --- |
| R.IEHAHPINR.I | Y | 56.51 | 1085.573 | 9 | 12.45 | 362.8675 | 3 | 9.50 | 0.7416 | 0.7489 | 10850 | 5.9e2 | 1 | 1 | 85 | 93 |  |  | DB Search |
| total 1 peptides |  |  |  |  |  |  |  |  |  |  |  |  |  |  |  |  |  |  |  |
| --- | --- | --- | --- | --- | --- | --- | --- | --- | --- | --- | --- | --- | --- | --- | --- | --- | --- | --- | --- |

Best Unique PSM (Scan Sample\_11\_A1\_1\_3648.d:10850, m/z=362.8675, z=3, RT=9.50, ppm=6.96):


TRINITY\_DN1172\_c0\_g1\_i3.p1

back to list

  

| Protein Coverage
| Supporting Peptides
| Best Unique PSM
|

Protein Coverage:

Supporting Peptides:

| Peptide | Uniq | -10lgP | Mass | Length | ppm | m/z | z | RT | 1/k0 Start | 1/k0 End | Precursor ID | Area Sample 2 | #Feature | #Feature Sample 2 | Start | End | PTM | AScore | Found By |
| --- | --- | --- | --- | --- | --- | --- | --- | --- | --- | --- | --- | --- | --- | --- | --- | --- | --- | --- | --- |
| K.VITSDDPAEKEK.K | Y | 58.87 | 1330.6616 | 12 | 2.64 | 666.3362 | 2 | 10.69 | 0.9848 | 0.992 | 17191 | 9.01e3 | 1 | 1 | 123 | 134 |  |  | DB Search |
| total 1 peptides |  |  |  |  |  |  |  |  |  |  |  |  |  |  |  |  |  |  |  |
| --- | --- | --- | --- | --- | --- | --- | --- | --- | --- | --- | --- | --- | --- | --- | --- | --- | --- | --- | --- |

Best Unique PSM (Scan Sample\_11\_A1\_1\_3648.d:17191, m/z=666.3362, z=2, RT=10.69, ppm=-2.84):


TRINITY\_DN22594\_c0\_g1\_i1.p2

back to list

  

| Protein Coverage
| Supporting Peptides
| Best Unique PSM
|

Protein Coverage:

Supporting Peptides:

| Peptide | Uniq | -10lgP | Mass | Length | ppm | m/z | z | RT | 1/k0 Start | 1/k0 End | Precursor ID | Area Sample 2 | #Feature | #Feature Sample 2 | Start | End | PTM | AScore | Found By |
| --- | --- | --- | --- | --- | --- | --- | --- | --- | --- | --- | --- | --- | --- | --- | --- | --- | --- | --- | --- |
| R.FRQLGLDFGK.L | Y | 40.24 | 1179.64 | 10 | -11.3 | 590.8174 | 2 | 19.48 | 0.9222 | 0.9295 | 64712 | 1.03e3 | 1 | 1 | 40 | 49 |  |  | DB Search |
| total 1 peptides |  |  |  |  |  |  |  |  |  |  |  |  |  |  |  |  |  |  |  |
| --- | --- | --- | --- | --- | --- | --- | --- | --- | --- | --- | --- | --- | --- | --- | --- | --- | --- | --- | --- |

Best Unique PSM (Scan Sample\_11\_A1\_1\_3648.d:64712, m/z=590.8174, z=2, RT=19.48, ppm=-16.78):


TRINITY\_DN982\_c0\_g1\_i2.p1

back to list

  

| Protein Coverage
| Supporting Peptides
| Best Unique PSM
|

Protein Coverage:

Supporting Peptides:

| Peptide | Uniq | -10lgP | Mass | Length | ppm | m/z | z | RT | 1/k0 Start | 1/k0 End | Precursor ID | Area Sample 2 | #Feature | #Feature Sample 2 | Start | End | PTM | AScore | Found By |
| --- | --- | --- | --- | --- | --- | --- | --- | --- | --- | --- | --- | --- | --- | --- | --- | --- | --- | --- | --- |
| K.VLLSQQIFYDKK.N | N | 26.23 | 1480.8289 | 12 | -0.12 | 741.4176 | 2 | 16.48 | 1.0608 | 1.068 | 48785 | 6.44e2 | 1 | 1 | 1479 | 1490 |  |  | DB Search |
| K.VLEVNDNKPK.F | Y | 26.02 | 1154.6295 | 10 | -0.56 | 578.3185 | 2 | 10.96 | 0.9234 | 0.9307 | 18881 | 0 | 0 | 0 | 459 | 468 |  |  | DB Search |
| total 2 peptides |  |  |  |  |  |  |  |  |  |  |  |  |  |  |  |  |  |  |  |
| --- | --- | --- | --- | --- | --- | --- | --- | --- | --- | --- | --- | --- | --- | --- | --- | --- | --- | --- | --- |

Best Unique PSM (Scan Sample\_11\_A1\_1\_3648.d:18881, m/z=578.3185, z=2, RT=10.96, ppm=-6.04):


TRINITY\_DN413\_c0\_g1\_i3.p1

back to list

  

| Protein Coverage
| Supporting Peptides
| Best Unique PSM
|

Protein Coverage:

Supporting Peptides:

| Peptide | Uniq | -10lgP | Mass | Length | ppm | m/z | z | RT | 1/k0 Start | 1/k0 End | Precursor ID | Area Sample 2 | #Feature | #Feature Sample 2 | Start | End | PTM | AScore | Found By |
| --- | --- | --- | --- | --- | --- | --- | --- | --- | --- | --- | --- | --- | --- | --- | --- | --- | --- | --- | --- |
| R.ADILC(+57.02)HDTKYGK.K | Y | 47.79 | 1419.6816 | 12 | 13.12 | 710.8535 | 2 | 12.07 | 1.0134 | 1.0206 | 25280 | 0 | 0 | 0 | 320 | 331 | Carbamidomethylation |  | DB Search |
| total 1 peptides |  |  |  |  |  |  |  |  |  |  |  |  |  |  |  |  |  |  |  |
| --- | --- | --- | --- | --- | --- | --- | --- | --- | --- | --- | --- | --- | --- | --- | --- | --- | --- | --- | --- |

Best Unique PSM (Scan Sample\_11\_A1\_1\_3648.d:25280, m/z=710.8535, z=2, RT=12.07, ppm=7.65):


TRINITY\_DN12629\_c0\_g1\_i1.p1

back to list

  

| Protein Coverage
| Supporting Peptides
| Best Unique PSM
|

Protein Coverage:

Supporting Peptides:

| Peptide | Uniq | -10lgP | Mass | Length | ppm | m/z | z | RT | 1/k0 Start | 1/k0 End | Precursor ID | Area Sample 2 | #Feature | #Feature Sample 2 | Start | End | PTM | AScore | Found By |
| --- | --- | --- | --- | --- | --- | --- | --- | --- | --- | --- | --- | --- | --- | --- | --- | --- | --- | --- | --- |
| R.IRVESLLVSAISK.A | Y | 54.29 | 1413.8555 | 13 | -0.42 | 707.9308 | 2 | 18.45 | 1.0311 | 1.0383 | 59251 | 0 | 0 | 0 | 386 | 398 |  |  | DB Search |
| total 1 peptides |  |  |  |  |  |  |  |  |  |  |  |  |  |  |  |  |  |  |  |
| --- | --- | --- | --- | --- | --- | --- | --- | --- | --- | --- | --- | --- | --- | --- | --- | --- | --- | --- | --- |

Best Unique PSM (Scan Sample\_11\_A1\_1\_3648.d:59251, m/z=707.9308, z=2, RT=18.45, ppm=-5.9):


TRINITY\_DN776\_c0\_g1\_i1.p1

back to list

  

| Protein Coverage
| Supporting Peptides
| Best Unique PSM
|

Protein Coverage:

Supporting Peptides:

| Peptide | Uniq | -10lgP | Mass | Length | ppm | m/z | z | RT | 1/k0 Start | 1/k0 End | Precursor ID | Area Sample 2 | #Feature | #Feature Sample 2 | Start | End | PTM | AScore | Found By |
| --- | --- | --- | --- | --- | --- | --- | --- | --- | --- | --- | --- | --- | --- | --- | --- | --- | --- | --- | --- |
| R.LFEGNALLRR.L | Y | 57.75 | 1187.6775 | 10 | 3.63 | 594.8449 | 2 | 15.37 | 0.9683 | 0.9755 | 43382 | 1.83e3 | 1 | 1 | 78 | 87 |  |  | DB Search |
| R.RLFEGNALLR.R | Y | 53.09 | 1187.6775 | 10 | 1.58 | 594.8437 | 2 | 15.84 | 0.9384 | 0.9457 | 45707 | 5.33e2 | 1 | 1 | 77 | 86 |  |  | DB Search |
| R.RLFEGNALLRR.L | Y | 20.39 | 1343.7786 | 11 | 6.92 | 448.9341 | 3 | 14.60 | 0.7538 | 0.7612 | 39347 | 0 | 0 | 0 | 77 | 87 |  |  | DB Search |
| total 3 peptides |  |  |  |  |  |  |  |  |  |  |  |  |  |  |  |  |  |  |  |
| --- | --- | --- | --- | --- | --- | --- | --- | --- | --- | --- | --- | --- | --- | --- | --- | --- | --- | --- | --- |

Best Unique PSM (Scan Sample\_11\_A1\_1\_3648.d:43382, m/z=594.8449, z=2, RT=15.37, ppm=-1.84):


TRINITY\_DN4523\_c0\_g1\_i2.p1

back to list

  

| Protein Coverage
| Supporting Peptides
| Best Unique PSM
|

Protein Coverage:

Supporting Peptides:

| Peptide | Uniq | -10lgP | Mass | Length | ppm | m/z | z | RT | 1/k0 Start | 1/k0 End | Precursor ID | Area Sample 2 | #Feature | #Feature Sample 2 | Start | End | PTM | AScore | Found By |
| --- | --- | --- | --- | --- | --- | --- | --- | --- | --- | --- | --- | --- | --- | --- | --- | --- | --- | --- | --- |
| R.VASDKFLTSFIK.S | Y | 52.71 | 1354.7496 | 12 | 1.41 | 678.3793 | 2 | 18.05 | 1.0234 | 1.0307 | 57158 | 3.75e2 | 1 | 1 | 1184 | 1195 |  |  | DB Search |
| total 1 peptides |  |  |  |  |  |  |  |  |  |  |  |  |  |  |  |  |  |  |  |
| --- | --- | --- | --- | --- | --- | --- | --- | --- | --- | --- | --- | --- | --- | --- | --- | --- | --- | --- | --- |

Best Unique PSM (Scan Sample\_11\_A1\_1\_3648.d:57158, m/z=678.3793, z=2, RT=18.05, ppm=-4.06):


TRINITY\_DN3986\_c0\_g1\_i2.p1

back to list

  

| Protein Coverage
| Supporting Peptides
| Best Unique PSM
|

Protein Coverage:

Supporting Peptides:

| Peptide | Uniq | -10lgP | Mass | Length | ppm | m/z | z | RT | 1/k0 Start | 1/k0 End | Precursor ID | Area Sample 2 | #Feature | #Feature Sample 2 | Start | End | PTM | AScore | Found By |
| --- | --- | --- | --- | --- | --- | --- | --- | --- | --- | --- | --- | --- | --- | --- | --- | --- | --- | --- | --- |
| R.FPGQLNADLRK.L | Y | 25.64 | 1257.6829 | 11 | -1.3 | 629.8445 | 2 | 14.18 | 0.9457 | 0.9529 | 37015 | 6.11e2 | 1 | 1 | 242 | 252 |  |  | DB Search |
| total 1 peptides |  |  |  |  |  |  |  |  |  |  |  |  |  |  |  |  |  |  |  |
| --- | --- | --- | --- | --- | --- | --- | --- | --- | --- | --- | --- | --- | --- | --- | --- | --- | --- | --- | --- |

Best Unique PSM (Scan Sample\_11\_A1\_1\_3648.d:37015, m/z=629.8445, z=2, RT=14.18, ppm=-6.78):


TRINITY\_DN11097\_c0\_g2\_i1.p1

back to list

  

| Protein Coverage
| Supporting Peptides
| Best Unique PSM
|

Protein Coverage:

Supporting Peptides:

| Peptide | Uniq | -10lgP | Mass | Length | ppm | m/z | z | RT | 1/k0 Start | 1/k0 End | Precursor ID | Area Sample 2 | #Feature | #Feature Sample 2 | Start | End | PTM | AScore | Found By |
| --- | --- | --- | --- | --- | --- | --- | --- | --- | --- | --- | --- | --- | --- | --- | --- | --- | --- | --- | --- |
| K.LRVDLSLFETGIK.V | Y | 57.58 | 1489.8504 | 13 | -1.87 | 745.927 | 2 | 19.61 | 1.0363 | 1.0435 | 65344 | 1.84e3 | 1 | 1 | 74 | 86 |  |  | DB Search |
| total 1 peptides |  |  |  |  |  |  |  |  |  |  |  |  |  |  |  |  |  |  |  |
| --- | --- | --- | --- | --- | --- | --- | --- | --- | --- | --- | --- | --- | --- | --- | --- | --- | --- | --- | --- |

Best Unique PSM (Scan Sample\_11\_A1\_1\_3648.d:65344, m/z=745.927, z=2, RT=19.61, ppm=-7.34):


TRINITY\_DN484\_c0\_g1\_i1.p1

back to list

  

| Protein Coverage
| Supporting Peptides
| Best Unique PSM
|

Protein Coverage:

Supporting Peptides:

| Peptide | Uniq | -10lgP | Mass | Length | ppm | m/z | z | RT | 1/k0 Start | 1/k0 End | Precursor ID | Area Sample 2 | #Feature | #Feature Sample 2 | Start | End | PTM | AScore | Found By |
| --- | --- | --- | --- | --- | --- | --- | --- | --- | --- | --- | --- | --- | --- | --- | --- | --- | --- | --- | --- |
| K.YFVEAGAM(+15.99)AVRR.I | Y | 52.04 | 1384.6921 | 12 | 2.12 | 693.351 | 2 | 13.54 | 1.0484 | 1.0556 | 33548 | 1.06e3 | 1 | 1 | 298 | 309 | Oxidation (M) | M8:Oxidation (M):1000 | DB Search |
| R.AYHNASQTQSDKAHLK.W | Y | 36.97 | 1797.8758 | 16 | -0.62 | 450.4735 | 4 | 9.45 | 0.7428 | 0.7502 | 10634 | 0 | 0 | 0 | 468 | 483 |  |  | DB Search |
| total 2 peptides |  |  |  |  |  |  |  |  |  |  |  |  |  |  |  |  |  |  |  |
| --- | --- | --- | --- | --- | --- | --- | --- | --- | --- | --- | --- | --- | --- | --- | --- | --- | --- | --- | --- |

Best Unique PSM (Scan Sample\_11\_A1\_1\_3648.d:33548, m/z=693.351, z=2, RT=13.54, ppm=-3.36):


TRINITY\_DN319\_c2\_g1\_i1.p1

back to list

  

| Protein Coverage
| Supporting Peptides
| Best Unique PSM
|

Protein Coverage:

Supporting Peptides:

| Peptide | Uniq | -10lgP | Mass | Length | ppm | m/z | z | RT | 1/k0 Start | 1/k0 End | Precursor ID | Area Sample 2 | #Feature | #Feature Sample 2 | Start | End | PTM | AScore | Found By |
| --- | --- | --- | --- | --- | --- | --- | --- | --- | --- | --- | --- | --- | --- | --- | --- | --- | --- | --- | --- |
| K.AQYYEAYETIIKR.H | Y | 58.88 | 1646.8304 | 13 | 5.56 | 824.4225 | 2 | 15.51 | 1.1025 | 1.1098 | 44074 | 4.92e2 | 1 | 1 | 2535 | 2547 |  |  | DB Search |
| K.KAQYYEAYETIIKR.H | Y | 58.38 | 1774.9254 | 14 | -0.12 | 592.6458 | 3 | 15.51 | 0.8644 | 0.8716 | 44111 | 1.84e3 | 2 | 2 | 2534 | 2547 |  |  | DB Search |
| total 2 peptides |  |  |  |  |  |  |  |  |  |  |  |  |  |  |  |  |  |  |  |
| --- | --- | --- | --- | --- | --- | --- | --- | --- | --- | --- | --- | --- | --- | --- | --- | --- | --- | --- | --- |

Best Unique PSM (Scan Sample\_11\_A1\_1\_3648.d:44074, m/z=822.9161, z=2, RT=15.56, ppm=0.09):


TRINITY\_DN649\_c0\_g1\_i2.p1

back to list

  

| Protein Coverage
| Supporting Peptides
| Best Unique PSM
|

Protein Coverage:

Supporting Peptides:

| Peptide | Uniq | -10lgP | Mass | Length | ppm | m/z | z | RT | 1/k0 Start | 1/k0 End | Precursor ID | Area Sample 2 | #Feature | #Feature Sample 2 | Start | End | PTM | AScore | Found By |
| --- | --- | --- | --- | --- | --- | --- | --- | --- | --- | --- | --- | --- | --- | --- | --- | --- | --- | --- | --- |
| K.FETQIATK.N | Y | 57.95 | 936.4916 | 8 | 1.49 | 937.4952 | 1 | 13.74 | 1.2208 | 1.228 | 34637 | 7.44e2 | 2 | 2 | 103 | 110 |  |  | DB Search |
| total 1 peptides |  |  |  |  |  |  |  |  |  |  |  |  |  |  |  |  |  |  |  |
| --- | --- | --- | --- | --- | --- | --- | --- | --- | --- | --- | --- | --- | --- | --- | --- | --- | --- | --- | --- |

Best Unique PSM (Scan Sample\_11\_A1\_1\_3648.d:34637, m/z=937.4952, z=1, RT=13.74, ppm=-3.98):


TRINITY\_DN9509\_c0\_g1\_i6.p1

back to list

  

| Protein Coverage
| Supporting Peptides
| Best Unique PSM
|

Protein Coverage:

Supporting Peptides:

| Peptide | Uniq | -10lgP | Mass | Length | ppm | m/z | z | RT | 1/k0 Start | 1/k0 End | Precursor ID | Area Sample 2 | #Feature | #Feature Sample 2 | Start | End | PTM | AScore | Found By |
| --- | --- | --- | --- | --- | --- | --- | --- | --- | --- | --- | --- | --- | --- | --- | --- | --- | --- | --- | --- |
| R.GQVIC(+57.02)APGATSTHTK.F | Y | 58 | 1526.7511 | 15 | 1.7 | 764.3799 | 2 | 11.76 | 1.0291 | 1.0363 | 23431 | 4.83e2 | 1 | 1 | 315 | 329 | Carbamidomethylation |  | DB Search |
| R.GITINASHIEYETSNR.H | Y | 57.62 | 1803.8751 | 16 | -0.09 | 902.9398 | 2 | 14.89 | 1.1674 | 1.1746 | 40938 | 0 | 0 | 0 | 74 | 89 |  |  | DB Search |
| total 2 peptides |  |  |  |  |  |  |  |  |  |  |  |  |  |  |  |  |  |  |  |
| --- | --- | --- | --- | --- | --- | --- | --- | --- | --- | --- | --- | --- | --- | --- | --- | --- | --- | --- | --- |

Best Unique PSM (Scan Sample\_11\_A1\_1\_3648.d:23431, m/z=764.3799, z=2, RT=11.76, ppm=-3.78):


TRINITY\_DN413\_c0\_g1\_i4.p1

back to list

  

| Protein Coverage
| Supporting Peptides
| Best Unique PSM
|

Protein Coverage:

Supporting Peptides:

| Peptide | Uniq | -10lgP | Mass | Length | ppm | m/z | z | RT | 1/k0 Start | 1/k0 End | Precursor ID | Area Sample 2 | #Feature | #Feature Sample 2 | Start | End | PTM | AScore | Found By |
| --- | --- | --- | --- | --- | --- | --- | --- | --- | --- | --- | --- | --- | --- | --- | --- | --- | --- | --- | --- |
| R.ADILC(+57.02)HDTKYGK.K | Y | 47.79 | 1419.6816 | 12 | 13.12 | 710.8535 | 2 | 12.07 | 1.0134 | 1.0206 | 25280 | 0 | 0 | 0 | 320 | 331 | Carbamidomethylation |  | DB Search |
| total 1 peptides |  |  |  |  |  |  |  |  |  |  |  |  |  |  |  |  |  |  |  |
| --- | --- | --- | --- | --- | --- | --- | --- | --- | --- | --- | --- | --- | --- | --- | --- | --- | --- | --- | --- |

Best Unique PSM (Scan Sample\_11\_A1\_1\_3648.d:25280, m/z=710.8535, z=2, RT=12.07, ppm=7.65):


TRINITY\_DN152\_c0\_g1\_i9.p1

back to list

  

| Protein Coverage
| Supporting Peptides
| Best Unique PSM
|

Protein Coverage:

Supporting Peptides:

| Peptide | Uniq | -10lgP | Mass | Length | ppm | m/z | z | RT | 1/k0 Start | 1/k0 End | Precursor ID | Area Sample 2 | #Feature | #Feature Sample 2 | Start | End | PTM | AScore | Found By |
| --- | --- | --- | --- | --- | --- | --- | --- | --- | --- | --- | --- | --- | --- | --- | --- | --- | --- | --- | --- |
| K.IQDKEGIPPDQQR.L | Y | 55.06 | 1522.7739 | 13 | 2.88 | 762.3923 | 2 | 11.00 | 1.0331 | 1.0403 | 18584 | 2.97e3 | 2 | 2 | 66 | 78 |  |  | DB Search |
| total 1 peptides |  |  |  |  |  |  |  |  |  |  |  |  |  |  |  |  |  |  |  |
| --- | --- | --- | --- | --- | --- | --- | --- | --- | --- | --- | --- | --- | --- | --- | --- | --- | --- | --- | --- |

Best Unique PSM (Scan Sample\_11\_A1\_1\_3648.d:18584, m/z=762.3923, z=2, RT=11.00, ppm=-2.59):


TRINITY\_DN2477\_c0\_g1\_i7.p1

back to list

  

| Protein Coverage
| Supporting Peptides
| Best Unique PSM
|

Protein Coverage:

Supporting Peptides:

| Peptide | Uniq | -10lgP | Mass | Length | ppm | m/z | z | RT | 1/k0 Start | 1/k0 End | Precursor ID | Area Sample 2 | #Feature | #Feature Sample 2 | Start | End | PTM | AScore | Found By |
| --- | --- | --- | --- | --- | --- | --- | --- | --- | --- | --- | --- | --- | --- | --- | --- | --- | --- | --- | --- |
| R.GYSFTTTAEREIVR.D | Y | 58.1 | 1628.8158 | 14 | 2.53 | 815.4128 | 2 | 15.79 | 1.121 | 1.1282 | 45442 | 6.41e3 | 1 | 1 | 101 | 114 |  |  | DB Search |
| total 1 peptides |  |  |  |  |  |  |  |  |  |  |  |  |  |  |  |  |  |  |  |
| --- | --- | --- | --- | --- | --- | --- | --- | --- | --- | --- | --- | --- | --- | --- | --- | --- | --- | --- | --- |

Best Unique PSM (Scan Sample\_11\_A1\_1\_3648.d:45442, m/z=815.4128, z=2, RT=15.79, ppm=-2.95):


TRINITY\_DN1172\_c0\_g2\_i2.p1

back to list

  

| Protein Coverage
| Supporting Peptides
| Best Unique PSM
|

Protein Coverage:

Supporting Peptides:

| Peptide | Uniq | -10lgP | Mass | Length | ppm | m/z | z | RT | 1/k0 Start | 1/k0 End | Precursor ID | Area Sample 2 | #Feature | #Feature Sample 2 | Start | End | PTM | AScore | Found By |
| --- | --- | --- | --- | --- | --- | --- | --- | --- | --- | --- | --- | --- | --- | --- | --- | --- | --- | --- | --- |
| K.VLTSSDPAEKEK.K | Y | 58.73 | 1302.6667 | 12 | -2.55 | 652.3354 | 2 | 10.56 | 0.9969 | 1.0041 | 16353 | 2.66e3 | 1 | 1 | 7 | 18 |  |  | DB Search |
| K.K(+42.01)LAAELANGR.L | Y | 57.57 | 1083.6036 | 10 | 2.65 | 542.8076 | 2 | 15.11 | 0.8668 | 0.8741 | 42049 | 7.44e2 | 1 | 1 | 19 | 28 | Acetylation (Protein N-term) | K1:Acetylation (Protein N-term):1000 | DB Search |
| total 2 peptides |  |  |  |  |  |  |  |  |  |  |  |  |  |  |  |  |  |  |  |
| --- | --- | --- | --- | --- | --- | --- | --- | --- | --- | --- | --- | --- | --- | --- | --- | --- | --- | --- | --- |

Best Unique PSM (Scan Sample\_11\_A1\_1\_3648.d:16353, m/z=652.3354, z=2, RT=10.56, ppm=-8.03):


TRINITY\_DN4595\_c0\_g1\_i4.p1

back to list

  

| Protein Coverage
| Supporting Peptides
| Best Unique PSM
|

Protein Coverage:

Supporting Peptides:

| Peptide | Uniq | -10lgP | Mass | Length | ppm | m/z | z | RT | 1/k0 Start | 1/k0 End | Precursor ID | Area Sample 2 | #Feature | #Feature Sample 2 | Start | End | PTM | AScore | Found By |
| --- | --- | --- | --- | --- | --- | --- | --- | --- | --- | --- | --- | --- | --- | --- | --- | --- | --- | --- | --- |
| R.AFNRVETR.D | Y | 25.84 | 991.5199 | 8 | -0.1 | 496.7645 | 2 | 10.70 | 0.8477 | 0.855 | 17302 | 3.57e3 | 1 | 1 | 282 | 289 |  |  | DB Search |
| total 1 peptides |  |  |  |  |  |  |  |  |  |  |  |  |  |  |  |  |  |  |  |
| --- | --- | --- | --- | --- | --- | --- | --- | --- | --- | --- | --- | --- | --- | --- | --- | --- | --- | --- | --- |

Best Unique PSM (Scan Sample\_11\_A1\_1\_3648.d:17302, m/z=496.7645, z=2, RT=10.70, ppm=-5.58):


TRINITY\_DN15599\_c0\_g1\_i2.p1

back to list

  

| Protein Coverage
| Supporting Peptides
| Best Unique PSM
|

Protein Coverage:

Supporting Peptides:

| Peptide | Uniq | -10lgP | Mass | Length | ppm | m/z | z | RT | 1/k0 Start | 1/k0 End | Precursor ID | Area Sample 2 | #Feature | #Feature Sample 2 | Start | End | PTM | AScore | Found By |
| --- | --- | --- | --- | --- | --- | --- | --- | --- | --- | --- | --- | --- | --- | --- | --- | --- | --- | --- | --- |
| K.KLQAELANGR.L | Y | 58.46 | 1098.6145 | 10 | 6.54 | 550.3151 | 2 | 11.42 | 0.8907 | 0.898 | 21463 | 3.82e3 | 1 | 1 | 184 | 193 |  |  | DB Search |
| total 1 peptides |  |  |  |  |  |  |  |  |  |  |  |  |  |  |  |  |  |  |  |
| --- | --- | --- | --- | --- | --- | --- | --- | --- | --- | --- | --- | --- | --- | --- | --- | --- | --- | --- | --- |

Best Unique PSM (Scan Sample\_11\_A1\_1\_3648.d:21463, m/z=550.3151, z=2, RT=11.42, ppm=1.06):


TRINITY\_DN79\_c0\_g1\_i1.p1

back to list

  

| Protein Coverage
| Supporting Peptides
| Best Unique PSM
|

Protein Coverage:

Supporting Peptides:

| Peptide | Uniq | -10lgP | Mass | Length | ppm | m/z | z | RT | 1/k0 Start | 1/k0 End | Precursor ID | Area Sample 2 | #Feature | #Feature Sample 2 | Start | End | PTM | AScore | Found By |
| --- | --- | --- | --- | --- | --- | --- | --- | --- | --- | --- | --- | --- | --- | --- | --- | --- | --- | --- | --- |
| K.IFGGDIKNHLLLFISK.K | Y | 58.85 | 1814.0454 | 16 | -0.66 | 605.6854 | 3 | 19.80 | 0.8611 | 0.8684 | 66221 | 1.45e3 | 1 | 1 | 209 | 224 |  |  | DB Search |
| K.KAENFAELKNTFQEAAK.G | Y | 55.22 | 1937.9846 | 17 | 1.66 | 646.9997 | 3 | 15.88 | 0.9194 | 0.9267 | 45985 | 0 | 0 | 0 | 225 | 241 |  |  | DB Search |
| total 2 peptides |  |  |  |  |  |  |  |  |  |  |  |  |  |  |  |  |  |  |  |
| --- | --- | --- | --- | --- | --- | --- | --- | --- | --- | --- | --- | --- | --- | --- | --- | --- | --- | --- | --- |

Best Unique PSM (Scan Sample\_11\_A1\_1\_3648.d:66221, m/z=605.6854, z=3, RT=19.80, ppm=-6.13):


TRINITY\_DN98420\_c0\_g1\_i1.p1

back to list

  

| Protein Coverage
| Supporting Peptides
| Best Unique PSM
|

Protein Coverage:

Supporting Peptides:

| Peptide | Uniq | -10lgP | Mass | Length | ppm | m/z | z | RT | 1/k0 Start | 1/k0 End | Precursor ID | Area Sample 2 | #Feature | #Feature Sample 2 | Start | End | PTM | AScore | Found By |
| --- | --- | --- | --- | --- | --- | --- | --- | --- | --- | --- | --- | --- | --- | --- | --- | --- | --- | --- | --- |
| R.AVIFDRFR.G | Y | 58.8 | 1022.5661 | 8 | 1.91 | 512.2885 | 2 | 15.68 | 0.8704 | 0.8777 | 44863 | 2.49e3 | 1 | 1 | 48 | 55 |  |  | DB Search |
| total 1 peptides |  |  |  |  |  |  |  |  |  |  |  |  |  |  |  |  |  |  |  |
| --- | --- | --- | --- | --- | --- | --- | --- | --- | --- | --- | --- | --- | --- | --- | --- | --- | --- | --- | --- |

Best Unique PSM (Scan Sample\_11\_A1\_1\_3648.d:44863, m/z=512.2885, z=2, RT=15.68, ppm=-3.56):


TRINITY\_DN585\_c0\_g1\_i2.p1

back to list

  

| Protein Coverage
| Supporting Peptides
| Best Unique PSM
|

Protein Coverage:

Supporting Peptides:

| Peptide | Uniq | -10lgP | Mass | Length | ppm | m/z | z | RT | 1/k0 Start | 1/k0 End | Precursor ID | Area Sample 2 | #Feature | #Feature Sample 2 | Start | End | PTM | AScore | Found By |
| --- | --- | --- | --- | --- | --- | --- | --- | --- | --- | --- | --- | --- | --- | --- | --- | --- | --- | --- | --- |
| K.IQDKEGIPPDQQR.L | Y | 55.06 | 1522.7739 | 13 | 2.88 | 762.3923 | 2 | 11.00 | 1.0331 | 1.0403 | 18584 | 2.97e3 | 2 | 2 | 2 | 14 |  |  | DB Search |
| total 1 peptides |  |  |  |  |  |  |  |  |  |  |  |  |  |  |  |  |  |  |  |
| --- | --- | --- | --- | --- | --- | --- | --- | --- | --- | --- | --- | --- | --- | --- | --- | --- | --- | --- | --- |

Best Unique PSM (Scan Sample\_11\_A1\_1\_3648.d:18584, m/z=762.3923, z=2, RT=11.00, ppm=-2.59):


TRINITY\_DN36\_c0\_g1\_i8.p1

back to list

  

| Protein Coverage
| Supporting Peptides
| Best Unique PSM
|

Protein Coverage:

Supporting Peptides:

| Peptide | Uniq | -10lgP | Mass | Length | ppm | m/z | z | RT | 1/k0 Start | 1/k0 End | Precursor ID | Area Sample 2 | #Feature | #Feature Sample 2 | Start | End | PTM | AScore | Found By |
| --- | --- | --- | --- | --- | --- | --- | --- | --- | --- | --- | --- | --- | --- | --- | --- | --- | --- | --- | --- |
| R.LGGIEVTQGNC(+57.02)ARK.Y | Y | 58.27 | 1501.7671 | 14 | 1.48 | 751.8878 | 2 | 12.59 | 1.0616 | 1.0688 | 28229 | 7.18e2 | 1 | 1 | 58 | 71 | Carbamidomethylation |  | DB Search |
| K.FETQIATK.D | Y | 57.95 | 936.4916 | 8 | 1.49 | 937.4952 | 1 | 13.74 | 1.2208 | 1.228 | 34637 | 7.44e2 | 2 | 2 | 167 | 174 |  |  | DB Search |
| total 2 peptides |  |  |  |  |  |  |  |  |  |  |  |  |  |  |  |  |  |  |  |
| --- | --- | --- | --- | --- | --- | --- | --- | --- | --- | --- | --- | --- | --- | --- | --- | --- | --- | --- | --- |

Best Unique PSM (Scan Sample\_11\_A1\_1\_3648.d:28229, m/z=751.8878, z=2, RT=12.59, ppm=-4):


TRINITY\_DN55\_c0\_g1\_i1.p1

back to list

  

| Protein Coverage
| Supporting Peptides
| Best Unique PSM
|

Protein Coverage:

Supporting Peptides:

| Peptide | Uniq | -10lgP | Mass | Length | ppm | m/z | z | RT | 1/k0 Start | 1/k0 End | Precursor ID | Area Sample 2 | #Feature | #Feature Sample 2 | Start | End | PTM | AScore | Found By |
| --- | --- | --- | --- | --- | --- | --- | --- | --- | --- | --- | --- | --- | --- | --- | --- | --- | --- | --- | --- |
| K.ATC(+57.02)C(+57.02)EGDNC(+57.02)NAGGR.D | Y | 58.87 | 1540.5453 | 14 | 5.37 | 771.2798 | 2 | 9.36 | 0.9553 | 0.9626 | 10231 | 2.8e2 | 1 | 1 | 87 | 100 | Carbamidomethylation, Carbamidomethylation, Carbamidomethylation |  | DB Search |
| total 1 peptides |  |  |  |  |  |  |  |  |  |  |  |  |  |  |  |  |  |  |  |
| --- | --- | --- | --- | --- | --- | --- | --- | --- | --- | --- | --- | --- | --- | --- | --- | --- | --- | --- | --- |

Best Unique PSM (Scan Sample\_11\_A1\_1\_3648.d:10231, m/z=771.2798, z=2, RT=9.36, ppm=-0.11):


TRINITY\_DN1616\_c0\_g1\_i6.p1

back to list

  

| Protein Coverage
| Supporting Peptides
| Best Unique PSM
|

Protein Coverage:

Supporting Peptides:

| Peptide | Uniq | -10lgP | Mass | Length | ppm | m/z | z | RT | 1/k0 Start | 1/k0 End | Precursor ID | Area Sample 2 | #Feature | #Feature Sample 2 | Start | End | PTM | AScore | Found By |
| --- | --- | --- | --- | --- | --- | --- | --- | --- | --- | --- | --- | --- | --- | --- | --- | --- | --- | --- | --- |
| K.YVVMLQYYHDSDTAK.Y | Y | 58.58 | 1831.8451 | 15 | 5.67 | 916.93 | 2 | 16.32 | 1.1813 | 1.1885 | 48049 | 0 | 0 | 0 | 1071 | 1085 |  |  | DB Search |
| K.VVAEC(+57.02)NNGGGR.F | Y | 57.96 | 1131.5091 | 11 | -8.94 | 566.7537 | 2 | 9.41 | 0.8234 | 0.8307 | 10484 | 0 | 0 | 0 | 3736 | 3746 | Carbamidomethylation |  | DB Search |
| total 2 peptides |  |  |  |  |  |  |  |  |  |  |  |  |  |  |  |  |  |  |  |
| --- | --- | --- | --- | --- | --- | --- | --- | --- | --- | --- | --- | --- | --- | --- | --- | --- | --- | --- | --- |

Best Unique PSM (Scan Sample\_11\_A1\_1\_3648.d:48049, m/z=916.93, z=2, RT=16.32, ppm=0.2):


TRINITY\_DN265\_c0\_g1\_i7.p1

back to list

  

| Protein Coverage
| Supporting Peptides
| Best Unique PSM
|

Protein Coverage:

Supporting Peptides:

| Peptide | Uniq | -10lgP | Mass | Length | ppm | m/z | z | RT | 1/k0 Start | 1/k0 End | Precursor ID | Area Sample 2 | #Feature | #Feature Sample 2 | Start | End | PTM | AScore | Found By |
| --- | --- | --- | --- | --- | --- | --- | --- | --- | --- | --- | --- | --- | --- | --- | --- | --- | --- | --- | --- |
| R.LTHDFHTNKR.I | Y | 58.75 | 1267.6421 | 10 | 2.44 | 423.5534 | 3 | 9.64 | 0.7441 | 0.7514 | 11479 | 1.15e3 | 1 | 1 | 24 | 33 |  |  | DB Search |
| total 1 peptides |  |  |  |  |  |  |  |  |  |  |  |  |  |  |  |  |  |  |  |
| --- | --- | --- | --- | --- | --- | --- | --- | --- | --- | --- | --- | --- | --- | --- | --- | --- | --- | --- | --- |

Best Unique PSM (Scan Sample\_11\_A1\_1\_3648.d:11479, m/z=423.5534, z=3, RT=9.64, ppm=-3.04):


TRINITY\_DN195948\_c0\_g1\_i1.p1

back to list

  

| Protein Coverage
| Supporting Peptides
| Best Unique PSM
|

Protein Coverage:

Supporting Peptides:

| Peptide | Uniq | -10lgP | Mass | Length | ppm | m/z | z | RT | 1/k0 Start | 1/k0 End | Precursor ID | Area Sample 2 | #Feature | #Feature Sample 2 | Start | End | PTM | AScore | Found By |
| --- | --- | --- | --- | --- | --- | --- | --- | --- | --- | --- | --- | --- | --- | --- | --- | --- | --- | --- | --- |
| R.HVYLRPNVGVGAVR.K | Y | 52.14 | 1535.8684 | 14 | -2.59 | 512.9593 | 3 | 15.00 | 0.8307 | 0.838 | 41514 | 0 | 0 | 0 | 88 | 101 |  |  | DB Search |
| R.KVM(+15.99)QSLEGVK.I | Y | 46.14 | 1133.6114 | 10 | 3 | 567.8116 | 2 | 11.20 | 0.904 | 0.9113 | 20184 | 2.1e2 | 1 | 1 | 127 | 136 | Oxidation (M) | M3:Oxidation (M):1000 | DB Search |
| total 2 peptides |  |  |  |  |  |  |  |  |  |  |  |  |  |  |  |  |  |  |  |
| --- | --- | --- | --- | --- | --- | --- | --- | --- | --- | --- | --- | --- | --- | --- | --- | --- | --- | --- | --- |

Best Unique PSM (Scan Sample\_11\_A1\_1\_3648.d:41514, m/z=512.9593, z=3, RT=15.00, ppm=-8.07):


TRINITY\_DN2805\_c0\_g1\_i1.p1

back to list

  

| Protein Coverage
| Supporting Peptides
| Best Unique PSM
|

Protein Coverage:

Supporting Peptides:

| Peptide | Uniq | -10lgP | Mass | Length | ppm | m/z | z | RT | 1/k0 Start | 1/k0 End | Precursor ID | Area Sample 2 | #Feature | #Feature Sample 2 | Start | End | PTM | AScore | Found By |
| --- | --- | --- | --- | --- | --- | --- | --- | --- | --- | --- | --- | --- | --- | --- | --- | --- | --- | --- | --- |
| K.VVIIDGKGHLM(+15.99)GR.L | Y | 29.79 | 1409.7813 | 13 | 3.46 | 705.8965 | 2 | 12.92 | 1.023 | 1.0303 | 30102 | 0 | 0 | 0 | 6 | 18 | Oxidation (M) | M11:Oxidation (M):1000 | DB Search |
| total 1 peptides |  |  |  |  |  |  |  |  |  |  |  |  |  |  |  |  |  |  |  |
| --- | --- | --- | --- | --- | --- | --- | --- | --- | --- | --- | --- | --- | --- | --- | --- | --- | --- | --- | --- |

Best Unique PSM (Scan Sample\_11\_A1\_1\_3648.d:30102, m/z=705.8965, z=2, RT=12.92, ppm=-2.02):


TRINITY\_DN1283\_c0\_g1\_i1.p1

back to list

  

| Protein Coverage
| Supporting Peptides
| Best Unique PSM
|

Protein Coverage:

Supporting Peptides:

| Peptide | Uniq | -10lgP | Mass | Length | ppm | m/z | z | RT | 1/k0 Start | 1/k0 End | Precursor ID | Area Sample 2 | #Feature | #Feature Sample 2 | Start | End | PTM | AScore | Found By |
| --- | --- | --- | --- | --- | --- | --- | --- | --- | --- | --- | --- | --- | --- | --- | --- | --- | --- | --- | --- |
| K.LDISDEFRR.C | Y | 25.79 | 1149.5778 | 9 | -5.84 | 575.7897 | 2 | 15.31 | 0.9093 | 0.9166 | 43114 | 0 | 0 | 0 | 190 | 198 |  |  | DB Search |
| total 1 peptides |  |  |  |  |  |  |  |  |  |  |  |  |  |  |  |  |  |  |  |
| --- | --- | --- | --- | --- | --- | --- | --- | --- | --- | --- | --- | --- | --- | --- | --- | --- | --- | --- | --- |

Best Unique PSM (Scan Sample\_11\_A1\_1\_3648.d:43114, m/z=575.7897, z=2, RT=15.31, ppm=-11.32):


TRINITY\_DN761\_c0\_g1\_i3.p1

back to list

  

| Protein Coverage
| Supporting Peptides
| Best Unique PSM
|

Protein Coverage:

Supporting Peptides:

| Peptide | Uniq | -10lgP | Mass | Length | ppm | m/z | z | RT | 1/k0 Start | 1/k0 End | Precursor ID | Area Sample 2 | #Feature | #Feature Sample 2 | Start | End | PTM | AScore | Found By |
| --- | --- | --- | --- | --- | --- | --- | --- | --- | --- | --- | --- | --- | --- | --- | --- | --- | --- | --- | --- |
| K.TRIEDLETVISQTQDHR.H | Y | 55.76 | 2040.0236 | 17 | -2.78 | 681.0095 | 3 | 17.11 | 0.9558 | 0.963 | 52031 | 0 | 0 | 0 | 275 | 291 |  |  | DB Search |
| total 1 peptides |  |  |  |  |  |  |  |  |  |  |  |  |  |  |  |  |  |  |  |
| --- | --- | --- | --- | --- | --- | --- | --- | --- | --- | --- | --- | --- | --- | --- | --- | --- | --- | --- | --- |

Best Unique PSM (Scan Sample\_11\_A1\_1\_3648.d:52031, m/z=681.0095, z=3, RT=17.11, ppm=-8.25):


TRINITY\_DN982\_c0\_g1\_i1.p1

back to list

  

| Protein Coverage
| Supporting Peptides
| Best Unique PSM
|

Protein Coverage:

Supporting Peptides:

| Peptide | Uniq | -10lgP | Mass | Length | ppm | m/z | z | RT | 1/k0 Start | 1/k0 End | Precursor ID | Area Sample 2 | #Feature | #Feature Sample 2 | Start | End | PTM | AScore | Found By |
| --- | --- | --- | --- | --- | --- | --- | --- | --- | --- | --- | --- | --- | --- | --- | --- | --- | --- | --- | --- |
| R.LVQNTPHNSIVTK.I | N | 58.89 | 1449.794 | 13 | 3.18 | 725.9026 | 2 | 11.65 | 1.0628 | 1.0701 | 22758 | 1.18e3 | 1 | 1 | 5129 | 5141 |  |  | DB Search |
| K.VLLSQQIFYDKK.N | N | 26.23 | 1480.8289 | 12 | -0.12 | 741.4176 | 2 | 16.48 | 1.0608 | 1.068 | 48785 | 6.44e2 | 1 | 1 | 1479 | 1490 |  |  | DB Search |
| K.VLEVNDNKPK.F | Y | 26.02 | 1154.6295 | 10 | -0.56 | 578.3185 | 2 | 10.96 | 0.9234 | 0.9307 | 18881 | 0 | 0 | 0 | 459 | 468 |  |  | DB Search |
| total 3 peptides |  |  |  |  |  |  |  |  |  |  |  |  |  |  |  |  |  |  |  |
| --- | --- | --- | --- | --- | --- | --- | --- | --- | --- | --- | --- | --- | --- | --- | --- | --- | --- | --- | --- |

Best Unique PSM (Scan Sample\_11\_A1\_1\_3648.d:18881, m/z=578.3185, z=2, RT=10.96, ppm=-6.04):


TRINITY\_DN2230\_c0\_g1\_i1.p1

back to list

  

| Protein Coverage
| Supporting Peptides
| Best Unique PSM
|

Protein Coverage:

Supporting Peptides:

| Peptide | Uniq | -10lgP | Mass | Length | ppm | m/z | z | RT | 1/k0 Start | 1/k0 End | Precursor ID | Area Sample 2 | #Feature | #Feature Sample 2 | Start | End | PTM | AScore | Found By |
| --- | --- | --- | --- | --- | --- | --- | --- | --- | --- | --- | --- | --- | --- | --- | --- | --- | --- | --- | --- |
| K.KGDNEIPGLTDRVIPR.R | Y | 55.53 | 1778.9638 | 16 | 1.69 | 593.993 | 3 | 15.42 | 0.8955 | 0.9028 | 43603 | 3.5e2 | 1 | 1 | 116 | 131 |  |  | DB Search |
| total 1 peptides |  |  |  |  |  |  |  |  |  |  |  |  |  |  |  |  |  |  |  |
| --- | --- | --- | --- | --- | --- | --- | --- | --- | --- | --- | --- | --- | --- | --- | --- | --- | --- | --- | --- |

Best Unique PSM (Scan Sample\_11\_A1\_1\_3648.d:43603, m/z=593.993, z=3, RT=15.42, ppm=-3.78):


TRINITY\_DN1583\_c0\_g1\_i1.p1

back to list

  

| Protein Coverage
| Supporting Peptides
| Best Unique PSM
|

Protein Coverage:

Supporting Peptides:

| Peptide | Uniq | -10lgP | Mass | Length | ppm | m/z | z | RT | 1/k0 Start | 1/k0 End | Precursor ID | Area Sample 2 | #Feature | #Feature Sample 2 | Start | End | PTM | AScore | Found By |
| --- | --- | --- | --- | --- | --- | --- | --- | --- | --- | --- | --- | --- | --- | --- | --- | --- | --- | --- | --- |
| K.ADGIHIINLRK.T | Y | 43.17 | 1248.7302 | 11 | 2.43 | 625.3705 | 2 | 13.40 | 0.9646 | 0.9719 | 32870 | 0 | 0 | 0 | 43 | 53 |  |  | DB Search |
| total 1 peptides |  |  |  |  |  |  |  |  |  |  |  |  |  |  |  |  |  |  |  |
| --- | --- | --- | --- | --- | --- | --- | --- | --- | --- | --- | --- | --- | --- | --- | --- | --- | --- | --- | --- |

Best Unique PSM (Scan Sample\_11\_A1\_1\_3648.d:32870, m/z=625.3705, z=2, RT=13.40, ppm=-3.05):


TRINITY\_DN333\_c0\_g1\_i2.p1

back to list

  

| Protein Coverage
| Supporting Peptides
| Best Unique PSM
|

Protein Coverage:

Supporting Peptides:

| Peptide | Uniq | -10lgP | Mass | Length | ppm | m/z | z | RT | 1/k0 Start | 1/k0 End | Precursor ID | Area Sample 2 | #Feature | #Feature Sample 2 | Start | End | PTM | AScore | Found By |
| --- | --- | --- | --- | --- | --- | --- | --- | --- | --- | --- | --- | --- | --- | --- | --- | --- | --- | --- | --- |
| R.RRYPIIVTGR.L | Y | 56.14 | 1229.7356 | 10 | 4.66 | 410.9188 | 3 | 12.79 | 0.777 | 0.7844 | 29251 | 7.87e2 | 1 | 1 | 286 | 295 |  |  | DB Search |
| total 1 peptides |  |  |  |  |  |  |  |  |  |  |  |  |  |  |  |  |  |  |  |
| --- | --- | --- | --- | --- | --- | --- | --- | --- | --- | --- | --- | --- | --- | --- | --- | --- | --- | --- | --- |

Best Unique PSM (Scan Sample\_11\_A1\_1\_3648.d:29251, m/z=410.9188, z=3, RT=12.79, ppm=-0.82):


TRINITY\_DN6701\_c0\_g1\_i2.p2

back to list

  

| Protein Coverage
| Supporting Peptides
| Best Unique PSM
|

Protein Coverage:

Supporting Peptides:

| Peptide | Uniq | -10lgP | Mass | Length | ppm | m/z | z | RT | 1/k0 Start | 1/k0 End | Precursor ID | Area Sample 2 | #Feature | #Feature Sample 2 | Start | End | PTM | AScore | Found By |
| --- | --- | --- | --- | --- | --- | --- | --- | --- | --- | --- | --- | --- | --- | --- | --- | --- | --- | --- | --- |
| K.VITSDDPAEKEK.K | Y | 58.87 | 1330.6616 | 12 | 2.64 | 666.3362 | 2 | 10.69 | 0.9848 | 0.992 | 17191 | 9.01e3 | 1 | 1 | 114 | 125 |  |  | DB Search |
| total 1 peptides |  |  |  |  |  |  |  |  |  |  |  |  |  |  |  |  |  |  |  |
| --- | --- | --- | --- | --- | --- | --- | --- | --- | --- | --- | --- | --- | --- | --- | --- | --- | --- | --- | --- |

Best Unique PSM (Scan Sample\_11\_A1\_1\_3648.d:17191, m/z=666.3362, z=2, RT=10.69, ppm=-2.84):


TRINITY\_DN4595\_c0\_g1\_i7.p1

back to list

  

| Protein Coverage
| Supporting Peptides
| Best Unique PSM
|

Protein Coverage:

Supporting Peptides:

| Peptide | Uniq | -10lgP | Mass | Length | ppm | m/z | z | RT | 1/k0 Start | 1/k0 End | Precursor ID | Area Sample 2 | #Feature | #Feature Sample 2 | Start | End | PTM | AScore | Found By |
| --- | --- | --- | --- | --- | --- | --- | --- | --- | --- | --- | --- | --- | --- | --- | --- | --- | --- | --- | --- |
| R.AFNRVETR.D | Y | 25.84 | 991.5199 | 8 | -0.1 | 496.7645 | 2 | 10.70 | 0.8477 | 0.855 | 17302 | 3.57e3 | 1 | 1 | 282 | 289 |  |  | DB Search |
| total 1 peptides |  |  |  |  |  |  |  |  |  |  |  |  |  |  |  |  |  |  |  |
| --- | --- | --- | --- | --- | --- | --- | --- | --- | --- | --- | --- | --- | --- | --- | --- | --- | --- | --- | --- |

Best Unique PSM (Scan Sample\_11\_A1\_1\_3648.d:17302, m/z=496.7645, z=2, RT=10.70, ppm=-5.58):


TRINITY\_DN6294\_c0\_g3\_i1.p1

back to list

  

| Protein Coverage
| Supporting Peptides
| Best Unique PSM
|

Protein Coverage:

Supporting Peptides:

| Peptide | Uniq | -10lgP | Mass | Length | ppm | m/z | z | RT | 1/k0 Start | 1/k0 End | Precursor ID | Area Sample 2 | #Feature | #Feature Sample 2 | Start | End | PTM | AScore | Found By |
| --- | --- | --- | --- | --- | --- | --- | --- | --- | --- | --- | --- | --- | --- | --- | --- | --- | --- | --- | --- |
| K.LVVIADIVDQKR.L | Y | 57.66 | 1367.8136 | 12 | 0.99 | 684.911 | 2 | 17.26 | 1.0419 | 1.0492 | 52731 | 1.74e3 | 1 | 1 | 24 | 35 |  |  | DB Search |
| total 1 peptides |  |  |  |  |  |  |  |  |  |  |  |  |  |  |  |  |  |  |  |
| --- | --- | --- | --- | --- | --- | --- | --- | --- | --- | --- | --- | --- | --- | --- | --- | --- | --- | --- | --- |

Best Unique PSM (Scan Sample\_11\_A1\_1\_3648.d:52731, m/z=684.911, z=2, RT=17.26, ppm=-4.49):


TRINITY\_DN2301\_c0\_g1\_i1.p1

back to list

  

| Protein Coverage
| Supporting Peptides
| Best Unique PSM
|

Protein Coverage:

Supporting Peptides:

| Peptide | Uniq | -10lgP | Mass | Length | ppm | m/z | z | RT | 1/k0 Start | 1/k0 End | Precursor ID | Area Sample 2 | #Feature | #Feature Sample 2 | Start | End | PTM | AScore | Found By |
| --- | --- | --- | --- | --- | --- | --- | --- | --- | --- | --- | --- | --- | --- | --- | --- | --- | --- | --- | --- |
| R.VGYTHLFSSTQVSVRPK.Q | Y | 57.32 | 1905.0108 | 17 | 9.78 | 953.5168 | 2 | 15.02 | 1.2479 | 1.2551 | 41579 | 9.02e1 | 1 | 1 | 390 | 406 |  |  | DB Search |
| total 1 peptides |  |  |  |  |  |  |  |  |  |  |  |  |  |  |  |  |  |  |  |
| --- | --- | --- | --- | --- | --- | --- | --- | --- | --- | --- | --- | --- | --- | --- | --- | --- | --- | --- | --- |

Best Unique PSM (Scan Sample\_11\_A1\_1\_3648.d:41579, m/z=953.5168, z=2, RT=15.02, ppm=4.3):


TRINITY\_DN20323\_c0\_g1\_i1.p1

back to list

  

| Protein Coverage
| Supporting Peptides
| Best Unique PSM
|

Protein Coverage:

Supporting Peptides:

| Peptide | Uniq | -10lgP | Mass | Length | ppm | m/z | z | RT | 1/k0 Start | 1/k0 End | Precursor ID | Area Sample 2 | #Feature | #Feature Sample 2 | Start | End | PTM | AScore | Found By |
| --- | --- | --- | --- | --- | --- | --- | --- | --- | --- | --- | --- | --- | --- | --- | --- | --- | --- | --- | --- |
| R.SGTLTYEAVHQTTK.V | Y | 57.82 | 1534.7627 | 14 | 1.94 | 768.3859 | 2 | 12.71 | 1.0701 | 1.0773 | 28882 | 4.08e2 | 1 | 1 | 193 | 206 |  |  | DB Search |
| total 1 peptides |  |  |  |  |  |  |  |  |  |  |  |  |  |  |  |  |  |  |  |
| --- | --- | --- | --- | --- | --- | --- | --- | --- | --- | --- | --- | --- | --- | --- | --- | --- | --- | --- | --- |

Best Unique PSM (Scan Sample\_11\_A1\_1\_3648.d:28882, m/z=768.3859, z=2, RT=12.71, ppm=-3.53):


TRINITY\_DN1933\_c0\_g1\_i7.p1

back to list

  

| Protein Coverage
| Supporting Peptides
| Best Unique PSM
|

Protein Coverage:

Supporting Peptides:

| Peptide | Uniq | -10lgP | Mass | Length | ppm | m/z | z | RT | 1/k0 Start | 1/k0 End | Precursor ID | Area Sample 2 | #Feature | #Feature Sample 2 | Start | End | PTM | AScore | Found By |
| --- | --- | --- | --- | --- | --- | --- | --- | --- | --- | --- | --- | --- | --- | --- | --- | --- | --- | --- | --- |
| R.RNLDIERPTYTNLNR.L | Y | 46.58 | 1873.9758 | 15 | -2.43 | 625.6609 | 3 | 13.94 | 0.8858 | 0.8931 | 35770 | 1.17e3 | 1 | 1 | 75 | 89 |  |  | DB Search |
| total 1 peptides |  |  |  |  |  |  |  |  |  |  |  |  |  |  |  |  |  |  |  |
| --- | --- | --- | --- | --- | --- | --- | --- | --- | --- | --- | --- | --- | --- | --- | --- | --- | --- | --- | --- |

Best Unique PSM (Scan Sample\_11\_A1\_1\_3648.d:35770, m/z=625.6609, z=3, RT=13.94, ppm=-7.91):


TRINITY\_DN471\_c0\_g1\_i10.p1

back to list

  

| Protein Coverage
| Supporting Peptides
| Best Unique PSM
|

Protein Coverage:

Supporting Peptides:

| Peptide | Uniq | -10lgP | Mass | Length | ppm | m/z | z | RT | 1/k0 Start | 1/k0 End | Precursor ID | Area Sample 2 | #Feature | #Feature Sample 2 | Start | End | PTM | AScore | Found By |
| --- | --- | --- | --- | --- | --- | --- | --- | --- | --- | --- | --- | --- | --- | --- | --- | --- | --- | --- | --- |
| R.TIQFVDWC(+57.02)PTGFK.C | Y | 54.82 | 1597.7599 | 13 | -6.53 | 799.8776 | 2 | 19.88 | 1.0351 | 1.0423 | 66701 | 3.31e2 | 1 | 1 | 378 | 390 | Carbamidomethylation |  | DB Search |
| R.RNLDIERPTYTNLNR.L | Y | 46.58 | 1873.9758 | 15 | -2.43 | 625.6609 | 3 | 13.94 | 0.8858 | 0.8931 | 35770 | 1.17e3 | 1 | 1 | 253 | 267 |  |  | DB Search |
| total 2 peptides |  |  |  |  |  |  |  |  |  |  |  |  |  |  |  |  |  |  |  |
| --- | --- | --- | --- | --- | --- | --- | --- | --- | --- | --- | --- | --- | --- | --- | --- | --- | --- | --- | --- |

Best Unique PSM (Scan Sample\_11\_A1\_1\_3648.d:66701, m/z=799.3823, z=2, RT=19.90, ppm=-12):


TRINITY\_DN649\_c0\_g1\_i1.p1

back to list

  

| Protein Coverage
| Supporting Peptides
| Best Unique PSM
|

Protein Coverage:

Supporting Peptides:

| Peptide | Uniq | -10lgP | Mass | Length | ppm | m/z | z | RT | 1/k0 Start | 1/k0 End | Precursor ID | Area Sample 2 | #Feature | #Feature Sample 2 | Start | End | PTM | AScore | Found By |
| --- | --- | --- | --- | --- | --- | --- | --- | --- | --- | --- | --- | --- | --- | --- | --- | --- | --- | --- | --- |
| K.FETQIATK.N | Y | 57.95 | 936.4916 | 8 | 1.49 | 937.4952 | 1 | 13.74 | 1.2208 | 1.228 | 34637 | 7.44e2 | 2 | 2 | 102 | 109 |  |  | DB Search |
| total 1 peptides |  |  |  |  |  |  |  |  |  |  |  |  |  |  |  |  |  |  |  |
| --- | --- | --- | --- | --- | --- | --- | --- | --- | --- | --- | --- | --- | --- | --- | --- | --- | --- | --- | --- |

Best Unique PSM (Scan Sample\_11\_A1\_1\_3648.d:34637, m/z=937.4952, z=1, RT=13.74, ppm=-3.98):


TRINITY\_DN6701\_c0\_g1\_i1.p1

back to list

  

| Protein Coverage
| Supporting Peptides
| Best Unique PSM
|

Protein Coverage:

Supporting Peptides:

| Peptide | Uniq | -10lgP | Mass | Length | ppm | m/z | z | RT | 1/k0 Start | 1/k0 End | Precursor ID | Area Sample 2 | #Feature | #Feature Sample 2 | Start | End | PTM | AScore | Found By |
| --- | --- | --- | --- | --- | --- | --- | --- | --- | --- | --- | --- | --- | --- | --- | --- | --- | --- | --- | --- |
| K.VITSDDPAEKEK.K | Y | 58.87 | 1330.6616 | 12 | 2.64 | 666.3362 | 2 | 10.69 | 0.9848 | 0.992 | 17191 | 9.01e3 | 1 | 1 | 158 | 169 |  |  | DB Search |
| total 1 peptides |  |  |  |  |  |  |  |  |  |  |  |  |  |  |  |  |  |  |  |
| --- | --- | --- | --- | --- | --- | --- | --- | --- | --- | --- | --- | --- | --- | --- | --- | --- | --- | --- | --- |

Best Unique PSM (Scan Sample\_11\_A1\_1\_3648.d:17191, m/z=666.3362, z=2, RT=10.69, ppm=-2.84):


TRINITY\_DN1505\_c0\_g1\_i1.p1

back to list

  

| Protein Coverage
| Supporting Peptides
| Best Unique PSM
|

Protein Coverage:

Supporting Peptides:

| Peptide | Uniq | -10lgP | Mass | Length | ppm | m/z | z | RT | 1/k0 Start | 1/k0 End | Precursor ID | Area Sample 2 | #Feature | #Feature Sample 2 | Start | End | PTM | AScore | Found By |
| --- | --- | --- | --- | --- | --- | --- | --- | --- | --- | --- | --- | --- | --- | --- | --- | --- | --- | --- | --- |
| M.S(+42.01)LVIPEKFQHILR.V | Y | 58.87 | 1620.9351 | 13 | -2.12 | 541.3149 | 3 | 19.23 | 0.8368 | 0.8441 | 63378 | 4.49e4 | 2 | 2 | 2 | 14 | Acetylation (Protein N-term) | S1:Acetylation (Protein N-term):1000 | DB Search |
| total 1 peptides |  |  |  |  |  |  |  |  |  |  |  |  |  |  |  |  |  |  |  |
| --- | --- | --- | --- | --- | --- | --- | --- | --- | --- | --- | --- | --- | --- | --- | --- | --- | --- | --- | --- |

Best Unique PSM (Scan Sample\_11\_A1\_1\_3648.d:63378, m/z=541.3149, z=3, RT=19.23, ppm=-7.6):


TRINITY\_DN119673\_c0\_g1\_i1.p1

back to list

  

| Protein Coverage
| Supporting Peptides
| Best Unique PSM
|

Protein Coverage:

Supporting Peptides:

| Peptide | Uniq | -10lgP | Mass | Length | ppm | m/z | z | RT | 1/k0 Start | 1/k0 End | Precursor ID | Area Sample 2 | #Feature | #Feature Sample 2 | Start | End | PTM | AScore | Found By |
| --- | --- | --- | --- | --- | --- | --- | --- | --- | --- | --- | --- | --- | --- | --- | --- | --- | --- | --- | --- |
| K.VLTSDDPAEKEK | Y | 58.87 | 1330.6616 | 12 | 2.64 | 666.3362 | 2 | 10.69 | 0.9848 | 0.992 | 17191 | 9.01e3 | 1 | 1 | 117 | 128 |  |  | DB Search |
| total 1 peptides |  |  |  |  |  |  |  |  |  |  |  |  |  |  |  |  |  |  |  |
| --- | --- | --- | --- | --- | --- | --- | --- | --- | --- | --- | --- | --- | --- | --- | --- | --- | --- | --- | --- |

Best Unique PSM (Scan Sample\_11\_A1\_1\_3648.d:17191, m/z=666.3362, z=2, RT=10.69, ppm=-2.84):


TRINITY\_DN36\_c0\_g1\_i11.p1

back to list

  

| Protein Coverage
| Supporting Peptides
| Best Unique PSM
|

Protein Coverage:

Supporting Peptides:

| Peptide | Uniq | -10lgP | Mass | Length | ppm | m/z | z | RT | 1/k0 Start | 1/k0 End | Precursor ID | Area Sample 2 | #Feature | #Feature Sample 2 | Start | End | PTM | AScore | Found By |
| --- | --- | --- | --- | --- | --- | --- | --- | --- | --- | --- | --- | --- | --- | --- | --- | --- | --- | --- | --- |
| R.LGGIEVTQGNC(+57.02)ARK.Y | Y | 58.27 | 1501.7671 | 14 | 1.48 | 751.8878 | 2 | 12.59 | 1.0616 | 1.0688 | 28229 | 7.18e2 | 1 | 1 | 58 | 71 | Carbamidomethylation |  | DB Search |
| total 1 peptides |  |  |  |  |  |  |  |  |  |  |  |  |  |  |  |  |  |  |  |
| --- | --- | --- | --- | --- | --- | --- | --- | --- | --- | --- | --- | --- | --- | --- | --- | --- | --- | --- | --- |

Best Unique PSM (Scan Sample\_11\_A1\_1\_3648.d:28229, m/z=751.8878, z=2, RT=12.59, ppm=-4):


TRINITY\_DN6397\_c0\_g2\_i4.p1

back to list

  

| Protein Coverage
| Supporting Peptides
| Best Unique PSM
|

Protein Coverage:

Supporting Peptides:

| Peptide | Uniq | -10lgP | Mass | Length | ppm | m/z | z | RT | 1/k0 Start | 1/k0 End | Precursor ID | Area Sample 2 | #Feature | #Feature Sample 2 | Start | End | PTM | AScore | Found By |
| --- | --- | --- | --- | --- | --- | --- | --- | --- | --- | --- | --- | --- | --- | --- | --- | --- | --- | --- | --- |
| K.K(+42.01)LSAEVANGR.L | Y | 58.78 | 1085.5829 | 10 | 5.56 | 543.7988 | 2 | 12.85 | 0.8538 | 0.8611 | 29679 | 1.02e3 | 1 | 1 | 12 | 21 | Acetylation (Protein N-term) | K1:Acetylation (Protein N-term):1000 | DB Search |
| total 1 peptides |  |  |  |  |  |  |  |  |  |  |  |  |  |  |  |  |  |  |  |
| --- | --- | --- | --- | --- | --- | --- | --- | --- | --- | --- | --- | --- | --- | --- | --- | --- | --- | --- | --- |

Best Unique PSM (Scan Sample\_11\_A1\_1\_3648.d:29679, m/z=543.7988, z=2, RT=12.85, ppm=0.08):


TRINITY\_DN1080\_c0\_g1\_i2.p1

back to list

  

| Protein Coverage
| Supporting Peptides
| Best Unique PSM
|

Protein Coverage:

Supporting Peptides:

| Peptide | Uniq | -10lgP | Mass | Length | ppm | m/z | z | RT | 1/k0 Start | 1/k0 End | Precursor ID | Area Sample 2 | #Feature | #Feature Sample 2 | Start | End | PTM | AScore | Found By |
| --- | --- | --- | --- | --- | --- | --- | --- | --- | --- | --- | --- | --- | --- | --- | --- | --- | --- | --- | --- |
| R.RPVPIVTRR.R | Y | 57.63 | 1092.688 | 9 | 6 | 365.2368 | 3 | 10.84 | 0.7045 | 0.7118 | 18035 | 1.06e3 | 1 | 1 | 281 | 289 |  |  | DB Search |
| R.RSITTSVKR.V | Y | 55.92 | 1046.6196 | 9 | 6.21 | 349.8807 | 3 | 9.78 | 0.7037 | 0.711 | 12172 | 0 | 0 | 0 | 193 | 201 |  |  | DB Search |
| total 2 peptides |  |  |  |  |  |  |  |  |  |  |  |  |  |  |  |  |  |  |  |
| --- | --- | --- | --- | --- | --- | --- | --- | --- | --- | --- | --- | --- | --- | --- | --- | --- | --- | --- | --- |

Best Unique PSM (Scan Sample\_11\_A1\_1\_3648.d:18035, m/z=365.2368, z=3, RT=10.84, ppm=0.51):


TRINITY\_DN384\_c0\_g1\_i1.p1

back to list

  

| Protein Coverage
| Supporting Peptides
| Best Unique PSM
|

Protein Coverage:

Supporting Peptides:

| Peptide | Uniq | -10lgP | Mass | Length | ppm | m/z | z | RT | 1/k0 Start | 1/k0 End | Precursor ID | Area Sample 2 | #Feature | #Feature Sample 2 | Start | End | PTM | AScore | Found By |
| --- | --- | --- | --- | --- | --- | --- | --- | --- | --- | --- | --- | --- | --- | --- | --- | --- | --- | --- | --- |
| K.FVLHGDVSHGSR.E | Y | 57.97 | 1309.6527 | 12 | -8.24 | 655.8246 | 2 | 11.96 | 0.9872 | 0.9945 | 24686 | 0 | 0 | 0 | 274 | 285 |  |  | DB Search |
| total 1 peptides |  |  |  |  |  |  |  |  |  |  |  |  |  |  |  |  |  |  |  |
| --- | --- | --- | --- | --- | --- | --- | --- | --- | --- | --- | --- | --- | --- | --- | --- | --- | --- | --- | --- |

Best Unique PSM (Scan Sample\_11\_A1\_1\_3648.d:24686, m/z=655.8246, z=2, RT=11.96, ppm=-13.71):


TRINITY\_DN36\_c0\_g1\_i19.p1

back to list

  

| Protein Coverage
| Supporting Peptides
| Best Unique PSM
|

Protein Coverage:

Supporting Peptides:

| Peptide | Uniq | -10lgP | Mass | Length | ppm | m/z | z | RT | 1/k0 Start | 1/k0 End | Precursor ID | Area Sample 2 | #Feature | #Feature Sample 2 | Start | End | PTM | AScore | Found By |
| --- | --- | --- | --- | --- | --- | --- | --- | --- | --- | --- | --- | --- | --- | --- | --- | --- | --- | --- | --- |
| K.GFDYC(+57.02)TTIKYR.L | Y | 58.87 | 1422.6602 | 11 | 2.87 | 712.3355 | 2 | 14.94 | 1.0242 | 1.0315 | 41159 | 9.5e2 | 1 | 1 | 46 | 56 | Carbamidomethylation |  | DB Search |
| R.LGGIEVTQGNC(+57.02)ARK.Y | Y | 58.27 | 1501.7671 | 14 | 1.48 | 751.8878 | 2 | 12.59 | 1.0616 | 1.0688 | 28229 | 7.18e2 | 1 | 1 | 57 | 70 | Carbamidomethylation |  | DB Search |
| K.FETQIATK.D | Y | 57.95 | 936.4916 | 8 | 1.49 | 937.4952 | 1 | 13.74 | 1.2208 | 1.228 | 34637 | 7.44e2 | 2 | 2 | 166 | 173 |  |  | DB Search |
| total 3 peptides |  |  |  |  |  |  |  |  |  |  |  |  |  |  |  |  |  |  |  |
| --- | --- | --- | --- | --- | --- | --- | --- | --- | --- | --- | --- | --- | --- | --- | --- | --- | --- | --- | --- |

Best Unique PSM (Scan Sample\_11\_A1\_1\_3648.d:41159, m/z=712.3355, z=2, RT=14.94, ppm=-2.6):


TRINITY\_DN36\_c0\_g1\_i5.p1

back to list

  

| Protein Coverage
| Supporting Peptides
| Best Unique PSM
|

Protein Coverage:

Supporting Peptides:

| Peptide | Uniq | -10lgP | Mass | Length | ppm | m/z | z | RT | 1/k0 Start | 1/k0 End | Precursor ID | Area Sample 2 | #Feature | #Feature Sample 2 | Start | End | PTM | AScore | Found By |
| --- | --- | --- | --- | --- | --- | --- | --- | --- | --- | --- | --- | --- | --- | --- | --- | --- | --- | --- | --- |
| K.GFDYC(+57.02)TTIKYR.L | Y | 58.87 | 1422.6602 | 11 | 2.87 | 712.3355 | 2 | 14.94 | 1.0242 | 1.0315 | 41159 | 9.5e2 | 1 | 1 | 46 | 56 | Carbamidomethylation |  | DB Search |
| R.LGGIEVTQGNC(+57.02)ARK.Y | Y | 58.27 | 1501.7671 | 14 | 1.48 | 751.8878 | 2 | 12.59 | 1.0616 | 1.0688 | 28229 | 7.18e2 | 1 | 1 | 57 | 70 | Carbamidomethylation |  | DB Search |
| total 2 peptides |  |  |  |  |  |  |  |  |  |  |  |  |  |  |  |  |  |  |  |
| --- | --- | --- | --- | --- | --- | --- | --- | --- | --- | --- | --- | --- | --- | --- | --- | --- | --- | --- | --- |

Best Unique PSM (Scan Sample\_11\_A1\_1\_3648.d:41159, m/z=712.3355, z=2, RT=14.94, ppm=-2.6):


TRINITY\_DN7214\_c0\_g1\_i1.p1

back to list

  

| Protein Coverage
| Supporting Peptides
| Best Unique PSM
|

Protein Coverage:

Supporting Peptides:

| Peptide | Uniq | -10lgP | Mass | Length | ppm | m/z | z | RT | 1/k0 Start | 1/k0 End | Precursor ID | Area Sample 2 | #Feature | #Feature Sample 2 | Start | End | PTM | AScore | Found By |
| --- | --- | --- | --- | --- | --- | --- | --- | --- | --- | --- | --- | --- | --- | --- | --- | --- | --- | --- | --- |
| R.LAADLVITHSSK.K | Y | 57.93 | 1253.6979 | 12 | -0.91 | 627.8522 | 2 | 14.11 | 0.9969 | 1.0041 | 36628 | 1.76e3 | 1 | 1 | 167 | 178 |  |  | DB Search |
| total 1 peptides |  |  |  |  |  |  |  |  |  |  |  |  |  |  |  |  |  |  |  |
| --- | --- | --- | --- | --- | --- | --- | --- | --- | --- | --- | --- | --- | --- | --- | --- | --- | --- | --- | --- |

Best Unique PSM (Scan Sample\_11\_A1\_1\_3648.d:36628, m/z=627.8522, z=2, RT=14.11, ppm=-6.38):


TRINITY\_DN95987\_c0\_g1\_i1.p1

back to list

  

| Protein Coverage
| Supporting Peptides
| Best Unique PSM
|

Protein Coverage:

Supporting Peptides:

| Peptide | Uniq | -10lgP | Mass | Length | ppm | m/z | z | RT | 1/k0 Start | 1/k0 End | Precursor ID | Area Sample 2 | #Feature | #Feature Sample 2 | Start | End | PTM | AScore | Found By |
| --- | --- | --- | --- | --- | --- | --- | --- | --- | --- | --- | --- | --- | --- | --- | --- | --- | --- | --- | --- |
| K.LISSDGFEFIVKR.E | Y | 58.16 | 1509.8191 | 13 | 0.29 | 755.9129 | 2 | 18.04 | 1.0969 | 1.1041 | 57047 | 2.11e2 | 1 | 1 | 21 | 33 |  |  | DB Search |
| total 1 peptides |  |  |  |  |  |  |  |  |  |  |  |  |  |  |  |  |  |  |  |
| --- | --- | --- | --- | --- | --- | --- | --- | --- | --- | --- | --- | --- | --- | --- | --- | --- | --- | --- | --- |

Best Unique PSM (Scan Sample\_11\_A1\_1\_3648.d:57047, m/z=755.9129, z=2, RT=18.04, ppm=-5.19):


TRINITY\_DN817\_c0\_g1\_i2.p1

back to list

  

| Protein Coverage
| Supporting Peptides
| Best Unique PSM
|

Protein Coverage:

Supporting Peptides:

| Peptide | Uniq | -10lgP | Mass | Length | ppm | m/z | z | RT | 1/k0 Start | 1/k0 End | Precursor ID | Area Sample 2 | #Feature | #Feature Sample 2 | Start | End | PTM | AScore | Found By |
| --- | --- | --- | --- | --- | --- | --- | --- | --- | --- | --- | --- | --- | --- | --- | --- | --- | --- | --- | --- |
| R.LC(+57.02)QPGYSTC(+57.02)ENHC(+57.02)R.R | Y | 38.48 | 1780.708 | 14 | 5.76 | 891.3615 | 2 | 11.39 | 1.0877 | 1.0949 | 21326 | 1.4e2 | 1 | 1 | 145 | 158 | Carbamidomethylation, Carbamidomethylation, Carbamidomethylation |  | DB Search |
| total 1 peptides |  |  |  |  |  |  |  |  |  |  |  |  |  |  |  |  |  |  |  |
| --- | --- | --- | --- | --- | --- | --- | --- | --- | --- | --- | --- | --- | --- | --- | --- | --- | --- | --- | --- |

Best Unique PSM (Scan Sample\_11\_A1\_1\_3648.d:21326, m/z=891.3615, z=2, RT=11.39, ppm=0.28):


TRINITY\_DN925\_c0\_g1\_i1.p1

back to list

  

| Protein Coverage
| Supporting Peptides
| Best Unique PSM
|

Protein Coverage:

Supporting Peptides:

| Peptide | Uniq | -10lgP | Mass | Length | ppm | m/z | z | RT | 1/k0 Start | 1/k0 End | Precursor ID | Area Sample 2 | #Feature | #Feature Sample 2 | Start | End | PTM | AScore | Found By |
| --- | --- | --- | --- | --- | --- | --- | --- | --- | --- | --- | --- | --- | --- | --- | --- | --- | --- | --- | --- |
| R.ASGDYATVVSHNR.D | Y | 58.13 | 1375.648 | 13 | 5.41 | 688.8312 | 2 | 12.05 | 0.9937 | 1.0009 | 25145 | 0 | 0 | 0 | 129 | 141 |  |  | DB Search |
| total 1 peptides |  |  |  |  |  |  |  |  |  |  |  |  |  |  |  |  |  |  |  |
| --- | --- | --- | --- | --- | --- | --- | --- | --- | --- | --- | --- | --- | --- | --- | --- | --- | --- | --- | --- |

Best Unique PSM (Scan Sample\_11\_A1\_1\_3648.d:25145, m/z=688.8312, z=2, RT=12.05, ppm=-0.07):


TRINITY\_DN152\_c0\_g1\_i2.p1

back to list

  

| Protein Coverage
| Supporting Peptides
| Best Unique PSM
|

Protein Coverage:

Supporting Peptides:

| Peptide | Uniq | -10lgP | Mass | Length | ppm | m/z | z | RT | 1/k0 Start | 1/k0 End | Precursor ID | Area Sample 2 | #Feature | #Feature Sample 2 | Start | End | PTM | AScore | Found By |
| --- | --- | --- | --- | --- | --- | --- | --- | --- | --- | --- | --- | --- | --- | --- | --- | --- | --- | --- | --- |
| K.IQDKEGIPPDQQR.L | Y | 55.06 | 1522.7739 | 13 | 2.88 | 762.3923 | 2 | 11.00 | 1.0331 | 1.0403 | 18584 | 2.97e3 | 2 | 2 | 66 | 78 |  |  | DB Search |
| total 1 peptides |  |  |  |  |  |  |  |  |  |  |  |  |  |  |  |  |  |  |  |
| --- | --- | --- | --- | --- | --- | --- | --- | --- | --- | --- | --- | --- | --- | --- | --- | --- | --- | --- | --- |

Best Unique PSM (Scan Sample\_11\_A1\_1\_3648.d:18584, m/z=762.3923, z=2, RT=11.00, ppm=-2.59):


TRINITY\_DN5906\_c3\_g1\_i1.p2

back to list

  

| Protein Coverage
| Supporting Peptides
| Best Unique PSM
|

Protein Coverage:

Supporting Peptides:

| Peptide | Uniq | -10lgP | Mass | Length | ppm | m/z | z | RT | 1/k0 Start | 1/k0 End | Precursor ID | Area Sample 2 | #Feature | #Feature Sample 2 | Start | End | PTM | AScore | Found By |
| --- | --- | --- | --- | --- | --- | --- | --- | --- | --- | --- | --- | --- | --- | --- | --- | --- | --- | --- | --- |
| K.AGM(+15.99)THIVR.D | Y | 38.19 | 899.4647 | 8 | 6.96 | 450.7403 | 2 | 9.74 | 0.8254 | 0.8327 | 11994 | 0 | 0 | 0 | 76 | 83 | Oxidation (M) | M3:Oxidation (M):1000 | DB Search |
| R.HGSLGFLPR.K | Y | 32.83 | 982.5348 | 9 | -0.04 | 492.272 | 2 | 16.93 | 0.8408 | 0.8481 | 51108 | 4.36e2 | 1 | 1 | 36 | 44 |  |  | DB Search |
| total 2 peptides |  |  |  |  |  |  |  |  |  |  |  |  |  |  |  |  |  |  |  |
| --- | --- | --- | --- | --- | --- | --- | --- | --- | --- | --- | --- | --- | --- | --- | --- | --- | --- | --- | --- |

Best Unique PSM (Scan Sample\_11\_A1\_1\_3648.d:11994, m/z=450.7403, z=2, RT=9.74, ppm=1.48):


TRINITY\_DN651\_c0\_g1\_i1.p1

back to list

  

| Protein Coverage
| Supporting Peptides
| Best Unique PSM
|

Protein Coverage:

Supporting Peptides:

| Peptide | Uniq | -10lgP | Mass | Length | ppm | m/z | z | RT | 1/k0 Start | 1/k0 End | Precursor ID | Area Sample 2 | #Feature | #Feature Sample 2 | Start | End | PTM | AScore | Found By |
| --- | --- | --- | --- | --- | --- | --- | --- | --- | --- | --- | --- | --- | --- | --- | --- | --- | --- | --- | --- |
| R.VVAISGNEDEIVNLLHK.I | Y | 55.96 | 1848.9945 | 17 | 2.17 | 925.5015 | 2 | 18.50 | 1.2109 | 1.2181 | 59519 | 0 | 0 | 0 | 160 | 176 |  |  | DB Search |
| total 1 peptides |  |  |  |  |  |  |  |  |  |  |  |  |  |  |  |  |  |  |  |
| --- | --- | --- | --- | --- | --- | --- | --- | --- | --- | --- | --- | --- | --- | --- | --- | --- | --- | --- | --- |

Best Unique PSM (Scan Sample\_11\_A1\_1\_3648.d:59519, m/z=925.5015, z=2, RT=18.50, ppm=-3.3):


TRINITY\_DN4595\_c0\_g1\_i10.p2

back to list

  

| Protein Coverage
| Supporting Peptides
| Best Unique PSM
|

Protein Coverage:

Supporting Peptides:

| Peptide | Uniq | -10lgP | Mass | Length | ppm | m/z | z | RT | 1/k0 Start | 1/k0 End | Precursor ID | Area Sample 2 | #Feature | #Feature Sample 2 | Start | End | PTM | AScore | Found By |
| --- | --- | --- | --- | --- | --- | --- | --- | --- | --- | --- | --- | --- | --- | --- | --- | --- | --- | --- | --- |
| R.AFNRVETR.D | Y | 25.84 | 991.5199 | 8 | -0.1 | 496.7645 | 2 | 10.70 | 0.8477 | 0.855 | 17302 | 3.57e3 | 1 | 1 | 152 | 159 |  |  | DB Search |
| total 1 peptides |  |  |  |  |  |  |  |  |  |  |  |  |  |  |  |  |  |  |  |
| --- | --- | --- | --- | --- | --- | --- | --- | --- | --- | --- | --- | --- | --- | --- | --- | --- | --- | --- | --- |

Best Unique PSM (Scan Sample\_11\_A1\_1\_3648.d:17302, m/z=496.7645, z=2, RT=10.70, ppm=-5.58):


TRINITY\_DN36\_c0\_g1\_i17.p1

back to list

  

| Protein Coverage
| Supporting Peptides
| Best Unique PSM
|

Protein Coverage:

Supporting Peptides:

| Peptide | Uniq | -10lgP | Mass | Length | ppm | m/z | z | RT | 1/k0 Start | 1/k0 End | Precursor ID | Area Sample 2 | #Feature | #Feature Sample 2 | Start | End | PTM | AScore | Found By |
| --- | --- | --- | --- | --- | --- | --- | --- | --- | --- | --- | --- | --- | --- | --- | --- | --- | --- | --- | --- |
| R.LGGIEVTQGNC(+57.02)ARK.Y | Y | 58.27 | 1501.7671 | 14 | 1.48 | 751.8878 | 2 | 12.59 | 1.0616 | 1.0688 | 28229 | 7.18e2 | 1 | 1 | 58 | 71 | Carbamidomethylation |  | DB Search |
| K.FETQIATK.D | Y | 57.95 | 936.4916 | 8 | 1.49 | 937.4952 | 1 | 13.74 | 1.2208 | 1.228 | 34637 | 7.44e2 | 2 | 2 | 167 | 174 |  |  | DB Search |
| total 2 peptides |  |  |  |  |  |  |  |  |  |  |  |  |  |  |  |  |  |  |  |
| --- | --- | --- | --- | --- | --- | --- | --- | --- | --- | --- | --- | --- | --- | --- | --- | --- | --- | --- | --- |

Best Unique PSM (Scan Sample\_11\_A1\_1\_3648.d:28229, m/z=751.8878, z=2, RT=12.59, ppm=-4):


TRINITY\_DN3991\_c0\_g1\_i1.p1

back to list

  

| Protein Coverage
| Supporting Peptides
| Best Unique PSM
|

Protein Coverage:

Supporting Peptides:

| Peptide | Uniq | -10lgP | Mass | Length | ppm | m/z | z | RT | 1/k0 Start | 1/k0 End | Precursor ID | Area Sample 2 | #Feature | #Feature Sample 2 | Start | End | PTM | AScore | Found By |
| --- | --- | --- | --- | --- | --- | --- | --- | --- | --- | --- | --- | --- | --- | --- | --- | --- | --- | --- | --- |
| R.AKLSQC(+57.02)TAASGK.H | Y | 59.12 | 1220.6183 | 12 | 6.64 | 611.3171 | 2 | 9.69 | 0.9529 | 0.9602 | 11719 | 0 | 0 | 0 | 128 | 139 | Carbamidomethylation |  | DB Search |
| K.LSQC(+57.02)TAASGK.H | Y | 58.87 | 1021.4862 | 10 | -0.07 | 511.7476 | 2 | 9.83 | 0.838 | 0.8453 | 11562 | 6.46e4 | 1 | 1 | 130 | 139 | Carbamidomethylation |  | DB Search |
| total 2 peptides |  |  |  |  |  |  |  |  |  |  |  |  |  |  |  |  |  |  |  |
| --- | --- | --- | --- | --- | --- | --- | --- | --- | --- | --- | --- | --- | --- | --- | --- | --- | --- | --- | --- |

Best Unique PSM (Scan Sample\_11\_A1\_1\_3648.d:11719, m/z=611.3171, z=2, RT=9.69, ppm=1.16):


TRINITY\_DN152\_c0\_g1\_i15.p1

back to list

  

| Protein Coverage
| Supporting Peptides
| Best Unique PSM
|

Protein Coverage:

Supporting Peptides:

| Peptide | Uniq | -10lgP | Mass | Length | ppm | m/z | z | RT | 1/k0 Start | 1/k0 End | Precursor ID | Area Sample 2 | #Feature | #Feature Sample 2 | Start | End | PTM | AScore | Found By |
| --- | --- | --- | --- | --- | --- | --- | --- | --- | --- | --- | --- | --- | --- | --- | --- | --- | --- | --- | --- |
| K.IQDKEGIPPDQQR.L | Y | 55.06 | 1522.7739 | 13 | 2.88 | 762.3923 | 2 | 11.00 | 1.0331 | 1.0403 | 18584 | 2.97e3 | 2 | 2 | 60 | 72 |  |  | DB Search |
| total 1 peptides |  |  |  |  |  |  |  |  |  |  |  |  |  |  |  |  |  |  |  |
| --- | --- | --- | --- | --- | --- | --- | --- | --- | --- | --- | --- | --- | --- | --- | --- | --- | --- | --- | --- |

Best Unique PSM (Scan Sample\_11\_A1\_1\_3648.d:18584, m/z=762.3923, z=2, RT=11.00, ppm=-2.59):


TRINITY\_DN4523\_c0\_g1\_i1.p1

back to list

  

| Protein Coverage
| Supporting Peptides
| Best Unique PSM
|

Protein Coverage:

Supporting Peptides:

| Peptide | Uniq | -10lgP | Mass | Length | ppm | m/z | z | RT | 1/k0 Start | 1/k0 End | Precursor ID | Area Sample 2 | #Feature | #Feature Sample 2 | Start | End | PTM | AScore | Found By |
| --- | --- | --- | --- | --- | --- | --- | --- | --- | --- | --- | --- | --- | --- | --- | --- | --- | --- | --- | --- |
| R.VASDKFLTSFIK.S | Y | 52.71 | 1354.7496 | 12 | 1.41 | 678.3793 | 2 | 18.05 | 1.0234 | 1.0307 | 57158 | 3.75e2 | 1 | 1 | 1119 | 1130 |  |  | DB Search |
| total 1 peptides |  |  |  |  |  |  |  |  |  |  |  |  |  |  |  |  |  |  |  |
| --- | --- | --- | --- | --- | --- | --- | --- | --- | --- | --- | --- | --- | --- | --- | --- | --- | --- | --- | --- |

Best Unique PSM (Scan Sample\_11\_A1\_1\_3648.d:57158, m/z=678.3793, z=2, RT=18.05, ppm=-4.06):


TRINITY\_DN9509\_c0\_g1\_i10.p1

back to list

  

| Protein Coverage
| Supporting Peptides
| Best Unique PSM
|

Protein Coverage:

Supporting Peptides:

| Peptide | Uniq | -10lgP | Mass | Length | ppm | m/z | z | RT | 1/k0 Start | 1/k0 End | Precursor ID | Area Sample 2 | #Feature | #Feature Sample 2 | Start | End | PTM | AScore | Found By |
| --- | --- | --- | --- | --- | --- | --- | --- | --- | --- | --- | --- | --- | --- | --- | --- | --- | --- | --- | --- |
| R.GQVIC(+57.02)APGATSTHTK.F | Y | 58 | 1526.7511 | 15 | 1.7 | 764.3799 | 2 | 11.76 | 1.0291 | 1.0363 | 23431 | 4.83e2 | 1 | 1 | 315 | 329 | Carbamidomethylation |  | DB Search |
| R.GITINASHIEYETSNR.H | Y | 57.62 | 1803.8751 | 16 | -0.09 | 902.9398 | 2 | 14.89 | 1.1674 | 1.1746 | 40938 | 0 | 0 | 0 | 74 | 89 |  |  | DB Search |
| total 2 peptides |  |  |  |  |  |  |  |  |  |  |  |  |  |  |  |  |  |  |  |
| --- | --- | --- | --- | --- | --- | --- | --- | --- | --- | --- | --- | --- | --- | --- | --- | --- | --- | --- | --- |

Best Unique PSM (Scan Sample\_11\_A1\_1\_3648.d:23431, m/z=764.3799, z=2, RT=11.76, ppm=-3.78):


TRINITY\_DN2885\_c0\_g1\_i4.p1

back to list

  

| Protein Coverage
| Supporting Peptides
| Best Unique PSM
|

Protein Coverage:

Supporting Peptides:

| Peptide | Uniq | -10lgP | Mass | Length | ppm | m/z | z | RT | 1/k0 Start | 1/k0 End | Precursor ID | Area Sample 2 | #Feature | #Feature Sample 2 | Start | End | PTM | AScore | Found By |
| --- | --- | --- | --- | --- | --- | --- | --- | --- | --- | --- | --- | --- | --- | --- | --- | --- | --- | --- | --- |
| K.VTRPHGASGVVR.A | Y | 58.91 | 1234.6894 | 12 | 6.71 | 412.5709 | 3 | 10.04 | 0.8209 | 0.8283 | 13404 | 2.81e4 | 3 | 3 | 72 | 83 |  |  | DB Search |
| total 1 peptides |  |  |  |  |  |  |  |  |  |  |  |  |  |  |  |  |  |  |  |
| --- | --- | --- | --- | --- | --- | --- | --- | --- | --- | --- | --- | --- | --- | --- | --- | --- | --- | --- | --- |

Best Unique PSM (Scan Sample\_11\_A1\_1\_3648.d:13404, m/z=412.5709, z=3, RT=10.04, ppm=1.23):


TRINITY\_DN2477\_c0\_g1\_i2.p1

back to list

  

| Protein Coverage
| Supporting Peptides
| Best Unique PSM
|

Protein Coverage:

Supporting Peptides:

| Peptide | Uniq | -10lgP | Mass | Length | ppm | m/z | z | RT | 1/k0 Start | 1/k0 End | Precursor ID | Area Sample 2 | #Feature | #Feature Sample 2 | Start | End | PTM | AScore | Found By |
| --- | --- | --- | --- | --- | --- | --- | --- | --- | --- | --- | --- | --- | --- | --- | --- | --- | --- | --- | --- |
| K.DSYVGDEAQSKR.G | Y | 59.21 | 1353.616 | 12 | 5.76 | 677.8155 | 2 | 10.93 | 1.0037 | 1.011 | 18642 | 9.97e3 | 1 | 1 | 19 | 30 |  |  | DB Search |
| R.VAPEEHPVLLTEAPLNPK.A | Y | 58.19 | 1953.0571 | 18 | -0.07 | 977.5304 | 2 | 16.19 | 1.2089 | 1.2161 | 47306 | 2.52e2 | 1 | 1 | 64 | 81 |  |  | DB Search |
| R.GYSFTTTAEREIVR.D | Y | 58.1 | 1628.8158 | 14 | 2.53 | 815.4128 | 2 | 15.79 | 1.121 | 1.1282 | 45442 | 6.41e3 | 1 | 1 | 165 | 178 |  |  | DB Search |
| total 3 peptides |  |  |  |  |  |  |  |  |  |  |  |  |  |  |  |  |  |  |  |
| --- | --- | --- | --- | --- | --- | --- | --- | --- | --- | --- | --- | --- | --- | --- | --- | --- | --- | --- | --- |

Best Unique PSM (Scan Sample\_11\_A1\_1\_3648.d:18642, m/z=677.8155, z=2, RT=10.93, ppm=0.28):


TRINITY\_DN152\_c0\_g1\_i10.p1

back to list

  

| Protein Coverage
| Supporting Peptides
| Best Unique PSM
|

Protein Coverage:

Supporting Peptides:

| Peptide | Uniq | -10lgP | Mass | Length | ppm | m/z | z | RT | 1/k0 Start | 1/k0 End | Precursor ID | Area Sample 2 | #Feature | #Feature Sample 2 | Start | End | PTM | AScore | Found By |
| --- | --- | --- | --- | --- | --- | --- | --- | --- | --- | --- | --- | --- | --- | --- | --- | --- | --- | --- | --- |
| K.IQDKEGIPPDQQR.L | Y | 55.06 | 1522.7739 | 13 | 2.88 | 762.3923 | 2 | 11.00 | 1.0331 | 1.0403 | 18584 | 2.97e3 | 2 | 2 | 141 | 153 |  |  | DB Search |
| total 1 peptides |  |  |  |  |  |  |  |  |  |  |  |  |  |  |  |  |  |  |  |
| --- | --- | --- | --- | --- | --- | --- | --- | --- | --- | --- | --- | --- | --- | --- | --- | --- | --- | --- | --- |

Best Unique PSM (Scan Sample\_11\_A1\_1\_3648.d:18584, m/z=762.3923, z=2, RT=11.00, ppm=-2.59):


TRINITY\_DN1428\_c0\_g1\_i1.p1

back to list

  

| Protein Coverage
| Supporting Peptides
| Best Unique PSM
|

Protein Coverage:

Supporting Peptides:

| Peptide | Uniq | -10lgP | Mass | Length | ppm | m/z | z | RT | 1/k0 Start | 1/k0 End | Precursor ID | Area Sample 2 | #Feature | #Feature Sample 2 | Start | End | PTM | AScore | Found By |
| --- | --- | --- | --- | --- | --- | --- | --- | --- | --- | --- | --- | --- | --- | --- | --- | --- | --- | --- | --- |
| R.IAYAQGYATGDPSKR.M | Y | 58.88 | 1596.7896 | 15 | 3.91 | 799.4008 | 2 | 12.65 | 1.1069 | 1.1142 | 28431 | 1.68e3 | 1 | 1 | 122 | 136 |  |  | DB Search |
| total 1 peptides |  |  |  |  |  |  |  |  |  |  |  |  |  |  |  |  |  |  |  |
| --- | --- | --- | --- | --- | --- | --- | --- | --- | --- | --- | --- | --- | --- | --- | --- | --- | --- | --- | --- |

Best Unique PSM (Scan Sample\_11\_A1\_1\_3648.d:28431, m/z=799.4008, z=2, RT=12.65, ppm=-1.57):


TRINITY\_DN471\_c0\_g1\_i6.p1

back to list

  

| Protein Coverage
| Supporting Peptides
| Best Unique PSM
|

Protein Coverage:

Supporting Peptides:

| Peptide | Uniq | -10lgP | Mass | Length | ppm | m/z | z | RT | 1/k0 Start | 1/k0 End | Precursor ID | Area Sample 2 | #Feature | #Feature Sample 2 | Start | End | PTM | AScore | Found By |
| --- | --- | --- | --- | --- | --- | --- | --- | --- | --- | --- | --- | --- | --- | --- | --- | --- | --- | --- | --- |
| R.RNLDIERPTYTNLNR.L | Y | 46.58 | 1873.9758 | 15 | -2.43 | 625.6609 | 3 | 13.94 | 0.8858 | 0.8931 | 35770 | 1.17e3 | 1 | 1 | 85 | 99 |  |  | DB Search |
| total 1 peptides |  |  |  |  |  |  |  |  |  |  |  |  |  |  |  |  |  |  |  |
| --- | --- | --- | --- | --- | --- | --- | --- | --- | --- | --- | --- | --- | --- | --- | --- | --- | --- | --- | --- |

Best Unique PSM (Scan Sample\_11\_A1\_1\_3648.d:35770, m/z=625.6609, z=3, RT=13.94, ppm=-7.91):


TRINITY\_DN189323\_c0\_g1\_i1.p1

back to list

  

| Protein Coverage
| Supporting Peptides
| Best Unique PSM
|

Protein Coverage:

Supporting Peptides:

| Peptide | Uniq | -10lgP | Mass | Length | ppm | m/z | z | RT | 1/k0 Start | 1/k0 End | Precursor ID | Area Sample 2 | #Feature | #Feature Sample 2 | Start | End | PTM | AScore | Found By |
| --- | --- | --- | --- | --- | --- | --- | --- | --- | --- | --- | --- | --- | --- | --- | --- | --- | --- | --- | --- |
| R.LFEGNALLRR.L | Y | 57.75 | 1187.6775 | 10 | 3.63 | 594.8449 | 2 | 15.37 | 0.9683 | 0.9755 | 43382 | 1.83e3 | 1 | 1 | 32 | 41 |  |  | DB Search |
| K.RLFEGNALLR.R | Y | 53.09 | 1187.6775 | 10 | 1.58 | 594.8437 | 2 | 15.84 | 0.9384 | 0.9457 | 45707 | 5.33e2 | 1 | 1 | 31 | 40 |  |  | DB Search |
| K.RLFEGNALLRR.L | Y | 20.39 | 1343.7786 | 11 | 6.92 | 448.9341 | 3 | 14.60 | 0.7538 | 0.7612 | 39347 | 0 | 0 | 0 | 31 | 41 |  |  | DB Search |
| total 3 peptides |  |  |  |  |  |  |  |  |  |  |  |  |  |  |  |  |  |  |  |
| --- | --- | --- | --- | --- | --- | --- | --- | --- | --- | --- | --- | --- | --- | --- | --- | --- | --- | --- | --- |

Best Unique PSM (Scan Sample\_11\_A1\_1\_3648.d:43382, m/z=594.8449, z=2, RT=15.37, ppm=-1.84):


TRINITY\_DN200\_c0\_g1\_i2.p1

back to list

  

| Protein Coverage
| Supporting Peptides
| Best Unique PSM
|

Protein Coverage:

Supporting Peptides:

| Peptide | Uniq | -10lgP | Mass | Length | ppm | m/z | z | RT | 1/k0 Start | 1/k0 End | Precursor ID | Area Sample 2 | #Feature | #Feature Sample 2 | Start | End | PTM | AScore | Found By |
| --- | --- | --- | --- | --- | --- | --- | --- | --- | --- | --- | --- | --- | --- | --- | --- | --- | --- | --- | --- |
| K.TAIAIDTIINQKR.F | Y | 58.72 | 1455.8409 | 13 | 0.37 | 728.924 | 2 | 16.37 | 1.0604 | 1.0676 | 48298 | 0 | 0 | 0 | 217 | 229 |  |  | DB Search |
| total 1 peptides |  |  |  |  |  |  |  |  |  |  |  |  |  |  |  |  |  |  |  |
| --- | --- | --- | --- | --- | --- | --- | --- | --- | --- | --- | --- | --- | --- | --- | --- | --- | --- | --- | --- |

Best Unique PSM (Scan Sample\_11\_A1\_1\_3648.d:48298, m/z=728.924, z=2, RT=16.37, ppm=-5.1):


TRINITY\_DN2941\_c0\_g1\_i4.p1

back to list

  

| Protein Coverage
| Supporting Peptides
| Best Unique PSM
|

Protein Coverage:

Supporting Peptides:

| Peptide | Uniq | -10lgP | Mass | Length | ppm | m/z | z | RT | 1/k0 Start | 1/k0 End | Precursor ID | Area Sample 2 | #Feature | #Feature Sample 2 | Start | End | PTM | AScore | Found By |
| --- | --- | --- | --- | --- | --- | --- | --- | --- | --- | --- | --- | --- | --- | --- | --- | --- | --- | --- | --- |
| R.VAPEEHPVLLTEAPLNPK.A | Y | 58.19 | 1953.0571 | 18 | -0.07 | 977.5304 | 2 | 16.19 | 1.2089 | 1.2161 | 47306 | 2.52e2 | 1 | 1 | 30 | 47 |  |  | DB Search |
| total 1 peptides |  |  |  |  |  |  |  |  |  |  |  |  |  |  |  |  |  |  |  |
| --- | --- | --- | --- | --- | --- | --- | --- | --- | --- | --- | --- | --- | --- | --- | --- | --- | --- | --- | --- |

Best Unique PSM (Scan Sample\_11\_A1\_1\_3648.d:47306, m/z=977.5304, z=2, RT=16.19, ppm=-5.54):


TRINITY\_DN1172\_c0\_g1\_i5.p1

back to list

  

| Protein Coverage
| Supporting Peptides
| Best Unique PSM
|

Protein Coverage:

Supporting Peptides:

| Peptide | Uniq | -10lgP | Mass | Length | ppm | m/z | z | RT | 1/k0 Start | 1/k0 End | Precursor ID | Area Sample 2 | #Feature | #Feature Sample 2 | Start | End | PTM | AScore | Found By |
| --- | --- | --- | --- | --- | --- | --- | --- | --- | --- | --- | --- | --- | --- | --- | --- | --- | --- | --- | --- |
| K.VITSDDPAEKEK.K | Y | 58.87 | 1330.6616 | 12 | 2.64 | 666.3362 | 2 | 10.69 | 0.9848 | 0.992 | 17191 | 9.01e3 | 1 | 1 | 123 | 134 |  |  | DB Search |
| total 1 peptides |  |  |  |  |  |  |  |  |  |  |  |  |  |  |  |  |  |  |  |
| --- | --- | --- | --- | --- | --- | --- | --- | --- | --- | --- | --- | --- | --- | --- | --- | --- | --- | --- | --- |

Best Unique PSM (Scan Sample\_11\_A1\_1\_3648.d:17191, m/z=666.3362, z=2, RT=10.69, ppm=-2.84):


TRINITY\_DN152\_c0\_g1\_i14.p2

back to list

  

| Protein Coverage
| Supporting Peptides
| Best Unique PSM
|

Protein Coverage:

Supporting Peptides:

| Peptide | Uniq | -10lgP | Mass | Length | ppm | m/z | z | RT | 1/k0 Start | 1/k0 End | Precursor ID | Area Sample 2 | #Feature | #Feature Sample 2 | Start | End | PTM | AScore | Found By |
| --- | --- | --- | --- | --- | --- | --- | --- | --- | --- | --- | --- | --- | --- | --- | --- | --- | --- | --- | --- |
| K.IQDKEGIPPDQQR.S | Y | 55.06 | 1522.7739 | 13 | 2.88 | 762.3923 | 2 | 11.00 | 1.0331 | 1.0403 | 18584 | 2.97e3 | 2 | 2 | 40 | 52 |  |  | DB Search |
| total 1 peptides |  |  |  |  |  |  |  |  |  |  |  |  |  |  |  |  |  |  |  |
| --- | --- | --- | --- | --- | --- | --- | --- | --- | --- | --- | --- | --- | --- | --- | --- | --- | --- | --- | --- |

Best Unique PSM (Scan Sample\_11\_A1\_1\_3648.d:18584, m/z=762.3923, z=2, RT=11.00, ppm=-2.59):


TRINITY\_DN598\_c1\_g1\_i2.p1

back to list

  

| Protein Coverage
| Supporting Peptides
| Best Unique PSM
|

Protein Coverage:

Supporting Peptides:

| Peptide | Uniq | -10lgP | Mass | Length | ppm | m/z | z | RT | 1/k0 Start | 1/k0 End | Precursor ID | Area Sample 2 | #Feature | #Feature Sample 2 | Start | End | PTM | AScore | Found By |
| --- | --- | --- | --- | --- | --- | --- | --- | --- | --- | --- | --- | --- | --- | --- | --- | --- | --- | --- | --- |
| R.ASFVTDIDRLR.A | Y | 58.82 | 1291.6884 | 11 | 5.03 | 646.8512 | 2 | 16.77 | 0.9888 | 0.9961 | 50096 | 2.99e3 | 1 | 1 | 302 | 312 |  |  | DB Search |
| total 1 peptides |  |  |  |  |  |  |  |  |  |  |  |  |  |  |  |  |  |  |  |
| --- | --- | --- | --- | --- | --- | --- | --- | --- | --- | --- | --- | --- | --- | --- | --- | --- | --- | --- | --- |

Best Unique PSM (Scan Sample\_11\_A1\_1\_3648.d:50096, m/z=646.8512, z=2, RT=16.77, ppm=-0.45):


TRINITY\_DN1339\_c6\_g1\_i1.p1

back to list

  

| Protein Coverage
| Supporting Peptides
| Best Unique PSM
|

Protein Coverage:

Supporting Peptides:

| Peptide | Uniq | -10lgP | Mass | Length | ppm | m/z | z | RT | 1/k0 Start | 1/k0 End | Precursor ID | Area Sample 2 | #Feature | #Feature Sample 2 | Start | End | PTM | AScore | Found By |
| --- | --- | --- | --- | --- | --- | --- | --- | --- | --- | --- | --- | --- | --- | --- | --- | --- | --- | --- | --- |
| K.VITSDDPAEKEK.K | Y | 58.87 | 1330.6616 | 12 | 2.64 | 666.3362 | 2 | 10.69 | 0.9848 | 0.992 | 17191 | 9.01e3 | 1 | 1 | 158 | 169 |  |  | DB Search |
| total 1 peptides |  |  |  |  |  |  |  |  |  |  |  |  |  |  |  |  |  |  |  |
| --- | --- | --- | --- | --- | --- | --- | --- | --- | --- | --- | --- | --- | --- | --- | --- | --- | --- | --- | --- |

Best Unique PSM (Scan Sample\_11\_A1\_1\_3648.d:17191, m/z=666.3362, z=2, RT=10.69, ppm=-2.84):


TRINITY\_DN15847\_c0\_g1\_i1.p1

back to list

  

| Protein Coverage
| Supporting Peptides
| Best Unique PSM
|

Protein Coverage:

Supporting Peptides:

| Peptide | Uniq | -10lgP | Mass | Length | ppm | m/z | z | RT | 1/k0 Start | 1/k0 End | Precursor ID | Area Sample 2 | #Feature | #Feature Sample 2 | Start | End | PTM | AScore | Found By |
| --- | --- | --- | --- | --- | --- | --- | --- | --- | --- | --- | --- | --- | --- | --- | --- | --- | --- | --- | --- |
| R.DVQLGDLVTVGQC(+57.02)RPLSK.T | Y | 55.96 | 1984.0411 | 18 | 3.74 | 993.0261 | 2 | 17.77 | 1.2766 | 1.2837 | 55652 | 0 | 0 | 0 | 126 | 143 | Carbamidomethylation |  | DB Search |
| total 1 peptides |  |  |  |  |  |  |  |  |  |  |  |  |  |  |  |  |  |  |  |
| --- | --- | --- | --- | --- | --- | --- | --- | --- | --- | --- | --- | --- | --- | --- | --- | --- | --- | --- | --- |

Best Unique PSM (Scan Sample\_11\_A1\_1\_3648.d:55652, m/z=993.0261, z=2, RT=17.77, ppm=-1.73):


TRINITY\_DN1933\_c0\_g1\_i8.p2

back to list

  

| Protein Coverage
| Supporting Peptides
| Best Unique PSM
|

Protein Coverage:

Supporting Peptides:

| Peptide | Uniq | -10lgP | Mass | Length | ppm | m/z | z | RT | 1/k0 Start | 1/k0 End | Precursor ID | Area Sample 2 | #Feature | #Feature Sample 2 | Start | End | PTM | AScore | Found By |
| --- | --- | --- | --- | --- | --- | --- | --- | --- | --- | --- | --- | --- | --- | --- | --- | --- | --- | --- | --- |
| R.RNLDIERPTYTNLNR.L | Y | 46.58 | 1873.9758 | 15 | -2.43 | 625.6609 | 3 | 13.94 | 0.8858 | 0.8931 | 35770 | 1.17e3 | 1 | 1 | 75 | 89 |  |  | DB Search |
| total 1 peptides |  |  |  |  |  |  |  |  |  |  |  |  |  |  |  |  |  |  |  |
| --- | --- | --- | --- | --- | --- | --- | --- | --- | --- | --- | --- | --- | --- | --- | --- | --- | --- | --- | --- |

Best Unique PSM (Scan Sample\_11\_A1\_1\_3648.d:35770, m/z=625.6609, z=3, RT=13.94, ppm=-7.91):


TRINITY\_DN554\_c0\_g1\_i3.p2

back to list

  

| Protein Coverage
| Supporting Peptides
| Best Unique PSM
|

Protein Coverage:

Supporting Peptides:

| Peptide | Uniq | -10lgP | Mass | Length | ppm | m/z | z | RT | 1/k0 Start | 1/k0 End | Precursor ID | Area Sample 2 | #Feature | #Feature Sample 2 | Start | End | PTM | AScore | Found By |
| --- | --- | --- | --- | --- | --- | --- | --- | --- | --- | --- | --- | --- | --- | --- | --- | --- | --- | --- | --- |
| K.IQDKEGIPPDQQR.L | Y | 55.06 | 1522.7739 | 13 | 2.88 | 762.3923 | 2 | 11.00 | 1.0331 | 1.0403 | 18584 | 2.97e3 | 2 | 2 | 30 | 42 |  |  | DB Search |
| total 1 peptides |  |  |  |  |  |  |  |  |  |  |  |  |  |  |  |  |  |  |  |
| --- | --- | --- | --- | --- | --- | --- | --- | --- | --- | --- | --- | --- | --- | --- | --- | --- | --- | --- | --- |

Best Unique PSM (Scan Sample\_11\_A1\_1\_3648.d:18584, m/z=762.3923, z=2, RT=11.00, ppm=-2.59):


TRINITY\_DN8101\_c0\_g1\_i8.p1

back to list

  

| Protein Coverage
| Supporting Peptides
| Best Unique PSM
|

Protein Coverage:

Supporting Peptides:

| Peptide | Uniq | -10lgP | Mass | Length | ppm | m/z | z | RT | 1/k0 Start | 1/k0 End | Precursor ID | Area Sample 2 | #Feature | #Feature Sample 2 | Start | End | PTM | AScore | Found By |
| --- | --- | --- | --- | --- | --- | --- | --- | --- | --- | --- | --- | --- | --- | --- | --- | --- | --- | --- | --- |
| R.SIGFIGHYLDQLR.L | Y | 54.32 | 1517.799 | 13 | 1.06 | 759.9034 | 2 | 19.56 | 1.0849 | 1.0921 | 65092 | 4.69e2 | 1 | 1 | 1077 | 1089 |  |  | DB Search |
| total 1 peptides |  |  |  |  |  |  |  |  |  |  |  |  |  |  |  |  |  |  |  |
| --- | --- | --- | --- | --- | --- | --- | --- | --- | --- | --- | --- | --- | --- | --- | --- | --- | --- | --- | --- |

Best Unique PSM (Scan Sample\_11\_A1\_1\_3648.d:65092, m/z=759.9034, z=2, RT=19.56, ppm=-4.41):


TRINITY\_DN3036\_c0\_g1\_i16.p1

back to list

  

| Protein Coverage
| Supporting Peptides
| Best Unique PSM
|

Protein Coverage:

Supporting Peptides:

| Peptide | Uniq | -10lgP | Mass | Length | ppm | m/z | z | RT | 1/k0 Start | 1/k0 End | Precursor ID | Area Sample 2 | #Feature | #Feature Sample 2 | Start | End | PTM | AScore | Found By |
| --- | --- | --- | --- | --- | --- | --- | --- | --- | --- | --- | --- | --- | --- | --- | --- | --- | --- | --- | --- |
| R.AGHGAVTSPQTQR.G | N | 58.95 | 1308.6534 | 13 | 4.15 | 655.3331 | 2 | 9.70 | 0.9146 | 0.9218 | 11889 | 1.61e4 | 2 | 2 | 135 | 147 |  |  | DB Search |
| R.VIGASGIHVGTM(+15.99)SFGK.M | N | 58.84 | 1575.8079 | 16 | 2.27 | 788.9087 | 2 | 14.96 | 1.1306 | 1.1378 | 41219 | 1.68e3 | 1 | 1 | 160 | 175 | Oxidation (M) | M12:Oxidation (M):1000 | DB Search |
| SNGGLVVGTIIKPK.L | Y | 56.21 | 1381.8293 | 14 | 0.75 | 691.9186 | 2 | 15.49 | 1.0528 | 1.06 | 43994 | 1.43e3 | 1 | 1 | 1 | 14 |  |  | DB Search |
| total 3 peptides |  |  |  |  |  |  |  |  |  |  |  |  |  |  |  |  |  |  |  |
| --- | --- | --- | --- | --- | --- | --- | --- | --- | --- | --- | --- | --- | --- | --- | --- | --- | --- | --- | --- |

Best Unique PSM (Scan Sample\_11\_A1\_1\_3648.d:43994, m/z=691.9186, z=2, RT=15.49, ppm=-4.73):


TRINITY\_DN22492\_c0\_g1\_i2.p1

back to list

  

| Protein Coverage
| Supporting Peptides
| Best Unique PSM
|

Protein Coverage:

Supporting Peptides:

| Peptide | Uniq | -10lgP | Mass | Length | ppm | m/z | z | RT | 1/k0 Start | 1/k0 End | Precursor ID | Area Sample 2 | #Feature | #Feature Sample 2 | Start | End | PTM | AScore | Found By |
| --- | --- | --- | --- | --- | --- | --- | --- | --- | --- | --- | --- | --- | --- | --- | --- | --- | --- | --- | --- |
| R.HC(+57.02)DPELSSFHTFWR.L | Y | 58.51 | 1817.7944 | 14 | -2.5 | 606.9339 | 3 | 18.48 | 0.8862 | 0.8935 | 59444 | 3.29e2 | 1 | 1 | 151 | 164 | Carbamidomethylation |  | DB Search |
| total 1 peptides |  |  |  |  |  |  |  |  |  |  |  |  |  |  |  |  |  |  |  |
| --- | --- | --- | --- | --- | --- | --- | --- | --- | --- | --- | --- | --- | --- | --- | --- | --- | --- | --- | --- |

Best Unique PSM (Scan Sample\_11\_A1\_1\_3648.d:59444, m/z=606.9339, z=3, RT=18.48, ppm=-7.97):


TRINITY\_DN240\_c0\_g1\_i6.p1

back to list

  

| Protein Coverage
| Supporting Peptides
| Best Unique PSM
|

Protein Coverage:

Supporting Peptides:

| Peptide | Uniq | -10lgP | Mass | Length | ppm | m/z | z | RT | 1/k0 Start | 1/k0 End | Precursor ID | Area Sample 2 | #Feature | #Feature Sample 2 | Start | End | PTM | AScore | Found By |
| --- | --- | --- | --- | --- | --- | --- | --- | --- | --- | --- | --- | --- | --- | --- | --- | --- | --- | --- | --- |
| K.AYAAFLEFKDVVK.A | Y | 58.73 | 1499.8024 | 13 | -0.24 | 750.9042 | 2 | 18.72 | 1.0941 | 1.1013 | 60609 | 3.07e3 | 1 | 1 | 217 | 229 |  |  | DB Search |
| total 1 peptides |  |  |  |  |  |  |  |  |  |  |  |  |  |  |  |  |  |  |  |
| --- | --- | --- | --- | --- | --- | --- | --- | --- | --- | --- | --- | --- | --- | --- | --- | --- | --- | --- | --- |

Best Unique PSM (Scan Sample\_11\_A1\_1\_3648.d:60609, m/z=750.9042, z=2, RT=18.72, ppm=-5.71):


TRINITY\_DN4101\_c0\_g1\_i1.p3

back to list

  

| Protein Coverage
| Supporting Peptides
| Best Unique PSM
|

Protein Coverage:

Supporting Peptides:

| Peptide | Uniq | -10lgP | Mass | Length | ppm | m/z | z | RT | 1/k0 Start | 1/k0 End | Precursor ID | Area Sample 2 | #Feature | #Feature Sample 2 | Start | End | PTM | AScore | Found By |
| --- | --- | --- | --- | --- | --- | --- | --- | --- | --- | --- | --- | --- | --- | --- | --- | --- | --- | --- | --- |
| R.GVDEDAYTLEVLHYK.A | Y | 58.84 | 1750.8413 | 15 | 4.4 | 876.427 | 2 | 17.48 | 1.1802 | 1.1873 | 54020 | 1.93e2 | 1 | 1 | 66 | 80 |  |  | DB Search |
| total 1 peptides |  |  |  |  |  |  |  |  |  |  |  |  |  |  |  |  |  |  |  |
| --- | --- | --- | --- | --- | --- | --- | --- | --- | --- | --- | --- | --- | --- | --- | --- | --- | --- | --- | --- |

Best Unique PSM (Scan Sample\_11\_A1\_1\_3648.d:54020, m/z=876.427, z=2, RT=17.48, ppm=-1.08):


TRINITY\_DN7112\_c0\_g1\_i2.p1

back to list

  

| Protein Coverage
| Supporting Peptides
| Best Unique PSM
|

Protein Coverage:

Supporting Peptides:

| Peptide | Uniq | -10lgP | Mass | Length | ppm | m/z | z | RT | 1/k0 Start | 1/k0 End | Precursor ID | Area Sample 2 | #Feature | #Feature Sample 2 | Start | End | PTM | AScore | Found By |
| --- | --- | --- | --- | --- | --- | --- | --- | --- | --- | --- | --- | --- | --- | --- | --- | --- | --- | --- | --- |
| K.AYDQSVSVLLGRFEK.Y | Y | 58.37 | 1710.894 | 15 | -2.61 | 856.4474 | 2 | 18.83 | 1.1482 | 1.1554 | 61287 | 0 | 0 | 0 | 130 | 144 |  |  | DB Search |
| total 1 peptides |  |  |  |  |  |  |  |  |  |  |  |  |  |  |  |  |  |  |  |
| --- | --- | --- | --- | --- | --- | --- | --- | --- | --- | --- | --- | --- | --- | --- | --- | --- | --- | --- | --- |

Best Unique PSM (Scan Sample\_11\_A1\_1\_3648.d:61287, m/z=856.4474, z=2, RT=18.83, ppm=-8.08):


TRINITY\_DN250\_c0\_g1\_i5.p1

back to list

  

| Protein Coverage
| Supporting Peptides
| Best Unique PSM
|

Protein Coverage:

Supporting Peptides:

| Peptide | Uniq | -10lgP | Mass | Length | ppm | m/z | z | RT | 1/k0 Start | 1/k0 End | Precursor ID | Area Sample 2 | #Feature | #Feature Sample 2 | Start | End | PTM | AScore | Found By |
| --- | --- | --- | --- | --- | --- | --- | --- | --- | --- | --- | --- | --- | --- | --- | --- | --- | --- | --- | --- |
| K.ELVDQVLDRIR.K | Y | 58.86 | 1354.7568 | 11 | 2.67 | 678.3838 | 2 | 18.48 | 1.009 | 1.0162 | 59338 | 1.45e3 | 1 | 1 | 113 | 123 |  |  | DB Search |
| R.TIQFVDWC(+57.02)PTGFK.V | Y | 54.82 | 1597.7599 | 13 | -6.53 | 799.8776 | 2 | 19.88 | 1.0351 | 1.0423 | 66701 | 3.31e2 | 1 | 1 | 340 | 352 | Carbamidomethylation |  | DB Search |
| R.RNLDIERPTYTNLNR.L | Y | 46.58 | 1873.9758 | 15 | -2.43 | 625.6609 | 3 | 13.94 | 0.8858 | 0.8931 | 35770 | 1.17e3 | 1 | 1 | 215 | 229 |  |  | DB Search |
| R.LDHKFDLM(+15.99)YAKR.A | Y | 40.99 | 1551.7868 | 12 | 3.09 | 776.8988 | 2 | 13.19 | 1.0881 | 1.0953 | 31591 | 1.18e2 | 1 | 1 | 391 | 402 | Oxidation (M) | M8:Oxidation (M):1000 | DB Search |
| total 4 peptides |  |  |  |  |  |  |  |  |  |  |  |  |  |  |  |  |  |  |  |
| --- | --- | --- | --- | --- | --- | --- | --- | --- | --- | --- | --- | --- | --- | --- | --- | --- | --- | --- | --- |

Best Unique PSM (Scan Sample\_11\_A1\_1\_3648.d:59338, m/z=678.3838, z=2, RT=18.48, ppm=-2.81):


TRINITY\_DN477\_c0\_g1\_i7.p1

back to list

  

| Protein Coverage
| Supporting Peptides
| Best Unique PSM
|

Protein Coverage:

Supporting Peptides:

| Peptide | Uniq | -10lgP | Mass | Length | ppm | m/z | z | RT | 1/k0 Start | 1/k0 End | Precursor ID | Area Sample 2 | #Feature | #Feature Sample 2 | Start | End | PTM | AScore | Found By |
| --- | --- | --- | --- | --- | --- | --- | --- | --- | --- | --- | --- | --- | --- | --- | --- | --- | --- | --- | --- |
| R.ILQC(+57.02)NQYANVEEAR.N | Y | 59.1 | 1706.8046 | 14 | -0.86 | 854.4042 | 2 | 14.11 | 1.0873 | 1.0945 | 36622 | 2.02e3 | 1 | 1 | 86 | 99 | Carbamidomethylation |  | DB Search |
| K.DKNILVLTLGVGPQVNR.N | Y | 55.18 | 1835.0628 | 17 | -2.24 | 918.5316 | 2 | 19.07 | 1.1314 | 1.1386 | 62691 | 0 | 0 | 0 | 168 | 184 |  |  | DB Search |
| total 2 peptides |  |  |  |  |  |  |  |  |  |  |  |  |  |  |  |  |  |  |  |
| --- | --- | --- | --- | --- | --- | --- | --- | --- | --- | --- | --- | --- | --- | --- | --- | --- | --- | --- | --- |

Best Unique PSM (Scan Sample\_11\_A1\_1\_3648.d:36622, m/z=854.4042, z=2, RT=14.11, ppm=-6.33):


TRINITY\_DN3036\_c0\_g1\_i11.p1

back to list

  

| Protein Coverage
| Supporting Peptides
| Best Unique PSM
|

Protein Coverage:

Supporting Peptides:

| Peptide | Uniq | -10lgP | Mass | Length | ppm | m/z | z | RT | 1/k0 Start | 1/k0 End | Precursor ID | Area Sample 2 | #Feature | #Feature Sample 2 | Start | End | PTM | AScore | Found By |
| --- | --- | --- | --- | --- | --- | --- | --- | --- | --- | --- | --- | --- | --- | --- | --- | --- | --- | --- | --- |
| R.AGHGAVTSPQTQR.G | N | 58.95 | 1308.6534 | 13 | 4.15 | 655.3331 | 2 | 9.70 | 0.9146 | 0.9218 | 11889 | 1.61e4 | 2 | 2 | 135 | 147 |  |  | DB Search |
| R.VIGASGIHVGTM(+15.99)SFGK.M | N | 58.84 | 1575.8079 | 16 | 2.27 | 788.9087 | 2 | 14.96 | 1.1306 | 1.1378 | 41219 | 1.68e3 | 1 | 1 | 160 | 175 | Oxidation (M) | M12:Oxidation (M):1000 | DB Search |
| SNGGLVVGTIIKPK.L | Y | 56.21 | 1381.8293 | 14 | 0.75 | 691.9186 | 2 | 15.49 | 1.0528 | 1.06 | 43994 | 1.43e3 | 1 | 1 | 1 | 14 |  |  | DB Search |
| total 3 peptides |  |  |  |  |  |  |  |  |  |  |  |  |  |  |  |  |  |  |  |
| --- | --- | --- | --- | --- | --- | --- | --- | --- | --- | --- | --- | --- | --- | --- | --- | --- | --- | --- | --- |

Best Unique PSM (Scan Sample\_11\_A1\_1\_3648.d:43994, m/z=691.9186, z=2, RT=15.49, ppm=-4.73):


TRINITY\_DN3036\_c0\_g1\_i2.p2

back to list

  

| Protein Coverage
| Supporting Peptides
| Best Unique PSM
|

Protein Coverage:

Supporting Peptides:

| Peptide | Uniq | -10lgP | Mass | Length | ppm | m/z | z | RT | 1/k0 Start | 1/k0 End | Precursor ID | Area Sample 2 | #Feature | #Feature Sample 2 | Start | End | PTM | AScore | Found By |
| --- | --- | --- | --- | --- | --- | --- | --- | --- | --- | --- | --- | --- | --- | --- | --- | --- | --- | --- | --- |
| R.AGHGAVTSPQTQR.G | N | 58.95 | 1308.6534 | 13 | 4.15 | 655.3331 | 2 | 9.70 | 0.9146 | 0.9218 | 11889 | 1.61e4 | 2 | 2 | 309 | 321 |  |  | DB Search |
| R.GTSNGGLVVGTIIKPK.L | Y | 58.87 | 1539.8984 | 16 | 2.71 | 770.9543 | 2 | 15.49 | 1.1254 | 1.1326 | 43901 | 6.84e3 | 1 | 1 | 173 | 188 |  |  | DB Search |
| R.VIGASGIHVGTM(+15.99)SFGK.M | N | 58.84 | 1575.8079 | 16 | 2.27 | 788.9087 | 2 | 14.96 | 1.1306 | 1.1378 | 41219 | 1.68e3 | 1 | 1 | 334 | 349 | Oxidation (M) | M12:Oxidation (M):1000 | DB Search |
| total 3 peptides |  |  |  |  |  |  |  |  |  |  |  |  |  |  |  |  |  |  |  |
| --- | --- | --- | --- | --- | --- | --- | --- | --- | --- | --- | --- | --- | --- | --- | --- | --- | --- | --- | --- |

Best Unique PSM (Scan Sample\_11\_A1\_1\_3648.d:43901, m/z=770.9543, z=2, RT=15.49, ppm=-2.77):


TRINITY\_DN1071\_c0\_g1\_i2.p1

back to list

  

| Protein Coverage
| Supporting Peptides
| Best Unique PSM
|

Protein Coverage:

Supporting Peptides:

| Peptide | Uniq | -10lgP | Mass | Length | ppm | m/z | z | RT | 1/k0 Start | 1/k0 End | Precursor ID | Area Sample 2 | #Feature | #Feature Sample 2 | Start | End | PTM | AScore | Found By |
| --- | --- | --- | --- | --- | --- | --- | --- | --- | --- | --- | --- | --- | --- | --- | --- | --- | --- | --- | --- |
| R.IQGHPTDKKFTK.N | Y | 55.25 | 1398.7619 | 12 | 4.46 | 467.2608 | 3 | 9.57 | 0.7677 | 0.775 | 11206 | 0 | 0 | 0 | 241 | 252 |  |  | DB Search |
| K.AVQIHPFNDVSVGDPK.V | Y | 31.99 | 1721.8736 | 16 | -1.26 | 861.9383 | 2 | 15.56 | 1.1158 | 1.123 | 44381 | 1.32e2 | 1 | 1 | 293 | 308 |  |  | DB Search |
| total 2 peptides |  |  |  |  |  |  |  |  |  |  |  |  |  |  |  |  |  |  |  |
| --- | --- | --- | --- | --- | --- | --- | --- | --- | --- | --- | --- | --- | --- | --- | --- | --- | --- | --- | --- |

Best Unique PSM (Scan Sample\_11\_A1\_1\_3648.d:11206, m/z=467.2608, z=3, RT=9.57, ppm=-1.02):


TRINITY\_DN2477\_c0\_g1\_i8.p1

back to list

  

| Protein Coverage
| Supporting Peptides
| Best Unique PSM
|

Protein Coverage:

Supporting Peptides:

| Peptide | Uniq | -10lgP | Mass | Length | ppm | m/z | z | RT | 1/k0 Start | 1/k0 End | Precursor ID | Area Sample 2 | #Feature | #Feature Sample 2 | Start | End | PTM | AScore | Found By |
| --- | --- | --- | --- | --- | --- | --- | --- | --- | --- | --- | --- | --- | --- | --- | --- | --- | --- | --- | --- |
| R.VAPEEHPVLLTEAPLNPK.A | Y | 58.19 | 1953.0571 | 18 | -0.07 | 977.5304 | 2 | 16.19 | 1.2089 | 1.2161 | 47306 | 2.52e2 | 1 | 1 | 29 | 46 |  |  | DB Search |
| R.GYSFTTTAEREIVR.D | Y | 58.1 | 1628.8158 | 14 | 2.53 | 815.4128 | 2 | 15.79 | 1.121 | 1.1282 | 45442 | 6.41e3 | 1 | 1 | 130 | 143 |  |  | DB Search |
| total 2 peptides |  |  |  |  |  |  |  |  |  |  |  |  |  |  |  |  |  |  |  |
| --- | --- | --- | --- | --- | --- | --- | --- | --- | --- | --- | --- | --- | --- | --- | --- | --- | --- | --- | --- |

Best Unique PSM (Scan Sample\_11\_A1\_1\_3648.d:47306, m/z=977.5304, z=2, RT=16.19, ppm=-5.54):


TRINITY\_DN691\_c0\_g1\_i1.p1

back to list

  

| Protein Coverage
| Supporting Peptides
| Best Unique PSM
|

Protein Coverage:

Supporting Peptides:

| Peptide | Uniq | -10lgP | Mass | Length | ppm | m/z | z | RT | 1/k0 Start | 1/k0 End | Precursor ID | Area Sample 2 | #Feature | #Feature Sample 2 | Start | End | PTM | AScore | Found By |
| --- | --- | --- | --- | --- | --- | --- | --- | --- | --- | --- | --- | --- | --- | --- | --- | --- | --- | --- | --- |
| R.AYGQDLGQPGSC(+57.02)LKR.F | Y | 57.94 | 1648.7991 | 15 | 3.8 | 825.4055 | 2 | 13.30 | 1.0721 | 1.0793 | 32177 | 9.59e2 | 1 | 1 | 1533 | 1547 | Carbamidomethylation |  | DB Search |
| total 1 peptides |  |  |  |  |  |  |  |  |  |  |  |  |  |  |  |  |  |  |  |
| --- | --- | --- | --- | --- | --- | --- | --- | --- | --- | --- | --- | --- | --- | --- | --- | --- | --- | --- | --- |

Best Unique PSM (Scan Sample\_11\_A1\_1\_3648.d:32177, m/z=825.4055, z=2, RT=13.30, ppm=-1.67):


TRINITY\_DN7659\_c0\_g1\_i1.p1

back to list

  

| Protein Coverage
| Supporting Peptides
| Best Unique PSM
|

Protein Coverage:

Supporting Peptides:

| Peptide | Uniq | -10lgP | Mass | Length | ppm | m/z | z | RT | 1/k0 Start | 1/k0 End | Precursor ID | Area Sample 2 | #Feature | #Feature Sample 2 | Start | End | PTM | AScore | Found By |
| --- | --- | --- | --- | --- | --- | --- | --- | --- | --- | --- | --- | --- | --- | --- | --- | --- | --- | --- | --- |
| M.A(+42.01)SAATDNRK.V | Y | 58.7 | 974.4781 | 9 | 5.12 | 488.2462 | 2 | 9.48 | 0.827 | 0.8343 | 10772 | 6.6e2 | 1 | 1 | 2 | 10 | Acetylation (Protein N-term) | A1:Acetylation (Protein N-term):1000 | DB Search |
| K.AQPTEIYNLGAQSHVK.V | Y | 50.58 | 1754.8951 | 16 | 0.53 | 878.4505 | 2 | 14.56 | 1.173 | 1.1802 | 39052 | 1.18e2 | 1 | 1 | 86 | 101 |  |  | DB Search |
| R.RGETFVTR.K | Y | 39.93 | 964.509 | 8 | -4.2 | 483.2571 | 2 | 11.39 | 0.8201 | 0.8274 | 21342 | 0 | 0 | 0 | 200 | 207 |  |  | DB Search |
| total 3 peptides |  |  |  |  |  |  |  |  |  |  |  |  |  |  |  |  |  |  |  |
| --- | --- | --- | --- | --- | --- | --- | --- | --- | --- | --- | --- | --- | --- | --- | --- | --- | --- | --- | --- |

Best Unique PSM (Scan Sample\_11\_A1\_1\_3648.d:10772, m/z=488.2462, z=2, RT=9.48, ppm=-0.36):


TRINITY\_DN1465\_c1\_g1\_i1.p1

back to list

  

| Protein Coverage
| Supporting Peptides
| Best Unique PSM
|

Protein Coverage:

Supporting Peptides:

| Peptide | Uniq | -10lgP | Mass | Length | ppm | m/z | z | RT | 1/k0 Start | 1/k0 End | Precursor ID | Area Sample 2 | #Feature | #Feature Sample 2 | Start | End | PTM | AScore | Found By |
| --- | --- | --- | --- | --- | --- | --- | --- | --- | --- | --- | --- | --- | --- | --- | --- | --- | --- | --- | --- |
| K.IQDKEGIPPDQQR.L | Y | 55.06 | 1522.7739 | 13 | 2.88 | 762.3923 | 2 | 11.00 | 1.0331 | 1.0403 | 18584 | 2.97e3 | 2 | 2 | 34 | 46 |  |  | DB Search |
| total 1 peptides |  |  |  |  |  |  |  |  |  |  |  |  |  |  |  |  |  |  |  |
| --- | --- | --- | --- | --- | --- | --- | --- | --- | --- | --- | --- | --- | --- | --- | --- | --- | --- | --- | --- |

Best Unique PSM (Scan Sample\_11\_A1\_1\_3648.d:18584, m/z=762.3923, z=2, RT=11.00, ppm=-2.59):


TRINITY\_DN286\_c0\_g1\_i2.p1

back to list

  

| Protein Coverage
| Supporting Peptides
| Best Unique PSM
|

Protein Coverage:

Supporting Peptides:

| Peptide | Uniq | -10lgP | Mass | Length | ppm | m/z | z | RT | 1/k0 Start | 1/k0 End | Precursor ID | Area Sample 2 | #Feature | #Feature Sample 2 | Start | End | PTM | AScore | Found By |
| --- | --- | --- | --- | --- | --- | --- | --- | --- | --- | --- | --- | --- | --- | --- | --- | --- | --- | --- | --- |
| K.RYGQAMC(+57.02)QFMR.N | Y | 58.88 | 1446.6319 | 11 | -6.73 | 724.3144 | 2 | 17.00 | 0.9836 | 0.9908 | 51341 | 4.96e3 | 1 | 1 | 71 | 81 | Carbamidomethylation |  | DB Search |
| K.RYGQAM(+15.99)C(+57.02)QFMR.N | Y | 56 | 1462.6268 | 11 | -6.37 | 732.312 | 2 | 14.39 | 0.9755 | 0.9828 | 37937 | 1.17e3 | 1 | 1 | 71 | 81 | Oxidation (M), Carbamidomethylation | M6:Oxidation (M):38.03 | DB Search |
| K.RYGQAM(+15.99)C(+57.02)QFM(+15.99)R.N | Y | 55.34 | 1478.6217 | 11 | -6.44 | 740.3093 | 2 | 12.85 | 0.9739 | 0.9812 | 29490 | 4.93e3 | 1 | 1 | 71 | 81 | Oxidation (M), Carbamidomethylation, Oxidation (M) | M6:Oxidation (M):1000 M10:Oxidation (M):1000 | DB Search |
| total 3 peptides |  |  |  |  |  |  |  |  |  |  |  |  |  |  |  |  |  |  |  |
| --- | --- | --- | --- | --- | --- | --- | --- | --- | --- | --- | --- | --- | --- | --- | --- | --- | --- | --- | --- |

Best Unique PSM (Scan Sample\_11\_A1\_1\_3648.d:51341, m/z=724.3144, z=2, RT=17.00, ppm=-12.2):


TRINITY\_DN8101\_c0\_g1\_i11.p2

back to list

  

| Protein Coverage
| Supporting Peptides
| Best Unique PSM
|

Protein Coverage:

Supporting Peptides:

| Peptide | Uniq | -10lgP | Mass | Length | ppm | m/z | z | RT | 1/k0 Start | 1/k0 End | Precursor ID | Area Sample 2 | #Feature | #Feature Sample 2 | Start | End | PTM | AScore | Found By |
| --- | --- | --- | --- | --- | --- | --- | --- | --- | --- | --- | --- | --- | --- | --- | --- | --- | --- | --- | --- |
| R.SIGFIGHYLDQLR.L | Y | 54.32 | 1517.799 | 13 | 1.06 | 759.9034 | 2 | 19.56 | 1.0849 | 1.0921 | 65092 | 4.69e2 | 1 | 1 | 1077 | 1089 |  |  | DB Search |
| total 1 peptides |  |  |  |  |  |  |  |  |  |  |  |  |  |  |  |  |  |  |  |
| --- | --- | --- | --- | --- | --- | --- | --- | --- | --- | --- | --- | --- | --- | --- | --- | --- | --- | --- | --- |

Best Unique PSM (Scan Sample\_11\_A1\_1\_3648.d:65092, m/z=759.9034, z=2, RT=19.56, ppm=-4.41):


TRINITY\_DN172571\_c0\_g1\_i1.p1

back to list

  

| Protein Coverage
| Supporting Peptides
| Best Unique PSM
|

Protein Coverage:

Supporting Peptides:

| Peptide | Uniq | -10lgP | Mass | Length | ppm | m/z | z | RT | 1/k0 Start | 1/k0 End | Precursor ID | Area Sample 2 | #Feature | #Feature Sample 2 | Start | End | PTM | AScore | Found By |
| --- | --- | --- | --- | --- | --- | --- | --- | --- | --- | --- | --- | --- | --- | --- | --- | --- | --- | --- | --- |
| R.DNIQGITKPAIR.R | Y | 58.88 | 1324.7463 | 12 | 3.85 | 663.3793 | 2 | 13.67 | 0.9747 | 0.982 | 34267 | 5.79e3 | 2 | 2 | 25 | 36 |  |  | DB Search |
| K.RISGLIYEETR.G | Y | 58.85 | 1335.7146 | 11 | 5.98 | 668.8649 | 2 | 14.62 | 0.9695 | 0.9767 | 39433 | 6.26e3 | 1 | 1 | 46 | 56 |  |  | DB Search |
| R.DNIQGITKPAIRR.L | Y | 58.42 | 1480.8474 | 13 | 5.31 | 494.623 | 3 | 12.73 | 0.7852 | 0.7925 | 28785 | 1.32e3 | 1 | 1 | 25 | 37 |  |  | DB Search |
| R.ISGLIYEETRGVLK.V | Y | 57.65 | 1576.8824 | 14 | 4.01 | 789.4473 | 2 | 17.11 | 1.0945 | 1.1017 | 51906 | 3.57e3 | 1 | 1 | 47 | 60 |  |  | DB Search |
| K.RISGLIYEETRGVLK.V | Y | 55.36 | 1732.9835 | 15 | 1.37 | 578.6661 | 3 | 16.41 | 0.8911 | 0.8984 | 48406 | 2.02e3 | 1 | 1 | 46 | 60 |  |  | DB Search |
| total 5 peptides |  |  |  |  |  |  |  |  |  |  |  |  |  |  |  |  |  |  |  |
| --- | --- | --- | --- | --- | --- | --- | --- | --- | --- | --- | --- | --- | --- | --- | --- | --- | --- | --- | --- |

Best Unique PSM (Scan Sample\_11\_A1\_1\_3648.d:34267, m/z=663.3793, z=2, RT=13.67, ppm=-1.63):


TRINITY\_DN6390\_c0\_g1\_i1.p1

back to list

  

| Protein Coverage
| Supporting Peptides
| Best Unique PSM
|

Protein Coverage:

Supporting Peptides:

| Peptide | Uniq | -10lgP | Mass | Length | ppm | m/z | z | RT | 1/k0 Start | 1/k0 End | Precursor ID | Area Sample 2 | #Feature | #Feature Sample 2 | Start | End | PTM | AScore | Found By |
| --- | --- | --- | --- | --- | --- | --- | --- | --- | --- | --- | --- | --- | --- | --- | --- | --- | --- | --- | --- |
| R.VGNWRDETLSC(+57.02)K.Q | Y | 57.99 | 1463.6827 | 12 | 3.82 | 732.8474 | 2 | 13.58 | 1.0114 | 1.0186 | 33747 | 3.4e2 | 1 | 1 | 1047 | 1058 | Carbamidomethylation |  | DB Search |
| total 1 peptides |  |  |  |  |  |  |  |  |  |  |  |  |  |  |  |  |  |  |  |
| --- | --- | --- | --- | --- | --- | --- | --- | --- | --- | --- | --- | --- | --- | --- | --- | --- | --- | --- | --- |

Best Unique PSM (Scan Sample\_11\_A1\_1\_3648.d:33747, m/z=732.8474, z=2, RT=13.58, ppm=-1.65):


TRINITY\_DN24414\_c1\_g1\_i2.p2

back to list

  

| Protein Coverage
| Supporting Peptides
| Best Unique PSM
|

Protein Coverage:

Supporting Peptides:

| Peptide | Uniq | -10lgP | Mass | Length | ppm | m/z | z | RT | 1/k0 Start | 1/k0 End | Precursor ID | Area Sample 2 | #Feature | #Feature Sample 2 | Start | End | PTM | AScore | Found By |
| --- | --- | --- | --- | --- | --- | --- | --- | --- | --- | --- | --- | --- | --- | --- | --- | --- | --- | --- | --- |
| K.VLTSDDPAEKEK.K | Y | 58.87 | 1330.6616 | 12 | 2.64 | 666.3362 | 2 | 10.69 | 0.9848 | 0.992 | 17191 | 9.01e3 | 1 | 1 | 95 | 106 |  |  | DB Search |
| total 1 peptides |  |  |  |  |  |  |  |  |  |  |  |  |  |  |  |  |  |  |  |
| --- | --- | --- | --- | --- | --- | --- | --- | --- | --- | --- | --- | --- | --- | --- | --- | --- | --- | --- | --- |

Best Unique PSM (Scan Sample\_11\_A1\_1\_3648.d:17191, m/z=666.3362, z=2, RT=10.69, ppm=-2.84):


TRINITY\_DN1933\_c0\_g1\_i1.p2

back to list

  

| Protein Coverage
| Supporting Peptides
| Best Unique PSM
|

Protein Coverage:

Supporting Peptides:

| Peptide | Uniq | -10lgP | Mass | Length | ppm | m/z | z | RT | 1/k0 Start | 1/k0 End | Precursor ID | Area Sample 2 | #Feature | #Feature Sample 2 | Start | End | PTM | AScore | Found By |
| --- | --- | --- | --- | --- | --- | --- | --- | --- | --- | --- | --- | --- | --- | --- | --- | --- | --- | --- | --- |
| R.RNLDIERPTYTNLNR.L | Y | 46.58 | 1873.9758 | 15 | -2.43 | 625.6609 | 3 | 13.94 | 0.8858 | 0.8931 | 35770 | 1.17e3 | 1 | 1 | 75 | 89 |  |  | DB Search |
| total 1 peptides |  |  |  |  |  |  |  |  |  |  |  |  |  |  |  |  |  |  |  |
| --- | --- | --- | --- | --- | --- | --- | --- | --- | --- | --- | --- | --- | --- | --- | --- | --- | --- | --- | --- |

Best Unique PSM (Scan Sample\_11\_A1\_1\_3648.d:35770, m/z=625.6609, z=3, RT=13.94, ppm=-7.91):


TRINITY\_DN240\_c0\_g1\_i1.p1

back to list

  

| Protein Coverage
| Supporting Peptides
| Best Unique PSM
|

Protein Coverage:

Supporting Peptides:

| Peptide | Uniq | -10lgP | Mass | Length | ppm | m/z | z | RT | 1/k0 Start | 1/k0 End | Precursor ID | Area Sample 2 | #Feature | #Feature Sample 2 | Start | End | PTM | AScore | Found By |
| --- | --- | --- | --- | --- | --- | --- | --- | --- | --- | --- | --- | --- | --- | --- | --- | --- | --- | --- | --- |
| K.AYAAFLEFKDVVK.A | Y | 58.73 | 1499.8024 | 13 | -0.24 | 750.9042 | 2 | 18.72 | 1.0941 | 1.1013 | 60609 | 3.07e3 | 1 | 1 | 217 | 229 |  |  | DB Search |
| total 1 peptides |  |  |  |  |  |  |  |  |  |  |  |  |  |  |  |  |  |  |  |
| --- | --- | --- | --- | --- | --- | --- | --- | --- | --- | --- | --- | --- | --- | --- | --- | --- | --- | --- | --- |

Best Unique PSM (Scan Sample\_11\_A1\_1\_3648.d:60609, m/z=750.9042, z=2, RT=18.72, ppm=-5.71):


TRINITY\_DN902\_c1\_g1\_i1.p1

back to list

  

| Protein Coverage
| Supporting Peptides
| Best Unique PSM
|

Protein Coverage:

Supporting Peptides:

| Peptide | Uniq | -10lgP | Mass | Length | ppm | m/z | z | RT | 1/k0 Start | 1/k0 End | Precursor ID | Area Sample 2 | #Feature | #Feature Sample 2 | Start | End | PTM | AScore | Found By |
| --- | --- | --- | --- | --- | --- | --- | --- | --- | --- | --- | --- | --- | --- | --- | --- | --- | --- | --- | --- |
| K.FGQATC(+57.02)QFMR.N | Y | 58.88 | 1244.543 | 10 | -15.5 | 623.2657 | 2 | 13.50 | 0.9194 | 0.9267 | 33267 | 2.65e3 | 1 | 1 | 71 | 80 | Carbamidomethylation |  | DB Search |
| total 1 peptides |  |  |  |  |  |  |  |  |  |  |  |  |  |  |  |  |  |  |  |
| --- | --- | --- | --- | --- | --- | --- | --- | --- | --- | --- | --- | --- | --- | --- | --- | --- | --- | --- | --- |

Best Unique PSM (Scan Sample\_11\_A1\_1\_3648.d:33267, m/z=623.2657, z=2, RT=13.50, ppm=-20.97):


TRINITY\_DN168534\_c0\_g1\_i1.p1

back to list

  

| Protein Coverage
| Supporting Peptides
| Best Unique PSM
|

Protein Coverage:

Supporting Peptides:

| Peptide | Uniq | -10lgP | Mass | Length | ppm | m/z | z | RT | 1/k0 Start | 1/k0 End | Precursor ID | Area Sample 2 | #Feature | #Feature Sample 2 | Start | End | PTM | AScore | Found By |
| --- | --- | --- | --- | --- | --- | --- | --- | --- | --- | --- | --- | --- | --- | --- | --- | --- | --- | --- | --- |
| K.AM(+15.99)TDC(+57.02)KETLC(+57.02)R.G | Y | 57.92 | 1399.5894 | 11 | 4.81 | 700.8015 | 2 | 10.58 | 0.9993 | 1.0065 | 16561 | 0 | 0 | 0 | 85 | 95 | Oxidation (M), Carbamidomethylation, Carbamidomethylation | M2:Oxidation (M):1000 | DB Search |
| R.GDYC(+57.02)NSALIEAEQKER.K | Y | 55.62 | 1881.8527 | 16 | 4.16 | 941.9324 | 2 | 14.25 | 1.2069 | 1.2141 | 37442 | 0 | 0 | 0 | 96 | 111 | Carbamidomethylation |  | DB Search |
| total 2 peptides |  |  |  |  |  |  |  |  |  |  |  |  |  |  |  |  |  |  |  |
| --- | --- | --- | --- | --- | --- | --- | --- | --- | --- | --- | --- | --- | --- | --- | --- | --- | --- | --- | --- |

Best Unique PSM (Scan Sample\_11\_A1\_1\_3648.d:16561, m/z=700.8015, z=2, RT=10.58, ppm=-0.66):


TRINITY\_DN3448\_c0\_g2\_i1.p1

back to list

  

| Protein Coverage
| Supporting Peptides
| Best Unique PSM
|

Protein Coverage:

Supporting Peptides:

| Peptide | Uniq | -10lgP | Mass | Length | ppm | m/z | z | RT | 1/k0 Start | 1/k0 End | Precursor ID | Area Sample 2 | #Feature | #Feature Sample 2 | Start | End | PTM | AScore | Found By |
| --- | --- | --- | --- | --- | --- | --- | --- | --- | --- | --- | --- | --- | --- | --- | --- | --- | --- | --- | --- |
| K.AGM(+15.99)THIVR.D | Y | 38.19 | 899.4647 | 8 | 6.96 | 450.7403 | 2 | 9.74 | 0.8254 | 0.8327 | 11994 | 0 | 0 | 0 | 31 | 38 | Oxidation (M) | M3:Oxidation (M):1000 | DB Search |
| total 1 peptides |  |  |  |  |  |  |  |  |  |  |  |  |  |  |  |  |  |  |  |
| --- | --- | --- | --- | --- | --- | --- | --- | --- | --- | --- | --- | --- | --- | --- | --- | --- | --- | --- | --- |

Best Unique PSM (Scan Sample\_11\_A1\_1\_3648.d:11994, m/z=450.7403, z=2, RT=9.74, ppm=1.48):


TRINITY\_DN138\_c0\_g1\_i1.p1

back to list

  

| Protein Coverage
| Supporting Peptides
| Best Unique PSM
|

Protein Coverage:

Supporting Peptides:

| Peptide | Uniq | -10lgP | Mass | Length | ppm | m/z | z | RT | 1/k0 Start | 1/k0 End | Precursor ID | Area Sample 2 | #Feature | #Feature Sample 2 | Start | End | PTM | AScore | Found By |
| --- | --- | --- | --- | --- | --- | --- | --- | --- | --- | --- | --- | --- | --- | --- | --- | --- | --- | --- | --- |
| K.DMGVNVISVGAGHTINHR.E | Y | 55.26 | 1875.9373 | 18 | 0.67 | 938.9714 | 2 | 15.08 | 1.2451 | 1.2523 | 41936 | 0 | 0 | 0 | 349 | 366 |  |  | DB Search |
| total 1 peptides |  |  |  |  |  |  |  |  |  |  |  |  |  |  |  |  |  |  |  |
| --- | --- | --- | --- | --- | --- | --- | --- | --- | --- | --- | --- | --- | --- | --- | --- | --- | --- | --- | --- |

Best Unique PSM (Scan Sample\_11\_A1\_1\_3648.d:41936, m/z=938.9714, z=2, RT=15.08, ppm=-4.8):


TRINITY\_DN10907\_c0\_g1\_i1.p1

back to list

  

| Protein Coverage
| Supporting Peptides
| Best Unique PSM
|

Protein Coverage:

Supporting Peptides:

| Peptide | Uniq | -10lgP | Mass | Length | ppm | m/z | z | RT | 1/k0 Start | 1/k0 End | Precursor ID | Area Sample 2 | #Feature | #Feature Sample 2 | Start | End | PTM | AScore | Found By |
| --- | --- | --- | --- | --- | --- | --- | --- | --- | --- | --- | --- | --- | --- | --- | --- | --- | --- | --- | --- |
| R.NIDEHASEGVER.M | Y | 58.02 | 1354.6113 | 12 | 8.37 | 678.3149 | 2 | 11.02 | 0.9695 | 0.9767 | 19141 | 1.13e3 | 1 | 1 | 106 | 117 |  |  | DB Search |
| total 1 peptides |  |  |  |  |  |  |  |  |  |  |  |  |  |  |  |  |  |  |  |
| --- | --- | --- | --- | --- | --- | --- | --- | --- | --- | --- | --- | --- | --- | --- | --- | --- | --- | --- | --- |

Best Unique PSM (Scan Sample\_11\_A1\_1\_3648.d:19141, m/z=678.3149, z=2, RT=11.02, ppm=2.89):


TRINITY\_DN471\_c0\_g1\_i4.p1

back to list

  

| Protein Coverage
| Supporting Peptides
| Best Unique PSM
|

Protein Coverage:

Supporting Peptides:

| Peptide | Uniq | -10lgP | Mass | Length | ppm | m/z | z | RT | 1/k0 Start | 1/k0 End | Precursor ID | Area Sample 2 | #Feature | #Feature Sample 2 | Start | End | PTM | AScore | Found By |
| --- | --- | --- | --- | --- | --- | --- | --- | --- | --- | --- | --- | --- | --- | --- | --- | --- | --- | --- | --- |
| R.TIQFVDWC(+57.02)PTGFK.C | Y | 54.82 | 1597.7599 | 13 | -6.53 | 799.8776 | 2 | 19.88 | 1.0351 | 1.0423 | 66701 | 3.31e2 | 1 | 1 | 378 | 390 | Carbamidomethylation |  | DB Search |
| R.RNLDIERPTYTNLNR.L | Y | 46.58 | 1873.9758 | 15 | -2.43 | 625.6609 | 3 | 13.94 | 0.8858 | 0.8931 | 35770 | 1.17e3 | 1 | 1 | 253 | 267 |  |  | DB Search |
| total 2 peptides |  |  |  |  |  |  |  |  |  |  |  |  |  |  |  |  |  |  |  |
| --- | --- | --- | --- | --- | --- | --- | --- | --- | --- | --- | --- | --- | --- | --- | --- | --- | --- | --- | --- |

Best Unique PSM (Scan Sample\_11\_A1\_1\_3648.d:66701, m/z=799.3823, z=2, RT=19.90, ppm=-12):


TRINITY\_DN161\_c0\_g1\_i3.p1

back to list

  

| Protein Coverage
| Supporting Peptides
| Best Unique PSM
|

Protein Coverage:

Supporting Peptides:

| Peptide | Uniq | -10lgP | Mass | Length | ppm | m/z | z | RT | 1/k0 Start | 1/k0 End | Precursor ID | Area Sample 2 | #Feature | #Feature Sample 2 | Start | End | PTM | AScore | Found By |
| --- | --- | --- | --- | --- | --- | --- | --- | --- | --- | --- | --- | --- | --- | --- | --- | --- | --- | --- | --- |
| K.DSYVGDEAQSKR.G | Y | 59.21 | 1353.616 | 12 | 5.76 | 677.8155 | 2 | 10.93 | 1.0037 | 1.011 | 18642 | 9.97e3 | 1 | 1 | 74 | 85 |  |  | DB Search |
| R.LDLAGRDLTDYLM(+15.99)K.I | Y | 58.87 | 1638.8287 | 14 | 0.58 | 820.4176 | 2 | 17.97 | 1.1438 | 1.151 | 56587 | 5.44e3 | 1 | 1 | 201 | 214 | Oxidation (M) | M13:Oxidation (M):1000 | DB Search |
| R.AVFPSIVGRPR.H | Y | 58.86 | 1197.6982 | 11 | -0.21 | 599.853 | 2 | 15.44 | 0.9586 | 0.9658 | 43471 | 2.65e4 | 1 | 1 | 52 | 62 |  |  | DB Search |
| R.LDLAGRDLTDYLMK.I | Y | 58.52 | 1622.8338 | 14 | 2.44 | 812.4217 | 2 | 18.99 | 1.0961 | 1.1033 | 62153 | 2.2e3 | 1 | 1 | 201 | 214 |  |  | DB Search |
| R.VAPEEHPVLLTEAPLNPK.A | Y | 58.19 | 1953.0571 | 18 | -0.07 | 977.5304 | 2 | 16.19 | 1.2089 | 1.2161 | 47306 | 2.52e2 | 1 | 1 | 119 | 136 |  |  | DB Search |
| R.GYSFTTTAEREIVR.D | Y | 58.1 | 1628.8158 | 14 | 2.53 | 815.4128 | 2 | 15.79 | 1.121 | 1.1282 | 45442 | 6.41e3 | 1 | 1 | 220 | 233 |  |  | DB Search |
| R.HQGVM(+15.99)VGM(+15.99)GQK.D | Y | 55.12 | 1202.5536 | 11 | 3.32 | 602.2828 | 2 | 9.73 | 0.9105 | 0.9178 | 11903 | 0 | 0 | 0 | 63 | 73 | Oxidation (M), Oxidation (M) | M5:Oxidation (M):1000 M8:Oxidation (M):1000 | DB Search |
| R.TTGIVLDSGDGVSHTVPIYEGYALPHAIIR.L | Y | 52.38 | 3150.6349 | 30 | -8.99 | 788.6546 | 4 | 18.79 | 1.0693 | 1.0765 | 61107 | 0 | 0 | 0 | 171 | 200 |  |  | DB Search |
| total 8 peptides |  |  |  |  |  |  |  |  |  |  |  |  |  |  |  |  |  |  |  |
| --- | --- | --- | --- | --- | --- | --- | --- | --- | --- | --- | --- | --- | --- | --- | --- | --- | --- | --- | --- |

Best Unique PSM (Scan Sample\_11\_A1\_1\_3648.d:18642, m/z=677.8155, z=2, RT=10.93, ppm=0.28):


TRINITY\_DN36\_c0\_g1\_i4.p1

back to list

  

| Protein Coverage
| Supporting Peptides
| Best Unique PSM
|

Protein Coverage:

Supporting Peptides:

| Peptide | Uniq | -10lgP | Mass | Length | ppm | m/z | z | RT | 1/k0 Start | 1/k0 End | Precursor ID | Area Sample 2 | #Feature | #Feature Sample 2 | Start | End | PTM | AScore | Found By |
| --- | --- | --- | --- | --- | --- | --- | --- | --- | --- | --- | --- | --- | --- | --- | --- | --- | --- | --- | --- |
| R.LGGIEVTQGNC(+57.02)ARK.Y | Y | 58.27 | 1501.7671 | 14 | 1.48 | 751.8878 | 2 | 12.59 | 1.0616 | 1.0688 | 28229 | 7.18e2 | 1 | 1 | 58 | 71 | Carbamidomethylation |  | DB Search |
| total 1 peptides |  |  |  |  |  |  |  |  |  |  |  |  |  |  |  |  |  |  |  |
| --- | --- | --- | --- | --- | --- | --- | --- | --- | --- | --- | --- | --- | --- | --- | --- | --- | --- | --- | --- |

Best Unique PSM (Scan Sample\_11\_A1\_1\_3648.d:28229, m/z=751.8878, z=2, RT=12.59, ppm=-4):


TRINITY\_DN902\_c1\_g1\_i2.p1

back to list

  

| Protein Coverage
| Supporting Peptides
| Best Unique PSM
|

Protein Coverage:

Supporting Peptides:

| Peptide | Uniq | -10lgP | Mass | Length | ppm | m/z | z | RT | 1/k0 Start | 1/k0 End | Precursor ID | Area Sample 2 | #Feature | #Feature Sample 2 | Start | End | PTM | AScore | Found By |
| --- | --- | --- | --- | --- | --- | --- | --- | --- | --- | --- | --- | --- | --- | --- | --- | --- | --- | --- | --- |
| K.FGQATC(+57.02)QFMR.N | Y | 58.88 | 1244.543 | 10 | -15.5 | 623.2657 | 2 | 13.50 | 0.9194 | 0.9267 | 33267 | 2.65e3 | 1 | 1 | 71 | 80 | Carbamidomethylation |  | DB Search |
| total 1 peptides |  |  |  |  |  |  |  |  |  |  |  |  |  |  |  |  |  |  |  |
| --- | --- | --- | --- | --- | --- | --- | --- | --- | --- | --- | --- | --- | --- | --- | --- | --- | --- | --- | --- |

Best Unique PSM (Scan Sample\_11\_A1\_1\_3648.d:33267, m/z=623.2657, z=2, RT=13.50, ppm=-20.97):


TRINITY\_DN3036\_c0\_g1\_i19.p1

back to list

  

| Protein Coverage
| Supporting Peptides
| Best Unique PSM
|

Protein Coverage:

Supporting Peptides:

| Peptide | Uniq | -10lgP | Mass | Length | ppm | m/z | z | RT | 1/k0 Start | 1/k0 End | Precursor ID | Area Sample 2 | #Feature | #Feature Sample 2 | Start | End | PTM | AScore | Found By |
| --- | --- | --- | --- | --- | --- | --- | --- | --- | --- | --- | --- | --- | --- | --- | --- | --- | --- | --- | --- |
| R.AGHGAVTSPQTQR.G | N | 58.95 | 1308.6534 | 13 | 4.15 | 655.3331 | 2 | 9.70 | 0.9146 | 0.9218 | 11889 | 1.61e4 | 2 | 2 | 135 | 147 |  |  | DB Search |
| R.VIGASGIHVGTM(+15.99)SFGK.M | N | 58.84 | 1575.8079 | 16 | 2.27 | 788.9087 | 2 | 14.96 | 1.1306 | 1.1378 | 41219 | 1.68e3 | 1 | 1 | 160 | 175 | Oxidation (M) | M12:Oxidation (M):1000 | DB Search |
| SNGGLVVGTIIKPK.L | Y | 56.21 | 1381.8293 | 14 | 0.75 | 691.9186 | 2 | 15.49 | 1.0528 | 1.06 | 43994 | 1.43e3 | 1 | 1 | 1 | 14 |  |  | DB Search |
| total 3 peptides |  |  |  |  |  |  |  |  |  |  |  |  |  |  |  |  |  |  |  |
| --- | --- | --- | --- | --- | --- | --- | --- | --- | --- | --- | --- | --- | --- | --- | --- | --- | --- | --- | --- |

Best Unique PSM (Scan Sample\_11\_A1\_1\_3648.d:43994, m/z=691.9186, z=2, RT=15.49, ppm=-4.73):


TRINITY\_DN477\_c0\_g1\_i9.p1

back to list

  

| Protein Coverage
| Supporting Peptides
| Best Unique PSM
|

Protein Coverage:

Supporting Peptides:

| Peptide | Uniq | -10lgP | Mass | Length | ppm | m/z | z | RT | 1/k0 Start | 1/k0 End | Precursor ID | Area Sample 2 | #Feature | #Feature Sample 2 | Start | End | PTM | AScore | Found By |
| --- | --- | --- | --- | --- | --- | --- | --- | --- | --- | --- | --- | --- | --- | --- | --- | --- | --- | --- | --- |
| R.TQAVLDRLNIEKR.V | Y | 57.97 | 1554.8842 | 13 | -4.75 | 519.2967 | 3 | 14.41 | 0.8031 | 0.8104 | 38344 | 0 | 0 | 0 | 220 | 232 |  |  | DB Search |
| R.TQAVLDRLNIEK.R | Y | 53.78 | 1398.783 | 12 | 2.78 | 700.3969 | 2 | 15.15 | 1.009 | 1.0162 | 42294 | 0 | 0 | 0 | 220 | 231 |  |  | DB Search |
| K.MRFDTAITAC(+57.02)R.S | Y | 50.32 | 1340.6329 | 11 | 4.02 | 671.3228 | 2 | 14.75 | 0.9622 | 0.9695 | 40083 | 6.01e2 | 1 | 1 | 276 | 286 | Carbamidomethylation |  | DB Search |
| total 3 peptides |  |  |  |  |  |  |  |  |  |  |  |  |  |  |  |  |  |  |  |
| --- | --- | --- | --- | --- | --- | --- | --- | --- | --- | --- | --- | --- | --- | --- | --- | --- | --- | --- | --- |

Best Unique PSM (Scan Sample\_11\_A1\_1\_3648.d:38344, m/z=519.2967, z=3, RT=14.41, ppm=-10.23):


TRINITY\_DN3271\_c0\_g1\_i2.p1

back to list

  

| Protein Coverage
| Supporting Peptides
| Best Unique PSM
|

Protein Coverage:

Supporting Peptides:

| Peptide | Uniq | -10lgP | Mass | Length | ppm | m/z | z | RT | 1/k0 Start | 1/k0 End | Precursor ID | Area Sample 2 | #Feature | #Feature Sample 2 | Start | End | PTM | AScore | Found By |
| --- | --- | --- | --- | --- | --- | --- | --- | --- | --- | --- | --- | --- | --- | --- | --- | --- | --- | --- | --- |
| K.TIESEAAHGTVTR.H | Y | 58.89 | 1370.679 | 13 | 4.55 | 686.3461 | 2 | 11.86 | 1.021 | 1.0283 | 23944 | 8.27e2 | 1 | 1 | 354 | 366 |  |  | DB Search |
| total 1 peptides |  |  |  |  |  |  |  |  |  |  |  |  |  |  |  |  |  |  |  |
| --- | --- | --- | --- | --- | --- | --- | --- | --- | --- | --- | --- | --- | --- | --- | --- | --- | --- | --- | --- |

Best Unique PSM (Scan Sample\_11\_A1\_1\_3648.d:23944, m/z=686.3461, z=2, RT=11.86, ppm=-0.93):


TRINITY\_DN250\_c0\_g1\_i1.p1

back to list

  

| Protein Coverage
| Supporting Peptides
| Best Unique PSM
|

Protein Coverage:

Supporting Peptides:

| Peptide | Uniq | -10lgP | Mass | Length | ppm | m/z | z | RT | 1/k0 Start | 1/k0 End | Precursor ID | Area Sample 2 | #Feature | #Feature Sample 2 | Start | End | PTM | AScore | Found By |
| --- | --- | --- | --- | --- | --- | --- | --- | --- | --- | --- | --- | --- | --- | --- | --- | --- | --- | --- | --- |
| K.ELVDQVLDRIR.K | Y | 58.86 | 1354.7568 | 11 | 2.67 | 678.3838 | 2 | 18.48 | 1.009 | 1.0162 | 59338 | 1.45e3 | 1 | 1 | 113 | 123 |  |  | DB Search |
| R.TIQFVDWC(+57.02)PTGFK.V | Y | 54.82 | 1597.7599 | 13 | -6.53 | 799.8776 | 2 | 19.88 | 1.0351 | 1.0423 | 66701 | 3.31e2 | 1 | 1 | 340 | 352 | Carbamidomethylation |  | DB Search |
| R.RNLDIERPTYTNLNR.L | Y | 46.58 | 1873.9758 | 15 | -2.43 | 625.6609 | 3 | 13.94 | 0.8858 | 0.8931 | 35770 | 1.17e3 | 1 | 1 | 215 | 229 |  |  | DB Search |
| total 3 peptides |  |  |  |  |  |  |  |  |  |  |  |  |  |  |  |  |  |  |  |
| --- | --- | --- | --- | --- | --- | --- | --- | --- | --- | --- | --- | --- | --- | --- | --- | --- | --- | --- | --- |

Best Unique PSM (Scan Sample\_11\_A1\_1\_3648.d:59338, m/z=678.3838, z=2, RT=18.48, ppm=-2.81):


TRINITY\_DN6920\_c0\_g1\_i2.p1

back to list

  

| Protein Coverage
| Supporting Peptides
| Best Unique PSM
|

Protein Coverage:

Supporting Peptides:

| Peptide | Uniq | -10lgP | Mass | Length | ppm | m/z | z | RT | 1/k0 Start | 1/k0 End | Precursor ID | Area Sample 2 | #Feature | #Feature Sample 2 | Start | End | PTM | AScore | Found By |
| --- | --- | --- | --- | --- | --- | --- | --- | --- | --- | --- | --- | --- | --- | --- | --- | --- | --- | --- | --- |
| R.VLVHTQQK.L | Y | 57.79 | 951.5501 | 8 | -1.07 | 476.7792 | 2 | 9.55 | 0.8352 | 0.8425 | 11014 | 5.75e2 | 1 | 1 | 169 | 176 |  |  | DB Search |
| K.AGM(+15.99)THIVR.E | Y | 38.19 | 899.4647 | 8 | 6.96 | 450.7403 | 2 | 9.74 | 0.8254 | 0.8327 | 11994 | 0 | 0 | 0 | 58 | 65 | Oxidation (M) | M3:Oxidation (M):1000 | DB Search |
| R.HGSLGFLPR.K | Y | 32.83 | 982.5348 | 9 | -0.04 | 492.272 | 2 | 16.93 | 0.8408 | 0.8481 | 51108 | 4.36e2 | 1 | 1 | 18 | 26 |  |  | DB Search |
| total 3 peptides |  |  |  |  |  |  |  |  |  |  |  |  |  |  |  |  |  |  |  |
| --- | --- | --- | --- | --- | --- | --- | --- | --- | --- | --- | --- | --- | --- | --- | --- | --- | --- | --- | --- |

Best Unique PSM (Scan Sample\_11\_A1\_1\_3648.d:11014, m/z=477.7582, z=2, RT=9.56, ppm=-6.55):


TRINITY\_DN2799\_c0\_g1\_i4.p2

back to list

  

| Protein Coverage
| Supporting Peptides
| Best Unique PSM
|

Protein Coverage:

Supporting Peptides:

| Peptide | Uniq | -10lgP | Mass | Length | ppm | m/z | z | RT | 1/k0 Start | 1/k0 End | Precursor ID | Area Sample 2 | #Feature | #Feature Sample 2 | Start | End | PTM | AScore | Found By |
| --- | --- | --- | --- | --- | --- | --- | --- | --- | --- | --- | --- | --- | --- | --- | --- | --- | --- | --- | --- |
| K.AGM(+15.99)THIVR.D | Y | 38.19 | 899.4647 | 8 | 6.96 | 450.7403 | 2 | 9.74 | 0.8254 | 0.8327 | 11994 | 0 | 0 | 0 | 68 | 75 | Oxidation (M) | M3:Oxidation (M):1000 | DB Search |
| R.HGSLGFLPR.K | Y | 32.83 | 982.5348 | 9 | -0.04 | 492.272 | 2 | 16.93 | 0.8408 | 0.8481 | 51108 | 4.36e2 | 1 | 1 | 28 | 36 |  |  | DB Search |
| total 2 peptides |  |  |  |  |  |  |  |  |  |  |  |  |  |  |  |  |  |  |  |
| --- | --- | --- | --- | --- | --- | --- | --- | --- | --- | --- | --- | --- | --- | --- | --- | --- | --- | --- | --- |

Best Unique PSM (Scan Sample\_11\_A1\_1\_3648.d:11994, m/z=450.7403, z=2, RT=9.74, ppm=1.48):


TRINITY\_DN2799\_c0\_g1\_i2.p1

back to list

  

| Protein Coverage
| Supporting Peptides
| Best Unique PSM
|

Protein Coverage:

Supporting Peptides:

| Peptide | Uniq | -10lgP | Mass | Length | ppm | m/z | z | RT | 1/k0 Start | 1/k0 End | Precursor ID | Area Sample 2 | #Feature | #Feature Sample 2 | Start | End | PTM | AScore | Found By |
| --- | --- | --- | --- | --- | --- | --- | --- | --- | --- | --- | --- | --- | --- | --- | --- | --- | --- | --- | --- |
| K.AGM(+15.99)THIVR.D | Y | 38.19 | 899.4647 | 8 | 6.96 | 450.7403 | 2 | 9.74 | 0.8254 | 0.8327 | 11994 | 0 | 0 | 0 | 68 | 75 | Oxidation (M) | M3:Oxidation (M):1000 | DB Search |
| R.HGSLGFLPR.K | Y | 32.83 | 982.5348 | 9 | -0.04 | 492.272 | 2 | 16.93 | 0.8408 | 0.8481 | 51108 | 4.36e2 | 1 | 1 | 28 | 36 |  |  | DB Search |
| total 2 peptides |  |  |  |  |  |  |  |  |  |  |  |  |  |  |  |  |  |  |  |
| --- | --- | --- | --- | --- | --- | --- | --- | --- | --- | --- | --- | --- | --- | --- | --- | --- | --- | --- | --- |

Best Unique PSM (Scan Sample\_11\_A1\_1\_3648.d:11994, m/z=450.7403, z=2, RT=9.74, ppm=1.48):


TRINITY\_DN276\_c0\_g1\_i2.p1

back to list

  

| Protein Coverage
| Supporting Peptides
| Best Unique PSM
|

Protein Coverage:

Supporting Peptides:

| Peptide | Uniq | -10lgP | Mass | Length | ppm | m/z | z | RT | 1/k0 Start | 1/k0 End | Precursor ID | Area Sample 2 | #Feature | #Feature Sample 2 | Start | End | PTM | AScore | Found By |
| --- | --- | --- | --- | --- | --- | --- | --- | --- | --- | --- | --- | --- | --- | --- | --- | --- | --- | --- | --- |
| R.VTFEEFIPLYHTQSK.K | Y | 53.33 | 1837.925 | 15 | -7.94 | 919.9575 | 2 | 19.63 | 1.1482 | 1.1554 | 65543 | 0 | 0 | 0 | 60 | 74 |  |  | DB Search |
| total 1 peptides |  |  |  |  |  |  |  |  |  |  |  |  |  |  |  |  |  |  |  |
| --- | --- | --- | --- | --- | --- | --- | --- | --- | --- | --- | --- | --- | --- | --- | --- | --- | --- | --- | --- |

Best Unique PSM (Scan Sample\_11\_A1\_1\_3648.d:65543, m/z=919.9575, z=2, RT=19.63, ppm=-13.41):


TRINITY\_DN1280\_c0\_g1\_i1.p1

back to list

  

| Protein Coverage
| Supporting Peptides
| Best Unique PSM
|

Protein Coverage:

Supporting Peptides:

| Peptide | Uniq | -10lgP | Mass | Length | ppm | m/z | z | RT | 1/k0 Start | 1/k0 End | Precursor ID | Area Sample 2 | #Feature | #Feature Sample 2 | Start | End | PTM | AScore | Found By |
| --- | --- | --- | --- | --- | --- | --- | --- | --- | --- | --- | --- | --- | --- | --- | --- | --- | --- | --- | --- |
| K.AEAEAEVIQKK.A | Y | 58.85 | 1214.6506 | 11 | -1.98 | 608.3281 | 2 | 11.12 | 0.9558 | 0.963 | 19778 | 1.01e3 | 1 | 1 | 321 | 331 |  |  | DB Search |
| K.AEYENKTEVAR.S | Y | 58.43 | 1308.631 | 11 | 6.12 | 655.3232 | 2 | 10.97 | 0.9771 | 0.9844 | 18941 | 1.06e3 | 1 | 1 | 202 | 212 |  |  | DB Search |
| R.IKAEYENKTEVAR.S | Y | 58.27 | 1549.81 | 13 | 1.64 | 775.9093 | 2 | 10.97 | 1.056 | 1.0632 | 18831 | 5.28e2 | 1 | 1 | 200 | 212 |  |  | DB Search |
| R.KFVWEFFQK.V | Y | 57.89 | 1257.6546 | 9 | -4.78 | 629.8281 | 2 | 19.49 | 0.9461 | 0.9533 | 64710 | 2.12e3 | 1 | 1 | 30 | 38 |  |  | DB Search |
| R.RIAEVQC(+57.02)EAR.I | Y | 57.78 | 1230.6139 | 10 | 3.28 | 616.3129 | 2 | 10.93 | 0.923 | 0.9303 | 18587 | 2.26e3 | 1 | 1 | 169 | 178 | Carbamidomethylation |  | DB Search |
| K.FVWEFFQK.V | Y | 57.31 | 1129.5596 | 8 | -6.33 | 1130.5536 | 1 | 19.47 | 1.2833 | 1.2905 | 64690 | 0 | 0 | 0 | 31 | 38 |  |  | DB Search |
| R.NIALETLEGHQR.A | Y | 57.29 | 1379.7157 | 12 | -4.63 | 690.8582 | 2 | 15.05 | 1.017 | 1.0242 | 41788 | 0 | 0 | 0 | 99 | 110 |  |  | DB Search |
| total 7 peptides |  |  |  |  |  |  |  |  |  |  |  |  |  |  |  |  |  |  |  |
| --- | --- | --- | --- | --- | --- | --- | --- | --- | --- | --- | --- | --- | --- | --- | --- | --- | --- | --- | --- |

Best Unique PSM (Scan Sample\_11\_A1\_1\_3648.d:19778, m/z=608.3281, z=2, RT=11.12, ppm=-7.45):


TRINITY\_DN2799\_c0\_g1\_i3.p1

back to list

  

| Protein Coverage
| Supporting Peptides
| Best Unique PSM
|

Protein Coverage:

Supporting Peptides:

| Peptide | Uniq | -10lgP | Mass | Length | ppm | m/z | z | RT | 1/k0 Start | 1/k0 End | Precursor ID | Area Sample 2 | #Feature | #Feature Sample 2 | Start | End | PTM | AScore | Found By |
| --- | --- | --- | --- | --- | --- | --- | --- | --- | --- | --- | --- | --- | --- | --- | --- | --- | --- | --- | --- |
| K.AGM(+15.99)THIVR.D | Y | 38.19 | 899.4647 | 8 | 6.96 | 450.7403 | 2 | 9.74 | 0.8254 | 0.8327 | 11994 | 0 | 0 | 0 | 69 | 76 | Oxidation (M) | M3:Oxidation (M):1000 | DB Search |
| R.HGSLGFLPR.K | Y | 32.83 | 982.5348 | 9 | -0.04 | 492.272 | 2 | 16.93 | 0.8408 | 0.8481 | 51108 | 4.36e2 | 1 | 1 | 29 | 37 |  |  | DB Search |
| total 2 peptides |  |  |  |  |  |  |  |  |  |  |  |  |  |  |  |  |  |  |  |
| --- | --- | --- | --- | --- | --- | --- | --- | --- | --- | --- | --- | --- | --- | --- | --- | --- | --- | --- | --- |

Best Unique PSM (Scan Sample\_11\_A1\_1\_3648.d:11994, m/z=450.7403, z=2, RT=9.74, ppm=1.48):


TRINITY\_DN535\_c0\_g1\_i2.p1

back to list

  

| Protein Coverage
| Supporting Peptides
| Best Unique PSM
|

Protein Coverage:

Supporting Peptides:

| Peptide | Uniq | -10lgP | Mass | Length | ppm | m/z | z | RT | 1/k0 Start | 1/k0 End | Precursor ID | Area Sample 2 | #Feature | #Feature Sample 2 | Start | End | PTM | AScore | Found By |
| --- | --- | --- | --- | --- | --- | --- | --- | --- | --- | --- | --- | --- | --- | --- | --- | --- | --- | --- | --- |
| R.GLFIIDDKGILR.Q | Y | 58.85 | 1358.7921 | 12 | -0.67 | 680.3992 | 2 | 18.97 | 1.0106 | 1.0178 | 62014 | 1.63e3 | 1 | 1 | 94 | 105 |  |  | DB Search |
| R.ALGC(+57.02)DVLAC(+57.02)SVDSHFSHLAWTQQSR.K | Y | 58.16 | 2844.3071 | 25 | -3.91 | 712.0774 | 4 | 18.53 | 0.9238 | 0.9311 | 59620 | 3.52e2 | 1 | 1 | 33 | 57 | Carbamidomethylation, Carbamidomethylation |  | DB Search |
| total 2 peptides |  |  |  |  |  |  |  |  |  |  |  |  |  |  |  |  |  |  |  |
| --- | --- | --- | --- | --- | --- | --- | --- | --- | --- | --- | --- | --- | --- | --- | --- | --- | --- | --- | --- |

Best Unique PSM (Scan Sample\_11\_A1\_1\_3648.d:62014, m/z=680.3992, z=2, RT=18.97, ppm=-6.15):


TRINITY\_DN15599\_c0\_g1\_i1.p1

back to list

  

| Protein Coverage
| Supporting Peptides
| Best Unique PSM
|

Protein Coverage:

Supporting Peptides:

| Peptide | Uniq | -10lgP | Mass | Length | ppm | m/z | z | RT | 1/k0 Start | 1/k0 End | Precursor ID | Area Sample 2 | #Feature | #Feature Sample 2 | Start | End | PTM | AScore | Found By |
| --- | --- | --- | --- | --- | --- | --- | --- | --- | --- | --- | --- | --- | --- | --- | --- | --- | --- | --- | --- |
| K.KLQAELANGR.L | Y | 58.46 | 1098.6145 | 10 | 6.54 | 550.3151 | 2 | 11.42 | 0.8907 | 0.898 | 21463 | 3.82e3 | 1 | 1 | 14 | 23 |  |  | DB Search |
| K.K(+42.01)LQAELANGR.L | Y | 55.01 | 1140.6251 | 10 | -7.15 | 571.3126 | 2 | 14.17 | 0.8927 | 0.9 | 37018 | 0 | 0 | 0 | 14 | 23 | Acetylation (Protein N-term) | K1:Acetylation (Protein N-term):1000 | DB Search |
| total 2 peptides |  |  |  |  |  |  |  |  |  |  |  |  |  |  |  |  |  |  |  |
| --- | --- | --- | --- | --- | --- | --- | --- | --- | --- | --- | --- | --- | --- | --- | --- | --- | --- | --- | --- |

Best Unique PSM (Scan Sample\_11\_A1\_1\_3648.d:21463, m/z=550.3151, z=2, RT=11.42, ppm=1.06):


TRINITY\_DN164753\_c0\_g1\_i1.p1

back to list

  

| Protein Coverage
| Supporting Peptides
| Best Unique PSM
|

Protein Coverage:

Supporting Peptides:

| Peptide | Uniq | -10lgP | Mass | Length | ppm | m/z | z | RT | 1/k0 Start | 1/k0 End | Precursor ID | Area Sample 2 | #Feature | #Feature Sample 2 | Start | End | PTM | AScore | Found By |
| --- | --- | --- | --- | --- | --- | --- | --- | --- | --- | --- | --- | --- | --- | --- | --- | --- | --- | --- | --- |
| K.FVDEASKK.E | Y | 58.37 | 922.476 | 8 | 2.26 | 462.2438 | 2 | 9.81 | 0.8128 | 0.8201 | 12343 | 2.44e3 | 1 | 1 | 95 | 102 |  |  | DB Search |
| total 1 peptides |  |  |  |  |  |  |  |  |  |  |  |  |  |  |  |  |  |  |  |
| --- | --- | --- | --- | --- | --- | --- | --- | --- | --- | --- | --- | --- | --- | --- | --- | --- | --- | --- | --- |

Best Unique PSM (Scan Sample\_11\_A1\_1\_3648.d:12343, m/z=462.2438, z=2, RT=9.81, ppm=-3.22):


TRINITY\_DN2477\_c0\_g1\_i9.p1

back to list

  

| Protein Coverage
| Supporting Peptides
| Best Unique PSM
|

Protein Coverage:

Supporting Peptides:

| Peptide | Uniq | -10lgP | Mass | Length | ppm | m/z | z | RT | 1/k0 Start | 1/k0 End | Precursor ID | Area Sample 2 | #Feature | #Feature Sample 2 | Start | End | PTM | AScore | Found By |
| --- | --- | --- | --- | --- | --- | --- | --- | --- | --- | --- | --- | --- | --- | --- | --- | --- | --- | --- | --- |
| K.DSYVGDEAQSKR.G | Y | 59.21 | 1353.616 | 12 | 5.76 | 677.8155 | 2 | 10.93 | 1.0037 | 1.011 | 18642 | 9.97e3 | 1 | 1 | 52 | 63 |  |  | DB Search |
| R.VAPEEHPVLLTEAPLNPK.A | Y | 58.19 | 1953.0571 | 18 | -0.07 | 977.5304 | 2 | 16.19 | 1.2089 | 1.2161 | 47306 | 2.52e2 | 1 | 1 | 97 | 114 |  |  | DB Search |
| R.GYSFTTTAEREIVR.D | Y | 58.1 | 1628.8158 | 14 | 2.53 | 815.4128 | 2 | 15.79 | 1.121 | 1.1282 | 45442 | 6.41e3 | 1 | 1 | 198 | 211 |  |  | DB Search |
| total 3 peptides |  |  |  |  |  |  |  |  |  |  |  |  |  |  |  |  |  |  |  |
| --- | --- | --- | --- | --- | --- | --- | --- | --- | --- | --- | --- | --- | --- | --- | --- | --- | --- | --- | --- |

Best Unique PSM (Scan Sample\_11\_A1\_1\_3648.d:18642, m/z=677.8155, z=2, RT=10.93, ppm=0.28):


TRINITY\_DN250\_c0\_g1\_i4.p1

back to list

  

| Protein Coverage
| Supporting Peptides
| Best Unique PSM
|

Protein Coverage:

Supporting Peptides:

| Peptide | Uniq | -10lgP | Mass | Length | ppm | m/z | z | RT | 1/k0 Start | 1/k0 End | Precursor ID | Area Sample 2 | #Feature | #Feature Sample 2 | Start | End | PTM | AScore | Found By |
| --- | --- | --- | --- | --- | --- | --- | --- | --- | --- | --- | --- | --- | --- | --- | --- | --- | --- | --- | --- |
| K.ELVDQVLDRIR.K | Y | 58.86 | 1354.7568 | 11 | 2.67 | 678.3838 | 2 | 18.48 | 1.009 | 1.0162 | 59338 | 1.45e3 | 1 | 1 | 113 | 123 |  |  | DB Search |
| R.TIQFVDWC(+57.02)PTGFK.V | Y | 54.82 | 1597.7599 | 13 | -6.53 | 799.8776 | 2 | 19.88 | 1.0351 | 1.0423 | 66701 | 3.31e2 | 1 | 1 | 340 | 352 | Carbamidomethylation |  | DB Search |
| R.RNLDIERPTYTNLNR.L | Y | 46.58 | 1873.9758 | 15 | -2.43 | 625.6609 | 3 | 13.94 | 0.8858 | 0.8931 | 35770 | 1.17e3 | 1 | 1 | 215 | 229 |  |  | DB Search |
| R.LDHKFDLM(+15.99)YAKR.A | Y | 40.99 | 1551.7868 | 12 | 3.09 | 776.8988 | 2 | 13.19 | 1.0881 | 1.0953 | 31591 | 1.18e2 | 1 | 1 | 391 | 402 | Oxidation (M) | M8:Oxidation (M):1000 | DB Search |
| total 4 peptides |  |  |  |  |  |  |  |  |  |  |  |  |  |  |  |  |  |  |  |
| --- | --- | --- | --- | --- | --- | --- | --- | --- | --- | --- | --- | --- | --- | --- | --- | --- | --- | --- | --- |

Best Unique PSM (Scan Sample\_11\_A1\_1\_3648.d:59338, m/z=678.3838, z=2, RT=18.48, ppm=-2.81):


TRINITY\_DN1080\_c0\_g1\_i3.p1

back to list

  

| Protein Coverage
| Supporting Peptides
| Best Unique PSM
|

Protein Coverage:

Supporting Peptides:

| Peptide | Uniq | -10lgP | Mass | Length | ppm | m/z | z | RT | 1/k0 Start | 1/k0 End | Precursor ID | Area Sample 2 | #Feature | #Feature Sample 2 | Start | End | PTM | AScore | Found By |
| --- | --- | --- | --- | --- | --- | --- | --- | --- | --- | --- | --- | --- | --- | --- | --- | --- | --- | --- | --- |
| R.RPVPIVTRR.R | Y | 57.63 | 1092.688 | 9 | 6 | 365.2368 | 3 | 10.84 | 0.7045 | 0.7118 | 18035 | 1.06e3 | 1 | 1 | 281 | 289 |  |  | DB Search |
| R.RSITTSVKR.V | Y | 55.92 | 1046.6196 | 9 | 6.21 | 349.8807 | 3 | 9.78 | 0.7037 | 0.711 | 12172 | 0 | 0 | 0 | 193 | 201 |  |  | DB Search |
| total 2 peptides |  |  |  |  |  |  |  |  |  |  |  |  |  |  |  |  |  |  |  |
| --- | --- | --- | --- | --- | --- | --- | --- | --- | --- | --- | --- | --- | --- | --- | --- | --- | --- | --- | --- |

Best Unique PSM (Scan Sample\_11\_A1\_1\_3648.d:18035, m/z=365.2368, z=3, RT=10.84, ppm=0.51):


TRINITY\_DN1320\_c0\_g2\_i1.p1

back to list

  

| Protein Coverage
| Supporting Peptides
| Best Unique PSM
|

Protein Coverage:

Supporting Peptides:

| Peptide | Uniq | -10lgP | Mass | Length | ppm | m/z | z | RT | 1/k0 Start | 1/k0 End | Precursor ID | Area Sample 2 | #Feature | #Feature Sample 2 | Start | End | PTM | AScore | Found By |
| --- | --- | --- | --- | --- | --- | --- | --- | --- | --- | --- | --- | --- | --- | --- | --- | --- | --- | --- | --- |
| R.SVNEGDVQDKKR.D | Y | 46.88 | 1373.6899 | 12 | -2.59 | 458.9002 | 3 | 9.40 | 0.7453 | 0.7526 | 10436 | 0 | 0 | 0 | 209 | 220 |  |  | DB Search |
| total 1 peptides |  |  |  |  |  |  |  |  |  |  |  |  |  |  |  |  |  |  |  |
| --- | --- | --- | --- | --- | --- | --- | --- | --- | --- | --- | --- | --- | --- | --- | --- | --- | --- | --- | --- |

Best Unique PSM (Scan Sample\_11\_A1\_1\_3648.d:10436, m/z=458.9002, z=3, RT=9.40, ppm=-8.07):


TRINITY\_DN902\_c1\_g2\_i3.p1

back to list

  

| Protein Coverage
| Supporting Peptides
| Best Unique PSM
|

Protein Coverage:

Supporting Peptides:

| Peptide | Uniq | -10lgP | Mass | Length | ppm | m/z | z | RT | 1/k0 Start | 1/k0 End | Precursor ID | Area Sample 2 | #Feature | #Feature Sample 2 | Start | End | PTM | AScore | Found By |
| --- | --- | --- | --- | --- | --- | --- | --- | --- | --- | --- | --- | --- | --- | --- | --- | --- | --- | --- | --- |
| K.GLTHC(+57.02)NTLSYK.L | Y | 58.83 | 1292.6183 | 11 | 4.02 | 647.3155 | 2 | 12.36 | 0.9695 | 0.9767 | 26924 | 0 | 0 | 0 | 62 | 72 | Carbamidomethylation |  | DB Search |
| total 1 peptides |  |  |  |  |  |  |  |  |  |  |  |  |  |  |  |  |  |  |  |
| --- | --- | --- | --- | --- | --- | --- | --- | --- | --- | --- | --- | --- | --- | --- | --- | --- | --- | --- | --- |

Best Unique PSM (Scan Sample\_11\_A1\_1\_3648.d:26924, m/z=647.3155, z=2, RT=12.36, ppm=-1.46):


TRINITY\_DN265\_c0\_g1\_i1.p1

back to list

  

| Protein Coverage
| Supporting Peptides
| Best Unique PSM
|

Protein Coverage:

Supporting Peptides:

| Peptide | Uniq | -10lgP | Mass | Length | ppm | m/z | z | RT | 1/k0 Start | 1/k0 End | Precursor ID | Area Sample 2 | #Feature | #Feature Sample 2 | Start | End | PTM | AScore | Found By |
| --- | --- | --- | --- | --- | --- | --- | --- | --- | --- | --- | --- | --- | --- | --- | --- | --- | --- | --- | --- |
| R.LTHDFHTNKR.M | Y | 58.75 | 1267.6421 | 10 | 2.44 | 423.5534 | 3 | 9.64 | 0.7441 | 0.7514 | 11479 | 1.15e3 | 1 | 1 | 24 | 33 |  |  | DB Search |
| total 1 peptides |  |  |  |  |  |  |  |  |  |  |  |  |  |  |  |  |  |  |  |
| --- | --- | --- | --- | --- | --- | --- | --- | --- | --- | --- | --- | --- | --- | --- | --- | --- | --- | --- | --- |

Best Unique PSM (Scan Sample\_11\_A1\_1\_3648.d:11479, m/z=423.5534, z=3, RT=9.64, ppm=-3.04):


TRINITY\_DN4\_c0\_g1\_i1.p1

back to list

  

| Protein Coverage
| Supporting Peptides
| Best Unique PSM
|

Protein Coverage:

Supporting Peptides:

| Peptide | Uniq | -10lgP | Mass | Length | ppm | m/z | z | RT | 1/k0 Start | 1/k0 End | Precursor ID | Area Sample 2 | #Feature | #Feature Sample 2 | Start | End | PTM | AScore | Found By |
| --- | --- | --- | --- | --- | --- | --- | --- | --- | --- | --- | --- | --- | --- | --- | --- | --- | --- | --- | --- |
| K.AFFTAEEPSAKPTVILAK.T | Y | 55.13 | 1919.0404 | 18 | -0.05 | 960.5222 | 2 | 16.95 | 1.2228 | 1.23 | 51036 | 1.07e2 | 1 | 1 | 185 | 202 |  |  | DB Search |
| total 1 peptides |  |  |  |  |  |  |  |  |  |  |  |  |  |  |  |  |  |  |  |
| --- | --- | --- | --- | --- | --- | --- | --- | --- | --- | --- | --- | --- | --- | --- | --- | --- | --- | --- | --- |

Best Unique PSM (Scan Sample\_11\_A1\_1\_3648.d:51036, m/z=960.5222, z=2, RT=16.95, ppm=-5.52):


TRINITY\_DN15584\_c0\_g1\_i1.p1

back to list

  

| Protein Coverage
| Supporting Peptides
| Best Unique PSM
|

Protein Coverage:

Supporting Peptides:

| Peptide | Uniq | -10lgP | Mass | Length | ppm | m/z | z | RT | 1/k0 Start | 1/k0 End | Precursor ID | Area Sample 2 | #Feature | #Feature Sample 2 | Start | End | PTM | AScore | Found By |
| --- | --- | --- | --- | --- | --- | --- | --- | --- | --- | --- | --- | --- | --- | --- | --- | --- | --- | --- | --- |
| K.AFVAIGDYGGHVGLGVK.C | Y | 58.87 | 1658.878 | 17 | 0.35 | 830.442 | 2 | 17.35 | 1.1626 | 1.1698 | 53157 | 4.11e3 | 1 | 1 | 109 | 125 |  |  | DB Search |
| K.IGKPHTVPC(+57.02)K.V | Y | 58.56 | 1135.6172 | 10 | 9.01 | 379.5477 | 3 | 9.76 | 0.7709 | 0.7783 | 11898 | 7.38e3 | 2 | 2 | 157 | 166 | Carbamidomethylation |  | DB Search |
| K.LSIIPVRR.G | Y | 26.05 | 952.6182 | 8 | 2.32 | 477.3148 | 2 | 14.61 | 0.8441 | 0.8514 | 39163 | 2.05e3 | 1 | 1 | 143 | 150 |  |  | DB Search |
| total 3 peptides |  |  |  |  |  |  |  |  |  |  |  |  |  |  |  |  |  |  |  |
| --- | --- | --- | --- | --- | --- | --- | --- | --- | --- | --- | --- | --- | --- | --- | --- | --- | --- | --- | --- |

Best Unique PSM (Scan Sample\_11\_A1\_1\_3648.d:53157, m/z=830.442, z=2, RT=17.35, ppm=-5.12):


TRINITY\_DN598\_c1\_g1\_i1.p1

back to list

  

| Protein Coverage
| Supporting Peptides
| Best Unique PSM
|

Protein Coverage:

Supporting Peptides:

| Peptide | Uniq | -10lgP | Mass | Length | ppm | m/z | z | RT | 1/k0 Start | 1/k0 End | Precursor ID | Area Sample 2 | #Feature | #Feature Sample 2 | Start | End | PTM | AScore | Found By |
| --- | --- | --- | --- | --- | --- | --- | --- | --- | --- | --- | --- | --- | --- | --- | --- | --- | --- | --- | --- |
| R.ASFVSDIDRLR.A | Y | 58.87 | 1277.6728 | 11 | -0.78 | 639.8397 | 2 | 16.62 | 0.986 | 0.9932 | 49505 | 2.05e3 | 1 | 1 | 302 | 312 |  |  | DB Search |
| total 1 peptides |  |  |  |  |  |  |  |  |  |  |  |  |  |  |  |  |  |  |  |
| --- | --- | --- | --- | --- | --- | --- | --- | --- | --- | --- | --- | --- | --- | --- | --- | --- | --- | --- | --- |

Best Unique PSM (Scan Sample\_11\_A1\_1\_3648.d:49505, m/z=639.8397, z=2, RT=16.62, ppm=-6.26):


TRINITY\_DN471\_c0\_g1\_i5.p1

back to list

  

| Protein Coverage
| Supporting Peptides
| Best Unique PSM
|

Protein Coverage:

Supporting Peptides:

| Peptide | Uniq | -10lgP | Mass | Length | ppm | m/z | z | RT | 1/k0 Start | 1/k0 End | Precursor ID | Area Sample 2 | #Feature | #Feature Sample 2 | Start | End | PTM | AScore | Found By |
| --- | --- | --- | --- | --- | --- | --- | --- | --- | --- | --- | --- | --- | --- | --- | --- | --- | --- | --- | --- |
| R.TIQFVDWC(+57.02)PTGFK.C | Y | 54.82 | 1597.7599 | 13 | -6.53 | 799.8776 | 2 | 19.88 | 1.0351 | 1.0423 | 66701 | 3.31e2 | 1 | 1 | 340 | 352 | Carbamidomethylation |  | DB Search |
| R.RNLDIERPTYTNLNR.L | Y | 46.58 | 1873.9758 | 15 | -2.43 | 625.6609 | 3 | 13.94 | 0.8858 | 0.8931 | 35770 | 1.17e3 | 1 | 1 | 215 | 229 |  |  | DB Search |
| total 2 peptides |  |  |  |  |  |  |  |  |  |  |  |  |  |  |  |  |  |  |  |
| --- | --- | --- | --- | --- | --- | --- | --- | --- | --- | --- | --- | --- | --- | --- | --- | --- | --- | --- | --- |

Best Unique PSM (Scan Sample\_11\_A1\_1\_3648.d:66701, m/z=799.3823, z=2, RT=19.90, ppm=-12):


TRINITY\_DN265\_c0\_g1\_i4.p1

back to list

  

| Protein Coverage
| Supporting Peptides
| Best Unique PSM
|

Protein Coverage:

Supporting Peptides:

| Peptide | Uniq | -10lgP | Mass | Length | ppm | m/z | z | RT | 1/k0 Start | 1/k0 End | Precursor ID | Area Sample 2 | #Feature | #Feature Sample 2 | Start | End | PTM | AScore | Found By |
| --- | --- | --- | --- | --- | --- | --- | --- | --- | --- | --- | --- | --- | --- | --- | --- | --- | --- | --- | --- |
| R.LTHDFHTNKR.M | Y | 58.75 | 1267.6421 | 10 | 2.44 | 423.5534 | 3 | 9.64 | 0.7441 | 0.7514 | 11479 | 1.15e3 | 1 | 1 | 24 | 33 |  |  | DB Search |
| total 1 peptides |  |  |  |  |  |  |  |  |  |  |  |  |  |  |  |  |  |  |  |
| --- | --- | --- | --- | --- | --- | --- | --- | --- | --- | --- | --- | --- | --- | --- | --- | --- | --- | --- | --- |

Best Unique PSM (Scan Sample\_11\_A1\_1\_3648.d:11479, m/z=423.5534, z=3, RT=9.64, ppm=-3.04):


TRINITY\_DN250\_c0\_g1\_i3.p1

back to list

  

| Protein Coverage
| Supporting Peptides
| Best Unique PSM
|

Protein Coverage:

Supporting Peptides:

| Peptide | Uniq | -10lgP | Mass | Length | ppm | m/z | z | RT | 1/k0 Start | 1/k0 End | Precursor ID | Area Sample 2 | #Feature | #Feature Sample 2 | Start | End | PTM | AScore | Found By |
| --- | --- | --- | --- | --- | --- | --- | --- | --- | --- | --- | --- | --- | --- | --- | --- | --- | --- | --- | --- |
| K.ELVDQVLDRIR.K | Y | 58.86 | 1354.7568 | 11 | 2.67 | 678.3838 | 2 | 18.48 | 1.009 | 1.0162 | 59338 | 1.45e3 | 1 | 1 | 113 | 123 |  |  | DB Search |
| R.TIQFVDWC(+57.02)PTGFK.V | Y | 54.82 | 1597.7599 | 13 | -6.53 | 799.8776 | 2 | 19.88 | 1.0351 | 1.0423 | 66701 | 3.31e2 | 1 | 1 | 340 | 352 | Carbamidomethylation |  | DB Search |
| R.RNLDIERPTYTNLNR.L | Y | 46.58 | 1873.9758 | 15 | -2.43 | 625.6609 | 3 | 13.94 | 0.8858 | 0.8931 | 35770 | 1.17e3 | 1 | 1 | 215 | 229 |  |  | DB Search |
| total 3 peptides |  |  |  |  |  |  |  |  |  |  |  |  |  |  |  |  |  |  |  |
| --- | --- | --- | --- | --- | --- | --- | --- | --- | --- | --- | --- | --- | --- | --- | --- | --- | --- | --- | --- |

Best Unique PSM (Scan Sample\_11\_A1\_1\_3648.d:59338, m/z=678.3838, z=2, RT=18.48, ppm=-2.81):


TRINITY\_DN158421\_c0\_g1\_i1.p1

back to list

  

| Protein Coverage
| Supporting Peptides
| Best Unique PSM
|

Protein Coverage:

Supporting Peptides:

| Peptide | Uniq | -10lgP | Mass | Length | ppm | m/z | z | RT | 1/k0 Start | 1/k0 End | Precursor ID | Area Sample 2 | #Feature | #Feature Sample 2 | Start | End | PTM | AScore | Found By |
| --- | --- | --- | --- | --- | --- | --- | --- | --- | --- | --- | --- | --- | --- | --- | --- | --- | --- | --- | --- |
| R.SSC(+57.02)RPILSVIK.I | Y | 27.61 | 1258.7067 | 11 | -7.39 | 630.3525 | 2 | 16.61 | 0.9687 | 0.9759 | 49383 | 6.05e2 | 1 | 1 | 90 | 100 | Carbamidomethylation |  | DB Search |
| total 1 peptides |  |  |  |  |  |  |  |  |  |  |  |  |  |  |  |  |  |  |  |
| --- | --- | --- | --- | --- | --- | --- | --- | --- | --- | --- | --- | --- | --- | --- | --- | --- | --- | --- | --- |

Best Unique PSM (Scan Sample\_11\_A1\_1\_3648.d:49383, m/z=630.3525, z=2, RT=16.61, ppm=-12.87):


TRINITY\_DN11545\_c0\_g1\_i1.p1

back to list

  

| Protein Coverage
| Supporting Peptides
| Best Unique PSM
|

Protein Coverage:

Supporting Peptides:

| Peptide | Uniq | -10lgP | Mass | Length | ppm | m/z | z | RT | 1/k0 Start | 1/k0 End | Precursor ID | Area Sample 2 | #Feature | #Feature Sample 2 | Start | End | PTM | AScore | Found By |
| --- | --- | --- | --- | --- | --- | --- | --- | --- | --- | --- | --- | --- | --- | --- | --- | --- | --- | --- | --- |
| K.VTDAQIEKIEK.L | Y | 22.24 | 1272.6925 | 11 | 12.94 | 637.3583 | 2 | 12.56 | 0.9622 | 0.9695 | 28055 | 1.37e3 | 1 | 1 | 86 | 96 |  |  | DB Search |
| total 1 peptides |  |  |  |  |  |  |  |  |  |  |  |  |  |  |  |  |  |  |  |
| --- | --- | --- | --- | --- | --- | --- | --- | --- | --- | --- | --- | --- | --- | --- | --- | --- | --- | --- | --- |

Best Unique PSM (Scan Sample\_11\_A1\_1\_3648.d:28055, m/z=637.3583, z=2, RT=12.56, ppm=7.47):


TRINITY\_DN3036\_c0\_g1\_i13.p1

back to list

  

| Protein Coverage
| Supporting Peptides
| Best Unique PSM
|

Protein Coverage:

Supporting Peptides:

| Peptide | Uniq | -10lgP | Mass | Length | ppm | m/z | z | RT | 1/k0 Start | 1/k0 End | Precursor ID | Area Sample 2 | #Feature | #Feature Sample 2 | Start | End | PTM | AScore | Found By |
| --- | --- | --- | --- | --- | --- | --- | --- | --- | --- | --- | --- | --- | --- | --- | --- | --- | --- | --- | --- |
| R.AGHGAVTSPQTQR.G | N | 58.95 | 1308.6534 | 13 | 4.15 | 655.3331 | 2 | 9.70 | 0.9146 | 0.9218 | 11889 | 1.61e4 | 2 | 2 | 135 | 147 |  |  | DB Search |
| R.VIGASGIHVGTM(+15.99)SFGK.M | N | 58.84 | 1575.8079 | 16 | 2.27 | 788.9087 | 2 | 14.96 | 1.1306 | 1.1378 | 41219 | 1.68e3 | 1 | 1 | 160 | 175 | Oxidation (M) | M12:Oxidation (M):1000 | DB Search |
| SNGGLVVGTIIKPK.L | Y | 56.21 | 1381.8293 | 14 | 0.75 | 691.9186 | 2 | 15.49 | 1.0528 | 1.06 | 43994 | 1.43e3 | 1 | 1 | 1 | 14 |  |  | DB Search |
| total 3 peptides |  |  |  |  |  |  |  |  |  |  |  |  |  |  |  |  |  |  |  |
| --- | --- | --- | --- | --- | --- | --- | --- | --- | --- | --- | --- | --- | --- | --- | --- | --- | --- | --- | --- |

Best Unique PSM (Scan Sample\_11\_A1\_1\_3648.d:43994, m/z=691.9186, z=2, RT=15.49, ppm=-4.73):


TRINITY\_DN189999\_c0\_g1\_i1.p1

back to list

  

| Protein Coverage
| Supporting Peptides
| Best Unique PSM
|

Protein Coverage:

Supporting Peptides:

| Peptide | Uniq | -10lgP | Mass | Length | ppm | m/z | z | RT | 1/k0 Start | 1/k0 End | Precursor ID | Area Sample 2 | #Feature | #Feature Sample 2 | Start | End | PTM | AScore | Found By |
| --- | --- | --- | --- | --- | --- | --- | --- | --- | --- | --- | --- | --- | --- | --- | --- | --- | --- | --- | --- |
| K.LEGLFTVTVSHPGAR.R | Y | 58.13 | 1582.8467 | 15 | -1.11 | 792.4254 | 2 | 17.01 | 1.0937 | 1.1009 | 51427 | 1.13e2 | 1 | 1 | 123 | 137 |  |  | DB Search |
| R.NGEDLSKLEGLFTVTVSHPGAR.R | Y | 55.71 | 2326.1917 | 22 | 0.49 | 776.4006 | 3 | 19.69 | 1.0267 | 1.0339 | 65768 | 5.58e2 | 1 | 1 | 116 | 137 |  |  | DB Search |
| total 2 peptides |  |  |  |  |  |  |  |  |  |  |  |  |  |  |  |  |  |  |  |
| --- | --- | --- | --- | --- | --- | --- | --- | --- | --- | --- | --- | --- | --- | --- | --- | --- | --- | --- | --- |

Best Unique PSM (Scan Sample\_11\_A1\_1\_3648.d:51427, m/z=792.4254, z=2, RT=17.01, ppm=-6.58):


TRINITY\_DN761\_c0\_g1\_i1.p1

back to list

  

| Protein Coverage
| Supporting Peptides
| Best Unique PSM
|

Protein Coverage:

Supporting Peptides:

| Peptide | Uniq | -10lgP | Mass | Length | ppm | m/z | z | RT | 1/k0 Start | 1/k0 End | Precursor ID | Area Sample 2 | #Feature | #Feature Sample 2 | Start | End | PTM | AScore | Found By |
| --- | --- | --- | --- | --- | --- | --- | --- | --- | --- | --- | --- | --- | --- | --- | --- | --- | --- | --- | --- |
| K.TRIEDLETVISQTQDHR.H | Y | 55.76 | 2040.0236 | 17 | -2.78 | 681.0095 | 3 | 17.11 | 0.9558 | 0.963 | 52031 | 0 | 0 | 0 | 260 | 276 |  |  | DB Search |
| total 1 peptides |  |  |  |  |  |  |  |  |  |  |  |  |  |  |  |  |  |  |  |
| --- | --- | --- | --- | --- | --- | --- | --- | --- | --- | --- | --- | --- | --- | --- | --- | --- | --- | --- | --- |

Best Unique PSM (Scan Sample\_11\_A1\_1\_3648.d:52031, m/z=681.0095, z=3, RT=17.11, ppm=-8.25):


TRINITY\_DN18187\_c0\_g1\_i5.p1

back to list

  

| Protein Coverage
| Supporting Peptides
| Best Unique PSM
|

Protein Coverage:

Supporting Peptides:

| Peptide | Uniq | -10lgP | Mass | Length | ppm | m/z | z | RT | 1/k0 Start | 1/k0 End | Precursor ID | Area Sample 2 | #Feature | #Feature Sample 2 | Start | End | PTM | AScore | Found By |
| --- | --- | --- | --- | --- | --- | --- | --- | --- | --- | --- | --- | --- | --- | --- | --- | --- | --- | --- | --- |
| R.YRPGTVALR.E | Y | 44.05 | 1031.5876 | 9 | -1.83 | 516.7973 | 2 | 12.44 | 0.8575 | 0.8648 | 27332 | 1.61e3 | 1 | 1 | 87 | 95 |  |  | DB Search |
| total 1 peptides |  |  |  |  |  |  |  |  |  |  |  |  |  |  |  |  |  |  |  |
| --- | --- | --- | --- | --- | --- | --- | --- | --- | --- | --- | --- | --- | --- | --- | --- | --- | --- | --- | --- |

Best Unique PSM (Scan Sample\_11\_A1\_1\_3648.d:27332, m/z=516.7973, z=2, RT=12.44, ppm=-7.31):


TRINITY\_DN1789\_c0\_g1\_i1.p1

back to list

  

| Protein Coverage
| Supporting Peptides
| Best Unique PSM
|

Protein Coverage:

Supporting Peptides:

| Peptide | Uniq | -10lgP | Mass | Length | ppm | m/z | z | RT | 1/k0 Start | 1/k0 End | Precursor ID | Area Sample 2 | #Feature | #Feature Sample 2 | Start | End | PTM | AScore | Found By |
| --- | --- | --- | --- | --- | --- | --- | --- | --- | --- | --- | --- | --- | --- | --- | --- | --- | --- | --- | --- |
| K.VVSSAIVDKYIGESAR.L | Y | 39.14 | 1692.9046 | 16 | 3.9 | 847.4583 | 2 | 16.24 | 1.1626 | 1.1698 | 47705 | 3.83e2 | 1 | 1 | 201 | 216 |  |  | DB Search |
| total 1 peptides |  |  |  |  |  |  |  |  |  |  |  |  |  |  |  |  |  |  |  |
| --- | --- | --- | --- | --- | --- | --- | --- | --- | --- | --- | --- | --- | --- | --- | --- | --- | --- | --- | --- |

Best Unique PSM (Scan Sample\_11\_A1\_1\_3648.d:47705, m/z=847.4583, z=2, RT=16.24, ppm=-1.57):


TRINITY\_DN3986\_c0\_g1\_i1.p1

back to list

  

| Protein Coverage
| Supporting Peptides
| Best Unique PSM
|

Protein Coverage:

Supporting Peptides:

| Peptide | Uniq | -10lgP | Mass | Length | ppm | m/z | z | RT | 1/k0 Start | 1/k0 End | Precursor ID | Area Sample 2 | #Feature | #Feature Sample 2 | Start | End | PTM | AScore | Found By |
| --- | --- | --- | --- | --- | --- | --- | --- | --- | --- | --- | --- | --- | --- | --- | --- | --- | --- | --- | --- |
| K.IREEYPDR.I | Y | 58.88 | 1076.525 | 8 | 2.94 | 539.2684 | 2 | 10.94 | 0.866 | 0.8733 | 18650 | 6.17e3 | 1 | 1 | 155 | 162 |  |  | DB Search |
| K.M(+15.99)SATFIGNSTAIQELFKR.I | Y | 58.33 | 2029.0302 | 18 | -2.43 | 677.3453 | 3 | 19.58 | 0.9344 | 0.9416 | 65134 | 3.85e3 | 1 | 1 | 363 | 380 | Oxidation (M) | M1:Oxidation (M):1000 | DB Search |
| K.NM(+15.99)M(+15.99)AAC(+57.02)DPR.H | Y | 58.21 | 1096.41 | 9 | 1.94 | 549.2103 | 2 | 9.53 | 0.8627 | 0.87 | 10857 | 1.37e3 | 1 | 1 | 298 | 306 | Oxidation (M), Oxidation (M), Carbamidomethylation | M2:Oxidation (M):1000 M3:Oxidation (M):1000 | DB Search |
| K.RISEQFTAM(+15.99)FR.R | Y | 39.68 | 1400.6871 | 11 | 1.59 | 701.3481 | 2 | 15.05 | 0.9848 | 0.992 | 41767 | 1.88e3 | 1 | 1 | 380 | 390 | Oxidation (M) | M9:Oxidation (M):1000 | DB Search |
| R.FPGQLNADLRK.L | Y | 25.64 | 1257.6829 | 11 | -1.3 | 629.8445 | 2 | 14.18 | 0.9457 | 0.9529 | 37015 | 6.11e2 | 1 | 1 | 242 | 252 |  |  | DB Search |
| total 5 peptides |  |  |  |  |  |  |  |  |  |  |  |  |  |  |  |  |  |  |  |
| --- | --- | --- | --- | --- | --- | --- | --- | --- | --- | --- | --- | --- | --- | --- | --- | --- | --- | --- | --- |

Best Unique PSM (Scan Sample\_11\_A1\_1\_3648.d:18650, m/z=539.8521, z=2, RT=10.95, ppm=-2.54):


TRINITY\_DN36\_c0\_g1\_i20.p1

back to list

  

| Protein Coverage
| Supporting Peptides
| Best Unique PSM
|

Protein Coverage:

Supporting Peptides:

| Peptide | Uniq | -10lgP | Mass | Length | ppm | m/z | z | RT | 1/k0 Start | 1/k0 End | Precursor ID | Area Sample 2 | #Feature | #Feature Sample 2 | Start | End | PTM | AScore | Found By |
| --- | --- | --- | --- | --- | --- | --- | --- | --- | --- | --- | --- | --- | --- | --- | --- | --- | --- | --- | --- |
| K.GFDYC(+57.02)TTIKYR.L | Y | 58.87 | 1422.6602 | 11 | 2.87 | 712.3355 | 2 | 14.94 | 1.0242 | 1.0315 | 41159 | 9.5e2 | 1 | 1 | 46 | 56 | Carbamidomethylation |  | DB Search |
| R.LGGIEVTQGNC(+57.02)ARK.Y | Y | 58.27 | 1501.7671 | 14 | 1.48 | 751.8878 | 2 | 12.59 | 1.0616 | 1.0688 | 28229 | 7.18e2 | 1 | 1 | 57 | 70 | Carbamidomethylation |  | DB Search |
| total 2 peptides |  |  |  |  |  |  |  |  |  |  |  |  |  |  |  |  |  |  |  |
| --- | --- | --- | --- | --- | --- | --- | --- | --- | --- | --- | --- | --- | --- | --- | --- | --- | --- | --- | --- |

Best Unique PSM (Scan Sample\_11\_A1\_1\_3648.d:41159, m/z=712.3355, z=2, RT=14.94, ppm=-2.6):


TRINITY\_DN3052\_c0\_g1\_i1.p1

back to list

  

| Protein Coverage
| Supporting Peptides
| Best Unique PSM
|

Protein Coverage:

Supporting Peptides:

| Peptide | Uniq | -10lgP | Mass | Length | ppm | m/z | z | RT | 1/k0 Start | 1/k0 End | Precursor ID | Area Sample 2 | #Feature | #Feature Sample 2 | Start | End | PTM | AScore | Found By |
| --- | --- | --- | --- | --- | --- | --- | --- | --- | --- | --- | --- | --- | --- | --- | --- | --- | --- | --- | --- |
| M.A(+42.01)HLSQEQRNELKK.N | Y | 57.66 | 1621.8536 | 13 | 3.86 | 541.6243 | 3 | 11.22 | 0.8071 | 0.8144 | 20267 | 8.15e2 | 1 | 1 | 2 | 14 | Acetylation (Protein N-term) | A1:Acetylation (Protein N-term):1000 | DB Search |
| total 1 peptides |  |  |  |  |  |  |  |  |  |  |  |  |  |  |  |  |  |  |  |
| --- | --- | --- | --- | --- | --- | --- | --- | --- | --- | --- | --- | --- | --- | --- | --- | --- | --- | --- | --- |

Best Unique PSM (Scan Sample\_11\_A1\_1\_3648.d:20267, m/z=541.6243, z=3, RT=11.22, ppm=-1.62):


TRINITY\_DN11662\_c0\_g1\_i1.p1

back to list

  

| Protein Coverage
| Supporting Peptides
| Best Unique PSM
|

Protein Coverage:

Supporting Peptides:

| Peptide | Uniq | -10lgP | Mass | Length | ppm | m/z | z | RT | 1/k0 Start | 1/k0 End | Precursor ID | Area Sample 2 | #Feature | #Feature Sample 2 | Start | End | PTM | AScore | Found By |
| --- | --- | --- | --- | --- | --- | --- | --- | --- | --- | --- | --- | --- | --- | --- | --- | --- | --- | --- | --- |
| M.A(+42.01)LSRPVYIIAAK.R | Y | 55.54 | 1342.7972 | 12 | -0.08 | 672.4022 | 2 | 17.65 | 1.0532 | 1.0604 | 54904 | 5.44e3 | 1 | 1 | 2 | 13 | Acetylation (Protein N-term) | A1:Acetylation (Protein N-term):1000 | DB Search |
| total 1 peptides |  |  |  |  |  |  |  |  |  |  |  |  |  |  |  |  |  |  |  |
| --- | --- | --- | --- | --- | --- | --- | --- | --- | --- | --- | --- | --- | --- | --- | --- | --- | --- | --- | --- |

Best Unique PSM (Scan Sample\_11\_A1\_1\_3648.d:54904, m/z=672.4022, z=2, RT=17.65, ppm=-5.56):


TRINITY\_DN5633\_c0\_g1\_i1.p1

back to list

  

| Protein Coverage
| Supporting Peptides
| Best Unique PSM
|

Protein Coverage:

Supporting Peptides:

| Peptide | Uniq | -10lgP | Mass | Length | ppm | m/z | z | RT | 1/k0 Start | 1/k0 End | Precursor ID | Area Sample 2 | #Feature | #Feature Sample 2 | Start | End | PTM | AScore | Found By |
| --- | --- | --- | --- | --- | --- | --- | --- | --- | --- | --- | --- | --- | --- | --- | --- | --- | --- | --- | --- |
| R.ELISNSSDALDKIR.F | Y | 55.36 | 1559.8155 | 14 | 3.71 | 780.9136 | 2 | 15.32 | 1.0745 | 1.0817 | 43130 | 3.07e2 | 1 | 1 | 108 | 121 |  |  | DB Search |
| K.LLEINVYHPLIQELQK.R | Y | 51.52 | 1949.0986 | 16 | 3.87 | 650.7057 | 3 | 19.28 | 0.9852 | 0.9924 | 63703 | 0 | 0 | 0 | 706 | 721 |  |  | DB Search |
| K.KLLEINVYHPLIQELQKR.V | Y | 30.15 | 2233.2946 | 18 | -0.32 | 559.3277 | 4 | 17.67 | 0.866 | 0.8733 | 55108 | 0 | 0 | 0 | 705 | 722 |  |  | DB Search |
| total 3 peptides |  |  |  |  |  |  |  |  |  |  |  |  |  |  |  |  |  |  |  |
| --- | --- | --- | --- | --- | --- | --- | --- | --- | --- | --- | --- | --- | --- | --- | --- | --- | --- | --- | --- |

Best Unique PSM (Scan Sample\_11\_A1\_1\_3648.d:43130, m/z=780.9136, z=2, RT=15.32, ppm=-1.76):


TRINITY\_DN2477\_c0\_g1\_i10.p1

back to list

  

| Protein Coverage
| Supporting Peptides
| Best Unique PSM
|

Protein Coverage:

Supporting Peptides:

| Peptide | Uniq | -10lgP | Mass | Length | ppm | m/z | z | RT | 1/k0 Start | 1/k0 End | Precursor ID | Area Sample 2 | #Feature | #Feature Sample 2 | Start | End | PTM | AScore | Found By |
| --- | --- | --- | --- | --- | --- | --- | --- | --- | --- | --- | --- | --- | --- | --- | --- | --- | --- | --- | --- |
| K.DSYVGDEAQSKR.G | Y | 59.21 | 1353.616 | 12 | 5.76 | 677.8155 | 2 | 10.93 | 1.0037 | 1.011 | 18642 | 9.97e3 | 1 | 1 | 19 | 30 |  |  | DB Search |
| R.VAPEEHPVLLTEAPLNPK.A | Y | 58.19 | 1953.0571 | 18 | -0.07 | 977.5304 | 2 | 16.19 | 1.2089 | 1.2161 | 47306 | 2.52e2 | 1 | 1 | 64 | 81 |  |  | DB Search |
| R.GYSFTTTAEREIVR.D | Y | 58.1 | 1628.8158 | 14 | 2.53 | 815.4128 | 2 | 15.79 | 1.121 | 1.1282 | 45442 | 6.41e3 | 1 | 1 | 165 | 178 |  |  | DB Search |
| total 3 peptides |  |  |  |  |  |  |  |  |  |  |  |  |  |  |  |  |  |  |  |
| --- | --- | --- | --- | --- | --- | --- | --- | --- | --- | --- | --- | --- | --- | --- | --- | --- | --- | --- | --- |

Best Unique PSM (Scan Sample\_11\_A1\_1\_3648.d:18642, m/z=677.8155, z=2, RT=10.93, ppm=0.28):


TRINITY\_DN309\_c0\_g1\_i1.p1

back to list

  

| Protein Coverage
| Supporting Peptides
| Best Unique PSM
|

Protein Coverage:

Supporting Peptides:

| Peptide | Uniq | -10lgP | Mass | Length | ppm | m/z | z | RT | 1/k0 Start | 1/k0 End | Precursor ID | Area Sample 2 | #Feature | #Feature Sample 2 | Start | End | PTM | AScore | Found By |
| --- | --- | --- | --- | --- | --- | --- | --- | --- | --- | --- | --- | --- | --- | --- | --- | --- | --- | --- | --- |
| K.VTSQREEDEYVK.A | Y | 57.91 | 1481.6998 | 12 | 3.51 | 741.8557 | 2 | 11.18 | 1.0488 | 1.056 | 20043 | 1.4e3 | 1 | 1 | 20 | 31 |  |  | DB Search |
| total 1 peptides |  |  |  |  |  |  |  |  |  |  |  |  |  |  |  |  |  |  |  |
| --- | --- | --- | --- | --- | --- | --- | --- | --- | --- | --- | --- | --- | --- | --- | --- | --- | --- | --- | --- |

Best Unique PSM (Scan Sample\_11\_A1\_1\_3648.d:20043, m/z=741.8557, z=2, RT=11.18, ppm=-1.96):


TRINITY\_DN161\_c0\_g1\_i1.p1

back to list

  

| Protein Coverage
| Supporting Peptides
| Best Unique PSM
|

Protein Coverage:

Supporting Peptides:

| Peptide | Uniq | -10lgP | Mass | Length | ppm | m/z | z | RT | 1/k0 Start | 1/k0 End | Precursor ID | Area Sample 2 | #Feature | #Feature Sample 2 | Start | End | PTM | AScore | Found By |
| --- | --- | --- | --- | --- | --- | --- | --- | --- | --- | --- | --- | --- | --- | --- | --- | --- | --- | --- | --- |
| K.DSYVGDEAQSKR.G | Y | 59.21 | 1353.616 | 12 | 5.76 | 677.8155 | 2 | 10.93 | 1.0037 | 1.011 | 18642 | 9.97e3 | 1 | 1 | 52 | 63 |  |  | DB Search |
| R.LDLAGRDLTDYLM(+15.99)K.I | Y | 58.87 | 1638.8287 | 14 | 0.58 | 820.4176 | 2 | 17.97 | 1.1438 | 1.151 | 56587 | 5.44e3 | 1 | 1 | 179 | 192 | Oxidation (M) | M13:Oxidation (M):1000 | DB Search |
| R.AVFPSIVGRPR.H | Y | 58.86 | 1197.6982 | 11 | -0.21 | 599.853 | 2 | 15.44 | 0.9586 | 0.9658 | 43471 | 2.65e4 | 1 | 1 | 30 | 40 |  |  | DB Search |
| R.LDLAGRDLTDYLMK.I | Y | 58.52 | 1622.8338 | 14 | 2.44 | 812.4217 | 2 | 18.99 | 1.0961 | 1.1033 | 62153 | 2.2e3 | 1 | 1 | 179 | 192 |  |  | DB Search |
| R.VAPEEHPVLLTEAPLNPK.A | Y | 58.19 | 1953.0571 | 18 | -0.07 | 977.5304 | 2 | 16.19 | 1.2089 | 1.2161 | 47306 | 2.52e2 | 1 | 1 | 97 | 114 |  |  | DB Search |
| R.GYSFTTTAEREIVR.D | Y | 58.1 | 1628.8158 | 14 | 2.53 | 815.4128 | 2 | 15.79 | 1.121 | 1.1282 | 45442 | 6.41e3 | 1 | 1 | 198 | 211 |  |  | DB Search |
| R.HQGVM(+15.99)VGM(+15.99)GQK.D | Y | 55.12 | 1202.5536 | 11 | 3.32 | 602.2828 | 2 | 9.73 | 0.9105 | 0.9178 | 11903 | 0 | 0 | 0 | 41 | 51 | Oxidation (M), Oxidation (M) | M5:Oxidation (M):1000 M8:Oxidation (M):1000 | DB Search |
| R.TTGIVLDSGDGVSHTVPIYEGYALPHAIIR.L | Y | 52.38 | 3150.6349 | 30 | -8.99 | 788.6546 | 4 | 18.79 | 1.0693 | 1.0765 | 61107 | 0 | 0 | 0 | 149 | 178 |  |  | DB Search |
| total 8 peptides |  |  |  |  |  |  |  |  |  |  |  |  |  |  |  |  |  |  |  |
| --- | --- | --- | --- | --- | --- | --- | --- | --- | --- | --- | --- | --- | --- | --- | --- | --- | --- | --- | --- |

Best Unique PSM (Scan Sample\_11\_A1\_1\_3648.d:18642, m/z=677.8155, z=2, RT=10.93, ppm=0.28):


TRINITY\_DN3078\_c0\_g1\_i1.p1

back to list

  

| Protein Coverage
| Supporting Peptides
| Best Unique PSM
|

Protein Coverage:

Supporting Peptides:

| Peptide | Uniq | -10lgP | Mass | Length | ppm | m/z | z | RT | 1/k0 Start | 1/k0 End | Precursor ID | Area Sample 2 | #Feature | #Feature Sample 2 | Start | End | PTM | AScore | Found By |
| --- | --- | --- | --- | --- | --- | --- | --- | --- | --- | --- | --- | --- | --- | --- | --- | --- | --- | --- | --- |
| R.KVSQTC(+57.02)AEHVFR.V | Y | 56.73 | 1460.7194 | 12 | 9.26 | 487.9156 | 3 | 11.84 | 0.7982 | 0.8055 | 23975 | 0 | 0 | 0 | 546 | 557 | Carbamidomethylation |  | DB Search |
| K.VSQTC(+57.02)AEHVFR.V | Y | 56.5 | 1332.6245 | 11 | -12.91 | 667.3073 | 2 | 11.84 | 0.9868 | 0.9941 | 23983 | 0 | 0 | 0 | 547 | 557 | Carbamidomethylation |  | DB Search |
| total 2 peptides |  |  |  |  |  |  |  |  |  |  |  |  |  |  |  |  |  |  |  |
| --- | --- | --- | --- | --- | --- | --- | --- | --- | --- | --- | --- | --- | --- | --- | --- | --- | --- | --- | --- |

Best Unique PSM (Scan Sample\_11\_A1\_1\_3648.d:23975, m/z=487.9156, z=3, RT=11.84, ppm=3.78):


TRINITY\_DN118520\_c0\_g1\_i1.p1

back to list

  

| Protein Coverage
| Supporting Peptides
| Best Unique PSM
|

Protein Coverage:

Supporting Peptides:

| Peptide | Uniq | -10lgP | Mass | Length | ppm | m/z | z | RT | 1/k0 Start | 1/k0 End | Precursor ID | Area Sample 2 | #Feature | #Feature Sample 2 | Start | End | PTM | AScore | Found By |
| --- | --- | --- | --- | --- | --- | --- | --- | --- | --- | --- | --- | --- | --- | --- | --- | --- | --- | --- | --- |
| K.ISNVQDIVPVLELANQHK.R | Y | 57.22 | 2016.1003 | 18 | -0.45 | 673.0367 | 3 | 19.27 | 0.9509 | 0.9582 | 63623 | 2.54e2 | 1 | 1 | 258 | 275 |  |  | DB Search |
| total 1 peptides |  |  |  |  |  |  |  |  |  |  |  |  |  |  |  |  |  |  |  |
| --- | --- | --- | --- | --- | --- | --- | --- | --- | --- | --- | --- | --- | --- | --- | --- | --- | --- | --- | --- |

Best Unique PSM (Scan Sample\_11\_A1\_1\_3648.d:63623, m/z=673.0367, z=3, RT=19.27, ppm=-5.93):


TRINITY\_DN8101\_c0\_g1\_i6.p2

back to list

  

| Protein Coverage
| Supporting Peptides
| Best Unique PSM
|

Protein Coverage:

Supporting Peptides:

| Peptide | Uniq | -10lgP | Mass | Length | ppm | m/z | z | RT | 1/k0 Start | 1/k0 End | Precursor ID | Area Sample 2 | #Feature | #Feature Sample 2 | Start | End | PTM | AScore | Found By |
| --- | --- | --- | --- | --- | --- | --- | --- | --- | --- | --- | --- | --- | --- | --- | --- | --- | --- | --- | --- |
| R.SIGFIGHYLDQLR.L | Y | 54.32 | 1517.799 | 13 | 1.06 | 759.9034 | 2 | 19.56 | 1.0849 | 1.0921 | 65092 | 4.69e2 | 1 | 1 | 784 | 796 |  |  | DB Search |
| total 1 peptides |  |  |  |  |  |  |  |  |  |  |  |  |  |  |  |  |  |  |  |
| --- | --- | --- | --- | --- | --- | --- | --- | --- | --- | --- | --- | --- | --- | --- | --- | --- | --- | --- | --- |

Best Unique PSM (Scan Sample\_11\_A1\_1\_3648.d:65092, m/z=759.9034, z=2, RT=19.56, ppm=-4.41):


TRINITY\_DN554\_c0\_g1\_i5.p1

back to list

  

| Protein Coverage
| Supporting Peptides
| Best Unique PSM
|

Protein Coverage:

Supporting Peptides:

| Peptide | Uniq | -10lgP | Mass | Length | ppm | m/z | z | RT | 1/k0 Start | 1/k0 End | Precursor ID | Area Sample 2 | #Feature | #Feature Sample 2 | Start | End | PTM | AScore | Found By |
| --- | --- | --- | --- | --- | --- | --- | --- | --- | --- | --- | --- | --- | --- | --- | --- | --- | --- | --- | --- |
| K.IQDKEGIPPDQQR.L | Y | 55.06 | 1522.7739 | 13 | 2.88 | 762.3923 | 2 | 11.00 | 1.0331 | 1.0403 | 18584 | 2.97e3 | 2 | 2 | 30 | 42 |  |  | DB Search |
| total 1 peptides |  |  |  |  |  |  |  |  |  |  |  |  |  |  |  |  |  |  |  |
| --- | --- | --- | --- | --- | --- | --- | --- | --- | --- | --- | --- | --- | --- | --- | --- | --- | --- | --- | --- |

Best Unique PSM (Scan Sample\_11\_A1\_1\_3648.d:18584, m/z=762.3923, z=2, RT=11.00, ppm=-2.59):


TRINITY\_DN118208\_c0\_g1\_i1.p1

back to list

  

| Protein Coverage
| Supporting Peptides
| Best Unique PSM
|

Protein Coverage:

Supporting Peptides:

| Peptide | Uniq | -10lgP | Mass | Length | ppm | m/z | z | RT | 1/k0 Start | 1/k0 End | Precursor ID | Area Sample 2 | #Feature | #Feature Sample 2 | Start | End | PTM | AScore | Found By |
| --- | --- | --- | --- | --- | --- | --- | --- | --- | --- | --- | --- | --- | --- | --- | --- | --- | --- | --- | --- |
| K.SDKEEHFC(+57.02)LR.G | Y | 57.1 | 1319.5928 | 10 | 6.18 | 440.8719 | 3 | 12.41 | 0.7844 | 0.7917 | 27277 | 0 | 0 | 0 | 347 | 356 | Carbamidomethylation |  | DB Search |
| total 1 peptides |  |  |  |  |  |  |  |  |  |  |  |  |  |  |  |  |  |  |  |
| --- | --- | --- | --- | --- | --- | --- | --- | --- | --- | --- | --- | --- | --- | --- | --- | --- | --- | --- | --- |

Best Unique PSM (Scan Sample\_11\_A1\_1\_3648.d:27277, m/z=440.8719, z=3, RT=12.41, ppm=0.7):


TRINITY\_DN10497\_c0\_g1\_i1.p1

back to list

  

| Protein Coverage
| Supporting Peptides
| Best Unique PSM
|

Protein Coverage:

Supporting Peptides:

| Peptide | Uniq | -10lgP | Mass | Length | ppm | m/z | z | RT | 1/k0 Start | 1/k0 End | Precursor ID | Area Sample 2 | #Feature | #Feature Sample 2 | Start | End | PTM | AScore | Found By |
| --- | --- | --- | --- | --- | --- | --- | --- | --- | --- | --- | --- | --- | --- | --- | --- | --- | --- | --- | --- |
| R.RVTILENELDYQHR.V | Y | 58.2 | 1784.9169 | 14 | 1.12 | 595.977 | 3 | 15.12 | 0.8895 | 0.8968 | 42087 | 3.46e2 | 1 | 1 | 164 | 177 |  |  | DB Search |
| total 1 peptides |  |  |  |  |  |  |  |  |  |  |  |  |  |  |  |  |  |  |  |
| --- | --- | --- | --- | --- | --- | --- | --- | --- | --- | --- | --- | --- | --- | --- | --- | --- | --- | --- | --- |

Best Unique PSM (Scan Sample\_11\_A1\_1\_3648.d:42087, m/z=595.977, z=3, RT=15.12, ppm=-4.36):


TRINITY\_DN16120\_c0\_g1\_i1.p1

back to list

  

| Protein Coverage
| Supporting Peptides
| Best Unique PSM
|

Protein Coverage:

Supporting Peptides:

| Peptide | Uniq | -10lgP | Mass | Length | ppm | m/z | z | RT | 1/k0 Start | 1/k0 End | Precursor ID | Area Sample 2 | #Feature | #Feature Sample 2 | Start | End | PTM | AScore | Found By |
| --- | --- | --- | --- | --- | --- | --- | --- | --- | --- | --- | --- | --- | --- | --- | --- | --- | --- | --- | --- |
| R.LC(+57.02)ETNTANTEAK.V | Y | 57.96 | 1350.6085 | 12 | -0.88 | 676.3073 | 2 | 10.37 | 0.9537 | 0.961 | 15355 | 0 | 0 | 0 | 138 | 149 | Carbamidomethylation |  | DB Search |
| total 1 peptides |  |  |  |  |  |  |  |  |  |  |  |  |  |  |  |  |  |  |  |
| --- | --- | --- | --- | --- | --- | --- | --- | --- | --- | --- | --- | --- | --- | --- | --- | --- | --- | --- | --- |

Best Unique PSM (Scan Sample\_11\_A1\_1\_3648.d:15355, m/z=676.3073, z=2, RT=10.37, ppm=-6.36):


TRINITY\_DN5153\_c0\_g1\_i1.p1

back to list

  

| Protein Coverage
| Supporting Peptides
| Best Unique PSM
|

Protein Coverage:

Supporting Peptides:

| Peptide | Uniq | -10lgP | Mass | Length | ppm | m/z | z | RT | 1/k0 Start | 1/k0 End | Precursor ID | Area Sample 2 | #Feature | #Feature Sample 2 | Start | End | PTM | AScore | Found By |
| --- | --- | --- | --- | --- | --- | --- | --- | --- | --- | --- | --- | --- | --- | --- | --- | --- | --- | --- | --- |
| R.ALELDALSNSHPIEVK.V | Y | 33.06 | 1734.9152 | 16 | 3.63 | 868.4633 | 2 | 16.96 | 1.1706 | 1.1778 | 51172 | 0 | 0 | 0 | 362 | 377 |  |  | DB Search |
| total 1 peptides |  |  |  |  |  |  |  |  |  |  |  |  |  |  |  |  |  |  |  |
| --- | --- | --- | --- | --- | --- | --- | --- | --- | --- | --- | --- | --- | --- | --- | --- | --- | --- | --- | --- |

Best Unique PSM (Scan Sample\_11\_A1\_1\_3648.d:51172, m/z=868.4633, z=2, RT=16.96, ppm=-1.85):


TRINITY\_DN36\_c0\_g1\_i12.p1

back to list

  

| Protein Coverage
| Supporting Peptides
| Best Unique PSM
|

Protein Coverage:

Supporting Peptides:

| Peptide | Uniq | -10lgP | Mass | Length | ppm | m/z | z | RT | 1/k0 Start | 1/k0 End | Precursor ID | Area Sample 2 | #Feature | #Feature Sample 2 | Start | End | PTM | AScore | Found By |
| --- | --- | --- | --- | --- | --- | --- | --- | --- | --- | --- | --- | --- | --- | --- | --- | --- | --- | --- | --- |
| R.DVEC(+57.02)GKQFDHC(+57.02)TTIK.Y | Y | 36.83 | 1836.8134 | 15 | 4.81 | 613.278 | 3 | 12.65 | 0.8571 | 0.8644 | 28563 | 3.3e2 | 1 | 1 | 40 | 54 | Carbamidomethylation, Carbamidomethylation |  | DB Search |
| total 1 peptides |  |  |  |  |  |  |  |  |  |  |  |  |  |  |  |  |  |  |  |
| --- | --- | --- | --- | --- | --- | --- | --- | --- | --- | --- | --- | --- | --- | --- | --- | --- | --- | --- | --- |

Best Unique PSM (Scan Sample\_11\_A1\_1\_3648.d:28563, m/z=613.278, z=3, RT=12.65, ppm=-0.67):


TRINITY\_DN9509\_c0\_g1\_i8.p1

back to list

  

| Protein Coverage
| Supporting Peptides
| Best Unique PSM
|

Protein Coverage:

Supporting Peptides:

| Peptide | Uniq | -10lgP | Mass | Length | ppm | m/z | z | RT | 1/k0 Start | 1/k0 End | Precursor ID | Area Sample 2 | #Feature | #Feature Sample 2 | Start | End | PTM | AScore | Found By |
| --- | --- | --- | --- | --- | --- | --- | --- | --- | --- | --- | --- | --- | --- | --- | --- | --- | --- | --- | --- |
| R.GQVIC(+57.02)APGATSTHTK.F | Y | 58 | 1526.7511 | 15 | 1.7 | 764.3799 | 2 | 11.76 | 1.0291 | 1.0363 | 23431 | 4.83e2 | 1 | 1 | 109 | 123 | Carbamidomethylation |  | DB Search |
| total 1 peptides |  |  |  |  |  |  |  |  |  |  |  |  |  |  |  |  |  |  |  |
| --- | --- | --- | --- | --- | --- | --- | --- | --- | --- | --- | --- | --- | --- | --- | --- | --- | --- | --- | --- |

Best Unique PSM (Scan Sample\_11\_A1\_1\_3648.d:23431, m/z=764.3799, z=2, RT=11.76, ppm=-3.78):


TRINITY\_DN21847\_c0\_g1\_i1.p1

back to list

  

| Protein Coverage
| Supporting Peptides
| Best Unique PSM
|

Protein Coverage:

Supporting Peptides:

| Peptide | Uniq | -10lgP | Mass | Length | ppm | m/z | z | RT | 1/k0 Start | 1/k0 End | Precursor ID | Area Sample 2 | #Feature | #Feature Sample 2 | Start | End | PTM | AScore | Found By |
| --- | --- | --- | --- | --- | --- | --- | --- | --- | --- | --- | --- | --- | --- | --- | --- | --- | --- | --- | --- |
| K.VLTSSDPAEKEK.K | Y | 58.73 | 1302.6667 | 12 | -2.55 | 652.3354 | 2 | 10.56 | 0.9969 | 1.0041 | 16353 | 2.66e3 | 1 | 1 | 101 | 112 |  |  | DB Search |
| total 1 peptides |  |  |  |  |  |  |  |  |  |  |  |  |  |  |  |  |  |  |  |
| --- | --- | --- | --- | --- | --- | --- | --- | --- | --- | --- | --- | --- | --- | --- | --- | --- | --- | --- | --- |

Best Unique PSM (Scan Sample\_11\_A1\_1\_3648.d:16353, m/z=652.3354, z=2, RT=10.56, ppm=-8.03):


TRINITY\_DN1080\_c0\_g2\_i1.p1

back to list

  

| Protein Coverage
| Supporting Peptides
| Best Unique PSM
|

Protein Coverage:

Supporting Peptides:

| Peptide | Uniq | -10lgP | Mass | Length | ppm | m/z | z | RT | 1/k0 Start | 1/k0 End | Precursor ID | Area Sample 2 | #Feature | #Feature Sample 2 | Start | End | PTM | AScore | Found By |
| --- | --- | --- | --- | --- | --- | --- | --- | --- | --- | --- | --- | --- | --- | --- | --- | --- | --- | --- | --- |
| R.RPVPIVTRR.R | Y | 57.63 | 1092.688 | 9 | 6 | 365.2368 | 3 | 10.84 | 0.7045 | 0.7118 | 18035 | 1.06e3 | 1 | 1 | 9 | 17 |  |  | DB Search |
| total 1 peptides |  |  |  |  |  |  |  |  |  |  |  |  |  |  |  |  |  |  |  |
| --- | --- | --- | --- | --- | --- | --- | --- | --- | --- | --- | --- | --- | --- | --- | --- | --- | --- | --- | --- |

Best Unique PSM (Scan Sample\_11\_A1\_1\_3648.d:18035, m/z=365.2368, z=3, RT=10.84, ppm=0.51):


TRINITY\_DN22077\_c0\_g1\_i1.p3

back to list

  

| Protein Coverage
| Supporting Peptides
| Best Unique PSM
|

Protein Coverage:

Supporting Peptides:

| Peptide | Uniq | -10lgP | Mass | Length | ppm | m/z | z | RT | 1/k0 Start | 1/k0 End | Precursor ID | Area Sample 2 | #Feature | #Feature Sample 2 | Start | End | PTM | AScore | Found By |
| --- | --- | --- | --- | --- | --- | --- | --- | --- | --- | --- | --- | --- | --- | --- | --- | --- | --- | --- | --- |
| K.RISGLIYEETR.G | Y | 58.85 | 1335.7146 | 11 | 5.98 | 668.8649 | 2 | 14.62 | 0.9695 | 0.9767 | 39433 | 6.26e3 | 1 | 1 | 93 | 103 |  |  | DB Search |
| R.ISGLIYEETRGVLK.S | Y | 57.65 | 1576.8824 | 14 | 4.01 | 789.4473 | 2 | 17.11 | 1.0945 | 1.1017 | 51906 | 3.57e3 | 1 | 1 | 94 | 107 |  |  | DB Search |
| K.RISGLIYEETRGVLK.S | Y | 55.36 | 1732.9835 | 15 | 1.37 | 578.6661 | 3 | 16.41 | 0.8911 | 0.8984 | 48406 | 2.02e3 | 1 | 1 | 93 | 107 |  |  | DB Search |
| total 3 peptides |  |  |  |  |  |  |  |  |  |  |  |  |  |  |  |  |  |  |  |
| --- | --- | --- | --- | --- | --- | --- | --- | --- | --- | --- | --- | --- | --- | --- | --- | --- | --- | --- | --- |

Best Unique PSM (Scan Sample\_11\_A1\_1\_3648.d:39433, m/z=668.8649, z=2, RT=14.62, ppm=0.5):


TRINITY\_DN10995\_c0\_g1\_i2.p3

back to list

  

| Protein Coverage
| Supporting Peptides
| Best Unique PSM
|

Protein Coverage:

Supporting Peptides:

| Peptide | Uniq | -10lgP | Mass | Length | ppm | m/z | z | RT | 1/k0 Start | 1/k0 End | Precursor ID | Area Sample 2 | #Feature | #Feature Sample 2 | Start | End | PTM | AScore | Found By |
| --- | --- | --- | --- | --- | --- | --- | --- | --- | --- | --- | --- | --- | --- | --- | --- | --- | --- | --- | --- |
| R.AGKDSLDWFFR.A | Y | 36.4 | 1340.6513 | 11 | -0.05 | 671.3292 | 2 | 19.38 | 0.963 | 0.9703 | 64246 | 0 | 0 | 0 | 77 | 87 |  |  | DB Search |
| total 1 peptides |  |  |  |  |  |  |  |  |  |  |  |  |  |  |  |  |  |  |  |
| --- | --- | --- | --- | --- | --- | --- | --- | --- | --- | --- | --- | --- | --- | --- | --- | --- | --- | --- | --- |

Best Unique PSM (Scan Sample\_11\_A1\_1\_3648.d:64246, m/z=671.3292, z=2, RT=19.38, ppm=-5.52):


TRINITY\_DN165536\_c0\_g1\_i1.p1

back to list

  

| Protein Coverage
| Supporting Peptides
| Best Unique PSM
|

Protein Coverage:

Supporting Peptides:

| Peptide | Uniq | -10lgP | Mass | Length | ppm | m/z | z | RT | 1/k0 Start | 1/k0 End | Precursor ID | Area Sample 2 | #Feature | #Feature Sample 2 | Start | End | PTM | AScore | Found By |
| --- | --- | --- | --- | --- | --- | --- | --- | --- | --- | --- | --- | --- | --- | --- | --- | --- | --- | --- | --- |
| K.ATETATGLATTHPIK.L | Y | 59 | 1510.7991 | 15 | 3.59 | 756.4054 | 2 | 12.84 | 1.1053 | 1.1126 | 29583 | 1.89e3 | 1 | 1 | 161 | 175 |  |  | DB Search |
| total 1 peptides |  |  |  |  |  |  |  |  |  |  |  |  |  |  |  |  |  |  |  |
| --- | --- | --- | --- | --- | --- | --- | --- | --- | --- | --- | --- | --- | --- | --- | --- | --- | --- | --- | --- |

Best Unique PSM (Scan Sample\_11\_A1\_1\_3648.d:29583, m/z=756.4054, z=2, RT=12.84, ppm=-1.89):


TRINITY\_DN1339\_c0\_g2\_i1.p2

back to list

  

| Protein Coverage
| Supporting Peptides
| Best Unique PSM
|

Protein Coverage:

Supporting Peptides:

| Peptide | Uniq | -10lgP | Mass | Length | ppm | m/z | z | RT | 1/k0 Start | 1/k0 End | Precursor ID | Area Sample 2 | #Feature | #Feature Sample 2 | Start | End | PTM | AScore | Found By |
| --- | --- | --- | --- | --- | --- | --- | --- | --- | --- | --- | --- | --- | --- | --- | --- | --- | --- | --- | --- |
| K.LLTSSDQAELTKK.L | Y | 58.87 | 1432.7773 | 13 | 4.21 | 717.395 | 2 | 12.69 | 1.0496 | 1.0568 | 28711 | 7.94e2 | 1 | 1 | 155 | 167 |  |  | DB Search |
| total 1 peptides |  |  |  |  |  |  |  |  |  |  |  |  |  |  |  |  |  |  |  |
| --- | --- | --- | --- | --- | --- | --- | --- | --- | --- | --- | --- | --- | --- | --- | --- | --- | --- | --- | --- |

Best Unique PSM (Scan Sample\_11\_A1\_1\_3648.d:28711, m/z=717.395, z=2, RT=12.69, ppm=-1.26):


TRINITY\_DN477\_c0\_g1\_i8.p1

back to list

  

| Protein Coverage
| Supporting Peptides
| Best Unique PSM
|

Protein Coverage:

Supporting Peptides:

| Peptide | Uniq | -10lgP | Mass | Length | ppm | m/z | z | RT | 1/k0 Start | 1/k0 End | Precursor ID | Area Sample 2 | #Feature | #Feature Sample 2 | Start | End | PTM | AScore | Found By |
| --- | --- | --- | --- | --- | --- | --- | --- | --- | --- | --- | --- | --- | --- | --- | --- | --- | --- | --- | --- |
| R.ILQC(+57.02)NQYANVEEAR.N | Y | 59.1 | 1706.8046 | 14 | -0.86 | 854.4042 | 2 | 14.11 | 1.0873 | 1.0945 | 36622 | 2.02e3 | 1 | 1 | 86 | 99 | Carbamidomethylation |  | DB Search |
| K.DKNILVLTLGVGPQVNR.N | Y | 55.18 | 1835.0628 | 17 | -2.24 | 918.5316 | 2 | 19.07 | 1.1314 | 1.1386 | 62691 | 0 | 0 | 0 | 168 | 184 |  |  | DB Search |
| total 2 peptides |  |  |  |  |  |  |  |  |  |  |  |  |  |  |  |  |  |  |  |
| --- | --- | --- | --- | --- | --- | --- | --- | --- | --- | --- | --- | --- | --- | --- | --- | --- | --- | --- | --- |

Best Unique PSM (Scan Sample\_11\_A1\_1\_3648.d:36622, m/z=854.4042, z=2, RT=14.11, ppm=-6.33):


TRINITY\_DN10940\_c0\_g1\_i1.p1

back to list

  

| Protein Coverage
| Supporting Peptides
| Best Unique PSM
|

Protein Coverage:

Supporting Peptides:

| Peptide | Uniq | -10lgP | Mass | Length | ppm | m/z | z | RT | 1/k0 Start | 1/k0 End | Precursor ID | Area Sample 2 | #Feature | #Feature Sample 2 | Start | End | PTM | AScore | Found By |
| --- | --- | --- | --- | --- | --- | --- | --- | --- | --- | --- | --- | --- | --- | --- | --- | --- | --- | --- | --- |
| K.KKGEGSLSLQC(+57.02)TNTAVVLAHTIEGK.S | Y | 56.21 | 2640.3905 | 25 | -9.74 | 661.0948 | 4 | 17.15 | 0.9339 | 0.9412 | 52200 | 0 | 0 | 0 | 89 | 113 | Carbamidomethylation |  | DB Search |
| total 1 peptides |  |  |  |  |  |  |  |  |  |  |  |  |  |  |  |  |  |  |  |
| --- | --- | --- | --- | --- | --- | --- | --- | --- | --- | --- | --- | --- | --- | --- | --- | --- | --- | --- | --- |

Best Unique PSM (Scan Sample\_11\_A1\_1\_3648.d:52200, m/z=661.0948, z=4, RT=17.15, ppm=-15.21):


TRINITY\_DN6920\_c0\_g1\_i4.p1

back to list

  

| Protein Coverage
| Supporting Peptides
| Best Unique PSM
|

Protein Coverage:

Supporting Peptides:

| Peptide | Uniq | -10lgP | Mass | Length | ppm | m/z | z | RT | 1/k0 Start | 1/k0 End | Precursor ID | Area Sample 2 | #Feature | #Feature Sample 2 | Start | End | PTM | AScore | Found By |
| --- | --- | --- | --- | --- | --- | --- | --- | --- | --- | --- | --- | --- | --- | --- | --- | --- | --- | --- | --- |
| R.VLVHTQQK.L | Y | 57.79 | 951.5501 | 8 | -1.07 | 476.7792 | 2 | 9.55 | 0.8352 | 0.8425 | 11014 | 5.75e2 | 1 | 1 | 40 | 47 |  |  | DB Search |
| total 1 peptides |  |  |  |  |  |  |  |  |  |  |  |  |  |  |  |  |  |  |  |
| --- | --- | --- | --- | --- | --- | --- | --- | --- | --- | --- | --- | --- | --- | --- | --- | --- | --- | --- | --- |

Best Unique PSM (Scan Sample\_11\_A1\_1\_3648.d:11014, m/z=477.7582, z=2, RT=9.56, ppm=-6.55):


TRINITY\_DN7333\_c0\_g1\_i1.p1

back to list

  

| Protein Coverage
| Supporting Peptides
| Best Unique PSM
|

Protein Coverage:

Supporting Peptides:

| Peptide | Uniq | -10lgP | Mass | Length | ppm | m/z | z | RT | 1/k0 Start | 1/k0 End | Precursor ID | Area Sample 2 | #Feature | #Feature Sample 2 | Start | End | PTM | AScore | Found By |
| --- | --- | --- | --- | --- | --- | --- | --- | --- | --- | --- | --- | --- | --- | --- | --- | --- | --- | --- | --- |
| K.TPFLLVGTQVDLRDDAAIVEK.L | Y | 56.85 | 2299.2423 | 21 | 2.14 | 767.4188 | 3 | 19.81 | 1.0319 | 1.0391 | 66139 | 4.65e2 | 1 | 1 | 108 | 128 |  |  | DB Search |
| total 1 peptides |  |  |  |  |  |  |  |  |  |  |  |  |  |  |  |  |  |  |  |
| --- | --- | --- | --- | --- | --- | --- | --- | --- | --- | --- | --- | --- | --- | --- | --- | --- | --- | --- | --- |

Best Unique PSM (Scan Sample\_11\_A1\_1\_3648.d:66139, m/z=767.4188, z=3, RT=19.81, ppm=-3.33):


TRINITY\_DN200\_c0\_g1\_i1.p1

back to list

  

| Protein Coverage
| Supporting Peptides
| Best Unique PSM
|

Protein Coverage:

Supporting Peptides:

| Peptide | Uniq | -10lgP | Mass | Length | ppm | m/z | z | RT | 1/k0 Start | 1/k0 End | Precursor ID | Area Sample 2 | #Feature | #Feature Sample 2 | Start | End | PTM | AScore | Found By |
| --- | --- | --- | --- | --- | --- | --- | --- | --- | --- | --- | --- | --- | --- | --- | --- | --- | --- | --- | --- |
| K.TAIAIDTIINQKR.F | Y | 58.72 | 1455.8409 | 13 | 0.37 | 728.924 | 2 | 16.37 | 1.0604 | 1.0676 | 48298 | 0 | 0 | 0 | 217 | 229 |  |  | DB Search |
| total 1 peptides |  |  |  |  |  |  |  |  |  |  |  |  |  |  |  |  |  |  |  |
| --- | --- | --- | --- | --- | --- | --- | --- | --- | --- | --- | --- | --- | --- | --- | --- | --- | --- | --- | --- |

Best Unique PSM (Scan Sample\_11\_A1\_1\_3648.d:48298, m/z=728.924, z=2, RT=16.37, ppm=-5.1):


TRINITY\_DN433\_c0\_g2\_i1.p1

back to list

  

| Protein Coverage
| Supporting Peptides
| Best Unique PSM
|

Protein Coverage:

Supporting Peptides:

| Peptide | Uniq | -10lgP | Mass | Length | ppm | m/z | z | RT | 1/k0 Start | 1/k0 End | Precursor ID | Area Sample 2 | #Feature | #Feature Sample 2 | Start | End | PTM | AScore | Found By |
| --- | --- | --- | --- | --- | --- | --- | --- | --- | --- | --- | --- | --- | --- | --- | --- | --- | --- | --- | --- |
| K.AVLTDLRR.M | Y | 19.88 | 942.561 | 8 | 2.21 | 472.2863 | 2 | 13.09 | 0.8408 | 0.8481 | 31027 | 0 | 0 | 1 | 113 | 120 |  |  | DB Search |
| total 1 peptides |  |  |  |  |  |  |  |  |  |  |  |  |  |  |  |  |  |  |  |
| --- | --- | --- | --- | --- | --- | --- | --- | --- | --- | --- | --- | --- | --- | --- | --- | --- | --- | --- | --- |

Best Unique PSM (Scan Sample\_11\_A1\_1\_3648.d:31027, m/z=472.2863, z=2, RT=13.09, ppm=-3.27):


TRINITY\_DN4497\_c0\_g2\_i1.p1

back to list

  

| Protein Coverage
| Supporting Peptides
| Best Unique PSM
|

Protein Coverage:

Supporting Peptides:

| Peptide | Uniq | -10lgP | Mass | Length | ppm | m/z | z | RT | 1/k0 Start | 1/k0 End | Precursor ID | Area Sample 2 | #Feature | #Feature Sample 2 | Start | End | PTM | AScore | Found By |
| --- | --- | --- | --- | --- | --- | --- | --- | --- | --- | --- | --- | --- | --- | --- | --- | --- | --- | --- | --- |
| K.VLTSSDPAEKEK.K | Y | 58.73 | 1302.6667 | 12 | -2.55 | 652.3354 | 2 | 10.56 | 0.9969 | 1.0041 | 16353 | 2.66e3 | 1 | 1 | 126 | 137 |  |  | DB Search |
| total 1 peptides |  |  |  |  |  |  |  |  |  |  |  |  |  |  |  |  |  |  |  |
| --- | --- | --- | --- | --- | --- | --- | --- | --- | --- | --- | --- | --- | --- | --- | --- | --- | --- | --- | --- |

Best Unique PSM (Scan Sample\_11\_A1\_1\_3648.d:16353, m/z=652.3354, z=2, RT=10.56, ppm=-8.03):


TRINITY\_DN173718\_c0\_g1\_i1.p1

back to list

  

| Protein Coverage
| Supporting Peptides
| Best Unique PSM
|

Protein Coverage:

Supporting Peptides:

| Peptide | Uniq | -10lgP | Mass | Length | ppm | m/z | z | RT | 1/k0 Start | 1/k0 End | Precursor ID | Area Sample 2 | #Feature | #Feature Sample 2 | Start | End | PTM | AScore | Found By |
| --- | --- | --- | --- | --- | --- | --- | --- | --- | --- | --- | --- | --- | --- | --- | --- | --- | --- | --- | --- |
| K.IQDKEGIPPDQQR.L | Y | 55.06 | 1522.7739 | 13 | 2.88 | 762.3923 | 2 | 11.00 | 1.0331 | 1.0403 | 18584 | 2.97e3 | 2 | 2 | 30 | 42 |  |  | DB Search |
| total 1 peptides |  |  |  |  |  |  |  |  |  |  |  |  |  |  |  |  |  |  |  |
| --- | --- | --- | --- | --- | --- | --- | --- | --- | --- | --- | --- | --- | --- | --- | --- | --- | --- | --- | --- |

Best Unique PSM (Scan Sample\_11\_A1\_1\_3648.d:18584, m/z=762.3923, z=2, RT=11.00, ppm=-2.59):


TRINITY\_DN1339\_c0\_g2\_i5.p1

back to list

  

| Protein Coverage
| Supporting Peptides
| Best Unique PSM
|

Protein Coverage:

Supporting Peptides:

| Peptide | Uniq | -10lgP | Mass | Length | ppm | m/z | z | RT | 1/k0 Start | 1/k0 End | Precursor ID | Area Sample 2 | #Feature | #Feature Sample 2 | Start | End | PTM | AScore | Found By |
| --- | --- | --- | --- | --- | --- | --- | --- | --- | --- | --- | --- | --- | --- | --- | --- | --- | --- | --- | --- |
| K.LLTSSDQGELTKK.L | Y | 50.28 | 1418.7616 | 13 | -0.77 | 710.3837 | 2 | 12.33 | 1.0391 | 1.0464 | 26741 | 3.62e2 | 1 | 1 | 155 | 167 |  |  | DB Search |
| total 1 peptides |  |  |  |  |  |  |  |  |  |  |  |  |  |  |  |  |  |  |  |
| --- | --- | --- | --- | --- | --- | --- | --- | --- | --- | --- | --- | --- | --- | --- | --- | --- | --- | --- | --- |

Best Unique PSM (Scan Sample\_11\_A1\_1\_3648.d:26741, m/z=710.3837, z=2, RT=12.33, ppm=-6.25):


TRINITY\_DN1339\_c6\_g1\_i2.p1

back to list

  

| Protein Coverage
| Supporting Peptides
| Best Unique PSM
|

Protein Coverage:

Supporting Peptides:

| Peptide | Uniq | -10lgP | Mass | Length | ppm | m/z | z | RT | 1/k0 Start | 1/k0 End | Precursor ID | Area Sample 2 | #Feature | #Feature Sample 2 | Start | End | PTM | AScore | Found By |
| --- | --- | --- | --- | --- | --- | --- | --- | --- | --- | --- | --- | --- | --- | --- | --- | --- | --- | --- | --- |
| K.VITSDDPAEKEK.K | Y | 58.87 | 1330.6616 | 12 | 2.64 | 666.3362 | 2 | 10.69 | 0.9848 | 0.992 | 17191 | 9.01e3 | 1 | 1 | 355 | 366 |  |  | DB Search |
| total 1 peptides |  |  |  |  |  |  |  |  |  |  |  |  |  |  |  |  |  |  |  |
| --- | --- | --- | --- | --- | --- | --- | --- | --- | --- | --- | --- | --- | --- | --- | --- | --- | --- | --- | --- |

Best Unique PSM (Scan Sample\_11\_A1\_1\_3648.d:17191, m/z=666.3362, z=2, RT=10.69, ppm=-2.84):


TRINITY\_DN2217\_c0\_g1\_i3.p1

back to list

  

| Protein Coverage
| Supporting Peptides
| Best Unique PSM
|

Protein Coverage:

Supporting Peptides:

| Peptide | Uniq | -10lgP | Mass | Length | ppm | m/z | z | RT | 1/k0 Start | 1/k0 End | Precursor ID | Area Sample 2 | #Feature | #Feature Sample 2 | Start | End | PTM | AScore | Found By |
| --- | --- | --- | --- | --- | --- | --- | --- | --- | --- | --- | --- | --- | --- | --- | --- | --- | --- | --- | --- |
| K.ATGC(+57.02)EVIWNQHNR.G | Y | 58.87 | 1583.7263 | 13 | 1.47 | 792.8672 | 2 | 13.74 | 1.0773 | 1.0845 | 34668 | 9.8e2 | 1 | 1 | 431 | 443 | Carbamidomethylation |  | DB Search |
| total 1 peptides |  |  |  |  |  |  |  |  |  |  |  |  |  |  |  |  |  |  |  |
| --- | --- | --- | --- | --- | --- | --- | --- | --- | --- | --- | --- | --- | --- | --- | --- | --- | --- | --- | --- |

Best Unique PSM (Scan Sample\_11\_A1\_1\_3648.d:34668, m/z=792.8672, z=2, RT=13.74, ppm=-4.01):


TRINITY\_DN4101\_c0\_g1\_i3.p2

back to list

  

| Protein Coverage
| Supporting Peptides
| Best Unique PSM
|

Protein Coverage:

Supporting Peptides:

| Peptide | Uniq | -10lgP | Mass | Length | ppm | m/z | z | RT | 1/k0 Start | 1/k0 End | Precursor ID | Area Sample 2 | #Feature | #Feature Sample 2 | Start | End | PTM | AScore | Found By |
| --- | --- | --- | --- | --- | --- | --- | --- | --- | --- | --- | --- | --- | --- | --- | --- | --- | --- | --- | --- |
| R.GVDEDAYTLEVLHYK.A | Y | 58.84 | 1750.8413 | 15 | 4.4 | 876.427 | 2 | 17.48 | 1.1802 | 1.1873 | 54020 | 1.93e2 | 1 | 1 | 81 | 95 |  |  | DB Search |
| total 1 peptides |  |  |  |  |  |  |  |  |  |  |  |  |  |  |  |  |  |  |  |
| --- | --- | --- | --- | --- | --- | --- | --- | --- | --- | --- | --- | --- | --- | --- | --- | --- | --- | --- | --- |

Best Unique PSM (Scan Sample\_11\_A1\_1\_3648.d:54020, m/z=876.427, z=2, RT=17.48, ppm=-1.08):


TRINITY\_DN5023\_c0\_g1\_i3.p2

back to list

  

| Protein Coverage
| Supporting Peptides
| Best Unique PSM
|

Protein Coverage:

Supporting Peptides:

| Peptide | Uniq | -10lgP | Mass | Length | ppm | m/z | z | RT | 1/k0 Start | 1/k0 End | Precursor ID | Area Sample 2 | #Feature | #Feature Sample 2 | Start | End | PTM | AScore | Found By |
| --- | --- | --- | --- | --- | --- | --- | --- | --- | --- | --- | --- | --- | --- | --- | --- | --- | --- | --- | --- |
| R.YRPGTVALR.E | Y | 44.05 | 1031.5876 | 9 | -1.83 | 516.7973 | 2 | 12.44 | 0.8575 | 0.8648 | 27332 | 1.61e3 | 1 | 1 | 78 | 86 |  |  | DB Search |
| total 1 peptides |  |  |  |  |  |  |  |  |  |  |  |  |  |  |  |  |  |  |  |
| --- | --- | --- | --- | --- | --- | --- | --- | --- | --- | --- | --- | --- | --- | --- | --- | --- | --- | --- | --- |

Best Unique PSM (Scan Sample\_11\_A1\_1\_3648.d:27332, m/z=516.7973, z=2, RT=12.44, ppm=-7.31):


TRINITY\_DN11097\_c0\_g1\_i1.p1

back to list

  

| Protein Coverage
| Supporting Peptides
| Best Unique PSM
|

Protein Coverage:

Supporting Peptides:

| Peptide | Uniq | -10lgP | Mass | Length | ppm | m/z | z | RT | 1/k0 Start | 1/k0 End | Precursor ID | Area Sample 2 | #Feature | #Feature Sample 2 | Start | End | PTM | AScore | Found By |
| --- | --- | --- | --- | --- | --- | --- | --- | --- | --- | --- | --- | --- | --- | --- | --- | --- | --- | --- | --- |
| K.LRVDLSLFETGIK.V | Y | 57.58 | 1489.8504 | 13 | -1.87 | 745.927 | 2 | 19.61 | 1.0363 | 1.0435 | 65344 | 1.84e3 | 1 | 1 | 362 | 374 |  |  | DB Search |
| total 1 peptides |  |  |  |  |  |  |  |  |  |  |  |  |  |  |  |  |  |  |  |
| --- | --- | --- | --- | --- | --- | --- | --- | --- | --- | --- | --- | --- | --- | --- | --- | --- | --- | --- | --- |

Best Unique PSM (Scan Sample\_11\_A1\_1\_3648.d:65344, m/z=745.927, z=2, RT=19.61, ppm=-7.34):


TRINITY\_DN123\_c1\_g1\_i1.p1

back to list

  

| Protein Coverage
| Supporting Peptides
| Best Unique PSM
|

Protein Coverage:

Supporting Peptides:

| Peptide | Uniq | -10lgP | Mass | Length | ppm | m/z | z | RT | 1/k0 Start | 1/k0 End | Precursor ID | Area Sample 2 | #Feature | #Feature Sample 2 | Start | End | PTM | AScore | Found By |
| --- | --- | --- | --- | --- | --- | --- | --- | --- | --- | --- | --- | --- | --- | --- | --- | --- | --- | --- | --- |
| R.YTVGDVVTC(+57.02)YIDLDARPR.A | Y | 26.69 | 2112.031 | 18 | 0.25 | 705.0139 | 3 | 19.89 | 0.94 | 0.9473 | 66734 | 0 | 0 | 0 | 338 | 355 | Carbamidomethylation |  | DB Search |
| total 1 peptides |  |  |  |  |  |  |  |  |  |  |  |  |  |  |  |  |  |  |  |
| --- | --- | --- | --- | --- | --- | --- | --- | --- | --- | --- | --- | --- | --- | --- | --- | --- | --- | --- | --- |

Best Unique PSM (Scan Sample\_11\_A1\_1\_3648.d:66734, m/z=705.0139, z=3, RT=19.89, ppm=-5.22):


TRINITY\_DN2772\_c0\_g1\_i3.p1

back to list

  

| Protein Coverage
| Supporting Peptides
| Best Unique PSM
|

Protein Coverage:

Supporting Peptides:

| Peptide | Uniq | -10lgP | Mass | Length | ppm | m/z | z | RT | 1/k0 Start | 1/k0 End | Precursor ID | Area Sample 2 | #Feature | #Feature Sample 2 | Start | End | PTM | AScore | Found By |
| --- | --- | --- | --- | --- | --- | --- | --- | --- | --- | --- | --- | --- | --- | --- | --- | --- | --- | --- | --- |
| K.LIEALC(+57.02)AEHEIK.L | Y | 58.73 | 1424.7333 | 12 | 3.46 | 713.3725 | 2 | 14.93 | 1.0717 | 1.0789 | 41037 | 2.22e3 | 1 | 1 | 73 | 84 | Carbamidomethylation |  | DB Search |
| total 1 peptides |  |  |  |  |  |  |  |  |  |  |  |  |  |  |  |  |  |  |  |
| --- | --- | --- | --- | --- | --- | --- | --- | --- | --- | --- | --- | --- | --- | --- | --- | --- | --- | --- | --- |

Best Unique PSM (Scan Sample\_11\_A1\_1\_3648.d:41037, m/z=712.3854, z=1, RT=14.93, ppm=-2.01):


TRINITY\_DN187823\_c0\_g1\_i1.p1

back to list

  

| Protein Coverage
| Supporting Peptides
| Best Unique PSM
|

Protein Coverage:

Supporting Peptides:

| Peptide | Uniq | -10lgP | Mass | Length | ppm | m/z | z | RT | 1/k0 Start | 1/k0 End | Precursor ID | Area Sample 2 | #Feature | #Feature Sample 2 | Start | End | PTM | AScore | Found By |
| --- | --- | --- | --- | --- | --- | --- | --- | --- | --- | --- | --- | --- | --- | --- | --- | --- | --- | --- | --- |
| R.YLDLILNEKVR.Q | Y | 57.31 | 1374.7871 | 11 | 3.44 | 688.3994 | 2 | 17.46 | 1.0407 | 1.048 | 53939 | 3.63e2 | 1 | 1 | 248 | 258 |  |  | DB Search |
| total 1 peptides |  |  |  |  |  |  |  |  |  |  |  |  |  |  |  |  |  |  |  |
| --- | --- | --- | --- | --- | --- | --- | --- | --- | --- | --- | --- | --- | --- | --- | --- | --- | --- | --- | --- |

Best Unique PSM (Scan Sample\_11\_A1\_1\_3648.d:53939, m/z=688.3994, z=2, RT=17.46, ppm=-2.03):


TRINITY\_DN585\_c0\_g1\_i3.p1

back to list

  

| Protein Coverage
| Supporting Peptides
| Best Unique PSM
|

Protein Coverage:

Supporting Peptides:

| Peptide | Uniq | -10lgP | Mass | Length | ppm | m/z | z | RT | 1/k0 Start | 1/k0 End | Precursor ID | Area Sample 2 | #Feature | #Feature Sample 2 | Start | End | PTM | AScore | Found By |
| --- | --- | --- | --- | --- | --- | --- | --- | --- | --- | --- | --- | --- | --- | --- | --- | --- | --- | --- | --- |
| K.IQDKEGIPPDQQR.L | Y | 55.06 | 1522.7739 | 13 | 2.88 | 762.3923 | 2 | 11.00 | 1.0331 | 1.0403 | 18584 | 2.97e3 | 2 | 2 | 2 | 14 |  |  | DB Search |
| total 1 peptides |  |  |  |  |  |  |  |  |  |  |  |  |  |  |  |  |  |  |  |
| --- | --- | --- | --- | --- | --- | --- | --- | --- | --- | --- | --- | --- | --- | --- | --- | --- | --- | --- | --- |

Best Unique PSM (Scan Sample\_11\_A1\_1\_3648.d:18584, m/z=762.3923, z=2, RT=11.00, ppm=-2.59):


TRINITY\_DN60705\_c0\_g1\_i1.p1

back to list

  

| Protein Coverage
| Supporting Peptides
| Best Unique PSM
|

Protein Coverage:

Supporting Peptides:

| Peptide | Uniq | -10lgP | Mass | Length | ppm | m/z | z | RT | 1/k0 Start | 1/k0 End | Precursor ID | Area Sample 2 | #Feature | #Feature Sample 2 | Start | End | PTM | AScore | Found By |
| --- | --- | --- | --- | --- | --- | --- | --- | --- | --- | --- | --- | --- | --- | --- | --- | --- | --- | --- | --- |
| K.VAVALREEENLHLLER.N | Y | 34.22 | 1890.0323 | 16 | -0.18 | 631.0145 | 3 | 16.17 | 0.8927 | 0.9 | 47345 | 1.03e2 | 1 | 1 | 83 | 98 |  |  | DB Search |
| total 1 peptides |  |  |  |  |  |  |  |  |  |  |  |  |  |  |  |  |  |  |  |
| --- | --- | --- | --- | --- | --- | --- | --- | --- | --- | --- | --- | --- | --- | --- | --- | --- | --- | --- | --- |

Best Unique PSM (Scan Sample\_11\_A1\_1\_3648.d:47345, m/z=631.0145, z=3, RT=16.17, ppm=-5.66):


TRINITY\_DN5263\_c0\_g1\_i1.p1

back to list

  

| Protein Coverage
| Supporting Peptides
| Best Unique PSM
|

Protein Coverage:

Supporting Peptides:

| Peptide | Uniq | -10lgP | Mass | Length | ppm | m/z | z | RT | 1/k0 Start | 1/k0 End | Precursor ID | Area Sample 2 | #Feature | #Feature Sample 2 | Start | End | PTM | AScore | Found By |
| --- | --- | --- | --- | --- | --- | --- | --- | --- | --- | --- | --- | --- | --- | --- | --- | --- | --- | --- | --- |
| R.IRELTSVVQKR.F | Y | 53.19 | 1327.7936 | 11 | 12.38 | 443.6082 | 3 | 12.34 | 0.7522 | 0.7595 | 26874 | 0 | 0 | 0 | 66 | 76 |  |  | DB Search |
| total 1 peptides |  |  |  |  |  |  |  |  |  |  |  |  |  |  |  |  |  |  |  |
| --- | --- | --- | --- | --- | --- | --- | --- | --- | --- | --- | --- | --- | --- | --- | --- | --- | --- | --- | --- |

Best Unique PSM (Scan Sample\_11\_A1\_1\_3648.d:26874, m/z=443.6082, z=3, RT=12.34, ppm=6.9):


TRINITY\_DN2028\_c0\_g1\_i1.p1

back to list

  

| Protein Coverage
| Supporting Peptides
| Best Unique PSM
|

Protein Coverage:

Supporting Peptides:

| Peptide | Uniq | -10lgP | Mass | Length | ppm | m/z | z | RT | 1/k0 Start | 1/k0 End | Precursor ID | Area Sample 2 | #Feature | #Feature Sample 2 | Start | End | PTM | AScore | Found By |
| --- | --- | --- | --- | --- | --- | --- | --- | --- | --- | --- | --- | --- | --- | --- | --- | --- | --- | --- | --- |
| K.LIQYIENKPGLTR.L | Y | 58.44 | 1543.8722 | 13 | 3.24 | 772.9417 | 2 | 14.62 | 1.0761 | 1.0833 | 39327 | 2e3 | 1 | 1 | 81 | 93 |  |  | DB Search |
| R.DFREFLYNSETGKVM(+15.99)GR.S | Y | 39.36 | 2063.9734 | 17 | -3.15 | 688.9925 | 3 | 17.26 | 0.9271 | 0.9344 | 52789 | 3.31e2 | 1 | 1 | 21 | 37 | Oxidation (M) | M15:Oxidation (M):1000 | DB Search |
| total 2 peptides |  |  |  |  |  |  |  |  |  |  |  |  |  |  |  |  |  |  |  |
| --- | --- | --- | --- | --- | --- | --- | --- | --- | --- | --- | --- | --- | --- | --- | --- | --- | --- | --- | --- |

Best Unique PSM (Scan Sample\_11\_A1\_1\_3648.d:39327, m/z=772.9417, z=2, RT=14.62, ppm=-2.23):


TRINITY\_DN6920\_c0\_g1\_i1.p1

back to list

  

| Protein Coverage
| Supporting Peptides
| Best Unique PSM
|

Protein Coverage:

Supporting Peptides:

| Peptide | Uniq | -10lgP | Mass | Length | ppm | m/z | z | RT | 1/k0 Start | 1/k0 End | Precursor ID | Area Sample 2 | #Feature | #Feature Sample 2 | Start | End | PTM | AScore | Found By |
| --- | --- | --- | --- | --- | --- | --- | --- | --- | --- | --- | --- | --- | --- | --- | --- | --- | --- | --- | --- |
| R.VLVHTQQK.L | Y | 57.79 | 951.5501 | 8 | -1.07 | 476.7792 | 2 | 9.55 | 0.8352 | 0.8425 | 11014 | 5.75e2 | 1 | 1 | 45 | 52 |  |  | DB Search |
| total 1 peptides |  |  |  |  |  |  |  |  |  |  |  |  |  |  |  |  |  |  |  |
| --- | --- | --- | --- | --- | --- | --- | --- | --- | --- | --- | --- | --- | --- | --- | --- | --- | --- | --- | --- |

Best Unique PSM (Scan Sample\_11\_A1\_1\_3648.d:11014, m/z=477.7582, z=2, RT=9.56, ppm=-6.55):


TRINITY\_DN1616\_c0\_g1\_i2.p1

back to list

  

| Protein Coverage
| Supporting Peptides
| Best Unique PSM
|

Protein Coverage:

Supporting Peptides:

| Peptide | Uniq | -10lgP | Mass | Length | ppm | m/z | z | RT | 1/k0 Start | 1/k0 End | Precursor ID | Area Sample 2 | #Feature | #Feature Sample 2 | Start | End | PTM | AScore | Found By |
| --- | --- | --- | --- | --- | --- | --- | --- | --- | --- | --- | --- | --- | --- | --- | --- | --- | --- | --- | --- |
| K.YVVMLQYYHDSDTAK.Y | Y | 58.58 | 1831.8451 | 15 | 5.67 | 916.93 | 2 | 16.32 | 1.1813 | 1.1885 | 48049 | 0 | 0 | 0 | 1071 | 1085 |  |  | DB Search |
| total 1 peptides |  |  |  |  |  |  |  |  |  |  |  |  |  |  |  |  |  |  |  |
| --- | --- | --- | --- | --- | --- | --- | --- | --- | --- | --- | --- | --- | --- | --- | --- | --- | --- | --- | --- |

Best Unique PSM (Scan Sample\_11\_A1\_1\_3648.d:48049, m/z=916.93, z=2, RT=16.32, ppm=0.2):


TRINITY\_DN1797\_c0\_g1\_i2.p1

back to list

  

| Protein Coverage
| Supporting Peptides
| Best Unique PSM
|

Protein Coverage:

Supporting Peptides:

| Peptide | Uniq | -10lgP | Mass | Length | ppm | m/z | z | RT | 1/k0 Start | 1/k0 End | Precursor ID | Area Sample 2 | #Feature | #Feature Sample 2 | Start | End | PTM | AScore | Found By |
| --- | --- | --- | --- | --- | --- | --- | --- | --- | --- | --- | --- | --- | --- | --- | --- | --- | --- | --- | --- |
| K.AVVIITDGKDDSTSR.L | Y | 31.58 | 1575.8104 | 15 | -9 | 788.9011 | 2 | 13.19 | 1.0937 | 1.1009 | 31695 | 0 | 0 | 0 | 872 | 886 |  |  | DB Search |
| total 1 peptides |  |  |  |  |  |  |  |  |  |  |  |  |  |  |  |  |  |  |  |
| --- | --- | --- | --- | --- | --- | --- | --- | --- | --- | --- | --- | --- | --- | --- | --- | --- | --- | --- | --- |

Best Unique PSM (Scan Sample\_11\_A1\_1\_3648.d:31695, m/z=788.9011, z=2, RT=13.19, ppm=-14.47):


TRINITY\_DN15847\_c0\_g1\_i2.p1

back to list

  

| Protein Coverage
| Supporting Peptides
| Best Unique PSM
|

Protein Coverage:

Supporting Peptides:

| Peptide | Uniq | -10lgP | Mass | Length | ppm | m/z | z | RT | 1/k0 Start | 1/k0 End | Precursor ID | Area Sample 2 | #Feature | #Feature Sample 2 | Start | End | PTM | AScore | Found By |
| --- | --- | --- | --- | --- | --- | --- | --- | --- | --- | --- | --- | --- | --- | --- | --- | --- | --- | --- | --- |
| R.DVQLGDLVTVGQC(+57.02)RPLSK.T | Y | 55.96 | 1984.0411 | 18 | 3.74 | 993.0261 | 2 | 17.77 | 1.2766 | 1.2837 | 55652 | 0 | 0 | 0 | 126 | 143 | Carbamidomethylation |  | DB Search |
| total 1 peptides |  |  |  |  |  |  |  |  |  |  |  |  |  |  |  |  |  |  |  |
| --- | --- | --- | --- | --- | --- | --- | --- | --- | --- | --- | --- | --- | --- | --- | --- | --- | --- | --- | --- |

Best Unique PSM (Scan Sample\_11\_A1\_1\_3648.d:55652, m/z=993.0261, z=2, RT=17.77, ppm=-1.73):


TRINITY\_DN471\_c0\_g1\_i3.p1

back to list

  

| Protein Coverage
| Supporting Peptides
| Best Unique PSM
|

Protein Coverage:

Supporting Peptides:

| Peptide | Uniq | -10lgP | Mass | Length | ppm | m/z | z | RT | 1/k0 Start | 1/k0 End | Precursor ID | Area Sample 2 | #Feature | #Feature Sample 2 | Start | End | PTM | AScore | Found By |
| --- | --- | --- | --- | --- | --- | --- | --- | --- | --- | --- | --- | --- | --- | --- | --- | --- | --- | --- | --- |
| R.TIQFVDWC(+57.02)PTGFK.C | Y | 54.82 | 1597.7599 | 13 | -6.53 | 799.8776 | 2 | 19.88 | 1.0351 | 1.0423 | 66701 | 3.31e2 | 1 | 1 | 210 | 222 | Carbamidomethylation |  | DB Search |
| R.RNLDIERPTYTNLNR.L | Y | 46.58 | 1873.9758 | 15 | -2.43 | 625.6609 | 3 | 13.94 | 0.8858 | 0.8931 | 35770 | 1.17e3 | 1 | 1 | 85 | 99 |  |  | DB Search |
| total 2 peptides |  |  |  |  |  |  |  |  |  |  |  |  |  |  |  |  |  |  |  |
| --- | --- | --- | --- | --- | --- | --- | --- | --- | --- | --- | --- | --- | --- | --- | --- | --- | --- | --- | --- |

Best Unique PSM (Scan Sample\_11\_A1\_1\_3648.d:66701, m/z=799.3823, z=2, RT=19.90, ppm=-12):


TRINITY\_DN276\_c0\_g1\_i1.p1

back to list

  

| Protein Coverage
| Supporting Peptides
| Best Unique PSM
|

Protein Coverage:

Supporting Peptides:

| Peptide | Uniq | -10lgP | Mass | Length | ppm | m/z | z | RT | 1/k0 Start | 1/k0 End | Precursor ID | Area Sample 2 | #Feature | #Feature Sample 2 | Start | End | PTM | AScore | Found By |
| --- | --- | --- | --- | --- | --- | --- | --- | --- | --- | --- | --- | --- | --- | --- | --- | --- | --- | --- | --- |
| R.VTFEEFIPLYHTQSK.K | Y | 53.33 | 1837.925 | 15 | -7.94 | 919.9575 | 2 | 19.63 | 1.1482 | 1.1554 | 65543 | 0 | 0 | 0 | 64 | 78 |  |  | DB Search |
| total 1 peptides |  |  |  |  |  |  |  |  |  |  |  |  |  |  |  |  |  |  |  |
| --- | --- | --- | --- | --- | --- | --- | --- | --- | --- | --- | --- | --- | --- | --- | --- | --- | --- | --- | --- |

Best Unique PSM (Scan Sample\_11\_A1\_1\_3648.d:65543, m/z=919.9575, z=2, RT=19.63, ppm=-13.41):


TRINITY\_DN58867\_c0\_g1\_i2.p1

back to list

  

| Protein Coverage
| Supporting Peptides
| Best Unique PSM
|

Protein Coverage:

Supporting Peptides:

| Peptide | Uniq | -10lgP | Mass | Length | ppm | m/z | z | RT | 1/k0 Start | 1/k0 End | Precursor ID | Area Sample 2 | #Feature | #Feature Sample 2 | Start | End | PTM | AScore | Found By |
| --- | --- | --- | --- | --- | --- | --- | --- | --- | --- | --- | --- | --- | --- | --- | --- | --- | --- | --- | --- |
| K.TILQFEADRR.A | Y | 53.68 | 1247.6622 | 10 | 2.15 | 624.8363 | 2 | 14.91 | 0.9505 | 0.9578 | 40952 | 9.36e2 | 1 | 1 | 211 | 220 |  |  | DB Search |
| total 1 peptides |  |  |  |  |  |  |  |  |  |  |  |  |  |  |  |  |  |  |  |
| --- | --- | --- | --- | --- | --- | --- | --- | --- | --- | --- | --- | --- | --- | --- | --- | --- | --- | --- | --- |

Best Unique PSM (Scan Sample\_11\_A1\_1\_3648.d:40952, m/z=624.8363, z=2, RT=14.91, ppm=-3.33):


TRINITY\_DN8101\_c0\_g1\_i4.p1

back to list

  

| Protein Coverage
| Supporting Peptides
| Best Unique PSM
|

Protein Coverage:

Supporting Peptides:

| Peptide | Uniq | -10lgP | Mass | Length | ppm | m/z | z | RT | 1/k0 Start | 1/k0 End | Precursor ID | Area Sample 2 | #Feature | #Feature Sample 2 | Start | End | PTM | AScore | Found By |
| --- | --- | --- | --- | --- | --- | --- | --- | --- | --- | --- | --- | --- | --- | --- | --- | --- | --- | --- | --- |
| R.SIGFIGHYLDQLR.L | Y | 54.32 | 1517.799 | 13 | 1.06 | 759.9034 | 2 | 19.56 | 1.0849 | 1.0921 | 65092 | 4.69e2 | 1 | 1 | 475 | 487 |  |  | DB Search |
| total 1 peptides |  |  |  |  |  |  |  |  |  |  |  |  |  |  |  |  |  |  |  |
| --- | --- | --- | --- | --- | --- | --- | --- | --- | --- | --- | --- | --- | --- | --- | --- | --- | --- | --- | --- |

Best Unique PSM (Scan Sample\_11\_A1\_1\_3648.d:65092, m/z=759.9034, z=2, RT=19.56, ppm=-4.41):


TRINITY\_DN1907\_c0\_g1\_i1.p1

back to list

  

| Protein Coverage
| Supporting Peptides
| Best Unique PSM
|

Protein Coverage:

Supporting Peptides:

| Peptide | Uniq | -10lgP | Mass | Length | ppm | m/z | z | RT | 1/k0 Start | 1/k0 End | Precursor ID | Area Sample 2 | #Feature | #Feature Sample 2 | Start | End | PTM | AScore | Found By |
| --- | --- | --- | --- | --- | --- | --- | --- | --- | --- | --- | --- | --- | --- | --- | --- | --- | --- | --- | --- |
| K.VIIDYQTHHR.L | Y | 58.05 | 1280.6625 | 10 | -2.89 | 641.3332 | 2 | 12.03 | 0.9533 | 0.9606 | 25056 | 0 | 0 | 0 | 151 | 160 |  |  | DB Search |
| total 1 peptides |  |  |  |  |  |  |  |  |  |  |  |  |  |  |  |  |  |  |  |
| --- | --- | --- | --- | --- | --- | --- | --- | --- | --- | --- | --- | --- | --- | --- | --- | --- | --- | --- | --- |

Best Unique PSM (Scan Sample\_11\_A1\_1\_3648.d:25056, m/z=641.3332, z=2, RT=12.03, ppm=-8.37):


TRINITY\_DN126137\_c0\_g1\_i1.p1

back to list

  

| Protein Coverage
| Supporting Peptides
| Best Unique PSM
|

Protein Coverage:

Supporting Peptides:

| Peptide | Uniq | -10lgP | Mass | Length | ppm | m/z | z | RT | 1/k0 Start | 1/k0 End | Precursor ID | Area Sample 2 | #Feature | #Feature Sample 2 | Start | End | PTM | AScore | Found By |
| --- | --- | --- | --- | --- | --- | --- | --- | --- | --- | --- | --- | --- | --- | --- | --- | --- | --- | --- | --- |
| K.DFVKM(+15.99)LLLLM(+15.99)LSK.I | Y | 57.98 | 1581.8874 | 13 | 1.83 | 528.3011 | 3 | 19.45 | 0.7742 | 0.7815 | 64632 | 0 | 0 | 0 | 190 | 202 | Oxidation (M), Oxidation (M) | M5:Oxidation (M):1000 M10:Oxidation (M):1000 | DB Search |
| total 1 peptides |  |  |  |  |  |  |  |  |  |  |  |  |  |  |  |  |  |  |  |
| --- | --- | --- | --- | --- | --- | --- | --- | --- | --- | --- | --- | --- | --- | --- | --- | --- | --- | --- | --- |

Best Unique PSM (Scan Sample\_11\_A1\_1\_3648.d:64632, m/z=528.3011, z=3, RT=19.45, ppm=-3.64):


TRINITY\_DN36\_c0\_g1\_i2.p1

back to list

  

| Protein Coverage
| Supporting Peptides
| Best Unique PSM
|

Protein Coverage:

Supporting Peptides:

| Peptide | Uniq | -10lgP | Mass | Length | ppm | m/z | z | RT | 1/k0 Start | 1/k0 End | Precursor ID | Area Sample 2 | #Feature | #Feature Sample 2 | Start | End | PTM | AScore | Found By |
| --- | --- | --- | --- | --- | --- | --- | --- | --- | --- | --- | --- | --- | --- | --- | --- | --- | --- | --- | --- |
| R.LGGIEVTQGNC(+57.02)ARK.Y | Y | 58.27 | 1501.7671 | 14 | 1.48 | 751.8878 | 2 | 12.59 | 1.0616 | 1.0688 | 28229 | 7.18e2 | 1 | 1 | 58 | 71 | Carbamidomethylation |  | DB Search |
| total 1 peptides |  |  |  |  |  |  |  |  |  |  |  |  |  |  |  |  |  |  |  |
| --- | --- | --- | --- | --- | --- | --- | --- | --- | --- | --- | --- | --- | --- | --- | --- | --- | --- | --- | --- |

Best Unique PSM (Scan Sample\_11\_A1\_1\_3648.d:28229, m/z=751.8878, z=2, RT=12.59, ppm=-4):


TRINITY\_DN535\_c0\_g1\_i1.p1

back to list

  

| Protein Coverage
| Supporting Peptides
| Best Unique PSM
|

Protein Coverage:

Supporting Peptides:

| Peptide | Uniq | -10lgP | Mass | Length | ppm | m/z | z | RT | 1/k0 Start | 1/k0 End | Precursor ID | Area Sample 2 | #Feature | #Feature Sample 2 | Start | End | PTM | AScore | Found By |
| --- | --- | --- | --- | --- | --- | --- | --- | --- | --- | --- | --- | --- | --- | --- | --- | --- | --- | --- | --- |
| R.GLFIIDDKGILR.Q | Y | 58.85 | 1358.7921 | 12 | -0.67 | 680.3992 | 2 | 18.97 | 1.0106 | 1.0178 | 62014 | 1.63e3 | 1 | 1 | 94 | 105 |  |  | DB Search |
| R.ALGC(+57.02)DVLAC(+57.02)SVDSHFSHLAWTQQSR.K | Y | 58.16 | 2844.3071 | 25 | -3.91 | 712.0774 | 4 | 18.53 | 0.9238 | 0.9311 | 59620 | 3.52e2 | 1 | 1 | 33 | 57 | Carbamidomethylation, Carbamidomethylation |  | DB Search |
| total 2 peptides |  |  |  |  |  |  |  |  |  |  |  |  |  |  |  |  |  |  |  |
| --- | --- | --- | --- | --- | --- | --- | --- | --- | --- | --- | --- | --- | --- | --- | --- | --- | --- | --- | --- |

Best Unique PSM (Scan Sample\_11\_A1\_1\_3648.d:62014, m/z=680.3992, z=2, RT=18.97, ppm=-6.15):


TRINITY\_DN2217\_c0\_g1\_i2.p1

back to list

  

| Protein Coverage
| Supporting Peptides
| Best Unique PSM
|

Protein Coverage:

Supporting Peptides:

| Peptide | Uniq | -10lgP | Mass | Length | ppm | m/z | z | RT | 1/k0 Start | 1/k0 End | Precursor ID | Area Sample 2 | #Feature | #Feature Sample 2 | Start | End | PTM | AScore | Found By |
| --- | --- | --- | --- | --- | --- | --- | --- | --- | --- | --- | --- | --- | --- | --- | --- | --- | --- | --- | --- |
| K.ATGC(+57.02)EVIWNQHNR.G | Y | 58.87 | 1583.7263 | 13 | 1.47 | 792.8672 | 2 | 13.74 | 1.0773 | 1.0845 | 34668 | 9.8e2 | 1 | 1 | 119 | 131 | Carbamidomethylation |  | DB Search |
| total 1 peptides |  |  |  |  |  |  |  |  |  |  |  |  |  |  |  |  |  |  |  |
| --- | --- | --- | --- | --- | --- | --- | --- | --- | --- | --- | --- | --- | --- | --- | --- | --- | --- | --- | --- |

Best Unique PSM (Scan Sample\_11\_A1\_1\_3648.d:34668, m/z=792.8672, z=2, RT=13.74, ppm=-4.01):


TRINITY\_DN200\_c0\_g1\_i5.p1

back to list

  

| Protein Coverage
| Supporting Peptides
| Best Unique PSM
|

Protein Coverage:

Supporting Peptides:

| Peptide | Uniq | -10lgP | Mass | Length | ppm | m/z | z | RT | 1/k0 Start | 1/k0 End | Precursor ID | Area Sample 2 | #Feature | #Feature Sample 2 | Start | End | PTM | AScore | Found By |
| --- | --- | --- | --- | --- | --- | --- | --- | --- | --- | --- | --- | --- | --- | --- | --- | --- | --- | --- | --- |
| K.TAIAIDTIINQKR.F | Y | 58.72 | 1455.8409 | 13 | 0.37 | 728.924 | 2 | 16.37 | 1.0604 | 1.0676 | 48298 | 0 | 0 | 0 | 217 | 229 |  |  | DB Search |
| total 1 peptides |  |  |  |  |  |  |  |  |  |  |  |  |  |  |  |  |  |  |  |
| --- | --- | --- | --- | --- | --- | --- | --- | --- | --- | --- | --- | --- | --- | --- | --- | --- | --- | --- | --- |

Best Unique PSM (Scan Sample\_11\_A1\_1\_3648.d:48298, m/z=728.924, z=2, RT=16.37, ppm=-5.1):


TRINITY\_DN98968\_c0\_g1\_i1.p1

back to list

  

| Protein Coverage
| Supporting Peptides
| Best Unique PSM
|

Protein Coverage:

Supporting Peptides:

| Peptide | Uniq | -10lgP | Mass | Length | ppm | m/z | z | RT | 1/k0 Start | 1/k0 End | Precursor ID | Area Sample 2 | #Feature | #Feature Sample 2 | Start | End | PTM | AScore | Found By |
| --- | --- | --- | --- | --- | --- | --- | --- | --- | --- | --- | --- | --- | --- | --- | --- | --- | --- | --- | --- |
| R.IESTAC(+57.02)TR.R | Y | 34.92 | 936.4335 | 8 | 2.84 | 469.2228 | 2 | 9.65 | 0.8136 | 0.8209 | 11495 | 1.18e3 | 1 | 1 | 204 | 211 | Carbamidomethylation |  | DB Search |
| total 1 peptides |  |  |  |  |  |  |  |  |  |  |  |  |  |  |  |  |  |  |  |
| --- | --- | --- | --- | --- | --- | --- | --- | --- | --- | --- | --- | --- | --- | --- | --- | --- | --- | --- | --- |

Best Unique PSM (Scan Sample\_11\_A1\_1\_3648.d:11495, m/z=469.2228, z=2, RT=9.65, ppm=-2.64):


TRINITY\_DN956\_c0\_g1\_i1.p1

back to list

  

| Protein Coverage
| Supporting Peptides
| Best Unique PSM
|

Protein Coverage:

Supporting Peptides:

| Peptide | Uniq | -10lgP | Mass | Length | ppm | m/z | z | RT | 1/k0 Start | 1/k0 End | Precursor ID | Area Sample 2 | #Feature | #Feature Sample 2 | Start | End | PTM | AScore | Found By |
| --- | --- | --- | --- | --- | --- | --- | --- | --- | --- | --- | --- | --- | --- | --- | --- | --- | --- | --- | --- |
| K.AGYGFVIFDDRR.D | Y | 41.74 | 1414.6993 | 12 | 1.94 | 708.3544 | 2 | 18.09 | 1.0379 | 1.0452 | 57312 | 3.6e2 | 1 | 1 | 40 | 51 |  |  | DB Search |
| total 1 peptides |  |  |  |  |  |  |  |  |  |  |  |  |  |  |  |  |  |  |  |
| --- | --- | --- | --- | --- | --- | --- | --- | --- | --- | --- | --- | --- | --- | --- | --- | --- | --- | --- | --- |

Best Unique PSM (Scan Sample\_11\_A1\_1\_3648.d:57312, m/z=708.3544, z=2, RT=18.09, ppm=-3.53):


TRINITY\_DN1797\_c0\_g1\_i3.p1

back to list

  

| Protein Coverage
| Supporting Peptides
| Best Unique PSM
|

Protein Coverage:

Supporting Peptides:

| Peptide | Uniq | -10lgP | Mass | Length | ppm | m/z | z | RT | 1/k0 Start | 1/k0 End | Precursor ID | Area Sample 2 | #Feature | #Feature Sample 2 | Start | End | PTM | AScore | Found By |
| --- | --- | --- | --- | --- | --- | --- | --- | --- | --- | --- | --- | --- | --- | --- | --- | --- | --- | --- | --- |
| K.AVVIITDGKDDSTSR.L | Y | 31.58 | 1575.8104 | 15 | -9 | 788.9011 | 2 | 13.19 | 1.0937 | 1.1009 | 31695 | 0 | 0 | 0 | 872 | 886 |  |  | DB Search |
| total 1 peptides |  |  |  |  |  |  |  |  |  |  |  |  |  |  |  |  |  |  |  |
| --- | --- | --- | --- | --- | --- | --- | --- | --- | --- | --- | --- | --- | --- | --- | --- | --- | --- | --- | --- |

Best Unique PSM (Scan Sample\_11\_A1\_1\_3648.d:31695, m/z=788.9011, z=2, RT=13.19, ppm=-14.47):


TRINITY\_DN265\_c0\_g1\_i5.p1

back to list

  

| Protein Coverage
| Supporting Peptides
| Best Unique PSM
|

Protein Coverage:

Supporting Peptides:

| Peptide | Uniq | -10lgP | Mass | Length | ppm | m/z | z | RT | 1/k0 Start | 1/k0 End | Precursor ID | Area Sample 2 | #Feature | #Feature Sample 2 | Start | End | PTM | AScore | Found By |
| --- | --- | --- | --- | --- | --- | --- | --- | --- | --- | --- | --- | --- | --- | --- | --- | --- | --- | --- | --- |
| R.LTHDFHTNKR.I | Y | 58.75 | 1267.6421 | 10 | 2.44 | 423.5534 | 3 | 9.64 | 0.7441 | 0.7514 | 11479 | 1.15e3 | 1 | 1 | 24 | 33 |  |  | DB Search |
| total 1 peptides |  |  |  |  |  |  |  |  |  |  |  |  |  |  |  |  |  |  |  |
| --- | --- | --- | --- | --- | --- | --- | --- | --- | --- | --- | --- | --- | --- | --- | --- | --- | --- | --- | --- |

Best Unique PSM (Scan Sample\_11\_A1\_1\_3648.d:11479, m/z=423.5534, z=3, RT=9.64, ppm=-3.04):


TRINITY\_DN1631\_c0\_g1\_i1.p1

back to list

  

| Protein Coverage
| Supporting Peptides
| Best Unique PSM
|

Protein Coverage:

Supporting Peptides:

| Peptide | Uniq | -10lgP | Mass | Length | ppm | m/z | z | RT | 1/k0 Start | 1/k0 End | Precursor ID | Area Sample 2 | #Feature | #Feature Sample 2 | Start | End | PTM | AScore | Found By |
| --- | --- | --- | --- | --- | --- | --- | --- | --- | --- | --- | --- | --- | --- | --- | --- | --- | --- | --- | --- |
| M.A(+42.01)EAPKPKPAPK.S | Y | 58.73 | 1174.671 | 11 | 4.56 | 392.5639 | 3 | 9.99 | 0.7665 | 0.7738 | 13134 | 4.33e3 | 2 | 2 | 2 | 12 | Acetylation (Protein N-term) | A1:Acetylation (Protein N-term):1000 | DB Search |
| total 1 peptides |  |  |  |  |  |  |  |  |  |  |  |  |  |  |  |  |  |  |  |
| --- | --- | --- | --- | --- | --- | --- | --- | --- | --- | --- | --- | --- | --- | --- | --- | --- | --- | --- | --- |

Best Unique PSM (Scan Sample\_11\_A1\_1\_3648.d:13134, m/z=392.5639, z=3, RT=9.99, ppm=-0.92):


TRINITY\_DN902\_c1\_g2\_i4.p1

back to list

  

| Protein Coverage
| Supporting Peptides
| Best Unique PSM
|

Protein Coverage:

Supporting Peptides:

| Peptide | Uniq | -10lgP | Mass | Length | ppm | m/z | z | RT | 1/k0 Start | 1/k0 End | Precursor ID | Area Sample 2 | #Feature | #Feature Sample 2 | Start | End | PTM | AScore | Found By |
| --- | --- | --- | --- | --- | --- | --- | --- | --- | --- | --- | --- | --- | --- | --- | --- | --- | --- | --- | --- |
| K.GLTHC(+57.02)NTLSYK.L | Y | 58.83 | 1292.6183 | 11 | 4.02 | 647.3155 | 2 | 12.36 | 0.9695 | 0.9767 | 26924 | 0 | 0 | 0 | 62 | 72 | Carbamidomethylation |  | DB Search |
| total 1 peptides |  |  |  |  |  |  |  |  |  |  |  |  |  |  |  |  |  |  |  |
| --- | --- | --- | --- | --- | --- | --- | --- | --- | --- | --- | --- | --- | --- | --- | --- | --- | --- | --- | --- |

Best Unique PSM (Scan Sample\_11\_A1\_1\_3648.d:26924, m/z=647.3155, z=2, RT=12.36, ppm=-1.46):


TRINITY\_DN3448\_c0\_g1\_i5.p2

back to list

  

| Protein Coverage
| Supporting Peptides
| Best Unique PSM
|

Protein Coverage:

Supporting Peptides:

| Peptide | Uniq | -10lgP | Mass | Length | ppm | m/z | z | RT | 1/k0 Start | 1/k0 End | Precursor ID | Area Sample 2 | #Feature | #Feature Sample 2 | Start | End | PTM | AScore | Found By |
| --- | --- | --- | --- | --- | --- | --- | --- | --- | --- | --- | --- | --- | --- | --- | --- | --- | --- | --- | --- |
| K.AGM(+15.99)THIVR.D | Y | 38.19 | 899.4647 | 8 | 6.96 | 450.7403 | 2 | 9.74 | 0.8254 | 0.8327 | 11994 | 0 | 0 | 0 | 27 | 34 | Oxidation (M) | M3:Oxidation (M):1000 | DB Search |
| total 1 peptides |  |  |  |  |  |  |  |  |  |  |  |  |  |  |  |  |  |  |  |
| --- | --- | --- | --- | --- | --- | --- | --- | --- | --- | --- | --- | --- | --- | --- | --- | --- | --- | --- | --- |

Best Unique PSM (Scan Sample\_11\_A1\_1\_3648.d:11994, m/z=450.7403, z=2, RT=9.74, ppm=1.48):


TRINITY\_DN773\_c0\_g1\_i1.p1

back to list

  

| Protein Coverage
| Supporting Peptides
| Best Unique PSM
|

Protein Coverage:

Supporting Peptides:

| Peptide | Uniq | -10lgP | Mass | Length | ppm | m/z | z | RT | 1/k0 Start | 1/k0 End | Precursor ID | Area Sample 2 | #Feature | #Feature Sample 2 | Start | End | PTM | AScore | Found By |
| --- | --- | --- | --- | --- | --- | --- | --- | --- | --- | --- | --- | --- | --- | --- | --- | --- | --- | --- | --- |
| R.GVSEETTTGVHNLYK.M | Y | 55.11 | 1633.7947 | 15 | 0.86 | 817.9009 | 2 | 13.48 | 1.0684 | 1.0757 | 33215 | 5.53e2 | 1 | 1 | 160 | 174 |  |  | DB Search |
| K.RLDEEVAR.L | Y | 48.47 | 986.5145 | 8 | -5.51 | 494.2591 | 2 | 11.06 | 0.8481 | 0.8554 | 19512 | 0 | 0 | 0 | 396 | 403 |  |  | DB Search |
| total 2 peptides |  |  |  |  |  |  |  |  |  |  |  |  |  |  |  |  |  |  |  |
| --- | --- | --- | --- | --- | --- | --- | --- | --- | --- | --- | --- | --- | --- | --- | --- | --- | --- | --- | --- |

Best Unique PSM (Scan Sample\_11\_A1\_1\_3648.d:33215, m/z=817.9009, z=2, RT=13.48, ppm=-4.61):


TRINITY\_DN164351\_c0\_g1\_i1.p1

back to list

  

| Protein Coverage
| Supporting Peptides
| Best Unique PSM
|

Protein Coverage:

Supporting Peptides:

| Peptide | Uniq | -10lgP | Mass | Length | ppm | m/z | z | RT | 1/k0 Start | 1/k0 End | Precursor ID | Area Sample 2 | #Feature | #Feature Sample 2 | Start | End | PTM | AScore | Found By |
| --- | --- | --- | --- | --- | --- | --- | --- | --- | --- | --- | --- | --- | --- | --- | --- | --- | --- | --- | --- |
| R.LTNSLM(+15.99)M(+15.99)HGR.N | Y | 58.85 | 1190.5536 | 10 | 1.85 | 596.2819 | 2 | 10.72 | 0.9105 | 0.9178 | 17299 | 2.75e3 | 1 | 1 | 78 | 87 | Oxidation (M), Oxidation (M) | M6:Oxidation (M):1000 M7:Oxidation (M):1000 | DB Search |
| total 1 peptides |  |  |  |  |  |  |  |  |  |  |  |  |  |  |  |  |  |  |  |
| --- | --- | --- | --- | --- | --- | --- | --- | --- | --- | --- | --- | --- | --- | --- | --- | --- | --- | --- | --- |

Best Unique PSM (Scan Sample\_11\_A1\_1\_3648.d:17299, m/z=596.2819, z=2, RT=10.72, ppm=-3.63):


TRINITY\_DN97809\_c0\_g1\_i1.p1

back to list

  

| Protein Coverage
| Supporting Peptides
| Best Unique PSM
|

Protein Coverage:

Supporting Peptides:

| Peptide | Uniq | -10lgP | Mass | Length | ppm | m/z | z | RT | 1/k0 Start | 1/k0 End | Precursor ID | Area Sample 2 | #Feature | #Feature Sample 2 | Start | End | PTM | AScore | Found By |
| --- | --- | --- | --- | --- | --- | --- | --- | --- | --- | --- | --- | --- | --- | --- | --- | --- | --- | --- | --- |
| LGPDQVDVAGAGR.D | Y | 20.68 | 1253.6364 | 13 | 1.35 | 627.8229 | 2 | 13.51 | 0.9327 | 0.94 | 33440 | 0 | 0 | 0 | 1 | 13 |  |  | DB Search |
| total 1 peptides |  |  |  |  |  |  |  |  |  |  |  |  |  |  |  |  |  |  |  |
| --- | --- | --- | --- | --- | --- | --- | --- | --- | --- | --- | --- | --- | --- | --- | --- | --- | --- | --- | --- |

Best Unique PSM (Scan Sample\_11\_A1\_1\_3648.d:33440, m/z=627.8229, z=2, RT=13.51, ppm=-4.12):


TRINITY\_DN571\_c0\_g1\_i2.p1

back to list

  

| Protein Coverage
| Supporting Peptides
| Best Unique PSM
|

Protein Coverage:

Supporting Peptides:

| Peptide | Uniq | -10lgP | Mass | Length | ppm | m/z | z | RT | 1/k0 Start | 1/k0 End | Precursor ID | Area Sample 2 | #Feature | #Feature Sample 2 | Start | End | PTM | AScore | Found By |
| --- | --- | --- | --- | --- | --- | --- | --- | --- | --- | --- | --- | --- | --- | --- | --- | --- | --- | --- | --- |
| R.VVGKDQGQSLAK.Q | Y | 55.18 | 1228.6775 | 12 | -1.12 | 615.342 | 2 | 10.28 | 0.9255 | 0.9327 | 14860 | 0 | 0 | 0 | 125 | 136 |  |  | DB Search |
| total 1 peptides |  |  |  |  |  |  |  |  |  |  |  |  |  |  |  |  |  |  |  |
| --- | --- | --- | --- | --- | --- | --- | --- | --- | --- | --- | --- | --- | --- | --- | --- | --- | --- | --- | --- |

Best Unique PSM (Scan Sample\_11\_A1\_1\_3648.d:14860, m/z=615.342, z=2, RT=10.28, ppm=-6.6):


TRINITY\_DN1339\_c0\_g2\_i2.p1

back to list

  

| Protein Coverage
| Supporting Peptides
| Best Unique PSM
|

Protein Coverage:

Supporting Peptides:

| Peptide | Uniq | -10lgP | Mass | Length | ppm | m/z | z | RT | 1/k0 Start | 1/k0 End | Precursor ID | Area Sample 2 | #Feature | #Feature Sample 2 | Start | End | PTM | AScore | Found By |
| --- | --- | --- | --- | --- | --- | --- | --- | --- | --- | --- | --- | --- | --- | --- | --- | --- | --- | --- | --- |
| K.LLTSSDQAELTKK.L | Y | 58.87 | 1432.7773 | 13 | 4.21 | 717.395 | 2 | 12.69 | 1.0496 | 1.0568 | 28711 | 7.94e2 | 1 | 1 | 155 | 167 |  |  | DB Search |
| total 1 peptides |  |  |  |  |  |  |  |  |  |  |  |  |  |  |  |  |  |  |  |
| --- | --- | --- | --- | --- | --- | --- | --- | --- | --- | --- | --- | --- | --- | --- | --- | --- | --- | --- | --- |

Best Unique PSM (Scan Sample\_11\_A1\_1\_3648.d:28711, m/z=717.395, z=2, RT=12.69, ppm=-1.26):


TRINITY\_DN2772\_c0\_g1\_i2.p1

back to list

  

| Protein Coverage
| Supporting Peptides
| Best Unique PSM
|

Protein Coverage:

Supporting Peptides:

| Peptide | Uniq | -10lgP | Mass | Length | ppm | m/z | z | RT | 1/k0 Start | 1/k0 End | Precursor ID | Area Sample 2 | #Feature | #Feature Sample 2 | Start | End | PTM | AScore | Found By |
| --- | --- | --- | --- | --- | --- | --- | --- | --- | --- | --- | --- | --- | --- | --- | --- | --- | --- | --- | --- |
| K.LIEALC(+57.02)AEHEIK.L | Y | 58.73 | 1424.7333 | 12 | 3.46 | 713.3725 | 2 | 14.93 | 1.0717 | 1.0789 | 41037 | 2.22e3 | 1 | 1 | 73 | 84 | Carbamidomethylation |  | DB Search |
| total 1 peptides |  |  |  |  |  |  |  |  |  |  |  |  |  |  |  |  |  |  |  |
| --- | --- | --- | --- | --- | --- | --- | --- | --- | --- | --- | --- | --- | --- | --- | --- | --- | --- | --- | --- |

Best Unique PSM (Scan Sample\_11\_A1\_1\_3648.d:41037, m/z=712.3854, z=1, RT=14.93, ppm=-2.01):


TRINITY\_DN1130\_c0\_g1\_i1.p1

back to list

  

| Protein Coverage
| Supporting Peptides
| Best Unique PSM
|

Protein Coverage:

Supporting Peptides:

| Peptide | Uniq | -10lgP | Mass | Length | ppm | m/z | z | RT | 1/k0 Start | 1/k0 End | Precursor ID | Area Sample 2 | #Feature | #Feature Sample 2 | Start | End | PTM | AScore | Found By |
| --- | --- | --- | --- | --- | --- | --- | --- | --- | --- | --- | --- | --- | --- | --- | --- | --- | --- | --- | --- |
| K.VQSGNYRPVLALNGR.S | Y | 29.19 | 1642.8903 | 15 | 5.65 | 822.4526 | 2 | 14.70 | 1.0837 | 1.0909 | 39738 | 6.08e3 | 1 | 1 | 269 | 283 |  |  | DB Search |
| total 1 peptides |  |  |  |  |  |  |  |  |  |  |  |  |  |  |  |  |  |  |  |
| --- | --- | --- | --- | --- | --- | --- | --- | --- | --- | --- | --- | --- | --- | --- | --- | --- | --- | --- | --- |

Best Unique PSM (Scan Sample\_11\_A1\_1\_3648.d:39738, m/z=821.3915, z=2, RT=14.72, ppm=0.18):


TRINITY\_DN34191\_c0\_g1\_i1.p1

back to list

  

| Protein Coverage
| Supporting Peptides
| Best Unique PSM
|

Protein Coverage:

Supporting Peptides:

| Peptide | Uniq | -10lgP | Mass | Length | ppm | m/z | z | RT | 1/k0 Start | 1/k0 End | Precursor ID | Area Sample 2 | #Feature | #Feature Sample 2 | Start | End | PTM | AScore | Found By |
| --- | --- | --- | --- | --- | --- | --- | --- | --- | --- | --- | --- | --- | --- | --- | --- | --- | --- | --- | --- |
| R.ARPHTM(+15.99)TSLTGSR.D | Y | 50.91 | 1429.7096 | 13 | 5.95 | 477.5774 | 3 | 9.99 | 0.8506 | 0.8579 | 13238 | 0 | 0 | 0 | 100 | 112 | Oxidation (M) | M6:Oxidation (M):1000 | DB Search |
| total 1 peptides |  |  |  |  |  |  |  |  |  |  |  |  |  |  |  |  |  |  |  |
| --- | --- | --- | --- | --- | --- | --- | --- | --- | --- | --- | --- | --- | --- | --- | --- | --- | --- | --- | --- |

Best Unique PSM (Scan Sample\_11\_A1\_1\_3648.d:13238, m/z=477.5774, z=3, RT=9.99, ppm=0.47):


TRINITY\_DN413\_c0\_g1\_i1.p1

back to list

  

| Protein Coverage
| Supporting Peptides
| Best Unique PSM
|

Protein Coverage:

Supporting Peptides:

| Peptide | Uniq | -10lgP | Mass | Length | ppm | m/z | z | RT | 1/k0 Start | 1/k0 End | Precursor ID | Area Sample 2 | #Feature | #Feature Sample 2 | Start | End | PTM | AScore | Found By |
| --- | --- | --- | --- | --- | --- | --- | --- | --- | --- | --- | --- | --- | --- | --- | --- | --- | --- | --- | --- |
| R.ADILC(+57.02)HDTKYGK.K | Y | 47.79 | 1419.6816 | 12 | 13.12 | 710.8535 | 2 | 12.07 | 1.0134 | 1.0206 | 25280 | 0 | 0 | 0 | 329 | 340 | Carbamidomethylation |  | DB Search |
| total 1 peptides |  |  |  |  |  |  |  |  |  |  |  |  |  |  |  |  |  |  |  |
| --- | --- | --- | --- | --- | --- | --- | --- | --- | --- | --- | --- | --- | --- | --- | --- | --- | --- | --- | --- |

Best Unique PSM (Scan Sample\_11\_A1\_1\_3648.d:25280, m/z=710.8535, z=2, RT=12.07, ppm=7.65):


TRINITY\_DN3448\_c0\_g1\_i7.p2

back to list

  

| Protein Coverage
| Supporting Peptides
| Best Unique PSM
|

Protein Coverage:

Supporting Peptides:

| Peptide | Uniq | -10lgP | Mass | Length | ppm | m/z | z | RT | 1/k0 Start | 1/k0 End | Precursor ID | Area Sample 2 | #Feature | #Feature Sample 2 | Start | End | PTM | AScore | Found By |
| --- | --- | --- | --- | --- | --- | --- | --- | --- | --- | --- | --- | --- | --- | --- | --- | --- | --- | --- | --- |
| K.AGM(+15.99)THIVR.D | Y | 38.19 | 899.4647 | 8 | 6.96 | 450.7403 | 2 | 9.74 | 0.8254 | 0.8327 | 11994 | 0 | 0 | 0 | 27 | 34 | Oxidation (M) | M3:Oxidation (M):1000 | DB Search |
| total 1 peptides |  |  |  |  |  |  |  |  |  |  |  |  |  |  |  |  |  |  |  |
| --- | --- | --- | --- | --- | --- | --- | --- | --- | --- | --- | --- | --- | --- | --- | --- | --- | --- | --- | --- |

Best Unique PSM (Scan Sample\_11\_A1\_1\_3648.d:11994, m/z=450.7403, z=2, RT=9.74, ppm=1.48):


TRINITY\_DN1933\_c0\_g1\_i4.p1

back to list

  

| Protein Coverage
| Supporting Peptides
| Best Unique PSM
|

Protein Coverage:

Supporting Peptides:

| Peptide | Uniq | -10lgP | Mass | Length | ppm | m/z | z | RT | 1/k0 Start | 1/k0 End | Precursor ID | Area Sample 2 | #Feature | #Feature Sample 2 | Start | End | PTM | AScore | Found By |
| --- | --- | --- | --- | --- | --- | --- | --- | --- | --- | --- | --- | --- | --- | --- | --- | --- | --- | --- | --- |
| R.RNLDIERPTYTNLNR.L | Y | 46.58 | 1873.9758 | 15 | -2.43 | 625.6609 | 3 | 13.94 | 0.8858 | 0.8931 | 35770 | 1.17e3 | 1 | 1 | 75 | 89 |  |  | DB Search |
| total 1 peptides |  |  |  |  |  |  |  |  |  |  |  |  |  |  |  |  |  |  |  |
| --- | --- | --- | --- | --- | --- | --- | --- | --- | --- | --- | --- | --- | --- | --- | --- | --- | --- | --- | --- |

Best Unique PSM (Scan Sample\_11\_A1\_1\_3648.d:35770, m/z=625.6609, z=3, RT=13.94, ppm=-7.91):


TRINITY\_DN817\_c0\_g1\_i6.p1

back to list

  

| Protein Coverage
| Supporting Peptides
| Best Unique PSM
|

Protein Coverage:

Supporting Peptides:

| Peptide | Uniq | -10lgP | Mass | Length | ppm | m/z | z | RT | 1/k0 Start | 1/k0 End | Precursor ID | Area Sample 2 | #Feature | #Feature Sample 2 | Start | End | PTM | AScore | Found By |
| --- | --- | --- | --- | --- | --- | --- | --- | --- | --- | --- | --- | --- | --- | --- | --- | --- | --- | --- | --- |
| R.LC(+57.02)QPGYSTC(+57.02)ENHC(+57.02)R.R | Y | 38.48 | 1780.708 | 14 | 5.76 | 891.3615 | 2 | 11.39 | 1.0877 | 1.0949 | 21326 | 1.4e2 | 1 | 1 | 147 | 160 | Carbamidomethylation, Carbamidomethylation, Carbamidomethylation |  | DB Search |
| total 1 peptides |  |  |  |  |  |  |  |  |  |  |  |  |  |  |  |  |  |  |  |
| --- | --- | --- | --- | --- | --- | --- | --- | --- | --- | --- | --- | --- | --- | --- | --- | --- | --- | --- | --- |

Best Unique PSM (Scan Sample\_11\_A1\_1\_3648.d:21326, m/z=891.3615, z=2, RT=11.39, ppm=0.28):


TRINITY\_DN614\_c0\_g1\_i1.p1

back to list

  

| Protein Coverage
| Supporting Peptides
| Best Unique PSM
|

Protein Coverage:

Supporting Peptides:

| Peptide | Uniq | -10lgP | Mass | Length | ppm | m/z | z | RT | 1/k0 Start | 1/k0 End | Precursor ID | Area Sample 2 | #Feature | #Feature Sample 2 | Start | End | PTM | AScore | Found By |
| --- | --- | --- | --- | --- | --- | --- | --- | --- | --- | --- | --- | --- | --- | --- | --- | --- | --- | --- | --- |
| R.VTEAKGELAK.M | Y | 57.99 | 1044.5815 | 10 | 1.77 | 523.2961 | 2 | 10.00 | 0.8947 | 0.902 | 13309 | 9.44e2 | 1 | 1 | 100 | 109 |  |  | DB Search |
| total 1 peptides |  |  |  |  |  |  |  |  |  |  |  |  |  |  |  |  |  |  |  |
| --- | --- | --- | --- | --- | --- | --- | --- | --- | --- | --- | --- | --- | --- | --- | --- | --- | --- | --- | --- |

Best Unique PSM (Scan Sample\_11\_A1\_1\_3648.d:13309, m/z=523.2961, z=2, RT=10.00, ppm=-3.71):


TRINITY\_DN7504\_c0\_g1\_i1.p1

back to list

  

| Protein Coverage
| Supporting Peptides
| Best Unique PSM
|

Protein Coverage:

Supporting Peptides:

| Peptide | Uniq | -10lgP | Mass | Length | ppm | m/z | z | RT | 1/k0 Start | 1/k0 End | Precursor ID | Area Sample 2 | #Feature | #Feature Sample 2 | Start | End | PTM | AScore | Found By |
| --- | --- | --- | --- | --- | --- | --- | --- | --- | --- | --- | --- | --- | --- | --- | --- | --- | --- | --- | --- |
| K.TIEAEAAHGTVTR.H | Y | 58.87 | 1354.6841 | 13 | 3.13 | 678.3477 | 2 | 11.77 | 1.0134 | 1.0206 | 23572 | 0 | 0 | 0 | 301 | 313 |  |  | DB Search |
| total 1 peptides |  |  |  |  |  |  |  |  |  |  |  |  |  |  |  |  |  |  |  |
| --- | --- | --- | --- | --- | --- | --- | --- | --- | --- | --- | --- | --- | --- | --- | --- | --- | --- | --- | --- |

Best Unique PSM (Scan Sample\_11\_A1\_1\_3648.d:23572, m/z=678.3477, z=2, RT=11.77, ppm=-2.34):


TRINITY\_DN3870\_c0\_g1\_i1.p1

back to list

  

| Protein Coverage
| Supporting Peptides
| Best Unique PSM
|

Protein Coverage:

Supporting Peptides:

| Peptide | Uniq | -10lgP | Mass | Length | ppm | m/z | z | RT | 1/k0 Start | 1/k0 End | Precursor ID | Area Sample 2 | #Feature | #Feature Sample 2 | Start | End | PTM | AScore | Found By |
| --- | --- | --- | --- | --- | --- | --- | --- | --- | --- | --- | --- | --- | --- | --- | --- | --- | --- | --- | --- |
| R.AYLTLC(+57.02)VDLKR.S | Y | 58.15 | 1350.7329 | 11 | -2.24 | 676.3685 | 2 | 17.00 | 1.019 | 1.0263 | 51369 | 5.99e2 | 1 | 1 | 53 | 63 | Carbamidomethylation |  | DB Search |
| total 1 peptides |  |  |  |  |  |  |  |  |  |  |  |  |  |  |  |  |  |  |  |
| --- | --- | --- | --- | --- | --- | --- | --- | --- | --- | --- | --- | --- | --- | --- | --- | --- | --- | --- | --- |

Best Unique PSM (Scan Sample\_11\_A1\_1\_3648.d:51369, m/z=676.3685, z=2, RT=17.00, ppm=-7.72):


TRINITY\_DN1339\_c0\_g2\_i4.p2

back to list

  

| Protein Coverage
| Supporting Peptides
| Best Unique PSM
|

Protein Coverage:

Supporting Peptides:

| Peptide | Uniq | -10lgP | Mass | Length | ppm | m/z | z | RT | 1/k0 Start | 1/k0 End | Precursor ID | Area Sample 2 | #Feature | #Feature Sample 2 | Start | End | PTM | AScore | Found By |
| --- | --- | --- | --- | --- | --- | --- | --- | --- | --- | --- | --- | --- | --- | --- | --- | --- | --- | --- | --- |
| K.LLTSSDQAELTKK.L | Y | 58.87 | 1432.7773 | 13 | 4.21 | 717.395 | 2 | 12.69 | 1.0496 | 1.0568 | 28711 | 7.94e2 | 1 | 1 | 155 | 167 |  |  | DB Search |
| total 1 peptides |  |  |  |  |  |  |  |  |  |  |  |  |  |  |  |  |  |  |  |
| --- | --- | --- | --- | --- | --- | --- | --- | --- | --- | --- | --- | --- | --- | --- | --- | --- | --- | --- | --- |

Best Unique PSM (Scan Sample\_11\_A1\_1\_3648.d:28711, m/z=717.395, z=2, RT=12.69, ppm=-1.26):


TRINITY\_DN128406\_c0\_g1\_i1.p1

back to list

  

| Protein Coverage
| Supporting Peptides
| Best Unique PSM
|

Protein Coverage:

Supporting Peptides:

| Peptide | Uniq | -10lgP | Mass | Length | ppm | m/z | z | RT | 1/k0 Start | 1/k0 End | Precursor ID | Area Sample 2 | #Feature | #Feature Sample 2 | Start | End | PTM | AScore | Found By |
| --- | --- | --- | --- | --- | --- | --- | --- | --- | --- | --- | --- | --- | --- | --- | --- | --- | --- | --- | --- |
| K.EIRDILVQYDR.S | Y | 58.36 | 1418.7517 | 11 | -6.7 | 710.3745 | 2 | 19.40 | 1.0339 | 1.0411 | 64335 | 5.13e3 | 1 | 1 | 110 | 120 |  |  | DB Search |
| total 1 peptides |  |  |  |  |  |  |  |  |  |  |  |  |  |  |  |  |  |  |  |
| --- | --- | --- | --- | --- | --- | --- | --- | --- | --- | --- | --- | --- | --- | --- | --- | --- | --- | --- | --- |

Best Unique PSM (Scan Sample\_11\_A1\_1\_3648.d:64335, m/z=710.3745, z=2, RT=19.40, ppm=-12.18):


TRINITY\_DN9509\_c0\_g1\_i2.p1

back to list

  

| Protein Coverage
| Supporting Peptides
| Best Unique PSM
|

Protein Coverage:

Supporting Peptides:

| Peptide | Uniq | -10lgP | Mass | Length | ppm | m/z | z | RT | 1/k0 Start | 1/k0 End | Precursor ID | Area Sample 2 | #Feature | #Feature Sample 2 | Start | End | PTM | AScore | Found By |
| --- | --- | --- | --- | --- | --- | --- | --- | --- | --- | --- | --- | --- | --- | --- | --- | --- | --- | --- | --- |
| R.GITINASHIEYETSNR.H | Y | 57.62 | 1803.8751 | 16 | -0.09 | 902.9398 | 2 | 14.89 | 1.1674 | 1.1746 | 40938 | 0 | 0 | 0 | 74 | 89 |  |  | DB Search |
| total 1 peptides |  |  |  |  |  |  |  |  |  |  |  |  |  |  |  |  |  |  |  |
| --- | --- | --- | --- | --- | --- | --- | --- | --- | --- | --- | --- | --- | --- | --- | --- | --- | --- | --- | --- |

Best Unique PSM (Scan Sample\_11\_A1\_1\_3648.d:40938, m/z=902.9398, z=2, RT=14.89, ppm=-5.56):


TRINITY\_DN265\_c0\_g1\_i8.p1

back to list

  

| Protein Coverage
| Supporting Peptides
| Best Unique PSM
|

Protein Coverage:

Supporting Peptides:

| Peptide | Uniq | -10lgP | Mass | Length | ppm | m/z | z | RT | 1/k0 Start | 1/k0 End | Precursor ID | Area Sample 2 | #Feature | #Feature Sample 2 | Start | End | PTM | AScore | Found By |
| --- | --- | --- | --- | --- | --- | --- | --- | --- | --- | --- | --- | --- | --- | --- | --- | --- | --- | --- | --- |
| R.LTHDFHTNKR.M | Y | 58.75 | 1267.6421 | 10 | 2.44 | 423.5534 | 3 | 9.64 | 0.7441 | 0.7514 | 11479 | 1.15e3 | 1 | 1 | 24 | 33 |  |  | DB Search |
| total 1 peptides |  |  |  |  |  |  |  |  |  |  |  |  |  |  |  |  |  |  |  |
| --- | --- | --- | --- | --- | --- | --- | --- | --- | --- | --- | --- | --- | --- | --- | --- | --- | --- | --- | --- |

Best Unique PSM (Scan Sample\_11\_A1\_1\_3648.d:11479, m/z=423.5534, z=3, RT=9.64, ppm=-3.04):


TRINITY\_DN1339\_c0\_g1\_i1.p1

back to list

  

| Protein Coverage
| Supporting Peptides
| Best Unique PSM
|

Protein Coverage:

Supporting Peptides:

| Peptide | Uniq | -10lgP | Mass | Length | ppm | m/z | z | RT | 1/k0 Start | 1/k0 End | Precursor ID | Area Sample 2 | #Feature | #Feature Sample 2 | Start | End | PTM | AScore | Found By |
| --- | --- | --- | --- | --- | --- | --- | --- | --- | --- | --- | --- | --- | --- | --- | --- | --- | --- | --- | --- |
| K.VITSDDPAEKEK.K | Y | 58.87 | 1330.6616 | 12 | 2.64 | 666.3362 | 2 | 10.69 | 0.9848 | 0.992 | 17191 | 9.01e3 | 1 | 1 | 160 | 171 |  |  | DB Search |
| total 1 peptides |  |  |  |  |  |  |  |  |  |  |  |  |  |  |  |  |  |  |  |
| --- | --- | --- | --- | --- | --- | --- | --- | --- | --- | --- | --- | --- | --- | --- | --- | --- | --- | --- | --- |

Best Unique PSM (Scan Sample\_11\_A1\_1\_3648.d:17191, m/z=666.3362, z=2, RT=10.69, ppm=-2.84):


TRINITY\_DN468\_c1\_g1\_i1.p1

back to list

  

| Protein Coverage
| Supporting Peptides
| Best Unique PSM
|

Protein Coverage:

Supporting Peptides:

| Peptide | Uniq | -10lgP | Mass | Length | ppm | m/z | z | RT | 1/k0 Start | 1/k0 End | Precursor ID | Area Sample 2 | #Feature | #Feature Sample 2 | Start | End | PTM | AScore | Found By |
| --- | --- | --- | --- | --- | --- | --- | --- | --- | --- | --- | --- | --- | --- | --- | --- | --- | --- | --- | --- |
| K.LGPVVDGKYDYTVITTDYK.A | Y | 58.12 | 2146.0834 | 19 | 7.52 | 1074.0511 | 2 | 17.25 | 1.2428 | 1.2499 | 52770 | 0 | 0 | 0 | 123 | 141 |  |  | DB Search |
| total 1 peptides |  |  |  |  |  |  |  |  |  |  |  |  |  |  |  |  |  |  |  |
| --- | --- | --- | --- | --- | --- | --- | --- | --- | --- | --- | --- | --- | --- | --- | --- | --- | --- | --- | --- |

Best Unique PSM (Scan Sample\_11\_A1\_1\_3648.d:52770, m/z=1074.0511, z=2, RT=17.25, ppm=2.04):


TRINITY\_DN585\_c0\_g1\_i4.p1

back to list

  

| Protein Coverage
| Supporting Peptides
| Best Unique PSM
|

Protein Coverage:

Supporting Peptides:

| Peptide | Uniq | -10lgP | Mass | Length | ppm | m/z | z | RT | 1/k0 Start | 1/k0 End | Precursor ID | Area Sample 2 | #Feature | #Feature Sample 2 | Start | End | PTM | AScore | Found By |
| --- | --- | --- | --- | --- | --- | --- | --- | --- | --- | --- | --- | --- | --- | --- | --- | --- | --- | --- | --- |
| K.IQDKEGIPPDQQR.L | Y | 55.06 | 1522.7739 | 13 | 2.88 | 762.3923 | 2 | 11.00 | 1.0331 | 1.0403 | 18584 | 2.97e3 | 2 | 2 | 30 | 42 |  |  | DB Search |
| total 1 peptides |  |  |  |  |  |  |  |  |  |  |  |  |  |  |  |  |  |  |  |
| --- | --- | --- | --- | --- | --- | --- | --- | --- | --- | --- | --- | --- | --- | --- | --- | --- | --- | --- | --- |

Best Unique PSM (Scan Sample\_11\_A1\_1\_3648.d:18584, m/z=762.3923, z=2, RT=11.00, ppm=-2.59):


TRINITY\_DN3034\_c0\_g1\_i1.p1

back to list

  

| Protein Coverage
| Supporting Peptides
| Best Unique PSM
|

Protein Coverage:

Supporting Peptides:

| Peptide | Uniq | -10lgP | Mass | Length | ppm | m/z | z | RT | 1/k0 Start | 1/k0 End | Precursor ID | Area Sample 2 | #Feature | #Feature Sample 2 | Start | End | PTM | AScore | Found By |
| --- | --- | --- | --- | --- | --- | --- | --- | --- | --- | --- | --- | --- | --- | --- | --- | --- | --- | --- | --- |
| K.TPGLVEGTLILHQLR.C | Y | 58.52 | 1645.9515 | 15 | -1.31 | 549.6541 | 3 | 18.53 | 0.8644 | 0.8716 | 59662 | 7.05e2 | 1 | 1 | 671 | 685 |  |  | DB Search |
| total 1 peptides |  |  |  |  |  |  |  |  |  |  |  |  |  |  |  |  |  |  |  |
| --- | --- | --- | --- | --- | --- | --- | --- | --- | --- | --- | --- | --- | --- | --- | --- | --- | --- | --- | --- |

Best Unique PSM (Scan Sample\_11\_A1\_1\_3648.d:59662, m/z=549.6541, z=3, RT=18.53, ppm=-6.79):


TRINITY\_DN2636\_c0\_g1\_i2.p1

back to list

  

| Protein Coverage
| Supporting Peptides
| Best Unique PSM
|

Protein Coverage:

Supporting Peptides:

| Peptide | Uniq | -10lgP | Mass | Length | ppm | m/z | z | RT | 1/k0 Start | 1/k0 End | Precursor ID | Area Sample 2 | #Feature | #Feature Sample 2 | Start | End | PTM | AScore | Found By |
| --- | --- | --- | --- | --- | --- | --- | --- | --- | --- | --- | --- | --- | --- | --- | --- | --- | --- | --- | --- |
| K.VVEKEAETER.K | Y | 58.8 | 1188.5986 | 10 | 7.97 | 397.2078 | 3 | 9.54 | 0.7322 | 0.7396 | 11020 | 5.76e2 | 1 | 1 | 199 | 208 |  |  | DB Search |
| total 1 peptides |  |  |  |  |  |  |  |  |  |  |  |  |  |  |  |  |  |  |  |
| --- | --- | --- | --- | --- | --- | --- | --- | --- | --- | --- | --- | --- | --- | --- | --- | --- | --- | --- | --- |

Best Unique PSM (Scan Sample\_11\_A1\_1\_3648.d:11020, m/z=397.2078, z=3, RT=9.54, ppm=2.49):


TRINITY\_DN817\_c0\_g1\_i4.p1

back to list

  

| Protein Coverage
| Supporting Peptides
| Best Unique PSM
|

Protein Coverage:

Supporting Peptides:

| Peptide | Uniq | -10lgP | Mass | Length | ppm | m/z | z | RT | 1/k0 Start | 1/k0 End | Precursor ID | Area Sample 2 | #Feature | #Feature Sample 2 | Start | End | PTM | AScore | Found By |
| --- | --- | --- | --- | --- | --- | --- | --- | --- | --- | --- | --- | --- | --- | --- | --- | --- | --- | --- | --- |
| R.LC(+57.02)QPGYSTC(+57.02)ENHC(+57.02)R.R | Y | 38.48 | 1780.708 | 14 | 5.76 | 891.3615 | 2 | 11.39 | 1.0877 | 1.0949 | 21326 | 1.4e2 | 1 | 1 | 145 | 158 | Carbamidomethylation, Carbamidomethylation, Carbamidomethylation |  | DB Search |
| total 1 peptides |  |  |  |  |  |  |  |  |  |  |  |  |  |  |  |  |  |  |  |
| --- | --- | --- | --- | --- | --- | --- | --- | --- | --- | --- | --- | --- | --- | --- | --- | --- | --- | --- | --- |

Best Unique PSM (Scan Sample\_11\_A1\_1\_3648.d:21326, m/z=891.3615, z=2, RT=11.39, ppm=0.28):


TRINITY\_DN2799\_c0\_g1\_i1.p1

back to list

  

| Protein Coverage
| Supporting Peptides
| Best Unique PSM
|

Protein Coverage:

Supporting Peptides:

| Peptide | Uniq | -10lgP | Mass | Length | ppm | m/z | z | RT | 1/k0 Start | 1/k0 End | Precursor ID | Area Sample 2 | #Feature | #Feature Sample 2 | Start | End | PTM | AScore | Found By |
| --- | --- | --- | --- | --- | --- | --- | --- | --- | --- | --- | --- | --- | --- | --- | --- | --- | --- | --- | --- |
| K.AGM(+15.99)THIVR.D | Y | 38.19 | 899.4647 | 8 | 6.96 | 450.7403 | 2 | 9.74 | 0.8254 | 0.8327 | 11994 | 0 | 0 | 0 | 74 | 81 | Oxidation (M) | M3:Oxidation (M):1000 | DB Search |
| R.HGSLGFLPR.K | Y | 32.83 | 982.5348 | 9 | -0.04 | 492.272 | 2 | 16.93 | 0.8408 | 0.8481 | 51108 | 4.36e2 | 1 | 1 | 34 | 42 |  |  | DB Search |
| total 2 peptides |  |  |  |  |  |  |  |  |  |  |  |  |  |  |  |  |  |  |  |
| --- | --- | --- | --- | --- | --- | --- | --- | --- | --- | --- | --- | --- | --- | --- | --- | --- | --- | --- | --- |

Best Unique PSM (Scan Sample\_11\_A1\_1\_3648.d:11994, m/z=450.7403, z=2, RT=9.74, ppm=1.48):


TRINITY\_DN1065\_c0\_g1\_i1.p1

back to list

  

| Protein Coverage
| Supporting Peptides
| Best Unique PSM
|

Protein Coverage:

Supporting Peptides:

| Peptide | Uniq | -10lgP | Mass | Length | ppm | m/z | z | RT | 1/k0 Start | 1/k0 End | Precursor ID | Area Sample 2 | #Feature | #Feature Sample 2 | Start | End | PTM | AScore | Found By |
| --- | --- | --- | --- | --- | --- | --- | --- | --- | --- | --- | --- | --- | --- | --- | --- | --- | --- | --- | --- |
| R.HILLAIANDEELNKLLR.N | Y | 58.87 | 1974.1262 | 17 | -3.82 | 659.0432 | 3 | 19.18 | 0.9856 | 0.9928 | 63150 | 5.97e2 | 1 | 1 | 79 | 95 |  |  | DB Search |
| total 1 peptides |  |  |  |  |  |  |  |  |  |  |  |  |  |  |  |  |  |  |  |
| --- | --- | --- | --- | --- | --- | --- | --- | --- | --- | --- | --- | --- | --- | --- | --- | --- | --- | --- | --- |

Best Unique PSM (Scan Sample\_11\_A1\_1\_3648.d:63150, m/z=659.0432, z=3, RT=19.18, ppm=-9.29):


TRINITY\_DN163801\_c0\_g1\_i1.p1

back to list

  

| Protein Coverage
| Supporting Peptides
| Best Unique PSM
|

Protein Coverage:

Supporting Peptides:

| Peptide | Uniq | -10lgP | Mass | Length | ppm | m/z | z | RT | 1/k0 Start | 1/k0 End | Precursor ID | Area Sample 2 | #Feature | #Feature Sample 2 | Start | End | PTM | AScore | Found By |
| --- | --- | --- | --- | --- | --- | --- | --- | --- | --- | --- | --- | --- | --- | --- | --- | --- | --- | --- | --- |
| M.A(+42.01)NPLKDIISR.F | Y | 58.83 | 1167.6611 | 10 | -5.53 | 584.8314 | 2 | 18.34 | 0.919 | 0.9263 | 58511 | 1.06e3 | 1 | 1 | 2 | 11 | Acetylation (Protein N-term) | A1:Acetylation (Protein N-term):1000 | DB Search |
| total 1 peptides |  |  |  |  |  |  |  |  |  |  |  |  |  |  |  |  |  |  |  |
| --- | --- | --- | --- | --- | --- | --- | --- | --- | --- | --- | --- | --- | --- | --- | --- | --- | --- | --- | --- |

Best Unique PSM (Scan Sample\_11\_A1\_1\_3648.d:58511, m/z=584.8314, z=2, RT=18.34, ppm=-11.01):


TRINITY\_DN95248\_c0\_g1\_i1.p1

back to list

  

| Protein Coverage
| Supporting Peptides
| Best Unique PSM
|

Protein Coverage:

Supporting Peptides:

| Peptide | Uniq | -10lgP | Mass | Length | ppm | m/z | z | RT | 1/k0 Start | 1/k0 End | Precursor ID | Area Sample 2 | #Feature | #Feature Sample 2 | Start | End | PTM | AScore | Found By |
| --- | --- | --- | --- | --- | --- | --- | --- | --- | --- | --- | --- | --- | --- | --- | --- | --- | --- | --- | --- |
| R.VVASSHTDYELR.Y | Y | 59.14 | 1375.6732 | 12 | 4.96 | 688.8435 | 2 | 12.47 | 1.0094 | 1.0166 | 27423 | 6.35e3 | 1 | 1 | 96 | 107 |  |  | DB Search |
| K.TKVTLVSERPFSK.R | Y | 58.85 | 1490.8456 | 13 | 0.25 | 746.4262 | 2 | 13.79 | 1.0632 | 1.0705 | 34922 | 3.55e2 | 1 | 1 | 62 | 74 |  |  | DB Search |
| K.VTLVSERPFSK.R | Y | 58.25 | 1261.703 | 11 | 2.16 | 631.8567 | 2 | 15.09 | 0.9602 | 0.9675 | 41854 | 1.01e3 | 1 | 1 | 64 | 74 |  |  | DB Search |
| K.IKVDDKLNNLDQITLEVAK.T | Y | 55.27 | 2168.2052 | 19 | -2.25 | 723.7368 | 3 | 17.58 | 0.9125 | 0.9198 | 54654 | 0 | 0 | 0 | 43 | 61 |  |  | DB Search |
| total 4 peptides |  |  |  |  |  |  |  |  |  |  |  |  |  |  |  |  |  |  |  |
| --- | --- | --- | --- | --- | --- | --- | --- | --- | --- | --- | --- | --- | --- | --- | --- | --- | --- | --- | --- |

Best Unique PSM (Scan Sample\_11\_A1\_1\_3648.d:27423, m/z=688.8435, z=2, RT=12.47, ppm=-0.52):


TRINITY\_DN30365\_c0\_g1\_i3.p1

back to list

  

| Protein Coverage
| Supporting Peptides
| Best Unique PSM
|

Protein Coverage:

Supporting Peptides:

| Peptide | Uniq | -10lgP | Mass | Length | ppm | m/z | z | RT | 1/k0 Start | 1/k0 End | Precursor ID | Area Sample 2 | #Feature | #Feature Sample 2 | Start | End | PTM | AScore | Found By |
| --- | --- | --- | --- | --- | --- | --- | --- | --- | --- | --- | --- | --- | --- | --- | --- | --- | --- | --- | --- |
| K.AVAHHTTASFIR.V | Y | 58.76 | 1309.6891 | 12 | -1.72 | 655.8471 | 2 | 11.68 | 0.9703 | 0.9775 | 22954 | 1.45e3 | 1 | 1 | 185 | 196 |  |  | DB Search |
| total 1 peptides |  |  |  |  |  |  |  |  |  |  |  |  |  |  |  |  |  |  |  |
| --- | --- | --- | --- | --- | --- | --- | --- | --- | --- | --- | --- | --- | --- | --- | --- | --- | --- | --- | --- |

Best Unique PSM (Scan Sample\_11\_A1\_1\_3648.d:22954, m/z=655.8471, z=2, RT=11.68, ppm=-7.2):


TRINITY\_DN32727\_c0\_g1\_i2.p1

back to list

  

| Protein Coverage
| Supporting Peptides
| Best Unique PSM
|

Protein Coverage:

Supporting Peptides:

| Peptide | Uniq | -10lgP | Mass | Length | ppm | m/z | z | RT | 1/k0 Start | 1/k0 End | Precursor ID | Area Sample 2 | #Feature | #Feature Sample 2 | Start | End | PTM | AScore | Found By |
| --- | --- | --- | --- | --- | --- | --- | --- | --- | --- | --- | --- | --- | --- | --- | --- | --- | --- | --- | --- |
| K.AGM(+15.99)THIVR.D | Y | 38.19 | 899.4647 | 8 | 6.96 | 450.7403 | 2 | 9.74 | 0.8254 | 0.8327 | 11994 | 0 | 0 | 0 | 40 | 47 | Oxidation (M) | M3:Oxidation (M):1000 | DB Search |
| total 1 peptides |  |  |  |  |  |  |  |  |  |  |  |  |  |  |  |  |  |  |  |
| --- | --- | --- | --- | --- | --- | --- | --- | --- | --- | --- | --- | --- | --- | --- | --- | --- | --- | --- | --- |

Best Unique PSM (Scan Sample\_11\_A1\_1\_3648.d:11994, m/z=450.7403, z=2, RT=9.74, ppm=1.48):


TRINITY\_DN4101\_c0\_g1\_i4.p2

back to list

  

| Protein Coverage
| Supporting Peptides
| Best Unique PSM
|

Protein Coverage:

Supporting Peptides:

| Peptide | Uniq | -10lgP | Mass | Length | ppm | m/z | z | RT | 1/k0 Start | 1/k0 End | Precursor ID | Area Sample 2 | #Feature | #Feature Sample 2 | Start | End | PTM | AScore | Found By |
| --- | --- | --- | --- | --- | --- | --- | --- | --- | --- | --- | --- | --- | --- | --- | --- | --- | --- | --- | --- |
| R.GVDEDAYTLEVLHYK.A | Y | 58.84 | 1750.8413 | 15 | 4.4 | 876.427 | 2 | 17.48 | 1.1802 | 1.1873 | 54020 | 1.93e2 | 1 | 1 | 81 | 95 |  |  | DB Search |
| total 1 peptides |  |  |  |  |  |  |  |  |  |  |  |  |  |  |  |  |  |  |  |
| --- | --- | --- | --- | --- | --- | --- | --- | --- | --- | --- | --- | --- | --- | --- | --- | --- | --- | --- | --- |

Best Unique PSM (Scan Sample\_11\_A1\_1\_3648.d:54020, m/z=876.427, z=2, RT=17.48, ppm=-1.08):


TRINITY\_DN77\_c1\_g1\_i1.p1

back to list

  

| Protein Coverage
| Supporting Peptides
| Best Unique PSM
|

Protein Coverage:

Supporting Peptides:

| Peptide | Uniq | -10lgP | Mass | Length | ppm | m/z | z | RT | 1/k0 Start | 1/k0 End | Precursor ID | Area Sample 2 | #Feature | #Feature Sample 2 | Start | End | PTM | AScore | Found By |
| --- | --- | --- | --- | --- | --- | --- | --- | --- | --- | --- | --- | --- | --- | --- | --- | --- | --- | --- | --- |
| R.AVDIHFALDASQNVGR.E | Y | 57.77 | 1711.8641 | 16 | -0.25 | 856.9344 | 2 | 17.17 | 1.1033 | 1.1106 | 52342 | 2.27e2 | 1 | 1 | 56 | 71 |  |  | DB Search |
| total 1 peptides |  |  |  |  |  |  |  |  |  |  |  |  |  |  |  |  |  |  |  |
| --- | --- | --- | --- | --- | --- | --- | --- | --- | --- | --- | --- | --- | --- | --- | --- | --- | --- | --- | --- |

Best Unique PSM (Scan Sample\_11\_A1\_1\_3648.d:52342, m/z=856.9344, z=2, RT=17.17, ppm=-5.72):


TRINITY\_DN932\_c0\_g1\_i1.p1

back to list

  

| Protein Coverage
| Supporting Peptides
| Best Unique PSM
|

Protein Coverage:

Supporting Peptides:

| Peptide | Uniq | -10lgP | Mass | Length | ppm | m/z | z | RT | 1/k0 Start | 1/k0 End | Precursor ID | Area Sample 2 | #Feature | #Feature Sample 2 | Start | End | PTM | AScore | Found By |
| --- | --- | --- | --- | --- | --- | --- | --- | --- | --- | --- | --- | --- | --- | --- | --- | --- | --- | --- | --- |
| R.EKSM(+15.99)IHELNR.Y | Y | 56.52 | 1271.6292 | 10 | 3.88 | 636.8209 | 2 | 10.41 | 0.9202 | 0.9275 | 15609 | 4.13e2 | 1 | 1 | 404 | 413 | Oxidation (M) | M4:Oxidation (M):1000 | DB Search |
| total 1 peptides |  |  |  |  |  |  |  |  |  |  |  |  |  |  |  |  |  |  |  |
| --- | --- | --- | --- | --- | --- | --- | --- | --- | --- | --- | --- | --- | --- | --- | --- | --- | --- | --- | --- |

Best Unique PSM (Scan Sample\_11\_A1\_1\_3648.d:15609, m/z=636.8209, z=2, RT=10.41, ppm=-1.6):


TRINITY\_DN5023\_c0\_g1\_i2.p2

back to list

  

| Protein Coverage
| Supporting Peptides
| Best Unique PSM
|

Protein Coverage:

Supporting Peptides:

| Peptide | Uniq | -10lgP | Mass | Length | ppm | m/z | z | RT | 1/k0 Start | 1/k0 End | Precursor ID | Area Sample 2 | #Feature | #Feature Sample 2 | Start | End | PTM | AScore | Found By |
| --- | --- | --- | --- | --- | --- | --- | --- | --- | --- | --- | --- | --- | --- | --- | --- | --- | --- | --- | --- |
| R.YRPGTVALR.E | Y | 44.05 | 1031.5876 | 9 | -1.83 | 516.7973 | 2 | 12.44 | 0.8575 | 0.8648 | 27332 | 1.61e3 | 1 | 1 | 78 | 86 |  |  | DB Search |
| total 1 peptides |  |  |  |  |  |  |  |  |  |  |  |  |  |  |  |  |  |  |  |
| --- | --- | --- | --- | --- | --- | --- | --- | --- | --- | --- | --- | --- | --- | --- | --- | --- | --- | --- | --- |

Best Unique PSM (Scan Sample\_11\_A1\_1\_3648.d:27332, m/z=516.7973, z=2, RT=12.44, ppm=-7.31):


TRINITY\_DN1989\_c0\_g1\_i1.p1

back to list

  

| Protein Coverage
| Supporting Peptides
| Best Unique PSM
|

Protein Coverage:

Supporting Peptides:

| Peptide | Uniq | -10lgP | Mass | Length | ppm | m/z | z | RT | 1/k0 Start | 1/k0 End | Precursor ID | Area Sample 2 | #Feature | #Feature Sample 2 | Start | End | PTM | AScore | Found By |
| --- | --- | --- | --- | --- | --- | --- | --- | --- | --- | --- | --- | --- | --- | --- | --- | --- | --- | --- | --- |
| R.FATHASALEIHGLDR.F | Y | 58.7 | 1636.8321 | 15 | 6.31 | 819.424 | 2 | 14.70 | 1.1366 | 1.1438 | 39824 | 2.68e2 | 1 | 1 | 227 | 241 |  |  | DB Search |
| total 1 peptides |  |  |  |  |  |  |  |  |  |  |  |  |  |  |  |  |  |  |  |
| --- | --- | --- | --- | --- | --- | --- | --- | --- | --- | --- | --- | --- | --- | --- | --- | --- | --- | --- | --- |

Best Unique PSM (Scan Sample\_11\_A1\_1\_3648.d:39824, m/z=819.424, z=2, RT=14.70, ppm=0.83):


TRINITY\_DN2885\_c0\_g1\_i5.p1

back to list

  

| Protein Coverage
| Supporting Peptides
| Best Unique PSM
|

Protein Coverage:

Supporting Peptides:

| Peptide | Uniq | -10lgP | Mass | Length | ppm | m/z | z | RT | 1/k0 Start | 1/k0 End | Precursor ID | Area Sample 2 | #Feature | #Feature Sample 2 | Start | End | PTM | AScore | Found By |
| --- | --- | --- | --- | --- | --- | --- | --- | --- | --- | --- | --- | --- | --- | --- | --- | --- | --- | --- | --- |
| K.VTRPHGASGVVR.A | Y | 58.91 | 1234.6894 | 12 | 6.71 | 412.5709 | 3 | 10.04 | 0.8209 | 0.8283 | 13404 | 2.81e4 | 3 | 3 | 77 | 88 |  |  | DB Search |
| total 1 peptides |  |  |  |  |  |  |  |  |  |  |  |  |  |  |  |  |  |  |  |
| --- | --- | --- | --- | --- | --- | --- | --- | --- | --- | --- | --- | --- | --- | --- | --- | --- | --- | --- | --- |

Best Unique PSM (Scan Sample\_11\_A1\_1\_3648.d:13404, m/z=412.5709, z=3, RT=10.04, ppm=1.23):


TRINITY\_DN8227\_c0\_g1\_i1.p1

back to list

  

| Protein Coverage
| Supporting Peptides
| Best Unique PSM
|

Protein Coverage:

Supporting Peptides:

| Peptide | Uniq | -10lgP | Mass | Length | ppm | m/z | z | RT | 1/k0 Start | 1/k0 End | Precursor ID | Area Sample 2 | #Feature | #Feature Sample 2 | Start | End | PTM | AScore | Found By |
| --- | --- | --- | --- | --- | --- | --- | --- | --- | --- | --- | --- | --- | --- | --- | --- | --- | --- | --- | --- |
| K.IQDKEGIPPDQQR.L | Y | 55.06 | 1522.7739 | 13 | 2.88 | 762.3923 | 2 | 11.00 | 1.0331 | 1.0403 | 18584 | 2.97e3 | 2 | 2 | 54 | 66 |  |  | DB Search |
| total 1 peptides |  |  |  |  |  |  |  |  |  |  |  |  |  |  |  |  |  |  |  |
| --- | --- | --- | --- | --- | --- | --- | --- | --- | --- | --- | --- | --- | --- | --- | --- | --- | --- | --- | --- |

Best Unique PSM (Scan Sample\_11\_A1\_1\_3648.d:18584, m/z=762.3923, z=2, RT=11.00, ppm=-2.59):


TRINITY\_DN36\_c0\_g1\_i9.p1

back to list

  

| Protein Coverage
| Supporting Peptides
| Best Unique PSM
|

Protein Coverage:

Supporting Peptides:

| Peptide | Uniq | -10lgP | Mass | Length | ppm | m/z | z | RT | 1/k0 Start | 1/k0 End | Precursor ID | Area Sample 2 | #Feature | #Feature Sample 2 | Start | End | PTM | AScore | Found By |
| --- | --- | --- | --- | --- | --- | --- | --- | --- | --- | --- | --- | --- | --- | --- | --- | --- | --- | --- | --- |
| R.LGGIEVTQGNC(+57.02)ARK.Y | Y | 58.27 | 1501.7671 | 14 | 1.48 | 751.8878 | 2 | 12.59 | 1.0616 | 1.0688 | 28229 | 7.18e2 | 1 | 1 | 58 | 71 | Carbamidomethylation |  | DB Search |
| total 1 peptides |  |  |  |  |  |  |  |  |  |  |  |  |  |  |  |  |  |  |  |
| --- | --- | --- | --- | --- | --- | --- | --- | --- | --- | --- | --- | --- | --- | --- | --- | --- | --- | --- | --- |

Best Unique PSM (Scan Sample\_11\_A1\_1\_3648.d:28229, m/z=751.8878, z=2, RT=12.59, ppm=-4):


TRINITY\_DN197343\_c0\_g1\_i1.p1

back to list

  

| Protein Coverage
| Supporting Peptides
| Best Unique PSM
|

Protein Coverage:

Supporting Peptides:

| Peptide | Uniq | -10lgP | Mass | Length | ppm | m/z | z | RT | 1/k0 Start | 1/k0 End | Precursor ID | Area Sample 2 | #Feature | #Feature Sample 2 | Start | End | PTM | AScore | Found By |
| --- | --- | --- | --- | --- | --- | --- | --- | --- | --- | --- | --- | --- | --- | --- | --- | --- | --- | --- | --- |
| R.VLTVISHNQR.E | Y | 58.24 | 1165.6567 | 10 | 5.45 | 583.8356 | 2 | 12.20 | 0.9315 | 0.9388 | 25932 | 5.23e2 | 1 | 1 | 57 | 66 |  |  | DB Search |
| R.KEELC(+57.02)HQLTELKTELQQLHVAK.V | Y | 58.14 | 2674.4112 | 22 | -1.57 | 535.8857 | 5 | 18.20 | 0.8408 | 0.8481 | 57883 | 8.64e2 | 2 | 2 | 14 | 35 | Carbamidomethylation |  | DB Search |
| total 2 peptides |  |  |  |  |  |  |  |  |  |  |  |  |  |  |  |  |  |  |  |
| --- | --- | --- | --- | --- | --- | --- | --- | --- | --- | --- | --- | --- | --- | --- | --- | --- | --- | --- | --- |

Best Unique PSM (Scan Sample\_11\_A1\_1\_3648.d:25932, m/z=583.8356, z=2, RT=12.20, ppm=-0.02):


TRINITY\_DN1616\_c0\_g1\_i7.p1

back to list

  

| Protein Coverage
| Supporting Peptides
| Best Unique PSM
|

Protein Coverage:

Supporting Peptides:

| Peptide | Uniq | -10lgP | Mass | Length | ppm | m/z | z | RT | 1/k0 Start | 1/k0 End | Precursor ID | Area Sample 2 | #Feature | #Feature Sample 2 | Start | End | PTM | AScore | Found By |
| --- | --- | --- | --- | --- | --- | --- | --- | --- | --- | --- | --- | --- | --- | --- | --- | --- | --- | --- | --- |
| K.YVVMLQYYHDSDTAK.Y | Y | 58.58 | 1831.8451 | 15 | 5.67 | 916.93 | 2 | 16.32 | 1.1813 | 1.1885 | 48049 | 0 | 0 | 0 | 1071 | 1085 |  |  | DB Search |
| K.VVAEC(+57.02)NNGGGR.F | Y | 57.96 | 1131.5091 | 11 | -8.94 | 566.7537 | 2 | 9.41 | 0.8234 | 0.8307 | 10484 | 0 | 0 | 0 | 3736 | 3746 | Carbamidomethylation |  | DB Search |
| total 2 peptides |  |  |  |  |  |  |  |  |  |  |  |  |  |  |  |  |  |  |  |
| --- | --- | --- | --- | --- | --- | --- | --- | --- | --- | --- | --- | --- | --- | --- | --- | --- | --- | --- | --- |

Best Unique PSM (Scan Sample\_11\_A1\_1\_3648.d:48049, m/z=916.93, z=2, RT=16.32, ppm=0.2):


TRINITY\_DN3448\_c0\_g1\_i9.p3

back to list

  

| Protein Coverage
| Supporting Peptides
| Best Unique PSM
|

Protein Coverage:

Supporting Peptides:

| Peptide | Uniq | -10lgP | Mass | Length | ppm | m/z | z | RT | 1/k0 Start | 1/k0 End | Precursor ID | Area Sample 2 | #Feature | #Feature Sample 2 | Start | End | PTM | AScore | Found By |
| --- | --- | --- | --- | --- | --- | --- | --- | --- | --- | --- | --- | --- | --- | --- | --- | --- | --- | --- | --- |
| K.AGM(+15.99)THIVR.D | Y | 38.19 | 899.4647 | 8 | 6.96 | 450.7403 | 2 | 9.74 | 0.8254 | 0.8327 | 11994 | 0 | 0 | 0 | 27 | 34 | Oxidation (M) | M3:Oxidation (M):1000 | DB Search |
| total 1 peptides |  |  |  |  |  |  |  |  |  |  |  |  |  |  |  |  |  |  |  |
| --- | --- | --- | --- | --- | --- | --- | --- | --- | --- | --- | --- | --- | --- | --- | --- | --- | --- | --- | --- |

Best Unique PSM (Scan Sample\_11\_A1\_1\_3648.d:11994, m/z=450.7403, z=2, RT=9.74, ppm=1.48):


TRINITY\_DN3545\_c0\_g1\_i2.p1

back to list

  

| Protein Coverage
| Supporting Peptides
| Best Unique PSM
|

Protein Coverage:

Supporting Peptides:

| Peptide | Uniq | -10lgP | Mass | Length | ppm | m/z | z | RT | 1/k0 Start | 1/k0 End | Precursor ID | Area Sample 2 | #Feature | #Feature Sample 2 | Start | End | PTM | AScore | Found By |
| --- | --- | --- | --- | --- | --- | --- | --- | --- | --- | --- | --- | --- | --- | --- | --- | --- | --- | --- | --- |
| R.SSC(+57.02)RPILSVIK.I | Y | 27.61 | 1258.7067 | 11 | -7.39 | 630.3525 | 2 | 16.61 | 0.9687 | 0.9759 | 49383 | 6.05e2 | 1 | 1 | 6 | 16 | Carbamidomethylation |  | DB Search |
| total 1 peptides |  |  |  |  |  |  |  |  |  |  |  |  |  |  |  |  |  |  |  |
| --- | --- | --- | --- | --- | --- | --- | --- | --- | --- | --- | --- | --- | --- | --- | --- | --- | --- | --- | --- |

Best Unique PSM (Scan Sample\_11\_A1\_1\_3648.d:49383, m/z=630.3525, z=2, RT=16.61, ppm=-12.87):


TRINITY\_DN118\_c1\_g1\_i3.p1

back to list

  

| Protein Coverage
| Supporting Peptides
| Best Unique PSM
|

Protein Coverage:

Supporting Peptides:

| Peptide | Uniq | -10lgP | Mass | Length | ppm | m/z | z | RT | 1/k0 Start | 1/k0 End | Precursor ID | Area Sample 2 | #Feature | #Feature Sample 2 | Start | End | PTM | AScore | Found By |
| --- | --- | --- | --- | --- | --- | --- | --- | --- | --- | --- | --- | --- | --- | --- | --- | --- | --- | --- | --- |
| R.ETC(+57.02)SDIEKR.E | Y | 58.04 | 1136.5132 | 9 | 2.35 | 569.2621 | 2 | 10.08 | 0.8947 | 0.902 | 13694 | 6.3e3 | 1 | 1 | 186 | 194 | Carbamidomethylation |  | DB Search |
| K.VC(+57.02)EDVKR.C | Y | 57.57 | 904.4436 | 7 | 2.45 | 453.2277 | 2 | 9.38 | 0.7722 | 0.7795 | 10303 | 3.95e3 | 2 | 2 | 122 | 128 | Carbamidomethylation |  | DB Search |
| K.FSYEKVC(+57.02)EDVKR.C | Y | 40.84 | 1558.745 | 12 | 0 | 780.3755 | 2 | 13.41 | 1.0709 | 1.0781 | 32836 | 5.73e2 | 1 | 1 | 117 | 128 | Carbamidomethylation |  | DB Search |
| total 3 peptides |  |  |  |  |  |  |  |  |  |  |  |  |  |  |  |  |  |  |  |
| --- | --- | --- | --- | --- | --- | --- | --- | --- | --- | --- | --- | --- | --- | --- | --- | --- | --- | --- | --- |

Best Unique PSM (Scan Sample\_11\_A1\_1\_3648.d:13694, m/z=569.2621, z=2, RT=10.08, ppm=-3.13):


TRINITY\_DN165469\_c0\_g1\_i1.p1

back to list

  

| Protein Coverage
| Supporting Peptides
| Best Unique PSM
|

Protein Coverage:

Supporting Peptides:

| Peptide | Uniq | -10lgP | Mass | Length | ppm | m/z | z | RT | 1/k0 Start | 1/k0 End | Precursor ID | Area Sample 2 | #Feature | #Feature Sample 2 | Start | End | PTM | AScore | Found By |
| --- | --- | --- | --- | --- | --- | --- | --- | --- | --- | --- | --- | --- | --- | --- | --- | --- | --- | --- | --- |
| K.RLDPSEQLQHLSSLR.Q | Y | 53.55 | 1777.9435 | 15 | 4.12 | 593.6543 | 3 | 16.18 | 0.9065 | 0.9137 | 47359 | 1.79e2 | 1 | 1 | 2221 | 2235 |  |  | DB Search |
| total 1 peptides |  |  |  |  |  |  |  |  |  |  |  |  |  |  |  |  |  |  |  |
| --- | --- | --- | --- | --- | --- | --- | --- | --- | --- | --- | --- | --- | --- | --- | --- | --- | --- | --- | --- |

Best Unique PSM (Scan Sample\_11\_A1\_1\_3648.d:47359, m/z=593.6543, z=3, RT=16.18, ppm=-1.35):


TRINITY\_DN18187\_c0\_g1\_i3.p4

back to list

  

| Protein Coverage
| Supporting Peptides
| Best Unique PSM
|

Protein Coverage:

Supporting Peptides:

| Peptide | Uniq | -10lgP | Mass | Length | ppm | m/z | z | RT | 1/k0 Start | 1/k0 End | Precursor ID | Area Sample 2 | #Feature | #Feature Sample 2 | Start | End | PTM | AScore | Found By |
| --- | --- | --- | --- | --- | --- | --- | --- | --- | --- | --- | --- | --- | --- | --- | --- | --- | --- | --- | --- |
| R.YRPGTVALR.E | Y | 44.05 | 1031.5876 | 9 | -1.83 | 516.7973 | 2 | 12.44 | 0.8575 | 0.8648 | 27332 | 1.61e3 | 1 | 1 | 48 | 56 |  |  | DB Search |
| total 1 peptides |  |  |  |  |  |  |  |  |  |  |  |  |  |  |  |  |  |  |  |
| --- | --- | --- | --- | --- | --- | --- | --- | --- | --- | --- | --- | --- | --- | --- | --- | --- | --- | --- | --- |

Best Unique PSM (Scan Sample\_11\_A1\_1\_3648.d:27332, m/z=516.7973, z=2, RT=12.44, ppm=-7.31):


TRINITY\_DN5276\_c0\_g1\_i12.p2

back to list

  

| Protein Coverage
| Supporting Peptides
| Best Unique PSM
|

Protein Coverage:

Supporting Peptides:

| Peptide | Uniq | -10lgP | Mass | Length | ppm | m/z | z | RT | 1/k0 Start | 1/k0 End | Precursor ID | Area Sample 2 | #Feature | #Feature Sample 2 | Start | End | PTM | AScore | Found By |
| --- | --- | --- | --- | --- | --- | --- | --- | --- | --- | --- | --- | --- | --- | --- | --- | --- | --- | --- | --- |
| R.APGDEGWRPPLLATDDAELK.K | Y | 23.14 | 2150.0643 | 20 | -9.02 | 717.685 | 3 | 18.26 | 0.917 | 0.9243 | 58289 | 0 | 0 | 0 | 37 | 56 |  |  | DB Search |
| total 1 peptides |  |  |  |  |  |  |  |  |  |  |  |  |  |  |  |  |  |  |  |
| --- | --- | --- | --- | --- | --- | --- | --- | --- | --- | --- | --- | --- | --- | --- | --- | --- | --- | --- | --- |

Best Unique PSM (Scan Sample\_11\_A1\_1\_3648.d:58289, m/z=717.685, z=3, RT=18.26, ppm=-14.49):


TRINITY\_DN585\_c0\_g1\_i5.p1

back to list

  

| Protein Coverage
| Supporting Peptides
| Best Unique PSM
|

Protein Coverage:

Supporting Peptides:

| Peptide | Uniq | -10lgP | Mass | Length | ppm | m/z | z | RT | 1/k0 Start | 1/k0 End | Precursor ID | Area Sample 2 | #Feature | #Feature Sample 2 | Start | End | PTM | AScore | Found By |
| --- | --- | --- | --- | --- | --- | --- | --- | --- | --- | --- | --- | --- | --- | --- | --- | --- | --- | --- | --- |
| K.IQDKEGIPPDQQR.L | Y | 55.06 | 1522.7739 | 13 | 2.88 | 762.3923 | 2 | 11.00 | 1.0331 | 1.0403 | 18584 | 2.97e3 | 2 | 2 | 2 | 14 |  |  | DB Search |
| total 1 peptides |  |  |  |  |  |  |  |  |  |  |  |  |  |  |  |  |  |  |  |
| --- | --- | --- | --- | --- | --- | --- | --- | --- | --- | --- | --- | --- | --- | --- | --- | --- | --- | --- | --- |

Best Unique PSM (Scan Sample\_11\_A1\_1\_3648.d:18584, m/z=762.3923, z=2, RT=11.00, ppm=-2.59):


TRINITY\_DN40728\_c1\_g1\_i1.p2

back to list

  

| Protein Coverage
| Supporting Peptides
| Best Unique PSM
|

Protein Coverage:

Supporting Peptides:

| Peptide | Uniq | -10lgP | Mass | Length | ppm | m/z | z | RT | 1/k0 Start | 1/k0 End | Precursor ID | Area Sample 2 | #Feature | #Feature Sample 2 | Start | End | PTM | AScore | Found By |
| --- | --- | --- | --- | --- | --- | --- | --- | --- | --- | --- | --- | --- | --- | --- | --- | --- | --- | --- | --- |
| K.IQDKEGIPPDQQR.L | Y | 55.06 | 1522.7739 | 13 | 2.88 | 762.3923 | 2 | 11.00 | 1.0331 | 1.0403 | 18584 | 2.97e3 | 2 | 2 | 25 | 37 |  |  | DB Search |
| total 1 peptides |  |  |  |  |  |  |  |  |  |  |  |  |  |  |  |  |  |  |  |
| --- | --- | --- | --- | --- | --- | --- | --- | --- | --- | --- | --- | --- | --- | --- | --- | --- | --- | --- | --- |

Best Unique PSM (Scan Sample\_11\_A1\_1\_3648.d:18584, m/z=762.3923, z=2, RT=11.00, ppm=-2.59):


TRINITY\_DN141338\_c2\_g1\_i1.p1

back to list

  

| Protein Coverage
| Supporting Peptides
| Best Unique PSM
|

Protein Coverage:

Supporting Peptides:

| Peptide | Uniq | -10lgP | Mass | Length | ppm | m/z | z | RT | 1/k0 Start | 1/k0 End | Precursor ID | Area Sample 2 | #Feature | #Feature Sample 2 | Start | End | PTM | AScore | Found By |
| --- | --- | --- | --- | --- | --- | --- | --- | --- | --- | --- | --- | --- | --- | --- | --- | --- | --- | --- | --- |
| R.ELISNSSDALDKIR.Y | Y | 55.36 | 1559.8155 | 14 | 3.71 | 780.9136 | 2 | 15.32 | 1.0745 | 1.0817 | 43130 | 3.07e2 | 1 | 1 | 37 | 50 |  |  | DB Search |
| total 1 peptides |  |  |  |  |  |  |  |  |  |  |  |  |  |  |  |  |  |  |  |
| --- | --- | --- | --- | --- | --- | --- | --- | --- | --- | --- | --- | --- | --- | --- | --- | --- | --- | --- | --- |

Best Unique PSM (Scan Sample\_11\_A1\_1\_3648.d:43130, m/z=780.9136, z=2, RT=15.32, ppm=-1.76):


TRINITY\_DN89\_c0\_g1\_i1.p1

back to list

  

| Protein Coverage
| Supporting Peptides
| Best Unique PSM
|

Protein Coverage:

Supporting Peptides:

| Peptide | Uniq | -10lgP | Mass | Length | ppm | m/z | z | RT | 1/k0 Start | 1/k0 End | Precursor ID | Area Sample 2 | #Feature | #Feature Sample 2 | Start | End | PTM | AScore | Found By |
| --- | --- | --- | --- | --- | --- | --- | --- | --- | --- | --- | --- | --- | --- | --- | --- | --- | --- | --- | --- |
| K.TTC(+57.02)GVC(+57.02)NNNYC(+57.02)EDDPR.F | Y | 52.33 | 1973.7302 | 16 | -1.89 | 987.8651 | 2 | 12.26 | 1.127 | 1.1342 | 26302 | 0 | 0 | 0 | 328 | 343 | Carbamidomethylation, Carbamidomethylation, Carbamidomethylation |  | DB Search |
| total 1 peptides |  |  |  |  |  |  |  |  |  |  |  |  |  |  |  |  |  |  |  |
| --- | --- | --- | --- | --- | --- | --- | --- | --- | --- | --- | --- | --- | --- | --- | --- | --- | --- | --- | --- |

Best Unique PSM (Scan Sample\_11\_A1\_1\_3648.d:26302, m/z=987.8651, z=2, RT=12.26, ppm=-7.37):


TRINITY\_DN8101\_c0\_g1\_i9.p2

back to list

  

| Protein Coverage
| Supporting Peptides
| Best Unique PSM
|

Protein Coverage:

Supporting Peptides:

| Peptide | Uniq | -10lgP | Mass | Length | ppm | m/z | z | RT | 1/k0 Start | 1/k0 End | Precursor ID | Area Sample 2 | #Feature | #Feature Sample 2 | Start | End | PTM | AScore | Found By |
| --- | --- | --- | --- | --- | --- | --- | --- | --- | --- | --- | --- | --- | --- | --- | --- | --- | --- | --- | --- |
| R.SIGFIGHYLDQLR.L | Y | 54.32 | 1517.799 | 13 | 1.06 | 759.9034 | 2 | 19.56 | 1.0849 | 1.0921 | 65092 | 4.69e2 | 1 | 1 | 1077 | 1089 |  |  | DB Search |
| total 1 peptides |  |  |  |  |  |  |  |  |  |  |  |  |  |  |  |  |  |  |  |
| --- | --- | --- | --- | --- | --- | --- | --- | --- | --- | --- | --- | --- | --- | --- | --- | --- | --- | --- | --- |

Best Unique PSM (Scan Sample\_11\_A1\_1\_3648.d:65092, m/z=759.9034, z=2, RT=19.56, ppm=-4.41):


TRINITY\_DN2477\_c0\_g1\_i6.p1

back to list

  

| Protein Coverage
| Supporting Peptides
| Best Unique PSM
|

Protein Coverage:

Supporting Peptides:

| Peptide | Uniq | -10lgP | Mass | Length | ppm | m/z | z | RT | 1/k0 Start | 1/k0 End | Precursor ID | Area Sample 2 | #Feature | #Feature Sample 2 | Start | End | PTM | AScore | Found By |
| --- | --- | --- | --- | --- | --- | --- | --- | --- | --- | --- | --- | --- | --- | --- | --- | --- | --- | --- | --- |
| K.DSYVGDEAQSKR.G | Y | 59.21 | 1353.616 | 12 | 5.76 | 677.8155 | 2 | 10.93 | 1.0037 | 1.011 | 18642 | 9.97e3 | 1 | 1 | 52 | 63 |  |  | DB Search |
| R.VAPEEHPVLLTEAPLNPK.A | Y | 58.19 | 1953.0571 | 18 | -0.07 | 977.5304 | 2 | 16.19 | 1.2089 | 1.2161 | 47306 | 2.52e2 | 1 | 1 | 97 | 114 |  |  | DB Search |
| R.GYSFTTTAEREIVR.D | Y | 58.1 | 1628.8158 | 14 | 2.53 | 815.4128 | 2 | 15.79 | 1.121 | 1.1282 | 45442 | 6.41e3 | 1 | 1 | 198 | 211 |  |  | DB Search |
| total 3 peptides |  |  |  |  |  |  |  |  |  |  |  |  |  |  |  |  |  |  |  |
| --- | --- | --- | --- | --- | --- | --- | --- | --- | --- | --- | --- | --- | --- | --- | --- | --- | --- | --- | --- |

Best Unique PSM (Scan Sample\_11\_A1\_1\_3648.d:18642, m/z=677.8155, z=2, RT=10.93, ppm=0.28):


TRINITY\_DN8903\_c0\_g1\_i1.p1

back to list

  

| Protein Coverage
| Supporting Peptides
| Best Unique PSM
|

Protein Coverage:

Supporting Peptides:

| Peptide | Uniq | -10lgP | Mass | Length | ppm | m/z | z | RT | 1/k0 Start | 1/k0 End | Precursor ID | Area Sample 2 | #Feature | #Feature Sample 2 | Start | End | PTM | AScore | Found By |
| --- | --- | --- | --- | --- | --- | --- | --- | --- | --- | --- | --- | --- | --- | --- | --- | --- | --- | --- | --- |
| K.DLHIIEDYVLK.H | Y | 58.51 | 1356.7289 | 11 | 1.5 | 679.369 | 2 | 19.23 | 0.9932 | 1.0005 | 63393 | 3.95e2 | 1 | 1 | 417 | 427 |  |  | DB Search |
| total 1 peptides |  |  |  |  |  |  |  |  |  |  |  |  |  |  |  |  |  |  |  |
| --- | --- | --- | --- | --- | --- | --- | --- | --- | --- | --- | --- | --- | --- | --- | --- | --- | --- | --- | --- |

Best Unique PSM (Scan Sample\_11\_A1\_1\_3648.d:63393, m/z=679.369, z=2, RT=19.23, ppm=-3.98):


TRINITY\_DN649\_c0\_g1\_i5.p1

back to list

  

| Protein Coverage
| Supporting Peptides
| Best Unique PSM
|

Protein Coverage:

Supporting Peptides:

| Peptide | Uniq | -10lgP | Mass | Length | ppm | m/z | z | RT | 1/k0 Start | 1/k0 End | Precursor ID | Area Sample 2 | #Feature | #Feature Sample 2 | Start | End | PTM | AScore | Found By |
| --- | --- | --- | --- | --- | --- | --- | --- | --- | --- | --- | --- | --- | --- | --- | --- | --- | --- | --- | --- |
| K.FETQIATK.N | Y | 57.95 | 936.4916 | 8 | 1.49 | 937.4952 | 1 | 13.74 | 1.2208 | 1.228 | 34637 | 7.44e2 | 2 | 2 | 102 | 109 |  |  | DB Search |
| total 1 peptides |  |  |  |  |  |  |  |  |  |  |  |  |  |  |  |  |  |  |  |
| --- | --- | --- | --- | --- | --- | --- | --- | --- | --- | --- | --- | --- | --- | --- | --- | --- | --- | --- | --- |

Best Unique PSM (Scan Sample\_11\_A1\_1\_3648.d:34637, m/z=937.4952, z=1, RT=13.74, ppm=-3.98):


TRINITY\_DN47849\_c0\_g1\_i1.p1

back to list

  

| Protein Coverage
| Supporting Peptides
| Best Unique PSM
|

Protein Coverage:

Supporting Peptides:

| Peptide | Uniq | -10lgP | Mass | Length | ppm | m/z | z | RT | 1/k0 Start | 1/k0 End | Precursor ID | Area Sample 2 | #Feature | #Feature Sample 2 | Start | End | PTM | AScore | Found By |
| --- | --- | --- | --- | --- | --- | --- | --- | --- | --- | --- | --- | --- | --- | --- | --- | --- | --- | --- | --- |
| R.RYILETLVNGLKR.L | Y | 52.1 | 1573.9304 | 13 | 3.66 | 525.6498 | 3 | 18.46 | 0.8384 | 0.8457 | 59376 | 0 | 0 | 0 | 17 | 29 |  |  | DB Search |
| total 1 peptides |  |  |  |  |  |  |  |  |  |  |  |  |  |  |  |  |  |  |  |
| --- | --- | --- | --- | --- | --- | --- | --- | --- | --- | --- | --- | --- | --- | --- | --- | --- | --- | --- | --- |

Best Unique PSM (Scan Sample\_11\_A1\_1\_3648.d:59376, m/z=525.6498, z=3, RT=18.46, ppm=-1.82):


TRINITY\_DN902\_c1\_g2\_i1.p1

back to list

  

| Protein Coverage
| Supporting Peptides
| Best Unique PSM
|

Protein Coverage:

Supporting Peptides:

| Peptide | Uniq | -10lgP | Mass | Length | ppm | m/z | z | RT | 1/k0 Start | 1/k0 End | Precursor ID | Area Sample 2 | #Feature | #Feature Sample 2 | Start | End | PTM | AScore | Found By |
| --- | --- | --- | --- | --- | --- | --- | --- | --- | --- | --- | --- | --- | --- | --- | --- | --- | --- | --- | --- |
| K.GLTHC(+57.02)NTLSYK.L | Y | 58.83 | 1292.6183 | 11 | 4.02 | 647.3155 | 2 | 12.36 | 0.9695 | 0.9767 | 26924 | 0 | 0 | 0 | 62 | 72 | Carbamidomethylation |  | DB Search |
| total 1 peptides |  |  |  |  |  |  |  |  |  |  |  |  |  |  |  |  |  |  |  |
| --- | --- | --- | --- | --- | --- | --- | --- | --- | --- | --- | --- | --- | --- | --- | --- | --- | --- | --- | --- |

Best Unique PSM (Scan Sample\_11\_A1\_1\_3648.d:26924, m/z=647.3155, z=2, RT=12.36, ppm=-1.46):


TRINITY\_DN121866\_c0\_g1\_i1.p1

back to list

  

| Protein Coverage
| Supporting Peptides
| Best Unique PSM
|

Protein Coverage:

Supporting Peptides:

| Peptide | Uniq | -10lgP | Mass | Length | ppm | m/z | z | RT | 1/k0 Start | 1/k0 End | Precursor ID | Area Sample 2 | #Feature | #Feature Sample 2 | Start | End | PTM | AScore | Found By |
| --- | --- | --- | --- | --- | --- | --- | --- | --- | --- | --- | --- | --- | --- | --- | --- | --- | --- | --- | --- |
| K.FSQHTDYVIAR.A | Y | 58.61 | 1335.6571 | 11 | 2.7 | 668.834 | 2 | 13.36 | 0.9759 | 0.9832 | 32542 | 1.21e3 | 1 | 1 | 194 | 204 |  |  | DB Search |
| total 1 peptides |  |  |  |  |  |  |  |  |  |  |  |  |  |  |  |  |  |  |  |
| --- | --- | --- | --- | --- | --- | --- | --- | --- | --- | --- | --- | --- | --- | --- | --- | --- | --- | --- | --- |

Best Unique PSM (Scan Sample\_11\_A1\_1\_3648.d:32542, m/z=668.834, z=2, RT=13.36, ppm=-2.78):


TRINITY\_DN2113\_c0\_g1\_i2.p1

back to list

  

| Protein Coverage
| Supporting Peptides
| Best Unique PSM
|

Protein Coverage:

Supporting Peptides:

| Peptide | Uniq | -10lgP | Mass | Length | ppm | m/z | z | RT | 1/k0 Start | 1/k0 End | Precursor ID | Area Sample 2 | #Feature | #Feature Sample 2 | Start | End | PTM | AScore | Found By |
| --- | --- | --- | --- | --- | --- | --- | --- | --- | --- | --- | --- | --- | --- | --- | --- | --- | --- | --- | --- |
| R.ALEHYSDIYDIKR.A | Y | 58.43 | 1621.81 | 13 | -3.12 | 541.606 | 3 | 14.46 | 0.8238 | 0.8311 | 38607 | 5.17e2 | 1 | 1 | 626 | 638 |  |  | DB Search |
| K.KVGFEPDYVSLLR.N | Y | 49.91 | 1521.8191 | 13 | -1.44 | 761.9116 | 2 | 18.24 | 1.0435 | 1.0508 | 58056 | 8.15e2 | 1 | 1 | 507 | 519 |  |  | DB Search |
| total 2 peptides |  |  |  |  |  |  |  |  |  |  |  |  |  |  |  |  |  |  |  |
| --- | --- | --- | --- | --- | --- | --- | --- | --- | --- | --- | --- | --- | --- | --- | --- | --- | --- | --- | --- |

Best Unique PSM (Scan Sample\_11\_A1\_1\_3648.d:38607, m/z=541.606, z=3, RT=14.46, ppm=-8.6):


TRINITY\_DN1614\_c0\_g1\_i1.p1

back to list

  

| Protein Coverage
| Supporting Peptides
| Best Unique PSM
|

Protein Coverage:

Supporting Peptides:

| Peptide | Uniq | -10lgP | Mass | Length | ppm | m/z | z | RT | 1/k0 Start | 1/k0 End | Precursor ID | Area Sample 2 | #Feature | #Feature Sample 2 | Start | End | PTM | AScore | Found By |
| --- | --- | --- | --- | --- | --- | --- | --- | --- | --- | --- | --- | --- | --- | --- | --- | --- | --- | --- | --- |
| K.GLDVDSLVIDHIQVNR.A | Y | 58.87 | 1791.9479 | 16 | 3.22 | 896.9792 | 2 | 18.76 | 1.1794 | 1.1865 | 60798 | 1.04e3 | 1 | 1 | 140 | 155 |  |  | DB Search |
| K.KSAQFLLQLLK.N | Y | 58.85 | 1287.7914 | 11 | -0.17 | 644.8994 | 2 | 19.31 | 0.9775 | 0.9848 | 63852 | 4.01e2 | 1 | 1 | 120 | 130 |  |  | DB Search |
| K.AHGC(+57.02)SQGRWPK.K | Y | 56.78 | 1282.5989 | 11 | 7.43 | 428.5411 | 3 | 10.35 | 0.7591 | 0.7665 | 15181 | 5.1e2 | 1 | 1 | 109 | 119 | Carbamidomethylation |  | DB Search |
| total 3 peptides |  |  |  |  |  |  |  |  |  |  |  |  |  |  |  |  |  |  |  |
| --- | --- | --- | --- | --- | --- | --- | --- | --- | --- | --- | --- | --- | --- | --- | --- | --- | --- | --- | --- |

Best Unique PSM (Scan Sample\_11\_A1\_1\_3648.d:60798, m/z=896.9792, z=2, RT=18.76, ppm=-2.26):


TRINITY\_DN1910\_c0\_g1\_i1.p1

back to list

  

| Protein Coverage
| Supporting Peptides
| Best Unique PSM
|

Protein Coverage:

Supporting Peptides:

| Peptide | Uniq | -10lgP | Mass | Length | ppm | m/z | z | RT | 1/k0 Start | 1/k0 End | Precursor ID | Area Sample 2 | #Feature | #Feature Sample 2 | Start | End | PTM | AScore | Found By |
| --- | --- | --- | --- | --- | --- | --- | --- | --- | --- | --- | --- | --- | --- | --- | --- | --- | --- | --- | --- |
| K.NM(+15.99)M(+15.99)AAC(+57.02)DPR.H | Y | 58.21 | 1096.41 | 9 | 1.94 | 549.2103 | 2 | 9.53 | 0.8627 | 0.87 | 10857 | 1.37e3 | 1 | 1 | 298 | 306 | Oxidation (M), Oxidation (M), Carbamidomethylation | M2:Oxidation (M):1000 M3:Oxidation (M):1000 | DB Search |
| K.RISEQFTAM(+15.99)FR.R | Y | 39.68 | 1400.6871 | 11 | 1.59 | 701.3481 | 2 | 15.05 | 0.9848 | 0.992 | 41767 | 1.88e3 | 1 | 1 | 380 | 390 | Oxidation (M) | M9:Oxidation (M):1000 | DB Search |
| R.FPGQLNADLRK.L | Y | 25.64 | 1257.6829 | 11 | -1.3 | 629.8445 | 2 | 14.18 | 0.9457 | 0.9529 | 37015 | 6.11e2 | 1 | 1 | 242 | 252 |  |  | DB Search |
| total 3 peptides |  |  |  |  |  |  |  |  |  |  |  |  |  |  |  |  |  |  |  |
| --- | --- | --- | --- | --- | --- | --- | --- | --- | --- | --- | --- | --- | --- | --- | --- | --- | --- | --- | --- |

Best Unique PSM (Scan Sample\_11\_A1\_1\_3648.d:10857, m/z=549.2103, z=2, RT=9.53, ppm=-3.54):


TRINITY\_DN123\_c0\_g1\_i1.p1

back to list

  

| Protein Coverage
| Supporting Peptides
| Best Unique PSM
|

Protein Coverage:

Supporting Peptides:

| Peptide | Uniq | -10lgP | Mass | Length | ppm | m/z | z | RT | 1/k0 Start | 1/k0 End | Precursor ID | Area Sample 2 | #Feature | #Feature Sample 2 | Start | End | PTM | AScore | Found By |
| --- | --- | --- | --- | --- | --- | --- | --- | --- | --- | --- | --- | --- | --- | --- | --- | --- | --- | --- | --- |
| K.IAAVIINKPDVLASR.T | Y | 57.88 | 1578.9457 | 15 | -5.91 | 790.4711 | 2 | 15.76 | 1.0989 | 1.1061 | 45377 | 0 | 0 | 0 | 484 | 498 |  |  | DB Search |
| total 1 peptides |  |  |  |  |  |  |  |  |  |  |  |  |  |  |  |  |  |  |  |
| --- | --- | --- | --- | --- | --- | --- | --- | --- | --- | --- | --- | --- | --- | --- | --- | --- | --- | --- | --- |

Best Unique PSM (Scan Sample\_11\_A1\_1\_3648.d:45377, m/z=790.4711, z=2, RT=15.76, ppm=-11.38):


TRINITY\_DN1744\_c0\_g1\_i2.p1

back to list

  

| Protein Coverage
| Supporting Peptides
| Best Unique PSM
|

Protein Coverage:

Supporting Peptides:

| Peptide | Uniq | -10lgP | Mass | Length | ppm | m/z | z | RT | 1/k0 Start | 1/k0 End | Precursor ID | Area Sample 2 | #Feature | #Feature Sample 2 | Start | End | PTM | AScore | Found By |
| --- | --- | --- | --- | --- | --- | --- | --- | --- | --- | --- | --- | --- | --- | --- | --- | --- | --- | --- | --- |
| R.ALFFKEIENIR.S | Y | 39.5 | 1378.7608 | 11 | 6.8 | 690.3886 | 2 | 18.36 | 1.0279 | 1.0351 | 58836 | 0 | 0 | 0 | 86 | 96 |  |  | DB Search |
| total 1 peptides |  |  |  |  |  |  |  |  |  |  |  |  |  |  |  |  |  |  |  |
| --- | --- | --- | --- | --- | --- | --- | --- | --- | --- | --- | --- | --- | --- | --- | --- | --- | --- | --- | --- |

Best Unique PSM (Scan Sample\_11\_A1\_1\_3648.d:58836, m/z=689.8637, z=2, RT=18.38, ppm=1.32):


TRINITY\_DN167010\_c0\_g1\_i1.p1

back to list

  

| Protein Coverage
| Supporting Peptides
| Best Unique PSM
|

Protein Coverage:

Supporting Peptides:

| Peptide | Uniq | -10lgP | Mass | Length | ppm | m/z | z | RT | 1/k0 Start | 1/k0 End | Precursor ID | Area Sample 2 | #Feature | #Feature Sample 2 | Start | End | PTM | AScore | Found By |
| --- | --- | --- | --- | --- | --- | --- | --- | --- | --- | --- | --- | --- | --- | --- | --- | --- | --- | --- | --- |
| R.IC(+57.02)RM(+15.99)LK.H | Y | 21.14 | 835.4408 | 6 | -1.07 | 418.7249 | 2 | 10.37 | 0.7884 | 0.7958 | 15283 | 1.3e3 | 1 | 1 | 86 | 91 | Carbamidomethylation, Oxidation (M) | M4:Oxidation (M):1000 | DB Search |
| total 1 peptides |  |  |  |  |  |  |  |  |  |  |  |  |  |  |  |  |  |  |  |
| --- | --- | --- | --- | --- | --- | --- | --- | --- | --- | --- | --- | --- | --- | --- | --- | --- | --- | --- | --- |

Best Unique PSM (Scan Sample\_11\_A1\_1\_3648.d:15283, m/z=418.7249, z=2, RT=10.37, ppm=-6.55):


TRINITY\_DN36\_c0\_g1\_i16.p1

back to list

  

| Protein Coverage
| Supporting Peptides
| Best Unique PSM
|

Protein Coverage:

Supporting Peptides:

| Peptide | Uniq | -10lgP | Mass | Length | ppm | m/z | z | RT | 1/k0 Start | 1/k0 End | Precursor ID | Area Sample 2 | #Feature | #Feature Sample 2 | Start | End | PTM | AScore | Found By |
| --- | --- | --- | --- | --- | --- | --- | --- | --- | --- | --- | --- | --- | --- | --- | --- | --- | --- | --- | --- |
| R.LGGIEVTQGNC(+57.02)ARK.Y | Y | 58.27 | 1501.7671 | 14 | 1.48 | 751.8878 | 2 | 12.59 | 1.0616 | 1.0688 | 28229 | 7.18e2 | 1 | 1 | 58 | 71 | Carbamidomethylation |  | DB Search |
| K.FETQIATK.D | Y | 57.95 | 936.4916 | 8 | 1.49 | 937.4952 | 1 | 13.74 | 1.2208 | 1.228 | 34637 | 7.44e2 | 2 | 2 | 167 | 174 |  |  | DB Search |
| total 2 peptides |  |  |  |  |  |  |  |  |  |  |  |  |  |  |  |  |  |  |  |
| --- | --- | --- | --- | --- | --- | --- | --- | --- | --- | --- | --- | --- | --- | --- | --- | --- | --- | --- | --- |

Best Unique PSM (Scan Sample\_11\_A1\_1\_3648.d:28229, m/z=751.8878, z=2, RT=12.59, ppm=-4):


TRINITY\_DN106952\_c0\_g1\_i1.p1

back to list

  

| Protein Coverage
| Supporting Peptides
| Best Unique PSM
|

Protein Coverage:

Supporting Peptides:

| Peptide | Uniq | -10lgP | Mass | Length | ppm | m/z | z | RT | 1/k0 Start | 1/k0 End | Precursor ID | Area Sample 2 | #Feature | #Feature Sample 2 | Start | End | PTM | AScore | Found By |
| --- | --- | --- | --- | --- | --- | --- | --- | --- | --- | --- | --- | --- | --- | --- | --- | --- | --- | --- | --- |
| K.AM(+15.99)SIM(+15.99)NSFVNDIFER.I | Y | 58.87 | 1804.8124 | 15 | 0.11 | 903.4086 | 2 | 19.92 | 1.0508 | 1.058 | 66700 | 2.51e4 | 4 | 4 | 68 | 82 | Oxidation (M), Oxidation (M) | M2:Oxidation (M):1000 M5:Oxidation (M):1000 | DB Search |
| total 1 peptides |  |  |  |  |  |  |  |  |  |  |  |  |  |  |  |  |  |  |  |
| --- | --- | --- | --- | --- | --- | --- | --- | --- | --- | --- | --- | --- | --- | --- | --- | --- | --- | --- | --- |

Best Unique PSM (Scan Sample\_11\_A1\_1\_3648.d:66700, m/z=903.4086, z=2, RT=19.92, ppm=-5.36):


TRINITY\_DN286\_c0\_g1\_i4.p1

back to list

  

| Protein Coverage
| Supporting Peptides
| Best Unique PSM
|

Protein Coverage:

Supporting Peptides:

| Peptide | Uniq | -10lgP | Mass | Length | ppm | m/z | z | RT | 1/k0 Start | 1/k0 End | Precursor ID | Area Sample 2 | #Feature | #Feature Sample 2 | Start | End | PTM | AScore | Found By |
| --- | --- | --- | --- | --- | --- | --- | --- | --- | --- | --- | --- | --- | --- | --- | --- | --- | --- | --- | --- |
| K.RYGQAMC(+57.02)QFMR.N | Y | 58.88 | 1446.6319 | 11 | -6.73 | 724.3144 | 2 | 17.00 | 0.9836 | 0.9908 | 51341 | 4.96e3 | 1 | 1 | 71 | 81 | Carbamidomethylation |  | DB Search |
| K.RYGQAM(+15.99)C(+57.02)QFMR.N | Y | 56 | 1462.6268 | 11 | -6.37 | 732.312 | 2 | 14.39 | 0.9755 | 0.9828 | 37937 | 1.17e3 | 1 | 1 | 71 | 81 | Oxidation (M), Carbamidomethylation | M6:Oxidation (M):38.03 | DB Search |
| K.RYGQAM(+15.99)C(+57.02)QFM(+15.99)R.N | Y | 55.34 | 1478.6217 | 11 | -6.44 | 740.3093 | 2 | 12.85 | 0.9739 | 0.9812 | 29490 | 4.93e3 | 1 | 1 | 71 | 81 | Oxidation (M), Carbamidomethylation, Oxidation (M) | M6:Oxidation (M):1000 M10:Oxidation (M):1000 | DB Search |
| total 3 peptides |  |  |  |  |  |  |  |  |  |  |  |  |  |  |  |  |  |  |  |
| --- | --- | --- | --- | --- | --- | --- | --- | --- | --- | --- | --- | --- | --- | --- | --- | --- | --- | --- | --- |

Best Unique PSM (Scan Sample\_11\_A1\_1\_3648.d:51341, m/z=724.3144, z=2, RT=17.00, ppm=-12.2):


TRINITY\_DN135129\_c0\_g1\_i1.p1

back to list

  

| Protein Coverage
| Supporting Peptides
| Best Unique PSM
|

Protein Coverage:

Supporting Peptides:

| Peptide | Uniq | -10lgP | Mass | Length | ppm | m/z | z | RT | 1/k0 Start | 1/k0 End | Precursor ID | Area Sample 2 | #Feature | #Feature Sample 2 | Start | End | PTM | AScore | Found By |
| --- | --- | --- | --- | --- | --- | --- | --- | --- | --- | --- | --- | --- | --- | --- | --- | --- | --- | --- | --- |
| K.SDSSDIEKR.A | Y | 58.12 | 1035.4832 | 9 | -14.56 | 518.7385 | 2 | 11.06 | 0.8567 | 0.864 | 19468 | 4.49e3 | 1 | 1 | 96 | 104 |  |  | DB Search |
| total 1 peptides |  |  |  |  |  |  |  |  |  |  |  |  |  |  |  |  |  |  |  |
| --- | --- | --- | --- | --- | --- | --- | --- | --- | --- | --- | --- | --- | --- | --- | --- | --- | --- | --- | --- |

Best Unique PSM (Scan Sample\_11\_A1\_1\_3648.d:19468, m/z=518.7385, z=2, RT=11.06, ppm=-20.04):


TRINITY\_DN265\_c0\_g1\_i6.p1

back to list

  

| Protein Coverage
| Supporting Peptides
| Best Unique PSM
|

Protein Coverage:

Supporting Peptides:

| Peptide | Uniq | -10lgP | Mass | Length | ppm | m/z | z | RT | 1/k0 Start | 1/k0 End | Precursor ID | Area Sample 2 | #Feature | #Feature Sample 2 | Start | End | PTM | AScore | Found By |
| --- | --- | --- | --- | --- | --- | --- | --- | --- | --- | --- | --- | --- | --- | --- | --- | --- | --- | --- | --- |
| R.LTHDFHTNKR.I | Y | 58.75 | 1267.6421 | 10 | 2.44 | 423.5534 | 3 | 9.64 | 0.7441 | 0.7514 | 11479 | 1.15e3 | 1 | 1 | 24 | 33 |  |  | DB Search |
| total 1 peptides |  |  |  |  |  |  |  |  |  |  |  |  |  |  |  |  |  |  |  |
| --- | --- | --- | --- | --- | --- | --- | --- | --- | --- | --- | --- | --- | --- | --- | --- | --- | --- | --- | --- |

Best Unique PSM (Scan Sample\_11\_A1\_1\_3648.d:11479, m/z=423.5534, z=3, RT=9.64, ppm=-3.04):


TRINITY\_DN7314\_c0\_g2\_i1.p1

back to list

  

| Protein Coverage
| Supporting Peptides
| Best Unique PSM
|

Protein Coverage:

Supporting Peptides:

| Peptide | Uniq | -10lgP | Mass | Length | ppm | m/z | z | RT | 1/k0 Start | 1/k0 End | Precursor ID | Area Sample 2 | #Feature | #Feature Sample 2 | Start | End | PTM | AScore | Found By |
| --- | --- | --- | --- | --- | --- | --- | --- | --- | --- | --- | --- | --- | --- | --- | --- | --- | --- | --- | --- |
| K.YVESAADKR.L | Y | 58.39 | 1037.5141 | 9 | 8.99 | 519.7662 | 2 | 9.59 | 0.8575 | 0.8648 | 11233 | 0 | 0 | 0 | 10 | 18 |  |  | DB Search |
| total 1 peptides |  |  |  |  |  |  |  |  |  |  |  |  |  |  |  |  |  |  |  |
| --- | --- | --- | --- | --- | --- | --- | --- | --- | --- | --- | --- | --- | --- | --- | --- | --- | --- | --- | --- |

Best Unique PSM (Scan Sample\_11\_A1\_1\_3648.d:11233, m/z=519.7662, z=2, RT=9.59, ppm=3.52):


TRINITY\_DN982\_c0\_g1\_i3.p1

back to list

  

| Protein Coverage
| Supporting Peptides
| Best Unique PSM
|

Protein Coverage:

Supporting Peptides:

| Peptide | Uniq | -10lgP | Mass | Length | ppm | m/z | z | RT | 1/k0 Start | 1/k0 End | Precursor ID | Area Sample 2 | #Feature | #Feature Sample 2 | Start | End | PTM | AScore | Found By |
| --- | --- | --- | --- | --- | --- | --- | --- | --- | --- | --- | --- | --- | --- | --- | --- | --- | --- | --- | --- |
| R.LVQNTPHNSIVTK.I | N | 58.89 | 1449.794 | 13 | 3.18 | 725.9026 | 2 | 11.65 | 1.0628 | 1.0701 | 22758 | 1.18e3 | 1 | 1 | 8569 | 8581 |  |  | DB Search |
| R.VLATDKDTLSSNK.Q | Y | 58.86 | 1390.7303 | 13 | 0.63 | 696.3691 | 2 | 11.49 | 1.015 | 1.0222 | 21855 | 2.04e3 | 1 | 1 | 3827 | 3839 |  |  | DB Search |
| K.SAIIVDGKNEEGR.I | Y | 58.54 | 1386.7103 | 13 | 3.91 | 694.3613 | 2 | 11.82 | 0.9961 | 1.0033 | 23812 | 1.85e3 | 1 | 1 | 95 | 107 |  |  | DB Search |
| K.VLLSQQIFYDKK.N | N | 26.23 | 1480.8289 | 12 | -0.12 | 741.4176 | 2 | 16.48 | 1.0608 | 1.068 | 48785 | 6.44e2 | 1 | 1 | 4919 | 4930 |  |  | DB Search |
| total 4 peptides |  |  |  |  |  |  |  |  |  |  |  |  |  |  |  |  |  |  |  |
| --- | --- | --- | --- | --- | --- | --- | --- | --- | --- | --- | --- | --- | --- | --- | --- | --- | --- | --- | --- |

Best Unique PSM (Scan Sample\_11\_A1\_1\_3648.d:21855, m/z=696.3691, z=2, RT=11.49, ppm=-4.85):


TRINITY\_DN332\_c0\_g1\_i1.p1

back to list

  

| Protein Coverage
| Supporting Peptides
| Best Unique PSM
|

Protein Coverage:

Supporting Peptides:

| Peptide | Uniq | -10lgP | Mass | Length | ppm | m/z | z | RT | 1/k0 Start | 1/k0 End | Precursor ID | Area Sample 2 | #Feature | #Feature Sample 2 | Start | End | PTM | AScore | Found By |
| --- | --- | --- | --- | --- | --- | --- | --- | --- | --- | --- | --- | --- | --- | --- | --- | --- | --- | --- | --- |
| K.DYGVLLEDAGHTLR.G | Y | 58.88 | 1557.7787 | 14 | -3.16 | 779.8899 | 2 | 18.01 | 1.1286 | 1.1358 | 56870 | 4.46e2 | 1 | 1 | 158 | 171 |  |  | DB Search |
| R.GLFIIDDKGVLR.Q | Y | 58.59 | 1344.7765 | 12 | 1.55 | 673.3929 | 2 | 18.40 | 1.0009 | 1.0081 | 58995 | 0 | 0 | 0 | 172 | 183 |  |  | DB Search |
| total 2 peptides |  |  |  |  |  |  |  |  |  |  |  |  |  |  |  |  |  |  |  |
| --- | --- | --- | --- | --- | --- | --- | --- | --- | --- | --- | --- | --- | --- | --- | --- | --- | --- | --- | --- |

Best Unique PSM (Scan Sample\_11\_A1\_1\_3648.d:56870, m/z=779.8899, z=2, RT=18.01, ppm=-8.63):


TRINITY\_DN160\_c0\_g1\_i1.p1

back to list

  

| Protein Coverage
| Supporting Peptides
| Best Unique PSM
|

Protein Coverage:

Supporting Peptides:

| Peptide | Uniq | -10lgP | Mass | Length | ppm | m/z | z | RT | 1/k0 Start | 1/k0 End | Precursor ID | Area Sample 2 | #Feature | #Feature Sample 2 | Start | End | PTM | AScore | Found By |
| --- | --- | --- | --- | --- | --- | --- | --- | --- | --- | --- | --- | --- | --- | --- | --- | --- | --- | --- | --- |
| K.LRLDFDHEAKLNMIFR.F | Y | 49.33 | 2017.0567 | 16 | 6.27 | 505.2719 | 4 | 18.22 | 0.8627 | 0.87 | 58067 | 0 | 0 | 0 | 1007 | 1022 |  |  | DB Search |
| total 1 peptides |  |  |  |  |  |  |  |  |  |  |  |  |  |  |  |  |  |  |  |
| --- | --- | --- | --- | --- | --- | --- | --- | --- | --- | --- | --- | --- | --- | --- | --- | --- | --- | --- | --- |

Best Unique PSM (Scan Sample\_11\_A1\_1\_3648.d:58067, m/z=505.2719, z=4, RT=18.22, ppm=0.79):


TRINITY\_DN1534\_c2\_g1\_i1.p1

back to list

  

| Protein Coverage
| Supporting Peptides
| Best Unique PSM
|

Protein Coverage:

Supporting Peptides:

| Peptide | Uniq | -10lgP | Mass | Length | ppm | m/z | z | RT | 1/k0 Start | 1/k0 End | Precursor ID | Area Sample 2 | #Feature | #Feature Sample 2 | Start | End | PTM | AScore | Found By |
| --- | --- | --- | --- | --- | --- | --- | --- | --- | --- | --- | --- | --- | --- | --- | --- | --- | --- | --- | --- |
| K.TFIVQGFGNVGLHSC(+57.02)R.Y | Y | 58.86 | 1790.8886 | 16 | 4.75 | 896.4509 | 2 | 17.62 | 1.2236 | 1.2308 | 54796 | 1.16e2 | 1 | 1 | 294 | 309 | Carbamidomethylation |  | DB Search |
| total 1 peptides |  |  |  |  |  |  |  |  |  |  |  |  |  |  |  |  |  |  |  |
| --- | --- | --- | --- | --- | --- | --- | --- | --- | --- | --- | --- | --- | --- | --- | --- | --- | --- | --- | --- |

Best Unique PSM (Scan Sample\_11\_A1\_1\_3648.d:54796, m/z=896.4509, z=2, RT=17.62, ppm=-0.72):


TRINITY\_DN453\_c0\_g1\_i2.p1

back to list

  

| Protein Coverage
| Supporting Peptides
| Best Unique PSM
|

Protein Coverage:

Supporting Peptides:

| Peptide | Uniq | -10lgP | Mass | Length | ppm | m/z | z | RT | 1/k0 Start | 1/k0 End | Precursor ID | Area Sample 2 | #Feature | #Feature Sample 2 | Start | End | PTM | AScore | Found By |
| --- | --- | --- | --- | --- | --- | --- | --- | --- | --- | --- | --- | --- | --- | --- | --- | --- | --- | --- | --- |
| K.SAATQTSIKENK.S | Y | 58.38 | 1276.6623 | 12 | -2.61 | 639.3333 | 2 | 9.66 | 0.9646 | 0.9719 | 11591 | 0 | 0 | 0 | 69 | 80 |  |  | DB Search |
| total 1 peptides |  |  |  |  |  |  |  |  |  |  |  |  |  |  |  |  |  |  |  |
| --- | --- | --- | --- | --- | --- | --- | --- | --- | --- | --- | --- | --- | --- | --- | --- | --- | --- | --- | --- |

Best Unique PSM (Scan Sample\_11\_A1\_1\_3648.d:11591, m/z=639.3333, z=2, RT=9.66, ppm=-8.09):


TRINITY\_DN79126\_c0\_g1\_i1.p1

back to list

  

| Protein Coverage
| Supporting Peptides
| Best Unique PSM
|

Protein Coverage:

Supporting Peptides:

| Peptide | Uniq | -10lgP | Mass | Length | ppm | m/z | z | RT | 1/k0 Start | 1/k0 End | Precursor ID | Area Sample 2 | #Feature | #Feature Sample 2 | Start | End | PTM | AScore | Found By |
| --- | --- | --- | --- | --- | --- | --- | --- | --- | --- | --- | --- | --- | --- | --- | --- | --- | --- | --- | --- |
| R.AFLIEEQKLVVR.V | Y | 58.34 | 1443.8449 | 12 | 1.95 | 722.9272 | 2 | 17.10 | 1.0492 | 1.0564 | 51842 | 4.02e3 | 1 | 1 | 92 | 103 |  |  | DB Search |
| total 1 peptides |  |  |  |  |  |  |  |  |  |  |  |  |  |  |  |  |  |  |  |
| --- | --- | --- | --- | --- | --- | --- | --- | --- | --- | --- | --- | --- | --- | --- | --- | --- | --- | --- | --- |

Best Unique PSM (Scan Sample\_11\_A1\_1\_3648.d:51842, m/z=722.9272, z=2, RT=17.10, ppm=-3.53):


TRINITY\_DN1616\_c0\_g1\_i3.p1

back to list

  

| Protein Coverage
| Supporting Peptides
| Best Unique PSM
|

Protein Coverage:

Supporting Peptides:

| Peptide | Uniq | -10lgP | Mass | Length | ppm | m/z | z | RT | 1/k0 Start | 1/k0 End | Precursor ID | Area Sample 2 | #Feature | #Feature Sample 2 | Start | End | PTM | AScore | Found By |
| --- | --- | --- | --- | --- | --- | --- | --- | --- | --- | --- | --- | --- | --- | --- | --- | --- | --- | --- | --- |
| K.YVVMLQYYHDSDTAK.Y | Y | 58.58 | 1831.8451 | 15 | 5.67 | 916.93 | 2 | 16.32 | 1.1813 | 1.1885 | 48049 | 0 | 0 | 0 | 1071 | 1085 |  |  | DB Search |
| K.VVAEC(+57.02)NNGGGR.F | Y | 57.96 | 1131.5091 | 11 | -8.94 | 566.7537 | 2 | 9.41 | 0.8234 | 0.8307 | 10484 | 0 | 0 | 0 | 3736 | 3746 | Carbamidomethylation |  | DB Search |
| total 2 peptides |  |  |  |  |  |  |  |  |  |  |  |  |  |  |  |  |  |  |  |
| --- | --- | --- | --- | --- | --- | --- | --- | --- | --- | --- | --- | --- | --- | --- | --- | --- | --- | --- | --- |

Best Unique PSM (Scan Sample\_11\_A1\_1\_3648.d:48049, m/z=916.93, z=2, RT=16.32, ppm=0.2):


TRINITY\_DN1319\_c0\_g1\_i1.p1

back to list

  

| Protein Coverage
| Supporting Peptides
| Best Unique PSM
|

Protein Coverage:

Supporting Peptides:

| Peptide | Uniq | -10lgP | Mass | Length | ppm | m/z | z | RT | 1/k0 Start | 1/k0 End | Precursor ID | Area Sample 2 | #Feature | #Feature Sample 2 | Start | End | PTM | AScore | Found By |
| --- | --- | --- | --- | --- | --- | --- | --- | --- | --- | --- | --- | --- | --- | --- | --- | --- | --- | --- | --- |
| K.GTHAAEIAHC(+57.02)VSR.R | Y | 59.14 | 1407.6677 | 13 | 2.78 | 704.8392 | 2 | 11.02 | 1.0263 | 1.0335 | 19137 | 2.25e3 | 1 | 1 | 91 | 103 | Carbamidomethylation |  | DB Search |
| total 1 peptides |  |  |  |  |  |  |  |  |  |  |  |  |  |  |  |  |  |  |  |
| --- | --- | --- | --- | --- | --- | --- | --- | --- | --- | --- | --- | --- | --- | --- | --- | --- | --- | --- | --- |

Best Unique PSM (Scan Sample\_11\_A1\_1\_3648.d:19137, m/z=704.8392, z=2, RT=11.02, ppm=-2.69):


TRINITY\_DN1378\_c0\_g1\_i1.p1

back to list

  

| Protein Coverage
| Supporting Peptides
| Best Unique PSM
|

Protein Coverage:

Supporting Peptides:

| Peptide | Uniq | -10lgP | Mass | Length | ppm | m/z | z | RT | 1/k0 Start | 1/k0 End | Precursor ID | Area Sample 2 | #Feature | #Feature Sample 2 | Start | End | PTM | AScore | Found By |
| --- | --- | --- | --- | --- | --- | --- | --- | --- | --- | --- | --- | --- | --- | --- | --- | --- | --- | --- | --- |
| K.LAALDATVHGQTASR.F | Y | 58.87 | 1509.7899 | 15 | 1.9 | 755.8995 | 2 | 13.35 | 1.0713 | 1.0785 | 32461 | 7.36e2 | 1 | 1 | 213 | 227 |  |  | DB Search |
| total 1 peptides |  |  |  |  |  |  |  |  |  |  |  |  |  |  |  |  |  |  |  |
| --- | --- | --- | --- | --- | --- | --- | --- | --- | --- | --- | --- | --- | --- | --- | --- | --- | --- | --- | --- |

Best Unique PSM (Scan Sample\_11\_A1\_1\_3648.d:32461, m/z=755.8995, z=2, RT=13.35, ppm=-3.58):


TRINITY\_DN319\_c2\_g1\_i3.p1

back to list

  

| Protein Coverage
| Supporting Peptides
| Best Unique PSM
|

Protein Coverage:

Supporting Peptides:

| Peptide | Uniq | -10lgP | Mass | Length | ppm | m/z | z | RT | 1/k0 Start | 1/k0 End | Precursor ID | Area Sample 2 | #Feature | #Feature Sample 2 | Start | End | PTM | AScore | Found By |
| --- | --- | --- | --- | --- | --- | --- | --- | --- | --- | --- | --- | --- | --- | --- | --- | --- | --- | --- | --- |
| K.AQYYEAYETIIKR.H | Y | 58.88 | 1646.8304 | 13 | 5.56 | 824.4225 | 2 | 15.51 | 1.1025 | 1.1098 | 44074 | 4.92e2 | 1 | 1 | 2535 | 2547 |  |  | DB Search |
| K.KAQYYEAYETIIKR.H | Y | 58.38 | 1774.9254 | 14 | -0.12 | 592.6458 | 3 | 15.51 | 0.8644 | 0.8716 | 44111 | 1.84e3 | 2 | 2 | 2534 | 2547 |  |  | DB Search |
| total 2 peptides |  |  |  |  |  |  |  |  |  |  |  |  |  |  |  |  |  |  |  |
| --- | --- | --- | --- | --- | --- | --- | --- | --- | --- | --- | --- | --- | --- | --- | --- | --- | --- | --- | --- |

Best Unique PSM (Scan Sample\_11\_A1\_1\_3648.d:44074, m/z=822.9161, z=2, RT=15.56, ppm=0.09):


TRINITY\_DN691\_c0\_g1\_i2.p1

back to list

  

| Protein Coverage
| Supporting Peptides
| Best Unique PSM
|

Protein Coverage:

Supporting Peptides:

| Peptide | Uniq | -10lgP | Mass | Length | ppm | m/z | z | RT | 1/k0 Start | 1/k0 End | Precursor ID | Area Sample 2 | #Feature | #Feature Sample 2 | Start | End | PTM | AScore | Found By |
| --- | --- | --- | --- | --- | --- | --- | --- | --- | --- | --- | --- | --- | --- | --- | --- | --- | --- | --- | --- |
| R.AYGQDLGQPGSC(+57.02)LKR.F | Y | 57.94 | 1648.7991 | 15 | 3.8 | 825.4055 | 2 | 13.30 | 1.0721 | 1.0793 | 32177 | 9.59e2 | 1 | 1 | 1480 | 1494 | Carbamidomethylation |  | DB Search |
| total 1 peptides |  |  |  |  |  |  |  |  |  |  |  |  |  |  |  |  |  |  |  |
| --- | --- | --- | --- | --- | --- | --- | --- | --- | --- | --- | --- | --- | --- | --- | --- | --- | --- | --- | --- |

Best Unique PSM (Scan Sample\_11\_A1\_1\_3648.d:32177, m/z=825.4055, z=2, RT=13.30, ppm=-1.67):


TRINITY\_DN8707\_c0\_g1\_i2.p1

back to list

  

| Protein Coverage
| Supporting Peptides
| Best Unique PSM
|

Protein Coverage:

Supporting Peptides:

| Peptide | Uniq | -10lgP | Mass | Length | ppm | m/z | z | RT | 1/k0 Start | 1/k0 End | Precursor ID | Area Sample 2 | #Feature | #Feature Sample 2 | Start | End | PTM | AScore | Found By |
| --- | --- | --- | --- | --- | --- | --- | --- | --- | --- | --- | --- | --- | --- | --- | --- | --- | --- | --- | --- |
| K.ALLSLEYHEKR.C | Y | 58.7 | 1357.7354 | 11 | 1.49 | 679.8723 | 2 | 14.29 | 1.0271 | 1.0343 | 37627 | 2.25e3 | 1 | 1 | 124 | 134 |  |  | DB Search |
| total 1 peptides |  |  |  |  |  |  |  |  |  |  |  |  |  |  |  |  |  |  |  |
| --- | --- | --- | --- | --- | --- | --- | --- | --- | --- | --- | --- | --- | --- | --- | --- | --- | --- | --- | --- |

Best Unique PSM (Scan Sample\_11\_A1\_1\_3648.d:37627, m/z=679.8723, z=2, RT=14.29, ppm=-3.99):


TRINITY\_DN436\_c0\_g1\_i1.p1

back to list

  

| Protein Coverage
| Supporting Peptides
| Best Unique PSM
|

Protein Coverage:

Supporting Peptides:

| Peptide | Uniq | -10lgP | Mass | Length | ppm | m/z | z | RT | 1/k0 Start | 1/k0 End | Precursor ID | Area Sample 2 | #Feature | #Feature Sample 2 | Start | End | PTM | AScore | Found By |
| --- | --- | --- | --- | --- | --- | --- | --- | --- | --- | --- | --- | --- | --- | --- | --- | --- | --- | --- | --- |
| K.RLPEEDQYLR.I | Y | 58.24 | 1317.6677 | 10 | 3.82 | 659.84 | 2 | 13.95 | 0.9711 | 0.9783 | 35764 | 3.56e3 | 1 | 1 | 54 | 63 |  |  | DB Search |
| total 1 peptides |  |  |  |  |  |  |  |  |  |  |  |  |  |  |  |  |  |  |  |
| --- | --- | --- | --- | --- | --- | --- | --- | --- | --- | --- | --- | --- | --- | --- | --- | --- | --- | --- | --- |

Best Unique PSM (Scan Sample\_11\_A1\_1\_3648.d:35764, m/z=659.84, z=2, RT=13.95, ppm=-1.66):


TRINITY\_DN1084\_c0\_g1\_i1.p1

back to list

  

| Protein Coverage
| Supporting Peptides
| Best Unique PSM
|

Protein Coverage:

Supporting Peptides:

| Peptide | Uniq | -10lgP | Mass | Length | ppm | m/z | z | RT | 1/k0 Start | 1/k0 End | Precursor ID | Area Sample 2 | #Feature | #Feature Sample 2 | Start | End | PTM | AScore | Found By |
| --- | --- | --- | --- | --- | --- | --- | --- | --- | --- | --- | --- | --- | --- | --- | --- | --- | --- | --- | --- |
| R.IVAINAHHFLK.N | Y | 58.09 | 1261.7295 | 11 | 2.94 | 631.8704 | 2 | 14.34 | 1.0166 | 1.0238 | 37934 | 0 | 0 | 0 | 224 | 234 |  |  | DB Search |
| total 1 peptides |  |  |  |  |  |  |  |  |  |  |  |  |  |  |  |  |  |  |  |
| --- | --- | --- | --- | --- | --- | --- | --- | --- | --- | --- | --- | --- | --- | --- | --- | --- | --- | --- | --- |

Best Unique PSM (Scan Sample\_11\_A1\_1\_3648.d:37934, m/z=631.8704, z=2, RT=14.34, ppm=-2.53):


TRINITY\_DN147947\_c0\_g1\_i1.p1

back to list

  

| Protein Coverage
| Supporting Peptides
| Best Unique PSM
|

Protein Coverage:

Supporting Peptides:

| Peptide | Uniq | -10lgP | Mass | Length | ppm | m/z | z | RT | 1/k0 Start | 1/k0 End | Precursor ID | Area Sample 2 | #Feature | #Feature Sample 2 | Start | End | PTM | AScore | Found By |
| --- | --- | --- | --- | --- | --- | --- | --- | --- | --- | --- | --- | --- | --- | --- | --- | --- | --- | --- | --- |
| R.SSFDEIHKFHTQILR.V | Y | 57.72 | 1856.9533 | 15 | 0.93 | 619.9889 | 3 | 15.24 | 0.8708 | 0.8781 | 42768 | 0 | 0 | 0 | 75 | 89 |  |  | DB Search |
| total 1 peptides |  |  |  |  |  |  |  |  |  |  |  |  |  |  |  |  |  |  |  |
| --- | --- | --- | --- | --- | --- | --- | --- | --- | --- | --- | --- | --- | --- | --- | --- | --- | --- | --- | --- |

Best Unique PSM (Scan Sample\_11\_A1\_1\_3648.d:42768, m/z=619.9889, z=3, RT=15.24, ppm=-4.55):


TRINITY\_DN687\_c0\_g1\_i1.p1

back to list

  

| Protein Coverage
| Supporting Peptides
| Best Unique PSM
|

Protein Coverage:

Supporting Peptides:

| Peptide | Uniq | -10lgP | Mass | Length | ppm | m/z | z | RT | 1/k0 Start | 1/k0 End | Precursor ID | Area Sample 2 | #Feature | #Feature Sample 2 | Start | End | PTM | AScore | Found By |
| --- | --- | --- | --- | --- | --- | --- | --- | --- | --- | --- | --- | --- | --- | --- | --- | --- | --- | --- | --- |
| R.TIQFVDWC(+57.02)PTGFK.V | Y | 54.82 | 1597.7599 | 13 | -6.53 | 799.8776 | 2 | 19.88 | 1.0351 | 1.0423 | 66701 | 3.31e2 | 1 | 1 | 340 | 352 | Carbamidomethylation |  | DB Search |
| R.LDHKFDLM(+15.99)YAKR.A | Y | 40.99 | 1551.7868 | 12 | 3.09 | 776.8988 | 2 | 13.19 | 1.0881 | 1.0953 | 31591 | 1.18e2 | 1 | 1 | 391 | 402 | Oxidation (M) | M8:Oxidation (M):1000 | DB Search |
| total 2 peptides |  |  |  |  |  |  |  |  |  |  |  |  |  |  |  |  |  |  |  |
| --- | --- | --- | --- | --- | --- | --- | --- | --- | --- | --- | --- | --- | --- | --- | --- | --- | --- | --- | --- |

Best Unique PSM (Scan Sample\_11\_A1\_1\_3648.d:66701, m/z=799.3823, z=2, RT=19.90, ppm=-12):


TRINITY\_DN6920\_c0\_g1\_i3.p1

back to list

  

| Protein Coverage
| Supporting Peptides
| Best Unique PSM
|

Protein Coverage:

Supporting Peptides:

| Peptide | Uniq | -10lgP | Mass | Length | ppm | m/z | z | RT | 1/k0 Start | 1/k0 End | Precursor ID | Area Sample 2 | #Feature | #Feature Sample 2 | Start | End | PTM | AScore | Found By |
| --- | --- | --- | --- | --- | --- | --- | --- | --- | --- | --- | --- | --- | --- | --- | --- | --- | --- | --- | --- |
| R.VLVHTQQK.L | Y | 57.79 | 951.5501 | 8 | -1.07 | 476.7792 | 2 | 9.55 | 0.8352 | 0.8425 | 11014 | 5.75e2 | 1 | 1 | 169 | 176 |  |  | DB Search |
| K.AGM(+15.99)THIVR.E | Y | 38.19 | 899.4647 | 8 | 6.96 | 450.7403 | 2 | 9.74 | 0.8254 | 0.8327 | 11994 | 0 | 0 | 0 | 58 | 65 | Oxidation (M) | M3:Oxidation (M):1000 | DB Search |
| R.HGSLGFLPR.K | Y | 32.83 | 982.5348 | 9 | -0.04 | 492.272 | 2 | 16.93 | 0.8408 | 0.8481 | 51108 | 4.36e2 | 1 | 1 | 18 | 26 |  |  | DB Search |
| total 3 peptides |  |  |  |  |  |  |  |  |  |  |  |  |  |  |  |  |  |  |  |
| --- | --- | --- | --- | --- | --- | --- | --- | --- | --- | --- | --- | --- | --- | --- | --- | --- | --- | --- | --- |

Best Unique PSM (Scan Sample\_11\_A1\_1\_3648.d:11014, m/z=477.7582, z=2, RT=9.56, ppm=-6.55):


TRINITY\_DN2885\_c0\_g1\_i2.p1

back to list

  

| Protein Coverage
| Supporting Peptides
| Best Unique PSM
|

Protein Coverage:

Supporting Peptides:

| Peptide | Uniq | -10lgP | Mass | Length | ppm | m/z | z | RT | 1/k0 Start | 1/k0 End | Precursor ID | Area Sample 2 | #Feature | #Feature Sample 2 | Start | End | PTM | AScore | Found By |
| --- | --- | --- | --- | --- | --- | --- | --- | --- | --- | --- | --- | --- | --- | --- | --- | --- | --- | --- | --- |
| K.VTRPHGASGVVR.A | Y | 58.91 | 1234.6894 | 12 | 6.71 | 412.5709 | 3 | 10.04 | 0.8209 | 0.8283 | 13404 | 2.81e4 | 3 | 3 | 77 | 88 |  |  | DB Search |
| total 1 peptides |  |  |  |  |  |  |  |  |  |  |  |  |  |  |  |  |  |  |  |
| --- | --- | --- | --- | --- | --- | --- | --- | --- | --- | --- | --- | --- | --- | --- | --- | --- | --- | --- | --- |

Best Unique PSM (Scan Sample\_11\_A1\_1\_3648.d:13404, m/z=412.5709, z=3, RT=10.04, ppm=1.23):


TRINITY\_DN6294\_c0\_g3\_i2.p1

back to list

  

| Protein Coverage
| Supporting Peptides
| Best Unique PSM
|

Protein Coverage:

Supporting Peptides:

| Peptide | Uniq | -10lgP | Mass | Length | ppm | m/z | z | RT | 1/k0 Start | 1/k0 End | Precursor ID | Area Sample 2 | #Feature | #Feature Sample 2 | Start | End | PTM | AScore | Found By |
| --- | --- | --- | --- | --- | --- | --- | --- | --- | --- | --- | --- | --- | --- | --- | --- | --- | --- | --- | --- |
| K.LVVIADIVDQKR.L | Y | 57.66 | 1367.8136 | 12 | 0.99 | 684.911 | 2 | 17.26 | 1.0419 | 1.0492 | 52731 | 1.74e3 | 1 | 1 | 24 | 35 |  |  | DB Search |
| total 1 peptides |  |  |  |  |  |  |  |  |  |  |  |  |  |  |  |  |  |  |  |
| --- | --- | --- | --- | --- | --- | --- | --- | --- | --- | --- | --- | --- | --- | --- | --- | --- | --- | --- | --- |

Best Unique PSM (Scan Sample\_11\_A1\_1\_3648.d:52731, m/z=684.911, z=2, RT=17.26, ppm=-4.49):


TRINITY\_DN32522\_c0\_g1\_i1.p1

back to list

  

| Protein Coverage
| Supporting Peptides
| Best Unique PSM
|

Protein Coverage:

Supporting Peptides:

| Peptide | Uniq | -10lgP | Mass | Length | ppm | m/z | z | RT | 1/k0 Start | 1/k0 End | Precursor ID | Area Sample 2 | #Feature | #Feature Sample 2 | Start | End | PTM | AScore | Found By |
| --- | --- | --- | --- | --- | --- | --- | --- | --- | --- | --- | --- | --- | --- | --- | --- | --- | --- | --- | --- |
| R.VDNKVNATER.K | Y | 58.41 | 1144.5836 | 10 | 1.18 | 573.2966 | 2 | 9.57 | 0.8862 | 0.8935 | 11155 | 1.2e3 | 1 | 1 | 191 | 200 |  |  | DB Search |
| total 1 peptides |  |  |  |  |  |  |  |  |  |  |  |  |  |  |  |  |  |  |  |
| --- | --- | --- | --- | --- | --- | --- | --- | --- | --- | --- | --- | --- | --- | --- | --- | --- | --- | --- | --- |

Best Unique PSM (Scan Sample\_11\_A1\_1\_3648.d:11155, m/z=573.2966, z=2, RT=9.57, ppm=-4.3):


TRINITY\_DN6397\_c0\_g2\_i3.p1

back to list

  

| Protein Coverage
| Supporting Peptides
| Best Unique PSM
|

Protein Coverage:

Supporting Peptides:

| Peptide | Uniq | -10lgP | Mass | Length | ppm | m/z | z | RT | 1/k0 Start | 1/k0 End | Precursor ID | Area Sample 2 | #Feature | #Feature Sample 2 | Start | End | PTM | AScore | Found By |
| --- | --- | --- | --- | --- | --- | --- | --- | --- | --- | --- | --- | --- | --- | --- | --- | --- | --- | --- | --- |
| K.K(+42.01)LSAEVANGR.L | Y | 58.78 | 1085.5829 | 10 | 5.56 | 543.7988 | 2 | 12.85 | 0.8538 | 0.8611 | 29679 | 1.02e3 | 1 | 1 | 12 | 21 | Acetylation (Protein N-term) | K1:Acetylation (Protein N-term):1000 | DB Search |
| total 1 peptides |  |  |  |  |  |  |  |  |  |  |  |  |  |  |  |  |  |  |  |
| --- | --- | --- | --- | --- | --- | --- | --- | --- | --- | --- | --- | --- | --- | --- | --- | --- | --- | --- | --- |

Best Unique PSM (Scan Sample\_11\_A1\_1\_3648.d:29679, m/z=543.7988, z=2, RT=12.85, ppm=0.08):


TRINITY\_DN16548\_c0\_g1\_i4.p1

back to list

  

| Protein Coverage
| Supporting Peptides
| Best Unique PSM
|

Protein Coverage:

Supporting Peptides:

| Peptide | Uniq | -10lgP | Mass | Length | ppm | m/z | z | RT | 1/k0 Start | 1/k0 End | Precursor ID | Area Sample 2 | #Feature | #Feature Sample 2 | Start | End | PTM | AScore | Found By |
| --- | --- | --- | --- | --- | --- | --- | --- | --- | --- | --- | --- | --- | --- | --- | --- | --- | --- | --- | --- |
| K.APGDYGWRPPLLATDDAALK.T | Y | 55.58 | 2126.0796 | 20 | 0.95 | 709.6973 | 3 | 18.70 | 0.8919 | 0.8992 | 60584 | 2.4e3 | 1 | 1 | 125 | 144 |  |  | DB Search |
| total 1 peptides |  |  |  |  |  |  |  |  |  |  |  |  |  |  |  |  |  |  |  |
| --- | --- | --- | --- | --- | --- | --- | --- | --- | --- | --- | --- | --- | --- | --- | --- | --- | --- | --- | --- |

Best Unique PSM (Scan Sample\_11\_A1\_1\_3648.d:60584, m/z=709.6973, z=3, RT=18.70, ppm=-4.52):


TRINITY\_DN2113\_c0\_g1\_i1.p1

back to list

  

| Protein Coverage
| Supporting Peptides
| Best Unique PSM
|

Protein Coverage:

Supporting Peptides:

| Peptide | Uniq | -10lgP | Mass | Length | ppm | m/z | z | RT | 1/k0 Start | 1/k0 End | Precursor ID | Area Sample 2 | #Feature | #Feature Sample 2 | Start | End | PTM | AScore | Found By |
| --- | --- | --- | --- | --- | --- | --- | --- | --- | --- | --- | --- | --- | --- | --- | --- | --- | --- | --- | --- |
| R.ALEHYSDIYDIKR.A | Y | 58.43 | 1621.81 | 13 | -3.12 | 541.606 | 3 | 14.46 | 0.8238 | 0.8311 | 38607 | 5.17e2 | 1 | 1 | 626 | 638 |  |  | DB Search |
| K.KVGFEPDYVSLLR.N | Y | 49.91 | 1521.8191 | 13 | -1.44 | 761.9116 | 2 | 18.24 | 1.0435 | 1.0508 | 58056 | 8.15e2 | 1 | 1 | 507 | 519 |  |  | DB Search |
| total 2 peptides |  |  |  |  |  |  |  |  |  |  |  |  |  |  |  |  |  |  |  |
| --- | --- | --- | --- | --- | --- | --- | --- | --- | --- | --- | --- | --- | --- | --- | --- | --- | --- | --- | --- |

Best Unique PSM (Scan Sample\_11\_A1\_1\_3648.d:38607, m/z=541.606, z=3, RT=14.46, ppm=-8.6):


TRINITY\_DN5365\_c0\_g1\_i3.p1

back to list

  

| Protein Coverage
| Supporting Peptides
| Best Unique PSM
|

Protein Coverage:

Supporting Peptides:

| Peptide | Uniq | -10lgP | Mass | Length | ppm | m/z | z | RT | 1/k0 Start | 1/k0 End | Precursor ID | Area Sample 2 | #Feature | #Feature Sample 2 | Start | End | PTM | AScore | Found By |
| --- | --- | --- | --- | --- | --- | --- | --- | --- | --- | --- | --- | --- | --- | --- | --- | --- | --- | --- | --- |
| R.FPGQLNADIRK.L | Y | 25.64 | 1257.6829 | 11 | -1.3 | 629.8445 | 2 | 14.18 | 0.9457 | 0.9529 | 37015 | 6.11e2 | 1 | 1 | 244 | 254 |  |  | DB Search |
| total 1 peptides |  |  |  |  |  |  |  |  |  |  |  |  |  |  |  |  |  |  |  |
| --- | --- | --- | --- | --- | --- | --- | --- | --- | --- | --- | --- | --- | --- | --- | --- | --- | --- | --- | --- |

Best Unique PSM (Scan Sample\_11\_A1\_1\_3648.d:37015, m/z=629.8445, z=2, RT=14.18, ppm=-6.78):


TRINITY\_DN329\_c0\_g1\_i1.p1

back to list

  

| Protein Coverage
| Supporting Peptides
| Best Unique PSM
|

Protein Coverage:

Supporting Peptides:

| Peptide | Uniq | -10lgP | Mass | Length | ppm | m/z | z | RT | 1/k0 Start | 1/k0 End | Precursor ID | Area Sample 2 | #Feature | #Feature Sample 2 | Start | End | PTM | AScore | Found By |
| --- | --- | --- | --- | --- | --- | --- | --- | --- | --- | --- | --- | --- | --- | --- | --- | --- | --- | --- | --- |
| K.SFHFGVIKLEGK.T | Y | 58.86 | 1360.7503 | 12 | -0.4 | 681.3784 | 2 | 16.70 | 1.0291 | 1.0363 | 49881 | 5.45e2 | 1 | 1 | 20 | 31 |  |  | DB Search |
| K.GLKVDFDTTFAPVTGKK.T | Y | 58.14 | 1822.9828 | 17 | 1.48 | 912.4951 | 2 | 16.88 | 1.1802 | 1.1873 | 50684 | 2.54e2 | 1 | 1 | 93 | 109 |  |  | DB Search |
| K.LRPGVELTMSSLVNAK.S | Y | 50.37 | 1713.9447 | 16 | -0.1 | 857.9749 | 2 | 17.45 | 1.145 | 1.1522 | 53751 | 1.26e3 | 1 | 1 | 250 | 265 |  |  | DB Search |
| K.LRPGVELTM(+15.99)SSLVNAK.S | Y | 42.38 | 1729.9396 | 16 | 3.46 | 865.9753 | 2 | 15.03 | 1.1338 | 1.141 | 41582 | 4.17e3 | 1 | 1 | 250 | 265 | Oxidation (M) | M9:Oxidation (M):1000 | DB Search |
| K.EGDLVIHSGITDAVK.Y | Y | 21.65 | 1552.8096 | 15 | -3.94 | 777.4048 | 2 | 15.22 | 1.0644 | 1.0717 | 42664 | 0 | 0 | 0 | 174 | 188 |  |  | DB Search |
| total 5 peptides |  |  |  |  |  |  |  |  |  |  |  |  |  |  |  |  |  |  |  |
| --- | --- | --- | --- | --- | --- | --- | --- | --- | --- | --- | --- | --- | --- | --- | --- | --- | --- | --- | --- |

Best Unique PSM (Scan Sample\_11\_A1\_1\_3648.d:49881, m/z=681.3784, z=2, RT=16.70, ppm=-5.88):


TRINITY\_DN1559\_c0\_g1\_i1.p1

back to list

  

| Protein Coverage
| Supporting Peptides
| Best Unique PSM
|

Protein Coverage:

Supporting Peptides:

| Peptide | Uniq | -10lgP | Mass | Length | ppm | m/z | z | RT | 1/k0 Start | 1/k0 End | Precursor ID | Area Sample 2 | #Feature | #Feature Sample 2 | Start | End | PTM | AScore | Found By |
| --- | --- | --- | --- | --- | --- | --- | --- | --- | --- | --- | --- | --- | --- | --- | --- | --- | --- | --- | --- |
| R.GVAGAGVLAGFDKFK.E | Y | 58.4 | 1435.7823 | 15 | 1.33 | 718.8954 | 2 | 17.98 | 1.0873 | 1.0945 | 56680 | 2.72e3 | 1 | 1 | 282 | 296 |  |  | DB Search |
| total 1 peptides |  |  |  |  |  |  |  |  |  |  |  |  |  |  |  |  |  |  |  |
| --- | --- | --- | --- | --- | --- | --- | --- | --- | --- | --- | --- | --- | --- | --- | --- | --- | --- | --- | --- |

Best Unique PSM (Scan Sample\_11\_A1\_1\_3648.d:56680, m/z=718.8954, z=2, RT=17.98, ppm=-4.14):


TRINITY\_DN18187\_c0\_g1\_i6.p2

back to list

  

| Protein Coverage
| Supporting Peptides
| Best Unique PSM
|

Protein Coverage:

Supporting Peptides:

| Peptide | Uniq | -10lgP | Mass | Length | ppm | m/z | z | RT | 1/k0 Start | 1/k0 End | Precursor ID | Area Sample 2 | #Feature | #Feature Sample 2 | Start | End | PTM | AScore | Found By |
| --- | --- | --- | --- | --- | --- | --- | --- | --- | --- | --- | --- | --- | --- | --- | --- | --- | --- | --- | --- |
| R.YRPGTVALR.E | Y | 44.05 | 1031.5876 | 9 | -1.83 | 516.7973 | 2 | 12.44 | 0.8575 | 0.8648 | 27332 | 1.61e3 | 1 | 1 | 83 | 91 |  |  | DB Search |
| total 1 peptides |  |  |  |  |  |  |  |  |  |  |  |  |  |  |  |  |  |  |  |
| --- | --- | --- | --- | --- | --- | --- | --- | --- | --- | --- | --- | --- | --- | --- | --- | --- | --- | --- | --- |

Best Unique PSM (Scan Sample\_11\_A1\_1\_3648.d:27332, m/z=516.7973, z=2, RT=12.44, ppm=-7.31):


TRINITY\_DN250\_c0\_g1\_i6.p1

back to list

  

| Protein Coverage
| Supporting Peptides
| Best Unique PSM
|

Protein Coverage:

Supporting Peptides:

| Peptide | Uniq | -10lgP | Mass | Length | ppm | m/z | z | RT | 1/k0 Start | 1/k0 End | Precursor ID | Area Sample 2 | #Feature | #Feature Sample 2 | Start | End | PTM | AScore | Found By |
| --- | --- | --- | --- | --- | --- | --- | --- | --- | --- | --- | --- | --- | --- | --- | --- | --- | --- | --- | --- |
| R.TIQFVDWC(+57.02)PTGFK.V | Y | 54.82 | 1597.7599 | 13 | -6.53 | 799.8776 | 2 | 19.88 | 1.0351 | 1.0423 | 66701 | 3.31e2 | 1 | 1 | 340 | 352 | Carbamidomethylation |  | DB Search |
| R.RNLDIERPTYTNLNR.L | Y | 46.58 | 1873.9758 | 15 | -2.43 | 625.6609 | 3 | 13.94 | 0.8858 | 0.8931 | 35770 | 1.17e3 | 1 | 1 | 215 | 229 |  |  | DB Search |
| R.LDHKFDLM(+15.99)YAKR.A | Y | 40.99 | 1551.7868 | 12 | 3.09 | 776.8988 | 2 | 13.19 | 1.0881 | 1.0953 | 31591 | 1.18e2 | 1 | 1 | 391 | 402 | Oxidation (M) | M8:Oxidation (M):1000 | DB Search |
| total 3 peptides |  |  |  |  |  |  |  |  |  |  |  |  |  |  |  |  |  |  |  |
| --- | --- | --- | --- | --- | --- | --- | --- | --- | --- | --- | --- | --- | --- | --- | --- | --- | --- | --- | --- |

Best Unique PSM (Scan Sample\_11\_A1\_1\_3648.d:66701, m/z=799.3823, z=2, RT=19.90, ppm=-12):


TRINITY\_DN2963\_c0\_g1\_i1.p1

back to list

  

| Protein Coverage
| Supporting Peptides
| Best Unique PSM
|

Protein Coverage:

Supporting Peptides:

| Peptide | Uniq | -10lgP | Mass | Length | ppm | m/z | z | RT | 1/k0 Start | 1/k0 End | Precursor ID | Area Sample 2 | #Feature | #Feature Sample 2 | Start | End | PTM | AScore | Found By |
| --- | --- | --- | --- | --- | --- | --- | --- | --- | --- | --- | --- | --- | --- | --- | --- | --- | --- | --- | --- |
| K.LYDEYNDKTPSR.L | Y | 58.85 | 1499.6892 | 12 | 0.4 | 750.8481 | 2 | 13.35 | 1.0142 | 1.0214 | 32540 | 3.34e3 | 2 | 2 | 14 | 25 |  |  | DB Search |
| M.S(+42.01)ATGNLVTVLRK.L | Y | 58.51 | 1299.751 | 12 | 3.45 | 650.8815 | 2 | 16.84 | 0.9973 | 1.0045 | 50424 | 5.95e3 | 1 | 1 | 2 | 13 | Acetylation (Protein N-term) | S1:Acetylation (Protein N-term):1000 | DB Search |
| R.KLYDEYNDKTPSR.L | Y | 37.19 | 1627.7842 | 13 | -2.71 | 814.8927 | 2 | 11.79 | 1.0387 | 1.046 | 23644 | 0 | 0 | 0 | 13 | 25 |  |  | DB Search |
| total 3 peptides |  |  |  |  |  |  |  |  |  |  |  |  |  |  |  |  |  |  |  |
| --- | --- | --- | --- | --- | --- | --- | --- | --- | --- | --- | --- | --- | --- | --- | --- | --- | --- | --- | --- |

Best Unique PSM (Scan Sample\_11\_A1\_1\_3648.d:32540, m/z=750.8481, z=2, RT=13.35, ppm=-5.07):


TRINITY\_DN1071\_c0\_g1\_i1.p1

back to list

  

| Protein Coverage
| Supporting Peptides
| Best Unique PSM
|

Protein Coverage:

Supporting Peptides:

| Peptide | Uniq | -10lgP | Mass | Length | ppm | m/z | z | RT | 1/k0 Start | 1/k0 End | Precursor ID | Area Sample 2 | #Feature | #Feature Sample 2 | Start | End | PTM | AScore | Found By |
| --- | --- | --- | --- | --- | --- | --- | --- | --- | --- | --- | --- | --- | --- | --- | --- | --- | --- | --- | --- |
| R.IQGHPTDKKFTK.N | Y | 55.25 | 1398.7619 | 12 | 4.46 | 467.2608 | 3 | 9.57 | 0.7677 | 0.775 | 11206 | 0 | 0 | 0 | 241 | 252 |  |  | DB Search |
| K.AVQIHPFNDVSVGDPK.V | Y | 31.99 | 1721.8736 | 16 | -1.26 | 861.9383 | 2 | 15.56 | 1.1158 | 1.123 | 44381 | 1.32e2 | 1 | 1 | 293 | 308 |  |  | DB Search |
| total 2 peptides |  |  |  |  |  |  |  |  |  |  |  |  |  |  |  |  |  |  |  |
| --- | --- | --- | --- | --- | --- | --- | --- | --- | --- | --- | --- | --- | --- | --- | --- | --- | --- | --- | --- |

Best Unique PSM (Scan Sample\_11\_A1\_1\_3648.d:11206, m/z=467.2608, z=3, RT=9.57, ppm=-1.02):


TRINITY\_DN5276\_c0\_g1\_i14.p1

back to list

  

| Protein Coverage
| Supporting Peptides
| Best Unique PSM
|

Protein Coverage:

Supporting Peptides:

| Peptide | Uniq | -10lgP | Mass | Length | ppm | m/z | z | RT | 1/k0 Start | 1/k0 End | Precursor ID | Area Sample 2 | #Feature | #Feature Sample 2 | Start | End | PTM | AScore | Found By |
| --- | --- | --- | --- | --- | --- | --- | --- | --- | --- | --- | --- | --- | --- | --- | --- | --- | --- | --- | --- |
| R.APGDEGWRPPLLATDDAELK.K | Y | 23.14 | 2150.0643 | 20 | -9.02 | 717.685 | 3 | 18.26 | 0.917 | 0.9243 | 58289 | 0 | 0 | 0 | 99 | 118 |  |  | DB Search |
| total 1 peptides |  |  |  |  |  |  |  |  |  |  |  |  |  |  |  |  |  |  |  |
| --- | --- | --- | --- | --- | --- | --- | --- | --- | --- | --- | --- | --- | --- | --- | --- | --- | --- | --- | --- |

Best Unique PSM (Scan Sample\_11\_A1\_1\_3648.d:58289, m/z=717.685, z=3, RT=18.26, ppm=-14.49):


TRINITY\_DN2126\_c0\_g1\_i1.p1

back to list

  

| Protein Coverage
| Supporting Peptides
| Best Unique PSM
|

Protein Coverage:

Supporting Peptides:

| Peptide | Uniq | -10lgP | Mass | Length | ppm | m/z | z | RT | 1/k0 Start | 1/k0 End | Precursor ID | Area Sample 2 | #Feature | #Feature Sample 2 | Start | End | PTM | AScore | Found By |
| --- | --- | --- | --- | --- | --- | --- | --- | --- | --- | --- | --- | --- | --- | --- | --- | --- | --- | --- | --- |
| K.LVIISSNTPQLRK.S | Y | 58.88 | 1467.8773 | 13 | 1.32 | 734.9429 | 2 | 14.77 | 1.058 | 1.0652 | 40172 | 1.16e3 | 1 | 1 | 43 | 55 |  |  | DB Search |
| K.AKLVIISSNTPQLRK.S | Y | 57.08 | 1667.0094 | 15 | 0.8 | 834.5081 | 2 | 14.23 | 1.1362 | 1.1434 | 37393 | 0 | 0 | 0 | 41 | 55 |  |  | DB Search |
| K.TGVHHYTGNNIELGTAC(+57.02)GK.Y | Y | 55.14 | 2027.9483 | 19 | 3.09 | 1014.979 | 2 | 12.60 | 1.173 | 1.1802 | 28328 | 0 | 0 | 0 | 67 | 85 | Carbamidomethylation |  | DB Search |
| total 3 peptides |  |  |  |  |  |  |  |  |  |  |  |  |  |  |  |  |  |  |  |
| --- | --- | --- | --- | --- | --- | --- | --- | --- | --- | --- | --- | --- | --- | --- | --- | --- | --- | --- | --- |

Best Unique PSM (Scan Sample\_11\_A1\_1\_3648.d:40172, m/z=734.9429, z=2, RT=14.77, ppm=-4.15):


TRINITY\_DN3036\_c0\_g1\_i8.p1

back to list

  

| Protein Coverage
| Supporting Peptides
| Best Unique PSM
|

Protein Coverage:

Supporting Peptides:

| Peptide | Uniq | -10lgP | Mass | Length | ppm | m/z | z | RT | 1/k0 Start | 1/k0 End | Precursor ID | Area Sample 2 | #Feature | #Feature Sample 2 | Start | End | PTM | AScore | Found By |
| --- | --- | --- | --- | --- | --- | --- | --- | --- | --- | --- | --- | --- | --- | --- | --- | --- | --- | --- | --- |
| R.AGHGAVTSPQTQR.G | N | 58.95 | 1308.6534 | 13 | 4.15 | 655.3331 | 2 | 9.70 | 0.9146 | 0.9218 | 11889 | 1.61e4 | 2 | 2 | 309 | 321 |  |  | DB Search |
| R.GTSNGGLVVGTIIKPK.L | Y | 58.87 | 1539.8984 | 16 | 2.71 | 770.9543 | 2 | 15.49 | 1.1254 | 1.1326 | 43901 | 6.84e3 | 1 | 1 | 173 | 188 |  |  | DB Search |
| R.VIGASGIHVGTM(+15.99)SFGK.M | N | 58.84 | 1575.8079 | 16 | 2.27 | 788.9087 | 2 | 14.96 | 1.1306 | 1.1378 | 41219 | 1.68e3 | 1 | 1 | 334 | 349 | Oxidation (M) | M12:Oxidation (M):1000 | DB Search |
| total 3 peptides |  |  |  |  |  |  |  |  |  |  |  |  |  |  |  |  |  |  |  |
| --- | --- | --- | --- | --- | --- | --- | --- | --- | --- | --- | --- | --- | --- | --- | --- | --- | --- | --- | --- |

Best Unique PSM (Scan Sample\_11\_A1\_1\_3648.d:43901, m/z=770.9543, z=2, RT=15.49, ppm=-2.77):


TRINITY\_DN761\_c0\_g1\_i2.p1

back to list

  

| Protein Coverage
| Supporting Peptides
| Best Unique PSM
|

Protein Coverage:

Supporting Peptides:

| Peptide | Uniq | -10lgP | Mass | Length | ppm | m/z | z | RT | 1/k0 Start | 1/k0 End | Precursor ID | Area Sample 2 | #Feature | #Feature Sample 2 | Start | End | PTM | AScore | Found By |
| --- | --- | --- | --- | --- | --- | --- | --- | --- | --- | --- | --- | --- | --- | --- | --- | --- | --- | --- | --- |
| K.TRIEDLETVISQTQDHR.H | Y | 55.76 | 2040.0236 | 17 | -2.78 | 681.0095 | 3 | 17.11 | 0.9558 | 0.963 | 52031 | 0 | 0 | 0 | 260 | 276 |  |  | DB Search |
| total 1 peptides |  |  |  |  |  |  |  |  |  |  |  |  |  |  |  |  |  |  |  |
| --- | --- | --- | --- | --- | --- | --- | --- | --- | --- | --- | --- | --- | --- | --- | --- | --- | --- | --- | --- |

Best Unique PSM (Scan Sample\_11\_A1\_1\_3648.d:52031, m/z=681.0095, z=3, RT=17.11, ppm=-8.25):


TRINITY\_DN3773\_c0\_g1\_i1.p1

back to list

  

| Protein Coverage
| Supporting Peptides
| Best Unique PSM
|

Protein Coverage:

Supporting Peptides:

| Peptide | Uniq | -10lgP | Mass | Length | ppm | m/z | z | RT | 1/k0 Start | 1/k0 End | Precursor ID | Area Sample 2 | #Feature | #Feature Sample 2 | Start | End | PTM | AScore | Found By |
| --- | --- | --- | --- | --- | --- | --- | --- | --- | --- | --- | --- | --- | --- | --- | --- | --- | --- | --- | --- |
| K.IPIFSAAGLPHNEIAAQIC(+57.02)R.Q | Y | 57.77 | 2177.1415 | 20 | 1.64 | 726.7183 | 3 | 18.57 | 1.0086 | 1.0158 | 59833 | 6.81e2 | 1 | 1 | 178 | 197 | Carbamidomethylation |  | DB Search |
| total 1 peptides |  |  |  |  |  |  |  |  |  |  |  |  |  |  |  |  |  |  |  |
| --- | --- | --- | --- | --- | --- | --- | --- | --- | --- | --- | --- | --- | --- | --- | --- | --- | --- | --- | --- |

Best Unique PSM (Scan Sample\_11\_A1\_1\_3648.d:59833, m/z=726.7183, z=3, RT=18.57, ppm=-3.84):


TRINITY\_DN22\_c0\_g1\_i1.p1

back to list

  

| Protein Coverage
| Supporting Peptides
| Best Unique PSM
|

Protein Coverage:

Supporting Peptides:

| Peptide | Uniq | -10lgP | Mass | Length | ppm | m/z | z | RT | 1/k0 Start | 1/k0 End | Precursor ID | Area Sample 2 | #Feature | #Feature Sample 2 | Start | End | PTM | AScore | Found By |
| --- | --- | --- | --- | --- | --- | --- | --- | --- | --- | --- | --- | --- | --- | --- | --- | --- | --- | --- | --- |
| R.FVLHGDVSHGSR.Y | Y | 57.97 | 1309.6527 | 12 | -8.24 | 655.8246 | 2 | 11.96 | 0.9872 | 0.9945 | 24686 | 0 | 0 | 0 | 314 | 325 |  |  | DB Search |
| total 1 peptides |  |  |  |  |  |  |  |  |  |  |  |  |  |  |  |  |  |  |  |
| --- | --- | --- | --- | --- | --- | --- | --- | --- | --- | --- | --- | --- | --- | --- | --- | --- | --- | --- | --- |

Best Unique PSM (Scan Sample\_11\_A1\_1\_3648.d:24686, m/z=655.8246, z=2, RT=11.96, ppm=-13.71):


TRINITY\_DN195631\_c0\_g1\_i1.p2

back to list

  

| Protein Coverage
| Supporting Peptides
| Best Unique PSM
|

Protein Coverage:

Supporting Peptides:

| Peptide | Uniq | -10lgP | Mass | Length | ppm | m/z | z | RT | 1/k0 Start | 1/k0 End | Precursor ID | Area Sample 2 | #Feature | #Feature Sample 2 | Start | End | PTM | AScore | Found By |
| --- | --- | --- | --- | --- | --- | --- | --- | --- | --- | --- | --- | --- | --- | --- | --- | --- | --- | --- | --- |
| K.IQDKEGIPPDQQR.L | Y | 55.06 | 1522.7739 | 13 | 2.88 | 762.3923 | 2 | 11.00 | 1.0331 | 1.0403 | 18584 | 2.97e3 | 2 | 2 | 122 | 134 |  |  | DB Search |
| total 1 peptides |  |  |  |  |  |  |  |  |  |  |  |  |  |  |  |  |  |  |  |
| --- | --- | --- | --- | --- | --- | --- | --- | --- | --- | --- | --- | --- | --- | --- | --- | --- | --- | --- | --- |

Best Unique PSM (Scan Sample\_11\_A1\_1\_3648.d:18584, m/z=762.3923, z=2, RT=11.00, ppm=-2.59):


TRINITY\_DN1566\_c0\_g1\_i1.p1

back to list

  

| Protein Coverage
| Supporting Peptides
| Best Unique PSM
|

Protein Coverage:

Supporting Peptides:

| Peptide | Uniq | -10lgP | Mass | Length | ppm | m/z | z | RT | 1/k0 Start | 1/k0 End | Precursor ID | Area Sample 2 | #Feature | #Feature Sample 2 | Start | End | PTM | AScore | Found By |
| --- | --- | --- | --- | --- | --- | --- | --- | --- | --- | --- | --- | --- | --- | --- | --- | --- | --- | --- | --- |
| R.AERPEVDTKR.S | Y | 58.22 | 1199.6258 | 10 | 3.55 | 400.8818 | 3 | 9.63 | 0.7143 | 0.7216 | 11402 | 6.01e2 | 1 | 1 | 3689 | 3698 |  |  | DB Search |
| R.IKLEHLITELVHQR.D | Y | 42.73 | 1728.0046 | 14 | -6.21 | 577.0021 | 3 | 18.10 | 0.8672 | 0.8745 | 57432 | 0 | 0 | 0 | 1781 | 1794 |  |  | DB Search |
| R.ITSTLRDQLGTAK.N | Y | 32.95 | 1402.778 | 13 | 4.84 | 702.3958 | 2 | 13.11 | 1.0198 | 1.0271 | 31190 | 3.61e2 | 1 | 1 | 549 | 561 |  |  | DB Search |
| total 3 peptides |  |  |  |  |  |  |  |  |  |  |  |  |  |  |  |  |  |  |  |
| --- | --- | --- | --- | --- | --- | --- | --- | --- | --- | --- | --- | --- | --- | --- | --- | --- | --- | --- | --- |

Best Unique PSM (Scan Sample\_11\_A1\_1\_3648.d:11402, m/z=400.8818, z=3, RT=9.63, ppm=-1.94):


TRINITY\_DN571\_c0\_g1\_i1.p1

back to list

  

| Protein Coverage
| Supporting Peptides
| Best Unique PSM
|

Protein Coverage:

Supporting Peptides:

| Peptide | Uniq | -10lgP | Mass | Length | ppm | m/z | z | RT | 1/k0 Start | 1/k0 End | Precursor ID | Area Sample 2 | #Feature | #Feature Sample 2 | Start | End | PTM | AScore | Found By |
| --- | --- | --- | --- | --- | --- | --- | --- | --- | --- | --- | --- | --- | --- | --- | --- | --- | --- | --- | --- |
| R.VVGKDQGQSLAK.Q | Y | 55.18 | 1228.6775 | 12 | -1.12 | 615.342 | 2 | 10.28 | 0.9255 | 0.9327 | 14860 | 0 | 0 | 0 | 125 | 136 |  |  | DB Search |
| total 1 peptides |  |  |  |  |  |  |  |  |  |  |  |  |  |  |  |  |  |  |  |
| --- | --- | --- | --- | --- | --- | --- | --- | --- | --- | --- | --- | --- | --- | --- | --- | --- | --- | --- | --- |

Best Unique PSM (Scan Sample\_11\_A1\_1\_3648.d:14860, m/z=615.342, z=2, RT=10.28, ppm=-6.6):


TRINITY\_DN471\_c0\_g1\_i7.p1

back to list

  

| Protein Coverage
| Supporting Peptides
| Best Unique PSM
|

Protein Coverage:

Supporting Peptides:

| Peptide | Uniq | -10lgP | Mass | Length | ppm | m/z | z | RT | 1/k0 Start | 1/k0 End | Precursor ID | Area Sample 2 | #Feature | #Feature Sample 2 | Start | End | PTM | AScore | Found By |
| --- | --- | --- | --- | --- | --- | --- | --- | --- | --- | --- | --- | --- | --- | --- | --- | --- | --- | --- | --- |
| R.RNLDIERPTYTNLNR.L | Y | 46.58 | 1873.9758 | 15 | -2.43 | 625.6609 | 3 | 13.94 | 0.8858 | 0.8931 | 35770 | 1.17e3 | 1 | 1 | 85 | 99 |  |  | DB Search |
| total 1 peptides |  |  |  |  |  |  |  |  |  |  |  |  |  |  |  |  |  |  |  |
| --- | --- | --- | --- | --- | --- | --- | --- | --- | --- | --- | --- | --- | --- | --- | --- | --- | --- | --- | --- |

Best Unique PSM (Scan Sample\_11\_A1\_1\_3648.d:35770, m/z=625.6609, z=3, RT=13.94, ppm=-7.91):


TRINITY\_DN471\_c0\_g1\_i9.p1

back to list

  

| Protein Coverage
| Supporting Peptides
| Best Unique PSM
|

Protein Coverage:

Supporting Peptides:

| Peptide | Uniq | -10lgP | Mass | Length | ppm | m/z | z | RT | 1/k0 Start | 1/k0 End | Precursor ID | Area Sample 2 | #Feature | #Feature Sample 2 | Start | End | PTM | AScore | Found By |
| --- | --- | --- | --- | --- | --- | --- | --- | --- | --- | --- | --- | --- | --- | --- | --- | --- | --- | --- | --- |
| R.RNLDIERPTYTNLNR.L | Y | 46.58 | 1873.9758 | 15 | -2.43 | 625.6609 | 3 | 13.94 | 0.8858 | 0.8931 | 35770 | 1.17e3 | 1 | 1 | 215 | 229 |  |  | DB Search |
| total 1 peptides |  |  |  |  |  |  |  |  |  |  |  |  |  |  |  |  |  |  |  |
| --- | --- | --- | --- | --- | --- | --- | --- | --- | --- | --- | --- | --- | --- | --- | --- | --- | --- | --- | --- |

Best Unique PSM (Scan Sample\_11\_A1\_1\_3648.d:35770, m/z=625.6609, z=3, RT=13.94, ppm=-7.91):


TRINITY\_DN36\_c0\_g1\_i3.p1

back to list

  

| Protein Coverage
| Supporting Peptides
| Best Unique PSM
|

Protein Coverage:

Supporting Peptides:

| Peptide | Uniq | -10lgP | Mass | Length | ppm | m/z | z | RT | 1/k0 Start | 1/k0 End | Precursor ID | Area Sample 2 | #Feature | #Feature Sample 2 | Start | End | PTM | AScore | Found By |
| --- | --- | --- | --- | --- | --- | --- | --- | --- | --- | --- | --- | --- | --- | --- | --- | --- | --- | --- | --- |
| R.LGGIEVTQGNC(+57.02)ARK.Y | Y | 58.27 | 1501.7671 | 14 | 1.48 | 751.8878 | 2 | 12.59 | 1.0616 | 1.0688 | 28229 | 7.18e2 | 1 | 1 | 58 | 71 | Carbamidomethylation |  | DB Search |
| K.FETQIATK.D | Y | 57.95 | 936.4916 | 8 | 1.49 | 937.4952 | 1 | 13.74 | 1.2208 | 1.228 | 34637 | 7.44e2 | 2 | 2 | 167 | 174 |  |  | DB Search |
| total 2 peptides |  |  |  |  |  |  |  |  |  |  |  |  |  |  |  |  |  |  |  |
| --- | --- | --- | --- | --- | --- | --- | --- | --- | --- | --- | --- | --- | --- | --- | --- | --- | --- | --- | --- |

Best Unique PSM (Scan Sample\_11\_A1\_1\_3648.d:28229, m/z=751.8878, z=2, RT=12.59, ppm=-4):


TRINITY\_DN1071\_c0\_g1\_i3.p1

back to list

  

| Protein Coverage
| Supporting Peptides
| Best Unique PSM
|

Protein Coverage:

Supporting Peptides:

| Peptide | Uniq | -10lgP | Mass | Length | ppm | m/z | z | RT | 1/k0 Start | 1/k0 End | Precursor ID | Area Sample 2 | #Feature | #Feature Sample 2 | Start | End | PTM | AScore | Found By |
| --- | --- | --- | --- | --- | --- | --- | --- | --- | --- | --- | --- | --- | --- | --- | --- | --- | --- | --- | --- |
| R.IQGHPTDKKFTK.N | Y | 55.25 | 1398.7619 | 12 | 4.46 | 467.2608 | 3 | 9.57 | 0.7677 | 0.775 | 11206 | 0 | 0 | 0 | 277 | 288 |  |  | DB Search |
| K.AVQIHPFNDVSVGDPK.V | Y | 31.99 | 1721.8736 | 16 | -1.26 | 861.9383 | 2 | 15.56 | 1.1158 | 1.123 | 44381 | 1.32e2 | 1 | 1 | 329 | 344 |  |  | DB Search |
| total 2 peptides |  |  |  |  |  |  |  |  |  |  |  |  |  |  |  |  |  |  |  |
| --- | --- | --- | --- | --- | --- | --- | --- | --- | --- | --- | --- | --- | --- | --- | --- | --- | --- | --- | --- |

Best Unique PSM (Scan Sample\_11\_A1\_1\_3648.d:11206, m/z=467.2608, z=3, RT=9.57, ppm=-1.02):


TRINITY\_DN195934\_c0\_g1\_i1.p2

back to list

  

| Protein Coverage
| Supporting Peptides
| Best Unique PSM
|

Protein Coverage:

Supporting Peptides:

| Peptide | Uniq | -10lgP | Mass | Length | ppm | m/z | z | RT | 1/k0 Start | 1/k0 End | Precursor ID | Area Sample 2 | #Feature | #Feature Sample 2 | Start | End | PTM | AScore | Found By |
| --- | --- | --- | --- | --- | --- | --- | --- | --- | --- | --- | --- | --- | --- | --- | --- | --- | --- | --- | --- |
| K.DSYVGDEAQSKR.G | Y | 59.21 | 1353.616 | 12 | 5.76 | 677.8155 | 2 | 10.93 | 1.0037 | 1.011 | 18642 | 9.97e3 | 1 | 1 | 10 | 21 |  |  | DB Search |
| R.VAPEEHPVLLTEAPLNPK.A | Y | 58.19 | 1953.0571 | 18 | -0.07 | 977.5304 | 2 | 16.19 | 1.2089 | 1.2161 | 47306 | 2.52e2 | 1 | 1 | 55 | 72 |  |  | DB Search |
| total 2 peptides |  |  |  |  |  |  |  |  |  |  |  |  |  |  |  |  |  |  |  |
| --- | --- | --- | --- | --- | --- | --- | --- | --- | --- | --- | --- | --- | --- | --- | --- | --- | --- | --- | --- |

Best Unique PSM (Scan Sample\_11\_A1\_1\_3648.d:18642, m/z=677.8155, z=2, RT=10.93, ppm=0.28):


TRINITY\_DN265\_c0\_g1\_i3.p1

back to list

  

| Protein Coverage
| Supporting Peptides
| Best Unique PSM
|

Protein Coverage:

Supporting Peptides:

| Peptide | Uniq | -10lgP | Mass | Length | ppm | m/z | z | RT | 1/k0 Start | 1/k0 End | Precursor ID | Area Sample 2 | #Feature | #Feature Sample 2 | Start | End | PTM | AScore | Found By |
| --- | --- | --- | --- | --- | --- | --- | --- | --- | --- | --- | --- | --- | --- | --- | --- | --- | --- | --- | --- |
| R.LTHDFHTNKR.I | Y | 58.75 | 1267.6421 | 10 | 2.44 | 423.5534 | 3 | 9.64 | 0.7441 | 0.7514 | 11479 | 1.15e3 | 1 | 1 | 24 | 33 |  |  | DB Search |
| total 1 peptides |  |  |  |  |  |  |  |  |  |  |  |  |  |  |  |  |  |  |  |
| --- | --- | --- | --- | --- | --- | --- | --- | --- | --- | --- | --- | --- | --- | --- | --- | --- | --- | --- | --- |

Best Unique PSM (Scan Sample\_11\_A1\_1\_3648.d:11479, m/z=423.5534, z=3, RT=9.64, ppm=-3.04):


TRINITY\_DN8101\_c0\_g1\_i2.p2

back to list

  

| Protein Coverage
| Supporting Peptides
| Best Unique PSM
|

Protein Coverage:

Supporting Peptides:

| Peptide | Uniq | -10lgP | Mass | Length | ppm | m/z | z | RT | 1/k0 Start | 1/k0 End | Precursor ID | Area Sample 2 | #Feature | #Feature Sample 2 | Start | End | PTM | AScore | Found By |
| --- | --- | --- | --- | --- | --- | --- | --- | --- | --- | --- | --- | --- | --- | --- | --- | --- | --- | --- | --- |
| R.SIGFIGHYLDQLR.L | Y | 54.32 | 1517.799 | 13 | 1.06 | 759.9034 | 2 | 19.56 | 1.0849 | 1.0921 | 65092 | 4.69e2 | 1 | 1 | 784 | 796 |  |  | DB Search |
| total 1 peptides |  |  |  |  |  |  |  |  |  |  |  |  |  |  |  |  |  |  |  |
| --- | --- | --- | --- | --- | --- | --- | --- | --- | --- | --- | --- | --- | --- | --- | --- | --- | --- | --- | --- |

Best Unique PSM (Scan Sample\_11\_A1\_1\_3648.d:65092, m/z=759.9034, z=2, RT=19.56, ppm=-4.41):


TRINITY\_DN26679\_c0\_g1\_i1.p3

back to list

  

| Protein Coverage
| Supporting Peptides
| Best Unique PSM
|

Protein Coverage:

Supporting Peptides:

| Peptide | Uniq | -10lgP | Mass | Length | ppm | m/z | z | RT | 1/k0 Start | 1/k0 End | Precursor ID | Area Sample 2 | #Feature | #Feature Sample 2 | Start | End | PTM | AScore | Found By |
| --- | --- | --- | --- | --- | --- | --- | --- | --- | --- | --- | --- | --- | --- | --- | --- | --- | --- | --- | --- |
| R.L(+42.01)AAPAFR.A | Y | 21.67 | 786.4388 | 7 | 12.34 | 394.2294 | 2 | 14.83 | 0.7257 | 0.7331 | 40486 | 1.26e3 | 1 | 1 | 11 | 17 | Acetylation (Protein N-term) | L1:Acetylation (Protein N-term):1000 | DB Search |
| total 1 peptides |  |  |  |  |  |  |  |  |  |  |  |  |  |  |  |  |  |  |  |
| --- | --- | --- | --- | --- | --- | --- | --- | --- | --- | --- | --- | --- | --- | --- | --- | --- | --- | --- | --- |

Best Unique PSM (Scan Sample\_11\_A1\_1\_3648.d:40486, m/z=394.2294, z=2, RT=14.83, ppm=6.86):


TRINITY\_DN104063\_c0\_g1\_i1.p1

back to list

  

| Protein Coverage
| Supporting Peptides
| Best Unique PSM
|

Protein Coverage:

Supporting Peptides:

| Peptide | Uniq | -10lgP | Mass | Length | ppm | m/z | z | RT | 1/k0 Start | 1/k0 End | Precursor ID | Area Sample 2 | #Feature | #Feature Sample 2 | Start | End | PTM | AScore | Found By |
| --- | --- | --- | --- | --- | --- | --- | --- | --- | --- | --- | --- | --- | --- | --- | --- | --- | --- | --- | --- |
| R.V(+42.01)LGVAAVRR.V | Y | 22.14 | 981.6083 | 9 | -0.62 | 491.8084 | 2 | 18.27 | 0.8652 | 0.8725 | 58336 | 1.02e3 | 1 | 1 | 15 | 23 | Acetylation (Protein N-term) | V1:Acetylation (Protein N-term):1000 | DB Search |
| total 1 peptides |  |  |  |  |  |  |  |  |  |  |  |  |  |  |  |  |  |  |  |
| --- | --- | --- | --- | --- | --- | --- | --- | --- | --- | --- | --- | --- | --- | --- | --- | --- | --- | --- | --- |

Best Unique PSM (Scan Sample\_11\_A1\_1\_3648.d:58336, m/z=491.8084, z=2, RT=18.27, ppm=-6.1):


TRINITY\_DN471\_c0\_g1\_i2.p1

back to list

  

| Protein Coverage
| Supporting Peptides
| Best Unique PSM
|

Protein Coverage:

Supporting Peptides:

| Peptide | Uniq | -10lgP | Mass | Length | ppm | m/z | z | RT | 1/k0 Start | 1/k0 End | Precursor ID | Area Sample 2 | #Feature | #Feature Sample 2 | Start | End | PTM | AScore | Found By |
| --- | --- | --- | --- | --- | --- | --- | --- | --- | --- | --- | --- | --- | --- | --- | --- | --- | --- | --- | --- |
| R.TIQFVDWC(+57.02)PTGFK.C | Y | 54.82 | 1597.7599 | 13 | -6.53 | 799.8776 | 2 | 19.88 | 1.0351 | 1.0423 | 66701 | 3.31e2 | 1 | 1 | 378 | 390 | Carbamidomethylation |  | DB Search |
| R.RNLDIERPTYTNLNR.L | Y | 46.58 | 1873.9758 | 15 | -2.43 | 625.6609 | 3 | 13.94 | 0.8858 | 0.8931 | 35770 | 1.17e3 | 1 | 1 | 253 | 267 |  |  | DB Search |
| total 2 peptides |  |  |  |  |  |  |  |  |  |  |  |  |  |  |  |  |  |  |  |
| --- | --- | --- | --- | --- | --- | --- | --- | --- | --- | --- | --- | --- | --- | --- | --- | --- | --- | --- | --- |

Best Unique PSM (Scan Sample\_11\_A1\_1\_3648.d:66701, m/z=799.3823, z=2, RT=19.90, ppm=-12):


TRINITY\_DN34191\_c0\_g2\_i1.p2

back to list

  

| Protein Coverage
| Supporting Peptides
| Best Unique PSM
|

Protein Coverage:

Supporting Peptides:

| Peptide | Uniq | -10lgP | Mass | Length | ppm | m/z | z | RT | 1/k0 Start | 1/k0 End | Precursor ID | Area Sample 2 | #Feature | #Feature Sample 2 | Start | End | PTM | AScore | Found By |
| --- | --- | --- | --- | --- | --- | --- | --- | --- | --- | --- | --- | --- | --- | --- | --- | --- | --- | --- | --- |
| R.ARPHTM(+15.99)TSLTGSR.D | Y | 50.91 | 1429.7096 | 13 | 5.95 | 477.5774 | 3 | 9.99 | 0.8506 | 0.8579 | 13238 | 0 | 0 | 0 | 77 | 89 | Oxidation (M) | M6:Oxidation (M):1000 | DB Search |
| total 1 peptides |  |  |  |  |  |  |  |  |  |  |  |  |  |  |  |  |  |  |  |
| --- | --- | --- | --- | --- | --- | --- | --- | --- | --- | --- | --- | --- | --- | --- | --- | --- | --- | --- | --- |

Best Unique PSM (Scan Sample\_11\_A1\_1\_3648.d:13238, m/z=477.5774, z=3, RT=9.99, ppm=0.47):


TRINITY\_DN1933\_c0\_g1\_i3.p2

back to list

  

| Protein Coverage
| Supporting Peptides
| Best Unique PSM
|

Protein Coverage:

Supporting Peptides:

| Peptide | Uniq | -10lgP | Mass | Length | ppm | m/z | z | RT | 1/k0 Start | 1/k0 End | Precursor ID | Area Sample 2 | #Feature | #Feature Sample 2 | Start | End | PTM | AScore | Found By |
| --- | --- | --- | --- | --- | --- | --- | --- | --- | --- | --- | --- | --- | --- | --- | --- | --- | --- | --- | --- |
| R.RNLDIERPTYTNLNR.L | Y | 46.58 | 1873.9758 | 15 | -2.43 | 625.6609 | 3 | 13.94 | 0.8858 | 0.8931 | 35770 | 1.17e3 | 1 | 1 | 75 | 89 |  |  | DB Search |
| total 1 peptides |  |  |  |  |  |  |  |  |  |  |  |  |  |  |  |  |  |  |  |
| --- | --- | --- | --- | --- | --- | --- | --- | --- | --- | --- | --- | --- | --- | --- | --- | --- | --- | --- | --- |

Best Unique PSM (Scan Sample\_11\_A1\_1\_3648.d:35770, m/z=625.6609, z=3, RT=13.94, ppm=-7.91):


TRINITY\_DN165\_c0\_g1\_i1.p1

back to list

  

| Protein Coverage
| Supporting Peptides
| Best Unique PSM
|

Protein Coverage:

Supporting Peptides:

| Peptide | Uniq | -10lgP | Mass | Length | ppm | m/z | z | RT | 1/k0 Start | 1/k0 End | Precursor ID | Area Sample 2 | #Feature | #Feature Sample 2 | Start | End | PTM | AScore | Found By |
| --- | --- | --- | --- | --- | --- | --- | --- | --- | --- | --- | --- | --- | --- | --- | --- | --- | --- | --- | --- |
| K.TPFNFEDKPFIVQYEVR.F | Y | 53.86 | 2128.0629 | 17 | -6.4 | 710.3531 | 3 | 19.12 | 0.9453 | 0.9525 | 62983 | 0 | 0 | 0 | 114 | 130 |  |  | DB Search |
| total 1 peptides |  |  |  |  |  |  |  |  |  |  |  |  |  |  |  |  |  |  |  |
| --- | --- | --- | --- | --- | --- | --- | --- | --- | --- | --- | --- | --- | --- | --- | --- | --- | --- | --- | --- |

Best Unique PSM (Scan Sample\_11\_A1\_1\_3648.d:62983, m/z=710.3531, z=3, RT=19.12, ppm=-11.88):


TRINITY\_DN751\_c0\_g1\_i1.p1

back to list

  

| Protein Coverage
| Supporting Peptides
| Best Unique PSM
|

Protein Coverage:

Supporting Peptides:

| Peptide | Uniq | -10lgP | Mass | Length | ppm | m/z | z | RT | 1/k0 Start | 1/k0 End | Precursor ID | Area Sample 2 | #Feature | #Feature Sample 2 | Start | End | PTM | AScore | Found By |
| --- | --- | --- | --- | --- | --- | --- | --- | --- | --- | --- | --- | --- | --- | --- | --- | --- | --- | --- | --- |
| R.LAADLVITHSSK.S | Y | 57.93 | 1253.6979 | 12 | -0.91 | 627.8522 | 2 | 14.11 | 0.9969 | 1.0041 | 36628 | 1.76e3 | 1 | 1 | 166 | 177 |  |  | DB Search |
| total 1 peptides |  |  |  |  |  |  |  |  |  |  |  |  |  |  |  |  |  |  |  |
| --- | --- | --- | --- | --- | --- | --- | --- | --- | --- | --- | --- | --- | --- | --- | --- | --- | --- | --- | --- |

Best Unique PSM (Scan Sample\_11\_A1\_1\_3648.d:36628, m/z=627.8522, z=2, RT=14.11, ppm=-6.38):


TRINITY\_DN36\_c0\_g1\_i14.p1

back to list

  

| Protein Coverage
| Supporting Peptides
| Best Unique PSM
|

Protein Coverage:

Supporting Peptides:

| Peptide | Uniq | -10lgP | Mass | Length | ppm | m/z | z | RT | 1/k0 Start | 1/k0 End | Precursor ID | Area Sample 2 | #Feature | #Feature Sample 2 | Start | End | PTM | AScore | Found By |
| --- | --- | --- | --- | --- | --- | --- | --- | --- | --- | --- | --- | --- | --- | --- | --- | --- | --- | --- | --- |
| R.LGGIEVTQGNC(+57.02)ARK.Y | Y | 58.27 | 1501.7671 | 14 | 1.48 | 751.8878 | 2 | 12.59 | 1.0616 | 1.0688 | 28229 | 7.18e2 | 1 | 1 | 58 | 71 | Carbamidomethylation |  | DB Search |
| K.FETQIATK.D | Y | 57.95 | 936.4916 | 8 | 1.49 | 937.4952 | 1 | 13.74 | 1.2208 | 1.228 | 34637 | 7.44e2 | 2 | 2 | 167 | 174 |  |  | DB Search |
| total 2 peptides |  |  |  |  |  |  |  |  |  |  |  |  |  |  |  |  |  |  |  |
| --- | --- | --- | --- | --- | --- | --- | --- | --- | --- | --- | --- | --- | --- | --- | --- | --- | --- | --- | --- |

Best Unique PSM (Scan Sample\_11\_A1\_1\_3648.d:28229, m/z=751.8878, z=2, RT=12.59, ppm=-4):


TRINITY\_DN262\_c0\_g1\_i1.p1

back to list

  

| Protein Coverage
| Supporting Peptides
| Best Unique PSM
|

Protein Coverage:

Supporting Peptides:

| Peptide | Uniq | -10lgP | Mass | Length | ppm | m/z | z | RT | 1/k0 Start | 1/k0 End | Precursor ID | Area Sample 2 | #Feature | #Feature Sample 2 | Start | End | PTM | AScore | Found By |
| --- | --- | --- | --- | --- | --- | --- | --- | --- | --- | --- | --- | --- | --- | --- | --- | --- | --- | --- | --- |
| R.AEFRPNQEEEPILKR.E | Y | 57.91 | 1854.9588 | 15 | 1.99 | 619.3247 | 3 | 13.64 | 0.8761 | 0.8834 | 34085 | 5.81e2 | 1 | 1 | 439 | 453 |  |  | DB Search |
| R.DPVRDKLVNER.L | Y | 57.67 | 1339.7208 | 11 | 2.48 | 447.5795 | 3 | 11.98 | 0.7428 | 0.7502 | 24731 | 1.17e2 | 1 | 1 | 847 | 857 |  |  | DB Search |
| R.DANAAVVHGSHLK.D | Y | 51.68 | 1317.6789 | 13 | 1.05 | 659.8438 | 2 | 10.89 | 1.017 | 1.0242 | 18446 | 0 | 0 | 0 | 668 | 680 |  |  | DB Search |
| total 3 peptides |  |  |  |  |  |  |  |  |  |  |  |  |  |  |  |  |  |  |  |
| --- | --- | --- | --- | --- | --- | --- | --- | --- | --- | --- | --- | --- | --- | --- | --- | --- | --- | --- | --- |

Best Unique PSM (Scan Sample\_11\_A1\_1\_3648.d:34085, m/z=619.3247, z=3, RT=13.64, ppm=-3.49):


TRINITY\_DN1071\_c0\_g1\_i4.p1

back to list

  

| Protein Coverage
| Supporting Peptides
| Best Unique PSM
|

Protein Coverage:

Supporting Peptides:

| Peptide | Uniq | -10lgP | Mass | Length | ppm | m/z | z | RT | 1/k0 Start | 1/k0 End | Precursor ID | Area Sample 2 | #Feature | #Feature Sample 2 | Start | End | PTM | AScore | Found By |
| --- | --- | --- | --- | --- | --- | --- | --- | --- | --- | --- | --- | --- | --- | --- | --- | --- | --- | --- | --- |
| R.IQGHPTDKKFTK.N | Y | 55.25 | 1398.7619 | 12 | 4.46 | 467.2608 | 3 | 9.57 | 0.7677 | 0.775 | 11206 | 0 | 0 | 0 | 277 | 288 |  |  | DB Search |
| K.AVQIHPFNDVSVGDPK.V | Y | 31.99 | 1721.8736 | 16 | -1.26 | 861.9383 | 2 | 15.56 | 1.1158 | 1.123 | 44381 | 1.32e2 | 1 | 1 | 329 | 344 |  |  | DB Search |
| total 2 peptides |  |  |  |  |  |  |  |  |  |  |  |  |  |  |  |  |  |  |  |
| --- | --- | --- | --- | --- | --- | --- | --- | --- | --- | --- | --- | --- | --- | --- | --- | --- | --- | --- | --- |

Best Unique PSM (Scan Sample\_11\_A1\_1\_3648.d:11206, m/z=467.2608, z=3, RT=9.57, ppm=-1.02):


TRINITY\_DN119684\_c0\_g1\_i1.p1

back to list

  

| Protein Coverage
| Supporting Peptides
| Best Unique PSM
|

Protein Coverage:

Supporting Peptides:

| Peptide | Uniq | -10lgP | Mass | Length | ppm | m/z | z | RT | 1/k0 Start | 1/k0 End | Precursor ID | Area Sample 2 | #Feature | #Feature Sample 2 | Start | End | PTM | AScore | Found By |
| --- | --- | --- | --- | --- | --- | --- | --- | --- | --- | --- | --- | --- | --- | --- | --- | --- | --- | --- | --- |
| K.GKKPHVQVNVK.E | Y | 58.75 | 1232.7353 | 11 | 5.53 | 411.9191 | 3 | 9.78 | 0.7917 | 0.799 | 12069 | 7.14e2 | 1 | 1 | 125 | 135 |  |  | DB Search |
| R.LSPEDIER.M | Y | 20.37 | 957.4767 | 8 | 15.64 | 479.7505 | 2 | 14.28 | 0.8262 | 0.8335 | 37640 | 4.81e3 | 1 | 1 | 535 | 542 |  |  | DB Search |
| total 2 peptides |  |  |  |  |  |  |  |  |  |  |  |  |  |  |  |  |  |  |  |
| --- | --- | --- | --- | --- | --- | --- | --- | --- | --- | --- | --- | --- | --- | --- | --- | --- | --- | --- | --- |

Best Unique PSM (Scan Sample\_11\_A1\_1\_3648.d:12069, m/z=411.9191, z=3, RT=9.78, ppm=0.05):


TRINITY\_DN18187\_c0\_g1\_i4.p3

back to list

  

| Protein Coverage
| Supporting Peptides
| Best Unique PSM
|

Protein Coverage:

Supporting Peptides:

| Peptide | Uniq | -10lgP | Mass | Length | ppm | m/z | z | RT | 1/k0 Start | 1/k0 End | Precursor ID | Area Sample 2 | #Feature | #Feature Sample 2 | Start | End | PTM | AScore | Found By |
| --- | --- | --- | --- | --- | --- | --- | --- | --- | --- | --- | --- | --- | --- | --- | --- | --- | --- | --- | --- |
| R.YRPGTVALR.E | Y | 44.05 | 1031.5876 | 9 | -1.83 | 516.7973 | 2 | 12.44 | 0.8575 | 0.8648 | 27332 | 1.61e3 | 1 | 1 | 17 | 25 |  |  | DB Search |
| total 1 peptides |  |  |  |  |  |  |  |  |  |  |  |  |  |  |  |  |  |  |  |
| --- | --- | --- | --- | --- | --- | --- | --- | --- | --- | --- | --- | --- | --- | --- | --- | --- | --- | --- | --- |

Best Unique PSM (Scan Sample\_11\_A1\_1\_3648.d:27332, m/z=516.7973, z=2, RT=12.44, ppm=-7.31):


TRINITY\_DN73587\_c0\_g1\_i1.p1

back to list

  

| Protein Coverage
| Supporting Peptides
| Best Unique PSM
|

Protein Coverage:

Supporting Peptides:

| Peptide | Uniq | -10lgP | Mass | Length | ppm | m/z | z | RT | 1/k0 Start | 1/k0 End | Precursor ID | Area Sample 2 | #Feature | #Feature Sample 2 | Start | End | PTM | AScore | Found By |
| --- | --- | --- | --- | --- | --- | --- | --- | --- | --- | --- | --- | --- | --- | --- | --- | --- | --- | --- | --- |
| R.LVLEVAQHLGENVVR.T | Y | 58.88 | 1674.9417 | 15 | -1.69 | 838.4721 | 2 | 17.70 | 1.1418 | 1.149 | 55180 | 1.21e3 | 1 | 1 | 99 | 113 |  |  | DB Search |
| total 1 peptides |  |  |  |  |  |  |  |  |  |  |  |  |  |  |  |  |  |  |  |
| --- | --- | --- | --- | --- | --- | --- | --- | --- | --- | --- | --- | --- | --- | --- | --- | --- | --- | --- | --- |

Best Unique PSM (Scan Sample\_11\_A1\_1\_3648.d:55180, m/z=838.4721, z=2, RT=17.70, ppm=-7.16):


TRINITY\_DN64\_c0\_g1\_i1.p1

back to list

  

| Protein Coverage
| Supporting Peptides
| Best Unique PSM
|

Protein Coverage:

Supporting Peptides:

| Peptide | Uniq | -10lgP | Mass | Length | ppm | m/z | z | RT | 1/k0 Start | 1/k0 End | Precursor ID | Area Sample 2 | #Feature | #Feature Sample 2 | Start | End | PTM | AScore | Found By |
| --- | --- | --- | --- | --- | --- | --- | --- | --- | --- | --- | --- | --- | --- | --- | --- | --- | --- | --- | --- |
| K.YYVTIIDAPGHR.D | Y | 58.85 | 1403.7197 | 12 | 0.07 | 702.8633 | 2 | 15.65 | 1.0427 | 1.05 | 44823 | 2.81e2 | 1 | 1 | 88 | 99 |  |  | DB Search |
| R.AIEKFEKEAQEM(+15.99)GK.G | Y | 45.67 | 1652.8079 | 14 | 0.54 | 827.4072 | 2 | 12.02 | 1.1009 | 1.1082 | 24955 | 0 | 0 | 0 | 41 | 54 | Oxidation (M) | M12:Oxidation (M):1000 | DB Search |
| K.YYVTIIDAPGHRDFIK.N | Y | 42.12 | 1906.9941 | 16 | 6.13 | 954.5049 | 2 | 16.49 | 1.2037 | 1.2109 | 48851 | 0 | 0 | 0 | 88 | 103 |  |  | DB Search |
| total 3 peptides |  |  |  |  |  |  |  |  |  |  |  |  |  |  |  |  |  |  |  |
| --- | --- | --- | --- | --- | --- | --- | --- | --- | --- | --- | --- | --- | --- | --- | --- | --- | --- | --- | --- |

Best Unique PSM (Scan Sample\_11\_A1\_1\_3648.d:44823, m/z=702.8633, z=2, RT=15.65, ppm=-5.4):


TRINITY\_DN49493\_c0\_g1\_i1.p1

back to list

  

| Protein Coverage
| Supporting Peptides
| Best Unique PSM
|

Protein Coverage:

Supporting Peptides:

| Peptide | Uniq | -10lgP | Mass | Length | ppm | m/z | z | RT | 1/k0 Start | 1/k0 End | Precursor ID | Area Sample 2 | #Feature | #Feature Sample 2 | Start | End | PTM | AScore | Found By |
| --- | --- | --- | --- | --- | --- | --- | --- | --- | --- | --- | --- | --- | --- | --- | --- | --- | --- | --- | --- |
| K.LDISDEFRR.G | Y | 25.79 | 1149.5778 | 9 | -5.84 | 575.7897 | 2 | 15.31 | 0.9093 | 0.9166 | 43114 | 0 | 0 | 0 | 61 | 69 |  |  | DB Search |
| total 1 peptides |  |  |  |  |  |  |  |  |  |  |  |  |  |  |  |  |  |  |  |
| --- | --- | --- | --- | --- | --- | --- | --- | --- | --- | --- | --- | --- | --- | --- | --- | --- | --- | --- | --- |

Best Unique PSM (Scan Sample\_11\_A1\_1\_3648.d:43114, m/z=575.7897, z=2, RT=15.31, ppm=-11.32):


TRINITY\_DN146208\_c0\_g1\_i1.p1

back to list

  

| Protein Coverage
| Supporting Peptides
| Best Unique PSM
|

Protein Coverage:

Supporting Peptides:

| Peptide | Uniq | -10lgP | Mass | Length | ppm | m/z | z | RT | 1/k0 Start | 1/k0 End | Precursor ID | Area Sample 2 | #Feature | #Feature Sample 2 | Start | End | PTM | AScore | Found By |
| --- | --- | --- | --- | --- | --- | --- | --- | --- | --- | --- | --- | --- | --- | --- | --- | --- | --- | --- | --- |
| K.YGINHITNLVESKR.A | Y | 58.9 | 1642.8791 | 14 | 4.87 | 548.6333 | 3 | 14.82 | 0.823 | 0.8303 | 40480 | 6.96e2 | 1 | 1 | 158 | 171 |  |  | DB Search |
| K.YGINHITNLVESK.R | Y | 56.67 | 1486.778 | 13 | -4.57 | 744.3888 | 2 | 15.60 | 1.0371 | 1.0443 | 44593 | 0 | 0 | 0 | 158 | 170 |  |  | DB Search |
| total 2 peptides |  |  |  |  |  |  |  |  |  |  |  |  |  |  |  |  |  |  |  |
| --- | --- | --- | --- | --- | --- | --- | --- | --- | --- | --- | --- | --- | --- | --- | --- | --- | --- | --- | --- |

Best Unique PSM (Scan Sample\_11\_A1\_1\_3648.d:40480, m/z=548.6333, z=3, RT=14.82, ppm=-0.61):


TRINITY\_DN3271\_c0\_g1\_i1.p1

back to list

  

| Protein Coverage
| Supporting Peptides
| Best Unique PSM
|

Protein Coverage:

Supporting Peptides:

| Peptide | Uniq | -10lgP | Mass | Length | ppm | m/z | z | RT | 1/k0 Start | 1/k0 End | Precursor ID | Area Sample 2 | #Feature | #Feature Sample 2 | Start | End | PTM | AScore | Found By |
| --- | --- | --- | --- | --- | --- | --- | --- | --- | --- | --- | --- | --- | --- | --- | --- | --- | --- | --- | --- |
| K.TIESEAAHGTVTR.H | Y | 58.89 | 1370.679 | 13 | 4.55 | 686.3461 | 2 | 11.86 | 1.021 | 1.0283 | 23944 | 8.27e2 | 1 | 1 | 285 | 297 |  |  | DB Search |
| total 1 peptides |  |  |  |  |  |  |  |  |  |  |  |  |  |  |  |  |  |  |  |
| --- | --- | --- | --- | --- | --- | --- | --- | --- | --- | --- | --- | --- | --- | --- | --- | --- | --- | --- | --- |

Best Unique PSM (Scan Sample\_11\_A1\_1\_3648.d:23944, m/z=686.3461, z=2, RT=11.86, ppm=-0.93):


TRINITY\_DN265\_c0\_g1\_i9.p1

back to list

  

| Protein Coverage
| Supporting Peptides
| Best Unique PSM
|

Protein Coverage:

Supporting Peptides:

| Peptide | Uniq | -10lgP | Mass | Length | ppm | m/z | z | RT | 1/k0 Start | 1/k0 End | Precursor ID | Area Sample 2 | #Feature | #Feature Sample 2 | Start | End | PTM | AScore | Found By |
| --- | --- | --- | --- | --- | --- | --- | --- | --- | --- | --- | --- | --- | --- | --- | --- | --- | --- | --- | --- |
| R.LTHDFHTNKR.M | Y | 58.75 | 1267.6421 | 10 | 2.44 | 423.5534 | 3 | 9.64 | 0.7441 | 0.7514 | 11479 | 1.15e3 | 1 | 1 | 24 | 33 |  |  | DB Search |
| total 1 peptides |  |  |  |  |  |  |  |  |  |  |  |  |  |  |  |  |  |  |  |
| --- | --- | --- | --- | --- | --- | --- | --- | --- | --- | --- | --- | --- | --- | --- | --- | --- | --- | --- | --- |

Best Unique PSM (Scan Sample\_11\_A1\_1\_3648.d:11479, m/z=423.5534, z=3, RT=9.64, ppm=-3.04):


TRINITY\_DN61835\_c0\_g1\_i1.p1

back to list

  

| Protein Coverage
| Supporting Peptides
| Best Unique PSM
|

Protein Coverage:

Supporting Peptides:

| Peptide | Uniq | -10lgP | Mass | Length | ppm | m/z | z | RT | 1/k0 Start | 1/k0 End | Precursor ID | Area Sample 2 | #Feature | #Feature Sample 2 | Start | End | PTM | AScore | Found By |
| --- | --- | --- | --- | --- | --- | --- | --- | --- | --- | --- | --- | --- | --- | --- | --- | --- | --- | --- | --- |
| K.LDISDEFRR.T | Y | 25.79 | 1149.5778 | 9 | -5.84 | 575.7897 | 2 | 15.31 | 0.9093 | 0.9166 | 43114 | 0 | 0 | 0 | 194 | 202 |  |  | DB Search |
| total 1 peptides |  |  |  |  |  |  |  |  |  |  |  |  |  |  |  |  |  |  |  |
| --- | --- | --- | --- | --- | --- | --- | --- | --- | --- | --- | --- | --- | --- | --- | --- | --- | --- | --- | --- |

Best Unique PSM (Scan Sample\_11\_A1\_1\_3648.d:43114, m/z=575.7897, z=2, RT=15.31, ppm=-11.32):


TRINITY\_DN384\_c0\_g1\_i2.p1

back to list

  

| Protein Coverage
| Supporting Peptides
| Best Unique PSM
|

Protein Coverage:

Supporting Peptides:

| Peptide | Uniq | -10lgP | Mass | Length | ppm | m/z | z | RT | 1/k0 Start | 1/k0 End | Precursor ID | Area Sample 2 | #Feature | #Feature Sample 2 | Start | End | PTM | AScore | Found By |
| --- | --- | --- | --- | --- | --- | --- | --- | --- | --- | --- | --- | --- | --- | --- | --- | --- | --- | --- | --- |
| K.FVLHGDVSHGSR.E | Y | 57.97 | 1309.6527 | 12 | -8.24 | 655.8246 | 2 | 11.96 | 0.9872 | 0.9945 | 24686 | 0 | 0 | 0 | 275 | 286 |  |  | DB Search |
| total 1 peptides |  |  |  |  |  |  |  |  |  |  |  |  |  |  |  |  |  |  |  |
| --- | --- | --- | --- | --- | --- | --- | --- | --- | --- | --- | --- | --- | --- | --- | --- | --- | --- | --- | --- |

Best Unique PSM (Scan Sample\_11\_A1\_1\_3648.d:24686, m/z=655.8246, z=2, RT=11.96, ppm=-13.71):


TRINITY\_DN1339\_c0\_g2\_i3.p1

back to list

  

| Protein Coverage
| Supporting Peptides
| Best Unique PSM
|

Protein Coverage:

Supporting Peptides:

| Peptide | Uniq | -10lgP | Mass | Length | ppm | m/z | z | RT | 1/k0 Start | 1/k0 End | Precursor ID | Area Sample 2 | #Feature | #Feature Sample 2 | Start | End | PTM | AScore | Found By |
| --- | --- | --- | --- | --- | --- | --- | --- | --- | --- | --- | --- | --- | --- | --- | --- | --- | --- | --- | --- |
| K.LLTSSDQAELTKK.L | Y | 58.87 | 1432.7773 | 13 | 4.21 | 717.395 | 2 | 12.69 | 1.0496 | 1.0568 | 28711 | 7.94e2 | 1 | 1 | 155 | 167 |  |  | DB Search |
| total 1 peptides |  |  |  |  |  |  |  |  |  |  |  |  |  |  |  |  |  |  |  |
| --- | --- | --- | --- | --- | --- | --- | --- | --- | --- | --- | --- | --- | --- | --- | --- | --- | --- | --- | --- |

Best Unique PSM (Scan Sample\_11\_A1\_1\_3648.d:28711, m/z=717.395, z=2, RT=12.69, ppm=-1.26):


TRINITY\_DN329\_c0\_g1\_i2.p1

back to list

  

| Protein Coverage
| Supporting Peptides
| Best Unique PSM
|

Protein Coverage:

Supporting Peptides:

| Peptide | Uniq | -10lgP | Mass | Length | ppm | m/z | z | RT | 1/k0 Start | 1/k0 End | Precursor ID | Area Sample 2 | #Feature | #Feature Sample 2 | Start | End | PTM | AScore | Found By |
| --- | --- | --- | --- | --- | --- | --- | --- | --- | --- | --- | --- | --- | --- | --- | --- | --- | --- | --- | --- |
| K.SFHFGVIKLEGK.T | Y | 58.86 | 1360.7503 | 12 | -0.4 | 681.3784 | 2 | 16.70 | 1.0291 | 1.0363 | 49881 | 5.45e2 | 1 | 1 | 57 | 68 |  |  | DB Search |
| K.GLKVDFDTTFAPVTGKK.T | Y | 58.14 | 1822.9828 | 17 | 1.48 | 912.4951 | 2 | 16.88 | 1.1802 | 1.1873 | 50684 | 2.54e2 | 1 | 1 | 130 | 146 |  |  | DB Search |
| K.LRPGVELTMSSLVNAK.S | Y | 50.37 | 1713.9447 | 16 | -0.1 | 857.9749 | 2 | 17.45 | 1.145 | 1.1522 | 53751 | 1.26e3 | 1 | 1 | 287 | 302 |  |  | DB Search |
| K.LRPGVELTM(+15.99)SSLVNAK.S | Y | 42.38 | 1729.9396 | 16 | 3.46 | 865.9753 | 2 | 15.03 | 1.1338 | 1.141 | 41582 | 4.17e3 | 1 | 1 | 287 | 302 | Oxidation (M) | M9:Oxidation (M):1000 | DB Search |
| K.EGDLVIHSGITDAVK.Y | Y | 21.65 | 1552.8096 | 15 | -3.94 | 777.4048 | 2 | 15.22 | 1.0644 | 1.0717 | 42664 | 0 | 0 | 0 | 211 | 225 |  |  | DB Search |
| total 5 peptides |  |  |  |  |  |  |  |  |  |  |  |  |  |  |  |  |  |  |  |
| --- | --- | --- | --- | --- | --- | --- | --- | --- | --- | --- | --- | --- | --- | --- | --- | --- | --- | --- | --- |

Best Unique PSM (Scan Sample\_11\_A1\_1\_3648.d:49881, m/z=681.3784, z=2, RT=16.70, ppm=-5.88):


TRINITY\_DN1797\_c0\_g1\_i1.p1

back to list

  

| Protein Coverage
| Supporting Peptides
| Best Unique PSM
|

Protein Coverage:

Supporting Peptides:

| Peptide | Uniq | -10lgP | Mass | Length | ppm | m/z | z | RT | 1/k0 Start | 1/k0 End | Precursor ID | Area Sample 2 | #Feature | #Feature Sample 2 | Start | End | PTM | AScore | Found By |
| --- | --- | --- | --- | --- | --- | --- | --- | --- | --- | --- | --- | --- | --- | --- | --- | --- | --- | --- | --- |
| K.AVVIITDGKDDSTSR.L | Y | 31.58 | 1575.8104 | 15 | -9 | 788.9011 | 2 | 13.19 | 1.0937 | 1.1009 | 31695 | 0 | 0 | 0 | 878 | 892 |  |  | DB Search |
| total 1 peptides |  |  |  |  |  |  |  |  |  |  |  |  |  |  |  |  |  |  |  |
| --- | --- | --- | --- | --- | --- | --- | --- | --- | --- | --- | --- | --- | --- | --- | --- | --- | --- | --- | --- |

Best Unique PSM (Scan Sample\_11\_A1\_1\_3648.d:31695, m/z=788.9011, z=2, RT=13.19, ppm=-14.47):


TRINITY\_DN1319\_c0\_g1\_i2.p1

back to list

  

| Protein Coverage
| Supporting Peptides
| Best Unique PSM
|

Protein Coverage:

Supporting Peptides:

| Peptide | Uniq | -10lgP | Mass | Length | ppm | m/z | z | RT | 1/k0 Start | 1/k0 End | Precursor ID | Area Sample 2 | #Feature | #Feature Sample 2 | Start | End | PTM | AScore | Found By |
| --- | --- | --- | --- | --- | --- | --- | --- | --- | --- | --- | --- | --- | --- | --- | --- | --- | --- | --- | --- |
| K.GTHAAEIAHC(+57.02)VSR.R | Y | 59.14 | 1407.6677 | 13 | 2.78 | 704.8392 | 2 | 11.02 | 1.0263 | 1.0335 | 19137 | 2.25e3 | 1 | 1 | 91 | 103 | Carbamidomethylation |  | DB Search |
| total 1 peptides |  |  |  |  |  |  |  |  |  |  |  |  |  |  |  |  |  |  |  |
| --- | --- | --- | --- | --- | --- | --- | --- | --- | --- | --- | --- | --- | --- | --- | --- | --- | --- | --- | --- |

Best Unique PSM (Scan Sample\_11\_A1\_1\_3648.d:19137, m/z=704.8392, z=2, RT=11.02, ppm=-2.69):


TRINITY\_DN109115\_c0\_g1\_i1.p1

back to list

  

| Protein Coverage
| Supporting Peptides
| Best Unique PSM
|

Protein Coverage:

Supporting Peptides:

| Peptide | Uniq | -10lgP | Mass | Length | ppm | m/z | z | RT | 1/k0 Start | 1/k0 End | Precursor ID | Area Sample 2 | #Feature | #Feature Sample 2 | Start | End | PTM | AScore | Found By |
| --- | --- | --- | --- | --- | --- | --- | --- | --- | --- | --- | --- | --- | --- | --- | --- | --- | --- | --- | --- |
| R.IRELTSVVQKR.F | Y | 53.19 | 1327.7936 | 11 | 12.38 | 443.6082 | 3 | 12.34 | 0.7522 | 0.7595 | 26874 | 0 | 0 | 0 | 45 | 55 |  |  | DB Search |
| total 1 peptides |  |  |  |  |  |  |  |  |  |  |  |  |  |  |  |  |  |  |  |
| --- | --- | --- | --- | --- | --- | --- | --- | --- | --- | --- | --- | --- | --- | --- | --- | --- | --- | --- | --- |

Best Unique PSM (Scan Sample\_11\_A1\_1\_3648.d:26874, m/z=443.6082, z=3, RT=12.34, ppm=6.9):


TRINITY\_DN3545\_c0\_g1\_i1.p1

back to list

  

| Protein Coverage
| Supporting Peptides
| Best Unique PSM
|

Protein Coverage:

Supporting Peptides:

| Peptide | Uniq | -10lgP | Mass | Length | ppm | m/z | z | RT | 1/k0 Start | 1/k0 End | Precursor ID | Area Sample 2 | #Feature | #Feature Sample 2 | Start | End | PTM | AScore | Found By |
| --- | --- | --- | --- | --- | --- | --- | --- | --- | --- | --- | --- | --- | --- | --- | --- | --- | --- | --- | --- |
| R.SSC(+57.02)RPILSVIK.I | Y | 27.61 | 1258.7067 | 11 | -7.39 | 630.3525 | 2 | 16.61 | 0.9687 | 0.9759 | 49383 | 6.05e2 | 1 | 1 | 6 | 16 | Carbamidomethylation |  | DB Search |
| total 1 peptides |  |  |  |  |  |  |  |  |  |  |  |  |  |  |  |  |  |  |  |
| --- | --- | --- | --- | --- | --- | --- | --- | --- | --- | --- | --- | --- | --- | --- | --- | --- | --- | --- | --- |

Best Unique PSM (Scan Sample\_11\_A1\_1\_3648.d:49383, m/z=630.3525, z=2, RT=16.61, ppm=-12.87):


TRINITY\_DN3339\_c0\_g1\_i1.p1

back to list

  

| Protein Coverage
| Supporting Peptides
| Best Unique PSM
|

Protein Coverage:

Supporting Peptides:

| Peptide | Uniq | -10lgP | Mass | Length | ppm | m/z | z | RT | 1/k0 Start | 1/k0 End | Precursor ID | Area Sample 2 | #Feature | #Feature Sample 2 | Start | End | PTM | AScore | Found By |
| --- | --- | --- | --- | --- | --- | --- | --- | --- | --- | --- | --- | --- | --- | --- | --- | --- | --- | --- | --- |
| K.HGYIGEFEVIDDHR.S | Y | 58.64 | 1685.7797 | 14 | 0.78 | 562.9312 | 3 | 17.55 | 0.8725 | 0.8798 | 54397 | 3.69e2 | 1 | 1 | 44 | 57 |  |  | DB Search |
| M.VRVNVLNDALNSIC(+57.02)NAEKR.G | Y | 31.55 | 2184.1433 | 19 | -0.13 | 729.051 | 3 | 18.48 | 0.9824 | 0.9896 | 59403 | 5.64e2 | 1 | 1 | 2 | 20 | Carbamidomethylation |  | DB Search |
| total 2 peptides |  |  |  |  |  |  |  |  |  |  |  |  |  |  |  |  |  |  |  |
| --- | --- | --- | --- | --- | --- | --- | --- | --- | --- | --- | --- | --- | --- | --- | --- | --- | --- | --- | --- |

Best Unique PSM (Scan Sample\_11\_A1\_1\_3648.d:54397, m/z=562.9312, z=3, RT=17.55, ppm=-4.7):


TRINITY\_DN1907\_c0\_g1\_i2.p1

back to list

  

| Protein Coverage
| Supporting Peptides
| Best Unique PSM
|

Protein Coverage:

Supporting Peptides:

| Peptide | Uniq | -10lgP | Mass | Length | ppm | m/z | z | RT | 1/k0 Start | 1/k0 End | Precursor ID | Area Sample 2 | #Feature | #Feature Sample 2 | Start | End | PTM | AScore | Found By |
| --- | --- | --- | --- | --- | --- | --- | --- | --- | --- | --- | --- | --- | --- | --- | --- | --- | --- | --- | --- |
| K.VIIDYQTHHR.L | Y | 58.05 | 1280.6625 | 10 | -2.89 | 641.3332 | 2 | 12.03 | 0.9533 | 0.9606 | 25056 | 0 | 0 | 0 | 150 | 159 |  |  | DB Search |
| total 1 peptides |  |  |  |  |  |  |  |  |  |  |  |  |  |  |  |  |  |  |  |
| --- | --- | --- | --- | --- | --- | --- | --- | --- | --- | --- | --- | --- | --- | --- | --- | --- | --- | --- | --- |

Best Unique PSM (Scan Sample\_11\_A1\_1\_3648.d:25056, m/z=641.3332, z=2, RT=12.03, ppm=-8.37):


TRINITY\_DN1272\_c0\_g1\_i1.p1

back to list

  

| Protein Coverage
| Supporting Peptides
| Best Unique PSM
|

Protein Coverage:

Supporting Peptides:

| Peptide | Uniq | -10lgP | Mass | Length | ppm | m/z | z | RT | 1/k0 Start | 1/k0 End | Precursor ID | Area Sample 2 | #Feature | #Feature Sample 2 | Start | End | PTM | AScore | Found By |
| --- | --- | --- | --- | --- | --- | --- | --- | --- | --- | --- | --- | --- | --- | --- | --- | --- | --- | --- | --- |
| K.YIQGENEKQEK.I | Y | 52.47 | 1364.6572 | 11 | 1.28 | 683.333 | 2 | 9.57 | 0.9679 | 0.9751 | 11149 | 0 | 0 | 0 | 116 | 126 |  |  | DB Search |
| total 1 peptides |  |  |  |  |  |  |  |  |  |  |  |  |  |  |  |  |  |  |  |
| --- | --- | --- | --- | --- | --- | --- | --- | --- | --- | --- | --- | --- | --- | --- | --- | --- | --- | --- | --- |

Best Unique PSM (Scan Sample\_11\_A1\_1\_3648.d:11149, m/z=683.333, z=2, RT=9.57, ppm=-4.2):


TRINITY\_DN1112\_c0\_g1\_i1.p1

back to list

  

| Protein Coverage
| Supporting Peptides
| Best Unique PSM
|

Protein Coverage:

Supporting Peptides:

| Peptide | Uniq | -10lgP | Mass | Length | ppm | m/z | z | RT | 1/k0 Start | 1/k0 End | Precursor ID | Area Sample 2 | #Feature | #Feature Sample 2 | Start | End | PTM | AScore | Found By |
| --- | --- | --- | --- | --- | --- | --- | --- | --- | --- | --- | --- | --- | --- | --- | --- | --- | --- | --- | --- |
| K.KIDLHLYHELYM(+15.99)K.A | Y | 55.33 | 1717.8861 | 13 | 6.51 | 859.9512 | 2 | 15.43 | 1.1406 | 1.1478 | 43636 | 4.16e2 | 1 | 1 | 114 | 126 | Oxidation (M) | M12:Oxidation (M):1000 | DB Search |
| K.IDLHLYHELYM(+15.99)K.A | Y | 54.04 | 1589.7912 | 12 | 3.21 | 795.9011 | 2 | 15.41 | 1.0925 | 1.0997 | 43639 | 0 | 0 | 0 | 115 | 126 | Oxidation (M) | M11:Oxidation (M):1000 | DB Search |
| total 2 peptides |  |  |  |  |  |  |  |  |  |  |  |  |  |  |  |  |  |  |  |
| --- | --- | --- | --- | --- | --- | --- | --- | --- | --- | --- | --- | --- | --- | --- | --- | --- | --- | --- | --- |

Best Unique PSM (Scan Sample\_11\_A1\_1\_3648.d:43636, m/z=859.9512, z=2, RT=15.43, ppm=1.04):


TRINITY\_DN161\_c0\_g1\_i2.p1

back to list

  

| Protein Coverage
| Supporting Peptides
| Best Unique PSM
|

Protein Coverage:

Supporting Peptides:

| Peptide | Uniq | -10lgP | Mass | Length | ppm | m/z | z | RT | 1/k0 Start | 1/k0 End | Precursor ID | Area Sample 2 | #Feature | #Feature Sample 2 | Start | End | PTM | AScore | Found By |
| --- | --- | --- | --- | --- | --- | --- | --- | --- | --- | --- | --- | --- | --- | --- | --- | --- | --- | --- | --- |
| K.DSYVGDEAQSKR.G | Y | 59.21 | 1353.616 | 12 | 5.76 | 677.8155 | 2 | 10.93 | 1.0037 | 1.011 | 18642 | 9.97e3 | 1 | 1 | 74 | 85 |  |  | DB Search |
| R.LDLAGRDLTDYLM(+15.99)K.I | Y | 58.87 | 1638.8287 | 14 | 0.58 | 820.4176 | 2 | 17.97 | 1.1438 | 1.151 | 56587 | 5.44e3 | 1 | 1 | 201 | 214 | Oxidation (M) | M13:Oxidation (M):1000 | DB Search |
| R.AVFPSIVGRPR.H | Y | 58.86 | 1197.6982 | 11 | -0.21 | 599.853 | 2 | 15.44 | 0.9586 | 0.9658 | 43471 | 2.65e4 | 1 | 1 | 52 | 62 |  |  | DB Search |
| R.LDLAGRDLTDYLMK.I | Y | 58.52 | 1622.8338 | 14 | 2.44 | 812.4217 | 2 | 18.99 | 1.0961 | 1.1033 | 62153 | 2.2e3 | 1 | 1 | 201 | 214 |  |  | DB Search |
| R.VAPEEHPVLLTEAPLNPK.A | Y | 58.19 | 1953.0571 | 18 | -0.07 | 977.5304 | 2 | 16.19 | 1.2089 | 1.2161 | 47306 | 2.52e2 | 1 | 1 | 119 | 136 |  |  | DB Search |
| R.GYSFTTTAEREIVR.D | Y | 58.1 | 1628.8158 | 14 | 2.53 | 815.4128 | 2 | 15.79 | 1.121 | 1.1282 | 45442 | 6.41e3 | 1 | 1 | 220 | 233 |  |  | DB Search |
| R.HQGVM(+15.99)VGM(+15.99)GQK.D | Y | 55.12 | 1202.5536 | 11 | 3.32 | 602.2828 | 2 | 9.73 | 0.9105 | 0.9178 | 11903 | 0 | 0 | 0 | 63 | 73 | Oxidation (M), Oxidation (M) | M5:Oxidation (M):1000 M8:Oxidation (M):1000 | DB Search |
| R.TTGIVLDSGDGVSHTVPIYEGYALPHAIIR.L | Y | 52.38 | 3150.6349 | 30 | -8.99 | 788.6546 | 4 | 18.79 | 1.0693 | 1.0765 | 61107 | 0 | 0 | 0 | 171 | 200 |  |  | DB Search |
| total 8 peptides |  |  |  |  |  |  |  |  |  |  |  |  |  |  |  |  |  |  |  |
| --- | --- | --- | --- | --- | --- | --- | --- | --- | --- | --- | --- | --- | --- | --- | --- | --- | --- | --- | --- |

Best Unique PSM (Scan Sample\_11\_A1\_1\_3648.d:18642, m/z=677.8155, z=2, RT=10.93, ppm=0.28):


TRINITY\_DN2477\_c0\_g1\_i5.p1

back to list

  

| Protein Coverage
| Supporting Peptides
| Best Unique PSM
|

Protein Coverage:

Supporting Peptides:

| Peptide | Uniq | -10lgP | Mass | Length | ppm | m/z | z | RT | 1/k0 Start | 1/k0 End | Precursor ID | Area Sample 2 | #Feature | #Feature Sample 2 | Start | End | PTM | AScore | Found By |
| --- | --- | --- | --- | --- | --- | --- | --- | --- | --- | --- | --- | --- | --- | --- | --- | --- | --- | --- | --- |
| K.DSYVGDEAQSKR.G | Y | 59.21 | 1353.616 | 12 | 5.76 | 677.8155 | 2 | 10.93 | 1.0037 | 1.011 | 18642 | 9.97e3 | 1 | 1 | 8 | 19 |  |  | DB Search |
| R.VAPEEHPVLLTEAPLNPK.A | Y | 58.19 | 1953.0571 | 18 | -0.07 | 977.5304 | 2 | 16.19 | 1.2089 | 1.2161 | 47306 | 2.52e2 | 1 | 1 | 53 | 70 |  |  | DB Search |
| R.GYSFTTTAEREIVR.D | Y | 58.1 | 1628.8158 | 14 | 2.53 | 815.4128 | 2 | 15.79 | 1.121 | 1.1282 | 45442 | 6.41e3 | 1 | 1 | 154 | 167 |  |  | DB Search |
| total 3 peptides |  |  |  |  |  |  |  |  |  |  |  |  |  |  |  |  |  |  |  |
| --- | --- | --- | --- | --- | --- | --- | --- | --- | --- | --- | --- | --- | --- | --- | --- | --- | --- | --- | --- |

Best Unique PSM (Scan Sample\_11\_A1\_1\_3648.d:18642, m/z=677.8155, z=2, RT=10.93, ppm=0.28):


TRINITY\_DN7671\_c0\_g1\_i1.p1

back to list

  

| Protein Coverage
| Supporting Peptides
| Best Unique PSM
|

Protein Coverage:

Supporting Peptides:

| Peptide | Uniq | -10lgP | Mass | Length | ppm | m/z | z | RT | 1/k0 Start | 1/k0 End | Precursor ID | Area Sample 2 | #Feature | #Feature Sample 2 | Start | End | PTM | AScore | Found By |
| --- | --- | --- | --- | --- | --- | --- | --- | --- | --- | --- | --- | --- | --- | --- | --- | --- | --- | --- | --- |
| K.LSYC(+57.02)VSC(+57.02)AIHSK.V | Y | 58.81 | 1423.6588 | 12 | 2.72 | 712.8347 | 2 | 13.91 | 1.0303 | 1.0375 | 35578 | 0 | 0 | 0 | 71 | 82 | Carbamidomethylation, Carbamidomethylation |  | DB Search |
| total 1 peptides |  |  |  |  |  |  |  |  |  |  |  |  |  |  |  |  |  |  |  |
| --- | --- | --- | --- | --- | --- | --- | --- | --- | --- | --- | --- | --- | --- | --- | --- | --- | --- | --- | --- |

Best Unique PSM (Scan Sample\_11\_A1\_1\_3648.d:35578, m/z=712.8347, z=2, RT=13.91, ppm=-2.75):


TRINITY\_DN2472\_c0\_g1\_i1.p1

back to list

  

| Protein Coverage
| Supporting Peptides
| Best Unique PSM
|

Protein Coverage:

Supporting Peptides:

| Peptide | Uniq | -10lgP | Mass | Length | ppm | m/z | z | RT | 1/k0 Start | 1/k0 End | Precursor ID | Area Sample 2 | #Feature | #Feature Sample 2 | Start | End | PTM | AScore | Found By |
| --- | --- | --- | --- | --- | --- | --- | --- | --- | --- | --- | --- | --- | --- | --- | --- | --- | --- | --- | --- |
| M.S(+42.01)SGEIESLRQEAENLRNQIK.D | Y | 54.3 | 2342.1826 | 20 | -2.81 | 781.7283 | 3 | 19.42 | 1.0086 | 1.0158 | 64378 | 7.58e2 | 1 | 1 | 2 | 21 | Acetylation (Protein N-term) | S1:Acetylation (Protein N-term):1000 | DB Search |
| M.S(+42.01)SGEIESLRQEAENLR.N | Y | 54.25 | 1858.9021 | 16 | 3.78 | 930.4567 | 2 | 18.51 | 1.1985 | 1.2057 | 59475 | 1.24e3 | 1 | 1 | 2 | 17 | Acetylation (Protein N-term) | S1:Acetylation (Protein N-term):1000 | DB Search |
| total 2 peptides |  |  |  |  |  |  |  |  |  |  |  |  |  |  |  |  |  |  |  |
| --- | --- | --- | --- | --- | --- | --- | --- | --- | --- | --- | --- | --- | --- | --- | --- | --- | --- | --- | --- |

Best Unique PSM (Scan Sample\_11\_A1\_1\_3648.d:64378, m/z=781.7283, z=3, RT=19.42, ppm=-8.29):


TRINITY\_DN7659\_c0\_g1\_i2.p1

back to list

  

| Protein Coverage
| Supporting Peptides
| Best Unique PSM
|

Protein Coverage:

Supporting Peptides:

| Peptide | Uniq | -10lgP | Mass | Length | ppm | m/z | z | RT | 1/k0 Start | 1/k0 End | Precursor ID | Area Sample 2 | #Feature | #Feature Sample 2 | Start | End | PTM | AScore | Found By |
| --- | --- | --- | --- | --- | --- | --- | --- | --- | --- | --- | --- | --- | --- | --- | --- | --- | --- | --- | --- |
| M.A(+42.01)SAATDNRK.V | Y | 58.7 | 974.4781 | 9 | 5.12 | 488.2462 | 2 | 9.48 | 0.827 | 0.8343 | 10772 | 6.6e2 | 1 | 1 | 2 | 10 | Acetylation (Protein N-term) | A1:Acetylation (Protein N-term):1000 | DB Search |
| K.AQPTEIYNLGAQSHVK.V | Y | 50.58 | 1754.8951 | 16 | 0.53 | 878.4505 | 2 | 14.56 | 1.173 | 1.1802 | 39052 | 1.18e2 | 1 | 1 | 86 | 101 |  |  | DB Search |
| R.RGETFVTR.K | Y | 39.93 | 964.509 | 8 | -4.2 | 483.2571 | 2 | 11.39 | 0.8201 | 0.8274 | 21342 | 0 | 0 | 0 | 200 | 207 |  |  | DB Search |
| total 3 peptides |  |  |  |  |  |  |  |  |  |  |  |  |  |  |  |  |  |  |  |
| --- | --- | --- | --- | --- | --- | --- | --- | --- | --- | --- | --- | --- | --- | --- | --- | --- | --- | --- | --- |

Best Unique PSM (Scan Sample\_11\_A1\_1\_3648.d:10772, m/z=488.2462, z=2, RT=9.48, ppm=-0.36):


TRINITY\_DN36\_c0\_g1\_i13.p1

back to list

  

| Protein Coverage
| Supporting Peptides
| Best Unique PSM
|

Protein Coverage:

Supporting Peptides:

| Peptide | Uniq | -10lgP | Mass | Length | ppm | m/z | z | RT | 1/k0 Start | 1/k0 End | Precursor ID | Area Sample 2 | #Feature | #Feature Sample 2 | Start | End | PTM | AScore | Found By |
| --- | --- | --- | --- | --- | --- | --- | --- | --- | --- | --- | --- | --- | --- | --- | --- | --- | --- | --- | --- |
| K.GFDYC(+57.02)TTIKYR.L | Y | 58.87 | 1422.6602 | 11 | 2.87 | 712.3355 | 2 | 14.94 | 1.0242 | 1.0315 | 41159 | 9.5e2 | 1 | 1 | 46 | 56 | Carbamidomethylation |  | DB Search |
| R.LGGIEVTQGNC(+57.02)ARK.Y | Y | 58.27 | 1501.7671 | 14 | 1.48 | 751.8878 | 2 | 12.59 | 1.0616 | 1.0688 | 28229 | 7.18e2 | 1 | 1 | 57 | 70 | Carbamidomethylation |  | DB Search |
| K.FETQIATK.D | Y | 57.95 | 936.4916 | 8 | 1.49 | 937.4952 | 1 | 13.74 | 1.2208 | 1.228 | 34637 | 7.44e2 | 2 | 2 | 166 | 173 |  |  | DB Search |
| total 3 peptides |  |  |  |  |  |  |  |  |  |  |  |  |  |  |  |  |  |  |  |
| --- | --- | --- | --- | --- | --- | --- | --- | --- | --- | --- | --- | --- | --- | --- | --- | --- | --- | --- | --- |

Best Unique PSM (Scan Sample\_11\_A1\_1\_3648.d:41159, m/z=712.3355, z=2, RT=14.94, ppm=-2.6):


TRINITY\_DN96161\_c0\_g1\_i1.p1

back to list

  

| Protein Coverage
| Supporting Peptides
| Best Unique PSM
|

Protein Coverage:

Supporting Peptides:

| Peptide | Uniq | -10lgP | Mass | Length | ppm | m/z | z | RT | 1/k0 Start | 1/k0 End | Precursor ID | Area Sample 2 | #Feature | #Feature Sample 2 | Start | End | PTM | AScore | Found By |
| --- | --- | --- | --- | --- | --- | --- | --- | --- | --- | --- | --- | --- | --- | --- | --- | --- | --- | --- | --- |
| R.LDHKFDLM(+15.99)YAKR.A | Y | 40.99 | 1551.7868 | 12 | 3.09 | 776.8988 | 2 | 13.19 | 1.0881 | 1.0953 | 31591 | 1.18e2 | 1 | 1 | 391 | 402 | Oxidation (M) | M8:Oxidation (M):1000 | DB Search |
| total 1 peptides |  |  |  |  |  |  |  |  |  |  |  |  |  |  |  |  |  |  |  |
| --- | --- | --- | --- | --- | --- | --- | --- | --- | --- | --- | --- | --- | --- | --- | --- | --- | --- | --- | --- |

Best Unique PSM (Scan Sample\_11\_A1\_1\_3648.d:31591, m/z=776.8988, z=2, RT=13.19, ppm=-2.38):


TRINITY\_DN240\_c0\_g1\_i4.p1

back to list

  

| Protein Coverage
| Supporting Peptides
| Best Unique PSM
|

Protein Coverage:

Supporting Peptides:

| Peptide | Uniq | -10lgP | Mass | Length | ppm | m/z | z | RT | 1/k0 Start | 1/k0 End | Precursor ID | Area Sample 2 | #Feature | #Feature Sample 2 | Start | End | PTM | AScore | Found By |
| --- | --- | --- | --- | --- | --- | --- | --- | --- | --- | --- | --- | --- | --- | --- | --- | --- | --- | --- | --- |
| K.AYAAFLEFKDVVK.A | Y | 58.73 | 1499.8024 | 13 | -0.24 | 750.9042 | 2 | 18.72 | 1.0941 | 1.1013 | 60609 | 3.07e3 | 1 | 1 | 217 | 229 |  |  | DB Search |
| total 1 peptides |  |  |  |  |  |  |  |  |  |  |  |  |  |  |  |  |  |  |  |
| --- | --- | --- | --- | --- | --- | --- | --- | --- | --- | --- | --- | --- | --- | --- | --- | --- | --- | --- | --- |

Best Unique PSM (Scan Sample\_11\_A1\_1\_3648.d:60609, m/z=750.9042, z=2, RT=18.72, ppm=-5.71):


TRINITY\_DN471\_c0\_g1\_i1.p1

back to list

  

| Protein Coverage
| Supporting Peptides
| Best Unique PSM
|

Protein Coverage:

Supporting Peptides:

| Peptide | Uniq | -10lgP | Mass | Length | ppm | m/z | z | RT | 1/k0 Start | 1/k0 End | Precursor ID | Area Sample 2 | #Feature | #Feature Sample 2 | Start | End | PTM | AScore | Found By |
| --- | --- | --- | --- | --- | --- | --- | --- | --- | --- | --- | --- | --- | --- | --- | --- | --- | --- | --- | --- |
| R.RNLDIERPTYTNLNR.L | Y | 46.58 | 1873.9758 | 15 | -2.43 | 625.6609 | 3 | 13.94 | 0.8858 | 0.8931 | 35770 | 1.17e3 | 1 | 1 | 22 | 36 |  |  | DB Search |
| total 1 peptides |  |  |  |  |  |  |  |  |  |  |  |  |  |  |  |  |  |  |  |
| --- | --- | --- | --- | --- | --- | --- | --- | --- | --- | --- | --- | --- | --- | --- | --- | --- | --- | --- | --- |

Best Unique PSM (Scan Sample\_11\_A1\_1\_3648.d:35770, m/z=625.6609, z=3, RT=13.94, ppm=-7.91):


TRINITY\_DN585\_c0\_g1\_i10.p1

back to list

  

| Protein Coverage
| Supporting Peptides
| Best Unique PSM
|

Protein Coverage:

Supporting Peptides:

| Peptide | Uniq | -10lgP | Mass | Length | ppm | m/z | z | RT | 1/k0 Start | 1/k0 End | Precursor ID | Area Sample 2 | #Feature | #Feature Sample 2 | Start | End | PTM | AScore | Found By |
| --- | --- | --- | --- | --- | --- | --- | --- | --- | --- | --- | --- | --- | --- | --- | --- | --- | --- | --- | --- |
| K.IQDKEGIPPDQQR.L | Y | 55.06 | 1522.7739 | 13 | 2.88 | 762.3923 | 2 | 11.00 | 1.0331 | 1.0403 | 18584 | 2.97e3 | 2 | 2 | 30 | 42 |  |  | DB Search |
| total 1 peptides |  |  |  |  |  |  |  |  |  |  |  |  |  |  |  |  |  |  |  |
| --- | --- | --- | --- | --- | --- | --- | --- | --- | --- | --- | --- | --- | --- | --- | --- | --- | --- | --- | --- |

Best Unique PSM (Scan Sample\_11\_A1\_1\_3648.d:18584, m/z=762.3923, z=2, RT=11.00, ppm=-2.59):


TRINITY\_DN817\_c0\_g1\_i10.p1

back to list

  

| Protein Coverage
| Supporting Peptides
| Best Unique PSM
|

Protein Coverage:

Supporting Peptides:

| Peptide | Uniq | -10lgP | Mass | Length | ppm | m/z | z | RT | 1/k0 Start | 1/k0 End | Precursor ID | Area Sample 2 | #Feature | #Feature Sample 2 | Start | End | PTM | AScore | Found By |
| --- | --- | --- | --- | --- | --- | --- | --- | --- | --- | --- | --- | --- | --- | --- | --- | --- | --- | --- | --- |
| R.LC(+57.02)QPGYSTC(+57.02)ENHC(+57.02)R.R | Y | 38.48 | 1780.708 | 14 | 5.76 | 891.3615 | 2 | 11.39 | 1.0877 | 1.0949 | 21326 | 1.4e2 | 1 | 1 | 155 | 168 | Carbamidomethylation, Carbamidomethylation, Carbamidomethylation |  | DB Search |
| total 1 peptides |  |  |  |  |  |  |  |  |  |  |  |  |  |  |  |  |  |  |  |
| --- | --- | --- | --- | --- | --- | --- | --- | --- | --- | --- | --- | --- | --- | --- | --- | --- | --- | --- | --- |

Best Unique PSM (Scan Sample\_11\_A1\_1\_3648.d:21326, m/z=891.3615, z=2, RT=11.39, ppm=0.28):


TRINITY\_DN36\_c0\_g1\_i1.p1

back to list

  

| Protein Coverage
| Supporting Peptides
| Best Unique PSM
|

Protein Coverage:

Supporting Peptides:

| Peptide | Uniq | -10lgP | Mass | Length | ppm | m/z | z | RT | 1/k0 Start | 1/k0 End | Precursor ID | Area Sample 2 | #Feature | #Feature Sample 2 | Start | End | PTM | AScore | Found By |
| --- | --- | --- | --- | --- | --- | --- | --- | --- | --- | --- | --- | --- | --- | --- | --- | --- | --- | --- | --- |
| R.LGGIEVTQGNC(+57.02)ARK.Y | Y | 58.27 | 1501.7671 | 14 | 1.48 | 751.8878 | 2 | 12.59 | 1.0616 | 1.0688 | 28229 | 7.18e2 | 1 | 1 | 58 | 71 | Carbamidomethylation |  | DB Search |
| total 1 peptides |  |  |  |  |  |  |  |  |  |  |  |  |  |  |  |  |  |  |  |
| --- | --- | --- | --- | --- | --- | --- | --- | --- | --- | --- | --- | --- | --- | --- | --- | --- | --- | --- | --- |

Best Unique PSM (Scan Sample\_11\_A1\_1\_3648.d:28229, m/z=751.8878, z=2, RT=12.59, ppm=-4):


TRINITY\_DN2636\_c0\_g1\_i1.p1

back to list

  

| Protein Coverage
| Supporting Peptides
| Best Unique PSM
|

Protein Coverage:

Supporting Peptides:

| Peptide | Uniq | -10lgP | Mass | Length | ppm | m/z | z | RT | 1/k0 Start | 1/k0 End | Precursor ID | Area Sample 2 | #Feature | #Feature Sample 2 | Start | End | PTM | AScore | Found By |
| --- | --- | --- | --- | --- | --- | --- | --- | --- | --- | --- | --- | --- | --- | --- | --- | --- | --- | --- | --- |
| K.VVEKEAETER.K | Y | 58.8 | 1188.5986 | 10 | 7.97 | 397.2078 | 3 | 9.54 | 0.7322 | 0.7396 | 11020 | 5.76e2 | 1 | 1 | 199 | 208 |  |  | DB Search |
| total 1 peptides |  |  |  |  |  |  |  |  |  |  |  |  |  |  |  |  |  |  |  |
| --- | --- | --- | --- | --- | --- | --- | --- | --- | --- | --- | --- | --- | --- | --- | --- | --- | --- | --- | --- |

Best Unique PSM (Scan Sample\_11\_A1\_1\_3648.d:11020, m/z=397.2078, z=3, RT=9.54, ppm=2.49):


TRINITY\_DN1339\_c0\_g1\_i2.p1

back to list

  

| Protein Coverage
| Supporting Peptides
| Best Unique PSM
|

Protein Coverage:

Supporting Peptides:

| Peptide | Uniq | -10lgP | Mass | Length | ppm | m/z | z | RT | 1/k0 Start | 1/k0 End | Precursor ID | Area Sample 2 | #Feature | #Feature Sample 2 | Start | End | PTM | AScore | Found By |
| --- | --- | --- | --- | --- | --- | --- | --- | --- | --- | --- | --- | --- | --- | --- | --- | --- | --- | --- | --- |
| K.VITSDDPAEKEK.K | Y | 58.87 | 1330.6616 | 12 | 2.64 | 666.3362 | 2 | 10.69 | 0.9848 | 0.992 | 17191 | 9.01e3 | 1 | 1 | 160 | 171 |  |  | DB Search |
| total 1 peptides |  |  |  |  |  |  |  |  |  |  |  |  |  |  |  |  |  |  |  |
| --- | --- | --- | --- | --- | --- | --- | --- | --- | --- | --- | --- | --- | --- | --- | --- | --- | --- | --- | --- |

Best Unique PSM (Scan Sample\_11\_A1\_1\_3648.d:17191, m/z=666.3362, z=2, RT=10.69, ppm=-2.84):


TRINITY\_DN471\_c0\_g1\_i11.p1

back to list

  

| Protein Coverage
| Supporting Peptides
| Best Unique PSM
|

Protein Coverage:

Supporting Peptides:

| Peptide | Uniq | -10lgP | Mass | Length | ppm | m/z | z | RT | 1/k0 Start | 1/k0 End | Precursor ID | Area Sample 2 | #Feature | #Feature Sample 2 | Start | End | PTM | AScore | Found By |
| --- | --- | --- | --- | --- | --- | --- | --- | --- | --- | --- | --- | --- | --- | --- | --- | --- | --- | --- | --- |
| R.TIQFVDWC(+57.02)PTGFK.C | Y | 54.82 | 1597.7599 | 13 | -6.53 | 799.8776 | 2 | 19.88 | 1.0351 | 1.0423 | 66701 | 3.31e2 | 1 | 1 | 378 | 390 | Carbamidomethylation |  | DB Search |
| R.RNLDIERPTYTNLNR.L | Y | 46.58 | 1873.9758 | 15 | -2.43 | 625.6609 | 3 | 13.94 | 0.8858 | 0.8931 | 35770 | 1.17e3 | 1 | 1 | 253 | 267 |  |  | DB Search |
| total 2 peptides |  |  |  |  |  |  |  |  |  |  |  |  |  |  |  |  |  |  |  |
| --- | --- | --- | --- | --- | --- | --- | --- | --- | --- | --- | --- | --- | --- | --- | --- | --- | --- | --- | --- |

Best Unique PSM (Scan Sample\_11\_A1\_1\_3648.d:66701, m/z=799.3823, z=2, RT=19.90, ppm=-12):


TRINITY\_DN160\_c0\_g1\_i2.p1

back to list

  

| Protein Coverage
| Supporting Peptides
| Best Unique PSM
|

Protein Coverage:

Supporting Peptides:

| Peptide | Uniq | -10lgP | Mass | Length | ppm | m/z | z | RT | 1/k0 Start | 1/k0 End | Precursor ID | Area Sample 2 | #Feature | #Feature Sample 2 | Start | End | PTM | AScore | Found By |
| --- | --- | --- | --- | --- | --- | --- | --- | --- | --- | --- | --- | --- | --- | --- | --- | --- | --- | --- | --- |
| K.LRLDFDHEAKLNMIFR.F | Y | 49.33 | 2017.0567 | 16 | 6.27 | 505.2719 | 4 | 18.22 | 0.8627 | 0.87 | 58067 | 0 | 0 | 0 | 1007 | 1022 |  |  | DB Search |
| total 1 peptides |  |  |  |  |  |  |  |  |  |  |  |  |  |  |  |  |  |  |  |
| --- | --- | --- | --- | --- | --- | --- | --- | --- | --- | --- | --- | --- | --- | --- | --- | --- | --- | --- | --- |

Best Unique PSM (Scan Sample\_11\_A1\_1\_3648.d:58067, m/z=505.2719, z=4, RT=18.22, ppm=0.79):


TRINITY\_DN1910\_c0\_g1\_i5.p1

back to list

  

| Protein Coverage
| Supporting Peptides
| Best Unique PSM
|

Protein Coverage:

Supporting Peptides:

| Peptide | Uniq | -10lgP | Mass | Length | ppm | m/z | z | RT | 1/k0 Start | 1/k0 End | Precursor ID | Area Sample 2 | #Feature | #Feature Sample 2 | Start | End | PTM | AScore | Found By |
| --- | --- | --- | --- | --- | --- | --- | --- | --- | --- | --- | --- | --- | --- | --- | --- | --- | --- | --- | --- |
| K.IREEYPDR.I | Y | 58.88 | 1076.525 | 8 | 2.94 | 539.2684 | 2 | 10.94 | 0.866 | 0.8733 | 18650 | 6.17e3 | 1 | 1 | 155 | 162 |  |  | DB Search |
| K.NM(+15.99)M(+15.99)AAC(+57.02)DPR.H | Y | 58.21 | 1096.41 | 9 | 1.94 | 549.2103 | 2 | 9.53 | 0.8627 | 0.87 | 10857 | 1.37e3 | 1 | 1 | 298 | 306 | Oxidation (M), Oxidation (M), Carbamidomethylation | M2:Oxidation (M):1000 M3:Oxidation (M):1000 | DB Search |
| R.FPGQLNADLRK.L | Y | 25.64 | 1257.6829 | 11 | -1.3 | 629.8445 | 2 | 14.18 | 0.9457 | 0.9529 | 37015 | 6.11e2 | 1 | 1 | 242 | 252 |  |  | DB Search |
| total 3 peptides |  |  |  |  |  |  |  |  |  |  |  |  |  |  |  |  |  |  |  |
| --- | --- | --- | --- | --- | --- | --- | --- | --- | --- | --- | --- | --- | --- | --- | --- | --- | --- | --- | --- |

Best Unique PSM (Scan Sample\_11\_A1\_1\_3648.d:18650, m/z=539.8521, z=2, RT=10.95, ppm=-2.54):


TRINITY\_DN118\_c1\_g1\_i5.p1

back to list

  

| Protein Coverage
| Supporting Peptides
| Best Unique PSM
|

Protein Coverage:

Supporting Peptides:

| Peptide | Uniq | -10lgP | Mass | Length | ppm | m/z | z | RT | 1/k0 Start | 1/k0 End | Precursor ID | Area Sample 2 | #Feature | #Feature Sample 2 | Start | End | PTM | AScore | Found By |
| --- | --- | --- | --- | --- | --- | --- | --- | --- | --- | --- | --- | --- | --- | --- | --- | --- | --- | --- | --- |
| R.ETC(+57.02)SDIEKR.E | Y | 58.04 | 1136.5132 | 9 | 2.35 | 569.2621 | 2 | 10.08 | 0.8947 | 0.902 | 13694 | 6.3e3 | 1 | 1 | 186 | 194 | Carbamidomethylation |  | DB Search |
| K.VC(+57.02)EDVKR.C | Y | 57.57 | 904.4436 | 7 | 2.45 | 453.2277 | 2 | 9.38 | 0.7722 | 0.7795 | 10303 | 3.95e3 | 2 | 2 | 122 | 128 | Carbamidomethylation |  | DB Search |
| total 2 peptides |  |  |  |  |  |  |  |  |  |  |  |  |  |  |  |  |  |  |  |
| --- | --- | --- | --- | --- | --- | --- | --- | --- | --- | --- | --- | --- | --- | --- | --- | --- | --- | --- | --- |

Best Unique PSM (Scan Sample\_11\_A1\_1\_3648.d:13694, m/z=569.2621, z=2, RT=10.08, ppm=-3.13):


TRINITY\_DN250\_c0\_g1\_i2.p1

back to list

  

| Protein Coverage
| Supporting Peptides
| Best Unique PSM
|

Protein Coverage:

Supporting Peptides:

| Peptide | Uniq | -10lgP | Mass | Length | ppm | m/z | z | RT | 1/k0 Start | 1/k0 End | Precursor ID | Area Sample 2 | #Feature | #Feature Sample 2 | Start | End | PTM | AScore | Found By |
| --- | --- | --- | --- | --- | --- | --- | --- | --- | --- | --- | --- | --- | --- | --- | --- | --- | --- | --- | --- |
| K.ELVDQVLDRIR.K | Y | 58.86 | 1354.7568 | 11 | 2.67 | 678.3838 | 2 | 18.48 | 1.009 | 1.0162 | 59338 | 1.45e3 | 1 | 1 | 113 | 123 |  |  | DB Search |
| R.RNLDIERPTYTNLNR.L | Y | 46.58 | 1873.9758 | 15 | -2.43 | 625.6609 | 3 | 13.94 | 0.8858 | 0.8931 | 35770 | 1.17e3 | 1 | 1 | 215 | 229 |  |  | DB Search |
| total 2 peptides |  |  |  |  |  |  |  |  |  |  |  |  |  |  |  |  |  |  |  |
| --- | --- | --- | --- | --- | --- | --- | --- | --- | --- | --- | --- | --- | --- | --- | --- | --- | --- | --- | --- |

Best Unique PSM (Scan Sample\_11\_A1\_1\_3648.d:59338, m/z=678.3838, z=2, RT=18.48, ppm=-2.81):


TRINITY\_DN8101\_c0\_g1\_i10.p2

back to list

  

| Protein Coverage
| Supporting Peptides
| Best Unique PSM
|

Protein Coverage:

Supporting Peptides:

| Peptide | Uniq | -10lgP | Mass | Length | ppm | m/z | z | RT | 1/k0 Start | 1/k0 End | Precursor ID | Area Sample 2 | #Feature | #Feature Sample 2 | Start | End | PTM | AScore | Found By |
| --- | --- | --- | --- | --- | --- | --- | --- | --- | --- | --- | --- | --- | --- | --- | --- | --- | --- | --- | --- |
| R.SIGFIGHYLDQLR.L | Y | 54.32 | 1517.799 | 13 | 1.06 | 759.9034 | 2 | 19.56 | 1.0849 | 1.0921 | 65092 | 4.69e2 | 1 | 1 | 192 | 204 |  |  | DB Search |
| total 1 peptides |  |  |  |  |  |  |  |  |  |  |  |  |  |  |  |  |  |  |  |
| --- | --- | --- | --- | --- | --- | --- | --- | --- | --- | --- | --- | --- | --- | --- | --- | --- | --- | --- | --- |

Best Unique PSM (Scan Sample\_11\_A1\_1\_3648.d:65092, m/z=759.9034, z=2, RT=19.56, ppm=-4.41):


TRINITY\_DN3448\_c0\_g2\_i3.p2

back to list

  

| Protein Coverage
| Supporting Peptides
| Best Unique PSM
|

Protein Coverage:

Supporting Peptides:

| Peptide | Uniq | -10lgP | Mass | Length | ppm | m/z | z | RT | 1/k0 Start | 1/k0 End | Precursor ID | Area Sample 2 | #Feature | #Feature Sample 2 | Start | End | PTM | AScore | Found By |
| --- | --- | --- | --- | --- | --- | --- | --- | --- | --- | --- | --- | --- | --- | --- | --- | --- | --- | --- | --- |
| K.AGM(+15.99)THIVR.D | Y | 38.19 | 899.4647 | 8 | 6.96 | 450.7403 | 2 | 9.74 | 0.8254 | 0.8327 | 11994 | 0 | 0 | 0 | 31 | 38 | Oxidation (M) | M3:Oxidation (M):1000 | DB Search |
| total 1 peptides |  |  |  |  |  |  |  |  |  |  |  |  |  |  |  |  |  |  |  |
| --- | --- | --- | --- | --- | --- | --- | --- | --- | --- | --- | --- | --- | --- | --- | --- | --- | --- | --- | --- |

Best Unique PSM (Scan Sample\_11\_A1\_1\_3648.d:11994, m/z=450.7403, z=2, RT=9.74, ppm=1.48):


TRINITY\_DN166344\_c0\_g1\_i1.p1

back to list

  

| Protein Coverage
| Supporting Peptides
| Best Unique PSM
|

Protein Coverage:

Supporting Peptides:

| Peptide | Uniq | -10lgP | Mass | Length | ppm | m/z | z | RT | 1/k0 Start | 1/k0 End | Precursor ID | Area Sample 2 | #Feature | #Feature Sample 2 | Start | End | PTM | AScore | Found By |
| --- | --- | --- | --- | --- | --- | --- | --- | --- | --- | --- | --- | --- | --- | --- | --- | --- | --- | --- | --- |
| R.TQITC(+57.02)TQISLKR.G | Y | 57.65 | 1447.7817 | 12 | -4.63 | 724.8908 | 2 | 13.68 | 1.025 | 1.0323 | 34409 | 0 | 0 | 0 | 174 | 185 | Carbamidomethylation |  | DB Search |
| total 1 peptides |  |  |  |  |  |  |  |  |  |  |  |  |  |  |  |  |  |  |  |
| --- | --- | --- | --- | --- | --- | --- | --- | --- | --- | --- | --- | --- | --- | --- | --- | --- | --- | --- | --- |

Best Unique PSM (Scan Sample\_11\_A1\_1\_3648.d:34409, m/z=724.8908, z=2, RT=13.68, ppm=-10.1):


TRINITY\_DN1468\_c0\_g1\_i1.p1

back to list

  

| Protein Coverage
| Supporting Peptides
| Best Unique PSM
|

Protein Coverage:

Supporting Peptides:

| Peptide | Uniq | -10lgP | Mass | Length | ppm | m/z | z | RT | 1/k0 Start | 1/k0 End | Precursor ID | Area Sample 2 | #Feature | #Feature Sample 2 | Start | End | PTM | AScore | Found By |
| --- | --- | --- | --- | --- | --- | --- | --- | --- | --- | --- | --- | --- | --- | --- | --- | --- | --- | --- | --- |
| R.LRQIHNGVFVK.L | Y | 40.8 | 1309.7619 | 11 | 2.18 | 655.886 | 2 | 12.40 | 0.9763 | 0.9836 | 27123 | 3.3e2 | 1 | 1 | 110 | 120 |  |  | DB Search |
| total 1 peptides |  |  |  |  |  |  |  |  |  |  |  |  |  |  |  |  |  |  |  |
| --- | --- | --- | --- | --- | --- | --- | --- | --- | --- | --- | --- | --- | --- | --- | --- | --- | --- | --- | --- |

Best Unique PSM (Scan Sample\_11\_A1\_1\_3648.d:27123, m/z=655.886, z=2, RT=12.40, ppm=-3.3):


TRINITY\_DN8101\_c0\_g1\_i5.p1

back to list

  

| Protein Coverage
| Supporting Peptides
| Best Unique PSM
|

Protein Coverage:

Supporting Peptides:

| Peptide | Uniq | -10lgP | Mass | Length | ppm | m/z | z | RT | 1/k0 Start | 1/k0 End | Precursor ID | Area Sample 2 | #Feature | #Feature Sample 2 | Start | End | PTM | AScore | Found By |
| --- | --- | --- | --- | --- | --- | --- | --- | --- | --- | --- | --- | --- | --- | --- | --- | --- | --- | --- | --- |
| R.SIGFIGHYLDQLR.L | Y | 54.32 | 1517.799 | 13 | 1.06 | 759.9034 | 2 | 19.56 | 1.0849 | 1.0921 | 65092 | 4.69e2 | 1 | 1 | 784 | 796 |  |  | DB Search |
| total 1 peptides |  |  |  |  |  |  |  |  |  |  |  |  |  |  |  |  |  |  |  |
| --- | --- | --- | --- | --- | --- | --- | --- | --- | --- | --- | --- | --- | --- | --- | --- | --- | --- | --- | --- |

Best Unique PSM (Scan Sample\_11\_A1\_1\_3648.d:65092, m/z=759.9034, z=2, RT=19.56, ppm=-4.41):


TRINITY\_DN11643\_c0\_g1\_i1.p1

back to list

  

| Protein Coverage
| Supporting Peptides
| Best Unique PSM
|

Protein Coverage:

Supporting Peptides:

| Peptide | Uniq | -10lgP | Mass | Length | ppm | m/z | z | RT | 1/k0 Start | 1/k0 End | Precursor ID | Area Sample 2 | #Feature | #Feature Sample 2 | Start | End | PTM | AScore | Found By |
| --- | --- | --- | --- | --- | --- | --- | --- | --- | --- | --- | --- | --- | --- | --- | --- | --- | --- | --- | --- |
| R.EVNTELDSRPETK.K | Y | 58.3 | 1516.7369 | 13 | 4.85 | 759.3752 | 2 | 11.70 | 1.025 | 1.0323 | 23146 | 0 | 0 | 0 | 97 | 109 |  |  | DB Search |
| total 1 peptides |  |  |  |  |  |  |  |  |  |  |  |  |  |  |  |  |  |  |  |
| --- | --- | --- | --- | --- | --- | --- | --- | --- | --- | --- | --- | --- | --- | --- | --- | --- | --- | --- | --- |

Best Unique PSM (Scan Sample\_11\_A1\_1\_3648.d:23146, m/z=759.8266, z=2, RT=11.74, ppm=-0.63):


TRINITY\_DN200\_c0\_g1\_i3.p1

back to list

  

| Protein Coverage
| Supporting Peptides
| Best Unique PSM
|

Protein Coverage:

Supporting Peptides:

| Peptide | Uniq | -10lgP | Mass | Length | ppm | m/z | z | RT | 1/k0 Start | 1/k0 End | Precursor ID | Area Sample 2 | #Feature | #Feature Sample 2 | Start | End | PTM | AScore | Found By |
| --- | --- | --- | --- | --- | --- | --- | --- | --- | --- | --- | --- | --- | --- | --- | --- | --- | --- | --- | --- |
| K.TAIAIDTIINQKR.F | Y | 58.72 | 1455.8409 | 13 | 0.37 | 728.924 | 2 | 16.37 | 1.0604 | 1.0676 | 48298 | 0 | 0 | 0 | 217 | 229 |  |  | DB Search |
| total 1 peptides |  |  |  |  |  |  |  |  |  |  |  |  |  |  |  |  |  |  |  |
| --- | --- | --- | --- | --- | --- | --- | --- | --- | --- | --- | --- | --- | --- | --- | --- | --- | --- | --- | --- |

Best Unique PSM (Scan Sample\_11\_A1\_1\_3648.d:48298, m/z=728.924, z=2, RT=16.37, ppm=-5.1):


TRINITY\_DN4497\_c0\_g1\_i1.p1

back to list

  

| Protein Coverage
| Supporting Peptides
| Best Unique PSM
|

Protein Coverage:

Supporting Peptides:

| Peptide | Uniq | -10lgP | Mass | Length | ppm | m/z | z | RT | 1/k0 Start | 1/k0 End | Precursor ID | Area Sample 2 | #Feature | #Feature Sample 2 | Start | End | PTM | AScore | Found By |
| --- | --- | --- | --- | --- | --- | --- | --- | --- | --- | --- | --- | --- | --- | --- | --- | --- | --- | --- | --- |
| K.VLTSSDPAEKEK.K | Y | 58.73 | 1302.6667 | 12 | -2.55 | 652.3354 | 2 | 10.56 | 0.9969 | 1.0041 | 16353 | 2.66e3 | 1 | 1 | 117 | 128 |  |  | DB Search |
| total 1 peptides |  |  |  |  |  |  |  |  |  |  |  |  |  |  |  |  |  |  |  |
| --- | --- | --- | --- | --- | --- | --- | --- | --- | --- | --- | --- | --- | --- | --- | --- | --- | --- | --- | --- |

Best Unique PSM (Scan Sample\_11\_A1\_1\_3648.d:16353, m/z=652.3354, z=2, RT=10.56, ppm=-8.03):


TRINITY\_DN5365\_c0\_g1\_i2.p1

back to list

  

| Protein Coverage
| Supporting Peptides
| Best Unique PSM
|

Protein Coverage:

Supporting Peptides:

| Peptide | Uniq | -10lgP | Mass | Length | ppm | m/z | z | RT | 1/k0 Start | 1/k0 End | Precursor ID | Area Sample 2 | #Feature | #Feature Sample 2 | Start | End | PTM | AScore | Found By |
| --- | --- | --- | --- | --- | --- | --- | --- | --- | --- | --- | --- | --- | --- | --- | --- | --- | --- | --- | --- |
| R.FPGQLNADIRK.L | Y | 25.64 | 1257.6829 | 11 | -1.3 | 629.8445 | 2 | 14.18 | 0.9457 | 0.9529 | 37015 | 6.11e2 | 1 | 1 | 244 | 254 |  |  | DB Search |
| total 1 peptides |  |  |  |  |  |  |  |  |  |  |  |  |  |  |  |  |  |  |  |
| --- | --- | --- | --- | --- | --- | --- | --- | --- | --- | --- | --- | --- | --- | --- | --- | --- | --- | --- | --- |

Best Unique PSM (Scan Sample\_11\_A1\_1\_3648.d:37015, m/z=629.8445, z=2, RT=14.18, ppm=-6.78):


TRINITY\_DN428\_c0\_g1\_i1.p1

back to list

  

| Protein Coverage
| Supporting Peptides
| Best Unique PSM
|

Protein Coverage:

Supporting Peptides:

| Peptide | Uniq | -10lgP | Mass | Length | ppm | m/z | z | RT | 1/k0 Start | 1/k0 End | Precursor ID | Area Sample 2 | #Feature | #Feature Sample 2 | Start | End | PTM | AScore | Found By |
| --- | --- | --- | --- | --- | --- | --- | --- | --- | --- | --- | --- | --- | --- | --- | --- | --- | --- | --- | --- |
| R.LPIYTDKVINLYK.G | Y | 58.88 | 1578.9021 | 13 | -0.18 | 790.4539 | 2 | 17.48 | 1.1009 | 1.1082 | 53935 | 7.95e2 | 1 | 1 | 126 | 138 |  |  | DB Search |
| K.RIQQLSAIR.V | Y | 41.24 | 1083.6513 | 9 | 2.29 | 542.8312 | 2 | 13.06 | 0.9141 | 0.9214 | 30814 | 1.68e3 | 1 | 1 | 799 | 807 |  |  | DB Search |
| total 2 peptides |  |  |  |  |  |  |  |  |  |  |  |  |  |  |  |  |  |  |  |
| --- | --- | --- | --- | --- | --- | --- | --- | --- | --- | --- | --- | --- | --- | --- | --- | --- | --- | --- | --- |

Best Unique PSM (Scan Sample\_11\_A1\_1\_3648.d:53935, m/z=790.412, z=1, RT=17.50, ppm=-5.66):


TRINITY\_DN22492\_c0\_g1\_i1.p1

back to list

  

| Protein Coverage
| Supporting Peptides
| Best Unique PSM
|

Protein Coverage:

Supporting Peptides:

| Peptide | Uniq | -10lgP | Mass | Length | ppm | m/z | z | RT | 1/k0 Start | 1/k0 End | Precursor ID | Area Sample 2 | #Feature | #Feature Sample 2 | Start | End | PTM | AScore | Found By |
| --- | --- | --- | --- | --- | --- | --- | --- | --- | --- | --- | --- | --- | --- | --- | --- | --- | --- | --- | --- |
| R.HC(+57.02)DPELSSFHTFWR.L | Y | 58.51 | 1817.7944 | 14 | -2.5 | 606.9339 | 3 | 18.48 | 0.8862 | 0.8935 | 59444 | 3.29e2 | 1 | 1 | 151 | 164 | Carbamidomethylation |  | DB Search |
| total 1 peptides |  |  |  |  |  |  |  |  |  |  |  |  |  |  |  |  |  |  |  |
| --- | --- | --- | --- | --- | --- | --- | --- | --- | --- | --- | --- | --- | --- | --- | --- | --- | --- | --- | --- |

Best Unique PSM (Scan Sample\_11\_A1\_1\_3648.d:59444, m/z=606.9339, z=3, RT=18.48, ppm=-7.97):


TRINITY\_DN7467\_c0\_g1\_i2.p3

back to list

  

| Protein Coverage
| Supporting Peptides
| Best Unique PSM
|

Protein Coverage:

Supporting Peptides:

| Peptide | Uniq | -10lgP | Mass | Length | ppm | m/z | z | RT | 1/k0 Start | 1/k0 End | Precursor ID | Area Sample 2 | #Feature | #Feature Sample 2 | Start | End | PTM | AScore | Found By |
| --- | --- | --- | --- | --- | --- | --- | --- | --- | --- | --- | --- | --- | --- | --- | --- | --- | --- | --- | --- |
| R.TIQFVDWC(+57.02)PTGFK.C | Y | 54.82 | 1597.7599 | 13 | -6.53 | 799.8776 | 2 | 19.88 | 1.0351 | 1.0423 | 66701 | 3.31e2 | 1 | 1 | 61 | 73 | Carbamidomethylation |  | DB Search |
| total 1 peptides |  |  |  |  |  |  |  |  |  |  |  |  |  |  |  |  |  |  |  |
| --- | --- | --- | --- | --- | --- | --- | --- | --- | --- | --- | --- | --- | --- | --- | --- | --- | --- | --- | --- |

Best Unique PSM (Scan Sample\_11\_A1\_1\_3648.d:66701, m/z=799.3823, z=2, RT=19.90, ppm=-12):


TRINITY\_DN161\_c0\_g1\_i4.p1

back to list

  

| Protein Coverage
| Supporting Peptides
| Best Unique PSM
|

Protein Coverage:

Supporting Peptides:

| Peptide | Uniq | -10lgP | Mass | Length | ppm | m/z | z | RT | 1/k0 Start | 1/k0 End | Precursor ID | Area Sample 2 | #Feature | #Feature Sample 2 | Start | End | PTM | AScore | Found By |
| --- | --- | --- | --- | --- | --- | --- | --- | --- | --- | --- | --- | --- | --- | --- | --- | --- | --- | --- | --- |
| R.LDLAGRDLTDYLM(+15.99)K.I | Y | 58.87 | 1638.8287 | 14 | 0.58 | 820.4176 | 2 | 17.97 | 1.1438 | 1.151 | 56587 | 5.44e3 | 1 | 1 | 34 | 47 | Oxidation (M) | M13:Oxidation (M):1000 | DB Search |
| R.LDLAGRDLTDYLMK.I | Y | 58.52 | 1622.8338 | 14 | 2.44 | 812.4217 | 2 | 18.99 | 1.0961 | 1.1033 | 62153 | 2.2e3 | 1 | 1 | 34 | 47 |  |  | DB Search |
| R.GYSFTTTAEREIVR.D | Y | 58.1 | 1628.8158 | 14 | 2.53 | 815.4128 | 2 | 15.79 | 1.121 | 1.1282 | 45442 | 6.41e3 | 1 | 1 | 53 | 66 |  |  | DB Search |
| R.TTGIVLDSGDGVSHTVPIYEGYALPHAIIR.L | Y | 52.38 | 3150.6349 | 30 | -8.99 | 788.6546 | 4 | 18.79 | 1.0693 | 1.0765 | 61107 | 0 | 0 | 0 | 4 | 33 |  |  | DB Search |
| total 4 peptides |  |  |  |  |  |  |  |  |  |  |  |  |  |  |  |  |  |  |  |
| --- | --- | --- | --- | --- | --- | --- | --- | --- | --- | --- | --- | --- | --- | --- | --- | --- | --- | --- | --- |

Best Unique PSM (Scan Sample\_11\_A1\_1\_3648.d:56587, m/z=820.4176, z=2, RT=17.97, ppm=-4.89):


TRINITY\_DN2772\_c0\_g1\_i1.p1

back to list

  

| Protein Coverage
| Supporting Peptides
| Best Unique PSM
|

Protein Coverage:

Supporting Peptides:

| Peptide | Uniq | -10lgP | Mass | Length | ppm | m/z | z | RT | 1/k0 Start | 1/k0 End | Precursor ID | Area Sample 2 | #Feature | #Feature Sample 2 | Start | End | PTM | AScore | Found By |
| --- | --- | --- | --- | --- | --- | --- | --- | --- | --- | --- | --- | --- | --- | --- | --- | --- | --- | --- | --- |
| K.LIEALC(+57.02)AEHEIK.L | Y | 58.73 | 1424.7333 | 12 | 3.46 | 713.3725 | 2 | 14.93 | 1.0717 | 1.0789 | 41037 | 2.22e3 | 1 | 1 | 73 | 84 | Carbamidomethylation |  | DB Search |
| total 1 peptides |  |  |  |  |  |  |  |  |  |  |  |  |  |  |  |  |  |  |  |
| --- | --- | --- | --- | --- | --- | --- | --- | --- | --- | --- | --- | --- | --- | --- | --- | --- | --- | --- | --- |

Best Unique PSM (Scan Sample\_11\_A1\_1\_3648.d:41037, m/z=712.3854, z=1, RT=14.93, ppm=-2.01):


TRINITY\_DN333\_c0\_g1\_i1.p1

back to list

  

| Protein Coverage
| Supporting Peptides
| Best Unique PSM
|

Protein Coverage:

Supporting Peptides:

| Peptide | Uniq | -10lgP | Mass | Length | ppm | m/z | z | RT | 1/k0 Start | 1/k0 End | Precursor ID | Area Sample 2 | #Feature | #Feature Sample 2 | Start | End | PTM | AScore | Found By |
| --- | --- | --- | --- | --- | --- | --- | --- | --- | --- | --- | --- | --- | --- | --- | --- | --- | --- | --- | --- |
| R.RRYPIIVTGR.L | Y | 56.14 | 1229.7356 | 10 | 4.66 | 410.9188 | 3 | 12.79 | 0.777 | 0.7844 | 29251 | 7.87e2 | 1 | 1 | 286 | 295 |  |  | DB Search |
| total 1 peptides |  |  |  |  |  |  |  |  |  |  |  |  |  |  |  |  |  |  |  |
| --- | --- | --- | --- | --- | --- | --- | --- | --- | --- | --- | --- | --- | --- | --- | --- | --- | --- | --- | --- |

Best Unique PSM (Scan Sample\_11\_A1\_1\_3648.d:29251, m/z=410.9188, z=3, RT=12.79, ppm=-0.82):


TRINITY\_DN40728\_c0\_g1\_i1.p1

back to list

  

| Protein Coverage
| Supporting Peptides
| Best Unique PSM
|

Protein Coverage:

Supporting Peptides:

| Peptide | Uniq | -10lgP | Mass | Length | ppm | m/z | z | RT | 1/k0 Start | 1/k0 End | Precursor ID | Area Sample 2 | #Feature | #Feature Sample 2 | Start | End | PTM | AScore | Found By |
| --- | --- | --- | --- | --- | --- | --- | --- | --- | --- | --- | --- | --- | --- | --- | --- | --- | --- | --- | --- |
| K.IQDKEGIPPDQQR.L | Y | 55.06 | 1522.7739 | 13 | 2.88 | 762.3923 | 2 | 11.00 | 1.0331 | 1.0403 | 18584 | 2.97e3 | 2 | 2 | 53 | 65 |  |  | DB Search |
| total 1 peptides |  |  |  |  |  |  |  |  |  |  |  |  |  |  |  |  |  |  |  |
| --- | --- | --- | --- | --- | --- | --- | --- | --- | --- | --- | --- | --- | --- | --- | --- | --- | --- | --- | --- |

Best Unique PSM (Scan Sample\_11\_A1\_1\_3648.d:18584, m/z=762.3923, z=2, RT=11.00, ppm=-2.59):


TRINITY\_DN687\_c0\_g2\_i1.p1

back to list

  

| Protein Coverage
| Supporting Peptides
| Best Unique PSM
|

Protein Coverage:

Supporting Peptides:

| Peptide | Uniq | -10lgP | Mass | Length | ppm | m/z | z | RT | 1/k0 Start | 1/k0 End | Precursor ID | Area Sample 2 | #Feature | #Feature Sample 2 | Start | End | PTM | AScore | Found By |
| --- | --- | --- | --- | --- | --- | --- | --- | --- | --- | --- | --- | --- | --- | --- | --- | --- | --- | --- | --- |
| R.TIQFVDWC(+57.02)PTGFK.V | Y | 54.82 | 1597.7599 | 13 | -6.53 | 799.8776 | 2 | 19.88 | 1.0351 | 1.0423 | 66701 | 3.31e2 | 1 | 1 | 340 | 352 | Carbamidomethylation |  | DB Search |
| R.RNLDIERPTYTNLNR.L | Y | 46.58 | 1873.9758 | 15 | -2.43 | 625.6609 | 3 | 13.94 | 0.8858 | 0.8931 | 35770 | 1.17e3 | 1 | 1 | 215 | 229 |  |  | DB Search |
| R.LDHKFDLM(+15.99)YAKR.A | Y | 40.99 | 1551.7868 | 12 | 3.09 | 776.8988 | 2 | 13.19 | 1.0881 | 1.0953 | 31591 | 1.18e2 | 1 | 1 | 391 | 402 | Oxidation (M) | M8:Oxidation (M):1000 | DB Search |
| total 3 peptides |  |  |  |  |  |  |  |  |  |  |  |  |  |  |  |  |  |  |  |
| --- | --- | --- | --- | --- | --- | --- | --- | --- | --- | --- | --- | --- | --- | --- | --- | --- | --- | --- | --- |

Best Unique PSM (Scan Sample\_11\_A1\_1\_3648.d:66701, m/z=799.3823, z=2, RT=19.90, ppm=-12):


TRINITY\_DN1386\_c0\_g1\_i1.p1

back to list

  

| Protein Coverage
| Supporting Peptides
| Best Unique PSM
|

Protein Coverage:

Supporting Peptides:

| Peptide | Uniq | -10lgP | Mass | Length | ppm | m/z | z | RT | 1/k0 Start | 1/k0 End | Precursor ID | Area Sample 2 | #Feature | #Feature Sample 2 | Start | End | PTM | AScore | Found By |
| --- | --- | --- | --- | --- | --- | --- | --- | --- | --- | --- | --- | --- | --- | --- | --- | --- | --- | --- | --- |
| R.KVIVDQLVTLATR.V | Y | 58.15 | 1454.882 | 13 | -1.14 | 728.4435 | 2 | 18.03 | 1.0275 | 1.0347 | 57007 | 5.01e2 | 1 | 1 | 547 | 559 |  |  | DB Search |
| K.SFLLALEKDQEK.H | Y | 58 | 1419.7609 | 12 | 1.3 | 710.8848 | 2 | 17.09 | 1.0733 | 1.0805 | 51721 | 4.89e3 | 1 | 1 | 61 | 72 |  |  | DB Search |
| R.SPTHSVIK.S | Y | 27.03 | 867.4814 | 8 | -9.62 | 434.7414 | 2 | 9.92 | 0.7876 | 0.7949 | 12850 | 0 | 0 | 0 | 259 | 266 |  |  | DB Search |
| total 3 peptides |  |  |  |  |  |  |  |  |  |  |  |  |  |  |  |  |  |  |  |
| --- | --- | --- | --- | --- | --- | --- | --- | --- | --- | --- | --- | --- | --- | --- | --- | --- | --- | --- | --- |

Best Unique PSM (Scan Sample\_11\_A1\_1\_3648.d:57007, m/z=728.4435, z=2, RT=18.03, ppm=-6.61):


TRINITY\_DN116\_c0\_g1\_i1.p1

back to list

  

| Protein Coverage
| Supporting Peptides
| Best Unique PSM
|

Protein Coverage:

Supporting Peptides:

| Peptide | Uniq | -10lgP | Mass | Length | ppm | m/z | z | RT | 1/k0 Start | 1/k0 End | Precursor ID | Area Sample 2 | #Feature | #Feature Sample 2 | Start | End | PTM | AScore | Found By |
| --- | --- | --- | --- | --- | --- | --- | --- | --- | --- | --- | --- | --- | --- | --- | --- | --- | --- | --- | --- |
| K.IAGDVIVC(+57.02)AAYSHELPR.Y | Y | 58.33 | 1869.9407 | 17 | 6.24 | 935.9783 | 2 | 16.90 | 1.2033 | 1.2105 | 50851 | 1.42e2 | 1 | 1 | 69 | 85 | Carbamidomethylation |  | DB Search |
| total 1 peptides |  |  |  |  |  |  |  |  |  |  |  |  |  |  |  |  |  |  |  |
| --- | --- | --- | --- | --- | --- | --- | --- | --- | --- | --- | --- | --- | --- | --- | --- | --- | --- | --- | --- |

Best Unique PSM (Scan Sample\_11\_A1\_1\_3648.d:50851, m/z=935.9783, z=2, RT=16.90, ppm=0.77):


TRINITY\_DN124769\_c0\_g1\_i1.p1

back to list

  

| Protein Coverage
| Supporting Peptides
| Best Unique PSM
|

Protein Coverage:

Supporting Peptides:

| Peptide | Uniq | -10lgP | Mass | Length | ppm | m/z | z | RT | 1/k0 Start | 1/k0 End | Precursor ID | Area Sample 2 | #Feature | #Feature Sample 2 | Start | End | PTM | AScore | Found By |
| --- | --- | --- | --- | --- | --- | --- | --- | --- | --- | --- | --- | --- | --- | --- | --- | --- | --- | --- | --- |
| K.VITSDDPAEKEK.K | Y | 58.87 | 1330.6616 | 12 | 2.64 | 666.3362 | 2 | 10.69 | 0.9848 | 0.992 | 17191 | 9.01e3 | 1 | 1 | 91 | 102 |  |  | DB Search |
| total 1 peptides |  |  |  |  |  |  |  |  |  |  |  |  |  |  |  |  |  |  |  |
| --- | --- | --- | --- | --- | --- | --- | --- | --- | --- | --- | --- | --- | --- | --- | --- | --- | --- | --- | --- |

Best Unique PSM (Scan Sample\_11\_A1\_1\_3648.d:17191, m/z=666.3362, z=2, RT=10.69, ppm=-2.84):


TRINITY\_DN956\_c0\_g1\_i2.p1

back to list

  

| Protein Coverage
| Supporting Peptides
| Best Unique PSM
|

Protein Coverage:

Supporting Peptides:

| Peptide | Uniq | -10lgP | Mass | Length | ppm | m/z | z | RT | 1/k0 Start | 1/k0 End | Precursor ID | Area Sample 2 | #Feature | #Feature Sample 2 | Start | End | PTM | AScore | Found By |
| --- | --- | --- | --- | --- | --- | --- | --- | --- | --- | --- | --- | --- | --- | --- | --- | --- | --- | --- | --- |
| K.AGYGFVIFDDRR.D | Y | 41.74 | 1414.6993 | 12 | 1.94 | 708.3544 | 2 | 18.09 | 1.0379 | 1.0452 | 57312 | 3.6e2 | 1 | 1 | 40 | 51 |  |  | DB Search |
| total 1 peptides |  |  |  |  |  |  |  |  |  |  |  |  |  |  |  |  |  |  |  |
| --- | --- | --- | --- | --- | --- | --- | --- | --- | --- | --- | --- | --- | --- | --- | --- | --- | --- | --- | --- |

Best Unique PSM (Scan Sample\_11\_A1\_1\_3648.d:57312, m/z=708.3544, z=2, RT=18.09, ppm=-3.53):


TRINITY\_DN3036\_c0\_g1\_i3.p1

back to list

  

| Protein Coverage
| Supporting Peptides
| Best Unique PSM
|

Protein Coverage:

Supporting Peptides:

| Peptide | Uniq | -10lgP | Mass | Length | ppm | m/z | z | RT | 1/k0 Start | 1/k0 End | Precursor ID | Area Sample 2 | #Feature | #Feature Sample 2 | Start | End | PTM | AScore | Found By |
| --- | --- | --- | --- | --- | --- | --- | --- | --- | --- | --- | --- | --- | --- | --- | --- | --- | --- | --- | --- |
| R.AGHGAVTSPQTQR.G | N | 58.95 | 1308.6534 | 13 | 4.15 | 655.3331 | 2 | 9.70 | 0.9146 | 0.9218 | 11889 | 1.61e4 | 2 | 2 | 309 | 321 |  |  | DB Search |
| R.GTSNGGLVVGTIIKPK.L | Y | 58.87 | 1539.8984 | 16 | 2.71 | 770.9543 | 2 | 15.49 | 1.1254 | 1.1326 | 43901 | 6.84e3 | 1 | 1 | 173 | 188 |  |  | DB Search |
| R.VIGASGIHVGTM(+15.99)SFGK.M | N | 58.84 | 1575.8079 | 16 | 2.27 | 788.9087 | 2 | 14.96 | 1.1306 | 1.1378 | 41219 | 1.68e3 | 1 | 1 | 334 | 349 | Oxidation (M) | M12:Oxidation (M):1000 | DB Search |
| total 3 peptides |  |  |  |  |  |  |  |  |  |  |  |  |  |  |  |  |  |  |  |
| --- | --- | --- | --- | --- | --- | --- | --- | --- | --- | --- | --- | --- | --- | --- | --- | --- | --- | --- | --- |

Best Unique PSM (Scan Sample\_11\_A1\_1\_3648.d:43901, m/z=770.9543, z=2, RT=15.49, ppm=-2.77):


TRINITY\_DN131028\_c0\_g1\_i1.p2

back to list

  

| Protein Coverage
| Supporting Peptides
| Best Unique PSM
|

Protein Coverage:

Supporting Peptides:

| Peptide | Uniq | -10lgP | Mass | Length | ppm | m/z | z | RT | 1/k0 Start | 1/k0 End | Precursor ID | Area Sample 2 | #Feature | #Feature Sample 2 | Start | End | PTM | AScore | Found By |
| --- | --- | --- | --- | --- | --- | --- | --- | --- | --- | --- | --- | --- | --- | --- | --- | --- | --- | --- | --- |
| FDKYHPGYFGK.V | Y | 58.78 | 1357.6455 | 11 | -2.44 | 679.8246 | 2 | 17.74 | 0.9876 | 0.9949 | 55445 | 8.83e2 | 1 | 1 | 1 | 11 |  |  | DB Search |
| total 1 peptides |  |  |  |  |  |  |  |  |  |  |  |  |  |  |  |  |  |  |  |
| --- | --- | --- | --- | --- | --- | --- | --- | --- | --- | --- | --- | --- | --- | --- | --- | --- | --- | --- | --- |

Best Unique PSM (Scan Sample\_11\_A1\_1\_3648.d:55445, m/z=679.8246, z=2, RT=17.74, ppm=-7.92):


TRINITY\_DN651\_c0\_g1\_i2.p1

back to list

  

| Protein Coverage
| Supporting Peptides
| Best Unique PSM
|

Protein Coverage:

Supporting Peptides:

| Peptide | Uniq | -10lgP | Mass | Length | ppm | m/z | z | RT | 1/k0 Start | 1/k0 End | Precursor ID | Area Sample 2 | #Feature | #Feature Sample 2 | Start | End | PTM | AScore | Found By |
| --- | --- | --- | --- | --- | --- | --- | --- | --- | --- | --- | --- | --- | --- | --- | --- | --- | --- | --- | --- |
| R.VVAISGNEDEIVNLLHK.I | Y | 55.96 | 1848.9945 | 17 | 2.17 | 925.5015 | 2 | 18.50 | 1.2109 | 1.2181 | 59519 | 0 | 0 | 0 | 160 | 176 |  |  | DB Search |
| total 1 peptides |  |  |  |  |  |  |  |  |  |  |  |  |  |  |  |  |  |  |  |
| --- | --- | --- | --- | --- | --- | --- | --- | --- | --- | --- | --- | --- | --- | --- | --- | --- | --- | --- | --- |

Best Unique PSM (Scan Sample\_11\_A1\_1\_3648.d:59519, m/z=925.5015, z=2, RT=18.50, ppm=-3.3):


---

Prepared with PEAKS ™ (bioinfor.com)
