# Supplementary material for: Proteomic Analysis and Biochemical Characterization of the Nematocyst Extract of the Hydrozoan Velella velella
Source: Mar Drugs. 2024 Oct 12;22(10):468. doi: 10.3390/md22100468 (PMC11509761; doi:10.3390/md22100468)
Supplement: Supplementary file 1 [file marinedrugs-22-00468-s001.zip › Supplementary File S1.zip!/Sample_2_Vellela/db.peptides.html]

peptide list


Peptide List

  

| Peptide | -10lgP | Mass | Length | ppm | m/z | RT | 1/k0 Start | 1/k0 End | Precursor ID | Area Sample 4 | #Feature | #Feature Sample 4 | Accession | PTM | AScore | Found By |
| --- | --- | --- | --- | --- | --- | --- | --- | --- | --- | --- | --- | --- | --- | --- | --- | --- |
| LIDELTPEVTSALEC(+57.02)EGGPR | 58.41 | 2185.0571 | 20 | 2.45 | 729.3599 | 19.44 | 0.9824 | 0.9896 | 73811 | 1.6e4 | 2 | 2 | TRINITY\_DN244\_c0\_g1\_i1.p1 | Carbamidomethylation |  | DB Search |
| TVSPEPFIESC(+57.02)QQETC(+57.02)LC(+57.02)K | 58.41 | 2312.0122 | 19 | -0.66 | 771.6758 | 17.07 | 0.9243 | 0.9315 | 58393 | 2.54e3 | 3 | 3 | TRINITY\_DN6390\_c0\_g1\_i1.p1 | Carbamidomethylation, Carbamidomethylation, Carbamidomethylation |  | DB Search |
| FEC(+57.02)TDFDC(+57.02)VAHC(+57.02)ASK | 58.41 | 1845.712 | 15 | -3.34 | 616.2412 | 15.02 | 0.898 | 0.9052 | 45403 | 3.7e3 | 2 | 2 | TRINITY\_DN6390\_c0\_g1\_i1.p1 | Carbamidomethylation, Carbamidomethylation, Carbamidomethylation |  | DB Search |
| AVQIHPFNDVSVGDPK | 58.41 | 1721.8737 | 16 | 11.97 | 574.9708 | 15.99 | 0.8761 | 0.8834 | 51505 | 2.3e4 | 2 | 2 | TRINITY\_DN1071\_c0\_g1\_i2.p1:TRINITY\_DN1071\_c0\_g1\_i3.p1:TRINITY\_DN1071\_c0\_g1\_i4.p1:TRINITY\_DN1071\_c0\_g1\_i1.p1 |  |  | DB Search |
| AC(+57.02)GLENM(+15.99)C(+57.02)VGSYK | 58.41 | 1503.6156 | 13 | -0.67 | 752.8129 | 13.59 | 0.9989 | 1.0061 | 36764 | 0 | 0 | 0 | TRINITY\_DN8903\_c0\_g1\_i1.p1:TRINITY\_DN24268\_c0\_g1\_i2.p1:TRINITY\_DN24268\_c0\_g1\_i1.p1 | Carbamidomethylation, Oxidation (M), Carbamidomethylation | M7:Oxidation (M):1000 | DB Search |
| FGDIMDWTVHQGK | 58.41 | 1532.7081 | 13 | 0.12 | 511.9089 | 18.62 | 0.7982 | 0.8055 | 68637 | 0 | 0 | 0 | TRINITY\_DN4781\_c0\_g1\_i1.p1:TRINITY\_DN4781\_c0\_g1\_i2.p1 |  |  | DB Search |
| GFAFETEDPQNFPLEK | 58.41 | 1867.8628 | 16 | 3.55 | 934.9399 | 19.12 | 1.1366 | 1.1438 | 71825 | 5.59e2 | 1 | 1 | TRINITY\_DN262\_c0\_g1\_i1.p1 |  |  | DB Search |
| ILALAGQLQSARDELVK | 58.41 | 1824.0469 | 17 | 2.89 | 609.0233 | 18.84 | 0.904 | 0.9113 | 69982 | 1.41e3 | 1 | 1 | TRINITY\_DN1366\_c0\_g1\_i1.p1 |  |  | DB Search |
| LLAVTVGNNYNLDTLEK | 58.4 | 1875.9941 | 17 | -12.01 | 938.991 | 18.74 | 1.1414 | 1.1486 | 69418 | 0 | 0 | 0 | TRINITY\_DN77\_c1\_g1\_i1.p1 |  |  | DB Search |
| LLILITTHVSHVR | 58.4 | 1500.9139 | 13 | -2.48 | 501.3096 | 16.66 | 0.8063 | 0.8136 | 55744 | 3.08e2 | 1 | 1 | TRINITY\_DN69484\_c0\_g1\_i1.p1 |  |  | DB Search |
| LLQQHELDLTPLK | 58.4 | 1546.8718 | 13 | -0.89 | 516.6296 | 16.98 | 0.8356 | 0.8429 | 57805 | 1.52e3 | 2 | 2 | TRINITY\_DN1676\_c0\_g1\_i6.p1:TRINITY\_DN1676\_c0\_g1\_i12.p1 |  |  | DB Search |
| LLSPVFKPHGNK | 58.4 | 1335.7662 | 12 | 7.47 | 446.265 | 13.80 | 0.7982 | 0.8055 | 37938 | 3.05e2 | 1 | 1 | TRINITY\_DN4781\_c0\_g1\_i1.p1:TRINITY\_DN4781\_c0\_g1\_i2.p1 |  |  | DB Search |
| SGGDTSPFGEC(+57.02)KK | 58.4 | 1368.5979 | 13 | 8.57 | 457.2095 | 11.91 | 0.7559 | 0.7632 | 26366 | 1.73e3 | 1 | 1 | TRINITY\_DN1676\_c0\_g1\_i6.p1:TRINITY\_DN1676\_c0\_g1\_i12.p1 | Carbamidomethylation |  | DB Search |
| EQVSTFDSLQADLVK | 58.4 | 1678.8413 | 15 | 2.3 | 840.428 | 18.77 | 1.0917 | 1.0989 | 69421 | 6.52e2 | 1 | 1 | TRINITY\_DN1043\_c0\_g1\_i2.p1:TRINITY\_DN7059\_c0\_g1\_i1.p1 |  |  | DB Search |
| SNIDEVNVKHEQIEEFIK | 58.4 | 2170.0906 | 18 | 0.32 | 724.3694 | 16.43 | 0.9566 | 0.9638 | 54256 | 7.37e2 | 1 | 1 | TRINITY\_DN1616\_c0\_g1\_i3.p1:TRINITY\_DN1616\_c0\_g1\_i6.p1:TRINITY\_DN1616\_c0\_g1\_i2.p1:TRINITY\_DN1616\_c0\_g1\_i7.p1 |  |  | DB Search |
| IGEIVFQTM(+15.99)QDAQK | 58.4 | 1622.7974 | 14 | -0.17 | 812.4041 | 16.34 | 1.0476 | 1.0548 | 53509 | 1.12e3 | 1 | 1 | TRINITY\_DN3795\_c0\_g1\_i1.p1 | Oxidation (M) | M9:Oxidation (M):1000 | DB Search |
| GQTFSGDIAIDDIK | 58.4 | 1478.7252 | 14 | 0.51 | 740.3687 | 17.63 | 1.0073 | 1.0146 | 61922 | 1.42e3 | 1 | 1 | TRINITY\_DN3655\_c0\_g1\_i2.p1 |  |  | DB Search |
| LNNEINDLHQR | 58.39 | 1364.6797 | 11 | -0.04 | 683.3456 | 12.69 | 0.9949 | 1.0021 | 31202 | 0 | 0 | 0 | TRINITY\_DN1366\_c0\_g1\_i1.p1 |  |  | DB Search |
| VIM(+15.99)VTGDHPITAK | 58.39 | 1396.7384 | 13 | 6.43 | 466.5887 | 13.30 | 0.8079 | 0.8153 | 34845 | 1.64e3 | 1 | 1 | TRINITY\_DN262\_c0\_g1\_i1.p1 | Oxidation (M) | M3:Oxidation (M):1000 | DB Search |
| SYELPDGQVITIGNER | 58.39 | 1789.8846 | 16 | 1.59 | 895.949 | 18.70 | 1.111 | 1.1182 | 69190 | 4.17e3 | 1 | 1 | TRINITY\_DN161\_c0\_g1\_i1.p1:TRINITY\_DN161\_c0\_g1\_i4.p1:TRINITY\_DN161\_c0\_g1\_i3.p1:TRINITY\_DN161\_c0\_g1\_i2.p1 |  |  | DB Search |
| VIDINDNAPIFNK | 58.39 | 1471.7671 | 13 | 2.95 | 736.8914 | 17.55 | 1.0081 | 1.0154 | 61422 | 9.25e2 | 1 | 1 | TRINITY\_DN14015\_c0\_g1\_i3.p1:TRINITY\_DN14015\_c0\_g1\_i2.p1 |  |  | DB Search |
| NGDDGDQGPVGK | 58.39 | 1157.4949 | 12 | 2.29 | 579.7548 | 9.94 | 0.8579 | 0.8652 | 14586 | 1.13e3 | 2 | 2 | TRINITY\_DN244\_c0\_g1\_i1.p1 |  |  | DB Search |
| FAIQDITVDEFSAK | 58.39 | 1582.7878 | 14 | 3.87 | 792.4025 | 19.84 | 1.0367 | 1.0439 | 76475 | 1.54e3 | 1 | 1 | TRINITY\_DN624\_c0\_g1\_i1.p1 |  |  | DB Search |
| VLLSQQIFYDKK | 58.39 | 1480.829 | 12 | 0.31 | 741.4203 | 17.02 | 1.0616 | 1.0688 | 58002 | 1.76e3 | 2 | 2 | TRINITY\_DN982\_c0\_g1\_i1.p1:TRINITY\_DN982\_c0\_g1\_i3.p1:TRINITY\_DN982\_c0\_g1\_i2.p1 |  |  | DB Search |
| YLFMESSSPVK | 58.39 | 1286.6217 | 11 | 0.88 | 644.3173 | 17.73 | 0.9218 | 0.9291 | 62641 | 6.16e2 | 1 | 1 | TRINITY\_DN1936\_c0\_g2\_i1.p1 |  |  | DB Search |
| IVTTDGVM(+15.99)IELK | 58.39 | 1333.7163 | 12 | -0.18 | 667.8638 | 16.81 | 0.9707 | 0.9779 | 56545 | 2.41e3 | 1 | 1 | TRINITY\_DN477\_c0\_g1\_i9.p1 | Oxidation (M) | M8:Oxidation (M):1000 | DB Search |
| YVFAVAVDHTDLK | 58.39 | 1476.7612 | 13 | 1.84 | 739.3876 | 17.08 | 1.0568 | 1.064 | 58449 | 5.53e2 | 2 | 2 | TRINITY\_DN61668\_c0\_g1\_i1.p1 |  |  | DB Search |
| DVNC(+57.02)NNNQLK | 58.39 | 1217.5459 | 10 | 2.19 | 609.7802 | 10.68 | 0.9129 | 0.9202 | 18856 | 1.17e3 | 1 | 1 | TRINITY\_DN4815\_c0\_g1\_i2.p1:TRINITY\_DN4815\_c0\_g1\_i1.p1 | Carbamidomethylation |  | DB Search |
| AAGDLGIYENGC(+57.02)YDQLLK | 58.39 | 1998.9357 | 18 | 5.61 | 1000.4785 | 18.94 | 1.1694 | 1.1766 | 70657 | 2.93e3 | 1 | 1 | TRINITY\_DN661\_c0\_g1\_i10.p1 | Carbamidomethylation |  | DB Search |
| ALVILGDGFISGGGTPSK | 58.39 | 1687.9144 | 18 | 2.69 | 844.9649 | 19.82 | 1.0821 | 1.0893 | 76374 | 1.04e4 | 2 | 2 | TRINITY\_DN244\_c0\_g1\_i1.p1 |  |  | DB Search |
| FSNDIVDSFAVSQKK | 58.39 | 1683.8468 | 15 | 1.47 | 562.2891 | 17.32 | 0.8635 | 0.8708 | 59993 | 0 | 0 | 0 | TRINITY\_DN244\_c0\_g1\_i1.p1 |  |  | DB Search |
| EQTLTIGGSLLLQK | 58.39 | 1499.8558 | 14 | 4.83 | 750.9372 | 19.02 | 1.0327 | 1.0399 | 71181 | 1.06e3 | 1 | 1 | TRINITY\_DN982\_c0\_g1\_i3.p1 |  |  | DB Search |
| ADPFIVVQLGK | 58.39 | 1185.6757 | 11 | 0.68 | 593.8442 | 19.35 | 0.9004 | 0.9077 | 73310 | 6.47e2 | 1 | 1 | TRINITY\_DN140702\_c0\_g1\_i1.p1 |  |  | DB Search |
| VLILLADGQQTYVPGVTEPSEVAK | 58.39 | 2526.3582 | 24 | 2.36 | 843.1268 | 19.70 | 1.1314 | 1.1386 | 75615 | 5.51e3 | 1 | 1 | TRINITY\_DN244\_c0\_g1\_i1.p1 |  |  | DB Search |
| AEFRPNQEEEPILKR | 58.39 | 1854.9587 | 15 | 1.86 | 619.3267 | 13.94 | 0.8785 | 0.8858 | 38653 | 6.54e3 | 2 | 2 | TRINITY\_DN262\_c0\_g1\_i1.p1 |  |  | DB Search |
| TC(+57.02)GGGFFFETR | 58.39 | 1277.5499 | 11 | 10.24 | 639.7874 | 18.62 | 0.921 | 0.9283 | 68551 | 2.46e2 | 1 | 1 | TRINITY\_DN4595\_c0\_g1\_i4.p1 | Carbamidomethylation |  | DB Search |
| AFVAIGDYGGHVGLGVK | 58.39 | 1658.8779 | 17 | 1.44 | 553.9662 | 17.82 | 0.8526 | 0.8599 | 63242 | 9.03e2 | 2 | 2 | TRINITY\_DN15584\_c0\_g1\_i1.p1 |  |  | DB Search |
| LAVNMVPFPR | 58.39 | 1142.627 | 10 | 0.31 | 572.3197 | 18.72 | 0.8721 | 0.8793 | 69256 | 4.38e2 | 1 | 1 | TRINITY\_DN1910\_c0\_g1\_i1.p1:TRINITY\_DN1910\_c0\_g1\_i5.p1:TRINITY\_DN3986\_c0\_g1\_i1.p1 |  |  | DB Search |
| GIVDSDDLPLNVSR | 58.39 | 1498.7627 | 14 | 6.82 | 750.3921 | 17.63 | 1.0029 | 1.0102 | 62065 | 0 | 0 | 0 | TRINITY\_DN5633\_c0\_g1\_i1.p1 |  |  | DB Search |
| YVFAVAVDHTDLKADK | 58.39 | 1790.9203 | 16 | 4.17 | 597.9819 | 16.00 | 0.8984 | 0.9057 | 51609 | 0 | 0 | 0 | TRINITY\_DN61668\_c0\_g1\_i1.p1 |  |  | DB Search |
| FFFHDQSC(+57.02)LDVYK | 58.39 | 1704.7606 | 13 | 3.79 | 569.2617 | 17.83 | 0.801 | 0.8084 | 63353 | 5.61e2 | 1 | 1 | TRINITY\_DN10094\_c0\_g1\_i1.p1:TRINITY\_DN10094\_c0\_g1\_i2.p1 | Carbamidomethylation |  | DB Search |
| TQAVLDRLNIEKR | 58.39 | 1554.8842 | 13 | -0.15 | 519.3008 | 14.76 | 0.8075 | 0.8149 | 43833 | 7.97e3 | 1 | 1 | TRINITY\_DN477\_c0\_g1\_i9.p1 |  |  | DB Search |
| YQGDIAVDDVSLSSK | 58.39 | 1595.7678 | 15 | 3 | 798.8918 | 16.09 | 1.0676 | 1.0749 | 52110 | 0 | 0 | 0 | TRINITY\_DN1936\_c0\_g2\_i1.p1 |  |  | DB Search |
| YPNGLHHC(+57.02)PTK | 58.39 | 1322.619 | 11 | 4.65 | 441.8813 | 10.44 | 0.7701 | 0.7775 | 17571 | 2.83e3 | 1 | 1 | TRINITY\_DN1369\_c0\_g1\_i1.p1 | Carbamidomethylation |  | DB Search |
| LVSELFDPTSSTR | 58.39 | 1450.7303 | 13 | 1.5 | 726.3719 | 17.91 | 1.0013 | 1.0086 | 64032 | 6.47e3 | 1 | 1 | TRINITY\_DN4781\_c0\_g1\_i3.p1:TRINITY\_DN4781\_c0\_g1\_i1.p1:TRINITY\_DN4781\_c0\_g1\_i2.p1 |  |  | DB Search |
| VLIVISDGNEDFKEK | 58.39 | 1704.8933 | 15 | 2.18 | 569.3051 | 16.95 | 0.8412 | 0.8485 | 57552 | 5.5e3 | 2 | 2 | TRINITY\_DN195348\_c0\_g1\_i1.p1 |  |  | DB Search |
| LIYENQNTIHHVVR | 58.39 | 1734.9165 | 14 | 3.92 | 579.3138 | 13.39 | 0.8473 | 0.8546 | 35379 | 0 | 0 | 0 | TRINITY\_DN26249\_c0\_g1\_i1.p1 |  |  | DB Search |
| NYVVQFC(+57.02)DQFR | 58.39 | 1474.6664 | 11 | -1.26 | 738.3379 | 18.45 | 0.9896 | 0.9969 | 67466 | 0 | 0 | 0 | TRINITY\_DN71928\_c0\_g1\_i1.p1 | Carbamidomethylation |  | DB Search |
| IVGDDFTVGQSVTIK | 58.39 | 1577.8301 | 15 | 1.5 | 789.9218 | 17.37 | 1.0359 | 1.0431 | 60205 | 1.59e3 | 1 | 1 | TRINITY\_DN991\_c0\_g1\_i5.p1:TRINITY\_DN991\_c0\_g1\_i2.p1:TRINITY\_DN991\_c0\_g1\_i6.p1 |  |  | DB Search |
| GSLSGGLEGFYK | 58.39 | 1213.5979 | 12 | 3.01 | 607.8067 | 17.60 | 0.9073 | 0.9146 | 61815 | 0 | 0 | 0 | TRINITY\_DN5584\_c0\_g1\_i1.p1:TRINITY\_DN5584\_c0\_g1\_i3.p1 |  |  | DB Search |
| ALAAELQSVSEQLSEGGK | 58.39 | 1815.9214 | 18 | -4.81 | 908.9616 | 19.65 | 1.1033 | 1.1106 | 75300 | 1.62e3 | 2 | 2 | TRINITY\_DN3034\_c0\_g1\_i1.p1 |  |  | DB Search |
| IQVLKDPDFEFTR | 58.39 | 1606.8354 | 13 | 0.79 | 536.6183 | 17.74 | 0.8457 | 0.853 | 62712 | 2.19e3 | 1 | 1 | TRINITY\_DN982\_c0\_g1\_i1.p1:TRINITY\_DN982\_c0\_g1\_i3.p1 |  |  | DB Search |
| DANAAVVHGSHLK | 58.39 | 1317.679 | 13 | -6.06 | 440.2299 | 10.96 | 0.7449 | 0.7522 | 20600 | 1.04e3 | 1 | 1 | TRINITY\_DN262\_c0\_g1\_i1.p1 |  |  | DB Search |
| GQAGPAGQDGVDGAVGAR | 58.39 | 1581.7495 | 18 | 2.58 | 791.8823 | 12.45 | 1.0077 | 1.015 | 29579 | 1.37e4 | 5 | 5 | TRINITY\_DN1030\_c0\_g1\_i1.p1:TRINITY\_DN1030\_c0\_g1\_i2.p1 |  |  | DB Search |
| VITSDDPAEKEK | 58.39 | 1330.6616 | 12 | 3.3 | 666.3388 | 10.77 | 0.9856 | 0.9928 | 19482 | 3.46e2 | 1 | 1 | TRINITY\_DN1339\_c6\_g1\_i2.p1:TRINITY\_DN1339\_c6\_g1\_i1.p1:TRINITY\_DN1339\_c0\_g1\_i2.p1:TRINITY\_DN6701\_c0\_g1\_i2.p2:TRINITY\_DN6701\_c0\_g1\_i1.p1:TRINITY\_DN124769\_c0\_g1\_i1.p1:TRINITY\_DN1172\_c0\_g1\_i5.p1:TRINITY\_DN1172\_c0\_g1\_i3.p1:TRINITY\_DN1339\_c0\_g1\_i1.p1 |  |  | DB Search |
| VLTSDDPAEKEK | 58.39 | 1330.6616 | 12 | 3.3 | 666.3388 | 10.77 | 0.9856 | 0.9928 | 19482 | 3.46e2 | 1 | 1 | TRINITY\_DN24414\_c1\_g1\_i2.p2:TRINITY\_DN119673\_c0\_g1\_i1.p1 |  |  | DB Search |
| ANPFIEC(+57.02)HGR | 58.39 | 1199.5505 | 10 | 3.99 | 600.7836 | 13.37 | 0.8923 | 0.8996 | 35227 | 1.55e3 | 1 | 1 | TRINITY\_DN691\_c0\_g1\_i2.p1:TRINITY\_DN691\_c0\_g1\_i1.p1 | Carbamidomethylation |  | DB Search |
| SYFEHSFVDLHR | 58.39 | 1535.7157 | 12 | -0.97 | 512.9109 | 16.25 | 0.7693 | 0.7766 | 53061 | 8.88e2 | 1 | 1 | TRINITY\_DN59572\_c0\_g1\_i1.p1 |  |  | DB Search |
| GFEC(+57.02)IGLIEETETC(+57.02)K | 58.39 | 1784.796 | 15 | 3.79 | 595.9402 | 18.69 | 0.8627 | 0.87 | 69011 | 2.49e3 | 2 | 2 | TRINITY\_DN4595\_c0\_g1\_i10.p1:TRINITY\_DN4595\_c0\_g1\_i4.p1 | Carbamidomethylation, Carbamidomethylation |  | DB Search |
| AEFRPNQEEEPILK | 58.39 | 1698.8577 | 14 | 1.42 | 567.2927 | 14.78 | 0.8279 | 0.8352 | 44049 | 2.24e3 | 2 | 2 | TRINITY\_DN262\_c0\_g1\_i1.p1 |  |  | DB Search |
| YGGQC(+57.02)AC(+57.02)KPGIGGR | 58.39 | 1479.671 | 14 | 1.49 | 494.2306 | 11.25 | 0.786 | 0.7933 | 22354 | 4.8e2 | 1 | 1 | TRINITY\_DN38399\_c0\_g1\_i1.p1 | Carbamidomethylation, Carbamidomethylation |  | DB Search |
| DNAGTSSSGTGPR | 58.39 | 1205.5272 | 13 | 4.49 | 603.7723 | 9.17 | 0.8538 | 0.8611 | 10872 | 1.6e3 | 2 | 2 | TRINITY\_DN4781\_c0\_g1\_i3.p3 |  |  | DB Search |
| AGNPITIRFEGVR | 58.39 | 1428.7837 | 13 | -7.01 | 477.2641 | 16.75 | 0.7534 | 0.7608 | 56404 | 0 | 0 | 0 | TRINITY\_DN1936\_c0\_g1\_i3.p1 |  |  | DB Search |
| SGNVVLC(+57.02)LIDVHK | 58.39 | 1452.7759 | 13 | 0.7 | 485.2652 | 18.74 | 0.7351 | 0.7424 | 69444 | 0 | 0 | 0 | TRINITY\_DN3078\_c0\_g1\_i1.p1 | Carbamidomethylation |  | DB Search |
| GLQGQQGEQGSTGVQGSVGGR | 58.39 | 1985.9514 | 21 | 1.48 | 993.9823 | 12.15 | 1.1314 | 1.1386 | 27609 | 1.56e4 | 3 | 3 | TRINITY\_DN1030\_c0\_g1\_i1.p1:TRINITY\_DN1030\_c0\_g1\_i2.p1 |  |  | DB Search |
| VFDKGSYPQPK | 58.39 | 1264.6451 | 11 | 5.46 | 422.557 | 12.55 | 0.8023 | 0.8096 | 30062 | 2.92e3 | 2 | 2 | TRINITY\_DN6390\_c0\_g1\_i1.p1 |  |  | DB Search |
| LNQKVESILHK | 58.39 | 1307.7561 | 11 | 3.28 | 436.9265 | 13.42 | 0.7453 | 0.7526 | 35604 | 4.6e2 | 1 | 1 | TRINITY\_DN3182\_c0\_g1\_i2.p1:TRINITY\_DN3182\_c0\_g1\_i3.p1 |  |  | DB Search |
| A(+42.01)DPSSLETKEK | 58.39 | 1245.6089 | 11 | -2 | 623.8091 | 12.75 | 0.9449 | 0.9521 | 31533 | 4.46e2 | 1 | 1 | TRINITY\_DN262\_c0\_g1\_i1.p1 | Acetylation (Protein N-term) | A1:Acetylation (Protein N-term):1000 | DB Search |
| YC(+57.02)FHASC(+57.02)K | 58.39 | 1071.4266 | 8 | -3.12 | 358.1476 | 11.77 | 0.7229 | 0.7302 | 25566 | 9.3e2 | 1 | 1 | TRINITY\_DN24268\_c0\_g1\_i2.p1:TRINITY\_DN24268\_c0\_g1\_i1.p1 | Carbamidomethylation, Carbamidomethylation |  | DB Search |
| YVEM(+15.99)ATSNANAVLK | 58.39 | 1525.7446 | 14 | 1.62 | 763.8792 | 13.94 | 1.0395 | 1.0468 | 38859 | 0 | 0 | 0 | TRINITY\_DN177106\_c0\_g1\_i1.p1 | Oxidation (M) | M4:Oxidation (M):1000 | DB Search |
| DGNDGQTGLTGQR | 58.38 | 1317.5909 | 13 | 0.63 | 659.8017 | 11.17 | 0.9303 | 0.9376 | 21848 | 0 | 0 | 0 | TRINITY\_DN691\_c0\_g1\_i2.p1:TRINITY\_DN691\_c0\_g1\_i1.p1 |  |  | DB Search |
| VVDTEANDLTEEVKK | 58.38 | 1688.8468 | 15 | -0.58 | 563.9547 | 14.21 | 0.8392 | 0.8465 | 40438 | 5.49e3 | 2 | 2 | TRINITY\_DN1616\_c0\_g1\_i3.p1:TRINITY\_DN1616\_c0\_g1\_i6.p1:TRINITY\_DN1616\_c0\_g1\_i2.p1:TRINITY\_DN1616\_c0\_g1\_i7.p1 |  |  | DB Search |
| GFDC(+57.02)NFDKEFC(+57.02)K | 58.38 | 1565.6279 | 12 | 12.85 | 522.8888 | 16.23 | 0.7689 | 0.7762 | 53044 | 0 | 0 | 0 | TRINITY\_DN1936\_c0\_g1\_i3.p1 | Carbamidomethylation, Carbamidomethylation |  | DB Search |
| GTVNRLPGDALSIGFPSK | 58.38 | 1827.9843 | 18 | 3.55 | 610.3362 | 18.02 | 0.9052 | 0.9125 | 64542 | 1.66e2 | 1 | 1 | TRINITY\_DN149493\_c0\_g1\_i1.p1 |  |  | DB Search |
| SAIIVDGKNEEGR | 58.38 | 1386.7103 | 13 | 4.33 | 694.3639 | 12.00 | 0.9969 | 1.0041 | 26845 | 7.58e2 | 1 | 1 | TRINITY\_DN982\_c0\_g1\_i3.p1 |  |  | DB Search |
| GC(+57.02)ELC(+57.02)NC(+57.02)NATGSK | 58.38 | 1469.5697 | 13 | -3.08 | 735.7883 | 11.24 | 0.9711 | 0.9783 | 22299 | 0 | 0 | 0 | TRINITY\_DN1676\_c0\_g1\_i6.p1:TRINITY\_DN1676\_c0\_g1\_i12.p1 | Carbamidomethylation, Carbamidomethylation, Carbamidomethylation |  | DB Search |
| NSEC(+57.02)SSDNPLETR | 58.38 | 1507.6208 | 13 | -0.6 | 754.8156 | 13.30 | 1.0122 | 1.0194 | 34858 | 8.83e2 | 1 | 1 | TRINITY\_DN262\_c0\_g1\_i1.p1 | Carbamidomethylation |  | DB Search |
| VPIVGGPVIK | 58.38 | 977.6273 | 10 | 8.05 | 489.8238 | 16.07 | 0.8368 | 0.8441 | 52060 | 0 | 0 | 0 | TRINITY\_DN2532\_c1\_g1\_i1.p1 |  |  | DB Search |
| EIEDTC(+57.02)HNR | 58.38 | 1172.488 | 9 | 6.16 | 391.8382 | 9.47 | 0.7102 | 0.7176 | 12293 | 0 | 0 | 0 | TRINITY\_DN1369\_c0\_g1\_i1.p1 | Carbamidomethylation |  | DB Search |
| VC(+57.02)LDDDIKVC(+57.02)K | 58.38 | 1363.6476 | 11 | 2.8 | 455.5567 | 14.43 | 0.7978 | 0.8051 | 41739 | 9.3e2 | 1 | 1 | TRINITY\_DN4595\_c0\_g1\_i10.p1:TRINITY\_DN4595\_c0\_g1\_i4.p1 | Carbamidomethylation, Carbamidomethylation |  | DB Search |
| EITLNYHDTVVHMK | 58.38 | 1698.84 | 14 | 0.55 | 850.4258 | 15.51 | 1.1114 | 1.1186 | 48520 | 2.47e2 | 1 | 1 | TRINITY\_DN1369\_c0\_g1\_i1.p1 |  |  | DB Search |
| DPIC(+57.02)HC(+57.02)DTSK | 58.38 | 1231.4961 | 10 | 7 | 411.508 | 9.95 | 0.7355 | 0.7428 | 14664 | 1.19e4 | 2 | 2 | TRINITY\_DN319\_c2\_g1\_i3.p1:TRINITY\_DN319\_c2\_g1\_i1.p1:TRINITY\_DN319\_c2\_g1\_i2.p1 | Carbamidomethylation, Carbamidomethylation |  | DB Search |
| GSFSSHIC(+57.02)LC(+57.02)HDPAYK | 58.38 | 1877.8188 | 16 | 0.93 | 626.9461 | 14.52 | 0.8854 | 0.8927 | 42298 | 8.32e3 | 1 | 1 | TRINITY\_DN319\_c2\_g1\_i3.p1:TRINITY\_DN319\_c2\_g1\_i1.p1:TRINITY\_DN319\_c2\_g1\_i2.p1 | Carbamidomethylation, Carbamidomethylation |  | DB Search |
| AKNQALEIFK | 58.38 | 1160.6553 | 10 | -0.5 | 581.3334 | 15.99 | 0.9158 | 0.923 | 51477 | 0 | 0 | 0 | TRINITY\_DN5584\_c0\_g1\_i2.p1:TRINITY\_DN5584\_c0\_g1\_i1.p1:TRINITY\_DN5584\_c0\_g1\_i3.p1 |  |  | DB Search |
| LNTGDVQSLTNPK | 58.37 | 1385.7151 | 13 | 1.86 | 693.8646 | 14.77 | 0.9977 | 1.0049 | 43652 | 1.53e3 | 1 | 1 | TRINITY\_DN1616\_c0\_g1\_i3.p1:TRINITY\_DN1616\_c0\_g1\_i6.p1:TRINITY\_DN1616\_c0\_g1\_i2.p1:TRINITY\_DN1616\_c0\_g1\_i7.p1 |  |  | DB Search |
| VANDLSETIVWK | 58.37 | 1373.719 | 12 | 2.31 | 687.8669 | 18.08 | 0.9711 | 0.9783 | 64974 | 2.42e2 | 1 | 1 | TRINITY\_DN1936\_c0\_g1\_i3.p1:TRINITY\_DN101355\_c0\_g1\_i1.p1 |  |  | DB Search |
| LALHVLNNEDIIGHR | 58.37 | 1712.9321 | 15 | 0.41 | 571.9836 | 16.58 | 0.8615 | 0.8688 | 55276 | 2.56e3 | 2 | 2 | TRINITY\_DN140702\_c0\_g1\_i1.p1 |  |  | DB Search |
| TPGLVEGTLILHQLR | 58.37 | 1645.9515 | 15 | -0.36 | 549.6564 | 19.04 | 0.8554 | 0.8627 | 71412 | 0 | 0 | 0 | TRINITY\_DN3034\_c0\_g1\_i1.p1 |  |  | DB Search |
| YC(+57.02)NNPVAER | 58.37 | 1121.4924 | 9 | 8.64 | 561.7571 | 11.65 | 0.8619 | 0.8692 | 24790 | 8.18e1 | 1 | 1 | TRINITY\_DN8903\_c0\_g1\_i1.p1 | Carbamidomethylation |  | DB Search |
| SGDPLTIGNFIK | 58.37 | 1260.6714 | 12 | 1.03 | 631.3422 | 19.83 | 0.8955 | 0.9028 | 76410 | 4.42e2 | 1 | 1 | TRINITY\_DN2577\_c1\_g1\_i1.p1:TRINITY\_DN2577\_c1\_g1\_i2.p1 |  |  | DB Search |
| SGDPLTLGNFIK | 58.37 | 1260.6714 | 12 | 1.03 | 631.3422 | 19.83 | 0.8955 | 0.9028 | 76410 | 4.42e2 | 1 | 1 | TRINITY\_DN3795\_c0\_g1\_i1.p1 |  |  | DB Search |
| TTGVC(+57.02)M(+15.99)C(+57.02)K | 58.37 | 971.3875 | 8 | 0.65 | 486.7003 | 9.45 | 0.7827 | 0.7901 | 12123 | 2.26e2 | 1 | 1 | TRINITY\_DN15274\_c0\_g1\_i1.p1 | Carbamidomethylation, Oxidation (M), Carbamidomethylation | M6:Oxidation (M):1000 | DB Search |
| LSFC(+57.02)DTLTTPK | 58.37 | 1281.6274 | 11 | 1.48 | 641.8206 | 17.08 | 0.9654 | 0.9727 | 58298 | 1.14e3 | 1 | 1 | TRINITY\_DN991\_c0\_g1\_i5.p1:TRINITY\_DN991\_c0\_g1\_i2.p1:TRINITY\_DN991\_c0\_g1\_i6.p1 | Carbamidomethylation |  | DB Search |
| AVPAPILDIPK | 58.37 | 1132.6855 | 11 | 0.34 | 567.349 | 18.31 | 0.8955 | 0.9028 | 66444 | 1.41e3 | 1 | 1 | TRINITY\_DN8941\_c0\_g1\_i2.p1:TRINITY\_DN8941\_c0\_g1\_i12.p1 |  |  | DB Search |
| GVGIISEGNETVEDIAQR | 58.37 | 1885.9381 | 18 | 2.32 | 943.9764 | 17.95 | 1.1378 | 1.145 | 63996 | 6.11e3 | 2 | 2 | TRINITY\_DN262\_c0\_g1\_i1.p1 |  |  | DB Search |
| LAQPLDYEIKK | 58.37 | 1316.734 | 11 | 4.03 | 439.9194 | 14.78 | 0.751 | 0.7583 | 43862 | 2.11e2 | 1 | 1 | TRINITY\_DN982\_c0\_g1\_i3.p1 |  |  | DB Search |
| LGGIEVTQGNC(+57.02)AR | 58.36 | 1373.6721 | 13 | -2.89 | 687.8398 | 13.93 | 0.9662 | 0.9735 | 38726 | 2.09e3 | 1 | 1 | TRINITY\_DN36\_c0\_g1\_i9.p1:TRINITY\_DN36\_c0\_g1\_i14.p1:TRINITY\_DN36\_c0\_g1\_i13.p1:TRINITY\_DN36\_c0\_g1\_i2.p1:TRINITY\_DN36\_c0\_g1\_i3.p1:TRINITY\_DN36\_c0\_g1\_i16.p1:TRINITY\_DN36\_c0\_g1\_i17.p1:TRINITY\_DN36\_c0\_g1\_i4.p1:TRINITY\_DN36\_c0\_g1\_i11.p1:TRINITY\_DN36\_c0\_g1\_i1.p1:TRINITY\_DN36\_c0\_g1\_i8.p1:TRINITY\_DN36\_c0\_g1\_i19.p1:TRINITY\_DN36\_c0\_g1\_i5.p1:TRINITY\_DN36\_c0\_g1\_i20.p1 | Carbamidomethylation |  | DB Search |
| ITQLQNDDKEAR | 58.36 | 1429.7161 | 12 | 4.64 | 477.5805 | 10.94 | 0.8055 | 0.8128 | 20429 | 1.37e3 | 1 | 1 | TRINITY\_DN1676\_c0\_g1\_i6.p1:TRINITY\_DN1676\_c0\_g1\_i12.p1 |  |  | DB Search |
| LLHC(+57.02)SDSEVDHVEVC(+57.02)GK | 58.36 | 1982.8826 | 17 | 6.36 | 661.9709 | 13.31 | 0.9247 | 0.9319 | 34838 | 1.49e4 | 4 | 4 | TRINITY\_DN319\_c2\_g1\_i3.p1:TRINITY\_DN319\_c2\_g1\_i1.p1:TRINITY\_DN319\_c2\_g1\_i2.p1 | Carbamidomethylation, Carbamidomethylation |  | DB Search |
| GGRFEC(+57.02)TDFDC(+57.02)VAHC(+57.02)ASK | 58.36 | 2115.856 | 18 | -4.91 | 529.9675 | 14.50 | 0.8279 | 0.8352 | 42192 | 0 | 0 | 0 | TRINITY\_DN6390\_c0\_g1\_i1.p1 | Carbamidomethylation, Carbamidomethylation, Carbamidomethylation |  | DB Search |
| VQENAVVPSILQK | 58.36 | 1423.8035 | 13 | -2.87 | 712.9054 | 17.06 | 0.9989 | 1.0061 | 58423 | 5.55e2 | 1 | 1 | TRINITY\_DN5554\_c0\_g1\_i1.p1 |  |  | DB Search |
| YGAVNDNC(+57.02)ER | 58.36 | 1196.488 | 10 | 4.23 | 599.2525 | 10.91 | 0.8802 | 0.8874 | 20281 | 8.99e2 | 1 | 1 | TRINITY\_DN1616\_c0\_g1\_i3.p1:TRINITY\_DN1616\_c0\_g1\_i6.p1:TRINITY\_DN1616\_c0\_g1\_i2.p1:TRINITY\_DN1616\_c0\_g1\_i7.p1 | Carbamidomethylation |  | DB Search |
| GVNYQGDISLDDFVVK | 58.36 | 1767.8679 | 16 | 4.61 | 884.9434 | 19.90 | 1.1082 | 1.1154 | 76829 | 2.2e3 | 1 | 1 | TRINITY\_DN193641\_c0\_g1\_i1.p1 |  |  | DB Search |
| GQYISVKPPR | 58.35 | 1143.64 | 10 | -2.31 | 572.8247 | 13.47 | 0.9121 | 0.9194 | 36000 | 0 | 0 | 0 | TRINITY\_DN1676\_c0\_g1\_i6.p1:TRINITY\_DN1676\_c0\_g1\_i12.p1 |  |  | DB Search |
| LLC(+57.02)EC(+57.02)PC(+57.02)EQDK | 58.35 | 1450.5891 | 11 | 1.37 | 726.3012 | 12.80 | 0.9763 | 0.9836 | 31825 | 0 | 0 | 0 | TRINITY\_DN25375\_c0\_g1\_i7.p1 | Carbamidomethylation, Carbamidomethylation, Carbamidomethylation |  | DB Search |
| ESSFSIAQWSK | 58.35 | 1268.6038 | 11 | -2.2 | 635.3063 | 17.87 | 0.9174 | 0.9247 | 63581 | 7.35e2 | 1 | 1 | TRINITY\_DN982\_c0\_g1\_i3.p1 |  |  | DB Search |
| VAVDSLNLNILK | 58.34 | 1297.7605 | 12 | -2.45 | 649.8845 | 19.54 | 0.9622 | 0.9695 | 74618 | 0 | 0 | 0 | TRINITY\_DN45008\_c0\_g1\_i1.p1 |  |  | DB Search |
| LVHGDVFRPPR | 58.34 | 1291.7148 | 11 | 1.79 | 431.5787 | 13.55 | 0.823 | 0.8303 | 36380 | 1.06e3 | 1 | 1 | TRINITY\_DN1285\_c0\_g1\_i1.p1:TRINITY\_DN11595\_c0\_g1\_i2.p1:TRINITY\_DN11595\_c0\_g1\_i1.p1 |  |  | DB Search |
| STENC(+57.02)VPR | 58.34 | 961.4287 | 8 | -0.32 | 481.7204 | 10.55 | 0.7998 | 0.8071 | 18222 | 1.46e3 | 1 | 1 | TRINITY\_DN6390\_c0\_g1\_i1.p1 | Carbamidomethylation |  | DB Search |
| SNIC(+57.02)DPQNGK | 58.34 | 1131.4979 | 10 | -0.79 | 566.7545 | 10.56 | 0.8526 | 0.8599 | 18199 | 6.49e2 | 1 | 1 | TRINITY\_DN1616\_c0\_g1\_i3.p1:TRINITY\_DN1616\_c0\_g1\_i6.p1:TRINITY\_DN1616\_c0\_g1\_i2.p1:TRINITY\_DN1616\_c0\_g1\_i7.p1 | Carbamidomethylation |  | DB Search |
| MSEIAEEARK | 58.34 | 1162.5652 | 10 | 1.11 | 582.2892 | 11.43 | 0.9218 | 0.9291 | 23472 | 3.63e2 | 1 | 1 | TRINITY\_DN1676\_c0\_g1\_i6.p1:TRINITY\_DN1676\_c0\_g1\_i12.p1 |  |  | DB Search |
| VIDKFQNEFK | 58.33 | 1266.6608 | 10 | -0.56 | 634.3359 | 14.67 | 0.9699 | 0.9771 | 43298 | 0 | 0 | 0 | TRINITY\_DN794\_c0\_g1\_i2.p1 |  |  | DB Search |
| TEIPGIIFEK | 58.33 | 1145.6332 | 10 | 1.1 | 573.8232 | 18.83 | 0.8874 | 0.8947 | 69581 | 1.78e3 | 1 | 1 | TRINITY\_DN982\_c0\_g1\_i1.p1:TRINITY\_DN982\_c0\_g1\_i3.p1 |  |  | DB Search |
| DFNVIIEAVK | 58.33 | 1146.6284 | 10 | 0.88 | 574.3207 | 19.65 | 0.8996 | 0.9069 | 75314 | 3.22e3 | 1 | 1 | TRINITY\_DN4781\_c0\_g1\_i3.p1:TRINITY\_DN4781\_c0\_g1\_i1.p1:TRINITY\_DN4781\_c0\_g1\_i2.p1 |  |  | DB Search |
| LQGWWQSVNGR | 58.33 | 1329.6578 | 11 | 2.85 | 665.8366 | 18.29 | 0.9416 | 0.9489 | 66414 | 3.67e2 | 1 | 1 | TRINITY\_DN15274\_c0\_g1\_i1.p1 |  |  | DB Search |
| TRIEDLETVISQTQDHR | 58.33 | 2040.0236 | 17 | 1.3 | 681.0145 | 17.58 | 0.9473 | 0.9545 | 61608 | 1.62e3 | 1 | 1 | TRINITY\_DN761\_c0\_g1\_i3.p1:TRINITY\_DN761\_c0\_g1\_i1.p1:TRINITY\_DN761\_c0\_g1\_i2.p1 |  |  | DB Search |
| VGNWRDETLSC(+57.02)K | 58.32 | 1463.6827 | 12 | 0.66 | 488.9008 | 13.88 | 0.7628 | 0.7701 | 38433 | 5.84e3 | 2 | 2 | TRINITY\_DN6390\_c0\_g1\_i1.p1 | Carbamidomethylation |  | DB Search |
| ETC(+57.02)SDIEKR | 58.32 | 1136.5132 | 9 | 0.46 | 569.2629 | 10.09 | 0.8883 | 0.8955 | 15406 | 2.17e3 | 1 | 1 | TRINITY\_DN118\_c1\_g1\_i3.p1:TRINITY\_DN118\_c1\_g1\_i5.p1 | Carbamidomethylation |  | DB Search |
| VTVTEIKDEIKK | 58.32 | 1401.8079 | 12 | -3.05 | 468.2741 | 14.41 | 0.7445 | 0.7518 | 41705 | 0 | 0 | 0 | TRINITY\_DN4770\_c0\_g1\_i1.p1 |  |  | DB Search |
| GDFILNSSNVIENVTR | 58.32 | 1776.9006 | 16 | 4.19 | 889.4594 | 19.77 | 1.1138 | 1.121 | 76079 | 0 | 0 | 0 | TRINITY\_DN39385\_c0\_g1\_i2.p1:TRINITY\_DN39385\_c0\_g1\_i1.p1 |  |  | DB Search |
| LQQGIQDLEESLEEEETQR | 58.32 | 2273.0659 | 19 | -2.61 | 758.6923 | 19.57 | 0.9771 | 0.9844 | 74723 | 3.71e2 | 1 | 1 | TRINITY\_DN428\_c0\_g1\_i1.p1 |  |  | DB Search |
| LPGDALSIGFPSK | 58.32 | 1300.7026 | 13 | 0.56 | 651.3575 | 18.65 | 0.9517 | 0.959 | 68777 | 1.5e3 | 1 | 1 | TRINITY\_DN149493\_c0\_g1\_i1.p1 |  |  | DB Search |
| IPITC(+57.02)YYPTSGK | 58.32 | 1398.6853 | 12 | 0.68 | 700.3489 | 15.91 | 0.9856 | 0.9928 | 50752 | 4.02e3 | 1 | 1 | TRINITY\_DN581\_c1\_g1\_i1.p1 | Carbamidomethylation |  | DB Search |
| VGSADKC(+57.02)C(+57.02)EC(+57.02)VC(+57.02)EEC(+57.02)TVK | 58.32 | 2189.8521 | 18 | 1.96 | 730.9578 | 12.05 | 0.8777 | 0.885 | 27079 | 1.94e3 | 1 | 1 | TRINITY\_DN1071\_c0\_g1\_i4.p1:TRINITY\_DN1071\_c0\_g1\_i1.p1 | Carbamidomethylation, Carbamidomethylation, Carbamidomethylation, Carbamidomethylation, Carbamidomethylation |  | DB Search |
| GDIAIDDITVKK | 58.32 | 1286.7081 | 12 | 0.84 | 644.3605 | 15.29 | 0.9602 | 0.9675 | 47150 | 2.79e2 | 1 | 1 | TRINITY\_DN4781\_c0\_g1\_i1.p1:TRINITY\_DN4781\_c0\_g1\_i2.p1 |  |  | DB Search |
| TNENRPIFQFTNPK | 58.31 | 1704.8584 | 14 | -6.5 | 569.2885 | 16.42 | 0.8465 | 0.8538 | 54306 | 6.65e2 | 2 | 2 | TRINITY\_DN14015\_c0\_g1\_i3.p1:TRINITY\_DN14015\_c0\_g1\_i2.p1 |  |  | DB Search |
| VIMVTGDHPITAK | 58.31 | 1380.7435 | 13 | 0.17 | 691.3776 | 14.09 | 1.0174 | 1.0246 | 39702 | 2.49e3 | 1 | 1 | TRINITY\_DN262\_c0\_g1\_i1.p1 |  |  | DB Search |
| IHINIVVIGHVDSGK | 58.31 | 1599.9097 | 15 | 5.74 | 534.3124 | 16.79 | 0.8477 | 0.855 | 56506 | 4.37e2 | 1 | 1 | TRINITY\_DN64\_c0\_g1\_i1.p1 |  |  | DB Search |
| DYQINEEVVIR | 58.31 | 1376.6936 | 11 | -3.21 | 689.3503 | 16.87 | 0.9755 | 0.9828 | 57134 | 0 | 0 | 0 | TRINITY\_DN8597\_c0\_g1\_i1.p1 |  |  | DB Search |
| ANHIFAHTDPLSQK | 58.31 | 1577.795 | 14 | -10.88 | 526.9321 | 13.75 | 0.8189 | 0.8262 | 37740 | 6.57e2 | 1 | 1 | TRINITY\_DN3698\_c0\_g2\_i4.p1:TRINITY\_DN3698\_c0\_g2\_i3.p1:TRINITY\_DN3698\_c0\_g2\_i6.p1:TRINITY\_DN3698\_c0\_g2\_i2.p1:TRINITY\_DN3698\_c0\_g2\_i1.p1 |  |  | DB Search |
| VIFDDFR | 58.31 | 910.4548 | 7 | 1.49 | 456.2344 | 17.85 | 0.7962 | 0.8035 | 63326 | 1.34e3 | 1 | 1 | TRINITY\_DN57\_c0\_g1\_i1.p1 |  |  | DB Search |
| DTVIEKFEENITK | 58.3 | 1564.7985 | 13 | 1.27 | 783.4058 | 19.63 | 1.068 | 1.0753 | 75192 | 9.24e2 | 2 | 2 | TRINITY\_DN4900\_c0\_g1\_i1.p1:TRINITY\_DN4900\_c0\_g1\_i4.p1 |  |  | DB Search |
| VYESLSGFIK | 58.3 | 1141.6019 | 10 | 1.34 | 571.8077 | 18.14 | 0.9097 | 0.917 | 65369 | 1.71e3 | 2 | 2 | TRINITY\_DN982\_c0\_g1\_i3.p1 |  |  | DB Search |
| AELVFVNHC(+57.02)R | 58.3 | 1243.6132 | 10 | 2.08 | 622.8138 | 14.57 | 0.9578 | 0.965 | 42523 | 1.49e3 | 1 | 1 | TRINITY\_DN101992\_c0\_g1\_i1.p1 | Carbamidomethylation |  | DB Search |
| VFSEC(+57.02)GPAC(+57.02)SSVC(+57.02)SAIGSK | 58.3 | 2001.8594 | 19 | 6.31 | 1001.9411 | 15.43 | 1.1534 | 1.1606 | 48146 | 2.26e2 | 1 | 1 | TRINITY\_DN1369\_c0\_g1\_i1.p1 | Carbamidomethylation, Carbamidomethylation, Carbamidomethylation |  | DB Search |
| MRFDTAITAC(+57.02)R | 58.3 | 1340.6329 | 11 | 1.65 | 671.3234 | 15.12 | 0.9666 | 0.9739 | 46027 | 4.31e3 | 1 | 1 | TRINITY\_DN477\_c0\_g1\_i9.p1 | Carbamidomethylation |  | DB Search |
| ADIGVAMGIAGSDVSK | 58.3 | 1489.7446 | 16 | 0.4 | 745.8782 | 17.01 | 1.0214 | 1.0287 | 58004 | 0 | 0 | 0 | TRINITY\_DN262\_c0\_g1\_i1.p1 |  |  | DB Search |
| VIVPYTPVTGYETGK | 58.3 | 1622.8556 | 15 | 1.23 | 812.4343 | 17.26 | 1.046 | 1.0532 | 59415 | 4.54e3 | 1 | 1 | TRINITY\_DN195348\_c0\_g1\_i1.p1 |  |  | DB Search |
| IVTTDGVMIELK | 58.3 | 1317.7213 | 12 | -15.83 | 659.8561 | 17.84 | 0.94 | 0.9473 | 63519 | 2.16e4 | 3 | 3 | TRINITY\_DN477\_c0\_g1\_i9.p1 |  |  | DB Search |
| GGC(+57.02)FPATGDLLVGR | 58.29 | 1418.6976 | 14 | 2.98 | 710.3566 | 18.13 | 0.9892 | 0.9965 | 64986 | 1.86e3 | 1 | 1 | TRINITY\_DN15274\_c0\_g1\_i1.p1 | Carbamidomethylation |  | DB Search |
| C(+57.02)FDLIQVAVNK | 58.29 | 1305.675 | 11 | -2.9 | 653.8415 | 19.16 | 0.9582 | 0.9654 | 71943 | 1.33e3 | 1 | 1 | TRINITY\_DN1676\_c0\_g1\_i6.p1:TRINITY\_DN1676\_c0\_g1\_i12.p1 | Carbamidomethylation |  | DB Search |
| GYAVSFIDDKFVQK | 58.29 | 1615.8246 | 14 | 3.47 | 539.6161 | 17.87 | 0.8035 | 0.8108 | 63558 | 1.55e3 | 2 | 2 | TRINITY\_DN61668\_c0\_g1\_i1.p1 |  |  | DB Search |
| FGTC(+57.02)IDLIGDYR | 58.29 | 1428.6708 | 12 | 0.76 | 715.3416 | 19.11 | 1.0049 | 1.0122 | 71833 | 0 | 0 | 0 | TRINITY\_DN991\_c0\_g1\_i5.p1:TRINITY\_DN991\_c0\_g1\_i2.p1:TRINITY\_DN991\_c0\_g1\_i6.p1 | Carbamidomethylation |  | DB Search |
| DGLNGDRGDPGPVGAVGAR | 58.29 | 1778.866 | 19 | -2.01 | 593.9601 | 13.86 | 0.8323 | 0.8396 | 38286 | 4.13e2 | 1 | 1 | TRINITY\_DN106\_c0\_g1\_i12.p1:TRINITY\_DN106\_c0\_g1\_i10.p1:TRINITY\_DN106\_c0\_g1\_i4.p1:TRINITY\_DN106\_c0\_g1\_i11.p1 |  |  | DB Search |
| TC(+57.02)VKDEVTGLHEIK | 58.29 | 1627.824 | 14 | 1.04 | 543.6146 | 13.87 | 0.8579 | 0.8652 | 38340 | 2.6e3 | 1 | 1 | TRINITY\_DN1369\_c0\_g1\_i1.p1 | Carbamidomethylation |  | DB Search |
| KFSNDIVDSFAVSQKK | 58.29 | 1811.9418 | 16 | 5.74 | 604.99 | 16.35 | 0.8943 | 0.9016 | 53776 | 0 | 0 | 0 | TRINITY\_DN244\_c0\_g1\_i1.p1 |  |  | DB Search |
| TNFQMC(+57.02)HQR | 58.29 | 1220.5179 | 9 | 3.31 | 611.2669 | 11.65 | 0.921 | 0.9283 | 24815 | 0 | 0 | 0 | TRINITY\_DN1058\_c0\_g1\_i1.p1:TRINITY\_DN1058\_c0\_g1\_i3.p1:TRINITY\_DN1058\_c0\_g1\_i2.p2 | Carbamidomethylation |  | DB Search |
| DC(+57.02)PNEPTER | 58.29 | 1116.4506 | 9 | 1.83 | 559.2324 | 10.18 | 0.853 | 0.8603 | 15597 | 3.45e3 | 1 | 1 | TRINITY\_DN4815\_c0\_g1\_i2.p1:TRINITY\_DN4815\_c0\_g1\_i1.p1 | Carbamidomethylation |  | DB Search |
| KIDEAEELGDKVQGSVDK | 58.29 | 1958.9796 | 18 | -0.5 | 653.9987 | 14.55 | 0.8603 | 0.8676 | 42443 | 2.02e3 | 2 | 2 | TRINITY\_DN15274\_c0\_g1\_i1.p1 |  |  | DB Search |
| IEDLETVISQTQDHR | 58.29 | 1782.8748 | 15 | -2.39 | 595.2961 | 17.95 | 0.8963 | 0.9036 | 64199 | 0 | 0 | 0 | TRINITY\_DN761\_c0\_g1\_i3.p1:TRINITY\_DN761\_c0\_g1\_i1.p1:TRINITY\_DN761\_c0\_g1\_i2.p1 |  |  | DB Search |
| AVAEEFISVDPSVR | 58.29 | 1517.7726 | 14 | 0.44 | 759.8922 | 17.79 | 1.0287 | 1.0359 | 62940 | 2.17e3 | 1 | 1 | TRINITY\_DN4481\_c0\_g1\_i2.p1:TRINITY\_DN598\_c1\_g1\_i2.p1:TRINITY\_DN4481\_c0\_g1\_i3.p1 |  |  | DB Search |
| LSLDVNGNYQGGGVC(+57.02)K | 58.29 | 1679.7937 | 16 | -4.07 | 840.8989 | 15.59 | 1.0705 | 1.0777 | 49051 | 0 | 0 | 0 | TRINITY\_DN1616\_c0\_g1\_i3.p1:TRINITY\_DN1616\_c0\_g1\_i6.p1:TRINITY\_DN1616\_c0\_g1\_i2.p1:TRINITY\_DN1616\_c0\_g1\_i7.p1 | Carbamidomethylation |  | DB Search |
| AEGPTSTGVTEVIVR | 58.29 | 1514.7939 | 15 | 2.95 | 758.4048 | 15.62 | 1.0106 | 1.0178 | 49221 | 7.66e2 | 1 | 1 | TRINITY\_DN982\_c0\_g1\_i3.p1 |  |  | DB Search |
| ILQC(+57.02)NQYANVEEAR | 58.29 | 1706.8046 | 14 | -5.21 | 854.4033 | 14.34 | 1.0697 | 1.0769 | 41279 | 1.23e3 | 1 | 1 | TRINITY\_DN477\_c0\_g1\_i8.p1:TRINITY\_DN477\_c0\_g1\_i7.p1 | Carbamidomethylation |  | DB Search |
| LVQNTPHNSIVTK | 58.29 | 1449.7939 | 13 | 3.11 | 484.2724 | 11.79 | 0.8075 | 0.8149 | 25665 | 5.5e2 | 1 | 1 | TRINITY\_DN982\_c0\_g1\_i1.p1:TRINITY\_DN982\_c0\_g1\_i3.p1 |  |  | DB Search |
| VC(+57.02)NGEPC(+57.02)ILQER | 58.29 | 1473.6704 | 12 | 1.84 | 737.8422 | 15.04 | 0.9832 | 0.9904 | 45530 | 3e3 | 1 | 1 | TRINITY\_DN4595\_c0\_g1\_i4.p1:TRINITY\_DN4595\_c0\_g1\_i10.p2 | Carbamidomethylation, Carbamidomethylation |  | DB Search |
| NWHGMEDC(+57.02)VVVK | 58.29 | 1472.6541 | 12 | 8.38 | 491.895 | 15.15 | 0.7534 | 0.7608 | 46340 | 0 | 0 | 0 | TRINITY\_DN11248\_c0\_g1\_i1.p1:TRINITY\_DN11248\_c0\_g1\_i3.p1 | Carbamidomethylation |  | DB Search |
| YTSC(+57.02)QSVTVVASDR | 58.29 | 1571.725 | 14 | -2.24 | 786.8663 | 13.52 | 1.0504 | 1.0576 | 36260 | 0 | 0 | 0 | TRINITY\_DN982\_c0\_g1\_i1.p1:TRINITY\_DN982\_c0\_g1\_i3.p1 | Carbamidomethylation |  | DB Search |
| DVIIDSGDESKR | 58.29 | 1332.6521 | 12 | 2.92 | 667.3338 | 12.51 | 0.9876 | 0.9949 | 30033 | 6.02e2 | 1 | 1 | TRINITY\_DN1366\_c0\_g1\_i1.p1 |  |  | DB Search |
| FELDRVNTFGDEVFGDPK | 58.29 | 2083.9851 | 18 | 0.34 | 695.6677 | 19.31 | 0.9182 | 0.9255 | 73205 | 0 | 0 | 0 | TRINITY\_DN1676\_c0\_g1\_i6.p1:TRINITY\_DN1676\_c0\_g1\_i12.p1 |  |  | DB Search |
| LC(+57.02)DATC(+57.02)SVFGDPHYK | 58.29 | 1768.7549 | 15 | 1.29 | 885.3839 | 15.38 | 1.1354 | 1.1426 | 47761 | 1.99e2 | 1 | 1 | TRINITY\_DN1369\_c0\_g1\_i1.p1 | Carbamidomethylation, Carbamidomethylation |  | DB Search |
| ILILLTDGSQTQDAGAEDPVK | 58.29 | 2183.1321 | 21 | -2.24 | 1092.5685 | 19.12 | 1.2432 | 1.2503 | 71899 | 0 | 0 | 0 | TRINITY\_DN5003\_c0\_g1\_i2.p1 |  |  | DB Search |
| LLILLTDGSQTQDAGAEDPVK | 58.29 | 2183.1321 | 21 | -2.24 | 1092.5685 | 19.12 | 1.2432 | 1.2503 | 71899 | 0 | 0 | 0 | TRINITY\_DN12542\_c0\_g1\_i1.p1 |  |  | DB Search |
| SDELLNLKEEASTLR | 58.29 | 1716.8894 | 15 | 14.54 | 573.3108 | 17.82 | 0.8473 | 0.8546 | 63372 | 0 | 0 | 0 | TRINITY\_DN140702\_c0\_g1\_i1.p1 |  |  | DB Search |
| EGPAGPAGPDGR | 58.29 | 1079.4995 | 12 | 3.84 | 540.7579 | 10.78 | 0.8404 | 0.8477 | 19235 | 1.85e3 | 1 | 1 | TRINITY\_DN179\_c0\_g1\_i1.p1 |  |  | DB Search |
| LLILLTDGSQTQDAGAEDPAK | 58.29 | 2155.1008 | 21 | 2.82 | 1078.5583 | 18.86 | 1.2388 | 1.2459 | 70204 | 0 | 0 | 0 | TRINITY\_DN4494\_c1\_g1\_i2.p1:TRINITY\_DN4494\_c1\_g1\_i7.p1:TRINITY\_DN4494\_c1\_g1\_i8.p1:TRINITY\_DN4494\_c1\_g1\_i10.p1 |  |  | DB Search |
| YVAIYPSDHLTVNR | 58.29 | 1646.8417 | 14 | 3.39 | 824.4291 | 15.76 | 1.1082 | 1.1154 | 49960 | 1.45e3 | 1 | 1 | TRINITY\_DN982\_c0\_g1\_i1.p1:TRINITY\_DN982\_c0\_g1\_i3.p1 |  |  | DB Search |
| LAYPQNNSPLIVSK | 58.29 | 1542.8406 | 14 | -0.82 | 772.4252 | 15.95 | 1.0472 | 1.0544 | 51022 | 2.12e3 | 1 | 1 | TRINITY\_DN1936\_c0\_g2\_i1.p1 |  |  | DB Search |
| DKIFPHIELFGK | 58.29 | 1442.7921 | 12 | 2.07 | 481.9379 | 18.74 | 0.8688 | 0.8761 | 69351 | 4.71e2 | 1 | 1 | TRINITY\_DN2490\_c0\_g1\_i1.p1 |  |  | DB Search |
| GLLGPDGEVGATGER | 58.29 | 1426.7052 | 15 | 0.49 | 714.3586 | 15.54 | 0.9896 | 0.9969 | 48738 | 0 | 0 | 0 | TRINITY\_DN179\_c0\_g1\_i2.p1 |  |  | DB Search |
| FC(+57.02)NNPAPSNGGR | 58.29 | 1289.5571 | 12 | 10.24 | 645.791 | 10.91 | 0.9158 | 0.923 | 20335 | 6.39e3 | 1 | 1 | TRINITY\_DN4595\_c0\_g1\_i10.p1:TRINITY\_DN4595\_c0\_g1\_i4.p1 | Carbamidomethylation |  | DB Search |
| S(+42.01)C(+57.02)SNPSPAHGGKPC(+57.02)SGELSK | 58.29 | 2097.9207 | 20 | 2.04 | 700.3141 | 11.24 | 0.8716 | 0.8789 | 22349 | 4.91e2 | 1 | 1 | TRINITY\_DN1613\_c1\_g1\_i2.p1:TRINITY\_DN1613\_c1\_g1\_i1.p1 | Acetylation (Protein N-term), Carbamidomethylation, Carbamidomethylation | S1:Acetylation (Protein N-term):1000 | DB Search |
| VGINYQPPTVVPGGDLAK | 58.29 | 1823.9781 | 18 | -4.28 | 912.9904 | 17.44 | 1.1142 | 1.1214 | 60745 | 0 | 0 | 0 | TRINITY\_DN687\_c0\_g2\_i1.p1:TRINITY\_DN687\_c0\_g1\_i1.p1:TRINITY\_DN250\_c0\_g1\_i5.p1:TRINITY\_DN250\_c0\_g1\_i1.p1:TRINITY\_DN250\_c0\_g1\_i6.p1:TRINITY\_DN250\_c0\_g1\_i4.p1:TRINITY\_DN96161\_c0\_g1\_i1.p1:TRINITY\_DN250\_c0\_g1\_i3.p1 |  |  | DB Search |
| LSTVGGMSIAVPGNVK | 58.29 | 1528.8282 | 16 | 4.47 | 765.4232 | 17.55 | 1.0351 | 1.0423 | 61420 | 0 | 0 | 0 | TRINITY\_DN10485\_c0\_g1\_i1.p1 |  |  | DB Search |
| RTC(+57.02)ADTSPAIDFK | 58.29 | 1480.698 | 13 | 1.84 | 741.356 | 14.27 | 1.017 | 1.0242 | 40672 | 1.48e3 | 1 | 1 | TRINITY\_DN319\_c2\_g1\_i3.p1:TRINITY\_DN319\_c2\_g1\_i1.p1:TRINITY\_DN319\_c2\_g1\_i2.p1 | Carbamidomethylation |  | DB Search |
| IDKALLLASSNAFDTSNGAR | 58.28 | 2063.0647 | 20 | -0.57 | 688.6936 | 17.50 | 0.9113 | 0.9186 | 61143 | 3.2e2 | 1 | 1 | TRINITY\_DN244\_c0\_g1\_i1.p1 |  |  | DB Search |
| SEEYKPTLGR | 58.28 | 1178.5931 | 10 | 1.77 | 590.3036 | 12.35 | 0.9073 | 0.9146 | 29036 | 2.36e2 | 1 | 1 | TRINITY\_DN144508\_c0\_g1\_i1.p1 |  |  | DB Search |
| YYVTIIDAPGHR | 58.28 | 1403.7197 | 12 | -1.46 | 468.9121 | 16.14 | 0.8023 | 0.8096 | 52343 | 2.5e3 | 2 | 2 | TRINITY\_DN64\_c0\_g1\_i1.p1 |  |  | DB Search |
| APTHILNPANKR | 58.28 | 1330.7469 | 12 | 0.07 | 666.3793 | 11.22 | 1.0098 | 1.017 | 22322 | 5.04e3 | 2 | 2 | TRINITY\_DN1058\_c0\_g1\_i4.p1:TRINITY\_DN1058\_c0\_g1\_i1.p1:TRINITY\_DN1058\_c0\_g1\_i3.p1:TRINITY\_DN1058\_c0\_g1\_i2.p2 |  |  | DB Search |
| FSNLSNKGDEHISNLDTLKK | 58.28 | 2259.1494 | 20 | 1.82 | 565.7944 | 13.91 | 0.8311 | 0.8384 | 38634 | 0 | 0 | 0 | TRINITY\_DN1616\_c0\_g1\_i3.p1:TRINITY\_DN1616\_c0\_g1\_i6.p1:TRINITY\_DN1616\_c0\_g1\_i2.p1:TRINITY\_DN1616\_c0\_g1\_i7.p1 |  |  | DB Search |
| YSRPTPGSNNNVM(+15.99)VR | 58.28 | 1706.8158 | 15 | 1.72 | 569.9456 | 11.63 | 0.8319 | 0.8392 | 24566 | 1.7e3 | 1 | 1 | TRINITY\_DN15274\_c0\_g1\_i1.p1 | Oxidation (M) | M13:Oxidation (M):1000 | DB Search |
| EGPTGPQGETGR | 58.28 | 1184.5421 | 12 | -1.15 | 593.2764 | 10.36 | 0.866 | 0.8733 | 16960 | 6.73e4 | 1 | 1 | TRINITY\_DN984\_c0\_g1\_i2.p1:TRINITY\_DN984\_c0\_g1\_i1.p1 |  |  | DB Search |
| DFQVIFEAK | 58.28 | 1095.5601 | 9 | -2.26 | 548.7849 | 19.00 | 0.8579 | 0.8652 | 71160 | 0 | 0 | 0 | TRINITY\_DN122779\_c0\_g1\_i1.p1:TRINITY\_DN4781\_c0\_g1\_i1.p2:TRINITY\_DN4781\_c0\_g1\_i3.p2:TRINITY\_DN4781\_c0\_g1\_i2.p1 |  |  | DB Search |
| S(+42.01)ATGNLVTVLR | 58.28 | 1171.656 | 11 | 0.35 | 586.8342 | 18.95 | 0.9453 | 0.9525 | 70826 | 3.39e3 | 1 | 1 | TRINITY\_DN2963\_c0\_g1\_i1.p1 | Acetylation (Protein N-term) | S1:Acetylation (Protein N-term):1000 | DB Search |
| IVIDSEHC(+57.02)NSR | 58.28 | 1328.6143 | 11 | 4.73 | 665.3161 | 11.46 | 0.965 | 0.9723 | 23601 | 5.49e2 | 1 | 1 | TRINITY\_DN25375\_c0\_g1\_i7.p1 | Carbamidomethylation |  | DB Search |
| ADIGVAM(+15.99)GIAGSDVSK | 58.28 | 1505.7395 | 16 | -8.69 | 753.8688 | 14.84 | 1.019 | 1.0263 | 44397 | 0 | 0 | 0 | TRINITY\_DN262\_c0\_g1\_i1.p1 | Oxidation (M) | M7:Oxidation (M):1000 | DB Search |
| LLYTPGESSLSNQR | 58.28 | 1563.7893 | 14 | 5.64 | 782.9046 | 15.52 | 1.0484 | 1.0556 | 48763 | 2.91e2 | 1 | 1 | TRINITY\_DN27058\_c0\_g1\_i1.p1 |  |  | DB Search |
| IGEIVFQTMQDAQK | 58.28 | 1606.8025 | 14 | 2.72 | 804.4089 | 18.66 | 1.0757 | 1.0829 | 68737 | 1.56e3 | 1 | 1 | TRINITY\_DN3795\_c0\_g1\_i1.p1 |  |  | DB Search |
| SGEYKPTLGR | 58.28 | 1106.572 | 10 | 4.62 | 554.2946 | 11.99 | 0.8988 | 0.9061 | 26709 | 9.46e2 | 1 | 1 | TRINITY\_DN4781\_c0\_g1\_i3.p3 |  |  | DB Search |
| DFDLDKHLVDK | 58.28 | 1343.6721 | 11 | 1.6 | 448.8977 | 15.28 | 0.7249 | 0.7322 | 47163 | 7.72e2 | 1 | 1 | TRINITY\_DN3078\_c0\_g1\_i1.p1 |  |  | DB Search |
| GYLGDIAIDDFK | 58.28 | 1325.6503 | 12 | -2.17 | 663.8295 | 19.70 | 0.9566 | 0.9638 | 75625 | 0 | 0 | 0 | TRINITY\_DN1936\_c0\_g2\_i1.p1 |  |  | DB Search |
| GTM(+15.99)KPPFHDPK | 58.28 | 1269.6176 | 11 | 0.53 | 635.815 | 10.78 | 0.9574 | 0.9646 | 19136 | 1.46e3 | 1 | 1 | TRINITY\_DN1676\_c0\_g1\_i6.p1:TRINITY\_DN1676\_c0\_g1\_i12.p1 | Oxidation (M) | M3:Oxidation (M):1000 | DB Search |
| FGWTATEAGTGC(+57.02)IETR | 58.28 | 1755.7886 | 16 | 0.08 | 878.8997 | 17.08 | 1.1013 | 1.1086 | 58476 | 0 | 0 | 0 | TRINITY\_DN2720\_c0\_g1\_i6.p1 | Carbamidomethylation |  | DB Search |
| INNLEIQLDQEAK | 58.28 | 1526.7941 | 13 | -0.82 | 764.402 | 16.63 | 1.0407 | 1.048 | 55620 | 0 | 0 | 0 | TRINITY\_DN428\_c0\_g1\_i1.p1 |  |  | DB Search |
| VNTFGDEVFGDPK | 58.28 | 1423.6619 | 13 | -3.52 | 712.8342 | 17.48 | 1.0118 | 1.019 | 60886 | 1.49e3 | 1 | 1 | TRINITY\_DN1676\_c0\_g1\_i6.p1:TRINITY\_DN1676\_c0\_g1\_i12.p1 |  |  | DB Search |
| FVFPFK | 58.28 | 783.4319 | 6 | 4.07 | 392.724 | 19.74 | 0.7681 | 0.7754 | 76008 | 6.59e2 | 2 | 2 | TRINITY\_DN140702\_c0\_g1\_i1.p1 |  |  | DB Search |
| EVNTELDSRPETK | 58.28 | 1516.7369 | 13 | 5.19 | 759.378 | 11.86 | 1.0242 | 1.0315 | 25945 | 9e2 | 1 | 1 | TRINITY\_DN11643\_c0\_g1\_i1.p1 |  |  | DB Search |
| AC(+57.02)DSGFSAVLSR | 58.28 | 1268.5819 | 12 | 1.5 | 635.2978 | 16.57 | 0.9485 | 0.9558 | 55157 | 3.01e2 | 1 | 1 | TRINITY\_DN4595\_c0\_g1\_i4.p1:TRINITY\_DN4595\_c0\_g1\_i10.p2 | Carbamidomethylation |  | DB Search |
| TLMEFENNIAK | 58.28 | 1308.6384 | 11 | 4.09 | 655.3277 | 17.39 | 0.9634 | 0.9707 | 60532 | 5.46e3 | 1 | 1 | TRINITY\_DN1676\_c0\_g1\_i6.p1:TRINITY\_DN1676\_c0\_g1\_i12.p1 |  |  | DB Search |
| TSFPLITLK | 58.28 | 1018.6063 | 9 | 2.17 | 510.3104 | 19.47 | 0.8404 | 0.8477 | 74096 | 7.34e2 | 1 | 1 | TRINITY\_DN21554\_c1\_g1\_i1.p1 |  |  | DB Search |
| DFREFLYNSETGK | 58.28 | 1604.7471 | 13 | 1.19 | 535.9224 | 18.53 | 0.8055 | 0.8128 | 68013 | 8.32e2 | 1 | 1 | TRINITY\_DN2028\_c0\_g1\_i1.p1 |  |  | DB Search |
| ELVLNSAFC(+57.02)EQYIK | 58.28 | 1712.8444 | 14 | 6.97 | 857.4335 | 19.12 | 1.0989 | 1.1061 | 71794 | 2.94e2 | 1 | 1 | TRINITY\_DN7641\_c0\_g1\_i1.p1 | Carbamidomethylation |  | DB Search |
| EGPAGPAGPVGR | 58.28 | 1063.541 | 12 | -11.82 | 532.7703 | 11.84 | 0.8461 | 0.8534 | 25992 | 0 | 0 | 0 | TRINITY\_DN106\_c0\_g1\_i2.p1:TRINITY\_DN106\_c0\_g1\_i6.p1:TRINITY\_DN106\_c0\_g1\_i5.p1:TRINITY\_DN106\_c0\_g1\_i8.p1:TRINITY\_DN106\_c0\_g1\_i3.p1:TRINITY\_DN106\_c0\_g1\_i9.p1 |  |  | DB Search |
| NFATAVLNVQK | 58.28 | 1203.6611 | 11 | -3.72 | 602.8343 | 17.63 | 0.923 | 0.9303 | 62041 | 0 | 0 | 0 | TRINITY\_DN1616\_c0\_g1\_i3.p1:TRINITY\_DN1616\_c0\_g1\_i6.p1:TRINITY\_DN1616\_c0\_g1\_i2.p1:TRINITY\_DN1616\_c0\_g1\_i7.p1 |  |  | DB Search |
| TLKVDISDMK | 58.28 | 1148.6111 | 10 | -6.01 | 575.3081 | 15.17 | 0.917 | 0.9243 | 46490 | 0 | 0 | 0 | TRINITY\_DN1616\_c0\_g1\_i3.p1:TRINITY\_DN1616\_c0\_g1\_i6.p1:TRINITY\_DN1616\_c0\_g1\_i2.p1:TRINITY\_DN1616\_c0\_g1\_i7.p1 |  |  | DB Search |
| IQTEFTSSGGPLVSVHVSDDPR | 58.28 | 2327.1394 | 22 | 0.25 | 776.7189 | 17.17 | 0.9553 | 0.9626 | 58886 | 6.44e2 | 1 | 1 | TRINITY\_DN3182\_c0\_g1\_i2.p1:TRINITY\_DN3182\_c0\_g1\_i3.p1 |  |  | DB Search |
| LGIHEDTQNR | 58.28 | 1181.5789 | 10 | 2.61 | 394.8671 | 11.20 | 0.7122 | 0.7196 | 22065 | 0 | 0 | 0 | TRINITY\_DN141338\_c2\_g1\_i1.p1 |  |  | DB Search |
| FDFHSWIGLKK | 58.28 | 1376.7241 | 11 | 3.36 | 459.9158 | 18.47 | 0.7363 | 0.7437 | 67625 | 5.05e2 | 1 | 1 | TRINITY\_DN12627\_c0\_g1\_i1.p1 |  |  | DB Search |
| AGVLAHLEEER | 58.28 | 1222.6306 | 11 | 0.12 | 612.3213 | 15.07 | 0.9558 | 0.963 | 45833 | 3.44e2 | 1 | 1 | TRINITY\_DN428\_c0\_g1\_i1.p1 |  |  | DB Search |
| HIQNFEGGER | 58.28 | 1185.5526 | 10 | -1.78 | 593.7812 | 11.51 | 0.8802 | 0.8874 | 23962 | 0 | 0 | 0 | TRINITY\_DN32804\_c0\_g1\_i1.p1 |  |  | DB Search |
| VGTVNQAVTDAFK | 58.28 | 1348.6986 | 13 | 1.51 | 675.3561 | 16.68 | 0.9747 | 0.982 | 55945 | 7.49e2 | 1 | 1 | TRINITY\_DN1189\_c0\_g1\_i1.p1 |  |  | DB Search |
| NAFFIVTSK | 58.27 | 1025.5546 | 9 | -0.8 | 513.783 | 18.00 | 0.8388 | 0.8461 | 64505 | 0 | 0 | 0 | TRINITY\_DN4595\_c0\_g1\_i10.p1:TRINITY\_DN4595\_c0\_g1\_i4.p1 |  |  | DB Search |
| SFVLSGLSK | 58.27 | 936.528 | 9 | 0.16 | 469.2703 | 17.30 | 0.7953 | 0.8027 | 59795 | 1.32e3 | 1 | 1 | TRINITY\_DN194261\_c0\_g1\_i1.p1 |  |  | DB Search |
| TVMAAC(+57.02)GNENK | 58.27 | 1193.5168 | 11 | 1.94 | 597.7656 | 10.70 | 0.8919 | 0.8992 | 19067 | 0 | 0 | 0 | TRINITY\_DN1058\_c0\_g1\_i1.p1:TRINITY\_DN1058\_c0\_g1\_i3.p1 | Carbamidomethylation |  | DB Search |
| IC(+57.02)GQGSTIC(+57.02)DGTAR | 58.27 | 1494.6555 | 14 | 4.48 | 748.3367 | 12.01 | 1.0009 | 1.0081 | 26890 | 9.48e2 | 1 | 1 | TRINITY\_DN1058\_c3\_g1\_i1.p1 | Carbamidomethylation, Carbamidomethylation |  | DB Search |
| TIAIDNLQINYK | 58.27 | 1404.7612 | 12 | 1.9 | 703.3877 | 18.00 | 1.0013 | 1.0086 | 64416 | 8.31e2 | 1 | 1 | TRINITY\_DN7849\_c0\_g1\_i1.p1 |  |  | DB Search |
| TC(+57.02)TC(+57.02)ENVR | 58.27 | 1038.4222 | 8 | 5.2 | 520.22 | 9.83 | 0.8425 | 0.8498 | 14008 | 2.42e3 | 1 | 1 | TRINITY\_DN4815\_c0\_g1\_i2.p1:TRINITY\_DN4815\_c0\_g1\_i1.p1 | Carbamidomethylation, Carbamidomethylation |  | DB Search |
| SFFDFENSK | 58.27 | 1119.4873 | 9 | 0.57 | 560.75 | 18.77 | 0.8595 | 0.8668 | 69552 | 3.17e2 | 1 | 1 | TRINITY\_DN16079\_c0\_g2\_i1.p1 |  |  | DB Search |
| LVNFGFENK | 58.27 | 1066.5447 | 9 | 4.08 | 534.2806 | 17.31 | 0.8404 | 0.8477 | 59814 | 6.14e2 | 1 | 1 | TRINITY\_DN77\_c1\_g1\_i1.p1 |  |  | DB Search |
| LAVNM(+15.99)VPFPR | 58.27 | 1158.6219 | 10 | -3.71 | 580.3148 | 17.06 | 0.8798 | 0.887 | 57945 | 1.18e3 | 1 | 1 | TRINITY\_DN1910\_c0\_g1\_i1.p1:TRINITY\_DN1910\_c0\_g1\_i5.p1:TRINITY\_DN3986\_c0\_g1\_i1.p1 | Oxidation (M) | M5:Oxidation (M):1000 | DB Search |
| DVSPILLR | 58.27 | 911.544 | 8 | -1.43 | 456.7776 | 17.20 | 0.7962 | 0.8035 | 59159 | 5.49e2 | 1 | 1 | TRINITY\_DN194261\_c0\_g1\_i1.p1 |  |  | DB Search |
| LSNTETELQSIR | 58.27 | 1389.71 | 12 | 3.32 | 695.863 | 15.17 | 0.9795 | 0.9868 | 46436 | 0 | 0 | 0 | TRINITY\_DN1366\_c0\_g1\_i1.p1 |  |  | DB Search |
| LFWDAPDVSLSK | 58.27 | 1376.6976 | 12 | -3.64 | 689.3521 | 19.88 | 0.9683 | 0.9755 | 76730 | 1.12e3 | 1 | 1 | TRINITY\_DN61668\_c0\_g1\_i1.p1 |  |  | DB Search |
| FIETDVNYDVM(+15.99)VTR | 58.27 | 1716.8029 | 14 | 0.97 | 859.4077 | 16.71 | 1.0809 | 1.0881 | 56014 | 1.52e3 | 1 | 1 | TRINITY\_DN1616\_c0\_g1\_i3.p1:TRINITY\_DN1616\_c0\_g1\_i6.p1:TRINITY\_DN1616\_c0\_g1\_i2.p1:TRINITY\_DN1616\_c0\_g1\_i7.p1 | Oxidation (M) | M11:Oxidation (M):1000 | DB Search |
| YLEQVHIGIK | 58.27 | 1198.671 | 10 | -5.33 | 600.3383 | 15.67 | 0.9243 | 0.9315 | 49623 | 0 | 0 | 0 | TRINITY\_DN62874\_c0\_g1\_i1.p1 |  |  | DB Search |
| ANDILDGLELR | 58.27 | 1227.6459 | 11 | 0.98 | 614.8295 | 19.57 | 0.9218 | 0.9291 | 74777 | 2.49e3 | 1 | 1 | TRINITY\_DN15274\_c0\_g1\_i1.p1 |  |  | DB Search |
| TVHQALVAALQK | 58.27 | 1277.7455 | 12 | -1.69 | 639.8776 | 15.15 | 0.9614 | 0.9687 | 46326 | 0 | 0 | 0 | TRINITY\_DN564\_c0\_g1\_i1.p1 |  |  | DB Search |
| VTLTQTHFLKDQDKPAK | 58.27 | 1969.0632 | 17 | 3.13 | 657.3623 | 13.38 | 0.9113 | 0.9186 | 35272 | 5.96e2 | 2 | 2 | TRINITY\_DN5584\_c0\_g1\_i1.p1:TRINITY\_DN5584\_c0\_g1\_i3.p1 |  |  | DB Search |
| KTC(+57.02)VKDEVTGLHEIK | 58.26 | 1755.9189 | 15 | 3.59 | 439.9876 | 12.92 | 0.7795 | 0.7868 | 32586 | 1.06e3 | 1 | 1 | TRINITY\_DN1369\_c0\_g1\_i1.p1 | Carbamidomethylation |  | DB Search |
| VIDEGEKNIK | 58.26 | 1143.6135 | 10 | 3.03 | 572.8145 | 10.89 | 0.9271 | 0.9344 | 20199 | 3.83e3 | 2 | 2 | TRINITY\_DN1616\_c0\_g1\_i3.p1:TRINITY\_DN1616\_c0\_g1\_i6.p1:TRINITY\_DN1616\_c0\_g1\_i2.p1:TRINITY\_DN1616\_c0\_g1\_i7.p1 |  |  | DB Search |
| ADIEDVKDLLK | 58.26 | 1257.6816 | 11 | 3.28 | 420.2349 | 17.77 | 0.7489 | 0.7563 | 62925 | 2.25e4 | 2 | 2 | TRINITY\_DN15274\_c0\_g1\_i1.p1 |  |  | DB Search |
| AEIGFNFVR | 58.26 | 1051.545 | 9 | -0.62 | 526.7783 | 18.51 | 0.8595 | 0.8668 | 67749 | 1.14e3 | 1 | 1 | TRINITY\_DN244\_c0\_g1\_i1.p1 |  |  | DB Search |
| LNEELISFNSK | 58.26 | 1292.6613 | 11 | 2.31 | 647.338 | 17.35 | 0.938 | 0.9453 | 60098 | 6.81e2 | 1 | 1 | TRINITY\_DN1366\_c0\_g1\_i1.p1 |  |  | DB Search |
| VSGSLQLVWTR | 58.26 | 1244.6877 | 11 | 4.57 | 623.3526 | 18.55 | 0.9307 | 0.938 | 68053 | 5.74e2 | 1 | 1 | TRINITY\_DN3655\_c0\_g1\_i2.p1:TRINITY\_DN3655\_c0\_g1\_i1.p1 |  |  | DB Search |
| FVIGLTC(+57.02)SK | 58.26 | 1023.5423 | 9 | 1.29 | 512.778 | 16.97 | 0.8498 | 0.8571 | 57580 | 4.72e3 | 1 | 1 | TRINITY\_DN15274\_c0\_g1\_i1.p1 | Carbamidomethylation |  | DB Search |
| VLVILTDGIQAR | 58.26 | 1296.7765 | 12 | 4.33 | 649.3969 | 18.70 | 0.9723 | 0.9795 | 69087 | 3.67e2 | 1 | 1 | TRINITY\_DN5500\_c0\_g1\_i2.p1:TRINITY\_DN5500\_c0\_g1\_i1.p1 |  |  | DB Search |
| LELQSISDDILSK | 58.26 | 1459.777 | 13 | -3.91 | 730.8913 | 19.63 | 1.0025 | 1.0098 | 75107 | 4.55e2 | 1 | 1 | TRINITY\_DN1850\_c0\_g1\_i3.p1 |  |  | DB Search |
| IGFAAADAVTGLK | 58.26 | 1232.6764 | 13 | 2.63 | 617.3458 | 17.96 | 0.9469 | 0.9541 | 64240 | 0 | 0 | 0 | TRINITY\_DN107813\_c0\_g1\_i1.p1 |  |  | DB Search |
| YYEC(+57.02)GSAC(+57.02)PK | 58.26 | 1233.4795 | 10 | 3.8 | 617.748 | 11.31 | 0.9174 | 0.9247 | 22752 | 0 | 0 | 0 | TRINITY\_DN6390\_c0\_g1\_i1.p1 | Carbamidomethylation, Carbamidomethylation |  | DB Search |
| LSQC(+57.02)TAASGK | 58.26 | 1021.4862 | 10 | 0.53 | 511.7495 | 9.84 | 0.8307 | 0.838 | 13091 | 8.33e4 | 1 | 1 | TRINITY\_DN3991\_c0\_g1\_i1.p1 | Carbamidomethylation |  | DB Search |
| VPDLLFSK | 58.26 | 917.5222 | 8 | 0.47 | 459.7676 | 18.14 | 0.7884 | 0.7958 | 65377 | 0 | 0 | 0 | TRINITY\_DN33328\_c0\_g1\_i1.p1 |  |  | DB Search |
| APPKPNPC(+57.02)AIK | 58.26 | 1191.6434 | 11 | 5.82 | 398.2232 | 11.50 | 0.7783 | 0.7856 | 23835 | 2.21e3 | 2 | 2 | TRINITY\_DN4781\_c0\_g1\_i1.p1:TRINITY\_DN4781\_c0\_g1\_i2.p1 | Carbamidomethylation |  | DB Search |
| VC(+57.02)NAALC(+57.02)R | 58.26 | 962.4426 | 8 | 0.23 | 482.2276 | 11.36 | 0.812 | 0.8193 | 23031 | 0 | 0 | 0 | TRINITY\_DN4595\_c0\_g1\_i7.p1:TRINITY\_DN4595\_c0\_g1\_i4.p1:TRINITY\_DN4595\_c0\_g1\_i10.p2 | Carbamidomethylation, Carbamidomethylation |  | DB Search |
| TLSFEAVGHDVDK | 58.26 | 1416.6885 | 13 | -7.21 | 709.3448 | 15.67 | 1.0242 | 1.0315 | 49710 | 3.97e2 | 1 | 1 | TRINITY\_DN9655\_c0\_g1\_i1.p1 |  |  | DB Search |
| ADIEDVKDLLKK | 58.26 | 1385.7765 | 12 | 3.93 | 462.9336 | 16.48 | 0.7306 | 0.7379 | 54482 | 1.57e4 | 2 | 2 | TRINITY\_DN15274\_c0\_g1\_i1.p1 |  |  | DB Search |
| ALFHEFVSEK | 58.26 | 1205.608 | 10 | 0.45 | 603.8102 | 16.09 | 0.9384 | 0.9457 | 52118 | 3.31e2 | 1 | 1 | TRINITY\_DN244\_c0\_g1\_i1.p1 |  |  | DB Search |
| LIQYIENKPGLTR | 58.26 | 1543.8722 | 13 | 2.17 | 772.9434 | 14.98 | 1.0769 | 1.0841 | 45128 | 1.91e4 | 2 | 2 | TRINITY\_DN2028\_c0\_g1\_i1.p1 |  |  | DB Search |
| LGIEAIDENGLR | 58.26 | 1298.683 | 12 | 0.2 | 650.3475 | 17.28 | 0.9566 | 0.9638 | 59638 | 7.15e2 | 1 | 1 | TRINITY\_DN14015\_c0\_g1\_i3.p1:TRINITY\_DN14015\_c0\_g1\_i2.p1 |  |  | DB Search |
| DLLTLPEKDNRR | 58.26 | 1468.7998 | 12 | -3.01 | 490.6046 | 14.81 | 0.7888 | 0.7962 | 44185 | 3.24e2 | 1 | 1 | TRINITY\_DN776\_c0\_g1\_i1.p1 |  |  | DB Search |
| IYIDGVFIR | 58.26 | 1094.6124 | 9 | -0.14 | 548.3122 | 18.85 | 0.885 | 0.8923 | 70109 | 4.57e3 | 1 | 1 | TRINITY\_DN5500\_c0\_g1\_i2.p1:TRINITY\_DN5500\_c0\_g1\_i1.p1 |  |  | DB Search |
| VSQTC(+57.02)AEHVFR | 58.25 | 1332.6245 | 11 | 2.4 | 667.3196 | 12.94 | 0.9824 | 0.9896 | 32741 | 6.68e2 | 1 | 1 | TRINITY\_DN3078\_c0\_g1\_i1.p1 | Carbamidomethylation |  | DB Search |
| LFGSIYLR | 58.25 | 967.5491 | 8 | -0.04 | 484.7807 | 18.85 | 0.8311 | 0.8384 | 70112 | 9.36e2 | 1 | 1 | TRINITY\_DN982\_c0\_g1\_i3.p1 |  |  | DB Search |
| ITAIVLGPGDELPEK | 58.25 | 1550.8555 | 15 | -7.77 | 776.4273 | 17.93 | 1.0351 | 1.0423 | 64057 | 1.66e3 | 1 | 1 | TRINITY\_DN140702\_c0\_g1\_i1.p1 |  |  | DB Search |
| GDVIQVNEC(+57.02)TKK | 58.25 | 1389.6921 | 12 | -0.59 | 695.8514 | 12.20 | 1.0138 | 1.021 | 28037 | 1.96e3 | 1 | 1 | TRINITY\_DN1369\_c0\_g1\_i1.p1 | Carbamidomethylation |  | DB Search |
| VLDVGFLLDSSDKVK | 58.25 | 1633.8926 | 15 | -4.48 | 545.6345 | 19.79 | 0.8725 | 0.8798 | 76169 | 4.96e2 | 1 | 1 | TRINITY\_DN4494\_c1\_g1\_i1.p1:TRINITY\_DN4494\_c1\_g1\_i7.p1:TRINITY\_DN4494\_c1\_g1\_i5.p1 |  |  | DB Search |
| IDVALNIVTK | 58.25 | 1084.6492 | 10 | 1.83 | 543.3317 | 17.50 | 0.8571 | 0.8644 | 61036 | 3.63e2 | 1 | 1 | TRINITY\_DN1795\_c0\_g1\_i4.p1:TRINITY\_DN1795\_c0\_g1\_i1.p1 |  |  | DB Search |
| VPVTC(+57.02)C(+57.02)K | 58.25 | 862.4041 | 7 | 4.46 | 432.2103 | 10.18 | 0.7677 | 0.775 | 15728 | 1.45e4 | 1 | 1 | TRINITY\_DN661\_c0\_g1\_i10.p1 | Carbamidomethylation, Carbamidomethylation |  | DB Search |
| EGLEFIGTLK | 58.25 | 1105.6019 | 10 | 3.2 | 553.8088 | 19.04 | 0.8627 | 0.87 | 71397 | 0 | 0 | 0 | TRINITY\_DN2577\_c1\_g1\_i1.p1:TRINITY\_DN2577\_c1\_g1\_i2.p1 |  |  | DB Search |
| TLDFVVELSK | 58.25 | 1149.6281 | 10 | 0.64 | 575.8204 | 19.63 | 0.8834 | 0.8907 | 75086 | 4.01e3 | 1 | 1 | TRINITY\_DN138\_c0\_g1\_i1.p1 |  |  | DB Search |
| LLLGDFNGDKR | 58.25 | 1246.667 | 11 | 4.07 | 624.3419 | 16.32 | 0.9844 | 0.9916 | 53554 | 5.68e2 | 1 | 1 | TRINITY\_DN413\_c0\_g1\_i4.p1:TRINITY\_DN413\_c0\_g1\_i1.p1:TRINITY\_DN413\_c0\_g1\_i2.p1:TRINITY\_DN413\_c0\_g1\_i3.p1 |  |  | DB Search |
| DLPQEKLDDILK | 58.25 | 1425.7715 | 12 | -1.41 | 713.8904 | 17.90 | 1.0307 | 1.0379 | 63772 | 2.09e3 | 2 | 2 | TRINITY\_DN262\_c0\_g1\_i1.p1 |  |  | DB Search |
| LGANAILGVSLAAAK | 58.25 | 1367.8136 | 15 | 1.11 | 684.9133 | 19.13 | 1.058 | 1.0652 | 71938 | 4.36e2 | 1 | 1 | TRINITY\_DN2796\_c0\_g1\_i2.p1:TRINITY\_DN2796\_c0\_g1\_i1.p1 |  |  | DB Search |
| DIIVLEAENPVK | 58.25 | 1338.7394 | 12 | 1.86 | 670.3768 | 18.10 | 0.9844 | 0.9916 | 65198 | 4.87e2 | 1 | 1 | TRINITY\_DN1616\_c0\_g1\_i3.p1:TRINITY\_DN1616\_c0\_g1\_i6.p1:TRINITY\_DN1616\_c0\_g1\_i2.p1:TRINITY\_DN1616\_c0\_g1\_i7.p1 |  |  | DB Search |
| VIGHGTIYGEEVTLNLR | 58.24 | 1869.9949 | 17 | 4.33 | 624.3402 | 17.56 | 0.919 | 0.9263 | 61629 | 0 | 0 | 0 | TRINITY\_DN15542\_c0\_g1\_i2.p1:TRINITY\_DN15542\_c0\_g1\_i1.p1 |  |  | DB Search |
| DGPAGPAGR | 58.24 | 796.3828 | 9 | 3.73 | 399.1992 | 9.76 | 0.7061 | 0.7135 | 13439 | 1.18e4 | 2 | 2 | TRINITY\_DN106\_c0\_g1\_i2.p1:TRINITY\_DN106\_c0\_g1\_i6.p1:TRINITY\_DN106\_c0\_g1\_i12.p1:TRINITY\_DN106\_c0\_g1\_i5.p1:TRINITY\_DN106\_c0\_g1\_i10.p1:TRINITY\_DN106\_c0\_g1\_i8.p1:TRINITY\_DN106\_c0\_g1\_i3.p1:TRINITY\_DN106\_c0\_g1\_i9.p1:TRINITY\_DN106\_c0\_g1\_i11.p1 |  |  | DB Search |
| LTIIDALPR | 58.24 | 1010.6124 | 9 | 0.02 | 506.3124 | 19.00 | 0.8392 | 0.8465 | 71132 | 3.79e2 | 1 | 1 | TRINITY\_DN1783\_c0\_g1\_i8.p2:TRINITY\_DN1783\_c0\_g1\_i1.p2:TRINITY\_DN1783\_c0\_g1\_i3.p1:TRINITY\_DN1783\_c0\_g1\_i2.p1:TRINITY\_DN1783\_c0\_g1\_i6.p1:TRINITY\_DN105041\_c0\_g1\_i1.p1:TRINITY\_DN1783\_c1\_g1\_i1.p2 |  |  | DB Search |
| ALLLASSNAFDTSNGAR | 58.24 | 1706.8588 | 17 | 2.06 | 854.4365 | 17.87 | 1.0945 | 1.1017 | 63441 | 8.53e3 | 2 | 2 | TRINITY\_DN244\_c0\_g1\_i1.p1 |  |  | DB Search |
| IGC(+57.02)FHDR | 58.24 | 903.4021 | 7 | 6.62 | 452.7103 | 11.34 | 0.8014 | 0.8088 | 22951 | 0 | 0 | 0 | TRINITY\_DN138\_c0\_g1\_i1.p1 | Carbamidomethylation |  | DB Search |
| TAVVDGVEIFK | 58.24 | 1176.639 | 11 | -0.39 | 589.3253 | 18.09 | 0.9133 | 0.9206 | 64929 | 1.01e4 | 1 | 1 | TRINITY\_DN6390\_c0\_g1\_i1.p1 |  |  | DB Search |
| VIVDQLVTLATR | 58.24 | 1326.7871 | 12 | 1.65 | 664.4005 | 19.89 | 0.957 | 0.9642 | 76836 | 0 | 0 | 0 | TRINITY\_DN1386\_c0\_g1\_i1.p1 |  |  | DB Search |
| VDVGFILDSSGSLR | 58.24 | 1463.762 | 14 | 1.17 | 732.8875 | 19.97 | 1.0102 | 1.0174 | 77350 | 0 | 0 | 0 | TRINITY\_DN17725\_c0\_g1\_i1.p1:TRINITY\_DN4494\_c1\_g1\_i2.p1:TRINITY\_DN4494\_c1\_g1\_i15.p1:TRINITY\_DN4494\_c2\_g1\_i1.p1:TRINITY\_DN4494\_c1\_g1\_i1.p1:TRINITY\_DN4494\_c1\_g1\_i12.p1:TRINITY\_DN172523\_c0\_g1\_i1.p1:TRINITY\_DN12542\_c0\_g1\_i1.p1:TRINITY\_DN4494\_c1\_g1\_i7.p1:TRINITY\_DN4494\_c1\_g1\_i8.p1:TRINITY\_DN4494\_c1\_g1\_i10.p1:TRINITY\_DN4494\_c1\_g1\_i5.p1 |  |  | DB Search |
| VPAENNPK | 58.24 | 867.445 | 8 | 3.57 | 434.7304 | 9.23 | 0.7766 | 0.784 | 11210 | 2.03e3 | 1 | 1 | TRINITY\_DN48767\_c0\_g1\_i1.p1 |  |  | DB Search |
| GLYISAEFAIPEDC(+57.02)KK | 58.24 | 1839.9076 | 16 | 0.49 | 920.9595 | 18.42 | 1.2013 | 1.2085 | 67321 | 1.12e2 | 1 | 1 | TRINITY\_DN8155\_c0\_g1\_i1.p1 | Carbamidomethylation |  | DB Search |
| IPSWVPSLPKPK | 58.24 | 1347.7914 | 12 | -9.34 | 450.2659 | 18.14 | 0.7734 | 0.7807 | 65403 | 6.48e2 | 1 | 1 | TRINITY\_DN2576\_c3\_g1\_i1.p2 |  |  | DB Search |
| TC(+57.02)TNPAPAHGGK | 58.24 | 1209.556 | 12 | 4.26 | 404.1935 | 8.97 | 0.7322 | 0.7396 | 10252 | 1.06e3 | 2 | 2 | TRINITY\_DN1613\_c1\_g1\_i2.p1:TRINITY\_DN1613\_c1\_g1\_i1.p1 | Carbamidomethylation |  | DB Search |
| TAC(+57.02)VGM(+15.99)DR | 58.23 | 924.3793 | 8 | 4.29 | 463.1979 | 9.57 | 0.7827 | 0.7901 | 12695 | 9.24e2 | 1 | 1 | TRINITY\_DN4595\_c0\_g1\_i10.p1:TRINITY\_DN4595\_c0\_g1\_i4.p1 | Carbamidomethylation, Oxidation (M) | M6:Oxidation (M):1000 | DB Search |
| C(+57.02)MLATC(+57.02)EAGQLDPVAVC(+57.02)GK | 58.23 | 2078.9258 | 19 | -13.35 | 1040.454 | 17.81 | 1.1985 | 1.2057 | 63229 | 0 | 0 | 0 | TRINITY\_DN319\_c2\_g1\_i3.p1:TRINITY\_DN319\_c2\_g1\_i1.p1 | Carbamidomethylation, Carbamidomethylation, Carbamidomethylation |  | DB Search |
| GVTSITLELQDLQR | 58.23 | 1571.8518 | 14 | 1.83 | 786.9329 | 19.49 | 1.0556 | 1.0628 | 74149 | 1.11e3 | 1 | 1 | TRINITY\_DN51907\_c0\_g1\_i1.p1 |  |  | DB Search |
| NNPPTATR | 58.23 | 869.4355 | 8 | 2.62 | 435.7252 | 9.23 | 0.784 | 0.7913 | 11045 | 2.65e3 | 1 | 1 | TRINITY\_DN477\_c0\_g1\_i8.p1:TRINITY\_DN477\_c0\_g1\_i7.p1 |  |  | DB Search |
| VFIYGYDFQGGK | 58.23 | 1392.6714 | 12 | -6.4 | 697.337 | 18.59 | 0.986 | 0.9932 | 68452 | 0 | 0 | 0 | TRINITY\_DN2357\_c1\_g1\_i1.p1 |  |  | DB Search |
| LNQSSLNLNAAANDVVHAAK | 58.22 | 2049.0603 | 20 | 5.38 | 684.0295 | 17.53 | 0.9662 | 0.9735 | 61365 | 0 | 0 | 0 | TRINITY\_DN18348\_c0\_g1\_i1.p1 |  |  | DB Search |
| LELEFTKDER | 58.22 | 1278.6455 | 10 | -2.73 | 640.3269 | 15.54 | 0.9739 | 0.9812 | 48812 | 0 | 0 | 0 | TRINITY\_DN2577\_c1\_g1\_i1.p1:TRINITY\_DN2577\_c1\_g1\_i2.p1 |  |  | DB Search |
| TC(+57.02)ESGDLDLDFAC(+57.02)GR | 58.22 | 1714.6926 | 15 | -2.61 | 858.3495 | 17.67 | 1.0572 | 1.0644 | 62177 | 1.81e3 | 1 | 1 | TRINITY\_DN28257\_c0\_g1\_i1.p1:TRINITY\_DN28257\_c0\_g1\_i2.p1 | Carbamidomethylation, Carbamidomethylation |  | DB Search |
| LGTIEKDLAK | 58.22 | 1086.6284 | 10 | -3.8 | 544.3182 | 13.35 | 0.9044 | 0.9117 | 35185 | 0 | 0 | 0 | TRINITY\_DN101600\_c0\_g1\_i1.p1 |  |  | DB Search |
| LSQTAPTC(+57.02)K | 58.21 | 1004.4961 | 9 | 4.86 | 503.2567 | 10.24 | 0.825 | 0.8323 | 16211 | 1.14e3 | 1 | 1 | TRINITY\_DN28022\_c0\_g2\_i1.p1 | Carbamidomethylation |  | DB Search |
| DANSM(+15.99)PTQSR | 58.2 | 1121.4772 | 10 | 7.65 | 561.7489 | 8.79 | 0.8189 | 0.8262 | 9919 | 1.9e3 | 1 | 1 | TRINITY\_DN477\_c0\_g1\_i9.p1 | Oxidation (M) | M5:Oxidation (M):1000 | DB Search |
| ANVNIHVR | 58.2 | 921.5144 | 8 | 4.06 | 461.7654 | 11.91 | 0.8307 | 0.838 | 26250 | 1.78e3 | 1 | 1 | TRINITY\_DN6390\_c0\_g1\_i1.p1 |  |  | DB Search |
| VMLLYTDGEESFR | 58.2 | 1558.7338 | 13 | 9.8 | 780.3801 | 18.73 | 1.0721 | 1.0793 | 69339 | 0 | 0 | 0 | TRINITY\_DN74720\_c0\_g1\_i1.p1 |  |  | DB Search |
| ALAWGLPGYK | 58.2 | 1074.5862 | 10 | -1.57 | 538.2983 | 19.05 | 0.8599 | 0.8672 | 71305 | 1.33e3 | 1 | 1 | TRINITY\_DN2490\_c0\_g1\_i1.p1 |  |  | DB Search |
| SSLNHNIVFEGR | 58.2 | 1371.6895 | 12 | 1.43 | 458.2368 | 14.67 | 0.7583 | 0.7656 | 43307 | 0 | 0 | 0 | TRINITY\_DN144508\_c0\_g1\_i1.p1 |  |  | DB Search |
| DNIQGITKPAIR | 58.19 | 1324.7462 | 12 | 0.94 | 663.3796 | 13.94 | 0.9795 | 0.9868 | 38749 | 1.14e3 | 1 | 1 | TRINITY\_DN172571\_c0\_g1\_i1.p1 |  |  | DB Search |
| GPVGTSGLR | 58.19 | 842.461 | 9 | 3.7 | 422.2384 | 12.14 | 0.7787 | 0.786 | 27733 | 1.47e4 | 1 | 1 | TRINITY\_DN77\_c0\_g2\_i4.p1:TRINITY\_DN77\_c0\_g2\_i2.p1:TRINITY\_DN77\_c0\_g2\_i1.p1:TRINITY\_DN77\_c0\_g2\_i3.p1 |  |  | DB Search |
| VLVHTQQK | 58.19 | 951.5502 | 8 | 0.78 | 476.7817 | 9.51 | 0.8335 | 0.8408 | 12449 | 5.52e2 | 1 | 1 | TRINITY\_DN6920\_c0\_g1\_i1.p1:TRINITY\_DN6920\_c0\_g1\_i3.p1:TRINITY\_DN6920\_c0\_g1\_i4.p1:TRINITY\_DN6920\_c0\_g1\_i2.p1 |  |  | DB Search |
| EIAQDFKTDLR | 58.18 | 1334.683 | 11 | 0.79 | 668.3478 | 14.95 | 0.9924 | 0.9997 | 45098 | 2.55e2 | 1 | 1 | TRINITY\_DN72753\_c0\_g1\_i1.p1 |  |  | DB Search |
| DFDSLPYYPR | 58.18 | 1271.5823 | 10 | 1.54 | 636.798 | 18.28 | 0.9222 | 0.9295 | 66470 | 3.66e2 | 1 | 1 | TRINITY\_DN5500\_c0\_g1\_i2.p1:TRINITY\_DN5500\_c0\_g1\_i1.p1 |  |  | DB Search |
| GPVGAQGTR | 58.18 | 841.4406 | 9 | 6.48 | 421.7294 | 9.59 | 0.7599 | 0.7673 | 12867 | 1.07e4 | 1 | 1 | TRINITY\_DN113\_c2\_g1\_i1.p1 |  |  | DB Search |
| SAVNHNIIFEGR | 58.18 | 1355.6946 | 12 | 3.09 | 678.8552 | 14.84 | 0.9751 | 0.9824 | 44311 | 1.57e3 | 1 | 1 | TRINITY\_DN1936\_c0\_g1\_i3.p1 |  |  | DB Search |
| GISIGFEEIR | 58.18 | 1119.5924 | 10 | 2.84 | 560.8038 | 18.57 | 0.8959 | 0.9032 | 68197 | 2.85e3 | 1 | 1 | TRINITY\_DN1774\_c0\_g1\_i1.p1 |  |  | DB Search |
| LIFVGTVGK | 58.18 | 932.5695 | 9 | 3.01 | 467.2924 | 17.35 | 0.8124 | 0.8197 | 60219 | 6.55e3 | 1 | 1 | TRINITY\_DN4781\_c0\_g1\_i3.p1:TRINITY\_DN4781\_c0\_g1\_i1.p1:TRINITY\_DN4781\_c0\_g1\_i2.p1 |  |  | DB Search |
| LLTTEDDFILR | 58.17 | 1334.7081 | 11 | -1.86 | 668.3586 | 19.19 | 0.9634 | 0.9707 | 72323 | 9.12e2 | 1 | 1 | TRINITY\_DN982\_c0\_g1\_i1.p1:TRINITY\_DN982\_c0\_g1\_i3.p1 |  |  | DB Search |
| AVAC(+57.02)NSGPC(+57.02)LAGC(+57.02)PR | 58.17 | 1588.6908 | 15 | 1.54 | 795.3522 | 13.16 | 1.015 | 1.0222 | 33794 | 1.36e4 | 1 | 1 | TRINITY\_DN4815\_c0\_g1\_i2.p1:TRINITY\_DN4815\_c0\_g1\_i1.p1 | Carbamidomethylation, Carbamidomethylation, Carbamidomethylation |  | DB Search |
| LQTDIHGVALEITK | 58.16 | 1536.8511 | 14 | 0.96 | 769.4319 | 16.71 | 1.0544 | 1.0616 | 56016 | 5.98e2 | 1 | 1 | TRINITY\_DN99404\_c0\_g1\_i1.p1 |  |  | DB Search |
| GLRGPEGSSGPIGK | 58.15 | 1310.6942 | 14 | -0.77 | 437.9041 | 12.30 | 0.7176 | 0.7249 | 28421 | 2.77e3 | 2 | 2 | TRINITY\_DN77\_c1\_g1\_i1.p1 |  |  | DB Search |
| IKLDC(+57.02)NINIAK | 58.15 | 1300.7173 | 11 | 2.08 | 651.3658 | 15.03 | 0.9852 | 0.9924 | 45500 | 4.03e2 | 1 | 1 | TRINITY\_DN8903\_c0\_g1\_i1.p1 | Carbamidomethylation |  | DB Search |
| GLAGPTGQR | 58.14 | 855.4562 | 9 | 5.02 | 428.7366 | 10.98 | 0.7506 | 0.7579 | 20698 | 6.08e3 | 1 | 1 | TRINITY\_DN113\_c0\_g1\_i1.p1 |  |  | DB Search |
| KSEGAAETGSVTISK | 58.14 | 1463.7467 | 15 | 0.58 | 732.8795 | 11.50 | 0.9844 | 0.9916 | 23786 | 1.99e3 | 1 | 1 | TRINITY\_DN1676\_c0\_g1\_i6.p1:TRINITY\_DN1676\_c0\_g1\_i12.p1 |  |  | DB Search |
| NAILDQNLENREK | 58.14 | 1555.7954 | 13 | -1.51 | 778.9021 | 13.92 | 1.0568 | 1.064 | 38720 | 0 | 0 | 0 | TRINITY\_DN15274\_c0\_g1\_i1.p1 |  |  | DB Search |
| M(+15.99)QC(+57.02)VPAK | 58.14 | 848.3884 | 7 | -1.44 | 425.2 | 9.64 | 0.8254 | 0.8327 | 13010 | 5.89e2 | 1 | 1 | TRINITY\_DN319\_c2\_g1\_i3.p1:TRINITY\_DN319\_c2\_g1\_i1.p1 | Oxidation (M), Carbamidomethylation | M1:Oxidation (M):1000 | DB Search |
| GSSFM(+15.99)GDIALDDISVR | 58.13 | 1697.793 | 16 | 8.34 | 849.909 | 18.58 | 1.0797 | 1.0869 | 68392 | 9.18e2 | 1 | 1 | TRINITY\_DN1936\_c0\_g1\_i3.p1 | Oxidation (M) | M5:Oxidation (M):1000 | DB Search |
| SAM(+15.99)DQVQEK | 58.13 | 1050.4652 | 9 | -2.78 | 526.2372 | 9.49 | 0.8481 | 0.8554 | 12232 | 8.46e2 | 1 | 1 | TRINITY\_DN661\_c0\_g1\_i10.p1 | Oxidation (M) | M3:Oxidation (M):1000 | DB Search |
| ATLYPC(+57.02)PETPNER | 58.12 | 1546.7086 | 13 | 5.6 | 774.3642 | 14.56 | 1.0359 | 1.0431 | 42458 | 8e2 | 1 | 1 | TRINITY\_DN761\_c0\_g1\_i3.p1:TRINITY\_DN761\_c0\_g1\_i1.p1:TRINITY\_DN761\_c0\_g1\_i2.p1 | Carbamidomethylation |  | DB Search |
| LGPIPLWK | 58.11 | 922.564 | 8 | 0.42 | 462.2885 | 19.50 | 0.7795 | 0.7868 | 74214 | 9.3e2 | 1 | 1 | TRINITY\_DN36217\_c0\_g1\_i1.p1 |  |  | DB Search |
| DM(+15.99)ITC(+57.02)NENPYITDDLFK | 58.11 | 2103.9128 | 17 | 4.31 | 1052.9659 | 19.48 | 1.1945 | 1.2017 | 74139 | 7.17e2 | 1 | 1 | TRINITY\_DN477\_c0\_g1\_i9.p1 | Oxidation (M), Carbamidomethylation | M2:Oxidation (M):1000 | DB Search |
| GANGQNGVQGAR | 58.1 | 1127.5432 | 12 | 10.77 | 564.7837 | 9.29 | 0.8441 | 0.8514 | 11509 | 0 | 0 | 0 | TRINITY\_DN77\_c1\_g1\_i1.p1 |  |  | DB Search |
| AVNDNSHDLIHSIR | 58.09 | 1589.791 | 14 | -0.42 | 530.9362 | 14.03 | 0.8404 | 0.8477 | 39366 | 0 | 0 | 0 | TRINITY\_DN18348\_c0\_g1\_i1.p1 |  |  | DB Search |
| SC(+57.02)VLASC(+57.02)SANSDVAVAC(+57.02)GK | 58.08 | 1954.8546 | 19 | 3.15 | 978.4355 | 14.42 | 1.153 | 1.1602 | 41659 | 2.61e2 | 1 | 1 | TRINITY\_DN1058\_c0\_g1\_i1.p1:TRINITY\_DN1058\_c0\_g1\_i3.p1:TRINITY\_DN1058\_c0\_g1\_i2.p2 | Carbamidomethylation, Carbamidomethylation, Carbamidomethylation |  | DB Search |
| WLIAEGGSELPSNLR | 58.07 | 1640.8522 | 15 | -10.17 | 821.4232 | 19.05 | 1.0664 | 1.0737 | 71492 | 0 | 0 | 0 | TRINITY\_DN21554\_c2\_g1\_i1.p1 |  |  | DB Search |
| RPLLGDPSFVADVSK | 58.07 | 1599.8621 | 15 | -5.12 | 534.2907 | 17.53 | 0.7746 | 0.7819 | 61329 | 0 | 0 | 0 | TRINITY\_DN10485\_c0\_g1\_i1.p1 |  |  | DB Search |
| FLVELAC(+57.02)K | 58.06 | 978.5208 | 8 | -1.7 | 490.2658 | 17.31 | 0.8116 | 0.8189 | 59794 | 1.21e3 | 1 | 1 | TRINITY\_DN1119\_c0\_g1\_i1.p1 | Carbamidomethylation |  | DB Search |
| LYISSLPLK | 58.05 | 1032.6219 | 9 | 2.33 | 517.3183 | 18.15 | 0.8587 | 0.866 | 65559 | 0 | 0 | 0 | TRINITY\_DN61742\_c0\_g1\_i2.p1:TRINITY\_DN61742\_c0\_g1\_i1.p1:TRINITY\_DN61742\_c0\_g1\_i3.p1 |  |  | DB Search |
| LGTIVAGFLK | 58.05 | 1017.6223 | 10 | -3.2 | 509.8156 | 19.28 | 0.8254 | 0.8327 | 72854 | 3.28e2 | 1 | 1 | TRINITY\_DN7674\_c0\_g1\_i2.p1:TRINITY\_DN7674\_c0\_g1\_i1.p1 |  |  | DB Search |
| ILDNNNQEVIHLNRPLR | 58.05 | 2057.113 | 17 | -0.42 | 515.2842 | 15.14 | 0.8258 | 0.8331 | 46227 | 0 | 0 | 0 | TRINITY\_DN49617\_c0\_g1\_i2.p1 |  |  | DB Search |
| DILGLDSEGTSDAYAK | 58.05 | 1653.7733 | 16 | 1.81 | 827.8936 | 17.69 | 1.0733 | 1.0805 | 62313 | 3.6e2 | 1 | 1 | TRINITY\_DN140702\_c0\_g1\_i1.p1 |  |  | DB Search |
| VDDIIC(+57.02)IAGC(+57.02)ELKK | 58.04 | 1632.8215 | 14 | -6.17 | 545.2766 | 17.85 | 0.8388 | 0.8461 | 63424 | 2.04e2 | 1 | 1 | TRINITY\_DN2577\_c1\_g1\_i1.p1:TRINITY\_DN2577\_c1\_g1\_i2.p1 | Carbamidomethylation, Carbamidomethylation |  | DB Search |
| GPDGAVGPTGPIGAR | 58.04 | 1320.6786 | 15 | 1.83 | 661.3463 | 13.72 | 0.9558 | 0.963 | 37443 | 1.21e4 | 2 | 2 | TRINITY\_DN106\_c0\_g1\_i6.p1:TRINITY\_DN106\_c0\_g1\_i5.p1:TRINITY\_DN106\_c0\_g1\_i8.p1:TRINITY\_DN106\_c0\_g1\_i3.p1:TRINITY\_DN106\_c0\_g1\_i9.p1:TRINITY\_DN106\_c0\_g1\_i11.p1 |  |  | DB Search |
| HILPHLAR | 58.04 | 955.5715 | 8 | 7.97 | 319.533 | 12.62 | 0.7029 | 0.7102 | 30779 | 0 | 0 | 0 | TRINITY\_DN2356\_c0\_g1\_i1.p1 |  |  | DB Search |
| MGAIVAVTGDGVNDSPALK | 58.03 | 1813.9243 | 19 | 0.14 | 907.9676 | 16.99 | 1.127 | 1.1342 | 57933 | 5.24e2 | 1 | 1 | TRINITY\_DN262\_c0\_g1\_i1.p1 |  |  | DB Search |
| DLEQDLQELR | 58.02 | 1257.6201 | 10 | 1.15 | 629.8167 | 19.45 | 0.9263 | 0.9335 | 73935 | 3.17e2 | 1 | 1 | TRINITY\_DN428\_c0\_g1\_i1.p1 |  |  | DB Search |
| TC(+57.02)VTASC(+57.02)R | 58.02 | 953.4059 | 8 | 4.85 | 477.7115 | 10.04 | 0.7986 | 0.8059 | 15114 | 2.52e3 | 1 | 1 | TRINITY\_DN24268\_c0\_g1\_i2.p1:TRINITY\_DN24268\_c0\_g1\_i1.p1 | Carbamidomethylation, Carbamidomethylation |  | DB Search |
| GKPEQVQVK | 58.01 | 1011.5713 | 9 | 0.86 | 506.7922 | 9.91 | 0.8376 | 0.8449 | 14438 | 2.02e2 | 1 | 1 | TRINITY\_DN11522\_c0\_g1\_i1.p1:TRINITY\_DN11522\_c0\_g1\_i2.p1:TRINITY\_DN11522\_c0\_g1\_i4.p1:TRINITY\_DN11522\_c0\_g1\_i3.p1 |  |  | DB Search |
| TC(+57.02)IGC(+57.02)PETQIWR | 58.01 | 1519.6912 | 12 | -0.87 | 760.8505 | 16.92 | 1.0053 | 1.0126 | 57323 | 1.28e3 | 1 | 1 | TRINITY\_DN4781\_c0\_g1\_i1.p1:TRINITY\_DN4781\_c0\_g1\_i2.p1 | Carbamidomethylation, Carbamidomethylation |  | DB Search |
| AVFPSIVGRPR | 58 | 1197.6982 | 11 | 3.54 | 599.8572 | 15.96 | 0.9642 | 0.9715 | 51028 | 1.61e4 | 1 | 1 | TRINITY\_DN161\_c0\_g1\_i1.p1:TRINITY\_DN161\_c0\_g1\_i3.p1:TRINITY\_DN161\_c0\_g1\_i2.p1 |  |  | DB Search |
| FPGQLNADIRK | 57.99 | 1257.683 | 11 | -7.77 | 420.2307 | 14.50 | 0.7033 | 0.7106 | 42199 | 0 | 0 | 0 | TRINITY\_DN5365\_c0\_g1\_i3.p1:TRINITY\_DN5365\_c0\_g1\_i2.p1 |  |  | DB Search |
| FPGQLNADLRK | 57.99 | 1257.683 | 11 | -7.77 | 420.2307 | 14.50 | 0.7033 | 0.7106 | 42199 | 0 | 0 | 0 | TRINITY\_DN1910\_c0\_g1\_i1.p1:TRINITY\_DN1910\_c0\_g1\_i5.p1:TRINITY\_DN3986\_c0\_g1\_i2.p1:TRINITY\_DN3986\_c0\_g1\_i1.p1 |  |  | DB Search |
| IGLMSSFQK | 57.99 | 1009.5266 | 9 | 4.5 | 505.7718 | 17.20 | 0.8352 | 0.8425 | 59131 | 1.33e3 | 1 | 1 | TRINITY\_DN982\_c0\_g1\_i3.p1 |  |  | DB Search |
| LLDWAQQR | 57.97 | 1028.5403 | 8 | 6.66 | 515.2797 | 16.40 | 0.8485 | 0.8558 | 54058 | 6.82e2 | 1 | 1 | TRINITY\_DN199\_c0\_g1\_i1.p1 |  |  | DB Search |
| DLFDTLTK | 57.97 | 951.4913 | 8 | 4.2 | 476.7539 | 19.15 | 0.8063 | 0.8136 | 71926 | 2.59e3 | 1 | 1 | TRINITY\_DN7946\_c0\_g1\_i1.p1 |  |  | DB Search |
| NDVNNPPQAR | 57.97 | 1123.537 | 10 | 3.56 | 562.7766 | 10.00 | 0.8615 | 0.8688 | 15207 | 4.89e4 | 2 | 2 | TRINITY\_DN477\_c0\_g1\_i3.p1:TRINITY\_DN477\_c0\_g1\_i7.p1:TRINITY\_DN477\_c0\_g1\_i6.p1:TRINITY\_DN477\_c0\_g1\_i8.p2:TRINITY\_DN477\_c0\_g1\_i5.p1 |  |  | DB Search |
| DIQDFLATDFNHPNASK | 57.97 | 1931.9014 | 17 | 1.13 | 644.9737 | 19.20 | 0.8477 | 0.855 | 72443 | 2.25e2 | 1 | 1 | TRINITY\_DN15274\_c0\_g1\_i1.p1 |  |  | DB Search |
| DLTDYLMK | 57.96 | 997.479 | 8 | 6.32 | 499.7488 | 18.67 | 0.8319 | 0.8392 | 68987 | 0 | 0 | 0 | TRINITY\_DN161\_c0\_g1\_i1.p1:TRINITY\_DN161\_c0\_g1\_i4.p1:TRINITY\_DN161\_c0\_g1\_i3.p1:TRINITY\_DN161\_c0\_g1\_i2.p1 |  |  | DB Search |
| AQLLLEDVK | 57.95 | 1027.5913 | 9 | 4.05 | 514.8039 | 17.08 | 0.8721 | 0.8793 | 58461 | 1.87e2 | 1 | 1 | TRINITY\_DN942\_c0\_g1\_i5.p1:TRINITY\_DN942\_c0\_g1\_i2.p1:TRINITY\_DN942\_c0\_g1\_i3.p1 |  |  | DB Search |
| GVAGAGVLAGFDKFK | 57.94 | 1435.7823 | 15 | 1.38 | 479.601 | 18.49 | 0.8453 | 0.8526 | 67586 | 2.66e3 | 2 | 2 | TRINITY\_DN1559\_c0\_g1\_i1.p1 |  |  | DB Search |
| DNGDWAIDNLR | 57.94 | 1287.5844 | 11 | -1.8 | 644.7969 | 17.93 | 0.9206 | 0.9279 | 64010 | 0 | 0 | 0 | TRINITY\_DN4815\_c0\_g1\_i2.p1:TRINITY\_DN4815\_c0\_g1\_i1.p1 |  |  | DB Search |
| AGVLAHLEEERDIK | 57.94 | 1578.8365 | 14 | 0.03 | 527.285 | 15.08 | 0.7994 | 0.8067 | 45791 | 4.01e2 | 1 | 1 | TRINITY\_DN428\_c0\_g1\_i1.p1 |  |  | DB Search |
| ASNEVSM(+15.99)K | 57.93 | 880.396 | 8 | -7.43 | 441.201 | 8.74 | 0.7469 | 0.7542 | 9825 | 0 | 0 | 0 | TRINITY\_DN24268\_c0\_g1\_i2.p1:TRINITY\_DN24268\_c0\_g1\_i1.p1 | Oxidation (M) | M7:Oxidation (M):1000 | DB Search |
| DEVTGLHEIK | 57.91 | 1139.5823 | 10 | -4.42 | 570.7946 | 13.56 | 0.917 | 0.9243 | 36523 | 0 | 0 | 0 | TRINITY\_DN1369\_c0\_g1\_i1.p1 |  |  | DB Search |
| LEIASWPLR | 57.9 | 1083.6077 | 9 | -0.52 | 542.8096 | 19.41 | 0.8684 | 0.8757 | 73699 | 4.16e2 | 1 | 1 | TRINITY\_DN77\_c1\_g1\_i1.p1 |  |  | DB Search |
| GLKVDFDTTFAPVTGKK | 57.88 | 1822.9828 | 17 | -3.05 | 608.665 | 17.39 | 0.8984 | 0.9057 | 60425 | 2.02e2 | 1 | 1 | TRINITY\_DN329\_c0\_g1\_i1.p1:TRINITY\_DN329\_c0\_g1\_i2.p1 |  |  | DB Search |
| AETDIM(+15.99)KR | 57.87 | 978.4804 | 8 | 3.54 | 490.2481 | 9.48 | 0.8108 | 0.8181 | 12357 | 0 | 0 | 0 | TRINITY\_DN262\_c0\_g1\_i1.p1 | Oxidation (M) | M6:Oxidation (M):1000 | DB Search |
| VLQEREC(+57.02)EQDTC(+57.02)FDGNTR | 57.83 | 2255.9536 | 18 | -6.28 | 752.9854 | 13.04 | 0.9323 | 0.9396 | 33365 | 6e2 | 1 | 1 | TRINITY\_DN4595\_c0\_g1\_i10.p1:TRINITY\_DN4595\_c0\_g1\_i4.p1 | Carbamidomethylation, Carbamidomethylation |  | DB Search |
| VTRPHGASGVVR | 57.82 | 1234.6895 | 12 | 7.66 | 412.5727 | 10.07 | 0.8108 | 0.8181 | 15251 | 2.57e2 | 1 | 1 | TRINITY\_DN2885\_c0\_g1\_i2.p1:TRINITY\_DN2885\_c0\_g1\_i4.p1:TRINITY\_DN2885\_c0\_g1\_i5.p1 |  |  | DB Search |
| AQIDIWLQR | 57.82 | 1141.6244 | 9 | 1.55 | 571.8191 | 18.61 | 0.8887 | 0.8959 | 68405 | 8.34e2 | 1 | 1 | TRINITY\_DN8926\_c0\_g1\_i1.p1:TRINITY\_DN8926\_c0\_g1\_i2.p1 |  |  | DB Search |
| YVQNPR | 57.81 | 775.3977 | 6 | 8.26 | 388.7085 | 9.75 | 0.7705 | 0.7779 | 13543 | 5.55e3 | 1 | 1 | TRINITY\_DN8903\_c0\_g1\_i1.p1 |  |  | DB Search |
| LC(+57.02)YVALDFEQEM(+15.99)QTAASSSSLEK | 57.8 | 2622.1829 | 23 | 5.82 | 875.0714 | 19.02 | 1.0741 | 1.0813 | 71279 | 4.32e2 | 1 | 1 | TRINITY\_DN161\_c0\_g1\_i1.p1:TRINITY\_DN161\_c0\_g1\_i4.p1:TRINITY\_DN161\_c0\_g1\_i3.p1:TRINITY\_DN161\_c0\_g1\_i2.p1 | Carbamidomethylation, Oxidation (M) | M12:Oxidation (M):1000 | DB Search |
| MAVNIMIK | 57.78 | 918.5031 | 8 | 13.05 | 460.2638 | 16.99 | 0.8071 | 0.8144 | 57925 | 0 | 0 | 0 | TRINITY\_DN982\_c0\_g1\_i3.p1 |  |  | DB Search |
| IC(+57.02)GHGGIR | 57.78 | 868.4337 | 8 | -3.19 | 435.2218 | 9.87 | 0.7892 | 0.7966 | 14210 | 0 | 0 | 0 | TRINITY\_DN1058\_c0\_g1\_i2.p1:TRINITY\_DN1058\_c0\_g1\_i1.p1:TRINITY\_DN1058\_c0\_g1\_i3.p1 | Carbamidomethylation |  | DB Search |
| LFEGNALLR | 57.78 | 1031.5763 | 9 | 0.94 | 516.7948 | 17.48 | 0.8631 | 0.8704 | 60870 | 9.34e2 | 1 | 1 | TRINITY\_DN189323\_c0\_g1\_i1.p1:TRINITY\_DN776\_c0\_g1\_i1.p1 |  |  | DB Search |
| LPHETAIIGSETIPVSGSGK | 57.77 | 1992.0527 | 20 | 2.18 | 665.0248 | 15.95 | 0.9226 | 0.9299 | 51107 | 1.64e3 | 1 | 1 | TRINITY\_DN1936\_c0\_g1\_i3.p1 |  |  | DB Search |
| YIFIESSKPAIK | 57.72 | 1394.7809 | 12 | 0.75 | 698.3967 | 15.84 | 1.0086 | 1.0158 | 50604 | 4.26e3 | 2 | 2 | TRINITY\_DN1936\_c0\_g1\_i4.p1:TRINITY\_DN1936\_c0\_g1\_i5.p1 |  |  | DB Search |
| LKTDLEAQKK | 57.72 | 1172.6765 | 10 | 3.8 | 391.9001 | 10.36 | 0.7408 | 0.7481 | 16865 | 9.07e2 | 1 | 1 | TRINITY\_DN4513\_c0\_g1\_i12.p1:TRINITY\_DN4513\_c0\_g1\_i3.p1 |  |  | DB Search |
| AKDVTAAVK | 57.69 | 901.5233 | 9 | 1.23 | 451.7685 | 10.02 | 0.7974 | 0.8047 | 15046 | 0 | 0 | 0 | TRINITY\_DN1189\_c0\_g1\_i1.p1 |  |  | DB Search |
| GYTTDPTDVQTC(+57.02)K | 57.69 | 1484.6453 | 13 | 4.36 | 743.3315 | 12.56 | 1.0069 | 1.0142 | 30265 | 6.8e2 | 1 | 1 | TRINITY\_DN28257\_c0\_g1\_i1.p1:TRINITY\_DN28257\_c0\_g1\_i2.p1 | Carbamidomethylation |  | DB Search |
| GPTGPQGER | 57.67 | 897.4304 | 9 | 3.94 | 449.7233 | 9.46 | 0.7709 | 0.7783 | 12020 | 3.18e3 | 1 | 1 | TRINITY\_DN691\_c0\_g1\_i1.p1 |  |  | DB Search |
| GSSYTGDIALDDFR | 57.66 | 1515.6841 | 14 | -0.42 | 758.8474 | 18.14 | 1.0146 | 1.0218 | 65410 | 0 | 0 | 0 | TRINITY\_DN66856\_c0\_g1\_i1.p1 |  |  | DB Search |
| TC(+57.02)FYPIIGDK | 57.66 | 1212.5848 | 10 | -2.99 | 607.2966 | 18.26 | 0.9 | 0.9073 | 66111 | 5.95e2 | 1 | 1 | TRINITY\_DN16533\_c0\_g2\_i1.p1 | Carbamidomethylation |  | DB Search |
| LLLPGELAK | 57.63 | 952.5957 | 9 | 2.84 | 477.3054 | 17.63 | 0.8307 | 0.838 | 61790 | 4.61e3 | 1 | 1 | TRINITY\_DN106952\_c0\_g1\_i1.p1 |  |  | DB Search |
| LIDSLQLDQSEYR | 57.62 | 1578.7889 | 13 | -0.6 | 790.3995 | 17.23 | 1.0604 | 1.0676 | 59328 | 3.65e2 | 1 | 1 | TRINITY\_DN3034\_c0\_g1\_i1.p1 |  |  | DB Search |
| LKNEIEIMESK | 57.61 | 1332.6959 | 11 | 0.56 | 667.3541 | 15.12 | 0.967 | 0.9743 | 46046 | 8e2 | 1 | 1 | TRINITY\_DN1676\_c0\_g1\_i6.p1:TRINITY\_DN1676\_c0\_g1\_i12.p1 |  |  | DB Search |
| ADC(+57.02)GVENTC(+57.02)FGSTR | 57.59 | 1572.6296 | 14 | 1.45 | 787.3215 | 13.67 | 1.0053 | 1.0126 | 37114 | 4.6e3 | 1 | 1 | TRINITY\_DN319\_c2\_g1\_i3.p1:TRINITY\_DN319\_c2\_g1\_i1.p1:TRINITY\_DN319\_c2\_g1\_i2.p1 | Carbamidomethylation, Carbamidomethylation |  | DB Search |
| DSFISVINMR | 57.58 | 1180.5911 | 10 | 1.69 | 591.3025 | 19.62 | 0.9012 | 0.9085 | 75085 | 1.94e3 | 1 | 1 | TRINITY\_DN982\_c0\_g1\_i3.p1 |  |  | DB Search |
| YQIVIEAIR | 57.58 | 1103.6339 | 9 | -0 | 552.823 | 17.92 | 0.8789 | 0.8862 | 63893 | 1.03e3 | 1 | 1 | TRINITY\_DN3655\_c1\_g1\_i1.p1 |  |  | DB Search |
| KVSQTC(+57.02)AEHVFR | 57.57 | 1460.7194 | 12 | 0.9 | 487.9131 | 12.05 | 0.797 | 0.8043 | 27165 | 0 | 0 | 0 | TRINITY\_DN3078\_c0\_g1\_i1.p1 | Carbamidomethylation |  | DB Search |
| DLSLVVEPLK | 57.54 | 1111.6488 | 10 | 3.46 | 556.8324 | 18.96 | 0.8943 | 0.9016 | 70793 | 9.98e2 | 1 | 1 | TRINITY\_DN244\_c0\_g1\_i1.p1 |  |  | DB Search |
| INNNQFSVVLIK | 57.54 | 1387.7823 | 12 | -2.73 | 694.895 | 18.38 | 0.9848 | 0.992 | 66945 | 4.66e2 | 1 | 1 | TRINITY\_DN982\_c0\_g1\_i1.p1:TRINITY\_DN982\_c0\_g1\_i2.p1 |  |  | DB Search |
| LENLFNTLQTK | 57.51 | 1319.7085 | 11 | 2.17 | 660.8615 | 18.79 | 0.9578 | 0.965 | 69766 | 0 | 0 | 0 | TRINITY\_DN624\_c0\_g1\_i1.p1 |  |  | DB Search |
| AC(+57.02)VDIDEC(+57.02)SEDPNAC(+57.02)R | 57.49 | 1909.724 | 16 | -0.91 | 955.8663 | 13.56 | 1.1029 | 1.1102 | 36467 | 0 | 0 | 0 | TRINITY\_DN2720\_c0\_g1\_i6.p1:TRINITY\_DN2720\_c0\_g1\_i2.p1 | Carbamidomethylation, Carbamidomethylation, Carbamidomethylation |  | DB Search |
| LAEMLVSFGK | 57.47 | 1093.5841 | 10 | -0.31 | 547.798 | 18.75 | 0.8587 | 0.866 | 69489 | 1.49e2 | 1 | 1 | TRINITY\_DN71276\_c0\_g1\_i1.p1 |  |  | DB Search |
| VQPLLSIAR | 57.44 | 995.6127 | 9 | 0.94 | 498.813 | 17.21 | 0.8433 | 0.8506 | 59276 | 1.54e2 | 1 | 1 | TRINITY\_DN3034\_c0\_g1\_i1.p1:TRINITY\_DN1507\_c0\_g1\_i1.p1 |  |  | DB Search |
| FLQVFENYR | 57.44 | 1214.6084 | 9 | -2.94 | 608.3083 | 18.79 | 0.936 | 0.9432 | 69682 | 4.69e2 | 1 | 1 | TRINITY\_DN4295\_c0\_g1\_i1.p1 |  |  | DB Search |
| SVQIEVSTPVC(+57.02)QTC(+57.02)PK | 57.42 | 1831.8809 | 16 | 3.34 | 916.9487 | 15.16 | 1.1126 | 1.1198 | 46105 | 3.11e3 | 1 | 1 | TRINITY\_DN6221\_c0\_g1\_i8.p1:TRINITY\_DN6221\_c0\_g1\_i3.p1:TRINITY\_DN6221\_c0\_g1\_i2.p1:TRINITY\_DN6221\_c0\_g1\_i5.p1 | Carbamidomethylation, Carbamidomethylation |  | DB Search |
| MQIFVK | 57.42 | 764.4255 | 6 | 0.18 | 383.2192 | 15.73 | 0.7363 | 0.7437 | 49899 | 2.46e2 | 1 | 1 | TRINITY\_DN554\_c0\_g1\_i3.p2:TRINITY\_DN585\_c0\_g1\_i8.p1:TRINITY\_DN585\_c0\_g1\_i4.p1:TRINITY\_DN101\_c0\_g1\_i1.p1:TRINITY\_DN554\_c0\_g1\_i4.p1:TRINITY\_DN173718\_c0\_g1\_i1.p1:TRINITY\_DN585\_c0\_g1\_i10.p1:TRINITY\_DN554\_c0\_g1\_i5.p1 |  |  | DB Search |
| YAAAITPPPIIK | 57.42 | 1253.7383 | 12 | -0.19 | 627.8749 | 17.28 | 1.009 | 1.0162 | 59755 | 1.23e3 | 1 | 1 | TRINITY\_DN113\_c3\_g1\_i1.p1 |  |  | DB Search |
| DGETGEQGPQGQEGPTGPR | 57.4 | 1895.8245 | 19 | 11.29 | 948.9282 | 11.41 | 1.0721 | 1.0793 | 23364 | 5.54e2 | 1 | 1 | TRINITY\_DN113\_c0\_g1\_i1.p1 |  |  | DB Search |
| TRVC(+57.02)LDDDIKVC(+57.02)K | 57.39 | 1620.7964 | 13 | -1.68 | 541.2706 | 13.95 | 0.838 | 0.8453 | 38871 | 6.52e2 | 1 | 1 | TRINITY\_DN4595\_c0\_g1\_i10.p1:TRINITY\_DN4595\_c0\_g1\_i4.p1 | Carbamidomethylation, Carbamidomethylation |  | DB Search |
| FYSFEVQVIDTNNGK | 57.39 | 1759.8417 | 15 | 4.04 | 880.9297 | 18.90 | 1.0965 | 1.1037 | 70365 | 3.63e2 | 1 | 1 | TRINITY\_DN982\_c0\_g1\_i3.p1 |  |  | DB Search |
| VKFC(+57.02)SLSQHTK | 57.38 | 1333.6813 | 11 | 3.63 | 445.5683 | 11.98 | 0.7518 | 0.7591 | 26681 | 1.88e3 | 1 | 1 | TRINITY\_DN187759\_c0\_g1\_i1.p1 | Carbamidomethylation |  | DB Search |
| LTLGENIADNGGFK | 57.37 | 1447.7307 | 14 | 2.38 | 724.8727 | 17.25 | 0.9993 | 1.0065 | 59417 | 7.06e2 | 1 | 1 | TRINITY\_DN3182\_c0\_g1\_i2.p1:TRINITY\_DN3182\_c0\_g1\_i3.p1 |  |  | DB Search |
| TPGIIVSIDEK | 57.35 | 1170.6495 | 11 | 0.16 | 586.3309 | 16.69 | 0.921 | 0.9283 | 55817 | 1.1e3 | 1 | 1 | TRINITY\_DN982\_c0\_g1\_i3.p1 |  |  | DB Search |
| IYNEGEVVLYK | 57.35 | 1325.6866 | 11 | 3.06 | 663.8512 | 16.17 | 0.9755 | 0.9828 | 52557 | 4.08e3 | 1 | 1 | TRINITY\_DN1369\_c0\_g1\_i1.p1 |  |  | DB Search |
| YVKPGVNIINIR | 57.33 | 1384.8191 | 12 | 6.22 | 462.6155 | 16.27 | 0.8043 | 0.8116 | 53149 | 6.85e2 | 2 | 2 | TRINITY\_DN1616\_c0\_g1\_i3.p1:TRINITY\_DN1616\_c0\_g1\_i6.p1:TRINITY\_DN1616\_c0\_g1\_i2.p1:TRINITY\_DN1616\_c0\_g1\_i7.p1 |  |  | DB Search |
| FLDIHELQAVK | 57.33 | 1311.7186 | 11 | -7.43 | 438.2426 | 17.63 | 0.773 | 0.7803 | 62025 | 0 | 0 | 0 | TRINITY\_DN165469\_c0\_g1\_i1.p1 |  |  | DB Search |
| FYLETVIK | 57.32 | 1011.5641 | 8 | 1.05 | 506.7887 | 18.58 | 0.8518 | 0.8591 | 68296 | 6.97e2 | 1 | 1 | TRINITY\_DN98417\_c0\_g1\_i1.p1 |  |  | DB Search |
| LFNEALKPLK | 57.32 | 1171.6964 | 10 | 3.28 | 586.8561 | 15.90 | 0.9509 | 0.9582 | 50919 | 4.48e2 | 1 | 1 | TRINITY\_DN1616\_c0\_g1\_i3.p1:TRINITY\_DN1616\_c0\_g1\_i6.p1:TRINITY\_DN1616\_c0\_g1\_i7.p1 |  |  | DB Search |
| DLHIIEDYVLK | 57.31 | 1356.7289 | 11 | 1.64 | 679.3713 | 19.73 | 0.9868 | 0.9941 | 75736 | 7.86e2 | 1 | 1 | TRINITY\_DN8903\_c0\_g1\_i1.p1 |  |  | DB Search |
| TPYLQIDFGK | 57.3 | 1180.6128 | 10 | 2.79 | 591.314 | 18.49 | 0.9109 | 0.9182 | 67728 | 8.28e2 | 1 | 1 | TRINITY\_DN11248\_c0\_g1\_i1.p1:TRINITY\_DN11248\_c0\_g1\_i3.p1 |  |  | DB Search |
| DFNINNGVFR | 57.29 | 1194.5781 | 10 | 1.03 | 598.2957 | 18.08 | 0.8927 | 0.9 | 64841 | 3.11e3 | 1 | 1 | TRINITY\_DN982\_c0\_g1\_i3.p1 |  |  | DB Search |
| DSYVGDEAQSKR | 57.28 | 1353.6161 | 12 | 2.57 | 677.8156 | 11.01 | 0.9993 | 1.0065 | 20794 | 1.16e3 | 1 | 1 | TRINITY\_DN2477\_c0\_g1\_i5.p1:TRINITY\_DN2477\_c0\_g1\_i6.p1:TRINITY\_DN195934\_c0\_g1\_i1.p2:TRINITY\_DN161\_c0\_g1\_i1.p1:TRINITY\_DN2477\_c0\_g1\_i9.p1:TRINITY\_DN2477\_c0\_g1\_i10.p1:TRINITY\_DN2477\_c0\_g1\_i2.p1:TRINITY\_DN161\_c0\_g1\_i3.p1:TRINITY\_DN161\_c0\_g1\_i2.p1 |  |  | DB Search |
| TPGEC(+57.02)C(+57.02)PQC(+57.02)K | 57.28 | 1235.4733 | 10 | 5.1 | 618.7457 | 10.00 | 0.8878 | 0.8951 | 14830 | 4.63e3 | 1 | 1 | TRINITY\_DN1369\_c0\_g1\_i1.p1 | Carbamidomethylation, Carbamidomethylation, Carbamidomethylation |  | DB Search |
| ESTLHLVLR | 57.27 | 1066.6135 | 9 | 2.57 | 534.3142 | 15.80 | 0.8785 | 0.8858 | 50332 | 4.97e3 | 1 | 1 | TRINITY\_DN554\_c0\_g1\_i3.p2:TRINITY\_DN585\_c0\_g1\_i3.p1:TRINITY\_DN554\_c0\_g1\_i4.p1:TRINITY\_DN1580\_c0\_g1\_i3.p1:TRINITY\_DN40728\_c1\_g1\_i1.p2:TRINITY\_DN152\_c0\_g1\_i2.p1:TRINITY\_DN585\_c0\_g1\_i5.p1:TRINITY\_DN585\_c0\_g2\_i1.p1:TRINITY\_DN1465\_c0\_g1\_i1.p2:TRINITY\_DN585\_c0\_g1\_i4.p1:TRINITY\_DN152\_c0\_g1\_i15.p1:TRINITY\_DN103165\_c0\_g1\_i1.p1:TRINITY\_DN152\_c0\_g1\_i10.p1:TRINITY\_DN585\_c0\_g1\_i8.p1:TRINITY\_DN101\_c0\_g1\_i1.p1:TRINITY\_DN10545\_c0\_g1\_i1.p3:TRINITY\_DN173718\_c0\_g1\_i1.p1:TRINITY\_DN1465\_c1\_g1\_i1.p1:TRINITY\_DN40728\_c0\_g1\_i1.p1:TRINITY\_DN152\_c0\_g1\_i9.p1:TRINITY\_DN1580\_c0\_g1\_i1.p1:TRINITY\_DN110760\_c0\_g1\_i1.p1:TRINITY\_DN554\_c0\_g1\_i2.p1:TRINITY\_DN585\_c0\_g1\_i10.p1:TRINITY\_DN554\_c0\_g1\_i5.p1:TRINITY\_DN585\_c0\_g1\_i2.p1:TRINITY\_DN8227\_c0\_g1\_i1.p1 |  |  | DB Search |
| VNFQVVMEAR | 57.24 | 1191.6071 | 10 | 0.27 | 596.8096 | 17.59 | 0.8972 | 0.9044 | 61571 | 2.93e3 | 1 | 1 | TRINITY\_DN4781\_c0\_g1\_i1.p1:TRINITY\_DN4781\_c0\_g1\_i2.p1 |  |  | DB Search |
| IGGTC(+57.02)VNIVGTYR | 57.24 | 1408.7133 | 13 | 3.99 | 705.3652 | 15.98 | 0.9941 | 1.0013 | 51355 | 1.59e3 | 1 | 1 | TRINITY\_DN2720\_c0\_g1\_i6.p1:TRINITY\_DN2720\_c0\_g1\_i2.p1 | Carbamidomethylation |  | DB Search |
| VEDSKESDINFLLK | 57.24 | 1635.8356 | 14 | 0.37 | 546.2848 | 17.72 | 0.8092 | 0.8165 | 62515 | 5.45e2 | 1 | 1 | TRINITY\_DN1821\_c0\_g1\_i2.p1:TRINITY\_DN1821\_c0\_g1\_i3.p1:TRINITY\_DN1821\_c0\_g1\_i6.p1:TRINITY\_DN1821\_c0\_g1\_i5.p1:TRINITY\_DN1821\_c0\_g1\_i4.p1 |  |  | DB Search |
| SPFVIMFK | 57.23 | 967.5201 | 8 | 1.15 | 484.7668 | 19.95 | 0.8063 | 0.8136 | 76925 | 1.04e4 | 1 | 1 | TRINITY\_DN4595\_c0\_g1\_i10.p1:TRINITY\_DN4595\_c0\_g1\_i4.p1 |  |  | DB Search |
| ENDFISAQLKR | 57.23 | 1319.6833 | 11 | 3.57 | 660.8499 | 15.34 | 0.9791 | 0.9864 | 47439 | 4.82e2 | 1 | 1 | TRINITY\_DN11248\_c0\_g1\_i1.p1:TRINITY\_DN11248\_c0\_g1\_i3.p1 |  |  | DB Search |
| GPAGAPGDEGQTGMR | 57.23 | 1399.615 | 15 | -0.7 | 700.8127 | 11.55 | 0.9396 | 0.9469 | 24084 | 1.21e3 | 1 | 1 | TRINITY\_DN77\_c0\_g2\_i4.p1:TRINITY\_DN77\_c0\_g2\_i2.p1:TRINITY\_DN77\_c0\_g2\_i1.p1:TRINITY\_DN77\_c0\_g2\_i3.p1 |  |  | DB Search |
| GLMVAAMDLDKK | 57.22 | 1290.6676 | 12 | 1.46 | 646.3406 | 16.83 | 1.0013 | 1.0086 | 56757 | 1.36e3 | 1 | 1 | TRINITY\_DN1043\_c0\_g1\_i2.p1 |  |  | DB Search |
| VLNIALDKYQEGVAK | 57.22 | 1659.9196 | 15 | -3.66 | 554.3105 | 16.21 | 0.8088 | 0.8161 | 52938 | 0 | 0 | 0 | TRINITY\_DN942\_c0\_g1\_i5.p1:TRINITY\_DN942\_c0\_g1\_i2.p1:TRINITY\_DN942\_c0\_g1\_i3.p1 |  |  | DB Search |
| SFTGDIALDDFR | 57.22 | 1355.6357 | 12 | 3.82 | 678.8262 | 19.15 | 0.9545 | 0.9618 | 72030 | 5.21e2 | 1 | 1 | TRINITY\_DN1936\_c0\_g2\_i1.p1 |  |  | DB Search |
| TSDIVNEIFSR | 57.21 | 1279.6409 | 11 | 2.89 | 640.8281 | 19.68 | 0.9303 | 0.9376 | 75523 | 8.32e2 | 1 | 1 | TRINITY\_DN75609\_c0\_g1\_i1.p1 |  |  | DB Search |
| MKDSYIINEER | 57.2 | 1396.6656 | 11 | 0.34 | 699.3388 | 13.80 | 0.9735 | 0.9808 | 37987 | 0 | 0 | 0 | TRINITY\_DN15274\_c0\_g1\_i1.p1 |  |  | DB Search |
| ITVTVSPDIHPPAK | 57.2 | 1473.8191 | 14 | -2.38 | 737.9135 | 15.04 | 1.0652 | 1.0725 | 45475 | 1.12e3 | 1 | 1 | TRINITY\_DN991\_c0\_g1\_i5.p1:TRINITY\_DN991\_c0\_g1\_i2.p1:TRINITY\_DN991\_c0\_g1\_i6.p1 |  |  | DB Search |
| LFADELMTNINR | 57.18 | 1435.7129 | 12 | -2.44 | 718.8604 | 19.26 | 0.9928 | 1.0001 | 72722 | 4.52e2 | 1 | 1 | TRINITY\_DN16079\_c0\_g2\_i1.p1 |  |  | DB Search |
| TC(+57.02)ADTSPAIDFK | 57.18 | 1324.5969 | 12 | -1.21 | 663.3035 | 15.19 | 0.9469 | 0.9541 | 46506 | 1.08e3 | 1 | 1 | TRINITY\_DN319\_c2\_g1\_i3.p1:TRINITY\_DN319\_c2\_g1\_i1.p1:TRINITY\_DN319\_c2\_g1\_i2.p1 | Carbamidomethylation |  | DB Search |
| TNFQVVIEAKR | 57.17 | 1303.7249 | 11 | 3.05 | 652.8702 | 15.14 | 0.9872 | 0.9945 | 46265 | 1.76e2 | 1 | 1 | TRINITY\_DN28022\_c0\_g2\_i1.p1 |  |  | DB Search |
| YQVTGC(+57.02)GGFC(+57.02)ESSVK | 57.17 | 1677.7126 | 15 | -3.52 | 839.8588 | 14.63 | 1.068 | 1.0753 | 43042 | 0 | 0 | 0 | TRINITY\_DN6221\_c0\_g1\_i8.p1:TRINITY\_DN6221\_c0\_g1\_i3.p1:TRINITY\_DN6221\_c0\_g1\_i2.p1:TRINITY\_DN6221\_c0\_g1\_i5.p1 | Carbamidomethylation, Carbamidomethylation |  | DB Search |
| NYNNDDEPGAK | 57.14 | 1235.5055 | 11 | 0.05 | 618.7587 | 10.27 | 0.9129 | 0.9202 | 16311 | 2.19e3 | 1 | 1 | TRINITY\_DN199\_c0\_g1\_i1.p1 |  |  | DB Search |
| FC(+57.02)SLSQHTK | 57.12 | 1106.5178 | 9 | 5.55 | 554.2681 | 11.18 | 0.898 | 0.9052 | 21967 | 0 | 0 | 0 | TRINITY\_DN187759\_c0\_g1\_i1.p1 | Carbamidomethylation |  | DB Search |
| SQSAFPTRR | 57.12 | 1048.5414 | 9 | 2.06 | 525.2779 | 11.08 | 0.8542 | 0.8615 | 21210 | 5.83e3 | 1 | 1 | TRINITY\_DN477\_c0\_g1\_i8.p1:TRINITY\_DN477\_c0\_g1\_i7.p1 |  |  | DB Search |
| DLEDVNFVR | 57.12 | 1105.5404 | 9 | 3.32 | 553.7781 | 17.07 | 0.8757 | 0.883 | 58345 | 1.18e3 | 1 | 1 | TRINITY\_DN581\_c1\_g1\_i1.p1 |  |  | DB Search |
| AKIEALHC(+57.02)LSENIVK | 57.12 | 1723.9291 | 15 | 3.74 | 575.6512 | 15.83 | 0.8262 | 0.8335 | 50444 | 1.65e3 | 1 | 1 | TRINITY\_DN3078\_c0\_g1\_i1.p1 | Carbamidomethylation |  | DB Search |
| GLPLAIGVR | 57.12 | 894.5651 | 9 | 0.27 | 448.2889 | 17.73 | 0.8124 | 0.8197 | 62574 | 3.52e3 | 1 | 1 | TRINITY\_DN36\_c0\_g1\_i9.p1:TRINITY\_DN36\_c0\_g1\_i14.p1:TRINITY\_DN36\_c0\_g1\_i13.p1:TRINITY\_DN36\_c0\_g1\_i2.p1:TRINITY\_DN36\_c0\_g1\_i3.p1:TRINITY\_DN36\_c0\_g1\_i16.p1:TRINITY\_DN36\_c0\_g1\_i17.p1:TRINITY\_DN36\_c0\_g1\_i4.p1:TRINITY\_DN36\_c0\_g1\_i11.p1:TRINITY\_DN36\_c0\_g1\_i1.p1:TRINITY\_DN36\_c0\_g1\_i8.p1:TRINITY\_DN36\_c0\_g1\_i19.p1:TRINITY\_DN36\_c0\_g1\_i5.p1:TRINITY\_DN36\_c0\_g1\_i20.p1 |  |  | DB Search |
| YDNTITLDAVSSQK | 57.09 | 1553.7573 | 14 | 3.45 | 777.8869 | 15.83 | 1.06 | 1.0672 | 50430 | 4.17e2 | 1 | 1 | TRINITY\_DN160\_c0\_g1\_i2.p1:TRINITY\_DN160\_c0\_g1\_i1.p1 |  |  | DB Search |
| SKDYTITSVGGR | 57.09 | 1282.6517 | 12 | 0.86 | 642.3323 | 13.05 | 0.9384 | 0.9457 | 33313 | 1.65e3 | 1 | 1 | TRINITY\_DN6390\_c0\_g1\_i1.p1 |  |  | DB Search |
| SSNTIEENRLK | 57.06 | 1289.6575 | 11 | 4.02 | 645.8372 | 11.54 | 0.9598 | 0.967 | 24083 | 4.33e2 | 1 | 1 | TRINITY\_DN982\_c0\_g1\_i3.p1 |  |  | DB Search |
| YQGIKPPVSR | 57.06 | 1143.64 | 10 | -0.07 | 572.826 | 12.77 | 0.9311 | 0.9384 | 31696 | 3.82e2 | 1 | 1 | TRINITY\_DN1821\_c0\_g1\_i2.p1:TRINITY\_DN1821\_c0\_g1\_i6.p1:TRINITY\_DN1821\_c0\_g1\_i5.p1:TRINITY\_DN1821\_c0\_g1\_i4.p1 |  |  | DB Search |
| GSYIYIEASYPR | 57.03 | 1417.6877 | 12 | 0.36 | 709.8499 | 17.28 | 1.0399 | 1.0472 | 59633 | 2.78e3 | 1 | 1 | TRINITY\_DN4781\_c0\_g1\_i3.p1:TRINITY\_DN4781\_c0\_g1\_i1.p1:TRINITY\_DN4781\_c0\_g1\_i2.p1 |  |  | DB Search |
| SPSDTTTDEELK | 57.03 | 1321.5885 | 12 | -0.87 | 661.7995 | 11.82 | 0.959 | 0.9662 | 25774 | 1.05e3 | 1 | 1 | TRINITY\_DN74720\_c0\_g1\_i1.p1 |  |  | DB Search |
| NLEC(+57.02)QC(+57.02)DESK | 57.02 | 1281.4966 | 10 | 0.49 | 641.7545 | 10.48 | 0.9012 | 0.9085 | 17668 | 4.3e3 | 1 | 1 | TRINITY\_DN319\_c2\_g1\_i3.p1:TRINITY\_DN319\_c2\_g1\_i1.p1:TRINITY\_DN319\_c2\_g1\_i2.p1 | Carbamidomethylation, Carbamidomethylation |  | DB Search |
| DLTDYLM(+15.99)K | 57.01 | 1013.4739 | 8 | 0.97 | 507.7436 | 16.91 | 0.8388 | 0.8461 | 57197 | 1.01e3 | 1 | 1 | TRINITY\_DN161\_c0\_g1\_i1.p1:TRINITY\_DN161\_c0\_g1\_i4.p1:TRINITY\_DN161\_c0\_g1\_i3.p1:TRINITY\_DN161\_c0\_g1\_i2.p1 | Oxidation (M) | M7:Oxidation (M):1000 | DB Search |
| GSSYLGDIALDDISVK | 57.01 | 1651.8304 | 16 | 3.59 | 826.9236 | 19.81 | 1.0777 | 1.0849 | 76261 | 7.88e2 | 1 | 1 | TRINITY\_DN82766\_c0\_g1\_i1.p1:TRINITY\_DN143583\_c0\_g1\_i1.p1 |  |  | DB Search |
| EC(+57.02)EQDTC(+57.02)FDGNTR | 57.01 | 1630.5988 | 13 | -1.43 | 816.3037 | 12.29 | 1.0122 | 1.0194 | 28637 | 3.57e2 | 1 | 1 | TRINITY\_DN4595\_c0\_g1\_i10.p1:TRINITY\_DN4595\_c0\_g1\_i4.p1 | Carbamidomethylation, Carbamidomethylation |  | DB Search |
| KLLDWAQQR | 57.01 | 1156.6353 | 9 | 2.16 | 579.3249 | 15.60 | 0.9089 | 0.9162 | 49058 | 1.7e3 | 1 | 1 | TRINITY\_DN199\_c0\_g1\_i1.p1 |  |  | DB Search |
| LEFVFK | 57 | 781.4374 | 6 | 2.76 | 391.7262 | 18.35 | 0.7286 | 0.7359 | 66730 | 1.18e3 | 1 | 1 | TRINITY\_DN1936\_c0\_g1\_i1.p1:TRINITY\_DN1936\_c0\_g1\_i4.p1 |  |  | DB Search |
| GVHIISVGLGTVDHR | 57 | 1558.8579 | 15 | -7.55 | 520.6215 | 16.00 | 0.8404 | 0.8477 | 51630 | 0 | 0 | 0 | TRINITY\_DN8008\_c0\_g1\_i2.p1:TRINITY\_DN8008\_c0\_g1\_i3.p1 |  |  | DB Search |
| AFNKVETR | 57 | 963.5137 | 8 | 4.36 | 482.7652 | 10.61 | 0.8274 | 0.8348 | 18412 | 3.24e3 | 1 | 1 | TRINITY\_DN4595\_c0\_g1\_i7.p1:TRINITY\_DN98968\_c0\_g1\_i1.p1:TRINITY\_DN8597\_c0\_g1\_i1.p1:TRINITY\_DN4595\_c0\_g1\_i4.p1:TRINITY\_DN4595\_c0\_g1\_i10.p2 |  |  | DB Search |
| DAQDQELEIKR | 57 | 1343.6681 | 11 | 0.37 | 672.8401 | 12.83 | 0.9501 | 0.9574 | 32029 | 0 | 0 | 0 | TRINITY\_DN86076\_c0\_g1\_i1.p1 |  |  | DB Search |
| VSSDRFFVQVDGR | 56.99 | 1510.7528 | 13 | 0.15 | 504.5905 | 16.15 | 0.8059 | 0.8132 | 52408 | 1.09e3 | 1 | 1 | TRINITY\_DN6390\_c0\_g1\_i1.p1 |  |  | DB Search |
| IVFDLR | 56.98 | 761.4435 | 6 | 4.32 | 381.7299 | 17.48 | 0.7551 | 0.7624 | 60813 | 1.19e4 | 1 | 1 | TRINITY\_DN113\_c3\_g1\_i1.p1 |  |  | DB Search |
| IC(+57.02)LSLVSLK | 56.98 | 1031.6049 | 9 | -1.88 | 516.8076 | 18.96 | 0.8648 | 0.8721 | 70795 | 4.03e2 | 1 | 1 | TRINITY\_DN5115\_c0\_g1\_i2.p1 | Carbamidomethylation |  | DB Search |
| AYVDLGGLGER | 56.98 | 1148.5825 | 11 | 5.34 | 575.3004 | 16.88 | 0.8923 | 0.8996 | 57086 | 1.22e3 | 1 | 1 | TRINITY\_DN262\_c0\_g1\_i1.p1 |  |  | DB Search |
| GLSFSGDIAIDDIK | 56.97 | 1449.7351 | 14 | 2.03 | 725.8747 | 19.24 | 1.0041 | 1.0114 | 72590 | 2.71e3 | 1 | 1 | TRINITY\_DN3655\_c0\_g1\_i1.p1 |  |  | DB Search |
| DKITQLQNDDKEAR | 56.95 | 1672.838 | 14 | 4.92 | 558.6215 | 11.86 | 0.8416 | 0.849 | 25955 | 4.36e2 | 1 | 1 | TRINITY\_DN1676\_c0\_g1\_i6.p1:TRINITY\_DN1676\_c0\_g1\_i12.p1 |  |  | DB Search |
| KVFSNEEDLPK | 56.95 | 1304.6613 | 11 | 1.38 | 653.3373 | 13.40 | 0.9791 | 0.9864 | 35445 | 4.52e2 | 1 | 1 | TRINITY\_DN6049\_c0\_g1\_i1.p1 |  |  | DB Search |
| ASILLNELAK | 56.95 | 1070.6335 | 10 | -3.29 | 536.3211 | 18.12 | 0.8729 | 0.8802 | 65288 | 0 | 0 | 0 | TRINITY\_DN61742\_c0\_g1\_i2.p1:TRINITY\_DN61742\_c0\_g1\_i1.p1 |  |  | DB Search |
| VFLENVIR | 56.94 | 988.5706 | 8 | 3.98 | 495.2934 | 18.16 | 0.8558 | 0.8631 | 65372 | 1.37e3 | 1 | 1 | TRINITY\_DN172571\_c0\_g1\_i1.p1 |  |  | DB Search |
| AVDFQSIIDNAQK | 56.94 | 1447.7307 | 13 | 4.22 | 724.8741 | 19.06 | 0.9904 | 0.9977 | 71389 | 6.5e2 | 1 | 1 | TRINITY\_DN1616\_c0\_g1\_i3.p1:TRINITY\_DN1616\_c0\_g1\_i6.p1:TRINITY\_DN1616\_c0\_g1\_i2.p1:TRINITY\_DN1616\_c0\_g1\_i7.p1 |  |  | DB Search |
| TGSYIFIETSNPR | 56.93 | 1483.7307 | 13 | -18.37 | 742.8574 | 17.31 | 1.0295 | 1.0367 | 59591 | 1.28e4 | 1 | 1 | TRINITY\_DN66856\_c0\_g1\_i1.p1 |  |  | DB Search |
| NLETFTGC(+57.02)ITAVR | 56.93 | 1480.7344 | 13 | 2.99 | 741.3751 | 18.29 | 0.9993 | 1.0065 | 66297 | 1.31e3 | 1 | 1 | TRINITY\_DN1616\_c0\_g1\_i3.p1:TRINITY\_DN1616\_c0\_g1\_i6.p1:TRINITY\_DN1616\_c0\_g1\_i7.p1 | Carbamidomethylation |  | DB Search |
| GSC(+57.02)EC(+57.02)GTC(+57.02)VC(+57.02)R | 56.93 | 1344.4679 | 11 | -2.11 | 673.2383 | 10.52 | 0.9291 | 0.9364 | 17995 | 0 | 0 | 0 | TRINITY\_DN1535\_c0\_g1\_i1.p1 | Carbamidomethylation, Carbamidomethylation, Carbamidomethylation, Carbamidomethylation |  | DB Search |
| TGVEILNWGVR | 56.92 | 1242.672 | 11 | -1.98 | 622.3407 | 19.74 | 0.9069 | 0.9141 | 75875 | 4.24e2 | 1 | 1 | TRINITY\_DN140702\_c0\_g1\_i1.p1 |  |  | DB Search |
| DGEKVETLKVDISDINK | 56.92 | 1901.9945 | 17 | 3.6 | 635.0063 | 16.09 | 0.9113 | 0.9186 | 52017 | 1.36e3 | 1 | 1 | TRINITY\_DN1616\_c0\_g1\_i3.p1:TRINITY\_DN1616\_c0\_g1\_i6.p1:TRINITY\_DN1616\_c0\_g1\_i2.p1:TRINITY\_DN1616\_c0\_g1\_i7.p1 |  |  | DB Search |
| VETLKVDISDINK | 56.92 | 1472.8086 | 13 | -2.26 | 491.9413 | 16.17 | 0.8222 | 0.8295 | 52567 | 5.32e2 | 1 | 1 | TRINITY\_DN1616\_c0\_g1\_i3.p1:TRINITY\_DN1616\_c0\_g1\_i6.p1:TRINITY\_DN1616\_c0\_g1\_i2.p1:TRINITY\_DN1616\_c0\_g1\_i7.p1 |  |  | DB Search |
| ATGLAIDKIENR | 56.92 | 1299.7146 | 12 | 5.06 | 650.8665 | 14.58 | 0.9924 | 0.9997 | 42725 | 0 | 0 | 0 | TRINITY\_DN4770\_c0\_g1\_i1.p1 |  |  | DB Search |
| ESESRLDLALK | 56.92 | 1259.6721 | 11 | 4.03 | 630.8445 | 14.96 | 0.9731 | 0.9804 | 45033 | 1.43e3 | 1 | 1 | TRINITY\_DN244\_c0\_g1\_i1.p1 |  |  | DB Search |
| TVISQVQSLSIR | 56.91 | 1329.7616 | 12 | 2.63 | 665.8884 | 17.88 | 0.9574 | 0.9646 | 63579 | 2.04e3 | 1 | 1 | TRINITY\_DN4781\_c0\_g1\_i1.p1:TRINITY\_DN4781\_c0\_g1\_i2.p1 |  |  | DB Search |
| VC(+57.02)EDVKR | 56.84 | 904.4437 | 7 | 1.73 | 453.2289 | 9.35 | 0.7742 | 0.7815 | 11721 | 6.11e3 | 1 | 1 | TRINITY\_DN118\_c1\_g1\_i3.p1:TRINITY\_DN118\_c1\_g1\_i5.p1 | Carbamidomethylation |  | DB Search |
| IEDQIVLLGR | 56.8 | 1154.6659 | 10 | 0.49 | 578.3392 | 17.96 | 0.8887 | 0.8959 | 64127 | 3.55e3 | 1 | 1 | TRINITY\_DN244\_c0\_g1\_i1.p1 |  |  | DB Search |
| LLNEIENLR | 56.78 | 1112.6189 | 9 | -2.59 | 557.3141 | 17.15 | 0.8919 | 0.8992 | 58964 | 0 | 0 | 0 | TRINITY\_DN1366\_c0\_g1\_i1.p1 |  |  | DB Search |
| DEKANDILDGLELR | 56.73 | 1599.8104 | 14 | 1.29 | 534.2769 | 19.09 | 0.7966 | 0.8039 | 71698 | 0 | 0 | 0 | TRINITY\_DN15274\_c0\_g1\_i1.p1 |  |  | DB Search |
| DKVSNAC(+57.02)VK | 56.71 | 1019.507 | 9 | -4.3 | 510.7574 | 9.41 | 0.836 | 0.8433 | 12034 | 0 | 0 | 0 | TRINITY\_DN11248\_c0\_g1\_i1.p1:TRINITY\_DN11248\_c0\_g1\_i3.p1 | Carbamidomethylation |  | DB Search |
| DLEFKFR | 56.71 | 953.497 | 7 | 0.03 | 477.7548 | 16.91 | 0.8408 | 0.8481 | 57332 | 4.21e2 | 1 | 1 | TRINITY\_DN160\_c0\_g1\_i2.p1:TRINITY\_DN160\_c0\_g1\_i1.p1 |  |  | DB Search |
| GYTTDPTDVQTC(+57.02)KK | 56.69 | 1612.7402 | 14 | 11.46 | 807.3849 | 11.69 | 1.0395 | 1.0468 | 24808 | 9.83e2 | 1 | 1 | TRINITY\_DN28257\_c0\_g1\_i1.p1:TRINITY\_DN28257\_c0\_g1\_i2.p1 | Carbamidomethylation |  | DB Search |
| EAIVQGAQSTQDTLK | 56.66 | 1587.8104 | 15 | -1.8 | 794.9093 | 13.80 | 1.0608 | 1.068 | 37982 | 0 | 0 | 0 | TRINITY\_DN564\_c0\_g1\_i1.p1 |  |  | DB Search |
| EC(+57.02)SEVC(+57.02)GVNGVAEVTR | 56.61 | 1764.7771 | 16 | -2.02 | 883.3921 | 14.69 | 1.0705 | 1.0777 | 43395 | 0 | 0 | 0 | TRINITY\_DN319\_c2\_g1\_i3.p1:TRINITY\_DN319\_c2\_g1\_i1.p1 | Carbamidomethylation, Carbamidomethylation |  | DB Search |
| KTTLEGEIESLKR | 56.56 | 1502.8304 | 13 | 1.66 | 501.9505 | 16.19 | 0.7994 | 0.8067 | 52735 | 5.81e2 | 1 | 1 | TRINITY\_DN1366\_c0\_g1\_i1.p1 |  |  | DB Search |
| FFDFAK | 56.55 | 773.3748 | 6 | 8.5 | 387.6971 | 17.81 | 0.7473 | 0.7547 | 63170 | 0 | 0 | 0 | TRINITY\_DN195348\_c0\_g1\_i1.p1 |  |  | DB Search |
| LGFVAGVINR | 56.55 | 1044.608 | 10 | -2.1 | 523.309 | 17.50 | 0.8579 | 0.8652 | 61009 | 1.18e3 | 1 | 1 | TRINITY\_DN761\_c0\_g1\_i3.p1 |  |  | DB Search |
| AKDLYYEIR | 56.53 | 1169.608 | 9 | -0.76 | 585.8096 | 14.74 | 0.9206 | 0.9279 | 43604 | 1.31e3 | 1 | 1 | TRINITY\_DN15274\_c0\_g1\_i1.p1 |  |  | DB Search |
| HLSVNILQTNTR | 56.43 | 1394.7629 | 12 | -1.72 | 698.386 | 15.49 | 1.0009 | 1.0081 | 48422 | 1.9e3 | 1 | 1 | TRINITY\_DN6390\_c0\_g1\_i1.p1 |  |  | DB Search |
| GDEHISNLDTLKK | 56.42 | 1468.7522 | 13 | -2.32 | 490.5891 | 12.90 | 0.7791 | 0.7864 | 32456 | 0 | 0 | 0 | TRINITY\_DN1616\_c0\_g1\_i3.p1:TRINITY\_DN1616\_c0\_g1\_i6.p1:TRINITY\_DN1616\_c0\_g1\_i2.p1:TRINITY\_DN1616\_c0\_g1\_i7.p1 |  |  | DB Search |
| DKFFGIQVVHHLR | 56.39 | 1594.8732 | 13 | -2.09 | 532.6294 | 16.30 | 0.8546 | 0.8619 | 53474 | 0 | 0 | 0 | TRINITY\_DN20671\_c0\_g1\_i1.p1 |  |  | DB Search |
| RC(+57.02)TDLNIR | 56.27 | 1046.5291 | 8 | 2.47 | 524.272 | 12.07 | 0.8708 | 0.8781 | 27297 | 3.54e3 | 1 | 1 | TRINITY\_DN6390\_c0\_g1\_i1.p1 | Carbamidomethylation |  | DB Search |
| GPEGSSGPIGK | 56.24 | 984.4876 | 11 | 3.31 | 493.2516 | 10.98 | 0.8128 | 0.8201 | 20694 | 2.62e3 | 1 | 1 | TRINITY\_DN77\_c1\_g1\_i1.p1 |  |  | DB Search |
| GSC(+57.02)SC(+57.02)YGHAEQC(+57.02)IPTK | 56.22 | 1853.7495 | 16 | -0.38 | 618.9222 | 12.47 | 0.8498 | 0.8571 | 29856 | 2.96e2 | 1 | 1 | TRINITY\_DN15274\_c0\_g1\_i1.p1 | Carbamidomethylation, Carbamidomethylation, Carbamidomethylation |  | DB Search |
| GTTSTAGTGPSFDHTK | 56.22 | 1563.7164 | 16 | 6.12 | 782.8686 | 11.55 | 1.0552 | 1.0624 | 24218 | 7.26e2 | 1 | 1 | TRINITY\_DN1936\_c0\_g2\_i1.p1 |  |  | DB Search |
| YSRPTPGSNNNVMVR | 56.2 | 1690.8209 | 15 | -0.93 | 564.6125 | 12.56 | 0.8246 | 0.8319 | 30408 | 1.67e2 | 1 | 1 | TRINITY\_DN15274\_c0\_g1\_i1.p1 |  |  | DB Search |
| KKDLEDVNFVR | 56.18 | 1361.7302 | 11 | 1.3 | 454.917 | 14.05 | 0.7583 | 0.7656 | 39398 | 8.73e2 | 1 | 1 | TRINITY\_DN581\_c1\_g1\_i1.p1 |  |  | DB Search |
| TLSFEAVGHDVDKTISR | 56.16 | 1873.9534 | 17 | -1.82 | 625.6559 | 17.29 | 0.9016 | 0.9089 | 59762 | 0 | 0 | 0 | TRINITY\_DN9655\_c0\_g1\_i1.p1 |  |  | DB Search |
| TRVC(+57.02)LDDDIK | 56.08 | 1233.6023 | 10 | 4.44 | 617.8098 | 13.63 | 0.9485 | 0.9558 | 36912 | 1.78e3 | 1 | 1 | TRINITY\_DN4595\_c0\_g1\_i10.p1:TRINITY\_DN4595\_c0\_g1\_i4.p1 | Carbamidomethylation |  | DB Search |
| SWIC(+57.02)DC(+57.02)NSYGSILNPSDVKK | 56.06 | 2342.0671 | 20 | 3.26 | 781.6971 | 18.19 | 1.0077 | 1.015 | 65664 | 5.83e2 | 1 | 1 | TRINITY\_DN319\_c2\_g1\_i3.p1:TRINITY\_DN319\_c2\_g1\_i1.p1:TRINITY\_DN319\_c2\_g1\_i2.p1 | Carbamidomethylation, Carbamidomethylation |  | DB Search |
| IQFSISAEINNGALK | 56.04 | 1603.8569 | 15 | 2.48 | 802.936 | 18.88 | 1.0701 | 1.0773 | 70412 | 0 | 0 | 0 | TRINITY\_DN15274\_c0\_g1\_i1.p1 |  |  | DB Search |
| MLDHFLSTDVFVK | 56.02 | 1550.7803 | 13 | -5.03 | 517.9303 | 19.28 | 0.7746 | 0.7819 | 72920 | 0 | 0 | 0 | TRINITY\_DN7946\_c0\_g1\_i1.p1 |  |  | DB Search |
| TSVIADSVGQTIGHVGDVVDTAK | 56.02 | 2268.1597 | 23 | -3.24 | 757.0564 | 19.21 | 1.017 | 1.0242 | 72415 | 3.24e2 | 1 | 1 | TRINITY\_DN1795\_c0\_g1\_i4.p1:TRINITY\_DN1795\_c0\_g1\_i1.p1 |  |  | DB Search |
| AC(+57.02)PPSNSGTR | 55.99 | 1045.4611 | 10 | 2.5 | 523.738 | 8.88 | 0.8092 | 0.8165 | 10061 | 1.48e2 | 1 | 1 | TRINITY\_DN2720\_c0\_g1\_i6.p1:TRINITY\_DN2720\_c0\_g1\_i2.p1 | Carbamidomethylation |  | DB Search |
| DLYANTVLSGGTTM(+15.99)FPGIADR | 55.95 | 2214.0627 | 21 | -1.47 | 1108.0345 | 19.56 | 1.2432 | 1.2503 | 74741 | 0 | 0 | 0 | TRINITY\_DN161\_c0\_g1\_i1.p1:TRINITY\_DN161\_c0\_g1\_i4.p1:TRINITY\_DN161\_c0\_g1\_i3.p1:TRINITY\_DN161\_c0\_g1\_i2.p1 | Oxidation (M) | M14:Oxidation (M):1000 | DB Search |
| STSTGHLIYK | 55.95 | 1105.5768 | 10 | -3.77 | 553.7924 | 12.15 | 0.8635 | 0.8708 | 27830 | 0 | 0 | 0 | TRINITY\_DN64\_c0\_g1\_i1.p1 |  |  | DB Search |
| GSIGPSGEQGAQGLPGPTGR | 55.93 | 1821.8969 | 20 | 0.48 | 911.9542 | 14.23 | 1.0857 | 1.0929 | 40533 | 1.87e3 | 1 | 1 | TRINITY\_DN984\_c0\_g1\_i2.p1:TRINITY\_DN984\_c0\_g1\_i1.p1 |  |  | DB Search |
| KLPEWPGLKR | 55.92 | 1222.7186 | 10 | -6.56 | 408.5766 | 15.62 | 0.7453 | 0.7526 | 49336 | 0 | 0 | 0 | TRINITY\_DN172717\_c0\_g1\_i1.p1 |  |  | DB Search |
| EGDQFFDAGEVSPSDC(+57.02)SK | 55.89 | 1973.7949 | 18 | 2.52 | 987.905 | 16.58 | 1.1402 | 1.1474 | 55286 | 0 | 0 | 0 | TRINITY\_DN6390\_c0\_g1\_i1.p1 | Carbamidomethylation |  | DB Search |
| K(+42.01)LAAELANGR | 55.86 | 1083.6036 | 10 | 2.07 | 542.809 | 15.52 | 0.8704 | 0.8777 | 48534 | 1.49e3 | 1 | 1 | TRINITY\_DN1172\_c0\_g2\_i2.p1 | Acetylation (Protein N-term) | K1:Acetylation (Protein N-term):1000 | DB Search |
| EAIVEAIEDGANTSK | 55.85 | 1545.7522 | 15 | 1.08 | 773.8825 | 17.30 | 1.1049 | 1.1122 | 59776 | 2.04e2 | 1 | 1 | TRINITY\_DN557\_c0\_g1\_i2.p1:TRINITY\_DN557\_c0\_g1\_i5.p1:TRINITY\_DN557\_c0\_g1\_i3.p1:TRINITY\_DN557\_c0\_g1\_i4.p1 |  |  | DB Search |
| RLTELEHFLK | 55.84 | 1284.719 | 10 | -4.48 | 429.2441 | 16.54 | 0.7453 | 0.7526 | 54983 | 6.53e2 | 1 | 1 | TRINITY\_DN1119\_c0\_g1\_i1.p1 |  |  | DB Search |
| VNEGNLVGDMVLR | 55.82 | 1414.7239 | 13 | 1.01 | 708.3683 | 18.54 | 0.9856 | 0.9928 | 68049 | 8.64e2 | 1 | 1 | TRINITY\_DN982\_c0\_g1\_i1.p1:TRINITY\_DN982\_c0\_g1\_i3.p1 |  |  | DB Search |
| ILFQEFR | 55.78 | 951.5178 | 7 | -1.31 | 476.7645 | 18.37 | 0.8108 | 0.8181 | 66910 | 9.28e2 | 1 | 1 | TRINITY\_DN428\_c0\_g1\_i1.p1 |  |  | DB Search |
| IATGFESIVR | 55.77 | 1091.5975 | 10 | 5.35 | 546.8077 | 17.15 | 0.855 | 0.8623 | 58937 | 0 | 0 | 0 | TRINITY\_DN1795\_c0\_g1\_i4.p1:TRINITY\_DN1795\_c0\_g1\_i1.p1 |  |  | DB Search |
| DGTTPC(+57.02)NTR | 55.73 | 1020.4294 | 9 | 2.58 | 511.2222 | 9.62 | 0.8311 | 0.8384 | 12948 | 1.32e3 | 1 | 1 | TRINITY\_DN4595\_c0\_g1\_i4.p1:TRINITY\_DN4595\_c0\_g1\_i10.p2 | Carbamidomethylation |  | DB Search |
| RMEC(+57.02)PELDC(+57.02)PEER | 55.72 | 1719.7014 | 13 | -2.5 | 574.2384 | 13.63 | 0.8108 | 0.8181 | 36937 | 2.69e2 | 1 | 1 | TRINITY\_DN1369\_c0\_g1\_i1.p1 | Carbamidomethylation, Carbamidomethylation |  | DB Search |
| NWHGM(+15.99)EDC(+57.02)VVVK | 55.7 | 1488.6489 | 12 | -2.89 | 497.2211 | 13.35 | 0.7599 | 0.7673 | 35192 | 0 | 0 | 0 | TRINITY\_DN11248\_c0\_g1\_i1.p1:TRINITY\_DN11248\_c0\_g1\_i3.p1 | Oxidation (M), Carbamidomethylation | M5:Oxidation (M):1000 | DB Search |
| SYQATSC(+57.02)SC(+57.02)SK | 55.69 | 1277.5017 | 11 | 4.9 | 639.7598 | 9.83 | 0.9255 | 0.9327 | 14058 | 3.33e2 | 1 | 1 | TRINITY\_DN205\_c0\_g1\_i3.p1 | Carbamidomethylation, Carbamidomethylation |  | DB Search |
| IC(+57.02)STC(+57.02)PPNR | 55.69 | 1103.4852 | 9 | 4.63 | 552.7512 | 10.47 | 0.8437 | 0.851 | 17525 | 1.43e3 | 1 | 1 | TRINITY\_DN63421\_c0\_g1\_i1.p1 | Carbamidomethylation, Carbamidomethylation |  | DB Search |
| RPLLGQDYQGDVTDLVC(+57.02)R | 55.62 | 2104.0371 | 18 | 2.09 | 702.3529 | 17.83 | 0.9032 | 0.9105 | 63348 | 2.22e2 | 1 | 1 | TRINITY\_DN100850\_c0\_g1\_i1.p1 | Carbamidomethylation |  | DB Search |
| ATENPSGGFSPDDR | 55.58 | 1448.6168 | 14 | 6.68 | 725.3189 | 12.97 | 0.9759 | 0.9832 | 32890 | 0 | 0 | 0 | TRINITY\_DN3698\_c0\_g2\_i4.p1:TRINITY\_DN3698\_c0\_g2\_i3.p1:TRINITY\_DN3698\_c0\_g2\_i6.p1:TRINITY\_DN3698\_c0\_g2\_i2.p1:TRINITY\_DN3698\_c0\_g2\_i1.p1 |  |  | DB Search |
| VDNSSLTGESEPQSR | 55.55 | 1604.7278 | 15 | -1.25 | 803.3684 | 12.02 | 1.0263 | 1.0335 | 26970 | 5.39e3 | 1 | 1 | TRINITY\_DN262\_c0\_g1\_i1.p1 |  |  | DB Search |
| GDEGFIGFEGAR | 55.52 | 1253.5676 | 12 | -2.01 | 627.7885 | 17.53 | 0.9121 | 0.9194 | 61207 | 1.99e3 | 1 | 1 | TRINITY\_DN244\_c0\_g1\_i1.p1 |  |  | DB Search |
| IYSIEITDVENGGTR | 55.52 | 1665.8209 | 15 | 0.19 | 833.9161 | 17.47 | 1.064 | 1.0713 | 60999 | 1.2e3 | 1 | 1 | TRINITY\_DN121236\_c0\_g1\_i1.p1 |  |  | DB Search |
| ADEGYYPDSDGKTC(+57.02)VLAPC(+57.02)K | 55.5 | 2244.9668 | 20 | 6.79 | 749.3329 | 14.72 | 0.9089 | 0.9162 | 43605 | 0 | 0 | 0 | TRINITY\_DN28257\_c0\_g1\_i1.p1:TRINITY\_DN28257\_c0\_g1\_i2.p1 | Carbamidomethylation, Carbamidomethylation |  | DB Search |
| GKDGETGEQGPQGQEGPTGPR | 55.49 | 2080.9409 | 21 | 4.28 | 694.6557 | 10.78 | 0.851 | 0.8583 | 19197 | 5.2e3 | 1 | 1 | TRINITY\_DN113\_c0\_g1\_i1.p1 |  |  | DB Search |
| ASNEVSMK | 55.46 | 864.4011 | 8 | -4.31 | 433.205 | 10.65 | 0.762 | 0.7693 | 18751 | 4.71e2 | 1 | 1 | TRINITY\_DN24268\_c0\_g1\_i2.p1:TRINITY\_DN24268\_c0\_g1\_i1.p1 |  |  | DB Search |
| GPQGQVGK | 55.44 | 769.4082 | 8 | 6.49 | 385.713 | 8.99 | 0.7069 | 0.7143 | 10347 | 1.81e3 | 1 | 1 | TRINITY\_DN77\_c0\_g2\_i4.p1:TRINITY\_DN77\_c0\_g2\_i2.p1:TRINITY\_DN77\_c0\_g2\_i1.p1:TRINITY\_DN77\_c0\_g2\_i3.p1 |  |  | DB Search |
| M(+15.99)TQLDQSK | 55.4 | 965.4488 | 8 | 5.2 | 483.7331 | 9.84 | 0.8075 | 0.8149 | 14010 | 1.8e3 | 1 | 1 | TRINITY\_DN477\_c0\_g1\_i9.p1 | Oxidation (M) | M1:Oxidation (M):1000 | DB Search |
| DC(+57.02)ELVGEDLKFK | 55.34 | 1451.6967 | 12 | 2.39 | 726.8557 | 16.71 | 1.0415 | 1.0488 | 56131 | 3.56e2 | 1 | 1 | TRINITY\_DN25375\_c0\_g1\_i7.p1 | Carbamidomethylation |  | DB Search |
| EGPTGPQGETGRR | 55.33 | 1340.6433 | 13 | 3.71 | 447.889 | 9.89 | 0.7632 | 0.7705 | 13877 | 4.68e3 | 1 | 1 | TRINITY\_DN984\_c0\_g1\_i2.p1:TRINITY\_DN984\_c0\_g1\_i1.p1 |  |  | DB Search |
| GVAGAGVLAGFDK | 55.31 | 1160.6189 | 13 | -7.63 | 581.311 | 17.62 | 0.8883 | 0.8955 | 61949 | 0 | 0 | 0 | TRINITY\_DN1559\_c0\_g1\_i1.p1 |  |  | DB Search |
| LRLDFDHEAK | 55.3 | 1242.6356 | 10 | 5.7 | 415.2206 | 14.42 | 0.7359 | 0.7432 | 41684 | 1.12e3 | 1 | 1 | TRINITY\_DN160\_c0\_g1\_i2.p1:TRINITY\_DN160\_c0\_g1\_i1.p1 |  |  | DB Search |
| SVTAFTQSWVYQSSDKVQDPSK | 55.29 | 2487.1917 | 22 | 5.18 | 830.0737 | 17.80 | 0.9767 | 0.984 | 62980 | 4.77e2 | 1 | 1 | TRINITY\_DN6390\_c0\_g1\_i1.p1 |  |  | DB Search |
| LKDLENFLK | 55.28 | 1118.6335 | 9 | -1.2 | 560.3221 | 17.44 | 0.8968 | 0.904 | 60643 | 4.2e3 | 2 | 2 | TRINITY\_DN598\_c1\_g1\_i1.p1 |  |  | DB Search |
| LKDNAVIPLFAVTK | 55.28 | 1527.9025 | 14 | -4.47 | 510.3047 | 19.12 | 0.8226 | 0.8299 | 71843 | 1.2e3 | 2 | 2 | TRINITY\_DN1535\_c0\_g1\_i1.p1 |  |  | DB Search |
| SAPIWNLK | 55.26 | 927.5178 | 8 | -6.21 | 464.7623 | 17.22 | 0.8059 | 0.8132 | 59364 | 0 | 0 | 0 | TRINITY\_DN1936\_c0\_g1\_i3.p1 |  |  | DB Search |
| AIESERDELLEELNNNSALK | 55.22 | 2286.1338 | 20 | 1.76 | 763.0516 | 18.48 | 0.9965 | 1.0037 | 67692 | 0 | 0 | 0 | TRINITY\_DN428\_c0\_g1\_i1.p1 |  |  | DB Search |
| GQSSDLATLSSPVLNQASSTC(+57.02)K | 55.21 | 2250.0798 | 22 | 4.76 | 1126.05 | 17.18 | 1.2328 | 1.24 | 59109 | 0 | 0 | 0 | TRINITY\_DN4781\_c0\_g1\_i3.p1:TRINITY\_DN4781\_c0\_g1\_i1.p1:TRINITY\_DN4781\_c0\_g1\_i2.p1 | Carbamidomethylation |  | DB Search |
| TQAVLDRLNIEK | 55.19 | 1398.7831 | 12 | 4.64 | 700.4005 | 15.56 | 1.0057 | 1.013 | 48737 | 1.68e4 | 2 | 2 | TRINITY\_DN477\_c0\_g1\_i9.p1 |  |  | DB Search |
| SVWIGLNDR | 55.19 | 1058.5509 | 9 | 8.2 | 530.2859 | 17.42 | 0.8688 | 0.8761 | 60696 | 0 | 0 | 0 | TRINITY\_DN12627\_c0\_g1\_i1.p1 |  |  | DB Search |
| YLSFIK | 55.18 | 769.4374 | 6 | 1.01 | 385.7255 | 17.32 | 0.7445 | 0.7518 | 59913 | 5.56e2 | 1 | 1 | TRINITY\_DN5633\_c0\_g1\_i1.p1 |  |  | DB Search |
| ADHTYETDFGK | 55.18 | 1282.5465 | 11 | 8.7 | 642.2847 | 12.90 | 0.9453 | 0.9525 | 32449 | 0 | 0 | 0 | TRINITY\_DN4781\_c0\_g1\_i3.p1:TRINITY\_DN4781\_c0\_g1\_i1.p1:TRINITY\_DN4781\_c0\_g1\_i2.p1 |  |  | DB Search |
| MFGEQFGGR | 55.12 | 1027.4546 | 9 | -1.81 | 514.7325 | 15.93 | 0.8144 | 0.8218 | 51038 | 1.4e3 | 1 | 1 | TRINITY\_DN244\_c0\_g1\_i1.p1 |  |  | DB Search |
| KTTELTNNLK | 55.11 | 1160.6401 | 10 | 5.27 | 581.3291 | 11.78 | 0.9178 | 0.9251 | 25555 | 4.08e3 | 1 | 1 | TRINITY\_DN72625\_c0\_g1\_i1.p1 |  |  | DB Search |
| ASFVSDIDRLR | 55.08 | 1277.6727 | 11 | 3.1 | 639.8442 | 17.17 | 0.9896 | 0.9969 | 59018 | 6.66e2 | 1 | 1 | TRINITY\_DN598\_c1\_g1\_i1.p1 |  |  | DB Search |
| TGTLTQNR | 55.05 | 889.4617 | 8 | -2.51 | 445.7361 | 9.97 | 0.7844 | 0.7917 | 14750 | 0 | 0 | 0 | TRINITY\_DN262\_c0\_g1\_i1.p1 |  |  | DB Search |
| GFVAVNDQQPSVVVSINVPK | 55.03 | 2096.1265 | 20 | 0.1 | 1049.0684 | 18.45 | 1.2157 | 1.2228 | 67713 | 4.05e2 | 1 | 1 | TRINITY\_DN1616\_c0\_g1\_i3.p1:TRINITY\_DN1616\_c0\_g1\_i6.p1:TRINITY\_DN1616\_c0\_g1\_i2.p1:TRINITY\_DN1616\_c0\_g1\_i7.p1 |  |  | DB Search |
| INNKLEVKR | 55.03 | 1112.6666 | 9 | 7.92 | 371.8983 | 10.56 | 0.711 | 0.7184 | 18207 | 0 | 0 | 0 | TRINITY\_DN20282\_c0\_g1\_i1.p1 |  |  | DB Search |
| LASNMIIIR | 55.01 | 1029.6005 | 9 | 4.3 | 515.8086 | 17.03 | 0.8453 | 0.8526 | 58043 | 1.79e3 | 1 | 1 | TRINITY\_DN1043\_c0\_g1\_i2.p1 |  |  | DB Search |
| IQDKEGIPPDQQR | 55.01 | 1522.7739 | 13 | 0.25 | 762.3928 | 11.08 | 1.0343 | 1.0415 | 21018 | 1.78e3 | 2 | 2 | TRINITY\_DN554\_c0\_g1\_i3.p2:TRINITY\_DN585\_c0\_g1\_i8.p1:TRINITY\_DN101\_c0\_g1\_i1.p1:TRINITY\_DN585\_c0\_g1\_i3.p1:TRINITY\_DN554\_c0\_g1\_i4.p1:TRINITY\_DN173718\_c0\_g1\_i1.p1:TRINITY\_DN1465\_c1\_g1\_i1.p1:TRINITY\_DN40728\_c1\_g1\_i1.p2:TRINITY\_DN152\_c0\_g1\_i2.p1:TRINITY\_DN585\_c0\_g1\_i5.p1:TRINITY\_DN152\_c0\_g1\_i14.p2:TRINITY\_DN585\_c0\_g2\_i1.p1:TRINITY\_DN40728\_c0\_g1\_i1.p1:TRINITY\_DN1465\_c0\_g1\_i1.p2:TRINITY\_DN152\_c0\_g1\_i9.p1:TRINITY\_DN585\_c0\_g1\_i4.p1:TRINITY\_DN554\_c0\_g1\_i2.p1:TRINITY\_DN152\_c0\_g1\_i15.p1:TRINITY\_DN585\_c0\_g1\_i10.p1:TRINITY\_DN195631\_c0\_g1\_i1.p2:TRINITY\_DN585\_c0\_g1\_i2.p1:TRINITY\_DN8227\_c0\_g1\_i1.p1:TRINITY\_DN554\_c0\_g1\_i5.p1:TRINITY\_DN152\_c0\_g1\_i10.p1 |  |  | DB Search |
| AYGQDLGQPGSC(+57.02)LKR | 55 | 1648.7991 | 15 | 2.6 | 825.4072 | 13.56 | 1.0761 | 1.0833 | 36469 | 5.48e2 | 1 | 1 | TRINITY\_DN691\_c0\_g1\_i2.p1:TRINITY\_DN691\_c0\_g1\_i1.p1 | Carbamidomethylation |  | DB Search |
| AENDIGDWFR | 54.99 | 1221.5414 | 10 | 2.04 | 611.7779 | 19.39 | 0.8834 | 0.8907 | 73581 | 3.56e2 | 1 | 1 | TRINITY\_DN121418\_c0\_g1\_i1.p1 |  |  | DB Search |
| GIDIGLDEVK | 54.98 | 1057.5656 | 10 | -4.24 | 529.7866 | 17.24 | 0.8619 | 0.8692 | 59426 | 5.78e3 | 1 | 1 | TRINITY\_DN18\_c0\_g1\_i2.p1:TRINITY\_DN18\_c0\_g1\_i1.p1 |  |  | DB Search |
| RANLC(+57.02)PITC(+57.02)QR | 54.97 | 1387.6813 | 11 | 2.53 | 694.8481 | 12.62 | 0.9679 | 0.9751 | 30632 | 1.25e3 | 1 | 1 | TRINITY\_DN6390\_c0\_g1\_i1.p1 | Carbamidomethylation, Carbamidomethylation |  | DB Search |
| TC(+57.02)LC(+57.02)NPATSYPSK | 54.97 | 1497.6592 | 13 | 0.78 | 749.8358 | 13.69 | 0.9896 | 0.9969 | 37441 | 1.47e3 | 1 | 1 | TRINITY\_DN1058\_c0\_g1\_i2.p1:TRINITY\_DN1058\_c0\_g1\_i1.p1:TRINITY\_DN1058\_c0\_g1\_i3.p1 | Carbamidomethylation, Carbamidomethylation |  | DB Search |
| AFNKIETR | 54.97 | 977.5294 | 8 | 8.36 | 489.775 | 11.54 | 0.8339 | 0.8412 | 24067 | 2.69e3 | 1 | 1 | TRINITY\_DN4595\_c0\_g1\_i1.p1:TRINITY\_DN4595\_c0\_g1\_i7.p1:TRINITY\_DN4595\_c0\_g1\_i4.p1 |  |  | DB Search |
| TIKDVNC(+57.02)NNNQLK | 54.96 | 1559.7726 | 13 | 3.46 | 520.9321 | 11.33 | 0.7892 | 0.7966 | 22761 | 1.98e3 | 1 | 1 | TRINITY\_DN4815\_c0\_g1\_i2.p1:TRINITY\_DN4815\_c0\_g1\_i1.p1 | Carbamidomethylation |  | DB Search |
| GSVASLTC(+57.02)EC(+57.02)NNHK | 54.96 | 1575.677 | 14 | 1.58 | 788.8453 | 12.07 | 1.0624 | 1.0697 | 27198 | 7.99e2 | 1 | 1 | TRINITY\_DN1058\_c3\_g1\_i1.p1 | Carbamidomethylation, Carbamidomethylation |  | DB Search |
| SLVYNPLHNDQEVFER | 54.95 | 1958.9486 | 16 | -1.87 | 653.9875 | 17.53 | 0.866 | 0.8733 | 61164 | 3.14e3 | 1 | 1 | TRINITY\_DN6390\_c0\_g1\_i1.p1 |  |  | DB Search |
| LLETKNEYLDFEYK | 54.94 | 1803.8931 | 14 | 2.05 | 602.3049 | 17.54 | 0.8303 | 0.8376 | 61326 | 5.89e2 | 1 | 1 | TRINITY\_DN11377\_c0\_g1\_i1.p1 |  |  | DB Search |
| LDSNKDFQVIFEAK | 54.94 | 1652.8409 | 14 | -3.52 | 551.9511 | 18.03 | 0.8254 | 0.8327 | 64725 | 0 | 0 | 0 | TRINITY\_DN4781\_c0\_g1\_i3.p2:TRINITY\_DN4781\_c0\_g1\_i2.p1 |  |  | DB Search |
| AVAEEFISVDPYVR | 54.94 | 1593.8038 | 14 | 3.64 | 797.9103 | 19.17 | 1.0532 | 1.0604 | 72128 | 7.25e3 | 1 | 1 | TRINITY\_DN598\_c1\_g1\_i1.p1 |  |  | DB Search |
| GFGGNLVTITNSDIQK | 54.93 | 1662.8577 | 16 | -0.02 | 832.4343 | 17.83 | 1.0789 | 1.0861 | 63151 | 2.79e3 | 1 | 1 | TRINITY\_DN12627\_c0\_g1\_i1.p1 |  |  | DB Search |
| YLLVFTDGR | 54.93 | 1082.576 | 9 | -0.17 | 542.294 | 19.12 | 0.8684 | 0.8757 | 71808 | 1.15e3 | 1 | 1 | TRINITY\_DN138\_c0\_g1\_i1.p1 |  |  | DB Search |
| VTLTQTHFLK | 54.93 | 1186.671 | 10 | -0.58 | 594.3411 | 15.07 | 0.9646 | 0.9719 | 45810 | 6.69e2 | 1 | 1 | TRINITY\_DN5584\_c0\_g1\_i1.p1:TRINITY\_DN5584\_c0\_g1\_i3.p1 |  |  | DB Search |
| EC(+57.02)FC(+57.02)FGHGVSC(+57.02)DAIEGIGVK | 54.91 | 2240.9653 | 20 | 3.38 | 747.9966 | 18.10 | 0.9763 | 0.9836 | 65084 | 1.23e3 | 1 | 1 | TRINITY\_DN1676\_c0\_g1\_i6.p1:TRINITY\_DN1676\_c0\_g1\_i12.p1 | Carbamidomethylation, Carbamidomethylation, Carbamidomethylation |  | DB Search |
| KADIGVAMGIAGSDVSK | 54.91 | 1617.8396 | 17 | 0.24 | 809.9255 | 15.67 | 1.0656 | 1.0729 | 49555 | 0 | 0 | 0 | TRINITY\_DN262\_c0\_g1\_i1.p1 |  |  | DB Search |
| DGEKVETLK | 54.9 | 1017.5342 | 9 | -2.12 | 509.7722 | 11.49 | 0.8587 | 0.866 | 23862 | 1.28e3 | 1 | 1 | TRINITY\_DN1616\_c0\_g1\_i3.p1:TRINITY\_DN1616\_c0\_g1\_i6.p1:TRINITY\_DN1616\_c0\_g1\_i2.p1:TRINITY\_DN1616\_c0\_g1\_i7.p1 |  |  | DB Search |
| LISTFIPATGDTGGK | 54.9 | 1476.7823 | 15 | 1.83 | 739.3982 | 17.40 | 1.0041 | 1.0114 | 60313 | 2.76e3 | 1 | 1 | TRINITY\_DN1936\_c0\_g1\_i4.p1:TRINITY\_DN1936\_c0\_g1\_i5.p1 |  |  | DB Search |
| TAVDGISIISSGLTK | 54.9 | 1460.8086 | 15 | 3.18 | 731.4123 | 18.95 | 1.0214 | 1.0287 | 70784 | 3.02e2 | 1 | 1 | TRINITY\_DN7946\_c0\_g1\_i1.p1 |  |  | DB Search |
| ERLELFGK | 54.89 | 990.5498 | 8 | -4.6 | 496.2788 | 16.06 | 0.8437 | 0.851 | 51911 | 7.08e2 | 1 | 1 | TRINITY\_DN143676\_c0\_g1\_i1.p1 |  |  | DB Search |
| IDNVKEDVK | 54.89 | 1058.5608 | 9 | 5.78 | 530.2896 | 10.73 | 0.8716 | 0.8789 | 19180 | 1.58e3 | 1 | 1 | TRINITY\_DN1616\_c0\_g1\_i3.p1:TRINITY\_DN1616\_c0\_g1\_i6.p1:TRINITY\_DN1616\_c0\_g1\_i2.p1:TRINITY\_DN1616\_c0\_g1\_i7.p1 |  |  | DB Search |
| SDIAVDDFEFK | 54.89 | 1284.5874 | 11 | 6.87 | 643.304 | 18.47 | 0.9303 | 0.9376 | 67658 | 0 | 0 | 0 | TRINITY\_DN1936\_c0\_g1\_i4.p1:TRINITY\_DN1936\_c0\_g1\_i5.p1 |  |  | DB Search |
| GAVTAAFDKVR | 54.88 | 1133.6193 | 11 | 3.89 | 567.8179 | 14.48 | 0.9352 | 0.9424 | 42102 | 1.49e3 | 1 | 1 | TRINITY\_DN2577\_c1\_g1\_i1.p1:TRINITY\_DN2577\_c1\_g1\_i2.p1 |  |  | DB Search |
| RFEEAKDTIENK | 54.88 | 1478.7365 | 12 | 3.61 | 493.9201 | 11.68 | 0.8031 | 0.8104 | 24961 | 1.52e3 | 1 | 1 | TRINITY\_DN1676\_c0\_g1\_i6.p1:TRINITY\_DN1676\_c0\_g1\_i12.p1 |  |  | DB Search |
| ILALAGQLQSAR | 54.88 | 1239.7299 | 12 | 5.1 | 620.874 | 16.91 | 0.9675 | 0.9747 | 57298 | 9.69e2 | 1 | 1 | TRINITY\_DN1366\_c0\_g1\_i1.p1 |  |  | DB Search |
| TMNTNYVDKK | 54.87 | 1212.5808 | 10 | 4.95 | 607.2994 | 11.20 | 0.9299 | 0.9372 | 22052 | 0 | 0 | 0 | TRINITY\_DN252\_c0\_g1\_i1.p1 |  |  | DB Search |
| YAAYVGC(+57.02)EFQSVDFSSVAK | 54.86 | 2126.9619 | 19 | 2.95 | 1064.489 | 18.93 | 1.226 | 1.2332 | 70654 | 0 | 0 | 0 | TRINITY\_DN17714\_c0\_g1\_i1.p1 | Carbamidomethylation |  | DB Search |
| VISFDVPPQEILTR | 54.85 | 1612.8824 | 14 | 0.83 | 807.4474 | 19.60 | 1.0588 | 1.066 | 74865 | 6.05e2 | 1 | 1 | TRINITY\_DN909\_c0\_g1\_i1.p1 |  |  | DB Search |
| LLSTVLKPPSNR | 54.85 | 1323.7874 | 12 | 4.07 | 662.9022 | 15.03 | 1.0037 | 1.011 | 45450 | 8.14e2 | 1 | 1 | TRINITY\_DN28022\_c0\_g2\_i1.p1 |  |  | DB Search |
| NLASLSSPILNQASSR | 54.83 | 1656.8795 | 16 | -5.09 | 829.441 | 17.89 | 1.0761 | 1.0833 | 63844 | 0 | 0 | 0 | TRINITY\_DN28022\_c0\_g2\_i1.p1 |  |  | DB Search |
| LISLPVESRPGGNC(+57.02)QVR | 54.82 | 1880.989 | 17 | 0.61 | 628.0026 | 15.71 | 0.8481 | 0.8554 | 49525 | 1.61e3 | 1 | 1 | TRINITY\_DN4781\_c0\_g1\_i1.p2:TRINITY\_DN4781\_c0\_g1\_i3.p2:TRINITY\_DN4781\_c0\_g1\_i2.p1 | Carbamidomethylation |  | DB Search |
| IDHAVNLVQQELK | 54.81 | 1505.8202 | 13 | 5.36 | 753.9197 | 15.83 | 1.0379 | 1.0452 | 50504 | 5.31e2 | 1 | 1 | TRINITY\_DN191650\_c0\_g1\_i1.p1 |  |  | DB Search |
| LRPGVELTM(+15.99)SSLVNAK | 54.81 | 1729.9396 | 16 | -2.37 | 577.6512 | 15.40 | 0.8749 | 0.8822 | 47976 | 0 | 0 | 0 | TRINITY\_DN329\_c0\_g1\_i1.p1:TRINITY\_DN329\_c0\_g1\_i2.p1 | Oxidation (M) | M9:Oxidation (M):1000 | DB Search |
| GPAGAVGPAGR | 54.78 | 908.4828 | 11 | 4.27 | 455.2496 | 10.87 | 0.7921 | 0.7994 | 20030 | 1.32e3 | 1 | 1 | TRINITY\_DN711\_c0\_g1\_i2.p1 |  |  | DB Search |
| YYAAAC(+57.02)LDNIFQR | 54.78 | 1603.7452 | 13 | 6.71 | 802.8835 | 19.80 | 1.0624 | 1.0697 | 76262 | 2.45e2 | 1 | 1 | TRINITY\_DN71276\_c0\_g1\_i1.p1 | Carbamidomethylation |  | DB Search |
| AYGQDLGQPGSC(+57.02)LK | 54.78 | 1492.698 | 14 | -2.16 | 747.353 | 14.36 | 0.9977 | 1.0049 | 41400 | 3.43e3 | 1 | 1 | TRINITY\_DN691\_c0\_g1\_i2.p1:TRINITY\_DN691\_c0\_g1\_i1.p1 | Carbamidomethylation |  | DB Search |
| IDYALELAK | 54.77 | 1034.5648 | 9 | 1.74 | 518.2894 | 16.99 | 0.8631 | 0.8704 | 57966 | 0 | 0 | 0 | TRINITY\_DN244\_c0\_g1\_i1.p1 |  |  | DB Search |
| LVEVETQLKR | 54.77 | 1213.703 | 10 | 4.99 | 607.8605 | 13.63 | 0.9501 | 0.9574 | 36929 | 7.45e2 | 1 | 1 | TRINITY\_DN1366\_c0\_g1\_i1.p1 |  |  | DB Search |
| EIEDINAANDEALASTK | 54.75 | 1802.8534 | 17 | -3.96 | 902.4284 | 15.28 | 1.1009 | 1.1082 | 47115 | 0 | 0 | 0 | TRINITY\_DN3034\_c0\_g1\_i1.p1 |  |  | DB Search |
| YIFIEASSPR | 54.75 | 1181.608 | 10 | -9.2 | 591.8046 | 16.96 | 0.9315 | 0.9388 | 57711 | 0 | 0 | 0 | TRINITY\_DN193641\_c0\_g1\_i1.p1 |  |  | DB Search |
| LQIIVGDLR | 54.74 | 1025.6233 | 9 | 1.09 | 513.8184 | 18.10 | 0.8672 | 0.8745 | 65042 | 2.27e3 | 1 | 1 | TRINITY\_DN1366\_c0\_g1\_i1.p1 |  |  | DB Search |
| AVGEEFNSVDPSVK | 54.73 | 1476.7096 | 14 | 1.1 | 739.3613 | 15.15 | 1.0061 | 1.0134 | 46241 | 7.54e2 | 1 | 1 | TRINITY\_DN1119\_c0\_g1\_i1.p1 |  |  | DB Search |
| FNAQLSEAQEELESVKK | 54.73 | 1948.9741 | 17 | 4.67 | 650.6669 | 16.99 | 0.8854 | 0.8927 | 57921 | 0 | 0 | 0 | TRINITY\_DN3034\_c0\_g1\_i1.p1 |  |  | DB Search |
| YC(+57.02)EIDKETEAVTIR | 54.7 | 1725.8243 | 14 | -7.9 | 576.2762 | 14.76 | 0.8514 | 0.8587 | 43877 | 0 | 0 | 0 | TRINITY\_DN4770\_c0\_g1\_i1.p1 | Carbamidomethylation |  | DB Search |
| DLADRDHVIEELER | 54.67 | 1708.838 | 14 | -0.7 | 570.6183 | 16.26 | 0.8343 | 0.8416 | 53234 | 0 | 0 | 0 | TRINITY\_DN1366\_c0\_g1\_i1.p1 |  |  | DB Search |
| NFGLC(+57.02)TEHTGNLITNK | 54.67 | 1817.873 | 16 | 1.42 | 606.9645 | 15.88 | 0.8412 | 0.8485 | 50739 | 7.31e2 | 1 | 1 | TRINITY\_DN10094\_c0\_g1\_i1.p1:TRINITY\_DN10094\_c0\_g1\_i2.p1 | Carbamidomethylation |  | DB Search |
| EYNINEEVNVR | 54.67 | 1377.6525 | 11 | -5.34 | 689.8283 | 14.94 | 0.9787 | 0.986 | 45239 | 5.98e2 | 1 | 1 | TRINITY\_DN4595\_c0\_g1\_i4.p1:TRINITY\_DN4595\_c0\_g1\_i10.p2 |  |  | DB Search |
| LLEINVYHPLIQELQK | 54.62 | 1949.0985 | 16 | -4.49 | 650.7025 | 19.83 | 0.9735 | 0.9808 | 76478 | 0 | 0 | 0 | TRINITY\_DN5633\_c0\_g1\_i1.p1 |  |  | DB Search |
| TGPSLDNTK | 54.61 | 931.4611 | 9 | 0.07 | 466.7368 | 11.29 | 0.8039 | 0.8112 | 22423 | 5.2e2 | 1 | 1 | TRINITY\_DN4781\_c0\_g1\_i3.p1:TRINITY\_DN4781\_c0\_g1\_i1.p1:TRINITY\_DN4781\_c0\_g1\_i2.p1 |  |  | DB Search |
| YLEFEYEC(+57.02)APK | 54.6 | 1447.6329 | 11 | -0.11 | 724.8221 | 17.24 | 1.0387 | 1.046 | 59469 | 6.12e2 | 1 | 1 | TRINITY\_DN1214\_c0\_g1\_i1.p1 | Carbamidomethylation |  | DB Search |
| VLISSLLIK | 54.58 | 984.6583 | 9 | 0.22 | 493.3354 | 19.79 | 0.8506 | 0.8579 | 76091 | 1.17e3 | 1 | 1 | TRINITY\_DN1616\_c0\_g1\_i3.p1:TRINITY\_DN1616\_c0\_g1\_i6.p1:TRINITY\_DN1616\_c0\_g1\_i2.p1:TRINITY\_DN1616\_c0\_g1\_i7.p1 |  |  | DB Search |
| S(+42.01)ATGNLVTVLRK | 54.57 | 1299.751 | 12 | -5.84 | 650.8776 | 17.30 | 0.9924 | 0.9997 | 59899 | 0 | 0 | 0 | TRINITY\_DN2963\_c0\_g1\_i1.p1 | Acetylation (Protein N-term) | S1:Acetylation (Protein N-term):1000 | DB Search |
| DMGVNVISVGAGHTINHR | 54.57 | 1875.9374 | 18 | -2.32 | 626.3169 | 15.47 | 0.8826 | 0.8899 | 48327 | 2.64e2 | 1 | 1 | TRINITY\_DN138\_c0\_g1\_i1.p1 |  |  | DB Search |
| TITLEVEPSDTIENVK | 54.56 | 1786.92 | 16 | 4.26 | 894.4691 | 17.62 | 1.1442 | 1.1514 | 61769 | 1.48e4 | 1 | 1 | TRINITY\_DN554\_c0\_g1\_i3.p2:TRINITY\_DN585\_c0\_g1\_i8.p1:TRINITY\_DN585\_c0\_g1\_i4.p1:TRINITY\_DN585\_c0\_g1\_i3.p1:TRINITY\_DN101\_c0\_g1\_i1.p1:TRINITY\_DN554\_c0\_g1\_i4.p1:TRINITY\_DN554\_c0\_g1\_i2.p1:TRINITY\_DN585\_c0\_g1\_i10.p1:TRINITY\_DN585\_c0\_g1\_i2.p1:TRINITY\_DN554\_c0\_g1\_i5.p1:TRINITY\_DN585\_c0\_g1\_i5.p1:TRINITY\_DN585\_c0\_g2\_i1.p1 |  |  | DB Search |
| DKPALDVTSTLKK | 54.52 | 1414.8031 | 13 | 2.31 | 472.6084 | 13.78 | 0.801 | 0.8084 | 37913 | 0 | 0 | 0 | TRINITY\_DN1821\_c0\_g1\_i2.p1:TRINITY\_DN1821\_c0\_g1\_i3.p1:TRINITY\_DN1821\_c0\_g1\_i6.p1:TRINITY\_DN1821\_c0\_g1\_i5.p1:TRINITY\_DN1821\_c0\_g1\_i4.p1 |  |  | DB Search |
| TEGLAPFWR | 54.51 | 1075.545 | 9 | -1.01 | 538.7781 | 19.20 | 0.853 | 0.8603 | 72310 | 1.12e3 | 1 | 1 | TRINITY\_DN1559\_c0\_g1\_i1.p1 |  |  | DB Search |
| FKNPSNINDLNR | 54.49 | 1430.7266 | 12 | 3.59 | 477.9168 | 13.53 | 0.7522 | 0.7595 | 36237 | 4.48e2 | 1 | 1 | TRINITY\_DN6304\_c0\_g1\_i1.p1 |  |  | DB Search |
| AILGTLSVEAIYK | 54.49 | 1376.7915 | 13 | -1.17 | 689.4007 | 19.66 | 1.0508 | 1.058 | 75332 | 3.13e2 | 1 | 1 | TRINITY\_DN11435\_c0\_g1\_i1.p1 |  |  | DB Search |
| GLSGVFNIEVK | 54.48 | 1161.6393 | 11 | -3.55 | 581.8236 | 18.95 | 0.9089 | 0.9162 | 70828 | 0 | 0 | 0 | TRINITY\_DN193641\_c0\_g1\_i1.p1 |  |  | DB Search |
| REGDNALLFGPK | 54.47 | 1315.6884 | 12 | 0.28 | 658.8502 | 15.81 | 0.9654 | 0.9727 | 50453 | 0 | 0 | 0 | TRINITY\_DN168145\_c0\_g1\_i1.p1 |  |  | DB Search |
| GVDIVVLAVGSK | 54.46 | 1155.6863 | 12 | -0.99 | 578.8486 | 18.70 | 0.9178 | 0.9251 | 69234 | 5.37e2 | 1 | 1 | TRINITY\_DN74720\_c0\_g1\_i1.p1 |  |  | DB Search |
| LEPQLQQILR | 54.46 | 1236.719 | 10 | -0.59 | 619.3651 | 18.13 | 0.9206 | 0.9279 | 65310 | 6.16e2 | 1 | 1 | TRINITY\_DN1366\_c0\_g1\_i1.p1 |  |  | DB Search |
| SIEAIYLGEFGTC(+57.02)K | 54.46 | 1586.765 | 14 | 6.64 | 794.3933 | 19.71 | 1.0981 | 1.1053 | 75650 | 6.41e2 | 1 | 1 | TRINITY\_DN11248\_c0\_g1\_i1.p1:TRINITY\_DN11248\_c0\_g1\_i3.p1 | Carbamidomethylation |  | DB Search |
| ELHTFGDDILGDSPK | 54.43 | 1642.7838 | 15 | -1.51 | 548.5999 | 17.34 | 0.8315 | 0.8388 | 59995 | 1.59e3 | 1 | 1 | TRINITY\_DN15274\_c0\_g1\_i1.p1 |  |  | DB Search |
| IDQHGGIK | 54.38 | 866.461 | 8 | 5.07 | 434.239 | 9.74 | 0.7892 | 0.7966 | 13590 | 0 | 0 | 0 | TRINITY\_DN982\_c0\_g1\_i1.p1:TRINITY\_DN982\_c0\_g1\_i3.p1 |  |  | DB Search |
| IC(+57.02)HLLGIQVTDFTK | 54.38 | 1643.8705 | 14 | 0.98 | 548.9634 | 18.67 | 0.8104 | 0.8177 | 68936 | 5.07e2 | 1 | 1 | TRINITY\_DN428\_c0\_g1\_i1.p1 | Carbamidomethylation |  | DB Search |
| LLILLTDGSQTQDIGAEDPAK | 54.36 | 2197.1477 | 21 | 2.89 | 1099.5819 | 19.49 | 1.2511 | 1.2583 | 74311 | 0 | 0 | 0 | TRINITY\_DN4494\_c1\_g1\_i1.p1:TRINITY\_DN4494\_c1\_g1\_i7.p1 |  |  | DB Search |
| IKC(+57.02)DDPDVDYEK | 54.35 | 1495.65 | 12 | 1.08 | 499.5567 | 12.15 | 0.7901 | 0.7974 | 27860 | 0 | 0 | 0 | TRINITY\_DN28257\_c0\_g1\_i1.p1:TRINITY\_DN28257\_c0\_g1\_i2.p1 | Carbamidomethylation |  | DB Search |
| SSNTIEENR | 54.34 | 1048.4785 | 9 | 3.11 | 525.247 | 10.20 | 0.8303 | 0.8376 | 15999 | 0 | 0 | 0 | TRINITY\_DN982\_c0\_g1\_i3.p1 |  |  | DB Search |
| LPGNVIPIK | 54.32 | 949.596 | 9 | 3.06 | 475.8057 | 16.31 | 0.8214 | 0.8287 | 53518 | 3.4e2 | 1 | 1 | TRINITY\_DN5584\_c0\_g1\_i1.p1:TRINITY\_DN5584\_c0\_g1\_i3.p1 |  |  | DB Search |
| GVLAGVGAIVK | 54.31 | 982.6175 | 11 | -1.97 | 492.314 | 17.55 | 0.8631 | 0.8704 | 61324 | 3.41e3 | 1 | 1 | TRINITY\_DN2577\_c1\_g1\_i1.p1:TRINITY\_DN2577\_c1\_g1\_i2.p1 |  |  | DB Search |
| YLFDLESNC(+57.02)DK | 54.31 | 1402.6074 | 11 | -3.51 | 702.307 | 18.48 | 0.9787 | 0.986 | 67762 | 0 | 0 | 0 | TRINITY\_DN6390\_c0\_g1\_i1.p1 | Carbamidomethylation |  | DB Search |
| LPGLLSPSYGVR | 54.31 | 1257.7081 | 12 | -1.48 | 629.859 | 18.18 | 0.9541 | 0.9614 | 65571 | 5.89e2 | 1 | 1 | TRINITY\_DN144214\_c0\_g1\_i1.p1 |  |  | DB Search |
| GPAGAPGDEGQTGM(+15.99)RGER | 54.26 | 1757.775 | 18 | 2.12 | 586.9323 | 10.62 | 0.8818 | 0.8891 | 18471 | 3.52e3 | 1 | 1 | TRINITY\_DN77\_c0\_g2\_i4.p1:TRINITY\_DN77\_c0\_g2\_i2.p1:TRINITY\_DN77\_c0\_g2\_i1.p1:TRINITY\_DN77\_c0\_g2\_i3.p1 | Oxidation (M) | M14:Oxidation (M):1000 | DB Search |
| GPAGAPGDEGQTGM(+15.99)R | 54.2 | 1415.6099 | 15 | 4.13 | 708.8136 | 10.40 | 0.9453 | 0.9525 | 17281 | 0 | 0 | 0 | TRINITY\_DN77\_c0\_g2\_i4.p1:TRINITY\_DN77\_c0\_g2\_i2.p1:TRINITY\_DN77\_c0\_g2\_i1.p1:TRINITY\_DN77\_c0\_g2\_i3.p1 | Oxidation (M) | M14:Oxidation (M):1000 | DB Search |
| IGVIVYSDTASIEIR | 54.11 | 1634.8879 | 15 | -3.55 | 818.4465 | 18.74 | 1.1017 | 1.109 | 69514 | 0 | 0 | 0 | TRINITY\_DN244\_c0\_g1\_i1.p1 |  |  | DB Search |
| LIVINLIK | 54.1 | 924.6371 | 8 | 1.83 | 463.3257 | 19.76 | 0.8031 | 0.8104 | 75960 | 1.58e3 | 1 | 1 | TRINITY\_DN942\_c0\_g1\_i5.p1:TRINITY\_DN942\_c0\_g1\_i2.p1:TRINITY\_DN942\_c0\_g1\_i3.p1 |  |  | DB Search |
| AQC(+57.02)EFAIEAITK | 54.03 | 1379.6754 | 12 | 0.01 | 690.8435 | 17.77 | 0.9622 | 0.9695 | 62914 | 2.6e2 | 1 | 1 | TRINITY\_DN428\_c0\_g1\_i1.p1 | Carbamidomethylation |  | DB Search |
| NYNNDDEPGAKK | 53.98 | 1363.6003 | 12 | -1.27 | 682.8051 | 9.59 | 0.9384 | 0.9457 | 12858 | 0 | 0 | 0 | TRINITY\_DN199\_c0\_g1\_i1.p1 |  |  | DB Search |
| GKDGLQGIAGPR | 53.95 | 1167.636 | 12 | 2.28 | 584.8253 | 13.08 | 0.885 | 0.8923 | 33537 | 5.42e2 | 1 | 1 | TRINITY\_DN691\_c0\_g1\_i2.p1:TRINITY\_DN691\_c0\_g1\_i1.p1 |  |  | DB Search |
| FDDVPNGLGAFSK | 53.88 | 1365.6565 | 13 | 1.41 | 683.835 | 17.84 | 0.959 | 0.9662 | 63447 | 0 | 0 | 0 | TRINITY\_DN5897\_c0\_g1\_i2.p1:TRINITY\_DN5897\_c0\_g1\_i1.p2:TRINITY\_DN144214\_c0\_g1\_i1.p1:TRINITY\_DN5897\_c0\_g1\_i3.p1 |  |  | DB Search |
| GSDC(+57.02)NLQTGQC(+57.02)EC(+57.02)QDGVGGR | 53.87 | 2196.8582 | 20 | -5.84 | 733.2875 | 12.26 | 0.8733 | 0.8806 | 28552 | 0 | 0 | 0 | TRINITY\_DN1676\_c0\_g1\_i6.p1:TRINITY\_DN1676\_c0\_g1\_i12.p1 | Carbamidomethylation, Carbamidomethylation, Carbamidomethylation |  | DB Search |
| RC(+57.02)EEC(+57.02)AAGHYQFPFC(+57.02)K | 53.84 | 2058.8499 | 16 | 0.01 | 687.2891 | 15.74 | 0.9218 | 0.9291 | 49834 | 2.69e2 | 1 | 1 | TRINITY\_DN1616\_c0\_g1\_i3.p1:TRINITY\_DN1616\_c0\_g1\_i6.p1:TRINITY\_DN1616\_c0\_g1\_i2.p1:TRINITY\_DN1616\_c0\_g1\_i7.p1 | Carbamidomethylation, Carbamidomethylation, Carbamidomethylation |  | DB Search |
| KYTPC(+57.02)EEK | 53.79 | 1053.4801 | 8 | 5.43 | 527.749 | 9.67 | 0.8558 | 0.8631 | 13183 | 2.75e2 | 1 | 1 | TRINITY\_DN1369\_c0\_g1\_i1.p1 | Carbamidomethylation |  | DB Search |
| KALVILGDGFISGGGTPSK | 53.76 | 1816.0094 | 19 | 1.61 | 606.3434 | 18.24 | 0.9 | 0.9073 | 66111 | 0 | 0 | 0 | TRINITY\_DN244\_c0\_g1\_i1.p1 |  |  | DB Search |
| DGEKVTLTQTHFLK | 53.73 | 1615.8569 | 14 | -13.39 | 539.6179 | 14.95 | 0.8534 | 0.8607 | 45082 | 0 | 0 | 0 | TRINITY\_DN5584\_c0\_g1\_i1.p1:TRINITY\_DN5584\_c0\_g1\_i3.p1 |  |  | DB Search |
| AVSFLVNDVPNLK | 53.73 | 1414.782 | 13 | 3.82 | 708.3994 | 19.29 | 1.0021 | 1.0094 | 72907 | 2.09e3 | 1 | 1 | TRINITY\_DN1189\_c0\_g1\_i1.p1 |  |  | DB Search |
| KYVFAVAVDHTDLK | 53.73 | 1604.8562 | 14 | -1.1 | 535.9576 | 15.88 | 0.8676 | 0.8749 | 50838 | 0 | 0 | 0 | TRINITY\_DN61668\_c0\_g1\_i1.p1 |  |  | DB Search |
| VQEMEEALAAAESK | 53.73 | 1504.7079 | 14 | -3.91 | 753.3566 | 16.96 | 1.013 | 1.0202 | 57682 | 3.72e2 | 1 | 1 | TRINITY\_DN76282\_c0\_g1\_i1.p1:TRINITY\_DN3034\_c0\_g1\_i1.p1:TRINITY\_DN1507\_c0\_g1\_i1.p1:TRINITY\_DN825\_c0\_g1\_i1.p1 |  |  | DB Search |
| EKGVDIVVLAVGSK | 53.72 | 1412.8239 | 14 | -0.15 | 471.9474 | 17.40 | 0.7933 | 0.8006 | 60372 | 6.65e2 | 1 | 1 | TRINITY\_DN74720\_c0\_g1\_i1.p1 |  |  | DB Search |
| AHVSLSTFSEDVTSLTK | 53.71 | 1820.9155 | 17 | -2.35 | 607.9764 | 19.00 | 0.8266 | 0.8339 | 71162 | 0 | 0 | 0 | TRINITY\_DN113\_c3\_g1\_i1.p1 |  |  | DB Search |
| MGAHVATIHYQK | 53.7 | 1354.6815 | 12 | 4.59 | 452.5689 | 12.20 | 0.7836 | 0.7909 | 28050 | 1.16e3 | 1 | 1 | TRINITY\_DN11248\_c0\_g1\_i1.p1:TRINITY\_DN11248\_c0\_g1\_i3.p1 |  |  | DB Search |
| HLC(+57.02)VGMGSGGK | 53.7 | 1101.5059 | 11 | 0.68 | 551.7594 | 14.22 | 0.8757 | 0.883 | 40611 | 2.66e2 | 1 | 1 | TRINITY\_DN11248\_c0\_g1\_i1.p1:TRINITY\_DN11248\_c0\_g1\_i3.p1 | Carbamidomethylation |  | DB Search |
| GC(+57.02)VTLDLRR | 53.68 | 1088.576 | 9 | -7.35 | 545.2901 | 14.15 | 0.8862 | 0.8935 | 40124 | 2.54e2 | 1 | 1 | TRINITY\_DN3795\_c0\_g1\_i1.p1 | Carbamidomethylation |  | DB Search |
| FYYHM(+15.99)YGDHIGALSVK | 53.61 | 1915.8927 | 16 | 2.41 | 639.6383 | 15.79 | 0.9085 | 0.9158 | 50276 | 2.57e2 | 1 | 1 | TRINITY\_DN4781\_c0\_g1\_i1.p1:TRINITY\_DN4781\_c0\_g1\_i2.p1 | Oxidation (M) | M5:Oxidation (M):1000 | DB Search |
| YLSWTLDNDKIR | 53.6 | 1522.778 | 12 | -3.19 | 508.5972 | 17.66 | 0.8063 | 0.8136 | 62168 | 3.04e2 | 1 | 1 | TRINITY\_DN5584\_c0\_g1\_i2.p1:TRINITY\_DN5584\_c0\_g1\_i1.p1:TRINITY\_DN5584\_c0\_g1\_i3.p1 |  |  | DB Search |
| YYVTIIDAPGHRDFIK | 53.54 | 1906.9941 | 16 | -0.07 | 636.6705 | 17.01 | 0.8915 | 0.8988 | 58058 | 0 | 0 | 0 | TRINITY\_DN64\_c0\_g1\_i1.p1 |  |  | DB Search |
| ASFLIDLR | 53.54 | 933.5283 | 8 | 1.82 | 467.7713 | 19.39 | 0.8153 | 0.8226 | 73480 | 2.89e3 | 1 | 1 | TRINITY\_DN129928\_c0\_g1\_i1.p1 |  |  | DB Search |
| SKIDYEALQVK | 53.53 | 1292.6976 | 11 | -4.87 | 647.3515 | 14.82 | 0.9606 | 0.9679 | 44312 | 0 | 0 | 0 | TRINITY\_DN982\_c0\_g1\_i1.p1:TRINITY\_DN982\_c0\_g1\_i3.p1 |  |  | DB Search |
| ISEQFTAMFR | 53.53 | 1228.5911 | 10 | 8.26 | 615.3065 | 18.88 | 0.904 | 0.9113 | 70394 | 0 | 0 | 0 | TRINITY\_DN1910\_c0\_g1\_i1.p1:TRINITY\_DN3986\_c0\_g1\_i1.p1 |  |  | DB Search |
| GLSGVNGLRGETGDR | 53.51 | 1486.7488 | 15 | 1.6 | 744.3812 | 13.43 | 1.0508 | 1.058 | 35633 | 9.09e2 | 1 | 1 | TRINITY\_DN691\_c0\_g1\_i2.p1:TRINITY\_DN691\_c0\_g1\_i1.p1 |  |  | DB Search |
| SC(+57.02)IEASC(+57.02)SSK | 53.5 | 1127.4587 | 10 | 5.45 | 564.7385 | 10.24 | 0.8733 | 0.8806 | 16314 | 7.28e2 | 1 | 1 | TRINITY\_DN1058\_c0\_g1\_i2.p1:TRINITY\_DN1058\_c0\_g1\_i1.p1:TRINITY\_DN1058\_c0\_g1\_i3.p1 | Carbamidomethylation, Carbamidomethylation |  | DB Search |
| FVGIPSIDISYR | 53.5 | 1365.7292 | 12 | -0.66 | 683.8699 | 19.88 | 0.9675 | 0.9747 | 76591 | 1.07e3 | 1 | 1 | TRINITY\_DN33467\_c0\_g1\_i1.p1 |  |  | DB Search |
| DVQC(+57.02)TC(+57.02)NNELGYVHSPDGK | 53.44 | 2191.9263 | 19 | 2.69 | 731.6497 | 14.19 | 0.9634 | 0.9707 | 40373 | 5.07e2 | 1 | 1 | TRINITY\_DN319\_c2\_g1\_i3.p1:TRINITY\_DN319\_c2\_g1\_i1.p1:TRINITY\_DN319\_c2\_g1\_i2.p1 | Carbamidomethylation, Carbamidomethylation |  | DB Search |
| ITETC(+57.02)IEGSYMDKNEIK | 53.44 | 2029.9336 | 17 | 2.79 | 677.6522 | 15.03 | 0.9505 | 0.9578 | 45564 | 0 | 0 | 0 | TRINITY\_DN1043\_c0\_g1\_i2.p1 | Carbamidomethylation |  | DB Search |
| LGDLESANQILAMEK | 53.43 | 1630.8236 | 15 | 4.51 | 816.421 | 18.64 | 1.06 | 1.0672 | 68833 | 4.12e2 | 1 | 1 | TRINITY\_DN1366\_c0\_g1\_i1.p1 |  |  | DB Search |
| KVLILLADGQQTYVPGVTEPSEVAK | 53.41 | 2654.4531 | 25 | 2.54 | 885.8253 | 18.52 | 1.0371 | 1.0443 | 67689 | 3.47e3 | 2 | 2 | TRINITY\_DN244\_c0\_g1\_i1.p1 |  |  | DB Search |
| M(+15.99)GAIVAVTGDGVNDSPALK | 53.36 | 1829.9193 | 19 | -12.74 | 915.9532 | 15.85 | 1.1182 | 1.1254 | 50596 | 3.97e2 | 1 | 1 | TRINITY\_DN262\_c0\_g1\_i1.p1 | Oxidation (M) | M1:Oxidation (M):1000 | DB Search |
| LFAIGFTK | 53.35 | 895.5167 | 8 | -4.7 | 448.7625 | 18.76 | 0.7844 | 0.7917 | 69557 | 2.62e2 | 1 | 1 | TRINITY\_DN577\_c0\_g1\_i1.p1 |  |  | DB Search |
| ITTSQILDHEQQK | 53.33 | 1539.7893 | 13 | 1.49 | 770.9014 | 14.03 | 1.0969 | 1.1041 | 39378 | 0 | 0 | 0 | TRINITY\_DN982\_c0\_g1\_i1.p1:TRINITY\_DN982\_c0\_g1\_i3.p1 |  |  | DB Search |
| AQAINVQLQEIQR | 53.32 | 1509.8263 | 13 | 1.6 | 755.92 | 16.19 | 1.0295 | 1.0367 | 52841 | 0 | 0 | 0 | TRINITY\_DN1280\_c0\_g1\_i1.p1 |  |  | DB Search |
| LIFEGIVGK | 53.31 | 974.58 | 9 | 4.48 | 488.2984 | 18.60 | 0.8161 | 0.8234 | 68409 | 4.22e3 | 1 | 1 | TRINITY\_DN168145\_c0\_g1\_i1.p1 |  |  | DB Search |
| IEALHC(+57.02)LSENIVK | 53.3 | 1524.797 | 13 | -4.69 | 509.2694 | 16.48 | 0.8051 | 0.8124 | 54755 | 5.65e2 | 1 | 1 | TRINITY\_DN3078\_c0\_g1\_i1.p1 | Carbamidomethylation |  | DB Search |
| RFDGIFQYTK | 53.29 | 1273.6455 | 10 | -1.75 | 637.8275 | 17.11 | 0.959 | 0.9662 | 58503 | 2e3 | 1 | 1 | TRINITY\_DN244\_c0\_g1\_i1.p1 |  |  | DB Search |
| IEDFEESPPTDPHIGPVEQR | 53.29 | 2291.0706 | 20 | 5.46 | 764.7 | 15.86 | 0.9295 | 0.9368 | 50734 | 0 | 0 | 0 | TRINITY\_DN140702\_c0\_g1\_i1.p1 |  |  | DB Search |
| IIIELQGDLIK | 53.27 | 1253.7594 | 11 | 3.89 | 627.8881 | 19.42 | 0.9513 | 0.9586 | 73842 | 0 | 0 | 0 | TRINITY\_DN5\_c0\_g1\_i7.p2:TRINITY\_DN5\_c0\_g1\_i9.p1:TRINITY\_DN5\_c0\_g1\_i8.p1:TRINITY\_DN5\_c0\_g1\_i11.p1:TRINITY\_DN5\_c0\_g1\_i16.p1:TRINITY\_DN5\_c0\_g1\_i18.p1:TRINITY\_DN5\_c0\_g1\_i10.p1:TRINITY\_DN5\_c0\_g1\_i7.p4:TRINITY\_DN5\_c0\_g1\_i13.p1:TRINITY\_DN5\_c0\_g1\_i10.p2:TRINITY\_DN5\_c0\_g1\_i15.p4:TRINITY\_DN5\_c0\_g1\_i18.p3:TRINITY\_DN5\_c0\_g1\_i1.p1:TRINITY\_DN5\_c0\_g1\_i15.p1 |  |  | DB Search |
| YFTPSADGFSC(+57.02)IPASC(+57.02)K | 53.17 | 1906.823 | 17 | 11.55 | 954.4277 | 17.51 | 1.1222 | 1.1294 | 61270 | 0 | 0 | 0 | TRINITY\_DN24268\_c0\_g1\_i2.p1:TRINITY\_DN24268\_c0\_g1\_i1.p1 | Carbamidomethylation, Carbamidomethylation |  | DB Search |
| AAFPC(+57.02)FDEPAMK | 53.17 | 1382.5999 | 12 | -3.57 | 692.3032 | 18.01 | 0.9675 | 0.9747 | 64265 | 9.62e2 | 1 | 1 | TRINITY\_DN5584\_c0\_g1\_i1.p1:TRINITY\_DN5584\_c0\_g1\_i3.p1 | Carbamidomethylation |  | DB Search |
| NEINLASGDVFEGGVVGSLK | 53.15 | 2004.0164 | 20 | 1.4 | 1003.0146 | 19.85 | 1.1829 | 1.1901 | 76578 | 0 | 0 | 0 | TRINITY\_DN1616\_c0\_g1\_i3.p1:TRINITY\_DN1616\_c0\_g1\_i6.p1:TRINITY\_DN1616\_c0\_g1\_i2.p1:TRINITY\_DN1616\_c0\_g1\_i4.p1:TRINITY\_DN1616\_c0\_g1\_i7.p1 |  |  | DB Search |
| SQDDC(+57.02)ETNEEYVK | 53.15 | 1615.6307 | 13 | 5.63 | 808.8254 | 12.08 | 1.0636 | 1.0709 | 27312 | 4.96e2 | 1 | 1 | TRINITY\_DN1071\_c0\_g1\_i4.p1:TRINITY\_DN1071\_c0\_g1\_i1.p1 | Carbamidomethylation |  | DB Search |
| IITEINPTIQVFQPK | 53.04 | 1739.9822 | 15 | 2.13 | 870.9983 | 19.16 | 1.1242 | 1.1314 | 72245 | 3.87e2 | 1 | 1 | TRINITY\_DN71276\_c0\_g1\_i1.p1 |  |  | DB Search |
| LKPGDTIEFDTSIK | 53.02 | 1562.8191 | 14 | 1.32 | 521.9465 | 16.40 | 0.7734 | 0.7807 | 54132 | 0 | 0 | 0 | TRINITY\_DN75529\_c0\_g1\_i1.p1 |  |  | DB Search |
| TKVEC(+57.02)VAGINALK | 53.02 | 1401.765 | 13 | 2.74 | 701.8901 | 14.63 | 1.0045 | 1.0118 | 43116 | 0 | 0 | 0 | TRINITY\_DN76789\_c0\_g1\_i1.p1 | Carbamidomethylation |  | DB Search |
| TALENENAEIAADLQR | 52.91 | 1756.8591 | 16 | -4.14 | 879.4313 | 16.92 | 1.0985 | 1.1057 | 57445 | 0 | 0 | 0 | TRINITY\_DN428\_c0\_g1\_i1.p1 |  |  | DB Search |
| DKLC(+57.02)EWLK | 52.89 | 1090.5481 | 8 | -1.41 | 546.2794 | 16.40 | 0.8696 | 0.8769 | 54029 | 4.39e2 | 1 | 1 | TRINITY\_DN8155\_c0\_g1\_i1.p1 | Carbamidomethylation |  | DB Search |
| YC(+57.02)VGEKVEIQAC(+57.02)STK | 52.87 | 1770.828 | 15 | 1.74 | 591.283 | 13.94 | 0.8339 | 0.8412 | 38817 | 2.77e2 | 1 | 1 | TRINITY\_DN738\_c0\_g1\_i1.p1 | Carbamidomethylation, Carbamidomethylation |  | DB Search |
| DKNILVLTLGVGPQVNR | 52.82 | 1835.0629 | 17 | -4.3 | 612.6909 | 19.59 | 0.8368 | 0.8441 | 74795 | 1.35e3 | 2 | 2 | TRINITY\_DN477\_c0\_g1\_i8.p1:TRINITY\_DN477\_c0\_g1\_i7.p1 |  |  | DB Search |
| FYYHMYGDHIGALSVK | 52.72 | 1899.8977 | 16 | -1 | 634.3045 | 16.96 | 0.917 | 0.9243 | 57688 | 0 | 0 | 0 | TRINITY\_DN4781\_c0\_g1\_i1.p1:TRINITY\_DN4781\_c0\_g1\_i2.p1 |  |  | DB Search |
| VFSIVESDANDQFVIDSNSGALR | 52.69 | 2482.1975 | 23 | 3.57 | 828.4076 | 19.68 | 1.0464 | 1.0536 | 75408 | 5.65e2 | 1 | 1 | TRINITY\_DN982\_c0\_g1\_i1.p1:TRINITY\_DN982\_c0\_g1\_i3.p1 |  |  | DB Search |
| SPDLTSC(+57.02)IPSDC(+57.02)R | 52.65 | 1506.6443 | 13 | -4.7 | 754.3242 | 14.88 | 1.0001 | 1.0073 | 44608 | 0 | 0 | 0 | TRINITY\_DN24268\_c0\_g1\_i2.p1:TRINITY\_DN24268\_c0\_g1\_i1.p1 | Carbamidomethylation, Carbamidomethylation |  | DB Search |
| RIVLLATDALPK | 52.64 | 1308.8129 | 12 | 1.05 | 655.413 | 17.39 | 1.009 | 1.0162 | 60449 | 7.55e2 | 1 | 1 | TRINITY\_DN11377\_c0\_g1\_i1.p1 |  |  | DB Search |
| GHHC(+57.02)IC(+57.02)SAEGFEVTGTK | 52.63 | 1888.8196 | 17 | 2.72 | 630.6141 | 13.51 | 0.8757 | 0.883 | 36216 | 0 | 0 | 0 | TRINITY\_DN28257\_c0\_g1\_i1.p1:TRINITY\_DN28257\_c0\_g1\_i2.p1 | Carbamidomethylation, Carbamidomethylation |  | DB Search |
| YQGDIAVDDVSLSSKK | 52.63 | 1723.8628 | 16 | 2.95 | 862.9393 | 14.98 | 1.1698 | 1.177 | 45228 | 0 | 0 | 0 | TRINITY\_DN1936\_c0\_g2\_i1.p1 |  |  | DB Search |
| LGDLESANQILAM(+15.99)EK | 52.58 | 1646.8185 | 15 | 6.24 | 824.4199 | 17.53 | 1.0552 | 1.0624 | 61340 | 0 | 0 | 0 | TRINITY\_DN1366\_c0\_g1\_i1.p1 | Oxidation (M) | M13:Oxidation (M):1000 | DB Search |
| FQQLSDKNNLALK | 52.57 | 1517.8202 | 13 | 5.73 | 759.92 | 14.50 | 1.0556 | 1.0628 | 42228 | 0 | 0 | 0 | TRINITY\_DN95415\_c0\_g1\_i1.p1 |  |  | DB Search |
| AQYYEAYETIIKR | 52.57 | 1646.8304 | 13 | 5.31 | 549.9525 | 16.99 | 0.8364 | 0.8437 | 57968 | 0 | 0 | 0 | TRINITY\_DN319\_c2\_g1\_i3.p1:TRINITY\_DN319\_c2\_g1\_i1.p1 |  |  | DB Search |
| MSAIVFEDYR | 52.54 | 1229.5751 | 10 | 5.58 | 615.7969 | 18.05 | 0.9158 | 0.923 | 64811 | 0 | 0 | 0 | TRINITY\_DN3078\_c0\_g1\_i1.p1 |  |  | DB Search |
| NHYIFNINEK | 52.53 | 1290.6356 | 10 | 1.67 | 646.3248 | 15.74 | 0.9598 | 0.967 | 50017 | 0 | 0 | 0 | TRINITY\_DN982\_c0\_g1\_i1.p1:TRINITY\_DN982\_c0\_g1\_i3.p1 |  |  | DB Search |
| YQGREYNINEEVNVR | 52.5 | 1881.897 | 15 | 3.38 | 941.9568 | 15.71 | 1.1422 | 1.1494 | 49787 | 0 | 0 | 0 | TRINITY\_DN4595\_c0\_g1\_i4.p1:TRINITY\_DN4595\_c0\_g1\_i10.p2 |  |  | DB Search |
| YGEFSEK | 52.42 | 858.3759 | 7 | 1.16 | 430.1948 | 12.54 | 0.7652 | 0.7726 | 30256 | 6.91e2 | 1 | 1 | TRINITY\_DN738\_c0\_g1\_i4.p1:TRINITY\_DN738\_c0\_g1\_i2.p1:TRINITY\_DN738\_c0\_g1\_i1.p1:TRINITY\_DN738\_c0\_g1\_i3.p1 |  |  | DB Search |
| RGPNGAIGER | 52.38 | 1025.5366 | 10 | 2.78 | 513.7759 | 10.08 | 0.8441 | 0.8514 | 15228 | 2.75e3 | 1 | 1 | TRINITY\_DN113\_c2\_g1\_i1.p1 |  |  | DB Search |
| KNVDC(+57.02)TLK | 52.37 | 976.5012 | 8 | 2.18 | 489.2578 | 10.11 | 0.8343 | 0.8416 | 15536 | 0 | 0 | 0 | TRINITY\_DN2028\_c0\_g1\_i1.p1 | Carbamidomethylation |  | DB Search |
| KVAC(+57.02)IGAWHPAR | 52.34 | 1364.7135 | 12 | 0.83 | 455.9112 | 13.48 | 0.7766 | 0.784 | 36007 | 9.51e1 | 1 | 1 | TRINITY\_DN6920\_c0\_g1\_i1.p1:TRINITY\_DN6920\_c0\_g1\_i3.p1:TRINITY\_DN6920\_c0\_g1\_i4.p1:TRINITY\_DN6920\_c0\_g1\_i2.p1 | Carbamidomethylation |  | DB Search |
| KVIVDQLVTLATR | 52.34 | 1454.8821 | 13 | 1.14 | 485.9674 | 18.50 | 0.7953 | 0.8027 | 67825 | 1.76e2 | 1 | 1 | TRINITY\_DN1386\_c0\_g1\_i1.p1 |  |  | DB Search |
| KDTVIEKFEENITK | 52.33 | 1692.8934 | 14 | -2.27 | 565.3026 | 17.43 | 0.8485 | 0.8558 | 60646 | 1.07e2 | 1 | 1 | TRINITY\_DN4900\_c0\_g1\_i1.p1:TRINITY\_DN4900\_c0\_g1\_i4.p1 |  |  | DB Search |
| LASTLVHLGEYQNAVDGAR | 52.16 | 2013.028 | 19 | 5.59 | 672.0189 | 16.99 | 0.9525 | 0.9598 | 57891 | 0 | 0 | 0 | TRINITY\_DN2113\_c0\_g1\_i1.p1:TRINITY\_DN2113\_c0\_g1\_i2.p1 |  |  | DB Search |
| LC(+57.02)QEDWNKVVR | 52.13 | 1445.7085 | 11 | 6.21 | 482.912 | 14.86 | 0.7909 | 0.7982 | 44521 | 0 | 0 | 0 | TRINITY\_DN48767\_c0\_g1\_i1.p1 | Carbamidomethylation |  | DB Search |
| ILALAGQLQSARDELVKK | 52.09 | 1952.1418 | 18 | -4.21 | 651.717 | 18.01 | 0.9194 | 0.9267 | 64607 | 0 | 0 | 0 | TRINITY\_DN1366\_c0\_g1\_i1.p1 |  |  | DB Search |
| LIFDDVGNIAEK | 51.9 | 1332.6925 | 12 | 4.84 | 667.3553 | 18.51 | 0.9622 | 0.9695 | 67867 | 5.56e2 | 1 | 1 | TRINITY\_DN1795\_c0\_g1\_i4.p1:TRINITY\_DN1795\_c0\_g1\_i1.p1 |  |  | DB Search |
| KLSLDVNGNYQGGGVC(+57.02)K | 51.86 | 1807.8887 | 17 | 0.8 | 603.636 | 14.64 | 0.8193 | 0.8266 | 43107 | 2.56e2 | 1 | 1 | TRINITY\_DN1616\_c0\_g1\_i3.p1:TRINITY\_DN1616\_c0\_g1\_i6.p1:TRINITY\_DN1616\_c0\_g1\_i2.p1:TRINITY\_DN1616\_c0\_g1\_i7.p1 | Carbamidomethylation |  | DB Search |
| LLVQNQDEM(+15.99)IK | 51.85 | 1345.6912 | 11 | -10.5 | 673.8443 | 13.94 | 0.9735 | 0.9808 | 38862 | 0 | 0 | 0 | TRINITY\_DN1559\_c0\_g1\_i1.p1 | Oxidation (M) | M9:Oxidation (M):1000 | DB Search |
| TGDNTVM(+15.99)GR | 51.75 | 965.4236 | 9 | 0.47 | 483.7183 | 9.24 | 0.7803 | 0.7876 | 11341 | 0 | 0 | 0 | TRINITY\_DN262\_c0\_g1\_i1.p1 | Oxidation (M) | M7:Oxidation (M):1000 | DB Search |
| EAAAQIVVEIIKR | 51.74 | 1438.8507 | 13 | 6.13 | 480.6261 | 19.26 | 0.7925 | 0.7998 | 72803 | 0 | 0 | 0 | TRINITY\_DN71276\_c0\_g1\_i1.p1 |  |  | DB Search |
| VC(+57.02)EDVK | 51.73 | 748.3425 | 6 | 8.04 | 375.1807 | 9.73 | 0.7567 | 0.764 | 13508 | 0 | 0 | 0 | TRINITY\_DN118\_c1\_g1\_i3.p1:TRINITY\_DN118\_c1\_g1\_i5.p1 | Carbamidomethylation |  | DB Search |
| IGALC(+57.02)NR | 51.72 | 802.4119 | 7 | 1.46 | 402.213 | 11.84 | 0.7384 | 0.7457 | 25883 | 9.4e2 | 1 | 1 | TRINITY\_DN262\_c0\_g1\_i1.p1 | Carbamidomethylation |  | DB Search |
| KQQENEQLLDEKR | 51.65 | 1656.8431 | 13 | -0.41 | 553.2869 | 11.55 | 0.8254 | 0.8327 | 24229 | 0 | 0 | 0 | TRINITY\_DN1366\_c0\_g1\_i1.p1 |  |  | DB Search |
| VNELLWK | 51.65 | 900.5069 | 7 | 0.77 | 451.2601 | 17.15 | 0.7758 | 0.7831 | 58792 | 3.57e2 | 1 | 1 | TRINITY\_DN3078\_c0\_g1\_i1.p1 |  |  | DB Search |
| NGPFLIVDIDSEK | 51.58 | 1445.7401 | 13 | 7.04 | 723.8809 | 19.95 | 0.9775 | 0.9848 | 77105 | 2.24e2 | 1 | 1 | TRINITY\_DN2577\_c1\_g1\_i1.p1:TRINITY\_DN2577\_c1\_g1\_i2.p1 |  |  | DB Search |
| ALSLQNANEELEDRLAEAER | 51.53 | 2270.1138 | 20 | 4.58 | 757.7137 | 18.93 | 0.9767 | 0.984 | 70669 | 5.99e2 | 1 | 1 | TRINITY\_DN428\_c0\_g1\_i1.p1 |  |  | DB Search |
| DLTASLDGVFQK | 51.39 | 1292.6613 | 12 | 1.56 | 647.3375 | 19.57 | 0.9505 | 0.9578 | 74776 | 1.66e2 | 1 | 1 | TRINITY\_DN1795\_c0\_g1\_i4.p1:TRINITY\_DN1795\_c0\_g1\_i1.p1 |  |  | DB Search |
| DVANLYSPYYPGSK | 51.34 | 1572.746 | 14 | -1.72 | 787.3772 | 17.32 | 1.0242 | 1.0315 | 60052 | 5.66e2 | 1 | 1 | TRINITY\_DN4781\_c0\_g1\_i3.p1:TRINITY\_DN4781\_c0\_g1\_i1.p1:TRINITY\_DN4781\_c0\_g1\_i2.p1 |  |  | DB Search |
| TGISLDSLASSHPISQPVDHPK | 51.15 | 2285.165 | 22 | 18.69 | 572.308 | 16.56 | 0.866 | 0.8733 | 55162 | 0 | 0 | 0 | TRINITY\_DN27058\_c0\_g1\_i1.p1 |  |  | DB Search |
| SC(+57.02)TNPRPLFGGK | 50.98 | 1332.6609 | 12 | 4.64 | 445.2286 | 13.73 | 0.7831 | 0.7905 | 37561 | 4.17e2 | 1 | 1 | TRINITY\_DN1613\_c1\_g1\_i2.p1:TRINITY\_DN1613\_c1\_g1\_i1.p1 | Carbamidomethylation |  | DB Search |
| VNQAC(+57.02)VR | 50.6 | 845.4178 | 7 | 9.12 | 423.7191 | 9.74 | 0.7636 | 0.7709 | 13525 | 1.64e3 | 1 | 1 | TRINITY\_DN160\_c0\_g1\_i2.p1:TRINITY\_DN160\_c0\_g1\_i1.p1 | Carbamidomethylation |  | DB Search |
| NFEIDATLPQK | 50.33 | 1274.6506 | 11 | -8.55 | 638.3257 | 16.85 | 0.957 | 0.9642 | 57115 | 1.62e3 | 1 | 1 | TRINITY\_DN3795\_c0\_g1\_i1.p1 |  |  | DB Search |
| FKADTTIADAAR | 49.7 | 1278.6567 | 12 | -1.01 | 640.3336 | 13.58 | 0.9707 | 0.9779 | 36609 | 0 | 0 | 0 | TRINITY\_DN11435\_c0\_g1\_i1.p1 |  |  | DB Search |
| LNMIFR | 49.11 | 792.4316 | 6 | 5.74 | 397.2245 | 17.36 | 0.7396 | 0.7469 | 60223 | 7.35e2 | 1 | 1 | TRINITY\_DN160\_c0\_g1\_i2.p1:TRINITY\_DN160\_c0\_g1\_i1.p1 |  |  | DB Search |
| ATHQELLNAEFIQK | 48.93 | 1640.8522 | 14 | 6.04 | 821.4365 | 16.16 | 1.0777 | 1.0849 | 52550 | 2.72e2 | 1 | 1 | TRINITY\_DN113\_c3\_g1\_i1.p1 |  |  | DB Search |
| TGEC(+57.02)ISQELAC(+57.02)DQVK | 48.47 | 1736.7709 | 15 | 15.02 | 869.4039 | 15.28 | 1.0837 | 1.0909 | 47116 | 0 | 0 | 0 | TRINITY\_DN2532\_c2\_g1\_i1.p1 | Carbamidomethylation, Carbamidomethylation |  | DB Search |
| VAAFPNFR | 48.17 | 920.4868 | 8 | 3.3 | 461.2512 | 16.95 | 0.7815 | 0.7888 | 57556 | 8.33e2 | 1 | 1 | TRINITY\_DN6304\_c0\_g1\_i1.p1 |  |  | DB Search |
| FTQAGSEVSALLGR | 48.14 | 1434.7467 | 14 | 5.7 | 718.3831 | 18.43 | 0.986 | 0.9932 | 67409 | 0 | 0 | 0 | TRINITY\_DN3434\_c0\_g1\_i10.p1:TRINITY\_DN3434\_c0\_g1\_i1.p1:TRINITY\_DN73587\_c0\_g1\_i1.p1:TRINITY\_DN3434\_c0\_g1\_i5.p1 |  |  | DB Search |
| YALNQIQPVTATVTAFK | 47.96 | 1864.0094 | 17 | 1.71 | 933.0115 | 19.31 | 1.1478 | 1.155 | 73122 | 0 | 0 | 0 | TRINITY\_DN1616\_c0\_g1\_i3.p1:TRINITY\_DN1616\_c0\_g1\_i6.p1:TRINITY\_DN1616\_c0\_g1\_i2.p1:TRINITY\_DN1616\_c0\_g1\_i7.p1 |  |  | DB Search |
| VLSNLQVQLDQAK | 47.86 | 1454.8093 | 13 | 1.55 | 728.4114 | 16.38 | 1.0222 | 1.0295 | 54047 | 0 | 0 | 0 | TRINITY\_DN428\_c0\_g1\_i1.p1 |  |  | DB Search |
| TC(+57.02)IEASC(+57.02)RR | 47.82 | 1151.5176 | 9 | 3.72 | 384.847 | 10.13 | 0.7061 | 0.7135 | 15528 | 5.07e3 | 2 | 2 | TRINITY\_DN319\_c2\_g1\_i3.p1:TRINITY\_DN319\_c2\_g1\_i1.p1:TRINITY\_DN319\_c2\_g1\_i2.p1 | Carbamidomethylation, Carbamidomethylation |  | DB Search |
| FIFGVPENPR | 47.68 | 1174.6134 | 10 | -0.68 | 588.3123 | 18.49 | 0.9295 | 0.9368 | 67817 | 5.2e3 | 1 | 1 | TRINITY\_DN18\_c0\_g1\_i2.p1:TRINITY\_DN18\_c0\_g1\_i1.p1 |  |  | DB Search |
| LFEIKFNAE | 47.56 | 1109.5757 | 9 | 0.53 | 555.7942 | 19.11 | 0.8619 | 0.8692 | 71841 | 3.8e2 | 1 | 1 | TRINITY\_DN495\_c0\_g1\_i2.p1:TRINITY\_DN495\_c0\_g1\_i3.p1 |  |  | DB Search |
| RMSEIAEEARK | 47.21 | 1318.6663 | 11 | 5.81 | 440.5643 | 11.07 | 0.7628 | 0.7701 | 21258 | 4.45e2 | 1 | 1 | TRINITY\_DN1676\_c0\_g1\_i6.p1:TRINITY\_DN1676\_c0\_g1\_i12.p1 |  |  | DB Search |
| DEDHAAR | 46.97 | 812.3412 | 7 | -12.21 | 407.1721 | 9.21 | 0.7807 | 0.788 | 11224 | 1.78e2 | 1 | 1 | TRINITY\_DN124859\_c0\_g1\_i1.p3 |  |  | DB Search |
| SLIQEPLGKPR | 46.63 | 1236.719 | 11 | -0.29 | 619.3652 | 14.50 | 0.9945 | 1.0017 | 42231 | 0 | 0 | 0 | TRINITY\_DN17095\_c0\_g1\_i1.p1 |  |  | DB Search |
| LRPGQATTVDITVK | 45.97 | 1497.8514 | 14 | 0.37 | 749.9316 | 14.22 | 1.0327 | 1.0399 | 40537 | 0 | 0 | 0 | TRINITY\_DN25375\_c0\_g1\_i7.p1 |  |  | DB Search |
| GVGHQC(+57.02)INTIGSFK | 45.41 | 1516.7456 | 14 | 11.11 | 506.5937 | 14.17 | 0.7807 | 0.788 | 40291 | 0 | 0 | 0 | TRINITY\_DN991\_c0\_g1\_i5.p1:TRINITY\_DN991\_c0\_g1\_i2.p1:TRINITY\_DN991\_c0\_g1\_i6.p1 | Carbamidomethylation |  | DB Search |
| LLDQNILDEMNR | 44.8 | 1472.7292 | 12 | 16.14 | 737.3822 | 18.83 | 1.0073 | 1.0146 | 70012 | 0 | 0 | 0 | TRINITY\_DN14729\_c0\_g1\_i1.p1 |  |  | DB Search |
| AFLFDVVSK | 44.57 | 1024.5593 | 9 | -4.12 | 513.2837 | 19.84 | 0.836 | 0.8433 | 76555 | 1.97e2 | 1 | 1 | TRINITY\_DN65215\_c0\_g1\_i1.p1 |  |  | DB Search |
| ILLLGAGESGK | 43.84 | 1056.6179 | 11 | 6.84 | 529.3187 | 17.27 | 0.8761 | 0.8834 | 59698 | 0 | 0 | 0 | TRINITY\_DN191980\_c0\_g1\_i1.p1 |  |  | DB Search |
| LLLLGAGESGK | 43.84 | 1056.6179 | 11 | 6.84 | 529.3187 | 17.27 | 0.8761 | 0.8834 | 59698 | 0 | 0 | 0 | TRINITY\_DN912\_c0\_g1\_i1.p1:TRINITY\_DN912\_c0\_g1\_i2.p1:TRINITY\_DN912\_c0\_g1\_i4.p1:TRINITY\_DN12734\_c0\_g1\_i2.p1:TRINITY\_DN192883\_c0\_g1\_i1.p1:TRINITY\_DN12734\_c0\_g1\_i1.p1:TRINITY\_DN912\_c0\_g1\_i3.p1:TRINITY\_DN12734\_c0\_g1\_i4.p1 |  |  | DB Search |
| GDFLILANSNGK | 42.7 | 1247.651 | 12 | -8.76 | 624.8259 | 18.05 | 0.9501 | 0.9574 | 64900 | 0 | 0 | 0 | TRINITY\_DN991\_c0\_g1\_i5.p1:TRINITY\_DN991\_c0\_g1\_i2.p1:TRINITY\_DN991\_c0\_g1\_i6.p1 |  |  | DB Search |
| KVNQGEKEFLQRPC(+57.02)R | 42.62 | 1887.9738 | 15 | 3.41 | 473.0013 | 12.00 | 0.8128 | 0.8201 | 26878 | 0 | 0 | 0 | TRINITY\_DN4595\_c0\_g1\_i10.p1:TRINITY\_DN4595\_c0\_g1\_i4.p1 | Carbamidomethylation |  | DB Search |
| LARDELFTESVGAR | 42.61 | 1562.8052 | 14 | 1.79 | 521.9421 | 15.78 | 0.8352 | 0.8425 | 50248 | 4.04e2 | 1 | 1 | TRINITY\_DN244\_c0\_g1\_i1.p1 |  |  | DB Search |
| ELHNLEQENQQLK | 42.32 | 1621.806 | 13 | 1.72 | 541.609 | 12.99 | 0.8063 | 0.8136 | 33004 | 0 | 0 | 0 | TRINITY\_DN1366\_c0\_g1\_i1.p1 |  |  | DB Search |
| FFQIKK | 42.21 | 809.4799 | 6 | -0.5 | 405.7462 | 13.84 | 0.7498 | 0.7571 | 38133 | 4.89e2 | 1 | 1 | TRINITY\_DN477\_c0\_g1\_i9.p1 |  |  | DB Search |
| GSTHVIIDVTDVNDNNPK | 41.87 | 1936.949 | 18 | -7.16 | 969.4727 | 15.19 | 1.153 | 1.1602 | 46639 | 0 | 0 | 0 | TRINITY\_DN982\_c0\_g1\_i1.p1:TRINITY\_DN982\_c0\_g1\_i3.p1 |  |  | DB Search |
| SIFAGLPSLDNQNKPK | 41.77 | 1727.9207 | 16 | -1.26 | 576.9788 | 18.14 | 0.8542 | 0.8615 | 65398 | 4.01e2 | 1 | 1 | TRINITY\_DN2356\_c0\_g1\_i1.p1 |  |  | DB Search |
| NLKDDDFDFTR | 41.38 | 1384.6259 | 11 | 14.15 | 693.3285 | 15.93 | 0.9775 | 0.9848 | 51300 | 6.08e2 | 1 | 1 | TRINITY\_DN4781\_c0\_g1\_i3.p1:TRINITY\_DN4781\_c0\_g1\_i1.p1:TRINITY\_DN4781\_c0\_g1\_i2.p1 |  |  | DB Search |
| DYYASEK | 41.11 | 874.3708 | 7 | 18.08 | 438.1996 | 11.74 | 0.8067 | 0.814 | 25358 | 3e3 | 1 | 1 | TRINITY\_DN7674\_c0\_g1\_i2.p1:TRINITY\_DN7674\_c0\_g1\_i1.p1 |  |  | DB Search |
| SSC(+57.02)KPILSVIK | 41 | 1230.7006 | 11 | -0.71 | 616.3558 | 15.33 | 0.9529 | 0.9602 | 47491 | 3.99e2 | 1 | 1 | TRINITY\_DN495\_c0\_g1\_i2.p1:TRINITY\_DN495\_c0\_g1\_i3.p1 | Carbamidomethylation |  | DB Search |
| TTQDAMNEIESSRR | 40.35 | 1636.7474 | 14 | -1.03 | 546.588 | 13.88 | 0.8153 | 0.8226 | 38475 | 7.82e2 | 1 | 1 | TRINITY\_DN1676\_c0\_g1\_i6.p1:TRINITY\_DN1676\_c0\_g1\_i12.p1 |  |  | DB Search |
| TVVTIVSDDEYQHM(+15.99)FDR | 40.35 | 2069.9363 | 17 | 3.13 | 1035.9764 | 16.66 | 1.2491 | 1.2563 | 55691 | 2.94e1 | 1 | 1 | TRINITY\_DN31399\_c0\_g1\_i3.p1:TRINITY\_DN31399\_c0\_g1\_i1.p1:TRINITY\_DN31399\_c0\_g1\_i2.p1 | Oxidation (M) | M14:Oxidation (M):1000 | DB Search |
| IRSNIDEVNVK | 39.98 | 1285.699 | 11 | -7.71 | 643.8504 | 14.93 | 0.9638 | 0.9711 | 44969 | 0 | 0 | 0 | TRINITY\_DN1616\_c0\_g1\_i3.p1:TRINITY\_DN1616\_c0\_g1\_i6.p1:TRINITY\_DN1616\_c0\_g1\_i2.p1:TRINITY\_DN1616\_c0\_g1\_i7.p1 |  |  | DB Search |
| RGEMGVSGADGIR | 39.76 | 1303.6302 | 13 | -4.57 | 652.818 | 12.74 | 0.94 | 0.9473 | 31487 | 0 | 0 | 0 | TRINITY\_DN113\_c0\_g1\_i1.p1 |  |  | DB Search |
| A(+42.01)NPLKDIISR | 39.76 | 1167.6611 | 10 | -4.53 | 584.8339 | 18.80 | 0.9117 | 0.919 | 69656 | 5.36e1 | 1 | 1 | TRINITY\_DN163801\_c0\_g1\_i1.p1 | Acetylation (Protein N-term) | A1:Acetylation (Protein N-term):1000 | DB Search |
| ISYEC(+57.02)DEGYHLR | 39.73 | 1540.6616 | 12 | 1.5 | 514.5608 | 14.27 | 0.7962 | 0.8035 | 40907 | 0 | 0 | 0 | TRINITY\_DN991\_c0\_g1\_i5.p1:TRINITY\_DN991\_c0\_g1\_i2.p1:TRINITY\_DN991\_c0\_g1\_i6.p1 | Carbamidomethylation |  | DB Search |
| LTGGEELIGFKPLIK | 39.32 | 1613.9392 | 15 | 6.16 | 538.9891 | 18.85 | 0.8887 | 0.8959 | 70137 | 3.72e2 | 1 | 1 | TRINITY\_DN1628\_c0\_g1\_i2.p1 |  |  | DB Search |
| SEQDRLESQR | 38.76 | 1246.5902 | 10 | 0.63 | 416.5367 | 10.63 | 0.7249 | 0.7322 | 18636 | 0 | 0 | 0 | TRINITY\_DN1366\_c0\_g1\_i1.p1 |  |  | DB Search |
| YLLNALNR | 38.38 | 975.5502 | 8 | 2.88 | 488.7827 | 16.96 | 0.8461 | 0.8534 | 57693 | 3.06e2 | 1 | 1 | TRINITY\_DN3332\_c0\_g1\_i1.p1 |  |  | DB Search |
| ITLQDVVLHGK | 38.06 | 1221.7081 | 11 | 4.85 | 408.2444 | 17.32 | 0.7167 | 0.7241 | 60002 | 0 | 0 | 0 | TRINITY\_DN4523\_c0\_g1\_i2.p1:TRINITY\_DN4523\_c0\_g1\_i1.p1 |  |  | DB Search |
| KVNMPISIIALNHR | 37.77 | 1604.9185 | 14 | 6.56 | 535.9824 | 17.41 | 0.8441 | 0.8514 | 60598 | 0 | 0 | 0 | TRINITY\_DN2433\_c0\_g1\_i2.p1:TRINITY\_DN2433\_c0\_g1\_i1.p1 |  |  | DB Search |
| VNQGEKEFLQRPC(+57.02)R | 37.68 | 1759.8788 | 14 | 0.76 | 587.6327 | 12.79 | 0.8635 | 0.8708 | 31645 | 8.61e2 | 1 | 1 | TRINITY\_DN4595\_c0\_g1\_i10.p1:TRINITY\_DN4595\_c0\_g1\_i4.p1 | Carbamidomethylation |  | DB Search |
| SFNKVESR | 37.49 | 965.493 | 8 | 3.7 | 483.7545 | 10.29 | 0.8319 | 0.8392 | 16526 | 9.88e2 | 1 | 1 | TRINITY\_DN5397\_c0\_g1\_i1.p1 |  |  | DB Search |
| total 741 peptides |  |  |  |  |  |  |  |  |  |  |  |  |  |  |  |  |
| --- | --- | --- | --- | --- | --- | --- | --- | --- | --- | --- | --- | --- | --- | --- | --- | --- |
