# Supplementary material for: Proteomic Analysis and Biochemical Characterization of the Nematocyst Extract of the Hydrozoan Velella velella
Source: Mar Drugs. 2024 Oct 12;22(10):468. doi: 10.3390/md22100468 (PMC11509761; doi:10.3390/md22100468)
Supplement: Supplementary file 1 [file marinedrugs-22-00468-s001.zip › Supplementary File S1.zip!/Sample_2_Vellela/db.proteins.html]

proteins


Protein List

  

| Protein Group | Protein ID | Accession | -10lgP | Coverage (%) | Coverage (%) Sample 4 | Area Sample 4 | #Peptides | #Unique | #Spec Sample 4 | PTM | Avg. Mass | Description |
| --- | --- | --- | --- | --- | --- | --- | --- | --- | --- | --- | --- | --- |
| 1 | 1 | TRINITY\_DN982\_c0\_g1\_i3.p1 | 229.05 | 3 | 3 | 1.44e4 | 29 | 15 | 37 | Carbamidomethylation | 1090854.75 | type:complete len:9852 gc:universal TRINITY\_DN982\_c0\_g1\_i3:29697-142(-) |
| 2 | 2 | TRINITY\_DN1616\_c0\_g1\_i3.p1 | 226.22 | 8 | 8 | 2.51e4 | 28 | 28 | 36 | Oxidation (M), Carbamidomethylation | 423947.2812 | type:complete len:3855 gc:universal TRINITY\_DN1616\_c0\_g1\_i3:218-11782(+) |
| 2 | 3 | TRINITY\_DN1616\_c0\_g1\_i7.p1 | 226.22 | 8 | 8 | 2.51e4 | 28 | 28 | 36 | Oxidation (M), Carbamidomethylation | 423875.2188 | type:complete len:3855 gc:universal TRINITY\_DN1616\_c0\_g1\_i7:218-11782(+) |
| 2 | 4 | TRINITY\_DN1616\_c0\_g1\_i6.p1 | 226.22 | 8 | 8 | 2.51e4 | 28 | 28 | 36 | Oxidation (M), Carbamidomethylation | 423911.25 | type:complete len:3855 gc:universal TRINITY\_DN1616\_c0\_g1\_i6:218-11782(+) |
| 2 | 5 | TRINITY\_DN1616\_c0\_g1\_i2.p1 | 221.9 | 9 | 9 | 2.33e4 | 26 | 26 | 34 | Oxidation (M), Carbamidomethylation | 374396 | type:3prime\_partial len:3398 gc:universal TRINITY\_DN1616\_c0\_g1\_i2:218-10408(+) |
| 2 | 6 | TRINITY\_DN1616\_c0\_g1\_i4.p1 | 53.15 | 6 | 6 | 0 | 1 | 1 | 1 |  | 37581.3203 | type:internal len:350 gc:universal TRINITY\_DN1616\_c0\_g1\_i4:3-1049(+) |
| 3 | 7 | TRINITY\_DN4781\_c0\_g1\_i2.p1 | 206.96 | 7 | 7 | 3.16e4 | 20 | 20 | 29 | Carbamidomethylation, Oxidation (M) | 378619.8438 | type:5prime\_partial len:3388 gc:universal TRINITY\_DN4781\_c0\_g1\_i2:10182-19(-) |
| 3 | 8 | TRINITY\_DN4781\_c0\_g1\_i1.p1 | 197.79 | 8 | 8 | 3e4 | 17 | 17 | 26 | Carbamidomethylation, Oxidation (M) | 302042.125 | type:5prime\_partial len:2696 gc:universal TRINITY\_DN4781\_c0\_g1\_i1:10504-2417(-) |
| 3 | 9 | TRINITY\_DN4781\_c0\_g1\_i3.p1 | 160.26 | 6 | 6 | 2.07e4 | 9 | 9 | 15 | Carbamidomethylation | 208363.3438 | type:5prime\_partial len:1859 gc:universal TRINITY\_DN4781\_c0\_g1\_i3:8547-2971(-) |
| 3 | 10 | TRINITY\_DN4781\_c0\_g1\_i3.p2 | 104.02 | 4 | 4 | 1.61e3 | 3 | 3 | 3 | Carbamidomethylation | 88479.2891 | type:complete len:799 gc:universal TRINITY\_DN4781\_c0\_g1\_i3:2415-19(-) |
| 3 | 11 | TRINITY\_DN4781\_c0\_g1\_i1.p2 | 85.69 | 3 | 3 | 1.61e3 | 2 | 2 | 2 | Carbamidomethylation | 88826.9375 | type:complete len:799 gc:universal TRINITY\_DN4781\_c0\_g1\_i1:2415-19(-) |
| 3 | 12 | TRINITY\_DN122779\_c0\_g1\_i1.p1 | 58.28 | 6 | 6 | 0 | 1 | 1 | 1 |  | 15102.6719 | type:internal len:141 gc:universal TRINITY\_DN122779\_c0\_g1\_i1:421-2(-) |
| 4 | 13 | TRINITY\_DN262\_c0\_g1\_i1.p1 | 202.48 | 20 | 20 | 3.25e4 | 18 | 18 | 39 | Acetylation (Protein N-term), Oxidation (M), Carbamidomethylation | 114520.6406 | type:complete len:1033 gc:universal TRINITY\_DN262\_c0\_g1\_i1:111-3209(+) |
| 5 | 14 | TRINITY\_DN4595\_c0\_g1\_i4.p1 | 209.47 | 11 | 11 | 3.35e4 | 21 | 20 | 33 | Carbamidomethylation, Oxidation (M) | 183684.4844 | type:5prime\_partial len:1639 gc:universal TRINITY\_DN4595\_c0\_g1\_i4:5857-941(-) |
| 5 | 15 | TRINITY\_DN4595\_c0\_g1\_i10.p1 | 177.17 | 8 | 8 | 2.54e4 | 12 | 12 | 21 | Carbamidomethylation, Oxidation (M) | 133760.0625 | type:complete len:1188 gc:universal TRINITY\_DN4595\_c0\_g1\_i10:4405-842(-) |
| 5 | 16 | TRINITY\_DN4595\_c0\_g1\_i10.p2 | 148.86 | 18 | 18 | 5.21e3 | 7 | 6 | 10 | Carbamidomethylation | 39822.0156 | type:5prime\_partial len:358 gc:universal TRINITY\_DN4595\_c0\_g1\_i10:5369-4296(-) |
| 5 | 17 | TRINITY\_DN4595\_c0\_g1\_i7.p1 | 105.08 | 7 | 7 | 2.69e3 | 3 | 2 | 4 | Carbamidomethylation | 38387.582 | type:internal len:348 gc:universal TRINITY\_DN4595\_c0\_g1\_i7:1042-2(-) |
| 5 | 18 | TRINITY\_DN4595\_c0\_g1\_i1.p1 | 54.97 | 6 | 6 | 2.69e3 | 1 | 1 | 1 |  | 15339.0303 | type:internal len:138 gc:universal TRINITY\_DN4595\_c0\_g1\_i1:412-2(-) |
| 6 | 19 | TRINITY\_DN244\_c0\_g1\_i1.p1 | 210.06 | 8 | 8 | 5.86e4 | 21 | 21 | 49 | Carbamidomethylation | 313594.0938 | type:complete len:3019 gc:universal TRINITY\_DN244\_c0\_g1\_i1:9748-692(-) |
| 7 | 20 | TRINITY\_DN1676\_c0\_g1\_i12.p1 | 204.61 | 13 | 13 | 2.19e4 | 19 | 19 | 27 | Carbamidomethylation, Oxidation (M) | 180975.5156 | type:complete len:1631 gc:universal TRINITY\_DN1676\_c0\_g1\_i12:206-5098(+) |
| 7 | 21 | TRINITY\_DN1676\_c0\_g1\_i6.p1 | 204.61 | 13 | 13 | 2.19e4 | 19 | 19 | 27 | Carbamidomethylation, Oxidation (M) | 180975.5156 | type:complete len:1631 gc:universal TRINITY\_DN1676\_c0\_g1\_i6:206-5098(+) |
| 8 | 22 | TRINITY\_DN6390\_c0\_g1\_i1.p1 | 202.42 | 11 | 11 | 4.14e4 | 18 | 18 | 31 | Carbamidomethylation | 214480.2344 | type:3prime\_partial len:1941 gc:universal TRINITY\_DN6390\_c0\_g1\_i1:5820-1(-) |
| 9 | 23 | TRINITY\_DN15274\_c0\_g1\_i1.p1 | 199.69 | 11 | 11 | 5.52e4 | 17 | 17 | 32 | Carbamidomethylation, Oxidation (M) | 201897.7344 | type:complete len:1822 gc:universal TRINITY\_DN15274\_c0\_g1\_i1:87-5552(+) |
| 10 | 24 | TRINITY\_DN1366\_c0\_g1\_i1.p1 | 194.92 | 4 | 4 | 8.29e3 | 17 | 17 | 20 | Oxidation (M) | 558202.1875 | type:3prime\_partial len:4702 gc:universal TRINITY\_DN1366\_c0\_g1\_i1:236-14338(+) |
| 11 | 25 | TRINITY\_DN982\_c0\_g1\_i1.p1 | 190.02 | 3 | 3 | 4.66e2 | 15 | 1 | 20 | Carbamidomethylation | 712566.0625 | type:5prime\_partial len:6412 gc:universal TRINITY\_DN982\_c0\_g1\_i1:19377-142(-) |
| 11 | 26 | TRINITY\_DN982\_c0\_g1\_i2.p1 | 87.16 | 1 | 1 | 4.66e2 | 2 | 1 | 3 |  | 190367.1406 | type:internal len:1718 gc:universal TRINITY\_DN982\_c0\_g1\_i2:5151-1(-) |
| 12 | 27 | TRINITY\_DN319\_c2\_g1\_i3.p1 | 187.3 | 7 | 7 | 5.33e4 | 14 | 14 | 28 | Carbamidomethylation, Oxidation (M) | 279087.1875 | type:complete len:2588 gc:universal TRINITY\_DN319\_c2\_g1\_i3:165-7928(+) |
| 12 | 28 | TRINITY\_DN319\_c2\_g1\_i1.p1 | 187.3 | 7 | 7 | 5.33e4 | 14 | 14 | 28 | Carbamidomethylation, Oxidation (M) | 279168.25 | type:complete len:2588 gc:universal TRINITY\_DN319\_c2\_g1\_i1:165-7928(+) |
| 12 | 29 | TRINITY\_DN319\_c2\_g1\_i2.p1 | 168.51 | 6 | 6 | 5.27e4 | 10 | 10 | 24 | Carbamidomethylation | 215796.4062 | type:3prime\_partial len:2004 gc:universal TRINITY\_DN319\_c2\_g1\_i2:165-6173(+) |
| 13 | 30 | TRINITY\_DN1369\_c0\_g1\_i1.p1 | 184.75 | 9 | 9 | 1.84e4 | 13 | 13 | 18 | Carbamidomethylation | 174229.4219 | type:3prime\_partial len:1577 gc:universal TRINITY\_DN1369\_c0\_g1\_i1:93-4820(+) |
| 14 | 31 | TRINITY\_DN428\_c0\_g1\_i1.p1 | 176.93 | 8 | 8 | 3.73e3 | 12 | 12 | 12 | Carbamidomethylation | 225824.1719 | type:complete len:1949 gc:universal TRINITY\_DN428\_c0\_g1\_i1:96-5942(+) |
| 15 | 32 | TRINITY\_DN477\_c0\_g1\_i9.p1 | 155.59 | 25 | 25 | 5.8e4 | 8 | 8 | 19 | Oxidation (M), Carbamidomethylation | 34222.9453 | type:complete len:306 gc:universal TRINITY\_DN477\_c0\_g1\_i9:1840-923(-) |
| 16 | 33 | TRINITY\_DN11248\_c0\_g1\_i1.p1 | 147.71 | 5 | 5 | 3.38e3 | 7 | 7 | 8 | Carbamidomethylation, Oxidation (M) | 194116.9531 | type:3prime\_partial len:1700 gc:universal TRINITY\_DN11248\_c0\_g1\_i1:111-5207(+) |
| 16 | 34 | TRINITY\_DN11248\_c0\_g1\_i3.p1 | 147.71 | 5 | 5 | 3.38e3 | 7 | 7 | 8 | Carbamidomethylation, Oxidation (M) | 194116.9531 | type:3prime\_partial len:1700 gc:universal TRINITY\_DN11248\_c0\_g1\_i3:111-5207(+) |
| 17 | 35 | TRINITY\_DN5584\_c0\_g1\_i1.p1 | 154.85 | 8 | 8 | 2.87e3 | 8 | 8 | 9 | Carbamidomethylation | 110660.9297 | type:complete len:961 gc:universal TRINITY\_DN5584\_c0\_g1\_i1:2917-35(-) |
| 17 | 36 | TRINITY\_DN5584\_c0\_g1\_i3.p1 | 154.85 | 8 | 8 | 2.87e3 | 8 | 8 | 9 | Carbamidomethylation | 110603.7422 | type:complete len:962 gc:universal TRINITY\_DN5584\_c0\_g1\_i3:2920-35(-) |
| 17 | 37 | TRINITY\_DN5584\_c0\_g1\_i2.p1 | 85.17 | 7 | 7 | 3.04e2 | 2 | 2 | 2 |  | 36306.3672 | type:complete len:313 gc:universal TRINITY\_DN5584\_c0\_g1\_i2:973-35(-) |
| 18 | 38 | TRINITY\_DN3078\_c0\_g1\_i1.p1 | 155.61 | 6 | 6 | 4.01e3 | 8 | 8 | 13 | Carbamidomethylation | 127689.1797 | type:complete len:1109 gc:universal TRINITY\_DN3078\_c0\_g1\_i1:3628-302(-) |
| 19 | 39 | TRINITY\_DN140702\_c0\_g1\_i1.p1 | 157.7 | 5 | 5 | 6.31e3 | 8 | 8 | 14 |  | 231919.1562 | type:complete len:2036 gc:universal TRINITY\_DN140702\_c0\_g1\_i1:94-6201(+) |
| 20 | 40 | TRINITY\_DN1058\_c0\_g1\_i1.p1 | 149.74 | 3 | 3 | 7.51e3 | 7 | 7 | 13 | Carbamidomethylation | 284466.2812 | type:complete len:2639 gc:universal TRINITY\_DN1058\_c0\_g1\_i1:134-8050(+) |
| 20 | 41 | TRINITY\_DN1058\_c0\_g1\_i3.p1 | 149.74 | 3 | 3 | 7.51e3 | 7 | 7 | 13 | Carbamidomethylation | 277633.5312 | type:complete len:2581 gc:universal TRINITY\_DN1058\_c0\_g1\_i3:134-7876(+) |
| 20 | 42 | TRINITY\_DN1058\_c0\_g1\_i2.p2 | 106.79 | 3 | 3 | 5.31e3 | 3 | 3 | 7 | Carbamidomethylation | 140284.0938 | type:complete len:1300 gc:universal TRINITY\_DN1058\_c0\_g1\_i2:4090-7989(+) |
| 20 | 43 | TRINITY\_DN1058\_c0\_g1\_i2.p1 | 103.1 | 2 | 2 | 2.2e3 | 3 | 3 | 5 | Carbamidomethylation | 147804.3906 | type:complete len:1365 gc:universal TRINITY\_DN1058\_c0\_g1\_i2:134-4228(+) |
| 20 | 44 | TRINITY\_DN1058\_c0\_g1\_i4.p1 | 58.28 | 2 | 2 | 5.04e3 | 1 | 1 | 5 |  | 79375.5703 | type:complete len:723 gc:universal TRINITY\_DN1058\_c0\_g1\_i4:366-2534(+) |
| 21 | 45 | TRINITY\_DN991\_c0\_g1\_i2.p1 | 143.18 | 3 | 3 | 3.86e3 | 7 | 7 | 7 | Carbamidomethylation | 358764.375 | type:complete len:3229 gc:universal TRINITY\_DN991\_c0\_g1\_i2:278-9964(+) |
| 21 | 46 | TRINITY\_DN991\_c0\_g1\_i6.p1 | 143.18 | 3 | 3 | 3.86e3 | 7 | 7 | 7 | Carbamidomethylation | 358765.375 | type:complete len:3229 gc:universal TRINITY\_DN991\_c0\_g1\_i6:278-9964(+) |
| 21 | 47 | TRINITY\_DN991\_c0\_g1\_i5.p1 | 143.18 | 3 | 3 | 3.86e3 | 7 | 7 | 7 | Carbamidomethylation | 358764.375 | type:complete len:3229 gc:universal TRINITY\_DN991\_c0\_g1\_i5:278-9964(+) |
| 22 | 48 | TRINITY\_DN1936\_c0\_g1\_i3.p1 | 150.73 | 9 | 9 | 4.37e3 | 7 | 7 | 10 | Carbamidomethylation, Oxidation (M) | 119975.3906 | type:internal len:1070 gc:universal TRINITY\_DN1936\_c0\_g1\_i3:3208-2(-) |
| 22 | 49 | TRINITY\_DN101355\_c0\_g1\_i1.p1 | 58.37 | 5 | 5 | 2.42e2 | 1 | 1 | 1 |  | 25816.6582 | type:internal len:232 gc:universal TRINITY\_DN101355\_c0\_g1\_i1:693-1(-) |
| 23 | 50 | TRINITY\_DN3034\_c0\_g1\_i1.p1 | 148.89 | 5 | 5 | 2.51e3 | 7 | 7 | 10 |  | 222266.9844 | type:complete len:1941 gc:universal TRINITY\_DN3034\_c0\_g1\_i1:6289-467(-) |
| 23 | 51 | TRINITY\_DN1507\_c0\_g1\_i1.p1 | 84.3 | 4 | 4 | 5.27e2 | 2 | 2 | 2 |  | 72554.2422 | type:internal len:637 gc:universal TRINITY\_DN1507\_c0\_g1\_i1:3-1910(+) |
| 23 | 52 | TRINITY\_DN825\_c0\_g1\_i1.p1 | 53.73 | 2 | 2 | 3.72e2 | 1 | 1 | 1 |  | 65186.4219 | type:5prime\_partial len:580 gc:universal TRINITY\_DN825\_c0\_g1\_i1:1848-109(-) |
| 23 | 53 | TRINITY\_DN76282\_c0\_g1\_i1.p1 | 53.73 | 13 | 13 | 3.72e2 | 1 | 1 | 1 |  | 11860.0615 | type:internal len:105 gc:universal TRINITY\_DN76282\_c0\_g1\_i1:313-2(-) |
| 24 | 54 | TRINITY\_DN1936\_c0\_g2\_i1.p1 | 149.92 | 4 | 4 | 3.98e3 | 7 | 7 | 8 |  | 208525.625 | type:3prime\_partial len:1894 gc:universal TRINITY\_DN1936\_c0\_g2\_i1:5680-2(-) |
| 25 | 55 | TRINITY\_DN161\_c0\_g1\_i2.p1 | 141.95 | 23 | 23 | 2.29e4 | 6 | 6 | 9 | Oxidation (M), Carbamidomethylation | 44577.6484 | type:5prime\_partial len:399 gc:universal TRINITY\_DN161\_c0\_g1\_i2:1683-487(-) |
| 25 | 56 | TRINITY\_DN161\_c0\_g1\_i3.p1 | 141.95 | 23 | 23 | 2.29e4 | 6 | 6 | 9 | Oxidation (M), Carbamidomethylation | 44592.6602 | type:5prime\_partial len:399 gc:universal TRINITY\_DN161\_c0\_g1\_i3:1450-254(-) |
| 25 | 57 | TRINITY\_DN161\_c0\_g1\_i1.p1 | 141.95 | 24 | 24 | 2.29e4 | 6 | 6 | 9 | Oxidation (M), Carbamidomethylation | 41918.5938 | type:complete len:377 gc:universal TRINITY\_DN161\_c0\_g1\_i1:1384-254(-) |
| 25 | 58 | TRINITY\_DN161\_c0\_g1\_i4.p1 | 120.63 | 29 | 29 | 5.61e3 | 4 | 4 | 6 | Oxidation (M), Carbamidomethylation | 25887.2988 | type:5prime\_partial len:232 gc:universal TRINITY\_DN161\_c0\_g1\_i4:949-254(-) |
| 25 | 59 | TRINITY\_DN2477\_c0\_g1\_i10.p1 | 57.28 | 3 | 3 | 1.16e3 | 1 | 1 | 1 |  | 38566.9688 | type:5prime\_partial len:344 gc:universal TRINITY\_DN2477\_c0\_g1\_i10:1215-184(-) |
| 25 | 60 | TRINITY\_DN195934\_c0\_g1\_i1.p2 | 57.28 | 10 | 10 | 1.16e3 | 1 | 1 | 1 |  | 14094.0938 | type:internal len:127 gc:universal TRINITY\_DN195934\_c0\_g1\_i1:3-380(+) |
| 25 | 61 | TRINITY\_DN2477\_c0\_g1\_i5.p1 | 57.28 | 4 | 4 | 1.16e3 | 1 | 1 | 1 |  | 37416.5195 | type:complete len:333 gc:universal TRINITY\_DN2477\_c0\_g1\_i5:1182-184(-) |
| 25 | 62 | TRINITY\_DN2477\_c0\_g1\_i2.p1 | 57.28 | 3 | 3 | 1.16e3 | 1 | 1 | 1 |  | 38566.9688 | type:5prime\_partial len:344 gc:universal TRINITY\_DN2477\_c0\_g1\_i2:1215-184(-) |
| 25 | 63 | TRINITY\_DN2477\_c0\_g1\_i6.p1 | 57.28 | 3 | 3 | 1.16e3 | 1 | 1 | 1 |  | 41878.6875 | type:complete len:377 gc:universal TRINITY\_DN2477\_c0\_g1\_i6:1314-184(-) |
| 25 | 64 | TRINITY\_DN2477\_c0\_g1\_i9.p1 | 57.28 | 3 | 3 | 1.16e3 | 1 | 1 | 1 |  | 41878.6875 | type:complete len:377 gc:universal TRINITY\_DN2477\_c0\_g1\_i9:1314-184(-) |
| 26 | 65 | TRINITY\_DN691\_c0\_g1\_i1.p1 | 148.15 | 4 | 4 | 1.02e4 | 7 | 7 | 10 | Carbamidomethylation | 169920.1562 | type:complete len:1726 gc:universal TRINITY\_DN691\_c0\_g1\_i1:5662-485(-) |
| 26 | 66 | TRINITY\_DN691\_c0\_g1\_i2.p1 | 139.32 | 4 | 4 | 6.98e3 | 6 | 6 | 9 | Carbamidomethylation | 164625.2188 | type:complete len:1673 gc:universal TRINITY\_DN691\_c0\_g1\_i2:5503-485(-) |
| 27 | 67 | TRINITY\_DN24268\_c0\_g1\_i1.p1 | 140.84 | 3 | 3 | 3.92e3 | 6 | 5 | 8 | Carbamidomethylation, Oxidation (M) | 232931.6406 | type:3prime\_partial len:2183 gc:universal TRINITY\_DN24268\_c0\_g1\_i1:122-6667(+) |
| 27 | 68 | TRINITY\_DN24268\_c0\_g1\_i2.p1 | 140.84 | 3 | 3 | 3.92e3 | 6 | 5 | 8 | Carbamidomethylation, Oxidation (M) | 232903.625 | type:3prime\_partial len:2183 gc:universal TRINITY\_DN24268\_c0\_g1\_i2:122-6667(+) |
| 28 | 69 | TRINITY\_DN2577\_c1\_g1\_i2.p1 | 148.81 | 7 | 7 | 5.32e3 | 7 | 6 | 8 | Carbamidomethylation | 121869.7266 | type:5prime\_partial len:1087 gc:universal TRINITY\_DN2577\_c1\_g1\_i2:3532-272(-) |
| 28 | 70 | TRINITY\_DN2577\_c1\_g1\_i1.p1 | 148.81 | 9 | 9 | 5.32e3 | 7 | 6 | 8 | Carbamidomethylation | 105277.2734 | type:complete len:937 gc:universal TRINITY\_DN2577\_c1\_g1\_i1:3082-272(-) |
| 29 | 71 | TRINITY\_DN77\_c1\_g1\_i1.p1 | 142.4 | 3 | 3 | 6.43e3 | 6 | 6 | 7 |  | 198350.2344 | type:3prime\_partial len:1965 gc:universal TRINITY\_DN77\_c1\_g1\_i1:2-5893(+) |
| 30 | 72 | TRINITY\_DN28257\_c0\_g1\_i1.p1 | 139.48 | 5 | 5 | 3.47e3 | 6 | 6 | 6 | Carbamidomethylation | 155819.5469 | type:3prime\_partial len:1462 gc:universal TRINITY\_DN28257\_c0\_g1\_i1:4384-2(-) |
| 30 | 73 | TRINITY\_DN28257\_c0\_g1\_i2.p1 | 139.48 | 5 | 5 | 3.47e3 | 6 | 6 | 6 | Carbamidomethylation | 155434.1719 | type:3prime\_partial len:1458 gc:universal TRINITY\_DN28257\_c0\_g1\_i2:4372-2(-) |
| 31 | 74 | TRINITY\_DN4815\_c0\_g1\_i2.p1 | 142.25 | 9 | 9 | 2.26e4 | 6 | 6 | 11 | Carbamidomethylation | 70695.4688 | type:complete len:638 gc:universal TRINITY\_DN4815\_c0\_g1\_i2:79-1992(+) |
| 31 | 75 | TRINITY\_DN4815\_c0\_g1\_i1.p1 | 142.25 | 9 | 9 | 2.26e4 | 6 | 6 | 11 | Carbamidomethylation | 66133.5156 | type:complete len:595 gc:universal TRINITY\_DN4815\_c0\_g1\_i1:79-1863(+) |
| 32 | 76 | TRINITY\_DN3795\_c0\_g1\_i1.p1 | 118.06 | 5 | 5 | 4.56e3 | 4 | 3 | 6 | Carbamidomethylation, Oxidation (M) | 97717.9844 | type:5prime\_partial len:867 gc:universal TRINITY\_DN3795\_c0\_g1\_i1:2719-119(-) |
| 33 | 77 | TRINITY\_DN8903\_c0\_g1\_i1.p1 | 132.89 | 11 | 11 | 6.82e3 | 5 | 4 | 6 | Carbamidomethylation, Oxidation (M) | 50029.5586 | type:5prime\_partial len:447 gc:universal TRINITY\_DN8903\_c0\_g1\_i1:1546-206(-) |
| 34 | 78 | TRINITY\_DN160\_c0\_g1\_i1.p1 | 126.35 | 4 | 4 | 4.34e3 | 5 | 5 | 6 | Carbamidomethylation | 126611.5703 | type:complete len:1145 gc:universal TRINITY\_DN160\_c0\_g1\_i1:4810-1376(-) |
| 34 | 79 | TRINITY\_DN160\_c0\_g1\_i2.p1 | 126.35 | 4 | 4 | 4.34e3 | 5 | 5 | 6 | Carbamidomethylation | 126611.5703 | type:complete len:1145 gc:universal TRINITY\_DN160\_c0\_g1\_i2:4810-1376(-) |
| 35 | 80 | TRINITY\_DN77\_c0\_g2\_i3.p1 | 118.85 | 3 | 3 | 2.12e4 | 4 | 4 | 12 | Oxidation (M) | 133966.4375 | type:internal len:1381 gc:universal TRINITY\_DN77\_c0\_g2\_i3:4141-2(-) |
| 35 | 81 | TRINITY\_DN77\_c0\_g2\_i1.p1 | 118.85 | 3 | 3 | 2.12e4 | 4 | 4 | 12 | Oxidation (M) | 133184.5781 | type:internal len:1374 gc:universal TRINITY\_DN77\_c0\_g2\_i1:4120-2(-) |
| 35 | 82 | TRINITY\_DN77\_c0\_g2\_i4.p1 | 118.85 | 3 | 3 | 2.12e4 | 4 | 4 | 12 | Oxidation (M) | 133952.4219 | type:internal len:1381 gc:universal TRINITY\_DN77\_c0\_g2\_i4:4141-2(-) |
| 35 | 83 | TRINITY\_DN77\_c0\_g2\_i2.p1 | 118.85 | 3 | 3 | 2.12e4 | 4 | 4 | 12 | Oxidation (M) | 133198.6094 | type:internal len:1374 gc:universal TRINITY\_DN77\_c0\_g2\_i2:4120-2(-) |
| 36 | 84 | TRINITY\_DN1795\_c0\_g1\_i1.p1 | 128.1 | 2 | 2 | 1.41e3 | 5 | 5 | 6 |  | 357567.5938 | type:5prime\_partial len:3198 gc:universal TRINITY\_DN1795\_c0\_g1\_i1:9621-28(-) |
| 36 | 85 | TRINITY\_DN1795\_c0\_g1\_i4.p1 | 128.1 | 2 | 2 | 1.41e3 | 5 | 5 | 6 |  | 357567.5938 | type:5prime\_partial len:3198 gc:universal TRINITY\_DN1795\_c0\_g1\_i4:9621-28(-) |
| 37 | 86 | TRINITY\_DN61668\_c0\_g1\_i1.p1 | 132.33 | 9 | 9 | 3.22e3 | 5 | 5 | 8 |  | 51212.582 | type:5prime\_partial len:453 gc:universal TRINITY\_DN61668\_c0\_g1\_i1:2-1360(+) |
| 38 | 87 | TRINITY\_DN477\_c0\_g1\_i7.p1 | 131.57 | 5 | 5 | 5.99e4 | 5 | 5 | 11 | Carbamidomethylation | 122478.2578 | type:3prime\_partial len:1130 gc:universal TRINITY\_DN477\_c0\_g1\_i7:3387-1(-) |
| 38 | 88 | TRINITY\_DN477\_c0\_g1\_i8.p1 | 119.65 | 5 | 5 | 1.11e4 | 4 | 4 | 8 | Carbamidomethylation | 107165.2031 | type:complete len:985 gc:universal TRINITY\_DN477\_c0\_g1\_i8:3548-594(-) |
| 38 | 89 | TRINITY\_DN477\_c0\_g1\_i8.p2 | 57.97 | 5 | 5 | 4.89e4 | 1 | 1 | 3 |  | 20774.9902 | type:3prime\_partial len:194 gc:universal TRINITY\_DN477\_c0\_g1\_i8:581-3(-) |
| 38 | 90 | TRINITY\_DN477\_c0\_g1\_i6.p1 | 57.97 | 5 | 5 | 4.89e4 | 1 | 1 | 3 |  | 20745.9492 | type:3prime\_partial len:194 gc:universal TRINITY\_DN477\_c0\_g1\_i6:581-3(-) |
| 38 | 91 | TRINITY\_DN477\_c0\_g1\_i3.p1 | 57.97 | 4 | 4 | 4.89e4 | 1 | 1 | 3 |  | 24580.2559 | type:internal len:228 gc:universal TRINITY\_DN477\_c0\_g1\_i3:681-1(-) |
| 38 | 92 | TRINITY\_DN477\_c0\_g1\_i5.p1 | 57.97 | 4 | 4 | 4.89e4 | 1 | 1 | 3 |  | 28860.875 | type:internal len:276 gc:universal TRINITY\_DN477\_c0\_g1\_i5:827-3(-) |
| 39 | 93 | TRINITY\_DN554\_c0\_g1\_i4.p1 | 118.03 | 14 | 14 | 2.18e4 | 4 | 4 | 10 |  | 35966.3984 | type:3prime\_partial len:319 gc:universal TRINITY\_DN554\_c0\_g1\_i4:956-3(-) |
| 39 | 94 | TRINITY\_DN585\_c0\_g1\_i8.p1 | 118.03 | 13 | 13 | 2.18e4 | 4 | 4 | 10 |  | 37858.4844 | type:3prime\_partial len:337 gc:universal TRINITY\_DN585\_c0\_g1\_i8:1008-1(-) |
| 39 | 95 | TRINITY\_DN585\_c0\_g1\_i10.p1 | 118.03 | 19 | 19 | 2.18e4 | 4 | 4 | 10 |  | 26563.5332 | type:3prime\_partial len:236 gc:universal TRINITY\_DN585\_c0\_g1\_i10:705-1(-) |
| 39 | 96 | TRINITY\_DN554\_c0\_g1\_i3.p2 | 118.03 | 16 | 16 | 2.18e4 | 4 | 4 | 10 |  | 30419.4961 | type:complete len:268 gc:universal TRINITY\_DN554\_c0\_g1\_i3:1464-661(-) |
| 39 | 97 | TRINITY\_DN101\_c0\_g1\_i1.p1 | 118.03 | 34 | 34 | 2.18e4 | 4 | 4 | 10 |  | 14847.2393 | type:complete len:129 gc:universal TRINITY\_DN101\_c0\_g1\_i1:416-30(-) |
| 39 | 98 | TRINITY\_DN585\_c0\_g1\_i4.p1 | 118.03 | 19 | 19 | 2.18e4 | 4 | 4 | 10 |  | 26348.0742 | type:complete len:232 gc:universal TRINITY\_DN585\_c0\_g1\_i4:847-152(-) |
| 39 | 99 | TRINITY\_DN554\_c0\_g1\_i5.p1 | 118.03 | 12 | 12 | 2.18e4 | 4 | 4 | 10 |  | 42081.4141 | type:3prime\_partial len:373 gc:universal TRINITY\_DN554\_c0\_g1\_i5:1118-3(-) |
| 39 | 100 | TRINITY\_DN173718\_c0\_g1\_i1.p1 | 104.39 | 25 | 25 | 7e3 | 3 | 3 | 5 |  | 12865.0928 | type:3prime\_partial len:112 gc:universal TRINITY\_DN173718\_c0\_g1\_i1:37-369(+) |
| 39 | 101 | TRINITY\_DN585\_c0\_g2\_i1.p1 | 102.96 | 25 | 25 | 2.16e4 | 3 | 3 | 9 |  | 17391.9824 | type:5prime\_partial len:152 gc:universal TRINITY\_DN585\_c0\_g2\_i1:598-143(-) |
| 39 | 102 | TRINITY\_DN554\_c0\_g1\_i2.p1 | 102.96 | 14 | 14 | 2.16e4 | 3 | 3 | 9 |  | 29982.375 | type:internal len:266 gc:universal TRINITY\_DN554\_c0\_g1\_i2:797-3(-) |
| 39 | 103 | TRINITY\_DN585\_c0\_g1\_i2.p1 | 102.96 | 21 | 21 | 2.16e4 | 3 | 3 | 9 |  | 20558.6738 | type:internal len:184 gc:universal TRINITY\_DN585\_c0\_g1\_i2:549-1(-) |
| 39 | 104 | TRINITY\_DN585\_c0\_g1\_i5.p1 | 102.96 | 15 | 15 | 2.16e4 | 3 | 3 | 9 |  | 28098.1602 | type:internal len:250 gc:universal TRINITY\_DN585\_c0\_g1\_i5:747-1(-) |
| 39 | 105 | TRINITY\_DN585\_c0\_g1\_i3.p1 | 102.96 | 24 | 24 | 2.16e4 | 3 | 3 | 9 |  | 17628.2207 | type:internal len:157 gc:universal TRINITY\_DN585\_c0\_g1\_i3:468-1(-) |
| 39 | 106 | TRINITY\_DN40728\_c1\_g1\_i1.p2 | 84.78 | 18 | 18 | 6.75e3 | 2 | 2 | 4 |  | 14073.3066 | type:5prime\_partial len:124 gc:universal TRINITY\_DN40728\_c1\_g1\_i1:1-372(+) |
| 39 | 107 | TRINITY\_DN40728\_c0\_g1\_i1.p1 | 84.78 | 21 | 21 | 6.75e3 | 2 | 2 | 4 |  | 11225.8301 | type:internal len:105 gc:universal TRINITY\_DN40728\_c0\_g1\_i1:1-312(+) |
| 39 | 108 | TRINITY\_DN8227\_c0\_g1\_i1.p1 | 84.78 | 16 | 16 | 6.75e3 | 2 | 2 | 4 |  | 14513.8135 | type:internal len:135 gc:universal TRINITY\_DN8227\_c0\_g1\_i1:404-3(-) |
| 39 | 109 | TRINITY\_DN152\_c0\_g1\_i15.p1 | 84.78 | 16 | 16 | 6.75e3 | 2 | 2 | 4 |  | 15560.71 | type:internal len:141 gc:universal TRINITY\_DN152\_c0\_g1\_i15:2-421(+) |
| 39 | 110 | TRINITY\_DN152\_c0\_g1\_i2.p1 | 84.78 | 21 | 21 | 6.75e3 | 2 | 2 | 4 |  | 11754.375 | type:internal len:105 gc:universal TRINITY\_DN152\_c0\_g1\_i2:1-312(+) |
| 39 | 111 | TRINITY\_DN152\_c0\_g1\_i10.p1 | 84.78 | 12 | 12 | 6.75e3 | 2 | 2 | 4 |  | 20063.498 | type:internal len:180 gc:universal TRINITY\_DN152\_c0\_g1\_i10:1-537(+) |
| 39 | 112 | TRINITY\_DN1465\_c1\_g1\_i1.p1 | 84.78 | 22 | 22 | 6.75e3 | 2 | 2 | 4 |  | 11342.1572 | type:internal len:102 gc:universal TRINITY\_DN1465\_c1\_g1\_i1:2-304(+) |
| 39 | 113 | TRINITY\_DN152\_c0\_g1\_i9.p1 | 84.78 | 14 | 14 | 6.75e3 | 2 | 2 | 4 |  | 17213.1973 | type:5prime\_partial len:152 gc:universal TRINITY\_DN152\_c0\_g1\_i9:1-456(+) |
| 39 | 114 | TRINITY\_DN1465\_c0\_g1\_i1.p2 | 84.78 | 14 | 14 | 6.75e3 | 2 | 2 | 4 |  | 17271.9648 | type:5prime\_partial len:156 gc:universal TRINITY\_DN1465\_c0\_g1\_i1:498-31(-) |
| 39 | 115 | TRINITY\_DN10545\_c0\_g1\_i1.p3 | 57.27 | 6 | 6 | 4.97e3 | 1 | 1 | 2 |  | 17872.502 | type:5prime\_partial len:162 gc:universal TRINITY\_DN10545\_c0\_g1\_i1:3-488(+) |
| 39 | 116 | TRINITY\_DN1580\_c0\_g1\_i1.p1 | 57.27 | 5 | 5 | 4.97e3 | 1 | 1 | 2 |  | 19628.5645 | type:5prime\_partial len:178 gc:universal TRINITY\_DN1580\_c0\_g1\_i1:1-534(+) |
| 39 | 117 | TRINITY\_DN1580\_c0\_g1\_i3.p1 | 57.27 | 5 | 5 | 4.97e3 | 1 | 1 | 2 |  | 19665.5898 | type:5prime\_partial len:179 gc:universal TRINITY\_DN1580\_c0\_g1\_i3:1-537(+) |
| 39 | 118 | TRINITY\_DN110760\_c0\_g1\_i1.p1 | 57.27 | 9 | 9 | 4.97e3 | 1 | 1 | 2 |  | 11456.2695 | type:internal len:104 gc:universal TRINITY\_DN110760\_c0\_g1\_i1:2-310(+) |
| 39 | 119 | TRINITY\_DN103165\_c0\_g1\_i1.p1 | 57.27 | 4 | 4 | 4.97e3 | 1 | 1 | 2 |  | 26936.2734 | type:internal len:242 gc:universal TRINITY\_DN103165\_c0\_g1\_i1:723-1(-) |
| 39 | 120 | TRINITY\_DN195631\_c0\_g1\_i1.p2 | 55.01 | 9 | 9 | 1.78e3 | 1 | 1 | 2 |  | 15844.8984 | type:internal len:145 gc:universal TRINITY\_DN195631\_c0\_g1\_i1:2-433(+) |
| 39 | 121 | TRINITY\_DN152\_c0\_g1\_i14.p2 | 55.01 | 11 | 11 | 1.78e3 | 1 | 1 | 2 |  | 12547.4834 | type:5prime\_partial len:115 gc:universal TRINITY\_DN152\_c0\_g1\_i14:3-347(+) |
| 40 | 122 | TRINITY\_DN4494\_c1\_g1\_i7.p1 | 120.41 | 13 | 13 | 4.96e2 | 4 | 3 | 4 |  | 57635.2422 | type:internal len:536 gc:universal TRINITY\_DN4494\_c1\_g1\_i7:1607-3(-) |
| 40 | 123 | TRINITY\_DN4494\_c1\_g1\_i1.p1 | 105.49 | 13 | 13 | 4.96e2 | 3 | 2 | 3 |  | 43468.3906 | type:internal len:400 gc:universal TRINITY\_DN4494\_c1\_g1\_i1:1197-1(-) |
| 40 | 124 | TRINITY\_DN4494\_c1\_g1\_i10.p1 | 87.41 | 8 | 8 | 0 | 2 | 1 | 2 |  | 50408.1719 | type:5prime\_partial len:462 gc:universal TRINITY\_DN4494\_c1\_g1\_i10:2120-735(-) |
| 40 | 125 | TRINITY\_DN4494\_c1\_g1\_i2.p1 | 87.41 | 7 | 7 | 0 | 2 | 1 | 2 |  | 50327.457 | type:internal len:469 gc:universal TRINITY\_DN4494\_c1\_g1\_i2:1406-3(-) |
| 40 | 126 | TRINITY\_DN4494\_c1\_g1\_i8.p1 | 87.41 | 9 | 9 | 0 | 2 | 1 | 2 |  | 41552.7188 | type:internal len:390 gc:universal TRINITY\_DN4494\_c1\_g1\_i8:1169-3(-) |
| 40 | 127 | TRINITY\_DN4494\_c1\_g1\_i5.p1 | 87.37 | 7 | 7 | 4.96e2 | 2 | 1 | 2 |  | 47190.5742 | type:internal len:439 gc:universal TRINITY\_DN4494\_c1\_g1\_i5:1316-3(-) |
| 41 | 128 | TRINITY\_DN74720\_c0\_g1\_i1.p1 | 118.31 | 32 | 32 | 2.25e3 | 4 | 4 | 5 |  | 14079.2627 | type:internal len:124 gc:universal TRINITY\_DN74720\_c0\_g1\_i1:3-371(+) |
| 42 | 129 | TRINITY\_DN113\_c0\_g1\_i1.p1 | 115.28 | 3 | 3 | 1.18e4 | 4 | 4 | 7 |  | 139235.5938 | type:complete len:1417 gc:universal TRINITY\_DN113\_c0\_g1\_i1:4413-163(-) |
| 43 | 130 | TRINITY\_DN25375\_c0\_g1\_i7.p1 | 117.43 | 6 | 6 | 9.04e2 | 4 | 4 | 5 | Carbamidomethylation | 90646.6094 | type:complete len:811 gc:universal TRINITY\_DN25375\_c0\_g1\_i7:112-2544(+) |
| 44 | 131 | TRINITY\_DN199\_c0\_g1\_i1.p1 | 119.04 | 9 | 9 | 4.58e3 | 4 | 4 | 4 |  | 25170.3418 | type:complete len:229 gc:universal TRINITY\_DN199\_c0\_g1\_i1:207-893(+) |
| 45 | 132 | TRINITY\_DN1043\_c0\_g1\_i2.p1 | 118.7 | 8 | 8 | 3.81e3 | 4 | 4 | 5 | Carbamidomethylation | 78392.1016 | type:complete len:700 gc:universal TRINITY\_DN1043\_c0\_g1\_i2:2410-311(-) |
| 45 | 133 | TRINITY\_DN7059\_c0\_g1\_i1.p1 | 58.4 | 7 | 7 | 6.52e2 | 1 | 1 | 2 |  | 22777.5508 | type:internal len:203 gc:universal TRINITY\_DN7059\_c0\_g1\_i1:1-606(+) |
| 46 | 134 | TRINITY\_DN1910\_c0\_g1\_i1.p1 | 105.23 | 7 | 7 | 1.61e3 | 3 | 3 | 5 | Oxidation (M) | 50135.0117 | type:complete len:447 gc:universal TRINITY\_DN1910\_c0\_g1\_i1:2183-843(-) |
| 46 | 135 | TRINITY\_DN3986\_c0\_g1\_i1.p1 | 105.23 | 7 | 7 | 1.61e3 | 3 | 3 | 5 | Oxidation (M) | 50011.8867 | type:complete len:446 gc:universal TRINITY\_DN3986\_c0\_g1\_i1:252-1589(+) |
| 46 | 136 | TRINITY\_DN1910\_c0\_g1\_i5.p1 | 87.39 | 5 | 5 | 1.61e3 | 2 | 2 | 4 | Oxidation (M) | 50120.9062 | type:complete len:447 gc:universal TRINITY\_DN1910\_c0\_g1\_i5:2031-691(-) |
| 46 | 137 | TRINITY\_DN5365\_c0\_g1\_i2.p1 | 57.99 | 2 | 2 | 0 | 1 | 1 | 1 |  | 51878.4453 | type:complete len:458 gc:universal TRINITY\_DN5365\_c0\_g1\_i2:1448-75(-) |
| 46 | 138 | TRINITY\_DN3986\_c0\_g1\_i2.p1 | 57.99 | 2 | 2 | 0 | 1 | 1 | 1 |  | 50284.332 | type:complete len:446 gc:universal TRINITY\_DN3986\_c0\_g1\_i2:47-1384(+) |
| 46 | 139 | TRINITY\_DN5365\_c0\_g1\_i3.p1 | 57.99 | 2 | 2 | 0 | 1 | 1 | 1 |  | 51878.4453 | type:complete len:458 gc:universal TRINITY\_DN5365\_c0\_g1\_i3:1448-75(-) |
| 47 | 140 | TRINITY\_DN761\_c0\_g1\_i3.p1 | 120.98 | 5 | 5 | 3.6e3 | 4 | 4 | 6 | Carbamidomethylation | 96968.6562 | type:complete len:845 gc:universal TRINITY\_DN761\_c0\_g1\_i3:129-2663(+) |
| 47 | 141 | TRINITY\_DN761\_c0\_g1\_i1.p1 | 106.85 | 4 | 4 | 2.42e3 | 3 | 3 | 4 | Carbamidomethylation | 95330.7812 | type:complete len:830 gc:universal TRINITY\_DN761\_c0\_g1\_i1:129-2618(+) |
| 47 | 142 | TRINITY\_DN761\_c0\_g1\_i2.p1 | 106.85 | 4 | 4 | 2.42e3 | 3 | 3 | 4 | Carbamidomethylation | 95330.7812 | type:complete len:830 gc:universal TRINITY\_DN761\_c0\_g1\_i2:87-2576(+) |
| 48 | 143 | TRINITY\_DN71276\_c0\_g1\_i1.p1 | 115.48 | 4 | 4 | 7.81e2 | 4 | 4 | 6 | Carbamidomethylation | 139103.3125 | type:5prime\_partial len:1201 gc:universal TRINITY\_DN71276\_c0\_g1\_i1:1-3603(+) |
| 49 | 144 | TRINITY\_DN28022\_c0\_g2\_i1.p1 | 118.78 | 5 | 5 | 2.13e3 | 4 | 4 | 5 | Carbamidomethylation | 103242.3281 | type:internal len:915 gc:universal TRINITY\_DN28022\_c0\_g2\_i1:1-2742(+) |
| 50 | 145 | TRINITY\_DN1559\_c0\_g1\_i1.p1 | 116.73 | 11 | 11 | 3.78e3 | 4 | 4 | 5 | Oxidation (M) | 33672.0977 | type:complete len:311 gc:universal TRINITY\_DN1559\_c0\_g1\_i1:221-1153(+) |
| 51 | 146 | TRINITY\_DN64\_c0\_g1\_i1.p1 | 119.49 | 9 | 9 | 2.94e3 | 4 | 4 | 6 |  | 51450.1914 | type:complete len:468 gc:universal TRINITY\_DN64\_c0\_g1\_i1:1555-152(-) |
| 52 | 147 | TRINITY\_DN1936\_c0\_g1\_i4.p1 | 118.25 | 2 | 2 | 8.19e3 | 4 | 4 | 8 |  | 196066.9688 | type:3prime\_partial len:1762 gc:universal TRINITY\_DN1936\_c0\_g1\_i4:5283-1(-) |
| 52 | 148 | TRINITY\_DN1936\_c0\_g1\_i5.p1 | 103.47 | 5 | 5 | 7.01e3 | 3 | 3 | 5 |  | 90361.6875 | type:complete len:819 gc:universal TRINITY\_DN1936\_c0\_g1\_i5:2692-236(-) |
| 52 | 149 | TRINITY\_DN1936\_c0\_g1\_i1.p1 | 57 | 0 | 0 | 1.18e3 | 1 | 1 | 3 |  | 136661.0312 | type:3prime\_partial len:1224 gc:universal TRINITY\_DN1936\_c0\_g1\_i1:3669-1(-) |
| 53 | 150 | TRINITY\_DN2720\_c0\_g1\_i6.p1 | 120.1 | 2 | 2 | 1.74e3 | 4 | 4 | 6 | Carbamidomethylation | 293288 | type:5prime\_partial len:2710 gc:universal TRINITY\_DN2720\_c0\_g1\_i6:2-8131(+) |
| 53 | 151 | TRINITY\_DN2720\_c0\_g1\_i2.p1 | 104.78 | 2 | 2 | 1.74e3 | 3 | 3 | 5 | Carbamidomethylation | 249953.75 | type:complete len:2312 gc:universal TRINITY\_DN2720\_c0\_g1\_i2:19-6954(+) |
| 54 | 152 | TRINITY\_DN138\_c0\_g1\_i1.p1 | 119.32 | 11 | 11 | 5.42e3 | 4 | 4 | 5 | Carbamidomethylation | 46324.8945 | type:complete len:401 gc:universal TRINITY\_DN138\_c0\_g1\_i1:157-1359(+) |
| 55 | 153 | TRINITY\_DN113\_c3\_g1\_i1.p1 | 116.04 | 4 | 4 | 1.34e4 | 4 | 4 | 7 |  | 125422.4922 | type:3prime\_partial len:1162 gc:universal TRINITY\_DN113\_c3\_g1\_i1:3485-3(-) |
| 56 | 154 | TRINITY\_DN7946\_c0\_g1\_i1.p1 | 104.28 | 4 | 4 | 2.89e3 | 3 | 3 | 8 |  | 114914.6172 | type:complete len:995 gc:universal TRINITY\_DN7946\_c0\_g1\_i1:83-3067(+) |
| 57 | 155 | TRINITY\_DN661\_c0\_g1\_i10.p1 | 106.89 | 13 | 13 | 1.83e4 | 3 | 3 | 8 | Carbamidomethylation, Oxidation (M) | 28014.7773 | type:complete len:253 gc:universal TRINITY\_DN661\_c0\_g1\_i10:1712-2470(+) |
| 58 | 156 | TRINITY\_DN118\_c1\_g1\_i5.p1 | 103.99 | 8 | 8 | 8.28e3 | 3 | 3 | 6 | Carbamidomethylation | 23277.6309 | type:5prime\_partial len:203 gc:universal TRINITY\_DN118\_c1\_g1\_i5:620-12(-) |
| 58 | 157 | TRINITY\_DN118\_c1\_g1\_i3.p1 | 103.99 | 8 | 8 | 8.28e3 | 3 | 3 | 6 | Carbamidomethylation | 22860.4883 | type:internal len:200 gc:universal TRINITY\_DN118\_c1\_g1\_i3:599-3(-) |
| 59 | 158 | TRINITY\_DN106\_c0\_g1\_i9.p1 | 106.75 | 3 | 3 | 0 | 3 | 1 | 10 |  | 123769.2656 | type:3prime\_partial len:1309 gc:universal TRINITY\_DN106\_c0\_g1\_i9:137-4060(+) |
| 59 | 159 | TRINITY\_DN106\_c0\_g1\_i5.p1 | 106.75 | 3 | 3 | 0 | 3 | 1 | 10 |  | 109115.5859 | type:3prime\_partial len:1168 gc:universal TRINITY\_DN106\_c0\_g1\_i5:137-3637(+) |
| 59 | 160 | TRINITY\_DN106\_c0\_g1\_i8.p1 | 106.75 | 3 | 3 | 0 | 3 | 1 | 10 |  | 124232.0078 | type:3prime\_partial len:1312 gc:universal TRINITY\_DN106\_c0\_g1\_i8:137-4069(+) |
| 59 | 161 | TRINITY\_DN106\_c0\_g1\_i6.p1 | 106.75 | 3 | 3 | 0 | 3 | 1 | 10 |  | 109300.5156 | type:3prime\_partial len:1168 gc:universal TRINITY\_DN106\_c0\_g1\_i6:137-3637(+) |
| 59 | 162 | TRINITY\_DN106\_c0\_g1\_i3.p1 | 106.75 | 3 | 3 | 0 | 3 | 1 | 10 |  | 124293.9609 | type:3prime\_partial len:1312 gc:universal TRINITY\_DN106\_c0\_g1\_i3:137-4069(+) |
| 59 | 163 | TRINITY\_DN106\_c0\_g1\_i2.p1 | 87.4 | 2 | 2 | 0 | 2 | 1 | 5 |  | 123847.7578 | type:3prime\_partial len:1309 gc:universal TRINITY\_DN106\_c0\_g1\_i2:137-4060(+) |
| 60 | 164 | TRINITY\_DN12627\_c0\_g1\_i1.p1 | 104.18 | 3 | 3 | 3.3e3 | 3 | 3 | 3 |  | 120592.0703 | type:5prime\_partial len:1061 gc:universal TRINITY\_DN12627\_c0\_g1\_i1:3456-274(-) |
| 61 | 165 | TRINITY\_DN195348\_c0\_g1\_i1.p1 | 106.39 | 12 | 12 | 1e4 | 3 | 3 | 7 |  | 34993.6055 | type:internal len:311 gc:universal TRINITY\_DN195348\_c0\_g1\_i1:1-930(+) |
| 62 | 166 | TRINITY\_DN1119\_c0\_g1\_i1.p1 | 104.23 | 3 | 3 | 2.61e3 | 3 | 3 | 4 | Carbamidomethylation | 105284.3438 | type:5prime\_partial len:949 gc:universal TRINITY\_DN1119\_c0\_g1\_i1:2934-88(-) |
| 63 | 167 | TRINITY\_DN193641\_c0\_g1\_i1.p1 | 103.89 | 22 | 22 | 2.2e3 | 3 | 3 | 4 |  | 19024.373 | type:internal len:173 gc:universal TRINITY\_DN193641\_c0\_g1\_i1:2-517(+) |
| 64 | 168 | TRINITY\_DN1821\_c0\_g1\_i4.p1 | 103.94 | 6 | 6 | 9.27e2 | 3 | 3 | 3 |  | 73525.9219 | type:complete len:637 gc:universal TRINITY\_DN1821\_c0\_g1\_i4:304-2214(+) |
| 64 | 169 | TRINITY\_DN1821\_c0\_g1\_i5.p1 | 103.94 | 6 | 6 | 9.27e2 | 3 | 3 | 3 |  | 73657.1172 | type:complete len:638 gc:universal TRINITY\_DN1821\_c0\_g1\_i5:297-2210(+) |
| 64 | 170 | TRINITY\_DN1821\_c0\_g1\_i6.p1 | 103.94 | 6 | 6 | 9.27e2 | 3 | 3 | 3 |  | 73657.1172 | type:complete len:638 gc:universal TRINITY\_DN1821\_c0\_g1\_i6:297-2210(+) |
| 64 | 171 | TRINITY\_DN1821\_c0\_g1\_i2.p1 | 103.94 | 12 | 12 | 9.27e2 | 3 | 3 | 3 |  | 35059.2227 | type:internal len:299 gc:universal TRINITY\_DN1821\_c0\_g1\_i2:1-894(+) |
| 64 | 172 | TRINITY\_DN1821\_c0\_g1\_i3.p1 | 84.5 | 6 | 6 | 5.45e2 | 2 | 2 | 2 |  | 51658.0039 | type:3prime\_partial len:450 gc:universal TRINITY\_DN1821\_c0\_g1\_i3:300-1646(+) |
| 65 | 173 | TRINITY\_DN5500\_c0\_g1\_i2.p1 | 106.79 | 3 | 3 | 5.3e3 | 3 | 3 | 4 |  | 130974.4219 | type:3prime\_partial len:1183 gc:universal TRINITY\_DN5500\_c0\_g1\_i2:3546-1(-) |
| 65 | 174 | TRINITY\_DN5500\_c0\_g1\_i1.p1 | 106.79 | 3 | 3 | 5.3e3 | 3 | 3 | 4 |  | 132375.9688 | type:3prime\_partial len:1196 gc:universal TRINITY\_DN5500\_c0\_g1\_i1:3585-1(-) |
| 66 | 175 | TRINITY\_DN4770\_c0\_g1\_i1.p1 | 105.02 | 2 | 2 | 0 | 3 | 3 | 3 | Carbamidomethylation | 184315.625 | type:5prime\_partial len:1635 gc:universal TRINITY\_DN4770\_c0\_g1\_i1:5012-108(-) |
| 67 | 176 | TRINITY\_DN1071\_c0\_g1\_i1.p1 | 105.29 | 7 | 7 | 2.54e4 | 3 | 3 | 9 | Carbamidomethylation | 71848.4062 | type:internal len:660 gc:universal TRINITY\_DN1071\_c0\_g1\_i1:1977-1(-) |
| 67 | 177 | TRINITY\_DN1071\_c0\_g1\_i4.p1 | 105.29 | 7 | 7 | 2.54e4 | 3 | 3 | 9 | Carbamidomethylation | 75408.3359 | type:internal len:696 gc:universal TRINITY\_DN1071\_c0\_g1\_i4:2085-1(-) |
| 67 | 178 | TRINITY\_DN1071\_c0\_g1\_i3.p1 | 58.41 | 4 | 4 | 2.3e4 | 1 | 1 | 7 |  | 39803.1602 | type:internal len:375 gc:universal TRINITY\_DN1071\_c0\_g1\_i3:1124-3(-) |
| 67 | 179 | TRINITY\_DN1071\_c0\_g1\_i2.p1 | 58.41 | 5 | 5 | 2.3e4 | 1 | 1 | 7 |  | 36243.2383 | type:internal len:339 gc:universal TRINITY\_DN1071\_c0\_g1\_i2:1016-3(-) |
| 68 | 180 | TRINITY\_DN14015\_c0\_g1\_i3.p1 | 106.97 | 2 | 2 | 2.31e3 | 3 | 3 | 5 |  | 284708.625 | type:5prime\_partial len:2566 gc:universal TRINITY\_DN14015\_c0\_g1\_i3:2-7699(+) |
| 68 | 181 | TRINITY\_DN14015\_c0\_g1\_i2.p1 | 106.97 | 2 | 2 | 2.31e3 | 3 | 3 | 5 |  | 287707.9062 | type:5prime\_partial len:2592 gc:universal TRINITY\_DN14015\_c0\_g1\_i2:2-7777(+) |
| 69 | 182 | TRINITY\_DN942\_c0\_g1\_i5.p1 | 104.59 | 1 | 1 | 1.77e3 | 3 | 3 | 4 |  | 591926.25 | type:complete len:5116 gc:universal TRINITY\_DN942\_c0\_g1\_i5:15664-317(-) |
| 69 | 183 | TRINITY\_DN942\_c0\_g1\_i2.p1 | 104.59 | 1 | 1 | 1.77e3 | 3 | 3 | 4 |  | 591926.3125 | type:complete len:5116 gc:universal TRINITY\_DN942\_c0\_g1\_i2:15664-317(-) |
| 69 | 184 | TRINITY\_DN942\_c0\_g1\_i3.p1 | 104.59 | 1 | 1 | 1.77e3 | 3 | 3 | 4 |  | 590662.8125 | type:complete len:5105 gc:universal TRINITY\_DN942\_c0\_g1\_i3:15631-317(-) |
| 70 | 185 | TRINITY\_DN1189\_c0\_g1\_i1.p1 | 105.03 | 8 | 8 | 2.84e3 | 3 | 3 | 4 |  | 50239.8086 | type:5prime\_partial len:449 gc:universal TRINITY\_DN1189\_c0\_g1\_i1:1554-208(-) |
| 71 | 186 | TRINITY\_DN3182\_c0\_g1\_i3.p1 | 106.65 | 6 | 6 | 1.81e3 | 3 | 3 | 3 |  | 90137.1406 | type:complete len:784 gc:universal TRINITY\_DN3182\_c0\_g1\_i3:2869-518(-) |
| 71 | 187 | TRINITY\_DN3182\_c0\_g1\_i2.p1 | 106.65 | 6 | 6 | 1.81e3 | 3 | 3 | 3 |  | 90137.1406 | type:complete len:784 gc:universal TRINITY\_DN3182\_c0\_g1\_i2:2429-78(-) |
| 72 | 188 | TRINITY\_DN984\_c0\_g1\_i1.p1 | 104.69 | 2 | 2 | 7.38e4 | 3 | 3 | 10 |  | 146638.4062 | type:complete len:1485 gc:universal TRINITY\_DN984\_c0\_g1\_i1:4558-104(-) |
| 72 | 189 | TRINITY\_DN984\_c0\_g1\_i2.p1 | 104.69 | 2 | 2 | 7.38e4 | 3 | 3 | 10 |  | 146638.4062 | type:complete len:1485 gc:universal TRINITY\_DN984\_c0\_g1\_i2:4558-104(-) |
| 73 | 190 | TRINITY\_DN1613\_c1\_g1\_i2.p1 | 104.4 | 4 | 4 | 1.97e3 | 3 | 3 | 8 | Acetylation (Protein N-term), Carbamidomethylation | 111170.0312 | type:internal len:1068 gc:universal TRINITY\_DN1613\_c1\_g1\_i2:3201-1(-) |
| 73 | 191 | TRINITY\_DN1613\_c1\_g1\_i1.p1 | 104.4 | 4 | 4 | 1.97e3 | 3 | 3 | 8 | Acetylation (Protein N-term), Carbamidomethylation | 112791.8203 | type:internal len:1083 gc:universal TRINITY\_DN1613\_c1\_g1\_i1:3248-3(-) |
| 74 | 192 | TRINITY\_DN2028\_c0\_g1\_i1.p1 | 104.87 | 12 | 12 | 1.99e4 | 3 | 3 | 6 | Carbamidomethylation | 30106.9453 | type:complete len:274 gc:universal TRINITY\_DN2028\_c0\_g1\_i1:1608-787(-) |
| 75 | 193 | TRINITY\_DN5633\_c0\_g1\_i1.p1 | 104.19 | 4 | 4 | 5.56e2 | 3 | 3 | 3 |  | 97310.9922 | type:complete len:848 gc:universal TRINITY\_DN5633\_c0\_g1\_i1:2881-338(-) |
| 76 | 194 | TRINITY\_DN598\_c1\_g1\_i1.p1 | 101.14 | 5 | 5 | 1.21e4 | 3 | 3 | 10 |  | 81322.8672 | type:5prime\_partial len:745 gc:universal TRINITY\_DN598\_c1\_g1\_i1:2-2236(+) |
| 77 | 195 | TRINITY\_DN581\_c1\_g1\_i1.p1 | 105.6 | 5 | 5 | 6.07e3 | 3 | 3 | 4 | Carbamidomethylation | 53141.4414 | type:5prime\_partial len:481 gc:universal TRINITY\_DN581\_c1\_g1\_i1:2139-697(-) |
| 78 | 196 | TRINITY\_DN106\_c0\_g1\_i11.p1 | 106.76 | 7 | 7 | 4.13e2 | 3 | 1 | 10 |  | 57100.6445 | type:internal len:621 gc:universal TRINITY\_DN106\_c0\_g1\_i11:2-1861(+) |
| 78 | 197 | TRINITY\_DN106\_c0\_g1\_i10.p1 | 87.41 | 5 | 5 | 4.13e2 | 2 | 1 | 5 |  | 57108.7969 | type:internal len:621 gc:universal TRINITY\_DN106\_c0\_g1\_i10:2-1861(+) |
| 78 | 198 | TRINITY\_DN106\_c0\_g1\_i12.p1 | 87.41 | 5 | 5 | 4.13e2 | 2 | 1 | 5 |  | 57124.8438 | type:internal len:621 gc:universal TRINITY\_DN106\_c0\_g1\_i12:2-1861(+) |
| 78 | 199 | TRINITY\_DN106\_c0\_g1\_i4.p1 | 58.29 | 15 | 15 | 4.13e2 | 1 | 1 | 1 |  | 12128.8213 | type:internal len:130 gc:universal TRINITY\_DN106\_c0\_g1\_i4:2-388(+) |
| 79 | 200 | TRINITY\_DN36\_c0\_g1\_i16.p1 | 86.92 | 10 | 10 | 5.6e3 | 2 | 2 | 3 | Carbamidomethylation | 24953.5938 | type:complete len:224 gc:universal TRINITY\_DN36\_c0\_g1\_i16:829-158(-) |
| 79 | 201 | TRINITY\_DN36\_c0\_g1\_i9.p1 | 86.92 | 10 | 10 | 5.6e3 | 2 | 2 | 3 | Carbamidomethylation | 24680.2773 | type:complete len:223 gc:universal TRINITY\_DN36\_c0\_g1\_i9:934-266(-) |
| 79 | 202 | TRINITY\_DN36\_c0\_g1\_i3.p1 | 86.92 | 10 | 10 | 5.6e3 | 2 | 2 | 3 | Carbamidomethylation | 24953.5938 | type:complete len:224 gc:universal TRINITY\_DN36\_c0\_g1\_i3:829-158(-) |
| 79 | 203 | TRINITY\_DN36\_c0\_g1\_i14.p1 | 86.92 | 10 | 10 | 5.6e3 | 2 | 2 | 3 | Carbamidomethylation | 24953.5938 | type:complete len:224 gc:universal TRINITY\_DN36\_c0\_g1\_i14:819-148(-) |
| 79 | 204 | TRINITY\_DN36\_c0\_g1\_i8.p1 | 86.92 | 10 | 10 | 5.6e3 | 2 | 2 | 3 | Carbamidomethylation | 24953.5938 | type:complete len:224 gc:universal TRINITY\_DN36\_c0\_g1\_i8:819-148(-) |
| 79 | 205 | TRINITY\_DN36\_c0\_g1\_i1.p1 | 86.92 | 10 | 10 | 5.6e3 | 2 | 2 | 3 | Carbamidomethylation | 25006.7051 | type:complete len:224 gc:universal TRINITY\_DN36\_c0\_g1\_i1:1175-504(-) |
| 79 | 206 | TRINITY\_DN36\_c0\_g1\_i19.p1 | 86.92 | 10 | 10 | 5.6e3 | 2 | 2 | 3 | Carbamidomethylation | 24821.4141 | type:complete len:223 gc:universal TRINITY\_DN36\_c0\_g1\_i19:826-158(-) |
| 79 | 207 | TRINITY\_DN36\_c0\_g1\_i17.p1 | 86.92 | 10 | 10 | 5.6e3 | 2 | 2 | 3 | Carbamidomethylation | 24953.5938 | type:complete len:224 gc:universal TRINITY\_DN36\_c0\_g1\_i17:819-148(-) |
| 79 | 208 | TRINITY\_DN36\_c0\_g1\_i13.p1 | 86.92 | 10 | 10 | 5.6e3 | 2 | 2 | 3 | Carbamidomethylation | 24821.4141 | type:complete len:223 gc:universal TRINITY\_DN36\_c0\_g1\_i13:816-148(-) |
| 79 | 209 | TRINITY\_DN36\_c0\_g1\_i11.p1 | 86.92 | 10 | 10 | 5.6e3 | 2 | 2 | 3 | Carbamidomethylation | 25006.7051 | type:complete len:224 gc:universal TRINITY\_DN36\_c0\_g1\_i11:1175-504(-) |
| 79 | 210 | TRINITY\_DN36\_c0\_g1\_i4.p1 | 86.92 | 10 | 10 | 5.6e3 | 2 | 2 | 3 | Carbamidomethylation | 24680.2773 | type:complete len:223 gc:universal TRINITY\_DN36\_c0\_g1\_i4:932-264(-) |
| 79 | 211 | TRINITY\_DN36\_c0\_g1\_i2.p1 | 86.92 | 10 | 10 | 5.6e3 | 2 | 2 | 3 | Carbamidomethylation | 24680.2773 | type:complete len:223 gc:universal TRINITY\_DN36\_c0\_g1\_i2:934-266(-) |
| 79 | 212 | TRINITY\_DN36\_c0\_g1\_i5.p1 | 86.92 | 10 | 10 | 5.6e3 | 2 | 2 | 3 | Carbamidomethylation | 24548.0977 | type:complete len:222 gc:universal TRINITY\_DN36\_c0\_g1\_i5:931-266(-) |
| 79 | 213 | TRINITY\_DN36\_c0\_g1\_i20.p1 | 86.92 | 10 | 10 | 5.6e3 | 2 | 2 | 3 | Carbamidomethylation | 24874.5234 | type:complete len:223 gc:universal TRINITY\_DN36\_c0\_g1\_i20:1172-504(-) |
| 80 | 214 | TRINITY\_DN495\_c0\_g1\_i2.p1 | 68.06 | 3 | 3 | 7.79e2 | 2 | 2 | 2 | Carbamidomethylation | 72975.0312 | type:complete len:655 gc:universal TRINITY\_DN495\_c0\_g1\_i2:152-2116(+) |
| 80 | 215 | TRINITY\_DN495\_c0\_g1\_i3.p1 | 68.06 | 3 | 3 | 7.79e2 | 2 | 2 | 2 | Carbamidomethylation | 73673.9297 | type:complete len:659 gc:universal TRINITY\_DN495\_c0\_g1\_i3:152-2128(+) |
| 81 | 216 | TRINITY\_DN3698\_c0\_g2\_i3.p1 | 86.1 | 4 | 4 | 6.57e2 | 2 | 2 | 6 |  | 80426.7734 | type:complete len:710 gc:universal TRINITY\_DN3698\_c0\_g2\_i3:78-2207(+) |
| 81 | 217 | TRINITY\_DN3698\_c0\_g2\_i6.p1 | 86.1 | 4 | 4 | 6.57e2 | 2 | 2 | 6 |  | 80426.7734 | type:complete len:710 gc:universal TRINITY\_DN3698\_c0\_g2\_i6:78-2207(+) |
| 81 | 218 | TRINITY\_DN3698\_c0\_g2\_i1.p1 | 86.1 | 4 | 4 | 6.57e2 | 2 | 2 | 6 |  | 80839.2578 | type:complete len:714 gc:universal TRINITY\_DN3698\_c0\_g2\_i1:69-2210(+) |
| 81 | 219 | TRINITY\_DN3698\_c0\_g2\_i4.p1 | 86.1 | 4 | 4 | 6.57e2 | 2 | 2 | 6 |  | 80164.3906 | type:5prime\_partial len:708 gc:universal TRINITY\_DN3698\_c0\_g2\_i4:2-2125(+) |
| 81 | 220 | TRINITY\_DN3698\_c0\_g2\_i2.p1 | 86.1 | 4 | 4 | 6.57e2 | 2 | 2 | 6 |  | 80839.2578 | type:complete len:714 gc:universal TRINITY\_DN3698\_c0\_g2\_i2:69-2210(+) |
| 82 | 221 | TRINITY\_DN10485\_c0\_g1\_i1.p1 | 87.32 | 5 | 5 | 0 | 2 | 2 | 2 |  | 66716.9844 | type:5prime\_partial len:604 gc:universal TRINITY\_DN10485\_c0\_g1\_i1:2-1813(+) |
| 83 | 222 | TRINITY\_DN8155\_c0\_g1\_i1.p1 | 84.68 | 10 | 10 | 5.51e2 | 2 | 2 | 2 | Carbamidomethylation | 27519.8945 | type:internal len:249 gc:universal TRINITY\_DN8155\_c0\_g1\_i1:744-1(-) |
| 84 | 223 | TRINITY\_DN3655\_c0\_g1\_i1.p1 | 86.75 | 5 | 5 | 2.71e3 | 2 | 1 | 2 |  | 59978.0352 | type:internal len:536 gc:universal TRINITY\_DN3655\_c0\_g1\_i1:1605-1(-) |
| 85 | 224 | TRINITY\_DN1030\_c0\_g1\_i2.p1 | 87.58 | 3 | 3 | 2.93e4 | 2 | 2 | 15 |  | 140221.3281 | type:complete len:1426 gc:universal TRINITY\_DN1030\_c0\_g1\_i2:4483-206(-) |
| 85 | 225 | TRINITY\_DN1030\_c0\_g1\_i1.p1 | 87.58 | 3 | 3 | 2.93e4 | 2 | 2 | 15 |  | 141535.875 | type:complete len:1439 gc:universal TRINITY\_DN1030\_c0\_g1\_i1:4522-206(-) |
| 86 | 226 | TRINITY\_DN6221\_c0\_g1\_i8.p1 | 86 | 4 | 4 | 3.11e3 | 2 | 2 | 4 | Carbamidomethylation | 89013.0859 | type:5prime\_partial len:855 gc:universal TRINITY\_DN6221\_c0\_g1\_i8:1-2565(+) |
| 86 | 227 | TRINITY\_DN6221\_c0\_g1\_i5.p1 | 86 | 4 | 4 | 3.11e3 | 2 | 2 | 4 | Carbamidomethylation | 90134.25 | type:5prime\_partial len:866 gc:universal TRINITY\_DN6221\_c0\_g1\_i5:1-2598(+) |
| 86 | 228 | TRINITY\_DN6221\_c0\_g1\_i2.p1 | 86 | 5 | 5 | 3.11e3 | 2 | 2 | 4 | Carbamidomethylation | 70144.25 | type:complete len:667 gc:universal TRINITY\_DN6221\_c0\_g1\_i2:385-2385(+) |
| 86 | 229 | TRINITY\_DN6221\_c0\_g1\_i3.p1 | 86 | 4 | 4 | 3.11e3 | 2 | 2 | 4 | Carbamidomethylation | 88999.0625 | type:5prime\_partial len:855 gc:universal TRINITY\_DN6221\_c0\_g1\_i3:1-2565(+) |
| 87 | 230 | TRINITY\_DN27058\_c0\_g1\_i1.p1 | 83.86 | 19 | 19 | 2.91e2 | 2 | 2 | 3 |  | 21608.4434 | type:internal len:188 gc:universal TRINITY\_DN27058\_c0\_g1\_i1:2-562(+) |
| 88 | 231 | TRINITY\_DN149493\_c0\_g1\_i1.p1 | 87.54 | 12 | 12 | 1.67e3 | 2 | 2 | 2 |  | 16274.376 | type:internal len:148 gc:universal TRINITY\_DN149493\_c0\_g1\_i1:2-442(+) |
| 89 | 232 | TRINITY\_DN4900\_c0\_g1\_i1.p1 | 84.47 | 6 | 6 | 1.03e3 | 2 | 2 | 3 |  | 26790.1289 | type:complete len:246 gc:universal TRINITY\_DN4900\_c0\_g1\_i1:153-890(+) |
| 89 | 233 | TRINITY\_DN4900\_c0\_g1\_i4.p1 | 84.47 | 6 | 6 | 1.03e3 | 2 | 2 | 3 |  | 26790.1289 | type:complete len:246 gc:universal TRINITY\_DN4900\_c0\_g1\_i4:81-818(+) |
| 90 | 234 | TRINITY\_DN8597\_c0\_g1\_i1.p1 | 86.81 | 15 | 15 | 0 | 2 | 1 | 4 |  | 13587.9307 | type:internal len:124 gc:universal TRINITY\_DN8597\_c0\_g1\_i1:369-1(-) |
| 91 | 235 | TRINITY\_DN187759\_c0\_g1\_i1.p1 | 85.94 | 2 | 2 | 1.88e3 | 2 | 2 | 3 | Carbamidomethylation | 60653.0039 | type:internal len:544 gc:universal TRINITY\_DN187759\_c0\_g1\_i1:1629-1(-) |
| 92 | 236 | TRINITY\_DN18\_c0\_g1\_i1.p1 | 78.82 | 8 | 8 | 1.1e4 | 2 | 2 | 3 |  | 27237.457 | type:complete len:256 gc:universal TRINITY\_DN18\_c0\_g1\_i1:53-820(+) |
| 92 | 237 | TRINITY\_DN18\_c0\_g1\_i2.p1 | 78.82 | 8 | 8 | 1.1e4 | 2 | 2 | 3 |  | 27237.457 | type:complete len:256 gc:universal TRINITY\_DN18\_c0\_g1\_i2:53-820(+) |
| 93 | 238 | TRINITY\_DN168145\_c0\_g1\_i1.p1 | 81.12 | 11 | 11 | 4.22e3 | 2 | 2 | 2 |  | 21195.6367 | type:internal len:186 gc:universal TRINITY\_DN168145\_c0\_g1\_i1:556-2(-) |
| 94 | 239 | TRINITY\_DN1535\_c0\_g1\_i1.p1 | 84.57 | 3 | 3 | 1.2e3 | 2 | 2 | 4 | Carbamidomethylation | 86942.8516 | type:complete len:779 gc:universal TRINITY\_DN1535\_c0\_g1\_i1:86-2422(+) |
| 95 | 240 | TRINITY\_DN10094\_c0\_g1\_i1.p1 | 85.72 | 11 | 11 | 1.29e3 | 2 | 2 | 3 | Carbamidomethylation | 28035.0664 | type:complete len:264 gc:universal TRINITY\_DN10094\_c0\_g1\_i1:1210-419(-) |
| 95 | 241 | TRINITY\_DN10094\_c0\_g1\_i2.p1 | 85.72 | 11 | 11 | 1.29e3 | 2 | 2 | 3 | Carbamidomethylation | 28035.0664 | type:complete len:264 gc:universal TRINITY\_DN10094\_c0\_g1\_i2:1210-419(-) |
| 96 | 242 | TRINITY\_DN172571\_c0\_g1\_i1.p1 | 86.66 | 19 | 19 | 2.51e3 | 2 | 2 | 2 |  | 11500.2529 | type:complete len:104 gc:universal TRINITY\_DN172571\_c0\_g1\_i1:30-341(+) |
| 97 | 243 | TRINITY\_DN2490\_c0\_g1\_i1.p1 | 87.39 | 2 | 2 | 1.8e3 | 2 | 2 | 2 |  | 116032.3672 | type:complete len:1044 gc:universal TRINITY\_DN2490\_c0\_g1\_i1:354-3485(+) |
| 98 | 244 | TRINITY\_DN6920\_c0\_g1\_i3.p1 | 84.36 | 5 | 5 | 6.47e2 | 2 | 2 | 2 | Carbamidomethylation | 46932.582 | type:5prime\_partial len:411 gc:universal TRINITY\_DN6920\_c0\_g1\_i3:2124-892(-) |
| 98 | 245 | TRINITY\_DN6920\_c0\_g1\_i2.p1 | 84.36 | 5 | 5 | 6.47e2 | 2 | 2 | 2 | Carbamidomethylation | 46932.582 | type:5prime\_partial len:411 gc:universal TRINITY\_DN6920\_c0\_g1\_i2:2147-915(-) |
| 98 | 246 | TRINITY\_DN6920\_c0\_g1\_i4.p1 | 84.36 | 7 | 7 | 6.47e2 | 2 | 2 | 2 | Carbamidomethylation | 32195.3809 | type:5prime\_partial len:282 gc:universal TRINITY\_DN6920\_c0\_g1\_i4:1321-476(-) |
| 98 | 247 | TRINITY\_DN6920\_c0\_g1\_i1.p1 | 84.36 | 7 | 7 | 6.47e2 | 2 | 2 | 2 | Carbamidomethylation | 32882.168 | type:complete len:287 gc:universal TRINITY\_DN6920\_c0\_g1\_i1:1775-915(-) |
| 99 | 248 | TRINITY\_DN776\_c0\_g1\_i1.p1 | 87.15 | 10 | 10 | 1.26e3 | 2 | 2 | 2 |  | 23300.8359 | type:5prime\_partial len:201 gc:universal TRINITY\_DN776\_c0\_g1\_i1:692-90(-) |
| 99 | 249 | TRINITY\_DN189323\_c0\_g1\_i1.p1 | 57.78 | 6 | 6 | 9.34e2 | 1 | 1 | 1 |  | 18178.9902 | type:5prime\_partial len:156 gc:universal TRINITY\_DN189323\_c0\_g1\_i1:2-469(+) |
| 100 | 250 | TRINITY\_DN2963\_c0\_g1\_i1.p1 | 85.57 | 10 | 10 | 3.39e3 | 2 | 2 | 2 | Acetylation (Protein N-term) | 13082.1787 | type:complete len:116 gc:universal TRINITY\_DN2963\_c0\_g1\_i1:1072-725(-) |
| 101 | 251 | TRINITY\_DN61742\_c0\_g1\_i1.p1 | 86.53 | 5 | 5 | 0 | 2 | 2 | 2 |  | 40028.4102 | type:complete len:349 gc:universal TRINITY\_DN61742\_c0\_g1\_i1:1073-27(-) |
| 101 | 252 | TRINITY\_DN61742\_c0\_g1\_i2.p1 | 86.53 | 3 | 3 | 0 | 2 | 2 | 2 |  | 65941 | type:complete len:591 gc:universal TRINITY\_DN61742\_c0\_g1\_i2:1799-27(-) |
| 101 | 253 | TRINITY\_DN61742\_c0\_g1\_i3.p1 | 58.05 | 5 | 5 | 0 | 1 | 1 | 1 |  | 20296.2402 | type:complete len:178 gc:universal TRINITY\_DN61742\_c0\_g1\_i3:538-5(-) |
| 102 | 254 | TRINITY\_DN564\_c0\_g1\_i1.p1 | 86.59 | 5 | 5 | 0 | 2 | 2 | 2 |  | 62932.8672 | type:complete len:563 gc:universal TRINITY\_DN564\_c0\_g1\_i1:2018-330(-) |
| 103 | 255 | TRINITY\_DN4781\_c0\_g1\_i3.p3 | 87.53 | 13 | 13 | 2.55e3 | 2 | 2 | 5 |  | 20029.2422 | type:complete len:183 gc:universal TRINITY\_DN4781\_c0\_g1\_i3:2965-2417(-) |
| 104 | 256 | TRINITY\_DN18348\_c0\_g1\_i1.p1 | 87.27 | 1 | 1 | 0 | 2 | 2 | 2 |  | 274175.6562 | type:complete len:2565 gc:universal TRINITY\_DN18348\_c0\_g1\_i1:90-7784(+) |
| 105 | 257 | TRINITY\_DN194261\_c0\_g1\_i1.p1 | 87.41 | 14 | 14 | 1.86e3 | 2 | 2 | 3 |  | 14048.4268 | type:internal len:124 gc:universal TRINITY\_DN194261\_c0\_g1\_i1:1-369(+) |
| 106 | 258 | TRINITY\_DN12542\_c0\_g1\_i1.p1 | 87.41 | 16 | 16 | 0 | 2 | 1 | 3 |  | 23963.9648 | type:internal len:226 gc:universal TRINITY\_DN12542\_c0\_g1\_i1:676-2(-) |
| 106 | 259 | TRINITY\_DN5003\_c0\_g1\_i2.p1 | 58.29 | 20 | 20 | 0 | 1 | 1 | 2 |  | 11342.9131 | type:internal len:106 gc:universal TRINITY\_DN5003\_c0\_g1\_i2:317-3(-) |
| 107 | 260 | TRINITY\_DN624\_c0\_g1\_i1.p1 | 87.15 | 3 | 3 | 1.54e3 | 2 | 2 | 2 |  | 101743.5391 | type:complete len:884 gc:universal TRINITY\_DN624\_c0\_g1\_i1:218-2869(+) |
| 108 | 261 | TRINITY\_DN1058\_c3\_g1\_i1.p1 | 85.75 | 6 | 6 | 1.75e3 | 2 | 2 | 2 | Carbamidomethylation | 51455.6484 | type:internal len:485 gc:universal TRINITY\_DN1058\_c3\_g1\_i1:1452-1(-) |
| 109 | 262 | TRINITY\_DN329\_c0\_g1\_i2.p1 | 85.29 | 10 | 10 | 2.02e2 | 2 | 2 | 2 | Oxidation (M) | 34761.9609 | type:5prime\_partial len:320 gc:universal TRINITY\_DN329\_c0\_g1\_i2:1-960(+) |
| 109 | 263 | TRINITY\_DN329\_c0\_g1\_i1.p1 | 85.29 | 12 | 12 | 2.02e2 | 2 | 2 | 2 | Oxidation (M) | 30514.9648 | type:complete len:283 gc:universal TRINITY\_DN329\_c0\_g1\_i1:158-1006(+) |
| 110 | 264 | TRINITY\_DN11377\_c0\_g1\_i1.p1 | 81.26 | 3 | 3 | 1.34e3 | 2 | 2 | 2 |  | 89751.1094 | type:complete len:804 gc:universal TRINITY\_DN11377\_c0\_g1\_i1:3179-768(-) |
| 111 | 265 | TRINITY\_DN16079\_c0\_g2\_i1.p1 | 86.86 | 5 | 5 | 7.69e2 | 2 | 2 | 2 |  | 44791.4727 | type:5prime\_partial len:396 gc:universal TRINITY\_DN16079\_c0\_g2\_i1:1686-499(-) |
| 112 | 266 | TRINITY\_DN9655\_c0\_g1\_i1.p1 | 86.34 | 3 | 3 | 3.97e2 | 2 | 2 | 3 |  | 54707.5352 | type:complete len:497 gc:universal TRINITY\_DN9655\_c0\_g1\_i1:132-1622(+) |
| 113 | 267 | TRINITY\_DN48767\_c0\_g1\_i1.p1 | 84.3 | 4 | 4 | 2.03e3 | 2 | 2 | 3 | Carbamidomethylation | 60323.9297 | type:complete len:533 gc:universal TRINITY\_DN48767\_c0\_g1\_i1:2135-537(-) |
| 114 | 268 | TRINITY\_DN1386\_c0\_g1\_i1.p1 | 84.41 | 2 | 2 | 1.76e2 | 2 | 2 | 4 |  | 68692.1953 | type:complete len:596 gc:universal TRINITY\_DN1386\_c0\_g1\_i1:2419-632(-) |
| 115 | 269 | TRINITY\_DN144214\_c0\_g1\_i1.p1 | 81.25 | 16 | 16 | 5.89e2 | 2 | 2 | 3 |  | 16648.9531 | type:internal len:154 gc:universal TRINITY\_DN144214\_c0\_g1\_i1:461-3(-) |
| 115 | 270 | TRINITY\_DN5897\_c0\_g1\_i2.p1 | 53.88 | 9 | 9 | 0 | 1 | 1 | 1 |  | 15600.9961 | type:internal len:145 gc:universal TRINITY\_DN5897\_c0\_g1\_i2:434-3(-) |
| 115 | 271 | TRINITY\_DN5897\_c0\_g1\_i3.p1 | 53.88 | 12 | 12 | 0 | 1 | 1 | 1 |  | 11673.5254 | type:internal len:111 gc:universal TRINITY\_DN5897\_c0\_g1\_i3:332-3(-) |
| 115 | 272 | TRINITY\_DN5897\_c0\_g1\_i1.p2 | 53.88 | 10 | 10 | 0 | 1 | 1 | 1 |  | 13978.9805 | type:internal len:130 gc:universal TRINITY\_DN5897\_c0\_g1\_i1:387-1(-) |
| 116 | 273 | TRINITY\_DN11435\_c0\_g1\_i1.p1 | 79.34 | 6 | 6 | 3.13e2 | 2 | 2 | 2 |  | 47019.5312 | type:complete len:430 gc:universal TRINITY\_DN11435\_c0\_g1\_i1:1616-327(-) |
| 117 | 274 | TRINITY\_DN144508\_c0\_g1\_i1.p1 | 87.39 | 8 | 8 | 2.36e2 | 2 | 2 | 3 |  | 29446.1543 | type:internal len:265 gc:universal TRINITY\_DN144508\_c0\_g1\_i1:1-792(+) |
| 118 | 275 | TRINITY\_DN3655\_c0\_g1\_i2.p1 | 87.53 | 5 | 5 | 1.42e3 | 2 | 1 | 2 |  | 55166.5625 | type:internal len:494 gc:universal TRINITY\_DN3655\_c0\_g1\_i2:1481-3(-) |
| 119 | 276 | TRINITY\_DN113\_c2\_g1\_i1.p1 | 84.37 | 2 | 2 | 1.34e4 | 2 | 2 | 3 |  | 118094.7031 | type:5prime\_partial len:1207 gc:universal TRINITY\_DN113\_c2\_g1\_i1:4425-805(-) |
| 120 | 277 | TRINITY\_DN66856\_c0\_g1\_i1.p1 | 86.13 | 6 | 6 | 1.28e4 | 2 | 2 | 2 |  | 49950.4219 | type:internal len:443 gc:universal TRINITY\_DN66856\_c0\_g1\_i1:1327-2(-) |
| 121 | 278 | TRINITY\_DN6304\_c0\_g1\_i1.p1 | 78.58 | 6 | 6 | 1.28e3 | 2 | 2 | 2 |  | 39706.2383 | type:complete len:348 gc:universal TRINITY\_DN6304\_c0\_g1\_i1:1626-583(-) |
| 122 | 279 | TRINITY\_DN2356\_c0\_g1\_i1.p1 | 78.92 | 9 | 9 | 4.01e2 | 2 | 2 | 3 |  | 31004.7148 | type:complete len:270 gc:universal TRINITY\_DN2356\_c0\_g1\_i1:194-1003(+) |
| 123 | 280 | TRINITY\_DN7674\_c0\_g1\_i1.p1 | 78.61 | 3 | 3 | 3.33e3 | 2 | 2 | 3 |  | 73304.8672 | type:complete len:661 gc:universal TRINITY\_DN7674\_c0\_g1\_i1:117-2099(+) |
| 123 | 281 | TRINITY\_DN7674\_c0\_g1\_i2.p1 | 78.61 | 3 | 3 | 3.33e3 | 2 | 2 | 3 |  | 73304.8672 | type:complete len:661 gc:universal TRINITY\_DN7674\_c0\_g1\_i2:42-2024(+) |
| 124 | 282 | TRINITY\_DN738\_c0\_g1\_i1.p1 | 79.08 | 7 | 7 | 9.69e2 | 2 | 2 | 2 | Carbamidomethylation | 36613.5547 | type:3prime\_partial len:338 gc:universal TRINITY\_DN738\_c0\_g1\_i1:1011-1(-) |
| 124 | 283 | TRINITY\_DN738\_c0\_g1\_i3.p1 | 52.42 | 3 | 3 | 6.91e2 | 1 | 1 | 1 |  | 30566.7344 | type:3prime\_partial len:281 gc:universal TRINITY\_DN738\_c0\_g1\_i3:840-1(-) |
| 124 | 284 | TRINITY\_DN738\_c0\_g1\_i2.p1 | 52.42 | 2 | 2 | 6.91e2 | 1 | 1 | 1 |  | 43179.1172 | type:3prime\_partial len:398 gc:universal TRINITY\_DN738\_c0\_g1\_i2:1191-1(-) |
| 124 | 285 | TRINITY\_DN738\_c0\_g1\_i4.p1 | 52.42 | 2 | 2 | 6.91e2 | 1 | 1 | 1 |  | 36633.5039 | type:3prime\_partial len:338 gc:universal TRINITY\_DN738\_c0\_g1\_i4:1011-1(-) |
| 125 | 286 | TRINITY\_DN2796\_c0\_g1\_i1.p1 | 58.25 | 3 | 3 | 4.36e2 | 1 | 1 | 1 |  | 55798.6328 | type:5prime\_partial len:521 gc:universal TRINITY\_DN2796\_c0\_g1\_i1:1-1563(+) |
| 125 | 287 | TRINITY\_DN2796\_c0\_g1\_i2.p1 | 58.25 | 3 | 3 | 4.36e2 | 1 | 1 | 1 |  | 55728.4961 | type:5prime\_partial len:521 gc:universal TRINITY\_DN2796\_c0\_g1\_i2:1-1563(+) |
| 126 | 288 | TRINITY\_DN5\_c0\_g1\_i15.p1 | 53.27 | 2 | 2 | 0 | 1 | 1 | 1 |  | 64391.1406 | type:3prime\_partial len:578 gc:universal TRINITY\_DN5\_c0\_g1\_i15:1731-1(-) |
| 126 | 289 | TRINITY\_DN5\_c0\_g1\_i18.p1 | 53.27 | 5 | 5 | 0 | 1 | 1 | 1 |  | 24823.0371 | type:3prime\_partial len:224 gc:universal TRINITY\_DN5\_c0\_g1\_i18:669-1(-) |
| 126 | 290 | TRINITY\_DN5\_c0\_g1\_i15.p4 | 53.27 | 11 | 11 | 0 | 1 | 1 | 1 |  | 11402.1768 | type:5prime\_partial len:100 gc:universal TRINITY\_DN5\_c0\_g1\_i15:3245-2946(-) |
| 126 | 291 | TRINITY\_DN5\_c0\_g1\_i1.p1 | 53.27 | 11 | 11 | 0 | 1 | 1 | 1 |  | 11271.0371 | type:5prime\_partial len:100 gc:universal TRINITY\_DN5\_c0\_g1\_i1:783-484(-) |
| 126 | 292 | TRINITY\_DN5\_c0\_g1\_i7.p2 | 53.27 | 3 | 3 | 0 | 1 | 1 | 1 |  | 36245.6406 | type:3prime\_partial len:325 gc:universal TRINITY\_DN5\_c0\_g1\_i7:972-1(-) |
| 126 | 293 | TRINITY\_DN5\_c0\_g1\_i10.p2 | 53.27 | 11 | 11 | 0 | 1 | 1 | 1 |  | 11402.1768 | type:5prime\_partial len:100 gc:universal TRINITY\_DN5\_c0\_g1\_i10:2071-1772(-) |
| 126 | 294 | TRINITY\_DN5\_c0\_g1\_i11.p1 | 53.27 | 11 | 11 | 0 | 1 | 1 | 1 |  | 11368.1592 | type:5prime\_partial len:100 gc:universal TRINITY\_DN5\_c0\_g1\_i11:602-303(-) |
| 126 | 295 | TRINITY\_DN5\_c0\_g1\_i8.p1 | 53.27 | 6 | 6 | 0 | 1 | 1 | 1 |  | 20697.7246 | type:5prime\_partial len:185 gc:universal TRINITY\_DN5\_c0\_g1\_i8:685-131(-) |
| 126 | 296 | TRINITY\_DN5\_c0\_g1\_i18.p3 | 53.27 | 9 | 9 | 0 | 1 | 1 | 1 |  | 14522.8203 | type:5prime\_partial len:128 gc:universal TRINITY\_DN5\_c0\_g1\_i18:1036-653(-) |
| 126 | 297 | TRINITY\_DN5\_c0\_g1\_i7.p4 | 53.27 | 11 | 11 | 0 | 1 | 1 | 1 |  | 11402.1768 | type:5prime\_partial len:100 gc:universal TRINITY\_DN5\_c0\_g1\_i7:3180-2881(-) |
| 126 | 298 | TRINITY\_DN5\_c0\_g1\_i10.p1 | 53.27 | 2 | 2 | 0 | 1 | 1 | 1 |  | 64560.3203 | type:3prime\_partial len:580 gc:universal TRINITY\_DN5\_c0\_g1\_i10:1737-1(-) |
| 126 | 299 | TRINITY\_DN5\_c0\_g1\_i16.p1 | 53.27 | 2 | 2 | 0 | 1 | 1 | 1 |  | 61174.9492 | type:5prime\_partial len:548 gc:universal TRINITY\_DN5\_c0\_g1\_i16:2230-587(-) |
| 126 | 300 | TRINITY\_DN5\_c0\_g1\_i9.p1 | 53.27 | 4 | 4 | 0 | 1 | 1 | 1 |  | 31401.6055 | type:internal len:281 gc:universal TRINITY\_DN5\_c0\_g1\_i9:842-3(-) |
| 126 | 301 | TRINITY\_DN5\_c0\_g1\_i13.p1 | 53.27 | 3 | 3 | 0 | 1 | 1 | 1 |  | 36581.6836 | type:internal len:328 gc:universal TRINITY\_DN5\_c0\_g1\_i13:981-1(-) |
| 127 | 302 | TRINITY\_DN75609\_c0\_g1\_i1.p1 | 57.21 | 5 | 5 | 8.32e2 | 1 | 1 | 1 |  | 25602.9277 | type:internal len:232 gc:universal TRINITY\_DN75609\_c0\_g1\_i1:694-2(-) |
| 128 | 303 | TRINITY\_DN179\_c0\_g1\_i1.p1 | 58.29 | 5 | 5 | 1.85e3 | 1 | 1 | 1 |  | 21927.1758 | type:3prime\_partial len:223 gc:universal TRINITY\_DN179\_c0\_g1\_i1:84-749(+) |
| 129 | 304 | TRINITY\_DN65215\_c0\_g1\_i1.p1 | 44.57 | 2 | 2 | 1.97e2 | 1 | 1 | 1 |  | 43734.125 | type:5prime\_partial len:383 gc:universal TRINITY\_DN65215\_c0\_g1\_i1:1336-188(-) |
| 130 | 305 | TRINITY\_DN165469\_c0\_g1\_i1.p1 | 57.33 | 0 | 0 | 0 | 1 | 1 | 1 |  | 298128.9688 | type:complete len:2668 gc:universal TRINITY\_DN165469\_c0\_g1\_i1:41-8044(+) |
| 131 | 306 | TRINITY\_DN106952\_c0\_g1\_i1.p1 | 57.63 | 7 | 7 | 4.61e3 | 1 | 1 | 1 |  | 14528.8164 | type:3prime\_partial len:135 gc:universal TRINITY\_DN106952\_c0\_g1\_i1:18-419(+) |
| 132 | 307 | TRINITY\_DN4481\_c0\_g1\_i3.p1 | 58.29 | 4 | 4 | 2.17e3 | 1 | 1 | 2 |  | 40458.9258 | type:internal len:369 gc:universal TRINITY\_DN4481\_c0\_g1\_i3:1-1104(+) |
| 132 | 308 | TRINITY\_DN598\_c1\_g1\_i2.p1 | 58.29 | 2 | 2 | 2.17e3 | 1 | 1 | 2 |  | 81043.625 | type:5prime\_partial len:742 gc:universal TRINITY\_DN598\_c1\_g1\_i2:2-2227(+) |
| 132 | 309 | TRINITY\_DN4481\_c0\_g1\_i2.p1 | 58.29 | 4 | 4 | 2.17e3 | 1 | 1 | 2 |  | 40425.9414 | type:internal len:369 gc:universal TRINITY\_DN4481\_c0\_g1\_i2:1-1104(+) |
| 133 | 310 | TRINITY\_DN17095\_c0\_g1\_i1.p1 | 46.63 | 2 | 2 | 0 | 1 | 1 | 1 |  | 68063.4688 | type:internal len:607 gc:universal TRINITY\_DN17095\_c0\_g1\_i1:1820-3(-) |
| 134 | 311 | TRINITY\_DN912\_c0\_g1\_i2.p1 | 43.84 | 3 | 3 | 0 | 1 | 1 | 1 |  | 40580.2539 | type:complete len:355 gc:universal TRINITY\_DN912\_c0\_g1\_i2:1586-522(-) |
| 134 | 312 | TRINITY\_DN191980\_c0\_g1\_i1.p1 | 43.84 | 9 | 9 | 0 | 1 | 1 | 1 |  | 13430.666 | type:3prime\_partial len:117 gc:universal TRINITY\_DN191980\_c0\_g1\_i1:196-543(+) |
| 134 | 313 | TRINITY\_DN12734\_c0\_g1\_i4.p1 | 43.84 | 3 | 3 | 0 | 1 | 1 | 1 |  | 42294.3555 | type:complete len:365 gc:universal TRINITY\_DN12734\_c0\_g1\_i4:509-1603(+) |
| 134 | 314 | TRINITY\_DN912\_c0\_g1\_i4.p1 | 43.84 | 3 | 3 | 0 | 1 | 1 | 1 |  | 40580.2539 | type:complete len:355 gc:universal TRINITY\_DN912\_c0\_g1\_i4:1586-522(-) |
| 134 | 315 | TRINITY\_DN12734\_c0\_g1\_i2.p1 | 43.84 | 3 | 3 | 0 | 1 | 1 | 1 |  | 42302.4258 | type:complete len:365 gc:universal TRINITY\_DN12734\_c0\_g1\_i2:321-1415(+) |
| 134 | 316 | TRINITY\_DN912\_c0\_g1\_i1.p1 | 43.84 | 3 | 3 | 0 | 1 | 1 | 1 |  | 40524.2109 | type:complete len:355 gc:universal TRINITY\_DN912\_c0\_g1\_i1:1586-522(-) |
| 134 | 317 | TRINITY\_DN12734\_c0\_g1\_i1.p1 | 43.84 | 3 | 3 | 0 | 1 | 1 | 1 |  | 42294.3555 | type:complete len:365 gc:universal TRINITY\_DN12734\_c0\_g1\_i1:509-1603(+) |
| 134 | 318 | TRINITY\_DN912\_c0\_g1\_i3.p1 | 43.84 | 3 | 3 | 0 | 1 | 1 | 1 |  | 40580.2539 | type:complete len:355 gc:universal TRINITY\_DN912\_c0\_g1\_i3:1586-522(-) |
| 134 | 319 | TRINITY\_DN192883\_c0\_g1\_i1.p1 | 43.84 | 3 | 3 | 0 | 1 | 1 | 1 |  | 44067.5391 | type:complete len:374 gc:universal TRINITY\_DN192883\_c0\_g1\_i1:1619-498(-) |
| 135 | 320 | TRINITY\_DN59572\_c0\_g1\_i1.p1 | 58.39 | 2 | 2 | 8.88e2 | 1 | 1 | 1 |  | 82544.875 | type:internal len:725 gc:universal TRINITY\_DN59572\_c0\_g1\_i1:3-2174(+) |
| 136 | 321 | TRINITY\_DN11522\_c0\_g1\_i1.p1 | 58.01 | 1 | 1 | 2.02e2 | 1 | 1 | 1 |  | 93223.9141 | type:5prime\_partial len:849 gc:universal TRINITY\_DN11522\_c0\_g1\_i1:2860-314(-) |
| 136 | 322 | TRINITY\_DN11522\_c0\_g1\_i3.p1 | 58.01 | 1 | 1 | 2.02e2 | 1 | 1 | 1 |  | 93820.75 | type:5prime\_partial len:855 gc:universal TRINITY\_DN11522\_c0\_g1\_i3:2878-314(-) |
| 136 | 323 | TRINITY\_DN11522\_c0\_g1\_i4.p1 | 58.01 | 1 | 1 | 2.02e2 | 1 | 1 | 1 |  | 93423.2266 | type:5prime\_partial len:851 gc:universal TRINITY\_DN11522\_c0\_g1\_i4:2866-314(-) |
| 136 | 324 | TRINITY\_DN11522\_c0\_g1\_i2.p1 | 58.01 | 1 | 1 | 2.02e2 | 1 | 1 | 1 |  | 93621.4375 | type:5prime\_partial len:853 gc:universal TRINITY\_DN11522\_c0\_g1\_i2:2872-314(-) |
| 137 | 325 | TRINITY\_DN75529\_c0\_g1\_i1.p1 | 53.02 | 3 | 3 | 0 | 1 | 1 | 1 |  | 57370.4688 | type:complete len:517 gc:universal TRINITY\_DN75529\_c0\_g1\_i1:94-1644(+) |
| 138 | 326 | TRINITY\_DN711\_c0\_g1\_i2.p1 | 54.78 | 6 | 6 | 1.32e3 | 1 | 1 | 1 |  | 16690.084 | type:internal len:183 gc:universal TRINITY\_DN711\_c0\_g1\_i2:548-3(-) |
| 139 | 327 | TRINITY\_DN1783\_c0\_g1\_i1.p2 | 58.24 | 2 | 2 | 3.79e2 | 1 | 1 | 1 |  | 53273.0938 | type:5prime\_partial len:473 gc:universal TRINITY\_DN1783\_c0\_g1\_i1:1460-42(-) |
| 139 | 328 | TRINITY\_DN1783\_c0\_g1\_i8.p2 | 58.24 | 2 | 2 | 3.79e2 | 1 | 1 | 1 |  | 53393.4727 | type:5prime\_partial len:472 gc:universal TRINITY\_DN1783\_c0\_g1\_i8:1457-42(-) |
| 139 | 329 | TRINITY\_DN105041\_c0\_g1\_i1.p1 | 58.24 | 6 | 6 | 3.79e2 | 1 | 1 | 1 |  | 15973.1162 | type:internal len:141 gc:universal TRINITY\_DN105041\_c0\_g1\_i1:2-421(+) |
| 139 | 330 | TRINITY\_DN1783\_c0\_g1\_i2.p1 | 58.24 | 2 | 2 | 3.79e2 | 1 | 1 | 1 |  | 53273.0938 | type:5prime\_partial len:473 gc:universal TRINITY\_DN1783\_c0\_g1\_i2:1528-110(-) |
| 139 | 331 | TRINITY\_DN1783\_c0\_g1\_i3.p1 | 58.24 | 3 | 3 | 3.79e2 | 1 | 1 | 1 |  | 38855.5742 | type:internal len:346 gc:universal TRINITY\_DN1783\_c0\_g1\_i3:1037-3(-) |
| 139 | 332 | TRINITY\_DN1783\_c0\_g1\_i6.p1 | 58.24 | 4 | 4 | 3.79e2 | 1 | 1 | 1 |  | 25307.8594 | type:3prime\_partial len:225 gc:universal TRINITY\_DN1783\_c0\_g1\_i6:674-3(-) |
| 139 | 333 | TRINITY\_DN1783\_c1\_g1\_i1.p2 | 58.24 | 7 | 7 | 3.79e2 | 1 | 1 | 1 |  | 15461.7402 | type:internal len:136 gc:universal TRINITY\_DN1783\_c1\_g1\_i1:2-406(+) |
| 140 | 334 | TRINITY\_DN98417\_c0\_g1\_i1.p1 | 57.32 | 4 | 4 | 6.97e2 | 1 | 1 | 1 |  | 19555.8887 | type:internal len:180 gc:universal TRINITY\_DN98417\_c0\_g1\_i1:2-538(+) |
| 141 | 335 | TRINITY\_DN38399\_c0\_g1\_i1.p1 | 58.39 | 2 | 2 | 4.8e2 | 1 | 1 | 1 | Carbamidomethylation | 97673.3438 | type:internal len:876 gc:universal TRINITY\_DN38399\_c0\_g1\_i1:2625-1(-) |
| 142 | 336 | TRINITY\_DN577\_c0\_g1\_i1.p1 | 53.35 | 3 | 3 | 2.62e2 | 1 | 1 | 1 |  | 30281.0371 | type:5prime\_partial len:269 gc:universal TRINITY\_DN577\_c0\_g1\_i1:1086-280(-) |
| 143 | 337 | TRINITY\_DN11595\_c0\_g1\_i2.p1 | 58.34 | 2 | 2 | 1.06e3 | 1 | 1 | 1 |  | 70926.875 | type:complete len:615 gc:universal TRINITY\_DN11595\_c0\_g1\_i2:1952-108(-) |
| 143 | 338 | TRINITY\_DN1285\_c0\_g1\_i1.p1 | 58.34 | 2 | 2 | 1.06e3 | 1 | 1 | 1 |  | 74668.875 | type:complete len:658 gc:universal TRINITY\_DN1285\_c0\_g1\_i1:2316-343(-) |
| 143 | 339 | TRINITY\_DN11595\_c0\_g1\_i1.p1 | 58.34 | 2 | 2 | 1.06e3 | 1 | 1 | 1 |  | 58738.1367 | type:complete len:510 gc:universal TRINITY\_DN11595\_c0\_g1\_i1:1768-239(-) |
| 144 | 340 | TRINITY\_DN3434\_c0\_g1\_i1.p1 | 48.14 | 4 | 4 | 0 | 1 | 1 | 1 |  | 33647.6406 | type:internal len:317 gc:universal TRINITY\_DN3434\_c0\_g1\_i1:1-948(+) |
| 144 | 341 | TRINITY\_DN73587\_c0\_g1\_i1.p1 | 48.14 | 3 | 3 | 0 | 1 | 1 | 1 |  | 57376.2227 | type:complete len:533 gc:universal TRINITY\_DN73587\_c0\_g1\_i1:94-1692(+) |
| 144 | 342 | TRINITY\_DN3434\_c0\_g1\_i5.p1 | 48.14 | 3 | 3 | 0 | 1 | 1 | 1 |  | 52717.207 | type:internal len:493 gc:universal TRINITY\_DN3434\_c0\_g1\_i5:1-1476(+) |
| 144 | 343 | TRINITY\_DN3434\_c0\_g1\_i10.p1 | 48.14 | 3 | 3 | 0 | 1 | 1 | 1 |  | 52717.207 | type:internal len:493 gc:universal TRINITY\_DN3434\_c0\_g1\_i10:1-1476(+) |
| 145 | 344 | TRINITY\_DN15584\_c0\_g1\_i1.p1 | 58.39 | 6 | 6 | 9.03e2 | 1 | 1 | 3 |  | 29534.9648 | type:complete len:275 gc:universal TRINITY\_DN15584\_c0\_g1\_i1:874-50(-) |
| 146 | 345 | TRINITY\_DN5115\_c0\_g1\_i2.p1 | 56.98 | 5 | 5 | 4.03e2 | 1 | 1 | 1 | Carbamidomethylation | 20983.8535 | type:3prime\_partial len:180 gc:universal TRINITY\_DN5115\_c0\_g1\_i2:539-3(-) |
| 147 | 346 | TRINITY\_DN557\_c0\_g1\_i5.p1 | 55.85 | 3 | 3 | 2.04e2 | 1 | 1 | 1 |  | 64487.5039 | type:complete len:571 gc:universal TRINITY\_DN557\_c0\_g1\_i5:26-1738(+) |
| 147 | 347 | TRINITY\_DN557\_c0\_g1\_i3.p1 | 55.85 | 2 | 2 | 2.04e2 | 1 | 1 | 1 |  | 90213.7969 | type:complete len:823 gc:universal TRINITY\_DN557\_c0\_g1\_i3:26-2494(+) |
| 147 | 348 | TRINITY\_DN557\_c0\_g1\_i2.p1 | 55.85 | 2 | 2 | 2.04e2 | 1 | 1 | 1 |  | 81549.1797 | type:3prime\_partial len:748 gc:universal TRINITY\_DN557\_c0\_g1\_i2:26-2266(+) |
| 147 | 349 | TRINITY\_DN557\_c0\_g1\_i4.p1 | 55.85 | 2 | 2 | 2.04e2 | 1 | 1 | 1 |  | 78484.6875 | type:complete len:708 gc:universal TRINITY\_DN557\_c0\_g1\_i4:26-2149(+) |
| 148 | 350 | TRINITY\_DN2885\_c0\_g1\_i2.p1 | 57.82 | 11 | 11 | 2.57e2 | 1 | 1 | 1 |  | 12758.7783 | type:complete len:114 gc:universal TRINITY\_DN2885\_c0\_g1\_i2:31-372(+) |
| 148 | 351 | TRINITY\_DN2885\_c0\_g1\_i5.p1 | 57.82 | 11 | 11 | 2.57e2 | 1 | 1 | 1 |  | 12383.5439 | type:3prime\_partial len:112 gc:universal TRINITY\_DN2885\_c0\_g1\_i5:31-363(+) |
| 148 | 352 | TRINITY\_DN2885\_c0\_g1\_i4.p1 | 57.82 | 11 | 11 | 2.57e2 | 1 | 1 | 1 |  | 12245.125 | type:5prime\_partial len:109 gc:universal TRINITY\_DN2885\_c0\_g1\_i4:1-327(+) |
| 149 | 353 | TRINITY\_DN17714\_c0\_g1\_i1.p1 | 54.86 | 3 | 3 | 0 | 1 | 1 | 1 | Carbamidomethylation | 72261.3516 | type:complete len:644 gc:universal TRINITY\_DN17714\_c0\_g1\_i1:102-2033(+) |
| 150 | 354 | TRINITY\_DN250\_c0\_g1\_i6.p1 | 58.29 | 4 | 4 | 0 | 1 | 1 | 2 |  | 50263.3672 | type:complete len:452 gc:universal TRINITY\_DN250\_c0\_g1\_i6:131-1486(+) |
| 150 | 355 | TRINITY\_DN687\_c0\_g2\_i1.p1 | 58.29 | 4 | 4 | 0 | 1 | 1 | 2 |  | 50237.3242 | type:complete len:453 gc:universal TRINITY\_DN687\_c0\_g2\_i1:90-1448(+) |
| 150 | 356 | TRINITY\_DN96161\_c0\_g1\_i1.p1 | 58.29 | 4 | 4 | 0 | 1 | 1 | 2 |  | 50498.6562 | type:complete len:453 gc:universal TRINITY\_DN96161\_c0\_g1\_i1:1593-235(-) |
| 150 | 357 | TRINITY\_DN687\_c0\_g1\_i1.p1 | 58.29 | 4 | 4 | 0 | 1 | 1 | 2 |  | 50621.6914 | type:complete len:453 gc:universal TRINITY\_DN687\_c0\_g1\_i1:95-1453(+) |
| 150 | 358 | TRINITY\_DN250\_c0\_g1\_i5.p1 | 58.29 | 4 | 4 | 0 | 1 | 1 | 2 |  | 50305.4453 | type:complete len:452 gc:universal TRINITY\_DN250\_c0\_g1\_i5:131-1486(+) |
| 150 | 359 | TRINITY\_DN250\_c0\_g1\_i3.p1 | 58.29 | 4 | 4 | 0 | 1 | 1 | 2 |  | 44456.6445 | type:3prime\_partial len:402 gc:universal TRINITY\_DN250\_c0\_g1\_i3:131-1333(+) |
| 150 | 360 | TRINITY\_DN250\_c0\_g1\_i4.p1 | 58.29 | 4 | 4 | 0 | 1 | 1 | 2 |  | 50305.4453 | type:complete len:452 gc:universal TRINITY\_DN250\_c0\_g1\_i4:131-1486(+) |
| 150 | 361 | TRINITY\_DN250\_c0\_g1\_i1.p1 | 58.29 | 4 | 4 | 0 | 1 | 1 | 2 |  | 44454.6094 | type:3prime\_partial len:402 gc:universal TRINITY\_DN250\_c0\_g1\_i1:131-1333(+) |
| 151 | 362 | TRINITY\_DN20282\_c0\_g1\_i1.p1 | 55.03 | 4 | 4 | 0 | 1 | 1 | 1 |  | 22865.916 | type:internal len:202 gc:universal TRINITY\_DN20282\_c0\_g1\_i1:3-605(+) |
| 152 | 363 | TRINITY\_DN6049\_c0\_g1\_i1.p1 | 56.95 | 7 | 7 | 4.52e2 | 1 | 1 | 2 |  | 17984.209 | type:complete len:156 gc:universal TRINITY\_DN6049\_c0\_g1\_i1:1046-579(-) |
| 153 | 364 | TRINITY\_DN3332\_c0\_g1\_i1.p1 | 38.38 | 2 | 2 | 3.06e2 | 1 | 1 | 1 |  | 39074.8789 | type:complete len:336 gc:universal TRINITY\_DN3332\_c0\_g1\_i1:1457-450(-) |
| 154 | 365 | TRINITY\_DN8926\_c0\_g1\_i1.p1 | 57.82 | 3 | 3 | 8.34e2 | 1 | 1 | 1 |  | 34324.4102 | type:complete len:307 gc:universal TRINITY\_DN8926\_c0\_g1\_i1:1655-735(-) |
| 154 | 366 | TRINITY\_DN8926\_c0\_g1\_i2.p1 | 57.82 | 3 | 3 | 8.34e2 | 1 | 1 | 1 |  | 34324.4102 | type:complete len:307 gc:universal TRINITY\_DN8926\_c0\_g1\_i2:1696-776(-) |
| 155 | 367 | TRINITY\_DN163801\_c0\_g1\_i1.p1 | 39.76 | 3 | 3 | 5.36e1 | 1 | 1 | 1 | Acetylation (Protein N-term) | 33076.9062 | type:complete len:298 gc:universal TRINITY\_DN163801\_c0\_g1\_i1:55-948(+) |
| 156 | 368 | TRINITY\_DN124859\_c0\_g1\_i1.p3 | 46.97 | 3 | 3 | 1.78e2 | 1 | 1 | 1 |  | 26114.5156 | type:5prime\_partial len:237 gc:universal TRINITY\_DN124859\_c0\_g1\_i1:884-174(-) |
| 157 | 369 | TRINITY\_DN141338\_c2\_g1\_i1.p1 | 58.28 | 1 | 1 | 0 | 1 | 1 | 1 |  | 82716.2266 | type:complete len:720 gc:universal TRINITY\_DN141338\_c2\_g1\_i1:2850-691(-) |
| 158 | 370 | TRINITY\_DN2532\_c2\_g1\_i1.p1 | 48.47 | 3 | 3 | 0 | 1 | 1 | 1 | Carbamidomethylation | 52409.3047 | type:internal len:466 gc:universal TRINITY\_DN2532\_c2\_g1\_i1:1396-2(-) |
| 159 | 371 | TRINITY\_DN1214\_c0\_g1\_i1.p1 | 54.6 | 1 | 1 | 6.12e2 | 1 | 1 | 1 | Carbamidomethylation | 85049.8047 | type:complete len:752 gc:universal TRINITY\_DN1214\_c0\_g1\_i1:19-2274(+) |
| 160 | 372 | TRINITY\_DN121236\_c0\_g1\_i1.p1 | 55.52 | 3 | 3 | 1.2e3 | 1 | 1 | 1 |  | 64133.2812 | type:internal len:568 gc:universal TRINITY\_DN121236\_c0\_g1\_i1:1701-1(-) |
| 161 | 373 | TRINITY\_DN4523\_c0\_g1\_i1.p1 | 38.06 | 1 | 1 | 0 | 1 | 1 | 1 |  | 188936.4688 | type:complete len:1645 gc:universal TRINITY\_DN4523\_c0\_g1\_i1:5332-398(-) |
| 161 | 374 | TRINITY\_DN4523\_c0\_g1\_i2.p1 | 38.06 | 1 | 1 | 0 | 1 | 1 | 1 |  | 195964.2344 | type:5prime\_partial len:1710 gc:universal TRINITY\_DN4523\_c0\_g1\_i2:5527-398(-) |
| 162 | 375 | TRINITY\_DN8941\_c0\_g1\_i12.p1 | 58.37 | 1 | 1 | 1.41e3 | 1 | 1 | 2 |  | 83188.6016 | type:5prime\_partial len:771 gc:universal TRINITY\_DN8941\_c0\_g1\_i12:2-2314(+) |
| 162 | 376 | TRINITY\_DN8941\_c0\_g1\_i2.p1 | 58.37 | 2 | 2 | 1.41e3 | 1 | 1 | 2 |  | 55903.7969 | type:5prime\_partial len:520 gc:universal TRINITY\_DN8941\_c0\_g1\_i2:2-1561(+) |
| 163 | 377 | TRINITY\_DN1339\_c6\_g1\_i1.p1 | 58.39 | 2 | 2 | 3.46e2 | 1 | 1 | 1 |  | 61274.3594 | type:internal len:570 gc:universal TRINITY\_DN1339\_c6\_g1\_i1:1-1707(+) |
| 163 | 378 | TRINITY\_DN1339\_c0\_g1\_i2.p1 | 58.39 | 3 | 3 | 3.46e2 | 1 | 1 | 1 |  | 41222.6992 | type:internal len:384 gc:universal TRINITY\_DN1339\_c0\_g1\_i2:1151-3(-) |
| 163 | 379 | TRINITY\_DN124769\_c0\_g1\_i1.p1 | 58.39 | 11 | 11 | 3.46e2 | 1 | 1 | 1 |  | 12144.8828 | type:internal len:113 gc:universal TRINITY\_DN124769\_c0\_g1\_i1:1-336(+) |
| 163 | 380 | TRINITY\_DN24414\_c1\_g1\_i2.p2 | 58.39 | 8 | 8 | 3.46e2 | 1 | 1 | 1 |  | 15590.8623 | type:internal len:146 gc:universal TRINITY\_DN24414\_c1\_g1\_i2:435-1(-) |
| 163 | 381 | TRINITY\_DN1172\_c0\_g1\_i3.p1 | 58.39 | 7 | 7 | 3.46e2 | 1 | 1 | 1 |  | 19709.3613 | type:internal len:182 gc:universal TRINITY\_DN1172\_c0\_g1\_i3:545-3(-) |
| 163 | 382 | TRINITY\_DN119673\_c0\_g1\_i1.p1 | 58.39 | 9 | 9 | 3.46e2 | 1 | 1 | 1 |  | 13988.7559 | type:internal len:129 gc:universal TRINITY\_DN119673\_c0\_g1\_i1:2-385(+) |
| 163 | 383 | TRINITY\_DN1339\_c0\_g1\_i1.p1 | 58.39 | 5 | 5 | 3.46e2 | 1 | 1 | 1 |  | 24722.7773 | type:internal len:229 gc:universal TRINITY\_DN1339\_c0\_g1\_i1:684-1(-) |
| 163 | 384 | TRINITY\_DN6701\_c0\_g1\_i1.p1 | 58.39 | 6 | 6 | 3.46e2 | 1 | 1 | 1 |  | 21246.0547 | type:internal len:196 gc:universal TRINITY\_DN6701\_c0\_g1\_i1:1-585(+) |
| 163 | 385 | TRINITY\_DN1172\_c0\_g1\_i5.p1 | 58.39 | 5 | 5 | 3.46e2 | 1 | 1 | 1 |  | 24525.6855 | type:internal len:226 gc:universal TRINITY\_DN1172\_c0\_g1\_i5:677-3(-) |
| 163 | 386 | TRINITY\_DN6701\_c0\_g1\_i2.p2 | 58.39 | 8 | 8 | 3.46e2 | 1 | 1 | 1 |  | 16512.8535 | type:internal len:152 gc:universal TRINITY\_DN6701\_c0\_g1\_i2:3-455(+) |
| 163 | 387 | TRINITY\_DN1339\_c6\_g1\_i2.p1 | 58.39 | 3 | 3 | 3.46e2 | 1 | 1 | 1 |  | 43821.8359 | type:internal len:408 gc:universal TRINITY\_DN1339\_c6\_g1\_i2:3-1223(+) |
| 164 | 388 | TRINITY\_DN2357\_c1\_g1\_i1.p1 | 58.23 | 1 | 1 | 0 | 1 | 1 | 1 |  | 196914.375 | type:complete len:1733 gc:universal TRINITY\_DN2357\_c1\_g1\_i1:5452-254(-) |
| 165 | 389 | TRINITY\_DN7641\_c0\_g1\_i1.p1 | 58.28 | 1 | 1 | 2.94e2 | 1 | 1 | 1 | Carbamidomethylation | 139763.5156 | type:complete len:1239 gc:universal TRINITY\_DN7641\_c0\_g1\_i1:53-3769(+) |
| 166 | 390 | TRINITY\_DN11643\_c0\_g1\_i1.p1 | 58.28 | 7 | 7 | 9e2 | 1 | 1 | 1 |  | 21061.2832 | type:complete len:187 gc:universal TRINITY\_DN11643\_c0\_g1\_i1:1748-1188(-) |
| 167 | 391 | TRINITY\_DN121418\_c0\_g1\_i1.p1 | 54.99 | 4 | 4 | 3.56e2 | 1 | 1 | 1 |  | 29750.6836 | type:complete len:259 gc:universal TRINITY\_DN121418\_c0\_g1\_i1:1061-285(-) |
| 168 | 392 | TRINITY\_DN57\_c0\_g1\_i1.p1 | 58.31 | 1 | 1 | 1.34e3 | 1 | 1 | 1 |  | 81029.6719 | type:complete len:712 gc:universal TRINITY\_DN57\_c0\_g1\_i1:140-2275(+) |
| 169 | 393 | TRINITY\_DN36217\_c0\_g1\_i1.p1 | 58.11 | 1 | 1 | 9.3e2 | 1 | 1 | 2 |  | 68373.1172 | type:5prime\_partial len:605 gc:universal TRINITY\_DN36217\_c0\_g1\_i1:2-1816(+) |
| 170 | 394 | TRINITY\_DN26249\_c0\_g1\_i1.p1 | 58.39 | 3 | 3 | 0 | 1 | 1 | 1 |  | 54490.1055 | type:5prime\_partial len:477 gc:universal TRINITY\_DN26249\_c0\_g1\_i1:1733-303(-) |
| 171 | 395 | TRINITY\_DN2113\_c0\_g1\_i1.p1 | 52.16 | 1 | 1 | 0 | 1 | 1 | 1 |  | 193799.0156 | type:complete len:1704 gc:universal TRINITY\_DN2113\_c0\_g1\_i1:5257-146(-) |
| 171 | 396 | TRINITY\_DN2113\_c0\_g1\_i2.p1 | 52.16 | 1 | 1 | 0 | 1 | 1 | 1 |  | 193799.0156 | type:complete len:1704 gc:universal TRINITY\_DN2113\_c0\_g1\_i2:5257-146(-) |
| 172 | 397 | TRINITY\_DN39385\_c0\_g1\_i1.p1 | 58.32 | 10 | 10 | 0 | 1 | 1 | 1 |  | 17334.5918 | type:5prime\_partial len:154 gc:universal TRINITY\_DN39385\_c0\_g1\_i1:2-463(+) |
| 172 | 398 | TRINITY\_DN39385\_c0\_g1\_i2.p1 | 58.32 | 14 | 14 | 0 | 1 | 1 | 1 |  | 13149.8369 | type:5prime\_partial len:117 gc:universal TRINITY\_DN39385\_c0\_g1\_i2:2-352(+) |
| 173 | 399 | TRINITY\_DN205\_c0\_g1\_i3.p1 | 55.69 | 2 | 2 | 3.33e2 | 1 | 1 | 1 | Carbamidomethylation | 69293.7422 | type:5prime\_partial len:702 gc:universal TRINITY\_DN205\_c0\_g1\_i3:3651-1546(-) |
| 174 | 400 | TRINITY\_DN2532\_c1\_g1\_i1.p1 | 58.38 | 1 | 1 | 0 | 1 | 1 | 1 |  | 81643.5312 | type:5prime\_partial len:719 gc:universal TRINITY\_DN2532\_c1\_g1\_i1:3-2159(+) |
| 175 | 401 | TRINITY\_DN100850\_c0\_g1\_i1.p1 | 55.62 | 10 | 10 | 2.22e2 | 1 | 1 | 2 | Carbamidomethylation | 20555.9395 | type:internal len:182 gc:universal TRINITY\_DN100850\_c0\_g1\_i1:543-1(-) |
| 176 | 402 | TRINITY\_DN3655\_c1\_g1\_i1.p1 | 57.58 | 5 | 5 | 1.03e3 | 1 | 1 | 1 |  | 21701.7266 | type:internal len:191 gc:universal TRINITY\_DN3655\_c1\_g1\_i1:2-571(+) |
| 177 | 403 | TRINITY\_DN909\_c0\_g1\_i1.p1 | 54.85 | 4 | 4 | 6.05e2 | 1 | 1 | 1 |  | 35211.4883 | type:5prime\_partial len:313 gc:universal TRINITY\_DN909\_c0\_g1\_i1:1-939(+) |
| 178 | 404 | TRINITY\_DN1774\_c0\_g1\_i1.p1 | 58.18 | 5 | 5 | 2.85e3 | 1 | 1 | 1 |  | 21382.0469 | type:3prime\_partial len:208 gc:universal TRINITY\_DN1774\_c0\_g1\_i1:218-838(+) |
| 179 | 405 | TRINITY\_DN413\_c0\_g1\_i4.p1 | 58.25 | 2 | 2 | 5.68e2 | 1 | 1 | 1 |  | 52354.1758 | type:3prime\_partial len:463 gc:universal TRINITY\_DN413\_c0\_g1\_i4:1388-3(-) |
| 179 | 406 | TRINITY\_DN413\_c0\_g1\_i1.p1 | 58.25 | 3 | 3 | 5.68e2 | 1 | 1 | 1 |  | 40351.7656 | type:complete len:355 gc:universal TRINITY\_DN413\_c0\_g1\_i1:1352-288(-) |
| 179 | 407 | TRINITY\_DN413\_c0\_g1\_i2.p1 | 58.25 | 8 | 8 | 5.68e2 | 1 | 1 | 1 |  | 15739.6523 | type:3prime\_partial len:140 gc:universal TRINITY\_DN413\_c0\_g1\_i2:419-3(-) |
| 179 | 408 | TRINITY\_DN413\_c0\_g1\_i3.p1 | 58.25 | 3 | 3 | 5.68e2 | 1 | 1 | 1 |  | 39501.6133 | type:complete len:350 gc:universal TRINITY\_DN413\_c0\_g1\_i3:1286-237(-) |
| 180 | 409 | TRINITY\_DN101600\_c0\_g1\_i1.p1 | 58.22 | 3 | 3 | 0 | 1 | 1 | 1 |  | 35005.5742 | type:3prime\_partial len:312 gc:universal TRINITY\_DN101600\_c0\_g1\_i1:935-3(-) |
| 181 | 410 | TRINITY\_DN143676\_c0\_g1\_i1.p1 | 54.89 | 7 | 7 | 7.08e2 | 1 | 1 | 1 |  | 15009.208 | type:internal len:124 gc:universal TRINITY\_DN143676\_c0\_g1\_i1:3-371(+) |
| 182 | 411 | TRINITY\_DN33328\_c0\_g1\_i1.p1 | 58.26 | 1 | 1 | 0 | 1 | 1 | 1 |  | 156121.4062 | type:3prime\_partial len:1422 gc:universal TRINITY\_DN33328\_c0\_g1\_i1:4264-2(-) |
| 183 | 412 | TRINITY\_DN5554\_c0\_g1\_i1.p1 | 58.36 | 4 | 4 | 5.55e2 | 1 | 1 | 2 |  | 35530.5234 | type:complete len:316 gc:universal TRINITY\_DN5554\_c0\_g1\_i1:223-1170(+) |
| 184 | 413 | TRINITY\_DN2433\_c0\_g1\_i2.p1 | 37.77 | 10 | 10 | 0 | 1 | 1 | 1 |  | 17063.7168 | type:5prime\_partial len:147 gc:universal TRINITY\_DN2433\_c0\_g1\_i2:1-441(+) |
| 184 | 414 | TRINITY\_DN2433\_c0\_g1\_i1.p1 | 37.77 | 5 | 5 | 0 | 1 | 1 | 1 |  | 31350.3457 | type:5prime\_partial len:267 gc:universal TRINITY\_DN2433\_c0\_g1\_i1:1-801(+) |
| 185 | 415 | TRINITY\_DN69484\_c0\_g1\_i1.p1 | 58.4 | 9 | 9 | 3.08e2 | 1 | 1 | 1 |  | 16505.6328 | type:internal len:142 gc:universal TRINITY\_DN69484\_c0\_g1\_i1:1-423(+) |
| 186 | 416 | TRINITY\_DN4513\_c0\_g1\_i3.p1 | 57.72 | 9 | 9 | 9.07e2 | 1 | 1 | 1 |  | 13358.792 | type:5prime\_partial len:116 gc:universal TRINITY\_DN4513\_c0\_g1\_i3:652-305(-) |
| 186 | 417 | TRINITY\_DN4513\_c0\_g1\_i12.p1 | 57.72 | 9 | 9 | 9.07e2 | 1 | 1 | 1 |  | 13180.7021 | type:internal len:116 gc:universal TRINITY\_DN4513\_c0\_g1\_i12:346-2(-) |
| 187 | 418 | TRINITY\_DN31399\_c0\_g1\_i1.p1 | 40.35 | 4 | 4 | 2.94e1 | 1 | 1 | 1 | Oxidation (M) | 42043.4648 | type:5prime\_partial len:382 gc:universal TRINITY\_DN31399\_c0\_g1\_i1:2-1147(+) |
| 187 | 419 | TRINITY\_DN31399\_c0\_g1\_i2.p1 | 40.35 | 2 | 2 | 2.94e1 | 1 | 1 | 1 | Oxidation (M) | 89961.6641 | type:complete len:819 gc:universal TRINITY\_DN31399\_c0\_g1\_i2:85-2541(+) |
| 187 | 420 | TRINITY\_DN31399\_c0\_g1\_i3.p1 | 40.35 | 2 | 2 | 2.94e1 | 1 | 1 | 1 | Oxidation (M) | 89350.0078 | type:complete len:813 gc:universal TRINITY\_DN31399\_c0\_g1\_i3:85-2523(+) |
| 188 | 421 | TRINITY\_DN177106\_c0\_g1\_i1.p1 | 58.39 | 10 | 10 | 0 | 1 | 1 | 1 | Oxidation (M) | 15440.9473 | type:internal len:139 gc:universal TRINITY\_DN177106\_c0\_g1\_i1:1-414(+) |
| 189 | 422 | TRINITY\_DN2576\_c3\_g1\_i1.p2 | 58.24 | 8 | 8 | 6.48e2 | 1 | 1 | 2 |  | 16068.6924 | type:complete len:145 gc:universal TRINITY\_DN2576\_c3\_g1\_i1:807-373(-) |
| 190 | 423 | TRINITY\_DN143583\_c0\_g1\_i1.p1 | 57.01 | 16 | 16 | 7.88e2 | 1 | 1 | 1 |  | 11081.3594 | type:internal len:103 gc:universal TRINITY\_DN143583\_c0\_g1\_i1:308-3(-) |
| 190 | 424 | TRINITY\_DN82766\_c0\_g1\_i1.p1 | 57.01 | 11 | 11 | 7.88e2 | 1 | 1 | 1 |  | 15890.7988 | type:internal len:142 gc:universal TRINITY\_DN82766\_c0\_g1\_i1:425-3(-) |
| 191 | 425 | TRINITY\_DN3991\_c0\_g1\_i1.p1 | 58.26 | 5 | 5 | 8.33e4 | 1 | 1 | 1 | Carbamidomethylation | 24846.6543 | type:3prime\_partial len:219 gc:universal TRINITY\_DN3991\_c0\_g1\_i1:84-737(+) |
| 192 | 426 | TRINITY\_DN72753\_c0\_g1\_i1.p1 | 58.18 | 8 | 8 | 2.55e2 | 1 | 1 | 1 |  | 15430.793 | type:complete len:137 gc:universal TRINITY\_DN72753\_c0\_g1\_i1:95-505(+) |
| 193 | 427 | TRINITY\_DN49617\_c0\_g1\_i2.p1 | 58.05 | 17 | 17 | 0 | 1 | 1 | 1 |  | 11096.9736 | type:internal len:100 gc:universal TRINITY\_DN49617\_c0\_g1\_i2:299-3(-) |
| 194 | 428 | TRINITY\_DN20671\_c0\_g1\_i1.p1 | 56.39 | 1 | 1 | 0 | 1 | 1 | 1 |  | 144159.875 | type:5prime\_partial len:1266 gc:universal TRINITY\_DN20671\_c0\_g1\_i1:4791-994(-) |
| 195 | 429 | TRINITY\_DN101992\_c0\_g1\_i1.p1 | 58.3 | 7 | 7 | 1.49e3 | 1 | 1 | 1 | Carbamidomethylation | 15607.4678 | type:internal len:139 gc:universal TRINITY\_DN101992\_c0\_g1\_i1:414-1(-) |
| 196 | 430 | TRINITY\_DN191650\_c0\_g1\_i1.p1 | 54.81 | 5 | 5 | 5.31e2 | 1 | 1 | 2 |  | 26425.8496 | type:complete len:239 gc:universal TRINITY\_DN191650\_c0\_g1\_i1:957-241(-) |
| 197 | 431 | TRINITY\_DN21554\_c2\_g1\_i1.p1 | 58.07 | 4 | 4 | 0 | 1 | 1 | 1 |  | 40301.0508 | type:5prime\_partial len:348 gc:universal TRINITY\_DN21554\_c2\_g1\_i1:2-1045(+) |
| 198 | 432 | TRINITY\_DN72625\_c0\_g1\_i1.p1 | 55.11 | 1 | 1 | 4.08e3 | 1 | 1 | 2 |  | 214200.3906 | type:complete len:1964 gc:universal TRINITY\_DN72625\_c0\_g1\_i1:173-6064(+) |
| 199 | 433 | TRINITY\_DN1628\_c0\_g1\_i2.p1 | 39.32 | 3 | 3 | 3.72e2 | 1 | 1 | 1 |  | 61120.0469 | type:complete len:556 gc:universal TRINITY\_DN1628\_c0\_g1\_i2:81-1748(+) |
| 200 | 434 | TRINITY\_DN21554\_c1\_g1\_i1.p1 | 58.28 | 1 | 1 | 7.34e2 | 1 | 1 | 3 |  | 75420.7656 | type:3prime\_partial len:650 gc:universal TRINITY\_DN21554\_c1\_g1\_i1:1947-1(-) |
| 201 | 435 | TRINITY\_DN86076\_c0\_g1\_i1.p1 | 57 | 5 | 5 | 0 | 1 | 1 | 1 |  | 24944.2871 | type:internal len:210 gc:universal TRINITY\_DN86076\_c0\_g1\_i1:628-2(-) |
| 202 | 436 | TRINITY\_DN16533\_c0\_g2\_i1.p1 | 57.66 | 3 | 3 | 5.95e2 | 1 | 1 | 1 | Carbamidomethylation | 45506.2344 | type:3prime\_partial len:396 gc:universal TRINITY\_DN16533\_c0\_g2\_i1:1185-1(-) |
| 203 | 437 | TRINITY\_DN33467\_c0\_g1\_i1.p1 | 53.5 | 5 | 5 | 1.07e3 | 1 | 1 | 1 |  | 27953.0371 | type:5prime\_partial len:243 gc:universal TRINITY\_DN33467\_c0\_g1\_i1:788-60(-) |
| 204 | 438 | TRINITY\_DN15542\_c0\_g1\_i1.p1 | 58.24 | 9 | 9 | 0 | 1 | 1 | 1 |  | 21338.2832 | type:internal len:195 gc:universal TRINITY\_DN15542\_c0\_g1\_i1:584-3(-) |
| 204 | 439 | TRINITY\_DN15542\_c0\_g1\_i2.p1 | 58.24 | 7 | 7 | 0 | 1 | 1 | 1 |  | 28581.1797 | type:internal len:260 gc:universal TRINITY\_DN15542\_c0\_g1\_i2:777-1(-) |
| 205 | 440 | TRINITY\_DN107813\_c0\_g1\_i1.p1 | 58.26 | 7 | 7 | 0 | 1 | 1 | 1 |  | 19146.4668 | type:internal len:176 gc:universal TRINITY\_DN107813\_c0\_g1\_i1:525-1(-) |
| 206 | 441 | TRINITY\_DN179\_c0\_g1\_i2.p1 | 58.29 | 2 | 2 | 0 | 1 | 1 | 1 |  | 58576.75 | type:3prime\_partial len:619 gc:universal TRINITY\_DN179\_c0\_g1\_i2:84-1937(+) |
| 207 | 442 | TRINITY\_DN8008\_c0\_g1\_i2.p1 | 57 | 1 | 1 | 0 | 1 | 1 | 2 |  | 146342.75 | type:5prime\_partial len:1298 gc:universal TRINITY\_DN8008\_c0\_g1\_i2:4205-312(-) |
| 207 | 443 | TRINITY\_DN8008\_c0\_g1\_i3.p1 | 57 | 1 | 1 | 0 | 1 | 1 | 2 |  | 146351.7188 | type:5prime\_partial len:1298 gc:universal TRINITY\_DN8008\_c0\_g1\_i3:4205-312(-) |
| 208 | 444 | TRINITY\_DN252\_c0\_g1\_i1.p1 | 54.87 | 5 | 5 | 0 | 1 | 1 | 1 |  | 20771.6992 | type:complete len:182 gc:universal TRINITY\_DN252\_c0\_g1\_i1:1409-864(-) |
| 209 | 445 | TRINITY\_DN95415\_c0\_g1\_i1.p1 | 52.57 | 7 | 7 | 0 | 1 | 1 | 1 |  | 20894.9082 | type:internal len:183 gc:universal TRINITY\_DN95415\_c0\_g1\_i1:546-1(-) |
| 210 | 446 | TRINITY\_DN794\_c0\_g1\_i2.p1 | 58.33 | 4 | 4 | 0 | 1 | 1 | 1 |  | 27820.5742 | type:complete len:250 gc:universal TRINITY\_DN794\_c0\_g1\_i2:72-821(+) |
| 211 | 447 | TRINITY\_DN14729\_c0\_g1\_i1.p1 | 44.8 | 2 | 2 | 0 | 1 | 1 | 1 |  | 79209.8594 | type:complete len:698 gc:universal TRINITY\_DN14729\_c0\_g1\_i1:45-2138(+) |
| 212 | 448 | TRINITY\_DN51907\_c0\_g1\_i1.p1 | 58.23 | 3 | 3 | 1.11e3 | 1 | 1 | 1 |  | 47638.75 | type:3prime\_partial len:421 gc:universal TRINITY\_DN51907\_c0\_g1\_i1:1262-3(-) |
| 213 | 449 | TRINITY\_DN1850\_c0\_g1\_i3.p1 | 58.26 | 8 | 8 | 4.55e2 | 1 | 1 | 2 |  | 18428.4492 | type:5prime\_partial len:164 gc:universal TRINITY\_DN1850\_c0\_g1\_i3:659-168(-) |
| 214 | 450 | TRINITY\_DN32804\_c0\_g1\_i1.p1 | 58.28 | 1 | 1 | 0 | 1 | 1 | 1 |  | 83025.1641 | type:5prime\_partial len:746 gc:universal TRINITY\_DN32804\_c0\_g1\_i1:3-2240(+) |
| 215 | 451 | TRINITY\_DN7849\_c0\_g1\_i1.p1 | 58.27 | 5 | 5 | 8.31e2 | 1 | 1 | 1 |  | 27911.9336 | type:complete len:251 gc:universal TRINITY\_DN7849\_c0\_g1\_i1:1417-665(-) |
| 216 | 452 | TRINITY\_DN99404\_c0\_g1\_i1.p1 | 58.16 | 13 | 13 | 5.98e2 | 1 | 1 | 1 |  | 12615.5928 | type:internal len:112 gc:universal TRINITY\_DN99404\_c0\_g1\_i1:2-334(+) |
| 217 | 453 | TRINITY\_DN129928\_c0\_g1\_i1.p1 | 53.54 | 5 | 5 | 2.89e3 | 1 | 1 | 1 |  | 16045.6582 | type:internal len:154 gc:universal TRINITY\_DN129928\_c0\_g1\_i1:460-2(-) |
| 218 | 454 | TRINITY\_DN62874\_c0\_g1\_i1.p1 | 58.27 | 5 | 5 | 0 | 1 | 1 | 1 |  | 21615.5625 | type:internal len:197 gc:universal TRINITY\_DN62874\_c0\_g1\_i1:2-589(+) |
| 219 | 455 | TRINITY\_DN76789\_c0\_g1\_i1.p1 | 53.02 | 3 | 3 | 0 | 1 | 1 | 1 | Carbamidomethylation | 43284.8242 | type:internal len:381 gc:universal TRINITY\_DN76789\_c0\_g1\_i1:1140-1(-) |
| 220 | 456 | TRINITY\_DN4295\_c0\_g1\_i1.p1 | 57.44 | 4 | 4 | 4.69e2 | 1 | 1 | 1 |  | 27574.5312 | type:complete len:245 gc:universal TRINITY\_DN4295\_c0\_g1\_i1:1031-297(-) |
| 221 | 457 | TRINITY\_DN63421\_c0\_g1\_i1.p1 | 55.69 | 3 | 3 | 1.43e3 | 1 | 1 | 1 | Carbamidomethylation | 30242.6914 | type:internal len:278 gc:universal TRINITY\_DN63421\_c0\_g1\_i1:1-831(+) |
| 222 | 458 | TRINITY\_DN1280\_c0\_g1\_i1.p1 | 53.32 | 3 | 3 | 0 | 1 | 1 | 1 |  | 47818.6641 | type:complete len:426 gc:universal TRINITY\_DN1280\_c0\_g1\_i1:195-1472(+) |
| 223 | 459 | TRINITY\_DN1172\_c0\_g2\_i2.p1 | 55.86 | 4 | 4 | 1.49e3 | 1 | 1 | 1 | Acetylation (Protein N-term) | 27813.4355 | type:internal len:264 gc:universal TRINITY\_DN1172\_c0\_g2\_i2:790-2(-) |
| 224 | 460 | TRINITY\_DN172717\_c0\_g1\_i1.p1 | 55.92 | 5 | 5 | 0 | 1 | 1 | 1 |  | 23810.2109 | type:internal len:213 gc:universal TRINITY\_DN172717\_c0\_g1\_i1:1-636(+) |
| 225 | 461 | TRINITY\_DN45008\_c0\_g1\_i1.p1 | 58.34 | 3 | 3 | 0 | 1 | 1 | 1 |  | 42838.9297 | type:3prime\_partial len:388 gc:universal TRINITY\_DN45008\_c0\_g1\_i1:1161-1(-) |
| 226 | 462 | TRINITY\_DN5397\_c0\_g1\_i1.p1 | 37.49 | 5 | 5 | 9.88e2 | 1 | 1 | 1 |  | 17679.4688 | type:internal len:161 gc:universal TRINITY\_DN5397\_c0\_g1\_i1:2-481(+) |
| 227 | 463 | TRINITY\_DN71928\_c0\_g1\_i1.p1 | 58.39 | 5 | 5 | 0 | 1 | 1 | 1 | Carbamidomethylation | 23048.7402 | type:complete len:202 gc:universal TRINITY\_DN71928\_c0\_g1\_i1:73-678(+) |
| total 463 proteins |  |  |  |  |  |  |  |  |  |  |  |  |
| --- | --- | --- | --- | --- | --- | --- | --- | --- | --- | --- | --- | --- |

  

TRINITY\_DN11435\_c0\_g1\_i1.p1

back to list

  

| Protein Coverage
| Supporting Peptides
| Best Unique PSM
|

Protein Coverage:

Supporting Peptides:

| Peptide | Uniq | -10lgP | Mass | Length | ppm | m/z | z | RT | 1/k0 Start | 1/k0 End | Precursor ID | Area Sample 4 | #Feature | #Feature Sample 4 | Start | End | PTM | AScore | Found By |
| --- | --- | --- | --- | --- | --- | --- | --- | --- | --- | --- | --- | --- | --- | --- | --- | --- | --- | --- | --- |
| R.AILGTLSVEAIYK.D | Y | 54.49 | 1376.7915 | 13 | -1.17 | 689.4007 | 2 | 19.66 | 1.0508 | 1.058 | 75332 | 3.13e2 | 1 | 1 | 113 | 125 |  |  | DB Search |
| R.FKADTTIADAAR.A | Y | 49.7 | 1278.6568 | 12 | -1.01 | 640.3336 | 2 | 13.58 | 0.9707 | 0.9779 | 36609 | 0 | 0 | 0 | 206 | 217 |  |  | DB Search |
| total 2 peptides |  |  |  |  |  |  |  |  |  |  |  |  |  |  |  |  |  |  |  |
| --- | --- | --- | --- | --- | --- | --- | --- | --- | --- | --- | --- | --- | --- | --- | --- | --- | --- | --- | --- |

Best Unique PSM (Scan Sample\_2\_A1\_1\_3640.d:75332, m/z=689.4007, z=2, RT=19.66, ppm=-3.37):


TRINITY\_DN101992\_c0\_g1\_i1.p1

back to list

  

| Protein Coverage
| Supporting Peptides
| Best Unique PSM
|

Protein Coverage:

Supporting Peptides:

| Peptide | Uniq | -10lgP | Mass | Length | ppm | m/z | z | RT | 1/k0 Start | 1/k0 End | Precursor ID | Area Sample 4 | #Feature | #Feature Sample 4 | Start | End | PTM | AScore | Found By |
| --- | --- | --- | --- | --- | --- | --- | --- | --- | --- | --- | --- | --- | --- | --- | --- | --- | --- | --- | --- |
| K.AELVFVNHC(+57.02)R.E | Y | 58.3 | 1243.6132 | 10 | 2.08 | 622.8138 | 2 | 14.57 | 0.9578 | 0.965 | 42523 | 1.49e3 | 1 | 1 | 118 | 127 | Carbamidomethylation |  | DB Search |
| total 1 peptides |  |  |  |  |  |  |  |  |  |  |  |  |  |  |  |  |  |  |  |
| --- | --- | --- | --- | --- | --- | --- | --- | --- | --- | --- | --- | --- | --- | --- | --- | --- | --- | --- | --- |

Best Unique PSM (Scan Sample\_2\_A1\_1\_3640.d:42523, m/z=622.8138, z=2, RT=14.57, ppm=-0.11):


TRINITY\_DN11522\_c0\_g1\_i4.p1

back to list

  

| Protein Coverage
| Supporting Peptides
| Best Unique PSM
|

Protein Coverage:

Supporting Peptides:

| Peptide | Uniq | -10lgP | Mass | Length | ppm | m/z | z | RT | 1/k0 Start | 1/k0 End | Precursor ID | Area Sample 4 | #Feature | #Feature Sample 4 | Start | End | PTM | AScore | Found By |
| --- | --- | --- | --- | --- | --- | --- | --- | --- | --- | --- | --- | --- | --- | --- | --- | --- | --- | --- | --- |
| K.GKPEQVQVK.M | Y | 58.01 | 1011.5713 | 9 | 0.86 | 506.7922 | 2 | 9.91 | 0.8376 | 0.8449 | 14438 | 2.02e2 | 1 | 1 | 544 | 552 |  |  | DB Search |
| total 1 peptides |  |  |  |  |  |  |  |  |  |  |  |  |  |  |  |  |  |  |  |
| --- | --- | --- | --- | --- | --- | --- | --- | --- | --- | --- | --- | --- | --- | --- | --- | --- | --- | --- | --- |

Best Unique PSM (Scan Sample\_2\_A1\_1\_3640.d:14438, m/z=506.7922, z=2, RT=9.91, ppm=-1.33):


TRINITY\_DN72753\_c0\_g1\_i1.p1

back to list

  

| Protein Coverage
| Supporting Peptides
| Best Unique PSM
|

Protein Coverage:

Supporting Peptides:

| Peptide | Uniq | -10lgP | Mass | Length | ppm | m/z | z | RT | 1/k0 Start | 1/k0 End | Precursor ID | Area Sample 4 | #Feature | #Feature Sample 4 | Start | End | PTM | AScore | Found By |
| --- | --- | --- | --- | --- | --- | --- | --- | --- | --- | --- | --- | --- | --- | --- | --- | --- | --- | --- | --- |
| R.EIAQDFKTDLR.F | Y | 58.18 | 1334.683 | 11 | 0.79 | 668.3478 | 2 | 14.95 | 0.9924 | 0.9997 | 45098 | 2.55e2 | 1 | 1 | 74 | 84 |  |  | DB Search |
| total 1 peptides |  |  |  |  |  |  |  |  |  |  |  |  |  |  |  |  |  |  |  |
| --- | --- | --- | --- | --- | --- | --- | --- | --- | --- | --- | --- | --- | --- | --- | --- | --- | --- | --- | --- |

Best Unique PSM (Scan Sample\_2\_A1\_1\_3640.d:45098, m/z=668.3478, z=2, RT=14.95, ppm=-1.4):


TRINITY\_DN144214\_c0\_g1\_i1.p1

back to list

  

| Protein Coverage
| Supporting Peptides
| Best Unique PSM
|

Protein Coverage:

Supporting Peptides:

| Peptide | Uniq | -10lgP | Mass | Length | ppm | m/z | z | RT | 1/k0 Start | 1/k0 End | Precursor ID | Area Sample 4 | #Feature | #Feature Sample 4 | Start | End | PTM | AScore | Found By |
| --- | --- | --- | --- | --- | --- | --- | --- | --- | --- | --- | --- | --- | --- | --- | --- | --- | --- | --- | --- |
| R.LPGLLSPSYGVR.F | Y | 54.31 | 1257.7081 | 12 | -1.48 | 629.859 | 2 | 18.18 | 0.9541 | 0.9614 | 65571 | 5.89e2 | 1 | 1 | 63 | 74 |  |  | DB Search |
| R.FDDVPNGLGAFSK.V | Y | 53.88 | 1365.6564 | 13 | 1.41 | 683.835 | 2 | 17.84 | 0.959 | 0.9662 | 63447 | 0 | 0 | 0 | 75 | 87 |  |  | DB Search |
| total 2 peptides |  |  |  |  |  |  |  |  |  |  |  |  |  |  |  |  |  |  |  |
| --- | --- | --- | --- | --- | --- | --- | --- | --- | --- | --- | --- | --- | --- | --- | --- | --- | --- | --- | --- |

Best Unique PSM (Scan Sample\_2\_A1\_1\_3640.d:65571, m/z=629.859, z=2, RT=18.18, ppm=-3.68):


TRINITY\_DN49617\_c0\_g1\_i2.p1

back to list

  

| Protein Coverage
| Supporting Peptides
| Best Unique PSM
|

Protein Coverage:

Supporting Peptides:

| Peptide | Uniq | -10lgP | Mass | Length | ppm | m/z | z | RT | 1/k0 Start | 1/k0 End | Precursor ID | Area Sample 4 | #Feature | #Feature Sample 4 | Start | End | PTM | AScore | Found By |
| --- | --- | --- | --- | --- | --- | --- | --- | --- | --- | --- | --- | --- | --- | --- | --- | --- | --- | --- | --- |
| K.ILDNNNQEVIHLNRPLR.C | Y | 58.05 | 2057.113 | 17 | -0.42 | 515.2842 | 4 | 15.14 | 0.8258 | 0.8331 | 46227 | 0 | 0 | 0 | 16 | 32 |  |  | DB Search |
| total 1 peptides |  |  |  |  |  |  |  |  |  |  |  |  |  |  |  |  |  |  |  |
| --- | --- | --- | --- | --- | --- | --- | --- | --- | --- | --- | --- | --- | --- | --- | --- | --- | --- | --- | --- |

Best Unique PSM (Scan Sample\_2\_A1\_1\_3640.d:46227, m/z=515.2842, z=4, RT=15.14, ppm=-2.61):


TRINITY\_DN10094\_c0\_g1\_i2.p1

back to list

  

| Protein Coverage
| Supporting Peptides
| Best Unique PSM
|

Protein Coverage:

Supporting Peptides:

| Peptide | Uniq | -10lgP | Mass | Length | ppm | m/z | z | RT | 1/k0 Start | 1/k0 End | Precursor ID | Area Sample 4 | #Feature | #Feature Sample 4 | Start | End | PTM | AScore | Found By |
| --- | --- | --- | --- | --- | --- | --- | --- | --- | --- | --- | --- | --- | --- | --- | --- | --- | --- | --- | --- |
| K.FFFHDQSC(+57.02)LDVYK.Y | Y | 58.39 | 1704.7606 | 13 | 3.79 | 569.2617 | 3 | 17.83 | 0.801 | 0.8084 | 63353 | 5.61e2 | 1 | 1 | 221 | 233 | Carbamidomethylation |  | DB Search |
| R.NFGLC(+57.02)TEHTGNLITNK.Y | Y | 54.67 | 1817.873 | 16 | 1.42 | 606.9645 | 3 | 15.88 | 0.8412 | 0.8485 | 50739 | 7.31e2 | 1 | 1 | 177 | 192 | Carbamidomethylation |  | DB Search |
| total 2 peptides |  |  |  |  |  |  |  |  |  |  |  |  |  |  |  |  |  |  |  |
| --- | --- | --- | --- | --- | --- | --- | --- | --- | --- | --- | --- | --- | --- | --- | --- | --- | --- | --- | --- |

Best Unique PSM (Scan Sample\_2\_A1\_1\_3640.d:63353, m/z=569.2617, z=3, RT=17.83, ppm=1.6):


TRINITY\_DN122779\_c0\_g1\_i1.p1

back to list

  

| Protein Coverage
| Supporting Peptides
| Best Unique PSM
|

Protein Coverage:

Supporting Peptides:

| Peptide | Uniq | -10lgP | Mass | Length | ppm | m/z | z | RT | 1/k0 Start | 1/k0 End | Precursor ID | Area Sample 4 | #Feature | #Feature Sample 4 | Start | End | PTM | AScore | Found By |
| --- | --- | --- | --- | --- | --- | --- | --- | --- | --- | --- | --- | --- | --- | --- | --- | --- | --- | --- | --- |
| K.DFQVIFEAK.T | Y | 58.28 | 1095.56 | 9 | -2.26 | 548.7849 | 2 | 19.00 | 0.8579 | 0.8652 | 71160 | 0 | 0 | 0 | 12 | 20 |  |  | DB Search |
| total 1 peptides |  |  |  |  |  |  |  |  |  |  |  |  |  |  |  |  |  |  |  |
| --- | --- | --- | --- | --- | --- | --- | --- | --- | --- | --- | --- | --- | --- | --- | --- | --- | --- | --- | --- |

Best Unique PSM (Scan Sample\_2\_A1\_1\_3640.d:71160, m/z=548.7849, z=2, RT=19.00, ppm=-4.45):


TRINITY\_DN1821\_c0\_g1\_i2.p1

back to list

  

| Protein Coverage
| Supporting Peptides
| Best Unique PSM
|

Protein Coverage:

Supporting Peptides:

| Peptide | Uniq | -10lgP | Mass | Length | ppm | m/z | z | RT | 1/k0 Start | 1/k0 End | Precursor ID | Area Sample 4 | #Feature | #Feature Sample 4 | Start | End | PTM | AScore | Found By |
| --- | --- | --- | --- | --- | --- | --- | --- | --- | --- | --- | --- | --- | --- | --- | --- | --- | --- | --- | --- |
| K.VEDSKESDINFLLK.T | Y | 57.24 | 1635.8355 | 14 | 0.37 | 546.2848 | 3 | 17.72 | 0.8092 | 0.8165 | 62515 | 5.45e2 | 1 | 1 | 195 | 208 |  |  | DB Search |
| K.YQGIKPPVSR.S | Y | 57.06 | 1143.64 | 10 | -0.07 | 572.826 | 2 | 12.77 | 0.9311 | 0.9384 | 31696 | 3.82e2 | 1 | 1 | 251 | 260 |  |  | DB Search |
| K.DKPALDVTSTLKK.K | Y | 54.52 | 1414.8031 | 13 | 2.31 | 472.6084 | 3 | 13.78 | 0.801 | 0.8084 | 37913 | 0 | 0 | 0 | 52 | 64 |  |  | DB Search |
| total 3 peptides |  |  |  |  |  |  |  |  |  |  |  |  |  |  |  |  |  |  |  |
| --- | --- | --- | --- | --- | --- | --- | --- | --- | --- | --- | --- | --- | --- | --- | --- | --- | --- | --- | --- |

Best Unique PSM (Scan Sample\_2\_A1\_1\_3640.d:62515, m/z=546.2848, z=3, RT=17.72, ppm=-1.83):


TRINITY\_DN28022\_c0\_g2\_i1.p1

back to list

  

| Protein Coverage
| Supporting Peptides
| Best Unique PSM
|

Protein Coverage:

Supporting Peptides:

| Peptide | Uniq | -10lgP | Mass | Length | ppm | m/z | z | RT | 1/k0 Start | 1/k0 End | Precursor ID | Area Sample 4 | #Feature | #Feature Sample 4 | Start | End | PTM | AScore | Found By |
| --- | --- | --- | --- | --- | --- | --- | --- | --- | --- | --- | --- | --- | --- | --- | --- | --- | --- | --- | --- |
| K.LSQTAPTC(+57.02)K.V | Y | 58.21 | 1004.4961 | 9 | 4.86 | 503.2567 | 2 | 10.24 | 0.825 | 0.8323 | 16211 | 1.14e3 | 1 | 1 | 527 | 535 | Carbamidomethylation |  | DB Search |
| K.TNFQVVIEAKR.G | Y | 57.17 | 1303.7248 | 11 | 3.05 | 652.8702 | 2 | 15.14 | 0.9872 | 0.9945 | 46265 | 1.76e2 | 1 | 1 | 798 | 808 |  |  | DB Search |
| R.LLSTVLKPPSNR.W | Y | 54.85 | 1323.7874 | 12 | 4.07 | 662.9022 | 2 | 15.03 | 1.0037 | 1.011 | 45450 | 8.14e2 | 1 | 1 | 730 | 741 |  |  | DB Search |
| K.NLASLSSPILNQASSR.C | Y | 54.83 | 1656.8795 | 16 | -5.09 | 829.441 | 2 | 17.89 | 1.0761 | 1.0833 | 63844 | 0 | 0 | 0 | 92 | 107 |  |  | DB Search |
| total 4 peptides |  |  |  |  |  |  |  |  |  |  |  |  |  |  |  |  |  |  |  |
| --- | --- | --- | --- | --- | --- | --- | --- | --- | --- | --- | --- | --- | --- | --- | --- | --- | --- | --- | --- |

Best Unique PSM (Scan Sample\_2\_A1\_1\_3640.d:16211, m/z=503.2567, z=2, RT=10.24, ppm=2.67):


TRINITY\_DN12734\_c0\_g1\_i2.p1

back to list

  

| Protein Coverage
| Supporting Peptides
| Best Unique PSM
|

Protein Coverage:

Supporting Peptides:

| Peptide | Uniq | -10lgP | Mass | Length | ppm | m/z | z | RT | 1/k0 Start | 1/k0 End | Precursor ID | Area Sample 4 | #Feature | #Feature Sample 4 | Start | End | PTM | AScore | Found By |
| --- | --- | --- | --- | --- | --- | --- | --- | --- | --- | --- | --- | --- | --- | --- | --- | --- | --- | --- | --- |
| K.LLLLGAGESGK.S | Y | 43.84 | 1056.6179 | 11 | 6.84 | 529.3187 | 2 | 17.27 | 0.8761 | 0.8834 | 59698 | 0 | 0 | 0 | 46 | 56 |  |  | DB Search |
| total 1 peptides |  |  |  |  |  |  |  |  |  |  |  |  |  |  |  |  |  |  |  |
| --- | --- | --- | --- | --- | --- | --- | --- | --- | --- | --- | --- | --- | --- | --- | --- | --- | --- | --- | --- |

Best Unique PSM (Scan Sample\_2\_A1\_1\_3640.d:59698, m/z=529.3187, z=2, RT=17.27, ppm=4.65):


TRINITY\_DN942\_c0\_g1\_i2.p1

back to list

  

| Protein Coverage
| Supporting Peptides
| Best Unique PSM
|

Protein Coverage:

Supporting Peptides:

| Peptide | Uniq | -10lgP | Mass | Length | ppm | m/z | z | RT | 1/k0 Start | 1/k0 End | Precursor ID | Area Sample 4 | #Feature | #Feature Sample 4 | Start | End | PTM | AScore | Found By |
| --- | --- | --- | --- | --- | --- | --- | --- | --- | --- | --- | --- | --- | --- | --- | --- | --- | --- | --- | --- |
| K.AQLLLEDVK.A | Y | 57.95 | 1027.5913 | 9 | 4.05 | 514.8039 | 2 | 17.08 | 0.8721 | 0.8793 | 58461 | 1.87e2 | 1 | 1 | 3333 | 3341 |  |  | DB Search |
| K.VLNIALDKYQEGVAK.T | Y | 57.22 | 1659.9195 | 15 | -3.66 | 554.3105 | 3 | 16.21 | 0.8088 | 0.8161 | 52938 | 0 | 0 | 0 | 4557 | 4571 |  |  | DB Search |
| R.LIVINLIK.K | Y | 54.1 | 924.6372 | 8 | 1.83 | 463.3257 | 2 | 19.76 | 0.8031 | 0.8104 | 75960 | 1.58e3 | 1 | 1 | 3044 | 3051 |  |  | DB Search |
| total 3 peptides |  |  |  |  |  |  |  |  |  |  |  |  |  |  |  |  |  |  |  |
| --- | --- | --- | --- | --- | --- | --- | --- | --- | --- | --- | --- | --- | --- | --- | --- | --- | --- | --- | --- |

Best Unique PSM (Scan Sample\_2\_A1\_1\_3640.d:58461, m/z=514.8039, z=2, RT=17.08, ppm=1.86):


TRINITY\_DN1821\_c0\_g1\_i6.p1

back to list

  

| Protein Coverage
| Supporting Peptides
| Best Unique PSM
|

Protein Coverage:

Supporting Peptides:

| Peptide | Uniq | -10lgP | Mass | Length | ppm | m/z | z | RT | 1/k0 Start | 1/k0 End | Precursor ID | Area Sample 4 | #Feature | #Feature Sample 4 | Start | End | PTM | AScore | Found By |
| --- | --- | --- | --- | --- | --- | --- | --- | --- | --- | --- | --- | --- | --- | --- | --- | --- | --- | --- | --- |
| K.VEDSKESDINFLLK.T | Y | 57.24 | 1635.8355 | 14 | 0.37 | 546.2848 | 3 | 17.72 | 0.8092 | 0.8165 | 62515 | 5.45e2 | 1 | 1 | 423 | 436 |  |  | DB Search |
| K.YQGIKPPVSR.S | Y | 57.06 | 1143.64 | 10 | -0.07 | 572.826 | 2 | 12.77 | 0.9311 | 0.9384 | 31696 | 3.82e2 | 1 | 1 | 479 | 488 |  |  | DB Search |
| K.DKPALDVTSTLKK.K | Y | 54.52 | 1414.8031 | 13 | 2.31 | 472.6084 | 3 | 13.78 | 0.801 | 0.8084 | 37913 | 0 | 0 | 0 | 280 | 292 |  |  | DB Search |
| total 3 peptides |  |  |  |  |  |  |  |  |  |  |  |  |  |  |  |  |  |  |  |
| --- | --- | --- | --- | --- | --- | --- | --- | --- | --- | --- | --- | --- | --- | --- | --- | --- | --- | --- | --- |

Best Unique PSM (Scan Sample\_2\_A1\_1\_3640.d:62515, m/z=546.2848, z=3, RT=17.72, ppm=-1.83):


TRINITY\_DN7674\_c0\_g1\_i1.p1

back to list

  

| Protein Coverage
| Supporting Peptides
| Best Unique PSM
|

Protein Coverage:

Supporting Peptides:

| Peptide | Uniq | -10lgP | Mass | Length | ppm | m/z | z | RT | 1/k0 Start | 1/k0 End | Precursor ID | Area Sample 4 | #Feature | #Feature Sample 4 | Start | End | PTM | AScore | Found By |
| --- | --- | --- | --- | --- | --- | --- | --- | --- | --- | --- | --- | --- | --- | --- | --- | --- | --- | --- | --- |
| R.LGTIVAGFLK.L | Y | 58.05 | 1017.6222 | 10 | -3.2 | 509.8156 | 2 | 19.28 | 0.8254 | 0.8327 | 72854 | 3.28e2 | 1 | 1 | 326 | 335 |  |  | DB Search |
| M.DYYASEK.K | Y | 41.11 | 874.3708 | 7 | 18.08 | 438.1996 | 2 | 11.74 | 0.8067 | 0.814 | 25358 | 3e3 | 1 | 1 | 2 | 8 |  |  | DB Search |
| total 2 peptides |  |  |  |  |  |  |  |  |  |  |  |  |  |  |  |  |  |  |  |
| --- | --- | --- | --- | --- | --- | --- | --- | --- | --- | --- | --- | --- | --- | --- | --- | --- | --- | --- | --- |

Best Unique PSM (Scan Sample\_2\_A1\_1\_3640.d:72854, m/z=509.8156, z=2, RT=19.28, ppm=-5.4):


TRINITY\_DN1172\_c0\_g1\_i3.p1

back to list

  

| Protein Coverage
| Supporting Peptides
| Best Unique PSM
|

Protein Coverage:

Supporting Peptides:

| Peptide | Uniq | -10lgP | Mass | Length | ppm | m/z | z | RT | 1/k0 Start | 1/k0 End | Precursor ID | Area Sample 4 | #Feature | #Feature Sample 4 | Start | End | PTM | AScore | Found By |
| --- | --- | --- | --- | --- | --- | --- | --- | --- | --- | --- | --- | --- | --- | --- | --- | --- | --- | --- | --- |
| K.VITSDDPAEKEK.K | Y | 58.39 | 1330.6616 | 12 | 3.3 | 666.3388 | 2 | 10.77 | 0.9856 | 0.9928 | 19482 | 3.46e2 | 1 | 1 | 123 | 134 |  |  | DB Search |
| total 1 peptides |  |  |  |  |  |  |  |  |  |  |  |  |  |  |  |  |  |  |  |
| --- | --- | --- | --- | --- | --- | --- | --- | --- | --- | --- | --- | --- | --- | --- | --- | --- | --- | --- | --- |

Best Unique PSM (Scan Sample\_2\_A1\_1\_3640.d:19482, m/z=666.3388, z=2, RT=10.77, ppm=1.11):


TRINITY\_DN982\_c0\_g1\_i2.p1

back to list

  

| Protein Coverage
| Supporting Peptides
| Best Unique PSM
|

Protein Coverage:

Supporting Peptides:

| Peptide | Uniq | -10lgP | Mass | Length | ppm | m/z | z | RT | 1/k0 Start | 1/k0 End | Precursor ID | Area Sample 4 | #Feature | #Feature Sample 4 | Start | End | PTM | AScore | Found By |
| --- | --- | --- | --- | --- | --- | --- | --- | --- | --- | --- | --- | --- | --- | --- | --- | --- | --- | --- | --- |
| K.VLLSQQIFYDKK.N | N | 58.39 | 1480.8289 | 12 | 0.31 | 741.4203 | 2 | 17.02 | 1.0616 | 1.0688 | 58002 | 1.76e3 | 2 | 2 | 1479 | 1490 |  |  | DB Search |
| K.INNNQFSVVLIK.N | Y | 57.54 | 1387.7823 | 12 | -2.73 | 694.895 | 2 | 18.38 | 0.9848 | 0.992 | 66945 | 4.66e2 | 1 | 1 | 100 | 111 |  |  | DB Search |
| total 2 peptides |  |  |  |  |  |  |  |  |  |  |  |  |  |  |  |  |  |  |  |
| --- | --- | --- | --- | --- | --- | --- | --- | --- | --- | --- | --- | --- | --- | --- | --- | --- | --- | --- | --- |

Best Unique PSM (Scan Sample\_2\_A1\_1\_3640.d:66945, m/z=694.895, z=2, RT=18.38, ppm=-4.93):


TRINITY\_DN413\_c0\_g1\_i3.p1

back to list

  

| Protein Coverage
| Supporting Peptides
| Best Unique PSM
|

Protein Coverage:

Supporting Peptides:

| Peptide | Uniq | -10lgP | Mass | Length | ppm | m/z | z | RT | 1/k0 Start | 1/k0 End | Precursor ID | Area Sample 4 | #Feature | #Feature Sample 4 | Start | End | PTM | AScore | Found By |
| --- | --- | --- | --- | --- | --- | --- | --- | --- | --- | --- | --- | --- | --- | --- | --- | --- | --- | --- | --- |
| R.LLLGDFNGDKR.T | Y | 58.25 | 1246.6669 | 11 | 4.07 | 624.3419 | 2 | 16.32 | 0.9844 | 0.9916 | 53554 | 5.68e2 | 1 | 1 | 46 | 56 |  |  | DB Search |
| total 1 peptides |  |  |  |  |  |  |  |  |  |  |  |  |  |  |  |  |  |  |  |
| --- | --- | --- | --- | --- | --- | --- | --- | --- | --- | --- | --- | --- | --- | --- | --- | --- | --- | --- | --- |

Best Unique PSM (Scan Sample\_2\_A1\_1\_3640.d:53554, m/z=624.3419, z=2, RT=16.32, ppm=1.87):


TRINITY\_DN61742\_c0\_g1\_i2.p1

back to list

  

| Protein Coverage
| Supporting Peptides
| Best Unique PSM
|

Protein Coverage:

Supporting Peptides:

| Peptide | Uniq | -10lgP | Mass | Length | ppm | m/z | z | RT | 1/k0 Start | 1/k0 End | Precursor ID | Area Sample 4 | #Feature | #Feature Sample 4 | Start | End | PTM | AScore | Found By |
| --- | --- | --- | --- | --- | --- | --- | --- | --- | --- | --- | --- | --- | --- | --- | --- | --- | --- | --- | --- |
| K.LYISSLPLK.V | Y | 58.05 | 1032.6219 | 9 | 2.33 | 517.3183 | 2 | 18.15 | 0.8587 | 0.866 | 65559 | 0 | 0 | 0 | 78 | 86 |  |  | DB Search |
| K.ASILLNELAK.Y | Y | 56.95 | 1070.6335 | 10 | -3.29 | 536.3211 | 2 | 18.12 | 0.8729 | 0.8802 | 65288 | 0 | 0 | 0 | 452 | 461 |  |  | DB Search |
| total 2 peptides |  |  |  |  |  |  |  |  |  |  |  |  |  |  |  |  |  |  |  |
| --- | --- | --- | --- | --- | --- | --- | --- | --- | --- | --- | --- | --- | --- | --- | --- | --- | --- | --- | --- |

Best Unique PSM (Scan Sample\_2\_A1\_1\_3640.d:65559, m/z=517.3183, z=2, RT=18.15, ppm=0.13):


TRINITY\_DN3698\_c0\_g2\_i4.p1

back to list

  

| Protein Coverage
| Supporting Peptides
| Best Unique PSM
|

Protein Coverage:

Supporting Peptides:

| Peptide | Uniq | -10lgP | Mass | Length | ppm | m/z | z | RT | 1/k0 Start | 1/k0 End | Precursor ID | Area Sample 4 | #Feature | #Feature Sample 4 | Start | End | PTM | AScore | Found By |
| --- | --- | --- | --- | --- | --- | --- | --- | --- | --- | --- | --- | --- | --- | --- | --- | --- | --- | --- | --- |
| K.ANHIFAHTDPLSQK.S | Y | 58.31 | 1577.795 | 14 | -10.88 | 526.9321 | 3 | 13.75 | 0.8189 | 0.8262 | 37740 | 6.57e2 | 1 | 1 | 381 | 394 |  |  | DB Search |
| R.ATENPSGGFSPDDR.C | Y | 55.58 | 1448.6168 | 14 | 6.68 | 725.3189 | 2 | 12.97 | 0.9759 | 0.9832 | 32890 | 0 | 0 | 0 | 114 | 127 |  |  | DB Search |
| total 2 peptides |  |  |  |  |  |  |  |  |  |  |  |  |  |  |  |  |  |  |  |
| --- | --- | --- | --- | --- | --- | --- | --- | --- | --- | --- | --- | --- | --- | --- | --- | --- | --- | --- | --- |

Best Unique PSM (Scan Sample\_2\_A1\_1\_3640.d:37740, m/z=526.9321, z=3, RT=13.75, ppm=-13.08):


TRINITY\_DN2577\_c1\_g1\_i2.p1

back to list

  

| Protein Coverage
| Supporting Peptides
| Best Unique PSM
|

Protein Coverage:

Supporting Peptides:

| Peptide | Uniq | -10lgP | Mass | Length | ppm | m/z | z | RT | 1/k0 Start | 1/k0 End | Precursor ID | Area Sample 4 | #Feature | #Feature Sample 4 | Start | End | PTM | AScore | Found By |
| --- | --- | --- | --- | --- | --- | --- | --- | --- | --- | --- | --- | --- | --- | --- | --- | --- | --- | --- | --- |
| R.SGDPLTIGNFIK.N | N | 58.37 | 1260.6714 | 12 | 1.03 | 631.3422 | 2 | 19.83 | 0.8955 | 0.9028 | 76410 | 4.42e2 | 1 | 1 | 322 | 333 |  |  | DB Search |
| K.EGLEFIGTLK.V | Y | 58.25 | 1105.6019 | 10 | 3.2 | 553.8088 | 2 | 19.04 | 0.8627 | 0.87 | 71397 | 0 | 0 | 0 | 223 | 232 |  |  | DB Search |
| K.LELEFTKDER.S | Y | 58.22 | 1278.6455 | 10 | -2.73 | 640.3269 | 2 | 15.54 | 0.9739 | 0.9812 | 48812 | 0 | 0 | 0 | 195 | 204 |  |  | DB Search |
| K.VDDIIC(+57.02)IAGC(+57.02)ELKK.G | Y | 58.04 | 1632.8215 | 14 | -6.17 | 545.2766 | 3 | 17.85 | 0.8388 | 0.8461 | 63424 | 2.04e2 | 1 | 1 | 625 | 638 | Carbamidomethylation, Carbamidomethylation |  | DB Search |
| R.GAVTAAFDKVR.K | Y | 54.88 | 1133.6193 | 11 | 3.89 | 567.8179 | 2 | 14.48 | 0.9352 | 0.9424 | 42102 | 1.49e3 | 1 | 1 | 546 | 556 |  |  | DB Search |
| K.GVLAGVGAIVK.A | Y | 54.31 | 982.6175 | 11 | -1.97 | 492.314 | 2 | 17.55 | 0.8631 | 0.8704 | 61324 | 3.41e3 | 1 | 1 | 696 | 706 |  |  | DB Search |
| K.NGPFLIVDIDSEK.T | Y | 51.58 | 1445.7402 | 13 | 7.04 | 723.8809 | 2 | 19.95 | 0.9775 | 0.9848 | 77105 | 2.24e2 | 1 | 1 | 431 | 443 |  |  | DB Search |
| total 7 peptides |  |  |  |  |  |  |  |  |  |  |  |  |  |  |  |  |  |  |  |
| --- | --- | --- | --- | --- | --- | --- | --- | --- | --- | --- | --- | --- | --- | --- | --- | --- | --- | --- | --- |

Best Unique PSM (Scan Sample\_2\_A1\_1\_3640.d:71397, m/z=553.8088, z=2, RT=19.04, ppm=1.01):


TRINITY\_DN776\_c0\_g1\_i1.p1

back to list

  

| Protein Coverage
| Supporting Peptides
| Best Unique PSM
|

Protein Coverage:

Supporting Peptides:

| Peptide | Uniq | -10lgP | Mass | Length | ppm | m/z | z | RT | 1/k0 Start | 1/k0 End | Precursor ID | Area Sample 4 | #Feature | #Feature Sample 4 | Start | End | PTM | AScore | Found By |
| --- | --- | --- | --- | --- | --- | --- | --- | --- | --- | --- | --- | --- | --- | --- | --- | --- | --- | --- | --- |
| R.DLLTLPEKDNRR.L | Y | 58.26 | 1468.7998 | 12 | -3.01 | 490.6046 | 3 | 14.81 | 0.7888 | 0.7962 | 44185 | 3.24e2 | 1 | 1 | 66 | 77 |  |  | DB Search |
| R.LFEGNALLR.R | Y | 57.78 | 1031.5763 | 9 | 0.94 | 516.7948 | 2 | 17.48 | 0.8631 | 0.8704 | 60870 | 9.34e2 | 1 | 1 | 78 | 86 |  |  | DB Search |
| total 2 peptides |  |  |  |  |  |  |  |  |  |  |  |  |  |  |  |  |  |  |  |
| --- | --- | --- | --- | --- | --- | --- | --- | --- | --- | --- | --- | --- | --- | --- | --- | --- | --- | --- | --- |

Best Unique PSM (Scan Sample\_2\_A1\_1\_3640.d:44185, m/z=490.6046, z=3, RT=14.81, ppm=-5.21):


TRINITY\_DN4494\_c1\_g1\_i1.p1

back to list

  

| Protein Coverage
| Supporting Peptides
| Best Unique PSM
|

Protein Coverage:

Supporting Peptides:

| Peptide | Uniq | -10lgP | Mass | Length | ppm | m/z | z | RT | 1/k0 Start | 1/k0 End | Precursor ID | Area Sample 4 | #Feature | #Feature Sample 4 | Start | End | PTM | AScore | Found By |
| --- | --- | --- | --- | --- | --- | --- | --- | --- | --- | --- | --- | --- | --- | --- | --- | --- | --- | --- | --- |
| R.VLDVGFLLDSSDKVK.D | Y | 58.25 | 1633.8926 | 15 | -4.48 | 545.6345 | 3 | 19.79 | 0.8725 | 0.8798 | 76169 | 4.96e2 | 1 | 1 | 109 | 123 |  |  | DB Search |
| K.VDVGFILDSSGSLR.N | N | 58.24 | 1463.762 | 14 | 1.17 | 732.8875 | 2 | 19.97 | 1.0102 | 1.0174 | 77350 | 0 | 0 | 0 | 307 | 320 |  |  | DB Search |
| K.LLILLTDGSQTQDIGAEDPAK.I | Y | 54.36 | 2197.1477 | 21 | 2.89 | 1099.5819 | 2 | 19.49 | 1.2511 | 1.2583 | 74311 | 0 | 0 | 0 | 16 | 36 |  |  | DB Search |
| total 3 peptides |  |  |  |  |  |  |  |  |  |  |  |  |  |  |  |  |  |  |  |
| --- | --- | --- | --- | --- | --- | --- | --- | --- | --- | --- | --- | --- | --- | --- | --- | --- | --- | --- | --- |

Best Unique PSM (Scan Sample\_2\_A1\_1\_3640.d:76169, m/z=545.6345, z=3, RT=19.79, ppm=-6.67):


TRINITY\_DN5897\_c0\_g1\_i1.p2

back to list

  

| Protein Coverage
| Supporting Peptides
| Best Unique PSM
|

Protein Coverage:

Supporting Peptides:

| Peptide | Uniq | -10lgP | Mass | Length | ppm | m/z | z | RT | 1/k0 Start | 1/k0 End | Precursor ID | Area Sample 4 | #Feature | #Feature Sample 4 | Start | End | PTM | AScore | Found By |
| --- | --- | --- | --- | --- | --- | --- | --- | --- | --- | --- | --- | --- | --- | --- | --- | --- | --- | --- | --- |
| K.FDDVPNGLGAFSK.V | Y | 53.88 | 1365.6564 | 13 | 1.41 | 683.835 | 2 | 17.84 | 0.959 | 0.9662 | 63447 | 0 | 0 | 0 | 50 | 62 |  |  | DB Search |
| total 1 peptides |  |  |  |  |  |  |  |  |  |  |  |  |  |  |  |  |  |  |  |
| --- | --- | --- | --- | --- | --- | --- | --- | --- | --- | --- | --- | --- | --- | --- | --- | --- | --- | --- | --- |

Best Unique PSM (Scan Sample\_2\_A1\_1\_3640.d:63447, m/z=683.835, z=2, RT=17.84, ppm=-0.78):


TRINITY\_DN4523\_c0\_g1\_i2.p1

back to list

  

| Protein Coverage
| Supporting Peptides
| Best Unique PSM
|

Protein Coverage:

Supporting Peptides:

| Peptide | Uniq | -10lgP | Mass | Length | ppm | m/z | z | RT | 1/k0 Start | 1/k0 End | Precursor ID | Area Sample 4 | #Feature | #Feature Sample 4 | Start | End | PTM | AScore | Found By |
| --- | --- | --- | --- | --- | --- | --- | --- | --- | --- | --- | --- | --- | --- | --- | --- | --- | --- | --- | --- |
| R.ITLQDVVLHGK.K | Y | 38.06 | 1221.7081 | 11 | 4.85 | 408.2444 | 3 | 17.32 | 0.7167 | 0.7241 | 60002 | 0 | 0 | 0 | 973 | 983 |  |  | DB Search |
| total 1 peptides |  |  |  |  |  |  |  |  |  |  |  |  |  |  |  |  |  |  |  |
| --- | --- | --- | --- | --- | --- | --- | --- | --- | --- | --- | --- | --- | --- | --- | --- | --- | --- | --- | --- |

Best Unique PSM (Scan Sample\_2\_A1\_1\_3640.d:60002, m/z=408.2444, z=3, RT=17.32, ppm=2.65):


TRINITY\_DN1821\_c0\_g1\_i5.p1

back to list

  

| Protein Coverage
| Supporting Peptides
| Best Unique PSM
|

Protein Coverage:

Supporting Peptides:

| Peptide | Uniq | -10lgP | Mass | Length | ppm | m/z | z | RT | 1/k0 Start | 1/k0 End | Precursor ID | Area Sample 4 | #Feature | #Feature Sample 4 | Start | End | PTM | AScore | Found By |
| --- | --- | --- | --- | --- | --- | --- | --- | --- | --- | --- | --- | --- | --- | --- | --- | --- | --- | --- | --- |
| K.VEDSKESDINFLLK.T | Y | 57.24 | 1635.8355 | 14 | 0.37 | 546.2848 | 3 | 17.72 | 0.8092 | 0.8165 | 62515 | 5.45e2 | 1 | 1 | 423 | 436 |  |  | DB Search |
| K.YQGIKPPVSR.S | Y | 57.06 | 1143.64 | 10 | -0.07 | 572.826 | 2 | 12.77 | 0.9311 | 0.9384 | 31696 | 3.82e2 | 1 | 1 | 479 | 488 |  |  | DB Search |
| K.DKPALDVTSTLKK.K | Y | 54.52 | 1414.8031 | 13 | 2.31 | 472.6084 | 3 | 13.78 | 0.801 | 0.8084 | 37913 | 0 | 0 | 0 | 280 | 292 |  |  | DB Search |
| total 3 peptides |  |  |  |  |  |  |  |  |  |  |  |  |  |  |  |  |  |  |  |
| --- | --- | --- | --- | --- | --- | --- | --- | --- | --- | --- | --- | --- | --- | --- | --- | --- | --- | --- | --- |

Best Unique PSM (Scan Sample\_2\_A1\_1\_3640.d:62515, m/z=546.2848, z=3, RT=17.72, ppm=-1.83):


TRINITY\_DN106\_c0\_g1\_i8.p1

back to list

  

| Protein Coverage
| Supporting Peptides
| Best Unique PSM
|

Protein Coverage:

Supporting Peptides:

| Peptide | Uniq | -10lgP | Mass | Length | ppm | m/z | z | RT | 1/k0 Start | 1/k0 End | Precursor ID | Area Sample 4 | #Feature | #Feature Sample 4 | Start | End | PTM | AScore | Found By |
| --- | --- | --- | --- | --- | --- | --- | --- | --- | --- | --- | --- | --- | --- | --- | --- | --- | --- | --- | --- |
| K.EGPAGPAGPVGR.L | Y | 58.28 | 1063.541 | 12 | -11.82 | 532.7703 | 2 | 11.84 | 0.8461 | 0.8534 | 25992 | 0 | 0 | 0 | 191 | 202 |  |  | DB Search |
| R.DGPAGPAGR.D | N | 58.24 | 796.3827 | 9 | 3.73 | 399.1992 | 2 | 9.76 | 0.7061 | 0.7135 | 13439 | 1.18e4 | 2 | 2 | 488 | 496 |  |  | DB Search |
| R.GPDGAVGPTGPIGAR.G | N | 58.04 | 1320.6786 | 15 | 1.83 | 661.3463 | 2 | 13.72 | 0.9558 | 0.963 | 37443 | 1.21e4 | 2 | 2 | 327 | 341 |  |  | DB Search |
| total 3 peptides |  |  |  |  |  |  |  |  |  |  |  |  |  |  |  |  |  |  |  |
| --- | --- | --- | --- | --- | --- | --- | --- | --- | --- | --- | --- | --- | --- | --- | --- | --- | --- | --- | --- |

Best Unique PSM (Scan Sample\_2\_A1\_1\_3640.d:25992, m/z=532.7703, z=2, RT=11.84, ppm=-14.02):


TRINITY\_DN3986\_c0\_g1\_i2.p1

back to list

  

| Protein Coverage
| Supporting Peptides
| Best Unique PSM
|

Protein Coverage:

Supporting Peptides:

| Peptide | Uniq | -10lgP | Mass | Length | ppm | m/z | z | RT | 1/k0 Start | 1/k0 End | Precursor ID | Area Sample 4 | #Feature | #Feature Sample 4 | Start | End | PTM | AScore | Found By |
| --- | --- | --- | --- | --- | --- | --- | --- | --- | --- | --- | --- | --- | --- | --- | --- | --- | --- | --- | --- |
| R.FPGQLNADLRK.L | Y | 57.99 | 1257.6829 | 11 | -7.77 | 420.2307 | 3 | 14.50 | 0.7033 | 0.7106 | 42199 | 0 | 0 | 0 | 242 | 252 |  |  | DB Search |
| total 1 peptides |  |  |  |  |  |  |  |  |  |  |  |  |  |  |  |  |  |  |  |
| --- | --- | --- | --- | --- | --- | --- | --- | --- | --- | --- | --- | --- | --- | --- | --- | --- | --- | --- | --- |

Best Unique PSM (Scan Sample\_2\_A1\_1\_3640.d:42199, m/z=420.2307, z=3, RT=14.50, ppm=-9.97):


TRINITY\_DN3698\_c0\_g2\_i1.p1

back to list

  

| Protein Coverage
| Supporting Peptides
| Best Unique PSM
|

Protein Coverage:

Supporting Peptides:

| Peptide | Uniq | -10lgP | Mass | Length | ppm | m/z | z | RT | 1/k0 Start | 1/k0 End | Precursor ID | Area Sample 4 | #Feature | #Feature Sample 4 | Start | End | PTM | AScore | Found By |
| --- | --- | --- | --- | --- | --- | --- | --- | --- | --- | --- | --- | --- | --- | --- | --- | --- | --- | --- | --- |
| K.ANHIFAHTDPLSQK.S | Y | 58.31 | 1577.795 | 14 | -10.88 | 526.9321 | 3 | 13.75 | 0.8189 | 0.8262 | 37740 | 6.57e2 | 1 | 1 | 387 | 400 |  |  | DB Search |
| R.ATENPSGGFSPDDR.C | Y | 55.58 | 1448.6168 | 14 | 6.68 | 725.3189 | 2 | 12.97 | 0.9759 | 0.9832 | 32890 | 0 | 0 | 0 | 120 | 133 |  |  | DB Search |
| total 2 peptides |  |  |  |  |  |  |  |  |  |  |  |  |  |  |  |  |  |  |  |
| --- | --- | --- | --- | --- | --- | --- | --- | --- | --- | --- | --- | --- | --- | --- | --- | --- | --- | --- | --- |

Best Unique PSM (Scan Sample\_2\_A1\_1\_3640.d:37740, m/z=526.9321, z=3, RT=13.75, ppm=-13.08):


TRINITY\_DN3655\_c0\_g1\_i2.p1

back to list

  

| Protein Coverage
| Supporting Peptides
| Best Unique PSM
|

Protein Coverage:

Supporting Peptides:

| Peptide | Uniq | -10lgP | Mass | Length | ppm | m/z | z | RT | 1/k0 Start | 1/k0 End | Precursor ID | Area Sample 4 | #Feature | #Feature Sample 4 | Start | End | PTM | AScore | Found By |
| --- | --- | --- | --- | --- | --- | --- | --- | --- | --- | --- | --- | --- | --- | --- | --- | --- | --- | --- | --- |
| R.GQTFSGDIAIDDIK.T | Y | 58.4 | 1478.7252 | 14 | 0.51 | 740.3687 | 2 | 17.63 | 1.0073 | 1.0146 | 61922 | 1.42e3 | 1 | 1 | 221 | 234 |  |  | DB Search |
| K.VSGSLQLVWTR.V | N | 58.26 | 1244.6877 | 11 | 4.57 | 623.3526 | 2 | 18.55 | 0.9307 | 0.938 | 68053 | 5.74e2 | 1 | 1 | 19 | 29 |  |  | DB Search |
| total 2 peptides |  |  |  |  |  |  |  |  |  |  |  |  |  |  |  |  |  |  |  |
| --- | --- | --- | --- | --- | --- | --- | --- | --- | --- | --- | --- | --- | --- | --- | --- | --- | --- | --- | --- |

Best Unique PSM (Scan Sample\_2\_A1\_1\_3640.d:61922, m/z=740.3687, z=2, RT=17.63, ppm=-1.69):


TRINITY\_DN413\_c0\_g1\_i4.p1

back to list

  

| Protein Coverage
| Supporting Peptides
| Best Unique PSM
|

Protein Coverage:

Supporting Peptides:

| Peptide | Uniq | -10lgP | Mass | Length | ppm | m/z | z | RT | 1/k0 Start | 1/k0 End | Precursor ID | Area Sample 4 | #Feature | #Feature Sample 4 | Start | End | PTM | AScore | Found By |
| --- | --- | --- | --- | --- | --- | --- | --- | --- | --- | --- | --- | --- | --- | --- | --- | --- | --- | --- | --- |
| R.LLLGDFNGDKR.T | Y | 58.25 | 1246.6669 | 11 | 4.07 | 624.3419 | 2 | 16.32 | 0.9844 | 0.9916 | 53554 | 5.68e2 | 1 | 1 | 46 | 56 |  |  | DB Search |
| total 1 peptides |  |  |  |  |  |  |  |  |  |  |  |  |  |  |  |  |  |  |  |
| --- | --- | --- | --- | --- | --- | --- | --- | --- | --- | --- | --- | --- | --- | --- | --- | --- | --- | --- | --- |

Best Unique PSM (Scan Sample\_2\_A1\_1\_3640.d:53554, m/z=624.3419, z=2, RT=16.32, ppm=1.87):


TRINITY\_DN319\_c2\_g1\_i1.p1

back to list

  

| Protein Coverage
| Supporting Peptides
| Best Unique PSM
|

Protein Coverage:

Supporting Peptides:

| Peptide | Uniq | -10lgP | Mass | Length | ppm | m/z | z | RT | 1/k0 Start | 1/k0 End | Precursor ID | Area Sample 4 | #Feature | #Feature Sample 4 | Start | End | PTM | AScore | Found By |
| --- | --- | --- | --- | --- | --- | --- | --- | --- | --- | --- | --- | --- | --- | --- | --- | --- | --- | --- | --- |
| R.DPIC(+57.02)HC(+57.02)DTSK.Y | Y | 58.38 | 1231.4961 | 10 | 7 | 411.508 | 3 | 9.95 | 0.7355 | 0.7428 | 14664 | 1.19e4 | 2 | 2 | 1448 | 1457 | Carbamidomethylation, Carbamidomethylation |  | DB Search |
| R.GSFSSHIC(+57.02)LC(+57.02)HDPAYK.S | Y | 58.38 | 1877.8189 | 16 | 0.93 | 626.9461 | 3 | 14.52 | 0.8854 | 0.8927 | 42298 | 8.32e3 | 1 | 1 | 1011 | 1026 | Carbamidomethylation, Carbamidomethylation |  | DB Search |
| K.LLHC(+57.02)SDSEVDHVEVC(+57.02)GK.G | Y | 58.36 | 1982.8826 | 17 | 6.36 | 661.9709 | 3 | 13.31 | 0.9247 | 0.9319 | 34838 | 1.49e4 | 4 | 4 | 845 | 861 | Carbamidomethylation, Carbamidomethylation |  | DB Search |
| R.RTC(+57.02)ADTSPAIDFK.S | Y | 58.29 | 1480.698 | 13 | 1.84 | 741.356 | 2 | 14.27 | 1.017 | 1.0242 | 40672 | 1.48e3 | 1 | 1 | 274 | 286 | Carbamidomethylation |  | DB Search |
| K.C(+57.02)MLATC(+57.02)EAGQLDPVAVC(+57.02)GK.G | Y | 58.23 | 2078.9257 | 19 | -13.35 | 1040.454 | 2 | 17.81 | 1.1985 | 1.2057 | 63229 | 0 | 0 | 0 | 1612 | 1630 | Carbamidomethylation, Carbamidomethylation, Carbamidomethylation |  | DB Search |
| K.M(+15.99)QC(+57.02)VPAK.C | Y | 58.14 | 848.3884 | 7 | -1.44 | 425.2 | 2 | 9.64 | 0.8254 | 0.8327 | 13010 | 5.89e2 | 1 | 1 | 2271 | 2277 | Oxidation (M), Carbamidomethylation | M1:Oxidation (M):1000 | DB Search |
| R.ADC(+57.02)GVENTC(+57.02)FGSTR.S | Y | 57.59 | 1572.6297 | 14 | 1.45 | 787.3215 | 2 | 13.67 | 1.0053 | 1.0126 | 37114 | 4.6e3 | 1 | 1 | 1578 | 1591 | Carbamidomethylation, Carbamidomethylation |  | DB Search |
| R.TC(+57.02)ADTSPAIDFK.S | Y | 57.18 | 1324.5969 | 12 | -1.21 | 663.3035 | 2 | 15.19 | 0.9469 | 0.9541 | 46506 | 1.08e3 | 1 | 1 | 275 | 286 | Carbamidomethylation |  | DB Search |
| K.NLEC(+57.02)QC(+57.02)DESK.G | Y | 57.02 | 1281.4965 | 10 | 0.49 | 641.7545 | 2 | 10.48 | 0.9012 | 0.9085 | 17668 | 4.3e3 | 1 | 1 | 777 | 786 | Carbamidomethylation, Carbamidomethylation |  | DB Search |
| K.EC(+57.02)SEVC(+57.02)GVNGVAEVTR.F | Y | 56.61 | 1764.7771 | 16 | -2.02 | 883.3921 | 2 | 14.69 | 1.0705 | 1.0777 | 43395 | 0 | 0 | 0 | 2380 | 2395 | Carbamidomethylation, Carbamidomethylation |  | DB Search |
| R.SWIC(+57.02)DC(+57.02)NSYGSILNPSDVKK.C | Y | 56.06 | 2342.0671 | 20 | 3.26 | 781.6971 | 3 | 18.19 | 1.0077 | 1.015 | 65664 | 5.83e2 | 1 | 1 | 1592 | 1611 | Carbamidomethylation, Carbamidomethylation |  | DB Search |
| R.DVQC(+57.02)TC(+57.02)NNELGYVHSPDGK.K | Y | 53.44 | 2191.9262 | 19 | 2.69 | 731.6497 | 3 | 14.19 | 0.9634 | 0.9707 | 40373 | 5.07e2 | 1 | 1 | 1641 | 1659 | Carbamidomethylation, Carbamidomethylation |  | DB Search |
| K.AQYYEAYETIIKR.H | Y | 52.57 | 1646.8304 | 13 | 5.31 | 549.9525 | 3 | 16.99 | 0.8364 | 0.8437 | 57968 | 0 | 0 | 0 | 2535 | 2547 |  |  | DB Search |
| R.TC(+57.02)IEASC(+57.02)RR.N | Y | 47.82 | 1151.5176 | 9 | 3.72 | 384.847 | 3 | 10.13 | 0.7061 | 0.7135 | 15528 | 5.07e3 | 2 | 2 | 1564 | 1572 | Carbamidomethylation, Carbamidomethylation |  | DB Search |
| total 14 peptides |  |  |  |  |  |  |  |  |  |  |  |  |  |  |  |  |  |  |  |
| --- | --- | --- | --- | --- | --- | --- | --- | --- | --- | --- | --- | --- | --- | --- | --- | --- | --- | --- | --- |

Best Unique PSM (Scan Sample\_2\_A1\_1\_3640.d:14664, m/z=411.508, z=3, RT=9.95, ppm=4.81):


TRINITY\_DN101600\_c0\_g1\_i1.p1

back to list

  

| Protein Coverage
| Supporting Peptides
| Best Unique PSM
|

Protein Coverage:

Supporting Peptides:

| Peptide | Uniq | -10lgP | Mass | Length | ppm | m/z | z | RT | 1/k0 Start | 1/k0 End | Precursor ID | Area Sample 4 | #Feature | #Feature Sample 4 | Start | End | PTM | AScore | Found By |
| --- | --- | --- | --- | --- | --- | --- | --- | --- | --- | --- | --- | --- | --- | --- | --- | --- | --- | --- | --- |
| R.LGTIEKDLAK.V | Y | 58.22 | 1086.6284 | 10 | -3.8 | 544.3182 | 2 | 13.35 | 0.9044 | 0.9117 | 35185 | 0 | 0 | 0 | 210 | 219 |  |  | DB Search |
| total 1 peptides |  |  |  |  |  |  |  |  |  |  |  |  |  |  |  |  |  |  |  |
| --- | --- | --- | --- | --- | --- | --- | --- | --- | --- | --- | --- | --- | --- | --- | --- | --- | --- | --- | --- |

Best Unique PSM (Scan Sample\_2\_A1\_1\_3640.d:35185, m/z=544.3182, z=2, RT=13.35, ppm=-6):


TRINITY\_DN3182\_c0\_g1\_i2.p1

back to list

  

| Protein Coverage
| Supporting Peptides
| Best Unique PSM
|

Protein Coverage:

Supporting Peptides:

| Peptide | Uniq | -10lgP | Mass | Length | ppm | m/z | z | RT | 1/k0 Start | 1/k0 End | Precursor ID | Area Sample 4 | #Feature | #Feature Sample 4 | Start | End | PTM | AScore | Found By |
| --- | --- | --- | --- | --- | --- | --- | --- | --- | --- | --- | --- | --- | --- | --- | --- | --- | --- | --- | --- |
| K.LNQKVESILHK.I | Y | 58.39 | 1307.7561 | 11 | 3.28 | 436.9265 | 3 | 13.42 | 0.7453 | 0.7526 | 35604 | 4.6e2 | 1 | 1 | 146 | 156 |  |  | DB Search |
| K.IQTEFTSSGGPLVSVHVSDDPR.L | Y | 58.28 | 2327.1393 | 22 | 0.25 | 776.7189 | 3 | 17.17 | 0.9553 | 0.9626 | 58886 | 6.44e2 | 1 | 1 | 224 | 245 |  |  | DB Search |
| K.LTLGENIADNGGFK.T | Y | 57.37 | 1447.7307 | 14 | 2.38 | 724.8727 | 2 | 17.25 | 0.9993 | 1.0065 | 59417 | 7.06e2 | 1 | 1 | 669 | 682 |  |  | DB Search |
| total 3 peptides |  |  |  |  |  |  |  |  |  |  |  |  |  |  |  |  |  |  |  |
| --- | --- | --- | --- | --- | --- | --- | --- | --- | --- | --- | --- | --- | --- | --- | --- | --- | --- | --- | --- |

Best Unique PSM (Scan Sample\_2\_A1\_1\_3640.d:35604, m/z=436.9265, z=3, RT=13.42, ppm=1.09):


TRINITY\_DN152\_c0\_g1\_i9.p1

back to list

  

| Protein Coverage
| Supporting Peptides
| Best Unique PSM
|

Protein Coverage:

Supporting Peptides:

| Peptide | Uniq | -10lgP | Mass | Length | ppm | m/z | z | RT | 1/k0 Start | 1/k0 End | Precursor ID | Area Sample 4 | #Feature | #Feature Sample 4 | Start | End | PTM | AScore | Found By |
| --- | --- | --- | --- | --- | --- | --- | --- | --- | --- | --- | --- | --- | --- | --- | --- | --- | --- | --- | --- |
| K.ESTLHLVLR.L | Y | 57.27 | 1066.6135 | 9 | 2.57 | 534.3142 | 2 | 15.80 | 0.8785 | 0.8858 | 50332 | 4.97e3 | 1 | 1 | 24 | 32 |  |  | DB Search |
| K.IQDKEGIPPDQQR.L | Y | 55.01 | 1522.7739 | 13 | 0.25 | 762.3928 | 2 | 11.08 | 1.0343 | 1.0415 | 21018 | 1.78e3 | 2 | 2 | 66 | 78 |  |  | DB Search |
| total 2 peptides |  |  |  |  |  |  |  |  |  |  |  |  |  |  |  |  |  |  |  |
| --- | --- | --- | --- | --- | --- | --- | --- | --- | --- | --- | --- | --- | --- | --- | --- | --- | --- | --- | --- |

Best Unique PSM (Scan Sample\_2\_A1\_1\_3640.d:50332, m/z=534.7766, z=2, RT=15.80, ppm=0.37):


TRINITY\_DN1172\_c0\_g2\_i2.p1

back to list

  

| Protein Coverage
| Supporting Peptides
| Best Unique PSM
|

Protein Coverage:

Supporting Peptides:

| Peptide | Uniq | -10lgP | Mass | Length | ppm | m/z | z | RT | 1/k0 Start | 1/k0 End | Precursor ID | Area Sample 4 | #Feature | #Feature Sample 4 | Start | End | PTM | AScore | Found By |
| --- | --- | --- | --- | --- | --- | --- | --- | --- | --- | --- | --- | --- | --- | --- | --- | --- | --- | --- | --- |
| K.K(+42.01)LAAELANGR.L | Y | 55.86 | 1083.6036 | 10 | 2.07 | 542.809 | 2 | 15.52 | 0.8704 | 0.8777 | 48534 | 1.49e3 | 1 | 1 | 19 | 28 | Acetylation (Protein N-term) | K1:Acetylation (Protein N-term):1000 | DB Search |
| total 1 peptides |  |  |  |  |  |  |  |  |  |  |  |  |  |  |  |  |  |  |  |
| --- | --- | --- | --- | --- | --- | --- | --- | --- | --- | --- | --- | --- | --- | --- | --- | --- | --- | --- | --- |

Best Unique PSM (Scan Sample\_2\_A1\_1\_3640.d:48534, m/z=542.809, z=2, RT=15.52, ppm=-0.12):


TRINITY\_DN24268\_c0\_g1\_i1.p1

back to list

  

| Protein Coverage
| Supporting Peptides
| Best Unique PSM
|

Protein Coverage:

Supporting Peptides:

| Peptide | Uniq | -10lgP | Mass | Length | ppm | m/z | z | RT | 1/k0 Start | 1/k0 End | Precursor ID | Area Sample 4 | #Feature | #Feature Sample 4 | Start | End | PTM | AScore | Found By |
| --- | --- | --- | --- | --- | --- | --- | --- | --- | --- | --- | --- | --- | --- | --- | --- | --- | --- | --- | --- |
| K.AC(+57.02)GLENM(+15.99)C(+57.02)VGSYK.S | N | 58.41 | 1503.6156 | 13 | -0.67 | 752.8129 | 2 | 13.59 | 0.9989 | 1.0061 | 36764 | 0 | 0 | 0 | 2156 | 2168 | Carbamidomethylation, Oxidation (M), Carbamidomethylation | M7:Oxidation (M):1000 | DB Search |
| R.YC(+57.02)FHASC(+57.02)K.T | Y | 58.39 | 1071.4266 | 8 | -3.12 | 358.1476 | 3 | 11.77 | 0.7229 | 0.7302 | 25566 | 9.3e2 | 1 | 1 | 133 | 140 | Carbamidomethylation, Carbamidomethylation |  | DB Search |
| R.TC(+57.02)VTASC(+57.02)R.T | Y | 58.02 | 953.4059 | 8 | 4.85 | 477.7115 | 2 | 10.04 | 0.7986 | 0.8059 | 15114 | 2.52e3 | 1 | 1 | 1573 | 1580 | Carbamidomethylation, Carbamidomethylation |  | DB Search |
| K.ASNEVSM(+15.99)K.C | Y | 57.93 | 880.396 | 8 | -7.43 | 441.201 | 2 | 8.74 | 0.7469 | 0.7542 | 9825 | 0 | 0 | 0 | 713 | 720 | Oxidation (M) | M7:Oxidation (M):1000 | DB Search |
| K.ASNEVSMK.C | Y | 55.46 | 864.4011 | 8 | -4.31 | 433.205 | 2 | 10.65 | 0.762 | 0.7693 | 18751 | 4.71e2 | 1 | 1 | 713 | 720 |  |  | DB Search |
| K.YFTPSADGFSC(+57.02)IPASC(+57.02)K.S | Y | 53.17 | 1906.8229 | 17 | 11.55 | 954.4277 | 2 | 17.51 | 1.1222 | 1.1294 | 61270 | 0 | 0 | 0 | 1757 | 1773 | Carbamidomethylation, Carbamidomethylation |  | DB Search |
| R.SPDLTSC(+57.02)IPSDC(+57.02)R.G | Y | 52.65 | 1506.6443 | 13 | -4.7 | 754.3242 | 2 | 14.88 | 1.0001 | 1.0073 | 44608 | 0 | 0 | 0 | 509 | 521 | Carbamidomethylation, Carbamidomethylation |  | DB Search |
| total 7 peptides |  |  |  |  |  |  |  |  |  |  |  |  |  |  |  |  |  |  |  |
| --- | --- | --- | --- | --- | --- | --- | --- | --- | --- | --- | --- | --- | --- | --- | --- | --- | --- | --- | --- |

Best Unique PSM (Scan Sample\_2\_A1\_1\_3640.d:25566, m/z=358.1476, z=3, RT=11.77, ppm=-5.32):


TRINITY\_DN72625\_c0\_g1\_i1.p1

back to list

  

| Protein Coverage
| Supporting Peptides
| Best Unique PSM
|

Protein Coverage:

Supporting Peptides:

| Peptide | Uniq | -10lgP | Mass | Length | ppm | m/z | z | RT | 1/k0 Start | 1/k0 End | Precursor ID | Area Sample 4 | #Feature | #Feature Sample 4 | Start | End | PTM | AScore | Found By |
| --- | --- | --- | --- | --- | --- | --- | --- | --- | --- | --- | --- | --- | --- | --- | --- | --- | --- | --- | --- |
| R.KTTELTNNLK.A | Y | 55.11 | 1160.6401 | 10 | 5.27 | 581.3291 | 2 | 11.78 | 0.9178 | 0.9251 | 25555 | 4.08e3 | 1 | 1 | 1921 | 1930 |  |  | DB Search |
| total 1 peptides |  |  |  |  |  |  |  |  |  |  |  |  |  |  |  |  |  |  |  |
| --- | --- | --- | --- | --- | --- | --- | --- | --- | --- | --- | --- | --- | --- | --- | --- | --- | --- | --- | --- |

Best Unique PSM (Scan Sample\_2\_A1\_1\_3640.d:25555, m/z=581.3291, z=2, RT=11.78, ppm=3.07):


TRINITY\_DN36\_c0\_g1\_i8.p1

back to list

  

| Protein Coverage
| Supporting Peptides
| Best Unique PSM
|

Protein Coverage:

Supporting Peptides:

| Peptide | Uniq | -10lgP | Mass | Length | ppm | m/z | z | RT | 1/k0 Start | 1/k0 End | Precursor ID | Area Sample 4 | #Feature | #Feature Sample 4 | Start | End | PTM | AScore | Found By |
| --- | --- | --- | --- | --- | --- | --- | --- | --- | --- | --- | --- | --- | --- | --- | --- | --- | --- | --- | --- |
| R.LGGIEVTQGNC(+57.02)AR.K | Y | 58.36 | 1373.6721 | 13 | -2.89 | 687.8398 | 2 | 13.93 | 0.9662 | 0.9735 | 38726 | 2.09e3 | 1 | 1 | 58 | 70 | Carbamidomethylation |  | DB Search |
| K.GLPLAIGVR.D | Y | 57.12 | 894.565 | 9 | 0.27 | 448.2889 | 2 | 17.73 | 0.8124 | 0.8197 | 62574 | 3.52e3 | 1 | 1 | 32 | 40 |  |  | DB Search |
| total 2 peptides |  |  |  |  |  |  |  |  |  |  |  |  |  |  |  |  |  |  |  |
| --- | --- | --- | --- | --- | --- | --- | --- | --- | --- | --- | --- | --- | --- | --- | --- | --- | --- | --- | --- |

Best Unique PSM (Scan Sample\_2\_A1\_1\_3640.d:38726, m/z=687.8398, z=2, RT=13.93, ppm=-5.09):


TRINITY\_DN585\_c0\_g1\_i2.p1

back to list

  

| Protein Coverage
| Supporting Peptides
| Best Unique PSM
|

Protein Coverage:

Supporting Peptides:

| Peptide | Uniq | -10lgP | Mass | Length | ppm | m/z | z | RT | 1/k0 Start | 1/k0 End | Precursor ID | Area Sample 4 | #Feature | #Feature Sample 4 | Start | End | PTM | AScore | Found By |
| --- | --- | --- | --- | --- | --- | --- | --- | --- | --- | --- | --- | --- | --- | --- | --- | --- | --- | --- | --- |
| K.ESTLHLVLR.L | Y | 57.27 | 1066.6135 | 9 | 2.57 | 534.3142 | 2 | 15.80 | 0.8785 | 0.8858 | 50332 | 4.97e3 | 1 | 1 | 36 | 44 |  |  | DB Search |
| K.IQDKEGIPPDQQR.L | Y | 55.01 | 1522.7739 | 13 | 0.25 | 762.3928 | 2 | 11.08 | 1.0343 | 1.0415 | 21018 | 1.78e3 | 2 | 2 | 2 | 14 |  |  | DB Search |
| K.TITLEVEPSDTIENVK.S | Y | 54.56 | 1786.92 | 16 | 4.26 | 894.4691 | 2 | 17.62 | 1.1442 | 1.1514 | 61769 | 1.48e4 | 1 | 1 | 60 | 75 |  |  | DB Search |
| total 3 peptides |  |  |  |  |  |  |  |  |  |  |  |  |  |  |  |  |  |  |  |
| --- | --- | --- | --- | --- | --- | --- | --- | --- | --- | --- | --- | --- | --- | --- | --- | --- | --- | --- | --- |

Best Unique PSM (Scan Sample\_2\_A1\_1\_3640.d:50332, m/z=534.7766, z=2, RT=15.80, ppm=0.37):


TRINITY\_DN33467\_c0\_g1\_i1.p1

back to list

  

| Protein Coverage
| Supporting Peptides
| Best Unique PSM
|

Protein Coverage:

Supporting Peptides:

| Peptide | Uniq | -10lgP | Mass | Length | ppm | m/z | z | RT | 1/k0 Start | 1/k0 End | Precursor ID | Area Sample 4 | #Feature | #Feature Sample 4 | Start | End | PTM | AScore | Found By |
| --- | --- | --- | --- | --- | --- | --- | --- | --- | --- | --- | --- | --- | --- | --- | --- | --- | --- | --- | --- |
| K.FVGIPSIDISYR.Q | Y | 53.5 | 1365.7292 | 12 | -0.66 | 683.8699 | 2 | 19.88 | 0.9675 | 0.9747 | 76591 | 1.07e3 | 1 | 1 | 13 | 24 |  |  | DB Search |
| total 1 peptides |  |  |  |  |  |  |  |  |  |  |  |  |  |  |  |  |  |  |  |
| --- | --- | --- | --- | --- | --- | --- | --- | --- | --- | --- | --- | --- | --- | --- | --- | --- | --- | --- | --- |

Best Unique PSM (Scan Sample\_2\_A1\_1\_3640.d:76591, m/z=683.8699, z=2, RT=19.88, ppm=-2.86):


TRINITY\_DN4595\_c0\_g1\_i4.p1

back to list

  

| Protein Coverage
| Supporting Peptides
| Best Unique PSM
|

Protein Coverage:

Supporting Peptides:

| Peptide | Uniq | -10lgP | Mass | Length | ppm | m/z | z | RT | 1/k0 Start | 1/k0 End | Precursor ID | Area Sample 4 | #Feature | #Feature Sample 4 | Start | End | PTM | AScore | Found By |
| --- | --- | --- | --- | --- | --- | --- | --- | --- | --- | --- | --- | --- | --- | --- | --- | --- | --- | --- | --- |
| K.TC(+57.02)GGGFFFETR.T | Y | 58.39 | 1277.5499 | 11 | 10.24 | 639.7874 | 2 | 18.62 | 0.921 | 0.9283 | 68551 | 2.46e2 | 1 | 1 | 480 | 490 | Carbamidomethylation |  | DB Search |
| K.GFEC(+57.02)IGLIEETETC(+57.02)K.L | Y | 58.39 | 1784.7961 | 15 | 3.79 | 595.9402 | 3 | 18.69 | 0.8627 | 0.87 | 69011 | 2.49e3 | 2 | 2 | 544 | 558 | Carbamidomethylation, Carbamidomethylation |  | DB Search |
| R.VC(+57.02)LDDDIKVC(+57.02)K.M | Y | 58.38 | 1363.6475 | 11 | 2.8 | 455.5567 | 3 | 14.43 | 0.7978 | 0.8051 | 41739 | 9.3e2 | 1 | 1 | 1140 | 1150 | Carbamidomethylation, Carbamidomethylation |  | DB Search |
| R.VC(+57.02)NGEPC(+57.02)ILQER.S | Y | 58.29 | 1473.6704 | 12 | 1.84 | 737.8422 | 2 | 15.04 | 0.9832 | 0.9904 | 45530 | 3e3 | 1 | 1 | 436 | 447 | Carbamidomethylation, Carbamidomethylation |  | DB Search |
| R.FC(+57.02)NNPAPSNGGR.M | Y | 58.29 | 1289.5571 | 12 | 10.24 | 645.791 | 2 | 10.91 | 0.9158 | 0.923 | 20335 | 6.39e3 | 1 | 1 | 1196 | 1207 | Carbamidomethylation |  | DB Search |
| R.AC(+57.02)DSGFSAVLSR.N | Y | 58.28 | 1268.5819 | 12 | 1.5 | 635.2978 | 2 | 16.57 | 0.9485 | 0.9558 | 55157 | 3.01e2 | 1 | 1 | 405 | 416 | Carbamidomethylation |  | DB Search |
| R.NAFFIVTSK.D | Y | 58.27 | 1025.5546 | 9 | -0.8 | 513.783 | 2 | 18.00 | 0.8388 | 0.8461 | 64505 | 0 | 0 | 0 | 595 | 603 |  |  | DB Search |
| R.VC(+57.02)NAALC(+57.02)R.Y | Y | 58.26 | 962.4426 | 8 | 0.23 | 482.2276 | 2 | 11.36 | 0.812 | 0.8193 | 23031 | 0 | 0 | 0 | 224 | 231 | Carbamidomethylation, Carbamidomethylation |  | DB Search |
| R.TAC(+57.02)VGM(+15.99)DR.E | Y | 58.23 | 924.3793 | 8 | 4.29 | 463.1979 | 2 | 9.57 | 0.7827 | 0.7901 | 12695 | 9.24e2 | 1 | 1 | 796 | 803 | Carbamidomethylation, Oxidation (M) | M6:Oxidation (M):1000 | DB Search |
| R.VLQEREC(+57.02)EQDTC(+57.02)FDGNTR.F | Y | 57.83 | 2255.9535 | 18 | -6.28 | 752.9854 | 3 | 13.04 | 0.9323 | 0.9396 | 33365 | 6e2 | 1 | 1 | 1258 | 1275 | Carbamidomethylation, Carbamidomethylation |  | DB Search |
| R.TRVC(+57.02)LDDDIKVC(+57.02)K.M | Y | 57.39 | 1620.7963 | 13 | -1.68 | 541.2706 | 3 | 13.95 | 0.838 | 0.8453 | 38871 | 6.52e2 | 1 | 1 | 1138 | 1150 | Carbamidomethylation, Carbamidomethylation |  | DB Search |
| R.SPFVIMFK.G | Y | 57.23 | 967.5201 | 8 | 1.15 | 484.7668 | 2 | 19.95 | 0.8063 | 0.8136 | 76925 | 1.04e4 | 1 | 1 | 1081 | 1088 |  |  | DB Search |
| R.EC(+57.02)EQDTC(+57.02)FDGNTR.F | Y | 57.01 | 1630.5988 | 13 | -1.43 | 816.3037 | 2 | 12.29 | 1.0122 | 1.0194 | 28637 | 3.57e2 | 1 | 1 | 1263 | 1275 | Carbamidomethylation, Carbamidomethylation |  | DB Search |
| R.AFNKVETR.N | N | 57 | 963.5138 | 8 | 4.36 | 482.7652 | 2 | 10.61 | 0.8274 | 0.8348 | 18412 | 3.24e3 | 1 | 1 | 177 | 184 |  |  | DB Search |
| R.TRVC(+57.02)LDDDIK.V | Y | 56.08 | 1233.6023 | 10 | 4.44 | 617.8098 | 2 | 13.63 | 0.9485 | 0.9558 | 36912 | 1.78e3 | 1 | 1 | 1138 | 1147 | Carbamidomethylation |  | DB Search |
| R.DGTTPC(+57.02)NTR.S | Y | 55.73 | 1020.4295 | 9 | 2.58 | 511.2222 | 2 | 9.62 | 0.8311 | 0.8384 | 12948 | 1.32e3 | 1 | 1 | 290 | 298 | Carbamidomethylation |  | DB Search |
| R.AFNKIETR.N | Y | 54.97 | 977.5294 | 8 | 8.36 | 489.775 | 2 | 11.54 | 0.8339 | 0.8412 | 24067 | 2.69e3 | 1 | 1 | 72 | 79 |  |  | DB Search |
| R.EYNINEEVNVR.Q | Y | 54.67 | 1377.6524 | 11 | -5.34 | 689.8283 | 2 | 14.94 | 0.9787 | 0.986 | 45239 | 5.98e2 | 1 | 1 | 342 | 352 |  |  | DB Search |
| R.YQGREYNINEEVNVR.Q | Y | 52.5 | 1881.8969 | 15 | 3.38 | 941.9568 | 2 | 15.71 | 1.1422 | 1.1494 | 49787 | 0 | 0 | 0 | 338 | 352 |  |  | DB Search |
| K.KVNQGEKEFLQRPC(+57.02)R.Y | Y | 42.62 | 1887.9737 | 15 | 3.41 | 473.0013 | 4 | 12.00 | 0.8128 | 0.8201 | 26878 | 0 | 0 | 0 | 1611 | 1625 | Carbamidomethylation |  | DB Search |
| K.VNQGEKEFLQRPC(+57.02)R.Y | Y | 37.68 | 1759.8788 | 14 | 0.76 | 587.6327 | 3 | 12.79 | 0.8635 | 0.8708 | 31645 | 8.61e2 | 1 | 1 | 1612 | 1625 | Carbamidomethylation |  | DB Search |
| total 21 peptides |  |  |  |  |  |  |  |  |  |  |  |  |  |  |  |  |  |  |  |
| --- | --- | --- | --- | --- | --- | --- | --- | --- | --- | --- | --- | --- | --- | --- | --- | --- | --- | --- | --- |

Best Unique PSM (Scan Sample\_2\_A1\_1\_3640.d:68551, m/z=639.7874, z=2, RT=18.62, ppm=8.04):


TRINITY\_DN5397\_c0\_g1\_i1.p1

back to list

  

| Protein Coverage
| Supporting Peptides
| Best Unique PSM
|

Protein Coverage:

Supporting Peptides:

| Peptide | Uniq | -10lgP | Mass | Length | ppm | m/z | z | RT | 1/k0 Start | 1/k0 End | Precursor ID | Area Sample 4 | #Feature | #Feature Sample 4 | Start | End | PTM | AScore | Found By |
| --- | --- | --- | --- | --- | --- | --- | --- | --- | --- | --- | --- | --- | --- | --- | --- | --- | --- | --- | --- |
| R.SFNKVESR.E | Y | 37.49 | 965.493 | 8 | 3.7 | 483.7545 | 2 | 10.29 | 0.8319 | 0.8392 | 16526 | 0 | 0 | 1 | 33 | 40 |  |  | DB Search |
| total 1 peptides |  |  |  |  |  |  |  |  |  |  |  |  |  |  |  |  |  |  |  |
| --- | --- | --- | --- | --- | --- | --- | --- | --- | --- | --- | --- | --- | --- | --- | --- | --- | --- | --- | --- |

Best Unique PSM (Scan Sample\_2\_A1\_1\_3640.d:16526, m/z=483.7545, z=2, RT=10.29, ppm=1.5):


TRINITY\_DN5\_c0\_g1\_i9.p1

back to list

  

| Protein Coverage
| Supporting Peptides
| Best Unique PSM
|

Protein Coverage:

Supporting Peptides:

| Peptide | Uniq | -10lgP | Mass | Length | ppm | m/z | z | RT | 1/k0 Start | 1/k0 End | Precursor ID | Area Sample 4 | #Feature | #Feature Sample 4 | Start | End | PTM | AScore | Found By |
| --- | --- | --- | --- | --- | --- | --- | --- | --- | --- | --- | --- | --- | --- | --- | --- | --- | --- | --- | --- |
| K.IIIELQGDLIK.I | Y | 53.27 | 1253.7594 | 11 | 3.89 | 627.8881 | 2 | 19.42 | 0.9513 | 0.9586 | 73842 | 0 | 0 | 0 | 65 | 75 |  |  | DB Search |
| total 1 peptides |  |  |  |  |  |  |  |  |  |  |  |  |  |  |  |  |  |  |  |
| --- | --- | --- | --- | --- | --- | --- | --- | --- | --- | --- | --- | --- | --- | --- | --- | --- | --- | --- | --- |

Best Unique PSM (Scan Sample\_2\_A1\_1\_3640.d:73842, m/z=627.8881, z=2, RT=19.42, ppm=1.69):


TRINITY\_DN585\_c0\_g2\_i1.p1

back to list

  

| Protein Coverage
| Supporting Peptides
| Best Unique PSM
|

Protein Coverage:

Supporting Peptides:

| Peptide | Uniq | -10lgP | Mass | Length | ppm | m/z | z | RT | 1/k0 Start | 1/k0 End | Precursor ID | Area Sample 4 | #Feature | #Feature Sample 4 | Start | End | PTM | AScore | Found By |
| --- | --- | --- | --- | --- | --- | --- | --- | --- | --- | --- | --- | --- | --- | --- | --- | --- | --- | --- | --- |
| K.ESTLHLVLR.L | Y | 57.27 | 1066.6135 | 9 | 2.57 | 534.3142 | 2 | 15.80 | 0.8785 | 0.8858 | 50332 | 4.97e3 | 1 | 1 | 57 | 65 |  |  | DB Search |
| K.IQDKEGIPPDQQR.L | Y | 55.01 | 1522.7739 | 13 | 0.25 | 762.3928 | 2 | 11.08 | 1.0343 | 1.0415 | 21018 | 1.78e3 | 2 | 2 | 23 | 35 |  |  | DB Search |
| K.TITLEVEPSDTIENVK.S | Y | 54.56 | 1786.92 | 16 | 4.26 | 894.4691 | 2 | 17.62 | 1.1442 | 1.1514 | 61769 | 1.48e4 | 1 | 1 | 5 | 20 |  |  | DB Search |
| total 3 peptides |  |  |  |  |  |  |  |  |  |  |  |  |  |  |  |  |  |  |  |
| --- | --- | --- | --- | --- | --- | --- | --- | --- | --- | --- | --- | --- | --- | --- | --- | --- | --- | --- | --- |

Best Unique PSM (Scan Sample\_2\_A1\_1\_3640.d:50332, m/z=534.7766, z=2, RT=15.80, ppm=0.37):


TRINITY\_DN1616\_c0\_g1\_i6.p1

back to list

  

| Protein Coverage
| Supporting Peptides
| Best Unique PSM
|

Protein Coverage:

Supporting Peptides:

| Peptide | Uniq | -10lgP | Mass | Length | ppm | m/z | z | RT | 1/k0 Start | 1/k0 End | Precursor ID | Area Sample 4 | #Feature | #Feature Sample 4 | Start | End | PTM | AScore | Found By |
| --- | --- | --- | --- | --- | --- | --- | --- | --- | --- | --- | --- | --- | --- | --- | --- | --- | --- | --- | --- |
| R.SNIDEVNVKHEQIEEFIK.T | Y | 58.4 | 2170.0906 | 18 | 0.32 | 724.3694 | 3 | 16.43 | 0.9566 | 0.9638 | 54256 | 7.37e2 | 1 | 1 | 2671 | 2688 |  |  | DB Search |
| K.VVDTEANDLTEEVKK.T | Y | 58.38 | 1688.8468 | 15 | -0.58 | 563.9547 | 3 | 14.21 | 0.8392 | 0.8465 | 40438 | 5.49e3 | 2 | 2 | 2542 | 2556 |  |  | DB Search |
| K.LNTGDVQSLTNPK.F | Y | 58.37 | 1385.715 | 13 | 1.86 | 693.8646 | 2 | 14.77 | 0.9977 | 1.0049 | 43652 | 1.53e3 | 1 | 1 | 2783 | 2795 |  |  | DB Search |
| R.YGAVNDNC(+57.02)ER.D | Y | 58.36 | 1196.488 | 10 | 4.23 | 599.2525 | 2 | 10.91 | 0.8802 | 0.8874 | 20281 | 8.99e2 | 1 | 1 | 498 | 507 | Carbamidomethylation |  | DB Search |
| K.SNIC(+57.02)DPQNGK.C | Y | 58.34 | 1131.4979 | 10 | -0.79 | 566.7545 | 2 | 10.56 | 0.8526 | 0.8599 | 18199 | 6.49e2 | 1 | 1 | 1794 | 1803 | Carbamidomethylation |  | DB Search |
| K.LSLDVNGNYQGGGVC(+57.02)K.N | Y | 58.29 | 1679.7937 | 16 | -4.07 | 840.8989 | 2 | 15.59 | 1.0705 | 1.0777 | 49051 | 0 | 0 | 0 | 350 | 365 | Carbamidomethylation |  | DB Search |
| R.FSNLSNKGDEHISNLDTLKK.A | Y | 58.28 | 2259.1495 | 20 | 1.82 | 565.7944 | 4 | 13.91 | 0.8311 | 0.8384 | 38634 | 0 | 0 | 0 | 2121 | 2140 |  |  | DB Search |
| R.NFATAVLNVQK.S | Y | 58.28 | 1203.6611 | 11 | -3.72 | 602.8343 | 2 | 17.63 | 0.923 | 0.9303 | 62041 | 0 | 0 | 0 | 2810 | 2820 |  |  | DB Search |
| K.TLKVDISDMK.M | Y | 58.28 | 1148.6111 | 10 | -6.01 | 575.3081 | 2 | 15.17 | 0.917 | 0.9243 | 46490 | 0 | 0 | 0 | 2557 | 2566 |  |  | DB Search |
| K.FIETDVNYDVM(+15.99)VTR.N | Y | 58.27 | 1716.8029 | 14 | 0.97 | 859.4077 | 2 | 16.71 | 1.0809 | 1.0881 | 56014 | 1.52e3 | 1 | 1 | 2796 | 2809 | Oxidation (M) | M11:Oxidation (M):1000 | DB Search |
| R.VIDEGEKNIK.L | Y | 58.26 | 1143.6135 | 10 | 3.03 | 572.8145 | 2 | 10.89 | 0.9271 | 0.9344 | 20199 | 3.83e3 | 2 | 2 | 2218 | 2227 |  |  | DB Search |
| K.DIIVLEAENPVK.M | Y | 58.25 | 1338.7394 | 12 | 1.86 | 670.3768 | 2 | 18.10 | 0.9844 | 0.9916 | 65198 | 4.87e2 | 1 | 1 | 1013 | 1024 |  |  | DB Search |
| R.YVKPGVNIINIR.D | Y | 57.33 | 1384.819 | 12 | 6.22 | 462.6155 | 3 | 16.27 | 0.8043 | 0.8116 | 53149 | 6.85e2 | 2 | 2 | 1217 | 1228 |  |  | DB Search |
| K.LFNEALKPLK.E | Y | 57.32 | 1171.6965 | 10 | 3.28 | 586.8561 | 2 | 15.90 | 0.9509 | 0.9582 | 50919 | 4.48e2 | 1 | 1 | 3651 | 3660 |  |  | DB Search |
| K.AVDFQSIIDNAQK.L | Y | 56.94 | 1447.7307 | 13 | 4.22 | 724.8741 | 2 | 19.06 | 0.9904 | 0.9977 | 71389 | 6.5e2 | 1 | 1 | 2383 | 2395 |  |  | DB Search |
| K.NLETFTGC(+57.02)ITAVR.I | Y | 56.93 | 1480.7344 | 13 | 2.99 | 741.3751 | 2 | 18.29 | 0.9993 | 1.0065 | 66297 | 1.31e3 | 1 | 1 | 3814 | 3826 | Carbamidomethylation |  | DB Search |
| K.DGEKVETLKVDISDINK.A | Y | 56.92 | 1901.9945 | 17 | 3.6 | 635.0063 | 3 | 16.09 | 0.9113 | 0.9186 | 52017 | 1.36e3 | 1 | 1 | 2634 | 2650 |  |  | DB Search |
| K.VETLKVDISDINK.A | Y | 56.92 | 1472.8086 | 13 | -2.26 | 491.9413 | 3 | 16.17 | 0.8222 | 0.8295 | 52567 | 5.32e2 | 1 | 1 | 2638 | 2650 |  |  | DB Search |
| K.GDEHISNLDTLKK.A | Y | 56.42 | 1468.7521 | 13 | -2.32 | 490.5891 | 3 | 12.90 | 0.7791 | 0.7864 | 32456 | 0 | 0 | 0 | 2128 | 2140 |  |  | DB Search |
| K.GFVAVNDQQPSVVVSINVPK.T | Y | 55.03 | 2096.1266 | 20 | 0.1 | 1049.0684 | 2 | 18.45 | 1.2157 | 1.2228 | 67713 | 4.05e2 | 1 | 1 | 865 | 884 |  |  | DB Search |
| K.DGEKVETLK.V | Y | 54.9 | 1017.5342 | 9 | -2.12 | 509.7722 | 2 | 11.49 | 0.8587 | 0.866 | 23862 | 1.28e3 | 1 | 1 | 2634 | 2642 |  |  | DB Search |
| R.IDNVKEDVK.S | Y | 54.89 | 1058.5608 | 9 | 5.78 | 530.2896 | 2 | 10.73 | 0.8716 | 0.8789 | 19180 | 1.58e3 | 1 | 1 | 2583 | 2591 |  |  | DB Search |
| K.VLISSLLIK.F | Y | 54.58 | 984.6583 | 9 | 0.22 | 493.3354 | 2 | 19.79 | 0.8506 | 0.8579 | 76091 | 1.17e3 | 1 | 1 | 3009 | 3017 |  |  | DB Search |
| R.RC(+57.02)EEC(+57.02)AAGHYQFPFC(+57.02)K.E | Y | 53.84 | 2058.8499 | 16 | 0.01 | 687.2891 | 3 | 15.74 | 0.9218 | 0.9291 | 49834 | 2.69e2 | 1 | 1 | 522 | 537 | Carbamidomethylation, Carbamidomethylation, Carbamidomethylation |  | DB Search |
| K.NEINLASGDVFEGGVVGSLK.L | Y | 53.15 | 2004.0163 | 20 | 1.4 | 1003.0146 | 2 | 19.85 | 1.1829 | 1.1901 | 76578 | 0 | 0 | 0 | 3249 | 3268 |  |  | DB Search |
| R.KLSLDVNGNYQGGGVC(+57.02)K.N | Y | 51.86 | 1807.8887 | 17 | 0.8 | 603.636 | 3 | 14.64 | 0.8193 | 0.8266 | 43107 | 2.56e2 | 1 | 1 | 349 | 365 | Carbamidomethylation |  | DB Search |
| R.YALNQIQPVTATVTAFK.G | Y | 47.96 | 1864.0094 | 17 | 1.71 | 933.0115 | 2 | 19.31 | 1.1478 | 1.155 | 73122 | 0 | 0 | 0 | 894 | 910 |  |  | DB Search |
| K.IRSNIDEVNVK.H | Y | 39.98 | 1285.699 | 11 | -7.71 | 643.8504 | 2 | 14.93 | 0.9638 | 0.9711 | 44969 | 0 | 0 | 0 | 2669 | 2679 |  |  | DB Search |
| total 28 peptides |  |  |  |  |  |  |  |  |  |  |  |  |  |  |  |  |  |  |  |
| --- | --- | --- | --- | --- | --- | --- | --- | --- | --- | --- | --- | --- | --- | --- | --- | --- | --- | --- | --- |

Best Unique PSM (Scan Sample\_2\_A1\_1\_3640.d:54256, m/z=724.3694, z=3, RT=16.43, ppm=-1.87):


TRINITY\_DN5\_c0\_g1\_i7.p4

back to list

  

| Protein Coverage
| Supporting Peptides
| Best Unique PSM
|

Protein Coverage:

Supporting Peptides:

| Peptide | Uniq | -10lgP | Mass | Length | ppm | m/z | z | RT | 1/k0 Start | 1/k0 End | Precursor ID | Area Sample 4 | #Feature | #Feature Sample 4 | Start | End | PTM | AScore | Found By |
| --- | --- | --- | --- | --- | --- | --- | --- | --- | --- | --- | --- | --- | --- | --- | --- | --- | --- | --- | --- |
| K.IIIELQGDLIK.I | Y | 53.27 | 1253.7594 | 11 | 3.89 | 627.8881 | 2 | 19.42 | 0.9513 | 0.9586 | 73842 | 0 | 0 | 0 | 65 | 75 |  |  | DB Search |
| total 1 peptides |  |  |  |  |  |  |  |  |  |  |  |  |  |  |  |  |  |  |  |
| --- | --- | --- | --- | --- | --- | --- | --- | --- | --- | --- | --- | --- | --- | --- | --- | --- | --- | --- | --- |

Best Unique PSM (Scan Sample\_2\_A1\_1\_3640.d:73842, m/z=627.8881, z=2, RT=19.42, ppm=1.69):


TRINITY\_DN942\_c0\_g1\_i5.p1

back to list

  

| Protein Coverage
| Supporting Peptides
| Best Unique PSM
|

Protein Coverage:

Supporting Peptides:

| Peptide | Uniq | -10lgP | Mass | Length | ppm | m/z | z | RT | 1/k0 Start | 1/k0 End | Precursor ID | Area Sample 4 | #Feature | #Feature Sample 4 | Start | End | PTM | AScore | Found By |
| --- | --- | --- | --- | --- | --- | --- | --- | --- | --- | --- | --- | --- | --- | --- | --- | --- | --- | --- | --- |
| K.AQLLLEDVK.A | Y | 57.95 | 1027.5913 | 9 | 4.05 | 514.8039 | 2 | 17.08 | 0.8721 | 0.8793 | 58461 | 1.87e2 | 1 | 1 | 3333 | 3341 |  |  | DB Search |
| K.VLNIALDKYQEGVAK.T | Y | 57.22 | 1659.9195 | 15 | -3.66 | 554.3105 | 3 | 16.21 | 0.8088 | 0.8161 | 52938 | 0 | 0 | 0 | 4557 | 4571 |  |  | DB Search |
| R.LIVINLIK.K | Y | 54.1 | 924.6372 | 8 | 1.83 | 463.3257 | 2 | 19.76 | 0.8031 | 0.8104 | 75960 | 1.58e3 | 1 | 1 | 3044 | 3051 |  |  | DB Search |
| total 3 peptides |  |  |  |  |  |  |  |  |  |  |  |  |  |  |  |  |  |  |  |
| --- | --- | --- | --- | --- | --- | --- | --- | --- | --- | --- | --- | --- | --- | --- | --- | --- | --- | --- | --- |

Best Unique PSM (Scan Sample\_2\_A1\_1\_3640.d:58461, m/z=514.8039, z=2, RT=17.08, ppm=1.86):


TRINITY\_DN31399\_c0\_g1\_i1.p1

back to list

  

| Protein Coverage
| Supporting Peptides
| Best Unique PSM
|

Protein Coverage:

Supporting Peptides:

| Peptide | Uniq | -10lgP | Mass | Length | ppm | m/z | z | RT | 1/k0 Start | 1/k0 End | Precursor ID | Area Sample 4 | #Feature | #Feature Sample 4 | Start | End | PTM | AScore | Found By |
| --- | --- | --- | --- | --- | --- | --- | --- | --- | --- | --- | --- | --- | --- | --- | --- | --- | --- | --- | --- |
| R.TVVTIVSDDEYQHM(+15.99)FDR.V | Y | 40.35 | 2069.9364 | 17 | 3.13 | 1035.9764 | 2 | 16.66 | 1.2491 | 1.2563 | 55691 | 2.94e1 | 1 | 1 | 124 | 140 | Oxidation (M) | M14:Oxidation (M):1000 | DB Search |
| total 1 peptides |  |  |  |  |  |  |  |  |  |  |  |  |  |  |  |  |  |  |  |
| --- | --- | --- | --- | --- | --- | --- | --- | --- | --- | --- | --- | --- | --- | --- | --- | --- | --- | --- | --- |

Best Unique PSM (Scan Sample\_2\_A1\_1\_3640.d:55691, m/z=1035.9764, z=2, RT=16.66, ppm=0.93):


TRINITY\_DN761\_c0\_g1\_i3.p1

back to list

  

| Protein Coverage
| Supporting Peptides
| Best Unique PSM
|

Protein Coverage:

Supporting Peptides:

| Peptide | Uniq | -10lgP | Mass | Length | ppm | m/z | z | RT | 1/k0 Start | 1/k0 End | Precursor ID | Area Sample 4 | #Feature | #Feature Sample 4 | Start | End | PTM | AScore | Found By |
| --- | --- | --- | --- | --- | --- | --- | --- | --- | --- | --- | --- | --- | --- | --- | --- | --- | --- | --- | --- |
| K.TRIEDLETVISQTQDHR.H | Y | 58.33 | 2040.0236 | 17 | 1.3 | 681.0145 | 3 | 17.58 | 0.9473 | 0.9545 | 61608 | 1.62e3 | 1 | 1 | 275 | 291 |  |  | DB Search |
| R.IEDLETVISQTQDHR.H | Y | 58.29 | 1782.8748 | 15 | -2.39 | 595.2961 | 3 | 17.95 | 0.8963 | 0.9036 | 64199 | 0 | 0 | 0 | 277 | 291 |  |  | DB Search |
| R.ATLYPC(+57.02)PETPNER.R | Y | 58.12 | 1546.7086 | 13 | 5.6 | 774.3642 | 2 | 14.56 | 1.0359 | 1.0431 | 42458 | 8e2 | 1 | 1 | 254 | 266 | Carbamidomethylation |  | DB Search |
| R.LGFVAGVINR.E | Y | 56.55 | 1044.608 | 10 | -2.1 | 523.309 | 2 | 17.50 | 0.8579 | 0.8652 | 61009 | 1.18e3 | 1 | 1 | 180 | 189 |  |  | DB Search |
| total 4 peptides |  |  |  |  |  |  |  |  |  |  |  |  |  |  |  |  |  |  |  |
| --- | --- | --- | --- | --- | --- | --- | --- | --- | --- | --- | --- | --- | --- | --- | --- | --- | --- | --- | --- |

Best Unique PSM (Scan Sample\_2\_A1\_1\_3640.d:61608, m/z=681.0145, z=3, RT=17.58, ppm=-0.89):


TRINITY\_DN5500\_c0\_g1\_i1.p1

back to list

  

| Protein Coverage
| Supporting Peptides
| Best Unique PSM
|

Protein Coverage:

Supporting Peptides:

| Peptide | Uniq | -10lgP | Mass | Length | ppm | m/z | z | RT | 1/k0 Start | 1/k0 End | Precursor ID | Area Sample 4 | #Feature | #Feature Sample 4 | Start | End | PTM | AScore | Found By |
| --- | --- | --- | --- | --- | --- | --- | --- | --- | --- | --- | --- | --- | --- | --- | --- | --- | --- | --- | --- |
| K.VLVILTDGIQAR.W | Y | 58.26 | 1296.7765 | 12 | 4.33 | 649.3969 | 2 | 18.70 | 0.9723 | 0.9795 | 69087 | 3.67e2 | 1 | 1 | 137 | 148 |  |  | DB Search |
| K.IYIDGVFIR.S | Y | 58.26 | 1094.6124 | 9 | -0.14 | 548.3122 | 2 | 18.85 | 0.885 | 0.8923 | 70109 | 4.57e3 | 1 | 1 | 938 | 946 |  |  | DB Search |
| R.DFDSLPYYPR.G | Y | 58.18 | 1271.5822 | 10 | 1.54 | 636.798 | 2 | 18.28 | 0.9222 | 0.9295 | 66470 | 3.66e2 | 1 | 1 | 500 | 509 |  |  | DB Search |
| total 3 peptides |  |  |  |  |  |  |  |  |  |  |  |  |  |  |  |  |  |  |  |
| --- | --- | --- | --- | --- | --- | --- | --- | --- | --- | --- | --- | --- | --- | --- | --- | --- | --- | --- | --- |

Best Unique PSM (Scan Sample\_2\_A1\_1\_3640.d:69087, m/z=649.3969, z=2, RT=18.70, ppm=2.14):


TRINITY\_DN982\_c0\_g1\_i1.p1

back to list

  

| Protein Coverage
| Supporting Peptides
| Best Unique PSM
|

Protein Coverage:

Supporting Peptides:

| Peptide | Uniq | -10lgP | Mass | Length | ppm | m/z | z | RT | 1/k0 Start | 1/k0 End | Precursor ID | Area Sample 4 | #Feature | #Feature Sample 4 | Start | End | PTM | AScore | Found By |
| --- | --- | --- | --- | --- | --- | --- | --- | --- | --- | --- | --- | --- | --- | --- | --- | --- | --- | --- | --- |
| K.VLLSQQIFYDKK.N | N | 58.39 | 1480.8289 | 12 | 0.31 | 741.4203 | 2 | 17.02 | 1.0616 | 1.0688 | 58002 | 1.76e3 | 2 | 2 | 1479 | 1490 |  |  | DB Search |
| R.IQVLKDPDFEFTR.S | N | 58.39 | 1606.8355 | 13 | 0.79 | 536.6183 | 3 | 17.74 | 0.8457 | 0.853 | 62712 | 2.19e3 | 1 | 1 | 2727 | 2739 |  |  | DB Search |
| R.TEIPGIIFEK.N | N | 58.33 | 1145.6332 | 10 | 1.1 | 573.8232 | 2 | 18.83 | 0.8874 | 0.8947 | 69581 | 1.78e3 | 1 | 1 | 2559 | 2568 |  |  | DB Search |
| R.LVQNTPHNSIVTK.I | N | 58.29 | 1449.794 | 13 | 3.11 | 484.2724 | 3 | 11.79 | 0.8075 | 0.8149 | 25665 | 5.5e2 | 1 | 1 | 5129 | 5141 |  |  | DB Search |
| R.YTSC(+57.02)QSVTVVASDR.S | N | 58.29 | 1571.725 | 14 | -2.24 | 786.8663 | 2 | 13.52 | 1.0504 | 1.0576 | 36260 | 0 | 0 | 0 | 5923 | 5936 | Carbamidomethylation |  | DB Search |
| K.YVAIYPSDHLTVNR.D | N | 58.29 | 1646.8416 | 14 | 3.39 | 824.4291 | 2 | 15.76 | 1.1082 | 1.1154 | 49960 | 1.45e3 | 1 | 1 | 2814 | 2827 |  |  | DB Search |
| K.LLTTEDDFILR.R | N | 58.17 | 1334.7081 | 11 | -1.86 | 668.3586 | 2 | 19.19 | 0.9634 | 0.9707 | 72323 | 9.12e2 | 1 | 1 | 3457 | 3467 |  |  | DB Search |
| K.INNNQFSVVLIK.N | Y | 57.54 | 1387.7823 | 12 | -2.73 | 694.895 | 2 | 18.38 | 0.9848 | 0.992 | 66945 | 4.66e2 | 1 | 1 | 100 | 111 |  |  | DB Search |
| R.VNEGNLVGDMVLR.V | N | 55.82 | 1414.7238 | 13 | 1.01 | 708.3683 | 2 | 18.54 | 0.9856 | 0.9928 | 68049 | 8.64e2 | 1 | 1 | 3391 | 3403 |  |  | DB Search |
| R.IDQHGGIK.T | N | 54.38 | 866.461 | 8 | 5.07 | 434.239 | 2 | 9.74 | 0.7892 | 0.7966 | 13590 | 0 | 0 | 0 | 4373 | 4380 |  |  | DB Search |
| K.SKIDYEALQVK.H | N | 53.53 | 1292.6976 | 11 | -4.87 | 647.3515 | 2 | 14.82 | 0.9606 | 0.9679 | 44312 | 0 | 0 | 0 | 3373 | 3383 |  |  | DB Search |
| R.ITTSQILDHEQQK.L | N | 53.33 | 1539.7893 | 13 | 1.49 | 770.9014 | 2 | 14.03 | 1.0969 | 1.1041 | 39378 | 0 | 0 | 0 | 1994 | 2006 |  |  | DB Search |
| R.VFSIVESDANDQFVIDSNSGALR.L | N | 52.69 | 2482.1975 | 23 | 3.57 | 828.4076 | 3 | 19.68 | 1.0464 | 1.0536 | 75408 | 5.65e2 | 1 | 1 | 4663 | 4685 |  |  | DB Search |
| K.NHYIFNINEK.E | N | 52.53 | 1290.6357 | 10 | 1.67 | 646.3248 | 2 | 15.74 | 0.9598 | 0.967 | 50017 | 0 | 0 | 0 | 2569 | 2578 |  |  | DB Search |
| K.GSTHVIIDVTDVNDNNPK.F | N | 41.87 | 1936.949 | 18 | -7.16 | 969.4727 | 2 | 15.19 | 1.153 | 1.1602 | 46639 | 0 | 0 | 0 | 3714 | 3731 |  |  | DB Search |
| total 15 peptides |  |  |  |  |  |  |  |  |  |  |  |  |  |  |  |  |  |  |  |
| --- | --- | --- | --- | --- | --- | --- | --- | --- | --- | --- | --- | --- | --- | --- | --- | --- | --- | --- | --- |

Best Unique PSM (Scan Sample\_2\_A1\_1\_3640.d:66945, m/z=694.895, z=2, RT=18.38, ppm=-4.93):


TRINITY\_DN6701\_c0\_g1\_i2.p2

back to list

  

| Protein Coverage
| Supporting Peptides
| Best Unique PSM
|

Protein Coverage:

Supporting Peptides:

| Peptide | Uniq | -10lgP | Mass | Length | ppm | m/z | z | RT | 1/k0 Start | 1/k0 End | Precursor ID | Area Sample 4 | #Feature | #Feature Sample 4 | Start | End | PTM | AScore | Found By |
| --- | --- | --- | --- | --- | --- | --- | --- | --- | --- | --- | --- | --- | --- | --- | --- | --- | --- | --- | --- |
| K.VITSDDPAEKEK.K | Y | 58.39 | 1330.6616 | 12 | 3.3 | 666.3388 | 2 | 10.77 | 0.9856 | 0.9928 | 19482 | 3.46e2 | 1 | 1 | 114 | 125 |  |  | DB Search |
| total 1 peptides |  |  |  |  |  |  |  |  |  |  |  |  |  |  |  |  |  |  |  |
| --- | --- | --- | --- | --- | --- | --- | --- | --- | --- | --- | --- | --- | --- | --- | --- | --- | --- | --- | --- |

Best Unique PSM (Scan Sample\_2\_A1\_1\_3640.d:19482, m/z=666.3388, z=2, RT=10.77, ppm=1.11):


TRINITY\_DN1030\_c0\_g1\_i1.p1

back to list

  

| Protein Coverage
| Supporting Peptides
| Best Unique PSM
|

Protein Coverage:

Supporting Peptides:

| Peptide | Uniq | -10lgP | Mass | Length | ppm | m/z | z | RT | 1/k0 Start | 1/k0 End | Precursor ID | Area Sample 4 | #Feature | #Feature Sample 4 | Start | End | PTM | AScore | Found By |
| --- | --- | --- | --- | --- | --- | --- | --- | --- | --- | --- | --- | --- | --- | --- | --- | --- | --- | --- | --- |
| R.GQAGPAGQDGVDGAVGAR.G | Y | 58.39 | 1581.7495 | 18 | 2.58 | 791.8823 | 2 | 12.45 | 1.0077 | 1.015 | 29579 | 1.37e4 | 5 | 5 | 316 | 333 |  |  | DB Search |
| R.GLQGQQGEQGSTGVQGSVGGR.G | Y | 58.39 | 1985.9515 | 21 | 1.48 | 993.9823 | 2 | 12.15 | 1.1314 | 1.1386 | 27609 | 1.56e4 | 3 | 3 | 361 | 381 |  |  | DB Search |
| total 2 peptides |  |  |  |  |  |  |  |  |  |  |  |  |  |  |  |  |  |  |  |
| --- | --- | --- | --- | --- | --- | --- | --- | --- | --- | --- | --- | --- | --- | --- | --- | --- | --- | --- | --- |

Best Unique PSM (Scan Sample\_2\_A1\_1\_3640.d:29579, m/z=791.8823, z=2, RT=12.45, ppm=0.38):


TRINITY\_DN8008\_c0\_g1\_i3.p1

back to list

  

| Protein Coverage
| Supporting Peptides
| Best Unique PSM
|

Protein Coverage:

Supporting Peptides:

| Peptide | Uniq | -10lgP | Mass | Length | ppm | m/z | z | RT | 1/k0 Start | 1/k0 End | Precursor ID | Area Sample 4 | #Feature | #Feature Sample 4 | Start | End | PTM | AScore | Found By |
| --- | --- | --- | --- | --- | --- | --- | --- | --- | --- | --- | --- | --- | --- | --- | --- | --- | --- | --- | --- |
| K.GVHIISVGLGTVDHR.G | Y | 57 | 1558.8579 | 15 | -7.55 | 520.6215 | 3 | 16.00 | 0.8404 | 0.8477 | 51630 | 0 | 0 | 0 | 1055 | 1069 |  |  | DB Search |
| total 1 peptides |  |  |  |  |  |  |  |  |  |  |  |  |  |  |  |  |  |  |  |
| --- | --- | --- | --- | --- | --- | --- | --- | --- | --- | --- | --- | --- | --- | --- | --- | --- | --- | --- | --- |

Best Unique PSM (Scan Sample\_2\_A1\_1\_3640.d:51630, m/z=520.259, z=2, RT=16.02, ppm=-9.75):


TRINITY\_DN4595\_c0\_g1\_i7.p1

back to list

  

| Protein Coverage
| Supporting Peptides
| Best Unique PSM
|

Protein Coverage:

Supporting Peptides:

| Peptide | Uniq | -10lgP | Mass | Length | ppm | m/z | z | RT | 1/k0 Start | 1/k0 End | Precursor ID | Area Sample 4 | #Feature | #Feature Sample 4 | Start | End | PTM | AScore | Found By |
| --- | --- | --- | --- | --- | --- | --- | --- | --- | --- | --- | --- | --- | --- | --- | --- | --- | --- | --- | --- |
| R.VC(+57.02)NAALC(+57.02)R.Y | Y | 58.26 | 962.4426 | 8 | 0.23 | 482.2276 | 2 | 11.36 | 0.812 | 0.8193 | 23031 | 0 | 0 | 0 | 224 | 231 | Carbamidomethylation, Carbamidomethylation |  | DB Search |
| R.AFNKVETR.N | N | 57 | 963.5138 | 8 | 4.36 | 482.7652 | 2 | 10.61 | 0.8274 | 0.8348 | 18412 | 3.24e3 | 1 | 1 | 177 | 184 |  |  | DB Search |
| R.AFNKIETR.N | Y | 54.97 | 977.5294 | 8 | 8.36 | 489.775 | 2 | 11.54 | 0.8339 | 0.8412 | 24067 | 2.69e3 | 1 | 1 | 72 | 79 |  |  | DB Search |
| total 3 peptides |  |  |  |  |  |  |  |  |  |  |  |  |  |  |  |  |  |  |  |
| --- | --- | --- | --- | --- | --- | --- | --- | --- | --- | --- | --- | --- | --- | --- | --- | --- | --- | --- | --- |

Best Unique PSM (Scan Sample\_2\_A1\_1\_3640.d:23031, m/z=483.2261, z=2, RT=11.36, ppm=-1.96):


TRINITY\_DN113\_c3\_g1\_i1.p1

back to list

  

| Protein Coverage
| Supporting Peptides
| Best Unique PSM
|

Protein Coverage:

Supporting Peptides:

| Peptide | Uniq | -10lgP | Mass | Length | ppm | m/z | z | RT | 1/k0 Start | 1/k0 End | Precursor ID | Area Sample 4 | #Feature | #Feature Sample 4 | Start | End | PTM | AScore | Found By |
| --- | --- | --- | --- | --- | --- | --- | --- | --- | --- | --- | --- | --- | --- | --- | --- | --- | --- | --- | --- |
| K.YAAAITPPPIIK.Q | Y | 57.42 | 1253.7383 | 12 | -0.19 | 627.8749 | 2 | 17.28 | 1.009 | 1.0162 | 59755 | 1.23e3 | 1 | 1 | 973 | 984 |  |  | DB Search |
| R.IVFDLR.E | Y | 56.98 | 761.4435 | 6 | 4.32 | 381.7299 | 2 | 17.48 | 0.7551 | 0.7624 | 60813 | 1.19e4 | 1 | 1 | 1068 | 1073 |  |  | DB Search |
| K.AHVSLSTFSEDVTSLTK.F | Y | 53.71 | 1820.9156 | 17 | -2.35 | 607.9764 | 3 | 19.00 | 0.8266 | 0.8339 | 71162 | 0 | 0 | 0 | 568 | 584 |  |  | DB Search |
| R.ATHQELLNAEFIQK.V | Y | 48.93 | 1640.8522 | 14 | 6.04 | 821.4365 | 2 | 16.16 | 1.0777 | 1.0849 | 52550 | 2.72e2 | 1 | 1 | 457 | 470 |  |  | DB Search |
| total 4 peptides |  |  |  |  |  |  |  |  |  |  |  |  |  |  |  |  |  |  |  |
| --- | --- | --- | --- | --- | --- | --- | --- | --- | --- | --- | --- | --- | --- | --- | --- | --- | --- | --- | --- |

Best Unique PSM (Scan Sample\_2\_A1\_1\_3640.d:59755, m/z=627.8749, z=2, RT=17.28, ppm=-2.39):


TRINITY\_DN413\_c0\_g1\_i2.p1

back to list

  

| Protein Coverage
| Supporting Peptides
| Best Unique PSM
|

Protein Coverage:

Supporting Peptides:

| Peptide | Uniq | -10lgP | Mass | Length | ppm | m/z | z | RT | 1/k0 Start | 1/k0 End | Precursor ID | Area Sample 4 | #Feature | #Feature Sample 4 | Start | End | PTM | AScore | Found By |
| --- | --- | --- | --- | --- | --- | --- | --- | --- | --- | --- | --- | --- | --- | --- | --- | --- | --- | --- | --- |
| R.LLLGDFNGDKR.T | Y | 58.25 | 1246.6669 | 11 | 4.07 | 624.3419 | 2 | 16.32 | 0.9844 | 0.9916 | 53554 | 5.68e2 | 1 | 1 | 46 | 56 |  |  | DB Search |
| total 1 peptides |  |  |  |  |  |  |  |  |  |  |  |  |  |  |  |  |  |  |  |
| --- | --- | --- | --- | --- | --- | --- | --- | --- | --- | --- | --- | --- | --- | --- | --- | --- | --- | --- | --- |

Best Unique PSM (Scan Sample\_2\_A1\_1\_3640.d:53554, m/z=624.3419, z=2, RT=16.32, ppm=1.87):


TRINITY\_DN5\_c0\_g1\_i16.p1

back to list

  

| Protein Coverage
| Supporting Peptides
| Best Unique PSM
|

Protein Coverage:

Supporting Peptides:

| Peptide | Uniq | -10lgP | Mass | Length | ppm | m/z | z | RT | 1/k0 Start | 1/k0 End | Precursor ID | Area Sample 4 | #Feature | #Feature Sample 4 | Start | End | PTM | AScore | Found By |
| --- | --- | --- | --- | --- | --- | --- | --- | --- | --- | --- | --- | --- | --- | --- | --- | --- | --- | --- | --- |
| K.IIIELQGDLIK.I | Y | 53.27 | 1253.7594 | 11 | 3.89 | 627.8881 | 2 | 19.42 | 0.9513 | 0.9586 | 73842 | 0 | 0 | 0 | 65 | 75 |  |  | DB Search |
| total 1 peptides |  |  |  |  |  |  |  |  |  |  |  |  |  |  |  |  |  |  |  |
| --- | --- | --- | --- | --- | --- | --- | --- | --- | --- | --- | --- | --- | --- | --- | --- | --- | --- | --- | --- |

Best Unique PSM (Scan Sample\_2\_A1\_1\_3640.d:73842, m/z=627.8881, z=2, RT=19.42, ppm=1.69):


TRINITY\_DN4494\_c1\_g1\_i8.p1

back to list

  

| Protein Coverage
| Supporting Peptides
| Best Unique PSM
|

Protein Coverage:

Supporting Peptides:

| Peptide | Uniq | -10lgP | Mass | Length | ppm | m/z | z | RT | 1/k0 Start | 1/k0 End | Precursor ID | Area Sample 4 | #Feature | #Feature Sample 4 | Start | End | PTM | AScore | Found By |
| --- | --- | --- | --- | --- | --- | --- | --- | --- | --- | --- | --- | --- | --- | --- | --- | --- | --- | --- | --- |
| K.LLILLTDGSQTQDAGAEDPAK.I | Y | 58.29 | 2155.1008 | 21 | 2.82 | 1078.5583 | 2 | 18.86 | 1.2388 | 1.2459 | 70204 | 0 | 0 | 0 | 145 | 165 |  |  | DB Search |
| K.VDVGFILDSSGSLR.N | N | 58.24 | 1463.762 | 14 | 1.17 | 732.8875 | 2 | 19.97 | 1.0102 | 1.0174 | 77350 | 0 | 0 | 0 | 41 | 54 |  |  | DB Search |
| total 2 peptides |  |  |  |  |  |  |  |  |  |  |  |  |  |  |  |  |  |  |  |
| --- | --- | --- | --- | --- | --- | --- | --- | --- | --- | --- | --- | --- | --- | --- | --- | --- | --- | --- | --- |

Best Unique PSM (Scan Sample\_2\_A1\_1\_3640.d:70204, m/z=1078.5583, z=2, RT=18.86, ppm=0.63):


TRINITY\_DN557\_c0\_g1\_i4.p1

back to list

  

| Protein Coverage
| Supporting Peptides
| Best Unique PSM
|

Protein Coverage:

Supporting Peptides:

| Peptide | Uniq | -10lgP | Mass | Length | ppm | m/z | z | RT | 1/k0 Start | 1/k0 End | Precursor ID | Area Sample 4 | #Feature | #Feature Sample 4 | Start | End | PTM | AScore | Found By |
| --- | --- | --- | --- | --- | --- | --- | --- | --- | --- | --- | --- | --- | --- | --- | --- | --- | --- | --- | --- |
| K.EAIVEAIEDGANTSK.E | Y | 55.85 | 1545.7522 | 15 | 1.08 | 773.8825 | 2 | 17.30 | 1.1049 | 1.1122 | 59776 | 2.04e2 | 1 | 1 | 116 | 130 |  |  | DB Search |
| total 1 peptides |  |  |  |  |  |  |  |  |  |  |  |  |  |  |  |  |  |  |  |
| --- | --- | --- | --- | --- | --- | --- | --- | --- | --- | --- | --- | --- | --- | --- | --- | --- | --- | --- | --- |

Best Unique PSM (Scan Sample\_2\_A1\_1\_3640.d:59776, m/z=773.8825, z=2, RT=17.30, ppm=-1.12):


TRINITY\_DN1058\_c0\_g1\_i2.p2

back to list

  

| Protein Coverage
| Supporting Peptides
| Best Unique PSM
|

Protein Coverage:

Supporting Peptides:

| Peptide | Uniq | -10lgP | Mass | Length | ppm | m/z | z | RT | 1/k0 Start | 1/k0 End | Precursor ID | Area Sample 4 | #Feature | #Feature Sample 4 | Start | End | PTM | AScore | Found By |
| --- | --- | --- | --- | --- | --- | --- | --- | --- | --- | --- | --- | --- | --- | --- | --- | --- | --- | --- | --- |
| K.TNFQMC(+57.02)HQR.M | Y | 58.29 | 1220.5179 | 9 | 3.31 | 611.2669 | 2 | 11.65 | 0.921 | 0.9283 | 24815 | 0 | 0 | 0 | 128 | 136 | Carbamidomethylation |  | DB Search |
| R.APTHILNPANKR.S | Y | 58.28 | 1330.7469 | 12 | 0.07 | 666.3793 | 2 | 11.22 | 1.0098 | 1.017 | 22322 | 5.04e3 | 2 | 2 | 975 | 986 |  |  | DB Search |
| R.SC(+57.02)VLASC(+57.02)SANSDVAVAC(+57.02)GK.V | Y | 58.08 | 1954.8547 | 19 | 3.15 | 978.4355 | 2 | 14.42 | 1.153 | 1.1602 | 41659 | 2.61e2 | 1 | 1 | 277 | 295 | Carbamidomethylation, Carbamidomethylation, Carbamidomethylation |  | DB Search |
| total 3 peptides |  |  |  |  |  |  |  |  |  |  |  |  |  |  |  |  |  |  |  |
| --- | --- | --- | --- | --- | --- | --- | --- | --- | --- | --- | --- | --- | --- | --- | --- | --- | --- | --- | --- |

Best Unique PSM (Scan Sample\_2\_A1\_1\_3640.d:24815, m/z=611.2669, z=2, RT=11.65, ppm=1.11):


TRINITY\_DN6701\_c0\_g1\_i1.p1

back to list

  

| Protein Coverage
| Supporting Peptides
| Best Unique PSM
|

Protein Coverage:

Supporting Peptides:

| Peptide | Uniq | -10lgP | Mass | Length | ppm | m/z | z | RT | 1/k0 Start | 1/k0 End | Precursor ID | Area Sample 4 | #Feature | #Feature Sample 4 | Start | End | PTM | AScore | Found By |
| --- | --- | --- | --- | --- | --- | --- | --- | --- | --- | --- | --- | --- | --- | --- | --- | --- | --- | --- | --- |
| K.VITSDDPAEKEK.K | Y | 58.39 | 1330.6616 | 12 | 3.3 | 666.3388 | 2 | 10.77 | 0.9856 | 0.9928 | 19482 | 3.46e2 | 1 | 1 | 158 | 169 |  |  | DB Search |
| total 1 peptides |  |  |  |  |  |  |  |  |  |  |  |  |  |  |  |  |  |  |  |
| --- | --- | --- | --- | --- | --- | --- | --- | --- | --- | --- | --- | --- | --- | --- | --- | --- | --- | --- | --- |

Best Unique PSM (Scan Sample\_2\_A1\_1\_3640.d:19482, m/z=666.3388, z=2, RT=10.77, ppm=1.11):


TRINITY\_DN107813\_c0\_g1\_i1.p1

back to list

  

| Protein Coverage
| Supporting Peptides
| Best Unique PSM
|

Protein Coverage:

Supporting Peptides:

| Peptide | Uniq | -10lgP | Mass | Length | ppm | m/z | z | RT | 1/k0 Start | 1/k0 End | Precursor ID | Area Sample 4 | #Feature | #Feature Sample 4 | Start | End | PTM | AScore | Found By |
| --- | --- | --- | --- | --- | --- | --- | --- | --- | --- | --- | --- | --- | --- | --- | --- | --- | --- | --- | --- |
| K.IGFAAADAVTGLK.L | Y | 58.26 | 1232.6764 | 13 | 2.63 | 617.3458 | 2 | 17.96 | 0.9469 | 0.9541 | 64240 | 0 | 0 | 0 | 158 | 170 |  |  | DB Search |
| total 1 peptides |  |  |  |  |  |  |  |  |  |  |  |  |  |  |  |  |  |  |  |
| --- | --- | --- | --- | --- | --- | --- | --- | --- | --- | --- | --- | --- | --- | --- | --- | --- | --- | --- | --- |

Best Unique PSM (Scan Sample\_2\_A1\_1\_3640.d:64240, m/z=617.3458, z=2, RT=17.96, ppm=0.44):


TRINITY\_DN113\_c2\_g1\_i1.p1

back to list

  

| Protein Coverage
| Supporting Peptides
| Best Unique PSM
|

Protein Coverage:

Supporting Peptides:

| Peptide | Uniq | -10lgP | Mass | Length | ppm | m/z | z | RT | 1/k0 Start | 1/k0 End | Precursor ID | Area Sample 4 | #Feature | #Feature Sample 4 | Start | End | PTM | AScore | Found By |
| --- | --- | --- | --- | --- | --- | --- | --- | --- | --- | --- | --- | --- | --- | --- | --- | --- | --- | --- | --- |
| R.GPVGAQGTR.G | Y | 58.18 | 841.4406 | 9 | 6.48 | 421.7294 | 2 | 9.59 | 0.7599 | 0.7673 | 12867 | 1.07e4 | 1 | 1 | 559 | 567 |  |  | DB Search |
| R.RGPNGAIGER.G | Y | 52.38 | 1025.5366 | 10 | 2.78 | 513.7759 | 2 | 10.08 | 0.8441 | 0.8514 | 15228 | 2.75e3 | 1 | 1 | 354 | 363 |  |  | DB Search |
| total 2 peptides |  |  |  |  |  |  |  |  |  |  |  |  |  |  |  |  |  |  |  |
| --- | --- | --- | --- | --- | --- | --- | --- | --- | --- | --- | --- | --- | --- | --- | --- | --- | --- | --- | --- |

Best Unique PSM (Scan Sample\_2\_A1\_1\_3640.d:12867, m/z=421.7294, z=2, RT=9.59, ppm=4.28):


TRINITY\_DN179\_c0\_g1\_i2.p1

back to list

  

| Protein Coverage
| Supporting Peptides
| Best Unique PSM
|

Protein Coverage:

Supporting Peptides:

| Peptide | Uniq | -10lgP | Mass | Length | ppm | m/z | z | RT | 1/k0 Start | 1/k0 End | Precursor ID | Area Sample 4 | #Feature | #Feature Sample 4 | Start | End | PTM | AScore | Found By |
| --- | --- | --- | --- | --- | --- | --- | --- | --- | --- | --- | --- | --- | --- | --- | --- | --- | --- | --- | --- |
| R.GLLGPDGEVGATGER.G | Y | 58.29 | 1426.7052 | 15 | 0.49 | 714.3586 | 2 | 15.54 | 0.9896 | 0.9969 | 48738 | 0 | 0 | 0 | 414 | 428 |  |  | DB Search |
| total 1 peptides |  |  |  |  |  |  |  |  |  |  |  |  |  |  |  |  |  |  |  |
| --- | --- | --- | --- | --- | --- | --- | --- | --- | --- | --- | --- | --- | --- | --- | --- | --- | --- | --- | --- |

Best Unique PSM (Scan Sample\_2\_A1\_1\_3640.d:48738, m/z=714.3607, z=1, RT=15.56, ppm=-1.71):


TRINITY\_DN119673\_c0\_g1\_i1.p1

back to list

  

| Protein Coverage
| Supporting Peptides
| Best Unique PSM
|

Protein Coverage:

Supporting Peptides:

| Peptide | Uniq | -10lgP | Mass | Length | ppm | m/z | z | RT | 1/k0 Start | 1/k0 End | Precursor ID | Area Sample 4 | #Feature | #Feature Sample 4 | Start | End | PTM | AScore | Found By |
| --- | --- | --- | --- | --- | --- | --- | --- | --- | --- | --- | --- | --- | --- | --- | --- | --- | --- | --- | --- |
| K.VLTSDDPAEKEK | Y | 58.39 | 1330.6616 | 12 | 3.3 | 666.3388 | 2 | 10.77 | 0.9856 | 0.9928 | 19482 | 3.46e2 | 1 | 1 | 117 | 128 |  |  | DB Search |
| total 1 peptides |  |  |  |  |  |  |  |  |  |  |  |  |  |  |  |  |  |  |  |
| --- | --- | --- | --- | --- | --- | --- | --- | --- | --- | --- | --- | --- | --- | --- | --- | --- | --- | --- | --- |

Best Unique PSM (Scan Sample\_2\_A1\_1\_3640.d:19482, m/z=666.3388, z=2, RT=10.77, ppm=1.11):


TRINITY\_DN36\_c0\_g1\_i11.p1

back to list

  

| Protein Coverage
| Supporting Peptides
| Best Unique PSM
|

Protein Coverage:

Supporting Peptides:

| Peptide | Uniq | -10lgP | Mass | Length | ppm | m/z | z | RT | 1/k0 Start | 1/k0 End | Precursor ID | Area Sample 4 | #Feature | #Feature Sample 4 | Start | End | PTM | AScore | Found By |
| --- | --- | --- | --- | --- | --- | --- | --- | --- | --- | --- | --- | --- | --- | --- | --- | --- | --- | --- | --- |
| R.LGGIEVTQGNC(+57.02)AR.K | Y | 58.36 | 1373.6721 | 13 | -2.89 | 687.8398 | 2 | 13.93 | 0.9662 | 0.9735 | 38726 | 2.09e3 | 1 | 1 | 58 | 70 | Carbamidomethylation |  | DB Search |
| K.GLPLAIGVR.D | Y | 57.12 | 894.565 | 9 | 0.27 | 448.2889 | 2 | 17.73 | 0.8124 | 0.8197 | 62574 | 3.52e3 | 1 | 1 | 32 | 40 |  |  | DB Search |
| total 2 peptides |  |  |  |  |  |  |  |  |  |  |  |  |  |  |  |  |  |  |  |
| --- | --- | --- | --- | --- | --- | --- | --- | --- | --- | --- | --- | --- | --- | --- | --- | --- | --- | --- | --- |

Best Unique PSM (Scan Sample\_2\_A1\_1\_3640.d:38726, m/z=687.8398, z=2, RT=13.93, ppm=-5.09):


TRINITY\_DN738\_c0\_g1\_i2.p1

back to list

  

| Protein Coverage
| Supporting Peptides
| Best Unique PSM
|

Protein Coverage:

Supporting Peptides:

| Peptide | Uniq | -10lgP | Mass | Length | ppm | m/z | z | RT | 1/k0 Start | 1/k0 End | Precursor ID | Area Sample 4 | #Feature | #Feature Sample 4 | Start | End | PTM | AScore | Found By |
| --- | --- | --- | --- | --- | --- | --- | --- | --- | --- | --- | --- | --- | --- | --- | --- | --- | --- | --- | --- |
| R.YGEFSEK.L | Y | 52.42 | 858.3759 | 7 | 1.16 | 430.1948 | 2 | 12.54 | 0.7652 | 0.7726 | 30256 | 6.91e2 | 1 | 1 | 54 | 60 |  |  | DB Search |
| total 1 peptides |  |  |  |  |  |  |  |  |  |  |  |  |  |  |  |  |  |  |  |
| --- | --- | --- | --- | --- | --- | --- | --- | --- | --- | --- | --- | --- | --- | --- | --- | --- | --- | --- | --- |

Best Unique PSM (Scan Sample\_2\_A1\_1\_3640.d:30256, m/z=430.1948, z=2, RT=12.54, ppm=-1.04):


TRINITY\_DN36\_c0\_g1\_i19.p1

back to list

  

| Protein Coverage
| Supporting Peptides
| Best Unique PSM
|

Protein Coverage:

Supporting Peptides:

| Peptide | Uniq | -10lgP | Mass | Length | ppm | m/z | z | RT | 1/k0 Start | 1/k0 End | Precursor ID | Area Sample 4 | #Feature | #Feature Sample 4 | Start | End | PTM | AScore | Found By |
| --- | --- | --- | --- | --- | --- | --- | --- | --- | --- | --- | --- | --- | --- | --- | --- | --- | --- | --- | --- |
| R.LGGIEVTQGNC(+57.02)AR.K | Y | 58.36 | 1373.6721 | 13 | -2.89 | 687.8398 | 2 | 13.93 | 0.9662 | 0.9735 | 38726 | 2.09e3 | 1 | 1 | 57 | 69 | Carbamidomethylation |  | DB Search |
| K.GLPLAIGVR.D | Y | 57.12 | 894.565 | 9 | 0.27 | 448.2889 | 2 | 17.73 | 0.8124 | 0.8197 | 62574 | 3.52e3 | 1 | 1 | 31 | 39 |  |  | DB Search |
| total 2 peptides |  |  |  |  |  |  |  |  |  |  |  |  |  |  |  |  |  |  |  |
| --- | --- | --- | --- | --- | --- | --- | --- | --- | --- | --- | --- | --- | --- | --- | --- | --- | --- | --- | --- |

Best Unique PSM (Scan Sample\_2\_A1\_1\_3640.d:38726, m/z=687.8398, z=2, RT=13.93, ppm=-5.09):


TRINITY\_DN106\_c0\_g1\_i11.p1

back to list

  

| Protein Coverage
| Supporting Peptides
| Best Unique PSM
|

Protein Coverage:

Supporting Peptides:

| Peptide | Uniq | -10lgP | Mass | Length | ppm | m/z | z | RT | 1/k0 Start | 1/k0 End | Precursor ID | Area Sample 4 | #Feature | #Feature Sample 4 | Start | End | PTM | AScore | Found By |
| --- | --- | --- | --- | --- | --- | --- | --- | --- | --- | --- | --- | --- | --- | --- | --- | --- | --- | --- | --- |
| R.DGLNGDRGDPGPVGAVGAR.G | Y | 58.29 | 1778.8659 | 19 | -2.01 | 593.9601 | 3 | 13.86 | 0.8323 | 0.8396 | 38286 | 4.13e2 | 1 | 1 | 37 | 55 |  |  | DB Search |
| R.DGPAGPAGR.D | N | 58.24 | 796.3827 | 9 | 3.73 | 399.1992 | 2 | 9.76 | 0.7061 | 0.7135 | 13439 | 1.18e4 | 2 | 2 | 283 | 291 |  |  | DB Search |
| R.GPDGAVGPTGPIGAR.G | N | 58.04 | 1320.6786 | 15 | 1.83 | 661.3463 | 2 | 13.72 | 0.9558 | 0.963 | 37443 | 1.21e4 | 2 | 2 | 122 | 136 |  |  | DB Search |
| total 3 peptides |  |  |  |  |  |  |  |  |  |  |  |  |  |  |  |  |  |  |  |
| --- | --- | --- | --- | --- | --- | --- | --- | --- | --- | --- | --- | --- | --- | --- | --- | --- | --- | --- | --- |

Best Unique PSM (Scan Sample\_2\_A1\_1\_3640.d:38286, m/z=593.9601, z=3, RT=13.86, ppm=-4.21):


TRINITY\_DN36\_c0\_g1\_i5.p1

back to list

  

| Protein Coverage
| Supporting Peptides
| Best Unique PSM
|

Protein Coverage:

Supporting Peptides:

| Peptide | Uniq | -10lgP | Mass | Length | ppm | m/z | z | RT | 1/k0 Start | 1/k0 End | Precursor ID | Area Sample 4 | #Feature | #Feature Sample 4 | Start | End | PTM | AScore | Found By |
| --- | --- | --- | --- | --- | --- | --- | --- | --- | --- | --- | --- | --- | --- | --- | --- | --- | --- | --- | --- |
| R.LGGIEVTQGNC(+57.02)AR.K | Y | 58.36 | 1373.6721 | 13 | -2.89 | 687.8398 | 2 | 13.93 | 0.9662 | 0.9735 | 38726 | 2.09e3 | 1 | 1 | 57 | 69 | Carbamidomethylation |  | DB Search |
| K.GLPLAIGVR.D | Y | 57.12 | 894.565 | 9 | 0.27 | 448.2889 | 2 | 17.73 | 0.8124 | 0.8197 | 62574 | 3.52e3 | 1 | 1 | 31 | 39 |  |  | DB Search |
| total 2 peptides |  |  |  |  |  |  |  |  |  |  |  |  |  |  |  |  |  |  |  |
| --- | --- | --- | --- | --- | --- | --- | --- | --- | --- | --- | --- | --- | --- | --- | --- | --- | --- | --- | --- |

Best Unique PSM (Scan Sample\_2\_A1\_1\_3640.d:38726, m/z=687.8398, z=2, RT=13.93, ppm=-5.09):


TRINITY\_DN1043\_c0\_g1\_i2.p1

back to list

  

| Protein Coverage
| Supporting Peptides
| Best Unique PSM
|

Protein Coverage:

Supporting Peptides:

| Peptide | Uniq | -10lgP | Mass | Length | ppm | m/z | z | RT | 1/k0 Start | 1/k0 End | Precursor ID | Area Sample 4 | #Feature | #Feature Sample 4 | Start | End | PTM | AScore | Found By |
| --- | --- | --- | --- | --- | --- | --- | --- | --- | --- | --- | --- | --- | --- | --- | --- | --- | --- | --- | --- |
| R.EQVSTFDSLQADLVK.L | Y | 58.4 | 1678.8413 | 15 | 2.3 | 840.428 | 2 | 18.77 | 1.0917 | 1.0989 | 69421 | 6.52e2 | 1 | 1 | 137 | 151 |  |  | DB Search |
| K.GLMVAAMDLDKK.Q | Y | 57.22 | 1290.6675 | 12 | 1.46 | 646.3406 | 2 | 16.83 | 1.0013 | 1.0086 | 56757 | 1.36e3 | 1 | 1 | 504 | 515 |  |  | DB Search |
| R.LASNMIIIR.E | Y | 55.01 | 1029.6005 | 9 | 4.3 | 515.8086 | 2 | 17.03 | 0.8453 | 0.8526 | 58043 | 1.79e3 | 1 | 1 | 128 | 136 |  |  | DB Search |
| K.ITETC(+57.02)IEGSYMDKNEIK.G | Y | 53.44 | 2029.9336 | 17 | 2.79 | 677.6522 | 3 | 15.03 | 0.9505 | 0.9578 | 45564 | 0 | 0 | 0 | 487 | 503 | Carbamidomethylation |  | DB Search |
| total 4 peptides |  |  |  |  |  |  |  |  |  |  |  |  |  |  |  |  |  |  |  |
| --- | --- | --- | --- | --- | --- | --- | --- | --- | --- | --- | --- | --- | --- | --- | --- | --- | --- | --- | --- |

Best Unique PSM (Scan Sample\_2\_A1\_1\_3640.d:69421, m/z=840.428, z=2, RT=18.77, ppm=0.11):


TRINITY\_DN557\_c0\_g1\_i2.p1

back to list

  

| Protein Coverage
| Supporting Peptides
| Best Unique PSM
|

Protein Coverage:

Supporting Peptides:

| Peptide | Uniq | -10lgP | Mass | Length | ppm | m/z | z | RT | 1/k0 Start | 1/k0 End | Precursor ID | Area Sample 4 | #Feature | #Feature Sample 4 | Start | End | PTM | AScore | Found By |
| --- | --- | --- | --- | --- | --- | --- | --- | --- | --- | --- | --- | --- | --- | --- | --- | --- | --- | --- | --- |
| K.EAIVEAIEDGANTSK.E | Y | 55.85 | 1545.7522 | 15 | 1.08 | 773.8825 | 2 | 17.30 | 1.1049 | 1.1122 | 59776 | 2.04e2 | 1 | 1 | 116 | 130 |  |  | DB Search |
| total 1 peptides |  |  |  |  |  |  |  |  |  |  |  |  |  |  |  |  |  |  |  |
| --- | --- | --- | --- | --- | --- | --- | --- | --- | --- | --- | --- | --- | --- | --- | --- | --- | --- | --- | --- |

Best Unique PSM (Scan Sample\_2\_A1\_1\_3640.d:59776, m/z=773.8825, z=2, RT=17.30, ppm=-1.12):


TRINITY\_DN1189\_c0\_g1\_i1.p1

back to list

  

| Protein Coverage
| Supporting Peptides
| Best Unique PSM
|

Protein Coverage:

Supporting Peptides:

| Peptide | Uniq | -10lgP | Mass | Length | ppm | m/z | z | RT | 1/k0 Start | 1/k0 End | Precursor ID | Area Sample 4 | #Feature | #Feature Sample 4 | Start | End | PTM | AScore | Found By |
| --- | --- | --- | --- | --- | --- | --- | --- | --- | --- | --- | --- | --- | --- | --- | --- | --- | --- | --- | --- |
| K.VGTVNQAVTDAFK.H | Y | 58.28 | 1348.6986 | 13 | 1.51 | 675.3561 | 2 | 16.68 | 0.9747 | 0.982 | 55945 | 7.49e2 | 1 | 1 | 62 | 74 |  |  | DB Search |
| K.AKDVTAAVK.K | Y | 57.69 | 901.5232 | 9 | 1.23 | 451.7685 | 2 | 10.02 | 0.7974 | 0.8047 | 15046 | 0 | 0 | 0 | 185 | 193 |  |  | DB Search |
| R.AVSFLVNDVPNLK.C | Y | 53.73 | 1414.782 | 13 | 3.82 | 708.3994 | 2 | 19.29 | 1.0021 | 1.0094 | 72907 | 2.09e3 | 1 | 1 | 148 | 160 |  |  | DB Search |
| total 3 peptides |  |  |  |  |  |  |  |  |  |  |  |  |  |  |  |  |  |  |  |
| --- | --- | --- | --- | --- | --- | --- | --- | --- | --- | --- | --- | --- | --- | --- | --- | --- | --- | --- | --- |

Best Unique PSM (Scan Sample\_2\_A1\_1\_3640.d:55945, m/z=675.3561, z=2, RT=16.68, ppm=-0.68):


TRINITY\_DN4494\_c1\_g1\_i5.p1

back to list

  

| Protein Coverage
| Supporting Peptides
| Best Unique PSM
|

Protein Coverage:

Supporting Peptides:

| Peptide | Uniq | -10lgP | Mass | Length | ppm | m/z | z | RT | 1/k0 Start | 1/k0 End | Precursor ID | Area Sample 4 | #Feature | #Feature Sample 4 | Start | End | PTM | AScore | Found By |
| --- | --- | --- | --- | --- | --- | --- | --- | --- | --- | --- | --- | --- | --- | --- | --- | --- | --- | --- | --- |
| R.VLDVGFLLDSSDKVK.D | Y | 58.25 | 1633.8926 | 15 | -4.48 | 545.6345 | 3 | 19.79 | 0.8725 | 0.8798 | 76169 | 4.96e2 | 1 | 1 | 90 | 104 |  |  | DB Search |
| K.VDVGFILDSSGSLR.N | N | 58.24 | 1463.762 | 14 | 1.17 | 732.8875 | 2 | 19.97 | 1.0102 | 1.0174 | 77350 | 0 | 0 | 0 | 288 | 301 |  |  | DB Search |
| total 2 peptides |  |  |  |  |  |  |  |  |  |  |  |  |  |  |  |  |  |  |  |
| --- | --- | --- | --- | --- | --- | --- | --- | --- | --- | --- | --- | --- | --- | --- | --- | --- | --- | --- | --- |

Best Unique PSM (Scan Sample\_2\_A1\_1\_3640.d:76169, m/z=545.6345, z=3, RT=19.79, ppm=-6.67):


TRINITY\_DN152\_c0\_g1\_i2.p1

back to list

  

| Protein Coverage
| Supporting Peptides
| Best Unique PSM
|

Protein Coverage:

Supporting Peptides:

| Peptide | Uniq | -10lgP | Mass | Length | ppm | m/z | z | RT | 1/k0 Start | 1/k0 End | Precursor ID | Area Sample 4 | #Feature | #Feature Sample 4 | Start | End | PTM | AScore | Found By |
| --- | --- | --- | --- | --- | --- | --- | --- | --- | --- | --- | --- | --- | --- | --- | --- | --- | --- | --- | --- |
| K.ESTLHLVLR.L | Y | 57.27 | 1066.6135 | 9 | 2.57 | 534.3142 | 2 | 15.80 | 0.8785 | 0.8858 | 50332 | 4.97e3 | 1 | 1 | 24 | 32 |  |  | DB Search |
| K.IQDKEGIPPDQQR.L | Y | 55.01 | 1522.7739 | 13 | 0.25 | 762.3928 | 2 | 11.08 | 1.0343 | 1.0415 | 21018 | 1.78e3 | 2 | 2 | 66 | 78 |  |  | DB Search |
| total 2 peptides |  |  |  |  |  |  |  |  |  |  |  |  |  |  |  |  |  |  |  |
| --- | --- | --- | --- | --- | --- | --- | --- | --- | --- | --- | --- | --- | --- | --- | --- | --- | --- | --- | --- |

Best Unique PSM (Scan Sample\_2\_A1\_1\_3640.d:50332, m/z=534.7766, z=2, RT=15.80, ppm=0.37):


TRINITY\_DN2576\_c3\_g1\_i1.p2

back to list

  

| Protein Coverage
| Supporting Peptides
| Best Unique PSM
|

Protein Coverage:

Supporting Peptides:

| Peptide | Uniq | -10lgP | Mass | Length | ppm | m/z | z | RT | 1/k0 Start | 1/k0 End | Precursor ID | Area Sample 4 | #Feature | #Feature Sample 4 | Start | End | PTM | AScore | Found By |
| --- | --- | --- | --- | --- | --- | --- | --- | --- | --- | --- | --- | --- | --- | --- | --- | --- | --- | --- | --- |
| K.IPSWVPSLPKPK.S | Y | 58.24 | 1347.7914 | 12 | -9.34 | 450.2659 | 3 | 18.14 | 0.7734 | 0.7807 | 65403 | 6.48e2 | 1 | 1 | 34 | 45 |  |  | DB Search |
| total 1 peptides |  |  |  |  |  |  |  |  |  |  |  |  |  |  |  |  |  |  |  |
| --- | --- | --- | --- | --- | --- | --- | --- | --- | --- | --- | --- | --- | --- | --- | --- | --- | --- | --- | --- |

Best Unique PSM (Scan Sample\_2\_A1\_1\_3640.d:65403, m/z=450.2659, z=3, RT=18.14, ppm=-11.54):


TRINITY\_DN4595\_c0\_g1\_i10.p2

back to list

  

| Protein Coverage
| Supporting Peptides
| Best Unique PSM
|

Protein Coverage:

Supporting Peptides:

| Peptide | Uniq | -10lgP | Mass | Length | ppm | m/z | z | RT | 1/k0 Start | 1/k0 End | Precursor ID | Area Sample 4 | #Feature | #Feature Sample 4 | Start | End | PTM | AScore | Found By |
| --- | --- | --- | --- | --- | --- | --- | --- | --- | --- | --- | --- | --- | --- | --- | --- | --- | --- | --- | --- |
| R.VC(+57.02)NGEPC(+57.02)ILQER.S | Y | 58.29 | 1473.6704 | 12 | 1.84 | 737.8422 | 2 | 15.04 | 0.9832 | 0.9904 | 45530 | 3e3 | 1 | 1 | 306 | 317 | Carbamidomethylation, Carbamidomethylation |  | DB Search |
| R.AC(+57.02)DSGFSAVLSR.N | Y | 58.28 | 1268.5819 | 12 | 1.5 | 635.2978 | 2 | 16.57 | 0.9485 | 0.9558 | 55157 | 3.01e2 | 1 | 1 | 275 | 286 | Carbamidomethylation |  | DB Search |
| R.VC(+57.02)NAALC(+57.02)R.Y | Y | 58.26 | 962.4426 | 8 | 0.23 | 482.2276 | 2 | 11.36 | 0.812 | 0.8193 | 23031 | 0 | 0 | 0 | 94 | 101 | Carbamidomethylation, Carbamidomethylation |  | DB Search |
| R.AFNKVETR.N | N | 57 | 963.5138 | 8 | 4.36 | 482.7652 | 2 | 10.61 | 0.8274 | 0.8348 | 18412 | 3.24e3 | 1 | 1 | 47 | 54 |  |  | DB Search |
| R.DGTTPC(+57.02)NTR.S | Y | 55.73 | 1020.4295 | 9 | 2.58 | 511.2222 | 2 | 9.62 | 0.8311 | 0.8384 | 12948 | 1.32e3 | 1 | 1 | 160 | 168 | Carbamidomethylation |  | DB Search |
| R.EYNINEEVNVR.Q | Y | 54.67 | 1377.6524 | 11 | -5.34 | 689.8283 | 2 | 14.94 | 0.9787 | 0.986 | 45239 | 5.98e2 | 1 | 1 | 212 | 222 |  |  | DB Search |
| R.YQGREYNINEEVNVR.Q | Y | 52.5 | 1881.8969 | 15 | 3.38 | 941.9568 | 2 | 15.71 | 1.1422 | 1.1494 | 49787 | 0 | 0 | 0 | 208 | 222 |  |  | DB Search |
| total 7 peptides |  |  |  |  |  |  |  |  |  |  |  |  |  |  |  |  |  |  |  |
| --- | --- | --- | --- | --- | --- | --- | --- | --- | --- | --- | --- | --- | --- | --- | --- | --- | --- | --- | --- |

Best Unique PSM (Scan Sample\_2\_A1\_1\_3640.d:45530, m/z=737.8422, z=2, RT=15.04, ppm=-0.35):


TRINITY\_DN36\_c0\_g1\_i17.p1

back to list

  

| Protein Coverage
| Supporting Peptides
| Best Unique PSM
|

Protein Coverage:

Supporting Peptides:

| Peptide | Uniq | -10lgP | Mass | Length | ppm | m/z | z | RT | 1/k0 Start | 1/k0 End | Precursor ID | Area Sample 4 | #Feature | #Feature Sample 4 | Start | End | PTM | AScore | Found By |
| --- | --- | --- | --- | --- | --- | --- | --- | --- | --- | --- | --- | --- | --- | --- | --- | --- | --- | --- | --- |
| R.LGGIEVTQGNC(+57.02)AR.K | Y | 58.36 | 1373.6721 | 13 | -2.89 | 687.8398 | 2 | 13.93 | 0.9662 | 0.9735 | 38726 | 2.09e3 | 1 | 1 | 58 | 70 | Carbamidomethylation |  | DB Search |
| K.GLPLAIGVR.D | Y | 57.12 | 894.565 | 9 | 0.27 | 448.2889 | 2 | 17.73 | 0.8124 | 0.8197 | 62574 | 3.52e3 | 1 | 1 | 32 | 40 |  |  | DB Search |
| total 2 peptides |  |  |  |  |  |  |  |  |  |  |  |  |  |  |  |  |  |  |  |
| --- | --- | --- | --- | --- | --- | --- | --- | --- | --- | --- | --- | --- | --- | --- | --- | --- | --- | --- | --- |

Best Unique PSM (Scan Sample\_2\_A1\_1\_3640.d:38726, m/z=687.8398, z=2, RT=13.93, ppm=-5.09):


TRINITY\_DN3991\_c0\_g1\_i1.p1

back to list

  

| Protein Coverage
| Supporting Peptides
| Best Unique PSM
|

Protein Coverage:

Supporting Peptides:

| Peptide | Uniq | -10lgP | Mass | Length | ppm | m/z | z | RT | 1/k0 Start | 1/k0 End | Precursor ID | Area Sample 4 | #Feature | #Feature Sample 4 | Start | End | PTM | AScore | Found By |
| --- | --- | --- | --- | --- | --- | --- | --- | --- | --- | --- | --- | --- | --- | --- | --- | --- | --- | --- | --- |
| K.LSQC(+57.02)TAASGK.H | Y | 58.26 | 1021.4862 | 10 | 0.53 | 511.7495 | 2 | 9.84 | 0.8307 | 0.838 | 13091 | 8.33e4 | 1 | 1 | 130 | 139 | Carbamidomethylation |  | DB Search |
| total 1 peptides |  |  |  |  |  |  |  |  |  |  |  |  |  |  |  |  |  |  |  |
| --- | --- | --- | --- | --- | --- | --- | --- | --- | --- | --- | --- | --- | --- | --- | --- | --- | --- | --- | --- |

Best Unique PSM (Scan Sample\_2\_A1\_1\_3640.d:13091, m/z=511.7495, z=2, RT=9.84, ppm=-1.67):


TRINITY\_DN152\_c0\_g1\_i15.p1

back to list

  

| Protein Coverage
| Supporting Peptides
| Best Unique PSM
|

Protein Coverage:

Supporting Peptides:

| Peptide | Uniq | -10lgP | Mass | Length | ppm | m/z | z | RT | 1/k0 Start | 1/k0 End | Precursor ID | Area Sample 4 | #Feature | #Feature Sample 4 | Start | End | PTM | AScore | Found By |
| --- | --- | --- | --- | --- | --- | --- | --- | --- | --- | --- | --- | --- | --- | --- | --- | --- | --- | --- | --- |
| K.ESTLHLVLR.L | Y | 57.27 | 1066.6135 | 9 | 2.57 | 534.3142 | 2 | 15.80 | 0.8785 | 0.8858 | 50332 | 4.97e3 | 1 | 1 | 94 | 102 |  |  | DB Search |
| K.IQDKEGIPPDQQR.L | Y | 55.01 | 1522.7739 | 13 | 0.25 | 762.3928 | 2 | 11.08 | 1.0343 | 1.0415 | 21018 | 1.78e3 | 2 | 2 | 60 | 72 |  |  | DB Search |
| total 2 peptides |  |  |  |  |  |  |  |  |  |  |  |  |  |  |  |  |  |  |  |
| --- | --- | --- | --- | --- | --- | --- | --- | --- | --- | --- | --- | --- | --- | --- | --- | --- | --- | --- | --- |

Best Unique PSM (Scan Sample\_2\_A1\_1\_3640.d:50332, m/z=534.7766, z=2, RT=15.80, ppm=0.37):


TRINITY\_DN4523\_c0\_g1\_i1.p1

back to list

  

| Protein Coverage
| Supporting Peptides
| Best Unique PSM
|

Protein Coverage:

Supporting Peptides:

| Peptide | Uniq | -10lgP | Mass | Length | ppm | m/z | z | RT | 1/k0 Start | 1/k0 End | Precursor ID | Area Sample 4 | #Feature | #Feature Sample 4 | Start | End | PTM | AScore | Found By |
| --- | --- | --- | --- | --- | --- | --- | --- | --- | --- | --- | --- | --- | --- | --- | --- | --- | --- | --- | --- |
| R.ITLQDVVLHGK.K | Y | 38.06 | 1221.7081 | 11 | 4.85 | 408.2444 | 3 | 17.32 | 0.7167 | 0.7241 | 60002 | 0 | 0 | 0 | 908 | 918 |  |  | DB Search |
| total 1 peptides |  |  |  |  |  |  |  |  |  |  |  |  |  |  |  |  |  |  |  |
| --- | --- | --- | --- | --- | --- | --- | --- | --- | --- | --- | --- | --- | --- | --- | --- | --- | --- | --- | --- |

Best Unique PSM (Scan Sample\_2\_A1\_1\_3640.d:60002, m/z=408.2444, z=3, RT=17.32, ppm=2.65):


TRINITY\_DN2477\_c0\_g1\_i2.p1

back to list

  

| Protein Coverage
| Supporting Peptides
| Best Unique PSM
|

Protein Coverage:

Supporting Peptides:

| Peptide | Uniq | -10lgP | Mass | Length | ppm | m/z | z | RT | 1/k0 Start | 1/k0 End | Precursor ID | Area Sample 4 | #Feature | #Feature Sample 4 | Start | End | PTM | AScore | Found By |
| --- | --- | --- | --- | --- | --- | --- | --- | --- | --- | --- | --- | --- | --- | --- | --- | --- | --- | --- | --- |
| K.DSYVGDEAQSKR.G | Y | 57.28 | 1353.616 | 12 | 2.57 | 677.8156 | 2 | 11.01 | 0.9993 | 1.0065 | 20794 | 1.16e3 | 1 | 1 | 19 | 30 |  |  | DB Search |
| total 1 peptides |  |  |  |  |  |  |  |  |  |  |  |  |  |  |  |  |  |  |  |
| --- | --- | --- | --- | --- | --- | --- | --- | --- | --- | --- | --- | --- | --- | --- | --- | --- | --- | --- | --- |

Best Unique PSM (Scan Sample\_2\_A1\_1\_3640.d:20794, m/z=677.8156, z=2, RT=11.01, ppm=0.37):


TRINITY\_DN2885\_c0\_g1\_i4.p1

back to list

  

| Protein Coverage
| Supporting Peptides
| Best Unique PSM
|

Protein Coverage:

Supporting Peptides:

| Peptide | Uniq | -10lgP | Mass | Length | ppm | m/z | z | RT | 1/k0 Start | 1/k0 End | Precursor ID | Area Sample 4 | #Feature | #Feature Sample 4 | Start | End | PTM | AScore | Found By |
| --- | --- | --- | --- | --- | --- | --- | --- | --- | --- | --- | --- | --- | --- | --- | --- | --- | --- | --- | --- |
| K.VTRPHGASGVVR.A | Y | 57.82 | 1234.6894 | 12 | 7.66 | 412.5727 | 3 | 10.07 | 0.8108 | 0.8181 | 15251 | 2.57e2 | 1 | 1 | 72 | 83 |  |  | DB Search |
| total 1 peptides |  |  |  |  |  |  |  |  |  |  |  |  |  |  |  |  |  |  |  |
| --- | --- | --- | --- | --- | --- | --- | --- | --- | --- | --- | --- | --- | --- | --- | --- | --- | --- | --- | --- |

Best Unique PSM (Scan Sample\_2\_A1\_1\_3640.d:15251, m/z=412.5727, z=3, RT=10.07, ppm=5.46):


TRINITY\_DN152\_c0\_g1\_i10.p1

back to list

  

| Protein Coverage
| Supporting Peptides
| Best Unique PSM
|

Protein Coverage:

Supporting Peptides:

| Peptide | Uniq | -10lgP | Mass | Length | ppm | m/z | z | RT | 1/k0 Start | 1/k0 End | Precursor ID | Area Sample 4 | #Feature | #Feature Sample 4 | Start | End | PTM | AScore | Found By |
| --- | --- | --- | --- | --- | --- | --- | --- | --- | --- | --- | --- | --- | --- | --- | --- | --- | --- | --- | --- |
| K.ESTLHLVLR.L | Y | 57.27 | 1066.6135 | 9 | 2.57 | 534.3142 | 2 | 15.80 | 0.8785 | 0.8858 | 50332 | 4.97e3 | 1 | 1 | 52 | 60 |  |  | DB Search |
| K.IQDKEGIPPDQQR.L | Y | 55.01 | 1522.7739 | 13 | 0.25 | 762.3928 | 2 | 11.08 | 1.0343 | 1.0415 | 21018 | 1.78e3 | 2 | 2 | 141 | 153 |  |  | DB Search |
| total 2 peptides |  |  |  |  |  |  |  |  |  |  |  |  |  |  |  |  |  |  |  |
| --- | --- | --- | --- | --- | --- | --- | --- | --- | --- | --- | --- | --- | --- | --- | --- | --- | --- | --- | --- |

Best Unique PSM (Scan Sample\_2\_A1\_1\_3640.d:50332, m/z=534.7766, z=2, RT=15.80, ppm=0.37):


TRINITY\_DN106\_c0\_g1\_i6.p1

back to list

  

| Protein Coverage
| Supporting Peptides
| Best Unique PSM
|

Protein Coverage:

Supporting Peptides:

| Peptide | Uniq | -10lgP | Mass | Length | ppm | m/z | z | RT | 1/k0 Start | 1/k0 End | Precursor ID | Area Sample 4 | #Feature | #Feature Sample 4 | Start | End | PTM | AScore | Found By |
| --- | --- | --- | --- | --- | --- | --- | --- | --- | --- | --- | --- | --- | --- | --- | --- | --- | --- | --- | --- |
| K.EGPAGPAGPVGR.L | Y | 58.28 | 1063.541 | 12 | -11.82 | 532.7703 | 2 | 11.84 | 0.8461 | 0.8534 | 25992 | 0 | 0 | 0 | 191 | 202 |  |  | DB Search |
| R.DGPAGPAGR.D | N | 58.24 | 796.3827 | 9 | 3.73 | 399.1992 | 2 | 9.76 | 0.7061 | 0.7135 | 13439 | 1.18e4 | 2 | 2 | 488 | 496 |  |  | DB Search |
| R.GPDGAVGPTGPIGAR.G | N | 58.04 | 1320.6786 | 15 | 1.83 | 661.3463 | 2 | 13.72 | 0.9558 | 0.963 | 37443 | 1.21e4 | 2 | 2 | 327 | 341 |  |  | DB Search |
| total 3 peptides |  |  |  |  |  |  |  |  |  |  |  |  |  |  |  |  |  |  |  |
| --- | --- | --- | --- | --- | --- | --- | --- | --- | --- | --- | --- | --- | --- | --- | --- | --- | --- | --- | --- |

Best Unique PSM (Scan Sample\_2\_A1\_1\_3640.d:25992, m/z=532.7703, z=2, RT=11.84, ppm=-14.02):


TRINITY\_DN189323\_c0\_g1\_i1.p1

back to list

  

| Protein Coverage
| Supporting Peptides
| Best Unique PSM
|

Protein Coverage:

Supporting Peptides:

| Peptide | Uniq | -10lgP | Mass | Length | ppm | m/z | z | RT | 1/k0 Start | 1/k0 End | Precursor ID | Area Sample 4 | #Feature | #Feature Sample 4 | Start | End | PTM | AScore | Found By |
| --- | --- | --- | --- | --- | --- | --- | --- | --- | --- | --- | --- | --- | --- | --- | --- | --- | --- | --- | --- |
| R.LFEGNALLR.R | Y | 57.78 | 1031.5763 | 9 | 0.94 | 516.7948 | 2 | 17.48 | 0.8631 | 0.8704 | 60870 | 9.34e2 | 1 | 1 | 32 | 40 |  |  | DB Search |
| total 1 peptides |  |  |  |  |  |  |  |  |  |  |  |  |  |  |  |  |  |  |  |
| --- | --- | --- | --- | --- | --- | --- | --- | --- | --- | --- | --- | --- | --- | --- | --- | --- | --- | --- | --- |

Best Unique PSM (Scan Sample\_2\_A1\_1\_3640.d:60870, m/z=516.7948, z=2, RT=17.48, ppm=-1.26):


TRINITY\_DN106\_c0\_g1\_i4.p1

back to list

  

| Protein Coverage
| Supporting Peptides
| Best Unique PSM
|

Protein Coverage:

Supporting Peptides:

| Peptide | Uniq | -10lgP | Mass | Length | ppm | m/z | z | RT | 1/k0 Start | 1/k0 End | Precursor ID | Area Sample 4 | #Feature | #Feature Sample 4 | Start | End | PTM | AScore | Found By |
| --- | --- | --- | --- | --- | --- | --- | --- | --- | --- | --- | --- | --- | --- | --- | --- | --- | --- | --- | --- |
| R.DGLNGDRGDPGPVGAVGAR.G | Y | 58.29 | 1778.8659 | 19 | -2.01 | 593.9601 | 3 | 13.86 | 0.8323 | 0.8396 | 38286 | 4.13e2 | 1 | 1 | 37 | 55 |  |  | DB Search |
| total 1 peptides |  |  |  |  |  |  |  |  |  |  |  |  |  |  |  |  |  |  |  |
| --- | --- | --- | --- | --- | --- | --- | --- | --- | --- | --- | --- | --- | --- | --- | --- | --- | --- | --- | --- |

Best Unique PSM (Scan Sample\_2\_A1\_1\_3640.d:38286, m/z=593.9601, z=3, RT=13.86, ppm=-4.21):


TRINITY\_DN8941\_c0\_g1\_i12.p1

back to list

  

| Protein Coverage
| Supporting Peptides
| Best Unique PSM
|

Protein Coverage:

Supporting Peptides:

| Peptide | Uniq | -10lgP | Mass | Length | ppm | m/z | z | RT | 1/k0 Start | 1/k0 End | Precursor ID | Area Sample 4 | #Feature | #Feature Sample 4 | Start | End | PTM | AScore | Found By |
| --- | --- | --- | --- | --- | --- | --- | --- | --- | --- | --- | --- | --- | --- | --- | --- | --- | --- | --- | --- |
| K.AVPAPILDIPK.R | Y | 58.37 | 1132.6855 | 11 | 0.34 | 567.349 | 2 | 18.31 | 0.8955 | 0.9028 | 66444 | 1.41e3 | 1 | 1 | 715 | 725 |  |  | DB Search |
| total 1 peptides |  |  |  |  |  |  |  |  |  |  |  |  |  |  |  |  |  |  |  |
| --- | --- | --- | --- | --- | --- | --- | --- | --- | --- | --- | --- | --- | --- | --- | --- | --- | --- | --- | --- |

Best Unique PSM (Scan Sample\_2\_A1\_1\_3640.d:66444, m/z=567.349, z=2, RT=18.31, ppm=-1.86):


TRINITY\_DN1172\_c0\_g1\_i5.p1

back to list

  

| Protein Coverage
| Supporting Peptides
| Best Unique PSM
|

Protein Coverage:

Supporting Peptides:

| Peptide | Uniq | -10lgP | Mass | Length | ppm | m/z | z | RT | 1/k0 Start | 1/k0 End | Precursor ID | Area Sample 4 | #Feature | #Feature Sample 4 | Start | End | PTM | AScore | Found By |
| --- | --- | --- | --- | --- | --- | --- | --- | --- | --- | --- | --- | --- | --- | --- | --- | --- | --- | --- | --- |
| K.VITSDDPAEKEK.K | Y | 58.39 | 1330.6616 | 12 | 3.3 | 666.3388 | 2 | 10.77 | 0.9856 | 0.9928 | 19482 | 3.46e2 | 1 | 1 | 123 | 134 |  |  | DB Search |
| total 1 peptides |  |  |  |  |  |  |  |  |  |  |  |  |  |  |  |  |  |  |  |
| --- | --- | --- | --- | --- | --- | --- | --- | --- | --- | --- | --- | --- | --- | --- | --- | --- | --- | --- | --- |

Best Unique PSM (Scan Sample\_2\_A1\_1\_3640.d:19482, m/z=666.3388, z=2, RT=10.77, ppm=1.11):


TRINITY\_DN152\_c0\_g1\_i14.p2

back to list

  

| Protein Coverage
| Supporting Peptides
| Best Unique PSM
|

Protein Coverage:

Supporting Peptides:

| Peptide | Uniq | -10lgP | Mass | Length | ppm | m/z | z | RT | 1/k0 Start | 1/k0 End | Precursor ID | Area Sample 4 | #Feature | #Feature Sample 4 | Start | End | PTM | AScore | Found By |
| --- | --- | --- | --- | --- | --- | --- | --- | --- | --- | --- | --- | --- | --- | --- | --- | --- | --- | --- | --- |
| K.IQDKEGIPPDQQR.S | Y | 55.01 | 1522.7739 | 13 | 0.25 | 762.3928 | 2 | 11.08 | 1.0343 | 1.0415 | 21018 | 1.78e3 | 2 | 2 | 40 | 52 |  |  | DB Search |
| total 1 peptides |  |  |  |  |  |  |  |  |  |  |  |  |  |  |  |  |  |  |  |
| --- | --- | --- | --- | --- | --- | --- | --- | --- | --- | --- | --- | --- | --- | --- | --- | --- | --- | --- | --- |

Best Unique PSM (Scan Sample\_2\_A1\_1\_3640.d:21018, m/z=762.3928, z=2, RT=11.08, ppm=-1.95):


TRINITY\_DN1339\_c6\_g1\_i1.p1

back to list

  

| Protein Coverage
| Supporting Peptides
| Best Unique PSM
|

Protein Coverage:

Supporting Peptides:

| Peptide | Uniq | -10lgP | Mass | Length | ppm | m/z | z | RT | 1/k0 Start | 1/k0 End | Precursor ID | Area Sample 4 | #Feature | #Feature Sample 4 | Start | End | PTM | AScore | Found By |
| --- | --- | --- | --- | --- | --- | --- | --- | --- | --- | --- | --- | --- | --- | --- | --- | --- | --- | --- | --- |
| K.VITSDDPAEKEK.K | Y | 58.39 | 1330.6616 | 12 | 3.3 | 666.3388 | 2 | 10.77 | 0.9856 | 0.9928 | 19482 | 3.46e2 | 1 | 1 | 158 | 169 |  |  | DB Search |
| total 1 peptides |  |  |  |  |  |  |  |  |  |  |  |  |  |  |  |  |  |  |  |
| --- | --- | --- | --- | --- | --- | --- | --- | --- | --- | --- | --- | --- | --- | --- | --- | --- | --- | --- | --- |

Best Unique PSM (Scan Sample\_2\_A1\_1\_3640.d:19482, m/z=666.3388, z=2, RT=10.77, ppm=1.11):


TRINITY\_DN598\_c1\_g1\_i2.p1

back to list

  

| Protein Coverage
| Supporting Peptides
| Best Unique PSM
|

Protein Coverage:

Supporting Peptides:

| Peptide | Uniq | -10lgP | Mass | Length | ppm | m/z | z | RT | 1/k0 Start | 1/k0 End | Precursor ID | Area Sample 4 | #Feature | #Feature Sample 4 | Start | End | PTM | AScore | Found By |
| --- | --- | --- | --- | --- | --- | --- | --- | --- | --- | --- | --- | --- | --- | --- | --- | --- | --- | --- | --- |
| K.AVAEEFISVDPSVR.G | Y | 58.29 | 1517.7725 | 14 | 0.44 | 759.8922 | 2 | 17.79 | 1.0287 | 1.0359 | 62940 | 2.17e3 | 1 | 1 | 148 | 161 |  |  | DB Search |
| total 1 peptides |  |  |  |  |  |  |  |  |  |  |  |  |  |  |  |  |  |  |  |
| --- | --- | --- | --- | --- | --- | --- | --- | --- | --- | --- | --- | --- | --- | --- | --- | --- | --- | --- | --- |

Best Unique PSM (Scan Sample\_2\_A1\_1\_3640.d:62940, m/z=759.8922, z=2, RT=17.79, ppm=-1.75):


TRINITY\_DN1580\_c0\_g1\_i1.p1

back to list

  

| Protein Coverage
| Supporting Peptides
| Best Unique PSM
|

Protein Coverage:

Supporting Peptides:

| Peptide | Uniq | -10lgP | Mass | Length | ppm | m/z | z | RT | 1/k0 Start | 1/k0 End | Precursor ID | Area Sample 4 | #Feature | #Feature Sample 4 | Start | End | PTM | AScore | Found By |
| --- | --- | --- | --- | --- | --- | --- | --- | --- | --- | --- | --- | --- | --- | --- | --- | --- | --- | --- | --- |
| K.ESTLHLVLR.L | Y | 57.27 | 1066.6135 | 9 | 2.57 | 534.3142 | 2 | 15.80 | 0.8785 | 0.8858 | 50332 | 4.97e3 | 1 | 1 | 23 | 31 |  |  | DB Search |
| total 1 peptides |  |  |  |  |  |  |  |  |  |  |  |  |  |  |  |  |  |  |  |
| --- | --- | --- | --- | --- | --- | --- | --- | --- | --- | --- | --- | --- | --- | --- | --- | --- | --- | --- | --- |

Best Unique PSM (Scan Sample\_2\_A1\_1\_3640.d:50332, m/z=534.7766, z=2, RT=15.80, ppm=0.37):


TRINITY\_DN554\_c0\_g1\_i3.p2

back to list

  

| Protein Coverage
| Supporting Peptides
| Best Unique PSM
|

Protein Coverage:

Supporting Peptides:

| Peptide | Uniq | -10lgP | Mass | Length | ppm | m/z | z | RT | 1/k0 Start | 1/k0 End | Precursor ID | Area Sample 4 | #Feature | #Feature Sample 4 | Start | End | PTM | AScore | Found By |
| --- | --- | --- | --- | --- | --- | --- | --- | --- | --- | --- | --- | --- | --- | --- | --- | --- | --- | --- | --- |
| MQIFVK.T | Y | 57.42 | 764.4255 | 6 | 0.18 | 383.2192 | 2 | 15.73 | 0.7363 | 0.7437 | 49899 | 2.46e2 | 1 | 1 | 1 | 6 |  |  | DB Search |
| K.ESTLHLVLR.L | Y | 57.27 | 1066.6135 | 9 | 2.57 | 534.3142 | 2 | 15.80 | 0.8785 | 0.8858 | 50332 | 4.97e3 | 1 | 1 | 64 | 72 |  |  | DB Search |
| K.IQDKEGIPPDQQR.L | Y | 55.01 | 1522.7739 | 13 | 0.25 | 762.3928 | 2 | 11.08 | 1.0343 | 1.0415 | 21018 | 1.78e3 | 2 | 2 | 30 | 42 |  |  | DB Search |
| K.TITLEVEPSDTIENVK.S | Y | 54.56 | 1786.92 | 16 | 4.26 | 894.4691 | 2 | 17.62 | 1.1442 | 1.1514 | 61769 | 1.48e4 | 1 | 1 | 12 | 27 |  |  | DB Search |
| total 4 peptides |  |  |  |  |  |  |  |  |  |  |  |  |  |  |  |  |  |  |  |
| --- | --- | --- | --- | --- | --- | --- | --- | --- | --- | --- | --- | --- | --- | --- | --- | --- | --- | --- | --- |

Best Unique PSM (Scan Sample\_2\_A1\_1\_3640.d:49899, m/z=383.2192, z=2, RT=15.73, ppm=-2.02):


TRINITY\_DN7641\_c0\_g1\_i1.p1

back to list

  

| Protein Coverage
| Supporting Peptides
| Best Unique PSM
|

Protein Coverage:

Supporting Peptides:

| Peptide | Uniq | -10lgP | Mass | Length | ppm | m/z | z | RT | 1/k0 Start | 1/k0 End | Precursor ID | Area Sample 4 | #Feature | #Feature Sample 4 | Start | End | PTM | AScore | Found By |
| --- | --- | --- | --- | --- | --- | --- | --- | --- | --- | --- | --- | --- | --- | --- | --- | --- | --- | --- | --- |
| R.ELVLNSAFC(+57.02)EQYIK.F | Y | 58.28 | 1712.8443 | 14 | 6.97 | 857.4335 | 2 | 19.12 | 1.0989 | 1.1061 | 71794 | 2.94e2 | 1 | 1 | 532 | 545 | Carbamidomethylation |  | DB Search |
| total 1 peptides |  |  |  |  |  |  |  |  |  |  |  |  |  |  |  |  |  |  |  |
| --- | --- | --- | --- | --- | --- | --- | --- | --- | --- | --- | --- | --- | --- | --- | --- | --- | --- | --- | --- |

Best Unique PSM (Scan Sample\_2\_A1\_1\_3640.d:71794, m/z=857.4335, z=2, RT=19.12, ppm=4.78):


TRINITY\_DN205\_c0\_g1\_i3.p1

back to list

  

| Protein Coverage
| Supporting Peptides
| Best Unique PSM
|

Protein Coverage:

Supporting Peptides:

| Peptide | Uniq | -10lgP | Mass | Length | ppm | m/z | z | RT | 1/k0 Start | 1/k0 End | Precursor ID | Area Sample 4 | #Feature | #Feature Sample 4 | Start | End | PTM | AScore | Found By |
| --- | --- | --- | --- | --- | --- | --- | --- | --- | --- | --- | --- | --- | --- | --- | --- | --- | --- | --- | --- |
| R.SYQATSC(+57.02)SC(+57.02)SK.D | Y | 55.69 | 1277.5016 | 11 | 4.9 | 639.7598 | 2 | 9.83 | 0.9255 | 0.9327 | 14058 | 3.33e2 | 1 | 1 | 648 | 658 | Carbamidomethylation, Carbamidomethylation |  | DB Search |
| total 1 peptides |  |  |  |  |  |  |  |  |  |  |  |  |  |  |  |  |  |  |  |
| --- | --- | --- | --- | --- | --- | --- | --- | --- | --- | --- | --- | --- | --- | --- | --- | --- | --- | --- | --- |

Best Unique PSM (Scan Sample\_2\_A1\_1\_3640.d:14058, m/z=639.7598, z=2, RT=9.83, ppm=2.7):


TRINITY\_DN4595\_c0\_g1\_i10.p1

back to list

  

| Protein Coverage
| Supporting Peptides
| Best Unique PSM
|

Protein Coverage:

Supporting Peptides:

| Peptide | Uniq | -10lgP | Mass | Length | ppm | m/z | z | RT | 1/k0 Start | 1/k0 End | Precursor ID | Area Sample 4 | #Feature | #Feature Sample 4 | Start | End | PTM | AScore | Found By |
| --- | --- | --- | --- | --- | --- | --- | --- | --- | --- | --- | --- | --- | --- | --- | --- | --- | --- | --- | --- |
| K.GFEC(+57.02)IGLIEETETC(+57.02)K.L | Y | 58.39 | 1784.7961 | 15 | 3.79 | 595.9402 | 3 | 18.69 | 0.8627 | 0.87 | 69011 | 2.49e3 | 2 | 2 | 93 | 107 | Carbamidomethylation, Carbamidomethylation |  | DB Search |
| R.VC(+57.02)LDDDIKVC(+57.02)K.M | Y | 58.38 | 1363.6475 | 11 | 2.8 | 455.5567 | 3 | 14.43 | 0.7978 | 0.8051 | 41739 | 9.3e2 | 1 | 1 | 689 | 699 | Carbamidomethylation, Carbamidomethylation |  | DB Search |
| R.FC(+57.02)NNPAPSNGGR.M | Y | 58.29 | 1289.5571 | 12 | 10.24 | 645.791 | 2 | 10.91 | 0.9158 | 0.923 | 20335 | 6.39e3 | 1 | 1 | 745 | 756 | Carbamidomethylation |  | DB Search |
| R.NAFFIVTSK.D | Y | 58.27 | 1025.5546 | 9 | -0.8 | 513.783 | 2 | 18.00 | 0.8388 | 0.8461 | 64505 | 0 | 0 | 0 | 144 | 152 |  |  | DB Search |
| R.TAC(+57.02)VGM(+15.99)DR.E | Y | 58.23 | 924.3793 | 8 | 4.29 | 463.1979 | 2 | 9.57 | 0.7827 | 0.7901 | 12695 | 9.24e2 | 1 | 1 | 345 | 352 | Carbamidomethylation, Oxidation (M) | M6:Oxidation (M):1000 | DB Search |
| R.VLQEREC(+57.02)EQDTC(+57.02)FDGNTR.F | Y | 57.83 | 2255.9535 | 18 | -6.28 | 752.9854 | 3 | 13.04 | 0.9323 | 0.9396 | 33365 | 6e2 | 1 | 1 | 807 | 824 | Carbamidomethylation, Carbamidomethylation |  | DB Search |
| R.TRVC(+57.02)LDDDIKVC(+57.02)K.M | Y | 57.39 | 1620.7963 | 13 | -1.68 | 541.2706 | 3 | 13.95 | 0.838 | 0.8453 | 38871 | 6.52e2 | 1 | 1 | 687 | 699 | Carbamidomethylation, Carbamidomethylation |  | DB Search |
| R.SPFVIMFK.G | Y | 57.23 | 967.5201 | 8 | 1.15 | 484.7668 | 2 | 19.95 | 0.8063 | 0.8136 | 76925 | 1.04e4 | 1 | 1 | 630 | 637 |  |  | DB Search |
| R.EC(+57.02)EQDTC(+57.02)FDGNTR.F | Y | 57.01 | 1630.5988 | 13 | -1.43 | 816.3037 | 2 | 12.29 | 1.0122 | 1.0194 | 28637 | 3.57e2 | 1 | 1 | 812 | 824 | Carbamidomethylation, Carbamidomethylation |  | DB Search |
| R.TRVC(+57.02)LDDDIK.V | Y | 56.08 | 1233.6023 | 10 | 4.44 | 617.8098 | 2 | 13.63 | 0.9485 | 0.9558 | 36912 | 1.78e3 | 1 | 1 | 687 | 696 | Carbamidomethylation |  | DB Search |
| K.KVNQGEKEFLQRPC(+57.02)R.Y | Y | 42.62 | 1887.9737 | 15 | 3.41 | 473.0013 | 4 | 12.00 | 0.8128 | 0.8201 | 26878 | 0 | 0 | 0 | 1160 | 1174 | Carbamidomethylation |  | DB Search |
| K.VNQGEKEFLQRPC(+57.02)R.Y | Y | 37.68 | 1759.8788 | 14 | 0.76 | 587.6327 | 3 | 12.79 | 0.8635 | 0.8708 | 31645 | 8.61e2 | 1 | 1 | 1161 | 1174 | Carbamidomethylation |  | DB Search |
| total 12 peptides |  |  |  |  |  |  |  |  |  |  |  |  |  |  |  |  |  |  |  |
| --- | --- | --- | --- | --- | --- | --- | --- | --- | --- | --- | --- | --- | --- | --- | --- | --- | --- | --- | --- |

Best Unique PSM (Scan Sample\_2\_A1\_1\_3640.d:69011, m/z=595.9402, z=3, RT=18.69, ppm=1.6):


TRINITY\_DN1580\_c0\_g1\_i3.p1

back to list

  

| Protein Coverage
| Supporting Peptides
| Best Unique PSM
|

Protein Coverage:

Supporting Peptides:

| Peptide | Uniq | -10lgP | Mass | Length | ppm | m/z | z | RT | 1/k0 Start | 1/k0 End | Precursor ID | Area Sample 4 | #Feature | #Feature Sample 4 | Start | End | PTM | AScore | Found By |
| --- | --- | --- | --- | --- | --- | --- | --- | --- | --- | --- | --- | --- | --- | --- | --- | --- | --- | --- | --- |
| K.ESTLHLVLR.L | Y | 57.27 | 1066.6135 | 9 | 2.57 | 534.3142 | 2 | 15.80 | 0.8785 | 0.8858 | 50332 | 4.97e3 | 1 | 1 | 23 | 31 |  |  | DB Search |
| total 1 peptides |  |  |  |  |  |  |  |  |  |  |  |  |  |  |  |  |  |  |  |
| --- | --- | --- | --- | --- | --- | --- | --- | --- | --- | --- | --- | --- | --- | --- | --- | --- | --- | --- | --- |

Best Unique PSM (Scan Sample\_2\_A1\_1\_3640.d:50332, m/z=534.7766, z=2, RT=15.80, ppm=0.37):


TRINITY\_DN250\_c0\_g1\_i5.p1

back to list

  

| Protein Coverage
| Supporting Peptides
| Best Unique PSM
|

Protein Coverage:

Supporting Peptides:

| Peptide | Uniq | -10lgP | Mass | Length | ppm | m/z | z | RT | 1/k0 Start | 1/k0 End | Precursor ID | Area Sample 4 | #Feature | #Feature Sample 4 | Start | End | PTM | AScore | Found By |
| --- | --- | --- | --- | --- | --- | --- | --- | --- | --- | --- | --- | --- | --- | --- | --- | --- | --- | --- | --- |
| K.VGINYQPPTVVPGGDLAK.V | Y | 58.29 | 1823.9781 | 18 | -4.28 | 912.9904 | 2 | 17.44 | 1.1142 | 1.1214 | 60745 | 0 | 0 | 0 | 353 | 370 |  |  | DB Search |
| total 1 peptides |  |  |  |  |  |  |  |  |  |  |  |  |  |  |  |  |  |  |  |
| --- | --- | --- | --- | --- | --- | --- | --- | --- | --- | --- | --- | --- | --- | --- | --- | --- | --- | --- | --- |

Best Unique PSM (Scan Sample\_2\_A1\_1\_3640.d:60745, m/z=912.9904, z=2, RT=17.44, ppm=-6.48):


TRINITY\_DN9655\_c0\_g1\_i1.p1

back to list

  

| Protein Coverage
| Supporting Peptides
| Best Unique PSM
|

Protein Coverage:

Supporting Peptides:

| Peptide | Uniq | -10lgP | Mass | Length | ppm | m/z | z | RT | 1/k0 Start | 1/k0 End | Precursor ID | Area Sample 4 | #Feature | #Feature Sample 4 | Start | End | PTM | AScore | Found By |
| --- | --- | --- | --- | --- | --- | --- | --- | --- | --- | --- | --- | --- | --- | --- | --- | --- | --- | --- | --- |
| K.TLSFEAVGHDVDK.T | Y | 58.26 | 1416.6885 | 13 | -7.21 | 709.3448 | 2 | 15.67 | 1.0242 | 1.0315 | 49710 | 3.97e2 | 1 | 1 | 212 | 224 |  |  | DB Search |
| K.TLSFEAVGHDVDKTISR.S | Y | 56.16 | 1873.9533 | 17 | -1.82 | 625.6559 | 3 | 17.29 | 0.9016 | 0.9089 | 59762 | 0 | 0 | 0 | 212 | 228 |  |  | DB Search |
| total 2 peptides |  |  |  |  |  |  |  |  |  |  |  |  |  |  |  |  |  |  |  |
| --- | --- | --- | --- | --- | --- | --- | --- | --- | --- | --- | --- | --- | --- | --- | --- | --- | --- | --- | --- |

Best Unique PSM (Scan Sample\_2\_A1\_1\_3640.d:49710, m/z=709.3448, z=2, RT=15.67, ppm=-9.4):


TRINITY\_DN1936\_c0\_g1\_i4.p1

back to list

  

| Protein Coverage
| Supporting Peptides
| Best Unique PSM
|

Protein Coverage:

Supporting Peptides:

| Peptide | Uniq | -10lgP | Mass | Length | ppm | m/z | z | RT | 1/k0 Start | 1/k0 End | Precursor ID | Area Sample 4 | #Feature | #Feature Sample 4 | Start | End | PTM | AScore | Found By |
| --- | --- | --- | --- | --- | --- | --- | --- | --- | --- | --- | --- | --- | --- | --- | --- | --- | --- | --- | --- |
| R.YIFIESSKPAIK.G | Y | 57.72 | 1394.7809 | 12 | 0.75 | 698.3967 | 2 | 15.84 | 1.0086 | 1.0158 | 50604 | 4.26e3 | 2 | 2 | 470 | 481 |  |  | DB Search |
| R.LEFVFK.Y | Y | 57 | 781.4374 | 6 | 2.76 | 391.7262 | 2 | 18.35 | 0.7286 | 0.7359 | 66730 | 1.18e3 | 1 | 1 | 1232 | 1237 |  |  | DB Search |
| R.LISTFIPATGDTGGK.C | Y | 54.9 | 1476.7824 | 15 | 1.83 | 739.3982 | 2 | 17.40 | 1.0041 | 1.0114 | 60313 | 2.76e3 | 1 | 1 | 677 | 691 |  |  | DB Search |
| R.SDIAVDDFEFK.S | Y | 54.89 | 1284.5874 | 11 | 6.87 | 643.304 | 2 | 18.47 | 0.9303 | 0.9376 | 67658 | 0 | 0 | 0 | 566 | 576 |  |  | DB Search |
| total 4 peptides |  |  |  |  |  |  |  |  |  |  |  |  |  |  |  |  |  |  |  |
| --- | --- | --- | --- | --- | --- | --- | --- | --- | --- | --- | --- | --- | --- | --- | --- | --- | --- | --- | --- |

Best Unique PSM (Scan Sample\_2\_A1\_1\_3640.d:50604, m/z=698.3967, z=2, RT=15.84, ppm=-1.44):


TRINITY\_DN477\_c0\_g1\_i7.p1

back to list

  

| Protein Coverage
| Supporting Peptides
| Best Unique PSM
|

Protein Coverage:

Supporting Peptides:

| Peptide | Uniq | -10lgP | Mass | Length | ppm | m/z | z | RT | 1/k0 Start | 1/k0 End | Precursor ID | Area Sample 4 | #Feature | #Feature Sample 4 | Start | End | PTM | AScore | Found By |
| --- | --- | --- | --- | --- | --- | --- | --- | --- | --- | --- | --- | --- | --- | --- | --- | --- | --- | --- | --- |
| R.ILQC(+57.02)NQYANVEEAR.N | Y | 58.29 | 1706.8046 | 14 | -5.21 | 854.4033 | 2 | 14.34 | 1.0697 | 1.0769 | 41279 | 1.23e3 | 1 | 1 | 86 | 99 | Carbamidomethylation |  | DB Search |
| R.NNPPTATR.T | Y | 58.23 | 869.4355 | 8 | 2.62 | 435.7252 | 2 | 9.23 | 0.784 | 0.7913 | 11045 | 2.65e3 | 1 | 1 | 925 | 932 |  |  | DB Search |
| R.NDVNNPPQAR.R | Y | 57.97 | 1123.537 | 10 | 3.56 | 562.7766 | 2 | 10.00 | 0.8615 | 0.8688 | 15207 | 4.89e4 | 2 | 2 | 1049 | 1058 |  |  | DB Search |
| R.SQSAFPTRR.R | Y | 57.12 | 1048.5414 | 9 | 2.06 | 525.2779 | 2 | 11.08 | 0.8542 | 0.8615 | 21210 | 5.83e3 | 1 | 1 | 856 | 864 |  |  | DB Search |
| K.DKNILVLTLGVGPQVNR.N | Y | 52.82 | 1835.0628 | 17 | -4.3 | 612.6909 | 3 | 19.59 | 0.8368 | 0.8441 | 74795 | 1.35e3 | 2 | 2 | 168 | 184 |  |  | DB Search |
| total 5 peptides |  |  |  |  |  |  |  |  |  |  |  |  |  |  |  |  |  |  |  |
| --- | --- | --- | --- | --- | --- | --- | --- | --- | --- | --- | --- | --- | --- | --- | --- | --- | --- | --- | --- |

Best Unique PSM (Scan Sample\_2\_A1\_1\_3640.d:41279, m/z=854.4033, z=2, RT=14.34, ppm=-7.4):


TRINITY\_DN477\_c0\_g1\_i6.p1

back to list

  

| Protein Coverage
| Supporting Peptides
| Best Unique PSM
|

Protein Coverage:

Supporting Peptides:

| Peptide | Uniq | -10lgP | Mass | Length | ppm | m/z | z | RT | 1/k0 Start | 1/k0 End | Precursor ID | Area Sample 4 | #Feature | #Feature Sample 4 | Start | End | PTM | AScore | Found By |
| --- | --- | --- | --- | --- | --- | --- | --- | --- | --- | --- | --- | --- | --- | --- | --- | --- | --- | --- | --- |
| R.NDVNNPPQAR.R | Y | 57.97 | 1123.537 | 10 | 3.56 | 562.7766 | 2 | 10.00 | 0.8615 | 0.8688 | 15207 | 4.89e4 | 2 | 2 | 86 | 95 |  |  | DB Search |
| total 1 peptides |  |  |  |  |  |  |  |  |  |  |  |  |  |  |  |  |  |  |  |
| --- | --- | --- | --- | --- | --- | --- | --- | --- | --- | --- | --- | --- | --- | --- | --- | --- | --- | --- | --- |

Best Unique PSM (Scan Sample\_2\_A1\_1\_3640.d:15207, m/z=562.7766, z=2, RT=10.00, ppm=1.36):


TRINITY\_DN1071\_c0\_g1\_i2.p1

back to list

  

| Protein Coverage
| Supporting Peptides
| Best Unique PSM
|

Protein Coverage:

Supporting Peptides:

| Peptide | Uniq | -10lgP | Mass | Length | ppm | m/z | z | RT | 1/k0 Start | 1/k0 End | Precursor ID | Area Sample 4 | #Feature | #Feature Sample 4 | Start | End | PTM | AScore | Found By |
| --- | --- | --- | --- | --- | --- | --- | --- | --- | --- | --- | --- | --- | --- | --- | --- | --- | --- | --- | --- |
| K.AVQIHPFNDVSVGDPK.V | Y | 58.41 | 1721.8736 | 16 | 11.97 | 574.9708 | 3 | 15.99 | 0.8761 | 0.8834 | 51505 | 2.3e4 | 2 | 2 | 293 | 308 |  |  | DB Search |
| total 1 peptides |  |  |  |  |  |  |  |  |  |  |  |  |  |  |  |  |  |  |  |
| --- | --- | --- | --- | --- | --- | --- | --- | --- | --- | --- | --- | --- | --- | --- | --- | --- | --- | --- | --- |

Best Unique PSM (Scan Sample\_2\_A1\_1\_3640.d:51505, m/z=574.9708, z=3, RT=15.99, ppm=9.77):


TRINITY\_DN140702\_c0\_g1\_i1.p1

back to list

  

| Protein Coverage
| Supporting Peptides
| Best Unique PSM
|

Protein Coverage:

Supporting Peptides:

| Peptide | Uniq | -10lgP | Mass | Length | ppm | m/z | z | RT | 1/k0 Start | 1/k0 End | Precursor ID | Area Sample 4 | #Feature | #Feature Sample 4 | Start | End | PTM | AScore | Found By |
| --- | --- | --- | --- | --- | --- | --- | --- | --- | --- | --- | --- | --- | --- | --- | --- | --- | --- | --- | --- |
| K.ADPFIVVQLGK.K | Y | 58.39 | 1185.6757 | 11 | 0.68 | 593.8442 | 2 | 19.35 | 0.9004 | 0.9077 | 73310 | 6.47e2 | 1 | 1 | 1554 | 1564 |  |  | DB Search |
| R.LALHVLNNEDIIGHR.L | Y | 58.37 | 1712.9322 | 15 | 0.41 | 571.9836 | 3 | 16.58 | 0.8615 | 0.8688 | 55276 | 2.56e3 | 2 | 2 | 1702 | 1716 |  |  | DB Search |
| R.SDELLNLKEEASTLR.E | Y | 58.29 | 1716.8894 | 15 | 14.54 | 573.3108 | 3 | 17.82 | 0.8473 | 0.8546 | 63372 | 0 | 0 | 0 | 729 | 743 |  |  | DB Search |
| R.FVFPFK.Y | Y | 58.28 | 783.4319 | 6 | 4.07 | 392.724 | 2 | 19.74 | 0.7681 | 0.7754 | 76008 | 6.59e2 | 2 | 2 | 1828 | 1833 |  |  | DB Search |
| K.ITAIVLGPGDELPEK.A | Y | 58.25 | 1550.8555 | 15 | -7.77 | 776.4273 | 2 | 17.93 | 1.0351 | 1.0423 | 64057 | 1.66e3 | 1 | 1 | 340 | 354 |  |  | DB Search |
| R.DILGLDSEGTSDAYAK.V | Y | 58.05 | 1653.7733 | 16 | 1.81 | 827.8936 | 2 | 17.69 | 1.0733 | 1.0805 | 62313 | 3.6e2 | 1 | 1 | 1135 | 1150 |  |  | DB Search |
| K.TGVEILNWGVR.N | Y | 56.92 | 1242.672 | 11 | -1.98 | 622.3407 | 2 | 19.74 | 0.9069 | 0.9141 | 75875 | 4.24e2 | 1 | 1 | 1288 | 1298 |  |  | DB Search |
| K.IEDFEESPPTDPHIGPVEQR.L | Y | 53.29 | 2291.0705 | 20 | 5.46 | 764.7 | 3 | 15.86 | 0.9295 | 0.9368 | 50734 | 0 | 0 | 0 | 1682 | 1701 |  |  | DB Search |
| total 8 peptides |  |  |  |  |  |  |  |  |  |  |  |  |  |  |  |  |  |  |  |
| --- | --- | --- | --- | --- | --- | --- | --- | --- | --- | --- | --- | --- | --- | --- | --- | --- | --- | --- | --- |

Best Unique PSM (Scan Sample\_2\_A1\_1\_3640.d:73310, m/z=593.8442, z=2, RT=19.35, ppm=-1.51):


TRINITY\_DN99404\_c0\_g1\_i1.p1

back to list

  

| Protein Coverage
| Supporting Peptides
| Best Unique PSM
|

Protein Coverage:

Supporting Peptides:

| Peptide | Uniq | -10lgP | Mass | Length | ppm | m/z | z | RT | 1/k0 Start | 1/k0 End | Precursor ID | Area Sample 4 | #Feature | #Feature Sample 4 | Start | End | PTM | AScore | Found By |
| --- | --- | --- | --- | --- | --- | --- | --- | --- | --- | --- | --- | --- | --- | --- | --- | --- | --- | --- | --- |
| K.LQTDIHGVALEITK.L | Y | 58.16 | 1536.8511 | 14 | 0.96 | 769.4319 | 2 | 16.71 | 1.0544 | 1.0616 | 56016 | 5.98e2 | 1 | 1 | 24 | 37 |  |  | DB Search |
| total 1 peptides |  |  |  |  |  |  |  |  |  |  |  |  |  |  |  |  |  |  |  |
| --- | --- | --- | --- | --- | --- | --- | --- | --- | --- | --- | --- | --- | --- | --- | --- | --- | --- | --- | --- |

Best Unique PSM (Scan Sample\_2\_A1\_1\_3640.d:56016, m/z=769.4319, z=2, RT=16.71, ppm=-1.24):


TRINITY\_DN4481\_c0\_g1\_i2.p1

back to list

  

| Protein Coverage
| Supporting Peptides
| Best Unique PSM
|

Protein Coverage:

Supporting Peptides:

| Peptide | Uniq | -10lgP | Mass | Length | ppm | m/z | z | RT | 1/k0 Start | 1/k0 End | Precursor ID | Area Sample 4 | #Feature | #Feature Sample 4 | Start | End | PTM | AScore | Found By |
| --- | --- | --- | --- | --- | --- | --- | --- | --- | --- | --- | --- | --- | --- | --- | --- | --- | --- | --- | --- |
| K.AVAEEFISVDPSVR.G | Y | 58.29 | 1517.7725 | 14 | 0.44 | 759.8922 | 2 | 17.79 | 1.0287 | 1.0359 | 62940 | 2.17e3 | 1 | 1 | 351 | 364 |  |  | DB Search |
| total 1 peptides |  |  |  |  |  |  |  |  |  |  |  |  |  |  |  |  |  |  |  |
| --- | --- | --- | --- | --- | --- | --- | --- | --- | --- | --- | --- | --- | --- | --- | --- | --- | --- | --- | --- |

Best Unique PSM (Scan Sample\_2\_A1\_1\_3640.d:62940, m/z=759.8922, z=2, RT=17.79, ppm=-1.75):


TRINITY\_DN691\_c0\_g1\_i1.p1

back to list

  

| Protein Coverage
| Supporting Peptides
| Best Unique PSM
|

Protein Coverage:

Supporting Peptides:

| Peptide | Uniq | -10lgP | Mass | Length | ppm | m/z | z | RT | 1/k0 Start | 1/k0 End | Precursor ID | Area Sample 4 | #Feature | #Feature Sample 4 | Start | End | PTM | AScore | Found By |
| --- | --- | --- | --- | --- | --- | --- | --- | --- | --- | --- | --- | --- | --- | --- | --- | --- | --- | --- | --- |
| R.ANPFIEC(+57.02)HGR.G | Y | 58.39 | 1199.5506 | 10 | 3.99 | 600.7836 | 2 | 13.37 | 0.8923 | 0.8996 | 35227 | 1.55e3 | 1 | 1 | 1660 | 1669 | Carbamidomethylation |  | DB Search |
| K.DGNDGQTGLTGQR.G | Y | 58.38 | 1317.5909 | 13 | 0.63 | 659.8017 | 2 | 11.17 | 0.9303 | 0.9376 | 21848 | 0 | 0 | 0 | 321 | 333 |  |  | DB Search |
| R.GPTGPQGER.G | Y | 57.67 | 897.4304 | 9 | 3.94 | 449.7233 | 2 | 9.46 | 0.7709 | 0.7783 | 12020 | 3.18e3 | 1 | 1 | 1209 | 1217 |  |  | DB Search |
| R.AYGQDLGQPGSC(+57.02)LKR.F | Y | 55 | 1648.7991 | 15 | 2.6 | 825.4072 | 2 | 13.56 | 1.0761 | 1.0833 | 36469 | 5.48e2 | 1 | 1 | 1533 | 1547 | Carbamidomethylation |  | DB Search |
| R.AYGQDLGQPGSC(+57.02)LK.R | Y | 54.78 | 1492.698 | 14 | -2.16 | 747.353 | 2 | 14.36 | 0.9977 | 1.0049 | 41400 | 3.43e3 | 1 | 1 | 1533 | 1546 | Carbamidomethylation |  | DB Search |
| R.GKDGLQGIAGPR.G | Y | 53.95 | 1167.636 | 12 | 2.28 | 584.8253 | 2 | 13.08 | 0.885 | 0.8923 | 33537 | 5.42e2 | 1 | 1 | 1073 | 1084 |  |  | DB Search |
| R.GLSGVNGLRGETGDR.G | Y | 53.51 | 1486.7488 | 15 | 1.6 | 744.3812 | 2 | 13.43 | 1.0508 | 1.058 | 35633 | 9.09e2 | 1 | 1 | 629 | 643 |  |  | DB Search |
| total 7 peptides |  |  |  |  |  |  |  |  |  |  |  |  |  |  |  |  |  |  |  |
| --- | --- | --- | --- | --- | --- | --- | --- | --- | --- | --- | --- | --- | --- | --- | --- | --- | --- | --- | --- |

Best Unique PSM (Scan Sample\_2\_A1\_1\_3640.d:35227, m/z=600.7836, z=2, RT=13.37, ppm=1.8):


TRINITY\_DN106\_c0\_g1\_i5.p1

back to list

  

| Protein Coverage
| Supporting Peptides
| Best Unique PSM
|

Protein Coverage:

Supporting Peptides:

| Peptide | Uniq | -10lgP | Mass | Length | ppm | m/z | z | RT | 1/k0 Start | 1/k0 End | Precursor ID | Area Sample 4 | #Feature | #Feature Sample 4 | Start | End | PTM | AScore | Found By |
| --- | --- | --- | --- | --- | --- | --- | --- | --- | --- | --- | --- | --- | --- | --- | --- | --- | --- | --- | --- |
| K.EGPAGPAGPVGR.L | Y | 58.28 | 1063.541 | 12 | -11.82 | 532.7703 | 2 | 11.84 | 0.8461 | 0.8534 | 25992 | 0 | 0 | 0 | 191 | 202 |  |  | DB Search |
| R.DGPAGPAGR.D | N | 58.24 | 796.3827 | 9 | 3.73 | 399.1992 | 2 | 9.76 | 0.7061 | 0.7135 | 13439 | 1.18e4 | 2 | 2 | 488 | 496 |  |  | DB Search |
| R.GPDGAVGPTGPIGAR.G | N | 58.04 | 1320.6786 | 15 | 1.83 | 661.3463 | 2 | 13.72 | 0.9558 | 0.963 | 37443 | 1.21e4 | 2 | 2 | 327 | 341 |  |  | DB Search |
| total 3 peptides |  |  |  |  |  |  |  |  |  |  |  |  |  |  |  |  |  |  |  |
| --- | --- | --- | --- | --- | --- | --- | --- | --- | --- | --- | --- | --- | --- | --- | --- | --- | --- | --- | --- |

Best Unique PSM (Scan Sample\_2\_A1\_1\_3640.d:25992, m/z=532.7703, z=2, RT=11.84, ppm=-14.02):


TRINITY\_DN21554\_c2\_g1\_i1.p1

back to list

  

| Protein Coverage
| Supporting Peptides
| Best Unique PSM
|

Protein Coverage:

Supporting Peptides:

| Peptide | Uniq | -10lgP | Mass | Length | ppm | m/z | z | RT | 1/k0 Start | 1/k0 End | Precursor ID | Area Sample 4 | #Feature | #Feature Sample 4 | Start | End | PTM | AScore | Found By |
| --- | --- | --- | --- | --- | --- | --- | --- | --- | --- | --- | --- | --- | --- | --- | --- | --- | --- | --- | --- |
| K.WLIAEGGSELPSNLR.S | Y | 58.07 | 1640.8522 | 15 | -10.17 | 821.4232 | 2 | 19.05 | 1.0664 | 1.0737 | 71492 | 0 | 0 | 0 | 155 | 169 |  |  | DB Search |
| total 1 peptides |  |  |  |  |  |  |  |  |  |  |  |  |  |  |  |  |  |  |  |
| --- | --- | --- | --- | --- | --- | --- | --- | --- | --- | --- | --- | --- | --- | --- | --- | --- | --- | --- | --- |

Best Unique PSM (Scan Sample\_2\_A1\_1\_3640.d:71492, m/z=821.4232, z=2, RT=19.05, ppm=-12.37):


TRINITY\_DN1465\_c1\_g1\_i1.p1

back to list

  

| Protein Coverage
| Supporting Peptides
| Best Unique PSM
|

Protein Coverage:

Supporting Peptides:

| Peptide | Uniq | -10lgP | Mass | Length | ppm | m/z | z | RT | 1/k0 Start | 1/k0 End | Precursor ID | Area Sample 4 | #Feature | #Feature Sample 4 | Start | End | PTM | AScore | Found By |
| --- | --- | --- | --- | --- | --- | --- | --- | --- | --- | --- | --- | --- | --- | --- | --- | --- | --- | --- | --- |
| K.ESTLHLVLR.L | Y | 57.27 | 1066.6135 | 9 | 2.57 | 534.3142 | 2 | 15.80 | 0.8785 | 0.8858 | 50332 | 4.97e3 | 1 | 1 | 68 | 76 |  |  | DB Search |
| K.IQDKEGIPPDQQR.L | Y | 55.01 | 1522.7739 | 13 | 0.25 | 762.3928 | 2 | 11.08 | 1.0343 | 1.0415 | 21018 | 1.78e3 | 2 | 2 | 34 | 46 |  |  | DB Search |
| total 2 peptides |  |  |  |  |  |  |  |  |  |  |  |  |  |  |  |  |  |  |  |
| --- | --- | --- | --- | --- | --- | --- | --- | --- | --- | --- | --- | --- | --- | --- | --- | --- | --- | --- | --- |

Best Unique PSM (Scan Sample\_2\_A1\_1\_3640.d:50332, m/z=534.7766, z=2, RT=15.80, ppm=0.37):


TRINITY\_DN1821\_c0\_g1\_i4.p1

back to list

  

| Protein Coverage
| Supporting Peptides
| Best Unique PSM
|

Protein Coverage:

Supporting Peptides:

| Peptide | Uniq | -10lgP | Mass | Length | ppm | m/z | z | RT | 1/k0 Start | 1/k0 End | Precursor ID | Area Sample 4 | #Feature | #Feature Sample 4 | Start | End | PTM | AScore | Found By |
| --- | --- | --- | --- | --- | --- | --- | --- | --- | --- | --- | --- | --- | --- | --- | --- | --- | --- | --- | --- |
| K.VEDSKESDINFLLK.T | Y | 57.24 | 1635.8355 | 14 | 0.37 | 546.2848 | 3 | 17.72 | 0.8092 | 0.8165 | 62515 | 5.45e2 | 1 | 1 | 422 | 435 |  |  | DB Search |
| K.YQGIKPPVSR.S | Y | 57.06 | 1143.64 | 10 | -0.07 | 572.826 | 2 | 12.77 | 0.9311 | 0.9384 | 31696 | 3.82e2 | 1 | 1 | 478 | 487 |  |  | DB Search |
| K.DKPALDVTSTLKK.K | Y | 54.52 | 1414.8031 | 13 | 2.31 | 472.6084 | 3 | 13.78 | 0.801 | 0.8084 | 37913 | 0 | 0 | 0 | 279 | 291 |  |  | DB Search |
| total 3 peptides |  |  |  |  |  |  |  |  |  |  |  |  |  |  |  |  |  |  |  |
| --- | --- | --- | --- | --- | --- | --- | --- | --- | --- | --- | --- | --- | --- | --- | --- | --- | --- | --- | --- |

Best Unique PSM (Scan Sample\_2\_A1\_1\_3640.d:62515, m/z=546.2848, z=3, RT=17.72, ppm=-1.83):


TRINITY\_DN477\_c0\_g1\_i5.p1

back to list

  

| Protein Coverage
| Supporting Peptides
| Best Unique PSM
|

Protein Coverage:

Supporting Peptides:

| Peptide | Uniq | -10lgP | Mass | Length | ppm | m/z | z | RT | 1/k0 Start | 1/k0 End | Precursor ID | Area Sample 4 | #Feature | #Feature Sample 4 | Start | End | PTM | AScore | Found By |
| --- | --- | --- | --- | --- | --- | --- | --- | --- | --- | --- | --- | --- | --- | --- | --- | --- | --- | --- | --- |
| R.NDVNNPPQAR.R | Y | 57.97 | 1123.537 | 10 | 3.56 | 562.7766 | 2 | 10.00 | 0.8615 | 0.8688 | 15207 | 4.89e4 | 2 | 2 | 168 | 177 |  |  | DB Search |
| total 1 peptides |  |  |  |  |  |  |  |  |  |  |  |  |  |  |  |  |  |  |  |
| --- | --- | --- | --- | --- | --- | --- | --- | --- | --- | --- | --- | --- | --- | --- | --- | --- | --- | --- | --- |

Best Unique PSM (Scan Sample\_2\_A1\_1\_3640.d:15207, m/z=562.7766, z=2, RT=10.00, ppm=1.36):


TRINITY\_DN2532\_c1\_g1\_i1.p1

back to list

  

| Protein Coverage
| Supporting Peptides
| Best Unique PSM
|

Protein Coverage:

Supporting Peptides:

| Peptide | Uniq | -10lgP | Mass | Length | ppm | m/z | z | RT | 1/k0 Start | 1/k0 End | Precursor ID | Area Sample 4 | #Feature | #Feature Sample 4 | Start | End | PTM | AScore | Found By |
| --- | --- | --- | --- | --- | --- | --- | --- | --- | --- | --- | --- | --- | --- | --- | --- | --- | --- | --- | --- |
| K.VPIVGGPVIK.L | Y | 58.38 | 977.6273 | 10 | 8.05 | 489.8238 | 2 | 16.07 | 0.8368 | 0.8441 | 52060 | 0 | 0 | 0 | 675 | 684 |  |  | DB Search |
| total 1 peptides |  |  |  |  |  |  |  |  |  |  |  |  |  |  |  |  |  |  |  |
| --- | --- | --- | --- | --- | --- | --- | --- | --- | --- | --- | --- | --- | --- | --- | --- | --- | --- | --- | --- |

Best Unique PSM (Scan Sample\_2\_A1\_1\_3640.d:52060, m/z=489.8238, z=2, RT=16.07, ppm=5.85):


TRINITY\_DN172571\_c0\_g1\_i1.p1

back to list

  

| Protein Coverage
| Supporting Peptides
| Best Unique PSM
|

Protein Coverage:

Supporting Peptides:

| Peptide | Uniq | -10lgP | Mass | Length | ppm | m/z | z | RT | 1/k0 Start | 1/k0 End | Precursor ID | Area Sample 4 | #Feature | #Feature Sample 4 | Start | End | PTM | AScore | Found By |
| --- | --- | --- | --- | --- | --- | --- | --- | --- | --- | --- | --- | --- | --- | --- | --- | --- | --- | --- | --- |
| R.DNIQGITKPAIR.R | Y | 58.19 | 1324.7463 | 12 | 0.94 | 663.3796 | 2 | 13.94 | 0.9795 | 0.9868 | 38749 | 1.14e3 | 1 | 1 | 25 | 36 |  |  | DB Search |
| K.VFLENVIR.D | Y | 56.94 | 988.5705 | 8 | 3.98 | 495.2934 | 2 | 18.16 | 0.8558 | 0.8631 | 65372 | 1.37e3 | 1 | 1 | 61 | 68 |  |  | DB Search |
| total 2 peptides |  |  |  |  |  |  |  |  |  |  |  |  |  |  |  |  |  |  |  |
| --- | --- | --- | --- | --- | --- | --- | --- | --- | --- | --- | --- | --- | --- | --- | --- | --- | --- | --- | --- |

Best Unique PSM (Scan Sample\_2\_A1\_1\_3640.d:38749, m/z=663.3796, z=2, RT=13.94, ppm=-1.26):


TRINITY\_DN2490\_c0\_g1\_i1.p1

back to list

  

| Protein Coverage
| Supporting Peptides
| Best Unique PSM
|

Protein Coverage:

Supporting Peptides:

| Peptide | Uniq | -10lgP | Mass | Length | ppm | m/z | z | RT | 1/k0 Start | 1/k0 End | Precursor ID | Area Sample 4 | #Feature | #Feature Sample 4 | Start | End | PTM | AScore | Found By |
| --- | --- | --- | --- | --- | --- | --- | --- | --- | --- | --- | --- | --- | --- | --- | --- | --- | --- | --- | --- |
| K.DKIFPHIELFGK.E | Y | 58.29 | 1442.7921 | 12 | 2.07 | 481.9379 | 3 | 18.74 | 0.8688 | 0.8761 | 69351 | 4.71e2 | 1 | 1 | 519 | 530 |  |  | DB Search |
| K.ALAWGLPGYK.S | Y | 58.2 | 1074.5862 | 10 | -1.57 | 538.2983 | 2 | 19.05 | 0.8599 | 0.8672 | 71305 | 1.33e3 | 1 | 1 | 355 | 364 |  |  | DB Search |
| total 2 peptides |  |  |  |  |  |  |  |  |  |  |  |  |  |  |  |  |  |  |  |
| --- | --- | --- | --- | --- | --- | --- | --- | --- | --- | --- | --- | --- | --- | --- | --- | --- | --- | --- | --- |

Best Unique PSM (Scan Sample\_2\_A1\_1\_3640.d:69351, m/z=481.9379, z=3, RT=18.74, ppm=-0.13):


TRINITY\_DN24414\_c1\_g1\_i2.p2

back to list

  

| Protein Coverage
| Supporting Peptides
| Best Unique PSM
|

Protein Coverage:

Supporting Peptides:

| Peptide | Uniq | -10lgP | Mass | Length | ppm | m/z | z | RT | 1/k0 Start | 1/k0 End | Precursor ID | Area Sample 4 | #Feature | #Feature Sample 4 | Start | End | PTM | AScore | Found By |
| --- | --- | --- | --- | --- | --- | --- | --- | --- | --- | --- | --- | --- | --- | --- | --- | --- | --- | --- | --- |
| K.VLTSDDPAEKEK.K | Y | 58.39 | 1330.6616 | 12 | 3.3 | 666.3388 | 2 | 10.77 | 0.9856 | 0.9928 | 19482 | 3.46e2 | 1 | 1 | 95 | 106 |  |  | DB Search |
| total 1 peptides |  |  |  |  |  |  |  |  |  |  |  |  |  |  |  |  |  |  |  |
| --- | --- | --- | --- | --- | --- | --- | --- | --- | --- | --- | --- | --- | --- | --- | --- | --- | --- | --- | --- |

Best Unique PSM (Scan Sample\_2\_A1\_1\_3640.d:19482, m/z=666.3388, z=2, RT=10.77, ppm=1.11):


TRINITY\_DN554\_c0\_g1\_i2.p1

back to list

  

| Protein Coverage
| Supporting Peptides
| Best Unique PSM
|

Protein Coverage:

Supporting Peptides:

| Peptide | Uniq | -10lgP | Mass | Length | ppm | m/z | z | RT | 1/k0 Start | 1/k0 End | Precursor ID | Area Sample 4 | #Feature | #Feature Sample 4 | Start | End | PTM | AScore | Found By |
| --- | --- | --- | --- | --- | --- | --- | --- | --- | --- | --- | --- | --- | --- | --- | --- | --- | --- | --- | --- |
| K.ESTLHLVLR.L | Y | 57.27 | 1066.6135 | 9 | 2.57 | 534.3142 | 2 | 15.80 | 0.8785 | 0.8858 | 50332 | 4.97e3 | 1 | 1 | 29 | 37 |  |  | DB Search |
| K.IQDKEGIPPDQQR.L | Y | 55.01 | 1522.7739 | 13 | 0.25 | 762.3928 | 2 | 11.08 | 1.0343 | 1.0415 | 21018 | 1.78e3 | 2 | 2 | 71 | 83 |  |  | DB Search |
| K.TITLEVEPSDTIENVK.S | Y | 54.56 | 1786.92 | 16 | 4.26 | 894.4691 | 2 | 17.62 | 1.1442 | 1.1514 | 61769 | 1.48e4 | 1 | 1 | 53 | 68 |  |  | DB Search |
| total 3 peptides |  |  |  |  |  |  |  |  |  |  |  |  |  |  |  |  |  |  |  |
| --- | --- | --- | --- | --- | --- | --- | --- | --- | --- | --- | --- | --- | --- | --- | --- | --- | --- | --- | --- |

Best Unique PSM (Scan Sample\_2\_A1\_1\_3640.d:50332, m/z=534.7766, z=2, RT=15.80, ppm=0.37):


TRINITY\_DN1613\_c1\_g1\_i1.p1

back to list

  

| Protein Coverage
| Supporting Peptides
| Best Unique PSM
|

Protein Coverage:

Supporting Peptides:

| Peptide | Uniq | -10lgP | Mass | Length | ppm | m/z | z | RT | 1/k0 Start | 1/k0 End | Precursor ID | Area Sample 4 | #Feature | #Feature Sample 4 | Start | End | PTM | AScore | Found By |
| --- | --- | --- | --- | --- | --- | --- | --- | --- | --- | --- | --- | --- | --- | --- | --- | --- | --- | --- | --- |
| R.S(+42.01)C(+57.02)SNPSPAHGGKPC(+57.02)SGELSK.T | Y | 58.29 | 2097.9208 | 20 | 2.04 | 700.3141 | 3 | 11.24 | 0.8716 | 0.8789 | 22349 | 4.91e2 | 1 | 1 | 14 | 33 | Acetylation (Protein N-term), Carbamidomethylation, Carbamidomethylation | S1:Acetylation (Protein N-term):1000 | DB Search |
| R.TC(+57.02)TNPAPAHGGK.S | Y | 58.24 | 1209.556 | 12 | 4.26 | 404.1935 | 3 | 8.97 | 0.7322 | 0.7396 | 10252 | 1.06e3 | 2 | 2 | 413 | 424 | Carbamidomethylation |  | DB Search |
| R.SC(+57.02)TNPRPLFGGK.A | Y | 50.98 | 1332.6608 | 12 | 4.64 | 445.2286 | 3 | 13.73 | 0.7831 | 0.7905 | 37561 | 4.17e2 | 1 | 1 | 356 | 367 | Carbamidomethylation |  | DB Search |
| total 3 peptides |  |  |  |  |  |  |  |  |  |  |  |  |  |  |  |  |  |  |  |
| --- | --- | --- | --- | --- | --- | --- | --- | --- | --- | --- | --- | --- | --- | --- | --- | --- | --- | --- | --- |

Best Unique PSM (Scan Sample\_2\_A1\_1\_3640.d:22349, m/z=700.3141, z=3, RT=11.24, ppm=-0.16):


TRINITY\_DN6390\_c0\_g1\_i1.p1

back to list

  

| Protein Coverage
| Supporting Peptides
| Best Unique PSM
|

Protein Coverage:

Supporting Peptides:

| Peptide | Uniq | -10lgP | Mass | Length | ppm | m/z | z | RT | 1/k0 Start | 1/k0 End | Precursor ID | Area Sample 4 | #Feature | #Feature Sample 4 | Start | End | PTM | AScore | Found By |
| --- | --- | --- | --- | --- | --- | --- | --- | --- | --- | --- | --- | --- | --- | --- | --- | --- | --- | --- | --- |
| K.TVSPEPFIESC(+57.02)QQETC(+57.02)LC(+57.02)K.S | Y | 58.41 | 2312.0123 | 19 | -0.66 | 771.6758 | 3 | 17.07 | 0.9243 | 0.9315 | 58393 | 2.54e3 | 3 | 3 | 1499 | 1517 | Carbamidomethylation, Carbamidomethylation, Carbamidomethylation |  | DB Search |
| R.FEC(+57.02)TDFDC(+57.02)VAHC(+57.02)ASK.G | Y | 58.41 | 1845.712 | 15 | -3.34 | 616.2412 | 3 | 15.02 | 0.898 | 0.9052 | 45403 | 3.7e3 | 2 | 2 | 1159 | 1173 | Carbamidomethylation, Carbamidomethylation, Carbamidomethylation |  | DB Search |
| K.VFDKGSYPQPK.T | Y | 58.39 | 1264.6452 | 11 | 5.46 | 422.557 | 3 | 12.55 | 0.8023 | 0.8096 | 30062 | 2.92e3 | 2 | 2 | 1363 | 1373 |  |  | DB Search |
| K.GGRFEC(+57.02)TDFDC(+57.02)VAHC(+57.02)ASK.G | Y | 58.36 | 2115.8561 | 18 | -4.91 | 529.9675 | 4 | 14.50 | 0.8279 | 0.8352 | 42192 | 0 | 0 | 0 | 1156 | 1173 | Carbamidomethylation, Carbamidomethylation, Carbamidomethylation |  | DB Search |
| K.STENC(+57.02)VPR.N | Y | 58.34 | 961.4287 | 8 | -0.32 | 481.7204 | 2 | 10.55 | 0.7998 | 0.8071 | 18222 | 1.46e3 | 1 | 1 | 725 | 732 | Carbamidomethylation |  | DB Search |
| R.VGNWRDETLSC(+57.02)K.Q | Y | 58.32 | 1463.6827 | 12 | 0.66 | 488.9008 | 3 | 13.88 | 0.7628 | 0.7701 | 38433 | 5.84e3 | 2 | 2 | 1047 | 1058 | Carbamidomethylation |  | DB Search |
| K.YYEC(+57.02)GSAC(+57.02)PK.T | Y | 58.26 | 1233.4794 | 10 | 3.8 | 617.748 | 2 | 11.31 | 0.9174 | 0.9247 | 22752 | 0 | 0 | 0 | 686 | 695 | Carbamidomethylation, Carbamidomethylation |  | DB Search |
| K.TAVVDGVEIFK.S | Y | 58.24 | 1176.639 | 11 | -0.39 | 589.3253 | 2 | 18.09 | 0.9133 | 0.9206 | 64929 | 1.01e4 | 1 | 1 | 1374 | 1384 |  |  | DB Search |
| K.ANVNIHVR.N | Y | 58.2 | 921.5144 | 8 | 4.06 | 461.7654 | 2 | 11.91 | 0.8307 | 0.838 | 26250 | 1.78e3 | 1 | 1 | 1309 | 1316 |  |  | DB Search |
| R.SKDYTITSVGGR.N | Y | 57.09 | 1282.6517 | 12 | 0.86 | 642.3323 | 2 | 13.05 | 0.9384 | 0.9457 | 33313 | 1.65e3 | 1 | 1 | 876 | 887 |  |  | DB Search |
| K.VSSDRFFVQVDGR.S | Y | 56.99 | 1510.7528 | 13 | 0.15 | 504.5905 | 3 | 16.15 | 0.8059 | 0.8132 | 52408 | 1.09e3 | 1 | 1 | 474 | 486 |  |  | DB Search |
| R.HLSVNILQTNTR.V | Y | 56.43 | 1394.763 | 12 | -1.72 | 698.386 | 2 | 15.49 | 1.0009 | 1.0081 | 48422 | 1.9e3 | 1 | 1 | 836 | 847 |  |  | DB Search |
| K.RC(+57.02)TDLNIR.V | Y | 56.27 | 1046.5291 | 8 | 2.47 | 524.272 | 2 | 12.07 | 0.8708 | 0.8781 | 27297 | 3.54e3 | 1 | 1 | 1039 | 1046 | Carbamidomethylation |  | DB Search |
| K.EGDQFFDAGEVSPSDC(+57.02)SK.V | Y | 55.89 | 1973.7949 | 18 | 2.52 | 987.905 | 2 | 16.58 | 1.1402 | 1.1474 | 55286 | 0 | 0 | 0 | 1225 | 1242 | Carbamidomethylation |  | DB Search |
| K.SVTAFTQSWVYQSSDKVQDPSK.C | Y | 55.29 | 2487.1918 | 22 | 5.18 | 830.0737 | 3 | 17.80 | 0.9767 | 0.984 | 62980 | 4.77e2 | 1 | 1 | 944 | 965 |  |  | DB Search |
| R.RANLC(+57.02)PITC(+57.02)QR.N | Y | 54.97 | 1387.6813 | 11 | 2.53 | 694.8481 | 2 | 12.62 | 0.9679 | 0.9751 | 30632 | 1.25e3 | 1 | 1 | 672 | 682 | Carbamidomethylation, Carbamidomethylation |  | DB Search |
| K.SLVYNPLHNDQEVFER.A | Y | 54.95 | 1958.9486 | 16 | -1.87 | 653.9875 | 3 | 17.53 | 0.866 | 0.8733 | 61164 | 3.14e3 | 1 | 1 | 590 | 605 |  |  | DB Search |
| K.YLFDLESNC(+57.02)DK.Q | Y | 54.31 | 1402.6075 | 11 | -3.51 | 702.307 | 2 | 18.48 | 0.9787 | 0.986 | 67762 | 0 | 0 | 0 | 303 | 313 | Carbamidomethylation |  | DB Search |
| total 18 peptides |  |  |  |  |  |  |  |  |  |  |  |  |  |  |  |  |  |  |  |
| --- | --- | --- | --- | --- | --- | --- | --- | --- | --- | --- | --- | --- | --- | --- | --- | --- | --- | --- | --- |

Best Unique PSM (Scan Sample\_2\_A1\_1\_3640.d:58393, m/z=771.6758, z=3, RT=17.07, ppm=-2.85):


TRINITY\_DN39385\_c0\_g1\_i1.p1

back to list

  

| Protein Coverage
| Supporting Peptides
| Best Unique PSM
|

Protein Coverage:

Supporting Peptides:

| Peptide | Uniq | -10lgP | Mass | Length | ppm | m/z | z | RT | 1/k0 Start | 1/k0 End | Precursor ID | Area Sample 4 | #Feature | #Feature Sample 4 | Start | End | PTM | AScore | Found By |
| --- | --- | --- | --- | --- | --- | --- | --- | --- | --- | --- | --- | --- | --- | --- | --- | --- | --- | --- | --- |
| K.GDFILNSSNVIENVTR.L | Y | 58.32 | 1776.9006 | 16 | 4.19 | 889.4594 | 2 | 19.77 | 1.1138 | 1.121 | 76079 | 0 | 0 | 0 | 81 | 96 |  |  | DB Search |
| total 1 peptides |  |  |  |  |  |  |  |  |  |  |  |  |  |  |  |  |  |  |  |
| --- | --- | --- | --- | --- | --- | --- | --- | --- | --- | --- | --- | --- | --- | --- | --- | --- | --- | --- | --- |

Best Unique PSM (Scan Sample\_2\_A1\_1\_3640.d:76079, m/z=889.4594, z=2, RT=19.77, ppm=2):


TRINITY\_DN26249\_c0\_g1\_i1.p1

back to list

  

| Protein Coverage
| Supporting Peptides
| Best Unique PSM
|

Protein Coverage:

Supporting Peptides:

| Peptide | Uniq | -10lgP | Mass | Length | ppm | m/z | z | RT | 1/k0 Start | 1/k0 End | Precursor ID | Area Sample 4 | #Feature | #Feature Sample 4 | Start | End | PTM | AScore | Found By |
| --- | --- | --- | --- | --- | --- | --- | --- | --- | --- | --- | --- | --- | --- | --- | --- | --- | --- | --- | --- |
| R.LIYENQNTIHHVVR.A | Y | 58.39 | 1734.9165 | 14 | 3.92 | 579.3138 | 3 | 13.39 | 0.8473 | 0.8546 | 35379 | 0 | 0 | 0 | 322 | 335 |  |  | DB Search |
| total 1 peptides |  |  |  |  |  |  |  |  |  |  |  |  |  |  |  |  |  |  |  |
| --- | --- | --- | --- | --- | --- | --- | --- | --- | --- | --- | --- | --- | --- | --- | --- | --- | --- | --- | --- |

Best Unique PSM (Scan Sample\_2\_A1\_1\_3640.d:35379, m/z=579.3138, z=3, RT=13.39, ppm=1.72):


TRINITY\_DN6304\_c0\_g1\_i1.p1

back to list

  

| Protein Coverage
| Supporting Peptides
| Best Unique PSM
|

Protein Coverage:

Supporting Peptides:

| Peptide | Uniq | -10lgP | Mass | Length | ppm | m/z | z | RT | 1/k0 Start | 1/k0 End | Precursor ID | Area Sample 4 | #Feature | #Feature Sample 4 | Start | End | PTM | AScore | Found By |
| --- | --- | --- | --- | --- | --- | --- | --- | --- | --- | --- | --- | --- | --- | --- | --- | --- | --- | --- | --- |
| K.FKNPSNINDLNR.Y | Y | 54.49 | 1430.7266 | 12 | 3.59 | 477.9168 | 3 | 13.53 | 0.7522 | 0.7595 | 36237 | 4.48e2 | 1 | 1 | 205 | 216 |  |  | DB Search |
| R.VAAFPNFR.K | Y | 48.17 | 920.4868 | 8 | 3.3 | 461.2512 | 2 | 16.95 | 0.7815 | 0.7888 | 57556 | 8.33e2 | 1 | 1 | 251 | 258 |  |  | DB Search |
| total 2 peptides |  |  |  |  |  |  |  |  |  |  |  |  |  |  |  |  |  |  |  |
| --- | --- | --- | --- | --- | --- | --- | --- | --- | --- | --- | --- | --- | --- | --- | --- | --- | --- | --- | --- |

Best Unique PSM (Scan Sample\_2\_A1\_1\_3640.d:36237, m/z=477.9168, z=3, RT=13.53, ppm=1.39):


TRINITY\_DN21554\_c1\_g1\_i1.p1

back to list

  

| Protein Coverage
| Supporting Peptides
| Best Unique PSM
|

Protein Coverage:

Supporting Peptides:

| Peptide | Uniq | -10lgP | Mass | Length | ppm | m/z | z | RT | 1/k0 Start | 1/k0 End | Precursor ID | Area Sample 4 | #Feature | #Feature Sample 4 | Start | End | PTM | AScore | Found By |
| --- | --- | --- | --- | --- | --- | --- | --- | --- | --- | --- | --- | --- | --- | --- | --- | --- | --- | --- | --- |
| K.TSFPLITLK.I | Y | 58.28 | 1018.6063 | 9 | 2.17 | 510.3104 | 2 | 19.47 | 0.8404 | 0.8477 | 74096 | 7.34e2 | 1 | 1 | 572 | 580 |  |  | DB Search |
| total 1 peptides |  |  |  |  |  |  |  |  |  |  |  |  |  |  |  |  |  |  |  |
| --- | --- | --- | --- | --- | --- | --- | --- | --- | --- | --- | --- | --- | --- | --- | --- | --- | --- | --- | --- |

Best Unique PSM (Scan Sample\_2\_A1\_1\_3640.d:74096, m/z=510.3104, z=2, RT=19.47, ppm=-0.02):


TRINITY\_DN4781\_c0\_g1\_i3.p1

back to list

  

| Protein Coverage
| Supporting Peptides
| Best Unique PSM
|

Protein Coverage:

Supporting Peptides:

| Peptide | Uniq | -10lgP | Mass | Length | ppm | m/z | z | RT | 1/k0 Start | 1/k0 End | Precursor ID | Area Sample 4 | #Feature | #Feature Sample 4 | Start | End | PTM | AScore | Found By |
| --- | --- | --- | --- | --- | --- | --- | --- | --- | --- | --- | --- | --- | --- | --- | --- | --- | --- | --- | --- |
| R.LVSELFDPTSSTR.G | Y | 58.39 | 1450.7303 | 13 | 1.5 | 726.3719 | 2 | 17.91 | 1.0013 | 1.0086 | 64032 | 6.47e3 | 1 | 1 | 265 | 277 |  |  | DB Search |
| K.DFNVIIEAVK.G | Y | 58.33 | 1146.6284 | 10 | 0.88 | 574.3207 | 2 | 19.65 | 0.8996 | 0.9069 | 75314 | 3.22e3 | 1 | 1 | 851 | 860 |  |  | DB Search |
| Q.LIFVGTVGK.S | Y | 58.18 | 932.5695 | 9 | 3.01 | 467.2924 | 2 | 17.35 | 0.8124 | 0.8197 | 60219 | 6.55e3 | 1 | 1 | 168 | 176 |  |  | DB Search |
| K.GSYIYIEASYPR.K | Y | 57.03 | 1417.6878 | 12 | 0.36 | 709.8499 | 2 | 17.28 | 1.0399 | 1.0472 | 59633 | 2.78e3 | 1 | 1 | 434 | 445 |  |  | DB Search |
| K.GQSSDLATLSSPVLNQASSTC(+57.02)K.M | Y | 55.21 | 2250.0798 | 22 | 4.76 | 1126.05 | 2 | 17.18 | 1.2328 | 1.24 | 59109 | 0 | 0 | 0 | 1601 | 1622 | Carbamidomethylation |  | DB Search |
| K.ADHTYETDFGK.Y | Y | 55.18 | 1282.5466 | 11 | 8.7 | 642.2847 | 2 | 12.90 | 0.9453 | 0.9525 | 32449 | 0 | 0 | 0 | 1196 | 1206 |  |  | DB Search |
| R.TGPSLDNTK.K | Y | 54.61 | 931.4611 | 9 | 0.07 | 466.7368 | 2 | 11.29 | 0.8039 | 0.8112 | 22423 | 5.2e2 | 1 | 1 | 233 | 241 |  |  | DB Search |
| R.DVANLYSPYYPGSK.T | Y | 51.34 | 1572.746 | 14 | -1.72 | 787.3772 | 2 | 17.32 | 1.0242 | 1.0315 | 60052 | 5.66e2 | 1 | 1 | 613 | 626 |  |  | DB Search |
| R.NLKDDDFDFTR.Q | Y | 41.38 | 1384.6259 | 11 | 14.15 | 693.3285 | 2 | 15.93 | 0.9775 | 0.9848 | 51300 | 6.08e2 | 1 | 1 | 1766 | 1776 |  |  | DB Search |
| total 9 peptides |  |  |  |  |  |  |  |  |  |  |  |  |  |  |  |  |  |  |  |
| --- | --- | --- | --- | --- | --- | --- | --- | --- | --- | --- | --- | --- | --- | --- | --- | --- | --- | --- | --- |

Best Unique PSM (Scan Sample\_2\_A1\_1\_3640.d:64032, m/z=726.3719, z=2, RT=17.91, ppm=-0.69):


TRINITY\_DN4295\_c0\_g1\_i1.p1

back to list

  

| Protein Coverage
| Supporting Peptides
| Best Unique PSM
|

Protein Coverage:

Supporting Peptides:

| Peptide | Uniq | -10lgP | Mass | Length | ppm | m/z | z | RT | 1/k0 Start | 1/k0 End | Precursor ID | Area Sample 4 | #Feature | #Feature Sample 4 | Start | End | PTM | AScore | Found By |
| --- | --- | --- | --- | --- | --- | --- | --- | --- | --- | --- | --- | --- | --- | --- | --- | --- | --- | --- | --- |
| R.FLQVFENYR.N | Y | 57.44 | 1214.6084 | 9 | -2.94 | 608.3083 | 2 | 18.79 | 0.936 | 0.9432 | 69682 | 4.69e2 | 1 | 1 | 169 | 177 |  |  | DB Search |
| total 1 peptides |  |  |  |  |  |  |  |  |  |  |  |  |  |  |  |  |  |  |  |
| --- | --- | --- | --- | --- | --- | --- | --- | --- | --- | --- | --- | --- | --- | --- | --- | --- | --- | --- | --- |

Best Unique PSM (Scan Sample\_2\_A1\_1\_3640.d:69682, m/z=608.3083, z=2, RT=18.79, ppm=-5.14):


TRINITY\_DN161\_c0\_g1\_i3.p1

back to list

  

| Protein Coverage
| Supporting Peptides
| Best Unique PSM
|

Protein Coverage:

Supporting Peptides:

| Peptide | Uniq | -10lgP | Mass | Length | ppm | m/z | z | RT | 1/k0 Start | 1/k0 End | Precursor ID | Area Sample 4 | #Feature | #Feature Sample 4 | Start | End | PTM | AScore | Found By |
| --- | --- | --- | --- | --- | --- | --- | --- | --- | --- | --- | --- | --- | --- | --- | --- | --- | --- | --- | --- |
| K.SYELPDGQVITIGNER.F | Y | 58.39 | 1789.8846 | 16 | 1.59 | 895.949 | 2 | 18.70 | 1.111 | 1.1182 | 69190 | 4.17e3 | 1 | 1 | 262 | 277 |  |  | DB Search |
| R.AVFPSIVGRPR.H | Y | 58 | 1197.6982 | 11 | 3.54 | 599.8572 | 2 | 15.96 | 0.9642 | 0.9715 | 51028 | 1.61e4 | 1 | 1 | 52 | 62 |  |  | DB Search |
| R.DLTDYLMK.I | Y | 57.96 | 997.479 | 8 | 6.32 | 499.7488 | 2 | 18.67 | 0.8319 | 0.8392 | 68987 | 0 | 0 | 0 | 207 | 214 |  |  | DB Search |
| K.LC(+57.02)YVALDFEQEM(+15.99)QTAASSSSLEK.S | Y | 57.8 | 2622.1829 | 23 | 5.82 | 875.0714 | 3 | 19.02 | 1.0741 | 1.0813 | 71279 | 4.32e2 | 1 | 1 | 239 | 261 | Carbamidomethylation, Oxidation (M) | M12:Oxidation (M):1000 | DB Search |
| K.DSYVGDEAQSKR.G | Y | 57.28 | 1353.616 | 12 | 2.57 | 677.8156 | 2 | 11.01 | 0.9993 | 1.0065 | 20794 | 1.16e3 | 1 | 1 | 74 | 85 |  |  | DB Search |
| R.DLTDYLM(+15.99)K.I | Y | 57.01 | 1013.4739 | 8 | 0.97 | 507.7436 | 2 | 16.91 | 0.8388 | 0.8461 | 57197 | 1.01e3 | 1 | 1 | 207 | 214 | Oxidation (M) | M7:Oxidation (M):1000 | DB Search |
| K.DLYANTVLSGGTTM(+15.99)FPGIADR.M | Y | 55.95 | 2214.0626 | 21 | -1.47 | 1108.0345 | 2 | 19.56 | 1.2432 | 1.2503 | 74741 | 0 | 0 | 0 | 315 | 335 | Oxidation (M) | M14:Oxidation (M):1000 | DB Search |
| total 7 peptides |  |  |  |  |  |  |  |  |  |  |  |  |  |  |  |  |  |  |  |
| --- | --- | --- | --- | --- | --- | --- | --- | --- | --- | --- | --- | --- | --- | --- | --- | --- | --- | --- | --- |

Best Unique PSM (Scan Sample\_2\_A1\_1\_3640.d:69190, m/z=895.949, z=2, RT=18.70, ppm=-0.61):


TRINITY\_DN138\_c0\_g1\_i1.p1

back to list

  

| Protein Coverage
| Supporting Peptides
| Best Unique PSM
|

Protein Coverage:

Supporting Peptides:

| Peptide | Uniq | -10lgP | Mass | Length | ppm | m/z | z | RT | 1/k0 Start | 1/k0 End | Precursor ID | Area Sample 4 | #Feature | #Feature Sample 4 | Start | End | PTM | AScore | Found By |
| --- | --- | --- | --- | --- | --- | --- | --- | --- | --- | --- | --- | --- | --- | --- | --- | --- | --- | --- | --- |
| R.TLDFVVELSK.G | Y | 58.25 | 1149.6281 | 10 | 0.64 | 575.8204 | 2 | 19.63 | 0.8834 | 0.8907 | 75086 | 4.01e3 | 1 | 1 | 237 | 246 |  |  | DB Search |
| K.IGC(+57.02)FHDR.I | Y | 58.24 | 903.4021 | 7 | 6.62 | 452.7103 | 2 | 11.34 | 0.8014 | 0.8088 | 22951 | 0 | 0 | 0 | 33 | 39 | Carbamidomethylation |  | DB Search |
| K.YLLVFTDGR.S | Y | 54.93 | 1082.576 | 9 | -0.17 | 542.294 | 2 | 19.12 | 0.8684 | 0.8757 | 71808 | 1.15e3 | 1 | 1 | 324 | 332 |  |  | DB Search |
| K.DMGVNVISVGAGHTINHR.E | Y | 54.57 | 1875.9373 | 18 | -2.32 | 626.3169 | 3 | 15.47 | 0.8826 | 0.8899 | 48327 | 2.64e2 | 1 | 1 | 349 | 366 |  |  | DB Search |
| total 4 peptides |  |  |  |  |  |  |  |  |  |  |  |  |  |  |  |  |  |  |  |
| --- | --- | --- | --- | --- | --- | --- | --- | --- | --- | --- | --- | --- | --- | --- | --- | --- | --- | --- | --- |

Best Unique PSM (Scan Sample\_2\_A1\_1\_3640.d:75086, m/z=575.8204, z=2, RT=19.63, ppm=-1.56):


TRINITY\_DN36\_c0\_g1\_i4.p1

back to list

  

| Protein Coverage
| Supporting Peptides
| Best Unique PSM
|

Protein Coverage:

Supporting Peptides:

| Peptide | Uniq | -10lgP | Mass | Length | ppm | m/z | z | RT | 1/k0 Start | 1/k0 End | Precursor ID | Area Sample 4 | #Feature | #Feature Sample 4 | Start | End | PTM | AScore | Found By |
| --- | --- | --- | --- | --- | --- | --- | --- | --- | --- | --- | --- | --- | --- | --- | --- | --- | --- | --- | --- |
| R.LGGIEVTQGNC(+57.02)AR.K | Y | 58.36 | 1373.6721 | 13 | -2.89 | 687.8398 | 2 | 13.93 | 0.9662 | 0.9735 | 38726 | 2.09e3 | 1 | 1 | 58 | 70 | Carbamidomethylation |  | DB Search |
| K.GLPLAIGVR.D | Y | 57.12 | 894.565 | 9 | 0.27 | 448.2889 | 2 | 17.73 | 0.8124 | 0.8197 | 62574 | 3.52e3 | 1 | 1 | 32 | 40 |  |  | DB Search |
| total 2 peptides |  |  |  |  |  |  |  |  |  |  |  |  |  |  |  |  |  |  |  |
| --- | --- | --- | --- | --- | --- | --- | --- | --- | --- | --- | --- | --- | --- | --- | --- | --- | --- | --- | --- |

Best Unique PSM (Scan Sample\_2\_A1\_1\_3640.d:38726, m/z=687.8398, z=2, RT=13.93, ppm=-5.09):


TRINITY\_DN2356\_c0\_g1\_i1.p1

back to list

  

| Protein Coverage
| Supporting Peptides
| Best Unique PSM
|

Protein Coverage:

Supporting Peptides:

| Peptide | Uniq | -10lgP | Mass | Length | ppm | m/z | z | RT | 1/k0 Start | 1/k0 End | Precursor ID | Area Sample 4 | #Feature | #Feature Sample 4 | Start | End | PTM | AScore | Found By |
| --- | --- | --- | --- | --- | --- | --- | --- | --- | --- | --- | --- | --- | --- | --- | --- | --- | --- | --- | --- |
| K.HILPHLAR.F | Y | 58.04 | 955.5715 | 8 | 7.97 | 319.533 | 3 | 12.62 | 0.7029 | 0.7102 | 30779 | 0 | 0 | 0 | 23 | 30 |  |  | DB Search |
| R.SIFAGLPSLDNQNKPK.T | Y | 41.77 | 1727.9206 | 16 | -1.26 | 576.9788 | 3 | 18.14 | 0.8542 | 0.8615 | 65398 | 4.01e2 | 1 | 1 | 140 | 155 |  |  | DB Search |
| total 2 peptides |  |  |  |  |  |  |  |  |  |  |  |  |  |  |  |  |  |  |  |
| --- | --- | --- | --- | --- | --- | --- | --- | --- | --- | --- | --- | --- | --- | --- | --- | --- | --- | --- | --- |

Best Unique PSM (Scan Sample\_2\_A1\_1\_3640.d:30779, m/z=319.533, z=3, RT=12.62, ppm=5.77):


TRINITY\_DN477\_c0\_g1\_i9.p1

back to list

  

| Protein Coverage
| Supporting Peptides
| Best Unique PSM
|

Protein Coverage:

Supporting Peptides:

| Peptide | Uniq | -10lgP | Mass | Length | ppm | m/z | z | RT | 1/k0 Start | 1/k0 End | Precursor ID | Area Sample 4 | #Feature | #Feature Sample 4 | Start | End | PTM | AScore | Found By |
| --- | --- | --- | --- | --- | --- | --- | --- | --- | --- | --- | --- | --- | --- | --- | --- | --- | --- | --- | --- |
| R.IVTTDGVM(+15.99)IELK.A | Y | 58.39 | 1333.7163 | 12 | -0.18 | 667.8638 | 2 | 16.81 | 0.9707 | 0.9779 | 56545 | 2.41e3 | 1 | 1 | 261 | 272 | Oxidation (M) | M8:Oxidation (M):1000 | DB Search |
| R.TQAVLDRLNIEKR.V | Y | 58.39 | 1554.8842 | 13 | -0.15 | 519.3008 | 3 | 14.76 | 0.8075 | 0.8149 | 43833 | 7.97e3 | 1 | 1 | 220 | 232 |  |  | DB Search |
| K.MRFDTAITAC(+57.02)R.S | Y | 58.3 | 1340.6329 | 11 | 1.65 | 671.3234 | 2 | 15.12 | 0.9666 | 0.9739 | 46027 | 4.31e3 | 1 | 1 | 276 | 286 | Carbamidomethylation |  | DB Search |
| R.IVTTDGVMIELK.A | Y | 58.3 | 1317.7214 | 12 | -15.83 | 659.8561 | 2 | 17.84 | 0.94 | 0.9473 | 63519 | 2.16e4 | 3 | 3 | 261 | 272 |  |  | DB Search |
| K.DANSM(+15.99)PTQSR.Q | Y | 58.2 | 1121.4771 | 10 | 7.65 | 561.7489 | 2 | 8.79 | 0.8189 | 0.8262 | 9919 | 1.9e3 | 1 | 1 | 51 | 60 | Oxidation (M) | M5:Oxidation (M):1000 | DB Search |
| R.DM(+15.99)ITC(+57.02)NENPYITDDLFK.V | Y | 58.11 | 2103.9129 | 17 | 4.31 | 1052.9659 | 2 | 19.48 | 1.1945 | 1.2017 | 74139 | 7.17e2 | 1 | 1 | 236 | 252 | Oxidation (M), Carbamidomethylation | M2:Oxidation (M):1000 | DB Search |
| R.M(+15.99)TQLDQSK.A | Y | 55.4 | 965.4488 | 8 | 5.2 | 483.7331 | 2 | 9.84 | 0.8075 | 0.8149 | 14010 | 1.8e3 | 1 | 1 | 203 | 210 | Oxidation (M) | M1:Oxidation (M):1000 | DB Search |
| R.TQAVLDRLNIEK.R | Y | 55.19 | 1398.783 | 12 | 4.64 | 700.4005 | 2 | 15.56 | 1.0057 | 1.013 | 48737 | 1.68e4 | 2 | 2 | 220 | 231 |  |  | DB Search |
| R.FFQIKK.S | Y | 42.21 | 809.4799 | 6 | -0.5 | 405.7462 | 2 | 13.84 | 0.7498 | 0.7571 | 38133 | 4.89e2 | 1 | 1 | 298 | 303 |  |  | DB Search |
| total 9 peptides |  |  |  |  |  |  |  |  |  |  |  |  |  |  |  |  |  |  |  |
| --- | --- | --- | --- | --- | --- | --- | --- | --- | --- | --- | --- | --- | --- | --- | --- | --- | --- | --- | --- |

Best Unique PSM (Scan Sample\_2\_A1\_1\_3640.d:56545, m/z=667.8638, z=2, RT=16.81, ppm=-2.37):


TRINITY\_DN250\_c0\_g1\_i1.p1

back to list

  

| Protein Coverage
| Supporting Peptides
| Best Unique PSM
|

Protein Coverage:

Supporting Peptides:

| Peptide | Uniq | -10lgP | Mass | Length | ppm | m/z | z | RT | 1/k0 Start | 1/k0 End | Precursor ID | Area Sample 4 | #Feature | #Feature Sample 4 | Start | End | PTM | AScore | Found By |
| --- | --- | --- | --- | --- | --- | --- | --- | --- | --- | --- | --- | --- | --- | --- | --- | --- | --- | --- | --- |
| K.VGINYQPPTVVPGGDLAK.V | Y | 58.29 | 1823.9781 | 18 | -4.28 | 912.9904 | 2 | 17.44 | 1.1142 | 1.1214 | 60745 | 0 | 0 | 0 | 353 | 370 |  |  | DB Search |
| total 1 peptides |  |  |  |  |  |  |  |  |  |  |  |  |  |  |  |  |  |  |  |
| --- | --- | --- | --- | --- | --- | --- | --- | --- | --- | --- | --- | --- | --- | --- | --- | --- | --- | --- | --- |

Best Unique PSM (Scan Sample\_2\_A1\_1\_3640.d:60745, m/z=912.9904, z=2, RT=17.44, ppm=-6.48):


TRINITY\_DN557\_c0\_g1\_i5.p1

back to list

  

| Protein Coverage
| Supporting Peptides
| Best Unique PSM
|

Protein Coverage:

Supporting Peptides:

| Peptide | Uniq | -10lgP | Mass | Length | ppm | m/z | z | RT | 1/k0 Start | 1/k0 End | Precursor ID | Area Sample 4 | #Feature | #Feature Sample 4 | Start | End | PTM | AScore | Found By |
| --- | --- | --- | --- | --- | --- | --- | --- | --- | --- | --- | --- | --- | --- | --- | --- | --- | --- | --- | --- |
| K.EAIVEAIEDGANTSK.E | Y | 55.85 | 1545.7522 | 15 | 1.08 | 773.8825 | 2 | 17.30 | 1.1049 | 1.1122 | 59776 | 2.04e2 | 1 | 1 | 116 | 130 |  |  | DB Search |
| total 1 peptides |  |  |  |  |  |  |  |  |  |  |  |  |  |  |  |  |  |  |  |
| --- | --- | --- | --- | --- | --- | --- | --- | --- | --- | --- | --- | --- | --- | --- | --- | --- | --- | --- | --- |

Best Unique PSM (Scan Sample\_2\_A1\_1\_3640.d:59776, m/z=773.8825, z=2, RT=17.30, ppm=-1.12):


TRINITY\_DN6920\_c0\_g1\_i2.p1

back to list

  

| Protein Coverage
| Supporting Peptides
| Best Unique PSM
|

Protein Coverage:

Supporting Peptides:

| Peptide | Uniq | -10lgP | Mass | Length | ppm | m/z | z | RT | 1/k0 Start | 1/k0 End | Precursor ID | Area Sample 4 | #Feature | #Feature Sample 4 | Start | End | PTM | AScore | Found By |
| --- | --- | --- | --- | --- | --- | --- | --- | --- | --- | --- | --- | --- | --- | --- | --- | --- | --- | --- | --- |
| R.VLVHTQQK.L | Y | 58.19 | 951.5501 | 8 | 0.78 | 476.7817 | 2 | 9.51 | 0.8335 | 0.8408 | 12449 | 5.52e2 | 1 | 1 | 169 | 176 |  |  | DB Search |
| R.KVAC(+57.02)IGAWHPAR.V | Y | 52.34 | 1364.7135 | 12 | 0.83 | 455.9112 | 3 | 13.48 | 0.7766 | 0.784 | 36007 | 9.51e1 | 1 | 1 | 257 | 268 | Carbamidomethylation |  | DB Search |
| total 2 peptides |  |  |  |  |  |  |  |  |  |  |  |  |  |  |  |  |  |  |  |
| --- | --- | --- | --- | --- | --- | --- | --- | --- | --- | --- | --- | --- | --- | --- | --- | --- | --- | --- | --- |

Best Unique PSM (Scan Sample\_2\_A1\_1\_3640.d:12449, m/z=476.7817, z=2, RT=9.51, ppm=-1.41):


TRINITY\_DN3434\_c0\_g1\_i5.p1

back to list

  

| Protein Coverage
| Supporting Peptides
| Best Unique PSM
|

Protein Coverage:

Supporting Peptides:

| Peptide | Uniq | -10lgP | Mass | Length | ppm | m/z | z | RT | 1/k0 Start | 1/k0 End | Precursor ID | Area Sample 4 | #Feature | #Feature Sample 4 | Start | End | PTM | AScore | Found By |
| --- | --- | --- | --- | --- | --- | --- | --- | --- | --- | --- | --- | --- | --- | --- | --- | --- | --- | --- | --- |
| R.FTQAGSEVSALLGR.I | Y | 48.14 | 1434.7467 | 14 | 5.7 | 718.3831 | 2 | 18.43 | 0.986 | 0.9932 | 67409 | 0 | 0 | 0 | 274 | 287 |  |  | DB Search |
| total 1 peptides |  |  |  |  |  |  |  |  |  |  |  |  |  |  |  |  |  |  |  |
| --- | --- | --- | --- | --- | --- | --- | --- | --- | --- | --- | --- | --- | --- | --- | --- | --- | --- | --- | --- |

Best Unique PSM (Scan Sample\_2\_A1\_1\_3640.d:67409, m/z=718.3831, z=2, RT=18.43, ppm=3.5):


TRINITY\_DN45008\_c0\_g1\_i1.p1

back to list

  

| Protein Coverage
| Supporting Peptides
| Best Unique PSM
|

Protein Coverage:

Supporting Peptides:

| Peptide | Uniq | -10lgP | Mass | Length | ppm | m/z | z | RT | 1/k0 Start | 1/k0 End | Precursor ID | Area Sample 4 | #Feature | #Feature Sample 4 | Start | End | PTM | AScore | Found By |
| --- | --- | --- | --- | --- | --- | --- | --- | --- | --- | --- | --- | --- | --- | --- | --- | --- | --- | --- | --- |
| K.VAVDSLNLNILK.G | Y | 58.34 | 1297.7605 | 12 | -2.45 | 649.8845 | 2 | 19.54 | 0.9622 | 0.9695 | 74618 | 0 | 0 | 0 | 249 | 260 |  |  | DB Search |
| total 1 peptides |  |  |  |  |  |  |  |  |  |  |  |  |  |  |  |  |  |  |  |
| --- | --- | --- | --- | --- | --- | --- | --- | --- | --- | --- | --- | --- | --- | --- | --- | --- | --- | --- | --- |

Best Unique PSM (Scan Sample\_2\_A1\_1\_3640.d:74618, m/z=649.8845, z=2, RT=19.54, ppm=-4.64):


TRINITY\_DN82766\_c0\_g1\_i1.p1

back to list

  

| Protein Coverage
| Supporting Peptides
| Best Unique PSM
|

Protein Coverage:

Supporting Peptides:

| Peptide | Uniq | -10lgP | Mass | Length | ppm | m/z | z | RT | 1/k0 Start | 1/k0 End | Precursor ID | Area Sample 4 | #Feature | #Feature Sample 4 | Start | End | PTM | AScore | Found By |
| --- | --- | --- | --- | --- | --- | --- | --- | --- | --- | --- | --- | --- | --- | --- | --- | --- | --- | --- | --- |
| R.GSSYLGDIALDDISVK.Y | Y | 57.01 | 1651.8304 | 16 | 3.59 | 826.9236 | 2 | 19.81 | 1.0777 | 1.0849 | 76261 | 7.88e2 | 1 | 1 | 7 | 22 |  |  | DB Search |
| total 1 peptides |  |  |  |  |  |  |  |  |  |  |  |  |  |  |  |  |  |  |  |
| --- | --- | --- | --- | --- | --- | --- | --- | --- | --- | --- | --- | --- | --- | --- | --- | --- | --- | --- | --- |

Best Unique PSM (Scan Sample\_2\_A1\_1\_3640.d:76261, m/z=826.9236, z=2, RT=19.81, ppm=1.39):


TRINITY\_DN557\_c0\_g1\_i3.p1

back to list

  

| Protein Coverage
| Supporting Peptides
| Best Unique PSM
|

Protein Coverage:

Supporting Peptides:

| Peptide | Uniq | -10lgP | Mass | Length | ppm | m/z | z | RT | 1/k0 Start | 1/k0 End | Precursor ID | Area Sample 4 | #Feature | #Feature Sample 4 | Start | End | PTM | AScore | Found By |
| --- | --- | --- | --- | --- | --- | --- | --- | --- | --- | --- | --- | --- | --- | --- | --- | --- | --- | --- | --- |
| K.EAIVEAIEDGANTSK.E | Y | 55.85 | 1545.7522 | 15 | 1.08 | 773.8825 | 2 | 17.30 | 1.1049 | 1.1122 | 59776 | 2.04e2 | 1 | 1 | 116 | 130 |  |  | DB Search |
| total 1 peptides |  |  |  |  |  |  |  |  |  |  |  |  |  |  |  |  |  |  |  |
| --- | --- | --- | --- | --- | --- | --- | --- | --- | --- | --- | --- | --- | --- | --- | --- | --- | --- | --- | --- |

Best Unique PSM (Scan Sample\_2\_A1\_1\_3640.d:59776, m/z=773.8825, z=2, RT=17.30, ppm=-1.12):


TRINITY\_DN1280\_c0\_g1\_i1.p1

back to list

  

| Protein Coverage
| Supporting Peptides
| Best Unique PSM
|

Protein Coverage:

Supporting Peptides:

| Peptide | Uniq | -10lgP | Mass | Length | ppm | m/z | z | RT | 1/k0 Start | 1/k0 End | Precursor ID | Area Sample 4 | #Feature | #Feature Sample 4 | Start | End | PTM | AScore | Found By |
| --- | --- | --- | --- | --- | --- | --- | --- | --- | --- | --- | --- | --- | --- | --- | --- | --- | --- | --- | --- |
| R.AQAINVQLQEIQR.R | Y | 53.32 | 1509.8263 | 13 | 1.6 | 755.92 | 2 | 16.19 | 1.0295 | 1.0367 | 52841 | 0 | 0 | 0 | 263 | 275 |  |  | DB Search |
| total 1 peptides |  |  |  |  |  |  |  |  |  |  |  |  |  |  |  |  |  |  |  |
| --- | --- | --- | --- | --- | --- | --- | --- | --- | --- | --- | --- | --- | --- | --- | --- | --- | --- | --- | --- |

Best Unique PSM (Scan Sample\_2\_A1\_1\_3640.d:52841, m/z=755.92, z=2, RT=16.19, ppm=-0.6):


TRINITY\_DN101\_c0\_g1\_i1.p1

back to list

  

| Protein Coverage
| Supporting Peptides
| Best Unique PSM
|

Protein Coverage:

Supporting Peptides:

| Peptide | Uniq | -10lgP | Mass | Length | ppm | m/z | z | RT | 1/k0 Start | 1/k0 End | Precursor ID | Area Sample 4 | #Feature | #Feature Sample 4 | Start | End | PTM | AScore | Found By |
| --- | --- | --- | --- | --- | --- | --- | --- | --- | --- | --- | --- | --- | --- | --- | --- | --- | --- | --- | --- |
| MQIFVK.T | Y | 57.42 | 764.4255 | 6 | 0.18 | 383.2192 | 2 | 15.73 | 0.7363 | 0.7437 | 49899 | 2.46e2 | 1 | 1 | 1 | 6 |  |  | DB Search |
| K.ESTLHLVLR.L | Y | 57.27 | 1066.6135 | 9 | 2.57 | 534.3142 | 2 | 15.80 | 0.8785 | 0.8858 | 50332 | 4.97e3 | 1 | 1 | 64 | 72 |  |  | DB Search |
| K.IQDKEGIPPDQQR.L | Y | 55.01 | 1522.7739 | 13 | 0.25 | 762.3928 | 2 | 11.08 | 1.0343 | 1.0415 | 21018 | 1.78e3 | 2 | 2 | 30 | 42 |  |  | DB Search |
| K.TITLEVEPSDTIENVK.A | Y | 54.56 | 1786.92 | 16 | 4.26 | 894.4691 | 2 | 17.62 | 1.1442 | 1.1514 | 61769 | 1.48e4 | 1 | 1 | 12 | 27 |  |  | DB Search |
| total 4 peptides |  |  |  |  |  |  |  |  |  |  |  |  |  |  |  |  |  |  |  |
| --- | --- | --- | --- | --- | --- | --- | --- | --- | --- | --- | --- | --- | --- | --- | --- | --- | --- | --- | --- |

Best Unique PSM (Scan Sample\_2\_A1\_1\_3640.d:49899, m/z=383.2192, z=2, RT=15.73, ppm=-2.02):


TRINITY\_DN15542\_c0\_g1\_i2.p1

back to list

  

| Protein Coverage
| Supporting Peptides
| Best Unique PSM
|

Protein Coverage:

Supporting Peptides:

| Peptide | Uniq | -10lgP | Mass | Length | ppm | m/z | z | RT | 1/k0 Start | 1/k0 End | Precursor ID | Area Sample 4 | #Feature | #Feature Sample 4 | Start | End | PTM | AScore | Found By |
| --- | --- | --- | --- | --- | --- | --- | --- | --- | --- | --- | --- | --- | --- | --- | --- | --- | --- | --- | --- |
| K.VIGHGTIYGEEVTLNLR.E | Y | 58.24 | 1869.9948 | 17 | 4.33 | 624.3402 | 3 | 17.56 | 0.919 | 0.9263 | 61629 | 0 | 0 | 0 | 80 | 96 |  |  | DB Search |
| total 1 peptides |  |  |  |  |  |  |  |  |  |  |  |  |  |  |  |  |  |  |  |
| --- | --- | --- | --- | --- | --- | --- | --- | --- | --- | --- | --- | --- | --- | --- | --- | --- | --- | --- | --- |

Best Unique PSM (Scan Sample\_2\_A1\_1\_3640.d:61629, m/z=624.3402, z=3, RT=17.56, ppm=2.13):


TRINITY\_DN86076\_c0\_g1\_i1.p1

back to list

  

| Protein Coverage
| Supporting Peptides
| Best Unique PSM
|

Protein Coverage:

Supporting Peptides:

| Peptide | Uniq | -10lgP | Mass | Length | ppm | m/z | z | RT | 1/k0 Start | 1/k0 End | Precursor ID | Area Sample 4 | #Feature | #Feature Sample 4 | Start | End | PTM | AScore | Found By |
| --- | --- | --- | --- | --- | --- | --- | --- | --- | --- | --- | --- | --- | --- | --- | --- | --- | --- | --- | --- |
| K.DAQDQELEIKR.D | Y | 57 | 1343.6681 | 11 | 0.37 | 672.8401 | 2 | 12.83 | 0.9501 | 0.9574 | 32029 | 0 | 0 | 0 | 22 | 32 |  |  | DB Search |
| total 1 peptides |  |  |  |  |  |  |  |  |  |  |  |  |  |  |  |  |  |  |  |
| --- | --- | --- | --- | --- | --- | --- | --- | --- | --- | --- | --- | --- | --- | --- | --- | --- | --- | --- | --- |

Best Unique PSM (Scan Sample\_2\_A1\_1\_3640.d:32029, m/z=672.8401, z=2, RT=12.83, ppm=-1.83):


TRINITY\_DN2477\_c0\_g1\_i9.p1

back to list

  

| Protein Coverage
| Supporting Peptides
| Best Unique PSM
|

Protein Coverage:

Supporting Peptides:

| Peptide | Uniq | -10lgP | Mass | Length | ppm | m/z | z | RT | 1/k0 Start | 1/k0 End | Precursor ID | Area Sample 4 | #Feature | #Feature Sample 4 | Start | End | PTM | AScore | Found By |
| --- | --- | --- | --- | --- | --- | --- | --- | --- | --- | --- | --- | --- | --- | --- | --- | --- | --- | --- | --- |
| K.DSYVGDEAQSKR.G | Y | 57.28 | 1353.616 | 12 | 2.57 | 677.8156 | 2 | 11.01 | 0.9993 | 1.0065 | 20794 | 1.16e3 | 1 | 1 | 52 | 63 |  |  | DB Search |
| total 1 peptides |  |  |  |  |  |  |  |  |  |  |  |  |  |  |  |  |  |  |  |
| --- | --- | --- | --- | --- | --- | --- | --- | --- | --- | --- | --- | --- | --- | --- | --- | --- | --- | --- | --- |

Best Unique PSM (Scan Sample\_2\_A1\_1\_3640.d:20794, m/z=677.8156, z=2, RT=11.01, ppm=0.37):


TRINITY\_DN5584\_c0\_g1\_i1.p1

back to list

  

| Protein Coverage
| Supporting Peptides
| Best Unique PSM
|

Protein Coverage:

Supporting Peptides:

| Peptide | Uniq | -10lgP | Mass | Length | ppm | m/z | z | RT | 1/k0 Start | 1/k0 End | Precursor ID | Area Sample 4 | #Feature | #Feature Sample 4 | Start | End | PTM | AScore | Found By |
| --- | --- | --- | --- | --- | --- | --- | --- | --- | --- | --- | --- | --- | --- | --- | --- | --- | --- | --- | --- |
| K.GSLSGGLEGFYK.S | Y | 58.39 | 1213.5979 | 12 | 3.01 | 607.8067 | 2 | 17.60 | 0.9073 | 0.9146 | 61815 | 0 | 0 | 0 | 192 | 203 |  |  | DB Search |
| K.AKNQALEIFK.D | Y | 58.38 | 1160.6553 | 10 | -0.5 | 581.3334 | 2 | 15.99 | 0.9158 | 0.923 | 51477 | 0 | 0 | 0 | 761 | 770 |  |  | DB Search |
| K.VTLTQTHFLKDQDKPAK.S | Y | 58.27 | 1969.0632 | 17 | 3.13 | 657.3623 | 3 | 13.38 | 0.9113 | 0.9186 | 35272 | 5.96e2 | 2 | 2 | 559 | 575 |  |  | DB Search |
| K.VTLTQTHFLK.D | Y | 54.93 | 1186.671 | 10 | -0.58 | 594.3411 | 2 | 15.07 | 0.9646 | 0.9719 | 45810 | 6.69e2 | 1 | 1 | 559 | 568 |  |  | DB Search |
| R.LPGNVIPIK.Y | Y | 54.32 | 949.596 | 9 | 3.06 | 475.8057 | 2 | 16.31 | 0.8214 | 0.8287 | 53518 | 3.4e2 | 1 | 1 | 82 | 90 |  |  | DB Search |
| R.DGEKVTLTQTHFLK.D | Y | 53.73 | 1615.8569 | 14 | -13.39 | 539.6179 | 3 | 14.95 | 0.8534 | 0.8607 | 45082 | 0 | 0 | 0 | 555 | 568 |  |  | DB Search |
| K.YLSWTLDNDKIR.S | Y | 53.6 | 1522.778 | 12 | -3.19 | 508.5972 | 3 | 17.66 | 0.8063 | 0.8136 | 62168 | 3.04e2 | 1 | 1 | 835 | 846 |  |  | DB Search |
| R.AAFPC(+57.02)FDEPAMK.A | Y | 53.17 | 1382.5999 | 12 | -3.57 | 692.3032 | 2 | 18.01 | 0.9675 | 0.9747 | 64265 | 9.62e2 | 1 | 1 | 228 | 239 | Carbamidomethylation |  | DB Search |
| total 8 peptides |  |  |  |  |  |  |  |  |  |  |  |  |  |  |  |  |  |  |  |
| --- | --- | --- | --- | --- | --- | --- | --- | --- | --- | --- | --- | --- | --- | --- | --- | --- | --- | --- | --- |

Best Unique PSM (Scan Sample\_2\_A1\_1\_3640.d:61815, m/z=607.8067, z=2, RT=17.60, ppm=0.81):


TRINITY\_DN250\_c0\_g1\_i4.p1

back to list

  

| Protein Coverage
| Supporting Peptides
| Best Unique PSM
|

Protein Coverage:

Supporting Peptides:

| Peptide | Uniq | -10lgP | Mass | Length | ppm | m/z | z | RT | 1/k0 Start | 1/k0 End | Precursor ID | Area Sample 4 | #Feature | #Feature Sample 4 | Start | End | PTM | AScore | Found By |
| --- | --- | --- | --- | --- | --- | --- | --- | --- | --- | --- | --- | --- | --- | --- | --- | --- | --- | --- | --- |
| K.VGINYQPPTVVPGGDLAK.V | Y | 58.29 | 1823.9781 | 18 | -4.28 | 912.9904 | 2 | 17.44 | 1.1142 | 1.1214 | 60745 | 0 | 0 | 0 | 353 | 370 |  |  | DB Search |
| total 1 peptides |  |  |  |  |  |  |  |  |  |  |  |  |  |  |  |  |  |  |  |
| --- | --- | --- | --- | --- | --- | --- | --- | --- | --- | --- | --- | --- | --- | --- | --- | --- | --- | --- | --- |

Best Unique PSM (Scan Sample\_2\_A1\_1\_3640.d:60745, m/z=912.9904, z=2, RT=17.44, ppm=-6.48):


TRINITY\_DN2433\_c0\_g1\_i1.p1

back to list

  

| Protein Coverage
| Supporting Peptides
| Best Unique PSM
|

Protein Coverage:

Supporting Peptides:

| Peptide | Uniq | -10lgP | Mass | Length | ppm | m/z | z | RT | 1/k0 Start | 1/k0 End | Precursor ID | Area Sample 4 | #Feature | #Feature Sample 4 | Start | End | PTM | AScore | Found By |
| --- | --- | --- | --- | --- | --- | --- | --- | --- | --- | --- | --- | --- | --- | --- | --- | --- | --- | --- | --- |
| R.KVNMPISIIALNHR.K | Y | 37.77 | 1604.9184 | 14 | 6.56 | 535.9824 | 3 | 17.41 | 0.8441 | 0.8514 | 60598 | 0 | 0 | 0 | 131 | 144 |  |  | DB Search |
| total 1 peptides |  |  |  |  |  |  |  |  |  |  |  |  |  |  |  |  |  |  |  |
| --- | --- | --- | --- | --- | --- | --- | --- | --- | --- | --- | --- | --- | --- | --- | --- | --- | --- | --- | --- |

Best Unique PSM (Scan Sample\_2\_A1\_1\_3640.d:60598, m/z=535.9824, z=3, RT=17.41, ppm=4.36):


TRINITY\_DN1628\_c0\_g1\_i2.p1

back to list

  

| Protein Coverage
| Supporting Peptides
| Best Unique PSM
|

Protein Coverage:

Supporting Peptides:

| Peptide | Uniq | -10lgP | Mass | Length | ppm | m/z | z | RT | 1/k0 Start | 1/k0 End | Precursor ID | Area Sample 4 | #Feature | #Feature Sample 4 | Start | End | PTM | AScore | Found By |
| --- | --- | --- | --- | --- | --- | --- | --- | --- | --- | --- | --- | --- | --- | --- | --- | --- | --- | --- | --- |
| R.LTGGEELIGFKPLIK.Y | Y | 39.32 | 1613.9392 | 15 | 6.16 | 538.9891 | 3 | 18.85 | 0.8887 | 0.8959 | 70137 | 3.72e2 | 1 | 1 | 447 | 461 |  |  | DB Search |
| total 1 peptides |  |  |  |  |  |  |  |  |  |  |  |  |  |  |  |  |  |  |  |
| --- | --- | --- | --- | --- | --- | --- | --- | --- | --- | --- | --- | --- | --- | --- | --- | --- | --- | --- | --- |

Best Unique PSM (Scan Sample\_2\_A1\_1\_3640.d:70137, m/z=538.9891, z=3, RT=18.85, ppm=3.96):


TRINITY\_DN1366\_c0\_g1\_i1.p1

back to list

  

| Protein Coverage
| Supporting Peptides
| Best Unique PSM
|

Protein Coverage:

Supporting Peptides:

| Peptide | Uniq | -10lgP | Mass | Length | ppm | m/z | z | RT | 1/k0 Start | 1/k0 End | Precursor ID | Area Sample 4 | #Feature | #Feature Sample 4 | Start | End | PTM | AScore | Found By |
| --- | --- | --- | --- | --- | --- | --- | --- | --- | --- | --- | --- | --- | --- | --- | --- | --- | --- | --- | --- |
| K.ILALAGQLQSARDELVK.K | Y | 58.41 | 1824.0468 | 17 | 2.89 | 609.0233 | 3 | 18.84 | 0.904 | 0.9113 | 69982 | 1.41e3 | 1 | 1 | 1580 | 1596 |  |  | DB Search |
| K.LNNEINDLHQR.L | Y | 58.39 | 1364.6797 | 11 | -0.04 | 683.3456 | 2 | 12.69 | 0.9949 | 1.0021 | 31202 | 0 | 0 | 0 | 3176 | 3186 |  |  | DB Search |
| R.DVIIDSGDESKR.D | Y | 58.29 | 1332.6521 | 12 | 2.92 | 667.3338 | 2 | 12.51 | 0.9876 | 0.9949 | 30033 | 6.02e2 | 1 | 1 | 2032 | 2043 |  |  | DB Search |
| K.LSNTETELQSIR.H | Y | 58.27 | 1389.71 | 12 | 3.32 | 695.863 | 2 | 15.17 | 0.9795 | 0.9868 | 46436 | 0 | 0 | 0 | 1412 | 1423 |  |  | DB Search |
| R.LNEELISFNSK.Y | Y | 58.26 | 1292.6612 | 11 | 2.31 | 647.338 | 2 | 17.35 | 0.938 | 0.9453 | 60098 | 6.81e2 | 1 | 1 | 1639 | 1649 |  |  | DB Search |
| R.LLNEIENLR.N | Y | 56.78 | 1112.6189 | 9 | -2.59 | 557.3141 | 2 | 17.15 | 0.8919 | 0.8992 | 58964 | 0 | 0 | 0 | 3372 | 3380 |  |  | DB Search |
| K.KTTLEGEIESLKR.D | Y | 56.56 | 1502.8304 | 13 | 1.66 | 501.9505 | 3 | 16.19 | 0.7994 | 0.8067 | 52735 | 5.81e2 | 1 | 1 | 3745 | 3757 |  |  | DB Search |
| K.ILALAGQLQSAR.D | Y | 54.88 | 1239.7299 | 12 | 5.1 | 620.874 | 2 | 16.91 | 0.9675 | 0.9747 | 57298 | 9.69e2 | 1 | 1 | 1580 | 1591 |  |  | DB Search |
| K.LVEVETQLKR.S | Y | 54.77 | 1213.703 | 10 | 4.99 | 607.8605 | 2 | 13.63 | 0.9501 | 0.9574 | 36929 | 7.45e2 | 1 | 1 | 2224 | 2233 |  |  | DB Search |
| K.LQIIVGDLR.A | Y | 54.74 | 1025.6233 | 9 | 1.09 | 513.8184 | 2 | 18.10 | 0.8672 | 0.8745 | 65042 | 2.27e3 | 1 | 1 | 2850 | 2858 |  |  | DB Search |
| R.DLADRDHVIEELER.E | Y | 54.67 | 1708.838 | 14 | -0.7 | 570.6183 | 3 | 16.26 | 0.8343 | 0.8416 | 53234 | 0 | 0 | 0 | 3464 | 3477 |  |  | DB Search |
| K.LEPQLQQILR.E | Y | 54.46 | 1236.719 | 10 | -0.59 | 619.3651 | 2 | 18.13 | 0.9206 | 0.9279 | 65310 | 6.16e2 | 1 | 1 | 1681 | 1690 |  |  | DB Search |
| K.LGDLESANQILAMEK.E | Y | 53.43 | 1630.8236 | 15 | 4.51 | 816.421 | 2 | 18.64 | 1.06 | 1.0672 | 68833 | 4.12e2 | 1 | 1 | 1622 | 1636 |  |  | DB Search |
| K.LGDLESANQILAM(+15.99)EK.E | Y | 52.58 | 1646.8185 | 15 | 6.24 | 824.4199 | 2 | 17.53 | 1.0552 | 1.0624 | 61340 | 0 | 0 | 0 | 1622 | 1636 | Oxidation (M) | M13:Oxidation (M):1000 | DB Search |
| K.ILALAGQLQSARDELVKK.Q | Y | 52.09 | 1952.1418 | 18 | -4.21 | 651.717 | 3 | 18.01 | 0.9194 | 0.9267 | 64607 | 0 | 0 | 0 | 1580 | 1597 |  |  | DB Search |
| K.KQQENEQLLDEKR.L | Y | 51.65 | 1656.8431 | 13 | -0.41 | 553.2869 | 3 | 11.55 | 0.8254 | 0.8327 | 24229 | 0 | 0 | 0 | 1597 | 1609 |  |  | DB Search |
| R.ELHNLEQENQQLK.A | Y | 42.32 | 1621.806 | 13 | 1.72 | 541.609 | 3 | 12.99 | 0.8063 | 0.8136 | 33004 | 0 | 0 | 0 | 2909 | 2921 |  |  | DB Search |
| K.SEQDRLESQR.Q | Y | 38.76 | 1246.5902 | 10 | 0.63 | 416.5367 | 3 | 10.63 | 0.7249 | 0.7322 | 18636 | 0 | 0 | 0 | 1660 | 1669 |  |  | DB Search |
| total 18 peptides |  |  |  |  |  |  |  |  |  |  |  |  |  |  |  |  |  |  |  |
| --- | --- | --- | --- | --- | --- | --- | --- | --- | --- | --- | --- | --- | --- | --- | --- | --- | --- | --- | --- |

Best Unique PSM (Scan Sample\_2\_A1\_1\_3640.d:69982, m/z=609.0233, z=3, RT=18.84, ppm=0.7):


TRINITY\_DN5\_c0\_g1\_i18.p3

back to list

  

| Protein Coverage
| Supporting Peptides
| Best Unique PSM
|

Protein Coverage:

Supporting Peptides:

| Peptide | Uniq | -10lgP | Mass | Length | ppm | m/z | z | RT | 1/k0 Start | 1/k0 End | Precursor ID | Area Sample 4 | #Feature | #Feature Sample 4 | Start | End | PTM | AScore | Found By |
| --- | --- | --- | --- | --- | --- | --- | --- | --- | --- | --- | --- | --- | --- | --- | --- | --- | --- | --- | --- |
| K.IIIELQGDLIK.I | Y | 53.27 | 1253.7594 | 11 | 3.89 | 627.8881 | 2 | 19.42 | 0.9513 | 0.9586 | 73842 | 0 | 0 | 0 | 65 | 75 |  |  | DB Search |
| total 1 peptides |  |  |  |  |  |  |  |  |  |  |  |  |  |  |  |  |  |  |  |
| --- | --- | --- | --- | --- | --- | --- | --- | --- | --- | --- | --- | --- | --- | --- | --- | --- | --- | --- | --- |

Best Unique PSM (Scan Sample\_2\_A1\_1\_3640.d:73842, m/z=627.8881, z=2, RT=19.42, ppm=1.69):


TRINITY\_DN15584\_c0\_g1\_i1.p1

back to list

  

| Protein Coverage
| Supporting Peptides
| Best Unique PSM
|

Protein Coverage:

Supporting Peptides:

| Peptide | Uniq | -10lgP | Mass | Length | ppm | m/z | z | RT | 1/k0 Start | 1/k0 End | Precursor ID | Area Sample 4 | #Feature | #Feature Sample 4 | Start | End | PTM | AScore | Found By |
| --- | --- | --- | --- | --- | --- | --- | --- | --- | --- | --- | --- | --- | --- | --- | --- | --- | --- | --- | --- |
| K.AFVAIGDYGGHVGLGVK.C | Y | 58.39 | 1658.878 | 17 | 1.44 | 553.9662 | 3 | 17.82 | 0.8526 | 0.8599 | 63242 | 9.03e2 | 2 | 2 | 109 | 125 |  |  | DB Search |
| total 1 peptides |  |  |  |  |  |  |  |  |  |  |  |  |  |  |  |  |  |  |  |
| --- | --- | --- | --- | --- | --- | --- | --- | --- | --- | --- | --- | --- | --- | --- | --- | --- | --- | --- | --- |

Best Unique PSM (Scan Sample\_2\_A1\_1\_3640.d:63242, m/z=553.9662, z=3, RT=17.82, ppm=-0.75):


TRINITY\_DN598\_c1\_g1\_i1.p1

back to list

  

| Protein Coverage
| Supporting Peptides
| Best Unique PSM
|

Protein Coverage:

Supporting Peptides:

| Peptide | Uniq | -10lgP | Mass | Length | ppm | m/z | z | RT | 1/k0 Start | 1/k0 End | Precursor ID | Area Sample 4 | #Feature | #Feature Sample 4 | Start | End | PTM | AScore | Found By |
| --- | --- | --- | --- | --- | --- | --- | --- | --- | --- | --- | --- | --- | --- | --- | --- | --- | --- | --- | --- |
| R.LKDLENFLK.V | Y | 55.28 | 1118.6335 | 9 | -1.2 | 560.3221 | 2 | 17.44 | 0.8968 | 0.904 | 60643 | 4.2e3 | 2 | 2 | 169 | 177 |  |  | DB Search |
| R.ASFVSDIDRLR.A | Y | 55.08 | 1277.6728 | 11 | 3.1 | 639.8442 | 2 | 17.17 | 0.9896 | 0.9969 | 59018 | 6.66e2 | 1 | 1 | 302 | 312 |  |  | DB Search |
| K.AVAEEFISVDPYVR.S | Y | 54.94 | 1593.8038 | 14 | 3.64 | 797.9103 | 2 | 19.17 | 1.0532 | 1.0604 | 72128 | 7.25e3 | 1 | 1 | 148 | 161 |  |  | DB Search |
| total 3 peptides |  |  |  |  |  |  |  |  |  |  |  |  |  |  |  |  |  |  |  |
| --- | --- | --- | --- | --- | --- | --- | --- | --- | --- | --- | --- | --- | --- | --- | --- | --- | --- | --- | --- |

Best Unique PSM (Scan Sample\_2\_A1\_1\_3640.d:60643, m/z=560.3221, z=2, RT=17.44, ppm=-3.39):


TRINITY\_DN250\_c0\_g1\_i3.p1

back to list

  

| Protein Coverage
| Supporting Peptides
| Best Unique PSM
|

Protein Coverage:

Supporting Peptides:

| Peptide | Uniq | -10lgP | Mass | Length | ppm | m/z | z | RT | 1/k0 Start | 1/k0 End | Precursor ID | Area Sample 4 | #Feature | #Feature Sample 4 | Start | End | PTM | AScore | Found By |
| --- | --- | --- | --- | --- | --- | --- | --- | --- | --- | --- | --- | --- | --- | --- | --- | --- | --- | --- | --- |
| K.VGINYQPPTVVPGGDLAK.V | Y | 58.29 | 1823.9781 | 18 | -4.28 | 912.9904 | 2 | 17.44 | 1.1142 | 1.1214 | 60745 | 0 | 0 | 0 | 353 | 370 |  |  | DB Search |
| total 1 peptides |  |  |  |  |  |  |  |  |  |  |  |  |  |  |  |  |  |  |  |
| --- | --- | --- | --- | --- | --- | --- | --- | --- | --- | --- | --- | --- | --- | --- | --- | --- | --- | --- | --- |

Best Unique PSM (Scan Sample\_2\_A1\_1\_3640.d:60745, m/z=912.9904, z=2, RT=17.44, ppm=-6.48):


TRINITY\_DN3434\_c0\_g1\_i10.p1

back to list

  

| Protein Coverage
| Supporting Peptides
| Best Unique PSM
|

Protein Coverage:

Supporting Peptides:

| Peptide | Uniq | -10lgP | Mass | Length | ppm | m/z | z | RT | 1/k0 Start | 1/k0 End | Precursor ID | Area Sample 4 | #Feature | #Feature Sample 4 | Start | End | PTM | AScore | Found By |
| --- | --- | --- | --- | --- | --- | --- | --- | --- | --- | --- | --- | --- | --- | --- | --- | --- | --- | --- | --- |
| R.FTQAGSEVSALLGR.I | Y | 48.14 | 1434.7467 | 14 | 5.7 | 718.3831 | 2 | 18.43 | 0.986 | 0.9932 | 67409 | 0 | 0 | 0 | 274 | 287 |  |  | DB Search |
| total 1 peptides |  |  |  |  |  |  |  |  |  |  |  |  |  |  |  |  |  |  |  |
| --- | --- | --- | --- | --- | --- | --- | --- | --- | --- | --- | --- | --- | --- | --- | --- | --- | --- | --- | --- |

Best Unique PSM (Scan Sample\_2\_A1\_1\_3640.d:67409, m/z=718.3831, z=2, RT=18.43, ppm=3.5):


TRINITY\_DN113\_c0\_g1\_i1.p1

back to list

  

| Protein Coverage
| Supporting Peptides
| Best Unique PSM
|

Protein Coverage:

Supporting Peptides:

| Peptide | Uniq | -10lgP | Mass | Length | ppm | m/z | z | RT | 1/k0 Start | 1/k0 End | Precursor ID | Area Sample 4 | #Feature | #Feature Sample 4 | Start | End | PTM | AScore | Found By |
| --- | --- | --- | --- | --- | --- | --- | --- | --- | --- | --- | --- | --- | --- | --- | --- | --- | --- | --- | --- |
| R.GLAGPTGQR.G | Y | 58.14 | 855.4562 | 9 | 5.02 | 428.7366 | 2 | 10.98 | 0.7506 | 0.7579 | 20698 | 6.08e3 | 1 | 1 | 546 | 554 |  |  | DB Search |
| K.DGETGEQGPQGQEGPTGPR.G | Y | 57.4 | 1895.8245 | 19 | 11.29 | 948.9282 | 2 | 11.41 | 1.0721 | 1.0793 | 23364 | 5.54e2 | 1 | 1 | 695 | 713 |  |  | DB Search |
| R.GKDGETGEQGPQGQEGPTGPR.G | Y | 55.49 | 2080.9409 | 21 | 4.28 | 694.6557 | 3 | 10.78 | 0.851 | 0.8583 | 19197 | 5.2e3 | 1 | 1 | 693 | 713 |  |  | DB Search |
| K.RGEMGVSGADGIR.G | Y | 39.76 | 1303.6302 | 13 | -4.57 | 652.818 | 2 | 12.74 | 0.94 | 0.9473 | 31487 | 0 | 0 | 0 | 932 | 944 |  |  | DB Search |
| total 4 peptides |  |  |  |  |  |  |  |  |  |  |  |  |  |  |  |  |  |  |  |
| --- | --- | --- | --- | --- | --- | --- | --- | --- | --- | --- | --- | --- | --- | --- | --- | --- | --- | --- | --- |

Best Unique PSM (Scan Sample\_2\_A1\_1\_3640.d:20698, m/z=428.7366, z=2, RT=10.98, ppm=2.82):


TRINITY\_DN1850\_c0\_g1\_i3.p1

back to list

  

| Protein Coverage
| Supporting Peptides
| Best Unique PSM
|

Protein Coverage:

Supporting Peptides:

| Peptide | Uniq | -10lgP | Mass | Length | ppm | m/z | z | RT | 1/k0 Start | 1/k0 End | Precursor ID | Area Sample 4 | #Feature | #Feature Sample 4 | Start | End | PTM | AScore | Found By |
| --- | --- | --- | --- | --- | --- | --- | --- | --- | --- | --- | --- | --- | --- | --- | --- | --- | --- | --- | --- |
| R.LELQSISDDILSK.F | Y | 58.26 | 1459.777 | 13 | -3.91 | 730.8913 | 2 | 19.63 | 1.0025 | 1.0098 | 75107 | 4.55e2 | 1 | 1 | 12 | 24 |  |  | DB Search |
| total 1 peptides |  |  |  |  |  |  |  |  |  |  |  |  |  |  |  |  |  |  |  |
| --- | --- | --- | --- | --- | --- | --- | --- | --- | --- | --- | --- | --- | --- | --- | --- | --- | --- | --- | --- |

Best Unique PSM (Scan Sample\_2\_A1\_1\_3640.d:75107, m/z=730.8913, z=2, RT=19.63, ppm=-6.11):


TRINITY\_DN3986\_c0\_g1\_i1.p1

back to list

  

| Protein Coverage
| Supporting Peptides
| Best Unique PSM
|

Protein Coverage:

Supporting Peptides:

| Peptide | Uniq | -10lgP | Mass | Length | ppm | m/z | z | RT | 1/k0 Start | 1/k0 End | Precursor ID | Area Sample 4 | #Feature | #Feature Sample 4 | Start | End | PTM | AScore | Found By |
| --- | --- | --- | --- | --- | --- | --- | --- | --- | --- | --- | --- | --- | --- | --- | --- | --- | --- | --- | --- |
| K.LAVNMVPFPR.L | Y | 58.39 | 1142.627 | 10 | 0.31 | 572.3197 | 2 | 18.72 | 0.8721 | 0.8793 | 69256 | 4.38e2 | 1 | 1 | 253 | 262 |  |  | DB Search |
| K.LAVNM(+15.99)VPFPR.L | Y | 58.27 | 1158.6219 | 10 | -3.71 | 580.3148 | 2 | 17.06 | 0.8798 | 0.887 | 57945 | 1.18e3 | 1 | 1 | 253 | 262 | Oxidation (M) | M5:Oxidation (M):1000 | DB Search |
| R.FPGQLNADLRK.L | Y | 57.99 | 1257.6829 | 11 | -7.77 | 420.2307 | 3 | 14.50 | 0.7033 | 0.7106 | 42199 | 0 | 0 | 0 | 242 | 252 |  |  | DB Search |
| R.ISEQFTAMFR.R | Y | 53.53 | 1228.591 | 10 | 8.26 | 615.3065 | 2 | 18.88 | 0.904 | 0.9113 | 70394 | 0 | 0 | 0 | 381 | 390 |  |  | DB Search |
| total 4 peptides |  |  |  |  |  |  |  |  |  |  |  |  |  |  |  |  |  |  |  |
| --- | --- | --- | --- | --- | --- | --- | --- | --- | --- | --- | --- | --- | --- | --- | --- | --- | --- | --- | --- |

Best Unique PSM (Scan Sample\_2\_A1\_1\_3640.d:69256, m/z=572.3197, z=2, RT=18.72, ppm=-1.89):


TRINITY\_DN1058\_c0\_g1\_i3.p1

back to list

  

| Protein Coverage
| Supporting Peptides
| Best Unique PSM
|

Protein Coverage:

Supporting Peptides:

| Peptide | Uniq | -10lgP | Mass | Length | ppm | m/z | z | RT | 1/k0 Start | 1/k0 End | Precursor ID | Area Sample 4 | #Feature | #Feature Sample 4 | Start | End | PTM | AScore | Found By |
| --- | --- | --- | --- | --- | --- | --- | --- | --- | --- | --- | --- | --- | --- | --- | --- | --- | --- | --- | --- |
| K.TNFQMC(+57.02)HQR.M | Y | 58.29 | 1220.5179 | 9 | 3.31 | 611.2669 | 2 | 11.65 | 0.921 | 0.9283 | 24815 | 0 | 0 | 0 | 1467 | 1475 | Carbamidomethylation |  | DB Search |
| R.APTHILNPANKR.S | Y | 58.28 | 1330.7469 | 12 | 0.07 | 666.3793 | 2 | 11.22 | 1.0098 | 1.017 | 22322 | 5.04e3 | 2 | 2 | 2314 | 2325 |  |  | DB Search |
| K.TVMAAC(+57.02)GNENK.C | Y | 58.27 | 1193.5169 | 11 | 1.94 | 597.7656 | 2 | 10.70 | 0.8919 | 0.8992 | 19067 | 0 | 0 | 0 | 1338 | 1348 | Carbamidomethylation |  | DB Search |
| R.SC(+57.02)VLASC(+57.02)SANSDVAVAC(+57.02)GK.V | Y | 58.08 | 1954.8547 | 19 | 3.15 | 978.4355 | 2 | 14.42 | 1.153 | 1.1602 | 41659 | 2.61e2 | 1 | 1 | 1616 | 1634 | Carbamidomethylation, Carbamidomethylation, Carbamidomethylation |  | DB Search |
| R.IC(+57.02)GHGGIR.C | Y | 57.78 | 868.4337 | 8 | -3.19 | 435.2218 | 2 | 9.87 | 0.7892 | 0.7966 | 14210 | 0 | 0 | 0 | 1008 | 1015 | Carbamidomethylation |  | DB Search |
| R.TC(+57.02)LC(+57.02)NPATSYPSK.D | Y | 54.97 | 1497.6592 | 13 | 0.78 | 749.8358 | 2 | 13.69 | 0.9896 | 0.9969 | 37441 | 1.47e3 | 1 | 1 | 157 | 169 | Carbamidomethylation, Carbamidomethylation |  | DB Search |
| K.SC(+57.02)IEASC(+57.02)SSK.A | Y | 53.5 | 1127.4587 | 10 | 5.45 | 564.7385 | 2 | 10.24 | 0.8733 | 0.8806 | 16314 | 7.28e2 | 1 | 1 | 465 | 474 | Carbamidomethylation, Carbamidomethylation |  | DB Search |
| total 7 peptides |  |  |  |  |  |  |  |  |  |  |  |  |  |  |  |  |  |  |  |
| --- | --- | --- | --- | --- | --- | --- | --- | --- | --- | --- | --- | --- | --- | --- | --- | --- | --- | --- | --- |

Best Unique PSM (Scan Sample\_2\_A1\_1\_3640.d:24815, m/z=611.2669, z=2, RT=11.65, ppm=1.11):


TRINITY\_DN71928\_c0\_g1\_i1.p1

back to list

  

| Protein Coverage
| Supporting Peptides
| Best Unique PSM
|

Protein Coverage:

Supporting Peptides:

| Peptide | Uniq | -10lgP | Mass | Length | ppm | m/z | z | RT | 1/k0 Start | 1/k0 End | Precursor ID | Area Sample 4 | #Feature | #Feature Sample 4 | Start | End | PTM | AScore | Found By |
| --- | --- | --- | --- | --- | --- | --- | --- | --- | --- | --- | --- | --- | --- | --- | --- | --- | --- | --- | --- |
| K.NYVVQFC(+57.02)DQFR.T | Y | 58.39 | 1474.6663 | 11 | -1.26 | 738.3379 | 2 | 18.45 | 0.9896 | 0.9969 | 67466 | 0 | 0 | 0 | 142 | 152 | Carbamidomethylation |  | DB Search |
| total 1 peptides |  |  |  |  |  |  |  |  |  |  |  |  |  |  |  |  |  |  |  |
| --- | --- | --- | --- | --- | --- | --- | --- | --- | --- | --- | --- | --- | --- | --- | --- | --- | --- | --- | --- |

Best Unique PSM (Scan Sample\_2\_A1\_1\_3640.d:67466, m/z=737.7029, z=3, RT=18.45, ppm=-3.45):


TRINITY\_DN581\_c1\_g1\_i1.p1

back to list

  

| Protein Coverage
| Supporting Peptides
| Best Unique PSM
|

Protein Coverage:

Supporting Peptides:

| Peptide | Uniq | -10lgP | Mass | Length | ppm | m/z | z | RT | 1/k0 Start | 1/k0 End | Precursor ID | Area Sample 4 | #Feature | #Feature Sample 4 | Start | End | PTM | AScore | Found By |
| --- | --- | --- | --- | --- | --- | --- | --- | --- | --- | --- | --- | --- | --- | --- | --- | --- | --- | --- | --- |
| R.IPITC(+57.02)YYPTSGK.A | Y | 58.32 | 1398.6853 | 12 | 0.68 | 700.3489 | 2 | 15.91 | 0.9856 | 0.9928 | 50752 | 4.02e3 | 1 | 1 | 222 | 233 | Carbamidomethylation |  | DB Search |
| K.DLEDVNFVR.F | Y | 57.12 | 1105.5404 | 9 | 3.32 | 553.7781 | 2 | 17.07 | 0.8757 | 0.883 | 58345 | 1.18e3 | 1 | 1 | 149 | 157 |  |  | DB Search |
| K.KKDLEDVNFVR.F | Y | 56.18 | 1361.7303 | 11 | 1.3 | 454.917 | 3 | 14.05 | 0.7583 | 0.7656 | 39398 | 8.73e2 | 1 | 1 | 147 | 157 |  |  | DB Search |
| total 3 peptides |  |  |  |  |  |  |  |  |  |  |  |  |  |  |  |  |  |  |  |
| --- | --- | --- | --- | --- | --- | --- | --- | --- | --- | --- | --- | --- | --- | --- | --- | --- | --- | --- | --- |

Best Unique PSM (Scan Sample\_2\_A1\_1\_3640.d:50752, m/z=700.3489, z=2, RT=15.91, ppm=-1.52):


TRINITY\_DN1285\_c0\_g1\_i1.p1

back to list

  

| Protein Coverage
| Supporting Peptides
| Best Unique PSM
|

Protein Coverage:

Supporting Peptides:

| Peptide | Uniq | -10lgP | Mass | Length | ppm | m/z | z | RT | 1/k0 Start | 1/k0 End | Precursor ID | Area Sample 4 | #Feature | #Feature Sample 4 | Start | End | PTM | AScore | Found By |
| --- | --- | --- | --- | --- | --- | --- | --- | --- | --- | --- | --- | --- | --- | --- | --- | --- | --- | --- | --- |
| K.LVHGDVFRPPR.A | Y | 58.34 | 1291.7149 | 11 | 1.79 | 431.5787 | 3 | 13.55 | 0.823 | 0.8303 | 36380 | 1.06e3 | 1 | 1 | 344 | 354 |  |  | DB Search |
| total 1 peptides |  |  |  |  |  |  |  |  |  |  |  |  |  |  |  |  |  |  |  |
| --- | --- | --- | --- | --- | --- | --- | --- | --- | --- | --- | --- | --- | --- | --- | --- | --- | --- | --- | --- |

Best Unique PSM (Scan Sample\_2\_A1\_1\_3640.d:36380, m/z=431.5787, z=3, RT=13.55, ppm=-0.4):


TRINITY\_DN195348\_c0\_g1\_i1.p1

back to list

  

| Protein Coverage
| Supporting Peptides
| Best Unique PSM
|

Protein Coverage:

Supporting Peptides:

| Peptide | Uniq | -10lgP | Mass | Length | ppm | m/z | z | RT | 1/k0 Start | 1/k0 End | Precursor ID | Area Sample 4 | #Feature | #Feature Sample 4 | Start | End | PTM | AScore | Found By |
| --- | --- | --- | --- | --- | --- | --- | --- | --- | --- | --- | --- | --- | --- | --- | --- | --- | --- | --- | --- |
| K.VLIVISDGNEDFKEK.Q | Y | 58.39 | 1704.8934 | 15 | 2.18 | 569.3051 | 3 | 16.95 | 0.8412 | 0.8485 | 57552 | 5.5e3 | 2 | 2 | 132 | 146 |  |  | DB Search |
| R.VIVPYTPVTGYETGK.A | Y | 58.3 | 1622.8555 | 15 | 1.23 | 812.4343 | 2 | 17.26 | 1.046 | 1.0532 | 59415 | 4.54e3 | 1 | 1 | 90 | 104 |  |  | DB Search |
| R.FFDFAK.M | Y | 56.55 | 773.3748 | 6 | 8.5 | 387.6971 | 2 | 17.81 | 0.7473 | 0.7547 | 63170 | 0 | 0 | 0 | 201 | 206 |  |  | DB Search |
| total 3 peptides |  |  |  |  |  |  |  |  |  |  |  |  |  |  |  |  |  |  |  |
| --- | --- | --- | --- | --- | --- | --- | --- | --- | --- | --- | --- | --- | --- | --- | --- | --- | --- | --- | --- |

Best Unique PSM (Scan Sample\_2\_A1\_1\_3640.d:57552, m/z=569.3051, z=3, RT=16.95, ppm=-0.02):


TRINITY\_DN6221\_c0\_g1\_i3.p1

back to list

  

| Protein Coverage
| Supporting Peptides
| Best Unique PSM
|

Protein Coverage:

Supporting Peptides:

| Peptide | Uniq | -10lgP | Mass | Length | ppm | m/z | z | RT | 1/k0 Start | 1/k0 End | Precursor ID | Area Sample 4 | #Feature | #Feature Sample 4 | Start | End | PTM | AScore | Found By |
| --- | --- | --- | --- | --- | --- | --- | --- | --- | --- | --- | --- | --- | --- | --- | --- | --- | --- | --- | --- |
| K.SVQIEVSTPVC(+57.02)QTC(+57.02)PK.G | Y | 57.42 | 1831.8808 | 16 | 3.34 | 916.9487 | 2 | 15.16 | 1.1126 | 1.1198 | 46105 | 3.11e3 | 1 | 1 | 667 | 682 | Carbamidomethylation, Carbamidomethylation |  | DB Search |
| K.YQVTGC(+57.02)GGFC(+57.02)ESSVK.A | Y | 57.17 | 1677.7127 | 15 | -3.52 | 839.8588 | 2 | 14.63 | 1.068 | 1.0753 | 43042 | 0 | 0 | 0 | 733 | 747 | Carbamidomethylation, Carbamidomethylation |  | DB Search |
| total 2 peptides |  |  |  |  |  |  |  |  |  |  |  |  |  |  |  |  |  |  |  |
| --- | --- | --- | --- | --- | --- | --- | --- | --- | --- | --- | --- | --- | --- | --- | --- | --- | --- | --- | --- |

Best Unique PSM (Scan Sample\_2\_A1\_1\_3640.d:46105, m/z=916.9487, z=2, RT=15.16, ppm=1.15):


TRINITY\_DN28257\_c0\_g1\_i2.p1

back to list

  

| Protein Coverage
| Supporting Peptides
| Best Unique PSM
|

Protein Coverage:

Supporting Peptides:

| Peptide | Uniq | -10lgP | Mass | Length | ppm | m/z | z | RT | 1/k0 Start | 1/k0 End | Precursor ID | Area Sample 4 | #Feature | #Feature Sample 4 | Start | End | PTM | AScore | Found By |
| --- | --- | --- | --- | --- | --- | --- | --- | --- | --- | --- | --- | --- | --- | --- | --- | --- | --- | --- | --- |
| K.TC(+57.02)ESGDLDLDFAC(+57.02)GR.G | Y | 58.22 | 1714.6927 | 15 | -2.61 | 858.3495 | 2 | 17.67 | 1.0572 | 1.0644 | 62177 | 1.81e3 | 1 | 1 | 513 | 527 | Carbamidomethylation, Carbamidomethylation |  | DB Search |
| R.GYTTDPTDVQTC(+57.02)K.K | Y | 57.69 | 1484.6453 | 13 | 4.36 | 743.3315 | 2 | 12.56 | 1.0069 | 1.0142 | 30265 | 6.8e2 | 1 | 1 | 308 | 320 | Carbamidomethylation |  | DB Search |
| R.GYTTDPTDVQTC(+57.02)KK.I | Y | 56.69 | 1612.7403 | 14 | 11.46 | 807.3849 | 2 | 11.69 | 1.0395 | 1.0468 | 24808 | 9.83e2 | 1 | 1 | 308 | 321 | Carbamidomethylation |  | DB Search |
| K.ADEGYYPDSDGKTC(+57.02)VLAPC(+57.02)K.S | Y | 55.5 | 2244.9667 | 20 | 6.79 | 749.3329 | 3 | 14.72 | 0.9089 | 0.9162 | 43605 | 0 | 0 | 0 | 449 | 468 | Carbamidomethylation, Carbamidomethylation |  | DB Search |
| K.IKC(+57.02)DDPDVDYEK.V | Y | 54.35 | 1495.65 | 12 | 1.08 | 499.5567 | 3 | 12.15 | 0.7901 | 0.7974 | 27860 | 0 | 0 | 0 | 704 | 715 | Carbamidomethylation |  | DB Search |
| K.GHHC(+57.02)IC(+57.02)SAEGFEVTGTK.C | Y | 52.63 | 1888.8196 | 17 | 2.72 | 630.6141 | 3 | 13.51 | 0.8757 | 0.883 | 36216 | 0 | 0 | 0 | 107 | 123 | Carbamidomethylation, Carbamidomethylation |  | DB Search |
| total 6 peptides |  |  |  |  |  |  |  |  |  |  |  |  |  |  |  |  |  |  |  |
| --- | --- | --- | --- | --- | --- | --- | --- | --- | --- | --- | --- | --- | --- | --- | --- | --- | --- | --- | --- |

Best Unique PSM (Scan Sample\_2\_A1\_1\_3640.d:62177, m/z=858.3495, z=2, RT=17.67, ppm=-4.8):


TRINITY\_DN7674\_c0\_g1\_i2.p1

back to list

  

| Protein Coverage
| Supporting Peptides
| Best Unique PSM
|

Protein Coverage:

Supporting Peptides:

| Peptide | Uniq | -10lgP | Mass | Length | ppm | m/z | z | RT | 1/k0 Start | 1/k0 End | Precursor ID | Area Sample 4 | #Feature | #Feature Sample 4 | Start | End | PTM | AScore | Found By |
| --- | --- | --- | --- | --- | --- | --- | --- | --- | --- | --- | --- | --- | --- | --- | --- | --- | --- | --- | --- |
| R.LGTIVAGFLK.L | Y | 58.05 | 1017.6222 | 10 | -3.2 | 509.8156 | 2 | 19.28 | 0.8254 | 0.8327 | 72854 | 3.28e2 | 1 | 1 | 326 | 335 |  |  | DB Search |
| M.DYYASEK.K | Y | 41.11 | 874.3708 | 7 | 18.08 | 438.1996 | 2 | 11.74 | 0.8067 | 0.814 | 25358 | 3e3 | 1 | 1 | 2 | 8 |  |  | DB Search |
| total 2 peptides |  |  |  |  |  |  |  |  |  |  |  |  |  |  |  |  |  |  |  |
| --- | --- | --- | --- | --- | --- | --- | --- | --- | --- | --- | --- | --- | --- | --- | --- | --- | --- | --- | --- |

Best Unique PSM (Scan Sample\_2\_A1\_1\_3640.d:72854, m/z=509.8156, z=2, RT=19.28, ppm=-5.4):


TRINITY\_DN761\_c0\_g1\_i1.p1

back to list

  

| Protein Coverage
| Supporting Peptides
| Best Unique PSM
|

Protein Coverage:

Supporting Peptides:

| Peptide | Uniq | -10lgP | Mass | Length | ppm | m/z | z | RT | 1/k0 Start | 1/k0 End | Precursor ID | Area Sample 4 | #Feature | #Feature Sample 4 | Start | End | PTM | AScore | Found By |
| --- | --- | --- | --- | --- | --- | --- | --- | --- | --- | --- | --- | --- | --- | --- | --- | --- | --- | --- | --- |
| K.TRIEDLETVISQTQDHR.H | Y | 58.33 | 2040.0236 | 17 | 1.3 | 681.0145 | 3 | 17.58 | 0.9473 | 0.9545 | 61608 | 1.62e3 | 1 | 1 | 260 | 276 |  |  | DB Search |
| R.IEDLETVISQTQDHR.H | Y | 58.29 | 1782.8748 | 15 | -2.39 | 595.2961 | 3 | 17.95 | 0.8963 | 0.9036 | 64199 | 0 | 0 | 0 | 262 | 276 |  |  | DB Search |
| R.ATLYPC(+57.02)PETPNER.R | Y | 58.12 | 1546.7086 | 13 | 5.6 | 774.3642 | 2 | 14.56 | 1.0359 | 1.0431 | 42458 | 8e2 | 1 | 1 | 239 | 251 | Carbamidomethylation |  | DB Search |
| total 3 peptides |  |  |  |  |  |  |  |  |  |  |  |  |  |  |  |  |  |  |  |
| --- | --- | --- | --- | --- | --- | --- | --- | --- | --- | --- | --- | --- | --- | --- | --- | --- | --- | --- | --- |

Best Unique PSM (Scan Sample\_2\_A1\_1\_3640.d:61608, m/z=681.0145, z=3, RT=17.58, ppm=-0.89):


TRINITY\_DN1616\_c0\_g1\_i4.p1

back to list

  

| Protein Coverage
| Supporting Peptides
| Best Unique PSM
|

Protein Coverage:

Supporting Peptides:

| Peptide | Uniq | -10lgP | Mass | Length | ppm | m/z | z | RT | 1/k0 Start | 1/k0 End | Precursor ID | Area Sample 4 | #Feature | #Feature Sample 4 | Start | End | PTM | AScore | Found By |
| --- | --- | --- | --- | --- | --- | --- | --- | --- | --- | --- | --- | --- | --- | --- | --- | --- | --- | --- | --- |
| K.NEINLASGDVFEGGVVGSLK.L | Y | 53.15 | 2004.0163 | 20 | 1.4 | 1003.0146 | 2 | 19.85 | 1.1829 | 1.1901 | 76578 | 0 | 0 | 0 | 201 | 220 |  |  | DB Search |
| total 1 peptides |  |  |  |  |  |  |  |  |  |  |  |  |  |  |  |  |  |  |  |
| --- | --- | --- | --- | --- | --- | --- | --- | --- | --- | --- | --- | --- | --- | --- | --- | --- | --- | --- | --- |

Best Unique PSM (Scan Sample\_2\_A1\_1\_3640.d:76578, m/z=1003.0146, z=2, RT=19.85, ppm=-0.79):


TRINITY\_DN11522\_c0\_g1\_i2.p1

back to list

  

| Protein Coverage
| Supporting Peptides
| Best Unique PSM
|

Protein Coverage:

Supporting Peptides:

| Peptide | Uniq | -10lgP | Mass | Length | ppm | m/z | z | RT | 1/k0 Start | 1/k0 End | Precursor ID | Area Sample 4 | #Feature | #Feature Sample 4 | Start | End | PTM | AScore | Found By |
| --- | --- | --- | --- | --- | --- | --- | --- | --- | --- | --- | --- | --- | --- | --- | --- | --- | --- | --- | --- |
| K.GKPEQVQVK.M | Y | 58.01 | 1011.5713 | 9 | 0.86 | 506.7922 | 2 | 9.91 | 0.8376 | 0.8449 | 14438 | 2.02e2 | 1 | 1 | 546 | 554 |  |  | DB Search |
| total 1 peptides |  |  |  |  |  |  |  |  |  |  |  |  |  |  |  |  |  |  |  |
| --- | --- | --- | --- | --- | --- | --- | --- | --- | --- | --- | --- | --- | --- | --- | --- | --- | --- | --- | --- |

Best Unique PSM (Scan Sample\_2\_A1\_1\_3640.d:14438, m/z=506.7922, z=2, RT=9.91, ppm=-1.33):


TRINITY\_DN2477\_c0\_g1\_i10.p1

back to list

  

| Protein Coverage
| Supporting Peptides
| Best Unique PSM
|

Protein Coverage:

Supporting Peptides:

| Peptide | Uniq | -10lgP | Mass | Length | ppm | m/z | z | RT | 1/k0 Start | 1/k0 End | Precursor ID | Area Sample 4 | #Feature | #Feature Sample 4 | Start | End | PTM | AScore | Found By |
| --- | --- | --- | --- | --- | --- | --- | --- | --- | --- | --- | --- | --- | --- | --- | --- | --- | --- | --- | --- |
| K.DSYVGDEAQSKR.G | Y | 57.28 | 1353.616 | 12 | 2.57 | 677.8156 | 2 | 11.01 | 0.9993 | 1.0065 | 20794 | 1.16e3 | 1 | 1 | 19 | 30 |  |  | DB Search |
| total 1 peptides |  |  |  |  |  |  |  |  |  |  |  |  |  |  |  |  |  |  |  |
| --- | --- | --- | --- | --- | --- | --- | --- | --- | --- | --- | --- | --- | --- | --- | --- | --- | --- | --- | --- |

Best Unique PSM (Scan Sample\_2\_A1\_1\_3640.d:20794, m/z=677.8156, z=2, RT=11.01, ppm=0.37):


TRINITY\_DN103165\_c0\_g1\_i1.p1

back to list

  

| Protein Coverage
| Supporting Peptides
| Best Unique PSM
|

Protein Coverage:

Supporting Peptides:

| Peptide | Uniq | -10lgP | Mass | Length | ppm | m/z | z | RT | 1/k0 Start | 1/k0 End | Precursor ID | Area Sample 4 | #Feature | #Feature Sample 4 | Start | End | PTM | AScore | Found By |
| --- | --- | --- | --- | --- | --- | --- | --- | --- | --- | --- | --- | --- | --- | --- | --- | --- | --- | --- | --- |
| K.ESTLHLVLR.L | Y | 57.27 | 1066.6135 | 9 | 2.57 | 534.3142 | 2 | 15.80 | 0.8785 | 0.8858 | 50332 | 4.97e3 | 1 | 1 | 16 | 24 |  |  | DB Search |
| total 1 peptides |  |  |  |  |  |  |  |  |  |  |  |  |  |  |  |  |  |  |  |
| --- | --- | --- | --- | --- | --- | --- | --- | --- | --- | --- | --- | --- | --- | --- | --- | --- | --- | --- | --- |

Best Unique PSM (Scan Sample\_2\_A1\_1\_3640.d:50332, m/z=534.7766, z=2, RT=15.80, ppm=0.37):


TRINITY\_DN36\_c0\_g1\_i20.p1

back to list

  

| Protein Coverage
| Supporting Peptides
| Best Unique PSM
|

Protein Coverage:

Supporting Peptides:

| Peptide | Uniq | -10lgP | Mass | Length | ppm | m/z | z | RT | 1/k0 Start | 1/k0 End | Precursor ID | Area Sample 4 | #Feature | #Feature Sample 4 | Start | End | PTM | AScore | Found By |
| --- | --- | --- | --- | --- | --- | --- | --- | --- | --- | --- | --- | --- | --- | --- | --- | --- | --- | --- | --- |
| R.LGGIEVTQGNC(+57.02)AR.K | Y | 58.36 | 1373.6721 | 13 | -2.89 | 687.8398 | 2 | 13.93 | 0.9662 | 0.9735 | 38726 | 2.09e3 | 1 | 1 | 57 | 69 | Carbamidomethylation |  | DB Search |
| K.GLPLAIGVR.D | Y | 57.12 | 894.565 | 9 | 0.27 | 448.2889 | 2 | 17.73 | 0.8124 | 0.8197 | 62574 | 3.52e3 | 1 | 1 | 31 | 39 |  |  | DB Search |
| total 2 peptides |  |  |  |  |  |  |  |  |  |  |  |  |  |  |  |  |  |  |  |
| --- | --- | --- | --- | --- | --- | --- | --- | --- | --- | --- | --- | --- | --- | --- | --- | --- | --- | --- | --- |

Best Unique PSM (Scan Sample\_2\_A1\_1\_3640.d:38726, m/z=687.8398, z=2, RT=13.93, ppm=-5.09):


TRINITY\_DN1119\_c0\_g1\_i1.p1

back to list

  

| Protein Coverage
| Supporting Peptides
| Best Unique PSM
|

Protein Coverage:

Supporting Peptides:

| Peptide | Uniq | -10lgP | Mass | Length | ppm | m/z | z | RT | 1/k0 Start | 1/k0 End | Precursor ID | Area Sample 4 | #Feature | #Feature Sample 4 | Start | End | PTM | AScore | Found By |
| --- | --- | --- | --- | --- | --- | --- | --- | --- | --- | --- | --- | --- | --- | --- | --- | --- | --- | --- | --- |
| K.FLVELAC(+57.02)K.C | Y | 58.06 | 978.5208 | 8 | -1.7 | 490.2658 | 2 | 17.31 | 0.8116 | 0.8189 | 59794 | 1.21e3 | 1 | 1 | 181 | 188 | Carbamidomethylation |  | DB Search |
| K.RLTELEHFLK.I | Y | 55.84 | 1284.719 | 10 | -4.48 | 429.2441 | 3 | 16.54 | 0.7453 | 0.7526 | 54983 | 6.53e2 | 1 | 1 | 376 | 385 |  |  | DB Search |
| K.AVGEEFNSVDPSVK.G | Y | 54.73 | 1476.7096 | 14 | 1.1 | 739.3613 | 2 | 15.15 | 1.0061 | 1.0134 | 46241 | 7.54e2 | 1 | 1 | 356 | 369 |  |  | DB Search |
| total 3 peptides |  |  |  |  |  |  |  |  |  |  |  |  |  |  |  |  |  |  |  |
| --- | --- | --- | --- | --- | --- | --- | --- | --- | --- | --- | --- | --- | --- | --- | --- | --- | --- | --- | --- |

Best Unique PSM (Scan Sample\_2\_A1\_1\_3640.d:59794, m/z=490.2658, z=2, RT=17.31, ppm=-3.89):


TRINITY\_DN15542\_c0\_g1\_i1.p1

back to list

  

| Protein Coverage
| Supporting Peptides
| Best Unique PSM
|

Protein Coverage:

Supporting Peptides:

| Peptide | Uniq | -10lgP | Mass | Length | ppm | m/z | z | RT | 1/k0 Start | 1/k0 End | Precursor ID | Area Sample 4 | #Feature | #Feature Sample 4 | Start | End | PTM | AScore | Found By |
| --- | --- | --- | --- | --- | --- | --- | --- | --- | --- | --- | --- | --- | --- | --- | --- | --- | --- | --- | --- |
| K.VIGHGTIYGEEVTLNLR.E | Y | 58.24 | 1869.9948 | 17 | 4.33 | 624.3402 | 3 | 17.56 | 0.919 | 0.9263 | 61629 | 0 | 0 | 0 | 80 | 96 |  |  | DB Search |
| total 1 peptides |  |  |  |  |  |  |  |  |  |  |  |  |  |  |  |  |  |  |  |
| --- | --- | --- | --- | --- | --- | --- | --- | --- | --- | --- | --- | --- | --- | --- | --- | --- | --- | --- | --- |

Best Unique PSM (Scan Sample\_2\_A1\_1\_3640.d:61629, m/z=624.3402, z=3, RT=17.56, ppm=2.13):


TRINITY\_DN5633\_c0\_g1\_i1.p1

back to list

  

| Protein Coverage
| Supporting Peptides
| Best Unique PSM
|

Protein Coverage:

Supporting Peptides:

| Peptide | Uniq | -10lgP | Mass | Length | ppm | m/z | z | RT | 1/k0 Start | 1/k0 End | Precursor ID | Area Sample 4 | #Feature | #Feature Sample 4 | Start | End | PTM | AScore | Found By |
| --- | --- | --- | --- | --- | --- | --- | --- | --- | --- | --- | --- | --- | --- | --- | --- | --- | --- | --- | --- |
| K.GIVDSDDLPLNVSR.E | Y | 58.39 | 1498.7627 | 14 | 6.82 | 750.3921 | 2 | 17.63 | 1.0029 | 1.0102 | 62065 | 0 | 0 | 0 | 457 | 470 |  |  | DB Search |
| K.YLSFIK.G | Y | 55.18 | 769.4374 | 6 | 1.01 | 385.7255 | 2 | 17.32 | 0.7445 | 0.7518 | 59913 | 5.56e2 | 1 | 1 | 451 | 456 |  |  | DB Search |
| K.LLEINVYHPLIQELQK.R | Y | 54.62 | 1949.0986 | 16 | -4.49 | 650.7025 | 3 | 19.83 | 0.9735 | 0.9808 | 76478 | 0 | 0 | 0 | 706 | 721 |  |  | DB Search |
| total 3 peptides |  |  |  |  |  |  |  |  |  |  |  |  |  |  |  |  |  |  |  |
| --- | --- | --- | --- | --- | --- | --- | --- | --- | --- | --- | --- | --- | --- | --- | --- | --- | --- | --- | --- |

Best Unique PSM (Scan Sample\_2\_A1\_1\_3640.d:62065, m/z=750.3921, z=2, RT=17.63, ppm=4.62):


TRINITY\_DN14015\_c0\_g1\_i2.p1

back to list

  

| Protein Coverage
| Supporting Peptides
| Best Unique PSM
|

Protein Coverage:

Supporting Peptides:

| Peptide | Uniq | -10lgP | Mass | Length | ppm | m/z | z | RT | 1/k0 Start | 1/k0 End | Precursor ID | Area Sample 4 | #Feature | #Feature Sample 4 | Start | End | PTM | AScore | Found By |
| --- | --- | --- | --- | --- | --- | --- | --- | --- | --- | --- | --- | --- | --- | --- | --- | --- | --- | --- | --- |
| K.VIDINDNAPIFNK.A | Y | 58.39 | 1471.7671 | 13 | 2.95 | 736.8914 | 2 | 17.55 | 1.0081 | 1.0154 | 61422 | 9.25e2 | 1 | 1 | 666 | 678 |  |  | DB Search |
| K.TNENRPIFQFTNPK.V | Y | 58.31 | 1704.8583 | 14 | -6.5 | 569.2885 | 3 | 16.42 | 0.8465 | 0.8538 | 54306 | 6.65e2 | 2 | 2 | 1891 | 1904 |  |  | DB Search |
| R.LGIEAIDENGLR.S | Y | 58.26 | 1298.683 | 12 | 0.2 | 650.3475 | 2 | 17.28 | 0.9566 | 0.9638 | 59638 | 7.15e2 | 1 | 1 | 956 | 967 |  |  | DB Search |
| total 3 peptides |  |  |  |  |  |  |  |  |  |  |  |  |  |  |  |  |  |  |  |
| --- | --- | --- | --- | --- | --- | --- | --- | --- | --- | --- | --- | --- | --- | --- | --- | --- | --- | --- | --- |

Best Unique PSM (Scan Sample\_2\_A1\_1\_3640.d:61422, m/z=736.8914, z=2, RT=17.55, ppm=0.75):


TRINITY\_DN77\_c0\_g2\_i2.p1

back to list

  

| Protein Coverage
| Supporting Peptides
| Best Unique PSM
|

Protein Coverage:

Supporting Peptides:

| Peptide | Uniq | -10lgP | Mass | Length | ppm | m/z | z | RT | 1/k0 Start | 1/k0 End | Precursor ID | Area Sample 4 | #Feature | #Feature Sample 4 | Start | End | PTM | AScore | Found By |
| --- | --- | --- | --- | --- | --- | --- | --- | --- | --- | --- | --- | --- | --- | --- | --- | --- | --- | --- | --- |
| R.GPVGTSGLR.G | Y | 58.19 | 842.461 | 9 | 3.7 | 422.2384 | 2 | 12.14 | 0.7787 | 0.786 | 27733 | 1.47e4 | 1 | 1 | 830 | 838 |  |  | DB Search |
| R.GPAGAPGDEGQTGMR.G | Y | 57.23 | 1399.615 | 15 | -0.7 | 700.8127 | 2 | 11.55 | 0.9396 | 0.9469 | 24084 | 1.21e3 | 1 | 1 | 626 | 640 |  |  | DB Search |
| R.GPQGQVGK.Q | Y | 55.44 | 769.4082 | 8 | 6.49 | 385.713 | 2 | 8.99 | 0.7069 | 0.7143 | 10347 | 1.81e3 | 1 | 1 | 473 | 480 |  |  | DB Search |
| R.GPAGAPGDEGQTGM(+15.99)RGER.G | Y | 54.26 | 1757.7751 | 18 | 2.12 | 586.9323 | 3 | 10.62 | 0.8818 | 0.8891 | 18471 | 3.52e3 | 1 | 1 | 626 | 643 | Oxidation (M) | M14:Oxidation (M):1000 | DB Search |
| R.GPAGAPGDEGQTGM(+15.99)R.G | Y | 54.2 | 1415.6099 | 15 | 4.13 | 708.8136 | 2 | 10.40 | 0.9453 | 0.9525 | 17281 | 0 | 0 | 0 | 626 | 640 | Oxidation (M) | M14:Oxidation (M):1000 | DB Search |
| total 5 peptides |  |  |  |  |  |  |  |  |  |  |  |  |  |  |  |  |  |  |  |
| --- | --- | --- | --- | --- | --- | --- | --- | --- | --- | --- | --- | --- | --- | --- | --- | --- | --- | --- | --- |

Best Unique PSM (Scan Sample\_2\_A1\_1\_3640.d:27733, m/z=422.2384, z=2, RT=12.14, ppm=1.5):


TRINITY\_DN1795\_c0\_g1\_i4.p1

back to list

  

| Protein Coverage
| Supporting Peptides
| Best Unique PSM
|

Protein Coverage:

Supporting Peptides:

| Peptide | Uniq | -10lgP | Mass | Length | ppm | m/z | z | RT | 1/k0 Start | 1/k0 End | Precursor ID | Area Sample 4 | #Feature | #Feature Sample 4 | Start | End | PTM | AScore | Found By |
| --- | --- | --- | --- | --- | --- | --- | --- | --- | --- | --- | --- | --- | --- | --- | --- | --- | --- | --- | --- |
| K.IDVALNIVTK.V | Y | 58.25 | 1084.6492 | 10 | 1.83 | 543.3317 | 2 | 17.50 | 0.8571 | 0.8644 | 61036 | 3.63e2 | 1 | 1 | 1881 | 1890 |  |  | DB Search |
| K.TSVIADSVGQTIGHVGDVVDTAK.L | Y | 56.02 | 2268.1597 | 23 | -3.24 | 757.0564 | 3 | 19.21 | 1.017 | 1.0242 | 72415 | 3.24e2 | 1 | 1 | 1840 | 1862 |  |  | DB Search |
| K.IATGFESIVR.G | Y | 55.77 | 1091.5975 | 10 | 5.35 | 546.8077 | 2 | 17.15 | 0.855 | 0.8623 | 58937 | 0 | 0 | 0 | 1794 | 1803 |  |  | DB Search |
| K.LIFDDVGNIAEK.L | Y | 51.9 | 1332.6925 | 12 | 4.84 | 667.3553 | 2 | 18.51 | 0.9622 | 0.9695 | 67867 | 5.56e2 | 1 | 1 | 1987 | 1998 |  |  | DB Search |
| K.DLTASLDGVFQK.T | Y | 51.39 | 1292.6612 | 12 | 1.56 | 647.3375 | 2 | 19.57 | 0.9505 | 0.9578 | 74776 | 1.66e2 | 1 | 1 | 1079 | 1090 |  |  | DB Search |
| total 5 peptides |  |  |  |  |  |  |  |  |  |  |  |  |  |  |  |  |  |  |  |
| --- | --- | --- | --- | --- | --- | --- | --- | --- | --- | --- | --- | --- | --- | --- | --- | --- | --- | --- | --- |

Best Unique PSM (Scan Sample\_2\_A1\_1\_3640.d:61036, m/z=543.3317, z=2, RT=17.50, ppm=-0.37):


TRINITY\_DN161\_c0\_g1\_i1.p1

back to list

  

| Protein Coverage
| Supporting Peptides
| Best Unique PSM
|

Protein Coverage:

Supporting Peptides:

| Peptide | Uniq | -10lgP | Mass | Length | ppm | m/z | z | RT | 1/k0 Start | 1/k0 End | Precursor ID | Area Sample 4 | #Feature | #Feature Sample 4 | Start | End | PTM | AScore | Found By |
| --- | --- | --- | --- | --- | --- | --- | --- | --- | --- | --- | --- | --- | --- | --- | --- | --- | --- | --- | --- |
| K.SYELPDGQVITIGNER.F | Y | 58.39 | 1789.8846 | 16 | 1.59 | 895.949 | 2 | 18.70 | 1.111 | 1.1182 | 69190 | 4.17e3 | 1 | 1 | 240 | 255 |  |  | DB Search |
| R.AVFPSIVGRPR.H | Y | 58 | 1197.6982 | 11 | 3.54 | 599.8572 | 2 | 15.96 | 0.9642 | 0.9715 | 51028 | 1.61e4 | 1 | 1 | 30 | 40 |  |  | DB Search |
| R.DLTDYLMK.I | Y | 57.96 | 997.479 | 8 | 6.32 | 499.7488 | 2 | 18.67 | 0.8319 | 0.8392 | 68987 | 0 | 0 | 0 | 185 | 192 |  |  | DB Search |
| K.LC(+57.02)YVALDFEQEM(+15.99)QTAASSSSLEK.S | Y | 57.8 | 2622.1829 | 23 | 5.82 | 875.0714 | 3 | 19.02 | 1.0741 | 1.0813 | 71279 | 4.32e2 | 1 | 1 | 217 | 239 | Carbamidomethylation, Oxidation (M) | M12:Oxidation (M):1000 | DB Search |
| K.DSYVGDEAQSKR.G | Y | 57.28 | 1353.616 | 12 | 2.57 | 677.8156 | 2 | 11.01 | 0.9993 | 1.0065 | 20794 | 1.16e3 | 1 | 1 | 52 | 63 |  |  | DB Search |
| R.DLTDYLM(+15.99)K.I | Y | 57.01 | 1013.4739 | 8 | 0.97 | 507.7436 | 2 | 16.91 | 0.8388 | 0.8461 | 57197 | 1.01e3 | 1 | 1 | 185 | 192 | Oxidation (M) | M7:Oxidation (M):1000 | DB Search |
| K.DLYANTVLSGGTTM(+15.99)FPGIADR.M | Y | 55.95 | 2214.0626 | 21 | -1.47 | 1108.0345 | 2 | 19.56 | 1.2432 | 1.2503 | 74741 | 0 | 0 | 0 | 293 | 313 | Oxidation (M) | M14:Oxidation (M):1000 | DB Search |
| total 7 peptides |  |  |  |  |  |  |  |  |  |  |  |  |  |  |  |  |  |  |  |
| --- | --- | --- | --- | --- | --- | --- | --- | --- | --- | --- | --- | --- | --- | --- | --- | --- | --- | --- | --- |

Best Unique PSM (Scan Sample\_2\_A1\_1\_3640.d:69190, m/z=895.949, z=2, RT=18.70, ppm=-0.61):


TRINITY\_DN1214\_c0\_g1\_i1.p1

back to list

  

| Protein Coverage
| Supporting Peptides
| Best Unique PSM
|

Protein Coverage:

Supporting Peptides:

| Peptide | Uniq | -10lgP | Mass | Length | ppm | m/z | z | RT | 1/k0 Start | 1/k0 End | Precursor ID | Area Sample 4 | #Feature | #Feature Sample 4 | Start | End | PTM | AScore | Found By |
| --- | --- | --- | --- | --- | --- | --- | --- | --- | --- | --- | --- | --- | --- | --- | --- | --- | --- | --- | --- |
| K.YLEFEYEC(+57.02)APK.G | Y | 54.6 | 1447.6329 | 11 | -0.11 | 724.8221 | 2 | 17.24 | 1.0387 | 1.046 | 59469 | 6.12e2 | 1 | 1 | 415 | 425 | Carbamidomethylation |  | DB Search |
| total 1 peptides |  |  |  |  |  |  |  |  |  |  |  |  |  |  |  |  |  |  |  |
| --- | --- | --- | --- | --- | --- | --- | --- | --- | --- | --- | --- | --- | --- | --- | --- | --- | --- | --- | --- |

Best Unique PSM (Scan Sample\_2\_A1\_1\_3640.d:59469, m/z=724.8221, z=2, RT=17.24, ppm=-2.3):


TRINITY\_DN5897\_c0\_g1\_i3.p1

back to list

  

| Protein Coverage
| Supporting Peptides
| Best Unique PSM
|

Protein Coverage:

Supporting Peptides:

| Peptide | Uniq | -10lgP | Mass | Length | ppm | m/z | z | RT | 1/k0 Start | 1/k0 End | Precursor ID | Area Sample 4 | #Feature | #Feature Sample 4 | Start | End | PTM | AScore | Found By |
| --- | --- | --- | --- | --- | --- | --- | --- | --- | --- | --- | --- | --- | --- | --- | --- | --- | --- | --- | --- |
| K.FDDVPNGLGAFSK.V | Y | 53.88 | 1365.6564 | 13 | 1.41 | 683.835 | 2 | 17.84 | 0.959 | 0.9662 | 63447 | 0 | 0 | 0 | 16 | 28 |  |  | DB Search |
| total 1 peptides |  |  |  |  |  |  |  |  |  |  |  |  |  |  |  |  |  |  |  |
| --- | --- | --- | --- | --- | --- | --- | --- | --- | --- | --- | --- | --- | --- | --- | --- | --- | --- | --- | --- |

Best Unique PSM (Scan Sample\_2\_A1\_1\_3640.d:63447, m/z=683.835, z=2, RT=17.84, ppm=-0.78):


TRINITY\_DN4900\_c0\_g1\_i4.p1

back to list

  

| Protein Coverage
| Supporting Peptides
| Best Unique PSM
|

Protein Coverage:

Supporting Peptides:

| Peptide | Uniq | -10lgP | Mass | Length | ppm | m/z | z | RT | 1/k0 Start | 1/k0 End | Precursor ID | Area Sample 4 | #Feature | #Feature Sample 4 | Start | End | PTM | AScore | Found By |
| --- | --- | --- | --- | --- | --- | --- | --- | --- | --- | --- | --- | --- | --- | --- | --- | --- | --- | --- | --- |
| K.DTVIEKFEENITK.G | Y | 58.3 | 1564.7984 | 13 | 1.27 | 783.4058 | 2 | 19.63 | 1.068 | 1.0753 | 75192 | 9.24e2 | 2 | 2 | 112 | 124 |  |  | DB Search |
| K.KDTVIEKFEENITK.G | Y | 52.33 | 1692.8934 | 14 | -2.27 | 565.3026 | 3 | 17.43 | 0.8485 | 0.8558 | 60646 | 1.07e2 | 1 | 1 | 111 | 124 |  |  | DB Search |
| total 2 peptides |  |  |  |  |  |  |  |  |  |  |  |  |  |  |  |  |  |  |  |
| --- | --- | --- | --- | --- | --- | --- | --- | --- | --- | --- | --- | --- | --- | --- | --- | --- | --- | --- | --- |

Best Unique PSM (Scan Sample\_2\_A1\_1\_3640.d:75192, m/z=783.4058, z=2, RT=19.63, ppm=-0.92):


TRINITY\_DN991\_c0\_g1\_i5.p1

back to list

  

| Protein Coverage
| Supporting Peptides
| Best Unique PSM
|

Protein Coverage:

Supporting Peptides:

| Peptide | Uniq | -10lgP | Mass | Length | ppm | m/z | z | RT | 1/k0 Start | 1/k0 End | Precursor ID | Area Sample 4 | #Feature | #Feature Sample 4 | Start | End | PTM | AScore | Found By |
| --- | --- | --- | --- | --- | --- | --- | --- | --- | --- | --- | --- | --- | --- | --- | --- | --- | --- | --- | --- |
| K.IVGDDFTVGQSVTIK.C | Y | 58.39 | 1577.83 | 15 | 1.5 | 789.9218 | 2 | 17.37 | 1.0359 | 1.0431 | 60205 | 1.59e3 | 1 | 1 | 688 | 702 |  |  | DB Search |
| K.LSFC(+57.02)DTLTTPK.N | Y | 58.37 | 1281.6275 | 11 | 1.48 | 641.8206 | 2 | 17.08 | 0.9654 | 0.9727 | 58298 | 1.14e3 | 1 | 1 | 674 | 684 | Carbamidomethylation |  | DB Search |
| K.FGTC(+57.02)IDLIGDYR.C | Y | 58.29 | 1428.6707 | 12 | 0.76 | 715.3416 | 2 | 19.11 | 1.0049 | 1.0122 | 71833 | 0 | 0 | 0 | 2005 | 2016 | Carbamidomethylation |  | DB Search |
| R.ITVTVSPDIHPPAK.L | Y | 57.2 | 1473.8191 | 14 | -2.38 | 737.9135 | 2 | 15.04 | 1.0652 | 1.0725 | 45475 | 1.12e3 | 1 | 1 | 1183 | 1196 |  |  | DB Search |
| K.GVGHQC(+57.02)INTIGSFK.C | Y | 45.41 | 1516.7456 | 14 | 11.11 | 506.5937 | 3 | 14.17 | 0.7807 | 0.788 | 40291 | 0 | 0 | 0 | 808 | 821 | Carbamidomethylation |  | DB Search |
| R.GDFLILANSNGK.V | Y | 42.7 | 1247.651 | 12 | -8.76 | 624.8259 | 2 | 18.05 | 0.9501 | 0.9574 | 64900 | 0 | 0 | 0 | 2267 | 2278 |  |  | DB Search |
| K.ISYEC(+57.02)DEGYHLR.N | Y | 39.73 | 1540.6616 | 12 | 1.5 | 514.5608 | 3 | 14.27 | 0.7962 | 0.8035 | 40907 | 0 | 0 | 0 | 579 | 590 | Carbamidomethylation |  | DB Search |
| total 7 peptides |  |  |  |  |  |  |  |  |  |  |  |  |  |  |  |  |  |  |  |
| --- | --- | --- | --- | --- | --- | --- | --- | --- | --- | --- | --- | --- | --- | --- | --- | --- | --- | --- | --- |

Best Unique PSM (Scan Sample\_2\_A1\_1\_3640.d:60205, m/z=789.9218, z=2, RT=17.37, ppm=-0.69):


TRINITY\_DN3078\_c0\_g1\_i1.p1

back to list

  

| Protein Coverage
| Supporting Peptides
| Best Unique PSM
|

Protein Coverage:

Supporting Peptides:

| Peptide | Uniq | -10lgP | Mass | Length | ppm | m/z | z | RT | 1/k0 Start | 1/k0 End | Precursor ID | Area Sample 4 | #Feature | #Feature Sample 4 | Start | End | PTM | AScore | Found By |
| --- | --- | --- | --- | --- | --- | --- | --- | --- | --- | --- | --- | --- | --- | --- | --- | --- | --- | --- | --- |
| K.SGNVVLC(+57.02)LIDVHK.A | Y | 58.39 | 1452.7759 | 13 | 0.7 | 485.2652 | 3 | 18.74 | 0.7351 | 0.7424 | 69444 | 0 | 0 | 0 | 1014 | 1026 | Carbamidomethylation |  | DB Search |
| K.DFDLDKHLVDK.C | Y | 58.28 | 1343.6721 | 11 | 1.6 | 448.8977 | 3 | 15.28 | 0.7249 | 0.7322 | 47163 | 7.72e2 | 1 | 1 | 623 | 633 |  |  | DB Search |
| K.VSQTC(+57.02)AEHVFR.V | Y | 58.25 | 1332.6245 | 11 | 2.4 | 667.3196 | 2 | 12.94 | 0.9824 | 0.9896 | 32741 | 6.68e2 | 1 | 1 | 547 | 557 | Carbamidomethylation |  | DB Search |
| R.KVSQTC(+57.02)AEHVFR.V | Y | 57.57 | 1460.7194 | 12 | 0.9 | 487.9131 | 3 | 12.05 | 0.797 | 0.8043 | 27165 | 0 | 0 | 0 | 546 | 557 | Carbamidomethylation |  | DB Search |
| K.AKIEALHC(+57.02)LSENIVK.D | Y | 57.12 | 1723.9291 | 15 | 3.74 | 575.6512 | 3 | 15.83 | 0.8262 | 0.8335 | 50444 | 1.65e3 | 1 | 1 | 709 | 723 | Carbamidomethylation |  | DB Search |
| K.IEALHC(+57.02)LSENIVK.D | Y | 53.3 | 1524.797 | 13 | -4.69 | 509.2694 | 3 | 16.48 | 0.8051 | 0.8124 | 54755 | 5.65e2 | 1 | 1 | 711 | 723 | Carbamidomethylation |  | DB Search |
| K.MSAIVFEDYR.L | Y | 52.54 | 1229.575 | 10 | 5.58 | 615.7969 | 2 | 18.05 | 0.9158 | 0.923 | 64811 | 0 | 0 | 0 | 168 | 177 |  |  | DB Search |
| K.VNELLWK.S | Y | 51.65 | 900.5069 | 7 | 0.77 | 451.2601 | 2 | 17.15 | 0.7758 | 0.7831 | 58792 | 3.57e2 | 1 | 1 | 305 | 311 |  |  | DB Search |
| total 8 peptides |  |  |  |  |  |  |  |  |  |  |  |  |  |  |  |  |  |  |  |
| --- | --- | --- | --- | --- | --- | --- | --- | --- | --- | --- | --- | --- | --- | --- | --- | --- | --- | --- | --- |

Best Unique PSM (Scan Sample\_2\_A1\_1\_3640.d:69444, m/z=485.2652, z=3, RT=18.74, ppm=-1.5):


TRINITY\_DN554\_c0\_g1\_i5.p1

back to list

  

| Protein Coverage
| Supporting Peptides
| Best Unique PSM
|

Protein Coverage:

Supporting Peptides:

| Peptide | Uniq | -10lgP | Mass | Length | ppm | m/z | z | RT | 1/k0 Start | 1/k0 End | Precursor ID | Area Sample 4 | #Feature | #Feature Sample 4 | Start | End | PTM | AScore | Found By |
| --- | --- | --- | --- | --- | --- | --- | --- | --- | --- | --- | --- | --- | --- | --- | --- | --- | --- | --- | --- |
| MQIFVK.T | Y | 57.42 | 764.4255 | 6 | 0.18 | 383.2192 | 2 | 15.73 | 0.7363 | 0.7437 | 49899 | 2.46e2 | 1 | 1 | 1 | 6 |  |  | DB Search |
| K.ESTLHLVLR.L | Y | 57.27 | 1066.6135 | 9 | 2.57 | 534.3142 | 2 | 15.80 | 0.8785 | 0.8858 | 50332 | 4.97e3 | 1 | 1 | 64 | 72 |  |  | DB Search |
| K.IQDKEGIPPDQQR.L | Y | 55.01 | 1522.7739 | 13 | 0.25 | 762.3928 | 2 | 11.08 | 1.0343 | 1.0415 | 21018 | 1.78e3 | 2 | 2 | 30 | 42 |  |  | DB Search |
| K.TITLEVEPSDTIENVK.S | Y | 54.56 | 1786.92 | 16 | 4.26 | 894.4691 | 2 | 17.62 | 1.1442 | 1.1514 | 61769 | 1.48e4 | 1 | 1 | 12 | 27 |  |  | DB Search |
| total 4 peptides |  |  |  |  |  |  |  |  |  |  |  |  |  |  |  |  |  |  |  |
| --- | --- | --- | --- | --- | --- | --- | --- | --- | --- | --- | --- | --- | --- | --- | --- | --- | --- | --- | --- |

Best Unique PSM (Scan Sample\_2\_A1\_1\_3640.d:49899, m/z=383.2192, z=2, RT=15.73, ppm=-2.02):


TRINITY\_DN5897\_c0\_g1\_i2.p1

back to list

  

| Protein Coverage
| Supporting Peptides
| Best Unique PSM
|

Protein Coverage:

Supporting Peptides:

| Peptide | Uniq | -10lgP | Mass | Length | ppm | m/z | z | RT | 1/k0 Start | 1/k0 End | Precursor ID | Area Sample 4 | #Feature | #Feature Sample 4 | Start | End | PTM | AScore | Found By |
| --- | --- | --- | --- | --- | --- | --- | --- | --- | --- | --- | --- | --- | --- | --- | --- | --- | --- | --- | --- |
| K.FDDVPNGLGAFSK.V | Y | 53.88 | 1365.6564 | 13 | 1.41 | 683.835 | 2 | 17.84 | 0.959 | 0.9662 | 63447 | 0 | 0 | 0 | 50 | 62 |  |  | DB Search |
| total 1 peptides |  |  |  |  |  |  |  |  |  |  |  |  |  |  |  |  |  |  |  |
| --- | --- | --- | --- | --- | --- | --- | --- | --- | --- | --- | --- | --- | --- | --- | --- | --- | --- | --- | --- |

Best Unique PSM (Scan Sample\_2\_A1\_1\_3640.d:63447, m/z=683.835, z=2, RT=17.84, ppm=-0.78):


TRINITY\_DN1936\_c0\_g2\_i1.p1

back to list

  

| Protein Coverage
| Supporting Peptides
| Best Unique PSM
|

Protein Coverage:

Supporting Peptides:

| Peptide | Uniq | -10lgP | Mass | Length | ppm | m/z | z | RT | 1/k0 Start | 1/k0 End | Precursor ID | Area Sample 4 | #Feature | #Feature Sample 4 | Start | End | PTM | AScore | Found By |
| --- | --- | --- | --- | --- | --- | --- | --- | --- | --- | --- | --- | --- | --- | --- | --- | --- | --- | --- | --- |
| K.YLFMESSSPVK.M | Y | 58.39 | 1286.6217 | 11 | 0.88 | 644.3173 | 2 | 17.73 | 0.9218 | 0.9291 | 62641 | 6.16e2 | 1 | 1 | 467 | 477 |  |  | DB Search |
| K.YQGDIAVDDVSLSSK.K | Y | 58.39 | 1595.7678 | 15 | 3 | 798.8918 | 2 | 16.09 | 1.0676 | 1.0749 | 52110 | 0 | 0 | 0 | 754 | 768 |  |  | DB Search |
| K.LAYPQNNSPLIVSK.D | Y | 58.29 | 1542.8406 | 14 | -0.82 | 772.4252 | 2 | 15.95 | 1.0472 | 1.0544 | 51022 | 2.12e3 | 1 | 1 | 1292 | 1305 |  |  | DB Search |
| K.GYLGDIAIDDFK.F | Y | 58.28 | 1325.6503 | 12 | -2.17 | 663.8295 | 2 | 19.70 | 0.9566 | 0.9638 | 75625 | 0 | 0 | 0 | 560 | 571 |  |  | DB Search |
| K.SFTGDIALDDFR.M | Y | 57.22 | 1355.6357 | 12 | 3.82 | 678.8262 | 2 | 19.15 | 0.9545 | 0.9618 | 72030 | 5.21e2 | 1 | 1 | 1584 | 1595 |  |  | DB Search |
| K.GTTSTAGTGPSFDHTK.G | Y | 56.22 | 1563.7165 | 16 | 6.12 | 782.8686 | 2 | 11.55 | 1.0552 | 1.0624 | 24218 | 7.26e2 | 1 | 1 | 636 | 651 |  |  | DB Search |
| K.YQGDIAVDDVSLSSKK.C | Y | 52.63 | 1723.8628 | 16 | 2.95 | 862.9393 | 2 | 14.98 | 1.1698 | 1.177 | 45228 | 0 | 0 | 0 | 754 | 769 |  |  | DB Search |
| total 7 peptides |  |  |  |  |  |  |  |  |  |  |  |  |  |  |  |  |  |  |  |
| --- | --- | --- | --- | --- | --- | --- | --- | --- | --- | --- | --- | --- | --- | --- | --- | --- | --- | --- | --- |

Best Unique PSM (Scan Sample\_2\_A1\_1\_3640.d:62641, m/z=644.3173, z=2, RT=17.73, ppm=-1.31):


TRINITY\_DN477\_c0\_g1\_i8.p1

back to list

  

| Protein Coverage
| Supporting Peptides
| Best Unique PSM
|

Protein Coverage:

Supporting Peptides:

| Peptide | Uniq | -10lgP | Mass | Length | ppm | m/z | z | RT | 1/k0 Start | 1/k0 End | Precursor ID | Area Sample 4 | #Feature | #Feature Sample 4 | Start | End | PTM | AScore | Found By |
| --- | --- | --- | --- | --- | --- | --- | --- | --- | --- | --- | --- | --- | --- | --- | --- | --- | --- | --- | --- |
| R.ILQC(+57.02)NQYANVEEAR.N | Y | 58.29 | 1706.8046 | 14 | -5.21 | 854.4033 | 2 | 14.34 | 1.0697 | 1.0769 | 41279 | 1.23e3 | 1 | 1 | 86 | 99 | Carbamidomethylation |  | DB Search |
| R.NNPPTATR.T | Y | 58.23 | 869.4355 | 8 | 2.62 | 435.7252 | 2 | 9.23 | 0.784 | 0.7913 | 11045 | 2.65e3 | 1 | 1 | 925 | 932 |  |  | DB Search |
| R.SQSAFPTRR.R | Y | 57.12 | 1048.5414 | 9 | 2.06 | 525.2779 | 2 | 11.08 | 0.8542 | 0.8615 | 21210 | 5.83e3 | 1 | 1 | 856 | 864 |  |  | DB Search |
| K.DKNILVLTLGVGPQVNR.N | Y | 52.82 | 1835.0628 | 17 | -4.3 | 612.6909 | 3 | 19.59 | 0.8368 | 0.8441 | 74795 | 1.35e3 | 2 | 2 | 168 | 184 |  |  | DB Search |
| total 4 peptides |  |  |  |  |  |  |  |  |  |  |  |  |  |  |  |  |  |  |  |
| --- | --- | --- | --- | --- | --- | --- | --- | --- | --- | --- | --- | --- | --- | --- | --- | --- | --- | --- | --- |

Best Unique PSM (Scan Sample\_2\_A1\_1\_3640.d:41279, m/z=854.4033, z=2, RT=14.34, ppm=-7.4):


TRINITY\_DN244\_c0\_g1\_i1.p1

back to list

  

| Protein Coverage
| Supporting Peptides
| Best Unique PSM
|

Protein Coverage:

Supporting Peptides:

| Peptide | Uniq | -10lgP | Mass | Length | ppm | m/z | z | RT | 1/k0 Start | 1/k0 End | Precursor ID | Area Sample 4 | #Feature | #Feature Sample 4 | Start | End | PTM | AScore | Found By |
| --- | --- | --- | --- | --- | --- | --- | --- | --- | --- | --- | --- | --- | --- | --- | --- | --- | --- | --- | --- |
| K.LIDELTPEVTSALEC(+57.02)EGGPR.G | Y | 58.41 | 2185.0572 | 20 | 2.45 | 729.3599 | 3 | 19.44 | 0.9824 | 0.9896 | 73811 | 1.6e4 | 2 | 2 | 2265 | 2284 | Carbamidomethylation |  | DB Search |
| R.NGDDGDQGPVGK.K | Y | 58.39 | 1157.4949 | 12 | 2.29 | 579.7548 | 2 | 9.94 | 0.8579 | 0.8652 | 14586 | 1.13e3 | 2 | 2 | 1242 | 1253 |  |  | DB Search |
| K.ALVILGDGFISGGGTPSK.T | Y | 58.39 | 1687.9144 | 18 | 2.69 | 844.9649 | 2 | 19.82 | 1.0821 | 1.0893 | 76374 | 1.04e4 | 2 | 2 | 1619 | 1636 |  |  | DB Search |
| K.FSNDIVDSFAVSQKK.T | Y | 58.39 | 1683.8468 | 15 | 1.47 | 562.2891 | 3 | 17.32 | 0.8635 | 0.8708 | 59993 | 0 | 0 | 0 | 295 | 309 |  |  | DB Search |
| K.VLILLADGQQTYVPGVTEPSEVAK.E | Y | 58.39 | 2526.3581 | 24 | 2.36 | 843.1268 | 3 | 19.70 | 1.1314 | 1.1386 | 75615 | 5.51e3 | 1 | 1 | 2200 | 2223 |  |  | DB Search |
| K.KFSNDIVDSFAVSQKK.T | Y | 58.29 | 1811.9417 | 16 | 5.74 | 604.99 | 3 | 16.35 | 0.8943 | 0.9016 | 53776 | 0 | 0 | 0 | 294 | 309 |  |  | DB Search |
| R.IDKALLLASSNAFDTSNGAR.E | Y | 58.28 | 2063.0647 | 20 | -0.57 | 688.6936 | 3 | 17.50 | 0.9113 | 0.9186 | 61143 | 3.2e2 | 1 | 1 | 1909 | 1928 |  |  | DB Search |
| R.AEIGFNFVR.F | Y | 58.26 | 1051.545 | 9 | -0.62 | 526.7783 | 2 | 18.51 | 0.8595 | 0.8668 | 67749 | 1.14e3 | 1 | 1 | 321 | 329 |  |  | DB Search |
| K.ALFHEFVSEK.E | Y | 58.26 | 1205.608 | 10 | 0.45 | 603.8102 | 2 | 16.09 | 0.9384 | 0.9457 | 52118 | 3.31e2 | 1 | 1 | 1668 | 1677 |  |  | DB Search |
| K.ALLLASSNAFDTSNGAR.E | Y | 58.24 | 1706.8587 | 17 | 2.06 | 854.4365 | 2 | 17.87 | 1.0945 | 1.1017 | 63441 | 8.53e3 | 2 | 2 | 1912 | 1928 |  |  | DB Search |
| K.DLSLVVEPLK.N | Y | 57.54 | 1111.6488 | 10 | 3.46 | 556.8324 | 2 | 18.96 | 0.8943 | 0.9016 | 70793 | 9.98e2 | 1 | 1 | 392 | 401 |  |  | DB Search |
| K.ESESRLDLALK.L | Y | 56.92 | 1259.6721 | 11 | 4.03 | 630.8445 | 2 | 14.96 | 0.9731 | 0.9804 | 45033 | 1.43e3 | 1 | 1 | 1588 | 1598 |  |  | DB Search |
| K.IEDQIVLLGR.V | Y | 56.8 | 1154.6659 | 10 | 0.49 | 578.3392 | 2 | 17.96 | 0.8887 | 0.8959 | 64127 | 3.55e3 | 1 | 1 | 2001 | 2010 |  |  | DB Search |
| R.GDEGFIGFEGAR.G | Y | 55.52 | 1253.5676 | 12 | -2.01 | 627.7885 | 2 | 17.53 | 0.9121 | 0.9194 | 61207 | 1.99e3 | 1 | 1 | 2371 | 2382 |  |  | DB Search |
| K.MFGEQFGGR.G | Y | 55.12 | 1027.4545 | 9 | -1.81 | 514.7325 | 2 | 15.93 | 0.8144 | 0.8218 | 51038 | 1.4e3 | 1 | 1 | 2186 | 2194 |  |  | DB Search |
| R.IDYALELAK.S | Y | 54.77 | 1034.5648 | 9 | 1.74 | 518.2894 | 2 | 16.99 | 0.8631 | 0.8704 | 57966 | 0 | 0 | 0 | 352 | 360 |  |  | DB Search |
| R.IGVIVYSDTASIEIR.F | Y | 54.11 | 1634.8879 | 15 | -3.55 | 818.4465 | 2 | 18.74 | 1.1017 | 1.109 | 69514 | 0 | 0 | 0 | 2136 | 2150 |  |  | DB Search |
| K.KALVILGDGFISGGGTPSK.T | Y | 53.76 | 1816.0094 | 19 | 1.61 | 606.3434 | 3 | 18.24 | 0.9 | 0.9073 | 66111 | 0 | 0 | 0 | 1618 | 1636 |  |  | DB Search |
| K.KVLILLADGQQTYVPGVTEPSEVAK.E | Y | 53.41 | 2654.453 | 25 | 2.54 | 885.8253 | 3 | 18.52 | 1.0371 | 1.0443 | 67689 | 3.47e3 | 2 | 2 | 2199 | 2223 |  |  | DB Search |
| K.RFDGIFQYTK.D | Y | 53.29 | 1273.6455 | 10 | -1.75 | 637.8275 | 2 | 17.11 | 0.959 | 0.9662 | 58503 | 2e3 | 1 | 1 | 678 | 687 |  |  | DB Search |
| K.LARDELFTESVGAR.G | Y | 42.61 | 1562.8052 | 14 | 1.79 | 521.9421 | 3 | 15.78 | 0.8352 | 0.8425 | 50248 | 4.04e2 | 1 | 1 | 1599 | 1612 |  |  | DB Search |
| total 21 peptides |  |  |  |  |  |  |  |  |  |  |  |  |  |  |  |  |  |  |  |
| --- | --- | --- | --- | --- | --- | --- | --- | --- | --- | --- | --- | --- | --- | --- | --- | --- | --- | --- | --- |

Best Unique PSM (Scan Sample\_2\_A1\_1\_3640.d:73811, m/z=729.3599, z=3, RT=19.44, ppm=0.25):


TRINITY\_DN2796\_c0\_g1\_i1.p1

back to list

  

| Protein Coverage
| Supporting Peptides
| Best Unique PSM
|

Protein Coverage:

Supporting Peptides:

| Peptide | Uniq | -10lgP | Mass | Length | ppm | m/z | z | RT | 1/k0 Start | 1/k0 End | Precursor ID | Area Sample 4 | #Feature | #Feature Sample 4 | Start | End | PTM | AScore | Found By |
| --- | --- | --- | --- | --- | --- | --- | --- | --- | --- | --- | --- | --- | --- | --- | --- | --- | --- | --- | --- |
| K.LGANAILGVSLAAAK.A | Y | 58.25 | 1367.8136 | 15 | 1.11 | 684.9133 | 2 | 19.13 | 1.058 | 1.0652 | 71938 | 4.36e2 | 1 | 1 | 168 | 182 |  |  | DB Search |
| total 1 peptides |  |  |  |  |  |  |  |  |  |  |  |  |  |  |  |  |  |  |  |
| --- | --- | --- | --- | --- | --- | --- | --- | --- | --- | --- | --- | --- | --- | --- | --- | --- | --- | --- | --- |

Best Unique PSM (Scan Sample\_2\_A1\_1\_3640.d:71938, m/z=684.9133, z=2, RT=19.13, ppm=-1.09):


TRINITY\_DN6920\_c0\_g1\_i4.p1

back to list

  

| Protein Coverage
| Supporting Peptides
| Best Unique PSM
|

Protein Coverage:

Supporting Peptides:

| Peptide | Uniq | -10lgP | Mass | Length | ppm | m/z | z | RT | 1/k0 Start | 1/k0 End | Precursor ID | Area Sample 4 | #Feature | #Feature Sample 4 | Start | End | PTM | AScore | Found By |
| --- | --- | --- | --- | --- | --- | --- | --- | --- | --- | --- | --- | --- | --- | --- | --- | --- | --- | --- | --- |
| R.VLVHTQQK.L | Y | 58.19 | 951.5501 | 8 | 0.78 | 476.7817 | 2 | 9.51 | 0.8335 | 0.8408 | 12449 | 5.52e2 | 1 | 1 | 40 | 47 |  |  | DB Search |
| R.KVAC(+57.02)IGAWHPAR.V | Y | 52.34 | 1364.7135 | 12 | 0.83 | 455.9112 | 3 | 13.48 | 0.7766 | 0.784 | 36007 | 9.51e1 | 1 | 1 | 128 | 139 | Carbamidomethylation |  | DB Search |
| total 2 peptides |  |  |  |  |  |  |  |  |  |  |  |  |  |  |  |  |  |  |  |
| --- | --- | --- | --- | --- | --- | --- | --- | --- | --- | --- | --- | --- | --- | --- | --- | --- | --- | --- | --- |

Best Unique PSM (Scan Sample\_2\_A1\_1\_3640.d:12449, m/z=476.7817, z=2, RT=9.51, ppm=-1.41):


TRINITY\_DN61742\_c0\_g1\_i3.p1

back to list

  

| Protein Coverage
| Supporting Peptides
| Best Unique PSM
|

Protein Coverage:

Supporting Peptides:

| Peptide | Uniq | -10lgP | Mass | Length | ppm | m/z | z | RT | 1/k0 Start | 1/k0 End | Precursor ID | Area Sample 4 | #Feature | #Feature Sample 4 | Start | End | PTM | AScore | Found By |
| --- | --- | --- | --- | --- | --- | --- | --- | --- | --- | --- | --- | --- | --- | --- | --- | --- | --- | --- | --- |
| K.LYISSLPLK.V | Y | 58.05 | 1032.6219 | 9 | 2.33 | 517.3183 | 2 | 18.15 | 0.8587 | 0.866 | 65559 | 0 | 0 | 0 | 78 | 86 |  |  | DB Search |
| total 1 peptides |  |  |  |  |  |  |  |  |  |  |  |  |  |  |  |  |  |  |  |
| --- | --- | --- | --- | --- | --- | --- | --- | --- | --- | --- | --- | --- | --- | --- | --- | --- | --- | --- | --- |

Best Unique PSM (Scan Sample\_2\_A1\_1\_3640.d:65559, m/z=517.3183, z=2, RT=18.15, ppm=0.13):


TRINITY\_DN129928\_c0\_g1\_i1.p1

back to list

  

| Protein Coverage
| Supporting Peptides
| Best Unique PSM
|

Protein Coverage:

Supporting Peptides:

| Peptide | Uniq | -10lgP | Mass | Length | ppm | m/z | z | RT | 1/k0 Start | 1/k0 End | Precursor ID | Area Sample 4 | #Feature | #Feature Sample 4 | Start | End | PTM | AScore | Found By |
| --- | --- | --- | --- | --- | --- | --- | --- | --- | --- | --- | --- | --- | --- | --- | --- | --- | --- | --- | --- |
| R.ASFLIDLR.N | Y | 53.54 | 933.5283 | 8 | 1.82 | 467.7713 | 2 | 19.39 | 0.8153 | 0.8226 | 73480 | 2.89e3 | 1 | 1 | 97 | 104 |  |  | DB Search |
| total 1 peptides |  |  |  |  |  |  |  |  |  |  |  |  |  |  |  |  |  |  |  |
| --- | --- | --- | --- | --- | --- | --- | --- | --- | --- | --- | --- | --- | --- | --- | --- | --- | --- | --- | --- |

Best Unique PSM (Scan Sample\_2\_A1\_1\_3640.d:73480, m/z=467.7713, z=2, RT=19.39, ppm=-0.37):


TRINITY\_DN173718\_c0\_g1\_i1.p1

back to list

  

| Protein Coverage
| Supporting Peptides
| Best Unique PSM
|

Protein Coverage:

Supporting Peptides:

| Peptide | Uniq | -10lgP | Mass | Length | ppm | m/z | z | RT | 1/k0 Start | 1/k0 End | Precursor ID | Area Sample 4 | #Feature | #Feature Sample 4 | Start | End | PTM | AScore | Found By |
| --- | --- | --- | --- | --- | --- | --- | --- | --- | --- | --- | --- | --- | --- | --- | --- | --- | --- | --- | --- |
| MQIFVK.T | Y | 57.42 | 764.4255 | 6 | 0.18 | 383.2192 | 2 | 15.73 | 0.7363 | 0.7437 | 49899 | 2.46e2 | 1 | 1 | 1 | 6 |  |  | DB Search |
| K.ESTLHLVLR.L | Y | 57.27 | 1066.6135 | 9 | 2.57 | 534.3142 | 2 | 15.80 | 0.8785 | 0.8858 | 50332 | 4.97e3 | 1 | 1 | 64 | 72 |  |  | DB Search |
| K.IQDKEGIPPDQQR.L | Y | 55.01 | 1522.7739 | 13 | 0.25 | 762.3928 | 2 | 11.08 | 1.0343 | 1.0415 | 21018 | 1.78e3 | 2 | 2 | 30 | 42 |  |  | DB Search |
| total 3 peptides |  |  |  |  |  |  |  |  |  |  |  |  |  |  |  |  |  |  |  |
| --- | --- | --- | --- | --- | --- | --- | --- | --- | --- | --- | --- | --- | --- | --- | --- | --- | --- | --- | --- |

Best Unique PSM (Scan Sample\_2\_A1\_1\_3640.d:49899, m/z=383.2192, z=2, RT=15.73, ppm=-2.02):


TRINITY\_DN11595\_c0\_g1\_i1.p1

back to list

  

| Protein Coverage
| Supporting Peptides
| Best Unique PSM
|

Protein Coverage:

Supporting Peptides:

| Peptide | Uniq | -10lgP | Mass | Length | ppm | m/z | z | RT | 1/k0 Start | 1/k0 End | Precursor ID | Area Sample 4 | #Feature | #Feature Sample 4 | Start | End | PTM | AScore | Found By |
| --- | --- | --- | --- | --- | --- | --- | --- | --- | --- | --- | --- | --- | --- | --- | --- | --- | --- | --- | --- |
| K.LVHGDVFRPPR.Y | Y | 58.34 | 1291.7149 | 11 | 1.79 | 431.5787 | 3 | 13.55 | 0.823 | 0.8303 | 36380 | 1.06e3 | 1 | 1 | 301 | 311 |  |  | DB Search |
| total 1 peptides |  |  |  |  |  |  |  |  |  |  |  |  |  |  |  |  |  |  |  |
| --- | --- | --- | --- | --- | --- | --- | --- | --- | --- | --- | --- | --- | --- | --- | --- | --- | --- | --- | --- |

Best Unique PSM (Scan Sample\_2\_A1\_1\_3640.d:36380, m/z=431.5787, z=3, RT=13.55, ppm=-0.4):


TRINITY\_DN984\_c0\_g1\_i1.p1

back to list

  

| Protein Coverage
| Supporting Peptides
| Best Unique PSM
|

Protein Coverage:

Supporting Peptides:

| Peptide | Uniq | -10lgP | Mass | Length | ppm | m/z | z | RT | 1/k0 Start | 1/k0 End | Precursor ID | Area Sample 4 | #Feature | #Feature Sample 4 | Start | End | PTM | AScore | Found By |
| --- | --- | --- | --- | --- | --- | --- | --- | --- | --- | --- | --- | --- | --- | --- | --- | --- | --- | --- | --- |
| R.EGPTGPQGETGR.R | Y | 58.28 | 1184.5421 | 12 | -1.15 | 593.2764 | 2 | 10.36 | 0.866 | 0.8733 | 16960 | 6.73e4 | 1 | 1 | 476 | 487 |  |  | DB Search |
| R.GSIGPSGEQGAQGLPGPTGR.R | Y | 55.93 | 1821.8969 | 20 | 0.48 | 911.9542 | 2 | 14.23 | 1.0857 | 1.0929 | 40533 | 1.87e3 | 1 | 1 | 867 | 886 |  |  | DB Search |
| R.EGPTGPQGETGRR.G | Y | 55.33 | 1340.6433 | 13 | 3.71 | 447.889 | 3 | 9.89 | 0.7632 | 0.7705 | 13877 | 4.68e3 | 1 | 1 | 476 | 488 |  |  | DB Search |
| total 3 peptides |  |  |  |  |  |  |  |  |  |  |  |  |  |  |  |  |  |  |  |
| --- | --- | --- | --- | --- | --- | --- | --- | --- | --- | --- | --- | --- | --- | --- | --- | --- | --- | --- | --- |

Best Unique PSM (Scan Sample\_2\_A1\_1\_3640.d:16960, m/z=593.2764, z=2, RT=10.36, ppm=-3.35):


TRINITY\_DN825\_c0\_g1\_i1.p1

back to list

  

| Protein Coverage
| Supporting Peptides
| Best Unique PSM
|

Protein Coverage:

Supporting Peptides:

| Peptide | Uniq | -10lgP | Mass | Length | ppm | m/z | z | RT | 1/k0 Start | 1/k0 End | Precursor ID | Area Sample 4 | #Feature | #Feature Sample 4 | Start | End | PTM | AScore | Found By |
| --- | --- | --- | --- | --- | --- | --- | --- | --- | --- | --- | --- | --- | --- | --- | --- | --- | --- | --- | --- |
| R.VQEMEEALAAAESK.A | Y | 53.73 | 1504.7079 | 14 | -3.91 | 753.3566 | 2 | 16.96 | 1.013 | 1.0202 | 57682 | 3.72e2 | 1 | 1 | 15 | 28 |  |  | DB Search |
| total 1 peptides |  |  |  |  |  |  |  |  |  |  |  |  |  |  |  |  |  |  |  |
| --- | --- | --- | --- | --- | --- | --- | --- | --- | --- | --- | --- | --- | --- | --- | --- | --- | --- | --- | --- |

Best Unique PSM (Scan Sample\_2\_A1\_1\_3640.d:57682, m/z=753.3566, z=2, RT=16.96, ppm=-6.1):


TRINITY\_DN32804\_c0\_g1\_i1.p1

back to list

  

| Protein Coverage
| Supporting Peptides
| Best Unique PSM
|

Protein Coverage:

Supporting Peptides:

| Peptide | Uniq | -10lgP | Mass | Length | ppm | m/z | z | RT | 1/k0 Start | 1/k0 End | Precursor ID | Area Sample 4 | #Feature | #Feature Sample 4 | Start | End | PTM | AScore | Found By |
| --- | --- | --- | --- | --- | --- | --- | --- | --- | --- | --- | --- | --- | --- | --- | --- | --- | --- | --- | --- |
| R.HIQNFEGGER.E | Y | 58.28 | 1185.5527 | 10 | -1.78 | 593.7812 | 2 | 11.51 | 0.8802 | 0.8874 | 23962 | 0 | 0 | 0 | 439 | 448 |  |  | DB Search |
| total 1 peptides |  |  |  |  |  |  |  |  |  |  |  |  |  |  |  |  |  |  |  |
| --- | --- | --- | --- | --- | --- | --- | --- | --- | --- | --- | --- | --- | --- | --- | --- | --- | --- | --- | --- |

Best Unique PSM (Scan Sample\_2\_A1\_1\_3640.d:23962, m/z=593.7812, z=2, RT=11.51, ppm=-3.97):


TRINITY\_DN1339\_c6\_g1\_i2.p1

back to list

  

| Protein Coverage
| Supporting Peptides
| Best Unique PSM
|

Protein Coverage:

Supporting Peptides:

| Peptide | Uniq | -10lgP | Mass | Length | ppm | m/z | z | RT | 1/k0 Start | 1/k0 End | Precursor ID | Area Sample 4 | #Feature | #Feature Sample 4 | Start | End | PTM | AScore | Found By |
| --- | --- | --- | --- | --- | --- | --- | --- | --- | --- | --- | --- | --- | --- | --- | --- | --- | --- | --- | --- |
| K.VITSDDPAEKEK.K | Y | 58.39 | 1330.6616 | 12 | 3.3 | 666.3388 | 2 | 10.77 | 0.9856 | 0.9928 | 19482 | 3.46e2 | 1 | 1 | 355 | 366 |  |  | DB Search |
| total 1 peptides |  |  |  |  |  |  |  |  |  |  |  |  |  |  |  |  |  |  |  |
| --- | --- | --- | --- | --- | --- | --- | --- | --- | --- | --- | --- | --- | --- | --- | --- | --- | --- | --- | --- |

Best Unique PSM (Scan Sample\_2\_A1\_1\_3640.d:19482, m/z=666.3388, z=2, RT=10.77, ppm=1.11):


TRINITY\_DN11377\_c0\_g1\_i1.p1

back to list

  

| Protein Coverage
| Supporting Peptides
| Best Unique PSM
|

Protein Coverage:

Supporting Peptides:

| Peptide | Uniq | -10lgP | Mass | Length | ppm | m/z | z | RT | 1/k0 Start | 1/k0 End | Precursor ID | Area Sample 4 | #Feature | #Feature Sample 4 | Start | End | PTM | AScore | Found By |
| --- | --- | --- | --- | --- | --- | --- | --- | --- | --- | --- | --- | --- | --- | --- | --- | --- | --- | --- | --- |
| R.LLETKNEYLDFEYK.Q | Y | 54.94 | 1803.893 | 14 | 2.05 | 602.3049 | 3 | 17.54 | 0.8303 | 0.8376 | 61326 | 5.89e2 | 1 | 1 | 386 | 399 |  |  | DB Search |
| R.RIVLLATDALPK.I | Y | 52.64 | 1308.8129 | 12 | 1.05 | 655.413 | 2 | 17.39 | 1.009 | 1.0162 | 60449 | 7.55e2 | 1 | 1 | 265 | 276 |  |  | DB Search |
| total 2 peptides |  |  |  |  |  |  |  |  |  |  |  |  |  |  |  |  |  |  |  |
| --- | --- | --- | --- | --- | --- | --- | --- | --- | --- | --- | --- | --- | --- | --- | --- | --- | --- | --- | --- |

Best Unique PSM (Scan Sample\_2\_A1\_1\_3640.d:61326, m/z=602.3049, z=3, RT=17.54, ppm=-0.15):


TRINITY\_DN738\_c0\_g1\_i4.p1

back to list

  

| Protein Coverage
| Supporting Peptides
| Best Unique PSM
|

Protein Coverage:

Supporting Peptides:

| Peptide | Uniq | -10lgP | Mass | Length | ppm | m/z | z | RT | 1/k0 Start | 1/k0 End | Precursor ID | Area Sample 4 | #Feature | #Feature Sample 4 | Start | End | PTM | AScore | Found By |
| --- | --- | --- | --- | --- | --- | --- | --- | --- | --- | --- | --- | --- | --- | --- | --- | --- | --- | --- | --- |
| R.YGEFSEK.L | Y | 52.42 | 858.3759 | 7 | 1.16 | 430.1948 | 2 | 12.54 | 0.7652 | 0.7726 | 30256 | 6.91e2 | 1 | 1 | 54 | 60 |  |  | DB Search |
| total 1 peptides |  |  |  |  |  |  |  |  |  |  |  |  |  |  |  |  |  |  |  |
| --- | --- | --- | --- | --- | --- | --- | --- | --- | --- | --- | --- | --- | --- | --- | --- | --- | --- | --- | --- |

Best Unique PSM (Scan Sample\_2\_A1\_1\_3640.d:30256, m/z=430.1948, z=2, RT=12.54, ppm=-1.04):


TRINITY\_DN4815\_c0\_g1\_i1.p1

back to list

  

| Protein Coverage
| Supporting Peptides
| Best Unique PSM
|

Protein Coverage:

Supporting Peptides:

| Peptide | Uniq | -10lgP | Mass | Length | ppm | m/z | z | RT | 1/k0 Start | 1/k0 End | Precursor ID | Area Sample 4 | #Feature | #Feature Sample 4 | Start | End | PTM | AScore | Found By |
| --- | --- | --- | --- | --- | --- | --- | --- | --- | --- | --- | --- | --- | --- | --- | --- | --- | --- | --- | --- |
| K.DVNC(+57.02)NNNQLK.S | Y | 58.39 | 1217.5459 | 10 | 2.19 | 609.7802 | 2 | 10.68 | 0.9129 | 0.9202 | 18856 | 1.17e3 | 1 | 1 | 493 | 502 | Carbamidomethylation |  | DB Search |
| R.DC(+57.02)PNEPTER.A | Y | 58.29 | 1116.4506 | 9 | 1.83 | 559.2324 | 2 | 10.18 | 0.853 | 0.8603 | 15597 | 3.45e3 | 1 | 1 | 416 | 424 | Carbamidomethylation |  | DB Search |
| K.TC(+57.02)TC(+57.02)ENVR.Q | Y | 58.27 | 1038.4223 | 8 | 5.2 | 520.22 | 2 | 9.83 | 0.8425 | 0.8498 | 14008 | 2.42e3 | 1 | 1 | 442 | 449 | Carbamidomethylation, Carbamidomethylation |  | DB Search |
| R.AVAC(+57.02)NSGPC(+57.02)LAGC(+57.02)PR.N | Y | 58.17 | 1588.6908 | 15 | 1.54 | 795.3522 | 2 | 13.16 | 1.015 | 1.0222 | 33794 | 1.36e4 | 1 | 1 | 425 | 439 | Carbamidomethylation, Carbamidomethylation, Carbamidomethylation |  | DB Search |
| K.DNGDWAIDNLR.I | Y | 57.94 | 1287.5843 | 11 | -1.8 | 644.7969 | 2 | 17.93 | 0.9206 | 0.9279 | 64010 | 0 | 0 | 0 | 186 | 196 |  |  | DB Search |
| K.TIKDVNC(+57.02)NNNQLK.S | Y | 54.96 | 1559.7726 | 13 | 3.46 | 520.9321 | 3 | 11.33 | 0.7892 | 0.7966 | 22761 | 1.98e3 | 1 | 1 | 490 | 502 | Carbamidomethylation |  | DB Search |
| total 6 peptides |  |  |  |  |  |  |  |  |  |  |  |  |  |  |  |  |  |  |  |
| --- | --- | --- | --- | --- | --- | --- | --- | --- | --- | --- | --- | --- | --- | --- | --- | --- | --- | --- | --- |

Best Unique PSM (Scan Sample\_2\_A1\_1\_3640.d:18856, m/z=609.7802, z=2, RT=10.68, ppm=-0.01):


TRINITY\_DN252\_c0\_g1\_i1.p1

back to list

  

| Protein Coverage
| Supporting Peptides
| Best Unique PSM
|

Protein Coverage:

Supporting Peptides:

| Peptide | Uniq | -10lgP | Mass | Length | ppm | m/z | z | RT | 1/k0 Start | 1/k0 End | Precursor ID | Area Sample 4 | #Feature | #Feature Sample 4 | Start | End | PTM | AScore | Found By |
| --- | --- | --- | --- | --- | --- | --- | --- | --- | --- | --- | --- | --- | --- | --- | --- | --- | --- | --- | --- |
| K.TMNTNYVDKK.T | Y | 54.87 | 1212.5809 | 10 | 4.95 | 607.2994 | 2 | 11.20 | 0.9299 | 0.9372 | 22052 | 0 | 0 | 0 | 101 | 110 |  |  | DB Search |
| total 1 peptides |  |  |  |  |  |  |  |  |  |  |  |  |  |  |  |  |  |  |  |
| --- | --- | --- | --- | --- | --- | --- | --- | --- | --- | --- | --- | --- | --- | --- | --- | --- | --- | --- | --- |

Best Unique PSM (Scan Sample\_2\_A1\_1\_3640.d:22052, m/z=607.2994, z=2, RT=11.20, ppm=2.75):


TRINITY\_DN912\_c0\_g1\_i2.p1

back to list

  

| Protein Coverage
| Supporting Peptides
| Best Unique PSM
|

Protein Coverage:

Supporting Peptides:

| Peptide | Uniq | -10lgP | Mass | Length | ppm | m/z | z | RT | 1/k0 Start | 1/k0 End | Precursor ID | Area Sample 4 | #Feature | #Feature Sample 4 | Start | End | PTM | AScore | Found By |
| --- | --- | --- | --- | --- | --- | --- | --- | --- | --- | --- | --- | --- | --- | --- | --- | --- | --- | --- | --- |
| K.LLLLGAGESGK.S | Y | 43.84 | 1056.6179 | 11 | 6.84 | 529.3187 | 2 | 17.27 | 0.8761 | 0.8834 | 59698 | 0 | 0 | 0 | 36 | 46 |  |  | DB Search |
| total 1 peptides |  |  |  |  |  |  |  |  |  |  |  |  |  |  |  |  |  |  |  |
| --- | --- | --- | --- | --- | --- | --- | --- | --- | --- | --- | --- | --- | --- | --- | --- | --- | --- | --- | --- |

Best Unique PSM (Scan Sample\_2\_A1\_1\_3640.d:59698, m/z=529.3187, z=2, RT=17.27, ppm=4.65):


TRINITY\_DN477\_c0\_g1\_i3.p1

back to list

  

| Protein Coverage
| Supporting Peptides
| Best Unique PSM
|

Protein Coverage:

Supporting Peptides:

| Peptide | Uniq | -10lgP | Mass | Length | ppm | m/z | z | RT | 1/k0 Start | 1/k0 End | Precursor ID | Area Sample 4 | #Feature | #Feature Sample 4 | Start | End | PTM | AScore | Found By |
| --- | --- | --- | --- | --- | --- | --- | --- | --- | --- | --- | --- | --- | --- | --- | --- | --- | --- | --- | --- |
| R.NDVNNPPQAR.R | Y | 57.97 | 1123.537 | 10 | 3.56 | 562.7766 | 2 | 10.00 | 0.8615 | 0.8688 | 15207 | 4.89e4 | 2 | 2 | 147 | 156 |  |  | DB Search |
| total 1 peptides |  |  |  |  |  |  |  |  |  |  |  |  |  |  |  |  |  |  |  |
| --- | --- | --- | --- | --- | --- | --- | --- | --- | --- | --- | --- | --- | --- | --- | --- | --- | --- | --- | --- |

Best Unique PSM (Scan Sample\_2\_A1\_1\_3640.d:15207, m/z=562.7766, z=2, RT=10.00, ppm=1.36):


TRINITY\_DN4481\_c0\_g1\_i3.p1

back to list

  

| Protein Coverage
| Supporting Peptides
| Best Unique PSM
|

Protein Coverage:

Supporting Peptides:

| Peptide | Uniq | -10lgP | Mass | Length | ppm | m/z | z | RT | 1/k0 Start | 1/k0 End | Precursor ID | Area Sample 4 | #Feature | #Feature Sample 4 | Start | End | PTM | AScore | Found By |
| --- | --- | --- | --- | --- | --- | --- | --- | --- | --- | --- | --- | --- | --- | --- | --- | --- | --- | --- | --- |
| K.AVAEEFISVDPSVR.G | Y | 58.29 | 1517.7725 | 14 | 0.44 | 759.8922 | 2 | 17.79 | 1.0287 | 1.0359 | 62940 | 2.17e3 | 1 | 1 | 351 | 364 |  |  | DB Search |
| total 1 peptides |  |  |  |  |  |  |  |  |  |  |  |  |  |  |  |  |  |  |  |
| --- | --- | --- | --- | --- | --- | --- | --- | --- | --- | --- | --- | --- | --- | --- | --- | --- | --- | --- | --- |

Best Unique PSM (Scan Sample\_2\_A1\_1\_3640.d:62940, m/z=759.8922, z=2, RT=17.79, ppm=-1.75):


TRINITY\_DN794\_c0\_g1\_i2.p1

back to list

  

| Protein Coverage
| Supporting Peptides
| Best Unique PSM
|

Protein Coverage:

Supporting Peptides:

| Peptide | Uniq | -10lgP | Mass | Length | ppm | m/z | z | RT | 1/k0 Start | 1/k0 End | Precursor ID | Area Sample 4 | #Feature | #Feature Sample 4 | Start | End | PTM | AScore | Found By |
| --- | --- | --- | --- | --- | --- | --- | --- | --- | --- | --- | --- | --- | --- | --- | --- | --- | --- | --- | --- |
| K.VIDKFQNEFK.C | Y | 58.33 | 1266.6608 | 10 | -0.56 | 634.3359 | 2 | 14.67 | 0.9699 | 0.9771 | 43298 | 0 | 0 | 0 | 138 | 147 |  |  | DB Search |
| total 1 peptides |  |  |  |  |  |  |  |  |  |  |  |  |  |  |  |  |  |  |  |
| --- | --- | --- | --- | --- | --- | --- | --- | --- | --- | --- | --- | --- | --- | --- | --- | --- | --- | --- | --- |

Best Unique PSM (Scan Sample\_2\_A1\_1\_3640.d:43298, m/z=634.3359, z=2, RT=14.67, ppm=-2.75):


TRINITY\_DN14729\_c0\_g1\_i1.p1

back to list

  

| Protein Coverage
| Supporting Peptides
| Best Unique PSM
|

Protein Coverage:

Supporting Peptides:

| Peptide | Uniq | -10lgP | Mass | Length | ppm | m/z | z | RT | 1/k0 Start | 1/k0 End | Precursor ID | Area Sample 4 | #Feature | #Feature Sample 4 | Start | End | PTM | AScore | Found By |
| --- | --- | --- | --- | --- | --- | --- | --- | --- | --- | --- | --- | --- | --- | --- | --- | --- | --- | --- | --- |
| K.LLDQNILDEMNR.L | Y | 44.8 | 1472.7293 | 12 | 16.14 | 737.3822 | 2 | 18.83 | 1.0073 | 1.0146 | 70012 | 0 | 0 | 0 | 151 | 162 |  |  | DB Search |
| total 1 peptides |  |  |  |  |  |  |  |  |  |  |  |  |  |  |  |  |  |  |  |
| --- | --- | --- | --- | --- | --- | --- | --- | --- | --- | --- | --- | --- | --- | --- | --- | --- | --- | --- | --- |

Best Unique PSM (Scan Sample\_2\_A1\_1\_3640.d:70012, m/z=737.3822, z=2, RT=18.83, ppm=13.95):


TRINITY\_DN51907\_c0\_g1\_i1.p1

back to list

  

| Protein Coverage
| Supporting Peptides
| Best Unique PSM
|

Protein Coverage:

Supporting Peptides:

| Peptide | Uniq | -10lgP | Mass | Length | ppm | m/z | z | RT | 1/k0 Start | 1/k0 End | Precursor ID | Area Sample 4 | #Feature | #Feature Sample 4 | Start | End | PTM | AScore | Found By |
| --- | --- | --- | --- | --- | --- | --- | --- | --- | --- | --- | --- | --- | --- | --- | --- | --- | --- | --- | --- |
| R.GVTSITLELQDLQR.N | Y | 58.23 | 1571.8519 | 14 | 1.83 | 786.9329 | 2 | 19.49 | 1.0556 | 1.0628 | 74149 | 1.11e3 | 1 | 1 | 5 | 18 |  |  | DB Search |
| total 1 peptides |  |  |  |  |  |  |  |  |  |  |  |  |  |  |  |  |  |  |  |
| --- | --- | --- | --- | --- | --- | --- | --- | --- | --- | --- | --- | --- | --- | --- | --- | --- | --- | --- | --- |

Best Unique PSM (Scan Sample\_2\_A1\_1\_3640.d:74149, m/z=786.9329, z=2, RT=19.49, ppm=-0.36):


TRINITY\_DN39385\_c0\_g1\_i2.p1

back to list

  

| Protein Coverage
| Supporting Peptides
| Best Unique PSM
|

Protein Coverage:

Supporting Peptides:

| Peptide | Uniq | -10lgP | Mass | Length | ppm | m/z | z | RT | 1/k0 Start | 1/k0 End | Precursor ID | Area Sample 4 | #Feature | #Feature Sample 4 | Start | End | PTM | AScore | Found By |
| --- | --- | --- | --- | --- | --- | --- | --- | --- | --- | --- | --- | --- | --- | --- | --- | --- | --- | --- | --- |
| K.GDFILNSSNVIENVTR.L | Y | 58.32 | 1776.9006 | 16 | 4.19 | 889.4594 | 2 | 19.77 | 1.1138 | 1.121 | 76079 | 0 | 0 | 0 | 81 | 96 |  |  | DB Search |
| total 1 peptides |  |  |  |  |  |  |  |  |  |  |  |  |  |  |  |  |  |  |  |
| --- | --- | --- | --- | --- | --- | --- | --- | --- | --- | --- | --- | --- | --- | --- | --- | --- | --- | --- | --- |

Best Unique PSM (Scan Sample\_2\_A1\_1\_3640.d:76079, m/z=889.4594, z=2, RT=19.77, ppm=2):


TRINITY\_DN1936\_c0\_g1\_i3.p1

back to list

  

| Protein Coverage
| Supporting Peptides
| Best Unique PSM
|

Protein Coverage:

Supporting Peptides:

| Peptide | Uniq | -10lgP | Mass | Length | ppm | m/z | z | RT | 1/k0 Start | 1/k0 End | Precursor ID | Area Sample 4 | #Feature | #Feature Sample 4 | Start | End | PTM | AScore | Found By |
| --- | --- | --- | --- | --- | --- | --- | --- | --- | --- | --- | --- | --- | --- | --- | --- | --- | --- | --- | --- |
| K.AGNPITIRFEGVR.G | Y | 58.39 | 1428.7837 | 13 | -7.01 | 477.2641 | 3 | 16.75 | 0.7534 | 0.7608 | 56404 | 0 | 0 | 0 | 925 | 937 |  |  | DB Search |
| K.GFDC(+57.02)NFDKEFC(+57.02)K.T | Y | 58.38 | 1565.6279 | 12 | 12.85 | 522.8888 | 3 | 16.23 | 0.7689 | 0.7762 | 53044 | 0 | 0 | 0 | 795 | 806 | Carbamidomethylation, Carbamidomethylation |  | DB Search |
| R.VANDLSETIVWK.L | Y | 58.37 | 1373.719 | 12 | 2.31 | 687.8669 | 2 | 18.08 | 0.9711 | 0.9783 | 64974 | 2.42e2 | 1 | 1 | 195 | 206 |  |  | DB Search |
| K.SAVNHNIIFEGR.T | Y | 58.18 | 1355.6946 | 12 | 3.09 | 678.8552 | 2 | 14.84 | 0.9751 | 0.9824 | 44311 | 1.57e3 | 1 | 1 | 59 | 70 |  |  | DB Search |
| R.GSSFM(+15.99)GDIALDDISVR.Y | Y | 58.13 | 1697.793 | 16 | 8.34 | 849.909 | 2 | 18.58 | 1.0797 | 1.0869 | 68392 | 9.18e2 | 1 | 1 | 938 | 953 | Oxidation (M) | M5:Oxidation (M):1000 | DB Search |
| R.LPHETAIIGSETIPVSGSGK.C | Y | 57.77 | 1992.0527 | 20 | 2.18 | 665.0248 | 3 | 15.95 | 0.9226 | 0.9299 | 51107 | 1.64e3 | 1 | 1 | 154 | 173 |  |  | DB Search |
| R.SAPIWNLK.G | Y | 55.26 | 927.5178 | 8 | -6.21 | 464.7623 | 2 | 17.22 | 0.8059 | 0.8132 | 59364 | 0 | 0 | 0 | 36 | 43 |  |  | DB Search |
| total 7 peptides |  |  |  |  |  |  |  |  |  |  |  |  |  |  |  |  |  |  |  |
| --- | --- | --- | --- | --- | --- | --- | --- | --- | --- | --- | --- | --- | --- | --- | --- | --- | --- | --- | --- |

Best Unique PSM (Scan Sample\_2\_A1\_1\_3640.d:56404, m/z=477.2641, z=3, RT=16.75, ppm=-9.2):


TRINITY\_DN585\_c0\_g1\_i3.p1

back to list

  

| Protein Coverage
| Supporting Peptides
| Best Unique PSM
|

Protein Coverage:

Supporting Peptides:

| Peptide | Uniq | -10lgP | Mass | Length | ppm | m/z | z | RT | 1/k0 Start | 1/k0 End | Precursor ID | Area Sample 4 | #Feature | #Feature Sample 4 | Start | End | PTM | AScore | Found By |
| --- | --- | --- | --- | --- | --- | --- | --- | --- | --- | --- | --- | --- | --- | --- | --- | --- | --- | --- | --- |
| K.ESTLHLVLR.L | Y | 57.27 | 1066.6135 | 9 | 2.57 | 534.3142 | 2 | 15.80 | 0.8785 | 0.8858 | 50332 | 4.97e3 | 1 | 1 | 36 | 44 |  |  | DB Search |
| K.IQDKEGIPPDQQR.L | Y | 55.01 | 1522.7739 | 13 | 0.25 | 762.3928 | 2 | 11.08 | 1.0343 | 1.0415 | 21018 | 1.78e3 | 2 | 2 | 2 | 14 |  |  | DB Search |
| K.TITLEVEPSDTIENVK.S | Y | 54.56 | 1786.92 | 16 | 4.26 | 894.4691 | 2 | 17.62 | 1.1442 | 1.1514 | 61769 | 1.48e4 | 1 | 1 | 60 | 75 |  |  | DB Search |
| total 3 peptides |  |  |  |  |  |  |  |  |  |  |  |  |  |  |  |  |  |  |  |
| --- | --- | --- | --- | --- | --- | --- | --- | --- | --- | --- | --- | --- | --- | --- | --- | --- | --- | --- | --- |

Best Unique PSM (Scan Sample\_2\_A1\_1\_3640.d:50332, m/z=534.7766, z=2, RT=15.80, ppm=0.37):


TRINITY\_DN48767\_c0\_g1\_i1.p1

back to list

  

| Protein Coverage
| Supporting Peptides
| Best Unique PSM
|

Protein Coverage:

Supporting Peptides:

| Peptide | Uniq | -10lgP | Mass | Length | ppm | m/z | z | RT | 1/k0 Start | 1/k0 End | Precursor ID | Area Sample 4 | #Feature | #Feature Sample 4 | Start | End | PTM | AScore | Found By |
| --- | --- | --- | --- | --- | --- | --- | --- | --- | --- | --- | --- | --- | --- | --- | --- | --- | --- | --- | --- |
| K.VPAENNPK.L | Y | 58.24 | 867.445 | 8 | 3.57 | 434.7304 | 2 | 9.23 | 0.7766 | 0.784 | 11210 | 2.03e3 | 1 | 1 | 214 | 221 |  |  | DB Search |
| K.LC(+57.02)QEDWNKVVR.E | Y | 52.13 | 1445.7085 | 11 | 6.21 | 482.912 | 3 | 14.86 | 0.7909 | 0.7982 | 44521 | 0 | 0 | 0 | 407 | 417 | Carbamidomethylation |  | DB Search |
| total 2 peptides |  |  |  |  |  |  |  |  |  |  |  |  |  |  |  |  |  |  |  |
| --- | --- | --- | --- | --- | --- | --- | --- | --- | --- | --- | --- | --- | --- | --- | --- | --- | --- | --- | --- |

Best Unique PSM (Scan Sample\_2\_A1\_1\_3640.d:11210, m/z=434.7304, z=2, RT=9.23, ppm=1.38):


TRINITY\_DN5\_c0\_g1\_i8.p1

back to list

  

| Protein Coverage
| Supporting Peptides
| Best Unique PSM
|

Protein Coverage:

Supporting Peptides:

| Peptide | Uniq | -10lgP | Mass | Length | ppm | m/z | z | RT | 1/k0 Start | 1/k0 End | Precursor ID | Area Sample 4 | #Feature | #Feature Sample 4 | Start | End | PTM | AScore | Found By |
| --- | --- | --- | --- | --- | --- | --- | --- | --- | --- | --- | --- | --- | --- | --- | --- | --- | --- | --- | --- |
| K.IIIELQGDLIK.I | Y | 53.27 | 1253.7594 | 11 | 3.89 | 627.8881 | 2 | 19.42 | 0.9513 | 0.9586 | 73842 | 0 | 0 | 0 | 65 | 75 |  |  | DB Search |
| total 1 peptides |  |  |  |  |  |  |  |  |  |  |  |  |  |  |  |  |  |  |  |
| --- | --- | --- | --- | --- | --- | --- | --- | --- | --- | --- | --- | --- | --- | --- | --- | --- | --- | --- | --- |

Best Unique PSM (Scan Sample\_2\_A1\_1\_3640.d:73842, m/z=627.8881, z=2, RT=19.42, ppm=1.69):


TRINITY\_DN106\_c0\_g1\_i3.p1

back to list

  

| Protein Coverage
| Supporting Peptides
| Best Unique PSM
|

Protein Coverage:

Supporting Peptides:

| Peptide | Uniq | -10lgP | Mass | Length | ppm | m/z | z | RT | 1/k0 Start | 1/k0 End | Precursor ID | Area Sample 4 | #Feature | #Feature Sample 4 | Start | End | PTM | AScore | Found By |
| --- | --- | --- | --- | --- | --- | --- | --- | --- | --- | --- | --- | --- | --- | --- | --- | --- | --- | --- | --- |
| K.EGPAGPAGPVGR.L | Y | 58.28 | 1063.541 | 12 | -11.82 | 532.7703 | 2 | 11.84 | 0.8461 | 0.8534 | 25992 | 0 | 0 | 0 | 191 | 202 |  |  | DB Search |
| R.DGPAGPAGR.D | N | 58.24 | 796.3827 | 9 | 3.73 | 399.1992 | 2 | 9.76 | 0.7061 | 0.7135 | 13439 | 1.18e4 | 2 | 2 | 488 | 496 |  |  | DB Search |
| R.GPDGAVGPTGPIGAR.G | N | 58.04 | 1320.6786 | 15 | 1.83 | 661.3463 | 2 | 13.72 | 0.9558 | 0.963 | 37443 | 1.21e4 | 2 | 2 | 327 | 341 |  |  | DB Search |
| total 3 peptides |  |  |  |  |  |  |  |  |  |  |  |  |  |  |  |  |  |  |  |
| --- | --- | --- | --- | --- | --- | --- | --- | --- | --- | --- | --- | --- | --- | --- | --- | --- | --- | --- | --- |

Best Unique PSM (Scan Sample\_2\_A1\_1\_3640.d:25992, m/z=532.7703, z=2, RT=11.84, ppm=-14.02):


TRINITY\_DN2028\_c0\_g1\_i1.p1

back to list

  

| Protein Coverage
| Supporting Peptides
| Best Unique PSM
|

Protein Coverage:

Supporting Peptides:

| Peptide | Uniq | -10lgP | Mass | Length | ppm | m/z | z | RT | 1/k0 Start | 1/k0 End | Precursor ID | Area Sample 4 | #Feature | #Feature Sample 4 | Start | End | PTM | AScore | Found By |
| --- | --- | --- | --- | --- | --- | --- | --- | --- | --- | --- | --- | --- | --- | --- | --- | --- | --- | --- | --- |
| R.DFREFLYNSETGK.V | Y | 58.28 | 1604.7471 | 13 | 1.19 | 535.9224 | 3 | 18.53 | 0.8055 | 0.8128 | 68013 | 8.32e2 | 1 | 1 | 21 | 33 |  |  | DB Search |
| K.LIQYIENKPGLTR.L | Y | 58.26 | 1543.8722 | 13 | 2.17 | 772.9434 | 2 | 14.98 | 1.0769 | 1.0841 | 45128 | 1.91e4 | 2 | 2 | 81 | 93 |  |  | DB Search |
| K.KNVDC(+57.02)TLK.G | Y | 52.37 | 976.5012 | 8 | 2.18 | 489.2578 | 2 | 10.11 | 0.8343 | 0.8416 | 15536 | 0 | 0 | 0 | 238 | 245 | Carbamidomethylation |  | DB Search |
| total 3 peptides |  |  |  |  |  |  |  |  |  |  |  |  |  |  |  |  |  |  |  |
| --- | --- | --- | --- | --- | --- | --- | --- | --- | --- | --- | --- | --- | --- | --- | --- | --- | --- | --- | --- |

Best Unique PSM (Scan Sample\_2\_A1\_1\_3640.d:68013, m/z=535.9224, z=3, RT=18.53, ppm=-1.01):


TRINITY\_DN6920\_c0\_g1\_i1.p1

back to list

  

| Protein Coverage
| Supporting Peptides
| Best Unique PSM
|

Protein Coverage:

Supporting Peptides:

| Peptide | Uniq | -10lgP | Mass | Length | ppm | m/z | z | RT | 1/k0 Start | 1/k0 End | Precursor ID | Area Sample 4 | #Feature | #Feature Sample 4 | Start | End | PTM | AScore | Found By |
| --- | --- | --- | --- | --- | --- | --- | --- | --- | --- | --- | --- | --- | --- | --- | --- | --- | --- | --- | --- |
| R.VLVHTQQK.L | Y | 58.19 | 951.5501 | 8 | 0.78 | 476.7817 | 2 | 9.51 | 0.8335 | 0.8408 | 12449 | 5.52e2 | 1 | 1 | 45 | 52 |  |  | DB Search |
| R.KVAC(+57.02)IGAWHPAR.V | Y | 52.34 | 1364.7135 | 12 | 0.83 | 455.9112 | 3 | 13.48 | 0.7766 | 0.784 | 36007 | 9.51e1 | 1 | 1 | 133 | 144 | Carbamidomethylation |  | DB Search |
| total 2 peptides |  |  |  |  |  |  |  |  |  |  |  |  |  |  |  |  |  |  |  |
| --- | --- | --- | --- | --- | --- | --- | --- | --- | --- | --- | --- | --- | --- | --- | --- | --- | --- | --- | --- |

Best Unique PSM (Scan Sample\_2\_A1\_1\_3640.d:12449, m/z=476.7817, z=2, RT=9.51, ppm=-1.41):


TRINITY\_DN5\_c0\_g1\_i10.p1

back to list

  

| Protein Coverage
| Supporting Peptides
| Best Unique PSM
|

Protein Coverage:

Supporting Peptides:

| Peptide | Uniq | -10lgP | Mass | Length | ppm | m/z | z | RT | 1/k0 Start | 1/k0 End | Precursor ID | Area Sample 4 | #Feature | #Feature Sample 4 | Start | End | PTM | AScore | Found By |
| --- | --- | --- | --- | --- | --- | --- | --- | --- | --- | --- | --- | --- | --- | --- | --- | --- | --- | --- | --- |
| K.IIIELQGDLIK.I | Y | 53.27 | 1253.7594 | 11 | 3.89 | 627.8881 | 2 | 19.42 | 0.9513 | 0.9586 | 73842 | 0 | 0 | 0 | 494 | 504 |  |  | DB Search |
| total 1 peptides |  |  |  |  |  |  |  |  |  |  |  |  |  |  |  |  |  |  |  |
| --- | --- | --- | --- | --- | --- | --- | --- | --- | --- | --- | --- | --- | --- | --- | --- | --- | --- | --- | --- |

Best Unique PSM (Scan Sample\_2\_A1\_1\_3640.d:73842, m/z=627.8881, z=2, RT=19.42, ppm=1.69):


TRINITY\_DN1465\_c0\_g1\_i1.p2

back to list

  

| Protein Coverage
| Supporting Peptides
| Best Unique PSM
|

Protein Coverage:

Supporting Peptides:

| Peptide | Uniq | -10lgP | Mass | Length | ppm | m/z | z | RT | 1/k0 Start | 1/k0 End | Precursor ID | Area Sample 4 | #Feature | #Feature Sample 4 | Start | End | PTM | AScore | Found By |
| --- | --- | --- | --- | --- | --- | --- | --- | --- | --- | --- | --- | --- | --- | --- | --- | --- | --- | --- | --- |
| K.ESTLHLVLR.L | Y | 57.27 | 1066.6135 | 9 | 2.57 | 534.3142 | 2 | 15.80 | 0.8785 | 0.8858 | 50332 | 4.97e3 | 1 | 1 | 91 | 99 |  |  | DB Search |
| K.IQDKEGIPPDQQR.L | Y | 55.01 | 1522.7739 | 13 | 0.25 | 762.3928 | 2 | 11.08 | 1.0343 | 1.0415 | 21018 | 1.78e3 | 2 | 2 | 57 | 69 |  |  | DB Search |
| total 2 peptides |  |  |  |  |  |  |  |  |  |  |  |  |  |  |  |  |  |  |  |
| --- | --- | --- | --- | --- | --- | --- | --- | --- | --- | --- | --- | --- | --- | --- | --- | --- | --- | --- | --- |

Best Unique PSM (Scan Sample\_2\_A1\_1\_3640.d:50332, m/z=534.7766, z=2, RT=15.80, ppm=0.37):


TRINITY\_DN74720\_c0\_g1\_i1.p1

back to list

  

| Protein Coverage
| Supporting Peptides
| Best Unique PSM
|

Protein Coverage:

Supporting Peptides:

| Peptide | Uniq | -10lgP | Mass | Length | ppm | m/z | z | RT | 1/k0 Start | 1/k0 End | Precursor ID | Area Sample 4 | #Feature | #Feature Sample 4 | Start | End | PTM | AScore | Found By |
| --- | --- | --- | --- | --- | --- | --- | --- | --- | --- | --- | --- | --- | --- | --- | --- | --- | --- | --- | --- |
| K.VMLLYTDGEESFR.S | Y | 58.2 | 1558.7337 | 13 | 9.8 | 780.3801 | 2 | 18.73 | 1.0721 | 1.0793 | 69339 | 0 | 0 | 0 | 33 | 45 |  |  | DB Search |
| R.SPSDTTTDEELK.M | Y | 57.03 | 1321.5885 | 12 | -0.87 | 661.7995 | 2 | 11.82 | 0.959 | 0.9662 | 25774 | 1.05e3 | 1 | 1 | 46 | 57 |  |  | DB Search |
| K.GVDIVVLAVGSK.I | Y | 54.46 | 1155.6863 | 12 | -0.99 | 578.8486 | 2 | 18.70 | 0.9178 | 0.9251 | 69234 | 5.37e2 | 1 | 1 | 63 | 74 |  |  | DB Search |
| R.EKGVDIVVLAVGSK.I | Y | 53.72 | 1412.8238 | 14 | -0.15 | 471.9474 | 3 | 17.40 | 0.7933 | 0.8006 | 60372 | 6.65e2 | 1 | 1 | 61 | 74 |  |  | DB Search |
| total 4 peptides |  |  |  |  |  |  |  |  |  |  |  |  |  |  |  |  |  |  |  |
| --- | --- | --- | --- | --- | --- | --- | --- | --- | --- | --- | --- | --- | --- | --- | --- | --- | --- | --- | --- |

Best Unique PSM (Scan Sample\_2\_A1\_1\_3640.d:69339, m/z=780.3801, z=2, RT=18.73, ppm=7.61):


TRINITY\_DN12734\_c0\_g1\_i1.p1

back to list

  

| Protein Coverage
| Supporting Peptides
| Best Unique PSM
|

Protein Coverage:

Supporting Peptides:

| Peptide | Uniq | -10lgP | Mass | Length | ppm | m/z | z | RT | 1/k0 Start | 1/k0 End | Precursor ID | Area Sample 4 | #Feature | #Feature Sample 4 | Start | End | PTM | AScore | Found By |
| --- | --- | --- | --- | --- | --- | --- | --- | --- | --- | --- | --- | --- | --- | --- | --- | --- | --- | --- | --- |
| K.LLLLGAGESGK.S | Y | 43.84 | 1056.6179 | 11 | 6.84 | 529.3187 | 2 | 17.27 | 0.8761 | 0.8834 | 59698 | 0 | 0 | 0 | 46 | 56 |  |  | DB Search |
| total 1 peptides |  |  |  |  |  |  |  |  |  |  |  |  |  |  |  |  |  |  |  |
| --- | --- | --- | --- | --- | --- | --- | --- | --- | --- | --- | --- | --- | --- | --- | --- | --- | --- | --- | --- |

Best Unique PSM (Scan Sample\_2\_A1\_1\_3640.d:59698, m/z=529.3187, z=2, RT=17.27, ppm=4.65):


TRINITY\_DN36\_c0\_g1\_i2.p1

back to list

  

| Protein Coverage
| Supporting Peptides
| Best Unique PSM
|

Protein Coverage:

Supporting Peptides:

| Peptide | Uniq | -10lgP | Mass | Length | ppm | m/z | z | RT | 1/k0 Start | 1/k0 End | Precursor ID | Area Sample 4 | #Feature | #Feature Sample 4 | Start | End | PTM | AScore | Found By |
| --- | --- | --- | --- | --- | --- | --- | --- | --- | --- | --- | --- | --- | --- | --- | --- | --- | --- | --- | --- |
| R.LGGIEVTQGNC(+57.02)AR.K | Y | 58.36 | 1373.6721 | 13 | -2.89 | 687.8398 | 2 | 13.93 | 0.9662 | 0.9735 | 38726 | 2.09e3 | 1 | 1 | 58 | 70 | Carbamidomethylation |  | DB Search |
| K.GLPLAIGVR.D | Y | 57.12 | 894.565 | 9 | 0.27 | 448.2889 | 2 | 17.73 | 0.8124 | 0.8197 | 62574 | 3.52e3 | 1 | 1 | 32 | 40 |  |  | DB Search |
| total 2 peptides |  |  |  |  |  |  |  |  |  |  |  |  |  |  |  |  |  |  |  |
| --- | --- | --- | --- | --- | --- | --- | --- | --- | --- | --- | --- | --- | --- | --- | --- | --- | --- | --- | --- |

Best Unique PSM (Scan Sample\_2\_A1\_1\_3640.d:38726, m/z=687.8398, z=2, RT=13.93, ppm=-5.09):


TRINITY\_DN912\_c0\_g1\_i3.p1

back to list

  

| Protein Coverage
| Supporting Peptides
| Best Unique PSM
|

Protein Coverage:

Supporting Peptides:

| Peptide | Uniq | -10lgP | Mass | Length | ppm | m/z | z | RT | 1/k0 Start | 1/k0 End | Precursor ID | Area Sample 4 | #Feature | #Feature Sample 4 | Start | End | PTM | AScore | Found By |
| --- | --- | --- | --- | --- | --- | --- | --- | --- | --- | --- | --- | --- | --- | --- | --- | --- | --- | --- | --- |
| K.LLLLGAGESGK.S | Y | 43.84 | 1056.6179 | 11 | 6.84 | 529.3187 | 2 | 17.27 | 0.8761 | 0.8834 | 59698 | 0 | 0 | 0 | 36 | 46 |  |  | DB Search |
| total 1 peptides |  |  |  |  |  |  |  |  |  |  |  |  |  |  |  |  |  |  |  |
| --- | --- | --- | --- | --- | --- | --- | --- | --- | --- | --- | --- | --- | --- | --- | --- | --- | --- | --- | --- |

Best Unique PSM (Scan Sample\_2\_A1\_1\_3640.d:59698, m/z=529.3187, z=2, RT=17.27, ppm=4.65):


TRINITY\_DN1783\_c1\_g1\_i1.p2

back to list

  

| Protein Coverage
| Supporting Peptides
| Best Unique PSM
|

Protein Coverage:

Supporting Peptides:

| Peptide | Uniq | -10lgP | Mass | Length | ppm | m/z | z | RT | 1/k0 Start | 1/k0 End | Precursor ID | Area Sample 4 | #Feature | #Feature Sample 4 | Start | End | PTM | AScore | Found By |
| --- | --- | --- | --- | --- | --- | --- | --- | --- | --- | --- | --- | --- | --- | --- | --- | --- | --- | --- | --- |
| K.LTIIDALPR.C | Y | 58.24 | 1010.6124 | 9 | 0.02 | 506.3124 | 2 | 19.00 | 0.8392 | 0.8465 | 71132 | 3.79e2 | 1 | 1 | 55 | 63 |  |  | DB Search |
| total 1 peptides |  |  |  |  |  |  |  |  |  |  |  |  |  |  |  |  |  |  |  |
| --- | --- | --- | --- | --- | --- | --- | --- | --- | --- | --- | --- | --- | --- | --- | --- | --- | --- | --- | --- |

Best Unique PSM (Scan Sample\_2\_A1\_1\_3640.d:71132, m/z=506.3124, z=2, RT=19.00, ppm=-2.17):


TRINITY\_DN1616\_c0\_g1\_i2.p1

back to list

  

| Protein Coverage
| Supporting Peptides
| Best Unique PSM
|

Protein Coverage:

Supporting Peptides:

| Peptide | Uniq | -10lgP | Mass | Length | ppm | m/z | z | RT | 1/k0 Start | 1/k0 End | Precursor ID | Area Sample 4 | #Feature | #Feature Sample 4 | Start | End | PTM | AScore | Found By |
| --- | --- | --- | --- | --- | --- | --- | --- | --- | --- | --- | --- | --- | --- | --- | --- | --- | --- | --- | --- |
| R.SNIDEVNVKHEQIEEFIK.T | Y | 58.4 | 2170.0906 | 18 | 0.32 | 724.3694 | 3 | 16.43 | 0.9566 | 0.9638 | 54256 | 7.37e2 | 1 | 1 | 2671 | 2688 |  |  | DB Search |
| K.VVDTEANDLTEEVKK.T | Y | 58.38 | 1688.8468 | 15 | -0.58 | 563.9547 | 3 | 14.21 | 0.8392 | 0.8465 | 40438 | 5.49e3 | 2 | 2 | 2542 | 2556 |  |  | DB Search |
| K.LNTGDVQSLTNPK.F | Y | 58.37 | 1385.715 | 13 | 1.86 | 693.8646 | 2 | 14.77 | 0.9977 | 1.0049 | 43652 | 1.53e3 | 1 | 1 | 2783 | 2795 |  |  | DB Search |
| R.YGAVNDNC(+57.02)ER.D | Y | 58.36 | 1196.488 | 10 | 4.23 | 599.2525 | 2 | 10.91 | 0.8802 | 0.8874 | 20281 | 8.99e2 | 1 | 1 | 498 | 507 | Carbamidomethylation |  | DB Search |
| K.SNIC(+57.02)DPQNGK.C | Y | 58.34 | 1131.4979 | 10 | -0.79 | 566.7545 | 2 | 10.56 | 0.8526 | 0.8599 | 18199 | 6.49e2 | 1 | 1 | 1794 | 1803 | Carbamidomethylation |  | DB Search |
| K.LSLDVNGNYQGGGVC(+57.02)K.N | Y | 58.29 | 1679.7937 | 16 | -4.07 | 840.8989 | 2 | 15.59 | 1.0705 | 1.0777 | 49051 | 0 | 0 | 0 | 350 | 365 | Carbamidomethylation |  | DB Search |
| R.FSNLSNKGDEHISNLDTLKK.A | Y | 58.28 | 2259.1495 | 20 | 1.82 | 565.7944 | 4 | 13.91 | 0.8311 | 0.8384 | 38634 | 0 | 0 | 0 | 2121 | 2140 |  |  | DB Search |
| R.NFATAVLNVQK.S | Y | 58.28 | 1203.6611 | 11 | -3.72 | 602.8343 | 2 | 17.63 | 0.923 | 0.9303 | 62041 | 0 | 0 | 0 | 2810 | 2820 |  |  | DB Search |
| K.TLKVDISDMK.M | Y | 58.28 | 1148.6111 | 10 | -6.01 | 575.3081 | 2 | 15.17 | 0.917 | 0.9243 | 46490 | 0 | 0 | 0 | 2557 | 2566 |  |  | DB Search |
| K.FIETDVNYDVM(+15.99)VTR.N | Y | 58.27 | 1716.8029 | 14 | 0.97 | 859.4077 | 2 | 16.71 | 1.0809 | 1.0881 | 56014 | 1.52e3 | 1 | 1 | 2796 | 2809 | Oxidation (M) | M11:Oxidation (M):1000 | DB Search |
| R.VIDEGEKNIK.L | Y | 58.26 | 1143.6135 | 10 | 3.03 | 572.8145 | 2 | 10.89 | 0.9271 | 0.9344 | 20199 | 3.83e3 | 2 | 2 | 2218 | 2227 |  |  | DB Search |
| K.DIIVLEAENPVK.M | Y | 58.25 | 1338.7394 | 12 | 1.86 | 670.3768 | 2 | 18.10 | 0.9844 | 0.9916 | 65198 | 4.87e2 | 1 | 1 | 1013 | 1024 |  |  | DB Search |
| R.YVKPGVNIINIR.D | Y | 57.33 | 1384.819 | 12 | 6.22 | 462.6155 | 3 | 16.27 | 0.8043 | 0.8116 | 53149 | 6.85e2 | 2 | 2 | 1217 | 1228 |  |  | DB Search |
| K.AVDFQSIIDNAQK.L | Y | 56.94 | 1447.7307 | 13 | 4.22 | 724.8741 | 2 | 19.06 | 0.9904 | 0.9977 | 71389 | 6.5e2 | 1 | 1 | 2383 | 2395 |  |  | DB Search |
| K.DGEKVETLKVDISDINK.A | Y | 56.92 | 1901.9945 | 17 | 3.6 | 635.0063 | 3 | 16.09 | 0.9113 | 0.9186 | 52017 | 1.36e3 | 1 | 1 | 2634 | 2650 |  |  | DB Search |
| K.VETLKVDISDINK.A | Y | 56.92 | 1472.8086 | 13 | -2.26 | 491.9413 | 3 | 16.17 | 0.8222 | 0.8295 | 52567 | 5.32e2 | 1 | 1 | 2638 | 2650 |  |  | DB Search |
| K.GDEHISNLDTLKK.A | Y | 56.42 | 1468.7521 | 13 | -2.32 | 490.5891 | 3 | 12.90 | 0.7791 | 0.7864 | 32456 | 0 | 0 | 0 | 2128 | 2140 |  |  | DB Search |
| K.GFVAVNDQQPSVVVSINVPK.T | Y | 55.03 | 2096.1266 | 20 | 0.1 | 1049.0684 | 2 | 18.45 | 1.2157 | 1.2228 | 67713 | 4.05e2 | 1 | 1 | 865 | 884 |  |  | DB Search |
| K.DGEKVETLK.V | Y | 54.9 | 1017.5342 | 9 | -2.12 | 509.7722 | 2 | 11.49 | 0.8587 | 0.866 | 23862 | 1.28e3 | 1 | 1 | 2634 | 2642 |  |  | DB Search |
| R.IDNVKEDVK.S | Y | 54.89 | 1058.5608 | 9 | 5.78 | 530.2896 | 2 | 10.73 | 0.8716 | 0.8789 | 19180 | 1.58e3 | 1 | 1 | 2583 | 2591 |  |  | DB Search |
| K.VLISSLLIK.F | Y | 54.58 | 984.6583 | 9 | 0.22 | 493.3354 | 2 | 19.79 | 0.8506 | 0.8579 | 76091 | 1.17e3 | 1 | 1 | 3009 | 3017 |  |  | DB Search |
| R.RC(+57.02)EEC(+57.02)AAGHYQFPFC(+57.02)K.E | Y | 53.84 | 2058.8499 | 16 | 0.01 | 687.2891 | 3 | 15.74 | 0.9218 | 0.9291 | 49834 | 2.69e2 | 1 | 1 | 522 | 537 | Carbamidomethylation, Carbamidomethylation, Carbamidomethylation |  | DB Search |
| K.NEINLASGDVFEGGVVGSLK.L | Y | 53.15 | 2004.0163 | 20 | 1.4 | 1003.0146 | 2 | 19.85 | 1.1829 | 1.1901 | 76578 | 0 | 0 | 0 | 3249 | 3268 |  |  | DB Search |
| R.KLSLDVNGNYQGGGVC(+57.02)K.N | Y | 51.86 | 1807.8887 | 17 | 0.8 | 603.636 | 3 | 14.64 | 0.8193 | 0.8266 | 43107 | 2.56e2 | 1 | 1 | 349 | 365 | Carbamidomethylation |  | DB Search |
| R.YALNQIQPVTATVTAFK.G | Y | 47.96 | 1864.0094 | 17 | 1.71 | 933.0115 | 2 | 19.31 | 1.1478 | 1.155 | 73122 | 0 | 0 | 0 | 894 | 910 |  |  | DB Search |
| K.IRSNIDEVNVK.H | Y | 39.98 | 1285.699 | 11 | -7.71 | 643.8504 | 2 | 14.93 | 0.9638 | 0.9711 | 44969 | 0 | 0 | 0 | 2669 | 2679 |  |  | DB Search |
| total 26 peptides |  |  |  |  |  |  |  |  |  |  |  |  |  |  |  |  |  |  |  |
| --- | --- | --- | --- | --- | --- | --- | --- | --- | --- | --- | --- | --- | --- | --- | --- | --- | --- | --- | --- |

Best Unique PSM (Scan Sample\_2\_A1\_1\_3640.d:54256, m/z=724.3694, z=3, RT=16.43, ppm=-1.87):


TRINITY\_DN71276\_c0\_g1\_i1.p1

back to list

  

| Protein Coverage
| Supporting Peptides
| Best Unique PSM
|

Protein Coverage:

Supporting Peptides:

| Peptide | Uniq | -10lgP | Mass | Length | ppm | m/z | z | RT | 1/k0 Start | 1/k0 End | Precursor ID | Area Sample 4 | #Feature | #Feature Sample 4 | Start | End | PTM | AScore | Found By |
| --- | --- | --- | --- | --- | --- | --- | --- | --- | --- | --- | --- | --- | --- | --- | --- | --- | --- | --- | --- |
| K.LAEMLVSFGK.I | Y | 57.47 | 1093.5841 | 10 | -0.31 | 547.798 | 2 | 18.75 | 0.8587 | 0.866 | 69489 | 1.49e2 | 1 | 1 | 353 | 362 |  |  | DB Search |
| R.YYAAAC(+57.02)LDNIFQR.K | Y | 54.78 | 1603.7453 | 13 | 6.71 | 802.8835 | 2 | 19.80 | 1.0624 | 1.0697 | 76262 | 2.45e2 | 1 | 1 | 293 | 305 | Carbamidomethylation |  | DB Search |
| K.IITEINPTIQVFQPK.C | Y | 53.04 | 1739.9821 | 15 | 2.13 | 870.9983 | 2 | 19.16 | 1.1242 | 1.1314 | 72245 | 3.87e2 | 1 | 1 | 820 | 834 |  |  | DB Search |
| K.EAAAQIVVEIIKR.T | Y | 51.74 | 1438.8507 | 13 | 6.13 | 480.6261 | 3 | 19.26 | 0.7925 | 0.7998 | 72803 | 0 | 0 | 0 | 149 | 161 |  |  | DB Search |
| total 4 peptides |  |  |  |  |  |  |  |  |  |  |  |  |  |  |  |  |  |  |  |
| --- | --- | --- | --- | --- | --- | --- | --- | --- | --- | --- | --- | --- | --- | --- | --- | --- | --- | --- | --- |

Best Unique PSM (Scan Sample\_2\_A1\_1\_3640.d:69489, m/z=547.798, z=2, RT=18.75, ppm=-2.51):


TRINITY\_DN38399\_c0\_g1\_i1.p1

back to list

  

| Protein Coverage
| Supporting Peptides
| Best Unique PSM
|

Protein Coverage:

Supporting Peptides:

| Peptide | Uniq | -10lgP | Mass | Length | ppm | m/z | z | RT | 1/k0 Start | 1/k0 End | Precursor ID | Area Sample 4 | #Feature | #Feature Sample 4 | Start | End | PTM | AScore | Found By |
| --- | --- | --- | --- | --- | --- | --- | --- | --- | --- | --- | --- | --- | --- | --- | --- | --- | --- | --- | --- |
| K.YGGQC(+57.02)AC(+57.02)KPGIGGR.D | Y | 58.39 | 1479.6711 | 14 | 1.49 | 494.2306 | 3 | 11.25 | 0.786 | 0.7933 | 22354 | 4.8e2 | 1 | 1 | 542 | 555 | Carbamidomethylation, Carbamidomethylation |  | DB Search |
| total 1 peptides |  |  |  |  |  |  |  |  |  |  |  |  |  |  |  |  |  |  |  |
| --- | --- | --- | --- | --- | --- | --- | --- | --- | --- | --- | --- | --- | --- | --- | --- | --- | --- | --- | --- |

Best Unique PSM (Scan Sample\_2\_A1\_1\_3640.d:22354, m/z=494.2306, z=3, RT=11.25, ppm=-0.7):


TRINITY\_DN7849\_c0\_g1\_i1.p1

back to list

  

| Protein Coverage
| Supporting Peptides
| Best Unique PSM
|

Protein Coverage:

Supporting Peptides:

| Peptide | Uniq | -10lgP | Mass | Length | ppm | m/z | z | RT | 1/k0 Start | 1/k0 End | Precursor ID | Area Sample 4 | #Feature | #Feature Sample 4 | Start | End | PTM | AScore | Found By |
| --- | --- | --- | --- | --- | --- | --- | --- | --- | --- | --- | --- | --- | --- | --- | --- | --- | --- | --- | --- |
| K.TIAIDNLQINYK.C | Y | 58.27 | 1404.7613 | 12 | 1.9 | 703.3877 | 2 | 18.00 | 1.0013 | 1.0086 | 64416 | 8.31e2 | 1 | 1 | 139 | 150 |  |  | DB Search |
| total 1 peptides |  |  |  |  |  |  |  |  |  |  |  |  |  |  |  |  |  |  |  |
| --- | --- | --- | --- | --- | --- | --- | --- | --- | --- | --- | --- | --- | --- | --- | --- | --- | --- | --- | --- |

Best Unique PSM (Scan Sample\_2\_A1\_1\_3640.d:64416, m/z=703.3877, z=2, RT=18.00, ppm=-0.3):


TRINITY\_DN5584\_c0\_g1\_i3.p1

back to list

  

| Protein Coverage
| Supporting Peptides
| Best Unique PSM
|

Protein Coverage:

Supporting Peptides:

| Peptide | Uniq | -10lgP | Mass | Length | ppm | m/z | z | RT | 1/k0 Start | 1/k0 End | Precursor ID | Area Sample 4 | #Feature | #Feature Sample 4 | Start | End | PTM | AScore | Found By |
| --- | --- | --- | --- | --- | --- | --- | --- | --- | --- | --- | --- | --- | --- | --- | --- | --- | --- | --- | --- |
| K.GSLSGGLEGFYK.S | Y | 58.39 | 1213.5979 | 12 | 3.01 | 607.8067 | 2 | 17.60 | 0.9073 | 0.9146 | 61815 | 0 | 0 | 0 | 192 | 203 |  |  | DB Search |
| K.AKNQALEIFK.D | Y | 58.38 | 1160.6553 | 10 | -0.5 | 581.3334 | 2 | 15.99 | 0.9158 | 0.923 | 51477 | 0 | 0 | 0 | 761 | 770 |  |  | DB Search |
| K.VTLTQTHFLKDQDKPAK.S | Y | 58.27 | 1969.0632 | 17 | 3.13 | 657.3623 | 3 | 13.38 | 0.9113 | 0.9186 | 35272 | 5.96e2 | 2 | 2 | 559 | 575 |  |  | DB Search |
| K.VTLTQTHFLK.D | Y | 54.93 | 1186.671 | 10 | -0.58 | 594.3411 | 2 | 15.07 | 0.9646 | 0.9719 | 45810 | 6.69e2 | 1 | 1 | 559 | 568 |  |  | DB Search |
| R.LPGNVIPIK.Y | Y | 54.32 | 949.596 | 9 | 3.06 | 475.8057 | 2 | 16.31 | 0.8214 | 0.8287 | 53518 | 3.4e2 | 1 | 1 | 82 | 90 |  |  | DB Search |
| R.DGEKVTLTQTHFLK.D | Y | 53.73 | 1615.8569 | 14 | -13.39 | 539.6179 | 3 | 14.95 | 0.8534 | 0.8607 | 45082 | 0 | 0 | 0 | 555 | 568 |  |  | DB Search |
| K.YLSWTLDNDKIR.S | Y | 53.6 | 1522.778 | 12 | -3.19 | 508.5972 | 3 | 17.66 | 0.8063 | 0.8136 | 62168 | 3.04e2 | 1 | 1 | 835 | 846 |  |  | DB Search |
| R.AAFPC(+57.02)FDEPAMK.A | Y | 53.17 | 1382.5999 | 12 | -3.57 | 692.3032 | 2 | 18.01 | 0.9675 | 0.9747 | 64265 | 9.62e2 | 1 | 1 | 228 | 239 | Carbamidomethylation |  | DB Search |
| total 8 peptides |  |  |  |  |  |  |  |  |  |  |  |  |  |  |  |  |  |  |  |
| --- | --- | --- | --- | --- | --- | --- | --- | --- | --- | --- | --- | --- | --- | --- | --- | --- | --- | --- | --- |

Best Unique PSM (Scan Sample\_2\_A1\_1\_3640.d:61815, m/z=607.8067, z=2, RT=17.60, ppm=0.81):


TRINITY\_DN62874\_c0\_g1\_i1.p1

back to list

  

| Protein Coverage
| Supporting Peptides
| Best Unique PSM
|

Protein Coverage:

Supporting Peptides:

| Peptide | Uniq | -10lgP | Mass | Length | ppm | m/z | z | RT | 1/k0 Start | 1/k0 End | Precursor ID | Area Sample 4 | #Feature | #Feature Sample 4 | Start | End | PTM | AScore | Found By |
| --- | --- | --- | --- | --- | --- | --- | --- | --- | --- | --- | --- | --- | --- | --- | --- | --- | --- | --- | --- |
| K.YLEQVHIGIK.G | Y | 58.27 | 1198.671 | 10 | -5.33 | 600.3383 | 2 | 15.67 | 0.9243 | 0.9315 | 49623 | 0 | 0 | 0 | 55 | 64 |  |  | DB Search |
| total 1 peptides |  |  |  |  |  |  |  |  |  |  |  |  |  |  |  |  |  |  |  |
| --- | --- | --- | --- | --- | --- | --- | --- | --- | --- | --- | --- | --- | --- | --- | --- | --- | --- | --- | --- |

Best Unique PSM (Scan Sample\_2\_A1\_1\_3640.d:49623, m/z=600.3383, z=2, RT=15.67, ppm=-7.52):


TRINITY\_DN76789\_c0\_g1\_i1.p1

back to list

  

| Protein Coverage
| Supporting Peptides
| Best Unique PSM
|

Protein Coverage:

Supporting Peptides:

| Peptide | Uniq | -10lgP | Mass | Length | ppm | m/z | z | RT | 1/k0 Start | 1/k0 End | Precursor ID | Area Sample 4 | #Feature | #Feature Sample 4 | Start | End | PTM | AScore | Found By |
| --- | --- | --- | --- | --- | --- | --- | --- | --- | --- | --- | --- | --- | --- | --- | --- | --- | --- | --- | --- |
| R.TKVEC(+57.02)VAGINALK.G | Y | 53.02 | 1401.765 | 13 | 2.74 | 701.8901 | 2 | 14.63 | 1.0045 | 1.0118 | 43116 | 0 | 0 | 0 | 262 | 274 | Carbamidomethylation |  | DB Search |
| total 1 peptides |  |  |  |  |  |  |  |  |  |  |  |  |  |  |  |  |  |  |  |
| --- | --- | --- | --- | --- | --- | --- | --- | --- | --- | --- | --- | --- | --- | --- | --- | --- | --- | --- | --- |

Best Unique PSM (Scan Sample\_2\_A1\_1\_3640.d:43116, m/z=701.8901, z=2, RT=14.63, ppm=0.55):


TRINITY\_DN5115\_c0\_g1\_i2.p1

back to list

  

| Protein Coverage
| Supporting Peptides
| Best Unique PSM
|

Protein Coverage:

Supporting Peptides:

| Peptide | Uniq | -10lgP | Mass | Length | ppm | m/z | z | RT | 1/k0 Start | 1/k0 End | Precursor ID | Area Sample 4 | #Feature | #Feature Sample 4 | Start | End | PTM | AScore | Found By |
| --- | --- | --- | --- | --- | --- | --- | --- | --- | --- | --- | --- | --- | --- | --- | --- | --- | --- | --- | --- |
| K.IC(+57.02)LSLVSLK.Q | Y | 56.98 | 1031.6049 | 9 | -1.88 | 516.8076 | 2 | 18.96 | 0.8648 | 0.8721 | 70795 | 4.03e2 | 1 | 1 | 61 | 69 | Carbamidomethylation |  | DB Search |
| total 1 peptides |  |  |  |  |  |  |  |  |  |  |  |  |  |  |  |  |  |  |  |
| --- | --- | --- | --- | --- | --- | --- | --- | --- | --- | --- | --- | --- | --- | --- | --- | --- | --- | --- | --- |

Best Unique PSM (Scan Sample\_2\_A1\_1\_3640.d:70795, m/z=516.8076, z=2, RT=18.96, ppm=-4.07):


TRINITY\_DN106\_c0\_g1\_i12.p1

back to list

  

| Protein Coverage
| Supporting Peptides
| Best Unique PSM
|

Protein Coverage:

Supporting Peptides:

| Peptide | Uniq | -10lgP | Mass | Length | ppm | m/z | z | RT | 1/k0 Start | 1/k0 End | Precursor ID | Area Sample 4 | #Feature | #Feature Sample 4 | Start | End | PTM | AScore | Found By |
| --- | --- | --- | --- | --- | --- | --- | --- | --- | --- | --- | --- | --- | --- | --- | --- | --- | --- | --- | --- |
| R.DGLNGDRGDPGPVGAVGAR.G | Y | 58.29 | 1778.8659 | 19 | -2.01 | 593.9601 | 3 | 13.86 | 0.8323 | 0.8396 | 38286 | 4.13e2 | 1 | 1 | 37 | 55 |  |  | DB Search |
| R.DGPAGPAGR.D | N | 58.24 | 796.3827 | 9 | 3.73 | 399.1992 | 2 | 9.76 | 0.7061 | 0.7135 | 13439 | 1.18e4 | 2 | 2 | 283 | 291 |  |  | DB Search |
| total 2 peptides |  |  |  |  |  |  |  |  |  |  |  |  |  |  |  |  |  |  |  |
| --- | --- | --- | --- | --- | --- | --- | --- | --- | --- | --- | --- | --- | --- | --- | --- | --- | --- | --- | --- |

Best Unique PSM (Scan Sample\_2\_A1\_1\_3640.d:38286, m/z=593.9601, z=3, RT=13.86, ppm=-4.21):


TRINITY\_DN192883\_c0\_g1\_i1.p1

back to list

  

| Protein Coverage
| Supporting Peptides
| Best Unique PSM
|

Protein Coverage:

Supporting Peptides:

| Peptide | Uniq | -10lgP | Mass | Length | ppm | m/z | z | RT | 1/k0 Start | 1/k0 End | Precursor ID | Area Sample 4 | #Feature | #Feature Sample 4 | Start | End | PTM | AScore | Found By |
| --- | --- | --- | --- | --- | --- | --- | --- | --- | --- | --- | --- | --- | --- | --- | --- | --- | --- | --- | --- |
| R.LLLLGAGESGK.S | Y | 43.84 | 1056.6179 | 11 | 6.84 | 529.3187 | 2 | 17.27 | 0.8761 | 0.8834 | 59698 | 0 | 0 | 0 | 36 | 46 |  |  | DB Search |
| total 1 peptides |  |  |  |  |  |  |  |  |  |  |  |  |  |  |  |  |  |  |  |
| --- | --- | --- | --- | --- | --- | --- | --- | --- | --- | --- | --- | --- | --- | --- | --- | --- | --- | --- | --- |

Best Unique PSM (Scan Sample\_2\_A1\_1\_3640.d:59698, m/z=529.3187, z=2, RT=17.27, ppm=4.65):


TRINITY\_DN6221\_c0\_g1\_i5.p1

back to list

  

| Protein Coverage
| Supporting Peptides
| Best Unique PSM
|

Protein Coverage:

Supporting Peptides:

| Peptide | Uniq | -10lgP | Mass | Length | ppm | m/z | z | RT | 1/k0 Start | 1/k0 End | Precursor ID | Area Sample 4 | #Feature | #Feature Sample 4 | Start | End | PTM | AScore | Found By |
| --- | --- | --- | --- | --- | --- | --- | --- | --- | --- | --- | --- | --- | --- | --- | --- | --- | --- | --- | --- |
| K.SVQIEVSTPVC(+57.02)QTC(+57.02)PK.G | Y | 57.42 | 1831.8808 | 16 | 3.34 | 916.9487 | 2 | 15.16 | 1.1126 | 1.1198 | 46105 | 3.11e3 | 1 | 1 | 678 | 693 | Carbamidomethylation, Carbamidomethylation |  | DB Search |
| K.YQVTGC(+57.02)GGFC(+57.02)ESSVK.A | Y | 57.17 | 1677.7127 | 15 | -3.52 | 839.8588 | 2 | 14.63 | 1.068 | 1.0753 | 43042 | 0 | 0 | 0 | 744 | 758 | Carbamidomethylation, Carbamidomethylation |  | DB Search |
| total 2 peptides |  |  |  |  |  |  |  |  |  |  |  |  |  |  |  |  |  |  |  |
| --- | --- | --- | --- | --- | --- | --- | --- | --- | --- | --- | --- | --- | --- | --- | --- | --- | --- | --- | --- |

Best Unique PSM (Scan Sample\_2\_A1\_1\_3640.d:46105, m/z=916.9487, z=2, RT=15.16, ppm=1.15):


TRINITY\_DN319\_c2\_g1\_i2.p1

back to list

  

| Protein Coverage
| Supporting Peptides
| Best Unique PSM
|

Protein Coverage:

Supporting Peptides:

| Peptide | Uniq | -10lgP | Mass | Length | ppm | m/z | z | RT | 1/k0 Start | 1/k0 End | Precursor ID | Area Sample 4 | #Feature | #Feature Sample 4 | Start | End | PTM | AScore | Found By |
| --- | --- | --- | --- | --- | --- | --- | --- | --- | --- | --- | --- | --- | --- | --- | --- | --- | --- | --- | --- |
| R.DPIC(+57.02)HC(+57.02)DTSK.Y | Y | 58.38 | 1231.4961 | 10 | 7 | 411.508 | 3 | 9.95 | 0.7355 | 0.7428 | 14664 | 1.19e4 | 2 | 2 | 1448 | 1457 | Carbamidomethylation, Carbamidomethylation |  | DB Search |
| R.GSFSSHIC(+57.02)LC(+57.02)HDPAYK.S | Y | 58.38 | 1877.8189 | 16 | 0.93 | 626.9461 | 3 | 14.52 | 0.8854 | 0.8927 | 42298 | 8.32e3 | 1 | 1 | 1011 | 1026 | Carbamidomethylation, Carbamidomethylation |  | DB Search |
| K.LLHC(+57.02)SDSEVDHVEVC(+57.02)GK.G | Y | 58.36 | 1982.8826 | 17 | 6.36 | 661.9709 | 3 | 13.31 | 0.9247 | 0.9319 | 34838 | 1.49e4 | 4 | 4 | 845 | 861 | Carbamidomethylation, Carbamidomethylation |  | DB Search |
| R.RTC(+57.02)ADTSPAIDFK.S | Y | 58.29 | 1480.698 | 13 | 1.84 | 741.356 | 2 | 14.27 | 1.017 | 1.0242 | 40672 | 1.48e3 | 1 | 1 | 274 | 286 | Carbamidomethylation |  | DB Search |
| R.ADC(+57.02)GVENTC(+57.02)FGSTR.S | Y | 57.59 | 1572.6297 | 14 | 1.45 | 787.3215 | 2 | 13.67 | 1.0053 | 1.0126 | 37114 | 4.6e3 | 1 | 1 | 1578 | 1591 | Carbamidomethylation, Carbamidomethylation |  | DB Search |
| R.TC(+57.02)ADTSPAIDFK.S | Y | 57.18 | 1324.5969 | 12 | -1.21 | 663.3035 | 2 | 15.19 | 0.9469 | 0.9541 | 46506 | 1.08e3 | 1 | 1 | 275 | 286 | Carbamidomethylation |  | DB Search |
| K.NLEC(+57.02)QC(+57.02)DESK.G | Y | 57.02 | 1281.4965 | 10 | 0.49 | 641.7545 | 2 | 10.48 | 0.9012 | 0.9085 | 17668 | 4.3e3 | 1 | 1 | 777 | 786 | Carbamidomethylation, Carbamidomethylation |  | DB Search |
| R.SWIC(+57.02)DC(+57.02)NSYGSILNPSDVKK.C | Y | 56.06 | 2342.0671 | 20 | 3.26 | 781.6971 | 3 | 18.19 | 1.0077 | 1.015 | 65664 | 5.83e2 | 1 | 1 | 1592 | 1611 | Carbamidomethylation, Carbamidomethylation |  | DB Search |
| R.DVQC(+57.02)TC(+57.02)NNELGYVHSPDGK.K | Y | 53.44 | 2191.9262 | 19 | 2.69 | 731.6497 | 3 | 14.19 | 0.9634 | 0.9707 | 40373 | 5.07e2 | 1 | 1 | 1641 | 1659 | Carbamidomethylation, Carbamidomethylation |  | DB Search |
| R.TC(+57.02)IEASC(+57.02)RR.N | Y | 47.82 | 1151.5176 | 9 | 3.72 | 384.847 | 3 | 10.13 | 0.7061 | 0.7135 | 15528 | 5.07e3 | 2 | 2 | 1564 | 1572 | Carbamidomethylation, Carbamidomethylation |  | DB Search |
| total 10 peptides |  |  |  |  |  |  |  |  |  |  |  |  |  |  |  |  |  |  |  |
| --- | --- | --- | --- | --- | --- | --- | --- | --- | --- | --- | --- | --- | --- | --- | --- | --- | --- | --- | --- |

Best Unique PSM (Scan Sample\_2\_A1\_1\_3640.d:14664, m/z=411.508, z=3, RT=9.95, ppm=4.81):


TRINITY\_DN4770\_c0\_g1\_i1.p1

back to list

  

| Protein Coverage
| Supporting Peptides
| Best Unique PSM
|

Protein Coverage:

Supporting Peptides:

| Peptide | Uniq | -10lgP | Mass | Length | ppm | m/z | z | RT | 1/k0 Start | 1/k0 End | Precursor ID | Area Sample 4 | #Feature | #Feature Sample 4 | Start | End | PTM | AScore | Found By |
| --- | --- | --- | --- | --- | --- | --- | --- | --- | --- | --- | --- | --- | --- | --- | --- | --- | --- | --- | --- |
| K.VTVTEIKDEIKK.S | Y | 58.32 | 1401.8079 | 12 | -3.05 | 468.2741 | 3 | 14.41 | 0.7445 | 0.7518 | 41705 | 0 | 0 | 0 | 1253 | 1264 |  |  | DB Search |
| R.ATGLAIDKIENR.L | Y | 56.92 | 1299.7146 | 12 | 5.06 | 650.8665 | 2 | 14.58 | 0.9924 | 0.9997 | 42725 | 0 | 0 | 0 | 47 | 58 |  |  | DB Search |
| R.YC(+57.02)EIDKETEAVTIR.L | Y | 54.7 | 1725.8243 | 14 | -7.9 | 576.2762 | 3 | 14.76 | 0.8514 | 0.8587 | 43877 | 0 | 0 | 0 | 1230 | 1243 | Carbamidomethylation |  | DB Search |
| total 3 peptides |  |  |  |  |  |  |  |  |  |  |  |  |  |  |  |  |  |  |  |
| --- | --- | --- | --- | --- | --- | --- | --- | --- | --- | --- | --- | --- | --- | --- | --- | --- | --- | --- | --- |

Best Unique PSM (Scan Sample\_2\_A1\_1\_3640.d:41705, m/z=468.2741, z=3, RT=14.41, ppm=-5.25):


TRINITY\_DN2796\_c0\_g1\_i2.p1

back to list

  

| Protein Coverage
| Supporting Peptides
| Best Unique PSM
|

Protein Coverage:

Supporting Peptides:

| Peptide | Uniq | -10lgP | Mass | Length | ppm | m/z | z | RT | 1/k0 Start | 1/k0 End | Precursor ID | Area Sample 4 | #Feature | #Feature Sample 4 | Start | End | PTM | AScore | Found By |
| --- | --- | --- | --- | --- | --- | --- | --- | --- | --- | --- | --- | --- | --- | --- | --- | --- | --- | --- | --- |
| K.LGANAILGVSLAAAK.A | Y | 58.25 | 1367.8136 | 15 | 1.11 | 684.9133 | 2 | 19.13 | 1.058 | 1.0652 | 71938 | 4.36e2 | 1 | 1 | 168 | 182 |  |  | DB Search |
| total 1 peptides |  |  |  |  |  |  |  |  |  |  |  |  |  |  |  |  |  |  |  |
| --- | --- | --- | --- | --- | --- | --- | --- | --- | --- | --- | --- | --- | --- | --- | --- | --- | --- | --- | --- |

Best Unique PSM (Scan Sample\_2\_A1\_1\_3640.d:71938, m/z=684.9133, z=2, RT=19.13, ppm=-1.09):


TRINITY\_DN984\_c0\_g1\_i2.p1

back to list

  

| Protein Coverage
| Supporting Peptides
| Best Unique PSM
|

Protein Coverage:

Supporting Peptides:

| Peptide | Uniq | -10lgP | Mass | Length | ppm | m/z | z | RT | 1/k0 Start | 1/k0 End | Precursor ID | Area Sample 4 | #Feature | #Feature Sample 4 | Start | End | PTM | AScore | Found By |
| --- | --- | --- | --- | --- | --- | --- | --- | --- | --- | --- | --- | --- | --- | --- | --- | --- | --- | --- | --- |
| R.EGPTGPQGETGR.R | Y | 58.28 | 1184.5421 | 12 | -1.15 | 593.2764 | 2 | 10.36 | 0.866 | 0.8733 | 16960 | 6.73e4 | 1 | 1 | 476 | 487 |  |  | DB Search |
| R.GSIGPSGEQGAQGLPGPTGR.R | Y | 55.93 | 1821.8969 | 20 | 0.48 | 911.9542 | 2 | 14.23 | 1.0857 | 1.0929 | 40533 | 1.87e3 | 1 | 1 | 867 | 886 |  |  | DB Search |
| R.EGPTGPQGETGRR.G | Y | 55.33 | 1340.6433 | 13 | 3.71 | 447.889 | 3 | 9.89 | 0.7632 | 0.7705 | 13877 | 4.68e3 | 1 | 1 | 476 | 488 |  |  | DB Search |
| total 3 peptides |  |  |  |  |  |  |  |  |  |  |  |  |  |  |  |  |  |  |  |
| --- | --- | --- | --- | --- | --- | --- | --- | --- | --- | --- | --- | --- | --- | --- | --- | --- | --- | --- | --- |

Best Unique PSM (Scan Sample\_2\_A1\_1\_3640.d:16960, m/z=593.2764, z=2, RT=10.36, ppm=-3.35):


TRINITY\_DN106\_c0\_g1\_i9.p1

back to list

  

| Protein Coverage
| Supporting Peptides
| Best Unique PSM
|

Protein Coverage:

Supporting Peptides:

| Peptide | Uniq | -10lgP | Mass | Length | ppm | m/z | z | RT | 1/k0 Start | 1/k0 End | Precursor ID | Area Sample 4 | #Feature | #Feature Sample 4 | Start | End | PTM | AScore | Found By |
| --- | --- | --- | --- | --- | --- | --- | --- | --- | --- | --- | --- | --- | --- | --- | --- | --- | --- | --- | --- |
| K.EGPAGPAGPVGR.L | Y | 58.28 | 1063.541 | 12 | -11.82 | 532.7703 | 2 | 11.84 | 0.8461 | 0.8534 | 25992 | 0 | 0 | 0 | 191 | 202 |  |  | DB Search |
| R.DGPAGPAGR.D | N | 58.24 | 796.3827 | 9 | 3.73 | 399.1992 | 2 | 9.76 | 0.7061 | 0.7135 | 13439 | 1.18e4 | 2 | 2 | 488 | 496 |  |  | DB Search |
| R.GPDGAVGPTGPIGAR.G | N | 58.04 | 1320.6786 | 15 | 1.83 | 661.3463 | 2 | 13.72 | 0.9558 | 0.963 | 37443 | 1.21e4 | 2 | 2 | 327 | 341 |  |  | DB Search |
| total 3 peptides |  |  |  |  |  |  |  |  |  |  |  |  |  |  |  |  |  |  |  |
| --- | --- | --- | --- | --- | --- | --- | --- | --- | --- | --- | --- | --- | --- | --- | --- | --- | --- | --- | --- |

Best Unique PSM (Scan Sample\_2\_A1\_1\_3640.d:25992, m/z=532.7703, z=2, RT=11.84, ppm=-14.02):


TRINITY\_DN738\_c0\_g1\_i3.p1

back to list

  

| Protein Coverage
| Supporting Peptides
| Best Unique PSM
|

Protein Coverage:

Supporting Peptides:

| Peptide | Uniq | -10lgP | Mass | Length | ppm | m/z | z | RT | 1/k0 Start | 1/k0 End | Precursor ID | Area Sample 4 | #Feature | #Feature Sample 4 | Start | End | PTM | AScore | Found By |
| --- | --- | --- | --- | --- | --- | --- | --- | --- | --- | --- | --- | --- | --- | --- | --- | --- | --- | --- | --- |
| R.YGEFSEK.L | Y | 52.42 | 858.3759 | 7 | 1.16 | 430.1948 | 2 | 12.54 | 0.7652 | 0.7726 | 30256 | 6.91e2 | 1 | 1 | 54 | 60 |  |  | DB Search |
| total 1 peptides |  |  |  |  |  |  |  |  |  |  |  |  |  |  |  |  |  |  |  |
| --- | --- | --- | --- | --- | --- | --- | --- | --- | --- | --- | --- | --- | --- | --- | --- | --- | --- | --- | --- |

Best Unique PSM (Scan Sample\_2\_A1\_1\_3640.d:30256, m/z=430.1948, z=2, RT=12.54, ppm=-1.04):


TRINITY\_DN413\_c0\_g1\_i1.p1

back to list

  

| Protein Coverage
| Supporting Peptides
| Best Unique PSM
|

Protein Coverage:

Supporting Peptides:

| Peptide | Uniq | -10lgP | Mass | Length | ppm | m/z | z | RT | 1/k0 Start | 1/k0 End | Precursor ID | Area Sample 4 | #Feature | #Feature Sample 4 | Start | End | PTM | AScore | Found By |
| --- | --- | --- | --- | --- | --- | --- | --- | --- | --- | --- | --- | --- | --- | --- | --- | --- | --- | --- | --- |
| R.LLLGDFNGDKR.T | Y | 58.25 | 1246.6669 | 11 | 4.07 | 624.3419 | 2 | 16.32 | 0.9844 | 0.9916 | 53554 | 5.68e2 | 1 | 1 | 46 | 56 |  |  | DB Search |
| total 1 peptides |  |  |  |  |  |  |  |  |  |  |  |  |  |  |  |  |  |  |  |
| --- | --- | --- | --- | --- | --- | --- | --- | --- | --- | --- | --- | --- | --- | --- | --- | --- | --- | --- | --- |

Best Unique PSM (Scan Sample\_2\_A1\_1\_3640.d:53554, m/z=624.3419, z=2, RT=16.32, ppm=1.87):


TRINITY\_DN6221\_c0\_g1\_i8.p1

back to list

  

| Protein Coverage
| Supporting Peptides
| Best Unique PSM
|

Protein Coverage:

Supporting Peptides:

| Peptide | Uniq | -10lgP | Mass | Length | ppm | m/z | z | RT | 1/k0 Start | 1/k0 End | Precursor ID | Area Sample 4 | #Feature | #Feature Sample 4 | Start | End | PTM | AScore | Found By |
| --- | --- | --- | --- | --- | --- | --- | --- | --- | --- | --- | --- | --- | --- | --- | --- | --- | --- | --- | --- |
| K.SVQIEVSTPVC(+57.02)QTC(+57.02)PK.G | Y | 57.42 | 1831.8808 | 16 | 3.34 | 916.9487 | 2 | 15.16 | 1.1126 | 1.1198 | 46105 | 3.11e3 | 1 | 1 | 667 | 682 | Carbamidomethylation, Carbamidomethylation |  | DB Search |
| K.YQVTGC(+57.02)GGFC(+57.02)ESSVK.A | Y | 57.17 | 1677.7127 | 15 | -3.52 | 839.8588 | 2 | 14.63 | 1.068 | 1.0753 | 43042 | 0 | 0 | 0 | 733 | 747 | Carbamidomethylation, Carbamidomethylation |  | DB Search |
| total 2 peptides |  |  |  |  |  |  |  |  |  |  |  |  |  |  |  |  |  |  |  |
| --- | --- | --- | --- | --- | --- | --- | --- | --- | --- | --- | --- | --- | --- | --- | --- | --- | --- | --- | --- |

Best Unique PSM (Scan Sample\_2\_A1\_1\_3640.d:46105, m/z=916.9487, z=2, RT=15.16, ppm=1.15):


TRINITY\_DN1783\_c0\_g1\_i8.p2

back to list

  

| Protein Coverage
| Supporting Peptides
| Best Unique PSM
|

Protein Coverage:

Supporting Peptides:

| Peptide | Uniq | -10lgP | Mass | Length | ppm | m/z | z | RT | 1/k0 Start | 1/k0 End | Precursor ID | Area Sample 4 | #Feature | #Feature Sample 4 | Start | End | PTM | AScore | Found By |
| --- | --- | --- | --- | --- | --- | --- | --- | --- | --- | --- | --- | --- | --- | --- | --- | --- | --- | --- | --- |
| K.LTIIDALPR.C | Y | 58.24 | 1010.6124 | 9 | 0.02 | 506.3124 | 2 | 19.00 | 0.8392 | 0.8465 | 71132 | 3.79e2 | 1 | 1 | 224 | 232 |  |  | DB Search |
| total 1 peptides |  |  |  |  |  |  |  |  |  |  |  |  |  |  |  |  |  |  |  |
| --- | --- | --- | --- | --- | --- | --- | --- | --- | --- | --- | --- | --- | --- | --- | --- | --- | --- | --- | --- |

Best Unique PSM (Scan Sample\_2\_A1\_1\_3640.d:71132, m/z=506.3124, z=2, RT=19.00, ppm=-2.17):


TRINITY\_DN912\_c0\_g1\_i4.p1

back to list

  

| Protein Coverage
| Supporting Peptides
| Best Unique PSM
|

Protein Coverage:

Supporting Peptides:

| Peptide | Uniq | -10lgP | Mass | Length | ppm | m/z | z | RT | 1/k0 Start | 1/k0 End | Precursor ID | Area Sample 4 | #Feature | #Feature Sample 4 | Start | End | PTM | AScore | Found By |
| --- | --- | --- | --- | --- | --- | --- | --- | --- | --- | --- | --- | --- | --- | --- | --- | --- | --- | --- | --- |
| K.LLLLGAGESGK.S | Y | 43.84 | 1056.6179 | 11 | 6.84 | 529.3187 | 2 | 17.27 | 0.8761 | 0.8834 | 59698 | 0 | 0 | 0 | 36 | 46 |  |  | DB Search |
| total 1 peptides |  |  |  |  |  |  |  |  |  |  |  |  |  |  |  |  |  |  |  |
| --- | --- | --- | --- | --- | --- | --- | --- | --- | --- | --- | --- | --- | --- | --- | --- | --- | --- | --- | --- |

Best Unique PSM (Scan Sample\_2\_A1\_1\_3640.d:59698, m/z=529.3187, z=2, RT=17.27, ppm=4.65):


TRINITY\_DN11522\_c0\_g1\_i1.p1

back to list

  

| Protein Coverage
| Supporting Peptides
| Best Unique PSM
|

Protein Coverage:

Supporting Peptides:

| Peptide | Uniq | -10lgP | Mass | Length | ppm | m/z | z | RT | 1/k0 Start | 1/k0 End | Precursor ID | Area Sample 4 | #Feature | #Feature Sample 4 | Start | End | PTM | AScore | Found By |
| --- | --- | --- | --- | --- | --- | --- | --- | --- | --- | --- | --- | --- | --- | --- | --- | --- | --- | --- | --- |
| K.GKPEQVQVK.M | Y | 58.01 | 1011.5713 | 9 | 0.86 | 506.7922 | 2 | 9.91 | 0.8376 | 0.8449 | 14438 | 2.02e2 | 1 | 1 | 542 | 550 |  |  | DB Search |
| total 1 peptides |  |  |  |  |  |  |  |  |  |  |  |  |  |  |  |  |  |  |  |
| --- | --- | --- | --- | --- | --- | --- | --- | --- | --- | --- | --- | --- | --- | --- | --- | --- | --- | --- | --- |

Best Unique PSM (Scan Sample\_2\_A1\_1\_3640.d:14438, m/z=506.7922, z=2, RT=9.91, ppm=-1.33):


TRINITY\_DN24268\_c0\_g1\_i2.p1

back to list

  

| Protein Coverage
| Supporting Peptides
| Best Unique PSM
|

Protein Coverage:

Supporting Peptides:

| Peptide | Uniq | -10lgP | Mass | Length | ppm | m/z | z | RT | 1/k0 Start | 1/k0 End | Precursor ID | Area Sample 4 | #Feature | #Feature Sample 4 | Start | End | PTM | AScore | Found By |
| --- | --- | --- | --- | --- | --- | --- | --- | --- | --- | --- | --- | --- | --- | --- | --- | --- | --- | --- | --- |
| K.AC(+57.02)GLENM(+15.99)C(+57.02)VGSYK.S | N | 58.41 | 1503.6156 | 13 | -0.67 | 752.8129 | 2 | 13.59 | 0.9989 | 1.0061 | 36764 | 0 | 0 | 0 | 2156 | 2168 | Carbamidomethylation, Oxidation (M), Carbamidomethylation | M7:Oxidation (M):1000 | DB Search |
| R.YC(+57.02)FHASC(+57.02)K.T | Y | 58.39 | 1071.4266 | 8 | -3.12 | 358.1476 | 3 | 11.77 | 0.7229 | 0.7302 | 25566 | 9.3e2 | 1 | 1 | 133 | 140 | Carbamidomethylation, Carbamidomethylation |  | DB Search |
| R.TC(+57.02)VTASC(+57.02)R.T | Y | 58.02 | 953.4059 | 8 | 4.85 | 477.7115 | 2 | 10.04 | 0.7986 | 0.8059 | 15114 | 2.52e3 | 1 | 1 | 1573 | 1580 | Carbamidomethylation, Carbamidomethylation |  | DB Search |
| K.ASNEVSM(+15.99)K.C | Y | 57.93 | 880.396 | 8 | -7.43 | 441.201 | 2 | 8.74 | 0.7469 | 0.7542 | 9825 | 0 | 0 | 0 | 713 | 720 | Oxidation (M) | M7:Oxidation (M):1000 | DB Search |
| K.ASNEVSMK.C | Y | 55.46 | 864.4011 | 8 | -4.31 | 433.205 | 2 | 10.65 | 0.762 | 0.7693 | 18751 | 4.71e2 | 1 | 1 | 713 | 720 |  |  | DB Search |
| K.YFTPSADGFSC(+57.02)IPASC(+57.02)K.S | Y | 53.17 | 1906.8229 | 17 | 11.55 | 954.4277 | 2 | 17.51 | 1.1222 | 1.1294 | 61270 | 0 | 0 | 0 | 1757 | 1773 | Carbamidomethylation, Carbamidomethylation |  | DB Search |
| R.SPDLTSC(+57.02)IPSDC(+57.02)R.G | Y | 52.65 | 1506.6443 | 13 | -4.7 | 754.3242 | 2 | 14.88 | 1.0001 | 1.0073 | 44608 | 0 | 0 | 0 | 509 | 521 | Carbamidomethylation, Carbamidomethylation |  | DB Search |
| total 7 peptides |  |  |  |  |  |  |  |  |  |  |  |  |  |  |  |  |  |  |  |
| --- | --- | --- | --- | --- | --- | --- | --- | --- | --- | --- | --- | --- | --- | --- | --- | --- | --- | --- | --- |

Best Unique PSM (Scan Sample\_2\_A1\_1\_3640.d:25566, m/z=358.1476, z=3, RT=11.77, ppm=-5.32):


TRINITY\_DN20282\_c0\_g1\_i1.p1

back to list

  

| Protein Coverage
| Supporting Peptides
| Best Unique PSM
|

Protein Coverage:

Supporting Peptides:

| Peptide | Uniq | -10lgP | Mass | Length | ppm | m/z | z | RT | 1/k0 Start | 1/k0 End | Precursor ID | Area Sample 4 | #Feature | #Feature Sample 4 | Start | End | PTM | AScore | Found By |
| --- | --- | --- | --- | --- | --- | --- | --- | --- | --- | --- | --- | --- | --- | --- | --- | --- | --- | --- | --- |
| R.INNKLEVKR.I | Y | 55.03 | 1112.6666 | 9 | 7.92 | 371.8983 | 3 | 10.56 | 0.711 | 0.7184 | 18207 | 0 | 0 | 0 | 29 | 37 |  |  | DB Search |
| total 1 peptides |  |  |  |  |  |  |  |  |  |  |  |  |  |  |  |  |  |  |  |
| --- | --- | --- | --- | --- | --- | --- | --- | --- | --- | --- | --- | --- | --- | --- | --- | --- | --- | --- | --- |

Best Unique PSM (Scan Sample\_2\_A1\_1\_3640.d:18207, m/z=371.8983, z=3, RT=10.56, ppm=5.72):


TRINITY\_DN10545\_c0\_g1\_i1.p3

back to list

  

| Protein Coverage
| Supporting Peptides
| Best Unique PSM
|

Protein Coverage:

Supporting Peptides:

| Peptide | Uniq | -10lgP | Mass | Length | ppm | m/z | z | RT | 1/k0 Start | 1/k0 End | Precursor ID | Area Sample 4 | #Feature | #Feature Sample 4 | Start | End | PTM | AScore | Found By |
| --- | --- | --- | --- | --- | --- | --- | --- | --- | --- | --- | --- | --- | --- | --- | --- | --- | --- | --- | --- |
| K.ESTLHLVLR.L | Y | 57.27 | 1066.6135 | 9 | 2.57 | 534.3142 | 2 | 15.80 | 0.8785 | 0.8858 | 50332 | 4.97e3 | 1 | 1 | 16 | 24 |  |  | DB Search |
| total 1 peptides |  |  |  |  |  |  |  |  |  |  |  |  |  |  |  |  |  |  |  |
| --- | --- | --- | --- | --- | --- | --- | --- | --- | --- | --- | --- | --- | --- | --- | --- | --- | --- | --- | --- |

Best Unique PSM (Scan Sample\_2\_A1\_1\_3640.d:50332, m/z=534.7766, z=2, RT=15.80, ppm=0.37):


TRINITY\_DN98417\_c0\_g1\_i1.p1

back to list

  

| Protein Coverage
| Supporting Peptides
| Best Unique PSM
|

Protein Coverage:

Supporting Peptides:

| Peptide | Uniq | -10lgP | Mass | Length | ppm | m/z | z | RT | 1/k0 Start | 1/k0 End | Precursor ID | Area Sample 4 | #Feature | #Feature Sample 4 | Start | End | PTM | AScore | Found By |
| --- | --- | --- | --- | --- | --- | --- | --- | --- | --- | --- | --- | --- | --- | --- | --- | --- | --- | --- | --- |
| K.FYLETVIK.S | Y | 57.32 | 1011.5641 | 8 | 1.05 | 506.7887 | 2 | 18.58 | 0.8518 | 0.8591 | 68296 | 6.97e2 | 1 | 1 | 161 | 168 |  |  | DB Search |
| total 1 peptides |  |  |  |  |  |  |  |  |  |  |  |  |  |  |  |  |  |  |  |
| --- | --- | --- | --- | --- | --- | --- | --- | --- | --- | --- | --- | --- | --- | --- | --- | --- | --- | --- | --- |

Best Unique PSM (Scan Sample\_2\_A1\_1\_3640.d:68296, m/z=506.7887, z=2, RT=18.58, ppm=-1.14):


TRINITY\_DN18348\_c0\_g1\_i1.p1

back to list

  

| Protein Coverage
| Supporting Peptides
| Best Unique PSM
|

Protein Coverage:

Supporting Peptides:

| Peptide | Uniq | -10lgP | Mass | Length | ppm | m/z | z | RT | 1/k0 Start | 1/k0 End | Precursor ID | Area Sample 4 | #Feature | #Feature Sample 4 | Start | End | PTM | AScore | Found By |
| --- | --- | --- | --- | --- | --- | --- | --- | --- | --- | --- | --- | --- | --- | --- | --- | --- | --- | --- | --- |
| K.LNQSSLNLNAAANDVVHAAK.G | Y | 58.22 | 2049.0603 | 20 | 5.38 | 684.0295 | 3 | 17.53 | 0.9662 | 0.9735 | 61365 | 0 | 0 | 0 | 1268 | 1287 |  |  | DB Search |
| K.AVNDNSHDLIHSIR.G | Y | 58.09 | 1589.791 | 14 | -0.42 | 530.9362 | 3 | 14.03 | 0.8404 | 0.8477 | 39366 | 0 | 0 | 0 | 982 | 995 |  |  | DB Search |
| total 2 peptides |  |  |  |  |  |  |  |  |  |  |  |  |  |  |  |  |  |  |  |
| --- | --- | --- | --- | --- | --- | --- | --- | --- | --- | --- | --- | --- | --- | --- | --- | --- | --- | --- | --- |

Best Unique PSM (Scan Sample\_2\_A1\_1\_3640.d:61365, m/z=683.8365, z=1, RT=17.56, ppm=3.18):


TRINITY\_DN6049\_c0\_g1\_i1.p1

back to list

  

| Protein Coverage
| Supporting Peptides
| Best Unique PSM
|

Protein Coverage:

Supporting Peptides:

| Peptide | Uniq | -10lgP | Mass | Length | ppm | m/z | z | RT | 1/k0 Start | 1/k0 End | Precursor ID | Area Sample 4 | #Feature | #Feature Sample 4 | Start | End | PTM | AScore | Found By |
| --- | --- | --- | --- | --- | --- | --- | --- | --- | --- | --- | --- | --- | --- | --- | --- | --- | --- | --- | --- |
| R.KVFSNEEDLPK.N | Y | 56.95 | 1304.6612 | 11 | 1.38 | 653.3373 | 2 | 13.40 | 0.9791 | 0.9864 | 35445 | 4.52e2 | 1 | 1 | 45 | 55 |  |  | DB Search |
| total 1 peptides |  |  |  |  |  |  |  |  |  |  |  |  |  |  |  |  |  |  |  |
| --- | --- | --- | --- | --- | --- | --- | --- | --- | --- | --- | --- | --- | --- | --- | --- | --- | --- | --- | --- |

Best Unique PSM (Scan Sample\_2\_A1\_1\_3640.d:35445, m/z=653.3373, z=2, RT=13.40, ppm=-0.82):


TRINITY\_DN12627\_c0\_g1\_i1.p1

back to list

  

| Protein Coverage
| Supporting Peptides
| Best Unique PSM
|

Protein Coverage:

Supporting Peptides:

| Peptide | Uniq | -10lgP | Mass | Length | ppm | m/z | z | RT | 1/k0 Start | 1/k0 End | Precursor ID | Area Sample 4 | #Feature | #Feature Sample 4 | Start | End | PTM | AScore | Found By |
| --- | --- | --- | --- | --- | --- | --- | --- | --- | --- | --- | --- | --- | --- | --- | --- | --- | --- | --- | --- |
| K.FDFHSWIGLKK.I | Y | 58.28 | 1376.7241 | 11 | 3.36 | 459.9158 | 3 | 18.47 | 0.7363 | 0.7437 | 67625 | 5.05e2 | 1 | 1 | 193 | 203 |  |  | DB Search |
| R.SVWIGLNDR.A | Y | 55.19 | 1058.5509 | 9 | 8.2 | 530.2859 | 2 | 17.42 | 0.8688 | 0.8761 | 60696 | 0 | 0 | 0 | 338 | 346 |  |  | DB Search |
| K.GFGGNLVTITNSDIQK.Q | Y | 54.93 | 1662.8577 | 16 | -0.02 | 832.4343 | 2 | 17.83 | 1.0789 | 1.0861 | 63151 | 2.79e3 | 1 | 1 | 311 | 326 |  |  | DB Search |
| total 3 peptides |  |  |  |  |  |  |  |  |  |  |  |  |  |  |  |  |  |  |  |
| --- | --- | --- | --- | --- | --- | --- | --- | --- | --- | --- | --- | --- | --- | --- | --- | --- | --- | --- | --- |

Best Unique PSM (Scan Sample\_2\_A1\_1\_3640.d:67625, m/z=459.9158, z=3, RT=18.47, ppm=1.16):


TRINITY\_DN495\_c0\_g1\_i3.p1

back to list

  

| Protein Coverage
| Supporting Peptides
| Best Unique PSM
|

Protein Coverage:

Supporting Peptides:

| Peptide | Uniq | -10lgP | Mass | Length | ppm | m/z | z | RT | 1/k0 Start | 1/k0 End | Precursor ID | Area Sample 4 | #Feature | #Feature Sample 4 | Start | End | PTM | AScore | Found By |
| --- | --- | --- | --- | --- | --- | --- | --- | --- | --- | --- | --- | --- | --- | --- | --- | --- | --- | --- | --- |
| R.LFEIKFNAE.X | Y | 47.56 | 1109.5757 | 9 | 0.53 | 555.7942 | 2 | 19.11 | 0.8619 | 0.8692 | 71841 | 3.8e2 | 1 | 1 | 650 | 658 |  |  | DB Search |
| R.SSC(+57.02)KPILSVIK.I | Y | 41 | 1230.7006 | 11 | -0.71 | 616.3558 | 2 | 15.33 | 0.9529 | 0.9602 | 47491 | 3.99e2 | 1 | 1 | 454 | 464 | Carbamidomethylation |  | DB Search |
| total 2 peptides |  |  |  |  |  |  |  |  |  |  |  |  |  |  |  |  |  |  |  |
| --- | --- | --- | --- | --- | --- | --- | --- | --- | --- | --- | --- | --- | --- | --- | --- | --- | --- | --- | --- |

Best Unique PSM (Scan Sample\_2\_A1\_1\_3640.d:71841, m/z=555.7942, z=2, RT=19.11, ppm=-1.67):


TRINITY\_DN1507\_c0\_g1\_i1.p1

back to list

  

| Protein Coverage
| Supporting Peptides
| Best Unique PSM
|

Protein Coverage:

Supporting Peptides:

| Peptide | Uniq | -10lgP | Mass | Length | ppm | m/z | z | RT | 1/k0 Start | 1/k0 End | Precursor ID | Area Sample 4 | #Feature | #Feature Sample 4 | Start | End | PTM | AScore | Found By |
| --- | --- | --- | --- | --- | --- | --- | --- | --- | --- | --- | --- | --- | --- | --- | --- | --- | --- | --- | --- |
| K.VQPLLSIAR.A | Y | 57.44 | 995.6127 | 9 | 0.94 | 498.813 | 2 | 17.21 | 0.8433 | 0.8506 | 59276 | 1.54e2 | 1 | 1 | 57 | 65 |  |  | DB Search |
| R.VQEMEEALAAAESK.A | Y | 53.73 | 1504.7079 | 14 | -3.91 | 753.3566 | 2 | 16.96 | 1.013 | 1.0202 | 57682 | 3.72e2 | 1 | 1 | 618 | 631 |  |  | DB Search |
| total 2 peptides |  |  |  |  |  |  |  |  |  |  |  |  |  |  |  |  |  |  |  |
| --- | --- | --- | --- | --- | --- | --- | --- | --- | --- | --- | --- | --- | --- | --- | --- | --- | --- | --- | --- |

Best Unique PSM (Scan Sample\_2\_A1\_1\_3640.d:59276, m/z=498.813, z=2, RT=17.21, ppm=-1.26):


TRINITY\_DN25375\_c0\_g1\_i7.p1

back to list

  

| Protein Coverage
| Supporting Peptides
| Best Unique PSM
|

Protein Coverage:

Supporting Peptides:

| Peptide | Uniq | -10lgP | Mass | Length | ppm | m/z | z | RT | 1/k0 Start | 1/k0 End | Precursor ID | Area Sample 4 | #Feature | #Feature Sample 4 | Start | End | PTM | AScore | Found By |
| --- | --- | --- | --- | --- | --- | --- | --- | --- | --- | --- | --- | --- | --- | --- | --- | --- | --- | --- | --- |
| K.LLC(+57.02)EC(+57.02)PC(+57.02)EQDK.S | Y | 58.35 | 1450.5891 | 11 | 1.37 | 726.3012 | 2 | 12.80 | 0.9763 | 0.9836 | 31825 | 0 | 0 | 0 | 454 | 464 | Carbamidomethylation, Carbamidomethylation, Carbamidomethylation |  | DB Search |
| K.IVIDSEHC(+57.02)NSR.G | Y | 58.28 | 1328.6143 | 11 | 4.73 | 665.3161 | 2 | 11.46 | 0.965 | 0.9723 | 23601 | 5.49e2 | 1 | 1 | 467 | 477 | Carbamidomethylation |  | DB Search |
| K.DC(+57.02)ELVGEDLKFK.I | Y | 55.34 | 1451.6966 | 12 | 2.39 | 726.8557 | 2 | 16.71 | 1.0415 | 1.0488 | 56131 | 3.56e2 | 1 | 1 | 429 | 440 | Carbamidomethylation |  | DB Search |
| K.LRPGQATTVDITVK.T | Y | 45.97 | 1497.8515 | 14 | 0.37 | 749.9316 | 2 | 14.22 | 1.0327 | 1.0399 | 40537 | 0 | 0 | 0 | 114 | 127 |  |  | DB Search |
| total 4 peptides |  |  |  |  |  |  |  |  |  |  |  |  |  |  |  |  |  |  |  |
| --- | --- | --- | --- | --- | --- | --- | --- | --- | --- | --- | --- | --- | --- | --- | --- | --- | --- | --- | --- |

Best Unique PSM (Scan Sample\_2\_A1\_1\_3640.d:31825, m/z=726.3012, z=2, RT=12.80, ppm=-0.83):


TRINITY\_DN564\_c0\_g1\_i1.p1

back to list

  

| Protein Coverage
| Supporting Peptides
| Best Unique PSM
|

Protein Coverage:

Supporting Peptides:

| Peptide | Uniq | -10lgP | Mass | Length | ppm | m/z | z | RT | 1/k0 Start | 1/k0 End | Precursor ID | Area Sample 4 | #Feature | #Feature Sample 4 | Start | End | PTM | AScore | Found By |
| --- | --- | --- | --- | --- | --- | --- | --- | --- | --- | --- | --- | --- | --- | --- | --- | --- | --- | --- | --- |
| K.TVHQALVAALQK.H | Y | 58.27 | 1277.7455 | 12 | -1.69 | 639.8776 | 2 | 15.15 | 0.9614 | 0.9687 | 46326 | 0 | 0 | 0 | 172 | 183 |  |  | DB Search |
| R.EAIVQGAQSTQDTLK.K | Y | 56.66 | 1587.8104 | 15 | -1.8 | 794.9093 | 2 | 13.80 | 1.0608 | 1.068 | 37982 | 0 | 0 | 0 | 301 | 315 |  |  | DB Search |
| total 2 peptides |  |  |  |  |  |  |  |  |  |  |  |  |  |  |  |  |  |  |  |
| --- | --- | --- | --- | --- | --- | --- | --- | --- | --- | --- | --- | --- | --- | --- | --- | --- | --- | --- | --- |

Best Unique PSM (Scan Sample\_2\_A1\_1\_3640.d:46326, m/z=640.8252, z=2, RT=15.16, ppm=-3.88):


TRINITY\_DN187759\_c0\_g1\_i1.p1

back to list

  

| Protein Coverage
| Supporting Peptides
| Best Unique PSM
|

Protein Coverage:

Supporting Peptides:

| Peptide | Uniq | -10lgP | Mass | Length | ppm | m/z | z | RT | 1/k0 Start | 1/k0 End | Precursor ID | Area Sample 4 | #Feature | #Feature Sample 4 | Start | End | PTM | AScore | Found By |
| --- | --- | --- | --- | --- | --- | --- | --- | --- | --- | --- | --- | --- | --- | --- | --- | --- | --- | --- | --- |
| R.VKFC(+57.02)SLSQHTK.E | Y | 57.38 | 1333.6812 | 11 | 3.63 | 445.5683 | 3 | 11.98 | 0.7518 | 0.7591 | 26681 | 1.88e3 | 1 | 1 | 305 | 315 | Carbamidomethylation |  | DB Search |
| K.FC(+57.02)SLSQHTK.E | Y | 57.12 | 1106.5179 | 9 | 5.55 | 554.2681 | 2 | 11.18 | 0.898 | 0.9052 | 21967 | 0 | 0 | 0 | 307 | 315 | Carbamidomethylation |  | DB Search |
| total 2 peptides |  |  |  |  |  |  |  |  |  |  |  |  |  |  |  |  |  |  |  |
| --- | --- | --- | --- | --- | --- | --- | --- | --- | --- | --- | --- | --- | --- | --- | --- | --- | --- | --- | --- |

Best Unique PSM (Scan Sample\_2\_A1\_1\_3640.d:26681, m/z=445.5683, z=3, RT=11.98, ppm=1.44):


TRINITY\_DN124859\_c0\_g1\_i1.p3

back to list

  

| Protein Coverage
| Supporting Peptides
| Best Unique PSM
|

Protein Coverage:

Supporting Peptides:

| Peptide | Uniq | -10lgP | Mass | Length | ppm | m/z | z | RT | 1/k0 Start | 1/k0 End | Precursor ID | Area Sample 4 | #Feature | #Feature Sample 4 | Start | End | PTM | AScore | Found By |
| --- | --- | --- | --- | --- | --- | --- | --- | --- | --- | --- | --- | --- | --- | --- | --- | --- | --- | --- | --- |
| R.DEDHAAR.Q | Y | 46.97 | 812.3413 | 7 | -12.21 | 407.1721 | 2 | 9.21 | 0.7807 | 0.788 | 11224 | 1.78e2 | 1 | 1 | 181 | 187 |  |  | DB Search |
| total 1 peptides |  |  |  |  |  |  |  |  |  |  |  |  |  |  |  |  |  |  |  |
| --- | --- | --- | --- | --- | --- | --- | --- | --- | --- | --- | --- | --- | --- | --- | --- | --- | --- | --- | --- |

Best Unique PSM (Scan Sample\_2\_A1\_1\_3640.d:11224, m/z=407.1721, z=2, RT=9.21, ppm=-14.41):


TRINITY\_DN738\_c0\_g1\_i1.p1

back to list

  

| Protein Coverage
| Supporting Peptides
| Best Unique PSM
|

Protein Coverage:

Supporting Peptides:

| Peptide | Uniq | -10lgP | Mass | Length | ppm | m/z | z | RT | 1/k0 Start | 1/k0 End | Precursor ID | Area Sample 4 | #Feature | #Feature Sample 4 | Start | End | PTM | AScore | Found By |
| --- | --- | --- | --- | --- | --- | --- | --- | --- | --- | --- | --- | --- | --- | --- | --- | --- | --- | --- | --- |
| R.YC(+57.02)VGEKVEIQAC(+57.02)STK.Y | Y | 52.87 | 1770.828 | 15 | 1.74 | 591.283 | 3 | 13.94 | 0.8339 | 0.8412 | 38817 | 2.77e2 | 1 | 1 | 294 | 308 | Carbamidomethylation, Carbamidomethylation |  | DB Search |
| R.YGEFSEK.L | Y | 52.42 | 858.3759 | 7 | 1.16 | 430.1948 | 2 | 12.54 | 0.7652 | 0.7726 | 30256 | 6.91e2 | 1 | 1 | 54 | 60 |  |  | DB Search |
| total 2 peptides |  |  |  |  |  |  |  |  |  |  |  |  |  |  |  |  |  |  |  |
| --- | --- | --- | --- | --- | --- | --- | --- | --- | --- | --- | --- | --- | --- | --- | --- | --- | --- | --- | --- |

Best Unique PSM (Scan Sample\_2\_A1\_1\_3640.d:38817, m/z=591.283, z=3, RT=13.94, ppm=-0.46):


TRINITY\_DN1339\_c0\_g1\_i1.p1

back to list

  

| Protein Coverage
| Supporting Peptides
| Best Unique PSM
|

Protein Coverage:

Supporting Peptides:

| Peptide | Uniq | -10lgP | Mass | Length | ppm | m/z | z | RT | 1/k0 Start | 1/k0 End | Precursor ID | Area Sample 4 | #Feature | #Feature Sample 4 | Start | End | PTM | AScore | Found By |
| --- | --- | --- | --- | --- | --- | --- | --- | --- | --- | --- | --- | --- | --- | --- | --- | --- | --- | --- | --- |
| K.VITSDDPAEKEK.K | Y | 58.39 | 1330.6616 | 12 | 3.3 | 666.3388 | 2 | 10.77 | 0.9856 | 0.9928 | 19482 | 3.46e2 | 1 | 1 | 160 | 171 |  |  | DB Search |
| total 1 peptides |  |  |  |  |  |  |  |  |  |  |  |  |  |  |  |  |  |  |  |
| --- | --- | --- | --- | --- | --- | --- | --- | --- | --- | --- | --- | --- | --- | --- | --- | --- | --- | --- | --- |

Best Unique PSM (Scan Sample\_2\_A1\_1\_3640.d:19482, m/z=666.3388, z=2, RT=10.77, ppm=1.11):


TRINITY\_DN172717\_c0\_g1\_i1.p1

back to list

  

| Protein Coverage
| Supporting Peptides
| Best Unique PSM
|

Protein Coverage:

Supporting Peptides:

| Peptide | Uniq | -10lgP | Mass | Length | ppm | m/z | z | RT | 1/k0 Start | 1/k0 End | Precursor ID | Area Sample 4 | #Feature | #Feature Sample 4 | Start | End | PTM | AScore | Found By |
| --- | --- | --- | --- | --- | --- | --- | --- | --- | --- | --- | --- | --- | --- | --- | --- | --- | --- | --- | --- |
| R.KLPEWPGLKR.S | Y | 55.92 | 1222.7186 | 10 | -6.56 | 408.5766 | 3 | 15.62 | 0.7453 | 0.7526 | 49336 | 0 | 0 | 0 | 28 | 37 |  |  | DB Search |
| total 1 peptides |  |  |  |  |  |  |  |  |  |  |  |  |  |  |  |  |  |  |  |
| --- | --- | --- | --- | --- | --- | --- | --- | --- | --- | --- | --- | --- | --- | --- | --- | --- | --- | --- | --- |

Best Unique PSM (Scan Sample\_2\_A1\_1\_3640.d:49336, m/z=408.5766, z=3, RT=15.62, ppm=-8.76):


TRINITY\_DN2433\_c0\_g1\_i2.p1

back to list

  

| Protein Coverage
| Supporting Peptides
| Best Unique PSM
|

Protein Coverage:

Supporting Peptides:

| Peptide | Uniq | -10lgP | Mass | Length | ppm | m/z | z | RT | 1/k0 Start | 1/k0 End | Precursor ID | Area Sample 4 | #Feature | #Feature Sample 4 | Start | End | PTM | AScore | Found By |
| --- | --- | --- | --- | --- | --- | --- | --- | --- | --- | --- | --- | --- | --- | --- | --- | --- | --- | --- | --- |
| R.KVNMPISIIALNHR.K | Y | 37.77 | 1604.9184 | 14 | 6.56 | 535.9824 | 3 | 17.41 | 0.8441 | 0.8514 | 60598 | 0 | 0 | 0 | 11 | 24 |  |  | DB Search |
| total 1 peptides |  |  |  |  |  |  |  |  |  |  |  |  |  |  |  |  |  |  |  |
| --- | --- | --- | --- | --- | --- | --- | --- | --- | --- | --- | --- | --- | --- | --- | --- | --- | --- | --- | --- |

Best Unique PSM (Scan Sample\_2\_A1\_1\_3640.d:60598, m/z=535.9824, z=3, RT=17.41, ppm=4.36):


TRINITY\_DN5\_c0\_g1\_i13.p1

back to list

  

| Protein Coverage
| Supporting Peptides
| Best Unique PSM
|

Protein Coverage:

Supporting Peptides:

| Peptide | Uniq | -10lgP | Mass | Length | ppm | m/z | z | RT | 1/k0 Start | 1/k0 End | Precursor ID | Area Sample 4 | #Feature | #Feature Sample 4 | Start | End | PTM | AScore | Found By |
| --- | --- | --- | --- | --- | --- | --- | --- | --- | --- | --- | --- | --- | --- | --- | --- | --- | --- | --- | --- |
| K.IIIELQGDLIK.I | Y | 53.27 | 1253.7594 | 11 | 3.89 | 627.8881 | 2 | 19.42 | 0.9513 | 0.9586 | 73842 | 0 | 0 | 0 | 65 | 75 |  |  | DB Search |
| total 1 peptides |  |  |  |  |  |  |  |  |  |  |  |  |  |  |  |  |  |  |  |
| --- | --- | --- | --- | --- | --- | --- | --- | --- | --- | --- | --- | --- | --- | --- | --- | --- | --- | --- | --- |

Best Unique PSM (Scan Sample\_2\_A1\_1\_3640.d:73842, m/z=627.8881, z=2, RT=19.42, ppm=1.69):


TRINITY\_DN585\_c0\_g1\_i4.p1

back to list

  

| Protein Coverage
| Supporting Peptides
| Best Unique PSM
|

Protein Coverage:

Supporting Peptides:

| Peptide | Uniq | -10lgP | Mass | Length | ppm | m/z | z | RT | 1/k0 Start | 1/k0 End | Precursor ID | Area Sample 4 | #Feature | #Feature Sample 4 | Start | End | PTM | AScore | Found By |
| --- | --- | --- | --- | --- | --- | --- | --- | --- | --- | --- | --- | --- | --- | --- | --- | --- | --- | --- | --- |
| MQIFVK.T | Y | 57.42 | 764.4255 | 6 | 0.18 | 383.2192 | 2 | 15.73 | 0.7363 | 0.7437 | 49899 | 2.46e2 | 1 | 1 | 1 | 6 |  |  | DB Search |
| K.ESTLHLVLR.L | Y | 57.27 | 1066.6135 | 9 | 2.57 | 534.3142 | 2 | 15.80 | 0.8785 | 0.8858 | 50332 | 4.97e3 | 1 | 1 | 64 | 72 |  |  | DB Search |
| K.IQDKEGIPPDQQR.L | Y | 55.01 | 1522.7739 | 13 | 0.25 | 762.3928 | 2 | 11.08 | 1.0343 | 1.0415 | 21018 | 1.78e3 | 2 | 2 | 30 | 42 |  |  | DB Search |
| K.TITLEVEPSDTIENVK.S | Y | 54.56 | 1786.92 | 16 | 4.26 | 894.4691 | 2 | 17.62 | 1.1442 | 1.1514 | 61769 | 1.48e4 | 1 | 1 | 12 | 27 |  |  | DB Search |
| total 4 peptides |  |  |  |  |  |  |  |  |  |  |  |  |  |  |  |  |  |  |  |
| --- | --- | --- | --- | --- | --- | --- | --- | --- | --- | --- | --- | --- | --- | --- | --- | --- | --- | --- | --- |

Best Unique PSM (Scan Sample\_2\_A1\_1\_3640.d:49899, m/z=383.2192, z=2, RT=15.73, ppm=-2.02):


TRINITY\_DN5584\_c0\_g1\_i2.p1

back to list

  

| Protein Coverage
| Supporting Peptides
| Best Unique PSM
|

Protein Coverage:

Supporting Peptides:

| Peptide | Uniq | -10lgP | Mass | Length | ppm | m/z | z | RT | 1/k0 Start | 1/k0 End | Precursor ID | Area Sample 4 | #Feature | #Feature Sample 4 | Start | End | PTM | AScore | Found By |
| --- | --- | --- | --- | --- | --- | --- | --- | --- | --- | --- | --- | --- | --- | --- | --- | --- | --- | --- | --- |
| K.AKNQALEIFK.D | Y | 58.38 | 1160.6553 | 10 | -0.5 | 581.3334 | 2 | 15.99 | 0.9158 | 0.923 | 51477 | 0 | 0 | 0 | 112 | 121 |  |  | DB Search |
| K.YLSWTLDNDKIR.S | Y | 53.6 | 1522.778 | 12 | -3.19 | 508.5972 | 3 | 17.66 | 0.8063 | 0.8136 | 62168 | 3.04e2 | 1 | 1 | 186 | 197 |  |  | DB Search |
| total 2 peptides |  |  |  |  |  |  |  |  |  |  |  |  |  |  |  |  |  |  |  |
| --- | --- | --- | --- | --- | --- | --- | --- | --- | --- | --- | --- | --- | --- | --- | --- | --- | --- | --- | --- |

Best Unique PSM (Scan Sample\_2\_A1\_1\_3640.d:51477, m/z=581.3334, z=2, RT=15.99, ppm=-2.7):


TRINITY\_DN3034\_c0\_g1\_i1.p1

back to list

  

| Protein Coverage
| Supporting Peptides
| Best Unique PSM
|

Protein Coverage:

Supporting Peptides:

| Peptide | Uniq | -10lgP | Mass | Length | ppm | m/z | z | RT | 1/k0 Start | 1/k0 End | Precursor ID | Area Sample 4 | #Feature | #Feature Sample 4 | Start | End | PTM | AScore | Found By |
| --- | --- | --- | --- | --- | --- | --- | --- | --- | --- | --- | --- | --- | --- | --- | --- | --- | --- | --- | --- |
| R.ALAAELQSVSEQLSEGGK.N | Y | 58.39 | 1815.9214 | 18 | -4.81 | 908.9616 | 2 | 19.65 | 1.1033 | 1.1106 | 75300 | 1.62e3 | 2 | 2 | 1494 | 1511 |  |  | DB Search |
| K.TPGLVEGTLILHQLR.C | Y | 58.37 | 1645.9515 | 15 | -0.36 | 549.6564 | 3 | 19.04 | 0.8554 | 0.8627 | 71412 | 0 | 0 | 0 | 671 | 685 |  |  | DB Search |
| K.LIDSLQLDQSEYR.V | Y | 57.62 | 1578.7889 | 13 | -0.6 | 790.3995 | 2 | 17.23 | 1.0604 | 1.0676 | 59328 | 3.65e2 | 1 | 1 | 735 | 747 |  |  | DB Search |
| K.VQPLLSIAR.A | Y | 57.44 | 995.6127 | 9 | 0.94 | 498.813 | 2 | 17.21 | 0.8433 | 0.8506 | 59276 | 1.54e2 | 1 | 1 | 826 | 834 |  |  | DB Search |
| K.EIEDINAANDEALASTK.H | Y | 54.75 | 1802.8534 | 17 | -3.96 | 902.4284 | 2 | 15.28 | 1.1009 | 1.1082 | 47115 | 0 | 0 | 0 | 1167 | 1183 |  |  | DB Search |
| K.FNAQLSEAQEELESVKK.A | Y | 54.73 | 1948.9741 | 17 | 4.67 | 650.6669 | 3 | 16.99 | 0.8854 | 0.8927 | 57921 | 0 | 0 | 0 | 1186 | 1202 |  |  | DB Search |
| R.VQEMEEALAAAESK.A | Y | 53.73 | 1504.7079 | 14 | -3.91 | 753.3566 | 2 | 16.96 | 1.013 | 1.0202 | 57682 | 3.72e2 | 1 | 1 | 1387 | 1400 |  |  | DB Search |
| total 7 peptides |  |  |  |  |  |  |  |  |  |  |  |  |  |  |  |  |  |  |  |
| --- | --- | --- | --- | --- | --- | --- | --- | --- | --- | --- | --- | --- | --- | --- | --- | --- | --- | --- | --- |

Best Unique PSM (Scan Sample\_2\_A1\_1\_3640.d:75300, m/z=908.9616, z=2, RT=19.65, ppm=-7):


TRINITY\_DN5\_c0\_g1\_i1.p1

back to list

  

| Protein Coverage
| Supporting Peptides
| Best Unique PSM
|

Protein Coverage:

Supporting Peptides:

| Peptide | Uniq | -10lgP | Mass | Length | ppm | m/z | z | RT | 1/k0 Start | 1/k0 End | Precursor ID | Area Sample 4 | #Feature | #Feature Sample 4 | Start | End | PTM | AScore | Found By |
| --- | --- | --- | --- | --- | --- | --- | --- | --- | --- | --- | --- | --- | --- | --- | --- | --- | --- | --- | --- |
| K.IIIELQGDLIK.I | Y | 53.27 | 1253.7594 | 11 | 3.89 | 627.8881 | 2 | 19.42 | 0.9513 | 0.9586 | 73842 | 0 | 0 | 0 | 65 | 75 |  |  | DB Search |
| total 1 peptides |  |  |  |  |  |  |  |  |  |  |  |  |  |  |  |  |  |  |  |
| --- | --- | --- | --- | --- | --- | --- | --- | --- | --- | --- | --- | --- | --- | --- | --- | --- | --- | --- | --- |

Best Unique PSM (Scan Sample\_2\_A1\_1\_3640.d:73842, m/z=627.8881, z=2, RT=19.42, ppm=1.69):


TRINITY\_DN3698\_c0\_g2\_i6.p1

back to list

  

| Protein Coverage
| Supporting Peptides
| Best Unique PSM
|

Protein Coverage:

Supporting Peptides:

| Peptide | Uniq | -10lgP | Mass | Length | ppm | m/z | z | RT | 1/k0 Start | 1/k0 End | Precursor ID | Area Sample 4 | #Feature | #Feature Sample 4 | Start | End | PTM | AScore | Found By |
| --- | --- | --- | --- | --- | --- | --- | --- | --- | --- | --- | --- | --- | --- | --- | --- | --- | --- | --- | --- |
| K.ANHIFAHTDPLSQK.S | Y | 58.31 | 1577.795 | 14 | -10.88 | 526.9321 | 3 | 13.75 | 0.8189 | 0.8262 | 37740 | 6.57e2 | 1 | 1 | 383 | 396 |  |  | DB Search |
| R.ATENPSGGFSPDDR.C | Y | 55.58 | 1448.6168 | 14 | 6.68 | 725.3189 | 2 | 12.97 | 0.9759 | 0.9832 | 32890 | 0 | 0 | 0 | 116 | 129 |  |  | DB Search |
| total 2 peptides |  |  |  |  |  |  |  |  |  |  |  |  |  |  |  |  |  |  |  |
| --- | --- | --- | --- | --- | --- | --- | --- | --- | --- | --- | --- | --- | --- | --- | --- | --- | --- | --- | --- |

Best Unique PSM (Scan Sample\_2\_A1\_1\_3640.d:37740, m/z=526.9321, z=3, RT=13.75, ppm=-13.08):


TRINITY\_DN3698\_c0\_g2\_i2.p1

back to list

  

| Protein Coverage
| Supporting Peptides
| Best Unique PSM
|

Protein Coverage:

Supporting Peptides:

| Peptide | Uniq | -10lgP | Mass | Length | ppm | m/z | z | RT | 1/k0 Start | 1/k0 End | Precursor ID | Area Sample 4 | #Feature | #Feature Sample 4 | Start | End | PTM | AScore | Found By |
| --- | --- | --- | --- | --- | --- | --- | --- | --- | --- | --- | --- | --- | --- | --- | --- | --- | --- | --- | --- |
| K.ANHIFAHTDPLSQK.S | Y | 58.31 | 1577.795 | 14 | -10.88 | 526.9321 | 3 | 13.75 | 0.8189 | 0.8262 | 37740 | 6.57e2 | 1 | 1 | 387 | 400 |  |  | DB Search |
| R.ATENPSGGFSPDDR.C | Y | 55.58 | 1448.6168 | 14 | 6.68 | 725.3189 | 2 | 12.97 | 0.9759 | 0.9832 | 32890 | 0 | 0 | 0 | 120 | 133 |  |  | DB Search |
| total 2 peptides |  |  |  |  |  |  |  |  |  |  |  |  |  |  |  |  |  |  |  |
| --- | --- | --- | --- | --- | --- | --- | --- | --- | --- | --- | --- | --- | --- | --- | --- | --- | --- | --- | --- |

Best Unique PSM (Scan Sample\_2\_A1\_1\_3640.d:37740, m/z=526.9321, z=3, RT=13.75, ppm=-13.08):


TRINITY\_DN4781\_c0\_g1\_i2.p1

back to list

  

| Protein Coverage
| Supporting Peptides
| Best Unique PSM
|

Protein Coverage:

Supporting Peptides:

| Peptide | Uniq | -10lgP | Mass | Length | ppm | m/z | z | RT | 1/k0 Start | 1/k0 End | Precursor ID | Area Sample 4 | #Feature | #Feature Sample 4 | Start | End | PTM | AScore | Found By |
| --- | --- | --- | --- | --- | --- | --- | --- | --- | --- | --- | --- | --- | --- | --- | --- | --- | --- | --- | --- |
| K.FGDIMDWTVHQGK.T | Y | 58.41 | 1532.7082 | 13 | 0.12 | 511.9089 | 3 | 18.62 | 0.7982 | 0.8055 | 68637 | 0 | 0 | 0 | 2412 | 2424 |  |  | DB Search |
| R.LLSPVFKPHGNK.K | Y | 58.4 | 1335.7663 | 12 | 7.47 | 446.265 | 3 | 13.80 | 0.7982 | 0.8055 | 37938 | 3.05e2 | 1 | 1 | 2241 | 2252 |  |  | DB Search |
| R.LVSELFDPTSSTR.G | Y | 58.39 | 1450.7303 | 13 | 1.5 | 726.3719 | 2 | 17.91 | 1.0013 | 1.0086 | 64032 | 6.47e3 | 1 | 1 | 265 | 277 |  |  | DB Search |
| K.DFNVIIEAVK.G | Y | 58.33 | 1146.6284 | 10 | 0.88 | 574.3207 | 2 | 19.65 | 0.8996 | 0.9069 | 75314 | 3.22e3 | 1 | 1 | 851 | 860 |  |  | DB Search |
| R.GDIAIDDITVKK.G | Y | 58.32 | 1286.7081 | 12 | 0.84 | 644.3605 | 2 | 15.29 | 0.9602 | 0.9675 | 47150 | 2.79e2 | 1 | 1 | 1480 | 1491 |  |  | DB Search |
| K.DFQVIFEAK.M | Y | 58.28 | 1095.56 | 9 | -2.26 | 548.7849 | 2 | 19.00 | 0.8579 | 0.8652 | 71160 | 0 | 0 | 0 | 2745 | 2753 |  |  | DB Search |
| K.APPKPNPC(+57.02)AIK.C | Y | 58.26 | 1191.6434 | 11 | 5.82 | 398.2232 | 3 | 11.50 | 0.7783 | 0.7856 | 23835 | 2.21e3 | 2 | 2 | 1499 | 1509 | Carbamidomethylation |  | DB Search |
| Q.LIFVGTVGK.S | Y | 58.18 | 932.5695 | 9 | 3.01 | 467.2924 | 2 | 17.35 | 0.8124 | 0.8197 | 60219 | 6.55e3 | 1 | 1 | 168 | 176 |  |  | DB Search |
| R.TC(+57.02)IGC(+57.02)PETQIWR.R | Y | 58.01 | 1519.6912 | 12 | -0.87 | 760.8505 | 2 | 16.92 | 1.0053 | 1.0126 | 57323 | 1.28e3 | 1 | 1 | 1852 | 1863 | Carbamidomethylation, Carbamidomethylation |  | DB Search |
| K.VNFQVVMEAR.R | Y | 57.24 | 1191.607 | 10 | 0.27 | 596.8096 | 2 | 17.59 | 0.8972 | 0.9044 | 61571 | 2.93e3 | 1 | 1 | 2309 | 2318 |  |  | DB Search |
| K.GSYIYIEASYPR.K | Y | 57.03 | 1417.6878 | 12 | 0.36 | 709.8499 | 2 | 17.28 | 1.0399 | 1.0472 | 59633 | 2.78e3 | 1 | 1 | 434 | 445 |  |  | DB Search |
| K.TVISQVQSLSIR.N | Y | 56.91 | 1329.7616 | 12 | 2.63 | 665.8884 | 2 | 17.88 | 0.9574 | 0.9646 | 63579 | 2.04e3 | 1 | 1 | 2075 | 2086 |  |  | DB Search |
| K.GQSSDLATLSSPVLNQASSTC(+57.02)K.M | Y | 55.21 | 2250.0798 | 22 | 4.76 | 1126.05 | 2 | 17.18 | 1.2328 | 1.24 | 59109 | 0 | 0 | 0 | 1600 | 1621 | Carbamidomethylation |  | DB Search |
| K.ADHTYETDFGK.Y | Y | 55.18 | 1282.5466 | 11 | 8.7 | 642.2847 | 2 | 12.90 | 0.9453 | 0.9525 | 32449 | 0 | 0 | 0 | 1196 | 1206 |  |  | DB Search |
| K.LDSNKDFQVIFEAK.M | Y | 54.94 | 1652.8409 | 14 | -3.52 | 551.9511 | 3 | 18.03 | 0.8254 | 0.8327 | 64725 | 0 | 0 | 0 | 2740 | 2753 |  |  | DB Search |
| R.LISLPVESRPGGNC(+57.02)QVR.F | Y | 54.82 | 1880.989 | 17 | 0.61 | 628.0026 | 3 | 15.71 | 0.8481 | 0.8554 | 49525 | 1.61e3 | 1 | 1 | 3102 | 3118 | Carbamidomethylation |  | DB Search |
| R.TGPSLDNTK.K | Y | 54.61 | 931.4611 | 9 | 0.07 | 466.7368 | 2 | 11.29 | 0.8039 | 0.8112 | 22423 | 5.2e2 | 1 | 1 | 233 | 241 |  |  | DB Search |
| R.FYYHM(+15.99)YGDHIGALSVK.L | Y | 53.61 | 1915.8927 | 16 | 2.41 | 639.6383 | 3 | 15.79 | 0.9085 | 0.9158 | 50276 | 2.57e2 | 1 | 1 | 1834 | 1849 | Oxidation (M) | M5:Oxidation (M):1000 | DB Search |
| R.FYYHMYGDHIGALSVK.L | Y | 52.72 | 1899.8978 | 16 | -1 | 634.3045 | 3 | 16.96 | 0.917 | 0.9243 | 57688 | 0 | 0 | 0 | 1834 | 1849 |  |  | DB Search |
| R.DVANLYSPYYPGSK.T | Y | 51.34 | 1572.746 | 14 | -1.72 | 787.3772 | 2 | 17.32 | 1.0242 | 1.0315 | 60052 | 5.66e2 | 1 | 1 | 613 | 626 |  |  | DB Search |
| R.NLKDDDFDFTR.Q | Y | 41.38 | 1384.6259 | 11 | 14.15 | 693.3285 | 2 | 15.93 | 0.9775 | 0.9848 | 51300 | 6.08e2 | 1 | 1 | 1765 | 1775 |  |  | DB Search |
| total 21 peptides |  |  |  |  |  |  |  |  |  |  |  |  |  |  |  |  |  |  |  |
| --- | --- | --- | --- | --- | --- | --- | --- | --- | --- | --- | --- | --- | --- | --- | --- | --- | --- | --- | --- |

Best Unique PSM (Scan Sample\_2\_A1\_1\_3640.d:68637, m/z=511.9089, z=3, RT=18.62, ppm=-2.08):


TRINITY\_DN8926\_c0\_g1\_i1.p1

back to list

  

| Protein Coverage
| Supporting Peptides
| Best Unique PSM
|

Protein Coverage:

Supporting Peptides:

| Peptide | Uniq | -10lgP | Mass | Length | ppm | m/z | z | RT | 1/k0 Start | 1/k0 End | Precursor ID | Area Sample 4 | #Feature | #Feature Sample 4 | Start | End | PTM | AScore | Found By |
| --- | --- | --- | --- | --- | --- | --- | --- | --- | --- | --- | --- | --- | --- | --- | --- | --- | --- | --- | --- |
| R.AQIDIWLQR.M | Y | 57.82 | 1141.6244 | 9 | 1.55 | 571.8191 | 2 | 18.61 | 0.8887 | 0.8959 | 68405 | 8.34e2 | 1 | 1 | 157 | 165 |  |  | DB Search |
| total 1 peptides |  |  |  |  |  |  |  |  |  |  |  |  |  |  |  |  |  |  |  |
| --- | --- | --- | --- | --- | --- | --- | --- | --- | --- | --- | --- | --- | --- | --- | --- | --- | --- | --- | --- |

Best Unique PSM (Scan Sample\_2\_A1\_1\_3640.d:68405, m/z=571.8191, z=2, RT=18.61, ppm=-0.65):


TRINITY\_DN163801\_c0\_g1\_i1.p1

back to list

  

| Protein Coverage
| Supporting Peptides
| Best Unique PSM
|

Protein Coverage:

Supporting Peptides:

| Peptide | Uniq | -10lgP | Mass | Length | ppm | m/z | z | RT | 1/k0 Start | 1/k0 End | Precursor ID | Area Sample 4 | #Feature | #Feature Sample 4 | Start | End | PTM | AScore | Found By |
| --- | --- | --- | --- | --- | --- | --- | --- | --- | --- | --- | --- | --- | --- | --- | --- | --- | --- | --- | --- |
| M.A(+42.01)NPLKDIISR.F | Y | 39.76 | 1167.6611 | 10 | -4.53 | 584.8339 | 2 | 18.80 | 0.9117 | 0.919 | 69656 | 5.36e1 | 1 | 1 | 2 | 11 | Acetylation (Protein N-term) | A1:Acetylation (Protein N-term):1000 | DB Search |
| total 1 peptides |  |  |  |  |  |  |  |  |  |  |  |  |  |  |  |  |  |  |  |
| --- | --- | --- | --- | --- | --- | --- | --- | --- | --- | --- | --- | --- | --- | --- | --- | --- | --- | --- | --- |

Best Unique PSM (Scan Sample\_2\_A1\_1\_3640.d:69656, m/z=584.8339, z=2, RT=18.80, ppm=-6.72):


TRINITY\_DN168145\_c0\_g1\_i1.p1

back to list

  

| Protein Coverage
| Supporting Peptides
| Best Unique PSM
|

Protein Coverage:

Supporting Peptides:

| Peptide | Uniq | -10lgP | Mass | Length | ppm | m/z | z | RT | 1/k0 Start | 1/k0 End | Precursor ID | Area Sample 4 | #Feature | #Feature Sample 4 | Start | End | PTM | AScore | Found By |
| --- | --- | --- | --- | --- | --- | --- | --- | --- | --- | --- | --- | --- | --- | --- | --- | --- | --- | --- | --- |
| R.REGDNALLFGPK.Y | Y | 54.47 | 1315.6884 | 12 | 0.28 | 658.8502 | 2 | 15.81 | 0.9654 | 0.9727 | 50453 | 0 | 0 | 0 | 52 | 63 |  |  | DB Search |
| R.LIFEGIVGK.N | Y | 53.31 | 974.58 | 9 | 4.48 | 488.2984 | 2 | 18.60 | 0.8161 | 0.8234 | 68409 | 4.22e3 | 1 | 1 | 125 | 133 |  |  | DB Search |
| total 2 peptides |  |  |  |  |  |  |  |  |  |  |  |  |  |  |  |  |  |  |  |
| --- | --- | --- | --- | --- | --- | --- | --- | --- | --- | --- | --- | --- | --- | --- | --- | --- | --- | --- | --- |

Best Unique PSM (Scan Sample\_2\_A1\_1\_3640.d:50453, m/z=658.8502, z=2, RT=15.81, ppm=-1.92):


TRINITY\_DN199\_c0\_g1\_i1.p1

back to list

  

| Protein Coverage
| Supporting Peptides
| Best Unique PSM
|

Protein Coverage:

Supporting Peptides:

| Peptide | Uniq | -10lgP | Mass | Length | ppm | m/z | z | RT | 1/k0 Start | 1/k0 End | Precursor ID | Area Sample 4 | #Feature | #Feature Sample 4 | Start | End | PTM | AScore | Found By |
| --- | --- | --- | --- | --- | --- | --- | --- | --- | --- | --- | --- | --- | --- | --- | --- | --- | --- | --- | --- |
| K.LLDWAQQR.F | Y | 57.97 | 1028.5403 | 8 | 6.66 | 515.2797 | 2 | 16.40 | 0.8485 | 0.8558 | 54058 | 6.82e2 | 1 | 1 | 136 | 143 |  |  | DB Search |
| K.NYNNDDEPGAK.K | Y | 57.14 | 1235.5054 | 11 | 0.05 | 618.7587 | 2 | 10.27 | 0.9129 | 0.9202 | 16311 | 2.19e3 | 1 | 1 | 124 | 134 |  |  | DB Search |
| K.KLLDWAQQR.F | Y | 57.01 | 1156.6353 | 9 | 2.16 | 579.3249 | 2 | 15.60 | 0.9089 | 0.9162 | 49058 | 1.7e3 | 1 | 1 | 135 | 143 |  |  | DB Search |
| K.NYNNDDEPGAKK.L | Y | 53.98 | 1363.6004 | 12 | -1.27 | 682.8051 | 2 | 9.59 | 0.9384 | 0.9457 | 12858 | 0 | 0 | 0 | 124 | 135 |  |  | DB Search |
| total 4 peptides |  |  |  |  |  |  |  |  |  |  |  |  |  |  |  |  |  |  |  |
| --- | --- | --- | --- | --- | --- | --- | --- | --- | --- | --- | --- | --- | --- | --- | --- | --- | --- | --- | --- |

Best Unique PSM (Scan Sample\_2\_A1\_1\_3640.d:54058, m/z=515.2797, z=2, RT=16.40, ppm=4.46):


TRINITY\_DN18\_c0\_g1\_i1.p1

back to list

  

| Protein Coverage
| Supporting Peptides
| Best Unique PSM
|

Protein Coverage:

Supporting Peptides:

| Peptide | Uniq | -10lgP | Mass | Length | ppm | m/z | z | RT | 1/k0 Start | 1/k0 End | Precursor ID | Area Sample 4 | #Feature | #Feature Sample 4 | Start | End | PTM | AScore | Found By |
| --- | --- | --- | --- | --- | --- | --- | --- | --- | --- | --- | --- | --- | --- | --- | --- | --- | --- | --- | --- |
| M.GIDIGLDEVK.T | Y | 54.98 | 1057.5655 | 10 | -4.24 | 529.7866 | 2 | 17.24 | 0.8619 | 0.8692 | 59426 | 5.78e3 | 1 | 1 | 2 | 11 |  |  | DB Search |
| R.FIFGVPENPR.H | Y | 47.68 | 1174.6135 | 10 | -0.68 | 588.3123 | 2 | 18.49 | 0.9295 | 0.9368 | 67817 | 5.2e3 | 1 | 1 | 229 | 238 |  |  | DB Search |
| total 2 peptides |  |  |  |  |  |  |  |  |  |  |  |  |  |  |  |  |  |  |  |
| --- | --- | --- | --- | --- | --- | --- | --- | --- | --- | --- | --- | --- | --- | --- | --- | --- | --- | --- | --- |

Best Unique PSM (Scan Sample\_2\_A1\_1\_3640.d:59426, m/z=529.7866, z=2, RT=17.24, ppm=-6.44):


TRINITY\_DN4781\_c0\_g1\_i3.p2

back to list

  

| Protein Coverage
| Supporting Peptides
| Best Unique PSM
|

Protein Coverage:

Supporting Peptides:

| Peptide | Uniq | -10lgP | Mass | Length | ppm | m/z | z | RT | 1/k0 Start | 1/k0 End | Precursor ID | Area Sample 4 | #Feature | #Feature Sample 4 | Start | End | PTM | AScore | Found By |
| --- | --- | --- | --- | --- | --- | --- | --- | --- | --- | --- | --- | --- | --- | --- | --- | --- | --- | --- | --- |
| K.DFQVIFEAK.M | Y | 58.28 | 1095.56 | 9 | -2.26 | 548.7849 | 2 | 19.00 | 0.8579 | 0.8652 | 71160 | 0 | 0 | 0 | 156 | 164 |  |  | DB Search |
| K.LDSNKDFQVIFEAK.M | Y | 54.94 | 1652.8409 | 14 | -3.52 | 551.9511 | 3 | 18.03 | 0.8254 | 0.8327 | 64725 | 0 | 0 | 0 | 151 | 164 |  |  | DB Search |
| R.LISLPVESRPGGNC(+57.02)QVR.F | Y | 54.82 | 1880.989 | 17 | 0.61 | 628.0026 | 3 | 15.71 | 0.8481 | 0.8554 | 49525 | 1.61e3 | 1 | 1 | 513 | 529 | Carbamidomethylation |  | DB Search |
| total 3 peptides |  |  |  |  |  |  |  |  |  |  |  |  |  |  |  |  |  |  |  |
| --- | --- | --- | --- | --- | --- | --- | --- | --- | --- | --- | --- | --- | --- | --- | --- | --- | --- | --- | --- |

Best Unique PSM (Scan Sample\_2\_A1\_1\_3640.d:71160, m/z=548.7849, z=2, RT=19.00, ppm=-4.45):


TRINITY\_DN2532\_c2\_g1\_i1.p1

back to list

  

| Protein Coverage
| Supporting Peptides
| Best Unique PSM
|

Protein Coverage:

Supporting Peptides:

| Peptide | Uniq | -10lgP | Mass | Length | ppm | m/z | z | RT | 1/k0 Start | 1/k0 End | Precursor ID | Area Sample 4 | #Feature | #Feature Sample 4 | Start | End | PTM | AScore | Found By |
| --- | --- | --- | --- | --- | --- | --- | --- | --- | --- | --- | --- | --- | --- | --- | --- | --- | --- | --- | --- |
| K.TGEC(+57.02)ISQELAC(+57.02)DQVK.D | Y | 48.47 | 1736.7709 | 15 | 15.02 | 869.4039 | 2 | 15.28 | 1.0837 | 1.0909 | 47116 | 0 | 0 | 0 | 297 | 311 | Carbamidomethylation, Carbamidomethylation |  | DB Search |
| total 1 peptides |  |  |  |  |  |  |  |  |  |  |  |  |  |  |  |  |  |  |  |
| --- | --- | --- | --- | --- | --- | --- | --- | --- | --- | --- | --- | --- | --- | --- | --- | --- | --- | --- | --- |

Best Unique PSM (Scan Sample\_2\_A1\_1\_3640.d:47116, m/z=869.4039, z=2, RT=15.28, ppm=12.82):


TRINITY\_DN14015\_c0\_g1\_i3.p1

back to list

  

| Protein Coverage
| Supporting Peptides
| Best Unique PSM
|

Protein Coverage:

Supporting Peptides:

| Peptide | Uniq | -10lgP | Mass | Length | ppm | m/z | z | RT | 1/k0 Start | 1/k0 End | Precursor ID | Area Sample 4 | #Feature | #Feature Sample 4 | Start | End | PTM | AScore | Found By |
| --- | --- | --- | --- | --- | --- | --- | --- | --- | --- | --- | --- | --- | --- | --- | --- | --- | --- | --- | --- |
| K.VIDINDNAPIFNK.A | Y | 58.39 | 1471.7671 | 13 | 2.95 | 736.8914 | 2 | 17.55 | 1.0081 | 1.0154 | 61422 | 9.25e2 | 1 | 1 | 666 | 678 |  |  | DB Search |
| K.TNENRPIFQFTNPK.V | Y | 58.31 | 1704.8583 | 14 | -6.5 | 569.2885 | 3 | 16.42 | 0.8465 | 0.8538 | 54306 | 6.65e2 | 2 | 2 | 1891 | 1904 |  |  | DB Search |
| R.LGIEAIDENGLR.S | Y | 58.26 | 1298.683 | 12 | 0.2 | 650.3475 | 2 | 17.28 | 0.9566 | 0.9638 | 59638 | 7.15e2 | 1 | 1 | 956 | 967 |  |  | DB Search |
| total 3 peptides |  |  |  |  |  |  |  |  |  |  |  |  |  |  |  |  |  |  |  |
| --- | --- | --- | --- | --- | --- | --- | --- | --- | --- | --- | --- | --- | --- | --- | --- | --- | --- | --- | --- |

Best Unique PSM (Scan Sample\_2\_A1\_1\_3640.d:61422, m/z=736.8914, z=2, RT=17.55, ppm=0.75):


TRINITY\_DN7946\_c0\_g1\_i1.p1

back to list

  

| Protein Coverage
| Supporting Peptides
| Best Unique PSM
|

Protein Coverage:

Supporting Peptides:

| Peptide | Uniq | -10lgP | Mass | Length | ppm | m/z | z | RT | 1/k0 Start | 1/k0 End | Precursor ID | Area Sample 4 | #Feature | #Feature Sample 4 | Start | End | PTM | AScore | Found By |
| --- | --- | --- | --- | --- | --- | --- | --- | --- | --- | --- | --- | --- | --- | --- | --- | --- | --- | --- | --- |
| K.DLFDTLTK.V | Y | 57.97 | 951.4913 | 8 | 4.2 | 476.7539 | 2 | 19.15 | 0.8063 | 0.8136 | 71926 | 2.59e3 | 1 | 1 | 531 | 538 |  |  | DB Search |
| R.MLDHFLSTDVFVK.G | Y | 56.02 | 1550.7803 | 13 | -5.03 | 517.9303 | 3 | 19.28 | 0.7746 | 0.7819 | 72920 | 0 | 0 | 0 | 501 | 513 |  |  | DB Search |
| K.TAVDGISIISSGLTK.T | Y | 54.9 | 1460.8086 | 15 | 3.18 | 731.4123 | 2 | 18.95 | 1.0214 | 1.0287 | 70784 | 3.02e2 | 1 | 1 | 728 | 742 |  |  | DB Search |
| total 3 peptides |  |  |  |  |  |  |  |  |  |  |  |  |  |  |  |  |  |  |  |
| --- | --- | --- | --- | --- | --- | --- | --- | --- | --- | --- | --- | --- | --- | --- | --- | --- | --- | --- | --- |

Best Unique PSM (Scan Sample\_2\_A1\_1\_3640.d:71926, m/z=476.7539, z=2, RT=19.15, ppm=2.01):


TRINITY\_DN77\_c1\_g1\_i1.p1

back to list

  

| Protein Coverage
| Supporting Peptides
| Best Unique PSM
|

Protein Coverage:

Supporting Peptides:

| Peptide | Uniq | -10lgP | Mass | Length | ppm | m/z | z | RT | 1/k0 Start | 1/k0 End | Precursor ID | Area Sample 4 | #Feature | #Feature Sample 4 | Start | End | PTM | AScore | Found By |
| --- | --- | --- | --- | --- | --- | --- | --- | --- | --- | --- | --- | --- | --- | --- | --- | --- | --- | --- | --- |
| R.LLAVTVGNNYNLDTLEK.V | Y | 58.4 | 1875.9942 | 17 | -12.01 | 938.991 | 2 | 18.74 | 1.1414 | 1.1486 | 69418 | 0 | 0 | 0 | 693 | 709 |  |  | DB Search |
| R.LVNFGFENK.I | Y | 58.27 | 1066.5447 | 9 | 4.08 | 534.2806 | 2 | 17.31 | 0.8404 | 0.8477 | 59814 | 6.14e2 | 1 | 1 | 471 | 479 |  |  | DB Search |
| R.GLRGPEGSSGPIGK.T | Y | 58.15 | 1310.6942 | 14 | -0.77 | 437.9041 | 3 | 12.30 | 0.7176 | 0.7249 | 28421 | 2.77e3 | 2 | 2 | 985 | 998 |  |  | DB Search |
| K.GANGQNGVQGAR.G | Y | 58.1 | 1127.5432 | 12 | 10.77 | 564.7837 | 2 | 9.29 | 0.8441 | 0.8514 | 11509 | 0 | 0 | 0 | 1417 | 1428 |  |  | DB Search |
| R.LEIASWPLR.K | Y | 57.9 | 1083.6076 | 9 | -0.52 | 542.8096 | 2 | 19.41 | 0.8684 | 0.8757 | 73699 | 4.16e2 | 1 | 1 | 669 | 677 |  |  | DB Search |
| R.GPEGSSGPIGK.T | Y | 56.24 | 984.4876 | 11 | 3.31 | 493.2516 | 2 | 10.98 | 0.8128 | 0.8201 | 20694 | 2.62e3 | 1 | 1 | 988 | 998 |  |  | DB Search |
| total 6 peptides |  |  |  |  |  |  |  |  |  |  |  |  |  |  |  |  |  |  |  |
| --- | --- | --- | --- | --- | --- | --- | --- | --- | --- | --- | --- | --- | --- | --- | --- | --- | --- | --- | --- |

Best Unique PSM (Scan Sample\_2\_A1\_1\_3640.d:69418, m/z=938.991, z=2, RT=18.74, ppm=-14.21):


TRINITY\_DN12542\_c0\_g1\_i1.p1

back to list

  

| Protein Coverage
| Supporting Peptides
| Best Unique PSM
|

Protein Coverage:

Supporting Peptides:

| Peptide | Uniq | -10lgP | Mass | Length | ppm | m/z | z | RT | 1/k0 Start | 1/k0 End | Precursor ID | Area Sample 4 | #Feature | #Feature Sample 4 | Start | End | PTM | AScore | Found By |
| --- | --- | --- | --- | --- | --- | --- | --- | --- | --- | --- | --- | --- | --- | --- | --- | --- | --- | --- | --- |
| K.LLILLTDGSQTQDAGAEDPVK.L | Y | 58.29 | 2183.1321 | 21 | -2.24 | 1092.5685 | 2 | 19.12 | 1.2432 | 1.2503 | 71899 | 0 | 0 | 0 | 101 | 121 |  |  | DB Search |
| K.VDVGFILDSSGSLR.N | N | 58.24 | 1463.762 | 14 | 1.17 | 732.8875 | 2 | 19.97 | 1.0102 | 1.0174 | 77350 | 0 | 0 | 0 | 195 | 208 |  |  | DB Search |
| total 2 peptides |  |  |  |  |  |  |  |  |  |  |  |  |  |  |  |  |  |  |  |
| --- | --- | --- | --- | --- | --- | --- | --- | --- | --- | --- | --- | --- | --- | --- | --- | --- | --- | --- | --- |

Best Unique PSM (Scan Sample\_2\_A1\_1\_3640.d:71899, m/z=1092.5685, z=2, RT=19.12, ppm=-4.43):


TRINITY\_DN31399\_c0\_g1\_i2.p1

back to list

  

| Protein Coverage
| Supporting Peptides
| Best Unique PSM
|

Protein Coverage:

Supporting Peptides:

| Peptide | Uniq | -10lgP | Mass | Length | ppm | m/z | z | RT | 1/k0 Start | 1/k0 End | Precursor ID | Area Sample 4 | #Feature | #Feature Sample 4 | Start | End | PTM | AScore | Found By |
| --- | --- | --- | --- | --- | --- | --- | --- | --- | --- | --- | --- | --- | --- | --- | --- | --- | --- | --- | --- |
| R.TVVTIVSDDEYQHM(+15.99)FDR.V | Y | 40.35 | 2069.9364 | 17 | 3.13 | 1035.9764 | 2 | 16.66 | 1.2491 | 1.2563 | 55691 | 2.94e1 | 1 | 1 | 561 | 577 | Oxidation (M) | M14:Oxidation (M):1000 | DB Search |
| total 1 peptides |  |  |  |  |  |  |  |  |  |  |  |  |  |  |  |  |  |  |  |
| --- | --- | --- | --- | --- | --- | --- | --- | --- | --- | --- | --- | --- | --- | --- | --- | --- | --- | --- | --- |

Best Unique PSM (Scan Sample\_2\_A1\_1\_3640.d:55691, m/z=1035.9764, z=2, RT=16.66, ppm=0.93):


TRINITY\_DN2885\_c0\_g1\_i5.p1

back to list

  

| Protein Coverage
| Supporting Peptides
| Best Unique PSM
|

Protein Coverage:

Supporting Peptides:

| Peptide | Uniq | -10lgP | Mass | Length | ppm | m/z | z | RT | 1/k0 Start | 1/k0 End | Precursor ID | Area Sample 4 | #Feature | #Feature Sample 4 | Start | End | PTM | AScore | Found By |
| --- | --- | --- | --- | --- | --- | --- | --- | --- | --- | --- | --- | --- | --- | --- | --- | --- | --- | --- | --- |
| K.VTRPHGASGVVR.A | Y | 57.82 | 1234.6894 | 12 | 7.66 | 412.5727 | 3 | 10.07 | 0.8108 | 0.8181 | 15251 | 2.57e2 | 1 | 1 | 77 | 88 |  |  | DB Search |
| total 1 peptides |  |  |  |  |  |  |  |  |  |  |  |  |  |  |  |  |  |  |  |
| --- | --- | --- | --- | --- | --- | --- | --- | --- | --- | --- | --- | --- | --- | --- | --- | --- | --- | --- | --- |

Best Unique PSM (Scan Sample\_2\_A1\_1\_3640.d:15251, m/z=412.5727, z=3, RT=10.07, ppm=5.46):


TRINITY\_DN63421\_c0\_g1\_i1.p1

back to list

  

| Protein Coverage
| Supporting Peptides
| Best Unique PSM
|

Protein Coverage:

Supporting Peptides:

| Peptide | Uniq | -10lgP | Mass | Length | ppm | m/z | z | RT | 1/k0 Start | 1/k0 End | Precursor ID | Area Sample 4 | #Feature | #Feature Sample 4 | Start | End | PTM | AScore | Found By |
| --- | --- | --- | --- | --- | --- | --- | --- | --- | --- | --- | --- | --- | --- | --- | --- | --- | --- | --- | --- |
| R.IC(+57.02)STC(+57.02)PPNR.F | Y | 55.69 | 1103.4852 | 9 | 4.63 | 552.7512 | 2 | 10.47 | 0.8437 | 0.851 | 17525 | 1.43e3 | 1 | 1 | 100 | 108 | Carbamidomethylation, Carbamidomethylation |  | DB Search |
| total 1 peptides |  |  |  |  |  |  |  |  |  |  |  |  |  |  |  |  |  |  |  |
| --- | --- | --- | --- | --- | --- | --- | --- | --- | --- | --- | --- | --- | --- | --- | --- | --- | --- | --- | --- |

Best Unique PSM (Scan Sample\_2\_A1\_1\_3640.d:17525, m/z=552.7512, z=2, RT=10.47, ppm=2.44):


TRINITY\_DN8227\_c0\_g1\_i1.p1

back to list

  

| Protein Coverage
| Supporting Peptides
| Best Unique PSM
|

Protein Coverage:

Supporting Peptides:

| Peptide | Uniq | -10lgP | Mass | Length | ppm | m/z | z | RT | 1/k0 Start | 1/k0 End | Precursor ID | Area Sample 4 | #Feature | #Feature Sample 4 | Start | End | PTM | AScore | Found By |
| --- | --- | --- | --- | --- | --- | --- | --- | --- | --- | --- | --- | --- | --- | --- | --- | --- | --- | --- | --- |
| K.ESTLHLVLR.L | Y | 57.27 | 1066.6135 | 9 | 2.57 | 534.3142 | 2 | 15.80 | 0.8785 | 0.8858 | 50332 | 4.97e3 | 1 | 1 | 88 | 96 |  |  | DB Search |
| K.IQDKEGIPPDQQR.L | Y | 55.01 | 1522.7739 | 13 | 0.25 | 762.3928 | 2 | 11.08 | 1.0343 | 1.0415 | 21018 | 1.78e3 | 2 | 2 | 54 | 66 |  |  | DB Search |
| total 2 peptides |  |  |  |  |  |  |  |  |  |  |  |  |  |  |  |  |  |  |  |
| --- | --- | --- | --- | --- | --- | --- | --- | --- | --- | --- | --- | --- | --- | --- | --- | --- | --- | --- | --- |

Best Unique PSM (Scan Sample\_2\_A1\_1\_3640.d:50332, m/z=534.7766, z=2, RT=15.80, ppm=0.37):


TRINITY\_DN36\_c0\_g1\_i9.p1

back to list

  

| Protein Coverage
| Supporting Peptides
| Best Unique PSM
|

Protein Coverage:

Supporting Peptides:

| Peptide | Uniq | -10lgP | Mass | Length | ppm | m/z | z | RT | 1/k0 Start | 1/k0 End | Precursor ID | Area Sample 4 | #Feature | #Feature Sample 4 | Start | End | PTM | AScore | Found By |
| --- | --- | --- | --- | --- | --- | --- | --- | --- | --- | --- | --- | --- | --- | --- | --- | --- | --- | --- | --- |
| R.LGGIEVTQGNC(+57.02)AR.K | Y | 58.36 | 1373.6721 | 13 | -2.89 | 687.8398 | 2 | 13.93 | 0.9662 | 0.9735 | 38726 | 2.09e3 | 1 | 1 | 58 | 70 | Carbamidomethylation |  | DB Search |
| K.GLPLAIGVR.D | Y | 57.12 | 894.565 | 9 | 0.27 | 448.2889 | 2 | 17.73 | 0.8124 | 0.8197 | 62574 | 3.52e3 | 1 | 1 | 32 | 40 |  |  | DB Search |
| total 2 peptides |  |  |  |  |  |  |  |  |  |  |  |  |  |  |  |  |  |  |  |
| --- | --- | --- | --- | --- | --- | --- | --- | --- | --- | --- | --- | --- | --- | --- | --- | --- | --- | --- | --- |

Best Unique PSM (Scan Sample\_2\_A1\_1\_3640.d:38726, m/z=687.8398, z=2, RT=13.93, ppm=-5.09):


TRINITY\_DN3434\_c0\_g1\_i1.p1

back to list

  

| Protein Coverage
| Supporting Peptides
| Best Unique PSM
|

Protein Coverage:

Supporting Peptides:

| Peptide | Uniq | -10lgP | Mass | Length | ppm | m/z | z | RT | 1/k0 Start | 1/k0 End | Precursor ID | Area Sample 4 | #Feature | #Feature Sample 4 | Start | End | PTM | AScore | Found By |
| --- | --- | --- | --- | --- | --- | --- | --- | --- | --- | --- | --- | --- | --- | --- | --- | --- | --- | --- | --- |
| R.FTQAGSEVSALLGR.I | Y | 48.14 | 1434.7467 | 14 | 5.7 | 718.3831 | 2 | 18.43 | 0.986 | 0.9932 | 67409 | 0 | 0 | 0 | 274 | 287 |  |  | DB Search |
| total 1 peptides |  |  |  |  |  |  |  |  |  |  |  |  |  |  |  |  |  |  |  |
| --- | --- | --- | --- | --- | --- | --- | --- | --- | --- | --- | --- | --- | --- | --- | --- | --- | --- | --- | --- |

Best Unique PSM (Scan Sample\_2\_A1\_1\_3640.d:67409, m/z=718.3831, z=2, RT=18.43, ppm=3.5):


TRINITY\_DN1616\_c0\_g1\_i7.p1

back to list

  

| Protein Coverage
| Supporting Peptides
| Best Unique PSM
|

Protein Coverage:

Supporting Peptides:

| Peptide | Uniq | -10lgP | Mass | Length | ppm | m/z | z | RT | 1/k0 Start | 1/k0 End | Precursor ID | Area Sample 4 | #Feature | #Feature Sample 4 | Start | End | PTM | AScore | Found By |
| --- | --- | --- | --- | --- | --- | --- | --- | --- | --- | --- | --- | --- | --- | --- | --- | --- | --- | --- | --- |
| R.SNIDEVNVKHEQIEEFIK.T | Y | 58.4 | 2170.0906 | 18 | 0.32 | 724.3694 | 3 | 16.43 | 0.9566 | 0.9638 | 54256 | 7.37e2 | 1 | 1 | 2671 | 2688 |  |  | DB Search |
| K.VVDTEANDLTEEVKK.T | Y | 58.38 | 1688.8468 | 15 | -0.58 | 563.9547 | 3 | 14.21 | 0.8392 | 0.8465 | 40438 | 5.49e3 | 2 | 2 | 2542 | 2556 |  |  | DB Search |
| K.LNTGDVQSLTNPK.F | Y | 58.37 | 1385.715 | 13 | 1.86 | 693.8646 | 2 | 14.77 | 0.9977 | 1.0049 | 43652 | 1.53e3 | 1 | 1 | 2783 | 2795 |  |  | DB Search |
| R.YGAVNDNC(+57.02)ER.D | Y | 58.36 | 1196.488 | 10 | 4.23 | 599.2525 | 2 | 10.91 | 0.8802 | 0.8874 | 20281 | 8.99e2 | 1 | 1 | 498 | 507 | Carbamidomethylation |  | DB Search |
| K.SNIC(+57.02)DPQNGK.C | Y | 58.34 | 1131.4979 | 10 | -0.79 | 566.7545 | 2 | 10.56 | 0.8526 | 0.8599 | 18199 | 6.49e2 | 1 | 1 | 1794 | 1803 | Carbamidomethylation |  | DB Search |
| K.LSLDVNGNYQGGGVC(+57.02)K.N | Y | 58.29 | 1679.7937 | 16 | -4.07 | 840.8989 | 2 | 15.59 | 1.0705 | 1.0777 | 49051 | 0 | 0 | 0 | 350 | 365 | Carbamidomethylation |  | DB Search |
| R.FSNLSNKGDEHISNLDTLKK.A | Y | 58.28 | 2259.1495 | 20 | 1.82 | 565.7944 | 4 | 13.91 | 0.8311 | 0.8384 | 38634 | 0 | 0 | 0 | 2121 | 2140 |  |  | DB Search |
| R.NFATAVLNVQK.S | Y | 58.28 | 1203.6611 | 11 | -3.72 | 602.8343 | 2 | 17.63 | 0.923 | 0.9303 | 62041 | 0 | 0 | 0 | 2810 | 2820 |  |  | DB Search |
| K.TLKVDISDMK.M | Y | 58.28 | 1148.6111 | 10 | -6.01 | 575.3081 | 2 | 15.17 | 0.917 | 0.9243 | 46490 | 0 | 0 | 0 | 2557 | 2566 |  |  | DB Search |
| K.FIETDVNYDVM(+15.99)VTR.N | Y | 58.27 | 1716.8029 | 14 | 0.97 | 859.4077 | 2 | 16.71 | 1.0809 | 1.0881 | 56014 | 1.52e3 | 1 | 1 | 2796 | 2809 | Oxidation (M) | M11:Oxidation (M):1000 | DB Search |
| R.VIDEGEKNIK.L | Y | 58.26 | 1143.6135 | 10 | 3.03 | 572.8145 | 2 | 10.89 | 0.9271 | 0.9344 | 20199 | 3.83e3 | 2 | 2 | 2218 | 2227 |  |  | DB Search |
| K.DIIVLEAENPVK.M | Y | 58.25 | 1338.7394 | 12 | 1.86 | 670.3768 | 2 | 18.10 | 0.9844 | 0.9916 | 65198 | 4.87e2 | 1 | 1 | 1013 | 1024 |  |  | DB Search |
| R.YVKPGVNIINIR.D | Y | 57.33 | 1384.819 | 12 | 6.22 | 462.6155 | 3 | 16.27 | 0.8043 | 0.8116 | 53149 | 6.85e2 | 2 | 2 | 1217 | 1228 |  |  | DB Search |
| K.LFNEALKPLK.E | Y | 57.32 | 1171.6965 | 10 | 3.28 | 586.8561 | 2 | 15.90 | 0.9509 | 0.9582 | 50919 | 4.48e2 | 1 | 1 | 3651 | 3660 |  |  | DB Search |
| K.AVDFQSIIDNAQK.L | Y | 56.94 | 1447.7307 | 13 | 4.22 | 724.8741 | 2 | 19.06 | 0.9904 | 0.9977 | 71389 | 6.5e2 | 1 | 1 | 2383 | 2395 |  |  | DB Search |
| K.NLETFTGC(+57.02)ITAVR.I | Y | 56.93 | 1480.7344 | 13 | 2.99 | 741.3751 | 2 | 18.29 | 0.9993 | 1.0065 | 66297 | 1.31e3 | 1 | 1 | 3814 | 3826 | Carbamidomethylation |  | DB Search |
| K.DGEKVETLKVDISDINK.A | Y | 56.92 | 1901.9945 | 17 | 3.6 | 635.0063 | 3 | 16.09 | 0.9113 | 0.9186 | 52017 | 1.36e3 | 1 | 1 | 2634 | 2650 |  |  | DB Search |
| K.VETLKVDISDINK.A | Y | 56.92 | 1472.8086 | 13 | -2.26 | 491.9413 | 3 | 16.17 | 0.8222 | 0.8295 | 52567 | 5.32e2 | 1 | 1 | 2638 | 2650 |  |  | DB Search |
| K.GDEHISNLDTLKK.A | Y | 56.42 | 1468.7521 | 13 | -2.32 | 490.5891 | 3 | 12.90 | 0.7791 | 0.7864 | 32456 | 0 | 0 | 0 | 2128 | 2140 |  |  | DB Search |
| K.GFVAVNDQQPSVVVSINVPK.T | Y | 55.03 | 2096.1266 | 20 | 0.1 | 1049.0684 | 2 | 18.45 | 1.2157 | 1.2228 | 67713 | 4.05e2 | 1 | 1 | 865 | 884 |  |  | DB Search |
| K.DGEKVETLK.V | Y | 54.9 | 1017.5342 | 9 | -2.12 | 509.7722 | 2 | 11.49 | 0.8587 | 0.866 | 23862 | 1.28e3 | 1 | 1 | 2634 | 2642 |  |  | DB Search |
| R.IDNVKEDVK.S | Y | 54.89 | 1058.5608 | 9 | 5.78 | 530.2896 | 2 | 10.73 | 0.8716 | 0.8789 | 19180 | 1.58e3 | 1 | 1 | 2583 | 2591 |  |  | DB Search |
| K.VLISSLLIK.F | Y | 54.58 | 984.6583 | 9 | 0.22 | 493.3354 | 2 | 19.79 | 0.8506 | 0.8579 | 76091 | 1.17e3 | 1 | 1 | 3009 | 3017 |  |  | DB Search |
| R.RC(+57.02)EEC(+57.02)AAGHYQFPFC(+57.02)K.E | Y | 53.84 | 2058.8499 | 16 | 0.01 | 687.2891 | 3 | 15.74 | 0.9218 | 0.9291 | 49834 | 2.69e2 | 1 | 1 | 522 | 537 | Carbamidomethylation, Carbamidomethylation, Carbamidomethylation |  | DB Search |
| K.NEINLASGDVFEGGVVGSLK.L | Y | 53.15 | 2004.0163 | 20 | 1.4 | 1003.0146 | 2 | 19.85 | 1.1829 | 1.1901 | 76578 | 0 | 0 | 0 | 3249 | 3268 |  |  | DB Search |
| R.KLSLDVNGNYQGGGVC(+57.02)K.N | Y | 51.86 | 1807.8887 | 17 | 0.8 | 603.636 | 3 | 14.64 | 0.8193 | 0.8266 | 43107 | 2.56e2 | 1 | 1 | 349 | 365 | Carbamidomethylation |  | DB Search |
| R.YALNQIQPVTATVTAFK.G | Y | 47.96 | 1864.0094 | 17 | 1.71 | 933.0115 | 2 | 19.31 | 1.1478 | 1.155 | 73122 | 0 | 0 | 0 | 894 | 910 |  |  | DB Search |
| K.IRSNIDEVNVK.H | Y | 39.98 | 1285.699 | 11 | -7.71 | 643.8504 | 2 | 14.93 | 0.9638 | 0.9711 | 44969 | 0 | 0 | 0 | 2669 | 2679 |  |  | DB Search |
| total 28 peptides |  |  |  |  |  |  |  |  |  |  |  |  |  |  |  |  |  |  |  |
| --- | --- | --- | --- | --- | --- | --- | --- | --- | --- | --- | --- | --- | --- | --- | --- | --- | --- | --- | --- |

Best Unique PSM (Scan Sample\_2\_A1\_1\_3640.d:54256, m/z=724.3694, z=3, RT=16.43, ppm=-1.87):


TRINITY\_DN3655\_c0\_g1\_i1.p1

back to list

  

| Protein Coverage
| Supporting Peptides
| Best Unique PSM
|

Protein Coverage:

Supporting Peptides:

| Peptide | Uniq | -10lgP | Mass | Length | ppm | m/z | z | RT | 1/k0 Start | 1/k0 End | Precursor ID | Area Sample 4 | #Feature | #Feature Sample 4 | Start | End | PTM | AScore | Found By |
| --- | --- | --- | --- | --- | --- | --- | --- | --- | --- | --- | --- | --- | --- | --- | --- | --- | --- | --- | --- |
| K.VSGSLQLVWTR.V | N | 58.26 | 1244.6877 | 11 | 4.57 | 623.3526 | 2 | 18.55 | 0.9307 | 0.938 | 68053 | 5.74e2 | 1 | 1 | 19 | 29 |  |  | DB Search |
| R.GLSFSGDIAIDDIK.T | Y | 56.97 | 1449.7351 | 14 | 2.03 | 725.8747 | 2 | 19.24 | 1.0041 | 1.0114 | 72590 | 2.71e3 | 1 | 1 | 221 | 234 |  |  | DB Search |
| total 2 peptides |  |  |  |  |  |  |  |  |  |  |  |  |  |  |  |  |  |  |  |
| --- | --- | --- | --- | --- | --- | --- | --- | --- | --- | --- | --- | --- | --- | --- | --- | --- | --- | --- | --- |

Best Unique PSM (Scan Sample\_2\_A1\_1\_3640.d:72590, m/z=725.8747, z=2, RT=19.24, ppm=-0.17):


TRINITY\_DN118\_c1\_g1\_i3.p1

back to list

  

| Protein Coverage
| Supporting Peptides
| Best Unique PSM
|

Protein Coverage:

Supporting Peptides:

| Peptide | Uniq | -10lgP | Mass | Length | ppm | m/z | z | RT | 1/k0 Start | 1/k0 End | Precursor ID | Area Sample 4 | #Feature | #Feature Sample 4 | Start | End | PTM | AScore | Found By |
| --- | --- | --- | --- | --- | --- | --- | --- | --- | --- | --- | --- | --- | --- | --- | --- | --- | --- | --- | --- |
| R.ETC(+57.02)SDIEKR.E | Y | 58.32 | 1136.5132 | 9 | 0.46 | 569.2629 | 2 | 10.09 | 0.8883 | 0.8955 | 15406 | 2.17e3 | 1 | 1 | 186 | 194 | Carbamidomethylation |  | DB Search |
| K.VC(+57.02)EDVKR.C | Y | 56.84 | 904.4436 | 7 | 1.73 | 453.2289 | 2 | 9.35 | 0.7742 | 0.7815 | 11721 | 6.11e3 | 1 | 1 | 122 | 128 | Carbamidomethylation |  | DB Search |
| K.VC(+57.02)EDVK.R | Y | 51.73 | 748.3425 | 6 | 8.04 | 375.1807 | 2 | 9.73 | 0.7567 | 0.764 | 13508 | 0 | 0 | 0 | 122 | 127 | Carbamidomethylation |  | DB Search |
| total 3 peptides |  |  |  |  |  |  |  |  |  |  |  |  |  |  |  |  |  |  |  |
| --- | --- | --- | --- | --- | --- | --- | --- | --- | --- | --- | --- | --- | --- | --- | --- | --- | --- | --- | --- |

Best Unique PSM (Scan Sample\_2\_A1\_1\_3640.d:15406, m/z=569.2629, z=2, RT=10.09, ppm=-1.73):


TRINITY\_DN5\_c0\_g1\_i18.p1

back to list

  

| Protein Coverage
| Supporting Peptides
| Best Unique PSM
|

Protein Coverage:

Supporting Peptides:

| Peptide | Uniq | -10lgP | Mass | Length | ppm | m/z | z | RT | 1/k0 Start | 1/k0 End | Precursor ID | Area Sample 4 | #Feature | #Feature Sample 4 | Start | End | PTM | AScore | Found By |
| --- | --- | --- | --- | --- | --- | --- | --- | --- | --- | --- | --- | --- | --- | --- | --- | --- | --- | --- | --- |
| K.IIIELQGDLIK.I | Y | 53.27 | 1253.7594 | 11 | 3.89 | 627.8881 | 2 | 19.42 | 0.9513 | 0.9586 | 73842 | 0 | 0 | 0 | 138 | 148 |  |  | DB Search |
| total 1 peptides |  |  |  |  |  |  |  |  |  |  |  |  |  |  |  |  |  |  |  |
| --- | --- | --- | --- | --- | --- | --- | --- | --- | --- | --- | --- | --- | --- | --- | --- | --- | --- | --- | --- |

Best Unique PSM (Scan Sample\_2\_A1\_1\_3640.d:73842, m/z=627.8881, z=2, RT=19.42, ppm=1.69):


TRINITY\_DN1369\_c0\_g1\_i1.p1

back to list

  

| Protein Coverage
| Supporting Peptides
| Best Unique PSM
|

Protein Coverage:

Supporting Peptides:

| Peptide | Uniq | -10lgP | Mass | Length | ppm | m/z | z | RT | 1/k0 Start | 1/k0 End | Precursor ID | Area Sample 4 | #Feature | #Feature Sample 4 | Start | End | PTM | AScore | Found By |
| --- | --- | --- | --- | --- | --- | --- | --- | --- | --- | --- | --- | --- | --- | --- | --- | --- | --- | --- | --- |
| K.YPNGLHHC(+57.02)PTK.Y | Y | 58.39 | 1322.619 | 11 | 4.65 | 441.8813 | 3 | 10.44 | 0.7701 | 0.7775 | 17571 | 2.83e3 | 1 | 1 | 1140 | 1150 | Carbamidomethylation |  | DB Search |
| K.EIEDTC(+57.02)HNR.K | Y | 58.38 | 1172.488 | 9 | 6.16 | 391.8382 | 3 | 9.47 | 0.7102 | 0.7176 | 12293 | 0 | 0 | 0 | 1029 | 1037 | Carbamidomethylation |  | DB Search |
| R.EITLNYHDTVVHMK.R | Y | 58.38 | 1698.8399 | 14 | 0.55 | 850.4258 | 2 | 15.51 | 1.1114 | 1.1186 | 48520 | 2.47e2 | 1 | 1 | 911 | 924 |  |  | DB Search |
| K.VFSEC(+57.02)GPAC(+57.02)SSVC(+57.02)SAIGSK.S | Y | 58.3 | 2001.8594 | 19 | 6.31 | 1001.9411 | 2 | 15.43 | 1.1534 | 1.1606 | 48146 | 2.26e2 | 1 | 1 | 303 | 321 | Carbamidomethylation, Carbamidomethylation, Carbamidomethylation |  | DB Search |
| K.TC(+57.02)VKDEVTGLHEIK.V | Y | 58.29 | 1627.8239 | 14 | 1.04 | 543.6146 | 3 | 13.87 | 0.8579 | 0.8652 | 38340 | 2.6e3 | 1 | 1 | 1346 | 1359 | Carbamidomethylation |  | DB Search |
| K.LC(+57.02)DATC(+57.02)SVFGDPHYK.T | Y | 58.29 | 1768.7549 | 15 | 1.29 | 885.3839 | 2 | 15.38 | 1.1354 | 1.1426 | 47761 | 1.99e2 | 1 | 1 | 846 | 860 | Carbamidomethylation, Carbamidomethylation |  | DB Search |
| K.KTC(+57.02)VKDEVTGLHEIK.V | Y | 58.26 | 1755.9189 | 15 | 3.59 | 439.9876 | 4 | 12.92 | 0.7795 | 0.7868 | 32586 | 1.06e3 | 1 | 1 | 1345 | 1359 | Carbamidomethylation |  | DB Search |
| R.GDVIQVNEC(+57.02)TKK.T | Y | 58.25 | 1389.6922 | 12 | -0.59 | 695.8514 | 2 | 12.20 | 1.0138 | 1.021 | 28037 | 1.96e3 | 1 | 1 | 1334 | 1345 | Carbamidomethylation |  | DB Search |
| K.DEVTGLHEIK.V | Y | 57.91 | 1139.5822 | 10 | -4.42 | 570.7946 | 2 | 13.56 | 0.917 | 0.9243 | 36523 | 0 | 0 | 0 | 1350 | 1359 |  |  | DB Search |
| K.IYNEGEVVLYK.C | Y | 57.35 | 1325.6867 | 11 | 3.06 | 663.8512 | 2 | 16.17 | 0.9755 | 0.9828 | 52557 | 4.08e3 | 1 | 1 | 1188 | 1198 |  |  | DB Search |
| K.TPGEC(+57.02)C(+57.02)PQC(+57.02)K.K | Y | 57.28 | 1235.4733 | 10 | 5.1 | 618.7457 | 2 | 10.00 | 0.8878 | 0.8951 | 14830 | 4.63e3 | 1 | 1 | 1387 | 1396 | Carbamidomethylation, Carbamidomethylation, Carbamidomethylation |  | DB Search |
| R.RMEC(+57.02)PELDC(+57.02)PEER.Q | Y | 55.72 | 1719.7015 | 13 | -2.5 | 574.2384 | 3 | 13.63 | 0.8108 | 0.8181 | 36937 | 2.69e2 | 1 | 1 | 1474 | 1486 | Carbamidomethylation, Carbamidomethylation |  | DB Search |
| R.KYTPC(+57.02)EEK.C | Y | 53.79 | 1053.4801 | 8 | 5.43 | 527.749 | 2 | 9.67 | 0.8558 | 0.8631 | 13183 | 2.75e2 | 1 | 1 | 649 | 656 | Carbamidomethylation |  | DB Search |
| total 13 peptides |  |  |  |  |  |  |  |  |  |  |  |  |  |  |  |  |  |  |  |
| --- | --- | --- | --- | --- | --- | --- | --- | --- | --- | --- | --- | --- | --- | --- | --- | --- | --- | --- | --- |

Best Unique PSM (Scan Sample\_2\_A1\_1\_3640.d:17571, m/z=441.8813, z=3, RT=10.44, ppm=2.45):


TRINITY\_DN165469\_c0\_g1\_i1.p1

back to list

  

| Protein Coverage
| Supporting Peptides
| Best Unique PSM
|

Protein Coverage:

Supporting Peptides:

| Peptide | Uniq | -10lgP | Mass | Length | ppm | m/z | z | RT | 1/k0 Start | 1/k0 End | Precursor ID | Area Sample 4 | #Feature | #Feature Sample 4 | Start | End | PTM | AScore | Found By |
| --- | --- | --- | --- | --- | --- | --- | --- | --- | --- | --- | --- | --- | --- | --- | --- | --- | --- | --- | --- |
| K.FLDIHELQAVK.C | Y | 57.33 | 1311.7186 | 11 | -7.43 | 438.2426 | 3 | 17.63 | 0.773 | 0.7803 | 62025 | 0 | 0 | 0 | 84 | 94 |  |  | DB Search |
| total 1 peptides |  |  |  |  |  |  |  |  |  |  |  |  |  |  |  |  |  |  |  |
| --- | --- | --- | --- | --- | --- | --- | --- | --- | --- | --- | --- | --- | --- | --- | --- | --- | --- | --- | --- |

Best Unique PSM (Scan Sample\_2\_A1\_1\_3640.d:62025, m/z=437.7822, z=2, RT=17.65, ppm=-9.63):


TRINITY\_DN40728\_c1\_g1\_i1.p2

back to list

  

| Protein Coverage
| Supporting Peptides
| Best Unique PSM
|

Protein Coverage:

Supporting Peptides:

| Peptide | Uniq | -10lgP | Mass | Length | ppm | m/z | z | RT | 1/k0 Start | 1/k0 End | Precursor ID | Area Sample 4 | #Feature | #Feature Sample 4 | Start | End | PTM | AScore | Found By |
| --- | --- | --- | --- | --- | --- | --- | --- | --- | --- | --- | --- | --- | --- | --- | --- | --- | --- | --- | --- |
| K.ESTLHLVLR.L | Y | 57.27 | 1066.6135 | 9 | 2.57 | 534.3142 | 2 | 15.80 | 0.8785 | 0.8858 | 50332 | 4.97e3 | 1 | 1 | 59 | 67 |  |  | DB Search |
| K.IQDKEGIPPDQQR.L | Y | 55.01 | 1522.7739 | 13 | 0.25 | 762.3928 | 2 | 11.08 | 1.0343 | 1.0415 | 21018 | 1.78e3 | 2 | 2 | 25 | 37 |  |  | DB Search |
| total 2 peptides |  |  |  |  |  |  |  |  |  |  |  |  |  |  |  |  |  |  |  |
| --- | --- | --- | --- | --- | --- | --- | --- | --- | --- | --- | --- | --- | --- | --- | --- | --- | --- | --- | --- |

Best Unique PSM (Scan Sample\_2\_A1\_1\_3640.d:50332, m/z=534.7766, z=2, RT=15.80, ppm=0.37):


TRINITY\_DN10485\_c0\_g1\_i1.p1

back to list

  

| Protein Coverage
| Supporting Peptides
| Best Unique PSM
|

Protein Coverage:

Supporting Peptides:

| Peptide | Uniq | -10lgP | Mass | Length | ppm | m/z | z | RT | 1/k0 Start | 1/k0 End | Precursor ID | Area Sample 4 | #Feature | #Feature Sample 4 | Start | End | PTM | AScore | Found By |
| --- | --- | --- | --- | --- | --- | --- | --- | --- | --- | --- | --- | --- | --- | --- | --- | --- | --- | --- | --- |
| R.LSTVGGMSIAVPGNVK.G | Y | 58.29 | 1528.8283 | 16 | 4.47 | 765.4232 | 2 | 17.55 | 1.0351 | 1.0423 | 61420 | 0 | 0 | 0 | 153 | 168 |  |  | DB Search |
| R.RPLLGDPSFVADVSK.V | Y | 58.07 | 1599.862 | 15 | -5.12 | 534.2907 | 3 | 17.53 | 0.7746 | 0.7819 | 61329 | 0 | 0 | 0 | 356 | 370 |  |  | DB Search |
| total 2 peptides |  |  |  |  |  |  |  |  |  |  |  |  |  |  |  |  |  |  |  |
| --- | --- | --- | --- | --- | --- | --- | --- | --- | --- | --- | --- | --- | --- | --- | --- | --- | --- | --- | --- |

Best Unique PSM (Scan Sample\_2\_A1\_1\_3640.d:61420, m/z=765.4232, z=2, RT=17.55, ppm=2.28):


TRINITY\_DN991\_c0\_g1\_i6.p1

back to list

  

| Protein Coverage
| Supporting Peptides
| Best Unique PSM
|

Protein Coverage:

Supporting Peptides:

| Peptide | Uniq | -10lgP | Mass | Length | ppm | m/z | z | RT | 1/k0 Start | 1/k0 End | Precursor ID | Area Sample 4 | #Feature | #Feature Sample 4 | Start | End | PTM | AScore | Found By |
| --- | --- | --- | --- | --- | --- | --- | --- | --- | --- | --- | --- | --- | --- | --- | --- | --- | --- | --- | --- |
| K.IVGDDFTVGQSVTIK.C | Y | 58.39 | 1577.83 | 15 | 1.5 | 789.9218 | 2 | 17.37 | 1.0359 | 1.0431 | 60205 | 1.59e3 | 1 | 1 | 688 | 702 |  |  | DB Search |
| K.LSFC(+57.02)DTLTTPK.N | Y | 58.37 | 1281.6275 | 11 | 1.48 | 641.8206 | 2 | 17.08 | 0.9654 | 0.9727 | 58298 | 1.14e3 | 1 | 1 | 674 | 684 | Carbamidomethylation |  | DB Search |
| K.FGTC(+57.02)IDLIGDYR.C | Y | 58.29 | 1428.6707 | 12 | 0.76 | 715.3416 | 2 | 19.11 | 1.0049 | 1.0122 | 71833 | 0 | 0 | 0 | 2005 | 2016 | Carbamidomethylation |  | DB Search |
| R.ITVTVSPDIHPPAK.L | Y | 57.2 | 1473.8191 | 14 | -2.38 | 737.9135 | 2 | 15.04 | 1.0652 | 1.0725 | 45475 | 1.12e3 | 1 | 1 | 1183 | 1196 |  |  | DB Search |
| K.GVGHQC(+57.02)INTIGSFK.C | Y | 45.41 | 1516.7456 | 14 | 11.11 | 506.5937 | 3 | 14.17 | 0.7807 | 0.788 | 40291 | 0 | 0 | 0 | 808 | 821 | Carbamidomethylation |  | DB Search |
| R.GDFLILANSNGK.V | Y | 42.7 | 1247.651 | 12 | -8.76 | 624.8259 | 2 | 18.05 | 0.9501 | 0.9574 | 64900 | 0 | 0 | 0 | 2267 | 2278 |  |  | DB Search |
| K.ISYEC(+57.02)DEGYHLR.N | Y | 39.73 | 1540.6616 | 12 | 1.5 | 514.5608 | 3 | 14.27 | 0.7962 | 0.8035 | 40907 | 0 | 0 | 0 | 579 | 590 | Carbamidomethylation |  | DB Search |
| total 7 peptides |  |  |  |  |  |  |  |  |  |  |  |  |  |  |  |  |  |  |  |
| --- | --- | --- | --- | --- | --- | --- | --- | --- | --- | --- | --- | --- | --- | --- | --- | --- | --- | --- | --- |

Best Unique PSM (Scan Sample\_2\_A1\_1\_3640.d:60205, m/z=789.9218, z=2, RT=17.37, ppm=-0.69):


TRINITY\_DN585\_c0\_g1\_i5.p1

back to list

  

| Protein Coverage
| Supporting Peptides
| Best Unique PSM
|

Protein Coverage:

Supporting Peptides:

| Peptide | Uniq | -10lgP | Mass | Length | ppm | m/z | z | RT | 1/k0 Start | 1/k0 End | Precursor ID | Area Sample 4 | #Feature | #Feature Sample 4 | Start | End | PTM | AScore | Found By |
| --- | --- | --- | --- | --- | --- | --- | --- | --- | --- | --- | --- | --- | --- | --- | --- | --- | --- | --- | --- |
| K.ESTLHLVLR.L | Y | 57.27 | 1066.6135 | 9 | 2.57 | 534.3142 | 2 | 15.80 | 0.8785 | 0.8858 | 50332 | 4.97e3 | 1 | 1 | 36 | 44 |  |  | DB Search |
| K.IQDKEGIPPDQQR.L | Y | 55.01 | 1522.7739 | 13 | 0.25 | 762.3928 | 2 | 11.08 | 1.0343 | 1.0415 | 21018 | 1.78e3 | 2 | 2 | 2 | 14 |  |  | DB Search |
| K.TITLEVEPSDTIENVK.S | Y | 54.56 | 1786.92 | 16 | 4.26 | 894.4691 | 2 | 17.62 | 1.1442 | 1.1514 | 61769 | 1.48e4 | 1 | 1 | 60 | 75 |  |  | DB Search |
| total 3 peptides |  |  |  |  |  |  |  |  |  |  |  |  |  |  |  |  |  |  |  |
| --- | --- | --- | --- | --- | --- | --- | --- | --- | --- | --- | --- | --- | --- | --- | --- | --- | --- | --- | --- |

Best Unique PSM (Scan Sample\_2\_A1\_1\_3640.d:50332, m/z=534.7766, z=2, RT=15.80, ppm=0.37):


TRINITY\_DN8155\_c0\_g1\_i1.p1

back to list

  

| Protein Coverage
| Supporting Peptides
| Best Unique PSM
|

Protein Coverage:

Supporting Peptides:

| Peptide | Uniq | -10lgP | Mass | Length | ppm | m/z | z | RT | 1/k0 Start | 1/k0 End | Precursor ID | Area Sample 4 | #Feature | #Feature Sample 4 | Start | End | PTM | AScore | Found By |
| --- | --- | --- | --- | --- | --- | --- | --- | --- | --- | --- | --- | --- | --- | --- | --- | --- | --- | --- | --- |
| R.GLYISAEFAIPEDC(+57.02)KK.D | Y | 58.24 | 1839.9076 | 16 | 0.49 | 920.9595 | 2 | 18.42 | 1.2013 | 1.2085 | 67321 | 1.12e2 | 1 | 1 | 123 | 138 | Carbamidomethylation |  | DB Search |
| K.DKLC(+57.02)EWLK.N | Y | 52.89 | 1090.5481 | 8 | -1.41 | 546.2794 | 2 | 16.40 | 0.8696 | 0.8769 | 54029 | 4.39e2 | 1 | 1 | 139 | 146 | Carbamidomethylation |  | DB Search |
| total 2 peptides |  |  |  |  |  |  |  |  |  |  |  |  |  |  |  |  |  |  |  |
| --- | --- | --- | --- | --- | --- | --- | --- | --- | --- | --- | --- | --- | --- | --- | --- | --- | --- | --- | --- |

Best Unique PSM (Scan Sample\_2\_A1\_1\_3640.d:67321, m/z=920.9595, z=2, RT=18.42, ppm=-1.7):


TRINITY\_DN65215\_c0\_g1\_i1.p1

back to list

  

| Protein Coverage
| Supporting Peptides
| Best Unique PSM
|

Protein Coverage:

Supporting Peptides:

| Peptide | Uniq | -10lgP | Mass | Length | ppm | m/z | z | RT | 1/k0 Start | 1/k0 End | Precursor ID | Area Sample 4 | #Feature | #Feature Sample 4 | Start | End | PTM | AScore | Found By |
| --- | --- | --- | --- | --- | --- | --- | --- | --- | --- | --- | --- | --- | --- | --- | --- | --- | --- | --- | --- |
| K.AFLFDVVSK.I | Y | 44.57 | 1024.5593 | 9 | -4.12 | 513.2837 | 2 | 19.84 | 0.836 | 0.8433 | 76555 | 1.97e2 | 1 | 1 | 248 | 256 |  |  | DB Search |
| total 1 peptides |  |  |  |  |  |  |  |  |  |  |  |  |  |  |  |  |  |  |  |
| --- | --- | --- | --- | --- | --- | --- | --- | --- | --- | --- | --- | --- | --- | --- | --- | --- | --- | --- | --- |

Best Unique PSM (Scan Sample\_2\_A1\_1\_3640.d:76555, m/z=513.2837, z=2, RT=19.84, ppm=-6.31):


TRINITY\_DN141338\_c2\_g1\_i1.p1

back to list

  

| Protein Coverage
| Supporting Peptides
| Best Unique PSM
|

Protein Coverage:

Supporting Peptides:

| Peptide | Uniq | -10lgP | Mass | Length | ppm | m/z | z | RT | 1/k0 Start | 1/k0 End | Precursor ID | Area Sample 4 | #Feature | #Feature Sample 4 | Start | End | PTM | AScore | Found By |
| --- | --- | --- | --- | --- | --- | --- | --- | --- | --- | --- | --- | --- | --- | --- | --- | --- | --- | --- | --- |
| K.LGIHEDTQNR.N | Y | 58.28 | 1181.5789 | 10 | 2.61 | 394.8671 | 3 | 11.20 | 0.7122 | 0.7196 | 22065 | 0 | 0 | 0 | 434 | 443 |  |  | DB Search |
| total 1 peptides |  |  |  |  |  |  |  |  |  |  |  |  |  |  |  |  |  |  |  |
| --- | --- | --- | --- | --- | --- | --- | --- | --- | --- | --- | --- | --- | --- | --- | --- | --- | --- | --- | --- |

Best Unique PSM (Scan Sample\_2\_A1\_1\_3640.d:22065, m/z=394.8671, z=3, RT=11.20, ppm=0.41):


TRINITY\_DN912\_c0\_g1\_i1.p1

back to list

  

| Protein Coverage
| Supporting Peptides
| Best Unique PSM
|

Protein Coverage:

Supporting Peptides:

| Peptide | Uniq | -10lgP | Mass | Length | ppm | m/z | z | RT | 1/k0 Start | 1/k0 End | Precursor ID | Area Sample 4 | #Feature | #Feature Sample 4 | Start | End | PTM | AScore | Found By |
| --- | --- | --- | --- | --- | --- | --- | --- | --- | --- | --- | --- | --- | --- | --- | --- | --- | --- | --- | --- |
| K.LLLLGAGESGK.S | Y | 43.84 | 1056.6179 | 11 | 6.84 | 529.3187 | 2 | 17.27 | 0.8761 | 0.8834 | 59698 | 0 | 0 | 0 | 36 | 46 |  |  | DB Search |
| total 1 peptides |  |  |  |  |  |  |  |  |  |  |  |  |  |  |  |  |  |  |  |
| --- | --- | --- | --- | --- | --- | --- | --- | --- | --- | --- | --- | --- | --- | --- | --- | --- | --- | --- | --- |

Best Unique PSM (Scan Sample\_2\_A1\_1\_3640.d:59698, m/z=529.3187, z=2, RT=17.27, ppm=4.65):


TRINITY\_DN2477\_c0\_g1\_i6.p1

back to list

  

| Protein Coverage
| Supporting Peptides
| Best Unique PSM
|

Protein Coverage:

Supporting Peptides:

| Peptide | Uniq | -10lgP | Mass | Length | ppm | m/z | z | RT | 1/k0 Start | 1/k0 End | Precursor ID | Area Sample 4 | #Feature | #Feature Sample 4 | Start | End | PTM | AScore | Found By |
| --- | --- | --- | --- | --- | --- | --- | --- | --- | --- | --- | --- | --- | --- | --- | --- | --- | --- | --- | --- |
| K.DSYVGDEAQSKR.G | Y | 57.28 | 1353.616 | 12 | 2.57 | 677.8156 | 2 | 11.01 | 0.9993 | 1.0065 | 20794 | 1.16e3 | 1 | 1 | 52 | 63 |  |  | DB Search |
| total 1 peptides |  |  |  |  |  |  |  |  |  |  |  |  |  |  |  |  |  |  |  |
| --- | --- | --- | --- | --- | --- | --- | --- | --- | --- | --- | --- | --- | --- | --- | --- | --- | --- | --- | --- |

Best Unique PSM (Scan Sample\_2\_A1\_1\_3640.d:20794, m/z=677.8156, z=2, RT=11.01, ppm=0.37):


TRINITY\_DN8903\_c0\_g1\_i1.p1

back to list

  

| Protein Coverage
| Supporting Peptides
| Best Unique PSM
|

Protein Coverage:

Supporting Peptides:

| Peptide | Uniq | -10lgP | Mass | Length | ppm | m/z | z | RT | 1/k0 Start | 1/k0 End | Precursor ID | Area Sample 4 | #Feature | #Feature Sample 4 | Start | End | PTM | AScore | Found By |
| --- | --- | --- | --- | --- | --- | --- | --- | --- | --- | --- | --- | --- | --- | --- | --- | --- | --- | --- | --- |
| K.AC(+57.02)GLENM(+15.99)C(+57.02)VGSYK.S | N | 58.41 | 1503.6156 | 13 | -0.67 | 752.8129 | 2 | 13.59 | 0.9989 | 1.0061 | 36764 | 0 | 0 | 0 | 6 | 18 | Carbamidomethylation, Oxidation (M), Carbamidomethylation | M7:Oxidation (M):1000 | DB Search |
| R.YC(+57.02)NNPVAER.F | Y | 58.37 | 1121.4924 | 9 | 8.64 | 561.7571 | 2 | 11.65 | 0.8619 | 0.8692 | 24790 | 8.18e1 | 1 | 1 | 255 | 263 | Carbamidomethylation |  | DB Search |
| R.IKLDC(+57.02)NINIAK.K | Y | 58.15 | 1300.7173 | 11 | 2.08 | 651.3658 | 2 | 15.03 | 0.9852 | 0.9924 | 45500 | 4.03e2 | 1 | 1 | 383 | 393 | Carbamidomethylation |  | DB Search |
| K.YVQNPR.N | Y | 57.81 | 775.3977 | 6 | 8.26 | 388.7085 | 2 | 9.75 | 0.7705 | 0.7779 | 13543 | 5.55e3 | 1 | 1 | 125 | 130 |  |  | DB Search |
| K.DLHIIEDYVLK.H | Y | 57.31 | 1356.7289 | 11 | 1.64 | 679.3713 | 2 | 19.73 | 0.9868 | 0.9941 | 75736 | 7.86e2 | 1 | 1 | 417 | 427 |  |  | DB Search |
| total 5 peptides |  |  |  |  |  |  |  |  |  |  |  |  |  |  |  |  |  |  |  |
| --- | --- | --- | --- | --- | --- | --- | --- | --- | --- | --- | --- | --- | --- | --- | --- | --- | --- | --- | --- |

Best Unique PSM (Scan Sample\_2\_A1\_1\_3640.d:24790, m/z=561.7571, z=2, RT=11.65, ppm=6.44):


TRINITY\_DN5003\_c0\_g1\_i2.p1

back to list

  

| Protein Coverage
| Supporting Peptides
| Best Unique PSM
|

Protein Coverage:

Supporting Peptides:

| Peptide | Uniq | -10lgP | Mass | Length | ppm | m/z | z | RT | 1/k0 Start | 1/k0 End | Precursor ID | Area Sample 4 | #Feature | #Feature Sample 4 | Start | End | PTM | AScore | Found By |
| --- | --- | --- | --- | --- | --- | --- | --- | --- | --- | --- | --- | --- | --- | --- | --- | --- | --- | --- | --- |
| K.ILILLTDGSQTQDAGAEDPVK.I | Y | 58.29 | 2183.1321 | 21 | -2.24 | 1092.5685 | 2 | 19.12 | 1.2432 | 1.2503 | 71899 | 0 | 0 | 0 | 64 | 84 |  |  | DB Search |
| total 1 peptides |  |  |  |  |  |  |  |  |  |  |  |  |  |  |  |  |  |  |  |
| --- | --- | --- | --- | --- | --- | --- | --- | --- | --- | --- | --- | --- | --- | --- | --- | --- | --- | --- | --- |

Best Unique PSM (Scan Sample\_2\_A1\_1\_3640.d:71899, m/z=1092.5685, z=2, RT=19.12, ppm=-4.43):


TRINITY\_DN3795\_c0\_g1\_i1.p1

back to list

  

| Protein Coverage
| Supporting Peptides
| Best Unique PSM
|

Protein Coverage:

Supporting Peptides:

| Peptide | Uniq | -10lgP | Mass | Length | ppm | m/z | z | RT | 1/k0 Start | 1/k0 End | Precursor ID | Area Sample 4 | #Feature | #Feature Sample 4 | Start | End | PTM | AScore | Found By |
| --- | --- | --- | --- | --- | --- | --- | --- | --- | --- | --- | --- | --- | --- | --- | --- | --- | --- | --- | --- |
| R.IGEIVFQTM(+15.99)QDAQK.A | Y | 58.4 | 1622.7974 | 14 | -0.17 | 812.4041 | 2 | 16.34 | 1.0476 | 1.0548 | 53509 | 1.12e3 | 1 | 1 | 293 | 306 | Oxidation (M) | M9:Oxidation (M):1000 | DB Search |
| R.SGDPLTLGNFIK.N | N | 58.37 | 1260.6714 | 12 | 1.03 | 631.3422 | 2 | 19.83 | 0.8955 | 0.9028 | 76410 | 4.42e2 | 1 | 1 | 100 | 111 |  |  | DB Search |
| R.IGEIVFQTMQDAQK.A | Y | 58.28 | 1606.8025 | 14 | 2.72 | 804.4089 | 2 | 18.66 | 1.0757 | 1.0829 | 68737 | 1.56e3 | 1 | 1 | 293 | 306 |  |  | DB Search |
| R.GC(+57.02)VTLDLRR.I | Y | 53.68 | 1088.576 | 9 | -7.35 | 545.2901 | 2 | 14.15 | 0.8862 | 0.8935 | 40124 | 2.54e2 | 1 | 1 | 284 | 292 | Carbamidomethylation |  | DB Search |
| K.NFEIDATLPQK.I | Y | 50.33 | 1274.6506 | 11 | -8.55 | 638.3257 | 2 | 16.85 | 0.957 | 0.9642 | 57115 | 1.62e3 | 1 | 1 | 112 | 122 |  |  | DB Search |
| total 5 peptides |  |  |  |  |  |  |  |  |  |  |  |  |  |  |  |  |  |  |  |
| --- | --- | --- | --- | --- | --- | --- | --- | --- | --- | --- | --- | --- | --- | --- | --- | --- | --- | --- | --- |

Best Unique PSM (Scan Sample\_2\_A1\_1\_3640.d:53509, m/z=812.4041, z=2, RT=16.34, ppm=-2.36):


TRINITY\_DN1058\_c0\_g1\_i2.p1

back to list

  

| Protein Coverage
| Supporting Peptides
| Best Unique PSM
|

Protein Coverage:

Supporting Peptides:

| Peptide | Uniq | -10lgP | Mass | Length | ppm | m/z | z | RT | 1/k0 Start | 1/k0 End | Precursor ID | Area Sample 4 | #Feature | #Feature Sample 4 | Start | End | PTM | AScore | Found By |
| --- | --- | --- | --- | --- | --- | --- | --- | --- | --- | --- | --- | --- | --- | --- | --- | --- | --- | --- | --- |
| R.IC(+57.02)GHGGIR.C | Y | 57.78 | 868.4337 | 8 | -3.19 | 435.2218 | 2 | 9.87 | 0.7892 | 0.7966 | 14210 | 0 | 0 | 0 | 1008 | 1015 | Carbamidomethylation |  | DB Search |
| R.TC(+57.02)LC(+57.02)NPATSYPSK.D | Y | 54.97 | 1497.6592 | 13 | 0.78 | 749.8358 | 2 | 13.69 | 0.9896 | 0.9969 | 37441 | 1.47e3 | 1 | 1 | 157 | 169 | Carbamidomethylation, Carbamidomethylation |  | DB Search |
| K.SC(+57.02)IEASC(+57.02)SSK.A | Y | 53.5 | 1127.4587 | 10 | 5.45 | 564.7385 | 2 | 10.24 | 0.8733 | 0.8806 | 16314 | 7.28e2 | 1 | 1 | 465 | 474 | Carbamidomethylation, Carbamidomethylation |  | DB Search |
| total 3 peptides |  |  |  |  |  |  |  |  |  |  |  |  |  |  |  |  |  |  |  |
| --- | --- | --- | --- | --- | --- | --- | --- | --- | --- | --- | --- | --- | --- | --- | --- | --- | --- | --- | --- |

Best Unique PSM (Scan Sample\_2\_A1\_1\_3640.d:14210, m/z=435.2218, z=2, RT=9.87, ppm=-5.39):


TRINITY\_DN75609\_c0\_g1\_i1.p1

back to list

  

| Protein Coverage
| Supporting Peptides
| Best Unique PSM
|

Protein Coverage:

Supporting Peptides:

| Peptide | Uniq | -10lgP | Mass | Length | ppm | m/z | z | RT | 1/k0 Start | 1/k0 End | Precursor ID | Area Sample 4 | #Feature | #Feature Sample 4 | Start | End | PTM | AScore | Found By |
| --- | --- | --- | --- | --- | --- | --- | --- | --- | --- | --- | --- | --- | --- | --- | --- | --- | --- | --- | --- |
| R.TSDIVNEIFSR.F | Y | 57.21 | 1279.6408 | 11 | 2.89 | 640.8281 | 2 | 19.68 | 0.9303 | 0.9376 | 75523 | 8.32e2 | 1 | 1 | 3 | 13 |  |  | DB Search |
| total 1 peptides |  |  |  |  |  |  |  |  |  |  |  |  |  |  |  |  |  |  |  |
| --- | --- | --- | --- | --- | --- | --- | --- | --- | --- | --- | --- | --- | --- | --- | --- | --- | --- | --- | --- |

Best Unique PSM (Scan Sample\_2\_A1\_1\_3640.d:75523, m/z=640.8281, z=2, RT=19.68, ppm=0.7):


TRINITY\_DN95415\_c0\_g1\_i1.p1

back to list

  

| Protein Coverage
| Supporting Peptides
| Best Unique PSM
|

Protein Coverage:

Supporting Peptides:

| Peptide | Uniq | -10lgP | Mass | Length | ppm | m/z | z | RT | 1/k0 Start | 1/k0 End | Precursor ID | Area Sample 4 | #Feature | #Feature Sample 4 | Start | End | PTM | AScore | Found By |
| --- | --- | --- | --- | --- | --- | --- | --- | --- | --- | --- | --- | --- | --- | --- | --- | --- | --- | --- | --- |
| R.FQQLSDKNNLALK.Q | Y | 52.57 | 1517.8202 | 13 | 5.73 | 759.92 | 2 | 14.50 | 1.0556 | 1.0628 | 42228 | 0 | 0 | 0 | 47 | 59 |  |  | DB Search |
| total 1 peptides |  |  |  |  |  |  |  |  |  |  |  |  |  |  |  |  |  |  |  |
| --- | --- | --- | --- | --- | --- | --- | --- | --- | --- | --- | --- | --- | --- | --- | --- | --- | --- | --- | --- |

Best Unique PSM (Scan Sample\_2\_A1\_1\_3640.d:42228, m/z=759.92, z=2, RT=14.50, ppm=3.54):


TRINITY\_DN179\_c0\_g1\_i1.p1

back to list

  

| Protein Coverage
| Supporting Peptides
| Best Unique PSM
|

Protein Coverage:

Supporting Peptides:

| Peptide | Uniq | -10lgP | Mass | Length | ppm | m/z | z | RT | 1/k0 Start | 1/k0 End | Precursor ID | Area Sample 4 | #Feature | #Feature Sample 4 | Start | End | PTM | AScore | Found By |
| --- | --- | --- | --- | --- | --- | --- | --- | --- | --- | --- | --- | --- | --- | --- | --- | --- | --- | --- | --- |
| K.EGPAGPAGPDGR.Q | Y | 58.29 | 1079.4995 | 12 | 3.84 | 540.7579 | 2 | 10.78 | 0.8404 | 0.8477 | 19235 | 1.85e3 | 1 | 1 | 176 | 187 |  |  | DB Search |
| total 1 peptides |  |  |  |  |  |  |  |  |  |  |  |  |  |  |  |  |  |  |  |
| --- | --- | --- | --- | --- | --- | --- | --- | --- | --- | --- | --- | --- | --- | --- | --- | --- | --- | --- | --- |

Best Unique PSM (Scan Sample\_2\_A1\_1\_3640.d:19235, m/z=540.7579, z=2, RT=10.78, ppm=1.65):


TRINITY\_DN2113\_c0\_g1\_i2.p1

back to list

  

| Protein Coverage
| Supporting Peptides
| Best Unique PSM
|

Protein Coverage:

Supporting Peptides:

| Peptide | Uniq | -10lgP | Mass | Length | ppm | m/z | z | RT | 1/k0 Start | 1/k0 End | Precursor ID | Area Sample 4 | #Feature | #Feature Sample 4 | Start | End | PTM | AScore | Found By |
| --- | --- | --- | --- | --- | --- | --- | --- | --- | --- | --- | --- | --- | --- | --- | --- | --- | --- | --- | --- |
| R.LASTLVHLGEYQNAVDGAR.K | Y | 52.16 | 2013.0279 | 19 | 5.59 | 672.0189 | 3 | 16.99 | 0.9525 | 0.9598 | 57891 | 0 | 0 | 0 | 1227 | 1245 |  |  | DB Search |
| total 1 peptides |  |  |  |  |  |  |  |  |  |  |  |  |  |  |  |  |  |  |  |
| --- | --- | --- | --- | --- | --- | --- | --- | --- | --- | --- | --- | --- | --- | --- | --- | --- | --- | --- | --- |

Best Unique PSM (Scan Sample\_2\_A1\_1\_3640.d:57891, m/z=672.0189, z=3, RT=16.99, ppm=3.4):


TRINITY\_DN17095\_c0\_g1\_i1.p1

back to list

  

| Protein Coverage
| Supporting Peptides
| Best Unique PSM
|

Protein Coverage:

Supporting Peptides:

| Peptide | Uniq | -10lgP | Mass | Length | ppm | m/z | z | RT | 1/k0 Start | 1/k0 End | Precursor ID | Area Sample 4 | #Feature | #Feature Sample 4 | Start | End | PTM | AScore | Found By |
| --- | --- | --- | --- | --- | --- | --- | --- | --- | --- | --- | --- | --- | --- | --- | --- | --- | --- | --- | --- |
| K.SLIQEPLGKPR.G | Y | 46.63 | 1236.719 | 11 | -0.29 | 619.3652 | 2 | 14.50 | 0.9945 | 1.0017 | 42231 | 0 | 0 | 0 | 559 | 569 |  |  | DB Search |
| total 1 peptides |  |  |  |  |  |  |  |  |  |  |  |  |  |  |  |  |  |  |  |
| --- | --- | --- | --- | --- | --- | --- | --- | --- | --- | --- | --- | --- | --- | --- | --- | --- | --- | --- | --- |

Best Unique PSM (Scan Sample\_2\_A1\_1\_3640.d:42231, m/z=619.3652, z=2, RT=14.50, ppm=-2.48):


TRINITY\_DN1910\_c0\_g1\_i1.p1

back to list

  

| Protein Coverage
| Supporting Peptides
| Best Unique PSM
|

Protein Coverage:

Supporting Peptides:

| Peptide | Uniq | -10lgP | Mass | Length | ppm | m/z | z | RT | 1/k0 Start | 1/k0 End | Precursor ID | Area Sample 4 | #Feature | #Feature Sample 4 | Start | End | PTM | AScore | Found By |
| --- | --- | --- | --- | --- | --- | --- | --- | --- | --- | --- | --- | --- | --- | --- | --- | --- | --- | --- | --- |
| K.LAVNMVPFPR.L | Y | 58.39 | 1142.627 | 10 | 0.31 | 572.3197 | 2 | 18.72 | 0.8721 | 0.8793 | 69256 | 4.38e2 | 1 | 1 | 253 | 262 |  |  | DB Search |
| K.LAVNM(+15.99)VPFPR.L | Y | 58.27 | 1158.6219 | 10 | -3.71 | 580.3148 | 2 | 17.06 | 0.8798 | 0.887 | 57945 | 1.18e3 | 1 | 1 | 253 | 262 | Oxidation (M) | M5:Oxidation (M):1000 | DB Search |
| R.FPGQLNADLRK.L | Y | 57.99 | 1257.6829 | 11 | -7.77 | 420.2307 | 3 | 14.50 | 0.7033 | 0.7106 | 42199 | 0 | 0 | 0 | 242 | 252 |  |  | DB Search |
| R.ISEQFTAMFR.R | Y | 53.53 | 1228.591 | 10 | 8.26 | 615.3065 | 2 | 18.88 | 0.904 | 0.9113 | 70394 | 0 | 0 | 0 | 381 | 390 |  |  | DB Search |
| total 4 peptides |  |  |  |  |  |  |  |  |  |  |  |  |  |  |  |  |  |  |  |
| --- | --- | --- | --- | --- | --- | --- | --- | --- | --- | --- | --- | --- | --- | --- | --- | --- | --- | --- | --- |

Best Unique PSM (Scan Sample\_2\_A1\_1\_3640.d:69256, m/z=572.3197, z=2, RT=18.72, ppm=-1.89):


TRINITY\_DN1058\_c0\_g1\_i4.p1

back to list

  

| Protein Coverage
| Supporting Peptides
| Best Unique PSM
|

Protein Coverage:

Supporting Peptides:

| Peptide | Uniq | -10lgP | Mass | Length | ppm | m/z | z | RT | 1/k0 Start | 1/k0 End | Precursor ID | Area Sample 4 | #Feature | #Feature Sample 4 | Start | End | PTM | AScore | Found By |
| --- | --- | --- | --- | --- | --- | --- | --- | --- | --- | --- | --- | --- | --- | --- | --- | --- | --- | --- | --- |
| R.APTHILNPANKR.S | Y | 58.28 | 1330.7469 | 12 | 0.07 | 666.3793 | 2 | 11.22 | 1.0098 | 1.017 | 22322 | 5.04e3 | 2 | 2 | 398 | 409 |  |  | DB Search |
| total 1 peptides |  |  |  |  |  |  |  |  |  |  |  |  |  |  |  |  |  |  |  |
| --- | --- | --- | --- | --- | --- | --- | --- | --- | --- | --- | --- | --- | --- | --- | --- | --- | --- | --- | --- |

Best Unique PSM (Scan Sample\_2\_A1\_1\_3640.d:22322, m/z=666.3793, z=2, RT=11.22, ppm=-2.12):


TRINITY\_DN1030\_c0\_g1\_i2.p1

back to list

  

| Protein Coverage
| Supporting Peptides
| Best Unique PSM
|

Protein Coverage:

Supporting Peptides:

| Peptide | Uniq | -10lgP | Mass | Length | ppm | m/z | z | RT | 1/k0 Start | 1/k0 End | Precursor ID | Area Sample 4 | #Feature | #Feature Sample 4 | Start | End | PTM | AScore | Found By |
| --- | --- | --- | --- | --- | --- | --- | --- | --- | --- | --- | --- | --- | --- | --- | --- | --- | --- | --- | --- |
| R.GQAGPAGQDGVDGAVGAR.G | Y | 58.39 | 1581.7495 | 18 | 2.58 | 791.8823 | 2 | 12.45 | 1.0077 | 1.015 | 29579 | 1.37e4 | 5 | 5 | 303 | 320 |  |  | DB Search |
| R.GLQGQQGEQGSTGVQGSVGGR.G | Y | 58.39 | 1985.9515 | 21 | 1.48 | 993.9823 | 2 | 12.15 | 1.1314 | 1.1386 | 27609 | 1.56e4 | 3 | 3 | 348 | 368 |  |  | DB Search |
| total 2 peptides |  |  |  |  |  |  |  |  |  |  |  |  |  |  |  |  |  |  |  |
| --- | --- | --- | --- | --- | --- | --- | --- | --- | --- | --- | --- | --- | --- | --- | --- | --- | --- | --- | --- |

Best Unique PSM (Scan Sample\_2\_A1\_1\_3640.d:29579, m/z=791.8823, z=2, RT=12.45, ppm=0.38):


TRINITY\_DN1936\_c0\_g1\_i5.p1

back to list

  

| Protein Coverage
| Supporting Peptides
| Best Unique PSM
|

Protein Coverage:

Supporting Peptides:

| Peptide | Uniq | -10lgP | Mass | Length | ppm | m/z | z | RT | 1/k0 Start | 1/k0 End | Precursor ID | Area Sample 4 | #Feature | #Feature Sample 4 | Start | End | PTM | AScore | Found By |
| --- | --- | --- | --- | --- | --- | --- | --- | --- | --- | --- | --- | --- | --- | --- | --- | --- | --- | --- | --- |
| R.YIFIESSKPAIK.G | Y | 57.72 | 1394.7809 | 12 | 0.75 | 698.3967 | 2 | 15.84 | 1.0086 | 1.0158 | 50604 | 4.26e3 | 2 | 2 | 470 | 481 |  |  | DB Search |
| R.LISTFIPATGDTGGK.C | Y | 54.9 | 1476.7824 | 15 | 1.83 | 739.3982 | 2 | 17.40 | 1.0041 | 1.0114 | 60313 | 2.76e3 | 1 | 1 | 677 | 691 |  |  | DB Search |
| R.SDIAVDDFEFK.S | Y | 54.89 | 1284.5874 | 11 | 6.87 | 643.304 | 2 | 18.47 | 0.9303 | 0.9376 | 67658 | 0 | 0 | 0 | 566 | 576 |  |  | DB Search |
| total 3 peptides |  |  |  |  |  |  |  |  |  |  |  |  |  |  |  |  |  |  |  |
| --- | --- | --- | --- | --- | --- | --- | --- | --- | --- | --- | --- | --- | --- | --- | --- | --- | --- | --- | --- |

Best Unique PSM (Scan Sample\_2\_A1\_1\_3640.d:50604, m/z=698.3967, z=2, RT=15.84, ppm=-1.44):


TRINITY\_DN11248\_c0\_g1\_i3.p1

back to list

  

| Protein Coverage
| Supporting Peptides
| Best Unique PSM
|

Protein Coverage:

Supporting Peptides:

| Peptide | Uniq | -10lgP | Mass | Length | ppm | m/z | z | RT | 1/k0 Start | 1/k0 End | Precursor ID | Area Sample 4 | #Feature | #Feature Sample 4 | Start | End | PTM | AScore | Found By |
| --- | --- | --- | --- | --- | --- | --- | --- | --- | --- | --- | --- | --- | --- | --- | --- | --- | --- | --- | --- |
| K.NWHGMEDC(+57.02)VVVK.S | Y | 58.29 | 1472.654 | 12 | 8.38 | 491.895 | 3 | 15.15 | 0.7534 | 0.7608 | 46340 | 0 | 0 | 0 | 1630 | 1641 | Carbamidomethylation |  | DB Search |
| K.TPYLQIDFGK.M | Y | 57.3 | 1180.6128 | 10 | 2.79 | 591.314 | 2 | 18.49 | 0.9109 | 0.9182 | 67728 | 8.28e2 | 1 | 1 | 514 | 523 |  |  | DB Search |
| K.ENDFISAQLKR.M | Y | 57.23 | 1319.6833 | 11 | 3.57 | 660.8499 | 2 | 15.34 | 0.9791 | 0.9864 | 47439 | 4.82e2 | 1 | 1 | 1581 | 1591 |  |  | DB Search |
| K.DKVSNAC(+57.02)VK.E | Y | 56.71 | 1019.507 | 9 | -4.3 | 510.7574 | 2 | 9.41 | 0.836 | 0.8433 | 12034 | 0 | 0 | 0 | 1176 | 1184 | Carbamidomethylation |  | DB Search |
| K.NWHGM(+15.99)EDC(+57.02)VVVK.S | Y | 55.7 | 1488.6489 | 12 | -2.89 | 497.2211 | 3 | 13.35 | 0.7599 | 0.7673 | 35192 | 0 | 0 | 0 | 1630 | 1641 | Oxidation (M), Carbamidomethylation | M5:Oxidation (M):1000 | DB Search |
| K.SIEAIYLGEFGTC(+57.02)K.S | Y | 54.46 | 1586.765 | 14 | 6.64 | 794.3933 | 2 | 19.71 | 1.0981 | 1.1053 | 75650 | 6.41e2 | 1 | 1 | 982 | 995 | Carbamidomethylation |  | DB Search |
| K.MGAHVATIHYQK.E | Y | 53.7 | 1354.6816 | 12 | 4.59 | 452.5689 | 3 | 12.20 | 0.7836 | 0.7909 | 28050 | 1.16e3 | 1 | 1 | 1569 | 1580 |  |  | DB Search |
| R.HLC(+57.02)VGMGSGGK.W | Y | 53.7 | 1101.5059 | 11 | 0.68 | 551.7594 | 2 | 14.22 | 0.8757 | 0.883 | 40611 | 2.66e2 | 1 | 1 | 1486 | 1496 | Carbamidomethylation |  | DB Search |
| total 8 peptides |  |  |  |  |  |  |  |  |  |  |  |  |  |  |  |  |  |  |  |
| --- | --- | --- | --- | --- | --- | --- | --- | --- | --- | --- | --- | --- | --- | --- | --- | --- | --- | --- | --- |

Best Unique PSM (Scan Sample\_2\_A1\_1\_3640.d:46340, m/z=491.895, z=3, RT=15.15, ppm=6.18):


TRINITY\_DN106952\_c0\_g1\_i1.p1

back to list

  

| Protein Coverage
| Supporting Peptides
| Best Unique PSM
|

Protein Coverage:

Supporting Peptides:

| Peptide | Uniq | -10lgP | Mass | Length | ppm | m/z | z | RT | 1/k0 Start | 1/k0 End | Precursor ID | Area Sample 4 | #Feature | #Feature Sample 4 | Start | End | PTM | AScore | Found By |
| --- | --- | --- | --- | --- | --- | --- | --- | --- | --- | --- | --- | --- | --- | --- | --- | --- | --- | --- | --- |
| R.LLLPGELAK.H | Y | 57.63 | 952.5957 | 9 | 2.84 | 477.3054 | 2 | 17.63 | 0.8307 | 0.838 | 61790 | 4.61e3 | 1 | 1 | 110 | 118 |  |  | DB Search |
| total 1 peptides |  |  |  |  |  |  |  |  |  |  |  |  |  |  |  |  |  |  |  |
| --- | --- | --- | --- | --- | --- | --- | --- | --- | --- | --- | --- | --- | --- | --- | --- | --- | --- | --- | --- |

Best Unique PSM (Scan Sample\_2\_A1\_1\_3640.d:61790, m/z=477.3054, z=2, RT=17.63, ppm=0.64):


TRINITY\_DN36\_c0\_g1\_i16.p1

back to list

  

| Protein Coverage
| Supporting Peptides
| Best Unique PSM
|

Protein Coverage:

Supporting Peptides:

| Peptide | Uniq | -10lgP | Mass | Length | ppm | m/z | z | RT | 1/k0 Start | 1/k0 End | Precursor ID | Area Sample 4 | #Feature | #Feature Sample 4 | Start | End | PTM | AScore | Found By |
| --- | --- | --- | --- | --- | --- | --- | --- | --- | --- | --- | --- | --- | --- | --- | --- | --- | --- | --- | --- |
| R.LGGIEVTQGNC(+57.02)AR.K | Y | 58.36 | 1373.6721 | 13 | -2.89 | 687.8398 | 2 | 13.93 | 0.9662 | 0.9735 | 38726 | 2.09e3 | 1 | 1 | 58 | 70 | Carbamidomethylation |  | DB Search |
| K.GLPLAIGVR.D | Y | 57.12 | 894.565 | 9 | 0.27 | 448.2889 | 2 | 17.73 | 0.8124 | 0.8197 | 62574 | 3.52e3 | 1 | 1 | 32 | 40 |  |  | DB Search |
| total 2 peptides |  |  |  |  |  |  |  |  |  |  |  |  |  |  |  |  |  |  |  |
| --- | --- | --- | --- | --- | --- | --- | --- | --- | --- | --- | --- | --- | --- | --- | --- | --- | --- | --- | --- |

Best Unique PSM (Scan Sample\_2\_A1\_1\_3640.d:38726, m/z=687.8398, z=2, RT=13.93, ppm=-5.09):


TRINITY\_DN110760\_c0\_g1\_i1.p1

back to list

  

| Protein Coverage
| Supporting Peptides
| Best Unique PSM
|

Protein Coverage:

Supporting Peptides:

| Peptide | Uniq | -10lgP | Mass | Length | ppm | m/z | z | RT | 1/k0 Start | 1/k0 End | Precursor ID | Area Sample 4 | #Feature | #Feature Sample 4 | Start | End | PTM | AScore | Found By |
| --- | --- | --- | --- | --- | --- | --- | --- | --- | --- | --- | --- | --- | --- | --- | --- | --- | --- | --- | --- |
| K.ESTLHLVLR.L | Y | 57.27 | 1066.6135 | 9 | 2.57 | 534.3142 | 2 | 15.80 | 0.8785 | 0.8858 | 50332 | 4.97e3 | 1 | 1 | 18 | 26 |  |  | DB Search |
| total 1 peptides |  |  |  |  |  |  |  |  |  |  |  |  |  |  |  |  |  |  |  |
| --- | --- | --- | --- | --- | --- | --- | --- | --- | --- | --- | --- | --- | --- | --- | --- | --- | --- | --- | --- |

Best Unique PSM (Scan Sample\_2\_A1\_1\_3640.d:50332, m/z=534.7766, z=2, RT=15.80, ppm=0.37):


TRINITY\_DN1613\_c1\_g1\_i2.p1

back to list

  

| Protein Coverage
| Supporting Peptides
| Best Unique PSM
|

Protein Coverage:

Supporting Peptides:

| Peptide | Uniq | -10lgP | Mass | Length | ppm | m/z | z | RT | 1/k0 Start | 1/k0 End | Precursor ID | Area Sample 4 | #Feature | #Feature Sample 4 | Start | End | PTM | AScore | Found By |
| --- | --- | --- | --- | --- | --- | --- | --- | --- | --- | --- | --- | --- | --- | --- | --- | --- | --- | --- | --- |
| R.S(+42.01)C(+57.02)SNPSPAHGGKPC(+57.02)SGELSK.T | Y | 58.29 | 2097.9208 | 20 | 2.04 | 700.3141 | 3 | 11.24 | 0.8716 | 0.8789 | 22349 | 4.91e2 | 1 | 1 | 14 | 33 | Acetylation (Protein N-term), Carbamidomethylation, Carbamidomethylation | S1:Acetylation (Protein N-term):1000 | DB Search |
| R.TC(+57.02)TNPAPAHGGK.S | Y | 58.24 | 1209.556 | 12 | 4.26 | 404.1935 | 3 | 8.97 | 0.7322 | 0.7396 | 10252 | 1.06e3 | 2 | 2 | 413 | 424 | Carbamidomethylation |  | DB Search |
| R.SC(+57.02)TNPRPLFGGK.A | Y | 50.98 | 1332.6608 | 12 | 4.64 | 445.2286 | 3 | 13.73 | 0.7831 | 0.7905 | 37561 | 4.17e2 | 1 | 1 | 356 | 367 | Carbamidomethylation |  | DB Search |
| total 3 peptides |  |  |  |  |  |  |  |  |  |  |  |  |  |  |  |  |  |  |  |
| --- | --- | --- | --- | --- | --- | --- | --- | --- | --- | --- | --- | --- | --- | --- | --- | --- | --- | --- | --- |

Best Unique PSM (Scan Sample\_2\_A1\_1\_3640.d:22349, m/z=700.3141, z=3, RT=11.24, ppm=-0.16):


TRINITY\_DN76282\_c0\_g1\_i1.p1

back to list

  

| Protein Coverage
| Supporting Peptides
| Best Unique PSM
|

Protein Coverage:

Supporting Peptides:

| Peptide | Uniq | -10lgP | Mass | Length | ppm | m/z | z | RT | 1/k0 Start | 1/k0 End | Precursor ID | Area Sample 4 | #Feature | #Feature Sample 4 | Start | End | PTM | AScore | Found By |
| --- | --- | --- | --- | --- | --- | --- | --- | --- | --- | --- | --- | --- | --- | --- | --- | --- | --- | --- | --- |
| R.VQEMEEALAAAESK.A | Y | 53.73 | 1504.7079 | 14 | -3.91 | 753.3566 | 2 | 16.96 | 1.013 | 1.0202 | 57682 | 3.72e2 | 1 | 1 | 79 | 92 |  |  | DB Search |
| total 1 peptides |  |  |  |  |  |  |  |  |  |  |  |  |  |  |  |  |  |  |  |
| --- | --- | --- | --- | --- | --- | --- | --- | --- | --- | --- | --- | --- | --- | --- | --- | --- | --- | --- | --- |

Best Unique PSM (Scan Sample\_2\_A1\_1\_3640.d:57682, m/z=753.3566, z=2, RT=16.96, ppm=-6.1):


TRINITY\_DN711\_c0\_g1\_i2.p1

back to list

  

| Protein Coverage
| Supporting Peptides
| Best Unique PSM
|

Protein Coverage:

Supporting Peptides:

| Peptide | Uniq | -10lgP | Mass | Length | ppm | m/z | z | RT | 1/k0 Start | 1/k0 End | Precursor ID | Area Sample 4 | #Feature | #Feature Sample 4 | Start | End | PTM | AScore | Found By |
| --- | --- | --- | --- | --- | --- | --- | --- | --- | --- | --- | --- | --- | --- | --- | --- | --- | --- | --- | --- |
| R.GPAGAVGPAGR.D | Y | 54.78 | 908.4828 | 11 | 4.27 | 455.2496 | 2 | 10.87 | 0.7921 | 0.7994 | 20030 | 1.32e3 | 1 | 1 | 113 | 123 |  |  | DB Search |
| total 1 peptides |  |  |  |  |  |  |  |  |  |  |  |  |  |  |  |  |  |  |  |
| --- | --- | --- | --- | --- | --- | --- | --- | --- | --- | --- | --- | --- | --- | --- | --- | --- | --- | --- | --- |

Best Unique PSM (Scan Sample\_2\_A1\_1\_3640.d:20030, m/z=454.7148, z=2, RT=10.87, ppm=2.08):


TRINITY\_DN2720\_c0\_g1\_i6.p1

back to list

  

| Protein Coverage
| Supporting Peptides
| Best Unique PSM
|

Protein Coverage:

Supporting Peptides:

| Peptide | Uniq | -10lgP | Mass | Length | ppm | m/z | z | RT | 1/k0 Start | 1/k0 End | Precursor ID | Area Sample 4 | #Feature | #Feature Sample 4 | Start | End | PTM | AScore | Found By |
| --- | --- | --- | --- | --- | --- | --- | --- | --- | --- | --- | --- | --- | --- | --- | --- | --- | --- | --- | --- |
| R.FGWTATEAGTGC(+57.02)IETR.I | Y | 58.28 | 1755.7886 | 16 | 0.08 | 878.8997 | 2 | 17.08 | 1.1013 | 1.1086 | 58476 | 0 | 0 | 0 | 274 | 289 | Carbamidomethylation |  | DB Search |
| R.AC(+57.02)VDIDEC(+57.02)SEDPNAC(+57.02)R.N | Y | 57.49 | 1909.724 | 16 | -0.91 | 955.8663 | 2 | 13.56 | 1.1029 | 1.1102 | 36467 | 0 | 0 | 0 | 909 | 924 | Carbamidomethylation, Carbamidomethylation, Carbamidomethylation |  | DB Search |
| R.IGGTC(+57.02)VNIVGTYR.C | Y | 57.24 | 1408.7133 | 13 | 3.99 | 705.3652 | 2 | 15.98 | 0.9941 | 1.0013 | 51355 | 1.59e3 | 1 | 1 | 1839 | 1851 | Carbamidomethylation |  | DB Search |
| R.AC(+57.02)PPSNSGTR.Q | Y | 55.99 | 1045.4611 | 10 | 2.5 | 523.738 | 2 | 8.88 | 0.8092 | 0.8165 | 10061 | 1.48e2 | 1 | 1 | 1374 | 1383 | Carbamidomethylation |  | DB Search |
| total 4 peptides |  |  |  |  |  |  |  |  |  |  |  |  |  |  |  |  |  |  |  |
| --- | --- | --- | --- | --- | --- | --- | --- | --- | --- | --- | --- | --- | --- | --- | --- | --- | --- | --- | --- |

Best Unique PSM (Scan Sample\_2\_A1\_1\_3640.d:58476, m/z=878.8997, z=2, RT=17.08, ppm=-2.12):


TRINITY\_DN554\_c0\_g1\_i4.p1

back to list

  

| Protein Coverage
| Supporting Peptides
| Best Unique PSM
|

Protein Coverage:

Supporting Peptides:

| Peptide | Uniq | -10lgP | Mass | Length | ppm | m/z | z | RT | 1/k0 Start | 1/k0 End | Precursor ID | Area Sample 4 | #Feature | #Feature Sample 4 | Start | End | PTM | AScore | Found By |
| --- | --- | --- | --- | --- | --- | --- | --- | --- | --- | --- | --- | --- | --- | --- | --- | --- | --- | --- | --- |
| MQIFVK.T | Y | 57.42 | 764.4255 | 6 | 0.18 | 383.2192 | 2 | 15.73 | 0.7363 | 0.7437 | 49899 | 2.46e2 | 1 | 1 | 1 | 6 |  |  | DB Search |
| K.ESTLHLVLR.L | Y | 57.27 | 1066.6135 | 9 | 2.57 | 534.3142 | 2 | 15.80 | 0.8785 | 0.8858 | 50332 | 4.97e3 | 1 | 1 | 64 | 72 |  |  | DB Search |
| K.IQDKEGIPPDQQR.L | Y | 55.01 | 1522.7739 | 13 | 0.25 | 762.3928 | 2 | 11.08 | 1.0343 | 1.0415 | 21018 | 1.78e3 | 2 | 2 | 30 | 42 |  |  | DB Search |
| K.TITLEVEPSDTIENVK.S | Y | 54.56 | 1786.92 | 16 | 4.26 | 894.4691 | 2 | 17.62 | 1.1442 | 1.1514 | 61769 | 1.48e4 | 1 | 1 | 12 | 27 |  |  | DB Search |
| total 4 peptides |  |  |  |  |  |  |  |  |  |  |  |  |  |  |  |  |  |  |  |
| --- | --- | --- | --- | --- | --- | --- | --- | --- | --- | --- | --- | --- | --- | --- | --- | --- | --- | --- | --- |

Best Unique PSM (Scan Sample\_2\_A1\_1\_3640.d:49899, m/z=383.2192, z=2, RT=15.73, ppm=-2.02):


TRINITY\_DN75529\_c0\_g1\_i1.p1

back to list

  

| Protein Coverage
| Supporting Peptides
| Best Unique PSM
|

Protein Coverage:

Supporting Peptides:

| Peptide | Uniq | -10lgP | Mass | Length | ppm | m/z | z | RT | 1/k0 Start | 1/k0 End | Precursor ID | Area Sample 4 | #Feature | #Feature Sample 4 | Start | End | PTM | AScore | Found By |
| --- | --- | --- | --- | --- | --- | --- | --- | --- | --- | --- | --- | --- | --- | --- | --- | --- | --- | --- | --- |
| K.LKPGDTIEFDTSIK.T | Y | 53.02 | 1562.8191 | 14 | 1.32 | 521.9465 | 3 | 16.40 | 0.7734 | 0.7807 | 54132 | 0 | 0 | 0 | 74 | 87 |  |  | DB Search |
| total 1 peptides |  |  |  |  |  |  |  |  |  |  |  |  |  |  |  |  |  |  |  |
| --- | --- | --- | --- | --- | --- | --- | --- | --- | --- | --- | --- | --- | --- | --- | --- | --- | --- | --- | --- |

Best Unique PSM (Scan Sample\_2\_A1\_1\_3640.d:54132, m/z=521.9465, z=3, RT=16.40, ppm=-0.88):


TRINITY\_DN2577\_c1\_g1\_i1.p1

back to list

  

| Protein Coverage
| Supporting Peptides
| Best Unique PSM
|

Protein Coverage:

Supporting Peptides:

| Peptide | Uniq | -10lgP | Mass | Length | ppm | m/z | z | RT | 1/k0 Start | 1/k0 End | Precursor ID | Area Sample 4 | #Feature | #Feature Sample 4 | Start | End | PTM | AScore | Found By |
| --- | --- | --- | --- | --- | --- | --- | --- | --- | --- | --- | --- | --- | --- | --- | --- | --- | --- | --- | --- |
| R.SGDPLTIGNFIK.N | N | 58.37 | 1260.6714 | 12 | 1.03 | 631.3422 | 2 | 19.83 | 0.8955 | 0.9028 | 76410 | 4.42e2 | 1 | 1 | 172 | 183 |  |  | DB Search |
| K.EGLEFIGTLK.V | Y | 58.25 | 1105.6019 | 10 | 3.2 | 553.8088 | 2 | 19.04 | 0.8627 | 0.87 | 71397 | 0 | 0 | 0 | 73 | 82 |  |  | DB Search |
| K.LELEFTKDER.S | Y | 58.22 | 1278.6455 | 10 | -2.73 | 640.3269 | 2 | 15.54 | 0.9739 | 0.9812 | 48812 | 0 | 0 | 0 | 45 | 54 |  |  | DB Search |
| K.VDDIIC(+57.02)IAGC(+57.02)ELKK.G | Y | 58.04 | 1632.8215 | 14 | -6.17 | 545.2766 | 3 | 17.85 | 0.8388 | 0.8461 | 63424 | 2.04e2 | 1 | 1 | 475 | 488 | Carbamidomethylation, Carbamidomethylation |  | DB Search |
| R.GAVTAAFDKVR.K | Y | 54.88 | 1133.6193 | 11 | 3.89 | 567.8179 | 2 | 14.48 | 0.9352 | 0.9424 | 42102 | 1.49e3 | 1 | 1 | 396 | 406 |  |  | DB Search |
| K.GVLAGVGAIVK.A | Y | 54.31 | 982.6175 | 11 | -1.97 | 492.314 | 2 | 17.55 | 0.8631 | 0.8704 | 61324 | 3.41e3 | 1 | 1 | 546 | 556 |  |  | DB Search |
| K.NGPFLIVDIDSEK.T | Y | 51.58 | 1445.7402 | 13 | 7.04 | 723.8809 | 2 | 19.95 | 0.9775 | 0.9848 | 77105 | 2.24e2 | 1 | 1 | 281 | 293 |  |  | DB Search |
| total 7 peptides |  |  |  |  |  |  |  |  |  |  |  |  |  |  |  |  |  |  |  |
| --- | --- | --- | --- | --- | --- | --- | --- | --- | --- | --- | --- | --- | --- | --- | --- | --- | --- | --- | --- |

Best Unique PSM (Scan Sample\_2\_A1\_1\_3640.d:71397, m/z=553.8088, z=2, RT=19.04, ppm=1.01):


TRINITY\_DN942\_c0\_g1\_i3.p1

back to list

  

| Protein Coverage
| Supporting Peptides
| Best Unique PSM
|

Protein Coverage:

Supporting Peptides:

| Peptide | Uniq | -10lgP | Mass | Length | ppm | m/z | z | RT | 1/k0 Start | 1/k0 End | Precursor ID | Area Sample 4 | #Feature | #Feature Sample 4 | Start | End | PTM | AScore | Found By |
| --- | --- | --- | --- | --- | --- | --- | --- | --- | --- | --- | --- | --- | --- | --- | --- | --- | --- | --- | --- |
| K.AQLLLEDVK.A | Y | 57.95 | 1027.5913 | 9 | 4.05 | 514.8039 | 2 | 17.08 | 0.8721 | 0.8793 | 58461 | 1.87e2 | 1 | 1 | 3322 | 3330 |  |  | DB Search |
| K.VLNIALDKYQEGVAK.T | Y | 57.22 | 1659.9195 | 15 | -3.66 | 554.3105 | 3 | 16.21 | 0.8088 | 0.8161 | 52938 | 0 | 0 | 0 | 4546 | 4560 |  |  | DB Search |
| R.LIVINLIK.K | Y | 54.1 | 924.6372 | 8 | 1.83 | 463.3257 | 2 | 19.76 | 0.8031 | 0.8104 | 75960 | 1.58e3 | 1 | 1 | 3044 | 3051 |  |  | DB Search |
| total 3 peptides |  |  |  |  |  |  |  |  |  |  |  |  |  |  |  |  |  |  |  |
| --- | --- | --- | --- | --- | --- | --- | --- | --- | --- | --- | --- | --- | --- | --- | --- | --- | --- | --- | --- |

Best Unique PSM (Scan Sample\_2\_A1\_1\_3640.d:58461, m/z=514.8039, z=2, RT=17.08, ppm=1.86):


TRINITY\_DN4494\_c1\_g1\_i7.p1

back to list

  

| Protein Coverage
| Supporting Peptides
| Best Unique PSM
|

Protein Coverage:

Supporting Peptides:

| Peptide | Uniq | -10lgP | Mass | Length | ppm | m/z | z | RT | 1/k0 Start | 1/k0 End | Precursor ID | Area Sample 4 | #Feature | #Feature Sample 4 | Start | End | PTM | AScore | Found By |
| --- | --- | --- | --- | --- | --- | --- | --- | --- | --- | --- | --- | --- | --- | --- | --- | --- | --- | --- | --- |
| K.LLILLTDGSQTQDAGAEDPAK.I | Y | 58.29 | 2155.1008 | 21 | 2.82 | 1078.5583 | 2 | 18.86 | 1.2388 | 1.2459 | 70204 | 0 | 0 | 0 | 411 | 431 |  |  | DB Search |
| R.VLDVGFLLDSSDKVK.D | Y | 58.25 | 1633.8926 | 15 | -4.48 | 545.6345 | 3 | 19.79 | 0.8725 | 0.8798 | 76169 | 4.96e2 | 1 | 1 | 109 | 123 |  |  | DB Search |
| K.VDVGFILDSSGSLR.N | N | 58.24 | 1463.762 | 14 | 1.17 | 732.8875 | 2 | 19.97 | 1.0102 | 1.0174 | 77350 | 0 | 0 | 0 | 307 | 320 |  |  | DB Search |
| K.LLILLTDGSQTQDIGAEDPAK.I | Y | 54.36 | 2197.1477 | 21 | 2.89 | 1099.5819 | 2 | 19.49 | 1.2511 | 1.2583 | 74311 | 0 | 0 | 0 | 16 | 36 |  |  | DB Search |
| total 4 peptides |  |  |  |  |  |  |  |  |  |  |  |  |  |  |  |  |  |  |  |
| --- | --- | --- | --- | --- | --- | --- | --- | --- | --- | --- | --- | --- | --- | --- | --- | --- | --- | --- | --- |

Best Unique PSM (Scan Sample\_2\_A1\_1\_3640.d:70204, m/z=1078.5583, z=2, RT=18.86, ppm=0.63):


TRINITY\_DN4494\_c1\_g1\_i10.p1

back to list

  

| Protein Coverage
| Supporting Peptides
| Best Unique PSM
|

Protein Coverage:

Supporting Peptides:

| Peptide | Uniq | -10lgP | Mass | Length | ppm | m/z | z | RT | 1/k0 Start | 1/k0 End | Precursor ID | Area Sample 4 | #Feature | #Feature Sample 4 | Start | End | PTM | AScore | Found By |
| --- | --- | --- | --- | --- | --- | --- | --- | --- | --- | --- | --- | --- | --- | --- | --- | --- | --- | --- | --- |
| K.LLILLTDGSQTQDAGAEDPAK.I | Y | 58.29 | 2155.1008 | 21 | 2.82 | 1078.5583 | 2 | 18.86 | 1.2388 | 1.2459 | 70204 | 0 | 0 | 0 | 27 | 47 |  |  | DB Search |
| K.VDVGFILDSSGSLR.N | N | 58.24 | 1463.762 | 14 | 1.17 | 732.8875 | 2 | 19.97 | 1.0102 | 1.0174 | 77350 | 0 | 0 | 0 | 318 | 331 |  |  | DB Search |
| total 2 peptides |  |  |  |  |  |  |  |  |  |  |  |  |  |  |  |  |  |  |  |
| --- | --- | --- | --- | --- | --- | --- | --- | --- | --- | --- | --- | --- | --- | --- | --- | --- | --- | --- | --- |

Best Unique PSM (Scan Sample\_2\_A1\_1\_3640.d:70204, m/z=1078.5583, z=2, RT=18.86, ppm=0.63):


TRINITY\_DN31399\_c0\_g1\_i3.p1

back to list

  

| Protein Coverage
| Supporting Peptides
| Best Unique PSM
|

Protein Coverage:

Supporting Peptides:

| Peptide | Uniq | -10lgP | Mass | Length | ppm | m/z | z | RT | 1/k0 Start | 1/k0 End | Precursor ID | Area Sample 4 | #Feature | #Feature Sample 4 | Start | End | PTM | AScore | Found By |
| --- | --- | --- | --- | --- | --- | --- | --- | --- | --- | --- | --- | --- | --- | --- | --- | --- | --- | --- | --- |
| R.TVVTIVSDDEYQHM(+15.99)FDR.V | Y | 40.35 | 2069.9364 | 17 | 3.13 | 1035.9764 | 2 | 16.66 | 1.2491 | 1.2563 | 55691 | 2.94e1 | 1 | 1 | 555 | 571 | Oxidation (M) | M14:Oxidation (M):1000 | DB Search |
| total 1 peptides |  |  |  |  |  |  |  |  |  |  |  |  |  |  |  |  |  |  |  |
| --- | --- | --- | --- | --- | --- | --- | --- | --- | --- | --- | --- | --- | --- | --- | --- | --- | --- | --- | --- |

Best Unique PSM (Scan Sample\_2\_A1\_1\_3640.d:55691, m/z=1035.9764, z=2, RT=16.66, ppm=0.93):


TRINITY\_DN160\_c0\_g1\_i1.p1

back to list

  

| Protein Coverage
| Supporting Peptides
| Best Unique PSM
|

Protein Coverage:

Supporting Peptides:

| Peptide | Uniq | -10lgP | Mass | Length | ppm | m/z | z | RT | 1/k0 Start | 1/k0 End | Precursor ID | Area Sample 4 | #Feature | #Feature Sample 4 | Start | End | PTM | AScore | Found By |
| --- | --- | --- | --- | --- | --- | --- | --- | --- | --- | --- | --- | --- | --- | --- | --- | --- | --- | --- | --- |
| K.YDNTITLDAVSSQK.L | Y | 57.09 | 1553.7573 | 14 | 3.45 | 777.8869 | 2 | 15.83 | 1.06 | 1.0672 | 50430 | 4.17e2 | 1 | 1 | 318 | 331 |  |  | DB Search |
| K.DLEFKFR.E | Y | 56.71 | 953.497 | 7 | 0.03 | 477.7548 | 2 | 16.91 | 0.8408 | 0.8481 | 57332 | 4.21e2 | 1 | 1 | 1031 | 1037 |  |  | DB Search |
| K.LRLDFDHEAK.L | Y | 55.3 | 1242.6356 | 10 | 5.7 | 415.2206 | 3 | 14.42 | 0.7359 | 0.7432 | 41684 | 1.12e3 | 1 | 1 | 1007 | 1016 |  |  | DB Search |
| K.VNQAC(+57.02)VR.T | Y | 50.6 | 845.4178 | 7 | 9.12 | 423.7191 | 2 | 9.74 | 0.7636 | 0.7709 | 13525 | 1.64e3 | 1 | 1 | 871 | 877 | Carbamidomethylation |  | DB Search |
| K.LNMIFR.F | Y | 49.11 | 792.4316 | 6 | 5.74 | 397.2245 | 2 | 17.36 | 0.7396 | 0.7469 | 60223 | 7.35e2 | 1 | 1 | 1017 | 1022 |  |  | DB Search |
| total 5 peptides |  |  |  |  |  |  |  |  |  |  |  |  |  |  |  |  |  |  |  |
| --- | --- | --- | --- | --- | --- | --- | --- | --- | --- | --- | --- | --- | --- | --- | --- | --- | --- | --- | --- |

Best Unique PSM (Scan Sample\_2\_A1\_1\_3640.d:50430, m/z=777.8869, z=2, RT=15.83, ppm=1.26):


TRINITY\_DN982\_c0\_g1\_i3.p1

back to list

  

| Protein Coverage
| Supporting Peptides
| Best Unique PSM
|

Protein Coverage:

Supporting Peptides:

| Peptide | Uniq | -10lgP | Mass | Length | ppm | m/z | z | RT | 1/k0 Start | 1/k0 End | Precursor ID | Area Sample 4 | #Feature | #Feature Sample 4 | Start | End | PTM | AScore | Found By |
| --- | --- | --- | --- | --- | --- | --- | --- | --- | --- | --- | --- | --- | --- | --- | --- | --- | --- | --- | --- |
| K.VLLSQQIFYDKK.N | N | 58.39 | 1480.8289 | 12 | 0.31 | 741.4203 | 2 | 17.02 | 1.0616 | 1.0688 | 58002 | 1.76e3 | 2 | 2 | 4919 | 4930 |  |  | DB Search |
| R.EQTLTIGGSLLLQK.F | Y | 58.39 | 1499.8559 | 14 | 4.83 | 750.9372 | 2 | 19.02 | 1.0327 | 1.0399 | 71181 | 1.06e3 | 1 | 1 | 1336 | 1349 |  |  | DB Search |
| R.IQVLKDPDFEFTR.S | N | 58.39 | 1606.8355 | 13 | 0.79 | 536.6183 | 3 | 17.74 | 0.8457 | 0.853 | 62712 | 2.19e3 | 1 | 1 | 6167 | 6179 |  |  | DB Search |
| K.SAIIVDGKNEEGR.I | Y | 58.38 | 1386.7103 | 13 | 4.33 | 694.3639 | 2 | 12.00 | 0.9969 | 1.0041 | 26845 | 7.58e2 | 1 | 1 | 95 | 107 |  |  | DB Search |
| K.LAQPLDYEIKK.Q | Y | 58.37 | 1316.734 | 11 | 4.03 | 439.9194 | 3 | 14.78 | 0.751 | 0.7583 | 43862 | 2.11e2 | 1 | 1 | 2824 | 2834 |  |  | DB Search |
| K.ESSFSIAQWSK.S | Y | 58.35 | 1268.6037 | 11 | -2.2 | 635.3063 | 2 | 17.87 | 0.9174 | 0.9247 | 63581 | 7.35e2 | 1 | 1 | 772 | 782 |  |  | DB Search |
| R.TEIPGIIFEK.N | N | 58.33 | 1145.6332 | 10 | 1.1 | 573.8232 | 2 | 18.83 | 0.8874 | 0.8947 | 69581 | 1.78e3 | 1 | 1 | 5999 | 6008 |  |  | DB Search |
| R.VYESLSGFIK.N | Y | 58.3 | 1141.6019 | 10 | 1.34 | 571.8077 | 2 | 18.14 | 0.9097 | 0.917 | 65369 | 1.71e3 | 2 | 2 | 202 | 211 |  |  | DB Search |
| K.AEGPTSTGVTEVIVR.V | Y | 58.29 | 1514.794 | 15 | 2.95 | 758.4048 | 2 | 15.62 | 1.0106 | 1.0178 | 49221 | 7.66e2 | 1 | 1 | 3358 | 3372 |  |  | DB Search |
| R.LVQNTPHNSIVTK.I | N | 58.29 | 1449.794 | 13 | 3.11 | 484.2724 | 3 | 11.79 | 0.8075 | 0.8149 | 25665 | 5.5e2 | 1 | 1 | 8569 | 8581 |  |  | DB Search |
| R.YTSC(+57.02)QSVTVVASDR.S | N | 58.29 | 1571.725 | 14 | -2.24 | 786.8663 | 2 | 13.52 | 1.0504 | 1.0576 | 36260 | 0 | 0 | 0 | 9363 | 9376 | Carbamidomethylation |  | DB Search |
| K.YVAIYPSDHLTVNR.D | N | 58.29 | 1646.8416 | 14 | 3.39 | 824.4291 | 2 | 15.76 | 1.1082 | 1.1154 | 49960 | 1.45e3 | 1 | 1 | 6254 | 6267 |  |  | DB Search |
| K.LFGSIYLR.A | Y | 58.25 | 967.5491 | 8 | -0.04 | 484.7807 | 2 | 18.85 | 0.8311 | 0.8384 | 70112 | 9.36e2 | 1 | 1 | 578 | 585 |  |  | DB Search |
| K.LLTTEDDFILR.R | N | 58.17 | 1334.7081 | 11 | -1.86 | 668.3586 | 2 | 19.19 | 0.9634 | 0.9707 | 72323 | 9.12e2 | 1 | 1 | 6897 | 6907 |  |  | DB Search |
| R.IGLMSSFQK.T | Y | 57.99 | 1009.5266 | 9 | 4.5 | 505.7718 | 2 | 17.20 | 0.8352 | 0.8425 | 59131 | 1.33e3 | 1 | 1 | 108 | 116 |  |  | DB Search |
| K.MAVNIMIK.A | Y | 57.78 | 918.5031 | 8 | 13.05 | 460.2638 | 2 | 16.99 | 0.8071 | 0.8144 | 57925 | 0 | 0 | 0 | 226 | 233 |  |  | DB Search |
| K.DSFISVINMR.Y | Y | 57.58 | 1180.591 | 10 | 1.69 | 591.3025 | 2 | 19.62 | 0.9012 | 0.9085 | 75085 | 1.94e3 | 1 | 1 | 459 | 468 |  |  | DB Search |
| R.FYSFEVQVIDTNNGK.T | Y | 57.39 | 1759.8417 | 15 | 4.04 | 880.9297 | 2 | 18.90 | 1.0965 | 1.1037 | 70365 | 3.63e2 | 1 | 1 | 3148 | 3162 |  |  | DB Search |
| K.TPGIIVSIDEK.I | Y | 57.35 | 1170.6496 | 11 | 0.16 | 586.3309 | 2 | 16.69 | 0.921 | 0.9283 | 55817 | 1.1e3 | 1 | 1 | 958 | 968 |  |  | DB Search |
| K.DFNINNGVFR.T | Y | 57.29 | 1194.5781 | 10 | 1.03 | 598.2957 | 2 | 18.08 | 0.8927 | 0.9 | 64841 | 3.11e3 | 1 | 1 | 509 | 518 |  |  | DB Search |
| K.SSNTIEENRLK.Y | Y | 57.06 | 1289.6575 | 11 | 4.02 | 645.8372 | 2 | 11.54 | 0.9598 | 0.967 | 24083 | 4.33e2 | 1 | 1 | 783 | 793 |  |  | DB Search |
| R.VNEGNLVGDMVLR.V | N | 55.82 | 1414.7238 | 13 | 1.01 | 708.3683 | 2 | 18.54 | 0.9856 | 0.9928 | 68049 | 8.64e2 | 1 | 1 | 6831 | 6843 |  |  | DB Search |
| R.IDQHGGIK.T | N | 54.38 | 866.461 | 8 | 5.07 | 434.239 | 2 | 9.74 | 0.7892 | 0.7966 | 13590 | 0 | 0 | 0 | 7813 | 7820 |  |  | DB Search |
| K.SSNTIEENR.L | Y | 54.34 | 1048.4785 | 9 | 3.11 | 525.247 | 2 | 10.20 | 0.8303 | 0.8376 | 15999 | 0 | 0 | 0 | 783 | 791 |  |  | DB Search |
| K.SKIDYEALQVK.H | N | 53.53 | 1292.6976 | 11 | -4.87 | 647.3515 | 2 | 14.82 | 0.9606 | 0.9679 | 44312 | 0 | 0 | 0 | 6813 | 6823 |  |  | DB Search |
| R.ITTSQILDHEQQK.L | N | 53.33 | 1539.7893 | 13 | 1.49 | 770.9014 | 2 | 14.03 | 1.0969 | 1.1041 | 39378 | 0 | 0 | 0 | 5434 | 5446 |  |  | DB Search |
| R.VFSIVESDANDQFVIDSNSGALR.L | N | 52.69 | 2482.1975 | 23 | 3.57 | 828.4076 | 3 | 19.68 | 1.0464 | 1.0536 | 75408 | 5.65e2 | 1 | 1 | 8103 | 8125 |  |  | DB Search |
| K.NHYIFNINEK.E | N | 52.53 | 1290.6357 | 10 | 1.67 | 646.3248 | 2 | 15.74 | 0.9598 | 0.967 | 50017 | 0 | 0 | 0 | 6009 | 6018 |  |  | DB Search |
| K.GSTHVIIDVTDVNDNNPK.F | N | 41.87 | 1936.949 | 18 | -7.16 | 969.4727 | 2 | 15.19 | 1.153 | 1.1602 | 46639 | 0 | 0 | 0 | 7154 | 7171 |  |  | DB Search |
| total 29 peptides |  |  |  |  |  |  |  |  |  |  |  |  |  |  |  |  |  |  |  |
| --- | --- | --- | --- | --- | --- | --- | --- | --- | --- | --- | --- | --- | --- | --- | --- | --- | --- | --- | --- |

Best Unique PSM (Scan Sample\_2\_A1\_1\_3640.d:71181, m/z=750.9372, z=2, RT=19.02, ppm=2.64):


TRINITY\_DN585\_c0\_g1\_i8.p1

back to list

  

| Protein Coverage
| Supporting Peptides
| Best Unique PSM
|

Protein Coverage:

Supporting Peptides:

| Peptide | Uniq | -10lgP | Mass | Length | ppm | m/z | z | RT | 1/k0 Start | 1/k0 End | Precursor ID | Area Sample 4 | #Feature | #Feature Sample 4 | Start | End | PTM | AScore | Found By |
| --- | --- | --- | --- | --- | --- | --- | --- | --- | --- | --- | --- | --- | --- | --- | --- | --- | --- | --- | --- |
| MQIFVK.T | Y | 57.42 | 764.4255 | 6 | 0.18 | 383.2192 | 2 | 15.73 | 0.7363 | 0.7437 | 49899 | 2.46e2 | 1 | 1 | 1 | 6 |  |  | DB Search |
| K.ESTLHLVLR.L | Y | 57.27 | 1066.6135 | 9 | 2.57 | 534.3142 | 2 | 15.80 | 0.8785 | 0.8858 | 50332 | 4.97e3 | 1 | 1 | 64 | 72 |  |  | DB Search |
| K.IQDKEGIPPDQQR.L | Y | 55.01 | 1522.7739 | 13 | 0.25 | 762.3928 | 2 | 11.08 | 1.0343 | 1.0415 | 21018 | 1.78e3 | 2 | 2 | 30 | 42 |  |  | DB Search |
| K.TITLEVEPSDTIENVK.S | Y | 54.56 | 1786.92 | 16 | 4.26 | 894.4691 | 2 | 17.62 | 1.1442 | 1.1514 | 61769 | 1.48e4 | 1 | 1 | 12 | 27 |  |  | DB Search |
| total 4 peptides |  |  |  |  |  |  |  |  |  |  |  |  |  |  |  |  |  |  |  |
| --- | --- | --- | --- | --- | --- | --- | --- | --- | --- | --- | --- | --- | --- | --- | --- | --- | --- | --- | --- |

Best Unique PSM (Scan Sample\_2\_A1\_1\_3640.d:49899, m/z=383.2192, z=2, RT=15.73, ppm=-2.02):


TRINITY\_DN4900\_c0\_g1\_i1.p1

back to list

  

| Protein Coverage
| Supporting Peptides
| Best Unique PSM
|

Protein Coverage:

Supporting Peptides:

| Peptide | Uniq | -10lgP | Mass | Length | ppm | m/z | z | RT | 1/k0 Start | 1/k0 End | Precursor ID | Area Sample 4 | #Feature | #Feature Sample 4 | Start | End | PTM | AScore | Found By |
| --- | --- | --- | --- | --- | --- | --- | --- | --- | --- | --- | --- | --- | --- | --- | --- | --- | --- | --- | --- |
| K.DTVIEKFEENITK.G | Y | 58.3 | 1564.7984 | 13 | 1.27 | 783.4058 | 2 | 19.63 | 1.068 | 1.0753 | 75192 | 9.24e2 | 2 | 2 | 112 | 124 |  |  | DB Search |
| K.KDTVIEKFEENITK.G | Y | 52.33 | 1692.8934 | 14 | -2.27 | 565.3026 | 3 | 17.43 | 0.8485 | 0.8558 | 60646 | 1.07e2 | 1 | 1 | 111 | 124 |  |  | DB Search |
| total 2 peptides |  |  |  |  |  |  |  |  |  |  |  |  |  |  |  |  |  |  |  |
| --- | --- | --- | --- | --- | --- | --- | --- | --- | --- | --- | --- | --- | --- | --- | --- | --- | --- | --- | --- |

Best Unique PSM (Scan Sample\_2\_A1\_1\_3640.d:75192, m/z=783.4058, z=2, RT=19.63, ppm=-0.92):


TRINITY\_DN77\_c0\_g2\_i3.p1

back to list

  

| Protein Coverage
| Supporting Peptides
| Best Unique PSM
|

Protein Coverage:

Supporting Peptides:

| Peptide | Uniq | -10lgP | Mass | Length | ppm | m/z | z | RT | 1/k0 Start | 1/k0 End | Precursor ID | Area Sample 4 | #Feature | #Feature Sample 4 | Start | End | PTM | AScore | Found By |
| --- | --- | --- | --- | --- | --- | --- | --- | --- | --- | --- | --- | --- | --- | --- | --- | --- | --- | --- | --- |
| R.GPVGTSGLR.G | Y | 58.19 | 842.461 | 9 | 3.7 | 422.2384 | 2 | 12.14 | 0.7787 | 0.786 | 27733 | 1.47e4 | 1 | 1 | 837 | 845 |  |  | DB Search |
| R.GPAGAPGDEGQTGMR.G | Y | 57.23 | 1399.615 | 15 | -0.7 | 700.8127 | 2 | 11.55 | 0.9396 | 0.9469 | 24084 | 1.21e3 | 1 | 1 | 633 | 647 |  |  | DB Search |
| R.GPQGQVGK.Q | Y | 55.44 | 769.4082 | 8 | 6.49 | 385.713 | 2 | 8.99 | 0.7069 | 0.7143 | 10347 | 1.81e3 | 1 | 1 | 480 | 487 |  |  | DB Search |
| R.GPAGAPGDEGQTGM(+15.99)RGER.G | Y | 54.26 | 1757.7751 | 18 | 2.12 | 586.9323 | 3 | 10.62 | 0.8818 | 0.8891 | 18471 | 3.52e3 | 1 | 1 | 633 | 650 | Oxidation (M) | M14:Oxidation (M):1000 | DB Search |
| R.GPAGAPGDEGQTGM(+15.99)R.G | Y | 54.2 | 1415.6099 | 15 | 4.13 | 708.8136 | 2 | 10.40 | 0.9453 | 0.9525 | 17281 | 0 | 0 | 0 | 633 | 647 | Oxidation (M) | M14:Oxidation (M):1000 | DB Search |
| total 5 peptides |  |  |  |  |  |  |  |  |  |  |  |  |  |  |  |  |  |  |  |
| --- | --- | --- | --- | --- | --- | --- | --- | --- | --- | --- | --- | --- | --- | --- | --- | --- | --- | --- | --- |

Best Unique PSM (Scan Sample\_2\_A1\_1\_3640.d:27733, m/z=422.2384, z=2, RT=12.14, ppm=1.5):


TRINITY\_DN17714\_c0\_g1\_i1.p1

back to list

  

| Protein Coverage
| Supporting Peptides
| Best Unique PSM
|

Protein Coverage:

Supporting Peptides:

| Peptide | Uniq | -10lgP | Mass | Length | ppm | m/z | z | RT | 1/k0 Start | 1/k0 End | Precursor ID | Area Sample 4 | #Feature | #Feature Sample 4 | Start | End | PTM | AScore | Found By |
| --- | --- | --- | --- | --- | --- | --- | --- | --- | --- | --- | --- | --- | --- | --- | --- | --- | --- | --- | --- |
| K.YAAYVGC(+57.02)EFQSVDFSSVAK.G | Y | 54.86 | 2126.9619 | 19 | 2.95 | 1064.489 | 2 | 18.93 | 1.226 | 1.2332 | 70654 | 0 | 0 | 0 | 537 | 555 | Carbamidomethylation |  | DB Search |
| total 1 peptides |  |  |  |  |  |  |  |  |  |  |  |  |  |  |  |  |  |  |  |
| --- | --- | --- | --- | --- | --- | --- | --- | --- | --- | --- | --- | --- | --- | --- | --- | --- | --- | --- | --- |

Best Unique PSM (Scan Sample\_2\_A1\_1\_3640.d:70654, m/z=1064.489, z=2, RT=18.93, ppm=0.75):


TRINITY\_DN1616\_c0\_g1\_i3.p1

back to list

  

| Protein Coverage
| Supporting Peptides
| Best Unique PSM
|

Protein Coverage:

Supporting Peptides:

| Peptide | Uniq | -10lgP | Mass | Length | ppm | m/z | z | RT | 1/k0 Start | 1/k0 End | Precursor ID | Area Sample 4 | #Feature | #Feature Sample 4 | Start | End | PTM | AScore | Found By |
| --- | --- | --- | --- | --- | --- | --- | --- | --- | --- | --- | --- | --- | --- | --- | --- | --- | --- | --- | --- |
| R.SNIDEVNVKHEQIEEFIK.T | Y | 58.4 | 2170.0906 | 18 | 0.32 | 724.3694 | 3 | 16.43 | 0.9566 | 0.9638 | 54256 | 7.37e2 | 1 | 1 | 2671 | 2688 |  |  | DB Search |
| K.VVDTEANDLTEEVKK.T | Y | 58.38 | 1688.8468 | 15 | -0.58 | 563.9547 | 3 | 14.21 | 0.8392 | 0.8465 | 40438 | 5.49e3 | 2 | 2 | 2542 | 2556 |  |  | DB Search |
| K.LNTGDVQSLTNPK.F | Y | 58.37 | 1385.715 | 13 | 1.86 | 693.8646 | 2 | 14.77 | 0.9977 | 1.0049 | 43652 | 1.53e3 | 1 | 1 | 2783 | 2795 |  |  | DB Search |
| R.YGAVNDNC(+57.02)ER.D | Y | 58.36 | 1196.488 | 10 | 4.23 | 599.2525 | 2 | 10.91 | 0.8802 | 0.8874 | 20281 | 8.99e2 | 1 | 1 | 498 | 507 | Carbamidomethylation |  | DB Search |
| K.SNIC(+57.02)DPQNGK.C | Y | 58.34 | 1131.4979 | 10 | -0.79 | 566.7545 | 2 | 10.56 | 0.8526 | 0.8599 | 18199 | 6.49e2 | 1 | 1 | 1794 | 1803 | Carbamidomethylation |  | DB Search |
| K.LSLDVNGNYQGGGVC(+57.02)K.N | Y | 58.29 | 1679.7937 | 16 | -4.07 | 840.8989 | 2 | 15.59 | 1.0705 | 1.0777 | 49051 | 0 | 0 | 0 | 350 | 365 | Carbamidomethylation |  | DB Search |
| R.FSNLSNKGDEHISNLDTLKK.A | Y | 58.28 | 2259.1495 | 20 | 1.82 | 565.7944 | 4 | 13.91 | 0.8311 | 0.8384 | 38634 | 0 | 0 | 0 | 2121 | 2140 |  |  | DB Search |
| R.NFATAVLNVQK.S | Y | 58.28 | 1203.6611 | 11 | -3.72 | 602.8343 | 2 | 17.63 | 0.923 | 0.9303 | 62041 | 0 | 0 | 0 | 2810 | 2820 |  |  | DB Search |
| K.TLKVDISDMK.M | Y | 58.28 | 1148.6111 | 10 | -6.01 | 575.3081 | 2 | 15.17 | 0.917 | 0.9243 | 46490 | 0 | 0 | 0 | 2557 | 2566 |  |  | DB Search |
| K.FIETDVNYDVM(+15.99)VTR.N | Y | 58.27 | 1716.8029 | 14 | 0.97 | 859.4077 | 2 | 16.71 | 1.0809 | 1.0881 | 56014 | 1.52e3 | 1 | 1 | 2796 | 2809 | Oxidation (M) | M11:Oxidation (M):1000 | DB Search |
| R.VIDEGEKNIK.L | Y | 58.26 | 1143.6135 | 10 | 3.03 | 572.8145 | 2 | 10.89 | 0.9271 | 0.9344 | 20199 | 3.83e3 | 2 | 2 | 2218 | 2227 |  |  | DB Search |
| K.DIIVLEAENPVK.M | Y | 58.25 | 1338.7394 | 12 | 1.86 | 670.3768 | 2 | 18.10 | 0.9844 | 0.9916 | 65198 | 4.87e2 | 1 | 1 | 1013 | 1024 |  |  | DB Search |
| R.YVKPGVNIINIR.D | Y | 57.33 | 1384.819 | 12 | 6.22 | 462.6155 | 3 | 16.27 | 0.8043 | 0.8116 | 53149 | 6.85e2 | 2 | 2 | 1217 | 1228 |  |  | DB Search |
| K.LFNEALKPLK.E | Y | 57.32 | 1171.6965 | 10 | 3.28 | 586.8561 | 2 | 15.90 | 0.9509 | 0.9582 | 50919 | 4.48e2 | 1 | 1 | 3651 | 3660 |  |  | DB Search |
| K.AVDFQSIIDNAQK.L | Y | 56.94 | 1447.7307 | 13 | 4.22 | 724.8741 | 2 | 19.06 | 0.9904 | 0.9977 | 71389 | 6.5e2 | 1 | 1 | 2383 | 2395 |  |  | DB Search |
| K.NLETFTGC(+57.02)ITAVR.I | Y | 56.93 | 1480.7344 | 13 | 2.99 | 741.3751 | 2 | 18.29 | 0.9993 | 1.0065 | 66297 | 1.31e3 | 1 | 1 | 3814 | 3826 | Carbamidomethylation |  | DB Search |
| K.DGEKVETLKVDISDINK.A | Y | 56.92 | 1901.9945 | 17 | 3.6 | 635.0063 | 3 | 16.09 | 0.9113 | 0.9186 | 52017 | 1.36e3 | 1 | 1 | 2634 | 2650 |  |  | DB Search |
| K.VETLKVDISDINK.A | Y | 56.92 | 1472.8086 | 13 | -2.26 | 491.9413 | 3 | 16.17 | 0.8222 | 0.8295 | 52567 | 5.32e2 | 1 | 1 | 2638 | 2650 |  |  | DB Search |
| K.GDEHISNLDTLKK.A | Y | 56.42 | 1468.7521 | 13 | -2.32 | 490.5891 | 3 | 12.90 | 0.7791 | 0.7864 | 32456 | 0 | 0 | 0 | 2128 | 2140 |  |  | DB Search |
| K.GFVAVNDQQPSVVVSINVPK.T | Y | 55.03 | 2096.1266 | 20 | 0.1 | 1049.0684 | 2 | 18.45 | 1.2157 | 1.2228 | 67713 | 4.05e2 | 1 | 1 | 865 | 884 |  |  | DB Search |
| K.DGEKVETLK.V | Y | 54.9 | 1017.5342 | 9 | -2.12 | 509.7722 | 2 | 11.49 | 0.8587 | 0.866 | 23862 | 1.28e3 | 1 | 1 | 2634 | 2642 |  |  | DB Search |
| R.IDNVKEDVK.S | Y | 54.89 | 1058.5608 | 9 | 5.78 | 530.2896 | 2 | 10.73 | 0.8716 | 0.8789 | 19180 | 1.58e3 | 1 | 1 | 2583 | 2591 |  |  | DB Search |
| K.VLISSLLIK.F | Y | 54.58 | 984.6583 | 9 | 0.22 | 493.3354 | 2 | 19.79 | 0.8506 | 0.8579 | 76091 | 1.17e3 | 1 | 1 | 3009 | 3017 |  |  | DB Search |
| R.RC(+57.02)EEC(+57.02)AAGHYQFPFC(+57.02)K.E | Y | 53.84 | 2058.8499 | 16 | 0.01 | 687.2891 | 3 | 15.74 | 0.9218 | 0.9291 | 49834 | 2.69e2 | 1 | 1 | 522 | 537 | Carbamidomethylation, Carbamidomethylation, Carbamidomethylation |  | DB Search |
| K.NEINLASGDVFEGGVVGSLK.L | Y | 53.15 | 2004.0163 | 20 | 1.4 | 1003.0146 | 2 | 19.85 | 1.1829 | 1.1901 | 76578 | 0 | 0 | 0 | 3249 | 3268 |  |  | DB Search |
| R.KLSLDVNGNYQGGGVC(+57.02)K.N | Y | 51.86 | 1807.8887 | 17 | 0.8 | 603.636 | 3 | 14.64 | 0.8193 | 0.8266 | 43107 | 2.56e2 | 1 | 1 | 349 | 365 | Carbamidomethylation |  | DB Search |
| R.YALNQIQPVTATVTAFK.G | Y | 47.96 | 1864.0094 | 17 | 1.71 | 933.0115 | 2 | 19.31 | 1.1478 | 1.155 | 73122 | 0 | 0 | 0 | 894 | 910 |  |  | DB Search |
| K.IRSNIDEVNVK.H | Y | 39.98 | 1285.699 | 11 | -7.71 | 643.8504 | 2 | 14.93 | 0.9638 | 0.9711 | 44969 | 0 | 0 | 0 | 2669 | 2679 |  |  | DB Search |
| total 28 peptides |  |  |  |  |  |  |  |  |  |  |  |  |  |  |  |  |  |  |  |
| --- | --- | --- | --- | --- | --- | --- | --- | --- | --- | --- | --- | --- | --- | --- | --- | --- | --- | --- | --- |

Best Unique PSM (Scan Sample\_2\_A1\_1\_3640.d:54256, m/z=724.3694, z=3, RT=16.43, ppm=-1.87):


TRINITY\_DN4781\_c0\_g1\_i1.p1

back to list

  

| Protein Coverage
| Supporting Peptides
| Best Unique PSM
|

Protein Coverage:

Supporting Peptides:

| Peptide | Uniq | -10lgP | Mass | Length | ppm | m/z | z | RT | 1/k0 Start | 1/k0 End | Precursor ID | Area Sample 4 | #Feature | #Feature Sample 4 | Start | End | PTM | AScore | Found By |
| --- | --- | --- | --- | --- | --- | --- | --- | --- | --- | --- | --- | --- | --- | --- | --- | --- | --- | --- | --- |
| K.FGDIMDWTVHQGK.T | Y | 58.41 | 1532.7082 | 13 | 0.12 | 511.9089 | 3 | 18.62 | 0.7982 | 0.8055 | 68637 | 0 | 0 | 0 | 2412 | 2424 |  |  | DB Search |
| R.LLSPVFKPHGNK.K | Y | 58.4 | 1335.7663 | 12 | 7.47 | 446.265 | 3 | 13.80 | 0.7982 | 0.8055 | 37938 | 3.05e2 | 1 | 1 | 2241 | 2252 |  |  | DB Search |
| R.LVSELFDPTSSTR.G | Y | 58.39 | 1450.7303 | 13 | 1.5 | 726.3719 | 2 | 17.91 | 1.0013 | 1.0086 | 64032 | 6.47e3 | 1 | 1 | 265 | 277 |  |  | DB Search |
| K.DFNVIIEAVK.G | Y | 58.33 | 1146.6284 | 10 | 0.88 | 574.3207 | 2 | 19.65 | 0.8996 | 0.9069 | 75314 | 3.22e3 | 1 | 1 | 851 | 860 |  |  | DB Search |
| R.GDIAIDDITVKK.G | Y | 58.32 | 1286.7081 | 12 | 0.84 | 644.3605 | 2 | 15.29 | 0.9602 | 0.9675 | 47150 | 2.79e2 | 1 | 1 | 1480 | 1491 |  |  | DB Search |
| K.APPKPNPC(+57.02)AIK.C | Y | 58.26 | 1191.6434 | 11 | 5.82 | 398.2232 | 3 | 11.50 | 0.7783 | 0.7856 | 23835 | 2.21e3 | 2 | 2 | 1499 | 1509 | Carbamidomethylation |  | DB Search |
| Q.LIFVGTVGK.S | Y | 58.18 | 932.5695 | 9 | 3.01 | 467.2924 | 2 | 17.35 | 0.8124 | 0.8197 | 60219 | 6.55e3 | 1 | 1 | 168 | 176 |  |  | DB Search |
| R.TC(+57.02)IGC(+57.02)PETQIWR.R | Y | 58.01 | 1519.6912 | 12 | -0.87 | 760.8505 | 2 | 16.92 | 1.0053 | 1.0126 | 57323 | 1.28e3 | 1 | 1 | 1852 | 1863 | Carbamidomethylation, Carbamidomethylation |  | DB Search |
| K.VNFQVVMEAR.R | Y | 57.24 | 1191.607 | 10 | 0.27 | 596.8096 | 2 | 17.59 | 0.8972 | 0.9044 | 61571 | 2.93e3 | 1 | 1 | 2309 | 2318 |  |  | DB Search |
| K.GSYIYIEASYPR.K | Y | 57.03 | 1417.6878 | 12 | 0.36 | 709.8499 | 2 | 17.28 | 1.0399 | 1.0472 | 59633 | 2.78e3 | 1 | 1 | 434 | 445 |  |  | DB Search |
| K.TVISQVQSLSIR.N | Y | 56.91 | 1329.7616 | 12 | 2.63 | 665.8884 | 2 | 17.88 | 0.9574 | 0.9646 | 63579 | 2.04e3 | 1 | 1 | 2075 | 2086 |  |  | DB Search |
| K.GQSSDLATLSSPVLNQASSTC(+57.02)K.M | Y | 55.21 | 2250.0798 | 22 | 4.76 | 1126.05 | 2 | 17.18 | 1.2328 | 1.24 | 59109 | 0 | 0 | 0 | 1600 | 1621 | Carbamidomethylation |  | DB Search |
| K.ADHTYETDFGK.Y | Y | 55.18 | 1282.5466 | 11 | 8.7 | 642.2847 | 2 | 12.90 | 0.9453 | 0.9525 | 32449 | 0 | 0 | 0 | 1196 | 1206 |  |  | DB Search |
| R.TGPSLDNTK.K | Y | 54.61 | 931.4611 | 9 | 0.07 | 466.7368 | 2 | 11.29 | 0.8039 | 0.8112 | 22423 | 5.2e2 | 1 | 1 | 233 | 241 |  |  | DB Search |
| R.FYYHM(+15.99)YGDHIGALSVK.L | Y | 53.61 | 1915.8927 | 16 | 2.41 | 639.6383 | 3 | 15.79 | 0.9085 | 0.9158 | 50276 | 2.57e2 | 1 | 1 | 1834 | 1849 | Oxidation (M) | M5:Oxidation (M):1000 | DB Search |
| R.FYYHMYGDHIGALSVK.L | Y | 52.72 | 1899.8978 | 16 | -1 | 634.3045 | 3 | 16.96 | 0.917 | 0.9243 | 57688 | 0 | 0 | 0 | 1834 | 1849 |  |  | DB Search |
| R.DVANLYSPYYPGSK.T | Y | 51.34 | 1572.746 | 14 | -1.72 | 787.3772 | 2 | 17.32 | 1.0242 | 1.0315 | 60052 | 5.66e2 | 1 | 1 | 613 | 626 |  |  | DB Search |
| R.NLKDDDFDFTR.Q | Y | 41.38 | 1384.6259 | 11 | 14.15 | 693.3285 | 2 | 15.93 | 0.9775 | 0.9848 | 51300 | 6.08e2 | 1 | 1 | 1765 | 1775 |  |  | DB Search |
| total 18 peptides |  |  |  |  |  |  |  |  |  |  |  |  |  |  |  |  |  |  |  |
| --- | --- | --- | --- | --- | --- | --- | --- | --- | --- | --- | --- | --- | --- | --- | --- | --- | --- | --- | --- |

Best Unique PSM (Scan Sample\_2\_A1\_1\_3640.d:68637, m/z=511.9089, z=3, RT=18.62, ppm=-2.08):


TRINITY\_DN495\_c0\_g1\_i2.p1

back to list

  

| Protein Coverage
| Supporting Peptides
| Best Unique PSM
|

Protein Coverage:

Supporting Peptides:

| Peptide | Uniq | -10lgP | Mass | Length | ppm | m/z | z | RT | 1/k0 Start | 1/k0 End | Precursor ID | Area Sample 4 | #Feature | #Feature Sample 4 | Start | End | PTM | AScore | Found By |
| --- | --- | --- | --- | --- | --- | --- | --- | --- | --- | --- | --- | --- | --- | --- | --- | --- | --- | --- | --- |
| R.LFEIKFNAE.X | Y | 47.56 | 1109.5757 | 9 | 0.53 | 555.7942 | 2 | 19.11 | 0.8619 | 0.8692 | 71841 | 3.8e2 | 1 | 1 | 646 | 654 |  |  | DB Search |
| R.SSC(+57.02)KPILSVIK.I | Y | 41 | 1230.7006 | 11 | -0.71 | 616.3558 | 2 | 15.33 | 0.9529 | 0.9602 | 47491 | 3.99e2 | 1 | 1 | 450 | 460 | Carbamidomethylation |  | DB Search |
| total 2 peptides |  |  |  |  |  |  |  |  |  |  |  |  |  |  |  |  |  |  |  |
| --- | --- | --- | --- | --- | --- | --- | --- | --- | --- | --- | --- | --- | --- | --- | --- | --- | --- | --- | --- |

Best Unique PSM (Scan Sample\_2\_A1\_1\_3640.d:71841, m/z=555.7942, z=2, RT=19.11, ppm=-1.67):


TRINITY\_DN1783\_c0\_g1\_i1.p2

back to list

  

| Protein Coverage
| Supporting Peptides
| Best Unique PSM
|

Protein Coverage:

Supporting Peptides:

| Peptide | Uniq | -10lgP | Mass | Length | ppm | m/z | z | RT | 1/k0 Start | 1/k0 End | Precursor ID | Area Sample 4 | #Feature | #Feature Sample 4 | Start | End | PTM | AScore | Found By |
| --- | --- | --- | --- | --- | --- | --- | --- | --- | --- | --- | --- | --- | --- | --- | --- | --- | --- | --- | --- |
| K.LTIIDALPR.C | Y | 58.24 | 1010.6124 | 9 | 0.02 | 506.3124 | 2 | 19.00 | 0.8392 | 0.8465 | 71132 | 3.79e2 | 1 | 1 | 225 | 233 |  |  | DB Search |
| total 1 peptides |  |  |  |  |  |  |  |  |  |  |  |  |  |  |  |  |  |  |  |
| --- | --- | --- | --- | --- | --- | --- | --- | --- | --- | --- | --- | --- | --- | --- | --- | --- | --- | --- | --- |

Best Unique PSM (Scan Sample\_2\_A1\_1\_3640.d:71132, m/z=506.3124, z=2, RT=19.00, ppm=-2.17):


TRINITY\_DN691\_c0\_g1\_i2.p1

back to list

  

| Protein Coverage
| Supporting Peptides
| Best Unique PSM
|

Protein Coverage:

Supporting Peptides:

| Peptide | Uniq | -10lgP | Mass | Length | ppm | m/z | z | RT | 1/k0 Start | 1/k0 End | Precursor ID | Area Sample 4 | #Feature | #Feature Sample 4 | Start | End | PTM | AScore | Found By |
| --- | --- | --- | --- | --- | --- | --- | --- | --- | --- | --- | --- | --- | --- | --- | --- | --- | --- | --- | --- |
| R.ANPFIEC(+57.02)HGR.G | Y | 58.39 | 1199.5506 | 10 | 3.99 | 600.7836 | 2 | 13.37 | 0.8923 | 0.8996 | 35227 | 1.55e3 | 1 | 1 | 1607 | 1616 | Carbamidomethylation |  | DB Search |
| K.DGNDGQTGLTGQR.G | Y | 58.38 | 1317.5909 | 13 | 0.63 | 659.8017 | 2 | 11.17 | 0.9303 | 0.9376 | 21848 | 0 | 0 | 0 | 321 | 333 |  |  | DB Search |
| R.AYGQDLGQPGSC(+57.02)LKR.F | Y | 55 | 1648.7991 | 15 | 2.6 | 825.4072 | 2 | 13.56 | 1.0761 | 1.0833 | 36469 | 5.48e2 | 1 | 1 | 1480 | 1494 | Carbamidomethylation |  | DB Search |
| R.AYGQDLGQPGSC(+57.02)LK.R | Y | 54.78 | 1492.698 | 14 | -2.16 | 747.353 | 2 | 14.36 | 0.9977 | 1.0049 | 41400 | 3.43e3 | 1 | 1 | 1480 | 1493 | Carbamidomethylation |  | DB Search |
| R.GKDGLQGIAGPR.G | Y | 53.95 | 1167.636 | 12 | 2.28 | 584.8253 | 2 | 13.08 | 0.885 | 0.8923 | 33537 | 5.42e2 | 1 | 1 | 1073 | 1084 |  |  | DB Search |
| R.GLSGVNGLRGETGDR.G | Y | 53.51 | 1486.7488 | 15 | 1.6 | 744.3812 | 2 | 13.43 | 1.0508 | 1.058 | 35633 | 9.09e2 | 1 | 1 | 629 | 643 |  |  | DB Search |
| total 6 peptides |  |  |  |  |  |  |  |  |  |  |  |  |  |  |  |  |  |  |  |
| --- | --- | --- | --- | --- | --- | --- | --- | --- | --- | --- | --- | --- | --- | --- | --- | --- | --- | --- | --- |

Best Unique PSM (Scan Sample\_2\_A1\_1\_3640.d:35227, m/z=600.7836, z=2, RT=13.37, ppm=1.8):


TRINITY\_DN319\_c2\_g1\_i3.p1

back to list

  

| Protein Coverage
| Supporting Peptides
| Best Unique PSM
|

Protein Coverage:

Supporting Peptides:

| Peptide | Uniq | -10lgP | Mass | Length | ppm | m/z | z | RT | 1/k0 Start | 1/k0 End | Precursor ID | Area Sample 4 | #Feature | #Feature Sample 4 | Start | End | PTM | AScore | Found By |
| --- | --- | --- | --- | --- | --- | --- | --- | --- | --- | --- | --- | --- | --- | --- | --- | --- | --- | --- | --- |
| R.DPIC(+57.02)HC(+57.02)DTSK.Y | Y | 58.38 | 1231.4961 | 10 | 7 | 411.508 | 3 | 9.95 | 0.7355 | 0.7428 | 14664 | 1.19e4 | 2 | 2 | 1448 | 1457 | Carbamidomethylation, Carbamidomethylation |  | DB Search |
| R.GSFSSHIC(+57.02)LC(+57.02)HDPAYK.S | Y | 58.38 | 1877.8189 | 16 | 0.93 | 626.9461 | 3 | 14.52 | 0.8854 | 0.8927 | 42298 | 8.32e3 | 1 | 1 | 1011 | 1026 | Carbamidomethylation, Carbamidomethylation |  | DB Search |
| K.LLHC(+57.02)SDSEVDHVEVC(+57.02)GK.G | Y | 58.36 | 1982.8826 | 17 | 6.36 | 661.9709 | 3 | 13.31 | 0.9247 | 0.9319 | 34838 | 1.49e4 | 4 | 4 | 845 | 861 | Carbamidomethylation, Carbamidomethylation |  | DB Search |
| R.RTC(+57.02)ADTSPAIDFK.S | Y | 58.29 | 1480.698 | 13 | 1.84 | 741.356 | 2 | 14.27 | 1.017 | 1.0242 | 40672 | 1.48e3 | 1 | 1 | 274 | 286 | Carbamidomethylation |  | DB Search |
| K.C(+57.02)MLATC(+57.02)EAGQLDPVAVC(+57.02)GK.G | Y | 58.23 | 2078.9257 | 19 | -13.35 | 1040.454 | 2 | 17.81 | 1.1985 | 1.2057 | 63229 | 0 | 0 | 0 | 1612 | 1630 | Carbamidomethylation, Carbamidomethylation, Carbamidomethylation |  | DB Search |
| K.M(+15.99)QC(+57.02)VPAK.C | Y | 58.14 | 848.3884 | 7 | -1.44 | 425.2 | 2 | 9.64 | 0.8254 | 0.8327 | 13010 | 5.89e2 | 1 | 1 | 2271 | 2277 | Oxidation (M), Carbamidomethylation | M1:Oxidation (M):1000 | DB Search |
| R.ADC(+57.02)GVENTC(+57.02)FGSTR.S | Y | 57.59 | 1572.6297 | 14 | 1.45 | 787.3215 | 2 | 13.67 | 1.0053 | 1.0126 | 37114 | 4.6e3 | 1 | 1 | 1578 | 1591 | Carbamidomethylation, Carbamidomethylation |  | DB Search |
| R.TC(+57.02)ADTSPAIDFK.S | Y | 57.18 | 1324.5969 | 12 | -1.21 | 663.3035 | 2 | 15.19 | 0.9469 | 0.9541 | 46506 | 1.08e3 | 1 | 1 | 275 | 286 | Carbamidomethylation |  | DB Search |
| K.NLEC(+57.02)QC(+57.02)DESK.G | Y | 57.02 | 1281.4965 | 10 | 0.49 | 641.7545 | 2 | 10.48 | 0.9012 | 0.9085 | 17668 | 4.3e3 | 1 | 1 | 777 | 786 | Carbamidomethylation, Carbamidomethylation |  | DB Search |
| K.EC(+57.02)SEVC(+57.02)GVNGVAEVTR.F | Y | 56.61 | 1764.7771 | 16 | -2.02 | 883.3921 | 2 | 14.69 | 1.0705 | 1.0777 | 43395 | 0 | 0 | 0 | 2380 | 2395 | Carbamidomethylation, Carbamidomethylation |  | DB Search |
| R.SWIC(+57.02)DC(+57.02)NSYGSILNPSDVKK.C | Y | 56.06 | 2342.0671 | 20 | 3.26 | 781.6971 | 3 | 18.19 | 1.0077 | 1.015 | 65664 | 5.83e2 | 1 | 1 | 1592 | 1611 | Carbamidomethylation, Carbamidomethylation |  | DB Search |
| R.DVQC(+57.02)TC(+57.02)NNELGYVHSPDGK.K | Y | 53.44 | 2191.9262 | 19 | 2.69 | 731.6497 | 3 | 14.19 | 0.9634 | 0.9707 | 40373 | 5.07e2 | 1 | 1 | 1641 | 1659 | Carbamidomethylation, Carbamidomethylation |  | DB Search |
| K.AQYYEAYETIIKR.H | Y | 52.57 | 1646.8304 | 13 | 5.31 | 549.9525 | 3 | 16.99 | 0.8364 | 0.8437 | 57968 | 0 | 0 | 0 | 2535 | 2547 |  |  | DB Search |
| R.TC(+57.02)IEASC(+57.02)RR.N | Y | 47.82 | 1151.5176 | 9 | 3.72 | 384.847 | 3 | 10.13 | 0.7061 | 0.7135 | 15528 | 5.07e3 | 2 | 2 | 1564 | 1572 | Carbamidomethylation, Carbamidomethylation |  | DB Search |
| total 14 peptides |  |  |  |  |  |  |  |  |  |  |  |  |  |  |  |  |  |  |  |
| --- | --- | --- | --- | --- | --- | --- | --- | --- | --- | --- | --- | --- | --- | --- | --- | --- | --- | --- | --- |

Best Unique PSM (Scan Sample\_2\_A1\_1\_3640.d:14664, m/z=411.508, z=3, RT=9.95, ppm=4.81):


TRINITY\_DN11595\_c0\_g1\_i2.p1

back to list

  

| Protein Coverage
| Supporting Peptides
| Best Unique PSM
|

Protein Coverage:

Supporting Peptides:

| Peptide | Uniq | -10lgP | Mass | Length | ppm | m/z | z | RT | 1/k0 Start | 1/k0 End | Precursor ID | Area Sample 4 | #Feature | #Feature Sample 4 | Start | End | PTM | AScore | Found By |
| --- | --- | --- | --- | --- | --- | --- | --- | --- | --- | --- | --- | --- | --- | --- | --- | --- | --- | --- | --- |
| K.LVHGDVFRPPR.Y | Y | 58.34 | 1291.7149 | 11 | 1.79 | 431.5787 | 3 | 13.55 | 0.823 | 0.8303 | 36380 | 1.06e3 | 1 | 1 | 301 | 311 |  |  | DB Search |
| total 1 peptides |  |  |  |  |  |  |  |  |  |  |  |  |  |  |  |  |  |  |  |
| --- | --- | --- | --- | --- | --- | --- | --- | --- | --- | --- | --- | --- | --- | --- | --- | --- | --- | --- | --- |

Best Unique PSM (Scan Sample\_2\_A1\_1\_3640.d:36380, m/z=431.5787, z=3, RT=13.55, ppm=-0.4):


TRINITY\_DN149493\_c0\_g1\_i1.p1

back to list

  

| Protein Coverage
| Supporting Peptides
| Best Unique PSM
|

Protein Coverage:

Supporting Peptides:

| Peptide | Uniq | -10lgP | Mass | Length | ppm | m/z | z | RT | 1/k0 Start | 1/k0 End | Precursor ID | Area Sample 4 | #Feature | #Feature Sample 4 | Start | End | PTM | AScore | Found By |
| --- | --- | --- | --- | --- | --- | --- | --- | --- | --- | --- | --- | --- | --- | --- | --- | --- | --- | --- | --- |
| R.GTVNRLPGDALSIGFPSK.D | Y | 58.38 | 1827.9842 | 18 | 3.55 | 610.3362 | 3 | 18.02 | 0.9052 | 0.9125 | 64542 | 1.66e2 | 1 | 1 | 68 | 85 |  |  | DB Search |
| R.LPGDALSIGFPSK.D | Y | 58.32 | 1300.7027 | 13 | 0.56 | 651.3575 | 2 | 18.65 | 0.9517 | 0.959 | 68777 | 1.5e3 | 1 | 1 | 73 | 85 |  |  | DB Search |
| total 2 peptides |  |  |  |  |  |  |  |  |  |  |  |  |  |  |  |  |  |  |  |
| --- | --- | --- | --- | --- | --- | --- | --- | --- | --- | --- | --- | --- | --- | --- | --- | --- | --- | --- | --- |

Best Unique PSM (Scan Sample\_2\_A1\_1\_3640.d:64542, m/z=610.3362, z=3, RT=18.02, ppm=1.35):


TRINITY\_DN577\_c0\_g1\_i1.p1

back to list

  

| Protein Coverage
| Supporting Peptides
| Best Unique PSM
|

Protein Coverage:

Supporting Peptides:

| Peptide | Uniq | -10lgP | Mass | Length | ppm | m/z | z | RT | 1/k0 Start | 1/k0 End | Precursor ID | Area Sample 4 | #Feature | #Feature Sample 4 | Start | End | PTM | AScore | Found By |
| --- | --- | --- | --- | --- | --- | --- | --- | --- | --- | --- | --- | --- | --- | --- | --- | --- | --- | --- | --- |
| R.LFAIGFTK.K | Y | 53.35 | 895.5167 | 8 | -4.7 | 448.7625 | 2 | 18.76 | 0.7844 | 0.7917 | 69557 | 2.62e2 | 1 | 1 | 143 | 150 |  |  | DB Search |
| total 1 peptides |  |  |  |  |  |  |  |  |  |  |  |  |  |  |  |  |  |  |  |
| --- | --- | --- | --- | --- | --- | --- | --- | --- | --- | --- | --- | --- | --- | --- | --- | --- | --- | --- | --- |

Best Unique PSM (Scan Sample\_2\_A1\_1\_3640.d:69557, m/z=448.7625, z=2, RT=18.76, ppm=-6.9):


TRINITY\_DN5500\_c0\_g1\_i2.p1

back to list

  

| Protein Coverage
| Supporting Peptides
| Best Unique PSM
|

Protein Coverage:

Supporting Peptides:

| Peptide | Uniq | -10lgP | Mass | Length | ppm | m/z | z | RT | 1/k0 Start | 1/k0 End | Precursor ID | Area Sample 4 | #Feature | #Feature Sample 4 | Start | End | PTM | AScore | Found By |
| --- | --- | --- | --- | --- | --- | --- | --- | --- | --- | --- | --- | --- | --- | --- | --- | --- | --- | --- | --- |
| K.VLVILTDGIQAR.W | Y | 58.26 | 1296.7765 | 12 | 4.33 | 649.3969 | 2 | 18.70 | 0.9723 | 0.9795 | 69087 | 3.67e2 | 1 | 1 | 137 | 148 |  |  | DB Search |
| K.IYIDGVFIR.S | Y | 58.26 | 1094.6124 | 9 | -0.14 | 548.3122 | 2 | 18.85 | 0.885 | 0.8923 | 70109 | 4.57e3 | 1 | 1 | 938 | 946 |  |  | DB Search |
| R.DFDSLPYYPR.G | Y | 58.18 | 1271.5822 | 10 | 1.54 | 636.798 | 2 | 18.28 | 0.9222 | 0.9295 | 66470 | 3.66e2 | 1 | 1 | 500 | 509 |  |  | DB Search |
| total 3 peptides |  |  |  |  |  |  |  |  |  |  |  |  |  |  |  |  |  |  |  |
| --- | --- | --- | --- | --- | --- | --- | --- | --- | --- | --- | --- | --- | --- | --- | --- | --- | --- | --- | --- |

Best Unique PSM (Scan Sample\_2\_A1\_1\_3640.d:69087, m/z=649.3969, z=2, RT=18.70, ppm=2.14):


TRINITY\_DN687\_c0\_g1\_i1.p1

back to list

  

| Protein Coverage
| Supporting Peptides
| Best Unique PSM
|

Protein Coverage:

Supporting Peptides:

| Peptide | Uniq | -10lgP | Mass | Length | ppm | m/z | z | RT | 1/k0 Start | 1/k0 End | Precursor ID | Area Sample 4 | #Feature | #Feature Sample 4 | Start | End | PTM | AScore | Found By |
| --- | --- | --- | --- | --- | --- | --- | --- | --- | --- | --- | --- | --- | --- | --- | --- | --- | --- | --- | --- |
| K.VGINYQPPTVVPGGDLAK.V | Y | 58.29 | 1823.9781 | 18 | -4.28 | 912.9904 | 2 | 17.44 | 1.1142 | 1.1214 | 60745 | 0 | 0 | 0 | 353 | 370 |  |  | DB Search |
| total 1 peptides |  |  |  |  |  |  |  |  |  |  |  |  |  |  |  |  |  |  |  |
| --- | --- | --- | --- | --- | --- | --- | --- | --- | --- | --- | --- | --- | --- | --- | --- | --- | --- | --- | --- |

Best Unique PSM (Scan Sample\_2\_A1\_1\_3640.d:60745, m/z=912.9904, z=2, RT=17.44, ppm=-6.48):


TRINITY\_DN6920\_c0\_g1\_i3.p1

back to list

  

| Protein Coverage
| Supporting Peptides
| Best Unique PSM
|

Protein Coverage:

Supporting Peptides:

| Peptide | Uniq | -10lgP | Mass | Length | ppm | m/z | z | RT | 1/k0 Start | 1/k0 End | Precursor ID | Area Sample 4 | #Feature | #Feature Sample 4 | Start | End | PTM | AScore | Found By |
| --- | --- | --- | --- | --- | --- | --- | --- | --- | --- | --- | --- | --- | --- | --- | --- | --- | --- | --- | --- |
| R.VLVHTQQK.L | Y | 58.19 | 951.5501 | 8 | 0.78 | 476.7817 | 2 | 9.51 | 0.8335 | 0.8408 | 12449 | 5.52e2 | 1 | 1 | 169 | 176 |  |  | DB Search |
| R.KVAC(+57.02)IGAWHPAR.V | Y | 52.34 | 1364.7135 | 12 | 0.83 | 455.9112 | 3 | 13.48 | 0.7766 | 0.784 | 36007 | 9.51e1 | 1 | 1 | 257 | 268 | Carbamidomethylation |  | DB Search |
| total 2 peptides |  |  |  |  |  |  |  |  |  |  |  |  |  |  |  |  |  |  |  |
| --- | --- | --- | --- | --- | --- | --- | --- | --- | --- | --- | --- | --- | --- | --- | --- | --- | --- | --- | --- |

Best Unique PSM (Scan Sample\_2\_A1\_1\_3640.d:12449, m/z=476.7817, z=2, RT=9.51, ppm=-1.41):


TRINITY\_DN1783\_c0\_g1\_i3.p1

back to list

  

| Protein Coverage
| Supporting Peptides
| Best Unique PSM
|

Protein Coverage:

Supporting Peptides:

| Peptide | Uniq | -10lgP | Mass | Length | ppm | m/z | z | RT | 1/k0 Start | 1/k0 End | Precursor ID | Area Sample 4 | #Feature | #Feature Sample 4 | Start | End | PTM | AScore | Found By |
| --- | --- | --- | --- | --- | --- | --- | --- | --- | --- | --- | --- | --- | --- | --- | --- | --- | --- | --- | --- |
| K.LTIIDALPR.C | Y | 58.24 | 1010.6124 | 9 | 0.02 | 506.3124 | 2 | 19.00 | 0.8392 | 0.8465 | 71132 | 3.79e2 | 1 | 1 | 225 | 233 |  |  | DB Search |
| total 1 peptides |  |  |  |  |  |  |  |  |  |  |  |  |  |  |  |  |  |  |  |
| --- | --- | --- | --- | --- | --- | --- | --- | --- | --- | --- | --- | --- | --- | --- | --- | --- | --- | --- | --- |

Best Unique PSM (Scan Sample\_2\_A1\_1\_3640.d:71132, m/z=506.3124, z=2, RT=19.00, ppm=-2.17):


TRINITY\_DN2885\_c0\_g1\_i2.p1

back to list

  

| Protein Coverage
| Supporting Peptides
| Best Unique PSM
|

Protein Coverage:

Supporting Peptides:

| Peptide | Uniq | -10lgP | Mass | Length | ppm | m/z | z | RT | 1/k0 Start | 1/k0 End | Precursor ID | Area Sample 4 | #Feature | #Feature Sample 4 | Start | End | PTM | AScore | Found By |
| --- | --- | --- | --- | --- | --- | --- | --- | --- | --- | --- | --- | --- | --- | --- | --- | --- | --- | --- | --- |
| K.VTRPHGASGVVR.A | Y | 57.82 | 1234.6894 | 12 | 7.66 | 412.5727 | 3 | 10.07 | 0.8108 | 0.8181 | 15251 | 2.57e2 | 1 | 1 | 77 | 88 |  |  | DB Search |
| total 1 peptides |  |  |  |  |  |  |  |  |  |  |  |  |  |  |  |  |  |  |  |
| --- | --- | --- | --- | --- | --- | --- | --- | --- | --- | --- | --- | --- | --- | --- | --- | --- | --- | --- | --- |

Best Unique PSM (Scan Sample\_2\_A1\_1\_3640.d:15251, m/z=412.5727, z=3, RT=10.07, ppm=5.46):


TRINITY\_DN61742\_c0\_g1\_i1.p1

back to list

  

| Protein Coverage
| Supporting Peptides
| Best Unique PSM
|

Protein Coverage:

Supporting Peptides:

| Peptide | Uniq | -10lgP | Mass | Length | ppm | m/z | z | RT | 1/k0 Start | 1/k0 End | Precursor ID | Area Sample 4 | #Feature | #Feature Sample 4 | Start | End | PTM | AScore | Found By |
| --- | --- | --- | --- | --- | --- | --- | --- | --- | --- | --- | --- | --- | --- | --- | --- | --- | --- | --- | --- |
| K.LYISSLPLK.V | Y | 58.05 | 1032.6219 | 9 | 2.33 | 517.3183 | 2 | 18.15 | 0.8587 | 0.866 | 65559 | 0 | 0 | 0 | 78 | 86 |  |  | DB Search |
| K.ASILLNELAK.Y | Y | 56.95 | 1070.6335 | 10 | -3.29 | 536.3211 | 2 | 18.12 | 0.8729 | 0.8802 | 65288 | 0 | 0 | 0 | 210 | 219 |  |  | DB Search |
| total 2 peptides |  |  |  |  |  |  |  |  |  |  |  |  |  |  |  |  |  |  |  |
| --- | --- | --- | --- | --- | --- | --- | --- | --- | --- | --- | --- | --- | --- | --- | --- | --- | --- | --- | --- |

Best Unique PSM (Scan Sample\_2\_A1\_1\_3640.d:65559, m/z=517.3183, z=2, RT=18.15, ppm=0.13):


TRINITY\_DN101355\_c0\_g1\_i1.p1

back to list

  

| Protein Coverage
| Supporting Peptides
| Best Unique PSM
|

Protein Coverage:

Supporting Peptides:

| Peptide | Uniq | -10lgP | Mass | Length | ppm | m/z | z | RT | 1/k0 Start | 1/k0 End | Precursor ID | Area Sample 4 | #Feature | #Feature Sample 4 | Start | End | PTM | AScore | Found By |
| --- | --- | --- | --- | --- | --- | --- | --- | --- | --- | --- | --- | --- | --- | --- | --- | --- | --- | --- | --- |
| R.VANDLSETIVWK.L | Y | 58.37 | 1373.719 | 12 | 2.31 | 687.8669 | 2 | 18.08 | 0.9711 | 0.9783 | 64974 | 2.42e2 | 1 | 1 | 127 | 138 |  |  | DB Search |
| total 1 peptides |  |  |  |  |  |  |  |  |  |  |  |  |  |  |  |  |  |  |  |
| --- | --- | --- | --- | --- | --- | --- | --- | --- | --- | --- | --- | --- | --- | --- | --- | --- | --- | --- | --- |

Best Unique PSM (Scan Sample\_2\_A1\_1\_3640.d:64974, m/z=687.8669, z=2, RT=18.08, ppm=0.12):


TRINITY\_DN3655\_c1\_g1\_i1.p1

back to list

  

| Protein Coverage
| Supporting Peptides
| Best Unique PSM
|

Protein Coverage:

Supporting Peptides:

| Peptide | Uniq | -10lgP | Mass | Length | ppm | m/z | z | RT | 1/k0 Start | 1/k0 End | Precursor ID | Area Sample 4 | #Feature | #Feature Sample 4 | Start | End | PTM | AScore | Found By |
| --- | --- | --- | --- | --- | --- | --- | --- | --- | --- | --- | --- | --- | --- | --- | --- | --- | --- | --- | --- |
| K.YQIVIEAIR.G | Y | 57.58 | 1103.6339 | 9 | -0 | 552.823 | 2 | 17.92 | 0.8789 | 0.8862 | 63893 | 1.03e3 | 1 | 1 | 39 | 47 |  |  | DB Search |
| total 1 peptides |  |  |  |  |  |  |  |  |  |  |  |  |  |  |  |  |  |  |  |
| --- | --- | --- | --- | --- | --- | --- | --- | --- | --- | --- | --- | --- | --- | --- | --- | --- | --- | --- | --- |

Best Unique PSM (Scan Sample\_2\_A1\_1\_3640.d:63893, m/z=552.823, z=2, RT=17.92, ppm=-2.2):


TRINITY\_DN2113\_c0\_g1\_i1.p1

back to list

  

| Protein Coverage
| Supporting Peptides
| Best Unique PSM
|

Protein Coverage:

Supporting Peptides:

| Peptide | Uniq | -10lgP | Mass | Length | ppm | m/z | z | RT | 1/k0 Start | 1/k0 End | Precursor ID | Area Sample 4 | #Feature | #Feature Sample 4 | Start | End | PTM | AScore | Found By |
| --- | --- | --- | --- | --- | --- | --- | --- | --- | --- | --- | --- | --- | --- | --- | --- | --- | --- | --- | --- |
| R.LASTLVHLGEYQNAVDGAR.K | Y | 52.16 | 2013.0279 | 19 | 5.59 | 672.0189 | 3 | 16.99 | 0.9525 | 0.9598 | 57891 | 0 | 0 | 0 | 1227 | 1245 |  |  | DB Search |
| total 1 peptides |  |  |  |  |  |  |  |  |  |  |  |  |  |  |  |  |  |  |  |
| --- | --- | --- | --- | --- | --- | --- | --- | --- | --- | --- | --- | --- | --- | --- | --- | --- | --- | --- | --- |

Best Unique PSM (Scan Sample\_2\_A1\_1\_3640.d:57891, m/z=672.0189, z=3, RT=16.99, ppm=3.4):


TRINITY\_DN5365\_c0\_g1\_i3.p1

back to list

  

| Protein Coverage
| Supporting Peptides
| Best Unique PSM
|

Protein Coverage:

Supporting Peptides:

| Peptide | Uniq | -10lgP | Mass | Length | ppm | m/z | z | RT | 1/k0 Start | 1/k0 End | Precursor ID | Area Sample 4 | #Feature | #Feature Sample 4 | Start | End | PTM | AScore | Found By |
| --- | --- | --- | --- | --- | --- | --- | --- | --- | --- | --- | --- | --- | --- | --- | --- | --- | --- | --- | --- |
| R.FPGQLNADIRK.L | Y | 57.99 | 1257.6829 | 11 | -7.77 | 420.2307 | 3 | 14.50 | 0.7033 | 0.7106 | 42199 | 0 | 0 | 0 | 244 | 254 |  |  | DB Search |
| total 1 peptides |  |  |  |  |  |  |  |  |  |  |  |  |  |  |  |  |  |  |  |
| --- | --- | --- | --- | --- | --- | --- | --- | --- | --- | --- | --- | --- | --- | --- | --- | --- | --- | --- | --- |

Best Unique PSM (Scan Sample\_2\_A1\_1\_3640.d:42199, m/z=420.2307, z=3, RT=14.50, ppm=-9.97):


TRINITY\_DN5\_c0\_g1\_i10.p2

back to list

  

| Protein Coverage
| Supporting Peptides
| Best Unique PSM
|

Protein Coverage:

Supporting Peptides:

| Peptide | Uniq | -10lgP | Mass | Length | ppm | m/z | z | RT | 1/k0 Start | 1/k0 End | Precursor ID | Area Sample 4 | #Feature | #Feature Sample 4 | Start | End | PTM | AScore | Found By |
| --- | --- | --- | --- | --- | --- | --- | --- | --- | --- | --- | --- | --- | --- | --- | --- | --- | --- | --- | --- |
| K.IIIELQGDLIK.I | Y | 53.27 | 1253.7594 | 11 | 3.89 | 627.8881 | 2 | 19.42 | 0.9513 | 0.9586 | 73842 | 0 | 0 | 0 | 65 | 75 |  |  | DB Search |
| total 1 peptides |  |  |  |  |  |  |  |  |  |  |  |  |  |  |  |  |  |  |  |
| --- | --- | --- | --- | --- | --- | --- | --- | --- | --- | --- | --- | --- | --- | --- | --- | --- | --- | --- | --- |

Best Unique PSM (Scan Sample\_2\_A1\_1\_3640.d:73842, m/z=627.8881, z=2, RT=19.42, ppm=1.69):


TRINITY\_DN8941\_c0\_g1\_i2.p1

back to list

  

| Protein Coverage
| Supporting Peptides
| Best Unique PSM
|

Protein Coverage:

Supporting Peptides:

| Peptide | Uniq | -10lgP | Mass | Length | ppm | m/z | z | RT | 1/k0 Start | 1/k0 End | Precursor ID | Area Sample 4 | #Feature | #Feature Sample 4 | Start | End | PTM | AScore | Found By |
| --- | --- | --- | --- | --- | --- | --- | --- | --- | --- | --- | --- | --- | --- | --- | --- | --- | --- | --- | --- |
| K.AVPAPILDIPK.R | Y | 58.37 | 1132.6855 | 11 | 0.34 | 567.349 | 2 | 18.31 | 0.8955 | 0.9028 | 66444 | 1.41e3 | 1 | 1 | 464 | 474 |  |  | DB Search |
| total 1 peptides |  |  |  |  |  |  |  |  |  |  |  |  |  |  |  |  |  |  |  |
| --- | --- | --- | --- | --- | --- | --- | --- | --- | --- | --- | --- | --- | --- | --- | --- | --- | --- | --- | --- |

Best Unique PSM (Scan Sample\_2\_A1\_1\_3640.d:66444, m/z=567.349, z=2, RT=18.31, ppm=-1.86):


TRINITY\_DN191650\_c0\_g1\_i1.p1

back to list

  

| Protein Coverage
| Supporting Peptides
| Best Unique PSM
|

Protein Coverage:

Supporting Peptides:

| Peptide | Uniq | -10lgP | Mass | Length | ppm | m/z | z | RT | 1/k0 Start | 1/k0 End | Precursor ID | Area Sample 4 | #Feature | #Feature Sample 4 | Start | End | PTM | AScore | Found By |
| --- | --- | --- | --- | --- | --- | --- | --- | --- | --- | --- | --- | --- | --- | --- | --- | --- | --- | --- | --- |
| K.IDHAVNLVQQELK.C | Y | 54.81 | 1505.8202 | 13 | 5.36 | 753.9197 | 2 | 15.83 | 1.0379 | 1.0452 | 50504 | 5.31e2 | 1 | 1 | 134 | 146 |  |  | DB Search |
| total 1 peptides |  |  |  |  |  |  |  |  |  |  |  |  |  |  |  |  |  |  |  |
| --- | --- | --- | --- | --- | --- | --- | --- | --- | --- | --- | --- | --- | --- | --- | --- | --- | --- | --- | --- |

Best Unique PSM (Scan Sample\_2\_A1\_1\_3640.d:50504, m/z=753.9197, z=2, RT=15.83, ppm=3.17):


TRINITY\_DN3332\_c0\_g1\_i1.p1

back to list

  

| Protein Coverage
| Supporting Peptides
| Best Unique PSM
|

Protein Coverage:

Supporting Peptides:

| Peptide | Uniq | -10lgP | Mass | Length | ppm | m/z | z | RT | 1/k0 Start | 1/k0 End | Precursor ID | Area Sample 4 | #Feature | #Feature Sample 4 | Start | End | PTM | AScore | Found By |
| --- | --- | --- | --- | --- | --- | --- | --- | --- | --- | --- | --- | --- | --- | --- | --- | --- | --- | --- | --- |
| K.YLLNALNR.C | Y | 38.38 | 975.5501 | 8 | 2.88 | 488.7827 | 2 | 16.96 | 0.8461 | 0.8534 | 57693 | 3.06e2 | 1 | 1 | 62 | 69 |  |  | DB Search |
| total 1 peptides |  |  |  |  |  |  |  |  |  |  |  |  |  |  |  |  |  |  |  |
| --- | --- | --- | --- | --- | --- | --- | --- | --- | --- | --- | --- | --- | --- | --- | --- | --- | --- | --- | --- |

Best Unique PSM (Scan Sample\_2\_A1\_1\_3640.d:57693, m/z=488.7827, z=2, RT=16.96, ppm=0.68):


TRINITY\_DN329\_c0\_g1\_i1.p1

back to list

  

| Protein Coverage
| Supporting Peptides
| Best Unique PSM
|

Protein Coverage:

Supporting Peptides:

| Peptide | Uniq | -10lgP | Mass | Length | ppm | m/z | z | RT | 1/k0 Start | 1/k0 End | Precursor ID | Area Sample 4 | #Feature | #Feature Sample 4 | Start | End | PTM | AScore | Found By |
| --- | --- | --- | --- | --- | --- | --- | --- | --- | --- | --- | --- | --- | --- | --- | --- | --- | --- | --- | --- |
| K.GLKVDFDTTFAPVTGKK.T | Y | 57.88 | 1822.9828 | 17 | -3.05 | 608.665 | 3 | 17.39 | 0.8984 | 0.9057 | 60425 | 2.02e2 | 1 | 1 | 93 | 109 |  |  | DB Search |
| K.LRPGVELTM(+15.99)SSLVNAK.S | Y | 54.81 | 1729.9396 | 16 | -2.37 | 577.6512 | 3 | 15.40 | 0.8749 | 0.8822 | 47976 | 0 | 0 | 0 | 250 | 265 | Oxidation (M) | M9:Oxidation (M):1000 | DB Search |
| total 2 peptides |  |  |  |  |  |  |  |  |  |  |  |  |  |  |  |  |  |  |  |
| --- | --- | --- | --- | --- | --- | --- | --- | --- | --- | --- | --- | --- | --- | --- | --- | --- | --- | --- | --- |

Best Unique PSM (Scan Sample\_2\_A1\_1\_3640.d:60425, m/z=608.665, z=3, RT=17.39, ppm=-5.24):


TRINITY\_DN33328\_c0\_g1\_i1.p1

back to list

  

| Protein Coverage
| Supporting Peptides
| Best Unique PSM
|

Protein Coverage:

Supporting Peptides:

| Peptide | Uniq | -10lgP | Mass | Length | ppm | m/z | z | RT | 1/k0 Start | 1/k0 End | Precursor ID | Area Sample 4 | #Feature | #Feature Sample 4 | Start | End | PTM | AScore | Found By |
| --- | --- | --- | --- | --- | --- | --- | --- | --- | --- | --- | --- | --- | --- | --- | --- | --- | --- | --- | --- |
| K.VPDLLFSK.V | Y | 58.26 | 917.5222 | 8 | 0.47 | 459.7676 | 2 | 18.14 | 0.7884 | 0.7958 | 65377 | 0 | 0 | 0 | 491 | 498 |  |  | DB Search |
| total 1 peptides |  |  |  |  |  |  |  |  |  |  |  |  |  |  |  |  |  |  |  |
| --- | --- | --- | --- | --- | --- | --- | --- | --- | --- | --- | --- | --- | --- | --- | --- | --- | --- | --- | --- |

Best Unique PSM (Scan Sample\_2\_A1\_1\_3640.d:65377, m/z=459.7676, z=2, RT=18.14, ppm=-1.73):


TRINITY\_DN1559\_c0\_g1\_i1.p1

back to list

  

| Protein Coverage
| Supporting Peptides
| Best Unique PSM
|

Protein Coverage:

Supporting Peptides:

| Peptide | Uniq | -10lgP | Mass | Length | ppm | m/z | z | RT | 1/k0 Start | 1/k0 End | Precursor ID | Area Sample 4 | #Feature | #Feature Sample 4 | Start | End | PTM | AScore | Found By |
| --- | --- | --- | --- | --- | --- | --- | --- | --- | --- | --- | --- | --- | --- | --- | --- | --- | --- | --- | --- |
| R.GVAGAGVLAGFDKFK.E | Y | 57.94 | 1435.7823 | 15 | 1.38 | 479.601 | 3 | 18.49 | 0.8453 | 0.8526 | 67586 | 2.66e3 | 2 | 2 | 282 | 296 |  |  | DB Search |
| R.GVAGAGVLAGFDK.F | Y | 55.31 | 1160.6189 | 13 | -7.63 | 581.311 | 2 | 17.62 | 0.8883 | 0.8955 | 61949 | 0 | 0 | 0 | 282 | 294 |  |  | DB Search |
| K.TEGLAPFWR.G | Y | 54.51 | 1075.545 | 9 | -1.01 | 538.7781 | 2 | 19.20 | 0.853 | 0.8603 | 72310 | 1.12e3 | 1 | 1 | 69 | 77 |  |  | DB Search |
| K.LLVQNQDEM(+15.99)IK.Q | Y | 51.85 | 1345.6911 | 11 | -10.5 | 673.8443 | 2 | 13.94 | 0.9735 | 0.9808 | 38862 | 0 | 0 | 0 | 38 | 48 | Oxidation (M) | M9:Oxidation (M):1000 | DB Search |
| total 4 peptides |  |  |  |  |  |  |  |  |  |  |  |  |  |  |  |  |  |  |  |
| --- | --- | --- | --- | --- | --- | --- | --- | --- | --- | --- | --- | --- | --- | --- | --- | --- | --- | --- | --- |

Best Unique PSM (Scan Sample\_2\_A1\_1\_3640.d:67586, m/z=479.601, z=3, RT=18.49, ppm=-0.81):


TRINITY\_DN250\_c0\_g1\_i6.p1

back to list

  

| Protein Coverage
| Supporting Peptides
| Best Unique PSM
|

Protein Coverage:

Supporting Peptides:

| Peptide | Uniq | -10lgP | Mass | Length | ppm | m/z | z | RT | 1/k0 Start | 1/k0 End | Precursor ID | Area Sample 4 | #Feature | #Feature Sample 4 | Start | End | PTM | AScore | Found By |
| --- | --- | --- | --- | --- | --- | --- | --- | --- | --- | --- | --- | --- | --- | --- | --- | --- | --- | --- | --- |
| K.VGINYQPPTVVPGGDLAK.V | Y | 58.29 | 1823.9781 | 18 | -4.28 | 912.9904 | 2 | 17.44 | 1.1142 | 1.1214 | 60745 | 0 | 0 | 0 | 353 | 370 |  |  | DB Search |
| total 1 peptides |  |  |  |  |  |  |  |  |  |  |  |  |  |  |  |  |  |  |  |
| --- | --- | --- | --- | --- | --- | --- | --- | --- | --- | --- | --- | --- | --- | --- | --- | --- | --- | --- | --- |

Best Unique PSM (Scan Sample\_2\_A1\_1\_3640.d:60745, m/z=912.9904, z=2, RT=17.44, ppm=-6.48):


TRINITY\_DN2963\_c0\_g1\_i1.p1

back to list

  

| Protein Coverage
| Supporting Peptides
| Best Unique PSM
|

Protein Coverage:

Supporting Peptides:

| Peptide | Uniq | -10lgP | Mass | Length | ppm | m/z | z | RT | 1/k0 Start | 1/k0 End | Precursor ID | Area Sample 4 | #Feature | #Feature Sample 4 | Start | End | PTM | AScore | Found By |
| --- | --- | --- | --- | --- | --- | --- | --- | --- | --- | --- | --- | --- | --- | --- | --- | --- | --- | --- | --- |
| M.S(+42.01)ATGNLVTVLR.K | Y | 58.28 | 1171.6561 | 11 | 0.35 | 586.8342 | 2 | 18.95 | 0.9453 | 0.9525 | 70826 | 3.39e3 | 1 | 1 | 2 | 12 | Acetylation (Protein N-term) | S1:Acetylation (Protein N-term):1000 | DB Search |
| M.S(+42.01)ATGNLVTVLRK.L | Y | 54.57 | 1299.751 | 12 | -5.84 | 650.8776 | 2 | 17.30 | 0.9924 | 0.9997 | 59899 | 0 | 0 | 0 | 2 | 13 | Acetylation (Protein N-term) | S1:Acetylation (Protein N-term):1000 | DB Search |
| total 2 peptides |  |  |  |  |  |  |  |  |  |  |  |  |  |  |  |  |  |  |  |
| --- | --- | --- | --- | --- | --- | --- | --- | --- | --- | --- | --- | --- | --- | --- | --- | --- | --- | --- | --- |

Best Unique PSM (Scan Sample\_2\_A1\_1\_3640.d:70826, m/z=586.8342, z=2, RT=18.95, ppm=-1.84):


TRINITY\_DN1071\_c0\_g1\_i1.p1

back to list

  

| Protein Coverage
| Supporting Peptides
| Best Unique PSM
|

Protein Coverage:

Supporting Peptides:

| Peptide | Uniq | -10lgP | Mass | Length | ppm | m/z | z | RT | 1/k0 Start | 1/k0 End | Precursor ID | Area Sample 4 | #Feature | #Feature Sample 4 | Start | End | PTM | AScore | Found By |
| --- | --- | --- | --- | --- | --- | --- | --- | --- | --- | --- | --- | --- | --- | --- | --- | --- | --- | --- | --- |
| K.AVQIHPFNDVSVGDPK.V | Y | 58.41 | 1721.8736 | 16 | 11.97 | 574.9708 | 3 | 15.99 | 0.8761 | 0.8834 | 51505 | 2.3e4 | 2 | 2 | 293 | 308 |  |  | DB Search |
| R.VGSADKC(+57.02)C(+57.02)EC(+57.02)VC(+57.02)EEC(+57.02)TVK.S | Y | 58.32 | 2189.852 | 18 | 1.96 | 730.9578 | 3 | 12.05 | 0.8777 | 0.885 | 27079 | 1.94e3 | 1 | 1 | 540 | 557 | Carbamidomethylation, Carbamidomethylation, Carbamidomethylation, Carbamidomethylation, Carbamidomethylation |  | DB Search |
| K.SQDDC(+57.02)ETNEEYVK.A | Y | 53.15 | 1615.6308 | 13 | 5.63 | 808.8254 | 2 | 12.08 | 1.0636 | 1.0709 | 27312 | 4.96e2 | 1 | 1 | 558 | 570 | Carbamidomethylation |  | DB Search |
| total 3 peptides |  |  |  |  |  |  |  |  |  |  |  |  |  |  |  |  |  |  |  |
| --- | --- | --- | --- | --- | --- | --- | --- | --- | --- | --- | --- | --- | --- | --- | --- | --- | --- | --- | --- |

Best Unique PSM (Scan Sample\_2\_A1\_1\_3640.d:51505, m/z=574.9708, z=3, RT=15.99, ppm=9.77):


TRINITY\_DN8597\_c0\_g1\_i1.p1

back to list

  

| Protein Coverage
| Supporting Peptides
| Best Unique PSM
|

Protein Coverage:

Supporting Peptides:

| Peptide | Uniq | -10lgP | Mass | Length | ppm | m/z | z | RT | 1/k0 Start | 1/k0 End | Precursor ID | Area Sample 4 | #Feature | #Feature Sample 4 | Start | End | PTM | AScore | Found By |
| --- | --- | --- | --- | --- | --- | --- | --- | --- | --- | --- | --- | --- | --- | --- | --- | --- | --- | --- | --- |
| R.DYQINEEVVIR.V | Y | 58.31 | 1376.6936 | 11 | -3.21 | 689.3503 | 2 | 16.87 | 0.9755 | 0.9828 | 57134 | 0 | 0 | 0 | 78 | 88 |  |  | DB Search |
| R.AFNKVETR.N | N | 57 | 963.5138 | 8 | 4.36 | 482.7652 | 2 | 10.61 | 0.8274 | 0.8348 | 18412 | 3.24e3 | 1 | 1 | 15 | 22 |  |  | DB Search |
| total 2 peptides |  |  |  |  |  |  |  |  |  |  |  |  |  |  |  |  |  |  |  |
| --- | --- | --- | --- | --- | --- | --- | --- | --- | --- | --- | --- | --- | --- | --- | --- | --- | --- | --- | --- |

Best Unique PSM (Scan Sample\_2\_A1\_1\_3640.d:57134, m/z=689.3503, z=2, RT=16.87, ppm=-5.4):


TRINITY\_DN761\_c0\_g1\_i2.p1

back to list

  

| Protein Coverage
| Supporting Peptides
| Best Unique PSM
|

Protein Coverage:

Supporting Peptides:

| Peptide | Uniq | -10lgP | Mass | Length | ppm | m/z | z | RT | 1/k0 Start | 1/k0 End | Precursor ID | Area Sample 4 | #Feature | #Feature Sample 4 | Start | End | PTM | AScore | Found By |
| --- | --- | --- | --- | --- | --- | --- | --- | --- | --- | --- | --- | --- | --- | --- | --- | --- | --- | --- | --- |
| K.TRIEDLETVISQTQDHR.H | Y | 58.33 | 2040.0236 | 17 | 1.3 | 681.0145 | 3 | 17.58 | 0.9473 | 0.9545 | 61608 | 1.62e3 | 1 | 1 | 260 | 276 |  |  | DB Search |
| R.IEDLETVISQTQDHR.H | Y | 58.29 | 1782.8748 | 15 | -2.39 | 595.2961 | 3 | 17.95 | 0.8963 | 0.9036 | 64199 | 0 | 0 | 0 | 262 | 276 |  |  | DB Search |
| R.ATLYPC(+57.02)PETPNER.R | Y | 58.12 | 1546.7086 | 13 | 5.6 | 774.3642 | 2 | 14.56 | 1.0359 | 1.0431 | 42458 | 8e2 | 1 | 1 | 239 | 251 | Carbamidomethylation |  | DB Search |
| total 3 peptides |  |  |  |  |  |  |  |  |  |  |  |  |  |  |  |  |  |  |  |
| --- | --- | --- | --- | --- | --- | --- | --- | --- | --- | --- | --- | --- | --- | --- | --- | --- | --- | --- | --- |

Best Unique PSM (Scan Sample\_2\_A1\_1\_3640.d:61608, m/z=681.0145, z=3, RT=17.58, ppm=-0.89):


TRINITY\_DN1936\_c0\_g1\_i1.p1

back to list

  

| Protein Coverage
| Supporting Peptides
| Best Unique PSM
|

Protein Coverage:

Supporting Peptides:

| Peptide | Uniq | -10lgP | Mass | Length | ppm | m/z | z | RT | 1/k0 Start | 1/k0 End | Precursor ID | Area Sample 4 | #Feature | #Feature Sample 4 | Start | End | PTM | AScore | Found By |
| --- | --- | --- | --- | --- | --- | --- | --- | --- | --- | --- | --- | --- | --- | --- | --- | --- | --- | --- | --- |
| R.LEFVFK.Y | Y | 57 | 781.4374 | 6 | 2.76 | 391.7262 | 2 | 18.35 | 0.7286 | 0.7359 | 66730 | 1.18e3 | 1 | 1 | 694 | 699 |  |  | DB Search |
| total 1 peptides |  |  |  |  |  |  |  |  |  |  |  |  |  |  |  |  |  |  |  |
| --- | --- | --- | --- | --- | --- | --- | --- | --- | --- | --- | --- | --- | --- | --- | --- | --- | --- | --- | --- |

Best Unique PSM (Scan Sample\_2\_A1\_1\_3640.d:66730, m/z=391.7262, z=2, RT=18.35, ppm=0.57):


TRINITY\_DN1676\_c0\_g1\_i6.p1

back to list

  

| Protein Coverage
| Supporting Peptides
| Best Unique PSM
|

Protein Coverage:

Supporting Peptides:

| Peptide | Uniq | -10lgP | Mass | Length | ppm | m/z | z | RT | 1/k0 Start | 1/k0 End | Precursor ID | Area Sample 4 | #Feature | #Feature Sample 4 | Start | End | PTM | AScore | Found By |
| --- | --- | --- | --- | --- | --- | --- | --- | --- | --- | --- | --- | --- | --- | --- | --- | --- | --- | --- | --- |
| K.LLQQHELDLTPLK.E | Y | 58.4 | 1546.8719 | 13 | -0.89 | 516.6296 | 3 | 16.98 | 0.8356 | 0.8429 | 57805 | 1.52e3 | 2 | 2 | 1589 | 1601 |  |  | DB Search |
| R.SGGDTSPFGEC(+57.02)KK.C | Y | 58.4 | 1368.598 | 13 | 8.57 | 457.2095 | 3 | 11.91 | 0.7559 | 0.7632 | 26366 | 1.73e3 | 1 | 1 | 717 | 729 | Carbamidomethylation |  | DB Search |
| K.GC(+57.02)ELC(+57.02)NC(+57.02)NATGSK.G | Y | 58.38 | 1469.5697 | 13 | -3.08 | 735.7883 | 2 | 11.24 | 0.9711 | 0.9783 | 22299 | 0 | 0 | 0 | 943 | 955 | Carbamidomethylation, Carbamidomethylation, Carbamidomethylation |  | DB Search |
| K.ITQLQNDDKEAR.E | Y | 58.36 | 1429.7161 | 12 | 4.64 | 477.5805 | 3 | 10.94 | 0.8055 | 0.8128 | 20429 | 1.37e3 | 1 | 1 | 1329 | 1340 |  |  | DB Search |
| R.GQYISVKPPR.N | Y | 58.35 | 1143.64 | 10 | -2.31 | 572.8247 | 2 | 13.47 | 0.9121 | 0.9194 | 36000 | 0 | 0 | 0 | 550 | 559 |  |  | DB Search |
| R.MSEIAEEARK.H | Y | 58.34 | 1162.5652 | 10 | 1.11 | 582.2892 | 2 | 11.43 | 0.9218 | 0.9291 | 23472 | 3.63e2 | 1 | 1 | 1193 | 1202 |  |  | DB Search |
| R.C(+57.02)FDLIQVAVNK.L | Y | 58.29 | 1305.6751 | 11 | -2.9 | 653.8415 | 2 | 19.16 | 0.9582 | 0.9654 | 71943 | 1.33e3 | 1 | 1 | 1044 | 1054 | Carbamidomethylation |  | DB Search |
| R.FELDRVNTFGDEVFGDPK.V | Y | 58.29 | 2083.985 | 18 | 0.34 | 695.6677 | 3 | 19.31 | 0.9182 | 0.9255 | 73205 | 0 | 0 | 0 | 252 | 269 |  |  | DB Search |
| K.GTM(+15.99)KPPFHDPK.L | Y | 58.28 | 1269.6176 | 11 | 0.53 | 635.815 | 2 | 10.78 | 0.9574 | 0.9646 | 19136 | 1.46e3 | 1 | 1 | 896 | 906 | Oxidation (M) | M3:Oxidation (M):1000 | DB Search |
| R.VNTFGDEVFGDPK.V | Y | 58.28 | 1423.6619 | 13 | -3.52 | 712.8342 | 2 | 17.48 | 1.0118 | 1.019 | 60886 | 1.49e3 | 1 | 1 | 257 | 269 |  |  | DB Search |
| K.TLMEFENNIAK.S | Y | 58.28 | 1308.6384 | 11 | 4.09 | 655.3277 | 2 | 17.39 | 0.9634 | 0.9707 | 60532 | 5.46e3 | 1 | 1 | 1387 | 1397 |  |  | DB Search |
| K.KSEGAAETGSVTISK.A | Y | 58.14 | 1463.7467 | 15 | 0.58 | 732.8795 | 2 | 11.50 | 0.9844 | 0.9916 | 23786 | 1.99e3 | 1 | 1 | 1135 | 1149 |  |  | DB Search |
| K.LKNEIEIMESK.L | Y | 57.61 | 1332.6959 | 11 | 0.56 | 667.3541 | 2 | 15.12 | 0.967 | 0.9743 | 46046 | 8e2 | 1 | 1 | 1572 | 1582 |  |  | DB Search |
| K.DKITQLQNDDKEAR.E | Y | 56.95 | 1672.838 | 14 | 4.92 | 558.6215 | 3 | 11.86 | 0.8416 | 0.849 | 25955 | 4.36e2 | 1 | 1 | 1327 | 1340 |  |  | DB Search |
| K.EC(+57.02)FC(+57.02)FGHGVSC(+57.02)DAIEGIGVK.E | Y | 54.91 | 2240.9653 | 20 | 3.38 | 747.9966 | 3 | 18.10 | 0.9763 | 0.9836 | 65084 | 1.23e3 | 1 | 1 | 498 | 517 | Carbamidomethylation, Carbamidomethylation, Carbamidomethylation |  | DB Search |
| K.RFEEAKDTIENK.L | Y | 54.88 | 1478.7365 | 12 | 3.61 | 493.9201 | 3 | 11.68 | 0.8031 | 0.8104 | 24961 | 1.52e3 | 1 | 1 | 1560 | 1571 |  |  | DB Search |
| K.GSDC(+57.02)NLQTGQC(+57.02)EC(+57.02)QDGVGGR.K | Y | 53.87 | 2196.8583 | 20 | -5.84 | 733.2875 | 3 | 12.26 | 0.8733 | 0.8806 | 28552 | 0 | 0 | 0 | 956 | 975 | Carbamidomethylation, Carbamidomethylation, Carbamidomethylation |  | DB Search |
| K.RMSEIAEEARK.H | Y | 47.21 | 1318.6663 | 11 | 5.81 | 440.5643 | 3 | 11.07 | 0.7628 | 0.7701 | 21258 | 4.45e2 | 1 | 1 | 1192 | 1202 |  |  | DB Search |
| K.TTQDAMNEIESSRR.E | Y | 40.35 | 1636.7475 | 14 | -1.03 | 546.588 | 3 | 13.88 | 0.8153 | 0.8226 | 38475 | 7.82e2 | 1 | 1 | 1450 | 1463 |  |  | DB Search |
| total 19 peptides |  |  |  |  |  |  |  |  |  |  |  |  |  |  |  |  |  |  |  |
| --- | --- | --- | --- | --- | --- | --- | --- | --- | --- | --- | --- | --- | --- | --- | --- | --- | --- | --- | --- |

Best Unique PSM (Scan Sample\_2\_A1\_1\_3640.d:57805, m/z=516.6296, z=3, RT=16.98, ppm=-3.08):


TRINITY\_DN77\_c0\_g2\_i1.p1

back to list

  

| Protein Coverage
| Supporting Peptides
| Best Unique PSM
|

Protein Coverage:

Supporting Peptides:

| Peptide | Uniq | -10lgP | Mass | Length | ppm | m/z | z | RT | 1/k0 Start | 1/k0 End | Precursor ID | Area Sample 4 | #Feature | #Feature Sample 4 | Start | End | PTM | AScore | Found By |
| --- | --- | --- | --- | --- | --- | --- | --- | --- | --- | --- | --- | --- | --- | --- | --- | --- | --- | --- | --- |
| R.GPVGTSGLR.G | Y | 58.19 | 842.461 | 9 | 3.7 | 422.2384 | 2 | 12.14 | 0.7787 | 0.786 | 27733 | 1.47e4 | 1 | 1 | 830 | 838 |  |  | DB Search |
| R.GPAGAPGDEGQTGMR.G | Y | 57.23 | 1399.615 | 15 | -0.7 | 700.8127 | 2 | 11.55 | 0.9396 | 0.9469 | 24084 | 1.21e3 | 1 | 1 | 626 | 640 |  |  | DB Search |
| R.GPQGQVGK.Q | Y | 55.44 | 769.4082 | 8 | 6.49 | 385.713 | 2 | 8.99 | 0.7069 | 0.7143 | 10347 | 1.81e3 | 1 | 1 | 473 | 480 |  |  | DB Search |
| R.GPAGAPGDEGQTGM(+15.99)RGER.G | Y | 54.26 | 1757.7751 | 18 | 2.12 | 586.9323 | 3 | 10.62 | 0.8818 | 0.8891 | 18471 | 3.52e3 | 1 | 1 | 626 | 643 | Oxidation (M) | M14:Oxidation (M):1000 | DB Search |
| R.GPAGAPGDEGQTGM(+15.99)R.G | Y | 54.2 | 1415.6099 | 15 | 4.13 | 708.8136 | 2 | 10.40 | 0.9453 | 0.9525 | 17281 | 0 | 0 | 0 | 626 | 640 | Oxidation (M) | M14:Oxidation (M):1000 | DB Search |
| total 5 peptides |  |  |  |  |  |  |  |  |  |  |  |  |  |  |  |  |  |  |  |
| --- | --- | --- | --- | --- | --- | --- | --- | --- | --- | --- | --- | --- | --- | --- | --- | --- | --- | --- | --- |

Best Unique PSM (Scan Sample\_2\_A1\_1\_3640.d:27733, m/z=422.2384, z=2, RT=12.14, ppm=1.5):


TRINITY\_DN144508\_c0\_g1\_i1.p1

back to list

  

| Protein Coverage
| Supporting Peptides
| Best Unique PSM
|

Protein Coverage:

Supporting Peptides:

| Peptide | Uniq | -10lgP | Mass | Length | ppm | m/z | z | RT | 1/k0 Start | 1/k0 End | Precursor ID | Area Sample 4 | #Feature | #Feature Sample 4 | Start | End | PTM | AScore | Found By |
| --- | --- | --- | --- | --- | --- | --- | --- | --- | --- | --- | --- | --- | --- | --- | --- | --- | --- | --- | --- |
| K.SEEYKPTLGR.C | Y | 58.28 | 1178.5931 | 10 | 1.77 | 590.3036 | 2 | 12.35 | 0.9073 | 0.9146 | 29036 | 2.36e2 | 1 | 1 | 174 | 183 |  |  | DB Search |
| K.SSLNHNIVFEGR.T | Y | 58.2 | 1371.6895 | 12 | 1.43 | 458.2368 | 3 | 14.67 | 0.7583 | 0.7656 | 43307 | 0 | 0 | 0 | 233 | 244 |  |  | DB Search |
| total 2 peptides |  |  |  |  |  |  |  |  |  |  |  |  |  |  |  |  |  |  |  |
| --- | --- | --- | --- | --- | --- | --- | --- | --- | --- | --- | --- | --- | --- | --- | --- | --- | --- | --- | --- |

Best Unique PSM (Scan Sample\_2\_A1\_1\_3640.d:29036, m/z=590.3036, z=2, RT=12.35, ppm=-0.43):


TRINITY\_DN909\_c0\_g1\_i1.p1

back to list

  

| Protein Coverage
| Supporting Peptides
| Best Unique PSM
|

Protein Coverage:

Supporting Peptides:

| Peptide | Uniq | -10lgP | Mass | Length | ppm | m/z | z | RT | 1/k0 Start | 1/k0 End | Precursor ID | Area Sample 4 | #Feature | #Feature Sample 4 | Start | End | PTM | AScore | Found By |
| --- | --- | --- | --- | --- | --- | --- | --- | --- | --- | --- | --- | --- | --- | --- | --- | --- | --- | --- | --- |
| R.VISFDVPPQEILTR.D | Y | 54.85 | 1612.8824 | 14 | 0.83 | 807.4474 | 2 | 19.60 | 1.0588 | 1.066 | 74865 | 6.05e2 | 1 | 1 | 128 | 141 |  |  | DB Search |
| total 1 peptides |  |  |  |  |  |  |  |  |  |  |  |  |  |  |  |  |  |  |  |
| --- | --- | --- | --- | --- | --- | --- | --- | --- | --- | --- | --- | --- | --- | --- | --- | --- | --- | --- | --- |

Best Unique PSM (Scan Sample\_2\_A1\_1\_3640.d:74865, m/z=807.4474, z=2, RT=19.60, ppm=-1.36):


TRINITY\_DN195631\_c0\_g1\_i1.p2

back to list

  

| Protein Coverage
| Supporting Peptides
| Best Unique PSM
|

Protein Coverage:

Supporting Peptides:

| Peptide | Uniq | -10lgP | Mass | Length | ppm | m/z | z | RT | 1/k0 Start | 1/k0 End | Precursor ID | Area Sample 4 | #Feature | #Feature Sample 4 | Start | End | PTM | AScore | Found By |
| --- | --- | --- | --- | --- | --- | --- | --- | --- | --- | --- | --- | --- | --- | --- | --- | --- | --- | --- | --- |
| K.IQDKEGIPPDQQR.L | Y | 55.01 | 1522.7739 | 13 | 0.25 | 762.3928 | 2 | 11.08 | 1.0343 | 1.0415 | 21018 | 1.78e3 | 2 | 2 | 122 | 134 |  |  | DB Search |
| total 1 peptides |  |  |  |  |  |  |  |  |  |  |  |  |  |  |  |  |  |  |  |
| --- | --- | --- | --- | --- | --- | --- | --- | --- | --- | --- | --- | --- | --- | --- | --- | --- | --- | --- | --- |

Best Unique PSM (Scan Sample\_2\_A1\_1\_3640.d:21018, m/z=762.3928, z=2, RT=11.08, ppm=-1.95):


TRINITY\_DN20671\_c0\_g1\_i1.p1

back to list

  

| Protein Coverage
| Supporting Peptides
| Best Unique PSM
|

Protein Coverage:

Supporting Peptides:

| Peptide | Uniq | -10lgP | Mass | Length | ppm | m/z | z | RT | 1/k0 Start | 1/k0 End | Precursor ID | Area Sample 4 | #Feature | #Feature Sample 4 | Start | End | PTM | AScore | Found By |
| --- | --- | --- | --- | --- | --- | --- | --- | --- | --- | --- | --- | --- | --- | --- | --- | --- | --- | --- | --- |
| K.DKFFGIQVVHHLR.Q | Y | 56.39 | 1594.8732 | 13 | -2.09 | 532.6294 | 3 | 16.30 | 0.8546 | 0.8619 | 53474 | 0 | 0 | 0 | 56 | 68 |  |  | DB Search |
| total 1 peptides |  |  |  |  |  |  |  |  |  |  |  |  |  |  |  |  |  |  |  |
| --- | --- | --- | --- | --- | --- | --- | --- | --- | --- | --- | --- | --- | --- | --- | --- | --- | --- | --- | --- |

Best Unique PSM (Scan Sample\_2\_A1\_1\_3640.d:53474, m/z=532.6294, z=3, RT=16.30, ppm=-4.28):


TRINITY\_DN1774\_c0\_g1\_i1.p1

back to list

  

| Protein Coverage
| Supporting Peptides
| Best Unique PSM
|

Protein Coverage:

Supporting Peptides:

| Peptide | Uniq | -10lgP | Mass | Length | ppm | m/z | z | RT | 1/k0 Start | 1/k0 End | Precursor ID | Area Sample 4 | #Feature | #Feature Sample 4 | Start | End | PTM | AScore | Found By |
| --- | --- | --- | --- | --- | --- | --- | --- | --- | --- | --- | --- | --- | --- | --- | --- | --- | --- | --- | --- |
| M.GISIGFEEIR.N | Y | 58.18 | 1119.5924 | 10 | 2.84 | 560.8038 | 2 | 18.57 | 0.8959 | 0.9032 | 68197 | 2.85e3 | 1 | 1 | 2 | 11 |  |  | DB Search |
| total 1 peptides |  |  |  |  |  |  |  |  |  |  |  |  |  |  |  |  |  |  |  |
| --- | --- | --- | --- | --- | --- | --- | --- | --- | --- | --- | --- | --- | --- | --- | --- | --- | --- | --- | --- |

Best Unique PSM (Scan Sample\_2\_A1\_1\_3640.d:68197, m/z=560.8038, z=2, RT=18.57, ppm=0.65):


TRINITY\_DN191980\_c0\_g1\_i1.p1

back to list

  

| Protein Coverage
| Supporting Peptides
| Best Unique PSM
|

Protein Coverage:

Supporting Peptides:

| Peptide | Uniq | -10lgP | Mass | Length | ppm | m/z | z | RT | 1/k0 Start | 1/k0 End | Precursor ID | Area Sample 4 | #Feature | #Feature Sample 4 | Start | End | PTM | AScore | Found By |
| --- | --- | --- | --- | --- | --- | --- | --- | --- | --- | --- | --- | --- | --- | --- | --- | --- | --- | --- | --- |
| K.ILLLGAGESGK.T | Y | 43.84 | 1056.6179 | 11 | 6.84 | 529.3187 | 2 | 17.27 | 0.8761 | 0.8834 | 59698 | 0 | 0 | 0 | 38 | 48 |  |  | DB Search |
| total 1 peptides |  |  |  |  |  |  |  |  |  |  |  |  |  |  |  |  |  |  |  |
| --- | --- | --- | --- | --- | --- | --- | --- | --- | --- | --- | --- | --- | --- | --- | --- | --- | --- | --- | --- |

Best Unique PSM (Scan Sample\_2\_A1\_1\_3640.d:59698, m/z=529.3187, z=2, RT=17.27, ppm=4.65):


TRINITY\_DN4781\_c0\_g1\_i3.p3

back to list

  

| Protein Coverage
| Supporting Peptides
| Best Unique PSM
|

Protein Coverage:

Supporting Peptides:

| Peptide | Uniq | -10lgP | Mass | Length | ppm | m/z | z | RT | 1/k0 Start | 1/k0 End | Precursor ID | Area Sample 4 | #Feature | #Feature Sample 4 | Start | End | PTM | AScore | Found By |
| --- | --- | --- | --- | --- | --- | --- | --- | --- | --- | --- | --- | --- | --- | --- | --- | --- | --- | --- | --- |
| R.DNAGTSSSGTGPR.F | Y | 58.39 | 1205.5272 | 13 | 4.49 | 603.7723 | 2 | 9.17 | 0.8538 | 0.8611 | 10872 | 1.6e3 | 2 | 2 | 126 | 138 |  |  | DB Search |
| K.SGEYKPTLGR.C | Y | 58.28 | 1106.572 | 10 | 4.62 | 554.2946 | 2 | 11.99 | 0.8988 | 0.9061 | 26709 | 9.46e2 | 1 | 1 | 169 | 178 |  |  | DB Search |
| total 2 peptides |  |  |  |  |  |  |  |  |  |  |  |  |  |  |  |  |  |  |  |
| --- | --- | --- | --- | --- | --- | --- | --- | --- | --- | --- | --- | --- | --- | --- | --- | --- | --- | --- | --- |

Best Unique PSM (Scan Sample\_2\_A1\_1\_3640.d:10872, m/z=603.7723, z=2, RT=9.17, ppm=2.29):


TRINITY\_DN16533\_c0\_g2\_i1.p1

back to list

  

| Protein Coverage
| Supporting Peptides
| Best Unique PSM
|

Protein Coverage:

Supporting Peptides:

| Peptide | Uniq | -10lgP | Mass | Length | ppm | m/z | z | RT | 1/k0 Start | 1/k0 End | Precursor ID | Area Sample 4 | #Feature | #Feature Sample 4 | Start | End | PTM | AScore | Found By |
| --- | --- | --- | --- | --- | --- | --- | --- | --- | --- | --- | --- | --- | --- | --- | --- | --- | --- | --- | --- |
| R.TC(+57.02)FYPIIGDK.I | Y | 57.66 | 1212.5849 | 10 | -2.99 | 607.2966 | 2 | 18.26 | 0.9 | 0.9073 | 66111 | 5.95e2 | 1 | 1 | 217 | 226 | Carbamidomethylation |  | DB Search |
| total 1 peptides |  |  |  |  |  |  |  |  |  |  |  |  |  |  |  |  |  |  |  |
| --- | --- | --- | --- | --- | --- | --- | --- | --- | --- | --- | --- | --- | --- | --- | --- | --- | --- | --- | --- |

Best Unique PSM (Scan Sample\_2\_A1\_1\_3640.d:66111, m/z=607.2966, z=2, RT=18.26, ppm=-5.18):


TRINITY\_DN61668\_c0\_g1\_i1.p1

back to list

  

| Protein Coverage
| Supporting Peptides
| Best Unique PSM
|

Protein Coverage:

Supporting Peptides:

| Peptide | Uniq | -10lgP | Mass | Length | ppm | m/z | z | RT | 1/k0 Start | 1/k0 End | Precursor ID | Area Sample 4 | #Feature | #Feature Sample 4 | Start | End | PTM | AScore | Found By |
| --- | --- | --- | --- | --- | --- | --- | --- | --- | --- | --- | --- | --- | --- | --- | --- | --- | --- | --- | --- |
| K.YVFAVAVDHTDLK.A | Y | 58.39 | 1476.7612 | 13 | 1.84 | 739.3876 | 2 | 17.08 | 1.0568 | 1.064 | 58449 | 5.53e2 | 2 | 2 | 220 | 232 |  |  | DB Search |
| K.YVFAVAVDHTDLKADK.L | Y | 58.39 | 1790.9202 | 16 | 4.17 | 597.9819 | 3 | 16.00 | 0.8984 | 0.9057 | 51609 | 0 | 0 | 0 | 220 | 235 |  |  | DB Search |
| K.GYAVSFIDDKFVQK.A | Y | 58.29 | 1615.8246 | 14 | 3.47 | 539.6161 | 3 | 17.87 | 0.8035 | 0.8108 | 63558 | 1.55e3 | 2 | 2 | 185 | 198 |  |  | DB Search |
| R.LFWDAPDVSLSK.V | Y | 58.27 | 1376.6976 | 12 | -3.64 | 689.3521 | 2 | 19.88 | 0.9683 | 0.9755 | 76730 | 1.12e3 | 1 | 1 | 171 | 182 |  |  | DB Search |
| R.KYVFAVAVDHTDLK.A | Y | 53.73 | 1604.8562 | 14 | -1.1 | 535.9576 | 3 | 15.88 | 0.8676 | 0.8749 | 50838 | 0 | 0 | 0 | 219 | 232 |  |  | DB Search |
| total 5 peptides |  |  |  |  |  |  |  |  |  |  |  |  |  |  |  |  |  |  |  |
| --- | --- | --- | --- | --- | --- | --- | --- | --- | --- | --- | --- | --- | --- | --- | --- | --- | --- | --- | --- |

Best Unique PSM (Scan Sample\_2\_A1\_1\_3640.d:58449, m/z=739.3876, z=2, RT=17.08, ppm=-0.35):


TRINITY\_DN12734\_c0\_g1\_i4.p1

back to list

  

| Protein Coverage
| Supporting Peptides
| Best Unique PSM
|

Protein Coverage:

Supporting Peptides:

| Peptide | Uniq | -10lgP | Mass | Length | ppm | m/z | z | RT | 1/k0 Start | 1/k0 End | Precursor ID | Area Sample 4 | #Feature | #Feature Sample 4 | Start | End | PTM | AScore | Found By |
| --- | --- | --- | --- | --- | --- | --- | --- | --- | --- | --- | --- | --- | --- | --- | --- | --- | --- | --- | --- |
| K.LLLLGAGESGK.S | Y | 43.84 | 1056.6179 | 11 | 6.84 | 529.3187 | 2 | 17.27 | 0.8761 | 0.8834 | 59698 | 0 | 0 | 0 | 46 | 56 |  |  | DB Search |
| total 1 peptides |  |  |  |  |  |  |  |  |  |  |  |  |  |  |  |  |  |  |  |
| --- | --- | --- | --- | --- | --- | --- | --- | --- | --- | --- | --- | --- | --- | --- | --- | --- | --- | --- | --- |

Best Unique PSM (Scan Sample\_2\_A1\_1\_3640.d:59698, m/z=529.3187, z=2, RT=17.27, ppm=4.65):


TRINITY\_DN143676\_c0\_g1\_i1.p1

back to list

  

| Protein Coverage
| Supporting Peptides
| Best Unique PSM
|

Protein Coverage:

Supporting Peptides:

| Peptide | Uniq | -10lgP | Mass | Length | ppm | m/z | z | RT | 1/k0 Start | 1/k0 End | Precursor ID | Area Sample 4 | #Feature | #Feature Sample 4 | Start | End | PTM | AScore | Found By |
| --- | --- | --- | --- | --- | --- | --- | --- | --- | --- | --- | --- | --- | --- | --- | --- | --- | --- | --- | --- |
| K.ERLELFGK.L | Y | 54.89 | 990.5498 | 8 | -4.6 | 496.2788 | 2 | 16.06 | 0.8437 | 0.851 | 51911 | 7.08e2 | 1 | 1 | 53 | 60 |  |  | DB Search |
| total 1 peptides |  |  |  |  |  |  |  |  |  |  |  |  |  |  |  |  |  |  |  |
| --- | --- | --- | --- | --- | --- | --- | --- | --- | --- | --- | --- | --- | --- | --- | --- | --- | --- | --- | --- |

Best Unique PSM (Scan Sample\_2\_A1\_1\_3640.d:51911, m/z=496.2788, z=2, RT=16.06, ppm=-6.8):


TRINITY\_DN36\_c0\_g1\_i3.p1

back to list

  

| Protein Coverage
| Supporting Peptides
| Best Unique PSM
|

Protein Coverage:

Supporting Peptides:

| Peptide | Uniq | -10lgP | Mass | Length | ppm | m/z | z | RT | 1/k0 Start | 1/k0 End | Precursor ID | Area Sample 4 | #Feature | #Feature Sample 4 | Start | End | PTM | AScore | Found By |
| --- | --- | --- | --- | --- | --- | --- | --- | --- | --- | --- | --- | --- | --- | --- | --- | --- | --- | --- | --- |
| R.LGGIEVTQGNC(+57.02)AR.K | Y | 58.36 | 1373.6721 | 13 | -2.89 | 687.8398 | 2 | 13.93 | 0.9662 | 0.9735 | 38726 | 2.09e3 | 1 | 1 | 58 | 70 | Carbamidomethylation |  | DB Search |
| K.GLPLAIGVR.D | Y | 57.12 | 894.565 | 9 | 0.27 | 448.2889 | 2 | 17.73 | 0.8124 | 0.8197 | 62574 | 3.52e3 | 1 | 1 | 32 | 40 |  |  | DB Search |
| total 2 peptides |  |  |  |  |  |  |  |  |  |  |  |  |  |  |  |  |  |  |  |
| --- | --- | --- | --- | --- | --- | --- | --- | --- | --- | --- | --- | --- | --- | --- | --- | --- | --- | --- | --- |

Best Unique PSM (Scan Sample\_2\_A1\_1\_3640.d:38726, m/z=687.8398, z=2, RT=13.93, ppm=-5.09):


TRINITY\_DN1071\_c0\_g1\_i3.p1

back to list

  

| Protein Coverage
| Supporting Peptides
| Best Unique PSM
|

Protein Coverage:

Supporting Peptides:

| Peptide | Uniq | -10lgP | Mass | Length | ppm | m/z | z | RT | 1/k0 Start | 1/k0 End | Precursor ID | Area Sample 4 | #Feature | #Feature Sample 4 | Start | End | PTM | AScore | Found By |
| --- | --- | --- | --- | --- | --- | --- | --- | --- | --- | --- | --- | --- | --- | --- | --- | --- | --- | --- | --- |
| K.AVQIHPFNDVSVGDPK.V | Y | 58.41 | 1721.8736 | 16 | 11.97 | 574.9708 | 3 | 15.99 | 0.8761 | 0.8834 | 51505 | 2.3e4 | 2 | 2 | 329 | 344 |  |  | DB Search |
| total 1 peptides |  |  |  |  |  |  |  |  |  |  |  |  |  |  |  |  |  |  |  |
| --- | --- | --- | --- | --- | --- | --- | --- | --- | --- | --- | --- | --- | --- | --- | --- | --- | --- | --- | --- |

Best Unique PSM (Scan Sample\_2\_A1\_1\_3640.d:51505, m/z=574.9708, z=3, RT=15.99, ppm=9.77):


TRINITY\_DN195934\_c0\_g1\_i1.p2

back to list

  

| Protein Coverage
| Supporting Peptides
| Best Unique PSM
|

Protein Coverage:

Supporting Peptides:

| Peptide | Uniq | -10lgP | Mass | Length | ppm | m/z | z | RT | 1/k0 Start | 1/k0 End | Precursor ID | Area Sample 4 | #Feature | #Feature Sample 4 | Start | End | PTM | AScore | Found By |
| --- | --- | --- | --- | --- | --- | --- | --- | --- | --- | --- | --- | --- | --- | --- | --- | --- | --- | --- | --- |
| K.DSYVGDEAQSKR.G | Y | 57.28 | 1353.616 | 12 | 2.57 | 677.8156 | 2 | 11.01 | 0.9993 | 1.0065 | 20794 | 1.16e3 | 1 | 1 | 10 | 21 |  |  | DB Search |
| total 1 peptides |  |  |  |  |  |  |  |  |  |  |  |  |  |  |  |  |  |  |  |
| --- | --- | --- | --- | --- | --- | --- | --- | --- | --- | --- | --- | --- | --- | --- | --- | --- | --- | --- | --- |

Best Unique PSM (Scan Sample\_2\_A1\_1\_3640.d:20794, m/z=677.8156, z=2, RT=11.01, ppm=0.37):


TRINITY\_DN1821\_c0\_g1\_i3.p1

back to list

  

| Protein Coverage
| Supporting Peptides
| Best Unique PSM
|

Protein Coverage:

Supporting Peptides:

| Peptide | Uniq | -10lgP | Mass | Length | ppm | m/z | z | RT | 1/k0 Start | 1/k0 End | Precursor ID | Area Sample 4 | #Feature | #Feature Sample 4 | Start | End | PTM | AScore | Found By |
| --- | --- | --- | --- | --- | --- | --- | --- | --- | --- | --- | --- | --- | --- | --- | --- | --- | --- | --- | --- |
| K.VEDSKESDINFLLK.T | Y | 57.24 | 1635.8355 | 14 | 0.37 | 546.2848 | 3 | 17.72 | 0.8092 | 0.8165 | 62515 | 5.45e2 | 1 | 1 | 423 | 436 |  |  | DB Search |
| K.DKPALDVTSTLKK.K | Y | 54.52 | 1414.8031 | 13 | 2.31 | 472.6084 | 3 | 13.78 | 0.801 | 0.8084 | 37913 | 0 | 0 | 0 | 280 | 292 |  |  | DB Search |
| total 2 peptides |  |  |  |  |  |  |  |  |  |  |  |  |  |  |  |  |  |  |  |
| --- | --- | --- | --- | --- | --- | --- | --- | --- | --- | --- | --- | --- | --- | --- | --- | --- | --- | --- | --- |

Best Unique PSM (Scan Sample\_2\_A1\_1\_3640.d:62515, m/z=546.2848, z=3, RT=17.72, ppm=-1.83):


TRINITY\_DN66856\_c0\_g1\_i1.p1

back to list

  

| Protein Coverage
| Supporting Peptides
| Best Unique PSM
|

Protein Coverage:

Supporting Peptides:

| Peptide | Uniq | -10lgP | Mass | Length | ppm | m/z | z | RT | 1/k0 Start | 1/k0 End | Precursor ID | Area Sample 4 | #Feature | #Feature Sample 4 | Start | End | PTM | AScore | Found By |
| --- | --- | --- | --- | --- | --- | --- | --- | --- | --- | --- | --- | --- | --- | --- | --- | --- | --- | --- | --- |
| R.GSSYTGDIALDDFR.M | Y | 57.66 | 1515.6841 | 14 | -0.42 | 758.8474 | 2 | 18.14 | 1.0146 | 1.0218 | 65410 | 0 | 0 | 0 | 123 | 136 |  |  | DB Search |
| K.TGSYIFIETSNPR.K | Y | 56.93 | 1483.7307 | 13 | -18.37 | 742.8574 | 2 | 17.31 | 1.0295 | 1.0367 | 59591 | 1.28e4 | 1 | 1 | 385 | 397 |  |  | DB Search |
| total 2 peptides |  |  |  |  |  |  |  |  |  |  |  |  |  |  |  |  |  |  |  |
| --- | --- | --- | --- | --- | --- | --- | --- | --- | --- | --- | --- | --- | --- | --- | --- | --- | --- | --- | --- |

Best Unique PSM (Scan Sample\_2\_A1\_1\_3640.d:65410, m/z=758.8474, z=2, RT=18.14, ppm=-2.62):


TRINITY\_DN5554\_c0\_g1\_i1.p1

back to list

  

| Protein Coverage
| Supporting Peptides
| Best Unique PSM
|

Protein Coverage:

Supporting Peptides:

| Peptide | Uniq | -10lgP | Mass | Length | ppm | m/z | z | RT | 1/k0 Start | 1/k0 End | Precursor ID | Area Sample 4 | #Feature | #Feature Sample 4 | Start | End | PTM | AScore | Found By |
| --- | --- | --- | --- | --- | --- | --- | --- | --- | --- | --- | --- | --- | --- | --- | --- | --- | --- | --- | --- |
| K.VQENAVVPSILQK.F | Y | 58.36 | 1423.8034 | 13 | -2.87 | 712.9054 | 2 | 17.06 | 0.9989 | 1.0061 | 58423 | 5.55e2 | 1 | 1 | 167 | 179 |  |  | DB Search |
| total 1 peptides |  |  |  |  |  |  |  |  |  |  |  |  |  |  |  |  |  |  |  |
| --- | --- | --- | --- | --- | --- | --- | --- | --- | --- | --- | --- | --- | --- | --- | --- | --- | --- | --- | --- |

Best Unique PSM (Scan Sample\_2\_A1\_1\_3640.d:58423, m/z=712.9054, z=2, RT=17.06, ppm=-5.06):


TRINITY\_DN5\_c0\_g1\_i7.p2

back to list

  

| Protein Coverage
| Supporting Peptides
| Best Unique PSM
|

Protein Coverage:

Supporting Peptides:

| Peptide | Uniq | -10lgP | Mass | Length | ppm | m/z | z | RT | 1/k0 Start | 1/k0 End | Precursor ID | Area Sample 4 | #Feature | #Feature Sample 4 | Start | End | PTM | AScore | Found By |
| --- | --- | --- | --- | --- | --- | --- | --- | --- | --- | --- | --- | --- | --- | --- | --- | --- | --- | --- | --- |
| K.IIIELQGDLIK.I | Y | 53.27 | 1253.7594 | 11 | 3.89 | 627.8881 | 2 | 19.42 | 0.9513 | 0.9586 | 73842 | 0 | 0 | 0 | 239 | 249 |  |  | DB Search |
| total 1 peptides |  |  |  |  |  |  |  |  |  |  |  |  |  |  |  |  |  |  |  |
| --- | --- | --- | --- | --- | --- | --- | --- | --- | --- | --- | --- | --- | --- | --- | --- | --- | --- | --- | --- |

Best Unique PSM (Scan Sample\_2\_A1\_1\_3640.d:73842, m/z=627.8881, z=2, RT=19.42, ppm=1.69):


TRINITY\_DN36\_c0\_g1\_i14.p1

back to list

  

| Protein Coverage
| Supporting Peptides
| Best Unique PSM
|

Protein Coverage:

Supporting Peptides:

| Peptide | Uniq | -10lgP | Mass | Length | ppm | m/z | z | RT | 1/k0 Start | 1/k0 End | Precursor ID | Area Sample 4 | #Feature | #Feature Sample 4 | Start | End | PTM | AScore | Found By |
| --- | --- | --- | --- | --- | --- | --- | --- | --- | --- | --- | --- | --- | --- | --- | --- | --- | --- | --- | --- |
| R.LGGIEVTQGNC(+57.02)AR.K | Y | 58.36 | 1373.6721 | 13 | -2.89 | 687.8398 | 2 | 13.93 | 0.9662 | 0.9735 | 38726 | 2.09e3 | 1 | 1 | 58 | 70 | Carbamidomethylation |  | DB Search |
| K.GLPLAIGVR.D | Y | 57.12 | 894.565 | 9 | 0.27 | 448.2889 | 2 | 17.73 | 0.8124 | 0.8197 | 62574 | 3.52e3 | 1 | 1 | 32 | 40 |  |  | DB Search |
| total 2 peptides |  |  |  |  |  |  |  |  |  |  |  |  |  |  |  |  |  |  |  |
| --- | --- | --- | --- | --- | --- | --- | --- | --- | --- | --- | --- | --- | --- | --- | --- | --- | --- | --- | --- |

Best Unique PSM (Scan Sample\_2\_A1\_1\_3640.d:38726, m/z=687.8398, z=2, RT=13.93, ppm=-5.09):


TRINITY\_DN1071\_c0\_g1\_i4.p1

back to list

  

| Protein Coverage
| Supporting Peptides
| Best Unique PSM
|

Protein Coverage:

Supporting Peptides:

| Peptide | Uniq | -10lgP | Mass | Length | ppm | m/z | z | RT | 1/k0 Start | 1/k0 End | Precursor ID | Area Sample 4 | #Feature | #Feature Sample 4 | Start | End | PTM | AScore | Found By |
| --- | --- | --- | --- | --- | --- | --- | --- | --- | --- | --- | --- | --- | --- | --- | --- | --- | --- | --- | --- |
| K.AVQIHPFNDVSVGDPK.V | Y | 58.41 | 1721.8736 | 16 | 11.97 | 574.9708 | 3 | 15.99 | 0.8761 | 0.8834 | 51505 | 2.3e4 | 2 | 2 | 329 | 344 |  |  | DB Search |
| R.VGSADKC(+57.02)C(+57.02)EC(+57.02)VC(+57.02)EEC(+57.02)TVK.S | Y | 58.32 | 2189.852 | 18 | 1.96 | 730.9578 | 3 | 12.05 | 0.8777 | 0.885 | 27079 | 1.94e3 | 1 | 1 | 576 | 593 | Carbamidomethylation, Carbamidomethylation, Carbamidomethylation, Carbamidomethylation, Carbamidomethylation |  | DB Search |
| K.SQDDC(+57.02)ETNEEYVK.A | Y | 53.15 | 1615.6308 | 13 | 5.63 | 808.8254 | 2 | 12.08 | 1.0636 | 1.0709 | 27312 | 4.96e2 | 1 | 1 | 594 | 606 | Carbamidomethylation |  | DB Search |
| total 3 peptides |  |  |  |  |  |  |  |  |  |  |  |  |  |  |  |  |  |  |  |
| --- | --- | --- | --- | --- | --- | --- | --- | --- | --- | --- | --- | --- | --- | --- | --- | --- | --- | --- | --- |

Best Unique PSM (Scan Sample\_2\_A1\_1\_3640.d:51505, m/z=574.9708, z=3, RT=15.99, ppm=9.77):


TRINITY\_DN4513\_c0\_g1\_i3.p1

back to list

  

| Protein Coverage
| Supporting Peptides
| Best Unique PSM
|

Protein Coverage:

Supporting Peptides:

| Peptide | Uniq | -10lgP | Mass | Length | ppm | m/z | z | RT | 1/k0 Start | 1/k0 End | Precursor ID | Area Sample 4 | #Feature | #Feature Sample 4 | Start | End | PTM | AScore | Found By |
| --- | --- | --- | --- | --- | --- | --- | --- | --- | --- | --- | --- | --- | --- | --- | --- | --- | --- | --- | --- |
| K.LKTDLEAQKK.E | Y | 57.72 | 1172.6765 | 10 | 3.8 | 391.9001 | 3 | 10.36 | 0.7408 | 0.7481 | 16865 | 9.07e2 | 1 | 1 | 9 | 18 |  |  | DB Search |
| total 1 peptides |  |  |  |  |  |  |  |  |  |  |  |  |  |  |  |  |  |  |  |
| --- | --- | --- | --- | --- | --- | --- | --- | --- | --- | --- | --- | --- | --- | --- | --- | --- | --- | --- | --- |

Best Unique PSM (Scan Sample\_2\_A1\_1\_3640.d:16865, m/z=391.9001, z=3, RT=10.36, ppm=1.6):


TRINITY\_DN1535\_c0\_g1\_i1.p1

back to list

  

| Protein Coverage
| Supporting Peptides
| Best Unique PSM
|

Protein Coverage:

Supporting Peptides:

| Peptide | Uniq | -10lgP | Mass | Length | ppm | m/z | z | RT | 1/k0 Start | 1/k0 End | Precursor ID | Area Sample 4 | #Feature | #Feature Sample 4 | Start | End | PTM | AScore | Found By |
| --- | --- | --- | --- | --- | --- | --- | --- | --- | --- | --- | --- | --- | --- | --- | --- | --- | --- | --- | --- |
| R.GSC(+57.02)EC(+57.02)GTC(+57.02)VC(+57.02)R.E | Y | 56.93 | 1344.4679 | 11 | -2.11 | 673.2383 | 2 | 10.52 | 0.9291 | 0.9364 | 17995 | 0 | 0 | 0 | 558 | 568 | Carbamidomethylation, Carbamidomethylation, Carbamidomethylation, Carbamidomethylation |  | DB Search |
| K.LKDNAVIPLFAVTK.N | Y | 55.28 | 1527.9024 | 14 | -4.47 | 510.3047 | 3 | 19.12 | 0.8226 | 0.8299 | 71843 | 1.2e3 | 2 | 2 | 306 | 319 |  |  | DB Search |
| total 2 peptides |  |  |  |  |  |  |  |  |  |  |  |  |  |  |  |  |  |  |  |
| --- | --- | --- | --- | --- | --- | --- | --- | --- | --- | --- | --- | --- | --- | --- | --- | --- | --- | --- | --- |

Best Unique PSM (Scan Sample\_2\_A1\_1\_3640.d:17995, m/z=673.2383, z=2, RT=10.52, ppm=-4.3):


TRINITY\_DN5\_c0\_g1\_i15.p4

back to list

  

| Protein Coverage
| Supporting Peptides
| Best Unique PSM
|

Protein Coverage:

Supporting Peptides:

| Peptide | Uniq | -10lgP | Mass | Length | ppm | m/z | z | RT | 1/k0 Start | 1/k0 End | Precursor ID | Area Sample 4 | #Feature | #Feature Sample 4 | Start | End | PTM | AScore | Found By |
| --- | --- | --- | --- | --- | --- | --- | --- | --- | --- | --- | --- | --- | --- | --- | --- | --- | --- | --- | --- |
| K.IIIELQGDLIK.I | Y | 53.27 | 1253.7594 | 11 | 3.89 | 627.8881 | 2 | 19.42 | 0.9513 | 0.9586 | 73842 | 0 | 0 | 0 | 65 | 75 |  |  | DB Search |
| total 1 peptides |  |  |  |  |  |  |  |  |  |  |  |  |  |  |  |  |  |  |  |
| --- | --- | --- | --- | --- | --- | --- | --- | --- | --- | --- | --- | --- | --- | --- | --- | --- | --- | --- | --- |

Best Unique PSM (Scan Sample\_2\_A1\_1\_3640.d:73842, m/z=627.8881, z=2, RT=19.42, ppm=1.69):


TRINITY\_DN77\_c0\_g2\_i4.p1

back to list

  

| Protein Coverage
| Supporting Peptides
| Best Unique PSM
|

Protein Coverage:

Supporting Peptides:

| Peptide | Uniq | -10lgP | Mass | Length | ppm | m/z | z | RT | 1/k0 Start | 1/k0 End | Precursor ID | Area Sample 4 | #Feature | #Feature Sample 4 | Start | End | PTM | AScore | Found By |
| --- | --- | --- | --- | --- | --- | --- | --- | --- | --- | --- | --- | --- | --- | --- | --- | --- | --- | --- | --- |
| R.GPVGTSGLR.G | Y | 58.19 | 842.461 | 9 | 3.7 | 422.2384 | 2 | 12.14 | 0.7787 | 0.786 | 27733 | 1.47e4 | 1 | 1 | 837 | 845 |  |  | DB Search |
| R.GPAGAPGDEGQTGMR.G | Y | 57.23 | 1399.615 | 15 | -0.7 | 700.8127 | 2 | 11.55 | 0.9396 | 0.9469 | 24084 | 1.21e3 | 1 | 1 | 633 | 647 |  |  | DB Search |
| R.GPQGQVGK.Q | Y | 55.44 | 769.4082 | 8 | 6.49 | 385.713 | 2 | 8.99 | 0.7069 | 0.7143 | 10347 | 1.81e3 | 1 | 1 | 480 | 487 |  |  | DB Search |
| R.GPAGAPGDEGQTGM(+15.99)RGER.G | Y | 54.26 | 1757.7751 | 18 | 2.12 | 586.9323 | 3 | 10.62 | 0.8818 | 0.8891 | 18471 | 3.52e3 | 1 | 1 | 633 | 650 | Oxidation (M) | M14:Oxidation (M):1000 | DB Search |
| R.GPAGAPGDEGQTGM(+15.99)R.G | Y | 54.2 | 1415.6099 | 15 | 4.13 | 708.8136 | 2 | 10.40 | 0.9453 | 0.9525 | 17281 | 0 | 0 | 0 | 633 | 647 | Oxidation (M) | M14:Oxidation (M):1000 | DB Search |
| total 5 peptides |  |  |  |  |  |  |  |  |  |  |  |  |  |  |  |  |  |  |  |
| --- | --- | --- | --- | --- | --- | --- | --- | --- | --- | --- | --- | --- | --- | --- | --- | --- | --- | --- | --- |

Best Unique PSM (Scan Sample\_2\_A1\_1\_3640.d:27733, m/z=422.2384, z=2, RT=12.14, ppm=1.5):


TRINITY\_DN262\_c0\_g1\_i1.p1

back to list

  

| Protein Coverage
| Supporting Peptides
| Best Unique PSM
|

Protein Coverage:

Supporting Peptides:

| Peptide | Uniq | -10lgP | Mass | Length | ppm | m/z | z | RT | 1/k0 Start | 1/k0 End | Precursor ID | Area Sample 4 | #Feature | #Feature Sample 4 | Start | End | PTM | AScore | Found By |
| --- | --- | --- | --- | --- | --- | --- | --- | --- | --- | --- | --- | --- | --- | --- | --- | --- | --- | --- | --- |
| K.GFAFETEDPQNFPLEK.L | Y | 58.41 | 1867.8628 | 16 | 3.55 | 934.9399 | 2 | 19.12 | 1.1366 | 1.1438 | 71825 | 5.59e2 | 1 | 1 | 576 | 591 |  |  | DB Search |
| K.VIM(+15.99)VTGDHPITAK.A | Y | 58.39 | 1396.7384 | 13 | 6.43 | 466.5887 | 3 | 13.30 | 0.8079 | 0.8153 | 34845 | 1.64e3 | 1 | 1 | 622 | 634 | Oxidation (M) | M3:Oxidation (M):1000 | DB Search |
| R.AEFRPNQEEEPILKR.E | Y | 58.39 | 1854.9588 | 15 | 1.86 | 619.3267 | 3 | 13.94 | 0.8785 | 0.8858 | 38653 | 6.54e3 | 2 | 2 | 439 | 453 |  |  | DB Search |
| R.DANAAVVHGSHLK.D | Y | 58.39 | 1317.6789 | 13 | -6.06 | 440.2299 | 3 | 10.96 | 0.7449 | 0.7522 | 20600 | 1.04e3 | 1 | 1 | 668 | 680 |  |  | DB Search |
| R.AEFRPNQEEEPILK.R | Y | 58.39 | 1698.8577 | 14 | 1.42 | 567.2927 | 3 | 14.78 | 0.8279 | 0.8352 | 44049 | 2.24e3 | 2 | 2 | 439 | 452 |  |  | DB Search |
| M.A(+42.01)DPSSLETKEK.D | Y | 58.39 | 1245.6088 | 11 | -2 | 623.8091 | 2 | 12.75 | 0.9449 | 0.9521 | 31533 | 4.46e2 | 1 | 1 | 2 | 12 | Acetylation (Protein N-term) | A1:Acetylation (Protein N-term):1000 | DB Search |
| R.NSEC(+57.02)SSDNPLETR.N | Y | 58.38 | 1507.6209 | 13 | -0.6 | 754.8156 | 2 | 13.30 | 1.0122 | 1.0194 | 34858 | 8.83e2 | 1 | 1 | 236 | 248 | Carbamidomethylation |  | DB Search |
| K.GVGIISEGNETVEDIAQR.L | Y | 58.37 | 1885.9381 | 18 | 2.32 | 943.9764 | 2 | 17.95 | 1.1378 | 1.145 | 63996 | 6.11e3 | 2 | 2 | 639 | 656 |  |  | DB Search |
| K.VIMVTGDHPITAK.A | Y | 58.31 | 1380.7435 | 13 | 0.17 | 691.3776 | 2 | 14.09 | 1.0174 | 1.0246 | 39702 | 2.49e3 | 1 | 1 | 622 | 634 |  |  | DB Search |
| K.ADIGVAMGIAGSDVSK.Q | Y | 58.3 | 1489.7446 | 16 | 0.4 | 745.8782 | 2 | 17.01 | 1.0214 | 1.0287 | 58004 | 0 | 0 | 0 | 737 | 752 |  |  | DB Search |
| K.ADIGVAM(+15.99)GIAGSDVSK.Q | Y | 58.28 | 1505.7395 | 16 | -8.69 | 753.8688 | 2 | 14.84 | 1.019 | 1.0263 | 44397 | 0 | 0 | 0 | 737 | 752 | Oxidation (M) | M7:Oxidation (M):1000 | DB Search |
| K.DLPQEKLDDILK.Y | Y | 58.25 | 1425.7715 | 12 | -1.41 | 713.8904 | 2 | 17.90 | 1.0307 | 1.0379 | 63772 | 2.09e3 | 2 | 2 | 681 | 692 |  |  | DB Search |
| R.MGAIVAVTGDGVNDSPALK.K | Y | 58.03 | 1813.9243 | 19 | 0.14 | 907.9676 | 2 | 16.99 | 1.127 | 1.1342 | 57933 | 5.24e2 | 1 | 1 | 717 | 735 |  |  | DB Search |
| K.AETDIM(+15.99)KR.K | Y | 57.87 | 978.4804 | 8 | 3.54 | 490.2481 | 2 | 9.48 | 0.8108 | 0.8181 | 12357 | 0 | 0 | 0 | 836 | 843 | Oxidation (M) | M6:Oxidation (M):1000 | DB Search |
| K.AYVDLGGLGER.V | Y | 56.98 | 1148.5825 | 11 | 5.34 | 575.3004 | 2 | 16.88 | 0.8923 | 0.8996 | 57086 | 1.22e3 | 1 | 1 | 549 | 559 |  |  | DB Search |
| K.VDNSSLTGESEPQSR.N | Y | 55.55 | 1604.7278 | 15 | -1.25 | 803.3684 | 2 | 12.02 | 1.0263 | 1.0335 | 26970 | 5.39e3 | 1 | 1 | 221 | 235 |  |  | DB Search |
| K.TGTLTQNR.M | Y | 55.05 | 889.4617 | 8 | -2.51 | 445.7361 | 2 | 9.97 | 0.7844 | 0.7917 | 14750 | 0 | 0 | 0 | 386 | 393 |  |  | DB Search |
| K.KADIGVAMGIAGSDVSK.Q | Y | 54.91 | 1617.8396 | 17 | 0.24 | 809.9255 | 2 | 15.67 | 1.0656 | 1.0729 | 49555 | 0 | 0 | 0 | 736 | 752 |  |  | DB Search |
| R.M(+15.99)GAIVAVTGDGVNDSPALK.K | Y | 53.36 | 1829.9193 | 19 | -12.74 | 915.9532 | 2 | 15.85 | 1.1182 | 1.1254 | 50596 | 3.97e2 | 1 | 1 | 717 | 735 | Oxidation (M) | M1:Oxidation (M):1000 | DB Search |
| K.TGDNTVM(+15.99)GR.I | Y | 51.75 | 965.4236 | 9 | 0.47 | 483.7183 | 2 | 9.24 | 0.7803 | 0.7876 | 11341 | 0 | 0 | 0 | 269 | 277 | Oxidation (M) | M7:Oxidation (M):1000 | DB Search |
| K.IGALC(+57.02)NR.A | Y | 51.72 | 802.4119 | 7 | 1.46 | 402.213 | 2 | 11.84 | 0.7384 | 0.7457 | 25883 | 9.4e2 | 1 | 1 | 432 | 438 | Carbamidomethylation |  | DB Search |
| total 21 peptides |  |  |  |  |  |  |  |  |  |  |  |  |  |  |  |  |  |  |  |
| --- | --- | --- | --- | --- | --- | --- | --- | --- | --- | --- | --- | --- | --- | --- | --- | --- | --- | --- | --- |

Best Unique PSM (Scan Sample\_2\_A1\_1\_3640.d:71825, m/z=934.9399, z=2, RT=19.12, ppm=1.36):


TRINITY\_DN8008\_c0\_g1\_i2.p1

back to list

  

| Protein Coverage
| Supporting Peptides
| Best Unique PSM
|

Protein Coverage:

Supporting Peptides:

| Peptide | Uniq | -10lgP | Mass | Length | ppm | m/z | z | RT | 1/k0 Start | 1/k0 End | Precursor ID | Area Sample 4 | #Feature | #Feature Sample 4 | Start | End | PTM | AScore | Found By |
| --- | --- | --- | --- | --- | --- | --- | --- | --- | --- | --- | --- | --- | --- | --- | --- | --- | --- | --- | --- |
| K.GVHIISVGLGTVDHR.G | Y | 57 | 1558.8579 | 15 | -7.55 | 520.6215 | 3 | 16.00 | 0.8404 | 0.8477 | 51630 | 0 | 0 | 0 | 1055 | 1069 |  |  | DB Search |
| total 1 peptides |  |  |  |  |  |  |  |  |  |  |  |  |  |  |  |  |  |  |  |
| --- | --- | --- | --- | --- | --- | --- | --- | --- | --- | --- | --- | --- | --- | --- | --- | --- | --- | --- | --- |

Best Unique PSM (Scan Sample\_2\_A1\_1\_3640.d:51630, m/z=520.259, z=2, RT=16.02, ppm=-9.75):


TRINITY\_DN1783\_c0\_g1\_i6.p1

back to list

  

| Protein Coverage
| Supporting Peptides
| Best Unique PSM
|

Protein Coverage:

Supporting Peptides:

| Peptide | Uniq | -10lgP | Mass | Length | ppm | m/z | z | RT | 1/k0 Start | 1/k0 End | Precursor ID | Area Sample 4 | #Feature | #Feature Sample 4 | Start | End | PTM | AScore | Found By |
| --- | --- | --- | --- | --- | --- | --- | --- | --- | --- | --- | --- | --- | --- | --- | --- | --- | --- | --- | --- |
| K.LTIIDALPR.C | Y | 58.24 | 1010.6124 | 9 | 0.02 | 506.3124 | 2 | 19.00 | 0.8392 | 0.8465 | 71132 | 3.79e2 | 1 | 1 | 104 | 112 |  |  | DB Search |
| total 1 peptides |  |  |  |  |  |  |  |  |  |  |  |  |  |  |  |  |  |  |  |
| --- | --- | --- | --- | --- | --- | --- | --- | --- | --- | --- | --- | --- | --- | --- | --- | --- | --- | --- | --- |

Best Unique PSM (Scan Sample\_2\_A1\_1\_3640.d:71132, m/z=506.3124, z=2, RT=19.00, ppm=-2.17):


TRINITY\_DN1795\_c0\_g1\_i1.p1

back to list

  

| Protein Coverage
| Supporting Peptides
| Best Unique PSM
|

Protein Coverage:

Supporting Peptides:

| Peptide | Uniq | -10lgP | Mass | Length | ppm | m/z | z | RT | 1/k0 Start | 1/k0 End | Precursor ID | Area Sample 4 | #Feature | #Feature Sample 4 | Start | End | PTM | AScore | Found By |
| --- | --- | --- | --- | --- | --- | --- | --- | --- | --- | --- | --- | --- | --- | --- | --- | --- | --- | --- | --- |
| K.IDVALNIVTK.V | Y | 58.25 | 1084.6492 | 10 | 1.83 | 543.3317 | 2 | 17.50 | 0.8571 | 0.8644 | 61036 | 3.63e2 | 1 | 1 | 1881 | 1890 |  |  | DB Search |
| K.TSVIADSVGQTIGHVGDVVDTAK.L | Y | 56.02 | 2268.1597 | 23 | -3.24 | 757.0564 | 3 | 19.21 | 1.017 | 1.0242 | 72415 | 3.24e2 | 1 | 1 | 1840 | 1862 |  |  | DB Search |
| K.IATGFESIVR.G | Y | 55.77 | 1091.5975 | 10 | 5.35 | 546.8077 | 2 | 17.15 | 0.855 | 0.8623 | 58937 | 0 | 0 | 0 | 1794 | 1803 |  |  | DB Search |
| K.LIFDDVGNIAEK.L | Y | 51.9 | 1332.6925 | 12 | 4.84 | 667.3553 | 2 | 18.51 | 0.9622 | 0.9695 | 67867 | 5.56e2 | 1 | 1 | 1987 | 1998 |  |  | DB Search |
| K.DLTASLDGVFQK.T | Y | 51.39 | 1292.6612 | 12 | 1.56 | 647.3375 | 2 | 19.57 | 0.9505 | 0.9578 | 74776 | 1.66e2 | 1 | 1 | 1079 | 1090 |  |  | DB Search |
| total 5 peptides |  |  |  |  |  |  |  |  |  |  |  |  |  |  |  |  |  |  |  |
| --- | --- | --- | --- | --- | --- | --- | --- | --- | --- | --- | --- | --- | --- | --- | --- | --- | --- | --- | --- |

Best Unique PSM (Scan Sample\_2\_A1\_1\_3640.d:61036, m/z=543.3317, z=2, RT=17.50, ppm=-0.37):


TRINITY\_DN64\_c0\_g1\_i1.p1

back to list

  

| Protein Coverage
| Supporting Peptides
| Best Unique PSM
|

Protein Coverage:

Supporting Peptides:

| Peptide | Uniq | -10lgP | Mass | Length | ppm | m/z | z | RT | 1/k0 Start | 1/k0 End | Precursor ID | Area Sample 4 | #Feature | #Feature Sample 4 | Start | End | PTM | AScore | Found By |
| --- | --- | --- | --- | --- | --- | --- | --- | --- | --- | --- | --- | --- | --- | --- | --- | --- | --- | --- | --- |
| K.IHINIVVIGHVDSGK.S | Y | 58.31 | 1599.9096 | 15 | 5.74 | 534.3124 | 3 | 16.79 | 0.8477 | 0.855 | 56506 | 4.37e2 | 1 | 1 | 9 | 23 |  |  | DB Search |
| K.YYVTIIDAPGHR.D | Y | 58.28 | 1403.7197 | 12 | -1.46 | 468.9121 | 3 | 16.14 | 0.8023 | 0.8096 | 52343 | 2.5e3 | 2 | 2 | 88 | 99 |  |  | DB Search |
| K.STSTGHLIYK.C | Y | 55.95 | 1105.5768 | 10 | -3.77 | 553.7924 | 2 | 12.15 | 0.8635 | 0.8708 | 27830 | 0 | 0 | 0 | 24 | 33 |  |  | DB Search |
| K.YYVTIIDAPGHRDFIK.N | Y | 53.54 | 1906.9941 | 16 | -0.07 | 636.6705 | 3 | 17.01 | 0.8915 | 0.8988 | 58058 | 0 | 0 | 0 | 88 | 103 |  |  | DB Search |
| total 4 peptides |  |  |  |  |  |  |  |  |  |  |  |  |  |  |  |  |  |  |  |
| --- | --- | --- | --- | --- | --- | --- | --- | --- | --- | --- | --- | --- | --- | --- | --- | --- | --- | --- | --- |

Best Unique PSM (Scan Sample\_2\_A1\_1\_3640.d:56506, m/z=534.3124, z=3, RT=16.79, ppm=3.55):


TRINITY\_DN73587\_c0\_g1\_i1.p1

back to list

  

| Protein Coverage
| Supporting Peptides
| Best Unique PSM
|

Protein Coverage:

Supporting Peptides:

| Peptide | Uniq | -10lgP | Mass | Length | ppm | m/z | z | RT | 1/k0 Start | 1/k0 End | Precursor ID | Area Sample 4 | #Feature | #Feature Sample 4 | Start | End | PTM | AScore | Found By |
| --- | --- | --- | --- | --- | --- | --- | --- | --- | --- | --- | --- | --- | --- | --- | --- | --- | --- | --- | --- |
| R.FTQAGSEVSALLGR.I | Y | 48.14 | 1434.7467 | 14 | 5.7 | 718.3831 | 2 | 18.43 | 0.986 | 0.9932 | 67409 | 0 | 0 | 0 | 315 | 328 |  |  | DB Search |
| total 1 peptides |  |  |  |  |  |  |  |  |  |  |  |  |  |  |  |  |  |  |  |
| --- | --- | --- | --- | --- | --- | --- | --- | --- | --- | --- | --- | --- | --- | --- | --- | --- | --- | --- | --- |

Best Unique PSM (Scan Sample\_2\_A1\_1\_3640.d:67409, m/z=718.3831, z=2, RT=18.43, ppm=3.5):


TRINITY\_DN69484\_c0\_g1\_i1.p1

back to list

  

| Protein Coverage
| Supporting Peptides
| Best Unique PSM
|

Protein Coverage:

Supporting Peptides:

| Peptide | Uniq | -10lgP | Mass | Length | ppm | m/z | z | RT | 1/k0 Start | 1/k0 End | Precursor ID | Area Sample 4 | #Feature | #Feature Sample 4 | Start | End | PTM | AScore | Found By |
| --- | --- | --- | --- | --- | --- | --- | --- | --- | --- | --- | --- | --- | --- | --- | --- | --- | --- | --- | --- |
| K.LLILITTHVSHVR.Q | Y | 58.4 | 1500.914 | 13 | -2.48 | 501.3096 | 3 | 16.66 | 0.8063 | 0.8136 | 55744 | 3.08e2 | 1 | 1 | 61 | 73 |  |  | DB Search |
| total 1 peptides |  |  |  |  |  |  |  |  |  |  |  |  |  |  |  |  |  |  |  |
| --- | --- | --- | --- | --- | --- | --- | --- | --- | --- | --- | --- | --- | --- | --- | --- | --- | --- | --- | --- |

Best Unique PSM (Scan Sample\_2\_A1\_1\_3640.d:55744, m/z=501.3096, z=3, RT=16.66, ppm=-4.68):


TRINITY\_DN3698\_c0\_g2\_i3.p1

back to list

  

| Protein Coverage
| Supporting Peptides
| Best Unique PSM
|

Protein Coverage:

Supporting Peptides:

| Peptide | Uniq | -10lgP | Mass | Length | ppm | m/z | z | RT | 1/k0 Start | 1/k0 End | Precursor ID | Area Sample 4 | #Feature | #Feature Sample 4 | Start | End | PTM | AScore | Found By |
| --- | --- | --- | --- | --- | --- | --- | --- | --- | --- | --- | --- | --- | --- | --- | --- | --- | --- | --- | --- |
| K.ANHIFAHTDPLSQK.S | Y | 58.31 | 1577.795 | 14 | -10.88 | 526.9321 | 3 | 13.75 | 0.8189 | 0.8262 | 37740 | 6.57e2 | 1 | 1 | 383 | 396 |  |  | DB Search |
| R.ATENPSGGFSPDDR.C | Y | 55.58 | 1448.6168 | 14 | 6.68 | 725.3189 | 2 | 12.97 | 0.9759 | 0.9832 | 32890 | 0 | 0 | 0 | 116 | 129 |  |  | DB Search |
| total 2 peptides |  |  |  |  |  |  |  |  |  |  |  |  |  |  |  |  |  |  |  |
| --- | --- | --- | --- | --- | --- | --- | --- | --- | --- | --- | --- | --- | --- | --- | --- | --- | --- | --- | --- |

Best Unique PSM (Scan Sample\_2\_A1\_1\_3640.d:37740, m/z=526.9321, z=3, RT=13.75, ppm=-13.08):


TRINITY\_DN624\_c0\_g1\_i1.p1

back to list

  

| Protein Coverage
| Supporting Peptides
| Best Unique PSM
|

Protein Coverage:

Supporting Peptides:

| Peptide | Uniq | -10lgP | Mass | Length | ppm | m/z | z | RT | 1/k0 Start | 1/k0 End | Precursor ID | Area Sample 4 | #Feature | #Feature Sample 4 | Start | End | PTM | AScore | Found By |
| --- | --- | --- | --- | --- | --- | --- | --- | --- | --- | --- | --- | --- | --- | --- | --- | --- | --- | --- | --- |
| R.FAIQDITVDEFSAK.E | Y | 58.39 | 1582.7878 | 14 | 3.87 | 792.4025 | 2 | 19.84 | 1.0367 | 1.0439 | 76475 | 1.54e3 | 1 | 1 | 132 | 145 |  |  | DB Search |
| K.LENLFNTLQTK.L | Y | 57.51 | 1319.7085 | 11 | 2.17 | 660.8615 | 2 | 18.79 | 0.9578 | 0.965 | 69766 | 0 | 0 | 0 | 332 | 342 |  |  | DB Search |
| total 2 peptides |  |  |  |  |  |  |  |  |  |  |  |  |  |  |  |  |  |  |  |
| --- | --- | --- | --- | --- | --- | --- | --- | --- | --- | --- | --- | --- | --- | --- | --- | --- | --- | --- | --- |

Best Unique PSM (Scan Sample\_2\_A1\_1\_3640.d:76475, m/z=792.4025, z=2, RT=19.84, ppm=1.68):


TRINITY\_DN15274\_c0\_g1\_i1.p1

back to list

  

| Protein Coverage
| Supporting Peptides
| Best Unique PSM
|

Protein Coverage:

Supporting Peptides:

| Peptide | Uniq | -10lgP | Mass | Length | ppm | m/z | z | RT | 1/k0 Start | 1/k0 End | Precursor ID | Area Sample 4 | #Feature | #Feature Sample 4 | Start | End | PTM | AScore | Found By |
| --- | --- | --- | --- | --- | --- | --- | --- | --- | --- | --- | --- | --- | --- | --- | --- | --- | --- | --- | --- |
| R.TTGVC(+57.02)M(+15.99)C(+57.02)K.D | Y | 58.37 | 971.3875 | 8 | 0.65 | 486.7003 | 2 | 9.45 | 0.7827 | 0.7901 | 12123 | 2.26e2 | 1 | 1 | 1150 | 1157 | Carbamidomethylation, Oxidation (M), Carbamidomethylation | M6:Oxidation (M):1000 | DB Search |
| R.LQGWWQSVNGR.Q | Y | 58.33 | 1329.6578 | 11 | 2.85 | 665.8366 | 2 | 18.29 | 0.9416 | 0.9489 | 66414 | 3.67e2 | 1 | 1 | 108 | 118 |  |  | DB Search |
| R.GGC(+57.02)FPATGDLLVGR.E | Y | 58.29 | 1418.6976 | 14 | 2.98 | 710.3566 | 2 | 18.13 | 0.9892 | 0.9965 | 64986 | 1.86e3 | 1 | 1 | 31 | 44 | Carbamidomethylation |  | DB Search |
| K.KIDEAEELGDKVQGSVDK.M | Y | 58.29 | 1958.9796 | 18 | -0.5 | 653.9987 | 3 | 14.55 | 0.8603 | 0.8676 | 42443 | 2.02e3 | 2 | 2 | 1673 | 1690 |  |  | DB Search |
| R.YSRPTPGSNNNVM(+15.99)VR.I | Y | 58.28 | 1706.8158 | 15 | 1.72 | 569.9456 | 3 | 11.63 | 0.8319 | 0.8392 | 24566 | 1.7e3 | 1 | 1 | 621 | 635 | Oxidation (M) | M13:Oxidation (M):1000 | DB Search |
| K.ANDILDGLELR.L | Y | 58.27 | 1227.6459 | 11 | 0.98 | 614.8295 | 2 | 19.57 | 0.9218 | 0.9291 | 74777 | 2.49e3 | 1 | 1 | 1286 | 1296 |  |  | DB Search |
| R.ADIEDVKDLLK.K | Y | 58.26 | 1257.6816 | 11 | 3.28 | 420.2349 | 3 | 17.77 | 0.7489 | 0.7563 | 62925 | 2.25e4 | 2 | 2 | 1616 | 1626 |  |  | DB Search |
| R.FVIGLTC(+57.02)SK.C | Y | 58.26 | 1023.5423 | 9 | 1.29 | 512.778 | 2 | 16.97 | 0.8498 | 0.8571 | 57580 | 4.72e3 | 1 | 1 | 486 | 494 | Carbamidomethylation |  | DB Search |
| R.ADIEDVKDLLKK.L | Y | 58.26 | 1385.7765 | 12 | 3.93 | 462.9336 | 3 | 16.48 | 0.7306 | 0.7379 | 54482 | 1.57e4 | 2 | 2 | 1616 | 1627 |  |  | DB Search |
| R.NAILDQNLENREK.L | Y | 58.14 | 1555.7954 | 13 | -1.51 | 778.9021 | 2 | 13.92 | 1.0568 | 1.064 | 38720 | 0 | 0 | 0 | 1654 | 1666 |  |  | DB Search |
| K.DIQDFLATDFNHPNASK.D | Y | 57.97 | 1931.9013 | 17 | 1.13 | 644.9737 | 3 | 19.20 | 0.8477 | 0.855 | 72443 | 2.25e2 | 1 | 1 | 1504 | 1520 |  |  | DB Search |
| K.MKDSYIINEER.A | Y | 57.2 | 1396.6656 | 11 | 0.34 | 699.3388 | 2 | 13.80 | 0.9735 | 0.9808 | 37987 | 0 | 0 | 0 | 1740 | 1750 |  |  | DB Search |
| R.DEKANDILDGLELR.L | Y | 56.73 | 1599.8104 | 14 | 1.29 | 534.2769 | 3 | 19.09 | 0.7966 | 0.8039 | 71698 | 0 | 0 | 0 | 1283 | 1296 |  |  | DB Search |
| R.AKDLYYEIR.R | Y | 56.53 | 1169.608 | 9 | -0.76 | 585.8096 | 2 | 14.74 | 0.9206 | 0.9279 | 43604 | 1.31e3 | 1 | 1 | 1751 | 1759 |  |  | DB Search |
| R.GSC(+57.02)SC(+57.02)YGHAEQC(+57.02)IPTK.G | Y | 56.22 | 1853.7495 | 16 | -0.38 | 618.9222 | 3 | 12.47 | 0.8498 | 0.8571 | 29856 | 2.96e2 | 1 | 1 | 270 | 285 | Carbamidomethylation, Carbamidomethylation, Carbamidomethylation |  | DB Search |
| R.YSRPTPGSNNNVMVR.I | Y | 56.2 | 1690.8209 | 15 | -0.93 | 564.6125 | 3 | 12.56 | 0.8246 | 0.8319 | 30408 | 1.67e2 | 1 | 1 | 621 | 635 |  |  | DB Search |
| K.IQFSISAEINNGALK.C | Y | 56.04 | 1603.8569 | 15 | 2.48 | 802.936 | 2 | 18.88 | 1.0701 | 1.0773 | 70412 | 0 | 0 | 0 | 759 | 773 |  |  | DB Search |
| K.ELHTFGDDILGDSPK.V | Y | 54.43 | 1642.7838 | 15 | -1.51 | 548.5999 | 3 | 17.34 | 0.8315 | 0.8388 | 59995 | 1.59e3 | 1 | 1 | 240 | 254 |  |  | DB Search |
| total 18 peptides |  |  |  |  |  |  |  |  |  |  |  |  |  |  |  |  |  |  |  |
| --- | --- | --- | --- | --- | --- | --- | --- | --- | --- | --- | --- | --- | --- | --- | --- | --- | --- | --- | --- |

Best Unique PSM (Scan Sample\_2\_A1\_1\_3640.d:12123, m/z=486.7003, z=2, RT=9.45, ppm=-1.55):


TRINITY\_DN5\_c0\_g1\_i11.p1

back to list

  

| Protein Coverage
| Supporting Peptides
| Best Unique PSM
|

Protein Coverage:

Supporting Peptides:

| Peptide | Uniq | -10lgP | Mass | Length | ppm | m/z | z | RT | 1/k0 Start | 1/k0 End | Precursor ID | Area Sample 4 | #Feature | #Feature Sample 4 | Start | End | PTM | AScore | Found By |
| --- | --- | --- | --- | --- | --- | --- | --- | --- | --- | --- | --- | --- | --- | --- | --- | --- | --- | --- | --- |
| K.IIIELQGDLIK.I | Y | 53.27 | 1253.7594 | 11 | 3.89 | 627.8881 | 2 | 19.42 | 0.9513 | 0.9586 | 73842 | 0 | 0 | 0 | 65 | 75 |  |  | DB Search |
| total 1 peptides |  |  |  |  |  |  |  |  |  |  |  |  |  |  |  |  |  |  |  |
| --- | --- | --- | --- | --- | --- | --- | --- | --- | --- | --- | --- | --- | --- | --- | --- | --- | --- | --- | --- |

Best Unique PSM (Scan Sample\_2\_A1\_1\_3640.d:73842, m/z=627.8881, z=2, RT=19.42, ppm=1.69):


TRINITY\_DN11248\_c0\_g1\_i1.p1

back to list

  

| Protein Coverage
| Supporting Peptides
| Best Unique PSM
|

Protein Coverage:

Supporting Peptides:

| Peptide | Uniq | -10lgP | Mass | Length | ppm | m/z | z | RT | 1/k0 Start | 1/k0 End | Precursor ID | Area Sample 4 | #Feature | #Feature Sample 4 | Start | End | PTM | AScore | Found By |
| --- | --- | --- | --- | --- | --- | --- | --- | --- | --- | --- | --- | --- | --- | --- | --- | --- | --- | --- | --- |
| K.NWHGMEDC(+57.02)VVVK.S | Y | 58.29 | 1472.654 | 12 | 8.38 | 491.895 | 3 | 15.15 | 0.7534 | 0.7608 | 46340 | 0 | 0 | 0 | 1630 | 1641 | Carbamidomethylation |  | DB Search |
| K.TPYLQIDFGK.M | Y | 57.3 | 1180.6128 | 10 | 2.79 | 591.314 | 2 | 18.49 | 0.9109 | 0.9182 | 67728 | 8.28e2 | 1 | 1 | 514 | 523 |  |  | DB Search |
| K.ENDFISAQLKR.M | Y | 57.23 | 1319.6833 | 11 | 3.57 | 660.8499 | 2 | 15.34 | 0.9791 | 0.9864 | 47439 | 4.82e2 | 1 | 1 | 1581 | 1591 |  |  | DB Search |
| K.DKVSNAC(+57.02)VK.E | Y | 56.71 | 1019.507 | 9 | -4.3 | 510.7574 | 2 | 9.41 | 0.836 | 0.8433 | 12034 | 0 | 0 | 0 | 1176 | 1184 | Carbamidomethylation |  | DB Search |
| K.NWHGM(+15.99)EDC(+57.02)VVVK.S | Y | 55.7 | 1488.6489 | 12 | -2.89 | 497.2211 | 3 | 13.35 | 0.7599 | 0.7673 | 35192 | 0 | 0 | 0 | 1630 | 1641 | Oxidation (M), Carbamidomethylation | M5:Oxidation (M):1000 | DB Search |
| K.SIEAIYLGEFGTC(+57.02)K.S | Y | 54.46 | 1586.765 | 14 | 6.64 | 794.3933 | 2 | 19.71 | 1.0981 | 1.1053 | 75650 | 6.41e2 | 1 | 1 | 982 | 995 | Carbamidomethylation |  | DB Search |
| K.MGAHVATIHYQK.E | Y | 53.7 | 1354.6816 | 12 | 4.59 | 452.5689 | 3 | 12.20 | 0.7836 | 0.7909 | 28050 | 1.16e3 | 1 | 1 | 1569 | 1580 |  |  | DB Search |
| R.HLC(+57.02)VGMGSGGK.W | Y | 53.7 | 1101.5059 | 11 | 0.68 | 551.7594 | 2 | 14.22 | 0.8757 | 0.883 | 40611 | 2.66e2 | 1 | 1 | 1486 | 1496 | Carbamidomethylation |  | DB Search |
| total 8 peptides |  |  |  |  |  |  |  |  |  |  |  |  |  |  |  |  |  |  |  |
| --- | --- | --- | --- | --- | --- | --- | --- | --- | --- | --- | --- | --- | --- | --- | --- | --- | --- | --- | --- |

Best Unique PSM (Scan Sample\_2\_A1\_1\_3640.d:46340, m/z=491.895, z=3, RT=15.15, ppm=6.18):


TRINITY\_DN329\_c0\_g1\_i2.p1

back to list

  

| Protein Coverage
| Supporting Peptides
| Best Unique PSM
|

Protein Coverage:

Supporting Peptides:

| Peptide | Uniq | -10lgP | Mass | Length | ppm | m/z | z | RT | 1/k0 Start | 1/k0 End | Precursor ID | Area Sample 4 | #Feature | #Feature Sample 4 | Start | End | PTM | AScore | Found By |
| --- | --- | --- | --- | --- | --- | --- | --- | --- | --- | --- | --- | --- | --- | --- | --- | --- | --- | --- | --- |
| K.GLKVDFDTTFAPVTGKK.T | Y | 57.88 | 1822.9828 | 17 | -3.05 | 608.665 | 3 | 17.39 | 0.8984 | 0.9057 | 60425 | 2.02e2 | 1 | 1 | 130 | 146 |  |  | DB Search |
| K.LRPGVELTM(+15.99)SSLVNAK.S | Y | 54.81 | 1729.9396 | 16 | -2.37 | 577.6512 | 3 | 15.40 | 0.8749 | 0.8822 | 47976 | 0 | 0 | 0 | 287 | 302 | Oxidation (M) | M9:Oxidation (M):1000 | DB Search |
| total 2 peptides |  |  |  |  |  |  |  |  |  |  |  |  |  |  |  |  |  |  |  |
| --- | --- | --- | --- | --- | --- | --- | --- | --- | --- | --- | --- | --- | --- | --- | --- | --- | --- | --- | --- |

Best Unique PSM (Scan Sample\_2\_A1\_1\_3640.d:60425, m/z=608.665, z=3, RT=17.39, ppm=-5.24):


TRINITY\_DN1058\_c0\_g1\_i1.p1

back to list

  

| Protein Coverage
| Supporting Peptides
| Best Unique PSM
|

Protein Coverage:

Supporting Peptides:

| Peptide | Uniq | -10lgP | Mass | Length | ppm | m/z | z | RT | 1/k0 Start | 1/k0 End | Precursor ID | Area Sample 4 | #Feature | #Feature Sample 4 | Start | End | PTM | AScore | Found By |
| --- | --- | --- | --- | --- | --- | --- | --- | --- | --- | --- | --- | --- | --- | --- | --- | --- | --- | --- | --- |
| K.TNFQMC(+57.02)HQR.M | Y | 58.29 | 1220.5179 | 9 | 3.31 | 611.2669 | 2 | 11.65 | 0.921 | 0.9283 | 24815 | 0 | 0 | 0 | 1467 | 1475 | Carbamidomethylation |  | DB Search |
| R.APTHILNPANKR.S | Y | 58.28 | 1330.7469 | 12 | 0.07 | 666.3793 | 2 | 11.22 | 1.0098 | 1.017 | 22322 | 5.04e3 | 2 | 2 | 2314 | 2325 |  |  | DB Search |
| K.TVMAAC(+57.02)GNENK.C | Y | 58.27 | 1193.5169 | 11 | 1.94 | 597.7656 | 2 | 10.70 | 0.8919 | 0.8992 | 19067 | 0 | 0 | 0 | 1338 | 1348 | Carbamidomethylation |  | DB Search |
| R.SC(+57.02)VLASC(+57.02)SANSDVAVAC(+57.02)GK.V | Y | 58.08 | 1954.8547 | 19 | 3.15 | 978.4355 | 2 | 14.42 | 1.153 | 1.1602 | 41659 | 2.61e2 | 1 | 1 | 1616 | 1634 | Carbamidomethylation, Carbamidomethylation, Carbamidomethylation |  | DB Search |
| R.IC(+57.02)GHGGIR.C | Y | 57.78 | 868.4337 | 8 | -3.19 | 435.2218 | 2 | 9.87 | 0.7892 | 0.7966 | 14210 | 0 | 0 | 0 | 1008 | 1015 | Carbamidomethylation |  | DB Search |
| R.TC(+57.02)LC(+57.02)NPATSYPSK.D | Y | 54.97 | 1497.6592 | 13 | 0.78 | 749.8358 | 2 | 13.69 | 0.9896 | 0.9969 | 37441 | 1.47e3 | 1 | 1 | 157 | 169 | Carbamidomethylation, Carbamidomethylation |  | DB Search |
| K.SC(+57.02)IEASC(+57.02)SSK.A | Y | 53.5 | 1127.4587 | 10 | 5.45 | 564.7385 | 2 | 10.24 | 0.8733 | 0.8806 | 16314 | 7.28e2 | 1 | 1 | 465 | 474 | Carbamidomethylation, Carbamidomethylation |  | DB Search |
| total 7 peptides |  |  |  |  |  |  |  |  |  |  |  |  |  |  |  |  |  |  |  |
| --- | --- | --- | --- | --- | --- | --- | --- | --- | --- | --- | --- | --- | --- | --- | --- | --- | --- | --- | --- |

Best Unique PSM (Scan Sample\_2\_A1\_1\_3640.d:24815, m/z=611.2669, z=2, RT=11.65, ppm=1.11):


TRINITY\_DN4513\_c0\_g1\_i12.p1

back to list

  

| Protein Coverage
| Supporting Peptides
| Best Unique PSM
|

Protein Coverage:

Supporting Peptides:

| Peptide | Uniq | -10lgP | Mass | Length | ppm | m/z | z | RT | 1/k0 Start | 1/k0 End | Precursor ID | Area Sample 4 | #Feature | #Feature Sample 4 | Start | End | PTM | AScore | Found By |
| --- | --- | --- | --- | --- | --- | --- | --- | --- | --- | --- | --- | --- | --- | --- | --- | --- | --- | --- | --- |
| K.LKTDLEAQKK.E | Y | 57.72 | 1172.6765 | 10 | 3.8 | 391.9001 | 3 | 10.36 | 0.7408 | 0.7481 | 16865 | 9.07e2 | 1 | 1 | 22 | 31 |  |  | DB Search |
| total 1 peptides |  |  |  |  |  |  |  |  |  |  |  |  |  |  |  |  |  |  |  |
| --- | --- | --- | --- | --- | --- | --- | --- | --- | --- | --- | --- | --- | --- | --- | --- | --- | --- | --- | --- |

Best Unique PSM (Scan Sample\_2\_A1\_1\_3640.d:16865, m/z=391.9001, z=3, RT=10.36, ppm=1.6):


TRINITY\_DN1676\_c0\_g1\_i12.p1

back to list

  

| Protein Coverage
| Supporting Peptides
| Best Unique PSM
|

Protein Coverage:

Supporting Peptides:

| Peptide | Uniq | -10lgP | Mass | Length | ppm | m/z | z | RT | 1/k0 Start | 1/k0 End | Precursor ID | Area Sample 4 | #Feature | #Feature Sample 4 | Start | End | PTM | AScore | Found By |
| --- | --- | --- | --- | --- | --- | --- | --- | --- | --- | --- | --- | --- | --- | --- | --- | --- | --- | --- | --- |
| K.LLQQHELDLTPLK.E | Y | 58.4 | 1546.8719 | 13 | -0.89 | 516.6296 | 3 | 16.98 | 0.8356 | 0.8429 | 57805 | 1.52e3 | 2 | 2 | 1589 | 1601 |  |  | DB Search |
| R.SGGDTSPFGEC(+57.02)KK.C | Y | 58.4 | 1368.598 | 13 | 8.57 | 457.2095 | 3 | 11.91 | 0.7559 | 0.7632 | 26366 | 1.73e3 | 1 | 1 | 717 | 729 | Carbamidomethylation |  | DB Search |
| K.GC(+57.02)ELC(+57.02)NC(+57.02)NATGSK.G | Y | 58.38 | 1469.5697 | 13 | -3.08 | 735.7883 | 2 | 11.24 | 0.9711 | 0.9783 | 22299 | 0 | 0 | 0 | 943 | 955 | Carbamidomethylation, Carbamidomethylation, Carbamidomethylation |  | DB Search |
| K.ITQLQNDDKEAR.E | Y | 58.36 | 1429.7161 | 12 | 4.64 | 477.5805 | 3 | 10.94 | 0.8055 | 0.8128 | 20429 | 1.37e3 | 1 | 1 | 1329 | 1340 |  |  | DB Search |
| R.GQYISVKPPR.N | Y | 58.35 | 1143.64 | 10 | -2.31 | 572.8247 | 2 | 13.47 | 0.9121 | 0.9194 | 36000 | 0 | 0 | 0 | 550 | 559 |  |  | DB Search |
| R.MSEIAEEARK.H | Y | 58.34 | 1162.5652 | 10 | 1.11 | 582.2892 | 2 | 11.43 | 0.9218 | 0.9291 | 23472 | 3.63e2 | 1 | 1 | 1193 | 1202 |  |  | DB Search |
| R.C(+57.02)FDLIQVAVNK.L | Y | 58.29 | 1305.6751 | 11 | -2.9 | 653.8415 | 2 | 19.16 | 0.9582 | 0.9654 | 71943 | 1.33e3 | 1 | 1 | 1044 | 1054 | Carbamidomethylation |  | DB Search |
| R.FELDRVNTFGDEVFGDPK.V | Y | 58.29 | 2083.985 | 18 | 0.34 | 695.6677 | 3 | 19.31 | 0.9182 | 0.9255 | 73205 | 0 | 0 | 0 | 252 | 269 |  |  | DB Search |
| K.GTM(+15.99)KPPFHDPK.L | Y | 58.28 | 1269.6176 | 11 | 0.53 | 635.815 | 2 | 10.78 | 0.9574 | 0.9646 | 19136 | 1.46e3 | 1 | 1 | 896 | 906 | Oxidation (M) | M3:Oxidation (M):1000 | DB Search |
| R.VNTFGDEVFGDPK.V | Y | 58.28 | 1423.6619 | 13 | -3.52 | 712.8342 | 2 | 17.48 | 1.0118 | 1.019 | 60886 | 1.49e3 | 1 | 1 | 257 | 269 |  |  | DB Search |
| K.TLMEFENNIAK.S | Y | 58.28 | 1308.6384 | 11 | 4.09 | 655.3277 | 2 | 17.39 | 0.9634 | 0.9707 | 60532 | 5.46e3 | 1 | 1 | 1387 | 1397 |  |  | DB Search |
| K.KSEGAAETGSVTISK.A | Y | 58.14 | 1463.7467 | 15 | 0.58 | 732.8795 | 2 | 11.50 | 0.9844 | 0.9916 | 23786 | 1.99e3 | 1 | 1 | 1135 | 1149 |  |  | DB Search |
| K.LKNEIEIMESK.L | Y | 57.61 | 1332.6959 | 11 | 0.56 | 667.3541 | 2 | 15.12 | 0.967 | 0.9743 | 46046 | 8e2 | 1 | 1 | 1572 | 1582 |  |  | DB Search |
| K.DKITQLQNDDKEAR.E | Y | 56.95 | 1672.838 | 14 | 4.92 | 558.6215 | 3 | 11.86 | 0.8416 | 0.849 | 25955 | 4.36e2 | 1 | 1 | 1327 | 1340 |  |  | DB Search |
| K.EC(+57.02)FC(+57.02)FGHGVSC(+57.02)DAIEGIGVK.E | Y | 54.91 | 2240.9653 | 20 | 3.38 | 747.9966 | 3 | 18.10 | 0.9763 | 0.9836 | 65084 | 1.23e3 | 1 | 1 | 498 | 517 | Carbamidomethylation, Carbamidomethylation, Carbamidomethylation |  | DB Search |
| K.RFEEAKDTIENK.L | Y | 54.88 | 1478.7365 | 12 | 3.61 | 493.9201 | 3 | 11.68 | 0.8031 | 0.8104 | 24961 | 1.52e3 | 1 | 1 | 1560 | 1571 |  |  | DB Search |
| K.GSDC(+57.02)NLQTGQC(+57.02)EC(+57.02)QDGVGGR.K | Y | 53.87 | 2196.8583 | 20 | -5.84 | 733.2875 | 3 | 12.26 | 0.8733 | 0.8806 | 28552 | 0 | 0 | 0 | 956 | 975 | Carbamidomethylation, Carbamidomethylation, Carbamidomethylation |  | DB Search |
| K.RMSEIAEEARK.H | Y | 47.21 | 1318.6663 | 11 | 5.81 | 440.5643 | 3 | 11.07 | 0.7628 | 0.7701 | 21258 | 4.45e2 | 1 | 1 | 1192 | 1202 |  |  | DB Search |
| K.TTQDAMNEIESSRR.E | Y | 40.35 | 1636.7475 | 14 | -1.03 | 546.588 | 3 | 13.88 | 0.8153 | 0.8226 | 38475 | 7.82e2 | 1 | 1 | 1450 | 1463 |  |  | DB Search |
| total 19 peptides |  |  |  |  |  |  |  |  |  |  |  |  |  |  |  |  |  |  |  |
| --- | --- | --- | --- | --- | --- | --- | --- | --- | --- | --- | --- | --- | --- | --- | --- | --- | --- | --- | --- |

Best Unique PSM (Scan Sample\_2\_A1\_1\_3640.d:57805, m/z=516.6296, z=3, RT=16.98, ppm=-3.08):


TRINITY\_DN8926\_c0\_g1\_i2.p1

back to list

  

| Protein Coverage
| Supporting Peptides
| Best Unique PSM
|

Protein Coverage:

Supporting Peptides:

| Peptide | Uniq | -10lgP | Mass | Length | ppm | m/z | z | RT | 1/k0 Start | 1/k0 End | Precursor ID | Area Sample 4 | #Feature | #Feature Sample 4 | Start | End | PTM | AScore | Found By |
| --- | --- | --- | --- | --- | --- | --- | --- | --- | --- | --- | --- | --- | --- | --- | --- | --- | --- | --- | --- |
| R.AQIDIWLQR.M | Y | 57.82 | 1141.6244 | 9 | 1.55 | 571.8191 | 2 | 18.61 | 0.8887 | 0.8959 | 68405 | 8.34e2 | 1 | 1 | 157 | 165 |  |  | DB Search |
| total 1 peptides |  |  |  |  |  |  |  |  |  |  |  |  |  |  |  |  |  |  |  |
| --- | --- | --- | --- | --- | --- | --- | --- | --- | --- | --- | --- | --- | --- | --- | --- | --- | --- | --- | --- |

Best Unique PSM (Scan Sample\_2\_A1\_1\_3640.d:68405, m/z=571.8191, z=2, RT=18.61, ppm=-0.65):


TRINITY\_DN121236\_c0\_g1\_i1.p1

back to list

  

| Protein Coverage
| Supporting Peptides
| Best Unique PSM
|

Protein Coverage:

Supporting Peptides:

| Peptide | Uniq | -10lgP | Mass | Length | ppm | m/z | z | RT | 1/k0 Start | 1/k0 End | Precursor ID | Area Sample 4 | #Feature | #Feature Sample 4 | Start | End | PTM | AScore | Found By |
| --- | --- | --- | --- | --- | --- | --- | --- | --- | --- | --- | --- | --- | --- | --- | --- | --- | --- | --- | --- |
| K.IYSIEITDVENGGTR.Q | Y | 55.52 | 1665.8209 | 15 | 0.19 | 833.9161 | 2 | 17.47 | 1.064 | 1.0713 | 60999 | 1.2e3 | 1 | 1 | 256 | 270 |  |  | DB Search |
| total 1 peptides |  |  |  |  |  |  |  |  |  |  |  |  |  |  |  |  |  |  |  |
| --- | --- | --- | --- | --- | --- | --- | --- | --- | --- | --- | --- | --- | --- | --- | --- | --- | --- | --- | --- |

Best Unique PSM (Scan Sample\_2\_A1\_1\_3640.d:60999, m/z=833.9161, z=2, RT=17.47, ppm=-2.01):


TRINITY\_DN16079\_c0\_g2\_i1.p1

back to list

  

| Protein Coverage
| Supporting Peptides
| Best Unique PSM
|

Protein Coverage:

Supporting Peptides:

| Peptide | Uniq | -10lgP | Mass | Length | ppm | m/z | z | RT | 1/k0 Start | 1/k0 End | Precursor ID | Area Sample 4 | #Feature | #Feature Sample 4 | Start | End | PTM | AScore | Found By |
| --- | --- | --- | --- | --- | --- | --- | --- | --- | --- | --- | --- | --- | --- | --- | --- | --- | --- | --- | --- |
| K.SFFDFENSK.L | Y | 58.27 | 1119.4873 | 9 | 0.57 | 560.75 | 2 | 18.77 | 0.8595 | 0.8668 | 69552 | 3.17e2 | 1 | 1 | 49 | 57 |  |  | DB Search |
| K.LFADELMTNINR.C | Y | 57.18 | 1435.7129 | 12 | -2.44 | 718.8604 | 2 | 19.26 | 0.9928 | 1.0001 | 72722 | 4.52e2 | 1 | 1 | 58 | 69 |  |  | DB Search |
| total 2 peptides |  |  |  |  |  |  |  |  |  |  |  |  |  |  |  |  |  |  |  |
| --- | --- | --- | --- | --- | --- | --- | --- | --- | --- | --- | --- | --- | --- | --- | --- | --- | --- | --- | --- |

Best Unique PSM (Scan Sample\_2\_A1\_1\_3640.d:69552, m/z=560.75, z=2, RT=18.77, ppm=-1.63):


TRINITY\_DN105041\_c0\_g1\_i1.p1

back to list

  

| Protein Coverage
| Supporting Peptides
| Best Unique PSM
|

Protein Coverage:

Supporting Peptides:

| Peptide | Uniq | -10lgP | Mass | Length | ppm | m/z | z | RT | 1/k0 Start | 1/k0 End | Precursor ID | Area Sample 4 | #Feature | #Feature Sample 4 | Start | End | PTM | AScore | Found By |
| --- | --- | --- | --- | --- | --- | --- | --- | --- | --- | --- | --- | --- | --- | --- | --- | --- | --- | --- | --- |
| K.LTIIDALPR.C | Y | 58.24 | 1010.6124 | 9 | 0.02 | 506.3124 | 2 | 19.00 | 0.8392 | 0.8465 | 71132 | 3.79e2 | 1 | 1 | 58 | 66 |  |  | DB Search |
| total 1 peptides |  |  |  |  |  |  |  |  |  |  |  |  |  |  |  |  |  |  |  |
| --- | --- | --- | --- | --- | --- | --- | --- | --- | --- | --- | --- | --- | --- | --- | --- | --- | --- | --- | --- |

Best Unique PSM (Scan Sample\_2\_A1\_1\_3640.d:71132, m/z=506.3124, z=2, RT=19.00, ppm=-2.17):


TRINITY\_DN5\_c0\_g1\_i15.p1

back to list

  

| Protein Coverage
| Supporting Peptides
| Best Unique PSM
|

Protein Coverage:

Supporting Peptides:

| Peptide | Uniq | -10lgP | Mass | Length | ppm | m/z | z | RT | 1/k0 Start | 1/k0 End | Precursor ID | Area Sample 4 | #Feature | #Feature Sample 4 | Start | End | PTM | AScore | Found By |
| --- | --- | --- | --- | --- | --- | --- | --- | --- | --- | --- | --- | --- | --- | --- | --- | --- | --- | --- | --- |
| K.IIIELQGDLIK.I | Y | 53.27 | 1253.7594 | 11 | 3.89 | 627.8881 | 2 | 19.42 | 0.9513 | 0.9586 | 73842 | 0 | 0 | 0 | 492 | 502 |  |  | DB Search |
| total 1 peptides |  |  |  |  |  |  |  |  |  |  |  |  |  |  |  |  |  |  |  |
| --- | --- | --- | --- | --- | --- | --- | --- | --- | --- | --- | --- | --- | --- | --- | --- | --- | --- | --- | --- |

Best Unique PSM (Scan Sample\_2\_A1\_1\_3640.d:73842, m/z=627.8881, z=2, RT=19.42, ppm=1.69):


TRINITY\_DN1058\_c3\_g1\_i1.p1

back to list

  

| Protein Coverage
| Supporting Peptides
| Best Unique PSM
|

Protein Coverage:

Supporting Peptides:

| Peptide | Uniq | -10lgP | Mass | Length | ppm | m/z | z | RT | 1/k0 Start | 1/k0 End | Precursor ID | Area Sample 4 | #Feature | #Feature Sample 4 | Start | End | PTM | AScore | Found By |
| --- | --- | --- | --- | --- | --- | --- | --- | --- | --- | --- | --- | --- | --- | --- | --- | --- | --- | --- | --- |
| K.IC(+57.02)GQGSTIC(+57.02)DGTAR.K | Y | 58.27 | 1494.6555 | 14 | 4.48 | 748.3367 | 2 | 12.01 | 1.0009 | 1.0081 | 26890 | 9.48e2 | 1 | 1 | 425 | 438 | Carbamidomethylation, Carbamidomethylation |  | DB Search |
| K.GSVASLTC(+57.02)EC(+57.02)NNHK.G | Y | 54.96 | 1575.677 | 14 | 1.58 | 788.8453 | 2 | 12.07 | 1.0624 | 1.0697 | 27198 | 7.99e2 | 1 | 1 | 243 | 256 | Carbamidomethylation, Carbamidomethylation |  | DB Search |
| total 2 peptides |  |  |  |  |  |  |  |  |  |  |  |  |  |  |  |  |  |  |  |
| --- | --- | --- | --- | --- | --- | --- | --- | --- | --- | --- | --- | --- | --- | --- | --- | --- | --- | --- | --- |

Best Unique PSM (Scan Sample\_2\_A1\_1\_3640.d:26890, m/z=748.3367, z=2, RT=12.01, ppm=2.28):


TRINITY\_DN161\_c0\_g1\_i2.p1

back to list

  

| Protein Coverage
| Supporting Peptides
| Best Unique PSM
|

Protein Coverage:

Supporting Peptides:

| Peptide | Uniq | -10lgP | Mass | Length | ppm | m/z | z | RT | 1/k0 Start | 1/k0 End | Precursor ID | Area Sample 4 | #Feature | #Feature Sample 4 | Start | End | PTM | AScore | Found By |
| --- | --- | --- | --- | --- | --- | --- | --- | --- | --- | --- | --- | --- | --- | --- | --- | --- | --- | --- | --- |
| K.SYELPDGQVITIGNER.F | Y | 58.39 | 1789.8846 | 16 | 1.59 | 895.949 | 2 | 18.70 | 1.111 | 1.1182 | 69190 | 4.17e3 | 1 | 1 | 262 | 277 |  |  | DB Search |
| R.AVFPSIVGRPR.H | Y | 58 | 1197.6982 | 11 | 3.54 | 599.8572 | 2 | 15.96 | 0.9642 | 0.9715 | 51028 | 1.61e4 | 1 | 1 | 52 | 62 |  |  | DB Search |
| R.DLTDYLMK.I | Y | 57.96 | 997.479 | 8 | 6.32 | 499.7488 | 2 | 18.67 | 0.8319 | 0.8392 | 68987 | 0 | 0 | 0 | 207 | 214 |  |  | DB Search |
| K.LC(+57.02)YVALDFEQEM(+15.99)QTAASSSSLEK.S | Y | 57.8 | 2622.1829 | 23 | 5.82 | 875.0714 | 3 | 19.02 | 1.0741 | 1.0813 | 71279 | 4.32e2 | 1 | 1 | 239 | 261 | Carbamidomethylation, Oxidation (M) | M12:Oxidation (M):1000 | DB Search |
| K.DSYVGDEAQSKR.G | Y | 57.28 | 1353.616 | 12 | 2.57 | 677.8156 | 2 | 11.01 | 0.9993 | 1.0065 | 20794 | 1.16e3 | 1 | 1 | 74 | 85 |  |  | DB Search |
| R.DLTDYLM(+15.99)K.I | Y | 57.01 | 1013.4739 | 8 | 0.97 | 507.7436 | 2 | 16.91 | 0.8388 | 0.8461 | 57197 | 1.01e3 | 1 | 1 | 207 | 214 | Oxidation (M) | M7:Oxidation (M):1000 | DB Search |
| K.DLYANTVLSGGTTM(+15.99)FPGIADR.M | Y | 55.95 | 2214.0626 | 21 | -1.47 | 1108.0345 | 2 | 19.56 | 1.2432 | 1.2503 | 74741 | 0 | 0 | 0 | 315 | 335 | Oxidation (M) | M14:Oxidation (M):1000 | DB Search |
| total 7 peptides |  |  |  |  |  |  |  |  |  |  |  |  |  |  |  |  |  |  |  |
| --- | --- | --- | --- | --- | --- | --- | --- | --- | --- | --- | --- | --- | --- | --- | --- | --- | --- | --- | --- |

Best Unique PSM (Scan Sample\_2\_A1\_1\_3640.d:69190, m/z=895.949, z=2, RT=18.70, ppm=-0.61):


TRINITY\_DN661\_c0\_g1\_i10.p1

back to list

  

| Protein Coverage
| Supporting Peptides
| Best Unique PSM
|

Protein Coverage:

Supporting Peptides:

| Peptide | Uniq | -10lgP | Mass | Length | ppm | m/z | z | RT | 1/k0 Start | 1/k0 End | Precursor ID | Area Sample 4 | #Feature | #Feature Sample 4 | Start | End | PTM | AScore | Found By |
| --- | --- | --- | --- | --- | --- | --- | --- | --- | --- | --- | --- | --- | --- | --- | --- | --- | --- | --- | --- |
| K.AAGDLGIYENGC(+57.02)YDQLLK.E | Y | 58.39 | 1998.9357 | 18 | 5.61 | 1000.4785 | 2 | 18.94 | 1.1694 | 1.1766 | 70657 | 2.93e3 | 1 | 1 | 192 | 209 | Carbamidomethylation |  | DB Search |
| R.VPVTC(+57.02)C(+57.02)K.S | Y | 58.25 | 862.4041 | 7 | 4.46 | 432.2103 | 2 | 10.18 | 0.7677 | 0.775 | 15728 | 1.45e4 | 1 | 1 | 171 | 177 | Carbamidomethylation, Carbamidomethylation |  | DB Search |
| K.SAM(+15.99)DQVQEK.I | Y | 58.13 | 1050.4652 | 9 | -2.78 | 526.2372 | 2 | 9.49 | 0.8481 | 0.8554 | 12232 | 8.46e2 | 1 | 1 | 133 | 141 | Oxidation (M) | M3:Oxidation (M):1000 | DB Search |
| total 3 peptides |  |  |  |  |  |  |  |  |  |  |  |  |  |  |  |  |  |  |  |
| --- | --- | --- | --- | --- | --- | --- | --- | --- | --- | --- | --- | --- | --- | --- | --- | --- | --- | --- | --- |

Best Unique PSM (Scan Sample\_2\_A1\_1\_3640.d:70657, m/z=1000.4785, z=2, RT=18.94, ppm=3.41):


TRINITY\_DN2477\_c0\_g1\_i5.p1

back to list

  

| Protein Coverage
| Supporting Peptides
| Best Unique PSM
|

Protein Coverage:

Supporting Peptides:

| Peptide | Uniq | -10lgP | Mass | Length | ppm | m/z | z | RT | 1/k0 Start | 1/k0 End | Precursor ID | Area Sample 4 | #Feature | #Feature Sample 4 | Start | End | PTM | AScore | Found By |
| --- | --- | --- | --- | --- | --- | --- | --- | --- | --- | --- | --- | --- | --- | --- | --- | --- | --- | --- | --- |
| K.DSYVGDEAQSKR.G | Y | 57.28 | 1353.616 | 12 | 2.57 | 677.8156 | 2 | 11.01 | 0.9993 | 1.0065 | 20794 | 1.16e3 | 1 | 1 | 8 | 19 |  |  | DB Search |
| total 1 peptides |  |  |  |  |  |  |  |  |  |  |  |  |  |  |  |  |  |  |  |
| --- | --- | --- | --- | --- | --- | --- | --- | --- | --- | --- | --- | --- | --- | --- | --- | --- | --- | --- | --- |

Best Unique PSM (Scan Sample\_2\_A1\_1\_3640.d:20794, m/z=677.8156, z=2, RT=11.01, ppm=0.37):


TRINITY\_DN121418\_c0\_g1\_i1.p1

back to list

  

| Protein Coverage
| Supporting Peptides
| Best Unique PSM
|

Protein Coverage:

Supporting Peptides:

| Peptide | Uniq | -10lgP | Mass | Length | ppm | m/z | z | RT | 1/k0 Start | 1/k0 End | Precursor ID | Area Sample 4 | #Feature | #Feature Sample 4 | Start | End | PTM | AScore | Found By |
| --- | --- | --- | --- | --- | --- | --- | --- | --- | --- | --- | --- | --- | --- | --- | --- | --- | --- | --- | --- |
| M.AENDIGDWFR.S | Y | 54.99 | 1221.5414 | 10 | 2.04 | 611.7779 | 2 | 19.39 | 0.8834 | 0.8907 | 73581 | 3.56e2 | 1 | 1 | 2 | 11 |  |  | DB Search |
| total 1 peptides |  |  |  |  |  |  |  |  |  |  |  |  |  |  |  |  |  |  |  |
| --- | --- | --- | --- | --- | --- | --- | --- | --- | --- | --- | --- | --- | --- | --- | --- | --- | --- | --- | --- |

Best Unique PSM (Scan Sample\_2\_A1\_1\_3640.d:73581, m/z=611.7779, z=2, RT=19.39, ppm=-0.15):


TRINITY\_DN3182\_c0\_g1\_i3.p1

back to list

  

| Protein Coverage
| Supporting Peptides
| Best Unique PSM
|

Protein Coverage:

Supporting Peptides:

| Peptide | Uniq | -10lgP | Mass | Length | ppm | m/z | z | RT | 1/k0 Start | 1/k0 End | Precursor ID | Area Sample 4 | #Feature | #Feature Sample 4 | Start | End | PTM | AScore | Found By |
| --- | --- | --- | --- | --- | --- | --- | --- | --- | --- | --- | --- | --- | --- | --- | --- | --- | --- | --- | --- |
| K.LNQKVESILHK.I | Y | 58.39 | 1307.7561 | 11 | 3.28 | 436.9265 | 3 | 13.42 | 0.7453 | 0.7526 | 35604 | 4.6e2 | 1 | 1 | 146 | 156 |  |  | DB Search |
| K.IQTEFTSSGGPLVSVHVSDDPR.L | Y | 58.28 | 2327.1393 | 22 | 0.25 | 776.7189 | 3 | 17.17 | 0.9553 | 0.9626 | 58886 | 6.44e2 | 1 | 1 | 224 | 245 |  |  | DB Search |
| K.LTLGENIADNGGFK.T | Y | 57.37 | 1447.7307 | 14 | 2.38 | 724.8727 | 2 | 17.25 | 0.9993 | 1.0065 | 59417 | 7.06e2 | 1 | 1 | 669 | 682 |  |  | DB Search |
| total 3 peptides |  |  |  |  |  |  |  |  |  |  |  |  |  |  |  |  |  |  |  |
| --- | --- | --- | --- | --- | --- | --- | --- | --- | --- | --- | --- | --- | --- | --- | --- | --- | --- | --- | --- |

Best Unique PSM (Scan Sample\_2\_A1\_1\_3640.d:35604, m/z=436.9265, z=3, RT=13.42, ppm=1.09):


TRINITY\_DN36\_c0\_g1\_i13.p1

back to list

  

| Protein Coverage
| Supporting Peptides
| Best Unique PSM
|

Protein Coverage:

Supporting Peptides:

| Peptide | Uniq | -10lgP | Mass | Length | ppm | m/z | z | RT | 1/k0 Start | 1/k0 End | Precursor ID | Area Sample 4 | #Feature | #Feature Sample 4 | Start | End | PTM | AScore | Found By |
| --- | --- | --- | --- | --- | --- | --- | --- | --- | --- | --- | --- | --- | --- | --- | --- | --- | --- | --- | --- |
| R.LGGIEVTQGNC(+57.02)AR.K | Y | 58.36 | 1373.6721 | 13 | -2.89 | 687.8398 | 2 | 13.93 | 0.9662 | 0.9735 | 38726 | 2.09e3 | 1 | 1 | 57 | 69 | Carbamidomethylation |  | DB Search |
| K.GLPLAIGVR.D | Y | 57.12 | 894.565 | 9 | 0.27 | 448.2889 | 2 | 17.73 | 0.8124 | 0.8197 | 62574 | 3.52e3 | 1 | 1 | 31 | 39 |  |  | DB Search |
| total 2 peptides |  |  |  |  |  |  |  |  |  |  |  |  |  |  |  |  |  |  |  |
| --- | --- | --- | --- | --- | --- | --- | --- | --- | --- | --- | --- | --- | --- | --- | --- | --- | --- | --- | --- |

Best Unique PSM (Scan Sample\_2\_A1\_1\_3640.d:38726, m/z=687.8398, z=2, RT=13.93, ppm=-5.09):


TRINITY\_DN106\_c0\_g1\_i10.p1

back to list

  

| Protein Coverage
| Supporting Peptides
| Best Unique PSM
|

Protein Coverage:

Supporting Peptides:

| Peptide | Uniq | -10lgP | Mass | Length | ppm | m/z | z | RT | 1/k0 Start | 1/k0 End | Precursor ID | Area Sample 4 | #Feature | #Feature Sample 4 | Start | End | PTM | AScore | Found By |
| --- | --- | --- | --- | --- | --- | --- | --- | --- | --- | --- | --- | --- | --- | --- | --- | --- | --- | --- | --- |
| R.DGLNGDRGDPGPVGAVGAR.G | Y | 58.29 | 1778.8659 | 19 | -2.01 | 593.9601 | 3 | 13.86 | 0.8323 | 0.8396 | 38286 | 4.13e2 | 1 | 1 | 37 | 55 |  |  | DB Search |
| R.DGPAGPAGR.D | N | 58.24 | 796.3827 | 9 | 3.73 | 399.1992 | 2 | 9.76 | 0.7061 | 0.7135 | 13439 | 1.18e4 | 2 | 2 | 283 | 291 |  |  | DB Search |
| total 2 peptides |  |  |  |  |  |  |  |  |  |  |  |  |  |  |  |  |  |  |  |
| --- | --- | --- | --- | --- | --- | --- | --- | --- | --- | --- | --- | --- | --- | --- | --- | --- | --- | --- | --- |

Best Unique PSM (Scan Sample\_2\_A1\_1\_3640.d:38286, m/z=593.9601, z=3, RT=13.86, ppm=-4.21):


TRINITY\_DN57\_c0\_g1\_i1.p1

back to list

  

| Protein Coverage
| Supporting Peptides
| Best Unique PSM
|

Protein Coverage:

Supporting Peptides:

| Peptide | Uniq | -10lgP | Mass | Length | ppm | m/z | z | RT | 1/k0 Start | 1/k0 End | Precursor ID | Area Sample 4 | #Feature | #Feature Sample 4 | Start | End | PTM | AScore | Found By |
| --- | --- | --- | --- | --- | --- | --- | --- | --- | --- | --- | --- | --- | --- | --- | --- | --- | --- | --- | --- |
| R.VIFDDFR.E | Y | 58.31 | 910.4548 | 7 | 1.49 | 456.2344 | 2 | 17.85 | 0.7962 | 0.8035 | 63326 | 1.34e3 | 1 | 1 | 509 | 515 |  |  | DB Search |
| total 1 peptides |  |  |  |  |  |  |  |  |  |  |  |  |  |  |  |  |  |  |  |
| --- | --- | --- | --- | --- | --- | --- | --- | --- | --- | --- | --- | --- | --- | --- | --- | --- | --- | --- | --- |

Best Unique PSM (Scan Sample\_2\_A1\_1\_3640.d:63326, m/z=456.2344, z=2, RT=17.85, ppm=-0.7):


TRINITY\_DN96161\_c0\_g1\_i1.p1

back to list

  

| Protein Coverage
| Supporting Peptides
| Best Unique PSM
|

Protein Coverage:

Supporting Peptides:

| Peptide | Uniq | -10lgP | Mass | Length | ppm | m/z | z | RT | 1/k0 Start | 1/k0 End | Precursor ID | Area Sample 4 | #Feature | #Feature Sample 4 | Start | End | PTM | AScore | Found By |
| --- | --- | --- | --- | --- | --- | --- | --- | --- | --- | --- | --- | --- | --- | --- | --- | --- | --- | --- | --- |
| K.VGINYQPPTVVPGGDLAK.V | Y | 58.29 | 1823.9781 | 18 | -4.28 | 912.9904 | 2 | 17.44 | 1.1142 | 1.1214 | 60745 | 0 | 0 | 0 | 353 | 370 |  |  | DB Search |
| total 1 peptides |  |  |  |  |  |  |  |  |  |  |  |  |  |  |  |  |  |  |  |
| --- | --- | --- | --- | --- | --- | --- | --- | --- | --- | --- | --- | --- | --- | --- | --- | --- | --- | --- | --- |

Best Unique PSM (Scan Sample\_2\_A1\_1\_3640.d:60745, m/z=912.9904, z=2, RT=17.44, ppm=-6.48):


TRINITY\_DN7059\_c0\_g1\_i1.p1

back to list

  

| Protein Coverage
| Supporting Peptides
| Best Unique PSM
|

Protein Coverage:

Supporting Peptides:

| Peptide | Uniq | -10lgP | Mass | Length | ppm | m/z | z | RT | 1/k0 Start | 1/k0 End | Precursor ID | Area Sample 4 | #Feature | #Feature Sample 4 | Start | End | PTM | AScore | Found By |
| --- | --- | --- | --- | --- | --- | --- | --- | --- | --- | --- | --- | --- | --- | --- | --- | --- | --- | --- | --- |
| R.EQVSTFDSLQADLVK.L | Y | 58.4 | 1678.8413 | 15 | 2.3 | 840.428 | 2 | 18.77 | 1.0917 | 1.0989 | 69421 | 6.52e2 | 1 | 1 | 3 | 17 |  |  | DB Search |
| total 1 peptides |  |  |  |  |  |  |  |  |  |  |  |  |  |  |  |  |  |  |  |
| --- | --- | --- | --- | --- | --- | --- | --- | --- | --- | --- | --- | --- | --- | --- | --- | --- | --- | --- | --- |

Best Unique PSM (Scan Sample\_2\_A1\_1\_3640.d:69421, m/z=840.428, z=2, RT=18.77, ppm=0.11):


TRINITY\_DN194261\_c0\_g1\_i1.p1

back to list

  

| Protein Coverage
| Supporting Peptides
| Best Unique PSM
|

Protein Coverage:

Supporting Peptides:

| Peptide | Uniq | -10lgP | Mass | Length | ppm | m/z | z | RT | 1/k0 Start | 1/k0 End | Precursor ID | Area Sample 4 | #Feature | #Feature Sample 4 | Start | End | PTM | AScore | Found By |
| --- | --- | --- | --- | --- | --- | --- | --- | --- | --- | --- | --- | --- | --- | --- | --- | --- | --- | --- | --- |
| R.SFVLSGLSK.D | Y | 58.27 | 936.528 | 9 | 0.16 | 469.2703 | 2 | 17.30 | 0.7953 | 0.8027 | 59795 | 1.32e3 | 1 | 1 | 86 | 94 |  |  | DB Search |
| K.DVSPILLR.M | Y | 58.27 | 911.544 | 8 | -1.43 | 456.7776 | 2 | 17.20 | 0.7962 | 0.8035 | 59159 | 5.49e2 | 1 | 1 | 67 | 74 |  |  | DB Search |
| total 2 peptides |  |  |  |  |  |  |  |  |  |  |  |  |  |  |  |  |  |  |  |
| --- | --- | --- | --- | --- | --- | --- | --- | --- | --- | --- | --- | --- | --- | --- | --- | --- | --- | --- | --- |

Best Unique PSM (Scan Sample\_2\_A1\_1\_3640.d:59795, m/z=469.2703, z=2, RT=17.30, ppm=-2.04):


TRINITY\_DN4494\_c1\_g1\_i2.p1

back to list

  

| Protein Coverage
| Supporting Peptides
| Best Unique PSM
|

Protein Coverage:

Supporting Peptides:

| Peptide | Uniq | -10lgP | Mass | Length | ppm | m/z | z | RT | 1/k0 Start | 1/k0 End | Precursor ID | Area Sample 4 | #Feature | #Feature Sample 4 | Start | End | PTM | AScore | Found By |
| --- | --- | --- | --- | --- | --- | --- | --- | --- | --- | --- | --- | --- | --- | --- | --- | --- | --- | --- | --- |
| K.LLILLTDGSQTQDAGAEDPAK.I | Y | 58.29 | 2155.1008 | 21 | 2.82 | 1078.5583 | 2 | 18.86 | 1.2388 | 1.2459 | 70204 | 0 | 0 | 0 | 27 | 47 |  |  | DB Search |
| K.VDVGFILDSSGSLR.N | N | 58.24 | 1463.762 | 14 | 1.17 | 732.8875 | 2 | 19.97 | 1.0102 | 1.0174 | 77350 | 0 | 0 | 0 | 318 | 331 |  |  | DB Search |
| total 2 peptides |  |  |  |  |  |  |  |  |  |  |  |  |  |  |  |  |  |  |  |
| --- | --- | --- | --- | --- | --- | --- | --- | --- | --- | --- | --- | --- | --- | --- | --- | --- | --- | --- | --- |

Best Unique PSM (Scan Sample\_2\_A1\_1\_3640.d:70204, m/z=1078.5583, z=2, RT=18.86, ppm=0.63):


TRINITY\_DN59572\_c0\_g1\_i1.p1

back to list

  

| Protein Coverage
| Supporting Peptides
| Best Unique PSM
|

Protein Coverage:

Supporting Peptides:

| Peptide | Uniq | -10lgP | Mass | Length | ppm | m/z | z | RT | 1/k0 Start | 1/k0 End | Precursor ID | Area Sample 4 | #Feature | #Feature Sample 4 | Start | End | PTM | AScore | Found By |
| --- | --- | --- | --- | --- | --- | --- | --- | --- | --- | --- | --- | --- | --- | --- | --- | --- | --- | --- | --- |
| K.SYFEHSFVDLHR.I | Y | 58.39 | 1535.7157 | 12 | -0.97 | 512.9109 | 3 | 16.25 | 0.7693 | 0.7766 | 53061 | 8.88e2 | 1 | 1 | 107 | 118 |  |  | DB Search |
| total 1 peptides |  |  |  |  |  |  |  |  |  |  |  |  |  |  |  |  |  |  |  |
| --- | --- | --- | --- | --- | --- | --- | --- | --- | --- | --- | --- | --- | --- | --- | --- | --- | --- | --- | --- |

Best Unique PSM (Scan Sample\_2\_A1\_1\_3640.d:53061, m/z=512.9109, z=3, RT=16.25, ppm=-3.16):


TRINITY\_DN177106\_c0\_g1\_i1.p1

back to list

  

| Protein Coverage
| Supporting Peptides
| Best Unique PSM
|

Protein Coverage:

Supporting Peptides:

| Peptide | Uniq | -10lgP | Mass | Length | ppm | m/z | z | RT | 1/k0 Start | 1/k0 End | Precursor ID | Area Sample 4 | #Feature | #Feature Sample 4 | Start | End | PTM | AScore | Found By |
| --- | --- | --- | --- | --- | --- | --- | --- | --- | --- | --- | --- | --- | --- | --- | --- | --- | --- | --- | --- |
| K.YVEM(+15.99)ATSNANAVLK.K | Y | 58.39 | 1525.7446 | 14 | 1.62 | 763.8792 | 2 | 13.94 | 1.0395 | 1.0468 | 38859 | 0 | 0 | 0 | 28 | 41 | Oxidation (M) | M4:Oxidation (M):1000 | DB Search |
| total 1 peptides |  |  |  |  |  |  |  |  |  |  |  |  |  |  |  |  |  |  |  |
| --- | --- | --- | --- | --- | --- | --- | --- | --- | --- | --- | --- | --- | --- | --- | --- | --- | --- | --- | --- |

Best Unique PSM (Scan Sample\_2\_A1\_1\_3640.d:38859, m/z=763.8792, z=2, RT=13.94, ppm=-0.57):


TRINITY\_DN18\_c0\_g1\_i2.p1

back to list

  

| Protein Coverage
| Supporting Peptides
| Best Unique PSM
|

Protein Coverage:

Supporting Peptides:

| Peptide | Uniq | -10lgP | Mass | Length | ppm | m/z | z | RT | 1/k0 Start | 1/k0 End | Precursor ID | Area Sample 4 | #Feature | #Feature Sample 4 | Start | End | PTM | AScore | Found By |
| --- | --- | --- | --- | --- | --- | --- | --- | --- | --- | --- | --- | --- | --- | --- | --- | --- | --- | --- | --- |
| M.GIDIGLDEVK.T | Y | 54.98 | 1057.5655 | 10 | -4.24 | 529.7866 | 2 | 17.24 | 0.8619 | 0.8692 | 59426 | 5.78e3 | 1 | 1 | 2 | 11 |  |  | DB Search |
| R.FIFGVPENPR.H | Y | 47.68 | 1174.6135 | 10 | -0.68 | 588.3123 | 2 | 18.49 | 0.9295 | 0.9368 | 67817 | 5.2e3 | 1 | 1 | 229 | 238 |  |  | DB Search |
| total 2 peptides |  |  |  |  |  |  |  |  |  |  |  |  |  |  |  |  |  |  |  |
| --- | --- | --- | --- | --- | --- | --- | --- | --- | --- | --- | --- | --- | --- | --- | --- | --- | --- | --- | --- |

Best Unique PSM (Scan Sample\_2\_A1\_1\_3640.d:59426, m/z=529.7866, z=2, RT=17.24, ppm=-6.44):


TRINITY\_DN585\_c0\_g1\_i10.p1

back to list

  

| Protein Coverage
| Supporting Peptides
| Best Unique PSM
|

Protein Coverage:

Supporting Peptides:

| Peptide | Uniq | -10lgP | Mass | Length | ppm | m/z | z | RT | 1/k0 Start | 1/k0 End | Precursor ID | Area Sample 4 | #Feature | #Feature Sample 4 | Start | End | PTM | AScore | Found By |
| --- | --- | --- | --- | --- | --- | --- | --- | --- | --- | --- | --- | --- | --- | --- | --- | --- | --- | --- | --- |
| MQIFVK.T | Y | 57.42 | 764.4255 | 6 | 0.18 | 383.2192 | 2 | 15.73 | 0.7363 | 0.7437 | 49899 | 2.46e2 | 1 | 1 | 1 | 6 |  |  | DB Search |
| K.ESTLHLVLR.L | Y | 57.27 | 1066.6135 | 9 | 2.57 | 534.3142 | 2 | 15.80 | 0.8785 | 0.8858 | 50332 | 4.97e3 | 1 | 1 | 64 | 72 |  |  | DB Search |
| K.IQDKEGIPPDQQR.L | Y | 55.01 | 1522.7739 | 13 | 0.25 | 762.3928 | 2 | 11.08 | 1.0343 | 1.0415 | 21018 | 1.78e3 | 2 | 2 | 30 | 42 |  |  | DB Search |
| K.TITLEVEPSDTIENVK.S | Y | 54.56 | 1786.92 | 16 | 4.26 | 894.4691 | 2 | 17.62 | 1.1442 | 1.1514 | 61769 | 1.48e4 | 1 | 1 | 12 | 27 |  |  | DB Search |
| total 4 peptides |  |  |  |  |  |  |  |  |  |  |  |  |  |  |  |  |  |  |  |
| --- | --- | --- | --- | --- | --- | --- | --- | --- | --- | --- | --- | --- | --- | --- | --- | --- | --- | --- | --- |

Best Unique PSM (Scan Sample\_2\_A1\_1\_3640.d:49899, m/z=383.2192, z=2, RT=15.73, ppm=-2.02):


TRINITY\_DN10094\_c0\_g1\_i1.p1

back to list

  

| Protein Coverage
| Supporting Peptides
| Best Unique PSM
|

Protein Coverage:

Supporting Peptides:

| Peptide | Uniq | -10lgP | Mass | Length | ppm | m/z | z | RT | 1/k0 Start | 1/k0 End | Precursor ID | Area Sample 4 | #Feature | #Feature Sample 4 | Start | End | PTM | AScore | Found By |
| --- | --- | --- | --- | --- | --- | --- | --- | --- | --- | --- | --- | --- | --- | --- | --- | --- | --- | --- | --- |
| K.FFFHDQSC(+57.02)LDVYK.Y | Y | 58.39 | 1704.7606 | 13 | 3.79 | 569.2617 | 3 | 17.83 | 0.801 | 0.8084 | 63353 | 5.61e2 | 1 | 1 | 221 | 233 | Carbamidomethylation |  | DB Search |
| R.NFGLC(+57.02)TEHTGNLITNK.Y | Y | 54.67 | 1817.873 | 16 | 1.42 | 606.9645 | 3 | 15.88 | 0.8412 | 0.8485 | 50739 | 7.31e2 | 1 | 1 | 177 | 192 | Carbamidomethylation |  | DB Search |
| total 2 peptides |  |  |  |  |  |  |  |  |  |  |  |  |  |  |  |  |  |  |  |
| --- | --- | --- | --- | --- | --- | --- | --- | --- | --- | --- | --- | --- | --- | --- | --- | --- | --- | --- | --- |

Best Unique PSM (Scan Sample\_2\_A1\_1\_3640.d:63353, m/z=569.2617, z=3, RT=17.83, ppm=1.6):


TRINITY\_DN1339\_c0\_g1\_i2.p1

back to list

  

| Protein Coverage
| Supporting Peptides
| Best Unique PSM
|

Protein Coverage:

Supporting Peptides:

| Peptide | Uniq | -10lgP | Mass | Length | ppm | m/z | z | RT | 1/k0 Start | 1/k0 End | Precursor ID | Area Sample 4 | #Feature | #Feature Sample 4 | Start | End | PTM | AScore | Found By |
| --- | --- | --- | --- | --- | --- | --- | --- | --- | --- | --- | --- | --- | --- | --- | --- | --- | --- | --- | --- |
| K.VITSDDPAEKEK.K | Y | 58.39 | 1330.6616 | 12 | 3.3 | 666.3388 | 2 | 10.77 | 0.9856 | 0.9928 | 19482 | 3.46e2 | 1 | 1 | 160 | 171 |  |  | DB Search |
| total 1 peptides |  |  |  |  |  |  |  |  |  |  |  |  |  |  |  |  |  |  |  |
| --- | --- | --- | --- | --- | --- | --- | --- | --- | --- | --- | --- | --- | --- | --- | --- | --- | --- | --- | --- |

Best Unique PSM (Scan Sample\_2\_A1\_1\_3640.d:19482, m/z=666.3388, z=2, RT=10.77, ppm=1.11):


TRINITY\_DN36\_c0\_g1\_i1.p1

back to list

  

| Protein Coverage
| Supporting Peptides
| Best Unique PSM
|

Protein Coverage:

Supporting Peptides:

| Peptide | Uniq | -10lgP | Mass | Length | ppm | m/z | z | RT | 1/k0 Start | 1/k0 End | Precursor ID | Area Sample 4 | #Feature | #Feature Sample 4 | Start | End | PTM | AScore | Found By |
| --- | --- | --- | --- | --- | --- | --- | --- | --- | --- | --- | --- | --- | --- | --- | --- | --- | --- | --- | --- |
| R.LGGIEVTQGNC(+57.02)AR.K | Y | 58.36 | 1373.6721 | 13 | -2.89 | 687.8398 | 2 | 13.93 | 0.9662 | 0.9735 | 38726 | 2.09e3 | 1 | 1 | 58 | 70 | Carbamidomethylation |  | DB Search |
| K.GLPLAIGVR.D | Y | 57.12 | 894.565 | 9 | 0.27 | 448.2889 | 2 | 17.73 | 0.8124 | 0.8197 | 62574 | 3.52e3 | 1 | 1 | 32 | 40 |  |  | DB Search |
| total 2 peptides |  |  |  |  |  |  |  |  |  |  |  |  |  |  |  |  |  |  |  |
| --- | --- | --- | --- | --- | --- | --- | --- | --- | --- | --- | --- | --- | --- | --- | --- | --- | --- | --- | --- |

Best Unique PSM (Scan Sample\_2\_A1\_1\_3640.d:38726, m/z=687.8398, z=2, RT=13.93, ppm=-5.09):


TRINITY\_DN193641\_c0\_g1\_i1.p1

back to list

  

| Protein Coverage
| Supporting Peptides
| Best Unique PSM
|

Protein Coverage:

Supporting Peptides:

| Peptide | Uniq | -10lgP | Mass | Length | ppm | m/z | z | RT | 1/k0 Start | 1/k0 End | Precursor ID | Area Sample 4 | #Feature | #Feature Sample 4 | Start | End | PTM | AScore | Found By |
| --- | --- | --- | --- | --- | --- | --- | --- | --- | --- | --- | --- | --- | --- | --- | --- | --- | --- | --- | --- |
| R.GVNYQGDISLDDFVVK.D | Y | 58.36 | 1767.8679 | 16 | 4.61 | 884.9434 | 2 | 19.90 | 1.1082 | 1.1154 | 76829 | 2.2e3 | 1 | 1 | 110 | 125 |  |  | DB Search |
| K.YIFIEASSPR.K | Y | 54.75 | 1181.608 | 10 | -9.2 | 591.8046 | 2 | 16.96 | 0.9315 | 0.9388 | 57711 | 0 | 0 | 0 | 18 | 27 |  |  | DB Search |
| R.GLSGVFNIEVK.G | Y | 54.48 | 1161.6393 | 11 | -3.55 | 581.8236 | 2 | 18.95 | 0.9089 | 0.9162 | 70828 | 0 | 0 | 0 | 96 | 106 |  |  | DB Search |
| total 3 peptides |  |  |  |  |  |  |  |  |  |  |  |  |  |  |  |  |  |  |  |
| --- | --- | --- | --- | --- | --- | --- | --- | --- | --- | --- | --- | --- | --- | --- | --- | --- | --- | --- | --- |

Best Unique PSM (Scan Sample\_2\_A1\_1\_3640.d:76829, m/z=884.9434, z=2, RT=19.90, ppm=2.42):


TRINITY\_DN1783\_c0\_g1\_i2.p1

back to list

  

| Protein Coverage
| Supporting Peptides
| Best Unique PSM
|

Protein Coverage:

Supporting Peptides:

| Peptide | Uniq | -10lgP | Mass | Length | ppm | m/z | z | RT | 1/k0 Start | 1/k0 End | Precursor ID | Area Sample 4 | #Feature | #Feature Sample 4 | Start | End | PTM | AScore | Found By |
| --- | --- | --- | --- | --- | --- | --- | --- | --- | --- | --- | --- | --- | --- | --- | --- | --- | --- | --- | --- |
| K.LTIIDALPR.C | Y | 58.24 | 1010.6124 | 9 | 0.02 | 506.3124 | 2 | 19.00 | 0.8392 | 0.8465 | 71132 | 3.79e2 | 1 | 1 | 225 | 233 |  |  | DB Search |
| total 1 peptides |  |  |  |  |  |  |  |  |  |  |  |  |  |  |  |  |  |  |  |
| --- | --- | --- | --- | --- | --- | --- | --- | --- | --- | --- | --- | --- | --- | --- | --- | --- | --- | --- | --- |

Best Unique PSM (Scan Sample\_2\_A1\_1\_3640.d:71132, m/z=506.3124, z=2, RT=19.00, ppm=-2.17):


TRINITY\_DN160\_c0\_g1\_i2.p1

back to list

  

| Protein Coverage
| Supporting Peptides
| Best Unique PSM
|

Protein Coverage:

Supporting Peptides:

| Peptide | Uniq | -10lgP | Mass | Length | ppm | m/z | z | RT | 1/k0 Start | 1/k0 End | Precursor ID | Area Sample 4 | #Feature | #Feature Sample 4 | Start | End | PTM | AScore | Found By |
| --- | --- | --- | --- | --- | --- | --- | --- | --- | --- | --- | --- | --- | --- | --- | --- | --- | --- | --- | --- |
| K.YDNTITLDAVSSQK.L | Y | 57.09 | 1553.7573 | 14 | 3.45 | 777.8869 | 2 | 15.83 | 1.06 | 1.0672 | 50430 | 4.17e2 | 1 | 1 | 318 | 331 |  |  | DB Search |
| K.DLEFKFR.E | Y | 56.71 | 953.497 | 7 | 0.03 | 477.7548 | 2 | 16.91 | 0.8408 | 0.8481 | 57332 | 4.21e2 | 1 | 1 | 1031 | 1037 |  |  | DB Search |
| K.LRLDFDHEAK.L | Y | 55.3 | 1242.6356 | 10 | 5.7 | 415.2206 | 3 | 14.42 | 0.7359 | 0.7432 | 41684 | 1.12e3 | 1 | 1 | 1007 | 1016 |  |  | DB Search |
| K.VNQAC(+57.02)VR.T | Y | 50.6 | 845.4178 | 7 | 9.12 | 423.7191 | 2 | 9.74 | 0.7636 | 0.7709 | 13525 | 1.64e3 | 1 | 1 | 871 | 877 | Carbamidomethylation |  | DB Search |
| K.LNMIFR.F | Y | 49.11 | 792.4316 | 6 | 5.74 | 397.2245 | 2 | 17.36 | 0.7396 | 0.7469 | 60223 | 7.35e2 | 1 | 1 | 1017 | 1022 |  |  | DB Search |
| total 5 peptides |  |  |  |  |  |  |  |  |  |  |  |  |  |  |  |  |  |  |  |
| --- | --- | --- | --- | --- | --- | --- | --- | --- | --- | --- | --- | --- | --- | --- | --- | --- | --- | --- | --- |

Best Unique PSM (Scan Sample\_2\_A1\_1\_3640.d:50430, m/z=777.8869, z=2, RT=15.83, ppm=1.26):


TRINITY\_DN1910\_c0\_g1\_i5.p1

back to list

  

| Protein Coverage
| Supporting Peptides
| Best Unique PSM
|

Protein Coverage:

Supporting Peptides:

| Peptide | Uniq | -10lgP | Mass | Length | ppm | m/z | z | RT | 1/k0 Start | 1/k0 End | Precursor ID | Area Sample 4 | #Feature | #Feature Sample 4 | Start | End | PTM | AScore | Found By |
| --- | --- | --- | --- | --- | --- | --- | --- | --- | --- | --- | --- | --- | --- | --- | --- | --- | --- | --- | --- |
| K.LAVNMVPFPR.L | Y | 58.39 | 1142.627 | 10 | 0.31 | 572.3197 | 2 | 18.72 | 0.8721 | 0.8793 | 69256 | 4.38e2 | 1 | 1 | 253 | 262 |  |  | DB Search |
| K.LAVNM(+15.99)VPFPR.L | Y | 58.27 | 1158.6219 | 10 | -3.71 | 580.3148 | 2 | 17.06 | 0.8798 | 0.887 | 57945 | 1.18e3 | 1 | 1 | 253 | 262 | Oxidation (M) | M5:Oxidation (M):1000 | DB Search |
| R.FPGQLNADLRK.L | Y | 57.99 | 1257.6829 | 11 | -7.77 | 420.2307 | 3 | 14.50 | 0.7033 | 0.7106 | 42199 | 0 | 0 | 0 | 242 | 252 |  |  | DB Search |
| total 3 peptides |  |  |  |  |  |  |  |  |  |  |  |  |  |  |  |  |  |  |  |
| --- | --- | --- | --- | --- | --- | --- | --- | --- | --- | --- | --- | --- | --- | --- | --- | --- | --- | --- | --- |

Best Unique PSM (Scan Sample\_2\_A1\_1\_3640.d:69256, m/z=572.3197, z=2, RT=18.72, ppm=-1.89):


TRINITY\_DN118\_c1\_g1\_i5.p1

back to list

  

| Protein Coverage
| Supporting Peptides
| Best Unique PSM
|

Protein Coverage:

Supporting Peptides:

| Peptide | Uniq | -10lgP | Mass | Length | ppm | m/z | z | RT | 1/k0 Start | 1/k0 End | Precursor ID | Area Sample 4 | #Feature | #Feature Sample 4 | Start | End | PTM | AScore | Found By |
| --- | --- | --- | --- | --- | --- | --- | --- | --- | --- | --- | --- | --- | --- | --- | --- | --- | --- | --- | --- |
| R.ETC(+57.02)SDIEKR.E | Y | 58.32 | 1136.5132 | 9 | 0.46 | 569.2629 | 2 | 10.09 | 0.8883 | 0.8955 | 15406 | 2.17e3 | 1 | 1 | 186 | 194 | Carbamidomethylation |  | DB Search |
| K.VC(+57.02)EDVKR.C | Y | 56.84 | 904.4436 | 7 | 1.73 | 453.2289 | 2 | 9.35 | 0.7742 | 0.7815 | 11721 | 6.11e3 | 1 | 1 | 122 | 128 | Carbamidomethylation |  | DB Search |
| K.VC(+57.02)EDVK.R | Y | 51.73 | 748.3425 | 6 | 8.04 | 375.1807 | 2 | 9.73 | 0.7567 | 0.764 | 13508 | 0 | 0 | 0 | 122 | 127 | Carbamidomethylation |  | DB Search |
| total 3 peptides |  |  |  |  |  |  |  |  |  |  |  |  |  |  |  |  |  |  |  |
| --- | --- | --- | --- | --- | --- | --- | --- | --- | --- | --- | --- | --- | --- | --- | --- | --- | --- | --- | --- |

Best Unique PSM (Scan Sample\_2\_A1\_1\_3640.d:15406, m/z=569.2629, z=2, RT=10.09, ppm=-1.73):


TRINITY\_DN143583\_c0\_g1\_i1.p1

back to list

  

| Protein Coverage
| Supporting Peptides
| Best Unique PSM
|

Protein Coverage:

Supporting Peptides:

| Peptide | Uniq | -10lgP | Mass | Length | ppm | m/z | z | RT | 1/k0 Start | 1/k0 End | Precursor ID | Area Sample 4 | #Feature | #Feature Sample 4 | Start | End | PTM | AScore | Found By |
| --- | --- | --- | --- | --- | --- | --- | --- | --- | --- | --- | --- | --- | --- | --- | --- | --- | --- | --- | --- |
| R.GSSYLGDIALDDISVK | Y | 57.01 | 1651.8304 | 16 | 3.59 | 826.9236 | 2 | 19.81 | 1.0777 | 1.0849 | 76261 | 7.88e2 | 1 | 1 | 87 | 102 |  |  | DB Search |
| total 1 peptides |  |  |  |  |  |  |  |  |  |  |  |  |  |  |  |  |  |  |  |
| --- | --- | --- | --- | --- | --- | --- | --- | --- | --- | --- | --- | --- | --- | --- | --- | --- | --- | --- | --- |

Best Unique PSM (Scan Sample\_2\_A1\_1\_3640.d:76261, m/z=826.9236, z=2, RT=19.81, ppm=1.39):


TRINITY\_DN106\_c0\_g1\_i2.p1

back to list

  

| Protein Coverage
| Supporting Peptides
| Best Unique PSM
|

Protein Coverage:

Supporting Peptides:

| Peptide | Uniq | -10lgP | Mass | Length | ppm | m/z | z | RT | 1/k0 Start | 1/k0 End | Precursor ID | Area Sample 4 | #Feature | #Feature Sample 4 | Start | End | PTM | AScore | Found By |
| --- | --- | --- | --- | --- | --- | --- | --- | --- | --- | --- | --- | --- | --- | --- | --- | --- | --- | --- | --- |
| K.EGPAGPAGPVGR.L | Y | 58.28 | 1063.541 | 12 | -11.82 | 532.7703 | 2 | 11.84 | 0.8461 | 0.8534 | 25992 | 0 | 0 | 0 | 191 | 202 |  |  | DB Search |
| R.DGPAGPAGR.D | N | 58.24 | 796.3827 | 9 | 3.73 | 399.1992 | 2 | 9.76 | 0.7061 | 0.7135 | 13439 | 1.18e4 | 2 | 2 | 488 | 496 |  |  | DB Search |
| total 2 peptides |  |  |  |  |  |  |  |  |  |  |  |  |  |  |  |  |  |  |  |
| --- | --- | --- | --- | --- | --- | --- | --- | --- | --- | --- | --- | --- | --- | --- | --- | --- | --- | --- | --- |

Best Unique PSM (Scan Sample\_2\_A1\_1\_3640.d:25992, m/z=532.7703, z=2, RT=11.84, ppm=-14.02):


TRINITY\_DN28257\_c0\_g1\_i1.p1

back to list

  

| Protein Coverage
| Supporting Peptides
| Best Unique PSM
|

Protein Coverage:

Supporting Peptides:

| Peptide | Uniq | -10lgP | Mass | Length | ppm | m/z | z | RT | 1/k0 Start | 1/k0 End | Precursor ID | Area Sample 4 | #Feature | #Feature Sample 4 | Start | End | PTM | AScore | Found By |
| --- | --- | --- | --- | --- | --- | --- | --- | --- | --- | --- | --- | --- | --- | --- | --- | --- | --- | --- | --- |
| K.TC(+57.02)ESGDLDLDFAC(+57.02)GR.G | Y | 58.22 | 1714.6927 | 15 | -2.61 | 858.3495 | 2 | 17.67 | 1.0572 | 1.0644 | 62177 | 1.81e3 | 1 | 1 | 517 | 531 | Carbamidomethylation, Carbamidomethylation |  | DB Search |
| R.GYTTDPTDVQTC(+57.02)K.K | Y | 57.69 | 1484.6453 | 13 | 4.36 | 743.3315 | 2 | 12.56 | 1.0069 | 1.0142 | 30265 | 6.8e2 | 1 | 1 | 312 | 324 | Carbamidomethylation |  | DB Search |
| R.GYTTDPTDVQTC(+57.02)KK.I | Y | 56.69 | 1612.7403 | 14 | 11.46 | 807.3849 | 2 | 11.69 | 1.0395 | 1.0468 | 24808 | 9.83e2 | 1 | 1 | 312 | 325 | Carbamidomethylation |  | DB Search |
| K.ADEGYYPDSDGKTC(+57.02)VLAPC(+57.02)K.S | Y | 55.5 | 2244.9667 | 20 | 6.79 | 749.3329 | 3 | 14.72 | 0.9089 | 0.9162 | 43605 | 0 | 0 | 0 | 453 | 472 | Carbamidomethylation, Carbamidomethylation |  | DB Search |
| K.IKC(+57.02)DDPDVDYEK.V | Y | 54.35 | 1495.65 | 12 | 1.08 | 499.5567 | 3 | 12.15 | 0.7901 | 0.7974 | 27860 | 0 | 0 | 0 | 708 | 719 | Carbamidomethylation |  | DB Search |
| K.GHHC(+57.02)IC(+57.02)SAEGFEVTGTK.C | Y | 52.63 | 1888.8196 | 17 | 2.72 | 630.6141 | 3 | 13.51 | 0.8757 | 0.883 | 36216 | 0 | 0 | 0 | 111 | 127 | Carbamidomethylation, Carbamidomethylation |  | DB Search |
| total 6 peptides |  |  |  |  |  |  |  |  |  |  |  |  |  |  |  |  |  |  |  |
| --- | --- | --- | --- | --- | --- | --- | --- | --- | --- | --- | --- | --- | --- | --- | --- | --- | --- | --- | --- |

Best Unique PSM (Scan Sample\_2\_A1\_1\_3640.d:62177, m/z=858.3495, z=2, RT=17.67, ppm=-4.8):


TRINITY\_DN11643\_c0\_g1\_i1.p1

back to list

  

| Protein Coverage
| Supporting Peptides
| Best Unique PSM
|

Protein Coverage:

Supporting Peptides:

| Peptide | Uniq | -10lgP | Mass | Length | ppm | m/z | z | RT | 1/k0 Start | 1/k0 End | Precursor ID | Area Sample 4 | #Feature | #Feature Sample 4 | Start | End | PTM | AScore | Found By |
| --- | --- | --- | --- | --- | --- | --- | --- | --- | --- | --- | --- | --- | --- | --- | --- | --- | --- | --- | --- |
| R.EVNTELDSRPETK.K | Y | 58.28 | 1516.7369 | 13 | 5.19 | 759.378 | 2 | 11.86 | 1.0242 | 1.0315 | 25945 | 9e2 | 1 | 1 | 97 | 109 |  |  | DB Search |
| total 1 peptides |  |  |  |  |  |  |  |  |  |  |  |  |  |  |  |  |  |  |  |
| --- | --- | --- | --- | --- | --- | --- | --- | --- | --- | --- | --- | --- | --- | --- | --- | --- | --- | --- | --- |

Best Unique PSM (Scan Sample\_2\_A1\_1\_3640.d:25945, m/z=759.378, z=2, RT=11.86, ppm=2.99):


TRINITY\_DN477\_c0\_g1\_i8.p2

back to list

  

| Protein Coverage
| Supporting Peptides
| Best Unique PSM
|

Protein Coverage:

Supporting Peptides:

| Peptide | Uniq | -10lgP | Mass | Length | ppm | m/z | z | RT | 1/k0 Start | 1/k0 End | Precursor ID | Area Sample 4 | #Feature | #Feature Sample 4 | Start | End | PTM | AScore | Found By |
| --- | --- | --- | --- | --- | --- | --- | --- | --- | --- | --- | --- | --- | --- | --- | --- | --- | --- | --- | --- |
| R.NDVNNPPQAR.R | Y | 57.97 | 1123.537 | 10 | 3.56 | 562.7766 | 2 | 10.00 | 0.8615 | 0.8688 | 15207 | 4.89e4 | 2 | 2 | 86 | 95 |  |  | DB Search |
| total 1 peptides |  |  |  |  |  |  |  |  |  |  |  |  |  |  |  |  |  |  |  |
| --- | --- | --- | --- | --- | --- | --- | --- | --- | --- | --- | --- | --- | --- | --- | --- | --- | --- | --- | --- |

Best Unique PSM (Scan Sample\_2\_A1\_1\_3640.d:15207, m/z=562.7766, z=2, RT=10.00, ppm=1.36):


TRINITY\_DN36217\_c0\_g1\_i1.p1

back to list

  

| Protein Coverage
| Supporting Peptides
| Best Unique PSM
|

Protein Coverage:

Supporting Peptides:

| Peptide | Uniq | -10lgP | Mass | Length | ppm | m/z | z | RT | 1/k0 Start | 1/k0 End | Precursor ID | Area Sample 4 | #Feature | #Feature Sample 4 | Start | End | PTM | AScore | Found By |
| --- | --- | --- | --- | --- | --- | --- | --- | --- | --- | --- | --- | --- | --- | --- | --- | --- | --- | --- | --- |
| R.LGPIPLWK.K | Y | 58.11 | 922.564 | 8 | 0.42 | 462.2885 | 2 | 19.50 | 0.7795 | 0.7868 | 74214 | 9.3e2 | 1 | 1 | 124 | 131 |  |  | DB Search |
| total 1 peptides |  |  |  |  |  |  |  |  |  |  |  |  |  |  |  |  |  |  |  |
| --- | --- | --- | --- | --- | --- | --- | --- | --- | --- | --- | --- | --- | --- | --- | --- | --- | --- | --- | --- |

Best Unique PSM (Scan Sample\_2\_A1\_1\_3640.d:74214, m/z=462.2885, z=2, RT=19.50, ppm=-1.77):


TRINITY\_DN2357\_c1\_g1\_i1.p1

back to list

  

| Protein Coverage
| Supporting Peptides
| Best Unique PSM
|

Protein Coverage:

Supporting Peptides:

| Peptide | Uniq | -10lgP | Mass | Length | ppm | m/z | z | RT | 1/k0 Start | 1/k0 End | Precursor ID | Area Sample 4 | #Feature | #Feature Sample 4 | Start | End | PTM | AScore | Found By |
| --- | --- | --- | --- | --- | --- | --- | --- | --- | --- | --- | --- | --- | --- | --- | --- | --- | --- | --- | --- |
| K.VFIYGYDFQGGK.F | Y | 58.23 | 1392.6714 | 12 | -6.4 | 697.337 | 2 | 18.59 | 0.986 | 0.9932 | 68452 | 0 | 0 | 0 | 1218 | 1229 |  |  | DB Search |
| total 1 peptides |  |  |  |  |  |  |  |  |  |  |  |  |  |  |  |  |  |  |  |
| --- | --- | --- | --- | --- | --- | --- | --- | --- | --- | --- | --- | --- | --- | --- | --- | --- | --- | --- | --- |

Best Unique PSM (Scan Sample\_2\_A1\_1\_3640.d:68452, m/z=697.337, z=2, RT=18.59, ppm=-8.6):


TRINITY\_DN11522\_c0\_g1\_i3.p1

back to list

  

| Protein Coverage
| Supporting Peptides
| Best Unique PSM
|

Protein Coverage:

Supporting Peptides:

| Peptide | Uniq | -10lgP | Mass | Length | ppm | m/z | z | RT | 1/k0 Start | 1/k0 End | Precursor ID | Area Sample 4 | #Feature | #Feature Sample 4 | Start | End | PTM | AScore | Found By |
| --- | --- | --- | --- | --- | --- | --- | --- | --- | --- | --- | --- | --- | --- | --- | --- | --- | --- | --- | --- |
| K.GKPEQVQVK.M | Y | 58.01 | 1011.5713 | 9 | 0.86 | 506.7922 | 2 | 9.91 | 0.8376 | 0.8449 | 14438 | 2.02e2 | 1 | 1 | 548 | 556 |  |  | DB Search |
| total 1 peptides |  |  |  |  |  |  |  |  |  |  |  |  |  |  |  |  |  |  |  |
| --- | --- | --- | --- | --- | --- | --- | --- | --- | --- | --- | --- | --- | --- | --- | --- | --- | --- | --- | --- |

Best Unique PSM (Scan Sample\_2\_A1\_1\_3640.d:14438, m/z=506.7922, z=2, RT=9.91, ppm=-1.33):


TRINITY\_DN5365\_c0\_g1\_i2.p1

back to list

  

| Protein Coverage
| Supporting Peptides
| Best Unique PSM
|

Protein Coverage:

Supporting Peptides:

| Peptide | Uniq | -10lgP | Mass | Length | ppm | m/z | z | RT | 1/k0 Start | 1/k0 End | Precursor ID | Area Sample 4 | #Feature | #Feature Sample 4 | Start | End | PTM | AScore | Found By |
| --- | --- | --- | --- | --- | --- | --- | --- | --- | --- | --- | --- | --- | --- | --- | --- | --- | --- | --- | --- |
| R.FPGQLNADIRK.L | Y | 57.99 | 1257.6829 | 11 | -7.77 | 420.2307 | 3 | 14.50 | 0.7033 | 0.7106 | 42199 | 0 | 0 | 0 | 244 | 254 |  |  | DB Search |
| total 1 peptides |  |  |  |  |  |  |  |  |  |  |  |  |  |  |  |  |  |  |  |
| --- | --- | --- | --- | --- | --- | --- | --- | --- | --- | --- | --- | --- | --- | --- | --- | --- | --- | --- | --- |

Best Unique PSM (Scan Sample\_2\_A1\_1\_3640.d:42199, m/z=420.2307, z=3, RT=14.50, ppm=-9.97):


TRINITY\_DN428\_c0\_g1\_i1.p1

back to list

  

| Protein Coverage
| Supporting Peptides
| Best Unique PSM
|

Protein Coverage:

Supporting Peptides:

| Peptide | Uniq | -10lgP | Mass | Length | ppm | m/z | z | RT | 1/k0 Start | 1/k0 End | Precursor ID | Area Sample 4 | #Feature | #Feature Sample 4 | Start | End | PTM | AScore | Found By |
| --- | --- | --- | --- | --- | --- | --- | --- | --- | --- | --- | --- | --- | --- | --- | --- | --- | --- | --- | --- |
| K.LQQGIQDLEESLEEEETQR.Q | Y | 58.32 | 2273.0659 | 19 | -2.61 | 758.6923 | 3 | 19.57 | 0.9771 | 0.9844 | 74723 | 3.71e2 | 1 | 1 | 938 | 956 |  |  | DB Search |
| K.INNLEIQLDQEAK.E | Y | 58.28 | 1526.794 | 13 | -0.82 | 764.402 | 2 | 16.63 | 1.0407 | 1.048 | 55620 | 0 | 0 | 0 | 1813 | 1825 |  |  | DB Search |
| R.AGVLAHLEEER.D | Y | 58.28 | 1222.6306 | 11 | 0.12 | 612.3213 | 2 | 15.07 | 0.9558 | 0.963 | 45833 | 3.44e2 | 1 | 1 | 762 | 772 |  |  | DB Search |
| R.DLEQDLQELR.S | Y | 58.02 | 1257.6201 | 10 | 1.15 | 629.8167 | 2 | 19.45 | 0.9263 | 0.9335 | 73935 | 3.17e2 | 1 | 1 | 1133 | 1142 |  |  | DB Search |
| R.AGVLAHLEEERDIK.L | Y | 57.94 | 1578.8365 | 14 | 0.03 | 527.285 | 3 | 15.08 | 0.7994 | 0.8067 | 45791 | 4.01e2 | 1 | 1 | 762 | 775 |  |  | DB Search |
| R.ILFQEFR.Q | Y | 55.78 | 951.5178 | 7 | -1.31 | 476.7645 | 2 | 18.37 | 0.8108 | 0.8181 | 66910 | 9.28e2 | 1 | 1 | 709 | 715 |  |  | DB Search |
| R.AIESERDELLEELNNNSALK.N | Y | 55.22 | 2286.1339 | 20 | 1.76 | 763.0516 | 3 | 18.48 | 0.9965 | 1.0037 | 67692 | 0 | 0 | 0 | 1695 | 1714 |  |  | DB Search |
| K.IC(+57.02)HLLGIQVTDFTK.S | Y | 54.38 | 1643.8705 | 14 | 0.98 | 548.9634 | 3 | 18.67 | 0.8104 | 0.8177 | 68936 | 5.07e2 | 1 | 1 | 377 | 390 | Carbamidomethylation |  | DB Search |
| K.AQC(+57.02)EFAIEAITK.A | Y | 54.03 | 1379.6755 | 12 | 0.01 | 690.8435 | 2 | 17.77 | 0.9622 | 0.9695 | 62914 | 2.6e2 | 1 | 1 | 411 | 422 | Carbamidomethylation |  | DB Search |
| K.TALENENAEIAADLQR.I | Y | 52.91 | 1756.8591 | 16 | -4.14 | 879.4313 | 2 | 16.92 | 1.0985 | 1.1057 | 57445 | 0 | 0 | 0 | 1217 | 1232 |  |  | DB Search |
| K.ALSLQNANEELEDRLAEAER.Q | Y | 51.53 | 2270.1138 | 20 | 4.58 | 757.7137 | 3 | 18.93 | 0.9767 | 0.984 | 70669 | 5.99e2 | 1 | 1 | 1475 | 1494 |  |  | DB Search |
| K.VLSNLQVQLDQAK.K | Y | 47.86 | 1454.8093 | 13 | 1.55 | 728.4114 | 2 | 16.38 | 1.0222 | 1.0295 | 54047 | 0 | 0 | 0 | 1355 | 1367 |  |  | DB Search |
| total 12 peptides |  |  |  |  |  |  |  |  |  |  |  |  |  |  |  |  |  |  |  |
| --- | --- | --- | --- | --- | --- | --- | --- | --- | --- | --- | --- | --- | --- | --- | --- | --- | --- | --- | --- |

Best Unique PSM (Scan Sample\_2\_A1\_1\_3640.d:74723, m/z=758.6923, z=3, RT=19.57, ppm=-4.81):


TRINITY\_DN4781\_c0\_g1\_i1.p2

back to list

  

| Protein Coverage
| Supporting Peptides
| Best Unique PSM
|

Protein Coverage:

Supporting Peptides:

| Peptide | Uniq | -10lgP | Mass | Length | ppm | m/z | z | RT | 1/k0 Start | 1/k0 End | Precursor ID | Area Sample 4 | #Feature | #Feature Sample 4 | Start | End | PTM | AScore | Found By |
| --- | --- | --- | --- | --- | --- | --- | --- | --- | --- | --- | --- | --- | --- | --- | --- | --- | --- | --- | --- |
| K.DFQVIFEAK.M | Y | 58.28 | 1095.56 | 9 | -2.26 | 548.7849 | 2 | 19.00 | 0.8579 | 0.8652 | 71160 | 0 | 0 | 0 | 156 | 164 |  |  | DB Search |
| R.LISLPVESRPGGNC(+57.02)QVR.F | Y | 54.82 | 1880.989 | 17 | 0.61 | 628.0026 | 3 | 15.71 | 0.8481 | 0.8554 | 49525 | 1.61e3 | 1 | 1 | 513 | 529 | Carbamidomethylation |  | DB Search |
| total 2 peptides |  |  |  |  |  |  |  |  |  |  |  |  |  |  |  |  |  |  |  |
| --- | --- | --- | --- | --- | --- | --- | --- | --- | --- | --- | --- | --- | --- | --- | --- | --- | --- | --- | --- |

Best Unique PSM (Scan Sample\_2\_A1\_1\_3640.d:71160, m/z=548.7849, z=2, RT=19.00, ppm=-4.45):


TRINITY\_DN6221\_c0\_g1\_i2.p1

back to list

  

| Protein Coverage
| Supporting Peptides
| Best Unique PSM
|

Protein Coverage:

Supporting Peptides:

| Peptide | Uniq | -10lgP | Mass | Length | ppm | m/z | z | RT | 1/k0 Start | 1/k0 End | Precursor ID | Area Sample 4 | #Feature | #Feature Sample 4 | Start | End | PTM | AScore | Found By |
| --- | --- | --- | --- | --- | --- | --- | --- | --- | --- | --- | --- | --- | --- | --- | --- | --- | --- | --- | --- |
| K.SVQIEVSTPVC(+57.02)QTC(+57.02)PK.G | Y | 57.42 | 1831.8808 | 16 | 3.34 | 916.9487 | 2 | 15.16 | 1.1126 | 1.1198 | 46105 | 3.11e3 | 1 | 1 | 479 | 494 | Carbamidomethylation, Carbamidomethylation |  | DB Search |
| K.YQVTGC(+57.02)GGFC(+57.02)ESSVK.A | Y | 57.17 | 1677.7127 | 15 | -3.52 | 839.8588 | 2 | 14.63 | 1.068 | 1.0753 | 43042 | 0 | 0 | 0 | 545 | 559 | Carbamidomethylation, Carbamidomethylation |  | DB Search |
| total 2 peptides |  |  |  |  |  |  |  |  |  |  |  |  |  |  |  |  |  |  |  |
| --- | --- | --- | --- | --- | --- | --- | --- | --- | --- | --- | --- | --- | --- | --- | --- | --- | --- | --- | --- |

Best Unique PSM (Scan Sample\_2\_A1\_1\_3640.d:46105, m/z=916.9487, z=2, RT=15.16, ppm=1.15):


TRINITY\_DN991\_c0\_g1\_i2.p1

back to list

  

| Protein Coverage
| Supporting Peptides
| Best Unique PSM
|

Protein Coverage:

Supporting Peptides:

| Peptide | Uniq | -10lgP | Mass | Length | ppm | m/z | z | RT | 1/k0 Start | 1/k0 End | Precursor ID | Area Sample 4 | #Feature | #Feature Sample 4 | Start | End | PTM | AScore | Found By |
| --- | --- | --- | --- | --- | --- | --- | --- | --- | --- | --- | --- | --- | --- | --- | --- | --- | --- | --- | --- |
| K.IVGDDFTVGQSVTIK.C | Y | 58.39 | 1577.83 | 15 | 1.5 | 789.9218 | 2 | 17.37 | 1.0359 | 1.0431 | 60205 | 1.59e3 | 1 | 1 | 688 | 702 |  |  | DB Search |
| K.LSFC(+57.02)DTLTTPK.N | Y | 58.37 | 1281.6275 | 11 | 1.48 | 641.8206 | 2 | 17.08 | 0.9654 | 0.9727 | 58298 | 1.14e3 | 1 | 1 | 674 | 684 | Carbamidomethylation |  | DB Search |
| K.FGTC(+57.02)IDLIGDYR.C | Y | 58.29 | 1428.6707 | 12 | 0.76 | 715.3416 | 2 | 19.11 | 1.0049 | 1.0122 | 71833 | 0 | 0 | 0 | 2005 | 2016 | Carbamidomethylation |  | DB Search |
| R.ITVTVSPDIHPPAK.L | Y | 57.2 | 1473.8191 | 14 | -2.38 | 737.9135 | 2 | 15.04 | 1.0652 | 1.0725 | 45475 | 1.12e3 | 1 | 1 | 1183 | 1196 |  |  | DB Search |
| K.GVGHQC(+57.02)INTIGSFK.C | Y | 45.41 | 1516.7456 | 14 | 11.11 | 506.5937 | 3 | 14.17 | 0.7807 | 0.788 | 40291 | 0 | 0 | 0 | 808 | 821 | Carbamidomethylation |  | DB Search |
| R.GDFLILANSNGK.V | Y | 42.7 | 1247.651 | 12 | -8.76 | 624.8259 | 2 | 18.05 | 0.9501 | 0.9574 | 64900 | 0 | 0 | 0 | 2267 | 2278 |  |  | DB Search |
| K.ISYEC(+57.02)DEGYHLR.N | Y | 39.73 | 1540.6616 | 12 | 1.5 | 514.5608 | 3 | 14.27 | 0.7962 | 0.8035 | 40907 | 0 | 0 | 0 | 579 | 590 | Carbamidomethylation |  | DB Search |
| total 7 peptides |  |  |  |  |  |  |  |  |  |  |  |  |  |  |  |  |  |  |  |
| --- | --- | --- | --- | --- | --- | --- | --- | --- | --- | --- | --- | --- | --- | --- | --- | --- | --- | --- | --- |

Best Unique PSM (Scan Sample\_2\_A1\_1\_3640.d:60205, m/z=789.9218, z=2, RT=17.37, ppm=-0.69):


TRINITY\_DN161\_c0\_g1\_i4.p1

back to list

  

| Protein Coverage
| Supporting Peptides
| Best Unique PSM
|

Protein Coverage:

Supporting Peptides:

| Peptide | Uniq | -10lgP | Mass | Length | ppm | m/z | z | RT | 1/k0 Start | 1/k0 End | Precursor ID | Area Sample 4 | #Feature | #Feature Sample 4 | Start | End | PTM | AScore | Found By |
| --- | --- | --- | --- | --- | --- | --- | --- | --- | --- | --- | --- | --- | --- | --- | --- | --- | --- | --- | --- |
| K.SYELPDGQVITIGNER.F | Y | 58.39 | 1789.8846 | 16 | 1.59 | 895.949 | 2 | 18.70 | 1.111 | 1.1182 | 69190 | 4.17e3 | 1 | 1 | 95 | 110 |  |  | DB Search |
| R.DLTDYLMK.I | Y | 57.96 | 997.479 | 8 | 6.32 | 499.7488 | 2 | 18.67 | 0.8319 | 0.8392 | 68987 | 0 | 0 | 0 | 40 | 47 |  |  | DB Search |
| K.LC(+57.02)YVALDFEQEM(+15.99)QTAASSSSLEK.S | Y | 57.8 | 2622.1829 | 23 | 5.82 | 875.0714 | 3 | 19.02 | 1.0741 | 1.0813 | 71279 | 4.32e2 | 1 | 1 | 72 | 94 | Carbamidomethylation, Oxidation (M) | M12:Oxidation (M):1000 | DB Search |
| R.DLTDYLM(+15.99)K.I | Y | 57.01 | 1013.4739 | 8 | 0.97 | 507.7436 | 2 | 16.91 | 0.8388 | 0.8461 | 57197 | 1.01e3 | 1 | 1 | 40 | 47 | Oxidation (M) | M7:Oxidation (M):1000 | DB Search |
| K.DLYANTVLSGGTTM(+15.99)FPGIADR.M | Y | 55.95 | 2214.0626 | 21 | -1.47 | 1108.0345 | 2 | 19.56 | 1.2432 | 1.2503 | 74741 | 0 | 0 | 0 | 148 | 168 | Oxidation (M) | M14:Oxidation (M):1000 | DB Search |
| total 5 peptides |  |  |  |  |  |  |  |  |  |  |  |  |  |  |  |  |  |  |  |
| --- | --- | --- | --- | --- | --- | --- | --- | --- | --- | --- | --- | --- | --- | --- | --- | --- | --- | --- | --- |

Best Unique PSM (Scan Sample\_2\_A1\_1\_3640.d:69190, m/z=895.949, z=2, RT=18.70, ppm=-0.61):


TRINITY\_DN40728\_c0\_g1\_i1.p1

back to list

  

| Protein Coverage
| Supporting Peptides
| Best Unique PSM
|

Protein Coverage:

Supporting Peptides:

| Peptide | Uniq | -10lgP | Mass | Length | ppm | m/z | z | RT | 1/k0 Start | 1/k0 End | Precursor ID | Area Sample 4 | #Feature | #Feature Sample 4 | Start | End | PTM | AScore | Found By |
| --- | --- | --- | --- | --- | --- | --- | --- | --- | --- | --- | --- | --- | --- | --- | --- | --- | --- | --- | --- |
| K.ESTLHLVLR.L | Y | 57.27 | 1066.6135 | 9 | 2.57 | 534.3142 | 2 | 15.80 | 0.8785 | 0.8858 | 50332 | 4.97e3 | 1 | 1 | 87 | 95 |  |  | DB Search |
| K.IQDKEGIPPDQQR.L | Y | 55.01 | 1522.7739 | 13 | 0.25 | 762.3928 | 2 | 11.08 | 1.0343 | 1.0415 | 21018 | 1.78e3 | 2 | 2 | 53 | 65 |  |  | DB Search |
| total 2 peptides |  |  |  |  |  |  |  |  |  |  |  |  |  |  |  |  |  |  |  |
| --- | --- | --- | --- | --- | --- | --- | --- | --- | --- | --- | --- | --- | --- | --- | --- | --- | --- | --- | --- |

Best Unique PSM (Scan Sample\_2\_A1\_1\_3640.d:50332, m/z=534.7766, z=2, RT=15.80, ppm=0.37):


TRINITY\_DN687\_c0\_g2\_i1.p1

back to list

  

| Protein Coverage
| Supporting Peptides
| Best Unique PSM
|

Protein Coverage:

Supporting Peptides:

| Peptide | Uniq | -10lgP | Mass | Length | ppm | m/z | z | RT | 1/k0 Start | 1/k0 End | Precursor ID | Area Sample 4 | #Feature | #Feature Sample 4 | Start | End | PTM | AScore | Found By |
| --- | --- | --- | --- | --- | --- | --- | --- | --- | --- | --- | --- | --- | --- | --- | --- | --- | --- | --- | --- |
| K.VGINYQPPTVVPGGDLAK.V | Y | 58.29 | 1823.9781 | 18 | -4.28 | 912.9904 | 2 | 17.44 | 1.1142 | 1.1214 | 60745 | 0 | 0 | 0 | 353 | 370 |  |  | DB Search |
| total 1 peptides |  |  |  |  |  |  |  |  |  |  |  |  |  |  |  |  |  |  |  |
| --- | --- | --- | --- | --- | --- | --- | --- | --- | --- | --- | --- | --- | --- | --- | --- | --- | --- | --- | --- |

Best Unique PSM (Scan Sample\_2\_A1\_1\_3640.d:60745, m/z=912.9904, z=2, RT=17.44, ppm=-6.48):


TRINITY\_DN1386\_c0\_g1\_i1.p1

back to list

  

| Protein Coverage
| Supporting Peptides
| Best Unique PSM
|

Protein Coverage:

Supporting Peptides:

| Peptide | Uniq | -10lgP | Mass | Length | ppm | m/z | z | RT | 1/k0 Start | 1/k0 End | Precursor ID | Area Sample 4 | #Feature | #Feature Sample 4 | Start | End | PTM | AScore | Found By |
| --- | --- | --- | --- | --- | --- | --- | --- | --- | --- | --- | --- | --- | --- | --- | --- | --- | --- | --- | --- |
| K.VIVDQLVTLATR.V | Y | 58.24 | 1326.7871 | 12 | 1.65 | 664.4005 | 2 | 19.89 | 0.957 | 0.9642 | 76836 | 0 | 0 | 0 | 548 | 559 |  |  | DB Search |
| R.KVIVDQLVTLATR.V | Y | 52.34 | 1454.882 | 13 | 1.14 | 485.9674 | 3 | 18.50 | 0.7953 | 0.8027 | 67825 | 1.76e2 | 1 | 1 | 547 | 559 |  |  | DB Search |
| total 2 peptides |  |  |  |  |  |  |  |  |  |  |  |  |  |  |  |  |  |  |  |
| --- | --- | --- | --- | --- | --- | --- | --- | --- | --- | --- | --- | --- | --- | --- | --- | --- | --- | --- | --- |

Best Unique PSM (Scan Sample\_2\_A1\_1\_3640.d:76836, m/z=664.4005, z=2, RT=19.89, ppm=-0.54):


TRINITY\_DN100850\_c0\_g1\_i1.p1

back to list

  

| Protein Coverage
| Supporting Peptides
| Best Unique PSM
|

Protein Coverage:

Supporting Peptides:

| Peptide | Uniq | -10lgP | Mass | Length | ppm | m/z | z | RT | 1/k0 Start | 1/k0 End | Precursor ID | Area Sample 4 | #Feature | #Feature Sample 4 | Start | End | PTM | AScore | Found By |
| --- | --- | --- | --- | --- | --- | --- | --- | --- | --- | --- | --- | --- | --- | --- | --- | --- | --- | --- | --- |
| K.RPLLGQDYQGDVTDLVC(+57.02)R.C | Y | 55.62 | 2104.0371 | 18 | 2.09 | 702.3529 | 3 | 17.83 | 0.9032 | 0.9105 | 63348 | 2.22e2 | 1 | 1 | 104 | 121 | Carbamidomethylation |  | DB Search |
| total 1 peptides |  |  |  |  |  |  |  |  |  |  |  |  |  |  |  |  |  |  |  |
| --- | --- | --- | --- | --- | --- | --- | --- | --- | --- | --- | --- | --- | --- | --- | --- | --- | --- | --- | --- |

Best Unique PSM (Scan Sample\_2\_A1\_1\_3640.d:63348, m/z=702.3529, z=3, RT=17.83, ppm=-0.1):


TRINITY\_DN4815\_c0\_g1\_i2.p1

back to list

  

| Protein Coverage
| Supporting Peptides
| Best Unique PSM
|

Protein Coverage:

Supporting Peptides:

| Peptide | Uniq | -10lgP | Mass | Length | ppm | m/z | z | RT | 1/k0 Start | 1/k0 End | Precursor ID | Area Sample 4 | #Feature | #Feature Sample 4 | Start | End | PTM | AScore | Found By |
| --- | --- | --- | --- | --- | --- | --- | --- | --- | --- | --- | --- | --- | --- | --- | --- | --- | --- | --- | --- |
| K.DVNC(+57.02)NNNQLK.S | Y | 58.39 | 1217.5459 | 10 | 2.19 | 609.7802 | 2 | 10.68 | 0.9129 | 0.9202 | 18856 | 1.17e3 | 1 | 1 | 536 | 545 | Carbamidomethylation |  | DB Search |
| R.DC(+57.02)PNEPTER.A | Y | 58.29 | 1116.4506 | 9 | 1.83 | 559.2324 | 2 | 10.18 | 0.853 | 0.8603 | 15597 | 3.45e3 | 1 | 1 | 459 | 467 | Carbamidomethylation |  | DB Search |
| K.TC(+57.02)TC(+57.02)ENVR.Q | Y | 58.27 | 1038.4223 | 8 | 5.2 | 520.22 | 2 | 9.83 | 0.8425 | 0.8498 | 14008 | 2.42e3 | 1 | 1 | 485 | 492 | Carbamidomethylation, Carbamidomethylation |  | DB Search |
| R.AVAC(+57.02)NSGPC(+57.02)LAGC(+57.02)PR.N | Y | 58.17 | 1588.6908 | 15 | 1.54 | 795.3522 | 2 | 13.16 | 1.015 | 1.0222 | 33794 | 1.36e4 | 1 | 1 | 468 | 482 | Carbamidomethylation, Carbamidomethylation, Carbamidomethylation |  | DB Search |
| K.DNGDWAIDNLR.I | Y | 57.94 | 1287.5843 | 11 | -1.8 | 644.7969 | 2 | 17.93 | 0.9206 | 0.9279 | 64010 | 0 | 0 | 0 | 186 | 196 |  |  | DB Search |
| K.TIKDVNC(+57.02)NNNQLK.S | Y | 54.96 | 1559.7726 | 13 | 3.46 | 520.9321 | 3 | 11.33 | 0.7892 | 0.7966 | 22761 | 1.98e3 | 1 | 1 | 533 | 545 | Carbamidomethylation |  | DB Search |
| total 6 peptides |  |  |  |  |  |  |  |  |  |  |  |  |  |  |  |  |  |  |  |
| --- | --- | --- | --- | --- | --- | --- | --- | --- | --- | --- | --- | --- | --- | --- | --- | --- | --- | --- | --- |

Best Unique PSM (Scan Sample\_2\_A1\_1\_3640.d:18856, m/z=609.7802, z=2, RT=10.68, ppm=-0.01):


TRINITY\_DN4595\_c0\_g1\_i1.p1

back to list

  

| Protein Coverage
| Supporting Peptides
| Best Unique PSM
|

Protein Coverage:

Supporting Peptides:

| Peptide | Uniq | -10lgP | Mass | Length | ppm | m/z | z | RT | 1/k0 Start | 1/k0 End | Precursor ID | Area Sample 4 | #Feature | #Feature Sample 4 | Start | End | PTM | AScore | Found By |
| --- | --- | --- | --- | --- | --- | --- | --- | --- | --- | --- | --- | --- | --- | --- | --- | --- | --- | --- | --- |
| R.AFNKIETR.N | Y | 54.97 | 977.5294 | 8 | 8.36 | 489.775 | 2 | 11.54 | 0.8339 | 0.8412 | 24067 | 2.69e3 | 1 | 1 | 72 | 79 |  |  | DB Search |
| total 1 peptides |  |  |  |  |  |  |  |  |  |  |  |  |  |  |  |  |  |  |  |
| --- | --- | --- | --- | --- | --- | --- | --- | --- | --- | --- | --- | --- | --- | --- | --- | --- | --- | --- | --- |

Best Unique PSM (Scan Sample\_2\_A1\_1\_3640.d:24067, m/z=489.775, z=2, RT=11.54, ppm=6.17):


TRINITY\_DN124769\_c0\_g1\_i1.p1

back to list

  

| Protein Coverage
| Supporting Peptides
| Best Unique PSM
|

Protein Coverage:

Supporting Peptides:

| Peptide | Uniq | -10lgP | Mass | Length | ppm | m/z | z | RT | 1/k0 Start | 1/k0 End | Precursor ID | Area Sample 4 | #Feature | #Feature Sample 4 | Start | End | PTM | AScore | Found By |
| --- | --- | --- | --- | --- | --- | --- | --- | --- | --- | --- | --- | --- | --- | --- | --- | --- | --- | --- | --- |
| K.VITSDDPAEKEK.K | Y | 58.39 | 1330.6616 | 12 | 3.3 | 666.3388 | 2 | 10.77 | 0.9856 | 0.9928 | 19482 | 3.46e2 | 1 | 1 | 91 | 102 |  |  | DB Search |
| total 1 peptides |  |  |  |  |  |  |  |  |  |  |  |  |  |  |  |  |  |  |  |
| --- | --- | --- | --- | --- | --- | --- | --- | --- | --- | --- | --- | --- | --- | --- | --- | --- | --- | --- | --- |

Best Unique PSM (Scan Sample\_2\_A1\_1\_3640.d:19482, m/z=666.3388, z=2, RT=10.77, ppm=1.11):


TRINITY\_DN2720\_c0\_g1\_i2.p1

back to list

  

| Protein Coverage
| Supporting Peptides
| Best Unique PSM
|

Protein Coverage:

Supporting Peptides:

| Peptide | Uniq | -10lgP | Mass | Length | ppm | m/z | z | RT | 1/k0 Start | 1/k0 End | Precursor ID | Area Sample 4 | #Feature | #Feature Sample 4 | Start | End | PTM | AScore | Found By |
| --- | --- | --- | --- | --- | --- | --- | --- | --- | --- | --- | --- | --- | --- | --- | --- | --- | --- | --- | --- |
| R.AC(+57.02)VDIDEC(+57.02)SEDPNAC(+57.02)R.N | Y | 57.49 | 1909.724 | 16 | -0.91 | 955.8663 | 2 | 13.56 | 1.1029 | 1.1102 | 36467 | 0 | 0 | 0 | 511 | 526 | Carbamidomethylation, Carbamidomethylation, Carbamidomethylation |  | DB Search |
| R.IGGTC(+57.02)VNIVGTYR.C | Y | 57.24 | 1408.7133 | 13 | 3.99 | 705.3652 | 2 | 15.98 | 0.9941 | 1.0013 | 51355 | 1.59e3 | 1 | 1 | 1441 | 1453 | Carbamidomethylation |  | DB Search |
| R.AC(+57.02)PPSNSGTR.Q | Y | 55.99 | 1045.4611 | 10 | 2.5 | 523.738 | 2 | 8.88 | 0.8092 | 0.8165 | 10061 | 1.48e2 | 1 | 1 | 976 | 985 | Carbamidomethylation |  | DB Search |
| total 3 peptides |  |  |  |  |  |  |  |  |  |  |  |  |  |  |  |  |  |  |  |
| --- | --- | --- | --- | --- | --- | --- | --- | --- | --- | --- | --- | --- | --- | --- | --- | --- | --- | --- | --- |

Best Unique PSM (Scan Sample\_2\_A1\_1\_3640.d:36467, m/z=955.8663, z=2, RT=13.56, ppm=-3.11):


TRINITY\_DN27058\_c0\_g1\_i1.p1

back to list

  

| Protein Coverage
| Supporting Peptides
| Best Unique PSM
|

Protein Coverage:

Supporting Peptides:

| Peptide | Uniq | -10lgP | Mass | Length | ppm | m/z | z | RT | 1/k0 Start | 1/k0 End | Precursor ID | Area Sample 4 | #Feature | #Feature Sample 4 | Start | End | PTM | AScore | Found By |
| --- | --- | --- | --- | --- | --- | --- | --- | --- | --- | --- | --- | --- | --- | --- | --- | --- | --- | --- | --- |
| R.LLYTPGESSLSNQR.S | Y | 58.28 | 1563.7893 | 14 | 5.64 | 782.9046 | 2 | 15.52 | 1.0484 | 1.0556 | 48763 | 2.91e2 | 1 | 1 | 41 | 54 |  |  | DB Search |
| K.TGISLDSLASSHPISQPVDHPK.Q | Y | 51.15 | 2285.1651 | 22 | 18.69 | 572.308 | 4 | 16.56 | 0.866 | 0.8733 | 55162 | 0 | 0 | 0 | 116 | 137 |  |  | DB Search |
| total 2 peptides |  |  |  |  |  |  |  |  |  |  |  |  |  |  |  |  |  |  |  |
| --- | --- | --- | --- | --- | --- | --- | --- | --- | --- | --- | --- | --- | --- | --- | --- | --- | --- | --- | --- |

Best Unique PSM (Scan Sample\_2\_A1\_1\_3640.d:48763, m/z=782.9046, z=2, RT=15.52, ppm=3.44):


---

Prepared with PEAKS ™ (bioinfor.com)
