# Supplementary material for: Proteomic Analysis and Biochemical Characterization of the Nematocyst Extract of the Hydrozoan Velella velella
Source: Mar Drugs. 2024 Oct 12;22(10):468. doi: 10.3390/md22100468 (PMC11509761; doi:10.3390/md22100468)
Supplement: Supplementary file 1 [file marinedrugs-22-00468-s001.zip › Supplementary File S1.zip!/Sample_3_Vellela/db.peptides.html]

peptide list


Peptide List

  

| Peptide | -10lgP | Mass | Length | ppm | m/z | RT | 1/k0 Start | 1/k0 End | Precursor ID | Area Sample 5 | #Feature | #Feature Sample 5 | Accession | PTM | AScore | Found By |
| --- | --- | --- | --- | --- | --- | --- | --- | --- | --- | --- | --- | --- | --- | --- | --- | --- |
| GC(+57.02)STC(+57.02)GC(+57.02)NGAGTNNGVK | 31.94 | 1712.6665 | 17 | 1.85 | 857.3384 | 9.80 | 1.0448 | 1.052 | 14171 | 0 | 0 | 0 | TRINITY\_DN1616\_c0\_g1\_i3.p1:TRINITY\_DN1616\_c0\_g1\_i6.p1:TRINITY\_DN1616\_c0\_g1\_i2.p1:TRINITY\_DN1616\_c0\_g1\_i7.p1 | Carbamidomethylation, Carbamidomethylation, Carbamidomethylation |  | DB Search |
| GLQGQQGEQGSTGVQGSVGGR | 31.94 | 1985.9514 | 21 | 4.67 | 993.9834 | 12.00 | 1.1326 | 1.1398 | 27213 | 3e3 | 1 | 1 | TRINITY\_DN1030\_c0\_g1\_i1.p1:TRINITY\_DN1030\_c0\_g1\_i2.p1 |  |  | DB Search |
| VLIVISDGNEDFKEK | 31.93 | 1704.8933 | 15 | -1.64 | 853.4489 | 16.67 | 1.1702 | 1.1774 | 51996 | 2.43e2 | 1 | 1 | TRINITY\_DN195348\_c0\_g1\_i1.p1 |  |  | DB Search |
| GSC(+57.02)SC(+57.02)YGHAEQC(+57.02)IPTK | 31.92 | 1853.7495 | 16 | 1.05 | 927.879 | 12.32 | 1.1869 | 1.1941 | 29052 | 1.68e2 | 1 | 1 | TRINITY\_DN15274\_c0\_g1\_i1.p1 | Carbamidomethylation, Carbamidomethylation, Carbamidomethylation |  | DB Search |
| YVAIYPSDHLTVNR | 31.92 | 1646.8417 | 14 | 0.07 | 824.4246 | 15.53 | 1.1061 | 1.1134 | 46351 | 1.08e3 | 1 | 1 | TRINITY\_DN982\_c0\_g1\_i3.p1:TRINITY\_DN982\_c0\_g1\_i1.p1 |  |  | DB Search |
| VSQTC(+57.02)AEHVFR | 31.91 | 1332.6245 | 11 | 4.3 | 667.3195 | 12.77 | 0.9848 | 0.992 | 31588 | 5.38e3 | 3 | 3 | TRINITY\_DN3078\_c0\_g1\_i1.p1 | Carbamidomethylation |  | DB Search |
| AVQIHPFNDVSVGDPK | 31.9 | 1721.8737 | 16 | 2.29 | 861.9424 | 15.73 | 1.1186 | 1.1258 | 47418 | 2.88e3 | 1 | 1 | TRINITY\_DN1071\_c0\_g1\_i2.p1:TRINITY\_DN1071\_c0\_g1\_i3.p1:TRINITY\_DN1071\_c0\_g1\_i1.p1:TRINITY\_DN1071\_c0\_g1\_i4.p1 |  |  | DB Search |
| GSVASLTC(+57.02)EC(+57.02)NNHK | 31.9 | 1575.677 | 14 | 5.83 | 788.847 | 11.91 | 1.064 | 1.0713 | 26802 | 0 | 0 | 0 | TRINITY\_DN1058\_c3\_g1\_i1.p1 | Carbamidomethylation, Carbamidomethylation |  | DB Search |
| AFVAIGDYGGHVGLGVK | 31.89 | 1658.8779 | 17 | 4.75 | 830.4467 | 17.61 | 1.1622 | 1.1694 | 57032 | 1.04e3 | 1 | 1 | TRINITY\_DN15584\_c0\_g1\_i1.p1 |  |  | DB Search |
| LTHQIEVTVSDR | 31.87 | 1396.7311 | 12 | -2.08 | 699.3683 | 13.43 | 0.9912 | 0.9985 | 35251 | 5.62e2 | 1 | 1 | TRINITY\_DN123121\_c0\_g1\_i1.p1 |  |  | DB Search |
| IVIDSEHC(+57.02)NSR | 31.84 | 1328.6143 | 11 | 4.43 | 665.3145 | 11.35 | 0.961 | 0.9683 | 23595 | 3.61e3 | 1 | 1 | TRINITY\_DN25375\_c0\_g1\_i7.p1 | Carbamidomethylation |  | DB Search |
| ALLLASSNAFDTSNGAR | 31.84 | 1706.8588 | 17 | 2.57 | 854.4352 | 17.65 | 1.0937 | 1.1009 | 57325 | 6.66e2 | 1 | 1 | TRINITY\_DN244\_c0\_g1\_i1.p1 |  |  | DB Search |
| TQAVLDRLNIEKR | 31.84 | 1554.8842 | 13 | 3.94 | 519.3018 | 14.55 | 0.7978 | 0.8051 | 41371 | 3.63e3 | 1 | 1 | TRINITY\_DN477\_c0\_g1\_i9.p1 |  |  | DB Search |
| LGVM(+15.99)EDHSNR | 31.83 | 1172.5244 | 10 | -12.49 | 587.2596 | 10.26 | 0.9012 | 0.9085 | 17065 | 0 | 0 | 0 | TRINITY\_DN5633\_c0\_g1\_i1.p1 | Oxidation (M) | M4:Oxidation (M):1000 | DB Search |
| AQLKNELIEQSENIK | 31.8 | 1755.9366 | 15 | 1.69 | 878.9733 | 15.24 | 1.1001 | 1.1074 | 44891 | 4.49e2 | 1 | 1 | TRINITY\_DN3078\_c0\_g1\_i1.p1 |  |  | DB Search |
| AVFPSIVGRPR | 31.8 | 1197.6982 | 11 | 3.08 | 599.8557 | 15.68 | 0.9574 | 0.9646 | 46487 | 1.74e4 | 1 | 1 | TRINITY\_DN161\_c0\_g1\_i1.p1:TRINITY\_DN161\_c0\_g1\_i3.p1:TRINITY\_DN161\_c0\_g1\_i2.p1 |  |  | DB Search |
| TPGEC(+57.02)C(+57.02)PQC(+57.02)K | 31.8 | 1235.4733 | 10 | 3.13 | 618.7432 | 9.97 | 0.8923 | 0.8996 | 15133 | 1.64e3 | 1 | 1 | TRINITY\_DN1369\_c0\_g1\_i1.p1 | Carbamidomethylation, Carbamidomethylation, Carbamidomethylation |  | DB Search |
| FSNDIVDSFAVSQKK | 31.79 | 1683.8468 | 15 | 6.6 | 562.2908 | 17.06 | 0.8664 | 0.8737 | 53931 | 0 | 0 | 0 | TRINITY\_DN244\_c0\_g1\_i1.p1 |  |  | DB Search |
| ALVILGDGFISGGGTPSK | 31.78 | 1687.9144 | 18 | 3.47 | 844.9638 | 19.74 | 1.167 | 1.1742 | 69284 | 6.7e3 | 1 | 1 | TRINITY\_DN244\_c0\_g1\_i1.p1 |  |  | DB Search |
| ILQC(+57.02)NQYANVEEAR | 31.75 | 1706.8046 | 14 | 3.31 | 854.4088 | 14.22 | 1.0813 | 1.0885 | 39617 | 7.69e2 | 1 | 1 | TRINITY\_DN477\_c0\_g1\_i8.p1:TRINITY\_DN477\_c0\_g1\_i7.p1 | Carbamidomethylation |  | DB Search |
| AINIHDLVGIDTKK | 31.74 | 1535.8671 | 14 | 2.57 | 768.9395 | 16.45 | 1.0753 | 1.0825 | 50998 | 1.15e3 | 1 | 1 | TRINITY\_DN61\_c0\_g1\_i1.p1 |  |  | DB Search |
| MRFDTAITAC(+57.02)R | 31.74 | 1340.6329 | 11 | 4.37 | 671.3238 | 14.91 | 0.9646 | 0.9719 | 43169 | 1.84e3 | 1 | 1 | TRINITY\_DN477\_c0\_g1\_i9.p1 | Carbamidomethylation |  | DB Search |
| LVQNTPHNSIVTK | 31.71 | 1449.7939 | 13 | 2.57 | 725.903 | 11.68 | 1.0572 | 1.0644 | 25401 | 4.01e3 | 1 | 1 | TRINITY\_DN982\_c0\_g1\_i3.p1:TRINITY\_DN982\_c0\_g1\_i1.p1 |  |  | DB Search |
| ASMDEGAKPK | 31.71 | 1032.491 | 10 | 6.03 | 517.2537 | 9.35 | 0.8704 | 0.8777 | 11527 | 2.53e2 | 1 | 1 | TRINITY\_DN2003\_c0\_g1\_i1.p1 |  |  | DB Search |
| APTHILNPANKR | 31.7 | 1330.7469 | 12 | 1.15 | 666.3787 | 11.11 | 1.0098 | 1.017 | 22167 | 1.2e4 | 2 | 2 | TRINITY\_DN1058\_c0\_g1\_i4.p1:TRINITY\_DN1058\_c0\_g1\_i1.p1:TRINITY\_DN1058\_c0\_g1\_i3.p1:TRINITY\_DN1058\_c0\_g1\_i2.p2 |  |  | DB Search |
| YC(+57.02)ILSFLDKTDK | 31.7 | 1501.7487 | 12 | -1.53 | 501.5872 | 19.67 | 0.7966 | 0.8039 | 69637 | 7.8e2 | 1 | 1 | TRINITY\_DN15274\_c0\_g1\_i1.p1 | Carbamidomethylation |  | DB Search |
| DPIC(+57.02)HC(+57.02)DTSK | 31.69 | 1231.4961 | 10 | 5.21 | 616.7559 | 9.93 | 0.904 | 0.9113 | 14979 | 3.6e3 | 1 | 1 | TRINITY\_DN319\_c2\_g1\_i3.p1:TRINITY\_DN319\_c2\_g1\_i1.p1:TRINITY\_DN319\_c2\_g1\_i2.p1 | Carbamidomethylation, Carbamidomethylation |  | DB Search |
| ASFVTDIDRLR | 31.69 | 1291.6885 | 11 | 4.02 | 646.8513 | 17.03 | 0.9868 | 0.9941 | 53636 | 3.39e3 | 1 | 1 | TRINITY\_DN598\_c1\_g1\_i2.p1 |  |  | DB Search |
| KAQYYEAYETIIKR | 31.69 | 1774.9254 | 14 | -12.66 | 592.639 | 15.71 | 0.8607 | 0.868 | 47344 | 1.05e3 | 1 | 1 | TRINITY\_DN319\_c2\_g1\_i3.p1:TRINITY\_DN319\_c2\_g1\_i1.p1 |  |  | DB Search |
| GYAVSFIDDKFVQK | 31.69 | 1615.8246 | 14 | 2.16 | 808.9178 | 17.66 | 1.1206 | 1.1278 | 57369 | 5.97e2 | 1 | 1 | TRINITY\_DN61668\_c0\_g1\_i1.p1 |  |  | DB Search |
| SISC(+57.02)SKDEEIK | 31.69 | 1294.6074 | 11 | 1.4 | 648.3091 | 10.84 | 0.9795 | 0.9868 | 20521 | 8.87e2 | 1 | 1 | TRINITY\_DN495\_c0\_g1\_i5.p1:TRINITY\_DN495\_c0\_g1\_i6.p1 | Carbamidomethylation |  | DB Search |
| DLHIIEDYVLK | 31.69 | 1356.7289 | 11 | -0.23 | 679.3687 | 19.54 | 0.9932 | 1.0005 | 68824 | 1.13e3 | 1 | 1 | TRINITY\_DN8903\_c0\_g1\_i1.p1 |  |  | DB Search |
| DSYVGDEAQSKR | 31.69 | 1353.6161 | 12 | 1.77 | 677.8136 | 10.94 | 1.0017 | 1.009 | 21026 | 1.85e3 | 1 | 1 | TRINITY\_DN2477\_c0\_g1\_i5.p1:TRINITY\_DN2477\_c0\_g1\_i6.p1:TRINITY\_DN195934\_c0\_g1\_i1.p2:TRINITY\_DN161\_c0\_g1\_i1.p1:TRINITY\_DN2477\_c0\_g1\_i9.p1:TRINITY\_DN2477\_c0\_g1\_i10.p1:TRINITY\_DN2477\_c0\_g1\_i2.p1:TRINITY\_DN161\_c0\_g1\_i3.p1:TRINITY\_DN161\_c0\_g1\_i2.p1 |  |  | DB Search |
| ASFVSDIDRLR | 31.69 | 1277.6727 | 11 | 1.75 | 639.842 | 16.90 | 0.9812 | 0.9884 | 53092 | 3.28e3 | 1 | 1 | TRINITY\_DN598\_c1\_g1\_i1.p1 |  |  | DB Search |
| YLADVVLTLDHTK | 31.69 | 1486.8031 | 13 | -2.87 | 496.6048 | 18.62 | 0.8307 | 0.838 | 63167 | 7.62e2 | 1 | 1 | TRINITY\_DN22240\_c0\_g1\_i1.p1 |  |  | DB Search |
| AELVFVNHC(+57.02)R | 31.69 | 1243.6132 | 10 | 3.87 | 622.8136 | 14.34 | 0.9566 | 0.9638 | 40177 | 2.27e3 | 1 | 1 | TRINITY\_DN101992\_c0\_g1\_i1.p1 | Carbamidomethylation |  | DB Search |
| GKDGETGEQGPQGQEGPTGPR | 31.69 | 2080.9409 | 21 | 3.9 | 694.654 | 10.71 | 0.849 | 0.8562 | 19412 | 7.08e3 | 1 | 1 | TRINITY\_DN113\_c0\_g1\_i1.p1 |  |  | DB Search |
| DFREFLYNSETGKVM(+15.99)GR | 31.69 | 2063.9734 | 17 | -1.15 | 688.9947 | 17.54 | 0.9287 | 0.936 | 56508 | 2.29e3 | 1 | 1 | TRINITY\_DN2028\_c0\_g1\_i1.p1 | Oxidation (M) | M15:Oxidation (M):1000 | DB Search |
| TNNALDYEAKR | 31.69 | 1293.6313 | 11 | 5.9 | 647.824 | 11.99 | 0.9618 | 0.9691 | 27271 | 3.6e2 | 1 | 1 | TRINITY\_DN982\_c0\_g1\_i3.p1:TRINITY\_DN982\_c0\_g1\_i1.p1 |  |  | DB Search |
| TRIEDLETVISQTQDHR | 31.69 | 2040.0236 | 17 | 6.44 | 681.0166 | 17.36 | 0.9614 | 0.9687 | 55514 | 1.11e3 | 1 | 1 | TRINITY\_DN761\_c0\_g1\_i1.p1:TRINITY\_DN761\_c0\_g1\_i3.p1:TRINITY\_DN761\_c0\_g1\_i2.p1 |  |  | DB Search |
| DANAAVVHGSHLK | 31.68 | 1317.679 | 13 | 5.41 | 659.8475 | 10.89 | 1.017 | 1.0242 | 20821 | 1.84e3 | 1 | 1 | TRINITY\_DN262\_c0\_g1\_i1.p1 |  |  | DB Search |
| SSLNHNIVFEGR | 31.68 | 1371.6895 | 12 | 6.28 | 686.8534 | 14.46 | 0.9816 | 0.9888 | 40919 | 0 | 0 | 0 | TRINITY\_DN144508\_c0\_g1\_i1.p1 |  |  | DB Search |
| FDFHSWIGLKK | 31.68 | 1376.7241 | 11 | -0.47 | 459.9131 | 18.24 | 0.7355 | 0.7428 | 60734 | 1.82e3 | 1 | 1 | TRINITY\_DN12627\_c0\_g1\_i1.p1 |  |  | DB Search |
| HQNIVC(+57.02)GGPER | 31.68 | 1265.5935 | 11 | 4.37 | 633.8041 | 12.40 | 0.9073 | 0.9146 | 29482 | 5.93e2 | 1 | 1 | TRINITY\_DN1535\_c0\_g1\_i1.p1 | Carbamidomethylation |  | DB Search |
| IKDTYLLTLEAR | 31.68 | 1434.8082 | 12 | -1.93 | 718.4069 | 16.86 | 1.0347 | 1.0419 | 52952 | 9.73e2 | 1 | 1 | TRINITY\_DN72625\_c0\_g1\_i1.p1 |  |  | DB Search |
| IEALHC(+57.02)LSENIVK | 31.68 | 1524.797 | 13 | 5.71 | 763.4069 | 15.59 | 1.0472 | 1.0544 | 46684 | 1.5e3 | 1 | 1 | TRINITY\_DN3078\_c0\_g1\_i1.p1 | Carbamidomethylation |  | DB Search |
| ATGNTSGGVC(+57.02)DDC(+57.02)NHNTAGR | 31.68 | 2062.8181 | 20 | 5.22 | 688.614 | 9.94 | 0.8562 | 0.8635 | 14982 | 8.15e2 | 1 | 1 | TRINITY\_DN15274\_c0\_g1\_i1.p1 | Carbamidomethylation, Carbamidomethylation |  | DB Search |
| GFGGNLVTITNSDIQK | 31.68 | 1662.8577 | 16 | -4.47 | 832.4288 | 17.63 | 1.0773 | 1.0845 | 57262 | 0 | 0 | 0 | TRINITY\_DN12627\_c0\_g1\_i1.p1 |  |  | DB Search |
| LALHVLNNEDIIGHR | 31.68 | 1712.9321 | 15 | 0.25 | 571.9824 | 16.32 | 0.853 | 0.8603 | 50289 | 4.27e3 | 2 | 2 | TRINITY\_DN140702\_c0\_g1\_i1.p1 |  |  | DB Search |
| VLDVGFLLDSSDKVK | 31.68 | 1633.8926 | 15 | 1.24 | 817.9511 | 19.58 | 1.1694 | 1.1766 | 69063 | 8.63e2 | 2 | 2 | TRINITY\_DN4494\_c1\_g1\_i1.p1:TRINITY\_DN4494\_c1\_g1\_i7.p1:TRINITY\_DN4494\_c1\_g1\_i5.p1 |  |  | DB Search |
| VVDTEANDLTEEVKK | 31.68 | 1688.8468 | 15 | 3.24 | 845.4298 | 14.05 | 1.1286 | 1.1358 | 38709 | 1.51e3 | 1 | 1 | TRINITY\_DN1616\_c0\_g1\_i3.p1:TRINITY\_DN1616\_c0\_g1\_i6.p1:TRINITY\_DN1616\_c0\_g1\_i2.p1:TRINITY\_DN1616\_c0\_g1\_i7.p1 |  |  | DB Search |
| AEFRPNQEEEPILKR | 31.68 | 1854.9587 | 15 | 3.55 | 619.3264 | 13.76 | 0.8773 | 0.8846 | 37073 | 6e3 | 1 | 1 | TRINITY\_DN262\_c0\_g1\_i1.p1 |  |  | DB Search |
| YYVTIIDAPGHR | 31.68 | 1403.7197 | 12 | 0.62 | 702.8646 | 15.88 | 1.0387 | 1.046 | 48209 | 0 | 0 | 0 | TRINITY\_DN64\_c0\_g1\_i1.p1 |  |  | DB Search |
| FFFHDQSC(+57.02)LDVYK | 31.68 | 1704.7606 | 13 | 0.29 | 569.2585 | 17.60 | 0.8075 | 0.8149 | 57111 | 0 | 0 | 0 | TRINITY\_DN10094\_c0\_g1\_i1.p1:TRINITY\_DN10094\_c0\_g1\_i2.p1 | Carbamidomethylation |  | DB Search |
| FELDRVNTFGDEVFGDPK | 31.68 | 2083.9851 | 18 | 4.09 | 695.6688 | 19.17 | 0.9198 | 0.9271 | 66516 | 5.06e2 | 1 | 1 | TRINITY\_DN1676\_c0\_g1\_i6.p1:TRINITY\_DN1676\_c0\_g1\_i12.p1 |  |  | DB Search |
| VLLSQQIFYDKK | 31.68 | 1480.829 | 12 | -0.33 | 741.4183 | 16.72 | 1.0572 | 1.0644 | 52159 | 3.15e3 | 1 | 1 | TRINITY\_DN982\_c0\_g1\_i3.p1:TRINITY\_DN982\_c0\_g1\_i1.p1:TRINITY\_DN982\_c0\_g1\_i2.p1 |  |  | DB Search |
| TQAVLDRLNIEK | 31.68 | 1398.7831 | 12 | 4.72 | 700.3991 | 15.32 | 1.0045 | 1.0118 | 45318 | 4.75e3 | 1 | 1 | TRINITY\_DN477\_c0\_g1\_i9.p1 |  |  | DB Search |
| VGNWRDETLSC(+57.02)K | 31.68 | 1463.6827 | 12 | 0.79 | 732.8461 | 13.70 | 1.0186 | 1.0259 | 36784 | 3.87e3 | 2 | 2 | TRINITY\_DN6390\_c0\_g1\_i1.p1 | Carbamidomethylation |  | DB Search |
| AVAC(+57.02)NSGPC(+57.02)LAGC(+57.02)PR | 31.68 | 1588.6908 | 15 | 4.47 | 795.3528 | 12.99 | 1.0178 | 1.025 | 32815 | 5.35e2 | 1 | 1 | TRINITY\_DN4815\_c0\_g1\_i2.p1:TRINITY\_DN4815\_c0\_g1\_i1.p1 | Carbamidomethylation, Carbamidomethylation, Carbamidomethylation |  | DB Search |
| TGAISLLASVDFETDRTHTAVVK | 31.68 | 2430.2754 | 23 | 0.72 | 811.0962 | 18.85 | 1.0367 | 1.0439 | 64505 | 1.34e3 | 2 | 2 | TRINITY\_DN982\_c0\_g1\_i3.p1 |  |  | DB Search |
| GVAGAGVLAGFDKFK | 31.68 | 1435.7823 | 15 | 2.01 | 718.8968 | 18.26 | 1.0849 | 1.0921 | 60846 | 1.07e3 | 1 | 1 | TRINITY\_DN1559\_c0\_g1\_i1.p1 |  |  | DB Search |
| DLPQEKLDDILK | 31.67 | 1425.7715 | 12 | 4.1 | 713.8929 | 17.71 | 1.0375 | 1.0448 | 57677 | 1.52e3 | 1 | 1 | TRINITY\_DN262\_c0\_g1\_i1.p1 |  |  | DB Search |
| GSFSSHIC(+57.02)LC(+57.02)HDPAYK | 31.67 | 1877.8188 | 16 | 3.89 | 626.9467 | 14.33 | 0.8838 | 0.8911 | 40207 | 1.11e3 | 1 | 1 | TRINITY\_DN319\_c2\_g1\_i3.p1:TRINITY\_DN319\_c2\_g1\_i1.p1:TRINITY\_DN319\_c2\_g1\_i2.p1 | Carbamidomethylation, Carbamidomethylation |  | DB Search |
| DNIQGITKPAIR | 31.67 | 1324.7462 | 12 | 3.85 | 663.3801 | 13.78 | 0.9808 | 0.988 | 37251 | 4.19e2 | 1 | 1 | TRINITY\_DN172571\_c0\_g1\_i1.p1 |  |  | DB Search |
| ITVTVSPDIHPPAK | 31.67 | 1473.8191 | 14 | 3.85 | 737.9165 | 14.85 | 1.064 | 1.0713 | 42886 | 6.83e2 | 1 | 1 | TRINITY\_DN991\_c0\_g1\_i5.p1:TRINITY\_DN991\_c0\_g1\_i2.p1:TRINITY\_DN991\_c0\_g1\_i6.p1 |  |  | DB Search |
| DLEDYKVTEREPVTTNVK | 31.67 | 2135.0747 | 18 | 1.09 | 712.6965 | 15.11 | 0.915 | 0.9222 | 44293 | 4.57e3 | 1 | 1 | TRINITY\_DN10485\_c0\_g1\_i1.p1 |  |  | DB Search |
| FLDIHELQAVK | 31.67 | 1311.7186 | 11 | 0.8 | 656.8643 | 17.39 | 1.0077 | 1.015 | 55792 | 0 | 0 | 0 | TRINITY\_DN165469\_c0\_g1\_i1.p1 |  |  | DB Search |
| VQNEITNPNTQFPVNGLFDLHR | 31.66 | 2552.2771 | 22 | 2.02 | 851.7644 | 19.92 | 1.0632 | 1.0705 | 71061 | 1.2e3 | 1 | 1 | TRINITY\_DN72625\_c0\_g1\_i1.p1 |  |  | DB Search |
| VDVGFILDSSGSLR | 31.66 | 1463.762 | 14 | -1.83 | 732.8838 | 19.80 | 1.0126 | 1.0198 | 70509 | 0 | 0 | 0 | TRINITY\_DN17725\_c0\_g1\_i1.p1:TRINITY\_DN4494\_c1\_g1\_i2.p1:TRINITY\_DN4494\_c1\_g1\_i15.p1:TRINITY\_DN4494\_c2\_g1\_i1.p1:TRINITY\_DN4494\_c1\_g1\_i12.p1:TRINITY\_DN4494\_c1\_g1\_i1.p1:TRINITY\_DN172523\_c0\_g1\_i1.p1:TRINITY\_DN12542\_c0\_g1\_i1.p1:TRINITY\_DN4494\_c1\_g1\_i7.p1:TRINITY\_DN4494\_c1\_g1\_i8.p1:TRINITY\_DN4494\_c1\_g1\_i5.p1:TRINITY\_DN4494\_c1\_g1\_i10.p1 |  |  | DB Search |
| APIGQSIVTVSAR | 31.66 | 1297.7354 | 13 | 3.68 | 649.8746 | 14.17 | 0.9598 | 0.967 | 39287 | 2.49e2 | 1 | 1 | TRINITY\_DN982\_c0\_g1\_i3.p1 |  |  | DB Search |
| GLMVAAMDLDKK | 31.65 | 1290.6676 | 12 | 2.51 | 646.3399 | 16.54 | 0.9985 | 1.0057 | 51403 | 5.13e2 | 1 | 1 | TRINITY\_DN1043\_c0\_g1\_i2.p1 |  |  | DB Search |
| FLLHLFDKELK | 31.65 | 1401.802 | 11 | 1.86 | 468.2735 | 19.03 | 0.7693 | 0.7766 | 65648 | 5.29e2 | 1 | 1 | TRINITY\_DN55094\_c0\_g1\_i1.p1 |  |  | DB Search |
| S(+42.01)ATGNLVTVLRK | 31.65 | 1299.751 | 12 | 5.44 | 650.8835 | 17.09 | 0.9969 | 1.0041 | 54070 | 7.16e2 | 1 | 1 | TRINITY\_DN2963\_c0\_g1\_i1.p1 | Acetylation (Protein N-term) | S1:Acetylation (Protein N-term):1000 | DB Search |
| IQFSISAEINNGALK | 31.65 | 1603.8569 | 15 | -1.52 | 802.9311 | 18.71 | 1.0737 | 1.0809 | 63761 | 0 | 0 | 0 | TRINITY\_DN15274\_c0\_g1\_i1.p1 |  |  | DB Search |
| VLNIALDKYQEGVAK | 31.63 | 1659.9196 | 15 | -0.5 | 830.9631 | 15.97 | 1.1546 | 1.1618 | 48647 | 0 | 0 | 0 | TRINITY\_DN942\_c0\_g1\_i5.p1:TRINITY\_DN942\_c0\_g1\_i2.p1:TRINITY\_DN942\_c0\_g1\_i3.p1 |  |  | DB Search |
| LLHC(+57.02)SDSEVDHVEVC(+57.02)GK | 31.63 | 1982.8826 | 17 | 4.93 | 661.9686 | 13.16 | 0.9226 | 0.9299 | 33774 | 0 | 0 | 0 | TRINITY\_DN319\_c2\_g1\_i3.p1:TRINITY\_DN319\_c2\_g1\_i1.p1:TRINITY\_DN319\_c2\_g1\_i2.p1 | Carbamidomethylation, Carbamidomethylation |  | DB Search |
| VLDVNDKQPVFVK | 31.63 | 1499.8347 | 13 | 5.63 | 750.9257 | 14.98 | 1.048 | 1.0552 | 43639 | 0 | 0 | 0 | TRINITY\_DN982\_c0\_g1\_i1.p1:TRINITY\_DN982\_c0\_g1\_i2.p1 |  |  | DB Search |
| AVAEEFISVDPYVR | 31.58 | 1593.8038 | 14 | 2.58 | 797.9078 | 18.98 | 1.0604 | 1.0676 | 65194 | 1.77e3 | 1 | 1 | TRINITY\_DN598\_c1\_g1\_i1.p1 |  |  | DB Search |
| DANSM(+15.99)PTQSR | 31.58 | 1121.4772 | 10 | 2.01 | 561.7446 | 8.83 | 0.8258 | 0.8331 | 9387 | 1.05e3 | 1 | 1 | TRINITY\_DN477\_c0\_g1\_i9.p1 | Oxidation (M) | M5:Oxidation (M):1000 | DB Search |
| A(+42.01)TDKLEGLIAR | 31.56 | 1227.6823 | 11 | 0.47 | 614.8461 | 17.84 | 0.9525 | 0.9598 | 58434 | 4.22e2 | 1 | 1 | TRINITY\_DN540\_c0\_g1\_i1.p1 | Acetylation (Protein N-term) | A1:Acetylation (Protein N-term):1000 | DB Search |
| VIDINDNIPIFSK | 31.55 | 1486.8031 | 13 | -0.75 | 744.4051 | 19.54 | 1.0198 | 1.0271 | 68788 | 8.64e2 | 1 | 1 | TRINITY\_DN69276\_c0\_g1\_i1.p1 |  |  | DB Search |
| IDNYIEFLDEDLKK | 31.54 | 1753.8774 | 14 | -5.87 | 585.6271 | 19.85 | 0.8441 | 0.8514 | 70732 | 0 | 0 | 0 | TRINITY\_DN278\_c0\_g1\_i1.p1 |  |  | DB Search |
| RVHQFLVEALDGGQPR | 31.54 | 1820.9645 | 16 | -3.51 | 607.9907 | 19.35 | 0.8692 | 0.8765 | 67626 | 6.55e2 | 1 | 1 | TRINITY\_DN71243\_c0\_g1\_i1.p1 |  |  | DB Search |
| APEDAPNREQEFEEK | 31.53 | 1787.7961 | 15 | 1.71 | 596.9378 | 12.11 | 0.823 | 0.8303 | 27908 | 7.39e2 | 1 | 1 | TRINITY\_DN30613\_c0\_g1\_i1.p1 |  |  | DB Search |
| LIQYIENKPGLTR | 31.53 | 1543.8722 | 13 | 5.29 | 772.9442 | 14.78 | 1.0753 | 1.0825 | 42347 | 2.27e4 | 1 | 1 | TRINITY\_DN2028\_c0\_g1\_i1.p1 |  |  | DB Search |
| LADLEEFISSPNHANISQVGER | 31.5 | 2425.1873 | 22 | 2.2 | 809.4014 | 18.76 | 0.9255 | 0.9327 | 63995 | 1.04e3 | 1 | 1 | TRINITY\_DN2113\_c0\_g1\_i1.p1:TRINITY\_DN2113\_c0\_g1\_i2.p1 |  |  | DB Search |
| LGPDQVDVAGAGR | 31.49 | 1253.6364 | 13 | -7.34 | 627.8182 | 13.58 | 0.9331 | 0.9404 | 36091 | 0 | 0 | 0 | TRINITY\_DN97809\_c0\_g1\_i1.p1 |  |  | DB Search |
| SPFVIMFK | 31.43 | 967.5201 | 8 | 3.55 | 484.767 | 19.71 | 0.8096 | 0.8169 | 69837 | 4.12e3 | 1 | 1 | TRINITY\_DN4595\_c0\_g1\_i10.p1:TRINITY\_DN4595\_c0\_g1\_i4.p1 |  |  | DB Search |
| ILLAVGGWNHER | 31.42 | 1363.7361 | 12 | 2.78 | 682.8743 | 16.92 | 1.0387 | 1.046 | 53358 | 3.81e2 | 1 | 1 | TRINITY\_DN6542\_c0\_g1\_i4.p1:TRINITY\_DN6542\_c0\_g1\_i1.p1:TRINITY\_DN6542\_c0\_g1\_i3.p1 |  |  | DB Search |
| LQTDIHGVALEITK | 31.4 | 1536.8511 | 14 | -1.96 | 769.428 | 16.47 | 1.052 | 1.0592 | 51159 | 0 | 0 | 0 | TRINITY\_DN99404\_c0\_g1\_i1.p1 |  |  | DB Search |
| AEFRPNQEEEPILK | 31.38 | 1698.8577 | 14 | 3.69 | 850.4356 | 14.58 | 1.0825 | 1.0897 | 41449 | 1.23e3 | 1 | 1 | TRINITY\_DN262\_c0\_g1\_i1.p1 |  |  | DB Search |
| ADLDIQKPIPIVSK | 31.36 | 1535.8922 | 14 | 2.25 | 768.9518 | 15.99 | 1.0705 | 1.0777 | 48786 | 2.79e2 | 1 | 1 | TRINITY\_DN26392\_c0\_g1\_i1.p1 |  |  | DB Search |
| YNHYWIGLNDIKK | 31.35 | 1662.8518 | 13 | -3 | 555.2872 | 16.29 | 0.8368 | 0.8441 | 50223 | 2.59e2 | 1 | 1 | TRINITY\_DN11248\_c0\_g1\_i1.p1:TRINITY\_DN11248\_c0\_g1\_i3.p1 |  |  | DB Search |
| VSSDRFFVQVDGR | 31.34 | 1510.7528 | 13 | 7.9 | 756.3864 | 15.89 | 1.0287 | 1.0359 | 48167 | 4.59e2 | 1 | 1 | TRINITY\_DN6390\_c0\_g1\_i1.p1 |  |  | DB Search |
| LLLGDFNGDKR | 31.33 | 1246.667 | 11 | 4.3 | 624.3408 | 16.04 | 0.9836 | 0.9908 | 48938 | 1.07e3 | 1 | 1 | TRINITY\_DN413\_c0\_g1\_i1.p1:TRINITY\_DN413\_c0\_g1\_i4.p1:TRINITY\_DN413\_c0\_g1\_i2.p1:TRINITY\_DN413\_c0\_g1\_i3.p1 |  |  | DB Search |
| LKDNAVIPLFAVTK | 31.32 | 1527.9025 | 14 | -0.82 | 764.9546 | 18.85 | 1.0331 | 1.0403 | 64545 | 4.93e2 | 1 | 1 | TRINITY\_DN1535\_c0\_g1\_i1.p1 |  |  | DB Search |
| DAPIYGLQWHPEK | 31.3 | 1552.7673 | 13 | -0.41 | 777.3873 | 17.24 | 1.0725 | 1.0797 | 54902 | 1.07e3 | 1 | 1 | TRINITY\_DN247\_c0\_g1\_i1.p1 |  |  | DB Search |
| VIM(+15.99)VTGDHPITAK | 31.28 | 1396.7384 | 13 | 3.65 | 699.376 | 13.17 | 1.023 | 1.0303 | 33679 | 7.16e2 | 1 | 1 | TRINITY\_DN262\_c0\_g1\_i1.p1 | Oxidation (M) | M3:Oxidation (M):1000 | DB Search |
| ALHEADKEWEVK | 31.26 | 1453.7201 | 12 | 5.69 | 727.8683 | 12.83 | 1.0379 | 1.0452 | 31955 | 0 | 0 | 0 | TRINITY\_DN13467\_c0\_g1\_i1.p1 |  |  | DB Search |
| IPSWVPSLPKPK | 31.25 | 1347.7914 | 12 | -0.39 | 450.269 | 17.94 | 0.7791 | 0.7864 | 58876 | 5.85e3 | 2 | 2 | TRINITY\_DN2576\_c3\_g1\_i1.p2 |  |  | DB Search |
| EAAAQIVVEIIKR | 31.24 | 1438.8507 | 13 | -1.6 | 720.4284 | 19.02 | 1.0512 | 1.0584 | 65681 | 0 | 0 | 0 | TRINITY\_DN71276\_c0\_g1\_i1.p1 |  |  | DB Search |
| GVGIISEGNETVEDIAQR | 31.23 | 1885.9381 | 18 | -3.75 | 943.9688 | 17.80 | 1.141 | 1.1482 | 58158 | 4.58e2 | 1 | 1 | TRINITY\_DN262\_c0\_g1\_i1.p1 |  |  | DB Search |
| IC(+57.02)HLLGIQVTDFTK | 31.2 | 1643.8705 | 14 | 2.62 | 548.9632 | 18.46 | 0.8197 | 0.827 | 62230 | 0 | 0 | 0 | TRINITY\_DN428\_c0\_g1\_i1.p1 | Carbamidomethylation |  | DB Search |
| VTLTQTHFLK | 31.18 | 1186.671 | 10 | 4.18 | 594.3427 | 14.84 | 0.9638 | 0.9711 | 42932 | 0 | 0 | 0 | TRINITY\_DN5584\_c0\_g1\_i1.p1:TRINITY\_DN5584\_c0\_g1\_i3.p1 |  |  | DB Search |
| IEDLETVISQTQDHR | 31.15 | 1782.8748 | 15 | -3.8 | 595.2941 | 17.75 | 0.9048 | 0.9121 | 58019 | 0 | 0 | 0 | TRINITY\_DN761\_c0\_g1\_i1.p1:TRINITY\_DN761\_c0\_g1\_i3.p1:TRINITY\_DN761\_c0\_g1\_i2.p1 |  |  | DB Search |
| GPTGPQGER | 31.15 | 897.4304 | 9 | 8.54 | 449.7244 | 9.46 | 0.7819 | 0.7892 | 12185 | 1.55e3 | 1 | 1 | TRINITY\_DN691\_c0\_g1\_i1.p1 |  |  | DB Search |
| DNAGTSSSGTGPR | 31.14 | 1205.5272 | 13 | 19.43 | 603.78 | 9.14 | 0.8635 | 0.8708 | 10555 | 0 | 0 | 0 | TRINITY\_DN4781\_c0\_g1\_i3.p3 |  |  | DB Search |
| GGRFEC(+57.02)TDFDC(+57.02)VAHC(+57.02)ASK | 31.13 | 2115.856 | 18 | 2.43 | 706.2913 | 14.33 | 0.9331 | 0.9404 | 40205 | 4.08e2 | 1 | 1 | TRINITY\_DN6390\_c0\_g1\_i1.p1 | Carbamidomethylation, Carbamidomethylation, Carbamidomethylation |  | DB Search |
| VGYLFITDWGGKPK | 31.13 | 1579.8398 | 14 | 2.42 | 527.6196 | 19.47 | 0.8047 | 0.812 | 68400 | 4.43e2 | 1 | 1 | TRINITY\_DN17095\_c0\_g1\_i1.p1 |  |  | DB Search |
| LLAVTVGNNYNLDTLEK | 31.11 | 1875.9941 | 17 | 0.03 | 939.0004 | 18.60 | 1.1454 | 1.1526 | 62944 | 7.3e2 | 1 | 1 | TRINITY\_DN77\_c1\_g1\_i1.p1 |  |  | DB Search |
| FSYEKVC(+57.02)EDVKR | 31.1 | 1558.745 | 12 | -0.77 | 520.5863 | 13.51 | 0.7888 | 0.7962 | 35740 | 0 | 0 | 0 | TRINITY\_DN118\_c1\_g1\_i3.p1 | Carbamidomethylation |  | DB Search |
| ATHQELLNAEFIQK | 31.06 | 1640.8522 | 14 | 2.54 | 821.4319 | 15.90 | 1.0777 | 1.0849 | 48321 | 4.04e2 | 1 | 1 | TRINITY\_DN113\_c3\_g1\_i1.p1 |  |  | DB Search |
| YGAVNDNC(+57.02)ERDNGQC(+57.02)SC(+57.02)K | 31.05 | 2145.8262 | 18 | 2.18 | 716.2812 | 10.55 | 0.8587 | 0.866 | 18796 | 7.2e2 | 1 | 1 | TRINITY\_DN1616\_c0\_g1\_i3.p1:TRINITY\_DN1616\_c0\_g1\_i6.p1:TRINITY\_DN1616\_c0\_g1\_i2.p1:TRINITY\_DN1616\_c0\_g1\_i7.p1 | Carbamidomethylation, Carbamidomethylation, Carbamidomethylation |  | DB Search |
| KALVILGDGFISGGGTPSK | 31.05 | 1816.0094 | 19 | 1.25 | 909.0092 | 18.01 | 1.1774 | 1.1845 | 59478 | 0 | 0 | 0 | TRINITY\_DN244\_c0\_g1\_i1.p1 |  |  | DB Search |
| DKNILVLTLGVGPQVNR | 31.03 | 1835.0629 | 17 | 0.02 | 918.5348 | 19.37 | 1.1334 | 1.1406 | 67784 | 1.18e3 | 1 | 1 | TRINITY\_DN477\_c0\_g1\_i8.p1:TRINITY\_DN477\_c0\_g1\_i7.p1 |  |  | DB Search |
| IQVLKDPDFEFTR | 31.02 | 1606.8354 | 13 | 5.47 | 804.426 | 17.53 | 1.0837 | 1.0909 | 56499 | 1.44e3 | 1 | 1 | TRINITY\_DN982\_c0\_g1\_i3.p1:TRINITY\_DN982\_c0\_g1\_i1.p1 |  |  | DB Search |
| ERIDYALELAK | 30.99 | 1319.7085 | 11 | 3.16 | 660.8608 | 16.11 | 0.9824 | 0.9896 | 49311 | 0 | 0 | 0 | TRINITY\_DN244\_c0\_g1\_i1.p1 |  |  | DB Search |
| LNNEINDLHQR | 30.95 | 1364.6797 | 11 | 5.8 | 683.3481 | 12.54 | 0.9949 | 1.0021 | 30348 | 0 | 0 | 0 | TRINITY\_DN1366\_c0\_g1\_i1.p1 |  |  | DB Search |
| SDELLNLKEEASTLR | 30.91 | 1716.8894 | 15 | -3.25 | 859.4455 | 17.63 | 1.1094 | 1.1166 | 57260 | 0 | 0 | 0 | TRINITY\_DN140702\_c0\_g1\_i1.p1 |  |  | DB Search |
| LIDELTPEVTSALEC(+57.02)EGGPR | 30.9 | 2185.0571 | 20 | -17.73 | 1093.5118 | 19.30 | 1.2173 | 1.2244 | 67386 | 0 | 0 | 0 | TRINITY\_DN244\_c0\_g1\_i1.p1 | Carbamidomethylation |  | DB Search |
| NC(+57.02)QHNTLGINC(+57.02)EK | 30.89 | 1586.693 | 13 | 4.28 | 794.3538 | 11.89 | 1.0371 | 1.0443 | 26586 | 7.24e2 | 1 | 1 | TRINITY\_DN1616\_c0\_g1\_i3.p1:TRINITY\_DN1616\_c0\_g1\_i6.p1:TRINITY\_DN1616\_c0\_g1\_i2.p1:TRINITY\_DN1616\_c0\_g1\_i7.p1 | Carbamidomethylation, Carbamidomethylation |  | DB Search |
| HIISDRDVMIER | 30.84 | 1482.7612 | 12 | -2.42 | 495.2577 | 15.36 | 0.8023 | 0.8096 | 45585 | 0 | 0 | 0 | TRINITY\_DN1366\_c0\_g1\_i1.p1 |  |  | DB Search |
| AYGQDLGQPGSC(+57.02)LKR | 30.81 | 1648.7991 | 15 | 2.46 | 825.4053 | 13.40 | 1.0733 | 1.0805 | 35031 | 2.16e3 | 1 | 1 | TRINITY\_DN691\_c0\_g1\_i2.p1:TRINITY\_DN691\_c0\_g1\_i1.p1 | Carbamidomethylation |  | DB Search |
| AIESERDELLEELNNNSALK | 30.81 | 2286.1338 | 20 | 3.61 | 763.0514 | 18.31 | 0.9989 | 1.0061 | 61258 | 0 | 0 | 0 | TRINITY\_DN428\_c0\_g1\_i1.p1 |  |  | DB Search |
| EGPTGPQGETGR | 30.8 | 1184.5421 | 12 | 6.09 | 593.2794 | 10.30 | 0.8806 | 0.8878 | 17329 | 0 | 0 | 0 | TRINITY\_DN984\_c0\_g1\_i2.p1:TRINITY\_DN984\_c0\_g1\_i1.p1 |  |  | DB Search |
| DKELSNVIESGFR | 30.71 | 1492.7522 | 13 | 4.38 | 747.3834 | 17.20 | 1.0419 | 1.0492 | 54715 | 0 | 0 | 0 | TRINITY\_DN775\_c0\_g1\_i2.p1:TRINITY\_DN775\_c0\_g1\_i1.p1 |  |  | DB Search |
| AEYENKTEVAR | 30.7 | 1308.631 | 11 | 4.55 | 655.3229 | 13.11 | 0.9812 | 0.9884 | 33509 | 0 | 0 | 0 | TRINITY\_DN1280\_c0\_g1\_i1.p1 |  |  | DB Search |
| HLAISNILK | 30.67 | 1007.6127 | 9 | 1.34 | 504.8122 | 18.76 | 0.8676 | 0.8749 | 63978 | 5.96e2 | 1 | 1 | TRINITY\_DN319\_c2\_g1\_i3.p1:TRINITY\_DN319\_c2\_g1\_i1.p1 |  |  | DB Search |
| V(+42.01)KEFLLSK | 30.66 | 1004.5906 | 8 | -8.96 | 503.2959 | 19.18 | 0.8319 | 0.8392 | 66669 | 0 | 0 | 0 | TRINITY\_DN1682\_c8\_g1\_i3.p1 | Acetylation (Protein N-term) | V1:Acetylation (Protein N-term):1000 | DB Search |
| EQVSTFDSLQADLVK | 30.64 | 1678.8413 | 15 | 4.31 | 840.428 | 18.59 | 1.0969 | 1.1041 | 62996 | 0 | 0 | 0 | TRINITY\_DN1043\_c0\_g1\_i2.p1 |  |  | DB Search |
| ADIEDVKDLLK | 30.63 | 1257.6816 | 11 | 4.2 | 629.848 | 17.58 | 0.9884 | 0.9957 | 56818 | 3.25e3 | 1 | 1 | TRINITY\_DN15274\_c0\_g1\_i1.p1 |  |  | DB Search |
| ATGC(+57.02)EVIWNQHNR | 30.62 | 1583.7263 | 13 | 2.43 | 792.869 | 13.87 | 1.0797 | 1.0869 | 37737 | 0 | 0 | 0 | TRINITY\_DN2217\_c0\_g1\_i3.p1:TRINITY\_DN2217\_c0\_g1\_i2.p1 | Carbamidomethylation |  | DB Search |
| KQQENEQLLDEKR | 30.62 | 1656.8431 | 13 | -3.85 | 553.2838 | 11.43 | 0.8222 | 0.8295 | 23996 | 3.61e2 | 1 | 1 | TRINITY\_DN1366\_c0\_g1\_i1.p1 |  |  | DB Search |
| AGDIAILESQQFNAVGGK | 30.61 | 1816.9319 | 18 | -19.18 | 909.4519 | 18.85 | 1.1853 | 1.1925 | 64620 | 3.67e2 | 1 | 1 | TRINITY\_DN1936\_c0\_g1\_i1.p1:TRINITY\_DN1936\_c0\_g1\_i4.p1 |  |  | DB Search |
| HLSVNILQTNTR | 30.59 | 1394.7629 | 12 | 0.11 | 698.3859 | 15.24 | 1.0037 | 1.011 | 44984 | 0 | 0 | 0 | TRINITY\_DN6390\_c0\_g1\_i1.p1 |  |  | DB Search |
| SGGQC(+57.02)PC(+57.02)KPGVVGR | 30.57 | 1457.6868 | 14 | 2.45 | 729.8493 | 10.68 | 0.98 | 0.9872 | 19470 | 8.78e2 | 1 | 1 | TRINITY\_DN15274\_c0\_g1\_i1.p1 | Carbamidomethylation, Carbamidomethylation |  | DB Search |
| FAIQDITVDEFSAK | 30.57 | 1582.7878 | 14 | 1.41 | 792.3989 | 19.69 | 1.0419 | 1.0492 | 69716 | 4.2e2 | 1 | 1 | TRINITY\_DN624\_c0\_g1\_i1.p1 |  |  | DB Search |
| DGEKVETLKVDISDINK | 30.55 | 1901.9945 | 17 | 2.61 | 635.0044 | 15.87 | 0.9065 | 0.9137 | 48130 | 2.66e2 | 1 | 1 | TRINITY\_DN1616\_c0\_g1\_i3.p1:TRINITY\_DN1616\_c0\_g1\_i6.p1:TRINITY\_DN1616\_c0\_g1\_i2.p1:TRINITY\_DN1616\_c0\_g1\_i7.p1 |  |  | DB Search |
| VVISDTC(+57.02)TNKWK | 30.5 | 1449.7286 | 12 | 0.59 | 725.8689 | 13.58 | 1.0379 | 1.0452 | 36084 | 0 | 0 | 0 | TRINITY\_DN1936\_c0\_g2\_i1.p1 | Carbamidomethylation |  | DB Search |
| IHGGAQAPKPK | 30.42 | 1102.6246 | 11 | 17.79 | 368.5538 | 8.91 | 0.7078 | 0.7151 | 9675 | 0 | 0 | 0 | TRINITY\_DN141561\_c0\_g1\_i1.p1 |  |  | DB Search |
| total 140 peptides |  |  |  |  |  |  |  |  |  |  |  |  |  |  |  |  |
| --- | --- | --- | --- | --- | --- | --- | --- | --- | --- | --- | --- | --- | --- | --- | --- | --- |
