# Supplementary material for: Proteomic Analysis and Biochemical Characterization of the Nematocyst Extract of the Hydrozoan Velella velella
Source: Mar Drugs. 2024 Oct 12;22(10):468. doi: 10.3390/md22100468 (PMC11509761; doi:10.3390/md22100468)
Supplement: Supplementary file 1 [file marinedrugs-22-00468-s001.zip › Supplementary File S1.zip!/Sample_3_Vellela/db.proteins.html]

proteins


Protein List

  

| Protein Group | Protein ID | Accession | -10lgP | Coverage (%) | Coverage (%) Sample 5 | Area Sample 5 | #Peptides | #Unique | #Spec Sample 5 | PTM | Avg. Mass | Description |
| --- | --- | --- | --- | --- | --- | --- | --- | --- | --- | --- | --- | --- |
| 1 | 1 | TRINITY\_DN982\_c0\_g1\_i3.p1 | 82.3 | 1 | 1 | 1.59e3 | 7 | 2 | 10 |  | 1090854.75 | type:complete len:9852 gc:universal TRINITY\_DN982\_c0\_g1\_i3:29697-142(-) |
| 2 | 2 | TRINITY\_DN244\_c0\_g1\_i1.p1 | 77.44 | 3 | 3 | 7.36e3 | 6 | 6 | 8 | Carbamidomethylation | 313594.0938 | type:complete len:3019 gc:universal TRINITY\_DN244\_c0\_g1\_i1:9748-692(-) |
| 3 | 3 | TRINITY\_DN262\_c0\_g1\_i1.p1 | 77.39 | 7 | 7 | 1.18e4 | 6 | 6 | 8 | Oxidation (M) | 114520.6406 | type:complete len:1033 gc:universal TRINITY\_DN262\_c0\_g1\_i1:111-3209(+) |
| 4 | 4 | TRINITY\_DN982\_c0\_g1\_i1.p1 | 77.75 | 1 | 1 | 0 | 6 | 1 | 8 |  | 712566.0625 | type:5prime\_partial len:6412 gc:universal TRINITY\_DN982\_c0\_g1\_i1:19377-142(-) |
| 4 | 5 | TRINITY\_DN982\_c0\_g1\_i2.p1 | 47.49 | 1 | 1 | 0 | 2 | 1 | 2 |  | 190367.1406 | type:internal len:1718 gc:universal TRINITY\_DN982\_c0\_g1\_i2:5151-1(-) |
| 5 | 6 | TRINITY\_DN15274\_c0\_g1\_i1.p1 | 77.46 | 5 | 5 | 5.89e3 | 6 | 6 | 9 | Carbamidomethylation | 201897.7344 | type:complete len:1822 gc:universal TRINITY\_DN15274\_c0\_g1\_i1:87-5552(+) |
| 6 | 7 | TRINITY\_DN1616\_c0\_g1\_i2.p1 | 71.97 | 2 | 2 | 3.22e3 | 5 | 5 | 5 | Carbamidomethylation | 374396 | type:3prime\_partial len:3398 gc:universal TRINITY\_DN1616\_c0\_g1\_i2:218-10408(+) |
| 6 | 8 | TRINITY\_DN1616\_c0\_g1\_i3.p1 | 71.97 | 2 | 2 | 3.22e3 | 5 | 5 | 5 | Carbamidomethylation | 423947.2812 | type:complete len:3855 gc:universal TRINITY\_DN1616\_c0\_g1\_i3:218-11782(+) |
| 6 | 9 | TRINITY\_DN1616\_c0\_g1\_i7.p1 | 71.97 | 2 | 2 | 3.22e3 | 5 | 5 | 5 | Carbamidomethylation | 423875.2188 | type:complete len:3855 gc:universal TRINITY\_DN1616\_c0\_g1\_i7:218-11782(+) |
| 6 | 10 | TRINITY\_DN1616\_c0\_g1\_i6.p1 | 71.97 | 2 | 2 | 3.22e3 | 5 | 5 | 5 | Carbamidomethylation | 423911.25 | type:complete len:3855 gc:universal TRINITY\_DN1616\_c0\_g1\_i6:218-11782(+) |
| 7 | 11 | TRINITY\_DN319\_c2\_g1\_i3.p1 | 72.13 | 3 | 3 | 6.35e3 | 5 | 5 | 7 | Carbamidomethylation | 279087.1875 | type:complete len:2588 gc:universal TRINITY\_DN319\_c2\_g1\_i3:165-7928(+) |
| 7 | 12 | TRINITY\_DN319\_c2\_g1\_i1.p1 | 72.13 | 3 | 3 | 6.35e3 | 5 | 5 | 7 | Carbamidomethylation | 279168.25 | type:complete len:2588 gc:universal TRINITY\_DN319\_c2\_g1\_i1:165-7928(+) |
| 7 | 13 | TRINITY\_DN319\_c2\_g1\_i2.p1 | 58.07 | 2 | 2 | 4.71e3 | 3 | 3 | 4 | Carbamidomethylation | 215796.4062 | type:3prime\_partial len:2004 gc:universal TRINITY\_DN319\_c2\_g1\_i2:165-6173(+) |
| 8 | 14 | TRINITY\_DN6390\_c0\_g1\_i1.p1 | 65.37 | 3 | 3 | 4.74e3 | 4 | 4 | 5 | Carbamidomethylation | 214480.2344 | type:3prime\_partial len:1941 gc:universal TRINITY\_DN6390\_c0\_g1\_i1:5820-1(-) |
| 9 | 15 | TRINITY\_DN477\_c0\_g1\_i9.p1 | 66.16 | 11 | 11 | 1.13e4 | 4 | 4 | 6 | Oxidation (M), Carbamidomethylation | 34222.9453 | type:complete len:306 gc:universal TRINITY\_DN477\_c0\_g1\_i9:1840-923(-) |
| 10 | 16 | TRINITY\_DN1366\_c0\_g1\_i1.p1 | 56.57 | 1 | 1 | 3.61e2 | 3 | 3 | 3 |  | 558202.1875 | type:3prime\_partial len:4702 gc:universal TRINITY\_DN1366\_c0\_g1\_i1:236-14338(+) |
| 11 | 17 | TRINITY\_DN3078\_c0\_g1\_i1.p1 | 58.37 | 4 | 4 | 7.32e3 | 3 | 3 | 5 | Carbamidomethylation | 127689.1797 | type:complete len:1109 gc:universal TRINITY\_DN3078\_c0\_g1\_i1:3628-302(-) |
| 12 | 18 | TRINITY\_DN4494\_c1\_g1\_i5.p1 | 47.51 | 7 | 7 | 8.63e2 | 2 | 2 | 3 |  | 47190.5742 | type:internal len:439 gc:universal TRINITY\_DN4494\_c1\_g1\_i5:1316-3(-) |
| 12 | 19 | TRINITY\_DN4494\_c1\_g1\_i7.p1 | 47.51 | 5 | 5 | 8.63e2 | 2 | 2 | 3 |  | 57635.2422 | type:internal len:536 gc:universal TRINITY\_DN4494\_c1\_g1\_i7:1607-3(-) |
| 12 | 20 | TRINITY\_DN4494\_c1\_g1\_i1.p1 | 47.51 | 7 | 7 | 8.63e2 | 2 | 2 | 3 |  | 43468.3906 | type:internal len:400 gc:universal TRINITY\_DN4494\_c1\_g1\_i1:1197-1(-) |
| 12 | 21 | TRINITY\_DN4494\_c1\_g1\_i10.p1 | 31.66 | 3 | 3 | 0 | 1 | 1 | 1 |  | 50408.1719 | type:5prime\_partial len:462 gc:universal TRINITY\_DN4494\_c1\_g1\_i10:2120-735(-) |
| 12 | 22 | TRINITY\_DN4494\_c1\_g1\_i12.p1 | 31.66 | 7 | 7 | 0 | 1 | 1 | 1 |  | 20465.0039 | type:internal len:192 gc:universal TRINITY\_DN4494\_c1\_g1\_i12:575-3(-) |
| 12 | 23 | TRINITY\_DN17725\_c0\_g1\_i1.p1 | 31.66 | 13 | 13 | 0 | 1 | 1 | 1 |  | 11833.5479 | type:3prime\_partial len:109 gc:universal TRINITY\_DN17725\_c0\_g1\_i1:103-426(+) |
| 12 | 24 | TRINITY\_DN172523\_c0\_g1\_i1.p1 | 31.66 | 7 | 7 | 0 | 1 | 1 | 1 |  | 21902.9141 | type:internal len:201 gc:universal TRINITY\_DN172523\_c0\_g1\_i1:600-1(-) |
| 12 | 25 | TRINITY\_DN4494\_c2\_g1\_i1.p1 | 31.66 | 13 | 13 | 0 | 1 | 1 | 1 |  | 11226.4531 | type:internal len:107 gc:universal TRINITY\_DN4494\_c2\_g1\_i1:2-319(+) |
| 12 | 26 | TRINITY\_DN4494\_c1\_g1\_i2.p1 | 31.66 | 3 | 3 | 0 | 1 | 1 | 1 |  | 50327.457 | type:internal len:469 gc:universal TRINITY\_DN4494\_c1\_g1\_i2:1406-3(-) |
| 12 | 27 | TRINITY\_DN4494\_c1\_g1\_i15.p1 | 31.66 | 11 | 11 | 0 | 1 | 1 | 1 |  | 14719.415 | type:internal len:134 gc:universal TRINITY\_DN4494\_c1\_g1\_i15:399-1(-) |
| 12 | 28 | TRINITY\_DN4494\_c1\_g1\_i8.p1 | 31.66 | 4 | 4 | 0 | 1 | 1 | 1 |  | 41552.7188 | type:internal len:390 gc:universal TRINITY\_DN4494\_c1\_g1\_i8:1169-3(-) |
| 12 | 29 | TRINITY\_DN12542\_c0\_g1\_i1.p1 | 31.66 | 6 | 6 | 0 | 1 | 1 | 1 |  | 23963.9648 | type:internal len:226 gc:universal TRINITY\_DN12542\_c0\_g1\_i1:676-2(-) |
| 13 | 30 | TRINITY\_DN1535\_c0\_g1\_i1.p1 | 47.34 | 3 | 3 | 1.09e3 | 2 | 2 | 2 | Carbamidomethylation | 86942.8516 | type:complete len:779 gc:universal TRINITY\_DN1535\_c0\_g1\_i1:86-2422(+) |
| 14 | 31 | TRINITY\_DN1043\_c0\_g1\_i2.p1 | 46.97 | 4 | 4 | 5.13e2 | 2 | 2 | 2 |  | 78392.1016 | type:complete len:700 gc:universal TRINITY\_DN1043\_c0\_g1\_i2:2410-311(-) |
| 15 | 32 | TRINITY\_DN161\_c0\_g1\_i2.p1 | 47.64 | 6 | 6 | 1.92e4 | 2 | 2 | 4 |  | 44577.6484 | type:5prime\_partial len:399 gc:universal TRINITY\_DN161\_c0\_g1\_i2:1683-487(-) |
| 15 | 33 | TRINITY\_DN161\_c0\_g1\_i3.p1 | 47.64 | 6 | 6 | 1.92e4 | 2 | 2 | 4 |  | 44592.6602 | type:5prime\_partial len:399 gc:universal TRINITY\_DN161\_c0\_g1\_i3:1450-254(-) |
| 15 | 34 | TRINITY\_DN161\_c0\_g1\_i1.p1 | 47.64 | 6 | 6 | 1.92e4 | 2 | 2 | 4 |  | 41918.5938 | type:complete len:377 gc:universal TRINITY\_DN161\_c0\_g1\_i1:1384-254(-) |
| 15 | 35 | TRINITY\_DN2477\_c0\_g1\_i2.p1 | 31.69 | 3 | 3 | 1.85e3 | 1 | 1 | 1 |  | 38566.9688 | type:5prime\_partial len:344 gc:universal TRINITY\_DN2477\_c0\_g1\_i2:1215-184(-) |
| 15 | 36 | TRINITY\_DN195934\_c0\_g1\_i1.p2 | 31.69 | 10 | 10 | 1.85e3 | 1 | 1 | 1 |  | 14094.0938 | type:internal len:127 gc:universal TRINITY\_DN195934\_c0\_g1\_i1:3-380(+) |
| 15 | 37 | TRINITY\_DN2477\_c0\_g1\_i6.p1 | 31.69 | 3 | 3 | 1.85e3 | 1 | 1 | 1 |  | 41878.6875 | type:complete len:377 gc:universal TRINITY\_DN2477\_c0\_g1\_i6:1314-184(-) |
| 15 | 38 | TRINITY\_DN2477\_c0\_g1\_i5.p1 | 31.69 | 4 | 4 | 1.85e3 | 1 | 1 | 1 |  | 37416.5195 | type:complete len:333 gc:universal TRINITY\_DN2477\_c0\_g1\_i5:1182-184(-) |
| 15 | 39 | TRINITY\_DN2477\_c0\_g1\_i9.p1 | 31.69 | 3 | 3 | 1.85e3 | 1 | 1 | 1 |  | 41878.6875 | type:complete len:377 gc:universal TRINITY\_DN2477\_c0\_g1\_i9:1314-184(-) |
| 15 | 40 | TRINITY\_DN2477\_c0\_g1\_i10.p1 | 31.69 | 3 | 3 | 1.85e3 | 1 | 1 | 1 |  | 38566.9688 | type:5prime\_partial len:344 gc:universal TRINITY\_DN2477\_c0\_g1\_i10:1215-184(-) |
| 16 | 41 | TRINITY\_DN691\_c0\_g1\_i1.p1 | 46.56 | 1 | 1 | 3.71e3 | 2 | 2 | 2 | Carbamidomethylation | 169920.1562 | type:complete len:1726 gc:universal TRINITY\_DN691\_c0\_g1\_i1:5662-485(-) |
| 16 | 42 | TRINITY\_DN691\_c0\_g1\_i2.p1 | 30.81 | 1 | 1 | 2.16e3 | 1 | 1 | 1 | Carbamidomethylation | 164625.2188 | type:complete len:1673 gc:universal TRINITY\_DN691\_c0\_g1\_i2:5503-485(-) |
| 17 | 43 | TRINITY\_DN477\_c0\_g1\_i8.p1 | 47.27 | 3 | 3 | 1.94e3 | 2 | 2 | 4 | Carbamidomethylation | 107165.2031 | type:complete len:985 gc:universal TRINITY\_DN477\_c0\_g1\_i8:3548-594(-) |
| 17 | 44 | TRINITY\_DN477\_c0\_g1\_i7.p1 | 47.27 | 3 | 3 | 1.94e3 | 2 | 2 | 4 | Carbamidomethylation | 122478.2578 | type:3prime\_partial len:1130 gc:universal TRINITY\_DN477\_c0\_g1\_i7:3387-1(-) |
| 18 | 45 | TRINITY\_DN140702\_c0\_g1\_i1.p1 | 47.14 | 1 | 1 | 4.27e3 | 2 | 2 | 4 |  | 231919.1562 | type:complete len:2036 gc:universal TRINITY\_DN140702\_c0\_g1\_i1:94-6201(+) |
| 19 | 46 | TRINITY\_DN2028\_c0\_g1\_i1.p1 | 47.45 | 11 | 11 | 2.5e4 | 2 | 2 | 3 | Oxidation (M) | 30106.9453 | type:complete len:274 gc:universal TRINITY\_DN2028\_c0\_g1\_i1:1608-787(-) |
| 20 | 47 | TRINITY\_DN428\_c0\_g1\_i1.p1 | 46.61 | 2 | 2 | 0 | 2 | 2 | 2 | Carbamidomethylation | 225824.1719 | type:complete len:1949 gc:universal TRINITY\_DN428\_c0\_g1\_i1:96-5942(+) |
| 21 | 48 | TRINITY\_DN72625\_c0\_g1\_i1.p1 | 47.52 | 2 | 2 | 2.17e3 | 2 | 2 | 2 |  | 214200.3906 | type:complete len:1964 gc:universal TRINITY\_DN72625\_c0\_g1\_i1:173-6064(+) |
| 22 | 49 | TRINITY\_DN12627\_c0\_g1\_i1.p1 | 47.53 | 3 | 3 | 1.82e3 | 2 | 2 | 3 |  | 120592.0703 | type:5prime\_partial len:1061 gc:universal TRINITY\_DN12627\_c0\_g1\_i1:3456-274(-) |
| 23 | 50 | TRINITY\_DN598\_c1\_g1\_i1.p1 | 47.48 | 3 | 3 | 5.05e3 | 2 | 2 | 2 |  | 81322.8672 | type:5prime\_partial len:745 gc:universal TRINITY\_DN598\_c1\_g1\_i1:2-2236(+) |
| 24 | 51 | TRINITY\_DN761\_c0\_g1\_i2.p1 | 47.26 | 2 | 2 | 1.11e3 | 2 | 2 | 2 |  | 95330.7812 | type:complete len:830 gc:universal TRINITY\_DN761\_c0\_g1\_i2:87-2576(+) |
| 24 | 52 | TRINITY\_DN761\_c0\_g1\_i1.p1 | 47.26 | 2 | 2 | 1.11e3 | 2 | 2 | 2 |  | 95330.7812 | type:complete len:830 gc:universal TRINITY\_DN761\_c0\_g1\_i1:129-2618(+) |
| 24 | 53 | TRINITY\_DN761\_c0\_g1\_i3.p1 | 47.26 | 2 | 2 | 1.11e3 | 2 | 2 | 2 |  | 96968.6562 | type:complete len:845 gc:universal TRINITY\_DN761\_c0\_g1\_i3:129-2663(+) |
| 25 | 54 | TRINITY\_DN1936\_c0\_g2\_i1.p1 | 30.5 | 1 | 1 | 0 | 1 | 1 | 1 | Carbamidomethylation | 208525.625 | type:3prime\_partial len:1894 gc:universal TRINITY\_DN1936\_c0\_g2\_i1:5680-2(-) |
| 26 | 55 | TRINITY\_DN624\_c0\_g1\_i1.p1 | 30.57 | 2 | 2 | 4.2e2 | 1 | 1 | 1 |  | 101743.5391 | type:complete len:884 gc:universal TRINITY\_DN624\_c0\_g1\_i1:218-2869(+) |
| 27 | 56 | TRINITY\_DN1058\_c3\_g1\_i1.p1 | 31.9 | 3 | 3 | 0 | 1 | 1 | 1 | Carbamidomethylation | 51455.6484 | type:internal len:485 gc:universal TRINITY\_DN1058\_c3\_g1\_i1:1452-1(-) |
| 28 | 57 | TRINITY\_DN1369\_c0\_g1\_i1.p1 | 31.8 | 1 | 1 | 1.64e3 | 1 | 1 | 1 | Carbamidomethylation | 174229.4219 | type:3prime\_partial len:1577 gc:universal TRINITY\_DN1369\_c0\_g1\_i1:93-4820(+) |
| 29 | 58 | TRINITY\_DN11248\_c0\_g1\_i1.p1 | 31.35 | 1 | 1 | 2.59e2 | 1 | 1 | 1 |  | 194116.9531 | type:3prime\_partial len:1700 gc:universal TRINITY\_DN11248\_c0\_g1\_i1:111-5207(+) |
| 29 | 59 | TRINITY\_DN11248\_c0\_g1\_i3.p1 | 31.35 | 1 | 1 | 2.59e2 | 1 | 1 | 1 |  | 194116.9531 | type:3prime\_partial len:1700 gc:universal TRINITY\_DN11248\_c0\_g1\_i3:111-5207(+) |
| 30 | 60 | TRINITY\_DN10485\_c0\_g1\_i1.p1 | 31.67 | 3 | 3 | 4.57e3 | 1 | 1 | 1 |  | 66716.9844 | type:5prime\_partial len:604 gc:universal TRINITY\_DN10485\_c0\_g1\_i1:2-1813(+) |
| 31 | 61 | TRINITY\_DN1058\_c0\_g1\_i1.p1 | 31.7 | 0 | 0 | 1.2e4 | 1 | 1 | 5 |  | 284466.2812 | type:complete len:2639 gc:universal TRINITY\_DN1058\_c0\_g1\_i1:134-8050(+) |
| 31 | 62 | TRINITY\_DN1058\_c0\_g1\_i3.p1 | 31.7 | 0 | 0 | 1.2e4 | 1 | 1 | 5 |  | 277633.5312 | type:complete len:2581 gc:universal TRINITY\_DN1058\_c0\_g1\_i3:134-7876(+) |
| 31 | 63 | TRINITY\_DN1058\_c0\_g1\_i4.p1 | 31.7 | 2 | 2 | 1.2e4 | 1 | 1 | 5 |  | 79375.5703 | type:complete len:723 gc:universal TRINITY\_DN1058\_c0\_g1\_i4:366-2534(+) |
| 31 | 64 | TRINITY\_DN1058\_c0\_g1\_i2.p2 | 31.7 | 1 | 1 | 1.2e4 | 1 | 1 | 5 |  | 140284.0938 | type:complete len:1300 gc:universal TRINITY\_DN1058\_c0\_g1\_i2:4090-7989(+) |
| 32 | 65 | TRINITY\_DN1676\_c0\_g1\_i12.p1 | 31.68 | 1 | 1 | 5.06e2 | 1 | 1 | 1 |  | 180975.5156 | type:complete len:1631 gc:universal TRINITY\_DN1676\_c0\_g1\_i12:206-5098(+) |
| 32 | 66 | TRINITY\_DN1676\_c0\_g1\_i6.p1 | 31.68 | 1 | 1 | 5.06e2 | 1 | 1 | 1 |  | 180975.5156 | type:complete len:1631 gc:universal TRINITY\_DN1676\_c0\_g1\_i6:206-5098(+) |
| 33 | 67 | TRINITY\_DN991\_c0\_g1\_i2.p1 | 31.67 | 0 | 0 | 6.83e2 | 1 | 1 | 1 |  | 358764.375 | type:complete len:3229 gc:universal TRINITY\_DN991\_c0\_g1\_i2:278-9964(+) |
| 33 | 68 | TRINITY\_DN991\_c0\_g1\_i6.p1 | 31.67 | 0 | 0 | 6.83e2 | 1 | 1 | 1 |  | 358765.375 | type:complete len:3229 gc:universal TRINITY\_DN991\_c0\_g1\_i6:278-9964(+) |
| 33 | 69 | TRINITY\_DN991\_c0\_g1\_i5.p1 | 31.67 | 0 | 0 | 6.83e2 | 1 | 1 | 1 |  | 358764.375 | type:complete len:3229 gc:universal TRINITY\_DN991\_c0\_g1\_i5:278-9964(+) |
| 34 | 70 | TRINITY\_DN165469\_c0\_g1\_i1.p1 | 31.67 | 0 | 0 | 0 | 1 | 1 | 1 |  | 298128.9688 | type:complete len:2668 gc:universal TRINITY\_DN165469\_c0\_g1\_i1:41-8044(+) |
| 35 | 71 | TRINITY\_DN2003\_c0\_g1\_i1.p1 | 31.71 | 3 | 3 | 2.53e2 | 1 | 1 | 1 |  | 42076.3203 | type:complete len:370 gc:universal TRINITY\_DN2003\_c0\_g1\_i1:89-1198(+) |
| 36 | 72 | TRINITY\_DN984\_c0\_g1\_i1.p1 | 30.8 | 1 | 1 | 0 | 1 | 1 | 1 |  | 146638.4062 | type:complete len:1485 gc:universal TRINITY\_DN984\_c0\_g1\_i1:4558-104(-) |
| 36 | 73 | TRINITY\_DN984\_c0\_g1\_i2.p1 | 30.8 | 1 | 1 | 0 | 1 | 1 | 1 |  | 146638.4062 | type:complete len:1485 gc:universal TRINITY\_DN984\_c0\_g1\_i2:4558-104(-) |
| 37 | 74 | TRINITY\_DN8903\_c0\_g1\_i1.p1 | 31.69 | 2 | 2 | 1.13e3 | 1 | 1 | 1 |  | 50029.5586 | type:5prime\_partial len:447 gc:universal TRINITY\_DN8903\_c0\_g1\_i1:1546-206(-) |
| 38 | 75 | TRINITY\_DN598\_c1\_g1\_i2.p1 | 31.69 | 1 | 1 | 3.39e3 | 1 | 1 | 2 |  | 81043.625 | type:5prime\_partial len:742 gc:universal TRINITY\_DN598\_c1\_g1\_i2:2-2227(+) |
| 39 | 76 | TRINITY\_DN69276\_c0\_g1\_i1.p1 | 31.55 | 1 | 1 | 8.64e2 | 1 | 1 | 1 |  | 97474.1719 | type:internal len:887 gc:universal TRINITY\_DN69276\_c0\_g1\_i1:2659-2(-) |
| 40 | 77 | TRINITY\_DN22240\_c0\_g1\_i1.p1 | 31.69 | 3 | 3 | 7.62e2 | 1 | 1 | 2 |  | 59687.8438 | type:complete len:513 gc:universal TRINITY\_DN22240\_c0\_g1\_i1:155-1693(+) |
| 41 | 78 | TRINITY\_DN495\_c0\_g1\_i5.p1 | 31.69 | 3 | 3 | 8.87e2 | 1 | 1 | 1 | Carbamidomethylation | 45678.0781 | type:3prime\_partial len:407 gc:universal TRINITY\_DN495\_c0\_g1\_i5:152-1369(+) |
| 41 | 79 | TRINITY\_DN495\_c0\_g1\_i6.p1 | 31.69 | 3 | 3 | 8.87e2 | 1 | 1 | 1 | Carbamidomethylation | 42984.9375 | type:3prime\_partial len:382 gc:universal TRINITY\_DN495\_c0\_g1\_i6:152-1294(+) |
| 42 | 80 | TRINITY\_DN2576\_c3\_g1\_i1.p2 | 31.25 | 8 | 8 | 5.85e3 | 1 | 1 | 2 |  | 16068.6924 | type:complete len:145 gc:universal TRINITY\_DN2576\_c3\_g1\_i1:807-373(-) |
| 43 | 81 | TRINITY\_DN10094\_c0\_g1\_i1.p1 | 31.68 | 5 | 5 | 0 | 1 | 1 | 1 | Carbamidomethylation | 28035.0664 | type:complete len:264 gc:universal TRINITY\_DN10094\_c0\_g1\_i1:1210-419(-) |
| 43 | 82 | TRINITY\_DN10094\_c0\_g1\_i2.p1 | 31.68 | 5 | 5 | 0 | 1 | 1 | 1 | Carbamidomethylation | 28035.0664 | type:complete len:264 gc:universal TRINITY\_DN10094\_c0\_g1\_i2:1210-419(-) |
| 44 | 83 | TRINITY\_DN1071\_c0\_g1\_i3.p1 | 31.9 | 4 | 4 | 2.88e3 | 1 | 1 | 2 |  | 39803.1602 | type:internal len:375 gc:universal TRINITY\_DN1071\_c0\_g1\_i3:1124-3(-) |
| 44 | 84 | TRINITY\_DN1071\_c0\_g1\_i2.p1 | 31.9 | 5 | 5 | 2.88e3 | 1 | 1 | 2 |  | 36243.2383 | type:internal len:339 gc:universal TRINITY\_DN1071\_c0\_g1\_i2:1016-3(-) |
| 44 | 85 | TRINITY\_DN1071\_c0\_g1\_i1.p1 | 31.9 | 2 | 2 | 2.88e3 | 1 | 1 | 2 |  | 71848.4062 | type:internal len:660 gc:universal TRINITY\_DN1071\_c0\_g1\_i1:1977-1(-) |
| 44 | 86 | TRINITY\_DN1071\_c0\_g1\_i4.p1 | 31.9 | 2 | 2 | 2.88e3 | 1 | 1 | 2 |  | 75408.3359 | type:internal len:696 gc:universal TRINITY\_DN1071\_c0\_g1\_i4:2085-1(-) |
| 45 | 87 | TRINITY\_DN2217\_c0\_g1\_i3.p1 | 30.62 | 2 | 2 | 0 | 1 | 1 | 1 | Carbamidomethylation | 63077.0508 | type:complete len:566 gc:universal TRINITY\_DN2217\_c0\_g1\_i3:1820-123(-) |
| 45 | 88 | TRINITY\_DN2217\_c0\_g1\_i2.p1 | 30.62 | 5 | 5 | 0 | 1 | 1 | 1 | Carbamidomethylation | 28618.9531 | type:complete len:254 gc:universal TRINITY\_DN2217\_c0\_g1\_i2:884-123(-) |
| 46 | 89 | TRINITY\_DN123121\_c0\_g1\_i1.p1 | 31.87 | 10 | 10 | 5.62e2 | 1 | 1 | 1 |  | 14215.7539 | type:internal len:127 gc:universal TRINITY\_DN123121\_c0\_g1\_i1:3-380(+) |
| 47 | 90 | TRINITY\_DN17095\_c0\_g1\_i1.p1 | 31.13 | 2 | 2 | 4.43e2 | 1 | 1 | 1 |  | 68063.4688 | type:internal len:607 gc:universal TRINITY\_DN17095\_c0\_g1\_i1:1820-3(-) |
| 48 | 91 | TRINITY\_DN30613\_c0\_g1\_i1.p1 | 31.53 | 2 | 2 | 7.39e2 | 1 | 1 | 1 |  | 106630.8516 | type:complete len:975 gc:universal TRINITY\_DN30613\_c0\_g1\_i1:154-3078(+) |
| 49 | 92 | TRINITY\_DN2113\_c0\_g1\_i2.p1 | 31.5 | 1 | 1 | 1.04e3 | 1 | 1 | 1 |  | 193799.0156 | type:complete len:1704 gc:universal TRINITY\_DN2113\_c0\_g1\_i2:5257-146(-) |
| 49 | 93 | TRINITY\_DN2113\_c0\_g1\_i1.p1 | 31.5 | 1 | 1 | 1.04e3 | 1 | 1 | 1 |  | 193799.0156 | type:complete len:1704 gc:universal TRINITY\_DN2113\_c0\_g1\_i1:5257-146(-) |
| 50 | 94 | TRINITY\_DN26392\_c0\_g1\_i1.p1 | 31.36 | 3 | 3 | 2.79e2 | 1 | 1 | 1 |  | 52063.2266 | type:internal len:469 gc:universal TRINITY\_DN26392\_c0\_g1\_i1:2-1405(+) |
| 51 | 95 | TRINITY\_DN4815\_c0\_g1\_i1.p1 | 31.68 | 3 | 3 | 5.35e2 | 1 | 1 | 1 | Carbamidomethylation | 66133.5156 | type:complete len:595 gc:universal TRINITY\_DN4815\_c0\_g1\_i1:79-1863(+) |
| 51 | 96 | TRINITY\_DN4815\_c0\_g1\_i2.p1 | 31.68 | 2 | 2 | 5.35e2 | 1 | 1 | 1 | Carbamidomethylation | 70695.4688 | type:complete len:638 gc:universal TRINITY\_DN4815\_c0\_g1\_i2:79-1992(+) |
| 52 | 97 | TRINITY\_DN1030\_c0\_g1\_i2.p1 | 31.94 | 1 | 1 | 3e3 | 1 | 1 | 1 |  | 140221.3281 | type:complete len:1426 gc:universal TRINITY\_DN1030\_c0\_g1\_i2:4483-206(-) |
| 52 | 98 | TRINITY\_DN1030\_c0\_g1\_i1.p1 | 31.94 | 1 | 1 | 3e3 | 1 | 1 | 1 |  | 141535.875 | type:complete len:1439 gc:universal TRINITY\_DN1030\_c0\_g1\_i1:4522-206(-) |
| 53 | 99 | TRINITY\_DN13467\_c0\_g1\_i1.p1 | 31.26 | 2 | 2 | 0 | 1 | 1 | 1 |  | 90166.0547 | type:5prime\_partial len:782 gc:universal TRINITY\_DN13467\_c0\_g1\_i1:2472-127(-) |
| 54 | 100 | TRINITY\_DN1936\_c0\_g1\_i4.p1 | 30.61 | 1 | 1 | 3.67e2 | 1 | 1 | 1 |  | 196066.9688 | type:3prime\_partial len:1762 gc:universal TRINITY\_DN1936\_c0\_g1\_i4:5283-1(-) |
| 54 | 101 | TRINITY\_DN1936\_c0\_g1\_i1.p1 | 30.61 | 1 | 1 | 3.67e2 | 1 | 1 | 1 |  | 136661.0312 | type:3prime\_partial len:1224 gc:universal TRINITY\_DN1936\_c0\_g1\_i1:3669-1(-) |
| 55 | 102 | TRINITY\_DN61\_c0\_g1\_i1.p1 | 31.74 | 2 | 2 | 1.15e3 | 1 | 1 | 1 |  | 71696.4219 | type:complete len:625 gc:universal TRINITY\_DN61\_c0\_g1\_i1:2929-1055(-) |
| 56 | 103 | TRINITY\_DN4595\_c0\_g1\_i4.p1 | 31.43 | 0 | 0 | 4.12e3 | 1 | 1 | 1 |  | 183684.4844 | type:5prime\_partial len:1639 gc:universal TRINITY\_DN4595\_c0\_g1\_i4:5857-941(-) |
| 56 | 104 | TRINITY\_DN4595\_c0\_g1\_i10.p1 | 31.43 | 1 | 1 | 4.12e3 | 1 | 1 | 1 |  | 133760.0625 | type:complete len:1188 gc:universal TRINITY\_DN4595\_c0\_g1\_i10:4405-842(-) |
| 57 | 105 | TRINITY\_DN99404\_c0\_g1\_i1.p1 | 31.4 | 13 | 13 | 0 | 1 | 1 | 1 |  | 12615.5928 | type:internal len:112 gc:universal TRINITY\_DN99404\_c0\_g1\_i1:2-334(+) |
| 58 | 106 | TRINITY\_DN942\_c0\_g1\_i3.p1 | 31.63 | 0 | 0 | 0 | 1 | 1 | 1 |  | 590662.8125 | type:complete len:5105 gc:universal TRINITY\_DN942\_c0\_g1\_i3:15631-317(-) |
| 58 | 107 | TRINITY\_DN942\_c0\_g1\_i2.p1 | 31.63 | 0 | 0 | 0 | 1 | 1 | 1 |  | 591926.3125 | type:complete len:5116 gc:universal TRINITY\_DN942\_c0\_g1\_i2:15664-317(-) |
| 58 | 108 | TRINITY\_DN942\_c0\_g1\_i5.p1 | 31.63 | 0 | 0 | 0 | 1 | 1 | 1 |  | 591926.25 | type:complete len:5116 gc:universal TRINITY\_DN942\_c0\_g1\_i5:15664-317(-) |
| 59 | 109 | TRINITY\_DN6542\_c0\_g1\_i3.p1 | 31.42 | 2 | 2 | 3.81e2 | 1 | 1 | 1 |  | 58403.6289 | type:5prime\_partial len:545 gc:universal TRINITY\_DN6542\_c0\_g1\_i3:2-1636(+) |
| 59 | 110 | TRINITY\_DN6542\_c0\_g1\_i1.p1 | 31.42 | 2 | 2 | 3.81e2 | 1 | 1 | 1 |  | 65866.8516 | type:internal len:628 gc:universal TRINITY\_DN6542\_c0\_g1\_i1:2-1882(+) |
| 59 | 111 | TRINITY\_DN6542\_c0\_g1\_i4.p1 | 31.42 | 2 | 2 | 3.81e2 | 1 | 1 | 1 |  | 58168.5312 | type:internal len:550 gc:universal TRINITY\_DN6542\_c0\_g1\_i4:2-1648(+) |
| 60 | 112 | TRINITY\_DN5584\_c0\_g1\_i3.p1 | 31.18 | 1 | 1 | 0 | 1 | 1 | 1 |  | 110603.7422 | type:complete len:962 gc:universal TRINITY\_DN5584\_c0\_g1\_i3:2920-35(-) |
| 60 | 113 | TRINITY\_DN5584\_c0\_g1\_i1.p1 | 31.18 | 1 | 1 | 0 | 1 | 1 | 1 |  | 110660.9297 | type:complete len:961 gc:universal TRINITY\_DN5584\_c0\_g1\_i1:2917-35(-) |
| 61 | 114 | TRINITY\_DN172571\_c0\_g1\_i1.p1 | 31.67 | 12 | 12 | 4.19e2 | 1 | 1 | 1 |  | 11500.2529 | type:complete len:104 gc:universal TRINITY\_DN172571\_c0\_g1\_i1:30-341(+) |
| 62 | 115 | TRINITY\_DN71276\_c0\_g1\_i1.p1 | 31.24 | 1 | 1 | 0 | 1 | 1 | 1 |  | 139103.3125 | type:5prime\_partial len:1201 gc:universal TRINITY\_DN71276\_c0\_g1\_i1:1-3603(+) |
| 63 | 116 | TRINITY\_DN118\_c1\_g1\_i3.p1 | 31.1 | 6 | 6 | 0 | 1 | 1 | 1 | Carbamidomethylation | 22860.4883 | type:internal len:200 gc:universal TRINITY\_DN118\_c1\_g1\_i3:599-3(-) |
| 64 | 117 | TRINITY\_DN540\_c0\_g1\_i1.p1 | 31.56 | 2 | 2 | 4.22e2 | 1 | 1 | 1 | Acetylation (Protein N-term) | 52183.2109 | type:complete len:480 gc:universal TRINITY\_DN540\_c0\_g1\_i1:1503-64(-) |
| 65 | 118 | TRINITY\_DN15584\_c0\_g1\_i1.p1 | 31.89 | 6 | 6 | 1.04e3 | 1 | 1 | 1 |  | 29534.9648 | type:complete len:275 gc:universal TRINITY\_DN15584\_c0\_g1\_i1:874-50(-) |
| 66 | 119 | TRINITY\_DN101992\_c0\_g1\_i1.p1 | 31.69 | 7 | 7 | 2.27e3 | 1 | 1 | 1 | Carbamidomethylation | 15607.4678 | type:internal len:139 gc:universal TRINITY\_DN101992\_c0\_g1\_i1:414-1(-) |
| 67 | 120 | TRINITY\_DN413\_c0\_g1\_i3.p1 | 31.33 | 3 | 3 | 1.07e3 | 1 | 1 | 1 |  | 39501.6133 | type:complete len:350 gc:universal TRINITY\_DN413\_c0\_g1\_i3:1286-237(-) |
| 67 | 121 | TRINITY\_DN413\_c0\_g1\_i4.p1 | 31.33 | 2 | 2 | 1.07e3 | 1 | 1 | 1 |  | 52354.1758 | type:3prime\_partial len:463 gc:universal TRINITY\_DN413\_c0\_g1\_i4:1388-3(-) |
| 67 | 122 | TRINITY\_DN413\_c0\_g1\_i1.p1 | 31.33 | 3 | 3 | 1.07e3 | 1 | 1 | 1 |  | 40351.7656 | type:complete len:355 gc:universal TRINITY\_DN413\_c0\_g1\_i1:1352-288(-) |
| 67 | 123 | TRINITY\_DN413\_c0\_g1\_i2.p1 | 31.33 | 8 | 8 | 1.07e3 | 1 | 1 | 1 |  | 15739.6523 | type:3prime\_partial len:140 gc:universal TRINITY\_DN413\_c0\_g1\_i2:419-3(-) |
| 68 | 124 | TRINITY\_DN97809\_c0\_g1\_i1.p1 | 31.49 | 7 | 7 | 0 | 1 | 1 | 1 |  | 20461.1211 | type:internal len:184 gc:universal TRINITY\_DN97809\_c0\_g1\_i1:2-550(+) |
| 69 | 125 | TRINITY\_DN1280\_c0\_g1\_i1.p1 | 30.7 | 3 | 3 | 0 | 1 | 1 | 1 |  | 47818.6641 | type:complete len:426 gc:universal TRINITY\_DN1280\_c0\_g1\_i1:195-1472(+) |
| 70 | 126 | TRINITY\_DN5633\_c0\_g1\_i1.p1 | 31.83 | 1 | 1 | 0 | 1 | 1 | 1 | Oxidation (M) | 97310.9922 | type:complete len:848 gc:universal TRINITY\_DN5633\_c0\_g1\_i1:2881-338(-) |
| 71 | 127 | TRINITY\_DN775\_c0\_g1\_i1.p1 | 30.71 | 2 | 2 | 0 | 1 | 1 | 1 |  | 72884.4609 | type:complete len:635 gc:universal TRINITY\_DN775\_c0\_g1\_i1:1927-23(-) |
| 71 | 128 | TRINITY\_DN775\_c0\_g1\_i2.p1 | 30.71 | 2 | 2 | 0 | 1 | 1 | 1 |  | 74657.6406 | type:complete len:647 gc:universal TRINITY\_DN775\_c0\_g1\_i2:1963-23(-) |
| 72 | 129 | TRINITY\_DN144508\_c0\_g1\_i1.p1 | 31.68 | 5 | 5 | 0 | 1 | 1 | 1 |  | 29446.1543 | type:internal len:265 gc:universal TRINITY\_DN144508\_c0\_g1\_i1:1-792(+) |
| 73 | 130 | TRINITY\_DN71243\_c0\_g1\_i1.p1 | 31.54 | 4 | 4 | 6.55e2 | 1 | 1 | 1 |  | 46628.2539 | type:internal len:422 gc:universal TRINITY\_DN71243\_c0\_g1\_i1:1265-3(-) |
| 74 | 131 | TRINITY\_DN278\_c0\_g1\_i1.p1 | 31.54 | 3 | 3 | 0 | 1 | 1 | 1 |  | 55197.2969 | type:complete len:470 gc:universal TRINITY\_DN278\_c0\_g1\_i1:3091-1682(-) |
| 75 | 132 | TRINITY\_DN2963\_c0\_g1\_i1.p1 | 31.65 | 10 | 10 | 7.16e2 | 1 | 1 | 1 | Acetylation (Protein N-term) | 13082.1787 | type:complete len:116 gc:universal TRINITY\_DN2963\_c0\_g1\_i1:1072-725(-) |
| 76 | 133 | TRINITY\_DN1559\_c0\_g1\_i1.p1 | 31.68 | 5 | 5 | 1.07e3 | 1 | 1 | 2 |  | 33672.0977 | type:complete len:311 gc:universal TRINITY\_DN1559\_c0\_g1\_i1:221-1153(+) |
| 77 | 134 | TRINITY\_DN1682\_c8\_g1\_i3.p1 | 30.66 | 7 | 7 | 0 | 1 | 1 | 1 | Acetylation (Protein N-term) | 13843.7529 | type:internal len:117 gc:universal TRINITY\_DN1682\_c8\_g1\_i3:348-1(-) |
| 78 | 135 | TRINITY\_DN195348\_c0\_g1\_i1.p1 | 31.93 | 5 | 5 | 2.43e2 | 1 | 1 | 1 |  | 34993.6055 | type:internal len:311 gc:universal TRINITY\_DN195348\_c0\_g1\_i1:1-930(+) |
| 79 | 136 | TRINITY\_DN113\_c0\_g1\_i1.p1 | 31.69 | 1 | 1 | 7.08e3 | 1 | 1 | 3 |  | 139235.5938 | type:complete len:1417 gc:universal TRINITY\_DN113\_c0\_g1\_i1:4413-163(-) |
| 80 | 137 | TRINITY\_DN4781\_c0\_g1\_i3.p3 | 31.14 | 7 | 7 | 0 | 1 | 1 | 1 |  | 20029.2422 | type:complete len:183 gc:universal TRINITY\_DN4781\_c0\_g1\_i3:2965-2417(-) |
| 81 | 138 | TRINITY\_DN113\_c3\_g1\_i1.p1 | 31.06 | 1 | 1 | 4.04e2 | 1 | 1 | 1 |  | 125422.4922 | type:3prime\_partial len:1162 gc:universal TRINITY\_DN113\_c3\_g1\_i1:3485-3(-) |
| 82 | 139 | TRINITY\_DN61668\_c0\_g1\_i1.p1 | 31.69 | 3 | 3 | 5.97e2 | 1 | 1 | 2 |  | 51212.582 | type:5prime\_partial len:453 gc:universal TRINITY\_DN61668\_c0\_g1\_i1:2-1360(+) |
| 83 | 140 | TRINITY\_DN25375\_c0\_g1\_i7.p1 | 31.84 | 1 | 1 | 3.61e3 | 1 | 1 | 1 | Carbamidomethylation | 90646.6094 | type:complete len:811 gc:universal TRINITY\_DN25375\_c0\_g1\_i7:112-2544(+) |
| 84 | 141 | TRINITY\_DN77\_c1\_g1\_i1.p1 | 31.11 | 1 | 1 | 7.3e2 | 1 | 1 | 1 |  | 198350.2344 | type:3prime\_partial len:1965 gc:universal TRINITY\_DN77\_c1\_g1\_i1:2-5893(+) |
| 85 | 142 | TRINITY\_DN64\_c0\_g1\_i1.p1 | 31.68 | 3 | 3 | 0 | 1 | 1 | 1 |  | 51450.1914 | type:complete len:468 gc:universal TRINITY\_DN64\_c0\_g1\_i1:1555-152(-) |
| 86 | 143 | TRINITY\_DN55094\_c0\_g1\_i1.p1 | 31.65 | 10 | 10 | 5.29e2 | 1 | 1 | 1 |  | 12167.1719 | type:internal len:107 gc:universal TRINITY\_DN55094\_c0\_g1\_i1:319-2(-) |
| 87 | 144 | TRINITY\_DN247\_c0\_g1\_i1.p1 | 31.3 | 3 | 3 | 1.07e3 | 1 | 1 | 1 |  | 42079.6172 | type:complete len:373 gc:universal TRINITY\_DN247\_c0\_g1\_i1:38-1156(+) |
| 88 | 145 | TRINITY\_DN141561\_c0\_g1\_i1.p1 | 30.42 | 4 | 4 | 0 | 1 | 1 | 1 |  | 28167.5371 | type:internal len:248 gc:universal TRINITY\_DN141561\_c0\_g1\_i1:742-2(-) |
| total 145 proteins |  |  |  |  |  |  |  |  |  |  |  |  |
| --- | --- | --- | --- | --- | --- | --- | --- | --- | --- | --- | --- | --- |

  

TRINITY\_DN319\_c2\_g1\_i2.p1

back to list

  

| Protein Coverage
| Supporting Peptides
| Best Unique PSM
|

Protein Coverage:

Supporting Peptides:

| Peptide | Uniq | -10lgP | Mass | Length | ppm | m/z | z | RT | 1/k0 Start | 1/k0 End | Precursor ID | Area Sample 5 | #Feature | #Feature Sample 5 | Start | End | PTM | AScore | Found By |
| --- | --- | --- | --- | --- | --- | --- | --- | --- | --- | --- | --- | --- | --- | --- | --- | --- | --- | --- | --- |
| R.DPIC(+57.02)HC(+57.02)DTSK.Y | Y | 31.69 | 1231.4961 | 10 | 5.21 | 616.7559 | 2 | 9.93 | 0.904 | 0.9113 | 14979 | 4.42e3 | 2 | 1 | 1448 | 1457 | Carbamidomethylation, Carbamidomethylation |  | DB Search |
| R.GSFSSHIC(+57.02)LC(+57.02)HDPAYK.S | Y | 31.67 | 1877.8189 | 16 | 3.89 | 626.9467 | 3 | 14.33 | 0.8838 | 0.8911 | 40207 | 1.11e3 | 1 | 1 | 1011 | 1026 | Carbamidomethylation, Carbamidomethylation |  | DB Search |
| K.LLHC(+57.02)SDSEVDHVEVC(+57.02)GK.G | Y | 31.63 | 1982.8826 | 17 | 4.93 | 661.9686 | 3 | 13.16 | 0.9226 | 0.9299 | 33774 | 0 | 0 | 0 | 845 | 861 | Carbamidomethylation, Carbamidomethylation |  | DB Search |
| total 3 peptides |  |  |  |  |  |  |  |  |  |  |  |  |  |  |  |  |  |  |  |
| --- | --- | --- | --- | --- | --- | --- | --- | --- | --- | --- | --- | --- | --- | --- | --- | --- | --- | --- | --- |

Best Unique PSM (Scan Sample\_3\_A1\_1\_3641.d:14979, m/z=616.7559, z=2, RT=9.93, ppm=0.93):


TRINITY\_DN97809\_c0\_g1\_i1.p1

back to list

  

| Protein Coverage
| Supporting Peptides
| Best Unique PSM
|

Protein Coverage:

Supporting Peptides:

| Peptide | Uniq | -10lgP | Mass | Length | ppm | m/z | z | RT | 1/k0 Start | 1/k0 End | Precursor ID | Area Sample 5 | #Feature | #Feature Sample 5 | Start | End | PTM | AScore | Found By |
| --- | --- | --- | --- | --- | --- | --- | --- | --- | --- | --- | --- | --- | --- | --- | --- | --- | --- | --- | --- |
| LGPDQVDVAGAGR.D | Y | 31.49 | 1253.6364 | 13 | -7.34 | 627.8182 | 2 | 13.58 | 0.9331 | 0.9404 | 36091 | 0 | 0 | 0 | 1 | 13 |  |  | DB Search |
| total 1 peptides |  |  |  |  |  |  |  |  |  |  |  |  |  |  |  |  |  |  |  |
| --- | --- | --- | --- | --- | --- | --- | --- | --- | --- | --- | --- | --- | --- | --- | --- | --- | --- | --- | --- |

Best Unique PSM (Scan Sample\_3\_A1\_1\_3641.d:36091, m/z=627.8182, z=2, RT=13.58, ppm=-11.62):


TRINITY\_DN101992\_c0\_g1\_i1.p1

back to list

  

| Protein Coverage
| Supporting Peptides
| Best Unique PSM
|

Protein Coverage:

Supporting Peptides:

| Peptide | Uniq | -10lgP | Mass | Length | ppm | m/z | z | RT | 1/k0 Start | 1/k0 End | Precursor ID | Area Sample 5 | #Feature | #Feature Sample 5 | Start | End | PTM | AScore | Found By |
| --- | --- | --- | --- | --- | --- | --- | --- | --- | --- | --- | --- | --- | --- | --- | --- | --- | --- | --- | --- |
| K.AELVFVNHC(+57.02)R.E | Y | 31.69 | 1243.6132 | 10 | 3.87 | 622.8136 | 2 | 14.34 | 0.9566 | 0.9638 | 40177 | 2.27e3 | 1 | 1 | 118 | 127 | Carbamidomethylation |  | DB Search |
| total 1 peptides |  |  |  |  |  |  |  |  |  |  |  |  |  |  |  |  |  |  |  |
| --- | --- | --- | --- | --- | --- | --- | --- | --- | --- | --- | --- | --- | --- | --- | --- | --- | --- | --- | --- |

Best Unique PSM (Scan Sample\_3\_A1\_1\_3641.d:40177, m/z=622.8136, z=2, RT=14.34, ppm=-0.41):


TRINITY\_DN161\_c0\_g1\_i3.p1

back to list

  

| Protein Coverage
| Supporting Peptides
| Best Unique PSM
|

Protein Coverage:

Supporting Peptides:

| Peptide | Uniq | -10lgP | Mass | Length | ppm | m/z | z | RT | 1/k0 Start | 1/k0 End | Precursor ID | Area Sample 5 | #Feature | #Feature Sample 5 | Start | End | PTM | AScore | Found By |
| --- | --- | --- | --- | --- | --- | --- | --- | --- | --- | --- | --- | --- | --- | --- | --- | --- | --- | --- | --- |
| R.AVFPSIVGRPR.H | Y | 31.8 | 1197.6982 | 11 | 3.08 | 599.8557 | 2 | 15.68 | 0.9574 | 0.9646 | 46487 | 1.74e4 | 1 | 1 | 52 | 62 |  |  | DB Search |
| K.DSYVGDEAQSKR.G | Y | 31.69 | 1353.616 | 12 | 1.77 | 677.8136 | 2 | 10.94 | 1.0017 | 1.009 | 21026 | 1.85e3 | 1 | 1 | 74 | 85 |  |  | DB Search |
| total 2 peptides |  |  |  |  |  |  |  |  |  |  |  |  |  |  |  |  |  |  |  |
| --- | --- | --- | --- | --- | --- | --- | --- | --- | --- | --- | --- | --- | --- | --- | --- | --- | --- | --- | --- |

Best Unique PSM (Scan Sample\_3\_A1\_1\_3641.d:46487, m/z=599.8557, z=2, RT=15.68, ppm=-1.2):


TRINITY\_DN2113\_c0\_g1\_i1.p1

back to list

  

| Protein Coverage
| Supporting Peptides
| Best Unique PSM
|

Protein Coverage:

Supporting Peptides:

| Peptide | Uniq | -10lgP | Mass | Length | ppm | m/z | z | RT | 1/k0 Start | 1/k0 End | Precursor ID | Area Sample 5 | #Feature | #Feature Sample 5 | Start | End | PTM | AScore | Found By |
| --- | --- | --- | --- | --- | --- | --- | --- | --- | --- | --- | --- | --- | --- | --- | --- | --- | --- | --- | --- |
| R.LADLEEFISSPNHANISQVGER.C | Y | 31.5 | 2425.1873 | 22 | 2.2 | 809.4014 | 3 | 18.76 | 0.9255 | 0.9327 | 63995 | 1.04e3 | 1 | 1 | 1183 | 1204 |  |  | DB Search |
| total 1 peptides |  |  |  |  |  |  |  |  |  |  |  |  |  |  |  |  |  |  |  |
| --- | --- | --- | --- | --- | --- | --- | --- | --- | --- | --- | --- | --- | --- | --- | --- | --- | --- | --- | --- |

Best Unique PSM (Scan Sample\_3\_A1\_1\_3641.d:63995, m/z=809.4014, z=3, RT=18.76, ppm=-2.08):


TRINITY\_DN10094\_c0\_g1\_i2.p1

back to list

  

| Protein Coverage
| Supporting Peptides
| Best Unique PSM
|

Protein Coverage:

Supporting Peptides:

| Peptide | Uniq | -10lgP | Mass | Length | ppm | m/z | z | RT | 1/k0 Start | 1/k0 End | Precursor ID | Area Sample 5 | #Feature | #Feature Sample 5 | Start | End | PTM | AScore | Found By |
| --- | --- | --- | --- | --- | --- | --- | --- | --- | --- | --- | --- | --- | --- | --- | --- | --- | --- | --- | --- |
| K.FFFHDQSC(+57.02)LDVYK.Y | Y | 31.68 | 1704.7606 | 13 | 0.29 | 569.2585 | 3 | 17.60 | 0.8075 | 0.8149 | 57111 | 0 | 0 | 0 | 221 | 233 | Carbamidomethylation |  | DB Search |
| total 1 peptides |  |  |  |  |  |  |  |  |  |  |  |  |  |  |  |  |  |  |  |
| --- | --- | --- | --- | --- | --- | --- | --- | --- | --- | --- | --- | --- | --- | --- | --- | --- | --- | --- | --- |

Best Unique PSM (Scan Sample\_3\_A1\_1\_3641.d:57111, m/z=569.2585, z=3, RT=17.60, ppm=-3.99):


TRINITY\_DN942\_c0\_g1\_i2.p1

back to list

  

| Protein Coverage
| Supporting Peptides
| Best Unique PSM
|

Protein Coverage:

Supporting Peptides:

| Peptide | Uniq | -10lgP | Mass | Length | ppm | m/z | z | RT | 1/k0 Start | 1/k0 End | Precursor ID | Area Sample 5 | #Feature | #Feature Sample 5 | Start | End | PTM | AScore | Found By |
| --- | --- | --- | --- | --- | --- | --- | --- | --- | --- | --- | --- | --- | --- | --- | --- | --- | --- | --- | --- |
| K.VLNIALDKYQEGVAK.T | Y | 31.63 | 1659.9195 | 15 | -0.5 | 830.9631 | 2 | 15.97 | 1.1546 | 1.1618 | 48647 | 0 | 0 | 0 | 4557 | 4571 |  |  | DB Search |
| total 1 peptides |  |  |  |  |  |  |  |  |  |  |  |  |  |  |  |  |  |  |  |
| --- | --- | --- | --- | --- | --- | --- | --- | --- | --- | --- | --- | --- | --- | --- | --- | --- | --- | --- | --- |

Best Unique PSM (Scan Sample\_3\_A1\_1\_3641.d:48647, m/z=830.9631, z=2, RT=15.97, ppm=-4.78):


TRINITY\_DN6542\_c0\_g1\_i4.p1

back to list

  

| Protein Coverage
| Supporting Peptides
| Best Unique PSM
|

Protein Coverage:

Supporting Peptides:

| Peptide | Uniq | -10lgP | Mass | Length | ppm | m/z | z | RT | 1/k0 Start | 1/k0 End | Precursor ID | Area Sample 5 | #Feature | #Feature Sample 5 | Start | End | PTM | AScore | Found By |
| --- | --- | --- | --- | --- | --- | --- | --- | --- | --- | --- | --- | --- | --- | --- | --- | --- | --- | --- | --- |
| K.ILLAVGGWNHER.Q | Y | 31.42 | 1363.736 | 12 | 2.78 | 682.8743 | 2 | 16.92 | 1.0387 | 1.046 | 53358 | 3.81e2 | 1 | 1 | 140 | 151 |  |  | DB Search |
| total 1 peptides |  |  |  |  |  |  |  |  |  |  |  |  |  |  |  |  |  |  |  |
| --- | --- | --- | --- | --- | --- | --- | --- | --- | --- | --- | --- | --- | --- | --- | --- | --- | --- | --- | --- |

Best Unique PSM (Scan Sample\_3\_A1\_1\_3641.d:53358, m/z=682.8743, z=2, RT=16.92, ppm=-1.5):


TRINITY\_DN984\_c0\_g1\_i2.p1

back to list

  

| Protein Coverage
| Supporting Peptides
| Best Unique PSM
|

Protein Coverage:

Supporting Peptides:

| Peptide | Uniq | -10lgP | Mass | Length | ppm | m/z | z | RT | 1/k0 Start | 1/k0 End | Precursor ID | Area Sample 5 | #Feature | #Feature Sample 5 | Start | End | PTM | AScore | Found By |
| --- | --- | --- | --- | --- | --- | --- | --- | --- | --- | --- | --- | --- | --- | --- | --- | --- | --- | --- | --- |
| R.EGPTGPQGETGR.R | Y | 30.8 | 1184.5421 | 12 | 6.09 | 593.2794 | 2 | 10.30 | 0.8806 | 0.8878 | 17329 | 0 | 0 | 0 | 476 | 487 |  |  | DB Search |
| total 1 peptides |  |  |  |  |  |  |  |  |  |  |  |  |  |  |  |  |  |  |  |
| --- | --- | --- | --- | --- | --- | --- | --- | --- | --- | --- | --- | --- | --- | --- | --- | --- | --- | --- | --- |

Best Unique PSM (Scan Sample\_3\_A1\_1\_3641.d:17329, m/z=593.2794, z=2, RT=10.30, ppm=1.8):


TRINITY\_DN477\_c0\_g1\_i9.p1

back to list

  

| Protein Coverage
| Supporting Peptides
| Best Unique PSM
|

Protein Coverage:

Supporting Peptides:

| Peptide | Uniq | -10lgP | Mass | Length | ppm | m/z | z | RT | 1/k0 Start | 1/k0 End | Precursor ID | Area Sample 5 | #Feature | #Feature Sample 5 | Start | End | PTM | AScore | Found By |
| --- | --- | --- | --- | --- | --- | --- | --- | --- | --- | --- | --- | --- | --- | --- | --- | --- | --- | --- | --- |
| R.TQAVLDRLNIEKR.V | Y | 31.84 | 1554.8842 | 13 | 3.94 | 519.3018 | 3 | 14.55 | 0.7978 | 0.8051 | 41371 | 3.63e3 | 1 | 1 | 220 | 232 |  |  | DB Search |
| K.MRFDTAITAC(+57.02)R.S | Y | 31.74 | 1340.6329 | 11 | 4.37 | 671.3238 | 2 | 14.91 | 0.9646 | 0.9719 | 43169 | 1.84e3 | 1 | 1 | 276 | 286 | Carbamidomethylation |  | DB Search |
| R.TQAVLDRLNIEK.R | Y | 31.68 | 1398.783 | 12 | 4.72 | 700.3991 | 2 | 15.32 | 1.0045 | 1.0118 | 45318 | 4.75e3 | 1 | 1 | 220 | 231 |  |  | DB Search |
| K.DANSM(+15.99)PTQSR.Q | Y | 31.58 | 1121.4771 | 10 | 2.01 | 561.7446 | 2 | 8.83 | 0.8258 | 0.8331 | 9387 | 1.05e3 | 1 | 1 | 51 | 60 | Oxidation (M) | M5:Oxidation (M):1000 | DB Search |
| total 4 peptides |  |  |  |  |  |  |  |  |  |  |  |  |  |  |  |  |  |  |  |
| --- | --- | --- | --- | --- | --- | --- | --- | --- | --- | --- | --- | --- | --- | --- | --- | --- | --- | --- | --- |

Best Unique PSM (Scan Sample\_3\_A1\_1\_3641.d:41371, m/z=519.3018, z=3, RT=14.55, ppm=-0.34):


TRINITY\_DN982\_c0\_g1\_i2.p1

back to list

  

| Protein Coverage
| Supporting Peptides
| Best Unique PSM
|

Protein Coverage:

Supporting Peptides:

| Peptide | Uniq | -10lgP | Mass | Length | ppm | m/z | z | RT | 1/k0 Start | 1/k0 End | Precursor ID | Area Sample 5 | #Feature | #Feature Sample 5 | Start | End | PTM | AScore | Found By |
| --- | --- | --- | --- | --- | --- | --- | --- | --- | --- | --- | --- | --- | --- | --- | --- | --- | --- | --- | --- |
| K.VLLSQQIFYDKK.N | N | 31.68 | 1480.8289 | 12 | -0.33 | 741.4183 | 2 | 16.72 | 1.0572 | 1.0644 | 52159 | 3.15e3 | 1 | 1 | 1479 | 1490 |  |  | DB Search |
| R.VLDVNDKQPVFVK.S | Y | 31.63 | 1499.8347 | 13 | 5.63 | 750.9257 | 2 | 14.98 | 1.048 | 1.0552 | 43639 | 0 | 0 | 0 | 670 | 682 |  |  | DB Search |
| total 2 peptides |  |  |  |  |  |  |  |  |  |  |  |  |  |  |  |  |  |  |  |
| --- | --- | --- | --- | --- | --- | --- | --- | --- | --- | --- | --- | --- | --- | --- | --- | --- | --- | --- | --- |

Best Unique PSM (Scan Sample\_3\_A1\_1\_3641.d:43639, m/z=751.8621, z=2, RT=15.01, ppm=1.35):


TRINITY\_DN71243\_c0\_g1\_i1.p1

back to list

  

| Protein Coverage
| Supporting Peptides
| Best Unique PSM
|

Protein Coverage:

Supporting Peptides:

| Peptide | Uniq | -10lgP | Mass | Length | ppm | m/z | z | RT | 1/k0 Start | 1/k0 End | Precursor ID | Area Sample 5 | #Feature | #Feature Sample 5 | Start | End | PTM | AScore | Found By |
| --- | --- | --- | --- | --- | --- | --- | --- | --- | --- | --- | --- | --- | --- | --- | --- | --- | --- | --- | --- |
| K.RVHQFLVEALDGGQPR.R | Y | 31.54 | 1820.9645 | 16 | -3.51 | 607.9907 | 3 | 19.35 | 0.8692 | 0.8765 | 67626 | 6.55e2 | 1 | 1 | 129 | 144 |  |  | DB Search |
| total 1 peptides |  |  |  |  |  |  |  |  |  |  |  |  |  |  |  |  |  |  |  |
| --- | --- | --- | --- | --- | --- | --- | --- | --- | --- | --- | --- | --- | --- | --- | --- | --- | --- | --- | --- |

Best Unique PSM (Scan Sample\_3\_A1\_1\_3641.d:67626, m/z=607.9907, z=3, RT=19.35, ppm=-7.79):


TRINITY\_DN413\_c0\_g1\_i3.p1

back to list

  

| Protein Coverage
| Supporting Peptides
| Best Unique PSM
|

Protein Coverage:

Supporting Peptides:

| Peptide | Uniq | -10lgP | Mass | Length | ppm | m/z | z | RT | 1/k0 Start | 1/k0 End | Precursor ID | Area Sample 5 | #Feature | #Feature Sample 5 | Start | End | PTM | AScore | Found By |
| --- | --- | --- | --- | --- | --- | --- | --- | --- | --- | --- | --- | --- | --- | --- | --- | --- | --- | --- | --- |
| R.LLLGDFNGDKR.T | Y | 31.33 | 1246.6669 | 11 | 4.3 | 624.3408 | 2 | 16.04 | 0.9836 | 0.9908 | 48938 | 1.07e3 | 1 | 1 | 46 | 56 |  |  | DB Search |
| total 1 peptides |  |  |  |  |  |  |  |  |  |  |  |  |  |  |  |  |  |  |  |
| --- | --- | --- | --- | --- | --- | --- | --- | --- | --- | --- | --- | --- | --- | --- | --- | --- | --- | --- | --- |

Best Unique PSM (Scan Sample\_3\_A1\_1\_3641.d:48938, m/z=624.3408, z=2, RT=16.04, ppm=0.01):


TRINITY\_DN17725\_c0\_g1\_i1.p1

back to list

  

| Protein Coverage
| Supporting Peptides
| Best Unique PSM
|

Protein Coverage:

Supporting Peptides:

| Peptide | Uniq | -10lgP | Mass | Length | ppm | m/z | z | RT | 1/k0 Start | 1/k0 End | Precursor ID | Area Sample 5 | #Feature | #Feature Sample 5 | Start | End | PTM | AScore | Found By |
| --- | --- | --- | --- | --- | --- | --- | --- | --- | --- | --- | --- | --- | --- | --- | --- | --- | --- | --- | --- |
| K.VDVGFILDSSGSLR.N | Y | 31.66 | 1463.762 | 14 | -1.83 | 732.8838 | 2 | 19.80 | 1.0126 | 1.0198 | 70509 | 0 | 0 | 0 | 35 | 48 |  |  | DB Search |
| total 1 peptides |  |  |  |  |  |  |  |  |  |  |  |  |  |  |  |  |  |  |  |
| --- | --- | --- | --- | --- | --- | --- | --- | --- | --- | --- | --- | --- | --- | --- | --- | --- | --- | --- | --- |

Best Unique PSM (Scan Sample\_3\_A1\_1\_3641.d:70509, m/z=732.8838, z=2, RT=19.80, ppm=-6.11):


TRINITY\_DN413\_c0\_g1\_i1.p1

back to list

  

| Protein Coverage
| Supporting Peptides
| Best Unique PSM
|

Protein Coverage:

Supporting Peptides:

| Peptide | Uniq | -10lgP | Mass | Length | ppm | m/z | z | RT | 1/k0 Start | 1/k0 End | Precursor ID | Area Sample 5 | #Feature | #Feature Sample 5 | Start | End | PTM | AScore | Found By |
| --- | --- | --- | --- | --- | --- | --- | --- | --- | --- | --- | --- | --- | --- | --- | --- | --- | --- | --- | --- |
| R.LLLGDFNGDKR.T | Y | 31.33 | 1246.6669 | 11 | 4.3 | 624.3408 | 2 | 16.04 | 0.9836 | 0.9908 | 48938 | 1.07e3 | 1 | 1 | 46 | 56 |  |  | DB Search |
| total 1 peptides |  |  |  |  |  |  |  |  |  |  |  |  |  |  |  |  |  |  |  |
| --- | --- | --- | --- | --- | --- | --- | --- | --- | --- | --- | --- | --- | --- | --- | --- | --- | --- | --- | --- |

Best Unique PSM (Scan Sample\_3\_A1\_1\_3641.d:48938, m/z=624.3408, z=2, RT=16.04, ppm=0.01):


TRINITY\_DN1280\_c0\_g1\_i1.p1

back to list

  

| Protein Coverage
| Supporting Peptides
| Best Unique PSM
|

Protein Coverage:

Supporting Peptides:

| Peptide | Uniq | -10lgP | Mass | Length | ppm | m/z | z | RT | 1/k0 Start | 1/k0 End | Precursor ID | Area Sample 5 | #Feature | #Feature Sample 5 | Start | End | PTM | AScore | Found By |
| --- | --- | --- | --- | --- | --- | --- | --- | --- | --- | --- | --- | --- | --- | --- | --- | --- | --- | --- | --- |
| K.AEYENKTEVAR.S | Y | 30.7 | 1308.631 | 11 | 4.55 | 655.3229 | 2 | 13.11 | 0.9812 | 0.9884 | 33509 | 0 | 0 | 0 | 202 | 212 |  |  | DB Search |
| total 1 peptides |  |  |  |  |  |  |  |  |  |  |  |  |  |  |  |  |  |  |  |
| --- | --- | --- | --- | --- | --- | --- | --- | --- | --- | --- | --- | --- | --- | --- | --- | --- | --- | --- | --- |

Best Unique PSM (Scan Sample\_3\_A1\_1\_3641.d:33509, m/z=655.3229, z=2, RT=13.11, ppm=0.27):


TRINITY\_DN1559\_c0\_g1\_i1.p1

back to list

  

| Protein Coverage
| Supporting Peptides
| Best Unique PSM
|

Protein Coverage:

Supporting Peptides:

| Peptide | Uniq | -10lgP | Mass | Length | ppm | m/z | z | RT | 1/k0 Start | 1/k0 End | Precursor ID | Area Sample 5 | #Feature | #Feature Sample 5 | Start | End | PTM | AScore | Found By |
| --- | --- | --- | --- | --- | --- | --- | --- | --- | --- | --- | --- | --- | --- | --- | --- | --- | --- | --- | --- |
| R.GVAGAGVLAGFDKFK.E | Y | 31.68 | 1435.7823 | 15 | 2.01 | 718.8968 | 2 | 18.26 | 1.0849 | 1.0921 | 60846 | 1.07e3 | 1 | 1 | 282 | 296 |  |  | DB Search |
| total 1 peptides |  |  |  |  |  |  |  |  |  |  |  |  |  |  |  |  |  |  |  |
| --- | --- | --- | --- | --- | --- | --- | --- | --- | --- | --- | --- | --- | --- | --- | --- | --- | --- | --- | --- |

Best Unique PSM (Scan Sample\_3\_A1\_1\_3641.d:60846, m/z=718.8968, z=2, RT=18.26, ppm=-2.27):


TRINITY\_DN5584\_c0\_g1\_i1.p1

back to list

  

| Protein Coverage
| Supporting Peptides
| Best Unique PSM
|

Protein Coverage:

Supporting Peptides:

| Peptide | Uniq | -10lgP | Mass | Length | ppm | m/z | z | RT | 1/k0 Start | 1/k0 End | Precursor ID | Area Sample 5 | #Feature | #Feature Sample 5 | Start | End | PTM | AScore | Found By |
| --- | --- | --- | --- | --- | --- | --- | --- | --- | --- | --- | --- | --- | --- | --- | --- | --- | --- | --- | --- |
| K.VTLTQTHFLK.D | Y | 31.18 | 1186.671 | 10 | 4.18 | 594.3427 | 2 | 14.84 | 0.9638 | 0.9711 | 42932 | 0 | 0 | 0 | 559 | 568 |  |  | DB Search |
| total 1 peptides |  |  |  |  |  |  |  |  |  |  |  |  |  |  |  |  |  |  |  |
| --- | --- | --- | --- | --- | --- | --- | --- | --- | --- | --- | --- | --- | --- | --- | --- | --- | --- | --- | --- |

Best Unique PSM (Scan Sample\_3\_A1\_1\_3641.d:42932, m/z=594.3427, z=2, RT=14.84, ppm=-0.1):


TRINITY\_DN4494\_c1\_g1\_i1.p1

back to list

  

| Protein Coverage
| Supporting Peptides
| Best Unique PSM
|

Protein Coverage:

Supporting Peptides:

| Peptide | Uniq | -10lgP | Mass | Length | ppm | m/z | z | RT | 1/k0 Start | 1/k0 End | Precursor ID | Area Sample 5 | #Feature | #Feature Sample 5 | Start | End | PTM | AScore | Found By |
| --- | --- | --- | --- | --- | --- | --- | --- | --- | --- | --- | --- | --- | --- | --- | --- | --- | --- | --- | --- |
| R.VLDVGFLLDSSDKVK.D | Y | 31.68 | 1633.8926 | 15 | 1.24 | 817.9511 | 2 | 19.58 | 1.1694 | 1.1766 | 69063 | 8.63e2 | 2 | 2 | 109 | 123 |  |  | DB Search |
| K.VDVGFILDSSGSLR.N | Y | 31.66 | 1463.762 | 14 | -1.83 | 732.8838 | 2 | 19.80 | 1.0126 | 1.0198 | 70509 | 0 | 0 | 0 | 307 | 320 |  |  | DB Search |
| total 2 peptides |  |  |  |  |  |  |  |  |  |  |  |  |  |  |  |  |  |  |  |
| --- | --- | --- | --- | --- | --- | --- | --- | --- | --- | --- | --- | --- | --- | --- | --- | --- | --- | --- | --- |

Best Unique PSM (Scan Sample\_3\_A1\_1\_3641.d:69063, m/z=817.9511, z=2, RT=19.58, ppm=-3.04):


TRINITY\_DN2963\_c0\_g1\_i1.p1

back to list

  

| Protein Coverage
| Supporting Peptides
| Best Unique PSM
|

Protein Coverage:

Supporting Peptides:

| Peptide | Uniq | -10lgP | Mass | Length | ppm | m/z | z | RT | 1/k0 Start | 1/k0 End | Precursor ID | Area Sample 5 | #Feature | #Feature Sample 5 | Start | End | PTM | AScore | Found By |
| --- | --- | --- | --- | --- | --- | --- | --- | --- | --- | --- | --- | --- | --- | --- | --- | --- | --- | --- | --- |
| M.S(+42.01)ATGNLVTVLRK.L | Y | 31.65 | 1299.751 | 12 | 5.44 | 650.8835 | 2 | 17.09 | 0.9969 | 1.0041 | 54070 | 7.16e2 | 1 | 1 | 2 | 13 | Acetylation (Protein N-term) | S1:Acetylation (Protein N-term):1000 | DB Search |
| total 1 peptides |  |  |  |  |  |  |  |  |  |  |  |  |  |  |  |  |  |  |  |
| --- | --- | --- | --- | --- | --- | --- | --- | --- | --- | --- | --- | --- | --- | --- | --- | --- | --- | --- | --- |

Best Unique PSM (Scan Sample\_3\_A1\_1\_3641.d:54070, m/z=650.8835, z=2, RT=17.09, ppm=1.16):


TRINITY\_DN2477\_c0\_g1\_i9.p1

back to list

  

| Protein Coverage
| Supporting Peptides
| Best Unique PSM
|

Protein Coverage:

Supporting Peptides:

| Peptide | Uniq | -10lgP | Mass | Length | ppm | m/z | z | RT | 1/k0 Start | 1/k0 End | Precursor ID | Area Sample 5 | #Feature | #Feature Sample 5 | Start | End | PTM | AScore | Found By |
| --- | --- | --- | --- | --- | --- | --- | --- | --- | --- | --- | --- | --- | --- | --- | --- | --- | --- | --- | --- |
| K.DSYVGDEAQSKR.G | Y | 31.69 | 1353.616 | 12 | 1.77 | 677.8136 | 2 | 10.94 | 1.0017 | 1.009 | 21026 | 1.85e3 | 1 | 1 | 52 | 63 |  |  | DB Search |
| total 1 peptides |  |  |  |  |  |  |  |  |  |  |  |  |  |  |  |  |  |  |  |
| --- | --- | --- | --- | --- | --- | --- | --- | --- | --- | --- | --- | --- | --- | --- | --- | --- | --- | --- | --- |

Best Unique PSM (Scan Sample\_3\_A1\_1\_3641.d:21026, m/z=677.8136, z=2, RT=10.94, ppm=-2.51):


TRINITY\_DN775\_c0\_g1\_i2.p1

back to list

  

| Protein Coverage
| Supporting Peptides
| Best Unique PSM
|

Protein Coverage:

Supporting Peptides:

| Peptide | Uniq | -10lgP | Mass | Length | ppm | m/z | z | RT | 1/k0 Start | 1/k0 End | Precursor ID | Area Sample 5 | #Feature | #Feature Sample 5 | Start | End | PTM | AScore | Found By |
| --- | --- | --- | --- | --- | --- | --- | --- | --- | --- | --- | --- | --- | --- | --- | --- | --- | --- | --- | --- |
| K.DKELSNVIESGFR.T | Y | 30.71 | 1492.7521 | 13 | 4.38 | 747.3834 | 2 | 17.20 | 1.0419 | 1.0492 | 54715 | 0 | 0 | 0 | 489 | 501 |  |  | DB Search |
| total 1 peptides |  |  |  |  |  |  |  |  |  |  |  |  |  |  |  |  |  |  |  |
| --- | --- | --- | --- | --- | --- | --- | --- | --- | --- | --- | --- | --- | --- | --- | --- | --- | --- | --- | --- |

Best Unique PSM (Scan Sample\_3\_A1\_1\_3641.d:54715, m/z=747.3834, z=2, RT=17.20, ppm=0.1):


TRINITY\_DN1071\_c0\_g1\_i1.p1

back to list

  

| Protein Coverage
| Supporting Peptides
| Best Unique PSM
|

Protein Coverage:

Supporting Peptides:

| Peptide | Uniq | -10lgP | Mass | Length | ppm | m/z | z | RT | 1/k0 Start | 1/k0 End | Precursor ID | Area Sample 5 | #Feature | #Feature Sample 5 | Start | End | PTM | AScore | Found By |
| --- | --- | --- | --- | --- | --- | --- | --- | --- | --- | --- | --- | --- | --- | --- | --- | --- | --- | --- | --- |
| K.AVQIHPFNDVSVGDPK.V | Y | 31.9 | 1721.8736 | 16 | 2.29 | 861.9424 | 2 | 15.73 | 1.1186 | 1.1258 | 47418 | 2.88e3 | 1 | 1 | 293 | 308 |  |  | DB Search |
| total 1 peptides |  |  |  |  |  |  |  |  |  |  |  |  |  |  |  |  |  |  |  |
| --- | --- | --- | --- | --- | --- | --- | --- | --- | --- | --- | --- | --- | --- | --- | --- | --- | --- | --- | --- |

Best Unique PSM (Scan Sample\_3\_A1\_1\_3641.d:47418, m/z=861.9424, z=2, RT=15.73, ppm=-1.99):


TRINITY\_DN1676\_c0\_g1\_i6.p1

back to list

  

| Protein Coverage
| Supporting Peptides
| Best Unique PSM
|

Protein Coverage:

Supporting Peptides:

| Peptide | Uniq | -10lgP | Mass | Length | ppm | m/z | z | RT | 1/k0 Start | 1/k0 End | Precursor ID | Area Sample 5 | #Feature | #Feature Sample 5 | Start | End | PTM | AScore | Found By |
| --- | --- | --- | --- | --- | --- | --- | --- | --- | --- | --- | --- | --- | --- | --- | --- | --- | --- | --- | --- |
| R.FELDRVNTFGDEVFGDPK.V | Y | 31.68 | 2083.985 | 18 | 4.09 | 695.6688 | 3 | 19.17 | 0.9198 | 0.9271 | 66516 | 5.06e2 | 1 | 1 | 252 | 269 |  |  | DB Search |
| total 1 peptides |  |  |  |  |  |  |  |  |  |  |  |  |  |  |  |  |  |  |  |
| --- | --- | --- | --- | --- | --- | --- | --- | --- | --- | --- | --- | --- | --- | --- | --- | --- | --- | --- | --- |

Best Unique PSM (Scan Sample\_3\_A1\_1\_3641.d:66516, m/z=695.6688, z=3, RT=19.17, ppm=-0.19):


TRINITY\_DN1366\_c0\_g1\_i1.p1

back to list

  

| Protein Coverage
| Supporting Peptides
| Best Unique PSM
|

Protein Coverage:

Supporting Peptides:

| Peptide | Uniq | -10lgP | Mass | Length | ppm | m/z | z | RT | 1/k0 Start | 1/k0 End | Precursor ID | Area Sample 5 | #Feature | #Feature Sample 5 | Start | End | PTM | AScore | Found By |
| --- | --- | --- | --- | --- | --- | --- | --- | --- | --- | --- | --- | --- | --- | --- | --- | --- | --- | --- | --- |
| K.LNNEINDLHQR.L | Y | 30.95 | 1364.6797 | 11 | 5.8 | 683.3481 | 2 | 12.54 | 0.9949 | 1.0021 | 30348 | 0 | 0 | 0 | 3176 | 3186 |  |  | DB Search |
| R.HIISDRDVMIER.L | Y | 30.84 | 1482.7613 | 12 | -2.42 | 495.2577 | 3 | 15.36 | 0.8023 | 0.8096 | 45585 | 0 | 0 | 0 | 1424 | 1435 |  |  | DB Search |
| K.KQQENEQLLDEKR.L | Y | 30.62 | 1656.8431 | 13 | -3.85 | 553.2838 | 3 | 11.43 | 0.8222 | 0.8295 | 23996 | 3.61e2 | 1 | 1 | 1597 | 1609 |  |  | DB Search |
| total 3 peptides |  |  |  |  |  |  |  |  |  |  |  |  |  |  |  |  |  |  |  |
| --- | --- | --- | --- | --- | --- | --- | --- | --- | --- | --- | --- | --- | --- | --- | --- | --- | --- | --- | --- |

Best Unique PSM (Scan Sample\_3\_A1\_1\_3641.d:30348, m/z=683.3481, z=2, RT=12.54, ppm=1.52):


TRINITY\_DN319\_c2\_g1\_i1.p1

back to list

  

| Protein Coverage
| Supporting Peptides
| Best Unique PSM
|

Protein Coverage:

Supporting Peptides:

| Peptide | Uniq | -10lgP | Mass | Length | ppm | m/z | z | RT | 1/k0 Start | 1/k0 End | Precursor ID | Area Sample 5 | #Feature | #Feature Sample 5 | Start | End | PTM | AScore | Found By |
| --- | --- | --- | --- | --- | --- | --- | --- | --- | --- | --- | --- | --- | --- | --- | --- | --- | --- | --- | --- |
| R.DPIC(+57.02)HC(+57.02)DTSK.Y | Y | 31.69 | 1231.4961 | 10 | 5.21 | 616.7559 | 2 | 9.93 | 0.904 | 0.9113 | 14979 | 4.42e3 | 2 | 1 | 1448 | 1457 | Carbamidomethylation, Carbamidomethylation |  | DB Search |
| K.KAQYYEAYETIIKR.H | Y | 31.69 | 1774.9254 | 14 | -12.66 | 592.639 | 3 | 15.71 | 0.8607 | 0.868 | 47344 | 1.05e3 | 1 | 1 | 2534 | 2547 |  |  | DB Search |
| R.GSFSSHIC(+57.02)LC(+57.02)HDPAYK.S | Y | 31.67 | 1877.8189 | 16 | 3.89 | 626.9467 | 3 | 14.33 | 0.8838 | 0.8911 | 40207 | 1.11e3 | 1 | 1 | 1011 | 1026 | Carbamidomethylation, Carbamidomethylation |  | DB Search |
| K.LLHC(+57.02)SDSEVDHVEVC(+57.02)GK.G | Y | 31.63 | 1982.8826 | 17 | 4.93 | 661.9686 | 3 | 13.16 | 0.9226 | 0.9299 | 33774 | 0 | 0 | 0 | 845 | 861 | Carbamidomethylation, Carbamidomethylation |  | DB Search |
| R.HLAISNILK.D | Y | 30.67 | 1007.6127 | 9 | 1.34 | 504.8122 | 2 | 18.76 | 0.8676 | 0.8749 | 63978 | 5.96e2 | 1 | 1 | 2548 | 2556 |  |  | DB Search |
| total 5 peptides |  |  |  |  |  |  |  |  |  |  |  |  |  |  |  |  |  |  |  |
| --- | --- | --- | --- | --- | --- | --- | --- | --- | --- | --- | --- | --- | --- | --- | --- | --- | --- | --- | --- |

Best Unique PSM (Scan Sample\_3\_A1\_1\_3641.d:14979, m/z=616.7559, z=2, RT=9.93, ppm=0.93):


TRINITY\_DN761\_c0\_g1\_i2.p1

back to list

  

| Protein Coverage
| Supporting Peptides
| Best Unique PSM
|

Protein Coverage:

Supporting Peptides:

| Peptide | Uniq | -10lgP | Mass | Length | ppm | m/z | z | RT | 1/k0 Start | 1/k0 End | Precursor ID | Area Sample 5 | #Feature | #Feature Sample 5 | Start | End | PTM | AScore | Found By |
| --- | --- | --- | --- | --- | --- | --- | --- | --- | --- | --- | --- | --- | --- | --- | --- | --- | --- | --- | --- |
| K.TRIEDLETVISQTQDHR.H | Y | 31.69 | 2040.0236 | 17 | 6.44 | 681.0166 | 3 | 17.36 | 0.9614 | 0.9687 | 55514 | 1.11e3 | 1 | 1 | 260 | 276 |  |  | DB Search |
| R.IEDLETVISQTQDHR.H | Y | 31.15 | 1782.8748 | 15 | -3.8 | 595.2941 | 3 | 17.75 | 0.9048 | 0.9121 | 58019 | 0 | 0 | 0 | 262 | 276 |  |  | DB Search |
| total 2 peptides |  |  |  |  |  |  |  |  |  |  |  |  |  |  |  |  |  |  |  |
| --- | --- | --- | --- | --- | --- | --- | --- | --- | --- | --- | --- | --- | --- | --- | --- | --- | --- | --- | --- |

Best Unique PSM (Scan Sample\_3\_A1\_1\_3641.d:55514, m/z=681.0166, z=3, RT=17.36, ppm=2.16):


TRINITY\_DN1936\_c0\_g1\_i1.p1

back to list

  

| Protein Coverage
| Supporting Peptides
| Best Unique PSM
|

Protein Coverage:

Supporting Peptides:

| Peptide | Uniq | -10lgP | Mass | Length | ppm | m/z | z | RT | 1/k0 Start | 1/k0 End | Precursor ID | Area Sample 5 | #Feature | #Feature Sample 5 | Start | End | PTM | AScore | Found By |
| --- | --- | --- | --- | --- | --- | --- | --- | --- | --- | --- | --- | --- | --- | --- | --- | --- | --- | --- | --- |
| K.AGDIAILESQQFNAVGGK.T | Y | 30.61 | 1816.9319 | 18 | -19.18 | 909.4519 | 2 | 18.85 | 1.1853 | 1.1925 | 64620 | 3.67e2 | 1 | 1 | 464 | 481 |  |  | DB Search |
| total 1 peptides |  |  |  |  |  |  |  |  |  |  |  |  |  |  |  |  |  |  |  |
| --- | --- | --- | --- | --- | --- | --- | --- | --- | --- | --- | --- | --- | --- | --- | --- | --- | --- | --- | --- |

Best Unique PSM (Scan Sample\_3\_A1\_1\_3641.d:64620, m/z=909.4519, z=2, RT=18.85, ppm=-23.46):


TRINITY\_DN413\_c0\_g1\_i4.p1

back to list

  

| Protein Coverage
| Supporting Peptides
| Best Unique PSM
|

Protein Coverage:

Supporting Peptides:

| Peptide | Uniq | -10lgP | Mass | Length | ppm | m/z | z | RT | 1/k0 Start | 1/k0 End | Precursor ID | Area Sample 5 | #Feature | #Feature Sample 5 | Start | End | PTM | AScore | Found By |
| --- | --- | --- | --- | --- | --- | --- | --- | --- | --- | --- | --- | --- | --- | --- | --- | --- | --- | --- | --- |
| R.LLLGDFNGDKR.T | Y | 31.33 | 1246.6669 | 11 | 4.3 | 624.3408 | 2 | 16.04 | 0.9836 | 0.9908 | 48938 | 1.07e3 | 1 | 1 | 46 | 56 |  |  | DB Search |
| total 1 peptides |  |  |  |  |  |  |  |  |  |  |  |  |  |  |  |  |  |  |  |
| --- | --- | --- | --- | --- | --- | --- | --- | --- | --- | --- | --- | --- | --- | --- | --- | --- | --- | --- | --- |

Best Unique PSM (Scan Sample\_3\_A1\_1\_3641.d:48938, m/z=624.3408, z=2, RT=16.04, ppm=0.01):


TRINITY\_DN278\_c0\_g1\_i1.p1

back to list

  

| Protein Coverage
| Supporting Peptides
| Best Unique PSM
|

Protein Coverage:

Supporting Peptides:

| Peptide | Uniq | -10lgP | Mass | Length | ppm | m/z | z | RT | 1/k0 Start | 1/k0 End | Precursor ID | Area Sample 5 | #Feature | #Feature Sample 5 | Start | End | PTM | AScore | Found By |
| --- | --- | --- | --- | --- | --- | --- | --- | --- | --- | --- | --- | --- | --- | --- | --- | --- | --- | --- | --- |
| K.IDNYIEFLDEDLKK.K | Y | 31.54 | 1753.8774 | 14 | -5.87 | 585.6271 | 3 | 19.85 | 0.8441 | 0.8514 | 70732 | 0 | 0 | 0 | 102 | 115 |  |  | DB Search |
| total 1 peptides |  |  |  |  |  |  |  |  |  |  |  |  |  |  |  |  |  |  |  |
| --- | --- | --- | --- | --- | --- | --- | --- | --- | --- | --- | --- | --- | --- | --- | --- | --- | --- | --- | --- |

Best Unique PSM (Scan Sample\_3\_A1\_1\_3641.d:70732, m/z=585.6271, z=3, RT=19.85, ppm=-10.15):


TRINITY\_DN12627\_c0\_g1\_i1.p1

back to list

  

| Protein Coverage
| Supporting Peptides
| Best Unique PSM
|

Protein Coverage:

Supporting Peptides:

| Peptide | Uniq | -10lgP | Mass | Length | ppm | m/z | z | RT | 1/k0 Start | 1/k0 End | Precursor ID | Area Sample 5 | #Feature | #Feature Sample 5 | Start | End | PTM | AScore | Found By |
| --- | --- | --- | --- | --- | --- | --- | --- | --- | --- | --- | --- | --- | --- | --- | --- | --- | --- | --- | --- |
| K.FDFHSWIGLKK.I | Y | 31.68 | 1376.7241 | 11 | -0.47 | 459.9131 | 3 | 18.24 | 0.7355 | 0.7428 | 60734 | 1.82e3 | 1 | 1 | 193 | 203 |  |  | DB Search |
| K.GFGGNLVTITNSDIQK.Q | Y | 31.68 | 1662.8577 | 16 | -4.47 | 832.4288 | 2 | 17.63 | 1.0773 | 1.0845 | 57262 | 0 | 0 | 0 | 311 | 326 |  |  | DB Search |
| total 2 peptides |  |  |  |  |  |  |  |  |  |  |  |  |  |  |  |  |  |  |  |
| --- | --- | --- | --- | --- | --- | --- | --- | --- | --- | --- | --- | --- | --- | --- | --- | --- | --- | --- | --- |

Best Unique PSM (Scan Sample\_3\_A1\_1\_3641.d:60734, m/z=459.9131, z=3, RT=18.24, ppm=-4.76):


TRINITY\_DN144508\_c0\_g1\_i1.p1

back to list

  

| Protein Coverage
| Supporting Peptides
| Best Unique PSM
|

Protein Coverage:

Supporting Peptides:

| Peptide | Uniq | -10lgP | Mass | Length | ppm | m/z | z | RT | 1/k0 Start | 1/k0 End | Precursor ID | Area Sample 5 | #Feature | #Feature Sample 5 | Start | End | PTM | AScore | Found By |
| --- | --- | --- | --- | --- | --- | --- | --- | --- | --- | --- | --- | --- | --- | --- | --- | --- | --- | --- | --- |
| K.SSLNHNIVFEGR.T | Y | 31.68 | 1371.6895 | 12 | 6.28 | 686.8534 | 2 | 14.46 | 0.9816 | 0.9888 | 40919 | 0 | 0 | 0 | 233 | 244 |  |  | DB Search |
| total 1 peptides |  |  |  |  |  |  |  |  |  |  |  |  |  |  |  |  |  |  |  |
| --- | --- | --- | --- | --- | --- | --- | --- | --- | --- | --- | --- | --- | --- | --- | --- | --- | --- | --- | --- |

Best Unique PSM (Scan Sample\_3\_A1\_1\_3641.d:40919, m/z=686.8534, z=2, RT=14.46, ppm=2):


TRINITY\_DN4595\_c0\_g1\_i4.p1

back to list

  

| Protein Coverage
| Supporting Peptides
| Best Unique PSM
|

Protein Coverage:

Supporting Peptides:

| Peptide | Uniq | -10lgP | Mass | Length | ppm | m/z | z | RT | 1/k0 Start | 1/k0 End | Precursor ID | Area Sample 5 | #Feature | #Feature Sample 5 | Start | End | PTM | AScore | Found By |
| --- | --- | --- | --- | --- | --- | --- | --- | --- | --- | --- | --- | --- | --- | --- | --- | --- | --- | --- | --- |
| R.SPFVIMFK.G | Y | 31.43 | 967.5201 | 8 | 3.55 | 484.767 | 2 | 19.71 | 0.8096 | 0.8169 | 69837 | 4.12e3 | 1 | 1 | 1081 | 1088 |  |  | DB Search |
| total 1 peptides |  |  |  |  |  |  |  |  |  |  |  |  |  |  |  |  |  |  |  |
| --- | --- | --- | --- | --- | --- | --- | --- | --- | --- | --- | --- | --- | --- | --- | --- | --- | --- | --- | --- |

Best Unique PSM (Scan Sample\_3\_A1\_1\_3641.d:69837, m/z=484.767, z=2, RT=19.71, ppm=-0.73):


TRINITY\_DN15584\_c0\_g1\_i1.p1

back to list

  

| Protein Coverage
| Supporting Peptides
| Best Unique PSM
|

Protein Coverage:

Supporting Peptides:

| Peptide | Uniq | -10lgP | Mass | Length | ppm | m/z | z | RT | 1/k0 Start | 1/k0 End | Precursor ID | Area Sample 5 | #Feature | #Feature Sample 5 | Start | End | PTM | AScore | Found By |
| --- | --- | --- | --- | --- | --- | --- | --- | --- | --- | --- | --- | --- | --- | --- | --- | --- | --- | --- | --- |
| K.AFVAIGDYGGHVGLGVK.C | Y | 31.89 | 1658.878 | 17 | 4.75 | 830.4467 | 2 | 17.61 | 1.1622 | 1.1694 | 57032 | 1.04e3 | 1 | 1 | 109 | 125 |  |  | DB Search |
| total 1 peptides |  |  |  |  |  |  |  |  |  |  |  |  |  |  |  |  |  |  |  |
| --- | --- | --- | --- | --- | --- | --- | --- | --- | --- | --- | --- | --- | --- | --- | --- | --- | --- | --- | --- |

Best Unique PSM (Scan Sample\_3\_A1\_1\_3641.d:57032, m/z=830.4467, z=2, RT=17.61, ppm=0.47):


TRINITY\_DN72625\_c0\_g1\_i1.p1

back to list

  

| Protein Coverage
| Supporting Peptides
| Best Unique PSM
|

Protein Coverage:

Supporting Peptides:

| Peptide | Uniq | -10lgP | Mass | Length | ppm | m/z | z | RT | 1/k0 Start | 1/k0 End | Precursor ID | Area Sample 5 | #Feature | #Feature Sample 5 | Start | End | PTM | AScore | Found By |
| --- | --- | --- | --- | --- | --- | --- | --- | --- | --- | --- | --- | --- | --- | --- | --- | --- | --- | --- | --- |
| R.IKDTYLLTLEAR.D | Y | 31.68 | 1434.8082 | 12 | -1.93 | 718.4069 | 2 | 16.86 | 1.0347 | 1.0419 | 52952 | 9.73e2 | 1 | 1 | 393 | 404 |  |  | DB Search |
| K.VQNEITNPNTQFPVNGLFDLHR.K | Y | 31.66 | 2552.2771 | 22 | 2.02 | 851.7644 | 3 | 19.92 | 1.0632 | 1.0705 | 71061 | 1.2e3 | 1 | 1 | 1880 | 1901 |  |  | DB Search |
| total 2 peptides |  |  |  |  |  |  |  |  |  |  |  |  |  |  |  |  |  |  |  |
| --- | --- | --- | --- | --- | --- | --- | --- | --- | --- | --- | --- | --- | --- | --- | --- | --- | --- | --- | --- |

Best Unique PSM (Scan Sample\_3\_A1\_1\_3641.d:52952, m/z=718.4069, z=2, RT=16.86, ppm=-6.21):


TRINITY\_DN598\_c1\_g1\_i1.p1

back to list

  

| Protein Coverage
| Supporting Peptides
| Best Unique PSM
|

Protein Coverage:

Supporting Peptides:

| Peptide | Uniq | -10lgP | Mass | Length | ppm | m/z | z | RT | 1/k0 Start | 1/k0 End | Precursor ID | Area Sample 5 | #Feature | #Feature Sample 5 | Start | End | PTM | AScore | Found By |
| --- | --- | --- | --- | --- | --- | --- | --- | --- | --- | --- | --- | --- | --- | --- | --- | --- | --- | --- | --- |
| R.ASFVSDIDRLR.A | Y | 31.69 | 1277.6728 | 11 | 1.75 | 639.842 | 2 | 16.90 | 0.9812 | 0.9884 | 53092 | 3.28e3 | 1 | 1 | 302 | 312 |  |  | DB Search |
| K.AVAEEFISVDPYVR.S | Y | 31.58 | 1593.8038 | 14 | 2.58 | 797.9078 | 2 | 18.98 | 1.0604 | 1.0676 | 65194 | 1.77e3 | 1 | 1 | 148 | 161 |  |  | DB Search |
| total 2 peptides |  |  |  |  |  |  |  |  |  |  |  |  |  |  |  |  |  |  |  |
| --- | --- | --- | --- | --- | --- | --- | --- | --- | --- | --- | --- | --- | --- | --- | --- | --- | --- | --- | --- |

Best Unique PSM (Scan Sample\_3\_A1\_1\_3641.d:53092, m/z=639.842, z=2, RT=16.90, ppm=-2.53):


TRINITY\_DN775\_c0\_g1\_i1.p1

back to list

  

| Protein Coverage
| Supporting Peptides
| Best Unique PSM
|

Protein Coverage:

Supporting Peptides:

| Peptide | Uniq | -10lgP | Mass | Length | ppm | m/z | z | RT | 1/k0 Start | 1/k0 End | Precursor ID | Area Sample 5 | #Feature | #Feature Sample 5 | Start | End | PTM | AScore | Found By |
| --- | --- | --- | --- | --- | --- | --- | --- | --- | --- | --- | --- | --- | --- | --- | --- | --- | --- | --- | --- |
| K.DKELSNVIESGFR.T | Y | 30.71 | 1492.7521 | 13 | 4.38 | 747.3834 | 2 | 17.20 | 1.0419 | 1.0492 | 54715 | 0 | 0 | 0 | 477 | 489 |  |  | DB Search |
| total 1 peptides |  |  |  |  |  |  |  |  |  |  |  |  |  |  |  |  |  |  |  |
| --- | --- | --- | --- | --- | --- | --- | --- | --- | --- | --- | --- | --- | --- | --- | --- | --- | --- | --- | --- |

Best Unique PSM (Scan Sample\_3\_A1\_1\_3641.d:54715, m/z=747.3834, z=2, RT=17.20, ppm=0.1):


TRINITY\_DN113\_c0\_g1\_i1.p1

back to list

  

| Protein Coverage
| Supporting Peptides
| Best Unique PSM
|

Protein Coverage:

Supporting Peptides:

| Peptide | Uniq | -10lgP | Mass | Length | ppm | m/z | z | RT | 1/k0 Start | 1/k0 End | Precursor ID | Area Sample 5 | #Feature | #Feature Sample 5 | Start | End | PTM | AScore | Found By |
| --- | --- | --- | --- | --- | --- | --- | --- | --- | --- | --- | --- | --- | --- | --- | --- | --- | --- | --- | --- |
| R.GKDGETGEQGPQGQEGPTGPR.G | Y | 31.69 | 2080.9409 | 21 | 3.9 | 694.654 | 3 | 10.71 | 0.849 | 0.8562 | 19412 | 7.08e3 | 1 | 1 | 693 | 713 |  |  | DB Search |
| total 1 peptides |  |  |  |  |  |  |  |  |  |  |  |  |  |  |  |  |  |  |  |
| --- | --- | --- | --- | --- | --- | --- | --- | --- | --- | --- | --- | --- | --- | --- | --- | --- | --- | --- | --- |

Best Unique PSM (Scan Sample\_3\_A1\_1\_3641.d:19412, m/z=694.654, z=3, RT=10.71, ppm=-0.38):


TRINITY\_DN4781\_c0\_g1\_i3.p3

back to list

  

| Protein Coverage
| Supporting Peptides
| Best Unique PSM
|

Protein Coverage:

Supporting Peptides:

| Peptide | Uniq | -10lgP | Mass | Length | ppm | m/z | z | RT | 1/k0 Start | 1/k0 End | Precursor ID | Area Sample 5 | #Feature | #Feature Sample 5 | Start | End | PTM | AScore | Found By |
| --- | --- | --- | --- | --- | --- | --- | --- | --- | --- | --- | --- | --- | --- | --- | --- | --- | --- | --- | --- |
| R.DNAGTSSSGTGPR.F | Y | 31.14 | 1205.5272 | 13 | 19.43 | 603.78 | 2 | 9.14 | 0.8635 | 0.8708 | 10555 | 6.72e2 | 1 | 0 | 126 | 138 |  |  | DB Search |
| total 1 peptides |  |  |  |  |  |  |  |  |  |  |  |  |  |  |  |  |  |  |  |
| --- | --- | --- | --- | --- | --- | --- | --- | --- | --- | --- | --- | --- | --- | --- | --- | --- | --- | --- | --- |

Best Unique PSM (Scan Sample\_3\_A1\_1\_3641.d:10555, m/z=603.78, z=2, RT=9.14, ppm=15.15):


TRINITY\_DN1616\_c0\_g1\_i6.p1

back to list

  

| Protein Coverage
| Supporting Peptides
| Best Unique PSM
|

Protein Coverage:

Supporting Peptides:

| Peptide | Uniq | -10lgP | Mass | Length | ppm | m/z | z | RT | 1/k0 Start | 1/k0 End | Precursor ID | Area Sample 5 | #Feature | #Feature Sample 5 | Start | End | PTM | AScore | Found By |
| --- | --- | --- | --- | --- | --- | --- | --- | --- | --- | --- | --- | --- | --- | --- | --- | --- | --- | --- | --- |
| K.GC(+57.02)STC(+57.02)GC(+57.02)NGAGTNNGVK.S | Y | 31.94 | 1712.6665 | 17 | 1.85 | 857.3384 | 2 | 9.80 | 1.0448 | 1.052 | 14171 | 0 | 0 | 0 | 732 | 748 | Carbamidomethylation, Carbamidomethylation, Carbamidomethylation |  | DB Search |
| K.VVDTEANDLTEEVKK.T | Y | 31.68 | 1688.8468 | 15 | 3.24 | 845.4298 | 2 | 14.05 | 1.1286 | 1.1358 | 38709 | 1.51e3 | 1 | 1 | 2542 | 2556 |  |  | DB Search |
| R.YGAVNDNC(+57.02)ERDNGQC(+57.02)SC(+57.02)K.V | Y | 31.05 | 2145.8262 | 18 | 2.18 | 716.2812 | 3 | 10.55 | 0.8587 | 0.866 | 18796 | 7.2e2 | 1 | 1 | 498 | 515 | Carbamidomethylation, Carbamidomethylation, Carbamidomethylation |  | DB Search |
| K.NC(+57.02)QHNTLGINC(+57.02)EK.C | Y | 30.89 | 1586.693 | 13 | 4.28 | 794.3538 | 2 | 11.89 | 1.0371 | 1.0443 | 26586 | 7.24e2 | 1 | 1 | 366 | 378 | Carbamidomethylation, Carbamidomethylation |  | DB Search |
| K.DGEKVETLKVDISDINK.A | Y | 30.55 | 1901.9945 | 17 | 2.61 | 635.0044 | 3 | 15.87 | 0.9065 | 0.9137 | 48130 | 2.66e2 | 1 | 1 | 2634 | 2650 |  |  | DB Search |
| total 5 peptides |  |  |  |  |  |  |  |  |  |  |  |  |  |  |  |  |  |  |  |
| --- | --- | --- | --- | --- | --- | --- | --- | --- | --- | --- | --- | --- | --- | --- | --- | --- | --- | --- | --- |

Best Unique PSM (Scan Sample\_3\_A1\_1\_3641.d:14171, m/z=857.3384, z=2, RT=9.80, ppm=-2.43):


TRINITY\_DN25375\_c0\_g1\_i7.p1

back to list

  

| Protein Coverage
| Supporting Peptides
| Best Unique PSM
|

Protein Coverage:

Supporting Peptides:

| Peptide | Uniq | -10lgP | Mass | Length | ppm | m/z | z | RT | 1/k0 Start | 1/k0 End | Precursor ID | Area Sample 5 | #Feature | #Feature Sample 5 | Start | End | PTM | AScore | Found By |
| --- | --- | --- | --- | --- | --- | --- | --- | --- | --- | --- | --- | --- | --- | --- | --- | --- | --- | --- | --- |
| K.IVIDSEHC(+57.02)NSR.G | Y | 31.84 | 1328.6143 | 11 | 4.43 | 665.3145 | 2 | 11.35 | 0.961 | 0.9683 | 23595 | 3.61e3 | 1 | 1 | 467 | 477 | Carbamidomethylation |  | DB Search |
| total 1 peptides |  |  |  |  |  |  |  |  |  |  |  |  |  |  |  |  |  |  |  |
| --- | --- | --- | --- | --- | --- | --- | --- | --- | --- | --- | --- | --- | --- | --- | --- | --- | --- | --- | --- |

Best Unique PSM (Scan Sample\_3\_A1\_1\_3641.d:23595, m/z=665.3145, z=2, RT=11.35, ppm=0.14):


TRINITY\_DN4494\_c1\_g1\_i15.p1

back to list

  

| Protein Coverage
| Supporting Peptides
| Best Unique PSM
|

Protein Coverage:

Supporting Peptides:

| Peptide | Uniq | -10lgP | Mass | Length | ppm | m/z | z | RT | 1/k0 Start | 1/k0 End | Precursor ID | Area Sample 5 | #Feature | #Feature Sample 5 | Start | End | PTM | AScore | Found By |
| --- | --- | --- | --- | --- | --- | --- | --- | --- | --- | --- | --- | --- | --- | --- | --- | --- | --- | --- | --- |
| K.VDVGFILDSSGSLR.N | Y | 31.66 | 1463.762 | 14 | -1.83 | 732.8838 | 2 | 19.80 | 1.0126 | 1.0198 | 70509 | 0 | 0 | 0 | 41 | 54 |  |  | DB Search |
| total 1 peptides |  |  |  |  |  |  |  |  |  |  |  |  |  |  |  |  |  |  |  |
| --- | --- | --- | --- | --- | --- | --- | --- | --- | --- | --- | --- | --- | --- | --- | --- | --- | --- | --- | --- |

Best Unique PSM (Scan Sample\_3\_A1\_1\_3641.d:70509, m/z=732.8838, z=2, RT=19.80, ppm=-6.11):


TRINITY\_DN61668\_c0\_g1\_i1.p1

back to list

  

| Protein Coverage
| Supporting Peptides
| Best Unique PSM
|

Protein Coverage:

Supporting Peptides:

| Peptide | Uniq | -10lgP | Mass | Length | ppm | m/z | z | RT | 1/k0 Start | 1/k0 End | Precursor ID | Area Sample 5 | #Feature | #Feature Sample 5 | Start | End | PTM | AScore | Found By |
| --- | --- | --- | --- | --- | --- | --- | --- | --- | --- | --- | --- | --- | --- | --- | --- | --- | --- | --- | --- |
| K.GYAVSFIDDKFVQK.A | Y | 31.69 | 1615.8246 | 14 | 2.16 | 808.9178 | 2 | 17.66 | 1.1206 | 1.1278 | 57369 | 5.97e2 | 1 | 1 | 185 | 198 |  |  | DB Search |
| total 1 peptides |  |  |  |  |  |  |  |  |  |  |  |  |  |  |  |  |  |  |  |
| --- | --- | --- | --- | --- | --- | --- | --- | --- | --- | --- | --- | --- | --- | --- | --- | --- | --- | --- | --- |

Best Unique PSM (Scan Sample\_3\_A1\_1\_3641.d:57369, m/z=808.9178, z=2, RT=17.66, ppm=-2.12):


TRINITY\_DN1058\_c0\_g1\_i3.p1

back to list

  

| Protein Coverage
| Supporting Peptides
| Best Unique PSM
|

Protein Coverage:

Supporting Peptides:

| Peptide | Uniq | -10lgP | Mass | Length | ppm | m/z | z | RT | 1/k0 Start | 1/k0 End | Precursor ID | Area Sample 5 | #Feature | #Feature Sample 5 | Start | End | PTM | AScore | Found By |
| --- | --- | --- | --- | --- | --- | --- | --- | --- | --- | --- | --- | --- | --- | --- | --- | --- | --- | --- | --- |
| R.APTHILNPANKR.S | Y | 31.7 | 1330.7469 | 12 | 1.15 | 666.3787 | 2 | 11.11 | 1.0098 | 1.017 | 22167 | 1.2e4 | 2 | 2 | 2314 | 2325 |  |  | DB Search |
| total 1 peptides |  |  |  |  |  |  |  |  |  |  |  |  |  |  |  |  |  |  |  |
| --- | --- | --- | --- | --- | --- | --- | --- | --- | --- | --- | --- | --- | --- | --- | --- | --- | --- | --- | --- |

Best Unique PSM (Scan Sample\_3\_A1\_1\_3641.d:22167, m/z=666.3787, z=2, RT=11.11, ppm=-3.13):


TRINITY\_DN1682\_c8\_g1\_i3.p1

back to list

  

| Protein Coverage
| Supporting Peptides
| Best Unique PSM
|

Protein Coverage:

Supporting Peptides:

| Peptide | Uniq | -10lgP | Mass | Length | ppm | m/z | z | RT | 1/k0 Start | 1/k0 End | Precursor ID | Area Sample 5 | #Feature | #Feature Sample 5 | Start | End | PTM | AScore | Found By |
| --- | --- | --- | --- | --- | --- | --- | --- | --- | --- | --- | --- | --- | --- | --- | --- | --- | --- | --- | --- |
| V(+42.01)KEFLLSK.K | Y | 30.66 | 1004.5906 | 8 | -8.96 | 503.2959 | 2 | 19.18 | 0.8319 | 0.8392 | 66669 | 0 | 0 | 0 | 1 | 8 | Acetylation (Protein N-term) | V1:Acetylation (Protein N-term):1000 | DB Search |
| total 1 peptides |  |  |  |  |  |  |  |  |  |  |  |  |  |  |  |  |  |  |  |
| --- | --- | --- | --- | --- | --- | --- | --- | --- | --- | --- | --- | --- | --- | --- | --- | --- | --- | --- | --- |

Best Unique PSM (Scan Sample\_3\_A1\_1\_3641.d:66669, m/z=503.2959, z=2, RT=19.18, ppm=-13.24):


TRINITY\_DN942\_c0\_g1\_i5.p1

back to list

  

| Protein Coverage
| Supporting Peptides
| Best Unique PSM
|

Protein Coverage:

Supporting Peptides:

| Peptide | Uniq | -10lgP | Mass | Length | ppm | m/z | z | RT | 1/k0 Start | 1/k0 End | Precursor ID | Area Sample 5 | #Feature | #Feature Sample 5 | Start | End | PTM | AScore | Found By |
| --- | --- | --- | --- | --- | --- | --- | --- | --- | --- | --- | --- | --- | --- | --- | --- | --- | --- | --- | --- |
| K.VLNIALDKYQEGVAK.T | Y | 31.63 | 1659.9195 | 15 | -0.5 | 830.9631 | 2 | 15.97 | 1.1546 | 1.1618 | 48647 | 0 | 0 | 0 | 4557 | 4571 |  |  | DB Search |
| total 1 peptides |  |  |  |  |  |  |  |  |  |  |  |  |  |  |  |  |  |  |  |
| --- | --- | --- | --- | --- | --- | --- | --- | --- | --- | --- | --- | --- | --- | --- | --- | --- | --- | --- | --- |

Best Unique PSM (Scan Sample\_3\_A1\_1\_3641.d:48647, m/z=830.9631, z=2, RT=15.97, ppm=-4.78):


TRINITY\_DN195348\_c0\_g1\_i1.p1

back to list

  

| Protein Coverage
| Supporting Peptides
| Best Unique PSM
|

Protein Coverage:

Supporting Peptides:

| Peptide | Uniq | -10lgP | Mass | Length | ppm | m/z | z | RT | 1/k0 Start | 1/k0 End | Precursor ID | Area Sample 5 | #Feature | #Feature Sample 5 | Start | End | PTM | AScore | Found By |
| --- | --- | --- | --- | --- | --- | --- | --- | --- | --- | --- | --- | --- | --- | --- | --- | --- | --- | --- | --- |
| K.VLIVISDGNEDFKEK.Q | Y | 31.93 | 1704.8934 | 15 | -1.64 | 853.4489 | 2 | 16.67 | 1.1702 | 1.1774 | 51996 | 2.43e2 | 1 | 1 | 132 | 146 |  |  | DB Search |
| total 1 peptides |  |  |  |  |  |  |  |  |  |  |  |  |  |  |  |  |  |  |  |
| --- | --- | --- | --- | --- | --- | --- | --- | --- | --- | --- | --- | --- | --- | --- | --- | --- | --- | --- | --- |

Best Unique PSM (Scan Sample\_3\_A1\_1\_3641.d:51996, m/z=853.4489, z=2, RT=16.67, ppm=-5.92):


TRINITY\_DN1071\_c0\_g1\_i3.p1

back to list

  

| Protein Coverage
| Supporting Peptides
| Best Unique PSM
|

Protein Coverage:

Supporting Peptides:

| Peptide | Uniq | -10lgP | Mass | Length | ppm | m/z | z | RT | 1/k0 Start | 1/k0 End | Precursor ID | Area Sample 5 | #Feature | #Feature Sample 5 | Start | End | PTM | AScore | Found By |
| --- | --- | --- | --- | --- | --- | --- | --- | --- | --- | --- | --- | --- | --- | --- | --- | --- | --- | --- | --- |
| K.AVQIHPFNDVSVGDPK.V | Y | 31.9 | 1721.8736 | 16 | 2.29 | 861.9424 | 2 | 15.73 | 1.1186 | 1.1258 | 47418 | 2.88e3 | 1 | 1 | 329 | 344 |  |  | DB Search |
| total 1 peptides |  |  |  |  |  |  |  |  |  |  |  |  |  |  |  |  |  |  |  |
| --- | --- | --- | --- | --- | --- | --- | --- | --- | --- | --- | --- | --- | --- | --- | --- | --- | --- | --- | --- |

Best Unique PSM (Scan Sample\_3\_A1\_1\_3641.d:47418, m/z=861.9424, z=2, RT=15.73, ppm=-1.99):


TRINITY\_DN195934\_c0\_g1\_i1.p2

back to list

  

| Protein Coverage
| Supporting Peptides
| Best Unique PSM
|

Protein Coverage:

Supporting Peptides:

| Peptide | Uniq | -10lgP | Mass | Length | ppm | m/z | z | RT | 1/k0 Start | 1/k0 End | Precursor ID | Area Sample 5 | #Feature | #Feature Sample 5 | Start | End | PTM | AScore | Found By |
| --- | --- | --- | --- | --- | --- | --- | --- | --- | --- | --- | --- | --- | --- | --- | --- | --- | --- | --- | --- |
| K.DSYVGDEAQSKR.G | Y | 31.69 | 1353.616 | 12 | 1.77 | 677.8136 | 2 | 10.94 | 1.0017 | 1.009 | 21026 | 1.85e3 | 1 | 1 | 10 | 21 |  |  | DB Search |
| total 1 peptides |  |  |  |  |  |  |  |  |  |  |  |  |  |  |  |  |  |  |  |
| --- | --- | --- | --- | --- | --- | --- | --- | --- | --- | --- | --- | --- | --- | --- | --- | --- | --- | --- | --- |

Best Unique PSM (Scan Sample\_3\_A1\_1\_3641.d:21026, m/z=677.8136, z=2, RT=10.94, ppm=-2.51):


TRINITY\_DN172523\_c0\_g1\_i1.p1

back to list

  

| Protein Coverage
| Supporting Peptides
| Best Unique PSM
|

Protein Coverage:

Supporting Peptides:

| Peptide | Uniq | -10lgP | Mass | Length | ppm | m/z | z | RT | 1/k0 Start | 1/k0 End | Precursor ID | Area Sample 5 | #Feature | #Feature Sample 5 | Start | End | PTM | AScore | Found By |
| --- | --- | --- | --- | --- | --- | --- | --- | --- | --- | --- | --- | --- | --- | --- | --- | --- | --- | --- | --- |
| K.VDVGFILDSSGSLR.N | Y | 31.66 | 1463.762 | 14 | -1.83 | 732.8838 | 2 | 19.80 | 1.0126 | 1.0198 | 70509 | 0 | 0 | 0 | 109 | 122 |  |  | DB Search |
| total 1 peptides |  |  |  |  |  |  |  |  |  |  |  |  |  |  |  |  |  |  |  |
| --- | --- | --- | --- | --- | --- | --- | --- | --- | --- | --- | --- | --- | --- | --- | --- | --- | --- | --- | --- |

Best Unique PSM (Scan Sample\_3\_A1\_1\_3641.d:70509, m/z=732.8838, z=2, RT=19.80, ppm=-6.11):


TRINITY\_DN761\_c0\_g1\_i3.p1

back to list

  

| Protein Coverage
| Supporting Peptides
| Best Unique PSM
|

Protein Coverage:

Supporting Peptides:

| Peptide | Uniq | -10lgP | Mass | Length | ppm | m/z | z | RT | 1/k0 Start | 1/k0 End | Precursor ID | Area Sample 5 | #Feature | #Feature Sample 5 | Start | End | PTM | AScore | Found By |
| --- | --- | --- | --- | --- | --- | --- | --- | --- | --- | --- | --- | --- | --- | --- | --- | --- | --- | --- | --- |
| K.TRIEDLETVISQTQDHR.H | Y | 31.69 | 2040.0236 | 17 | 6.44 | 681.0166 | 3 | 17.36 | 0.9614 | 0.9687 | 55514 | 1.11e3 | 1 | 1 | 275 | 291 |  |  | DB Search |
| R.IEDLETVISQTQDHR.H | Y | 31.15 | 1782.8748 | 15 | -3.8 | 595.2941 | 3 | 17.75 | 0.9048 | 0.9121 | 58019 | 0 | 0 | 0 | 277 | 291 |  |  | DB Search |
| total 2 peptides |  |  |  |  |  |  |  |  |  |  |  |  |  |  |  |  |  |  |  |
| --- | --- | --- | --- | --- | --- | --- | --- | --- | --- | --- | --- | --- | --- | --- | --- | --- | --- | --- | --- |

Best Unique PSM (Scan Sample\_3\_A1\_1\_3641.d:55514, m/z=681.0166, z=3, RT=17.36, ppm=2.16):


TRINITY\_DN761\_c0\_g1\_i1.p1

back to list

  

| Protein Coverage
| Supporting Peptides
| Best Unique PSM
|

Protein Coverage:

Supporting Peptides:

| Peptide | Uniq | -10lgP | Mass | Length | ppm | m/z | z | RT | 1/k0 Start | 1/k0 End | Precursor ID | Area Sample 5 | #Feature | #Feature Sample 5 | Start | End | PTM | AScore | Found By |
| --- | --- | --- | --- | --- | --- | --- | --- | --- | --- | --- | --- | --- | --- | --- | --- | --- | --- | --- | --- |
| K.TRIEDLETVISQTQDHR.H | Y | 31.69 | 2040.0236 | 17 | 6.44 | 681.0166 | 3 | 17.36 | 0.9614 | 0.9687 | 55514 | 1.11e3 | 1 | 1 | 260 | 276 |  |  | DB Search |
| R.IEDLETVISQTQDHR.H | Y | 31.15 | 1782.8748 | 15 | -3.8 | 595.2941 | 3 | 17.75 | 0.9048 | 0.9121 | 58019 | 0 | 0 | 0 | 262 | 276 |  |  | DB Search |
| total 2 peptides |  |  |  |  |  |  |  |  |  |  |  |  |  |  |  |  |  |  |  |
| --- | --- | --- | --- | --- | --- | --- | --- | --- | --- | --- | --- | --- | --- | --- | --- | --- | --- | --- | --- |

Best Unique PSM (Scan Sample\_3\_A1\_1\_3641.d:55514, m/z=681.0166, z=3, RT=17.36, ppm=2.16):


TRINITY\_DN982\_c0\_g1\_i1.p1

back to list

  

| Protein Coverage
| Supporting Peptides
| Best Unique PSM
|

Protein Coverage:

Supporting Peptides:

| Peptide | Uniq | -10lgP | Mass | Length | ppm | m/z | z | RT | 1/k0 Start | 1/k0 End | Precursor ID | Area Sample 5 | #Feature | #Feature Sample 5 | Start | End | PTM | AScore | Found By |
| --- | --- | --- | --- | --- | --- | --- | --- | --- | --- | --- | --- | --- | --- | --- | --- | --- | --- | --- | --- |
| K.YVAIYPSDHLTVNR.D | N | 31.92 | 1646.8416 | 14 | 0.07 | 824.4246 | 2 | 15.53 | 1.1061 | 1.1134 | 46351 | 1.08e3 | 1 | 1 | 2814 | 2827 |  |  | DB Search |
| R.LVQNTPHNSIVTK.I | N | 31.71 | 1449.794 | 13 | 2.57 | 725.903 | 2 | 11.68 | 1.0572 | 1.0644 | 25401 | 4.01e3 | 1 | 1 | 5129 | 5141 |  |  | DB Search |
| R.TNNALDYEAKR.Q | N | 31.69 | 1293.6313 | 11 | 5.9 | 647.824 | 2 | 11.99 | 0.9618 | 0.9691 | 27271 | 3.6e2 | 1 | 1 | 4285 | 4295 |  |  | DB Search |
| K.VLLSQQIFYDKK.N | N | 31.68 | 1480.8289 | 12 | -0.33 | 741.4183 | 2 | 16.72 | 1.0572 | 1.0644 | 52159 | 3.15e3 | 1 | 1 | 1479 | 1490 |  |  | DB Search |
| R.VLDVNDKQPVFVK.S | Y | 31.63 | 1499.8347 | 13 | 5.63 | 750.9257 | 2 | 14.98 | 1.048 | 1.0552 | 43639 | 0 | 0 | 0 | 670 | 682 |  |  | DB Search |
| R.IQVLKDPDFEFTR.S | N | 31.02 | 1606.8355 | 13 | 5.47 | 804.426 | 2 | 17.53 | 1.0837 | 1.0909 | 56499 | 1.44e3 | 1 | 1 | 2727 | 2739 |  |  | DB Search |
| total 6 peptides |  |  |  |  |  |  |  |  |  |  |  |  |  |  |  |  |  |  |  |
| --- | --- | --- | --- | --- | --- | --- | --- | --- | --- | --- | --- | --- | --- | --- | --- | --- | --- | --- | --- |

Best Unique PSM (Scan Sample\_3\_A1\_1\_3641.d:43639, m/z=751.8621, z=2, RT=15.01, ppm=1.35):


TRINITY\_DN1030\_c0\_g1\_i1.p1

back to list

  

| Protein Coverage
| Supporting Peptides
| Best Unique PSM
|

Protein Coverage:

Supporting Peptides:

| Peptide | Uniq | -10lgP | Mass | Length | ppm | m/z | z | RT | 1/k0 Start | 1/k0 End | Precursor ID | Area Sample 5 | #Feature | #Feature Sample 5 | Start | End | PTM | AScore | Found By |
| --- | --- | --- | --- | --- | --- | --- | --- | --- | --- | --- | --- | --- | --- | --- | --- | --- | --- | --- | --- |
| R.GLQGQQGEQGSTGVQGSVGGR.G | Y | 31.94 | 1985.9515 | 21 | 4.67 | 993.9834 | 2 | 12.00 | 1.1326 | 1.1398 | 27213 | 3e3 | 1 | 1 | 361 | 381 |  |  | DB Search |
| total 1 peptides |  |  |  |  |  |  |  |  |  |  |  |  |  |  |  |  |  |  |  |
| --- | --- | --- | --- | --- | --- | --- | --- | --- | --- | --- | --- | --- | --- | --- | --- | --- | --- | --- | --- |

Best Unique PSM (Scan Sample\_3\_A1\_1\_3641.d:27213, m/z=993.9834, z=2, RT=12.00, ppm=0.4):


TRINITY\_DN247\_c0\_g1\_i1.p1

back to list

  

| Protein Coverage
| Supporting Peptides
| Best Unique PSM
|

Protein Coverage:

Supporting Peptides:

| Peptide | Uniq | -10lgP | Mass | Length | ppm | m/z | z | RT | 1/k0 Start | 1/k0 End | Precursor ID | Area Sample 5 | #Feature | #Feature Sample 5 | Start | End | PTM | AScore | Found By |
| --- | --- | --- | --- | --- | --- | --- | --- | --- | --- | --- | --- | --- | --- | --- | --- | --- | --- | --- | --- |
| R.DAPIYGLQWHPEK.S | Y | 31.3 | 1552.7674 | 13 | -0.41 | 777.3873 | 2 | 17.24 | 1.0725 | 1.0797 | 54902 | 1.07e3 | 1 | 1 | 258 | 270 |  |  | DB Search |
| total 1 peptides |  |  |  |  |  |  |  |  |  |  |  |  |  |  |  |  |  |  |  |
| --- | --- | --- | --- | --- | --- | --- | --- | --- | --- | --- | --- | --- | --- | --- | --- | --- | --- | --- | --- |

Best Unique PSM (Scan Sample\_3\_A1\_1\_3641.d:54902, m/z=777.3873, z=2, RT=17.24, ppm=-4.69):


TRINITY\_DN1071\_c0\_g1\_i4.p1

back to list

  

| Protein Coverage
| Supporting Peptides
| Best Unique PSM
|

Protein Coverage:

Supporting Peptides:

| Peptide | Uniq | -10lgP | Mass | Length | ppm | m/z | z | RT | 1/k0 Start | 1/k0 End | Precursor ID | Area Sample 5 | #Feature | #Feature Sample 5 | Start | End | PTM | AScore | Found By |
| --- | --- | --- | --- | --- | --- | --- | --- | --- | --- | --- | --- | --- | --- | --- | --- | --- | --- | --- | --- |
| K.AVQIHPFNDVSVGDPK.V | Y | 31.9 | 1721.8736 | 16 | 2.29 | 861.9424 | 2 | 15.73 | 1.1186 | 1.1258 | 47418 | 2.88e3 | 1 | 1 | 329 | 344 |  |  | DB Search |
| total 1 peptides |  |  |  |  |  |  |  |  |  |  |  |  |  |  |  |  |  |  |  |
| --- | --- | --- | --- | --- | --- | --- | --- | --- | --- | --- | --- | --- | --- | --- | --- | --- | --- | --- | --- |

Best Unique PSM (Scan Sample\_3\_A1\_1\_3641.d:47418, m/z=861.9424, z=2, RT=15.73, ppm=-1.99):


TRINITY\_DN262\_c0\_g1\_i1.p1

back to list

  

| Protein Coverage
| Supporting Peptides
| Best Unique PSM
|

Protein Coverage:

Supporting Peptides:

| Peptide | Uniq | -10lgP | Mass | Length | ppm | m/z | z | RT | 1/k0 Start | 1/k0 End | Precursor ID | Area Sample 5 | #Feature | #Feature Sample 5 | Start | End | PTM | AScore | Found By |
| --- | --- | --- | --- | --- | --- | --- | --- | --- | --- | --- | --- | --- | --- | --- | --- | --- | --- | --- | --- |
| R.DANAAVVHGSHLK.D | Y | 31.68 | 1317.6789 | 13 | 5.41 | 659.8475 | 2 | 10.89 | 1.017 | 1.0242 | 20821 | 1.84e3 | 1 | 1 | 668 | 680 |  |  | DB Search |
| R.AEFRPNQEEEPILKR.E | Y | 31.68 | 1854.9588 | 15 | 3.55 | 619.3264 | 3 | 13.76 | 0.8773 | 0.8846 | 37073 | 6e3 | 1 | 1 | 439 | 453 |  |  | DB Search |
| K.DLPQEKLDDILK.Y | Y | 31.67 | 1425.7715 | 12 | 4.1 | 713.8929 | 2 | 17.71 | 1.0375 | 1.0448 | 57677 | 1.52e3 | 1 | 1 | 681 | 692 |  |  | DB Search |
| R.AEFRPNQEEEPILK.R | Y | 31.38 | 1698.8577 | 14 | 3.69 | 850.4356 | 2 | 14.58 | 1.0825 | 1.0897 | 41449 | 1.23e3 | 1 | 1 | 439 | 452 |  |  | DB Search |
| K.VIM(+15.99)VTGDHPITAK.A | Y | 31.28 | 1396.7384 | 13 | 3.65 | 699.376 | 2 | 13.17 | 1.023 | 1.0303 | 33679 | 7.16e2 | 1 | 1 | 622 | 634 | Oxidation (M) | M3:Oxidation (M):1000 | DB Search |
| K.GVGIISEGNETVEDIAQR.L | Y | 31.23 | 1885.9381 | 18 | -3.75 | 943.9688 | 2 | 17.80 | 1.141 | 1.1482 | 58158 | 4.58e2 | 1 | 1 | 639 | 656 |  |  | DB Search |
| total 6 peptides |  |  |  |  |  |  |  |  |  |  |  |  |  |  |  |  |  |  |  |
| --- | --- | --- | --- | --- | --- | --- | --- | --- | --- | --- | --- | --- | --- | --- | --- | --- | --- | --- | --- |

Best Unique PSM (Scan Sample\_3\_A1\_1\_3641.d:20821, m/z=659.8475, z=2, RT=10.89, ppm=1.13):


TRINITY\_DN5633\_c0\_g1\_i1.p1

back to list

  

| Protein Coverage
| Supporting Peptides
| Best Unique PSM
|

Protein Coverage:

Supporting Peptides:

| Peptide | Uniq | -10lgP | Mass | Length | ppm | m/z | z | RT | 1/k0 Start | 1/k0 End | Precursor ID | Area Sample 5 | #Feature | #Feature Sample 5 | Start | End | PTM | AScore | Found By |
| --- | --- | --- | --- | --- | --- | --- | --- | --- | --- | --- | --- | --- | --- | --- | --- | --- | --- | --- | --- |
| K.LGVM(+15.99)EDHSNR.T | Y | 31.83 | 1172.5244 | 10 | -12.49 | 587.2596 | 2 | 10.26 | 0.9012 | 0.9085 | 17065 | 0 | 0 | 0 | 516 | 525 | Oxidation (M) | M4:Oxidation (M):1000 | DB Search |
| total 1 peptides |  |  |  |  |  |  |  |  |  |  |  |  |  |  |  |  |  |  |  |
| --- | --- | --- | --- | --- | --- | --- | --- | --- | --- | --- | --- | --- | --- | --- | --- | --- | --- | --- | --- |

Best Unique PSM (Scan Sample\_3\_A1\_1\_3641.d:17065, m/z=587.2596, z=2, RT=10.26, ppm=-16.77):


TRINITY\_DN2477\_c0\_g1\_i10.p1

back to list

  

| Protein Coverage
| Supporting Peptides
| Best Unique PSM
|

Protein Coverage:

Supporting Peptides:

| Peptide | Uniq | -10lgP | Mass | Length | ppm | m/z | z | RT | 1/k0 Start | 1/k0 End | Precursor ID | Area Sample 5 | #Feature | #Feature Sample 5 | Start | End | PTM | AScore | Found By |
| --- | --- | --- | --- | --- | --- | --- | --- | --- | --- | --- | --- | --- | --- | --- | --- | --- | --- | --- | --- |
| K.DSYVGDEAQSKR.G | Y | 31.69 | 1353.616 | 12 | 1.77 | 677.8136 | 2 | 10.94 | 1.0017 | 1.009 | 21026 | 1.85e3 | 1 | 1 | 19 | 30 |  |  | DB Search |
| total 1 peptides |  |  |  |  |  |  |  |  |  |  |  |  |  |  |  |  |  |  |  |
| --- | --- | --- | --- | --- | --- | --- | --- | --- | --- | --- | --- | --- | --- | --- | --- | --- | --- | --- | --- |

Best Unique PSM (Scan Sample\_3\_A1\_1\_3641.d:21026, m/z=677.8136, z=2, RT=10.94, ppm=-2.51):


TRINITY\_DN113\_c3\_g1\_i1.p1

back to list

  

| Protein Coverage
| Supporting Peptides
| Best Unique PSM
|

Protein Coverage:

Supporting Peptides:

| Peptide | Uniq | -10lgP | Mass | Length | ppm | m/z | z | RT | 1/k0 Start | 1/k0 End | Precursor ID | Area Sample 5 | #Feature | #Feature Sample 5 | Start | End | PTM | AScore | Found By |
| --- | --- | --- | --- | --- | --- | --- | --- | --- | --- | --- | --- | --- | --- | --- | --- | --- | --- | --- | --- |
| R.ATHQELLNAEFIQK.V | Y | 31.06 | 1640.8522 | 14 | 2.54 | 821.4319 | 2 | 15.90 | 1.0777 | 1.0849 | 48321 | 4.04e2 | 1 | 1 | 457 | 470 |  |  | DB Search |
| total 1 peptides |  |  |  |  |  |  |  |  |  |  |  |  |  |  |  |  |  |  |  |
| --- | --- | --- | --- | --- | --- | --- | --- | --- | --- | --- | --- | --- | --- | --- | --- | --- | --- | --- | --- |

Best Unique PSM (Scan Sample\_3\_A1\_1\_3641.d:48321, m/z=821.4319, z=2, RT=15.90, ppm=-1.74):


TRINITY\_DN413\_c0\_g1\_i2.p1

back to list

  

| Protein Coverage
| Supporting Peptides
| Best Unique PSM
|

Protein Coverage:

Supporting Peptides:

| Peptide | Uniq | -10lgP | Mass | Length | ppm | m/z | z | RT | 1/k0 Start | 1/k0 End | Precursor ID | Area Sample 5 | #Feature | #Feature Sample 5 | Start | End | PTM | AScore | Found By |
| --- | --- | --- | --- | --- | --- | --- | --- | --- | --- | --- | --- | --- | --- | --- | --- | --- | --- | --- | --- |
| R.LLLGDFNGDKR.T | Y | 31.33 | 1246.6669 | 11 | 4.3 | 624.3408 | 2 | 16.04 | 0.9836 | 0.9908 | 48938 | 1.07e3 | 1 | 1 | 46 | 56 |  |  | DB Search |
| total 1 peptides |  |  |  |  |  |  |  |  |  |  |  |  |  |  |  |  |  |  |  |
| --- | --- | --- | --- | --- | --- | --- | --- | --- | --- | --- | --- | --- | --- | --- | --- | --- | --- | --- | --- |

Best Unique PSM (Scan Sample\_3\_A1\_1\_3641.d:48938, m/z=624.3408, z=2, RT=16.04, ppm=0.01):


TRINITY\_DN4494\_c1\_g1\_i8.p1

back to list

  

| Protein Coverage
| Supporting Peptides
| Best Unique PSM
|

Protein Coverage:

Supporting Peptides:

| Peptide | Uniq | -10lgP | Mass | Length | ppm | m/z | z | RT | 1/k0 Start | 1/k0 End | Precursor ID | Area Sample 5 | #Feature | #Feature Sample 5 | Start | End | PTM | AScore | Found By |
| --- | --- | --- | --- | --- | --- | --- | --- | --- | --- | --- | --- | --- | --- | --- | --- | --- | --- | --- | --- |
| K.VDVGFILDSSGSLR.N | Y | 31.66 | 1463.762 | 14 | -1.83 | 732.8838 | 2 | 19.80 | 1.0126 | 1.0198 | 70509 | 0 | 0 | 0 | 41 | 54 |  |  | DB Search |
| total 1 peptides |  |  |  |  |  |  |  |  |  |  |  |  |  |  |  |  |  |  |  |
| --- | --- | --- | --- | --- | --- | --- | --- | --- | --- | --- | --- | --- | --- | --- | --- | --- | --- | --- | --- |

Best Unique PSM (Scan Sample\_3\_A1\_1\_3641.d:70509, m/z=732.8838, z=2, RT=19.80, ppm=-6.11):


TRINITY\_DN1058\_c0\_g1\_i2.p2

back to list

  

| Protein Coverage
| Supporting Peptides
| Best Unique PSM
|

Protein Coverage:

Supporting Peptides:

| Peptide | Uniq | -10lgP | Mass | Length | ppm | m/z | z | RT | 1/k0 Start | 1/k0 End | Precursor ID | Area Sample 5 | #Feature | #Feature Sample 5 | Start | End | PTM | AScore | Found By |
| --- | --- | --- | --- | --- | --- | --- | --- | --- | --- | --- | --- | --- | --- | --- | --- | --- | --- | --- | --- |
| R.APTHILNPANKR.S | Y | 31.7 | 1330.7469 | 12 | 1.15 | 666.3787 | 2 | 11.11 | 1.0098 | 1.017 | 22167 | 1.2e4 | 2 | 2 | 975 | 986 |  |  | DB Search |
| total 1 peptides |  |  |  |  |  |  |  |  |  |  |  |  |  |  |  |  |  |  |  |
| --- | --- | --- | --- | --- | --- | --- | --- | --- | --- | --- | --- | --- | --- | --- | --- | --- | --- | --- | --- |

Best Unique PSM (Scan Sample\_3\_A1\_1\_3641.d:22167, m/z=666.3787, z=2, RT=11.11, ppm=-3.13):


TRINITY\_DN141561\_c0\_g1\_i1.p1

back to list

  

| Protein Coverage
| Supporting Peptides
| Best Unique PSM
|

Protein Coverage:

Supporting Peptides:

| Peptide | Uniq | -10lgP | Mass | Length | ppm | m/z | z | RT | 1/k0 Start | 1/k0 End | Precursor ID | Area Sample 5 | #Feature | #Feature Sample 5 | Start | End | PTM | AScore | Found By |
| --- | --- | --- | --- | --- | --- | --- | --- | --- | --- | --- | --- | --- | --- | --- | --- | --- | --- | --- | --- |
| R.IHGGAQAPKPK.S | Y | 30.42 | 1102.6247 | 11 | 17.79 | 368.5538 | 3 | 8.91 | 0.7078 | 0.7151 | 9675 | 0 | 0 | 0 | 171 | 181 |  |  | DB Search |
| total 1 peptides |  |  |  |  |  |  |  |  |  |  |  |  |  |  |  |  |  |  |  |
| --- | --- | --- | --- | --- | --- | --- | --- | --- | --- | --- | --- | --- | --- | --- | --- | --- | --- | --- | --- |

Best Unique PSM (Scan Sample\_3\_A1\_1\_3641.d:9675, m/z=368.5538, z=3, RT=8.91, ppm=13.51):


TRINITY\_DN1535\_c0\_g1\_i1.p1

back to list

  

| Protein Coverage
| Supporting Peptides
| Best Unique PSM
|

Protein Coverage:

Supporting Peptides:

| Peptide | Uniq | -10lgP | Mass | Length | ppm | m/z | z | RT | 1/k0 Start | 1/k0 End | Precursor ID | Area Sample 5 | #Feature | #Feature Sample 5 | Start | End | PTM | AScore | Found By |
| --- | --- | --- | --- | --- | --- | --- | --- | --- | --- | --- | --- | --- | --- | --- | --- | --- | --- | --- | --- |
| R.HQNIVC(+57.02)GGPER.G | Y | 31.68 | 1265.5935 | 11 | 4.37 | 633.8041 | 2 | 12.40 | 0.9073 | 0.9146 | 29482 | 5.93e2 | 1 | 1 | 547 | 557 | Carbamidomethylation |  | DB Search |
| K.LKDNAVIPLFAVTK.N | Y | 31.32 | 1527.9024 | 14 | -0.82 | 764.9546 | 2 | 18.85 | 1.0331 | 1.0403 | 64545 | 4.93e2 | 1 | 1 | 306 | 319 |  |  | DB Search |
| total 2 peptides |  |  |  |  |  |  |  |  |  |  |  |  |  |  |  |  |  |  |  |
| --- | --- | --- | --- | --- | --- | --- | --- | --- | --- | --- | --- | --- | --- | --- | --- | --- | --- | --- | --- |

Best Unique PSM (Scan Sample\_3\_A1\_1\_3641.d:29482, m/z=633.8041, z=2, RT=12.40, ppm=0.09):


TRINITY\_DN4494\_c2\_g1\_i1.p1

back to list

  

| Protein Coverage
| Supporting Peptides
| Best Unique PSM
|

Protein Coverage:

Supporting Peptides:

| Peptide | Uniq | -10lgP | Mass | Length | ppm | m/z | z | RT | 1/k0 Start | 1/k0 End | Precursor ID | Area Sample 5 | #Feature | #Feature Sample 5 | Start | End | PTM | AScore | Found By |
| --- | --- | --- | --- | --- | --- | --- | --- | --- | --- | --- | --- | --- | --- | --- | --- | --- | --- | --- | --- |
| K.VDVGFILDSSGSLR.N | Y | 31.66 | 1463.762 | 14 | -1.83 | 732.8838 | 2 | 19.80 | 1.0126 | 1.0198 | 70509 | 0 | 0 | 0 | 57 | 70 |  |  | DB Search |
| total 1 peptides |  |  |  |  |  |  |  |  |  |  |  |  |  |  |  |  |  |  |  |
| --- | --- | --- | --- | --- | --- | --- | --- | --- | --- | --- | --- | --- | --- | --- | --- | --- | --- | --- | --- |

Best Unique PSM (Scan Sample\_3\_A1\_1\_3641.d:70509, m/z=732.8838, z=2, RT=19.80, ppm=-6.11):


TRINITY\_DN77\_c1\_g1\_i1.p1

back to list

  

| Protein Coverage
| Supporting Peptides
| Best Unique PSM
|

Protein Coverage:

Supporting Peptides:

| Peptide | Uniq | -10lgP | Mass | Length | ppm | m/z | z | RT | 1/k0 Start | 1/k0 End | Precursor ID | Area Sample 5 | #Feature | #Feature Sample 5 | Start | End | PTM | AScore | Found By |
| --- | --- | --- | --- | --- | --- | --- | --- | --- | --- | --- | --- | --- | --- | --- | --- | --- | --- | --- | --- |
| R.LLAVTVGNNYNLDTLEK.V | Y | 31.11 | 1875.9942 | 17 | 0.03 | 939.0004 | 2 | 18.60 | 1.1454 | 1.1526 | 62944 | 7.3e2 | 1 | 1 | 693 | 709 |  |  | DB Search |
| total 1 peptides |  |  |  |  |  |  |  |  |  |  |  |  |  |  |  |  |  |  |  |
| --- | --- | --- | --- | --- | --- | --- | --- | --- | --- | --- | --- | --- | --- | --- | --- | --- | --- | --- | --- |

Best Unique PSM (Scan Sample\_3\_A1\_1\_3641.d:62944, m/z=939.0004, z=2, RT=18.60, ppm=-4.25):


TRINITY\_DN161\_c0\_g1\_i1.p1

back to list

  

| Protein Coverage
| Supporting Peptides
| Best Unique PSM
|

Protein Coverage:

Supporting Peptides:

| Peptide | Uniq | -10lgP | Mass | Length | ppm | m/z | z | RT | 1/k0 Start | 1/k0 End | Precursor ID | Area Sample 5 | #Feature | #Feature Sample 5 | Start | End | PTM | AScore | Found By |
| --- | --- | --- | --- | --- | --- | --- | --- | --- | --- | --- | --- | --- | --- | --- | --- | --- | --- | --- | --- |
| R.AVFPSIVGRPR.H | Y | 31.8 | 1197.6982 | 11 | 3.08 | 599.8557 | 2 | 15.68 | 0.9574 | 0.9646 | 46487 | 1.74e4 | 1 | 1 | 30 | 40 |  |  | DB Search |
| K.DSYVGDEAQSKR.G | Y | 31.69 | 1353.616 | 12 | 1.77 | 677.8136 | 2 | 10.94 | 1.0017 | 1.009 | 21026 | 1.85e3 | 1 | 1 | 52 | 63 |  |  | DB Search |
| total 2 peptides |  |  |  |  |  |  |  |  |  |  |  |  |  |  |  |  |  |  |  |
| --- | --- | --- | --- | --- | --- | --- | --- | --- | --- | --- | --- | --- | --- | --- | --- | --- | --- | --- | --- |

Best Unique PSM (Scan Sample\_3\_A1\_1\_3641.d:46487, m/z=599.8557, z=2, RT=15.68, ppm=-1.2):


TRINITY\_DN64\_c0\_g1\_i1.p1

back to list

  

| Protein Coverage
| Supporting Peptides
| Best Unique PSM
|

Protein Coverage:

Supporting Peptides:

| Peptide | Uniq | -10lgP | Mass | Length | ppm | m/z | z | RT | 1/k0 Start | 1/k0 End | Precursor ID | Area Sample 5 | #Feature | #Feature Sample 5 | Start | End | PTM | AScore | Found By |
| --- | --- | --- | --- | --- | --- | --- | --- | --- | --- | --- | --- | --- | --- | --- | --- | --- | --- | --- | --- |
| K.YYVTIIDAPGHR.D | Y | 31.68 | 1403.7197 | 12 | 0.62 | 702.8646 | 2 | 15.88 | 1.0387 | 1.046 | 48209 | 0 | 0 | 0 | 88 | 99 |  |  | DB Search |
| total 1 peptides |  |  |  |  |  |  |  |  |  |  |  |  |  |  |  |  |  |  |  |
| --- | --- | --- | --- | --- | --- | --- | --- | --- | --- | --- | --- | --- | --- | --- | --- | --- | --- | --- | --- |

Best Unique PSM (Scan Sample\_3\_A1\_1\_3641.d:48209, m/z=702.8646, z=2, RT=15.88, ppm=-3.66):


TRINITY\_DN12542\_c0\_g1\_i1.p1

back to list

  

| Protein Coverage
| Supporting Peptides
| Best Unique PSM
|

Protein Coverage:

Supporting Peptides:

| Peptide | Uniq | -10lgP | Mass | Length | ppm | m/z | z | RT | 1/k0 Start | 1/k0 End | Precursor ID | Area Sample 5 | #Feature | #Feature Sample 5 | Start | End | PTM | AScore | Found By |
| --- | --- | --- | --- | --- | --- | --- | --- | --- | --- | --- | --- | --- | --- | --- | --- | --- | --- | --- | --- |
| K.VDVGFILDSSGSLR.N | Y | 31.66 | 1463.762 | 14 | -1.83 | 732.8838 | 2 | 19.80 | 1.0126 | 1.0198 | 70509 | 0 | 0 | 0 | 195 | 208 |  |  | DB Search |
| total 1 peptides |  |  |  |  |  |  |  |  |  |  |  |  |  |  |  |  |  |  |  |
| --- | --- | --- | --- | --- | --- | --- | --- | --- | --- | --- | --- | --- | --- | --- | --- | --- | --- | --- | --- |

Best Unique PSM (Scan Sample\_3\_A1\_1\_3641.d:70509, m/z=732.8838, z=2, RT=19.80, ppm=-6.11):


TRINITY\_DN991\_c0\_g1\_i5.p1

back to list

  

| Protein Coverage
| Supporting Peptides
| Best Unique PSM
|

Protein Coverage:

Supporting Peptides:

| Peptide | Uniq | -10lgP | Mass | Length | ppm | m/z | z | RT | 1/k0 Start | 1/k0 End | Precursor ID | Area Sample 5 | #Feature | #Feature Sample 5 | Start | End | PTM | AScore | Found By |
| --- | --- | --- | --- | --- | --- | --- | --- | --- | --- | --- | --- | --- | --- | --- | --- | --- | --- | --- | --- |
| R.ITVTVSPDIHPPAK.L | Y | 31.67 | 1473.8191 | 14 | 3.85 | 737.9165 | 2 | 14.85 | 1.064 | 1.0713 | 42886 | 6.83e2 | 1 | 1 | 1183 | 1196 |  |  | DB Search |
| total 1 peptides |  |  |  |  |  |  |  |  |  |  |  |  |  |  |  |  |  |  |  |
| --- | --- | --- | --- | --- | --- | --- | --- | --- | --- | --- | --- | --- | --- | --- | --- | --- | --- | --- | --- |

Best Unique PSM (Scan Sample\_3\_A1\_1\_3641.d:42886, m/z=737.9165, z=2, RT=14.85, ppm=-0.43):


TRINITY\_DN3078\_c0\_g1\_i1.p1

back to list

  

| Protein Coverage
| Supporting Peptides
| Best Unique PSM
|

Protein Coverage:

Supporting Peptides:

| Peptide | Uniq | -10lgP | Mass | Length | ppm | m/z | z | RT | 1/k0 Start | 1/k0 End | Precursor ID | Area Sample 5 | #Feature | #Feature Sample 5 | Start | End | PTM | AScore | Found By |
| --- | --- | --- | --- | --- | --- | --- | --- | --- | --- | --- | --- | --- | --- | --- | --- | --- | --- | --- | --- |
| K.VSQTC(+57.02)AEHVFR.V | Y | 31.91 | 1332.6245 | 11 | 4.3 | 667.3195 | 2 | 12.77 | 0.9848 | 0.992 | 31588 | 5.38e3 | 3 | 3 | 547 | 557 | Carbamidomethylation |  | DB Search |
| R.AQLKNELIEQSENIK.L | Y | 31.8 | 1755.9366 | 15 | 1.69 | 878.9733 | 2 | 15.24 | 1.1001 | 1.1074 | 44891 | 4.49e2 | 1 | 1 | 734 | 748 |  |  | DB Search |
| K.IEALHC(+57.02)LSENIVK.D | Y | 31.68 | 1524.797 | 13 | 5.71 | 763.4069 | 2 | 15.59 | 1.0472 | 1.0544 | 46684 | 1.5e3 | 1 | 1 | 711 | 723 | Carbamidomethylation |  | DB Search |
| total 3 peptides |  |  |  |  |  |  |  |  |  |  |  |  |  |  |  |  |  |  |  |
| --- | --- | --- | --- | --- | --- | --- | --- | --- | --- | --- | --- | --- | --- | --- | --- | --- | --- | --- | --- |

Best Unique PSM (Scan Sample\_3\_A1\_1\_3641.d:31588, m/z=667.3195, z=2, RT=12.77, ppm=0.02):


TRINITY\_DN1616\_c0\_g1\_i7.p1

back to list

  

| Protein Coverage
| Supporting Peptides
| Best Unique PSM
|

Protein Coverage:

Supporting Peptides:

| Peptide | Uniq | -10lgP | Mass | Length | ppm | m/z | z | RT | 1/k0 Start | 1/k0 End | Precursor ID | Area Sample 5 | #Feature | #Feature Sample 5 | Start | End | PTM | AScore | Found By |
| --- | --- | --- | --- | --- | --- | --- | --- | --- | --- | --- | --- | --- | --- | --- | --- | --- | --- | --- | --- |
| K.GC(+57.02)STC(+57.02)GC(+57.02)NGAGTNNGVK.S | Y | 31.94 | 1712.6665 | 17 | 1.85 | 857.3384 | 2 | 9.80 | 1.0448 | 1.052 | 14171 | 0 | 0 | 0 | 732 | 748 | Carbamidomethylation, Carbamidomethylation, Carbamidomethylation |  | DB Search |
| K.VVDTEANDLTEEVKK.T | Y | 31.68 | 1688.8468 | 15 | 3.24 | 845.4298 | 2 | 14.05 | 1.1286 | 1.1358 | 38709 | 1.51e3 | 1 | 1 | 2542 | 2556 |  |  | DB Search |
| R.YGAVNDNC(+57.02)ERDNGQC(+57.02)SC(+57.02)K.V | Y | 31.05 | 2145.8262 | 18 | 2.18 | 716.2812 | 3 | 10.55 | 0.8587 | 0.866 | 18796 | 7.2e2 | 1 | 1 | 498 | 515 | Carbamidomethylation, Carbamidomethylation, Carbamidomethylation |  | DB Search |
| K.NC(+57.02)QHNTLGINC(+57.02)EK.C | Y | 30.89 | 1586.693 | 13 | 4.28 | 794.3538 | 2 | 11.89 | 1.0371 | 1.0443 | 26586 | 7.24e2 | 1 | 1 | 366 | 378 | Carbamidomethylation, Carbamidomethylation |  | DB Search |
| K.DGEKVETLKVDISDINK.A | Y | 30.55 | 1901.9945 | 17 | 2.61 | 635.0044 | 3 | 15.87 | 0.9065 | 0.9137 | 48130 | 2.66e2 | 1 | 1 | 2634 | 2650 |  |  | DB Search |
| total 5 peptides |  |  |  |  |  |  |  |  |  |  |  |  |  |  |  |  |  |  |  |
| --- | --- | --- | --- | --- | --- | --- | --- | --- | --- | --- | --- | --- | --- | --- | --- | --- | --- | --- | --- |

Best Unique PSM (Scan Sample\_3\_A1\_1\_3641.d:14171, m/z=857.3384, z=2, RT=9.80, ppm=-2.43):


TRINITY\_DN1369\_c0\_g1\_i1.p1

back to list

  

| Protein Coverage
| Supporting Peptides
| Best Unique PSM
|

Protein Coverage:

Supporting Peptides:

| Peptide | Uniq | -10lgP | Mass | Length | ppm | m/z | z | RT | 1/k0 Start | 1/k0 End | Precursor ID | Area Sample 5 | #Feature | #Feature Sample 5 | Start | End | PTM | AScore | Found By |
| --- | --- | --- | --- | --- | --- | --- | --- | --- | --- | --- | --- | --- | --- | --- | --- | --- | --- | --- | --- |
| K.TPGEC(+57.02)C(+57.02)PQC(+57.02)K.K | Y | 31.8 | 1235.4733 | 10 | 3.13 | 618.7432 | 2 | 9.97 | 0.8923 | 0.8996 | 15133 | 1.64e3 | 1 | 1 | 1387 | 1396 | Carbamidomethylation, Carbamidomethylation, Carbamidomethylation |  | DB Search |
| total 1 peptides |  |  |  |  |  |  |  |  |  |  |  |  |  |  |  |  |  |  |  |
| --- | --- | --- | --- | --- | --- | --- | --- | --- | --- | --- | --- | --- | --- | --- | --- | --- | --- | --- | --- |

Best Unique PSM (Scan Sample\_3\_A1\_1\_3641.d:15133, m/z=618.7432, z=2, RT=9.97, ppm=-1.15):


TRINITY\_DN118\_c1\_g1\_i3.p1

back to list

  

| Protein Coverage
| Supporting Peptides
| Best Unique PSM
|

Protein Coverage:

Supporting Peptides:

| Peptide | Uniq | -10lgP | Mass | Length | ppm | m/z | z | RT | 1/k0 Start | 1/k0 End | Precursor ID | Area Sample 5 | #Feature | #Feature Sample 5 | Start | End | PTM | AScore | Found By |
| --- | --- | --- | --- | --- | --- | --- | --- | --- | --- | --- | --- | --- | --- | --- | --- | --- | --- | --- | --- |
| K.FSYEKVC(+57.02)EDVKR.C | Y | 31.1 | 1558.745 | 12 | -0.77 | 520.5863 | 3 | 13.51 | 0.7888 | 0.7962 | 35740 | 0 | 0 | 0 | 117 | 128 | Carbamidomethylation |  | DB Search |
| total 1 peptides |  |  |  |  |  |  |  |  |  |  |  |  |  |  |  |  |  |  |  |
| --- | --- | --- | --- | --- | --- | --- | --- | --- | --- | --- | --- | --- | --- | --- | --- | --- | --- | --- | --- |

Best Unique PSM (Scan Sample\_3\_A1\_1\_3641.d:35740, m/z=519.928, z=3, RT=13.54, ppm=-5.05):


TRINITY\_DN15274\_c0\_g1\_i1.p1

back to list

  

| Protein Coverage
| Supporting Peptides
| Best Unique PSM
|

Protein Coverage:

Supporting Peptides:

| Peptide | Uniq | -10lgP | Mass | Length | ppm | m/z | z | RT | 1/k0 Start | 1/k0 End | Precursor ID | Area Sample 5 | #Feature | #Feature Sample 5 | Start | End | PTM | AScore | Found By |
| --- | --- | --- | --- | --- | --- | --- | --- | --- | --- | --- | --- | --- | --- | --- | --- | --- | --- | --- | --- |
| R.GSC(+57.02)SC(+57.02)YGHAEQC(+57.02)IPTK.G | Y | 31.92 | 1853.7495 | 16 | 1.05 | 927.879 | 2 | 12.32 | 1.1869 | 1.1941 | 29052 | 1.68e2 | 1 | 1 | 270 | 285 | Carbamidomethylation, Carbamidomethylation, Carbamidomethylation |  | DB Search |
| K.YC(+57.02)ILSFLDKTDK.C | Y | 31.7 | 1501.7486 | 12 | -1.53 | 501.5872 | 3 | 19.67 | 0.7966 | 0.8039 | 69637 | 7.8e2 | 1 | 1 | 62 | 73 | Carbamidomethylation |  | DB Search |
| K.ATGNTSGGVC(+57.02)DDC(+57.02)NHNTAGR.Q | Y | 31.68 | 2062.8181 | 20 | 5.22 | 688.614 | 3 | 9.94 | 0.8562 | 0.8635 | 14982 | 8.15e2 | 1 | 1 | 353 | 372 | Carbamidomethylation, Carbamidomethylation |  | DB Search |
| K.IQFSISAEINNGALK.C | Y | 31.65 | 1603.8569 | 15 | -1.52 | 802.9311 | 2 | 18.71 | 1.0737 | 1.0809 | 63761 | 0 | 0 | 0 | 759 | 773 |  |  | DB Search |
| R.ADIEDVKDLLK.K | Y | 30.63 | 1257.6816 | 11 | 4.2 | 629.848 | 2 | 17.58 | 0.9884 | 0.9957 | 56818 | 3.25e3 | 1 | 1 | 1616 | 1626 |  |  | DB Search |
| K.SGGQC(+57.02)PC(+57.02)KPGVVGR.R | Y | 30.57 | 1457.6867 | 14 | 2.45 | 729.8493 | 2 | 10.68 | 0.98 | 0.9872 | 19470 | 8.78e2 | 1 | 1 | 795 | 808 | Carbamidomethylation, Carbamidomethylation |  | DB Search |
| total 6 peptides |  |  |  |  |  |  |  |  |  |  |  |  |  |  |  |  |  |  |  |
| --- | --- | --- | --- | --- | --- | --- | --- | --- | --- | --- | --- | --- | --- | --- | --- | --- | --- | --- | --- |

Best Unique PSM (Scan Sample\_3\_A1\_1\_3641.d:29052, m/z=927.879, z=2, RT=12.32, ppm=-3.23):


TRINITY\_DN624\_c0\_g1\_i1.p1

back to list

  

| Protein Coverage
| Supporting Peptides
| Best Unique PSM
|

Protein Coverage:

Supporting Peptides:

| Peptide | Uniq | -10lgP | Mass | Length | ppm | m/z | z | RT | 1/k0 Start | 1/k0 End | Precursor ID | Area Sample 5 | #Feature | #Feature Sample 5 | Start | End | PTM | AScore | Found By |
| --- | --- | --- | --- | --- | --- | --- | --- | --- | --- | --- | --- | --- | --- | --- | --- | --- | --- | --- | --- |
| R.FAIQDITVDEFSAK.E | Y | 30.57 | 1582.7878 | 14 | 1.41 | 792.3989 | 2 | 19.69 | 1.0419 | 1.0492 | 69716 | 4.2e2 | 1 | 1 | 132 | 145 |  |  | DB Search |
| total 1 peptides |  |  |  |  |  |  |  |  |  |  |  |  |  |  |  |  |  |  |  |
| --- | --- | --- | --- | --- | --- | --- | --- | --- | --- | --- | --- | --- | --- | --- | --- | --- | --- | --- | --- |

Best Unique PSM (Scan Sample\_3\_A1\_1\_3641.d:69716, m/z=792.3989, z=2, RT=19.69, ppm=-2.87):


TRINITY\_DN1043\_c0\_g1\_i2.p1

back to list

  

| Protein Coverage
| Supporting Peptides
| Best Unique PSM
|

Protein Coverage:

Supporting Peptides:

| Peptide | Uniq | -10lgP | Mass | Length | ppm | m/z | z | RT | 1/k0 Start | 1/k0 End | Precursor ID | Area Sample 5 | #Feature | #Feature Sample 5 | Start | End | PTM | AScore | Found By |
| --- | --- | --- | --- | --- | --- | --- | --- | --- | --- | --- | --- | --- | --- | --- | --- | --- | --- | --- | --- |
| K.GLMVAAMDLDKK.Q | Y | 31.65 | 1290.6675 | 12 | 2.51 | 646.3399 | 2 | 16.54 | 0.9985 | 1.0057 | 51403 | 5.13e2 | 1 | 1 | 504 | 515 |  |  | DB Search |
| R.EQVSTFDSLQADLVK.L | Y | 30.64 | 1678.8413 | 15 | 4.31 | 840.428 | 2 | 18.59 | 1.0969 | 1.1041 | 62996 | 0 | 0 | 0 | 137 | 151 |  |  | DB Search |
| total 2 peptides |  |  |  |  |  |  |  |  |  |  |  |  |  |  |  |  |  |  |  |
| --- | --- | --- | --- | --- | --- | --- | --- | --- | --- | --- | --- | --- | --- | --- | --- | --- | --- | --- | --- |

Best Unique PSM (Scan Sample\_3\_A1\_1\_3641.d:51403, m/z=646.3399, z=2, RT=16.54, ppm=-1.77):


TRINITY\_DN11248\_c0\_g1\_i1.p1

back to list

  

| Protein Coverage
| Supporting Peptides
| Best Unique PSM
|

Protein Coverage:

Supporting Peptides:

| Peptide | Uniq | -10lgP | Mass | Length | ppm | m/z | z | RT | 1/k0 Start | 1/k0 End | Precursor ID | Area Sample 5 | #Feature | #Feature Sample 5 | Start | End | PTM | AScore | Found By |
| --- | --- | --- | --- | --- | --- | --- | --- | --- | --- | --- | --- | --- | --- | --- | --- | --- | --- | --- | --- |
| K.YNHYWIGLNDIKK.E | Y | 31.35 | 1662.8518 | 13 | -3 | 555.2872 | 3 | 16.29 | 0.8368 | 0.8441 | 50223 | 2.59e2 | 1 | 1 | 84 | 96 |  |  | DB Search |
| total 1 peptides |  |  |  |  |  |  |  |  |  |  |  |  |  |  |  |  |  |  |  |
| --- | --- | --- | --- | --- | --- | --- | --- | --- | --- | --- | --- | --- | --- | --- | --- | --- | --- | --- | --- |

Best Unique PSM (Scan Sample\_3\_A1\_1\_3641.d:50223, m/z=555.2872, z=3, RT=16.29, ppm=-7.29):


TRINITY\_DN4494\_c1\_g1\_i12.p1

back to list

  

| Protein Coverage
| Supporting Peptides
| Best Unique PSM
|

Protein Coverage:

Supporting Peptides:

| Peptide | Uniq | -10lgP | Mass | Length | ppm | m/z | z | RT | 1/k0 Start | 1/k0 End | Precursor ID | Area Sample 5 | #Feature | #Feature Sample 5 | Start | End | PTM | AScore | Found By |
| --- | --- | --- | --- | --- | --- | --- | --- | --- | --- | --- | --- | --- | --- | --- | --- | --- | --- | --- | --- |
| K.VDVGFILDSSGSLR.N | Y | 31.66 | 1463.762 | 14 | -1.83 | 732.8838 | 2 | 19.80 | 1.0126 | 1.0198 | 70509 | 0 | 0 | 0 | 41 | 54 |  |  | DB Search |
| total 1 peptides |  |  |  |  |  |  |  |  |  |  |  |  |  |  |  |  |  |  |  |
| --- | --- | --- | --- | --- | --- | --- | --- | --- | --- | --- | --- | --- | --- | --- | --- | --- | --- | --- | --- |

Best Unique PSM (Scan Sample\_3\_A1\_1\_3641.d:70509, m/z=732.8838, z=2, RT=19.80, ppm=-6.11):


TRINITY\_DN540\_c0\_g1\_i1.p1

back to list

  

| Protein Coverage
| Supporting Peptides
| Best Unique PSM
|

Protein Coverage:

Supporting Peptides:

| Peptide | Uniq | -10lgP | Mass | Length | ppm | m/z | z | RT | 1/k0 Start | 1/k0 End | Precursor ID | Area Sample 5 | #Feature | #Feature Sample 5 | Start | End | PTM | AScore | Found By |
| --- | --- | --- | --- | --- | --- | --- | --- | --- | --- | --- | --- | --- | --- | --- | --- | --- | --- | --- | --- |
| M.A(+42.01)TDKLEGLIAR.L | Y | 31.56 | 1227.6823 | 11 | 0.47 | 614.8461 | 2 | 17.84 | 0.9525 | 0.9598 | 58434 | 4.22e2 | 1 | 1 | 2 | 12 | Acetylation (Protein N-term) | A1:Acetylation (Protein N-term):1000 | DB Search |
| total 1 peptides |  |  |  |  |  |  |  |  |  |  |  |  |  |  |  |  |  |  |  |
| --- | --- | --- | --- | --- | --- | --- | --- | --- | --- | --- | --- | --- | --- | --- | --- | --- | --- | --- | --- |

Best Unique PSM (Scan Sample\_3\_A1\_1\_3641.d:58434, m/z=614.8461, z=2, RT=17.84, ppm=-3.81):


TRINITY\_DN55094\_c0\_g1\_i1.p1

back to list

  

| Protein Coverage
| Supporting Peptides
| Best Unique PSM
|

Protein Coverage:

Supporting Peptides:

| Peptide | Uniq | -10lgP | Mass | Length | ppm | m/z | z | RT | 1/k0 Start | 1/k0 End | Precursor ID | Area Sample 5 | #Feature | #Feature Sample 5 | Start | End | PTM | AScore | Found By |
| --- | --- | --- | --- | --- | --- | --- | --- | --- | --- | --- | --- | --- | --- | --- | --- | --- | --- | --- | --- |
| K.FLLHLFDKELK.M | Y | 31.65 | 1401.802 | 11 | 1.86 | 468.2735 | 3 | 19.03 | 0.7693 | 0.7766 | 65648 | 5.29e2 | 1 | 1 | 95 | 105 |  |  | DB Search |
| total 1 peptides |  |  |  |  |  |  |  |  |  |  |  |  |  |  |  |  |  |  |  |
| --- | --- | --- | --- | --- | --- | --- | --- | --- | --- | --- | --- | --- | --- | --- | --- | --- | --- | --- | --- |

Best Unique PSM (Scan Sample\_3\_A1\_1\_3641.d:65648, m/z=468.2735, z=3, RT=19.03, ppm=-2.43):


TRINITY\_DN1936\_c0\_g2\_i1.p1

back to list

  

| Protein Coverage
| Supporting Peptides
| Best Unique PSM
|

Protein Coverage:

Supporting Peptides:

| Peptide | Uniq | -10lgP | Mass | Length | ppm | m/z | z | RT | 1/k0 Start | 1/k0 End | Precursor ID | Area Sample 5 | #Feature | #Feature Sample 5 | Start | End | PTM | AScore | Found By |
| --- | --- | --- | --- | --- | --- | --- | --- | --- | --- | --- | --- | --- | --- | --- | --- | --- | --- | --- | --- |
| K.VVISDTC(+57.02)TNKWK.M | Y | 30.5 | 1449.7286 | 12 | 0.59 | 725.8689 | 2 | 13.58 | 1.0379 | 1.0452 | 36084 | 0 | 0 | 0 | 1259 | 1270 | Carbamidomethylation |  | DB Search |
| total 1 peptides |  |  |  |  |  |  |  |  |  |  |  |  |  |  |  |  |  |  |  |
| --- | --- | --- | --- | --- | --- | --- | --- | --- | --- | --- | --- | --- | --- | --- | --- | --- | --- | --- | --- |

Best Unique PSM (Scan Sample\_3\_A1\_1\_3641.d:36084, m/z=725.8689, z=2, RT=13.58, ppm=-3.69):


TRINITY\_DN1058\_c0\_g1\_i1.p1

back to list

  

| Protein Coverage
| Supporting Peptides
| Best Unique PSM
|

Protein Coverage:

Supporting Peptides:

| Peptide | Uniq | -10lgP | Mass | Length | ppm | m/z | z | RT | 1/k0 Start | 1/k0 End | Precursor ID | Area Sample 5 | #Feature | #Feature Sample 5 | Start | End | PTM | AScore | Found By |
| --- | --- | --- | --- | --- | --- | --- | --- | --- | --- | --- | --- | --- | --- | --- | --- | --- | --- | --- | --- |
| R.APTHILNPANKR.S | Y | 31.7 | 1330.7469 | 12 | 1.15 | 666.3787 | 2 | 11.11 | 1.0098 | 1.017 | 22167 | 1.2e4 | 2 | 2 | 2314 | 2325 |  |  | DB Search |
| total 1 peptides |  |  |  |  |  |  |  |  |  |  |  |  |  |  |  |  |  |  |  |
| --- | --- | --- | --- | --- | --- | --- | --- | --- | --- | --- | --- | --- | --- | --- | --- | --- | --- | --- | --- |

Best Unique PSM (Scan Sample\_3\_A1\_1\_3641.d:22167, m/z=666.3787, z=2, RT=11.11, ppm=-3.13):


TRINITY\_DN165469\_c0\_g1\_i1.p1

back to list

  

| Protein Coverage
| Supporting Peptides
| Best Unique PSM
|

Protein Coverage:

Supporting Peptides:

| Peptide | Uniq | -10lgP | Mass | Length | ppm | m/z | z | RT | 1/k0 Start | 1/k0 End | Precursor ID | Area Sample 5 | #Feature | #Feature Sample 5 | Start | End | PTM | AScore | Found By |
| --- | --- | --- | --- | --- | --- | --- | --- | --- | --- | --- | --- | --- | --- | --- | --- | --- | --- | --- | --- |
| K.FLDIHELQAVK.C | Y | 31.67 | 1311.7186 | 11 | 0.8 | 656.8643 | 2 | 17.39 | 1.0077 | 1.015 | 55792 | 0 | 0 | 0 | 84 | 94 |  |  | DB Search |
| total 1 peptides |  |  |  |  |  |  |  |  |  |  |  |  |  |  |  |  |  |  |  |
| --- | --- | --- | --- | --- | --- | --- | --- | --- | --- | --- | --- | --- | --- | --- | --- | --- | --- | --- | --- |

Best Unique PSM (Scan Sample\_3\_A1\_1\_3641.d:55792, m/z=656.8643, z=2, RT=17.39, ppm=-3.48):


TRINITY\_DN1676\_c0\_g1\_i12.p1

back to list

  

| Protein Coverage
| Supporting Peptides
| Best Unique PSM
|

Protein Coverage:

Supporting Peptides:

| Peptide | Uniq | -10lgP | Mass | Length | ppm | m/z | z | RT | 1/k0 Start | 1/k0 End | Precursor ID | Area Sample 5 | #Feature | #Feature Sample 5 | Start | End | PTM | AScore | Found By |
| --- | --- | --- | --- | --- | --- | --- | --- | --- | --- | --- | --- | --- | --- | --- | --- | --- | --- | --- | --- |
| R.FELDRVNTFGDEVFGDPK.V | Y | 31.68 | 2083.985 | 18 | 4.09 | 695.6688 | 3 | 19.17 | 0.9198 | 0.9271 | 66516 | 5.06e2 | 1 | 1 | 252 | 269 |  |  | DB Search |
| total 1 peptides |  |  |  |  |  |  |  |  |  |  |  |  |  |  |  |  |  |  |  |
| --- | --- | --- | --- | --- | --- | --- | --- | --- | --- | --- | --- | --- | --- | --- | --- | --- | --- | --- | --- |

Best Unique PSM (Scan Sample\_3\_A1\_1\_3641.d:66516, m/z=695.6688, z=3, RT=19.17, ppm=-0.19):


TRINITY\_DN4494\_c1\_g1\_i5.p1

back to list

  

| Protein Coverage
| Supporting Peptides
| Best Unique PSM
|

Protein Coverage:

Supporting Peptides:

| Peptide | Uniq | -10lgP | Mass | Length | ppm | m/z | z | RT | 1/k0 Start | 1/k0 End | Precursor ID | Area Sample 5 | #Feature | #Feature Sample 5 | Start | End | PTM | AScore | Found By |
| --- | --- | --- | --- | --- | --- | --- | --- | --- | --- | --- | --- | --- | --- | --- | --- | --- | --- | --- | --- |
| R.VLDVGFLLDSSDKVK.D | Y | 31.68 | 1633.8926 | 15 | 1.24 | 817.9511 | 2 | 19.58 | 1.1694 | 1.1766 | 69063 | 8.63e2 | 2 | 2 | 90 | 104 |  |  | DB Search |
| K.VDVGFILDSSGSLR.N | Y | 31.66 | 1463.762 | 14 | -1.83 | 732.8838 | 2 | 19.80 | 1.0126 | 1.0198 | 70509 | 0 | 0 | 0 | 288 | 301 |  |  | DB Search |
| total 2 peptides |  |  |  |  |  |  |  |  |  |  |  |  |  |  |  |  |  |  |  |
| --- | --- | --- | --- | --- | --- | --- | --- | --- | --- | --- | --- | --- | --- | --- | --- | --- | --- | --- | --- |

Best Unique PSM (Scan Sample\_3\_A1\_1\_3641.d:69063, m/z=817.9511, z=2, RT=19.58, ppm=-3.04):


TRINITY\_DN991\_c0\_g1\_i6.p1

back to list

  

| Protein Coverage
| Supporting Peptides
| Best Unique PSM
|

Protein Coverage:

Supporting Peptides:

| Peptide | Uniq | -10lgP | Mass | Length | ppm | m/z | z | RT | 1/k0 Start | 1/k0 End | Precursor ID | Area Sample 5 | #Feature | #Feature Sample 5 | Start | End | PTM | AScore | Found By |
| --- | --- | --- | --- | --- | --- | --- | --- | --- | --- | --- | --- | --- | --- | --- | --- | --- | --- | --- | --- |
| R.ITVTVSPDIHPPAK.L | Y | 31.67 | 1473.8191 | 14 | 3.85 | 737.9165 | 2 | 14.85 | 1.064 | 1.0713 | 42886 | 6.83e2 | 1 | 1 | 1183 | 1196 |  |  | DB Search |
| total 1 peptides |  |  |  |  |  |  |  |  |  |  |  |  |  |  |  |  |  |  |  |
| --- | --- | --- | --- | --- | --- | --- | --- | --- | --- | --- | --- | --- | --- | --- | --- | --- | --- | --- | --- |

Best Unique PSM (Scan Sample\_3\_A1\_1\_3641.d:42886, m/z=737.9165, z=2, RT=14.85, ppm=-0.43):


TRINITY\_DN10485\_c0\_g1\_i1.p1

back to list

  

| Protein Coverage
| Supporting Peptides
| Best Unique PSM
|

Protein Coverage:

Supporting Peptides:

| Peptide | Uniq | -10lgP | Mass | Length | ppm | m/z | z | RT | 1/k0 Start | 1/k0 End | Precursor ID | Area Sample 5 | #Feature | #Feature Sample 5 | Start | End | PTM | AScore | Found By |
| --- | --- | --- | --- | --- | --- | --- | --- | --- | --- | --- | --- | --- | --- | --- | --- | --- | --- | --- | --- |
| K.DLEDYKVTEREPVTTNVK.G | Y | 31.67 | 2135.0746 | 18 | 1.09 | 712.6965 | 3 | 15.11 | 0.915 | 0.9222 | 44293 | 4.57e3 | 1 | 1 | 282 | 299 |  |  | DB Search |
| total 1 peptides |  |  |  |  |  |  |  |  |  |  |  |  |  |  |  |  |  |  |  |
| --- | --- | --- | --- | --- | --- | --- | --- | --- | --- | --- | --- | --- | --- | --- | --- | --- | --- | --- | --- |

Best Unique PSM (Scan Sample\_3\_A1\_1\_3641.d:44293, m/z=712.6965, z=3, RT=15.11, ppm=-3.19):


TRINITY\_DN2477\_c0\_g1\_i6.p1

back to list

  

| Protein Coverage
| Supporting Peptides
| Best Unique PSM
|

Protein Coverage:

Supporting Peptides:

| Peptide | Uniq | -10lgP | Mass | Length | ppm | m/z | z | RT | 1/k0 Start | 1/k0 End | Precursor ID | Area Sample 5 | #Feature | #Feature Sample 5 | Start | End | PTM | AScore | Found By |
| --- | --- | --- | --- | --- | --- | --- | --- | --- | --- | --- | --- | --- | --- | --- | --- | --- | --- | --- | --- |
| K.DSYVGDEAQSKR.G | Y | 31.69 | 1353.616 | 12 | 1.77 | 677.8136 | 2 | 10.94 | 1.0017 | 1.009 | 21026 | 1.85e3 | 1 | 1 | 52 | 63 |  |  | DB Search |
| total 1 peptides |  |  |  |  |  |  |  |  |  |  |  |  |  |  |  |  |  |  |  |
| --- | --- | --- | --- | --- | --- | --- | --- | --- | --- | --- | --- | --- | --- | --- | --- | --- | --- | --- | --- |

Best Unique PSM (Scan Sample\_3\_A1\_1\_3641.d:21026, m/z=677.8136, z=2, RT=10.94, ppm=-2.51):


TRINITY\_DN8903\_c0\_g1\_i1.p1

back to list

  

| Protein Coverage
| Supporting Peptides
| Best Unique PSM
|

Protein Coverage:

Supporting Peptides:

| Peptide | Uniq | -10lgP | Mass | Length | ppm | m/z | z | RT | 1/k0 Start | 1/k0 End | Precursor ID | Area Sample 5 | #Feature | #Feature Sample 5 | Start | End | PTM | AScore | Found By |
| --- | --- | --- | --- | --- | --- | --- | --- | --- | --- | --- | --- | --- | --- | --- | --- | --- | --- | --- | --- |
| K.DLHIIEDYVLK.H | Y | 31.69 | 1356.7289 | 11 | -0.23 | 679.3687 | 2 | 19.54 | 0.9932 | 1.0005 | 68824 | 1.13e3 | 1 | 1 | 417 | 427 |  |  | DB Search |
| total 1 peptides |  |  |  |  |  |  |  |  |  |  |  |  |  |  |  |  |  |  |  |
| --- | --- | --- | --- | --- | --- | --- | --- | --- | --- | --- | --- | --- | --- | --- | --- | --- | --- | --- | --- |

Best Unique PSM (Scan Sample\_3\_A1\_1\_3641.d:68824, m/z=679.3687, z=2, RT=19.54, ppm=-4.52):


TRINITY\_DN2576\_c3\_g1\_i1.p2

back to list

  

| Protein Coverage
| Supporting Peptides
| Best Unique PSM
|

Protein Coverage:

Supporting Peptides:

| Peptide | Uniq | -10lgP | Mass | Length | ppm | m/z | z | RT | 1/k0 Start | 1/k0 End | Precursor ID | Area Sample 5 | #Feature | #Feature Sample 5 | Start | End | PTM | AScore | Found By |
| --- | --- | --- | --- | --- | --- | --- | --- | --- | --- | --- | --- | --- | --- | --- | --- | --- | --- | --- | --- |
| K.IPSWVPSLPKPK.S | Y | 31.25 | 1347.7914 | 12 | -0.39 | 450.269 | 3 | 17.94 | 0.7791 | 0.7864 | 58876 | 7.07e3 | 3 | 2 | 34 | 45 |  |  | DB Search |
| total 1 peptides |  |  |  |  |  |  |  |  |  |  |  |  |  |  |  |  |  |  |  |
| --- | --- | --- | --- | --- | --- | --- | --- | --- | --- | --- | --- | --- | --- | --- | --- | --- | --- | --- | --- |

Best Unique PSM (Scan Sample\_3\_A1\_1\_3641.d:58876, m/z=450.269, z=3, RT=17.94, ppm=-4.67):


TRINITY\_DN2003\_c0\_g1\_i1.p1

back to list

  

| Protein Coverage
| Supporting Peptides
| Best Unique PSM
|

Protein Coverage:

Supporting Peptides:

| Peptide | Uniq | -10lgP | Mass | Length | ppm | m/z | z | RT | 1/k0 Start | 1/k0 End | Precursor ID | Area Sample 5 | #Feature | #Feature Sample 5 | Start | End | PTM | AScore | Found By |
| --- | --- | --- | --- | --- | --- | --- | --- | --- | --- | --- | --- | --- | --- | --- | --- | --- | --- | --- | --- |
| R.ASMDEGAKPK.E | Y | 31.71 | 1032.491 | 10 | 6.03 | 517.2537 | 2 | 9.35 | 0.8704 | 0.8777 | 11527 | 2.53e2 | 1 | 1 | 133 | 142 |  |  | DB Search |
| total 1 peptides |  |  |  |  |  |  |  |  |  |  |  |  |  |  |  |  |  |  |  |
| --- | --- | --- | --- | --- | --- | --- | --- | --- | --- | --- | --- | --- | --- | --- | --- | --- | --- | --- | --- |

Best Unique PSM (Scan Sample\_3\_A1\_1\_3641.d:11527, m/z=517.2537, z=2, RT=9.35, ppm=1.75):


TRINITY\_DN244\_c0\_g1\_i1.p1

back to list

  

| Protein Coverage
| Supporting Peptides
| Best Unique PSM
|

Protein Coverage:

Supporting Peptides:

| Peptide | Uniq | -10lgP | Mass | Length | ppm | m/z | z | RT | 1/k0 Start | 1/k0 End | Precursor ID | Area Sample 5 | #Feature | #Feature Sample 5 | Start | End | PTM | AScore | Found By |
| --- | --- | --- | --- | --- | --- | --- | --- | --- | --- | --- | --- | --- | --- | --- | --- | --- | --- | --- | --- |
| K.ALLLASSNAFDTSNGAR.E | Y | 31.84 | 1706.8587 | 17 | 2.57 | 854.4352 | 2 | 17.65 | 1.0937 | 1.1009 | 57325 | 6.66e2 | 1 | 1 | 1912 | 1928 |  |  | DB Search |
| K.FSNDIVDSFAVSQKK.T | Y | 31.79 | 1683.8468 | 15 | 6.6 | 562.2908 | 3 | 17.06 | 0.8664 | 0.8737 | 53931 | 0 | 0 | 0 | 295 | 309 |  |  | DB Search |
| K.ALVILGDGFISGGGTPSK.T | Y | 31.78 | 1687.9144 | 18 | 3.47 | 844.9638 | 2 | 19.74 | 1.167 | 1.1742 | 69284 | 6.7e3 | 1 | 1 | 1619 | 1636 |  |  | DB Search |
| K.KALVILGDGFISGGGTPSK.T | Y | 31.05 | 1816.0094 | 19 | 1.25 | 909.0092 | 2 | 18.01 | 1.1774 | 1.1845 | 59478 | 0 | 0 | 0 | 1618 | 1636 |  |  | DB Search |
| K.ERIDYALELAK.S | Y | 30.99 | 1319.7085 | 11 | 3.16 | 660.8608 | 2 | 16.11 | 0.9824 | 0.9896 | 49311 | 0 | 0 | 0 | 350 | 360 |  |  | DB Search |
| K.LIDELTPEVTSALEC(+57.02)EGGPR.G | Y | 30.9 | 2185.0572 | 20 | -17.73 | 1093.5118 | 2 | 19.30 | 1.2173 | 1.2244 | 67386 | 0 | 0 | 0 | 2265 | 2284 | Carbamidomethylation |  | DB Search |
| total 6 peptides |  |  |  |  |  |  |  |  |  |  |  |  |  |  |  |  |  |  |  |
| --- | --- | --- | --- | --- | --- | --- | --- | --- | --- | --- | --- | --- | --- | --- | --- | --- | --- | --- | --- |

Best Unique PSM (Scan Sample\_3\_A1\_1\_3641.d:57325, m/z=854.4352, z=2, RT=17.65, ppm=-1.71):


TRINITY\_DN477\_c0\_g1\_i8.p1

back to list

  

| Protein Coverage
| Supporting Peptides
| Best Unique PSM
|

Protein Coverage:

Supporting Peptides:

| Peptide | Uniq | -10lgP | Mass | Length | ppm | m/z | z | RT | 1/k0 Start | 1/k0 End | Precursor ID | Area Sample 5 | #Feature | #Feature Sample 5 | Start | End | PTM | AScore | Found By |
| --- | --- | --- | --- | --- | --- | --- | --- | --- | --- | --- | --- | --- | --- | --- | --- | --- | --- | --- | --- |
| R.ILQC(+57.02)NQYANVEEAR.N | Y | 31.75 | 1706.8046 | 14 | 3.31 | 854.4088 | 2 | 14.22 | 1.0813 | 1.0885 | 39617 | 7.69e2 | 1 | 1 | 86 | 99 | Carbamidomethylation |  | DB Search |
| K.DKNILVLTLGVGPQVNR.N | Y | 31.03 | 1835.0628 | 17 | 0.02 | 918.5348 | 2 | 19.37 | 1.1334 | 1.1406 | 67784 | 1.18e3 | 1 | 1 | 168 | 184 |  |  | DB Search |
| total 2 peptides |  |  |  |  |  |  |  |  |  |  |  |  |  |  |  |  |  |  |  |
| --- | --- | --- | --- | --- | --- | --- | --- | --- | --- | --- | --- | --- | --- | --- | --- | --- | --- | --- | --- |

Best Unique PSM (Scan Sample\_3\_A1\_1\_3641.d:39617, m/z=854.4088, z=2, RT=14.22, ppm=-0.97):


TRINITY\_DN2477\_c0\_g1\_i2.p1

back to list

  

| Protein Coverage
| Supporting Peptides
| Best Unique PSM
|

Protein Coverage:

Supporting Peptides:

| Peptide | Uniq | -10lgP | Mass | Length | ppm | m/z | z | RT | 1/k0 Start | 1/k0 End | Precursor ID | Area Sample 5 | #Feature | #Feature Sample 5 | Start | End | PTM | AScore | Found By |
| --- | --- | --- | --- | --- | --- | --- | --- | --- | --- | --- | --- | --- | --- | --- | --- | --- | --- | --- | --- |
| K.DSYVGDEAQSKR.G | Y | 31.69 | 1353.616 | 12 | 1.77 | 677.8136 | 2 | 10.94 | 1.0017 | 1.009 | 21026 | 1.85e3 | 1 | 1 | 19 | 30 |  |  | DB Search |
| total 1 peptides |  |  |  |  |  |  |  |  |  |  |  |  |  |  |  |  |  |  |  |
| --- | --- | --- | --- | --- | --- | --- | --- | --- | --- | --- | --- | --- | --- | --- | --- | --- | --- | --- | --- |

Best Unique PSM (Scan Sample\_3\_A1\_1\_3641.d:21026, m/z=677.8136, z=2, RT=10.94, ppm=-2.51):


TRINITY\_DN1058\_c3\_g1\_i1.p1

back to list

  

| Protein Coverage
| Supporting Peptides
| Best Unique PSM
|

Protein Coverage:

Supporting Peptides:

| Peptide | Uniq | -10lgP | Mass | Length | ppm | m/z | z | RT | 1/k0 Start | 1/k0 End | Precursor ID | Area Sample 5 | #Feature | #Feature Sample 5 | Start | End | PTM | AScore | Found By |
| --- | --- | --- | --- | --- | --- | --- | --- | --- | --- | --- | --- | --- | --- | --- | --- | --- | --- | --- | --- |
| K.GSVASLTC(+57.02)EC(+57.02)NNHK.G | Y | 31.9 | 1575.677 | 14 | 5.83 | 788.847 | 2 | 11.91 | 1.064 | 1.0713 | 26802 | 0 | 0 | 0 | 243 | 256 | Carbamidomethylation, Carbamidomethylation |  | DB Search |
| total 1 peptides |  |  |  |  |  |  |  |  |  |  |  |  |  |  |  |  |  |  |  |
| --- | --- | --- | --- | --- | --- | --- | --- | --- | --- | --- | --- | --- | --- | --- | --- | --- | --- | --- | --- |

Best Unique PSM (Scan Sample\_3\_A1\_1\_3641.d:26802, m/z=788.847, z=2, RT=11.91, ppm=1.55):


TRINITY\_DN161\_c0\_g1\_i2.p1

back to list

  

| Protein Coverage
| Supporting Peptides
| Best Unique PSM
|

Protein Coverage:

Supporting Peptides:

| Peptide | Uniq | -10lgP | Mass | Length | ppm | m/z | z | RT | 1/k0 Start | 1/k0 End | Precursor ID | Area Sample 5 | #Feature | #Feature Sample 5 | Start | End | PTM | AScore | Found By |
| --- | --- | --- | --- | --- | --- | --- | --- | --- | --- | --- | --- | --- | --- | --- | --- | --- | --- | --- | --- |
| R.AVFPSIVGRPR.H | Y | 31.8 | 1197.6982 | 11 | 3.08 | 599.8557 | 2 | 15.68 | 0.9574 | 0.9646 | 46487 | 1.74e4 | 1 | 1 | 52 | 62 |  |  | DB Search |
| K.DSYVGDEAQSKR.G | Y | 31.69 | 1353.616 | 12 | 1.77 | 677.8136 | 2 | 10.94 | 1.0017 | 1.009 | 21026 | 1.85e3 | 1 | 1 | 74 | 85 |  |  | DB Search |
| total 2 peptides |  |  |  |  |  |  |  |  |  |  |  |  |  |  |  |  |  |  |  |
| --- | --- | --- | --- | --- | --- | --- | --- | --- | --- | --- | --- | --- | --- | --- | --- | --- | --- | --- | --- |

Best Unique PSM (Scan Sample\_3\_A1\_1\_3641.d:46487, m/z=599.8557, z=2, RT=15.68, ppm=-1.2):


TRINITY\_DN2113\_c0\_g1\_i2.p1

back to list

  

| Protein Coverage
| Supporting Peptides
| Best Unique PSM
|

Protein Coverage:

Supporting Peptides:

| Peptide | Uniq | -10lgP | Mass | Length | ppm | m/z | z | RT | 1/k0 Start | 1/k0 End | Precursor ID | Area Sample 5 | #Feature | #Feature Sample 5 | Start | End | PTM | AScore | Found By |
| --- | --- | --- | --- | --- | --- | --- | --- | --- | --- | --- | --- | --- | --- | --- | --- | --- | --- | --- | --- |
| R.LADLEEFISSPNHANISQVGER.C | Y | 31.5 | 2425.1873 | 22 | 2.2 | 809.4014 | 3 | 18.76 | 0.9255 | 0.9327 | 63995 | 1.04e3 | 1 | 1 | 1183 | 1204 |  |  | DB Search |
| total 1 peptides |  |  |  |  |  |  |  |  |  |  |  |  |  |  |  |  |  |  |  |
| --- | --- | --- | --- | --- | --- | --- | --- | --- | --- | --- | --- | --- | --- | --- | --- | --- | --- | --- | --- |

Best Unique PSM (Scan Sample\_3\_A1\_1\_3641.d:63995, m/z=809.4014, z=3, RT=18.76, ppm=-2.08):


TRINITY\_DN2477\_c0\_g1\_i5.p1

back to list

  

| Protein Coverage
| Supporting Peptides
| Best Unique PSM
|

Protein Coverage:

Supporting Peptides:

| Peptide | Uniq | -10lgP | Mass | Length | ppm | m/z | z | RT | 1/k0 Start | 1/k0 End | Precursor ID | Area Sample 5 | #Feature | #Feature Sample 5 | Start | End | PTM | AScore | Found By |
| --- | --- | --- | --- | --- | --- | --- | --- | --- | --- | --- | --- | --- | --- | --- | --- | --- | --- | --- | --- |
| K.DSYVGDEAQSKR.G | Y | 31.69 | 1353.616 | 12 | 1.77 | 677.8136 | 2 | 10.94 | 1.0017 | 1.009 | 21026 | 1.85e3 | 1 | 1 | 8 | 19 |  |  | DB Search |
| total 1 peptides |  |  |  |  |  |  |  |  |  |  |  |  |  |  |  |  |  |  |  |
| --- | --- | --- | --- | --- | --- | --- | --- | --- | --- | --- | --- | --- | --- | --- | --- | --- | --- | --- | --- |

Best Unique PSM (Scan Sample\_3\_A1\_1\_3641.d:21026, m/z=677.8136, z=2, RT=10.94, ppm=-2.51):


TRINITY\_DN26392\_c0\_g1\_i1.p1

back to list

  

| Protein Coverage
| Supporting Peptides
| Best Unique PSM
|

Protein Coverage:

Supporting Peptides:

| Peptide | Uniq | -10lgP | Mass | Length | ppm | m/z | z | RT | 1/k0 Start | 1/k0 End | Precursor ID | Area Sample 5 | #Feature | #Feature Sample 5 | Start | End | PTM | AScore | Found By |
| --- | --- | --- | --- | --- | --- | --- | --- | --- | --- | --- | --- | --- | --- | --- | --- | --- | --- | --- | --- |
| K.ADLDIQKPIPIVSK.Y | Y | 31.36 | 1535.8922 | 14 | 2.25 | 768.9518 | 2 | 15.99 | 1.0705 | 1.0777 | 48786 | 2.79e2 | 1 | 1 | 127 | 140 |  |  | DB Search |
| total 1 peptides |  |  |  |  |  |  |  |  |  |  |  |  |  |  |  |  |  |  |  |
| --- | --- | --- | --- | --- | --- | --- | --- | --- | --- | --- | --- | --- | --- | --- | --- | --- | --- | --- | --- |

Best Unique PSM (Scan Sample\_3\_A1\_1\_3641.d:48786, m/z=769.4517, z=2, RT=15.99, ppm=-2.03):


TRINITY\_DN17095\_c0\_g1\_i1.p1

back to list

  

| Protein Coverage
| Supporting Peptides
| Best Unique PSM
|

Protein Coverage:

Supporting Peptides:

| Peptide | Uniq | -10lgP | Mass | Length | ppm | m/z | z | RT | 1/k0 Start | 1/k0 End | Precursor ID | Area Sample 5 | #Feature | #Feature Sample 5 | Start | End | PTM | AScore | Found By |
| --- | --- | --- | --- | --- | --- | --- | --- | --- | --- | --- | --- | --- | --- | --- | --- | --- | --- | --- | --- |
| R.VGYLFITDWGGKPK.I | Y | 31.13 | 1579.8398 | 14 | 2.42 | 527.6196 | 3 | 19.47 | 0.8047 | 0.812 | 68400 | 4.43e2 | 1 | 1 | 260 | 273 |  |  | DB Search |
| total 1 peptides |  |  |  |  |  |  |  |  |  |  |  |  |  |  |  |  |  |  |  |
| --- | --- | --- | --- | --- | --- | --- | --- | --- | --- | --- | --- | --- | --- | --- | --- | --- | --- | --- | --- |

Best Unique PSM (Scan Sample\_3\_A1\_1\_3641.d:68400, m/z=527.6196, z=3, RT=19.47, ppm=-1.86):


TRINITY\_DN13467\_c0\_g1\_i1.p1

back to list

  

| Protein Coverage
| Supporting Peptides
| Best Unique PSM
|

Protein Coverage:

Supporting Peptides:

| Peptide | Uniq | -10lgP | Mass | Length | ppm | m/z | z | RT | 1/k0 Start | 1/k0 End | Precursor ID | Area Sample 5 | #Feature | #Feature Sample 5 | Start | End | PTM | AScore | Found By |
| --- | --- | --- | --- | --- | --- | --- | --- | --- | --- | --- | --- | --- | --- | --- | --- | --- | --- | --- | --- |
| K.ALHEADKEWEVK.T | Y | 31.26 | 1453.7201 | 12 | 5.69 | 727.8683 | 2 | 12.83 | 1.0379 | 1.0452 | 31955 | 0 | 0 | 0 | 110 | 121 |  |  | DB Search |
| total 1 peptides |  |  |  |  |  |  |  |  |  |  |  |  |  |  |  |  |  |  |  |
| --- | --- | --- | --- | --- | --- | --- | --- | --- | --- | --- | --- | --- | --- | --- | --- | --- | --- | --- | --- |

Best Unique PSM (Scan Sample\_3\_A1\_1\_3641.d:31955, m/z=727.8683, z=2, RT=12.83, ppm=1.41):


TRINITY\_DN1058\_c0\_g1\_i4.p1

back to list

  

| Protein Coverage
| Supporting Peptides
| Best Unique PSM
|

Protein Coverage:

Supporting Peptides:

| Peptide | Uniq | -10lgP | Mass | Length | ppm | m/z | z | RT | 1/k0 Start | 1/k0 End | Precursor ID | Area Sample 5 | #Feature | #Feature Sample 5 | Start | End | PTM | AScore | Found By |
| --- | --- | --- | --- | --- | --- | --- | --- | --- | --- | --- | --- | --- | --- | --- | --- | --- | --- | --- | --- |
| R.APTHILNPANKR.S | Y | 31.7 | 1330.7469 | 12 | 1.15 | 666.3787 | 2 | 11.11 | 1.0098 | 1.017 | 22167 | 1.2e4 | 2 | 2 | 398 | 409 |  |  | DB Search |
| total 1 peptides |  |  |  |  |  |  |  |  |  |  |  |  |  |  |  |  |  |  |  |
| --- | --- | --- | --- | --- | --- | --- | --- | --- | --- | --- | --- | --- | --- | --- | --- | --- | --- | --- | --- |

Best Unique PSM (Scan Sample\_3\_A1\_1\_3641.d:22167, m/z=666.3787, z=2, RT=11.11, ppm=-3.13):


TRINITY\_DN1030\_c0\_g1\_i2.p1

back to list

  

| Protein Coverage
| Supporting Peptides
| Best Unique PSM
|

Protein Coverage:

Supporting Peptides:

| Peptide | Uniq | -10lgP | Mass | Length | ppm | m/z | z | RT | 1/k0 Start | 1/k0 End | Precursor ID | Area Sample 5 | #Feature | #Feature Sample 5 | Start | End | PTM | AScore | Found By |
| --- | --- | --- | --- | --- | --- | --- | --- | --- | --- | --- | --- | --- | --- | --- | --- | --- | --- | --- | --- |
| R.GLQGQQGEQGSTGVQGSVGGR.G | Y | 31.94 | 1985.9515 | 21 | 4.67 | 993.9834 | 2 | 12.00 | 1.1326 | 1.1398 | 27213 | 3e3 | 1 | 1 | 348 | 368 |  |  | DB Search |
| total 1 peptides |  |  |  |  |  |  |  |  |  |  |  |  |  |  |  |  |  |  |  |
| --- | --- | --- | --- | --- | --- | --- | --- | --- | --- | --- | --- | --- | --- | --- | --- | --- | --- | --- | --- |

Best Unique PSM (Scan Sample\_3\_A1\_1\_3641.d:27213, m/z=993.9834, z=2, RT=12.00, ppm=0.4):


TRINITY\_DN11248\_c0\_g1\_i3.p1

back to list

  

| Protein Coverage
| Supporting Peptides
| Best Unique PSM
|

Protein Coverage:

Supporting Peptides:

| Peptide | Uniq | -10lgP | Mass | Length | ppm | m/z | z | RT | 1/k0 Start | 1/k0 End | Precursor ID | Area Sample 5 | #Feature | #Feature Sample 5 | Start | End | PTM | AScore | Found By |
| --- | --- | --- | --- | --- | --- | --- | --- | --- | --- | --- | --- | --- | --- | --- | --- | --- | --- | --- | --- |
| K.YNHYWIGLNDIKK.E | Y | 31.35 | 1662.8518 | 13 | -3 | 555.2872 | 3 | 16.29 | 0.8368 | 0.8441 | 50223 | 2.59e2 | 1 | 1 | 84 | 96 |  |  | DB Search |
| total 1 peptides |  |  |  |  |  |  |  |  |  |  |  |  |  |  |  |  |  |  |  |
| --- | --- | --- | --- | --- | --- | --- | --- | --- | --- | --- | --- | --- | --- | --- | --- | --- | --- | --- | --- |

Best Unique PSM (Scan Sample\_3\_A1\_1\_3641.d:50223, m/z=555.2872, z=3, RT=16.29, ppm=-7.29):


TRINITY\_DN984\_c0\_g1\_i1.p1

back to list

  

| Protein Coverage
| Supporting Peptides
| Best Unique PSM
|

Protein Coverage:

Supporting Peptides:

| Peptide | Uniq | -10lgP | Mass | Length | ppm | m/z | z | RT | 1/k0 Start | 1/k0 End | Precursor ID | Area Sample 5 | #Feature | #Feature Sample 5 | Start | End | PTM | AScore | Found By |
| --- | --- | --- | --- | --- | --- | --- | --- | --- | --- | --- | --- | --- | --- | --- | --- | --- | --- | --- | --- |
| R.EGPTGPQGETGR.R | Y | 30.8 | 1184.5421 | 12 | 6.09 | 593.2794 | 2 | 10.30 | 0.8806 | 0.8878 | 17329 | 0 | 0 | 0 | 476 | 487 |  |  | DB Search |
| total 1 peptides |  |  |  |  |  |  |  |  |  |  |  |  |  |  |  |  |  |  |  |
| --- | --- | --- | --- | --- | --- | --- | --- | --- | --- | --- | --- | --- | --- | --- | --- | --- | --- | --- | --- |

Best Unique PSM (Scan Sample\_3\_A1\_1\_3641.d:17329, m/z=593.2794, z=2, RT=10.30, ppm=1.8):


TRINITY\_DN4815\_c0\_g1\_i1.p1

back to list

  

| Protein Coverage
| Supporting Peptides
| Best Unique PSM
|

Protein Coverage:

Supporting Peptides:

| Peptide | Uniq | -10lgP | Mass | Length | ppm | m/z | z | RT | 1/k0 Start | 1/k0 End | Precursor ID | Area Sample 5 | #Feature | #Feature Sample 5 | Start | End | PTM | AScore | Found By |
| --- | --- | --- | --- | --- | --- | --- | --- | --- | --- | --- | --- | --- | --- | --- | --- | --- | --- | --- | --- |
| R.AVAC(+57.02)NSGPC(+57.02)LAGC(+57.02)PR.N | Y | 31.68 | 1588.6908 | 15 | 4.47 | 795.3528 | 2 | 12.99 | 1.0178 | 1.025 | 32815 | 5.35e2 | 1 | 1 | 425 | 439 | Carbamidomethylation, Carbamidomethylation, Carbamidomethylation |  | DB Search |
| total 1 peptides |  |  |  |  |  |  |  |  |  |  |  |  |  |  |  |  |  |  |  |
| --- | --- | --- | --- | --- | --- | --- | --- | --- | --- | --- | --- | --- | --- | --- | --- | --- | --- | --- | --- |

Best Unique PSM (Scan Sample\_3\_A1\_1\_3641.d:32815, m/z=795.3528, z=2, RT=12.99, ppm=0.19):


TRINITY\_DN2217\_c0\_g1\_i3.p1

back to list

  

| Protein Coverage
| Supporting Peptides
| Best Unique PSM
|

Protein Coverage:

Supporting Peptides:

| Peptide | Uniq | -10lgP | Mass | Length | ppm | m/z | z | RT | 1/k0 Start | 1/k0 End | Precursor ID | Area Sample 5 | #Feature | #Feature Sample 5 | Start | End | PTM | AScore | Found By |
| --- | --- | --- | --- | --- | --- | --- | --- | --- | --- | --- | --- | --- | --- | --- | --- | --- | --- | --- | --- |
| K.ATGC(+57.02)EVIWNQHNR.G | Y | 30.62 | 1583.7263 | 13 | 2.43 | 792.869 | 2 | 13.87 | 1.0797 | 1.0869 | 37737 | 0 | 0 | 0 | 431 | 443 | Carbamidomethylation |  | DB Search |
| total 1 peptides |  |  |  |  |  |  |  |  |  |  |  |  |  |  |  |  |  |  |  |
| --- | --- | --- | --- | --- | --- | --- | --- | --- | --- | --- | --- | --- | --- | --- | --- | --- | --- | --- | --- |

Best Unique PSM (Scan Sample\_3\_A1\_1\_3641.d:37737, m/z=792.869, z=2, RT=13.87, ppm=-1.85):


TRINITY\_DN598\_c1\_g1\_i2.p1

back to list

  

| Protein Coverage
| Supporting Peptides
| Best Unique PSM
|

Protein Coverage:

Supporting Peptides:

| Peptide | Uniq | -10lgP | Mass | Length | ppm | m/z | z | RT | 1/k0 Start | 1/k0 End | Precursor ID | Area Sample 5 | #Feature | #Feature Sample 5 | Start | End | PTM | AScore | Found By |
| --- | --- | --- | --- | --- | --- | --- | --- | --- | --- | --- | --- | --- | --- | --- | --- | --- | --- | --- | --- |
| R.ASFVTDIDRLR.A | Y | 31.69 | 1291.6884 | 11 | 4.02 | 646.8513 | 2 | 17.03 | 0.9868 | 0.9941 | 53636 | 3.39e3 | 1 | 1 | 302 | 312 |  |  | DB Search |
| total 1 peptides |  |  |  |  |  |  |  |  |  |  |  |  |  |  |  |  |  |  |  |
| --- | --- | --- | --- | --- | --- | --- | --- | --- | --- | --- | --- | --- | --- | --- | --- | --- | --- | --- | --- |

Best Unique PSM (Scan Sample\_3\_A1\_1\_3641.d:53636, m/z=646.8513, z=2, RT=17.03, ppm=-0.26):


TRINITY\_DN4494\_c1\_g1\_i2.p1

back to list

  

| Protein Coverage
| Supporting Peptides
| Best Unique PSM
|

Protein Coverage:

Supporting Peptides:

| Peptide | Uniq | -10lgP | Mass | Length | ppm | m/z | z | RT | 1/k0 Start | 1/k0 End | Precursor ID | Area Sample 5 | #Feature | #Feature Sample 5 | Start | End | PTM | AScore | Found By |
| --- | --- | --- | --- | --- | --- | --- | --- | --- | --- | --- | --- | --- | --- | --- | --- | --- | --- | --- | --- |
| K.VDVGFILDSSGSLR.N | Y | 31.66 | 1463.762 | 14 | -1.83 | 732.8838 | 2 | 19.80 | 1.0126 | 1.0198 | 70509 | 0 | 0 | 0 | 318 | 331 |  |  | DB Search |
| total 1 peptides |  |  |  |  |  |  |  |  |  |  |  |  |  |  |  |  |  |  |  |
| --- | --- | --- | --- | --- | --- | --- | --- | --- | --- | --- | --- | --- | --- | --- | --- | --- | --- | --- | --- |

Best Unique PSM (Scan Sample\_3\_A1\_1\_3641.d:70509, m/z=732.8838, z=2, RT=19.80, ppm=-6.11):


TRINITY\_DN69276\_c0\_g1\_i1.p1

back to list

  

| Protein Coverage
| Supporting Peptides
| Best Unique PSM
|

Protein Coverage:

Supporting Peptides:

| Peptide | Uniq | -10lgP | Mass | Length | ppm | m/z | z | RT | 1/k0 Start | 1/k0 End | Precursor ID | Area Sample 5 | #Feature | #Feature Sample 5 | Start | End | PTM | AScore | Found By |
| --- | --- | --- | --- | --- | --- | --- | --- | --- | --- | --- | --- | --- | --- | --- | --- | --- | --- | --- | --- |
| K.VIDINDNIPIFSK.S | Y | 31.55 | 1486.8031 | 13 | -0.75 | 744.4051 | 2 | 19.54 | 1.0198 | 1.0271 | 68788 | 8.64e2 | 1 | 1 | 301 | 313 |  |  | DB Search |
| total 1 peptides |  |  |  |  |  |  |  |  |  |  |  |  |  |  |  |  |  |  |  |
| --- | --- | --- | --- | --- | --- | --- | --- | --- | --- | --- | --- | --- | --- | --- | --- | --- | --- | --- | --- |

Best Unique PSM (Scan Sample\_3\_A1\_1\_3641.d:68788, m/z=744.4051, z=2, RT=19.54, ppm=-5.03):


TRINITY\_DN30613\_c0\_g1\_i1.p1

back to list

  

| Protein Coverage
| Supporting Peptides
| Best Unique PSM
|

Protein Coverage:

Supporting Peptides:

| Peptide | Uniq | -10lgP | Mass | Length | ppm | m/z | z | RT | 1/k0 Start | 1/k0 End | Precursor ID | Area Sample 5 | #Feature | #Feature Sample 5 | Start | End | PTM | AScore | Found By |
| --- | --- | --- | --- | --- | --- | --- | --- | --- | --- | --- | --- | --- | --- | --- | --- | --- | --- | --- | --- |
| K.APEDAPNREQEFEEK.A | Y | 31.53 | 1787.7962 | 15 | 1.71 | 596.9378 | 3 | 12.11 | 0.823 | 0.8303 | 27908 | 7.39e2 | 1 | 1 | 502 | 516 |  |  | DB Search |
| total 1 peptides |  |  |  |  |  |  |  |  |  |  |  |  |  |  |  |  |  |  |  |
| --- | --- | --- | --- | --- | --- | --- | --- | --- | --- | --- | --- | --- | --- | --- | --- | --- | --- | --- | --- |

Best Unique PSM (Scan Sample\_3\_A1\_1\_3641.d:27908, m/z=596.9378, z=3, RT=12.11, ppm=-2.57):


TRINITY\_DN495\_c0\_g1\_i5.p1

back to list

  

| Protein Coverage
| Supporting Peptides
| Best Unique PSM
|

Protein Coverage:

Supporting Peptides:

| Peptide | Uniq | -10lgP | Mass | Length | ppm | m/z | z | RT | 1/k0 Start | 1/k0 End | Precursor ID | Area Sample 5 | #Feature | #Feature Sample 5 | Start | End | PTM | AScore | Found By |
| --- | --- | --- | --- | --- | --- | --- | --- | --- | --- | --- | --- | --- | --- | --- | --- | --- | --- | --- | --- |
| C.SISC(+57.02)SKDEEIK.V | Y | 31.69 | 1294.6075 | 11 | 1.4 | 648.3091 | 2 | 10.84 | 0.9795 | 0.9868 | 20521 | 8.87e2 | 1 | 1 | 273 | 283 | Carbamidomethylation |  | DB Search |
| total 1 peptides |  |  |  |  |  |  |  |  |  |  |  |  |  |  |  |  |  |  |  |
| --- | --- | --- | --- | --- | --- | --- | --- | --- | --- | --- | --- | --- | --- | --- | --- | --- | --- | --- | --- |

Best Unique PSM (Scan Sample\_3\_A1\_1\_3641.d:20521, m/z=648.3091, z=2, RT=10.84, ppm=-2.88):


TRINITY\_DN22240\_c0\_g1\_i1.p1

back to list

  

| Protein Coverage
| Supporting Peptides
| Best Unique PSM
|

Protein Coverage:

Supporting Peptides:

| Peptide | Uniq | -10lgP | Mass | Length | ppm | m/z | z | RT | 1/k0 Start | 1/k0 End | Precursor ID | Area Sample 5 | #Feature | #Feature Sample 5 | Start | End | PTM | AScore | Found By |
| --- | --- | --- | --- | --- | --- | --- | --- | --- | --- | --- | --- | --- | --- | --- | --- | --- | --- | --- | --- |
| K.YLADVVLTLDHTK.M | Y | 31.69 | 1486.8031 | 13 | -2.87 | 496.6048 | 3 | 18.62 | 0.8307 | 0.838 | 63167 | 7.62e2 | 1 | 1 | 19 | 31 |  |  | DB Search |
| total 1 peptides |  |  |  |  |  |  |  |  |  |  |  |  |  |  |  |  |  |  |  |
| --- | --- | --- | --- | --- | --- | --- | --- | --- | --- | --- | --- | --- | --- | --- | --- | --- | --- | --- | --- |

Best Unique PSM (Scan Sample\_3\_A1\_1\_3641.d:63167, m/z=496.6048, z=3, RT=18.62, ppm=-7.15):


TRINITY\_DN10094\_c0\_g1\_i1.p1

back to list

  

| Protein Coverage
| Supporting Peptides
| Best Unique PSM
|

Protein Coverage:

Supporting Peptides:

| Peptide | Uniq | -10lgP | Mass | Length | ppm | m/z | z | RT | 1/k0 Start | 1/k0 End | Precursor ID | Area Sample 5 | #Feature | #Feature Sample 5 | Start | End | PTM | AScore | Found By |
| --- | --- | --- | --- | --- | --- | --- | --- | --- | --- | --- | --- | --- | --- | --- | --- | --- | --- | --- | --- |
| K.FFFHDQSC(+57.02)LDVYK.Y | Y | 31.68 | 1704.7606 | 13 | 0.29 | 569.2585 | 3 | 17.60 | 0.8075 | 0.8149 | 57111 | 0 | 0 | 0 | 221 | 233 | Carbamidomethylation |  | DB Search |
| total 1 peptides |  |  |  |  |  |  |  |  |  |  |  |  |  |  |  |  |  |  |  |
| --- | --- | --- | --- | --- | --- | --- | --- | --- | --- | --- | --- | --- | --- | --- | --- | --- | --- | --- | --- |

Best Unique PSM (Scan Sample\_3\_A1\_1\_3641.d:57111, m/z=569.2585, z=3, RT=17.60, ppm=-3.99):


TRINITY\_DN123121\_c0\_g1\_i1.p1

back to list

  

| Protein Coverage
| Supporting Peptides
| Best Unique PSM
|

Protein Coverage:

Supporting Peptides:

| Peptide | Uniq | -10lgP | Mass | Length | ppm | m/z | z | RT | 1/k0 Start | 1/k0 End | Precursor ID | Area Sample 5 | #Feature | #Feature Sample 5 | Start | End | PTM | AScore | Found By |
| --- | --- | --- | --- | --- | --- | --- | --- | --- | --- | --- | --- | --- | --- | --- | --- | --- | --- | --- | --- |
| R.LTHQIEVTVSDR.E | Y | 31.87 | 1396.731 | 12 | -2.08 | 699.3683 | 2 | 13.43 | 0.9912 | 0.9985 | 35251 | 5.62e2 | 1 | 1 | 94 | 105 |  |  | DB Search |
| total 1 peptides |  |  |  |  |  |  |  |  |  |  |  |  |  |  |  |  |  |  |  |
| --- | --- | --- | --- | --- | --- | --- | --- | --- | --- | --- | --- | --- | --- | --- | --- | --- | --- | --- | --- |

Best Unique PSM (Scan Sample\_3\_A1\_1\_3641.d:35251, m/z=699.3683, z=2, RT=13.43, ppm=-6.35):


TRINITY\_DN61\_c0\_g1\_i1.p1

back to list

  

| Protein Coverage
| Supporting Peptides
| Best Unique PSM
|

Protein Coverage:

Supporting Peptides:

| Peptide | Uniq | -10lgP | Mass | Length | ppm | m/z | z | RT | 1/k0 Start | 1/k0 End | Precursor ID | Area Sample 5 | #Feature | #Feature Sample 5 | Start | End | PTM | AScore | Found By |
| --- | --- | --- | --- | --- | --- | --- | --- | --- | --- | --- | --- | --- | --- | --- | --- | --- | --- | --- | --- |
| R.AINIHDLVGIDTKK.E | Y | 31.74 | 1535.8671 | 14 | 2.57 | 768.9395 | 2 | 16.45 | 1.0753 | 1.0825 | 50998 | 1.15e3 | 1 | 1 | 339 | 352 |  |  | DB Search |
| total 1 peptides |  |  |  |  |  |  |  |  |  |  |  |  |  |  |  |  |  |  |  |
| --- | --- | --- | --- | --- | --- | --- | --- | --- | --- | --- | --- | --- | --- | --- | --- | --- | --- | --- | --- |

Best Unique PSM (Scan Sample\_3\_A1\_1\_3641.d:50998, m/z=768.9395, z=2, RT=16.45, ppm=-1.71):


TRINITY\_DN4595\_c0\_g1\_i10.p1

back to list

  

| Protein Coverage
| Supporting Peptides
| Best Unique PSM
|

Protein Coverage:

Supporting Peptides:

| Peptide | Uniq | -10lgP | Mass | Length | ppm | m/z | z | RT | 1/k0 Start | 1/k0 End | Precursor ID | Area Sample 5 | #Feature | #Feature Sample 5 | Start | End | PTM | AScore | Found By |
| --- | --- | --- | --- | --- | --- | --- | --- | --- | --- | --- | --- | --- | --- | --- | --- | --- | --- | --- | --- |
| R.SPFVIMFK.G | Y | 31.43 | 967.5201 | 8 | 3.55 | 484.767 | 2 | 19.71 | 0.8096 | 0.8169 | 69837 | 4.12e3 | 1 | 1 | 630 | 637 |  |  | DB Search |
| total 1 peptides |  |  |  |  |  |  |  |  |  |  |  |  |  |  |  |  |  |  |  |
| --- | --- | --- | --- | --- | --- | --- | --- | --- | --- | --- | --- | --- | --- | --- | --- | --- | --- | --- | --- |

Best Unique PSM (Scan Sample\_3\_A1\_1\_3641.d:69837, m/z=484.767, z=2, RT=19.71, ppm=-0.73):


TRINITY\_DN942\_c0\_g1\_i3.p1

back to list

  

| Protein Coverage
| Supporting Peptides
| Best Unique PSM
|

Protein Coverage:

Supporting Peptides:

| Peptide | Uniq | -10lgP | Mass | Length | ppm | m/z | z | RT | 1/k0 Start | 1/k0 End | Precursor ID | Area Sample 5 | #Feature | #Feature Sample 5 | Start | End | PTM | AScore | Found By |
| --- | --- | --- | --- | --- | --- | --- | --- | --- | --- | --- | --- | --- | --- | --- | --- | --- | --- | --- | --- |
| K.VLNIALDKYQEGVAK.T | Y | 31.63 | 1659.9195 | 15 | -0.5 | 830.9631 | 2 | 15.97 | 1.1546 | 1.1618 | 48647 | 0 | 0 | 0 | 4546 | 4560 |  |  | DB Search |
| total 1 peptides |  |  |  |  |  |  |  |  |  |  |  |  |  |  |  |  |  |  |  |
| --- | --- | --- | --- | --- | --- | --- | --- | --- | --- | --- | --- | --- | --- | --- | --- | --- | --- | --- | --- |

Best Unique PSM (Scan Sample\_3\_A1\_1\_3641.d:48647, m/z=830.9631, z=2, RT=15.97, ppm=-4.78):


TRINITY\_DN982\_c0\_g1\_i3.p1

back to list

  

| Protein Coverage
| Supporting Peptides
| Best Unique PSM
|

Protein Coverage:

Supporting Peptides:

| Peptide | Uniq | -10lgP | Mass | Length | ppm | m/z | z | RT | 1/k0 Start | 1/k0 End | Precursor ID | Area Sample 5 | #Feature | #Feature Sample 5 | Start | End | PTM | AScore | Found By |
| --- | --- | --- | --- | --- | --- | --- | --- | --- | --- | --- | --- | --- | --- | --- | --- | --- | --- | --- | --- |
| K.YVAIYPSDHLTVNR.D | N | 31.92 | 1646.8416 | 14 | 0.07 | 824.4246 | 2 | 15.53 | 1.1061 | 1.1134 | 46351 | 1.08e3 | 1 | 1 | 6254 | 6267 |  |  | DB Search |
| R.LVQNTPHNSIVTK.I | N | 31.71 | 1449.794 | 13 | 2.57 | 725.903 | 2 | 11.68 | 1.0572 | 1.0644 | 25401 | 4.01e3 | 1 | 1 | 8569 | 8581 |  |  | DB Search |
| R.TNNALDYEAKR.Q | N | 31.69 | 1293.6313 | 11 | 5.9 | 647.824 | 2 | 11.99 | 0.9618 | 0.9691 | 27271 | 3.6e2 | 1 | 1 | 7725 | 7735 |  |  | DB Search |
| K.VLLSQQIFYDKK.N | N | 31.68 | 1480.8289 | 12 | -0.33 | 741.4183 | 2 | 16.72 | 1.0572 | 1.0644 | 52159 | 3.15e3 | 1 | 1 | 4919 | 4930 |  |  | DB Search |
| K.TGAISLLASVDFETDRTHTAVVK.A | Y | 31.68 | 2430.2754 | 23 | 0.72 | 811.0962 | 3 | 18.85 | 1.0367 | 1.0439 | 64505 | 1.34e3 | 2 | 2 | 3335 | 3357 |  |  | DB Search |
| K.APIGQSIVTVSAR.D | Y | 31.66 | 1297.7354 | 13 | 3.68 | 649.8746 | 2 | 14.17 | 0.9598 | 0.967 | 39287 | 2.49e2 | 1 | 1 | 4029 | 4041 |  |  | DB Search |
| R.IQVLKDPDFEFTR.S | N | 31.02 | 1606.8355 | 13 | 5.47 | 804.426 | 2 | 17.53 | 1.0837 | 1.0909 | 56499 | 1.44e3 | 1 | 1 | 6167 | 6179 |  |  | DB Search |
| total 7 peptides |  |  |  |  |  |  |  |  |  |  |  |  |  |  |  |  |  |  |  |
| --- | --- | --- | --- | --- | --- | --- | --- | --- | --- | --- | --- | --- | --- | --- | --- | --- | --- | --- | --- |

Best Unique PSM (Scan Sample\_3\_A1\_1\_3641.d:64505, m/z=811.0962, z=3, RT=18.85, ppm=-3.56):


TRINITY\_DN4494\_c1\_g1\_i7.p1

back to list

  

| Protein Coverage
| Supporting Peptides
| Best Unique PSM
|

Protein Coverage:

Supporting Peptides:

| Peptide | Uniq | -10lgP | Mass | Length | ppm | m/z | z | RT | 1/k0 Start | 1/k0 End | Precursor ID | Area Sample 5 | #Feature | #Feature Sample 5 | Start | End | PTM | AScore | Found By |
| --- | --- | --- | --- | --- | --- | --- | --- | --- | --- | --- | --- | --- | --- | --- | --- | --- | --- | --- | --- |
| R.VLDVGFLLDSSDKVK.D | Y | 31.68 | 1633.8926 | 15 | 1.24 | 817.9511 | 2 | 19.58 | 1.1694 | 1.1766 | 69063 | 8.63e2 | 2 | 2 | 109 | 123 |  |  | DB Search |
| K.VDVGFILDSSGSLR.N | Y | 31.66 | 1463.762 | 14 | -1.83 | 732.8838 | 2 | 19.80 | 1.0126 | 1.0198 | 70509 | 0 | 0 | 0 | 307 | 320 |  |  | DB Search |
| total 2 peptides |  |  |  |  |  |  |  |  |  |  |  |  |  |  |  |  |  |  |  |
| --- | --- | --- | --- | --- | --- | --- | --- | --- | --- | --- | --- | --- | --- | --- | --- | --- | --- | --- | --- |

Best Unique PSM (Scan Sample\_3\_A1\_1\_3641.d:69063, m/z=817.9511, z=2, RT=19.58, ppm=-3.04):


TRINITY\_DN4494\_c1\_g1\_i10.p1

back to list

  

| Protein Coverage
| Supporting Peptides
| Best Unique PSM
|

Protein Coverage:

Supporting Peptides:

| Peptide | Uniq | -10lgP | Mass | Length | ppm | m/z | z | RT | 1/k0 Start | 1/k0 End | Precursor ID | Area Sample 5 | #Feature | #Feature Sample 5 | Start | End | PTM | AScore | Found By |
| --- | --- | --- | --- | --- | --- | --- | --- | --- | --- | --- | --- | --- | --- | --- | --- | --- | --- | --- | --- |
| K.VDVGFILDSSGSLR.N | Y | 31.66 | 1463.762 | 14 | -1.83 | 732.8838 | 2 | 19.80 | 1.0126 | 1.0198 | 70509 | 0 | 0 | 0 | 318 | 331 |  |  | DB Search |
| total 1 peptides |  |  |  |  |  |  |  |  |  |  |  |  |  |  |  |  |  |  |  |
| --- | --- | --- | --- | --- | --- | --- | --- | --- | --- | --- | --- | --- | --- | --- | --- | --- | --- | --- | --- |

Best Unique PSM (Scan Sample\_3\_A1\_1\_3641.d:70509, m/z=732.8838, z=2, RT=19.80, ppm=-6.11):


TRINITY\_DN2028\_c0\_g1\_i1.p1

back to list

  

| Protein Coverage
| Supporting Peptides
| Best Unique PSM
|

Protein Coverage:

Supporting Peptides:

| Peptide | Uniq | -10lgP | Mass | Length | ppm | m/z | z | RT | 1/k0 Start | 1/k0 End | Precursor ID | Area Sample 5 | #Feature | #Feature Sample 5 | Start | End | PTM | AScore | Found By |
| --- | --- | --- | --- | --- | --- | --- | --- | --- | --- | --- | --- | --- | --- | --- | --- | --- | --- | --- | --- |
| R.DFREFLYNSETGKVM(+15.99)GR.S | Y | 31.69 | 2063.9734 | 17 | -1.15 | 688.9947 | 3 | 17.54 | 0.9287 | 0.936 | 56508 | 2.29e3 | 1 | 1 | 21 | 37 | Oxidation (M) | M15:Oxidation (M):1000 | DB Search |
| K.LIQYIENKPGLTR.L | Y | 31.53 | 1543.8722 | 13 | 5.29 | 772.9442 | 2 | 14.78 | 1.0753 | 1.0825 | 42347 | 2.27e4 | 1 | 1 | 81 | 93 |  |  | DB Search |
| total 2 peptides |  |  |  |  |  |  |  |  |  |  |  |  |  |  |  |  |  |  |  |
| --- | --- | --- | --- | --- | --- | --- | --- | --- | --- | --- | --- | --- | --- | --- | --- | --- | --- | --- | --- |

Best Unique PSM (Scan Sample\_3\_A1\_1\_3641.d:56508, m/z=688.9947, z=3, RT=17.54, ppm=-5.43):


TRINITY\_DN495\_c0\_g1\_i6.p1

back to list

  

| Protein Coverage
| Supporting Peptides
| Best Unique PSM
|

Protein Coverage:

Supporting Peptides:

| Peptide | Uniq | -10lgP | Mass | Length | ppm | m/z | z | RT | 1/k0 Start | 1/k0 End | Precursor ID | Area Sample 5 | #Feature | #Feature Sample 5 | Start | End | PTM | AScore | Found By |
| --- | --- | --- | --- | --- | --- | --- | --- | --- | --- | --- | --- | --- | --- | --- | --- | --- | --- | --- | --- |
| K.SISC(+57.02)SKDEEIK.V | Y | 31.69 | 1294.6075 | 11 | 1.4 | 648.3091 | 2 | 10.84 | 0.9795 | 0.9868 | 20521 | 8.87e2 | 1 | 1 | 248 | 258 | Carbamidomethylation |  | DB Search |
| total 1 peptides |  |  |  |  |  |  |  |  |  |  |  |  |  |  |  |  |  |  |  |
| --- | --- | --- | --- | --- | --- | --- | --- | --- | --- | --- | --- | --- | --- | --- | --- | --- | --- | --- | --- |

Best Unique PSM (Scan Sample\_3\_A1\_1\_3641.d:20521, m/z=648.3091, z=2, RT=10.84, ppm=-2.88):


TRINITY\_DN6542\_c0\_g1\_i1.p1

back to list

  

| Protein Coverage
| Supporting Peptides
| Best Unique PSM
|

Protein Coverage:

Supporting Peptides:

| Peptide | Uniq | -10lgP | Mass | Length | ppm | m/z | z | RT | 1/k0 Start | 1/k0 End | Precursor ID | Area Sample 5 | #Feature | #Feature Sample 5 | Start | End | PTM | AScore | Found By |
| --- | --- | --- | --- | --- | --- | --- | --- | --- | --- | --- | --- | --- | --- | --- | --- | --- | --- | --- | --- |
| K.ILLAVGGWNHER.Q | Y | 31.42 | 1363.736 | 12 | 2.78 | 682.8743 | 2 | 16.92 | 1.0387 | 1.046 | 53358 | 3.81e2 | 1 | 1 | 140 | 151 |  |  | DB Search |
| total 1 peptides |  |  |  |  |  |  |  |  |  |  |  |  |  |  |  |  |  |  |  |
| --- | --- | --- | --- | --- | --- | --- | --- | --- | --- | --- | --- | --- | --- | --- | --- | --- | --- | --- | --- |

Best Unique PSM (Scan Sample\_3\_A1\_1\_3641.d:53358, m/z=682.8743, z=2, RT=16.92, ppm=-1.5):


TRINITY\_DN1936\_c0\_g1\_i4.p1

back to list

  

| Protein Coverage
| Supporting Peptides
| Best Unique PSM
|

Protein Coverage:

Supporting Peptides:

| Peptide | Uniq | -10lgP | Mass | Length | ppm | m/z | z | RT | 1/k0 Start | 1/k0 End | Precursor ID | Area Sample 5 | #Feature | #Feature Sample 5 | Start | End | PTM | AScore | Found By |
| --- | --- | --- | --- | --- | --- | --- | --- | --- | --- | --- | --- | --- | --- | --- | --- | --- | --- | --- | --- |
| K.AGDIAILESQQFNAVGGK.T | Y | 30.61 | 1816.9319 | 18 | -19.18 | 909.4519 | 2 | 18.85 | 1.1853 | 1.1925 | 64620 | 3.67e2 | 1 | 1 | 1002 | 1019 |  |  | DB Search |
| total 1 peptides |  |  |  |  |  |  |  |  |  |  |  |  |  |  |  |  |  |  |  |
| --- | --- | --- | --- | --- | --- | --- | --- | --- | --- | --- | --- | --- | --- | --- | --- | --- | --- | --- | --- |

Best Unique PSM (Scan Sample\_3\_A1\_1\_3641.d:64620, m/z=909.4519, z=2, RT=18.85, ppm=-23.46):


TRINITY\_DN1616\_c0\_g1\_i2.p1

back to list

  

| Protein Coverage
| Supporting Peptides
| Best Unique PSM
|

Protein Coverage:

Supporting Peptides:

| Peptide | Uniq | -10lgP | Mass | Length | ppm | m/z | z | RT | 1/k0 Start | 1/k0 End | Precursor ID | Area Sample 5 | #Feature | #Feature Sample 5 | Start | End | PTM | AScore | Found By |
| --- | --- | --- | --- | --- | --- | --- | --- | --- | --- | --- | --- | --- | --- | --- | --- | --- | --- | --- | --- |
| K.GC(+57.02)STC(+57.02)GC(+57.02)NGAGTNNGVK.S | Y | 31.94 | 1712.6665 | 17 | 1.85 | 857.3384 | 2 | 9.80 | 1.0448 | 1.052 | 14171 | 0 | 0 | 0 | 732 | 748 | Carbamidomethylation, Carbamidomethylation, Carbamidomethylation |  | DB Search |
| K.VVDTEANDLTEEVKK.T | Y | 31.68 | 1688.8468 | 15 | 3.24 | 845.4298 | 2 | 14.05 | 1.1286 | 1.1358 | 38709 | 1.51e3 | 1 | 1 | 2542 | 2556 |  |  | DB Search |
| R.YGAVNDNC(+57.02)ERDNGQC(+57.02)SC(+57.02)K.V | Y | 31.05 | 2145.8262 | 18 | 2.18 | 716.2812 | 3 | 10.55 | 0.8587 | 0.866 | 18796 | 7.2e2 | 1 | 1 | 498 | 515 | Carbamidomethylation, Carbamidomethylation, Carbamidomethylation |  | DB Search |
| K.NC(+57.02)QHNTLGINC(+57.02)EK.C | Y | 30.89 | 1586.693 | 13 | 4.28 | 794.3538 | 2 | 11.89 | 1.0371 | 1.0443 | 26586 | 7.24e2 | 1 | 1 | 366 | 378 | Carbamidomethylation, Carbamidomethylation |  | DB Search |
| K.DGEKVETLKVDISDINK.A | Y | 30.55 | 1901.9945 | 17 | 2.61 | 635.0044 | 3 | 15.87 | 0.9065 | 0.9137 | 48130 | 2.66e2 | 1 | 1 | 2634 | 2650 |  |  | DB Search |
| total 5 peptides |  |  |  |  |  |  |  |  |  |  |  |  |  |  |  |  |  |  |  |
| --- | --- | --- | --- | --- | --- | --- | --- | --- | --- | --- | --- | --- | --- | --- | --- | --- | --- | --- | --- |

Best Unique PSM (Scan Sample\_3\_A1\_1\_3641.d:14171, m/z=857.3384, z=2, RT=9.80, ppm=-2.43):


TRINITY\_DN477\_c0\_g1\_i7.p1

back to list

  

| Protein Coverage
| Supporting Peptides
| Best Unique PSM
|

Protein Coverage:

Supporting Peptides:

| Peptide | Uniq | -10lgP | Mass | Length | ppm | m/z | z | RT | 1/k0 Start | 1/k0 End | Precursor ID | Area Sample 5 | #Feature | #Feature Sample 5 | Start | End | PTM | AScore | Found By |
| --- | --- | --- | --- | --- | --- | --- | --- | --- | --- | --- | --- | --- | --- | --- | --- | --- | --- | --- | --- |
| R.ILQC(+57.02)NQYANVEEAR.N | Y | 31.75 | 1706.8046 | 14 | 3.31 | 854.4088 | 2 | 14.22 | 1.0813 | 1.0885 | 39617 | 7.69e2 | 1 | 1 | 86 | 99 | Carbamidomethylation |  | DB Search |
| K.DKNILVLTLGVGPQVNR.N | Y | 31.03 | 1835.0628 | 17 | 0.02 | 918.5348 | 2 | 19.37 | 1.1334 | 1.1406 | 67784 | 1.18e3 | 1 | 1 | 168 | 184 |  |  | DB Search |
| total 2 peptides |  |  |  |  |  |  |  |  |  |  |  |  |  |  |  |  |  |  |  |
| --- | --- | --- | --- | --- | --- | --- | --- | --- | --- | --- | --- | --- | --- | --- | --- | --- | --- | --- | --- |

Best Unique PSM (Scan Sample\_3\_A1\_1\_3641.d:39617, m/z=854.4088, z=2, RT=14.22, ppm=-0.97):


TRINITY\_DN140702\_c0\_g1\_i1.p1

back to list

  

| Protein Coverage
| Supporting Peptides
| Best Unique PSM
|

Protein Coverage:

Supporting Peptides:

| Peptide | Uniq | -10lgP | Mass | Length | ppm | m/z | z | RT | 1/k0 Start | 1/k0 End | Precursor ID | Area Sample 5 | #Feature | #Feature Sample 5 | Start | End | PTM | AScore | Found By |
| --- | --- | --- | --- | --- | --- | --- | --- | --- | --- | --- | --- | --- | --- | --- | --- | --- | --- | --- | --- |
| R.LALHVLNNEDIIGHR.L | Y | 31.68 | 1712.9322 | 15 | 0.25 | 571.9824 | 3 | 16.32 | 0.853 | 0.8603 | 50289 | 4.27e3 | 2 | 2 | 1702 | 1716 |  |  | DB Search |
| R.SDELLNLKEEASTLR.E | Y | 30.91 | 1716.8894 | 15 | -3.25 | 859.4455 | 2 | 17.63 | 1.1094 | 1.1166 | 57260 | 0 | 0 | 0 | 729 | 743 |  |  | DB Search |
| total 2 peptides |  |  |  |  |  |  |  |  |  |  |  |  |  |  |  |  |  |  |  |
| --- | --- | --- | --- | --- | --- | --- | --- | --- | --- | --- | --- | --- | --- | --- | --- | --- | --- | --- | --- |

Best Unique PSM (Scan Sample\_3\_A1\_1\_3641.d:50289, m/z=571.9824, z=3, RT=16.32, ppm=-4.03):


TRINITY\_DN1071\_c0\_g1\_i2.p1

back to list

  

| Protein Coverage
| Supporting Peptides
| Best Unique PSM
|

Protein Coverage:

Supporting Peptides:

| Peptide | Uniq | -10lgP | Mass | Length | ppm | m/z | z | RT | 1/k0 Start | 1/k0 End | Precursor ID | Area Sample 5 | #Feature | #Feature Sample 5 | Start | End | PTM | AScore | Found By |
| --- | --- | --- | --- | --- | --- | --- | --- | --- | --- | --- | --- | --- | --- | --- | --- | --- | --- | --- | --- |
| K.AVQIHPFNDVSVGDPK.V | Y | 31.9 | 1721.8736 | 16 | 2.29 | 861.9424 | 2 | 15.73 | 1.1186 | 1.1258 | 47418 | 2.88e3 | 1 | 1 | 293 | 308 |  |  | DB Search |
| total 1 peptides |  |  |  |  |  |  |  |  |  |  |  |  |  |  |  |  |  |  |  |
| --- | --- | --- | --- | --- | --- | --- | --- | --- | --- | --- | --- | --- | --- | --- | --- | --- | --- | --- | --- |

Best Unique PSM (Scan Sample\_3\_A1\_1\_3641.d:47418, m/z=861.9424, z=2, RT=15.73, ppm=-1.99):


TRINITY\_DN99404\_c0\_g1\_i1.p1

back to list

  

| Protein Coverage
| Supporting Peptides
| Best Unique PSM
|

Protein Coverage:

Supporting Peptides:

| Peptide | Uniq | -10lgP | Mass | Length | ppm | m/z | z | RT | 1/k0 Start | 1/k0 End | Precursor ID | Area Sample 5 | #Feature | #Feature Sample 5 | Start | End | PTM | AScore | Found By |
| --- | --- | --- | --- | --- | --- | --- | --- | --- | --- | --- | --- | --- | --- | --- | --- | --- | --- | --- | --- |
| K.LQTDIHGVALEITK.L | Y | 31.4 | 1536.8511 | 14 | -1.96 | 769.428 | 2 | 16.47 | 1.052 | 1.0592 | 51159 | 0 | 0 | 0 | 24 | 37 |  |  | DB Search |
| total 1 peptides |  |  |  |  |  |  |  |  |  |  |  |  |  |  |  |  |  |  |  |
| --- | --- | --- | --- | --- | --- | --- | --- | --- | --- | --- | --- | --- | --- | --- | --- | --- | --- | --- | --- |

Best Unique PSM (Scan Sample\_3\_A1\_1\_3641.d:51159, m/z=769.428, z=2, RT=16.47, ppm=-6.24):


TRINITY\_DN428\_c0\_g1\_i1.p1

back to list

  

| Protein Coverage
| Supporting Peptides
| Best Unique PSM
|

Protein Coverage:

Supporting Peptides:

| Peptide | Uniq | -10lgP | Mass | Length | ppm | m/z | z | RT | 1/k0 Start | 1/k0 End | Precursor ID | Area Sample 5 | #Feature | #Feature Sample 5 | Start | End | PTM | AScore | Found By |
| --- | --- | --- | --- | --- | --- | --- | --- | --- | --- | --- | --- | --- | --- | --- | --- | --- | --- | --- | --- |
| K.IC(+57.02)HLLGIQVTDFTK.S | Y | 31.2 | 1643.8705 | 14 | 2.62 | 548.9632 | 3 | 18.46 | 0.8197 | 0.827 | 62230 | 0 | 0 | 0 | 377 | 390 | Carbamidomethylation |  | DB Search |
| R.AIESERDELLEELNNNSALK.N | Y | 30.81 | 2286.1339 | 20 | 3.61 | 763.0514 | 3 | 18.31 | 0.9989 | 1.0061 | 61258 | 0 | 0 | 0 | 1695 | 1714 |  |  | DB Search |
| total 2 peptides |  |  |  |  |  |  |  |  |  |  |  |  |  |  |  |  |  |  |  |
| --- | --- | --- | --- | --- | --- | --- | --- | --- | --- | --- | --- | --- | --- | --- | --- | --- | --- | --- | --- |

Best Unique PSM (Scan Sample\_3\_A1\_1\_3641.d:62230, m/z=548.9632, z=3, RT=18.46, ppm=-1.66):


TRINITY\_DN1616\_c0\_g1\_i3.p1

back to list

  

| Protein Coverage
| Supporting Peptides
| Best Unique PSM
|

Protein Coverage:

Supporting Peptides:

| Peptide | Uniq | -10lgP | Mass | Length | ppm | m/z | z | RT | 1/k0 Start | 1/k0 End | Precursor ID | Area Sample 5 | #Feature | #Feature Sample 5 | Start | End | PTM | AScore | Found By |
| --- | --- | --- | --- | --- | --- | --- | --- | --- | --- | --- | --- | --- | --- | --- | --- | --- | --- | --- | --- |
| K.GC(+57.02)STC(+57.02)GC(+57.02)NGAGTNNGVK.S | Y | 31.94 | 1712.6665 | 17 | 1.85 | 857.3384 | 2 | 9.80 | 1.0448 | 1.052 | 14171 | 0 | 0 | 0 | 732 | 748 | Carbamidomethylation, Carbamidomethylation, Carbamidomethylation |  | DB Search |
| K.VVDTEANDLTEEVKK.T | Y | 31.68 | 1688.8468 | 15 | 3.24 | 845.4298 | 2 | 14.05 | 1.1286 | 1.1358 | 38709 | 1.51e3 | 1 | 1 | 2542 | 2556 |  |  | DB Search |
| R.YGAVNDNC(+57.02)ERDNGQC(+57.02)SC(+57.02)K.V | Y | 31.05 | 2145.8262 | 18 | 2.18 | 716.2812 | 3 | 10.55 | 0.8587 | 0.866 | 18796 | 7.2e2 | 1 | 1 | 498 | 515 | Carbamidomethylation, Carbamidomethylation, Carbamidomethylation |  | DB Search |
| K.NC(+57.02)QHNTLGINC(+57.02)EK.C | Y | 30.89 | 1586.693 | 13 | 4.28 | 794.3538 | 2 | 11.89 | 1.0371 | 1.0443 | 26586 | 7.24e2 | 1 | 1 | 366 | 378 | Carbamidomethylation, Carbamidomethylation |  | DB Search |
| K.DGEKVETLKVDISDINK.A | Y | 30.55 | 1901.9945 | 17 | 2.61 | 635.0044 | 3 | 15.87 | 0.9065 | 0.9137 | 48130 | 2.66e2 | 1 | 1 | 2634 | 2650 |  |  | DB Search |
| total 5 peptides |  |  |  |  |  |  |  |  |  |  |  |  |  |  |  |  |  |  |  |
| --- | --- | --- | --- | --- | --- | --- | --- | --- | --- | --- | --- | --- | --- | --- | --- | --- | --- | --- | --- |

Best Unique PSM (Scan Sample\_3\_A1\_1\_3641.d:14171, m/z=857.3384, z=2, RT=9.80, ppm=-2.43):


TRINITY\_DN691\_c0\_g1\_i1.p1

back to list

  

| Protein Coverage
| Supporting Peptides
| Best Unique PSM
|

Protein Coverage:

Supporting Peptides:

| Peptide | Uniq | -10lgP | Mass | Length | ppm | m/z | z | RT | 1/k0 Start | 1/k0 End | Precursor ID | Area Sample 5 | #Feature | #Feature Sample 5 | Start | End | PTM | AScore | Found By |
| --- | --- | --- | --- | --- | --- | --- | --- | --- | --- | --- | --- | --- | --- | --- | --- | --- | --- | --- | --- |
| R.GPTGPQGER.G | Y | 31.15 | 897.4304 | 9 | 8.54 | 449.7244 | 2 | 9.46 | 0.7819 | 0.7892 | 12185 | 1.55e3 | 1 | 1 | 1209 | 1217 |  |  | DB Search |
| R.AYGQDLGQPGSC(+57.02)LKR.F | Y | 30.81 | 1648.7991 | 15 | 2.46 | 825.4053 | 2 | 13.40 | 1.0733 | 1.0805 | 35031 | 2.16e3 | 1 | 1 | 1533 | 1547 | Carbamidomethylation |  | DB Search |
| total 2 peptides |  |  |  |  |  |  |  |  |  |  |  |  |  |  |  |  |  |  |  |
| --- | --- | --- | --- | --- | --- | --- | --- | --- | --- | --- | --- | --- | --- | --- | --- | --- | --- | --- | --- |

Best Unique PSM (Scan Sample\_3\_A1\_1\_3641.d:12185, m/z=449.7244, z=2, RT=9.46, ppm=4.26):


TRINITY\_DN991\_c0\_g1\_i2.p1

back to list

  

| Protein Coverage
| Supporting Peptides
| Best Unique PSM
|

Protein Coverage:

Supporting Peptides:

| Peptide | Uniq | -10lgP | Mass | Length | ppm | m/z | z | RT | 1/k0 Start | 1/k0 End | Precursor ID | Area Sample 5 | #Feature | #Feature Sample 5 | Start | End | PTM | AScore | Found By |
| --- | --- | --- | --- | --- | --- | --- | --- | --- | --- | --- | --- | --- | --- | --- | --- | --- | --- | --- | --- |
| R.ITVTVSPDIHPPAK.L | Y | 31.67 | 1473.8191 | 14 | 3.85 | 737.9165 | 2 | 14.85 | 1.064 | 1.0713 | 42886 | 6.83e2 | 1 | 1 | 1183 | 1196 |  |  | DB Search |
| total 1 peptides |  |  |  |  |  |  |  |  |  |  |  |  |  |  |  |  |  |  |  |
| --- | --- | --- | --- | --- | --- | --- | --- | --- | --- | --- | --- | --- | --- | --- | --- | --- | --- | --- | --- |

Best Unique PSM (Scan Sample\_3\_A1\_1\_3641.d:42886, m/z=737.9165, z=2, RT=14.85, ppm=-0.43):


TRINITY\_DN6542\_c0\_g1\_i3.p1

back to list

  

| Protein Coverage
| Supporting Peptides
| Best Unique PSM
|

Protein Coverage:

Supporting Peptides:

| Peptide | Uniq | -10lgP | Mass | Length | ppm | m/z | z | RT | 1/k0 Start | 1/k0 End | Precursor ID | Area Sample 5 | #Feature | #Feature Sample 5 | Start | End | PTM | AScore | Found By |
| --- | --- | --- | --- | --- | --- | --- | --- | --- | --- | --- | --- | --- | --- | --- | --- | --- | --- | --- | --- |
| K.ILLAVGGWNHER.Q | Y | 31.42 | 1363.736 | 12 | 2.78 | 682.8743 | 2 | 16.92 | 1.0387 | 1.046 | 53358 | 3.81e2 | 1 | 1 | 140 | 151 |  |  | DB Search |
| total 1 peptides |  |  |  |  |  |  |  |  |  |  |  |  |  |  |  |  |  |  |  |
| --- | --- | --- | --- | --- | --- | --- | --- | --- | --- | --- | --- | --- | --- | --- | --- | --- | --- | --- | --- |

Best Unique PSM (Scan Sample\_3\_A1\_1\_3641.d:53358, m/z=682.8743, z=2, RT=16.92, ppm=-1.5):


TRINITY\_DN71276\_c0\_g1\_i1.p1

back to list

  

| Protein Coverage
| Supporting Peptides
| Best Unique PSM
|

Protein Coverage:

Supporting Peptides:

| Peptide | Uniq | -10lgP | Mass | Length | ppm | m/z | z | RT | 1/k0 Start | 1/k0 End | Precursor ID | Area Sample 5 | #Feature | #Feature Sample 5 | Start | End | PTM | AScore | Found By |
| --- | --- | --- | --- | --- | --- | --- | --- | --- | --- | --- | --- | --- | --- | --- | --- | --- | --- | --- | --- |
| K.EAAAQIVVEIIKR.T | Y | 31.24 | 1438.8507 | 13 | -1.6 | 720.4284 | 2 | 19.02 | 1.0512 | 1.0584 | 65681 | 0 | 0 | 0 | 149 | 161 |  |  | DB Search |
| total 1 peptides |  |  |  |  |  |  |  |  |  |  |  |  |  |  |  |  |  |  |  |
| --- | --- | --- | --- | --- | --- | --- | --- | --- | --- | --- | --- | --- | --- | --- | --- | --- | --- | --- | --- |

Best Unique PSM (Scan Sample\_3\_A1\_1\_3641.d:65681, m/z=720.4284, z=2, RT=19.02, ppm=-5.88):


TRINITY\_DN172571\_c0\_g1\_i1.p1

back to list

  

| Protein Coverage
| Supporting Peptides
| Best Unique PSM
|

Protein Coverage:

Supporting Peptides:

| Peptide | Uniq | -10lgP | Mass | Length | ppm | m/z | z | RT | 1/k0 Start | 1/k0 End | Precursor ID | Area Sample 5 | #Feature | #Feature Sample 5 | Start | End | PTM | AScore | Found By |
| --- | --- | --- | --- | --- | --- | --- | --- | --- | --- | --- | --- | --- | --- | --- | --- | --- | --- | --- | --- |
| R.DNIQGITKPAIR.R | Y | 31.67 | 1324.7463 | 12 | 3.85 | 663.3801 | 2 | 13.78 | 0.9808 | 0.988 | 37251 | 4.19e2 | 1 | 1 | 25 | 36 |  |  | DB Search |
| total 1 peptides |  |  |  |  |  |  |  |  |  |  |  |  |  |  |  |  |  |  |  |
| --- | --- | --- | --- | --- | --- | --- | --- | --- | --- | --- | --- | --- | --- | --- | --- | --- | --- | --- | --- |

Best Unique PSM (Scan Sample\_3\_A1\_1\_3641.d:37251, m/z=663.3801, z=2, RT=13.78, ppm=-0.43):


TRINITY\_DN319\_c2\_g1\_i3.p1

back to list

  

| Protein Coverage
| Supporting Peptides
| Best Unique PSM
|

Protein Coverage:

Supporting Peptides:

| Peptide | Uniq | -10lgP | Mass | Length | ppm | m/z | z | RT | 1/k0 Start | 1/k0 End | Precursor ID | Area Sample 5 | #Feature | #Feature Sample 5 | Start | End | PTM | AScore | Found By |
| --- | --- | --- | --- | --- | --- | --- | --- | --- | --- | --- | --- | --- | --- | --- | --- | --- | --- | --- | --- |
| R.DPIC(+57.02)HC(+57.02)DTSK.Y | Y | 31.69 | 1231.4961 | 10 | 5.21 | 616.7559 | 2 | 9.93 | 0.904 | 0.9113 | 14979 | 4.42e3 | 2 | 1 | 1448 | 1457 | Carbamidomethylation, Carbamidomethylation |  | DB Search |
| K.KAQYYEAYETIIKR.H | Y | 31.69 | 1774.9254 | 14 | -12.66 | 592.639 | 3 | 15.71 | 0.8607 | 0.868 | 47344 | 1.05e3 | 1 | 1 | 2534 | 2547 |  |  | DB Search |
| R.GSFSSHIC(+57.02)LC(+57.02)HDPAYK.S | Y | 31.67 | 1877.8189 | 16 | 3.89 | 626.9467 | 3 | 14.33 | 0.8838 | 0.8911 | 40207 | 1.11e3 | 1 | 1 | 1011 | 1026 | Carbamidomethylation, Carbamidomethylation |  | DB Search |
| K.LLHC(+57.02)SDSEVDHVEVC(+57.02)GK.G | Y | 31.63 | 1982.8826 | 17 | 4.93 | 661.9686 | 3 | 13.16 | 0.9226 | 0.9299 | 33774 | 0 | 0 | 0 | 845 | 861 | Carbamidomethylation, Carbamidomethylation |  | DB Search |
| R.HLAISNILK.D | Y | 30.67 | 1007.6127 | 9 | 1.34 | 504.8122 | 2 | 18.76 | 0.8676 | 0.8749 | 63978 | 5.96e2 | 1 | 1 | 2548 | 2556 |  |  | DB Search |
| total 5 peptides |  |  |  |  |  |  |  |  |  |  |  |  |  |  |  |  |  |  |  |
| --- | --- | --- | --- | --- | --- | --- | --- | --- | --- | --- | --- | --- | --- | --- | --- | --- | --- | --- | --- |

Best Unique PSM (Scan Sample\_3\_A1\_1\_3641.d:14979, m/z=616.7559, z=2, RT=9.93, ppm=0.93):


TRINITY\_DN691\_c0\_g1\_i2.p1

back to list

  

| Protein Coverage
| Supporting Peptides
| Best Unique PSM
|

Protein Coverage:

Supporting Peptides:

| Peptide | Uniq | -10lgP | Mass | Length | ppm | m/z | z | RT | 1/k0 Start | 1/k0 End | Precursor ID | Area Sample 5 | #Feature | #Feature Sample 5 | Start | End | PTM | AScore | Found By |
| --- | --- | --- | --- | --- | --- | --- | --- | --- | --- | --- | --- | --- | --- | --- | --- | --- | --- | --- | --- |
| R.AYGQDLGQPGSC(+57.02)LKR.F | Y | 30.81 | 1648.7991 | 15 | 2.46 | 825.4053 | 2 | 13.40 | 1.0733 | 1.0805 | 35031 | 2.16e3 | 1 | 1 | 1480 | 1494 | Carbamidomethylation |  | DB Search |
| total 1 peptides |  |  |  |  |  |  |  |  |  |  |  |  |  |  |  |  |  |  |  |
| --- | --- | --- | --- | --- | --- | --- | --- | --- | --- | --- | --- | --- | --- | --- | --- | --- | --- | --- | --- |

Best Unique PSM (Scan Sample\_3\_A1\_1\_3641.d:35031, m/z=825.4053, z=2, RT=13.40, ppm=-1.82):


TRINITY\_DN2217\_c0\_g1\_i2.p1

back to list

  

| Protein Coverage
| Supporting Peptides
| Best Unique PSM
|

Protein Coverage:

Supporting Peptides:

| Peptide | Uniq | -10lgP | Mass | Length | ppm | m/z | z | RT | 1/k0 Start | 1/k0 End | Precursor ID | Area Sample 5 | #Feature | #Feature Sample 5 | Start | End | PTM | AScore | Found By |
| --- | --- | --- | --- | --- | --- | --- | --- | --- | --- | --- | --- | --- | --- | --- | --- | --- | --- | --- | --- |
| K.ATGC(+57.02)EVIWNQHNR.G | Y | 30.62 | 1583.7263 | 13 | 2.43 | 792.869 | 2 | 13.87 | 1.0797 | 1.0869 | 37737 | 0 | 0 | 0 | 119 | 131 | Carbamidomethylation |  | DB Search |
| total 1 peptides |  |  |  |  |  |  |  |  |  |  |  |  |  |  |  |  |  |  |  |
| --- | --- | --- | --- | --- | --- | --- | --- | --- | --- | --- | --- | --- | --- | --- | --- | --- | --- | --- | --- |

Best Unique PSM (Scan Sample\_3\_A1\_1\_3641.d:37737, m/z=792.869, z=2, RT=13.87, ppm=-1.85):


TRINITY\_DN4815\_c0\_g1\_i2.p1

back to list

  

| Protein Coverage
| Supporting Peptides
| Best Unique PSM
|

Protein Coverage:

Supporting Peptides:

| Peptide | Uniq | -10lgP | Mass | Length | ppm | m/z | z | RT | 1/k0 Start | 1/k0 End | Precursor ID | Area Sample 5 | #Feature | #Feature Sample 5 | Start | End | PTM | AScore | Found By |
| --- | --- | --- | --- | --- | --- | --- | --- | --- | --- | --- | --- | --- | --- | --- | --- | --- | --- | --- | --- |
| R.AVAC(+57.02)NSGPC(+57.02)LAGC(+57.02)PR.N | Y | 31.68 | 1588.6908 | 15 | 4.47 | 795.3528 | 2 | 12.99 | 1.0178 | 1.025 | 32815 | 5.35e2 | 1 | 1 | 468 | 482 | Carbamidomethylation, Carbamidomethylation, Carbamidomethylation |  | DB Search |
| total 1 peptides |  |  |  |  |  |  |  |  |  |  |  |  |  |  |  |  |  |  |  |
| --- | --- | --- | --- | --- | --- | --- | --- | --- | --- | --- | --- | --- | --- | --- | --- | --- | --- | --- | --- |

Best Unique PSM (Scan Sample\_3\_A1\_1\_3641.d:32815, m/z=795.3528, z=2, RT=12.99, ppm=0.19):


TRINITY\_DN6390\_c0\_g1\_i1.p1

back to list

  

| Protein Coverage
| Supporting Peptides
| Best Unique PSM
|

Protein Coverage:

Supporting Peptides:

| Peptide | Uniq | -10lgP | Mass | Length | ppm | m/z | z | RT | 1/k0 Start | 1/k0 End | Precursor ID | Area Sample 5 | #Feature | #Feature Sample 5 | Start | End | PTM | AScore | Found By |
| --- | --- | --- | --- | --- | --- | --- | --- | --- | --- | --- | --- | --- | --- | --- | --- | --- | --- | --- | --- |
| R.VGNWRDETLSC(+57.02)K.Q | Y | 31.68 | 1463.6827 | 12 | 0.79 | 732.8461 | 2 | 13.70 | 1.0186 | 1.0259 | 36784 | 3.87e3 | 2 | 2 | 1047 | 1058 | Carbamidomethylation |  | DB Search |
| K.VSSDRFFVQVDGR.S | Y | 31.34 | 1510.7528 | 13 | 7.9 | 756.3864 | 2 | 15.89 | 1.0287 | 1.0359 | 48167 | 4.59e2 | 1 | 1 | 474 | 486 |  |  | DB Search |
| K.GGRFEC(+57.02)TDFDC(+57.02)VAHC(+57.02)ASK.G | Y | 31.13 | 2115.8561 | 18 | 2.43 | 706.2913 | 3 | 14.33 | 0.9331 | 0.9404 | 40205 | 4.08e2 | 1 | 1 | 1156 | 1173 | Carbamidomethylation, Carbamidomethylation, Carbamidomethylation |  | DB Search |
| R.HLSVNILQTNTR.V | Y | 30.59 | 1394.763 | 12 | 0.11 | 698.3859 | 2 | 15.24 | 1.0037 | 1.011 | 44984 | 3.19e2 | 1 | 0 | 836 | 847 |  |  | DB Search |
| total 4 peptides |  |  |  |  |  |  |  |  |  |  |  |  |  |  |  |  |  |  |  |
| --- | --- | --- | --- | --- | --- | --- | --- | --- | --- | --- | --- | --- | --- | --- | --- | --- | --- | --- | --- |

Best Unique PSM (Scan Sample\_3\_A1\_1\_3641.d:36784, m/z=732.8461, z=2, RT=13.70, ppm=-3.49):


TRINITY\_DN5584\_c0\_g1\_i3.p1

back to list

  

| Protein Coverage
| Supporting Peptides
| Best Unique PSM
|

Protein Coverage:

Supporting Peptides:

| Peptide | Uniq | -10lgP | Mass | Length | ppm | m/z | z | RT | 1/k0 Start | 1/k0 End | Precursor ID | Area Sample 5 | #Feature | #Feature Sample 5 | Start | End | PTM | AScore | Found By |
| --- | --- | --- | --- | --- | --- | --- | --- | --- | --- | --- | --- | --- | --- | --- | --- | --- | --- | --- | --- |
| K.VTLTQTHFLK.D | Y | 31.18 | 1186.671 | 10 | 4.18 | 594.3427 | 2 | 14.84 | 0.9638 | 0.9711 | 42932 | 0 | 0 | 0 | 559 | 568 |  |  | DB Search |
| total 1 peptides |  |  |  |  |  |  |  |  |  |  |  |  |  |  |  |  |  |  |  |
| --- | --- | --- | --- | --- | --- | --- | --- | --- | --- | --- | --- | --- | --- | --- | --- | --- | --- | --- | --- |

Best Unique PSM (Scan Sample\_3\_A1\_1\_3641.d:42932, m/z=594.3427, z=2, RT=14.84, ppm=-0.1):


---

Prepared with PEAKS ™ (bioinfor.com)
