# Supplementary material for: Proteomic Analysis and Biochemical Characterization of the Nematocyst Extract of the Hydrozoan Velella velella
Source: Mar Drugs. 2024 Oct 12;22(10):468. doi: 10.3390/md22100468 (PMC11509761; doi:10.3390/md22100468)
Supplement: Supplementary file 1 [file marinedrugs-22-00468-s001.zip › Supplementary File S1.zip!/Sample_3_Vellela/db.summary.html]

Protein ID Summary


# 1. Notes

# 2. Result Statistics

**Figure 1.**
False discovery rate (FDR) curve. X axis is the number of peptides being kept. Y axis is the corresponding FDR.

  


  

**Figure 2.**
Score distribution.  **(a)**
Distribution of PEAKS peptide score;  **(b)**
Scatterplot of PEAKS peptide score versus precursor mass error.

**(a)**

**(b)**

**Table 1.**
Statistics of data.

|  |  |  |  |  |  |  |  |  |  |  |  |  |  |
| --- | --- | --- | --- | --- | --- | --- | --- | --- | --- | --- | --- | --- | --- |
|  | #Frames | | #Features | #Precursors | | Identified | | | #Peptides | #Sequences | #Proteins^\*^ | | |
|  | MS1 | MS/MS |  | #Chimera | #Original | #PSMs | #Scans | #Features^\*\*^ |  |  | Groups | All | Top |
| Total | 1038 | 5181 | 52544 | 5174 | 71444 | 178 | 178 | 108 | 140 | 140 | 88 | 145 | 127 |
| Sample 5 | 1038 | 5181 | 52544 | 5174 | 71444 | 178 | 178 | 108 | 140 | 140 | 88 | 145 | 127 |

^\* proteins with significant peptides are used in counts.^  
^\*\* features are identified by DB search only.^

**Figure 3.**
Sample overlap for Proteins and Peptides (up to 8 samples).  **(a)**
All Proteins;  **(b)**
Top Proteins;  **(c)**
Peptides;

**(a)**

**Not applicable to only one sample**

**(b)**

**Not applicable to only one sample**

**(c)**

**Not applicable to only one sample**


**Figure 4.**
Distribution of peptide feature detection.  **(a)**
Feature m/z distribution;  **(b)**
Feature RT distribution.

**(a)**

**(b)**

**Figure 5.**
Distribution of identified peptide features.  **(a)**
Feature abundance distribution;

**(a)**

**Table 2.**
Result filtration parameters.

|  |  |
| --- | --- |
| Peptide -10lgP | ≥ 25.26 |
| Protein -10lgP | ≥ 15 |
| Proteins unique peptides | ≥ 1 |
| De novo score(%) | ≥ 50% |

**Table 3.**
Statistics of filtered result.

|  |  |
| --- | --- |
| FDR (Peptide-Spectrum Matches) | 0.6% |
| FDR (Peptide Sequences) | 1.0% |
| FDR (Protein Group) | 1.0% |
| De Novo Only Spectra | 13444 |

**Table 4.**
PTM profile.

| Name | ∆Mass | Position | # peptides | # proteins | # modification  sites || Carbamidomethylation | 57.02 | C | 30 | 30 | 86 (86) |
| Oxidation (M) | 15.99 | M | 4 | 4 | 2 (4) |
| Acetylation (Protein N-term) | 42.01 | Protein N-term | 3 | 3 | 1 (3) |

# 3. Experiment Control

**Figure 6.**
Precursor mass error of peptide-spectrum matches (PSM) in filtered result.  **(a)**
Distribution of precursor mass error in ppm;  **(b)**
Scatterplot of precursor m/z versus precursor mass error in ppm.

**(a)**

**(b)**

**Table 5.**
Number of identified peptides in each sample by the number of missed cleavages.

|  |  |  |  |  |  |
| --- | --- | --- | --- | --- | --- |
| Missed Cleavages | 0 | 1 | 2 | 3 | 4+ |
| Sample 5 | 76 | 57 | 7 | 0 | 0 |

# 4. Other Information

**Table 6.**
Search parameters.

Search Engine Name: PEAKS  
Parent Mass Error Tolerance: 20.0 ppm  
Fragment Mass Error Tolerance: 0.05 Da  
Precursor Mass Search Type: monoisotopic  
Enzyme: Trypsin  
Max Missed Cleavages: 2  
Digest Mode: Specific  
Peptide Length Range: 6 - 55  
Fixed Modifications:   
  Carbamidomethylation (+57.02)  
Variable Modifications:   
  Acetylation (Protein N-term) (+42.01)  
  Oxidation (M) (+15.99)  
Max Variable PTM Per Peptide: 2  
Database: Velella\_singlebestonly\_minlen100\_2STEP  
Taxon: all species  
Searched Entries: 194010  
Deep Learning Boost: Yes  
FDR Estimation: Enabled
