# Supplementary material for: Proteomic Analysis and Biochemical Characterization of the Nematocyst Extract of the Hydrozoan Velella velella
Source: Mar Drugs. 2024 Oct 12;22(10):468. doi: 10.3390/md22100468 (PMC11509761; doi:10.3390/md22100468)
Supplement: Supplementary file 1 [file marinedrugs-22-00468-s001.zip › Supplementary File S1.zip!/Sample_4_Vellela/db.peptides.html]

peptide list


Peptide List

  

| Peptide | -10lgP | Mass | Length | ppm | m/z | RT | 1/k0 Start | 1/k0 End | Precursor ID | Area Sample 6 | #Feature | #Feature Sample 6 | Accession | PTM | AScore | Found By |
| --- | --- | --- | --- | --- | --- | --- | --- | --- | --- | --- | --- | --- | --- | --- | --- | --- |
| LIEDTEYTAHEGAK | 47.51 | 1575.7416 | 14 | -1.61 | 788.8734 | 12.79 | 1.0773 | 1.0845 | 31281 | 0 | 0 | 0 | TRINITY\_DN12632\_c1\_g1\_i1.p1 |  |  | DB Search |
| LLAVTVGNNYNLDTLEK | 47.51 | 1875.9941 | 17 | -4.15 | 938.9963 | 18.69 | 1.147 | 1.1542 | 67001 | 0 | 0 | 0 | TRINITY\_DN77\_c1\_g1\_i1.p1 |  |  | DB Search |
| GSC(+57.02)EC(+57.02)GTC(+57.02)VC(+57.02)R | 47.49 | 1344.4679 | 11 | -0.08 | 673.2382 | 10.53 | 0.9323 | 0.9396 | 17626 | 7.83e2 | 1 | 1 | TRINITY\_DN1535\_c0\_g1\_i1.p1 | Carbamidomethylation, Carbamidomethylation, Carbamidomethylation, Carbamidomethylation |  | DB Search |
| EGSTPC(+57.02)NTQAC(+57.02)GSGLSEER | 47.45 | 2038.832 | 19 | 1.22 | 1020.42 | 12.08 | 1.1382 | 1.1454 | 27081 | 0 | 0 | 0 | TRINITY\_DN5397\_c0\_g1\_i1.p1 | Carbamidomethylation, Carbamidomethylation |  | DB Search |
| LKLQMFDWDR | 47.4 | 1350.6754 | 10 | 2.3 | 676.3436 | 19.46 | 0.9727 | 0.98 | 71904 | 6.52e3 | 1 | 1 | TRINITY\_DN140702\_c0\_g1\_i1.p1 |  |  | DB Search |
| DPIC(+57.02)HC(+57.02)DTSK | 47.35 | 1231.4961 | 10 | 6.31 | 411.5068 | 9.96 | 0.7351 | 0.7424 | 14092 | 1.65e3 | 2 | 2 | TRINITY\_DN319\_c2\_g1\_i3.p1:TRINITY\_DN319\_c2\_g1\_i1.p1:TRINITY\_DN319\_c2\_g1\_i2.p1 | Carbamidomethylation, Carbamidomethylation |  | DB Search |
| SFASSLLDHFRVEPAK | 47.34 | 1802.9315 | 16 | 2.06 | 601.983 | 19.97 | 0.9057 | 0.9129 | 75829 | 0 | 0 | 0 | TRINITY\_DN77\_c1\_g1\_i1.p1 |  |  | DB Search |
| ASISSNDFKDFLR | 47.32 | 1498.7416 | 13 | 1.94 | 750.3762 | 18.00 | 1.0881 | 1.0953 | 62229 | 0 | 0 | 0 | TRINITY\_DN11635\_c0\_g1\_i1.p1 |  |  | DB Search |
| YVAIYPSDHLTVNR | 47.28 | 1646.8417 | 14 | 3.15 | 824.4271 | 15.69 | 1.1053 | 1.1126 | 48025 | 9.15e2 | 1 | 1 | TRINITY\_DN982\_c0\_g1\_i1.p1:TRINITY\_DN982\_c0\_g1\_i3.p1 |  |  | DB Search |
| YTSC(+57.02)QSVTVVASDR | 47.27 | 1571.725 | 14 | 6.1 | 786.8711 | 13.47 | 1.05 | 1.0572 | 35313 | 0 | 0 | 0 | TRINITY\_DN982\_c0\_g1\_i1.p1:TRINITY\_DN982\_c0\_g1\_i3.p1 | Carbamidomethylation |  | DB Search |
| LIYENQNTIHHVVR | 47.27 | 1734.9165 | 14 | 4.54 | 579.3129 | 13.33 | 0.8445 | 0.8518 | 34380 | 7.41e2 | 1 | 1 | TRINITY\_DN26249\_c0\_g1\_i1.p1 |  |  | DB Search |
| DNAGTSSSGTGPR | 47.27 | 1205.5272 | 13 | 3.05 | 603.7701 | 9.17 | 0.8587 | 0.866 | 10272 | 4.31e2 | 1 | 1 | TRINITY\_DN4781\_c0\_g1\_i3.p3 |  |  | DB Search |
| GGAGDM(+15.99)GAEGPR | 47.26 | 1089.4509 | 12 | 1.17 | 545.731 | 9.59 | 0.8323 | 0.8396 | 12176 | 6.75e2 | 1 | 1 | TRINITY\_DN113\_c2\_g1\_i1.p1 | Oxidation (M) | M6:Oxidation (M):1000 | DB Search |
| SVNEGDVQDKKR | 47.26 | 1373.6898 | 12 | 8.6 | 458.9058 | 9.38 | 0.7392 | 0.7465 | 11053 | 6.7e2 | 1 | 1 | TRINITY\_DN1320\_c0\_g2\_i1.p1 |  |  | DB Search |
| GVGIISEGNETVEDIAQR | 47.26 | 1885.9381 | 18 | -1.95 | 943.9703 | 17.89 | 1.1414 | 1.1486 | 61431 | 7.95e2 | 1 | 1 | TRINITY\_DN262\_c0\_g1\_i1.p1 |  |  | DB Search |
| EGPTGPQGETGR | 47.26 | 1184.5421 | 12 | 0.95 | 593.2763 | 10.36 | 0.8798 | 0.887 | 16389 | 1.9e3 | 1 | 1 | TRINITY\_DN984\_c0\_g1\_i2.p1:TRINITY\_DN984\_c0\_g1\_i1.p1 |  |  | DB Search |
| VIM(+15.99)VTGDHPITAK | 47.26 | 1396.7384 | 13 | 1.06 | 699.3741 | 13.28 | 1.0202 | 1.0275 | 34024 | 1.25e3 | 1 | 1 | TRINITY\_DN262\_c0\_g1\_i1.p1 | Oxidation (M) | M3:Oxidation (M):1000 | DB Search |
| LVGQSIISYLQK | 47.26 | 1347.7761 | 12 | 10.68 | 674.8996 | 19.69 | 0.9783 | 0.9856 | 73873 | 2.42e2 | 1 | 1 | TRINITY\_DN187147\_c0\_g1\_i1.p1 |  |  | DB Search |
| GYVIWQEVVDNQVK | 47.25 | 1675.8569 | 14 | -0.86 | 838.9313 | 19.92 | 1.1314 | 1.1386 | 75433 | 1.33e3 | 1 | 1 | TRINITY\_DN3156\_c0\_g1\_i1.p1:TRINITY\_DN3156\_c0\_g1\_i2.p1 |  |  | DB Search |
| AFLIEEQKLVVR | 47.25 | 1443.8448 | 12 | -1.32 | 722.9256 | 17.51 | 1.0452 | 1.0524 | 58803 | 0 | 0 | 0 | TRINITY\_DN79126\_c0\_g1\_i1.p1 |  |  | DB Search |
| IGLGNIRDPSISLTR | 47.25 | 1610.9104 | 15 | 2.96 | 537.9766 | 17.36 | 0.797 | 0.8043 | 57658 | 1.69e3 | 1 | 1 | TRINITY\_DN11522\_c0\_g1\_i1.p1:TRINITY\_DN11522\_c0\_g1\_i2.p1:TRINITY\_DN11522\_c0\_g1\_i4.p1:TRINITY\_DN11522\_c0\_g1\_i3.p1 |  |  | DB Search |
| DM(+15.99)GVNVISVGAGHTINHR | 47.25 | 1891.9323 | 18 | 13.94 | 946.9824 | 14.29 | 1.2292 | 1.2364 | 40084 | 0 | 0 | 0 | TRINITY\_DN138\_c0\_g1\_i1.p1 | Oxidation (M) | M2:Oxidation (M):1000 | DB Search |
| KVIVDQLVTLATR | 47.25 | 1454.8821 | 13 | 0.73 | 728.4456 | 18.43 | 1.0283 | 1.0355 | 65218 | 4.09e2 | 1 | 1 | TRINITY\_DN1386\_c0\_g1\_i1.p1 |  |  | DB Search |
| GLQGQQGEQGSTGVQGSVGGR | 47.24 | 1985.9514 | 21 | 2.4 | 993.981 | 12.07 | 1.133 | 1.1402 | 26902 | 2.07e3 | 1 | 1 | TRINITY\_DN1030\_c0\_g1\_i1.p1:TRINITY\_DN1030\_c0\_g1\_i2.p1 |  |  | DB Search |
| NDVNNPPQAR | 47.24 | 1123.537 | 10 | 0.77 | 562.7737 | 9.95 | 0.866 | 0.8733 | 13858 | 3.59e3 | 2 | 2 | TRINITY\_DN477\_c0\_g1\_i3.p1:TRINITY\_DN477\_c0\_g1\_i7.p1:TRINITY\_DN477\_c0\_g1\_i6.p1:TRINITY\_DN477\_c0\_g1\_i5.p1:TRINITY\_DN477\_c0\_g1\_i8.p2 |  |  | DB Search |
| FC(+57.02)NNPAPSNGGR | 47.24 | 1289.5571 | 12 | 0.71 | 645.7834 | 10.87 | 0.9416 | 0.9489 | 19820 | 2.76e2 | 1 | 1 | TRINITY\_DN4595\_c0\_g1\_i10.p1:TRINITY\_DN4595\_c0\_g1\_i4.p1 | Carbamidomethylation |  | DB Search |
| TPGEC(+57.02)C(+57.02)PQC(+57.02)K | 47.23 | 1235.4733 | 10 | 1.97 | 618.7424 | 9.99 | 0.8939 | 0.9012 | 14287 | 6.05e2 | 1 | 1 | TRINITY\_DN1369\_c0\_g1\_i1.p1 | Carbamidomethylation, Carbamidomethylation, Carbamidomethylation |  | DB Search |
| NYNNDDEPGAK | 47.22 | 1235.5055 | 11 | 6.2 | 618.7611 | 10.14 | 0.8919 | 0.8992 | 15177 | 1.07e3 | 1 | 1 | TRINITY\_DN199\_c0\_g1\_i1.p1 |  |  | DB Search |
| SC(+57.02)TNPAPANGGK | 47.22 | 1172.5244 | 12 | 1.68 | 587.2679 | 9.40 | 0.8429 | 0.8502 | 11161 | 2.16e2 | 1 | 1 | TRINITY\_DN495\_c0\_g1\_i2.p1:TRINITY\_DN3545\_c0\_g1\_i2.p1:TRINITY\_DN3545\_c0\_g1\_i1.p1:TRINITY\_DN495\_c0\_g1\_i3.p1 | Carbamidomethylation |  | DB Search |
| VGNWRDETLSC(+57.02)K | 47.22 | 1463.6827 | 12 | 6.62 | 488.9026 | 13.82 | 0.7624 | 0.7697 | 37393 | 0 | 0 | 0 | TRINITY\_DN6390\_c0\_g1\_i1.p1 | Carbamidomethylation |  | DB Search |
| LSTDYC(+57.02)QLGLFK | 47.21 | 1443.7068 | 12 | -0.18 | 722.8574 | 18.83 | 1.0057 | 1.013 | 67864 | 8.93e2 | 1 | 1 | TRINITY\_DN875\_c0\_g1\_i1.p1:TRINITY\_DN875\_c0\_g1\_i2.p1:TRINITY\_DN875\_c0\_g1\_i3.p1 | Carbamidomethylation |  | DB Search |
| LLVHNWEFLGK | 47.21 | 1354.7397 | 11 | 5.1 | 452.5875 | 18.51 | 0.7628 | 0.7701 | 65747 | 6.02e2 | 1 | 1 | TRINITY\_DN2390\_c0\_g1\_i1.p1:TRINITY\_DN2390\_c0\_g1\_i2.p1 |  |  | DB Search |
| AESIALVTEFTDEAAPK | 47.21 | 1790.8938 | 17 | -2.34 | 896.4481 | 19.93 | 1.1214 | 1.1286 | 75448 | 1.02e3 | 1 | 1 | TRINITY\_DN79\_c0\_g1\_i1.p1 |  |  | DB Search |
| GNQGEQGPAGPVGPK | 47.2 | 1391.6793 | 15 | 3.52 | 696.8463 | 11.46 | 0.9461 | 0.9533 | 23184 | 1.15e3 | 1 | 1 | TRINITY\_DN5987\_c0\_g1\_i2.p1:TRINITY\_DN5987\_c0\_g1\_i4.p1:TRINITY\_DN5987\_c0\_g1\_i3.p2 |  |  | DB Search |
| LGIHEDTQNR | 47.2 | 1181.5789 | 10 | 7.67 | 591.7986 | 11.15 | 0.9012 | 0.9085 | 21461 | 0 | 0 | 0 | TRINITY\_DN141338\_c2\_g1\_i1.p1 |  |  | DB Search |
| AFVAIGDYGGHVGLGVK | 47.2 | 1658.8779 | 17 | 4.43 | 830.4463 | 17.74 | 1.163 | 1.1702 | 60353 | 7.45e2 | 1 | 1 | TRINITY\_DN15584\_c0\_g1\_i1.p1 |  |  | DB Search |
| YLFDLESNC(+57.02)DK | 47.2 | 1402.6074 | 11 | -1.48 | 702.3069 | 18.41 | 0.9844 | 0.9916 | 65101 | 0 | 0 | 0 | TRINITY\_DN6390\_c0\_g1\_i1.p1 | Carbamidomethylation |  | DB Search |
| SYELPDGQVITIGNER | 47.19 | 1789.8846 | 16 | -1.46 | 895.9443 | 18.64 | 1.1122 | 1.1194 | 66524 | 6.05e2 | 1 | 1 | TRINITY\_DN161\_c0\_g1\_i1.p1:TRINITY\_DN161\_c0\_g1\_i4.p1:TRINITY\_DN161\_c0\_g1\_i3.p1:TRINITY\_DN161\_c0\_g1\_i2.p1 |  |  | DB Search |
| ILQC(+57.02)NQYANVEEAR | 47.19 | 1706.8046 | 14 | 1.57 | 854.4072 | 14.32 | 1.0821 | 1.0893 | 40295 | 6.26e3 | 1 | 1 | TRINITY\_DN477\_c0\_g1\_i8.p1:TRINITY\_DN477\_c0\_g1\_i7.p1 | Carbamidomethylation |  | DB Search |
| AVQIHPFNDVSVGDPK | 47.19 | 1721.8737 | 16 | 3.05 | 861.9429 | 15.94 | 1.1162 | 1.1234 | 49403 | 4.56e3 | 1 | 1 | TRINITY\_DN1071\_c0\_g1\_i2.p1:TRINITY\_DN1071\_c0\_g1\_i3.p1:TRINITY\_DN1071\_c0\_g1\_i1.p1:TRINITY\_DN1071\_c0\_g1\_i4.p1 |  |  | DB Search |
| INNNQFSVVLIK | 47.19 | 1387.7823 | 12 | 4.05 | 694.8982 | 18.30 | 0.9928 | 1.0001 | 64280 | 6.59e2 | 1 | 1 | TRINITY\_DN982\_c0\_g1\_i1.p1:TRINITY\_DN982\_c0\_g1\_i2.p1 |  |  | DB Search |
| AEGPTSTGVTEVIVR | 47.19 | 1514.7939 | 15 | -18.54 | 758.3869 | 15.55 | 1.0102 | 1.0174 | 47407 | 0 | 0 | 0 | TRINITY\_DN982\_c0\_g1\_i3.p1 |  |  | DB Search |
| AVFPSIVGRPR | 47.19 | 1197.6982 | 11 | 2.39 | 599.8552 | 15.88 | 0.9444 | 0.9517 | 49107 | 3.41e4 | 1 | 1 | TRINITY\_DN161\_c0\_g1\_i1.p1:TRINITY\_DN161\_c0\_g1\_i3.p1:TRINITY\_DN161\_c0\_g1\_i2.p1 |  |  | DB Search |
| AVAEEFISVDPYVR | 47.19 | 1593.8038 | 14 | -4.8 | 797.9019 | 19.09 | 1.1069 | 1.1142 | 69857 | 2.96e3 | 1 | 1 | TRINITY\_DN598\_c1\_g1\_i1.p1 |  |  | DB Search |
| DMGVNVISVGAGHTINHR | 47.18 | 1875.9374 | 18 | 0.83 | 938.9726 | 15.42 | 1.2483 | 1.2555 | 46480 | 8.69e2 | 2 | 2 | TRINITY\_DN138\_c0\_g1\_i1.p1 |  |  | DB Search |
| SESESKELIDKLK | 47.18 | 1504.7985 | 13 | -0.34 | 502.6044 | 14.64 | 0.7742 | 0.7815 | 42112 | 2.93e2 | 1 | 1 | TRINITY\_DN5500\_c0\_g1\_i2.p1:TRINITY\_DN5500\_c0\_g1\_i1.p1 |  |  | DB Search |
| LVQNTPHNSIVTK | 47.18 | 1449.7939 | 13 | -3.54 | 725.8985 | 11.74 | 1.0568 | 1.064 | 24940 | 0 | 0 | 0 | TRINITY\_DN982\_c0\_g1\_i1.p1:TRINITY\_DN982\_c0\_g1\_i3.p1 |  |  | DB Search |
| TQAVLDRLNIEKR | 47.18 | 1554.8842 | 13 | 1.35 | 519.3004 | 14.70 | 0.8019 | 0.8092 | 42401 | 1.64e3 | 1 | 1 | TRINITY\_DN477\_c0\_g1\_i9.p1 |  |  | DB Search |
| GC(+57.02)STC(+57.02)GC(+57.02)NGAGTNNGVK | 47.18 | 1712.6665 | 17 | 14.87 | 857.3495 | 9.81 | 1.0411 | 1.0484 | 13314 | 0 | 0 | 0 | TRINITY\_DN1616\_c0\_g1\_i3.p1:TRINITY\_DN1616\_c0\_g1\_i6.p1:TRINITY\_DN1616\_c0\_g1\_i2.p1:TRINITY\_DN1616\_c0\_g1\_i7.p1 | Carbamidomethylation, Carbamidomethylation, Carbamidomethylation |  | DB Search |
| AKIEALHC(+57.02)LSENIVK | 47.18 | 1723.9291 | 15 | 4.04 | 575.6501 | 15.76 | 0.8226 | 0.8299 | 48472 | 1e3 | 2 | 2 | TRINITY\_DN3078\_c0\_g1\_i1.p1 | Carbamidomethylation |  | DB Search |
| DLPQEKLDDILK | 47.18 | 1425.7715 | 12 | -2.46 | 713.8881 | 17.82 | 1.0367 | 1.0439 | 60992 | 0 | 0 | 0 | TRINITY\_DN262\_c0\_g1\_i1.p1 |  |  | DB Search |
| DAPIYGLQWHPEK | 47.18 | 1552.7673 | 13 | 1.36 | 777.3886 | 17.41 | 1.0741 | 1.0813 | 58076 | 0 | 0 | 0 | TRINITY\_DN247\_c0\_g1\_i1.p1 |  |  | DB Search |
| YGGQC(+57.02)AC(+57.02)KPGIGGR | 47.17 | 1479.671 | 14 | -0.82 | 494.2284 | 11.19 | 0.7897 | 0.797 | 21658 | 6.62e2 | 1 | 1 | TRINITY\_DN38399\_c0\_g1\_i1.p1 | Carbamidomethylation, Carbamidomethylation |  | DB Search |
| VSQTC(+57.02)AEHVFR | 47.17 | 1332.6245 | 11 | 3.6 | 667.319 | 13.74 | 0.984 | 0.9912 | 36845 | 6.47e2 | 1 | 1 | TRINITY\_DN3078\_c0\_g1\_i1.p1 | Carbamidomethylation |  | DB Search |
| LMLLLFQHESM(+15.99)K | 47.16 | 1504.7782 | 12 | -4.04 | 502.5958 | 19.47 | 0.8116 | 0.8189 | 72453 | 0 | 0 | 0 | TRINITY\_DN71276\_c0\_g1\_i1.p1 | Oxidation (M) | M11:Oxidation (M):90.01 | DB Search |
| LLLGDFNGDKR | 47.16 | 1246.667 | 11 | 0.01 | 624.338 | 16.23 | 0.9844 | 0.9916 | 51088 | 9.22e2 | 1 | 1 | TRINITY\_DN413\_c0\_g1\_i1.p1:TRINITY\_DN413\_c0\_g1\_i4.p1:TRINITY\_DN413\_c0\_g1\_i2.p1:TRINITY\_DN413\_c0\_g1\_i3.p1 |  |  | DB Search |
| LQGWWQSVNGR | 47.16 | 1329.6578 | 11 | -1.01 | 665.8326 | 18.21 | 0.9449 | 0.9521 | 63598 | 6.83e2 | 1 | 1 | TRINITY\_DN15274\_c0\_g1\_i1.p1 |  |  | DB Search |
| FANQKLENVSLVLR | 47.16 | 1629.9202 | 14 | 9.03 | 544.3165 | 17.19 | 0.8234 | 0.8307 | 56634 | 1.4e2 | 1 | 1 | TRINITY\_DN1653\_c0\_g1\_i3.p1:TRINITY\_DN1653\_c0\_g1\_i1.p1 |  |  | DB Search |
| AVAC(+57.02)NSGPC(+57.02)LAGC(+57.02)PR | 47.15 | 1588.6908 | 15 | 1.52 | 795.3504 | 13.09 | 1.0178 | 1.025 | 33019 | 7.5e2 | 1 | 1 | TRINITY\_DN4815\_c0\_g1\_i2.p1:TRINITY\_DN4815\_c0\_g1\_i1.p1 | Carbamidomethylation, Carbamidomethylation, Carbamidomethylation |  | DB Search |
| AVFC(+57.02)EHQGVK | 47.14 | 1173.5601 | 10 | 0.43 | 587.785 | 11.65 | 0.8984 | 0.9057 | 24435 | 0 | 0 | 0 | TRINITY\_DN875\_c0\_g1\_i1.p1:TRINITY\_DN875\_c0\_g1\_i2.p1:TRINITY\_DN875\_c0\_g1\_i3.p1 | Carbamidomethylation |  | DB Search |
| LALHVLNNEDIIGHR | 47.14 | 1712.9321 | 15 | 3.94 | 857.473 | 16.50 | 1.1362 | 1.1434 | 52619 | 5.01e3 | 2 | 2 | TRINITY\_DN140702\_c0\_g1\_i1.p1 |  |  | DB Search |
| LLEINVYHPLIQELQK | 47.14 | 1949.0985 | 16 | 2.04 | 650.7053 | 19.77 | 0.9808 | 0.988 | 74434 | 4.48e2 | 1 | 1 | TRINITY\_DN5633\_c0\_g1\_i1.p1 |  |  | DB Search |
| AGDIAILESQQFNAVGGK | 47.14 | 1816.9319 | 18 | -16.1 | 909.4546 | 18.91 | 1.1845 | 1.1917 | 68601 | 1.41e3 | 1 | 1 | TRINITY\_DN1936\_c0\_g1\_i1.p1:TRINITY\_DN1936\_c0\_g1\_i4.p1 |  |  | DB Search |
| YYVTIIDAPGHR | 47.14 | 1403.7197 | 12 | 0.82 | 702.8646 | 16.06 | 1.0403 | 1.0476 | 50166 | 5.77e2 | 1 | 1 | TRINITY\_DN64\_c0\_g1\_i1.p1 |  |  | DB Search |
| LINLFEAVSKK | 47.13 | 1260.7441 | 11 | -0.85 | 631.376 | 19.03 | 0.9832 | 0.9904 | 69384 | 1.61e3 | 2 | 2 | TRINITY\_DN1653\_c0\_g1\_i3.p1:TRINITY\_DN1653\_c0\_g1\_i1.p1 |  |  | DB Search |
| AGAIAEEVISSIR | 47.12 | 1314.7142 | 13 | -2.53 | 658.3599 | 19.95 | 0.9715 | 0.9787 | 75736 | 0 | 0 | 0 | TRINITY\_DN7102\_c0\_g1\_i1.p1 |  |  | DB Search |
| IC(+57.02)HLLGIQVTDFTK | 47.12 | 1643.8705 | 14 | -3.6 | 548.9597 | 18.60 | 0.8185 | 0.8258 | 66312 | 1.88e3 | 1 | 1 | TRINITY\_DN428\_c0\_g1\_i1.p1 | Carbamidomethylation |  | DB Search |
| IAAVIINKPDVLASR | 47.12 | 1578.9457 | 15 | 3.53 | 790.4794 | 16.15 | 1.1021 | 1.1094 | 50690 | 2.9e2 | 1 | 1 | TRINITY\_DN123\_c0\_g1\_i1.p1 |  |  | DB Search |
| DTFSADLNHVIAQSR | 47.12 | 1672.8169 | 15 | 6.59 | 837.4175 | 18.41 | 1.1262 | 1.1334 | 65092 | 0 | 0 | 0 | TRINITY\_DN55094\_c0\_g1\_i1.p1 |  |  | DB Search |
| GSC(+57.02)SC(+57.02)YGHAEQC(+57.02)IPTK | 47.11 | 1853.7495 | 16 | 8.42 | 927.8857 | 12.41 | 1.1813 | 1.1885 | 29006 | 8.23e2 | 2 | 2 | TRINITY\_DN15274\_c0\_g1\_i1.p1 | Carbamidomethylation, Carbamidomethylation, Carbamidomethylation |  | DB Search |
| AYGQDLGQPGSC(+57.02)LKR | 47.11 | 1648.7991 | 15 | 3.54 | 550.6065 | 13.51 | 0.8348 | 0.8421 | 35593 | 7.17e2 | 1 | 1 | TRINITY\_DN691\_c0\_g1\_i2.p1:TRINITY\_DN691\_c0\_g1\_i1.p1 | Carbamidomethylation |  | DB Search |
| LIQYIENKPGLTR | 47.09 | 1543.8722 | 13 | 0.19 | 772.9401 | 14.92 | 1.0769 | 1.0841 | 43527 | 6.02e3 | 1 | 1 | TRINITY\_DN2028\_c0\_g1\_i1.p1 |  |  | DB Search |
| HQNIVC(+57.02)GGPER | 47.07 | 1265.5935 | 11 | 3.24 | 633.8033 | 11.10 | 0.9081 | 0.9154 | 21116 | 0 | 0 | 0 | TRINITY\_DN1535\_c0\_g1\_i1.p1 | Carbamidomethylation |  | DB Search |
| EGFYVHEALAAGIVK | 47.06 | 1602.8406 | 15 | -1.99 | 802.4224 | 17.69 | 1.0837 | 1.0909 | 59893 | 9.17e2 | 1 | 1 | TRINITY\_DN602\_c0\_g1\_i4.p1:TRINITY\_DN602\_c0\_g1\_i3.p1:TRINITY\_DN602\_c0\_g1\_i1.p1 |  |  | DB Search |
| IEALHC(+57.02)LSENIVK | 47.04 | 1524.797 | 13 | 2.71 | 763.4045 | 15.75 | 1.0504 | 1.0576 | 48457 | 7.46e2 | 1 | 1 | TRINITY\_DN3078\_c0\_g1\_i1.p1 | Carbamidomethylation |  | DB Search |
| IGIVNEFYVR | 47.03 | 1208.6553 | 10 | 2.52 | 605.3338 | 18.83 | 0.9444 | 0.9517 | 67867 | 1.06e3 | 1 | 1 | TRINITY\_DN1653\_c0\_g1\_i3.p1:TRINITY\_DN1653\_c0\_g1\_i1.p1 |  |  | DB Search |
| NYNNDDEPGAKK | 47.02 | 1363.6003 | 12 | 2.39 | 455.5398 | 9.57 | 0.7485 | 0.7559 | 12066 | 1.03e3 | 1 | 1 | TRINITY\_DN199\_c0\_g1\_i1.p1 |  |  | DB Search |
| AQLTLTIFLHK | 46.99 | 1283.7601 | 11 | 3.95 | 428.9271 | 19.57 | 0.7489 | 0.7563 | 73093 | 6.45e2 | 1 | 1 | TRINITY\_DN982\_c0\_g1\_i1.p1:TRINITY\_DN982\_c0\_g1\_i3.p1:TRINITY\_DN982\_c0\_g1\_i2.p1 |  |  | DB Search |
| A(+42.01)DPSSLETKEK | 46.98 | 1245.6089 | 11 | 15.01 | 623.8183 | 12.69 | 0.9449 | 0.9521 | 30683 | 0 | 0 | 0 | TRINITY\_DN262\_c0\_g1\_i1.p1 | Acetylation (Protein N-term) | A1:Acetylation (Protein N-term):1000 | DB Search |
| VDNSSLTGESEPQSR | 46.98 | 1604.7278 | 15 | 5.37 | 803.3719 | 11.95 | 1.0198 | 1.0271 | 26200 | 0 | 0 | 0 | TRINITY\_DN262\_c0\_g1\_i1.p1 |  |  | DB Search |
| S(+42.01)SGEIESLRQEAENLR | 46.98 | 1858.9021 | 16 | -0.12 | 930.4541 | 18.84 | 1.1961 | 1.2033 | 67975 | 1.99e3 | 1 | 1 | TRINITY\_DN2472\_c0\_g1\_i1.p1 | Acetylation (Protein N-term) | S1:Acetylation (Protein N-term):1000 | DB Search |
| KVSQTC(+57.02)AEHVFR | 46.98 | 1460.7194 | 12 | 2.73 | 487.9129 | 11.99 | 0.801 | 0.8084 | 26442 | 8.32e2 | 1 | 1 | TRINITY\_DN3078\_c0\_g1\_i1.p1 | Carbamidomethylation |  | DB Search |
| AQAINVQLQEIQR | 46.95 | 1509.8263 | 13 | 3.56 | 755.9198 | 16.14 | 1.0279 | 1.0351 | 50668 | 0 | 0 | 0 | TRINITY\_DN1280\_c0\_g1\_i1.p1 |  |  | DB Search |
| ASFVSDIDRLR | 46.94 | 1277.6727 | 11 | 1.29 | 639.8417 | 17.08 | 0.9844 | 0.9916 | 56001 | 9.19e2 | 1 | 1 | TRINITY\_DN598\_c1\_g1\_i1.p1 |  |  | DB Search |
| APTHILNPANKR | 46.93 | 1330.7469 | 12 | 2.71 | 444.5888 | 11.17 | 0.7559 | 0.7632 | 21058 | 5.5e3 | 2 | 2 | TRINITY\_DN1058\_c0\_g1\_i4.p1:TRINITY\_DN1058\_c0\_g1\_i1.p1:TRINITY\_DN1058\_c0\_g1\_i3.p1:TRINITY\_DN1058\_c0\_g1\_i2.p2 |  |  | DB Search |
| DANSM(+15.99)PTQSR | 46.92 | 1121.4772 | 10 | -7.45 | 561.7392 | 8.81 | 0.8234 | 0.8307 | 9288 | 0 | 0 | 0 | TRINITY\_DN477\_c0\_g1\_i9.p1 | Oxidation (M) | M5:Oxidation (M):1000 | DB Search |
| FVFPFK | 46.92 | 783.4319 | 6 | -0.19 | 392.7214 | 19.66 | 0.7722 | 0.7795 | 73690 | 7.97e2 | 1 | 1 | TRINITY\_DN140702\_c0\_g1\_i1.p1 |  |  | DB Search |
| LGPIPLWK | 46.9 | 922.564 | 8 | 3.89 | 462.289 | 19.42 | 0.7909 | 0.7982 | 71845 | 3.48e2 | 1 | 1 | TRINITY\_DN36217\_c0\_g1\_i1.p1 |  |  | DB Search |
| GGGGHPER | 46.89 | 765.3517 | 8 | -11.05 | 383.6772 | 9.02 | 0.7122 | 0.7196 | 9725 | 2.35e2 | 1 | 1 | TRINITY\_DN109148\_c0\_g1\_i1.p2 |  |  | DB Search |
| YLADVVLTLDHTK | 46.89 | 1486.8031 | 13 | 1.62 | 496.6069 | 18.76 | 0.8343 | 0.8416 | 67561 | 0 | 0 | 0 | TRINITY\_DN22240\_c0\_g1\_i1.p1 |  |  | DB Search |
| IYIDGVFIR | 46.89 | 1094.6124 | 9 | 1.62 | 548.312 | 18.78 | 0.8895 | 0.8968 | 67582 | 8.72e2 | 1 | 1 | TRINITY\_DN5500\_c0\_g1\_i2.p1:TRINITY\_DN5500\_c0\_g1\_i1.p1 |  |  | DB Search |
| AINIHDLVGIDTKK | 46.89 | 1535.8671 | 14 | 1.14 | 512.9613 | 16.65 | 0.7913 | 0.7986 | 53503 | 2.9e3 | 1 | 1 | TRINITY\_DN61\_c0\_g1\_i1.p1 |  |  | DB Search |
| LDLAGRDLTDYLMK | 46.87 | 1622.8337 | 14 | -1.16 | 541.9489 | 19.39 | 0.7966 | 0.8039 | 71871 | 4.27e2 | 1 | 1 | TRINITY\_DN161\_c0\_g1\_i1.p1:TRINITY\_DN161\_c0\_g1\_i4.p1:TRINITY\_DN161\_c0\_g1\_i3.p1:TRINITY\_DN161\_c0\_g1\_i2.p1 |  |  | DB Search |
| DKNILVLTLGVGPQVNR | 46.87 | 1835.0629 | 17 | 2.5 | 612.6937 | 19.53 | 0.8408 | 0.8481 | 72572 | 1.06e4 | 3 | 3 | TRINITY\_DN477\_c0\_g1\_i8.p1:TRINITY\_DN477\_c0\_g1\_i7.p1 |  |  | DB Search |
| SIEAIYLGEFGTC(+57.02)K | 46.86 | 1586.765 | 14 | 5.15 | 794.3904 | 19.64 | 1.1029 | 1.1102 | 73637 | 0 | 0 | 0 | TRINITY\_DN11248\_c0\_g1\_i1.p1:TRINITY\_DN11248\_c0\_g1\_i3.p1 | Carbamidomethylation |  | DB Search |
| EITLNYHDTVVHMK | 46.86 | 1698.84 | 14 | -1.84 | 850.4219 | 15.44 | 1.111 | 1.1182 | 46709 | 5.77e2 | 1 | 1 | TRINITY\_DN1369\_c0\_g1\_i1.p1 |  |  | DB Search |
| SYFEHSFVDLHR | 46.86 | 1535.7157 | 12 | 4.7 | 512.9127 | 16.16 | 0.7673 | 0.7746 | 50725 | 1.42e3 | 1 | 1 | TRINITY\_DN59572\_c0\_g1\_i1.p1 |  |  | DB Search |
| TQAVLDRLNIEK | 46.84 | 1398.7831 | 12 | 4.58 | 700.3989 | 15.49 | 1.0073 | 1.0146 | 46990 | 2.49e3 | 1 | 1 | TRINITY\_DN477\_c0\_g1\_i9.p1 |  |  | DB Search |
| RLDPSEQLQHLSSLR | 46.82 | 1777.9435 | 15 | 3.26 | 593.6544 | 16.59 | 0.9077 | 0.915 | 53199 | 0 | 0 | 0 | TRINITY\_DN165469\_c0\_g1\_i1.p1 |  |  | DB Search |
| ADIEDVKDLLK | 46.82 | 1257.6816 | 11 | 3.16 | 629.8473 | 17.70 | 0.99 | 0.9973 | 60061 | 2.93e2 | 1 | 1 | TRINITY\_DN15274\_c0\_g1\_i1.p1 |  |  | DB Search |
| FYSFEVQVIDTNNGK | 46.82 | 1759.8417 | 15 | 2.16 | 880.9261 | 18.83 | 1.1005 | 1.1078 | 67916 | 4.09e2 | 1 | 1 | TRINITY\_DN982\_c0\_g1\_i3.p1 |  |  | DB Search |
| DANAAVVHGSHLK | 46.79 | 1317.679 | 13 | 0.06 | 659.8439 | 10.92 | 1.0158 | 1.023 | 20063 | 0 | 0 | 0 | TRINITY\_DN262\_c0\_g1\_i1.p1 |  |  | DB Search |
| ALLLASSNAFDTSNGAR | 46.77 | 1706.8588 | 17 | 2.69 | 854.4352 | 17.79 | 1.0977 | 1.1049 | 60767 | 2.78e3 | 2 | 2 | TRINITY\_DN244\_c0\_g1\_i1.p1 |  |  | DB Search |
| YLTTAVITNKR | 46.75 | 1278.7296 | 11 | -5.61 | 640.3657 | 13.99 | 1.0005 | 1.0077 | 38385 | 0 | 0 | 0 | TRINITY\_DN923\_c0\_g1\_i1.p1 |  |  | DB Search |
| KVDIIFAVDVSKR | 46.75 | 1488.8663 | 13 | 6.22 | 497.297 | 16.89 | 0.8222 | 0.8295 | 54835 | 0 | 0 | 0 | TRINITY\_DN8008\_c0\_g1\_i2.p1:TRINITY\_DN8008\_c0\_g1\_i3.p1 |  |  | DB Search |
| YLSWTLDNDKIR | 46.73 | 1522.778 | 12 | 4.13 | 508.5998 | 17.56 | 0.8108 | 0.8181 | 59147 | 0 | 0 | 0 | TRINITY\_DN5584\_c0\_g1\_i2.p1:TRINITY\_DN5584\_c0\_g1\_i1.p1:TRINITY\_DN5584\_c0\_g1\_i3.p1 |  |  | DB Search |
| ITNLYC(+57.02)DLSIYR | 46.72 | 1529.7548 | 12 | 3.24 | 765.8838 | 19.27 | 1.0371 | 1.0443 | 70930 | 3.14e2 | 1 | 1 | TRINITY\_DN4726\_c0\_g1\_i2.p1:TRINITY\_DN4726\_c0\_g1\_i1.p1:TRINITY\_DN4726\_c0\_g1\_i3.p1 | Carbamidomethylation |  | DB Search |
| AFSFSDKPAITFEIHDFK | 46.72 | 2099.0364 | 18 | 7.34 | 700.6881 | 19.02 | 0.9808 | 0.988 | 69353 | 0 | 0 | 0 | TRINITY\_DN33065\_c0\_g2\_i1.p1 |  |  | DB Search |
| A(+42.01)TDKLEGLIAR | 46.72 | 1227.6823 | 11 | 5.76 | 614.8492 | 17.96 | 0.9505 | 0.9578 | 61874 | 1.15e3 | 1 | 1 | TRINITY\_DN540\_c0\_g1\_i1.p1 | Acetylation (Protein N-term) | A1:Acetylation (Protein N-term):1000 | DB Search |
| DLYANTVLSGGTTM(+15.99)FPGIADR | 46.72 | 2214.0627 | 21 | 10.66 | 1108.0455 | 19.50 | 1.2475 | 1.2547 | 72698 | 0 | 0 | 0 | TRINITY\_DN161\_c0\_g1\_i1.p1:TRINITY\_DN161\_c0\_g1\_i4.p1:TRINITY\_DN161\_c0\_g1\_i3.p1:TRINITY\_DN161\_c0\_g1\_i2.p1 | Oxidation (M) | M14:Oxidation (M):1000 | DB Search |
| IQFSISAEINNGALK | 46.71 | 1603.8569 | 15 | -0.57 | 802.9318 | 18.83 | 1.0737 | 1.0809 | 68043 | 0 | 0 | 0 | TRINITY\_DN15274\_c0\_g1\_i1.p1 |  |  | DB Search |
| YVKPGVNIINIR | 46.71 | 1384.8191 | 12 | 3.8 | 693.4164 | 16.18 | 1.0009 | 1.0081 | 50795 | 7.11e2 | 1 | 1 | TRINITY\_DN1616\_c0\_g1\_i3.p1:TRINITY\_DN1616\_c0\_g1\_i6.p1:TRINITY\_DN1616\_c0\_g1\_i2.p1:TRINITY\_DN1616\_c0\_g1\_i7.p1 |  |  | DB Search |
| DPINNHWDGNLINWR | 46.7 | 1862.8812 | 15 | 2.3 | 621.9664 | 19.05 | 0.814 | 0.8214 | 69560 | 4.43e2 | 1 | 1 | TRINITY\_DN138\_c0\_g1\_i1.p1 |  |  | DB Search |
| IEDFEESPPTDPHIGPVEQR | 46.7 | 2291.0706 | 20 | 6.71 | 764.6992 | 15.81 | 0.9303 | 0.9376 | 48644 | 1.97e3 | 1 | 1 | TRINITY\_DN140702\_c0\_g1\_i1.p1 |  |  | DB Search |
| TITLEVEPSDTIENVK | 46.69 | 1786.92 | 16 | 0.39 | 894.4637 | 17.56 | 1.1442 | 1.1514 | 58994 | 8.78e2 | 1 | 1 | TRINITY\_DN554\_c0\_g1\_i3.p2:TRINITY\_DN585\_c0\_g1\_i8.p1:TRINITY\_DN585\_c0\_g1\_i4.p1:TRINITY\_DN101\_c0\_g1\_i1.p1:TRINITY\_DN585\_c0\_g1\_i3.p1:TRINITY\_DN554\_c0\_g1\_i4.p1:TRINITY\_DN554\_c0\_g1\_i2.p1:TRINITY\_DN585\_c0\_g1\_i10.p1:TRINITY\_DN585\_c0\_g1\_i2.p1:TRINITY\_DN554\_c0\_g1\_i5.p1:TRINITY\_DN585\_c0\_g1\_i5.p1:TRINITY\_DN585\_c0\_g2\_i1.p1 |  |  | DB Search |
| LADLEEFISSPNHANISQVGER | 46.68 | 2425.1873 | 22 | -0.55 | 809.399 | 18.89 | 0.9243 | 0.9315 | 68335 | 1.48e3 | 1 | 1 | TRINITY\_DN2113\_c0\_g1\_i1.p1:TRINITY\_DN2113\_c0\_g1\_i2.p1 |  |  | DB Search |
| LLTSPAPSSGSLM(+15.99)ALALK | 46.68 | 1771.9753 | 18 | 0.26 | 886.9913 | 18.27 | 1.1354 | 1.1426 | 64205 | 0 | 0 | 0 | TRINITY\_DN602\_c0\_g1\_i4.p1:TRINITY\_DN602\_c0\_g1\_i3.p1:TRINITY\_DN602\_c0\_g1\_i1.p1 | Oxidation (M) | M13:Oxidation (M):1000 | DB Search |
| SYYFAISDITVGGR | 46.66 | 1547.762 | 14 | -2.17 | 774.8832 | 19.95 | 1.0472 | 1.0544 | 75627 | 4.27e2 | 1 | 1 | TRINITY\_DN1676\_c0\_g1\_i6.p1:TRINITY\_DN1676\_c0\_g1\_i12.p1 |  |  | DB Search |
| DTVIEKFEENITK | 46.65 | 1564.7985 | 13 | 3.48 | 783.4058 | 19.56 | 1.0717 | 1.0789 | 73037 | 0 | 0 | 0 | TRINITY\_DN4900\_c0\_g1\_i1.p1:TRINITY\_DN4900\_c0\_g1\_i4.p1 |  |  | DB Search |
| APIGQSIVTVSAR | 46.62 | 1297.7354 | 13 | -6.54 | 649.8679 | 14.25 | 0.967 | 0.9743 | 39958 | 0 | 0 | 0 | TRINITY\_DN982\_c0\_g1\_i3.p1 |  |  | DB Search |
| SC(+57.02)ENPKPLNGGK | 46.61 | 1299.6241 | 12 | 2.25 | 650.8179 | 11.07 | 0.9505 | 0.9578 | 20781 | 2.84e3 | 1 | 1 | TRINITY\_DN6428\_c0\_g1\_i1.p1:TRINITY\_DN6428\_c0\_g1\_i2.p1 | Carbamidomethylation |  | DB Search |
| LLTTEDDFILR | 46.58 | 1334.7081 | 11 | 1.62 | 668.3595 | 19.11 | 0.9735 | 0.9808 | 69947 | 3e2 | 1 | 1 | TRINITY\_DN982\_c0\_g1\_i1.p1:TRINITY\_DN982\_c0\_g1\_i3.p1 |  |  | DB Search |
| ASMDEGAKPK | 46.57 | 1032.491 | 10 | 5.09 | 517.2531 | 9.36 | 0.8575 | 0.8648 | 10976 | 2.73e2 | 1 | 1 | TRINITY\_DN2003\_c0\_g1\_i1.p1 |  |  | DB Search |
| AELVFVNHC(+57.02)R | 46.56 | 1243.6132 | 10 | 2.23 | 622.8125 | 14.50 | 0.9574 | 0.9646 | 41298 | 1.35e3 | 1 | 1 | TRINITY\_DN101992\_c0\_g1\_i1.p1 | Carbamidomethylation |  | DB Search |
| AEFRPNQEEEPILK | 46.54 | 1698.8577 | 14 | 4.96 | 850.4366 | 14.72 | 1.0849 | 1.0921 | 42598 | 0 | 0 | 0 | TRINITY\_DN262\_c0\_g1\_i1.p1 |  |  | DB Search |
| AEYENKTEVAR | 46.54 | 1308.631 | 11 | 0.66 | 655.3203 | 13.19 | 0.9812 | 0.9884 | 33744 | 0 | 0 | 0 | TRINITY\_DN1280\_c0\_g1\_i1.p1 |  |  | DB Search |
| ATGC(+57.02)EVIWNQHNR | 46.53 | 1583.7263 | 13 | 3.17 | 792.8694 | 14.02 | 1.0785 | 1.0857 | 38485 | 7.79e2 | 1 | 1 | TRINITY\_DN2217\_c0\_g1\_i3.p1:TRINITY\_DN2217\_c0\_g1\_i2.p1 | Carbamidomethylation |  | DB Search |
| DKELSNVIESGFR | 46.52 | 1492.7522 | 13 | 2.71 | 747.3821 | 17.35 | 1.0411 | 1.0484 | 57734 | 0 | 0 | 0 | TRINITY\_DN775\_c0\_g1\_i2.p1:TRINITY\_DN775\_c0\_g1\_i1.p1 |  |  | DB Search |
| DGPAGPAGR | 46.52 | 796.3828 | 9 | 7.09 | 399.1997 | 9.76 | 0.7233 | 0.7306 | 13098 | 1.65e3 | 1 | 1 | TRINITY\_DN106\_c0\_g1\_i2.p1:TRINITY\_DN106\_c0\_g1\_i6.p1:TRINITY\_DN106\_c0\_g1\_i12.p1:TRINITY\_DN106\_c0\_g1\_i5.p1:TRINITY\_DN106\_c0\_g1\_i10.p1:TRINITY\_DN106\_c0\_g1\_i8.p1:TRINITY\_DN106\_c0\_g1\_i3.p1:TRINITY\_DN106\_c0\_g1\_i9.p1:TRINITY\_DN106\_c0\_g1\_i11.p1 |  |  | DB Search |
| YLLVFTDGR | 46.52 | 1082.576 | 9 | 0.01 | 542.2929 | 19.04 | 0.8696 | 0.8769 | 69183 | 6.93e3 | 1 | 1 | TRINITY\_DN138\_c0\_g1\_i1.p1 |  |  | DB Search |
| ATDIDQEVKEK | 46.52 | 1274.6354 | 11 | 0.55 | 638.3225 | 11.59 | 0.9634 | 0.9707 | 24012 | 8.17e2 | 1 | 1 | TRINITY\_DN3347\_c0\_g1\_i1.p1 |  |  | DB Search |
| LGTNSATSDSC(+57.02)DR | 46.51 | 1382.5732 | 13 | 0.7 | 692.2913 | 10.49 | 0.9566 | 0.9638 | 17428 | 0 | 0 | 0 | TRINITY\_DN15274\_c0\_g1\_i1.p1 | Carbamidomethylation |  | DB Search |
| DSYVGDEAQSKR | 46.51 | 1353.6161 | 12 | 5.22 | 677.8159 | 10.97 | 1.0005 | 1.0077 | 20266 | 2.51e3 | 1 | 1 | TRINITY\_DN2477\_c0\_g1\_i6.p1:TRINITY\_DN2477\_c0\_g1\_i5.p1:TRINITY\_DN195934\_c0\_g1\_i1.p2:TRINITY\_DN161\_c0\_g1\_i1.p1:TRINITY\_DN2477\_c0\_g1\_i9.p1:TRINITY\_DN2477\_c0\_g1\_i10.p1:TRINITY\_DN2477\_c0\_g1\_i2.p1:TRINITY\_DN161\_c0\_g1\_i3.p1:TRINITY\_DN161\_c0\_g1\_i2.p1 |  |  | DB Search |
| AVDIHFALDASQNVGR | 46.51 | 1711.8641 | 16 | 2.6 | 571.6276 | 17.54 | 0.8587 | 0.866 | 58896 | 1.61e3 | 2 | 2 | TRINITY\_DN77\_c1\_g1\_i1.p1 |  |  | DB Search |
| VYLAQAEAEKIK | 46.51 | 1361.7554 | 12 | 2.91 | 681.884 | 14.42 | 1.0347 | 1.0419 | 40879 | 0 | 0 | 0 | TRINITY\_DN11435\_c0\_g1\_i1.p1 |  |  | DB Search |
| VLLSQQIFYDKK | 46.51 | 1480.829 | 12 | 0.37 | 494.6149 | 16.93 | 0.7616 | 0.7689 | 55019 | 1.05e4 | 2 | 2 | TRINITY\_DN982\_c0\_g1\_i1.p1:TRINITY\_DN982\_c0\_g1\_i3.p1:TRINITY\_DN982\_c0\_g1\_i2.p1 |  |  | DB Search |
| FDFHSWIGLKK | 46.51 | 1376.7241 | 11 | -6.14 | 459.9105 | 18.38 | 0.7384 | 0.7457 | 64918 | 0 | 0 | 0 | TRINITY\_DN12627\_c0\_g1\_i1.p1 |  |  | DB Search |
| GVHIISVGLGTVDHR | 46.51 | 1558.8579 | 15 | -9.41 | 780.4255 | 15.93 | 1.0893 | 1.0965 | 49497 | 0 | 0 | 0 | TRINITY\_DN8008\_c0\_g1\_i2.p1:TRINITY\_DN8008\_c0\_g1\_i3.p1 |  |  | DB Search |
| KAQYYEAYETIIKR | 46.51 | 1774.9254 | 14 | -1.09 | 592.6458 | 15.90 | 0.8656 | 0.8729 | 49338 | 0 | 0 | 0 | TRINITY\_DN319\_c2\_g1\_i3.p1:TRINITY\_DN319\_c2\_g1\_i1.p1 |  |  | DB Search |
| LTHQIEVTVSDREGK | 46.51 | 1710.89 | 15 | 0.23 | 571.3016 | 12.83 | 0.8652 | 0.8725 | 31549 | 0 | 0 | 0 | TRINITY\_DN123121\_c0\_g1\_i1.p1 |  |  | DB Search |
| AYGQDLGQPGSC(+57.02)LK | 46.51 | 1492.698 | 14 | 2.01 | 747.3545 | 14.33 | 1.0049 | 1.0122 | 40326 | 6.56e2 | 1 | 1 | TRINITY\_DN691\_c0\_g1\_i2.p1:TRINITY\_DN691\_c0\_g1\_i1.p1 | Carbamidomethylation |  | DB Search |
| TRIEDLETVISQTQDHR | 46.51 | 2040.0236 | 17 | 3.42 | 681.0145 | 17.49 | 0.963 | 0.9703 | 58651 | 0 | 0 | 0 | TRINITY\_DN761\_c0\_g1\_i3.p1:TRINITY\_DN761\_c0\_g1\_i1.p1:TRINITY\_DN761\_c0\_g1\_i2.p1 |  |  | DB Search |
| AEFRPNQEEEPILKR | 46.51 | 1854.9587 | 15 | 3.18 | 619.3261 | 13.89 | 0.8802 | 0.8874 | 37756 | 4.58e3 | 1 | 1 | TRINITY\_DN262\_c0\_g1\_i1.p1 |  |  | DB Search |
| DLEDYKVTEREPVTTNVK | 46.5 | 2135.0747 | 18 | 4.9 | 712.6992 | 15.24 | 0.9178 | 0.9251 | 45519 | 1.88e3 | 1 | 1 | TRINITY\_DN10485\_c0\_g1\_i1.p1 |  |  | DB Search |
| YSRPTPGSNNNVM(+15.99)VR | 46.5 | 1706.8158 | 15 | 0.06 | 569.9434 | 11.58 | 0.8311 | 0.8384 | 23889 | 1.61e3 | 1 | 1 | TRINITY\_DN15274\_c0\_g1\_i1.p1 | Oxidation (M) | M13:Oxidation (M):1000 | DB Search |
| VTRPHGASGVVR | 46.49 | 1234.6895 | 12 | 7.12 | 412.5715 | 10.06 | 0.8169 | 0.8242 | 14707 | 3.57e2 | 1 | 1 | TRINITY\_DN2885\_c0\_g1\_i2.p1:TRINITY\_DN2885\_c0\_g1\_i5.p1:TRINITY\_DN2885\_c0\_g1\_i4.p1 |  |  | DB Search |
| FDFGLSDKTYWNSESFEK | 46.49 | 2198.9797 | 18 | 1.41 | 733.9983 | 19.59 | 0.8947 | 0.902 | 73255 | 7.9e2 | 1 | 1 | TRINITY\_DN138\_c0\_g1\_i1.p1 |  |  | DB Search |
| LTIIDALPR | 46.48 | 1010.6124 | 9 | 5.92 | 506.3142 | 18.91 | 0.8445 | 0.8518 | 68779 | 8.74e2 | 1 | 1 | TRINITY\_DN1783\_c0\_g1\_i8.p2:TRINITY\_DN1783\_c0\_g1\_i1.p2:TRINITY\_DN1783\_c0\_g1\_i3.p1:TRINITY\_DN1783\_c0\_g1\_i2.p1:TRINITY\_DN1783\_c0\_g1\_i6.p1:TRINITY\_DN105041\_c0\_g1\_i1.p1:TRINITY\_DN1783\_c1\_g1\_i1.p2 |  |  | DB Search |
| SIEHC(+57.02)IDTK | 46.48 | 1101.5125 | 9 | 3.06 | 551.7628 | 11.27 | 0.8773 | 0.8846 | 22178 | 0 | 0 | 0 | TRINITY\_DN1119\_c0\_g1\_i1.p1 | Carbamidomethylation |  | DB Search |
| YLLVFTDGK | 46.48 | 1054.5698 | 9 | 2.83 | 528.2914 | 18.82 | 0.8579 | 0.8652 | 67996 | 2.92e3 | 1 | 1 | TRINITY\_DN4040\_c0\_g1\_i1.p1 |  |  | DB Search |
| VVDTEANDLTEEVKK | 46.47 | 1688.8468 | 15 | 4.23 | 845.4305 | 14.16 | 1.1298 | 1.137 | 39294 | 3.14e2 | 1 | 1 | TRINITY\_DN1616\_c0\_g1\_i3.p1:TRINITY\_DN1616\_c0\_g1\_i6.p1:TRINITY\_DN1616\_c0\_g1\_i2.p1:TRINITY\_DN1616\_c0\_g1\_i7.p1 |  |  | DB Search |
| TC(+57.02)TNPAPAHGGK | 46.47 | 1209.556 | 12 | 7.45 | 404.1938 | 8.98 | 0.731 | 0.7384 | 9664 | 0 | 0 | 0 | TRINITY\_DN1613\_c1\_g1\_i2.p1:TRINITY\_DN1613\_c1\_g1\_i1.p1 | Carbamidomethylation |  | DB Search |
| GC(+57.02)YIHTQEIAR | 46.47 | 1346.6401 | 11 | -0.76 | 674.3239 | 13.04 | 1.0158 | 1.023 | 32770 | 0 | 0 | 0 | TRINITY\_DN2217\_c0\_g1\_i3.p1:TRINITY\_DN38397\_c0\_g1\_i1.p1:TRINITY\_DN2217\_c0\_g1\_i2.p1 | Carbamidomethylation |  | DB Search |
| VLEVNDNKPK | 46.46 | 1154.6295 | 10 | 4.39 | 578.322 | 11.01 | 0.9263 | 0.9335 | 20603 | 6.84e2 | 1 | 1 | TRINITY\_DN982\_c0\_g1\_i1.p1:TRINITY\_DN982\_c0\_g1\_i2.p1 |  |  | DB Search |
| VAPEEHPVLLTEAPLNPK | 46.46 | 1953.0571 | 18 | 9.42 | 977.5407 | 16.61 | 1.2085 | 1.2157 | 53280 | 0 | 0 | 0 | TRINITY\_DN2477\_c0\_g1\_i6.p1:TRINITY\_DN2477\_c0\_g1\_i5.p1:TRINITY\_DN2477\_c0\_g1\_i8.p1:TRINITY\_DN195934\_c0\_g1\_i1.p2:TRINITY\_DN161\_c0\_g1\_i1.p1:TRINITY\_DN2477\_c0\_g1\_i9.p1:TRINITY\_DN2477\_c0\_g1\_i10.p1:TRINITY\_DN2477\_c0\_g1\_i2.p1:TRINITY\_DN161\_c0\_g1\_i3.p1:TRINITY\_DN2941\_c0\_g1\_i4.p1:TRINITY\_DN161\_c0\_g1\_i2.p1 |  |  | DB Search |
| ATNPDVEK | 46.45 | 872.424 | 8 | 5.44 | 437.2197 | 9.69 | 0.7945 | 0.8019 | 12496 | 5.14e3 | 1 | 1 | TRINITY\_DN72625\_c0\_g1\_i1.p1 |  |  | DB Search |
| DRHVLTIQSR | 46.4 | 1223.6735 | 10 | 4.59 | 408.8985 | 12.00 | 0.7559 | 0.7632 | 26560 | 0 | 0 | 0 | TRINITY\_DN982\_c0\_g1\_i1.p1:TRINITY\_DN982\_c0\_g1\_i3.p1:TRINITY\_DN982\_c0\_g1\_i2.p1 |  |  | DB Search |
| YGNQC(+57.02)PAPR | 46.39 | 1061.4713 | 9 | 9.38 | 531.7455 | 10.33 | 0.8518 | 0.8591 | 16370 | 2.83e2 | 1 | 1 | TRINITY\_DN1936\_c0\_g1\_i3.p1 | Carbamidomethylation |  | DB Search |
| LM(+15.99)GQIVSSITASLR | 46.37 | 1490.8126 | 14 | 3.71 | 746.4131 | 18.78 | 1.0347 | 1.0419 | 67549 | 8.43e1 | 1 | 1 | TRINITY\_DN250\_c0\_g1\_i2.p1:TRINITY\_DN250\_c0\_g1\_i3.p1 | Oxidation (M) | M2:Oxidation (M):1000 | DB Search |
| SISC(+57.02)SKDEEIK | 46.33 | 1294.6074 | 11 | 8.03 | 648.3134 | 10.87 | 0.9723 | 0.9795 | 19739 | 0 | 0 | 0 | TRINITY\_DN495\_c0\_g1\_i5.p1:TRINITY\_DN495\_c0\_g1\_i6.p1 | Carbamidomethylation |  | DB Search |
| VLVHTQQK | 46.31 | 951.5502 | 8 | 1.77 | 476.7811 | 9.52 | 0.8331 | 0.8404 | 11777 | 3.85e2 | 1 | 1 | TRINITY\_DN6920\_c0\_g1\_i1.p1:TRINITY\_DN6920\_c0\_g1\_i3.p1:TRINITY\_DN6920\_c0\_g1\_i4.p1:TRINITY\_DN6920\_c0\_g1\_i2.p1 |  |  | DB Search |
| IQVLKDPDFEFTR | 46.31 | 1606.8354 | 13 | -1.78 | 804.42 | 17.65 | 1.0801 | 1.0873 | 59732 | 1.21e3 | 1 | 1 | TRINITY\_DN982\_c0\_g1\_i1.p1:TRINITY\_DN982\_c0\_g1\_i3.p1 |  |  | DB Search |
| ISNVQDIVPVLELANQHK | 46.31 | 2016.1003 | 18 | 1.11 | 673.0385 | 19.73 | 0.9461 | 0.9533 | 74326 | 3.57e3 | 1 | 1 | TRINITY\_DN118520\_c0\_g1\_i1.p1 |  |  | DB Search |
| FC(+57.02)SNPEPENGGQTC(+57.02)EGEAR | 46.3 | 2137.843 | 19 | -0.42 | 1069.9236 | 12.15 | 1.1566 | 1.1638 | 27281 | 5.66e2 | 1 | 1 | TRINITY\_DN138\_c0\_g1\_i1.p1 | Carbamidomethylation, Carbamidomethylation |  | DB Search |
| ELEITVLESKPVNSFVIR | 46.29 | 2072.1516 | 18 | 4.54 | 691.7246 | 19.86 | 0.961 | 0.9683 | 74982 | 5.9e2 | 1 | 1 | TRINITY\_DN982\_c0\_g1\_i1.p1:TRINITY\_DN982\_c0\_g1\_i2.p1 |  |  | DB Search |
| S(+42.01)LVIPEKFQHILR | 46.28 | 1620.9352 | 13 | 4.83 | 541.3192 | 19.72 | 0.8421 | 0.8494 | 73912 | 4.82e2 | 1 | 1 | TRINITY\_DN1505\_c0\_g1\_i1.p1 | Acetylation (Protein N-term) | S1:Acetylation (Protein N-term):1000 | DB Search |
| GVSEETTTGVHNLYK | 46.25 | 1633.7948 | 15 | 1.81 | 817.9025 | 13.69 | 1.0664 | 1.0737 | 36539 | 4.48e2 | 1 | 1 | TRINITY\_DN773\_c0\_g1\_i1.p1 |  |  | DB Search |
| FFFHDQSC(+57.02)LDVYK | 46.21 | 1704.7606 | 13 | 2.99 | 569.26 | 17.76 | 0.8067 | 0.814 | 60434 | 5.36e2 | 1 | 1 | TRINITY\_DN10094\_c0\_g1\_i1.p1:TRINITY\_DN10094\_c0\_g1\_i2.p1 | Carbamidomethylation |  | DB Search |
| EAAAQIVVEIIKR | 46.21 | 1438.8507 | 13 | 2.76 | 720.4315 | 19.19 | 1.0524 | 1.0596 | 70467 | 2.59e2 | 1 | 1 | TRINITY\_DN71276\_c0\_g1\_i1.p1 |  |  | DB Search |
| SPFVIMFK | 46.2 | 967.5201 | 8 | -0.36 | 484.765 | 19.87 | 0.8079 | 0.8153 | 75042 | 1.61e3 | 1 | 1 | TRINITY\_DN4595\_c0\_g1\_i10.p1:TRINITY\_DN4595\_c0\_g1\_i4.p1 |  |  | DB Search |
| IDKALLLASSNAFDTSNGAR | 46.2 | 2063.0647 | 20 | -3.87 | 688.6898 | 17.41 | 0.921 | 0.9283 | 58153 | 2.44e2 | 1 | 1 | TRINITY\_DN244\_c0\_g1\_i1.p1 |  |  | DB Search |
| GLMVAAMDLDKK | 46.19 | 1290.6676 | 12 | 2.34 | 646.3397 | 16.73 | 0.9997 | 1.0069 | 53872 | 9.71e2 | 1 | 1 | TRINITY\_DN1043\_c0\_g1\_i2.p1 |  |  | DB Search |
| ATGNTSGGVC(+57.02)DDC(+57.02)NHNTAGR | 46.19 | 2062.8181 | 20 | 7.38 | 688.6154 | 9.96 | 0.8579 | 0.8652 | 14102 | 8.31e2 | 1 | 1 | TRINITY\_DN15274\_c0\_g1\_i1.p1 | Carbamidomethylation, Carbamidomethylation |  | DB Search |
| GSFSSHIC(+57.02)LC(+57.02)HDPAYK | 46.19 | 1877.8188 | 16 | 4.79 | 626.9471 | 14.44 | 0.887 | 0.8943 | 41053 | 0 | 0 | 0 | TRINITY\_DN319\_c2\_g1\_i3.p1:TRINITY\_DN319\_c2\_g1\_i1.p1:TRINITY\_DN319\_c2\_g1\_i2.p1 | Carbamidomethylation, Carbamidomethylation |  | DB Search |
| DLHIIEDYVLK | 46.18 | 1356.7289 | 11 | 2.32 | 679.3703 | 19.66 | 0.9928 | 1.0001 | 73679 | 1.43e3 | 1 | 1 | TRINITY\_DN8903\_c0\_g1\_i1.p1 |  |  | DB Search |
| IITEINPTIQVFQPK | 46.17 | 1739.9822 | 15 | 0.13 | 870.9946 | 19.11 | 1.131 | 1.1382 | 69937 | 3.91e2 | 1 | 1 | TRINITY\_DN71276\_c0\_g1\_i1.p1 |  |  | DB Search |
| ASFVTDIDRLR | 46.17 | 1291.6885 | 11 | 5.09 | 646.8519 | 17.22 | 0.9904 | 0.9977 | 56760 | 8.37e2 | 1 | 1 | TRINITY\_DN598\_c1\_g1\_i2.p1 |  |  | DB Search |
| TLDFVVELSK | 46.17 | 1149.6281 | 10 | -1.62 | 575.8179 | 19.58 | 0.8854 | 0.8927 | 72942 | 1.16e4 | 1 | 1 | TRINITY\_DN138\_c0\_g1\_i1.p1 |  |  | DB Search |
| RVHQFLVEALDGGQPR | 46.14 | 1820.9645 | 16 | -9.01 | 607.9873 | 19.50 | 0.868 | 0.8753 | 72483 | 1.07e3 | 1 | 1 | TRINITY\_DN71243\_c0\_g1\_i1.p1 |  |  | DB Search |
| LLEFVGHDVLR | 46.14 | 1296.719 | 11 | 6.13 | 433.2477 | 17.91 | 0.7713 | 0.7787 | 61665 | 0 | 0 | 0 | TRINITY\_DN464\_c0\_g1\_i4.p1 |  |  | DB Search |
| YTGAC(+57.02)NANGNTDK | 46.13 | 1384.5677 | 13 | 10.57 | 693.2954 | 9.52 | 0.9428 | 0.9501 | 11788 | 0 | 0 | 0 | TRINITY\_DN4595\_c0\_g1\_i10.p1:TRINITY\_DN4595\_c0\_g1\_i4.p1 | Carbamidomethylation |  | DB Search |
| APEDAPNREQEFEEK | 46.1 | 1787.7961 | 15 | 5.21 | 596.9398 | 12.20 | 0.825 | 0.8323 | 27725 | 7.16e2 | 1 | 1 | TRINITY\_DN30613\_c0\_g1\_i1.p1 |  |  | DB Search |
| AC(+57.02)SNVNQC(+57.02)GVNTGTQTVYK | 46.08 | 2099.9365 | 19 | 6.81 | 1050.978 | 12.97 | 1.1746 | 1.1817 | 32306 | 0 | 0 | 0 | TRINITY\_DN8597\_c0\_g1\_i1.p1 | Carbamidomethylation, Carbamidomethylation |  | DB Search |
| SDELLNLKEEASTLR | 46.08 | 1716.8894 | 15 | 3.13 | 859.4509 | 17.75 | 1.1114 | 1.1186 | 60489 | 0 | 0 | 0 | TRINITY\_DN140702\_c0\_g1\_i1.p1 |  |  | DB Search |
| YVVMLQYYHDSDTAK | 46.08 | 1831.8451 | 15 | 8.86 | 916.9339 | 16.78 | 1.1825 | 1.1897 | 54165 | 6.38e1 | 1 | 1 | TRINITY\_DN1616\_c0\_g1\_i3.p1:TRINITY\_DN1616\_c0\_g1\_i6.p1:TRINITY\_DN1616\_c0\_g1\_i2.p1:TRINITY\_DN1616\_c0\_g1\_i7.p1 |  |  | DB Search |
| TC(+57.02)GGGFFFETR | 46.05 | 1277.5499 | 11 | 0.02 | 639.7794 | 18.54 | 0.9267 | 0.9339 | 65970 | 3.17e2 | 1 | 1 | TRINITY\_DN4595\_c0\_g1\_i4.p1 | Carbamidomethylation |  | DB Search |
| AHVSLSTFSEDVTSLTK | 46.04 | 1820.9155 | 17 | 4.83 | 911.4655 | 18.94 | 1.1398 | 1.147 | 68762 | 2.56e3 | 2 | 2 | TRINITY\_DN113\_c3\_g1\_i1.p1 |  |  | DB Search |
| LRPGQATTVDITVK | 46.03 | 1497.8514 | 14 | 3.07 | 749.932 | 14.17 | 1.0331 | 1.0403 | 39382 | 6.12e2 | 1 | 1 | TRINITY\_DN25375\_c0\_g1\_i7.p1 |  |  | DB Search |
| KYTPC(+57.02)EEK | 46.01 | 1053.4801 | 8 | 0.34 | 527.7452 | 9.67 | 0.8619 | 0.8692 | 12492 | 6.29e2 | 1 | 1 | TRINITY\_DN1369\_c0\_g1\_i1.p1 | Carbamidomethylation |  | DB Search |
| ANKEFQEIVQNTK | 46.01 | 1547.7943 | 13 | 2.47 | 774.903 | 14.63 | 1.0676 | 1.0749 | 42038 | 4.72e2 | 1 | 1 | TRINITY\_DN4726\_c0\_g1\_i1.p1 |  |  | DB Search |
| AC(+57.02)AELHQDVNVK | 46.01 | 1382.6613 | 12 | 2.82 | 692.3368 | 11.95 | 1.0065 | 1.0138 | 26229 | 0 | 0 | 0 | TRINITY\_DN34481\_c0\_g1\_i1.p1 | Carbamidomethylation |  | DB Search |
| GFEC(+57.02)IGLIEETETC(+57.02)K | 45.99 | 1784.796 | 15 | 4.3 | 893.4052 | 18.62 | 1.0981 | 1.1053 | 66554 | 4.49e2 | 1 | 1 | TRINITY\_DN4595\_c0\_g1\_i10.p1:TRINITY\_DN4595\_c0\_g1\_i4.p1 | Carbamidomethylation, Carbamidomethylation |  | DB Search |
| SGGQC(+57.02)PC(+57.02)KPGVVGR | 45.97 | 1457.6868 | 14 | 1.98 | 486.9017 | 10.72 | 0.7864 | 0.7937 | 18818 | 1.41e3 | 2 | 2 | TRINITY\_DN15274\_c0\_g1\_i1.p1 | Carbamidomethylation, Carbamidomethylation |  | DB Search |
| MLVYYAEKEC(+57.02)EILR | 45.96 | 1815.8899 | 14 | 0.28 | 908.9485 | 17.53 | 1.1654 | 1.1726 | 58763 | 8.89e2 | 1 | 1 | TRINITY\_DN1369\_c0\_g1\_i1.p1 | Carbamidomethylation |  | DB Search |
| SKDYTITSVGGR | 45.96 | 1282.6517 | 12 | 4.88 | 642.3334 | 12.98 | 0.9457 | 0.9529 | 32411 | 5.09e2 | 1 | 1 | TRINITY\_DN6390\_c0\_g1\_i1.p1 |  |  | DB Search |
| AVDRGNPPQDSVNK | 45.95 | 1495.7379 | 14 | 6.38 | 499.5876 | 10.19 | 0.7868 | 0.7941 | 15626 | 0 | 0 | 0 | TRINITY\_DN982\_c0\_g1\_i1.p1:TRINITY\_DN982\_c0\_g1\_i3.p1 |  |  | DB Search |
| GKDGETGEQGPQGQEGPTGPR | 45.95 | 2080.9409 | 21 | 2.44 | 694.6529 | 10.76 | 0.8494 | 0.8567 | 18980 | 2.66e3 | 1 | 1 | TRINITY\_DN113\_c0\_g1\_i1.p1 |  |  | DB Search |
| VLIVISDGNEDFKEK | 45.95 | 1704.8933 | 15 | 0.7 | 853.4508 | 16.86 | 1.1702 | 1.1774 | 54589 | 4.23e2 | 1 | 1 | TRINITY\_DN195348\_c0\_g1\_i1.p1 |  |  | DB Search |
| IKDTYLLTLEAR | 45.94 | 1434.8082 | 12 | 1.17 | 718.4091 | 17.05 | 1.0379 | 1.0452 | 55684 | 2.02e3 | 1 | 1 | TRINITY\_DN72625\_c0\_g1\_i1.p1 |  |  | DB Search |
| TNNALDYEAKR | 45.94 | 1293.6313 | 11 | 0.07 | 647.8201 | 12.09 | 0.9618 | 0.9691 | 27037 | 8.23e2 | 1 | 1 | TRINITY\_DN982\_c0\_g1\_i1.p1:TRINITY\_DN982\_c0\_g1\_i3.p1 |  |  | DB Search |
| ALSLQNANEELEDRLAEAER | 45.94 | 2270.1138 | 20 | 0.89 | 757.7092 | 18.86 | 0.9836 | 0.9908 | 68234 | 0 | 0 | 0 | TRINITY\_DN428\_c0\_g1\_i1.p1 |  |  | DB Search |
| FAIQDITVDEFSAK | 45.94 | 1582.7878 | 14 | 3.22 | 792.4003 | 19.79 | 1.0448 | 1.052 | 74660 | 7.64e2 | 1 | 1 | TRINITY\_DN624\_c0\_g1\_i1.p1 |  |  | DB Search |
| DTLVFEIDEIRK | 45.93 | 1476.7823 | 12 | -0.51 | 739.3948 | 18.60 | 1.0343 | 1.0415 | 66411 | 0 | 0 | 0 | TRINITY\_DN31399\_c0\_g1\_i3.p1:TRINITY\_DN31399\_c0\_g1\_i2.p1 |  |  | DB Search |
| DVIIDSGDESKR | 45.93 | 1332.6521 | 12 | -17.59 | 667.3187 | 12.42 | 0.9864 | 0.9937 | 29240 | 2.37e3 | 1 | 1 | TRINITY\_DN1366\_c0\_g1\_i1.p1 |  |  | DB Search |
| GFGGNLVTITNSDIQK | 45.92 | 1662.8577 | 16 | 0.13 | 832.4326 | 17.74 | 1.0813 | 1.0885 | 60461 | 6.04e2 | 1 | 1 | TRINITY\_DN12627\_c0\_g1\_i1.p1 |  |  | DB Search |
| VHFGVLSFSHR | 45.92 | 1284.6727 | 11 | 1.26 | 643.3416 | 16.41 | 0.9791 | 0.9864 | 52149 | 6.37e2 | 2 | 2 | TRINITY\_DN138\_c0\_g1\_i1.p1 |  |  | DB Search |
| FLLHLFDKELK | 45.88 | 1401.802 | 11 | 9.16 | 468.2768 | 19.19 | 0.7677 | 0.775 | 70553 | 0 | 0 | 0 | TRINITY\_DN55094\_c0\_g1\_i1.p1 |  |  | DB Search |
| RPAFQIVFQGVR | 45.87 | 1416.799 | 12 | -0.91 | 473.2711 | 18.12 | 0.7477 | 0.7551 | 63094 | 0 | 0 | 0 | TRINITY\_DN4781\_c0\_g1\_i3.p1:TRINITY\_DN4781\_c0\_g1\_i1.p1:TRINITY\_DN4781\_c0\_g1\_i2.p1 |  |  | DB Search |
| TILIHGFGTIQR | 45.83 | 1354.7721 | 12 | -0.03 | 678.3903 | 16.94 | 1.0102 | 1.0174 | 55113 | 0 | 0 | 0 | TRINITY\_DN73457\_c0\_g1\_i1.p1 |  |  | DB Search |
| VC(+57.02)NGEPC(+57.02)ILQER | 45.81 | 1473.6704 | 12 | 3.3 | 737.8417 | 14.98 | 0.9876 | 0.9949 | 44106 | 8.93e2 | 1 | 1 | TRINITY\_DN4595\_c0\_g1\_i4.p1:TRINITY\_DN4595\_c0\_g1\_i10.p2 | Carbamidomethylation, Carbamidomethylation |  | DB Search |
| LAALDATVHGQTASR | 45.78 | 1509.7899 | 15 | -1.6 | 755.8977 | 13.57 | 1.0721 | 1.0793 | 35881 | 2.4e2 | 1 | 1 | TRINITY\_DN1378\_c0\_g1\_i1.p1 |  |  | DB Search |
| NYFHYENQLELK | 45.77 | 1596.7572 | 12 | -0.53 | 799.382 | 16.59 | 1.0849 | 1.0921 | 53009 | 7.6e2 | 1 | 1 | TRINITY\_DN1320\_c0\_g2\_i1.p1 |  |  | DB Search |
| IVNTDLQEIKDIIK | 45.77 | 1640.9348 | 14 | -1.11 | 547.9825 | 18.81 | 0.8177 | 0.825 | 67904 | 0 | 0 | 0 | TRINITY\_DN3016\_c0\_g1\_i17.p1:TRINITY\_DN3016\_c0\_g1\_i11.p1:TRINITY\_DN3016\_c0\_g1\_i6.p1:TRINITY\_DN3016\_c0\_g1\_i18.p1:TRINITY\_DN3016\_c0\_g1\_i10.p1:TRINITY\_DN3016\_c0\_g1\_i8.p1:TRINITY\_DN3016\_c0\_g1\_i13.p1:TRINITY\_DN3016\_c0\_g1\_i2.p1:TRINITY\_DN3016\_c0\_g1\_i4.p1:TRINITY\_DN3016\_c0\_g1\_i5.p1 |  |  | DB Search |
| VDVGFILDSSGSLR | 45.76 | 1463.762 | 14 | -1.21 | 732.8842 | 19.93 | 1.0102 | 1.0174 | 75559 | 4.99e2 | 1 | 1 | TRINITY\_DN17725\_c0\_g1\_i1.p1:TRINITY\_DN4494\_c1\_g1\_i2.p1:TRINITY\_DN4494\_c1\_g1\_i15.p1:TRINITY\_DN4494\_c2\_g1\_i1.p1:TRINITY\_DN4494\_c1\_g1\_i12.p1:TRINITY\_DN4494\_c1\_g1\_i1.p1:TRINITY\_DN172523\_c0\_g1\_i1.p1:TRINITY\_DN12542\_c0\_g1\_i1.p1:TRINITY\_DN4494\_c1\_g1\_i7.p1:TRINITY\_DN4494\_c1\_g1\_i8.p1:TRINITY\_DN4494\_c1\_g1\_i5.p1:TRINITY\_DN4494\_c1\_g1\_i10.p1 |  |  | DB Search |
| KEFFC(+57.02)DSC(+57.02)GNRK | 45.74 | 1546.6656 | 12 | 5.94 | 516.5633 | 12.01 | 0.7929 | 0.8002 | 26624 | 4.96e2 | 1 | 1 | TRINITY\_DN4040\_c0\_g1\_i1.p1 | Carbamidomethylation, Carbamidomethylation |  | DB Search |
| VFSEC(+57.02)GPAC(+57.02)SSVC(+57.02)SAIGSK | 45.7 | 2001.8594 | 19 | 9.61 | 1001.9422 | 15.38 | 1.1518 | 1.159 | 46383 | 0 | 0 | 0 | TRINITY\_DN1369\_c0\_g1\_i1.p1 | Carbamidomethylation, Carbamidomethylation, Carbamidomethylation |  | DB Search |
| DPVRDKLVNER | 45.69 | 1339.7208 | 11 | 7.42 | 447.5822 | 12.08 | 0.7449 | 0.7522 | 27124 | 0 | 0 | 0 | TRINITY\_DN262\_c0\_g1\_i1.p1 |  |  | DB Search |
| LQTDIHGVALEITK | 45.64 | 1536.8511 | 14 | 2.93 | 769.4317 | 16.63 | 1.0556 | 1.0628 | 53420 | 0 | 0 | 0 | TRINITY\_DN99404\_c0\_g1\_i1.p1 |  |  | DB Search |
| DNIQGITKPAIR | 45.63 | 1324.7462 | 12 | -2.11 | 663.3761 | 13.89 | 0.9808 | 0.988 | 37776 | 0 | 0 | 0 | TRINITY\_DN172571\_c0\_g1\_i1.p1 |  |  | DB Search |
| NIALETLEGHQR | 45.63 | 1379.7157 | 12 | 6.86 | 690.8668 | 15.42 | 1.0178 | 1.025 | 46588 | 3.18e2 | 1 | 1 | TRINITY\_DN1280\_c0\_g1\_i1.p1 |  |  | DB Search |
| YQGREYNINEEVNVR | 45.61 | 1881.897 | 15 | -1.16 | 941.9505 | 15.67 | 1.1394 | 1.1466 | 47924 | 1.31e3 | 1 | 1 | TRINITY\_DN4595\_c0\_g1\_i4.p1:TRINITY\_DN4595\_c0\_g1\_i10.p2 |  |  | DB Search |
| VPAENNPK | 45.57 | 867.445 | 8 | 1.28 | 434.7284 | 9.23 | 0.7738 | 0.7811 | 10441 | 5.64e2 | 1 | 1 | TRINITY\_DN48767\_c0\_g1\_i1.p1 |  |  | DB Search |
| SQSAFPTRR | 45.57 | 1048.5414 | 9 | 1.94 | 525.2767 | 11.05 | 0.8514 | 0.8587 | 20691 | 6.92e3 | 1 | 1 | TRINITY\_DN477\_c0\_g1\_i8.p1:TRINITY\_DN477\_c0\_g1\_i7.p1 |  |  | DB Search |
| LGGIEVTQGNC(+57.02)AR | 45.45 | 1373.6721 | 13 | 2.96 | 687.8423 | 13.87 | 0.9687 | 0.9759 | 37701 | 0 | 0 | 0 | TRINITY\_DN36\_c0\_g1\_i16.p1:TRINITY\_DN36\_c0\_g1\_i3.p1:TRINITY\_DN36\_c0\_g1\_i14.p1:TRINITY\_DN36\_c0\_g1\_i17.p1:TRINITY\_DN36\_c0\_g1\_i13.p1:TRINITY\_DN36\_c0\_g1\_i8.p1:TRINITY\_DN36\_c0\_g1\_i19.p1 | Carbamidomethylation |  | DB Search |
| AYDFRETLPK | 45.42 | 1238.6295 | 10 | 5.77 | 620.3229 | 15.38 | 0.9497 | 0.957 | 46320 | 7.87e2 | 1 | 1 | TRINITY\_DN602\_c0\_g1\_i4.p1:TRINITY\_DN602\_c0\_g2\_i1.p1:TRINITY\_DN602\_c0\_g1\_i3.p1:TRINITY\_DN602\_c0\_g1\_i1.p1 |  |  | DB Search |
| TGVEILNWGVR | 45.34 | 1242.672 | 11 | 1.79 | 622.3417 | 19.66 | 0.9065 | 0.9137 | 73704 | 5.55e2 | 1 | 1 | TRINITY\_DN140702\_c0\_g1\_i1.p1 |  |  | DB Search |
| TIESEAAHGTVTR | 45.3 | 1370.679 | 13 | -1.69 | 686.3426 | 11.96 | 1.0142 | 1.0214 | 26346 | 0 | 0 | 0 | TRINITY\_DN3271\_c0\_g1\_i1.p1:TRINITY\_DN3271\_c0\_g1\_i2.p1 |  |  | DB Search |
| RYGQAMC(+57.02)QFMR | 45.29 | 1446.6318 | 11 | -3.75 | 724.3173 | 17.41 | 0.9856 | 0.9928 | 58107 | 7.27e2 | 1 | 1 | TRINITY\_DN286\_c0\_g1\_i4.p1:TRINITY\_DN286\_c0\_g1\_i2.p1 | Carbamidomethylation |  | DB Search |
| IFPNRPYPFELVNHR | 45.28 | 1897.9951 | 15 | 3.81 | 475.5058 | 17.41 | 0.7844 | 0.7917 | 58138 | 0 | 0 | 0 | TRINITY\_DN138\_c0\_g1\_i1.p1 |  |  | DB Search |
| VLDVNDKQPVFVK | 45.16 | 1499.8347 | 13 | 0.06 | 750.9214 | 15.14 | 1.0705 | 1.0777 | 45016 | 7.57e2 | 1 | 1 | TRINITY\_DN982\_c0\_g1\_i1.p1:TRINITY\_DN982\_c0\_g1\_i2.p1 |  |  | DB Search |
| ASGDYATVVSHNR | 45.13 | 1375.6481 | 13 | -4.36 | 688.8253 | 12.17 | 0.9953 | 1.0025 | 27659 | 0 | 0 | 0 | TRINITY\_DN925\_c0\_g1\_i1.p1 |  |  | DB Search |
| AGHGAVTSPQTQR | 45.07 | 1308.6534 | 13 | 6.45 | 437.226 | 9.72 | 0.7041 | 0.7114 | 12869 | 7.9e2 | 1 | 1 | TRINITY\_DN3036\_c0\_g1\_i2.p2:TRINITY\_DN3036\_c0\_g1\_i8.p1 |  |  | DB Search |
| KIDDVIC(+57.02)EER | 44.84 | 1275.6129 | 10 | 10.25 | 638.8174 | 13.59 | 0.9295 | 0.9368 | 36142 | 1.97e3 | 1 | 1 | TRINITY\_DN15274\_c0\_g1\_i1.p1 | Carbamidomethylation |  | DB Search |
| FETQIATK | 44.83 | 936.4916 | 8 | 7 | 937.5013 | 13.97 | 1.1802 | 1.1873 | 38247 | 0 | 0 | 0 | TRINITY\_DN649\_c0\_g1\_i2.p1:TRINITY\_DN36\_c0\_g1\_i16.p1:TRINITY\_DN36\_c0\_g1\_i3.p1:TRINITY\_DN36\_c0\_g1\_i14.p1:TRINITY\_DN36\_c0\_g1\_i17.p1:TRINITY\_DN649\_c0\_g1\_i5.p1:TRINITY\_DN36\_c0\_g1\_i13.p1:TRINITY\_DN36\_c0\_g1\_i8.p1:TRINITY\_DN36\_c0\_g1\_i19.p1:TRINITY\_DN649\_c0\_g1\_i1.p1 |  |  | DB Search |
| ANYVLTIEASDNFETPKK | 44.76 | 2039.0211 | 18 | -2.13 | 1020.5112 | 17.53 | 1.271 | 1.2782 | 58757 | 1.41e2 | 1 | 1 | TRINITY\_DN14015\_c0\_g1\_i3.p1:TRINITY\_DN14015\_c0\_g1\_i2.p1 |  |  | DB Search |
| TGAISLLASVDFETDRTHTAVVK | 44.6 | 2430.2754 | 23 | -2.1 | 811.0938 | 18.98 | 1.0427 | 1.05 | 69102 | 6.22e2 | 1 | 1 | TRINITY\_DN982\_c0\_g1\_i3.p1 |  |  | DB Search |
| VLLAVGGWNHENGNGQGK | 44.58 | 1848.9231 | 18 | 4.74 | 925.4691 | 16.16 | 1.171 | 1.1782 | 50811 | 0 | 0 | 0 | TRINITY\_DN67\_c0\_g1\_i2.p1 |  |  | DB Search |
| ADKFDWLR | 44.56 | 1049.5294 | 8 | -0.14 | 525.7696 | 17.03 | 0.8603 | 0.8676 | 55648 | 3.58e2 | 1 | 1 | TRINITY\_DN1936\_c0\_g1\_i3.p1 |  |  | DB Search |
| RGPNGAIGER | 44.52 | 1025.5366 | 10 | 3.56 | 513.7751 | 10.07 | 0.8465 | 0.8538 | 14705 | 1.66e3 | 1 | 1 | TRINITY\_DN113\_c2\_g1\_i1.p1 |  |  | DB Search |
| DLEFKFR | 44.49 | 953.497 | 7 | 5.18 | 477.7562 | 16.82 | 0.8425 | 0.8498 | 54418 | 0 | 0 | 0 | TRINITY\_DN160\_c0\_g1\_i2.p1:TRINITY\_DN160\_c0\_g1\_i1.p1 |  |  | DB Search |
| AANAGGVAVSGLEM(+15.99)SQNSTR | 44.37 | 1934.9116 | 20 | 10.47 | 968.4689 | 14.29 | 1.2778 | 1.2849 | 40083 | 0 | 0 | 0 | TRINITY\_DN360\_c0\_g1\_i1.p1 | Oxidation (M) | M14:Oxidation (M):1000 | DB Search |
| S(+42.01)C(+57.02)SNPSPAHGGKPC(+57.02)SGELSK | 44.35 | 2097.9207 | 20 | -0.21 | 700.311 | 11.19 | 0.887 | 0.8943 | 21775 | 7.11e2 | 1 | 1 | TRINITY\_DN1613\_c1\_g1\_i2.p1:TRINITY\_DN1613\_c1\_g1\_i1.p1 | Acetylation (Protein N-term), Carbamidomethylation, Carbamidomethylation | S1:Acetylation (Protein N-term):1000 | DB Search |
| AFNKVETR | 44.29 | 963.5137 | 8 | -5.34 | 482.7595 | 10.58 | 0.8283 | 0.8356 | 17963 | 0 | 0 | 0 | TRINITY\_DN4595\_c0\_g1\_i7.p1:TRINITY\_DN98968\_c0\_g1\_i1.p1:TRINITY\_DN8597\_c0\_g1\_i1.p1:TRINITY\_DN4595\_c0\_g1\_i4.p1:TRINITY\_DN4595\_c0\_g1\_i10.p2 |  |  | DB Search |
| DGTTPC(+57.02)NTR | 44.18 | 1020.4294 | 9 | 6.58 | 511.2231 | 9.57 | 0.8295 | 0.8368 | 12093 | 0 | 0 | 0 | TRINITY\_DN4595\_c0\_g1\_i4.p1:TRINITY\_DN4595\_c0\_g1\_i10.p2 | Carbamidomethylation |  | DB Search |
| ALEHYSDIYDIKR | 43.91 | 1621.8099 | 13 | 7.89 | 541.6125 | 14.74 | 0.825 | 0.8323 | 42743 | 0 | 0 | 0 | TRINITY\_DN2113\_c0\_g1\_i1.p1:TRINITY\_DN2113\_c0\_g1\_i2.p1 |  |  | DB Search |
| GTTSTAGTGPSFDHTK | 43.88 | 1563.7164 | 16 | 5.67 | 782.8665 | 11.49 | 1.0536 | 1.0608 | 23479 | 0 | 0 | 0 | TRINITY\_DN1936\_c0\_g2\_i1.p1 |  |  | DB Search |
| AFLFDVVSK | 43.87 | 1024.5593 | 9 | 2.62 | 513.286 | 19.76 | 0.8388 | 0.8461 | 74385 | 7.51e2 | 1 | 1 | TRINITY\_DN65215\_c0\_g1\_i1.p1 |  |  | DB Search |
| ADLDIQKPIPIVSK | 43.74 | 1535.8922 | 14 | 3.09 | 768.9524 | 16.14 | 1.0753 | 1.0825 | 50729 | 0 | 0 | 0 | TRINITY\_DN26392\_c0\_g1\_i1.p1 |  |  | DB Search |
| GFVAVNDQQPSVVVSINVPK | 43.72 | 2096.1265 | 20 | 10.57 | 1049.077 | 18.41 | 1.2185 | 1.2256 | 65167 | 0 | 0 | 0 | TRINITY\_DN1616\_c0\_g1\_i3.p1:TRINITY\_DN1616\_c0\_g1\_i6.p1:TRINITY\_DN1616\_c0\_g1\_i2.p1:TRINITY\_DN1616\_c0\_g1\_i7.p1 |  |  | DB Search |
| IDNYIEFLDEDLKK | 43.58 | 1753.8774 | 14 | -1.78 | 585.6295 | 19.97 | 0.8437 | 0.851 | 75833 | 0 | 0 | 0 | TRINITY\_DN278\_c0\_g1\_i1.p1 |  |  | DB Search |
| LPAGSLSYLDKPENTNR | 43.58 | 1873.9534 | 17 | -5.38 | 625.6523 | 15.41 | 0.868 | 0.8753 | 46646 | 0 | 0 | 0 | TRINITY\_DN11096\_c0\_g1\_i1.p1 |  |  | DB Search |
| AGKPC(+57.02)DIAIDARK | 43.41 | 1413.7397 | 13 | -2.72 | 472.2505 | 11.95 | 0.7746 | 0.7819 | 26267 | 0 | 0 | 0 | TRINITY\_DN941\_c0\_g1\_i9.p1 | Carbamidomethylation |  | DB Search |
| HQGVM(+15.99)VGM(+15.99)GQK | 43.4 | 1202.5536 | 11 | 4.58 | 602.2842 | 9.74 | 0.9081 | 0.9154 | 12838 | 6.82e2 | 1 | 1 | TRINITY\_DN161\_c0\_g1\_i1.p1:TRINITY\_DN161\_c0\_g1\_i3.p1:TRINITY\_DN161\_c0\_g1\_i2.p1 | Oxidation (M), Oxidation (M) | M5:Oxidation (M):1000 M8:Oxidation (M):1000 | DB Search |
| ITTSQILDHEQQK | 43.4 | 1539.7893 | 13 | 3.46 | 770.9012 | 13.97 | 1.0949 | 1.1021 | 38279 | 0 | 0 | 0 | TRINITY\_DN982\_c0\_g1\_i1.p1:TRINITY\_DN982\_c0\_g1\_i3.p1 |  |  | DB Search |
| ATHQELLNAEFIQK | 43.35 | 1640.8522 | 14 | -1.87 | 821.4282 | 16.09 | 1.0757 | 1.0829 | 50251 | 1.12e3 | 1 | 1 | TRINITY\_DN113\_c3\_g1\_i1.p1 |  |  | DB Search |
| LSQC(+57.02)TAASGK | 43.29 | 1021.4862 | 10 | -0.25 | 511.748 | 9.82 | 0.8364 | 0.8437 | 12382 | 2.03e5 | 1 | 1 | TRINITY\_DN3991\_c0\_g1\_i1.p1 | Carbamidomethylation |  | DB Search |
| LRPGVELTMSSLVNAK | 43.26 | 1713.9447 | 16 | 0.82 | 572.3201 | 17.86 | 0.9097 | 0.917 | 61340 | 0 | 0 | 0 | TRINITY\_DN329\_c0\_g1\_i1.p1:TRINITY\_DN329\_c0\_g1\_i2.p1 |  |  | DB Search |
| LVFHNGDISYAR | 43.26 | 1390.6993 | 12 | -2.27 | 696.3523 | 14.91 | 1.0053 | 1.0126 | 43739 | 0 | 0 | 0 | TRINITY\_DN27379\_c0\_g1\_i1.p1 |  |  | DB Search |
| EAIVQGAQSTQDTLKK | 43.22 | 1715.9054 | 16 | 4.98 | 858.9604 | 12.79 | 1.1514 | 1.1586 | 31276 | 0 | 0 | 0 | TRINITY\_DN564\_c0\_g1\_i1.p1 |  |  | DB Search |
| AIESERDELLEELNNNSALK | 43.21 | 2286.1338 | 20 | 3.65 | 763.0513 | 18.41 | 1.0029 | 1.0102 | 65156 | 0 | 0 | 0 | TRINITY\_DN428\_c0\_g1\_i1.p1 |  |  | DB Search |
| VIMVTGDHPITAK | 43.21 | 1380.7435 | 13 | -0.18 | 691.3759 | 14.04 | 1.0182 | 1.0255 | 38661 | 1.06e3 | 1 | 1 | TRINITY\_DN262\_c0\_g1\_i1.p1 |  |  | DB Search |
| IVNAEWNILYEKLEK | 43.15 | 1860.9985 | 15 | 0.82 | 621.3379 | 19.64 | 0.8947 | 0.902 | 73650 | 0 | 0 | 0 | TRINITY\_DN1194\_c0\_g1\_i1.p1 |  |  | DB Search |
| LGPDQVDVAGAGR | 43.15 | 1253.6364 | 13 | 15.86 | 627.8326 | 13.68 | 0.9335 | 0.9408 | 36571 | 6.32e2 | 1 | 1 | TRINITY\_DN97809\_c0\_g1\_i1.p1 |  |  | DB Search |
| RIVLLATDALPK | 43.02 | 1308.8129 | 12 | 3.07 | 655.4128 | 17.30 | 1.0142 | 1.0214 | 57454 | 1.74e2 | 1 | 1 | TRINITY\_DN11377\_c0\_g1\_i1.p1 |  |  | DB Search |
| AVC(+57.02)M(+15.99)LSNTTAIAEAWAR | 43.01 | 1879.8921 | 17 | 2.28 | 940.9513 | 18.79 | 1.147 | 1.1542 | 67649 | 2.71e2 | 1 | 1 | TRINITY\_DN687\_c0\_g2\_i1.p1:TRINITY\_DN250\_c0\_g1\_i5.p1:TRINITY\_DN687\_c0\_g1\_i1.p1:TRINITY\_DN250\_c0\_g1\_i1.p1:TRINITY\_DN250\_c0\_g1\_i3.p1 | Carbamidomethylation, Oxidation (M) | M4:Oxidation (M):1000 | DB Search |
| AKQEAFIAELEDQIAKGDK | 42.99 | 2103.0847 | 19 | -0.47 | 702.0321 | 19.58 | 0.9428 | 0.9501 | 73252 | 2.03e2 | 1 | 1 | TRINITY\_DN428\_c0\_g1\_i1.p1 |  |  | DB Search |
| TVSDSIKDMGVNVISVGAGHTINHR | 42.95 | 2606.3235 | 25 | 3.28 | 652.5874 | 17.67 | 0.9141 | 0.9214 | 59942 | 2.01e2 | 1 | 1 | TRINITY\_DN138\_c0\_g1\_i1.p1 |  |  | DB Search |
| LNFKGDVLQC(+57.02)LER | 42.85 | 1590.8187 | 13 | -4.9 | 531.2753 | 18.12 | 0.7673 | 0.7746 | 63093 | 0 | 0 | 0 | TRINITY\_DN278\_c0\_g1\_i1.p1 | Carbamidomethylation |  | DB Search |
| ALTEITFAHELGHSFGAQHDPNTK | 42.4 | 2620.2668 | 24 | -0.69 | 656.0707 | 17.24 | 0.9093 | 0.9166 | 56979 | 7.14e2 | 1 | 1 | TRINITY\_DN4066\_c0\_g1\_i1.p1 |  |  | DB Search |
| GVAGAGVLAGFDKFK | 42.3 | 1435.7823 | 15 | -1.19 | 479.5987 | 18.41 | 0.8498 | 0.8571 | 65109 | 0 | 0 | 0 | TRINITY\_DN1559\_c0\_g1\_i1.p1 |  |  | DB Search |
| RFDGIFQYTK | 42.23 | 1273.6455 | 10 | 4.96 | 637.8304 | 17.01 | 0.9594 | 0.9666 | 55510 | 0 | 0 | 0 | TRINITY\_DN244\_c0\_g1\_i1.p1 |  |  | DB Search |
| AFNKIETR | 41.96 | 977.5294 | 8 | 2.83 | 489.7712 | 11.48 | 0.8343 | 0.8416 | 23389 | 1.84e3 | 1 | 1 | TRINITY\_DN4595\_c0\_g1\_i1.p1:TRINITY\_DN4595\_c0\_g1\_i7.p1:TRINITY\_DN4595\_c0\_g1\_i4.p1 |  |  | DB Search |
| AQEVSDKLNQK | 41.26 | 1258.6517 | 11 | -1.32 | 630.3295 | 10.77 | 0.9372 | 0.9444 | 19179 | 0 | 0 | 0 | TRINITY\_DN3034\_c0\_g1\_i1.p1 |  |  | DB Search |
| IGC(+57.02)FHDR | 41.14 | 903.4021 | 7 | 9.24 | 452.7105 | 11.29 | 0.8039 | 0.8112 | 22280 | 0 | 0 | 0 | TRINITY\_DN138\_c0\_g1\_i1.p1 | Carbamidomethylation |  | DB Search |
| GLKVDFDTTFAPVTGK | 40.84 | 1694.8879 | 16 | -12.98 | 565.9601 | 18.27 | 0.8339 | 0.8412 | 64168 | 0 | 0 | 0 | TRINITY\_DN329\_c0\_g1\_i1.p1:TRINITY\_DN329\_c0\_g1\_i2.p1 |  |  | DB Search |
| SDSSDIEKR | 40.54 | 1035.4833 | 9 | -16.22 | 518.7382 | 11.11 | 0.8571 | 0.8644 | 21120 | 2.69e3 | 1 | 1 | TRINITY\_DN135129\_c0\_g1\_i1.p1 |  |  | DB Search |
| AVAAGMNPMDIKR | 40.45 | 1372.6956 | 13 | -2.78 | 687.3501 | 14.90 | 1.0102 | 1.0174 | 43658 | 5.01e2 | 1 | 1 | TRINITY\_DN15151\_c0\_g1\_i2.p1:TRINITY\_DN15151\_c0\_g1\_i3.p1:TRINITY\_DN15151\_c0\_g1\_i1.p1 |  |  | DB Search |
| VVAEC(+57.02)NNGGGR | 40.05 | 1131.509 | 11 | 3.26 | 566.7612 | 9.40 | 0.825 | 0.8323 | 11191 | 0 | 0 | 0 | TRINITY\_DN1616\_c0\_g1\_i3.p1:TRINITY\_DN1616\_c0\_g1\_i6.p1:TRINITY\_DN1616\_c0\_g1\_i7.p1 | Carbamidomethylation |  | DB Search |
| VKADGPGLKPGNK | 40.05 | 1279.7247 | 13 | 11.94 | 427.5854 | 10.11 | 0.7734 | 0.7807 | 15039 | 0 | 0 | 0 | TRINITY\_DN941\_c0\_g1\_i9.p1 |  |  | DB Search |
| MTSC(+57.02)EANHQVAK | 39.54 | 1374.6021 | 12 | -0.33 | 459.2058 | 9.67 | 0.7726 | 0.7799 | 12570 | 0 | 0 | 0 | TRINITY\_DN1369\_c0\_g1\_i1.p1 | Carbamidomethylation |  | DB Search |
| FSNDIVDSFAVSQKK | 39.48 | 1683.8468 | 15 | -4.48 | 562.2845 | 17.25 | 0.8688 | 0.8761 | 57087 | 0 | 0 | 0 | TRINITY\_DN244\_c0\_g1\_i1.p1 |  |  | DB Search |
| AGAQGVDGR | 39.31 | 829.4042 | 9 | 0.74 | 415.7079 | 9.36 | 0.7184 | 0.7257 | 10966 | 2.27e2 | 1 | 1 | TRINITY\_DN113\_c0\_g1\_i1.p1 |  |  | DB Search |
| YRVSQDIGVTDFNLVDPK | 39.31 | 2065.0481 | 18 | 0.36 | 1033.5271 | 19.75 | 1.1905 | 1.1977 | 74388 | 0 | 0 | 0 | TRINITY\_DN2357\_c1\_g1\_i1.p1 |  |  | DB Search |
| YEC(+57.02)NC(+57.02)QYNTEGPR | 39.28 | 1689.6511 | 13 | 9.13 | 845.8369 | 12.04 | 1.0323 | 1.0395 | 26880 | 1.68e2 | 1 | 1 | TRINITY\_DN1616\_c0\_g1\_i3.p1:TRINITY\_DN1616\_c0\_g1\_i6.p1:TRINITY\_DN1616\_c0\_g1\_i2.p1:TRINITY\_DN1616\_c0\_g1\_i7.p1 | Carbamidomethylation, Carbamidomethylation |  | DB Search |
| KKLEPQAAPR | 39.03 | 1136.6665 | 10 | -0.45 | 379.8943 | 9.66 | 0.7485 | 0.7559 | 12515 | 8.42e2 | 1 | 1 | TRINITY\_DN22492\_c0\_g1\_i1.p1:TRINITY\_DN22492\_c0\_g1\_i2.p1 |  |  | DB Search |
| LSFAVNFR | 39.01 | 952.513 | 8 | 7.62 | 477.2653 | 18.46 | 0.8214 | 0.8287 | 65548 | 0 | 0 | 0 | TRINITY\_DN17292\_c0\_g1\_i1.p1 |  |  | DB Search |
| AKQEAFIAELEDQIAK | 38.87 | 1802.9414 | 16 | -2.92 | 601.9833 | 19.97 | 0.881 | 0.8883 | 75797 | 0 | 0 | 0 | TRINITY\_DN428\_c0\_g1\_i1.p1 |  |  | DB Search |
| SPFVIM(+15.99)FK | 38.78 | 983.515 | 8 | -5.11 | 492.7601 | 18.60 | 0.8128 | 0.8201 | 66496 | 4.32e2 | 1 | 1 | TRINITY\_DN4595\_c0\_g1\_i10.p1:TRINITY\_DN4595\_c0\_g1\_i4.p1 | Oxidation (M) | M6:Oxidation (M):1000 | DB Search |
| ADLTRPSGK | 38.69 | 943.5087 | 9 | -2.87 | 472.7582 | 10.10 | 0.8189 | 0.8262 | 15057 | 1.13e3 | 1 | 1 | TRINITY\_DN941\_c0\_g1\_i15.p1:TRINITY\_DN941\_c0\_g1\_i3.p1 |  |  | DB Search |
| K(+42.01)LAAELANGR | 38.62 | 1083.6036 | 10 | 4.95 | 542.8094 | 15.45 | 0.8648 | 0.8721 | 46842 | 3.78e2 | 1 | 1 | TRINITY\_DN1172\_c0\_g2\_i2.p1 | Acetylation (Protein N-term) | K1:Acetylation (Protein N-term):1000 | DB Search |
| TPYLQIDFGK | 38.54 | 1180.6128 | 10 | -1.83 | 591.31 | 18.42 | 0.9198 | 0.9271 | 65300 | 4.11e2 | 1 | 1 | TRINITY\_DN11248\_c0\_g1\_i1.p1:TRINITY\_DN11248\_c0\_g1\_i3.p1 |  |  | DB Search |
| KTTELTNNLK | 38.39 | 1160.6401 | 10 | 1.48 | 581.3256 | 11.73 | 0.9182 | 0.9255 | 24848 | 1.03e3 | 1 | 1 | TRINITY\_DN72625\_c0\_g1\_i1.p1 |  |  | DB Search |
| LASDLIILK | 37.83 | 984.6219 | 9 | 0.65 | 493.3164 | 18.35 | 0.8287 | 0.836 | 64664 | 5.03e2 | 1 | 1 | TRINITY\_DN118327\_c0\_g1\_i1.p1 |  |  | DB Search |
| RLTELEHFLK | 37.74 | 1284.719 | 10 | -0.56 | 429.2448 | 16.44 | 0.7445 | 0.7518 | 52334 | 1.1e3 | 1 | 1 | TRINITY\_DN1119\_c0\_g1\_i1.p1 |  |  | DB Search |
| LC(+57.02)FVGLM(+15.99)SM(+15.99)IDPPR | 37.39 | 1666.7881 | 14 | 11.56 | 834.4073 | 18.83 | 1.0761 | 1.0833 | 67962 | 2.82e2 | 1 | 1 | TRINITY\_DN262\_c0\_g1\_i1.p1 | Carbamidomethylation, Oxidation (M), Oxidation (M) | M7:Oxidation (M):1000 M9:Oxidation (M):1000 | DB Search |
| AMQSLPPGPQHR | 37.32 | 1317.6611 | 12 | -2.22 | 659.8335 | 12.94 | 0.965 | 0.9723 | 32063 | 2.43e3 | 1 | 1 | TRINITY\_DN165490\_c0\_g1\_i1.p1 |  |  | DB Search |
| ATETATGLATTHPIK | 37.3 | 1510.7991 | 15 | 4.21 | 756.4067 | 13.04 | 1.1001 | 1.1074 | 32766 | 0 | 0 | 0 | TRINITY\_DN165536\_c0\_g1\_i1.p1 |  |  | DB Search |
| FELDRVNTFGDEVFGDPK | 37.28 | 2083.9851 | 18 | 5.01 | 695.6694 | 19.27 | 0.9178 | 0.9251 | 71114 | 5.19e2 | 1 | 1 | TRINITY\_DN1676\_c0\_g1\_i6.p1:TRINITY\_DN1676\_c0\_g1\_i12.p1 |  |  | DB Search |
| ADILC(+57.02)HDTK | 37.17 | 1071.5018 | 9 | 4.34 | 536.7582 | 11.78 | 0.8716 | 0.8789 | 25234 | 2.05e2 | 1 | 1 | TRINITY\_DN413\_c0\_g1\_i1.p1:TRINITY\_DN413\_c0\_g1\_i4.p1:TRINITY\_DN413\_c0\_g1\_i3.p1 | Carbamidomethylation |  | DB Search |
| DLTDYLMK | 37.13 | 997.479 | 8 | 2.04 | 499.7456 | 18.64 | 0.8372 | 0.8445 | 66608 | 3.54e2 | 1 | 1 | TRINITY\_DN161\_c0\_g1\_i1.p1:TRINITY\_DN161\_c0\_g1\_i4.p1:TRINITY\_DN161\_c0\_g1\_i3.p1:TRINITY\_DN161\_c0\_g1\_i2.p1 |  |  | DB Search |
| VGINYQPPTVVPGGDLAK | 37.08 | 1823.9781 | 18 | 0.87 | 912.9931 | 17.39 | 1.1218 | 1.129 | 57959 | 0 | 0 | 0 | TRINITY\_DN687\_c0\_g2\_i1.p1:TRINITY\_DN250\_c0\_g1\_i5.p1:TRINITY\_DN687\_c0\_g1\_i1.p1:TRINITY\_DN250\_c0\_g1\_i1.p1:TRINITY\_DN250\_c0\_g1\_i6.p1:TRINITY\_DN250\_c0\_g1\_i4.p1:TRINITY\_DN96161\_c0\_g1\_i1.p1:TRINITY\_DN250\_c0\_g1\_i3.p1 |  |  | DB Search |
| HGADLFYR | 37.07 | 977.4719 | 8 | 0.29 | 489.7412 | 16.16 | 0.8372 | 0.8445 | 50704 | 4.61e2 | 1 | 1 | TRINITY\_DN602\_c0\_g1\_i4.p1:TRINITY\_DN602\_c0\_g1\_i3.p1:TRINITY\_DN602\_c0\_g1\_i1.p1 |  |  | DB Search |
| FLVELAC(+57.02)K | 36.88 | 978.5208 | 8 | 0.57 | 490.2658 | 17.23 | 0.8242 | 0.8315 | 56835 | 2.47e3 | 1 | 1 | TRINITY\_DN1119\_c0\_g1\_i1.p1 | Carbamidomethylation |  | DB Search |
| total 303 peptides |  |  |  |  |  |  |  |  |  |  |  |  |  |  |  |  |
| --- | --- | --- | --- | --- | --- | --- | --- | --- | --- | --- | --- | --- | --- | --- | --- | --- |
