# Supplementary material for: Proteomic Analysis and Biochemical Characterization of the Nematocyst Extract of the Hydrozoan Velella velella
Source: Mar Drugs. 2024 Oct 12;22(10):468. doi: 10.3390/md22100468 (PMC11509761; doi:10.3390/md22100468)
Supplement: Supplementary file 1 [file marinedrugs-22-00468-s001.zip › Supplementary File S1.zip!/Sample_4_Vellela/db.proteins.html]

proteins


Protein List

  

| Protein Group | Protein ID | Accession | -10lgP | Coverage (%) | Coverage (%) Sample 6 | Area Sample 6 | #Peptides | #Unique | #Spec Sample 6 | PTM | Avg. Mass | Description |
| --- | --- | --- | --- | --- | --- | --- | --- | --- | --- | --- | --- | --- |
| 1 | 1 | TRINITY\_DN982\_c0\_g1\_i3.p1 | 155.58 | 2 | 2 | 1.03e3 | 15 | 4 | 27 | Carbamidomethylation | 1090854.75 | type:complete len:9852 gc:universal TRINITY\_DN982\_c0\_g1\_i3:29697-142(-) |
| 2 | 2 | TRINITY\_DN982\_c0\_g1\_i1.p1 | 155.53 | 3 | 3 | 2.69e3 | 15 | 4 | 28 | Carbamidomethylation | 712566.0625 | type:5prime\_partial len:6412 gc:universal TRINITY\_DN982\_c0\_g1\_i1:19377-142(-) |
| 2 | 3 | TRINITY\_DN982\_c0\_g1\_i2.p1 | 121.25 | 5 | 5 | 2.69e3 | 7 | 4 | 13 |  | 190367.1406 | type:internal len:1718 gc:universal TRINITY\_DN982\_c0\_g1\_i2:5151-1(-) |
| 3 | 4 | TRINITY\_DN4595\_c0\_g1\_i4.p1 | 135.22 | 7 | 7 | 7.12e3 | 10 | 9 | 12 | Carbamidomethylation, Oxidation (M) | 183684.4844 | type:5prime\_partial len:1639 gc:universal TRINITY\_DN4595\_c0\_g1\_i4:5857-941(-) |
| 3 | 5 | TRINITY\_DN4595\_c0\_g1\_i10.p1 | 97.21 | 4 | 4 | 2.77e3 | 4 | 4 | 6 | Carbamidomethylation, Oxidation (M) | 133760.0625 | type:complete len:1188 gc:universal TRINITY\_DN4595\_c0\_g1\_i10:4405-842(-) |
| 3 | 6 | TRINITY\_DN4595\_c0\_g1\_i10.p2 | 94.42 | 12 | 12 | 2.2e3 | 4 | 3 | 4 | Carbamidomethylation | 39822.0156 | type:5prime\_partial len:358 gc:universal TRINITY\_DN4595\_c0\_g1\_i10:5369-4296(-) |
| 3 | 7 | TRINITY\_DN4595\_c0\_g1\_i7.p1 | 65.27 | 5 | 5 | 1.84e3 | 2 | 1 | 2 |  | 38387.582 | type:internal len:348 gc:universal TRINITY\_DN4595\_c0\_g1\_i7:1042-2(-) |
| 3 | 8 | TRINITY\_DN4595\_c0\_g1\_i1.p1 | 41.96 | 6 | 6 | 1.84e3 | 1 | 1 | 1 |  | 15339.0303 | type:internal len:138 gc:universal TRINITY\_DN4595\_c0\_g1\_i1:412-2(-) |
| 4 | 9 | TRINITY\_DN138\_c0\_g1\_i1.p1 | 135.79 | 32 | 32 | 2.2e4 | 10 | 10 | 18 | Oxidation (M), Carbamidomethylation | 46324.8945 | type:complete len:401 gc:universal TRINITY\_DN138\_c0\_g1\_i1:157-1359(+) |
| 5 | 10 | TRINITY\_DN262\_c0\_g1\_i1.p1 | 136.83 | 12 | 12 | 7.97e3 | 10 | 10 | 13 | Acetylation (Protein N-term), Carbamidomethylation, Oxidation (M) | 114520.6406 | type:complete len:1033 gc:universal TRINITY\_DN262\_c0\_g1\_i1:111-3209(+) |
| 6 | 11 | TRINITY\_DN15274\_c0\_g1\_i1.p1 | 132.38 | 7 | 7 | 7.62e3 | 9 | 9 | 11 | Carbamidomethylation, Oxidation (M) | 201897.7344 | type:complete len:1822 gc:universal TRINITY\_DN15274\_c0\_g1\_i1:87-5552(+) |
| 7 | 12 | TRINITY\_DN161\_c0\_g1\_i2.p1 | 125.98 | 26 | 26 | 3.87e4 | 8 | 8 | 11 | Oxidation (M) | 44577.6484 | type:5prime\_partial len:399 gc:universal TRINITY\_DN161\_c0\_g1\_i2:1683-487(-) |
| 7 | 13 | TRINITY\_DN161\_c0\_g1\_i3.p1 | 125.98 | 26 | 26 | 3.87e4 | 8 | 8 | 11 | Oxidation (M) | 44592.6602 | type:5prime\_partial len:399 gc:universal TRINITY\_DN161\_c0\_g1\_i3:1450-254(-) |
| 7 | 14 | TRINITY\_DN161\_c0\_g1\_i1.p1 | 125.98 | 27 | 27 | 3.87e4 | 8 | 8 | 11 | Oxidation (M) | 41918.5938 | type:complete len:377 gc:universal TRINITY\_DN161\_c0\_g1\_i1:1384-254(-) |
| 7 | 15 | TRINITY\_DN161\_c0\_g1\_i4.p1 | 95.49 | 22 | 22 | 1.39e3 | 4 | 4 | 4 | Oxidation (M) | 25887.2988 | type:5prime\_partial len:232 gc:universal TRINITY\_DN161\_c0\_g1\_i4:949-254(-) |
| 7 | 16 | TRINITY\_DN2477\_c0\_g1\_i5.p1 | 69.74 | 9 | 9 | 2.51e3 | 2 | 2 | 2 |  | 37416.5195 | type:complete len:333 gc:universal TRINITY\_DN2477\_c0\_g1\_i5:1182-184(-) |
| 7 | 17 | TRINITY\_DN2477\_c0\_g1\_i10.p1 | 69.74 | 9 | 9 | 2.51e3 | 2 | 2 | 2 |  | 38566.9688 | type:5prime\_partial len:344 gc:universal TRINITY\_DN2477\_c0\_g1\_i10:1215-184(-) |
| 7 | 18 | TRINITY\_DN2477\_c0\_g1\_i2.p1 | 69.74 | 9 | 9 | 2.51e3 | 2 | 2 | 2 |  | 38566.9688 | type:5prime\_partial len:344 gc:universal TRINITY\_DN2477\_c0\_g1\_i2:1215-184(-) |
| 7 | 19 | TRINITY\_DN195934\_c0\_g1\_i1.p2 | 69.74 | 24 | 24 | 2.51e3 | 2 | 2 | 2 |  | 14094.0938 | type:internal len:127 gc:universal TRINITY\_DN195934\_c0\_g1\_i1:3-380(+) |
| 7 | 20 | TRINITY\_DN2477\_c0\_g1\_i6.p1 | 69.74 | 8 | 8 | 2.51e3 | 2 | 2 | 2 |  | 41878.6875 | type:complete len:377 gc:universal TRINITY\_DN2477\_c0\_g1\_i6:1314-184(-) |
| 7 | 21 | TRINITY\_DN2477\_c0\_g1\_i9.p1 | 69.74 | 8 | 8 | 2.51e3 | 2 | 2 | 2 |  | 41878.6875 | type:complete len:377 gc:universal TRINITY\_DN2477\_c0\_g1\_i9:1314-184(-) |
| 7 | 22 | TRINITY\_DN2941\_c0\_g1\_i4.p1 | 46.46 | 16 | 16 | 0 | 1 | 1 | 1 |  | 13034.9365 | type:internal len:116 gc:universal TRINITY\_DN2941\_c0\_g1\_i4:2-346(+) |
| 7 | 23 | TRINITY\_DN2477\_c0\_g1\_i8.p1 | 46.46 | 6 | 6 | 0 | 1 | 1 | 1 |  | 34820.5938 | type:5prime\_partial len:309 gc:universal TRINITY\_DN2477\_c0\_g1\_i8:1110-184(-) |
| 8 | 24 | TRINITY\_DN1616\_c0\_g1\_i3.p1 | 118.57 | 3 | 3 | 1.26e3 | 7 | 7 | 7 | Carbamidomethylation | 423947.2812 | type:complete len:3855 gc:universal TRINITY\_DN1616\_c0\_g1\_i3:218-11782(+) |
| 8 | 25 | TRINITY\_DN1616\_c0\_g1\_i7.p1 | 118.57 | 3 | 3 | 1.26e3 | 7 | 7 | 7 | Carbamidomethylation | 423875.2188 | type:complete len:3855 gc:universal TRINITY\_DN1616\_c0\_g1\_i7:218-11782(+) |
| 8 | 26 | TRINITY\_DN1616\_c0\_g1\_i6.p1 | 118.57 | 3 | 3 | 1.26e3 | 7 | 7 | 7 | Carbamidomethylation | 423911.25 | type:complete len:3855 gc:universal TRINITY\_DN1616\_c0\_g1\_i6:218-11782(+) |
| 8 | 27 | TRINITY\_DN1616\_c0\_g1\_i2.p1 | 112.83 | 3 | 3 | 1.26e3 | 6 | 6 | 6 | Carbamidomethylation | 374396 | type:3prime\_partial len:3398 gc:universal TRINITY\_DN1616\_c0\_g1\_i2:218-10408(+) |
| 9 | 28 | TRINITY\_DN1369\_c0\_g1\_i1.p1 | 113.21 | 5 | 5 | 2.7e3 | 6 | 6 | 7 | Carbamidomethylation | 174229.4219 | type:3prime\_partial len:1577 gc:universal TRINITY\_DN1369\_c0\_g1\_i1:93-4820(+) |
| 10 | 29 | TRINITY\_DN140702\_c0\_g1\_i1.p1 | 115.06 | 4 | 4 | 1.49e4 | 6 | 6 | 9 |  | 231919.1562 | type:complete len:2036 gc:universal TRINITY\_DN140702\_c0\_g1\_i1:94-6201(+) |
| 11 | 30 | TRINITY\_DN428\_c0\_g1\_i1.p1 | 103.02 | 4 | 4 | 2.08e3 | 5 | 5 | 5 | Carbamidomethylation | 225824.1719 | type:complete len:1949 gc:universal TRINITY\_DN428\_c0\_g1\_i1:96-5942(+) |
| 12 | 31 | TRINITY\_DN602\_c0\_g1\_i3.p1 | 94.81 | 6 | 6 | 2.17e3 | 4 | 4 | 4 | Oxidation (M) | 92279.0078 | type:complete len:851 gc:universal TRINITY\_DN602\_c0\_g1\_i3:3046-494(-) |
| 12 | 32 | TRINITY\_DN602\_c0\_g1\_i4.p1 | 94.81 | 9 | 9 | 2.17e3 | 4 | 4 | 4 | Oxidation (M) | 61175.9766 | type:5prime\_partial len:552 gc:universal TRINITY\_DN602\_c0\_g1\_i4:3498-1843(-) |
| 12 | 33 | TRINITY\_DN602\_c0\_g1\_i1.p1 | 94.81 | 9 | 9 | 2.17e3 | 4 | 4 | 4 | Oxidation (M) | 65701.2422 | type:complete len:593 gc:universal TRINITY\_DN602\_c0\_g1\_i1:3622-1844(-) |
| 12 | 34 | TRINITY\_DN602\_c0\_g2\_i1.p1 | 45.42 | 2 | 2 | 7.87e2 | 1 | 1 | 1 |  | 62057.1875 | type:5prime\_partial len:561 gc:universal TRINITY\_DN602\_c0\_g2\_i1:1994-312(-) |
| 13 | 35 | TRINITY\_DN477\_c0\_g1\_i7.p1 | 97.86 | 4 | 4 | 2.74e4 | 4 | 4 | 11 | Carbamidomethylation | 122478.2578 | type:3prime\_partial len:1130 gc:universal TRINITY\_DN477\_c0\_g1\_i7:3387-1(-) |
| 13 | 36 | TRINITY\_DN477\_c0\_g1\_i8.p1 | 85.82 | 4 | 4 | 2.38e4 | 3 | 3 | 9 | Carbamidomethylation | 107165.2031 | type:complete len:985 gc:universal TRINITY\_DN477\_c0\_g1\_i8:3548-594(-) |
| 13 | 37 | TRINITY\_DN477\_c0\_g1\_i3.p1 | 47.24 | 4 | 4 | 3.59e3 | 1 | 1 | 2 |  | 24580.2559 | type:internal len:228 gc:universal TRINITY\_DN477\_c0\_g1\_i3:681-1(-) |
| 13 | 38 | TRINITY\_DN477\_c0\_g1\_i8.p2 | 47.24 | 5 | 5 | 3.59e3 | 1 | 1 | 2 |  | 20774.9902 | type:3prime\_partial len:194 gc:universal TRINITY\_DN477\_c0\_g1\_i8:581-3(-) |
| 13 | 39 | TRINITY\_DN477\_c0\_g1\_i6.p1 | 47.24 | 5 | 5 | 3.59e3 | 1 | 1 | 2 |  | 20745.9492 | type:3prime\_partial len:194 gc:universal TRINITY\_DN477\_c0\_g1\_i6:581-3(-) |
| 13 | 40 | TRINITY\_DN477\_c0\_g1\_i5.p1 | 47.24 | 4 | 4 | 3.59e3 | 1 | 1 | 2 |  | 28860.875 | type:internal len:276 gc:universal TRINITY\_DN477\_c0\_g1\_i5:827-3(-) |
| 14 | 41 | TRINITY\_DN3078\_c0\_g1\_i1.p1 | 98.19 | 2 | 2 | 3.23e3 | 4 | 4 | 6 | Carbamidomethylation | 127689.1797 | type:complete len:1109 gc:universal TRINITY\_DN3078\_c0\_g1\_i1:3628-302(-) |
| 15 | 42 | TRINITY\_DN244\_c0\_g1\_i1.p1 | 93.81 | 1 | 1 | 3.02e3 | 4 | 4 | 8 |  | 313594.0938 | type:complete len:3019 gc:universal TRINITY\_DN244\_c0\_g1\_i1:9748-692(-) |
| 16 | 43 | TRINITY\_DN71276\_c0\_g1\_i1.p1 | 85.66 | 3 | 3 | 6.5e2 | 3 | 3 | 4 | Oxidation (M) | 139103.3125 | type:5prime\_partial len:1201 gc:universal TRINITY\_DN71276\_c0\_g1\_i1:1-3603(+) |
| 17 | 44 | TRINITY\_DN72625\_c0\_g1\_i1.p1 | 82.22 | 2 | 2 | 8.19e3 | 3 | 3 | 4 |  | 214200.3906 | type:complete len:1964 gc:universal TRINITY\_DN72625\_c0\_g1\_i1:173-6064(+) |
| 18 | 45 | TRINITY\_DN1119\_c0\_g1\_i1.p1 | 77.64 | 3 | 3 | 3.56e3 | 3 | 3 | 3 | Carbamidomethylation | 105284.3438 | type:5prime\_partial len:949 gc:universal TRINITY\_DN1119\_c0\_g1\_i1:2934-88(-) |
| 19 | 46 | TRINITY\_DN77\_c1\_g1\_i1.p1 | 86.68 | 2 | 2 | 1.61e3 | 3 | 3 | 4 |  | 198350.2344 | type:3prime\_partial len:1965 gc:universal TRINITY\_DN77\_c1\_g1\_i1:2-5893(+) |
| 20 | 47 | TRINITY\_DN1653\_c0\_g1\_i3.p1 | 86.4 | 3 | 3 | 2.82e3 | 3 | 3 | 6 |  | 114336.4531 | type:complete len:1023 gc:universal TRINITY\_DN1653\_c0\_g1\_i3:180-3248(+) |
| 20 | 48 | TRINITY\_DN1653\_c0\_g1\_i1.p1 | 86.4 | 3 | 3 | 2.82e3 | 3 | 3 | 6 |  | 125115.2109 | type:complete len:1122 gc:universal TRINITY\_DN1653\_c0\_g1\_i1:180-3545(+) |
| 21 | 49 | TRINITY\_DN250\_c0\_g1\_i3.p1 | 80.23 | 12 | 12 | 3.55e2 | 3 | 3 | 3 | Carbamidomethylation, Oxidation (M) | 44456.6445 | type:3prime\_partial len:402 gc:universal TRINITY\_DN250\_c0\_g1\_i3:131-1333(+) |
| 21 | 50 | TRINITY\_DN250\_c0\_g1\_i5.p1 | 61.55 | 8 | 8 | 2.71e2 | 2 | 2 | 2 | Carbamidomethylation, Oxidation (M) | 50305.4453 | type:complete len:452 gc:universal TRINITY\_DN250\_c0\_g1\_i5:131-1486(+) |
| 21 | 51 | TRINITY\_DN687\_c0\_g2\_i1.p1 | 61.55 | 8 | 8 | 2.71e2 | 2 | 2 | 2 | Carbamidomethylation, Oxidation (M) | 50237.3242 | type:complete len:453 gc:universal TRINITY\_DN687\_c0\_g2\_i1:90-1448(+) |
| 21 | 52 | TRINITY\_DN250\_c0\_g1\_i1.p1 | 61.55 | 9 | 9 | 2.71e2 | 2 | 2 | 2 | Carbamidomethylation, Oxidation (M) | 44454.6094 | type:3prime\_partial len:402 gc:universal TRINITY\_DN250\_c0\_g1\_i1:131-1333(+) |
| 21 | 53 | TRINITY\_DN687\_c0\_g1\_i1.p1 | 61.55 | 8 | 8 | 2.71e2 | 2 | 2 | 2 | Carbamidomethylation, Oxidation (M) | 50621.6914 | type:complete len:453 gc:universal TRINITY\_DN687\_c0\_g1\_i1:95-1453(+) |
| 21 | 54 | TRINITY\_DN250\_c0\_g1\_i2.p1 | 46.37 | 4 | 4 | 8.43e1 | 1 | 1 | 1 | Oxidation (M) | 35668.3789 | type:3prime\_partial len:322 gc:universal TRINITY\_DN250\_c0\_g1\_i2:131-1093(+) |
| 21 | 55 | TRINITY\_DN250\_c0\_g1\_i6.p1 | 37.08 | 4 | 4 | 0 | 1 | 1 | 1 |  | 50263.3672 | type:complete len:452 gc:universal TRINITY\_DN250\_c0\_g1\_i6:131-1486(+) |
| 21 | 56 | TRINITY\_DN96161\_c0\_g1\_i1.p1 | 37.08 | 4 | 4 | 0 | 1 | 1 | 1 |  | 50498.6562 | type:complete len:453 gc:universal TRINITY\_DN96161\_c0\_g1\_i1:1593-235(-) |
| 21 | 57 | TRINITY\_DN250\_c0\_g1\_i4.p1 | 37.08 | 4 | 4 | 0 | 1 | 1 | 1 |  | 50305.4453 | type:complete len:452 gc:universal TRINITY\_DN250\_c0\_g1\_i4:131-1486(+) |
| 22 | 58 | TRINITY\_DN6390\_c0\_g1\_i1.p1 | 86.14 | 2 | 2 | 5.09e2 | 3 | 3 | 6 | Carbamidomethylation | 214480.2344 | type:3prime\_partial len:1941 gc:universal TRINITY\_DN6390\_c0\_g1\_i1:5820-1(-) |
| 23 | 59 | TRINITY\_DN319\_c2\_g1\_i3.p1 | 86 | 2 | 2 | 1.65e3 | 3 | 3 | 4 | Carbamidomethylation | 279087.1875 | type:complete len:2588 gc:universal TRINITY\_DN319\_c2\_g1\_i3:165-7928(+) |
| 23 | 60 | TRINITY\_DN319\_c2\_g1\_i1.p1 | 86 | 2 | 2 | 1.65e3 | 3 | 3 | 4 | Carbamidomethylation | 279168.25 | type:complete len:2588 gc:universal TRINITY\_DN319\_c2\_g1\_i1:165-7928(+) |
| 23 | 61 | TRINITY\_DN319\_c2\_g1\_i2.p1 | 70.44 | 1 | 1 | 1.65e3 | 2 | 2 | 3 | Carbamidomethylation | 215796.4062 | type:3prime\_partial len:2004 gc:universal TRINITY\_DN319\_c2\_g1\_i2:165-6173(+) |
| 24 | 62 | TRINITY\_DN477\_c0\_g1\_i9.p1 | 86.25 | 8 | 8 | 4.13e3 | 3 | 3 | 5 | Oxidation (M) | 34222.9453 | type:complete len:306 gc:universal TRINITY\_DN477\_c0\_g1\_i9:1840-923(-) |
| 25 | 63 | TRINITY\_DN1280\_c0\_g1\_i1.p1 | 85.43 | 8 | 8 | 3.18e2 | 3 | 3 | 3 |  | 47818.6641 | type:complete len:426 gc:universal TRINITY\_DN1280\_c0\_g1\_i1:195-1472(+) |
| 26 | 64 | TRINITY\_DN36\_c0\_g1\_i16.p1 | 67.87 | 9 | 9 | 0 | 2 | 2 | 2 | Carbamidomethylation | 24953.5938 | type:complete len:224 gc:universal TRINITY\_DN36\_c0\_g1\_i16:829-158(-) |
| 26 | 65 | TRINITY\_DN36\_c0\_g1\_i19.p1 | 67.87 | 9 | 9 | 0 | 2 | 2 | 2 | Carbamidomethylation | 24821.4141 | type:complete len:223 gc:universal TRINITY\_DN36\_c0\_g1\_i19:826-158(-) |
| 26 | 66 | TRINITY\_DN36\_c0\_g1\_i17.p1 | 67.87 | 9 | 9 | 0 | 2 | 2 | 2 | Carbamidomethylation | 24953.5938 | type:complete len:224 gc:universal TRINITY\_DN36\_c0\_g1\_i17:819-148(-) |
| 26 | 67 | TRINITY\_DN36\_c0\_g1\_i13.p1 | 67.87 | 9 | 9 | 0 | 2 | 2 | 2 | Carbamidomethylation | 24821.4141 | type:complete len:223 gc:universal TRINITY\_DN36\_c0\_g1\_i13:816-148(-) |
| 26 | 68 | TRINITY\_DN36\_c0\_g1\_i3.p1 | 67.87 | 9 | 9 | 0 | 2 | 2 | 2 | Carbamidomethylation | 24953.5938 | type:complete len:224 gc:universal TRINITY\_DN36\_c0\_g1\_i3:829-158(-) |
| 26 | 69 | TRINITY\_DN36\_c0\_g1\_i14.p1 | 67.87 | 9 | 9 | 0 | 2 | 2 | 2 | Carbamidomethylation | 24953.5938 | type:complete len:224 gc:universal TRINITY\_DN36\_c0\_g1\_i14:819-148(-) |
| 26 | 70 | TRINITY\_DN36\_c0\_g1\_i8.p1 | 67.87 | 9 | 9 | 0 | 2 | 2 | 2 | Carbamidomethylation | 24953.5938 | type:complete len:224 gc:universal TRINITY\_DN36\_c0\_g1\_i8:819-148(-) |
| 26 | 71 | TRINITY\_DN649\_c0\_g1\_i5.p1 | 44.83 | 7 | 7 | 0 | 1 | 1 | 1 |  | 13746.6875 | type:internal len:124 gc:universal TRINITY\_DN649\_c0\_g1\_i5:371-3(-) |
| 26 | 72 | TRINITY\_DN649\_c0\_g1\_i2.p1 | 44.83 | 6 | 6 | 0 | 1 | 1 | 1 |  | 13819.7393 | type:internal len:125 gc:universal TRINITY\_DN649\_c0\_g1\_i2:374-3(-) |
| 26 | 73 | TRINITY\_DN649\_c0\_g1\_i1.p1 | 44.83 | 7 | 7 | 0 | 1 | 1 | 1 |  | 13690.5801 | type:internal len:124 gc:universal TRINITY\_DN649\_c0\_g1\_i1:371-3(-) |
| 27 | 74 | TRINITY\_DN11248\_c0\_g1\_i1.p1 | 66.14 | 1 | 1 | 4.11e2 | 2 | 2 | 2 | Carbamidomethylation | 194116.9531 | type:3prime\_partial len:1700 gc:universal TRINITY\_DN11248\_c0\_g1\_i1:111-5207(+) |
| 27 | 75 | TRINITY\_DN11248\_c0\_g1\_i3.p1 | 66.14 | 1 | 1 | 4.11e2 | 2 | 2 | 2 | Carbamidomethylation | 194116.9531 | type:3prime\_partial len:1700 gc:universal TRINITY\_DN11248\_c0\_g1\_i3:111-5207(+) |
| 28 | 76 | TRINITY\_DN1676\_c0\_g1\_i12.p1 | 65.3 | 2 | 2 | 9.47e2 | 2 | 2 | 2 |  | 180975.5156 | type:complete len:1631 gc:universal TRINITY\_DN1676\_c0\_g1\_i12:206-5098(+) |
| 28 | 77 | TRINITY\_DN1676\_c0\_g1\_i6.p1 | 65.3 | 2 | 2 | 9.47e2 | 2 | 2 | 2 |  | 180975.5156 | type:complete len:1631 gc:universal TRINITY\_DN1676\_c0\_g1\_i6:206-5098(+) |
| 29 | 78 | TRINITY\_DN691\_c0\_g1\_i1.p1 | 70.36 | 1 | 1 | 1.37e3 | 2 | 2 | 3 | Carbamidomethylation | 169920.1562 | type:complete len:1726 gc:universal TRINITY\_DN691\_c0\_g1\_i1:5662-485(-) |
| 29 | 79 | TRINITY\_DN691\_c0\_g1\_i2.p1 | 70.36 | 1 | 1 | 1.37e3 | 2 | 2 | 3 | Carbamidomethylation | 164625.2188 | type:complete len:1673 gc:universal TRINITY\_DN691\_c0\_g1\_i2:5503-485(-) |
| 30 | 80 | TRINITY\_DN4726\_c0\_g1\_i1.p1 | 69.73 | 4 | 4 | 7.86e2 | 2 | 2 | 2 | Carbamidomethylation | 75463.25 | type:5prime\_partial len:670 gc:universal TRINITY\_DN4726\_c0\_g1\_i1:2906-897(-) |
| 30 | 81 | TRINITY\_DN4726\_c0\_g1\_i2.p1 | 46.72 | 2 | 2 | 3.14e2 | 1 | 1 | 1 | Carbamidomethylation | 86431.8438 | type:5prime\_partial len:763 gc:universal TRINITY\_DN4726\_c0\_g1\_i2:3186-898(-) |
| 30 | 82 | TRINITY\_DN4726\_c0\_g1\_i3.p1 | 46.72 | 2 | 2 | 3.14e2 | 1 | 1 | 1 | Carbamidomethylation | 84416.5469 | type:5prime\_partial len:746 gc:universal TRINITY\_DN4726\_c0\_g1\_i3:3135-898(-) |
| 31 | 83 | TRINITY\_DN329\_c0\_g1\_i2.p1 | 63.67 | 10 | 10 | 0 | 2 | 2 | 2 |  | 34761.9609 | type:5prime\_partial len:320 gc:universal TRINITY\_DN329\_c0\_g1\_i2:1-960(+) |
| 31 | 84 | TRINITY\_DN329\_c0\_g1\_i1.p1 | 63.67 | 11 | 11 | 0 | 2 | 2 | 2 |  | 30514.9648 | type:complete len:283 gc:universal TRINITY\_DN329\_c0\_g1\_i1:158-1006(+) |
| 32 | 85 | TRINITY\_DN1936\_c0\_g1\_i3.p1 | 68.67 | 2 | 2 | 6.41e2 | 2 | 2 | 2 | Carbamidomethylation | 119975.3906 | type:internal len:1070 gc:universal TRINITY\_DN1936\_c0\_g1\_i3:3208-2(-) |
| 33 | 86 | TRINITY\_DN875\_c0\_g1\_i1.p1 | 70.78 | 4 | 4 | 8.93e2 | 2 | 2 | 2 | Carbamidomethylation | 67023.6094 | type:complete len:606 gc:universal TRINITY\_DN875\_c0\_g1\_i1:2207-390(-) |
| 33 | 87 | TRINITY\_DN875\_c0\_g1\_i2.p1 | 70.78 | 4 | 4 | 8.93e2 | 2 | 2 | 2 | Carbamidomethylation | 67023.6094 | type:complete len:606 gc:universal TRINITY\_DN875\_c0\_g1\_i2:2136-319(-) |
| 33 | 88 | TRINITY\_DN875\_c0\_g1\_i3.p1 | 70.78 | 4 | 4 | 8.93e2 | 2 | 2 | 2 | Carbamidomethylation | 67023.6094 | type:complete len:606 gc:universal TRINITY\_DN875\_c0\_g1\_i3:2042-225(-) |
| 34 | 89 | TRINITY\_DN2217\_c0\_g1\_i2.p1 | 69.76 | 9 | 9 | 7.79e2 | 2 | 2 | 2 | Carbamidomethylation | 28618.9531 | type:complete len:254 gc:universal TRINITY\_DN2217\_c0\_g1\_i2:884-123(-) |
| 34 | 90 | TRINITY\_DN2217\_c0\_g1\_i3.p1 | 69.76 | 4 | 4 | 7.79e2 | 2 | 2 | 2 | Carbamidomethylation | 63077.0508 | type:complete len:566 gc:universal TRINITY\_DN2217\_c0\_g1\_i3:1820-123(-) |
| 34 | 91 | TRINITY\_DN38397\_c0\_g1\_i1.p1 | 46.47 | 3 | 3 | 0 | 1 | 1 | 1 | Carbamidomethylation | 40487.2539 | type:internal len:364 gc:universal TRINITY\_DN38397\_c0\_g1\_i1:3-1091(+) |
| 35 | 92 | TRINITY\_DN941\_c0\_g1\_i9.p1 | 63.43 | 1 | 1 | 0 | 2 | 2 | 2 | Carbamidomethylation | 340219.4688 | type:complete len:3186 gc:universal TRINITY\_DN941\_c0\_g1\_i9:86-9643(+) |
| 36 | 93 | TRINITY\_DN12627\_c0\_g1\_i1.p1 | 69.47 | 3 | 3 | 6.04e2 | 2 | 2 | 3 |  | 120592.0703 | type:5prime\_partial len:1061 gc:universal TRINITY\_DN12627\_c0\_g1\_i1:3456-274(-) |
| 37 | 94 | TRINITY\_DN8597\_c0\_g1\_i1.p1 | 68.23 | 22 | 22 | 0 | 2 | 1 | 2 | Carbamidomethylation | 13587.9307 | type:internal len:124 gc:universal TRINITY\_DN8597\_c0\_g1\_i1:369-1(-) |
| 38 | 95 | TRINITY\_DN113\_c2\_g1\_i1.p1 | 69.52 | 2 | 2 | 2.33e3 | 2 | 2 | 2 | Oxidation (M) | 118094.7031 | type:5prime\_partial len:1207 gc:universal TRINITY\_DN113\_c2\_g1\_i1:4425-805(-) |
| 39 | 96 | TRINITY\_DN113\_c0\_g1\_i1.p1 | 65.61 | 2 | 2 | 2.89e3 | 2 | 2 | 2 |  | 139235.5938 | type:complete len:1417 gc:universal TRINITY\_DN113\_c0\_g1\_i1:4413-163(-) |
| 40 | 97 | TRINITY\_DN199\_c0\_g1\_i1.p1 | 70.73 | 5 | 5 | 2.09e3 | 2 | 2 | 4 |  | 25170.3418 | type:complete len:229 gc:universal TRINITY\_DN199\_c0\_g1\_i1:207-893(+) |
| 41 | 98 | TRINITY\_DN55094\_c0\_g1\_i1.p1 | 70.06 | 25 | 25 | 0 | 2 | 2 | 2 |  | 12167.1719 | type:internal len:107 gc:universal TRINITY\_DN55094\_c0\_g1\_i1:319-2(-) |
| 42 | 99 | TRINITY\_DN8008\_c0\_g1\_i2.p1 | 70 | 2 | 2 | 0 | 2 | 2 | 2 |  | 146342.75 | type:5prime\_partial len:1298 gc:universal TRINITY\_DN8008\_c0\_g1\_i2:4205-312(-) |
| 42 | 100 | TRINITY\_DN8008\_c0\_g1\_i3.p1 | 70 | 2 | 2 | 0 | 2 | 2 | 2 |  | 146351.7188 | type:5prime\_partial len:1298 gc:universal TRINITY\_DN8008\_c0\_g1\_i3:4205-312(-) |
| 43 | 101 | TRINITY\_DN1535\_c0\_g1\_i1.p1 | 71.02 | 3 | 3 | 7.83e2 | 2 | 2 | 2 | Carbamidomethylation | 86942.8516 | type:complete len:779 gc:universal TRINITY\_DN1535\_c0\_g1\_i1:86-2422(+) |
| 44 | 102 | TRINITY\_DN1613\_c1\_g1\_i2.p1 | 68.64 | 3 | 3 | 7.11e2 | 2 | 2 | 3 | Acetylation (Protein N-term), Carbamidomethylation | 111170.0312 | type:internal len:1068 gc:universal TRINITY\_DN1613\_c1\_g1\_i2:3201-1(-) |
| 44 | 103 | TRINITY\_DN1613\_c1\_g1\_i1.p1 | 68.64 | 3 | 3 | 7.11e2 | 2 | 2 | 3 | Acetylation (Protein N-term), Carbamidomethylation | 112791.8203 | type:internal len:1083 gc:universal TRINITY\_DN1613\_c1\_g1\_i1:3248-3(-) |
| 45 | 104 | TRINITY\_DN2113\_c0\_g1\_i2.p1 | 68.64 | 2 | 2 | 1.48e3 | 2 | 2 | 2 |  | 193799.0156 | type:complete len:1704 gc:universal TRINITY\_DN2113\_c0\_g1\_i2:5257-146(-) |
| 45 | 105 | TRINITY\_DN2113\_c0\_g1\_i1.p1 | 68.64 | 2 | 2 | 1.48e3 | 2 | 2 | 2 |  | 193799.0156 | type:complete len:1704 gc:universal TRINITY\_DN2113\_c0\_g1\_i1:5257-146(-) |
| 46 | 106 | TRINITY\_DN5500\_c0\_g1\_i2.p1 | 70.63 | 2 | 2 | 1.17e3 | 2 | 2 | 2 |  | 130974.4219 | type:3prime\_partial len:1183 gc:universal TRINITY\_DN5500\_c0\_g1\_i2:3546-1(-) |
| 46 | 107 | TRINITY\_DN5500\_c0\_g1\_i1.p1 | 70.63 | 2 | 2 | 1.17e3 | 2 | 2 | 2 |  | 132375.9688 | type:3prime\_partial len:1196 gc:universal TRINITY\_DN5500\_c0\_g1\_i1:3585-1(-) |
| 47 | 108 | TRINITY\_DN4040\_c0\_g1\_i1.p1 | 69.35 | 5 | 5 | 3.42e3 | 2 | 2 | 2 | Carbamidomethylation | 45832.1406 | type:complete len:402 gc:universal TRINITY\_DN4040\_c0\_g1\_i1:1386-181(-) |
| 48 | 109 | TRINITY\_DN1320\_c0\_g2\_i1.p1 | 70.15 | 9 | 9 | 1.43e3 | 2 | 2 | 2 |  | 28787.1875 | type:complete len:260 gc:universal TRINITY\_DN1320\_c0\_g2\_i1:97-876(+) |
| 49 | 110 | TRINITY\_DN413\_c0\_g1\_i3.p1 | 65.74 | 6 | 6 | 1.13e3 | 2 | 2 | 2 | Carbamidomethylation | 39501.6133 | type:complete len:350 gc:universal TRINITY\_DN413\_c0\_g1\_i3:1286-237(-) |
| 49 | 111 | TRINITY\_DN413\_c0\_g1\_i4.p1 | 65.74 | 4 | 4 | 1.13e3 | 2 | 2 | 2 | Carbamidomethylation | 52354.1758 | type:3prime\_partial len:463 gc:universal TRINITY\_DN413\_c0\_g1\_i4:1388-3(-) |
| 49 | 112 | TRINITY\_DN413\_c0\_g1\_i1.p1 | 65.74 | 6 | 6 | 1.13e3 | 2 | 2 | 2 | Carbamidomethylation | 40351.7656 | type:complete len:355 gc:universal TRINITY\_DN413\_c0\_g1\_i1:1352-288(-) |
| 49 | 113 | TRINITY\_DN413\_c0\_g1\_i2.p1 | 47.16 | 8 | 8 | 9.22e2 | 1 | 1 | 1 |  | 15739.6523 | type:3prime\_partial len:140 gc:universal TRINITY\_DN413\_c0\_g1\_i2:419-3(-) |
| 50 | 114 | TRINITY\_DN278\_c0\_g1\_i1.p1 | 65.01 | 6 | 6 | 0 | 2 | 2 | 3 | Carbamidomethylation | 55197.2969 | type:complete len:470 gc:universal TRINITY\_DN278\_c0\_g1\_i1:3091-1682(-) |
| 51 | 115 | TRINITY\_DN598\_c1\_g1\_i1.p1 | 70.66 | 3 | 3 | 3.88e3 | 2 | 2 | 3 |  | 81322.8672 | type:5prime\_partial len:745 gc:universal TRINITY\_DN598\_c1\_g1\_i1:2-2236(+) |
| 52 | 116 | TRINITY\_DN113\_c3\_g1\_i1.p1 | 67.72 | 3 | 3 | 3.68e3 | 2 | 2 | 3 |  | 125422.4922 | type:3prime\_partial len:1162 gc:universal TRINITY\_DN113\_c3\_g1\_i1:3485-3(-) |
| 53 | 117 | TRINITY\_DN165490\_c0\_g1\_i1.p1 | 37.32 | 7 | 7 | 2.43e3 | 1 | 1 | 1 |  | 19653.5332 | type:internal len:171 gc:universal TRINITY\_DN165490\_c0\_g1\_i1:2-511(+) |
| 54 | 118 | TRINITY\_DN1505\_c0\_g1\_i1.p1 | 46.28 | 8 | 8 | 4.82e2 | 1 | 1 | 1 | Acetylation (Protein N-term) | 17930.752 | type:complete len:154 gc:universal TRINITY\_DN1505\_c0\_g1\_i1:98-559(+) |
| 55 | 119 | TRINITY\_DN1936\_c0\_g2\_i1.p1 | 43.88 | 1 | 1 | 0 | 1 | 1 | 1 |  | 208525.625 | type:3prime\_partial len:1894 gc:universal TRINITY\_DN1936\_c0\_g2\_i1:5680-2(-) |
| 56 | 120 | TRINITY\_DN925\_c0\_g1\_i1.p1 | 45.13 | 5 | 5 | 0 | 1 | 1 | 1 |  | 28336.7676 | type:complete len:261 gc:universal TRINITY\_DN925\_c0\_g1\_i1:826-44(-) |
| 57 | 121 | TRINITY\_DN624\_c0\_g1\_i1.p1 | 45.94 | 2 | 2 | 7.64e2 | 1 | 1 | 1 |  | 101743.5391 | type:complete len:884 gc:universal TRINITY\_DN624\_c0\_g1\_i1:218-2869(+) |
| 58 | 122 | TRINITY\_DN495\_c0\_g1\_i2.p1 | 47.22 | 2 | 2 | 2.16e2 | 1 | 1 | 2 | Carbamidomethylation | 72975.0312 | type:complete len:655 gc:universal TRINITY\_DN495\_c0\_g1\_i2:152-2116(+) |
| 58 | 123 | TRINITY\_DN3545\_c0\_g1\_i2.p1 | 47.22 | 5 | 5 | 2.16e2 | 1 | 1 | 2 | Carbamidomethylation | 25563.4785 | type:5prime\_partial len:236 gc:universal TRINITY\_DN3545\_c0\_g1\_i2:3-710(+) |
| 58 | 124 | TRINITY\_DN495\_c0\_g1\_i3.p1 | 47.22 | 2 | 2 | 2.16e2 | 1 | 1 | 2 | Carbamidomethylation | 73673.9297 | type:complete len:659 gc:universal TRINITY\_DN495\_c0\_g1\_i3:152-2128(+) |
| 58 | 125 | TRINITY\_DN3545\_c0\_g1\_i1.p1 | 47.22 | 4 | 4 | 2.16e2 | 1 | 1 | 2 | Carbamidomethylation | 31576.2656 | type:5prime\_partial len:293 gc:universal TRINITY\_DN3545\_c0\_g1\_i1:3-881(+) |
| 59 | 126 | TRINITY\_DN10485\_c0\_g1\_i1.p1 | 46.5 | 3 | 3 | 1.88e3 | 1 | 1 | 1 |  | 66716.9844 | type:5prime\_partial len:604 gc:universal TRINITY\_DN10485\_c0\_g1\_i1:2-1813(+) |
| 60 | 127 | TRINITY\_DN585\_c0\_g1\_i10.p1 | 46.69 | 7 | 7 | 8.78e2 | 1 | 1 | 1 |  | 26563.5332 | type:3prime\_partial len:236 gc:universal TRINITY\_DN585\_c0\_g1\_i10:705-1(-) |
| 60 | 128 | TRINITY\_DN585\_c0\_g2\_i1.p1 | 46.69 | 11 | 11 | 8.78e2 | 1 | 1 | 1 |  | 17391.9824 | type:5prime\_partial len:152 gc:universal TRINITY\_DN585\_c0\_g2\_i1:598-143(-) |
| 60 | 129 | TRINITY\_DN554\_c0\_g1\_i2.p1 | 46.69 | 6 | 6 | 8.78e2 | 1 | 1 | 1 |  | 29982.375 | type:internal len:266 gc:universal TRINITY\_DN554\_c0\_g1\_i2:797-3(-) |
| 60 | 130 | TRINITY\_DN554\_c0\_g1\_i3.p2 | 46.69 | 6 | 6 | 8.78e2 | 1 | 1 | 1 |  | 30419.4961 | type:complete len:268 gc:universal TRINITY\_DN554\_c0\_g1\_i3:1464-661(-) |
| 60 | 131 | TRINITY\_DN554\_c0\_g1\_i4.p1 | 46.69 | 5 | 5 | 8.78e2 | 1 | 1 | 1 |  | 35966.3984 | type:3prime\_partial len:319 gc:universal TRINITY\_DN554\_c0\_g1\_i4:956-3(-) |
| 60 | 132 | TRINITY\_DN585\_c0\_g1\_i8.p1 | 46.69 | 5 | 5 | 8.78e2 | 1 | 1 | 1 |  | 37858.4844 | type:3prime\_partial len:337 gc:universal TRINITY\_DN585\_c0\_g1\_i8:1008-1(-) |
| 60 | 133 | TRINITY\_DN585\_c0\_g1\_i2.p1 | 46.69 | 9 | 9 | 8.78e2 | 1 | 1 | 1 |  | 20558.6738 | type:internal len:184 gc:universal TRINITY\_DN585\_c0\_g1\_i2:549-1(-) |
| 60 | 134 | TRINITY\_DN585\_c0\_g1\_i5.p1 | 46.69 | 6 | 6 | 8.78e2 | 1 | 1 | 1 |  | 28098.1602 | type:internal len:250 gc:universal TRINITY\_DN585\_c0\_g1\_i5:747-1(-) |
| 60 | 135 | TRINITY\_DN585\_c0\_g1\_i3.p1 | 46.69 | 10 | 10 | 8.78e2 | 1 | 1 | 1 |  | 17628.2207 | type:internal len:157 gc:universal TRINITY\_DN585\_c0\_g1\_i3:468-1(-) |
| 60 | 136 | TRINITY\_DN101\_c0\_g1\_i1.p1 | 46.69 | 12 | 12 | 8.78e2 | 1 | 1 | 1 |  | 14847.2393 | type:complete len:129 gc:universal TRINITY\_DN101\_c0\_g1\_i1:416-30(-) |
| 60 | 137 | TRINITY\_DN585\_c0\_g1\_i4.p1 | 46.69 | 7 | 7 | 8.78e2 | 1 | 1 | 1 |  | 26348.0742 | type:complete len:232 gc:universal TRINITY\_DN585\_c0\_g1\_i4:847-152(-) |
| 60 | 138 | TRINITY\_DN554\_c0\_g1\_i5.p1 | 46.69 | 4 | 4 | 8.78e2 | 1 | 1 | 1 |  | 42081.4141 | type:3prime\_partial len:373 gc:universal TRINITY\_DN554\_c0\_g1\_i5:1118-3(-) |
| 61 | 139 | TRINITY\_DN12632\_c1\_g1\_i1.p1 | 47.51 | 3 | 3 | 0 | 1 | 1 | 1 |  | 56568.082 | type:complete len:504 gc:universal TRINITY\_DN12632\_c1\_g1\_i1:165-1676(+) |
| 62 | 140 | TRINITY\_DN1058\_c0\_g1\_i1.p1 | 46.93 | 0 | 0 | 5.5e3 | 1 | 1 | 3 |  | 284466.2812 | type:complete len:2639 gc:universal TRINITY\_DN1058\_c0\_g1\_i1:134-8050(+) |
| 62 | 141 | TRINITY\_DN1058\_c0\_g1\_i3.p1 | 46.93 | 0 | 0 | 5.5e3 | 1 | 1 | 3 |  | 277633.5312 | type:complete len:2581 gc:universal TRINITY\_DN1058\_c0\_g1\_i3:134-7876(+) |
| 62 | 142 | TRINITY\_DN1058\_c0\_g1\_i4.p1 | 46.93 | 2 | 2 | 5.5e3 | 1 | 1 | 3 |  | 79375.5703 | type:complete len:723 gc:universal TRINITY\_DN1058\_c0\_g1\_i4:366-2534(+) |
| 62 | 143 | TRINITY\_DN1058\_c0\_g1\_i2.p2 | 46.93 | 1 | 1 | 5.5e3 | 1 | 1 | 3 |  | 140284.0938 | type:complete len:1300 gc:universal TRINITY\_DN1058\_c0\_g1\_i2:4090-7989(+) |
| 63 | 144 | TRINITY\_DN65215\_c0\_g1\_i1.p1 | 43.87 | 2 | 2 | 7.51e2 | 1 | 1 | 1 |  | 43734.125 | type:5prime\_partial len:383 gc:universal TRINITY\_DN65215\_c0\_g1\_i1:1336-188(-) |
| 64 | 145 | TRINITY\_DN165536\_c0\_g1\_i1.p1 | 37.3 | 6 | 6 | 0 | 1 | 1 | 1 |  | 28339.4531 | type:complete len:249 gc:universal TRINITY\_DN165536\_c0\_g1\_i1:851-105(-) |
| 65 | 146 | TRINITY\_DN165469\_c0\_g1\_i1.p1 | 46.82 | 1 | 1 | 0 | 1 | 1 | 2 |  | 298128.9688 | type:complete len:2668 gc:universal TRINITY\_DN165469\_c0\_g1\_i1:41-8044(+) |
| 66 | 147 | TRINITY\_DN2003\_c0\_g1\_i1.p1 | 46.57 | 3 | 3 | 2.73e2 | 1 | 1 | 1 |  | 42076.3203 | type:complete len:370 gc:universal TRINITY\_DN2003\_c0\_g1\_i1:89-1198(+) |
| 67 | 148 | TRINITY\_DN8903\_c0\_g1\_i1.p1 | 46.18 | 2 | 2 | 1.43e3 | 1 | 1 | 2 |  | 50029.5586 | type:5prime\_partial len:447 gc:universal TRINITY\_DN8903\_c0\_g1\_i1:1546-206(-) |
| 68 | 149 | TRINITY\_DN11377\_c0\_g1\_i1.p1 | 43.02 | 1 | 1 | 1.74e2 | 1 | 1 | 1 |  | 89751.1094 | type:complete len:804 gc:universal TRINITY\_DN11377\_c0\_g1\_i1:3179-768(-) |
| 69 | 150 | TRINITY\_DN2472\_c0\_g1\_i1.p1 | 46.98 | 5 | 5 | 1.99e3 | 1 | 1 | 2 | Acetylation (Protein N-term) | 37676.8203 | type:complete len:344 gc:universal TRINITY\_DN2472\_c0\_g1\_i1:311-1342(+) |
| 70 | 151 | TRINITY\_DN123121\_c0\_g1\_i1.p1 | 46.51 | 12 | 12 | 0 | 1 | 1 | 1 |  | 14215.7539 | type:internal len:127 gc:universal TRINITY\_DN123121\_c0\_g1\_i1:3-380(+) |
| 71 | 152 | TRINITY\_DN26392\_c0\_g1\_i1.p1 | 43.74 | 3 | 3 | 0 | 1 | 1 | 1 |  | 52063.2266 | type:internal len:469 gc:universal TRINITY\_DN26392\_c0\_g1\_i1:2-1405(+) |
| 72 | 153 | TRINITY\_DN1030\_c0\_g1\_i2.p1 | 47.24 | 1 | 1 | 2.07e3 | 1 | 1 | 3 |  | 140221.3281 | type:complete len:1426 gc:universal TRINITY\_DN1030\_c0\_g1\_i2:4483-206(-) |
| 72 | 154 | TRINITY\_DN1030\_c0\_g1\_i1.p1 | 47.24 | 1 | 1 | 2.07e3 | 1 | 1 | 3 |  | 141535.875 | type:complete len:1439 gc:universal TRINITY\_DN1030\_c0\_g1\_i1:4522-206(-) |
| 73 | 155 | TRINITY\_DN4494\_c1\_g1\_i10.p1 | 45.76 | 3 | 3 | 4.99e2 | 1 | 1 | 1 |  | 50408.1719 | type:5prime\_partial len:462 gc:universal TRINITY\_DN4494\_c1\_g1\_i10:2120-735(-) |
| 73 | 156 | TRINITY\_DN4494\_c1\_g1\_i7.p1 | 45.76 | 3 | 3 | 4.99e2 | 1 | 1 | 1 |  | 57635.2422 | type:internal len:536 gc:universal TRINITY\_DN4494\_c1\_g1\_i7:1607-3(-) |
| 73 | 157 | TRINITY\_DN4494\_c1\_g1\_i1.p1 | 45.76 | 4 | 4 | 4.99e2 | 1 | 1 | 1 |  | 43468.3906 | type:internal len:400 gc:universal TRINITY\_DN4494\_c1\_g1\_i1:1197-1(-) |
| 73 | 158 | TRINITY\_DN17725\_c0\_g1\_i1.p1 | 45.76 | 13 | 13 | 4.99e2 | 1 | 1 | 1 |  | 11833.5479 | type:3prime\_partial len:109 gc:universal TRINITY\_DN17725\_c0\_g1\_i1:103-426(+) |
| 73 | 159 | TRINITY\_DN4494\_c2\_g1\_i1.p1 | 45.76 | 13 | 13 | 4.99e2 | 1 | 1 | 1 |  | 11226.4531 | type:internal len:107 gc:universal TRINITY\_DN4494\_c2\_g1\_i1:2-319(+) |
| 73 | 160 | TRINITY\_DN172523\_c0\_g1\_i1.p1 | 45.76 | 7 | 7 | 4.99e2 | 1 | 1 | 1 |  | 21902.9141 | type:internal len:201 gc:universal TRINITY\_DN172523\_c0\_g1\_i1:600-1(-) |
| 73 | 161 | TRINITY\_DN4494\_c1\_g1\_i8.p1 | 45.76 | 4 | 4 | 4.99e2 | 1 | 1 | 1 |  | 41552.7188 | type:internal len:390 gc:universal TRINITY\_DN4494\_c1\_g1\_i8:1169-3(-) |
| 73 | 162 | TRINITY\_DN4494\_c1\_g1\_i5.p1 | 45.76 | 3 | 3 | 4.99e2 | 1 | 1 | 1 |  | 47190.5742 | type:internal len:439 gc:universal TRINITY\_DN4494\_c1\_g1\_i5:1316-3(-) |
| 73 | 163 | TRINITY\_DN4494\_c1\_g1\_i12.p1 | 45.76 | 7 | 7 | 4.99e2 | 1 | 1 | 1 |  | 20465.0039 | type:internal len:192 gc:universal TRINITY\_DN4494\_c1\_g1\_i12:575-3(-) |
| 73 | 164 | TRINITY\_DN4494\_c1\_g1\_i2.p1 | 45.76 | 3 | 3 | 4.99e2 | 1 | 1 | 1 |  | 50327.457 | type:internal len:469 gc:universal TRINITY\_DN4494\_c1\_g1\_i2:1406-3(-) |
| 73 | 165 | TRINITY\_DN4494\_c1\_g1\_i15.p1 | 45.76 | 11 | 11 | 4.99e2 | 1 | 1 | 1 |  | 14719.415 | type:internal len:134 gc:universal TRINITY\_DN4494\_c1\_g1\_i15:399-1(-) |
| 73 | 166 | TRINITY\_DN12542\_c0\_g1\_i1.p1 | 45.76 | 6 | 6 | 4.99e2 | 1 | 1 | 1 |  | 23963.9648 | type:internal len:226 gc:universal TRINITY\_DN12542\_c0\_g1\_i1:676-2(-) |
| 74 | 167 | TRINITY\_DN3347\_c0\_g1\_i1.p1 | 46.52 | 1 | 1 | 8.17e2 | 1 | 1 | 1 |  | 104268.7266 | type:5prime\_partial len:924 gc:universal TRINITY\_DN3347\_c0\_g1\_i1:2-2773(+) |
| 75 | 168 | TRINITY\_DN59572\_c0\_g1\_i1.p1 | 46.86 | 2 | 2 | 1.42e3 | 1 | 1 | 1 |  | 82544.875 | type:internal len:725 gc:universal TRINITY\_DN59572\_c0\_g1\_i1:3-2174(+) |
| 76 | 169 | TRINITY\_DN160\_c0\_g1\_i1.p1 | 44.49 | 1 | 1 | 0 | 1 | 1 | 1 |  | 126611.5703 | type:complete len:1145 gc:universal TRINITY\_DN160\_c0\_g1\_i1:4810-1376(-) |
| 76 | 170 | TRINITY\_DN160\_c0\_g1\_i2.p1 | 44.49 | 1 | 1 | 0 | 1 | 1 | 1 |  | 126611.5703 | type:complete len:1145 gc:universal TRINITY\_DN160\_c0\_g1\_i2:4810-1376(-) |
| 77 | 171 | TRINITY\_DN11635\_c0\_g1\_i1.p1 | 47.32 | 2 | 2 | 0 | 1 | 1 | 1 |  | 79052.75 | type:complete len:705 gc:universal TRINITY\_DN11635\_c0\_g1\_i1:2148-34(-) |
| 78 | 172 | TRINITY\_DN48767\_c0\_g1\_i1.p1 | 45.57 | 2 | 2 | 5.64e2 | 1 | 1 | 1 |  | 60323.9297 | type:complete len:533 gc:universal TRINITY\_DN48767\_c0\_g1\_i1:2135-537(-) |
| 79 | 173 | TRINITY\_DN3016\_c0\_g1\_i5.p1 | 45.77 | 3 | 3 | 0 | 1 | 1 | 1 |  | 46762.2305 | type:complete len:406 gc:universal TRINITY\_DN3016\_c0\_g1\_i5:2713-1496(-) |
| 79 | 174 | TRINITY\_DN3016\_c0\_g1\_i10.p1 | 45.77 | 3 | 3 | 0 | 1 | 1 | 1 |  | 46762.2305 | type:complete len:406 gc:universal TRINITY\_DN3016\_c0\_g1\_i10:2760-1543(-) |
| 79 | 175 | TRINITY\_DN3016\_c0\_g1\_i17.p1 | 45.77 | 3 | 3 | 0 | 1 | 1 | 1 |  | 46762.2305 | type:complete len:406 gc:universal TRINITY\_DN3016\_c0\_g1\_i17:2691-1474(-) |
| 79 | 176 | TRINITY\_DN3016\_c0\_g1\_i8.p1 | 45.77 | 3 | 3 | 0 | 1 | 1 | 1 |  | 46762.2305 | type:complete len:406 gc:universal TRINITY\_DN3016\_c0\_g1\_i8:2713-1496(-) |
| 79 | 177 | TRINITY\_DN3016\_c0\_g1\_i13.p1 | 45.77 | 3 | 3 | 0 | 1 | 1 | 1 |  | 46762.2305 | type:complete len:406 gc:universal TRINITY\_DN3016\_c0\_g1\_i13:2760-1543(-) |
| 79 | 178 | TRINITY\_DN3016\_c0\_g1\_i4.p1 | 45.77 | 3 | 3 | 0 | 1 | 1 | 1 |  | 46762.2305 | type:complete len:406 gc:universal TRINITY\_DN3016\_c0\_g1\_i4:1529-312(-) |
| 79 | 179 | TRINITY\_DN3016\_c0\_g1\_i18.p1 | 45.77 | 3 | 3 | 0 | 1 | 1 | 1 |  | 46762.2305 | type:complete len:406 gc:universal TRINITY\_DN3016\_c0\_g1\_i18:2766-1549(-) |
| 79 | 180 | TRINITY\_DN3016\_c0\_g1\_i2.p1 | 45.77 | 3 | 3 | 0 | 1 | 1 | 1 |  | 46762.2305 | type:complete len:406 gc:universal TRINITY\_DN3016\_c0\_g1\_i2:2691-1474(-) |
| 79 | 181 | TRINITY\_DN3016\_c0\_g1\_i11.p1 | 45.77 | 3 | 3 | 0 | 1 | 1 | 1 |  | 46762.2305 | type:complete len:406 gc:universal TRINITY\_DN3016\_c0\_g1\_i11:2541-1324(-) |
| 79 | 182 | TRINITY\_DN3016\_c0\_g1\_i6.p1 | 45.77 | 3 | 3 | 0 | 1 | 1 | 1 |  | 46762.2305 | type:complete len:406 gc:universal TRINITY\_DN3016\_c0\_g1\_i6:2760-1543(-) |
| 80 | 183 | TRINITY\_DN79126\_c0\_g1\_i1.p1 | 47.25 | 10 | 10 | 0 | 1 | 1 | 1 |  | 13015.4727 | type:complete len:115 gc:universal TRINITY\_DN79126\_c0\_g1\_i1:397-53(-) |
| 81 | 184 | TRINITY\_DN11522\_c0\_g1\_i3.p1 | 47.25 | 2 | 2 | 1.69e3 | 1 | 1 | 2 |  | 93820.75 | type:5prime\_partial len:855 gc:universal TRINITY\_DN11522\_c0\_g1\_i3:2878-314(-) |
| 81 | 185 | TRINITY\_DN11522\_c0\_g1\_i1.p1 | 47.25 | 2 | 2 | 1.69e3 | 1 | 1 | 2 |  | 93223.9141 | type:5prime\_partial len:849 gc:universal TRINITY\_DN11522\_c0\_g1\_i1:2860-314(-) |
| 81 | 186 | TRINITY\_DN11522\_c0\_g1\_i4.p1 | 47.25 | 2 | 2 | 1.69e3 | 1 | 1 | 2 |  | 93423.2266 | type:5prime\_partial len:851 gc:universal TRINITY\_DN11522\_c0\_g1\_i4:2866-314(-) |
| 81 | 187 | TRINITY\_DN11522\_c0\_g1\_i2.p1 | 47.25 | 2 | 2 | 1.69e3 | 1 | 1 | 2 |  | 93621.4375 | type:5prime\_partial len:853 gc:universal TRINITY\_DN11522\_c0\_g1\_i2:2872-314(-) |
| 82 | 188 | TRINITY\_DN1194\_c0\_g1\_i1.p1 | 43.15 | 3 | 3 | 0 | 1 | 1 | 1 |  | 61081.3672 | type:complete len:555 gc:universal TRINITY\_DN1194\_c0\_g1\_i1:101-1765(+) |
| 83 | 189 | TRINITY\_DN1783\_c0\_g1\_i1.p2 | 46.48 | 2 | 2 | 8.74e2 | 1 | 1 | 1 |  | 53273.0938 | type:5prime\_partial len:473 gc:universal TRINITY\_DN1783\_c0\_g1\_i1:1460-42(-) |
| 83 | 190 | TRINITY\_DN1783\_c0\_g1\_i8.p2 | 46.48 | 2 | 2 | 8.74e2 | 1 | 1 | 1 |  | 53393.4727 | type:5prime\_partial len:472 gc:universal TRINITY\_DN1783\_c0\_g1\_i8:1457-42(-) |
| 83 | 191 | TRINITY\_DN1783\_c0\_g1\_i6.p1 | 46.48 | 4 | 4 | 8.74e2 | 1 | 1 | 1 |  | 25307.8594 | type:3prime\_partial len:225 gc:universal TRINITY\_DN1783\_c0\_g1\_i6:674-3(-) |
| 83 | 192 | TRINITY\_DN105041\_c0\_g1\_i1.p1 | 46.48 | 6 | 6 | 8.74e2 | 1 | 1 | 1 |  | 15973.1162 | type:internal len:141 gc:universal TRINITY\_DN105041\_c0\_g1\_i1:2-421(+) |
| 83 | 193 | TRINITY\_DN1783\_c0\_g1\_i2.p1 | 46.48 | 2 | 2 | 8.74e2 | 1 | 1 | 1 |  | 53273.0938 | type:5prime\_partial len:473 gc:universal TRINITY\_DN1783\_c0\_g1\_i2:1528-110(-) |
| 83 | 194 | TRINITY\_DN1783\_c1\_g1\_i1.p2 | 46.48 | 7 | 7 | 8.74e2 | 1 | 1 | 1 |  | 15461.7402 | type:internal len:136 gc:universal TRINITY\_DN1783\_c1\_g1\_i1:2-406(+) |
| 83 | 195 | TRINITY\_DN1783\_c0\_g1\_i3.p1 | 46.48 | 3 | 3 | 8.74e2 | 1 | 1 | 1 |  | 38855.5742 | type:internal len:346 gc:universal TRINITY\_DN1783\_c0\_g1\_i3:1037-3(-) |
| 84 | 196 | TRINITY\_DN38399\_c0\_g1\_i1.p1 | 47.17 | 2 | 2 | 6.62e2 | 1 | 1 | 1 | Carbamidomethylation | 97673.3438 | type:internal len:876 gc:universal TRINITY\_DN38399\_c0\_g1\_i1:2625-1(-) |
| 85 | 197 | TRINITY\_DN3991\_c0\_g1\_i1.p1 | 43.29 | 5 | 5 | 2.03e5 | 1 | 1 | 1 | Carbamidomethylation | 24846.6543 | type:3prime\_partial len:219 gc:universal TRINITY\_DN3991\_c0\_g1\_i1:84-737(+) |
| 86 | 198 | TRINITY\_DN5584\_c0\_g1\_i3.p1 | 46.73 | 1 | 1 | 0 | 1 | 1 | 1 |  | 110603.7422 | type:complete len:962 gc:universal TRINITY\_DN5584\_c0\_g1\_i3:2920-35(-) |
| 86 | 199 | TRINITY\_DN5584\_c0\_g1\_i1.p1 | 46.73 | 1 | 1 | 0 | 1 | 1 | 1 |  | 110660.9297 | type:complete len:961 gc:universal TRINITY\_DN5584\_c0\_g1\_i1:2917-35(-) |
| 86 | 200 | TRINITY\_DN5584\_c0\_g1\_i2.p1 | 46.73 | 4 | 4 | 0 | 1 | 1 | 1 |  | 36306.3672 | type:complete len:313 gc:universal TRINITY\_DN5584\_c0\_g1\_i2:973-35(-) |
| 87 | 201 | TRINITY\_DN941\_c0\_g1\_i3.p1 | 38.69 | 2 | 2 | 1.13e3 | 1 | 1 | 1 |  | 39175.6641 | type:internal len:363 gc:universal TRINITY\_DN941\_c0\_g1\_i3:3-1088(+) |
| 87 | 202 | TRINITY\_DN941\_c0\_g1\_i15.p1 | 38.69 | 1 | 1 | 1.13e3 | 1 | 1 | 1 |  | 127030.5156 | type:5prime\_partial len:1194 gc:universal TRINITY\_DN941\_c0\_g1\_i15:3-3584(+) |
| 88 | 203 | TRINITY\_DN106\_c0\_g1\_i2.p1 | 46.52 | 1 | 1 | 1.65e3 | 1 | 1 | 2 |  | 123847.7578 | type:3prime\_partial len:1309 gc:universal TRINITY\_DN106\_c0\_g1\_i2:137-4060(+) |
| 88 | 204 | TRINITY\_DN106\_c0\_g1\_i9.p1 | 46.52 | 1 | 1 | 1.65e3 | 1 | 1 | 2 |  | 123769.2656 | type:3prime\_partial len:1309 gc:universal TRINITY\_DN106\_c0\_g1\_i9:137-4060(+) |
| 88 | 205 | TRINITY\_DN106\_c0\_g1\_i5.p1 | 46.52 | 1 | 1 | 1.65e3 | 1 | 1 | 2 |  | 109115.5859 | type:3prime\_partial len:1168 gc:universal TRINITY\_DN106\_c0\_g1\_i5:137-3637(+) |
| 88 | 206 | TRINITY\_DN106\_c0\_g1\_i8.p1 | 46.52 | 1 | 1 | 1.65e3 | 1 | 1 | 2 |  | 124232.0078 | type:3prime\_partial len:1312 gc:universal TRINITY\_DN106\_c0\_g1\_i8:137-4069(+) |
| 88 | 207 | TRINITY\_DN106\_c0\_g1\_i6.p1 | 46.52 | 1 | 1 | 1.65e3 | 1 | 1 | 2 |  | 109300.5156 | type:3prime\_partial len:1168 gc:universal TRINITY\_DN106\_c0\_g1\_i6:137-3637(+) |
| 88 | 208 | TRINITY\_DN106\_c0\_g1\_i3.p1 | 46.52 | 1 | 1 | 1.65e3 | 1 | 1 | 2 |  | 124293.9609 | type:3prime\_partial len:1312 gc:universal TRINITY\_DN106\_c0\_g1\_i3:137-4069(+) |
| 88 | 209 | TRINITY\_DN106\_c0\_g1\_i10.p1 | 46.52 | 1 | 1 | 1.65e3 | 1 | 1 | 2 |  | 57108.7969 | type:internal len:621 gc:universal TRINITY\_DN106\_c0\_g1\_i10:2-1861(+) |
| 88 | 210 | TRINITY\_DN106\_c0\_g1\_i12.p1 | 46.52 | 1 | 1 | 1.65e3 | 1 | 1 | 2 |  | 57124.8438 | type:internal len:621 gc:universal TRINITY\_DN106\_c0\_g1\_i12:2-1861(+) |
| 88 | 211 | TRINITY\_DN106\_c0\_g1\_i11.p1 | 46.52 | 1 | 1 | 1.65e3 | 1 | 1 | 2 |  | 57100.6445 | type:internal len:621 gc:universal TRINITY\_DN106\_c0\_g1\_i11:2-1861(+) |
| 89 | 212 | TRINITY\_DN1386\_c0\_g1\_i1.p1 | 47.25 | 2 | 2 | 4.09e2 | 1 | 1 | 1 |  | 68692.1953 | type:complete len:596 gc:universal TRINITY\_DN1386\_c0\_g1\_i1:2419-632(-) |
| 90 | 213 | TRINITY\_DN6428\_c0\_g1\_i2.p1 | 46.61 | 4 | 4 | 2.84e3 | 1 | 1 | 1 | Carbamidomethylation | 33956.7344 | type:3prime\_partial len:303 gc:universal TRINITY\_DN6428\_c0\_g1\_i2:906-1(-) |
| 90 | 214 | TRINITY\_DN6428\_c0\_g1\_i1.p1 | 46.61 | 3 | 3 | 2.84e3 | 1 | 1 | 1 | Carbamidomethylation | 41179.0664 | type:internal len:367 gc:universal TRINITY\_DN6428\_c0\_g1\_i1:1098-1(-) |
| 91 | 215 | TRINITY\_DN2390\_c0\_g1\_i2.p1 | 47.21 | 2 | 2 | 6.02e2 | 1 | 1 | 1 |  | 54292.3164 | type:complete len:467 gc:universal TRINITY\_DN2390\_c0\_g1\_i2:82-1482(+) |
| 91 | 216 | TRINITY\_DN2390\_c0\_g1\_i1.p1 | 47.21 | 2 | 2 | 6.02e2 | 1 | 1 | 1 |  | 54292.3164 | type:complete len:467 gc:universal TRINITY\_DN2390\_c0\_g1\_i1:82-1482(+) |
| 92 | 217 | TRINITY\_DN1071\_c0\_g1\_i2.p1 | 47.19 | 5 | 5 | 4.56e3 | 1 | 1 | 3 |  | 36243.2383 | type:internal len:339 gc:universal TRINITY\_DN1071\_c0\_g1\_i2:1016-3(-) |
| 92 | 218 | TRINITY\_DN1071\_c0\_g1\_i3.p1 | 47.19 | 4 | 4 | 4.56e3 | 1 | 1 | 3 |  | 39803.1602 | type:internal len:375 gc:universal TRINITY\_DN1071\_c0\_g1\_i3:1124-3(-) |
| 92 | 219 | TRINITY\_DN1071\_c0\_g1\_i1.p1 | 47.19 | 2 | 2 | 4.56e3 | 1 | 1 | 3 |  | 71848.4062 | type:internal len:660 gc:universal TRINITY\_DN1071\_c0\_g1\_i1:1977-1(-) |
| 92 | 220 | TRINITY\_DN1071\_c0\_g1\_i4.p1 | 47.19 | 2 | 2 | 4.56e3 | 1 | 1 | 3 |  | 75408.3359 | type:internal len:696 gc:universal TRINITY\_DN1071\_c0\_g1\_i4:2085-1(-) |
| 93 | 221 | TRINITY\_DN4815\_c0\_g1\_i2.p1 | 47.15 | 2 | 2 | 7.5e2 | 1 | 1 | 2 | Carbamidomethylation | 70695.4688 | type:complete len:638 gc:universal TRINITY\_DN4815\_c0\_g1\_i2:79-1992(+) |
| 93 | 222 | TRINITY\_DN4815\_c0\_g1\_i1.p1 | 47.15 | 3 | 3 | 7.5e2 | 1 | 1 | 2 | Carbamidomethylation | 66133.5156 | type:complete len:595 gc:universal TRINITY\_DN4815\_c0\_g1\_i1:79-1863(+) |
| 94 | 223 | TRINITY\_DN15584\_c0\_g1\_i1.p1 | 47.2 | 6 | 6 | 7.45e2 | 1 | 1 | 1 |  | 29534.9648 | type:complete len:275 gc:universal TRINITY\_DN15584\_c0\_g1\_i1:874-50(-) |
| 95 | 224 | TRINITY\_DN187147\_c0\_g1\_i1.p1 | 47.26 | 1 | 1 | 2.42e2 | 1 | 1 | 1 |  | 138182.3125 | type:complete len:1223 gc:universal TRINITY\_DN187147\_c0\_g1\_i1:3708-40(-) |
| 96 | 225 | TRINITY\_DN101992\_c0\_g1\_i1.p1 | 46.56 | 7 | 7 | 1.35e3 | 1 | 1 | 1 | Carbamidomethylation | 15607.4678 | type:internal len:139 gc:universal TRINITY\_DN101992\_c0\_g1\_i1:414-1(-) |
| 97 | 226 | TRINITY\_DN286\_c0\_g1\_i2.p1 | 45.29 | 10 | 10 | 7.27e2 | 1 | 1 | 1 | Carbamidomethylation | 11368.1738 | type:3prime\_partial len:106 gc:universal TRINITY\_DN286\_c0\_g1\_i2:40-354(+) |
| 97 | 227 | TRINITY\_DN286\_c0\_g1\_i4.p1 | 45.29 | 5 | 5 | 7.27e2 | 1 | 1 | 1 | Carbamidomethylation | 25084.5293 | type:complete len:224 gc:universal TRINITY\_DN286\_c0\_g1\_i4:40-711(+) |
| 98 | 228 | TRINITY\_DN4900\_c0\_g1\_i1.p1 | 46.65 | 5 | 5 | 0 | 1 | 1 | 1 |  | 26790.1289 | type:complete len:246 gc:universal TRINITY\_DN4900\_c0\_g1\_i1:153-890(+) |
| 98 | 229 | TRINITY\_DN4900\_c0\_g1\_i4.p1 | 46.65 | 5 | 5 | 0 | 1 | 1 | 1 |  | 26790.1289 | type:complete len:246 gc:universal TRINITY\_DN4900\_c0\_g1\_i4:81-818(+) |
| 99 | 230 | TRINITY\_DN11435\_c0\_g1\_i1.p1 | 46.51 | 3 | 3 | 0 | 1 | 1 | 1 |  | 47019.5312 | type:complete len:430 gc:universal TRINITY\_DN11435\_c0\_g1\_i1:1616-327(-) |
| 100 | 231 | TRINITY\_DN2885\_c0\_g1\_i2.p1 | 46.49 | 11 | 11 | 3.57e2 | 1 | 1 | 1 |  | 12758.7783 | type:complete len:114 gc:universal TRINITY\_DN2885\_c0\_g1\_i2:31-372(+) |
| 100 | 232 | TRINITY\_DN2885\_c0\_g1\_i4.p1 | 46.49 | 11 | 11 | 3.57e2 | 1 | 1 | 1 |  | 12245.125 | type:5prime\_partial len:109 gc:universal TRINITY\_DN2885\_c0\_g1\_i4:1-327(+) |
| 100 | 233 | TRINITY\_DN2885\_c0\_g1\_i5.p1 | 46.49 | 11 | 11 | 3.57e2 | 1 | 1 | 1 |  | 12383.5439 | type:3prime\_partial len:112 gc:universal TRINITY\_DN2885\_c0\_g1\_i5:31-363(+) |
| 101 | 234 | TRINITY\_DN6920\_c0\_g1\_i2.p1 | 46.31 | 2 | 2 | 3.85e2 | 1 | 1 | 1 |  | 46932.582 | type:5prime\_partial len:411 gc:universal TRINITY\_DN6920\_c0\_g1\_i2:2147-915(-) |
| 101 | 235 | TRINITY\_DN6920\_c0\_g1\_i4.p1 | 46.31 | 3 | 3 | 3.85e2 | 1 | 1 | 1 |  | 32195.3809 | type:5prime\_partial len:282 gc:universal TRINITY\_DN6920\_c0\_g1\_i4:1321-476(-) |
| 101 | 236 | TRINITY\_DN6920\_c0\_g1\_i1.p1 | 46.31 | 3 | 3 | 3.85e2 | 1 | 1 | 1 |  | 32882.168 | type:complete len:287 gc:universal TRINITY\_DN6920\_c0\_g1\_i1:1775-915(-) |
| 101 | 237 | TRINITY\_DN6920\_c0\_g1\_i3.p1 | 46.31 | 2 | 2 | 3.85e2 | 1 | 1 | 1 |  | 46932.582 | type:5prime\_partial len:411 gc:universal TRINITY\_DN6920\_c0\_g1\_i3:2124-892(-) |
| 102 | 238 | TRINITY\_DN4781\_c0\_g1\_i3.p1 | 45.87 | 1 | 1 | 0 | 1 | 1 | 2 |  | 208363.3438 | type:5prime\_partial len:1859 gc:universal TRINITY\_DN4781\_c0\_g1\_i3:8547-2971(-) |
| 102 | 239 | TRINITY\_DN4781\_c0\_g1\_i2.p1 | 45.87 | 0 | 0 | 0 | 1 | 1 | 2 |  | 378619.8438 | type:5prime\_partial len:3388 gc:universal TRINITY\_DN4781\_c0\_g1\_i2:10182-19(-) |
| 102 | 240 | TRINITY\_DN4781\_c0\_g1\_i1.p1 | 45.87 | 0 | 0 | 0 | 1 | 1 | 2 |  | 302042.125 | type:5prime\_partial len:2696 gc:universal TRINITY\_DN4781\_c0\_g1\_i1:10504-2417(-) |
| 103 | 241 | TRINITY\_DN923\_c0\_g1\_i1.p1 | 46.75 | 2 | 2 | 0 | 1 | 1 | 1 |  | 51773.8242 | type:complete len:442 gc:universal TRINITY\_DN923\_c0\_g1\_i1:53-1378(+) |
| 104 | 242 | TRINITY\_DN10094\_c0\_g1\_i2.p1 | 46.21 | 5 | 5 | 5.36e2 | 1 | 1 | 1 | Carbamidomethylation | 28035.0664 | type:complete len:264 gc:universal TRINITY\_DN10094\_c0\_g1\_i2:1210-419(-) |
| 104 | 243 | TRINITY\_DN10094\_c0\_g1\_i1.p1 | 46.21 | 5 | 5 | 5.36e2 | 1 | 1 | 1 | Carbamidomethylation | 28035.0664 | type:complete len:264 gc:universal TRINITY\_DN10094\_c0\_g1\_i1:1210-419(-) |
| 105 | 244 | TRINITY\_DN79\_c0\_g1\_i1.p1 | 47.21 | 4 | 4 | 1.02e3 | 1 | 1 | 2 |  | 52590.9805 | type:5prime\_partial len:478 gc:universal TRINITY\_DN79\_c0\_g1\_i1:2132-699(-) |
| 106 | 245 | TRINITY\_DN118327\_c0\_g1\_i1.p1 | 37.83 | 1 | 1 | 5.03e2 | 1 | 1 | 1 |  | 79756.7109 | type:5prime\_partial len:702 gc:universal TRINITY\_DN118327\_c0\_g1\_i1:3-2108(+) |
| 107 | 246 | TRINITY\_DN3156\_c0\_g1\_i1.p1 | 47.25 | 3 | 3 | 1.33e3 | 1 | 1 | 1 |  | 57939.9062 | type:complete len:501 gc:universal TRINITY\_DN3156\_c0\_g1\_i1:39-1541(+) |
| 107 | 247 | TRINITY\_DN3156\_c0\_g1\_i2.p1 | 47.25 | 3 | 3 | 1.33e3 | 1 | 1 | 1 |  | 63479.1914 | type:complete len:548 gc:universal TRINITY\_DN3156\_c0\_g1\_i2:39-1682(+) |
| 108 | 248 | TRINITY\_DN1936\_c0\_g1\_i1.p1 | 47.14 | 1 | 1 | 1.41e3 | 1 | 1 | 1 |  | 136661.0312 | type:3prime\_partial len:1224 gc:universal TRINITY\_DN1936\_c0\_g1\_i1:3669-1(-) |
| 108 | 249 | TRINITY\_DN1936\_c0\_g1\_i4.p1 | 47.14 | 1 | 1 | 1.41e3 | 1 | 1 | 1 |  | 196066.9688 | type:3prime\_partial len:1762 gc:universal TRINITY\_DN1936\_c0\_g1\_i4:5283-1(-) |
| 109 | 250 | TRINITY\_DN1559\_c0\_g1\_i1.p1 | 42.3 | 5 | 5 | 0 | 1 | 1 | 1 |  | 33672.0977 | type:complete len:311 gc:universal TRINITY\_DN1559\_c0\_g1\_i1:221-1153(+) |
| 110 | 251 | TRINITY\_DN195348\_c0\_g1\_i1.p1 | 45.95 | 5 | 5 | 4.23e2 | 1 | 1 | 1 |  | 34993.6055 | type:internal len:311 gc:universal TRINITY\_DN195348\_c0\_g1\_i1:1-930(+) |
| 111 | 252 | TRINITY\_DN3034\_c0\_g1\_i1.p1 | 41.26 | 1 | 1 | 0 | 1 | 1 | 1 |  | 222266.9844 | type:complete len:1941 gc:universal TRINITY\_DN3034\_c0\_g1\_i1:6289-467(-) |
| 112 | 253 | TRINITY\_DN5987\_c0\_g1\_i4.p1 | 47.2 | 4 | 4 | 1.15e3 | 1 | 1 | 1 |  | 32201.8711 | type:complete len:336 gc:universal TRINITY\_DN5987\_c0\_g1\_i4:136-1143(+) |
| 112 | 254 | TRINITY\_DN5987\_c0\_g1\_i3.p2 | 47.2 | 9 | 9 | 1.15e3 | 1 | 1 | 1 |  | 14553.1807 | type:5prime\_partial len:158 gc:universal TRINITY\_DN5987\_c0\_g1\_i3:2-475(+) |
| 112 | 255 | TRINITY\_DN5987\_c0\_g1\_i2.p1 | 47.2 | 5 | 5 | 1.15e3 | 1 | 1 | 1 |  | 29022.416 | type:complete len:300 gc:universal TRINITY\_DN5987\_c0\_g1\_i2:136-1035(+) |
| 113 | 256 | TRINITY\_DN360\_c0\_g1\_i1.p1 | 44.37 | 4 | 4 | 0 | 1 | 1 | 1 | Oxidation (M) | 52389.3047 | type:5prime\_partial len:476 gc:universal TRINITY\_DN360\_c0\_g1\_i1:2237-810(-) |
| 114 | 257 | TRINITY\_DN761\_c0\_g1\_i1.p1 | 46.51 | 2 | 2 | 0 | 1 | 1 | 1 |  | 95330.7812 | type:complete len:830 gc:universal TRINITY\_DN761\_c0\_g1\_i1:129-2618(+) |
| 114 | 258 | TRINITY\_DN761\_c0\_g1\_i2.p1 | 46.51 | 2 | 2 | 0 | 1 | 1 | 1 |  | 95330.7812 | type:complete len:830 gc:universal TRINITY\_DN761\_c0\_g1\_i2:87-2576(+) |
| 114 | 259 | TRINITY\_DN761\_c0\_g1\_i3.p1 | 46.51 | 2 | 2 | 0 | 1 | 1 | 1 |  | 96968.6562 | type:complete len:845 gc:universal TRINITY\_DN761\_c0\_g1\_i3:129-2663(+) |
| 115 | 260 | TRINITY\_DN25375\_c0\_g1\_i7.p1 | 46.03 | 2 | 2 | 6.12e2 | 1 | 1 | 1 |  | 90646.6094 | type:complete len:811 gc:universal TRINITY\_DN25375\_c0\_g1\_i7:112-2544(+) |
| 116 | 261 | TRINITY\_DN141338\_c2\_g1\_i1.p1 | 47.2 | 1 | 1 | 0 | 1 | 1 | 1 |  | 82716.2266 | type:complete len:720 gc:universal TRINITY\_DN141338\_c2\_g1\_i1:2850-691(-) |
| 117 | 262 | TRINITY\_DN11096\_c0\_g1\_i1.p1 | 43.58 | 3 | 3 | 0 | 1 | 1 | 1 |  | 61992.0312 | type:complete len:557 gc:universal TRINITY\_DN11096\_c0\_g1\_i1:1858-188(-) |
| 118 | 263 | TRINITY\_DN64\_c0\_g1\_i1.p1 | 47.14 | 3 | 3 | 5.77e2 | 1 | 1 | 1 |  | 51450.1914 | type:complete len:468 gc:universal TRINITY\_DN64\_c0\_g1\_i1:1555-152(-) |
| 119 | 264 | TRINITY\_DN15151\_c0\_g1\_i3.p1 | 40.45 | 2 | 2 | 5.01e2 | 1 | 1 | 1 |  | 58451.293 | type:internal len:560 gc:universal TRINITY\_DN15151\_c0\_g1\_i3:1-1677(+) |
| 119 | 265 | TRINITY\_DN15151\_c0\_g1\_i1.p1 | 40.45 | 2 | 2 | 5.01e2 | 1 | 1 | 1 |  | 62118.2383 | type:5prime\_partial len:592 gc:universal TRINITY\_DN15151\_c0\_g1\_i1:1-1776(+) |
| 119 | 266 | TRINITY\_DN15151\_c0\_g1\_i2.p1 | 40.45 | 2 | 2 | 5.01e2 | 1 | 1 | 1 |  | 58324.2109 | type:internal len:560 gc:universal TRINITY\_DN15151\_c0\_g1\_i2:1-1677(+) |
| 120 | 267 | TRINITY\_DN7102\_c0\_g1\_i1.p1 | 47.12 | 1 | 1 | 0 | 1 | 1 | 1 |  | 139040.8594 | type:5prime\_partial len:1263 gc:universal TRINITY\_DN7102\_c0\_g1\_i1:3-3791(+) |
| 121 | 268 | TRINITY\_DN34481\_c0\_g1\_i1.p1 | 46.01 | 1 | 1 | 0 | 1 | 1 | 1 | Carbamidomethylation | 92307.5469 | type:complete len:802 gc:universal TRINITY\_DN34481\_c0\_g1\_i1:2487-82(-) |
| 122 | 269 | TRINITY\_DN118520\_c0\_g1\_i1.p1 | 46.31 | 3 | 3 | 3.57e3 | 1 | 1 | 1 |  | 61587.4766 | type:complete len:581 gc:universal TRINITY\_DN118520\_c0\_g1\_i1:1895-153(-) |
| 123 | 270 | TRINITY\_DN3271\_c0\_g1\_i1.p1 | 45.3 | 3 | 3 | 0 | 1 | 1 | 1 |  | 45246.3555 | type:complete len:399 gc:universal TRINITY\_DN3271\_c0\_g1\_i1:218-1414(+) |
| 123 | 271 | TRINITY\_DN3271\_c0\_g1\_i2.p1 | 45.3 | 3 | 3 | 0 | 1 | 1 | 1 |  | 53020.332 | type:5prime\_partial len:468 gc:universal TRINITY\_DN3271\_c0\_g1\_i2:2-1405(+) |
| 124 | 272 | TRINITY\_DN1043\_c0\_g1\_i2.p1 | 46.19 | 2 | 2 | 9.71e2 | 1 | 1 | 1 |  | 78392.1016 | type:complete len:700 gc:universal TRINITY\_DN1043\_c0\_g1\_i2:2410-311(-) |
| 125 | 273 | TRINITY\_DN31399\_c0\_g1\_i2.p1 | 45.93 | 1 | 1 | 0 | 1 | 1 | 2 |  | 89961.6641 | type:complete len:819 gc:universal TRINITY\_DN31399\_c0\_g1\_i2:85-2541(+) |
| 125 | 274 | TRINITY\_DN31399\_c0\_g1\_i3.p1 | 45.93 | 1 | 1 | 0 | 1 | 1 | 2 |  | 89350.0078 | type:complete len:813 gc:universal TRINITY\_DN31399\_c0\_g1\_i3:85-2523(+) |
| 126 | 275 | TRINITY\_DN33065\_c0\_g2\_i1.p1 | 46.72 | 2 | 2 | 0 | 1 | 1 | 1 |  | 89122.9766 | type:3prime\_partial len:804 gc:universal TRINITY\_DN33065\_c0\_g2\_i1:2409-1(-) |
| 127 | 276 | TRINITY\_DN984\_c0\_g1\_i1.p1 | 47.26 | 1 | 1 | 1.9e3 | 1 | 1 | 2 |  | 146638.4062 | type:complete len:1485 gc:universal TRINITY\_DN984\_c0\_g1\_i1:4558-104(-) |
| 127 | 277 | TRINITY\_DN984\_c0\_g1\_i2.p1 | 47.26 | 1 | 1 | 1.9e3 | 1 | 1 | 2 |  | 146638.4062 | type:complete len:1485 gc:universal TRINITY\_DN984\_c0\_g1\_i2:4558-104(-) |
| 128 | 278 | TRINITY\_DN598\_c1\_g1\_i2.p1 | 46.17 | 1 | 1 | 8.37e2 | 1 | 1 | 1 |  | 81043.625 | type:5prime\_partial len:742 gc:universal TRINITY\_DN598\_c1\_g1\_i2:2-2227(+) |
| 129 | 279 | TRINITY\_DN22240\_c0\_g1\_i1.p1 | 46.89 | 3 | 3 | 0 | 1 | 1 | 2 |  | 59687.8438 | type:complete len:513 gc:universal TRINITY\_DN22240\_c0\_g1\_i1:155-1693(+) |
| 130 | 280 | TRINITY\_DN123\_c0\_g1\_i1.p1 | 47.12 | 1 | 1 | 2.9e2 | 1 | 1 | 2 |  | 136822.0312 | type:5prime\_partial len:1231 gc:universal TRINITY\_DN123\_c0\_g1\_i1:2-3694(+) |
| 131 | 281 | TRINITY\_DN2357\_c1\_g1\_i1.p1 | 39.31 | 1 | 1 | 0 | 1 | 1 | 1 |  | 196914.375 | type:complete len:1733 gc:universal TRINITY\_DN2357\_c1\_g1\_i1:5452-254(-) |
| 132 | 282 | TRINITY\_DN495\_c0\_g1\_i5.p1 | 46.33 | 3 | 3 | 0 | 1 | 1 | 1 | Carbamidomethylation | 45678.0781 | type:3prime\_partial len:407 gc:universal TRINITY\_DN495\_c0\_g1\_i5:152-1369(+) |
| 132 | 283 | TRINITY\_DN495\_c0\_g1\_i6.p1 | 46.33 | 3 | 3 | 0 | 1 | 1 | 1 | Carbamidomethylation | 42984.9375 | type:3prime\_partial len:382 gc:universal TRINITY\_DN495\_c0\_g1\_i6:152-1294(+) |
| 133 | 284 | TRINITY\_DN135129\_c0\_g1\_i1.p1 | 40.54 | 6 | 6 | 2.69e3 | 1 | 1 | 1 |  | 17174.043 | type:internal len:155 gc:universal TRINITY\_DN135129\_c0\_g1\_i1:1-462(+) |
| 134 | 285 | TRINITY\_DN30613\_c0\_g1\_i1.p1 | 46.1 | 2 | 2 | 7.16e2 | 1 | 1 | 1 |  | 106630.8516 | type:complete len:975 gc:universal TRINITY\_DN30613\_c0\_g1\_i1:154-3078(+) |
| 135 | 286 | TRINITY\_DN4066\_c0\_g1\_i1.p1 | 42.4 | 3 | 3 | 7.14e2 | 1 | 1 | 1 |  | 82921.5625 | type:complete len:726 gc:universal TRINITY\_DN4066\_c0\_g1\_i1:143-2320(+) |
| 136 | 287 | TRINITY\_DN22492\_c0\_g1\_i2.p1 | 39.03 | 1 | 1 | 8.42e2 | 1 | 1 | 2 |  | 115355.5859 | type:complete len:1026 gc:universal TRINITY\_DN22492\_c0\_g1\_i2:39-3116(+) |
| 136 | 288 | TRINITY\_DN22492\_c0\_g1\_i1.p1 | 39.03 | 1 | 1 | 8.42e2 | 1 | 1 | 2 |  | 115296.5156 | type:complete len:1026 gc:universal TRINITY\_DN22492\_c0\_g1\_i1:171-3248(+) |
| 137 | 289 | TRINITY\_DN61\_c0\_g1\_i1.p1 | 46.89 | 2 | 2 | 2.9e3 | 1 | 1 | 3 |  | 71696.4219 | type:complete len:625 gc:universal TRINITY\_DN61\_c0\_g1\_i1:2929-1055(-) |
| 138 | 290 | TRINITY\_DN3036\_c0\_g1\_i2.p2 | 45.07 | 2 | 2 | 7.9e2 | 1 | 1 | 1 |  | 59586.3086 | type:internal len:548 gc:universal TRINITY\_DN3036\_c0\_g1\_i2:1643-3(-) |
| 138 | 291 | TRINITY\_DN3036\_c0\_g1\_i8.p1 | 45.07 | 2 | 2 | 7.9e2 | 1 | 1 | 1 |  | 59630.4023 | type:internal len:548 gc:universal TRINITY\_DN3036\_c0\_g1\_i8:1643-3(-) |
| 139 | 292 | TRINITY\_DN99404\_c0\_g1\_i1.p1 | 45.64 | 13 | 13 | 0 | 1 | 1 | 1 |  | 12615.5928 | type:internal len:112 gc:universal TRINITY\_DN99404\_c0\_g1\_i1:2-334(+) |
| 140 | 293 | TRINITY\_DN1366\_c0\_g1\_i1.p1 | 45.93 | 0 | 0 | 2.37e3 | 1 | 1 | 1 |  | 558202.1875 | type:3prime\_partial len:4702 gc:universal TRINITY\_DN1366\_c0\_g1\_i1:236-14338(+) |
| 141 | 294 | TRINITY\_DN2028\_c0\_g1\_i1.p1 | 47.09 | 5 | 5 | 6.02e3 | 1 | 1 | 3 |  | 30106.9453 | type:complete len:274 gc:universal TRINITY\_DN2028\_c0\_g1\_i1:1608-787(-) |
| 142 | 295 | TRINITY\_DN36217\_c0\_g1\_i1.p1 | 46.9 | 1 | 1 | 3.48e2 | 1 | 1 | 1 |  | 68373.1172 | type:5prime\_partial len:605 gc:universal TRINITY\_DN36217\_c0\_g1\_i1:2-1816(+) |
| 143 | 296 | TRINITY\_DN26249\_c0\_g1\_i1.p1 | 47.27 | 3 | 3 | 7.41e2 | 1 | 1 | 1 |  | 54490.1055 | type:5prime\_partial len:477 gc:universal TRINITY\_DN26249\_c0\_g1\_i1:1733-303(-) |
| 144 | 297 | TRINITY\_DN172571\_c0\_g1\_i1.p1 | 45.63 | 12 | 12 | 0 | 1 | 1 | 1 |  | 11500.2529 | type:complete len:104 gc:universal TRINITY\_DN172571\_c0\_g1\_i1:30-341(+) |
| 145 | 298 | TRINITY\_DN1378\_c0\_g1\_i1.p1 | 45.78 | 3 | 3 | 2.4e2 | 1 | 1 | 1 |  | 47978.9219 | type:complete len:443 gc:universal TRINITY\_DN1378\_c0\_g1\_i1:2151-823(-) |
| 146 | 299 | TRINITY\_DN540\_c0\_g1\_i1.p1 | 46.72 | 2 | 2 | 1.15e3 | 1 | 1 | 1 | Acetylation (Protein N-term) | 52183.2109 | type:complete len:480 gc:universal TRINITY\_DN540\_c0\_g1\_i1:1503-64(-) |
| 147 | 300 | TRINITY\_DN109148\_c0\_g1\_i1.p2 | 46.89 | 6 | 6 | 2.35e2 | 1 | 1 | 2 |  | 14395.7793 | type:internal len:131 gc:universal TRINITY\_DN109148\_c0\_g1\_i1:391-2(-) |
| 148 | 301 | TRINITY\_DN97809\_c0\_g1\_i1.p1 | 43.15 | 7 | 7 | 6.32e2 | 1 | 1 | 3 |  | 20461.1211 | type:internal len:184 gc:universal TRINITY\_DN97809\_c0\_g1\_i1:2-550(+) |
| 149 | 302 | TRINITY\_DN773\_c0\_g1\_i1.p1 | 46.25 | 3 | 3 | 4.48e2 | 1 | 1 | 1 |  | 48865.75 | type:complete len:440 gc:universal TRINITY\_DN773\_c0\_g1\_i1:1608-289(-) |
| 150 | 303 | TRINITY\_DN5633\_c0\_g1\_i1.p1 | 47.14 | 2 | 2 | 4.48e2 | 1 | 1 | 1 |  | 97310.9922 | type:complete len:848 gc:universal TRINITY\_DN5633\_c0\_g1\_i1:2881-338(-) |
| 151 | 304 | TRINITY\_DN775\_c0\_g1\_i1.p1 | 46.52 | 2 | 2 | 0 | 1 | 1 | 1 |  | 72884.4609 | type:complete len:635 gc:universal TRINITY\_DN775\_c0\_g1\_i1:1927-23(-) |
| 151 | 305 | TRINITY\_DN775\_c0\_g1\_i2.p1 | 46.52 | 2 | 2 | 0 | 1 | 1 | 1 |  | 74657.6406 | type:complete len:647 gc:universal TRINITY\_DN775\_c0\_g1\_i2:1963-23(-) |
| 152 | 306 | TRINITY\_DN71243\_c0\_g1\_i1.p1 | 46.14 | 4 | 4 | 1.07e3 | 1 | 1 | 2 |  | 46628.2539 | type:internal len:422 gc:universal TRINITY\_DN71243\_c0\_g1\_i1:1265-3(-) |
| 153 | 307 | TRINITY\_DN1172\_c0\_g2\_i2.p1 | 38.62 | 4 | 4 | 3.78e2 | 1 | 1 | 1 | Acetylation (Protein N-term) | 27813.4355 | type:internal len:264 gc:universal TRINITY\_DN1172\_c0\_g2\_i2:790-2(-) |
| 154 | 308 | TRINITY\_DN564\_c0\_g1\_i1.p1 | 43.22 | 3 | 3 | 0 | 1 | 1 | 1 |  | 62932.8672 | type:complete len:563 gc:universal TRINITY\_DN564\_c0\_g1\_i1:2018-330(-) |
| 155 | 309 | TRINITY\_DN14015\_c0\_g1\_i3.p1 | 44.76 | 1 | 1 | 1.41e2 | 1 | 1 | 2 |  | 284708.625 | type:5prime\_partial len:2566 gc:universal TRINITY\_DN14015\_c0\_g1\_i3:2-7699(+) |
| 155 | 310 | TRINITY\_DN14015\_c0\_g1\_i2.p1 | 44.76 | 1 | 1 | 1.41e2 | 1 | 1 | 2 |  | 287707.9062 | type:5prime\_partial len:2592 gc:universal TRINITY\_DN14015\_c0\_g1\_i2:2-7777(+) |
| 156 | 311 | TRINITY\_DN4781\_c0\_g1\_i3.p3 | 47.27 | 7 | 7 | 4.31e2 | 1 | 1 | 4 |  | 20029.2422 | type:complete len:183 gc:universal TRINITY\_DN4781\_c0\_g1\_i3:2965-2417(-) |
| 157 | 312 | TRINITY\_DN73457\_c0\_g1\_i1.p1 | 45.83 | 3 | 3 | 0 | 1 | 1 | 1 |  | 54003.8125 | type:complete len:467 gc:universal TRINITY\_DN73457\_c0\_g1\_i1:1435-35(-) |
| 158 | 313 | TRINITY\_DN464\_c0\_g1\_i4.p1 | 46.14 | 2 | 2 | 0 | 1 | 1 | 1 |  | 82057.25 | type:complete len:717 gc:universal TRINITY\_DN464\_c0\_g1\_i4:2167-17(-) |
| 159 | 314 | TRINITY\_DN67\_c0\_g1\_i2.p1 | 44.58 | 4 | 4 | 0 | 1 | 1 | 1 |  | 52170.8203 | type:5prime\_partial len:474 gc:universal TRINITY\_DN67\_c0\_g1\_i2:1488-67(-) |
| 160 | 315 | TRINITY\_DN27379\_c0\_g1\_i1.p1 | 43.26 | 2 | 2 | 0 | 1 | 1 | 1 |  | 67596.9375 | type:complete len:583 gc:universal TRINITY\_DN27379\_c0\_g1\_i1:133-1881(+) |
| 161 | 316 | TRINITY\_DN5397\_c0\_g1\_i1.p1 | 47.45 | 12 | 12 | 0 | 1 | 1 | 1 | Carbamidomethylation | 17679.4688 | type:internal len:161 gc:universal TRINITY\_DN5397\_c0\_g1\_i1:2-481(+) |
| 162 | 317 | TRINITY\_DN247\_c0\_g1\_i1.p1 | 47.18 | 3 | 3 | 0 | 1 | 1 | 1 |  | 42079.6172 | type:complete len:373 gc:universal TRINITY\_DN247\_c0\_g1\_i1:38-1156(+) |
| 163 | 318 | TRINITY\_DN17292\_c0\_g1\_i1.p1 | 39.01 | 2 | 2 | 0 | 1 | 1 | 1 |  | 59018.2578 | type:3prime\_partial len:534 gc:universal TRINITY\_DN17292\_c0\_g1\_i1:38-1636(+) |
| total 318 proteins |  |  |  |  |  |  |  |  |  |  |  |  |
| --- | --- | --- | --- | --- | --- | --- | --- | --- | --- | --- | --- | --- |

  

TRINITY\_DN11435\_c0\_g1\_i1.p1

back to list

  

| Protein Coverage
| Supporting Peptides
| Best Unique PSM
|

Protein Coverage:

Supporting Peptides:

| Peptide | Uniq | -10lgP | Mass | Length | ppm | m/z | z | RT | 1/k0 Start | 1/k0 End | Precursor ID | Area Sample 6 | #Feature | #Feature Sample 6 | Start | End | PTM | AScore | Found By |
| --- | --- | --- | --- | --- | --- | --- | --- | --- | --- | --- | --- | --- | --- | --- | --- | --- | --- | --- | --- |
| K.VYLAQAEAEKIK.M | Y | 46.51 | 1361.7554 | 12 | 2.91 | 681.884 | 2 | 14.42 | 1.0347 | 1.0419 | 40879 | 0 | 0 | 0 | 309 | 320 |  |  | DB Search |
| total 1 peptides |  |  |  |  |  |  |  |  |  |  |  |  |  |  |  |  |  |  |  |
| --- | --- | --- | --- | --- | --- | --- | --- | --- | --- | --- | --- | --- | --- | --- | --- | --- | --- | --- | --- |

Best Unique PSM (Scan Sample\_4\_A1\_1\_3642.d:40879, m/z=681.884, z=2, RT=14.42, ppm=-1.5):


TRINITY\_DN11522\_c0\_g1\_i4.p1

back to list

  

| Protein Coverage
| Supporting Peptides
| Best Unique PSM
|

Protein Coverage:

Supporting Peptides:

| Peptide | Uniq | -10lgP | Mass | Length | ppm | m/z | z | RT | 1/k0 Start | 1/k0 End | Precursor ID | Area Sample 6 | #Feature | #Feature Sample 6 | Start | End | PTM | AScore | Found By |
| --- | --- | --- | --- | --- | --- | --- | --- | --- | --- | --- | --- | --- | --- | --- | --- | --- | --- | --- | --- |
| R.IGLGNIRDPSISLTR.G | Y | 47.25 | 1610.9104 | 15 | 2.96 | 537.9766 | 3 | 17.36 | 0.797 | 0.8043 | 57658 | 1.69e3 | 1 | 1 | 27 | 41 |  |  | DB Search |
| total 1 peptides |  |  |  |  |  |  |  |  |  |  |  |  |  |  |  |  |  |  |  |
| --- | --- | --- | --- | --- | --- | --- | --- | --- | --- | --- | --- | --- | --- | --- | --- | --- | --- | --- | --- |

Best Unique PSM (Scan Sample\_4\_A1\_1\_3642.d:57658, m/z=537.9766, z=3, RT=17.36, ppm=-1.44):


TRINITY\_DN319\_c2\_g1\_i2.p1

back to list

  

| Protein Coverage
| Supporting Peptides
| Best Unique PSM
|

Protein Coverage:

Supporting Peptides:

| Peptide | Uniq | -10lgP | Mass | Length | ppm | m/z | z | RT | 1/k0 Start | 1/k0 End | Precursor ID | Area Sample 6 | #Feature | #Feature Sample 6 | Start | End | PTM | AScore | Found By |
| --- | --- | --- | --- | --- | --- | --- | --- | --- | --- | --- | --- | --- | --- | --- | --- | --- | --- | --- | --- |
| R.DPIC(+57.02)HC(+57.02)DTSK.Y | Y | 47.35 | 1231.4961 | 10 | 6.31 | 411.5068 | 3 | 9.96 | 0.7351 | 0.7424 | 14092 | 1.65e3 | 2 | 2 | 1448 | 1457 | Carbamidomethylation, Carbamidomethylation |  | DB Search |
| R.GSFSSHIC(+57.02)LC(+57.02)HDPAYK.S | Y | 46.19 | 1877.8189 | 16 | 4.79 | 626.9471 | 3 | 14.44 | 0.887 | 0.8943 | 41053 | 0 | 0 | 0 | 1011 | 1026 | Carbamidomethylation, Carbamidomethylation |  | DB Search |
| total 2 peptides |  |  |  |  |  |  |  |  |  |  |  |  |  |  |  |  |  |  |  |
| --- | --- | --- | --- | --- | --- | --- | --- | --- | --- | --- | --- | --- | --- | --- | --- | --- | --- | --- | --- |

Best Unique PSM (Scan Sample\_4\_A1\_1\_3642.d:14092, m/z=411.5068, z=3, RT=9.96, ppm=1.91):


TRINITY\_DN773\_c0\_g1\_i1.p1

back to list

  

| Protein Coverage
| Supporting Peptides
| Best Unique PSM
|

Protein Coverage:

Supporting Peptides:

| Peptide | Uniq | -10lgP | Mass | Length | ppm | m/z | z | RT | 1/k0 Start | 1/k0 End | Precursor ID | Area Sample 6 | #Feature | #Feature Sample 6 | Start | End | PTM | AScore | Found By |
| --- | --- | --- | --- | --- | --- | --- | --- | --- | --- | --- | --- | --- | --- | --- | --- | --- | --- | --- | --- |
| R.GVSEETTTGVHNLYK.M | Y | 46.25 | 1633.7947 | 15 | 1.81 | 817.9025 | 2 | 13.69 | 1.0664 | 1.0737 | 36539 | 4.48e2 | 1 | 1 | 160 | 174 |  |  | DB Search |
| total 1 peptides |  |  |  |  |  |  |  |  |  |  |  |  |  |  |  |  |  |  |  |
| --- | --- | --- | --- | --- | --- | --- | --- | --- | --- | --- | --- | --- | --- | --- | --- | --- | --- | --- | --- |

Best Unique PSM (Scan Sample\_4\_A1\_1\_3642.d:36539, m/z=817.9025, z=2, RT=13.69, ppm=-2.59):


TRINITY\_DN10094\_c0\_g1\_i2.p1

back to list

  

| Protein Coverage
| Supporting Peptides
| Best Unique PSM
|

Protein Coverage:

Supporting Peptides:

| Peptide | Uniq | -10lgP | Mass | Length | ppm | m/z | z | RT | 1/k0 Start | 1/k0 End | Precursor ID | Area Sample 6 | #Feature | #Feature Sample 6 | Start | End | PTM | AScore | Found By |
| --- | --- | --- | --- | --- | --- | --- | --- | --- | --- | --- | --- | --- | --- | --- | --- | --- | --- | --- | --- |
| K.FFFHDQSC(+57.02)LDVYK.Y | Y | 46.21 | 1704.7606 | 13 | 2.99 | 569.26 | 3 | 17.76 | 0.8067 | 0.814 | 60434 | 5.36e2 | 1 | 1 | 221 | 233 | Carbamidomethylation |  | DB Search |
| total 1 peptides |  |  |  |  |  |  |  |  |  |  |  |  |  |  |  |  |  |  |  |
| --- | --- | --- | --- | --- | --- | --- | --- | --- | --- | --- | --- | --- | --- | --- | --- | --- | --- | --- | --- |

Best Unique PSM (Scan Sample\_4\_A1\_1\_3642.d:60434, m/z=569.26, z=3, RT=17.76, ppm=-1.41):


TRINITY\_DN106\_c0\_g1\_i12.p1

back to list

  

| Protein Coverage
| Supporting Peptides
| Best Unique PSM
|

Protein Coverage:

Supporting Peptides:

| Peptide | Uniq | -10lgP | Mass | Length | ppm | m/z | z | RT | 1/k0 Start | 1/k0 End | Precursor ID | Area Sample 6 | #Feature | #Feature Sample 6 | Start | End | PTM | AScore | Found By |
| --- | --- | --- | --- | --- | --- | --- | --- | --- | --- | --- | --- | --- | --- | --- | --- | --- | --- | --- | --- |
| R.DGPAGPAGR.D | Y | 46.52 | 796.3827 | 9 | 7.09 | 399.1997 | 2 | 9.76 | 0.7233 | 0.7306 | 13098 | 1.65e3 | 1 | 1 | 283 | 291 |  |  | DB Search |
| total 1 peptides |  |  |  |  |  |  |  |  |  |  |  |  |  |  |  |  |  |  |  |
| --- | --- | --- | --- | --- | --- | --- | --- | --- | --- | --- | --- | --- | --- | --- | --- | --- | --- | --- | --- |

Best Unique PSM (Scan Sample\_4\_A1\_1\_3642.d:13098, m/z=399.1997, z=2, RT=9.76, ppm=2.68):


TRINITY\_DN101992\_c0\_g1\_i1.p1

back to list

  

| Protein Coverage
| Supporting Peptides
| Best Unique PSM
|

Protein Coverage:

Supporting Peptides:

| Peptide | Uniq | -10lgP | Mass | Length | ppm | m/z | z | RT | 1/k0 Start | 1/k0 End | Precursor ID | Area Sample 6 | #Feature | #Feature Sample 6 | Start | End | PTM | AScore | Found By |
| --- | --- | --- | --- | --- | --- | --- | --- | --- | --- | --- | --- | --- | --- | --- | --- | --- | --- | --- | --- |
| K.AELVFVNHC(+57.02)R.E | Y | 46.56 | 1243.6132 | 10 | 2.23 | 622.8125 | 2 | 14.50 | 0.9574 | 0.9646 | 41298 | 1.35e3 | 1 | 1 | 118 | 127 | Carbamidomethylation |  | DB Search |
| total 1 peptides |  |  |  |  |  |  |  |  |  |  |  |  |  |  |  |  |  |  |  |
| --- | --- | --- | --- | --- | --- | --- | --- | --- | --- | --- | --- | --- | --- | --- | --- | --- | --- | --- | --- |

Best Unique PSM (Scan Sample\_4\_A1\_1\_3642.d:41298, m/z=622.8125, z=2, RT=14.50, ppm=-2.18):


TRINITY\_DN923\_c0\_g1\_i1.p1

back to list

  

| Protein Coverage
| Supporting Peptides
| Best Unique PSM
|

Protein Coverage:

Supporting Peptides:

| Peptide | Uniq | -10lgP | Mass | Length | ppm | m/z | z | RT | 1/k0 Start | 1/k0 End | Precursor ID | Area Sample 6 | #Feature | #Feature Sample 6 | Start | End | PTM | AScore | Found By |
| --- | --- | --- | --- | --- | --- | --- | --- | --- | --- | --- | --- | --- | --- | --- | --- | --- | --- | --- | --- |
| R.YLTTAVITNKR.R | Y | 46.75 | 1278.7296 | 11 | -5.61 | 640.3657 | 2 | 13.99 | 1.0005 | 1.0077 | 38385 | 0 | 0 | 0 | 258 | 268 |  |  | DB Search |
| total 1 peptides |  |  |  |  |  |  |  |  |  |  |  |  |  |  |  |  |  |  |  |
| --- | --- | --- | --- | --- | --- | --- | --- | --- | --- | --- | --- | --- | --- | --- | --- | --- | --- | --- | --- |

Best Unique PSM (Scan Sample\_4\_A1\_1\_3642.d:38385, m/z=640.3657, z=2, RT=13.99, ppm=-10.01):


TRINITY\_DN97809\_c0\_g1\_i1.p1

back to list

  

| Protein Coverage
| Supporting Peptides
| Best Unique PSM
|

Protein Coverage:

Supporting Peptides:

| Peptide | Uniq | -10lgP | Mass | Length | ppm | m/z | z | RT | 1/k0 Start | 1/k0 End | Precursor ID | Area Sample 6 | #Feature | #Feature Sample 6 | Start | End | PTM | AScore | Found By |
| --- | --- | --- | --- | --- | --- | --- | --- | --- | --- | --- | --- | --- | --- | --- | --- | --- | --- | --- | --- |
| LGPDQVDVAGAGR.D | Y | 43.15 | 1253.6364 | 13 | 15.86 | 627.8326 | 2 | 13.68 | 0.9335 | 0.9408 | 36571 | 6.32e2 | 1 | 1 | 1 | 13 |  |  | DB Search |
| total 1 peptides |  |  |  |  |  |  |  |  |  |  |  |  |  |  |  |  |  |  |  |
| --- | --- | --- | --- | --- | --- | --- | --- | --- | --- | --- | --- | --- | --- | --- | --- | --- | --- | --- | --- |

Best Unique PSM (Scan Sample\_4\_A1\_1\_3642.d:36571, m/z=627.8326, z=2, RT=13.68, ppm=11.46):


TRINITY\_DN984\_c0\_g1\_i2.p1

back to list

  

| Protein Coverage
| Supporting Peptides
| Best Unique PSM
|

Protein Coverage:

Supporting Peptides:

| Peptide | Uniq | -10lgP | Mass | Length | ppm | m/z | z | RT | 1/k0 Start | 1/k0 End | Precursor ID | Area Sample 6 | #Feature | #Feature Sample 6 | Start | End | PTM | AScore | Found By |
| --- | --- | --- | --- | --- | --- | --- | --- | --- | --- | --- | --- | --- | --- | --- | --- | --- | --- | --- | --- |
| R.EGPTGPQGETGR.R | Y | 47.26 | 1184.5421 | 12 | 0.95 | 593.2763 | 2 | 10.36 | 0.8798 | 0.887 | 16389 | 1.9e3 | 1 | 1 | 476 | 487 |  |  | DB Search |
| total 1 peptides |  |  |  |  |  |  |  |  |  |  |  |  |  |  |  |  |  |  |  |
| --- | --- | --- | --- | --- | --- | --- | --- | --- | --- | --- | --- | --- | --- | --- | --- | --- | --- | --- | --- |

Best Unique PSM (Scan Sample\_4\_A1\_1\_3642.d:16389, m/z=593.2763, z=2, RT=10.36, ppm=-3.45):


TRINITY\_DN3156\_c0\_g1\_i1.p1

back to list

  

| Protein Coverage
| Supporting Peptides
| Best Unique PSM
|

Protein Coverage:

Supporting Peptides:

| Peptide | Uniq | -10lgP | Mass | Length | ppm | m/z | z | RT | 1/k0 Start | 1/k0 End | Precursor ID | Area Sample 6 | #Feature | #Feature Sample 6 | Start | End | PTM | AScore | Found By |
| --- | --- | --- | --- | --- | --- | --- | --- | --- | --- | --- | --- | --- | --- | --- | --- | --- | --- | --- | --- |
| R.GYVIWQEVVDNQVK.Y | Y | 47.25 | 1675.8569 | 14 | -0.86 | 838.9313 | 2 | 19.92 | 1.1314 | 1.1386 | 75433 | 1.33e3 | 1 | 1 | 388 | 401 |  |  | DB Search |
| total 1 peptides |  |  |  |  |  |  |  |  |  |  |  |  |  |  |  |  |  |  |  |
| --- | --- | --- | --- | --- | --- | --- | --- | --- | --- | --- | --- | --- | --- | --- | --- | --- | --- | --- | --- |

Best Unique PSM (Scan Sample\_4\_A1\_1\_3642.d:75433, m/z=838.9313, z=2, RT=19.92, ppm=-5.26):


TRINITY\_DN106\_c0\_g1\_i9.p1

back to list

  

| Protein Coverage
| Supporting Peptides
| Best Unique PSM
|

Protein Coverage:

Supporting Peptides:

| Peptide | Uniq | -10lgP | Mass | Length | ppm | m/z | z | RT | 1/k0 Start | 1/k0 End | Precursor ID | Area Sample 6 | #Feature | #Feature Sample 6 | Start | End | PTM | AScore | Found By |
| --- | --- | --- | --- | --- | --- | --- | --- | --- | --- | --- | --- | --- | --- | --- | --- | --- | --- | --- | --- |
| R.DGPAGPAGR.D | Y | 46.52 | 796.3827 | 9 | 7.09 | 399.1997 | 2 | 9.76 | 0.7233 | 0.7306 | 13098 | 1.65e3 | 1 | 1 | 488 | 496 |  |  | DB Search |
| total 1 peptides |  |  |  |  |  |  |  |  |  |  |  |  |  |  |  |  |  |  |  |
| --- | --- | --- | --- | --- | --- | --- | --- | --- | --- | --- | --- | --- | --- | --- | --- | --- | --- | --- | --- |

Best Unique PSM (Scan Sample\_4\_A1\_1\_3642.d:13098, m/z=399.1997, z=2, RT=9.76, ppm=2.68):


TRINITY\_DN982\_c0\_g1\_i2.p1

back to list

  

| Protein Coverage
| Supporting Peptides
| Best Unique PSM
|

Protein Coverage:

Supporting Peptides:

| Peptide | Uniq | -10lgP | Mass | Length | ppm | m/z | z | RT | 1/k0 Start | 1/k0 End | Precursor ID | Area Sample 6 | #Feature | #Feature Sample 6 | Start | End | PTM | AScore | Found By |
| --- | --- | --- | --- | --- | --- | --- | --- | --- | --- | --- | --- | --- | --- | --- | --- | --- | --- | --- | --- |
| K.INNNQFSVVLIK.N | Y | 47.19 | 1387.7823 | 12 | 4.05 | 694.8982 | 2 | 18.30 | 0.9928 | 1.0001 | 64280 | 6.59e2 | 1 | 1 | 100 | 111 |  |  | DB Search |
| K.AQLTLTIFLHK.S | N | 46.99 | 1283.7601 | 11 | 3.95 | 428.9271 | 3 | 19.57 | 0.7489 | 0.7563 | 73093 | 6.45e2 | 1 | 1 | 1509 | 1519 |  |  | DB Search |
| K.VLLSQQIFYDKK.N | N | 46.51 | 1480.8289 | 12 | 0.37 | 494.6149 | 3 | 16.93 | 0.7616 | 0.7689 | 55019 | 1.05e4 | 2 | 2 | 1479 | 1490 |  |  | DB Search |
| K.VLEVNDNKPK.F | Y | 46.46 | 1154.6295 | 10 | 4.39 | 578.322 | 2 | 11.01 | 0.9263 | 0.9335 | 20603 | 6.84e2 | 1 | 1 | 459 | 468 |  |  | DB Search |
| R.DRHVLTIQSR.G | N | 46.4 | 1223.6734 | 10 | 4.59 | 408.8985 | 3 | 12.00 | 0.7559 | 0.7632 | 26560 | 0 | 0 | 0 | 1590 | 1599 |  |  | DB Search |
| K.ELEITVLESKPVNSFVIR.V | Y | 46.29 | 2072.1517 | 18 | 4.54 | 691.7246 | 3 | 19.86 | 0.961 | 0.9683 | 74982 | 5.9e2 | 1 | 1 | 368 | 385 |  |  | DB Search |
| R.VLDVNDKQPVFVK.S | Y | 45.16 | 1499.8347 | 13 | 0.06 | 750.9214 | 2 | 15.14 | 1.0705 | 1.0777 | 45016 | 7.57e2 | 1 | 1 | 670 | 682 |  |  | DB Search |
| total 7 peptides |  |  |  |  |  |  |  |  |  |  |  |  |  |  |  |  |  |  |  |
| --- | --- | --- | --- | --- | --- | --- | --- | --- | --- | --- | --- | --- | --- | --- | --- | --- | --- | --- | --- |

Best Unique PSM (Scan Sample\_4\_A1\_1\_3642.d:64280, m/z=694.8982, z=2, RT=18.30, ppm=-0.36):


TRINITY\_DN17725\_c0\_g1\_i1.p1

back to list

  

| Protein Coverage
| Supporting Peptides
| Best Unique PSM
|

Protein Coverage:

Supporting Peptides:

| Peptide | Uniq | -10lgP | Mass | Length | ppm | m/z | z | RT | 1/k0 Start | 1/k0 End | Precursor ID | Area Sample 6 | #Feature | #Feature Sample 6 | Start | End | PTM | AScore | Found By |
| --- | --- | --- | --- | --- | --- | --- | --- | --- | --- | --- | --- | --- | --- | --- | --- | --- | --- | --- | --- |
| K.VDVGFILDSSGSLR.N | Y | 45.76 | 1463.762 | 14 | -1.21 | 732.8842 | 2 | 19.93 | 1.0102 | 1.0174 | 75559 | 4.99e2 | 1 | 1 | 35 | 48 |  |  | DB Search |
| total 1 peptides |  |  |  |  |  |  |  |  |  |  |  |  |  |  |  |  |  |  |  |
| --- | --- | --- | --- | --- | --- | --- | --- | --- | --- | --- | --- | --- | --- | --- | --- | --- | --- | --- | --- |

Best Unique PSM (Scan Sample\_4\_A1\_1\_3642.d:75559, m/z=732.8842, z=2, RT=19.93, ppm=-5.61):


TRINITY\_DN413\_c0\_g1\_i3.p1

back to list

  

| Protein Coverage
| Supporting Peptides
| Best Unique PSM
|

Protein Coverage:

Supporting Peptides:

| Peptide | Uniq | -10lgP | Mass | Length | ppm | m/z | z | RT | 1/k0 Start | 1/k0 End | Precursor ID | Area Sample 6 | #Feature | #Feature Sample 6 | Start | End | PTM | AScore | Found By |
| --- | --- | --- | --- | --- | --- | --- | --- | --- | --- | --- | --- | --- | --- | --- | --- | --- | --- | --- | --- |
| R.LLLGDFNGDKR.T | Y | 47.16 | 1246.6669 | 11 | 0.01 | 624.338 | 2 | 16.23 | 0.9844 | 0.9916 | 51088 | 9.22e2 | 1 | 1 | 46 | 56 |  |  | DB Search |
| R.ADILC(+57.02)HDTK.Y | Y | 37.17 | 1071.5019 | 9 | 4.34 | 536.7582 | 2 | 11.78 | 0.8716 | 0.8789 | 25234 | 2.05e2 | 1 | 1 | 320 | 328 | Carbamidomethylation |  | DB Search |
| total 2 peptides |  |  |  |  |  |  |  |  |  |  |  |  |  |  |  |  |  |  |  |
| --- | --- | --- | --- | --- | --- | --- | --- | --- | --- | --- | --- | --- | --- | --- | --- | --- | --- | --- | --- |

Best Unique PSM (Scan Sample\_4\_A1\_1\_3642.d:51088, m/z=624.338, z=2, RT=16.23, ppm=-4.39):


TRINITY\_DN413\_c0\_g1\_i1.p1

back to list

  

| Protein Coverage
| Supporting Peptides
| Best Unique PSM
|

Protein Coverage:

Supporting Peptides:

| Peptide | Uniq | -10lgP | Mass | Length | ppm | m/z | z | RT | 1/k0 Start | 1/k0 End | Precursor ID | Area Sample 6 | #Feature | #Feature Sample 6 | Start | End | PTM | AScore | Found By |
| --- | --- | --- | --- | --- | --- | --- | --- | --- | --- | --- | --- | --- | --- | --- | --- | --- | --- | --- | --- |
| R.LLLGDFNGDKR.T | Y | 47.16 | 1246.6669 | 11 | 0.01 | 624.338 | 2 | 16.23 | 0.9844 | 0.9916 | 51088 | 9.22e2 | 1 | 1 | 46 | 56 |  |  | DB Search |
| R.ADILC(+57.02)HDTK.Y | Y | 37.17 | 1071.5019 | 9 | 4.34 | 536.7582 | 2 | 11.78 | 0.8716 | 0.8789 | 25234 | 2.05e2 | 1 | 1 | 329 | 337 | Carbamidomethylation |  | DB Search |
| total 2 peptides |  |  |  |  |  |  |  |  |  |  |  |  |  |  |  |  |  |  |  |
| --- | --- | --- | --- | --- | --- | --- | --- | --- | --- | --- | --- | --- | --- | --- | --- | --- | --- | --- | --- |

Best Unique PSM (Scan Sample\_4\_A1\_1\_3642.d:51088, m/z=624.338, z=2, RT=16.23, ppm=-4.39):


TRINITY\_DN1783\_c0\_g1\_i8.p2

back to list

  

| Protein Coverage
| Supporting Peptides
| Best Unique PSM
|

Protein Coverage:

Supporting Peptides:

| Peptide | Uniq | -10lgP | Mass | Length | ppm | m/z | z | RT | 1/k0 Start | 1/k0 End | Precursor ID | Area Sample 6 | #Feature | #Feature Sample 6 | Start | End | PTM | AScore | Found By |
| --- | --- | --- | --- | --- | --- | --- | --- | --- | --- | --- | --- | --- | --- | --- | --- | --- | --- | --- | --- |
| K.LTIIDALPR.C | Y | 46.48 | 1010.6124 | 9 | 5.92 | 506.3142 | 2 | 18.91 | 0.8445 | 0.8518 | 68779 | 8.74e2 | 1 | 1 | 224 | 232 |  |  | DB Search |
| total 1 peptides |  |  |  |  |  |  |  |  |  |  |  |  |  |  |  |  |  |  |  |
| --- | --- | --- | --- | --- | --- | --- | --- | --- | --- | --- | --- | --- | --- | --- | --- | --- | --- | --- | --- |

Best Unique PSM (Scan Sample\_4\_A1\_1\_3642.d:68779, m/z=506.3142, z=2, RT=18.91, ppm=1.51):


TRINITY\_DN11522\_c0\_g1\_i1.p1

back to list

  

| Protein Coverage
| Supporting Peptides
| Best Unique PSM
|

Protein Coverage:

Supporting Peptides:

| Peptide | Uniq | -10lgP | Mass | Length | ppm | m/z | z | RT | 1/k0 Start | 1/k0 End | Precursor ID | Area Sample 6 | #Feature | #Feature Sample 6 | Start | End | PTM | AScore | Found By |
| --- | --- | --- | --- | --- | --- | --- | --- | --- | --- | --- | --- | --- | --- | --- | --- | --- | --- | --- | --- |
| R.IGLGNIRDPSISLTR.G | Y | 47.25 | 1610.9104 | 15 | 2.96 | 537.9766 | 3 | 17.36 | 0.797 | 0.8043 | 57658 | 1.69e3 | 1 | 1 | 25 | 39 |  |  | DB Search |
| total 1 peptides |  |  |  |  |  |  |  |  |  |  |  |  |  |  |  |  |  |  |  |
| --- | --- | --- | --- | --- | --- | --- | --- | --- | --- | --- | --- | --- | --- | --- | --- | --- | --- | --- | --- |

Best Unique PSM (Scan Sample\_4\_A1\_1\_3642.d:57658, m/z=537.9766, z=3, RT=17.36, ppm=-1.44):


TRINITY\_DN4494\_c1\_g1\_i1.p1

back to list

  

| Protein Coverage
| Supporting Peptides
| Best Unique PSM
|

Protein Coverage:

Supporting Peptides:

| Peptide | Uniq | -10lgP | Mass | Length | ppm | m/z | z | RT | 1/k0 Start | 1/k0 End | Precursor ID | Area Sample 6 | #Feature | #Feature Sample 6 | Start | End | PTM | AScore | Found By |
| --- | --- | --- | --- | --- | --- | --- | --- | --- | --- | --- | --- | --- | --- | --- | --- | --- | --- | --- | --- |
| K.VDVGFILDSSGSLR.N | Y | 45.76 | 1463.762 | 14 | -1.21 | 732.8842 | 2 | 19.93 | 1.0102 | 1.0174 | 75559 | 4.99e2 | 1 | 1 | 307 | 320 |  |  | DB Search |
| total 1 peptides |  |  |  |  |  |  |  |  |  |  |  |  |  |  |  |  |  |  |  |
| --- | --- | --- | --- | --- | --- | --- | --- | --- | --- | --- | --- | --- | --- | --- | --- | --- | --- | --- | --- |

Best Unique PSM (Scan Sample\_4\_A1\_1\_3642.d:75559, m/z=732.8842, z=2, RT=19.93, ppm=-5.61):


TRINITY\_DN775\_c0\_g1\_i2.p1

back to list

  

| Protein Coverage
| Supporting Peptides
| Best Unique PSM
|

Protein Coverage:

Supporting Peptides:

| Peptide | Uniq | -10lgP | Mass | Length | ppm | m/z | z | RT | 1/k0 Start | 1/k0 End | Precursor ID | Area Sample 6 | #Feature | #Feature Sample 6 | Start | End | PTM | AScore | Found By |
| --- | --- | --- | --- | --- | --- | --- | --- | --- | --- | --- | --- | --- | --- | --- | --- | --- | --- | --- | --- |
| K.DKELSNVIESGFR.T | Y | 46.52 | 1492.7521 | 13 | 2.71 | 747.3821 | 2 | 17.35 | 1.0411 | 1.0484 | 57734 | 0 | 0 | 0 | 489 | 501 |  |  | DB Search |
| total 1 peptides |  |  |  |  |  |  |  |  |  |  |  |  |  |  |  |  |  |  |  |
| --- | --- | --- | --- | --- | --- | --- | --- | --- | --- | --- | --- | --- | --- | --- | --- | --- | --- | --- | --- |

Best Unique PSM (Scan Sample\_4\_A1\_1\_3642.d:57734, m/z=747.3821, z=2, RT=17.35, ppm=-1.7):


TRINITY\_DN106\_c0\_g1\_i8.p1

back to list

  

| Protein Coverage
| Supporting Peptides
| Best Unique PSM
|

Protein Coverage:

Supporting Peptides:

| Peptide | Uniq | -10lgP | Mass | Length | ppm | m/z | z | RT | 1/k0 Start | 1/k0 End | Precursor ID | Area Sample 6 | #Feature | #Feature Sample 6 | Start | End | PTM | AScore | Found By |
| --- | --- | --- | --- | --- | --- | --- | --- | --- | --- | --- | --- | --- | --- | --- | --- | --- | --- | --- | --- |
| R.DGPAGPAGR.D | Y | 46.52 | 796.3827 | 9 | 7.09 | 399.1997 | 2 | 9.76 | 0.7233 | 0.7306 | 13098 | 1.65e3 | 1 | 1 | 488 | 496 |  |  | DB Search |
| total 1 peptides |  |  |  |  |  |  |  |  |  |  |  |  |  |  |  |  |  |  |  |
| --- | --- | --- | --- | --- | --- | --- | --- | --- | --- | --- | --- | --- | --- | --- | --- | --- | --- | --- | --- |

Best Unique PSM (Scan Sample\_4\_A1\_1\_3642.d:13098, m/z=399.1997, z=2, RT=9.76, ppm=2.68):


TRINITY\_DN649\_c0\_g1\_i2.p1

back to list

  

| Protein Coverage
| Supporting Peptides
| Best Unique PSM
|

Protein Coverage:

Supporting Peptides:

| Peptide | Uniq | -10lgP | Mass | Length | ppm | m/z | z | RT | 1/k0 Start | 1/k0 End | Precursor ID | Area Sample 6 | #Feature | #Feature Sample 6 | Start | End | PTM | AScore | Found By |
| --- | --- | --- | --- | --- | --- | --- | --- | --- | --- | --- | --- | --- | --- | --- | --- | --- | --- | --- | --- |
| K.FETQIATK.N | Y | 44.83 | 936.4916 | 8 | 7 | 937.5013 | 1 | 13.97 | 1.1802 | 1.1873 | 38247 | 0 | 0 | 0 | 103 | 110 |  |  | DB Search |
| total 1 peptides |  |  |  |  |  |  |  |  |  |  |  |  |  |  |  |  |  |  |  |
| --- | --- | --- | --- | --- | --- | --- | --- | --- | --- | --- | --- | --- | --- | --- | --- | --- | --- | --- | --- |

Best Unique PSM (Scan Sample\_4\_A1\_1\_3642.d:38247, m/z=937.5013, z=1, RT=13.97, ppm=2.6):


TRINITY\_DN319\_c2\_g1\_i1.p1

back to list

  

| Protein Coverage
| Supporting Peptides
| Best Unique PSM
|

Protein Coverage:

Supporting Peptides:

| Peptide | Uniq | -10lgP | Mass | Length | ppm | m/z | z | RT | 1/k0 Start | 1/k0 End | Precursor ID | Area Sample 6 | #Feature | #Feature Sample 6 | Start | End | PTM | AScore | Found By |
| --- | --- | --- | --- | --- | --- | --- | --- | --- | --- | --- | --- | --- | --- | --- | --- | --- | --- | --- | --- |
| R.DPIC(+57.02)HC(+57.02)DTSK.Y | Y | 47.35 | 1231.4961 | 10 | 6.31 | 411.5068 | 3 | 9.96 | 0.7351 | 0.7424 | 14092 | 1.65e3 | 2 | 2 | 1448 | 1457 | Carbamidomethylation, Carbamidomethylation |  | DB Search |
| K.KAQYYEAYETIIKR.H | Y | 46.51 | 1774.9254 | 14 | -1.09 | 592.6458 | 3 | 15.90 | 0.8656 | 0.8729 | 49338 | 0 | 0 | 0 | 2534 | 2547 |  |  | DB Search |
| R.GSFSSHIC(+57.02)LC(+57.02)HDPAYK.S | Y | 46.19 | 1877.8189 | 16 | 4.79 | 626.9471 | 3 | 14.44 | 0.887 | 0.8943 | 41053 | 0 | 0 | 0 | 1011 | 1026 | Carbamidomethylation, Carbamidomethylation |  | DB Search |
| total 3 peptides |  |  |  |  |  |  |  |  |  |  |  |  |  |  |  |  |  |  |  |
| --- | --- | --- | --- | --- | --- | --- | --- | --- | --- | --- | --- | --- | --- | --- | --- | --- | --- | --- | --- |

Best Unique PSM (Scan Sample\_4\_A1\_1\_3642.d:14092, m/z=411.5068, z=3, RT=9.96, ppm=1.91):


TRINITY\_DN4040\_c0\_g1\_i1.p1

back to list

  

| Protein Coverage
| Supporting Peptides
| Best Unique PSM
|

Protein Coverage:

Supporting Peptides:

| Peptide | Uniq | -10lgP | Mass | Length | ppm | m/z | z | RT | 1/k0 Start | 1/k0 End | Precursor ID | Area Sample 6 | #Feature | #Feature Sample 6 | Start | End | PTM | AScore | Found By |
| --- | --- | --- | --- | --- | --- | --- | --- | --- | --- | --- | --- | --- | --- | --- | --- | --- | --- | --- | --- |
| K.YLLVFTDGK.S | Y | 46.48 | 1054.5699 | 9 | 2.83 | 528.2914 | 2 | 18.82 | 0.8579 | 0.8652 | 67996 | 2.92e3 | 1 | 1 | 324 | 332 |  |  | DB Search |
| K.KEFFC(+57.02)DSC(+57.02)GNRK.G | Y | 45.74 | 1546.6657 | 12 | 5.94 | 516.5633 | 3 | 12.01 | 0.7929 | 0.8002 | 26624 | 4.96e2 | 1 | 1 | 308 | 319 | Carbamidomethylation, Carbamidomethylation |  | DB Search |
| total 2 peptides |  |  |  |  |  |  |  |  |  |  |  |  |  |  |  |  |  |  |  |
| --- | --- | --- | --- | --- | --- | --- | --- | --- | --- | --- | --- | --- | --- | --- | --- | --- | --- | --- | --- |

Best Unique PSM (Scan Sample\_4\_A1\_1\_3642.d:67996, m/z=528.2914, z=2, RT=18.82, ppm=-1.57):


TRINITY\_DN413\_c0\_g1\_i4.p1

back to list

  

| Protein Coverage
| Supporting Peptides
| Best Unique PSM
|

Protein Coverage:

Supporting Peptides:

| Peptide | Uniq | -10lgP | Mass | Length | ppm | m/z | z | RT | 1/k0 Start | 1/k0 End | Precursor ID | Area Sample 6 | #Feature | #Feature Sample 6 | Start | End | PTM | AScore | Found By |
| --- | --- | --- | --- | --- | --- | --- | --- | --- | --- | --- | --- | --- | --- | --- | --- | --- | --- | --- | --- |
| R.LLLGDFNGDKR.T | Y | 47.16 | 1246.6669 | 11 | 0.01 | 624.338 | 2 | 16.23 | 0.9844 | 0.9916 | 51088 | 9.22e2 | 1 | 1 | 46 | 56 |  |  | DB Search |
| R.ADILC(+57.02)HDTK.Y | Y | 37.17 | 1071.5019 | 9 | 4.34 | 536.7582 | 2 | 11.78 | 0.8716 | 0.8789 | 25234 | 2.05e2 | 1 | 1 | 320 | 328 | Carbamidomethylation |  | DB Search |
| total 2 peptides |  |  |  |  |  |  |  |  |  |  |  |  |  |  |  |  |  |  |  |
| --- | --- | --- | --- | --- | --- | --- | --- | --- | --- | --- | --- | --- | --- | --- | --- | --- | --- | --- | --- |

Best Unique PSM (Scan Sample\_4\_A1\_1\_3642.d:51088, m/z=624.338, z=2, RT=16.23, ppm=-4.39):


TRINITY\_DN278\_c0\_g1\_i1.p1

back to list

  

| Protein Coverage
| Supporting Peptides
| Best Unique PSM
|

Protein Coverage:

Supporting Peptides:

| Peptide | Uniq | -10lgP | Mass | Length | ppm | m/z | z | RT | 1/k0 Start | 1/k0 End | Precursor ID | Area Sample 6 | #Feature | #Feature Sample 6 | Start | End | PTM | AScore | Found By |
| --- | --- | --- | --- | --- | --- | --- | --- | --- | --- | --- | --- | --- | --- | --- | --- | --- | --- | --- | --- |
| K.IDNYIEFLDEDLKK.K | Y | 43.58 | 1753.8774 | 14 | -1.78 | 585.6295 | 3 | 19.97 | 0.8437 | 0.851 | 75833 | 0 | 0 | 0 | 102 | 115 |  |  | DB Search |
| K.LNFKGDVLQC(+57.02)LER.R | Y | 42.85 | 1590.8188 | 13 | -4.9 | 531.2753 | 3 | 18.12 | 0.7673 | 0.7746 | 63093 | 0 | 0 | 0 | 136 | 148 | Carbamidomethylation |  | DB Search |
| total 2 peptides |  |  |  |  |  |  |  |  |  |  |  |  |  |  |  |  |  |  |  |
| --- | --- | --- | --- | --- | --- | --- | --- | --- | --- | --- | --- | --- | --- | --- | --- | --- | --- | --- | --- |

Best Unique PSM (Scan Sample\_4\_A1\_1\_3642.d:75833, m/z=585.6295, z=3, RT=19.97, ppm=-6.19):


TRINITY\_DN602\_c0\_g2\_i1.p1

back to list

  

| Protein Coverage
| Supporting Peptides
| Best Unique PSM
|

Protein Coverage:

Supporting Peptides:

| Peptide | Uniq | -10lgP | Mass | Length | ppm | m/z | z | RT | 1/k0 Start | 1/k0 End | Precursor ID | Area Sample 6 | #Feature | #Feature Sample 6 | Start | End | PTM | AScore | Found By |
| --- | --- | --- | --- | --- | --- | --- | --- | --- | --- | --- | --- | --- | --- | --- | --- | --- | --- | --- | --- |
| R.AYDFRETLPK.K | Y | 45.42 | 1238.6295 | 10 | 5.77 | 620.3229 | 2 | 15.38 | 0.9497 | 0.957 | 46320 | 7.87e2 | 1 | 1 | 94 | 103 |  |  | DB Search |
| total 1 peptides |  |  |  |  |  |  |  |  |  |  |  |  |  |  |  |  |  |  |  |
| --- | --- | --- | --- | --- | --- | --- | --- | --- | --- | --- | --- | --- | --- | --- | --- | --- | --- | --- | --- |

Best Unique PSM (Scan Sample\_4\_A1\_1\_3642.d:46320, m/z=620.3229, z=2, RT=15.38, ppm=1.37):


TRINITY\_DN12627\_c0\_g1\_i1.p1

back to list

  

| Protein Coverage
| Supporting Peptides
| Best Unique PSM
|

Protein Coverage:

Supporting Peptides:

| Peptide | Uniq | -10lgP | Mass | Length | ppm | m/z | z | RT | 1/k0 Start | 1/k0 End | Precursor ID | Area Sample 6 | #Feature | #Feature Sample 6 | Start | End | PTM | AScore | Found By |
| --- | --- | --- | --- | --- | --- | --- | --- | --- | --- | --- | --- | --- | --- | --- | --- | --- | --- | --- | --- |
| K.FDFHSWIGLKK.I | Y | 46.51 | 1376.7241 | 11 | -6.14 | 459.9105 | 3 | 18.38 | 0.7384 | 0.7457 | 64918 | 0 | 0 | 0 | 193 | 203 |  |  | DB Search |
| K.GFGGNLVTITNSDIQK.Q | Y | 45.92 | 1662.8577 | 16 | 0.13 | 832.4326 | 2 | 17.74 | 1.0813 | 1.0885 | 60461 | 6.04e2 | 1 | 1 | 311 | 326 |  |  | DB Search |
| total 2 peptides |  |  |  |  |  |  |  |  |  |  |  |  |  |  |  |  |  |  |  |
| --- | --- | --- | --- | --- | --- | --- | --- | --- | --- | --- | --- | --- | --- | --- | --- | --- | --- | --- | --- |

Best Unique PSM (Scan Sample\_4\_A1\_1\_3642.d:64918, m/z=459.9105, z=3, RT=18.38, ppm=-10.54):


TRINITY\_DN495\_c0\_g1\_i3.p1

back to list

  

| Protein Coverage
| Supporting Peptides
| Best Unique PSM
|

Protein Coverage:

Supporting Peptides:

| Peptide | Uniq | -10lgP | Mass | Length | ppm | m/z | z | RT | 1/k0 Start | 1/k0 End | Precursor ID | Area Sample 6 | #Feature | #Feature Sample 6 | Start | End | PTM | AScore | Found By |
| --- | --- | --- | --- | --- | --- | --- | --- | --- | --- | --- | --- | --- | --- | --- | --- | --- | --- | --- | --- |
| R.SC(+57.02)TNPAPANGGK.A | Y | 47.22 | 1172.5244 | 12 | 1.68 | 587.2679 | 2 | 9.40 | 0.8429 | 0.8502 | 11161 | 2.16e2 | 1 | 1 | 503 | 514 | Carbamidomethylation |  | DB Search |
| total 1 peptides |  |  |  |  |  |  |  |  |  |  |  |  |  |  |  |  |  |  |  |
| --- | --- | --- | --- | --- | --- | --- | --- | --- | --- | --- | --- | --- | --- | --- | --- | --- | --- | --- | --- |

Best Unique PSM (Scan Sample\_4\_A1\_1\_3642.d:11161, m/z=587.2679, z=2, RT=9.40, ppm=-2.72):


TRINITY\_DN1172\_c0\_g2\_i2.p1

back to list

  

| Protein Coverage
| Supporting Peptides
| Best Unique PSM
|

Protein Coverage:

Supporting Peptides:

| Peptide | Uniq | -10lgP | Mass | Length | ppm | m/z | z | RT | 1/k0 Start | 1/k0 End | Precursor ID | Area Sample 6 | #Feature | #Feature Sample 6 | Start | End | PTM | AScore | Found By |
| --- | --- | --- | --- | --- | --- | --- | --- | --- | --- | --- | --- | --- | --- | --- | --- | --- | --- | --- | --- |
| K.K(+42.01)LAAELANGR.L | Y | 38.62 | 1083.6036 | 10 | 4.95 | 542.8094 | 2 | 15.45 | 0.8648 | 0.8721 | 46842 | 3.78e2 | 1 | 1 | 19 | 28 | Acetylation (Protein N-term) | K1:Acetylation (Protein N-term):1000 | DB Search |
| total 1 peptides |  |  |  |  |  |  |  |  |  |  |  |  |  |  |  |  |  |  |  |
| --- | --- | --- | --- | --- | --- | --- | --- | --- | --- | --- | --- | --- | --- | --- | --- | --- | --- | --- | --- |

Best Unique PSM (Scan Sample\_4\_A1\_1\_3642.d:46842, m/z=542.8094, z=2, RT=15.45, ppm=0.55):


TRINITY\_DN72625\_c0\_g1\_i1.p1

back to list

  

| Protein Coverage
| Supporting Peptides
| Best Unique PSM
|

Protein Coverage:

Supporting Peptides:

| Peptide | Uniq | -10lgP | Mass | Length | ppm | m/z | z | RT | 1/k0 Start | 1/k0 End | Precursor ID | Area Sample 6 | #Feature | #Feature Sample 6 | Start | End | PTM | AScore | Found By |
| --- | --- | --- | --- | --- | --- | --- | --- | --- | --- | --- | --- | --- | --- | --- | --- | --- | --- | --- | --- |
| R.ATNPDVEK.I | Y | 46.45 | 872.4239 | 8 | 5.44 | 437.2197 | 2 | 9.69 | 0.7945 | 0.8019 | 12496 | 5.14e3 | 1 | 1 | 302 | 309 |  |  | DB Search |
| R.IKDTYLLTLEAR.D | Y | 45.94 | 1434.8082 | 12 | 1.17 | 718.4091 | 2 | 17.05 | 1.0379 | 1.0452 | 55684 | 2.02e3 | 1 | 1 | 393 | 404 |  |  | DB Search |
| R.KTTELTNNLK.A | Y | 38.39 | 1160.6401 | 10 | 1.48 | 581.3256 | 2 | 11.73 | 0.9182 | 0.9255 | 24848 | 1.03e3 | 1 | 1 | 1921 | 1930 |  |  | DB Search |
| total 3 peptides |  |  |  |  |  |  |  |  |  |  |  |  |  |  |  |  |  |  |  |
| --- | --- | --- | --- | --- | --- | --- | --- | --- | --- | --- | --- | --- | --- | --- | --- | --- | --- | --- | --- |

Best Unique PSM (Scan Sample\_4\_A1\_1\_3642.d:12496, m/z=437.2197, z=2, RT=9.69, ppm=1.03):


TRINITY\_DN775\_c0\_g1\_i1.p1

back to list

  

| Protein Coverage
| Supporting Peptides
| Best Unique PSM
|

Protein Coverage:

Supporting Peptides:

| Peptide | Uniq | -10lgP | Mass | Length | ppm | m/z | z | RT | 1/k0 Start | 1/k0 End | Precursor ID | Area Sample 6 | #Feature | #Feature Sample 6 | Start | End | PTM | AScore | Found By |
| --- | --- | --- | --- | --- | --- | --- | --- | --- | --- | --- | --- | --- | --- | --- | --- | --- | --- | --- | --- |
| K.DKELSNVIESGFR.T | Y | 46.52 | 1492.7521 | 13 | 2.71 | 747.3821 | 2 | 17.35 | 1.0411 | 1.0484 | 57734 | 0 | 0 | 0 | 477 | 489 |  |  | DB Search |
| total 1 peptides |  |  |  |  |  |  |  |  |  |  |  |  |  |  |  |  |  |  |  |
| --- | --- | --- | --- | --- | --- | --- | --- | --- | --- | --- | --- | --- | --- | --- | --- | --- | --- | --- | --- |

Best Unique PSM (Scan Sample\_4\_A1\_1\_3642.d:57734, m/z=747.3821, z=2, RT=17.35, ppm=-1.7):


TRINITY\_DN4595\_c0\_g1\_i4.p1

back to list

  

| Protein Coverage
| Supporting Peptides
| Best Unique PSM
|

Protein Coverage:

Supporting Peptides:

| Peptide | Uniq | -10lgP | Mass | Length | ppm | m/z | z | RT | 1/k0 Start | 1/k0 End | Precursor ID | Area Sample 6 | #Feature | #Feature Sample 6 | Start | End | PTM | AScore | Found By |
| --- | --- | --- | --- | --- | --- | --- | --- | --- | --- | --- | --- | --- | --- | --- | --- | --- | --- | --- | --- |
| R.FC(+57.02)NNPAPSNGGR.M | Y | 47.24 | 1289.5571 | 12 | 0.71 | 645.7834 | 2 | 10.87 | 0.9416 | 0.9489 | 19820 | 2.76e2 | 1 | 1 | 1196 | 1207 | Carbamidomethylation |  | DB Search |
| R.SPFVIMFK.G | Y | 46.2 | 967.5201 | 8 | -0.36 | 484.765 | 2 | 19.87 | 0.8079 | 0.8153 | 75042 | 1.61e3 | 1 | 1 | 1081 | 1088 |  |  | DB Search |
| K.YTGAC(+57.02)NANGNTDK.T | Y | 46.13 | 1384.5677 | 13 | 10.57 | 693.2954 | 2 | 9.52 | 0.9428 | 0.9501 | 11788 | 0 | 0 | 0 | 1492 | 1504 | Carbamidomethylation |  | DB Search |
| K.TC(+57.02)GGGFFFETR.T | Y | 46.05 | 1277.5499 | 11 | 0.02 | 639.7794 | 2 | 18.54 | 0.9267 | 0.9339 | 65970 | 3.17e2 | 1 | 1 | 480 | 490 | Carbamidomethylation |  | DB Search |
| K.GFEC(+57.02)IGLIEETETC(+57.02)K.L | Y | 45.99 | 1784.7961 | 15 | 4.3 | 893.4052 | 2 | 18.62 | 1.0981 | 1.1053 | 66554 | 4.49e2 | 1 | 1 | 544 | 558 | Carbamidomethylation, Carbamidomethylation |  | DB Search |
| R.VC(+57.02)NGEPC(+57.02)ILQER.S | Y | 45.81 | 1473.6704 | 12 | 3.3 | 737.8417 | 2 | 14.98 | 0.9876 | 0.9949 | 44106 | 8.93e2 | 1 | 1 | 436 | 447 | Carbamidomethylation, Carbamidomethylation |  | DB Search |
| R.YQGREYNINEEVNVR.Q | Y | 45.61 | 1881.8969 | 15 | -1.16 | 941.9505 | 2 | 15.67 | 1.1394 | 1.1466 | 47924 | 1.31e3 | 1 | 1 | 338 | 352 |  |  | DB Search |
| R.AFNKVETR.N | N | 44.29 | 963.5138 | 8 | -5.34 | 482.7595 | 2 | 10.58 | 0.8283 | 0.8356 | 17963 | 0 | 0 | 0 | 177 | 184 |  |  | DB Search |
| R.DGTTPC(+57.02)NTR.S | Y | 44.18 | 1020.4295 | 9 | 6.58 | 511.2231 | 2 | 9.57 | 0.8295 | 0.8368 | 12093 | 0 | 0 | 0 | 290 | 298 | Carbamidomethylation |  | DB Search |
| R.AFNKIETR.N | Y | 41.96 | 977.5294 | 8 | 2.83 | 489.7712 | 2 | 11.48 | 0.8343 | 0.8416 | 23389 | 1.84e3 | 1 | 1 | 72 | 79 |  |  | DB Search |
| R.SPFVIM(+15.99)FK.G | Y | 38.78 | 983.515 | 8 | -5.11 | 492.7601 | 2 | 18.60 | 0.8128 | 0.8201 | 66496 | 4.32e2 | 1 | 1 | 1081 | 1088 | Oxidation (M) | M6:Oxidation (M):1000 | DB Search |
| total 11 peptides |  |  |  |  |  |  |  |  |  |  |  |  |  |  |  |  |  |  |  |
| --- | --- | --- | --- | --- | --- | --- | --- | --- | --- | --- | --- | --- | --- | --- | --- | --- | --- | --- | --- |

Best Unique PSM (Scan Sample\_4\_A1\_1\_3642.d:19820, m/z=645.7834, z=2, RT=10.87, ppm=-3.69):


TRINITY\_DN79\_c0\_g1\_i1.p1

back to list

  

| Protein Coverage
| Supporting Peptides
| Best Unique PSM
|

Protein Coverage:

Supporting Peptides:

| Peptide | Uniq | -10lgP | Mass | Length | ppm | m/z | z | RT | 1/k0 Start | 1/k0 End | Precursor ID | Area Sample 6 | #Feature | #Feature Sample 6 | Start | End | PTM | AScore | Found By |
| --- | --- | --- | --- | --- | --- | --- | --- | --- | --- | --- | --- | --- | --- | --- | --- | --- | --- | --- | --- |
| R.AESIALVTEFTDEAAPK.I | Y | 47.21 | 1790.8938 | 17 | -2.34 | 896.4481 | 2 | 19.93 | 1.1214 | 1.1286 | 75448 | 1.02e3 | 1 | 1 | 192 | 208 |  |  | DB Search |
| total 1 peptides |  |  |  |  |  |  |  |  |  |  |  |  |  |  |  |  |  |  |  |
| --- | --- | --- | --- | --- | --- | --- | --- | --- | --- | --- | --- | --- | --- | --- | --- | --- | --- | --- | --- |

Best Unique PSM (Scan Sample\_4\_A1\_1\_3642.d:75448, m/z=896.4481, z=2, RT=19.93, ppm=-6.74):


TRINITY\_DN585\_c0\_g1\_i2.p1

back to list

  

| Protein Coverage
| Supporting Peptides
| Best Unique PSM
|

Protein Coverage:

Supporting Peptides:

| Peptide | Uniq | -10lgP | Mass | Length | ppm | m/z | z | RT | 1/k0 Start | 1/k0 End | Precursor ID | Area Sample 6 | #Feature | #Feature Sample 6 | Start | End | PTM | AScore | Found By |
| --- | --- | --- | --- | --- | --- | --- | --- | --- | --- | --- | --- | --- | --- | --- | --- | --- | --- | --- | --- |
| K.TITLEVEPSDTIENVK.S | Y | 46.69 | 1786.92 | 16 | 0.39 | 894.4637 | 2 | 17.56 | 1.1442 | 1.1514 | 58994 | 8.78e2 | 1 | 1 | 60 | 75 |  |  | DB Search |
| total 1 peptides |  |  |  |  |  |  |  |  |  |  |  |  |  |  |  |  |  |  |  |
| --- | --- | --- | --- | --- | --- | --- | --- | --- | --- | --- | --- | --- | --- | --- | --- | --- | --- | --- | --- |

Best Unique PSM (Scan Sample\_4\_A1\_1\_3642.d:58994, m/z=894.4637, z=2, RT=17.56, ppm=-4.01):


TRINITY\_DN36\_c0\_g1\_i8.p1

back to list

  

| Protein Coverage
| Supporting Peptides
| Best Unique PSM
|

Protein Coverage:

Supporting Peptides:

| Peptide | Uniq | -10lgP | Mass | Length | ppm | m/z | z | RT | 1/k0 Start | 1/k0 End | Precursor ID | Area Sample 6 | #Feature | #Feature Sample 6 | Start | End | PTM | AScore | Found By |
| --- | --- | --- | --- | --- | --- | --- | --- | --- | --- | --- | --- | --- | --- | --- | --- | --- | --- | --- | --- |
| R.LGGIEVTQGNC(+57.02)AR.K | Y | 45.45 | 1373.6721 | 13 | 2.96 | 687.8423 | 2 | 13.87 | 0.9687 | 0.9759 | 37701 | 0 | 0 | 0 | 58 | 70 | Carbamidomethylation |  | DB Search |
| K.FETQIATK.D | Y | 44.83 | 936.4916 | 8 | 7 | 937.5013 | 1 | 13.97 | 1.1802 | 1.1873 | 38247 | 0 | 0 | 0 | 167 | 174 |  |  | DB Search |
| total 2 peptides |  |  |  |  |  |  |  |  |  |  |  |  |  |  |  |  |  |  |  |
| --- | --- | --- | --- | --- | --- | --- | --- | --- | --- | --- | --- | --- | --- | --- | --- | --- | --- | --- | --- |

Best Unique PSM (Scan Sample\_4\_A1\_1\_3642.d:37701, m/z=687.8423, z=2, RT=13.87, ppm=-1.44):


TRINITY\_DN73457\_c0\_g1\_i1.p1

back to list

  

| Protein Coverage
| Supporting Peptides
| Best Unique PSM
|

Protein Coverage:

Supporting Peptides:

| Peptide | Uniq | -10lgP | Mass | Length | ppm | m/z | z | RT | 1/k0 Start | 1/k0 End | Precursor ID | Area Sample 6 | #Feature | #Feature Sample 6 | Start | End | PTM | AScore | Found By |
| --- | --- | --- | --- | --- | --- | --- | --- | --- | --- | --- | --- | --- | --- | --- | --- | --- | --- | --- | --- |
| K.TILIHGFGTIQR.M | Y | 45.83 | 1354.7721 | 12 | -0.03 | 678.3903 | 2 | 16.94 | 1.0102 | 1.0174 | 55113 | 0 | 0 | 0 | 230 | 241 |  |  | DB Search |
| total 1 peptides |  |  |  |  |  |  |  |  |  |  |  |  |  |  |  |  |  |  |  |
| --- | --- | --- | --- | --- | --- | --- | --- | --- | --- | --- | --- | --- | --- | --- | --- | --- | --- | --- | --- |

Best Unique PSM (Scan Sample\_4\_A1\_1\_3642.d:55113, m/z=678.3903, z=2, RT=16.94, ppm=-4.43):


TRINITY\_DN5987\_c0\_g1\_i4.p1

back to list

  

| Protein Coverage
| Supporting Peptides
| Best Unique PSM
|

Protein Coverage:

Supporting Peptides:

| Peptide | Uniq | -10lgP | Mass | Length | ppm | m/z | z | RT | 1/k0 Start | 1/k0 End | Precursor ID | Area Sample 6 | #Feature | #Feature Sample 6 | Start | End | PTM | AScore | Found By |
| --- | --- | --- | --- | --- | --- | --- | --- | --- | --- | --- | --- | --- | --- | --- | --- | --- | --- | --- | --- |
| R.GNQGEQGPAGPVGPK.G | Y | 47.2 | 1391.6793 | 15 | 3.52 | 696.8463 | 2 | 11.46 | 0.9461 | 0.9533 | 23184 | 1.15e3 | 1 | 1 | 237 | 251 |  |  | DB Search |
| total 1 peptides |  |  |  |  |  |  |  |  |  |  |  |  |  |  |  |  |  |  |  |
| --- | --- | --- | --- | --- | --- | --- | --- | --- | --- | --- | --- | --- | --- | --- | --- | --- | --- | --- | --- |

Best Unique PSM (Scan Sample\_4\_A1\_1\_3642.d:23184, m/z=696.8463, z=2, RT=11.46, ppm=-0.88):


TRINITY\_DN1616\_c0\_g1\_i6.p1

back to list

  

| Protein Coverage
| Supporting Peptides
| Best Unique PSM
|

Protein Coverage:

Supporting Peptides:

| Peptide | Uniq | -10lgP | Mass | Length | ppm | m/z | z | RT | 1/k0 Start | 1/k0 End | Precursor ID | Area Sample 6 | #Feature | #Feature Sample 6 | Start | End | PTM | AScore | Found By |
| --- | --- | --- | --- | --- | --- | --- | --- | --- | --- | --- | --- | --- | --- | --- | --- | --- | --- | --- | --- |
| K.GC(+57.02)STC(+57.02)GC(+57.02)NGAGTNNGVK.S | Y | 47.18 | 1712.6665 | 17 | 14.87 | 857.3495 | 2 | 9.81 | 1.0411 | 1.0484 | 13314 | 0 | 0 | 0 | 732 | 748 | Carbamidomethylation, Carbamidomethylation, Carbamidomethylation |  | DB Search |
| R.YVKPGVNIINIR.D | Y | 46.71 | 1384.819 | 12 | 3.8 | 693.4164 | 2 | 16.18 | 1.0009 | 1.0081 | 50795 | 7.11e2 | 1 | 1 | 1217 | 1228 |  |  | DB Search |
| K.VVDTEANDLTEEVKK.T | Y | 46.47 | 1688.8468 | 15 | 4.23 | 845.4305 | 2 | 14.16 | 1.1298 | 1.137 | 39294 | 3.14e2 | 1 | 1 | 2542 | 2556 |  |  | DB Search |
| K.YVVMLQYYHDSDTAK.Y | Y | 46.08 | 1831.8451 | 15 | 8.86 | 916.9339 | 2 | 16.78 | 1.1825 | 1.1897 | 54165 | 6.38e1 | 1 | 1 | 1071 | 1085 |  |  | DB Search |
| K.GFVAVNDQQPSVVVSINVPK.T | Y | 43.72 | 2096.1266 | 20 | 10.57 | 1049.077 | 2 | 18.41 | 1.2185 | 1.2256 | 65167 | 0 | 0 | 0 | 865 | 884 |  |  | DB Search |
| K.VVAEC(+57.02)NNGGGR.F | Y | 40.05 | 1131.5091 | 11 | 3.26 | 566.7612 | 2 | 9.40 | 0.825 | 0.8323 | 11191 | 0 | 0 | 0 | 3736 | 3746 | Carbamidomethylation |  | DB Search |
| K.YEC(+57.02)NC(+57.02)QYNTEGPR.C | Y | 39.28 | 1689.6512 | 13 | 9.13 | 845.8369 | 2 | 12.04 | 1.0323 | 1.0395 | 26880 | 1.68e2 | 1 | 1 | 291 | 303 | Carbamidomethylation, Carbamidomethylation |  | DB Search |
| total 7 peptides |  |  |  |  |  |  |  |  |  |  |  |  |  |  |  |  |  |  |  |
| --- | --- | --- | --- | --- | --- | --- | --- | --- | --- | --- | --- | --- | --- | --- | --- | --- | --- | --- | --- |

Best Unique PSM (Scan Sample\_4\_A1\_1\_3642.d:13314, m/z=857.3495, z=2, RT=9.81, ppm=10.47):


TRINITY\_DN25375\_c0\_g1\_i7.p1

back to list

  

| Protein Coverage
| Supporting Peptides
| Best Unique PSM
|

Protein Coverage:

Supporting Peptides:

| Peptide | Uniq | -10lgP | Mass | Length | ppm | m/z | z | RT | 1/k0 Start | 1/k0 End | Precursor ID | Area Sample 6 | #Feature | #Feature Sample 6 | Start | End | PTM | AScore | Found By |
| --- | --- | --- | --- | --- | --- | --- | --- | --- | --- | --- | --- | --- | --- | --- | --- | --- | --- | --- | --- |
| K.LRPGQATTVDITVK.T | Y | 46.03 | 1497.8515 | 14 | 3.07 | 749.932 | 2 | 14.17 | 1.0331 | 1.0403 | 39382 | 6.12e2 | 1 | 1 | 114 | 127 |  |  | DB Search |
| total 1 peptides |  |  |  |  |  |  |  |  |  |  |  |  |  |  |  |  |  |  |  |
| --- | --- | --- | --- | --- | --- | --- | --- | --- | --- | --- | --- | --- | --- | --- | --- | --- | --- | --- | --- |

Best Unique PSM (Scan Sample\_4\_A1\_1\_3642.d:39382, m/z=749.932, z=2, RT=14.17, ppm=-1.33):


TRINITY\_DN5397\_c0\_g1\_i1.p1

back to list

  

| Protein Coverage
| Supporting Peptides
| Best Unique PSM
|

Protein Coverage:

Supporting Peptides:

| Peptide | Uniq | -10lgP | Mass | Length | ppm | m/z | z | RT | 1/k0 Start | 1/k0 End | Precursor ID | Area Sample 6 | #Feature | #Feature Sample 6 | Start | End | PTM | AScore | Found By |
| --- | --- | --- | --- | --- | --- | --- | --- | --- | --- | --- | --- | --- | --- | --- | --- | --- | --- | --- | --- |
| R.EGSTPC(+57.02)NTQAC(+57.02)GSGLSEER.F | Y | 47.45 | 2038.832 | 19 | 1.22 | 1020.42 | 2 | 12.08 | 1.1382 | 1.1454 | 27081 | 0 | 0 | 0 | 41 | 59 | Carbamidomethylation, Carbamidomethylation |  | DB Search |
| total 1 peptides |  |  |  |  |  |  |  |  |  |  |  |  |  |  |  |  |  |  |  |
| --- | --- | --- | --- | --- | --- | --- | --- | --- | --- | --- | --- | --- | --- | --- | --- | --- | --- | --- | --- |

Best Unique PSM (Scan Sample\_4\_A1\_1\_3642.d:27081, m/z=1020.42, z=2, RT=12.08, ppm=-3.18):


TRINITY\_DN4494\_c1\_g1\_i15.p1

back to list

  

| Protein Coverage
| Supporting Peptides
| Best Unique PSM
|

Protein Coverage:

Supporting Peptides:

| Peptide | Uniq | -10lgP | Mass | Length | ppm | m/z | z | RT | 1/k0 Start | 1/k0 End | Precursor ID | Area Sample 6 | #Feature | #Feature Sample 6 | Start | End | PTM | AScore | Found By |
| --- | --- | --- | --- | --- | --- | --- | --- | --- | --- | --- | --- | --- | --- | --- | --- | --- | --- | --- | --- |
| K.VDVGFILDSSGSLR.N | Y | 45.76 | 1463.762 | 14 | -1.21 | 732.8842 | 2 | 19.93 | 1.0102 | 1.0174 | 75559 | 4.99e2 | 1 | 1 | 41 | 54 |  |  | DB Search |
| total 1 peptides |  |  |  |  |  |  |  |  |  |  |  |  |  |  |  |  |  |  |  |
| --- | --- | --- | --- | --- | --- | --- | --- | --- | --- | --- | --- | --- | --- | --- | --- | --- | --- | --- | --- |

Best Unique PSM (Scan Sample\_4\_A1\_1\_3642.d:75559, m/z=732.8842, z=2, RT=19.93, ppm=-5.61):


TRINITY\_DN564\_c0\_g1\_i1.p1

back to list

  

| Protein Coverage
| Supporting Peptides
| Best Unique PSM
|

Protein Coverage:

Supporting Peptides:

| Peptide | Uniq | -10lgP | Mass | Length | ppm | m/z | z | RT | 1/k0 Start | 1/k0 End | Precursor ID | Area Sample 6 | #Feature | #Feature Sample 6 | Start | End | PTM | AScore | Found By |
| --- | --- | --- | --- | --- | --- | --- | --- | --- | --- | --- | --- | --- | --- | --- | --- | --- | --- | --- | --- |
| R.EAIVQGAQSTQDTLKK.A | Y | 43.22 | 1715.9053 | 16 | 4.98 | 858.9604 | 2 | 12.79 | 1.1514 | 1.1586 | 31276 | 0 | 0 | 0 | 301 | 316 |  |  | DB Search |
| total 1 peptides |  |  |  |  |  |  |  |  |  |  |  |  |  |  |  |  |  |  |  |
| --- | --- | --- | --- | --- | --- | --- | --- | --- | --- | --- | --- | --- | --- | --- | --- | --- | --- | --- | --- |

Best Unique PSM (Scan Sample\_4\_A1\_1\_3642.d:31276, m/z=858.9604, z=2, RT=12.79, ppm=0.58):


TRINITY\_DN585\_c0\_g2\_i1.p1

back to list

  

| Protein Coverage
| Supporting Peptides
| Best Unique PSM
|

Protein Coverage:

Supporting Peptides:

| Peptide | Uniq | -10lgP | Mass | Length | ppm | m/z | z | RT | 1/k0 Start | 1/k0 End | Precursor ID | Area Sample 6 | #Feature | #Feature Sample 6 | Start | End | PTM | AScore | Found By |
| --- | --- | --- | --- | --- | --- | --- | --- | --- | --- | --- | --- | --- | --- | --- | --- | --- | --- | --- | --- |
| K.TITLEVEPSDTIENVK.S | Y | 46.69 | 1786.92 | 16 | 0.39 | 894.4637 | 2 | 17.56 | 1.1442 | 1.1514 | 58994 | 8.78e2 | 1 | 1 | 5 | 20 |  |  | DB Search |
| total 1 peptides |  |  |  |  |  |  |  |  |  |  |  |  |  |  |  |  |  |  |  |
| --- | --- | --- | --- | --- | --- | --- | --- | --- | --- | --- | --- | --- | --- | --- | --- | --- | --- | --- | --- |

Best Unique PSM (Scan Sample\_4\_A1\_1\_3642.d:58994, m/z=894.4637, z=2, RT=17.56, ppm=-4.01):


TRINITY\_DN585\_c0\_g1\_i4.p1

back to list

  

| Protein Coverage
| Supporting Peptides
| Best Unique PSM
|

Protein Coverage:

Supporting Peptides:

| Peptide | Uniq | -10lgP | Mass | Length | ppm | m/z | z | RT | 1/k0 Start | 1/k0 End | Precursor ID | Area Sample 6 | #Feature | #Feature Sample 6 | Start | End | PTM | AScore | Found By |
| --- | --- | --- | --- | --- | --- | --- | --- | --- | --- | --- | --- | --- | --- | --- | --- | --- | --- | --- | --- |
| K.TITLEVEPSDTIENVK.S | Y | 46.69 | 1786.92 | 16 | 0.39 | 894.4637 | 2 | 17.56 | 1.1442 | 1.1514 | 58994 | 8.78e2 | 1 | 1 | 12 | 27 |  |  | DB Search |
| total 1 peptides |  |  |  |  |  |  |  |  |  |  |  |  |  |  |  |  |  |  |  |
| --- | --- | --- | --- | --- | --- | --- | --- | --- | --- | --- | --- | --- | --- | --- | --- | --- | --- | --- | --- |

Best Unique PSM (Scan Sample\_4\_A1\_1\_3642.d:58994, m/z=894.4637, z=2, RT=17.56, ppm=-4.01):


TRINITY\_DN5584\_c0\_g1\_i2.p1

back to list

  

| Protein Coverage
| Supporting Peptides
| Best Unique PSM
|

Protein Coverage:

Supporting Peptides:

| Peptide | Uniq | -10lgP | Mass | Length | ppm | m/z | z | RT | 1/k0 Start | 1/k0 End | Precursor ID | Area Sample 6 | #Feature | #Feature Sample 6 | Start | End | PTM | AScore | Found By |
| --- | --- | --- | --- | --- | --- | --- | --- | --- | --- | --- | --- | --- | --- | --- | --- | --- | --- | --- | --- |
| K.YLSWTLDNDKIR.S | Y | 46.73 | 1522.778 | 12 | 4.13 | 508.5998 | 3 | 17.56 | 0.8108 | 0.8181 | 59147 | 0 | 0 | 0 | 186 | 197 |  |  | DB Search |
| total 1 peptides |  |  |  |  |  |  |  |  |  |  |  |  |  |  |  |  |  |  |  |
| --- | --- | --- | --- | --- | --- | --- | --- | --- | --- | --- | --- | --- | --- | --- | --- | --- | --- | --- | --- |

Best Unique PSM (Scan Sample\_4\_A1\_1\_3642.d:59147, m/z=508.5998, z=3, RT=17.56, ppm=-0.27):


TRINITY\_DN38397\_c0\_g1\_i1.p1

back to list

  

| Protein Coverage
| Supporting Peptides
| Best Unique PSM
|

Protein Coverage:

Supporting Peptides:

| Peptide | Uniq | -10lgP | Mass | Length | ppm | m/z | z | RT | 1/k0 Start | 1/k0 End | Precursor ID | Area Sample 6 | #Feature | #Feature Sample 6 | Start | End | PTM | AScore | Found By |
| --- | --- | --- | --- | --- | --- | --- | --- | --- | --- | --- | --- | --- | --- | --- | --- | --- | --- | --- | --- |
| R.GC(+57.02)YIHTQEIAR.G | Y | 46.47 | 1346.6401 | 11 | -0.76 | 674.3239 | 2 | 13.04 | 1.0158 | 1.023 | 32770 | 0 | 0 | 0 | 202 | 212 | Carbamidomethylation |  | DB Search |
| total 1 peptides |  |  |  |  |  |  |  |  |  |  |  |  |  |  |  |  |  |  |  |
| --- | --- | --- | --- | --- | --- | --- | --- | --- | --- | --- | --- | --- | --- | --- | --- | --- | --- | --- | --- |

Best Unique PSM (Scan Sample\_4\_A1\_1\_3642.d:32770, m/z=674.3239, z=2, RT=13.04, ppm=-5.16):


TRINITY\_DN761\_c0\_g1\_i3.p1

back to list

  

| Protein Coverage
| Supporting Peptides
| Best Unique PSM
|

Protein Coverage:

Supporting Peptides:

| Peptide | Uniq | -10lgP | Mass | Length | ppm | m/z | z | RT | 1/k0 Start | 1/k0 End | Precursor ID | Area Sample 6 | #Feature | #Feature Sample 6 | Start | End | PTM | AScore | Found By |
| --- | --- | --- | --- | --- | --- | --- | --- | --- | --- | --- | --- | --- | --- | --- | --- | --- | --- | --- | --- |
| K.TRIEDLETVISQTQDHR.H | Y | 46.51 | 2040.0236 | 17 | 3.42 | 681.0145 | 3 | 17.49 | 0.963 | 0.9703 | 58651 | 0 | 0 | 0 | 275 | 291 |  |  | DB Search |
| total 1 peptides |  |  |  |  |  |  |  |  |  |  |  |  |  |  |  |  |  |  |  |
| --- | --- | --- | --- | --- | --- | --- | --- | --- | --- | --- | --- | --- | --- | --- | --- | --- | --- | --- | --- |

Best Unique PSM (Scan Sample\_4\_A1\_1\_3642.d:58651, m/z=681.0145, z=3, RT=17.49, ppm=-0.98):


TRINITY\_DN3034\_c0\_g1\_i1.p1

back to list

  

| Protein Coverage
| Supporting Peptides
| Best Unique PSM
|

Protein Coverage:

Supporting Peptides:

| Peptide | Uniq | -10lgP | Mass | Length | ppm | m/z | z | RT | 1/k0 Start | 1/k0 End | Precursor ID | Area Sample 6 | #Feature | #Feature Sample 6 | Start | End | PTM | AScore | Found By |
| --- | --- | --- | --- | --- | --- | --- | --- | --- | --- | --- | --- | --- | --- | --- | --- | --- | --- | --- | --- |
| R.AQEVSDKLNQK.M | Y | 41.26 | 1258.6517 | 11 | -1.32 | 630.3295 | 2 | 10.77 | 0.9372 | 0.9444 | 19179 | 0 | 0 | 0 | 1854 | 1864 |  |  | DB Search |
| total 1 peptides |  |  |  |  |  |  |  |  |  |  |  |  |  |  |  |  |  |  |  |
| --- | --- | --- | --- | --- | --- | --- | --- | --- | --- | --- | --- | --- | --- | --- | --- | --- | --- | --- | --- |

Best Unique PSM (Scan Sample\_4\_A1\_1\_3642.d:19179, m/z=630.3295, z=2, RT=10.77, ppm=-5.72):


TRINITY\_DN5500\_c0\_g1\_i1.p1

back to list

  

| Protein Coverage
| Supporting Peptides
| Best Unique PSM
|

Protein Coverage:

Supporting Peptides:

| Peptide | Uniq | -10lgP | Mass | Length | ppm | m/z | z | RT | 1/k0 Start | 1/k0 End | Precursor ID | Area Sample 6 | #Feature | #Feature Sample 6 | Start | End | PTM | AScore | Found By |
| --- | --- | --- | --- | --- | --- | --- | --- | --- | --- | --- | --- | --- | --- | --- | --- | --- | --- | --- | --- |
| K.SESESKELIDKLK.A | Y | 47.18 | 1504.7984 | 13 | -0.34 | 502.6044 | 3 | 14.64 | 0.7742 | 0.7815 | 42112 | 2.93e2 | 1 | 1 | 549 | 561 |  |  | DB Search |
| K.IYIDGVFIR.S | Y | 46.89 | 1094.6124 | 9 | 1.62 | 548.312 | 2 | 18.78 | 0.8895 | 0.8968 | 67582 | 8.72e2 | 1 | 1 | 938 | 946 |  |  | DB Search |
| total 2 peptides |  |  |  |  |  |  |  |  |  |  |  |  |  |  |  |  |  |  |  |
| --- | --- | --- | --- | --- | --- | --- | --- | --- | --- | --- | --- | --- | --- | --- | --- | --- | --- | --- | --- |

Best Unique PSM (Scan Sample\_4\_A1\_1\_3642.d:42112, m/z=502.6044, z=3, RT=14.64, ppm=-4.74):


TRINITY\_DN4781\_c0\_g1\_i2.p1

back to list

  

| Protein Coverage
| Supporting Peptides
| Best Unique PSM
|

Protein Coverage:

Supporting Peptides:

| Peptide | Uniq | -10lgP | Mass | Length | ppm | m/z | z | RT | 1/k0 Start | 1/k0 End | Precursor ID | Area Sample 6 | #Feature | #Feature Sample 6 | Start | End | PTM | AScore | Found By |
| --- | --- | --- | --- | --- | --- | --- | --- | --- | --- | --- | --- | --- | --- | --- | --- | --- | --- | --- | --- |
| R.RPAFQIVFQGVR.G | Y | 45.87 | 1416.799 | 12 | -0.91 | 473.2711 | 3 | 18.12 | 0.7477 | 0.7551 | 63094 | 0 | 0 | 0 | 1123 | 1134 |  |  | DB Search |
| total 1 peptides |  |  |  |  |  |  |  |  |  |  |  |  |  |  |  |  |  |  |  |
| --- | --- | --- | --- | --- | --- | --- | --- | --- | --- | --- | --- | --- | --- | --- | --- | --- | --- | --- | --- |

Best Unique PSM (Scan Sample\_4\_A1\_1\_3642.d:63094, m/z=473.2711, z=3, RT=18.12, ppm=-5.32):


TRINITY\_DN982\_c0\_g1\_i1.p1

back to list

  

| Protein Coverage
| Supporting Peptides
| Best Unique PSM
|

Protein Coverage:

Supporting Peptides:

| Peptide | Uniq | -10lgP | Mass | Length | ppm | m/z | z | RT | 1/k0 Start | 1/k0 End | Precursor ID | Area Sample 6 | #Feature | #Feature Sample 6 | Start | End | PTM | AScore | Found By |
| --- | --- | --- | --- | --- | --- | --- | --- | --- | --- | --- | --- | --- | --- | --- | --- | --- | --- | --- | --- |
| K.YVAIYPSDHLTVNR.D | N | 47.28 | 1646.8416 | 14 | 3.15 | 824.4271 | 2 | 15.69 | 1.1053 | 1.1126 | 48025 | 9.15e2 | 1 | 1 | 2814 | 2827 |  |  | DB Search |
| R.YTSC(+57.02)QSVTVVASDR.S | N | 47.27 | 1571.725 | 14 | 6.1 | 786.8711 | 2 | 13.47 | 1.05 | 1.0572 | 35313 | 0 | 0 | 0 | 5923 | 5936 | Carbamidomethylation |  | DB Search |
| K.INNNQFSVVLIK.N | Y | 47.19 | 1387.7823 | 12 | 4.05 | 694.8982 | 2 | 18.30 | 0.9928 | 1.0001 | 64280 | 6.59e2 | 1 | 1 | 100 | 111 |  |  | DB Search |
| R.LVQNTPHNSIVTK.I | N | 47.18 | 1449.794 | 13 | -3.54 | 725.8985 | 2 | 11.74 | 1.0568 | 1.064 | 24940 | 0 | 0 | 0 | 5129 | 5141 |  |  | DB Search |
| K.AQLTLTIFLHK.S | N | 46.99 | 1283.7601 | 11 | 3.95 | 428.9271 | 3 | 19.57 | 0.7489 | 0.7563 | 73093 | 6.45e2 | 1 | 1 | 1509 | 1519 |  |  | DB Search |
| K.LLTTEDDFILR.R | N | 46.58 | 1334.7081 | 11 | 1.62 | 668.3595 | 2 | 19.11 | 0.9735 | 0.9808 | 69947 | 3e2 | 1 | 1 | 3457 | 3467 |  |  | DB Search |
| K.VLLSQQIFYDKK.N | N | 46.51 | 1480.8289 | 12 | 0.37 | 494.6149 | 3 | 16.93 | 0.7616 | 0.7689 | 55019 | 1.05e4 | 2 | 2 | 1479 | 1490 |  |  | DB Search |
| K.VLEVNDNKPK.F | Y | 46.46 | 1154.6295 | 10 | 4.39 | 578.322 | 2 | 11.01 | 0.9263 | 0.9335 | 20603 | 6.84e2 | 1 | 1 | 459 | 468 |  |  | DB Search |
| R.DRHVLTIQSR.G | N | 46.4 | 1223.6734 | 10 | 4.59 | 408.8985 | 3 | 12.00 | 0.7559 | 0.7632 | 26560 | 0 | 0 | 0 | 1590 | 1599 |  |  | DB Search |
| R.IQVLKDPDFEFTR.S | N | 46.31 | 1606.8355 | 13 | -1.78 | 804.42 | 2 | 17.65 | 1.0801 | 1.0873 | 59732 | 1.21e3 | 1 | 1 | 2727 | 2739 |  |  | DB Search |
| K.ELEITVLESKPVNSFVIR.V | Y | 46.29 | 2072.1517 | 18 | 4.54 | 691.7246 | 3 | 19.86 | 0.961 | 0.9683 | 74982 | 5.9e2 | 1 | 1 | 368 | 385 |  |  | DB Search |
| K.AVDRGNPPQDSVNK.A | N | 45.95 | 1495.7379 | 14 | 6.38 | 499.5876 | 3 | 10.19 | 0.7868 | 0.7941 | 15626 | 0 | 0 | 0 | 1699 | 1712 |  |  | DB Search |
| R.TNNALDYEAKR.Q | N | 45.94 | 1293.6313 | 11 | 0.07 | 647.8201 | 2 | 12.09 | 0.9618 | 0.9691 | 27037 | 8.23e2 | 1 | 1 | 4285 | 4295 |  |  | DB Search |
| R.VLDVNDKQPVFVK.S | Y | 45.16 | 1499.8347 | 13 | 0.06 | 750.9214 | 2 | 15.14 | 1.0705 | 1.0777 | 45016 | 7.57e2 | 1 | 1 | 670 | 682 |  |  | DB Search |
| R.ITTSQILDHEQQK.L | N | 43.4 | 1539.7893 | 13 | 3.46 | 770.9012 | 2 | 13.97 | 1.0949 | 1.1021 | 38279 | 0 | 0 | 0 | 1994 | 2006 |  |  | DB Search |
| total 15 peptides |  |  |  |  |  |  |  |  |  |  |  |  |  |  |  |  |  |  |  |
| --- | --- | --- | --- | --- | --- | --- | --- | --- | --- | --- | --- | --- | --- | --- | --- | --- | --- | --- | --- |

Best Unique PSM (Scan Sample\_4\_A1\_1\_3642.d:64280, m/z=694.8982, z=2, RT=18.30, ppm=-0.36):


TRINITY\_DN360\_c0\_g1\_i1.p1

back to list

  

| Protein Coverage
| Supporting Peptides
| Best Unique PSM
|

Protein Coverage:

Supporting Peptides:

| Peptide | Uniq | -10lgP | Mass | Length | ppm | m/z | z | RT | 1/k0 Start | 1/k0 End | Precursor ID | Area Sample 6 | #Feature | #Feature Sample 6 | Start | End | PTM | AScore | Found By |
| --- | --- | --- | --- | --- | --- | --- | --- | --- | --- | --- | --- | --- | --- | --- | --- | --- | --- | --- | --- |
| K.AANAGGVAVSGLEM(+15.99)SQNSTR.F | Y | 44.37 | 1934.9116 | 20 | 10.47 | 968.4689 | 2 | 14.29 | 1.2778 | 1.2849 | 40083 | 0 | 0 | 0 | 398 | 417 | Oxidation (M) | M14:Oxidation (M):1000 | DB Search |
| total 1 peptides |  |  |  |  |  |  |  |  |  |  |  |  |  |  |  |  |  |  |  |
| --- | --- | --- | --- | --- | --- | --- | --- | --- | --- | --- | --- | --- | --- | --- | --- | --- | --- | --- | --- |

Best Unique PSM (Scan Sample\_4\_A1\_1\_3642.d:40083, m/z=968.4689, z=2, RT=14.29, ppm=6.07):


TRINITY\_DN1030\_c0\_g1\_i1.p1

back to list

  

| Protein Coverage
| Supporting Peptides
| Best Unique PSM
|

Protein Coverage:

Supporting Peptides:

| Peptide | Uniq | -10lgP | Mass | Length | ppm | m/z | z | RT | 1/k0 Start | 1/k0 End | Precursor ID | Area Sample 6 | #Feature | #Feature Sample 6 | Start | End | PTM | AScore | Found By |
| --- | --- | --- | --- | --- | --- | --- | --- | --- | --- | --- | --- | --- | --- | --- | --- | --- | --- | --- | --- |
| R.GLQGQQGEQGSTGVQGSVGGR.G | Y | 47.24 | 1985.9515 | 21 | 2.4 | 993.981 | 2 | 12.07 | 1.133 | 1.1402 | 26902 | 2.07e3 | 1 | 1 | 361 | 381 |  |  | DB Search |
| total 1 peptides |  |  |  |  |  |  |  |  |  |  |  |  |  |  |  |  |  |  |  |
| --- | --- | --- | --- | --- | --- | --- | --- | --- | --- | --- | --- | --- | --- | --- | --- | --- | --- | --- | --- |

Best Unique PSM (Scan Sample\_4\_A1\_1\_3642.d:26902, m/z=993.981, z=2, RT=12.07, ppm=-2):


TRINITY\_DN8008\_c0\_g1\_i3.p1

back to list

  

| Protein Coverage
| Supporting Peptides
| Best Unique PSM
|

Protein Coverage:

Supporting Peptides:

| Peptide | Uniq | -10lgP | Mass | Length | ppm | m/z | z | RT | 1/k0 Start | 1/k0 End | Precursor ID | Area Sample 6 | #Feature | #Feature Sample 6 | Start | End | PTM | AScore | Found By |
| --- | --- | --- | --- | --- | --- | --- | --- | --- | --- | --- | --- | --- | --- | --- | --- | --- | --- | --- | --- |
| R.KVDIIFAVDVSKR.F | Y | 46.75 | 1488.8664 | 13 | 6.22 | 497.297 | 3 | 16.89 | 0.8222 | 0.8295 | 54835 | 0 | 0 | 0 | 918 | 930 |  |  | DB Search |
| K.GVHIISVGLGTVDHR.G | Y | 46.51 | 1558.8579 | 15 | -9.41 | 780.4255 | 2 | 15.93 | 1.0893 | 1.0965 | 49497 | 0 | 0 | 0 | 1055 | 1069 |  |  | DB Search |
| total 2 peptides |  |  |  |  |  |  |  |  |  |  |  |  |  |  |  |  |  |  |  |
| --- | --- | --- | --- | --- | --- | --- | --- | --- | --- | --- | --- | --- | --- | --- | --- | --- | --- | --- | --- |

Best Unique PSM (Scan Sample\_4\_A1\_1\_3642.d:54835, m/z=497.297, z=3, RT=16.89, ppm=1.82):


TRINITY\_DN109148\_c0\_g1\_i1.p2

back to list

  

| Protein Coverage
| Supporting Peptides
| Best Unique PSM
|

Protein Coverage:

Supporting Peptides:

| Peptide | Uniq | -10lgP | Mass | Length | ppm | m/z | z | RT | 1/k0 Start | 1/k0 End | Precursor ID | Area Sample 6 | #Feature | #Feature Sample 6 | Start | End | PTM | AScore | Found By |
| --- | --- | --- | --- | --- | --- | --- | --- | --- | --- | --- | --- | --- | --- | --- | --- | --- | --- | --- | --- |
| R.GGGGHPER.G | Y | 46.89 | 765.3518 | 8 | -11.05 | 383.6772 | 2 | 9.02 | 0.7122 | 0.7196 | 9725 | 2.35e2 | 1 | 1 | 97 | 104 |  |  | DB Search |
| total 1 peptides |  |  |  |  |  |  |  |  |  |  |  |  |  |  |  |  |  |  |  |
| --- | --- | --- | --- | --- | --- | --- | --- | --- | --- | --- | --- | --- | --- | --- | --- | --- | --- | --- | --- |

Best Unique PSM (Scan Sample\_4\_A1\_1\_3642.d:9725, m/z=383.6772, z=2, RT=9.02, ppm=-15.45):


TRINITY\_DN4595\_c0\_g1\_i7.p1

back to list

  

| Protein Coverage
| Supporting Peptides
| Best Unique PSM
|

Protein Coverage:

Supporting Peptides:

| Peptide | Uniq | -10lgP | Mass | Length | ppm | m/z | z | RT | 1/k0 Start | 1/k0 End | Precursor ID | Area Sample 6 | #Feature | #Feature Sample 6 | Start | End | PTM | AScore | Found By |
| --- | --- | --- | --- | --- | --- | --- | --- | --- | --- | --- | --- | --- | --- | --- | --- | --- | --- | --- | --- |
| R.AFNKVETR.N | N | 44.29 | 963.5138 | 8 | -5.34 | 482.7595 | 2 | 10.58 | 0.8283 | 0.8356 | 17963 | 0 | 0 | 0 | 177 | 184 |  |  | DB Search |
| R.AFNKIETR.N | Y | 41.96 | 977.5294 | 8 | 2.83 | 489.7712 | 2 | 11.48 | 0.8343 | 0.8416 | 23389 | 1.84e3 | 1 | 1 | 72 | 79 |  |  | DB Search |
| total 2 peptides |  |  |  |  |  |  |  |  |  |  |  |  |  |  |  |  |  |  |  |
| --- | --- | --- | --- | --- | --- | --- | --- | --- | --- | --- | --- | --- | --- | --- | --- | --- | --- | --- | --- |

Best Unique PSM (Scan Sample\_4\_A1\_1\_3642.d:23389, m/z=489.7712, z=2, RT=11.48, ppm=-1.58):


TRINITY\_DN113\_c3\_g1\_i1.p1

back to list

  

| Protein Coverage
| Supporting Peptides
| Best Unique PSM
|

Protein Coverage:

Supporting Peptides:

| Peptide | Uniq | -10lgP | Mass | Length | ppm | m/z | z | RT | 1/k0 Start | 1/k0 End | Precursor ID | Area Sample 6 | #Feature | #Feature Sample 6 | Start | End | PTM | AScore | Found By |
| --- | --- | --- | --- | --- | --- | --- | --- | --- | --- | --- | --- | --- | --- | --- | --- | --- | --- | --- | --- |
| K.AHVSLSTFSEDVTSLTK.F | Y | 46.04 | 1820.9156 | 17 | 4.83 | 911.4655 | 2 | 18.94 | 1.1398 | 1.147 | 68762 | 2.56e3 | 2 | 2 | 568 | 584 |  |  | DB Search |
| R.ATHQELLNAEFIQK.V | Y | 43.35 | 1640.8522 | 14 | -1.87 | 821.4282 | 2 | 16.09 | 1.0757 | 1.0829 | 50251 | 1.12e3 | 1 | 1 | 457 | 470 |  |  | DB Search |
| total 2 peptides |  |  |  |  |  |  |  |  |  |  |  |  |  |  |  |  |  |  |  |
| --- | --- | --- | --- | --- | --- | --- | --- | --- | --- | --- | --- | --- | --- | --- | --- | --- | --- | --- | --- |

Best Unique PSM (Scan Sample\_4\_A1\_1\_3642.d:68762, m/z=909.9528, z=2, RT=18.96, ppm=0.43):


TRINITY\_DN1653\_c0\_g1\_i3.p1

back to list

  

| Protein Coverage
| Supporting Peptides
| Best Unique PSM
|

Protein Coverage:

Supporting Peptides:

| Peptide | Uniq | -10lgP | Mass | Length | ppm | m/z | z | RT | 1/k0 Start | 1/k0 End | Precursor ID | Area Sample 6 | #Feature | #Feature Sample 6 | Start | End | PTM | AScore | Found By |
| --- | --- | --- | --- | --- | --- | --- | --- | --- | --- | --- | --- | --- | --- | --- | --- | --- | --- | --- | --- |
| K.FANQKLENVSLVLR.A | Y | 47.16 | 1629.9202 | 14 | 9.03 | 544.3165 | 3 | 17.19 | 0.8234 | 0.8307 | 56634 | 1.4e2 | 1 | 1 | 70 | 83 |  |  | DB Search |
| R.LINLFEAVSKK.S | Y | 47.13 | 1260.7441 | 11 | -0.85 | 631.376 | 2 | 19.03 | 0.9832 | 0.9904 | 69384 | 1.61e3 | 2 | 2 | 49 | 59 |  |  | DB Search |
| K.IGIVNEFYVR.S | Y | 47.03 | 1208.6553 | 10 | 2.52 | 605.3338 | 2 | 18.83 | 0.9444 | 0.9517 | 67867 | 1.06e3 | 1 | 1 | 354 | 363 |  |  | DB Search |
| total 3 peptides |  |  |  |  |  |  |  |  |  |  |  |  |  |  |  |  |  |  |  |
| --- | --- | --- | --- | --- | --- | --- | --- | --- | --- | --- | --- | --- | --- | --- | --- | --- | --- | --- | --- |

Best Unique PSM (Scan Sample\_4\_A1\_1\_3642.d:56634, m/z=544.3165, z=3, RT=17.19, ppm=4.63):


TRINITY\_DN199\_c0\_g1\_i1.p1

back to list

  

| Protein Coverage
| Supporting Peptides
| Best Unique PSM
|

Protein Coverage:

Supporting Peptides:

| Peptide | Uniq | -10lgP | Mass | Length | ppm | m/z | z | RT | 1/k0 Start | 1/k0 End | Precursor ID | Area Sample 6 | #Feature | #Feature Sample 6 | Start | End | PTM | AScore | Found By |
| --- | --- | --- | --- | --- | --- | --- | --- | --- | --- | --- | --- | --- | --- | --- | --- | --- | --- | --- | --- |
| K.NYNNDDEPGAK.K | Y | 47.22 | 1235.5054 | 11 | 6.2 | 618.7611 | 2 | 10.14 | 0.8919 | 0.8992 | 15177 | 1.07e3 | 1 | 1 | 124 | 134 |  |  | DB Search |
| K.NYNNDDEPGAKK.L | Y | 47.02 | 1363.6004 | 12 | 2.39 | 455.5398 | 3 | 9.57 | 0.7485 | 0.7559 | 12066 | 1.03e3 | 1 | 1 | 124 | 135 |  |  | DB Search |
| total 2 peptides |  |  |  |  |  |  |  |  |  |  |  |  |  |  |  |  |  |  |  |
| --- | --- | --- | --- | --- | --- | --- | --- | --- | --- | --- | --- | --- | --- | --- | --- | --- | --- | --- | --- |

Best Unique PSM (Scan Sample\_4\_A1\_1\_3642.d:15177, m/z=618.7611, z=2, RT=10.14, ppm=1.8):


TRINITY\_DN413\_c0\_g1\_i2.p1

back to list

  

| Protein Coverage
| Supporting Peptides
| Best Unique PSM
|

Protein Coverage:

Supporting Peptides:

| Peptide | Uniq | -10lgP | Mass | Length | ppm | m/z | z | RT | 1/k0 Start | 1/k0 End | Precursor ID | Area Sample 6 | #Feature | #Feature Sample 6 | Start | End | PTM | AScore | Found By |
| --- | --- | --- | --- | --- | --- | --- | --- | --- | --- | --- | --- | --- | --- | --- | --- | --- | --- | --- | --- |
| R.LLLGDFNGDKR.T | Y | 47.16 | 1246.6669 | 11 | 0.01 | 624.338 | 2 | 16.23 | 0.9844 | 0.9916 | 51088 | 9.22e2 | 1 | 1 | 46 | 56 |  |  | DB Search |
| total 1 peptides |  |  |  |  |  |  |  |  |  |  |  |  |  |  |  |  |  |  |  |
| --- | --- | --- | --- | --- | --- | --- | --- | --- | --- | --- | --- | --- | --- | --- | --- | --- | --- | --- | --- |

Best Unique PSM (Scan Sample\_4\_A1\_1\_3642.d:51088, m/z=624.338, z=2, RT=16.23, ppm=-4.39):


TRINITY\_DN4494\_c1\_g1\_i8.p1

back to list

  

| Protein Coverage
| Supporting Peptides
| Best Unique PSM
|

Protein Coverage:

Supporting Peptides:

| Peptide | Uniq | -10lgP | Mass | Length | ppm | m/z | z | RT | 1/k0 Start | 1/k0 End | Precursor ID | Area Sample 6 | #Feature | #Feature Sample 6 | Start | End | PTM | AScore | Found By |
| --- | --- | --- | --- | --- | --- | --- | --- | --- | --- | --- | --- | --- | --- | --- | --- | --- | --- | --- | --- |
| K.VDVGFILDSSGSLR.N | Y | 45.76 | 1463.762 | 14 | -1.21 | 732.8842 | 2 | 19.93 | 1.0102 | 1.0174 | 75559 | 4.99e2 | 1 | 1 | 41 | 54 |  |  | DB Search |
| total 1 peptides |  |  |  |  |  |  |  |  |  |  |  |  |  |  |  |  |  |  |  |
| --- | --- | --- | --- | --- | --- | --- | --- | --- | --- | --- | --- | --- | --- | --- | --- | --- | --- | --- | --- |

Best Unique PSM (Scan Sample\_4\_A1\_1\_3642.d:75559, m/z=732.8842, z=2, RT=19.93, ppm=-5.61):


TRINITY\_DN649\_c0\_g1\_i1.p1

back to list

  

| Protein Coverage
| Supporting Peptides
| Best Unique PSM
|

Protein Coverage:

Supporting Peptides:

| Peptide | Uniq | -10lgP | Mass | Length | ppm | m/z | z | RT | 1/k0 Start | 1/k0 End | Precursor ID | Area Sample 6 | #Feature | #Feature Sample 6 | Start | End | PTM | AScore | Found By |
| --- | --- | --- | --- | --- | --- | --- | --- | --- | --- | --- | --- | --- | --- | --- | --- | --- | --- | --- | --- |
| K.FETQIATK.N | Y | 44.83 | 936.4916 | 8 | 7 | 937.5013 | 1 | 13.97 | 1.1802 | 1.1873 | 38247 | 0 | 0 | 0 | 102 | 109 |  |  | DB Search |
| total 1 peptides |  |  |  |  |  |  |  |  |  |  |  |  |  |  |  |  |  |  |  |
| --- | --- | --- | --- | --- | --- | --- | --- | --- | --- | --- | --- | --- | --- | --- | --- | --- | --- | --- | --- |

Best Unique PSM (Scan Sample\_4\_A1\_1\_3642.d:38247, m/z=937.5013, z=1, RT=13.97, ppm=2.6):


TRINITY\_DN1058\_c0\_g1\_i2.p2

back to list

  

| Protein Coverage
| Supporting Peptides
| Best Unique PSM
|

Protein Coverage:

Supporting Peptides:

| Peptide | Uniq | -10lgP | Mass | Length | ppm | m/z | z | RT | 1/k0 Start | 1/k0 End | Precursor ID | Area Sample 6 | #Feature | #Feature Sample 6 | Start | End | PTM | AScore | Found By |
| --- | --- | --- | --- | --- | --- | --- | --- | --- | --- | --- | --- | --- | --- | --- | --- | --- | --- | --- | --- |
| R.APTHILNPANKR.S | Y | 46.93 | 1330.7469 | 12 | 2.71 | 444.5888 | 3 | 11.17 | 0.7559 | 0.7632 | 21058 | 5.5e3 | 2 | 2 | 975 | 986 |  |  | DB Search |
| total 1 peptides |  |  |  |  |  |  |  |  |  |  |  |  |  |  |  |  |  |  |  |
| --- | --- | --- | --- | --- | --- | --- | --- | --- | --- | --- | --- | --- | --- | --- | --- | --- | --- | --- | --- |

Best Unique PSM (Scan Sample\_4\_A1\_1\_3642.d:21058, m/z=444.5888, z=3, RT=11.17, ppm=-1.7):


TRINITY\_DN14015\_c0\_g1\_i3.p1

back to list

  

| Protein Coverage
| Supporting Peptides
| Best Unique PSM
|

Protein Coverage:

Supporting Peptides:

| Peptide | Uniq | -10lgP | Mass | Length | ppm | m/z | z | RT | 1/k0 Start | 1/k0 End | Precursor ID | Area Sample 6 | #Feature | #Feature Sample 6 | Start | End | PTM | AScore | Found By |
| --- | --- | --- | --- | --- | --- | --- | --- | --- | --- | --- | --- | --- | --- | --- | --- | --- | --- | --- | --- |
| R.ANYVLTIEASDNFETPKK.S | Y | 44.76 | 2039.0211 | 18 | -2.13 | 1020.5112 | 2 | 17.53 | 1.271 | 1.2782 | 58757 | 1.41e2 | 1 | 1 | 1766 | 1783 |  |  | DB Search |
| total 1 peptides |  |  |  |  |  |  |  |  |  |  |  |  |  |  |  |  |  |  |  |
| --- | --- | --- | --- | --- | --- | --- | --- | --- | --- | --- | --- | --- | --- | --- | --- | --- | --- | --- | --- |

Best Unique PSM (Scan Sample\_4\_A1\_1\_3642.d:58757, m/z=1020.5112, z=2, RT=17.53, ppm=-6.53):


TRINITY\_DN941\_c0\_g1\_i15.p1

back to list

  

| Protein Coverage
| Supporting Peptides
| Best Unique PSM
|

Protein Coverage:

Supporting Peptides:

| Peptide | Uniq | -10lgP | Mass | Length | ppm | m/z | z | RT | 1/k0 Start | 1/k0 End | Precursor ID | Area Sample 6 | #Feature | #Feature Sample 6 | Start | End | PTM | AScore | Found By |
| --- | --- | --- | --- | --- | --- | --- | --- | --- | --- | --- | --- | --- | --- | --- | --- | --- | --- | --- | --- |
| K.ADLTRPSGK.K | Y | 38.69 | 943.5087 | 9 | -2.87 | 472.7582 | 2 | 10.10 | 0.8189 | 0.8262 | 15057 | 1.13e3 | 1 | 1 | 114 | 122 |  |  | DB Search |
| total 1 peptides |  |  |  |  |  |  |  |  |  |  |  |  |  |  |  |  |  |  |  |
| --- | --- | --- | --- | --- | --- | --- | --- | --- | --- | --- | --- | --- | --- | --- | --- | --- | --- | --- | --- |

Best Unique PSM (Scan Sample\_4\_A1\_1\_3642.d:15057, m/z=472.7582, z=2, RT=10.10, ppm=-7.27):


TRINITY\_DN34481\_c0\_g1\_i1.p1

back to list

  

| Protein Coverage
| Supporting Peptides
| Best Unique PSM
|

Protein Coverage:

Supporting Peptides:

| Peptide | Uniq | -10lgP | Mass | Length | ppm | m/z | z | RT | 1/k0 Start | 1/k0 End | Precursor ID | Area Sample 6 | #Feature | #Feature Sample 6 | Start | End | PTM | AScore | Found By |
| --- | --- | --- | --- | --- | --- | --- | --- | --- | --- | --- | --- | --- | --- | --- | --- | --- | --- | --- | --- |
| K.AC(+57.02)AELHQDVNVK.N | Y | 46.01 | 1382.6612 | 12 | 2.82 | 692.3368 | 2 | 11.95 | 1.0065 | 1.0138 | 26229 | 0 | 0 | 0 | 282 | 293 | Carbamidomethylation |  | DB Search |
| total 1 peptides |  |  |  |  |  |  |  |  |  |  |  |  |  |  |  |  |  |  |  |
| --- | --- | --- | --- | --- | --- | --- | --- | --- | --- | --- | --- | --- | --- | --- | --- | --- | --- | --- | --- |

Best Unique PSM (Scan Sample\_4\_A1\_1\_3642.d:26229, m/z=692.3368, z=2, RT=11.95, ppm=-1.59):


TRINITY\_DN113\_c2\_g1\_i1.p1

back to list

  

| Protein Coverage
| Supporting Peptides
| Best Unique PSM
|

Protein Coverage:

Supporting Peptides:

| Peptide | Uniq | -10lgP | Mass | Length | ppm | m/z | z | RT | 1/k0 Start | 1/k0 End | Precursor ID | Area Sample 6 | #Feature | #Feature Sample 6 | Start | End | PTM | AScore | Found By |
| --- | --- | --- | --- | --- | --- | --- | --- | --- | --- | --- | --- | --- | --- | --- | --- | --- | --- | --- | --- |
| R.GGAGDM(+15.99)GAEGPR.G | Y | 47.26 | 1089.4509 | 12 | 1.17 | 545.731 | 2 | 9.59 | 0.8323 | 0.8396 | 12176 | 6.75e2 | 1 | 1 | 823 | 834 | Oxidation (M) | M6:Oxidation (M):1000 | DB Search |
| R.RGPNGAIGER.G | Y | 44.52 | 1025.5366 | 10 | 3.56 | 513.7751 | 2 | 10.07 | 0.8465 | 0.8538 | 14705 | 1.66e3 | 1 | 1 | 354 | 363 |  |  | DB Search |
| total 2 peptides |  |  |  |  |  |  |  |  |  |  |  |  |  |  |  |  |  |  |  |
| --- | --- | --- | --- | --- | --- | --- | --- | --- | --- | --- | --- | --- | --- | --- | --- | --- | --- | --- | --- |

Best Unique PSM (Scan Sample\_4\_A1\_1\_3642.d:12176, m/z=545.731, z=2, RT=9.59, ppm=-3.23):


TRINITY\_DN4494\_c2\_g1\_i1.p1

back to list

  

| Protein Coverage
| Supporting Peptides
| Best Unique PSM
|

Protein Coverage:

Supporting Peptides:

| Peptide | Uniq | -10lgP | Mass | Length | ppm | m/z | z | RT | 1/k0 Start | 1/k0 End | Precursor ID | Area Sample 6 | #Feature | #Feature Sample 6 | Start | End | PTM | AScore | Found By |
| --- | --- | --- | --- | --- | --- | --- | --- | --- | --- | --- | --- | --- | --- | --- | --- | --- | --- | --- | --- |
| K.VDVGFILDSSGSLR.N | Y | 45.76 | 1463.762 | 14 | -1.21 | 732.8842 | 2 | 19.93 | 1.0102 | 1.0174 | 75559 | 4.99e2 | 1 | 1 | 57 | 70 |  |  | DB Search |
| total 1 peptides |  |  |  |  |  |  |  |  |  |  |  |  |  |  |  |  |  |  |  |
| --- | --- | --- | --- | --- | --- | --- | --- | --- | --- | --- | --- | --- | --- | --- | --- | --- | --- | --- | --- |

Best Unique PSM (Scan Sample\_4\_A1\_1\_3642.d:75559, m/z=732.8842, z=2, RT=19.93, ppm=-5.61):


TRINITY\_DN1505\_c0\_g1\_i1.p1

back to list

  

| Protein Coverage
| Supporting Peptides
| Best Unique PSM
|

Protein Coverage:

Supporting Peptides:

| Peptide | Uniq | -10lgP | Mass | Length | ppm | m/z | z | RT | 1/k0 Start | 1/k0 End | Precursor ID | Area Sample 6 | #Feature | #Feature Sample 6 | Start | End | PTM | AScore | Found By |
| --- | --- | --- | --- | --- | --- | --- | --- | --- | --- | --- | --- | --- | --- | --- | --- | --- | --- | --- | --- |
| M.S(+42.01)LVIPEKFQHILR.V | Y | 46.28 | 1620.9351 | 13 | 4.83 | 541.3192 | 3 | 19.72 | 0.8421 | 0.8494 | 73912 | 4.82e2 | 1 | 1 | 2 | 14 | Acetylation (Protein N-term) | S1:Acetylation (Protein N-term):1000 | DB Search |
| total 1 peptides |  |  |  |  |  |  |  |  |  |  |  |  |  |  |  |  |  |  |  |
| --- | --- | --- | --- | --- | --- | --- | --- | --- | --- | --- | --- | --- | --- | --- | --- | --- | --- | --- | --- |

Best Unique PSM (Scan Sample\_4\_A1\_1\_3642.d:73912, m/z=541.3192, z=3, RT=19.72, ppm=0.42):


TRINITY\_DN77\_c1\_g1\_i1.p1

back to list

  

| Protein Coverage
| Supporting Peptides
| Best Unique PSM
|

Protein Coverage:

Supporting Peptides:

| Peptide | Uniq | -10lgP | Mass | Length | ppm | m/z | z | RT | 1/k0 Start | 1/k0 End | Precursor ID | Area Sample 6 | #Feature | #Feature Sample 6 | Start | End | PTM | AScore | Found By |
| --- | --- | --- | --- | --- | --- | --- | --- | --- | --- | --- | --- | --- | --- | --- | --- | --- | --- | --- | --- |
| R.LLAVTVGNNYNLDTLEK.V | Y | 47.51 | 1875.9942 | 17 | -4.15 | 938.9963 | 2 | 18.69 | 1.147 | 1.1542 | 67001 | 0 | 0 | 0 | 693 | 709 |  |  | DB Search |
| K.SFASSLLDHFRVEPAK.T | Y | 47.34 | 1802.9315 | 16 | 2.06 | 601.983 | 3 | 19.97 | 0.9057 | 0.9129 | 75829 | 0 | 0 | 0 | 318 | 333 |  |  | DB Search |
| R.AVDIHFALDASQNVGR.E | Y | 46.51 | 1711.8641 | 16 | 2.6 | 571.6276 | 3 | 17.54 | 0.8587 | 0.866 | 58896 | 1.61e3 | 2 | 2 | 56 | 71 |  |  | DB Search |
| total 3 peptides |  |  |  |  |  |  |  |  |  |  |  |  |  |  |  |  |  |  |  |
| --- | --- | --- | --- | --- | --- | --- | --- | --- | --- | --- | --- | --- | --- | --- | --- | --- | --- | --- | --- |

Best Unique PSM (Scan Sample\_4\_A1\_1\_3642.d:67001, m/z=938.9963, z=2, RT=18.69, ppm=-8.55):


TRINITY\_DN187147\_c0\_g1\_i1.p1

back to list

  

| Protein Coverage
| Supporting Peptides
| Best Unique PSM
|

Protein Coverage:

Supporting Peptides:

| Peptide | Uniq | -10lgP | Mass | Length | ppm | m/z | z | RT | 1/k0 Start | 1/k0 End | Precursor ID | Area Sample 6 | #Feature | #Feature Sample 6 | Start | End | PTM | AScore | Found By |
| --- | --- | --- | --- | --- | --- | --- | --- | --- | --- | --- | --- | --- | --- | --- | --- | --- | --- | --- | --- |
| K.LVGQSIISYLQK.K | Y | 47.26 | 1347.7762 | 12 | 10.68 | 674.8996 | 2 | 19.69 | 0.9783 | 0.9856 | 73873 | 2.42e2 | 1 | 1 | 627 | 638 |  |  | DB Search |
| total 1 peptides |  |  |  |  |  |  |  |  |  |  |  |  |  |  |  |  |  |  |  |
| --- | --- | --- | --- | --- | --- | --- | --- | --- | --- | --- | --- | --- | --- | --- | --- | --- | --- | --- | --- |

Best Unique PSM (Scan Sample\_4\_A1\_1\_3642.d:73873, m/z=674.8996, z=2, RT=19.69, ppm=6.28):


TRINITY\_DN12542\_c0\_g1\_i1.p1

back to list

  

| Protein Coverage
| Supporting Peptides
| Best Unique PSM
|

Protein Coverage:

Supporting Peptides:

| Peptide | Uniq | -10lgP | Mass | Length | ppm | m/z | z | RT | 1/k0 Start | 1/k0 End | Precursor ID | Area Sample 6 | #Feature | #Feature Sample 6 | Start | End | PTM | AScore | Found By |
| --- | --- | --- | --- | --- | --- | --- | --- | --- | --- | --- | --- | --- | --- | --- | --- | --- | --- | --- | --- |
| K.VDVGFILDSSGSLR.N | Y | 45.76 | 1463.762 | 14 | -1.21 | 732.8842 | 2 | 19.93 | 1.0102 | 1.0174 | 75559 | 4.99e2 | 1 | 1 | 195 | 208 |  |  | DB Search |
| total 1 peptides |  |  |  |  |  |  |  |  |  |  |  |  |  |  |  |  |  |  |  |
| --- | --- | --- | --- | --- | --- | --- | --- | --- | --- | --- | --- | --- | --- | --- | --- | --- | --- | --- | --- |

Best Unique PSM (Scan Sample\_4\_A1\_1\_3642.d:75559, m/z=732.8842, z=2, RT=19.93, ppm=-5.61):


TRINITY\_DN4726\_c0\_g1\_i3.p1

back to list

  

| Protein Coverage
| Supporting Peptides
| Best Unique PSM
|

Protein Coverage:

Supporting Peptides:

| Peptide | Uniq | -10lgP | Mass | Length | ppm | m/z | z | RT | 1/k0 Start | 1/k0 End | Precursor ID | Area Sample 6 | #Feature | #Feature Sample 6 | Start | End | PTM | AScore | Found By |
| --- | --- | --- | --- | --- | --- | --- | --- | --- | --- | --- | --- | --- | --- | --- | --- | --- | --- | --- | --- |
| R.ITNLYC(+57.02)DLSIYR.Q | Y | 46.72 | 1529.7548 | 12 | 3.24 | 765.8838 | 2 | 19.27 | 1.0371 | 1.0443 | 70930 | 3.14e2 | 1 | 1 | 352 | 363 | Carbamidomethylation |  | DB Search |
| total 1 peptides |  |  |  |  |  |  |  |  |  |  |  |  |  |  |  |  |  |  |  |
| --- | --- | --- | --- | --- | --- | --- | --- | --- | --- | --- | --- | --- | --- | --- | --- | --- | --- | --- | --- |

Best Unique PSM (Scan Sample\_4\_A1\_1\_3642.d:70930, m/z=765.8838, z=2, RT=19.27, ppm=-1.16):


TRINITY\_DN15151\_c0\_g1\_i3.p1

back to list

  

| Protein Coverage
| Supporting Peptides
| Best Unique PSM
|

Protein Coverage:

Supporting Peptides:

| Peptide | Uniq | -10lgP | Mass | Length | ppm | m/z | z | RT | 1/k0 Start | 1/k0 End | Precursor ID | Area Sample 6 | #Feature | #Feature Sample 6 | Start | End | PTM | AScore | Found By |
| --- | --- | --- | --- | --- | --- | --- | --- | --- | --- | --- | --- | --- | --- | --- | --- | --- | --- | --- | --- |
| K.AVAAGMNPMDIKR.G | Y | 40.45 | 1372.6955 | 13 | -2.78 | 687.3501 | 2 | 14.90 | 1.0102 | 1.0174 | 43658 | 5.01e2 | 1 | 1 | 145 | 157 |  |  | DB Search |
| total 1 peptides |  |  |  |  |  |  |  |  |  |  |  |  |  |  |  |  |  |  |  |
| --- | --- | --- | --- | --- | --- | --- | --- | --- | --- | --- | --- | --- | --- | --- | --- | --- | --- | --- | --- |

Best Unique PSM (Scan Sample\_4\_A1\_1\_3642.d:43658, m/z=687.3501, z=2, RT=14.90, ppm=-7.18):


TRINITY\_DN67\_c0\_g1\_i2.p1

back to list

  

| Protein Coverage
| Supporting Peptides
| Best Unique PSM
|

Protein Coverage:

Supporting Peptides:

| Peptide | Uniq | -10lgP | Mass | Length | ppm | m/z | z | RT | 1/k0 Start | 1/k0 End | Precursor ID | Area Sample 6 | #Feature | #Feature Sample 6 | Start | End | PTM | AScore | Found By |
| --- | --- | --- | --- | --- | --- | --- | --- | --- | --- | --- | --- | --- | --- | --- | --- | --- | --- | --- | --- |
| K.VLLAVGGWNHENGNGQGK.F | Y | 44.58 | 1848.923 | 18 | 4.74 | 925.4691 | 2 | 16.16 | 1.171 | 1.1782 | 50811 | 0 | 0 | 0 | 98 | 115 |  |  | DB Search |
| total 1 peptides |  |  |  |  |  |  |  |  |  |  |  |  |  |  |  |  |  |  |  |
| --- | --- | --- | --- | --- | --- | --- | --- | --- | --- | --- | --- | --- | --- | --- | --- | --- | --- | --- | --- |

Best Unique PSM (Scan Sample\_4\_A1\_1\_3642.d:50811, m/z=925.4691, z=2, RT=16.16, ppm=0.34):


TRINITY\_DN31399\_c0\_g1\_i2.p1

back to list

  

| Protein Coverage
| Supporting Peptides
| Best Unique PSM
|

Protein Coverage:

Supporting Peptides:

| Peptide | Uniq | -10lgP | Mass | Length | ppm | m/z | z | RT | 1/k0 Start | 1/k0 End | Precursor ID | Area Sample 6 | #Feature | #Feature Sample 6 | Start | End | PTM | AScore | Found By |
| --- | --- | --- | --- | --- | --- | --- | --- | --- | --- | --- | --- | --- | --- | --- | --- | --- | --- | --- | --- |
| K.DTLVFEIDEIRK.S | Y | 45.93 | 1476.7824 | 12 | -0.51 | 739.3948 | 2 | 18.60 | 1.0343 | 1.0415 | 66411 | 0 | 0 | 0 | 396 | 407 |  |  | DB Search |
| total 1 peptides |  |  |  |  |  |  |  |  |  |  |  |  |  |  |  |  |  |  |  |
| --- | --- | --- | --- | --- | --- | --- | --- | --- | --- | --- | --- | --- | --- | --- | --- | --- | --- | --- | --- |

Best Unique PSM (Scan Sample\_4\_A1\_1\_3642.d:66411, m/z=739.3948, z=2, RT=18.60, ppm=-4.91):


TRINITY\_DN2885\_c0\_g1\_i5.p1

back to list

  

| Protein Coverage
| Supporting Peptides
| Best Unique PSM
|

Protein Coverage:

Supporting Peptides:

| Peptide | Uniq | -10lgP | Mass | Length | ppm | m/z | z | RT | 1/k0 Start | 1/k0 End | Precursor ID | Area Sample 6 | #Feature | #Feature Sample 6 | Start | End | PTM | AScore | Found By |
| --- | --- | --- | --- | --- | --- | --- | --- | --- | --- | --- | --- | --- | --- | --- | --- | --- | --- | --- | --- |
| K.VTRPHGASGVVR.A | Y | 46.49 | 1234.6894 | 12 | 7.12 | 412.5715 | 3 | 10.06 | 0.8169 | 0.8242 | 14707 | 3.57e2 | 1 | 1 | 77 | 88 |  |  | DB Search |
| total 1 peptides |  |  |  |  |  |  |  |  |  |  |  |  |  |  |  |  |  |  |  |
| --- | --- | --- | --- | --- | --- | --- | --- | --- | --- | --- | --- | --- | --- | --- | --- | --- | --- | --- | --- |

Best Unique PSM (Scan Sample\_4\_A1\_1\_3642.d:14707, m/z=412.5715, z=3, RT=10.06, ppm=2.72):


TRINITY\_DN36\_c0\_g1\_i19.p1

back to list

  

| Protein Coverage
| Supporting Peptides
| Best Unique PSM
|

Protein Coverage:

Supporting Peptides:

| Peptide | Uniq | -10lgP | Mass | Length | ppm | m/z | z | RT | 1/k0 Start | 1/k0 End | Precursor ID | Area Sample 6 | #Feature | #Feature Sample 6 | Start | End | PTM | AScore | Found By |
| --- | --- | --- | --- | --- | --- | --- | --- | --- | --- | --- | --- | --- | --- | --- | --- | --- | --- | --- | --- |
| R.LGGIEVTQGNC(+57.02)AR.K | Y | 45.45 | 1373.6721 | 13 | 2.96 | 687.8423 | 2 | 13.87 | 0.9687 | 0.9759 | 37701 | 0 | 0 | 0 | 57 | 69 | Carbamidomethylation |  | DB Search |
| K.FETQIATK.D | Y | 44.83 | 936.4916 | 8 | 7 | 937.5013 | 1 | 13.97 | 1.1802 | 1.1873 | 38247 | 0 | 0 | 0 | 166 | 173 |  |  | DB Search |
| total 2 peptides |  |  |  |  |  |  |  |  |  |  |  |  |  |  |  |  |  |  |  |
| --- | --- | --- | --- | --- | --- | --- | --- | --- | --- | --- | --- | --- | --- | --- | --- | --- | --- | --- | --- |

Best Unique PSM (Scan Sample\_4\_A1\_1\_3642.d:37701, m/z=687.8423, z=2, RT=13.87, ppm=-1.44):


TRINITY\_DN106\_c0\_g1\_i11.p1

back to list

  

| Protein Coverage
| Supporting Peptides
| Best Unique PSM
|

Protein Coverage:

Supporting Peptides:

| Peptide | Uniq | -10lgP | Mass | Length | ppm | m/z | z | RT | 1/k0 Start | 1/k0 End | Precursor ID | Area Sample 6 | #Feature | #Feature Sample 6 | Start | End | PTM | AScore | Found By |
| --- | --- | --- | --- | --- | --- | --- | --- | --- | --- | --- | --- | --- | --- | --- | --- | --- | --- | --- | --- |
| R.DGPAGPAGR.D | Y | 46.52 | 796.3827 | 9 | 7.09 | 399.1997 | 2 | 9.76 | 0.7233 | 0.7306 | 13098 | 1.65e3 | 1 | 1 | 283 | 291 |  |  | DB Search |
| total 1 peptides |  |  |  |  |  |  |  |  |  |  |  |  |  |  |  |  |  |  |  |
| --- | --- | --- | --- | --- | --- | --- | --- | --- | --- | --- | --- | --- | --- | --- | --- | --- | --- | --- | --- |

Best Unique PSM (Scan Sample\_4\_A1\_1\_3642.d:13098, m/z=399.1997, z=2, RT=9.76, ppm=2.68):


TRINITY\_DN3545\_c0\_g1\_i2.p1

back to list

  

| Protein Coverage
| Supporting Peptides
| Best Unique PSM
|

Protein Coverage:

Supporting Peptides:

| Peptide | Uniq | -10lgP | Mass | Length | ppm | m/z | z | RT | 1/k0 Start | 1/k0 End | Precursor ID | Area Sample 6 | #Feature | #Feature Sample 6 | Start | End | PTM | AScore | Found By |
| --- | --- | --- | --- | --- | --- | --- | --- | --- | --- | --- | --- | --- | --- | --- | --- | --- | --- | --- | --- |
| R.SC(+57.02)TNPAPANGGK.H | Y | 47.22 | 1172.5244 | 12 | 1.68 | 587.2679 | 2 | 9.40 | 0.8429 | 0.8502 | 11161 | 2.16e2 | 1 | 1 | 55 | 66 | Carbamidomethylation |  | DB Search |
| total 1 peptides |  |  |  |  |  |  |  |  |  |  |  |  |  |  |  |  |  |  |  |
| --- | --- | --- | --- | --- | --- | --- | --- | --- | --- | --- | --- | --- | --- | --- | --- | --- | --- | --- | --- |

Best Unique PSM (Scan Sample\_4\_A1\_1\_3642.d:11161, m/z=587.2679, z=2, RT=9.40, ppm=-2.72):


TRINITY\_DN1043\_c0\_g1\_i2.p1

back to list

  

| Protein Coverage
| Supporting Peptides
| Best Unique PSM
|

Protein Coverage:

Supporting Peptides:

| Peptide | Uniq | -10lgP | Mass | Length | ppm | m/z | z | RT | 1/k0 Start | 1/k0 End | Precursor ID | Area Sample 6 | #Feature | #Feature Sample 6 | Start | End | PTM | AScore | Found By |
| --- | --- | --- | --- | --- | --- | --- | --- | --- | --- | --- | --- | --- | --- | --- | --- | --- | --- | --- | --- |
| K.GLMVAAMDLDKK.Q | Y | 46.19 | 1290.6675 | 12 | 2.34 | 646.3397 | 2 | 16.73 | 0.9997 | 1.0069 | 53872 | 9.71e2 | 1 | 1 | 504 | 515 |  |  | DB Search |
| total 1 peptides |  |  |  |  |  |  |  |  |  |  |  |  |  |  |  |  |  |  |  |
| --- | --- | --- | --- | --- | --- | --- | --- | --- | --- | --- | --- | --- | --- | --- | --- | --- | --- | --- | --- |

Best Unique PSM (Scan Sample\_4\_A1\_1\_3642.d:53872, m/z=646.3397, z=2, RT=16.73, ppm=-2.06):


TRINITY\_DN1616\_c0\_g1\_i7.p1

back to list

  

| Protein Coverage
| Supporting Peptides
| Best Unique PSM
|

Protein Coverage:

Supporting Peptides:

| Peptide | Uniq | -10lgP | Mass | Length | ppm | m/z | z | RT | 1/k0 Start | 1/k0 End | Precursor ID | Area Sample 6 | #Feature | #Feature Sample 6 | Start | End | PTM | AScore | Found By |
| --- | --- | --- | --- | --- | --- | --- | --- | --- | --- | --- | --- | --- | --- | --- | --- | --- | --- | --- | --- |
| K.GC(+57.02)STC(+57.02)GC(+57.02)NGAGTNNGVK.S | Y | 47.18 | 1712.6665 | 17 | 14.87 | 857.3495 | 2 | 9.81 | 1.0411 | 1.0484 | 13314 | 0 | 0 | 0 | 732 | 748 | Carbamidomethylation, Carbamidomethylation, Carbamidomethylation |  | DB Search |
| R.YVKPGVNIINIR.D | Y | 46.71 | 1384.819 | 12 | 3.8 | 693.4164 | 2 | 16.18 | 1.0009 | 1.0081 | 50795 | 7.11e2 | 1 | 1 | 1217 | 1228 |  |  | DB Search |
| K.VVDTEANDLTEEVKK.T | Y | 46.47 | 1688.8468 | 15 | 4.23 | 845.4305 | 2 | 14.16 | 1.1298 | 1.137 | 39294 | 3.14e2 | 1 | 1 | 2542 | 2556 |  |  | DB Search |
| K.YVVMLQYYHDSDTAK.Y | Y | 46.08 | 1831.8451 | 15 | 8.86 | 916.9339 | 2 | 16.78 | 1.1825 | 1.1897 | 54165 | 6.38e1 | 1 | 1 | 1071 | 1085 |  |  | DB Search |
| K.GFVAVNDQQPSVVVSINVPK.T | Y | 43.72 | 2096.1266 | 20 | 10.57 | 1049.077 | 2 | 18.41 | 1.2185 | 1.2256 | 65167 | 0 | 0 | 0 | 865 | 884 |  |  | DB Search |
| K.VVAEC(+57.02)NNGGGR.F | Y | 40.05 | 1131.5091 | 11 | 3.26 | 566.7612 | 2 | 9.40 | 0.825 | 0.8323 | 11191 | 0 | 0 | 0 | 3736 | 3746 | Carbamidomethylation |  | DB Search |
| K.YEC(+57.02)NC(+57.02)QYNTEGPR.C | Y | 39.28 | 1689.6512 | 13 | 9.13 | 845.8369 | 2 | 12.04 | 1.0323 | 1.0395 | 26880 | 1.68e2 | 1 | 1 | 291 | 303 | Carbamidomethylation, Carbamidomethylation |  | DB Search |
| total 7 peptides |  |  |  |  |  |  |  |  |  |  |  |  |  |  |  |  |  |  |  |
| --- | --- | --- | --- | --- | --- | --- | --- | --- | --- | --- | --- | --- | --- | --- | --- | --- | --- | --- | --- |

Best Unique PSM (Scan Sample\_4\_A1\_1\_3642.d:13314, m/z=857.3495, z=2, RT=9.81, ppm=10.47):


TRINITY\_DN4494\_c1\_g1\_i12.p1

back to list

  

| Protein Coverage
| Supporting Peptides
| Best Unique PSM
|

Protein Coverage:

Supporting Peptides:

| Peptide | Uniq | -10lgP | Mass | Length | ppm | m/z | z | RT | 1/k0 Start | 1/k0 End | Precursor ID | Area Sample 6 | #Feature | #Feature Sample 6 | Start | End | PTM | AScore | Found By |
| --- | --- | --- | --- | --- | --- | --- | --- | --- | --- | --- | --- | --- | --- | --- | --- | --- | --- | --- | --- |
| K.VDVGFILDSSGSLR.N | Y | 45.76 | 1463.762 | 14 | -1.21 | 732.8842 | 2 | 19.93 | 1.0102 | 1.0174 | 75559 | 4.99e2 | 1 | 1 | 41 | 54 |  |  | DB Search |
| total 1 peptides |  |  |  |  |  |  |  |  |  |  |  |  |  |  |  |  |  |  |  |
| --- | --- | --- | --- | --- | --- | --- | --- | --- | --- | --- | --- | --- | --- | --- | --- | --- | --- | --- | --- |

Best Unique PSM (Scan Sample\_4\_A1\_1\_3642.d:75559, m/z=732.8842, z=2, RT=19.93, ppm=-5.61):


TRINITY\_DN540\_c0\_g1\_i1.p1

back to list

  

| Protein Coverage
| Supporting Peptides
| Best Unique PSM
|

Protein Coverage:

Supporting Peptides:

| Peptide | Uniq | -10lgP | Mass | Length | ppm | m/z | z | RT | 1/k0 Start | 1/k0 End | Precursor ID | Area Sample 6 | #Feature | #Feature Sample 6 | Start | End | PTM | AScore | Found By |
| --- | --- | --- | --- | --- | --- | --- | --- | --- | --- | --- | --- | --- | --- | --- | --- | --- | --- | --- | --- |
| M.A(+42.01)TDKLEGLIAR.L | Y | 46.72 | 1227.6823 | 11 | 5.76 | 614.8492 | 2 | 17.96 | 0.9505 | 0.9578 | 61874 | 1.15e3 | 1 | 1 | 2 | 12 | Acetylation (Protein N-term) | A1:Acetylation (Protein N-term):1000 | DB Search |
| total 1 peptides |  |  |  |  |  |  |  |  |  |  |  |  |  |  |  |  |  |  |  |
| --- | --- | --- | --- | --- | --- | --- | --- | --- | --- | --- | --- | --- | --- | --- | --- | --- | --- | --- | --- |

Best Unique PSM (Scan Sample\_4\_A1\_1\_3642.d:61874, m/z=614.8492, z=2, RT=17.96, ppm=1.36):


TRINITY\_DN1369\_c0\_g1\_i1.p1

back to list

  

| Protein Coverage
| Supporting Peptides
| Best Unique PSM
|

Protein Coverage:

Supporting Peptides:

| Peptide | Uniq | -10lgP | Mass | Length | ppm | m/z | z | RT | 1/k0 Start | 1/k0 End | Precursor ID | Area Sample 6 | #Feature | #Feature Sample 6 | Start | End | PTM | AScore | Found By |
| --- | --- | --- | --- | --- | --- | --- | --- | --- | --- | --- | --- | --- | --- | --- | --- | --- | --- | --- | --- |
| K.TPGEC(+57.02)C(+57.02)PQC(+57.02)K.K | Y | 47.23 | 1235.4733 | 10 | 1.97 | 618.7424 | 2 | 9.99 | 0.8939 | 0.9012 | 14287 | 6.05e2 | 1 | 1 | 1387 | 1396 | Carbamidomethylation, Carbamidomethylation, Carbamidomethylation |  | DB Search |
| R.EITLNYHDTVVHMK.R | Y | 46.86 | 1698.8399 | 14 | -1.84 | 850.4219 | 2 | 15.44 | 1.111 | 1.1182 | 46709 | 5.77e2 | 1 | 1 | 911 | 924 |  |  | DB Search |
| R.KYTPC(+57.02)EEK.C | Y | 46.01 | 1053.4801 | 8 | 0.34 | 527.7452 | 2 | 9.67 | 0.8619 | 0.8692 | 12492 | 6.29e2 | 1 | 1 | 649 | 656 | Carbamidomethylation |  | DB Search |
| K.MLVYYAEKEC(+57.02)EILR.T | Y | 45.96 | 1815.8899 | 14 | 0.28 | 908.9485 | 2 | 17.53 | 1.1654 | 1.1726 | 58763 | 8.89e2 | 1 | 1 | 219 | 232 | Carbamidomethylation |  | DB Search |
| K.VFSEC(+57.02)GPAC(+57.02)SSVC(+57.02)SAIGSK.S | Y | 45.7 | 2001.8594 | 19 | 9.61 | 1001.9422 | 2 | 15.38 | 1.1518 | 1.159 | 46383 | 0 | 0 | 0 | 303 | 321 | Carbamidomethylation, Carbamidomethylation, Carbamidomethylation |  | DB Search |
| R.MTSC(+57.02)EANHQVAK.Y | Y | 39.54 | 1374.602 | 12 | -0.33 | 459.2058 | 3 | 9.67 | 0.7726 | 0.7799 | 12570 | 0 | 0 | 0 | 571 | 582 | Carbamidomethylation |  | DB Search |
| total 6 peptides |  |  |  |  |  |  |  |  |  |  |  |  |  |  |  |  |  |  |  |
| --- | --- | --- | --- | --- | --- | --- | --- | --- | --- | --- | --- | --- | --- | --- | --- | --- | --- | --- | --- |

Best Unique PSM (Scan Sample\_4\_A1\_1\_3642.d:14287, m/z=618.7424, z=2, RT=9.99, ppm=-2.43):


TRINITY\_DN55094\_c0\_g1\_i1.p1

back to list

  

| Protein Coverage
| Supporting Peptides
| Best Unique PSM
|

Protein Coverage:

Supporting Peptides:

| Peptide | Uniq | -10lgP | Mass | Length | ppm | m/z | z | RT | 1/k0 Start | 1/k0 End | Precursor ID | Area Sample 6 | #Feature | #Feature Sample 6 | Start | End | PTM | AScore | Found By |
| --- | --- | --- | --- | --- | --- | --- | --- | --- | --- | --- | --- | --- | --- | --- | --- | --- | --- | --- | --- |
| R.DTFSADLNHVIAQSR.C | Y | 47.12 | 1672.8169 | 15 | 6.59 | 837.4175 | 2 | 18.41 | 1.1262 | 1.1334 | 65092 | 0 | 0 | 0 | 78 | 92 |  |  | DB Search |
| K.FLLHLFDKELK.M | Y | 45.88 | 1401.802 | 11 | 9.16 | 468.2768 | 3 | 19.19 | 0.7677 | 0.775 | 70553 | 0 | 0 | 0 | 95 | 105 |  |  | DB Search |
| total 2 peptides |  |  |  |  |  |  |  |  |  |  |  |  |  |  |  |  |  |  |  |
| --- | --- | --- | --- | --- | --- | --- | --- | --- | --- | --- | --- | --- | --- | --- | --- | --- | --- | --- | --- |

Best Unique PSM (Scan Sample\_4\_A1\_1\_3642.d:65092, m/z=837.4175, z=2, RT=18.41, ppm=2.19):


TRINITY\_DN925\_c0\_g1\_i1.p1

back to list

  

| Protein Coverage
| Supporting Peptides
| Best Unique PSM
|

Protein Coverage:

Supporting Peptides:

| Peptide | Uniq | -10lgP | Mass | Length | ppm | m/z | z | RT | 1/k0 Start | 1/k0 End | Precursor ID | Area Sample 6 | #Feature | #Feature Sample 6 | Start | End | PTM | AScore | Found By |
| --- | --- | --- | --- | --- | --- | --- | --- | --- | --- | --- | --- | --- | --- | --- | --- | --- | --- | --- | --- |
| R.ASGDYATVVSHNR.D | Y | 45.13 | 1375.648 | 13 | -4.36 | 688.8253 | 2 | 12.17 | 0.9953 | 1.0025 | 27659 | 0 | 0 | 0 | 129 | 141 |  |  | DB Search |
| total 1 peptides |  |  |  |  |  |  |  |  |  |  |  |  |  |  |  |  |  |  |  |
| --- | --- | --- | --- | --- | --- | --- | --- | --- | --- | --- | --- | --- | --- | --- | --- | --- | --- | --- | --- |

Best Unique PSM (Scan Sample\_4\_A1\_1\_3642.d:27659, m/z=688.8253, z=2, RT=12.17, ppm=-8.77):


TRINITY\_DN165469\_c0\_g1\_i1.p1

back to list

  

| Protein Coverage
| Supporting Peptides
| Best Unique PSM
|

Protein Coverage:

Supporting Peptides:

| Peptide | Uniq | -10lgP | Mass | Length | ppm | m/z | z | RT | 1/k0 Start | 1/k0 End | Precursor ID | Area Sample 6 | #Feature | #Feature Sample 6 | Start | End | PTM | AScore | Found By |
| --- | --- | --- | --- | --- | --- | --- | --- | --- | --- | --- | --- | --- | --- | --- | --- | --- | --- | --- | --- |
| K.RLDPSEQLQHLSSLR.Q | Y | 46.82 | 1777.9435 | 15 | 3.26 | 593.6544 | 3 | 16.59 | 0.9077 | 0.915 | 53199 | 0 | 0 | 0 | 2221 | 2235 |  |  | DB Search |
| total 1 peptides |  |  |  |  |  |  |  |  |  |  |  |  |  |  |  |  |  |  |  |
| --- | --- | --- | --- | --- | --- | --- | --- | --- | --- | --- | --- | --- | --- | --- | --- | --- | --- | --- | --- |

Best Unique PSM (Scan Sample\_4\_A1\_1\_3642.d:53199, m/z=593.6544, z=3, RT=16.59, ppm=-1.15):


TRINITY\_DN4494\_c1\_g1\_i5.p1

back to list

  

| Protein Coverage
| Supporting Peptides
| Best Unique PSM
|

Protein Coverage:

Supporting Peptides:

| Peptide | Uniq | -10lgP | Mass | Length | ppm | m/z | z | RT | 1/k0 Start | 1/k0 End | Precursor ID | Area Sample 6 | #Feature | #Feature Sample 6 | Start | End | PTM | AScore | Found By |
| --- | --- | --- | --- | --- | --- | --- | --- | --- | --- | --- | --- | --- | --- | --- | --- | --- | --- | --- | --- |
| K.VDVGFILDSSGSLR.N | Y | 45.76 | 1463.762 | 14 | -1.21 | 732.8842 | 2 | 19.93 | 1.0102 | 1.0174 | 75559 | 4.99e2 | 1 | 1 | 288 | 301 |  |  | DB Search |
| total 1 peptides |  |  |  |  |  |  |  |  |  |  |  |  |  |  |  |  |  |  |  |
| --- | --- | --- | --- | --- | --- | --- | --- | --- | --- | --- | --- | --- | --- | --- | --- | --- | --- | --- | --- |

Best Unique PSM (Scan Sample\_4\_A1\_1\_3642.d:75559, m/z=732.8842, z=2, RT=19.93, ppm=-5.61):


TRINITY\_DN585\_c0\_g1\_i5.p1

back to list

  

| Protein Coverage
| Supporting Peptides
| Best Unique PSM
|

Protein Coverage:

Supporting Peptides:

| Peptide | Uniq | -10lgP | Mass | Length | ppm | m/z | z | RT | 1/k0 Start | 1/k0 End | Precursor ID | Area Sample 6 | #Feature | #Feature Sample 6 | Start | End | PTM | AScore | Found By |
| --- | --- | --- | --- | --- | --- | --- | --- | --- | --- | --- | --- | --- | --- | --- | --- | --- | --- | --- | --- |
| K.TITLEVEPSDTIENVK.S | Y | 46.69 | 1786.92 | 16 | 0.39 | 894.4637 | 2 | 17.56 | 1.1442 | 1.1514 | 58994 | 8.78e2 | 1 | 1 | 60 | 75 |  |  | DB Search |
| total 1 peptides |  |  |  |  |  |  |  |  |  |  |  |  |  |  |  |  |  |  |  |
| --- | --- | --- | --- | --- | --- | --- | --- | --- | --- | --- | --- | --- | --- | --- | --- | --- | --- | --- | --- |

Best Unique PSM (Scan Sample\_4\_A1\_1\_3642.d:58994, m/z=894.4637, z=2, RT=17.56, ppm=-4.01):


TRINITY\_DN10485\_c0\_g1\_i1.p1

back to list

  

| Protein Coverage
| Supporting Peptides
| Best Unique PSM
|

Protein Coverage:

Supporting Peptides:

| Peptide | Uniq | -10lgP | Mass | Length | ppm | m/z | z | RT | 1/k0 Start | 1/k0 End | Precursor ID | Area Sample 6 | #Feature | #Feature Sample 6 | Start | End | PTM | AScore | Found By |
| --- | --- | --- | --- | --- | --- | --- | --- | --- | --- | --- | --- | --- | --- | --- | --- | --- | --- | --- | --- |
| K.DLEDYKVTEREPVTTNVK.G | Y | 46.5 | 2135.0746 | 18 | 4.9 | 712.6992 | 3 | 15.24 | 0.9178 | 0.9251 | 45519 | 1.88e3 | 1 | 1 | 282 | 299 |  |  | DB Search |
| total 1 peptides |  |  |  |  |  |  |  |  |  |  |  |  |  |  |  |  |  |  |  |
| --- | --- | --- | --- | --- | --- | --- | --- | --- | --- | --- | --- | --- | --- | --- | --- | --- | --- | --- | --- |

Best Unique PSM (Scan Sample\_4\_A1\_1\_3642.d:45519, m/z=712.6992, z=3, RT=15.24, ppm=0.5):


TRINITY\_DN4595\_c0\_g1\_i10.p2

back to list

  

| Protein Coverage
| Supporting Peptides
| Best Unique PSM
|

Protein Coverage:

Supporting Peptides:

| Peptide | Uniq | -10lgP | Mass | Length | ppm | m/z | z | RT | 1/k0 Start | 1/k0 End | Precursor ID | Area Sample 6 | #Feature | #Feature Sample 6 | Start | End | PTM | AScore | Found By |
| --- | --- | --- | --- | --- | --- | --- | --- | --- | --- | --- | --- | --- | --- | --- | --- | --- | --- | --- | --- |
| R.VC(+57.02)NGEPC(+57.02)ILQER.S | Y | 45.81 | 1473.6704 | 12 | 3.3 | 737.8417 | 2 | 14.98 | 0.9876 | 0.9949 | 44106 | 8.93e2 | 1 | 1 | 306 | 317 | Carbamidomethylation, Carbamidomethylation |  | DB Search |
| R.YQGREYNINEEVNVR.Q | Y | 45.61 | 1881.8969 | 15 | -1.16 | 941.9505 | 2 | 15.67 | 1.1394 | 1.1466 | 47924 | 1.31e3 | 1 | 1 | 208 | 222 |  |  | DB Search |
| R.AFNKVETR.N | N | 44.29 | 963.5138 | 8 | -5.34 | 482.7595 | 2 | 10.58 | 0.8283 | 0.8356 | 17963 | 0 | 0 | 0 | 47 | 54 |  |  | DB Search |
| R.DGTTPC(+57.02)NTR.S | Y | 44.18 | 1020.4295 | 9 | 6.58 | 511.2231 | 2 | 9.57 | 0.8295 | 0.8368 | 12093 | 0 | 0 | 0 | 160 | 168 | Carbamidomethylation |  | DB Search |
| total 4 peptides |  |  |  |  |  |  |  |  |  |  |  |  |  |  |  |  |  |  |  |
| --- | --- | --- | --- | --- | --- | --- | --- | --- | --- | --- | --- | --- | --- | --- | --- | --- | --- | --- | --- |

Best Unique PSM (Scan Sample\_4\_A1\_1\_3642.d:44106, m/z=737.8417, z=2, RT=14.98, ppm=-1.1):


TRINITY\_DN65215\_c0\_g1\_i1.p1

back to list

  

| Protein Coverage
| Supporting Peptides
| Best Unique PSM
|

Protein Coverage:

Supporting Peptides:

| Peptide | Uniq | -10lgP | Mass | Length | ppm | m/z | z | RT | 1/k0 Start | 1/k0 End | Precursor ID | Area Sample 6 | #Feature | #Feature Sample 6 | Start | End | PTM | AScore | Found By |
| --- | --- | --- | --- | --- | --- | --- | --- | --- | --- | --- | --- | --- | --- | --- | --- | --- | --- | --- | --- |
| K.AFLFDVVSK.I | Y | 43.87 | 1024.5593 | 9 | 2.62 | 513.286 | 2 | 19.76 | 0.8388 | 0.8461 | 74385 | 7.51e2 | 1 | 1 | 248 | 256 |  |  | DB Search |
| total 1 peptides |  |  |  |  |  |  |  |  |  |  |  |  |  |  |  |  |  |  |  |
| --- | --- | --- | --- | --- | --- | --- | --- | --- | --- | --- | --- | --- | --- | --- | --- | --- | --- | --- | --- |

Best Unique PSM (Scan Sample\_4\_A1\_1\_3642.d:74385, m/z=513.286, z=2, RT=19.76, ppm=-1.79):


TRINITY\_DN141338\_c2\_g1\_i1.p1

back to list

  

| Protein Coverage
| Supporting Peptides
| Best Unique PSM
|

Protein Coverage:

Supporting Peptides:

| Peptide | Uniq | -10lgP | Mass | Length | ppm | m/z | z | RT | 1/k0 Start | 1/k0 End | Precursor ID | Area Sample 6 | #Feature | #Feature Sample 6 | Start | End | PTM | AScore | Found By |
| --- | --- | --- | --- | --- | --- | --- | --- | --- | --- | --- | --- | --- | --- | --- | --- | --- | --- | --- | --- |
| K.LGIHEDTQNR.N | Y | 47.2 | 1181.5789 | 10 | 7.67 | 591.7986 | 2 | 11.15 | 0.9012 | 0.9085 | 21461 | 0 | 0 | 0 | 434 | 443 |  |  | DB Search |
| total 1 peptides |  |  |  |  |  |  |  |  |  |  |  |  |  |  |  |  |  |  |  |
| --- | --- | --- | --- | --- | --- | --- | --- | --- | --- | --- | --- | --- | --- | --- | --- | --- | --- | --- | --- |

Best Unique PSM (Scan Sample\_4\_A1\_1\_3642.d:21461, m/z=591.7986, z=2, RT=11.15, ppm=3.27):


TRINITY\_DN33065\_c0\_g2\_i1.p1

back to list

  

| Protein Coverage
| Supporting Peptides
| Best Unique PSM
|

Protein Coverage:

Supporting Peptides:

| Peptide | Uniq | -10lgP | Mass | Length | ppm | m/z | z | RT | 1/k0 Start | 1/k0 End | Precursor ID | Area Sample 6 | #Feature | #Feature Sample 6 | Start | End | PTM | AScore | Found By |
| --- | --- | --- | --- | --- | --- | --- | --- | --- | --- | --- | --- | --- | --- | --- | --- | --- | --- | --- | --- |
| R.AFSFSDKPAITFEIHDFK.M | Y | 46.72 | 2099.0363 | 18 | 7.34 | 700.6881 | 3 | 19.02 | 0.9808 | 0.988 | 69353 | 0 | 0 | 0 | 595 | 612 |  |  | DB Search |
| total 1 peptides |  |  |  |  |  |  |  |  |  |  |  |  |  |  |  |  |  |  |  |
| --- | --- | --- | --- | --- | --- | --- | --- | --- | --- | --- | --- | --- | --- | --- | --- | --- | --- | --- | --- |

Best Unique PSM (Scan Sample\_4\_A1\_1\_3642.d:69353, m/z=700.6881, z=3, RT=19.02, ppm=2.94):


TRINITY\_DN2477\_c0\_g1\_i6.p1

back to list

  

| Protein Coverage
| Supporting Peptides
| Best Unique PSM
|

Protein Coverage:

Supporting Peptides:

| Peptide | Uniq | -10lgP | Mass | Length | ppm | m/z | z | RT | 1/k0 Start | 1/k0 End | Precursor ID | Area Sample 6 | #Feature | #Feature Sample 6 | Start | End | PTM | AScore | Found By |
| --- | --- | --- | --- | --- | --- | --- | --- | --- | --- | --- | --- | --- | --- | --- | --- | --- | --- | --- | --- |
| K.DSYVGDEAQSKR.G | Y | 46.51 | 1353.616 | 12 | 5.22 | 677.8159 | 2 | 10.97 | 1.0005 | 1.0077 | 20266 | 2.51e3 | 1 | 1 | 52 | 63 |  |  | DB Search |
| R.VAPEEHPVLLTEAPLNPK.A | Y | 46.46 | 1953.0571 | 18 | 9.42 | 977.5407 | 2 | 16.61 | 1.2085 | 1.2157 | 53280 | 0 | 0 | 0 | 97 | 114 |  |  | DB Search |
| total 2 peptides |  |  |  |  |  |  |  |  |  |  |  |  |  |  |  |  |  |  |  |
| --- | --- | --- | --- | --- | --- | --- | --- | --- | --- | --- | --- | --- | --- | --- | --- | --- | --- | --- | --- |

Best Unique PSM (Scan Sample\_4\_A1\_1\_3642.d:20266, m/z=677.8159, z=2, RT=10.97, ppm=0.82):


TRINITY\_DN8903\_c0\_g1\_i1.p1

back to list

  

| Protein Coverage
| Supporting Peptides
| Best Unique PSM
|

Protein Coverage:

Supporting Peptides:

| Peptide | Uniq | -10lgP | Mass | Length | ppm | m/z | z | RT | 1/k0 Start | 1/k0 End | Precursor ID | Area Sample 6 | #Feature | #Feature Sample 6 | Start | End | PTM | AScore | Found By |
| --- | --- | --- | --- | --- | --- | --- | --- | --- | --- | --- | --- | --- | --- | --- | --- | --- | --- | --- | --- |
| K.DLHIIEDYVLK.H | Y | 46.18 | 1356.7289 | 11 | 2.32 | 679.3703 | 2 | 19.66 | 0.9928 | 1.0001 | 73679 | 1.43e3 | 1 | 1 | 417 | 427 |  |  | DB Search |
| total 1 peptides |  |  |  |  |  |  |  |  |  |  |  |  |  |  |  |  |  |  |  |
| --- | --- | --- | --- | --- | --- | --- | --- | --- | --- | --- | --- | --- | --- | --- | --- | --- | --- | --- | --- |

Best Unique PSM (Scan Sample\_4\_A1\_1\_3642.d:73679, m/z=679.3703, z=2, RT=19.66, ppm=-2.09):


TRINITY\_DN11096\_c0\_g1\_i1.p1

back to list

  

| Protein Coverage
| Supporting Peptides
| Best Unique PSM
|

Protein Coverage:

Supporting Peptides:

| Peptide | Uniq | -10lgP | Mass | Length | ppm | m/z | z | RT | 1/k0 Start | 1/k0 End | Precursor ID | Area Sample 6 | #Feature | #Feature Sample 6 | Start | End | PTM | AScore | Found By |
| --- | --- | --- | --- | --- | --- | --- | --- | --- | --- | --- | --- | --- | --- | --- | --- | --- | --- | --- | --- |
| R.LPAGSLSYLDKPENTNR.G | Y | 43.58 | 1873.9534 | 17 | -5.38 | 625.6523 | 3 | 15.41 | 0.868 | 0.8753 | 46646 | 0 | 0 | 0 | 374 | 390 |  |  | DB Search |
| total 1 peptides |  |  |  |  |  |  |  |  |  |  |  |  |  |  |  |  |  |  |  |
| --- | --- | --- | --- | --- | --- | --- | --- | --- | --- | --- | --- | --- | --- | --- | --- | --- | --- | --- | --- |

Best Unique PSM (Scan Sample\_4\_A1\_1\_3642.d:46646, m/z=625.6523, z=3, RT=15.41, ppm=-9.79):


TRINITY\_DN36\_c0\_g1\_i17.p1

back to list

  

| Protein Coverage
| Supporting Peptides
| Best Unique PSM
|

Protein Coverage:

Supporting Peptides:

| Peptide | Uniq | -10lgP | Mass | Length | ppm | m/z | z | RT | 1/k0 Start | 1/k0 End | Precursor ID | Area Sample 6 | #Feature | #Feature Sample 6 | Start | End | PTM | AScore | Found By |
| --- | --- | --- | --- | --- | --- | --- | --- | --- | --- | --- | --- | --- | --- | --- | --- | --- | --- | --- | --- |
| R.LGGIEVTQGNC(+57.02)AR.K | Y | 45.45 | 1373.6721 | 13 | 2.96 | 687.8423 | 2 | 13.87 | 0.9687 | 0.9759 | 37701 | 0 | 0 | 0 | 58 | 70 | Carbamidomethylation |  | DB Search |
| K.FETQIATK.D | Y | 44.83 | 936.4916 | 8 | 7 | 937.5013 | 1 | 13.97 | 1.1802 | 1.1873 | 38247 | 0 | 0 | 0 | 167 | 174 |  |  | DB Search |
| total 2 peptides |  |  |  |  |  |  |  |  |  |  |  |  |  |  |  |  |  |  |  |
| --- | --- | --- | --- | --- | --- | --- | --- | --- | --- | --- | --- | --- | --- | --- | --- | --- | --- | --- | --- |

Best Unique PSM (Scan Sample\_4\_A1\_1\_3642.d:37701, m/z=687.8423, z=2, RT=13.87, ppm=-1.44):


TRINITY\_DN3991\_c0\_g1\_i1.p1

back to list

  

| Protein Coverage
| Supporting Peptides
| Best Unique PSM
|

Protein Coverage:

Supporting Peptides:

| Peptide | Uniq | -10lgP | Mass | Length | ppm | m/z | z | RT | 1/k0 Start | 1/k0 End | Precursor ID | Area Sample 6 | #Feature | #Feature Sample 6 | Start | End | PTM | AScore | Found By |
| --- | --- | --- | --- | --- | --- | --- | --- | --- | --- | --- | --- | --- | --- | --- | --- | --- | --- | --- | --- |
| K.LSQC(+57.02)TAASGK.H | Y | 43.29 | 1021.4862 | 10 | -0.25 | 511.748 | 2 | 9.82 | 0.8364 | 0.8437 | 12382 | 2.03e5 | 1 | 1 | 130 | 139 | Carbamidomethylation |  | DB Search |
| total 1 peptides |  |  |  |  |  |  |  |  |  |  |  |  |  |  |  |  |  |  |  |
| --- | --- | --- | --- | --- | --- | --- | --- | --- | --- | --- | --- | --- | --- | --- | --- | --- | --- | --- | --- |

Best Unique PSM (Scan Sample\_4\_A1\_1\_3642.d:12382, m/z=511.748, z=2, RT=9.82, ppm=-4.65):


TRINITY\_DN649\_c0\_g1\_i5.p1

back to list

  

| Protein Coverage
| Supporting Peptides
| Best Unique PSM
|

Protein Coverage:

Supporting Peptides:

| Peptide | Uniq | -10lgP | Mass | Length | ppm | m/z | z | RT | 1/k0 Start | 1/k0 End | Precursor ID | Area Sample 6 | #Feature | #Feature Sample 6 | Start | End | PTM | AScore | Found By |
| --- | --- | --- | --- | --- | --- | --- | --- | --- | --- | --- | --- | --- | --- | --- | --- | --- | --- | --- | --- |
| K.FETQIATK.N | Y | 44.83 | 936.4916 | 8 | 7 | 937.5013 | 1 | 13.97 | 1.1802 | 1.1873 | 38247 | 0 | 0 | 0 | 102 | 109 |  |  | DB Search |
| total 1 peptides |  |  |  |  |  |  |  |  |  |  |  |  |  |  |  |  |  |  |  |
| --- | --- | --- | --- | --- | --- | --- | --- | --- | --- | --- | --- | --- | --- | --- | --- | --- | --- | --- | --- |

Best Unique PSM (Scan Sample\_4\_A1\_1\_3642.d:38247, m/z=937.5013, z=1, RT=13.97, ppm=2.6):


TRINITY\_DN6428\_c0\_g1\_i1.p1

back to list

  

| Protein Coverage
| Supporting Peptides
| Best Unique PSM
|

Protein Coverage:

Supporting Peptides:

| Peptide | Uniq | -10lgP | Mass | Length | ppm | m/z | z | RT | 1/k0 Start | 1/k0 End | Precursor ID | Area Sample 6 | #Feature | #Feature Sample 6 | Start | End | PTM | AScore | Found By |
| --- | --- | --- | --- | --- | --- | --- | --- | --- | --- | --- | --- | --- | --- | --- | --- | --- | --- | --- | --- |
| R.SC(+57.02)ENPKPLNGGK.Q | Y | 46.61 | 1299.6241 | 12 | 2.25 | 650.8179 | 2 | 11.07 | 0.9505 | 0.9578 | 20781 | 2.84e3 | 1 | 1 | 245 | 256 | Carbamidomethylation |  | DB Search |
| total 1 peptides |  |  |  |  |  |  |  |  |  |  |  |  |  |  |  |  |  |  |  |
| --- | --- | --- | --- | --- | --- | --- | --- | --- | --- | --- | --- | --- | --- | --- | --- | --- | --- | --- | --- |

Best Unique PSM (Scan Sample\_4\_A1\_1\_3642.d:20781, m/z=650.8179, z=2, RT=11.07, ppm=-2.16):


TRINITY\_DN4726\_c0\_g1\_i1.p1

back to list

  

| Protein Coverage
| Supporting Peptides
| Best Unique PSM
|

Protein Coverage:

Supporting Peptides:

| Peptide | Uniq | -10lgP | Mass | Length | ppm | m/z | z | RT | 1/k0 Start | 1/k0 End | Precursor ID | Area Sample 6 | #Feature | #Feature Sample 6 | Start | End | PTM | AScore | Found By |
| --- | --- | --- | --- | --- | --- | --- | --- | --- | --- | --- | --- | --- | --- | --- | --- | --- | --- | --- | --- |
| R.ITNLYC(+57.02)DLSIYR.Q | Y | 46.72 | 1529.7548 | 12 | 3.24 | 765.8838 | 2 | 19.27 | 1.0371 | 1.0443 | 70930 | 3.14e2 | 1 | 1 | 276 | 287 | Carbamidomethylation |  | DB Search |
| K.ANKEFQEIVQNTK.Y | Y | 46.01 | 1547.7943 | 13 | 2.47 | 774.903 | 2 | 14.63 | 1.0676 | 1.0749 | 42038 | 4.72e2 | 1 | 1 | 364 | 376 |  |  | DB Search |
| total 2 peptides |  |  |  |  |  |  |  |  |  |  |  |  |  |  |  |  |  |  |  |
| --- | --- | --- | --- | --- | --- | --- | --- | --- | --- | --- | --- | --- | --- | --- | --- | --- | --- | --- | --- |

Best Unique PSM (Scan Sample\_4\_A1\_1\_3642.d:70930, m/z=765.8838, z=2, RT=19.27, ppm=-1.16):


TRINITY\_DN5987\_c0\_g1\_i3.p2

back to list

  

| Protein Coverage
| Supporting Peptides
| Best Unique PSM
|

Protein Coverage:

Supporting Peptides:

| Peptide | Uniq | -10lgP | Mass | Length | ppm | m/z | z | RT | 1/k0 Start | 1/k0 End | Precursor ID | Area Sample 6 | #Feature | #Feature Sample 6 | Start | End | PTM | AScore | Found By |
| --- | --- | --- | --- | --- | --- | --- | --- | --- | --- | --- | --- | --- | --- | --- | --- | --- | --- | --- | --- |
| R.GNQGEQGPAGPVGPK.G | Y | 47.2 | 1391.6793 | 15 | 3.52 | 696.8463 | 2 | 11.46 | 0.9461 | 0.9533 | 23184 | 1.15e3 | 1 | 1 | 5 | 19 |  |  | DB Search |
| total 1 peptides |  |  |  |  |  |  |  |  |  |  |  |  |  |  |  |  |  |  |  |
| --- | --- | --- | --- | --- | --- | --- | --- | --- | --- | --- | --- | --- | --- | --- | --- | --- | --- | --- | --- |

Best Unique PSM (Scan Sample\_4\_A1\_1\_3642.d:23184, m/z=696.8463, z=2, RT=11.46, ppm=-0.88):


TRINITY\_DN2477\_c0\_g1\_i2.p1

back to list

  

| Protein Coverage
| Supporting Peptides
| Best Unique PSM
|

Protein Coverage:

Supporting Peptides:

| Peptide | Uniq | -10lgP | Mass | Length | ppm | m/z | z | RT | 1/k0 Start | 1/k0 End | Precursor ID | Area Sample 6 | #Feature | #Feature Sample 6 | Start | End | PTM | AScore | Found By |
| --- | --- | --- | --- | --- | --- | --- | --- | --- | --- | --- | --- | --- | --- | --- | --- | --- | --- | --- | --- |
| K.DSYVGDEAQSKR.G | Y | 46.51 | 1353.616 | 12 | 5.22 | 677.8159 | 2 | 10.97 | 1.0005 | 1.0077 | 20266 | 2.51e3 | 1 | 1 | 19 | 30 |  |  | DB Search |
| R.VAPEEHPVLLTEAPLNPK.A | Y | 46.46 | 1953.0571 | 18 | 9.42 | 977.5407 | 2 | 16.61 | 1.2085 | 1.2157 | 53280 | 0 | 0 | 0 | 64 | 81 |  |  | DB Search |
| total 2 peptides |  |  |  |  |  |  |  |  |  |  |  |  |  |  |  |  |  |  |  |
| --- | --- | --- | --- | --- | --- | --- | --- | --- | --- | --- | --- | --- | --- | --- | --- | --- | --- | --- | --- |

Best Unique PSM (Scan Sample\_4\_A1\_1\_3642.d:20266, m/z=677.8159, z=2, RT=10.97, ppm=0.82):


TRINITY\_DN2885\_c0\_g1\_i4.p1

back to list

  

| Protein Coverage
| Supporting Peptides
| Best Unique PSM
|

Protein Coverage:

Supporting Peptides:

| Peptide | Uniq | -10lgP | Mass | Length | ppm | m/z | z | RT | 1/k0 Start | 1/k0 End | Precursor ID | Area Sample 6 | #Feature | #Feature Sample 6 | Start | End | PTM | AScore | Found By |
| --- | --- | --- | --- | --- | --- | --- | --- | --- | --- | --- | --- | --- | --- | --- | --- | --- | --- | --- | --- |
| K.VTRPHGASGVVR.A | Y | 46.49 | 1234.6894 | 12 | 7.12 | 412.5715 | 3 | 10.06 | 0.8169 | 0.8242 | 14707 | 3.57e2 | 1 | 1 | 72 | 83 |  |  | DB Search |
| total 1 peptides |  |  |  |  |  |  |  |  |  |  |  |  |  |  |  |  |  |  |  |
| --- | --- | --- | --- | --- | --- | --- | --- | --- | --- | --- | --- | --- | --- | --- | --- | --- | --- | --- | --- |

Best Unique PSM (Scan Sample\_4\_A1\_1\_3642.d:14707, m/z=412.5715, z=3, RT=10.06, ppm=2.72):


TRINITY\_DN165490\_c0\_g1\_i1.p1

back to list

  

| Protein Coverage
| Supporting Peptides
| Best Unique PSM
|

Protein Coverage:

Supporting Peptides:

| Peptide | Uniq | -10lgP | Mass | Length | ppm | m/z | z | RT | 1/k0 Start | 1/k0 End | Precursor ID | Area Sample 6 | #Feature | #Feature Sample 6 | Start | End | PTM | AScore | Found By |
| --- | --- | --- | --- | --- | --- | --- | --- | --- | --- | --- | --- | --- | --- | --- | --- | --- | --- | --- | --- |
| K.AMQSLPPGPQHR.A | Y | 37.32 | 1317.6612 | 12 | -2.22 | 659.8335 | 2 | 12.94 | 0.965 | 0.9723 | 32063 | 2.43e3 | 1 | 1 | 34 | 45 |  |  | DB Search |
| total 1 peptides |  |  |  |  |  |  |  |  |  |  |  |  |  |  |  |  |  |  |  |
| --- | --- | --- | --- | --- | --- | --- | --- | --- | --- | --- | --- | --- | --- | --- | --- | --- | --- | --- | --- |

Best Unique PSM (Scan Sample\_4\_A1\_1\_3642.d:32063, m/z=659.8335, z=2, RT=12.94, ppm=-6.62):


TRINITY\_DN4726\_c0\_g1\_i2.p1

back to list

  

| Protein Coverage
| Supporting Peptides
| Best Unique PSM
|

Protein Coverage:

Supporting Peptides:

| Peptide | Uniq | -10lgP | Mass | Length | ppm | m/z | z | RT | 1/k0 Start | 1/k0 End | Precursor ID | Area Sample 6 | #Feature | #Feature Sample 6 | Start | End | PTM | AScore | Found By |
| --- | --- | --- | --- | --- | --- | --- | --- | --- | --- | --- | --- | --- | --- | --- | --- | --- | --- | --- | --- |
| R.ITNLYC(+57.02)DLSIYR.Q | Y | 46.72 | 1529.7548 | 12 | 3.24 | 765.8838 | 2 | 19.27 | 1.0371 | 1.0443 | 70930 | 3.14e2 | 1 | 1 | 369 | 380 | Carbamidomethylation |  | DB Search |
| total 1 peptides |  |  |  |  |  |  |  |  |  |  |  |  |  |  |  |  |  |  |  |
| --- | --- | --- | --- | --- | --- | --- | --- | --- | --- | --- | --- | --- | --- | --- | --- | --- | --- | --- | --- |

Best Unique PSM (Scan Sample\_4\_A1\_1\_3642.d:70930, m/z=765.8838, z=2, RT=19.27, ppm=-1.16):


TRINITY\_DN106\_c0\_g1\_i6.p1

back to list

  

| Protein Coverage
| Supporting Peptides
| Best Unique PSM
|

Protein Coverage:

Supporting Peptides:

| Peptide | Uniq | -10lgP | Mass | Length | ppm | m/z | z | RT | 1/k0 Start | 1/k0 End | Precursor ID | Area Sample 6 | #Feature | #Feature Sample 6 | Start | End | PTM | AScore | Found By |
| --- | --- | --- | --- | --- | --- | --- | --- | --- | --- | --- | --- | --- | --- | --- | --- | --- | --- | --- | --- |
| R.DGPAGPAGR.D | Y | 46.52 | 796.3827 | 9 | 7.09 | 399.1997 | 2 | 9.76 | 0.7233 | 0.7306 | 13098 | 1.65e3 | 1 | 1 | 488 | 496 |  |  | DB Search |
| total 1 peptides |  |  |  |  |  |  |  |  |  |  |  |  |  |  |  |  |  |  |  |
| --- | --- | --- | --- | --- | --- | --- | --- | --- | --- | --- | --- | --- | --- | --- | --- | --- | --- | --- | --- |

Best Unique PSM (Scan Sample\_4\_A1\_1\_3642.d:13098, m/z=399.1997, z=2, RT=9.76, ppm=2.68):


TRINITY\_DN2113\_c0\_g1\_i2.p1

back to list

  

| Protein Coverage
| Supporting Peptides
| Best Unique PSM
|

Protein Coverage:

Supporting Peptides:

| Peptide | Uniq | -10lgP | Mass | Length | ppm | m/z | z | RT | 1/k0 Start | 1/k0 End | Precursor ID | Area Sample 6 | #Feature | #Feature Sample 6 | Start | End | PTM | AScore | Found By |
| --- | --- | --- | --- | --- | --- | --- | --- | --- | --- | --- | --- | --- | --- | --- | --- | --- | --- | --- | --- |
| R.LADLEEFISSPNHANISQVGER.C | Y | 46.68 | 2425.1873 | 22 | -0.55 | 809.399 | 3 | 18.89 | 0.9243 | 0.9315 | 68335 | 1.48e3 | 1 | 1 | 1183 | 1204 |  |  | DB Search |
| R.ALEHYSDIYDIKR.A | Y | 43.91 | 1621.81 | 13 | 7.89 | 541.6125 | 3 | 14.74 | 0.825 | 0.8323 | 42743 | 0 | 0 | 0 | 626 | 638 |  |  | DB Search |
| total 2 peptides |  |  |  |  |  |  |  |  |  |  |  |  |  |  |  |  |  |  |  |
| --- | --- | --- | --- | --- | --- | --- | --- | --- | --- | --- | --- | --- | --- | --- | --- | --- | --- | --- | --- |

Best Unique PSM (Scan Sample\_4\_A1\_1\_3642.d:68335, m/z=809.399, z=3, RT=18.89, ppm=-4.95):


TRINITY\_DN123\_c0\_g1\_i1.p1

back to list

  

| Protein Coverage
| Supporting Peptides
| Best Unique PSM
|

Protein Coverage:

Supporting Peptides:

| Peptide | Uniq | -10lgP | Mass | Length | ppm | m/z | z | RT | 1/k0 Start | 1/k0 End | Precursor ID | Area Sample 6 | #Feature | #Feature Sample 6 | Start | End | PTM | AScore | Found By |
| --- | --- | --- | --- | --- | --- | --- | --- | --- | --- | --- | --- | --- | --- | --- | --- | --- | --- | --- | --- |
| K.IAAVIINKPDVLASR.T | Y | 47.12 | 1578.9457 | 15 | 3.53 | 790.4794 | 2 | 16.15 | 1.1021 | 1.1094 | 50690 | 2.9e2 | 1 | 1 | 484 | 498 |  |  | DB Search |
| total 1 peptides |  |  |  |  |  |  |  |  |  |  |  |  |  |  |  |  |  |  |  |
| --- | --- | --- | --- | --- | --- | --- | --- | --- | --- | --- | --- | --- | --- | --- | --- | --- | --- | --- | --- |

Best Unique PSM (Scan Sample\_4\_A1\_1\_3642.d:50690, m/z=790.4794, z=2, RT=16.15, ppm=-0.87):


TRINITY\_DN1058\_c0\_g1\_i4.p1

back to list

  

| Protein Coverage
| Supporting Peptides
| Best Unique PSM
|

Protein Coverage:

Supporting Peptides:

| Peptide | Uniq | -10lgP | Mass | Length | ppm | m/z | z | RT | 1/k0 Start | 1/k0 End | Precursor ID | Area Sample 6 | #Feature | #Feature Sample 6 | Start | End | PTM | AScore | Found By |
| --- | --- | --- | --- | --- | --- | --- | --- | --- | --- | --- | --- | --- | --- | --- | --- | --- | --- | --- | --- |
| R.APTHILNPANKR.S | Y | 46.93 | 1330.7469 | 12 | 2.71 | 444.5888 | 3 | 11.17 | 0.7559 | 0.7632 | 21058 | 5.5e3 | 2 | 2 | 398 | 409 |  |  | DB Search |
| total 1 peptides |  |  |  |  |  |  |  |  |  |  |  |  |  |  |  |  |  |  |  |
| --- | --- | --- | --- | --- | --- | --- | --- | --- | --- | --- | --- | --- | --- | --- | --- | --- | --- | --- | --- |

Best Unique PSM (Scan Sample\_4\_A1\_1\_3642.d:21058, m/z=444.5888, z=3, RT=11.17, ppm=-1.7):


TRINITY\_DN1030\_c0\_g1\_i2.p1

back to list

  

| Protein Coverage
| Supporting Peptides
| Best Unique PSM
|

Protein Coverage:

Supporting Peptides:

| Peptide | Uniq | -10lgP | Mass | Length | ppm | m/z | z | RT | 1/k0 Start | 1/k0 End | Precursor ID | Area Sample 6 | #Feature | #Feature Sample 6 | Start | End | PTM | AScore | Found By |
| --- | --- | --- | --- | --- | --- | --- | --- | --- | --- | --- | --- | --- | --- | --- | --- | --- | --- | --- | --- |
| R.GLQGQQGEQGSTGVQGSVGGR.G | Y | 47.24 | 1985.9515 | 21 | 2.4 | 993.981 | 2 | 12.07 | 1.133 | 1.1402 | 26902 | 2.07e3 | 1 | 1 | 348 | 368 |  |  | DB Search |
| total 1 peptides |  |  |  |  |  |  |  |  |  |  |  |  |  |  |  |  |  |  |  |
| --- | --- | --- | --- | --- | --- | --- | --- | --- | --- | --- | --- | --- | --- | --- | --- | --- | --- | --- | --- |

Best Unique PSM (Scan Sample\_4\_A1\_1\_3642.d:26902, m/z=993.981, z=2, RT=12.07, ppm=-2):


TRINITY\_DN11248\_c0\_g1\_i3.p1

back to list

  

| Protein Coverage
| Supporting Peptides
| Best Unique PSM
|

Protein Coverage:

Supporting Peptides:

| Peptide | Uniq | -10lgP | Mass | Length | ppm | m/z | z | RT | 1/k0 Start | 1/k0 End | Precursor ID | Area Sample 6 | #Feature | #Feature Sample 6 | Start | End | PTM | AScore | Found By |
| --- | --- | --- | --- | --- | --- | --- | --- | --- | --- | --- | --- | --- | --- | --- | --- | --- | --- | --- | --- |
| K.SIEAIYLGEFGTC(+57.02)K.S | Y | 46.86 | 1586.765 | 14 | 5.15 | 794.3904 | 2 | 19.64 | 1.1029 | 1.1102 | 73637 | 0 | 0 | 0 | 982 | 995 | Carbamidomethylation |  | DB Search |
| K.TPYLQIDFGK.M | Y | 38.54 | 1180.6128 | 10 | -1.83 | 591.31 | 2 | 18.42 | 0.9198 | 0.9271 | 65300 | 4.11e2 | 1 | 1 | 514 | 523 |  |  | DB Search |
| total 2 peptides |  |  |  |  |  |  |  |  |  |  |  |  |  |  |  |  |  |  |  |
| --- | --- | --- | --- | --- | --- | --- | --- | --- | --- | --- | --- | --- | --- | --- | --- | --- | --- | --- | --- |

Best Unique PSM (Scan Sample\_4\_A1\_1\_3642.d:73637, m/z=794.3904, z=2, RT=19.64, ppm=0.75):


TRINITY\_DN2941\_c0\_g1\_i4.p1

back to list

  

| Protein Coverage
| Supporting Peptides
| Best Unique PSM
|

Protein Coverage:

Supporting Peptides:

| Peptide | Uniq | -10lgP | Mass | Length | ppm | m/z | z | RT | 1/k0 Start | 1/k0 End | Precursor ID | Area Sample 6 | #Feature | #Feature Sample 6 | Start | End | PTM | AScore | Found By |
| --- | --- | --- | --- | --- | --- | --- | --- | --- | --- | --- | --- | --- | --- | --- | --- | --- | --- | --- | --- |
| R.VAPEEHPVLLTEAPLNPK.A | Y | 46.46 | 1953.0571 | 18 | 9.42 | 977.5407 | 2 | 16.61 | 1.2085 | 1.2157 | 53280 | 0 | 0 | 0 | 30 | 47 |  |  | DB Search |
| total 1 peptides |  |  |  |  |  |  |  |  |  |  |  |  |  |  |  |  |  |  |  |
| --- | --- | --- | --- | --- | --- | --- | --- | --- | --- | --- | --- | --- | --- | --- | --- | --- | --- | --- | --- |

Best Unique PSM (Scan Sample\_4\_A1\_1\_3642.d:53280, m/z=977.5407, z=2, RT=16.61, ppm=5.02):


TRINITY\_DN602\_c0\_g1\_i1.p1

back to list

  

| Protein Coverage
| Supporting Peptides
| Best Unique PSM
|

Protein Coverage:

Supporting Peptides:

| Peptide | Uniq | -10lgP | Mass | Length | ppm | m/z | z | RT | 1/k0 Start | 1/k0 End | Precursor ID | Area Sample 6 | #Feature | #Feature Sample 6 | Start | End | PTM | AScore | Found By |
| --- | --- | --- | --- | --- | --- | --- | --- | --- | --- | --- | --- | --- | --- | --- | --- | --- | --- | --- | --- |
| K.EGFYVHEALAAGIVK.K | Y | 47.06 | 1602.8405 | 15 | -1.99 | 802.4224 | 2 | 17.69 | 1.0837 | 1.0909 | 59893 | 9.17e2 | 1 | 1 | 185 | 199 |  |  | DB Search |
| K.LLTSPAPSSGSLM(+15.99)ALALK.I | Y | 46.68 | 1771.9753 | 18 | 0.26 | 886.9913 | 2 | 18.27 | 1.1354 | 1.1426 | 64205 | 0 | 0 | 0 | 292 | 309 | Oxidation (M) | M13:Oxidation (M):1000 | DB Search |
| R.AYDFRETLPK.K | Y | 45.42 | 1238.6295 | 10 | 5.77 | 620.3229 | 2 | 15.38 | 0.9497 | 0.957 | 46320 | 7.87e2 | 1 | 1 | 128 | 137 |  |  | DB Search |
| K.HGADLFYR.G | Y | 37.07 | 977.4719 | 8 | 0.29 | 489.7412 | 2 | 16.16 | 0.8372 | 0.8445 | 50704 | 4.61e2 | 1 | 1 | 242 | 249 |  |  | DB Search |
| total 4 peptides |  |  |  |  |  |  |  |  |  |  |  |  |  |  |  |  |  |  |  |
| --- | --- | --- | --- | --- | --- | --- | --- | --- | --- | --- | --- | --- | --- | --- | --- | --- | --- | --- | --- |

Best Unique PSM (Scan Sample\_4\_A1\_1\_3642.d:59893, m/z=802.4224, z=2, RT=17.69, ppm=-6.39):


TRINITY\_DN36\_c0\_g1\_i16.p1

back to list

  

| Protein Coverage
| Supporting Peptides
| Best Unique PSM
|

Protein Coverage:

Supporting Peptides:

| Peptide | Uniq | -10lgP | Mass | Length | ppm | m/z | z | RT | 1/k0 Start | 1/k0 End | Precursor ID | Area Sample 6 | #Feature | #Feature Sample 6 | Start | End | PTM | AScore | Found By |
| --- | --- | --- | --- | --- | --- | --- | --- | --- | --- | --- | --- | --- | --- | --- | --- | --- | --- | --- | --- |
| R.LGGIEVTQGNC(+57.02)AR.K | Y | 45.45 | 1373.6721 | 13 | 2.96 | 687.8423 | 2 | 13.87 | 0.9687 | 0.9759 | 37701 | 0 | 0 | 0 | 58 | 70 | Carbamidomethylation |  | DB Search |
| K.FETQIATK.D | Y | 44.83 | 936.4916 | 8 | 7 | 937.5013 | 1 | 13.97 | 1.1802 | 1.1873 | 38247 | 0 | 0 | 0 | 167 | 174 |  |  | DB Search |
| total 2 peptides |  |  |  |  |  |  |  |  |  |  |  |  |  |  |  |  |  |  |  |
| --- | --- | --- | --- | --- | --- | --- | --- | --- | --- | --- | --- | --- | --- | --- | --- | --- | --- | --- | --- |

Best Unique PSM (Scan Sample\_4\_A1\_1\_3642.d:37701, m/z=687.8423, z=2, RT=13.87, ppm=-1.44):


TRINITY\_DN598\_c1\_g1\_i2.p1

back to list

  

| Protein Coverage
| Supporting Peptides
| Best Unique PSM
|

Protein Coverage:

Supporting Peptides:

| Peptide | Uniq | -10lgP | Mass | Length | ppm | m/z | z | RT | 1/k0 Start | 1/k0 End | Precursor ID | Area Sample 6 | #Feature | #Feature Sample 6 | Start | End | PTM | AScore | Found By |
| --- | --- | --- | --- | --- | --- | --- | --- | --- | --- | --- | --- | --- | --- | --- | --- | --- | --- | --- | --- |
| R.ASFVTDIDRLR.A | Y | 46.17 | 1291.6884 | 11 | 5.09 | 646.8519 | 2 | 17.22 | 0.9904 | 0.9977 | 56760 | 8.37e2 | 1 | 1 | 302 | 312 |  |  | DB Search |
| total 1 peptides |  |  |  |  |  |  |  |  |  |  |  |  |  |  |  |  |  |  |  |
| --- | --- | --- | --- | --- | --- | --- | --- | --- | --- | --- | --- | --- | --- | --- | --- | --- | --- | --- | --- |

Best Unique PSM (Scan Sample\_4\_A1\_1\_3642.d:56760, m/z=646.8519, z=2, RT=17.22, ppm=0.69):


TRINITY\_DN1613\_c1\_g1\_i2.p1

back to list

  

| Protein Coverage
| Supporting Peptides
| Best Unique PSM
|

Protein Coverage:

Supporting Peptides:

| Peptide | Uniq | -10lgP | Mass | Length | ppm | m/z | z | RT | 1/k0 Start | 1/k0 End | Precursor ID | Area Sample 6 | #Feature | #Feature Sample 6 | Start | End | PTM | AScore | Found By |
| --- | --- | --- | --- | --- | --- | --- | --- | --- | --- | --- | --- | --- | --- | --- | --- | --- | --- | --- | --- |
| R.TC(+57.02)TNPAPAHGGK.S | Y | 46.47 | 1209.556 | 12 | 7.45 | 404.1938 | 3 | 8.98 | 0.731 | 0.7384 | 9664 | 0 | 0 | 0 | 413 | 424 | Carbamidomethylation |  | DB Search |
| R.S(+42.01)C(+57.02)SNPSPAHGGKPC(+57.02)SGELSK.T | Y | 44.35 | 2097.9208 | 20 | -0.21 | 700.311 | 3 | 11.19 | 0.887 | 0.8943 | 21775 | 7.11e2 | 1 | 1 | 14 | 33 | Acetylation (Protein N-term), Carbamidomethylation, Carbamidomethylation | S1:Acetylation (Protein N-term):1000 | DB Search |
| total 2 peptides |  |  |  |  |  |  |  |  |  |  |  |  |  |  |  |  |  |  |  |
| --- | --- | --- | --- | --- | --- | --- | --- | --- | --- | --- | --- | --- | --- | --- | --- | --- | --- | --- | --- |

Best Unique PSM (Scan Sample\_4\_A1\_1\_3642.d:9664, m/z=404.1938, z=3, RT=8.98, ppm=3.04):


TRINITY\_DN286\_c0\_g1\_i4.p1

back to list

  

| Protein Coverage
| Supporting Peptides
| Best Unique PSM
|

Protein Coverage:

Supporting Peptides:

| Peptide | Uniq | -10lgP | Mass | Length | ppm | m/z | z | RT | 1/k0 Start | 1/k0 End | Precursor ID | Area Sample 6 | #Feature | #Feature Sample 6 | Start | End | PTM | AScore | Found By |
| --- | --- | --- | --- | --- | --- | --- | --- | --- | --- | --- | --- | --- | --- | --- | --- | --- | --- | --- | --- |
| K.RYGQAMC(+57.02)QFMR.N | Y | 45.29 | 1446.6319 | 11 | -3.75 | 724.3173 | 2 | 17.41 | 0.9856 | 0.9928 | 58107 | 7.27e2 | 1 | 1 | 71 | 81 | Carbamidomethylation |  | DB Search |
| total 1 peptides |  |  |  |  |  |  |  |  |  |  |  |  |  |  |  |  |  |  |  |
| --- | --- | --- | --- | --- | --- | --- | --- | --- | --- | --- | --- | --- | --- | --- | --- | --- | --- | --- | --- |

Best Unique PSM (Scan Sample\_4\_A1\_1\_3642.d:58107, m/z=724.3173, z=2, RT=17.41, ppm=-8.15):


TRINITY\_DN30613\_c0\_g1\_i1.p1

back to list

  

| Protein Coverage
| Supporting Peptides
| Best Unique PSM
|

Protein Coverage:

Supporting Peptides:

| Peptide | Uniq | -10lgP | Mass | Length | ppm | m/z | z | RT | 1/k0 Start | 1/k0 End | Precursor ID | Area Sample 6 | #Feature | #Feature Sample 6 | Start | End | PTM | AScore | Found By |
| --- | --- | --- | --- | --- | --- | --- | --- | --- | --- | --- | --- | --- | --- | --- | --- | --- | --- | --- | --- |
| K.APEDAPNREQEFEEK.A | Y | 46.1 | 1787.7962 | 15 | 5.21 | 596.9398 | 3 | 12.20 | 0.825 | 0.8323 | 27725 | 7.16e2 | 1 | 1 | 502 | 516 |  |  | DB Search |
| total 1 peptides |  |  |  |  |  |  |  |  |  |  |  |  |  |  |  |  |  |  |  |
| --- | --- | --- | --- | --- | --- | --- | --- | --- | --- | --- | --- | --- | --- | --- | --- | --- | --- | --- | --- |

Best Unique PSM (Scan Sample\_4\_A1\_1\_3642.d:27725, m/z=596.9398, z=3, RT=12.20, ppm=0.81):


TRINITY\_DN495\_c0\_g1\_i5.p1

back to list

  

| Protein Coverage
| Supporting Peptides
| Best Unique PSM
|

Protein Coverage:

Supporting Peptides:

| Peptide | Uniq | -10lgP | Mass | Length | ppm | m/z | z | RT | 1/k0 Start | 1/k0 End | Precursor ID | Area Sample 6 | #Feature | #Feature Sample 6 | Start | End | PTM | AScore | Found By |
| --- | --- | --- | --- | --- | --- | --- | --- | --- | --- | --- | --- | --- | --- | --- | --- | --- | --- | --- | --- |
| C.SISC(+57.02)SKDEEIK.V | Y | 46.33 | 1294.6075 | 11 | 8.03 | 648.3134 | 2 | 10.87 | 0.9723 | 0.9795 | 19739 | 0 | 0 | 0 | 273 | 283 | Carbamidomethylation |  | DB Search |
| total 1 peptides |  |  |  |  |  |  |  |  |  |  |  |  |  |  |  |  |  |  |  |
| --- | --- | --- | --- | --- | --- | --- | --- | --- | --- | --- | --- | --- | --- | --- | --- | --- | --- | --- | --- |

Best Unique PSM (Scan Sample\_4\_A1\_1\_3642.d:19739, m/z=648.3134, z=2, RT=10.87, ppm=3.63):


TRINITY\_DN135129\_c0\_g1\_i1.p1

back to list

  

| Protein Coverage
| Supporting Peptides
| Best Unique PSM
|

Protein Coverage:

Supporting Peptides:

| Peptide | Uniq | -10lgP | Mass | Length | ppm | m/z | z | RT | 1/k0 Start | 1/k0 End | Precursor ID | Area Sample 6 | #Feature | #Feature Sample 6 | Start | End | PTM | AScore | Found By |
| --- | --- | --- | --- | --- | --- | --- | --- | --- | --- | --- | --- | --- | --- | --- | --- | --- | --- | --- | --- |
| K.SDSSDIEKR.A | Y | 40.54 | 1035.4832 | 9 | -16.22 | 518.7382 | 2 | 11.11 | 0.8571 | 0.8644 | 21120 | 2.69e3 | 1 | 1 | 96 | 104 |  |  | DB Search |
| total 1 peptides |  |  |  |  |  |  |  |  |  |  |  |  |  |  |  |  |  |  |  |
| --- | --- | --- | --- | --- | --- | --- | --- | --- | --- | --- | --- | --- | --- | --- | --- | --- | --- | --- | --- |

Best Unique PSM (Scan Sample\_4\_A1\_1\_3642.d:21120, m/z=518.7382, z=2, RT=11.11, ppm=-20.63):


TRINITY\_DN22240\_c0\_g1\_i1.p1

back to list

  

| Protein Coverage
| Supporting Peptides
| Best Unique PSM
|

Protein Coverage:

Supporting Peptides:

| Peptide | Uniq | -10lgP | Mass | Length | ppm | m/z | z | RT | 1/k0 Start | 1/k0 End | Precursor ID | Area Sample 6 | #Feature | #Feature Sample 6 | Start | End | PTM | AScore | Found By |
| --- | --- | --- | --- | --- | --- | --- | --- | --- | --- | --- | --- | --- | --- | --- | --- | --- | --- | --- | --- |
| K.YLADVVLTLDHTK.M | Y | 46.89 | 1486.8031 | 13 | 1.62 | 496.6069 | 3 | 18.76 | 0.8343 | 0.8416 | 67561 | 0 | 0 | 0 | 19 | 31 |  |  | DB Search |
| total 1 peptides |  |  |  |  |  |  |  |  |  |  |  |  |  |  |  |  |  |  |  |
| --- | --- | --- | --- | --- | --- | --- | --- | --- | --- | --- | --- | --- | --- | --- | --- | --- | --- | --- | --- |

Best Unique PSM (Scan Sample\_4\_A1\_1\_3642.d:67561, m/z=496.6069, z=3, RT=18.76, ppm=-2.78):


TRINITY\_DN123121\_c0\_g1\_i1.p1

back to list

  

| Protein Coverage
| Supporting Peptides
| Best Unique PSM
|

Protein Coverage:

Supporting Peptides:

| Peptide | Uniq | -10lgP | Mass | Length | ppm | m/z | z | RT | 1/k0 Start | 1/k0 End | Precursor ID | Area Sample 6 | #Feature | #Feature Sample 6 | Start | End | PTM | AScore | Found By |
| --- | --- | --- | --- | --- | --- | --- | --- | --- | --- | --- | --- | --- | --- | --- | --- | --- | --- | --- | --- |
| R.LTHQIEVTVSDREGK.S | Y | 46.51 | 1710.89 | 15 | 0.23 | 571.3016 | 3 | 12.83 | 0.8652 | 0.8725 | 31549 | 0 | 0 | 0 | 94 | 108 |  |  | DB Search |
| total 1 peptides |  |  |  |  |  |  |  |  |  |  |  |  |  |  |  |  |  |  |  |
| --- | --- | --- | --- | --- | --- | --- | --- | --- | --- | --- | --- | --- | --- | --- | --- | --- | --- | --- | --- |

Best Unique PSM (Scan Sample\_4\_A1\_1\_3642.d:31549, m/z=571.3016, z=3, RT=12.83, ppm=-4.17):


TRINITY\_DN3016\_c0\_g1\_i5.p1

back to list

  

| Protein Coverage
| Supporting Peptides
| Best Unique PSM
|

Protein Coverage:

Supporting Peptides:

| Peptide | Uniq | -10lgP | Mass | Length | ppm | m/z | z | RT | 1/k0 Start | 1/k0 End | Precursor ID | Area Sample 6 | #Feature | #Feature Sample 6 | Start | End | PTM | AScore | Found By |
| --- | --- | --- | --- | --- | --- | --- | --- | --- | --- | --- | --- | --- | --- | --- | --- | --- | --- | --- | --- |
| R.IVNTDLQEIKDIIK.G | Y | 45.77 | 1640.9348 | 14 | -1.11 | 547.9825 | 3 | 18.81 | 0.8177 | 0.825 | 67904 | 0 | 0 | 0 | 154 | 167 |  |  | DB Search |
| total 1 peptides |  |  |  |  |  |  |  |  |  |  |  |  |  |  |  |  |  |  |  |
| --- | --- | --- | --- | --- | --- | --- | --- | --- | --- | --- | --- | --- | --- | --- | --- | --- | --- | --- | --- |

Best Unique PSM (Scan Sample\_4\_A1\_1\_3642.d:67904, m/z=547.9825, z=3, RT=18.81, ppm=-5.51):


TRINITY\_DN554\_c0\_g1\_i3.p2

back to list

  

| Protein Coverage
| Supporting Peptides
| Best Unique PSM
|

Protein Coverage:

Supporting Peptides:

| Peptide | Uniq | -10lgP | Mass | Length | ppm | m/z | z | RT | 1/k0 Start | 1/k0 End | Precursor ID | Area Sample 6 | #Feature | #Feature Sample 6 | Start | End | PTM | AScore | Found By |
| --- | --- | --- | --- | --- | --- | --- | --- | --- | --- | --- | --- | --- | --- | --- | --- | --- | --- | --- | --- |
| K.TITLEVEPSDTIENVK.S | Y | 46.69 | 1786.92 | 16 | 0.39 | 894.4637 | 2 | 17.56 | 1.1442 | 1.1514 | 58994 | 8.78e2 | 1 | 1 | 12 | 27 |  |  | DB Search |
| total 1 peptides |  |  |  |  |  |  |  |  |  |  |  |  |  |  |  |  |  |  |  |
| --- | --- | --- | --- | --- | --- | --- | --- | --- | --- | --- | --- | --- | --- | --- | --- | --- | --- | --- | --- |

Best Unique PSM (Scan Sample\_4\_A1\_1\_3642.d:58994, m/z=894.4637, z=2, RT=17.56, ppm=-4.01):


TRINITY\_DN554\_c0\_g1\_i4.p1

back to list

  

| Protein Coverage
| Supporting Peptides
| Best Unique PSM
|

Protein Coverage:

Supporting Peptides:

| Peptide | Uniq | -10lgP | Mass | Length | ppm | m/z | z | RT | 1/k0 Start | 1/k0 End | Precursor ID | Area Sample 6 | #Feature | #Feature Sample 6 | Start | End | PTM | AScore | Found By |
| --- | --- | --- | --- | --- | --- | --- | --- | --- | --- | --- | --- | --- | --- | --- | --- | --- | --- | --- | --- |
| K.TITLEVEPSDTIENVK.S | Y | 46.69 | 1786.92 | 16 | 0.39 | 894.4637 | 2 | 17.56 | 1.1442 | 1.1514 | 58994 | 8.78e2 | 1 | 1 | 12 | 27 |  |  | DB Search |
| total 1 peptides |  |  |  |  |  |  |  |  |  |  |  |  |  |  |  |  |  |  |  |
| --- | --- | --- | --- | --- | --- | --- | --- | --- | --- | --- | --- | --- | --- | --- | --- | --- | --- | --- | --- |

Best Unique PSM (Scan Sample\_4\_A1\_1\_3642.d:58994, m/z=894.4637, z=2, RT=17.56, ppm=-4.01):


TRINITY\_DN3016\_c0\_g1\_i10.p1

back to list

  

| Protein Coverage
| Supporting Peptides
| Best Unique PSM
|

Protein Coverage:

Supporting Peptides:

| Peptide | Uniq | -10lgP | Mass | Length | ppm | m/z | z | RT | 1/k0 Start | 1/k0 End | Precursor ID | Area Sample 6 | #Feature | #Feature Sample 6 | Start | End | PTM | AScore | Found By |
| --- | --- | --- | --- | --- | --- | --- | --- | --- | --- | --- | --- | --- | --- | --- | --- | --- | --- | --- | --- |
| R.IVNTDLQEIKDIIK.G | Y | 45.77 | 1640.9348 | 14 | -1.11 | 547.9825 | 3 | 18.81 | 0.8177 | 0.825 | 67904 | 0 | 0 | 0 | 154 | 167 |  |  | DB Search |
| total 1 peptides |  |  |  |  |  |  |  |  |  |  |  |  |  |  |  |  |  |  |  |
| --- | --- | --- | --- | --- | --- | --- | --- | --- | --- | --- | --- | --- | --- | --- | --- | --- | --- | --- | --- |

Best Unique PSM (Scan Sample\_4\_A1\_1\_3642.d:67904, m/z=547.9825, z=3, RT=18.81, ppm=-5.51):


TRINITY\_DN4595\_c0\_g1\_i10.p1

back to list

  

| Protein Coverage
| Supporting Peptides
| Best Unique PSM
|

Protein Coverage:

Supporting Peptides:

| Peptide | Uniq | -10lgP | Mass | Length | ppm | m/z | z | RT | 1/k0 Start | 1/k0 End | Precursor ID | Area Sample 6 | #Feature | #Feature Sample 6 | Start | End | PTM | AScore | Found By |
| --- | --- | --- | --- | --- | --- | --- | --- | --- | --- | --- | --- | --- | --- | --- | --- | --- | --- | --- | --- |
| R.FC(+57.02)NNPAPSNGGR.M | Y | 47.24 | 1289.5571 | 12 | 0.71 | 645.7834 | 2 | 10.87 | 0.9416 | 0.9489 | 19820 | 2.76e2 | 1 | 1 | 745 | 756 | Carbamidomethylation |  | DB Search |
| R.SPFVIMFK.G | Y | 46.2 | 967.5201 | 8 | -0.36 | 484.765 | 2 | 19.87 | 0.8079 | 0.8153 | 75042 | 1.61e3 | 1 | 1 | 630 | 637 |  |  | DB Search |
| K.YTGAC(+57.02)NANGNTDK.T | Y | 46.13 | 1384.5677 | 13 | 10.57 | 693.2954 | 2 | 9.52 | 0.9428 | 0.9501 | 11788 | 0 | 0 | 0 | 1041 | 1053 | Carbamidomethylation |  | DB Search |
| K.GFEC(+57.02)IGLIEETETC(+57.02)K.L | Y | 45.99 | 1784.7961 | 15 | 4.3 | 893.4052 | 2 | 18.62 | 1.0981 | 1.1053 | 66554 | 4.49e2 | 1 | 1 | 93 | 107 | Carbamidomethylation, Carbamidomethylation |  | DB Search |
| R.SPFVIM(+15.99)FK.G | Y | 38.78 | 983.515 | 8 | -5.11 | 492.7601 | 2 | 18.60 | 0.8128 | 0.8201 | 66496 | 4.32e2 | 1 | 1 | 630 | 637 | Oxidation (M) | M6:Oxidation (M):1000 | DB Search |
| total 5 peptides |  |  |  |  |  |  |  |  |  |  |  |  |  |  |  |  |  |  |  |
| --- | --- | --- | --- | --- | --- | --- | --- | --- | --- | --- | --- | --- | --- | --- | --- | --- | --- | --- | --- |

Best Unique PSM (Scan Sample\_4\_A1\_1\_3642.d:19820, m/z=645.7834, z=2, RT=10.87, ppm=-3.69):


TRINITY\_DN4494\_c1\_g1\_i7.p1

back to list

  

| Protein Coverage
| Supporting Peptides
| Best Unique PSM
|

Protein Coverage:

Supporting Peptides:

| Peptide | Uniq | -10lgP | Mass | Length | ppm | m/z | z | RT | 1/k0 Start | 1/k0 End | Precursor ID | Area Sample 6 | #Feature | #Feature Sample 6 | Start | End | PTM | AScore | Found By |
| --- | --- | --- | --- | --- | --- | --- | --- | --- | --- | --- | --- | --- | --- | --- | --- | --- | --- | --- | --- |
| K.VDVGFILDSSGSLR.N | Y | 45.76 | 1463.762 | 14 | -1.21 | 732.8842 | 2 | 19.93 | 1.0102 | 1.0174 | 75559 | 4.99e2 | 1 | 1 | 307 | 320 |  |  | DB Search |
| total 1 peptides |  |  |  |  |  |  |  |  |  |  |  |  |  |  |  |  |  |  |  |
| --- | --- | --- | --- | --- | --- | --- | --- | --- | --- | --- | --- | --- | --- | --- | --- | --- | --- | --- | --- |

Best Unique PSM (Scan Sample\_4\_A1\_1\_3642.d:75559, m/z=732.8842, z=2, RT=19.93, ppm=-5.61):


TRINITY\_DN22492\_c0\_g1\_i2.p1

back to list

  

| Protein Coverage
| Supporting Peptides
| Best Unique PSM
|

Protein Coverage:

Supporting Peptides:

| Peptide | Uniq | -10lgP | Mass | Length | ppm | m/z | z | RT | 1/k0 Start | 1/k0 End | Precursor ID | Area Sample 6 | #Feature | #Feature Sample 6 | Start | End | PTM | AScore | Found By |
| --- | --- | --- | --- | --- | --- | --- | --- | --- | --- | --- | --- | --- | --- | --- | --- | --- | --- | --- | --- |
| K.KKLEPQAAPR.E | Y | 39.03 | 1136.6666 | 10 | -0.45 | 379.8943 | 3 | 9.66 | 0.7485 | 0.7559 | 12515 | 8.42e2 | 1 | 1 | 425 | 434 |  |  | DB Search |
| total 1 peptides |  |  |  |  |  |  |  |  |  |  |  |  |  |  |  |  |  |  |  |
| --- | --- | --- | --- | --- | --- | --- | --- | --- | --- | --- | --- | --- | --- | --- | --- | --- | --- | --- | --- |

Best Unique PSM (Scan Sample\_4\_A1\_1\_3642.d:12515, m/z=379.8943, z=3, RT=9.66, ppm=-4.86):


TRINITY\_DN982\_c0\_g1\_i3.p1

back to list

  

| Protein Coverage
| Supporting Peptides
| Best Unique PSM
|

Protein Coverage:

Supporting Peptides:

| Peptide | Uniq | -10lgP | Mass | Length | ppm | m/z | z | RT | 1/k0 Start | 1/k0 End | Precursor ID | Area Sample 6 | #Feature | #Feature Sample 6 | Start | End | PTM | AScore | Found By |
| --- | --- | --- | --- | --- | --- | --- | --- | --- | --- | --- | --- | --- | --- | --- | --- | --- | --- | --- | --- |
| K.YVAIYPSDHLTVNR.D | N | 47.28 | 1646.8416 | 14 | 3.15 | 824.4271 | 2 | 15.69 | 1.1053 | 1.1126 | 48025 | 9.15e2 | 1 | 1 | 6254 | 6267 |  |  | DB Search |
| R.YTSC(+57.02)QSVTVVASDR.S | N | 47.27 | 1571.725 | 14 | 6.1 | 786.8711 | 2 | 13.47 | 1.05 | 1.0572 | 35313 | 0 | 0 | 0 | 9363 | 9376 | Carbamidomethylation |  | DB Search |
| K.AEGPTSTGVTEVIVR.V | Y | 47.19 | 1514.794 | 15 | -18.54 | 758.3869 | 2 | 15.55 | 1.0102 | 1.0174 | 47407 | 0 | 0 | 0 | 3358 | 3372 |  |  | DB Search |
| R.LVQNTPHNSIVTK.I | N | 47.18 | 1449.794 | 13 | -3.54 | 725.8985 | 2 | 11.74 | 1.0568 | 1.064 | 24940 | 0 | 0 | 0 | 8569 | 8581 |  |  | DB Search |
| K.AQLTLTIFLHK.S | N | 46.99 | 1283.7601 | 11 | 3.95 | 428.9271 | 3 | 19.57 | 0.7489 | 0.7563 | 73093 | 6.45e2 | 1 | 1 | 4949 | 4959 |  |  | DB Search |
| R.FYSFEVQVIDTNNGK.T | Y | 46.82 | 1759.8417 | 15 | 2.16 | 880.9261 | 2 | 18.83 | 1.1005 | 1.1078 | 67916 | 4.09e2 | 1 | 1 | 3148 | 3162 |  |  | DB Search |
| K.APIGQSIVTVSAR.D | Y | 46.62 | 1297.7354 | 13 | -6.54 | 649.8679 | 2 | 14.25 | 0.967 | 0.9743 | 39958 | 0 | 0 | 0 | 4029 | 4041 |  |  | DB Search |
| K.LLTTEDDFILR.R | N | 46.58 | 1334.7081 | 11 | 1.62 | 668.3595 | 2 | 19.11 | 0.9735 | 0.9808 | 69947 | 3e2 | 1 | 1 | 6897 | 6907 |  |  | DB Search |
| K.VLLSQQIFYDKK.N | N | 46.51 | 1480.8289 | 12 | 0.37 | 494.6149 | 3 | 16.93 | 0.7616 | 0.7689 | 55019 | 1.05e4 | 2 | 2 | 4919 | 4930 |  |  | DB Search |
| R.DRHVLTIQSR.G | N | 46.4 | 1223.6734 | 10 | 4.59 | 408.8985 | 3 | 12.00 | 0.7559 | 0.7632 | 26560 | 0 | 0 | 0 | 5030 | 5039 |  |  | DB Search |
| R.IQVLKDPDFEFTR.S | N | 46.31 | 1606.8355 | 13 | -1.78 | 804.42 | 2 | 17.65 | 1.0801 | 1.0873 | 59732 | 1.21e3 | 1 | 1 | 6167 | 6179 |  |  | DB Search |
| K.AVDRGNPPQDSVNK.A | N | 45.95 | 1495.7379 | 14 | 6.38 | 499.5876 | 3 | 10.19 | 0.7868 | 0.7941 | 15626 | 0 | 0 | 0 | 5139 | 5152 |  |  | DB Search |
| R.TNNALDYEAKR.Q | N | 45.94 | 1293.6313 | 11 | 0.07 | 647.8201 | 2 | 12.09 | 0.9618 | 0.9691 | 27037 | 8.23e2 | 1 | 1 | 7725 | 7735 |  |  | DB Search |
| K.TGAISLLASVDFETDRTHTAVVK.A | Y | 44.6 | 2430.2754 | 23 | -2.1 | 811.0938 | 3 | 18.98 | 1.0427 | 1.05 | 69102 | 6.22e2 | 1 | 1 | 3335 | 3357 |  |  | DB Search |
| R.ITTSQILDHEQQK.L | N | 43.4 | 1539.7893 | 13 | 3.46 | 770.9012 | 2 | 13.97 | 1.0949 | 1.1021 | 38279 | 0 | 0 | 0 | 5434 | 5446 |  |  | DB Search |
| total 15 peptides |  |  |  |  |  |  |  |  |  |  |  |  |  |  |  |  |  |  |  |
| --- | --- | --- | --- | --- | --- | --- | --- | --- | --- | --- | --- | --- | --- | --- | --- | --- | --- | --- | --- |

Best Unique PSM (Scan Sample\_4\_A1\_1\_3642.d:47407, m/z=757.369, z=2, RT=15.57, ppm=-22.94):


TRINITY\_DN4494\_c1\_g1\_i10.p1

back to list

  

| Protein Coverage
| Supporting Peptides
| Best Unique PSM
|

Protein Coverage:

Supporting Peptides:

| Peptide | Uniq | -10lgP | Mass | Length | ppm | m/z | z | RT | 1/k0 Start | 1/k0 End | Precursor ID | Area Sample 6 | #Feature | #Feature Sample 6 | Start | End | PTM | AScore | Found By |
| --- | --- | --- | --- | --- | --- | --- | --- | --- | --- | --- | --- | --- | --- | --- | --- | --- | --- | --- | --- |
| K.VDVGFILDSSGSLR.N | Y | 45.76 | 1463.762 | 14 | -1.21 | 732.8842 | 2 | 19.93 | 1.0102 | 1.0174 | 75559 | 4.99e2 | 1 | 1 | 318 | 331 |  |  | DB Search |
| total 1 peptides |  |  |  |  |  |  |  |  |  |  |  |  |  |  |  |  |  |  |  |
| --- | --- | --- | --- | --- | --- | --- | --- | --- | --- | --- | --- | --- | --- | --- | --- | --- | --- | --- | --- |

Best Unique PSM (Scan Sample\_4\_A1\_1\_3642.d:75559, m/z=732.8842, z=2, RT=19.93, ppm=-5.61):


TRINITY\_DN160\_c0\_g1\_i1.p1

back to list

  

| Protein Coverage
| Supporting Peptides
| Best Unique PSM
|

Protein Coverage:

Supporting Peptides:

| Peptide | Uniq | -10lgP | Mass | Length | ppm | m/z | z | RT | 1/k0 Start | 1/k0 End | Precursor ID | Area Sample 6 | #Feature | #Feature Sample 6 | Start | End | PTM | AScore | Found By |
| --- | --- | --- | --- | --- | --- | --- | --- | --- | --- | --- | --- | --- | --- | --- | --- | --- | --- | --- | --- |
| K.DLEFKFR.E | Y | 44.49 | 953.497 | 7 | 5.18 | 477.7562 | 2 | 16.82 | 0.8425 | 0.8498 | 54418 | 0 | 0 | 0 | 1031 | 1037 |  |  | DB Search |
| total 1 peptides |  |  |  |  |  |  |  |  |  |  |  |  |  |  |  |  |  |  |  |
| --- | --- | --- | --- | --- | --- | --- | --- | --- | --- | --- | --- | --- | --- | --- | --- | --- | --- | --- | --- |

Best Unique PSM (Scan Sample\_4\_A1\_1\_3642.d:54418, m/z=476.7815, z=2, RT=16.84, ppm=0.77):


TRINITY\_DN250\_c0\_g1\_i5.p1

back to list

  

| Protein Coverage
| Supporting Peptides
| Best Unique PSM
|

Protein Coverage:

Supporting Peptides:

| Peptide | Uniq | -10lgP | Mass | Length | ppm | m/z | z | RT | 1/k0 Start | 1/k0 End | Precursor ID | Area Sample 6 | #Feature | #Feature Sample 6 | Start | End | PTM | AScore | Found By |
| --- | --- | --- | --- | --- | --- | --- | --- | --- | --- | --- | --- | --- | --- | --- | --- | --- | --- | --- | --- |
| R.AVC(+57.02)M(+15.99)LSNTTAIAEAWAR.L | Y | 43.01 | 1879.892 | 17 | 2.28 | 940.9513 | 2 | 18.79 | 1.147 | 1.1542 | 67649 | 2.71e2 | 1 | 1 | 374 | 390 | Carbamidomethylation, Oxidation (M) | M4:Oxidation (M):1000 | DB Search |
| K.VGINYQPPTVVPGGDLAK.V | Y | 37.08 | 1823.9781 | 18 | 0.87 | 912.9931 | 2 | 17.39 | 1.1218 | 1.129 | 57959 | 0 | 0 | 0 | 353 | 370 |  |  | DB Search |
| total 2 peptides |  |  |  |  |  |  |  |  |  |  |  |  |  |  |  |  |  |  |  |
| --- | --- | --- | --- | --- | --- | --- | --- | --- | --- | --- | --- | --- | --- | --- | --- | --- | --- | --- | --- |

Best Unique PSM (Scan Sample\_4\_A1\_1\_3642.d:67649, m/z=940.9513, z=2, RT=18.79, ppm=-2.12):


TRINITY\_DN602\_c0\_g1\_i3.p1

back to list

  

| Protein Coverage
| Supporting Peptides
| Best Unique PSM
|

Protein Coverage:

Supporting Peptides:

| Peptide | Uniq | -10lgP | Mass | Length | ppm | m/z | z | RT | 1/k0 Start | 1/k0 End | Precursor ID | Area Sample 6 | #Feature | #Feature Sample 6 | Start | End | PTM | AScore | Found By |
| --- | --- | --- | --- | --- | --- | --- | --- | --- | --- | --- | --- | --- | --- | --- | --- | --- | --- | --- | --- |
| K.EGFYVHEALAAGIVK.K | Y | 47.06 | 1602.8405 | 15 | -1.99 | 802.4224 | 2 | 17.69 | 1.0837 | 1.0909 | 59893 | 9.17e2 | 1 | 1 | 443 | 457 |  |  | DB Search |
| K.LLTSPAPSSGSLM(+15.99)ALALK.I | Y | 46.68 | 1771.9753 | 18 | 0.26 | 886.9913 | 2 | 18.27 | 1.1354 | 1.1426 | 64205 | 0 | 0 | 0 | 550 | 567 | Oxidation (M) | M13:Oxidation (M):1000 | DB Search |
| R.AYDFRETLPK.K | Y | 45.42 | 1238.6295 | 10 | 5.77 | 620.3229 | 2 | 15.38 | 0.9497 | 0.957 | 46320 | 7.87e2 | 1 | 1 | 386 | 395 |  |  | DB Search |
| K.HGADLFYR.G | Y | 37.07 | 977.4719 | 8 | 0.29 | 489.7412 | 2 | 16.16 | 0.8372 | 0.8445 | 50704 | 4.61e2 | 1 | 1 | 500 | 507 |  |  | DB Search |
| total 4 peptides |  |  |  |  |  |  |  |  |  |  |  |  |  |  |  |  |  |  |  |
| --- | --- | --- | --- | --- | --- | --- | --- | --- | --- | --- | --- | --- | --- | --- | --- | --- | --- | --- | --- |

Best Unique PSM (Scan Sample\_4\_A1\_1\_3642.d:59893, m/z=802.4224, z=2, RT=17.69, ppm=-6.39):


TRINITY\_DN31399\_c0\_g1\_i3.p1

back to list

  

| Protein Coverage
| Supporting Peptides
| Best Unique PSM
|

Protein Coverage:

Supporting Peptides:

| Peptide | Uniq | -10lgP | Mass | Length | ppm | m/z | z | RT | 1/k0 Start | 1/k0 End | Precursor ID | Area Sample 6 | #Feature | #Feature Sample 6 | Start | End | PTM | AScore | Found By |
| --- | --- | --- | --- | --- | --- | --- | --- | --- | --- | --- | --- | --- | --- | --- | --- | --- | --- | --- | --- |
| K.DTLVFEIDEIRK.S | Y | 45.93 | 1476.7824 | 12 | -0.51 | 739.3948 | 2 | 18.60 | 1.0343 | 1.0415 | 66411 | 0 | 0 | 0 | 390 | 401 |  |  | DB Search |
| total 1 peptides |  |  |  |  |  |  |  |  |  |  |  |  |  |  |  |  |  |  |  |
| --- | --- | --- | --- | --- | --- | --- | --- | --- | --- | --- | --- | --- | --- | --- | --- | --- | --- | --- | --- |

Best Unique PSM (Scan Sample\_4\_A1\_1\_3642.d:66411, m/z=739.3948, z=2, RT=18.60, ppm=-4.91):


TRINITY\_DN1936\_c0\_g1\_i4.p1

back to list

  

| Protein Coverage
| Supporting Peptides
| Best Unique PSM
|

Protein Coverage:

Supporting Peptides:

| Peptide | Uniq | -10lgP | Mass | Length | ppm | m/z | z | RT | 1/k0 Start | 1/k0 End | Precursor ID | Area Sample 6 | #Feature | #Feature Sample 6 | Start | End | PTM | AScore | Found By |
| --- | --- | --- | --- | --- | --- | --- | --- | --- | --- | --- | --- | --- | --- | --- | --- | --- | --- | --- | --- |
| K.AGDIAILESQQFNAVGGK.T | Y | 47.14 | 1816.9319 | 18 | -16.1 | 909.4546 | 2 | 18.91 | 1.1845 | 1.1917 | 68601 | 1.41e3 | 1 | 1 | 1002 | 1019 |  |  | DB Search |
| total 1 peptides |  |  |  |  |  |  |  |  |  |  |  |  |  |  |  |  |  |  |  |
| --- | --- | --- | --- | --- | --- | --- | --- | --- | --- | --- | --- | --- | --- | --- | --- | --- | --- | --- | --- |

Best Unique PSM (Scan Sample\_4\_A1\_1\_3642.d:68601, m/z=909.4546, z=2, RT=18.91, ppm=-20.5):


TRINITY\_DN118327\_c0\_g1\_i1.p1

back to list

  

| Protein Coverage
| Supporting Peptides
| Best Unique PSM
|

Protein Coverage:

Supporting Peptides:

| Peptide | Uniq | -10lgP | Mass | Length | ppm | m/z | z | RT | 1/k0 Start | 1/k0 End | Precursor ID | Area Sample 6 | #Feature | #Feature Sample 6 | Start | End | PTM | AScore | Found By |
| --- | --- | --- | --- | --- | --- | --- | --- | --- | --- | --- | --- | --- | --- | --- | --- | --- | --- | --- | --- |
| R.LASDLIILK.G | Y | 37.83 | 984.6219 | 9 | 0.65 | 493.3164 | 2 | 18.35 | 0.8287 | 0.836 | 64664 | 5.03e2 | 1 | 1 | 132 | 140 |  |  | DB Search |
| total 1 peptides |  |  |  |  |  |  |  |  |  |  |  |  |  |  |  |  |  |  |  |
| --- | --- | --- | --- | --- | --- | --- | --- | --- | --- | --- | --- | --- | --- | --- | --- | --- | --- | --- | --- |

Best Unique PSM (Scan Sample\_4\_A1\_1\_3642.d:64664, m/z=493.3164, z=2, RT=18.35, ppm=-3.76):


TRINITY\_DN477\_c0\_g1\_i7.p1

back to list

  

| Protein Coverage
| Supporting Peptides
| Best Unique PSM
|

Protein Coverage:

Supporting Peptides:

| Peptide | Uniq | -10lgP | Mass | Length | ppm | m/z | z | RT | 1/k0 Start | 1/k0 End | Precursor ID | Area Sample 6 | #Feature | #Feature Sample 6 | Start | End | PTM | AScore | Found By |
| --- | --- | --- | --- | --- | --- | --- | --- | --- | --- | --- | --- | --- | --- | --- | --- | --- | --- | --- | --- |
| R.NDVNNPPQAR.R | Y | 47.24 | 1123.537 | 10 | 0.77 | 562.7737 | 2 | 9.95 | 0.866 | 0.8733 | 13858 | 3.59e3 | 2 | 2 | 1049 | 1058 |  |  | DB Search |
| R.ILQC(+57.02)NQYANVEEAR.N | Y | 47.19 | 1706.8046 | 14 | 1.57 | 854.4072 | 2 | 14.32 | 1.0821 | 1.0893 | 40295 | 6.26e3 | 1 | 1 | 86 | 99 | Carbamidomethylation |  | DB Search |
| K.DKNILVLTLGVGPQVNR.N | Y | 46.87 | 1835.0628 | 17 | 2.5 | 612.6937 | 3 | 19.53 | 0.8408 | 0.8481 | 72572 | 1.06e4 | 3 | 3 | 168 | 184 |  |  | DB Search |
| R.SQSAFPTRR.R | Y | 45.57 | 1048.5414 | 9 | 1.94 | 525.2767 | 2 | 11.05 | 0.8514 | 0.8587 | 20691 | 6.92e3 | 1 | 1 | 856 | 864 |  |  | DB Search |
| total 4 peptides |  |  |  |  |  |  |  |  |  |  |  |  |  |  |  |  |  |  |  |
| --- | --- | --- | --- | --- | --- | --- | --- | --- | --- | --- | --- | --- | --- | --- | --- | --- | --- | --- | --- |

Best Unique PSM (Scan Sample\_4\_A1\_1\_3642.d:13858, m/z=562.7737, z=2, RT=9.95, ppm=-3.64):


TRINITY\_DN6428\_c0\_g1\_i2.p1

back to list

  

| Protein Coverage
| Supporting Peptides
| Best Unique PSM
|

Protein Coverage:

Supporting Peptides:

| Peptide | Uniq | -10lgP | Mass | Length | ppm | m/z | z | RT | 1/k0 Start | 1/k0 End | Precursor ID | Area Sample 6 | #Feature | #Feature Sample 6 | Start | End | PTM | AScore | Found By |
| --- | --- | --- | --- | --- | --- | --- | --- | --- | --- | --- | --- | --- | --- | --- | --- | --- | --- | --- | --- |
| R.SC(+57.02)ENPKPLNGGK.Q | Y | 46.61 | 1299.6241 | 12 | 2.25 | 650.8179 | 2 | 11.07 | 0.9505 | 0.9578 | 20781 | 2.84e3 | 1 | 1 | 181 | 192 | Carbamidomethylation |  | DB Search |
| total 1 peptides |  |  |  |  |  |  |  |  |  |  |  |  |  |  |  |  |  |  |  |
| --- | --- | --- | --- | --- | --- | --- | --- | --- | --- | --- | --- | --- | --- | --- | --- | --- | --- | --- | --- |

Best Unique PSM (Scan Sample\_4\_A1\_1\_3642.d:20781, m/z=650.8179, z=2, RT=11.07, ppm=-2.16):


TRINITY\_DN477\_c0\_g1\_i6.p1

back to list

  

| Protein Coverage
| Supporting Peptides
| Best Unique PSM
|

Protein Coverage:

Supporting Peptides:

| Peptide | Uniq | -10lgP | Mass | Length | ppm | m/z | z | RT | 1/k0 Start | 1/k0 End | Precursor ID | Area Sample 6 | #Feature | #Feature Sample 6 | Start | End | PTM | AScore | Found By |
| --- | --- | --- | --- | --- | --- | --- | --- | --- | --- | --- | --- | --- | --- | --- | --- | --- | --- | --- | --- |
| R.NDVNNPPQAR.R | Y | 47.24 | 1123.537 | 10 | 0.77 | 562.7737 | 2 | 9.95 | 0.866 | 0.8733 | 13858 | 3.59e3 | 2 | 2 | 86 | 95 |  |  | DB Search |
| total 1 peptides |  |  |  |  |  |  |  |  |  |  |  |  |  |  |  |  |  |  |  |
| --- | --- | --- | --- | --- | --- | --- | --- | --- | --- | --- | --- | --- | --- | --- | --- | --- | --- | --- | --- |

Best Unique PSM (Scan Sample\_4\_A1\_1\_3642.d:13858, m/z=562.7737, z=2, RT=9.95, ppm=-3.64):


TRINITY\_DN3036\_c0\_g1\_i2.p2

back to list

  

| Protein Coverage
| Supporting Peptides
| Best Unique PSM
|

Protein Coverage:

Supporting Peptides:

| Peptide | Uniq | -10lgP | Mass | Length | ppm | m/z | z | RT | 1/k0 Start | 1/k0 End | Precursor ID | Area Sample 6 | #Feature | #Feature Sample 6 | Start | End | PTM | AScore | Found By |
| --- | --- | --- | --- | --- | --- | --- | --- | --- | --- | --- | --- | --- | --- | --- | --- | --- | --- | --- | --- |
| R.AGHGAVTSPQTQR.G | Y | 45.07 | 1308.6534 | 13 | 6.45 | 437.226 | 3 | 9.72 | 0.7041 | 0.7114 | 12869 | 7.9e2 | 1 | 1 | 309 | 321 |  |  | DB Search |
| total 1 peptides |  |  |  |  |  |  |  |  |  |  |  |  |  |  |  |  |  |  |  |
| --- | --- | --- | --- | --- | --- | --- | --- | --- | --- | --- | --- | --- | --- | --- | --- | --- | --- | --- | --- |

Best Unique PSM (Scan Sample\_4\_A1\_1\_3642.d:12869, m/z=437.226, z=3, RT=9.72, ppm=2.05):


TRINITY\_DN140702\_c0\_g1\_i1.p1

back to list

  

| Protein Coverage
| Supporting Peptides
| Best Unique PSM
|

Protein Coverage:

Supporting Peptides:

| Peptide | Uniq | -10lgP | Mass | Length | ppm | m/z | z | RT | 1/k0 Start | 1/k0 End | Precursor ID | Area Sample 6 | #Feature | #Feature Sample 6 | Start | End | PTM | AScore | Found By |
| --- | --- | --- | --- | --- | --- | --- | --- | --- | --- | --- | --- | --- | --- | --- | --- | --- | --- | --- | --- |
| R.LKLQMFDWDR.I | Y | 47.4 | 1350.6754 | 10 | 2.3 | 676.3436 | 2 | 19.46 | 0.9727 | 0.98 | 71904 | 6.52e3 | 1 | 1 | 463 | 472 |  |  | DB Search |
| R.LALHVLNNEDIIGHR.L | Y | 47.14 | 1712.9322 | 15 | 3.94 | 857.473 | 2 | 16.50 | 1.1362 | 1.1434 | 52619 | 5.01e3 | 2 | 2 | 1702 | 1716 |  |  | DB Search |
| R.FVFPFK.Y | Y | 46.92 | 783.4319 | 6 | -0.19 | 392.7214 | 2 | 19.66 | 0.7722 | 0.7795 | 73690 | 7.97e2 | 1 | 1 | 1828 | 1833 |  |  | DB Search |
| K.IEDFEESPPTDPHIGPVEQR.L | Y | 46.7 | 2291.0705 | 20 | 6.71 | 764.6992 | 3 | 15.81 | 0.9303 | 0.9376 | 48644 | 1.97e3 | 1 | 1 | 1682 | 1701 |  |  | DB Search |
| R.SDELLNLKEEASTLR.E | Y | 46.08 | 1716.8894 | 15 | 3.13 | 859.4509 | 2 | 17.75 | 1.1114 | 1.1186 | 60489 | 0 | 0 | 0 | 729 | 743 |  |  | DB Search |
| K.TGVEILNWGVR.N | Y | 45.34 | 1242.672 | 11 | 1.79 | 622.3417 | 2 | 19.66 | 0.9065 | 0.9137 | 73704 | 5.55e2 | 1 | 1 | 1288 | 1298 |  |  | DB Search |
| total 6 peptides |  |  |  |  |  |  |  |  |  |  |  |  |  |  |  |  |  |  |  |
| --- | --- | --- | --- | --- | --- | --- | --- | --- | --- | --- | --- | --- | --- | --- | --- | --- | --- | --- | --- |

Best Unique PSM (Scan Sample\_4\_A1\_1\_3642.d:71904, m/z=676.3436, z=2, RT=19.46, ppm=-2.1):


TRINITY\_DN1194\_c0\_g1\_i1.p1

back to list

  

| Protein Coverage
| Supporting Peptides
| Best Unique PSM
|

Protein Coverage:

Supporting Peptides:

| Peptide | Uniq | -10lgP | Mass | Length | ppm | m/z | z | RT | 1/k0 Start | 1/k0 End | Precursor ID | Area Sample 6 | #Feature | #Feature Sample 6 | Start | End | PTM | AScore | Found By |
| --- | --- | --- | --- | --- | --- | --- | --- | --- | --- | --- | --- | --- | --- | --- | --- | --- | --- | --- | --- |
| K.IVNAEWNILYEKLEK.I | Y | 43.15 | 1860.9985 | 15 | 0.82 | 621.3379 | 3 | 19.64 | 0.8947 | 0.902 | 73650 | 0 | 0 | 0 | 274 | 288 |  |  | DB Search |
| total 1 peptides |  |  |  |  |  |  |  |  |  |  |  |  |  |  |  |  |  |  |  |
| --- | --- | --- | --- | --- | --- | --- | --- | --- | --- | --- | --- | --- | --- | --- | --- | --- | --- | --- | --- |

Best Unique PSM (Scan Sample\_4\_A1\_1\_3642.d:73650, m/z=621.3379, z=3, RT=19.64, ppm=-3.58):


TRINITY\_DN79126\_c0\_g1\_i1.p1

back to list

  

| Protein Coverage
| Supporting Peptides
| Best Unique PSM
|

Protein Coverage:

Supporting Peptides:

| Peptide | Uniq | -10lgP | Mass | Length | ppm | m/z | z | RT | 1/k0 Start | 1/k0 End | Precursor ID | Area Sample 6 | #Feature | #Feature Sample 6 | Start | End | PTM | AScore | Found By |
| --- | --- | --- | --- | --- | --- | --- | --- | --- | --- | --- | --- | --- | --- | --- | --- | --- | --- | --- | --- |
| R.AFLIEEQKLVVR.V | Y | 47.25 | 1443.8449 | 12 | -1.32 | 722.9256 | 2 | 17.51 | 1.0452 | 1.0524 | 58803 | 0 | 0 | 0 | 92 | 103 |  |  | DB Search |
| total 1 peptides |  |  |  |  |  |  |  |  |  |  |  |  |  |  |  |  |  |  |  |
| --- | --- | --- | --- | --- | --- | --- | --- | --- | --- | --- | --- | --- | --- | --- | --- | --- | --- | --- | --- |

Best Unique PSM (Scan Sample\_4\_A1\_1\_3642.d:58803, m/z=722.9256, z=2, RT=17.51, ppm=-5.72):


TRINITY\_DN1071\_c0\_g1\_i2.p1

back to list

  

| Protein Coverage
| Supporting Peptides
| Best Unique PSM
|

Protein Coverage:

Supporting Peptides:

| Peptide | Uniq | -10lgP | Mass | Length | ppm | m/z | z | RT | 1/k0 Start | 1/k0 End | Precursor ID | Area Sample 6 | #Feature | #Feature Sample 6 | Start | End | PTM | AScore | Found By |
| --- | --- | --- | --- | --- | --- | --- | --- | --- | --- | --- | --- | --- | --- | --- | --- | --- | --- | --- | --- |
| K.AVQIHPFNDVSVGDPK.V | Y | 47.19 | 1721.8736 | 16 | 3.05 | 861.9429 | 2 | 15.94 | 1.1162 | 1.1234 | 49403 | 4.56e3 | 1 | 1 | 293 | 308 |  |  | DB Search |
| total 1 peptides |  |  |  |  |  |  |  |  |  |  |  |  |  |  |  |  |  |  |  |
| --- | --- | --- | --- | --- | --- | --- | --- | --- | --- | --- | --- | --- | --- | --- | --- | --- | --- | --- | --- |

Best Unique PSM (Scan Sample\_4\_A1\_1\_3642.d:49403, m/z=861.9429, z=2, RT=15.94, ppm=-1.35):


TRINITY\_DN99404\_c0\_g1\_i1.p1

back to list

  

| Protein Coverage
| Supporting Peptides
| Best Unique PSM
|

Protein Coverage:

Supporting Peptides:

| Peptide | Uniq | -10lgP | Mass | Length | ppm | m/z | z | RT | 1/k0 Start | 1/k0 End | Precursor ID | Area Sample 6 | #Feature | #Feature Sample 6 | Start | End | PTM | AScore | Found By |
| --- | --- | --- | --- | --- | --- | --- | --- | --- | --- | --- | --- | --- | --- | --- | --- | --- | --- | --- | --- |
| K.LQTDIHGVALEITK.L | Y | 45.64 | 1536.8511 | 14 | 2.93 | 769.4317 | 2 | 16.63 | 1.0556 | 1.0628 | 53420 | 0 | 0 | 0 | 24 | 37 |  |  | DB Search |
| total 1 peptides |  |  |  |  |  |  |  |  |  |  |  |  |  |  |  |  |  |  |  |
| --- | --- | --- | --- | --- | --- | --- | --- | --- | --- | --- | --- | --- | --- | --- | --- | --- | --- | --- | --- |

Best Unique PSM (Scan Sample\_4\_A1\_1\_3642.d:53420, m/z=769.4317, z=2, RT=16.63, ppm=-1.48):


TRINITY\_DN2477\_c0\_g1\_i8.p1

back to list

  

| Protein Coverage
| Supporting Peptides
| Best Unique PSM
|

Protein Coverage:

Supporting Peptides:

| Peptide | Uniq | -10lgP | Mass | Length | ppm | m/z | z | RT | 1/k0 Start | 1/k0 End | Precursor ID | Area Sample 6 | #Feature | #Feature Sample 6 | Start | End | PTM | AScore | Found By |
| --- | --- | --- | --- | --- | --- | --- | --- | --- | --- | --- | --- | --- | --- | --- | --- | --- | --- | --- | --- |
| R.VAPEEHPVLLTEAPLNPK.A | Y | 46.46 | 1953.0571 | 18 | 9.42 | 977.5407 | 2 | 16.61 | 1.2085 | 1.2157 | 53280 | 0 | 0 | 0 | 29 | 46 |  |  | DB Search |
| total 1 peptides |  |  |  |  |  |  |  |  |  |  |  |  |  |  |  |  |  |  |  |
| --- | --- | --- | --- | --- | --- | --- | --- | --- | --- | --- | --- | --- | --- | --- | --- | --- | --- | --- | --- |

Best Unique PSM (Scan Sample\_4\_A1\_1\_3642.d:53280, m/z=977.5407, z=2, RT=16.61, ppm=5.02):


TRINITY\_DN585\_c0\_g1\_i8.p1

back to list

  

| Protein Coverage
| Supporting Peptides
| Best Unique PSM
|

Protein Coverage:

Supporting Peptides:

| Peptide | Uniq | -10lgP | Mass | Length | ppm | m/z | z | RT | 1/k0 Start | 1/k0 End | Precursor ID | Area Sample 6 | #Feature | #Feature Sample 6 | Start | End | PTM | AScore | Found By |
| --- | --- | --- | --- | --- | --- | --- | --- | --- | --- | --- | --- | --- | --- | --- | --- | --- | --- | --- | --- |
| K.TITLEVEPSDTIENVK.S | Y | 46.69 | 1786.92 | 16 | 0.39 | 894.4637 | 2 | 17.56 | 1.1442 | 1.1514 | 58994 | 8.78e2 | 1 | 1 | 12 | 27 |  |  | DB Search |
| total 1 peptides |  |  |  |  |  |  |  |  |  |  |  |  |  |  |  |  |  |  |  |
| --- | --- | --- | --- | --- | --- | --- | --- | --- | --- | --- | --- | --- | --- | --- | --- | --- | --- | --- | --- |

Best Unique PSM (Scan Sample\_4\_A1\_1\_3642.d:58994, m/z=894.4637, z=2, RT=17.56, ppm=-4.01):


TRINITY\_DN602\_c0\_g1\_i4.p1

back to list

  

| Protein Coverage
| Supporting Peptides
| Best Unique PSM
|

Protein Coverage:

Supporting Peptides:

| Peptide | Uniq | -10lgP | Mass | Length | ppm | m/z | z | RT | 1/k0 Start | 1/k0 End | Precursor ID | Area Sample 6 | #Feature | #Feature Sample 6 | Start | End | PTM | AScore | Found By |
| --- | --- | --- | --- | --- | --- | --- | --- | --- | --- | --- | --- | --- | --- | --- | --- | --- | --- | --- | --- |
| K.EGFYVHEALAAGIVK.K | Y | 47.06 | 1602.8405 | 15 | -1.99 | 802.4224 | 2 | 17.69 | 1.0837 | 1.0909 | 59893 | 9.17e2 | 1 | 1 | 144 | 158 |  |  | DB Search |
| K.LLTSPAPSSGSLM(+15.99)ALALK.I | Y | 46.68 | 1771.9753 | 18 | 0.26 | 886.9913 | 2 | 18.27 | 1.1354 | 1.1426 | 64205 | 0 | 0 | 0 | 251 | 268 | Oxidation (M) | M13:Oxidation (M):1000 | DB Search |
| R.AYDFRETLPK.K | Y | 45.42 | 1238.6295 | 10 | 5.77 | 620.3229 | 2 | 15.38 | 0.9497 | 0.957 | 46320 | 7.87e2 | 1 | 1 | 87 | 96 |  |  | DB Search |
| K.HGADLFYR.G | Y | 37.07 | 977.4719 | 8 | 0.29 | 489.7412 | 2 | 16.16 | 0.8372 | 0.8445 | 50704 | 4.61e2 | 1 | 1 | 201 | 208 |  |  | DB Search |
| total 4 peptides |  |  |  |  |  |  |  |  |  |  |  |  |  |  |  |  |  |  |  |
| --- | --- | --- | --- | --- | --- | --- | --- | --- | --- | --- | --- | --- | --- | --- | --- | --- | --- | --- | --- |

Best Unique PSM (Scan Sample\_4\_A1\_1\_3642.d:59893, m/z=802.4224, z=2, RT=17.69, ppm=-6.39):


TRINITY\_DN1616\_c0\_g1\_i3.p1

back to list

  

| Protein Coverage
| Supporting Peptides
| Best Unique PSM
|

Protein Coverage:

Supporting Peptides:

| Peptide | Uniq | -10lgP | Mass | Length | ppm | m/z | z | RT | 1/k0 Start | 1/k0 End | Precursor ID | Area Sample 6 | #Feature | #Feature Sample 6 | Start | End | PTM | AScore | Found By |
| --- | --- | --- | --- | --- | --- | --- | --- | --- | --- | --- | --- | --- | --- | --- | --- | --- | --- | --- | --- |
| K.GC(+57.02)STC(+57.02)GC(+57.02)NGAGTNNGVK.S | Y | 47.18 | 1712.6665 | 17 | 14.87 | 857.3495 | 2 | 9.81 | 1.0411 | 1.0484 | 13314 | 0 | 0 | 0 | 732 | 748 | Carbamidomethylation, Carbamidomethylation, Carbamidomethylation |  | DB Search |
| R.YVKPGVNIINIR.D | Y | 46.71 | 1384.819 | 12 | 3.8 | 693.4164 | 2 | 16.18 | 1.0009 | 1.0081 | 50795 | 7.11e2 | 1 | 1 | 1217 | 1228 |  |  | DB Search |
| K.VVDTEANDLTEEVKK.T | Y | 46.47 | 1688.8468 | 15 | 4.23 | 845.4305 | 2 | 14.16 | 1.1298 | 1.137 | 39294 | 3.14e2 | 1 | 1 | 2542 | 2556 |  |  | DB Search |
| K.YVVMLQYYHDSDTAK.Y | Y | 46.08 | 1831.8451 | 15 | 8.86 | 916.9339 | 2 | 16.78 | 1.1825 | 1.1897 | 54165 | 6.38e1 | 1 | 1 | 1071 | 1085 |  |  | DB Search |
| K.GFVAVNDQQPSVVVSINVPK.T | Y | 43.72 | 2096.1266 | 20 | 10.57 | 1049.077 | 2 | 18.41 | 1.2185 | 1.2256 | 65167 | 0 | 0 | 0 | 865 | 884 |  |  | DB Search |
| K.VVAEC(+57.02)NNGGGR.F | Y | 40.05 | 1131.5091 | 11 | 3.26 | 566.7612 | 2 | 9.40 | 0.825 | 0.8323 | 11191 | 0 | 0 | 0 | 3736 | 3746 | Carbamidomethylation |  | DB Search |
| K.YEC(+57.02)NC(+57.02)QYNTEGPR.C | Y | 39.28 | 1689.6512 | 13 | 9.13 | 845.8369 | 2 | 12.04 | 1.0323 | 1.0395 | 26880 | 1.68e2 | 1 | 1 | 291 | 303 | Carbamidomethylation, Carbamidomethylation |  | DB Search |
| total 7 peptides |  |  |  |  |  |  |  |  |  |  |  |  |  |  |  |  |  |  |  |
| --- | --- | --- | --- | --- | --- | --- | --- | --- | --- | --- | --- | --- | --- | --- | --- | --- | --- | --- | --- |

Best Unique PSM (Scan Sample\_4\_A1\_1\_3642.d:13314, m/z=857.3495, z=2, RT=9.81, ppm=10.47):


TRINITY\_DN875\_c0\_g1\_i1.p1

back to list

  

| Protein Coverage
| Supporting Peptides
| Best Unique PSM
|

Protein Coverage:

Supporting Peptides:

| Peptide | Uniq | -10lgP | Mass | Length | ppm | m/z | z | RT | 1/k0 Start | 1/k0 End | Precursor ID | Area Sample 6 | #Feature | #Feature Sample 6 | Start | End | PTM | AScore | Found By |
| --- | --- | --- | --- | --- | --- | --- | --- | --- | --- | --- | --- | --- | --- | --- | --- | --- | --- | --- | --- |
| R.LSTDYC(+57.02)QLGLFK.Q | Y | 47.21 | 1443.7068 | 12 | -0.18 | 722.8574 | 2 | 18.83 | 1.0057 | 1.013 | 67864 | 8.93e2 | 1 | 1 | 542 | 553 | Carbamidomethylation |  | DB Search |
| R.AVFC(+57.02)EHQGVK.T | Y | 47.14 | 1173.5601 | 10 | 0.43 | 587.785 | 2 | 11.65 | 0.8984 | 0.9057 | 24435 | 0 | 0 | 0 | 512 | 521 | Carbamidomethylation |  | DB Search |
| total 2 peptides |  |  |  |  |  |  |  |  |  |  |  |  |  |  |  |  |  |  |  |
| --- | --- | --- | --- | --- | --- | --- | --- | --- | --- | --- | --- | --- | --- | --- | --- | --- | --- | --- | --- |

Best Unique PSM (Scan Sample\_4\_A1\_1\_3642.d:67864, m/z=722.8574, z=2, RT=18.83, ppm=-4.58):


TRINITY\_DN3016\_c0\_g1\_i8.p1

back to list

  

| Protein Coverage
| Supporting Peptides
| Best Unique PSM
|

Protein Coverage:

Supporting Peptides:

| Peptide | Uniq | -10lgP | Mass | Length | ppm | m/z | z | RT | 1/k0 Start | 1/k0 End | Precursor ID | Area Sample 6 | #Feature | #Feature Sample 6 | Start | End | PTM | AScore | Found By |
| --- | --- | --- | --- | --- | --- | --- | --- | --- | --- | --- | --- | --- | --- | --- | --- | --- | --- | --- | --- |
| R.IVNTDLQEIKDIIK.G | Y | 45.77 | 1640.9348 | 14 | -1.11 | 547.9825 | 3 | 18.81 | 0.8177 | 0.825 | 67904 | 0 | 0 | 0 | 154 | 167 |  |  | DB Search |
| total 1 peptides |  |  |  |  |  |  |  |  |  |  |  |  |  |  |  |  |  |  |  |
| --- | --- | --- | --- | --- | --- | --- | --- | --- | --- | --- | --- | --- | --- | --- | --- | --- | --- | --- | --- |

Best Unique PSM (Scan Sample\_4\_A1\_1\_3642.d:67904, m/z=547.9825, z=3, RT=18.81, ppm=-5.51):


TRINITY\_DN691\_c0\_g1\_i1.p1

back to list

  

| Protein Coverage
| Supporting Peptides
| Best Unique PSM
|

Protein Coverage:

Supporting Peptides:

| Peptide | Uniq | -10lgP | Mass | Length | ppm | m/z | z | RT | 1/k0 Start | 1/k0 End | Precursor ID | Area Sample 6 | #Feature | #Feature Sample 6 | Start | End | PTM | AScore | Found By |
| --- | --- | --- | --- | --- | --- | --- | --- | --- | --- | --- | --- | --- | --- | --- | --- | --- | --- | --- | --- |
| R.AYGQDLGQPGSC(+57.02)LKR.F | Y | 47.11 | 1648.7991 | 15 | 3.54 | 550.6065 | 3 | 13.51 | 0.8348 | 0.8421 | 35593 | 7.17e2 | 1 | 1 | 1533 | 1547 | Carbamidomethylation |  | DB Search |
| R.AYGQDLGQPGSC(+57.02)LK.R | Y | 46.51 | 1492.698 | 14 | 2.01 | 747.3545 | 2 | 14.33 | 1.0049 | 1.0122 | 40326 | 6.56e2 | 1 | 1 | 1533 | 1546 | Carbamidomethylation |  | DB Search |
| total 2 peptides |  |  |  |  |  |  |  |  |  |  |  |  |  |  |  |  |  |  |  |
| --- | --- | --- | --- | --- | --- | --- | --- | --- | --- | --- | --- | --- | --- | --- | --- | --- | --- | --- | --- |

Best Unique PSM (Scan Sample\_4\_A1\_1\_3642.d:35593, m/z=550.6065, z=3, RT=13.51, ppm=-0.86):


TRINITY\_DN106\_c0\_g1\_i5.p1

back to list

  

| Protein Coverage
| Supporting Peptides
| Best Unique PSM
|

Protein Coverage:

Supporting Peptides:

| Peptide | Uniq | -10lgP | Mass | Length | ppm | m/z | z | RT | 1/k0 Start | 1/k0 End | Precursor ID | Area Sample 6 | #Feature | #Feature Sample 6 | Start | End | PTM | AScore | Found By |
| --- | --- | --- | --- | --- | --- | --- | --- | --- | --- | --- | --- | --- | --- | --- | --- | --- | --- | --- | --- |
| R.DGPAGPAGR.D | Y | 46.52 | 796.3827 | 9 | 7.09 | 399.1997 | 2 | 9.76 | 0.7233 | 0.7306 | 13098 | 1.65e3 | 1 | 1 | 488 | 496 |  |  | DB Search |
| total 1 peptides |  |  |  |  |  |  |  |  |  |  |  |  |  |  |  |  |  |  |  |
| --- | --- | --- | --- | --- | --- | --- | --- | --- | --- | --- | --- | --- | --- | --- | --- | --- | --- | --- | --- |

Best Unique PSM (Scan Sample\_4\_A1\_1\_3642.d:13098, m/z=399.1997, z=2, RT=9.76, ppm=2.68):


TRINITY\_DN1378\_c0\_g1\_i1.p1

back to list

  

| Protein Coverage
| Supporting Peptides
| Best Unique PSM
|

Protein Coverage:

Supporting Peptides:

| Peptide | Uniq | -10lgP | Mass | Length | ppm | m/z | z | RT | 1/k0 Start | 1/k0 End | Precursor ID | Area Sample 6 | #Feature | #Feature Sample 6 | Start | End | PTM | AScore | Found By |
| --- | --- | --- | --- | --- | --- | --- | --- | --- | --- | --- | --- | --- | --- | --- | --- | --- | --- | --- | --- |
| K.LAALDATVHGQTASR.F | Y | 45.78 | 1509.7899 | 15 | -1.6 | 755.8977 | 2 | 13.57 | 1.0721 | 1.0793 | 35881 | 2.4e2 | 1 | 1 | 213 | 227 |  |  | DB Search |
| total 1 peptides |  |  |  |  |  |  |  |  |  |  |  |  |  |  |  |  |  |  |  |
| --- | --- | --- | --- | --- | --- | --- | --- | --- | --- | --- | --- | --- | --- | --- | --- | --- | --- | --- | --- |

Best Unique PSM (Scan Sample\_4\_A1\_1\_3642.d:35881, m/z=755.8977, z=2, RT=13.57, ppm=-6):


TRINITY\_DN4900\_c0\_g1\_i1.p1

back to list

  

| Protein Coverage
| Supporting Peptides
| Best Unique PSM
|

Protein Coverage:

Supporting Peptides:

| Peptide | Uniq | -10lgP | Mass | Length | ppm | m/z | z | RT | 1/k0 Start | 1/k0 End | Precursor ID | Area Sample 6 | #Feature | #Feature Sample 6 | Start | End | PTM | AScore | Found By |
| --- | --- | --- | --- | --- | --- | --- | --- | --- | --- | --- | --- | --- | --- | --- | --- | --- | --- | --- | --- |
| K.DTVIEKFEENITK.G | Y | 46.65 | 1564.7984 | 13 | 3.48 | 783.4058 | 2 | 19.56 | 1.0717 | 1.0789 | 73037 | 0 | 0 | 0 | 112 | 124 |  |  | DB Search |
| total 1 peptides |  |  |  |  |  |  |  |  |  |  |  |  |  |  |  |  |  |  |  |
| --- | --- | --- | --- | --- | --- | --- | --- | --- | --- | --- | --- | --- | --- | --- | --- | --- | --- | --- | --- |

Best Unique PSM (Scan Sample\_4\_A1\_1\_3642.d:73037, m/z=783.4058, z=2, RT=19.56, ppm=-0.92):


TRINITY\_DN286\_c0\_g1\_i2.p1

back to list

  

| Protein Coverage
| Supporting Peptides
| Best Unique PSM
|

Protein Coverage:

Supporting Peptides:

| Peptide | Uniq | -10lgP | Mass | Length | ppm | m/z | z | RT | 1/k0 Start | 1/k0 End | Precursor ID | Area Sample 6 | #Feature | #Feature Sample 6 | Start | End | PTM | AScore | Found By |
| --- | --- | --- | --- | --- | --- | --- | --- | --- | --- | --- | --- | --- | --- | --- | --- | --- | --- | --- | --- |
| K.RYGQAMC(+57.02)QFMR.N | Y | 45.29 | 1446.6319 | 11 | -3.75 | 724.3173 | 2 | 17.41 | 0.9856 | 0.9928 | 58107 | 7.27e2 | 1 | 1 | 71 | 81 | Carbamidomethylation |  | DB Search |
| total 1 peptides |  |  |  |  |  |  |  |  |  |  |  |  |  |  |  |  |  |  |  |
| --- | --- | --- | --- | --- | --- | --- | --- | --- | --- | --- | --- | --- | --- | --- | --- | --- | --- | --- | --- |

Best Unique PSM (Scan Sample\_4\_A1\_1\_3642.d:58107, m/z=724.3173, z=2, RT=17.41, ppm=-8.15):


TRINITY\_DN4781\_c0\_g1\_i1.p1

back to list

  

| Protein Coverage
| Supporting Peptides
| Best Unique PSM
|

Protein Coverage:

Supporting Peptides:

| Peptide | Uniq | -10lgP | Mass | Length | ppm | m/z | z | RT | 1/k0 Start | 1/k0 End | Precursor ID | Area Sample 6 | #Feature | #Feature Sample 6 | Start | End | PTM | AScore | Found By |
| --- | --- | --- | --- | --- | --- | --- | --- | --- | --- | --- | --- | --- | --- | --- | --- | --- | --- | --- | --- |
| R.RPAFQIVFQGVR.G | Y | 45.87 | 1416.799 | 12 | -0.91 | 473.2711 | 3 | 18.12 | 0.7477 | 0.7551 | 63094 | 0 | 0 | 0 | 1123 | 1134 |  |  | DB Search |
| total 1 peptides |  |  |  |  |  |  |  |  |  |  |  |  |  |  |  |  |  |  |  |
| --- | --- | --- | --- | --- | --- | --- | --- | --- | --- | --- | --- | --- | --- | --- | --- | --- | --- | --- | --- |

Best Unique PSM (Scan Sample\_4\_A1\_1\_3642.d:63094, m/z=473.2711, z=3, RT=18.12, ppm=-5.32):


TRINITY\_DN477\_c0\_g1\_i5.p1

back to list

  

| Protein Coverage
| Supporting Peptides
| Best Unique PSM
|

Protein Coverage:

Supporting Peptides:

| Peptide | Uniq | -10lgP | Mass | Length | ppm | m/z | z | RT | 1/k0 Start | 1/k0 End | Precursor ID | Area Sample 6 | #Feature | #Feature Sample 6 | Start | End | PTM | AScore | Found By |
| --- | --- | --- | --- | --- | --- | --- | --- | --- | --- | --- | --- | --- | --- | --- | --- | --- | --- | --- | --- |
| R.NDVNNPPQAR.R | Y | 47.24 | 1123.537 | 10 | 0.77 | 562.7737 | 2 | 9.95 | 0.866 | 0.8733 | 13858 | 3.59e3 | 2 | 2 | 168 | 177 |  |  | DB Search |
| total 1 peptides |  |  |  |  |  |  |  |  |  |  |  |  |  |  |  |  |  |  |  |
| --- | --- | --- | --- | --- | --- | --- | --- | --- | --- | --- | --- | --- | --- | --- | --- | --- | --- | --- | --- |

Best Unique PSM (Scan Sample\_4\_A1\_1\_3642.d:13858, m/z=562.7737, z=2, RT=9.95, ppm=-3.64):


TRINITY\_DN495\_c0\_g1\_i2.p1

back to list

  

| Protein Coverage
| Supporting Peptides
| Best Unique PSM
|

Protein Coverage:

Supporting Peptides:

| Peptide | Uniq | -10lgP | Mass | Length | ppm | m/z | z | RT | 1/k0 Start | 1/k0 End | Precursor ID | Area Sample 6 | #Feature | #Feature Sample 6 | Start | End | PTM | AScore | Found By |
| --- | --- | --- | --- | --- | --- | --- | --- | --- | --- | --- | --- | --- | --- | --- | --- | --- | --- | --- | --- |
| R.SC(+57.02)TNPAPANGGK.A | Y | 47.22 | 1172.5244 | 12 | 1.68 | 587.2679 | 2 | 9.40 | 0.8429 | 0.8502 | 11161 | 2.16e2 | 1 | 1 | 499 | 510 | Carbamidomethylation |  | DB Search |
| total 1 peptides |  |  |  |  |  |  |  |  |  |  |  |  |  |  |  |  |  |  |  |
| --- | --- | --- | --- | --- | --- | --- | --- | --- | --- | --- | --- | --- | --- | --- | --- | --- | --- | --- | --- |

Best Unique PSM (Scan Sample\_4\_A1\_1\_3642.d:11161, m/z=587.2679, z=2, RT=9.40, ppm=-2.72):


TRINITY\_DN3016\_c0\_g1\_i17.p1

back to list

  

| Protein Coverage
| Supporting Peptides
| Best Unique PSM
|

Protein Coverage:

Supporting Peptides:

| Peptide | Uniq | -10lgP | Mass | Length | ppm | m/z | z | RT | 1/k0 Start | 1/k0 End | Precursor ID | Area Sample 6 | #Feature | #Feature Sample 6 | Start | End | PTM | AScore | Found By |
| --- | --- | --- | --- | --- | --- | --- | --- | --- | --- | --- | --- | --- | --- | --- | --- | --- | --- | --- | --- |
| R.IVNTDLQEIKDIIK.G | Y | 45.77 | 1640.9348 | 14 | -1.11 | 547.9825 | 3 | 18.81 | 0.8177 | 0.825 | 67904 | 0 | 0 | 0 | 154 | 167 |  |  | DB Search |
| total 1 peptides |  |  |  |  |  |  |  |  |  |  |  |  |  |  |  |  |  |  |  |
| --- | --- | --- | --- | --- | --- | --- | --- | --- | --- | --- | --- | --- | --- | --- | --- | --- | --- | --- | --- |

Best Unique PSM (Scan Sample\_4\_A1\_1\_3642.d:67904, m/z=547.9825, z=3, RT=18.81, ppm=-5.51):


TRINITY\_DN319\_c2\_g1\_i3.p1

back to list

  

| Protein Coverage
| Supporting Peptides
| Best Unique PSM
|

Protein Coverage:

Supporting Peptides:

| Peptide | Uniq | -10lgP | Mass | Length | ppm | m/z | z | RT | 1/k0 Start | 1/k0 End | Precursor ID | Area Sample 6 | #Feature | #Feature Sample 6 | Start | End | PTM | AScore | Found By |
| --- | --- | --- | --- | --- | --- | --- | --- | --- | --- | --- | --- | --- | --- | --- | --- | --- | --- | --- | --- |
| R.DPIC(+57.02)HC(+57.02)DTSK.Y | Y | 47.35 | 1231.4961 | 10 | 6.31 | 411.5068 | 3 | 9.96 | 0.7351 | 0.7424 | 14092 | 1.65e3 | 2 | 2 | 1448 | 1457 | Carbamidomethylation, Carbamidomethylation |  | DB Search |
| K.KAQYYEAYETIIKR.H | Y | 46.51 | 1774.9254 | 14 | -1.09 | 592.6458 | 3 | 15.90 | 0.8656 | 0.8729 | 49338 | 0 | 0 | 0 | 2534 | 2547 |  |  | DB Search |
| R.GSFSSHIC(+57.02)LC(+57.02)HDPAYK.S | Y | 46.19 | 1877.8189 | 16 | 4.79 | 626.9471 | 3 | 14.44 | 0.887 | 0.8943 | 41053 | 0 | 0 | 0 | 1011 | 1026 | Carbamidomethylation, Carbamidomethylation |  | DB Search |
| total 3 peptides |  |  |  |  |  |  |  |  |  |  |  |  |  |  |  |  |  |  |  |
| --- | --- | --- | --- | --- | --- | --- | --- | --- | --- | --- | --- | --- | --- | --- | --- | --- | --- | --- | --- |

Best Unique PSM (Scan Sample\_4\_A1\_1\_3642.d:14092, m/z=411.5068, z=3, RT=9.96, ppm=1.91):


TRINITY\_DN172571\_c0\_g1\_i1.p1

back to list

  

| Protein Coverage
| Supporting Peptides
| Best Unique PSM
|

Protein Coverage:

Supporting Peptides:

| Peptide | Uniq | -10lgP | Mass | Length | ppm | m/z | z | RT | 1/k0 Start | 1/k0 End | Precursor ID | Area Sample 6 | #Feature | #Feature Sample 6 | Start | End | PTM | AScore | Found By |
| --- | --- | --- | --- | --- | --- | --- | --- | --- | --- | --- | --- | --- | --- | --- | --- | --- | --- | --- | --- |
| R.DNIQGITKPAIR.R | Y | 45.63 | 1324.7463 | 12 | -2.11 | 663.3761 | 2 | 13.89 | 0.9808 | 0.988 | 37776 | 0 | 0 | 0 | 25 | 36 |  |  | DB Search |
| total 1 peptides |  |  |  |  |  |  |  |  |  |  |  |  |  |  |  |  |  |  |  |
| --- | --- | --- | --- | --- | --- | --- | --- | --- | --- | --- | --- | --- | --- | --- | --- | --- | --- | --- | --- |

Best Unique PSM (Scan Sample\_4\_A1\_1\_3642.d:37776, m/z=663.3761, z=2, RT=13.89, ppm=-6.51):


TRINITY\_DN691\_c0\_g1\_i2.p1

back to list

  

| Protein Coverage
| Supporting Peptides
| Best Unique PSM
|

Protein Coverage:

Supporting Peptides:

| Peptide | Uniq | -10lgP | Mass | Length | ppm | m/z | z | RT | 1/k0 Start | 1/k0 End | Precursor ID | Area Sample 6 | #Feature | #Feature Sample 6 | Start | End | PTM | AScore | Found By |
| --- | --- | --- | --- | --- | --- | --- | --- | --- | --- | --- | --- | --- | --- | --- | --- | --- | --- | --- | --- |
| R.AYGQDLGQPGSC(+57.02)LKR.F | Y | 47.11 | 1648.7991 | 15 | 3.54 | 550.6065 | 3 | 13.51 | 0.8348 | 0.8421 | 35593 | 7.17e2 | 1 | 1 | 1480 | 1494 | Carbamidomethylation |  | DB Search |
| R.AYGQDLGQPGSC(+57.02)LK.R | Y | 46.51 | 1492.698 | 14 | 2.01 | 747.3545 | 2 | 14.33 | 1.0049 | 1.0122 | 40326 | 6.56e2 | 1 | 1 | 1480 | 1493 | Carbamidomethylation |  | DB Search |
| total 2 peptides |  |  |  |  |  |  |  |  |  |  |  |  |  |  |  |  |  |  |  |
| --- | --- | --- | --- | --- | --- | --- | --- | --- | --- | --- | --- | --- | --- | --- | --- | --- | --- | --- | --- |

Best Unique PSM (Scan Sample\_4\_A1\_1\_3642.d:35593, m/z=550.6065, z=3, RT=13.51, ppm=-0.86):


TRINITY\_DN6390\_c0\_g1\_i1.p1

back to list

  

| Protein Coverage
| Supporting Peptides
| Best Unique PSM
|

Protein Coverage:

Supporting Peptides:

| Peptide | Uniq | -10lgP | Mass | Length | ppm | m/z | z | RT | 1/k0 Start | 1/k0 End | Precursor ID | Area Sample 6 | #Feature | #Feature Sample 6 | Start | End | PTM | AScore | Found By |
| --- | --- | --- | --- | --- | --- | --- | --- | --- | --- | --- | --- | --- | --- | --- | --- | --- | --- | --- | --- |
| R.VGNWRDETLSC(+57.02)K.Q | Y | 47.22 | 1463.6827 | 12 | 6.62 | 488.9026 | 3 | 13.82 | 0.7624 | 0.7697 | 37393 | 0 | 0 | 0 | 1047 | 1058 | Carbamidomethylation |  | DB Search |
| K.YLFDLESNC(+57.02)DK.Q | Y | 47.2 | 1402.6075 | 11 | -1.48 | 702.3069 | 2 | 18.41 | 0.9844 | 0.9916 | 65101 | 0 | 0 | 0 | 303 | 313 | Carbamidomethylation |  | DB Search |
| R.SKDYTITSVGGR.N | Y | 45.96 | 1282.6517 | 12 | 4.88 | 642.3334 | 2 | 12.98 | 0.9457 | 0.9529 | 32411 | 5.09e2 | 1 | 1 | 876 | 887 |  |  | DB Search |
| total 3 peptides |  |  |  |  |  |  |  |  |  |  |  |  |  |  |  |  |  |  |  |
| --- | --- | --- | --- | --- | --- | --- | --- | --- | --- | --- | --- | --- | --- | --- | --- | --- | --- | --- | --- |

Best Unique PSM (Scan Sample\_4\_A1\_1\_3642.d:37393, m/z=488.9026, z=3, RT=13.82, ppm=2.22):


TRINITY\_DN1783\_c0\_g1\_i1.p2

back to list

  

| Protein Coverage
| Supporting Peptides
| Best Unique PSM
|

Protein Coverage:

Supporting Peptides:

| Peptide | Uniq | -10lgP | Mass | Length | ppm | m/z | z | RT | 1/k0 Start | 1/k0 End | Precursor ID | Area Sample 6 | #Feature | #Feature Sample 6 | Start | End | PTM | AScore | Found By |
| --- | --- | --- | --- | --- | --- | --- | --- | --- | --- | --- | --- | --- | --- | --- | --- | --- | --- | --- | --- |
| K.LTIIDALPR.C | Y | 46.48 | 1010.6124 | 9 | 5.92 | 506.3142 | 2 | 18.91 | 0.8445 | 0.8518 | 68779 | 8.74e2 | 1 | 1 | 225 | 233 |  |  | DB Search |
| total 1 peptides |  |  |  |  |  |  |  |  |  |  |  |  |  |  |  |  |  |  |  |
| --- | --- | --- | --- | --- | --- | --- | --- | --- | --- | --- | --- | --- | --- | --- | --- | --- | --- | --- | --- |

Best Unique PSM (Scan Sample\_4\_A1\_1\_3642.d:68779, m/z=506.3142, z=2, RT=18.91, ppm=1.51):


TRINITY\_DN554\_c0\_g1\_i2.p1

back to list

  

| Protein Coverage
| Supporting Peptides
| Best Unique PSM
|

Protein Coverage:

Supporting Peptides:

| Peptide | Uniq | -10lgP | Mass | Length | ppm | m/z | z | RT | 1/k0 Start | 1/k0 End | Precursor ID | Area Sample 6 | #Feature | #Feature Sample 6 | Start | End | PTM | AScore | Found By |
| --- | --- | --- | --- | --- | --- | --- | --- | --- | --- | --- | --- | --- | --- | --- | --- | --- | --- | --- | --- |
| K.TITLEVEPSDTIENVK.S | Y | 46.69 | 1786.92 | 16 | 0.39 | 894.4637 | 2 | 17.56 | 1.1442 | 1.1514 | 58994 | 8.78e2 | 1 | 1 | 53 | 68 |  |  | DB Search |
| total 1 peptides |  |  |  |  |  |  |  |  |  |  |  |  |  |  |  |  |  |  |  |
| --- | --- | --- | --- | --- | --- | --- | --- | --- | --- | --- | --- | --- | --- | --- | --- | --- | --- | --- | --- |

Best Unique PSM (Scan Sample\_4\_A1\_1\_3642.d:58994, m/z=894.4637, z=2, RT=17.56, ppm=-4.01):


TRINITY\_DN1613\_c1\_g1\_i1.p1

back to list

  

| Protein Coverage
| Supporting Peptides
| Best Unique PSM
|

Protein Coverage:

Supporting Peptides:

| Peptide | Uniq | -10lgP | Mass | Length | ppm | m/z | z | RT | 1/k0 Start | 1/k0 End | Precursor ID | Area Sample 6 | #Feature | #Feature Sample 6 | Start | End | PTM | AScore | Found By |
| --- | --- | --- | --- | --- | --- | --- | --- | --- | --- | --- | --- | --- | --- | --- | --- | --- | --- | --- | --- |
| R.TC(+57.02)TNPAPAHGGK.S | Y | 46.47 | 1209.556 | 12 | 7.45 | 404.1938 | 3 | 8.98 | 0.731 | 0.7384 | 9664 | 0 | 0 | 0 | 413 | 424 | Carbamidomethylation |  | DB Search |
| R.S(+42.01)C(+57.02)SNPSPAHGGKPC(+57.02)SGELSK.T | Y | 44.35 | 2097.9208 | 20 | -0.21 | 700.311 | 3 | 11.19 | 0.887 | 0.8943 | 21775 | 7.11e2 | 1 | 1 | 14 | 33 | Acetylation (Protein N-term), Carbamidomethylation, Carbamidomethylation | S1:Acetylation (Protein N-term):1000 | DB Search |
| total 2 peptides |  |  |  |  |  |  |  |  |  |  |  |  |  |  |  |  |  |  |  |
| --- | --- | --- | --- | --- | --- | --- | --- | --- | --- | --- | --- | --- | --- | --- | --- | --- | --- | --- | --- |

Best Unique PSM (Scan Sample\_4\_A1\_1\_3642.d:9664, m/z=404.1938, z=3, RT=8.98, ppm=3.04):


TRINITY\_DN3016\_c0\_g1\_i4.p1

back to list

  

| Protein Coverage
| Supporting Peptides
| Best Unique PSM
|

Protein Coverage:

Supporting Peptides:

| Peptide | Uniq | -10lgP | Mass | Length | ppm | m/z | z | RT | 1/k0 Start | 1/k0 End | Precursor ID | Area Sample 6 | #Feature | #Feature Sample 6 | Start | End | PTM | AScore | Found By |
| --- | --- | --- | --- | --- | --- | --- | --- | --- | --- | --- | --- | --- | --- | --- | --- | --- | --- | --- | --- |
| R.IVNTDLQEIKDIIK.G | Y | 45.77 | 1640.9348 | 14 | -1.11 | 547.9825 | 3 | 18.81 | 0.8177 | 0.825 | 67904 | 0 | 0 | 0 | 154 | 167 |  |  | DB Search |
| total 1 peptides |  |  |  |  |  |  |  |  |  |  |  |  |  |  |  |  |  |  |  |
| --- | --- | --- | --- | --- | --- | --- | --- | --- | --- | --- | --- | --- | --- | --- | --- | --- | --- | --- | --- |

Best Unique PSM (Scan Sample\_4\_A1\_1\_3642.d:67904, m/z=547.9825, z=3, RT=18.81, ppm=-5.51):


TRINITY\_DN875\_c0\_g1\_i2.p1

back to list

  

| Protein Coverage
| Supporting Peptides
| Best Unique PSM
|

Protein Coverage:

Supporting Peptides:

| Peptide | Uniq | -10lgP | Mass | Length | ppm | m/z | z | RT | 1/k0 Start | 1/k0 End | Precursor ID | Area Sample 6 | #Feature | #Feature Sample 6 | Start | End | PTM | AScore | Found By |
| --- | --- | --- | --- | --- | --- | --- | --- | --- | --- | --- | --- | --- | --- | --- | --- | --- | --- | --- | --- |
| R.LSTDYC(+57.02)QLGLFK.Q | Y | 47.21 | 1443.7068 | 12 | -0.18 | 722.8574 | 2 | 18.83 | 1.0057 | 1.013 | 67864 | 8.93e2 | 1 | 1 | 542 | 553 | Carbamidomethylation |  | DB Search |
| R.AVFC(+57.02)EHQGVK.T | Y | 47.14 | 1173.5601 | 10 | 0.43 | 587.785 | 2 | 11.65 | 0.8984 | 0.9057 | 24435 | 0 | 0 | 0 | 512 | 521 | Carbamidomethylation |  | DB Search |
| total 2 peptides |  |  |  |  |  |  |  |  |  |  |  |  |  |  |  |  |  |  |  |
| --- | --- | --- | --- | --- | --- | --- | --- | --- | --- | --- | --- | --- | --- | --- | --- | --- | --- | --- | --- |

Best Unique PSM (Scan Sample\_4\_A1\_1\_3642.d:67864, m/z=722.8574, z=2, RT=18.83, ppm=-4.58):


TRINITY\_DN2390\_c0\_g1\_i2.p1

back to list

  

| Protein Coverage
| Supporting Peptides
| Best Unique PSM
|

Protein Coverage:

Supporting Peptides:

| Peptide | Uniq | -10lgP | Mass | Length | ppm | m/z | z | RT | 1/k0 Start | 1/k0 End | Precursor ID | Area Sample 6 | #Feature | #Feature Sample 6 | Start | End | PTM | AScore | Found By |
| --- | --- | --- | --- | --- | --- | --- | --- | --- | --- | --- | --- | --- | --- | --- | --- | --- | --- | --- | --- |
| K.LLVHNWEFLGK.Q | Y | 47.21 | 1354.7397 | 11 | 5.1 | 452.5875 | 3 | 18.51 | 0.7628 | 0.7701 | 65747 | 6.02e2 | 1 | 1 | 451 | 461 |  |  | DB Search |
| total 1 peptides |  |  |  |  |  |  |  |  |  |  |  |  |  |  |  |  |  |  |  |
| --- | --- | --- | --- | --- | --- | --- | --- | --- | --- | --- | --- | --- | --- | --- | --- | --- | --- | --- | --- |

Best Unique PSM (Scan Sample\_4\_A1\_1\_3642.d:65747, m/z=452.5875, z=3, RT=18.51, ppm=0.69):


TRINITY\_DN26249\_c0\_g1\_i1.p1

back to list

  

| Protein Coverage
| Supporting Peptides
| Best Unique PSM
|

Protein Coverage:

Supporting Peptides:

| Peptide | Uniq | -10lgP | Mass | Length | ppm | m/z | z | RT | 1/k0 Start | 1/k0 End | Precursor ID | Area Sample 6 | #Feature | #Feature Sample 6 | Start | End | PTM | AScore | Found By |
| --- | --- | --- | --- | --- | --- | --- | --- | --- | --- | --- | --- | --- | --- | --- | --- | --- | --- | --- | --- |
| R.LIYENQNTIHHVVR.A | Y | 47.27 | 1734.9165 | 14 | 4.54 | 579.3129 | 3 | 13.33 | 0.8445 | 0.8518 | 34380 | 7.41e2 | 1 | 1 | 322 | 335 |  |  | DB Search |
| total 1 peptides |  |  |  |  |  |  |  |  |  |  |  |  |  |  |  |  |  |  |  |
| --- | --- | --- | --- | --- | --- | --- | --- | --- | --- | --- | --- | --- | --- | --- | --- | --- | --- | --- | --- |

Best Unique PSM (Scan Sample\_4\_A1\_1\_3642.d:34380, m/z=579.3129, z=3, RT=13.33, ppm=0.14):


TRINITY\_DN5500\_c0\_g1\_i2.p1

back to list

  

| Protein Coverage
| Supporting Peptides
| Best Unique PSM
|

Protein Coverage:

Supporting Peptides:

| Peptide | Uniq | -10lgP | Mass | Length | ppm | m/z | z | RT | 1/k0 Start | 1/k0 End | Precursor ID | Area Sample 6 | #Feature | #Feature Sample 6 | Start | End | PTM | AScore | Found By |
| --- | --- | --- | --- | --- | --- | --- | --- | --- | --- | --- | --- | --- | --- | --- | --- | --- | --- | --- | --- |
| K.SESESKELIDKLK.A | Y | 47.18 | 1504.7984 | 13 | -0.34 | 502.6044 | 3 | 14.64 | 0.7742 | 0.7815 | 42112 | 2.93e2 | 1 | 1 | 549 | 561 |  |  | DB Search |
| K.IYIDGVFIR.S | Y | 46.89 | 1094.6124 | 9 | 1.62 | 548.312 | 2 | 18.78 | 0.8895 | 0.8968 | 67582 | 8.72e2 | 1 | 1 | 938 | 946 |  |  | DB Search |
| total 2 peptides |  |  |  |  |  |  |  |  |  |  |  |  |  |  |  |  |  |  |  |
| --- | --- | --- | --- | --- | --- | --- | --- | --- | --- | --- | --- | --- | --- | --- | --- | --- | --- | --- | --- |

Best Unique PSM (Scan Sample\_4\_A1\_1\_3642.d:42112, m/z=502.6044, z=3, RT=14.64, ppm=-4.74):


TRINITY\_DN4781\_c0\_g1\_i3.p1

back to list

  

| Protein Coverage
| Supporting Peptides
| Best Unique PSM
|

Protein Coverage:

Supporting Peptides:

| Peptide | Uniq | -10lgP | Mass | Length | ppm | m/z | z | RT | 1/k0 Start | 1/k0 End | Precursor ID | Area Sample 6 | #Feature | #Feature Sample 6 | Start | End | PTM | AScore | Found By |
| --- | --- | --- | --- | --- | --- | --- | --- | --- | --- | --- | --- | --- | --- | --- | --- | --- | --- | --- | --- |
| R.RPAFQIVFQGVR.G | Y | 45.87 | 1416.799 | 12 | -0.91 | 473.2711 | 3 | 18.12 | 0.7477 | 0.7551 | 63094 | 0 | 0 | 0 | 1123 | 1134 |  |  | DB Search |
| total 1 peptides |  |  |  |  |  |  |  |  |  |  |  |  |  |  |  |  |  |  |  |
| --- | --- | --- | --- | --- | --- | --- | --- | --- | --- | --- | --- | --- | --- | --- | --- | --- | --- | --- | --- |

Best Unique PSM (Scan Sample\_4\_A1\_1\_3642.d:63094, m/z=473.2711, z=3, RT=18.12, ppm=-5.32):


TRINITY\_DN3016\_c0\_g1\_i11.p1

back to list

  

| Protein Coverage
| Supporting Peptides
| Best Unique PSM
|

Protein Coverage:

Supporting Peptides:

| Peptide | Uniq | -10lgP | Mass | Length | ppm | m/z | z | RT | 1/k0 Start | 1/k0 End | Precursor ID | Area Sample 6 | #Feature | #Feature Sample 6 | Start | End | PTM | AScore | Found By |
| --- | --- | --- | --- | --- | --- | --- | --- | --- | --- | --- | --- | --- | --- | --- | --- | --- | --- | --- | --- |
| R.IVNTDLQEIKDIIK.G | Y | 45.77 | 1640.9348 | 14 | -1.11 | 547.9825 | 3 | 18.81 | 0.8177 | 0.825 | 67904 | 0 | 0 | 0 | 154 | 167 |  |  | DB Search |
| total 1 peptides |  |  |  |  |  |  |  |  |  |  |  |  |  |  |  |  |  |  |  |
| --- | --- | --- | --- | --- | --- | --- | --- | --- | --- | --- | --- | --- | --- | --- | --- | --- | --- | --- | --- |

Best Unique PSM (Scan Sample\_4\_A1\_1\_3642.d:67904, m/z=547.9825, z=3, RT=18.81, ppm=-5.51):


TRINITY\_DN687\_c0\_g1\_i1.p1

back to list

  

| Protein Coverage
| Supporting Peptides
| Best Unique PSM
|

Protein Coverage:

Supporting Peptides:

| Peptide | Uniq | -10lgP | Mass | Length | ppm | m/z | z | RT | 1/k0 Start | 1/k0 End | Precursor ID | Area Sample 6 | #Feature | #Feature Sample 6 | Start | End | PTM | AScore | Found By |
| --- | --- | --- | --- | --- | --- | --- | --- | --- | --- | --- | --- | --- | --- | --- | --- | --- | --- | --- | --- |
| R.AVC(+57.02)M(+15.99)LSNTTAIAEAWAR.L | Y | 43.01 | 1879.892 | 17 | 2.28 | 940.9513 | 2 | 18.79 | 1.147 | 1.1542 | 67649 | 2.71e2 | 1 | 1 | 374 | 390 | Carbamidomethylation, Oxidation (M) | M4:Oxidation (M):1000 | DB Search |
| K.VGINYQPPTVVPGGDLAK.V | Y | 37.08 | 1823.9781 | 18 | 0.87 | 912.9931 | 2 | 17.39 | 1.1218 | 1.129 | 57959 | 0 | 0 | 0 | 353 | 370 |  |  | DB Search |
| total 2 peptides |  |  |  |  |  |  |  |  |  |  |  |  |  |  |  |  |  |  |  |
| --- | --- | --- | --- | --- | --- | --- | --- | --- | --- | --- | --- | --- | --- | --- | --- | --- | --- | --- | --- |

Best Unique PSM (Scan Sample\_4\_A1\_1\_3642.d:67649, m/z=940.9513, z=2, RT=18.79, ppm=-2.12):


TRINITY\_DN6920\_c0\_g1\_i3.p1

back to list

  

| Protein Coverage
| Supporting Peptides
| Best Unique PSM
|

Protein Coverage:

Supporting Peptides:

| Peptide | Uniq | -10lgP | Mass | Length | ppm | m/z | z | RT | 1/k0 Start | 1/k0 End | Precursor ID | Area Sample 6 | #Feature | #Feature Sample 6 | Start | End | PTM | AScore | Found By |
| --- | --- | --- | --- | --- | --- | --- | --- | --- | --- | --- | --- | --- | --- | --- | --- | --- | --- | --- | --- |
| R.VLVHTQQK.L | Y | 46.31 | 951.5501 | 8 | 1.77 | 476.7811 | 2 | 9.52 | 0.8331 | 0.8404 | 11777 | 3.85e2 | 1 | 1 | 169 | 176 |  |  | DB Search |
| total 1 peptides |  |  |  |  |  |  |  |  |  |  |  |  |  |  |  |  |  |  |  |
| --- | --- | --- | --- | --- | --- | --- | --- | --- | --- | --- | --- | --- | --- | --- | --- | --- | --- | --- | --- |

Best Unique PSM (Scan Sample\_4\_A1\_1\_3642.d:11777, m/z=476.7811, z=2, RT=9.52, ppm=-2.63):


TRINITY\_DN1783\_c0\_g1\_i3.p1

back to list

  

| Protein Coverage
| Supporting Peptides
| Best Unique PSM
|

Protein Coverage:

Supporting Peptides:

| Peptide | Uniq | -10lgP | Mass | Length | ppm | m/z | z | RT | 1/k0 Start | 1/k0 End | Precursor ID | Area Sample 6 | #Feature | #Feature Sample 6 | Start | End | PTM | AScore | Found By |
| --- | --- | --- | --- | --- | --- | --- | --- | --- | --- | --- | --- | --- | --- | --- | --- | --- | --- | --- | --- |
| K.LTIIDALPR.C | Y | 46.48 | 1010.6124 | 9 | 5.92 | 506.3142 | 2 | 18.91 | 0.8445 | 0.8518 | 68779 | 8.74e2 | 1 | 1 | 225 | 233 |  |  | DB Search |
| total 1 peptides |  |  |  |  |  |  |  |  |  |  |  |  |  |  |  |  |  |  |  |
| --- | --- | --- | --- | --- | --- | --- | --- | --- | --- | --- | --- | --- | --- | --- | --- | --- | --- | --- | --- |

Best Unique PSM (Scan Sample\_4\_A1\_1\_3642.d:68779, m/z=506.3142, z=2, RT=18.91, ppm=1.51):


TRINITY\_DN2885\_c0\_g1\_i2.p1

back to list

  

| Protein Coverage
| Supporting Peptides
| Best Unique PSM
|

Protein Coverage:

Supporting Peptides:

| Peptide | Uniq | -10lgP | Mass | Length | ppm | m/z | z | RT | 1/k0 Start | 1/k0 End | Precursor ID | Area Sample 6 | #Feature | #Feature Sample 6 | Start | End | PTM | AScore | Found By |
| --- | --- | --- | --- | --- | --- | --- | --- | --- | --- | --- | --- | --- | --- | --- | --- | --- | --- | --- | --- |
| K.VTRPHGASGVVR.A | Y | 46.49 | 1234.6894 | 12 | 7.12 | 412.5715 | 3 | 10.06 | 0.8169 | 0.8242 | 14707 | 3.57e2 | 1 | 1 | 77 | 88 |  |  | DB Search |
| total 1 peptides |  |  |  |  |  |  |  |  |  |  |  |  |  |  |  |  |  |  |  |
| --- | --- | --- | --- | --- | --- | --- | --- | --- | --- | --- | --- | --- | --- | --- | --- | --- | --- | --- | --- |

Best Unique PSM (Scan Sample\_4\_A1\_1\_3642.d:14707, m/z=412.5715, z=3, RT=10.06, ppm=2.72):


TRINITY\_DN161\_c0\_g1\_i3.p1

back to list

  

| Protein Coverage
| Supporting Peptides
| Best Unique PSM
|

Protein Coverage:

Supporting Peptides:

| Peptide | Uniq | -10lgP | Mass | Length | ppm | m/z | z | RT | 1/k0 Start | 1/k0 End | Precursor ID | Area Sample 6 | #Feature | #Feature Sample 6 | Start | End | PTM | AScore | Found By |
| --- | --- | --- | --- | --- | --- | --- | --- | --- | --- | --- | --- | --- | --- | --- | --- | --- | --- | --- | --- |
| K.SYELPDGQVITIGNER.F | Y | 47.19 | 1789.8846 | 16 | -1.46 | 895.9443 | 2 | 18.64 | 1.1122 | 1.1194 | 66524 | 6.05e2 | 1 | 1 | 262 | 277 |  |  | DB Search |
| R.AVFPSIVGRPR.H | Y | 47.19 | 1197.6982 | 11 | 2.39 | 599.8552 | 2 | 15.88 | 0.9444 | 0.9517 | 49107 | 3.41e4 | 1 | 1 | 52 | 62 |  |  | DB Search |
| R.LDLAGRDLTDYLMK.I | Y | 46.87 | 1622.8338 | 14 | -1.16 | 541.9489 | 3 | 19.39 | 0.7966 | 0.8039 | 71871 | 4.27e2 | 1 | 1 | 201 | 214 |  |  | DB Search |
| K.DLYANTVLSGGTTM(+15.99)FPGIADR.M | Y | 46.72 | 2214.0626 | 21 | 10.66 | 1108.0455 | 2 | 19.50 | 1.2475 | 1.2547 | 72698 | 0 | 0 | 0 | 315 | 335 | Oxidation (M) | M14:Oxidation (M):1000 | DB Search |
| K.DSYVGDEAQSKR.G | Y | 46.51 | 1353.616 | 12 | 5.22 | 677.8159 | 2 | 10.97 | 1.0005 | 1.0077 | 20266 | 2.51e3 | 1 | 1 | 74 | 85 |  |  | DB Search |
| R.VAPEEHPVLLTEAPLNPK.A | Y | 46.46 | 1953.0571 | 18 | 9.42 | 977.5407 | 2 | 16.61 | 1.2085 | 1.2157 | 53280 | 0 | 0 | 0 | 119 | 136 |  |  | DB Search |
| R.HQGVM(+15.99)VGM(+15.99)GQK.D | Y | 43.4 | 1202.5536 | 11 | 4.58 | 602.2842 | 2 | 9.74 | 0.9081 | 0.9154 | 12838 | 6.82e2 | 1 | 1 | 63 | 73 | Oxidation (M), Oxidation (M) | M5:Oxidation (M):1000 M8:Oxidation (M):1000 | DB Search |
| R.DLTDYLMK.I | Y | 37.13 | 997.479 | 8 | 2.04 | 499.7456 | 2 | 18.64 | 0.8372 | 0.8445 | 66608 | 3.54e2 | 1 | 1 | 207 | 214 |  |  | DB Search |
| total 8 peptides |  |  |  |  |  |  |  |  |  |  |  |  |  |  |  |  |  |  |  |
| --- | --- | --- | --- | --- | --- | --- | --- | --- | --- | --- | --- | --- | --- | --- | --- | --- | --- | --- | --- |

Best Unique PSM (Scan Sample\_4\_A1\_1\_3642.d:66524, m/z=895.9443, z=2, RT=18.64, ppm=-5.86):


TRINITY\_DN875\_c0\_g1\_i3.p1

back to list

  

| Protein Coverage
| Supporting Peptides
| Best Unique PSM
|

Protein Coverage:

Supporting Peptides:

| Peptide | Uniq | -10lgP | Mass | Length | ppm | m/z | z | RT | 1/k0 Start | 1/k0 End | Precursor ID | Area Sample 6 | #Feature | #Feature Sample 6 | Start | End | PTM | AScore | Found By |
| --- | --- | --- | --- | --- | --- | --- | --- | --- | --- | --- | --- | --- | --- | --- | --- | --- | --- | --- | --- |
| R.LSTDYC(+57.02)QLGLFK.Q | Y | 47.21 | 1443.7068 | 12 | -0.18 | 722.8574 | 2 | 18.83 | 1.0057 | 1.013 | 67864 | 8.93e2 | 1 | 1 | 542 | 553 | Carbamidomethylation |  | DB Search |
| R.AVFC(+57.02)EHQGVK.T | Y | 47.14 | 1173.5601 | 10 | 0.43 | 587.785 | 2 | 11.65 | 0.8984 | 0.9057 | 24435 | 0 | 0 | 0 | 512 | 521 | Carbamidomethylation |  | DB Search |
| total 2 peptides |  |  |  |  |  |  |  |  |  |  |  |  |  |  |  |  |  |  |  |
| --- | --- | --- | --- | --- | --- | --- | --- | --- | --- | --- | --- | --- | --- | --- | --- | --- | --- | --- | --- |

Best Unique PSM (Scan Sample\_4\_A1\_1\_3642.d:67864, m/z=722.8574, z=2, RT=18.83, ppm=-4.58):


TRINITY\_DN2113\_c0\_g1\_i1.p1

back to list

  

| Protein Coverage
| Supporting Peptides
| Best Unique PSM
|

Protein Coverage:

Supporting Peptides:

| Peptide | Uniq | -10lgP | Mass | Length | ppm | m/z | z | RT | 1/k0 Start | 1/k0 End | Precursor ID | Area Sample 6 | #Feature | #Feature Sample 6 | Start | End | PTM | AScore | Found By |
| --- | --- | --- | --- | --- | --- | --- | --- | --- | --- | --- | --- | --- | --- | --- | --- | --- | --- | --- | --- |
| R.LADLEEFISSPNHANISQVGER.C | Y | 46.68 | 2425.1873 | 22 | -0.55 | 809.399 | 3 | 18.89 | 0.9243 | 0.9315 | 68335 | 1.48e3 | 1 | 1 | 1183 | 1204 |  |  | DB Search |
| R.ALEHYSDIYDIKR.A | Y | 43.91 | 1621.81 | 13 | 7.89 | 541.6125 | 3 | 14.74 | 0.825 | 0.8323 | 42743 | 0 | 0 | 0 | 626 | 638 |  |  | DB Search |
| total 2 peptides |  |  |  |  |  |  |  |  |  |  |  |  |  |  |  |  |  |  |  |
| --- | --- | --- | --- | --- | --- | --- | --- | --- | --- | --- | --- | --- | --- | --- | --- | --- | --- | --- | --- |

Best Unique PSM (Scan Sample\_4\_A1\_1\_3642.d:68335, m/z=809.399, z=3, RT=18.89, ppm=-4.95):


TRINITY\_DN138\_c0\_g1\_i1.p1

back to list

  

| Protein Coverage
| Supporting Peptides
| Best Unique PSM
|

Protein Coverage:

Supporting Peptides:

| Peptide | Uniq | -10lgP | Mass | Length | ppm | m/z | z | RT | 1/k0 Start | 1/k0 End | Precursor ID | Area Sample 6 | #Feature | #Feature Sample 6 | Start | End | PTM | AScore | Found By |
| --- | --- | --- | --- | --- | --- | --- | --- | --- | --- | --- | --- | --- | --- | --- | --- | --- | --- | --- | --- |
| K.DM(+15.99)GVNVISVGAGHTINHR.E | Y | 47.25 | 1891.9322 | 18 | 13.94 | 946.9824 | 2 | 14.29 | 1.2292 | 1.2364 | 40084 | 0 | 0 | 0 | 349 | 366 | Oxidation (M) | M2:Oxidation (M):1000 | DB Search |
| K.DMGVNVISVGAGHTINHR.E | Y | 47.18 | 1875.9373 | 18 | 0.83 | 938.9726 | 2 | 15.42 | 1.2483 | 1.2555 | 46480 | 8.69e2 | 2 | 2 | 349 | 366 |  |  | DB Search |
| R.DPINNHWDGNLINWR.K | Y | 46.7 | 1862.8812 | 15 | 2.3 | 621.9664 | 3 | 19.05 | 0.814 | 0.8214 | 69560 | 4.43e2 | 1 | 1 | 55 | 69 |  |  | DB Search |
| K.YLLVFTDGR.S | Y | 46.52 | 1082.576 | 9 | 0.01 | 542.2929 | 2 | 19.04 | 0.8696 | 0.8769 | 69183 | 6.93e3 | 1 | 1 | 324 | 332 |  |  | DB Search |
| R.FDFGLSDKTYWNSESFEK.K | Y | 46.49 | 2198.9796 | 18 | 1.41 | 733.9983 | 3 | 19.59 | 0.8947 | 0.902 | 73255 | 7.9e2 | 1 | 1 | 268 | 285 |  |  | DB Search |
| R.FC(+57.02)SNPEPENGGQTC(+57.02)EGEAR.E | Y | 46.3 | 2137.8429 | 19 | -0.42 | 1069.9236 | 2 | 12.15 | 1.1566 | 1.1638 | 27281 | 5.66e2 | 1 | 1 | 181 | 199 | Carbamidomethylation, Carbamidomethylation |  | DB Search |
| R.TLDFVVELSK.G | Y | 46.17 | 1149.6281 | 10 | -1.62 | 575.8179 | 2 | 19.58 | 0.8854 | 0.8927 | 72942 | 1.16e4 | 1 | 1 | 237 | 246 |  |  | DB Search |
| K.VHFGVLSFSHR.T | Y | 45.92 | 1284.6727 | 11 | 1.26 | 643.3416 | 2 | 16.41 | 0.9791 | 0.9864 | 52149 | 6.37e2 | 2 | 2 | 255 | 265 |  |  | DB Search |
| R.IFPNRPYPFELVNHR.D | Y | 45.28 | 1897.9951 | 15 | 3.81 | 475.5058 | 4 | 17.41 | 0.7844 | 0.7917 | 58138 | 0 | 0 | 0 | 40 | 54 |  |  | DB Search |
| K.TVSDSIKDMGVNVISVGAGHTINHR.E | Y | 42.95 | 2606.3234 | 25 | 3.28 | 652.5874 | 4 | 17.67 | 0.9141 | 0.9214 | 59942 | 2.01e2 | 1 | 1 | 342 | 366 |  |  | DB Search |
| K.IGC(+57.02)FHDR.I | Y | 41.14 | 903.4021 | 7 | 9.24 | 452.7105 | 2 | 11.29 | 0.8039 | 0.8112 | 22280 | 0 | 0 | 0 | 33 | 39 | Carbamidomethylation |  | DB Search |
| total 11 peptides |  |  |  |  |  |  |  |  |  |  |  |  |  |  |  |  |  |  |  |
| --- | --- | --- | --- | --- | --- | --- | --- | --- | --- | --- | --- | --- | --- | --- | --- | --- | --- | --- | --- |

Best Unique PSM (Scan Sample\_4\_A1\_1\_3642.d:40084, m/z=946.9824, z=2, RT=14.29, ppm=9.54):


TRINITY\_DN6920\_c0\_g1\_i2.p1

back to list

  

| Protein Coverage
| Supporting Peptides
| Best Unique PSM
|

Protein Coverage:

Supporting Peptides:

| Peptide | Uniq | -10lgP | Mass | Length | ppm | m/z | z | RT | 1/k0 Start | 1/k0 End | Precursor ID | Area Sample 6 | #Feature | #Feature Sample 6 | Start | End | PTM | AScore | Found By |
| --- | --- | --- | --- | --- | --- | --- | --- | --- | --- | --- | --- | --- | --- | --- | --- | --- | --- | --- | --- |
| R.VLVHTQQK.L | Y | 46.31 | 951.5501 | 8 | 1.77 | 476.7811 | 2 | 9.52 | 0.8331 | 0.8404 | 11777 | 3.85e2 | 1 | 1 | 169 | 176 |  |  | DB Search |
| total 1 peptides |  |  |  |  |  |  |  |  |  |  |  |  |  |  |  |  |  |  |  |
| --- | --- | --- | --- | --- | --- | --- | --- | --- | --- | --- | --- | --- | --- | --- | --- | --- | --- | --- | --- |

Best Unique PSM (Scan Sample\_4\_A1\_1\_3642.d:11777, m/z=476.7811, z=2, RT=9.52, ppm=-2.63):


TRINITY\_DN477\_c0\_g1\_i9.p1

back to list

  

| Protein Coverage
| Supporting Peptides
| Best Unique PSM
|

Protein Coverage:

Supporting Peptides:

| Peptide | Uniq | -10lgP | Mass | Length | ppm | m/z | z | RT | 1/k0 Start | 1/k0 End | Precursor ID | Area Sample 6 | #Feature | #Feature Sample 6 | Start | End | PTM | AScore | Found By |
| --- | --- | --- | --- | --- | --- | --- | --- | --- | --- | --- | --- | --- | --- | --- | --- | --- | --- | --- | --- |
| R.TQAVLDRLNIEKR.V | Y | 47.18 | 1554.8842 | 13 | 1.35 | 519.3004 | 3 | 14.70 | 0.8019 | 0.8092 | 42401 | 1.64e3 | 1 | 1 | 220 | 232 |  |  | DB Search |
| K.DANSM(+15.99)PTQSR.Q | Y | 46.92 | 1121.4771 | 10 | -7.45 | 561.7392 | 2 | 8.81 | 0.8234 | 0.8307 | 9288 | 0 | 0 | 0 | 51 | 60 | Oxidation (M) | M5:Oxidation (M):1000 | DB Search |
| R.TQAVLDRLNIEK.R | Y | 46.84 | 1398.783 | 12 | 4.58 | 700.3989 | 2 | 15.49 | 1.0073 | 1.0146 | 46990 | 2.49e3 | 1 | 1 | 220 | 231 |  |  | DB Search |
| total 3 peptides |  |  |  |  |  |  |  |  |  |  |  |  |  |  |  |  |  |  |  |
| --- | --- | --- | --- | --- | --- | --- | --- | --- | --- | --- | --- | --- | --- | --- | --- | --- | --- | --- | --- |

Best Unique PSM (Scan Sample\_4\_A1\_1\_3642.d:42401, m/z=519.3004, z=3, RT=14.70, ppm=-3.05):


TRINITY\_DN3271\_c0\_g1\_i2.p1

back to list

  

| Protein Coverage
| Supporting Peptides
| Best Unique PSM
|

Protein Coverage:

Supporting Peptides:

| Peptide | Uniq | -10lgP | Mass | Length | ppm | m/z | z | RT | 1/k0 Start | 1/k0 End | Precursor ID | Area Sample 6 | #Feature | #Feature Sample 6 | Start | End | PTM | AScore | Found By |
| --- | --- | --- | --- | --- | --- | --- | --- | --- | --- | --- | --- | --- | --- | --- | --- | --- | --- | --- | --- |
| K.TIESEAAHGTVTR.H | Y | 45.3 | 1370.679 | 13 | -1.69 | 686.3426 | 2 | 11.96 | 1.0142 | 1.0214 | 26346 | 0 | 0 | 0 | 354 | 366 |  |  | DB Search |
| total 1 peptides |  |  |  |  |  |  |  |  |  |  |  |  |  |  |  |  |  |  |  |
| --- | --- | --- | --- | --- | --- | --- | --- | --- | --- | --- | --- | --- | --- | --- | --- | --- | --- | --- | --- |

Best Unique PSM (Scan Sample\_4\_A1\_1\_3642.d:26346, m/z=686.3426, z=2, RT=11.96, ppm=-6.09):


TRINITY\_DN250\_c0\_g1\_i1.p1

back to list

  

| Protein Coverage
| Supporting Peptides
| Best Unique PSM
|

Protein Coverage:

Supporting Peptides:

| Peptide | Uniq | -10lgP | Mass | Length | ppm | m/z | z | RT | 1/k0 Start | 1/k0 End | Precursor ID | Area Sample 6 | #Feature | #Feature Sample 6 | Start | End | PTM | AScore | Found By |
| --- | --- | --- | --- | --- | --- | --- | --- | --- | --- | --- | --- | --- | --- | --- | --- | --- | --- | --- | --- |
| R.AVC(+57.02)M(+15.99)LSNTTAIAEAWAR.L | Y | 43.01 | 1879.892 | 17 | 2.28 | 940.9513 | 2 | 18.79 | 1.147 | 1.1542 | 67649 | 2.71e2 | 1 | 1 | 374 | 390 | Carbamidomethylation, Oxidation (M) | M4:Oxidation (M):1000 | DB Search |
| K.VGINYQPPTVVPGGDLAK.V | Y | 37.08 | 1823.9781 | 18 | 0.87 | 912.9931 | 2 | 17.39 | 1.1218 | 1.129 | 57959 | 0 | 0 | 0 | 353 | 370 |  |  | DB Search |
| total 2 peptides |  |  |  |  |  |  |  |  |  |  |  |  |  |  |  |  |  |  |  |
| --- | --- | --- | --- | --- | --- | --- | --- | --- | --- | --- | --- | --- | --- | --- | --- | --- | --- | --- | --- |

Best Unique PSM (Scan Sample\_4\_A1\_1\_3642.d:67649, m/z=940.9513, z=2, RT=18.79, ppm=-2.12):


TRINITY\_DN71243\_c0\_g1\_i1.p1

back to list

  

| Protein Coverage
| Supporting Peptides
| Best Unique PSM
|

Protein Coverage:

Supporting Peptides:

| Peptide | Uniq | -10lgP | Mass | Length | ppm | m/z | z | RT | 1/k0 Start | 1/k0 End | Precursor ID | Area Sample 6 | #Feature | #Feature Sample 6 | Start | End | PTM | AScore | Found By |
| --- | --- | --- | --- | --- | --- | --- | --- | --- | --- | --- | --- | --- | --- | --- | --- | --- | --- | --- | --- |
| K.RVHQFLVEALDGGQPR.R | Y | 46.14 | 1820.9645 | 16 | -9.01 | 607.9873 | 3 | 19.50 | 0.868 | 0.8753 | 72483 | 1.07e3 | 1 | 1 | 129 | 144 |  |  | DB Search |
| total 1 peptides |  |  |  |  |  |  |  |  |  |  |  |  |  |  |  |  |  |  |  |
| --- | --- | --- | --- | --- | --- | --- | --- | --- | --- | --- | --- | --- | --- | --- | --- | --- | --- | --- | --- |

Best Unique PSM (Scan Sample\_4\_A1\_1\_3642.d:72483, m/z=607.9873, z=3, RT=19.50, ppm=-13.42):


TRINITY\_DN329\_c0\_g1\_i1.p1

back to list

  

| Protein Coverage
| Supporting Peptides
| Best Unique PSM
|

Protein Coverage:

Supporting Peptides:

| Peptide | Uniq | -10lgP | Mass | Length | ppm | m/z | z | RT | 1/k0 Start | 1/k0 End | Precursor ID | Area Sample 6 | #Feature | #Feature Sample 6 | Start | End | PTM | AScore | Found By |
| --- | --- | --- | --- | --- | --- | --- | --- | --- | --- | --- | --- | --- | --- | --- | --- | --- | --- | --- | --- |
| K.LRPGVELTMSSLVNAK.S | Y | 43.26 | 1713.9447 | 16 | 0.82 | 572.3201 | 3 | 17.86 | 0.9097 | 0.917 | 61340 | 0 | 0 | 0 | 250 | 265 |  |  | DB Search |
| K.GLKVDFDTTFAPVTGK.K | Y | 40.84 | 1694.8879 | 16 | -12.98 | 565.9601 | 3 | 18.27 | 0.8339 | 0.8412 | 64168 | 0 | 0 | 0 | 93 | 108 |  |  | DB Search |
| total 2 peptides |  |  |  |  |  |  |  |  |  |  |  |  |  |  |  |  |  |  |  |
| --- | --- | --- | --- | --- | --- | --- | --- | --- | --- | --- | --- | --- | --- | --- | --- | --- | --- | --- | --- |

Best Unique PSM (Scan Sample\_4\_A1\_1\_3642.d:61340, m/z=572.3201, z=3, RT=17.86, ppm=-3.59):


TRINITY\_DN1280\_c0\_g1\_i1.p1

back to list

  

| Protein Coverage
| Supporting Peptides
| Best Unique PSM
|

Protein Coverage:

Supporting Peptides:

| Peptide | Uniq | -10lgP | Mass | Length | ppm | m/z | z | RT | 1/k0 Start | 1/k0 End | Precursor ID | Area Sample 6 | #Feature | #Feature Sample 6 | Start | End | PTM | AScore | Found By |
| --- | --- | --- | --- | --- | --- | --- | --- | --- | --- | --- | --- | --- | --- | --- | --- | --- | --- | --- | --- |
| R.AQAINVQLQEIQR.R | Y | 46.95 | 1509.8263 | 13 | 3.56 | 755.9198 | 2 | 16.14 | 1.0279 | 1.0351 | 50668 | 0 | 0 | 0 | 263 | 275 |  |  | DB Search |
| K.AEYENKTEVAR.S | Y | 46.54 | 1308.631 | 11 | 0.66 | 655.3203 | 2 | 13.19 | 0.9812 | 0.9884 | 33744 | 0 | 0 | 0 | 202 | 212 |  |  | DB Search |
| R.NIALETLEGHQR.A | Y | 45.63 | 1379.7157 | 12 | 6.86 | 690.8668 | 2 | 15.42 | 1.0178 | 1.025 | 46588 | 3.18e2 | 1 | 1 | 99 | 110 |  |  | DB Search |
| total 3 peptides |  |  |  |  |  |  |  |  |  |  |  |  |  |  |  |  |  |  |  |
| --- | --- | --- | --- | --- | --- | --- | --- | --- | --- | --- | --- | --- | --- | --- | --- | --- | --- | --- | --- |

Best Unique PSM (Scan Sample\_4\_A1\_1\_3642.d:50668, m/z=755.9198, z=2, RT=16.14, ppm=-0.84):


TRINITY\_DN1559\_c0\_g1\_i1.p1

back to list

  

| Protein Coverage
| Supporting Peptides
| Best Unique PSM
|

Protein Coverage:

Supporting Peptides:

| Peptide | Uniq | -10lgP | Mass | Length | ppm | m/z | z | RT | 1/k0 Start | 1/k0 End | Precursor ID | Area Sample 6 | #Feature | #Feature Sample 6 | Start | End | PTM | AScore | Found By |
| --- | --- | --- | --- | --- | --- | --- | --- | --- | --- | --- | --- | --- | --- | --- | --- | --- | --- | --- | --- |
| R.GVAGAGVLAGFDKFK.E | Y | 42.3 | 1435.7823 | 15 | -1.19 | 479.5987 | 3 | 18.41 | 0.8498 | 0.8571 | 65109 | 0 | 0 | 0 | 282 | 296 |  |  | DB Search |
| total 1 peptides |  |  |  |  |  |  |  |  |  |  |  |  |  |  |  |  |  |  |  |
| --- | --- | --- | --- | --- | --- | --- | --- | --- | --- | --- | --- | --- | --- | --- | --- | --- | --- | --- | --- |

Best Unique PSM (Scan Sample\_4\_A1\_1\_3642.d:65109, m/z=479.5987, z=3, RT=18.41, ppm=-5.6):


TRINITY\_DN101\_c0\_g1\_i1.p1

back to list

  

| Protein Coverage
| Supporting Peptides
| Best Unique PSM
|

Protein Coverage:

Supporting Peptides:

| Peptide | Uniq | -10lgP | Mass | Length | ppm | m/z | z | RT | 1/k0 Start | 1/k0 End | Precursor ID | Area Sample 6 | #Feature | #Feature Sample 6 | Start | End | PTM | AScore | Found By |
| --- | --- | --- | --- | --- | --- | --- | --- | --- | --- | --- | --- | --- | --- | --- | --- | --- | --- | --- | --- |
| K.TITLEVEPSDTIENVK.A | Y | 46.69 | 1786.92 | 16 | 0.39 | 894.4637 | 2 | 17.56 | 1.1442 | 1.1514 | 58994 | 8.78e2 | 1 | 1 | 12 | 27 |  |  | DB Search |
| total 1 peptides |  |  |  |  |  |  |  |  |  |  |  |  |  |  |  |  |  |  |  |
| --- | --- | --- | --- | --- | --- | --- | --- | --- | --- | --- | --- | --- | --- | --- | --- | --- | --- | --- | --- |

Best Unique PSM (Scan Sample\_4\_A1\_1\_3642.d:58994, m/z=894.4637, z=2, RT=17.56, ppm=-4.01):


TRINITY\_DN5584\_c0\_g1\_i1.p1

back to list

  

| Protein Coverage
| Supporting Peptides
| Best Unique PSM
|

Protein Coverage:

Supporting Peptides:

| Peptide | Uniq | -10lgP | Mass | Length | ppm | m/z | z | RT | 1/k0 Start | 1/k0 End | Precursor ID | Area Sample 6 | #Feature | #Feature Sample 6 | Start | End | PTM | AScore | Found By |
| --- | --- | --- | --- | --- | --- | --- | --- | --- | --- | --- | --- | --- | --- | --- | --- | --- | --- | --- | --- |
| K.YLSWTLDNDKIR.S | Y | 46.73 | 1522.778 | 12 | 4.13 | 508.5998 | 3 | 17.56 | 0.8108 | 0.8181 | 59147 | 0 | 0 | 0 | 835 | 846 |  |  | DB Search |
| total 1 peptides |  |  |  |  |  |  |  |  |  |  |  |  |  |  |  |  |  |  |  |
| --- | --- | --- | --- | --- | --- | --- | --- | --- | --- | --- | --- | --- | --- | --- | --- | --- | --- | --- | --- |

Best Unique PSM (Scan Sample\_4\_A1\_1\_3642.d:59147, m/z=508.5998, z=3, RT=17.56, ppm=-0.27):


TRINITY\_DN250\_c0\_g1\_i6.p1

back to list

  

| Protein Coverage
| Supporting Peptides
| Best Unique PSM
|

Protein Coverage:

Supporting Peptides:

| Peptide | Uniq | -10lgP | Mass | Length | ppm | m/z | z | RT | 1/k0 Start | 1/k0 End | Precursor ID | Area Sample 6 | #Feature | #Feature Sample 6 | Start | End | PTM | AScore | Found By |
| --- | --- | --- | --- | --- | --- | --- | --- | --- | --- | --- | --- | --- | --- | --- | --- | --- | --- | --- | --- |
| K.VGINYQPPTVVPGGDLAK.V | Y | 37.08 | 1823.9781 | 18 | 0.87 | 912.9931 | 2 | 17.39 | 1.1218 | 1.129 | 57959 | 0 | 0 | 0 | 353 | 370 |  |  | DB Search |
| total 1 peptides |  |  |  |  |  |  |  |  |  |  |  |  |  |  |  |  |  |  |  |
| --- | --- | --- | --- | --- | --- | --- | --- | --- | --- | --- | --- | --- | --- | --- | --- | --- | --- | --- | --- |

Best Unique PSM (Scan Sample\_4\_A1\_1\_3642.d:57959, m/z=912.4467, z=1, RT=17.39, ppm=-3.53):


TRINITY\_DN2477\_c0\_g1\_i9.p1

back to list

  

| Protein Coverage
| Supporting Peptides
| Best Unique PSM
|

Protein Coverage:

Supporting Peptides:

| Peptide | Uniq | -10lgP | Mass | Length | ppm | m/z | z | RT | 1/k0 Start | 1/k0 End | Precursor ID | Area Sample 6 | #Feature | #Feature Sample 6 | Start | End | PTM | AScore | Found By |
| --- | --- | --- | --- | --- | --- | --- | --- | --- | --- | --- | --- | --- | --- | --- | --- | --- | --- | --- | --- |
| K.DSYVGDEAQSKR.G | Y | 46.51 | 1353.616 | 12 | 5.22 | 677.8159 | 2 | 10.97 | 1.0005 | 1.0077 | 20266 | 2.51e3 | 1 | 1 | 52 | 63 |  |  | DB Search |
| R.VAPEEHPVLLTEAPLNPK.A | Y | 46.46 | 1953.0571 | 18 | 9.42 | 977.5407 | 2 | 16.61 | 1.2085 | 1.2157 | 53280 | 0 | 0 | 0 | 97 | 114 |  |  | DB Search |
| total 2 peptides |  |  |  |  |  |  |  |  |  |  |  |  |  |  |  |  |  |  |  |
| --- | --- | --- | --- | --- | --- | --- | --- | --- | --- | --- | --- | --- | --- | --- | --- | --- | --- | --- | --- |

Best Unique PSM (Scan Sample\_4\_A1\_1\_3642.d:20266, m/z=677.8159, z=2, RT=10.97, ppm=0.82):


TRINITY\_DN1071\_c0\_g1\_i1.p1

back to list

  

| Protein Coverage
| Supporting Peptides
| Best Unique PSM
|

Protein Coverage:

Supporting Peptides:

| Peptide | Uniq | -10lgP | Mass | Length | ppm | m/z | z | RT | 1/k0 Start | 1/k0 End | Precursor ID | Area Sample 6 | #Feature | #Feature Sample 6 | Start | End | PTM | AScore | Found By |
| --- | --- | --- | --- | --- | --- | --- | --- | --- | --- | --- | --- | --- | --- | --- | --- | --- | --- | --- | --- |
| K.AVQIHPFNDVSVGDPK.V | Y | 47.19 | 1721.8736 | 16 | 3.05 | 861.9429 | 2 | 15.94 | 1.1162 | 1.1234 | 49403 | 4.56e3 | 1 | 1 | 293 | 308 |  |  | DB Search |
| total 1 peptides |  |  |  |  |  |  |  |  |  |  |  |  |  |  |  |  |  |  |  |
| --- | --- | --- | --- | --- | --- | --- | --- | --- | --- | --- | --- | --- | --- | --- | --- | --- | --- | --- | --- |

Best Unique PSM (Scan Sample\_4\_A1\_1\_3642.d:49403, m/z=861.9429, z=2, RT=15.94, ppm=-1.35):


TRINITY\_DN8597\_c0\_g1\_i1.p1

back to list

  

| Protein Coverage
| Supporting Peptides
| Best Unique PSM
|

Protein Coverage:

Supporting Peptides:

| Peptide | Uniq | -10lgP | Mass | Length | ppm | m/z | z | RT | 1/k0 Start | 1/k0 End | Precursor ID | Area Sample 6 | #Feature | #Feature Sample 6 | Start | End | PTM | AScore | Found By |
| --- | --- | --- | --- | --- | --- | --- | --- | --- | --- | --- | --- | --- | --- | --- | --- | --- | --- | --- | --- |
| R.AC(+57.02)SNVNQC(+57.02)GVNTGTQTVYK.V | Y | 46.08 | 2099.9364 | 19 | 6.81 | 1050.978 | 2 | 12.97 | 1.1746 | 1.1817 | 32306 | 0 | 0 | 0 | 47 | 65 | Carbamidomethylation, Carbamidomethylation |  | DB Search |
| R.AFNKVETR.N | N | 44.29 | 963.5138 | 8 | -5.34 | 482.7595 | 2 | 10.58 | 0.8283 | 0.8356 | 17963 | 0 | 0 | 0 | 15 | 22 |  |  | DB Search |
| total 2 peptides |  |  |  |  |  |  |  |  |  |  |  |  |  |  |  |  |  |  |  |
| --- | --- | --- | --- | --- | --- | --- | --- | --- | --- | --- | --- | --- | --- | --- | --- | --- | --- | --- | --- |

Best Unique PSM (Scan Sample\_4\_A1\_1\_3642.d:32306, m/z=1050.978, z=2, RT=12.97, ppm=2.41):


TRINITY\_DN250\_c0\_g1\_i4.p1

back to list

  

| Protein Coverage
| Supporting Peptides
| Best Unique PSM
|

Protein Coverage:

Supporting Peptides:

| Peptide | Uniq | -10lgP | Mass | Length | ppm | m/z | z | RT | 1/k0 Start | 1/k0 End | Precursor ID | Area Sample 6 | #Feature | #Feature Sample 6 | Start | End | PTM | AScore | Found By |
| --- | --- | --- | --- | --- | --- | --- | --- | --- | --- | --- | --- | --- | --- | --- | --- | --- | --- | --- | --- |
| K.VGINYQPPTVVPGGDLAK.V | Y | 37.08 | 1823.9781 | 18 | 0.87 | 912.9931 | 2 | 17.39 | 1.1218 | 1.129 | 57959 | 0 | 0 | 0 | 353 | 370 |  |  | DB Search |
| total 1 peptides |  |  |  |  |  |  |  |  |  |  |  |  |  |  |  |  |  |  |  |
| --- | --- | --- | --- | --- | --- | --- | --- | --- | --- | --- | --- | --- | --- | --- | --- | --- | --- | --- | --- |

Best Unique PSM (Scan Sample\_4\_A1\_1\_3642.d:57959, m/z=912.4467, z=1, RT=17.39, ppm=-3.53):


TRINITY\_DN1676\_c0\_g1\_i6.p1

back to list

  

| Protein Coverage
| Supporting Peptides
| Best Unique PSM
|

Protein Coverage:

Supporting Peptides:

| Peptide | Uniq | -10lgP | Mass | Length | ppm | m/z | z | RT | 1/k0 Start | 1/k0 End | Precursor ID | Area Sample 6 | #Feature | #Feature Sample 6 | Start | End | PTM | AScore | Found By |
| --- | --- | --- | --- | --- | --- | --- | --- | --- | --- | --- | --- | --- | --- | --- | --- | --- | --- | --- | --- |
| R.SYYFAISDITVGGR.C | Y | 46.66 | 1547.762 | 14 | -2.17 | 774.8832 | 2 | 19.95 | 1.0472 | 1.0544 | 75627 | 4.27e2 | 1 | 1 | 273 | 286 |  |  | DB Search |
| R.FELDRVNTFGDEVFGDPK.V | Y | 37.28 | 2083.985 | 18 | 5.01 | 695.6694 | 3 | 19.27 | 0.9178 | 0.9251 | 71114 | 5.19e2 | 1 | 1 | 252 | 269 |  |  | DB Search |
| total 2 peptides |  |  |  |  |  |  |  |  |  |  |  |  |  |  |  |  |  |  |  |
| --- | --- | --- | --- | --- | --- | --- | --- | --- | --- | --- | --- | --- | --- | --- | --- | --- | --- | --- | --- |

Best Unique PSM (Scan Sample\_4\_A1\_1\_3642.d:75627, m/z=774.8832, z=2, RT=19.95, ppm=-6.57):


TRINITY\_DN1366\_c0\_g1\_i1.p1

back to list

  

| Protein Coverage
| Supporting Peptides
| Best Unique PSM
|

Protein Coverage:

Supporting Peptides:

| Peptide | Uniq | -10lgP | Mass | Length | ppm | m/z | z | RT | 1/k0 Start | 1/k0 End | Precursor ID | Area Sample 6 | #Feature | #Feature Sample 6 | Start | End | PTM | AScore | Found By |
| --- | --- | --- | --- | --- | --- | --- | --- | --- | --- | --- | --- | --- | --- | --- | --- | --- | --- | --- | --- |
| R.DVIIDSGDESKR.D | Y | 45.93 | 1332.6521 | 12 | -17.59 | 667.3187 | 2 | 12.42 | 0.9864 | 0.9937 | 29240 | 2.37e3 | 1 | 1 | 2032 | 2043 |  |  | DB Search |
| total 1 peptides |  |  |  |  |  |  |  |  |  |  |  |  |  |  |  |  |  |  |  |
| --- | --- | --- | --- | --- | --- | --- | --- | --- | --- | --- | --- | --- | --- | --- | --- | --- | --- | --- | --- |

Best Unique PSM (Scan Sample\_4\_A1\_1\_3642.d:29240, m/z=667.3187, z=2, RT=12.42, ppm=-21.99):


TRINITY\_DN1320\_c0\_g2\_i1.p1

back to list

  

| Protein Coverage
| Supporting Peptides
| Best Unique PSM
|

Protein Coverage:

Supporting Peptides:

| Peptide | Uniq | -10lgP | Mass | Length | ppm | m/z | z | RT | 1/k0 Start | 1/k0 End | Precursor ID | Area Sample 6 | #Feature | #Feature Sample 6 | Start | End | PTM | AScore | Found By |
| --- | --- | --- | --- | --- | --- | --- | --- | --- | --- | --- | --- | --- | --- | --- | --- | --- | --- | --- | --- |
| R.SVNEGDVQDKKR.D | Y | 47.26 | 1373.6899 | 12 | 8.6 | 458.9058 | 3 | 9.38 | 0.7392 | 0.7465 | 11053 | 6.7e2 | 1 | 1 | 209 | 220 |  |  | DB Search |
| K.NYFHYENQLELK.F | Y | 45.77 | 1596.7572 | 12 | -0.53 | 799.382 | 2 | 16.59 | 1.0849 | 1.0921 | 53009 | 7.6e2 | 1 | 1 | 176 | 187 |  |  | DB Search |
| total 2 peptides |  |  |  |  |  |  |  |  |  |  |  |  |  |  |  |  |  |  |  |
| --- | --- | --- | --- | --- | --- | --- | --- | --- | --- | --- | --- | --- | --- | --- | --- | --- | --- | --- | --- |

Best Unique PSM (Scan Sample\_4\_A1\_1\_3642.d:11053, m/z=458.9058, z=3, RT=9.38, ppm=4.19):


TRINITY\_DN3036\_c0\_g1\_i8.p1

back to list

  

| Protein Coverage
| Supporting Peptides
| Best Unique PSM
|

Protein Coverage:

Supporting Peptides:

| Peptide | Uniq | -10lgP | Mass | Length | ppm | m/z | z | RT | 1/k0 Start | 1/k0 End | Precursor ID | Area Sample 6 | #Feature | #Feature Sample 6 | Start | End | PTM | AScore | Found By |
| --- | --- | --- | --- | --- | --- | --- | --- | --- | --- | --- | --- | --- | --- | --- | --- | --- | --- | --- | --- |
| R.AGHGAVTSPQTQR.G | Y | 45.07 | 1308.6534 | 13 | 6.45 | 437.226 | 3 | 9.72 | 0.7041 | 0.7114 | 12869 | 7.9e2 | 1 | 1 | 309 | 321 |  |  | DB Search |
| total 1 peptides |  |  |  |  |  |  |  |  |  |  |  |  |  |  |  |  |  |  |  |
| --- | --- | --- | --- | --- | --- | --- | --- | --- | --- | --- | --- | --- | --- | --- | --- | --- | --- | --- | --- |

Best Unique PSM (Scan Sample\_4\_A1\_1\_3642.d:12869, m/z=437.226, z=3, RT=9.72, ppm=2.05):


TRINITY\_DN761\_c0\_g1\_i2.p1

back to list

  

| Protein Coverage
| Supporting Peptides
| Best Unique PSM
|

Protein Coverage:

Supporting Peptides:

| Peptide | Uniq | -10lgP | Mass | Length | ppm | m/z | z | RT | 1/k0 Start | 1/k0 End | Precursor ID | Area Sample 6 | #Feature | #Feature Sample 6 | Start | End | PTM | AScore | Found By |
| --- | --- | --- | --- | --- | --- | --- | --- | --- | --- | --- | --- | --- | --- | --- | --- | --- | --- | --- | --- |
| K.TRIEDLETVISQTQDHR.H | Y | 46.51 | 2040.0236 | 17 | 3.42 | 681.0145 | 3 | 17.49 | 0.963 | 0.9703 | 58651 | 0 | 0 | 0 | 260 | 276 |  |  | DB Search |
| total 1 peptides |  |  |  |  |  |  |  |  |  |  |  |  |  |  |  |  |  |  |  |
| --- | --- | --- | --- | --- | --- | --- | --- | --- | --- | --- | --- | --- | --- | --- | --- | --- | --- | --- | --- |

Best Unique PSM (Scan Sample\_4\_A1\_1\_3642.d:58651, m/z=681.0145, z=3, RT=17.49, ppm=-0.98):


TRINITY\_DN1936\_c0\_g1\_i1.p1

back to list

  

| Protein Coverage
| Supporting Peptides
| Best Unique PSM
|

Protein Coverage:

Supporting Peptides:

| Peptide | Uniq | -10lgP | Mass | Length | ppm | m/z | z | RT | 1/k0 Start | 1/k0 End | Precursor ID | Area Sample 6 | #Feature | #Feature Sample 6 | Start | End | PTM | AScore | Found By |
| --- | --- | --- | --- | --- | --- | --- | --- | --- | --- | --- | --- | --- | --- | --- | --- | --- | --- | --- | --- |
| K.AGDIAILESQQFNAVGGK.T | Y | 47.14 | 1816.9319 | 18 | -16.1 | 909.4546 | 2 | 18.91 | 1.1845 | 1.1917 | 68601 | 1.41e3 | 1 | 1 | 464 | 481 |  |  | DB Search |
| total 1 peptides |  |  |  |  |  |  |  |  |  |  |  |  |  |  |  |  |  |  |  |
| --- | --- | --- | --- | --- | --- | --- | --- | --- | --- | --- | --- | --- | --- | --- | --- | --- | --- | --- | --- |

Best Unique PSM (Scan Sample\_4\_A1\_1\_3642.d:68601, m/z=909.4546, z=2, RT=18.91, ppm=-20.5):


TRINITY\_DN15584\_c0\_g1\_i1.p1

back to list

  

| Protein Coverage
| Supporting Peptides
| Best Unique PSM
|

Protein Coverage:

Supporting Peptides:

| Peptide | Uniq | -10lgP | Mass | Length | ppm | m/z | z | RT | 1/k0 Start | 1/k0 End | Precursor ID | Area Sample 6 | #Feature | #Feature Sample 6 | Start | End | PTM | AScore | Found By |
| --- | --- | --- | --- | --- | --- | --- | --- | --- | --- | --- | --- | --- | --- | --- | --- | --- | --- | --- | --- |
| K.AFVAIGDYGGHVGLGVK.C | Y | 47.2 | 1658.878 | 17 | 4.43 | 830.4463 | 2 | 17.74 | 1.163 | 1.1702 | 60353 | 7.45e2 | 1 | 1 | 109 | 125 |  |  | DB Search |
| total 1 peptides |  |  |  |  |  |  |  |  |  |  |  |  |  |  |  |  |  |  |  |
| --- | --- | --- | --- | --- | --- | --- | --- | --- | --- | --- | --- | --- | --- | --- | --- | --- | --- | --- | --- |

Best Unique PSM (Scan Sample\_4\_A1\_1\_3642.d:60353, m/z=830.4463, z=2, RT=17.74, ppm=0.03):


TRINITY\_DN598\_c1\_g1\_i1.p1

back to list

  

| Protein Coverage
| Supporting Peptides
| Best Unique PSM
|

Protein Coverage:

Supporting Peptides:

| Peptide | Uniq | -10lgP | Mass | Length | ppm | m/z | z | RT | 1/k0 Start | 1/k0 End | Precursor ID | Area Sample 6 | #Feature | #Feature Sample 6 | Start | End | PTM | AScore | Found By |
| --- | --- | --- | --- | --- | --- | --- | --- | --- | --- | --- | --- | --- | --- | --- | --- | --- | --- | --- | --- |
| K.AVAEEFISVDPYVR.S | Y | 47.19 | 1593.8038 | 14 | -4.8 | 797.9019 | 2 | 19.09 | 1.1069 | 1.1142 | 69857 | 2.96e3 | 1 | 1 | 148 | 161 |  |  | DB Search |
| R.ASFVSDIDRLR.A | Y | 46.94 | 1277.6728 | 11 | 1.29 | 639.8417 | 2 | 17.08 | 0.9844 | 0.9916 | 56001 | 9.19e2 | 1 | 1 | 302 | 312 |  |  | DB Search |
| total 2 peptides |  |  |  |  |  |  |  |  |  |  |  |  |  |  |  |  |  |  |  |
| --- | --- | --- | --- | --- | --- | --- | --- | --- | --- | --- | --- | --- | --- | --- | --- | --- | --- | --- | --- |

Best Unique PSM (Scan Sample\_4\_A1\_1\_3642.d:69857, m/z=797.9019, z=2, RT=19.09, ppm=-9.2):


TRINITY\_DN941\_c0\_g1\_i9.p1

back to list

  

| Protein Coverage
| Supporting Peptides
| Best Unique PSM
|

Protein Coverage:

Supporting Peptides:

| Peptide | Uniq | -10lgP | Mass | Length | ppm | m/z | z | RT | 1/k0 Start | 1/k0 End | Precursor ID | Area Sample 6 | #Feature | #Feature Sample 6 | Start | End | PTM | AScore | Found By |
| --- | --- | --- | --- | --- | --- | --- | --- | --- | --- | --- | --- | --- | --- | --- | --- | --- | --- | --- | --- |
| K.AGKPC(+57.02)DIAIDARK.A | Y | 43.41 | 1413.7398 | 13 | -2.72 | 472.2505 | 3 | 11.95 | 0.7746 | 0.7819 | 26267 | 0 | 0 | 0 | 850 | 862 | Carbamidomethylation |  | DB Search |
| K.VKADGPGLKPGNK.A | Y | 40.05 | 1279.7248 | 13 | 11.94 | 427.5854 | 3 | 10.11 | 0.7734 | 0.7807 | 15039 | 0 | 0 | 0 | 837 | 849 |  |  | DB Search |
| total 2 peptides |  |  |  |  |  |  |  |  |  |  |  |  |  |  |  |  |  |  |  |
| --- | --- | --- | --- | --- | --- | --- | --- | --- | --- | --- | --- | --- | --- | --- | --- | --- | --- | --- | --- |

Best Unique PSM (Scan Sample\_4\_A1\_1\_3642.d:26267, m/z=472.2505, z=3, RT=11.95, ppm=-7.12):


TRINITY\_DN250\_c0\_g1\_i3.p1

back to list

  

| Protein Coverage
| Supporting Peptides
| Best Unique PSM
|

Protein Coverage:

Supporting Peptides:

| Peptide | Uniq | -10lgP | Mass | Length | ppm | m/z | z | RT | 1/k0 Start | 1/k0 End | Precursor ID | Area Sample 6 | #Feature | #Feature Sample 6 | Start | End | PTM | AScore | Found By |
| --- | --- | --- | --- | --- | --- | --- | --- | --- | --- | --- | --- | --- | --- | --- | --- | --- | --- | --- | --- |
| R.LM(+15.99)GQIVSSITASLR.F | Y | 46.37 | 1490.8126 | 14 | 3.71 | 746.4131 | 2 | 18.78 | 1.0347 | 1.0419 | 67549 | 8.43e1 | 1 | 1 | 230 | 243 | Oxidation (M) | M2:Oxidation (M):1000 | DB Search |
| R.AVC(+57.02)M(+15.99)LSNTTAIAEAWAR.L | Y | 43.01 | 1879.892 | 17 | 2.28 | 940.9513 | 2 | 18.79 | 1.147 | 1.1542 | 67649 | 2.71e2 | 1 | 1 | 374 | 390 | Carbamidomethylation, Oxidation (M) | M4:Oxidation (M):1000 | DB Search |
| K.VGINYQPPTVVPGGDLAK.V | Y | 37.08 | 1823.9781 | 18 | 0.87 | 912.9931 | 2 | 17.39 | 1.1218 | 1.129 | 57959 | 0 | 0 | 0 | 353 | 370 |  |  | DB Search |
| total 3 peptides |  |  |  |  |  |  |  |  |  |  |  |  |  |  |  |  |  |  |  |
| --- | --- | --- | --- | --- | --- | --- | --- | --- | --- | --- | --- | --- | --- | --- | --- | --- | --- | --- | --- |

Best Unique PSM (Scan Sample\_4\_A1\_1\_3642.d:67549, m/z=746.4131, z=2, RT=18.78, ppm=-0.69):


TRINITY\_DN113\_c0\_g1\_i1.p1

back to list

  

| Protein Coverage
| Supporting Peptides
| Best Unique PSM
|

Protein Coverage:

Supporting Peptides:

| Peptide | Uniq | -10lgP | Mass | Length | ppm | m/z | z | RT | 1/k0 Start | 1/k0 End | Precursor ID | Area Sample 6 | #Feature | #Feature Sample 6 | Start | End | PTM | AScore | Found By |
| --- | --- | --- | --- | --- | --- | --- | --- | --- | --- | --- | --- | --- | --- | --- | --- | --- | --- | --- | --- |
| R.GKDGETGEQGPQGQEGPTGPR.G | Y | 45.95 | 2080.9409 | 21 | 2.44 | 694.6529 | 3 | 10.76 | 0.8494 | 0.8567 | 18980 | 2.66e3 | 1 | 1 | 693 | 713 |  |  | DB Search |
| R.AGAQGVDGR.L | Y | 39.31 | 829.4042 | 9 | 0.74 | 415.7079 | 2 | 9.36 | 0.7184 | 0.7257 | 10966 | 2.27e2 | 1 | 1 | 1010 | 1018 |  |  | DB Search |
| total 2 peptides |  |  |  |  |  |  |  |  |  |  |  |  |  |  |  |  |  |  |  |
| --- | --- | --- | --- | --- | --- | --- | --- | --- | --- | --- | --- | --- | --- | --- | --- | --- | --- | --- | --- |

Best Unique PSM (Scan Sample\_4\_A1\_1\_3642.d:18980, m/z=694.6529, z=3, RT=10.76, ppm=-1.96):


TRINITY\_DN4781\_c0\_g1\_i3.p3

back to list

  

| Protein Coverage
| Supporting Peptides
| Best Unique PSM
|

Protein Coverage:

Supporting Peptides:

| Peptide | Uniq | -10lgP | Mass | Length | ppm | m/z | z | RT | 1/k0 Start | 1/k0 End | Precursor ID | Area Sample 6 | #Feature | #Feature Sample 6 | Start | End | PTM | AScore | Found By |
| --- | --- | --- | --- | --- | --- | --- | --- | --- | --- | --- | --- | --- | --- | --- | --- | --- | --- | --- | --- |
| R.DNAGTSSSGTGPR.F | Y | 47.27 | 1205.5272 | 13 | 3.05 | 603.7701 | 2 | 9.17 | 0.8587 | 0.866 | 10272 | 4.31e2 | 1 | 1 | 126 | 138 |  |  | DB Search |
| total 1 peptides |  |  |  |  |  |  |  |  |  |  |  |  |  |  |  |  |  |  |  |
| --- | --- | --- | --- | --- | --- | --- | --- | --- | --- | --- | --- | --- | --- | --- | --- | --- | --- | --- | --- |

Best Unique PSM (Scan Sample\_4\_A1\_1\_3642.d:10272, m/z=603.7701, z=2, RT=9.17, ppm=-1.35):


TRINITY\_DN464\_c0\_g1\_i4.p1

back to list

  

| Protein Coverage
| Supporting Peptides
| Best Unique PSM
|

Protein Coverage:

Supporting Peptides:

| Peptide | Uniq | -10lgP | Mass | Length | ppm | m/z | z | RT | 1/k0 Start | 1/k0 End | Precursor ID | Area Sample 6 | #Feature | #Feature Sample 6 | Start | End | PTM | AScore | Found By |
| --- | --- | --- | --- | --- | --- | --- | --- | --- | --- | --- | --- | --- | --- | --- | --- | --- | --- | --- | --- |
| R.LLEFVGHDVLR.L | Y | 46.14 | 1296.719 | 11 | 6.13 | 433.2477 | 3 | 17.91 | 0.7713 | 0.7787 | 61665 | 0 | 0 | 0 | 279 | 289 |  |  | DB Search |
| total 1 peptides |  |  |  |  |  |  |  |  |  |  |  |  |  |  |  |  |  |  |  |
| --- | --- | --- | --- | --- | --- | --- | --- | --- | --- | --- | --- | --- | --- | --- | --- | --- | --- | --- | --- |

Best Unique PSM (Scan Sample\_4\_A1\_1\_3642.d:61665, m/z=433.2477, z=3, RT=17.91, ppm=1.72):


TRINITY\_DN3016\_c0\_g1\_i18.p1

back to list

  

| Protein Coverage
| Supporting Peptides
| Best Unique PSM
|

Protein Coverage:

Supporting Peptides:

| Peptide | Uniq | -10lgP | Mass | Length | ppm | m/z | z | RT | 1/k0 Start | 1/k0 End | Precursor ID | Area Sample 6 | #Feature | #Feature Sample 6 | Start | End | PTM | AScore | Found By |
| --- | --- | --- | --- | --- | --- | --- | --- | --- | --- | --- | --- | --- | --- | --- | --- | --- | --- | --- | --- |
| R.IVNTDLQEIKDIIK.G | Y | 45.77 | 1640.9348 | 14 | -1.11 | 547.9825 | 3 | 18.81 | 0.8177 | 0.825 | 67904 | 0 | 0 | 0 | 154 | 167 |  |  | DB Search |
| total 1 peptides |  |  |  |  |  |  |  |  |  |  |  |  |  |  |  |  |  |  |  |
| --- | --- | --- | --- | --- | --- | --- | --- | --- | --- | --- | --- | --- | --- | --- | --- | --- | --- | --- | --- |

Best Unique PSM (Scan Sample\_4\_A1\_1\_3642.d:67904, m/z=547.9825, z=3, RT=18.81, ppm=-5.51):


TRINITY\_DN3016\_c0\_g1\_i6.p1

back to list

  

| Protein Coverage
| Supporting Peptides
| Best Unique PSM
|

Protein Coverage:

Supporting Peptides:

| Peptide | Uniq | -10lgP | Mass | Length | ppm | m/z | z | RT | 1/k0 Start | 1/k0 End | Precursor ID | Area Sample 6 | #Feature | #Feature Sample 6 | Start | End | PTM | AScore | Found By |
| --- | --- | --- | --- | --- | --- | --- | --- | --- | --- | --- | --- | --- | --- | --- | --- | --- | --- | --- | --- |
| R.IVNTDLQEIKDIIK.G | Y | 45.77 | 1640.9348 | 14 | -1.11 | 547.9825 | 3 | 18.81 | 0.8177 | 0.825 | 67904 | 0 | 0 | 0 | 154 | 167 |  |  | DB Search |
| total 1 peptides |  |  |  |  |  |  |  |  |  |  |  |  |  |  |  |  |  |  |  |
| --- | --- | --- | --- | --- | --- | --- | --- | --- | --- | --- | --- | --- | --- | --- | --- | --- | --- | --- | --- |

Best Unique PSM (Scan Sample\_4\_A1\_1\_3642.d:67904, m/z=547.9825, z=3, RT=18.81, ppm=-5.51):


TRINITY\_DN1058\_c0\_g1\_i3.p1

back to list

  

| Protein Coverage
| Supporting Peptides
| Best Unique PSM
|

Protein Coverage:

Supporting Peptides:

| Peptide | Uniq | -10lgP | Mass | Length | ppm | m/z | z | RT | 1/k0 Start | 1/k0 End | Precursor ID | Area Sample 6 | #Feature | #Feature Sample 6 | Start | End | PTM | AScore | Found By |
| --- | --- | --- | --- | --- | --- | --- | --- | --- | --- | --- | --- | --- | --- | --- | --- | --- | --- | --- | --- |
| R.APTHILNPANKR.S | Y | 46.93 | 1330.7469 | 12 | 2.71 | 444.5888 | 3 | 11.17 | 0.7559 | 0.7632 | 21058 | 5.5e3 | 2 | 2 | 2314 | 2325 |  |  | DB Search |
| total 1 peptides |  |  |  |  |  |  |  |  |  |  |  |  |  |  |  |  |  |  |  |
| --- | --- | --- | --- | --- | --- | --- | --- | --- | --- | --- | --- | --- | --- | --- | --- | --- | --- | --- | --- |

Best Unique PSM (Scan Sample\_4\_A1\_1\_3642.d:21058, m/z=444.5888, z=3, RT=11.17, ppm=-1.7):


TRINITY\_DN17292\_c0\_g1\_i1.p1

back to list

  

| Protein Coverage
| Supporting Peptides
| Best Unique PSM
|

Protein Coverage:

Supporting Peptides:

| Peptide | Uniq | -10lgP | Mass | Length | ppm | m/z | z | RT | 1/k0 Start | 1/k0 End | Precursor ID | Area Sample 6 | #Feature | #Feature Sample 6 | Start | End | PTM | AScore | Found By |
| --- | --- | --- | --- | --- | --- | --- | --- | --- | --- | --- | --- | --- | --- | --- | --- | --- | --- | --- | --- |
| K.LSFAVNFR.S | Y | 39.01 | 952.513 | 8 | 7.62 | 477.2653 | 2 | 18.46 | 0.8214 | 0.8287 | 65548 | 0 | 0 | 0 | 296 | 303 |  |  | DB Search |
| total 1 peptides |  |  |  |  |  |  |  |  |  |  |  |  |  |  |  |  |  |  |  |
| --- | --- | --- | --- | --- | --- | --- | --- | --- | --- | --- | --- | --- | --- | --- | --- | --- | --- | --- | --- |

Best Unique PSM (Scan Sample\_4\_A1\_1\_3642.d:65548, m/z=477.2653, z=2, RT=18.46, ppm=3.21):


TRINITY\_DN36\_c0\_g1\_i3.p1

back to list

  

| Protein Coverage
| Supporting Peptides
| Best Unique PSM
|

Protein Coverage:

Supporting Peptides:

| Peptide | Uniq | -10lgP | Mass | Length | ppm | m/z | z | RT | 1/k0 Start | 1/k0 End | Precursor ID | Area Sample 6 | #Feature | #Feature Sample 6 | Start | End | PTM | AScore | Found By |
| --- | --- | --- | --- | --- | --- | --- | --- | --- | --- | --- | --- | --- | --- | --- | --- | --- | --- | --- | --- |
| R.LGGIEVTQGNC(+57.02)AR.K | Y | 45.45 | 1373.6721 | 13 | 2.96 | 687.8423 | 2 | 13.87 | 0.9687 | 0.9759 | 37701 | 0 | 0 | 0 | 58 | 70 | Carbamidomethylation |  | DB Search |
| K.FETQIATK.D | Y | 44.83 | 936.4916 | 8 | 7 | 937.5013 | 1 | 13.97 | 1.1802 | 1.1873 | 38247 | 0 | 0 | 0 | 167 | 174 |  |  | DB Search |
| total 2 peptides |  |  |  |  |  |  |  |  |  |  |  |  |  |  |  |  |  |  |  |
| --- | --- | --- | --- | --- | --- | --- | --- | --- | --- | --- | --- | --- | --- | --- | --- | --- | --- | --- | --- |

Best Unique PSM (Scan Sample\_4\_A1\_1\_3642.d:37701, m/z=687.8423, z=2, RT=13.87, ppm=-1.44):


TRINITY\_DN195348\_c0\_g1\_i1.p1

back to list

  

| Protein Coverage
| Supporting Peptides
| Best Unique PSM
|

Protein Coverage:

Supporting Peptides:

| Peptide | Uniq | -10lgP | Mass | Length | ppm | m/z | z | RT | 1/k0 Start | 1/k0 End | Precursor ID | Area Sample 6 | #Feature | #Feature Sample 6 | Start | End | PTM | AScore | Found By |
| --- | --- | --- | --- | --- | --- | --- | --- | --- | --- | --- | --- | --- | --- | --- | --- | --- | --- | --- | --- |
| K.VLIVISDGNEDFKEK.Q | Y | 45.95 | 1704.8934 | 15 | 0.7 | 853.4508 | 2 | 16.86 | 1.1702 | 1.1774 | 54589 | 4.23e2 | 1 | 1 | 132 | 146 |  |  | DB Search |
| total 1 peptides |  |  |  |  |  |  |  |  |  |  |  |  |  |  |  |  |  |  |  |
| --- | --- | --- | --- | --- | --- | --- | --- | --- | --- | --- | --- | --- | --- | --- | --- | --- | --- | --- | --- |

Best Unique PSM (Scan Sample\_4\_A1\_1\_3642.d:54589, m/z=853.4508, z=2, RT=16.86, ppm=-3.7):


TRINITY\_DN1071\_c0\_g1\_i3.p1

back to list

  

| Protein Coverage
| Supporting Peptides
| Best Unique PSM
|

Protein Coverage:

Supporting Peptides:

| Peptide | Uniq | -10lgP | Mass | Length | ppm | m/z | z | RT | 1/k0 Start | 1/k0 End | Precursor ID | Area Sample 6 | #Feature | #Feature Sample 6 | Start | End | PTM | AScore | Found By |
| --- | --- | --- | --- | --- | --- | --- | --- | --- | --- | --- | --- | --- | --- | --- | --- | --- | --- | --- | --- |
| K.AVQIHPFNDVSVGDPK.V | Y | 47.19 | 1721.8736 | 16 | 3.05 | 861.9429 | 2 | 15.94 | 1.1162 | 1.1234 | 49403 | 4.56e3 | 1 | 1 | 329 | 344 |  |  | DB Search |
| total 1 peptides |  |  |  |  |  |  |  |  |  |  |  |  |  |  |  |  |  |  |  |
| --- | --- | --- | --- | --- | --- | --- | --- | --- | --- | --- | --- | --- | --- | --- | --- | --- | --- | --- | --- |

Best Unique PSM (Scan Sample\_4\_A1\_1\_3642.d:49403, m/z=861.9429, z=2, RT=15.94, ppm=-1.35):


TRINITY\_DN195934\_c0\_g1\_i1.p2

back to list

  

| Protein Coverage
| Supporting Peptides
| Best Unique PSM
|

Protein Coverage:

Supporting Peptides:

| Peptide | Uniq | -10lgP | Mass | Length | ppm | m/z | z | RT | 1/k0 Start | 1/k0 End | Precursor ID | Area Sample 6 | #Feature | #Feature Sample 6 | Start | End | PTM | AScore | Found By |
| --- | --- | --- | --- | --- | --- | --- | --- | --- | --- | --- | --- | --- | --- | --- | --- | --- | --- | --- | --- |
| K.DSYVGDEAQSKR.G | Y | 46.51 | 1353.616 | 12 | 5.22 | 677.8159 | 2 | 10.97 | 1.0005 | 1.0077 | 20266 | 2.51e3 | 1 | 1 | 10 | 21 |  |  | DB Search |
| R.VAPEEHPVLLTEAPLNPK.A | Y | 46.46 | 1953.0571 | 18 | 9.42 | 977.5407 | 2 | 16.61 | 1.2085 | 1.2157 | 53280 | 0 | 0 | 0 | 55 | 72 |  |  | DB Search |
| total 2 peptides |  |  |  |  |  |  |  |  |  |  |  |  |  |  |  |  |  |  |  |
| --- | --- | --- | --- | --- | --- | --- | --- | --- | --- | --- | --- | --- | --- | --- | --- | --- | --- | --- | --- |

Best Unique PSM (Scan Sample\_4\_A1\_1\_3642.d:20266, m/z=677.8159, z=2, RT=10.97, ppm=0.82):


TRINITY\_DN172523\_c0\_g1\_i1.p1

back to list

  

| Protein Coverage
| Supporting Peptides
| Best Unique PSM
|

Protein Coverage:

Supporting Peptides:

| Peptide | Uniq | -10lgP | Mass | Length | ppm | m/z | z | RT | 1/k0 Start | 1/k0 End | Precursor ID | Area Sample 6 | #Feature | #Feature Sample 6 | Start | End | PTM | AScore | Found By |
| --- | --- | --- | --- | --- | --- | --- | --- | --- | --- | --- | --- | --- | --- | --- | --- | --- | --- | --- | --- |
| K.VDVGFILDSSGSLR.N | Y | 45.76 | 1463.762 | 14 | -1.21 | 732.8842 | 2 | 19.93 | 1.0102 | 1.0174 | 75559 | 4.99e2 | 1 | 1 | 109 | 122 |  |  | DB Search |
| total 1 peptides |  |  |  |  |  |  |  |  |  |  |  |  |  |  |  |  |  |  |  |
| --- | --- | --- | --- | --- | --- | --- | --- | --- | --- | --- | --- | --- | --- | --- | --- | --- | --- | --- | --- |

Best Unique PSM (Scan Sample\_4\_A1\_1\_3642.d:75559, m/z=732.8842, z=2, RT=19.93, ppm=-5.61):


TRINITY\_DN761\_c0\_g1\_i1.p1

back to list

  

| Protein Coverage
| Supporting Peptides
| Best Unique PSM
|

Protein Coverage:

Supporting Peptides:

| Peptide | Uniq | -10lgP | Mass | Length | ppm | m/z | z | RT | 1/k0 Start | 1/k0 End | Precursor ID | Area Sample 6 | #Feature | #Feature Sample 6 | Start | End | PTM | AScore | Found By |
| --- | --- | --- | --- | --- | --- | --- | --- | --- | --- | --- | --- | --- | --- | --- | --- | --- | --- | --- | --- |
| K.TRIEDLETVISQTQDHR.H | Y | 46.51 | 2040.0236 | 17 | 3.42 | 681.0145 | 3 | 17.49 | 0.963 | 0.9703 | 58651 | 0 | 0 | 0 | 260 | 276 |  |  | DB Search |
| total 1 peptides |  |  |  |  |  |  |  |  |  |  |  |  |  |  |  |  |  |  |  |
| --- | --- | --- | --- | --- | --- | --- | --- | --- | --- | --- | --- | --- | --- | --- | --- | --- | --- | --- | --- |

Best Unique PSM (Scan Sample\_4\_A1\_1\_3642.d:58651, m/z=681.0145, z=3, RT=17.49, ppm=-0.98):


TRINITY\_DN11522\_c0\_g1\_i2.p1

back to list

  

| Protein Coverage
| Supporting Peptides
| Best Unique PSM
|

Protein Coverage:

Supporting Peptides:

| Peptide | Uniq | -10lgP | Mass | Length | ppm | m/z | z | RT | 1/k0 Start | 1/k0 End | Precursor ID | Area Sample 6 | #Feature | #Feature Sample 6 | Start | End | PTM | AScore | Found By |
| --- | --- | --- | --- | --- | --- | --- | --- | --- | --- | --- | --- | --- | --- | --- | --- | --- | --- | --- | --- |
| R.IGLGNIRDPSISLTR.G | Y | 47.25 | 1610.9104 | 15 | 2.96 | 537.9766 | 3 | 17.36 | 0.797 | 0.8043 | 57658 | 1.69e3 | 1 | 1 | 25 | 39 |  |  | DB Search |
| total 1 peptides |  |  |  |  |  |  |  |  |  |  |  |  |  |  |  |  |  |  |  |
| --- | --- | --- | --- | --- | --- | --- | --- | --- | --- | --- | --- | --- | --- | --- | --- | --- | --- | --- | --- |

Best Unique PSM (Scan Sample\_4\_A1\_1\_3642.d:57658, m/z=537.9766, z=3, RT=17.36, ppm=-1.44):


TRINITY\_DN3156\_c0\_g1\_i2.p1

back to list

  

| Protein Coverage
| Supporting Peptides
| Best Unique PSM
|

Protein Coverage:

Supporting Peptides:

| Peptide | Uniq | -10lgP | Mass | Length | ppm | m/z | z | RT | 1/k0 Start | 1/k0 End | Precursor ID | Area Sample 6 | #Feature | #Feature Sample 6 | Start | End | PTM | AScore | Found By |
| --- | --- | --- | --- | --- | --- | --- | --- | --- | --- | --- | --- | --- | --- | --- | --- | --- | --- | --- | --- |
| R.GYVIWQEVVDNQVK.V | Y | 47.25 | 1675.8569 | 14 | -0.86 | 838.9313 | 2 | 19.92 | 1.1314 | 1.1386 | 75433 | 1.33e3 | 1 | 1 | 388 | 401 |  |  | DB Search |
| total 1 peptides |  |  |  |  |  |  |  |  |  |  |  |  |  |  |  |  |  |  |  |
| --- | --- | --- | --- | --- | --- | --- | --- | --- | --- | --- | --- | --- | --- | --- | --- | --- | --- | --- | --- |

Best Unique PSM (Scan Sample\_4\_A1\_1\_3642.d:75433, m/z=838.9313, z=2, RT=19.92, ppm=-5.26):


TRINITY\_DN1119\_c0\_g1\_i1.p1

back to list

  

| Protein Coverage
| Supporting Peptides
| Best Unique PSM
|

Protein Coverage:

Supporting Peptides:

| Peptide | Uniq | -10lgP | Mass | Length | ppm | m/z | z | RT | 1/k0 Start | 1/k0 End | Precursor ID | Area Sample 6 | #Feature | #Feature Sample 6 | Start | End | PTM | AScore | Found By |
| --- | --- | --- | --- | --- | --- | --- | --- | --- | --- | --- | --- | --- | --- | --- | --- | --- | --- | --- | --- |
| K.SIEHC(+57.02)IDTK.S | Y | 46.48 | 1101.5124 | 9 | 3.06 | 551.7628 | 2 | 11.27 | 0.8773 | 0.8846 | 22178 | 0 | 0 | 0 | 108 | 116 | Carbamidomethylation |  | DB Search |
| K.RLTELEHFLK.I | Y | 37.74 | 1284.719 | 10 | -0.56 | 429.2448 | 3 | 16.44 | 0.7445 | 0.7518 | 52334 | 1.1e3 | 1 | 1 | 376 | 385 |  |  | DB Search |
| K.FLVELAC(+57.02)K.C | Y | 36.88 | 978.5208 | 8 | 0.57 | 490.2658 | 2 | 17.23 | 0.8242 | 0.8315 | 56835 | 0 | 0 | 1 | 181 | 188 | Carbamidomethylation |  | DB Search |
| total 3 peptides |  |  |  |  |  |  |  |  |  |  |  |  |  |  |  |  |  |  |  |
| --- | --- | --- | --- | --- | --- | --- | --- | --- | --- | --- | --- | --- | --- | --- | --- | --- | --- | --- | --- |

Best Unique PSM (Scan Sample\_4\_A1\_1\_3642.d:22178, m/z=551.7628, z=2, RT=11.27, ppm=-1.35):


TRINITY\_DN8008\_c0\_g1\_i2.p1

back to list

  

| Protein Coverage
| Supporting Peptides
| Best Unique PSM
|

Protein Coverage:

Supporting Peptides:

| Peptide | Uniq | -10lgP | Mass | Length | ppm | m/z | z | RT | 1/k0 Start | 1/k0 End | Precursor ID | Area Sample 6 | #Feature | #Feature Sample 6 | Start | End | PTM | AScore | Found By |
| --- | --- | --- | --- | --- | --- | --- | --- | --- | --- | --- | --- | --- | --- | --- | --- | --- | --- | --- | --- |
| R.KVDIIFAVDVSKR.F | Y | 46.75 | 1488.8664 | 13 | 6.22 | 497.297 | 3 | 16.89 | 0.8222 | 0.8295 | 54835 | 0 | 0 | 0 | 918 | 930 |  |  | DB Search |
| K.GVHIISVGLGTVDHR.G | Y | 46.51 | 1558.8579 | 15 | -9.41 | 780.4255 | 2 | 15.93 | 1.0893 | 1.0965 | 49497 | 0 | 0 | 0 | 1055 | 1069 |  |  | DB Search |
| total 2 peptides |  |  |  |  |  |  |  |  |  |  |  |  |  |  |  |  |  |  |  |
| --- | --- | --- | --- | --- | --- | --- | --- | --- | --- | --- | --- | --- | --- | --- | --- | --- | --- | --- | --- |

Best Unique PSM (Scan Sample\_4\_A1\_1\_3642.d:54835, m/z=497.297, z=3, RT=16.89, ppm=1.82):


TRINITY\_DN36\_c0\_g1\_i14.p1

back to list

  

| Protein Coverage
| Supporting Peptides
| Best Unique PSM
|

Protein Coverage:

Supporting Peptides:

| Peptide | Uniq | -10lgP | Mass | Length | ppm | m/z | z | RT | 1/k0 Start | 1/k0 End | Precursor ID | Area Sample 6 | #Feature | #Feature Sample 6 | Start | End | PTM | AScore | Found By |
| --- | --- | --- | --- | --- | --- | --- | --- | --- | --- | --- | --- | --- | --- | --- | --- | --- | --- | --- | --- |
| R.LGGIEVTQGNC(+57.02)AR.K | Y | 45.45 | 1373.6721 | 13 | 2.96 | 687.8423 | 2 | 13.87 | 0.9687 | 0.9759 | 37701 | 0 | 0 | 0 | 58 | 70 | Carbamidomethylation |  | DB Search |
| K.FETQIATK.D | Y | 44.83 | 936.4916 | 8 | 7 | 937.5013 | 1 | 13.97 | 1.1802 | 1.1873 | 38247 | 0 | 0 | 0 | 167 | 174 |  |  | DB Search |
| total 2 peptides |  |  |  |  |  |  |  |  |  |  |  |  |  |  |  |  |  |  |  |
| --- | --- | --- | --- | --- | --- | --- | --- | --- | --- | --- | --- | --- | --- | --- | --- | --- | --- | --- | --- |

Best Unique PSM (Scan Sample\_4\_A1\_1\_3642.d:37701, m/z=687.8423, z=2, RT=13.87, ppm=-1.44):


TRINITY\_DN247\_c0\_g1\_i1.p1

back to list

  

| Protein Coverage
| Supporting Peptides
| Best Unique PSM
|

Protein Coverage:

Supporting Peptides:

| Peptide | Uniq | -10lgP | Mass | Length | ppm | m/z | z | RT | 1/k0 Start | 1/k0 End | Precursor ID | Area Sample 6 | #Feature | #Feature Sample 6 | Start | End | PTM | AScore | Found By |
| --- | --- | --- | --- | --- | --- | --- | --- | --- | --- | --- | --- | --- | --- | --- | --- | --- | --- | --- | --- |
| R.DAPIYGLQWHPEK.S | Y | 47.18 | 1552.7674 | 13 | 1.36 | 777.3886 | 2 | 17.41 | 1.0741 | 1.0813 | 58076 | 0 | 0 | 0 | 258 | 270 |  |  | DB Search |
| total 1 peptides |  |  |  |  |  |  |  |  |  |  |  |  |  |  |  |  |  |  |  |
| --- | --- | --- | --- | --- | --- | --- | --- | --- | --- | --- | --- | --- | --- | --- | --- | --- | --- | --- | --- |

Best Unique PSM (Scan Sample\_4\_A1\_1\_3642.d:58076, m/z=777.3886, z=2, RT=17.41, ppm=-3.04):


TRINITY\_DN15151\_c0\_g1\_i2.p1

back to list

  

| Protein Coverage
| Supporting Peptides
| Best Unique PSM
|

Protein Coverage:

Supporting Peptides:

| Peptide | Uniq | -10lgP | Mass | Length | ppm | m/z | z | RT | 1/k0 Start | 1/k0 End | Precursor ID | Area Sample 6 | #Feature | #Feature Sample 6 | Start | End | PTM | AScore | Found By |
| --- | --- | --- | --- | --- | --- | --- | --- | --- | --- | --- | --- | --- | --- | --- | --- | --- | --- | --- | --- |
| K.AVAAGMNPMDIKR.G | Y | 40.45 | 1372.6955 | 13 | -2.78 | 687.3501 | 2 | 14.90 | 1.0102 | 1.0174 | 43658 | 5.01e2 | 1 | 1 | 145 | 157 |  |  | DB Search |
| total 1 peptides |  |  |  |  |  |  |  |  |  |  |  |  |  |  |  |  |  |  |  |
| --- | --- | --- | --- | --- | --- | --- | --- | --- | --- | --- | --- | --- | --- | --- | --- | --- | --- | --- | --- |

Best Unique PSM (Scan Sample\_4\_A1\_1\_3642.d:43658, m/z=687.3501, z=2, RT=14.90, ppm=-7.18):


TRINITY\_DN1071\_c0\_g1\_i4.p1

back to list

  

| Protein Coverage
| Supporting Peptides
| Best Unique PSM
|

Protein Coverage:

Supporting Peptides:

| Peptide | Uniq | -10lgP | Mass | Length | ppm | m/z | z | RT | 1/k0 Start | 1/k0 End | Precursor ID | Area Sample 6 | #Feature | #Feature Sample 6 | Start | End | PTM | AScore | Found By |
| --- | --- | --- | --- | --- | --- | --- | --- | --- | --- | --- | --- | --- | --- | --- | --- | --- | --- | --- | --- |
| K.AVQIHPFNDVSVGDPK.V | Y | 47.19 | 1721.8736 | 16 | 3.05 | 861.9429 | 2 | 15.94 | 1.1162 | 1.1234 | 49403 | 4.56e3 | 1 | 1 | 329 | 344 |  |  | DB Search |
| total 1 peptides |  |  |  |  |  |  |  |  |  |  |  |  |  |  |  |  |  |  |  |
| --- | --- | --- | --- | --- | --- | --- | --- | --- | --- | --- | --- | --- | --- | --- | --- | --- | --- | --- | --- |

Best Unique PSM (Scan Sample\_4\_A1\_1\_3642.d:49403, m/z=861.9429, z=2, RT=15.94, ppm=-1.35):


TRINITY\_DN5633\_c0\_g1\_i1.p1

back to list

  

| Protein Coverage
| Supporting Peptides
| Best Unique PSM
|

Protein Coverage:

Supporting Peptides:

| Peptide | Uniq | -10lgP | Mass | Length | ppm | m/z | z | RT | 1/k0 Start | 1/k0 End | Precursor ID | Area Sample 6 | #Feature | #Feature Sample 6 | Start | End | PTM | AScore | Found By |
| --- | --- | --- | --- | --- | --- | --- | --- | --- | --- | --- | --- | --- | --- | --- | --- | --- | --- | --- | --- |
| K.LLEINVYHPLIQELQK.R | Y | 47.14 | 1949.0986 | 16 | 2.04 | 650.7053 | 3 | 19.77 | 0.9808 | 0.988 | 74434 | 4.48e2 | 1 | 1 | 706 | 721 |  |  | DB Search |
| total 1 peptides |  |  |  |  |  |  |  |  |  |  |  |  |  |  |  |  |  |  |  |
| --- | --- | --- | --- | --- | --- | --- | --- | --- | --- | --- | --- | --- | --- | --- | --- | --- | --- | --- | --- |

Best Unique PSM (Scan Sample\_4\_A1\_1\_3642.d:74434, m/z=650.7053, z=3, RT=19.77, ppm=-2.36):


TRINITY\_DN262\_c0\_g1\_i1.p1

back to list

  

| Protein Coverage
| Supporting Peptides
| Best Unique PSM
|

Protein Coverage:

Supporting Peptides:

| Peptide | Uniq | -10lgP | Mass | Length | ppm | m/z | z | RT | 1/k0 Start | 1/k0 End | Precursor ID | Area Sample 6 | #Feature | #Feature Sample 6 | Start | End | PTM | AScore | Found By |
| --- | --- | --- | --- | --- | --- | --- | --- | --- | --- | --- | --- | --- | --- | --- | --- | --- | --- | --- | --- |
| K.GVGIISEGNETVEDIAQR.L | Y | 47.26 | 1885.9381 | 18 | -1.95 | 943.9703 | 2 | 17.89 | 1.1414 | 1.1486 | 61431 | 7.95e2 | 1 | 1 | 639 | 656 |  |  | DB Search |
| K.VIM(+15.99)VTGDHPITAK.A | Y | 47.26 | 1396.7384 | 13 | 1.06 | 699.3741 | 2 | 13.28 | 1.0202 | 1.0275 | 34024 | 1.25e3 | 1 | 1 | 622 | 634 | Oxidation (M) | M3:Oxidation (M):1000 | DB Search |
| K.DLPQEKLDDILK.Y | Y | 47.18 | 1425.7715 | 12 | -2.46 | 713.8881 | 2 | 17.82 | 1.0367 | 1.0439 | 60992 | 0 | 0 | 0 | 681 | 692 |  |  | DB Search |
| M.A(+42.01)DPSSLETKEK.D | Y | 46.98 | 1245.6088 | 11 | 15.01 | 623.8183 | 2 | 12.69 | 0.9449 | 0.9521 | 30683 | 0 | 0 | 0 | 2 | 12 | Acetylation (Protein N-term) | A1:Acetylation (Protein N-term):1000 | DB Search |
| K.VDNSSLTGESEPQSR.N | Y | 46.98 | 1604.7278 | 15 | 5.37 | 803.3719 | 2 | 11.95 | 1.0198 | 1.0271 | 26200 | 0 | 0 | 0 | 221 | 235 |  |  | DB Search |
| R.DANAAVVHGSHLK.D | Y | 46.79 | 1317.6789 | 13 | 0.06 | 659.8439 | 2 | 10.92 | 1.0158 | 1.023 | 20063 | 0 | 0 | 0 | 668 | 680 |  |  | DB Search |
| R.AEFRPNQEEEPILK.R | Y | 46.54 | 1698.8577 | 14 | 4.96 | 850.4366 | 2 | 14.72 | 1.0849 | 1.0921 | 42598 | 0 | 0 | 0 | 439 | 452 |  |  | DB Search |
| R.AEFRPNQEEEPILKR.E | Y | 46.51 | 1854.9588 | 15 | 3.18 | 619.3261 | 3 | 13.89 | 0.8802 | 0.8874 | 37756 | 4.58e3 | 1 | 1 | 439 | 453 |  |  | DB Search |
| R.DPVRDKLVNER.L | Y | 45.69 | 1339.7208 | 11 | 7.42 | 447.5822 | 3 | 12.08 | 0.7449 | 0.7522 | 27124 | 0 | 0 | 0 | 847 | 857 |  |  | DB Search |
| K.VIMVTGDHPITAK.A | Y | 43.21 | 1380.7435 | 13 | -0.18 | 691.3759 | 2 | 14.04 | 1.0182 | 1.0255 | 38661 | 1.06e3 | 1 | 1 | 622 | 634 |  |  | DB Search |
| K.LC(+57.02)FVGLM(+15.99)SM(+15.99)IDPPR.A | Y | 37.39 | 1666.7881 | 14 | 11.56 | 834.4073 | 2 | 18.83 | 1.0761 | 1.0833 | 67962 | 2.82e2 | 1 | 1 | 592 | 605 | Carbamidomethylation, Oxidation (M), Oxidation (M) | M7:Oxidation (M):1000 M9:Oxidation (M):1000 | DB Search |
| total 11 peptides |  |  |  |  |  |  |  |  |  |  |  |  |  |  |  |  |  |  |  |
| --- | --- | --- | --- | --- | --- | --- | --- | --- | --- | --- | --- | --- | --- | --- | --- | --- | --- | --- | --- |

Best Unique PSM (Scan Sample\_4\_A1\_1\_3642.d:61431, m/z=943.9703, z=2, RT=17.89, ppm=-6.35):


TRINITY\_DN2477\_c0\_g1\_i10.p1

back to list

  

| Protein Coverage
| Supporting Peptides
| Best Unique PSM
|

Protein Coverage:

Supporting Peptides:

| Peptide | Uniq | -10lgP | Mass | Length | ppm | m/z | z | RT | 1/k0 Start | 1/k0 End | Precursor ID | Area Sample 6 | #Feature | #Feature Sample 6 | Start | End | PTM | AScore | Found By |
| --- | --- | --- | --- | --- | --- | --- | --- | --- | --- | --- | --- | --- | --- | --- | --- | --- | --- | --- | --- |
| K.DSYVGDEAQSKR.G | Y | 46.51 | 1353.616 | 12 | 5.22 | 677.8159 | 2 | 10.97 | 1.0005 | 1.0077 | 20266 | 2.51e3 | 1 | 1 | 19 | 30 |  |  | DB Search |
| R.VAPEEHPVLLTEAPLNPK.A | Y | 46.46 | 1953.0571 | 18 | 9.42 | 977.5407 | 2 | 16.61 | 1.2085 | 1.2157 | 53280 | 0 | 0 | 0 | 64 | 81 |  |  | DB Search |
| total 2 peptides |  |  |  |  |  |  |  |  |  |  |  |  |  |  |  |  |  |  |  |
| --- | --- | --- | --- | --- | --- | --- | --- | --- | --- | --- | --- | --- | --- | --- | --- | --- | --- | --- | --- |

Best Unique PSM (Scan Sample\_4\_A1\_1\_3642.d:20266, m/z=677.8159, z=2, RT=10.97, ppm=0.82):


TRINITY\_DN1653\_c0\_g1\_i1.p1

back to list

  

| Protein Coverage
| Supporting Peptides
| Best Unique PSM
|

Protein Coverage:

Supporting Peptides:

| Peptide | Uniq | -10lgP | Mass | Length | ppm | m/z | z | RT | 1/k0 Start | 1/k0 End | Precursor ID | Area Sample 6 | #Feature | #Feature Sample 6 | Start | End | PTM | AScore | Found By |
| --- | --- | --- | --- | --- | --- | --- | --- | --- | --- | --- | --- | --- | --- | --- | --- | --- | --- | --- | --- |
| K.FANQKLENVSLVLR.A | Y | 47.16 | 1629.9202 | 14 | 9.03 | 544.3165 | 3 | 17.19 | 0.8234 | 0.8307 | 56634 | 1.4e2 | 1 | 1 | 70 | 83 |  |  | DB Search |
| R.LINLFEAVSKK.S | Y | 47.13 | 1260.7441 | 11 | -0.85 | 631.376 | 2 | 19.03 | 0.9832 | 0.9904 | 69384 | 1.61e3 | 2 | 2 | 49 | 59 |  |  | DB Search |
| K.IGIVNEFYVR.S | Y | 47.03 | 1208.6553 | 10 | 2.52 | 605.3338 | 2 | 18.83 | 0.9444 | 0.9517 | 67867 | 1.06e3 | 1 | 1 | 354 | 363 |  |  | DB Search |
| total 3 peptides |  |  |  |  |  |  |  |  |  |  |  |  |  |  |  |  |  |  |  |
| --- | --- | --- | --- | --- | --- | --- | --- | --- | --- | --- | --- | --- | --- | --- | --- | --- | --- | --- | --- |

Best Unique PSM (Scan Sample\_4\_A1\_1\_3642.d:56634, m/z=544.3165, z=3, RT=17.19, ppm=4.63):


TRINITY\_DN14015\_c0\_g1\_i2.p1

back to list

  

| Protein Coverage
| Supporting Peptides
| Best Unique PSM
|

Protein Coverage:

Supporting Peptides:

| Peptide | Uniq | -10lgP | Mass | Length | ppm | m/z | z | RT | 1/k0 Start | 1/k0 End | Precursor ID | Area Sample 6 | #Feature | #Feature Sample 6 | Start | End | PTM | AScore | Found By |
| --- | --- | --- | --- | --- | --- | --- | --- | --- | --- | --- | --- | --- | --- | --- | --- | --- | --- | --- | --- |
| R.ANYVLTIEASDNFETPKK.S | Y | 44.76 | 2039.0211 | 18 | -2.13 | 1020.5112 | 2 | 17.53 | 1.271 | 1.2782 | 58757 | 1.41e2 | 1 | 1 | 1766 | 1783 |  |  | DB Search |
| total 1 peptides |  |  |  |  |  |  |  |  |  |  |  |  |  |  |  |  |  |  |  |
| --- | --- | --- | --- | --- | --- | --- | --- | --- | --- | --- | --- | --- | --- | --- | --- | --- | --- | --- | --- |

Best Unique PSM (Scan Sample\_4\_A1\_1\_3642.d:58757, m/z=1020.5112, z=2, RT=17.53, ppm=-6.53):


TRINITY\_DN27379\_c0\_g1\_i1.p1

back to list

  

| Protein Coverage
| Supporting Peptides
| Best Unique PSM
|

Protein Coverage:

Supporting Peptides:

| Peptide | Uniq | -10lgP | Mass | Length | ppm | m/z | z | RT | 1/k0 Start | 1/k0 End | Precursor ID | Area Sample 6 | #Feature | #Feature Sample 6 | Start | End | PTM | AScore | Found By |
| --- | --- | --- | --- | --- | --- | --- | --- | --- | --- | --- | --- | --- | --- | --- | --- | --- | --- | --- | --- |
| K.LVFHNGDISYAR.G | Y | 43.26 | 1390.6993 | 12 | -2.27 | 696.3523 | 2 | 14.91 | 1.0053 | 1.0126 | 43739 | 0 | 0 | 0 | 316 | 327 |  |  | DB Search |
| total 1 peptides |  |  |  |  |  |  |  |  |  |  |  |  |  |  |  |  |  |  |  |
| --- | --- | --- | --- | --- | --- | --- | --- | --- | --- | --- | --- | --- | --- | --- | --- | --- | --- | --- | --- |

Best Unique PSM (Scan Sample\_4\_A1\_1\_3642.d:43739, m/z=696.3523, z=2, RT=14.91, ppm=-6.67):


TRINITY\_DN1535\_c0\_g1\_i1.p1

back to list

  

| Protein Coverage
| Supporting Peptides
| Best Unique PSM
|

Protein Coverage:

Supporting Peptides:

| Peptide | Uniq | -10lgP | Mass | Length | ppm | m/z | z | RT | 1/k0 Start | 1/k0 End | Precursor ID | Area Sample 6 | #Feature | #Feature Sample 6 | Start | End | PTM | AScore | Found By |
| --- | --- | --- | --- | --- | --- | --- | --- | --- | --- | --- | --- | --- | --- | --- | --- | --- | --- | --- | --- |
| R.GSC(+57.02)EC(+57.02)GTC(+57.02)VC(+57.02)R.E | Y | 47.49 | 1344.4679 | 11 | -0.08 | 673.2382 | 2 | 10.53 | 0.9323 | 0.9396 | 17626 | 7.83e2 | 1 | 1 | 558 | 568 | Carbamidomethylation, Carbamidomethylation, Carbamidomethylation, Carbamidomethylation |  | DB Search |
| R.HQNIVC(+57.02)GGPER.G | Y | 47.07 | 1265.5935 | 11 | 3.24 | 633.8033 | 2 | 11.10 | 0.9081 | 0.9154 | 21116 | 0 | 0 | 0 | 547 | 557 | Carbamidomethylation |  | DB Search |
| total 2 peptides |  |  |  |  |  |  |  |  |  |  |  |  |  |  |  |  |  |  |  |
| --- | --- | --- | --- | --- | --- | --- | --- | --- | --- | --- | --- | --- | --- | --- | --- | --- | --- | --- | --- |

Best Unique PSM (Scan Sample\_4\_A1\_1\_3642.d:17626, m/z=673.2382, z=2, RT=10.53, ppm=-4.49):


TRINITY\_DN1783\_c0\_g1\_i6.p1

back to list

  

| Protein Coverage
| Supporting Peptides
| Best Unique PSM
|

Protein Coverage:

Supporting Peptides:

| Peptide | Uniq | -10lgP | Mass | Length | ppm | m/z | z | RT | 1/k0 Start | 1/k0 End | Precursor ID | Area Sample 6 | #Feature | #Feature Sample 6 | Start | End | PTM | AScore | Found By |
| --- | --- | --- | --- | --- | --- | --- | --- | --- | --- | --- | --- | --- | --- | --- | --- | --- | --- | --- | --- |
| K.LTIIDALPR.C | Y | 46.48 | 1010.6124 | 9 | 5.92 | 506.3142 | 2 | 18.91 | 0.8445 | 0.8518 | 68779 | 8.74e2 | 1 | 1 | 104 | 112 |  |  | DB Search |
| total 1 peptides |  |  |  |  |  |  |  |  |  |  |  |  |  |  |  |  |  |  |  |
| --- | --- | --- | --- | --- | --- | --- | --- | --- | --- | --- | --- | --- | --- | --- | --- | --- | --- | --- | --- |

Best Unique PSM (Scan Sample\_4\_A1\_1\_3642.d:68779, m/z=506.3142, z=2, RT=18.91, ppm=1.51):


TRINITY\_DN161\_c0\_g1\_i1.p1

back to list

  

| Protein Coverage
| Supporting Peptides
| Best Unique PSM
|

Protein Coverage:

Supporting Peptides:

| Peptide | Uniq | -10lgP | Mass | Length | ppm | m/z | z | RT | 1/k0 Start | 1/k0 End | Precursor ID | Area Sample 6 | #Feature | #Feature Sample 6 | Start | End | PTM | AScore | Found By |
| --- | --- | --- | --- | --- | --- | --- | --- | --- | --- | --- | --- | --- | --- | --- | --- | --- | --- | --- | --- |
| K.SYELPDGQVITIGNER.F | Y | 47.19 | 1789.8846 | 16 | -1.46 | 895.9443 | 2 | 18.64 | 1.1122 | 1.1194 | 66524 | 6.05e2 | 1 | 1 | 240 | 255 |  |  | DB Search |
| R.AVFPSIVGRPR.H | Y | 47.19 | 1197.6982 | 11 | 2.39 | 599.8552 | 2 | 15.88 | 0.9444 | 0.9517 | 49107 | 3.41e4 | 1 | 1 | 30 | 40 |  |  | DB Search |
| R.LDLAGRDLTDYLMK.I | Y | 46.87 | 1622.8338 | 14 | -1.16 | 541.9489 | 3 | 19.39 | 0.7966 | 0.8039 | 71871 | 4.27e2 | 1 | 1 | 179 | 192 |  |  | DB Search |
| K.DLYANTVLSGGTTM(+15.99)FPGIADR.M | Y | 46.72 | 2214.0626 | 21 | 10.66 | 1108.0455 | 2 | 19.50 | 1.2475 | 1.2547 | 72698 | 0 | 0 | 0 | 293 | 313 | Oxidation (M) | M14:Oxidation (M):1000 | DB Search |
| K.DSYVGDEAQSKR.G | Y | 46.51 | 1353.616 | 12 | 5.22 | 677.8159 | 2 | 10.97 | 1.0005 | 1.0077 | 20266 | 2.51e3 | 1 | 1 | 52 | 63 |  |  | DB Search |
| R.VAPEEHPVLLTEAPLNPK.A | Y | 46.46 | 1953.0571 | 18 | 9.42 | 977.5407 | 2 | 16.61 | 1.2085 | 1.2157 | 53280 | 0 | 0 | 0 | 97 | 114 |  |  | DB Search |
| R.HQGVM(+15.99)VGM(+15.99)GQK.D | Y | 43.4 | 1202.5536 | 11 | 4.58 | 602.2842 | 2 | 9.74 | 0.9081 | 0.9154 | 12838 | 6.82e2 | 1 | 1 | 41 | 51 | Oxidation (M), Oxidation (M) | M5:Oxidation (M):1000 M8:Oxidation (M):1000 | DB Search |
| R.DLTDYLMK.I | Y | 37.13 | 997.479 | 8 | 2.04 | 499.7456 | 2 | 18.64 | 0.8372 | 0.8445 | 66608 | 3.54e2 | 1 | 1 | 185 | 192 |  |  | DB Search |
| total 8 peptides |  |  |  |  |  |  |  |  |  |  |  |  |  |  |  |  |  |  |  |
| --- | --- | --- | --- | --- | --- | --- | --- | --- | --- | --- | --- | --- | --- | --- | --- | --- | --- | --- | --- |

Best Unique PSM (Scan Sample\_4\_A1\_1\_3642.d:66524, m/z=895.9443, z=2, RT=18.64, ppm=-5.86):


TRINITY\_DN64\_c0\_g1\_i1.p1

back to list

  

| Protein Coverage
| Supporting Peptides
| Best Unique PSM
|

Protein Coverage:

Supporting Peptides:

| Peptide | Uniq | -10lgP | Mass | Length | ppm | m/z | z | RT | 1/k0 Start | 1/k0 End | Precursor ID | Area Sample 6 | #Feature | #Feature Sample 6 | Start | End | PTM | AScore | Found By |
| --- | --- | --- | --- | --- | --- | --- | --- | --- | --- | --- | --- | --- | --- | --- | --- | --- | --- | --- | --- |
| K.YYVTIIDAPGHR.D | Y | 47.14 | 1403.7197 | 12 | 0.82 | 702.8646 | 2 | 16.06 | 1.0403 | 1.0476 | 50166 | 5.77e2 | 1 | 1 | 88 | 99 |  |  | DB Search |
| total 1 peptides |  |  |  |  |  |  |  |  |  |  |  |  |  |  |  |  |  |  |  |
| --- | --- | --- | --- | --- | --- | --- | --- | --- | --- | --- | --- | --- | --- | --- | --- | --- | --- | --- | --- |

Best Unique PSM (Scan Sample\_4\_A1\_1\_3642.d:50166, m/z=702.8646, z=2, RT=16.06, ppm=-3.58):


TRINITY\_DN3078\_c0\_g1\_i1.p1

back to list

  

| Protein Coverage
| Supporting Peptides
| Best Unique PSM
|

Protein Coverage:

Supporting Peptides:

| Peptide | Uniq | -10lgP | Mass | Length | ppm | m/z | z | RT | 1/k0 Start | 1/k0 End | Precursor ID | Area Sample 6 | #Feature | #Feature Sample 6 | Start | End | PTM | AScore | Found By |
| --- | --- | --- | --- | --- | --- | --- | --- | --- | --- | --- | --- | --- | --- | --- | --- | --- | --- | --- | --- |
| K.AKIEALHC(+57.02)LSENIVK.D | Y | 47.18 | 1723.9291 | 15 | 4.04 | 575.6501 | 3 | 15.76 | 0.8226 | 0.8299 | 48472 | 1e3 | 2 | 2 | 709 | 723 | Carbamidomethylation |  | DB Search |
| K.VSQTC(+57.02)AEHVFR.V | Y | 47.17 | 1332.6245 | 11 | 3.6 | 667.319 | 2 | 13.74 | 0.984 | 0.9912 | 36845 | 6.47e2 | 1 | 1 | 547 | 557 | Carbamidomethylation |  | DB Search |
| K.IEALHC(+57.02)LSENIVK.D | Y | 47.04 | 1524.797 | 13 | 2.71 | 763.4045 | 2 | 15.75 | 1.0504 | 1.0576 | 48457 | 7.46e2 | 1 | 1 | 711 | 723 | Carbamidomethylation |  | DB Search |
| R.KVSQTC(+57.02)AEHVFR.V | Y | 46.98 | 1460.7194 | 12 | 2.73 | 487.9129 | 3 | 11.99 | 0.801 | 0.8084 | 26442 | 8.32e2 | 1 | 1 | 546 | 557 | Carbamidomethylation |  | DB Search |
| total 4 peptides |  |  |  |  |  |  |  |  |  |  |  |  |  |  |  |  |  |  |  |
| --- | --- | --- | --- | --- | --- | --- | --- | --- | --- | --- | --- | --- | --- | --- | --- | --- | --- | --- | --- |

Best Unique PSM (Scan Sample\_4\_A1\_1\_3642.d:48472, m/z=575.6501, z=3, RT=15.76, ppm=-0.36):


TRINITY\_DN7102\_c0\_g1\_i1.p1

back to list

  

| Protein Coverage
| Supporting Peptides
| Best Unique PSM
|

Protein Coverage:

Supporting Peptides:

| Peptide | Uniq | -10lgP | Mass | Length | ppm | m/z | z | RT | 1/k0 Start | 1/k0 End | Precursor ID | Area Sample 6 | #Feature | #Feature Sample 6 | Start | End | PTM | AScore | Found By |
| --- | --- | --- | --- | --- | --- | --- | --- | --- | --- | --- | --- | --- | --- | --- | --- | --- | --- | --- | --- |
| K.AGAIAEEVISSIR.T | Y | 47.12 | 1314.7143 | 13 | -2.53 | 658.3599 | 2 | 19.95 | 0.9715 | 0.9787 | 75736 | 0 | 0 | 0 | 212 | 224 |  |  | DB Search |
| total 1 peptides |  |  |  |  |  |  |  |  |  |  |  |  |  |  |  |  |  |  |  |
| --- | --- | --- | --- | --- | --- | --- | --- | --- | --- | --- | --- | --- | --- | --- | --- | --- | --- | --- | --- |

Best Unique PSM (Scan Sample\_4\_A1\_1\_3642.d:75736, m/z=658.3599, z=2, RT=19.95, ppm=-6.93):


TRINITY\_DN4900\_c0\_g1\_i4.p1

back to list

  

| Protein Coverage
| Supporting Peptides
| Best Unique PSM
|

Protein Coverage:

Supporting Peptides:

| Peptide | Uniq | -10lgP | Mass | Length | ppm | m/z | z | RT | 1/k0 Start | 1/k0 End | Precursor ID | Area Sample 6 | #Feature | #Feature Sample 6 | Start | End | PTM | AScore | Found By |
| --- | --- | --- | --- | --- | --- | --- | --- | --- | --- | --- | --- | --- | --- | --- | --- | --- | --- | --- | --- |
| K.DTVIEKFEENITK.G | Y | 46.65 | 1564.7984 | 13 | 3.48 | 783.4058 | 2 | 19.56 | 1.0717 | 1.0789 | 73037 | 0 | 0 | 0 | 112 | 124 |  |  | DB Search |
| total 1 peptides |  |  |  |  |  |  |  |  |  |  |  |  |  |  |  |  |  |  |  |
| --- | --- | --- | --- | --- | --- | --- | --- | --- | --- | --- | --- | --- | --- | --- | --- | --- | --- | --- | --- |

Best Unique PSM (Scan Sample\_4\_A1\_1\_3642.d:73037, m/z=783.4058, z=2, RT=19.56, ppm=-0.92):


TRINITY\_DN118520\_c0\_g1\_i1.p1

back to list

  

| Protein Coverage
| Supporting Peptides
| Best Unique PSM
|

Protein Coverage:

Supporting Peptides:

| Peptide | Uniq | -10lgP | Mass | Length | ppm | m/z | z | RT | 1/k0 Start | 1/k0 End | Precursor ID | Area Sample 6 | #Feature | #Feature Sample 6 | Start | End | PTM | AScore | Found By |
| --- | --- | --- | --- | --- | --- | --- | --- | --- | --- | --- | --- | --- | --- | --- | --- | --- | --- | --- | --- |
| K.ISNVQDIVPVLELANQHK.R | Y | 46.31 | 2016.1003 | 18 | 1.11 | 673.0385 | 3 | 19.73 | 0.9461 | 0.9533 | 74326 | 3.57e3 | 1 | 1 | 258 | 275 |  |  | DB Search |
| total 1 peptides |  |  |  |  |  |  |  |  |  |  |  |  |  |  |  |  |  |  |  |
| --- | --- | --- | --- | --- | --- | --- | --- | --- | --- | --- | --- | --- | --- | --- | --- | --- | --- | --- | --- |

Best Unique PSM (Scan Sample\_4\_A1\_1\_3642.d:74326, m/z=673.0385, z=3, RT=19.73, ppm=-3.29):


TRINITY\_DN554\_c0\_g1\_i5.p1

back to list

  

| Protein Coverage
| Supporting Peptides
| Best Unique PSM
|

Protein Coverage:

Supporting Peptides:

| Peptide | Uniq | -10lgP | Mass | Length | ppm | m/z | z | RT | 1/k0 Start | 1/k0 End | Precursor ID | Area Sample 6 | #Feature | #Feature Sample 6 | Start | End | PTM | AScore | Found By |
| --- | --- | --- | --- | --- | --- | --- | --- | --- | --- | --- | --- | --- | --- | --- | --- | --- | --- | --- | --- |
| K.TITLEVEPSDTIENVK.S | Y | 46.69 | 1786.92 | 16 | 0.39 | 894.4637 | 2 | 17.56 | 1.1442 | 1.1514 | 58994 | 8.78e2 | 1 | 1 | 12 | 27 |  |  | DB Search |
| total 1 peptides |  |  |  |  |  |  |  |  |  |  |  |  |  |  |  |  |  |  |  |
| --- | --- | --- | --- | --- | --- | --- | --- | --- | --- | --- | --- | --- | --- | --- | --- | --- | --- | --- | --- |

Best Unique PSM (Scan Sample\_4\_A1\_1\_3642.d:58994, m/z=894.4637, z=2, RT=17.56, ppm=-4.01):


TRINITY\_DN3271\_c0\_g1\_i1.p1

back to list

  

| Protein Coverage
| Supporting Peptides
| Best Unique PSM
|

Protein Coverage:

Supporting Peptides:

| Peptide | Uniq | -10lgP | Mass | Length | ppm | m/z | z | RT | 1/k0 Start | 1/k0 End | Precursor ID | Area Sample 6 | #Feature | #Feature Sample 6 | Start | End | PTM | AScore | Found By |
| --- | --- | --- | --- | --- | --- | --- | --- | --- | --- | --- | --- | --- | --- | --- | --- | --- | --- | --- | --- |
| K.TIESEAAHGTVTR.H | Y | 45.3 | 1370.679 | 13 | -1.69 | 686.3426 | 2 | 11.96 | 1.0142 | 1.0214 | 26346 | 0 | 0 | 0 | 285 | 297 |  |  | DB Search |
| total 1 peptides |  |  |  |  |  |  |  |  |  |  |  |  |  |  |  |  |  |  |  |
| --- | --- | --- | --- | --- | --- | --- | --- | --- | --- | --- | --- | --- | --- | --- | --- | --- | --- | --- | --- |

Best Unique PSM (Scan Sample\_4\_A1\_1\_3642.d:26346, m/z=686.3426, z=2, RT=11.96, ppm=-6.09):


TRINITY\_DN624\_c0\_g1\_i1.p1

back to list

  

| Protein Coverage
| Supporting Peptides
| Best Unique PSM
|

Protein Coverage:

Supporting Peptides:

| Peptide | Uniq | -10lgP | Mass | Length | ppm | m/z | z | RT | 1/k0 Start | 1/k0 End | Precursor ID | Area Sample 6 | #Feature | #Feature Sample 6 | Start | End | PTM | AScore | Found By |
| --- | --- | --- | --- | --- | --- | --- | --- | --- | --- | --- | --- | --- | --- | --- | --- | --- | --- | --- | --- |
| R.FAIQDITVDEFSAK.E | Y | 45.94 | 1582.7878 | 14 | 3.22 | 792.4003 | 2 | 19.79 | 1.0448 | 1.052 | 74660 | 7.64e2 | 1 | 1 | 132 | 145 |  |  | DB Search |
| total 1 peptides |  |  |  |  |  |  |  |  |  |  |  |  |  |  |  |  |  |  |  |
| --- | --- | --- | --- | --- | --- | --- | --- | --- | --- | --- | --- | --- | --- | --- | --- | --- | --- | --- | --- |

Best Unique PSM (Scan Sample\_4\_A1\_1\_3642.d:74660, m/z=792.4003, z=2, RT=19.79, ppm=-1.18):


TRINITY\_DN15274\_c0\_g1\_i1.p1

back to list

  

| Protein Coverage
| Supporting Peptides
| Best Unique PSM
|

Protein Coverage:

Supporting Peptides:

| Peptide | Uniq | -10lgP | Mass | Length | ppm | m/z | z | RT | 1/k0 Start | 1/k0 End | Precursor ID | Area Sample 6 | #Feature | #Feature Sample 6 | Start | End | PTM | AScore | Found By |
| --- | --- | --- | --- | --- | --- | --- | --- | --- | --- | --- | --- | --- | --- | --- | --- | --- | --- | --- | --- |
| R.LQGWWQSVNGR.Q | Y | 47.16 | 1329.6578 | 11 | -1.01 | 665.8326 | 2 | 18.21 | 0.9449 | 0.9521 | 63598 | 6.83e2 | 1 | 1 | 108 | 118 |  |  | DB Search |
| R.GSC(+57.02)SC(+57.02)YGHAEQC(+57.02)IPTK.G | Y | 47.11 | 1853.7495 | 16 | 8.42 | 927.8857 | 2 | 12.41 | 1.1813 | 1.1885 | 29006 | 8.23e2 | 2 | 2 | 270 | 285 | Carbamidomethylation, Carbamidomethylation, Carbamidomethylation |  | DB Search |
| R.ADIEDVKDLLK.K | Y | 46.82 | 1257.6816 | 11 | 3.16 | 629.8473 | 2 | 17.70 | 0.99 | 0.9973 | 60061 | 2.93e2 | 1 | 1 | 1616 | 1626 |  |  | DB Search |
| K.IQFSISAEINNGALK.C | Y | 46.71 | 1603.8569 | 15 | -0.57 | 802.9318 | 2 | 18.83 | 1.0737 | 1.0809 | 68043 | 0 | 0 | 0 | 759 | 773 |  |  | DB Search |
| K.LGTNSATSDSC(+57.02)DR.E | Y | 46.51 | 1382.5732 | 13 | 0.7 | 692.2913 | 2 | 10.49 | 0.9566 | 0.9638 | 17428 | 0 | 0 | 0 | 1039 | 1051 | Carbamidomethylation |  | DB Search |
| R.YSRPTPGSNNNVM(+15.99)VR.I | Y | 46.5 | 1706.8158 | 15 | 0.06 | 569.9434 | 3 | 11.58 | 0.8311 | 0.8384 | 23889 | 1.61e3 | 1 | 1 | 621 | 635 | Oxidation (M) | M13:Oxidation (M):1000 | DB Search |
| K.ATGNTSGGVC(+57.02)DDC(+57.02)NHNTAGR.Q | Y | 46.19 | 2062.8181 | 20 | 7.38 | 688.6154 | 3 | 9.96 | 0.8579 | 0.8652 | 14102 | 8.31e2 | 1 | 1 | 353 | 372 | Carbamidomethylation, Carbamidomethylation |  | DB Search |
| K.SGGQC(+57.02)PC(+57.02)KPGVVGR.R | Y | 45.97 | 1457.6867 | 14 | 1.98 | 486.9017 | 3 | 10.72 | 0.7864 | 0.7937 | 18818 | 1.41e3 | 2 | 2 | 795 | 808 | Carbamidomethylation, Carbamidomethylation |  | DB Search |
| R.KIDDVIC(+57.02)EER.Y | Y | 44.84 | 1275.6129 | 10 | 10.25 | 638.8174 | 2 | 13.59 | 0.9295 | 0.9368 | 36142 | 1.97e3 | 1 | 1 | 184 | 193 | Carbamidomethylation |  | DB Search |
| total 9 peptides |  |  |  |  |  |  |  |  |  |  |  |  |  |  |  |  |  |  |  |
| --- | --- | --- | --- | --- | --- | --- | --- | --- | --- | --- | --- | --- | --- | --- | --- | --- | --- | --- | --- |

Best Unique PSM (Scan Sample\_4\_A1\_1\_3642.d:63598, m/z=665.8326, z=2, RT=18.21, ppm=-5.41):


TRINITY\_DN11248\_c0\_g1\_i1.p1

back to list

  

| Protein Coverage
| Supporting Peptides
| Best Unique PSM
|

Protein Coverage:

Supporting Peptides:

| Peptide | Uniq | -10lgP | Mass | Length | ppm | m/z | z | RT | 1/k0 Start | 1/k0 End | Precursor ID | Area Sample 6 | #Feature | #Feature Sample 6 | Start | End | PTM | AScore | Found By |
| --- | --- | --- | --- | --- | --- | --- | --- | --- | --- | --- | --- | --- | --- | --- | --- | --- | --- | --- | --- |
| K.SIEAIYLGEFGTC(+57.02)K.S | Y | 46.86 | 1586.765 | 14 | 5.15 | 794.3904 | 2 | 19.64 | 1.1029 | 1.1102 | 73637 | 0 | 0 | 0 | 982 | 995 | Carbamidomethylation |  | DB Search |
| K.TPYLQIDFGK.M | Y | 38.54 | 1180.6128 | 10 | -1.83 | 591.31 | 2 | 18.42 | 0.9198 | 0.9271 | 65300 | 4.11e2 | 1 | 1 | 514 | 523 |  |  | DB Search |
| total 2 peptides |  |  |  |  |  |  |  |  |  |  |  |  |  |  |  |  |  |  |  |
| --- | --- | --- | --- | --- | --- | --- | --- | --- | --- | --- | --- | --- | --- | --- | --- | --- | --- | --- | --- |

Best Unique PSM (Scan Sample\_4\_A1\_1\_3642.d:73637, m/z=794.3904, z=2, RT=19.64, ppm=0.75):


TRINITY\_DN1936\_c0\_g2\_i1.p1

back to list

  

| Protein Coverage
| Supporting Peptides
| Best Unique PSM
|

Protein Coverage:

Supporting Peptides:

| Peptide | Uniq | -10lgP | Mass | Length | ppm | m/z | z | RT | 1/k0 Start | 1/k0 End | Precursor ID | Area Sample 6 | #Feature | #Feature Sample 6 | Start | End | PTM | AScore | Found By |
| --- | --- | --- | --- | --- | --- | --- | --- | --- | --- | --- | --- | --- | --- | --- | --- | --- | --- | --- | --- |
| K.GTTSTAGTGPSFDHTK.G | Y | 43.88 | 1563.7165 | 16 | 5.67 | 782.8665 | 2 | 11.49 | 1.0536 | 1.0608 | 23479 | 0 | 0 | 0 | 636 | 651 |  |  | DB Search |
| total 1 peptides |  |  |  |  |  |  |  |  |  |  |  |  |  |  |  |  |  |  |  |
| --- | --- | --- | --- | --- | --- | --- | --- | --- | --- | --- | --- | --- | --- | --- | --- | --- | --- | --- | --- |

Best Unique PSM (Scan Sample\_4\_A1\_1\_3642.d:23479, m/z=782.8665, z=2, RT=11.49, ppm=1.27):


TRINITY\_DN165536\_c0\_g1\_i1.p1

back to list

  

| Protein Coverage
| Supporting Peptides
| Best Unique PSM
|

Protein Coverage:

Supporting Peptides:

| Peptide | Uniq | -10lgP | Mass | Length | ppm | m/z | z | RT | 1/k0 Start | 1/k0 End | Precursor ID | Area Sample 6 | #Feature | #Feature Sample 6 | Start | End | PTM | AScore | Found By |
| --- | --- | --- | --- | --- | --- | --- | --- | --- | --- | --- | --- | --- | --- | --- | --- | --- | --- | --- | --- |
| K.ATETATGLATTHPIK.L | Y | 37.3 | 1510.7991 | 15 | 4.21 | 756.4067 | 2 | 13.04 | 1.1001 | 1.1074 | 32766 | 0 | 0 | 0 | 161 | 175 |  |  | DB Search |
| total 1 peptides |  |  |  |  |  |  |  |  |  |  |  |  |  |  |  |  |  |  |  |
| --- | --- | --- | --- | --- | --- | --- | --- | --- | --- | --- | --- | --- | --- | --- | --- | --- | --- | --- | --- |

Best Unique PSM (Scan Sample\_4\_A1\_1\_3642.d:32766, m/z=756.4067, z=2, RT=13.04, ppm=-0.19):


TRINITY\_DN1058\_c0\_g1\_i1.p1

back to list

  

| Protein Coverage
| Supporting Peptides
| Best Unique PSM
|

Protein Coverage:

Supporting Peptides:

| Peptide | Uniq | -10lgP | Mass | Length | ppm | m/z | z | RT | 1/k0 Start | 1/k0 End | Precursor ID | Area Sample 6 | #Feature | #Feature Sample 6 | Start | End | PTM | AScore | Found By |
| --- | --- | --- | --- | --- | --- | --- | --- | --- | --- | --- | --- | --- | --- | --- | --- | --- | --- | --- | --- |
| R.APTHILNPANKR.S | Y | 46.93 | 1330.7469 | 12 | 2.71 | 444.5888 | 3 | 11.17 | 0.7559 | 0.7632 | 21058 | 5.5e3 | 2 | 2 | 2314 | 2325 |  |  | DB Search |
| total 1 peptides |  |  |  |  |  |  |  |  |  |  |  |  |  |  |  |  |  |  |  |
| --- | --- | --- | --- | --- | --- | --- | --- | --- | --- | --- | --- | --- | --- | --- | --- | --- | --- | --- | --- |

Best Unique PSM (Scan Sample\_4\_A1\_1\_3642.d:21058, m/z=444.5888, z=3, RT=11.17, ppm=-1.7):


TRINITY\_DN329\_c0\_g1\_i2.p1

back to list

  

| Protein Coverage
| Supporting Peptides
| Best Unique PSM
|

Protein Coverage:

Supporting Peptides:

| Peptide | Uniq | -10lgP | Mass | Length | ppm | m/z | z | RT | 1/k0 Start | 1/k0 End | Precursor ID | Area Sample 6 | #Feature | #Feature Sample 6 | Start | End | PTM | AScore | Found By |
| --- | --- | --- | --- | --- | --- | --- | --- | --- | --- | --- | --- | --- | --- | --- | --- | --- | --- | --- | --- |
| K.LRPGVELTMSSLVNAK.S | Y | 43.26 | 1713.9447 | 16 | 0.82 | 572.3201 | 3 | 17.86 | 0.9097 | 0.917 | 61340 | 0 | 0 | 0 | 287 | 302 |  |  | DB Search |
| K.GLKVDFDTTFAPVTGK.K | Y | 40.84 | 1694.8879 | 16 | -12.98 | 565.9601 | 3 | 18.27 | 0.8339 | 0.8412 | 64168 | 0 | 0 | 0 | 130 | 145 |  |  | DB Search |
| total 2 peptides |  |  |  |  |  |  |  |  |  |  |  |  |  |  |  |  |  |  |  |
| --- | --- | --- | --- | --- | --- | --- | --- | --- | --- | --- | --- | --- | --- | --- | --- | --- | --- | --- | --- |

Best Unique PSM (Scan Sample\_4\_A1\_1\_3642.d:61340, m/z=572.3201, z=3, RT=17.86, ppm=-3.59):


TRINITY\_DN1676\_c0\_g1\_i12.p1

back to list

  

| Protein Coverage
| Supporting Peptides
| Best Unique PSM
|

Protein Coverage:

Supporting Peptides:

| Peptide | Uniq | -10lgP | Mass | Length | ppm | m/z | z | RT | 1/k0 Start | 1/k0 End | Precursor ID | Area Sample 6 | #Feature | #Feature Sample 6 | Start | End | PTM | AScore | Found By |
| --- | --- | --- | --- | --- | --- | --- | --- | --- | --- | --- | --- | --- | --- | --- | --- | --- | --- | --- | --- |
| R.SYYFAISDITVGGR.C | Y | 46.66 | 1547.762 | 14 | -2.17 | 774.8832 | 2 | 19.95 | 1.0472 | 1.0544 | 75627 | 4.27e2 | 1 | 1 | 273 | 286 |  |  | DB Search |
| R.FELDRVNTFGDEVFGDPK.V | Y | 37.28 | 2083.985 | 18 | 5.01 | 695.6694 | 3 | 19.27 | 0.9178 | 0.9251 | 71114 | 5.19e2 | 1 | 1 | 252 | 269 |  |  | DB Search |
| total 2 peptides |  |  |  |  |  |  |  |  |  |  |  |  |  |  |  |  |  |  |  |
| --- | --- | --- | --- | --- | --- | --- | --- | --- | --- | --- | --- | --- | --- | --- | --- | --- | --- | --- | --- |

Best Unique PSM (Scan Sample\_4\_A1\_1\_3642.d:75627, m/z=774.8832, z=2, RT=19.95, ppm=-6.57):


TRINITY\_DN15151\_c0\_g1\_i1.p1

back to list

  

| Protein Coverage
| Supporting Peptides
| Best Unique PSM
|

Protein Coverage:

Supporting Peptides:

| Peptide | Uniq | -10lgP | Mass | Length | ppm | m/z | z | RT | 1/k0 Start | 1/k0 End | Precursor ID | Area Sample 6 | #Feature | #Feature Sample 6 | Start | End | PTM | AScore | Found By |
| --- | --- | --- | --- | --- | --- | --- | --- | --- | --- | --- | --- | --- | --- | --- | --- | --- | --- | --- | --- |
| K.AVAAGMNPMDIKR.G | Y | 40.45 | 1372.6955 | 13 | -2.78 | 687.3501 | 2 | 14.90 | 1.0102 | 1.0174 | 43658 | 5.01e2 | 1 | 1 | 145 | 157 |  |  | DB Search |
| total 1 peptides |  |  |  |  |  |  |  |  |  |  |  |  |  |  |  |  |  |  |  |
| --- | --- | --- | --- | --- | --- | --- | --- | --- | --- | --- | --- | --- | --- | --- | --- | --- | --- | --- | --- |

Best Unique PSM (Scan Sample\_4\_A1\_1\_3642.d:43658, m/z=687.3501, z=2, RT=14.90, ppm=-7.18):


TRINITY\_DN2003\_c0\_g1\_i1.p1

back to list

  

| Protein Coverage
| Supporting Peptides
| Best Unique PSM
|

Protein Coverage:

Supporting Peptides:

| Peptide | Uniq | -10lgP | Mass | Length | ppm | m/z | z | RT | 1/k0 Start | 1/k0 End | Precursor ID | Area Sample 6 | #Feature | #Feature Sample 6 | Start | End | PTM | AScore | Found By |
| --- | --- | --- | --- | --- | --- | --- | --- | --- | --- | --- | --- | --- | --- | --- | --- | --- | --- | --- | --- |
| R.ASMDEGAKPK.E | Y | 46.57 | 1032.491 | 10 | 5.09 | 517.2531 | 2 | 9.36 | 0.8575 | 0.8648 | 10976 | 2.73e2 | 1 | 1 | 133 | 142 |  |  | DB Search |
| total 1 peptides |  |  |  |  |  |  |  |  |  |  |  |  |  |  |  |  |  |  |  |
| --- | --- | --- | --- | --- | --- | --- | --- | --- | --- | --- | --- | --- | --- | --- | --- | --- | --- | --- | --- |

Best Unique PSM (Scan Sample\_4\_A1\_1\_3642.d:10976, m/z=517.2531, z=2, RT=9.36, ppm=0.68):


TRINITY\_DN244\_c0\_g1\_i1.p1

back to list

  

| Protein Coverage
| Supporting Peptides
| Best Unique PSM
|

Protein Coverage:

Supporting Peptides:

| Peptide | Uniq | -10lgP | Mass | Length | ppm | m/z | z | RT | 1/k0 Start | 1/k0 End | Precursor ID | Area Sample 6 | #Feature | #Feature Sample 6 | Start | End | PTM | AScore | Found By |
| --- | --- | --- | --- | --- | --- | --- | --- | --- | --- | --- | --- | --- | --- | --- | --- | --- | --- | --- | --- |
| K.ALLLASSNAFDTSNGAR.E | Y | 46.77 | 1706.8587 | 17 | 2.69 | 854.4352 | 2 | 17.79 | 1.0977 | 1.1049 | 60767 | 2.78e3 | 2 | 2 | 1912 | 1928 |  |  | DB Search |
| R.IDKALLLASSNAFDTSNGAR.E | Y | 46.2 | 2063.0647 | 20 | -3.87 | 688.6898 | 3 | 17.41 | 0.921 | 0.9283 | 58153 | 2.44e2 | 1 | 1 | 1909 | 1928 |  |  | DB Search |
| K.RFDGIFQYTK.D | Y | 42.23 | 1273.6455 | 10 | 4.96 | 637.8304 | 2 | 17.01 | 0.9594 | 0.9666 | 55510 | 0 | 0 | 0 | 678 | 687 |  |  | DB Search |
| K.FSNDIVDSFAVSQKK.T | Y | 39.48 | 1683.8468 | 15 | -4.48 | 562.2845 | 3 | 17.25 | 0.8688 | 0.8761 | 57087 | 0 | 0 | 0 | 295 | 309 |  |  | DB Search |
| total 4 peptides |  |  |  |  |  |  |  |  |  |  |  |  |  |  |  |  |  |  |  |
| --- | --- | --- | --- | --- | --- | --- | --- | --- | --- | --- | --- | --- | --- | --- | --- | --- | --- | --- | --- |

Best Unique PSM (Scan Sample\_4\_A1\_1\_3642.d:60767, m/z=854.4352, z=2, RT=17.79, ppm=-1.71):


TRINITY\_DN477\_c0\_g1\_i8.p1

back to list

  

| Protein Coverage
| Supporting Peptides
| Best Unique PSM
|

Protein Coverage:

Supporting Peptides:

| Peptide | Uniq | -10lgP | Mass | Length | ppm | m/z | z | RT | 1/k0 Start | 1/k0 End | Precursor ID | Area Sample 6 | #Feature | #Feature Sample 6 | Start | End | PTM | AScore | Found By |
| --- | --- | --- | --- | --- | --- | --- | --- | --- | --- | --- | --- | --- | --- | --- | --- | --- | --- | --- | --- |
| R.ILQC(+57.02)NQYANVEEAR.N | Y | 47.19 | 1706.8046 | 14 | 1.57 | 854.4072 | 2 | 14.32 | 1.0821 | 1.0893 | 40295 | 6.26e3 | 1 | 1 | 86 | 99 | Carbamidomethylation |  | DB Search |
| K.DKNILVLTLGVGPQVNR.N | Y | 46.87 | 1835.0628 | 17 | 2.5 | 612.6937 | 3 | 19.53 | 0.8408 | 0.8481 | 72572 | 1.06e4 | 3 | 3 | 168 | 184 |  |  | DB Search |
| R.SQSAFPTRR.R | Y | 45.57 | 1048.5414 | 9 | 1.94 | 525.2767 | 2 | 11.05 | 0.8514 | 0.8587 | 20691 | 6.92e3 | 1 | 1 | 856 | 864 |  |  | DB Search |
| total 3 peptides |  |  |  |  |  |  |  |  |  |  |  |  |  |  |  |  |  |  |  |
| --- | --- | --- | --- | --- | --- | --- | --- | --- | --- | --- | --- | --- | --- | --- | --- | --- | --- | --- | --- |

Best Unique PSM (Scan Sample\_4\_A1\_1\_3642.d:40295, m/z=854.4072, z=2, RT=14.32, ppm=-2.83):


TRINITY\_DN6920\_c0\_g1\_i4.p1

back to list

  

| Protein Coverage
| Supporting Peptides
| Best Unique PSM
|

Protein Coverage:

Supporting Peptides:

| Peptide | Uniq | -10lgP | Mass | Length | ppm | m/z | z | RT | 1/k0 Start | 1/k0 End | Precursor ID | Area Sample 6 | #Feature | #Feature Sample 6 | Start | End | PTM | AScore | Found By |
| --- | --- | --- | --- | --- | --- | --- | --- | --- | --- | --- | --- | --- | --- | --- | --- | --- | --- | --- | --- |
| R.VLVHTQQK.L | Y | 46.31 | 951.5501 | 8 | 1.77 | 476.7811 | 2 | 9.52 | 0.8331 | 0.8404 | 11777 | 3.85e2 | 1 | 1 | 40 | 47 |  |  | DB Search |
| total 1 peptides |  |  |  |  |  |  |  |  |  |  |  |  |  |  |  |  |  |  |  |
| --- | --- | --- | --- | --- | --- | --- | --- | --- | --- | --- | --- | --- | --- | --- | --- | --- | --- | --- | --- |

Best Unique PSM (Scan Sample\_4\_A1\_1\_3642.d:11777, m/z=476.7811, z=2, RT=9.52, ppm=-2.63):


TRINITY\_DN3545\_c0\_g1\_i1.p1

back to list

  

| Protein Coverage
| Supporting Peptides
| Best Unique PSM
|

Protein Coverage:

Supporting Peptides:

| Peptide | Uniq | -10lgP | Mass | Length | ppm | m/z | z | RT | 1/k0 Start | 1/k0 End | Precursor ID | Area Sample 6 | #Feature | #Feature Sample 6 | Start | End | PTM | AScore | Found By |
| --- | --- | --- | --- | --- | --- | --- | --- | --- | --- | --- | --- | --- | --- | --- | --- | --- | --- | --- | --- |
| R.SC(+57.02)TNPAPANGGK.P | Y | 47.22 | 1172.5244 | 12 | 1.68 | 587.2679 | 2 | 9.40 | 0.8429 | 0.8502 | 11161 | 2.16e2 | 1 | 1 | 55 | 66 | Carbamidomethylation |  | DB Search |
| total 1 peptides |  |  |  |  |  |  |  |  |  |  |  |  |  |  |  |  |  |  |  |
| --- | --- | --- | --- | --- | --- | --- | --- | --- | --- | --- | --- | --- | --- | --- | --- | --- | --- | --- | --- |

Best Unique PSM (Scan Sample\_4\_A1\_1\_3642.d:11161, m/z=587.2679, z=2, RT=9.40, ppm=-2.72):


TRINITY\_DN105041\_c0\_g1\_i1.p1

back to list

  

| Protein Coverage
| Supporting Peptides
| Best Unique PSM
|

Protein Coverage:

Supporting Peptides:

| Peptide | Uniq | -10lgP | Mass | Length | ppm | m/z | z | RT | 1/k0 Start | 1/k0 End | Precursor ID | Area Sample 6 | #Feature | #Feature Sample 6 | Start | End | PTM | AScore | Found By |
| --- | --- | --- | --- | --- | --- | --- | --- | --- | --- | --- | --- | --- | --- | --- | --- | --- | --- | --- | --- |
| K.LTIIDALPR.C | Y | 46.48 | 1010.6124 | 9 | 5.92 | 506.3142 | 2 | 18.91 | 0.8445 | 0.8518 | 68779 | 8.74e2 | 1 | 1 | 58 | 66 |  |  | DB Search |
| total 1 peptides |  |  |  |  |  |  |  |  |  |  |  |  |  |  |  |  |  |  |  |
| --- | --- | --- | --- | --- | --- | --- | --- | --- | --- | --- | --- | --- | --- | --- | --- | --- | --- | --- | --- |

Best Unique PSM (Scan Sample\_4\_A1\_1\_3642.d:68779, m/z=506.3142, z=2, RT=18.91, ppm=1.51):


TRINITY\_DN26392\_c0\_g1\_i1.p1

back to list

  

| Protein Coverage
| Supporting Peptides
| Best Unique PSM
|

Protein Coverage:

Supporting Peptides:

| Peptide | Uniq | -10lgP | Mass | Length | ppm | m/z | z | RT | 1/k0 Start | 1/k0 End | Precursor ID | Area Sample 6 | #Feature | #Feature Sample 6 | Start | End | PTM | AScore | Found By |
| --- | --- | --- | --- | --- | --- | --- | --- | --- | --- | --- | --- | --- | --- | --- | --- | --- | --- | --- | --- |
| K.ADLDIQKPIPIVSK.Y | Y | 43.74 | 1535.8922 | 14 | 3.09 | 768.9524 | 2 | 16.14 | 1.0753 | 1.0825 | 50729 | 0 | 0 | 0 | 127 | 140 |  |  | DB Search |
| total 1 peptides |  |  |  |  |  |  |  |  |  |  |  |  |  |  |  |  |  |  |  |
| --- | --- | --- | --- | --- | --- | --- | --- | --- | --- | --- | --- | --- | --- | --- | --- | --- | --- | --- | --- |

Best Unique PSM (Scan Sample\_4\_A1\_1\_3642.d:50729, m/z=768.9524, z=2, RT=16.14, ppm=-1.31):


TRINITY\_DN2477\_c0\_g1\_i5.p1

back to list

  

| Protein Coverage
| Supporting Peptides
| Best Unique PSM
|

Protein Coverage:

Supporting Peptides:

| Peptide | Uniq | -10lgP | Mass | Length | ppm | m/z | z | RT | 1/k0 Start | 1/k0 End | Precursor ID | Area Sample 6 | #Feature | #Feature Sample 6 | Start | End | PTM | AScore | Found By |
| --- | --- | --- | --- | --- | --- | --- | --- | --- | --- | --- | --- | --- | --- | --- | --- | --- | --- | --- | --- |
| K.DSYVGDEAQSKR.G | Y | 46.51 | 1353.616 | 12 | 5.22 | 677.8159 | 2 | 10.97 | 1.0005 | 1.0077 | 20266 | 2.51e3 | 1 | 1 | 8 | 19 |  |  | DB Search |
| R.VAPEEHPVLLTEAPLNPK.A | Y | 46.46 | 1953.0571 | 18 | 9.42 | 977.5407 | 2 | 16.61 | 1.2085 | 1.2157 | 53280 | 0 | 0 | 0 | 53 | 70 |  |  | DB Search |
| total 2 peptides |  |  |  |  |  |  |  |  |  |  |  |  |  |  |  |  |  |  |  |
| --- | --- | --- | --- | --- | --- | --- | --- | --- | --- | --- | --- | --- | --- | --- | --- | --- | --- | --- | --- |

Best Unique PSM (Scan Sample\_4\_A1\_1\_3642.d:20266, m/z=677.8159, z=2, RT=10.97, ppm=0.82):


TRINITY\_DN2472\_c0\_g1\_i1.p1

back to list

  

| Protein Coverage
| Supporting Peptides
| Best Unique PSM
|

Protein Coverage:

Supporting Peptides:

| Peptide | Uniq | -10lgP | Mass | Length | ppm | m/z | z | RT | 1/k0 Start | 1/k0 End | Precursor ID | Area Sample 6 | #Feature | #Feature Sample 6 | Start | End | PTM | AScore | Found By |
| --- | --- | --- | --- | --- | --- | --- | --- | --- | --- | --- | --- | --- | --- | --- | --- | --- | --- | --- | --- |
| M.S(+42.01)SGEIESLRQEAENLR.N | Y | 46.98 | 1858.9021 | 16 | -0.12 | 930.4541 | 2 | 18.84 | 1.1961 | 1.2033 | 67975 | 1.99e3 | 1 | 1 | 2 | 17 | Acetylation (Protein N-term) | S1:Acetylation (Protein N-term):1000 | DB Search |
| total 1 peptides |  |  |  |  |  |  |  |  |  |  |  |  |  |  |  |  |  |  |  |
| --- | --- | --- | --- | --- | --- | --- | --- | --- | --- | --- | --- | --- | --- | --- | --- | --- | --- | --- | --- |

Best Unique PSM (Scan Sample\_4\_A1\_1\_3642.d:67975, m/z=930.4541, z=2, RT=18.84, ppm=-4.52):


TRINITY\_DN161\_c0\_g1\_i2.p1

back to list

  

| Protein Coverage
| Supporting Peptides
| Best Unique PSM
|

Protein Coverage:

Supporting Peptides:

| Peptide | Uniq | -10lgP | Mass | Length | ppm | m/z | z | RT | 1/k0 Start | 1/k0 End | Precursor ID | Area Sample 6 | #Feature | #Feature Sample 6 | Start | End | PTM | AScore | Found By |
| --- | --- | --- | --- | --- | --- | --- | --- | --- | --- | --- | --- | --- | --- | --- | --- | --- | --- | --- | --- |
| K.SYELPDGQVITIGNER.F | Y | 47.19 | 1789.8846 | 16 | -1.46 | 895.9443 | 2 | 18.64 | 1.1122 | 1.1194 | 66524 | 6.05e2 | 1 | 1 | 262 | 277 |  |  | DB Search |
| R.AVFPSIVGRPR.H | Y | 47.19 | 1197.6982 | 11 | 2.39 | 599.8552 | 2 | 15.88 | 0.9444 | 0.9517 | 49107 | 3.41e4 | 1 | 1 | 52 | 62 |  |  | DB Search |
| R.LDLAGRDLTDYLMK.I | Y | 46.87 | 1622.8338 | 14 | -1.16 | 541.9489 | 3 | 19.39 | 0.7966 | 0.8039 | 71871 | 4.27e2 | 1 | 1 | 201 | 214 |  |  | DB Search |
| K.DLYANTVLSGGTTM(+15.99)FPGIADR.M | Y | 46.72 | 2214.0626 | 21 | 10.66 | 1108.0455 | 2 | 19.50 | 1.2475 | 1.2547 | 72698 | 0 | 0 | 0 | 315 | 335 | Oxidation (M) | M14:Oxidation (M):1000 | DB Search |
| K.DSYVGDEAQSKR.G | Y | 46.51 | 1353.616 | 12 | 5.22 | 677.8159 | 2 | 10.97 | 1.0005 | 1.0077 | 20266 | 2.51e3 | 1 | 1 | 74 | 85 |  |  | DB Search |
| R.VAPEEHPVLLTEAPLNPK.A | Y | 46.46 | 1953.0571 | 18 | 9.42 | 977.5407 | 2 | 16.61 | 1.2085 | 1.2157 | 53280 | 0 | 0 | 0 | 119 | 136 |  |  | DB Search |
| R.HQGVM(+15.99)VGM(+15.99)GQK.D | Y | 43.4 | 1202.5536 | 11 | 4.58 | 602.2842 | 2 | 9.74 | 0.9081 | 0.9154 | 12838 | 6.82e2 | 1 | 1 | 63 | 73 | Oxidation (M), Oxidation (M) | M5:Oxidation (M):1000 M8:Oxidation (M):1000 | DB Search |
| R.DLTDYLMK.I | Y | 37.13 | 997.479 | 8 | 2.04 | 499.7456 | 2 | 18.64 | 0.8372 | 0.8445 | 66608 | 3.54e2 | 1 | 1 | 207 | 214 |  |  | DB Search |
| total 8 peptides |  |  |  |  |  |  |  |  |  |  |  |  |  |  |  |  |  |  |  |
| --- | --- | --- | --- | --- | --- | --- | --- | --- | --- | --- | --- | --- | --- | --- | --- | --- | --- | --- | --- |

Best Unique PSM (Scan Sample\_4\_A1\_1\_3642.d:66524, m/z=895.9443, z=2, RT=18.64, ppm=-5.86):


TRINITY\_DN36\_c0\_g1\_i13.p1

back to list

  

| Protein Coverage
| Supporting Peptides
| Best Unique PSM
|

Protein Coverage:

Supporting Peptides:

| Peptide | Uniq | -10lgP | Mass | Length | ppm | m/z | z | RT | 1/k0 Start | 1/k0 End | Precursor ID | Area Sample 6 | #Feature | #Feature Sample 6 | Start | End | PTM | AScore | Found By |
| --- | --- | --- | --- | --- | --- | --- | --- | --- | --- | --- | --- | --- | --- | --- | --- | --- | --- | --- | --- |
| R.LGGIEVTQGNC(+57.02)AR.K | Y | 45.45 | 1373.6721 | 13 | 2.96 | 687.8423 | 2 | 13.87 | 0.9687 | 0.9759 | 37701 | 0 | 0 | 0 | 57 | 69 | Carbamidomethylation |  | DB Search |
| K.FETQIATK.D | Y | 44.83 | 936.4916 | 8 | 7 | 937.5013 | 1 | 13.97 | 1.1802 | 1.1873 | 38247 | 0 | 0 | 0 | 166 | 173 |  |  | DB Search |
| total 2 peptides |  |  |  |  |  |  |  |  |  |  |  |  |  |  |  |  |  |  |  |
| --- | --- | --- | --- | --- | --- | --- | --- | --- | --- | --- | --- | --- | --- | --- | --- | --- | --- | --- | --- |

Best Unique PSM (Scan Sample\_4\_A1\_1\_3642.d:37701, m/z=687.8423, z=2, RT=13.87, ppm=-1.44):


TRINITY\_DN106\_c0\_g1\_i10.p1

back to list

  

| Protein Coverage
| Supporting Peptides
| Best Unique PSM
|

Protein Coverage:

Supporting Peptides:

| Peptide | Uniq | -10lgP | Mass | Length | ppm | m/z | z | RT | 1/k0 Start | 1/k0 End | Precursor ID | Area Sample 6 | #Feature | #Feature Sample 6 | Start | End | PTM | AScore | Found By |
| --- | --- | --- | --- | --- | --- | --- | --- | --- | --- | --- | --- | --- | --- | --- | --- | --- | --- | --- | --- |
| R.DGPAGPAGR.D | Y | 46.52 | 796.3827 | 9 | 7.09 | 399.1997 | 2 | 9.76 | 0.7233 | 0.7306 | 13098 | 1.65e3 | 1 | 1 | 283 | 291 |  |  | DB Search |
| total 1 peptides |  |  |  |  |  |  |  |  |  |  |  |  |  |  |  |  |  |  |  |
| --- | --- | --- | --- | --- | --- | --- | --- | --- | --- | --- | --- | --- | --- | --- | --- | --- | --- | --- | --- |

Best Unique PSM (Scan Sample\_4\_A1\_1\_3642.d:13098, m/z=399.1997, z=2, RT=9.76, ppm=2.68):


TRINITY\_DN96161\_c0\_g1\_i1.p1

back to list

  

| Protein Coverage
| Supporting Peptides
| Best Unique PSM
|

Protein Coverage:

Supporting Peptides:

| Peptide | Uniq | -10lgP | Mass | Length | ppm | m/z | z | RT | 1/k0 Start | 1/k0 End | Precursor ID | Area Sample 6 | #Feature | #Feature Sample 6 | Start | End | PTM | AScore | Found By |
| --- | --- | --- | --- | --- | --- | --- | --- | --- | --- | --- | --- | --- | --- | --- | --- | --- | --- | --- | --- |
| K.VGINYQPPTVVPGGDLAK.V | Y | 37.08 | 1823.9781 | 18 | 0.87 | 912.9931 | 2 | 17.39 | 1.1218 | 1.129 | 57959 | 0 | 0 | 0 | 353 | 370 |  |  | DB Search |
| total 1 peptides |  |  |  |  |  |  |  |  |  |  |  |  |  |  |  |  |  |  |  |
| --- | --- | --- | --- | --- | --- | --- | --- | --- | --- | --- | --- | --- | --- | --- | --- | --- | --- | --- | --- |

Best Unique PSM (Scan Sample\_4\_A1\_1\_3642.d:57959, m/z=912.4467, z=1, RT=17.39, ppm=-3.53):


TRINITY\_DN984\_c0\_g1\_i1.p1

back to list

  

| Protein Coverage
| Supporting Peptides
| Best Unique PSM
|

Protein Coverage:

Supporting Peptides:

| Peptide | Uniq | -10lgP | Mass | Length | ppm | m/z | z | RT | 1/k0 Start | 1/k0 End | Precursor ID | Area Sample 6 | #Feature | #Feature Sample 6 | Start | End | PTM | AScore | Found By |
| --- | --- | --- | --- | --- | --- | --- | --- | --- | --- | --- | --- | --- | --- | --- | --- | --- | --- | --- | --- |
| R.EGPTGPQGETGR.R | Y | 47.26 | 1184.5421 | 12 | 0.95 | 593.2763 | 2 | 10.36 | 0.8798 | 0.887 | 16389 | 1.9e3 | 1 | 1 | 476 | 487 |  |  | DB Search |
| total 1 peptides |  |  |  |  |  |  |  |  |  |  |  |  |  |  |  |  |  |  |  |
| --- | --- | --- | --- | --- | --- | --- | --- | --- | --- | --- | --- | --- | --- | --- | --- | --- | --- | --- | --- |

Best Unique PSM (Scan Sample\_4\_A1\_1\_3642.d:16389, m/z=593.2763, z=2, RT=10.36, ppm=-3.45):


TRINITY\_DN4815\_c0\_g1\_i1.p1

back to list

  

| Protein Coverage
| Supporting Peptides
| Best Unique PSM
|

Protein Coverage:

Supporting Peptides:

| Peptide | Uniq | -10lgP | Mass | Length | ppm | m/z | z | RT | 1/k0 Start | 1/k0 End | Precursor ID | Area Sample 6 | #Feature | #Feature Sample 6 | Start | End | PTM | AScore | Found By |
| --- | --- | --- | --- | --- | --- | --- | --- | --- | --- | --- | --- | --- | --- | --- | --- | --- | --- | --- | --- |
| R.AVAC(+57.02)NSGPC(+57.02)LAGC(+57.02)PR.N | Y | 47.15 | 1588.6908 | 15 | 1.52 | 795.3504 | 2 | 13.09 | 1.0178 | 1.025 | 33019 | 7.5e2 | 1 | 1 | 425 | 439 | Carbamidomethylation, Carbamidomethylation, Carbamidomethylation |  | DB Search |
| total 1 peptides |  |  |  |  |  |  |  |  |  |  |  |  |  |  |  |  |  |  |  |
| --- | --- | --- | --- | --- | --- | --- | --- | --- | --- | --- | --- | --- | --- | --- | --- | --- | --- | --- | --- |

Best Unique PSM (Scan Sample\_4\_A1\_1\_3642.d:33019, m/z=795.3504, z=2, RT=13.09, ppm=-2.88):


TRINITY\_DN4066\_c0\_g1\_i1.p1

back to list

  

| Protein Coverage
| Supporting Peptides
| Best Unique PSM
|

Protein Coverage:

Supporting Peptides:

| Peptide | Uniq | -10lgP | Mass | Length | ppm | m/z | z | RT | 1/k0 Start | 1/k0 End | Precursor ID | Area Sample 6 | #Feature | #Feature Sample 6 | Start | End | PTM | AScore | Found By |
| --- | --- | --- | --- | --- | --- | --- | --- | --- | --- | --- | --- | --- | --- | --- | --- | --- | --- | --- | --- |
| R.ALTEITFAHELGHSFGAQHDPNTK.E | Y | 42.4 | 2620.267 | 24 | -0.69 | 656.0707 | 4 | 17.24 | 0.9093 | 0.9166 | 56979 | 7.14e2 | 1 | 1 | 372 | 395 |  |  | DB Search |
| total 1 peptides |  |  |  |  |  |  |  |  |  |  |  |  |  |  |  |  |  |  |  |
| --- | --- | --- | --- | --- | --- | --- | --- | --- | --- | --- | --- | --- | --- | --- | --- | --- | --- | --- | --- |

Best Unique PSM (Scan Sample\_4\_A1\_1\_3642.d:56979, m/z=656.0707, z=4, RT=17.24, ppm=-5.09):


TRINITY\_DN11377\_c0\_g1\_i1.p1

back to list

  

| Protein Coverage
| Supporting Peptides
| Best Unique PSM
|

Protein Coverage:

Supporting Peptides:

| Peptide | Uniq | -10lgP | Mass | Length | ppm | m/z | z | RT | 1/k0 Start | 1/k0 End | Precursor ID | Area Sample 6 | #Feature | #Feature Sample 6 | Start | End | PTM | AScore | Found By |
| --- | --- | --- | --- | --- | --- | --- | --- | --- | --- | --- | --- | --- | --- | --- | --- | --- | --- | --- | --- |
| R.RIVLLATDALPK.I | Y | 43.02 | 1308.8129 | 12 | 3.07 | 655.4128 | 2 | 17.30 | 1.0142 | 1.0214 | 57454 | 1.74e2 | 1 | 1 | 265 | 276 |  |  | DB Search |
| total 1 peptides |  |  |  |  |  |  |  |  |  |  |  |  |  |  |  |  |  |  |  |
| --- | --- | --- | --- | --- | --- | --- | --- | --- | --- | --- | --- | --- | --- | --- | --- | --- | --- | --- | --- |

Best Unique PSM (Scan Sample\_4\_A1\_1\_3642.d:57454, m/z=655.4128, z=2, RT=17.30, ppm=-1.33):


TRINITY\_DN2217\_c0\_g1\_i3.p1

back to list

  

| Protein Coverage
| Supporting Peptides
| Best Unique PSM
|

Protein Coverage:

Supporting Peptides:

| Peptide | Uniq | -10lgP | Mass | Length | ppm | m/z | z | RT | 1/k0 Start | 1/k0 End | Precursor ID | Area Sample 6 | #Feature | #Feature Sample 6 | Start | End | PTM | AScore | Found By |
| --- | --- | --- | --- | --- | --- | --- | --- | --- | --- | --- | --- | --- | --- | --- | --- | --- | --- | --- | --- |
| K.ATGC(+57.02)EVIWNQHNR.G | Y | 46.53 | 1583.7263 | 13 | 3.17 | 792.8694 | 2 | 14.02 | 1.0785 | 1.0857 | 38485 | 7.79e2 | 1 | 1 | 431 | 443 | Carbamidomethylation |  | DB Search |
| R.GC(+57.02)YIHTQEIAR.G | Y | 46.47 | 1346.6401 | 11 | -0.76 | 674.3239 | 2 | 13.04 | 1.0158 | 1.023 | 32770 | 0 | 0 | 0 | 538 | 548 | Carbamidomethylation |  | DB Search |
| total 2 peptides |  |  |  |  |  |  |  |  |  |  |  |  |  |  |  |  |  |  |  |
| --- | --- | --- | --- | --- | --- | --- | --- | --- | --- | --- | --- | --- | --- | --- | --- | --- | --- | --- | --- |

Best Unique PSM (Scan Sample\_4\_A1\_1\_3642.d:38485, m/z=792.8694, z=2, RT=14.02, ppm=-1.23):


TRINITY\_DN4494\_c1\_g1\_i2.p1

back to list

  

| Protein Coverage
| Supporting Peptides
| Best Unique PSM
|

Protein Coverage:

Supporting Peptides:

| Peptide | Uniq | -10lgP | Mass | Length | ppm | m/z | z | RT | 1/k0 Start | 1/k0 End | Precursor ID | Area Sample 6 | #Feature | #Feature Sample 6 | Start | End | PTM | AScore | Found By |
| --- | --- | --- | --- | --- | --- | --- | --- | --- | --- | --- | --- | --- | --- | --- | --- | --- | --- | --- | --- |
| K.VDVGFILDSSGSLR.N | Y | 45.76 | 1463.762 | 14 | -1.21 | 732.8842 | 2 | 19.93 | 1.0102 | 1.0174 | 75559 | 4.99e2 | 1 | 1 | 318 | 331 |  |  | DB Search |
| total 1 peptides |  |  |  |  |  |  |  |  |  |  |  |  |  |  |  |  |  |  |  |
| --- | --- | --- | --- | --- | --- | --- | --- | --- | --- | --- | --- | --- | --- | --- | --- | --- | --- | --- | --- |

Best Unique PSM (Scan Sample\_4\_A1\_1\_3642.d:75559, m/z=732.8842, z=2, RT=19.93, ppm=-5.61):


TRINITY\_DN477\_c0\_g1\_i3.p1

back to list

  

| Protein Coverage
| Supporting Peptides
| Best Unique PSM
|

Protein Coverage:

Supporting Peptides:

| Peptide | Uniq | -10lgP | Mass | Length | ppm | m/z | z | RT | 1/k0 Start | 1/k0 End | Precursor ID | Area Sample 6 | #Feature | #Feature Sample 6 | Start | End | PTM | AScore | Found By |
| --- | --- | --- | --- | --- | --- | --- | --- | --- | --- | --- | --- | --- | --- | --- | --- | --- | --- | --- | --- |
| R.NDVNNPPQAR.R | Y | 47.24 | 1123.537 | 10 | 0.77 | 562.7737 | 2 | 9.95 | 0.866 | 0.8733 | 13858 | 3.59e3 | 2 | 2 | 147 | 156 |  |  | DB Search |
| total 1 peptides |  |  |  |  |  |  |  |  |  |  |  |  |  |  |  |  |  |  |  |
| --- | --- | --- | --- | --- | --- | --- | --- | --- | --- | --- | --- | --- | --- | --- | --- | --- | --- | --- | --- |

Best Unique PSM (Scan Sample\_4\_A1\_1\_3642.d:13858, m/z=562.7737, z=2, RT=9.95, ppm=-3.64):


TRINITY\_DN3347\_c0\_g1\_i1.p1

back to list

  

| Protein Coverage
| Supporting Peptides
| Best Unique PSM
|

Protein Coverage:

Supporting Peptides:

| Peptide | Uniq | -10lgP | Mass | Length | ppm | m/z | z | RT | 1/k0 Start | 1/k0 End | Precursor ID | Area Sample 6 | #Feature | #Feature Sample 6 | Start | End | PTM | AScore | Found By |
| --- | --- | --- | --- | --- | --- | --- | --- | --- | --- | --- | --- | --- | --- | --- | --- | --- | --- | --- | --- |
| R.ATDIDQEVKEK.S | Y | 46.52 | 1274.6354 | 11 | 0.55 | 638.3225 | 2 | 11.59 | 0.9634 | 0.9707 | 24012 | 8.17e2 | 1 | 1 | 263 | 273 |  |  | DB Search |
| total 1 peptides |  |  |  |  |  |  |  |  |  |  |  |  |  |  |  |  |  |  |  |
| --- | --- | --- | --- | --- | --- | --- | --- | --- | --- | --- | --- | --- | --- | --- | --- | --- | --- | --- | --- |

Best Unique PSM (Scan Sample\_4\_A1\_1\_3642.d:24012, m/z=638.3225, z=2, RT=11.59, ppm=-3.85):


TRINITY\_DN59572\_c0\_g1\_i1.p1

back to list

  

| Protein Coverage
| Supporting Peptides
| Best Unique PSM
|

Protein Coverage:

Supporting Peptides:

| Peptide | Uniq | -10lgP | Mass | Length | ppm | m/z | z | RT | 1/k0 Start | 1/k0 End | Precursor ID | Area Sample 6 | #Feature | #Feature Sample 6 | Start | End | PTM | AScore | Found By |
| --- | --- | --- | --- | --- | --- | --- | --- | --- | --- | --- | --- | --- | --- | --- | --- | --- | --- | --- | --- |
| K.SYFEHSFVDLHR.I | Y | 46.86 | 1535.7157 | 12 | 4.7 | 512.9127 | 3 | 16.16 | 0.7673 | 0.7746 | 50725 | 1.42e3 | 1 | 1 | 107 | 118 |  |  | DB Search |
| total 1 peptides |  |  |  |  |  |  |  |  |  |  |  |  |  |  |  |  |  |  |  |
| --- | --- | --- | --- | --- | --- | --- | --- | --- | --- | --- | --- | --- | --- | --- | --- | --- | --- | --- | --- |

Best Unique PSM (Scan Sample\_4\_A1\_1\_3642.d:50725, m/z=512.9127, z=3, RT=16.16, ppm=0.29):


TRINITY\_DN585\_c0\_g1\_i10.p1

back to list

  

| Protein Coverage
| Supporting Peptides
| Best Unique PSM
|

Protein Coverage:

Supporting Peptides:

| Peptide | Uniq | -10lgP | Mass | Length | ppm | m/z | z | RT | 1/k0 Start | 1/k0 End | Precursor ID | Area Sample 6 | #Feature | #Feature Sample 6 | Start | End | PTM | AScore | Found By |
| --- | --- | --- | --- | --- | --- | --- | --- | --- | --- | --- | --- | --- | --- | --- | --- | --- | --- | --- | --- |
| K.TITLEVEPSDTIENVK.S | Y | 46.69 | 1786.92 | 16 | 0.39 | 894.4637 | 2 | 17.56 | 1.1442 | 1.1514 | 58994 | 8.78e2 | 1 | 1 | 12 | 27 |  |  | DB Search |
| total 1 peptides |  |  |  |  |  |  |  |  |  |  |  |  |  |  |  |  |  |  |  |
| --- | --- | --- | --- | --- | --- | --- | --- | --- | --- | --- | --- | --- | --- | --- | --- | --- | --- | --- | --- |

Best Unique PSM (Scan Sample\_4\_A1\_1\_3642.d:58994, m/z=894.4637, z=2, RT=17.56, ppm=-4.01):


TRINITY\_DN3016\_c0\_g1\_i2.p1

back to list

  

| Protein Coverage
| Supporting Peptides
| Best Unique PSM
|

Protein Coverage:

Supporting Peptides:

| Peptide | Uniq | -10lgP | Mass | Length | ppm | m/z | z | RT | 1/k0 Start | 1/k0 End | Precursor ID | Area Sample 6 | #Feature | #Feature Sample 6 | Start | End | PTM | AScore | Found By |
| --- | --- | --- | --- | --- | --- | --- | --- | --- | --- | --- | --- | --- | --- | --- | --- | --- | --- | --- | --- |
| R.IVNTDLQEIKDIIK.G | Y | 45.77 | 1640.9348 | 14 | -1.11 | 547.9825 | 3 | 18.81 | 0.8177 | 0.825 | 67904 | 0 | 0 | 0 | 154 | 167 |  |  | DB Search |
| total 1 peptides |  |  |  |  |  |  |  |  |  |  |  |  |  |  |  |  |  |  |  |
| --- | --- | --- | --- | --- | --- | --- | --- | --- | --- | --- | --- | --- | --- | --- | --- | --- | --- | --- | --- |

Best Unique PSM (Scan Sample\_4\_A1\_1\_3642.d:67904, m/z=547.9825, z=3, RT=18.81, ppm=-5.51):


TRINITY\_DN10094\_c0\_g1\_i1.p1

back to list

  

| Protein Coverage
| Supporting Peptides
| Best Unique PSM
|

Protein Coverage:

Supporting Peptides:

| Peptide | Uniq | -10lgP | Mass | Length | ppm | m/z | z | RT | 1/k0 Start | 1/k0 End | Precursor ID | Area Sample 6 | #Feature | #Feature Sample 6 | Start | End | PTM | AScore | Found By |
| --- | --- | --- | --- | --- | --- | --- | --- | --- | --- | --- | --- | --- | --- | --- | --- | --- | --- | --- | --- |
| K.FFFHDQSC(+57.02)LDVYK.Y | Y | 46.21 | 1704.7606 | 13 | 2.99 | 569.26 | 3 | 17.76 | 0.8067 | 0.814 | 60434 | 5.36e2 | 1 | 1 | 221 | 233 | Carbamidomethylation |  | DB Search |
| total 1 peptides |  |  |  |  |  |  |  |  |  |  |  |  |  |  |  |  |  |  |  |
| --- | --- | --- | --- | --- | --- | --- | --- | --- | --- | --- | --- | --- | --- | --- | --- | --- | --- | --- | --- |

Best Unique PSM (Scan Sample\_4\_A1\_1\_3642.d:60434, m/z=569.26, z=3, RT=17.76, ppm=-1.41):


TRINITY\_DN61\_c0\_g1\_i1.p1

back to list

  

| Protein Coverage
| Supporting Peptides
| Best Unique PSM
|

Protein Coverage:

Supporting Peptides:

| Peptide | Uniq | -10lgP | Mass | Length | ppm | m/z | z | RT | 1/k0 Start | 1/k0 End | Precursor ID | Area Sample 6 | #Feature | #Feature Sample 6 | Start | End | PTM | AScore | Found By |
| --- | --- | --- | --- | --- | --- | --- | --- | --- | --- | --- | --- | --- | --- | --- | --- | --- | --- | --- | --- |
| R.AINIHDLVGIDTKK.E | Y | 46.89 | 1535.8671 | 14 | 1.14 | 512.9613 | 3 | 16.65 | 0.7913 | 0.7986 | 53503 | 2.9e3 | 1 | 1 | 339 | 352 |  |  | DB Search |
| total 1 peptides |  |  |  |  |  |  |  |  |  |  |  |  |  |  |  |  |  |  |  |
| --- | --- | --- | --- | --- | --- | --- | --- | --- | --- | --- | --- | --- | --- | --- | --- | --- | --- | --- | --- |

Best Unique PSM (Scan Sample\_4\_A1\_1\_3642.d:53503, m/z=512.9613, z=3, RT=16.65, ppm=-3.27):


TRINITY\_DN1936\_c0\_g1\_i3.p1

back to list

  

| Protein Coverage
| Supporting Peptides
| Best Unique PSM
|

Protein Coverage:

Supporting Peptides:

| Peptide | Uniq | -10lgP | Mass | Length | ppm | m/z | z | RT | 1/k0 Start | 1/k0 End | Precursor ID | Area Sample 6 | #Feature | #Feature Sample 6 | Start | End | PTM | AScore | Found By |
| --- | --- | --- | --- | --- | --- | --- | --- | --- | --- | --- | --- | --- | --- | --- | --- | --- | --- | --- | --- |
| R.YGNQC(+57.02)PAPR.E | Y | 46.39 | 1061.4713 | 9 | 9.38 | 531.7455 | 2 | 10.33 | 0.8518 | 0.8591 | 16370 | 2.83e2 | 1 | 1 | 954 | 962 | Carbamidomethylation |  | DB Search |
| R.ADKFDWLR.N | Y | 44.56 | 1049.5294 | 8 | -0.14 | 525.7696 | 2 | 17.03 | 0.8603 | 0.8676 | 55648 | 3.58e2 | 1 | 1 | 285 | 292 |  |  | DB Search |
| total 2 peptides |  |  |  |  |  |  |  |  |  |  |  |  |  |  |  |  |  |  |  |
| --- | --- | --- | --- | --- | --- | --- | --- | --- | --- | --- | --- | --- | --- | --- | --- | --- | --- | --- | --- |

Best Unique PSM (Scan Sample\_4\_A1\_1\_3642.d:16370, m/z=531.7455, z=2, RT=10.33, ppm=4.98):


TRINITY\_DN585\_c0\_g1\_i3.p1

back to list

  

| Protein Coverage
| Supporting Peptides
| Best Unique PSM
|

Protein Coverage:

Supporting Peptides:

| Peptide | Uniq | -10lgP | Mass | Length | ppm | m/z | z | RT | 1/k0 Start | 1/k0 End | Precursor ID | Area Sample 6 | #Feature | #Feature Sample 6 | Start | End | PTM | AScore | Found By |
| --- | --- | --- | --- | --- | --- | --- | --- | --- | --- | --- | --- | --- | --- | --- | --- | --- | --- | --- | --- |
| K.TITLEVEPSDTIENVK.S | Y | 46.69 | 1786.92 | 16 | 0.39 | 894.4637 | 2 | 17.56 | 1.1442 | 1.1514 | 58994 | 8.78e2 | 1 | 1 | 60 | 75 |  |  | DB Search |
| total 1 peptides |  |  |  |  |  |  |  |  |  |  |  |  |  |  |  |  |  |  |  |
| --- | --- | --- | --- | --- | --- | --- | --- | --- | --- | --- | --- | --- | --- | --- | --- | --- | --- | --- | --- |

Best Unique PSM (Scan Sample\_4\_A1\_1\_3642.d:58994, m/z=894.4637, z=2, RT=17.56, ppm=-4.01):


TRINITY\_DN160\_c0\_g1\_i2.p1

back to list

  

| Protein Coverage
| Supporting Peptides
| Best Unique PSM
|

Protein Coverage:

Supporting Peptides:

| Peptide | Uniq | -10lgP | Mass | Length | ppm | m/z | z | RT | 1/k0 Start | 1/k0 End | Precursor ID | Area Sample 6 | #Feature | #Feature Sample 6 | Start | End | PTM | AScore | Found By |
| --- | --- | --- | --- | --- | --- | --- | --- | --- | --- | --- | --- | --- | --- | --- | --- | --- | --- | --- | --- |
| K.DLEFKFR.E | Y | 44.49 | 953.497 | 7 | 5.18 | 477.7562 | 2 | 16.82 | 0.8425 | 0.8498 | 54418 | 0 | 0 | 0 | 1031 | 1037 |  |  | DB Search |
| total 1 peptides |  |  |  |  |  |  |  |  |  |  |  |  |  |  |  |  |  |  |  |
| --- | --- | --- | --- | --- | --- | --- | --- | --- | --- | --- | --- | --- | --- | --- | --- | --- | --- | --- | --- |

Best Unique PSM (Scan Sample\_4\_A1\_1\_3642.d:54418, m/z=476.7815, z=2, RT=16.84, ppm=0.77):


TRINITY\_DN1783\_c0\_g1\_i2.p1

back to list

  

| Protein Coverage
| Supporting Peptides
| Best Unique PSM
|

Protein Coverage:

Supporting Peptides:

| Peptide | Uniq | -10lgP | Mass | Length | ppm | m/z | z | RT | 1/k0 Start | 1/k0 End | Precursor ID | Area Sample 6 | #Feature | #Feature Sample 6 | Start | End | PTM | AScore | Found By |
| --- | --- | --- | --- | --- | --- | --- | --- | --- | --- | --- | --- | --- | --- | --- | --- | --- | --- | --- | --- |
| K.LTIIDALPR.C | Y | 46.48 | 1010.6124 | 9 | 5.92 | 506.3142 | 2 | 18.91 | 0.8445 | 0.8518 | 68779 | 8.74e2 | 1 | 1 | 225 | 233 |  |  | DB Search |
| total 1 peptides |  |  |  |  |  |  |  |  |  |  |  |  |  |  |  |  |  |  |  |
| --- | --- | --- | --- | --- | --- | --- | --- | --- | --- | --- | --- | --- | --- | --- | --- | --- | --- | --- | --- |

Best Unique PSM (Scan Sample\_4\_A1\_1\_3642.d:68779, m/z=506.3142, z=2, RT=18.91, ppm=1.51):


TRINITY\_DN48767\_c0\_g1\_i1.p1

back to list

  

| Protein Coverage
| Supporting Peptides
| Best Unique PSM
|

Protein Coverage:

Supporting Peptides:

| Peptide | Uniq | -10lgP | Mass | Length | ppm | m/z | z | RT | 1/k0 Start | 1/k0 End | Precursor ID | Area Sample 6 | #Feature | #Feature Sample 6 | Start | End | PTM | AScore | Found By |
| --- | --- | --- | --- | --- | --- | --- | --- | --- | --- | --- | --- | --- | --- | --- | --- | --- | --- | --- | --- |
| K.VPAENNPK.L | Y | 45.57 | 867.445 | 8 | 1.28 | 434.7284 | 2 | 9.23 | 0.7738 | 0.7811 | 10441 | 5.64e2 | 1 | 1 | 214 | 221 |  |  | DB Search |
| total 1 peptides |  |  |  |  |  |  |  |  |  |  |  |  |  |  |  |  |  |  |  |
| --- | --- | --- | --- | --- | --- | --- | --- | --- | --- | --- | --- | --- | --- | --- | --- | --- | --- | --- | --- |

Best Unique PSM (Scan Sample\_4\_A1\_1\_3642.d:10441, m/z=434.7284, z=2, RT=9.23, ppm=-3.13):


TRINITY\_DN2390\_c0\_g1\_i1.p1

back to list

  

| Protein Coverage
| Supporting Peptides
| Best Unique PSM
|

Protein Coverage:

Supporting Peptides:

| Peptide | Uniq | -10lgP | Mass | Length | ppm | m/z | z | RT | 1/k0 Start | 1/k0 End | Precursor ID | Area Sample 6 | #Feature | #Feature Sample 6 | Start | End | PTM | AScore | Found By |
| --- | --- | --- | --- | --- | --- | --- | --- | --- | --- | --- | --- | --- | --- | --- | --- | --- | --- | --- | --- |
| K.LLVHNWEFLGK.Q | Y | 47.21 | 1354.7397 | 11 | 5.1 | 452.5875 | 3 | 18.51 | 0.7628 | 0.7701 | 65747 | 6.02e2 | 1 | 1 | 451 | 461 |  |  | DB Search |
| total 1 peptides |  |  |  |  |  |  |  |  |  |  |  |  |  |  |  |  |  |  |  |
| --- | --- | --- | --- | --- | --- | --- | --- | --- | --- | --- | --- | --- | --- | --- | --- | --- | --- | --- | --- |

Best Unique PSM (Scan Sample\_4\_A1\_1\_3642.d:65747, m/z=452.5875, z=3, RT=18.51, ppm=0.69):


TRINITY\_DN106\_c0\_g1\_i3.p1

back to list

  

| Protein Coverage
| Supporting Peptides
| Best Unique PSM
|

Protein Coverage:

Supporting Peptides:

| Peptide | Uniq | -10lgP | Mass | Length | ppm | m/z | z | RT | 1/k0 Start | 1/k0 End | Precursor ID | Area Sample 6 | #Feature | #Feature Sample 6 | Start | End | PTM | AScore | Found By |
| --- | --- | --- | --- | --- | --- | --- | --- | --- | --- | --- | --- | --- | --- | --- | --- | --- | --- | --- | --- |
| R.DGPAGPAGR.D | Y | 46.52 | 796.3827 | 9 | 7.09 | 399.1997 | 2 | 9.76 | 0.7233 | 0.7306 | 13098 | 1.65e3 | 1 | 1 | 488 | 496 |  |  | DB Search |
| total 1 peptides |  |  |  |  |  |  |  |  |  |  |  |  |  |  |  |  |  |  |  |
| --- | --- | --- | --- | --- | --- | --- | --- | --- | --- | --- | --- | --- | --- | --- | --- | --- | --- | --- | --- |

Best Unique PSM (Scan Sample\_4\_A1\_1\_3642.d:13098, m/z=399.1997, z=2, RT=9.76, ppm=2.68):


TRINITY\_DN250\_c0\_g1\_i2.p1

back to list

  

| Protein Coverage
| Supporting Peptides
| Best Unique PSM
|

Protein Coverage:

Supporting Peptides:

| Peptide | Uniq | -10lgP | Mass | Length | ppm | m/z | z | RT | 1/k0 Start | 1/k0 End | Precursor ID | Area Sample 6 | #Feature | #Feature Sample 6 | Start | End | PTM | AScore | Found By |
| --- | --- | --- | --- | --- | --- | --- | --- | --- | --- | --- | --- | --- | --- | --- | --- | --- | --- | --- | --- |
| R.LM(+15.99)GQIVSSITASLR.F | Y | 46.37 | 1490.8126 | 14 | 3.71 | 746.4131 | 2 | 18.78 | 1.0347 | 1.0419 | 67549 | 8.43e1 | 1 | 1 | 230 | 243 | Oxidation (M) | M2:Oxidation (M):1000 | DB Search |
| total 1 peptides |  |  |  |  |  |  |  |  |  |  |  |  |  |  |  |  |  |  |  |
| --- | --- | --- | --- | --- | --- | --- | --- | --- | --- | --- | --- | --- | --- | --- | --- | --- | --- | --- | --- |

Best Unique PSM (Scan Sample\_4\_A1\_1\_3642.d:67549, m/z=746.4131, z=2, RT=18.78, ppm=-0.69):


TRINITY\_DN2028\_c0\_g1\_i1.p1

back to list

  

| Protein Coverage
| Supporting Peptides
| Best Unique PSM
|

Protein Coverage:

Supporting Peptides:

| Peptide | Uniq | -10lgP | Mass | Length | ppm | m/z | z | RT | 1/k0 Start | 1/k0 End | Precursor ID | Area Sample 6 | #Feature | #Feature Sample 6 | Start | End | PTM | AScore | Found By |
| --- | --- | --- | --- | --- | --- | --- | --- | --- | --- | --- | --- | --- | --- | --- | --- | --- | --- | --- | --- |
| K.LIQYIENKPGLTR.L | Y | 47.09 | 1543.8722 | 13 | 0.19 | 772.9401 | 2 | 14.92 | 1.0769 | 1.0841 | 43527 | 6.02e3 | 1 | 1 | 81 | 93 |  |  | DB Search |
| total 1 peptides |  |  |  |  |  |  |  |  |  |  |  |  |  |  |  |  |  |  |  |
| --- | --- | --- | --- | --- | --- | --- | --- | --- | --- | --- | --- | --- | --- | --- | --- | --- | --- | --- | --- |

Best Unique PSM (Scan Sample\_4\_A1\_1\_3642.d:43527, m/z=772.9401, z=2, RT=14.92, ppm=-4.21):


TRINITY\_DN495\_c0\_g1\_i6.p1

back to list

  

| Protein Coverage
| Supporting Peptides
| Best Unique PSM
|

Protein Coverage:

Supporting Peptides:

| Peptide | Uniq | -10lgP | Mass | Length | ppm | m/z | z | RT | 1/k0 Start | 1/k0 End | Precursor ID | Area Sample 6 | #Feature | #Feature Sample 6 | Start | End | PTM | AScore | Found By |
| --- | --- | --- | --- | --- | --- | --- | --- | --- | --- | --- | --- | --- | --- | --- | --- | --- | --- | --- | --- |
| K.SISC(+57.02)SKDEEIK.V | Y | 46.33 | 1294.6075 | 11 | 8.03 | 648.3134 | 2 | 10.87 | 0.9723 | 0.9795 | 19739 | 0 | 0 | 0 | 248 | 258 | Carbamidomethylation |  | DB Search |
| total 1 peptides |  |  |  |  |  |  |  |  |  |  |  |  |  |  |  |  |  |  |  |
| --- | --- | --- | --- | --- | --- | --- | --- | --- | --- | --- | --- | --- | --- | --- | --- | --- | --- | --- | --- |

Best Unique PSM (Scan Sample\_4\_A1\_1\_3642.d:19739, m/z=648.3134, z=2, RT=10.87, ppm=3.63):


TRINITY\_DN6920\_c0\_g1\_i1.p1

back to list

  

| Protein Coverage
| Supporting Peptides
| Best Unique PSM
|

Protein Coverage:

Supporting Peptides:

| Peptide | Uniq | -10lgP | Mass | Length | ppm | m/z | z | RT | 1/k0 Start | 1/k0 End | Precursor ID | Area Sample 6 | #Feature | #Feature Sample 6 | Start | End | PTM | AScore | Found By |
| --- | --- | --- | --- | --- | --- | --- | --- | --- | --- | --- | --- | --- | --- | --- | --- | --- | --- | --- | --- |
| R.VLVHTQQK.L | Y | 46.31 | 951.5501 | 8 | 1.77 | 476.7811 | 2 | 9.52 | 0.8331 | 0.8404 | 11777 | 3.85e2 | 1 | 1 | 45 | 52 |  |  | DB Search |
| total 1 peptides |  |  |  |  |  |  |  |  |  |  |  |  |  |  |  |  |  |  |  |
| --- | --- | --- | --- | --- | --- | --- | --- | --- | --- | --- | --- | --- | --- | --- | --- | --- | --- | --- | --- |

Best Unique PSM (Scan Sample\_4\_A1\_1\_3642.d:11777, m/z=476.7811, z=2, RT=9.52, ppm=-2.63):


TRINITY\_DN106\_c0\_g1\_i2.p1

back to list

  

| Protein Coverage
| Supporting Peptides
| Best Unique PSM
|

Protein Coverage:

Supporting Peptides:

| Peptide | Uniq | -10lgP | Mass | Length | ppm | m/z | z | RT | 1/k0 Start | 1/k0 End | Precursor ID | Area Sample 6 | #Feature | #Feature Sample 6 | Start | End | PTM | AScore | Found By |
| --- | --- | --- | --- | --- | --- | --- | --- | --- | --- | --- | --- | --- | --- | --- | --- | --- | --- | --- | --- |
| R.DGPAGPAGR.D | Y | 46.52 | 796.3827 | 9 | 7.09 | 399.1997 | 2 | 9.76 | 0.7233 | 0.7306 | 13098 | 1.65e3 | 1 | 1 | 488 | 496 |  |  | DB Search |
| total 1 peptides |  |  |  |  |  |  |  |  |  |  |  |  |  |  |  |  |  |  |  |
| --- | --- | --- | --- | --- | --- | --- | --- | --- | --- | --- | --- | --- | --- | --- | --- | --- | --- | --- | --- |

Best Unique PSM (Scan Sample\_4\_A1\_1\_3642.d:13098, m/z=399.1997, z=2, RT=9.76, ppm=2.68):


TRINITY\_DN941\_c0\_g1\_i3.p1

back to list

  

| Protein Coverage
| Supporting Peptides
| Best Unique PSM
|

Protein Coverage:

Supporting Peptides:

| Peptide | Uniq | -10lgP | Mass | Length | ppm | m/z | z | RT | 1/k0 Start | 1/k0 End | Precursor ID | Area Sample 6 | #Feature | #Feature Sample 6 | Start | End | PTM | AScore | Found By |
| --- | --- | --- | --- | --- | --- | --- | --- | --- | --- | --- | --- | --- | --- | --- | --- | --- | --- | --- | --- |
| K.ADLTRPSGK.K | Y | 38.69 | 943.5087 | 9 | -2.87 | 472.7582 | 2 | 10.10 | 0.8189 | 0.8262 | 15057 | 1.13e3 | 1 | 1 | 114 | 122 |  |  | DB Search |
| total 1 peptides |  |  |  |  |  |  |  |  |  |  |  |  |  |  |  |  |  |  |  |
| --- | --- | --- | --- | --- | --- | --- | --- | --- | --- | --- | --- | --- | --- | --- | --- | --- | --- | --- | --- |

Best Unique PSM (Scan Sample\_4\_A1\_1\_3642.d:15057, m/z=472.7582, z=2, RT=10.10, ppm=-7.27):


TRINITY\_DN1616\_c0\_g1\_i2.p1

back to list

  

| Protein Coverage
| Supporting Peptides
| Best Unique PSM
|

Protein Coverage:

Supporting Peptides:

| Peptide | Uniq | -10lgP | Mass | Length | ppm | m/z | z | RT | 1/k0 Start | 1/k0 End | Precursor ID | Area Sample 6 | #Feature | #Feature Sample 6 | Start | End | PTM | AScore | Found By |
| --- | --- | --- | --- | --- | --- | --- | --- | --- | --- | --- | --- | --- | --- | --- | --- | --- | --- | --- | --- |
| K.GC(+57.02)STC(+57.02)GC(+57.02)NGAGTNNGVK.S | Y | 47.18 | 1712.6665 | 17 | 14.87 | 857.3495 | 2 | 9.81 | 1.0411 | 1.0484 | 13314 | 0 | 0 | 0 | 732 | 748 | Carbamidomethylation, Carbamidomethylation, Carbamidomethylation |  | DB Search |
| R.YVKPGVNIINIR.D | Y | 46.71 | 1384.819 | 12 | 3.8 | 693.4164 | 2 | 16.18 | 1.0009 | 1.0081 | 50795 | 7.11e2 | 1 | 1 | 1217 | 1228 |  |  | DB Search |
| K.VVDTEANDLTEEVKK.T | Y | 46.47 | 1688.8468 | 15 | 4.23 | 845.4305 | 2 | 14.16 | 1.1298 | 1.137 | 39294 | 3.14e2 | 1 | 1 | 2542 | 2556 |  |  | DB Search |
| K.YVVMLQYYHDSDTAK.Y | Y | 46.08 | 1831.8451 | 15 | 8.86 | 916.9339 | 2 | 16.78 | 1.1825 | 1.1897 | 54165 | 6.38e1 | 1 | 1 | 1071 | 1085 |  |  | DB Search |
| K.GFVAVNDQQPSVVVSINVPK.T | Y | 43.72 | 2096.1266 | 20 | 10.57 | 1049.077 | 2 | 18.41 | 1.2185 | 1.2256 | 65167 | 0 | 0 | 0 | 865 | 884 |  |  | DB Search |
| K.YEC(+57.02)NC(+57.02)QYNTEGPR.C | Y | 39.28 | 1689.6512 | 13 | 9.13 | 845.8369 | 2 | 12.04 | 1.0323 | 1.0395 | 26880 | 1.68e2 | 1 | 1 | 291 | 303 | Carbamidomethylation, Carbamidomethylation |  | DB Search |
| total 6 peptides |  |  |  |  |  |  |  |  |  |  |  |  |  |  |  |  |  |  |  |
| --- | --- | --- | --- | --- | --- | --- | --- | --- | --- | --- | --- | --- | --- | --- | --- | --- | --- | --- | --- |

Best Unique PSM (Scan Sample\_4\_A1\_1\_3642.d:13314, m/z=857.3495, z=2, RT=9.81, ppm=10.47):


TRINITY\_DN3016\_c0\_g1\_i13.p1

back to list

  

| Protein Coverage
| Supporting Peptides
| Best Unique PSM
|

Protein Coverage:

Supporting Peptides:

| Peptide | Uniq | -10lgP | Mass | Length | ppm | m/z | z | RT | 1/k0 Start | 1/k0 End | Precursor ID | Area Sample 6 | #Feature | #Feature Sample 6 | Start | End | PTM | AScore | Found By |
| --- | --- | --- | --- | --- | --- | --- | --- | --- | --- | --- | --- | --- | --- | --- | --- | --- | --- | --- | --- |
| R.IVNTDLQEIKDIIK.G | Y | 45.77 | 1640.9348 | 14 | -1.11 | 547.9825 | 3 | 18.81 | 0.8177 | 0.825 | 67904 | 0 | 0 | 0 | 154 | 167 |  |  | DB Search |
| total 1 peptides |  |  |  |  |  |  |  |  |  |  |  |  |  |  |  |  |  |  |  |
| --- | --- | --- | --- | --- | --- | --- | --- | --- | --- | --- | --- | --- | --- | --- | --- | --- | --- | --- | --- |

Best Unique PSM (Scan Sample\_4\_A1\_1\_3642.d:67904, m/z=547.9825, z=3, RT=18.81, ppm=-5.51):


TRINITY\_DN11635\_c0\_g1\_i1.p1

back to list

  

| Protein Coverage
| Supporting Peptides
| Best Unique PSM
|

Protein Coverage:

Supporting Peptides:

| Peptide | Uniq | -10lgP | Mass | Length | ppm | m/z | z | RT | 1/k0 Start | 1/k0 End | Precursor ID | Area Sample 6 | #Feature | #Feature Sample 6 | Start | End | PTM | AScore | Found By |
| --- | --- | --- | --- | --- | --- | --- | --- | --- | --- | --- | --- | --- | --- | --- | --- | --- | --- | --- | --- |
| K.ASISSNDFKDFLR.E | Y | 47.32 | 1498.7416 | 13 | 1.94 | 750.3762 | 2 | 18.00 | 1.0881 | 1.0953 | 62229 | 0 | 0 | 0 | 382 | 394 |  |  | DB Search |
| total 1 peptides |  |  |  |  |  |  |  |  |  |  |  |  |  |  |  |  |  |  |  |
| --- | --- | --- | --- | --- | --- | --- | --- | --- | --- | --- | --- | --- | --- | --- | --- | --- | --- | --- | --- |

Best Unique PSM (Scan Sample\_4\_A1\_1\_3642.d:62229, m/z=750.3762, z=2, RT=18.00, ppm=-2.46):


TRINITY\_DN477\_c0\_g1\_i8.p2

back to list

  

| Protein Coverage
| Supporting Peptides
| Best Unique PSM
|

Protein Coverage:

Supporting Peptides:

| Peptide | Uniq | -10lgP | Mass | Length | ppm | m/z | z | RT | 1/k0 Start | 1/k0 End | Precursor ID | Area Sample 6 | #Feature | #Feature Sample 6 | Start | End | PTM | AScore | Found By |
| --- | --- | --- | --- | --- | --- | --- | --- | --- | --- | --- | --- | --- | --- | --- | --- | --- | --- | --- | --- |
| R.NDVNNPPQAR.R | Y | 47.24 | 1123.537 | 10 | 0.77 | 562.7737 | 2 | 9.95 | 0.866 | 0.8733 | 13858 | 3.59e3 | 2 | 2 | 86 | 95 |  |  | DB Search |
| total 1 peptides |  |  |  |  |  |  |  |  |  |  |  |  |  |  |  |  |  |  |  |
| --- | --- | --- | --- | --- | --- | --- | --- | --- | --- | --- | --- | --- | --- | --- | --- | --- | --- | --- | --- |

Best Unique PSM (Scan Sample\_4\_A1\_1\_3642.d:13858, m/z=562.7737, z=2, RT=9.95, ppm=-3.64):


TRINITY\_DN36217\_c0\_g1\_i1.p1

back to list

  

| Protein Coverage
| Supporting Peptides
| Best Unique PSM
|

Protein Coverage:

Supporting Peptides:

| Peptide | Uniq | -10lgP | Mass | Length | ppm | m/z | z | RT | 1/k0 Start | 1/k0 End | Precursor ID | Area Sample 6 | #Feature | #Feature Sample 6 | Start | End | PTM | AScore | Found By |
| --- | --- | --- | --- | --- | --- | --- | --- | --- | --- | --- | --- | --- | --- | --- | --- | --- | --- | --- | --- |
| R.LGPIPLWK.K | Y | 46.9 | 922.564 | 8 | 3.89 | 462.289 | 2 | 19.42 | 0.7909 | 0.7982 | 71845 | 3.48e2 | 1 | 1 | 124 | 131 |  |  | DB Search |
| total 1 peptides |  |  |  |  |  |  |  |  |  |  |  |  |  |  |  |  |  |  |  |
| --- | --- | --- | --- | --- | --- | --- | --- | --- | --- | --- | --- | --- | --- | --- | --- | --- | --- | --- | --- |

Best Unique PSM (Scan Sample\_4\_A1\_1\_3642.d:71845, m/z=462.289, z=2, RT=19.42, ppm=-0.52):


TRINITY\_DN2357\_c1\_g1\_i1.p1

back to list

  

| Protein Coverage
| Supporting Peptides
| Best Unique PSM
|

Protein Coverage:

Supporting Peptides:

| Peptide | Uniq | -10lgP | Mass | Length | ppm | m/z | z | RT | 1/k0 Start | 1/k0 End | Precursor ID | Area Sample 6 | #Feature | #Feature Sample 6 | Start | End | PTM | AScore | Found By |
| --- | --- | --- | --- | --- | --- | --- | --- | --- | --- | --- | --- | --- | --- | --- | --- | --- | --- | --- | --- |
| R.YRVSQDIGVTDFNLVDPK.I | Y | 39.31 | 2065.048 | 18 | 0.36 | 1033.5271 | 2 | 19.75 | 1.1905 | 1.1977 | 74388 | 0 | 0 | 0 | 1112 | 1129 |  |  | DB Search |
| total 1 peptides |  |  |  |  |  |  |  |  |  |  |  |  |  |  |  |  |  |  |  |
| --- | --- | --- | --- | --- | --- | --- | --- | --- | --- | --- | --- | --- | --- | --- | --- | --- | --- | --- | --- |

Best Unique PSM (Scan Sample\_4\_A1\_1\_3642.d:74388, m/z=1033.5239, z=1, RT=19.79, ppm=-4.03):


TRINITY\_DN11522\_c0\_g1\_i3.p1

back to list

  

| Protein Coverage
| Supporting Peptides
| Best Unique PSM
|

Protein Coverage:

Supporting Peptides:

| Peptide | Uniq | -10lgP | Mass | Length | ppm | m/z | z | RT | 1/k0 Start | 1/k0 End | Precursor ID | Area Sample 6 | #Feature | #Feature Sample 6 | Start | End | PTM | AScore | Found By |
| --- | --- | --- | --- | --- | --- | --- | --- | --- | --- | --- | --- | --- | --- | --- | --- | --- | --- | --- | --- |
| R.IGLGNIRDPSISLTR.G | Y | 47.25 | 1610.9104 | 15 | 2.96 | 537.9766 | 3 | 17.36 | 0.797 | 0.8043 | 57658 | 1.69e3 | 1 | 1 | 27 | 41 |  |  | DB Search |
| total 1 peptides |  |  |  |  |  |  |  |  |  |  |  |  |  |  |  |  |  |  |  |
| --- | --- | --- | --- | --- | --- | --- | --- | --- | --- | --- | --- | --- | --- | --- | --- | --- | --- | --- | --- |

Best Unique PSM (Scan Sample\_4\_A1\_1\_3642.d:57658, m/z=537.9766, z=3, RT=17.36, ppm=-1.44):


TRINITY\_DN22492\_c0\_g1\_i1.p1

back to list

  

| Protein Coverage
| Supporting Peptides
| Best Unique PSM
|

Protein Coverage:

Supporting Peptides:

| Peptide | Uniq | -10lgP | Mass | Length | ppm | m/z | z | RT | 1/k0 Start | 1/k0 End | Precursor ID | Area Sample 6 | #Feature | #Feature Sample 6 | Start | End | PTM | AScore | Found By |
| --- | --- | --- | --- | --- | --- | --- | --- | --- | --- | --- | --- | --- | --- | --- | --- | --- | --- | --- | --- |
| K.KKLEPQAAPR.E | Y | 39.03 | 1136.6666 | 10 | -0.45 | 379.8943 | 3 | 9.66 | 0.7485 | 0.7559 | 12515 | 8.42e2 | 1 | 1 | 425 | 434 |  |  | DB Search |
| total 1 peptides |  |  |  |  |  |  |  |  |  |  |  |  |  |  |  |  |  |  |  |
| --- | --- | --- | --- | --- | --- | --- | --- | --- | --- | --- | --- | --- | --- | --- | --- | --- | --- | --- | --- |

Best Unique PSM (Scan Sample\_4\_A1\_1\_3642.d:12515, m/z=379.8943, z=3, RT=9.66, ppm=-4.86):


TRINITY\_DN428\_c0\_g1\_i1.p1

back to list

  

| Protein Coverage
| Supporting Peptides
| Best Unique PSM
|

Protein Coverage:

Supporting Peptides:

| Peptide | Uniq | -10lgP | Mass | Length | ppm | m/z | z | RT | 1/k0 Start | 1/k0 End | Precursor ID | Area Sample 6 | #Feature | #Feature Sample 6 | Start | End | PTM | AScore | Found By |
| --- | --- | --- | --- | --- | --- | --- | --- | --- | --- | --- | --- | --- | --- | --- | --- | --- | --- | --- | --- |
| K.IC(+57.02)HLLGIQVTDFTK.S | Y | 47.12 | 1643.8705 | 14 | -3.6 | 548.9597 | 3 | 18.60 | 0.8185 | 0.8258 | 66312 | 1.88e3 | 1 | 1 | 377 | 390 | Carbamidomethylation |  | DB Search |
| K.ALSLQNANEELEDRLAEAER.Q | Y | 45.94 | 2270.1138 | 20 | 0.89 | 757.7092 | 3 | 18.86 | 0.9836 | 0.9908 | 68234 | 0 | 0 | 0 | 1475 | 1494 |  |  | DB Search |
| R.AIESERDELLEELNNNSALK.N | Y | 43.21 | 2286.1339 | 20 | 3.65 | 763.0513 | 3 | 18.41 | 1.0029 | 1.0102 | 65156 | 0 | 0 | 0 | 1695 | 1714 |  |  | DB Search |
| K.AKQEAFIAELEDQIAKGDK.S | Y | 42.99 | 2103.0847 | 19 | -0.47 | 702.0321 | 3 | 19.58 | 0.9428 | 0.9501 | 73252 | 2.03e2 | 1 | 1 | 1020 | 1038 |  |  | DB Search |
| K.AKQEAFIAELEDQIAK.G | Y | 38.87 | 1802.9414 | 16 | -2.92 | 601.9833 | 3 | 19.97 | 0.881 | 0.8883 | 75797 | 0 | 0 | 0 | 1020 | 1035 |  |  | DB Search |
| total 5 peptides |  |  |  |  |  |  |  |  |  |  |  |  |  |  |  |  |  |  |  |
| --- | --- | --- | --- | --- | --- | --- | --- | --- | --- | --- | --- | --- | --- | --- | --- | --- | --- | --- | --- |

Best Unique PSM (Scan Sample\_4\_A1\_1\_3642.d:66312, m/z=548.9597, z=3, RT=18.60, ppm=-8.01):


TRINITY\_DN12632\_c1\_g1\_i1.p1

back to list

  

| Protein Coverage
| Supporting Peptides
| Best Unique PSM
|

Protein Coverage:

Supporting Peptides:

| Peptide | Uniq | -10lgP | Mass | Length | ppm | m/z | z | RT | 1/k0 Start | 1/k0 End | Precursor ID | Area Sample 6 | #Feature | #Feature Sample 6 | Start | End | PTM | AScore | Found By |
| --- | --- | --- | --- | --- | --- | --- | --- | --- | --- | --- | --- | --- | --- | --- | --- | --- | --- | --- | --- |
| R.LIEDTEYTAHEGAK.F | Y | 47.51 | 1575.7416 | 14 | -1.61 | 788.8734 | 2 | 12.79 | 1.0773 | 1.0845 | 31281 | 0 | 0 | 0 | 378 | 391 |  |  | DB Search |
| total 1 peptides |  |  |  |  |  |  |  |  |  |  |  |  |  |  |  |  |  |  |  |
| --- | --- | --- | --- | --- | --- | --- | --- | --- | --- | --- | --- | --- | --- | --- | --- | --- | --- | --- | --- |

Best Unique PSM (Scan Sample\_4\_A1\_1\_3642.d:31281, m/z=788.8734, z=2, RT=12.79, ppm=-6.01):


TRINITY\_DN161\_c0\_g1\_i4.p1

back to list

  

| Protein Coverage
| Supporting Peptides
| Best Unique PSM
|

Protein Coverage:

Supporting Peptides:

| Peptide | Uniq | -10lgP | Mass | Length | ppm | m/z | z | RT | 1/k0 Start | 1/k0 End | Precursor ID | Area Sample 6 | #Feature | #Feature Sample 6 | Start | End | PTM | AScore | Found By |
| --- | --- | --- | --- | --- | --- | --- | --- | --- | --- | --- | --- | --- | --- | --- | --- | --- | --- | --- | --- |
| K.SYELPDGQVITIGNER.F | Y | 47.19 | 1789.8846 | 16 | -1.46 | 895.9443 | 2 | 18.64 | 1.1122 | 1.1194 | 66524 | 6.05e2 | 1 | 1 | 95 | 110 |  |  | DB Search |
| R.LDLAGRDLTDYLMK.I | Y | 46.87 | 1622.8338 | 14 | -1.16 | 541.9489 | 3 | 19.39 | 0.7966 | 0.8039 | 71871 | 4.27e2 | 1 | 1 | 34 | 47 |  |  | DB Search |
| K.DLYANTVLSGGTTM(+15.99)FPGIADR.M | Y | 46.72 | 2214.0626 | 21 | 10.66 | 1108.0455 | 2 | 19.50 | 1.2475 | 1.2547 | 72698 | 0 | 0 | 0 | 148 | 168 | Oxidation (M) | M14:Oxidation (M):1000 | DB Search |
| R.DLTDYLMK.I | Y | 37.13 | 997.479 | 8 | 2.04 | 499.7456 | 2 | 18.64 | 0.8372 | 0.8445 | 66608 | 3.54e2 | 1 | 1 | 40 | 47 |  |  | DB Search |
| total 4 peptides |  |  |  |  |  |  |  |  |  |  |  |  |  |  |  |  |  |  |  |
| --- | --- | --- | --- | --- | --- | --- | --- | --- | --- | --- | --- | --- | --- | --- | --- | --- | --- | --- | --- |

Best Unique PSM (Scan Sample\_4\_A1\_1\_3642.d:66524, m/z=895.9443, z=2, RT=18.64, ppm=-5.86):


TRINITY\_DN1783\_c1\_g1\_i1.p2

back to list

  

| Protein Coverage
| Supporting Peptides
| Best Unique PSM
|

Protein Coverage:

Supporting Peptides:

| Peptide | Uniq | -10lgP | Mass | Length | ppm | m/z | z | RT | 1/k0 Start | 1/k0 End | Precursor ID | Area Sample 6 | #Feature | #Feature Sample 6 | Start | End | PTM | AScore | Found By |
| --- | --- | --- | --- | --- | --- | --- | --- | --- | --- | --- | --- | --- | --- | --- | --- | --- | --- | --- | --- |
| K.LTIIDALPR.C | Y | 46.48 | 1010.6124 | 9 | 5.92 | 506.3142 | 2 | 18.91 | 0.8445 | 0.8518 | 68779 | 8.74e2 | 1 | 1 | 55 | 63 |  |  | DB Search |
| total 1 peptides |  |  |  |  |  |  |  |  |  |  |  |  |  |  |  |  |  |  |  |
| --- | --- | --- | --- | --- | --- | --- | --- | --- | --- | --- | --- | --- | --- | --- | --- | --- | --- | --- | --- |

Best Unique PSM (Scan Sample\_4\_A1\_1\_3642.d:68779, m/z=506.3142, z=2, RT=18.91, ppm=1.51):


TRINITY\_DN2217\_c0\_g1\_i2.p1

back to list

  

| Protein Coverage
| Supporting Peptides
| Best Unique PSM
|

Protein Coverage:

Supporting Peptides:

| Peptide | Uniq | -10lgP | Mass | Length | ppm | m/z | z | RT | 1/k0 Start | 1/k0 End | Precursor ID | Area Sample 6 | #Feature | #Feature Sample 6 | Start | End | PTM | AScore | Found By |
| --- | --- | --- | --- | --- | --- | --- | --- | --- | --- | --- | --- | --- | --- | --- | --- | --- | --- | --- | --- |
| K.ATGC(+57.02)EVIWNQHNR.G | Y | 46.53 | 1583.7263 | 13 | 3.17 | 792.8694 | 2 | 14.02 | 1.0785 | 1.0857 | 38485 | 7.79e2 | 1 | 1 | 119 | 131 | Carbamidomethylation |  | DB Search |
| R.GC(+57.02)YIHTQEIAR.G | Y | 46.47 | 1346.6401 | 11 | -0.76 | 674.3239 | 2 | 13.04 | 1.0158 | 1.023 | 32770 | 0 | 0 | 0 | 226 | 236 | Carbamidomethylation |  | DB Search |
| total 2 peptides |  |  |  |  |  |  |  |  |  |  |  |  |  |  |  |  |  |  |  |
| --- | --- | --- | --- | --- | --- | --- | --- | --- | --- | --- | --- | --- | --- | --- | --- | --- | --- | --- | --- |

Best Unique PSM (Scan Sample\_4\_A1\_1\_3642.d:38485, m/z=792.8694, z=2, RT=14.02, ppm=-1.23):


TRINITY\_DN71276\_c0\_g1\_i1.p1

back to list

  

| Protein Coverage
| Supporting Peptides
| Best Unique PSM
|

Protein Coverage:

Supporting Peptides:

| Peptide | Uniq | -10lgP | Mass | Length | ppm | m/z | z | RT | 1/k0 Start | 1/k0 End | Precursor ID | Area Sample 6 | #Feature | #Feature Sample 6 | Start | End | PTM | AScore | Found By |
| --- | --- | --- | --- | --- | --- | --- | --- | --- | --- | --- | --- | --- | --- | --- | --- | --- | --- | --- | --- |
| R.LMLLLFQHESM(+15.99)K.L | Y | 47.16 | 1504.7782 | 12 | -4.04 | 502.5958 | 3 | 19.47 | 0.8116 | 0.8189 | 72453 | 0 | 0 | 0 | 388 | 399 | Oxidation (M) | M11:Oxidation (M):90.01 | DB Search |
| K.EAAAQIVVEIIKR.T | Y | 46.21 | 1438.8507 | 13 | 2.76 | 720.4315 | 2 | 19.19 | 1.0524 | 1.0596 | 70467 | 2.59e2 | 1 | 1 | 149 | 161 |  |  | DB Search |
| K.IITEINPTIQVFQPK.C | Y | 46.17 | 1739.9821 | 15 | 0.13 | 870.9946 | 2 | 19.11 | 1.131 | 1.1382 | 69937 | 3.91e2 | 1 | 1 | 820 | 834 |  |  | DB Search |
| total 3 peptides |  |  |  |  |  |  |  |  |  |  |  |  |  |  |  |  |  |  |  |
| --- | --- | --- | --- | --- | --- | --- | --- | --- | --- | --- | --- | --- | --- | --- | --- | --- | --- | --- | --- |

Best Unique PSM (Scan Sample\_4\_A1\_1\_3642.d:72453, m/z=502.5958, z=3, RT=19.47, ppm=-8.45):


TRINITY\_DN687\_c0\_g2\_i1.p1

back to list

  

| Protein Coverage
| Supporting Peptides
| Best Unique PSM
|

Protein Coverage:

Supporting Peptides:

| Peptide | Uniq | -10lgP | Mass | Length | ppm | m/z | z | RT | 1/k0 Start | 1/k0 End | Precursor ID | Area Sample 6 | #Feature | #Feature Sample 6 | Start | End | PTM | AScore | Found By |
| --- | --- | --- | --- | --- | --- | --- | --- | --- | --- | --- | --- | --- | --- | --- | --- | --- | --- | --- | --- |
| R.AVC(+57.02)M(+15.99)LSNTTAIAEAWAR.L | Y | 43.01 | 1879.892 | 17 | 2.28 | 940.9513 | 2 | 18.79 | 1.147 | 1.1542 | 67649 | 2.71e2 | 1 | 1 | 374 | 390 | Carbamidomethylation, Oxidation (M) | M4:Oxidation (M):1000 | DB Search |
| K.VGINYQPPTVVPGGDLAK.V | Y | 37.08 | 1823.9781 | 18 | 0.87 | 912.9931 | 2 | 17.39 | 1.1218 | 1.129 | 57959 | 0 | 0 | 0 | 353 | 370 |  |  | DB Search |
| total 2 peptides |  |  |  |  |  |  |  |  |  |  |  |  |  |  |  |  |  |  |  |
| --- | --- | --- | --- | --- | --- | --- | --- | --- | --- | --- | --- | --- | --- | --- | --- | --- | --- | --- | --- |

Best Unique PSM (Scan Sample\_4\_A1\_1\_3642.d:67649, m/z=940.9513, z=2, RT=18.79, ppm=-2.12):


TRINITY\_DN1386\_c0\_g1\_i1.p1

back to list

  

| Protein Coverage
| Supporting Peptides
| Best Unique PSM
|

Protein Coverage:

Supporting Peptides:

| Peptide | Uniq | -10lgP | Mass | Length | ppm | m/z | z | RT | 1/k0 Start | 1/k0 End | Precursor ID | Area Sample 6 | #Feature | #Feature Sample 6 | Start | End | PTM | AScore | Found By |
| --- | --- | --- | --- | --- | --- | --- | --- | --- | --- | --- | --- | --- | --- | --- | --- | --- | --- | --- | --- |
| R.KVIVDQLVTLATR.V | Y | 47.25 | 1454.882 | 13 | 0.73 | 728.4456 | 2 | 18.43 | 1.0283 | 1.0355 | 65218 | 4.09e2 | 1 | 1 | 547 | 559 |  |  | DB Search |
| total 1 peptides |  |  |  |  |  |  |  |  |  |  |  |  |  |  |  |  |  |  |  |
| --- | --- | --- | --- | --- | --- | --- | --- | --- | --- | --- | --- | --- | --- | --- | --- | --- | --- | --- | --- |

Best Unique PSM (Scan Sample\_4\_A1\_1\_3642.d:65218, m/z=728.4456, z=2, RT=18.43, ppm=-3.67):


TRINITY\_DN5987\_c0\_g1\_i2.p1

back to list

  

| Protein Coverage
| Supporting Peptides
| Best Unique PSM
|

Protein Coverage:

Supporting Peptides:

| Peptide | Uniq | -10lgP | Mass | Length | ppm | m/z | z | RT | 1/k0 Start | 1/k0 End | Precursor ID | Area Sample 6 | #Feature | #Feature Sample 6 | Start | End | PTM | AScore | Found By |
| --- | --- | --- | --- | --- | --- | --- | --- | --- | --- | --- | --- | --- | --- | --- | --- | --- | --- | --- | --- |
| R.GNQGEQGPAGPVGPK.G | Y | 47.2 | 1391.6793 | 15 | 3.52 | 696.8463 | 2 | 11.46 | 0.9461 | 0.9533 | 23184 | 1.15e3 | 1 | 1 | 237 | 251 |  |  | DB Search |
| total 1 peptides |  |  |  |  |  |  |  |  |  |  |  |  |  |  |  |  |  |  |  |
| --- | --- | --- | --- | --- | --- | --- | --- | --- | --- | --- | --- | --- | --- | --- | --- | --- | --- | --- | --- |

Best Unique PSM (Scan Sample\_4\_A1\_1\_3642.d:23184, m/z=696.8463, z=2, RT=11.46, ppm=-0.88):


TRINITY\_DN4815\_c0\_g1\_i2.p1

back to list

  

| Protein Coverage
| Supporting Peptides
| Best Unique PSM
|

Protein Coverage:

Supporting Peptides:

| Peptide | Uniq | -10lgP | Mass | Length | ppm | m/z | z | RT | 1/k0 Start | 1/k0 End | Precursor ID | Area Sample 6 | #Feature | #Feature Sample 6 | Start | End | PTM | AScore | Found By |
| --- | --- | --- | --- | --- | --- | --- | --- | --- | --- | --- | --- | --- | --- | --- | --- | --- | --- | --- | --- |
| R.AVAC(+57.02)NSGPC(+57.02)LAGC(+57.02)PR.N | Y | 47.15 | 1588.6908 | 15 | 1.52 | 795.3504 | 2 | 13.09 | 1.0178 | 1.025 | 33019 | 7.5e2 | 1 | 1 | 468 | 482 | Carbamidomethylation, Carbamidomethylation, Carbamidomethylation |  | DB Search |
| total 1 peptides |  |  |  |  |  |  |  |  |  |  |  |  |  |  |  |  |  |  |  |
| --- | --- | --- | --- | --- | --- | --- | --- | --- | --- | --- | --- | --- | --- | --- | --- | --- | --- | --- | --- |

Best Unique PSM (Scan Sample\_4\_A1\_1\_3642.d:33019, m/z=795.3504, z=2, RT=13.09, ppm=-2.88):


TRINITY\_DN4595\_c0\_g1\_i1.p1

back to list

  

| Protein Coverage
| Supporting Peptides
| Best Unique PSM
|

Protein Coverage:

Supporting Peptides:

| Peptide | Uniq | -10lgP | Mass | Length | ppm | m/z | z | RT | 1/k0 Start | 1/k0 End | Precursor ID | Area Sample 6 | #Feature | #Feature Sample 6 | Start | End | PTM | AScore | Found By |
| --- | --- | --- | --- | --- | --- | --- | --- | --- | --- | --- | --- | --- | --- | --- | --- | --- | --- | --- | --- |
| R.AFNKIETR.N | Y | 41.96 | 977.5294 | 8 | 2.83 | 489.7712 | 2 | 11.48 | 0.8343 | 0.8416 | 23389 | 1.84e3 | 1 | 1 | 72 | 79 |  |  | DB Search |
| total 1 peptides |  |  |  |  |  |  |  |  |  |  |  |  |  |  |  |  |  |  |  |
| --- | --- | --- | --- | --- | --- | --- | --- | --- | --- | --- | --- | --- | --- | --- | --- | --- | --- | --- | --- |

Best Unique PSM (Scan Sample\_4\_A1\_1\_3642.d:23389, m/z=489.7712, z=2, RT=11.48, ppm=-1.58):


TRINITY\_DN38399\_c0\_g1\_i1.p1

back to list

  

| Protein Coverage
| Supporting Peptides
| Best Unique PSM
|

Protein Coverage:

Supporting Peptides:

| Peptide | Uniq | -10lgP | Mass | Length | ppm | m/z | z | RT | 1/k0 Start | 1/k0 End | Precursor ID | Area Sample 6 | #Feature | #Feature Sample 6 | Start | End | PTM | AScore | Found By |
| --- | --- | --- | --- | --- | --- | --- | --- | --- | --- | --- | --- | --- | --- | --- | --- | --- | --- | --- | --- |
| K.YGGQC(+57.02)AC(+57.02)KPGIGGR.D | Y | 47.17 | 1479.6711 | 14 | -0.82 | 494.2284 | 3 | 11.19 | 0.7897 | 0.797 | 21658 | 6.62e2 | 1 | 1 | 542 | 555 | Carbamidomethylation, Carbamidomethylation |  | DB Search |
| total 1 peptides |  |  |  |  |  |  |  |  |  |  |  |  |  |  |  |  |  |  |  |
| --- | --- | --- | --- | --- | --- | --- | --- | --- | --- | --- | --- | --- | --- | --- | --- | --- | --- | --- | --- |

Best Unique PSM (Scan Sample\_4\_A1\_1\_3642.d:21658, m/z=494.2284, z=3, RT=11.19, ppm=-5.22):


TRINITY\_DN5584\_c0\_g1\_i3.p1

back to list

  

| Protein Coverage
| Supporting Peptides
| Best Unique PSM
|

Protein Coverage:

Supporting Peptides:

| Peptide | Uniq | -10lgP | Mass | Length | ppm | m/z | z | RT | 1/k0 Start | 1/k0 End | Precursor ID | Area Sample 6 | #Feature | #Feature Sample 6 | Start | End | PTM | AScore | Found By |
| --- | --- | --- | --- | --- | --- | --- | --- | --- | --- | --- | --- | --- | --- | --- | --- | --- | --- | --- | --- |
| K.YLSWTLDNDKIR.S | Y | 46.73 | 1522.778 | 12 | 4.13 | 508.5998 | 3 | 17.56 | 0.8108 | 0.8181 | 59147 | 0 | 0 | 0 | 835 | 846 |  |  | DB Search |
| total 1 peptides |  |  |  |  |  |  |  |  |  |  |  |  |  |  |  |  |  |  |  |
| --- | --- | --- | --- | --- | --- | --- | --- | --- | --- | --- | --- | --- | --- | --- | --- | --- | --- | --- | --- |

Best Unique PSM (Scan Sample\_4\_A1\_1\_3642.d:59147, m/z=508.5998, z=3, RT=17.56, ppm=-0.27):


---

Prepared with PEAKS ™ (bioinfor.com)
