# Supplementary material for: Proteomic Analysis and Biochemical Characterization of the Nematocyst Extract of the Hydrozoan Velella velella
Source: Mar Drugs. 2024 Oct 12;22(10):468. doi: 10.3390/md22100468 (PMC11509761; doi:10.3390/md22100468)
Supplement: Supplementary file 1 [file marinedrugs-22-00468-s001.zip › Supplementary File S1.zip!/Sample_5_Vellela/db.peptides.html]

peptide list


Peptide List

  

| Peptide | -10lgP | Mass | Length | ppm | m/z | RT | 1/k0 Start | 1/k0 End | Precursor ID | Area Sample 7 | #Feature | #Feature Sample 7 | Accession | PTM | AScore | Found By |
| --- | --- | --- | --- | --- | --- | --- | --- | --- | --- | --- | --- | --- | --- | --- | --- | --- |
| GSC(+57.02)SC(+57.02)YGHAEQC(+57.02)IPTK | 56.43 | 1853.7495 | 16 | 5.37 | 618.9242 | 12.44 | 0.8623 | 0.8696 | 27900 | 3.78e3 | 2 | 2 | TRINITY\_DN15274\_c0\_g1\_i1.p1 | Carbamidomethylation, Carbamidomethylation, Carbamidomethylation |  | DB Search |
| EITLNYHDTVVHMK | 56.41 | 1698.84 | 14 | -1.2 | 850.4222 | 15.44 | 1.1134 | 1.1206 | 46227 | 6.3e2 | 1 | 1 | TRINITY\_DN1369\_c0\_g1\_i1.p1 |  |  | DB Search |
| GLQGQQGEQGSTGVQGSVGGR | 56.41 | 1985.9514 | 21 | 4.71 | 993.983 | 12.10 | 1.139 | 1.1462 | 25849 | 1.24e3 | 1 | 1 | TRINITY\_DN1030\_c0\_g1\_i1.p1:TRINITY\_DN1030\_c0\_g1\_i2.p1 |  |  | DB Search |
| GSC(+57.02)EC(+57.02)GTC(+57.02)VC(+57.02)R | 56.41 | 1344.4679 | 11 | 6.01 | 673.2421 | 10.52 | 0.9299 | 0.9372 | 16603 | 0 | 0 | 0 | TRINITY\_DN1535\_c0\_g1\_i1.p1 | Carbamidomethylation, Carbamidomethylation, Carbamidomethylation, Carbamidomethylation |  | DB Search |
| SYFEHSFVDLHR | 56.4 | 1535.7157 | 12 | 4.62 | 512.9125 | 16.16 | 0.7705 | 0.7779 | 50408 | 5.95e2 | 1 | 1 | TRINITY\_DN59572\_c0\_g1\_i1.p1 |  |  | DB Search |
| ALLLASSNAFDTSNGAR | 56.4 | 1706.8588 | 17 | 2.83 | 854.4351 | 17.79 | 1.1642 | 1.1714 | 60238 | 4.39e2 | 1 | 1 | TRINITY\_DN244\_c0\_g1\_i1.p1 |  |  | DB Search |
| NYNNDDEPGAKK | 56.4 | 1363.6003 | 12 | 2.6 | 455.5398 | 9.60 | 0.7489 | 0.7563 | 11895 | 1.07e3 | 2 | 2 | TRINITY\_DN199\_c0\_g1\_i1.p1 |  |  | DB Search |
| VVDTEANDLTEEVKK | 56.4 | 1688.8468 | 15 | 6.39 | 845.4321 | 14.15 | 1.1318 | 1.139 | 38615 | 0 | 0 | 0 | TRINITY\_DN1616\_c0\_g1\_i3.p1:TRINITY\_DN1616\_c0\_g1\_i6.p1:TRINITY\_DN1616\_c0\_g1\_i2.p1:TRINITY\_DN1616\_c0\_g1\_i7.p1 |  |  | DB Search |
| LIYENQNTIHHVVR | 56.4 | 1734.9165 | 14 | 3.34 | 579.312 | 13.35 | 0.8485 | 0.8558 | 33666 | 6.97e2 | 1 | 1 | TRINITY\_DN26249\_c0\_g1\_i1.p1 |  |  | DB Search |
| ATGC(+57.02)EVIWNQHNR | 56.39 | 1583.7263 | 13 | 0.67 | 792.8672 | 14.03 | 1.0829 | 1.0901 | 37880 | 1.73e2 | 1 | 1 | TRINITY\_DN2217\_c0\_g1\_i3.p1:TRINITY\_DN2217\_c0\_g1\_i2.p1 | Carbamidomethylation |  | DB Search |
| S(+42.01)LVIPEKFQHILR | 56.39 | 1620.9352 | 13 | 1.15 | 541.3171 | 19.75 | 0.8425 | 0.8498 | 73247 | 9.92e2 | 1 | 1 | TRINITY\_DN1505\_c0\_g1\_i1.p1 | Acetylation (Protein N-term) | S1:Acetylation (Protein N-term):1000 | DB Search |
| ILQC(+57.02)NQYANVEEAR | 56.39 | 1706.8046 | 14 | 0.71 | 854.4062 | 14.33 | 1.0877 | 1.0949 | 39688 | 4.48e3 | 1 | 1 | TRINITY\_DN477\_c0\_g1\_i8.p1:TRINITY\_DN477\_c0\_g1\_i7.p1 | Carbamidomethylation |  | DB Search |
| INNNQFSVVLIK | 56.39 | 1387.7823 | 12 | 0.37 | 694.8954 | 18.30 | 0.9945 | 1.0017 | 63702 | 4.8e2 | 1 | 1 | TRINITY\_DN982\_c0\_g1\_i1.p1:TRINITY\_DN982\_c0\_g1\_i2.p1 |  |  | DB Search |
| AC(+57.02)SNVNQC(+57.02)GVNTGTQTVYK | 56.39 | 2099.9365 | 19 | 5.81 | 1050.9767 | 12.99 | 1.177 | 1.1841 | 31410 | 0 | 0 | 0 | TRINITY\_DN8597\_c0\_g1\_i1.p1 | Carbamidomethylation, Carbamidomethylation |  | DB Search |
| LALHVLNNEDIIGHR | 56.39 | 1712.9321 | 15 | 2.65 | 857.4716 | 16.49 | 1.1374 | 1.1446 | 52281 | 2.86e3 | 2 | 2 | TRINITY\_DN140702\_c0\_g1\_i1.p1 |  |  | DB Search |
| STAGDTHLGGEDFDNR | 56.39 | 1690.7183 | 16 | 2.27 | 564.5787 | 13.29 | 0.8014 | 0.8088 | 33297 | 4.2e3 | 2 | 2 | TRINITY\_DN167\_c0\_g1\_i8.p1:TRINITY\_DN167\_c0\_g1\_i7.p1:TRINITY\_DN167\_c0\_g1\_i1.p1:TRINITY\_DN167\_c0\_g1\_i4.p1:TRINITY\_DN167\_c0\_g1\_i3.p1:TRINITY\_DN167\_c0\_g1\_i10.p1:TRINITY\_DN167\_c0\_g2\_i1.p1:TRINITY\_DN167\_c0\_g1\_i6.p1 |  |  | DB Search |
| ATHQELLNAEFIQK | 56.39 | 1640.8522 | 14 | 6.81 | 821.4351 | 16.09 | 1.0773 | 1.0845 | 49865 | 4.64e2 | 1 | 1 | TRINITY\_DN113\_c3\_g1\_i1.p1 |  |  | DB Search |
| VSQTC(+57.02)AEHVFR | 56.39 | 1332.6245 | 11 | 4.52 | 667.3194 | 12.90 | 0.9868 | 0.9941 | 30882 | 0 | 0 | 0 | TRINITY\_DN3078\_c0\_g1\_i1.p1 | Carbamidomethylation |  | DB Search |
| AFVAIGDYGGHVGLGVK | 56.39 | 1658.8779 | 17 | -3.02 | 830.4399 | 17.74 | 1.1646 | 1.1718 | 59877 | 0 | 0 | 0 | TRINITY\_DN15584\_c0\_g1\_i1.p1 |  |  | DB Search |
| VIM(+15.99)VTGDHPITAK | 56.39 | 1396.7384 | 13 | 5.45 | 699.377 | 13.30 | 1.0263 | 1.0335 | 33329 | 8.77e2 | 1 | 1 | TRINITY\_DN262\_c0\_g1\_i1.p1 | Oxidation (M) | M3:Oxidation (M):1000 | DB Search |
| LVQNTPHNSIVTK | 56.38 | 1449.7939 | 13 | 5.15 | 484.2722 | 11.78 | 0.8132 | 0.8205 | 23867 | 5.7e3 | 2 | 2 | TRINITY\_DN982\_c0\_g1\_i3.p1:TRINITY\_DN982\_c0\_g1\_i1.p1 |  |  | DB Search |
| GSFSSHIC(+57.02)LC(+57.02)HDPAYK | 56.38 | 1877.8188 | 16 | 2.34 | 626.9454 | 14.46 | 0.8866 | 0.8939 | 40485 | 0 | 0 | 0 | TRINITY\_DN319\_c2\_g1\_i3.p1:TRINITY\_DN319\_c2\_g1\_i1.p1:TRINITY\_DN319\_c2\_g1\_i2.p1 | Carbamidomethylation, Carbamidomethylation |  | DB Search |
| YTSC(+57.02)QSVTVVASDR | 56.38 | 1571.725 | 14 | 0.63 | 786.8666 | 13.49 | 1.052 | 1.0592 | 34562 | 0 | 0 | 0 | TRINITY\_DN982\_c0\_g1\_i3.p1:TRINITY\_DN982\_c0\_g1\_i1.p1 | Carbamidomethylation |  | DB Search |
| VIDINDNIPIFSK | 56.38 | 1486.8031 | 13 | 0.06 | 744.4054 | 19.67 | 1.0238 | 1.0311 | 72860 | 1.48e3 | 1 | 1 | TRINITY\_DN69276\_c0\_g1\_i1.p1 |  |  | DB Search |
| YSRPTPGSNNNVMVR | 56.38 | 1690.8209 | 15 | 2.63 | 564.6131 | 12.53 | 0.8339 | 0.8412 | 28586 | 2.15e2 | 1 | 1 | TRINITY\_DN15274\_c0\_g1\_i1.p1 |  |  | DB Search |
| FSNDIVDSFAVSQKK | 56.38 | 1683.8468 | 15 | 3.19 | 562.2887 | 17.23 | 0.8688 | 0.8761 | 56580 | 0 | 0 | 0 | TRINITY\_DN244\_c0\_g1\_i1.p1 |  |  | DB Search |
| AEYENKTEVAR | 56.38 | 1308.631 | 11 | 4.95 | 655.3229 | 13.23 | 0.9824 | 0.9896 | 32963 | 9.59e2 | 1 | 1 | TRINITY\_DN1280\_c0\_g1\_i1.p1 |  |  | DB Search |
| SSSC(+57.02)QEFGGKC(+57.02)PC(+57.02)K | 56.37 | 1630.6538 | 14 | -16.59 | 816.3168 | 12.09 | 1.0335 | 1.0407 | 25828 | 0 | 0 | 0 | TRINITY\_DN1616\_c0\_g1\_i3.p1:TRINITY\_DN1616\_c0\_g1\_i6.p1:TRINITY\_DN1616\_c0\_g1\_i2.p1:TRINITY\_DN1616\_c0\_g1\_i7.p1 | Carbamidomethylation, Carbamidomethylation, Carbamidomethylation |  | DB Search |
| VTRPHGASGVVR | 56.37 | 1234.6895 | 12 | 3.4 | 412.5699 | 10.07 | 0.8104 | 0.8177 | 14175 | 0 | 0 | 0 | TRINITY\_DN2885\_c0\_g1\_i2.p1:TRINITY\_DN2885\_c0\_g1\_i4.p1:TRINITY\_DN2885\_c0\_g1\_i5.p1 |  |  | DB Search |
| LLTTEDDFILR | 56.37 | 1334.7081 | 11 | -1.75 | 668.3571 | 19.12 | 0.9751 | 0.9824 | 69298 | 0 | 0 | 0 | TRINITY\_DN982\_c0\_g1\_i3.p1:TRINITY\_DN982\_c0\_g1\_i1.p1 |  |  | DB Search |
| IIVADGAEYKK | 56.37 | 1205.6655 | 11 | 5.35 | 603.8405 | 12.62 | 0.982 | 0.9892 | 29075 | 4.32e2 | 1 | 1 | TRINITY\_DN33065\_c0\_g2\_i1.p1 |  |  | DB Search |
| AVAEEFISVDPYVR | 56.37 | 1593.8038 | 14 | 14.01 | 797.9166 | 19.08 | 1.0656 | 1.0729 | 69186 | 1.42e4 | 1 | 1 | TRINITY\_DN598\_c1\_g1\_i1.p1 |  |  | DB Search |
| YYEC(+57.02)GSAC(+57.02)PK | 56.36 | 1233.4795 | 10 | -0.44 | 617.7438 | 11.29 | 0.917 | 0.9243 | 21021 | 0 | 0 | 0 | TRINITY\_DN6390\_c0\_g1\_i1.p1 | Carbamidomethylation, Carbamidomethylation |  | DB Search |
| VKESYTLNIEAR | 56.36 | 1421.7515 | 12 | -2.77 | 711.8777 | 14.75 | 1.0013 | 1.0086 | 42302 | 0 | 0 | 0 | TRINITY\_DN72625\_c0\_g1\_i1.p1 |  |  | DB Search |
| EGFYVHEALAAGIVK | 56.36 | 1602.8406 | 15 | 7.5 | 802.4298 | 17.69 | 1.0873 | 1.0945 | 59517 | 5.28e2 | 1 | 1 | TRINITY\_DN602\_c0\_g1\_i4.p1:TRINITY\_DN602\_c0\_g1\_i3.p1:TRINITY\_DN602\_c0\_g1\_i1.p1 |  |  | DB Search |
| YLFDLESNC(+57.02)DK | 56.36 | 1402.6074 | 11 | -2.5 | 702.306 | 18.42 | 0.984 | 0.9912 | 64464 | 1.37e2 | 1 | 1 | TRINITY\_DN6390\_c0\_g1\_i1.p1 | Carbamidomethylation |  | DB Search |
| IVNTDLQEIKDIIK | 56.35 | 1640.9348 | 14 | -0.94 | 547.9825 | 18.84 | 0.8193 | 0.8266 | 67326 | 7.77e2 | 1 | 1 | TRINITY\_DN3016\_c0\_g1\_i17.p1:TRINITY\_DN3016\_c0\_g1\_i11.p1:TRINITY\_DN3016\_c0\_g1\_i10.p1:TRINITY\_DN3016\_c0\_g1\_i6.p1:TRINITY\_DN3016\_c0\_g1\_i18.p1:TRINITY\_DN3016\_c0\_g1\_i8.p1:TRINITY\_DN3016\_c0\_g1\_i13.p1:TRINITY\_DN3016\_c0\_g1\_i2.p1:TRINITY\_DN3016\_c0\_g1\_i4.p1:TRINITY\_DN3016\_c0\_g1\_i5.p1 |  |  | DB Search |
| DKELSNVIESGFR | 56.35 | 1492.7522 | 13 | 2.74 | 747.3819 | 17.35 | 1.0464 | 1.0536 | 57241 | 4.08e2 | 1 | 1 | TRINITY\_DN775\_c0\_g1\_i2.p1:TRINITY\_DN775\_c0\_g1\_i1.p1 |  |  | DB Search |
| YGDYTPEIHQK | 56.34 | 1349.6251 | 11 | 4.63 | 675.8198 | 12.99 | 0.9924 | 0.9997 | 31469 | 0 | 0 | 0 | TRINITY\_DN6188\_c0\_g1\_i1.p1 |  |  | DB Search |
| YGAVNDNC(+57.02)ER | 56.34 | 1196.488 | 10 | 3.96 | 599.2509 | 10.89 | 0.885 | 0.8923 | 18607 | 1.07e3 | 1 | 1 | TRINITY\_DN1616\_c0\_g1\_i3.p1:TRINITY\_DN1616\_c0\_g1\_i6.p1:TRINITY\_DN1616\_c0\_g1\_i2.p1:TRINITY\_DN1616\_c0\_g1\_i7.p1 | Carbamidomethylation |  | DB Search |
| SYYFAISDITVGGR | 56.33 | 1547.762 | 14 | -2.52 | 774.8827 | 19.97 | 1.046 | 1.0532 | 74887 | 4.98e2 | 1 | 1 | TRINITY\_DN1676\_c0\_g1\_i6.p1:TRINITY\_DN1676\_c0\_g1\_i12.p1 |  |  | DB Search |
| TQAVLDRLNIEKR | 56.32 | 1554.8842 | 13 | 2.69 | 519.301 | 14.70 | 0.8035 | 0.8108 | 41823 | 2.15e3 | 1 | 1 | TRINITY\_DN477\_c0\_g1\_i9.p1 |  |  | DB Search |
| LINLFEAVSKK | 56.32 | 1260.7441 | 11 | 0.6 | 631.3768 | 19.05 | 0.9836 | 0.9908 | 68790 | 7.03e2 | 2 | 2 | TRINITY\_DN1653\_c0\_g1\_i3.p1:TRINITY\_DN1653\_c0\_g1\_i1.p1 |  |  | DB Search |
| ASMDEGAKPK | 56.32 | 1032.491 | 10 | 1.35 | 517.251 | 9.36 | 0.8558 | 0.8631 | 10916 | 0 | 0 | 0 | TRINITY\_DN2003\_c0\_g1\_i1.p1 |  |  | DB Search |
| ATDIDQEVKEK | 56.31 | 1274.6354 | 11 | 0.83 | 638.3225 | 11.62 | 0.9642 | 0.9715 | 22970 | 0 | 0 | 0 | TRINITY\_DN3347\_c0\_g1\_i1.p1 |  |  | DB Search |
| AVDIHFALDASQNVGR | 56.28 | 1711.8641 | 16 | 1.27 | 571.6267 | 17.55 | 0.8591 | 0.8664 | 58465 | 7.9e2 | 1 | 1 | TRINITY\_DN77\_c1\_g1\_i1.p1 |  |  | DB Search |
| IC(+57.02)HLLGIQVTDFTK | 56.23 | 1643.8705 | 14 | -2.32 | 548.9603 | 18.61 | 0.8197 | 0.827 | 65792 | 1.09e3 | 1 | 1 | TRINITY\_DN428\_c0\_g1\_i1.p1 | Carbamidomethylation |  | DB Search |
| LTHQIEVTVSDREGK | 56.23 | 1710.89 | 15 | -5.27 | 571.2983 | 12.85 | 0.8696 | 0.8769 | 30546 | 0 | 0 | 0 | TRINITY\_DN123121\_c0\_g1\_i1.p1 |  |  | DB Search |
| LRLDFDHEAK | 56.21 | 1242.6356 | 10 | -2.49 | 415.2162 | 14.37 | 0.7326 | 0.74 | 39914 | 3.79e2 | 1 | 1 | TRINITY\_DN160\_c0\_g1\_i2.p1:TRINITY\_DN160\_c0\_g1\_i1.p1 |  |  | DB Search |
| LGIHEDTQNR | 56.21 | 1181.5789 | 10 | 4.79 | 394.8669 | 11.18 | 0.711 | 0.7184 | 20409 | 9.9e2 | 1 | 1 | TRINITY\_DN141338\_c2\_g1\_i1.p1 |  |  | DB Search |
| LLTSPAPSSGSLM(+15.99)ALALK | 56.18 | 1771.9753 | 18 | -1.19 | 886.9897 | 18.28 | 1.1498 | 1.157 | 63418 | 9.34e2 | 1 | 1 | TRINITY\_DN602\_c0\_g1\_i4.p1:TRINITY\_DN602\_c0\_g1\_i3.p1:TRINITY\_DN602\_c0\_g1\_i1.p1 | Oxidation (M) | M13:Oxidation (M):1000 | DB Search |
| AVQIHPFNDVSVGDPK | 56.18 | 1721.8737 | 16 | -1.14 | 861.9391 | 15.94 | 1.1178 | 1.125 | 48886 | 2.33e3 | 1 | 1 | TRINITY\_DN1071\_c0\_g1\_i2.p1:TRINITY\_DN1071\_c0\_g1\_i3.p1:TRINITY\_DN1071\_c0\_g1\_i1.p1:TRINITY\_DN1071\_c0\_g1\_i4.p1 |  |  | DB Search |
| AFSFSDKPAITFEIHDFK | 56.18 | 2099.0364 | 18 | 0.2 | 700.6829 | 19.06 | 0.9795 | 0.9868 | 68737 | 2.48e2 | 1 | 1 | TRINITY\_DN33065\_c0\_g2\_i1.p1 |  |  | DB Search |
| LQGWWQSVNGR | 56.18 | 1329.6578 | 11 | 3.31 | 665.8353 | 18.21 | 0.9444 | 0.9517 | 63090 | 0 | 0 | 0 | TRINITY\_DN15274\_c0\_g1\_i1.p1 |  |  | DB Search |
| AVFC(+57.02)EHQGVK | 56.17 | 1173.5601 | 10 | 2.17 | 587.7858 | 11.71 | 0.9036 | 0.9109 | 23391 | 2.96e3 | 1 | 1 | TRINITY\_DN875\_c0\_g1\_i1.p1:TRINITY\_DN875\_c0\_g1\_i3.p1:TRINITY\_DN875\_c0\_g1\_i2.p1 | Carbamidomethylation |  | DB Search |
| AVTIESTSNSVNGVFDTVEGK | 56.17 | 2153.0488 | 21 | -2.28 | 1077.5242 | 18.97 | 1.2196 | 1.2268 | 68255 | 3.01e2 | 1 | 1 | TRINITY\_DN33065\_c0\_g1\_i1.p1 |  |  | DB Search |
| AESIALVTEFTDEAAPK | 56.17 | 1790.8938 | 17 | 5.03 | 896.4545 | 19.96 | 1.1194 | 1.1266 | 74593 | 1.16e3 | 1 | 1 | TRINITY\_DN79\_c0\_g1\_i1.p1 |  |  | DB Search |
| NYNNDDEPGAK | 56.17 | 1235.5055 | 11 | 4.61 | 618.7599 | 10.13 | 0.8842 | 0.8915 | 14506 | 1.78e3 | 2 | 2 | TRINITY\_DN199\_c0\_g1\_i1.p1 |  |  | DB Search |
| DNAGTSSSGTGPR | 56.16 | 1205.5272 | 13 | 0.39 | 603.7683 | 9.17 | 0.866 | 0.8733 | 10097 | 2.6e2 | 1 | 1 | TRINITY\_DN4781\_c0\_g1\_i3.p3 |  |  | DB Search |
| KVIVDQLVTLATR | 56.14 | 1454.8821 | 13 | -0.17 | 728.4448 | 18.43 | 1.0299 | 1.0371 | 64581 | 0 | 0 | 0 | TRINITY\_DN1386\_c0\_g1\_i1.p1 |  |  | DB Search |
| SC(+57.02)ENPKPLNGGK | 56.12 | 1299.6241 | 12 | 2.81 | 650.8181 | 11.10 | 0.9469 | 0.9541 | 19780 | 7.77e2 | 1 | 1 | TRINITY\_DN6428\_c0\_g1\_i1.p1:TRINITY\_DN6428\_c0\_g1\_i2.p1 | Carbamidomethylation |  | DB Search |
| LGFGEEAQQHFR | 56.11 | 1417.6738 | 12 | 3.8 | 709.8436 | 15.03 | 1.0271 | 1.0343 | 43872 | 0 | 0 | 0 | TRINITY\_DN7299\_c0\_g1\_i1.p2 |  |  | DB Search |
| IYIDGVFIR | 56.04 | 1094.6124 | 9 | 2.12 | 548.3121 | 18.80 | 0.8903 | 0.8976 | 67021 | 1.29e3 | 1 | 1 | TRINITY\_DN5500\_c0\_g1\_i2.p1:TRINITY\_DN5500\_c0\_g1\_i1.p1 |  |  | DB Search |
| AITDNIKDILSVK | 56.03 | 1428.8187 | 13 | -0.47 | 477.2777 | 19.32 | 0.7929 | 0.8002 | 70439 | 6.5e2 | 2 | 2 | TRINITY\_DN1739\_c0\_g1\_i1.p1 |  |  | DB Search |
| DANSMPTQSR | 56.02 | 1105.4822 | 10 | 4.07 | 553.748 | 10.94 | 0.8583 | 0.8656 | 18894 | 7.52e2 | 1 | 1 | TRINITY\_DN477\_c0\_g1\_i9.p1 |  |  | DB Search |
| IQVLKDPDFEFTR | 55.75 | 1606.8354 | 13 | 2.82 | 536.6181 | 17.65 | 0.8518 | 0.8591 | 59377 | 0 | 0 | 0 | TRINITY\_DN982\_c0\_g1\_i3.p1:TRINITY\_DN982\_c0\_g1\_i1.p1 |  |  | DB Search |
| AELVFVNHC(+57.02)R | 55.56 | 1243.6132 | 10 | 1.33 | 622.8118 | 14.50 | 0.9602 | 0.9675 | 40700 | 1.63e3 | 1 | 1 | TRINITY\_DN101992\_c0\_g1\_i1.p1 | Carbamidomethylation |  | DB Search |
| AQAINVQLQEIQR | 55.46 | 1509.8263 | 13 | 1.57 | 755.9181 | 16.14 | 1.0295 | 1.0367 | 50268 | 0 | 0 | 0 | TRINITY\_DN1280\_c0\_g1\_i1.p1 |  |  | DB Search |
| LVPEHVESR | 55.38 | 1064.5614 | 9 | 0.73 | 533.2859 | 11.15 | 0.8911 | 0.8984 | 20102 | 7.51e2 | 1 | 1 | TRINITY\_DN140702\_c0\_g1\_i1.p1 |  |  | DB Search |
| AYGQDLGQPGSC(+57.02)LKR | 55.37 | 1648.7991 | 15 | 4.15 | 550.6067 | 13.53 | 0.8457 | 0.853 | 34711 | 1.5e3 | 1 | 1 | TRINITY\_DN691\_c0\_g1\_i2.p1:TRINITY\_DN691\_c0\_g1\_i1.p1 | Carbamidomethylation |  | DB Search |
| GNQGEQGPAGPVGPK | 55.37 | 1391.6793 | 15 | 2.49 | 696.8454 | 11.50 | 0.9469 | 0.9541 | 22230 | 5.82e3 | 1 | 1 | TRINITY\_DN5987\_c0\_g1\_i2.p1:TRINITY\_DN5987\_c0\_g1\_i4.p1:TRINITY\_DN5987\_c0\_g1\_i3.p2 |  |  | DB Search |
| SYELPDGQVITIGNER | 55.37 | 1789.8846 | 16 | 2.43 | 895.9476 | 18.67 | 1.117 | 1.1242 | 66156 | 3.51e2 | 1 | 1 | TRINITY\_DN161\_c0\_g1\_i1.p1:TRINITY\_DN161\_c0\_g1\_i4.p1:TRINITY\_DN161\_c0\_g1\_i3.p1:TRINITY\_DN161\_c0\_g1\_i2.p1 |  |  | DB Search |
| AEFRPNQEEEPILKR | 55.37 | 1854.9587 | 15 | 3.46 | 619.3261 | 13.89 | 0.8826 | 0.8899 | 36828 | 3.47e3 | 1 | 1 | TRINITY\_DN262\_c0\_g1\_i1.p1 |  |  | DB Search |
| AINIHDLVGIDTKK | 55.37 | 1535.8671 | 14 | 4.52 | 512.9629 | 16.65 | 0.7913 | 0.7986 | 53072 | 3.27e3 | 2 | 2 | TRINITY\_DN61\_c0\_g1\_i1.p1 |  |  | DB Search |
| KVSQTC(+57.02)AEHVFR | 55.37 | 1460.7194 | 12 | 3.01 | 487.9129 | 12.03 | 0.8027 | 0.81 | 25408 | 9.73e2 | 1 | 1 | TRINITY\_DN3078\_c0\_g1\_i1.p1 | Carbamidomethylation |  | DB Search |
| HQGVMVGMGQK | 55.37 | 1170.5637 | 11 | 3.68 | 586.2886 | 12.36 | 0.9105 | 0.9178 | 27450 | 5.73e2 | 1 | 1 | TRINITY\_DN161\_c0\_g1\_i1.p1:TRINITY\_DN161\_c0\_g1\_i3.p1:TRINITY\_DN161\_c0\_g1\_i2.p1 |  |  | DB Search |
| LIQYIENKPGLTR | 55.37 | 1543.8722 | 13 | 3.16 | 772.9422 | 14.92 | 1.0721 | 1.0793 | 43103 | 1.9e3 | 1 | 1 | TRINITY\_DN2028\_c0\_g1\_i1.p1 |  |  | DB Search |
| S(+42.01)SFLVVPLKK | 55.36 | 1158.7012 | 10 | -0.75 | 580.3547 | 19.06 | 0.9481 | 0.9553 | 68767 | 9.06e2 | 1 | 1 | TRINITY\_DN17358\_c0\_g1\_i1.p1 | Acetylation (Protein N-term) | S1:Acetylation (Protein N-term):1000 | DB Search |
| AKIEALHC(+57.02)LSENIVK | 55.36 | 1723.9291 | 15 | 5.81 | 575.6509 | 15.74 | 0.8258 | 0.8331 | 47999 | 0 | 0 | 0 | TRINITY\_DN3078\_c0\_g1\_i1.p1 | Carbamidomethylation |  | DB Search |
| VLLSQQIFYDKK | 55.36 | 1480.829 | 12 | 1.76 | 494.6155 | 16.92 | 0.7616 | 0.7689 | 54594 | 7.01e3 | 2 | 2 | TRINITY\_DN982\_c0\_g1\_i3.p1:TRINITY\_DN982\_c0\_g1\_i1.p1:TRINITY\_DN982\_c0\_g1\_i2.p1 |  |  | DB Search |
| KHLSELSEEAAK | 55.36 | 1340.6936 | 12 | -9.06 | 447.899 | 11.10 | 0.7856 | 0.7929 | 19897 | 0 | 0 | 0 | TRINITY\_DN61707\_c0\_g1\_i1.p1 |  |  | DB Search |
| LTHQIEVTVSDR | 55.36 | 1396.7311 | 12 | 1.03 | 699.3702 | 13.57 | 0.9965 | 1.0037 | 35021 | 1.59e3 | 1 | 1 | TRINITY\_DN123121\_c0\_g1\_i1.p1 |  |  | DB Search |
| ALVILGDGFISGGGTPSK | 55.36 | 1687.9144 | 18 | -3.94 | 844.9572 | 19.80 | 1.1694 | 1.1766 | 73673 | 1.43e3 | 1 | 1 | TRINITY\_DN244\_c0\_g1\_i1.p1 |  |  | DB Search |
| FPEFTLQGNK | 55.36 | 1179.5924 | 10 | 3.01 | 590.8025 | 17.68 | 0.8935 | 0.9008 | 59467 | 7.06e2 | 1 | 1 | TRINITY\_DN982\_c0\_g1\_i3.p1:TRINITY\_DN982\_c0\_g1\_i1.p1 |  |  | DB Search |
| IKDTYLLTLEAR | 55.35 | 1434.8082 | 12 | 3.07 | 718.4102 | 17.05 | 1.0379 | 1.0452 | 55307 | 1.14e3 | 1 | 1 | TRINITY\_DN72625\_c0\_g1\_i1.p1 |  |  | DB Search |
| LPGDALSIGFPSK | 55.35 | 1300.7026 | 13 | -4.93 | 651.3524 | 18.60 | 0.9691 | 0.9763 | 65620 | 9.17e2 | 1 | 1 | TRINITY\_DN149493\_c0\_g1\_i1.p1 |  |  | DB Search |
| IGLGNIRDPSISLTR | 55.35 | 1610.9104 | 15 | -3.69 | 537.9729 | 17.37 | 0.7986 | 0.8059 | 57304 | 1.32e3 | 1 | 1 | TRINITY\_DN11522\_c0\_g1\_i1.p1:TRINITY\_DN11522\_c0\_g1\_i2.p1:TRINITY\_DN11522\_c0\_g1\_i4.p1:TRINITY\_DN11522\_c0\_g1\_i3.p1 |  |  | DB Search |
| KFDTYLPGFIC(+57.02)R | 55.35 | 1515.7544 | 12 | -1.87 | 506.2554 | 18.73 | 0.7856 | 0.7929 | 66611 | 0 | 0 | 0 | TRINITY\_DN785\_c0\_g1\_i1.p1 | Carbamidomethylation |  | DB Search |
| VC(+57.02)NGEPC(+57.02)ILQER | 55.34 | 1473.6704 | 12 | 3.33 | 737.8415 | 14.98 | 0.9896 | 0.9969 | 43535 | 0 | 0 | 0 | TRINITY\_DN4595\_c0\_g1\_i4.p1:TRINITY\_DN4595\_c0\_g1\_i10.p2 | Carbamidomethylation, Carbamidomethylation |  | DB Search |
| VGNWRDETLSC(+57.02)K | 55.34 | 1463.6827 | 12 | -0.36 | 488.899 | 13.83 | 0.7608 | 0.7681 | 36637 | 1.03e3 | 1 | 1 | TRINITY\_DN6390\_c0\_g1\_i1.p1 | Carbamidomethylation |  | DB Search |
| YLTVAAMFR | 55.34 | 1070.5582 | 9 | -0.51 | 536.2836 | 19.90 | 0.8656 | 0.8729 | 74532 | 0 | 0 | 0 | TRINITY\_DN1910\_c0\_g1\_i1.p1:TRINITY\_DN3986\_c0\_g1\_i1.p1 |  |  | DB Search |
| EAIEGTYIDKK | 55.33 | 1265.6503 | 11 | 3.67 | 633.8318 | 12.92 | 0.9505 | 0.9578 | 30926 | 8.95e2 | 1 | 1 | TRINITY\_DN15847\_c0\_g1\_i1.p1:TRINITY\_DN15847\_c0\_g1\_i2.p1 |  |  | DB Search |
| ADILC(+57.02)HDTK | 55.33 | 1071.5018 | 9 | 3.94 | 536.7578 | 11.82 | 0.881 | 0.8883 | 24181 | 1.06e3 | 1 | 1 | TRINITY\_DN413\_c0\_g1\_i1.p1:TRINITY\_DN413\_c0\_g1\_i4.p1:TRINITY\_DN413\_c0\_g1\_i3.p1 | Carbamidomethylation |  | DB Search |
| STSTGHLIYK | 55.32 | 1105.5768 | 10 | -1.17 | 553.7924 | 12.14 | 0.8712 | 0.8785 | 26038 | 8.26e2 | 1 | 1 | TRINITY\_DN64\_c0\_g1\_i1.p1 |  |  | DB Search |
| YVAIYPSDHLTVNR | 55.32 | 1646.8417 | 14 | -3.91 | 824.421 | 15.67 | 1.1098 | 1.117 | 47583 | 0 | 0 | 0 | TRINITY\_DN982\_c0\_g1\_i3.p1:TRINITY\_DN982\_c0\_g1\_i1.p1 |  |  | DB Search |
| HQGVM(+15.99)VGMGQK | 55.3 | 1186.5587 | 11 | 10.45 | 594.29 | 11.22 | 0.9133 | 0.9206 | 20614 | 0 | 0 | 0 | TRINITY\_DN161\_c0\_g1\_i1.p1:TRINITY\_DN161\_c0\_g1\_i3.p1:TRINITY\_DN161\_c0\_g1\_i2.p1 | Oxidation (M) | M5:Oxidation (M):60 | DB Search |
| VQGSM(+15.99)TIVK | 55.29 | 977.5215 | 9 | 3.77 | 489.7676 | 11.21 | 0.8287 | 0.836 | 20591 | 1.12e3 | 1 | 1 | TRINITY\_DN199\_c0\_g1\_i1.p1 | Oxidation (M) | M5:Oxidation (M):1000 | DB Search |
| VLEVNDNKPK | 55.29 | 1154.6295 | 10 | 3.29 | 578.3212 | 11.02 | 0.9255 | 0.9327 | 19462 | 6.22e2 | 1 | 1 | TRINITY\_DN982\_c0\_g1\_i1.p1:TRINITY\_DN982\_c0\_g1\_i2.p1 |  |  | DB Search |
| NDVNNPPQAR | 55.29 | 1123.537 | 10 | 3.11 | 562.7749 | 10.15 | 0.8558 | 0.8631 | 13772 | 1.28e4 | 1 | 1 | TRINITY\_DN477\_c0\_g1\_i3.p1:TRINITY\_DN477\_c0\_g1\_i7.p1:TRINITY\_DN477\_c0\_g1\_i6.p1:TRINITY\_DN477\_c0\_g1\_i5.p1:TRINITY\_DN477\_c0\_g1\_i8.p2 |  |  | DB Search |
| ELEITVLESKPVNSFVIR | 55.28 | 2072.1516 | 18 | 0.84 | 691.7219 | 19.88 | 0.9634 | 0.9707 | 74309 | 3.41e2 | 1 | 1 | TRINITY\_DN982\_c0\_g1\_i1.p1:TRINITY\_DN982\_c0\_g1\_i2.p1 |  |  | DB Search |
| YSRPTPGSNNNVM(+15.99)VR | 55.28 | 1706.8158 | 15 | 5.92 | 569.9466 | 11.60 | 0.8388 | 0.8461 | 22687 | 2.26e3 | 1 | 1 | TRINITY\_DN15274\_c0\_g1\_i1.p1 | Oxidation (M) | M13:Oxidation (M):1000 | DB Search |
| DSHGVAQVR | 55.25 | 967.4835 | 9 | 5.54 | 484.7495 | 10.13 | 0.8144 | 0.8218 | 14459 | 0 | 0 | 0 | TRINITY\_DN80458\_c0\_g1\_i1.p1 |  |  | DB Search |
| LLLGDFNGDKR | 55.25 | 1246.667 | 11 | -0.59 | 624.3375 | 16.23 | 0.9856 | 0.9928 | 50739 | 5.27e2 | 1 | 1 | TRINITY\_DN413\_c0\_g1\_i1.p1:TRINITY\_DN413\_c0\_g1\_i4.p1:TRINITY\_DN413\_c0\_g1\_i2.p1:TRINITY\_DN413\_c0\_g1\_i3.p1 |  |  | DB Search |
| DSYVGDEAQSKR | 55.24 | 1353.6161 | 12 | 0.18 | 677.8123 | 10.99 | 0.9932 | 1.0005 | 19155 | 1.01e3 | 1 | 1 | TRINITY\_DN2477\_c0\_g1\_i5.p1:TRINITY\_DN2477\_c0\_g1\_i6.p1:TRINITY\_DN195934\_c0\_g1\_i1.p2:TRINITY\_DN161\_c0\_g1\_i1.p1:TRINITY\_DN2477\_c0\_g1\_i9.p1:TRINITY\_DN2477\_c0\_g1\_i10.p1:TRINITY\_DN2477\_c0\_g1\_i2.p1:TRINITY\_DN161\_c0\_g1\_i3.p1:TRINITY\_DN161\_c0\_g1\_i2.p1 |  |  | DB Search |
| SGNVVLC(+57.02)LIDVHK | 55.24 | 1452.7759 | 13 | 2.74 | 485.265 | 18.67 | 0.7416 | 0.7489 | 66263 | 0 | 0 | 0 | TRINITY\_DN3078\_c0\_g1\_i1.p1 | Carbamidomethylation |  | DB Search |
| AVFPSIVGRPR | 55.22 | 1197.6982 | 11 | 2.36 | 599.855 | 15.87 | 0.961 | 0.9683 | 48378 | 1.86e4 | 1 | 1 | TRINITY\_DN161\_c0\_g1\_i1.p1:TRINITY\_DN161\_c0\_g1\_i3.p1:TRINITY\_DN161\_c0\_g1\_i2.p1 |  |  | DB Search |
| YYAAAC(+57.02)LDNIFQR | 55.19 | 1603.7452 | 13 | -7.09 | 802.8705 | 19.75 | 1.0701 | 1.0773 | 73459 | 4.66e2 | 1 | 1 | TRINITY\_DN71276\_c0\_g1\_i1.p1 | Carbamidomethylation |  | DB Search |
| YTNQETGEINDVVSYVPK | 55.19 | 2054.9797 | 18 | -4.38 | 1028.4878 | 18.17 | 1.2041 | 1.2113 | 62839 | 0 | 0 | 0 | TRINITY\_DN1668\_c0\_g1\_i1.p1 |  |  | DB Search |
| IGC(+57.02)FHDR | 55.18 | 903.4021 | 7 | 2.15 | 452.7072 | 11.34 | 0.8031 | 0.8104 | 21250 | 4.1e3 | 1 | 1 | TRINITY\_DN138\_c0\_g1\_i1.p1 | Carbamidomethylation |  | DB Search |
| IEDFEESPPTDPHIGPVEQR | 55.18 | 2291.0706 | 20 | 5.47 | 764.6981 | 15.80 | 0.9319 | 0.9392 | 48175 | 1.66e3 | 1 | 1 | TRINITY\_DN140702\_c0\_g1\_i1.p1 |  |  | DB Search |
| KTTELTNNLK | 55.18 | 1160.6401 | 10 | 0.91 | 581.3251 | 11.77 | 0.9182 | 0.9255 | 23835 | 9.14e2 | 1 | 1 | TRINITY\_DN72625\_c0\_g1\_i1.p1 |  |  | DB Search |
| ALNVNQGNEK | 55.18 | 1085.5465 | 10 | 8.59 | 543.7827 | 10.95 | 0.8619 | 0.8692 | 18965 | 2.35e3 | 1 | 1 | TRINITY\_DN624\_c0\_g1\_i1.p1 |  |  | DB Search |
| AIQGATSHHLGQNFSK | 55.18 | 1694.8489 | 16 | 7.42 | 565.9584 | 12.02 | 0.8518 | 0.8591 | 25405 | 5.02e2 | 1 | 1 | TRINITY\_DN81013\_c0\_g1\_i1.p2:TRINITY\_DN5001\_c0\_g1\_i1.p1 |  |  | DB Search |
| APPKPNPC(+57.02)AIK | 55.16 | 1191.6434 | 11 | 6.69 | 398.2225 | 11.49 | 0.7791 | 0.7864 | 22133 | 1.06e3 | 1 | 1 | TRINITY\_DN4781\_c0\_g1\_i1.p1:TRINITY\_DN4781\_c0\_g1\_i2.p1 | Carbamidomethylation |  | DB Search |
| APIGQSIVTVSAR | 55.13 | 1297.7354 | 13 | 0.79 | 649.8724 | 14.27 | 0.9691 | 0.9763 | 39347 | 0 | 0 | 0 | TRINITY\_DN982\_c0\_g1\_i3.p1 |  |  | DB Search |
| TITLEVEPSDTIENVK | 55.12 | 1786.92 | 16 | 0.46 | 894.4635 | 17.55 | 1.1414 | 1.1486 | 58604 | 8.72e2 | 1 | 1 | TRINITY\_DN554\_c0\_g1\_i3.p2:TRINITY\_DN585\_c0\_g1\_i8.p1:TRINITY\_DN585\_c0\_g1\_i3.p1:TRINITY\_DN101\_c0\_g1\_i1.p1:TRINITY\_DN585\_c0\_g1\_i4.p1:TRINITY\_DN554\_c0\_g1\_i4.p1:TRINITY\_DN554\_c0\_g1\_i2.p1:TRINITY\_DN585\_c0\_g1\_i10.p1:TRINITY\_DN585\_c0\_g1\_i2.p1:TRINITY\_DN554\_c0\_g1\_i5.p1:TRINITY\_DN585\_c0\_g1\_i5.p1:TRINITY\_DN585\_c0\_g2\_i1.p1 |  |  | DB Search |
| VAPEEHPVLLTEAPLNPK | 55.12 | 1953.0571 | 18 | 2.07 | 977.5333 | 16.62 | 1.2101 | 1.2173 | 52945 | 1.41e2 | 1 | 1 | TRINITY\_DN2477\_c0\_g1\_i5.p1:TRINITY\_DN2477\_c0\_g1\_i6.p1:TRINITY\_DN2477\_c0\_g1\_i8.p1:TRINITY\_DN195934\_c0\_g1\_i1.p2:TRINITY\_DN161\_c0\_g1\_i1.p1:TRINITY\_DN2477\_c0\_g1\_i9.p1:TRINITY\_DN2477\_c0\_g1\_i10.p1:TRINITY\_DN2477\_c0\_g1\_i2.p1:TRINITY\_DN161\_c0\_g1\_i3.p1:TRINITY\_DN2941\_c0\_g1\_i4.p1:TRINITY\_DN161\_c0\_g1\_i2.p1 |  |  | DB Search |
| VLIVISDGNEDFKEK | 55.12 | 1704.8933 | 15 | 3.91 | 853.4533 | 16.85 | 1.1742 | 1.1813 | 54267 | 0 | 0 | 0 | TRINITY\_DN195348\_c0\_g1\_i1.p1 |  |  | DB Search |
| ATETATGLATTHPIK | 55.11 | 1510.7991 | 15 | 0.61 | 756.4037 | 13.07 | 1.1041 | 1.1114 | 31991 | 0 | 0 | 0 | TRINITY\_DN165536\_c0\_g1\_i1.p1 |  |  | DB Search |
| GVAGAGVLAGFDKFK | 55.1 | 1435.7823 | 15 | 1.47 | 718.8961 | 18.42 | 1.0889 | 1.0961 | 64513 | 2.84e2 | 1 | 1 | TRINITY\_DN1559\_c0\_g1\_i1.p1 |  |  | DB Search |
| YQGNDYTR | 55.1 | 1015.4359 | 8 | 1.65 | 508.7237 | 10.77 | 0.8469 | 0.8542 | 17934 | 1.34e3 | 1 | 1 | TRINITY\_DN4595\_c0\_g1\_i1.p1:TRINITY\_DN4595\_c0\_g1\_i7.p1:TRINITY\_DN4595\_c0\_g1\_i4.p1 |  |  | DB Search |
| LVSELFDPTSSTR | 55.08 | 1450.7303 | 13 | -1.65 | 726.3679 | 17.84 | 1.0033 | 1.0106 | 60692 | 0 | 0 | 0 | TRINITY\_DN4781\_c0\_g1\_i3.p1:TRINITY\_DN4781\_c0\_g1\_i1.p1:TRINITY\_DN4781\_c0\_g1\_i2.p1 |  |  | DB Search |
| A(+42.01)HLSQEQR | 55.07 | 1009.4941 | 8 | 7.42 | 505.7557 | 10.99 | 0.8684 | 0.8757 | 19264 | 0 | 0 | 0 | TRINITY\_DN3052\_c0\_g1\_i1.p1 | Acetylation (Protein N-term) | A1:Acetylation (Protein N-term):1000 | DB Search |
| TLDFVVELSK | 55.07 | 1149.6281 | 10 | -1.12 | 575.818 | 19.60 | 0.887 | 0.8943 | 72344 | 9.89e3 | 1 | 1 | TRINITY\_DN138\_c0\_g1\_i1.p1 |  |  | DB Search |
| DKNILVLTLGVGPQVNR | 54.98 | 1835.0629 | 17 | 0.89 | 918.5352 | 19.51 | 1.1354 | 1.1426 | 71919 | 8.54e3 | 2 | 2 | TRINITY\_DN477\_c0\_g1\_i8.p1:TRINITY\_DN477\_c0\_g1\_i7.p1 |  |  | DB Search |
| AGM(+15.99)THIVR | 54.97 | 899.4647 | 8 | 4.88 | 450.7397 | 9.72 | 0.8144 | 0.8218 | 12487 | 9.73e2 | 1 | 1 | TRINITY\_DN2799\_c0\_g1\_i3.p1:TRINITY\_DN6920\_c0\_g1\_i3.p1:TRINITY\_DN2799\_c0\_g1\_i1.p1:TRINITY\_DN3448\_c0\_g1\_i7.p2:TRINITY\_DN3448\_c0\_g1\_i9.p3:TRINITY\_DN3448\_c0\_g2\_i1.p1:TRINITY\_DN3448\_c0\_g1\_i5.p2:TRINITY\_DN5906\_c3\_g1\_i1.p2:TRINITY\_DN32727\_c0\_g1\_i2.p1:TRINITY\_DN3448\_c0\_g2\_i3.p2:TRINITY\_DN6920\_c0\_g1\_i2.p1:TRINITY\_DN2799\_c0\_g1\_i2.p1:TRINITY\_DN2799\_c0\_g1\_i4.p2 | Oxidation (M) | M3:Oxidation (M):1000 | DB Search |
| YYVTIIDAPGHR | 54.9 | 1403.7197 | 12 | -0.29 | 702.8636 | 16.07 | 1.0427 | 1.05 | 49774 | 7.1e2 | 1 | 1 | TRINITY\_DN64\_c0\_g1\_i1.p1 |  |  | DB Search |
| TGVEILNWGVR | 54.86 | 1242.672 | 11 | 2.47 | 622.3419 | 19.69 | 0.9101 | 0.9174 | 73072 | 3.26e2 | 1 | 1 | TRINITY\_DN140702\_c0\_g1\_i1.p1 |  |  | DB Search |
| FVFPFK | 54.68 | 783.4319 | 6 | 5.78 | 392.7237 | 19.69 | 0.7632 | 0.7705 | 73141 | 4.67e2 | 1 | 1 | TRINITY\_DN140702\_c0\_g1\_i1.p1 |  |  | DB Search |
| SPFVIMFK | 54.61 | 967.5201 | 8 | 4.71 | 484.7673 | 19.89 | 0.81 | 0.8173 | 74398 | 6.85e2 | 1 | 1 | TRINITY\_DN4595\_c0\_g1\_i10.p1:TRINITY\_DN4595\_c0\_g1\_i4.p1 |  |  | DB Search |
| HQNIVC(+57.02)GGPER | 54.58 | 1265.5935 | 11 | -0.53 | 633.8007 | 12.52 | 0.9109 | 0.9182 | 28563 | 4.18e2 | 1 | 1 | TRINITY\_DN1535\_c0\_g1\_i1.p1 | Carbamidomethylation |  | DB Search |
| GVSEETTTGVHNLYK | 54.51 | 1633.7948 | 15 | -1.65 | 817.8995 | 13.70 | 1.0701 | 1.0773 | 35859 | 0 | 0 | 0 | TRINITY\_DN773\_c0\_g1\_i1.p1 |  |  | DB Search |
| AGDIAILESQQFNAVGGK | 54.47 | 1816.9319 | 18 | -18.71 | 909.452 | 18.92 | 1.1813 | 1.1885 | 67826 | 1.32e3 | 1 | 1 | TRINITY\_DN1936\_c0\_g1\_i1.p1:TRINITY\_DN1936\_c0\_g1\_i4.p1 |  |  | DB Search |
| DSYVGDEAQSK | 54.42 | 1197.5149 | 11 | 5.23 | 599.7651 | 11.40 | 0.8968 | 0.904 | 21678 | 5.55e2 | 1 | 1 | TRINITY\_DN2477\_c0\_g1\_i5.p1:TRINITY\_DN2477\_c0\_g1\_i6.p1:TRINITY\_DN195934\_c0\_g1\_i1.p2:TRINITY\_DN161\_c0\_g1\_i1.p1:TRINITY\_DN2477\_c0\_g1\_i9.p1:TRINITY\_DN2477\_c0\_g1\_i10.p1:TRINITY\_DN2477\_c0\_g1\_i2.p1:TRINITY\_DN161\_c0\_g1\_i3.p1:TRINITY\_DN161\_c0\_g1\_i2.p1 |  |  | DB Search |
| DPIC(+57.02)HC(+57.02)DTSK | 54.33 | 1231.4961 | 10 | 7.69 | 616.7572 | 9.95 | 0.8951 | 0.9024 | 13586 | 0 | 0 | 0 | TRINITY\_DN319\_c2\_g1\_i3.p1:TRINITY\_DN319\_c2\_g1\_i1.p1:TRINITY\_DN319\_c2\_g1\_i2.p1 | Carbamidomethylation, Carbamidomethylation |  | DB Search |
| A(+42.01)NPLKDIISR | 54.32 | 1167.6611 | 10 | 0.57 | 584.8354 | 18.73 | 0.9206 | 0.9279 | 66485 | 8.04e2 | 1 | 1 | TRINITY\_DN163801\_c0\_g1\_i1.p1 | Acetylation (Protein N-term) | A1:Acetylation (Protein N-term):1000 | DB Search |
| DAVTYTEHAK | 54.29 | 1133.5353 | 10 | 7.89 | 567.7767 | 11.15 | 0.9004 | 0.9077 | 20203 | 0 | 0 | 0 | TRINITY\_DN172571\_c0\_g1\_i1.p1 |  |  | DB Search |
| KLLDWAQQR | 54.2 | 1156.6353 | 9 | 4.33 | 579.3247 | 15.52 | 0.9097 | 0.917 | 46753 | 0 | 0 | 0 | TRINITY\_DN199\_c0\_g1\_i1.p1 |  |  | DB Search |
| YLLVFTDGR | 54.18 | 1082.576 | 9 | -0.05 | 542.2927 | 19.06 | 0.8745 | 0.8818 | 68859 | 1.53e3 | 1 | 1 | TRINITY\_DN138\_c0\_g1\_i1.p1 |  |  | DB Search |
| HFGIC(+57.02)IGYNK | 54 | 1207.5808 | 10 | -0.18 | 604.7947 | 17.63 | 0.923 | 0.9303 | 59179 | 0 | 0 | 0 | TRINITY\_DN1119\_c0\_g1\_i1.p1 | Carbamidomethylation |  | DB Search |
| EIRDILVQYDR | 53.99 | 1418.7517 | 11 | -15.07 | 710.3691 | 19.87 | 1.0327 | 1.0399 | 74273 | 0 | 0 | 0 | TRINITY\_DN128406\_c0\_g1\_i1.p1 |  |  | DB Search |
| TQAVLDRLNIEK | 53.96 | 1398.7831 | 12 | 1.89 | 700.3969 | 15.48 | 1.009 | 1.0162 | 46512 | 0 | 0 | 0 | TRINITY\_DN477\_c0\_g1\_i9.p1 |  |  | DB Search |
| FETQIATK | 53.77 | 936.4916 | 8 | 12.43 | 937.5062 | 13.97 | 1.1614 | 1.1686 | 37568 | 0 | 0 | 0 | TRINITY\_DN649\_c0\_g1\_i2.p1:TRINITY\_DN36\_c0\_g1\_i3.p1:TRINITY\_DN36\_c0\_g1\_i16.p1:TRINITY\_DN36\_c0\_g1\_i14.p1:TRINITY\_DN36\_c0\_g1\_i17.p1:TRINITY\_DN649\_c0\_g1\_i5.p1:TRINITY\_DN36\_c0\_g1\_i13.p1:TRINITY\_DN36\_c0\_g1\_i8.p1:TRINITY\_DN649\_c0\_g1\_i1.p1:TRINITY\_DN36\_c0\_g1\_i19.p1 |  |  | DB Search |
| VLDVNDKQPVFVK | 53.63 | 1499.8347 | 13 | 1.56 | 750.9223 | 15.14 | 1.052 | 1.0592 | 44491 | 0 | 0 | 0 | TRINITY\_DN982\_c0\_g1\_i1.p1:TRINITY\_DN982\_c0\_g1\_i2.p1 |  |  | DB Search |
| SPTHSVIK | 53.63 | 867.4814 | 8 | 6.75 | 434.7489 | 9.91 | 0.777 | 0.7844 | 13342 | 3.42e2 | 1 | 1 | TRINITY\_DN1386\_c0\_g1\_i1.p1 |  |  | DB Search |
| YQGREYNINEEVNVR | 53.45 | 1881.897 | 15 | -4.84 | 941.9468 | 15.64 | 1.1442 | 1.1514 | 47466 | 0 | 0 | 0 | TRINITY\_DN4595\_c0\_g1\_i4.p1:TRINITY\_DN4595\_c0\_g1\_i10.p2 |  |  | DB Search |
| VDNSSLTGESEPQSR | 53.35 | 1604.7278 | 15 | -5.23 | 803.3632 | 12.02 | 1.0275 | 1.0347 | 25394 | 0 | 0 | 0 | TRINITY\_DN262\_c0\_g1\_i1.p1 |  |  | DB Search |
| HGADLFYR | 53.19 | 977.4719 | 8 | 0.63 | 489.7412 | 16.16 | 0.8376 | 0.8449 | 50305 | 4.17e2 | 1 | 1 | TRINITY\_DN602\_c0\_g1\_i4.p1:TRINITY\_DN602\_c0\_g1\_i3.p1:TRINITY\_DN602\_c0\_g1\_i1.p1 |  |  | DB Search |
| AVDRGNPPQDSVNK | 52.97 | 1495.7379 | 14 | -0.57 | 499.584 | 10.23 | 0.773 | 0.7803 | 15050 | 0 | 0 | 0 | TRINITY\_DN982\_c0\_g1\_i3.p1:TRINITY\_DN982\_c0\_g1\_i1.p1 |  |  | DB Search |
| HQGVM(+15.99)VGM(+15.99)GQK | 52.78 | 1202.5536 | 11 | 2.62 | 602.2828 | 9.74 | 0.8955 | 0.9028 | 12426 | 1.52e3 | 1 | 1 | TRINITY\_DN161\_c0\_g1\_i1.p1:TRINITY\_DN161\_c0\_g1\_i3.p1:TRINITY\_DN161\_c0\_g1\_i2.p1 | Oxidation (M), Oxidation (M) | M5:Oxidation (M):1000 M8:Oxidation (M):1000 | DB Search |
| AFLFDVVSK | 52.39 | 1024.5593 | 9 | -2.23 | 513.2834 | 19.79 | 0.8412 | 0.8485 | 73630 | 7e2 | 1 | 1 | TRINITY\_DN65215\_c0\_g1\_i1.p1 |  |  | DB Search |
| SEEYKPTLGR | 52.38 | 1178.5931 | 10 | -5.07 | 590.2981 | 12.31 | 0.9133 | 0.9206 | 27257 | 0 | 0 | 0 | TRINITY\_DN144508\_c0\_g1\_i1.p1 |  |  | DB Search |
| DGSTGLAGNR | 52.33 | 946.4468 | 10 | 13.34 | 474.2348 | 10.74 | 0.8189 | 0.8262 | 17706 | 5.15e3 | 1 | 1 | TRINITY\_DN77\_c0\_g2\_i4.p1:TRINITY\_DN77\_c0\_g2\_i2.p1:TRINITY\_DN77\_c0\_g2\_i1.p1:TRINITY\_DN77\_c0\_g2\_i3.p1 |  |  | DB Search |
| APTHILNPANKR | 52.25 | 1330.7469 | 12 | 5.05 | 444.5898 | 11.20 | 0.7481 | 0.7555 | 20091 | 3.03e3 | 1 | 1 | TRINITY\_DN1058\_c0\_g1\_i4.p1:TRINITY\_DN1058\_c0\_g1\_i1.p1:TRINITY\_DN1058\_c0\_g1\_i3.p1:TRINITY\_DN1058\_c0\_g1\_i2.p2 |  |  | DB Search |
| ALSLQNANEELEDRLAEAER | 52.19 | 2270.1138 | 20 | 0.29 | 757.7086 | 18.89 | 0.984 | 0.9912 | 67687 | 7.99e2 | 1 | 1 | TRINITY\_DN428\_c0\_g1\_i1.p1 |  |  | DB Search |
| FELDRVNTFGDEVFGDPK | 52.07 | 2083.9851 | 18 | 2.03 | 695.6671 | 19.30 | 0.9206 | 0.9279 | 70383 | 6.13e2 | 1 | 1 | TRINITY\_DN1676\_c0\_g1\_i6.p1:TRINITY\_DN1676\_c0\_g1\_i12.p1 |  |  | DB Search |
| LVGQSIISYLQK | 52.05 | 1347.7761 | 12 | 2.9 | 674.8942 | 19.71 | 0.9783 | 0.9856 | 73270 | 0 | 0 | 0 | TRINITY\_DN187147\_c0\_g1\_i1.p1 |  |  | DB Search |
| DNIQGITKPAIR | 52.04 | 1324.7462 | 12 | 0.94 | 663.3779 | 13.90 | 0.9844 | 0.9916 | 37014 | 7.98e2 | 1 | 1 | TRINITY\_DN172571\_c0\_g1\_i1.p1 |  |  | DB Search |
| TPYLQIDFGK | 52.02 | 1180.6128 | 10 | -2.38 | 591.3095 | 18.44 | 0.917 | 0.9243 | 64648 | 4.48e2 | 1 | 1 | TRINITY\_DN11248\_c0\_g1\_i1.p1:TRINITY\_DN11248\_c0\_g1\_i3.p1 |  |  | DB Search |
| RVHQFLVEALDGGQPR | 51.92 | 1820.9645 | 16 | -4.41 | 607.9899 | 19.51 | 0.8704 | 0.8777 | 71893 | 7.34e2 | 1 | 1 | TRINITY\_DN71243\_c0\_g1\_i1.p1 |  |  | DB Search |
| FSYEKVC(+57.02)EDVKR | 51.53 | 1558.745 | 12 | -1.54 | 520.5857 | 13.64 | 0.7978 | 0.8051 | 35482 | 2.86e2 | 1 | 1 | TRINITY\_DN118\_c1\_g1\_i3.p1 | Carbamidomethylation |  | DB Search |
| TRIEDLETVISQTQDHR | 51.48 | 2040.0236 | 17 | 6.93 | 681.0167 | 17.50 | 0.9638 | 0.9711 | 58233 | 3.05e2 | 1 | 1 | TRINITY\_DN761\_c0\_g1\_i1.p1:TRINITY\_DN761\_c0\_g1\_i3.p1:TRINITY\_DN761\_c0\_g1\_i2.p1 |  |  | DB Search |
| LTHLEEAYTTEHWLVR | 51.48 | 1997.0006 | 16 | 4.65 | 666.6741 | 17.32 | 0.9594 | 0.9666 | 57137 | 0 | 0 | 0 | TRINITY\_DN21998\_c0\_g1\_i1.p1 |  |  | DB Search |
| ISNVQDIVPVLELANQHK | 51.46 | 2016.1003 | 18 | 2.3 | 673.0391 | 19.75 | 0.9457 | 0.9529 | 73571 | 2.32e3 | 1 | 1 | TRINITY\_DN118520\_c0\_g1\_i1.p1 |  |  | DB Search |
| FC(+57.02)NNPAPSNGGR | 51.44 | 1289.5571 | 12 | 4.78 | 645.7859 | 10.87 | 0.944 | 0.9513 | 18646 | 0 | 0 | 0 | TRINITY\_DN4595\_c0\_g1\_i10.p1:TRINITY\_DN4595\_c0\_g1\_i4.p1 | Carbamidomethylation |  | DB Search |
| ILQLEEEFIKK | 51.41 | 1388.7915 | 11 | -3.68 | 463.9339 | 17.84 | 0.7506 | 0.7579 | 60678 | 0 | 0 | 0 | TRINITY\_DN120478\_c0\_g1\_i1.p1 |  |  | DB Search |
| AHVAQLC(+57.02)EK | 51.4 | 1054.5229 | 9 | 8.37 | 528.2707 | 10.76 | 0.8583 | 0.8656 | 17883 | 1.38e3 | 1 | 1 | TRINITY\_DN2113\_c0\_g1\_i1.p1:TRINITY\_DN2113\_c0\_g1\_i2.p1 | Carbamidomethylation |  | DB Search |
| LDHKFDLM(+15.99)YAK | 51.39 | 1395.6857 | 11 | 4.19 | 466.2356 | 14.23 | 0.7844 | 0.7917 | 39140 | 0 | 0 | 0 | TRINITY\_DN687\_c0\_g2\_i1.p1:TRINITY\_DN250\_c0\_g1\_i5.p1:TRINITY\_DN687\_c0\_g1\_i1.p1:TRINITY\_DN250\_c0\_g1\_i6.p1:TRINITY\_DN250\_c0\_g1\_i1.p1:TRINITY\_DN250\_c0\_g1\_i4.p1:TRINITY\_DN96161\_c0\_g1\_i1.p1:TRINITY\_DN250\_c0\_g1\_i3.p1 | Oxidation (M) | M8:Oxidation (M):1000 | DB Search |
| SIEHC(+57.02)IDTK | 51.35 | 1101.5125 | 9 | 3.22 | 551.7627 | 11.31 | 0.8834 | 0.8907 | 21126 | 0 | 0 | 0 | TRINITY\_DN1119\_c0\_g1\_i1.p1 | Carbamidomethylation |  | DB Search |
| LGPDQVDVAGAGR | 51.28 | 1253.6364 | 13 | 6.6 | 627.8267 | 13.74 | 0.9344 | 0.9416 | 35866 | 6.27e3 | 2 | 2 | TRINITY\_DN97809\_c0\_g1\_i1.p1 |  |  | DB Search |
| AFLTQAQEEFKK | 51.12 | 1438.7456 | 12 | 5.01 | 720.3803 | 15.74 | 1.0456 | 1.0528 | 48009 | 0 | 0 | 0 | TRINITY\_DN108993\_c0\_g1\_i1.p1 |  |  | DB Search |
| LC(+57.02)DATC(+57.02)SVFGDPHYK | 51.11 | 1768.7549 | 15 | 2.6 | 885.3829 | 15.31 | 1.1402 | 1.1474 | 45505 | 4.4e2 | 1 | 1 | TRINITY\_DN1369\_c0\_g1\_i1.p1 | Carbamidomethylation, Carbamidomethylation |  | DB Search |
| GFGGNLVTITNSDIQK | 51.11 | 1662.8577 | 16 | 0.85 | 832.4329 | 17.75 | 1.0813 | 1.0885 | 60042 | 0 | 0 | 0 | TRINITY\_DN12627\_c0\_g1\_i1.p1 |  |  | DB Search |
| TNNALDYEAKR | 51.04 | 1293.6313 | 11 | 1.86 | 647.8211 | 12.12 | 0.9622 | 0.9695 | 26057 | 0 | 0 | 0 | TRINITY\_DN982\_c0\_g1\_i3.p1:TRINITY\_DN982\_c0\_g1\_i1.p1 |  |  | DB Search |
| FIWGEVTELNGADR | 50.99 | 1605.7787 | 14 | -4.61 | 803.8892 | 19.74 | 1.0431 | 1.0504 | 73266 | 4.98e2 | 1 | 1 | TRINITY\_DN2626\_c0\_g1\_i2.p1:TRINITY\_DN2626\_c0\_g1\_i6.p1 |  |  | DB Search |
| HC(+57.02)VFGR | 50.91 | 774.3595 | 6 | 3.29 | 388.1865 | 13.39 | 0.7526 | 0.7599 | 33963 | 0 | 0 | 0 | TRINITY\_DN875\_c0\_g1\_i1.p1:TRINITY\_DN875\_c0\_g1\_i3.p1:TRINITY\_DN875\_c0\_g1\_i2.p1 | Carbamidomethylation |  | DB Search |
| NQGATGSAGTGPK | 50.58 | 1144.5472 | 13 | 3.6 | 573.2803 | 8.98 | 0.8708 | 0.8781 | 9648 | 0 | 0 | 0 | TRINITY\_DN1936\_c0\_g1\_i3.p1 |  |  | DB Search |
| AVC(+57.02)M(+15.99)LSNTTAIAEAWAR | 50.49 | 1879.8921 | 17 | -0.63 | 940.9483 | 18.78 | 1.1482 | 1.1554 | 66979 | 0 | 0 | 0 | TRINITY\_DN687\_c0\_g2\_i1.p1:TRINITY\_DN250\_c0\_g1\_i5.p1:TRINITY\_DN687\_c0\_g1\_i1.p1:TRINITY\_DN250\_c0\_g1\_i1.p1:TRINITY\_DN250\_c0\_g1\_i3.p1 | Carbamidomethylation, Oxidation (M) | M4:Oxidation (M):1000 | DB Search |
| RLTELEHFLK | 50.47 | 1284.719 | 10 | 3.5 | 429.2464 | 16.44 | 0.7453 | 0.7526 | 51972 | 4.72e2 | 1 | 1 | TRINITY\_DN1119\_c0\_g1\_i1.p1 |  |  | DB Search |
| GVGIISEGNETVEDIAQR | 50.29 | 1885.9381 | 18 | 4.42 | 943.9761 | 17.89 | 1.1446 | 1.1518 | 60967 | 0 | 0 | 0 | TRINITY\_DN262\_c0\_g1\_i1.p1 |  |  | DB Search |
| AVAC(+57.02)NSGPC(+57.02)LAGC(+57.02)PR | 50.26 | 1588.6908 | 15 | 1.49 | 795.3502 | 13.12 | 1.019 | 1.0263 | 32158 | 1.27e3 | 1 | 1 | TRINITY\_DN4815\_c0\_g1\_i2.p1:TRINITY\_DN4815\_c0\_g1\_i1.p1 | Carbamidomethylation, Carbamidomethylation, Carbamidomethylation |  | DB Search |
| DANAAVVHGSHLK | 50.26 | 1317.679 | 13 | 6.36 | 659.8478 | 10.94 | 1.0118 | 1.019 | 18954 | 0 | 0 | 0 | TRINITY\_DN262\_c0\_g1\_i1.p1 |  |  | DB Search |
| FC(+57.02)SNPEPENGGQTC(+57.02)EGEAR | 50.09 | 2137.843 | 19 | 2.83 | 1069.9268 | 12.17 | 1.1582 | 1.1654 | 26131 | 9.57e2 | 1 | 1 | TRINITY\_DN138\_c0\_g1\_i1.p1 | Carbamidomethylation, Carbamidomethylation |  | DB Search |
| DMGVNVISVGAGHTINHR | 50.02 | 1875.9374 | 18 | 2.35 | 938.9738 | 15.41 | 1.2495 | 1.2567 | 46101 | 0 | 0 | 0 | TRINITY\_DN138\_c0\_g1\_i1.p1 |  |  | DB Search |
| IAVHC(+57.02)TVR | 49.93 | 954.5069 | 8 | 4.46 | 478.2606 | 10.97 | 0.8502 | 0.8575 | 19084 | 8.91e2 | 1 | 1 | TRINITY\_DN1704\_c0\_g1\_i1.p1 | Carbamidomethylation |  | DB Search |
| LADLEEFISSPNHANISQVGER | 49.83 | 2425.1873 | 22 | 1.54 | 809.4005 | 18.91 | 0.9238 | 0.9311 | 67723 | 2.6e3 | 1 | 1 | TRINITY\_DN2113\_c0\_g1\_i1.p1:TRINITY\_DN2113\_c0\_g1\_i2.p1 |  |  | DB Search |
| AFNKIETR | 49.77 | 977.5294 | 8 | 3.42 | 489.7714 | 11.52 | 0.8388 | 0.8461 | 22323 | 8.13e3 | 1 | 1 | TRINITY\_DN4595\_c0\_g1\_i1.p1:TRINITY\_DN4595\_c0\_g1\_i7.p1:TRINITY\_DN4595\_c0\_g1\_i4.p1 |  |  | DB Search |
| ITQQQSHQEIADQLR | 49.73 | 1793.902 | 15 | 3.55 | 598.9739 | 13.30 | 0.8449 | 0.8522 | 33399 | 5.82e2 | 1 | 1 | TRINITY\_DN1377\_c0\_g1\_i1.p1 |  |  | DB Search |
| LGGIEVTQGNC(+57.02)AR | 49.67 | 1373.6721 | 13 | 2.61 | 687.8419 | 13.89 | 0.9695 | 0.9767 | 37015 | 0 | 0 | 0 | TRINITY\_DN36\_c0\_g1\_i9.p1:TRINITY\_DN36\_c0\_g1\_i14.p1:TRINITY\_DN36\_c0\_g1\_i13.p1:TRINITY\_DN36\_c0\_g1\_i2.p1:TRINITY\_DN36\_c0\_g1\_i3.p1:TRINITY\_DN36\_c0\_g1\_i16.p1:TRINITY\_DN36\_c0\_g1\_i17.p1:TRINITY\_DN36\_c0\_g1\_i4.p1:TRINITY\_DN36\_c0\_g1\_i11.p1:TRINITY\_DN36\_c0\_g1\_i8.p1:TRINITY\_DN36\_c0\_g1\_i1.p1:TRINITY\_DN36\_c0\_g1\_i19.p1:TRINITY\_DN36\_c0\_g1\_i5.p1:TRINITY\_DN36\_c0\_g1\_i20.p1 | Carbamidomethylation |  | DB Search |
| AMTEEILNERDTASK | 49.64 | 1706.8145 | 15 | 1.5 | 854.4118 | 14.35 | 1.1302 | 1.1374 | 39764 | 4.47e2 | 1 | 1 | TRINITY\_DN493\_c0\_g1\_i1.p1 |  |  | DB Search |
| EAIVQGAQSTQDTLKK | 49.64 | 1715.9054 | 16 | 10.53 | 858.965 | 12.81 | 1.1522 | 1.1594 | 30342 | 0 | 0 | 0 | TRINITY\_DN564\_c0\_g1\_i1.p1 |  |  | DB Search |
| V(+42.01)HDLVLR | 49.6 | 892.513 | 7 | -1.25 | 447.2611 | 18.00 | 0.8136 | 0.8209 | 61579 | 2.99e2 | 1 | 1 | TRINITY\_DN9819\_c0\_g1\_i6.p2:TRINITY\_DN9819\_c0\_g1\_i7.p2:TRINITY\_DN9819\_c0\_g1\_i1.p2 | Acetylation (Protein N-term) | V1:Acetylation (Protein N-term):1000 | DB Search |
| AHVSLSTFSEDVTSLTK | 49.58 | 1820.9155 | 17 | 2.15 | 607.9776 | 18.97 | 0.8327 | 0.84 | 68160 | 1.3e3 | 1 | 1 | TRINITY\_DN113\_c3\_g1\_i1.p1 |  |  | DB Search |
| AFNKVETR | 49.58 | 963.5137 | 8 | 6.34 | 482.765 | 10.59 | 0.8266 | 0.8339 | 16901 | 3.56e3 | 1 | 1 | TRINITY\_DN98968\_c0\_g1\_i1.p1:TRINITY\_DN4595\_c0\_g1\_i7.p1:TRINITY\_DN8597\_c0\_g1\_i1.p1:TRINITY\_DN4595\_c0\_g1\_i4.p1:TRINITY\_DN4595\_c0\_g1\_i10.p2 |  |  | DB Search |
| ILLAVGGWNHER | 49.52 | 1363.7361 | 12 | 4.17 | 682.8749 | 17.15 | 1.0423 | 1.0496 | 56044 | 3.26e2 | 1 | 1 | TRINITY\_DN6542\_c0\_g1\_i4.p1:TRINITY\_DN6542\_c0\_g1\_i1.p1:TRINITY\_DN6542\_c0\_g1\_i3.p1 |  |  | DB Search |
| LLAVTVGNNYNLDTLEK | 49.51 | 1875.9941 | 17 | -8.81 | 938.9917 | 18.74 | 1.1506 | 1.1578 | 66472 | 9.46e2 | 1 | 1 | TRINITY\_DN77\_c1\_g1\_i1.p1 |  |  | DB Search |
| EC(+57.02)SC(+57.02)NSYYC(+57.02)VGK | 49.5 | 1525.5636 | 12 | -0.7 | 763.785 | 11.90 | 1.0061 | 1.0134 | 24544 | 1.21e3 | 1 | 1 | TRINITY\_DN493\_c0\_g1\_i1.p1 | Carbamidomethylation, Carbamidomethylation, Carbamidomethylation |  | DB Search |
| LRPGVELTMSSLVNAK | 49.47 | 1713.9447 | 16 | -6.6 | 572.3157 | 17.86 | 0.9117 | 0.919 | 60815 | 0 | 0 | 0 | TRINITY\_DN329\_c0\_g1\_i1.p1:TRINITY\_DN329\_c0\_g1\_i2.p1 |  |  | DB Search |
| ADLDIQKPIPIVSK | 49.47 | 1535.8922 | 14 | 3.13 | 768.9522 | 16.14 | 1.0769 | 1.0841 | 50314 | 0 | 0 | 0 | TRINITY\_DN26392\_c0\_g1\_i1.p1 |  |  | DB Search |
| TLDTVC(+57.02)DSGTGVC(+57.02)QC(+57.02)K | 49.46 | 1799.7488 | 16 | -4.78 | 900.8732 | 13.15 | 1.0901 | 1.0973 | 32411 | 1.65e2 | 1 | 1 | TRINITY\_DN1616\_c0\_g1\_i3.p1:TRINITY\_DN1616\_c0\_g1\_i6.p1:TRINITY\_DN1616\_c0\_g1\_i2.p1:TRINITY\_DN1616\_c0\_g1\_i7.p1 | Carbamidomethylation, Carbamidomethylation, Carbamidomethylation |  | DB Search |
| TPGLVEGTLILHQLR | 49.43 | 1645.9515 | 15 | -9.67 | 549.6499 | 18.99 | 0.8631 | 0.8704 | 68418 | 0 | 0 | 0 | TRINITY\_DN3034\_c0\_g1\_i1.p1 |  |  | DB Search |
| VAHAGLTVAEFFR | 49.43 | 1416.7513 | 13 | -3.33 | 473.2539 | 19.07 | 0.751 | 0.7583 | 68989 | 0 | 0 | 0 | TRINITY\_DN11097\_c0\_g1\_i1.p1 |  |  | DB Search |
| DAPIYGLQWHPEK | 49.41 | 1552.7673 | 13 | 1.48 | 777.3885 | 17.41 | 1.0745 | 1.0817 | 57577 | 1.18e3 | 1 | 1 | TRINITY\_DN247\_c0\_g1\_i1.p1 |  |  | DB Search |
| KRFDEEPNPLC(+57.02)K | 49.41 | 1531.7452 | 12 | 3.63 | 511.5885 | 13.21 | 0.7685 | 0.7758 | 32789 | 2.49e3 | 2 | 2 | TRINITY\_DN840\_c0\_g1\_i1.p1 | Carbamidomethylation |  | DB Search |
| GLMVAAMDLDKK | 49.4 | 1290.6676 | 12 | 6.41 | 646.3422 | 16.72 | 1.0001 | 1.0073 | 53531 | 4.99e2 | 1 | 1 | TRINITY\_DN1043\_c0\_g1\_i2.p1 |  |  | DB Search |
| YTGAC(+57.02)NANGNTDK | 49.38 | 1384.5677 | 13 | 0 | 693.2879 | 9.54 | 0.9315 | 0.9388 | 11651 | 4.52e2 | 1 | 1 | TRINITY\_DN4595\_c0\_g1\_i10.p1:TRINITY\_DN4595\_c0\_g1\_i4.p1 | Carbamidomethylation |  | DB Search |
| TQLIAHDKEVYDIAFSR | 49.38 | 2005.0269 | 17 | 3.02 | 669.3484 | 16.77 | 0.9089 | 0.9162 | 53806 | 0 | 0 | 0 | TRINITY\_DN65211\_c0\_g1\_i2.p1:TRINITY\_DN65211\_c0\_g1\_i3.p1:TRINITY\_DN65211\_c0\_g1\_i1.p1 |  |  | DB Search |
| EAAAQIVVEIIKR | 49.37 | 1438.8507 | 13 | 8.13 | 720.4351 | 19.21 | 1.0544 | 1.0616 | 69860 | 0 | 0 | 0 | TRINITY\_DN71276\_c0\_g1\_i1.p1 |  |  | DB Search |
| FAIQDITVDEFSAK | 49.36 | 1582.7878 | 14 | 12.76 | 792.4076 | 19.82 | 1.046 | 1.0532 | 73951 | 0 | 0 | 0 | TRINITY\_DN624\_c0\_g1\_i1.p1 |  |  | DB Search |
| SGGQC(+57.02)PC(+57.02)KPGVVGR | 49.35 | 1457.6868 | 14 | 7.15 | 486.9041 | 10.75 | 0.7787 | 0.786 | 17784 | 3e2 | 1 | 1 | TRINITY\_DN15274\_c0\_g1\_i1.p1 | Carbamidomethylation, Carbamidomethylation |  | DB Search |
| EGSTPC(+57.02)NTQAC(+57.02)GSGLSEER | 49.28 | 2038.832 | 19 | 5.27 | 1020.4239 | 12.12 | 1.1354 | 1.1426 | 25919 | 1.95e2 | 1 | 1 | TRINITY\_DN5397\_c0\_g1\_i1.p1 | Carbamidomethylation, Carbamidomethylation |  | DB Search |
| ASFVTDIDRLR | 49.26 | 1291.6885 | 11 | -0.87 | 646.8479 | 17.21 | 0.992 | 0.9993 | 56353 | 6.03e2 | 1 | 1 | TRINITY\_DN598\_c1\_g1\_i2.p1 |  |  | DB Search |
| IGETVNFIVIDYADSAAGKK | 49.19 | 2110.0945 | 20 | 6.76 | 704.3736 | 19.80 | 0.9465 | 0.9537 | 73806 | 0 | 0 | 0 | TRINITY\_DN17117\_c0\_g1\_i1.p1 |  |  | DB Search |
| LLEINVYHPLIQELQK | 49.16 | 1949.0985 | 16 | 5.33 | 650.7072 | 19.80 | 0.9812 | 0.9884 | 73781 | 2.5e2 | 1 | 1 | TRINITY\_DN5633\_c0\_g1\_i1.p1 |  |  | DB Search |
| ITVTVSPDIHPPAK | 49.15 | 1473.8191 | 14 | -0.97 | 737.9127 | 14.98 | 1.0668 | 1.0741 | 43563 | 1.94e2 | 1 | 1 | TRINITY\_DN991\_c0\_g1\_i5.p1:TRINITY\_DN991\_c0\_g1\_i2.p1:TRINITY\_DN991\_c0\_g1\_i6.p1 |  |  | DB Search |
| SKIDYEALQVK | 48.81 | 1292.6976 | 11 | -2.48 | 647.3514 | 14.75 | 0.9618 | 0.9691 | 42265 | 0 | 0 | 0 | TRINITY\_DN982\_c0\_g1\_i3.p1:TRINITY\_DN982\_c0\_g1\_i1.p1 |  |  | DB Search |
| VIDINDNAPIFNK | 48.8 | 1471.7671 | 13 | 3.85 | 736.8902 | 17.47 | 1.0146 | 1.0218 | 57919 | 8.75e2 | 1 | 1 | TRINITY\_DN14015\_c0\_g1\_i3.p1:TRINITY\_DN14015\_c0\_g1\_i2.p1 |  |  | DB Search |
| KAQYYEAYETIIKR | 48.69 | 1774.9254 | 14 | 2.8 | 592.6479 | 15.92 | 0.8656 | 0.8729 | 49007 | 1.65e2 | 1 | 1 | TRINITY\_DN319\_c2\_g1\_i3.p1:TRINITY\_DN319\_c2\_g1\_i1.p1 |  |  | DB Search |
| VGINYQPPTVVPGGDLAK | 48.61 | 1823.9781 | 18 | -1.33 | 912.9908 | 17.37 | 1.1238 | 1.131 | 57429 | 0 | 0 | 0 | TRINITY\_DN687\_c0\_g2\_i1.p1:TRINITY\_DN250\_c0\_g1\_i5.p1:TRINITY\_DN687\_c0\_g1\_i1.p1:TRINITY\_DN250\_c0\_g1\_i6.p1:TRINITY\_DN250\_c0\_g1\_i1.p1:TRINITY\_DN250\_c0\_g1\_i4.p1:TRINITY\_DN96161\_c0\_g1\_i1.p1:TRINITY\_DN250\_c0\_g1\_i3.p1 |  |  | DB Search |
| ASDKFSASGPGLVEPK | 48.48 | 1588.8097 | 16 | 6.54 | 795.4136 | 14.23 | 1.0608 | 1.068 | 39146 | 0 | 0 | 0 | TRINITY\_DN1653\_c0\_g1\_i3.p1:TRINITY\_DN1653\_c0\_g1\_i1.p1 |  |  | DB Search |
| HQGVMVGM(+15.99)GQK | 48 | 1186.5587 | 11 | 4.48 | 594.2865 | 10.48 | 0.9052 | 0.9125 | 16419 | 1.61e3 | 1 | 1 | TRINITY\_DN161\_c0\_g1\_i1.p1:TRINITY\_DN161\_c0\_g1\_i3.p1:TRINITY\_DN161\_c0\_g1\_i2.p1 | Oxidation (M) | M8:Oxidation (M):32.97 | DB Search |
| GQFSTDDLVAEVEKR | 47.98 | 1692.8318 | 15 | -2.37 | 565.2806 | 17.82 | 0.8518 | 0.8591 | 60505 | 0 | 0 | 0 | TRINITY\_DN2113\_c0\_g1\_i1.p1:TRINITY\_DN2113\_c0\_g1\_i2.p1 |  |  | DB Search |
| RIIVADGAEYKK | 47.86 | 1361.7666 | 12 | 5.65 | 454.9299 | 11.98 | 0.8023 | 0.8096 | 25196 | 0 | 0 | 0 | TRINITY\_DN33065\_c0\_g2\_i1.p1 |  |  | DB Search |
| GEPGEIGEQGK | 47.83 | 1099.5145 | 11 | 4.88 | 550.7646 | 11.17 | 0.855 | 0.8623 | 20334 | 0 | 0 | 0 | TRINITY\_DN5987\_c0\_g1\_i2.p1:TRINITY\_DN5987\_c0\_g1\_i4.p1 |  |  | DB Search |
| S(+42.01)C(+57.02)SNPSPAHGGKPC(+57.02)SGELSK | 47.79 | 2097.9207 | 20 | 5.48 | 700.3148 | 11.22 | 0.8785 | 0.8858 | 20616 | 3.89e2 | 1 | 1 | TRINITY\_DN1613\_c1\_g1\_i2.p1:TRINITY\_DN1613\_c1\_g1\_i1.p1 | Acetylation (Protein N-term), Carbamidomethylation, Carbamidomethylation | S1:Acetylation (Protein N-term):1000 | DB Search |
| VLVIFANGDINDEQAVK | 47.03 | 1843.9679 | 17 | -10.38 | 922.9774 | 18.88 | 1.1845 | 1.1917 | 67755 | 0 | 0 | 0 | TRINITY\_DN113\_c3\_g1\_i1.p1 |  |  | DB Search |
| ALELDALSNSHPIEVK | 46.98 | 1734.9152 | 16 | 10.01 | 868.4695 | 17.34 | 1.1762 | 1.1833 | 57207 | 0 | 0 | 0 | TRINITY\_DN5153\_c0\_g1\_i1.p1 |  |  | DB Search |
| total 227 peptides |  |  |  |  |  |  |  |  |  |  |  |  |  |  |  |  |
| --- | --- | --- | --- | --- | --- | --- | --- | --- | --- | --- | --- | --- | --- | --- | --- | --- |
