# Supplementary material for: Proteomic Analysis and Biochemical Characterization of the Nematocyst Extract of the Hydrozoan Velella velella
Source: Mar Drugs. 2024 Oct 12;22(10):468. doi: 10.3390/md22100468 (PMC11509761; doi:10.3390/md22100468)
Supplement: Supplementary file 1 [file marinedrugs-22-00468-s001.zip › Supplementary File S1.zip!/Sample_5_Vellela/db.proteins.html]

proteins


Protein List

  

| Protein Group | Protein ID | Accession | -10lgP | Coverage (%) | Coverage (%) Sample 7 | Area Sample 7 | #Peptides | #Unique | #Spec Sample 7 | PTM | Avg. Mass | Description |
| --- | --- | --- | --- | --- | --- | --- | --- | --- | --- | --- | --- | --- |
| 1 | 1 | TRINITY\_DN982\_c0\_g1\_i1.p1 | 181.04 | 3 | 3 | 1.44e3 | 14 | 4 | 21 | Carbamidomethylation | 712566.0625 | type:5prime\_partial len:6412 gc:universal TRINITY\_DN982\_c0\_g1\_i1:19377-142(-) |
| 1 | 2 | TRINITY\_DN982\_c0\_g1\_i2.p1 | 127.04 | 4 | 4 | 1.44e3 | 5 | 4 | 6 |  | 190367.1406 | type:internal len:1718 gc:universal TRINITY\_DN982\_c0\_g1\_i2:5151-1(-) |
| 2 | 3 | TRINITY\_DN982\_c0\_g1\_i3.p1 | 167.82 | 1 | 1 | 0 | 11 | 1 | 18 | Carbamidomethylation | 1090854.75 | type:complete len:9852 gc:universal TRINITY\_DN982\_c0\_g1\_i3:29697-142(-) |
| 3 | 4 | TRINITY\_DN161\_c0\_g1\_i2.p1 | 135.37 | 17 | 17 | 2.44e4 | 6 | 6 | 10 | Oxidation (M) | 44577.6484 | type:5prime\_partial len:399 gc:universal TRINITY\_DN161\_c0\_g1\_i2:1683-487(-) |
| 3 | 5 | TRINITY\_DN161\_c0\_g1\_i3.p1 | 135.37 | 17 | 17 | 2.44e4 | 6 | 6 | 10 | Oxidation (M) | 44592.6602 | type:5prime\_partial len:399 gc:universal TRINITY\_DN161\_c0\_g1\_i3:1450-254(-) |
| 3 | 6 | TRINITY\_DN161\_c0\_g1\_i1.p1 | 135.37 | 18 | 18 | 2.44e4 | 6 | 6 | 10 | Oxidation (M) | 41918.5938 | type:complete len:377 gc:universal TRINITY\_DN161\_c0\_g1\_i1:1384-254(-) |
| 3 | 7 | TRINITY\_DN2477\_c0\_g1\_i5.p1 | 100.95 | 9 | 9 | 1.7e3 | 3 | 3 | 3 |  | 37416.5195 | type:complete len:333 gc:universal TRINITY\_DN2477\_c0\_g1\_i5:1182-184(-) |
| 3 | 8 | TRINITY\_DN2477\_c0\_g1\_i10.p1 | 100.95 | 9 | 9 | 1.7e3 | 3 | 3 | 3 |  | 38566.9688 | type:5prime\_partial len:344 gc:universal TRINITY\_DN2477\_c0\_g1\_i10:1215-184(-) |
| 3 | 9 | TRINITY\_DN2477\_c0\_g1\_i2.p1 | 100.95 | 9 | 9 | 1.7e3 | 3 | 3 | 3 |  | 38566.9688 | type:5prime\_partial len:344 gc:universal TRINITY\_DN2477\_c0\_g1\_i2:1215-184(-) |
| 3 | 10 | TRINITY\_DN195934\_c0\_g1\_i1.p2 | 100.95 | 24 | 24 | 1.7e3 | 3 | 3 | 3 |  | 14094.0938 | type:internal len:127 gc:universal TRINITY\_DN195934\_c0\_g1\_i1:3-380(+) |
| 3 | 11 | TRINITY\_DN2477\_c0\_g1\_i6.p1 | 100.95 | 8 | 8 | 1.7e3 | 3 | 3 | 3 |  | 41878.6875 | type:complete len:377 gc:universal TRINITY\_DN2477\_c0\_g1\_i6:1314-184(-) |
| 3 | 12 | TRINITY\_DN2477\_c0\_g1\_i9.p1 | 100.95 | 8 | 8 | 1.7e3 | 3 | 3 | 3 |  | 41878.6875 | type:complete len:377 gc:universal TRINITY\_DN2477\_c0\_g1\_i9:1314-184(-) |
| 3 | 13 | TRINITY\_DN161\_c0\_g1\_i4.p1 | 55.37 | 7 | 7 | 3.51e2 | 1 | 1 | 1 |  | 25887.2988 | type:5prime\_partial len:232 gc:universal TRINITY\_DN161\_c0\_g1\_i4:949-254(-) |
| 3 | 14 | TRINITY\_DN2941\_c0\_g1\_i4.p1 | 55.12 | 16 | 16 | 1.41e2 | 1 | 1 | 1 |  | 13034.9365 | type:internal len:116 gc:universal TRINITY\_DN2941\_c0\_g1\_i4:2-346(+) |
| 3 | 15 | TRINITY\_DN2477\_c0\_g1\_i8.p1 | 55.12 | 6 | 6 | 1.41e2 | 1 | 1 | 1 |  | 34820.5938 | type:5prime\_partial len:309 gc:universal TRINITY\_DN2477\_c0\_g1\_i8:1110-184(-) |
| 4 | 16 | TRINITY\_DN4595\_c0\_g1\_i4.p1 | 146.3 | 5 | 5 | 1.06e4 | 8 | 7 | 9 | Carbamidomethylation | 183684.4844 | type:5prime\_partial len:1639 gc:universal TRINITY\_DN4595\_c0\_g1\_i4:5857-941(-) |
| 4 | 17 | TRINITY\_DN4595\_c0\_g1\_i10.p2 | 98.59 | 10 | 10 | 0 | 3 | 2 | 4 | Carbamidomethylation | 39822.0156 | type:5prime\_partial len:358 gc:universal TRINITY\_DN4595\_c0\_g1\_i10:5369-4296(-) |
| 4 | 18 | TRINITY\_DN4595\_c0\_g1\_i10.p1 | 96.79 | 3 | 3 | 1.14e3 | 3 | 3 | 3 | Carbamidomethylation | 133760.0625 | type:complete len:1188 gc:universal TRINITY\_DN4595\_c0\_g1\_i10:4405-842(-) |
| 4 | 19 | TRINITY\_DN4595\_c0\_g1\_i7.p1 | 96.51 | 7 | 7 | 9.48e3 | 3 | 2 | 3 |  | 38387.582 | type:internal len:348 gc:universal TRINITY\_DN4595\_c0\_g1\_i7:1042-2(-) |
| 4 | 20 | TRINITY\_DN4595\_c0\_g1\_i1.p1 | 79.98 | 12 | 12 | 9.48e3 | 2 | 2 | 2 |  | 15339.0303 | type:internal len:138 gc:universal TRINITY\_DN4595\_c0\_g1\_i1:412-2(-) |
| 5 | 21 | TRINITY\_DN15274\_c0\_g1\_i1.p1 | 115.68 | 3 | 3 | 6.56e3 | 4 | 4 | 6 | Carbamidomethylation, Oxidation (M) | 201897.7344 | type:complete len:1822 gc:universal TRINITY\_DN15274\_c0\_g1\_i1:87-5552(+) |
| 6 | 22 | TRINITY\_DN140702\_c0\_g1\_i1.p1 | 127.12 | 3 | 3 | 6.06e3 | 5 | 5 | 9 |  | 231919.1562 | type:complete len:2036 gc:universal TRINITY\_DN140702\_c0\_g1\_i1:94-6201(+) |
| 7 | 23 | TRINITY\_DN138\_c0\_g1\_i1.p1 | 123.3 | 16 | 16 | 1.65e4 | 5 | 5 | 11 | Carbamidomethylation | 46324.8945 | type:complete len:401 gc:universal TRINITY\_DN138\_c0\_g1\_i1:157-1359(+) |
| 8 | 24 | TRINITY\_DN262\_c0\_g1\_i1.p1 | 124.48 | 7 | 7 | 4.35e3 | 5 | 5 | 9 | Oxidation (M) | 114520.6406 | type:complete len:1033 gc:universal TRINITY\_DN262\_c0\_g1\_i1:111-3209(+) |
| 9 | 25 | TRINITY\_DN1616\_c0\_g1\_i2.p1 | 115.73 | 2 | 2 | 1.24e3 | 4 | 4 | 4 | Carbamidomethylation | 374396 | type:3prime\_partial len:3398 gc:universal TRINITY\_DN1616\_c0\_g1\_i2:218-10408(+) |
| 9 | 26 | TRINITY\_DN1616\_c0\_g1\_i3.p1 | 115.73 | 1 | 1 | 1.24e3 | 4 | 4 | 4 | Carbamidomethylation | 423947.2812 | type:complete len:3855 gc:universal TRINITY\_DN1616\_c0\_g1\_i3:218-11782(+) |
| 9 | 27 | TRINITY\_DN1616\_c0\_g1\_i7.p1 | 115.73 | 1 | 1 | 1.24e3 | 4 | 4 | 4 | Carbamidomethylation | 423875.2188 | type:complete len:3855 gc:universal TRINITY\_DN1616\_c0\_g1\_i7:218-11782(+) |
| 9 | 28 | TRINITY\_DN1616\_c0\_g1\_i6.p1 | 115.73 | 1 | 1 | 1.24e3 | 4 | 4 | 4 | Carbamidomethylation | 423911.25 | type:complete len:3855 gc:universal TRINITY\_DN1616\_c0\_g1\_i6:218-11782(+) |
| 10 | 29 | TRINITY\_DN199\_c0\_g1\_i1.p1 | 116.46 | 13 | 13 | 3.97e3 | 4 | 4 | 6 | Oxidation (M) | 25170.3418 | type:complete len:229 gc:universal TRINITY\_DN199\_c0\_g1\_i1:207-893(+) |
| 11 | 30 | TRINITY\_DN3078\_c0\_g1\_i1.p1 | 116.33 | 4 | 4 | 9.73e2 | 4 | 4 | 5 | Carbamidomethylation | 127689.1797 | type:complete len:1109 gc:universal TRINITY\_DN3078\_c0\_g1\_i1:3628-302(-) |
| 12 | 31 | TRINITY\_DN602\_c0\_g1\_i3.p1 | 102.18 | 5 | 5 | 1.88e3 | 3 | 3 | 3 | Oxidation (M) | 92279.0078 | type:complete len:851 gc:universal TRINITY\_DN602\_c0\_g1\_i3:3046-494(-) |
| 12 | 32 | TRINITY\_DN602\_c0\_g1\_i4.p1 | 102.18 | 7 | 7 | 1.88e3 | 3 | 3 | 3 | Oxidation (M) | 61175.9766 | type:5prime\_partial len:552 gc:universal TRINITY\_DN602\_c0\_g1\_i4:3498-1843(-) |
| 12 | 33 | TRINITY\_DN602\_c0\_g1\_i1.p1 | 102.18 | 7 | 7 | 1.88e3 | 3 | 3 | 3 | Oxidation (M) | 65701.2422 | type:complete len:593 gc:universal TRINITY\_DN602\_c0\_g1\_i1:3622-1844(-) |
| 13 | 34 | TRINITY\_DN477\_c0\_g1\_i7.p1 | 102.36 | 4 | 4 | 2.59e4 | 3 | 3 | 10 | Carbamidomethylation | 122478.2578 | type:3prime\_partial len:1130 gc:universal TRINITY\_DN477\_c0\_g1\_i7:3387-1(-) |
| 13 | 35 | TRINITY\_DN477\_c0\_g1\_i8.p1 | 83.87 | 3 | 3 | 1.3e4 | 2 | 2 | 7 | Carbamidomethylation | 107165.2031 | type:complete len:985 gc:universal TRINITY\_DN477\_c0\_g1\_i8:3548-594(-) |
| 13 | 36 | TRINITY\_DN477\_c0\_g1\_i3.p1 | 55.29 | 4 | 4 | 1.28e4 | 1 | 1 | 3 |  | 24580.2559 | type:internal len:228 gc:universal TRINITY\_DN477\_c0\_g1\_i3:681-1(-) |
| 13 | 37 | TRINITY\_DN477\_c0\_g1\_i8.p2 | 55.29 | 5 | 5 | 1.28e4 | 1 | 1 | 3 |  | 20774.9902 | type:3prime\_partial len:194 gc:universal TRINITY\_DN477\_c0\_g1\_i8:581-3(-) |
| 13 | 38 | TRINITY\_DN477\_c0\_g1\_i6.p1 | 55.29 | 5 | 5 | 1.28e4 | 1 | 1 | 3 |  | 20745.9492 | type:3prime\_partial len:194 gc:universal TRINITY\_DN477\_c0\_g1\_i6:581-3(-) |
| 13 | 39 | TRINITY\_DN477\_c0\_g1\_i5.p1 | 55.29 | 4 | 4 | 1.28e4 | 1 | 1 | 3 |  | 28860.875 | type:internal len:276 gc:universal TRINITY\_DN477\_c0\_g1\_i5:827-3(-) |
| 14 | 40 | TRINITY\_DN72625\_c0\_g1\_i1.p1 | 102.43 | 2 | 2 | 2.05e3 | 3 | 3 | 3 |  | 214200.3906 | type:complete len:1964 gc:universal TRINITY\_DN72625\_c0\_g1\_i1:173-6064(+) |
| 15 | 41 | TRINITY\_DN250\_c0\_g1\_i5.p1 | 92.84 | 10 | 10 | 0 | 3 | 3 | 3 | Carbamidomethylation, Oxidation (M) | 50305.4453 | type:complete len:452 gc:universal TRINITY\_DN250\_c0\_g1\_i5:131-1486(+) |
| 15 | 42 | TRINITY\_DN687\_c0\_g2\_i1.p1 | 92.84 | 10 | 10 | 0 | 3 | 3 | 3 | Carbamidomethylation, Oxidation (M) | 50237.3242 | type:complete len:453 gc:universal TRINITY\_DN687\_c0\_g2\_i1:90-1448(+) |
| 15 | 43 | TRINITY\_DN250\_c0\_g1\_i3.p1 | 92.84 | 11 | 11 | 0 | 3 | 3 | 3 | Carbamidomethylation, Oxidation (M) | 44456.6445 | type:3prime\_partial len:402 gc:universal TRINITY\_DN250\_c0\_g1\_i3:131-1333(+) |
| 15 | 44 | TRINITY\_DN250\_c0\_g1\_i1.p1 | 92.84 | 11 | 11 | 0 | 3 | 3 | 3 | Carbamidomethylation, Oxidation (M) | 44454.6094 | type:3prime\_partial len:402 gc:universal TRINITY\_DN250\_c0\_g1\_i1:131-1333(+) |
| 15 | 45 | TRINITY\_DN687\_c0\_g1\_i1.p1 | 92.84 | 10 | 10 | 0 | 3 | 3 | 3 | Carbamidomethylation, Oxidation (M) | 50621.6914 | type:complete len:453 gc:universal TRINITY\_DN687\_c0\_g1\_i1:95-1453(+) |
| 15 | 46 | TRINITY\_DN250\_c0\_g1\_i6.p1 | 75.7 | 6 | 6 | 0 | 2 | 2 | 2 | Oxidation (M) | 50263.3672 | type:complete len:452 gc:universal TRINITY\_DN250\_c0\_g1\_i6:131-1486(+) |
| 15 | 47 | TRINITY\_DN96161\_c0\_g1\_i1.p1 | 75.7 | 6 | 6 | 0 | 2 | 2 | 2 | Oxidation (M) | 50498.6562 | type:complete len:453 gc:universal TRINITY\_DN96161\_c0\_g1\_i1:1593-235(-) |
| 15 | 48 | TRINITY\_DN250\_c0\_g1\_i4.p1 | 75.7 | 6 | 6 | 0 | 2 | 2 | 2 | Oxidation (M) | 50305.4453 | type:complete len:452 gc:universal TRINITY\_DN250\_c0\_g1\_i4:131-1486(+) |
| 16 | 49 | TRINITY\_DN1119\_c0\_g1\_i1.p1 | 96.5 | 3 | 3 | 4.72e2 | 3 | 3 | 3 | Carbamidomethylation | 105284.3438 | type:5prime\_partial len:949 gc:universal TRINITY\_DN1119\_c0\_g1\_i1:2934-88(-) |
| 17 | 50 | TRINITY\_DN33065\_c0\_g2\_i1.p1 | 100.41 | 4 | 4 | 6.8e2 | 3 | 3 | 3 |  | 89122.9766 | type:3prime\_partial len:804 gc:universal TRINITY\_DN33065\_c0\_g2\_i1:2409-1(-) |
| 18 | 51 | TRINITY\_DN244\_c0\_g1\_i1.p1 | 103.05 | 2 | 2 | 1.87e3 | 3 | 3 | 5 |  | 313594.0938 | type:complete len:3019 gc:universal TRINITY\_DN244\_c0\_g1\_i1:9748-692(-) |
| 19 | 52 | TRINITY\_DN2113\_c0\_g1\_i2.p1 | 92.3 | 3 | 3 | 3.97e3 | 3 | 3 | 3 | Carbamidomethylation | 193799.0156 | type:complete len:1704 gc:universal TRINITY\_DN2113\_c0\_g1\_i2:5257-146(-) |
| 19 | 53 | TRINITY\_DN2113\_c0\_g1\_i1.p1 | 92.3 | 3 | 3 | 3.97e3 | 3 | 3 | 3 | Carbamidomethylation | 193799.0156 | type:complete len:1704 gc:universal TRINITY\_DN2113\_c0\_g1\_i1:5257-146(-) |
| 20 | 54 | TRINITY\_DN6390\_c0\_g1\_i1.p1 | 102.99 | 2 | 2 | 1.17e3 | 3 | 3 | 3 | Carbamidomethylation | 214480.2344 | type:3prime\_partial len:1941 gc:universal TRINITY\_DN6390\_c0\_g1\_i1:5820-1(-) |
| 21 | 55 | TRINITY\_DN319\_c2\_g1\_i3.p1 | 99.78 | 2 | 2 | 1.65e2 | 3 | 3 | 3 | Carbamidomethylation | 279087.1875 | type:complete len:2588 gc:universal TRINITY\_DN319\_c2\_g1\_i3:165-7928(+) |
| 21 | 56 | TRINITY\_DN319\_c2\_g1\_i1.p1 | 99.78 | 2 | 2 | 1.65e2 | 3 | 3 | 3 | Carbamidomethylation | 279168.25 | type:complete len:2588 gc:universal TRINITY\_DN319\_c2\_g1\_i1:165-7928(+) |
| 21 | 57 | TRINITY\_DN319\_c2\_g1\_i2.p1 | 83.55 | 1 | 1 | 0 | 2 | 2 | 2 | Carbamidomethylation | 215796.4062 | type:3prime\_partial len:2004 gc:universal TRINITY\_DN319\_c2\_g1\_i2:165-6173(+) |
| 22 | 58 | TRINITY\_DN477\_c0\_g1\_i9.p1 | 102.32 | 8 | 8 | 2.91e3 | 3 | 3 | 4 |  | 34222.9453 | type:complete len:306 gc:universal TRINITY\_DN477\_c0\_g1\_i9:1840-923(-) |
| 23 | 59 | TRINITY\_DN113\_c3\_g1\_i1.p1 | 96.85 | 4 | 4 | 1.76e3 | 3 | 3 | 4 |  | 125422.4922 | type:3prime\_partial len:1162 gc:universal TRINITY\_DN113\_c3\_g1\_i1:3485-3(-) |
| 24 | 60 | TRINITY\_DN36\_c0\_g1\_i16.p1 | 78.6 | 9 | 9 | 0 | 2 | 2 | 2 | Carbamidomethylation | 24953.5938 | type:complete len:224 gc:universal TRINITY\_DN36\_c0\_g1\_i16:829-158(-) |
| 24 | 61 | TRINITY\_DN36\_c0\_g1\_i19.p1 | 78.6 | 9 | 9 | 0 | 2 | 2 | 2 | Carbamidomethylation | 24821.4141 | type:complete len:223 gc:universal TRINITY\_DN36\_c0\_g1\_i19:826-158(-) |
| 24 | 62 | TRINITY\_DN36\_c0\_g1\_i17.p1 | 78.6 | 9 | 9 | 0 | 2 | 2 | 2 | Carbamidomethylation | 24953.5938 | type:complete len:224 gc:universal TRINITY\_DN36\_c0\_g1\_i17:819-148(-) |
| 24 | 63 | TRINITY\_DN36\_c0\_g1\_i13.p1 | 78.6 | 9 | 9 | 0 | 2 | 2 | 2 | Carbamidomethylation | 24821.4141 | type:complete len:223 gc:universal TRINITY\_DN36\_c0\_g1\_i13:816-148(-) |
| 24 | 64 | TRINITY\_DN36\_c0\_g1\_i3.p1 | 78.6 | 9 | 9 | 0 | 2 | 2 | 2 | Carbamidomethylation | 24953.5938 | type:complete len:224 gc:universal TRINITY\_DN36\_c0\_g1\_i3:829-158(-) |
| 24 | 65 | TRINITY\_DN36\_c0\_g1\_i14.p1 | 78.6 | 9 | 9 | 0 | 2 | 2 | 2 | Carbamidomethylation | 24953.5938 | type:complete len:224 gc:universal TRINITY\_DN36\_c0\_g1\_i14:819-148(-) |
| 24 | 66 | TRINITY\_DN36\_c0\_g1\_i8.p1 | 78.6 | 9 | 9 | 0 | 2 | 2 | 2 | Carbamidomethylation | 24953.5938 | type:complete len:224 gc:universal TRINITY\_DN36\_c0\_g1\_i8:819-148(-) |
| 24 | 67 | TRINITY\_DN649\_c0\_g1\_i5.p1 | 53.77 | 7 | 7 | 0 | 1 | 1 | 1 |  | 13746.6875 | type:internal len:124 gc:universal TRINITY\_DN649\_c0\_g1\_i5:371-3(-) |
| 24 | 68 | TRINITY\_DN649\_c0\_g1\_i2.p1 | 53.77 | 6 | 6 | 0 | 1 | 1 | 1 |  | 13819.7393 | type:internal len:125 gc:universal TRINITY\_DN649\_c0\_g1\_i2:374-3(-) |
| 24 | 69 | TRINITY\_DN649\_c0\_g1\_i1.p1 | 53.77 | 7 | 7 | 0 | 1 | 1 | 1 |  | 13690.5801 | type:internal len:124 gc:universal TRINITY\_DN649\_c0\_g1\_i1:371-3(-) |
| 24 | 70 | TRINITY\_DN36\_c0\_g1\_i9.p1 | 49.67 | 6 | 6 | 0 | 1 | 1 | 1 | Carbamidomethylation | 24680.2773 | type:complete len:223 gc:universal TRINITY\_DN36\_c0\_g1\_i9:934-266(-) |
| 24 | 71 | TRINITY\_DN36\_c0\_g1\_i11.p1 | 49.67 | 6 | 6 | 0 | 1 | 1 | 1 | Carbamidomethylation | 25006.7051 | type:complete len:224 gc:universal TRINITY\_DN36\_c0\_g1\_i11:1175-504(-) |
| 24 | 72 | TRINITY\_DN36\_c0\_g1\_i4.p1 | 49.67 | 6 | 6 | 0 | 1 | 1 | 1 | Carbamidomethylation | 24680.2773 | type:complete len:223 gc:universal TRINITY\_DN36\_c0\_g1\_i4:932-264(-) |
| 24 | 73 | TRINITY\_DN36\_c0\_g1\_i2.p1 | 49.67 | 6 | 6 | 0 | 1 | 1 | 1 | Carbamidomethylation | 24680.2773 | type:complete len:223 gc:universal TRINITY\_DN36\_c0\_g1\_i2:934-266(-) |
| 24 | 74 | TRINITY\_DN36\_c0\_g1\_i5.p1 | 49.67 | 6 | 6 | 0 | 1 | 1 | 1 | Carbamidomethylation | 24548.0977 | type:complete len:222 gc:universal TRINITY\_DN36\_c0\_g1\_i5:931-266(-) |
| 24 | 75 | TRINITY\_DN36\_c0\_g1\_i20.p1 | 49.67 | 6 | 6 | 0 | 1 | 1 | 1 | Carbamidomethylation | 24874.5234 | type:complete len:223 gc:universal TRINITY\_DN36\_c0\_g1\_i20:1172-504(-) |
| 24 | 76 | TRINITY\_DN36\_c0\_g1\_i1.p1 | 49.67 | 6 | 6 | 0 | 1 | 1 | 1 | Carbamidomethylation | 25006.7051 | type:complete len:224 gc:universal TRINITY\_DN36\_c0\_g1\_i1:1175-504(-) |
| 25 | 77 | TRINITY\_DN624\_c0\_g1\_i1.p1 | 79.86 | 3 | 3 | 2.35e3 | 2 | 2 | 2 |  | 101743.5391 | type:complete len:884 gc:universal TRINITY\_DN624\_c0\_g1\_i1:218-2869(+) |
| 26 | 78 | TRINITY\_DN1369\_c0\_g1\_i1.p1 | 81.97 | 2 | 2 | 1.07e3 | 2 | 2 | 4 | Carbamidomethylation | 174229.4219 | type:3prime\_partial len:1577 gc:universal TRINITY\_DN1369\_c0\_g1\_i1:93-4820(+) |
| 27 | 79 | TRINITY\_DN1676\_c0\_g1\_i12.p1 | 82.36 | 2 | 2 | 1.11e3 | 2 | 2 | 3 |  | 180975.5156 | type:complete len:1631 gc:universal TRINITY\_DN1676\_c0\_g1\_i12:206-5098(+) |
| 27 | 80 | TRINITY\_DN1676\_c0\_g1\_i6.p1 | 82.36 | 2 | 2 | 1.11e3 | 2 | 2 | 3 |  | 180975.5156 | type:complete len:1631 gc:universal TRINITY\_DN1676\_c0\_g1\_i6:206-5098(+) |
| 28 | 81 | TRINITY\_DN123121\_c0\_g1\_i1.p1 | 83.91 | 12 | 12 | 1.59e3 | 2 | 2 | 2 |  | 14215.7539 | type:internal len:127 gc:universal TRINITY\_DN123121\_c0\_g1\_i1:3-380(+) |
| 29 | 82 | TRINITY\_DN71276\_c0\_g1\_i1.p1 | 79.88 | 2 | 2 | 4.66e2 | 2 | 2 | 2 | Carbamidomethylation | 139103.3125 | type:5prime\_partial len:1201 gc:universal TRINITY\_DN71276\_c0\_g1\_i1:1-3603(+) |
| 30 | 83 | TRINITY\_DN1386\_c0\_g1\_i1.p1 | 82.96 | 4 | 4 | 3.42e2 | 2 | 2 | 2 |  | 68692.1953 | type:complete len:596 gc:universal TRINITY\_DN1386\_c0\_g1\_i1:2419-632(-) |
| 31 | 84 | TRINITY\_DN428\_c0\_g1\_i1.p1 | 82.33 | 2 | 2 | 1.88e3 | 2 | 2 | 2 | Carbamidomethylation | 225824.1719 | type:complete len:1949 gc:universal TRINITY\_DN428\_c0\_g1\_i1:96-5942(+) |
| 32 | 85 | TRINITY\_DN875\_c0\_g1\_i1.p1 | 81.63 | 3 | 3 | 2.96e3 | 2 | 2 | 2 | Carbamidomethylation | 67023.6094 | type:complete len:606 gc:universal TRINITY\_DN875\_c0\_g1\_i1:2207-390(-) |
| 32 | 86 | TRINITY\_DN875\_c0\_g1\_i2.p1 | 81.63 | 3 | 3 | 2.96e3 | 2 | 2 | 2 | Carbamidomethylation | 67023.6094 | type:complete len:606 gc:universal TRINITY\_DN875\_c0\_g1\_i2:2136-319(-) |
| 32 | 87 | TRINITY\_DN875\_c0\_g1\_i3.p1 | 81.63 | 3 | 3 | 2.96e3 | 2 | 2 | 2 | Carbamidomethylation | 67023.6094 | type:complete len:606 gc:universal TRINITY\_DN875\_c0\_g1\_i3:2042-225(-) |
| 33 | 88 | TRINITY\_DN8597\_c0\_g1\_i1.p1 | 81.17 | 22 | 22 | 0 | 2 | 1 | 3 | Carbamidomethylation | 13587.9307 | type:internal len:124 gc:universal TRINITY\_DN8597\_c0\_g1\_i1:369-1(-) |
| 34 | 89 | TRINITY\_DN5987\_c0\_g1\_i4.p1 | 79.28 | 8 | 8 | 5.82e3 | 2 | 2 | 3 |  | 32201.8711 | type:complete len:336 gc:universal TRINITY\_DN5987\_c0\_g1\_i4:136-1143(+) |
| 34 | 90 | TRINITY\_DN5987\_c0\_g1\_i2.p1 | 79.28 | 9 | 9 | 5.82e3 | 2 | 2 | 3 |  | 29022.416 | type:complete len:300 gc:universal TRINITY\_DN5987\_c0\_g1\_i2:136-1035(+) |
| 34 | 91 | TRINITY\_DN5987\_c0\_g1\_i3.p2 | 55.37 | 9 | 9 | 5.82e3 | 1 | 1 | 2 |  | 14553.1807 | type:5prime\_partial len:158 gc:universal TRINITY\_DN5987\_c0\_g1\_i3:2-475(+) |
| 35 | 92 | TRINITY\_DN77\_c1\_g1\_i1.p1 | 81.03 | 2 | 2 | 1.74e3 | 2 | 2 | 2 |  | 198350.2344 | type:3prime\_partial len:1965 gc:universal TRINITY\_DN77\_c1\_g1\_i1:2-5893(+) |
| 36 | 93 | TRINITY\_DN4781\_c0\_g1\_i2.p1 | 82.7 | 1 | 1 | 1.06e3 | 2 | 2 | 2 | Carbamidomethylation | 378619.8438 | type:5prime\_partial len:3388 gc:universal TRINITY\_DN4781\_c0\_g1\_i2:10182-19(-) |
| 36 | 94 | TRINITY\_DN4781\_c0\_g1\_i1.p1 | 82.7 | 1 | 1 | 1.06e3 | 2 | 2 | 2 | Carbamidomethylation | 302042.125 | type:5prime\_partial len:2696 gc:universal TRINITY\_DN4781\_c0\_g1\_i1:10504-2417(-) |
| 36 | 95 | TRINITY\_DN4781\_c0\_g1\_i3.p1 | 55.08 | 1 | 1 | 0 | 1 | 1 | 1 |  | 208363.3438 | type:5prime\_partial len:1859 gc:universal TRINITY\_DN4781\_c0\_g1\_i3:8547-2971(-) |
| 37 | 96 | TRINITY\_DN64\_c0\_g1\_i1.p1 | 82.77 | 5 | 5 | 1.54e3 | 2 | 2 | 2 |  | 51450.1914 | type:complete len:468 gc:universal TRINITY\_DN64\_c0\_g1\_i1:1555-152(-) |
| 38 | 97 | TRINITY\_DN1653\_c0\_g1\_i3.p1 | 80.56 | 3 | 3 | 7.03e2 | 2 | 2 | 3 |  | 114336.4531 | type:complete len:1023 gc:universal TRINITY\_DN1653\_c0\_g1\_i3:180-3248(+) |
| 38 | 98 | TRINITY\_DN1653\_c0\_g1\_i1.p1 | 80.56 | 2 | 2 | 7.03e2 | 2 | 2 | 3 |  | 125115.2109 | type:complete len:1122 gc:universal TRINITY\_DN1653\_c0\_g1\_i1:180-3545(+) |
| 39 | 99 | TRINITY\_DN1535\_c0\_g1\_i1.p1 | 83.7 | 3 | 3 | 4.18e2 | 2 | 2 | 2 | Carbamidomethylation | 86942.8516 | type:complete len:779 gc:universal TRINITY\_DN1535\_c0\_g1\_i1:86-2422(+) |
| 40 | 100 | TRINITY\_DN172571\_c0\_g1\_i1.p1 | 80.31 | 21 | 21 | 7.98e2 | 2 | 2 | 2 |  | 11500.2529 | type:complete len:104 gc:universal TRINITY\_DN172571\_c0\_g1\_i1:30-341(+) |
| 41 | 101 | TRINITY\_DN413\_c0\_g1\_i3.p1 | 82.95 | 6 | 6 | 1.59e3 | 2 | 2 | 2 | Carbamidomethylation | 39501.6133 | type:complete len:350 gc:universal TRINITY\_DN413\_c0\_g1\_i3:1286-237(-) |
| 41 | 102 | TRINITY\_DN413\_c0\_g1\_i4.p1 | 82.95 | 4 | 4 | 1.59e3 | 2 | 2 | 2 | Carbamidomethylation | 52354.1758 | type:3prime\_partial len:463 gc:universal TRINITY\_DN413\_c0\_g1\_i4:1388-3(-) |
| 41 | 103 | TRINITY\_DN413\_c0\_g1\_i1.p1 | 82.95 | 6 | 6 | 1.59e3 | 2 | 2 | 2 | Carbamidomethylation | 40351.7656 | type:complete len:355 gc:universal TRINITY\_DN413\_c0\_g1\_i1:1352-288(-) |
| 41 | 104 | TRINITY\_DN413\_c0\_g1\_i2.p1 | 55.25 | 8 | 8 | 5.27e2 | 1 | 1 | 1 |  | 15739.6523 | type:3prime\_partial len:140 gc:universal TRINITY\_DN413\_c0\_g1\_i2:419-3(-) |
| 42 | 105 | TRINITY\_DN1280\_c0\_g1\_i1.p1 | 84.1 | 6 | 6 | 9.59e2 | 2 | 2 | 2 |  | 47818.6641 | type:complete len:426 gc:universal TRINITY\_DN1280\_c0\_g1\_i1:195-1472(+) |
| 43 | 106 | TRINITY\_DN493\_c0\_g1\_i1.p1 | 74.39 | 6 | 6 | 1.65e3 | 2 | 2 | 2 | Carbamidomethylation | 55371.7461 | type:complete len:473 gc:universal TRINITY\_DN493\_c0\_g1\_i1:2681-1263(-) |
| 44 | 107 | TRINITY\_DN1505\_c0\_g1\_i1.p1 | 56.39 | 8 | 8 | 9.92e2 | 1 | 1 | 1 | Acetylation (Protein N-term) | 17930.752 | type:complete len:154 gc:universal TRINITY\_DN1505\_c0\_g1\_i1:98-559(+) |
| 45 | 108 | TRINITY\_DN3448\_c0\_g1\_i9.p3 | 54.97 | 8 | 8 | 9.73e2 | 1 | 1 | 1 | Oxidation (M) | 11358.1816 | type:internal len:102 gc:universal TRINITY\_DN3448\_c0\_g1\_i9:303-1(-) |
| 45 | 109 | TRINITY\_DN5906\_c3\_g1\_i1.p2 | 54.97 | 6 | 6 | 9.73e2 | 1 | 1 | 1 | Oxidation (M) | 15031.4814 | type:internal len:137 gc:universal TRINITY\_DN5906\_c3\_g1\_i1:3-410(+) |
| 45 | 110 | TRINITY\_DN6920\_c0\_g1\_i2.p1 | 54.97 | 2 | 2 | 9.73e2 | 1 | 1 | 1 | Oxidation (M) | 46932.582 | type:5prime\_partial len:411 gc:universal TRINITY\_DN6920\_c0\_g1\_i2:2147-915(-) |
| 45 | 111 | TRINITY\_DN3448\_c0\_g2\_i1.p1 | 54.97 | 7 | 7 | 9.73e2 | 1 | 1 | 1 | Oxidation (M) | 12264.2549 | type:internal len:109 gc:universal TRINITY\_DN3448\_c0\_g2\_i1:326-3(-) |
| 45 | 112 | TRINITY\_DN2799\_c0\_g1\_i3.p1 | 54.97 | 7 | 7 | 9.73e2 | 1 | 1 | 1 | Oxidation (M) | 12365.4863 | type:internal len:112 gc:universal TRINITY\_DN2799\_c0\_g1\_i3:334-2(-) |
| 45 | 113 | TRINITY\_DN2799\_c0\_g1\_i2.p1 | 54.97 | 7 | 7 | 9.73e2 | 1 | 1 | 1 | Oxidation (M) | 12149.2383 | type:internal len:111 gc:universal TRINITY\_DN2799\_c0\_g1\_i2:331-2(-) |
| 45 | 114 | TRINITY\_DN2799\_c0\_g1\_i4.p2 | 54.97 | 7 | 7 | 9.73e2 | 1 | 1 | 1 | Oxidation (M) | 12149.2383 | type:internal len:111 gc:universal TRINITY\_DN2799\_c0\_g1\_i4:331-2(-) |
| 45 | 115 | TRINITY\_DN6920\_c0\_g1\_i3.p1 | 54.97 | 2 | 2 | 9.73e2 | 1 | 1 | 1 | Oxidation (M) | 46932.582 | type:5prime\_partial len:411 gc:universal TRINITY\_DN6920\_c0\_g1\_i3:2124-892(-) |
| 45 | 116 | TRINITY\_DN3448\_c0\_g1\_i5.p2 | 54.97 | 8 | 8 | 9.73e2 | 1 | 1 | 1 | Oxidation (M) | 11358.1816 | type:internal len:102 gc:universal TRINITY\_DN3448\_c0\_g1\_i5:303-1(-) |
| 45 | 117 | TRINITY\_DN3448\_c0\_g2\_i3.p2 | 54.97 | 8 | 8 | 9.73e2 | 1 | 1 | 1 | Oxidation (M) | 12003.9434 | type:internal len:107 gc:universal TRINITY\_DN3448\_c0\_g2\_i3:318-1(-) |
| 45 | 118 | TRINITY\_DN3448\_c0\_g1\_i7.p2 | 54.97 | 7 | 7 | 9.73e2 | 1 | 1 | 1 | Oxidation (M) | 12485.4922 | type:internal len:111 gc:universal TRINITY\_DN3448\_c0\_g1\_i7:330-1(-) |
| 45 | 119 | TRINITY\_DN32727\_c0\_g1\_i2.p1 | 54.97 | 5 | 5 | 9.73e2 | 1 | 1 | 1 | Oxidation (M) | 17342.1152 | type:internal len:154 gc:universal TRINITY\_DN32727\_c0\_g1\_i2:460-2(-) |
| 45 | 120 | TRINITY\_DN2799\_c0\_g1\_i1.p1 | 54.97 | 7 | 7 | 9.73e2 | 1 | 1 | 1 | Oxidation (M) | 12875.0586 | type:internal len:117 gc:universal TRINITY\_DN2799\_c0\_g1\_i1:349-2(-) |
| 46 | 121 | TRINITY\_DN11248\_c0\_g1\_i1.p1 | 52.02 | 1 | 1 | 4.48e2 | 1 | 1 | 1 |  | 194116.9531 | type:3prime\_partial len:1700 gc:universal TRINITY\_DN11248\_c0\_g1\_i1:111-5207(+) |
| 46 | 122 | TRINITY\_DN11248\_c0\_g1\_i3.p1 | 52.02 | 1 | 1 | 4.48e2 | 1 | 1 | 1 |  | 194116.9531 | type:3prime\_partial len:1700 gc:universal TRINITY\_DN11248\_c0\_g1\_i3:111-5207(+) |
| 47 | 123 | TRINITY\_DN9819\_c0\_g1\_i7.p2 | 49.6 | 1 | 1 | 2.99e2 | 1 | 1 | 1 | Acetylation (Protein N-term) | 53383.6875 | type:complete len:483 gc:universal TRINITY\_DN9819\_c0\_g1\_i7:1449-1(-) |
| 47 | 124 | TRINITY\_DN9819\_c0\_g1\_i6.p2 | 49.6 | 1 | 1 | 2.99e2 | 1 | 1 | 1 | Acetylation (Protein N-term) | 53129.3164 | type:complete len:483 gc:universal TRINITY\_DN9819\_c0\_g1\_i6:1449-1(-) |
| 47 | 125 | TRINITY\_DN9819\_c0\_g1\_i1.p2 | 49.6 | 1 | 1 | 2.99e2 | 1 | 1 | 1 | Acetylation (Protein N-term) | 53173.543 | type:complete len:483 gc:universal TRINITY\_DN9819\_c0\_g1\_i1:1449-1(-) |
| 48 | 126 | TRINITY\_DN585\_c0\_g1\_i10.p1 | 55.12 | 7 | 7 | 8.72e2 | 1 | 1 | 4 |  | 26563.5332 | type:3prime\_partial len:236 gc:universal TRINITY\_DN585\_c0\_g1\_i10:705-1(-) |
| 48 | 127 | TRINITY\_DN585\_c0\_g2\_i1.p1 | 55.12 | 11 | 11 | 8.72e2 | 1 | 1 | 4 |  | 17391.9824 | type:5prime\_partial len:152 gc:universal TRINITY\_DN585\_c0\_g2\_i1:598-143(-) |
| 48 | 128 | TRINITY\_DN554\_c0\_g1\_i2.p1 | 55.12 | 6 | 6 | 8.72e2 | 1 | 1 | 4 |  | 29982.375 | type:internal len:266 gc:universal TRINITY\_DN554\_c0\_g1\_i2:797-3(-) |
| 48 | 129 | TRINITY\_DN554\_c0\_g1\_i3.p2 | 55.12 | 6 | 6 | 8.72e2 | 1 | 1 | 4 |  | 30419.4961 | type:complete len:268 gc:universal TRINITY\_DN554\_c0\_g1\_i3:1464-661(-) |
| 48 | 130 | TRINITY\_DN554\_c0\_g1\_i4.p1 | 55.12 | 5 | 5 | 8.72e2 | 1 | 1 | 4 |  | 35966.3984 | type:3prime\_partial len:319 gc:universal TRINITY\_DN554\_c0\_g1\_i4:956-3(-) |
| 48 | 131 | TRINITY\_DN585\_c0\_g1\_i8.p1 | 55.12 | 5 | 5 | 8.72e2 | 1 | 1 | 4 |  | 37858.4844 | type:3prime\_partial len:337 gc:universal TRINITY\_DN585\_c0\_g1\_i8:1008-1(-) |
| 48 | 132 | TRINITY\_DN585\_c0\_g1\_i2.p1 | 55.12 | 9 | 9 | 8.72e2 | 1 | 1 | 4 |  | 20558.6738 | type:internal len:184 gc:universal TRINITY\_DN585\_c0\_g1\_i2:549-1(-) |
| 48 | 133 | TRINITY\_DN585\_c0\_g1\_i5.p1 | 55.12 | 6 | 6 | 8.72e2 | 1 | 1 | 4 |  | 28098.1602 | type:internal len:250 gc:universal TRINITY\_DN585\_c0\_g1\_i5:747-1(-) |
| 48 | 134 | TRINITY\_DN585\_c0\_g1\_i3.p1 | 55.12 | 10 | 10 | 8.72e2 | 1 | 1 | 4 |  | 17628.2207 | type:internal len:157 gc:universal TRINITY\_DN585\_c0\_g1\_i3:468-1(-) |
| 48 | 135 | TRINITY\_DN101\_c0\_g1\_i1.p1 | 55.12 | 12 | 12 | 8.72e2 | 1 | 1 | 4 |  | 14847.2393 | type:complete len:129 gc:universal TRINITY\_DN101\_c0\_g1\_i1:416-30(-) |
| 48 | 136 | TRINITY\_DN585\_c0\_g1\_i4.p1 | 55.12 | 7 | 7 | 8.72e2 | 1 | 1 | 4 |  | 26348.0742 | type:complete len:232 gc:universal TRINITY\_DN585\_c0\_g1\_i4:847-152(-) |
| 48 | 137 | TRINITY\_DN554\_c0\_g1\_i5.p1 | 55.12 | 4 | 4 | 8.72e2 | 1 | 1 | 4 |  | 42081.4141 | type:3prime\_partial len:373 gc:universal TRINITY\_DN554\_c0\_g1\_i5:1118-3(-) |
| 49 | 138 | TRINITY\_DN1058\_c0\_g1\_i1.p1 | 52.25 | 0 | 0 | 3.03e3 | 1 | 1 | 1 |  | 284466.2812 | type:complete len:2639 gc:universal TRINITY\_DN1058\_c0\_g1\_i1:134-8050(+) |
| 49 | 139 | TRINITY\_DN1058\_c0\_g1\_i3.p1 | 52.25 | 0 | 0 | 3.03e3 | 1 | 1 | 1 |  | 277633.5312 | type:complete len:2581 gc:universal TRINITY\_DN1058\_c0\_g1\_i3:134-7876(+) |
| 49 | 140 | TRINITY\_DN1058\_c0\_g1\_i4.p1 | 52.25 | 2 | 2 | 3.03e3 | 1 | 1 | 1 |  | 79375.5703 | type:complete len:723 gc:universal TRINITY\_DN1058\_c0\_g1\_i4:366-2534(+) |
| 49 | 141 | TRINITY\_DN1058\_c0\_g1\_i2.p2 | 52.25 | 1 | 1 | 3.03e3 | 1 | 1 | 1 |  | 140284.0938 | type:complete len:1300 gc:universal TRINITY\_DN1058\_c0\_g1\_i2:4090-7989(+) |
| 50 | 142 | TRINITY\_DN21998\_c0\_g1\_i1.p1 | 51.48 | 3 | 3 | 0 | 1 | 1 | 1 |  | 58159.8398 | type:5prime\_partial len:507 gc:universal TRINITY\_DN21998\_c0\_g1\_i1:1561-41(-) |
| 51 | 143 | TRINITY\_DN167\_c0\_g1\_i7.p1 | 56.39 | 3 | 3 | 4.2e3 | 1 | 1 | 2 |  | 54308.0703 | type:internal len:492 gc:universal TRINITY\_DN167\_c0\_g1\_i7:2-1474(+) |
| 51 | 144 | TRINITY\_DN167\_c0\_g1\_i3.p1 | 56.39 | 3 | 3 | 4.2e3 | 1 | 1 | 2 |  | 57150.8828 | type:5prime\_partial len:518 gc:universal TRINITY\_DN167\_c0\_g1\_i3:2-1555(+) |
| 51 | 145 | TRINITY\_DN167\_c0\_g1\_i8.p1 | 56.39 | 3 | 3 | 4.2e3 | 1 | 1 | 2 |  | 57208.9023 | type:5prime\_partial len:518 gc:universal TRINITY\_DN167\_c0\_g1\_i8:2-1555(+) |
| 51 | 146 | TRINITY\_DN167\_c0\_g1\_i4.p1 | 56.39 | 9 | 9 | 4.2e3 | 1 | 1 | 2 |  | 19659.3105 | type:internal len:179 gc:universal TRINITY\_DN167\_c0\_g1\_i4:2-535(+) |
| 51 | 147 | TRINITY\_DN167\_c0\_g2\_i1.p1 | 56.39 | 2 | 2 | 4.2e3 | 1 | 1 | 2 |  | 71610.8359 | type:complete len:658 gc:universal TRINITY\_DN167\_c0\_g2\_i1:165-2138(+) |
| 51 | 148 | TRINITY\_DN167\_c0\_g1\_i10.p1 | 56.39 | 3 | 3 | 4.2e3 | 1 | 1 | 2 |  | 57122.8281 | type:5prime\_partial len:518 gc:universal TRINITY\_DN167\_c0\_g1\_i10:2-1555(+) |
| 51 | 149 | TRINITY\_DN167\_c0\_g1\_i1.p1 | 56.39 | 11 | 11 | 4.2e3 | 1 | 1 | 2 |  | 15245.8467 | type:5prime\_partial len:141 gc:universal TRINITY\_DN167\_c0\_g1\_i1:2-424(+) |
| 51 | 150 | TRINITY\_DN167\_c0\_g1\_i6.p1 | 56.39 | 11 | 11 | 4.2e3 | 1 | 1 | 2 |  | 15245.8467 | type:5prime\_partial len:141 gc:universal TRINITY\_DN167\_c0\_g1\_i6:2-424(+) |
| 52 | 151 | TRINITY\_DN120478\_c0\_g1\_i1.p1 | 51.41 | 2 | 2 | 0 | 1 | 1 | 1 |  | 59788.2227 | type:complete len:544 gc:universal TRINITY\_DN120478\_c0\_g1\_i1:2674-1043(-) |
| 53 | 152 | TRINITY\_DN691\_c0\_g1\_i1.p1 | 55.37 | 1 | 1 | 1.5e3 | 1 | 1 | 2 | Carbamidomethylation | 169920.1562 | type:complete len:1726 gc:universal TRINITY\_DN691\_c0\_g1\_i1:5662-485(-) |
| 53 | 153 | TRINITY\_DN691\_c0\_g1\_i2.p1 | 55.37 | 1 | 1 | 1.5e3 | 1 | 1 | 2 | Carbamidomethylation | 164625.2188 | type:complete len:1673 gc:universal TRINITY\_DN691\_c0\_g1\_i2:5503-485(-) |
| 54 | 154 | TRINITY\_DN65215\_c0\_g1\_i1.p1 | 52.39 | 2 | 2 | 7e2 | 1 | 1 | 1 |  | 43734.125 | type:5prime\_partial len:383 gc:universal TRINITY\_DN65215\_c0\_g1\_i1:1336-188(-) |
| 55 | 155 | TRINITY\_DN991\_c0\_g1\_i2.p1 | 49.15 | 0 | 0 | 1.94e2 | 1 | 1 | 1 |  | 358764.375 | type:complete len:3229 gc:universal TRINITY\_DN991\_c0\_g1\_i2:278-9964(+) |
| 55 | 156 | TRINITY\_DN991\_c0\_g1\_i6.p1 | 49.15 | 0 | 0 | 1.94e2 | 1 | 1 | 1 |  | 358765.375 | type:complete len:3229 gc:universal TRINITY\_DN991\_c0\_g1\_i6:278-9964(+) |
| 55 | 157 | TRINITY\_DN991\_c0\_g1\_i5.p1 | 49.15 | 0 | 0 | 1.94e2 | 1 | 1 | 1 |  | 358764.375 | type:complete len:3229 gc:universal TRINITY\_DN991\_c0\_g1\_i5:278-9964(+) |
| 56 | 158 | TRINITY\_DN165536\_c0\_g1\_i1.p1 | 55.11 | 6 | 6 | 0 | 1 | 1 | 1 |  | 28339.4531 | type:complete len:249 gc:universal TRINITY\_DN165536\_c0\_g1\_i1:851-105(-) |
| 57 | 159 | TRINITY\_DN329\_c0\_g1\_i2.p1 | 49.47 | 5 | 5 | 0 | 1 | 1 | 1 |  | 34761.9609 | type:5prime\_partial len:320 gc:universal TRINITY\_DN329\_c0\_g1\_i2:1-960(+) |
| 57 | 160 | TRINITY\_DN329\_c0\_g1\_i1.p1 | 49.47 | 6 | 6 | 0 | 1 | 1 | 1 |  | 30514.9648 | type:complete len:283 gc:universal TRINITY\_DN329\_c0\_g1\_i1:158-1006(+) |
| 58 | 161 | TRINITY\_DN2003\_c0\_g1\_i1.p1 | 56.32 | 3 | 3 | 0 | 1 | 1 | 1 |  | 42076.3203 | type:complete len:370 gc:universal TRINITY\_DN2003\_c0\_g1\_i1:89-1198(+) |
| 59 | 162 | TRINITY\_DN6188\_c0\_g1\_i1.p1 | 56.34 | 2 | 2 | 0 | 1 | 1 | 1 |  | 66918.6719 | type:complete len:566 gc:universal TRINITY\_DN6188\_c0\_g1\_i1:2302-605(-) |
| 60 | 163 | TRINITY\_DN69276\_c0\_g1\_i1.p1 | 56.38 | 1 | 1 | 1.48e3 | 1 | 1 | 1 |  | 97474.1719 | type:internal len:887 gc:universal TRINITY\_DN69276\_c0\_g1\_i1:2659-2(-) |
| 61 | 164 | TRINITY\_DN65211\_c0\_g1\_i2.p1 | 49.38 | 5 | 5 | 0 | 1 | 1 | 1 |  | 38770.3633 | type:complete len:342 gc:universal TRINITY\_DN65211\_c0\_g1\_i2:1592-567(-) |
| 61 | 165 | TRINITY\_DN65211\_c0\_g1\_i1.p1 | 49.38 | 5 | 5 | 0 | 1 | 1 | 1 |  | 38740.3398 | type:complete len:342 gc:universal TRINITY\_DN65211\_c0\_g1\_i1:1473-448(-) |
| 61 | 166 | TRINITY\_DN65211\_c0\_g1\_i3.p1 | 49.38 | 5 | 5 | 0 | 1 | 1 | 1 |  | 38740.3398 | type:complete len:342 gc:universal TRINITY\_DN65211\_c0\_g1\_i3:1613-588(-) |
| 62 | 167 | TRINITY\_DN26392\_c0\_g1\_i1.p1 | 49.47 | 3 | 3 | 0 | 1 | 1 | 1 |  | 52063.2266 | type:internal len:469 gc:universal TRINITY\_DN26392\_c0\_g1\_i1:2-1405(+) |
| 63 | 168 | TRINITY\_DN1030\_c0\_g1\_i2.p1 | 56.41 | 1 | 1 | 1.24e3 | 1 | 1 | 3 |  | 140221.3281 | type:complete len:1426 gc:universal TRINITY\_DN1030\_c0\_g1\_i2:4483-206(-) |
| 63 | 169 | TRINITY\_DN1030\_c0\_g1\_i1.p1 | 56.41 | 1 | 1 | 1.24e3 | 1 | 1 | 3 |  | 141535.875 | type:complete len:1439 gc:universal TRINITY\_DN1030\_c0\_g1\_i1:4522-206(-) |
| 64 | 170 | TRINITY\_DN3347\_c0\_g1\_i1.p1 | 56.31 | 1 | 1 | 0 | 1 | 1 | 1 |  | 104268.7266 | type:5prime\_partial len:924 gc:universal TRINITY\_DN3347\_c0\_g1\_i1:2-2773(+) |
| 65 | 171 | TRINITY\_DN59572\_c0\_g1\_i1.p1 | 56.4 | 2 | 2 | 5.95e2 | 1 | 1 | 1 |  | 82544.875 | type:internal len:725 gc:universal TRINITY\_DN59572\_c0\_g1\_i1:3-2174(+) |
| 66 | 172 | TRINITY\_DN160\_c0\_g1\_i1.p1 | 56.21 | 1 | 1 | 3.79e2 | 1 | 1 | 1 |  | 126611.5703 | type:complete len:1145 gc:universal TRINITY\_DN160\_c0\_g1\_i1:4810-1376(-) |
| 66 | 173 | TRINITY\_DN160\_c0\_g1\_i2.p1 | 56.21 | 1 | 1 | 3.79e2 | 1 | 1 | 1 |  | 126611.5703 | type:complete len:1145 gc:universal TRINITY\_DN160\_c0\_g1\_i2:4810-1376(-) |
| 67 | 174 | TRINITY\_DN15847\_c0\_g1\_i2.p1 | 55.33 | 7 | 7 | 8.95e2 | 1 | 1 | 1 |  | 19271.252 | type:5prime\_partial len:166 gc:universal TRINITY\_DN15847\_c0\_g1\_i2:3-500(+) |
| 67 | 175 | TRINITY\_DN15847\_c0\_g1\_i1.p1 | 55.33 | 7 | 7 | 8.95e2 | 1 | 1 | 1 |  | 19271.252 | type:5prime\_partial len:166 gc:universal TRINITY\_DN15847\_c0\_g1\_i1:3-500(+) |
| 68 | 176 | TRINITY\_DN3016\_c0\_g1\_i5.p1 | 56.35 | 3 | 3 | 7.77e2 | 1 | 1 | 1 |  | 46762.2305 | type:complete len:406 gc:universal TRINITY\_DN3016\_c0\_g1\_i5:2713-1496(-) |
| 68 | 177 | TRINITY\_DN3016\_c0\_g1\_i10.p1 | 56.35 | 3 | 3 | 7.77e2 | 1 | 1 | 1 |  | 46762.2305 | type:complete len:406 gc:universal TRINITY\_DN3016\_c0\_g1\_i10:2760-1543(-) |
| 68 | 178 | TRINITY\_DN3016\_c0\_g1\_i17.p1 | 56.35 | 3 | 3 | 7.77e2 | 1 | 1 | 1 |  | 46762.2305 | type:complete len:406 gc:universal TRINITY\_DN3016\_c0\_g1\_i17:2691-1474(-) |
| 68 | 179 | TRINITY\_DN3016\_c0\_g1\_i8.p1 | 56.35 | 3 | 3 | 7.77e2 | 1 | 1 | 1 |  | 46762.2305 | type:complete len:406 gc:universal TRINITY\_DN3016\_c0\_g1\_i8:2713-1496(-) |
| 68 | 180 | TRINITY\_DN3016\_c0\_g1\_i13.p1 | 56.35 | 3 | 3 | 7.77e2 | 1 | 1 | 1 |  | 46762.2305 | type:complete len:406 gc:universal TRINITY\_DN3016\_c0\_g1\_i13:2760-1543(-) |
| 68 | 181 | TRINITY\_DN3016\_c0\_g1\_i4.p1 | 56.35 | 3 | 3 | 7.77e2 | 1 | 1 | 1 |  | 46762.2305 | type:complete len:406 gc:universal TRINITY\_DN3016\_c0\_g1\_i4:1529-312(-) |
| 68 | 182 | TRINITY\_DN3016\_c0\_g1\_i18.p1 | 56.35 | 3 | 3 | 7.77e2 | 1 | 1 | 1 |  | 46762.2305 | type:complete len:406 gc:universal TRINITY\_DN3016\_c0\_g1\_i18:2766-1549(-) |
| 68 | 183 | TRINITY\_DN3016\_c0\_g1\_i2.p1 | 56.35 | 3 | 3 | 7.77e2 | 1 | 1 | 1 |  | 46762.2305 | type:complete len:406 gc:universal TRINITY\_DN3016\_c0\_g1\_i2:2691-1474(-) |
| 68 | 184 | TRINITY\_DN3016\_c0\_g1\_i11.p1 | 56.35 | 3 | 3 | 7.77e2 | 1 | 1 | 1 |  | 46762.2305 | type:complete len:406 gc:universal TRINITY\_DN3016\_c0\_g1\_i11:2541-1324(-) |
| 68 | 185 | TRINITY\_DN3016\_c0\_g1\_i6.p1 | 56.35 | 3 | 3 | 7.77e2 | 1 | 1 | 1 |  | 46762.2305 | type:complete len:406 gc:universal TRINITY\_DN3016\_c0\_g1\_i6:2760-1543(-) |
| 69 | 186 | TRINITY\_DN11522\_c0\_g1\_i3.p1 | 55.35 | 2 | 2 | 1.32e3 | 1 | 1 | 2 |  | 93820.75 | type:5prime\_partial len:855 gc:universal TRINITY\_DN11522\_c0\_g1\_i3:2878-314(-) |
| 69 | 187 | TRINITY\_DN11522\_c0\_g1\_i1.p1 | 55.35 | 2 | 2 | 1.32e3 | 1 | 1 | 2 |  | 93223.9141 | type:5prime\_partial len:849 gc:universal TRINITY\_DN11522\_c0\_g1\_i1:2860-314(-) |
| 69 | 188 | TRINITY\_DN11522\_c0\_g1\_i4.p1 | 55.35 | 2 | 2 | 1.32e3 | 1 | 1 | 2 |  | 93423.2266 | type:5prime\_partial len:851 gc:universal TRINITY\_DN11522\_c0\_g1\_i4:2866-314(-) |
| 69 | 189 | TRINITY\_DN11522\_c0\_g1\_i2.p1 | 55.35 | 2 | 2 | 1.32e3 | 1 | 1 | 2 |  | 93621.4375 | type:5prime\_partial len:853 gc:universal TRINITY\_DN11522\_c0\_g1\_i2:2872-314(-) |
| 70 | 190 | TRINITY\_DN840\_c0\_g1\_i1.p1 | 49.41 | 3 | 3 | 2.49e3 | 1 | 1 | 2 | Carbamidomethylation | 47186.0312 | type:complete len:409 gc:universal TRINITY\_DN840\_c0\_g1\_i1:1437-211(-) |
| 71 | 191 | TRINITY\_DN1936\_c0\_g1\_i3.p1 | 50.58 | 1 | 1 | 0 | 1 | 1 | 1 |  | 119975.3906 | type:internal len:1070 gc:universal TRINITY\_DN1936\_c0\_g1\_i3:3208-2(-) |
| 72 | 192 | TRINITY\_DN6542\_c0\_g1\_i3.p1 | 49.52 | 2 | 2 | 3.26e2 | 1 | 1 | 1 |  | 58403.6289 | type:5prime\_partial len:545 gc:universal TRINITY\_DN6542\_c0\_g1\_i3:2-1636(+) |
| 72 | 193 | TRINITY\_DN6542\_c0\_g1\_i4.p1 | 49.52 | 2 | 2 | 3.26e2 | 1 | 1 | 1 |  | 58168.5312 | type:internal len:550 gc:universal TRINITY\_DN6542\_c0\_g1\_i4:2-1648(+) |
| 72 | 194 | TRINITY\_DN6542\_c0\_g1\_i1.p1 | 49.52 | 2 | 2 | 3.26e2 | 1 | 1 | 1 |  | 65866.8516 | type:internal len:628 gc:universal TRINITY\_DN6542\_c0\_g1\_i1:2-1882(+) |
| 73 | 195 | TRINITY\_DN80458\_c0\_g1\_i1.p1 | 55.25 | 6 | 6 | 0 | 1 | 1 | 2 |  | 17768.7109 | type:5prime\_partial len:157 gc:universal TRINITY\_DN80458\_c0\_g1\_i1:519-49(-) |
| 74 | 196 | TRINITY\_DN6428\_c0\_g1\_i2.p1 | 56.12 | 4 | 4 | 7.77e2 | 1 | 1 | 1 | Carbamidomethylation | 33956.7344 | type:3prime\_partial len:303 gc:universal TRINITY\_DN6428\_c0\_g1\_i2:906-1(-) |
| 74 | 197 | TRINITY\_DN6428\_c0\_g1\_i1.p1 | 56.12 | 3 | 3 | 7.77e2 | 1 | 1 | 1 | Carbamidomethylation | 41179.0664 | type:internal len:367 gc:universal TRINITY\_DN6428\_c0\_g1\_i1:1098-1(-) |
| 75 | 198 | TRINITY\_DN1377\_c0\_g1\_i1.p1 | 49.73 | 2 | 2 | 5.82e2 | 1 | 1 | 1 |  | 99936.9219 | type:complete len:878 gc:universal TRINITY\_DN1377\_c0\_g1\_i1:68-2701(+) |
| 76 | 199 | TRINITY\_DN118\_c1\_g1\_i3.p1 | 51.53 | 6 | 6 | 2.86e2 | 1 | 1 | 1 | Carbamidomethylation | 22860.4883 | type:internal len:200 gc:universal TRINITY\_DN118\_c1\_g1\_i3:599-3(-) |
| 77 | 200 | TRINITY\_DN149493\_c0\_g1\_i1.p1 | 55.35 | 9 | 9 | 9.17e2 | 1 | 1 | 1 |  | 16274.376 | type:internal len:148 gc:universal TRINITY\_DN149493\_c0\_g1\_i1:2-442(+) |
| 78 | 201 | TRINITY\_DN2217\_c0\_g1\_i2.p1 | 56.39 | 5 | 5 | 1.73e2 | 1 | 1 | 2 | Carbamidomethylation | 28618.9531 | type:complete len:254 gc:universal TRINITY\_DN2217\_c0\_g1\_i2:884-123(-) |
| 78 | 202 | TRINITY\_DN2217\_c0\_g1\_i3.p1 | 56.39 | 2 | 2 | 1.73e2 | 1 | 1 | 2 | Carbamidomethylation | 63077.0508 | type:complete len:566 gc:universal TRINITY\_DN2217\_c0\_g1\_i3:1820-123(-) |
| 79 | 203 | TRINITY\_DN1071\_c0\_g1\_i2.p1 | 56.18 | 5 | 5 | 2.33e3 | 1 | 1 | 2 |  | 36243.2383 | type:internal len:339 gc:universal TRINITY\_DN1071\_c0\_g1\_i2:1016-3(-) |
| 79 | 204 | TRINITY\_DN1071\_c0\_g1\_i3.p1 | 56.18 | 4 | 4 | 2.33e3 | 1 | 1 | 2 |  | 39803.1602 | type:internal len:375 gc:universal TRINITY\_DN1071\_c0\_g1\_i3:1124-3(-) |
| 79 | 205 | TRINITY\_DN1071\_c0\_g1\_i1.p1 | 56.18 | 2 | 2 | 2.33e3 | 1 | 1 | 2 |  | 71848.4062 | type:internal len:660 gc:universal TRINITY\_DN1071\_c0\_g1\_i1:1977-1(-) |
| 79 | 206 | TRINITY\_DN1071\_c0\_g1\_i4.p1 | 56.18 | 2 | 2 | 2.33e3 | 1 | 1 | 2 |  | 75408.3359 | type:internal len:696 gc:universal TRINITY\_DN1071\_c0\_g1\_i4:2085-1(-) |
| 80 | 207 | TRINITY\_DN77\_c0\_g2\_i3.p1 | 52.33 | 1 | 1 | 5.15e3 | 1 | 1 | 1 |  | 133966.4375 | type:internal len:1381 gc:universal TRINITY\_DN77\_c0\_g2\_i3:4141-2(-) |
| 80 | 208 | TRINITY\_DN77\_c0\_g2\_i2.p1 | 52.33 | 1 | 1 | 5.15e3 | 1 | 1 | 1 |  | 133198.6094 | type:internal len:1374 gc:universal TRINITY\_DN77\_c0\_g2\_i2:4120-2(-) |
| 80 | 209 | TRINITY\_DN77\_c0\_g2\_i1.p1 | 52.33 | 1 | 1 | 5.15e3 | 1 | 1 | 1 |  | 133184.5781 | type:internal len:1374 gc:universal TRINITY\_DN77\_c0\_g2\_i1:4120-2(-) |
| 80 | 210 | TRINITY\_DN77\_c0\_g2\_i4.p1 | 52.33 | 1 | 1 | 5.15e3 | 1 | 1 | 1 |  | 133952.4219 | type:internal len:1381 gc:universal TRINITY\_DN77\_c0\_g2\_i4:4141-2(-) |
| 81 | 211 | TRINITY\_DN4815\_c0\_g1\_i2.p1 | 50.26 | 2 | 2 | 1.27e3 | 1 | 1 | 1 | Carbamidomethylation | 70695.4688 | type:complete len:638 gc:universal TRINITY\_DN4815\_c0\_g1\_i2:79-1992(+) |
| 81 | 212 | TRINITY\_DN4815\_c0\_g1\_i1.p1 | 50.26 | 3 | 3 | 1.27e3 | 1 | 1 | 1 | Carbamidomethylation | 66133.5156 | type:complete len:595 gc:universal TRINITY\_DN4815\_c0\_g1\_i1:79-1863(+) |
| 82 | 213 | TRINITY\_DN15584\_c0\_g1\_i1.p1 | 56.39 | 6 | 6 | 0 | 1 | 1 | 1 |  | 29534.9648 | type:complete len:275 gc:universal TRINITY\_DN15584\_c0\_g1\_i1:874-50(-) |
| 83 | 214 | TRINITY\_DN187147\_c0\_g1\_i1.p1 | 52.05 | 1 | 1 | 0 | 1 | 1 | 1 |  | 138182.3125 | type:complete len:1223 gc:universal TRINITY\_DN187147\_c0\_g1\_i1:3708-40(-) |
| 84 | 215 | TRINITY\_DN101992\_c0\_g1\_i1.p1 | 55.56 | 7 | 7 | 1.63e3 | 1 | 1 | 2 | Carbamidomethylation | 15607.4678 | type:internal len:139 gc:universal TRINITY\_DN101992\_c0\_g1\_i1:414-1(-) |
| 85 | 216 | TRINITY\_DN2885\_c0\_g1\_i2.p1 | 56.37 | 11 | 11 | 0 | 1 | 1 | 1 |  | 12758.7783 | type:complete len:114 gc:universal TRINITY\_DN2885\_c0\_g1\_i2:31-372(+) |
| 85 | 217 | TRINITY\_DN2885\_c0\_g1\_i4.p1 | 56.37 | 11 | 11 | 0 | 1 | 1 | 1 |  | 12245.125 | type:5prime\_partial len:109 gc:universal TRINITY\_DN2885\_c0\_g1\_i4:1-327(+) |
| 85 | 218 | TRINITY\_DN2885\_c0\_g1\_i5.p1 | 56.37 | 11 | 11 | 0 | 1 | 1 | 1 |  | 12383.5439 | type:3prime\_partial len:112 gc:universal TRINITY\_DN2885\_c0\_g1\_i5:31-363(+) |
| 86 | 219 | TRINITY\_DN79\_c0\_g1\_i1.p1 | 56.17 | 4 | 4 | 1.16e3 | 1 | 1 | 2 |  | 52590.9805 | type:5prime\_partial len:478 gc:universal TRINITY\_DN79\_c0\_g1\_i1:2132-699(-) |
| 87 | 220 | TRINITY\_DN12627\_c0\_g1\_i1.p1 | 51.11 | 2 | 2 | 0 | 1 | 1 | 1 |  | 120592.0703 | type:5prime\_partial len:1061 gc:universal TRINITY\_DN12627\_c0\_g1\_i1:3456-274(-) |
| 88 | 221 | TRINITY\_DN1936\_c0\_g1\_i1.p1 | 54.47 | 1 | 1 | 1.32e3 | 1 | 1 | 2 |  | 136661.0312 | type:3prime\_partial len:1224 gc:universal TRINITY\_DN1936\_c0\_g1\_i1:3669-1(-) |
| 88 | 222 | TRINITY\_DN1936\_c0\_g1\_i4.p1 | 54.47 | 1 | 1 | 1.32e3 | 1 | 1 | 2 |  | 196066.9688 | type:3prime\_partial len:1762 gc:universal TRINITY\_DN1936\_c0\_g1\_i4:5283-1(-) |
| 89 | 223 | TRINITY\_DN144508\_c0\_g1\_i1.p1 | 52.38 | 4 | 4 | 0 | 1 | 1 | 1 |  | 29446.1543 | type:internal len:265 gc:universal TRINITY\_DN144508\_c0\_g1\_i1:1-792(+) |
| 90 | 224 | TRINITY\_DN2626\_c0\_g1\_i2.p1 | 50.99 | 6 | 6 | 4.98e2 | 1 | 1 | 1 |  | 24476.0684 | type:internal len:219 gc:universal TRINITY\_DN2626\_c0\_g1\_i2:2-655(+) |
| 90 | 225 | TRINITY\_DN2626\_c0\_g1\_i6.p1 | 50.99 | 6 | 6 | 4.98e2 | 1 | 1 | 1 |  | 24213.7539 | type:internal len:218 gc:universal TRINITY\_DN2626\_c0\_g1\_i6:1-651(+) |
| 91 | 226 | TRINITY\_DN1559\_c0\_g1\_i1.p1 | 55.1 | 5 | 5 | 2.84e2 | 1 | 1 | 1 |  | 33672.0977 | type:complete len:311 gc:universal TRINITY\_DN1559\_c0\_g1\_i1:221-1153(+) |
| 92 | 227 | TRINITY\_DN3986\_c0\_g1\_i1.p1 | 55.34 | 2 | 2 | 0 | 1 | 1 | 1 |  | 50011.8867 | type:complete len:446 gc:universal TRINITY\_DN3986\_c0\_g1\_i1:252-1589(+) |
| 92 | 228 | TRINITY\_DN1910\_c0\_g1\_i1.p1 | 55.34 | 2 | 2 | 0 | 1 | 1 | 1 |  | 50135.0117 | type:complete len:447 gc:universal TRINITY\_DN1910\_c0\_g1\_i1:2183-843(-) |
| 93 | 229 | TRINITY\_DN195348\_c0\_g1\_i1.p1 | 55.12 | 5 | 5 | 0 | 1 | 1 | 1 |  | 34993.6055 | type:internal len:311 gc:universal TRINITY\_DN195348\_c0\_g1\_i1:1-930(+) |
| 94 | 230 | TRINITY\_DN3034\_c0\_g1\_i1.p1 | 49.43 | 1 | 1 | 0 | 1 | 1 | 1 |  | 222266.9844 | type:complete len:1941 gc:universal TRINITY\_DN3034\_c0\_g1\_i1:6289-467(-) |
| 95 | 231 | TRINITY\_DN1704\_c0\_g1\_i1.p1 | 49.93 | 4 | 4 | 8.91e2 | 1 | 1 | 1 | Carbamidomethylation | 21614.0098 | type:5prime\_partial len:190 gc:universal TRINITY\_DN1704\_c0\_g1\_i1:1-570(+) |
| 96 | 232 | TRINITY\_DN163801\_c0\_g1\_i1.p1 | 54.32 | 3 | 3 | 8.04e2 | 1 | 1 | 1 | Acetylation (Protein N-term) | 33076.9062 | type:complete len:298 gc:universal TRINITY\_DN163801\_c0\_g1\_i1:55-948(+) |
| 97 | 233 | TRINITY\_DN761\_c0\_g1\_i1.p1 | 51.48 | 2 | 2 | 3.05e2 | 1 | 1 | 1 |  | 95330.7812 | type:complete len:830 gc:universal TRINITY\_DN761\_c0\_g1\_i1:129-2618(+) |
| 97 | 234 | TRINITY\_DN761\_c0\_g1\_i2.p1 | 51.48 | 2 | 2 | 3.05e2 | 1 | 1 | 1 |  | 95330.7812 | type:complete len:830 gc:universal TRINITY\_DN761\_c0\_g1\_i2:87-2576(+) |
| 97 | 235 | TRINITY\_DN761\_c0\_g1\_i3.p1 | 51.48 | 2 | 2 | 3.05e2 | 1 | 1 | 1 |  | 96968.6562 | type:complete len:845 gc:universal TRINITY\_DN761\_c0\_g1\_i3:129-2663(+) |
| 98 | 236 | TRINITY\_DN1739\_c0\_g1\_i1.p1 | 56.03 | 3 | 3 | 6.5e2 | 1 | 1 | 2 |  | 46350.6953 | type:complete len:400 gc:universal TRINITY\_DN1739\_c0\_g1\_i1:86-1285(+) |
| 99 | 237 | TRINITY\_DN141338\_c2\_g1\_i1.p1 | 56.21 | 1 | 1 | 9.9e2 | 1 | 1 | 1 |  | 82716.2266 | type:complete len:720 gc:universal TRINITY\_DN141338\_c2\_g1\_i1:2850-691(-) |
| 100 | 238 | TRINITY\_DN118520\_c0\_g1\_i1.p1 | 51.46 | 3 | 3 | 2.32e3 | 1 | 1 | 1 |  | 61587.4766 | type:complete len:581 gc:universal TRINITY\_DN118520\_c0\_g1\_i1:1895-153(-) |
| 101 | 239 | TRINITY\_DN1043\_c0\_g1\_i2.p1 | 49.4 | 2 | 2 | 4.99e2 | 1 | 1 | 1 |  | 78392.1016 | type:complete len:700 gc:universal TRINITY\_DN1043\_c0\_g1\_i2:2410-311(-) |
| 102 | 240 | TRINITY\_DN108993\_c0\_g1\_i1.p1 | 51.12 | 7 | 7 | 0 | 1 | 1 | 1 |  | 20892.0039 | type:3prime\_partial len:180 gc:universal TRINITY\_DN108993\_c0\_g1\_i1:538-2(-) |
| 103 | 241 | TRINITY\_DN1613\_c1\_g1\_i2.p1 | 47.79 | 2 | 2 | 3.89e2 | 1 | 1 | 1 | Acetylation (Protein N-term), Carbamidomethylation | 111170.0312 | type:internal len:1068 gc:universal TRINITY\_DN1613\_c1\_g1\_i2:3201-1(-) |
| 103 | 242 | TRINITY\_DN1613\_c1\_g1\_i1.p1 | 47.79 | 2 | 2 | 3.89e2 | 1 | 1 | 1 | Acetylation (Protein N-term), Carbamidomethylation | 112791.8203 | type:internal len:1083 gc:universal TRINITY\_DN1613\_c1\_g1\_i1:3248-3(-) |
| 104 | 243 | TRINITY\_DN598\_c1\_g1\_i2.p1 | 49.26 | 1 | 1 | 6.03e2 | 1 | 1 | 1 |  | 81043.625 | type:5prime\_partial len:742 gc:universal TRINITY\_DN598\_c1\_g1\_i2:2-2227(+) |
| 105 | 244 | TRINITY\_DN5001\_c0\_g1\_i1.p1 | 55.18 | 1 | 1 | 5.02e2 | 1 | 1 | 1 |  | 177237.6562 | type:complete len:1575 gc:universal TRINITY\_DN5001\_c0\_g1\_i1:40-4764(+) |
| 105 | 245 | TRINITY\_DN81013\_c0\_g1\_i1.p2 | 55.18 | 5 | 5 | 5.02e2 | 1 | 1 | 1 |  | 38269.6328 | type:5prime\_partial len:345 gc:universal TRINITY\_DN81013\_c0\_g1\_i1:2-1036(+) |
| 106 | 246 | TRINITY\_DN785\_c0\_g1\_i1.p1 | 55.35 | 4 | 4 | 0 | 1 | 1 | 1 | Carbamidomethylation | 33410.082 | type:complete len:289 gc:universal TRINITY\_DN785\_c0\_g1\_i1:1062-196(-) |
| 107 | 247 | TRINITY\_DN11097\_c0\_g1\_i1.p1 | 49.43 | 2 | 2 | 0 | 1 | 1 | 1 |  | 80111.1172 | type:complete len:730 gc:universal TRINITY\_DN11097\_c0\_g1\_i1:2348-159(-) |
| 108 | 248 | TRINITY\_DN61\_c0\_g1\_i1.p1 | 55.37 | 2 | 2 | 3.27e3 | 1 | 1 | 2 |  | 71696.4219 | type:complete len:625 gc:universal TRINITY\_DN61\_c0\_g1\_i1:2929-1055(-) |
| 109 | 249 | TRINITY\_DN2028\_c0\_g1\_i1.p1 | 55.37 | 5 | 5 | 1.9e3 | 1 | 1 | 2 |  | 30106.9453 | type:complete len:274 gc:universal TRINITY\_DN2028\_c0\_g1\_i1:1608-787(-) |
| 110 | 250 | TRINITY\_DN26249\_c0\_g1\_i1.p1 | 56.4 | 3 | 3 | 6.97e2 | 1 | 1 | 1 |  | 54490.1055 | type:5prime\_partial len:477 gc:universal TRINITY\_DN26249\_c0\_g1\_i1:1733-303(-) |
| 111 | 251 | TRINITY\_DN17117\_c0\_g1\_i1.p1 | 49.19 | 6 | 6 | 0 | 1 | 1 | 1 |  | 39329.6055 | type:5prime\_partial len:345 gc:universal TRINITY\_DN17117\_c0\_g1\_i1:1183-149(-) |
| 112 | 252 | TRINITY\_DN17358\_c0\_g1\_i1.p1 | 55.36 | 8 | 8 | 9.06e2 | 1 | 1 | 1 | Acetylation (Protein N-term) | 13936.9082 | type:3prime\_partial len:125 gc:universal TRINITY\_DN17358\_c0\_g1\_i1:42-413(+) |
| 113 | 253 | TRINITY\_DN7299\_c0\_g1\_i1.p2 | 56.11 | 3 | 3 | 0 | 1 | 1 | 1 |  | 36360.918 | type:5prime\_partial len:345 gc:universal TRINITY\_DN7299\_c0\_g1\_i1:1201-167(-) |
| 114 | 254 | TRINITY\_DN5500\_c0\_g1\_i2.p1 | 56.04 | 1 | 1 | 1.29e3 | 1 | 1 | 1 |  | 130974.4219 | type:3prime\_partial len:1183 gc:universal TRINITY\_DN5500\_c0\_g1\_i2:3546-1(-) |
| 114 | 255 | TRINITY\_DN5500\_c0\_g1\_i1.p1 | 56.04 | 1 | 1 | 1.29e3 | 1 | 1 | 1 |  | 132375.9688 | type:3prime\_partial len:1196 gc:universal TRINITY\_DN5500\_c0\_g1\_i1:3585-1(-) |
| 115 | 256 | TRINITY\_DN61707\_c0\_g1\_i1.p1 | 55.36 | 3 | 3 | 0 | 1 | 1 | 2 |  | 46897.5508 | type:3prime\_partial len:417 gc:universal TRINITY\_DN61707\_c0\_g1\_i1:1250-3(-) |
| 116 | 257 | TRINITY\_DN1668\_c0\_g1\_i1.p1 | 55.19 | 8 | 8 | 0 | 1 | 1 | 1 |  | 24764.1523 | type:complete len:220 gc:universal TRINITY\_DN1668\_c0\_g1\_i1:748-89(-) |
| 117 | 258 | TRINITY\_DN97809\_c0\_g1\_i1.p1 | 51.28 | 7 | 7 | 6.27e3 | 1 | 1 | 2 |  | 20461.1211 | type:internal len:184 gc:universal TRINITY\_DN97809\_c0\_g1\_i1:2-550(+) |
| 118 | 259 | TRINITY\_DN773\_c0\_g1\_i1.p1 | 54.51 | 3 | 3 | 0 | 1 | 1 | 1 |  | 48865.75 | type:complete len:440 gc:universal TRINITY\_DN773\_c0\_g1\_i1:1608-289(-) |
| 119 | 260 | TRINITY\_DN5633\_c0\_g1\_i1.p1 | 49.16 | 2 | 2 | 2.5e2 | 1 | 1 | 1 |  | 97310.9922 | type:complete len:848 gc:universal TRINITY\_DN5633\_c0\_g1\_i1:2881-338(-) |
| 120 | 261 | TRINITY\_DN775\_c0\_g1\_i1.p1 | 56.35 | 2 | 2 | 4.08e2 | 1 | 1 | 1 |  | 72884.4609 | type:complete len:635 gc:universal TRINITY\_DN775\_c0\_g1\_i1:1927-23(-) |
| 120 | 262 | TRINITY\_DN775\_c0\_g1\_i2.p1 | 56.35 | 2 | 2 | 4.08e2 | 1 | 1 | 1 |  | 74657.6406 | type:complete len:647 gc:universal TRINITY\_DN775\_c0\_g1\_i2:1963-23(-) |
| 121 | 263 | TRINITY\_DN71243\_c0\_g1\_i1.p1 | 51.92 | 4 | 4 | 7.34e2 | 1 | 1 | 1 |  | 46628.2539 | type:internal len:422 gc:universal TRINITY\_DN71243\_c0\_g1\_i1:1265-3(-) |
| 122 | 264 | TRINITY\_DN33065\_c0\_g1\_i1.p1 | 56.17 | 3 | 3 | 3.01e2 | 1 | 1 | 1 |  | 73381.4062 | type:3prime\_partial len:663 gc:universal TRINITY\_DN33065\_c0\_g1\_i1:1986-1(-) |
| 123 | 265 | TRINITY\_DN598\_c1\_g1\_i1.p1 | 56.37 | 2 | 2 | 1.42e4 | 1 | 1 | 1 |  | 81322.8672 | type:5prime\_partial len:745 gc:universal TRINITY\_DN598\_c1\_g1\_i1:2-2236(+) |
| 124 | 266 | TRINITY\_DN128406\_c0\_g1\_i1.p1 | 53.99 | 7 | 7 | 0 | 1 | 1 | 1 |  | 16958.5273 | type:5prime\_partial len:150 gc:universal TRINITY\_DN128406\_c0\_g1\_i1:474-25(-) |
| 125 | 267 | TRINITY\_DN564\_c0\_g1\_i1.p1 | 49.64 | 3 | 3 | 0 | 1 | 1 | 1 |  | 62932.8672 | type:complete len:563 gc:universal TRINITY\_DN564\_c0\_g1\_i1:2018-330(-) |
| 126 | 268 | TRINITY\_DN14015\_c0\_g1\_i3.p1 | 48.8 | 1 | 1 | 8.75e2 | 1 | 1 | 1 |  | 284708.625 | type:5prime\_partial len:2566 gc:universal TRINITY\_DN14015\_c0\_g1\_i3:2-7699(+) |
| 126 | 269 | TRINITY\_DN14015\_c0\_g1\_i2.p1 | 48.8 | 1 | 1 | 8.75e2 | 1 | 1 | 1 |  | 287707.9062 | type:5prime\_partial len:2592 gc:universal TRINITY\_DN14015\_c0\_g1\_i2:2-7777(+) |
| 127 | 270 | TRINITY\_DN4781\_c0\_g1\_i3.p3 | 56.16 | 7 | 7 | 2.6e2 | 1 | 1 | 1 |  | 20029.2422 | type:complete len:183 gc:universal TRINITY\_DN4781\_c0\_g1\_i3:2965-2417(-) |
| 128 | 271 | TRINITY\_DN3052\_c0\_g1\_i1.p1 | 55.07 | 2 | 2 | 0 | 1 | 1 | 1 | Acetylation (Protein N-term) | 39261.4453 | type:complete len:362 gc:universal TRINITY\_DN3052\_c0\_g1\_i1:45-1130(+) |
| 129 | 272 | TRINITY\_DN5397\_c0\_g1\_i1.p1 | 49.28 | 12 | 12 | 1.95e2 | 1 | 1 | 1 | Carbamidomethylation | 17679.4688 | type:internal len:161 gc:universal TRINITY\_DN5397\_c0\_g1\_i1:2-481(+) |
| 130 | 273 | TRINITY\_DN247\_c0\_g1\_i1.p1 | 49.41 | 3 | 3 | 1.18e3 | 1 | 1 | 1 |  | 42079.6172 | type:complete len:373 gc:universal TRINITY\_DN247\_c0\_g1\_i1:38-1156(+) |
| 131 | 274 | TRINITY\_DN5153\_c0\_g1\_i1.p1 | 46.98 | 2 | 2 | 0 | 1 | 1 | 1 |  | 98168.6016 | type:complete len:862 gc:universal TRINITY\_DN5153\_c0\_g1\_i1:61-2646(+) |
| total 274 proteins |  |  |  |  |  |  |  |  |  |  |  |  |
| --- | --- | --- | --- | --- | --- | --- | --- | --- | --- | --- | --- | --- |

  

TRINITY\_DN101992\_c0\_g1\_i1.p1

back to list

  

| Protein Coverage
| Supporting Peptides
| Best Unique PSM
|

Protein Coverage:

Supporting Peptides:

| Peptide | Uniq | -10lgP | Mass | Length | ppm | m/z | z | RT | 1/k0 Start | 1/k0 End | Precursor ID | Area Sample 7 | #Feature | #Feature Sample 7 | Start | End | PTM | AScore | Found By |
| --- | --- | --- | --- | --- | --- | --- | --- | --- | --- | --- | --- | --- | --- | --- | --- | --- | --- | --- | --- |
| K.AELVFVNHC(+57.02)R.E | Y | 55.56 | 1243.6132 | 10 | 1.33 | 622.8118 | 2 | 14.50 | 0.9602 | 0.9675 | 40700 | 1.63e3 | 1 | 1 | 118 | 127 | Carbamidomethylation |  | DB Search |
| total 1 peptides |  |  |  |  |  |  |  |  |  |  |  |  |  |  |  |  |  |  |  |
| --- | --- | --- | --- | --- | --- | --- | --- | --- | --- | --- | --- | --- | --- | --- | --- | --- | --- | --- | --- |

Best Unique PSM (Scan Sample\_5\_A1\_1\_3643.d:40700, m/z=622.8118, z=2, RT=14.50, ppm=-3.35):


TRINITY\_DN167\_c0\_g1\_i6.p1

back to list

  

| Protein Coverage
| Supporting Peptides
| Best Unique PSM
|

Protein Coverage:

Supporting Peptides:

| Peptide | Uniq | -10lgP | Mass | Length | ppm | m/z | z | RT | 1/k0 Start | 1/k0 End | Precursor ID | Area Sample 7 | #Feature | #Feature Sample 7 | Start | End | PTM | AScore | Found By |
| --- | --- | --- | --- | --- | --- | --- | --- | --- | --- | --- | --- | --- | --- | --- | --- | --- | --- | --- | --- |
| K.STAGDTHLGGEDFDNR.L | Y | 56.39 | 1690.7183 | 16 | 2.27 | 564.5787 | 3 | 13.29 | 0.8014 | 0.8088 | 33297 | 4.2e3 | 2 | 2 | 107 | 122 |  |  | DB Search |
| total 1 peptides |  |  |  |  |  |  |  |  |  |  |  |  |  |  |  |  |  |  |  |
| --- | --- | --- | --- | --- | --- | --- | --- | --- | --- | --- | --- | --- | --- | --- | --- | --- | --- | --- | --- |

Best Unique PSM (Scan Sample\_5\_A1\_1\_3643.d:33297, m/z=564.5787, z=3, RT=13.29, ppm=-2.41):


TRINITY\_DN11522\_c0\_g1\_i4.p1

back to list

  

| Protein Coverage
| Supporting Peptides
| Best Unique PSM
|

Protein Coverage:

Supporting Peptides:

| Peptide | Uniq | -10lgP | Mass | Length | ppm | m/z | z | RT | 1/k0 Start | 1/k0 End | Precursor ID | Area Sample 7 | #Feature | #Feature Sample 7 | Start | End | PTM | AScore | Found By |
| --- | --- | --- | --- | --- | --- | --- | --- | --- | --- | --- | --- | --- | --- | --- | --- | --- | --- | --- | --- |
| R.IGLGNIRDPSISLTR.G | Y | 55.35 | 1610.9104 | 15 | -3.69 | 537.9729 | 3 | 17.37 | 0.7986 | 0.8059 | 57304 | 1.32e3 | 1 | 1 | 27 | 41 |  |  | DB Search |
| total 1 peptides |  |  |  |  |  |  |  |  |  |  |  |  |  |  |  |  |  |  |  |
| --- | --- | --- | --- | --- | --- | --- | --- | --- | --- | --- | --- | --- | --- | --- | --- | --- | --- | --- | --- |

Best Unique PSM (Scan Sample\_5\_A1\_1\_3643.d:57304, m/z=537.9729, z=3, RT=17.37, ppm=-8.38):


TRINITY\_DN97809\_c0\_g1\_i1.p1

back to list

  

| Protein Coverage
| Supporting Peptides
| Best Unique PSM
|

Protein Coverage:

Supporting Peptides:

| Peptide | Uniq | -10lgP | Mass | Length | ppm | m/z | z | RT | 1/k0 Start | 1/k0 End | Precursor ID | Area Sample 7 | #Feature | #Feature Sample 7 | Start | End | PTM | AScore | Found By |
| --- | --- | --- | --- | --- | --- | --- | --- | --- | --- | --- | --- | --- | --- | --- | --- | --- | --- | --- | --- |
| LGPDQVDVAGAGR.D | Y | 51.28 | 1253.6364 | 13 | 6.6 | 627.8267 | 2 | 13.74 | 0.9344 | 0.9416 | 35866 | 6.27e3 | 2 | 2 | 1 | 13 |  |  | DB Search |
| total 1 peptides |  |  |  |  |  |  |  |  |  |  |  |  |  |  |  |  |  |  |  |
| --- | --- | --- | --- | --- | --- | --- | --- | --- | --- | --- | --- | --- | --- | --- | --- | --- | --- | --- | --- |

Best Unique PSM (Scan Sample\_5\_A1\_1\_3643.d:35866, m/z=627.8267, z=2, RT=13.74, ppm=1.91):


TRINITY\_DN3448\_c0\_g1\_i5.p2

back to list

  

| Protein Coverage
| Supporting Peptides
| Best Unique PSM
|

Protein Coverage:

Supporting Peptides:

| Peptide | Uniq | -10lgP | Mass | Length | ppm | m/z | z | RT | 1/k0 Start | 1/k0 End | Precursor ID | Area Sample 7 | #Feature | #Feature Sample 7 | Start | End | PTM | AScore | Found By |
| --- | --- | --- | --- | --- | --- | --- | --- | --- | --- | --- | --- | --- | --- | --- | --- | --- | --- | --- | --- |
| K.AGM(+15.99)THIVR.D | Y | 54.97 | 899.4647 | 8 | 4.88 | 450.7397 | 2 | 9.72 | 0.8144 | 0.8218 | 12487 | 9.73e2 | 1 | 1 | 27 | 34 | Oxidation (M) | M3:Oxidation (M):1000 | DB Search |
| total 1 peptides |  |  |  |  |  |  |  |  |  |  |  |  |  |  |  |  |  |  |  |
| --- | --- | --- | --- | --- | --- | --- | --- | --- | --- | --- | --- | --- | --- | --- | --- | --- | --- | --- | --- |

Best Unique PSM (Scan Sample\_5\_A1\_1\_3643.d:12487, m/z=450.7397, z=2, RT=9.72, ppm=0.2):


TRINITY\_DN773\_c0\_g1\_i1.p1

back to list

  

| Protein Coverage
| Supporting Peptides
| Best Unique PSM
|

Protein Coverage:

Supporting Peptides:

| Peptide | Uniq | -10lgP | Mass | Length | ppm | m/z | z | RT | 1/k0 Start | 1/k0 End | Precursor ID | Area Sample 7 | #Feature | #Feature Sample 7 | Start | End | PTM | AScore | Found By |
| --- | --- | --- | --- | --- | --- | --- | --- | --- | --- | --- | --- | --- | --- | --- | --- | --- | --- | --- | --- |
| R.GVSEETTTGVHNLYK.M | Y | 54.51 | 1633.7947 | 15 | -1.65 | 817.8995 | 2 | 13.70 | 1.0701 | 1.0773 | 35859 | 0 | 0 | 0 | 160 | 174 |  |  | DB Search |
| total 1 peptides |  |  |  |  |  |  |  |  |  |  |  |  |  |  |  |  |  |  |  |
| --- | --- | --- | --- | --- | --- | --- | --- | --- | --- | --- | --- | --- | --- | --- | --- | --- | --- | --- | --- |

Best Unique PSM (Scan Sample\_5\_A1\_1\_3643.d:35859, m/z=817.8995, z=2, RT=13.70, ppm=-6.33):


TRINITY\_DN319\_c2\_g1\_i2.p1

back to list

  

| Protein Coverage
| Supporting Peptides
| Best Unique PSM
|

Protein Coverage:

Supporting Peptides:

| Peptide | Uniq | -10lgP | Mass | Length | ppm | m/z | z | RT | 1/k0 Start | 1/k0 End | Precursor ID | Area Sample 7 | #Feature | #Feature Sample 7 | Start | End | PTM | AScore | Found By |
| --- | --- | --- | --- | --- | --- | --- | --- | --- | --- | --- | --- | --- | --- | --- | --- | --- | --- | --- | --- |
| R.GSFSSHIC(+57.02)LC(+57.02)HDPAYK.S | Y | 56.38 | 1877.8189 | 16 | 2.34 | 626.9454 | 3 | 14.46 | 0.8866 | 0.8939 | 40485 | 0 | 0 | 0 | 1011 | 1026 | Carbamidomethylation, Carbamidomethylation |  | DB Search |
| R.DPIC(+57.02)HC(+57.02)DTSK.Y | Y | 54.33 | 1231.4961 | 10 | 7.69 | 616.7572 | 2 | 9.95 | 0.8951 | 0.9024 | 13586 | 0 | 0 | 0 | 1448 | 1457 | Carbamidomethylation, Carbamidomethylation |  | DB Search |
| total 2 peptides |  |  |  |  |  |  |  |  |  |  |  |  |  |  |  |  |  |  |  |
| --- | --- | --- | --- | --- | --- | --- | --- | --- | --- | --- | --- | --- | --- | --- | --- | --- | --- | --- | --- |

Best Unique PSM (Scan Sample\_5\_A1\_1\_3643.d:40485, m/z=626.9454, z=3, RT=14.46, ppm=-2.34):


TRINITY\_DN1377\_c0\_g1\_i1.p1

back to list

  

| Protein Coverage
| Supporting Peptides
| Best Unique PSM
|

Protein Coverage:

Supporting Peptides:

| Peptide | Uniq | -10lgP | Mass | Length | ppm | m/z | z | RT | 1/k0 Start | 1/k0 End | Precursor ID | Area Sample 7 | #Feature | #Feature Sample 7 | Start | End | PTM | AScore | Found By |
| --- | --- | --- | --- | --- | --- | --- | --- | --- | --- | --- | --- | --- | --- | --- | --- | --- | --- | --- | --- |
| R.ITQQQSHQEIADQLR.V | Y | 49.73 | 1793.902 | 15 | 3.55 | 598.9739 | 3 | 13.30 | 0.8449 | 0.8522 | 33399 | 5.82e2 | 1 | 1 | 678 | 692 |  |  | DB Search |
| total 1 peptides |  |  |  |  |  |  |  |  |  |  |  |  |  |  |  |  |  |  |  |
| --- | --- | --- | --- | --- | --- | --- | --- | --- | --- | --- | --- | --- | --- | --- | --- | --- | --- | --- | --- |

Best Unique PSM (Scan Sample\_5\_A1\_1\_3643.d:33399, m/z=598.9739, z=3, RT=13.30, ppm=-1.13):


TRINITY\_DN982\_c0\_g1\_i2.p1

back to list

  

| Protein Coverage
| Supporting Peptides
| Best Unique PSM
|

Protein Coverage:

Supporting Peptides:

| Peptide | Uniq | -10lgP | Mass | Length | ppm | m/z | z | RT | 1/k0 Start | 1/k0 End | Precursor ID | Area Sample 7 | #Feature | #Feature Sample 7 | Start | End | PTM | AScore | Found By |
| --- | --- | --- | --- | --- | --- | --- | --- | --- | --- | --- | --- | --- | --- | --- | --- | --- | --- | --- | --- |
| K.INNNQFSVVLIK.N | Y | 56.39 | 1387.7823 | 12 | 0.37 | 694.8954 | 2 | 18.30 | 0.9945 | 1.0017 | 63702 | 4.8e2 | 1 | 1 | 100 | 111 |  |  | DB Search |
| K.VLLSQQIFYDKK.N | N | 55.36 | 1480.8289 | 12 | 1.76 | 494.6155 | 3 | 16.92 | 0.7616 | 0.7689 | 54594 | 7.01e3 | 2 | 2 | 1479 | 1490 |  |  | DB Search |
| K.VLEVNDNKPK.F | Y | 55.29 | 1154.6295 | 10 | 3.29 | 578.3212 | 2 | 11.02 | 0.9255 | 0.9327 | 19462 | 6.22e2 | 1 | 1 | 459 | 468 |  |  | DB Search |
| K.ELEITVLESKPVNSFVIR.V | Y | 55.28 | 2072.1517 | 18 | 0.84 | 691.7219 | 3 | 19.88 | 0.9634 | 0.9707 | 74309 | 3.41e2 | 1 | 1 | 368 | 385 |  |  | DB Search |
| R.VLDVNDKQPVFVK.S | Y | 53.63 | 1499.8347 | 13 | 1.56 | 750.9223 | 2 | 15.14 | 1.052 | 1.0592 | 44491 | 0 | 0 | 0 | 670 | 682 |  |  | DB Search |
| total 5 peptides |  |  |  |  |  |  |  |  |  |  |  |  |  |  |  |  |  |  |  |
| --- | --- | --- | --- | --- | --- | --- | --- | --- | --- | --- | --- | --- | --- | --- | --- | --- | --- | --- | --- |

Best Unique PSM (Scan Sample\_5\_A1\_1\_3643.d:63702, m/z=694.8954, z=2, RT=18.30, ppm=-4.31):


TRINITY\_DN413\_c0\_g1\_i3.p1

back to list

  

| Protein Coverage
| Supporting Peptides
| Best Unique PSM
|

Protein Coverage:

Supporting Peptides:

| Peptide | Uniq | -10lgP | Mass | Length | ppm | m/z | z | RT | 1/k0 Start | 1/k0 End | Precursor ID | Area Sample 7 | #Feature | #Feature Sample 7 | Start | End | PTM | AScore | Found By |
| --- | --- | --- | --- | --- | --- | --- | --- | --- | --- | --- | --- | --- | --- | --- | --- | --- | --- | --- | --- |
| R.ADILC(+57.02)HDTK.Y | Y | 55.33 | 1071.5019 | 9 | 3.94 | 536.7578 | 2 | 11.82 | 0.881 | 0.8883 | 24181 | 1.06e3 | 1 | 1 | 320 | 328 | Carbamidomethylation |  | DB Search |
| R.LLLGDFNGDKR.T | Y | 55.25 | 1246.6669 | 11 | -0.59 | 624.3375 | 2 | 16.23 | 0.9856 | 0.9928 | 50739 | 5.27e2 | 1 | 1 | 46 | 56 |  |  | DB Search |
| total 2 peptides |  |  |  |  |  |  |  |  |  |  |  |  |  |  |  |  |  |  |  |
| --- | --- | --- | --- | --- | --- | --- | --- | --- | --- | --- | --- | --- | --- | --- | --- | --- | --- | --- | --- |

Best Unique PSM (Scan Sample\_5\_A1\_1\_3643.d:24181, m/z=536.7578, z=2, RT=11.82, ppm=-0.75):


TRINITY\_DN12627\_c0\_g1\_i1.p1

back to list

  

| Protein Coverage
| Supporting Peptides
| Best Unique PSM
|

Protein Coverage:

Supporting Peptides:

| Peptide | Uniq | -10lgP | Mass | Length | ppm | m/z | z | RT | 1/k0 Start | 1/k0 End | Precursor ID | Area Sample 7 | #Feature | #Feature Sample 7 | Start | End | PTM | AScore | Found By |
| --- | --- | --- | --- | --- | --- | --- | --- | --- | --- | --- | --- | --- | --- | --- | --- | --- | --- | --- | --- |
| K.GFGGNLVTITNSDIQK.Q | Y | 51.11 | 1662.8577 | 16 | 0.85 | 832.4329 | 2 | 17.75 | 1.0813 | 1.0885 | 60042 | 0 | 0 | 0 | 311 | 326 |  |  | DB Search |
| total 1 peptides |  |  |  |  |  |  |  |  |  |  |  |  |  |  |  |  |  |  |  |
| --- | --- | --- | --- | --- | --- | --- | --- | --- | --- | --- | --- | --- | --- | --- | --- | --- | --- | --- | --- |

Best Unique PSM (Scan Sample\_5\_A1\_1\_3643.d:60042, m/z=832.4329, z=2, RT=17.75, ppm=-3.83):


TRINITY\_DN413\_c0\_g1\_i1.p1

back to list

  

| Protein Coverage
| Supporting Peptides
| Best Unique PSM
|

Protein Coverage:

Supporting Peptides:

| Peptide | Uniq | -10lgP | Mass | Length | ppm | m/z | z | RT | 1/k0 Start | 1/k0 End | Precursor ID | Area Sample 7 | #Feature | #Feature Sample 7 | Start | End | PTM | AScore | Found By |
| --- | --- | --- | --- | --- | --- | --- | --- | --- | --- | --- | --- | --- | --- | --- | --- | --- | --- | --- | --- |
| R.ADILC(+57.02)HDTK.Y | Y | 55.33 | 1071.5019 | 9 | 3.94 | 536.7578 | 2 | 11.82 | 0.881 | 0.8883 | 24181 | 1.06e3 | 1 | 1 | 329 | 337 | Carbamidomethylation |  | DB Search |
| R.LLLGDFNGDKR.T | Y | 55.25 | 1246.6669 | 11 | -0.59 | 624.3375 | 2 | 16.23 | 0.9856 | 0.9928 | 50739 | 5.27e2 | 1 | 1 | 46 | 56 |  |  | DB Search |
| total 2 peptides |  |  |  |  |  |  |  |  |  |  |  |  |  |  |  |  |  |  |  |
| --- | --- | --- | --- | --- | --- | --- | --- | --- | --- | --- | --- | --- | --- | --- | --- | --- | --- | --- | --- |

Best Unique PSM (Scan Sample\_5\_A1\_1\_3643.d:24181, m/z=536.7578, z=2, RT=11.82, ppm=-0.75):


TRINITY\_DN3448\_c0\_g1\_i7.p2

back to list

  

| Protein Coverage
| Supporting Peptides
| Best Unique PSM
|

Protein Coverage:

Supporting Peptides:

| Peptide | Uniq | -10lgP | Mass | Length | ppm | m/z | z | RT | 1/k0 Start | 1/k0 End | Precursor ID | Area Sample 7 | #Feature | #Feature Sample 7 | Start | End | PTM | AScore | Found By |
| --- | --- | --- | --- | --- | --- | --- | --- | --- | --- | --- | --- | --- | --- | --- | --- | --- | --- | --- | --- |
| K.AGM(+15.99)THIVR.D | Y | 54.97 | 899.4647 | 8 | 4.88 | 450.7397 | 2 | 9.72 | 0.8144 | 0.8218 | 12487 | 9.73e2 | 1 | 1 | 27 | 34 | Oxidation (M) | M3:Oxidation (M):1000 | DB Search |
| total 1 peptides |  |  |  |  |  |  |  |  |  |  |  |  |  |  |  |  |  |  |  |
| --- | --- | --- | --- | --- | --- | --- | --- | --- | --- | --- | --- | --- | --- | --- | --- | --- | --- | --- | --- |

Best Unique PSM (Scan Sample\_5\_A1\_1\_3643.d:12487, m/z=450.7397, z=2, RT=9.72, ppm=0.2):


TRINITY\_DN11522\_c0\_g1\_i1.p1

back to list

  

| Protein Coverage
| Supporting Peptides
| Best Unique PSM
|

Protein Coverage:

Supporting Peptides:

| Peptide | Uniq | -10lgP | Mass | Length | ppm | m/z | z | RT | 1/k0 Start | 1/k0 End | Precursor ID | Area Sample 7 | #Feature | #Feature Sample 7 | Start | End | PTM | AScore | Found By |
| --- | --- | --- | --- | --- | --- | --- | --- | --- | --- | --- | --- | --- | --- | --- | --- | --- | --- | --- | --- |
| R.IGLGNIRDPSISLTR.G | Y | 55.35 | 1610.9104 | 15 | -3.69 | 537.9729 | 3 | 17.37 | 0.7986 | 0.8059 | 57304 | 1.32e3 | 1 | 1 | 25 | 39 |  |  | DB Search |
| total 1 peptides |  |  |  |  |  |  |  |  |  |  |  |  |  |  |  |  |  |  |  |
| --- | --- | --- | --- | --- | --- | --- | --- | --- | --- | --- | --- | --- | --- | --- | --- | --- | --- | --- | --- |

Best Unique PSM (Scan Sample\_5\_A1\_1\_3643.d:57304, m/z=537.9729, z=3, RT=17.37, ppm=-8.38):


TRINITY\_DN775\_c0\_g1\_i2.p1

back to list

  

| Protein Coverage
| Supporting Peptides
| Best Unique PSM
|

Protein Coverage:

Supporting Peptides:

| Peptide | Uniq | -10lgP | Mass | Length | ppm | m/z | z | RT | 1/k0 Start | 1/k0 End | Precursor ID | Area Sample 7 | #Feature | #Feature Sample 7 | Start | End | PTM | AScore | Found By |
| --- | --- | --- | --- | --- | --- | --- | --- | --- | --- | --- | --- | --- | --- | --- | --- | --- | --- | --- | --- |
| K.DKELSNVIESGFR.T | Y | 56.35 | 1492.7521 | 13 | 2.74 | 747.3819 | 2 | 17.35 | 1.0464 | 1.0536 | 57241 | 4.08e2 | 1 | 1 | 489 | 501 |  |  | DB Search |
| total 1 peptides |  |  |  |  |  |  |  |  |  |  |  |  |  |  |  |  |  |  |  |
| --- | --- | --- | --- | --- | --- | --- | --- | --- | --- | --- | --- | --- | --- | --- | --- | --- | --- | --- | --- |

Best Unique PSM (Scan Sample\_5\_A1\_1\_3643.d:57241, m/z=747.3819, z=2, RT=17.35, ppm=-1.94):


TRINITY\_DN319\_c2\_g1\_i1.p1

back to list

  

| Protein Coverage
| Supporting Peptides
| Best Unique PSM
|

Protein Coverage:

Supporting Peptides:

| Peptide | Uniq | -10lgP | Mass | Length | ppm | m/z | z | RT | 1/k0 Start | 1/k0 End | Precursor ID | Area Sample 7 | #Feature | #Feature Sample 7 | Start | End | PTM | AScore | Found By |
| --- | --- | --- | --- | --- | --- | --- | --- | --- | --- | --- | --- | --- | --- | --- | --- | --- | --- | --- | --- |
| R.GSFSSHIC(+57.02)LC(+57.02)HDPAYK.S | Y | 56.38 | 1877.8189 | 16 | 2.34 | 626.9454 | 3 | 14.46 | 0.8866 | 0.8939 | 40485 | 0 | 0 | 0 | 1011 | 1026 | Carbamidomethylation, Carbamidomethylation |  | DB Search |
| R.DPIC(+57.02)HC(+57.02)DTSK.Y | Y | 54.33 | 1231.4961 | 10 | 7.69 | 616.7572 | 2 | 9.95 | 0.8951 | 0.9024 | 13586 | 0 | 0 | 0 | 1448 | 1457 | Carbamidomethylation, Carbamidomethylation |  | DB Search |
| K.KAQYYEAYETIIKR.H | Y | 48.69 | 1774.9254 | 14 | 2.8 | 592.6479 | 3 | 15.92 | 0.8656 | 0.8729 | 49007 | 1.65e2 | 1 | 1 | 2534 | 2547 |  |  | DB Search |
| total 3 peptides |  |  |  |  |  |  |  |  |  |  |  |  |  |  |  |  |  |  |  |
| --- | --- | --- | --- | --- | --- | --- | --- | --- | --- | --- | --- | --- | --- | --- | --- | --- | --- | --- | --- |

Best Unique PSM (Scan Sample\_5\_A1\_1\_3643.d:40485, m/z=626.9454, z=3, RT=14.46, ppm=-2.34):


TRINITY\_DN649\_c0\_g1\_i2.p1

back to list

  

| Protein Coverage
| Supporting Peptides
| Best Unique PSM
|

Protein Coverage:

Supporting Peptides:

| Peptide | Uniq | -10lgP | Mass | Length | ppm | m/z | z | RT | 1/k0 Start | 1/k0 End | Precursor ID | Area Sample 7 | #Feature | #Feature Sample 7 | Start | End | PTM | AScore | Found By |
| --- | --- | --- | --- | --- | --- | --- | --- | --- | --- | --- | --- | --- | --- | --- | --- | --- | --- | --- | --- |
| K.FETQIATK.N | Y | 53.77 | 936.4916 | 8 | 12.43 | 937.5062 | 1 | 13.97 | 1.1614 | 1.1686 | 37568 | 0 | 0 | 0 | 103 | 110 |  |  | DB Search |
| total 1 peptides |  |  |  |  |  |  |  |  |  |  |  |  |  |  |  |  |  |  |  |
| --- | --- | --- | --- | --- | --- | --- | --- | --- | --- | --- | --- | --- | --- | --- | --- | --- | --- | --- | --- |

Best Unique PSM (Scan Sample\_5\_A1\_1\_3643.d:37568, m/z=937.5062, z=1, RT=13.97, ppm=7.75):


TRINITY\_DN413\_c0\_g1\_i4.p1

back to list

  

| Protein Coverage
| Supporting Peptides
| Best Unique PSM
|

Protein Coverage:

Supporting Peptides:

| Peptide | Uniq | -10lgP | Mass | Length | ppm | m/z | z | RT | 1/k0 Start | 1/k0 End | Precursor ID | Area Sample 7 | #Feature | #Feature Sample 7 | Start | End | PTM | AScore | Found By |
| --- | --- | --- | --- | --- | --- | --- | --- | --- | --- | --- | --- | --- | --- | --- | --- | --- | --- | --- | --- |
| R.ADILC(+57.02)HDTK.Y | Y | 55.33 | 1071.5019 | 9 | 3.94 | 536.7578 | 2 | 11.82 | 0.881 | 0.8883 | 24181 | 1.06e3 | 1 | 1 | 320 | 328 | Carbamidomethylation |  | DB Search |
| R.LLLGDFNGDKR.T | Y | 55.25 | 1246.6669 | 11 | -0.59 | 624.3375 | 2 | 16.23 | 0.9856 | 0.9928 | 50739 | 5.27e2 | 1 | 1 | 46 | 56 |  |  | DB Search |
| total 2 peptides |  |  |  |  |  |  |  |  |  |  |  |  |  |  |  |  |  |  |  |
| --- | --- | --- | --- | --- | --- | --- | --- | --- | --- | --- | --- | --- | --- | --- | --- | --- | --- | --- | --- |

Best Unique PSM (Scan Sample\_5\_A1\_1\_3643.d:24181, m/z=536.7578, z=2, RT=11.82, ppm=-0.75):


TRINITY\_DN4595\_c0\_g1\_i4.p1

back to list

  

| Protein Coverage
| Supporting Peptides
| Best Unique PSM
|

Protein Coverage:

Supporting Peptides:

| Peptide | Uniq | -10lgP | Mass | Length | ppm | m/z | z | RT | 1/k0 Start | 1/k0 End | Precursor ID | Area Sample 7 | #Feature | #Feature Sample 7 | Start | End | PTM | AScore | Found By |
| --- | --- | --- | --- | --- | --- | --- | --- | --- | --- | --- | --- | --- | --- | --- | --- | --- | --- | --- | --- |
| R.VC(+57.02)NGEPC(+57.02)ILQER.S | Y | 55.34 | 1473.6704 | 12 | 3.33 | 737.8415 | 2 | 14.98 | 0.9896 | 0.9969 | 43535 | 0 | 0 | 0 | 436 | 447 | Carbamidomethylation, Carbamidomethylation |  | DB Search |
| K.YQGNDYTR.N | Y | 55.1 | 1015.4359 | 8 | 1.65 | 508.7237 | 2 | 10.77 | 0.8469 | 0.8542 | 17934 | 1.34e3 | 1 | 1 | 22 | 29 |  |  | DB Search |
| R.SPFVIMFK.G | Y | 54.61 | 967.5201 | 8 | 4.71 | 484.7673 | 2 | 19.89 | 0.81 | 0.8173 | 74398 | 6.85e2 | 1 | 1 | 1081 | 1088 |  |  | DB Search |
| R.YQGREYNINEEVNVR.Q | Y | 53.45 | 1881.8969 | 15 | -4.84 | 941.9468 | 2 | 15.64 | 1.1442 | 1.1514 | 47466 | 0 | 0 | 0 | 338 | 352 |  |  | DB Search |
| R.FC(+57.02)NNPAPSNGGR.M | Y | 51.44 | 1289.5571 | 12 | 4.78 | 645.7859 | 2 | 10.87 | 0.944 | 0.9513 | 18646 | 0 | 0 | 0 | 1196 | 1207 | Carbamidomethylation |  | DB Search |
| R.AFNKIETR.N | Y | 49.77 | 977.5294 | 8 | 3.42 | 489.7714 | 2 | 11.52 | 0.8388 | 0.8461 | 22323 | 8.13e3 | 1 | 1 | 72 | 79 |  |  | DB Search |
| R.AFNKVETR.N | N | 49.58 | 963.5138 | 8 | 6.34 | 482.765 | 2 | 10.59 | 0.8266 | 0.8339 | 16901 | 3.56e3 | 1 | 1 | 177 | 184 |  |  | DB Search |
| K.YTGAC(+57.02)NANGNTDK.T | Y | 49.38 | 1384.5677 | 13 | 0 | 693.2879 | 2 | 9.54 | 0.9315 | 0.9388 | 11651 | 4.52e2 | 1 | 1 | 1492 | 1504 | Carbamidomethylation |  | DB Search |
| total 8 peptides |  |  |  |  |  |  |  |  |  |  |  |  |  |  |  |  |  |  |  |
| --- | --- | --- | --- | --- | --- | --- | --- | --- | --- | --- | --- | --- | --- | --- | --- | --- | --- | --- | --- |

Best Unique PSM (Scan Sample\_5\_A1\_1\_3643.d:43535, m/z=737.8415, z=2, RT=14.98, ppm=-1.35):


TRINITY\_DN72625\_c0\_g1\_i1.p1

back to list

  

| Protein Coverage
| Supporting Peptides
| Best Unique PSM
|

Protein Coverage:

Supporting Peptides:

| Peptide | Uniq | -10lgP | Mass | Length | ppm | m/z | z | RT | 1/k0 Start | 1/k0 End | Precursor ID | Area Sample 7 | #Feature | #Feature Sample 7 | Start | End | PTM | AScore | Found By |
| --- | --- | --- | --- | --- | --- | --- | --- | --- | --- | --- | --- | --- | --- | --- | --- | --- | --- | --- | --- |
| R.VKESYTLNIEAR.D | Y | 56.36 | 1421.7514 | 12 | -2.77 | 711.8777 | 2 | 14.75 | 1.0013 | 1.0086 | 42302 | 0 | 0 | 0 | 709 | 720 |  |  | DB Search |
| R.IKDTYLLTLEAR.D | Y | 55.35 | 1434.8082 | 12 | 3.07 | 718.4102 | 2 | 17.05 | 1.0379 | 1.0452 | 55307 | 1.14e3 | 1 | 1 | 393 | 404 |  |  | DB Search |
| R.KTTELTNNLK.A | Y | 55.18 | 1160.6401 | 10 | 0.91 | 581.3251 | 2 | 11.77 | 0.9182 | 0.9255 | 23835 | 9.14e2 | 1 | 1 | 1921 | 1930 |  |  | DB Search |
| total 3 peptides |  |  |  |  |  |  |  |  |  |  |  |  |  |  |  |  |  |  |  |
| --- | --- | --- | --- | --- | --- | --- | --- | --- | --- | --- | --- | --- | --- | --- | --- | --- | --- | --- | --- |

Best Unique PSM (Scan Sample\_5\_A1\_1\_3643.d:42302, m/z=710.3586, z=2, RT=14.77, ppm=-7.46):


TRINITY\_DN775\_c0\_g1\_i1.p1

back to list

  

| Protein Coverage
| Supporting Peptides
| Best Unique PSM
|

Protein Coverage:

Supporting Peptides:

| Peptide | Uniq | -10lgP | Mass | Length | ppm | m/z | z | RT | 1/k0 Start | 1/k0 End | Precursor ID | Area Sample 7 | #Feature | #Feature Sample 7 | Start | End | PTM | AScore | Found By |
| --- | --- | --- | --- | --- | --- | --- | --- | --- | --- | --- | --- | --- | --- | --- | --- | --- | --- | --- | --- |
| K.DKELSNVIESGFR.T | Y | 56.35 | 1492.7521 | 13 | 2.74 | 747.3819 | 2 | 17.35 | 1.0464 | 1.0536 | 57241 | 4.08e2 | 1 | 1 | 477 | 489 |  |  | DB Search |
| total 1 peptides |  |  |  |  |  |  |  |  |  |  |  |  |  |  |  |  |  |  |  |
| --- | --- | --- | --- | --- | --- | --- | --- | --- | --- | --- | --- | --- | --- | --- | --- | --- | --- | --- | --- |

Best Unique PSM (Scan Sample\_5\_A1\_1\_3643.d:57241, m/z=747.3819, z=2, RT=17.35, ppm=-1.94):


TRINITY\_DN79\_c0\_g1\_i1.p1

back to list

  

| Protein Coverage
| Supporting Peptides
| Best Unique PSM
|

Protein Coverage:

Supporting Peptides:

| Peptide | Uniq | -10lgP | Mass | Length | ppm | m/z | z | RT | 1/k0 Start | 1/k0 End | Precursor ID | Area Sample 7 | #Feature | #Feature Sample 7 | Start | End | PTM | AScore | Found By |
| --- | --- | --- | --- | --- | --- | --- | --- | --- | --- | --- | --- | --- | --- | --- | --- | --- | --- | --- | --- |
| R.AESIALVTEFTDEAAPK.I | Y | 56.17 | 1790.8938 | 17 | 5.03 | 896.4545 | 2 | 19.96 | 1.1194 | 1.1266 | 74593 | 1.16e3 | 1 | 1 | 192 | 208 |  |  | DB Search |
| total 1 peptides |  |  |  |  |  |  |  |  |  |  |  |  |  |  |  |  |  |  |  |
| --- | --- | --- | --- | --- | --- | --- | --- | --- | --- | --- | --- | --- | --- | --- | --- | --- | --- | --- | --- |

Best Unique PSM (Scan Sample\_5\_A1\_1\_3643.d:74593, m/z=896.4545, z=2, RT=19.96, ppm=0.35):


TRINITY\_DN585\_c0\_g1\_i2.p1

back to list

  

| Protein Coverage
| Supporting Peptides
| Best Unique PSM
|

Protein Coverage:

Supporting Peptides:

| Peptide | Uniq | -10lgP | Mass | Length | ppm | m/z | z | RT | 1/k0 Start | 1/k0 End | Precursor ID | Area Sample 7 | #Feature | #Feature Sample 7 | Start | End | PTM | AScore | Found By |
| --- | --- | --- | --- | --- | --- | --- | --- | --- | --- | --- | --- | --- | --- | --- | --- | --- | --- | --- | --- |
| K.TITLEVEPSDTIENVK.S | Y | 55.12 | 1786.92 | 16 | 0.46 | 894.4635 | 2 | 17.55 | 1.1414 | 1.1486 | 58604 | 8.72e2 | 1 | 1 | 60 | 75 |  |  | DB Search |
| total 1 peptides |  |  |  |  |  |  |  |  |  |  |  |  |  |  |  |  |  |  |  |
| --- | --- | --- | --- | --- | --- | --- | --- | --- | --- | --- | --- | --- | --- | --- | --- | --- | --- | --- | --- |

Best Unique PSM (Scan Sample\_5\_A1\_1\_3643.d:58604, m/z=894.4635, z=2, RT=17.55, ppm=-4.22):


TRINITY\_DN36\_c0\_g1\_i8.p1

back to list

  

| Protein Coverage
| Supporting Peptides
| Best Unique PSM
|

Protein Coverage:

Supporting Peptides:

| Peptide | Uniq | -10lgP | Mass | Length | ppm | m/z | z | RT | 1/k0 Start | 1/k0 End | Precursor ID | Area Sample 7 | #Feature | #Feature Sample 7 | Start | End | PTM | AScore | Found By |
| --- | --- | --- | --- | --- | --- | --- | --- | --- | --- | --- | --- | --- | --- | --- | --- | --- | --- | --- | --- |
| K.FETQIATK.D | Y | 53.77 | 936.4916 | 8 | 12.43 | 937.5062 | 1 | 13.97 | 1.1614 | 1.1686 | 37568 | 0 | 0 | 0 | 167 | 174 |  |  | DB Search |
| R.LGGIEVTQGNC(+57.02)AR.K | Y | 49.67 | 1373.6721 | 13 | 2.61 | 687.8419 | 2 | 13.89 | 0.9695 | 0.9767 | 37015 | 0 | 0 | 0 | 58 | 70 | Carbamidomethylation |  | DB Search |
| total 2 peptides |  |  |  |  |  |  |  |  |  |  |  |  |  |  |  |  |  |  |  |
| --- | --- | --- | --- | --- | --- | --- | --- | --- | --- | --- | --- | --- | --- | --- | --- | --- | --- | --- | --- |

Best Unique PSM (Scan Sample\_5\_A1\_1\_3643.d:37568, m/z=937.5062, z=1, RT=13.97, ppm=7.75):


TRINITY\_DN128406\_c0\_g1\_i1.p1

back to list

  

| Protein Coverage
| Supporting Peptides
| Best Unique PSM
|

Protein Coverage:

Supporting Peptides:

| Peptide | Uniq | -10lgP | Mass | Length | ppm | m/z | z | RT | 1/k0 Start | 1/k0 End | Precursor ID | Area Sample 7 | #Feature | #Feature Sample 7 | Start | End | PTM | AScore | Found By |
| --- | --- | --- | --- | --- | --- | --- | --- | --- | --- | --- | --- | --- | --- | --- | --- | --- | --- | --- | --- |
| K.EIRDILVQYDR.S | Y | 53.99 | 1418.7517 | 11 | -15.07 | 710.3691 | 2 | 19.87 | 1.0327 | 1.0399 | 74273 | 0 | 0 | 0 | 110 | 120 |  |  | DB Search |
| total 1 peptides |  |  |  |  |  |  |  |  |  |  |  |  |  |  |  |  |  |  |  |
| --- | --- | --- | --- | --- | --- | --- | --- | --- | --- | --- | --- | --- | --- | --- | --- | --- | --- | --- | --- |

Best Unique PSM (Scan Sample\_5\_A1\_1\_3643.d:74273, m/z=710.3691, z=2, RT=19.87, ppm=-19.75):


TRINITY\_DN2626\_c0\_g1\_i2.p1

back to list

  

| Protein Coverage
| Supporting Peptides
| Best Unique PSM
|

Protein Coverage:

Supporting Peptides:

| Peptide | Uniq | -10lgP | Mass | Length | ppm | m/z | z | RT | 1/k0 Start | 1/k0 End | Precursor ID | Area Sample 7 | #Feature | #Feature Sample 7 | Start | End | PTM | AScore | Found By |
| --- | --- | --- | --- | --- | --- | --- | --- | --- | --- | --- | --- | --- | --- | --- | --- | --- | --- | --- | --- |
| K.FIWGEVTELNGADR.T | Y | 50.99 | 1605.7787 | 14 | -4.61 | 803.8892 | 2 | 19.74 | 1.0431 | 1.0504 | 73266 | 4.98e2 | 1 | 1 | 98 | 111 |  |  | DB Search |
| total 1 peptides |  |  |  |  |  |  |  |  |  |  |  |  |  |  |  |  |  |  |  |
| --- | --- | --- | --- | --- | --- | --- | --- | --- | --- | --- | --- | --- | --- | --- | --- | --- | --- | --- | --- |

Best Unique PSM (Scan Sample\_5\_A1\_1\_3643.d:73266, m/z=803.8892, z=2, RT=19.74, ppm=-9.29):


TRINITY\_DN1616\_c0\_g1\_i6.p1

back to list

  

| Protein Coverage
| Supporting Peptides
| Best Unique PSM
|

Protein Coverage:

Supporting Peptides:

| Peptide | Uniq | -10lgP | Mass | Length | ppm | m/z | z | RT | 1/k0 Start | 1/k0 End | Precursor ID | Area Sample 7 | #Feature | #Feature Sample 7 | Start | End | PTM | AScore | Found By |
| --- | --- | --- | --- | --- | --- | --- | --- | --- | --- | --- | --- | --- | --- | --- | --- | --- | --- | --- | --- |
| K.VVDTEANDLTEEVKK.T | Y | 56.4 | 1688.8468 | 15 | 6.39 | 845.4321 | 2 | 14.15 | 1.1318 | 1.139 | 38615 | 0 | 0 | 0 | 2542 | 2556 |  |  | DB Search |
| K.SSSC(+57.02)QEFGGKC(+57.02)PC(+57.02)K.D | Y | 56.37 | 1630.6538 | 14 | -16.59 | 816.3168 | 2 | 12.09 | 1.0335 | 1.0407 | 25828 | 0 | 0 | 0 | 1387 | 1400 | Carbamidomethylation, Carbamidomethylation, Carbamidomethylation |  | DB Search |
| R.YGAVNDNC(+57.02)ER.D | Y | 56.34 | 1196.488 | 10 | 3.96 | 599.2509 | 2 | 10.89 | 0.885 | 0.8923 | 18607 | 1.07e3 | 1 | 1 | 498 | 507 | Carbamidomethylation |  | DB Search |
| K.TLDTVC(+57.02)DSGTGVC(+57.02)QC(+57.02)K.S | Y | 49.46 | 1799.7488 | 16 | -4.78 | 900.8732 | 2 | 13.15 | 1.0901 | 1.0973 | 32411 | 1.65e2 | 1 | 1 | 546 | 561 | Carbamidomethylation, Carbamidomethylation, Carbamidomethylation |  | DB Search |
| total 4 peptides |  |  |  |  |  |  |  |  |  |  |  |  |  |  |  |  |  |  |  |
| --- | --- | --- | --- | --- | --- | --- | --- | --- | --- | --- | --- | --- | --- | --- | --- | --- | --- | --- | --- |

Best Unique PSM (Scan Sample\_5\_A1\_1\_3643.d:38615, m/z=845.4321, z=2, RT=14.15, ppm=1.71):


TRINITY\_DN5987\_c0\_g1\_i4.p1

back to list

  

| Protein Coverage
| Supporting Peptides
| Best Unique PSM
|

Protein Coverage:

Supporting Peptides:

| Peptide | Uniq | -10lgP | Mass | Length | ppm | m/z | z | RT | 1/k0 Start | 1/k0 End | Precursor ID | Area Sample 7 | #Feature | #Feature Sample 7 | Start | End | PTM | AScore | Found By |
| --- | --- | --- | --- | --- | --- | --- | --- | --- | --- | --- | --- | --- | --- | --- | --- | --- | --- | --- | --- |
| R.GNQGEQGPAGPVGPK.G | Y | 55.37 | 1391.6793 | 15 | 2.49 | 696.8454 | 2 | 11.50 | 0.9469 | 0.9541 | 22230 | 5.82e3 | 1 | 1 | 237 | 251 |  |  | DB Search |
| K.GEPGEIGEQGK.Q | Y | 47.83 | 1099.5145 | 11 | 4.88 | 550.7646 | 2 | 11.17 | 0.855 | 0.8623 | 20334 | 0 | 0 | 0 | 177 | 187 |  |  | DB Search |
| total 2 peptides |  |  |  |  |  |  |  |  |  |  |  |  |  |  |  |  |  |  |  |
| --- | --- | --- | --- | --- | --- | --- | --- | --- | --- | --- | --- | --- | --- | --- | --- | --- | --- | --- | --- |

Best Unique PSM (Scan Sample\_5\_A1\_1\_3643.d:22230, m/z=696.8454, z=2, RT=11.50, ppm=-2.19):


TRINITY\_DN5397\_c0\_g1\_i1.p1

back to list

  

| Protein Coverage
| Supporting Peptides
| Best Unique PSM
|

Protein Coverage:

Supporting Peptides:

| Peptide | Uniq | -10lgP | Mass | Length | ppm | m/z | z | RT | 1/k0 Start | 1/k0 End | Precursor ID | Area Sample 7 | #Feature | #Feature Sample 7 | Start | End | PTM | AScore | Found By |
| --- | --- | --- | --- | --- | --- | --- | --- | --- | --- | --- | --- | --- | --- | --- | --- | --- | --- | --- | --- |
| R.EGSTPC(+57.02)NTQAC(+57.02)GSGLSEER.F | Y | 49.28 | 2038.832 | 19 | 5.27 | 1020.4239 | 2 | 12.12 | 1.1354 | 1.1426 | 25919 | 1.95e2 | 1 | 1 | 41 | 59 | Carbamidomethylation, Carbamidomethylation |  | DB Search |
| total 1 peptides |  |  |  |  |  |  |  |  |  |  |  |  |  |  |  |  |  |  |  |
| --- | --- | --- | --- | --- | --- | --- | --- | --- | --- | --- | --- | --- | --- | --- | --- | --- | --- | --- | --- |

Best Unique PSM (Scan Sample\_5\_A1\_1\_3643.d:25919, m/z=1020.4239, z=2, RT=12.12, ppm=0.59):


TRINITY\_DN564\_c0\_g1\_i1.p1

back to list

  

| Protein Coverage
| Supporting Peptides
| Best Unique PSM
|

Protein Coverage:

Supporting Peptides:

| Peptide | Uniq | -10lgP | Mass | Length | ppm | m/z | z | RT | 1/k0 Start | 1/k0 End | Precursor ID | Area Sample 7 | #Feature | #Feature Sample 7 | Start | End | PTM | AScore | Found By |
| --- | --- | --- | --- | --- | --- | --- | --- | --- | --- | --- | --- | --- | --- | --- | --- | --- | --- | --- | --- |
| R.EAIVQGAQSTQDTLKK.A | Y | 49.64 | 1715.9053 | 16 | 10.53 | 858.965 | 2 | 12.81 | 1.1522 | 1.1594 | 30342 | 0 | 0 | 0 | 301 | 316 |  |  | DB Search |
| total 1 peptides |  |  |  |  |  |  |  |  |  |  |  |  |  |  |  |  |  |  |  |
| --- | --- | --- | --- | --- | --- | --- | --- | --- | --- | --- | --- | --- | --- | --- | --- | --- | --- | --- | --- |

Best Unique PSM (Scan Sample\_5\_A1\_1\_3643.d:30342, m/z=858.965, z=2, RT=12.81, ppm=5.85):


TRINITY\_DN1739\_c0\_g1\_i1.p1

back to list

  

| Protein Coverage
| Supporting Peptides
| Best Unique PSM
|

Protein Coverage:

Supporting Peptides:

| Peptide | Uniq | -10lgP | Mass | Length | ppm | m/z | z | RT | 1/k0 Start | 1/k0 End | Precursor ID | Area Sample 7 | #Feature | #Feature Sample 7 | Start | End | PTM | AScore | Found By |
| --- | --- | --- | --- | --- | --- | --- | --- | --- | --- | --- | --- | --- | --- | --- | --- | --- | --- | --- | --- |
| M.AITDNIKDILSVK.I | Y | 56.03 | 1428.8188 | 13 | -0.47 | 477.2777 | 3 | 19.32 | 0.7929 | 0.8002 | 70439 | 6.5e2 | 2 | 2 | 2 | 14 |  |  | DB Search |
| total 1 peptides |  |  |  |  |  |  |  |  |  |  |  |  |  |  |  |  |  |  |  |
| --- | --- | --- | --- | --- | --- | --- | --- | --- | --- | --- | --- | --- | --- | --- | --- | --- | --- | --- | --- |

Best Unique PSM (Scan Sample\_5\_A1\_1\_3643.d:70439, m/z=477.2777, z=3, RT=19.32, ppm=-5.15):


TRINITY\_DN65211\_c0\_g1\_i1.p1

back to list

  

| Protein Coverage
| Supporting Peptides
| Best Unique PSM
|

Protein Coverage:

Supporting Peptides:

| Peptide | Uniq | -10lgP | Mass | Length | ppm | m/z | z | RT | 1/k0 Start | 1/k0 End | Precursor ID | Area Sample 7 | #Feature | #Feature Sample 7 | Start | End | PTM | AScore | Found By |
| --- | --- | --- | --- | --- | --- | --- | --- | --- | --- | --- | --- | --- | --- | --- | --- | --- | --- | --- | --- |
| K.TQLIAHDKEVYDIAFSR.A | Y | 49.38 | 2005.0268 | 17 | 3.02 | 669.3484 | 3 | 16.77 | 0.9089 | 0.9162 | 53806 | 0 | 0 | 0 | 167 | 183 |  |  | DB Search |
| total 1 peptides |  |  |  |  |  |  |  |  |  |  |  |  |  |  |  |  |  |  |  |
| --- | --- | --- | --- | --- | --- | --- | --- | --- | --- | --- | --- | --- | --- | --- | --- | --- | --- | --- | --- |

Best Unique PSM (Scan Sample\_5\_A1\_1\_3643.d:53806, m/z=669.3484, z=3, RT=16.77, ppm=-1.66):


TRINITY\_DN585\_c0\_g2\_i1.p1

back to list

  

| Protein Coverage
| Supporting Peptides
| Best Unique PSM
|

Protein Coverage:

Supporting Peptides:

| Peptide | Uniq | -10lgP | Mass | Length | ppm | m/z | z | RT | 1/k0 Start | 1/k0 End | Precursor ID | Area Sample 7 | #Feature | #Feature Sample 7 | Start | End | PTM | AScore | Found By |
| --- | --- | --- | --- | --- | --- | --- | --- | --- | --- | --- | --- | --- | --- | --- | --- | --- | --- | --- | --- |
| K.TITLEVEPSDTIENVK.S | Y | 55.12 | 1786.92 | 16 | 0.46 | 894.4635 | 2 | 17.55 | 1.1414 | 1.1486 | 58604 | 8.72e2 | 1 | 1 | 5 | 20 |  |  | DB Search |
| total 1 peptides |  |  |  |  |  |  |  |  |  |  |  |  |  |  |  |  |  |  |  |
| --- | --- | --- | --- | --- | --- | --- | --- | --- | --- | --- | --- | --- | --- | --- | --- | --- | --- | --- | --- |

Best Unique PSM (Scan Sample\_5\_A1\_1\_3643.d:58604, m/z=894.4635, z=2, RT=17.55, ppm=-4.22):


TRINITY\_DN585\_c0\_g1\_i4.p1

back to list

  

| Protein Coverage
| Supporting Peptides
| Best Unique PSM
|

Protein Coverage:

Supporting Peptides:

| Peptide | Uniq | -10lgP | Mass | Length | ppm | m/z | z | RT | 1/k0 Start | 1/k0 End | Precursor ID | Area Sample 7 | #Feature | #Feature Sample 7 | Start | End | PTM | AScore | Found By |
| --- | --- | --- | --- | --- | --- | --- | --- | --- | --- | --- | --- | --- | --- | --- | --- | --- | --- | --- | --- |
| K.TITLEVEPSDTIENVK.S | Y | 55.12 | 1786.92 | 16 | 0.46 | 894.4635 | 2 | 17.55 | 1.1414 | 1.1486 | 58604 | 8.72e2 | 1 | 1 | 12 | 27 |  |  | DB Search |
| total 1 peptides |  |  |  |  |  |  |  |  |  |  |  |  |  |  |  |  |  |  |  |
| --- | --- | --- | --- | --- | --- | --- | --- | --- | --- | --- | --- | --- | --- | --- | --- | --- | --- | --- | --- |

Best Unique PSM (Scan Sample\_5\_A1\_1\_3643.d:58604, m/z=894.4635, z=2, RT=17.55, ppm=-4.22):


TRINITY\_DN761\_c0\_g1\_i3.p1

back to list

  

| Protein Coverage
| Supporting Peptides
| Best Unique PSM
|

Protein Coverage:

Supporting Peptides:

| Peptide | Uniq | -10lgP | Mass | Length | ppm | m/z | z | RT | 1/k0 Start | 1/k0 End | Precursor ID | Area Sample 7 | #Feature | #Feature Sample 7 | Start | End | PTM | AScore | Found By |
| --- | --- | --- | --- | --- | --- | --- | --- | --- | --- | --- | --- | --- | --- | --- | --- | --- | --- | --- | --- |
| K.TRIEDLETVISQTQDHR.H | Y | 51.48 | 2040.0236 | 17 | 6.93 | 681.0167 | 3 | 17.50 | 0.9638 | 0.9711 | 58233 | 3.05e2 | 1 | 1 | 275 | 291 |  |  | DB Search |
| total 1 peptides |  |  |  |  |  |  |  |  |  |  |  |  |  |  |  |  |  |  |  |
| --- | --- | --- | --- | --- | --- | --- | --- | --- | --- | --- | --- | --- | --- | --- | --- | --- | --- | --- | --- |

Best Unique PSM (Scan Sample\_5\_A1\_1\_3643.d:58233, m/z=681.0167, z=3, RT=17.50, ppm=2.25):


TRINITY\_DN3034\_c0\_g1\_i1.p1

back to list

  

| Protein Coverage
| Supporting Peptides
| Best Unique PSM
|

Protein Coverage:

Supporting Peptides:

| Peptide | Uniq | -10lgP | Mass | Length | ppm | m/z | z | RT | 1/k0 Start | 1/k0 End | Precursor ID | Area Sample 7 | #Feature | #Feature Sample 7 | Start | End | PTM | AScore | Found By |
| --- | --- | --- | --- | --- | --- | --- | --- | --- | --- | --- | --- | --- | --- | --- | --- | --- | --- | --- | --- |
| K.TPGLVEGTLILHQLR.C | Y | 49.43 | 1645.9515 | 15 | -9.67 | 549.6499 | 3 | 18.99 | 0.8631 | 0.8704 | 68418 | 0 | 0 | 0 | 671 | 685 |  |  | DB Search |
| total 1 peptides |  |  |  |  |  |  |  |  |  |  |  |  |  |  |  |  |  |  |  |
| --- | --- | --- | --- | --- | --- | --- | --- | --- | --- | --- | --- | --- | --- | --- | --- | --- | --- | --- | --- |

Best Unique PSM (Scan Sample\_5\_A1\_1\_3643.d:68418, m/z=549.6499, z=3, RT=18.99, ppm=-14.35):


TRINITY\_DN5500\_c0\_g1\_i1.p1

back to list

  

| Protein Coverage
| Supporting Peptides
| Best Unique PSM
|

Protein Coverage:

Supporting Peptides:

| Peptide | Uniq | -10lgP | Mass | Length | ppm | m/z | z | RT | 1/k0 Start | 1/k0 End | Precursor ID | Area Sample 7 | #Feature | #Feature Sample 7 | Start | End | PTM | AScore | Found By |
| --- | --- | --- | --- | --- | --- | --- | --- | --- | --- | --- | --- | --- | --- | --- | --- | --- | --- | --- | --- |
| K.IYIDGVFIR.S | Y | 56.04 | 1094.6124 | 9 | 2.12 | 548.3121 | 2 | 18.80 | 0.8903 | 0.8976 | 67021 | 1.29e3 | 1 | 1 | 938 | 946 |  |  | DB Search |
| total 1 peptides |  |  |  |  |  |  |  |  |  |  |  |  |  |  |  |  |  |  |  |
| --- | --- | --- | --- | --- | --- | --- | --- | --- | --- | --- | --- | --- | --- | --- | --- | --- | --- | --- | --- |

Best Unique PSM (Scan Sample\_5\_A1\_1\_3643.d:67021, m/z=548.3121, z=2, RT=18.80, ppm=-2.56):


TRINITY\_DN4781\_c0\_g1\_i2.p1

back to list

  

| Protein Coverage
| Supporting Peptides
| Best Unique PSM
|

Protein Coverage:

Supporting Peptides:

| Peptide | Uniq | -10lgP | Mass | Length | ppm | m/z | z | RT | 1/k0 Start | 1/k0 End | Precursor ID | Area Sample 7 | #Feature | #Feature Sample 7 | Start | End | PTM | AScore | Found By |
| --- | --- | --- | --- | --- | --- | --- | --- | --- | --- | --- | --- | --- | --- | --- | --- | --- | --- | --- | --- |
| K.APPKPNPC(+57.02)AIK.C | Y | 55.16 | 1191.6434 | 11 | 6.69 | 398.2225 | 3 | 11.49 | 0.7791 | 0.7864 | 22133 | 1.06e3 | 1 | 1 | 1499 | 1509 | Carbamidomethylation |  | DB Search |
| R.LVSELFDPTSSTR.G | Y | 55.08 | 1450.7303 | 13 | -1.65 | 726.3679 | 2 | 17.84 | 1.0033 | 1.0106 | 60692 | 0 | 0 | 0 | 265 | 277 |  |  | DB Search |
| total 2 peptides |  |  |  |  |  |  |  |  |  |  |  |  |  |  |  |  |  |  |  |
| --- | --- | --- | --- | --- | --- | --- | --- | --- | --- | --- | --- | --- | --- | --- | --- | --- | --- | --- | --- |

Best Unique PSM (Scan Sample\_5\_A1\_1\_3643.d:22133, m/z=398.2225, z=3, RT=11.49, ppm=2.01):


TRINITY\_DN1030\_c0\_g1\_i1.p1

back to list

  

| Protein Coverage
| Supporting Peptides
| Best Unique PSM
|

Protein Coverage:

Supporting Peptides:

| Peptide | Uniq | -10lgP | Mass | Length | ppm | m/z | z | RT | 1/k0 Start | 1/k0 End | Precursor ID | Area Sample 7 | #Feature | #Feature Sample 7 | Start | End | PTM | AScore | Found By |
| --- | --- | --- | --- | --- | --- | --- | --- | --- | --- | --- | --- | --- | --- | --- | --- | --- | --- | --- | --- |
| R.GLQGQQGEQGSTGVQGSVGGR.G | Y | 56.41 | 1985.9515 | 21 | 4.71 | 993.983 | 2 | 12.10 | 1.139 | 1.1462 | 25849 | 1.24e3 | 1 | 1 | 361 | 381 |  |  | DB Search |
| total 1 peptides |  |  |  |  |  |  |  |  |  |  |  |  |  |  |  |  |  |  |  |
| --- | --- | --- | --- | --- | --- | --- | --- | --- | --- | --- | --- | --- | --- | --- | --- | --- | --- | --- | --- |

Best Unique PSM (Scan Sample\_5\_A1\_1\_3643.d:25849, m/z=993.983, z=2, RT=12.10, ppm=0.03):


TRINITY\_DN982\_c0\_g1\_i1.p1

back to list

  

| Protein Coverage
| Supporting Peptides
| Best Unique PSM
|

Protein Coverage:

Supporting Peptides:

| Peptide | Uniq | -10lgP | Mass | Length | ppm | m/z | z | RT | 1/k0 Start | 1/k0 End | Precursor ID | Area Sample 7 | #Feature | #Feature Sample 7 | Start | End | PTM | AScore | Found By |
| --- | --- | --- | --- | --- | --- | --- | --- | --- | --- | --- | --- | --- | --- | --- | --- | --- | --- | --- | --- |
| K.INNNQFSVVLIK.N | Y | 56.39 | 1387.7823 | 12 | 0.37 | 694.8954 | 2 | 18.30 | 0.9945 | 1.0017 | 63702 | 4.8e2 | 1 | 1 | 100 | 111 |  |  | DB Search |
| R.LVQNTPHNSIVTK.I | N | 56.38 | 1449.794 | 13 | 5.15 | 484.2722 | 3 | 11.78 | 0.8132 | 0.8205 | 23867 | 5.7e3 | 2 | 2 | 5129 | 5141 |  |  | DB Search |
| R.YTSC(+57.02)QSVTVVASDR.S | N | 56.38 | 1571.725 | 14 | 0.63 | 786.8666 | 2 | 13.49 | 1.052 | 1.0592 | 34562 | 0 | 0 | 0 | 5923 | 5936 | Carbamidomethylation |  | DB Search |
| K.LLTTEDDFILR.R | N | 56.37 | 1334.7081 | 11 | -1.75 | 668.3571 | 2 | 19.12 | 0.9751 | 0.9824 | 69298 | 0 | 0 | 0 | 3457 | 3467 |  |  | DB Search |
| R.IQVLKDPDFEFTR.S | N | 55.75 | 1606.8355 | 13 | 2.82 | 536.6181 | 3 | 17.65 | 0.8518 | 0.8591 | 59377 | 0 | 0 | 0 | 2727 | 2739 |  |  | DB Search |
| K.VLLSQQIFYDKK.N | N | 55.36 | 1480.8289 | 12 | 1.76 | 494.6155 | 3 | 16.92 | 0.7616 | 0.7689 | 54594 | 7.01e3 | 2 | 2 | 1479 | 1490 |  |  | DB Search |
| K.FPEFTLQGNK.I | N | 55.36 | 1179.5924 | 10 | 3.01 | 590.8025 | 2 | 17.68 | 0.8935 | 0.9008 | 59467 | 7.06e2 | 1 | 1 | 3883 | 3892 |  |  | DB Search |
| K.YVAIYPSDHLTVNR.D | N | 55.32 | 1646.8416 | 14 | -3.91 | 824.421 | 2 | 15.67 | 1.1098 | 1.117 | 47583 | 0 | 0 | 0 | 2814 | 2827 |  |  | DB Search |
| K.VLEVNDNKPK.F | Y | 55.29 | 1154.6295 | 10 | 3.29 | 578.3212 | 2 | 11.02 | 0.9255 | 0.9327 | 19462 | 6.22e2 | 1 | 1 | 459 | 468 |  |  | DB Search |
| K.ELEITVLESKPVNSFVIR.V | Y | 55.28 | 2072.1517 | 18 | 0.84 | 691.7219 | 3 | 19.88 | 0.9634 | 0.9707 | 74309 | 3.41e2 | 1 | 1 | 368 | 385 |  |  | DB Search |
| R.VLDVNDKQPVFVK.S | Y | 53.63 | 1499.8347 | 13 | 1.56 | 750.9223 | 2 | 15.14 | 1.052 | 1.0592 | 44491 | 0 | 0 | 0 | 670 | 682 |  |  | DB Search |
| K.AVDRGNPPQDSVNK.A | N | 52.97 | 1495.7379 | 14 | -0.57 | 499.584 | 3 | 10.23 | 0.773 | 0.7803 | 15050 | 0 | 0 | 0 | 1699 | 1712 |  |  | DB Search |
| R.TNNALDYEAKR.Q | N | 51.04 | 1293.6313 | 11 | 1.86 | 647.8211 | 2 | 12.12 | 0.9622 | 0.9695 | 26057 | 0 | 0 | 0 | 4285 | 4295 |  |  | DB Search |
| K.SKIDYEALQVK.H | N | 48.81 | 1292.6976 | 11 | -2.48 | 647.3514 | 2 | 14.75 | 0.9618 | 0.9691 | 42265 | 0 | 0 | 0 | 3373 | 3383 |  |  | DB Search |
| total 14 peptides |  |  |  |  |  |  |  |  |  |  |  |  |  |  |  |  |  |  |  |
| --- | --- | --- | --- | --- | --- | --- | --- | --- | --- | --- | --- | --- | --- | --- | --- | --- | --- | --- | --- |

Best Unique PSM (Scan Sample\_5\_A1\_1\_3643.d:63702, m/z=694.8954, z=2, RT=18.30, ppm=-4.31):


TRINITY\_DN2799\_c0\_g1\_i1.p1

back to list

  

| Protein Coverage
| Supporting Peptides
| Best Unique PSM
|

Protein Coverage:

Supporting Peptides:

| Peptide | Uniq | -10lgP | Mass | Length | ppm | m/z | z | RT | 1/k0 Start | 1/k0 End | Precursor ID | Area Sample 7 | #Feature | #Feature Sample 7 | Start | End | PTM | AScore | Found By |
| --- | --- | --- | --- | --- | --- | --- | --- | --- | --- | --- | --- | --- | --- | --- | --- | --- | --- | --- | --- |
| K.AGM(+15.99)THIVR.D | Y | 54.97 | 899.4647 | 8 | 4.88 | 450.7397 | 2 | 9.72 | 0.8144 | 0.8218 | 12487 | 9.73e2 | 1 | 1 | 74 | 81 | Oxidation (M) | M3:Oxidation (M):1000 | DB Search |
| total 1 peptides |  |  |  |  |  |  |  |  |  |  |  |  |  |  |  |  |  |  |  |
| --- | --- | --- | --- | --- | --- | --- | --- | --- | --- | --- | --- | --- | --- | --- | --- | --- | --- | --- | --- |

Best Unique PSM (Scan Sample\_5\_A1\_1\_3643.d:12487, m/z=450.7397, z=2, RT=9.72, ppm=0.2):


TRINITY\_DN4595\_c0\_g1\_i7.p1

back to list

  

| Protein Coverage
| Supporting Peptides
| Best Unique PSM
|

Protein Coverage:

Supporting Peptides:

| Peptide | Uniq | -10lgP | Mass | Length | ppm | m/z | z | RT | 1/k0 Start | 1/k0 End | Precursor ID | Area Sample 7 | #Feature | #Feature Sample 7 | Start | End | PTM | AScore | Found By |
| --- | --- | --- | --- | --- | --- | --- | --- | --- | --- | --- | --- | --- | --- | --- | --- | --- | --- | --- | --- |
| K.YQGNDYTR.N | Y | 55.1 | 1015.4359 | 8 | 1.65 | 508.7237 | 2 | 10.77 | 0.8469 | 0.8542 | 17934 | 1.34e3 | 1 | 1 | 22 | 29 |  |  | DB Search |
| R.AFNKIETR.N | Y | 49.77 | 977.5294 | 8 | 3.42 | 489.7714 | 2 | 11.52 | 0.8388 | 0.8461 | 22323 | 8.13e3 | 1 | 1 | 72 | 79 |  |  | DB Search |
| R.AFNKVETR.N | N | 49.58 | 963.5138 | 8 | 6.34 | 482.765 | 2 | 10.59 | 0.8266 | 0.8339 | 16901 | 3.56e3 | 1 | 1 | 177 | 184 |  |  | DB Search |
| total 3 peptides |  |  |  |  |  |  |  |  |  |  |  |  |  |  |  |  |  |  |  |
| --- | --- | --- | --- | --- | --- | --- | --- | --- | --- | --- | --- | --- | --- | --- | --- | --- | --- | --- | --- |

Best Unique PSM (Scan Sample\_5\_A1\_1\_3643.d:17934, m/z=508.7237, z=2, RT=10.77, ppm=-3.04):


TRINITY\_DN163801\_c0\_g1\_i1.p1

back to list

  

| Protein Coverage
| Supporting Peptides
| Best Unique PSM
|

Protein Coverage:

Supporting Peptides:

| Peptide | Uniq | -10lgP | Mass | Length | ppm | m/z | z | RT | 1/k0 Start | 1/k0 End | Precursor ID | Area Sample 7 | #Feature | #Feature Sample 7 | Start | End | PTM | AScore | Found By |
| --- | --- | --- | --- | --- | --- | --- | --- | --- | --- | --- | --- | --- | --- | --- | --- | --- | --- | --- | --- |
| M.A(+42.01)NPLKDIISR.F | Y | 54.32 | 1167.6611 | 10 | 0.57 | 584.8354 | 2 | 18.73 | 0.9206 | 0.9279 | 66485 | 8.04e2 | 1 | 1 | 2 | 11 | Acetylation (Protein N-term) | A1:Acetylation (Protein N-term):1000 | DB Search |
| total 1 peptides |  |  |  |  |  |  |  |  |  |  |  |  |  |  |  |  |  |  |  |
| --- | --- | --- | --- | --- | --- | --- | --- | --- | --- | --- | --- | --- | --- | --- | --- | --- | --- | --- | --- |

Best Unique PSM (Scan Sample\_5\_A1\_1\_3643.d:66485, m/z=584.8354, z=2, RT=18.73, ppm=-4.11):


TRINITY\_DN113\_c3\_g1\_i1.p1

back to list

  

| Protein Coverage
| Supporting Peptides
| Best Unique PSM
|

Protein Coverage:

Supporting Peptides:

| Peptide | Uniq | -10lgP | Mass | Length | ppm | m/z | z | RT | 1/k0 Start | 1/k0 End | Precursor ID | Area Sample 7 | #Feature | #Feature Sample 7 | Start | End | PTM | AScore | Found By |
| --- | --- | --- | --- | --- | --- | --- | --- | --- | --- | --- | --- | --- | --- | --- | --- | --- | --- | --- | --- |
| R.ATHQELLNAEFIQK.V | Y | 56.39 | 1640.8522 | 14 | 6.81 | 821.4351 | 2 | 16.09 | 1.0773 | 1.0845 | 49865 | 4.64e2 | 1 | 1 | 457 | 470 |  |  | DB Search |
| K.AHVSLSTFSEDVTSLTK.F | Y | 49.58 | 1820.9156 | 17 | 2.15 | 607.9776 | 3 | 18.97 | 0.8327 | 0.84 | 68160 | 1.3e3 | 1 | 1 | 568 | 584 |  |  | DB Search |
| R.VLVIFANGDINDEQAVK.A | Y | 47.03 | 1843.9679 | 17 | -10.38 | 922.9774 | 2 | 18.88 | 1.1845 | 1.1917 | 67755 | 0 | 0 | 0 | 636 | 652 |  |  | DB Search |
| total 3 peptides |  |  |  |  |  |  |  |  |  |  |  |  |  |  |  |  |  |  |  |
| --- | --- | --- | --- | --- | --- | --- | --- | --- | --- | --- | --- | --- | --- | --- | --- | --- | --- | --- | --- |

Best Unique PSM (Scan Sample\_5\_A1\_1\_3643.d:49865, m/z=821.4351, z=2, RT=16.09, ppm=2.13):


TRINITY\_DN1653\_c0\_g1\_i3.p1

back to list

  

| Protein Coverage
| Supporting Peptides
| Best Unique PSM
|

Protein Coverage:

Supporting Peptides:

| Peptide | Uniq | -10lgP | Mass | Length | ppm | m/z | z | RT | 1/k0 Start | 1/k0 End | Precursor ID | Area Sample 7 | #Feature | #Feature Sample 7 | Start | End | PTM | AScore | Found By |
| --- | --- | --- | --- | --- | --- | --- | --- | --- | --- | --- | --- | --- | --- | --- | --- | --- | --- | --- | --- |
| R.LINLFEAVSKK.S | Y | 56.32 | 1260.7441 | 11 | 0.6 | 631.3768 | 2 | 19.05 | 0.9836 | 0.9908 | 68790 | 7.03e2 | 2 | 2 | 49 | 59 |  |  | DB Search |
| R.ASDKFSASGPGLVEPK.I | Y | 48.48 | 1588.8096 | 16 | 6.54 | 795.4136 | 2 | 14.23 | 1.0608 | 1.068 | 39146 | 0 | 0 | 0 | 338 | 353 |  |  | DB Search |
| total 2 peptides |  |  |  |  |  |  |  |  |  |  |  |  |  |  |  |  |  |  |  |
| --- | --- | --- | --- | --- | --- | --- | --- | --- | --- | --- | --- | --- | --- | --- | --- | --- | --- | --- | --- |

Best Unique PSM (Scan Sample\_5\_A1\_1\_3643.d:68790, m/z=631.3768, z=2, RT=19.05, ppm=-4.09):


TRINITY\_DN413\_c0\_g1\_i2.p1

back to list

  

| Protein Coverage
| Supporting Peptides
| Best Unique PSM
|

Protein Coverage:

Supporting Peptides:

| Peptide | Uniq | -10lgP | Mass | Length | ppm | m/z | z | RT | 1/k0 Start | 1/k0 End | Precursor ID | Area Sample 7 | #Feature | #Feature Sample 7 | Start | End | PTM | AScore | Found By |
| --- | --- | --- | --- | --- | --- | --- | --- | --- | --- | --- | --- | --- | --- | --- | --- | --- | --- | --- | --- |
| R.LLLGDFNGDKR.T | Y | 55.25 | 1246.6669 | 11 | -0.59 | 624.3375 | 2 | 16.23 | 0.9856 | 0.9928 | 50739 | 5.27e2 | 1 | 1 | 46 | 56 |  |  | DB Search |
| total 1 peptides |  |  |  |  |  |  |  |  |  |  |  |  |  |  |  |  |  |  |  |
| --- | --- | --- | --- | --- | --- | --- | --- | --- | --- | --- | --- | --- | --- | --- | --- | --- | --- | --- | --- |

Best Unique PSM (Scan Sample\_5\_A1\_1\_3643.d:50739, m/z=624.3375, z=2, RT=16.23, ppm=-5.27):


TRINITY\_DN199\_c0\_g1\_i1.p1

back to list

  

| Protein Coverage
| Supporting Peptides
| Best Unique PSM
|

Protein Coverage:

Supporting Peptides:

| Peptide | Uniq | -10lgP | Mass | Length | ppm | m/z | z | RT | 1/k0 Start | 1/k0 End | Precursor ID | Area Sample 7 | #Feature | #Feature Sample 7 | Start | End | PTM | AScore | Found By |
| --- | --- | --- | --- | --- | --- | --- | --- | --- | --- | --- | --- | --- | --- | --- | --- | --- | --- | --- | --- |
| K.NYNNDDEPGAKK.L | Y | 56.4 | 1363.6004 | 12 | 2.6 | 455.5398 | 3 | 9.60 | 0.7489 | 0.7563 | 11895 | 1.07e3 | 2 | 2 | 124 | 135 |  |  | DB Search |
| K.NYNNDDEPGAK.K | Y | 56.17 | 1235.5054 | 11 | 4.61 | 618.7599 | 2 | 10.13 | 0.8842 | 0.8915 | 14506 | 1.78e3 | 2 | 2 | 124 | 134 |  |  | DB Search |
| M.VQGSM(+15.99)TIVK.Y | Y | 55.29 | 977.5216 | 9 | 3.77 | 489.7676 | 2 | 11.21 | 0.8287 | 0.836 | 20591 | 1.12e3 | 1 | 1 | 2 | 10 | Oxidation (M) | M5:Oxidation (M):1000 | DB Search |
| K.KLLDWAQQR.F | Y | 54.2 | 1156.6353 | 9 | 4.33 | 579.3247 | 2 | 15.52 | 0.9097 | 0.917 | 46753 | 0 | 0 | 0 | 135 | 143 |  |  | DB Search |
| total 4 peptides |  |  |  |  |  |  |  |  |  |  |  |  |  |  |  |  |  |  |  |
| --- | --- | --- | --- | --- | --- | --- | --- | --- | --- | --- | --- | --- | --- | --- | --- | --- | --- | --- | --- |

Best Unique PSM (Scan Sample\_5\_A1\_1\_3643.d:11895, m/z=455.5398, z=3, RT=9.60, ppm=-2.08):


TRINITY\_DN649\_c0\_g1\_i1.p1

back to list

  

| Protein Coverage
| Supporting Peptides
| Best Unique PSM
|

Protein Coverage:

Supporting Peptides:

| Peptide | Uniq | -10lgP | Mass | Length | ppm | m/z | z | RT | 1/k0 Start | 1/k0 End | Precursor ID | Area Sample 7 | #Feature | #Feature Sample 7 | Start | End | PTM | AScore | Found By |
| --- | --- | --- | --- | --- | --- | --- | --- | --- | --- | --- | --- | --- | --- | --- | --- | --- | --- | --- | --- |
| K.FETQIATK.N | Y | 53.77 | 936.4916 | 8 | 12.43 | 937.5062 | 1 | 13.97 | 1.1614 | 1.1686 | 37568 | 0 | 0 | 0 | 102 | 109 |  |  | DB Search |
| total 1 peptides |  |  |  |  |  |  |  |  |  |  |  |  |  |  |  |  |  |  |  |
| --- | --- | --- | --- | --- | --- | --- | --- | --- | --- | --- | --- | --- | --- | --- | --- | --- | --- | --- | --- |

Best Unique PSM (Scan Sample\_5\_A1\_1\_3643.d:37568, m/z=937.5062, z=1, RT=13.97, ppm=7.75):


TRINITY\_DN1058\_c0\_g1\_i2.p2

back to list

  

| Protein Coverage
| Supporting Peptides
| Best Unique PSM
|

Protein Coverage:

Supporting Peptides:

| Peptide | Uniq | -10lgP | Mass | Length | ppm | m/z | z | RT | 1/k0 Start | 1/k0 End | Precursor ID | Area Sample 7 | #Feature | #Feature Sample 7 | Start | End | PTM | AScore | Found By |
| --- | --- | --- | --- | --- | --- | --- | --- | --- | --- | --- | --- | --- | --- | --- | --- | --- | --- | --- | --- |
| R.APTHILNPANKR.S | Y | 52.25 | 1330.7469 | 12 | 5.05 | 444.5898 | 3 | 11.20 | 0.7481 | 0.7555 | 20091 | 3.03e3 | 1 | 1 | 975 | 986 |  |  | DB Search |
| total 1 peptides |  |  |  |  |  |  |  |  |  |  |  |  |  |  |  |  |  |  |  |
| --- | --- | --- | --- | --- | --- | --- | --- | --- | --- | --- | --- | --- | --- | --- | --- | --- | --- | --- | --- |

Best Unique PSM (Scan Sample\_5\_A1\_1\_3643.d:20091, m/z=444.5898, z=3, RT=11.20, ppm=0.37):


TRINITY\_DN14015\_c0\_g1\_i3.p1

back to list

  

| Protein Coverage
| Supporting Peptides
| Best Unique PSM
|

Protein Coverage:

Supporting Peptides:

| Peptide | Uniq | -10lgP | Mass | Length | ppm | m/z | z | RT | 1/k0 Start | 1/k0 End | Precursor ID | Area Sample 7 | #Feature | #Feature Sample 7 | Start | End | PTM | AScore | Found By |
| --- | --- | --- | --- | --- | --- | --- | --- | --- | --- | --- | --- | --- | --- | --- | --- | --- | --- | --- | --- |
| K.VIDINDNAPIFNK.A | Y | 48.8 | 1471.7671 | 13 | 3.85 | 736.8902 | 2 | 17.47 | 1.0146 | 1.0218 | 57919 | 8.75e2 | 1 | 1 | 666 | 678 |  |  | DB Search |
| total 1 peptides |  |  |  |  |  |  |  |  |  |  |  |  |  |  |  |  |  |  |  |
| --- | --- | --- | --- | --- | --- | --- | --- | --- | --- | --- | --- | --- | --- | --- | --- | --- | --- | --- | --- |

Best Unique PSM (Scan Sample\_5\_A1\_1\_3643.d:57919, m/z=736.8902, z=2, RT=17.47, ppm=-0.83):


TRINITY\_DN32727\_c0\_g1\_i2.p1

back to list

  

| Protein Coverage
| Supporting Peptides
| Best Unique PSM
|

Protein Coverage:

Supporting Peptides:

| Peptide | Uniq | -10lgP | Mass | Length | ppm | m/z | z | RT | 1/k0 Start | 1/k0 End | Precursor ID | Area Sample 7 | #Feature | #Feature Sample 7 | Start | End | PTM | AScore | Found By |
| --- | --- | --- | --- | --- | --- | --- | --- | --- | --- | --- | --- | --- | --- | --- | --- | --- | --- | --- | --- |
| K.AGM(+15.99)THIVR.D | Y | 54.97 | 899.4647 | 8 | 4.88 | 450.7397 | 2 | 9.72 | 0.8144 | 0.8218 | 12487 | 9.73e2 | 1 | 1 | 40 | 47 | Oxidation (M) | M3:Oxidation (M):1000 | DB Search |
| total 1 peptides |  |  |  |  |  |  |  |  |  |  |  |  |  |  |  |  |  |  |  |
| --- | --- | --- | --- | --- | --- | --- | --- | --- | --- | --- | --- | --- | --- | --- | --- | --- | --- | --- | --- |

Best Unique PSM (Scan Sample\_5\_A1\_1\_3643.d:12487, m/z=450.7397, z=2, RT=9.72, ppm=0.2):


TRINITY\_DN77\_c1\_g1\_i1.p1

back to list

  

| Protein Coverage
| Supporting Peptides
| Best Unique PSM
|

Protein Coverage:

Supporting Peptides:

| Peptide | Uniq | -10lgP | Mass | Length | ppm | m/z | z | RT | 1/k0 Start | 1/k0 End | Precursor ID | Area Sample 7 | #Feature | #Feature Sample 7 | Start | End | PTM | AScore | Found By |
| --- | --- | --- | --- | --- | --- | --- | --- | --- | --- | --- | --- | --- | --- | --- | --- | --- | --- | --- | --- |
| R.AVDIHFALDASQNVGR.E | Y | 56.28 | 1711.8641 | 16 | 1.27 | 571.6267 | 3 | 17.55 | 0.8591 | 0.8664 | 58465 | 7.9e2 | 1 | 1 | 56 | 71 |  |  | DB Search |
| R.LLAVTVGNNYNLDTLEK.V | Y | 49.51 | 1875.9942 | 17 | -8.81 | 938.9917 | 2 | 18.74 | 1.1506 | 1.1578 | 66472 | 9.46e2 | 1 | 1 | 693 | 709 |  |  | DB Search |
| total 2 peptides |  |  |  |  |  |  |  |  |  |  |  |  |  |  |  |  |  |  |  |
| --- | --- | --- | --- | --- | --- | --- | --- | --- | --- | --- | --- | --- | --- | --- | --- | --- | --- | --- | --- |

Best Unique PSM (Scan Sample\_5\_A1\_1\_3643.d:58465, m/z=571.6267, z=3, RT=17.55, ppm=-3.41):


TRINITY\_DN1505\_c0\_g1\_i1.p1

back to list

  

| Protein Coverage
| Supporting Peptides
| Best Unique PSM
|

Protein Coverage:

Supporting Peptides:

| Peptide | Uniq | -10lgP | Mass | Length | ppm | m/z | z | RT | 1/k0 Start | 1/k0 End | Precursor ID | Area Sample 7 | #Feature | #Feature Sample 7 | Start | End | PTM | AScore | Found By |
| --- | --- | --- | --- | --- | --- | --- | --- | --- | --- | --- | --- | --- | --- | --- | --- | --- | --- | --- | --- |
| M.S(+42.01)LVIPEKFQHILR.V | Y | 56.39 | 1620.9351 | 13 | 1.15 | 541.3171 | 3 | 19.75 | 0.8425 | 0.8498 | 73247 | 9.92e2 | 1 | 1 | 2 | 14 | Acetylation (Protein N-term) | S1:Acetylation (Protein N-term):1000 | DB Search |
| total 1 peptides |  |  |  |  |  |  |  |  |  |  |  |  |  |  |  |  |  |  |  |
| --- | --- | --- | --- | --- | --- | --- | --- | --- | --- | --- | --- | --- | --- | --- | --- | --- | --- | --- | --- |

Best Unique PSM (Scan Sample\_5\_A1\_1\_3643.d:73247, m/z=541.3171, z=3, RT=19.75, ppm=-3.53):


TRINITY\_DN187147\_c0\_g1\_i1.p1

back to list

  

| Protein Coverage
| Supporting Peptides
| Best Unique PSM
|

Protein Coverage:

Supporting Peptides:

| Peptide | Uniq | -10lgP | Mass | Length | ppm | m/z | z | RT | 1/k0 Start | 1/k0 End | Precursor ID | Area Sample 7 | #Feature | #Feature Sample 7 | Start | End | PTM | AScore | Found By |
| --- | --- | --- | --- | --- | --- | --- | --- | --- | --- | --- | --- | --- | --- | --- | --- | --- | --- | --- | --- |
| K.LVGQSIISYLQK.K | Y | 52.05 | 1347.7762 | 12 | 2.9 | 674.8942 | 2 | 19.71 | 0.9783 | 0.9856 | 73270 | 0 | 0 | 0 | 627 | 638 |  |  | DB Search |
| total 1 peptides |  |  |  |  |  |  |  |  |  |  |  |  |  |  |  |  |  |  |  |
| --- | --- | --- | --- | --- | --- | --- | --- | --- | --- | --- | --- | --- | --- | --- | --- | --- | --- | --- | --- |

Best Unique PSM (Scan Sample\_5\_A1\_1\_3643.d:73270, m/z=674.8942, z=2, RT=19.71, ppm=-1.78):


TRINITY\_DN36\_c0\_g1\_i11.p1

back to list

  

| Protein Coverage
| Supporting Peptides
| Best Unique PSM
|

Protein Coverage:

Supporting Peptides:

| Peptide | Uniq | -10lgP | Mass | Length | ppm | m/z | z | RT | 1/k0 Start | 1/k0 End | Precursor ID | Area Sample 7 | #Feature | #Feature Sample 7 | Start | End | PTM | AScore | Found By |
| --- | --- | --- | --- | --- | --- | --- | --- | --- | --- | --- | --- | --- | --- | --- | --- | --- | --- | --- | --- |
| R.LGGIEVTQGNC(+57.02)AR.K | Y | 49.67 | 1373.6721 | 13 | 2.61 | 687.8419 | 2 | 13.89 | 0.9695 | 0.9767 | 37015 | 0 | 0 | 0 | 58 | 70 | Carbamidomethylation |  | DB Search |
| total 1 peptides |  |  |  |  |  |  |  |  |  |  |  |  |  |  |  |  |  |  |  |
| --- | --- | --- | --- | --- | --- | --- | --- | --- | --- | --- | --- | --- | --- | --- | --- | --- | --- | --- | --- |

Best Unique PSM (Scan Sample\_5\_A1\_1\_3643.d:37015, m/z=687.8419, z=2, RT=13.89, ppm=-2.07):


TRINITY\_DN2885\_c0\_g1\_i5.p1

back to list

  

| Protein Coverage
| Supporting Peptides
| Best Unique PSM
|

Protein Coverage:

Supporting Peptides:

| Peptide | Uniq | -10lgP | Mass | Length | ppm | m/z | z | RT | 1/k0 Start | 1/k0 End | Precursor ID | Area Sample 7 | #Feature | #Feature Sample 7 | Start | End | PTM | AScore | Found By |
| --- | --- | --- | --- | --- | --- | --- | --- | --- | --- | --- | --- | --- | --- | --- | --- | --- | --- | --- | --- |
| K.VTRPHGASGVVR.A | Y | 56.37 | 1234.6894 | 12 | 3.4 | 412.5699 | 3 | 10.07 | 0.8104 | 0.8177 | 14175 | 0 | 0 | 0 | 77 | 88 |  |  | DB Search |
| total 1 peptides |  |  |  |  |  |  |  |  |  |  |  |  |  |  |  |  |  |  |  |
| --- | --- | --- | --- | --- | --- | --- | --- | --- | --- | --- | --- | --- | --- | --- | --- | --- | --- | --- | --- |

Best Unique PSM (Scan Sample\_5\_A1\_1\_3643.d:14175, m/z=412.5699, z=3, RT=10.07, ppm=-1.29):


TRINITY\_DN36\_c0\_g1\_i19.p1

back to list

  

| Protein Coverage
| Supporting Peptides
| Best Unique PSM
|

Protein Coverage:

Supporting Peptides:

| Peptide | Uniq | -10lgP | Mass | Length | ppm | m/z | z | RT | 1/k0 Start | 1/k0 End | Precursor ID | Area Sample 7 | #Feature | #Feature Sample 7 | Start | End | PTM | AScore | Found By |
| --- | --- | --- | --- | --- | --- | --- | --- | --- | --- | --- | --- | --- | --- | --- | --- | --- | --- | --- | --- |
| K.FETQIATK.D | Y | 53.77 | 936.4916 | 8 | 12.43 | 937.5062 | 1 | 13.97 | 1.1614 | 1.1686 | 37568 | 0 | 0 | 0 | 166 | 173 |  |  | DB Search |
| R.LGGIEVTQGNC(+57.02)AR.K | Y | 49.67 | 1373.6721 | 13 | 2.61 | 687.8419 | 2 | 13.89 | 0.9695 | 0.9767 | 37015 | 0 | 0 | 0 | 57 | 69 | Carbamidomethylation |  | DB Search |
| total 2 peptides |  |  |  |  |  |  |  |  |  |  |  |  |  |  |  |  |  |  |  |
| --- | --- | --- | --- | --- | --- | --- | --- | --- | --- | --- | --- | --- | --- | --- | --- | --- | --- | --- | --- |

Best Unique PSM (Scan Sample\_5\_A1\_1\_3643.d:37568, m/z=937.5062, z=1, RT=13.97, ppm=7.75):


TRINITY\_DN36\_c0\_g1\_i5.p1

back to list

  

| Protein Coverage
| Supporting Peptides
| Best Unique PSM
|

Protein Coverage:

Supporting Peptides:

| Peptide | Uniq | -10lgP | Mass | Length | ppm | m/z | z | RT | 1/k0 Start | 1/k0 End | Precursor ID | Area Sample 7 | #Feature | #Feature Sample 7 | Start | End | PTM | AScore | Found By |
| --- | --- | --- | --- | --- | --- | --- | --- | --- | --- | --- | --- | --- | --- | --- | --- | --- | --- | --- | --- |
| R.LGGIEVTQGNC(+57.02)AR.K | Y | 49.67 | 1373.6721 | 13 | 2.61 | 687.8419 | 2 | 13.89 | 0.9695 | 0.9767 | 37015 | 0 | 0 | 0 | 57 | 69 | Carbamidomethylation |  | DB Search |
| total 1 peptides |  |  |  |  |  |  |  |  |  |  |  |  |  |  |  |  |  |  |  |
| --- | --- | --- | --- | --- | --- | --- | --- | --- | --- | --- | --- | --- | --- | --- | --- | --- | --- | --- | --- |

Best Unique PSM (Scan Sample\_5\_A1\_1\_3643.d:37015, m/z=687.8419, z=2, RT=13.89, ppm=-2.07):


TRINITY\_DN1616\_c0\_g1\_i7.p1

back to list

  

| Protein Coverage
| Supporting Peptides
| Best Unique PSM
|

Protein Coverage:

Supporting Peptides:

| Peptide | Uniq | -10lgP | Mass | Length | ppm | m/z | z | RT | 1/k0 Start | 1/k0 End | Precursor ID | Area Sample 7 | #Feature | #Feature Sample 7 | Start | End | PTM | AScore | Found By |
| --- | --- | --- | --- | --- | --- | --- | --- | --- | --- | --- | --- | --- | --- | --- | --- | --- | --- | --- | --- |
| K.VVDTEANDLTEEVKK.T | Y | 56.4 | 1688.8468 | 15 | 6.39 | 845.4321 | 2 | 14.15 | 1.1318 | 1.139 | 38615 | 0 | 0 | 0 | 2542 | 2556 |  |  | DB Search |
| K.SSSC(+57.02)QEFGGKC(+57.02)PC(+57.02)K.D | Y | 56.37 | 1630.6538 | 14 | -16.59 | 816.3168 | 2 | 12.09 | 1.0335 | 1.0407 | 25828 | 0 | 0 | 0 | 1387 | 1400 | Carbamidomethylation, Carbamidomethylation, Carbamidomethylation |  | DB Search |
| R.YGAVNDNC(+57.02)ER.D | Y | 56.34 | 1196.488 | 10 | 3.96 | 599.2509 | 2 | 10.89 | 0.885 | 0.8923 | 18607 | 1.07e3 | 1 | 1 | 498 | 507 | Carbamidomethylation |  | DB Search |
| K.TLDTVC(+57.02)DSGTGVC(+57.02)QC(+57.02)K.S | Y | 49.46 | 1799.7488 | 16 | -4.78 | 900.8732 | 2 | 13.15 | 1.0901 | 1.0973 | 32411 | 1.65e2 | 1 | 1 | 546 | 561 | Carbamidomethylation, Carbamidomethylation, Carbamidomethylation |  | DB Search |
| total 4 peptides |  |  |  |  |  |  |  |  |  |  |  |  |  |  |  |  |  |  |  |
| --- | --- | --- | --- | --- | --- | --- | --- | --- | --- | --- | --- | --- | --- | --- | --- | --- | --- | --- | --- |

Best Unique PSM (Scan Sample\_5\_A1\_1\_3643.d:38615, m/z=845.4321, z=2, RT=14.15, ppm=1.71):


TRINITY\_DN36\_c0\_g1\_i9.p1

back to list

  

| Protein Coverage
| Supporting Peptides
| Best Unique PSM
|

Protein Coverage:

Supporting Peptides:

| Peptide | Uniq | -10lgP | Mass | Length | ppm | m/z | z | RT | 1/k0 Start | 1/k0 End | Precursor ID | Area Sample 7 | #Feature | #Feature Sample 7 | Start | End | PTM | AScore | Found By |
| --- | --- | --- | --- | --- | --- | --- | --- | --- | --- | --- | --- | --- | --- | --- | --- | --- | --- | --- | --- |
| R.LGGIEVTQGNC(+57.02)AR.K | Y | 49.67 | 1373.6721 | 13 | 2.61 | 687.8419 | 2 | 13.89 | 0.9695 | 0.9767 | 37015 | 0 | 0 | 0 | 58 | 70 | Carbamidomethylation |  | DB Search |
| total 1 peptides |  |  |  |  |  |  |  |  |  |  |  |  |  |  |  |  |  |  |  |
| --- | --- | --- | --- | --- | --- | --- | --- | --- | --- | --- | --- | --- | --- | --- | --- | --- | --- | --- | --- |

Best Unique PSM (Scan Sample\_5\_A1\_1\_3643.d:37015, m/z=687.8419, z=2, RT=13.89, ppm=-2.07):


TRINITY\_DN108993\_c0\_g1\_i1.p1

back to list

  

| Protein Coverage
| Supporting Peptides
| Best Unique PSM
|

Protein Coverage:

Supporting Peptides:

| Peptide | Uniq | -10lgP | Mass | Length | ppm | m/z | z | RT | 1/k0 Start | 1/k0 End | Precursor ID | Area Sample 7 | #Feature | #Feature Sample 7 | Start | End | PTM | AScore | Found By |
| --- | --- | --- | --- | --- | --- | --- | --- | --- | --- | --- | --- | --- | --- | --- | --- | --- | --- | --- | --- |
| K.AFLTQAQEEFKK.K | Y | 51.12 | 1438.7456 | 12 | 5.01 | 720.3803 | 2 | 15.74 | 1.0456 | 1.0528 | 48009 | 0 | 0 | 0 | 18 | 29 |  |  | DB Search |
| total 1 peptides |  |  |  |  |  |  |  |  |  |  |  |  |  |  |  |  |  |  |  |
| --- | --- | --- | --- | --- | --- | --- | --- | --- | --- | --- | --- | --- | --- | --- | --- | --- | --- | --- | --- |

Best Unique PSM (Scan Sample\_5\_A1\_1\_3643.d:48009, m/z=720.3803, z=2, RT=15.74, ppm=0.33):


TRINITY\_DN3448\_c0\_g1\_i9.p3

back to list

  

| Protein Coverage
| Supporting Peptides
| Best Unique PSM
|

Protein Coverage:

Supporting Peptides:

| Peptide | Uniq | -10lgP | Mass | Length | ppm | m/z | z | RT | 1/k0 Start | 1/k0 End | Precursor ID | Area Sample 7 | #Feature | #Feature Sample 7 | Start | End | PTM | AScore | Found By |
| --- | --- | --- | --- | --- | --- | --- | --- | --- | --- | --- | --- | --- | --- | --- | --- | --- | --- | --- | --- |
| K.AGM(+15.99)THIVR.D | Y | 54.97 | 899.4647 | 8 | 4.88 | 450.7397 | 2 | 9.72 | 0.8144 | 0.8218 | 12487 | 9.73e2 | 1 | 1 | 27 | 34 | Oxidation (M) | M3:Oxidation (M):1000 | DB Search |
| total 1 peptides |  |  |  |  |  |  |  |  |  |  |  |  |  |  |  |  |  |  |  |
| --- | --- | --- | --- | --- | --- | --- | --- | --- | --- | --- | --- | --- | --- | --- | --- | --- | --- | --- | --- |

Best Unique PSM (Scan Sample\_5\_A1\_1\_3643.d:12487, m/z=450.7397, z=2, RT=9.72, ppm=0.2):


TRINITY\_DN1043\_c0\_g1\_i2.p1

back to list

  

| Protein Coverage
| Supporting Peptides
| Best Unique PSM
|

Protein Coverage:

Supporting Peptides:

| Peptide | Uniq | -10lgP | Mass | Length | ppm | m/z | z | RT | 1/k0 Start | 1/k0 End | Precursor ID | Area Sample 7 | #Feature | #Feature Sample 7 | Start | End | PTM | AScore | Found By |
| --- | --- | --- | --- | --- | --- | --- | --- | --- | --- | --- | --- | --- | --- | --- | --- | --- | --- | --- | --- |
| K.GLMVAAMDLDKK.Q | Y | 49.4 | 1290.6675 | 12 | 6.41 | 646.3422 | 2 | 16.72 | 1.0001 | 1.0073 | 53531 | 4.99e2 | 1 | 1 | 504 | 515 |  |  | DB Search |
| total 1 peptides |  |  |  |  |  |  |  |  |  |  |  |  |  |  |  |  |  |  |  |
| --- | --- | --- | --- | --- | --- | --- | --- | --- | --- | --- | --- | --- | --- | --- | --- | --- | --- | --- | --- |

Best Unique PSM (Scan Sample\_5\_A1\_1\_3643.d:53531, m/z=646.3422, z=2, RT=16.72, ppm=1.72):


TRINITY\_DN118\_c1\_g1\_i3.p1

back to list

  

| Protein Coverage
| Supporting Peptides
| Best Unique PSM
|

Protein Coverage:

Supporting Peptides:

| Peptide | Uniq | -10lgP | Mass | Length | ppm | m/z | z | RT | 1/k0 Start | 1/k0 End | Precursor ID | Area Sample 7 | #Feature | #Feature Sample 7 | Start | End | PTM | AScore | Found By |
| --- | --- | --- | --- | --- | --- | --- | --- | --- | --- | --- | --- | --- | --- | --- | --- | --- | --- | --- | --- |
| K.FSYEKVC(+57.02)EDVKR.C | Y | 51.53 | 1558.745 | 12 | -1.54 | 520.5857 | 3 | 13.64 | 0.7978 | 0.8051 | 35482 | 2.86e2 | 1 | 1 | 117 | 128 | Carbamidomethylation |  | DB Search |
| total 1 peptides |  |  |  |  |  |  |  |  |  |  |  |  |  |  |  |  |  |  |  |
| --- | --- | --- | --- | --- | --- | --- | --- | --- | --- | --- | --- | --- | --- | --- | --- | --- | --- | --- | --- |

Best Unique PSM (Scan Sample\_5\_A1\_1\_3643.d:35482, m/z=520.5857, z=3, RT=13.64, ppm=-6.22):


TRINITY\_DN1369\_c0\_g1\_i1.p1

back to list

  

| Protein Coverage
| Supporting Peptides
| Best Unique PSM
|

Protein Coverage:

Supporting Peptides:

| Peptide | Uniq | -10lgP | Mass | Length | ppm | m/z | z | RT | 1/k0 Start | 1/k0 End | Precursor ID | Area Sample 7 | #Feature | #Feature Sample 7 | Start | End | PTM | AScore | Found By |
| --- | --- | --- | --- | --- | --- | --- | --- | --- | --- | --- | --- | --- | --- | --- | --- | --- | --- | --- | --- |
| R.EITLNYHDTVVHMK.R | Y | 56.41 | 1698.8399 | 14 | -1.2 | 850.4222 | 2 | 15.44 | 1.1134 | 1.1206 | 46227 | 6.3e2 | 1 | 1 | 911 | 924 |  |  | DB Search |
| K.LC(+57.02)DATC(+57.02)SVFGDPHYK.T | Y | 51.11 | 1768.7549 | 15 | 2.6 | 885.3829 | 2 | 15.31 | 1.1402 | 1.1474 | 45505 | 4.4e2 | 1 | 1 | 846 | 860 | Carbamidomethylation, Carbamidomethylation |  | DB Search |
| total 2 peptides |  |  |  |  |  |  |  |  |  |  |  |  |  |  |  |  |  |  |  |
| --- | --- | --- | --- | --- | --- | --- | --- | --- | --- | --- | --- | --- | --- | --- | --- | --- | --- | --- | --- |

Best Unique PSM (Scan Sample\_5\_A1\_1\_3643.d:46227, m/z=850.4222, z=2, RT=15.44, ppm=-5.88):


TRINITY\_DN9819\_c0\_g1\_i6.p2

back to list

  

| Protein Coverage
| Supporting Peptides
| Best Unique PSM
|

Protein Coverage:

Supporting Peptides:

| Peptide | Uniq | -10lgP | Mass | Length | ppm | m/z | z | RT | 1/k0 Start | 1/k0 End | Precursor ID | Area Sample 7 | #Feature | #Feature Sample 7 | Start | End | PTM | AScore | Found By |
| --- | --- | --- | --- | --- | --- | --- | --- | --- | --- | --- | --- | --- | --- | --- | --- | --- | --- | --- | --- |
| M.V(+42.01)HDLVLR.Q | Y | 49.6 | 892.513 | 7 | -1.25 | 447.2611 | 2 | 18.00 | 0.8136 | 0.8209 | 61579 | 2.99e2 | 1 | 1 | 2 | 8 | Acetylation (Protein N-term) | V1:Acetylation (Protein N-term):1000 | DB Search |
| total 1 peptides |  |  |  |  |  |  |  |  |  |  |  |  |  |  |  |  |  |  |  |
| --- | --- | --- | --- | --- | --- | --- | --- | --- | --- | --- | --- | --- | --- | --- | --- | --- | --- | --- | --- |

Best Unique PSM (Scan Sample\_5\_A1\_1\_3643.d:61579, m/z=447.2611, z=2, RT=18.00, ppm=-5.94):


TRINITY\_DN585\_c0\_g1\_i5.p1

back to list

  

| Protein Coverage
| Supporting Peptides
| Best Unique PSM
|

Protein Coverage:

Supporting Peptides:

| Peptide | Uniq | -10lgP | Mass | Length | ppm | m/z | z | RT | 1/k0 Start | 1/k0 End | Precursor ID | Area Sample 7 | #Feature | #Feature Sample 7 | Start | End | PTM | AScore | Found By |
| --- | --- | --- | --- | --- | --- | --- | --- | --- | --- | --- | --- | --- | --- | --- | --- | --- | --- | --- | --- |
| K.TITLEVEPSDTIENVK.S | Y | 55.12 | 1786.92 | 16 | 0.46 | 894.4635 | 2 | 17.55 | 1.1414 | 1.1486 | 58604 | 8.72e2 | 1 | 1 | 60 | 75 |  |  | DB Search |
| total 1 peptides |  |  |  |  |  |  |  |  |  |  |  |  |  |  |  |  |  |  |  |
| --- | --- | --- | --- | --- | --- | --- | --- | --- | --- | --- | --- | --- | --- | --- | --- | --- | --- | --- | --- |

Best Unique PSM (Scan Sample\_5\_A1\_1\_3643.d:58604, m/z=894.4635, z=2, RT=17.55, ppm=-4.22):


TRINITY\_DN991\_c0\_g1\_i6.p1

back to list

  

| Protein Coverage
| Supporting Peptides
| Best Unique PSM
|

Protein Coverage:

Supporting Peptides:

| Peptide | Uniq | -10lgP | Mass | Length | ppm | m/z | z | RT | 1/k0 Start | 1/k0 End | Precursor ID | Area Sample 7 | #Feature | #Feature Sample 7 | Start | End | PTM | AScore | Found By |
| --- | --- | --- | --- | --- | --- | --- | --- | --- | --- | --- | --- | --- | --- | --- | --- | --- | --- | --- | --- |
| R.ITVTVSPDIHPPAK.L | Y | 49.15 | 1473.8191 | 14 | -0.97 | 737.9127 | 2 | 14.98 | 1.0668 | 1.0741 | 43563 | 1.94e2 | 1 | 1 | 1183 | 1196 |  |  | DB Search |
| total 1 peptides |  |  |  |  |  |  |  |  |  |  |  |  |  |  |  |  |  |  |  |
| --- | --- | --- | --- | --- | --- | --- | --- | --- | --- | --- | --- | --- | --- | --- | --- | --- | --- | --- | --- |

Best Unique PSM (Scan Sample\_5\_A1\_1\_3643.d:43563, m/z=736.8683, z=2, RT=15.00, ppm=-5.65):


TRINITY\_DN5906\_c3\_g1\_i1.p2

back to list

  

| Protein Coverage
| Supporting Peptides
| Best Unique PSM
|

Protein Coverage:

Supporting Peptides:

| Peptide | Uniq | -10lgP | Mass | Length | ppm | m/z | z | RT | 1/k0 Start | 1/k0 End | Precursor ID | Area Sample 7 | #Feature | #Feature Sample 7 | Start | End | PTM | AScore | Found By |
| --- | --- | --- | --- | --- | --- | --- | --- | --- | --- | --- | --- | --- | --- | --- | --- | --- | --- | --- | --- |
| K.AGM(+15.99)THIVR.D | Y | 54.97 | 899.4647 | 8 | 4.88 | 450.7397 | 2 | 9.72 | 0.8144 | 0.8218 | 12487 | 9.73e2 | 1 | 1 | 76 | 83 | Oxidation (M) | M3:Oxidation (M):1000 | DB Search |
| total 1 peptides |  |  |  |  |  |  |  |  |  |  |  |  |  |  |  |  |  |  |  |
| --- | --- | --- | --- | --- | --- | --- | --- | --- | --- | --- | --- | --- | --- | --- | --- | --- | --- | --- | --- |

Best Unique PSM (Scan Sample\_5\_A1\_1\_3643.d:12487, m/z=450.7397, z=2, RT=9.72, ppm=0.2):


TRINITY\_DN4595\_c0\_g1\_i10.p2

back to list

  

| Protein Coverage
| Supporting Peptides
| Best Unique PSM
|

Protein Coverage:

Supporting Peptides:

| Peptide | Uniq | -10lgP | Mass | Length | ppm | m/z | z | RT | 1/k0 Start | 1/k0 End | Precursor ID | Area Sample 7 | #Feature | #Feature Sample 7 | Start | End | PTM | AScore | Found By |
| --- | --- | --- | --- | --- | --- | --- | --- | --- | --- | --- | --- | --- | --- | --- | --- | --- | --- | --- | --- |
| R.VC(+57.02)NGEPC(+57.02)ILQER.S | Y | 55.34 | 1473.6704 | 12 | 3.33 | 737.8415 | 2 | 14.98 | 0.9896 | 0.9969 | 43535 | 0 | 0 | 0 | 306 | 317 | Carbamidomethylation, Carbamidomethylation |  | DB Search |
| R.YQGREYNINEEVNVR.Q | Y | 53.45 | 1881.8969 | 15 | -4.84 | 941.9468 | 2 | 15.64 | 1.1442 | 1.1514 | 47466 | 0 | 0 | 0 | 208 | 222 |  |  | DB Search |
| R.AFNKVETR.N | N | 49.58 | 963.5138 | 8 | 6.34 | 482.765 | 2 | 10.59 | 0.8266 | 0.8339 | 16901 | 3.56e3 | 1 | 1 | 47 | 54 |  |  | DB Search |
| total 3 peptides |  |  |  |  |  |  |  |  |  |  |  |  |  |  |  |  |  |  |  |
| --- | --- | --- | --- | --- | --- | --- | --- | --- | --- | --- | --- | --- | --- | --- | --- | --- | --- | --- | --- |

Best Unique PSM (Scan Sample\_5\_A1\_1\_3643.d:43535, m/z=737.8415, z=2, RT=14.98, ppm=-1.35):


TRINITY\_DN65215\_c0\_g1\_i1.p1

back to list

  

| Protein Coverage
| Supporting Peptides
| Best Unique PSM
|

Protein Coverage:

Supporting Peptides:

| Peptide | Uniq | -10lgP | Mass | Length | ppm | m/z | z | RT | 1/k0 Start | 1/k0 End | Precursor ID | Area Sample 7 | #Feature | #Feature Sample 7 | Start | End | PTM | AScore | Found By |
| --- | --- | --- | --- | --- | --- | --- | --- | --- | --- | --- | --- | --- | --- | --- | --- | --- | --- | --- | --- |
| K.AFLFDVVSK.I | Y | 52.39 | 1024.5593 | 9 | -2.23 | 513.2834 | 2 | 19.79 | 0.8412 | 0.8485 | 73630 | 7e2 | 1 | 1 | 248 | 256 |  |  | DB Search |
| total 1 peptides |  |  |  |  |  |  |  |  |  |  |  |  |  |  |  |  |  |  |  |
| --- | --- | --- | --- | --- | --- | --- | --- | --- | --- | --- | --- | --- | --- | --- | --- | --- | --- | --- | --- |

Best Unique PSM (Scan Sample\_5\_A1\_1\_3643.d:73630, m/z=513.2834, z=2, RT=19.79, ppm=-6.91):


TRINITY\_DN141338\_c2\_g1\_i1.p1

back to list

  

| Protein Coverage
| Supporting Peptides
| Best Unique PSM
|

Protein Coverage:

Supporting Peptides:

| Peptide | Uniq | -10lgP | Mass | Length | ppm | m/z | z | RT | 1/k0 Start | 1/k0 End | Precursor ID | Area Sample 7 | #Feature | #Feature Sample 7 | Start | End | PTM | AScore | Found By |
| --- | --- | --- | --- | --- | --- | --- | --- | --- | --- | --- | --- | --- | --- | --- | --- | --- | --- | --- | --- |
| K.LGIHEDTQNR.N | Y | 56.21 | 1181.5789 | 10 | 4.79 | 394.8669 | 3 | 11.18 | 0.711 | 0.7184 | 20409 | 9.9e2 | 1 | 1 | 434 | 443 |  |  | DB Search |
| total 1 peptides |  |  |  |  |  |  |  |  |  |  |  |  |  |  |  |  |  |  |  |
| --- | --- | --- | --- | --- | --- | --- | --- | --- | --- | --- | --- | --- | --- | --- | --- | --- | --- | --- | --- |

Best Unique PSM (Scan Sample\_5\_A1\_1\_3643.d:20409, m/z=394.8669, z=3, RT=11.18, ppm=0.1):


TRINITY\_DN33065\_c0\_g2\_i1.p1

back to list

  

| Protein Coverage
| Supporting Peptides
| Best Unique PSM
|

Protein Coverage:

Supporting Peptides:

| Peptide | Uniq | -10lgP | Mass | Length | ppm | m/z | z | RT | 1/k0 Start | 1/k0 End | Precursor ID | Area Sample 7 | #Feature | #Feature Sample 7 | Start | End | PTM | AScore | Found By |
| --- | --- | --- | --- | --- | --- | --- | --- | --- | --- | --- | --- | --- | --- | --- | --- | --- | --- | --- | --- |
| R.IIVADGAEYKK.T | Y | 56.37 | 1205.6655 | 11 | 5.35 | 603.8405 | 2 | 12.62 | 0.982 | 0.9892 | 29075 | 4.32e2 | 1 | 1 | 392 | 402 |  |  | DB Search |
| R.AFSFSDKPAITFEIHDFK.M | Y | 56.18 | 2099.0363 | 18 | 0.2 | 700.6829 | 3 | 19.06 | 0.9795 | 0.9868 | 68737 | 2.48e2 | 1 | 1 | 595 | 612 |  |  | DB Search |
| K.RIIVADGAEYKK.T | Y | 47.86 | 1361.7667 | 12 | 5.65 | 454.9299 | 3 | 11.98 | 0.8023 | 0.8096 | 25196 | 0 | 0 | 0 | 391 | 402 |  |  | DB Search |
| total 3 peptides |  |  |  |  |  |  |  |  |  |  |  |  |  |  |  |  |  |  |  |
| --- | --- | --- | --- | --- | --- | --- | --- | --- | --- | --- | --- | --- | --- | --- | --- | --- | --- | --- | --- |

Best Unique PSM (Scan Sample\_5\_A1\_1\_3643.d:29075, m/z=603.8405, z=2, RT=12.62, ppm=0.67):


TRINITY\_DN36\_c0\_g1\_i17.p1

back to list

  

| Protein Coverage
| Supporting Peptides
| Best Unique PSM
|

Protein Coverage:

Supporting Peptides:

| Peptide | Uniq | -10lgP | Mass | Length | ppm | m/z | z | RT | 1/k0 Start | 1/k0 End | Precursor ID | Area Sample 7 | #Feature | #Feature Sample 7 | Start | End | PTM | AScore | Found By |
| --- | --- | --- | --- | --- | --- | --- | --- | --- | --- | --- | --- | --- | --- | --- | --- | --- | --- | --- | --- |
| K.FETQIATK.D | Y | 53.77 | 936.4916 | 8 | 12.43 | 937.5062 | 1 | 13.97 | 1.1614 | 1.1686 | 37568 | 0 | 0 | 0 | 167 | 174 |  |  | DB Search |
| R.LGGIEVTQGNC(+57.02)AR.K | Y | 49.67 | 1373.6721 | 13 | 2.61 | 687.8419 | 2 | 13.89 | 0.9695 | 0.9767 | 37015 | 0 | 0 | 0 | 58 | 70 | Carbamidomethylation |  | DB Search |
| total 2 peptides |  |  |  |  |  |  |  |  |  |  |  |  |  |  |  |  |  |  |  |
| --- | --- | --- | --- | --- | --- | --- | --- | --- | --- | --- | --- | --- | --- | --- | --- | --- | --- | --- | --- |

Best Unique PSM (Scan Sample\_5\_A1\_1\_3643.d:37568, m/z=937.5062, z=1, RT=13.97, ppm=7.75):


TRINITY\_DN2477\_c0\_g1\_i6.p1

back to list

  

| Protein Coverage
| Supporting Peptides
| Best Unique PSM
|

Protein Coverage:

Supporting Peptides:

| Peptide | Uniq | -10lgP | Mass | Length | ppm | m/z | z | RT | 1/k0 Start | 1/k0 End | Precursor ID | Area Sample 7 | #Feature | #Feature Sample 7 | Start | End | PTM | AScore | Found By |
| --- | --- | --- | --- | --- | --- | --- | --- | --- | --- | --- | --- | --- | --- | --- | --- | --- | --- | --- | --- |
| K.DSYVGDEAQSKR.G | Y | 55.24 | 1353.616 | 12 | 0.18 | 677.8123 | 2 | 10.99 | 0.9932 | 1.0005 | 19155 | 1.01e3 | 1 | 1 | 52 | 63 |  |  | DB Search |
| R.VAPEEHPVLLTEAPLNPK.A | Y | 55.12 | 1953.0571 | 18 | 2.07 | 977.5333 | 2 | 16.62 | 1.2101 | 1.2173 | 52945 | 1.41e2 | 1 | 1 | 97 | 114 |  |  | DB Search |
| K.DSYVGDEAQSK.R | Y | 54.42 | 1197.5149 | 11 | 5.23 | 599.7651 | 2 | 11.40 | 0.8968 | 0.904 | 21678 | 5.55e2 | 1 | 1 | 52 | 62 |  |  | DB Search |
| total 3 peptides |  |  |  |  |  |  |  |  |  |  |  |  |  |  |  |  |  |  |  |
| --- | --- | --- | --- | --- | --- | --- | --- | --- | --- | --- | --- | --- | --- | --- | --- | --- | --- | --- | --- |

Best Unique PSM (Scan Sample\_5\_A1\_1\_3643.d:19155, m/z=677.8123, z=2, RT=10.99, ppm=-4.5):


TRINITY\_DN649\_c0\_g1\_i5.p1

back to list

  

| Protein Coverage
| Supporting Peptides
| Best Unique PSM
|

Protein Coverage:

Supporting Peptides:

| Peptide | Uniq | -10lgP | Mass | Length | ppm | m/z | z | RT | 1/k0 Start | 1/k0 End | Precursor ID | Area Sample 7 | #Feature | #Feature Sample 7 | Start | End | PTM | AScore | Found By |
| --- | --- | --- | --- | --- | --- | --- | --- | --- | --- | --- | --- | --- | --- | --- | --- | --- | --- | --- | --- |
| K.FETQIATK.N | Y | 53.77 | 936.4916 | 8 | 12.43 | 937.5062 | 1 | 13.97 | 1.1614 | 1.1686 | 37568 | 0 | 0 | 0 | 102 | 109 |  |  | DB Search |
| total 1 peptides |  |  |  |  |  |  |  |  |  |  |  |  |  |  |  |  |  |  |  |
| --- | --- | --- | --- | --- | --- | --- | --- | --- | --- | --- | --- | --- | --- | --- | --- | --- | --- | --- | --- |

Best Unique PSM (Scan Sample\_5\_A1\_1\_3643.d:37568, m/z=937.5062, z=1, RT=13.97, ppm=7.75):


TRINITY\_DN167\_c0\_g1\_i4.p1

back to list

  

| Protein Coverage
| Supporting Peptides
| Best Unique PSM
|

Protein Coverage:

Supporting Peptides:

| Peptide | Uniq | -10lgP | Mass | Length | ppm | m/z | z | RT | 1/k0 Start | 1/k0 End | Precursor ID | Area Sample 7 | #Feature | #Feature Sample 7 | Start | End | PTM | AScore | Found By |
| --- | --- | --- | --- | --- | --- | --- | --- | --- | --- | --- | --- | --- | --- | --- | --- | --- | --- | --- | --- |
| K.STAGDTHLGGEDFDNR.L | Y | 56.39 | 1690.7183 | 16 | 2.27 | 564.5787 | 3 | 13.29 | 0.8014 | 0.8088 | 33297 | 4.2e3 | 2 | 2 | 107 | 122 |  |  | DB Search |
| total 1 peptides |  |  |  |  |  |  |  |  |  |  |  |  |  |  |  |  |  |  |  |
| --- | --- | --- | --- | --- | --- | --- | --- | --- | --- | --- | --- | --- | --- | --- | --- | --- | --- | --- | --- |

Best Unique PSM (Scan Sample\_5\_A1\_1\_3643.d:33297, m/z=564.5787, z=3, RT=13.29, ppm=-2.41):


TRINITY\_DN5001\_c0\_g1\_i1.p1

back to list

  

| Protein Coverage
| Supporting Peptides
| Best Unique PSM
|

Protein Coverage:

Supporting Peptides:

| Peptide | Uniq | -10lgP | Mass | Length | ppm | m/z | z | RT | 1/k0 Start | 1/k0 End | Precursor ID | Area Sample 7 | #Feature | #Feature Sample 7 | Start | End | PTM | AScore | Found By |
| --- | --- | --- | --- | --- | --- | --- | --- | --- | --- | --- | --- | --- | --- | --- | --- | --- | --- | --- | --- |
| R.AIQGATSHHLGQNFSK.M | Y | 55.18 | 1694.8488 | 16 | 7.42 | 565.9584 | 3 | 12.02 | 0.8518 | 0.8591 | 25405 | 5.02e2 | 1 | 1 | 1293 | 1308 |  |  | DB Search |
| total 1 peptides |  |  |  |  |  |  |  |  |  |  |  |  |  |  |  |  |  |  |  |
| --- | --- | --- | --- | --- | --- | --- | --- | --- | --- | --- | --- | --- | --- | --- | --- | --- | --- | --- | --- |

Best Unique PSM (Scan Sample\_5\_A1\_1\_3643.d:25405, m/z=565.9584, z=3, RT=12.02, ppm=2.74):


TRINITY\_DN6428\_c0\_g1\_i1.p1

back to list

  

| Protein Coverage
| Supporting Peptides
| Best Unique PSM
|

Protein Coverage:

Supporting Peptides:

| Peptide | Uniq | -10lgP | Mass | Length | ppm | m/z | z | RT | 1/k0 Start | 1/k0 End | Precursor ID | Area Sample 7 | #Feature | #Feature Sample 7 | Start | End | PTM | AScore | Found By |
| --- | --- | --- | --- | --- | --- | --- | --- | --- | --- | --- | --- | --- | --- | --- | --- | --- | --- | --- | --- |
| R.SC(+57.02)ENPKPLNGGK.Q | Y | 56.12 | 1299.6241 | 12 | 2.81 | 650.8181 | 2 | 11.10 | 0.9469 | 0.9541 | 19780 | 7.77e2 | 1 | 1 | 245 | 256 | Carbamidomethylation |  | DB Search |
| total 1 peptides |  |  |  |  |  |  |  |  |  |  |  |  |  |  |  |  |  |  |  |
| --- | --- | --- | --- | --- | --- | --- | --- | --- | --- | --- | --- | --- | --- | --- | --- | --- | --- | --- | --- |

Best Unique PSM (Scan Sample\_5\_A1\_1\_3643.d:19780, m/z=650.8181, z=2, RT=11.10, ppm=-1.87):


TRINITY\_DN1058\_c0\_g1\_i4.p1

back to list

  

| Protein Coverage
| Supporting Peptides
| Best Unique PSM
|

Protein Coverage:

Supporting Peptides:

| Peptide | Uniq | -10lgP | Mass | Length | ppm | m/z | z | RT | 1/k0 Start | 1/k0 End | Precursor ID | Area Sample 7 | #Feature | #Feature Sample 7 | Start | End | PTM | AScore | Found By |
| --- | --- | --- | --- | --- | --- | --- | --- | --- | --- | --- | --- | --- | --- | --- | --- | --- | --- | --- | --- |
| R.APTHILNPANKR.S | Y | 52.25 | 1330.7469 | 12 | 5.05 | 444.5898 | 3 | 11.20 | 0.7481 | 0.7555 | 20091 | 3.03e3 | 1 | 1 | 398 | 409 |  |  | DB Search |
| total 1 peptides |  |  |  |  |  |  |  |  |  |  |  |  |  |  |  |  |  |  |  |
| --- | --- | --- | --- | --- | --- | --- | --- | --- | --- | --- | --- | --- | --- | --- | --- | --- | --- | --- | --- |

Best Unique PSM (Scan Sample\_5\_A1\_1\_3643.d:20091, m/z=444.5898, z=3, RT=11.20, ppm=0.37):


TRINITY\_DN120478\_c0\_g1\_i1.p1

back to list

  

| Protein Coverage
| Supporting Peptides
| Best Unique PSM
|

Protein Coverage:

Supporting Peptides:

| Peptide | Uniq | -10lgP | Mass | Length | ppm | m/z | z | RT | 1/k0 Start | 1/k0 End | Precursor ID | Area Sample 7 | #Feature | #Feature Sample 7 | Start | End | PTM | AScore | Found By |
| --- | --- | --- | --- | --- | --- | --- | --- | --- | --- | --- | --- | --- | --- | --- | --- | --- | --- | --- | --- |
| R.ILQLEEEFIKK.M | Y | 51.41 | 1388.7915 | 11 | -3.68 | 463.9339 | 3 | 17.84 | 0.7506 | 0.7579 | 60678 | 0 | 0 | 0 | 267 | 277 |  |  | DB Search |
| total 1 peptides |  |  |  |  |  |  |  |  |  |  |  |  |  |  |  |  |  |  |  |
| --- | --- | --- | --- | --- | --- | --- | --- | --- | --- | --- | --- | --- | --- | --- | --- | --- | --- | --- | --- |

Best Unique PSM (Scan Sample\_5\_A1\_1\_3643.d:60678, m/z=463.9339, z=3, RT=17.84, ppm=-8.36):


TRINITY\_DN5987\_c0\_g1\_i3.p2

back to list

  

| Protein Coverage
| Supporting Peptides
| Best Unique PSM
|

Protein Coverage:

Supporting Peptides:

| Peptide | Uniq | -10lgP | Mass | Length | ppm | m/z | z | RT | 1/k0 Start | 1/k0 End | Precursor ID | Area Sample 7 | #Feature | #Feature Sample 7 | Start | End | PTM | AScore | Found By |
| --- | --- | --- | --- | --- | --- | --- | --- | --- | --- | --- | --- | --- | --- | --- | --- | --- | --- | --- | --- |
| R.GNQGEQGPAGPVGPK.G | Y | 55.37 | 1391.6793 | 15 | 2.49 | 696.8454 | 2 | 11.50 | 0.9469 | 0.9541 | 22230 | 5.82e3 | 1 | 1 | 5 | 19 |  |  | DB Search |
| total 1 peptides |  |  |  |  |  |  |  |  |  |  |  |  |  |  |  |  |  |  |  |
| --- | --- | --- | --- | --- | --- | --- | --- | --- | --- | --- | --- | --- | --- | --- | --- | --- | --- | --- | --- |

Best Unique PSM (Scan Sample\_5\_A1\_1\_3643.d:22230, m/z=696.8454, z=2, RT=11.50, ppm=-2.19):


TRINITY\_DN9819\_c0\_g1\_i7.p2

back to list

  

| Protein Coverage
| Supporting Peptides
| Best Unique PSM
|

Protein Coverage:

Supporting Peptides:

| Peptide | Uniq | -10lgP | Mass | Length | ppm | m/z | z | RT | 1/k0 Start | 1/k0 End | Precursor ID | Area Sample 7 | #Feature | #Feature Sample 7 | Start | End | PTM | AScore | Found By |
| --- | --- | --- | --- | --- | --- | --- | --- | --- | --- | --- | --- | --- | --- | --- | --- | --- | --- | --- | --- |
| M.V(+42.01)HDLVLR.Q | Y | 49.6 | 892.513 | 7 | -1.25 | 447.2611 | 2 | 18.00 | 0.8136 | 0.8209 | 61579 | 2.99e2 | 1 | 1 | 2 | 8 | Acetylation (Protein N-term) | V1:Acetylation (Protein N-term):1000 | DB Search |
| total 1 peptides |  |  |  |  |  |  |  |  |  |  |  |  |  |  |  |  |  |  |  |
| --- | --- | --- | --- | --- | --- | --- | --- | --- | --- | --- | --- | --- | --- | --- | --- | --- | --- | --- | --- |

Best Unique PSM (Scan Sample\_5\_A1\_1\_3643.d:61579, m/z=447.2611, z=2, RT=18.00, ppm=-5.94):


TRINITY\_DN2477\_c0\_g1\_i2.p1

back to list

  

| Protein Coverage
| Supporting Peptides
| Best Unique PSM
|

Protein Coverage:

Supporting Peptides:

| Peptide | Uniq | -10lgP | Mass | Length | ppm | m/z | z | RT | 1/k0 Start | 1/k0 End | Precursor ID | Area Sample 7 | #Feature | #Feature Sample 7 | Start | End | PTM | AScore | Found By |
| --- | --- | --- | --- | --- | --- | --- | --- | --- | --- | --- | --- | --- | --- | --- | --- | --- | --- | --- | --- |
| K.DSYVGDEAQSKR.G | Y | 55.24 | 1353.616 | 12 | 0.18 | 677.8123 | 2 | 10.99 | 0.9932 | 1.0005 | 19155 | 1.01e3 | 1 | 1 | 19 | 30 |  |  | DB Search |
| R.VAPEEHPVLLTEAPLNPK.A | Y | 55.12 | 1953.0571 | 18 | 2.07 | 977.5333 | 2 | 16.62 | 1.2101 | 1.2173 | 52945 | 1.41e2 | 1 | 1 | 64 | 81 |  |  | DB Search |
| K.DSYVGDEAQSK.R | Y | 54.42 | 1197.5149 | 11 | 5.23 | 599.7651 | 2 | 11.40 | 0.8968 | 0.904 | 21678 | 5.55e2 | 1 | 1 | 19 | 29 |  |  | DB Search |
| total 3 peptides |  |  |  |  |  |  |  |  |  |  |  |  |  |  |  |  |  |  |  |
| --- | --- | --- | --- | --- | --- | --- | --- | --- | --- | --- | --- | --- | --- | --- | --- | --- | --- | --- | --- |

Best Unique PSM (Scan Sample\_5\_A1\_1\_3643.d:19155, m/z=677.8123, z=2, RT=10.99, ppm=-4.5):


TRINITY\_DN2885\_c0\_g1\_i4.p1

back to list

  

| Protein Coverage
| Supporting Peptides
| Best Unique PSM
|

Protein Coverage:

Supporting Peptides:

| Peptide | Uniq | -10lgP | Mass | Length | ppm | m/z | z | RT | 1/k0 Start | 1/k0 End | Precursor ID | Area Sample 7 | #Feature | #Feature Sample 7 | Start | End | PTM | AScore | Found By |
| --- | --- | --- | --- | --- | --- | --- | --- | --- | --- | --- | --- | --- | --- | --- | --- | --- | --- | --- | --- |
| K.VTRPHGASGVVR.A | Y | 56.37 | 1234.6894 | 12 | 3.4 | 412.5699 | 3 | 10.07 | 0.8104 | 0.8177 | 14175 | 0 | 0 | 0 | 72 | 83 |  |  | DB Search |
| total 1 peptides |  |  |  |  |  |  |  |  |  |  |  |  |  |  |  |  |  |  |  |
| --- | --- | --- | --- | --- | --- | --- | --- | --- | --- | --- | --- | --- | --- | --- | --- | --- | --- | --- | --- |

Best Unique PSM (Scan Sample\_5\_A1\_1\_3643.d:14175, m/z=412.5699, z=3, RT=10.07, ppm=-1.29):


TRINITY\_DN2113\_c0\_g1\_i2.p1

back to list

  

| Protein Coverage
| Supporting Peptides
| Best Unique PSM
|

Protein Coverage:

Supporting Peptides:

| Peptide | Uniq | -10lgP | Mass | Length | ppm | m/z | z | RT | 1/k0 Start | 1/k0 End | Precursor ID | Area Sample 7 | #Feature | #Feature Sample 7 | Start | End | PTM | AScore | Found By |
| --- | --- | --- | --- | --- | --- | --- | --- | --- | --- | --- | --- | --- | --- | --- | --- | --- | --- | --- | --- |
| R.AHVAQLC(+57.02)EK.A | Y | 51.4 | 1054.5229 | 9 | 8.37 | 528.2707 | 2 | 10.76 | 0.8583 | 0.8656 | 17883 | 1.38e3 | 1 | 1 | 611 | 619 | Carbamidomethylation |  | DB Search |
| R.LADLEEFISSPNHANISQVGER.C | Y | 49.83 | 2425.1873 | 22 | 1.54 | 809.4005 | 3 | 18.91 | 0.9238 | 0.9311 | 67723 | 2.6e3 | 1 | 1 | 1183 | 1204 |  |  | DB Search |
| K.GQFSTDDLVAEVEKR.N | Y | 47.98 | 1692.8318 | 15 | -2.37 | 565.2806 | 3 | 17.82 | 0.8518 | 0.8591 | 60505 | 0 | 0 | 0 | 838 | 852 |  |  | DB Search |
| total 3 peptides |  |  |  |  |  |  |  |  |  |  |  |  |  |  |  |  |  |  |  |
| --- | --- | --- | --- | --- | --- | --- | --- | --- | --- | --- | --- | --- | --- | --- | --- | --- | --- | --- | --- |

Best Unique PSM (Scan Sample\_5\_A1\_1\_3643.d:17883, m/z=528.2707, z=2, RT=10.76, ppm=3.69):


TRINITY\_DN1910\_c0\_g1\_i1.p1

back to list

  

| Protein Coverage
| Supporting Peptides
| Best Unique PSM
|

Protein Coverage:

Supporting Peptides:

| Peptide | Uniq | -10lgP | Mass | Length | ppm | m/z | z | RT | 1/k0 Start | 1/k0 End | Precursor ID | Area Sample 7 | #Feature | #Feature Sample 7 | Start | End | PTM | AScore | Found By |
| --- | --- | --- | --- | --- | --- | --- | --- | --- | --- | --- | --- | --- | --- | --- | --- | --- | --- | --- | --- |
| R.YLTVAAMFR.G | Y | 55.34 | 1070.5583 | 9 | -0.51 | 536.2836 | 2 | 19.90 | 0.8656 | 0.8729 | 74532 | 0 | 0 | 0 | 310 | 318 |  |  | DB Search |
| total 1 peptides |  |  |  |  |  |  |  |  |  |  |  |  |  |  |  |  |  |  |  |
| --- | --- | --- | --- | --- | --- | --- | --- | --- | --- | --- | --- | --- | --- | --- | --- | --- | --- | --- | --- |

Best Unique PSM (Scan Sample\_5\_A1\_1\_3643.d:74532, m/z=536.2836, z=2, RT=19.90, ppm=-5.19):


TRINITY\_DN1030\_c0\_g1\_i2.p1

back to list

  

| Protein Coverage
| Supporting Peptides
| Best Unique PSM
|

Protein Coverage:

Supporting Peptides:

| Peptide | Uniq | -10lgP | Mass | Length | ppm | m/z | z | RT | 1/k0 Start | 1/k0 End | Precursor ID | Area Sample 7 | #Feature | #Feature Sample 7 | Start | End | PTM | AScore | Found By |
| --- | --- | --- | --- | --- | --- | --- | --- | --- | --- | --- | --- | --- | --- | --- | --- | --- | --- | --- | --- |
| R.GLQGQQGEQGSTGVQGSVGGR.G | Y | 56.41 | 1985.9515 | 21 | 4.71 | 993.983 | 2 | 12.10 | 1.139 | 1.1462 | 25849 | 1.24e3 | 1 | 1 | 348 | 368 |  |  | DB Search |
| total 1 peptides |  |  |  |  |  |  |  |  |  |  |  |  |  |  |  |  |  |  |  |
| --- | --- | --- | --- | --- | --- | --- | --- | --- | --- | --- | --- | --- | --- | --- | --- | --- | --- | --- | --- |

Best Unique PSM (Scan Sample\_5\_A1\_1\_3643.d:25849, m/z=993.983, z=2, RT=12.10, ppm=0.03):


TRINITY\_DN11248\_c0\_g1\_i3.p1

back to list

  

| Protein Coverage
| Supporting Peptides
| Best Unique PSM
|

Protein Coverage:

Supporting Peptides:

| Peptide | Uniq | -10lgP | Mass | Length | ppm | m/z | z | RT | 1/k0 Start | 1/k0 End | Precursor ID | Area Sample 7 | #Feature | #Feature Sample 7 | Start | End | PTM | AScore | Found By |
| --- | --- | --- | --- | --- | --- | --- | --- | --- | --- | --- | --- | --- | --- | --- | --- | --- | --- | --- | --- |
| K.TPYLQIDFGK.M | Y | 52.02 | 1180.6128 | 10 | -2.38 | 591.3095 | 2 | 18.44 | 0.917 | 0.9243 | 64648 | 4.48e2 | 1 | 1 | 514 | 523 |  |  | DB Search |
| total 1 peptides |  |  |  |  |  |  |  |  |  |  |  |  |  |  |  |  |  |  |  |
| --- | --- | --- | --- | --- | --- | --- | --- | --- | --- | --- | --- | --- | --- | --- | --- | --- | --- | --- | --- |

Best Unique PSM (Scan Sample\_5\_A1\_1\_3643.d:64648, m/z=591.3095, z=2, RT=18.44, ppm=-7.06):


TRINITY\_DN785\_c0\_g1\_i1.p1

back to list

  

| Protein Coverage
| Supporting Peptides
| Best Unique PSM
|

Protein Coverage:

Supporting Peptides:

| Peptide | Uniq | -10lgP | Mass | Length | ppm | m/z | z | RT | 1/k0 Start | 1/k0 End | Precursor ID | Area Sample 7 | #Feature | #Feature Sample 7 | Start | End | PTM | AScore | Found By |
| --- | --- | --- | --- | --- | --- | --- | --- | --- | --- | --- | --- | --- | --- | --- | --- | --- | --- | --- | --- |
| K.KFDTYLPGFIC(+57.02)R.C | Y | 55.35 | 1515.7544 | 12 | -1.87 | 506.2554 | 3 | 18.73 | 0.7856 | 0.7929 | 66611 | 0 | 0 | 0 | 77 | 88 | Carbamidomethylation |  | DB Search |
| total 1 peptides |  |  |  |  |  |  |  |  |  |  |  |  |  |  |  |  |  |  |  |
| --- | --- | --- | --- | --- | --- | --- | --- | --- | --- | --- | --- | --- | --- | --- | --- | --- | --- | --- | --- |

Best Unique PSM (Scan Sample\_5\_A1\_1\_3643.d:66611, m/z=506.2554, z=3, RT=18.73, ppm=-6.55):


TRINITY\_DN2941\_c0\_g1\_i4.p1

back to list

  

| Protein Coverage
| Supporting Peptides
| Best Unique PSM
|

Protein Coverage:

Supporting Peptides:

| Peptide | Uniq | -10lgP | Mass | Length | ppm | m/z | z | RT | 1/k0 Start | 1/k0 End | Precursor ID | Area Sample 7 | #Feature | #Feature Sample 7 | Start | End | PTM | AScore | Found By |
| --- | --- | --- | --- | --- | --- | --- | --- | --- | --- | --- | --- | --- | --- | --- | --- | --- | --- | --- | --- |
| R.VAPEEHPVLLTEAPLNPK.A | Y | 55.12 | 1953.0571 | 18 | 2.07 | 977.5333 | 2 | 16.62 | 1.2101 | 1.2173 | 52945 | 1.41e2 | 1 | 1 | 30 | 47 |  |  | DB Search |
| total 1 peptides |  |  |  |  |  |  |  |  |  |  |  |  |  |  |  |  |  |  |  |
| --- | --- | --- | --- | --- | --- | --- | --- | --- | --- | --- | --- | --- | --- | --- | --- | --- | --- | --- | --- |

Best Unique PSM (Scan Sample\_5\_A1\_1\_3643.d:52945, m/z=977.5333, z=2, RT=16.62, ppm=-2.61):


TRINITY\_DN602\_c0\_g1\_i1.p1

back to list

  

| Protein Coverage
| Supporting Peptides
| Best Unique PSM
|

Protein Coverage:

Supporting Peptides:

| Peptide | Uniq | -10lgP | Mass | Length | ppm | m/z | z | RT | 1/k0 Start | 1/k0 End | Precursor ID | Area Sample 7 | #Feature | #Feature Sample 7 | Start | End | PTM | AScore | Found By |
| --- | --- | --- | --- | --- | --- | --- | --- | --- | --- | --- | --- | --- | --- | --- | --- | --- | --- | --- | --- |
| K.EGFYVHEALAAGIVK.K | Y | 56.36 | 1602.8405 | 15 | 7.5 | 802.4298 | 2 | 17.69 | 1.0873 | 1.0945 | 59517 | 5.28e2 | 1 | 1 | 185 | 199 |  |  | DB Search |
| K.LLTSPAPSSGSLM(+15.99)ALALK.I | Y | 56.18 | 1771.9753 | 18 | -1.19 | 886.9897 | 2 | 18.28 | 1.1498 | 1.157 | 63418 | 9.34e2 | 1 | 1 | 292 | 309 | Oxidation (M) | M13:Oxidation (M):1000 | DB Search |
| K.HGADLFYR.G | Y | 53.19 | 977.4719 | 8 | 0.63 | 489.7412 | 2 | 16.16 | 0.8376 | 0.8449 | 50305 | 4.17e2 | 1 | 1 | 242 | 249 |  |  | DB Search |
| total 3 peptides |  |  |  |  |  |  |  |  |  |  |  |  |  |  |  |  |  |  |  |
| --- | --- | --- | --- | --- | --- | --- | --- | --- | --- | --- | --- | --- | --- | --- | --- | --- | --- | --- | --- |

Best Unique PSM (Scan Sample\_5\_A1\_1\_3643.d:59517, m/z=802.4298, z=2, RT=17.69, ppm=2.83):


TRINITY\_DN21998\_c0\_g1\_i1.p1

back to list

  

| Protein Coverage
| Supporting Peptides
| Best Unique PSM
|

Protein Coverage:

Supporting Peptides:

| Peptide | Uniq | -10lgP | Mass | Length | ppm | m/z | z | RT | 1/k0 Start | 1/k0 End | Precursor ID | Area Sample 7 | #Feature | #Feature Sample 7 | Start | End | PTM | AScore | Found By |
| --- | --- | --- | --- | --- | --- | --- | --- | --- | --- | --- | --- | --- | --- | --- | --- | --- | --- | --- | --- |
| K.LTHLEEAYTTEHWLVR.I | Y | 51.48 | 1997.0006 | 16 | 4.65 | 666.6741 | 3 | 17.32 | 0.9594 | 0.9666 | 57137 | 0 | 0 | 0 | 421 | 436 |  |  | DB Search |
| total 1 peptides |  |  |  |  |  |  |  |  |  |  |  |  |  |  |  |  |  |  |  |
| --- | --- | --- | --- | --- | --- | --- | --- | --- | --- | --- | --- | --- | --- | --- | --- | --- | --- | --- | --- |

Best Unique PSM (Scan Sample\_5\_A1\_1\_3643.d:57137, m/z=666.6741, z=3, RT=17.32, ppm=-0.03):


TRINITY\_DN36\_c0\_g1\_i16.p1

back to list

  

| Protein Coverage
| Supporting Peptides
| Best Unique PSM
|

Protein Coverage:

Supporting Peptides:

| Peptide | Uniq | -10lgP | Mass | Length | ppm | m/z | z | RT | 1/k0 Start | 1/k0 End | Precursor ID | Area Sample 7 | #Feature | #Feature Sample 7 | Start | End | PTM | AScore | Found By |
| --- | --- | --- | --- | --- | --- | --- | --- | --- | --- | --- | --- | --- | --- | --- | --- | --- | --- | --- | --- |
| K.FETQIATK.D | Y | 53.77 | 936.4916 | 8 | 12.43 | 937.5062 | 1 | 13.97 | 1.1614 | 1.1686 | 37568 | 0 | 0 | 0 | 167 | 174 |  |  | DB Search |
| R.LGGIEVTQGNC(+57.02)AR.K | Y | 49.67 | 1373.6721 | 13 | 2.61 | 687.8419 | 2 | 13.89 | 0.9695 | 0.9767 | 37015 | 0 | 0 | 0 | 58 | 70 | Carbamidomethylation |  | DB Search |
| total 2 peptides |  |  |  |  |  |  |  |  |  |  |  |  |  |  |  |  |  |  |  |
| --- | --- | --- | --- | --- | --- | --- | --- | --- | --- | --- | --- | --- | --- | --- | --- | --- | --- | --- | --- |

Best Unique PSM (Scan Sample\_5\_A1\_1\_3643.d:37568, m/z=937.5062, z=1, RT=13.97, ppm=7.75):


TRINITY\_DN598\_c1\_g1\_i2.p1

back to list

  

| Protein Coverage
| Supporting Peptides
| Best Unique PSM
|

Protein Coverage:

Supporting Peptides:

| Peptide | Uniq | -10lgP | Mass | Length | ppm | m/z | z | RT | 1/k0 Start | 1/k0 End | Precursor ID | Area Sample 7 | #Feature | #Feature Sample 7 | Start | End | PTM | AScore | Found By |
| --- | --- | --- | --- | --- | --- | --- | --- | --- | --- | --- | --- | --- | --- | --- | --- | --- | --- | --- | --- |
| R.ASFVTDIDRLR.A | Y | 49.26 | 1291.6884 | 11 | -0.87 | 646.8479 | 2 | 17.21 | 0.992 | 0.9993 | 56353 | 6.03e2 | 1 | 1 | 302 | 312 |  |  | DB Search |
| total 1 peptides |  |  |  |  |  |  |  |  |  |  |  |  |  |  |  |  |  |  |  |
| --- | --- | --- | --- | --- | --- | --- | --- | --- | --- | --- | --- | --- | --- | --- | --- | --- | --- | --- | --- |

Best Unique PSM (Scan Sample\_5\_A1\_1\_3643.d:56353, m/z=646.8479, z=2, RT=17.21, ppm=-5.55):


TRINITY\_DN69276\_c0\_g1\_i1.p1

back to list

  

| Protein Coverage
| Supporting Peptides
| Best Unique PSM
|

Protein Coverage:

Supporting Peptides:

| Peptide | Uniq | -10lgP | Mass | Length | ppm | m/z | z | RT | 1/k0 Start | 1/k0 End | Precursor ID | Area Sample 7 | #Feature | #Feature Sample 7 | Start | End | PTM | AScore | Found By |
| --- | --- | --- | --- | --- | --- | --- | --- | --- | --- | --- | --- | --- | --- | --- | --- | --- | --- | --- | --- |
| K.VIDINDNIPIFSK.S | Y | 56.38 | 1486.8031 | 13 | 0.06 | 744.4054 | 2 | 19.67 | 1.0238 | 1.0311 | 72860 | 1.48e3 | 1 | 1 | 301 | 313 |  |  | DB Search |
| total 1 peptides |  |  |  |  |  |  |  |  |  |  |  |  |  |  |  |  |  |  |  |
| --- | --- | --- | --- | --- | --- | --- | --- | --- | --- | --- | --- | --- | --- | --- | --- | --- | --- | --- | --- |

Best Unique PSM (Scan Sample\_5\_A1\_1\_3643.d:72860, m/z=744.4054, z=2, RT=19.67, ppm=-4.62):


TRINITY\_DN1613\_c1\_g1\_i2.p1

back to list

  

| Protein Coverage
| Supporting Peptides
| Best Unique PSM
|

Protein Coverage:

Supporting Peptides:

| Peptide | Uniq | -10lgP | Mass | Length | ppm | m/z | z | RT | 1/k0 Start | 1/k0 End | Precursor ID | Area Sample 7 | #Feature | #Feature Sample 7 | Start | End | PTM | AScore | Found By |
| --- | --- | --- | --- | --- | --- | --- | --- | --- | --- | --- | --- | --- | --- | --- | --- | --- | --- | --- | --- |
| R.S(+42.01)C(+57.02)SNPSPAHGGKPC(+57.02)SGELSK.T | Y | 47.79 | 2097.9208 | 20 | 5.48 | 700.3148 | 3 | 11.22 | 0.8785 | 0.8858 | 20616 | 3.89e2 | 1 | 1 | 14 | 33 | Acetylation (Protein N-term), Carbamidomethylation, Carbamidomethylation | S1:Acetylation (Protein N-term):1000 | DB Search |
| total 1 peptides |  |  |  |  |  |  |  |  |  |  |  |  |  |  |  |  |  |  |  |
| --- | --- | --- | --- | --- | --- | --- | --- | --- | --- | --- | --- | --- | --- | --- | --- | --- | --- | --- | --- |

Best Unique PSM (Scan Sample\_5\_A1\_1\_3643.d:20616, m/z=700.3148, z=3, RT=11.22, ppm=0.8):


TRINITY\_DN123121\_c0\_g1\_i1.p1

back to list

  

| Protein Coverage
| Supporting Peptides
| Best Unique PSM
|

Protein Coverage:

Supporting Peptides:

| Peptide | Uniq | -10lgP | Mass | Length | ppm | m/z | z | RT | 1/k0 Start | 1/k0 End | Precursor ID | Area Sample 7 | #Feature | #Feature Sample 7 | Start | End | PTM | AScore | Found By |
| --- | --- | --- | --- | --- | --- | --- | --- | --- | --- | --- | --- | --- | --- | --- | --- | --- | --- | --- | --- |
| R.LTHQIEVTVSDREGK.S | Y | 56.23 | 1710.89 | 15 | -5.27 | 571.2983 | 3 | 12.85 | 0.8696 | 0.8769 | 30546 | 0 | 0 | 0 | 94 | 108 |  |  | DB Search |
| R.LTHQIEVTVSDR.E | Y | 55.36 | 1396.731 | 12 | 1.03 | 699.3702 | 2 | 13.57 | 0.9965 | 1.0037 | 35021 | 1.59e3 | 1 | 1 | 94 | 105 |  |  | DB Search |
| total 2 peptides |  |  |  |  |  |  |  |  |  |  |  |  |  |  |  |  |  |  |  |
| --- | --- | --- | --- | --- | --- | --- | --- | --- | --- | --- | --- | --- | --- | --- | --- | --- | --- | --- | --- |

Best Unique PSM (Scan Sample\_5\_A1\_1\_3643.d:30546, m/z=571.2983, z=3, RT=12.85, ppm=-9.95):


TRINITY\_DN3016\_c0\_g1\_i5.p1

back to list

  

| Protein Coverage
| Supporting Peptides
| Best Unique PSM
|

Protein Coverage:

Supporting Peptides:

| Peptide | Uniq | -10lgP | Mass | Length | ppm | m/z | z | RT | 1/k0 Start | 1/k0 End | Precursor ID | Area Sample 7 | #Feature | #Feature Sample 7 | Start | End | PTM | AScore | Found By |
| --- | --- | --- | --- | --- | --- | --- | --- | --- | --- | --- | --- | --- | --- | --- | --- | --- | --- | --- | --- |
| R.IVNTDLQEIKDIIK.G | Y | 56.35 | 1640.9348 | 14 | -0.94 | 547.9825 | 3 | 18.84 | 0.8193 | 0.8266 | 67326 | 7.77e2 | 1 | 1 | 154 | 167 |  |  | DB Search |
| total 1 peptides |  |  |  |  |  |  |  |  |  |  |  |  |  |  |  |  |  |  |  |
| --- | --- | --- | --- | --- | --- | --- | --- | --- | --- | --- | --- | --- | --- | --- | --- | --- | --- | --- | --- |

Best Unique PSM (Scan Sample\_5\_A1\_1\_3643.d:67326, m/z=547.9825, z=3, RT=18.84, ppm=-5.62):


TRINITY\_DN554\_c0\_g1\_i3.p2

back to list

  

| Protein Coverage
| Supporting Peptides
| Best Unique PSM
|

Protein Coverage:

Supporting Peptides:

| Peptide | Uniq | -10lgP | Mass | Length | ppm | m/z | z | RT | 1/k0 Start | 1/k0 End | Precursor ID | Area Sample 7 | #Feature | #Feature Sample 7 | Start | End | PTM | AScore | Found By |
| --- | --- | --- | --- | --- | --- | --- | --- | --- | --- | --- | --- | --- | --- | --- | --- | --- | --- | --- | --- |
| K.TITLEVEPSDTIENVK.S | Y | 55.12 | 1786.92 | 16 | 0.46 | 894.4635 | 2 | 17.55 | 1.1414 | 1.1486 | 58604 | 8.72e2 | 1 | 1 | 12 | 27 |  |  | DB Search |
| total 1 peptides |  |  |  |  |  |  |  |  |  |  |  |  |  |  |  |  |  |  |  |
| --- | --- | --- | --- | --- | --- | --- | --- | --- | --- | --- | --- | --- | --- | --- | --- | --- | --- | --- | --- |

Best Unique PSM (Scan Sample\_5\_A1\_1\_3643.d:58604, m/z=894.4635, z=2, RT=17.55, ppm=-4.22):


TRINITY\_DN15847\_c0\_g1\_i1.p1

back to list

  

| Protein Coverage
| Supporting Peptides
| Best Unique PSM
|

Protein Coverage:

Supporting Peptides:

| Peptide | Uniq | -10lgP | Mass | Length | ppm | m/z | z | RT | 1/k0 Start | 1/k0 End | Precursor ID | Area Sample 7 | #Feature | #Feature Sample 7 | Start | End | PTM | AScore | Found By |
| --- | --- | --- | --- | --- | --- | --- | --- | --- | --- | --- | --- | --- | --- | --- | --- | --- | --- | --- | --- |
| R.EAIEGTYIDKK.C | Y | 55.33 | 1265.6503 | 11 | 3.67 | 633.8318 | 2 | 12.92 | 0.9505 | 0.9578 | 30926 | 8.95e2 | 1 | 1 | 56 | 66 |  |  | DB Search |
| total 1 peptides |  |  |  |  |  |  |  |  |  |  |  |  |  |  |  |  |  |  |  |
| --- | --- | --- | --- | --- | --- | --- | --- | --- | --- | --- | --- | --- | --- | --- | --- | --- | --- | --- | --- |

Best Unique PSM (Scan Sample\_5\_A1\_1\_3643.d:30926, m/z=633.8318, z=2, RT=12.92, ppm=-1.01):


TRINITY\_DN4595\_c0\_g1\_i10.p1

back to list

  

| Protein Coverage
| Supporting Peptides
| Best Unique PSM
|

Protein Coverage:

Supporting Peptides:

| Peptide | Uniq | -10lgP | Mass | Length | ppm | m/z | z | RT | 1/k0 Start | 1/k0 End | Precursor ID | Area Sample 7 | #Feature | #Feature Sample 7 | Start | End | PTM | AScore | Found By |
| --- | --- | --- | --- | --- | --- | --- | --- | --- | --- | --- | --- | --- | --- | --- | --- | --- | --- | --- | --- |
| R.SPFVIMFK.G | Y | 54.61 | 967.5201 | 8 | 4.71 | 484.7673 | 2 | 19.89 | 0.81 | 0.8173 | 74398 | 6.85e2 | 1 | 1 | 630 | 637 |  |  | DB Search |
| R.FC(+57.02)NNPAPSNGGR.M | Y | 51.44 | 1289.5571 | 12 | 4.78 | 645.7859 | 2 | 10.87 | 0.944 | 0.9513 | 18646 | 0 | 0 | 0 | 745 | 756 | Carbamidomethylation |  | DB Search |
| K.YTGAC(+57.02)NANGNTDK.T | Y | 49.38 | 1384.5677 | 13 | 0 | 693.2879 | 2 | 9.54 | 0.9315 | 0.9388 | 11651 | 4.52e2 | 1 | 1 | 1041 | 1053 | Carbamidomethylation |  | DB Search |
| total 3 peptides |  |  |  |  |  |  |  |  |  |  |  |  |  |  |  |  |  |  |  |
| --- | --- | --- | --- | --- | --- | --- | --- | --- | --- | --- | --- | --- | --- | --- | --- | --- | --- | --- | --- |

Best Unique PSM (Scan Sample\_5\_A1\_1\_3643.d:74398, m/z=484.7673, z=2, RT=19.89, ppm=0.03):


TRINITY\_DN554\_c0\_g1\_i4.p1

back to list

  

| Protein Coverage
| Supporting Peptides
| Best Unique PSM
|

Protein Coverage:

Supporting Peptides:

| Peptide | Uniq | -10lgP | Mass | Length | ppm | m/z | z | RT | 1/k0 Start | 1/k0 End | Precursor ID | Area Sample 7 | #Feature | #Feature Sample 7 | Start | End | PTM | AScore | Found By |
| --- | --- | --- | --- | --- | --- | --- | --- | --- | --- | --- | --- | --- | --- | --- | --- | --- | --- | --- | --- |
| K.TITLEVEPSDTIENVK.S | Y | 55.12 | 1786.92 | 16 | 0.46 | 894.4635 | 2 | 17.55 | 1.1414 | 1.1486 | 58604 | 8.72e2 | 1 | 1 | 12 | 27 |  |  | DB Search |
| total 1 peptides |  |  |  |  |  |  |  |  |  |  |  |  |  |  |  |  |  |  |  |
| --- | --- | --- | --- | --- | --- | --- | --- | --- | --- | --- | --- | --- | --- | --- | --- | --- | --- | --- | --- |

Best Unique PSM (Scan Sample\_5\_A1\_1\_3643.d:58604, m/z=894.4635, z=2, RT=17.55, ppm=-4.22):


TRINITY\_DN3016\_c0\_g1\_i10.p1

back to list

  

| Protein Coverage
| Supporting Peptides
| Best Unique PSM
|

Protein Coverage:

Supporting Peptides:

| Peptide | Uniq | -10lgP | Mass | Length | ppm | m/z | z | RT | 1/k0 Start | 1/k0 End | Precursor ID | Area Sample 7 | #Feature | #Feature Sample 7 | Start | End | PTM | AScore | Found By |
| --- | --- | --- | --- | --- | --- | --- | --- | --- | --- | --- | --- | --- | --- | --- | --- | --- | --- | --- | --- |
| R.IVNTDLQEIKDIIK.G | Y | 56.35 | 1640.9348 | 14 | -0.94 | 547.9825 | 3 | 18.84 | 0.8193 | 0.8266 | 67326 | 7.77e2 | 1 | 1 | 154 | 167 |  |  | DB Search |
| total 1 peptides |  |  |  |  |  |  |  |  |  |  |  |  |  |  |  |  |  |  |  |
| --- | --- | --- | --- | --- | --- | --- | --- | --- | --- | --- | --- | --- | --- | --- | --- | --- | --- | --- | --- |

Best Unique PSM (Scan Sample\_5\_A1\_1\_3643.d:67326, m/z=547.9825, z=3, RT=18.84, ppm=-5.62):


TRINITY\_DN167\_c0\_g2\_i1.p1

back to list

  

| Protein Coverage
| Supporting Peptides
| Best Unique PSM
|

Protein Coverage:

Supporting Peptides:

| Peptide | Uniq | -10lgP | Mass | Length | ppm | m/z | z | RT | 1/k0 Start | 1/k0 End | Precursor ID | Area Sample 7 | #Feature | #Feature Sample 7 | Start | End | PTM | AScore | Found By |
| --- | --- | --- | --- | --- | --- | --- | --- | --- | --- | --- | --- | --- | --- | --- | --- | --- | --- | --- | --- |
| K.STAGDTHLGGEDFDNR.M | Y | 56.39 | 1690.7183 | 16 | 2.27 | 564.5787 | 3 | 13.29 | 0.8014 | 0.8088 | 33297 | 4.2e3 | 2 | 2 | 221 | 236 |  |  | DB Search |
| total 1 peptides |  |  |  |  |  |  |  |  |  |  |  |  |  |  |  |  |  |  |  |
| --- | --- | --- | --- | --- | --- | --- | --- | --- | --- | --- | --- | --- | --- | --- | --- | --- | --- | --- | --- |

Best Unique PSM (Scan Sample\_5\_A1\_1\_3643.d:33297, m/z=564.5787, z=3, RT=13.29, ppm=-2.41):


TRINITY\_DN167\_c0\_g1\_i10.p1

back to list

  

| Protein Coverage
| Supporting Peptides
| Best Unique PSM
|

Protein Coverage:

Supporting Peptides:

| Peptide | Uniq | -10lgP | Mass | Length | ppm | m/z | z | RT | 1/k0 Start | 1/k0 End | Precursor ID | Area Sample 7 | #Feature | #Feature Sample 7 | Start | End | PTM | AScore | Found By |
| --- | --- | --- | --- | --- | --- | --- | --- | --- | --- | --- | --- | --- | --- | --- | --- | --- | --- | --- | --- |
| K.STAGDTHLGGEDFDNR.L | Y | 56.39 | 1690.7183 | 16 | 2.27 | 564.5787 | 3 | 13.29 | 0.8014 | 0.8088 | 33297 | 4.2e3 | 2 | 2 | 107 | 122 |  |  | DB Search |
| total 1 peptides |  |  |  |  |  |  |  |  |  |  |  |  |  |  |  |  |  |  |  |
| --- | --- | --- | --- | --- | --- | --- | --- | --- | --- | --- | --- | --- | --- | --- | --- | --- | --- | --- | --- |

Best Unique PSM (Scan Sample\_5\_A1\_1\_3643.d:33297, m/z=564.5787, z=3, RT=13.29, ppm=-2.41):


TRINITY\_DN9819\_c0\_g1\_i1.p2

back to list

  

| Protein Coverage
| Supporting Peptides
| Best Unique PSM
|

Protein Coverage:

Supporting Peptides:

| Peptide | Uniq | -10lgP | Mass | Length | ppm | m/z | z | RT | 1/k0 Start | 1/k0 End | Precursor ID | Area Sample 7 | #Feature | #Feature Sample 7 | Start | End | PTM | AScore | Found By |
| --- | --- | --- | --- | --- | --- | --- | --- | --- | --- | --- | --- | --- | --- | --- | --- | --- | --- | --- | --- |
| M.V(+42.01)HDLVLR.Q | Y | 49.6 | 892.513 | 7 | -1.25 | 447.2611 | 2 | 18.00 | 0.8136 | 0.8209 | 61579 | 2.99e2 | 1 | 1 | 2 | 8 | Acetylation (Protein N-term) | V1:Acetylation (Protein N-term):1000 | DB Search |
| total 1 peptides |  |  |  |  |  |  |  |  |  |  |  |  |  |  |  |  |  |  |  |
| --- | --- | --- | --- | --- | --- | --- | --- | --- | --- | --- | --- | --- | --- | --- | --- | --- | --- | --- | --- |

Best Unique PSM (Scan Sample\_5\_A1\_1\_3643.d:61579, m/z=447.2611, z=2, RT=18.00, ppm=-5.94):


TRINITY\_DN7299\_c0\_g1\_i1.p2

back to list

  

| Protein Coverage
| Supporting Peptides
| Best Unique PSM
|

Protein Coverage:

Supporting Peptides:

| Peptide | Uniq | -10lgP | Mass | Length | ppm | m/z | z | RT | 1/k0 Start | 1/k0 End | Precursor ID | Area Sample 7 | #Feature | #Feature Sample 7 | Start | End | PTM | AScore | Found By |
| --- | --- | --- | --- | --- | --- | --- | --- | --- | --- | --- | --- | --- | --- | --- | --- | --- | --- | --- | --- |
| R.LGFGEEAQQHFR.Q | Y | 56.11 | 1417.6738 | 12 | 3.8 | 709.8436 | 2 | 15.03 | 1.0271 | 1.0343 | 43872 | 0 | 0 | 0 | 301 | 312 |  |  | DB Search |
| total 1 peptides |  |  |  |  |  |  |  |  |  |  |  |  |  |  |  |  |  |  |  |
| --- | --- | --- | --- | --- | --- | --- | --- | --- | --- | --- | --- | --- | --- | --- | --- | --- | --- | --- | --- |

Best Unique PSM (Scan Sample\_5\_A1\_1\_3643.d:43872, m/z=709.8436, z=2, RT=15.03, ppm=-0.88):


TRINITY\_DN160\_c0\_g1\_i1.p1

back to list

  

| Protein Coverage
| Supporting Peptides
| Best Unique PSM
|

Protein Coverage:

Supporting Peptides:

| Peptide | Uniq | -10lgP | Mass | Length | ppm | m/z | z | RT | 1/k0 Start | 1/k0 End | Precursor ID | Area Sample 7 | #Feature | #Feature Sample 7 | Start | End | PTM | AScore | Found By |
| --- | --- | --- | --- | --- | --- | --- | --- | --- | --- | --- | --- | --- | --- | --- | --- | --- | --- | --- | --- |
| K.LRLDFDHEAK.L | Y | 56.21 | 1242.6356 | 10 | -2.49 | 415.2162 | 3 | 14.37 | 0.7326 | 0.74 | 39914 | 3.79e2 | 1 | 1 | 1007 | 1016 |  |  | DB Search |
| total 1 peptides |  |  |  |  |  |  |  |  |  |  |  |  |  |  |  |  |  |  |  |
| --- | --- | --- | --- | --- | --- | --- | --- | --- | --- | --- | --- | --- | --- | --- | --- | --- | --- | --- | --- |

Best Unique PSM (Scan Sample\_5\_A1\_1\_3643.d:39914, m/z=415.2162, z=3, RT=14.37, ppm=-7.18):


TRINITY\_DN982\_c0\_g1\_i3.p1

back to list

  

| Protein Coverage
| Supporting Peptides
| Best Unique PSM
|

Protein Coverage:

Supporting Peptides:

| Peptide | Uniq | -10lgP | Mass | Length | ppm | m/z | z | RT | 1/k0 Start | 1/k0 End | Precursor ID | Area Sample 7 | #Feature | #Feature Sample 7 | Start | End | PTM | AScore | Found By |
| --- | --- | --- | --- | --- | --- | --- | --- | --- | --- | --- | --- | --- | --- | --- | --- | --- | --- | --- | --- |
| R.LVQNTPHNSIVTK.I | N | 56.38 | 1449.794 | 13 | 5.15 | 484.2722 | 3 | 11.78 | 0.8132 | 0.8205 | 23867 | 5.7e3 | 2 | 2 | 8569 | 8581 |  |  | DB Search |
| R.YTSC(+57.02)QSVTVVASDR.S | N | 56.38 | 1571.725 | 14 | 0.63 | 786.8666 | 2 | 13.49 | 1.052 | 1.0592 | 34562 | 0 | 0 | 0 | 9363 | 9376 | Carbamidomethylation |  | DB Search |
| K.LLTTEDDFILR.R | N | 56.37 | 1334.7081 | 11 | -1.75 | 668.3571 | 2 | 19.12 | 0.9751 | 0.9824 | 69298 | 0 | 0 | 0 | 6897 | 6907 |  |  | DB Search |
| R.IQVLKDPDFEFTR.S | N | 55.75 | 1606.8355 | 13 | 2.82 | 536.6181 | 3 | 17.65 | 0.8518 | 0.8591 | 59377 | 0 | 0 | 0 | 6167 | 6179 |  |  | DB Search |
| K.VLLSQQIFYDKK.N | N | 55.36 | 1480.8289 | 12 | 1.76 | 494.6155 | 3 | 16.92 | 0.7616 | 0.7689 | 54594 | 7.01e3 | 2 | 2 | 4919 | 4930 |  |  | DB Search |
| K.FPEFTLQGNK.I | N | 55.36 | 1179.5924 | 10 | 3.01 | 590.8025 | 2 | 17.68 | 0.8935 | 0.9008 | 59467 | 7.06e2 | 1 | 1 | 7323 | 7332 |  |  | DB Search |
| K.YVAIYPSDHLTVNR.D | N | 55.32 | 1646.8416 | 14 | -3.91 | 824.421 | 2 | 15.67 | 1.1098 | 1.117 | 47583 | 0 | 0 | 0 | 6254 | 6267 |  |  | DB Search |
| K.APIGQSIVTVSAR.D | Y | 55.13 | 1297.7354 | 13 | 0.79 | 649.8724 | 2 | 14.27 | 0.9691 | 0.9763 | 39347 | 0 | 0 | 0 | 4029 | 4041 |  |  | DB Search |
| K.AVDRGNPPQDSVNK.A | N | 52.97 | 1495.7379 | 14 | -0.57 | 499.584 | 3 | 10.23 | 0.773 | 0.7803 | 15050 | 0 | 0 | 0 | 5139 | 5152 |  |  | DB Search |
| R.TNNALDYEAKR.Q | N | 51.04 | 1293.6313 | 11 | 1.86 | 647.8211 | 2 | 12.12 | 0.9622 | 0.9695 | 26057 | 0 | 0 | 0 | 7725 | 7735 |  |  | DB Search |
| K.SKIDYEALQVK.H | N | 48.81 | 1292.6976 | 11 | -2.48 | 647.3514 | 2 | 14.75 | 0.9618 | 0.9691 | 42265 | 0 | 0 | 0 | 6813 | 6823 |  |  | DB Search |
| total 11 peptides |  |  |  |  |  |  |  |  |  |  |  |  |  |  |  |  |  |  |  |
| --- | --- | --- | --- | --- | --- | --- | --- | --- | --- | --- | --- | --- | --- | --- | --- | --- | --- | --- | --- |

Best Unique PSM (Scan Sample\_5\_A1\_1\_3643.d:39347, m/z=649.8724, z=2, RT=14.27, ppm=-3.89):


TRINITY\_DN17358\_c0\_g1\_i1.p1

back to list

  

| Protein Coverage
| Supporting Peptides
| Best Unique PSM
|

Protein Coverage:

Supporting Peptides:

| Peptide | Uniq | -10lgP | Mass | Length | ppm | m/z | z | RT | 1/k0 Start | 1/k0 End | Precursor ID | Area Sample 7 | #Feature | #Feature Sample 7 | Start | End | PTM | AScore | Found By |
| --- | --- | --- | --- | --- | --- | --- | --- | --- | --- | --- | --- | --- | --- | --- | --- | --- | --- | --- | --- |
| M.S(+42.01)SFLVVPLKK.T | Y | 55.36 | 1158.7012 | 10 | -0.75 | 580.3547 | 2 | 19.06 | 0.9481 | 0.9553 | 68767 | 9.06e2 | 1 | 1 | 2 | 11 | Acetylation (Protein N-term) | S1:Acetylation (Protein N-term):1000 | DB Search |
| total 1 peptides |  |  |  |  |  |  |  |  |  |  |  |  |  |  |  |  |  |  |  |
| --- | --- | --- | --- | --- | --- | --- | --- | --- | --- | --- | --- | --- | --- | --- | --- | --- | --- | --- | --- |

Best Unique PSM (Scan Sample\_5\_A1\_1\_3643.d:68767, m/z=580.3547, z=2, RT=19.06, ppm=-5.43):


TRINITY\_DN840\_c0\_g1\_i1.p1

back to list

  

| Protein Coverage
| Supporting Peptides
| Best Unique PSM
|

Protein Coverage:

Supporting Peptides:

| Peptide | Uniq | -10lgP | Mass | Length | ppm | m/z | z | RT | 1/k0 Start | 1/k0 End | Precursor ID | Area Sample 7 | #Feature | #Feature Sample 7 | Start | End | PTM | AScore | Found By |
| --- | --- | --- | --- | --- | --- | --- | --- | --- | --- | --- | --- | --- | --- | --- | --- | --- | --- | --- | --- |
| R.KRFDEEPNPLC(+57.02)K.E | Y | 49.41 | 1531.7453 | 12 | 3.63 | 511.5885 | 3 | 13.21 | 0.7685 | 0.7758 | 32789 | 2.49e3 | 2 | 2 | 114 | 125 | Carbamidomethylation |  | DB Search |
| total 1 peptides |  |  |  |  |  |  |  |  |  |  |  |  |  |  |  |  |  |  |  |
| --- | --- | --- | --- | --- | --- | --- | --- | --- | --- | --- | --- | --- | --- | --- | --- | --- | --- | --- | --- |

Best Unique PSM (Scan Sample\_5\_A1\_1\_3643.d:32789, m/z=511.5885, z=3, RT=13.21, ppm=-1.05):


TRINITY\_DN250\_c0\_g1\_i5.p1

back to list

  

| Protein Coverage
| Supporting Peptides
| Best Unique PSM
|

Protein Coverage:

Supporting Peptides:

| Peptide | Uniq | -10lgP | Mass | Length | ppm | m/z | z | RT | 1/k0 Start | 1/k0 End | Precursor ID | Area Sample 7 | #Feature | #Feature Sample 7 | Start | End | PTM | AScore | Found By |
| --- | --- | --- | --- | --- | --- | --- | --- | --- | --- | --- | --- | --- | --- | --- | --- | --- | --- | --- | --- |
| R.LDHKFDLM(+15.99)YAK.R | Y | 51.39 | 1395.6856 | 11 | 4.19 | 466.2356 | 3 | 14.23 | 0.7844 | 0.7917 | 39140 | 0 | 0 | 0 | 391 | 401 | Oxidation (M) | M8:Oxidation (M):1000 | DB Search |
| R.AVC(+57.02)M(+15.99)LSNTTAIAEAWAR.L | Y | 50.49 | 1879.892 | 17 | -0.63 | 940.9483 | 2 | 18.78 | 1.1482 | 1.1554 | 66979 | 0 | 0 | 0 | 374 | 390 | Carbamidomethylation, Oxidation (M) | M4:Oxidation (M):1000 | DB Search |
| K.VGINYQPPTVVPGGDLAK.V | Y | 48.61 | 1823.9781 | 18 | -1.33 | 912.9908 | 2 | 17.37 | 1.1238 | 1.131 | 57429 | 0 | 0 | 0 | 353 | 370 |  |  | DB Search |
| total 3 peptides |  |  |  |  |  |  |  |  |  |  |  |  |  |  |  |  |  |  |  |
| --- | --- | --- | --- | --- | --- | --- | --- | --- | --- | --- | --- | --- | --- | --- | --- | --- | --- | --- | --- |

Best Unique PSM (Scan Sample\_5\_A1\_1\_3643.d:39140, m/z=466.2356, z=3, RT=14.23, ppm=-0.49):


TRINITY\_DN1936\_c0\_g1\_i4.p1

back to list

  

| Protein Coverage
| Supporting Peptides
| Best Unique PSM
|

Protein Coverage:

Supporting Peptides:

| Peptide | Uniq | -10lgP | Mass | Length | ppm | m/z | z | RT | 1/k0 Start | 1/k0 End | Precursor ID | Area Sample 7 | #Feature | #Feature Sample 7 | Start | End | PTM | AScore | Found By |
| --- | --- | --- | --- | --- | --- | --- | --- | --- | --- | --- | --- | --- | --- | --- | --- | --- | --- | --- | --- |
| K.AGDIAILESQQFNAVGGK.T | Y | 54.47 | 1816.9319 | 18 | -18.71 | 909.452 | 2 | 18.92 | 1.1813 | 1.1885 | 67826 | 1.32e3 | 1 | 1 | 1002 | 1019 |  |  | DB Search |
| total 1 peptides |  |  |  |  |  |  |  |  |  |  |  |  |  |  |  |  |  |  |  |
| --- | --- | --- | --- | --- | --- | --- | --- | --- | --- | --- | --- | --- | --- | --- | --- | --- | --- | --- | --- |

Best Unique PSM (Scan Sample\_5\_A1\_1\_3643.d:67826, m/z=909.452, z=2, RT=18.92, ppm=-23.39):


TRINITY\_DN602\_c0\_g1\_i3.p1

back to list

  

| Protein Coverage
| Supporting Peptides
| Best Unique PSM
|

Protein Coverage:

Supporting Peptides:

| Peptide | Uniq | -10lgP | Mass | Length | ppm | m/z | z | RT | 1/k0 Start | 1/k0 End | Precursor ID | Area Sample 7 | #Feature | #Feature Sample 7 | Start | End | PTM | AScore | Found By |
| --- | --- | --- | --- | --- | --- | --- | --- | --- | --- | --- | --- | --- | --- | --- | --- | --- | --- | --- | --- |
| K.EGFYVHEALAAGIVK.K | Y | 56.36 | 1602.8405 | 15 | 7.5 | 802.4298 | 2 | 17.69 | 1.0873 | 1.0945 | 59517 | 5.28e2 | 1 | 1 | 443 | 457 |  |  | DB Search |
| K.LLTSPAPSSGSLM(+15.99)ALALK.I | Y | 56.18 | 1771.9753 | 18 | -1.19 | 886.9897 | 2 | 18.28 | 1.1498 | 1.157 | 63418 | 9.34e2 | 1 | 1 | 550 | 567 | Oxidation (M) | M13:Oxidation (M):1000 | DB Search |
| K.HGADLFYR.G | Y | 53.19 | 977.4719 | 8 | 0.63 | 489.7412 | 2 | 16.16 | 0.8376 | 0.8449 | 50305 | 4.17e2 | 1 | 1 | 500 | 507 |  |  | DB Search |
| total 3 peptides |  |  |  |  |  |  |  |  |  |  |  |  |  |  |  |  |  |  |  |
| --- | --- | --- | --- | --- | --- | --- | --- | --- | --- | --- | --- | --- | --- | --- | --- | --- | --- | --- | --- |

Best Unique PSM (Scan Sample\_5\_A1\_1\_3643.d:59517, m/z=802.4298, z=2, RT=17.69, ppm=2.83):


TRINITY\_DN477\_c0\_g1\_i7.p1

back to list

  

| Protein Coverage
| Supporting Peptides
| Best Unique PSM
|

Protein Coverage:

Supporting Peptides:

| Peptide | Uniq | -10lgP | Mass | Length | ppm | m/z | z | RT | 1/k0 Start | 1/k0 End | Precursor ID | Area Sample 7 | #Feature | #Feature Sample 7 | Start | End | PTM | AScore | Found By |
| --- | --- | --- | --- | --- | --- | --- | --- | --- | --- | --- | --- | --- | --- | --- | --- | --- | --- | --- | --- |
| R.ILQC(+57.02)NQYANVEEAR.N | Y | 56.39 | 1706.8046 | 14 | 0.71 | 854.4062 | 2 | 14.33 | 1.0877 | 1.0949 | 39688 | 4.48e3 | 1 | 1 | 86 | 99 | Carbamidomethylation |  | DB Search |
| R.NDVNNPPQAR.R | Y | 55.29 | 1123.537 | 10 | 3.11 | 562.7749 | 2 | 10.15 | 0.8558 | 0.8631 | 13772 | 1.28e4 | 1 | 1 | 1049 | 1058 |  |  | DB Search |
| K.DKNILVLTLGVGPQVNR.N | Y | 54.98 | 1835.0628 | 17 | 0.89 | 918.5352 | 2 | 19.51 | 1.1354 | 1.1426 | 71919 | 8.54e3 | 2 | 2 | 168 | 184 |  |  | DB Search |
| total 3 peptides |  |  |  |  |  |  |  |  |  |  |  |  |  |  |  |  |  |  |  |
| --- | --- | --- | --- | --- | --- | --- | --- | --- | --- | --- | --- | --- | --- | --- | --- | --- | --- | --- | --- |

Best Unique PSM (Scan Sample\_5\_A1\_1\_3643.d:39688, m/z=854.4062, z=2, RT=14.33, ppm=-3.97):


TRINITY\_DN6428\_c0\_g1\_i2.p1

back to list

  

| Protein Coverage
| Supporting Peptides
| Best Unique PSM
|

Protein Coverage:

Supporting Peptides:

| Peptide | Uniq | -10lgP | Mass | Length | ppm | m/z | z | RT | 1/k0 Start | 1/k0 End | Precursor ID | Area Sample 7 | #Feature | #Feature Sample 7 | Start | End | PTM | AScore | Found By |
| --- | --- | --- | --- | --- | --- | --- | --- | --- | --- | --- | --- | --- | --- | --- | --- | --- | --- | --- | --- |
| R.SC(+57.02)ENPKPLNGGK.Q | Y | 56.12 | 1299.6241 | 12 | 2.81 | 650.8181 | 2 | 11.10 | 0.9469 | 0.9541 | 19780 | 7.77e2 | 1 | 1 | 181 | 192 | Carbamidomethylation |  | DB Search |
| total 1 peptides |  |  |  |  |  |  |  |  |  |  |  |  |  |  |  |  |  |  |  |
| --- | --- | --- | --- | --- | --- | --- | --- | --- | --- | --- | --- | --- | --- | --- | --- | --- | --- | --- | --- |

Best Unique PSM (Scan Sample\_5\_A1\_1\_3643.d:19780, m/z=650.8181, z=2, RT=11.10, ppm=-1.87):


TRINITY\_DN477\_c0\_g1\_i6.p1

back to list

  

| Protein Coverage
| Supporting Peptides
| Best Unique PSM
|

Protein Coverage:

Supporting Peptides:

| Peptide | Uniq | -10lgP | Mass | Length | ppm | m/z | z | RT | 1/k0 Start | 1/k0 End | Precursor ID | Area Sample 7 | #Feature | #Feature Sample 7 | Start | End | PTM | AScore | Found By |
| --- | --- | --- | --- | --- | --- | --- | --- | --- | --- | --- | --- | --- | --- | --- | --- | --- | --- | --- | --- |
| R.NDVNNPPQAR.R | Y | 55.29 | 1123.537 | 10 | 3.11 | 562.7749 | 2 | 10.15 | 0.8558 | 0.8631 | 13772 | 1.28e4 | 1 | 1 | 86 | 95 |  |  | DB Search |
| total 1 peptides |  |  |  |  |  |  |  |  |  |  |  |  |  |  |  |  |  |  |  |
| --- | --- | --- | --- | --- | --- | --- | --- | --- | --- | --- | --- | --- | --- | --- | --- | --- | --- | --- | --- |

Best Unique PSM (Scan Sample\_5\_A1\_1\_3643.d:13772, m/z=562.7749, z=2, RT=10.15, ppm=-1.57):


TRINITY\_DN1071\_c0\_g1\_i2.p1

back to list

  

| Protein Coverage
| Supporting Peptides
| Best Unique PSM
|

Protein Coverage:

Supporting Peptides:

| Peptide | Uniq | -10lgP | Mass | Length | ppm | m/z | z | RT | 1/k0 Start | 1/k0 End | Precursor ID | Area Sample 7 | #Feature | #Feature Sample 7 | Start | End | PTM | AScore | Found By |
| --- | --- | --- | --- | --- | --- | --- | --- | --- | --- | --- | --- | --- | --- | --- | --- | --- | --- | --- | --- |
| K.AVQIHPFNDVSVGDPK.V | Y | 56.18 | 1721.8736 | 16 | -1.14 | 861.9391 | 2 | 15.94 | 1.1178 | 1.125 | 48886 | 2.33e3 | 1 | 1 | 293 | 308 |  |  | DB Search |
| total 1 peptides |  |  |  |  |  |  |  |  |  |  |  |  |  |  |  |  |  |  |  |
| --- | --- | --- | --- | --- | --- | --- | --- | --- | --- | --- | --- | --- | --- | --- | --- | --- | --- | --- | --- |

Best Unique PSM (Scan Sample\_5\_A1\_1\_3643.d:48886, m/z=861.9391, z=2, RT=15.94, ppm=-5.82):


TRINITY\_DN2477\_c0\_g1\_i8.p1

back to list

  

| Protein Coverage
| Supporting Peptides
| Best Unique PSM
|

Protein Coverage:

Supporting Peptides:

| Peptide | Uniq | -10lgP | Mass | Length | ppm | m/z | z | RT | 1/k0 Start | 1/k0 End | Precursor ID | Area Sample 7 | #Feature | #Feature Sample 7 | Start | End | PTM | AScore | Found By |
| --- | --- | --- | --- | --- | --- | --- | --- | --- | --- | --- | --- | --- | --- | --- | --- | --- | --- | --- | --- |
| R.VAPEEHPVLLTEAPLNPK.A | Y | 55.12 | 1953.0571 | 18 | 2.07 | 977.5333 | 2 | 16.62 | 1.2101 | 1.2173 | 52945 | 1.41e2 | 1 | 1 | 29 | 46 |  |  | DB Search |
| total 1 peptides |  |  |  |  |  |  |  |  |  |  |  |  |  |  |  |  |  |  |  |
| --- | --- | --- | --- | --- | --- | --- | --- | --- | --- | --- | --- | --- | --- | --- | --- | --- | --- | --- | --- |

Best Unique PSM (Scan Sample\_5\_A1\_1\_3643.d:52945, m/z=977.5333, z=2, RT=16.62, ppm=-2.61):


TRINITY\_DN140702\_c0\_g1\_i1.p1

back to list

  

| Protein Coverage
| Supporting Peptides
| Best Unique PSM
|

Protein Coverage:

Supporting Peptides:

| Peptide | Uniq | -10lgP | Mass | Length | ppm | m/z | z | RT | 1/k0 Start | 1/k0 End | Precursor ID | Area Sample 7 | #Feature | #Feature Sample 7 | Start | End | PTM | AScore | Found By |
| --- | --- | --- | --- | --- | --- | --- | --- | --- | --- | --- | --- | --- | --- | --- | --- | --- | --- | --- | --- |
| R.LALHVLNNEDIIGHR.L | Y | 56.39 | 1712.9322 | 15 | 2.65 | 857.4716 | 2 | 16.49 | 1.1374 | 1.1446 | 52281 | 2.86e3 | 2 | 2 | 1702 | 1716 |  |  | DB Search |
| R.LVPEHVESR.S | Y | 55.38 | 1064.5614 | 9 | 0.73 | 533.2859 | 2 | 11.15 | 0.8911 | 0.8984 | 20102 | 7.51e2 | 1 | 1 | 1717 | 1725 |  |  | DB Search |
| K.IEDFEESPPTDPHIGPVEQR.L | Y | 55.18 | 2291.0705 | 20 | 5.47 | 764.6981 | 3 | 15.80 | 0.9319 | 0.9392 | 48175 | 1.66e3 | 1 | 1 | 1682 | 1701 |  |  | DB Search |
| K.TGVEILNWGVR.N | Y | 54.86 | 1242.672 | 11 | 2.47 | 622.3419 | 2 | 19.69 | 0.9101 | 0.9174 | 73072 | 3.26e2 | 1 | 1 | 1288 | 1298 |  |  | DB Search |
| R.FVFPFK.Y | Y | 54.68 | 783.4319 | 6 | 5.78 | 392.7237 | 2 | 19.69 | 0.7632 | 0.7705 | 73141 | 4.67e2 | 1 | 1 | 1828 | 1833 |  |  | DB Search |
| total 5 peptides |  |  |  |  |  |  |  |  |  |  |  |  |  |  |  |  |  |  |  |
| --- | --- | --- | --- | --- | --- | --- | --- | --- | --- | --- | --- | --- | --- | --- | --- | --- | --- | --- | --- |

Best Unique PSM (Scan Sample\_5\_A1\_1\_3643.d:52281, m/z=857.4716, z=2, RT=16.49, ppm=-2.03):


TRINITY\_DN585\_c0\_g1\_i8.p1

back to list

  

| Protein Coverage
| Supporting Peptides
| Best Unique PSM
|

Protein Coverage:

Supporting Peptides:

| Peptide | Uniq | -10lgP | Mass | Length | ppm | m/z | z | RT | 1/k0 Start | 1/k0 End | Precursor ID | Area Sample 7 | #Feature | #Feature Sample 7 | Start | End | PTM | AScore | Found By |
| --- | --- | --- | --- | --- | --- | --- | --- | --- | --- | --- | --- | --- | --- | --- | --- | --- | --- | --- | --- |
| K.TITLEVEPSDTIENVK.S | Y | 55.12 | 1786.92 | 16 | 0.46 | 894.4635 | 2 | 17.55 | 1.1414 | 1.1486 | 58604 | 8.72e2 | 1 | 1 | 12 | 27 |  |  | DB Search |
| total 1 peptides |  |  |  |  |  |  |  |  |  |  |  |  |  |  |  |  |  |  |  |
| --- | --- | --- | --- | --- | --- | --- | --- | --- | --- | --- | --- | --- | --- | --- | --- | --- | --- | --- | --- |

Best Unique PSM (Scan Sample\_5\_A1\_1\_3643.d:58604, m/z=894.4635, z=2, RT=17.55, ppm=-4.22):


TRINITY\_DN1616\_c0\_g1\_i3.p1

back to list

  

| Protein Coverage
| Supporting Peptides
| Best Unique PSM
|

Protein Coverage:

Supporting Peptides:

| Peptide | Uniq | -10lgP | Mass | Length | ppm | m/z | z | RT | 1/k0 Start | 1/k0 End | Precursor ID | Area Sample 7 | #Feature | #Feature Sample 7 | Start | End | PTM | AScore | Found By |
| --- | --- | --- | --- | --- | --- | --- | --- | --- | --- | --- | --- | --- | --- | --- | --- | --- | --- | --- | --- |
| K.VVDTEANDLTEEVKK.T | Y | 56.4 | 1688.8468 | 15 | 6.39 | 845.4321 | 2 | 14.15 | 1.1318 | 1.139 | 38615 | 0 | 0 | 0 | 2542 | 2556 |  |  | DB Search |
| K.SSSC(+57.02)QEFGGKC(+57.02)PC(+57.02)K.D | Y | 56.37 | 1630.6538 | 14 | -16.59 | 816.3168 | 2 | 12.09 | 1.0335 | 1.0407 | 25828 | 0 | 0 | 0 | 1387 | 1400 | Carbamidomethylation, Carbamidomethylation, Carbamidomethylation |  | DB Search |
| R.YGAVNDNC(+57.02)ER.D | Y | 56.34 | 1196.488 | 10 | 3.96 | 599.2509 | 2 | 10.89 | 0.885 | 0.8923 | 18607 | 1.07e3 | 1 | 1 | 498 | 507 | Carbamidomethylation |  | DB Search |
| K.TLDTVC(+57.02)DSGTGVC(+57.02)QC(+57.02)K.S | Y | 49.46 | 1799.7488 | 16 | -4.78 | 900.8732 | 2 | 13.15 | 1.0901 | 1.0973 | 32411 | 1.65e2 | 1 | 1 | 546 | 561 | Carbamidomethylation, Carbamidomethylation, Carbamidomethylation |  | DB Search |
| total 4 peptides |  |  |  |  |  |  |  |  |  |  |  |  |  |  |  |  |  |  |  |
| --- | --- | --- | --- | --- | --- | --- | --- | --- | --- | --- | --- | --- | --- | --- | --- | --- | --- | --- | --- |

Best Unique PSM (Scan Sample\_5\_A1\_1\_3643.d:38615, m/z=845.4321, z=2, RT=14.15, ppm=1.71):


TRINITY\_DN602\_c0\_g1\_i4.p1

back to list

  

| Protein Coverage
| Supporting Peptides
| Best Unique PSM
|

Protein Coverage:

Supporting Peptides:

| Peptide | Uniq | -10lgP | Mass | Length | ppm | m/z | z | RT | 1/k0 Start | 1/k0 End | Precursor ID | Area Sample 7 | #Feature | #Feature Sample 7 | Start | End | PTM | AScore | Found By |
| --- | --- | --- | --- | --- | --- | --- | --- | --- | --- | --- | --- | --- | --- | --- | --- | --- | --- | --- | --- |
| K.EGFYVHEALAAGIVK.K | Y | 56.36 | 1602.8405 | 15 | 7.5 | 802.4298 | 2 | 17.69 | 1.0873 | 1.0945 | 59517 | 5.28e2 | 1 | 1 | 144 | 158 |  |  | DB Search |
| K.LLTSPAPSSGSLM(+15.99)ALALK.I | Y | 56.18 | 1771.9753 | 18 | -1.19 | 886.9897 | 2 | 18.28 | 1.1498 | 1.157 | 63418 | 9.34e2 | 1 | 1 | 251 | 268 | Oxidation (M) | M13:Oxidation (M):1000 | DB Search |
| K.HGADLFYR.G | Y | 53.19 | 977.4719 | 8 | 0.63 | 489.7412 | 2 | 16.16 | 0.8376 | 0.8449 | 50305 | 4.17e2 | 1 | 1 | 201 | 208 |  |  | DB Search |
| total 3 peptides |  |  |  |  |  |  |  |  |  |  |  |  |  |  |  |  |  |  |  |
| --- | --- | --- | --- | --- | --- | --- | --- | --- | --- | --- | --- | --- | --- | --- | --- | --- | --- | --- | --- |

Best Unique PSM (Scan Sample\_5\_A1\_1\_3643.d:59517, m/z=802.4298, z=2, RT=17.69, ppm=2.83):


TRINITY\_DN875\_c0\_g1\_i1.p1

back to list

  

| Protein Coverage
| Supporting Peptides
| Best Unique PSM
|

Protein Coverage:

Supporting Peptides:

| Peptide | Uniq | -10lgP | Mass | Length | ppm | m/z | z | RT | 1/k0 Start | 1/k0 End | Precursor ID | Area Sample 7 | #Feature | #Feature Sample 7 | Start | End | PTM | AScore | Found By |
| --- | --- | --- | --- | --- | --- | --- | --- | --- | --- | --- | --- | --- | --- | --- | --- | --- | --- | --- | --- |
| R.AVFC(+57.02)EHQGVK.T | Y | 56.17 | 1173.5601 | 10 | 2.17 | 587.7858 | 2 | 11.71 | 0.9036 | 0.9109 | 23391 | 2.96e3 | 1 | 1 | 512 | 521 | Carbamidomethylation |  | DB Search |
| K.HC(+57.02)VFGR.T | Y | 50.91 | 774.3595 | 6 | 3.29 | 388.1865 | 2 | 13.39 | 0.7526 | 0.7599 | 33963 | 0 | 0 | 0 | 246 | 251 | Carbamidomethylation |  | DB Search |
| total 2 peptides |  |  |  |  |  |  |  |  |  |  |  |  |  |  |  |  |  |  |  |
| --- | --- | --- | --- | --- | --- | --- | --- | --- | --- | --- | --- | --- | --- | --- | --- | --- | --- | --- | --- |

Best Unique PSM (Scan Sample\_5\_A1\_1\_3643.d:23391, m/z=587.7858, z=2, RT=11.71, ppm=-2.51):


TRINITY\_DN691\_c0\_g1\_i1.p1

back to list

  

| Protein Coverage
| Supporting Peptides
| Best Unique PSM
|

Protein Coverage:

Supporting Peptides:

| Peptide | Uniq | -10lgP | Mass | Length | ppm | m/z | z | RT | 1/k0 Start | 1/k0 End | Precursor ID | Area Sample 7 | #Feature | #Feature Sample 7 | Start | End | PTM | AScore | Found By |
| --- | --- | --- | --- | --- | --- | --- | --- | --- | --- | --- | --- | --- | --- | --- | --- | --- | --- | --- | --- |
| R.AYGQDLGQPGSC(+57.02)LKR.F | Y | 55.37 | 1648.7991 | 15 | 4.15 | 550.6067 | 3 | 13.53 | 0.8457 | 0.853 | 34711 | 1.5e3 | 1 | 1 | 1533 | 1547 | Carbamidomethylation |  | DB Search |
| total 1 peptides |  |  |  |  |  |  |  |  |  |  |  |  |  |  |  |  |  |  |  |
| --- | --- | --- | --- | --- | --- | --- | --- | --- | --- | --- | --- | --- | --- | --- | --- | --- | --- | --- | --- |

Best Unique PSM (Scan Sample\_5\_A1\_1\_3643.d:34711, m/z=550.6067, z=3, RT=13.53, ppm=-0.53):


TRINITY\_DN3016\_c0\_g1\_i8.p1

back to list

  

| Protein Coverage
| Supporting Peptides
| Best Unique PSM
|

Protein Coverage:

Supporting Peptides:

| Peptide | Uniq | -10lgP | Mass | Length | ppm | m/z | z | RT | 1/k0 Start | 1/k0 End | Precursor ID | Area Sample 7 | #Feature | #Feature Sample 7 | Start | End | PTM | AScore | Found By |
| --- | --- | --- | --- | --- | --- | --- | --- | --- | --- | --- | --- | --- | --- | --- | --- | --- | --- | --- | --- |
| R.IVNTDLQEIKDIIK.G | Y | 56.35 | 1640.9348 | 14 | -0.94 | 547.9825 | 3 | 18.84 | 0.8193 | 0.8266 | 67326 | 7.77e2 | 1 | 1 | 154 | 167 |  |  | DB Search |
| total 1 peptides |  |  |  |  |  |  |  |  |  |  |  |  |  |  |  |  |  |  |  |
| --- | --- | --- | --- | --- | --- | --- | --- | --- | --- | --- | --- | --- | --- | --- | --- | --- | --- | --- | --- |

Best Unique PSM (Scan Sample\_5\_A1\_1\_3643.d:67326, m/z=547.9825, z=3, RT=18.84, ppm=-5.62):


TRINITY\_DN77\_c0\_g2\_i3.p1

back to list

  

| Protein Coverage
| Supporting Peptides
| Best Unique PSM
|

Protein Coverage:

Supporting Peptides:

| Peptide | Uniq | -10lgP | Mass | Length | ppm | m/z | z | RT | 1/k0 Start | 1/k0 End | Precursor ID | Area Sample 7 | #Feature | #Feature Sample 7 | Start | End | PTM | AScore | Found By |
| --- | --- | --- | --- | --- | --- | --- | --- | --- | --- | --- | --- | --- | --- | --- | --- | --- | --- | --- | --- |
| R.DGSTGLAGNR.G | Y | 52.33 | 946.4468 | 10 | 13.34 | 474.2348 | 2 | 10.74 | 0.8189 | 0.8262 | 17706 | 5.15e3 | 1 | 1 | 1004 | 1013 |  |  | DB Search |
| total 1 peptides |  |  |  |  |  |  |  |  |  |  |  |  |  |  |  |  |  |  |  |
| --- | --- | --- | --- | --- | --- | --- | --- | --- | --- | --- | --- | --- | --- | --- | --- | --- | --- | --- | --- |

Best Unique PSM (Scan Sample\_5\_A1\_1\_3643.d:17706, m/z=474.2348, z=2, RT=10.74, ppm=8.66):


TRINITY\_DN4781\_c0\_g1\_i1.p1

back to list

  

| Protein Coverage
| Supporting Peptides
| Best Unique PSM
|

Protein Coverage:

Supporting Peptides:

| Peptide | Uniq | -10lgP | Mass | Length | ppm | m/z | z | RT | 1/k0 Start | 1/k0 End | Precursor ID | Area Sample 7 | #Feature | #Feature Sample 7 | Start | End | PTM | AScore | Found By |
| --- | --- | --- | --- | --- | --- | --- | --- | --- | --- | --- | --- | --- | --- | --- | --- | --- | --- | --- | --- |
| K.APPKPNPC(+57.02)AIK.C | Y | 55.16 | 1191.6434 | 11 | 6.69 | 398.2225 | 3 | 11.49 | 0.7791 | 0.7864 | 22133 | 1.06e3 | 1 | 1 | 1499 | 1509 | Carbamidomethylation |  | DB Search |
| R.LVSELFDPTSSTR.G | Y | 55.08 | 1450.7303 | 13 | -1.65 | 726.3679 | 2 | 17.84 | 1.0033 | 1.0106 | 60692 | 0 | 0 | 0 | 265 | 277 |  |  | DB Search |
| total 2 peptides |  |  |  |  |  |  |  |  |  |  |  |  |  |  |  |  |  |  |  |
| --- | --- | --- | --- | --- | --- | --- | --- | --- | --- | --- | --- | --- | --- | --- | --- | --- | --- | --- | --- |

Best Unique PSM (Scan Sample\_5\_A1\_1\_3643.d:22133, m/z=398.2225, z=3, RT=11.49, ppm=2.01):


TRINITY\_DN477\_c0\_g1\_i5.p1

back to list

  

| Protein Coverage
| Supporting Peptides
| Best Unique PSM
|

Protein Coverage:

Supporting Peptides:

| Peptide | Uniq | -10lgP | Mass | Length | ppm | m/z | z | RT | 1/k0 Start | 1/k0 End | Precursor ID | Area Sample 7 | #Feature | #Feature Sample 7 | Start | End | PTM | AScore | Found By |
| --- | --- | --- | --- | --- | --- | --- | --- | --- | --- | --- | --- | --- | --- | --- | --- | --- | --- | --- | --- |
| R.NDVNNPPQAR.R | Y | 55.29 | 1123.537 | 10 | 3.11 | 562.7749 | 2 | 10.15 | 0.8558 | 0.8631 | 13772 | 1.28e4 | 1 | 1 | 168 | 177 |  |  | DB Search |
| total 1 peptides |  |  |  |  |  |  |  |  |  |  |  |  |  |  |  |  |  |  |  |
| --- | --- | --- | --- | --- | --- | --- | --- | --- | --- | --- | --- | --- | --- | --- | --- | --- | --- | --- | --- |

Best Unique PSM (Scan Sample\_5\_A1\_1\_3643.d:13772, m/z=562.7749, z=2, RT=10.15, ppm=-1.57):


TRINITY\_DN3016\_c0\_g1\_i17.p1

back to list

  

| Protein Coverage
| Supporting Peptides
| Best Unique PSM
|

Protein Coverage:

Supporting Peptides:

| Peptide | Uniq | -10lgP | Mass | Length | ppm | m/z | z | RT | 1/k0 Start | 1/k0 End | Precursor ID | Area Sample 7 | #Feature | #Feature Sample 7 | Start | End | PTM | AScore | Found By |
| --- | --- | --- | --- | --- | --- | --- | --- | --- | --- | --- | --- | --- | --- | --- | --- | --- | --- | --- | --- |
| R.IVNTDLQEIKDIIK.G | Y | 56.35 | 1640.9348 | 14 | -0.94 | 547.9825 | 3 | 18.84 | 0.8193 | 0.8266 | 67326 | 7.77e2 | 1 | 1 | 154 | 167 |  |  | DB Search |
| total 1 peptides |  |  |  |  |  |  |  |  |  |  |  |  |  |  |  |  |  |  |  |
| --- | --- | --- | --- | --- | --- | --- | --- | --- | --- | --- | --- | --- | --- | --- | --- | --- | --- | --- | --- |

Best Unique PSM (Scan Sample\_5\_A1\_1\_3643.d:67326, m/z=547.9825, z=3, RT=18.84, ppm=-5.62):


TRINITY\_DN319\_c2\_g1\_i3.p1

back to list

  

| Protein Coverage
| Supporting Peptides
| Best Unique PSM
|

Protein Coverage:

Supporting Peptides:

| Peptide | Uniq | -10lgP | Mass | Length | ppm | m/z | z | RT | 1/k0 Start | 1/k0 End | Precursor ID | Area Sample 7 | #Feature | #Feature Sample 7 | Start | End | PTM | AScore | Found By |
| --- | --- | --- | --- | --- | --- | --- | --- | --- | --- | --- | --- | --- | --- | --- | --- | --- | --- | --- | --- |
| R.GSFSSHIC(+57.02)LC(+57.02)HDPAYK.S | Y | 56.38 | 1877.8189 | 16 | 2.34 | 626.9454 | 3 | 14.46 | 0.8866 | 0.8939 | 40485 | 0 | 0 | 0 | 1011 | 1026 | Carbamidomethylation, Carbamidomethylation |  | DB Search |
| R.DPIC(+57.02)HC(+57.02)DTSK.Y | Y | 54.33 | 1231.4961 | 10 | 7.69 | 616.7572 | 2 | 9.95 | 0.8951 | 0.9024 | 13586 | 0 | 0 | 0 | 1448 | 1457 | Carbamidomethylation, Carbamidomethylation |  | DB Search |
| K.KAQYYEAYETIIKR.H | Y | 48.69 | 1774.9254 | 14 | 2.8 | 592.6479 | 3 | 15.92 | 0.8656 | 0.8729 | 49007 | 1.65e2 | 1 | 1 | 2534 | 2547 |  |  | DB Search |
| total 3 peptides |  |  |  |  |  |  |  |  |  |  |  |  |  |  |  |  |  |  |  |
| --- | --- | --- | --- | --- | --- | --- | --- | --- | --- | --- | --- | --- | --- | --- | --- | --- | --- | --- | --- |

Best Unique PSM (Scan Sample\_5\_A1\_1\_3643.d:40485, m/z=626.9454, z=3, RT=14.46, ppm=-2.34):


TRINITY\_DN172571\_c0\_g1\_i1.p1

back to list

  

| Protein Coverage
| Supporting Peptides
| Best Unique PSM
|

Protein Coverage:

Supporting Peptides:

| Peptide | Uniq | -10lgP | Mass | Length | ppm | m/z | z | RT | 1/k0 Start | 1/k0 End | Precursor ID | Area Sample 7 | #Feature | #Feature Sample 7 | Start | End | PTM | AScore | Found By |
| --- | --- | --- | --- | --- | --- | --- | --- | --- | --- | --- | --- | --- | --- | --- | --- | --- | --- | --- | --- |
| R.DAVTYTEHAK.R | Y | 54.29 | 1133.5353 | 10 | 7.89 | 567.7767 | 2 | 11.15 | 0.9004 | 0.9077 | 20203 | 0 | 0 | 0 | 69 | 78 |  |  | DB Search |
| R.DNIQGITKPAIR.R | Y | 52.04 | 1324.7463 | 12 | 0.94 | 663.3779 | 2 | 13.90 | 0.9844 | 0.9916 | 37014 | 7.98e2 | 1 | 1 | 25 | 36 |  |  | DB Search |
| total 2 peptides |  |  |  |  |  |  |  |  |  |  |  |  |  |  |  |  |  |  |  |
| --- | --- | --- | --- | --- | --- | --- | --- | --- | --- | --- | --- | --- | --- | --- | --- | --- | --- | --- | --- |

Best Unique PSM (Scan Sample\_5\_A1\_1\_3643.d:20203, m/z=567.7767, z=2, RT=11.15, ppm=3.21):


TRINITY\_DN691\_c0\_g1\_i2.p1

back to list

  

| Protein Coverage
| Supporting Peptides
| Best Unique PSM
|

Protein Coverage:

Supporting Peptides:

| Peptide | Uniq | -10lgP | Mass | Length | ppm | m/z | z | RT | 1/k0 Start | 1/k0 End | Precursor ID | Area Sample 7 | #Feature | #Feature Sample 7 | Start | End | PTM | AScore | Found By |
| --- | --- | --- | --- | --- | --- | --- | --- | --- | --- | --- | --- | --- | --- | --- | --- | --- | --- | --- | --- |
| R.AYGQDLGQPGSC(+57.02)LKR.F | Y | 55.37 | 1648.7991 | 15 | 4.15 | 550.6067 | 3 | 13.53 | 0.8457 | 0.853 | 34711 | 1.5e3 | 1 | 1 | 1480 | 1494 | Carbamidomethylation |  | DB Search |
| total 1 peptides |  |  |  |  |  |  |  |  |  |  |  |  |  |  |  |  |  |  |  |
| --- | --- | --- | --- | --- | --- | --- | --- | --- | --- | --- | --- | --- | --- | --- | --- | --- | --- | --- | --- |

Best Unique PSM (Scan Sample\_5\_A1\_1\_3643.d:34711, m/z=550.6067, z=3, RT=13.53, ppm=-0.53):


TRINITY\_DN6390\_c0\_g1\_i1.p1

back to list

  

| Protein Coverage
| Supporting Peptides
| Best Unique PSM
|

Protein Coverage:

Supporting Peptides:

| Peptide | Uniq | -10lgP | Mass | Length | ppm | m/z | z | RT | 1/k0 Start | 1/k0 End | Precursor ID | Area Sample 7 | #Feature | #Feature Sample 7 | Start | End | PTM | AScore | Found By |
| --- | --- | --- | --- | --- | --- | --- | --- | --- | --- | --- | --- | --- | --- | --- | --- | --- | --- | --- | --- |
| K.YYEC(+57.02)GSAC(+57.02)PK.T | Y | 56.36 | 1233.4794 | 10 | -0.44 | 617.7438 | 2 | 11.29 | 0.917 | 0.9243 | 21021 | 0 | 0 | 0 | 686 | 695 | Carbamidomethylation, Carbamidomethylation |  | DB Search |
| K.YLFDLESNC(+57.02)DK.Q | Y | 56.36 | 1402.6075 | 11 | -2.5 | 702.306 | 2 | 18.42 | 0.984 | 0.9912 | 64464 | 1.37e2 | 1 | 1 | 303 | 313 | Carbamidomethylation |  | DB Search |
| R.VGNWRDETLSC(+57.02)K.Q | Y | 55.34 | 1463.6827 | 12 | -0.36 | 488.899 | 3 | 13.83 | 0.7608 | 0.7681 | 36637 | 1.03e3 | 1 | 1 | 1047 | 1058 | Carbamidomethylation |  | DB Search |
| total 3 peptides |  |  |  |  |  |  |  |  |  |  |  |  |  |  |  |  |  |  |  |
| --- | --- | --- | --- | --- | --- | --- | --- | --- | --- | --- | --- | --- | --- | --- | --- | --- | --- | --- | --- |

Best Unique PSM (Scan Sample\_5\_A1\_1\_3643.d:21021, m/z=617.7438, z=2, RT=11.29, ppm=-5.12):


TRINITY\_DN554\_c0\_g1\_i2.p1

back to list

  

| Protein Coverage
| Supporting Peptides
| Best Unique PSM
|

Protein Coverage:

Supporting Peptides:

| Peptide | Uniq | -10lgP | Mass | Length | ppm | m/z | z | RT | 1/k0 Start | 1/k0 End | Precursor ID | Area Sample 7 | #Feature | #Feature Sample 7 | Start | End | PTM | AScore | Found By |
| --- | --- | --- | --- | --- | --- | --- | --- | --- | --- | --- | --- | --- | --- | --- | --- | --- | --- | --- | --- |
| K.TITLEVEPSDTIENVK.S | Y | 55.12 | 1786.92 | 16 | 0.46 | 894.4635 | 2 | 17.55 | 1.1414 | 1.1486 | 58604 | 8.72e2 | 1 | 1 | 53 | 68 |  |  | DB Search |
| total 1 peptides |  |  |  |  |  |  |  |  |  |  |  |  |  |  |  |  |  |  |  |
| --- | --- | --- | --- | --- | --- | --- | --- | --- | --- | --- | --- | --- | --- | --- | --- | --- | --- | --- | --- |

Best Unique PSM (Scan Sample\_5\_A1\_1\_3643.d:58604, m/z=894.4635, z=2, RT=17.55, ppm=-4.22):


TRINITY\_DN1613\_c1\_g1\_i1.p1

back to list

  

| Protein Coverage
| Supporting Peptides
| Best Unique PSM
|

Protein Coverage:

Supporting Peptides:

| Peptide | Uniq | -10lgP | Mass | Length | ppm | m/z | z | RT | 1/k0 Start | 1/k0 End | Precursor ID | Area Sample 7 | #Feature | #Feature Sample 7 | Start | End | PTM | AScore | Found By |
| --- | --- | --- | --- | --- | --- | --- | --- | --- | --- | --- | --- | --- | --- | --- | --- | --- | --- | --- | --- |
| R.S(+42.01)C(+57.02)SNPSPAHGGKPC(+57.02)SGELSK.T | Y | 47.79 | 2097.9208 | 20 | 5.48 | 700.3148 | 3 | 11.22 | 0.8785 | 0.8858 | 20616 | 3.89e2 | 1 | 1 | 14 | 33 | Acetylation (Protein N-term), Carbamidomethylation, Carbamidomethylation | S1:Acetylation (Protein N-term):1000 | DB Search |
| total 1 peptides |  |  |  |  |  |  |  |  |  |  |  |  |  |  |  |  |  |  |  |
| --- | --- | --- | --- | --- | --- | --- | --- | --- | --- | --- | --- | --- | --- | --- | --- | --- | --- | --- | --- |

Best Unique PSM (Scan Sample\_5\_A1\_1\_3643.d:20616, m/z=700.3148, z=3, RT=11.22, ppm=0.8):


TRINITY\_DN3016\_c0\_g1\_i4.p1

back to list

  

| Protein Coverage
| Supporting Peptides
| Best Unique PSM
|

Protein Coverage:

Supporting Peptides:

| Peptide | Uniq | -10lgP | Mass | Length | ppm | m/z | z | RT | 1/k0 Start | 1/k0 End | Precursor ID | Area Sample 7 | #Feature | #Feature Sample 7 | Start | End | PTM | AScore | Found By |
| --- | --- | --- | --- | --- | --- | --- | --- | --- | --- | --- | --- | --- | --- | --- | --- | --- | --- | --- | --- |
| R.IVNTDLQEIKDIIK.G | Y | 56.35 | 1640.9348 | 14 | -0.94 | 547.9825 | 3 | 18.84 | 0.8193 | 0.8266 | 67326 | 7.77e2 | 1 | 1 | 154 | 167 |  |  | DB Search |
| total 1 peptides |  |  |  |  |  |  |  |  |  |  |  |  |  |  |  |  |  |  |  |
| --- | --- | --- | --- | --- | --- | --- | --- | --- | --- | --- | --- | --- | --- | --- | --- | --- | --- | --- | --- |

Best Unique PSM (Scan Sample\_5\_A1\_1\_3643.d:67326, m/z=547.9825, z=3, RT=18.84, ppm=-5.62):


TRINITY\_DN875\_c0\_g1\_i2.p1

back to list

  

| Protein Coverage
| Supporting Peptides
| Best Unique PSM
|

Protein Coverage:

Supporting Peptides:

| Peptide | Uniq | -10lgP | Mass | Length | ppm | m/z | z | RT | 1/k0 Start | 1/k0 End | Precursor ID | Area Sample 7 | #Feature | #Feature Sample 7 | Start | End | PTM | AScore | Found By |
| --- | --- | --- | --- | --- | --- | --- | --- | --- | --- | --- | --- | --- | --- | --- | --- | --- | --- | --- | --- |
| R.AVFC(+57.02)EHQGVK.T | Y | 56.17 | 1173.5601 | 10 | 2.17 | 587.7858 | 2 | 11.71 | 0.9036 | 0.9109 | 23391 | 2.96e3 | 1 | 1 | 512 | 521 | Carbamidomethylation |  | DB Search |
| K.HC(+57.02)VFGR.T | Y | 50.91 | 774.3595 | 6 | 3.29 | 388.1865 | 2 | 13.39 | 0.7526 | 0.7599 | 33963 | 0 | 0 | 0 | 246 | 251 | Carbamidomethylation |  | DB Search |
| total 2 peptides |  |  |  |  |  |  |  |  |  |  |  |  |  |  |  |  |  |  |  |
| --- | --- | --- | --- | --- | --- | --- | --- | --- | --- | --- | --- | --- | --- | --- | --- | --- | --- | --- | --- |

Best Unique PSM (Scan Sample\_5\_A1\_1\_3643.d:23391, m/z=587.7858, z=2, RT=11.71, ppm=-2.51):


TRINITY\_DN26249\_c0\_g1\_i1.p1

back to list

  

| Protein Coverage
| Supporting Peptides
| Best Unique PSM
|

Protein Coverage:

Supporting Peptides:

| Peptide | Uniq | -10lgP | Mass | Length | ppm | m/z | z | RT | 1/k0 Start | 1/k0 End | Precursor ID | Area Sample 7 | #Feature | #Feature Sample 7 | Start | End | PTM | AScore | Found By |
| --- | --- | --- | --- | --- | --- | --- | --- | --- | --- | --- | --- | --- | --- | --- | --- | --- | --- | --- | --- |
| R.LIYENQNTIHHVVR.A | Y | 56.4 | 1734.9165 | 14 | 3.34 | 579.312 | 3 | 13.35 | 0.8485 | 0.8558 | 33666 | 6.97e2 | 1 | 1 | 322 | 335 |  |  | DB Search |
| total 1 peptides |  |  |  |  |  |  |  |  |  |  |  |  |  |  |  |  |  |  |  |
| --- | --- | --- | --- | --- | --- | --- | --- | --- | --- | --- | --- | --- | --- | --- | --- | --- | --- | --- | --- |

Best Unique PSM (Scan Sample\_5\_A1\_1\_3643.d:33666, m/z=579.312, z=3, RT=13.35, ppm=-1.34):


TRINITY\_DN149493\_c0\_g1\_i1.p1

back to list

  

| Protein Coverage
| Supporting Peptides
| Best Unique PSM
|

Protein Coverage:

Supporting Peptides:

| Peptide | Uniq | -10lgP | Mass | Length | ppm | m/z | z | RT | 1/k0 Start | 1/k0 End | Precursor ID | Area Sample 7 | #Feature | #Feature Sample 7 | Start | End | PTM | AScore | Found By |
| --- | --- | --- | --- | --- | --- | --- | --- | --- | --- | --- | --- | --- | --- | --- | --- | --- | --- | --- | --- |
| R.LPGDALSIGFPSK.D | Y | 55.35 | 1300.7027 | 13 | -4.93 | 651.3524 | 2 | 18.60 | 0.9691 | 0.9763 | 65620 | 9.17e2 | 1 | 1 | 73 | 85 |  |  | DB Search |
| total 1 peptides |  |  |  |  |  |  |  |  |  |  |  |  |  |  |  |  |  |  |  |
| --- | --- | --- | --- | --- | --- | --- | --- | --- | --- | --- | --- | --- | --- | --- | --- | --- | --- | --- | --- |

Best Unique PSM (Scan Sample\_5\_A1\_1\_3643.d:65620, m/z=651.3524, z=2, RT=18.60, ppm=-9.61):


TRINITY\_DN5500\_c0\_g1\_i2.p1

back to list

  

| Protein Coverage
| Supporting Peptides
| Best Unique PSM
|

Protein Coverage:

Supporting Peptides:

| Peptide | Uniq | -10lgP | Mass | Length | ppm | m/z | z | RT | 1/k0 Start | 1/k0 End | Precursor ID | Area Sample 7 | #Feature | #Feature Sample 7 | Start | End | PTM | AScore | Found By |
| --- | --- | --- | --- | --- | --- | --- | --- | --- | --- | --- | --- | --- | --- | --- | --- | --- | --- | --- | --- |
| K.IYIDGVFIR.S | Y | 56.04 | 1094.6124 | 9 | 2.12 | 548.3121 | 2 | 18.80 | 0.8903 | 0.8976 | 67021 | 1.29e3 | 1 | 1 | 938 | 946 |  |  | DB Search |
| total 1 peptides |  |  |  |  |  |  |  |  |  |  |  |  |  |  |  |  |  |  |  |
| --- | --- | --- | --- | --- | --- | --- | --- | --- | --- | --- | --- | --- | --- | --- | --- | --- | --- | --- | --- |

Best Unique PSM (Scan Sample\_5\_A1\_1\_3643.d:67021, m/z=548.3121, z=2, RT=18.80, ppm=-2.56):


TRINITY\_DN4781\_c0\_g1\_i3.p1

back to list

  

| Protein Coverage
| Supporting Peptides
| Best Unique PSM
|

Protein Coverage:

Supporting Peptides:

| Peptide | Uniq | -10lgP | Mass | Length | ppm | m/z | z | RT | 1/k0 Start | 1/k0 End | Precursor ID | Area Sample 7 | #Feature | #Feature Sample 7 | Start | End | PTM | AScore | Found By |
| --- | --- | --- | --- | --- | --- | --- | --- | --- | --- | --- | --- | --- | --- | --- | --- | --- | --- | --- | --- |
| R.LVSELFDPTSSTR.G | Y | 55.08 | 1450.7303 | 13 | -1.65 | 726.3679 | 2 | 17.84 | 1.0033 | 1.0106 | 60692 | 0 | 0 | 0 | 265 | 277 |  |  | DB Search |
| total 1 peptides |  |  |  |  |  |  |  |  |  |  |  |  |  |  |  |  |  |  |  |
| --- | --- | --- | --- | --- | --- | --- | --- | --- | --- | --- | --- | --- | --- | --- | --- | --- | --- | --- | --- |

Best Unique PSM (Scan Sample\_5\_A1\_1\_3643.d:60692, m/z=726.3679, z=2, RT=17.84, ppm=-6.33):


TRINITY\_DN687\_c0\_g1\_i1.p1

back to list

  

| Protein Coverage
| Supporting Peptides
| Best Unique PSM
|

Protein Coverage:

Supporting Peptides:

| Peptide | Uniq | -10lgP | Mass | Length | ppm | m/z | z | RT | 1/k0 Start | 1/k0 End | Precursor ID | Area Sample 7 | #Feature | #Feature Sample 7 | Start | End | PTM | AScore | Found By |
| --- | --- | --- | --- | --- | --- | --- | --- | --- | --- | --- | --- | --- | --- | --- | --- | --- | --- | --- | --- |
| R.LDHKFDLM(+15.99)YAK.R | Y | 51.39 | 1395.6856 | 11 | 4.19 | 466.2356 | 3 | 14.23 | 0.7844 | 0.7917 | 39140 | 0 | 0 | 0 | 391 | 401 | Oxidation (M) | M8:Oxidation (M):1000 | DB Search |
| R.AVC(+57.02)M(+15.99)LSNTTAIAEAWAR.L | Y | 50.49 | 1879.892 | 17 | -0.63 | 940.9483 | 2 | 18.78 | 1.1482 | 1.1554 | 66979 | 0 | 0 | 0 | 374 | 390 | Carbamidomethylation, Oxidation (M) | M4:Oxidation (M):1000 | DB Search |
| K.VGINYQPPTVVPGGDLAK.V | Y | 48.61 | 1823.9781 | 18 | -1.33 | 912.9908 | 2 | 17.37 | 1.1238 | 1.131 | 57429 | 0 | 0 | 0 | 353 | 370 |  |  | DB Search |
| total 3 peptides |  |  |  |  |  |  |  |  |  |  |  |  |  |  |  |  |  |  |  |
| --- | --- | --- | --- | --- | --- | --- | --- | --- | --- | --- | --- | --- | --- | --- | --- | --- | --- | --- | --- |

Best Unique PSM (Scan Sample\_5\_A1\_1\_3643.d:39140, m/z=466.2356, z=3, RT=14.23, ppm=-0.49):


TRINITY\_DN3016\_c0\_g1\_i11.p1

back to list

  

| Protein Coverage
| Supporting Peptides
| Best Unique PSM
|

Protein Coverage:

Supporting Peptides:

| Peptide | Uniq | -10lgP | Mass | Length | ppm | m/z | z | RT | 1/k0 Start | 1/k0 End | Precursor ID | Area Sample 7 | #Feature | #Feature Sample 7 | Start | End | PTM | AScore | Found By |
| --- | --- | --- | --- | --- | --- | --- | --- | --- | --- | --- | --- | --- | --- | --- | --- | --- | --- | --- | --- |
| R.IVNTDLQEIKDIIK.G | Y | 56.35 | 1640.9348 | 14 | -0.94 | 547.9825 | 3 | 18.84 | 0.8193 | 0.8266 | 67326 | 7.77e2 | 1 | 1 | 154 | 167 |  |  | DB Search |
| total 1 peptides |  |  |  |  |  |  |  |  |  |  |  |  |  |  |  |  |  |  |  |
| --- | --- | --- | --- | --- | --- | --- | --- | --- | --- | --- | --- | --- | --- | --- | --- | --- | --- | --- | --- |

Best Unique PSM (Scan Sample\_5\_A1\_1\_3643.d:67326, m/z=547.9825, z=3, RT=18.84, ppm=-5.62):


TRINITY\_DN6920\_c0\_g1\_i3.p1

back to list

  

| Protein Coverage
| Supporting Peptides
| Best Unique PSM
|

Protein Coverage:

Supporting Peptides:

| Peptide | Uniq | -10lgP | Mass | Length | ppm | m/z | z | RT | 1/k0 Start | 1/k0 End | Precursor ID | Area Sample 7 | #Feature | #Feature Sample 7 | Start | End | PTM | AScore | Found By |
| --- | --- | --- | --- | --- | --- | --- | --- | --- | --- | --- | --- | --- | --- | --- | --- | --- | --- | --- | --- |
| K.AGM(+15.99)THIVR.E | Y | 54.97 | 899.4647 | 8 | 4.88 | 450.7397 | 2 | 9.72 | 0.8144 | 0.8218 | 12487 | 9.73e2 | 1 | 1 | 58 | 65 | Oxidation (M) | M3:Oxidation (M):1000 | DB Search |
| total 1 peptides |  |  |  |  |  |  |  |  |  |  |  |  |  |  |  |  |  |  |  |
| --- | --- | --- | --- | --- | --- | --- | --- | --- | --- | --- | --- | --- | --- | --- | --- | --- | --- | --- | --- |

Best Unique PSM (Scan Sample\_5\_A1\_1\_3643.d:12487, m/z=450.7397, z=2, RT=9.72, ppm=0.2):


TRINITY\_DN167\_c0\_g1\_i1.p1

back to list

  

| Protein Coverage
| Supporting Peptides
| Best Unique PSM
|

Protein Coverage:

Supporting Peptides:

| Peptide | Uniq | -10lgP | Mass | Length | ppm | m/z | z | RT | 1/k0 Start | 1/k0 End | Precursor ID | Area Sample 7 | #Feature | #Feature Sample 7 | Start | End | PTM | AScore | Found By |
| --- | --- | --- | --- | --- | --- | --- | --- | --- | --- | --- | --- | --- | --- | --- | --- | --- | --- | --- | --- |
| K.STAGDTHLGGEDFDNR.L | Y | 56.39 | 1690.7183 | 16 | 2.27 | 564.5787 | 3 | 13.29 | 0.8014 | 0.8088 | 33297 | 4.2e3 | 2 | 2 | 107 | 122 |  |  | DB Search |
| total 1 peptides |  |  |  |  |  |  |  |  |  |  |  |  |  |  |  |  |  |  |  |
| --- | --- | --- | --- | --- | --- | --- | --- | --- | --- | --- | --- | --- | --- | --- | --- | --- | --- | --- | --- |

Best Unique PSM (Scan Sample\_5\_A1\_1\_3643.d:33297, m/z=564.5787, z=3, RT=13.29, ppm=-2.41):


TRINITY\_DN61707\_c0\_g1\_i1.p1

back to list

  

| Protein Coverage
| Supporting Peptides
| Best Unique PSM
|

Protein Coverage:

Supporting Peptides:

| Peptide | Uniq | -10lgP | Mass | Length | ppm | m/z | z | RT | 1/k0 Start | 1/k0 End | Precursor ID | Area Sample 7 | #Feature | #Feature Sample 7 | Start | End | PTM | AScore | Found By |
| --- | --- | --- | --- | --- | --- | --- | --- | --- | --- | --- | --- | --- | --- | --- | --- | --- | --- | --- | --- |
| R.KHLSELSEEAAK.L | Y | 55.36 | 1340.6936 | 12 | -9.06 | 447.899 | 3 | 11.10 | 0.7856 | 0.7929 | 19897 | 0 | 0 | 0 | 264 | 275 |  |  | DB Search |
| total 1 peptides |  |  |  |  |  |  |  |  |  |  |  |  |  |  |  |  |  |  |  |
| --- | --- | --- | --- | --- | --- | --- | --- | --- | --- | --- | --- | --- | --- | --- | --- | --- | --- | --- | --- |

Best Unique PSM (Scan Sample\_5\_A1\_1\_3643.d:19897, m/z=447.899, z=3, RT=11.10, ppm=-13.74):


TRINITY\_DN3448\_c0\_g2\_i1.p1

back to list

  

| Protein Coverage
| Supporting Peptides
| Best Unique PSM
|

Protein Coverage:

Supporting Peptides:

| Peptide | Uniq | -10lgP | Mass | Length | ppm | m/z | z | RT | 1/k0 Start | 1/k0 End | Precursor ID | Area Sample 7 | #Feature | #Feature Sample 7 | Start | End | PTM | AScore | Found By |
| --- | --- | --- | --- | --- | --- | --- | --- | --- | --- | --- | --- | --- | --- | --- | --- | --- | --- | --- | --- |
| K.AGM(+15.99)THIVR.D | Y | 54.97 | 899.4647 | 8 | 4.88 | 450.7397 | 2 | 9.72 | 0.8144 | 0.8218 | 12487 | 9.73e2 | 1 | 1 | 31 | 38 | Oxidation (M) | M3:Oxidation (M):1000 | DB Search |
| total 1 peptides |  |  |  |  |  |  |  |  |  |  |  |  |  |  |  |  |  |  |  |
| --- | --- | --- | --- | --- | --- | --- | --- | --- | --- | --- | --- | --- | --- | --- | --- | --- | --- | --- | --- |

Best Unique PSM (Scan Sample\_5\_A1\_1\_3643.d:12487, m/z=450.7397, z=2, RT=9.72, ppm=0.2):


TRINITY\_DN2885\_c0\_g1\_i2.p1

back to list

  

| Protein Coverage
| Supporting Peptides
| Best Unique PSM
|

Protein Coverage:

Supporting Peptides:

| Peptide | Uniq | -10lgP | Mass | Length | ppm | m/z | z | RT | 1/k0 Start | 1/k0 End | Precursor ID | Area Sample 7 | #Feature | #Feature Sample 7 | Start | End | PTM | AScore | Found By |
| --- | --- | --- | --- | --- | --- | --- | --- | --- | --- | --- | --- | --- | --- | --- | --- | --- | --- | --- | --- |
| K.VTRPHGASGVVR.A | Y | 56.37 | 1234.6894 | 12 | 3.4 | 412.5699 | 3 | 10.07 | 0.8104 | 0.8177 | 14175 | 0 | 0 | 0 | 77 | 88 |  |  | DB Search |
| total 1 peptides |  |  |  |  |  |  |  |  |  |  |  |  |  |  |  |  |  |  |  |
| --- | --- | --- | --- | --- | --- | --- | --- | --- | --- | --- | --- | --- | --- | --- | --- | --- | --- | --- | --- |

Best Unique PSM (Scan Sample\_5\_A1\_1\_3643.d:14175, m/z=412.5699, z=3, RT=10.07, ppm=-1.29):


TRINITY\_DN875\_c0\_g1\_i3.p1

back to list

  

| Protein Coverage
| Supporting Peptides
| Best Unique PSM
|

Protein Coverage:

Supporting Peptides:

| Peptide | Uniq | -10lgP | Mass | Length | ppm | m/z | z | RT | 1/k0 Start | 1/k0 End | Precursor ID | Area Sample 7 | #Feature | #Feature Sample 7 | Start | End | PTM | AScore | Found By |
| --- | --- | --- | --- | --- | --- | --- | --- | --- | --- | --- | --- | --- | --- | --- | --- | --- | --- | --- | --- |
| R.AVFC(+57.02)EHQGVK.T | Y | 56.17 | 1173.5601 | 10 | 2.17 | 587.7858 | 2 | 11.71 | 0.9036 | 0.9109 | 23391 | 2.96e3 | 1 | 1 | 512 | 521 | Carbamidomethylation |  | DB Search |
| K.HC(+57.02)VFGR.T | Y | 50.91 | 774.3595 | 6 | 3.29 | 388.1865 | 2 | 13.39 | 0.7526 | 0.7599 | 33963 | 0 | 0 | 0 | 246 | 251 | Carbamidomethylation |  | DB Search |
| total 2 peptides |  |  |  |  |  |  |  |  |  |  |  |  |  |  |  |  |  |  |  |
| --- | --- | --- | --- | --- | --- | --- | --- | --- | --- | --- | --- | --- | --- | --- | --- | --- | --- | --- | --- |

Best Unique PSM (Scan Sample\_5\_A1\_1\_3643.d:23391, m/z=587.7858, z=2, RT=11.71, ppm=-2.51):


TRINITY\_DN161\_c0\_g1\_i3.p1

back to list

  

| Protein Coverage
| Supporting Peptides
| Best Unique PSM
|

Protein Coverage:

Supporting Peptides:

| Peptide | Uniq | -10lgP | Mass | Length | ppm | m/z | z | RT | 1/k0 Start | 1/k0 End | Precursor ID | Area Sample 7 | #Feature | #Feature Sample 7 | Start | End | PTM | AScore | Found By |
| --- | --- | --- | --- | --- | --- | --- | --- | --- | --- | --- | --- | --- | --- | --- | --- | --- | --- | --- | --- |
| K.SYELPDGQVITIGNER.F | Y | 55.37 | 1789.8846 | 16 | 2.43 | 895.9476 | 2 | 18.67 | 1.117 | 1.1242 | 66156 | 3.51e2 | 1 | 1 | 262 | 277 |  |  | DB Search |
| R.HQGVMVGMGQK.D | Y | 55.37 | 1170.5638 | 11 | 3.68 | 586.2886 | 2 | 12.36 | 0.9105 | 0.9178 | 27450 | 5.73e2 | 1 | 1 | 63 | 73 |  |  | DB Search |
| R.HQGVM(+15.99)VGMGQK.D | Y | 55.3 | 1186.5587 | 11 | 10.45 | 594.29 | 2 | 11.22 | 0.9133 | 0.9206 | 20614 | 0 | 0 | 0 | 63 | 73 | Oxidation (M) | M5:Oxidation (M):60 | DB Search |
| K.DSYVGDEAQSKR.G | Y | 55.24 | 1353.616 | 12 | 0.18 | 677.8123 | 2 | 10.99 | 0.9932 | 1.0005 | 19155 | 1.01e3 | 1 | 1 | 74 | 85 |  |  | DB Search |
| R.AVFPSIVGRPR.H | Y | 55.22 | 1197.6982 | 11 | 2.36 | 599.855 | 2 | 15.87 | 0.961 | 0.9683 | 48378 | 1.86e4 | 1 | 1 | 52 | 62 |  |  | DB Search |
| R.VAPEEHPVLLTEAPLNPK.A | Y | 55.12 | 1953.0571 | 18 | 2.07 | 977.5333 | 2 | 16.62 | 1.2101 | 1.2173 | 52945 | 1.41e2 | 1 | 1 | 119 | 136 |  |  | DB Search |
| K.DSYVGDEAQSK.R | Y | 54.42 | 1197.5149 | 11 | 5.23 | 599.7651 | 2 | 11.40 | 0.8968 | 0.904 | 21678 | 5.55e2 | 1 | 1 | 74 | 84 |  |  | DB Search |
| R.HQGVM(+15.99)VGM(+15.99)GQK.D | Y | 52.78 | 1202.5536 | 11 | 2.62 | 602.2828 | 2 | 9.74 | 0.8955 | 0.9028 | 12426 | 1.52e3 | 1 | 1 | 63 | 73 | Oxidation (M), Oxidation (M) | M5:Oxidation (M):1000 M8:Oxidation (M):1000 | DB Search |
| R.HQGVMVGM(+15.99)GQK.D | Y | 48 | 1186.5587 | 11 | 4.48 | 594.2865 | 2 | 10.48 | 0.9052 | 0.9125 | 16419 | 1.61e3 | 1 | 1 | 63 | 73 | Oxidation (M) | M8:Oxidation (M):32.97 | DB Search |
| total 9 peptides |  |  |  |  |  |  |  |  |  |  |  |  |  |  |  |  |  |  |  |
| --- | --- | --- | --- | --- | --- | --- | --- | --- | --- | --- | --- | --- | --- | --- | --- | --- | --- | --- | --- |

Best Unique PSM (Scan Sample\_5\_A1\_1\_3643.d:66156, m/z=895.9476, z=2, RT=18.67, ppm=-2.25):


TRINITY\_DN6542\_c0\_g1\_i4.p1

back to list

  

| Protein Coverage
| Supporting Peptides
| Best Unique PSM
|

Protein Coverage:

Supporting Peptides:

| Peptide | Uniq | -10lgP | Mass | Length | ppm | m/z | z | RT | 1/k0 Start | 1/k0 End | Precursor ID | Area Sample 7 | #Feature | #Feature Sample 7 | Start | End | PTM | AScore | Found By |
| --- | --- | --- | --- | --- | --- | --- | --- | --- | --- | --- | --- | --- | --- | --- | --- | --- | --- | --- | --- |
| K.ILLAVGGWNHER.Q | Y | 49.52 | 1363.736 | 12 | 4.17 | 682.8749 | 2 | 17.15 | 1.0423 | 1.0496 | 56044 | 3.26e2 | 1 | 1 | 140 | 151 |  |  | DB Search |
| total 1 peptides |  |  |  |  |  |  |  |  |  |  |  |  |  |  |  |  |  |  |  |
| --- | --- | --- | --- | --- | --- | --- | --- | --- | --- | --- | --- | --- | --- | --- | --- | --- | --- | --- | --- |

Best Unique PSM (Scan Sample\_5\_A1\_1\_3643.d:56044, m/z=682.8749, z=2, RT=17.15, ppm=-0.52):


TRINITY\_DN138\_c0\_g1\_i1.p1

back to list

  

| Protein Coverage
| Supporting Peptides
| Best Unique PSM
|

Protein Coverage:

Supporting Peptides:

| Peptide | Uniq | -10lgP | Mass | Length | ppm | m/z | z | RT | 1/k0 Start | 1/k0 End | Precursor ID | Area Sample 7 | #Feature | #Feature Sample 7 | Start | End | PTM | AScore | Found By |
| --- | --- | --- | --- | --- | --- | --- | --- | --- | --- | --- | --- | --- | --- | --- | --- | --- | --- | --- | --- |
| K.IGC(+57.02)FHDR.I | Y | 55.18 | 903.4021 | 7 | 2.15 | 452.7072 | 2 | 11.34 | 0.8031 | 0.8104 | 21250 | 4.1e3 | 1 | 1 | 33 | 39 | Carbamidomethylation |  | DB Search |
| R.TLDFVVELSK.G | Y | 55.07 | 1149.6281 | 10 | -1.12 | 575.818 | 2 | 19.60 | 0.887 | 0.8943 | 72344 | 9.89e3 | 1 | 1 | 237 | 246 |  |  | DB Search |
| K.YLLVFTDGR.S | Y | 54.18 | 1082.576 | 9 | -0.05 | 542.2927 | 2 | 19.06 | 0.8745 | 0.8818 | 68859 | 1.53e3 | 1 | 1 | 324 | 332 |  |  | DB Search |
| R.FC(+57.02)SNPEPENGGQTC(+57.02)EGEAR.E | Y | 50.09 | 2137.8429 | 19 | 2.83 | 1069.9268 | 2 | 12.17 | 1.1582 | 1.1654 | 26131 | 9.57e2 | 1 | 1 | 181 | 199 | Carbamidomethylation, Carbamidomethylation |  | DB Search |
| K.DMGVNVISVGAGHTINHR.E | Y | 50.02 | 1875.9373 | 18 | 2.35 | 938.9738 | 2 | 15.41 | 1.2495 | 1.2567 | 46101 | 0 | 0 | 0 | 349 | 366 |  |  | DB Search |
| total 5 peptides |  |  |  |  |  |  |  |  |  |  |  |  |  |  |  |  |  |  |  |
| --- | --- | --- | --- | --- | --- | --- | --- | --- | --- | --- | --- | --- | --- | --- | --- | --- | --- | --- | --- |

Best Unique PSM (Scan Sample\_5\_A1\_1\_3643.d:21250, m/z=452.7072, z=2, RT=11.34, ppm=-2.53):


TRINITY\_DN36\_c0\_g1\_i4.p1

back to list

  

| Protein Coverage
| Supporting Peptides
| Best Unique PSM
|

Protein Coverage:

Supporting Peptides:

| Peptide | Uniq | -10lgP | Mass | Length | ppm | m/z | z | RT | 1/k0 Start | 1/k0 End | Precursor ID | Area Sample 7 | #Feature | #Feature Sample 7 | Start | End | PTM | AScore | Found By |
| --- | --- | --- | --- | --- | --- | --- | --- | --- | --- | --- | --- | --- | --- | --- | --- | --- | --- | --- | --- |
| R.LGGIEVTQGNC(+57.02)AR.K | Y | 49.67 | 1373.6721 | 13 | 2.61 | 687.8419 | 2 | 13.89 | 0.9695 | 0.9767 | 37015 | 0 | 0 | 0 | 58 | 70 | Carbamidomethylation |  | DB Search |
| total 1 peptides |  |  |  |  |  |  |  |  |  |  |  |  |  |  |  |  |  |  |  |
| --- | --- | --- | --- | --- | --- | --- | --- | --- | --- | --- | --- | --- | --- | --- | --- | --- | --- | --- | --- |

Best Unique PSM (Scan Sample\_5\_A1\_1\_3643.d:37015, m/z=687.8419, z=2, RT=13.89, ppm=-2.07):


TRINITY\_DN2113\_c0\_g1\_i1.p1

back to list

  

| Protein Coverage
| Supporting Peptides
| Best Unique PSM
|

Protein Coverage:

Supporting Peptides:

| Peptide | Uniq | -10lgP | Mass | Length | ppm | m/z | z | RT | 1/k0 Start | 1/k0 End | Precursor ID | Area Sample 7 | #Feature | #Feature Sample 7 | Start | End | PTM | AScore | Found By |
| --- | --- | --- | --- | --- | --- | --- | --- | --- | --- | --- | --- | --- | --- | --- | --- | --- | --- | --- | --- |
| R.AHVAQLC(+57.02)EK.A | Y | 51.4 | 1054.5229 | 9 | 8.37 | 528.2707 | 2 | 10.76 | 0.8583 | 0.8656 | 17883 | 1.38e3 | 1 | 1 | 611 | 619 | Carbamidomethylation |  | DB Search |
| R.LADLEEFISSPNHANISQVGER.C | Y | 49.83 | 2425.1873 | 22 | 1.54 | 809.4005 | 3 | 18.91 | 0.9238 | 0.9311 | 67723 | 2.6e3 | 1 | 1 | 1183 | 1204 |  |  | DB Search |
| K.GQFSTDDLVAEVEKR.N | Y | 47.98 | 1692.8318 | 15 | -2.37 | 565.2806 | 3 | 17.82 | 0.8518 | 0.8591 | 60505 | 0 | 0 | 0 | 838 | 852 |  |  | DB Search |
| total 3 peptides |  |  |  |  |  |  |  |  |  |  |  |  |  |  |  |  |  |  |  |
| --- | --- | --- | --- | --- | --- | --- | --- | --- | --- | --- | --- | --- | --- | --- | --- | --- | --- | --- | --- |

Best Unique PSM (Scan Sample\_5\_A1\_1\_3643.d:17883, m/z=528.2707, z=2, RT=10.76, ppm=3.69):


TRINITY\_DN477\_c0\_g1\_i9.p1

back to list

  

| Protein Coverage
| Supporting Peptides
| Best Unique PSM
|

Protein Coverage:

Supporting Peptides:

| Peptide | Uniq | -10lgP | Mass | Length | ppm | m/z | z | RT | 1/k0 Start | 1/k0 End | Precursor ID | Area Sample 7 | #Feature | #Feature Sample 7 | Start | End | PTM | AScore | Found By |
| --- | --- | --- | --- | --- | --- | --- | --- | --- | --- | --- | --- | --- | --- | --- | --- | --- | --- | --- | --- |
| R.TQAVLDRLNIEKR.V | Y | 56.32 | 1554.8842 | 13 | 2.69 | 519.301 | 3 | 14.70 | 0.8035 | 0.8108 | 41823 | 2.15e3 | 1 | 1 | 220 | 232 |  |  | DB Search |
| K.DANSMPTQSR.Q | Y | 56.02 | 1105.4822 | 10 | 4.07 | 553.748 | 2 | 10.94 | 0.8583 | 0.8656 | 18894 | 7.52e2 | 1 | 1 | 51 | 60 |  |  | DB Search |
| R.TQAVLDRLNIEK.R | Y | 53.96 | 1398.783 | 12 | 1.89 | 700.3969 | 2 | 15.48 | 1.009 | 1.0162 | 46512 | 0 | 0 | 0 | 220 | 231 |  |  | DB Search |
| total 3 peptides |  |  |  |  |  |  |  |  |  |  |  |  |  |  |  |  |  |  |  |
| --- | --- | --- | --- | --- | --- | --- | --- | --- | --- | --- | --- | --- | --- | --- | --- | --- | --- | --- | --- |

Best Unique PSM (Scan Sample\_5\_A1\_1\_3643.d:41823, m/z=519.301, z=3, RT=14.70, ppm=-1.99):


TRINITY\_DN250\_c0\_g1\_i1.p1

back to list

  

| Protein Coverage
| Supporting Peptides
| Best Unique PSM
|

Protein Coverage:

Supporting Peptides:

| Peptide | Uniq | -10lgP | Mass | Length | ppm | m/z | z | RT | 1/k0 Start | 1/k0 End | Precursor ID | Area Sample 7 | #Feature | #Feature Sample 7 | Start | End | PTM | AScore | Found By |
| --- | --- | --- | --- | --- | --- | --- | --- | --- | --- | --- | --- | --- | --- | --- | --- | --- | --- | --- | --- |
| R.LDHKFDLM(+15.99)YAK | Y | 51.39 | 1395.6856 | 11 | 4.19 | 466.2356 | 3 | 14.23 | 0.7844 | 0.7917 | 39140 | 0 | 0 | 0 | 391 | 401 | Oxidation (M) | M8:Oxidation (M):1000 | DB Search |
| R.AVC(+57.02)M(+15.99)LSNTTAIAEAWAR.L | Y | 50.49 | 1879.892 | 17 | -0.63 | 940.9483 | 2 | 18.78 | 1.1482 | 1.1554 | 66979 | 0 | 0 | 0 | 374 | 390 | Carbamidomethylation, Oxidation (M) | M4:Oxidation (M):1000 | DB Search |
| K.VGINYQPPTVVPGGDLAK.V | Y | 48.61 | 1823.9781 | 18 | -1.33 | 912.9908 | 2 | 17.37 | 1.1238 | 1.131 | 57429 | 0 | 0 | 0 | 353 | 370 |  |  | DB Search |
| total 3 peptides |  |  |  |  |  |  |  |  |  |  |  |  |  |  |  |  |  |  |  |
| --- | --- | --- | --- | --- | --- | --- | --- | --- | --- | --- | --- | --- | --- | --- | --- | --- | --- | --- | --- |

Best Unique PSM (Scan Sample\_5\_A1\_1\_3643.d:39140, m/z=466.2356, z=3, RT=14.23, ppm=-0.49):


TRINITY\_DN6920\_c0\_g1\_i2.p1

back to list

  

| Protein Coverage
| Supporting Peptides
| Best Unique PSM
|

Protein Coverage:

Supporting Peptides:

| Peptide | Uniq | -10lgP | Mass | Length | ppm | m/z | z | RT | 1/k0 Start | 1/k0 End | Precursor ID | Area Sample 7 | #Feature | #Feature Sample 7 | Start | End | PTM | AScore | Found By |
| --- | --- | --- | --- | --- | --- | --- | --- | --- | --- | --- | --- | --- | --- | --- | --- | --- | --- | --- | --- |
| K.AGM(+15.99)THIVR.E | Y | 54.97 | 899.4647 | 8 | 4.88 | 450.7397 | 2 | 9.72 | 0.8144 | 0.8218 | 12487 | 9.73e2 | 1 | 1 | 58 | 65 | Oxidation (M) | M3:Oxidation (M):1000 | DB Search |
| total 1 peptides |  |  |  |  |  |  |  |  |  |  |  |  |  |  |  |  |  |  |  |
| --- | --- | --- | --- | --- | --- | --- | --- | --- | --- | --- | --- | --- | --- | --- | --- | --- | --- | --- | --- |

Best Unique PSM (Scan Sample\_5\_A1\_1\_3643.d:12487, m/z=450.7397, z=2, RT=9.72, ppm=0.2):


TRINITY\_DN1668\_c0\_g1\_i1.p1

back to list

  

| Protein Coverage
| Supporting Peptides
| Best Unique PSM
|

Protein Coverage:

Supporting Peptides:

| Peptide | Uniq | -10lgP | Mass | Length | ppm | m/z | z | RT | 1/k0 Start | 1/k0 End | Precursor ID | Area Sample 7 | #Feature | #Feature Sample 7 | Start | End | PTM | AScore | Found By |
| --- | --- | --- | --- | --- | --- | --- | --- | --- | --- | --- | --- | --- | --- | --- | --- | --- | --- | --- | --- |
| R.YTNQETGEINDVVSYVPK.E | Y | 55.19 | 2054.9796 | 18 | -4.38 | 1028.4878 | 2 | 18.17 | 1.2041 | 1.2113 | 62839 | 0 | 0 | 0 | 171 | 188 |  |  | DB Search |
| total 1 peptides |  |  |  |  |  |  |  |  |  |  |  |  |  |  |  |  |  |  |  |
| --- | --- | --- | --- | --- | --- | --- | --- | --- | --- | --- | --- | --- | --- | --- | --- | --- | --- | --- | --- |

Best Unique PSM (Scan Sample\_5\_A1\_1\_3643.d:62839, m/z=1028.4878, z=2, RT=18.17, ppm=-9.05):


TRINITY\_DN71243\_c0\_g1\_i1.p1

back to list

  

| Protein Coverage
| Supporting Peptides
| Best Unique PSM
|

Protein Coverage:

Supporting Peptides:

| Peptide | Uniq | -10lgP | Mass | Length | ppm | m/z | z | RT | 1/k0 Start | 1/k0 End | Precursor ID | Area Sample 7 | #Feature | #Feature Sample 7 | Start | End | PTM | AScore | Found By |
| --- | --- | --- | --- | --- | --- | --- | --- | --- | --- | --- | --- | --- | --- | --- | --- | --- | --- | --- | --- |
| K.RVHQFLVEALDGGQPR.R | Y | 51.92 | 1820.9645 | 16 | -4.41 | 607.9899 | 3 | 19.51 | 0.8704 | 0.8777 | 71893 | 7.34e2 | 1 | 1 | 129 | 144 |  |  | DB Search |
| total 1 peptides |  |  |  |  |  |  |  |  |  |  |  |  |  |  |  |  |  |  |  |
| --- | --- | --- | --- | --- | --- | --- | --- | --- | --- | --- | --- | --- | --- | --- | --- | --- | --- | --- | --- |

Best Unique PSM (Scan Sample\_5\_A1\_1\_3643.d:71893, m/z=607.9899, z=3, RT=19.51, ppm=-9.09):


TRINITY\_DN2799\_c0\_g1\_i3.p1

back to list

  

| Protein Coverage
| Supporting Peptides
| Best Unique PSM
|

Protein Coverage:

Supporting Peptides:

| Peptide | Uniq | -10lgP | Mass | Length | ppm | m/z | z | RT | 1/k0 Start | 1/k0 End | Precursor ID | Area Sample 7 | #Feature | #Feature Sample 7 | Start | End | PTM | AScore | Found By |
| --- | --- | --- | --- | --- | --- | --- | --- | --- | --- | --- | --- | --- | --- | --- | --- | --- | --- | --- | --- |
| K.AGM(+15.99)THIVR.D | Y | 54.97 | 899.4647 | 8 | 4.88 | 450.7397 | 2 | 9.72 | 0.8144 | 0.8218 | 12487 | 9.73e2 | 1 | 1 | 69 | 76 | Oxidation (M) | M3:Oxidation (M):1000 | DB Search |
| total 1 peptides |  |  |  |  |  |  |  |  |  |  |  |  |  |  |  |  |  |  |  |
| --- | --- | --- | --- | --- | --- | --- | --- | --- | --- | --- | --- | --- | --- | --- | --- | --- | --- | --- | --- |

Best Unique PSM (Scan Sample\_5\_A1\_1\_3643.d:12487, m/z=450.7397, z=2, RT=9.72, ppm=0.2):


TRINITY\_DN2799\_c0\_g1\_i4.p2

back to list

  

| Protein Coverage
| Supporting Peptides
| Best Unique PSM
|

Protein Coverage:

Supporting Peptides:

| Peptide | Uniq | -10lgP | Mass | Length | ppm | m/z | z | RT | 1/k0 Start | 1/k0 End | Precursor ID | Area Sample 7 | #Feature | #Feature Sample 7 | Start | End | PTM | AScore | Found By |
| --- | --- | --- | --- | --- | --- | --- | --- | --- | --- | --- | --- | --- | --- | --- | --- | --- | --- | --- | --- |
| K.AGM(+15.99)THIVR.D | Y | 54.97 | 899.4647 | 8 | 4.88 | 450.7397 | 2 | 9.72 | 0.8144 | 0.8218 | 12487 | 9.73e2 | 1 | 1 | 68 | 75 | Oxidation (M) | M3:Oxidation (M):1000 | DB Search |
| total 1 peptides |  |  |  |  |  |  |  |  |  |  |  |  |  |  |  |  |  |  |  |
| --- | --- | --- | --- | --- | --- | --- | --- | --- | --- | --- | --- | --- | --- | --- | --- | --- | --- | --- | --- |

Best Unique PSM (Scan Sample\_5\_A1\_1\_3643.d:12487, m/z=450.7397, z=2, RT=9.72, ppm=0.2):


TRINITY\_DN33065\_c0\_g1\_i1.p1

back to list

  

| Protein Coverage
| Supporting Peptides
| Best Unique PSM
|

Protein Coverage:

Supporting Peptides:

| Peptide | Uniq | -10lgP | Mass | Length | ppm | m/z | z | RT | 1/k0 Start | 1/k0 End | Precursor ID | Area Sample 7 | #Feature | #Feature Sample 7 | Start | End | PTM | AScore | Found By |
| --- | --- | --- | --- | --- | --- | --- | --- | --- | --- | --- | --- | --- | --- | --- | --- | --- | --- | --- | --- |
| K.AVTIESTSNSVNGVFDTVEGK.F | Y | 56.17 | 2153.0488 | 21 | -2.28 | 1077.5242 | 2 | 18.97 | 1.2196 | 1.2268 | 68255 | 3.01e2 | 1 | 1 | 281 | 301 |  |  | DB Search |
| total 1 peptides |  |  |  |  |  |  |  |  |  |  |  |  |  |  |  |  |  |  |  |
| --- | --- | --- | --- | --- | --- | --- | --- | --- | --- | --- | --- | --- | --- | --- | --- | --- | --- | --- | --- |

Best Unique PSM (Scan Sample\_5\_A1\_1\_3643.d:68255, m/z=1077.5242, z=2, RT=18.97, ppm=-6.96):


TRINITY\_DN329\_c0\_g1\_i1.p1

back to list

  

| Protein Coverage
| Supporting Peptides
| Best Unique PSM
|

Protein Coverage:

Supporting Peptides:

| Peptide | Uniq | -10lgP | Mass | Length | ppm | m/z | z | RT | 1/k0 Start | 1/k0 End | Precursor ID | Area Sample 7 | #Feature | #Feature Sample 7 | Start | End | PTM | AScore | Found By |
| --- | --- | --- | --- | --- | --- | --- | --- | --- | --- | --- | --- | --- | --- | --- | --- | --- | --- | --- | --- |
| K.LRPGVELTMSSLVNAK.S | Y | 49.47 | 1713.9447 | 16 | -6.6 | 572.3157 | 3 | 17.86 | 0.9117 | 0.919 | 60815 | 0 | 0 | 0 | 250 | 265 |  |  | DB Search |
| total 1 peptides |  |  |  |  |  |  |  |  |  |  |  |  |  |  |  |  |  |  |  |
| --- | --- | --- | --- | --- | --- | --- | --- | --- | --- | --- | --- | --- | --- | --- | --- | --- | --- | --- | --- |

Best Unique PSM (Scan Sample\_5\_A1\_1\_3643.d:60815, m/z=572.3157, z=3, RT=17.86, ppm=-11.28):


TRINITY\_DN2799\_c0\_g1\_i2.p1

back to list

  

| Protein Coverage
| Supporting Peptides
| Best Unique PSM
|

Protein Coverage:

Supporting Peptides:

| Peptide | Uniq | -10lgP | Mass | Length | ppm | m/z | z | RT | 1/k0 Start | 1/k0 End | Precursor ID | Area Sample 7 | #Feature | #Feature Sample 7 | Start | End | PTM | AScore | Found By |
| --- | --- | --- | --- | --- | --- | --- | --- | --- | --- | --- | --- | --- | --- | --- | --- | --- | --- | --- | --- |
| K.AGM(+15.99)THIVR.D | Y | 54.97 | 899.4647 | 8 | 4.88 | 450.7397 | 2 | 9.72 | 0.8144 | 0.8218 | 12487 | 9.73e2 | 1 | 1 | 68 | 75 | Oxidation (M) | M3:Oxidation (M):1000 | DB Search |
| total 1 peptides |  |  |  |  |  |  |  |  |  |  |  |  |  |  |  |  |  |  |  |
| --- | --- | --- | --- | --- | --- | --- | --- | --- | --- | --- | --- | --- | --- | --- | --- | --- | --- | --- | --- |

Best Unique PSM (Scan Sample\_5\_A1\_1\_3643.d:12487, m/z=450.7397, z=2, RT=9.72, ppm=0.2):


TRINITY\_DN1280\_c0\_g1\_i1.p1

back to list

  

| Protein Coverage
| Supporting Peptides
| Best Unique PSM
|

Protein Coverage:

Supporting Peptides:

| Peptide | Uniq | -10lgP | Mass | Length | ppm | m/z | z | RT | 1/k0 Start | 1/k0 End | Precursor ID | Area Sample 7 | #Feature | #Feature Sample 7 | Start | End | PTM | AScore | Found By |
| --- | --- | --- | --- | --- | --- | --- | --- | --- | --- | --- | --- | --- | --- | --- | --- | --- | --- | --- | --- |
| K.AEYENKTEVAR.S | Y | 56.38 | 1308.631 | 11 | 4.95 | 655.3229 | 2 | 13.23 | 0.9824 | 0.9896 | 32963 | 9.59e2 | 1 | 1 | 202 | 212 |  |  | DB Search |
| R.AQAINVQLQEIQR.R | Y | 55.46 | 1509.8263 | 13 | 1.57 | 755.9181 | 2 | 16.14 | 1.0295 | 1.0367 | 50268 | 0 | 0 | 0 | 263 | 275 |  |  | DB Search |
| total 2 peptides |  |  |  |  |  |  |  |  |  |  |  |  |  |  |  |  |  |  |  |
| --- | --- | --- | --- | --- | --- | --- | --- | --- | --- | --- | --- | --- | --- | --- | --- | --- | --- | --- | --- |

Best Unique PSM (Scan Sample\_5\_A1\_1\_3643.d:32963, m/z=655.3229, z=2, RT=13.23, ppm=0.27):


TRINITY\_DN1559\_c0\_g1\_i1.p1

back to list

  

| Protein Coverage
| Supporting Peptides
| Best Unique PSM
|

Protein Coverage:

Supporting Peptides:

| Peptide | Uniq | -10lgP | Mass | Length | ppm | m/z | z | RT | 1/k0 Start | 1/k0 End | Precursor ID | Area Sample 7 | #Feature | #Feature Sample 7 | Start | End | PTM | AScore | Found By |
| --- | --- | --- | --- | --- | --- | --- | --- | --- | --- | --- | --- | --- | --- | --- | --- | --- | --- | --- | --- |
| R.GVAGAGVLAGFDKFK.E | Y | 55.1 | 1435.7823 | 15 | 1.47 | 718.8961 | 2 | 18.42 | 1.0889 | 1.0961 | 64513 | 2.84e2 | 1 | 1 | 282 | 296 |  |  | DB Search |
| total 1 peptides |  |  |  |  |  |  |  |  |  |  |  |  |  |  |  |  |  |  |  |
| --- | --- | --- | --- | --- | --- | --- | --- | --- | --- | --- | --- | --- | --- | --- | --- | --- | --- | --- | --- |

Best Unique PSM (Scan Sample\_5\_A1\_1\_3643.d:64513, m/z=718.8961, z=2, RT=18.42, ppm=-3.21):


TRINITY\_DN101\_c0\_g1\_i1.p1

back to list

  

| Protein Coverage
| Supporting Peptides
| Best Unique PSM
|

Protein Coverage:

Supporting Peptides:

| Peptide | Uniq | -10lgP | Mass | Length | ppm | m/z | z | RT | 1/k0 Start | 1/k0 End | Precursor ID | Area Sample 7 | #Feature | #Feature Sample 7 | Start | End | PTM | AScore | Found By |
| --- | --- | --- | --- | --- | --- | --- | --- | --- | --- | --- | --- | --- | --- | --- | --- | --- | --- | --- | --- |
| K.TITLEVEPSDTIENVK.A | Y | 55.12 | 1786.92 | 16 | 0.46 | 894.4635 | 2 | 17.55 | 1.1414 | 1.1486 | 58604 | 8.72e2 | 1 | 1 | 12 | 27 |  |  | DB Search |
| total 1 peptides |  |  |  |  |  |  |  |  |  |  |  |  |  |  |  |  |  |  |  |
| --- | --- | --- | --- | --- | --- | --- | --- | --- | --- | --- | --- | --- | --- | --- | --- | --- | --- | --- | --- |

Best Unique PSM (Scan Sample\_5\_A1\_1\_3643.d:58604, m/z=894.4635, z=2, RT=17.55, ppm=-4.22):


TRINITY\_DN250\_c0\_g1\_i6.p1

back to list

  

| Protein Coverage
| Supporting Peptides
| Best Unique PSM
|

Protein Coverage:

Supporting Peptides:

| Peptide | Uniq | -10lgP | Mass | Length | ppm | m/z | z | RT | 1/k0 Start | 1/k0 End | Precursor ID | Area Sample 7 | #Feature | #Feature Sample 7 | Start | End | PTM | AScore | Found By |
| --- | --- | --- | --- | --- | --- | --- | --- | --- | --- | --- | --- | --- | --- | --- | --- | --- | --- | --- | --- |
| R.LDHKFDLM(+15.99)YAK.R | Y | 51.39 | 1395.6856 | 11 | 4.19 | 466.2356 | 3 | 14.23 | 0.7844 | 0.7917 | 39140 | 0 | 0 | 0 | 391 | 401 | Oxidation (M) | M8:Oxidation (M):1000 | DB Search |
| K.VGINYQPPTVVPGGDLAK.V | Y | 48.61 | 1823.9781 | 18 | -1.33 | 912.9908 | 2 | 17.37 | 1.1238 | 1.131 | 57429 | 0 | 0 | 0 | 353 | 370 |  |  | DB Search |
| total 2 peptides |  |  |  |  |  |  |  |  |  |  |  |  |  |  |  |  |  |  |  |
| --- | --- | --- | --- | --- | --- | --- | --- | --- | --- | --- | --- | --- | --- | --- | --- | --- | --- | --- | --- |

Best Unique PSM (Scan Sample\_5\_A1\_1\_3643.d:39140, m/z=466.2356, z=3, RT=14.23, ppm=-0.49):


TRINITY\_DN2477\_c0\_g1\_i9.p1

back to list

  

| Protein Coverage
| Supporting Peptides
| Best Unique PSM
|

Protein Coverage:

Supporting Peptides:

| Peptide | Uniq | -10lgP | Mass | Length | ppm | m/z | z | RT | 1/k0 Start | 1/k0 End | Precursor ID | Area Sample 7 | #Feature | #Feature Sample 7 | Start | End | PTM | AScore | Found By |
| --- | --- | --- | --- | --- | --- | --- | --- | --- | --- | --- | --- | --- | --- | --- | --- | --- | --- | --- | --- |
| K.DSYVGDEAQSKR.G | Y | 55.24 | 1353.616 | 12 | 0.18 | 677.8123 | 2 | 10.99 | 0.9932 | 1.0005 | 19155 | 1.01e3 | 1 | 1 | 52 | 63 |  |  | DB Search |
| R.VAPEEHPVLLTEAPLNPK.A | Y | 55.12 | 1953.0571 | 18 | 2.07 | 977.5333 | 2 | 16.62 | 1.2101 | 1.2173 | 52945 | 1.41e2 | 1 | 1 | 97 | 114 |  |  | DB Search |
| K.DSYVGDEAQSK.R | Y | 54.42 | 1197.5149 | 11 | 5.23 | 599.7651 | 2 | 11.40 | 0.8968 | 0.904 | 21678 | 5.55e2 | 1 | 1 | 52 | 62 |  |  | DB Search |
| total 3 peptides |  |  |  |  |  |  |  |  |  |  |  |  |  |  |  |  |  |  |  |
| --- | --- | --- | --- | --- | --- | --- | --- | --- | --- | --- | --- | --- | --- | --- | --- | --- | --- | --- | --- |

Best Unique PSM (Scan Sample\_5\_A1\_1\_3643.d:19155, m/z=677.8123, z=2, RT=10.99, ppm=-4.5):


TRINITY\_DN1071\_c0\_g1\_i1.p1

back to list

  

| Protein Coverage
| Supporting Peptides
| Best Unique PSM
|

Protein Coverage:

Supporting Peptides:

| Peptide | Uniq | -10lgP | Mass | Length | ppm | m/z | z | RT | 1/k0 Start | 1/k0 End | Precursor ID | Area Sample 7 | #Feature | #Feature Sample 7 | Start | End | PTM | AScore | Found By |
| --- | --- | --- | --- | --- | --- | --- | --- | --- | --- | --- | --- | --- | --- | --- | --- | --- | --- | --- | --- |
| K.AVQIHPFNDVSVGDPK.V | Y | 56.18 | 1721.8736 | 16 | -1.14 | 861.9391 | 2 | 15.94 | 1.1178 | 1.125 | 48886 | 2.33e3 | 1 | 1 | 293 | 308 |  |  | DB Search |
| total 1 peptides |  |  |  |  |  |  |  |  |  |  |  |  |  |  |  |  |  |  |  |
| --- | --- | --- | --- | --- | --- | --- | --- | --- | --- | --- | --- | --- | --- | --- | --- | --- | --- | --- | --- |

Best Unique PSM (Scan Sample\_5\_A1\_1\_3643.d:48886, m/z=861.9391, z=2, RT=15.94, ppm=-5.82):


TRINITY\_DN8597\_c0\_g1\_i1.p1

back to list

  

| Protein Coverage
| Supporting Peptides
| Best Unique PSM
|

Protein Coverage:

Supporting Peptides:

| Peptide | Uniq | -10lgP | Mass | Length | ppm | m/z | z | RT | 1/k0 Start | 1/k0 End | Precursor ID | Area Sample 7 | #Feature | #Feature Sample 7 | Start | End | PTM | AScore | Found By |
| --- | --- | --- | --- | --- | --- | --- | --- | --- | --- | --- | --- | --- | --- | --- | --- | --- | --- | --- | --- |
| R.AC(+57.02)SNVNQC(+57.02)GVNTGTQTVYK.V | Y | 56.39 | 2099.9364 | 19 | 5.81 | 1050.9767 | 2 | 12.99 | 1.177 | 1.1841 | 31410 | 0 | 0 | 0 | 47 | 65 | Carbamidomethylation, Carbamidomethylation |  | DB Search |
| R.AFNKVETR.N | N | 49.58 | 963.5138 | 8 | 6.34 | 482.765 | 2 | 10.59 | 0.8266 | 0.8339 | 16901 | 3.56e3 | 1 | 1 | 15 | 22 |  |  | DB Search |
| total 2 peptides |  |  |  |  |  |  |  |  |  |  |  |  |  |  |  |  |  |  |  |
| --- | --- | --- | --- | --- | --- | --- | --- | --- | --- | --- | --- | --- | --- | --- | --- | --- | --- | --- | --- |

Best Unique PSM (Scan Sample\_5\_A1\_1\_3643.d:31410, m/z=1050.9767, z=2, RT=12.99, ppm=1.13):


TRINITY\_DN1676\_c0\_g1\_i6.p1

back to list

  

| Protein Coverage
| Supporting Peptides
| Best Unique PSM
|

Protein Coverage:

Supporting Peptides:

| Peptide | Uniq | -10lgP | Mass | Length | ppm | m/z | z | RT | 1/k0 Start | 1/k0 End | Precursor ID | Area Sample 7 | #Feature | #Feature Sample 7 | Start | End | PTM | AScore | Found By |
| --- | --- | --- | --- | --- | --- | --- | --- | --- | --- | --- | --- | --- | --- | --- | --- | --- | --- | --- | --- |
| R.SYYFAISDITVGGR.C | Y | 56.33 | 1547.762 | 14 | -2.52 | 774.8827 | 2 | 19.97 | 1.046 | 1.0532 | 74887 | 4.98e2 | 1 | 1 | 273 | 286 |  |  | DB Search |
| R.FELDRVNTFGDEVFGDPK.V | Y | 52.07 | 2083.985 | 18 | 2.03 | 695.6671 | 3 | 19.30 | 0.9206 | 0.9279 | 70383 | 6.13e2 | 1 | 1 | 252 | 269 |  |  | DB Search |
| total 2 peptides |  |  |  |  |  |  |  |  |  |  |  |  |  |  |  |  |  |  |  |
| --- | --- | --- | --- | --- | --- | --- | --- | --- | --- | --- | --- | --- | --- | --- | --- | --- | --- | --- | --- |

Best Unique PSM (Scan Sample\_5\_A1\_1\_3643.d:74887, m/z=774.8827, z=2, RT=19.97, ppm=-7.2):


TRINITY\_DN250\_c0\_g1\_i4.p1

back to list

  

| Protein Coverage
| Supporting Peptides
| Best Unique PSM
|

Protein Coverage:

Supporting Peptides:

| Peptide | Uniq | -10lgP | Mass | Length | ppm | m/z | z | RT | 1/k0 Start | 1/k0 End | Precursor ID | Area Sample 7 | #Feature | #Feature Sample 7 | Start | End | PTM | AScore | Found By |
| --- | --- | --- | --- | --- | --- | --- | --- | --- | --- | --- | --- | --- | --- | --- | --- | --- | --- | --- | --- |
| R.LDHKFDLM(+15.99)YAK.R | Y | 51.39 | 1395.6856 | 11 | 4.19 | 466.2356 | 3 | 14.23 | 0.7844 | 0.7917 | 39140 | 0 | 0 | 0 | 391 | 401 | Oxidation (M) | M8:Oxidation (M):1000 | DB Search |
| K.VGINYQPPTVVPGGDLAK.V | Y | 48.61 | 1823.9781 | 18 | -1.33 | 912.9908 | 2 | 17.37 | 1.1238 | 1.131 | 57429 | 0 | 0 | 0 | 353 | 370 |  |  | DB Search |
| total 2 peptides |  |  |  |  |  |  |  |  |  |  |  |  |  |  |  |  |  |  |  |
| --- | --- | --- | --- | --- | --- | --- | --- | --- | --- | --- | --- | --- | --- | --- | --- | --- | --- | --- | --- |

Best Unique PSM (Scan Sample\_5\_A1\_1\_3643.d:39140, m/z=466.2356, z=3, RT=14.23, ppm=-0.49):


TRINITY\_DN761\_c0\_g1\_i2.p1

back to list

  

| Protein Coverage
| Supporting Peptides
| Best Unique PSM
|

Protein Coverage:

Supporting Peptides:

| Peptide | Uniq | -10lgP | Mass | Length | ppm | m/z | z | RT | 1/k0 Start | 1/k0 End | Precursor ID | Area Sample 7 | #Feature | #Feature Sample 7 | Start | End | PTM | AScore | Found By |
| --- | --- | --- | --- | --- | --- | --- | --- | --- | --- | --- | --- | --- | --- | --- | --- | --- | --- | --- | --- |
| K.TRIEDLETVISQTQDHR.H | Y | 51.48 | 2040.0236 | 17 | 6.93 | 681.0167 | 3 | 17.50 | 0.9638 | 0.9711 | 58233 | 3.05e2 | 1 | 1 | 260 | 276 |  |  | DB Search |
| total 1 peptides |  |  |  |  |  |  |  |  |  |  |  |  |  |  |  |  |  |  |  |
| --- | --- | --- | --- | --- | --- | --- | --- | --- | --- | --- | --- | --- | --- | --- | --- | --- | --- | --- | --- |

Best Unique PSM (Scan Sample\_5\_A1\_1\_3643.d:58233, m/z=681.0167, z=3, RT=17.50, ppm=2.25):


TRINITY\_DN4781\_c0\_g1\_i3.p3

back to list

  

| Protein Coverage
| Supporting Peptides
| Best Unique PSM
|

Protein Coverage:

Supporting Peptides:

| Peptide | Uniq | -10lgP | Mass | Length | ppm | m/z | z | RT | 1/k0 Start | 1/k0 End | Precursor ID | Area Sample 7 | #Feature | #Feature Sample 7 | Start | End | PTM | AScore | Found By |
| --- | --- | --- | --- | --- | --- | --- | --- | --- | --- | --- | --- | --- | --- | --- | --- | --- | --- | --- | --- |
| R.DNAGTSSSGTGPR.F | Y | 56.16 | 1205.5272 | 13 | 0.39 | 603.7683 | 2 | 9.17 | 0.866 | 0.8733 | 10097 | 2.6e2 | 1 | 1 | 126 | 138 |  |  | DB Search |
| total 1 peptides |  |  |  |  |  |  |  |  |  |  |  |  |  |  |  |  |  |  |  |
| --- | --- | --- | --- | --- | --- | --- | --- | --- | --- | --- | --- | --- | --- | --- | --- | --- | --- | --- | --- |

Best Unique PSM (Scan Sample\_5\_A1\_1\_3643.d:10097, m/z=603.7683, z=2, RT=9.17, ppm=-4.29):


TRINITY\_DN1936\_c0\_g1\_i1.p1

back to list

  

| Protein Coverage
| Supporting Peptides
| Best Unique PSM
|

Protein Coverage:

Supporting Peptides:

| Peptide | Uniq | -10lgP | Mass | Length | ppm | m/z | z | RT | 1/k0 Start | 1/k0 End | Precursor ID | Area Sample 7 | #Feature | #Feature Sample 7 | Start | End | PTM | AScore | Found By |
| --- | --- | --- | --- | --- | --- | --- | --- | --- | --- | --- | --- | --- | --- | --- | --- | --- | --- | --- | --- |
| K.AGDIAILESQQFNAVGGK.T | Y | 54.47 | 1816.9319 | 18 | -18.71 | 909.452 | 2 | 18.92 | 1.1813 | 1.1885 | 67826 | 1.32e3 | 1 | 1 | 464 | 481 |  |  | DB Search |
| total 1 peptides |  |  |  |  |  |  |  |  |  |  |  |  |  |  |  |  |  |  |  |
| --- | --- | --- | --- | --- | --- | --- | --- | --- | --- | --- | --- | --- | --- | --- | --- | --- | --- | --- | --- |

Best Unique PSM (Scan Sample\_5\_A1\_1\_3643.d:67826, m/z=909.452, z=2, RT=18.92, ppm=-23.39):


TRINITY\_DN77\_c0\_g2\_i1.p1

back to list

  

| Protein Coverage
| Supporting Peptides
| Best Unique PSM
|

Protein Coverage:

Supporting Peptides:

| Peptide | Uniq | -10lgP | Mass | Length | ppm | m/z | z | RT | 1/k0 Start | 1/k0 End | Precursor ID | Area Sample 7 | #Feature | #Feature Sample 7 | Start | End | PTM | AScore | Found By |
| --- | --- | --- | --- | --- | --- | --- | --- | --- | --- | --- | --- | --- | --- | --- | --- | --- | --- | --- | --- |
| R.DGSTGLAGNR.G | Y | 52.33 | 946.4468 | 10 | 13.34 | 474.2348 | 2 | 10.74 | 0.8189 | 0.8262 | 17706 | 5.15e3 | 1 | 1 | 997 | 1006 |  |  | DB Search |
| total 1 peptides |  |  |  |  |  |  |  |  |  |  |  |  |  |  |  |  |  |  |  |
| --- | --- | --- | --- | --- | --- | --- | --- | --- | --- | --- | --- | --- | --- | --- | --- | --- | --- | --- | --- |

Best Unique PSM (Scan Sample\_5\_A1\_1\_3643.d:17706, m/z=474.2348, z=2, RT=10.74, ppm=8.66):


TRINITY\_DN144508\_c0\_g1\_i1.p1

back to list

  

| Protein Coverage
| Supporting Peptides
| Best Unique PSM
|

Protein Coverage:

Supporting Peptides:

| Peptide | Uniq | -10lgP | Mass | Length | ppm | m/z | z | RT | 1/k0 Start | 1/k0 End | Precursor ID | Area Sample 7 | #Feature | #Feature Sample 7 | Start | End | PTM | AScore | Found By |
| --- | --- | --- | --- | --- | --- | --- | --- | --- | --- | --- | --- | --- | --- | --- | --- | --- | --- | --- | --- |
| K.SEEYKPTLGR.C | Y | 52.38 | 1178.5931 | 10 | -5.07 | 590.2981 | 2 | 12.31 | 0.9133 | 0.9206 | 27257 | 0 | 0 | 0 | 174 | 183 |  |  | DB Search |
| total 1 peptides |  |  |  |  |  |  |  |  |  |  |  |  |  |  |  |  |  |  |  |
| --- | --- | --- | --- | --- | --- | --- | --- | --- | --- | --- | --- | --- | --- | --- | --- | --- | --- | --- | --- |

Best Unique PSM (Scan Sample\_5\_A1\_1\_3643.d:27257, m/z=590.2981, z=2, RT=12.31, ppm=-9.75):


TRINITY\_DN15584\_c0\_g1\_i1.p1

back to list

  

| Protein Coverage
| Supporting Peptides
| Best Unique PSM
|

Protein Coverage:

Supporting Peptides:

| Peptide | Uniq | -10lgP | Mass | Length | ppm | m/z | z | RT | 1/k0 Start | 1/k0 End | Precursor ID | Area Sample 7 | #Feature | #Feature Sample 7 | Start | End | PTM | AScore | Found By |
| --- | --- | --- | --- | --- | --- | --- | --- | --- | --- | --- | --- | --- | --- | --- | --- | --- | --- | --- | --- |
| K.AFVAIGDYGGHVGLGVK.C | Y | 56.39 | 1658.878 | 17 | -3.02 | 830.4399 | 2 | 17.74 | 1.1646 | 1.1718 | 59877 | 0 | 0 | 0 | 109 | 125 |  |  | DB Search |
| total 1 peptides |  |  |  |  |  |  |  |  |  |  |  |  |  |  |  |  |  |  |  |
| --- | --- | --- | --- | --- | --- | --- | --- | --- | --- | --- | --- | --- | --- | --- | --- | --- | --- | --- | --- |

Best Unique PSM (Scan Sample\_5\_A1\_1\_3643.d:59877, m/z=830.4399, z=2, RT=17.74, ppm=-7.7):


TRINITY\_DN598\_c1\_g1\_i1.p1

back to list

  

| Protein Coverage
| Supporting Peptides
| Best Unique PSM
|

Protein Coverage:

Supporting Peptides:

| Peptide | Uniq | -10lgP | Mass | Length | ppm | m/z | z | RT | 1/k0 Start | 1/k0 End | Precursor ID | Area Sample 7 | #Feature | #Feature Sample 7 | Start | End | PTM | AScore | Found By |
| --- | --- | --- | --- | --- | --- | --- | --- | --- | --- | --- | --- | --- | --- | --- | --- | --- | --- | --- | --- |
| K.AVAEEFISVDPYVR.S | Y | 56.37 | 1593.8038 | 14 | 14.01 | 797.9166 | 2 | 19.08 | 1.0656 | 1.0729 | 69186 | 1.42e4 | 1 | 1 | 148 | 161 |  |  | DB Search |
| total 1 peptides |  |  |  |  |  |  |  |  |  |  |  |  |  |  |  |  |  |  |  |
| --- | --- | --- | --- | --- | --- | --- | --- | --- | --- | --- | --- | --- | --- | --- | --- | --- | --- | --- | --- |

Best Unique PSM (Scan Sample\_5\_A1\_1\_3643.d:69186, m/z=797.6552, z=2, RT=19.09, ppm=9.33):


TRINITY\_DN250\_c0\_g1\_i3.p1

back to list

  

| Protein Coverage
| Supporting Peptides
| Best Unique PSM
|

Protein Coverage:

Supporting Peptides:

| Peptide | Uniq | -10lgP | Mass | Length | ppm | m/z | z | RT | 1/k0 Start | 1/k0 End | Precursor ID | Area Sample 7 | #Feature | #Feature Sample 7 | Start | End | PTM | AScore | Found By |
| --- | --- | --- | --- | --- | --- | --- | --- | --- | --- | --- | --- | --- | --- | --- | --- | --- | --- | --- | --- |
| R.LDHKFDLM(+15.99)YAK | Y | 51.39 | 1395.6856 | 11 | 4.19 | 466.2356 | 3 | 14.23 | 0.7844 | 0.7917 | 39140 | 0 | 0 | 0 | 391 | 401 | Oxidation (M) | M8:Oxidation (M):1000 | DB Search |
| R.AVC(+57.02)M(+15.99)LSNTTAIAEAWAR.L | Y | 50.49 | 1879.892 | 17 | -0.63 | 940.9483 | 2 | 18.78 | 1.1482 | 1.1554 | 66979 | 0 | 0 | 0 | 374 | 390 | Carbamidomethylation, Oxidation (M) | M4:Oxidation (M):1000 | DB Search |
| K.VGINYQPPTVVPGGDLAK.V | Y | 48.61 | 1823.9781 | 18 | -1.33 | 912.9908 | 2 | 17.37 | 1.1238 | 1.131 | 57429 | 0 | 0 | 0 | 353 | 370 |  |  | DB Search |
| total 3 peptides |  |  |  |  |  |  |  |  |  |  |  |  |  |  |  |  |  |  |  |
| --- | --- | --- | --- | --- | --- | --- | --- | --- | --- | --- | --- | --- | --- | --- | --- | --- | --- | --- | --- |

Best Unique PSM (Scan Sample\_5\_A1\_1\_3643.d:39140, m/z=466.2356, z=3, RT=14.23, ppm=-0.49):


TRINITY\_DN1704\_c0\_g1\_i1.p1

back to list

  

| Protein Coverage
| Supporting Peptides
| Best Unique PSM
|

Protein Coverage:

Supporting Peptides:

| Peptide | Uniq | -10lgP | Mass | Length | ppm | m/z | z | RT | 1/k0 Start | 1/k0 End | Precursor ID | Area Sample 7 | #Feature | #Feature Sample 7 | Start | End | PTM | AScore | Found By |
| --- | --- | --- | --- | --- | --- | --- | --- | --- | --- | --- | --- | --- | --- | --- | --- | --- | --- | --- | --- |
| K.IAVHC(+57.02)TVR.G | Y | 49.93 | 954.5069 | 8 | 4.46 | 478.2606 | 2 | 10.97 | 0.8502 | 0.8575 | 19084 | 8.91e2 | 1 | 1 | 81 | 88 | Carbamidomethylation |  | DB Search |
| total 1 peptides |  |  |  |  |  |  |  |  |  |  |  |  |  |  |  |  |  |  |  |
| --- | --- | --- | --- | --- | --- | --- | --- | --- | --- | --- | --- | --- | --- | --- | --- | --- | --- | --- | --- |

Best Unique PSM (Scan Sample\_5\_A1\_1\_3643.d:19084, m/z=478.2606, z=2, RT=10.97, ppm=-0.23):


TRINITY\_DN3016\_c0\_g1\_i18.p1

back to list

  

| Protein Coverage
| Supporting Peptides
| Best Unique PSM
|

Protein Coverage:

Supporting Peptides:

| Peptide | Uniq | -10lgP | Mass | Length | ppm | m/z | z | RT | 1/k0 Start | 1/k0 End | Precursor ID | Area Sample 7 | #Feature | #Feature Sample 7 | Start | End | PTM | AScore | Found By |
| --- | --- | --- | --- | --- | --- | --- | --- | --- | --- | --- | --- | --- | --- | --- | --- | --- | --- | --- | --- |
| R.IVNTDLQEIKDIIK.G | Y | 56.35 | 1640.9348 | 14 | -0.94 | 547.9825 | 3 | 18.84 | 0.8193 | 0.8266 | 67326 | 7.77e2 | 1 | 1 | 154 | 167 |  |  | DB Search |
| total 1 peptides |  |  |  |  |  |  |  |  |  |  |  |  |  |  |  |  |  |  |  |
| --- | --- | --- | --- | --- | --- | --- | --- | --- | --- | --- | --- | --- | --- | --- | --- | --- | --- | --- | --- |

Best Unique PSM (Scan Sample\_5\_A1\_1\_3643.d:67326, m/z=547.9825, z=3, RT=18.84, ppm=-5.62):


TRINITY\_DN3016\_c0\_g1\_i6.p1

back to list

  

| Protein Coverage
| Supporting Peptides
| Best Unique PSM
|

Protein Coverage:

Supporting Peptides:

| Peptide | Uniq | -10lgP | Mass | Length | ppm | m/z | z | RT | 1/k0 Start | 1/k0 End | Precursor ID | Area Sample 7 | #Feature | #Feature Sample 7 | Start | End | PTM | AScore | Found By |
| --- | --- | --- | --- | --- | --- | --- | --- | --- | --- | --- | --- | --- | --- | --- | --- | --- | --- | --- | --- |
| R.IVNTDLQEIKDIIK.G | Y | 56.35 | 1640.9348 | 14 | -0.94 | 547.9825 | 3 | 18.84 | 0.8193 | 0.8266 | 67326 | 7.77e2 | 1 | 1 | 154 | 167 |  |  | DB Search |
| total 1 peptides |  |  |  |  |  |  |  |  |  |  |  |  |  |  |  |  |  |  |  |
| --- | --- | --- | --- | --- | --- | --- | --- | --- | --- | --- | --- | --- | --- | --- | --- | --- | --- | --- | --- |

Best Unique PSM (Scan Sample\_5\_A1\_1\_3643.d:67326, m/z=547.9825, z=3, RT=18.84, ppm=-5.62):


TRINITY\_DN3986\_c0\_g1\_i1.p1

back to list

  

| Protein Coverage
| Supporting Peptides
| Best Unique PSM
|

Protein Coverage:

Supporting Peptides:

| Peptide | Uniq | -10lgP | Mass | Length | ppm | m/z | z | RT | 1/k0 Start | 1/k0 End | Precursor ID | Area Sample 7 | #Feature | #Feature Sample 7 | Start | End | PTM | AScore | Found By |
| --- | --- | --- | --- | --- | --- | --- | --- | --- | --- | --- | --- | --- | --- | --- | --- | --- | --- | --- | --- |
| R.YLTVAAMFR.G | Y | 55.34 | 1070.5583 | 9 | -0.51 | 536.2836 | 2 | 19.90 | 0.8656 | 0.8729 | 74532 | 0 | 0 | 0 | 310 | 318 |  |  | DB Search |
| total 1 peptides |  |  |  |  |  |  |  |  |  |  |  |  |  |  |  |  |  |  |  |
| --- | --- | --- | --- | --- | --- | --- | --- | --- | --- | --- | --- | --- | --- | --- | --- | --- | --- | --- | --- |

Best Unique PSM (Scan Sample\_5\_A1\_1\_3643.d:74532, m/z=536.2836, z=2, RT=19.90, ppm=-5.19):


TRINITY\_DN1058\_c0\_g1\_i3.p1

back to list

  

| Protein Coverage
| Supporting Peptides
| Best Unique PSM
|

Protein Coverage:

Supporting Peptides:

| Peptide | Uniq | -10lgP | Mass | Length | ppm | m/z | z | RT | 1/k0 Start | 1/k0 End | Precursor ID | Area Sample 7 | #Feature | #Feature Sample 7 | Start | End | PTM | AScore | Found By |
| --- | --- | --- | --- | --- | --- | --- | --- | --- | --- | --- | --- | --- | --- | --- | --- | --- | --- | --- | --- |
| R.APTHILNPANKR.S | Y | 52.25 | 1330.7469 | 12 | 5.05 | 444.5898 | 3 | 11.20 | 0.7481 | 0.7555 | 20091 | 3.03e3 | 1 | 1 | 2314 | 2325 |  |  | DB Search |
| total 1 peptides |  |  |  |  |  |  |  |  |  |  |  |  |  |  |  |  |  |  |  |
| --- | --- | --- | --- | --- | --- | --- | --- | --- | --- | --- | --- | --- | --- | --- | --- | --- | --- | --- | --- |

Best Unique PSM (Scan Sample\_5\_A1\_1\_3643.d:20091, m/z=444.5898, z=3, RT=11.20, ppm=0.37):


TRINITY\_DN36\_c0\_g1\_i3.p1

back to list

  

| Protein Coverage
| Supporting Peptides
| Best Unique PSM
|

Protein Coverage:

Supporting Peptides:

| Peptide | Uniq | -10lgP | Mass | Length | ppm | m/z | z | RT | 1/k0 Start | 1/k0 End | Precursor ID | Area Sample 7 | #Feature | #Feature Sample 7 | Start | End | PTM | AScore | Found By |
| --- | --- | --- | --- | --- | --- | --- | --- | --- | --- | --- | --- | --- | --- | --- | --- | --- | --- | --- | --- |
| K.FETQIATK.D | Y | 53.77 | 936.4916 | 8 | 12.43 | 937.5062 | 1 | 13.97 | 1.1614 | 1.1686 | 37568 | 0 | 0 | 0 | 167 | 174 |  |  | DB Search |
| R.LGGIEVTQGNC(+57.02)AR.K | Y | 49.67 | 1373.6721 | 13 | 2.61 | 687.8419 | 2 | 13.89 | 0.9695 | 0.9767 | 37015 | 0 | 0 | 0 | 58 | 70 | Carbamidomethylation |  | DB Search |
| total 2 peptides |  |  |  |  |  |  |  |  |  |  |  |  |  |  |  |  |  |  |  |
| --- | --- | --- | --- | --- | --- | --- | --- | --- | --- | --- | --- | --- | --- | --- | --- | --- | --- | --- | --- |

Best Unique PSM (Scan Sample\_5\_A1\_1\_3643.d:37568, m/z=937.5062, z=1, RT=13.97, ppm=7.75):


TRINITY\_DN195348\_c0\_g1\_i1.p1

back to list

  

| Protein Coverage
| Supporting Peptides
| Best Unique PSM
|

Protein Coverage:

Supporting Peptides:

| Peptide | Uniq | -10lgP | Mass | Length | ppm | m/z | z | RT | 1/k0 Start | 1/k0 End | Precursor ID | Area Sample 7 | #Feature | #Feature Sample 7 | Start | End | PTM | AScore | Found By |
| --- | --- | --- | --- | --- | --- | --- | --- | --- | --- | --- | --- | --- | --- | --- | --- | --- | --- | --- | --- |
| K.VLIVISDGNEDFKEK.Q | Y | 55.12 | 1704.8934 | 15 | 3.91 | 853.4533 | 2 | 16.85 | 1.1742 | 1.1813 | 54267 | 0 | 0 | 0 | 132 | 146 |  |  | DB Search |
| total 1 peptides |  |  |  |  |  |  |  |  |  |  |  |  |  |  |  |  |  |  |  |
| --- | --- | --- | --- | --- | --- | --- | --- | --- | --- | --- | --- | --- | --- | --- | --- | --- | --- | --- | --- |

Best Unique PSM (Scan Sample\_5\_A1\_1\_3643.d:54267, m/z=853.4533, z=2, RT=16.85, ppm=-0.77):


TRINITY\_DN167\_c0\_g1\_i8.p1

back to list

  

| Protein Coverage
| Supporting Peptides
| Best Unique PSM
|

Protein Coverage:

Supporting Peptides:

| Peptide | Uniq | -10lgP | Mass | Length | ppm | m/z | z | RT | 1/k0 Start | 1/k0 End | Precursor ID | Area Sample 7 | #Feature | #Feature Sample 7 | Start | End | PTM | AScore | Found By |
| --- | --- | --- | --- | --- | --- | --- | --- | --- | --- | --- | --- | --- | --- | --- | --- | --- | --- | --- | --- |
| K.STAGDTHLGGEDFDNR.L | Y | 56.39 | 1690.7183 | 16 | 2.27 | 564.5787 | 3 | 13.29 | 0.8014 | 0.8088 | 33297 | 4.2e3 | 2 | 2 | 107 | 122 |  |  | DB Search |
| total 1 peptides |  |  |  |  |  |  |  |  |  |  |  |  |  |  |  |  |  |  |  |
| --- | --- | --- | --- | --- | --- | --- | --- | --- | --- | --- | --- | --- | --- | --- | --- | --- | --- | --- | --- |

Best Unique PSM (Scan Sample\_5\_A1\_1\_3643.d:33297, m/z=564.5787, z=3, RT=13.29, ppm=-2.41):


TRINITY\_DN195934\_c0\_g1\_i1.p2

back to list

  

| Protein Coverage
| Supporting Peptides
| Best Unique PSM
|

Protein Coverage:

Supporting Peptides:

| Peptide | Uniq | -10lgP | Mass | Length | ppm | m/z | z | RT | 1/k0 Start | 1/k0 End | Precursor ID | Area Sample 7 | #Feature | #Feature Sample 7 | Start | End | PTM | AScore | Found By |
| --- | --- | --- | --- | --- | --- | --- | --- | --- | --- | --- | --- | --- | --- | --- | --- | --- | --- | --- | --- |
| K.DSYVGDEAQSKR.G | Y | 55.24 | 1353.616 | 12 | 0.18 | 677.8123 | 2 | 10.99 | 0.9932 | 1.0005 | 19155 | 1.01e3 | 1 | 1 | 10 | 21 |  |  | DB Search |
| R.VAPEEHPVLLTEAPLNPK.A | Y | 55.12 | 1953.0571 | 18 | 2.07 | 977.5333 | 2 | 16.62 | 1.2101 | 1.2173 | 52945 | 1.41e2 | 1 | 1 | 55 | 72 |  |  | DB Search |
| K.DSYVGDEAQSK.R | Y | 54.42 | 1197.5149 | 11 | 5.23 | 599.7651 | 2 | 11.40 | 0.8968 | 0.904 | 21678 | 5.55e2 | 1 | 1 | 10 | 20 |  |  | DB Search |
| total 3 peptides |  |  |  |  |  |  |  |  |  |  |  |  |  |  |  |  |  |  |  |
| --- | --- | --- | --- | --- | --- | --- | --- | --- | --- | --- | --- | --- | --- | --- | --- | --- | --- | --- | --- |

Best Unique PSM (Scan Sample\_5\_A1\_1\_3643.d:19155, m/z=677.8123, z=2, RT=10.99, ppm=-4.5):


TRINITY\_DN493\_c0\_g1\_i1.p1

back to list

  

| Protein Coverage
| Supporting Peptides
| Best Unique PSM
|

Protein Coverage:

Supporting Peptides:

| Peptide | Uniq | -10lgP | Mass | Length | ppm | m/z | z | RT | 1/k0 Start | 1/k0 End | Precursor ID | Area Sample 7 | #Feature | #Feature Sample 7 | Start | End | PTM | AScore | Found By |
| --- | --- | --- | --- | --- | --- | --- | --- | --- | --- | --- | --- | --- | --- | --- | --- | --- | --- | --- | --- |
| R.AMTEEILNERDTASK.V | Y | 49.64 | 1706.8145 | 15 | 1.5 | 854.4118 | 2 | 14.35 | 1.1302 | 1.1374 | 39764 | 4.47e2 | 1 | 1 | 240 | 254 |  |  | DB Search |
| K.EC(+57.02)SC(+57.02)NSYYC(+57.02)VGK.T | Y | 49.5 | 1525.5636 | 12 | -0.7 | 763.785 | 2 | 11.90 | 1.0061 | 1.0134 | 24544 | 1.21e3 | 1 | 1 | 451 | 462 | Carbamidomethylation, Carbamidomethylation, Carbamidomethylation |  | DB Search |
| total 2 peptides |  |  |  |  |  |  |  |  |  |  |  |  |  |  |  |  |  |  |  |
| --- | --- | --- | --- | --- | --- | --- | --- | --- | --- | --- | --- | --- | --- | --- | --- | --- | --- | --- | --- |

Best Unique PSM (Scan Sample\_5\_A1\_1\_3643.d:39764, m/z=854.4118, z=2, RT=14.35, ppm=-3.18):


TRINITY\_DN1071\_c0\_g1\_i3.p1

back to list

  

| Protein Coverage
| Supporting Peptides
| Best Unique PSM
|

Protein Coverage:

Supporting Peptides:

| Peptide | Uniq | -10lgP | Mass | Length | ppm | m/z | z | RT | 1/k0 Start | 1/k0 End | Precursor ID | Area Sample 7 | #Feature | #Feature Sample 7 | Start | End | PTM | AScore | Found By |
| --- | --- | --- | --- | --- | --- | --- | --- | --- | --- | --- | --- | --- | --- | --- | --- | --- | --- | --- | --- |
| K.AVQIHPFNDVSVGDPK.V | Y | 56.18 | 1721.8736 | 16 | -1.14 | 861.9391 | 2 | 15.94 | 1.1178 | 1.125 | 48886 | 2.33e3 | 1 | 1 | 329 | 344 |  |  | DB Search |
| total 1 peptides |  |  |  |  |  |  |  |  |  |  |  |  |  |  |  |  |  |  |  |
| --- | --- | --- | --- | --- | --- | --- | --- | --- | --- | --- | --- | --- | --- | --- | --- | --- | --- | --- | --- |

Best Unique PSM (Scan Sample\_5\_A1\_1\_3643.d:48886, m/z=861.9391, z=2, RT=15.94, ppm=-5.82):


TRINITY\_DN761\_c0\_g1\_i1.p1

back to list

  

| Protein Coverage
| Supporting Peptides
| Best Unique PSM
|

Protein Coverage:

Supporting Peptides:

| Peptide | Uniq | -10lgP | Mass | Length | ppm | m/z | z | RT | 1/k0 Start | 1/k0 End | Precursor ID | Area Sample 7 | #Feature | #Feature Sample 7 | Start | End | PTM | AScore | Found By |
| --- | --- | --- | --- | --- | --- | --- | --- | --- | --- | --- | --- | --- | --- | --- | --- | --- | --- | --- | --- |
| K.TRIEDLETVISQTQDHR.H | Y | 51.48 | 2040.0236 | 17 | 6.93 | 681.0167 | 3 | 17.50 | 0.9638 | 0.9711 | 58233 | 3.05e2 | 1 | 1 | 260 | 276 |  |  | DB Search |
| total 1 peptides |  |  |  |  |  |  |  |  |  |  |  |  |  |  |  |  |  |  |  |
| --- | --- | --- | --- | --- | --- | --- | --- | --- | --- | --- | --- | --- | --- | --- | --- | --- | --- | --- | --- |

Best Unique PSM (Scan Sample\_5\_A1\_1\_3643.d:58233, m/z=681.0167, z=3, RT=17.50, ppm=2.25):


TRINITY\_DN11522\_c0\_g1\_i2.p1

back to list

  

| Protein Coverage
| Supporting Peptides
| Best Unique PSM
|

Protein Coverage:

Supporting Peptides:

| Peptide | Uniq | -10lgP | Mass | Length | ppm | m/z | z | RT | 1/k0 Start | 1/k0 End | Precursor ID | Area Sample 7 | #Feature | #Feature Sample 7 | Start | End | PTM | AScore | Found By |
| --- | --- | --- | --- | --- | --- | --- | --- | --- | --- | --- | --- | --- | --- | --- | --- | --- | --- | --- | --- |
| R.IGLGNIRDPSISLTR.G | Y | 55.35 | 1610.9104 | 15 | -3.69 | 537.9729 | 3 | 17.37 | 0.7986 | 0.8059 | 57304 | 1.32e3 | 1 | 1 | 25 | 39 |  |  | DB Search |
| total 1 peptides |  |  |  |  |  |  |  |  |  |  |  |  |  |  |  |  |  |  |  |
| --- | --- | --- | --- | --- | --- | --- | --- | --- | --- | --- | --- | --- | --- | --- | --- | --- | --- | --- | --- |

Best Unique PSM (Scan Sample\_5\_A1\_1\_3643.d:57304, m/z=537.9729, z=3, RT=17.37, ppm=-8.38):


TRINITY\_DN36\_c0\_g1\_i20.p1

back to list

  

| Protein Coverage
| Supporting Peptides
| Best Unique PSM
|

Protein Coverage:

Supporting Peptides:

| Peptide | Uniq | -10lgP | Mass | Length | ppm | m/z | z | RT | 1/k0 Start | 1/k0 End | Precursor ID | Area Sample 7 | #Feature | #Feature Sample 7 | Start | End | PTM | AScore | Found By |
| --- | --- | --- | --- | --- | --- | --- | --- | --- | --- | --- | --- | --- | --- | --- | --- | --- | --- | --- | --- |
| R.LGGIEVTQGNC(+57.02)AR.K | Y | 49.67 | 1373.6721 | 13 | 2.61 | 687.8419 | 2 | 13.89 | 0.9695 | 0.9767 | 37015 | 0 | 0 | 0 | 57 | 69 | Carbamidomethylation |  | DB Search |
| total 1 peptides |  |  |  |  |  |  |  |  |  |  |  |  |  |  |  |  |  |  |  |
| --- | --- | --- | --- | --- | --- | --- | --- | --- | --- | --- | --- | --- | --- | --- | --- | --- | --- | --- | --- |

Best Unique PSM (Scan Sample\_5\_A1\_1\_3643.d:37015, m/z=687.8419, z=2, RT=13.89, ppm=-2.07):


TRINITY\_DN1119\_c0\_g1\_i1.p1

back to list

  

| Protein Coverage
| Supporting Peptides
| Best Unique PSM
|

Protein Coverage:

Supporting Peptides:

| Peptide | Uniq | -10lgP | Mass | Length | ppm | m/z | z | RT | 1/k0 Start | 1/k0 End | Precursor ID | Area Sample 7 | #Feature | #Feature Sample 7 | Start | End | PTM | AScore | Found By |
| --- | --- | --- | --- | --- | --- | --- | --- | --- | --- | --- | --- | --- | --- | --- | --- | --- | --- | --- | --- |
| R.HFGIC(+57.02)IGYNK.I | Y | 54 | 1207.5808 | 10 | -0.18 | 604.7947 | 2 | 17.63 | 0.923 | 0.9303 | 59179 | 0 | 0 | 0 | 92 | 101 | Carbamidomethylation |  | DB Search |
| K.SIEHC(+57.02)IDTK.S | Y | 51.35 | 1101.5124 | 9 | 3.22 | 551.7627 | 2 | 11.31 | 0.8834 | 0.8907 | 21126 | 0 | 0 | 0 | 108 | 116 | Carbamidomethylation |  | DB Search |
| K.RLTELEHFLK.I | Y | 50.47 | 1284.719 | 10 | 3.5 | 429.2464 | 3 | 16.44 | 0.7453 | 0.7526 | 51972 | 4.72e2 | 1 | 1 | 376 | 385 |  |  | DB Search |
| total 3 peptides |  |  |  |  |  |  |  |  |  |  |  |  |  |  |  |  |  |  |  |
| --- | --- | --- | --- | --- | --- | --- | --- | --- | --- | --- | --- | --- | --- | --- | --- | --- | --- | --- | --- |

Best Unique PSM (Scan Sample\_5\_A1\_1\_3643.d:59179, m/z=604.7947, z=2, RT=17.63, ppm=-4.86):


TRINITY\_DN36\_c0\_g1\_i14.p1

back to list

  

| Protein Coverage
| Supporting Peptides
| Best Unique PSM
|

Protein Coverage:

Supporting Peptides:

| Peptide | Uniq | -10lgP | Mass | Length | ppm | m/z | z | RT | 1/k0 Start | 1/k0 End | Precursor ID | Area Sample 7 | #Feature | #Feature Sample 7 | Start | End | PTM | AScore | Found By |
| --- | --- | --- | --- | --- | --- | --- | --- | --- | --- | --- | --- | --- | --- | --- | --- | --- | --- | --- | --- |
| K.FETQIATK.D | Y | 53.77 | 936.4916 | 8 | 12.43 | 937.5062 | 1 | 13.97 | 1.1614 | 1.1686 | 37568 | 0 | 0 | 0 | 167 | 174 |  |  | DB Search |
| R.LGGIEVTQGNC(+57.02)AR.K | Y | 49.67 | 1373.6721 | 13 | 2.61 | 687.8419 | 2 | 13.89 | 0.9695 | 0.9767 | 37015 | 0 | 0 | 0 | 58 | 70 | Carbamidomethylation |  | DB Search |
| total 2 peptides |  |  |  |  |  |  |  |  |  |  |  |  |  |  |  |  |  |  |  |
| --- | --- | --- | --- | --- | --- | --- | --- | --- | --- | --- | --- | --- | --- | --- | --- | --- | --- | --- | --- |

Best Unique PSM (Scan Sample\_5\_A1\_1\_3643.d:37568, m/z=937.5062, z=1, RT=13.97, ppm=7.75):


TRINITY\_DN247\_c0\_g1\_i1.p1

back to list

  

| Protein Coverage
| Supporting Peptides
| Best Unique PSM
|

Protein Coverage:

Supporting Peptides:

| Peptide | Uniq | -10lgP | Mass | Length | ppm | m/z | z | RT | 1/k0 Start | 1/k0 End | Precursor ID | Area Sample 7 | #Feature | #Feature Sample 7 | Start | End | PTM | AScore | Found By |
| --- | --- | --- | --- | --- | --- | --- | --- | --- | --- | --- | --- | --- | --- | --- | --- | --- | --- | --- | --- |
| R.DAPIYGLQWHPEK.S | Y | 49.41 | 1552.7674 | 13 | 1.48 | 777.3885 | 2 | 17.41 | 1.0745 | 1.0817 | 57577 | 1.18e3 | 1 | 1 | 258 | 270 |  |  | DB Search |
| total 1 peptides |  |  |  |  |  |  |  |  |  |  |  |  |  |  |  |  |  |  |  |
| --- | --- | --- | --- | --- | --- | --- | --- | --- | --- | --- | --- | --- | --- | --- | --- | --- | --- | --- | --- |

Best Unique PSM (Scan Sample\_5\_A1\_1\_3643.d:57577, m/z=777.3885, z=2, RT=17.41, ppm=-3.2):


TRINITY\_DN81013\_c0\_g1\_i1.p2

back to list

  

| Protein Coverage
| Supporting Peptides
| Best Unique PSM
|

Protein Coverage:

Supporting Peptides:

| Peptide | Uniq | -10lgP | Mass | Length | ppm | m/z | z | RT | 1/k0 Start | 1/k0 End | Precursor ID | Area Sample 7 | #Feature | #Feature Sample 7 | Start | End | PTM | AScore | Found By |
| --- | --- | --- | --- | --- | --- | --- | --- | --- | --- | --- | --- | --- | --- | --- | --- | --- | --- | --- | --- |
| R.AIQGATSHHLGQNFSK.M | Y | 55.18 | 1694.8488 | 16 | 7.42 | 565.9584 | 3 | 12.02 | 0.8518 | 0.8591 | 25405 | 5.02e2 | 1 | 1 | 56 | 71 |  |  | DB Search |
| total 1 peptides |  |  |  |  |  |  |  |  |  |  |  |  |  |  |  |  |  |  |  |
| --- | --- | --- | --- | --- | --- | --- | --- | --- | --- | --- | --- | --- | --- | --- | --- | --- | --- | --- | --- |

Best Unique PSM (Scan Sample\_5\_A1\_1\_3643.d:25405, m/z=565.9584, z=3, RT=12.02, ppm=2.74):


TRINITY\_DN3052\_c0\_g1\_i1.p1

back to list

  

| Protein Coverage
| Supporting Peptides
| Best Unique PSM
|

Protein Coverage:

Supporting Peptides:

| Peptide | Uniq | -10lgP | Mass | Length | ppm | m/z | z | RT | 1/k0 Start | 1/k0 End | Precursor ID | Area Sample 7 | #Feature | #Feature Sample 7 | Start | End | PTM | AScore | Found By |
| --- | --- | --- | --- | --- | --- | --- | --- | --- | --- | --- | --- | --- | --- | --- | --- | --- | --- | --- | --- |
| M.A(+42.01)HLSQEQR.N | Y | 55.07 | 1009.4941 | 8 | 7.42 | 505.7557 | 2 | 10.99 | 0.8684 | 0.8757 | 19264 | 0 | 0 | 0 | 2 | 9 | Acetylation (Protein N-term) | A1:Acetylation (Protein N-term):1000 | DB Search |
| total 1 peptides |  |  |  |  |  |  |  |  |  |  |  |  |  |  |  |  |  |  |  |
| --- | --- | --- | --- | --- | --- | --- | --- | --- | --- | --- | --- | --- | --- | --- | --- | --- | --- | --- | --- |

Best Unique PSM (Scan Sample\_5\_A1\_1\_3643.d:19264, m/z=505.7557, z=2, RT=10.99, ppm=2.74):


TRINITY\_DN262\_c0\_g1\_i1.p1

back to list

  

| Protein Coverage
| Supporting Peptides
| Best Unique PSM
|

Protein Coverage:

Supporting Peptides:

| Peptide | Uniq | -10lgP | Mass | Length | ppm | m/z | z | RT | 1/k0 Start | 1/k0 End | Precursor ID | Area Sample 7 | #Feature | #Feature Sample 7 | Start | End | PTM | AScore | Found By |
| --- | --- | --- | --- | --- | --- | --- | --- | --- | --- | --- | --- | --- | --- | --- | --- | --- | --- | --- | --- |
| K.VIM(+15.99)VTGDHPITAK.A | Y | 56.39 | 1396.7384 | 13 | 5.45 | 699.377 | 2 | 13.30 | 1.0263 | 1.0335 | 33329 | 8.77e2 | 1 | 1 | 622 | 634 | Oxidation (M) | M3:Oxidation (M):1000 | DB Search |
| R.AEFRPNQEEEPILKR.E | Y | 55.37 | 1854.9588 | 15 | 3.46 | 619.3261 | 3 | 13.89 | 0.8826 | 0.8899 | 36828 | 3.47e3 | 1 | 1 | 439 | 453 |  |  | DB Search |
| K.VDNSSLTGESEPQSR.N | Y | 53.35 | 1604.7278 | 15 | -5.23 | 803.3632 | 2 | 12.02 | 1.0275 | 1.0347 | 25394 | 0 | 0 | 0 | 221 | 235 |  |  | DB Search |
| K.GVGIISEGNETVEDIAQR.L | Y | 50.29 | 1885.9381 | 18 | 4.42 | 943.9761 | 2 | 17.89 | 1.1446 | 1.1518 | 60967 | 0 | 0 | 0 | 639 | 656 |  |  | DB Search |
| R.DANAAVVHGSHLK.D | Y | 50.26 | 1317.6789 | 13 | 6.36 | 659.8478 | 2 | 10.94 | 1.0118 | 1.019 | 18954 | 0 | 0 | 0 | 668 | 680 |  |  | DB Search |
| total 5 peptides |  |  |  |  |  |  |  |  |  |  |  |  |  |  |  |  |  |  |  |
| --- | --- | --- | --- | --- | --- | --- | --- | --- | --- | --- | --- | --- | --- | --- | --- | --- | --- | --- | --- |

Best Unique PSM (Scan Sample\_5\_A1\_1\_3643.d:33329, m/z=699.377, z=2, RT=13.30, ppm=0.77):


TRINITY\_DN5633\_c0\_g1\_i1.p1

back to list

  

| Protein Coverage
| Supporting Peptides
| Best Unique PSM
|

Protein Coverage:

Supporting Peptides:

| Peptide | Uniq | -10lgP | Mass | Length | ppm | m/z | z | RT | 1/k0 Start | 1/k0 End | Precursor ID | Area Sample 7 | #Feature | #Feature Sample 7 | Start | End | PTM | AScore | Found By |
| --- | --- | --- | --- | --- | --- | --- | --- | --- | --- | --- | --- | --- | --- | --- | --- | --- | --- | --- | --- |
| K.LLEINVYHPLIQELQK.R | Y | 49.16 | 1949.0986 | 16 | 5.33 | 650.7072 | 3 | 19.80 | 0.9812 | 0.9884 | 73781 | 2.5e2 | 1 | 1 | 706 | 721 |  |  | DB Search |
| total 1 peptides |  |  |  |  |  |  |  |  |  |  |  |  |  |  |  |  |  |  |  |
| --- | --- | --- | --- | --- | --- | --- | --- | --- | --- | --- | --- | --- | --- | --- | --- | --- | --- | --- | --- |

Best Unique PSM (Scan Sample\_5\_A1\_1\_3643.d:73781, m/z=650.7072, z=3, RT=19.80, ppm=0.64):


TRINITY\_DN1071\_c0\_g1\_i4.p1

back to list

  

| Protein Coverage
| Supporting Peptides
| Best Unique PSM
|

Protein Coverage:

Supporting Peptides:

| Peptide | Uniq | -10lgP | Mass | Length | ppm | m/z | z | RT | 1/k0 Start | 1/k0 End | Precursor ID | Area Sample 7 | #Feature | #Feature Sample 7 | Start | End | PTM | AScore | Found By |
| --- | --- | --- | --- | --- | --- | --- | --- | --- | --- | --- | --- | --- | --- | --- | --- | --- | --- | --- | --- |
| K.AVQIHPFNDVSVGDPK.V | Y | 56.18 | 1721.8736 | 16 | -1.14 | 861.9391 | 2 | 15.94 | 1.1178 | 1.125 | 48886 | 2.33e3 | 1 | 1 | 329 | 344 |  |  | DB Search |
| total 1 peptides |  |  |  |  |  |  |  |  |  |  |  |  |  |  |  |  |  |  |  |
| --- | --- | --- | --- | --- | --- | --- | --- | --- | --- | --- | --- | --- | --- | --- | --- | --- | --- | --- | --- |

Best Unique PSM (Scan Sample\_5\_A1\_1\_3643.d:48886, m/z=861.9391, z=2, RT=15.94, ppm=-5.82):


TRINITY\_DN2477\_c0\_g1\_i10.p1

back to list

  

| Protein Coverage
| Supporting Peptides
| Best Unique PSM
|

Protein Coverage:

Supporting Peptides:

| Peptide | Uniq | -10lgP | Mass | Length | ppm | m/z | z | RT | 1/k0 Start | 1/k0 End | Precursor ID | Area Sample 7 | #Feature | #Feature Sample 7 | Start | End | PTM | AScore | Found By |
| --- | --- | --- | --- | --- | --- | --- | --- | --- | --- | --- | --- | --- | --- | --- | --- | --- | --- | --- | --- |
| K.DSYVGDEAQSKR.G | Y | 55.24 | 1353.616 | 12 | 0.18 | 677.8123 | 2 | 10.99 | 0.9932 | 1.0005 | 19155 | 1.01e3 | 1 | 1 | 19 | 30 |  |  | DB Search |
| R.VAPEEHPVLLTEAPLNPK.A | Y | 55.12 | 1953.0571 | 18 | 2.07 | 977.5333 | 2 | 16.62 | 1.2101 | 1.2173 | 52945 | 1.41e2 | 1 | 1 | 64 | 81 |  |  | DB Search |
| K.DSYVGDEAQSK.R | Y | 54.42 | 1197.5149 | 11 | 5.23 | 599.7651 | 2 | 11.40 | 0.8968 | 0.904 | 21678 | 5.55e2 | 1 | 1 | 19 | 29 |  |  | DB Search |
| total 3 peptides |  |  |  |  |  |  |  |  |  |  |  |  |  |  |  |  |  |  |  |
| --- | --- | --- | --- | --- | --- | --- | --- | --- | --- | --- | --- | --- | --- | --- | --- | --- | --- | --- | --- |

Best Unique PSM (Scan Sample\_5\_A1\_1\_3643.d:19155, m/z=677.8123, z=2, RT=10.99, ppm=-4.5):


TRINITY\_DN1653\_c0\_g1\_i1.p1

back to list

  

| Protein Coverage
| Supporting Peptides
| Best Unique PSM
|

Protein Coverage:

Supporting Peptides:

| Peptide | Uniq | -10lgP | Mass | Length | ppm | m/z | z | RT | 1/k0 Start | 1/k0 End | Precursor ID | Area Sample 7 | #Feature | #Feature Sample 7 | Start | End | PTM | AScore | Found By |
| --- | --- | --- | --- | --- | --- | --- | --- | --- | --- | --- | --- | --- | --- | --- | --- | --- | --- | --- | --- |
| R.LINLFEAVSKK.S | Y | 56.32 | 1260.7441 | 11 | 0.6 | 631.3768 | 2 | 19.05 | 0.9836 | 0.9908 | 68790 | 7.03e2 | 2 | 2 | 49 | 59 |  |  | DB Search |
| R.ASDKFSASGPGLVEPK.I | Y | 48.48 | 1588.8096 | 16 | 6.54 | 795.4136 | 2 | 14.23 | 1.0608 | 1.068 | 39146 | 0 | 0 | 0 | 338 | 353 |  |  | DB Search |
| total 2 peptides |  |  |  |  |  |  |  |  |  |  |  |  |  |  |  |  |  |  |  |
| --- | --- | --- | --- | --- | --- | --- | --- | --- | --- | --- | --- | --- | --- | --- | --- | --- | --- | --- | --- |

Best Unique PSM (Scan Sample\_5\_A1\_1\_3643.d:68790, m/z=631.3768, z=2, RT=19.05, ppm=-4.09):


TRINITY\_DN14015\_c0\_g1\_i2.p1

back to list

  

| Protein Coverage
| Supporting Peptides
| Best Unique PSM
|

Protein Coverage:

Supporting Peptides:

| Peptide | Uniq | -10lgP | Mass | Length | ppm | m/z | z | RT | 1/k0 Start | 1/k0 End | Precursor ID | Area Sample 7 | #Feature | #Feature Sample 7 | Start | End | PTM | AScore | Found By |
| --- | --- | --- | --- | --- | --- | --- | --- | --- | --- | --- | --- | --- | --- | --- | --- | --- | --- | --- | --- |
| K.VIDINDNAPIFNK.A | Y | 48.8 | 1471.7671 | 13 | 3.85 | 736.8902 | 2 | 17.47 | 1.0146 | 1.0218 | 57919 | 8.75e2 | 1 | 1 | 666 | 678 |  |  | DB Search |
| total 1 peptides |  |  |  |  |  |  |  |  |  |  |  |  |  |  |  |  |  |  |  |
| --- | --- | --- | --- | --- | --- | --- | --- | --- | --- | --- | --- | --- | --- | --- | --- | --- | --- | --- | --- |

Best Unique PSM (Scan Sample\_5\_A1\_1\_3643.d:57919, m/z=736.8902, z=2, RT=17.47, ppm=-0.83):


TRINITY\_DN77\_c0\_g2\_i2.p1

back to list

  

| Protein Coverage
| Supporting Peptides
| Best Unique PSM
|

Protein Coverage:

Supporting Peptides:

| Peptide | Uniq | -10lgP | Mass | Length | ppm | m/z | z | RT | 1/k0 Start | 1/k0 End | Precursor ID | Area Sample 7 | #Feature | #Feature Sample 7 | Start | End | PTM | AScore | Found By |
| --- | --- | --- | --- | --- | --- | --- | --- | --- | --- | --- | --- | --- | --- | --- | --- | --- | --- | --- | --- |
| R.DGSTGLAGNR.G | Y | 52.33 | 946.4468 | 10 | 13.34 | 474.2348 | 2 | 10.74 | 0.8189 | 0.8262 | 17706 | 5.15e3 | 1 | 1 | 997 | 1006 |  |  | DB Search |
| total 1 peptides |  |  |  |  |  |  |  |  |  |  |  |  |  |  |  |  |  |  |  |
| --- | --- | --- | --- | --- | --- | --- | --- | --- | --- | --- | --- | --- | --- | --- | --- | --- | --- | --- | --- |

Best Unique PSM (Scan Sample\_5\_A1\_1\_3643.d:17706, m/z=474.2348, z=2, RT=10.74, ppm=8.66):


TRINITY\_DN77\_c0\_g2\_i4.p1

back to list

  

| Protein Coverage
| Supporting Peptides
| Best Unique PSM
|

Protein Coverage:

Supporting Peptides:

| Peptide | Uniq | -10lgP | Mass | Length | ppm | m/z | z | RT | 1/k0 Start | 1/k0 End | Precursor ID | Area Sample 7 | #Feature | #Feature Sample 7 | Start | End | PTM | AScore | Found By |
| --- | --- | --- | --- | --- | --- | --- | --- | --- | --- | --- | --- | --- | --- | --- | --- | --- | --- | --- | --- |
| R.DGSTGLAGNR.G | Y | 52.33 | 946.4468 | 10 | 13.34 | 474.2348 | 2 | 10.74 | 0.8189 | 0.8262 | 17706 | 5.15e3 | 1 | 1 | 1004 | 1013 |  |  | DB Search |
| total 1 peptides |  |  |  |  |  |  |  |  |  |  |  |  |  |  |  |  |  |  |  |
| --- | --- | --- | --- | --- | --- | --- | --- | --- | --- | --- | --- | --- | --- | --- | --- | --- | --- | --- | --- |

Best Unique PSM (Scan Sample\_5\_A1\_1\_3643.d:17706, m/z=474.2348, z=2, RT=10.74, ppm=8.66):


TRINITY\_DN1535\_c0\_g1\_i1.p1

back to list

  

| Protein Coverage
| Supporting Peptides
| Best Unique PSM
|

Protein Coverage:

Supporting Peptides:

| Peptide | Uniq | -10lgP | Mass | Length | ppm | m/z | z | RT | 1/k0 Start | 1/k0 End | Precursor ID | Area Sample 7 | #Feature | #Feature Sample 7 | Start | End | PTM | AScore | Found By |
| --- | --- | --- | --- | --- | --- | --- | --- | --- | --- | --- | --- | --- | --- | --- | --- | --- | --- | --- | --- |
| R.GSC(+57.02)EC(+57.02)GTC(+57.02)VC(+57.02)R.E | Y | 56.41 | 1344.4679 | 11 | 6.01 | 673.2421 | 2 | 10.52 | 0.9299 | 0.9372 | 16603 | 0 | 0 | 0 | 558 | 568 | Carbamidomethylation, Carbamidomethylation, Carbamidomethylation, Carbamidomethylation |  | DB Search |
| R.HQNIVC(+57.02)GGPER.G | Y | 54.58 | 1265.5935 | 11 | -0.53 | 633.8007 | 2 | 12.52 | 0.9109 | 0.9182 | 28563 | 4.18e2 | 1 | 1 | 547 | 557 | Carbamidomethylation |  | DB Search |
| total 2 peptides |  |  |  |  |  |  |  |  |  |  |  |  |  |  |  |  |  |  |  |
| --- | --- | --- | --- | --- | --- | --- | --- | --- | --- | --- | --- | --- | --- | --- | --- | --- | --- | --- | --- |

Best Unique PSM (Scan Sample\_5\_A1\_1\_3643.d:16603, m/z=673.2421, z=2, RT=10.52, ppm=1.32):


TRINITY\_DN161\_c0\_g1\_i1.p1

back to list

  

| Protein Coverage
| Supporting Peptides
| Best Unique PSM
|

Protein Coverage:

Supporting Peptides:

| Peptide | Uniq | -10lgP | Mass | Length | ppm | m/z | z | RT | 1/k0 Start | 1/k0 End | Precursor ID | Area Sample 7 | #Feature | #Feature Sample 7 | Start | End | PTM | AScore | Found By |
| --- | --- | --- | --- | --- | --- | --- | --- | --- | --- | --- | --- | --- | --- | --- | --- | --- | --- | --- | --- |
| K.SYELPDGQVITIGNER.F | Y | 55.37 | 1789.8846 | 16 | 2.43 | 895.9476 | 2 | 18.67 | 1.117 | 1.1242 | 66156 | 3.51e2 | 1 | 1 | 240 | 255 |  |  | DB Search |
| R.HQGVMVGMGQK.D | Y | 55.37 | 1170.5638 | 11 | 3.68 | 586.2886 | 2 | 12.36 | 0.9105 | 0.9178 | 27450 | 5.73e2 | 1 | 1 | 41 | 51 |  |  | DB Search |
| R.HQGVM(+15.99)VGMGQK.D | Y | 55.3 | 1186.5587 | 11 | 10.45 | 594.29 | 2 | 11.22 | 0.9133 | 0.9206 | 20614 | 0 | 0 | 0 | 41 | 51 | Oxidation (M) | M5:Oxidation (M):60 | DB Search |
| K.DSYVGDEAQSKR.G | Y | 55.24 | 1353.616 | 12 | 0.18 | 677.8123 | 2 | 10.99 | 0.9932 | 1.0005 | 19155 | 1.01e3 | 1 | 1 | 52 | 63 |  |  | DB Search |
| R.AVFPSIVGRPR.H | Y | 55.22 | 1197.6982 | 11 | 2.36 | 599.855 | 2 | 15.87 | 0.961 | 0.9683 | 48378 | 1.86e4 | 1 | 1 | 30 | 40 |  |  | DB Search |
| R.VAPEEHPVLLTEAPLNPK.A | Y | 55.12 | 1953.0571 | 18 | 2.07 | 977.5333 | 2 | 16.62 | 1.2101 | 1.2173 | 52945 | 1.41e2 | 1 | 1 | 97 | 114 |  |  | DB Search |
| K.DSYVGDEAQSK.R | Y | 54.42 | 1197.5149 | 11 | 5.23 | 599.7651 | 2 | 11.40 | 0.8968 | 0.904 | 21678 | 5.55e2 | 1 | 1 | 52 | 62 |  |  | DB Search |
| R.HQGVM(+15.99)VGM(+15.99)GQK.D | Y | 52.78 | 1202.5536 | 11 | 2.62 | 602.2828 | 2 | 9.74 | 0.8955 | 0.9028 | 12426 | 1.52e3 | 1 | 1 | 41 | 51 | Oxidation (M), Oxidation (M) | M5:Oxidation (M):1000 M8:Oxidation (M):1000 | DB Search |
| R.HQGVMVGM(+15.99)GQK.D | Y | 48 | 1186.5587 | 11 | 4.48 | 594.2865 | 2 | 10.48 | 0.9052 | 0.9125 | 16419 | 1.61e3 | 1 | 1 | 41 | 51 | Oxidation (M) | M8:Oxidation (M):32.97 | DB Search |
| total 9 peptides |  |  |  |  |  |  |  |  |  |  |  |  |  |  |  |  |  |  |  |
| --- | --- | --- | --- | --- | --- | --- | --- | --- | --- | --- | --- | --- | --- | --- | --- | --- | --- | --- | --- |

Best Unique PSM (Scan Sample\_5\_A1\_1\_3643.d:66156, m/z=895.9476, z=2, RT=18.67, ppm=-2.25):


TRINITY\_DN64\_c0\_g1\_i1.p1

back to list

  

| Protein Coverage
| Supporting Peptides
| Best Unique PSM
|

Protein Coverage:

Supporting Peptides:

| Peptide | Uniq | -10lgP | Mass | Length | ppm | m/z | z | RT | 1/k0 Start | 1/k0 End | Precursor ID | Area Sample 7 | #Feature | #Feature Sample 7 | Start | End | PTM | AScore | Found By |
| --- | --- | --- | --- | --- | --- | --- | --- | --- | --- | --- | --- | --- | --- | --- | --- | --- | --- | --- | --- |
| K.STSTGHLIYK.C | Y | 55.32 | 1105.5768 | 10 | -1.17 | 553.7924 | 2 | 12.14 | 0.8712 | 0.8785 | 26038 | 8.26e2 | 1 | 1 | 24 | 33 |  |  | DB Search |
| K.YYVTIIDAPGHR.D | Y | 54.9 | 1403.7197 | 12 | -0.29 | 702.8636 | 2 | 16.07 | 1.0427 | 1.05 | 49774 | 7.1e2 | 1 | 1 | 88 | 99 |  |  | DB Search |
| total 2 peptides |  |  |  |  |  |  |  |  |  |  |  |  |  |  |  |  |  |  |  |
| --- | --- | --- | --- | --- | --- | --- | --- | --- | --- | --- | --- | --- | --- | --- | --- | --- | --- | --- | --- |

Best Unique PSM (Scan Sample\_5\_A1\_1\_3643.d:26038, m/z=553.7924, z=2, RT=12.14, ppm=-5.85):


TRINITY\_DN167\_c0\_g1\_i3.p1

back to list

  

| Protein Coverage
| Supporting Peptides
| Best Unique PSM
|

Protein Coverage:

Supporting Peptides:

| Peptide | Uniq | -10lgP | Mass | Length | ppm | m/z | z | RT | 1/k0 Start | 1/k0 End | Precursor ID | Area Sample 7 | #Feature | #Feature Sample 7 | Start | End | PTM | AScore | Found By |
| --- | --- | --- | --- | --- | --- | --- | --- | --- | --- | --- | --- | --- | --- | --- | --- | --- | --- | --- | --- |
| K.STAGDTHLGGEDFDNR.L | Y | 56.39 | 1690.7183 | 16 | 2.27 | 564.5787 | 3 | 13.29 | 0.8014 | 0.8088 | 33297 | 4.2e3 | 2 | 2 | 107 | 122 |  |  | DB Search |
| total 1 peptides |  |  |  |  |  |  |  |  |  |  |  |  |  |  |  |  |  |  |  |
| --- | --- | --- | --- | --- | --- | --- | --- | --- | --- | --- | --- | --- | --- | --- | --- | --- | --- | --- | --- |

Best Unique PSM (Scan Sample\_5\_A1\_1\_3643.d:33297, m/z=564.5787, z=3, RT=13.29, ppm=-2.41):


TRINITY\_DN991\_c0\_g1\_i5.p1

back to list

  

| Protein Coverage
| Supporting Peptides
| Best Unique PSM
|

Protein Coverage:

Supporting Peptides:

| Peptide | Uniq | -10lgP | Mass | Length | ppm | m/z | z | RT | 1/k0 Start | 1/k0 End | Precursor ID | Area Sample 7 | #Feature | #Feature Sample 7 | Start | End | PTM | AScore | Found By |
| --- | --- | --- | --- | --- | --- | --- | --- | --- | --- | --- | --- | --- | --- | --- | --- | --- | --- | --- | --- |
| R.ITVTVSPDIHPPAK.L | Y | 49.15 | 1473.8191 | 14 | -0.97 | 737.9127 | 2 | 14.98 | 1.0668 | 1.0741 | 43563 | 1.94e2 | 1 | 1 | 1183 | 1196 |  |  | DB Search |
| total 1 peptides |  |  |  |  |  |  |  |  |  |  |  |  |  |  |  |  |  |  |  |
| --- | --- | --- | --- | --- | --- | --- | --- | --- | --- | --- | --- | --- | --- | --- | --- | --- | --- | --- | --- |

Best Unique PSM (Scan Sample\_5\_A1\_1\_3643.d:43563, m/z=736.8683, z=2, RT=15.00, ppm=-5.65):


TRINITY\_DN3078\_c0\_g1\_i1.p1

back to list

  

| Protein Coverage
| Supporting Peptides
| Best Unique PSM
|

Protein Coverage:

Supporting Peptides:

| Peptide | Uniq | -10lgP | Mass | Length | ppm | m/z | z | RT | 1/k0 Start | 1/k0 End | Precursor ID | Area Sample 7 | #Feature | #Feature Sample 7 | Start | End | PTM | AScore | Found By |
| --- | --- | --- | --- | --- | --- | --- | --- | --- | --- | --- | --- | --- | --- | --- | --- | --- | --- | --- | --- |
| K.VSQTC(+57.02)AEHVFR.V | Y | 56.39 | 1332.6245 | 11 | 4.52 | 667.3194 | 2 | 12.90 | 0.9868 | 0.9941 | 30882 | 0 | 0 | 0 | 547 | 557 | Carbamidomethylation |  | DB Search |
| R.KVSQTC(+57.02)AEHVFR.V | Y | 55.37 | 1460.7194 | 12 | 3.01 | 487.9129 | 3 | 12.03 | 0.8027 | 0.81 | 25408 | 9.73e2 | 1 | 1 | 546 | 557 | Carbamidomethylation |  | DB Search |
| K.AKIEALHC(+57.02)LSENIVK.D | Y | 55.36 | 1723.9291 | 15 | 5.81 | 575.6509 | 3 | 15.74 | 0.8258 | 0.8331 | 47999 | 0 | 0 | 0 | 709 | 723 | Carbamidomethylation |  | DB Search |
| K.SGNVVLC(+57.02)LIDVHK.A | Y | 55.24 | 1452.7759 | 13 | 2.74 | 485.265 | 3 | 18.67 | 0.7416 | 0.7489 | 66263 | 0 | 0 | 0 | 1014 | 1026 | Carbamidomethylation |  | DB Search |
| total 4 peptides |  |  |  |  |  |  |  |  |  |  |  |  |  |  |  |  |  |  |  |
| --- | --- | --- | --- | --- | --- | --- | --- | --- | --- | --- | --- | --- | --- | --- | --- | --- | --- | --- | --- |

Best Unique PSM (Scan Sample\_5\_A1\_1\_3643.d:30882, m/z=667.3194, z=2, RT=12.90, ppm=-0.16):


TRINITY\_DN118520\_c0\_g1\_i1.p1

back to list

  

| Protein Coverage
| Supporting Peptides
| Best Unique PSM
|

Protein Coverage:

Supporting Peptides:

| Peptide | Uniq | -10lgP | Mass | Length | ppm | m/z | z | RT | 1/k0 Start | 1/k0 End | Precursor ID | Area Sample 7 | #Feature | #Feature Sample 7 | Start | End | PTM | AScore | Found By |
| --- | --- | --- | --- | --- | --- | --- | --- | --- | --- | --- | --- | --- | --- | --- | --- | --- | --- | --- | --- |
| K.ISNVQDIVPVLELANQHK.R | Y | 51.46 | 2016.1003 | 18 | 2.3 | 673.0391 | 3 | 19.75 | 0.9457 | 0.9529 | 73571 | 2.32e3 | 1 | 1 | 258 | 275 |  |  | DB Search |
| total 1 peptides |  |  |  |  |  |  |  |  |  |  |  |  |  |  |  |  |  |  |  |
| --- | --- | --- | --- | --- | --- | --- | --- | --- | --- | --- | --- | --- | --- | --- | --- | --- | --- | --- | --- |

Best Unique PSM (Scan Sample\_5\_A1\_1\_3643.d:73571, m/z=673.0391, z=3, RT=19.75, ppm=-2.38):


TRINITY\_DN554\_c0\_g1\_i5.p1

back to list

  

| Protein Coverage
| Supporting Peptides
| Best Unique PSM
|

Protein Coverage:

Supporting Peptides:

| Peptide | Uniq | -10lgP | Mass | Length | ppm | m/z | z | RT | 1/k0 Start | 1/k0 End | Precursor ID | Area Sample 7 | #Feature | #Feature Sample 7 | Start | End | PTM | AScore | Found By |
| --- | --- | --- | --- | --- | --- | --- | --- | --- | --- | --- | --- | --- | --- | --- | --- | --- | --- | --- | --- |
| K.TITLEVEPSDTIENVK.S | Y | 55.12 | 1786.92 | 16 | 0.46 | 894.4635 | 2 | 17.55 | 1.1414 | 1.1486 | 58604 | 8.72e2 | 1 | 1 | 12 | 27 |  |  | DB Search |
| total 1 peptides |  |  |  |  |  |  |  |  |  |  |  |  |  |  |  |  |  |  |  |
| --- | --- | --- | --- | --- | --- | --- | --- | --- | --- | --- | --- | --- | --- | --- | --- | --- | --- | --- | --- |

Best Unique PSM (Scan Sample\_5\_A1\_1\_3643.d:58604, m/z=894.4635, z=2, RT=17.55, ppm=-4.22):


TRINITY\_DN15274\_c0\_g1\_i1.p1

back to list

  

| Protein Coverage
| Supporting Peptides
| Best Unique PSM
|

Protein Coverage:

Supporting Peptides:

| Peptide | Uniq | -10lgP | Mass | Length | ppm | m/z | z | RT | 1/k0 Start | 1/k0 End | Precursor ID | Area Sample 7 | #Feature | #Feature Sample 7 | Start | End | PTM | AScore | Found By |
| --- | --- | --- | --- | --- | --- | --- | --- | --- | --- | --- | --- | --- | --- | --- | --- | --- | --- | --- | --- |
| R.GSC(+57.02)SC(+57.02)YGHAEQC(+57.02)IPTK.G | Y | 56.43 | 1853.7495 | 16 | 5.37 | 618.9242 | 3 | 12.44 | 0.8623 | 0.8696 | 27900 | 3.78e3 | 2 | 2 | 270 | 285 | Carbamidomethylation, Carbamidomethylation, Carbamidomethylation |  | DB Search |
| R.YSRPTPGSNNNVMVR.I | Y | 56.38 | 1690.8209 | 15 | 2.63 | 564.6131 | 3 | 12.53 | 0.8339 | 0.8412 | 28586 | 2.15e2 | 1 | 1 | 621 | 635 |  |  | DB Search |
| R.LQGWWQSVNGR.Q | Y | 56.18 | 1329.6578 | 11 | 3.31 | 665.8353 | 2 | 18.21 | 0.9444 | 0.9517 | 63090 | 0 | 0 | 0 | 108 | 118 |  |  | DB Search |
| R.YSRPTPGSNNNVM(+15.99)VR.I | Y | 55.28 | 1706.8158 | 15 | 5.92 | 569.9466 | 3 | 11.60 | 0.8388 | 0.8461 | 22687 | 2.26e3 | 1 | 1 | 621 | 635 | Oxidation (M) | M13:Oxidation (M):1000 | DB Search |
| K.SGGQC(+57.02)PC(+57.02)KPGVVGR.R | Y | 49.35 | 1457.6867 | 14 | 7.15 | 486.9041 | 3 | 10.75 | 0.7787 | 0.786 | 17784 | 3e2 | 1 | 1 | 795 | 808 | Carbamidomethylation, Carbamidomethylation |  | DB Search |
| total 5 peptides |  |  |  |  |  |  |  |  |  |  |  |  |  |  |  |  |  |  |  |
| --- | --- | --- | --- | --- | --- | --- | --- | --- | --- | --- | --- | --- | --- | --- | --- | --- | --- | --- | --- |

Best Unique PSM (Scan Sample\_5\_A1\_1\_3643.d:27900, m/z=618.9242, z=3, RT=12.44, ppm=0.69):


TRINITY\_DN624\_c0\_g1\_i1.p1

back to list

  

| Protein Coverage
| Supporting Peptides
| Best Unique PSM
|

Protein Coverage:

Supporting Peptides:

| Peptide | Uniq | -10lgP | Mass | Length | ppm | m/z | z | RT | 1/k0 Start | 1/k0 End | Precursor ID | Area Sample 7 | #Feature | #Feature Sample 7 | Start | End | PTM | AScore | Found By |
| --- | --- | --- | --- | --- | --- | --- | --- | --- | --- | --- | --- | --- | --- | --- | --- | --- | --- | --- | --- |
| K.ALNVNQGNEK.A | Y | 55.18 | 1085.5465 | 10 | 8.59 | 543.7827 | 2 | 10.95 | 0.8619 | 0.8692 | 18965 | 2.35e3 | 1 | 1 | 263 | 272 |  |  | DB Search |
| R.FAIQDITVDEFSAK.E | Y | 49.36 | 1582.7878 | 14 | 12.76 | 792.4076 | 2 | 19.82 | 1.046 | 1.0532 | 73951 | 0 | 0 | 0 | 132 | 145 |  |  | DB Search |
| total 2 peptides |  |  |  |  |  |  |  |  |  |  |  |  |  |  |  |  |  |  |  |
| --- | --- | --- | --- | --- | --- | --- | --- | --- | --- | --- | --- | --- | --- | --- | --- | --- | --- | --- | --- |

Best Unique PSM (Scan Sample\_5\_A1\_1\_3643.d:18965, m/z=543.7827, z=2, RT=10.95, ppm=3.9):


TRINITY\_DN11248\_c0\_g1\_i1.p1

back to list

  

| Protein Coverage
| Supporting Peptides
| Best Unique PSM
|

Protein Coverage:

Supporting Peptides:

| Peptide | Uniq | -10lgP | Mass | Length | ppm | m/z | z | RT | 1/k0 Start | 1/k0 End | Precursor ID | Area Sample 7 | #Feature | #Feature Sample 7 | Start | End | PTM | AScore | Found By |
| --- | --- | --- | --- | --- | --- | --- | --- | --- | --- | --- | --- | --- | --- | --- | --- | --- | --- | --- | --- |
| K.TPYLQIDFGK.M | Y | 52.02 | 1180.6128 | 10 | -2.38 | 591.3095 | 2 | 18.44 | 0.917 | 0.9243 | 64648 | 4.48e2 | 1 | 1 | 514 | 523 |  |  | DB Search |
| total 1 peptides |  |  |  |  |  |  |  |  |  |  |  |  |  |  |  |  |  |  |  |
| --- | --- | --- | --- | --- | --- | --- | --- | --- | --- | --- | --- | --- | --- | --- | --- | --- | --- | --- | --- |

Best Unique PSM (Scan Sample\_5\_A1\_1\_3643.d:64648, m/z=591.3095, z=2, RT=18.44, ppm=-7.06):


TRINITY\_DN1058\_c0\_g1\_i1.p1

back to list

  

| Protein Coverage
| Supporting Peptides
| Best Unique PSM
|

Protein Coverage:

Supporting Peptides:

| Peptide | Uniq | -10lgP | Mass | Length | ppm | m/z | z | RT | 1/k0 Start | 1/k0 End | Precursor ID | Area Sample 7 | #Feature | #Feature Sample 7 | Start | End | PTM | AScore | Found By |
| --- | --- | --- | --- | --- | --- | --- | --- | --- | --- | --- | --- | --- | --- | --- | --- | --- | --- | --- | --- |
| R.APTHILNPANKR.S | Y | 52.25 | 1330.7469 | 12 | 5.05 | 444.5898 | 3 | 11.20 | 0.7481 | 0.7555 | 20091 | 3.03e3 | 1 | 1 | 2314 | 2325 |  |  | DB Search |
| total 1 peptides |  |  |  |  |  |  |  |  |  |  |  |  |  |  |  |  |  |  |  |
| --- | --- | --- | --- | --- | --- | --- | --- | --- | --- | --- | --- | --- | --- | --- | --- | --- | --- | --- | --- |

Best Unique PSM (Scan Sample\_5\_A1\_1\_3643.d:20091, m/z=444.5898, z=3, RT=11.20, ppm=0.37):


TRINITY\_DN165536\_c0\_g1\_i1.p1

back to list

  

| Protein Coverage
| Supporting Peptides
| Best Unique PSM
|

Protein Coverage:

Supporting Peptides:

| Peptide | Uniq | -10lgP | Mass | Length | ppm | m/z | z | RT | 1/k0 Start | 1/k0 End | Precursor ID | Area Sample 7 | #Feature | #Feature Sample 7 | Start | End | PTM | AScore | Found By |
| --- | --- | --- | --- | --- | --- | --- | --- | --- | --- | --- | --- | --- | --- | --- | --- | --- | --- | --- | --- |
| K.ATETATGLATTHPIK.L | Y | 55.11 | 1510.7991 | 15 | 0.61 | 756.4037 | 2 | 13.07 | 1.1041 | 1.1114 | 31991 | 0 | 0 | 0 | 161 | 175 |  |  | DB Search |
| total 1 peptides |  |  |  |  |  |  |  |  |  |  |  |  |  |  |  |  |  |  |  |
| --- | --- | --- | --- | --- | --- | --- | --- | --- | --- | --- | --- | --- | --- | --- | --- | --- | --- | --- | --- |

Best Unique PSM (Scan Sample\_5\_A1\_1\_3643.d:31991, m/z=756.4037, z=2, RT=13.07, ppm=-4.07):


TRINITY\_DN65211\_c0\_g1\_i3.p1

back to list

  

| Protein Coverage
| Supporting Peptides
| Best Unique PSM
|

Protein Coverage:

Supporting Peptides:

| Peptide | Uniq | -10lgP | Mass | Length | ppm | m/z | z | RT | 1/k0 Start | 1/k0 End | Precursor ID | Area Sample 7 | #Feature | #Feature Sample 7 | Start | End | PTM | AScore | Found By |
| --- | --- | --- | --- | --- | --- | --- | --- | --- | --- | --- | --- | --- | --- | --- | --- | --- | --- | --- | --- |
| K.TQLIAHDKEVYDIAFSR.A | Y | 49.38 | 2005.0268 | 17 | 3.02 | 669.3484 | 3 | 16.77 | 0.9089 | 0.9162 | 53806 | 0 | 0 | 0 | 167 | 183 |  |  | DB Search |
| total 1 peptides |  |  |  |  |  |  |  |  |  |  |  |  |  |  |  |  |  |  |  |
| --- | --- | --- | --- | --- | --- | --- | --- | --- | --- | --- | --- | --- | --- | --- | --- | --- | --- | --- | --- |

Best Unique PSM (Scan Sample\_5\_A1\_1\_3643.d:53806, m/z=669.3484, z=3, RT=16.77, ppm=-1.66):


TRINITY\_DN329\_c0\_g1\_i2.p1

back to list

  

| Protein Coverage
| Supporting Peptides
| Best Unique PSM
|

Protein Coverage:

Supporting Peptides:

| Peptide | Uniq | -10lgP | Mass | Length | ppm | m/z | z | RT | 1/k0 Start | 1/k0 End | Precursor ID | Area Sample 7 | #Feature | #Feature Sample 7 | Start | End | PTM | AScore | Found By |
| --- | --- | --- | --- | --- | --- | --- | --- | --- | --- | --- | --- | --- | --- | --- | --- | --- | --- | --- | --- |
| K.LRPGVELTMSSLVNAK.S | Y | 49.47 | 1713.9447 | 16 | -6.6 | 572.3157 | 3 | 17.86 | 0.9117 | 0.919 | 60815 | 0 | 0 | 0 | 287 | 302 |  |  | DB Search |
| total 1 peptides |  |  |  |  |  |  |  |  |  |  |  |  |  |  |  |  |  |  |  |
| --- | --- | --- | --- | --- | --- | --- | --- | --- | --- | --- | --- | --- | --- | --- | --- | --- | --- | --- | --- |

Best Unique PSM (Scan Sample\_5\_A1\_1\_3643.d:60815, m/z=572.3157, z=3, RT=17.86, ppm=-11.28):


TRINITY\_DN1676\_c0\_g1\_i12.p1

back to list

  

| Protein Coverage
| Supporting Peptides
| Best Unique PSM
|

Protein Coverage:

Supporting Peptides:

| Peptide | Uniq | -10lgP | Mass | Length | ppm | m/z | z | RT | 1/k0 Start | 1/k0 End | Precursor ID | Area Sample 7 | #Feature | #Feature Sample 7 | Start | End | PTM | AScore | Found By |
| --- | --- | --- | --- | --- | --- | --- | --- | --- | --- | --- | --- | --- | --- | --- | --- | --- | --- | --- | --- |
| R.SYYFAISDITVGGR.C | Y | 56.33 | 1547.762 | 14 | -2.52 | 774.8827 | 2 | 19.97 | 1.046 | 1.0532 | 74887 | 4.98e2 | 1 | 1 | 273 | 286 |  |  | DB Search |
| R.FELDRVNTFGDEVFGDPK.V | Y | 52.07 | 2083.985 | 18 | 2.03 | 695.6671 | 3 | 19.30 | 0.9206 | 0.9279 | 70383 | 6.13e2 | 1 | 1 | 252 | 269 |  |  | DB Search |
| total 2 peptides |  |  |  |  |  |  |  |  |  |  |  |  |  |  |  |  |  |  |  |
| --- | --- | --- | --- | --- | --- | --- | --- | --- | --- | --- | --- | --- | --- | --- | --- | --- | --- | --- | --- |

Best Unique PSM (Scan Sample\_5\_A1\_1\_3643.d:74887, m/z=774.8827, z=2, RT=19.97, ppm=-7.2):


TRINITY\_DN2626\_c0\_g1\_i6.p1

back to list

  

| Protein Coverage
| Supporting Peptides
| Best Unique PSM
|

Protein Coverage:

Supporting Peptides:

| Peptide | Uniq | -10lgP | Mass | Length | ppm | m/z | z | RT | 1/k0 Start | 1/k0 End | Precursor ID | Area Sample 7 | #Feature | #Feature Sample 7 | Start | End | PTM | AScore | Found By |
| --- | --- | --- | --- | --- | --- | --- | --- | --- | --- | --- | --- | --- | --- | --- | --- | --- | --- | --- | --- |
| K.FIWGEVTELNGADR.T | Y | 50.99 | 1605.7787 | 14 | -4.61 | 803.8892 | 2 | 19.74 | 1.0431 | 1.0504 | 73266 | 4.98e2 | 1 | 1 | 97 | 110 |  |  | DB Search |
| total 1 peptides |  |  |  |  |  |  |  |  |  |  |  |  |  |  |  |  |  |  |  |
| --- | --- | --- | --- | --- | --- | --- | --- | --- | --- | --- | --- | --- | --- | --- | --- | --- | --- | --- | --- |

Best Unique PSM (Scan Sample\_5\_A1\_1\_3643.d:73266, m/z=803.8892, z=2, RT=19.74, ppm=-9.29):


TRINITY\_DN5153\_c0\_g1\_i1.p1

back to list

  

| Protein Coverage
| Supporting Peptides
| Best Unique PSM
|

Protein Coverage:

Supporting Peptides:

| Peptide | Uniq | -10lgP | Mass | Length | ppm | m/z | z | RT | 1/k0 Start | 1/k0 End | Precursor ID | Area Sample 7 | #Feature | #Feature Sample 7 | Start | End | PTM | AScore | Found By |
| --- | --- | --- | --- | --- | --- | --- | --- | --- | --- | --- | --- | --- | --- | --- | --- | --- | --- | --- | --- |
| R.ALELDALSNSHPIEVK.V | Y | 46.98 | 1734.9152 | 16 | 10.01 | 868.4695 | 2 | 17.34 | 1.1762 | 1.1833 | 57207 | 0 | 0 | 0 | 362 | 377 |  |  | DB Search |
| total 1 peptides |  |  |  |  |  |  |  |  |  |  |  |  |  |  |  |  |  |  |  |
| --- | --- | --- | --- | --- | --- | --- | --- | --- | --- | --- | --- | --- | --- | --- | --- | --- | --- | --- | --- |

Best Unique PSM (Scan Sample\_5\_A1\_1\_3643.d:57207, m/z=868.4695, z=2, RT=17.34, ppm=5.33):


TRINITY\_DN2003\_c0\_g1\_i1.p1

back to list

  

| Protein Coverage
| Supporting Peptides
| Best Unique PSM
|

Protein Coverage:

Supporting Peptides:

| Peptide | Uniq | -10lgP | Mass | Length | ppm | m/z | z | RT | 1/k0 Start | 1/k0 End | Precursor ID | Area Sample 7 | #Feature | #Feature Sample 7 | Start | End | PTM | AScore | Found By |
| --- | --- | --- | --- | --- | --- | --- | --- | --- | --- | --- | --- | --- | --- | --- | --- | --- | --- | --- | --- |
| R.ASMDEGAKPK.E | Y | 56.32 | 1032.491 | 10 | 1.35 | 517.251 | 2 | 9.36 | 0.8558 | 0.8631 | 10916 | 0 | 0 | 0 | 133 | 142 |  |  | DB Search |
| total 1 peptides |  |  |  |  |  |  |  |  |  |  |  |  |  |  |  |  |  |  |  |
| --- | --- | --- | --- | --- | --- | --- | --- | --- | --- | --- | --- | --- | --- | --- | --- | --- | --- | --- | --- |

Best Unique PSM (Scan Sample\_5\_A1\_1\_3643.d:10916, m/z=517.251, z=2, RT=9.36, ppm=-3.34):


TRINITY\_DN244\_c0\_g1\_i1.p1

back to list

  

| Protein Coverage
| Supporting Peptides
| Best Unique PSM
|

Protein Coverage:

Supporting Peptides:

| Peptide | Uniq | -10lgP | Mass | Length | ppm | m/z | z | RT | 1/k0 Start | 1/k0 End | Precursor ID | Area Sample 7 | #Feature | #Feature Sample 7 | Start | End | PTM | AScore | Found By |
| --- | --- | --- | --- | --- | --- | --- | --- | --- | --- | --- | --- | --- | --- | --- | --- | --- | --- | --- | --- |
| K.ALLLASSNAFDTSNGAR.E | Y | 56.4 | 1706.8587 | 17 | 2.83 | 854.4351 | 2 | 17.79 | 1.1642 | 1.1714 | 60238 | 4.39e2 | 1 | 1 | 1912 | 1928 |  |  | DB Search |
| K.FSNDIVDSFAVSQKK.T | Y | 56.38 | 1683.8468 | 15 | 3.19 | 562.2887 | 3 | 17.23 | 0.8688 | 0.8761 | 56580 | 0 | 0 | 0 | 295 | 309 |  |  | DB Search |
| K.ALVILGDGFISGGGTPSK.T | Y | 55.36 | 1687.9144 | 18 | -3.94 | 844.9572 | 2 | 19.80 | 1.1694 | 1.1766 | 73673 | 1.43e3 | 1 | 1 | 1619 | 1636 |  |  | DB Search |
| total 3 peptides |  |  |  |  |  |  |  |  |  |  |  |  |  |  |  |  |  |  |  |
| --- | --- | --- | --- | --- | --- | --- | --- | --- | --- | --- | --- | --- | --- | --- | --- | --- | --- | --- | --- |

Best Unique PSM (Scan Sample\_5\_A1\_1\_3643.d:60238, m/z=852.4237, z=2, RT=17.79, ppm=-1.85):


TRINITY\_DN65211\_c0\_g1\_i2.p1

back to list

  

| Protein Coverage
| Supporting Peptides
| Best Unique PSM
|

Protein Coverage:

Supporting Peptides:

| Peptide | Uniq | -10lgP | Mass | Length | ppm | m/z | z | RT | 1/k0 Start | 1/k0 End | Precursor ID | Area Sample 7 | #Feature | #Feature Sample 7 | Start | End | PTM | AScore | Found By |
| --- | --- | --- | --- | --- | --- | --- | --- | --- | --- | --- | --- | --- | --- | --- | --- | --- | --- | --- | --- |
| K.TQLIAHDKEVYDIAFSR.A | Y | 49.38 | 2005.0268 | 17 | 3.02 | 669.3484 | 3 | 16.77 | 0.9089 | 0.9162 | 53806 | 0 | 0 | 0 | 167 | 183 |  |  | DB Search |
| total 1 peptides |  |  |  |  |  |  |  |  |  |  |  |  |  |  |  |  |  |  |  |
| --- | --- | --- | --- | --- | --- | --- | --- | --- | --- | --- | --- | --- | --- | --- | --- | --- | --- | --- | --- |

Best Unique PSM (Scan Sample\_5\_A1\_1\_3643.d:53806, m/z=669.3484, z=3, RT=16.77, ppm=-1.66):


TRINITY\_DN477\_c0\_g1\_i8.p1

back to list

  

| Protein Coverage
| Supporting Peptides
| Best Unique PSM
|

Protein Coverage:

Supporting Peptides:

| Peptide | Uniq | -10lgP | Mass | Length | ppm | m/z | z | RT | 1/k0 Start | 1/k0 End | Precursor ID | Area Sample 7 | #Feature | #Feature Sample 7 | Start | End | PTM | AScore | Found By |
| --- | --- | --- | --- | --- | --- | --- | --- | --- | --- | --- | --- | --- | --- | --- | --- | --- | --- | --- | --- |
| R.ILQC(+57.02)NQYANVEEAR.N | Y | 56.39 | 1706.8046 | 14 | 0.71 | 854.4062 | 2 | 14.33 | 1.0877 | 1.0949 | 39688 | 4.48e3 | 1 | 1 | 86 | 99 | Carbamidomethylation |  | DB Search |
| K.DKNILVLTLGVGPQVNR.N | Y | 54.98 | 1835.0628 | 17 | 0.89 | 918.5352 | 2 | 19.51 | 1.1354 | 1.1426 | 71919 | 8.54e3 | 2 | 2 | 168 | 184 |  |  | DB Search |
| total 2 peptides |  |  |  |  |  |  |  |  |  |  |  |  |  |  |  |  |  |  |  |
| --- | --- | --- | --- | --- | --- | --- | --- | --- | --- | --- | --- | --- | --- | --- | --- | --- | --- | --- | --- |

Best Unique PSM (Scan Sample\_5\_A1\_1\_3643.d:39688, m/z=854.4062, z=2, RT=14.33, ppm=-3.97):


TRINITY\_DN161\_c0\_g1\_i2.p1

back to list

  

| Protein Coverage
| Supporting Peptides
| Best Unique PSM
|

Protein Coverage:

Supporting Peptides:

| Peptide | Uniq | -10lgP | Mass | Length | ppm | m/z | z | RT | 1/k0 Start | 1/k0 End | Precursor ID | Area Sample 7 | #Feature | #Feature Sample 7 | Start | End | PTM | AScore | Found By |
| --- | --- | --- | --- | --- | --- | --- | --- | --- | --- | --- | --- | --- | --- | --- | --- | --- | --- | --- | --- |
| K.SYELPDGQVITIGNER.F | Y | 55.37 | 1789.8846 | 16 | 2.43 | 895.9476 | 2 | 18.67 | 1.117 | 1.1242 | 66156 | 3.51e2 | 1 | 1 | 262 | 277 |  |  | DB Search |
| R.HQGVMVGMGQK.D | Y | 55.37 | 1170.5638 | 11 | 3.68 | 586.2886 | 2 | 12.36 | 0.9105 | 0.9178 | 27450 | 5.73e2 | 1 | 1 | 63 | 73 |  |  | DB Search |
| R.HQGVM(+15.99)VGMGQK.D | Y | 55.3 | 1186.5587 | 11 | 10.45 | 594.29 | 2 | 11.22 | 0.9133 | 0.9206 | 20614 | 0 | 0 | 0 | 63 | 73 | Oxidation (M) | M5:Oxidation (M):60 | DB Search |
| K.DSYVGDEAQSKR.G | Y | 55.24 | 1353.616 | 12 | 0.18 | 677.8123 | 2 | 10.99 | 0.9932 | 1.0005 | 19155 | 1.01e3 | 1 | 1 | 74 | 85 |  |  | DB Search |
| R.AVFPSIVGRPR.H | Y | 55.22 | 1197.6982 | 11 | 2.36 | 599.855 | 2 | 15.87 | 0.961 | 0.9683 | 48378 | 1.86e4 | 1 | 1 | 52 | 62 |  |  | DB Search |
| R.VAPEEHPVLLTEAPLNPK.A | Y | 55.12 | 1953.0571 | 18 | 2.07 | 977.5333 | 2 | 16.62 | 1.2101 | 1.2173 | 52945 | 1.41e2 | 1 | 1 | 119 | 136 |  |  | DB Search |
| K.DSYVGDEAQSK.R | Y | 54.42 | 1197.5149 | 11 | 5.23 | 599.7651 | 2 | 11.40 | 0.8968 | 0.904 | 21678 | 5.55e2 | 1 | 1 | 74 | 84 |  |  | DB Search |
| R.HQGVM(+15.99)VGM(+15.99)GQK.D | Y | 52.78 | 1202.5536 | 11 | 2.62 | 602.2828 | 2 | 9.74 | 0.8955 | 0.9028 | 12426 | 1.52e3 | 1 | 1 | 63 | 73 | Oxidation (M), Oxidation (M) | M5:Oxidation (M):1000 M8:Oxidation (M):1000 | DB Search |
| R.HQGVMVGM(+15.99)GQK.D | Y | 48 | 1186.5587 | 11 | 4.48 | 594.2865 | 2 | 10.48 | 0.9052 | 0.9125 | 16419 | 1.61e3 | 1 | 1 | 63 | 73 | Oxidation (M) | M8:Oxidation (M):32.97 | DB Search |
| total 9 peptides |  |  |  |  |  |  |  |  |  |  |  |  |  |  |  |  |  |  |  |
| --- | --- | --- | --- | --- | --- | --- | --- | --- | --- | --- | --- | --- | --- | --- | --- | --- | --- | --- | --- |

Best Unique PSM (Scan Sample\_5\_A1\_1\_3643.d:66156, m/z=895.9476, z=2, RT=18.67, ppm=-2.25):


TRINITY\_DN26392\_c0\_g1\_i1.p1

back to list

  

| Protein Coverage
| Supporting Peptides
| Best Unique PSM
|

Protein Coverage:

Supporting Peptides:

| Peptide | Uniq | -10lgP | Mass | Length | ppm | m/z | z | RT | 1/k0 Start | 1/k0 End | Precursor ID | Area Sample 7 | #Feature | #Feature Sample 7 | Start | End | PTM | AScore | Found By |
| --- | --- | --- | --- | --- | --- | --- | --- | --- | --- | --- | --- | --- | --- | --- | --- | --- | --- | --- | --- |
| K.ADLDIQKPIPIVSK.Y | Y | 49.47 | 1535.8922 | 14 | 3.13 | 768.9522 | 2 | 16.14 | 1.0769 | 1.0841 | 50314 | 0 | 0 | 0 | 127 | 140 |  |  | DB Search |
| total 1 peptides |  |  |  |  |  |  |  |  |  |  |  |  |  |  |  |  |  |  |  |
| --- | --- | --- | --- | --- | --- | --- | --- | --- | --- | --- | --- | --- | --- | --- | --- | --- | --- | --- | --- |

Best Unique PSM (Scan Sample\_5\_A1\_1\_3643.d:50314, m/z=768.9522, z=2, RT=16.14, ppm=-1.55):


TRINITY\_DN2477\_c0\_g1\_i5.p1

back to list

  

| Protein Coverage
| Supporting Peptides
| Best Unique PSM
|

Protein Coverage:

Supporting Peptides:

| Peptide | Uniq | -10lgP | Mass | Length | ppm | m/z | z | RT | 1/k0 Start | 1/k0 End | Precursor ID | Area Sample 7 | #Feature | #Feature Sample 7 | Start | End | PTM | AScore | Found By |
| --- | --- | --- | --- | --- | --- | --- | --- | --- | --- | --- | --- | --- | --- | --- | --- | --- | --- | --- | --- |
| K.DSYVGDEAQSKR.G | Y | 55.24 | 1353.616 | 12 | 0.18 | 677.8123 | 2 | 10.99 | 0.9932 | 1.0005 | 19155 | 1.01e3 | 1 | 1 | 8 | 19 |  |  | DB Search |
| R.VAPEEHPVLLTEAPLNPK.A | Y | 55.12 | 1953.0571 | 18 | 2.07 | 977.5333 | 2 | 16.62 | 1.2101 | 1.2173 | 52945 | 1.41e2 | 1 | 1 | 53 | 70 |  |  | DB Search |
| K.DSYVGDEAQSK.R | Y | 54.42 | 1197.5149 | 11 | 5.23 | 599.7651 | 2 | 11.40 | 0.8968 | 0.904 | 21678 | 5.55e2 | 1 | 1 | 8 | 18 |  |  | DB Search |
| total 3 peptides |  |  |  |  |  |  |  |  |  |  |  |  |  |  |  |  |  |  |  |
| --- | --- | --- | --- | --- | --- | --- | --- | --- | --- | --- | --- | --- | --- | --- | --- | --- | --- | --- | --- |

Best Unique PSM (Scan Sample\_5\_A1\_1\_3643.d:19155, m/z=677.8123, z=2, RT=10.99, ppm=-4.5):


TRINITY\_DN36\_c0\_g1\_i13.p1

back to list

  

| Protein Coverage
| Supporting Peptides
| Best Unique PSM
|

Protein Coverage:

Supporting Peptides:

| Peptide | Uniq | -10lgP | Mass | Length | ppm | m/z | z | RT | 1/k0 Start | 1/k0 End | Precursor ID | Area Sample 7 | #Feature | #Feature Sample 7 | Start | End | PTM | AScore | Found By |
| --- | --- | --- | --- | --- | --- | --- | --- | --- | --- | --- | --- | --- | --- | --- | --- | --- | --- | --- | --- |
| K.FETQIATK.D | Y | 53.77 | 936.4916 | 8 | 12.43 | 937.5062 | 1 | 13.97 | 1.1614 | 1.1686 | 37568 | 0 | 0 | 0 | 166 | 173 |  |  | DB Search |
| R.LGGIEVTQGNC(+57.02)AR.K | Y | 49.67 | 1373.6721 | 13 | 2.61 | 687.8419 | 2 | 13.89 | 0.9695 | 0.9767 | 37015 | 0 | 0 | 0 | 57 | 69 | Carbamidomethylation |  | DB Search |
| total 2 peptides |  |  |  |  |  |  |  |  |  |  |  |  |  |  |  |  |  |  |  |
| --- | --- | --- | --- | --- | --- | --- | --- | --- | --- | --- | --- | --- | --- | --- | --- | --- | --- | --- | --- |

Best Unique PSM (Scan Sample\_5\_A1\_1\_3643.d:37568, m/z=937.5062, z=1, RT=13.97, ppm=7.75):


TRINITY\_DN96161\_c0\_g1\_i1.p1

back to list

  

| Protein Coverage
| Supporting Peptides
| Best Unique PSM
|

Protein Coverage:

Supporting Peptides:

| Peptide | Uniq | -10lgP | Mass | Length | ppm | m/z | z | RT | 1/k0 Start | 1/k0 End | Precursor ID | Area Sample 7 | #Feature | #Feature Sample 7 | Start | End | PTM | AScore | Found By |
| --- | --- | --- | --- | --- | --- | --- | --- | --- | --- | --- | --- | --- | --- | --- | --- | --- | --- | --- | --- |
| R.LDHKFDLM(+15.99)YAK.R | Y | 51.39 | 1395.6856 | 11 | 4.19 | 466.2356 | 3 | 14.23 | 0.7844 | 0.7917 | 39140 | 0 | 0 | 0 | 391 | 401 | Oxidation (M) | M8:Oxidation (M):1000 | DB Search |
| K.VGINYQPPTVVPGGDLAK.V | Y | 48.61 | 1823.9781 | 18 | -1.33 | 912.9908 | 2 | 17.37 | 1.1238 | 1.131 | 57429 | 0 | 0 | 0 | 353 | 370 |  |  | DB Search |
| total 2 peptides |  |  |  |  |  |  |  |  |  |  |  |  |  |  |  |  |  |  |  |
| --- | --- | --- | --- | --- | --- | --- | --- | --- | --- | --- | --- | --- | --- | --- | --- | --- | --- | --- | --- |

Best Unique PSM (Scan Sample\_5\_A1\_1\_3643.d:39140, m/z=466.2356, z=3, RT=14.23, ppm=-0.49):


TRINITY\_DN6188\_c0\_g1\_i1.p1

back to list

  

| Protein Coverage
| Supporting Peptides
| Best Unique PSM
|

Protein Coverage:

Supporting Peptides:

| Peptide | Uniq | -10lgP | Mass | Length | ppm | m/z | z | RT | 1/k0 Start | 1/k0 End | Precursor ID | Area Sample 7 | #Feature | #Feature Sample 7 | Start | End | PTM | AScore | Found By |
| --- | --- | --- | --- | --- | --- | --- | --- | --- | --- | --- | --- | --- | --- | --- | --- | --- | --- | --- | --- |
| K.YGDYTPEIHQK.D | Y | 56.34 | 1349.6252 | 11 | 4.63 | 675.8198 | 2 | 12.99 | 0.9924 | 0.9997 | 31469 | 0 | 0 | 0 | 134 | 144 |  |  | DB Search |
| total 1 peptides |  |  |  |  |  |  |  |  |  |  |  |  |  |  |  |  |  |  |  |
| --- | --- | --- | --- | --- | --- | --- | --- | --- | --- | --- | --- | --- | --- | --- | --- | --- | --- | --- | --- |

Best Unique PSM (Scan Sample\_5\_A1\_1\_3643.d:31469, m/z=675.8198, z=2, RT=12.99, ppm=-0.05):


TRINITY\_DN4815\_c0\_g1\_i1.p1

back to list

  

| Protein Coverage
| Supporting Peptides
| Best Unique PSM
|

Protein Coverage:

Supporting Peptides:

| Peptide | Uniq | -10lgP | Mass | Length | ppm | m/z | z | RT | 1/k0 Start | 1/k0 End | Precursor ID | Area Sample 7 | #Feature | #Feature Sample 7 | Start | End | PTM | AScore | Found By |
| --- | --- | --- | --- | --- | --- | --- | --- | --- | --- | --- | --- | --- | --- | --- | --- | --- | --- | --- | --- |
| R.AVAC(+57.02)NSGPC(+57.02)LAGC(+57.02)PR.N | Y | 50.26 | 1588.6908 | 15 | 1.49 | 795.3502 | 2 | 13.12 | 1.019 | 1.0263 | 32158 | 1.27e3 | 1 | 1 | 425 | 439 | Carbamidomethylation, Carbamidomethylation, Carbamidomethylation |  | DB Search |
| total 1 peptides |  |  |  |  |  |  |  |  |  |  |  |  |  |  |  |  |  |  |  |
| --- | --- | --- | --- | --- | --- | --- | --- | --- | --- | --- | --- | --- | --- | --- | --- | --- | --- | --- | --- |

Best Unique PSM (Scan Sample\_5\_A1\_1\_3643.d:32158, m/z=795.3502, z=2, RT=13.12, ppm=-3.19):


TRINITY\_DN2217\_c0\_g1\_i3.p1

back to list

  

| Protein Coverage
| Supporting Peptides
| Best Unique PSM
|

Protein Coverage:

Supporting Peptides:

| Peptide | Uniq | -10lgP | Mass | Length | ppm | m/z | z | RT | 1/k0 Start | 1/k0 End | Precursor ID | Area Sample 7 | #Feature | #Feature Sample 7 | Start | End | PTM | AScore | Found By |
| --- | --- | --- | --- | --- | --- | --- | --- | --- | --- | --- | --- | --- | --- | --- | --- | --- | --- | --- | --- |
| K.ATGC(+57.02)EVIWNQHNR.G | Y | 56.39 | 1583.7263 | 13 | 0.67 | 792.8672 | 2 | 14.03 | 1.0829 | 1.0901 | 37880 | 1.73e2 | 1 | 1 | 431 | 443 | Carbamidomethylation |  | DB Search |
| total 1 peptides |  |  |  |  |  |  |  |  |  |  |  |  |  |  |  |  |  |  |  |
| --- | --- | --- | --- | --- | --- | --- | --- | --- | --- | --- | --- | --- | --- | --- | --- | --- | --- | --- | --- |

Best Unique PSM (Scan Sample\_5\_A1\_1\_3643.d:37880, m/z=792.8672, z=2, RT=14.03, ppm=-4.01):


TRINITY\_DN477\_c0\_g1\_i3.p1

back to list

  

| Protein Coverage
| Supporting Peptides
| Best Unique PSM
|

Protein Coverage:

Supporting Peptides:

| Peptide | Uniq | -10lgP | Mass | Length | ppm | m/z | z | RT | 1/k0 Start | 1/k0 End | Precursor ID | Area Sample 7 | #Feature | #Feature Sample 7 | Start | End | PTM | AScore | Found By |
| --- | --- | --- | --- | --- | --- | --- | --- | --- | --- | --- | --- | --- | --- | --- | --- | --- | --- | --- | --- |
| R.NDVNNPPQAR.R | Y | 55.29 | 1123.537 | 10 | 3.11 | 562.7749 | 2 | 10.15 | 0.8558 | 0.8631 | 13772 | 1.28e4 | 1 | 1 | 147 | 156 |  |  | DB Search |
| total 1 peptides |  |  |  |  |  |  |  |  |  |  |  |  |  |  |  |  |  |  |  |
| --- | --- | --- | --- | --- | --- | --- | --- | --- | --- | --- | --- | --- | --- | --- | --- | --- | --- | --- | --- |

Best Unique PSM (Scan Sample\_5\_A1\_1\_3643.d:13772, m/z=562.7749, z=2, RT=10.15, ppm=-1.57):


TRINITY\_DN3347\_c0\_g1\_i1.p1

back to list

  

| Protein Coverage
| Supporting Peptides
| Best Unique PSM
|

Protein Coverage:

Supporting Peptides:

| Peptide | Uniq | -10lgP | Mass | Length | ppm | m/z | z | RT | 1/k0 Start | 1/k0 End | Precursor ID | Area Sample 7 | #Feature | #Feature Sample 7 | Start | End | PTM | AScore | Found By |
| --- | --- | --- | --- | --- | --- | --- | --- | --- | --- | --- | --- | --- | --- | --- | --- | --- | --- | --- | --- |
| R.ATDIDQEVKEK.S | Y | 56.31 | 1274.6354 | 11 | 0.83 | 638.3225 | 2 | 11.62 | 0.9642 | 0.9715 | 22970 | 0 | 0 | 0 | 263 | 273 |  |  | DB Search |
| total 1 peptides |  |  |  |  |  |  |  |  |  |  |  |  |  |  |  |  |  |  |  |
| --- | --- | --- | --- | --- | --- | --- | --- | --- | --- | --- | --- | --- | --- | --- | --- | --- | --- | --- | --- |

Best Unique PSM (Scan Sample\_5\_A1\_1\_3643.d:22970, m/z=638.3225, z=2, RT=11.62, ppm=-3.85):


TRINITY\_DN167\_c0\_g1\_i7.p1

back to list

  

| Protein Coverage
| Supporting Peptides
| Best Unique PSM
|

Protein Coverage:

Supporting Peptides:

| Peptide | Uniq | -10lgP | Mass | Length | ppm | m/z | z | RT | 1/k0 Start | 1/k0 End | Precursor ID | Area Sample 7 | #Feature | #Feature Sample 7 | Start | End | PTM | AScore | Found By |
| --- | --- | --- | --- | --- | --- | --- | --- | --- | --- | --- | --- | --- | --- | --- | --- | --- | --- | --- | --- |
| K.STAGDTHLGGEDFDNR.L | Y | 56.39 | 1690.7183 | 16 | 2.27 | 564.5787 | 3 | 13.29 | 0.8014 | 0.8088 | 33297 | 4.2e3 | 2 | 2 | 107 | 122 |  |  | DB Search |
| total 1 peptides |  |  |  |  |  |  |  |  |  |  |  |  |  |  |  |  |  |  |  |
| --- | --- | --- | --- | --- | --- | --- | --- | --- | --- | --- | --- | --- | --- | --- | --- | --- | --- | --- | --- |

Best Unique PSM (Scan Sample\_5\_A1\_1\_3643.d:33297, m/z=564.5787, z=3, RT=13.29, ppm=-2.41):


TRINITY\_DN59572\_c0\_g1\_i1.p1

back to list

  

| Protein Coverage
| Supporting Peptides
| Best Unique PSM
|

Protein Coverage:

Supporting Peptides:

| Peptide | Uniq | -10lgP | Mass | Length | ppm | m/z | z | RT | 1/k0 Start | 1/k0 End | Precursor ID | Area Sample 7 | #Feature | #Feature Sample 7 | Start | End | PTM | AScore | Found By |
| --- | --- | --- | --- | --- | --- | --- | --- | --- | --- | --- | --- | --- | --- | --- | --- | --- | --- | --- | --- |
| K.SYFEHSFVDLHR.I | Y | 56.4 | 1535.7157 | 12 | 4.62 | 512.9125 | 3 | 16.16 | 0.7705 | 0.7779 | 50408 | 5.95e2 | 1 | 1 | 107 | 118 |  |  | DB Search |
| total 1 peptides |  |  |  |  |  |  |  |  |  |  |  |  |  |  |  |  |  |  |  |
| --- | --- | --- | --- | --- | --- | --- | --- | --- | --- | --- | --- | --- | --- | --- | --- | --- | --- | --- | --- |

Best Unique PSM (Scan Sample\_5\_A1\_1\_3643.d:50408, m/z=512.9125, z=3, RT=16.16, ppm=-0.06):


TRINITY\_DN11097\_c0\_g1\_i1.p1

back to list

  

| Protein Coverage
| Supporting Peptides
| Best Unique PSM
|

Protein Coverage:

Supporting Peptides:

| Peptide | Uniq | -10lgP | Mass | Length | ppm | m/z | z | RT | 1/k0 Start | 1/k0 End | Precursor ID | Area Sample 7 | #Feature | #Feature Sample 7 | Start | End | PTM | AScore | Found By |
| --- | --- | --- | --- | --- | --- | --- | --- | --- | --- | --- | --- | --- | --- | --- | --- | --- | --- | --- | --- |
| R.VAHAGLTVAEFFR.D | Y | 49.43 | 1416.7513 | 13 | -3.33 | 473.2539 | 3 | 19.07 | 0.751 | 0.7583 | 68989 | 0 | 0 | 0 | 493 | 505 |  |  | DB Search |
| total 1 peptides |  |  |  |  |  |  |  |  |  |  |  |  |  |  |  |  |  |  |  |
| --- | --- | --- | --- | --- | --- | --- | --- | --- | --- | --- | --- | --- | --- | --- | --- | --- | --- | --- | --- |

Best Unique PSM (Scan Sample\_5\_A1\_1\_3643.d:68989, m/z=473.2539, z=3, RT=19.07, ppm=-8.02):


TRINITY\_DN585\_c0\_g1\_i10.p1

back to list

  

| Protein Coverage
| Supporting Peptides
| Best Unique PSM
|

Protein Coverage:

Supporting Peptides:

| Peptide | Uniq | -10lgP | Mass | Length | ppm | m/z | z | RT | 1/k0 Start | 1/k0 End | Precursor ID | Area Sample 7 | #Feature | #Feature Sample 7 | Start | End | PTM | AScore | Found By |
| --- | --- | --- | --- | --- | --- | --- | --- | --- | --- | --- | --- | --- | --- | --- | --- | --- | --- | --- | --- |
| K.TITLEVEPSDTIENVK.S | Y | 55.12 | 1786.92 | 16 | 0.46 | 894.4635 | 2 | 17.55 | 1.1414 | 1.1486 | 58604 | 8.72e2 | 1 | 1 | 12 | 27 |  |  | DB Search |
| total 1 peptides |  |  |  |  |  |  |  |  |  |  |  |  |  |  |  |  |  |  |  |
| --- | --- | --- | --- | --- | --- | --- | --- | --- | --- | --- | --- | --- | --- | --- | --- | --- | --- | --- | --- |

Best Unique PSM (Scan Sample\_5\_A1\_1\_3643.d:58604, m/z=894.4635, z=2, RT=17.55, ppm=-4.22):


TRINITY\_DN3016\_c0\_g1\_i2.p1

back to list

  

| Protein Coverage
| Supporting Peptides
| Best Unique PSM
|

Protein Coverage:

Supporting Peptides:

| Peptide | Uniq | -10lgP | Mass | Length | ppm | m/z | z | RT | 1/k0 Start | 1/k0 End | Precursor ID | Area Sample 7 | #Feature | #Feature Sample 7 | Start | End | PTM | AScore | Found By |
| --- | --- | --- | --- | --- | --- | --- | --- | --- | --- | --- | --- | --- | --- | --- | --- | --- | --- | --- | --- |
| R.IVNTDLQEIKDIIK.G | Y | 56.35 | 1640.9348 | 14 | -0.94 | 547.9825 | 3 | 18.84 | 0.8193 | 0.8266 | 67326 | 7.77e2 | 1 | 1 | 154 | 167 |  |  | DB Search |
| total 1 peptides |  |  |  |  |  |  |  |  |  |  |  |  |  |  |  |  |  |  |  |
| --- | --- | --- | --- | --- | --- | --- | --- | --- | --- | --- | --- | --- | --- | --- | --- | --- | --- | --- | --- |

Best Unique PSM (Scan Sample\_5\_A1\_1\_3643.d:67326, m/z=547.9825, z=3, RT=18.84, ppm=-5.62):


TRINITY\_DN36\_c0\_g1\_i1.p1

back to list

  

| Protein Coverage
| Supporting Peptides
| Best Unique PSM
|

Protein Coverage:

Supporting Peptides:

| Peptide | Uniq | -10lgP | Mass | Length | ppm | m/z | z | RT | 1/k0 Start | 1/k0 End | Precursor ID | Area Sample 7 | #Feature | #Feature Sample 7 | Start | End | PTM | AScore | Found By |
| --- | --- | --- | --- | --- | --- | --- | --- | --- | --- | --- | --- | --- | --- | --- | --- | --- | --- | --- | --- |
| R.LGGIEVTQGNC(+57.02)AR.K | Y | 49.67 | 1373.6721 | 13 | 2.61 | 687.8419 | 2 | 13.89 | 0.9695 | 0.9767 | 37015 | 0 | 0 | 0 | 58 | 70 | Carbamidomethylation |  | DB Search |
| total 1 peptides |  |  |  |  |  |  |  |  |  |  |  |  |  |  |  |  |  |  |  |
| --- | --- | --- | --- | --- | --- | --- | --- | --- | --- | --- | --- | --- | --- | --- | --- | --- | --- | --- | --- |

Best Unique PSM (Scan Sample\_5\_A1\_1\_3643.d:37015, m/z=687.8419, z=2, RT=13.89, ppm=-2.07):


TRINITY\_DN61\_c0\_g1\_i1.p1

back to list

  

| Protein Coverage
| Supporting Peptides
| Best Unique PSM
|

Protein Coverage:

Supporting Peptides:

| Peptide | Uniq | -10lgP | Mass | Length | ppm | m/z | z | RT | 1/k0 Start | 1/k0 End | Precursor ID | Area Sample 7 | #Feature | #Feature Sample 7 | Start | End | PTM | AScore | Found By |
| --- | --- | --- | --- | --- | --- | --- | --- | --- | --- | --- | --- | --- | --- | --- | --- | --- | --- | --- | --- |
| R.AINIHDLVGIDTKK.E | Y | 55.37 | 1535.8671 | 14 | 4.52 | 512.9629 | 3 | 16.65 | 0.7913 | 0.7986 | 53072 | 3.27e3 | 2 | 2 | 339 | 352 |  |  | DB Search |
| total 1 peptides |  |  |  |  |  |  |  |  |  |  |  |  |  |  |  |  |  |  |  |
| --- | --- | --- | --- | --- | --- | --- | --- | --- | --- | --- | --- | --- | --- | --- | --- | --- | --- | --- | --- |

Best Unique PSM (Scan Sample\_5\_A1\_1\_3643.d:53072, m/z=512.9629, z=3, RT=16.65, ppm=-0.17):


TRINITY\_DN1936\_c0\_g1\_i3.p1

back to list

  

| Protein Coverage
| Supporting Peptides
| Best Unique PSM
|

Protein Coverage:

Supporting Peptides:

| Peptide | Uniq | -10lgP | Mass | Length | ppm | m/z | z | RT | 1/k0 Start | 1/k0 End | Precursor ID | Area Sample 7 | #Feature | #Feature Sample 7 | Start | End | PTM | AScore | Found By |
| --- | --- | --- | --- | --- | --- | --- | --- | --- | --- | --- | --- | --- | --- | --- | --- | --- | --- | --- | --- |
| R.NQGATGSAGTGPK.A | Y | 50.58 | 1144.5472 | 13 | 3.6 | 573.2803 | 2 | 8.98 | 0.8708 | 0.8781 | 9648 | 0 | 0 | 0 | 293 | 305 |  |  | DB Search |
| total 1 peptides |  |  |  |  |  |  |  |  |  |  |  |  |  |  |  |  |  |  |  |
| --- | --- | --- | --- | --- | --- | --- | --- | --- | --- | --- | --- | --- | --- | --- | --- | --- | --- | --- | --- |

Best Unique PSM (Scan Sample\_5\_A1\_1\_3643.d:9648, m/z=573.2803, z=2, RT=8.98, ppm=-1.09):


TRINITY\_DN585\_c0\_g1\_i3.p1

back to list

  

| Protein Coverage
| Supporting Peptides
| Best Unique PSM
|

Protein Coverage:

Supporting Peptides:

| Peptide | Uniq | -10lgP | Mass | Length | ppm | m/z | z | RT | 1/k0 Start | 1/k0 End | Precursor ID | Area Sample 7 | #Feature | #Feature Sample 7 | Start | End | PTM | AScore | Found By |
| --- | --- | --- | --- | --- | --- | --- | --- | --- | --- | --- | --- | --- | --- | --- | --- | --- | --- | --- | --- |
| K.TITLEVEPSDTIENVK.S | Y | 55.12 | 1786.92 | 16 | 0.46 | 894.4635 | 2 | 17.55 | 1.1414 | 1.1486 | 58604 | 8.72e2 | 1 | 1 | 60 | 75 |  |  | DB Search |
| total 1 peptides |  |  |  |  |  |  |  |  |  |  |  |  |  |  |  |  |  |  |  |
| --- | --- | --- | --- | --- | --- | --- | --- | --- | --- | --- | --- | --- | --- | --- | --- | --- | --- | --- | --- |

Best Unique PSM (Scan Sample\_5\_A1\_1\_3643.d:58604, m/z=894.4635, z=2, RT=17.55, ppm=-4.22):


TRINITY\_DN160\_c0\_g1\_i2.p1

back to list

  

| Protein Coverage
| Supporting Peptides
| Best Unique PSM
|

Protein Coverage:

Supporting Peptides:

| Peptide | Uniq | -10lgP | Mass | Length | ppm | m/z | z | RT | 1/k0 Start | 1/k0 End | Precursor ID | Area Sample 7 | #Feature | #Feature Sample 7 | Start | End | PTM | AScore | Found By |
| --- | --- | --- | --- | --- | --- | --- | --- | --- | --- | --- | --- | --- | --- | --- | --- | --- | --- | --- | --- |
| K.LRLDFDHEAK.L | Y | 56.21 | 1242.6356 | 10 | -2.49 | 415.2162 | 3 | 14.37 | 0.7326 | 0.74 | 39914 | 3.79e2 | 1 | 1 | 1007 | 1016 |  |  | DB Search |
| total 1 peptides |  |  |  |  |  |  |  |  |  |  |  |  |  |  |  |  |  |  |  |
| --- | --- | --- | --- | --- | --- | --- | --- | --- | --- | --- | --- | --- | --- | --- | --- | --- | --- | --- | --- |

Best Unique PSM (Scan Sample\_5\_A1\_1\_3643.d:39914, m/z=415.2162, z=3, RT=14.37, ppm=-7.18):


TRINITY\_DN2028\_c0\_g1\_i1.p1

back to list

  

| Protein Coverage
| Supporting Peptides
| Best Unique PSM
|

Protein Coverage:

Supporting Peptides:

| Peptide | Uniq | -10lgP | Mass | Length | ppm | m/z | z | RT | 1/k0 Start | 1/k0 End | Precursor ID | Area Sample 7 | #Feature | #Feature Sample 7 | Start | End | PTM | AScore | Found By |
| --- | --- | --- | --- | --- | --- | --- | --- | --- | --- | --- | --- | --- | --- | --- | --- | --- | --- | --- | --- |
| K.LIQYIENKPGLTR.L | Y | 55.37 | 1543.8722 | 13 | 3.16 | 772.9422 | 2 | 14.92 | 1.0721 | 1.0793 | 43103 | 1.9e3 | 1 | 1 | 81 | 93 |  |  | DB Search |
| total 1 peptides |  |  |  |  |  |  |  |  |  |  |  |  |  |  |  |  |  |  |  |
| --- | --- | --- | --- | --- | --- | --- | --- | --- | --- | --- | --- | --- | --- | --- | --- | --- | --- | --- | --- |

Best Unique PSM (Scan Sample\_5\_A1\_1\_3643.d:43103, m/z=772.9422, z=2, RT=14.92, ppm=-1.52):


TRINITY\_DN6542\_c0\_g1\_i1.p1

back to list

  

| Protein Coverage
| Supporting Peptides
| Best Unique PSM
|

Protein Coverage:

Supporting Peptides:

| Peptide | Uniq | -10lgP | Mass | Length | ppm | m/z | z | RT | 1/k0 Start | 1/k0 End | Precursor ID | Area Sample 7 | #Feature | #Feature Sample 7 | Start | End | PTM | AScore | Found By |
| --- | --- | --- | --- | --- | --- | --- | --- | --- | --- | --- | --- | --- | --- | --- | --- | --- | --- | --- | --- |
| K.ILLAVGGWNHER.Q | Y | 49.52 | 1363.736 | 12 | 4.17 | 682.8749 | 2 | 17.15 | 1.0423 | 1.0496 | 56044 | 3.26e2 | 1 | 1 | 140 | 151 |  |  | DB Search |
| total 1 peptides |  |  |  |  |  |  |  |  |  |  |  |  |  |  |  |  |  |  |  |
| --- | --- | --- | --- | --- | --- | --- | --- | --- | --- | --- | --- | --- | --- | --- | --- | --- | --- | --- | --- |

Best Unique PSM (Scan Sample\_5\_A1\_1\_3643.d:56044, m/z=682.8749, z=2, RT=17.15, ppm=-0.52):


TRINITY\_DN3448\_c0\_g2\_i3.p2

back to list

  

| Protein Coverage
| Supporting Peptides
| Best Unique PSM
|

Protein Coverage:

Supporting Peptides:

| Peptide | Uniq | -10lgP | Mass | Length | ppm | m/z | z | RT | 1/k0 Start | 1/k0 End | Precursor ID | Area Sample 7 | #Feature | #Feature Sample 7 | Start | End | PTM | AScore | Found By |
| --- | --- | --- | --- | --- | --- | --- | --- | --- | --- | --- | --- | --- | --- | --- | --- | --- | --- | --- | --- |
| K.AGM(+15.99)THIVR.D | Y | 54.97 | 899.4647 | 8 | 4.88 | 450.7397 | 2 | 9.72 | 0.8144 | 0.8218 | 12487 | 9.73e2 | 1 | 1 | 31 | 38 | Oxidation (M) | M3:Oxidation (M):1000 | DB Search |
| total 1 peptides |  |  |  |  |  |  |  |  |  |  |  |  |  |  |  |  |  |  |  |
| --- | --- | --- | --- | --- | --- | --- | --- | --- | --- | --- | --- | --- | --- | --- | --- | --- | --- | --- | --- |

Best Unique PSM (Scan Sample\_5\_A1\_1\_3643.d:12487, m/z=450.7397, z=2, RT=9.72, ppm=0.2):


TRINITY\_DN1616\_c0\_g1\_i2.p1

back to list

  

| Protein Coverage
| Supporting Peptides
| Best Unique PSM
|

Protein Coverage:

Supporting Peptides:

| Peptide | Uniq | -10lgP | Mass | Length | ppm | m/z | z | RT | 1/k0 Start | 1/k0 End | Precursor ID | Area Sample 7 | #Feature | #Feature Sample 7 | Start | End | PTM | AScore | Found By |
| --- | --- | --- | --- | --- | --- | --- | --- | --- | --- | --- | --- | --- | --- | --- | --- | --- | --- | --- | --- |
| K.VVDTEANDLTEEVKK.T | Y | 56.4 | 1688.8468 | 15 | 6.39 | 845.4321 | 2 | 14.15 | 1.1318 | 1.139 | 38615 | 0 | 0 | 0 | 2542 | 2556 |  |  | DB Search |
| K.SSSC(+57.02)QEFGGKC(+57.02)PC(+57.02)K.D | Y | 56.37 | 1630.6538 | 14 | -16.59 | 816.3168 | 2 | 12.09 | 1.0335 | 1.0407 | 25828 | 0 | 0 | 0 | 1387 | 1400 | Carbamidomethylation, Carbamidomethylation, Carbamidomethylation |  | DB Search |
| R.YGAVNDNC(+57.02)ER.D | Y | 56.34 | 1196.488 | 10 | 3.96 | 599.2509 | 2 | 10.89 | 0.885 | 0.8923 | 18607 | 1.07e3 | 1 | 1 | 498 | 507 | Carbamidomethylation |  | DB Search |
| K.TLDTVC(+57.02)DSGTGVC(+57.02)QC(+57.02)K.S | Y | 49.46 | 1799.7488 | 16 | -4.78 | 900.8732 | 2 | 13.15 | 1.0901 | 1.0973 | 32411 | 1.65e2 | 1 | 1 | 546 | 561 | Carbamidomethylation, Carbamidomethylation, Carbamidomethylation |  | DB Search |
| total 4 peptides |  |  |  |  |  |  |  |  |  |  |  |  |  |  |  |  |  |  |  |
| --- | --- | --- | --- | --- | --- | --- | --- | --- | --- | --- | --- | --- | --- | --- | --- | --- | --- | --- | --- |

Best Unique PSM (Scan Sample\_5\_A1\_1\_3643.d:38615, m/z=845.4321, z=2, RT=14.15, ppm=1.71):


TRINITY\_DN3016\_c0\_g1\_i13.p1

back to list

  

| Protein Coverage
| Supporting Peptides
| Best Unique PSM
|

Protein Coverage:

Supporting Peptides:

| Peptide | Uniq | -10lgP | Mass | Length | ppm | m/z | z | RT | 1/k0 Start | 1/k0 End | Precursor ID | Area Sample 7 | #Feature | #Feature Sample 7 | Start | End | PTM | AScore | Found By |
| --- | --- | --- | --- | --- | --- | --- | --- | --- | --- | --- | --- | --- | --- | --- | --- | --- | --- | --- | --- |
| R.IVNTDLQEIKDIIK.G | Y | 56.35 | 1640.9348 | 14 | -0.94 | 547.9825 | 3 | 18.84 | 0.8193 | 0.8266 | 67326 | 7.77e2 | 1 | 1 | 154 | 167 |  |  | DB Search |
| total 1 peptides |  |  |  |  |  |  |  |  |  |  |  |  |  |  |  |  |  |  |  |
| --- | --- | --- | --- | --- | --- | --- | --- | --- | --- | --- | --- | --- | --- | --- | --- | --- | --- | --- | --- |

Best Unique PSM (Scan Sample\_5\_A1\_1\_3643.d:67326, m/z=547.9825, z=3, RT=18.84, ppm=-5.62):


TRINITY\_DN477\_c0\_g1\_i8.p2

back to list

  

| Protein Coverage
| Supporting Peptides
| Best Unique PSM
|

Protein Coverage:

Supporting Peptides:

| Peptide | Uniq | -10lgP | Mass | Length | ppm | m/z | z | RT | 1/k0 Start | 1/k0 End | Precursor ID | Area Sample 7 | #Feature | #Feature Sample 7 | Start | End | PTM | AScore | Found By |
| --- | --- | --- | --- | --- | --- | --- | --- | --- | --- | --- | --- | --- | --- | --- | --- | --- | --- | --- | --- |
| R.NDVNNPPQAR.R | Y | 55.29 | 1123.537 | 10 | 3.11 | 562.7749 | 2 | 10.15 | 0.8558 | 0.8631 | 13772 | 1.28e4 | 1 | 1 | 86 | 95 |  |  | DB Search |
| total 1 peptides |  |  |  |  |  |  |  |  |  |  |  |  |  |  |  |  |  |  |  |
| --- | --- | --- | --- | --- | --- | --- | --- | --- | --- | --- | --- | --- | --- | --- | --- | --- | --- | --- | --- |

Best Unique PSM (Scan Sample\_5\_A1\_1\_3643.d:13772, m/z=562.7749, z=2, RT=10.15, ppm=-1.57):


TRINITY\_DN11522\_c0\_g1\_i3.p1

back to list

  

| Protein Coverage
| Supporting Peptides
| Best Unique PSM
|

Protein Coverage:

Supporting Peptides:

| Peptide | Uniq | -10lgP | Mass | Length | ppm | m/z | z | RT | 1/k0 Start | 1/k0 End | Precursor ID | Area Sample 7 | #Feature | #Feature Sample 7 | Start | End | PTM | AScore | Found By |
| --- | --- | --- | --- | --- | --- | --- | --- | --- | --- | --- | --- | --- | --- | --- | --- | --- | --- | --- | --- |
| R.IGLGNIRDPSISLTR.G | Y | 55.35 | 1610.9104 | 15 | -3.69 | 537.9729 | 3 | 17.37 | 0.7986 | 0.8059 | 57304 | 1.32e3 | 1 | 1 | 27 | 41 |  |  | DB Search |
| total 1 peptides |  |  |  |  |  |  |  |  |  |  |  |  |  |  |  |  |  |  |  |
| --- | --- | --- | --- | --- | --- | --- | --- | --- | --- | --- | --- | --- | --- | --- | --- | --- | --- | --- | --- |

Best Unique PSM (Scan Sample\_5\_A1\_1\_3643.d:57304, m/z=537.9729, z=3, RT=17.37, ppm=-8.38):


TRINITY\_DN15847\_c0\_g1\_i2.p1

back to list

  

| Protein Coverage
| Supporting Peptides
| Best Unique PSM
|

Protein Coverage:

Supporting Peptides:

| Peptide | Uniq | -10lgP | Mass | Length | ppm | m/z | z | RT | 1/k0 Start | 1/k0 End | Precursor ID | Area Sample 7 | #Feature | #Feature Sample 7 | Start | End | PTM | AScore | Found By |
| --- | --- | --- | --- | --- | --- | --- | --- | --- | --- | --- | --- | --- | --- | --- | --- | --- | --- | --- | --- |
| R.EAIEGTYIDKK.C | Y | 55.33 | 1265.6503 | 11 | 3.67 | 633.8318 | 2 | 12.92 | 0.9505 | 0.9578 | 30926 | 8.95e2 | 1 | 1 | 56 | 66 |  |  | DB Search |
| total 1 peptides |  |  |  |  |  |  |  |  |  |  |  |  |  |  |  |  |  |  |  |
| --- | --- | --- | --- | --- | --- | --- | --- | --- | --- | --- | --- | --- | --- | --- | --- | --- | --- | --- | --- |

Best Unique PSM (Scan Sample\_5\_A1\_1\_3643.d:30926, m/z=633.8318, z=2, RT=12.92, ppm=-1.01):


TRINITY\_DN428\_c0\_g1\_i1.p1

back to list

  

| Protein Coverage
| Supporting Peptides
| Best Unique PSM
|

Protein Coverage:

Supporting Peptides:

| Peptide | Uniq | -10lgP | Mass | Length | ppm | m/z | z | RT | 1/k0 Start | 1/k0 End | Precursor ID | Area Sample 7 | #Feature | #Feature Sample 7 | Start | End | PTM | AScore | Found By |
| --- | --- | --- | --- | --- | --- | --- | --- | --- | --- | --- | --- | --- | --- | --- | --- | --- | --- | --- | --- |
| K.IC(+57.02)HLLGIQVTDFTK.S | Y | 56.23 | 1643.8705 | 14 | -2.32 | 548.9603 | 3 | 18.61 | 0.8197 | 0.827 | 65792 | 1.09e3 | 1 | 1 | 377 | 390 | Carbamidomethylation |  | DB Search |
| K.ALSLQNANEELEDRLAEAER.Q | Y | 52.19 | 2270.1138 | 20 | 0.29 | 757.7086 | 3 | 18.89 | 0.984 | 0.9912 | 67687 | 7.99e2 | 1 | 1 | 1475 | 1494 |  |  | DB Search |
| total 2 peptides |  |  |  |  |  |  |  |  |  |  |  |  |  |  |  |  |  |  |  |
| --- | --- | --- | --- | --- | --- | --- | --- | --- | --- | --- | --- | --- | --- | --- | --- | --- | --- | --- | --- |

Best Unique PSM (Scan Sample\_5\_A1\_1\_3643.d:65792, m/z=548.9603, z=3, RT=18.61, ppm=-7):


TRINITY\_DN161\_c0\_g1\_i4.p1

back to list

  

| Protein Coverage
| Supporting Peptides
| Best Unique PSM
|

Protein Coverage:

Supporting Peptides:

| Peptide | Uniq | -10lgP | Mass | Length | ppm | m/z | z | RT | 1/k0 Start | 1/k0 End | Precursor ID | Area Sample 7 | #Feature | #Feature Sample 7 | Start | End | PTM | AScore | Found By |
| --- | --- | --- | --- | --- | --- | --- | --- | --- | --- | --- | --- | --- | --- | --- | --- | --- | --- | --- | --- |
| K.SYELPDGQVITIGNER.F | Y | 55.37 | 1789.8846 | 16 | 2.43 | 895.9476 | 2 | 18.67 | 1.117 | 1.1242 | 66156 | 3.51e2 | 1 | 1 | 95 | 110 |  |  | DB Search |
| total 1 peptides |  |  |  |  |  |  |  |  |  |  |  |  |  |  |  |  |  |  |  |
| --- | --- | --- | --- | --- | --- | --- | --- | --- | --- | --- | --- | --- | --- | --- | --- | --- | --- | --- | --- |

Best Unique PSM (Scan Sample\_5\_A1\_1\_3643.d:66156, m/z=895.9476, z=2, RT=18.67, ppm=-2.25):


TRINITY\_DN36\_c0\_g1\_i2.p1

back to list

  

| Protein Coverage
| Supporting Peptides
| Best Unique PSM
|

Protein Coverage:

Supporting Peptides:

| Peptide | Uniq | -10lgP | Mass | Length | ppm | m/z | z | RT | 1/k0 Start | 1/k0 End | Precursor ID | Area Sample 7 | #Feature | #Feature Sample 7 | Start | End | PTM | AScore | Found By |
| --- | --- | --- | --- | --- | --- | --- | --- | --- | --- | --- | --- | --- | --- | --- | --- | --- | --- | --- | --- |
| R.LGGIEVTQGNC(+57.02)AR.K | Y | 49.67 | 1373.6721 | 13 | 2.61 | 687.8419 | 2 | 13.89 | 0.9695 | 0.9767 | 37015 | 0 | 0 | 0 | 58 | 70 | Carbamidomethylation |  | DB Search |
| total 1 peptides |  |  |  |  |  |  |  |  |  |  |  |  |  |  |  |  |  |  |  |
| --- | --- | --- | --- | --- | --- | --- | --- | --- | --- | --- | --- | --- | --- | --- | --- | --- | --- | --- | --- |

Best Unique PSM (Scan Sample\_5\_A1\_1\_3643.d:37015, m/z=687.8419, z=2, RT=13.89, ppm=-2.07):


TRINITY\_DN991\_c0\_g1\_i2.p1

back to list

  

| Protein Coverage
| Supporting Peptides
| Best Unique PSM
|

Protein Coverage:

Supporting Peptides:

| Peptide | Uniq | -10lgP | Mass | Length | ppm | m/z | z | RT | 1/k0 Start | 1/k0 End | Precursor ID | Area Sample 7 | #Feature | #Feature Sample 7 | Start | End | PTM | AScore | Found By |
| --- | --- | --- | --- | --- | --- | --- | --- | --- | --- | --- | --- | --- | --- | --- | --- | --- | --- | --- | --- |
| R.ITVTVSPDIHPPAK.L | Y | 49.15 | 1473.8191 | 14 | -0.97 | 737.9127 | 2 | 14.98 | 1.0668 | 1.0741 | 43563 | 1.94e2 | 1 | 1 | 1183 | 1196 |  |  | DB Search |
| total 1 peptides |  |  |  |  |  |  |  |  |  |  |  |  |  |  |  |  |  |  |  |
| --- | --- | --- | --- | --- | --- | --- | --- | --- | --- | --- | --- | --- | --- | --- | --- | --- | --- | --- | --- |

Best Unique PSM (Scan Sample\_5\_A1\_1\_3643.d:43563, m/z=736.8683, z=2, RT=15.00, ppm=-5.65):


TRINITY\_DN2217\_c0\_g1\_i2.p1

back to list

  

| Protein Coverage
| Supporting Peptides
| Best Unique PSM
|

Protein Coverage:

Supporting Peptides:

| Peptide | Uniq | -10lgP | Mass | Length | ppm | m/z | z | RT | 1/k0 Start | 1/k0 End | Precursor ID | Area Sample 7 | #Feature | #Feature Sample 7 | Start | End | PTM | AScore | Found By |
| --- | --- | --- | --- | --- | --- | --- | --- | --- | --- | --- | --- | --- | --- | --- | --- | --- | --- | --- | --- |
| K.ATGC(+57.02)EVIWNQHNR.G | Y | 56.39 | 1583.7263 | 13 | 0.67 | 792.8672 | 2 | 14.03 | 1.0829 | 1.0901 | 37880 | 1.73e2 | 1 | 1 | 119 | 131 | Carbamidomethylation |  | DB Search |
| total 1 peptides |  |  |  |  |  |  |  |  |  |  |  |  |  |  |  |  |  |  |  |
| --- | --- | --- | --- | --- | --- | --- | --- | --- | --- | --- | --- | --- | --- | --- | --- | --- | --- | --- | --- |

Best Unique PSM (Scan Sample\_5\_A1\_1\_3643.d:37880, m/z=792.8672, z=2, RT=14.03, ppm=-4.01):


TRINITY\_DN6542\_c0\_g1\_i3.p1

back to list

  

| Protein Coverage
| Supporting Peptides
| Best Unique PSM
|

Protein Coverage:

Supporting Peptides:

| Peptide | Uniq | -10lgP | Mass | Length | ppm | m/z | z | RT | 1/k0 Start | 1/k0 End | Precursor ID | Area Sample 7 | #Feature | #Feature Sample 7 | Start | End | PTM | AScore | Found By |
| --- | --- | --- | --- | --- | --- | --- | --- | --- | --- | --- | --- | --- | --- | --- | --- | --- | --- | --- | --- |
| K.ILLAVGGWNHER.Q | Y | 49.52 | 1363.736 | 12 | 4.17 | 682.8749 | 2 | 17.15 | 1.0423 | 1.0496 | 56044 | 3.26e2 | 1 | 1 | 140 | 151 |  |  | DB Search |
| total 1 peptides |  |  |  |  |  |  |  |  |  |  |  |  |  |  |  |  |  |  |  |
| --- | --- | --- | --- | --- | --- | --- | --- | --- | --- | --- | --- | --- | --- | --- | --- | --- | --- | --- | --- |

Best Unique PSM (Scan Sample\_5\_A1\_1\_3643.d:56044, m/z=682.8749, z=2, RT=17.15, ppm=-0.52):


TRINITY\_DN71276\_c0\_g1\_i1.p1

back to list

  

| Protein Coverage
| Supporting Peptides
| Best Unique PSM
|

Protein Coverage:

Supporting Peptides:

| Peptide | Uniq | -10lgP | Mass | Length | ppm | m/z | z | RT | 1/k0 Start | 1/k0 End | Precursor ID | Area Sample 7 | #Feature | #Feature Sample 7 | Start | End | PTM | AScore | Found By |
| --- | --- | --- | --- | --- | --- | --- | --- | --- | --- | --- | --- | --- | --- | --- | --- | --- | --- | --- | --- |
| R.YYAAAC(+57.02)LDNIFQR.K | Y | 55.19 | 1603.7453 | 13 | -7.09 | 802.8705 | 2 | 19.75 | 1.0701 | 1.0773 | 73459 | 4.66e2 | 1 | 1 | 293 | 305 | Carbamidomethylation |  | DB Search |
| K.EAAAQIVVEIIKR.T | Y | 49.37 | 1438.8507 | 13 | 8.13 | 720.4351 | 2 | 19.21 | 1.0544 | 1.0616 | 69860 | 0 | 0 | 0 | 149 | 161 |  |  | DB Search |
| total 2 peptides |  |  |  |  |  |  |  |  |  |  |  |  |  |  |  |  |  |  |  |
| --- | --- | --- | --- | --- | --- | --- | --- | --- | --- | --- | --- | --- | --- | --- | --- | --- | --- | --- | --- |

Best Unique PSM (Scan Sample\_5\_A1\_1\_3643.d:73459, m/z=802.8705, z=2, RT=19.75, ppm=-11.77):


TRINITY\_DN687\_c0\_g2\_i1.p1

back to list

  

| Protein Coverage
| Supporting Peptides
| Best Unique PSM
|

Protein Coverage:

Supporting Peptides:

| Peptide | Uniq | -10lgP | Mass | Length | ppm | m/z | z | RT | 1/k0 Start | 1/k0 End | Precursor ID | Area Sample 7 | #Feature | #Feature Sample 7 | Start | End | PTM | AScore | Found By |
| --- | --- | --- | --- | --- | --- | --- | --- | --- | --- | --- | --- | --- | --- | --- | --- | --- | --- | --- | --- |
| R.LDHKFDLM(+15.99)YAK.R | Y | 51.39 | 1395.6856 | 11 | 4.19 | 466.2356 | 3 | 14.23 | 0.7844 | 0.7917 | 39140 | 0 | 0 | 0 | 391 | 401 | Oxidation (M) | M8:Oxidation (M):1000 | DB Search |
| R.AVC(+57.02)M(+15.99)LSNTTAIAEAWAR.L | Y | 50.49 | 1879.892 | 17 | -0.63 | 940.9483 | 2 | 18.78 | 1.1482 | 1.1554 | 66979 | 0 | 0 | 0 | 374 | 390 | Carbamidomethylation, Oxidation (M) | M4:Oxidation (M):1000 | DB Search |
| K.VGINYQPPTVVPGGDLAK.V | Y | 48.61 | 1823.9781 | 18 | -1.33 | 912.9908 | 2 | 17.37 | 1.1238 | 1.131 | 57429 | 0 | 0 | 0 | 353 | 370 |  |  | DB Search |
| total 3 peptides |  |  |  |  |  |  |  |  |  |  |  |  |  |  |  |  |  |  |  |
| --- | --- | --- | --- | --- | --- | --- | --- | --- | --- | --- | --- | --- | --- | --- | --- | --- | --- | --- | --- |

Best Unique PSM (Scan Sample\_5\_A1\_1\_3643.d:39140, m/z=466.2356, z=3, RT=14.23, ppm=-0.49):


TRINITY\_DN5987\_c0\_g1\_i2.p1

back to list

  

| Protein Coverage
| Supporting Peptides
| Best Unique PSM
|

Protein Coverage:

Supporting Peptides:

| Peptide | Uniq | -10lgP | Mass | Length | ppm | m/z | z | RT | 1/k0 Start | 1/k0 End | Precursor ID | Area Sample 7 | #Feature | #Feature Sample 7 | Start | End | PTM | AScore | Found By |
| --- | --- | --- | --- | --- | --- | --- | --- | --- | --- | --- | --- | --- | --- | --- | --- | --- | --- | --- | --- |
| R.GNQGEQGPAGPVGPK.G | Y | 55.37 | 1391.6793 | 15 | 2.49 | 696.8454 | 2 | 11.50 | 0.9469 | 0.9541 | 22230 | 5.82e3 | 1 | 1 | 237 | 251 |  |  | DB Search |
| K.GEPGEIGEQGK.Q | Y | 47.83 | 1099.5145 | 11 | 4.88 | 550.7646 | 2 | 11.17 | 0.855 | 0.8623 | 20334 | 0 | 0 | 0 | 177 | 187 |  |  | DB Search |
| total 2 peptides |  |  |  |  |  |  |  |  |  |  |  |  |  |  |  |  |  |  |  |
| --- | --- | --- | --- | --- | --- | --- | --- | --- | --- | --- | --- | --- | --- | --- | --- | --- | --- | --- | --- |

Best Unique PSM (Scan Sample\_5\_A1\_1\_3643.d:22230, m/z=696.8454, z=2, RT=11.50, ppm=-2.19):


TRINITY\_DN1386\_c0\_g1\_i1.p1

back to list

  

| Protein Coverage
| Supporting Peptides
| Best Unique PSM
|

Protein Coverage:

Supporting Peptides:

| Peptide | Uniq | -10lgP | Mass | Length | ppm | m/z | z | RT | 1/k0 Start | 1/k0 End | Precursor ID | Area Sample 7 | #Feature | #Feature Sample 7 | Start | End | PTM | AScore | Found By |
| --- | --- | --- | --- | --- | --- | --- | --- | --- | --- | --- | --- | --- | --- | --- | --- | --- | --- | --- | --- |
| R.KVIVDQLVTLATR.V | Y | 56.14 | 1454.882 | 13 | -0.17 | 728.4448 | 2 | 18.43 | 1.0299 | 1.0371 | 64581 | 0 | 0 | 0 | 547 | 559 |  |  | DB Search |
| R.SPTHSVIK.S | Y | 53.63 | 867.4814 | 8 | 6.75 | 434.7489 | 2 | 9.91 | 0.777 | 0.7844 | 13342 | 3.42e2 | 1 | 1 | 259 | 266 |  |  | DB Search |
| total 2 peptides |  |  |  |  |  |  |  |  |  |  |  |  |  |  |  |  |  |  |  |
| --- | --- | --- | --- | --- | --- | --- | --- | --- | --- | --- | --- | --- | --- | --- | --- | --- | --- | --- | --- |

Best Unique PSM (Scan Sample\_5\_A1\_1\_3643.d:64581, m/z=728.4448, z=2, RT=18.43, ppm=-4.85):


TRINITY\_DN80458\_c0\_g1\_i1.p1

back to list

  

| Protein Coverage
| Supporting Peptides
| Best Unique PSM
|

Protein Coverage:

Supporting Peptides:

| Peptide | Uniq | -10lgP | Mass | Length | ppm | m/z | z | RT | 1/k0 Start | 1/k0 End | Precursor ID | Area Sample 7 | #Feature | #Feature Sample 7 | Start | End | PTM | AScore | Found By |
| --- | --- | --- | --- | --- | --- | --- | --- | --- | --- | --- | --- | --- | --- | --- | --- | --- | --- | --- | --- |
| R.DSHGVAQVR.Y | Y | 55.25 | 967.4835 | 9 | 5.54 | 484.7495 | 2 | 10.13 | 0.8144 | 0.8218 | 14459 | 0 | 0 | 0 | 61 | 69 |  |  | DB Search |
| total 1 peptides |  |  |  |  |  |  |  |  |  |  |  |  |  |  |  |  |  |  |  |
| --- | --- | --- | --- | --- | --- | --- | --- | --- | --- | --- | --- | --- | --- | --- | --- | --- | --- | --- | --- |

Best Unique PSM (Scan Sample\_5\_A1\_1\_3643.d:14459, m/z=484.7495, z=2, RT=10.13, ppm=0.86):


TRINITY\_DN4815\_c0\_g1\_i2.p1

back to list

  

| Protein Coverage
| Supporting Peptides
| Best Unique PSM
|

Protein Coverage:

Supporting Peptides:

| Peptide | Uniq | -10lgP | Mass | Length | ppm | m/z | z | RT | 1/k0 Start | 1/k0 End | Precursor ID | Area Sample 7 | #Feature | #Feature Sample 7 | Start | End | PTM | AScore | Found By |
| --- | --- | --- | --- | --- | --- | --- | --- | --- | --- | --- | --- | --- | --- | --- | --- | --- | --- | --- | --- |
| R.AVAC(+57.02)NSGPC(+57.02)LAGC(+57.02)PR.N | Y | 50.26 | 1588.6908 | 15 | 1.49 | 795.3502 | 2 | 13.12 | 1.019 | 1.0263 | 32158 | 1.27e3 | 1 | 1 | 468 | 482 | Carbamidomethylation, Carbamidomethylation, Carbamidomethylation |  | DB Search |
| total 1 peptides |  |  |  |  |  |  |  |  |  |  |  |  |  |  |  |  |  |  |  |
| --- | --- | --- | --- | --- | --- | --- | --- | --- | --- | --- | --- | --- | --- | --- | --- | --- | --- | --- | --- |

Best Unique PSM (Scan Sample\_5\_A1\_1\_3643.d:32158, m/z=795.3502, z=2, RT=13.12, ppm=-3.19):


TRINITY\_DN4595\_c0\_g1\_i1.p1

back to list

  

| Protein Coverage
| Supporting Peptides
| Best Unique PSM
|

Protein Coverage:

Supporting Peptides:

| Peptide | Uniq | -10lgP | Mass | Length | ppm | m/z | z | RT | 1/k0 Start | 1/k0 End | Precursor ID | Area Sample 7 | #Feature | #Feature Sample 7 | Start | End | PTM | AScore | Found By |
| --- | --- | --- | --- | --- | --- | --- | --- | --- | --- | --- | --- | --- | --- | --- | --- | --- | --- | --- | --- |
| K.YQGNDYTR.N | Y | 55.1 | 1015.4359 | 8 | 1.65 | 508.7237 | 2 | 10.77 | 0.8469 | 0.8542 | 17934 | 1.34e3 | 1 | 1 | 22 | 29 |  |  | DB Search |
| R.AFNKIETR.N | Y | 49.77 | 977.5294 | 8 | 3.42 | 489.7714 | 2 | 11.52 | 0.8388 | 0.8461 | 22323 | 8.13e3 | 1 | 1 | 72 | 79 |  |  | DB Search |
| total 2 peptides |  |  |  |  |  |  |  |  |  |  |  |  |  |  |  |  |  |  |  |
| --- | --- | --- | --- | --- | --- | --- | --- | --- | --- | --- | --- | --- | --- | --- | --- | --- | --- | --- | --- |

Best Unique PSM (Scan Sample\_5\_A1\_1\_3643.d:17934, m/z=508.7237, z=2, RT=10.77, ppm=-3.04):


TRINITY\_DN17117\_c0\_g1\_i1.p1

back to list

  

| Protein Coverage
| Supporting Peptides
| Best Unique PSM
|

Protein Coverage:

Supporting Peptides:

| Peptide | Uniq | -10lgP | Mass | Length | ppm | m/z | z | RT | 1/k0 Start | 1/k0 End | Precursor ID | Area Sample 7 | #Feature | #Feature Sample 7 | Start | End | PTM | AScore | Found By |
| --- | --- | --- | --- | --- | --- | --- | --- | --- | --- | --- | --- | --- | --- | --- | --- | --- | --- | --- | --- |
| R.IGETVNFIVIDYADSAAGKK.Q | Y | 49.19 | 2110.0946 | 20 | 6.76 | 704.3736 | 3 | 19.80 | 0.9465 | 0.9537 | 73806 | 0 | 0 | 0 | 308 | 327 |  |  | DB Search |
| total 1 peptides |  |  |  |  |  |  |  |  |  |  |  |  |  |  |  |  |  |  |  |
| --- | --- | --- | --- | --- | --- | --- | --- | --- | --- | --- | --- | --- | --- | --- | --- | --- | --- | --- | --- |

Best Unique PSM (Scan Sample\_5\_A1\_1\_3643.d:73806, m/z=704.3736, z=3, RT=19.80, ppm=2.08):


---

Prepared with PEAKS ™ (bioinfor.com)
