# Supplementary material for: Proteomic Analysis and Biochemical Characterization of the Nematocyst Extract of the Hydrozoan Velella velella
Source: Mar Drugs. 2024 Oct 12;22(10):468. doi: 10.3390/md22100468 (PMC11509761; doi:10.3390/md22100468)
Supplement: Supplementary file 1 [file marinedrugs-22-00468-s001.zip › Supplementary File S1.zip!/Sample_7_Vellela/db.peptides.html]

peptide list


Peptide List

  

| Peptide | -10lgP | Mass | Length | ppm | m/z | RT | 1/k0 Start | 1/k0 End | Precursor ID | Area Sample 8 | #Feature | #Feature Sample 8 | Accession | PTM | AScore | Found By |
| --- | --- | --- | --- | --- | --- | --- | --- | --- | --- | --- | --- | --- | --- | --- | --- | --- |
| VVHNIC(+57.02)SSAITNSVTNK | 43.87 | 1842.9258 | 17 | 2.87 | 922.4687 | 13.93 | 1.1394 | 1.1466 | 37331 | 5.78e2 | 1 | 1 | TRINITY\_DN1199\_c0\_g1\_i1.p1:TRINITY\_DN1199\_c0\_g1\_i2.p1 | Carbamidomethylation |  | DB Search |
| LALHVLNNEDIIGHR | 43.86 | 1712.9321 | 15 | 1.95 | 857.4712 | 16.55 | 1.1378 | 1.145 | 52597 | 1.8e3 | 2 | 2 | TRINITY\_DN140702\_c0\_g1\_i1.p1 |  |  | DB Search |
| SYELPDGQVITIGNER | 43.86 | 1789.8846 | 16 | 3.32 | 895.9485 | 18.70 | 1.1126 | 1.1198 | 65490 | 4.79e3 | 2 | 2 | TRINITY\_DN161\_c0\_g1\_i1.p1:TRINITY\_DN161\_c0\_g1\_i4.p1:TRINITY\_DN161\_c0\_g1\_i3.p1:TRINITY\_DN161\_c0\_g1\_i2.p1 |  |  | DB Search |
| S(+42.01)EQEISNLVEQAK | 43.84 | 1515.7417 | 13 | 3.29 | 758.8772 | 19.50 | 1.0568 | 1.064 | 70956 | 0 | 0 | 0 | TRINITY\_DN165536\_c0\_g1\_i1.p1 | Acetylation (Protein N-term) | S1:Acetylation (Protein N-term):1000 | DB Search |
| NDVNNPPQAR | 43.84 | 1123.537 | 10 | 3.77 | 562.7754 | 10.18 | 0.8676 | 0.8749 | 15798 | 1.5e4 | 1 | 1 | TRINITY\_DN477\_c0\_g1\_i3.p1:TRINITY\_DN477\_c0\_g1\_i7.p1:TRINITY\_DN477\_c0\_g1\_i6.p1:TRINITY\_DN477\_c0\_g1\_i5.p1:TRINITY\_DN477\_c0\_g1\_i8.p2 |  |  | DB Search |
| NYNNDDEPGAK | 43.84 | 1235.5055 | 11 | 1.73 | 618.7583 | 10.30 | 0.9198 | 0.9271 | 16375 | 3.11e3 | 1 | 1 | TRINITY\_DN199\_c0\_g1\_i1.p1 |  |  | DB Search |
| ATHQELLNAEFIQK | 43.84 | 1640.8522 | 14 | 1.4 | 821.4308 | 16.12 | 1.0821 | 1.0893 | 50093 | 9.45e2 | 1 | 1 | TRINITY\_DN113\_c3\_g1\_i1.p1 |  |  | DB Search |
| ALLLASSNAFDTSNGAR | 43.83 | 1706.8588 | 17 | 2.98 | 854.4354 | 17.86 | 1.0977 | 1.1049 | 60419 | 5.82e2 | 1 | 1 | TRINITY\_DN244\_c0\_g1\_i1.p1 |  |  | DB Search |
| AEYENKTEVAR | 43.83 | 1308.631 | 11 | 1.95 | 655.3211 | 10.58 | 0.9727 | 0.98 | 17983 | 0 | 0 | 0 | TRINITY\_DN1280\_c0\_g1\_i1.p1 |  |  | DB Search |
| YYVTIIDAPGHR | 43.83 | 1403.7197 | 12 | 1.16 | 702.8648 | 16.09 | 1.0399 | 1.0472 | 49950 | 5.46e2 | 1 | 1 | TRINITY\_DN64\_c0\_g1\_i1.p1 |  |  | DB Search |
| SGGQC(+57.02)PC(+57.02)KPGVVGR | 43.83 | 1457.6868 | 14 | 2.89 | 729.8495 | 10.75 | 0.9751 | 0.9824 | 18858 | 2.96e3 | 2 | 2 | TRINITY\_DN15274\_c0\_g1\_i1.p1 | Carbamidomethylation, Carbamidomethylation |  | DB Search |
| EGDLVIHSGITDAVK | 43.83 | 1552.8097 | 15 | 0.72 | 777.4092 | 15.57 | 1.0576 | 1.0648 | 46999 | 6.3e3 | 3 | 3 | TRINITY\_DN329\_c0\_g1\_i1.p1:TRINITY\_DN329\_c0\_g1\_i2.p1 |  |  | DB Search |
| AEFRPNQEEEPILKR | 43.83 | 1854.9587 | 15 | -0 | 619.3241 | 13.90 | 0.8785 | 0.8858 | 36999 | 2.93e3 | 1 | 1 | TRINITY\_DN262\_c0\_g1\_i1.p1 |  |  | DB Search |
| HLSVNILQTNTR | 43.83 | 1394.7629 | 12 | 3.19 | 698.3879 | 15.45 | 1.0005 | 1.0077 | 46263 | 8.86e2 | 1 | 1 | TRINITY\_DN6390\_c0\_g1\_i1.p1 |  |  | DB Search |
| DNIQGITKPAIR | 43.83 | 1324.7462 | 12 | 2.3 | 663.379 | 13.91 | 0.9808 | 0.988 | 37136 | 4.46e2 | 1 | 1 | TRINITY\_DN172571\_c0\_g1\_i1.p1 |  |  | DB Search |
| KAQYYEAYETIIKR | 43.83 | 1774.9254 | 14 | -1.22 | 592.6457 | 15.96 | 0.8664 | 0.8737 | 49246 | 6.27e2 | 1 | 1 | TRINITY\_DN319\_c2\_g1\_i3.p1:TRINITY\_DN319\_c2\_g1\_i1.p1 |  |  | DB Search |
| TRIEDLETVISQTQDHR | 43.83 | 2040.0236 | 17 | 1.88 | 681.0134 | 17.57 | 0.9626 | 0.9699 | 58583 | 4.36e2 | 1 | 1 | TRINITY\_DN761\_c0\_g1\_i3.p1:TRINITY\_DN761\_c0\_g1\_i1.p1:TRINITY\_DN761\_c0\_g1\_i2.p1 |  |  | DB Search |
| FC(+57.02)NNPAPSNGGR | 43.83 | 1289.5571 | 12 | 3.53 | 645.7852 | 10.94 | 0.9449 | 0.9521 | 20039 | 4e2 | 1 | 1 | TRINITY\_DN4595\_c0\_g1\_i10.p1:TRINITY\_DN4595\_c0\_g1\_i4.p1 | Carbamidomethylation |  | DB Search |
| ILQC(+57.02)NQYANVEEAR | 43.82 | 1706.8046 | 14 | 3.36 | 854.4086 | 14.34 | 1.0941 | 1.1013 | 39739 | 5.68e3 | 1 | 1 | TRINITY\_DN477\_c0\_g1\_i8.p1:TRINITY\_DN477\_c0\_g1\_i7.p1 | Carbamidomethylation |  | DB Search |
| YGNQC(+57.02)PAPR | 43.82 | 1061.4713 | 9 | 4.39 | 531.7429 | 10.38 | 0.855 | 0.8623 | 16893 | 1.47e3 | 1 | 1 | TRINITY\_DN1936\_c0\_g1\_i3.p1 | Carbamidomethylation |  | DB Search |
| ASGDYATVVSHNR | 43.82 | 1375.6481 | 13 | -1.36 | 688.8273 | 12.19 | 0.9953 | 1.0025 | 27207 | 0 | 0 | 0 | TRINITY\_DN925\_c0\_g1\_i1.p1 |  |  | DB Search |
| VLVIFANGDINDEQAVK | 43.82 | 1843.9679 | 17 | 0.34 | 922.9874 | 18.93 | 1.1917 | 1.1989 | 67392 | 0 | 0 | 0 | TRINITY\_DN113\_c3\_g1\_i1.p1 |  |  | DB Search |
| VIIDYQTHHR | 43.82 | 1280.6626 | 10 | -5.51 | 641.3322 | 12.21 | 0.9574 | 0.9646 | 27303 | 0 | 0 | 0 | TRINITY\_DN1907\_c0\_g1\_i1.p1:TRINITY\_DN1907\_c0\_g1\_i2.p1 |  |  | DB Search |
| FAIQDITVDEFSAK | 43.82 | 1582.7878 | 14 | -0.64 | 792.3972 | 19.87 | 1.0443 | 1.0516 | 73247 | 7.51e2 | 1 | 1 | TRINITY\_DN624\_c0\_g1\_i1.p1 |  |  | DB Search |
| LGIHEDTQNR | 43.82 | 1181.5789 | 10 | 2.89 | 591.7958 | 11.20 | 0.9073 | 0.9146 | 21408 | 1.32e3 | 1 | 1 | TRINITY\_DN141338\_c2\_g1\_i1.p1 |  |  | DB Search |
| ELVMHEQSR | 43.82 | 1127.5393 | 9 | -5.85 | 564.7711 | 11.37 | 0.8988 | 0.9061 | 22493 | 0 | 0 | 0 | TRINITY\_DN160\_c0\_g1\_i2.p1:TRINITY\_DN160\_c0\_g1\_i1.p1 |  |  | DB Search |
| SVTTHQGNAIVIK | 43.82 | 1366.7568 | 13 | 3.74 | 684.3852 | 12.65 | 0.9949 | 1.0021 | 29797 | 4.39e2 | 1 | 1 | TRINITY\_DN1377\_c0\_g1\_i1.p1 |  |  | DB Search |
| ALVILGDGFISGGGTPSK | 43.81 | 1687.9144 | 18 | -0.24 | 844.9605 | 19.84 | 1.1622 | 1.1694 | 72833 | 2.32e3 | 1 | 1 | TRINITY\_DN244\_c0\_g1\_i1.p1 |  |  | DB Search |
| HKLEPVGC(+57.02)FK | 43.81 | 1213.6277 | 10 | 6.02 | 405.5505 | 12.58 | 0.7086 | 0.7159 | 29300 | 3.93e3 | 1 | 1 | TRINITY\_DN3991\_c0\_g1\_i1.p1 | Carbamidomethylation |  | DB Search |
| SC(+57.02)ENPKPLNGGK | 43.81 | 1299.6241 | 12 | 4.1 | 650.8191 | 11.12 | 0.9509 | 0.9582 | 20845 | 1.35e3 | 1 | 1 | TRINITY\_DN6428\_c0\_g1\_i1.p1:TRINITY\_DN6428\_c0\_g1\_i2.p1 | Carbamidomethylation |  | DB Search |
| SFHFGVIKLEGK | 43.81 | 1360.7502 | 12 | 3.09 | 681.3815 | 17.25 | 1.0303 | 1.0375 | 56659 | 1.05e4 | 3 | 3 | TRINITY\_DN329\_c0\_g1\_i1.p1:TRINITY\_DN329\_c0\_g1\_i2.p1 |  |  | DB Search |
| GLKVDFDTTFAPVTGK | 43.81 | 1694.8879 | 16 | -2.21 | 848.4456 | 18.33 | 1.0921 | 1.0993 | 63359 | 2.86e3 | 1 | 1 | TRINITY\_DN329\_c0\_g1\_i1.p1:TRINITY\_DN329\_c0\_g1\_i2.p1 |  |  | DB Search |
| TIEAEAAHGTVTR | 43.81 | 1354.6841 | 13 | 1.86 | 678.3475 | 11.89 | 1.0126 | 1.0198 | 25470 | 0 | 0 | 0 | TRINITY\_DN7504\_c0\_g1\_i1.p1 |  |  | DB Search |
| DSYVGDEAQSKR | 43.81 | 1353.6161 | 12 | 3.22 | 677.8145 | 11.01 | 0.9961 | 1.0033 | 20355 | 1.65e3 | 1 | 1 | TRINITY\_DN2477\_c0\_g1\_i6.p1:TRINITY\_DN2477\_c0\_g1\_i5.p1:TRINITY\_DN195934\_c0\_g1\_i1.p2:TRINITY\_DN161\_c0\_g1\_i1.p1:TRINITY\_DN2477\_c0\_g1\_i9.p1:TRINITY\_DN2477\_c0\_g1\_i10.p1:TRINITY\_DN2477\_c0\_g1\_i2.p1:TRINITY\_DN161\_c0\_g1\_i3.p1:TRINITY\_DN161\_c0\_g1\_i2.p1 |  |  | DB Search |
| SC(+57.02)TNPAPANGGK | 43.8 | 1172.5244 | 12 | 2.69 | 587.2684 | 9.47 | 0.838 | 0.8453 | 12375 | 4.7e3 | 1 | 1 | TRINITY\_DN495\_c0\_g1\_i2.p1:TRINITY\_DN3545\_c0\_g1\_i2.p1:TRINITY\_DN3545\_c0\_g1\_i1.p1:TRINITY\_DN495\_c0\_g1\_i3.p1 | Carbamidomethylation |  | DB Search |
| GLKVDFDTTFAPVTGKK | 43.8 | 1822.9828 | 17 | 0.54 | 912.4951 | 17.35 | 1.1825 | 1.1897 | 57165 | 5.55e3 | 2 | 2 | TRINITY\_DN329\_c0\_g1\_i1.p1:TRINITY\_DN329\_c0\_g1\_i2.p1 |  |  | DB Search |
| TPGEC(+57.02)C(+57.02)PQC(+57.02)K | 43.8 | 1235.4733 | 10 | -0.13 | 618.7411 | 10.03 | 0.8866 | 0.8939 | 14986 | 1.58e3 | 1 | 1 | TRINITY\_DN1369\_c0\_g1\_i1.p1 | Carbamidomethylation, Carbamidomethylation, Carbamidomethylation |  | DB Search |
| AYGQDLGQPGSC(+57.02)LKR | 43.8 | 1648.7991 | 15 | 0.73 | 825.4037 | 13.53 | 1.0749 | 1.0821 | 34989 | 5.46e2 | 1 | 1 | TRINITY\_DN691\_c0\_g1\_i2.p1:TRINITY\_DN691\_c0\_g1\_i1.p1 | Carbamidomethylation |  | DB Search |
| APTHILNPANKR | 43.8 | 1330.7469 | 12 | 0.61 | 666.3782 | 11.23 | 1.0057 | 1.013 | 21549 | 1.39e3 | 2 | 2 | TRINITY\_DN1058\_c0\_g1\_i4.p1:TRINITY\_DN1058\_c0\_g1\_i1.p1:TRINITY\_DN1058\_c0\_g1\_i3.p1:TRINITY\_DN1058\_c0\_g1\_i2.p2 |  |  | DB Search |
| DANAAVVHGSHLK | 43.8 | 1317.679 | 13 | 5.88 | 659.8477 | 10.96 | 1.0118 | 1.019 | 20131 | 0 | 0 | 0 | TRINITY\_DN262\_c0\_g1\_i1.p1 |  |  | DB Search |
| HKLEPVGC(+57.02)FKDR | 43.8 | 1484.7557 | 12 | -2.3 | 495.9225 | 11.84 | 0.8181 | 0.8254 | 25216 | 0 | 0 | 0 | TRINITY\_DN3991\_c0\_g1\_i1.p1 | Carbamidomethylation |  | DB Search |
| VLATDKDTLSSNK | 43.8 | 1390.7303 | 13 | -0.28 | 696.3691 | 11.58 | 1.0134 | 1.0206 | 23612 | 0 | 0 | 0 | TRINITY\_DN982\_c0\_g1\_i3.p1 |  |  | DB Search |
| SAVNHNIIFEGR | 43.8 | 1355.6946 | 12 | 4.46 | 678.8546 | 14.79 | 0.9731 | 0.9804 | 42450 | 7.31e2 | 1 | 1 | TRINITY\_DN1936\_c0\_g1\_i3.p1 |  |  | DB Search |
| M(+42.01)(+15.99)HPPNSAPVK | 43.8 | 1134.5492 | 10 | 4.48 | 568.2819 | 11.01 | 0.9174 | 0.9247 | 20310 | 4.98e3 | 1 | 1 | TRINITY\_DN5818\_c1\_g1\_i1.p1 | Acetylation (Protein N-term), Oxidation (M) | M1:Acetylation (Protein N-term):1000 M1:Oxidation (M):1000 | DB Search |
| SGEYKPTLGR | 43.8 | 1106.572 | 10 | 3.81 | 554.2929 | 11.95 | 0.904 | 0.9113 | 25715 | 8.34e2 | 1 | 1 | TRINITY\_DN4781\_c0\_g1\_i3.p3 |  |  | DB Search |
| EGFYVHEALAAGIVK | 43.8 | 1602.8406 | 15 | 3.49 | 802.4268 | 17.74 | 1.0861 | 1.0933 | 59699 | 0 | 0 | 0 | TRINITY\_DN602\_c0\_g1\_i4.p1:TRINITY\_DN602\_c0\_g1\_i3.p1:TRINITY\_DN602\_c0\_g1\_i1.p1 |  |  | DB Search |
| LIAFINDLHSGK | 43.8 | 1326.7295 | 12 | 3.13 | 664.3712 | 17.73 | 1.025 | 1.0323 | 59589 | 4.22e2 | 1 | 1 | TRINITY\_DN3452\_c0\_g1\_i1.p1 |  |  | DB Search |
| AYAAFLEFKDVVK | 43.79 | 1499.8024 | 13 | 3.07 | 750.9074 | 19.27 | 1.0953 | 1.1025 | 69440 | 1.07e4 | 2 | 2 | TRINITY\_DN240\_c0\_g1\_i6.p1:TRINITY\_DN240\_c0\_g1\_i1.p1:TRINITY\_DN240\_c0\_g1\_i4.p1 |  |  | DB Search |
| KYTPC(+57.02)EEK | 43.79 | 1053.4801 | 8 | 3.66 | 527.7469 | 9.73 | 0.855 | 0.8623 | 13599 | 0 | 0 | 0 | TRINITY\_DN1369\_c0\_g1\_i1.p1 | Carbamidomethylation |  | DB Search |
| LSQC(+57.02)TAASGK | 43.79 | 1021.4862 | 10 | 0.54 | 511.7484 | 9.85 | 0.8323 | 0.8396 | 13418 | 2.29e5 | 2 | 2 | TRINITY\_DN3991\_c0\_g1\_i1.p1 | Carbamidomethylation |  | DB Search |
| YSRPTPGSNNNVM(+15.99)VR | 43.79 | 1706.8158 | 15 | 0.89 | 569.9438 | 11.60 | 0.8352 | 0.8425 | 23563 | 1.16e3 | 1 | 1 | TRINITY\_DN15274\_c0\_g1\_i1.p1 | Oxidation (M) | M13:Oxidation (M):1000 | DB Search |
| LLAVTVGNNYNLDTLEK | 43.79 | 1875.9941 | 17 | -8.17 | 938.9925 | 18.79 | 1.1474 | 1.1546 | 66275 | 9.54e2 | 1 | 1 | TRINITY\_DN77\_c1\_g1\_i1.p1 |  |  | DB Search |
| NIALETLEGHQR | 43.79 | 1379.7157 | 12 | 3.84 | 690.8647 | 15.44 | 1.0166 | 1.0238 | 46287 | 6.11e2 | 1 | 1 | TRINITY\_DN1280\_c0\_g1\_i1.p1 |  |  | DB Search |
| GVHIISVGLGTVDHR | 43.79 | 1558.8579 | 15 | 0.84 | 780.4334 | 15.97 | 1.0917 | 1.0989 | 49325 | 0 | 0 | 0 | TRINITY\_DN8008\_c0\_g1\_i2.p1:TRINITY\_DN8008\_c0\_g1\_i3.p1 |  |  | DB Search |
| TDFLC(+57.02)HDSR | 43.78 | 1149.4873 | 9 | 2.13 | 575.7496 | 13.37 | 0.8684 | 0.8757 | 33932 | 8.97e2 | 1 | 1 | TRINITY\_DN413\_c0\_g1\_i4.p1:TRINITY\_DN413\_c0\_g1\_i2.p1 | Carbamidomethylation |  | DB Search |
| AMTEEILNERDTASK | 43.78 | 1706.8145 | 15 | 1.51 | 854.412 | 14.34 | 1.129 | 1.1362 | 39888 | 0 | 0 | 0 | TRINITY\_DN493\_c0\_g1\_i1.p1 |  |  | DB Search |
| A(+42.01)NPLKDIISR | 43.78 | 1167.6611 | 10 | 1.73 | 584.8362 | 18.79 | 0.9178 | 0.9251 | 66131 | 9.72e3 | 1 | 1 | TRINITY\_DN163801\_c0\_g1\_i1.p1 | Acetylation (Protein N-term) | A1:Acetylation (Protein N-term):1000 | DB Search |
| SIEAIYLGEFGTC(+57.02)K | 43.78 | 1586.765 | 14 | -3.85 | 794.3832 | 19.71 | 1.1009 | 1.1082 | 72286 | 0 | 0 | 0 | TRINITY\_DN11248\_c0\_g1\_i1.p1:TRINITY\_DN11248\_c0\_g1\_i3.p1 | Carbamidomethylation |  | DB Search |
| SGNVVLC(+57.02)LIDVHK | 43.78 | 1452.7759 | 13 | 4.56 | 727.3953 | 18.73 | 1.0391 | 1.0464 | 66055 | 4.92e3 | 2 | 2 | TRINITY\_DN3078\_c0\_g1\_i1.p1 | Carbamidomethylation |  | DB Search |
| IHINIVVIGHVDSGK | 43.78 | 1599.9097 | 15 | -1.01 | 800.9577 | 16.73 | 1.1001 | 1.1074 | 53733 | 6.87e2 | 2 | 2 | TRINITY\_DN64\_c0\_g1\_i1.p1 |  |  | DB Search |
| SPYHVSVTPAADR | 43.78 | 1398.6892 | 13 | 6.42 | 700.3532 | 13.00 | 0.9868 | 0.9941 | 31744 | 1.26e3 | 1 | 1 | TRINITY\_DN941\_c0\_g1\_i9.p1 |  |  | DB Search |
| AVFPSIVGRPR | 43.77 | 1197.6982 | 11 | 1.95 | 599.8549 | 15.90 | 0.959 | 0.9662 | 48470 | 4.43e4 | 1 | 1 | TRINITY\_DN161\_c0\_g1\_i1.p1:TRINITY\_DN161\_c0\_g1\_i3.p1:TRINITY\_DN161\_c0\_g1\_i2.p1 |  |  | DB Search |
| SC(+57.02)ASDFSAER | 43.77 | 1128.4506 | 10 | -0.21 | 565.2299 | 12.29 | 0.8546 | 0.8619 | 27814 | 0 | 0 | 0 | TRINITY\_DN4595\_c0\_g1\_i4.p1:TRINITY\_DN4595\_c0\_g1\_i10.p2 | Carbamidomethylation |  | DB Search |
| ASFVTDIDRLR | 43.77 | 1291.6885 | 11 | 1.29 | 646.8494 | 17.27 | 0.99 | 0.9973 | 56790 | 1.44e3 | 1 | 1 | TRINITY\_DN598\_c1\_g1\_i2.p1 |  |  | DB Search |
| LPNFFQYAGHIK | 43.77 | 1433.7455 | 12 | 1.54 | 478.9211 | 19.36 | 0.7567 | 0.764 | 70118 | 6.21e3 | 2 | 2 | TRINITY\_DN1199\_c0\_g1\_i1.p1:TRINITY\_DN1199\_c0\_g1\_i2.p1 |  |  | DB Search |
| ALLSLEYHEKR | 43.77 | 1357.7354 | 11 | -1.31 | 679.871 | 14.63 | 1.0283 | 1.0355 | 41504 | 1.16e3 | 1 | 1 | TRINITY\_DN8707\_c0\_g1\_i2.p1 |  |  | DB Search |
| DEDHAAR | 43.77 | 812.3412 | 7 | -12.56 | 407.171 | 9.29 | 0.7665 | 0.7738 | 11855 | 1.9e2 | 1 | 1 | TRINITY\_DN124859\_c0\_g1\_i1.p3 |  |  | DB Search |
| SPVGANLHEIR | 43.77 | 1191.636 | 11 | -2.1 | 596.8214 | 13.27 | 0.9533 | 0.9606 | 33347 | 6.88e2 | 1 | 1 | TRINITY\_DN32270\_c0\_g1\_i1.p2 |  |  | DB Search |
| ATETATGLATTHPIK | 43.77 | 1510.7991 | 15 | -0.08 | 756.4034 | 13.06 | 1.1041 | 1.1114 | 32134 | 2.41e3 | 1 | 1 | TRINITY\_DN165536\_c0\_g1\_i1.p1 |  |  | DB Search |
| TPNEVEFHQAVK | 43.77 | 1397.6938 | 12 | -4.4 | 699.848 | 13.04 | 0.9904 | 0.9977 | 32035 | 1.84e3 | 2 | 2 | TRINITY\_DN360\_c0\_g1\_i1.p1 |  |  | DB Search |
| GSC(+57.02)SC(+57.02)YGHAEQC(+57.02)IPTK | 43.77 | 1853.7495 | 16 | -0.77 | 618.9205 | 12.43 | 0.855 | 0.8623 | 28624 | 3.91e2 | 1 | 1 | TRINITY\_DN15274\_c0\_g1\_i1.p1 | Carbamidomethylation, Carbamidomethylation, Carbamidomethylation |  | DB Search |
| ATGC(+57.02)EVIWNQHNR | 43.77 | 1583.7263 | 13 | 0.77 | 792.8675 | 14.03 | 1.0805 | 1.0877 | 37976 | 0 | 0 | 0 | TRINITY\_DN2217\_c0\_g1\_i3.p1:TRINITY\_DN2217\_c0\_g1\_i2.p1 | Carbamidomethylation |  | DB Search |
| AISSASGPTGM(+15.99)TSK | 43.77 | 1309.6184 | 14 | 1.88 | 655.8148 | 10.56 | 0.9319 | 0.9392 | 17892 | 3.72e3 | 1 | 1 | TRINITY\_DN240\_c0\_g1\_i6.p1:TRINITY\_DN240\_c0\_g1\_i1.p1:TRINITY\_DN240\_c0\_g1\_i4.p1 | Oxidation (M) | M11:Oxidation (M):1000 | DB Search |
| VDFDTTFAPVTGKK | 43.77 | 1524.7823 | 14 | 1.56 | 763.3962 | 16.78 | 1.0524 | 1.0596 | 54009 | 4.02e3 | 2 | 2 | TRINITY\_DN329\_c0\_g1\_i1.p1:TRINITY\_DN329\_c0\_g1\_i2.p1 |  |  | DB Search |
| DLQGETSGSFKR | 43.77 | 1323.6418 | 12 | 0.47 | 662.8256 | 12.73 | 0.9598 | 0.967 | 30294 | 0 | 0 | 0 | TRINITY\_DN3190\_c1\_g1\_i1.p1 |  |  | DB Search |
| TQITC(+57.02)TQISLKR | 43.76 | 1447.7817 | 12 | 4.4 | 724.8981 | 13.95 | 1.0259 | 1.0331 | 37448 | 1.26e3 | 1 | 1 | TRINITY\_DN166344\_c0\_g1\_i1.p1 | Carbamidomethylation |  | DB Search |
| TC(+57.02)ALGNDEC(+57.02)PTNSK | 43.76 | 1565.645 | 14 | 2.52 | 783.8282 | 11.67 | 1.0118 | 1.019 | 24176 | 0 | 0 | 0 | TRINITY\_DN16079\_c0\_g2\_i1.p1 | Carbamidomethylation, Carbamidomethylation |  | DB Search |
| AHVSLSTFSEDVTSLTK | 43.76 | 1820.9155 | 17 | -1.27 | 911.4598 | 18.98 | 1.1354 | 1.1426 | 67769 | 1.47e3 | 1 | 1 | TRINITY\_DN113\_c3\_g1\_i1.p1 |  |  | DB Search |
| KVSQTC(+57.02)AEHVFR | 43.76 | 1460.7194 | 12 | -0.08 | 487.9115 | 12.02 | 0.8039 | 0.8112 | 26130 | 7.24e2 | 1 | 1 | TRINITY\_DN3078\_c0\_g1\_i1.p1 | Carbamidomethylation |  | DB Search |
| AFVAIGDYGGHVGLGVK | 43.76 | 1658.8779 | 17 | 4.21 | 830.446 | 17.81 | 1.1638 | 1.171 | 60022 | 7.23e3 | 1 | 1 | TRINITY\_DN15584\_c0\_g1\_i1.p1 |  |  | DB Search |
| LEPVGC(+57.02)FKDR | 43.76 | 1219.6019 | 10 | 3.27 | 610.8075 | 13.16 | 0.9416 | 0.9489 | 32775 | 2.03e3 | 2 | 2 | TRINITY\_DN3991\_c0\_g1\_i1.p1 | Carbamidomethylation |  | DB Search |
| GVAGAGVLAGFDKFK | 43.76 | 1435.7823 | 15 | 1.27 | 718.8961 | 18.47 | 1.0865 | 1.0937 | 64267 | 2.7e3 | 1 | 1 | TRINITY\_DN1559\_c0\_g1\_i1.p1 |  |  | DB Search |
| VLLSQQIFYDKK | 43.76 | 1480.829 | 12 | 0.2 | 741.4186 | 16.98 | 1.0632 | 1.0705 | 54998 | 6.1e3 | 2 | 2 | TRINITY\_DN982\_c0\_g1\_i1.p1:TRINITY\_DN982\_c0\_g1\_i3.p1:TRINITY\_DN982\_c0\_g1\_i2.p1 |  |  | DB Search |
| LLLGDFNGDKR | 43.76 | 1246.667 | 11 | -0.7 | 624.3375 | 16.26 | 0.9832 | 0.9904 | 50917 | 2.97e3 | 1 | 1 | TRINITY\_DN413\_c0\_g1\_i4.p1:TRINITY\_DN413\_c0\_g1\_i1.p1:TRINITY\_DN413\_c0\_g1\_i2.p1:TRINITY\_DN413\_c0\_g1\_i3.p1 |  |  | DB Search |
| LINLFEAVSKK | 43.76 | 1260.7441 | 11 | -5.13 | 631.3733 | 19.09 | 0.986 | 0.9932 | 68432 | 1.56e3 | 1 | 1 | TRINITY\_DN1653\_c0\_g1\_i3.p1:TRINITY\_DN1653\_c0\_g1\_i1.p1 |  |  | DB Search |
| ANQVSSAAAAPVPAAGGDK | 43.76 | 1680.843 | 19 | -0.14 | 841.4249 | 12.55 | 1.0656 | 1.0729 | 29279 | 4.54e3 | 1 | 1 | TRINITY\_DN240\_c0\_g1\_i6.p1:TRINITY\_DN240\_c0\_g1\_i1.p1:TRINITY\_DN240\_c0\_g1\_i4.p1 |  |  | DB Search |
| AGVLAHLEEER | 43.76 | 1222.6306 | 11 | 0.61 | 612.3202 | 15.03 | 0.9541 | 0.9614 | 43865 | 8.73e2 | 1 | 1 | TRINITY\_DN428\_c0\_g1\_i1.p1 |  |  | DB Search |
| GTSNGGLVVGTIIKPK | 43.76 | 1539.8984 | 16 | -3.44 | 770.9504 | 15.91 | 1.1262 | 1.1334 | 48868 | 9.21e2 | 1 | 1 | TRINITY\_DN3036\_c0\_g1\_i3.p1:TRINITY\_DN3036\_c0\_g1\_i2.p2:TRINITY\_DN3036\_c0\_g1\_i8.p1 |  |  | DB Search |
| SSC(+57.02)KPILSVIK | 43.76 | 1230.7006 | 11 | 2.76 | 616.3565 | 15.26 | 0.9529 | 0.9602 | 45369 | 2.7e3 | 1 | 1 | TRINITY\_DN495\_c0\_g1\_i2.p1:TRINITY\_DN495\_c0\_g1\_i3.p1 | Carbamidomethylation |  | DB Search |
| TQAVLDRLNIEK | 43.76 | 1398.7831 | 12 | 6.66 | 700.4003 | 15.50 | 1.0053 | 1.0126 | 46564 | 4.13e3 | 1 | 1 | TRINITY\_DN477\_c0\_g1\_i9.p1 |  |  | DB Search |
| YSRPTPGSNNNVMVR | 43.76 | 1690.8209 | 15 | -3.2 | 564.6099 | 12.52 | 0.8299 | 0.8372 | 29054 | 3.96e2 | 1 | 1 | TRINITY\_DN15274\_c0\_g1\_i1.p1 |  |  | DB Search |
| FKADTTIADAAR | 43.76 | 1278.6567 | 12 | -3.02 | 640.3309 | 13.52 | 0.9695 | 0.9767 | 34971 | 0 | 0 | 0 | TRINITY\_DN11435\_c0\_g1\_i1.p1 |  |  | DB Search |
| TSTDKFEC(+57.02)EK | 43.76 | 1243.5391 | 10 | -3.39 | 622.7719 | 10.75 | 0.9255 | 0.9327 | 18979 | 0 | 0 | 0 | TRINITY\_DN1616\_c0\_g1\_i3.p1:TRINITY\_DN1616\_c0\_g1\_i6.p1:TRINITY\_DN1616\_c0\_g1\_i2.p1:TRINITY\_DN1616\_c0\_g1\_i7.p1 | Carbamidomethylation |  | DB Search |
| IC(+57.02)HLLGIQVTDFTK | 43.76 | 1643.8705 | 14 | -0.97 | 548.9611 | 18.66 | 0.8226 | 0.8299 | 65627 | 2.22e3 | 2 | 2 | TRINITY\_DN428\_c0\_g1\_i1.p1 | Carbamidomethylation |  | DB Search |
| SLDLKQENSNR | 43.76 | 1302.6528 | 11 | 2.34 | 652.3323 | 11.51 | 0.9509 | 0.9582 | 23115 | 2.77e3 | 1 | 1 | TRINITY\_DN8341\_c0\_g1\_i2.p1:TRINITY\_DN8341\_c0\_g1\_i1.p1 |  |  | DB Search |
| SISC(+57.02)SKDEEIK | 43.76 | 1294.6074 | 11 | 2.25 | 648.3096 | 10.92 | 0.9755 | 0.9828 | 19848 | 1.92e3 | 1 | 1 | TRINITY\_DN495\_c0\_g1\_i5.p1:TRINITY\_DN495\_c0\_g1\_i6.p1 | Carbamidomethylation |  | DB Search |
| STSTGHLIYK | 43.76 | 1105.5768 | 10 | 4.14 | 553.7955 | 12.13 | 0.8664 | 0.8737 | 26715 | 2.71e3 | 1 | 1 | TRINITY\_DN64\_c0\_g1\_i1.p1 |  |  | DB Search |
| AEFDKEVQTK | 43.76 | 1193.5928 | 10 | 0.25 | 597.8011 | 11.72 | 0.9243 | 0.9315 | 24343 | 9.56e2 | 1 | 1 | TRINITY\_DN11435\_c0\_g1\_i1.p1 |  |  | DB Search |
| S(+42.01)ATGNLVTVLRK | 43.75 | 1299.751 | 12 | 3.1 | 650.8819 | 17.29 | 1.0005 | 1.0077 | 56892 | 0 | 0 | 0 | TRINITY\_DN2963\_c0\_g1\_i1.p1 | Acetylation (Protein N-term) | S1:Acetylation (Protein N-term):1000 | DB Search |
| KC(+57.02)TVAIEC(+57.02)K | 43.75 | 1107.5416 | 9 | 3.67 | 554.7776 | 11.08 | 0.8793 | 0.8866 | 20850 | 0 | 0 | 0 | TRINITY\_DN72625\_c0\_g1\_i1.p1 | Carbamidomethylation, Carbamidomethylation |  | DB Search |
| LEPVGC(+57.02)FK | 43.75 | 948.4739 | 8 | -18.5 | 949.4594 | 14.04 | 1.2857 | 1.2929 | 38072 | 3.42e2 | 1 | 1 | TRINITY\_DN3991\_c0\_g1\_i1.p1 | Carbamidomethylation |  | DB Search |
| AKLSQC(+57.02)TAASGK | 43.75 | 1220.6183 | 12 | 9.93 | 407.8823 | 9.74 | 0.7033 | 0.7106 | 13580 | 6.95e3 | 2 | 2 | TRINITY\_DN3991\_c0\_g1\_i1.p1 | Carbamidomethylation |  | DB Search |
| VTGHAGIQLK | 43.75 | 1022.5872 | 10 | -0.65 | 512.2983 | 13.00 | 0.8773 | 0.8846 | 31972 | 0 | 0 | 0 | TRINITY\_DN73067\_c0\_g1\_i1.p1 |  |  | DB Search |
| GTGIVAAGVSKK | 43.75 | 1086.6396 | 12 | 7.69 | 544.3289 | 11.37 | 0.9129 | 0.9202 | 22492 | 0 | 0 | 0 | TRINITY\_DN15584\_c0\_g1\_i1.p1 |  |  | DB Search |
| TIESEAAHGTVTR | 43.75 | 1370.679 | 13 | 3.55 | 686.3461 | 12.00 | 1.0174 | 1.0246 | 26077 | 5.62e2 | 1 | 1 | TRINITY\_DN3271\_c0\_g1\_i1.p1:TRINITY\_DN3271\_c0\_g1\_i2.p1 |  |  | DB Search |
| AVIFDRFR | 43.75 | 1022.5661 | 8 | 5.93 | 512.2911 | 16.16 | 0.87 | 0.8773 | 50308 | 2.24e3 | 1 | 1 | TRINITY\_DN98420\_c0\_g1\_i1.p1 |  |  | DB Search |
| SQSAFPTRR | 43.75 | 1048.5414 | 9 | 2.01 | 525.2767 | 11.08 | 0.8526 | 0.8599 | 20720 | 4.77e3 | 1 | 1 | TRINITY\_DN477\_c0\_g1\_i8.p1:TRINITY\_DN477\_c0\_g1\_i7.p1 |  |  | DB Search |
| IAVFDHISSATAVK | 43.74 | 1457.7878 | 14 | -4.3 | 729.8948 | 16.25 | 1.0218 | 1.0291 | 50841 | 1.56e3 | 1 | 1 | TRINITY\_DN144181\_c0\_g1\_i1.p1 |  |  | DB Search |
| K(+42.01)LSAEVANGR | 43.74 | 1085.5829 | 10 | -2.08 | 543.7952 | 13.04 | 0.8733 | 0.8806 | 32177 | 0 | 0 | 0 | TRINITY\_DN6397\_c0\_g2\_i4.p1:TRINITY\_DN6397\_c0\_g2\_i3.p1 | Acetylation (Protein N-term) | K1:Acetylation (Protein N-term):1000 | DB Search |
| TGDNTVM(+15.99)GR | 43.74 | 965.4236 | 9 | 2.88 | 483.7183 | 9.33 | 0.777 | 0.7844 | 11865 | 5.26e2 | 1 | 1 | TRINITY\_DN262\_c0\_g1\_i1.p1 | Oxidation (M) | M7:Oxidation (M):1000 | DB Search |
| LRPGVELTMSSLVNAK | 43.74 | 1713.9447 | 16 | 1.1 | 572.3203 | 17.93 | 0.9113 | 0.9186 | 60853 | 1.13e4 | 3 | 3 | TRINITY\_DN329\_c0\_g1\_i1.p1:TRINITY\_DN329\_c0\_g1\_i2.p1 |  |  | DB Search |
| YVAIYPSDHLTVNR | 43.74 | 1646.8417 | 14 | -4.41 | 824.4208 | 15.73 | 1.1094 | 1.1166 | 47842 | 6.9e2 | 1 | 1 | TRINITY\_DN982\_c0\_g1\_i1.p1:TRINITY\_DN982\_c0\_g1\_i3.p1 |  |  | DB Search |
| LLEFVGHDVLR | 43.74 | 1296.719 | 11 | 5.97 | 649.3677 | 17.98 | 1.0057 | 1.013 | 61273 | 0 | 0 | 0 | TRINITY\_DN464\_c0\_g1\_i4.p1 |  |  | DB Search |
| IGKPHTVPC(+57.02)K | 43.74 | 1135.6172 | 10 | 9.46 | 379.5482 | 9.79 | 0.7624 | 0.7697 | 13817 | 1.7e3 | 1 | 1 | TRINITY\_DN15584\_c0\_g1\_i1.p1 | Carbamidomethylation |  | DB Search |
| AFNKVETR | 43.74 | 963.5137 | 8 | 5.69 | 482.7647 | 10.62 | 0.8299 | 0.8372 | 18171 | 1.3e3 | 1 | 1 | TRINITY\_DN4595\_c0\_g1\_i7.p1:TRINITY\_DN98968\_c0\_g1\_i1.p1:TRINITY\_DN8597\_c0\_g1\_i1.p1:TRINITY\_DN4595\_c0\_g1\_i4.p1:TRINITY\_DN4595\_c0\_g1\_i10.p2 |  |  | DB Search |
| AHVAQLC(+57.02)EK | 43.74 | 1054.5229 | 9 | 0.64 | 528.2667 | 10.78 | 0.8603 | 0.8676 | 19105 | 9.91e2 | 1 | 1 | TRINITY\_DN2113\_c0\_g1\_i1.p1:TRINITY\_DN2113\_c0\_g1\_i2.p1 | Carbamidomethylation |  | DB Search |
| TVWAMTQAEFSAK | 43.74 | 1468.702 | 13 | 1.05 | 735.3558 | 18.57 | 1.0464 | 1.0536 | 65125 | 0 | 0 | 0 | TRINITY\_DN81944\_c0\_g1\_i1.p1 |  |  | DB Search |
| TLDFVVELSK | 43.74 | 1149.6281 | 10 | -2.5 | 575.8173 | 19.63 | 0.8874 | 0.8947 | 71820 | 0 | 0 | 0 | TRINITY\_DN138\_c0\_g1\_i1.p1 |  |  | DB Search |
| VIM(+15.99)VTGDHPITAK | 43.74 | 1396.7384 | 13 | 2.18 | 699.3749 | 13.28 | 1.023 | 1.0303 | 33438 | 7.1e2 | 1 | 1 | TRINITY\_DN262\_c0\_g1\_i1.p1 | Oxidation (M) | M3:Oxidation (M):1000 | DB Search |
| IGAAAQALADQSYPFLK | 43.73 | 1762.9253 | 17 | 0.62 | 882.4666 | 19.27 | 1.1574 | 1.1646 | 68962 | 5.17e3 | 2 | 2 | TRINITY\_DN240\_c0\_g1\_i6.p1:TRINITY\_DN240\_c0\_g1\_i1.p1:TRINITY\_DN240\_c0\_g1\_i4.p1 |  |  | DB Search |
| TILQFEADRR | 43.73 | 1247.6622 | 10 | 4.77 | 624.8386 | 15.30 | 0.9501 | 0.9574 | 45395 | 1.54e3 | 1 | 1 | TRINITY\_DN58867\_c0\_g1\_i2.p1 |  |  | DB Search |
| ATNPDVEK | 43.73 | 872.424 | 8 | 1.03 | 437.2177 | 9.75 | 0.7937 | 0.801 | 13513 | 8.88e3 | 1 | 1 | TRINITY\_DN72625\_c0\_g1\_i1.p1 |  |  | DB Search |
| AM(+15.99)AAEAESSR | 43.73 | 1037.4447 | 10 | 3.58 | 519.7292 | 9.13 | 0.8177 | 0.825 | 11333 | 3.21e2 | 1 | 1 | TRINITY\_DN909\_c0\_g1\_i1.p1 | Oxidation (M) | M2:Oxidation (M):1000 | DB Search |
| LIYENQNTIHHVVR | 43.73 | 1734.9165 | 14 | 3.14 | 579.312 | 13.35 | 0.8441 | 0.8514 | 33933 | 4.2e2 | 1 | 1 | TRINITY\_DN26249\_c0\_g1\_i1.p1 |  |  | DB Search |
| APIGQSIVTVSAR | 43.72 | 1297.7354 | 13 | 0.4 | 649.8723 | 14.27 | 0.9707 | 0.9779 | 39470 | 0 | 0 | 0 | TRINITY\_DN982\_c0\_g1\_i3.p1 |  |  | DB Search |
| RYGQAMC(+57.02)QFMR | 43.72 | 1446.6318 | 11 | -7.64 | 724.3145 | 17.46 | 0.988 | 0.9953 | 57985 | 5.65e2 | 1 | 1 | TRINITY\_DN286\_c0\_g1\_i4.p1 | Carbamidomethylation |  | DB Search |
| IGLGNIRDPSISLTR | 43.72 | 1610.9104 | 15 | 2.12 | 537.9761 | 17.42 | 0.8019 | 0.8092 | 57662 | 1.88e3 | 1 | 1 | TRINITY\_DN11522\_c0\_g1\_i1.p1:TRINITY\_DN11522\_c0\_g1\_i2.p1:TRINITY\_DN11522\_c0\_g1\_i4.p1:TRINITY\_DN11522\_c0\_g1\_i3.p1 |  |  | DB Search |
| LDLAGRDLTDYLMK | 43.72 | 1622.8337 | 14 | -2.1 | 541.9483 | 19.46 | 0.7998 | 0.8071 | 70651 | 1.99e3 | 2 | 2 | TRINITY\_DN161\_c0\_g1\_i1.p1:TRINITY\_DN161\_c0\_g1\_i4.p1:TRINITY\_DN161\_c0\_g1\_i3.p1:TRINITY\_DN161\_c0\_g1\_i2.p1 |  |  | DB Search |
| ALQDEDEDAFKR | 43.71 | 1435.658 | 12 | 7.39 | 718.8383 | 13.32 | 0.9957 | 1.0029 | 33731 | 1.03e3 | 1 | 1 | TRINITY\_DN116\_c0\_g1\_i1.p1 |  |  | DB Search |
| S(+42.01)SGEIESLRQEAENLR | 43.71 | 1858.9021 | 16 | 1.2 | 930.4553 | 18.91 | 1.1973 | 1.2045 | 67059 | 4.17e3 | 1 | 1 | TRINITY\_DN2472\_c0\_g1\_i1.p1 | Acetylation (Protein N-term) | S1:Acetylation (Protein N-term):1000 | DB Search |
| DLHIIEDYVLK | 43.71 | 1356.7289 | 11 | -2.47 | 679.367 | 19.75 | 0.9969 | 1.0041 | 72430 | 4.44e2 | 1 | 1 | TRINITY\_DN8903\_c0\_g1\_i1.p1 |  |  | DB Search |
| STSQGQALTK | 43.71 | 1019.5247 | 10 | 7.68 | 510.7713 | 10.06 | 0.8291 | 0.8364 | 15179 | 0 | 0 | 0 | TRINITY\_DN38657\_c0\_g1\_i3.p1:TRINITY\_DN38657\_c0\_g1\_i5.p1:TRINITY\_DN38657\_c0\_g1\_i4.p1:TRINITY\_DN38657\_c0\_g1\_i2.p1 |  |  | DB Search |
| VLVHTQQK | 43.71 | 951.5502 | 8 | -3.93 | 476.7784 | 9.59 | 0.8291 | 0.8364 | 12948 | 0 | 0 | 0 | TRINITY\_DN6920\_c0\_g1\_i1.p1:TRINITY\_DN6920\_c0\_g1\_i3.p1:TRINITY\_DN6920\_c0\_g1\_i4.p1:TRINITY\_DN6920\_c0\_g1\_i2.p1 |  |  | DB Search |
| AGM(+15.99)THIVR | 43.71 | 899.4647 | 8 | 5.69 | 450.7402 | 9.78 | 0.8161 | 0.8234 | 13845 | 0 | 0 | 0 | TRINITY\_DN2799\_c0\_g1\_i3.p1:TRINITY\_DN3448\_c0\_g1\_i7.p2:TRINITY\_DN6920\_c0\_g1\_i3.p1:TRINITY\_DN2799\_c0\_g1\_i1.p1:TRINITY\_DN3448\_c0\_g1\_i9.p3:TRINITY\_DN3448\_c0\_g2\_i1.p1:TRINITY\_DN3448\_c0\_g1\_i5.p2:TRINITY\_DN5906\_c3\_g1\_i1.p2:TRINITY\_DN32727\_c0\_g1\_i2.p1:TRINITY\_DN3448\_c0\_g2\_i3.p2:TRINITY\_DN6920\_c0\_g1\_i2.p1:TRINITY\_DN2799\_c0\_g1\_i2.p1:TRINITY\_DN2799\_c0\_g1\_i4.p2 | Oxidation (M) | M3:Oxidation (M):1000 | DB Search |
| YTGAC(+57.02)NANGNTDK | 43.7 | 1384.5677 | 13 | 5.35 | 693.2917 | 9.59 | 0.9287 | 0.936 | 12941 | 0 | 0 | 0 | TRINITY\_DN4595\_c0\_g1\_i10.p1:TRINITY\_DN4595\_c0\_g1\_i4.p1 | Carbamidomethylation |  | DB Search |
| V(+42.01)LLHSLK | 43.7 | 850.5276 | 7 | -3.41 | 426.2677 | 18.85 | 0.8266 | 0.8339 | 67000 | 3.43e2 | 1 | 1 | TRINITY\_DN97639\_c0\_g1\_i1.p1 | Acetylation (Protein N-term) | V1:Acetylation (Protein N-term):1000 | DB Search |
| IGC(+57.02)FHDR | 43.69 | 903.4021 | 7 | 2.82 | 452.7076 | 11.35 | 0.814 | 0.8214 | 22258 | 5.3e2 | 1 | 1 | TRINITY\_DN138\_c0\_g1\_i1.p1 | Carbamidomethylation |  | DB Search |
| KTTELTNNLK | 43.69 | 1160.6401 | 10 | 0.29 | 581.3249 | 11.75 | 0.915 | 0.9222 | 24554 | 2.48e3 | 1 | 1 | TRINITY\_DN72625\_c0\_g1\_i1.p1 |  |  | DB Search |
| ETC(+57.02)SDIEKR | 43.68 | 1136.5132 | 9 | 2.96 | 569.263 | 10.13 | 0.8895 | 0.8968 | 15523 | 2.26e3 | 1 | 1 | TRINITY\_DN118\_c1\_g1\_i3.p1:TRINITY\_DN118\_c1\_g1\_i5.p1 | Carbamidomethylation |  | DB Search |
| VAHAGLTVAEFFR | 43.68 | 1416.7513 | 13 | 2.73 | 473.2569 | 19.14 | 0.7502 | 0.7575 | 68636 | 3.46e2 | 1 | 1 | TRINITY\_DN11097\_c0\_g1\_i1.p1:TRINITY\_DN11097\_c0\_g2\_i1.p1 |  |  | DB Search |
| SKDYTITSVGGR | 43.68 | 1282.6517 | 12 | 1.14 | 642.331 | 13.00 | 0.9307 | 0.938 | 31872 | 1.2e3 | 1 | 1 | TRINITY\_DN6390\_c0\_g1\_i1.p1 |  |  | DB Search |
| GLMVAAMDLDKK | 43.68 | 1290.6676 | 12 | 2.7 | 646.3399 | 16.78 | 0.9985 | 1.0057 | 53932 | 3.84e2 | 1 | 1 | TRINITY\_DN1043\_c0\_g1\_i2.p1 |  |  | DB Search |
| TPNGIHGPVNTR | 43.67 | 1261.6527 | 12 | -0.19 | 631.8307 | 11.57 | 0.9372 | 0.9444 | 23558 | 0 | 0 | 0 | TRINITY\_DN9413\_c0\_g1\_i1.p1 |  |  | DB Search |
| LIQYIENKPGLTR | 43.67 | 1543.8722 | 13 | 1.61 | 772.9412 | 14.92 | 1.0773 | 1.0845 | 43262 | 1.11e3 | 1 | 1 | TRINITY\_DN2028\_c0\_g1\_i1.p1 |  |  | DB Search |
| EAAAQIVVEIIKR | 43.67 | 1438.8507 | 13 | 1.9 | 720.4308 | 19.26 | 1.0524 | 1.0596 | 69475 | 0 | 0 | 0 | TRINITY\_DN71276\_c0\_g1\_i1.p1 |  |  | DB Search |
| TC(+57.02)TNPEPK | 43.67 | 945.4225 | 8 | 1.26 | 473.717 | 9.52 | 0.7734 | 0.7807 | 12535 | 1.14e4 | 2 | 2 | TRINITY\_DN875\_c0\_g1\_i1.p1:TRINITY\_DN875\_c0\_g1\_i3.p1:TRINITY\_DN875\_c0\_g1\_i2.p1 | Carbamidomethylation |  | DB Search |
| TPGLVEGTLILHQLR | 43.67 | 1645.9515 | 15 | 1.25 | 549.656 | 19.04 | 0.8611 | 0.8684 | 67992 | 4.54e2 | 1 | 1 | TRINITY\_DN3034\_c0\_g1\_i1.p1 |  |  | DB Search |
| VNQAC(+57.02)VR | 43.66 | 845.4178 | 7 | 1.37 | 423.7148 | 9.80 | 0.7644 | 0.7718 | 13832 | 6.46e3 | 1 | 1 | TRINITY\_DN160\_c0\_g1\_i2.p1:TRINITY\_DN160\_c0\_g1\_i1.p1 | Carbamidomethylation |  | DB Search |
| A(+42.01)TVVDYPDFDAQADAETLRK | 43.66 | 2266.0752 | 20 | 1.77 | 1134.0419 | 18.50 | 1.2877 | 1.2948 | 64568 | 0 | 0 | 0 | TRINITY\_DN3190\_c1\_g1\_i1.p1 | Acetylation (Protein N-term) | A1:Acetylation (Protein N-term):1000 | DB Search |
| YLFDLESNC(+57.02)DK | 43.66 | 1402.6074 | 11 | -1.67 | 702.3067 | 18.46 | 0.9872 | 0.9945 | 64425 | 0 | 0 | 0 | TRINITY\_DN6390\_c0\_g1\_i1.p1 | Carbamidomethylation |  | DB Search |
| A(+42.01)TVVDYPDFDAQADAETLR | 43.66 | 2137.9802 | 19 | -0.79 | 1069.9918 | 19.84 | 1.2364 | 1.2436 | 72906 | 6.34e2 | 1 | 1 | TRINITY\_DN3190\_c1\_g1\_i1.p1 | Acetylation (Protein N-term) | A1:Acetylation (Protein N-term):1000 | DB Search |
| YTSC(+57.02)QSVTVVASDR | 43.65 | 1571.725 | 14 | -1.13 | 786.8654 | 13.49 | 1.05 | 1.0572 | 34821 | 0 | 0 | 0 | TRINITY\_DN982\_c0\_g1\_i1.p1:TRINITY\_DN982\_c0\_g1\_i3.p1 | Carbamidomethylation |  | DB Search |
| DKNILVLTLGVGPQVNR | 43.64 | 1835.0629 | 17 | 2.21 | 918.5366 | 19.60 | 1.1334 | 1.1406 | 71277 | 2.86e3 | 1 | 1 | TRINITY\_DN477\_c0\_g1\_i8.p1:TRINITY\_DN477\_c0\_g1\_i7.p1 |  |  | DB Search |
| ILQLEEEFIKK | 43.63 | 1388.7915 | 11 | -1.71 | 463.9349 | 17.91 | 0.7489 | 0.7563 | 60751 | 4.87e2 | 1 | 1 | TRINITY\_DN120478\_c0\_g1\_i1.p1 |  |  | DB Search |
| LLAQTTLRNELGTK | 43.63 | 1556.8885 | 14 | -0.19 | 779.4479 | 15.37 | 1.1098 | 1.117 | 45889 | 3.33e2 | 1 | 1 | TRINITY\_DN5818\_c1\_g1\_i2.p1:TRINITY\_DN41662\_c0\_g1\_i2.p1:TRINITY\_DN5818\_c1\_g1\_i1.p1:TRINITY\_DN909\_c0\_g1\_i1.p1 |  |  | DB Search |
| NM(+15.99)M(+15.99)AAC(+57.02)DPR | 43.62 | 1096.41 | 9 | -0.29 | 549.2097 | 9.58 | 0.8615 | 0.8688 | 12888 | 5.74e2 | 1 | 1 | TRINITY\_DN1910\_c0\_g1\_i1.p1:TRINITY\_DN1910\_c0\_g1\_i5.p1:TRINITY\_DN3986\_c0\_g1\_i1.p1 | Oxidation (M), Oxidation (M), Carbamidomethylation | M2:Oxidation (M):1000 M3:Oxidation (M):1000 | DB Search |
| YGAVNDNC(+57.02)ER | 43.62 | 1196.488 | 10 | -2.06 | 599.2474 | 10.92 | 0.8834 | 0.8907 | 19854 | 4.23e2 | 1 | 1 | TRINITY\_DN1616\_c0\_g1\_i3.p1:TRINITY\_DN1616\_c0\_g1\_i6.p1:TRINITY\_DN1616\_c0\_g1\_i2.p1:TRINITY\_DN1616\_c0\_g1\_i7.p1 | Carbamidomethylation |  | DB Search |
| A(+42.01)HLSQEQR | 43.62 | 1009.4941 | 8 | 6.92 | 505.7556 | 11.01 | 0.8708 | 0.8781 | 20435 | 0 | 0 | 0 | TRINITY\_DN3052\_c0\_g1\_i1.p1 | Acetylation (Protein N-term) | A1:Acetylation (Protein N-term):1000 | DB Search |
| GNHEC(+57.02)ASINR | 43.61 | 1156.5044 | 10 | 15.1 | 386.5128 | 9.81 | 0.7286 | 0.7359 | 14029 | 0 | 0 | 0 | TRINITY\_DN1355\_c0\_g1\_i1.p1:TRINITY\_DN8458\_c0\_g2\_i1.p1:TRINITY\_DN73788\_c0\_g1\_i1.p1 | Carbamidomethylation |  | DB Search |
| LVGQSIISYLQK | 43.61 | 1347.7761 | 12 | 0.16 | 674.8925 | 19.77 | 0.9804 | 0.9876 | 72610 | 0 | 0 | 0 | TRINITY\_DN187147\_c0\_g1\_i1.p1 |  |  | DB Search |
| ALADYIHSQDLK | 43.6 | 1372.6986 | 12 | 0.78 | 687.3541 | 15.82 | 1.0061 | 1.0134 | 48440 | 7.24e2 | 1 | 1 | TRINITY\_DN1819\_c0\_g1\_i5.p1:TRINITY\_DN1819\_c0\_g1\_i7.p1 |  |  | DB Search |
| YTNQETGEINDVVSYVPK | 43.6 | 2054.9797 | 18 | -0.78 | 1028.4917 | 18.22 | 1.1989 | 1.2061 | 62789 | 1.96e2 | 1 | 1 | TRINITY\_DN1668\_c0\_g1\_i1.p1 |  |  | DB Search |
| DANSM(+15.99)PTQSR | 43.59 | 1121.4772 | 10 | 5.25 | 561.7463 | 8.93 | 0.8201 | 0.8274 | 10974 | 0 | 0 | 0 | TRINITY\_DN477\_c0\_g1\_i9.p1 | Oxidation (M) | M5:Oxidation (M):1000 | DB Search |
| VVKPGEEATFR | 43.59 | 1231.656 | 11 | 2.64 | 616.8342 | 12.47 | 0.9315 | 0.9388 | 28694 | 1.17e3 | 2 | 2 | TRINITY\_DN310\_c0\_g1\_i1.p1 |  |  | DB Search |
| SYFEHSFVDLHR | 43.58 | 1535.7157 | 12 | 3.1 | 512.9118 | 16.20 | 0.7681 | 0.7754 | 50655 | 3.97e2 | 1 | 1 | TRINITY\_DN59572\_c0\_g1\_i1.p1 |  |  | DB Search |
| AC(+57.02)SNVNQC(+57.02)GVNTGTQTVYK | 43.58 | 2099.9365 | 19 | 4.79 | 1050.9758 | 12.97 | 1.1794 | 1.1865 | 31687 | 0 | 0 | 0 | TRINITY\_DN8597\_c0\_g1\_i1.p1 | Carbamidomethylation, Carbamidomethylation |  | DB Search |
| VGINYQPPTVVPGGDLAK | 43.58 | 1823.9781 | 18 | 1.41 | 912.9935 | 17.42 | 1.1214 | 1.1286 | 57750 | 0 | 0 | 0 | TRINITY\_DN687\_c0\_g2\_i1.p1:TRINITY\_DN687\_c0\_g1\_i1.p1:TRINITY\_DN250\_c0\_g1\_i5.p1:TRINITY\_DN250\_c0\_g1\_i6.p1:TRINITY\_DN250\_c0\_g1\_i1.p1:TRINITY\_DN250\_c0\_g1\_i4.p1:TRINITY\_DN96161\_c0\_g1\_i1.p1:TRINITY\_DN250\_c0\_g1\_i3.p1 |  |  | DB Search |
| AFNKIETR | 43.58 | 977.5294 | 8 | 6.21 | 489.7728 | 11.52 | 0.8372 | 0.8445 | 23205 | 2.22e3 | 1 | 1 | TRINITY\_DN4595\_c0\_g1\_i1.p1:TRINITY\_DN4595\_c0\_g1\_i7.p1:TRINITY\_DN4595\_c0\_g1\_i4.p1 |  |  | DB Search |
| AYGQDLGQPGSC(+57.02)LK | 43.57 | 1492.698 | 14 | 1.84 | 747.3543 | 14.35 | 1.0069 | 1.0142 | 39867 | 7.2e2 | 1 | 1 | TRINITY\_DN691\_c0\_g1\_i2.p1:TRINITY\_DN691\_c0\_g1\_i1.p1 | Carbamidomethylation |  | DB Search |
| TAGDVC(+57.02)GR | 43.57 | 834.3654 | 8 | 3.61 | 418.1896 | 9.57 | 0.7518 | 0.7591 | 12879 | 0 | 0 | 0 | TRINITY\_DN495\_c0\_g1\_i5.p1:TRINITY\_DN495\_c0\_g1\_i6.p1 | Carbamidomethylation |  | DB Search |
| AC(+57.02)AELHQDVNVK | 43.57 | 1382.6613 | 12 | 2.54 | 692.3365 | 11.96 | 1.0065 | 1.0138 | 25860 | 0 | 0 | 0 | TRINITY\_DN34481\_c0\_g1\_i1.p1 | Carbamidomethylation |  | DB Search |
| DMGVNVISVGAGHTINHR | 43.56 | 1875.9374 | 18 | 12.29 | 938.9833 | 15.86 | 1.2424 | 1.2495 | 48723 | 3.08e2 | 1 | 1 | TRINITY\_DN138\_c0\_g1\_i1.p1 |  |  | DB Search |
| YIDIEYVC(+57.02)KPPVK | 43.56 | 1622.8378 | 13 | -3.07 | 812.42 | 16.34 | 1.1282 | 1.1354 | 51406 | 7.33e2 | 1 | 1 | TRINITY\_DN374\_c0\_g1\_i1.p1:TRINITY\_DN374\_c0\_g1\_i2.p1 | Carbamidomethylation |  | DB Search |
| FETQIATK | 43.56 | 936.4916 | 8 | 1.93 | 937.4965 | 13.99 | 1.2029 | 1.2101 | 37753 | 0 | 0 | 0 | TRINITY\_DN649\_c0\_g1\_i2.p1:TRINITY\_DN36\_c0\_g1\_i16.p1:TRINITY\_DN36\_c0\_g1\_i3.p1:TRINITY\_DN36\_c0\_g1\_i14.p1:TRINITY\_DN36\_c0\_g1\_i17.p1:TRINITY\_DN649\_c0\_g1\_i5.p1:TRINITY\_DN36\_c0\_g1\_i13.p1:TRINITY\_DN36\_c0\_g1\_i8.p1:TRINITY\_DN649\_c0\_g1\_i1.p1:TRINITY\_DN36\_c0\_g1\_i19.p1 |  |  | DB Search |
| KVIVDQLVTLATR | 43.56 | 1454.8821 | 13 | -1.21 | 728.4442 | 18.49 | 1.0299 | 1.0371 | 64477 | 3.86e2 | 1 | 1 | TRINITY\_DN1386\_c0\_g1\_i1.p1 |  |  | DB Search |
| YC(+57.02)ILSFLDKTDK | 43.56 | 1501.7487 | 12 | -0.54 | 501.5876 | 19.89 | 0.7966 | 0.8039 | 73398 | 0 | 0 | 0 | TRINITY\_DN15274\_c0\_g1\_i1.p1 | Carbamidomethylation |  | DB Search |
| YVKPGVNIINIR | 43.56 | 1384.8191 | 12 | 3.35 | 693.416 | 16.23 | 1.0013 | 1.0086 | 50888 | 0 | 0 | 0 | TRINITY\_DN1616\_c0\_g1\_i3.p1:TRINITY\_DN1616\_c0\_g1\_i6.p1:TRINITY\_DN1616\_c0\_g1\_i2.p1:TRINITY\_DN1616\_c0\_g1\_i7.p1 |  |  | DB Search |
| SSNTIEENRLK | 43.56 | 1289.6575 | 11 | 2.52 | 645.8348 | 11.51 | 0.9574 | 0.9646 | 23219 | 0 | 0 | 0 | TRINITY\_DN982\_c0\_g1\_i3.p1 |  |  | DB Search |
| IEDFEESPPTDPHIGPVEQR | 43.56 | 2291.0706 | 20 | 0.78 | 764.6946 | 15.83 | 0.9319 | 0.9392 | 48664 | 2.31e2 | 1 | 1 | TRINITY\_DN140702\_c0\_g1\_i1.p1 |  |  | DB Search |
| FC(+57.02)SNPEPENGGQTC(+57.02)EGEAR | 43.56 | 2137.843 | 19 | -3.54 | 1069.9202 | 12.17 | 1.159 | 1.1662 | 27091 | 7.37e1 | 1 | 1 | TRINITY\_DN138\_c0\_g1\_i1.p1 | Carbamidomethylation, Carbamidomethylation |  | DB Search |
| AGHGAVTSPQTQR | 43.55 | 1308.6534 | 13 | 3.24 | 437.2245 | 9.76 | 0.7045 | 0.7118 | 13596 | 2.79e3 | 2 | 2 | TRINITY\_DN3036\_c0\_g1\_i19.p1:TRINITY\_DN3036\_c0\_g1\_i13.p1:TRINITY\_DN3036\_c0\_g1\_i11.p1:TRINITY\_DN3036\_c0\_g1\_i3.p1:TRINITY\_DN3036\_c0\_g1\_i16.p1:TRINITY\_DN3036\_c0\_g1\_i2.p2:TRINITY\_DN3036\_c0\_g1\_i8.p1 |  |  | DB Search |
| FLNRHDLDLIC(+57.02)R | 43.55 | 1570.8038 | 12 | 5.48 | 524.6091 | 16.23 | 0.8067 | 0.814 | 50829 | 2.25e2 | 1 | 1 | TRINITY\_DN1355\_c0\_g1\_i1.p1 | Carbamidomethylation |  | DB Search |
| DFILTAAHC(+57.02)FK | 43.55 | 1321.6488 | 11 | -2.89 | 661.8268 | 18.07 | 1.0094 | 1.0166 | 61842 | 4.05e2 | 1 | 1 | TRINITY\_DN8341\_c0\_g1\_i2.p1:TRINITY\_DN8341\_c0\_g1\_i1.p1 | Carbamidomethylation |  | DB Search |
| TNNALDYEAKR | 43.54 | 1293.6313 | 11 | 0.43 | 647.8203 | 12.12 | 0.965 | 0.9723 | 26731 | 1.84e3 | 1 | 1 | TRINITY\_DN982\_c0\_g1\_i1.p1:TRINITY\_DN982\_c0\_g1\_i3.p1 |  |  | DB Search |
| VVTSSTGPGEHMR | 43.54 | 1356.6456 | 13 | 0.36 | 679.3273 | 10.92 | 0.9783 | 0.9856 | 19750 | 1.32e3 | 1 | 1 | TRINITY\_DN73067\_c0\_g1\_i1.p1 |  |  | DB Search |
| TC(+57.02)TNPAPAHGGK | 43.53 | 1209.556 | 12 | -0.8 | 605.7821 | 9.09 | 0.8558 | 0.8631 | 11293 | 0 | 0 | 0 | TRINITY\_DN1613\_c1\_g1\_i2.p1:TRINITY\_DN1613\_c1\_g1\_i1.p1 | Carbamidomethylation |  | DB Search |
| RLLTSLVQGAR | 43.53 | 1212.7302 | 11 | 2.64 | 607.3713 | 15.40 | 0.9787 | 0.986 | 46092 | 6.47e2 | 1 | 1 | TRINITY\_DN3190\_c1\_g1\_i1.p1 |  |  | DB Search |
| LM(+15.99)GQIVSSITASLR | 43.53 | 1490.8126 | 14 | 3.71 | 746.413 | 18.84 | 1.0359 | 1.0431 | 66733 | 4.18e2 | 1 | 1 | TRINITY\_DN250\_c0\_g1\_i2.p1:TRINITY\_DN250\_c0\_g1\_i3.p1 | Oxidation (M) | M2:Oxidation (M):1000 | DB Search |
| ALEHYSDIYDIKR | 43.53 | 1621.8099 | 13 | -3.67 | 541.6062 | 14.76 | 0.8238 | 0.8311 | 42332 | 0 | 0 | 0 | TRINITY\_DN2113\_c0\_g1\_i1.p1:TRINITY\_DN2113\_c0\_g1\_i2.p1 |  |  | DB Search |
| ALNQQLTNHIR | 43.53 | 1306.7106 | 11 | -3.95 | 654.3571 | 13.42 | 0.9691 | 0.9763 | 34343 | 0 | 0 | 0 | TRINITY\_DN30094\_c0\_g1\_i1.p1 |  |  | DB Search |
| YVVMLQYYHDSDTAK | 43.52 | 1831.8451 | 15 | 8.47 | 916.9335 | 16.82 | 1.1857 | 1.1929 | 54206 | 0 | 0 | 0 | TRINITY\_DN1616\_c0\_g1\_i3.p1:TRINITY\_DN1616\_c0\_g1\_i6.p1:TRINITY\_DN1616\_c0\_g1\_i2.p1:TRINITY\_DN1616\_c0\_g1\_i7.p1 |  |  | DB Search |
| GVGIISEGNETVEDIAQR | 43.52 | 1885.9381 | 18 | 0.98 | 943.973 | 17.94 | 1.1446 | 1.1518 | 61086 | 0 | 0 | 0 | TRINITY\_DN262\_c0\_g1\_i1.p1 |  |  | DB Search |
| TQAVLDRLNIEKR | 43.51 | 1554.8842 | 13 | -5.4 | 519.2969 | 14.70 | 0.8047 | 0.812 | 42040 | 0 | 0 | 0 | TRINITY\_DN477\_c0\_g1\_i9.p1 |  |  | DB Search |
| AVC(+57.02)M(+15.99)LSNTTAIAEAWAR | 43.51 | 1879.8921 | 17 | 2.8 | 940.9517 | 18.84 | 1.1454 | 1.1526 | 66760 | 4.63e2 | 1 | 1 | TRINITY\_DN687\_c0\_g2\_i1.p1:TRINITY\_DN687\_c0\_g1\_i1.p1:TRINITY\_DN250\_c0\_g1\_i5.p1:TRINITY\_DN250\_c0\_g1\_i1.p1:TRINITY\_DN250\_c0\_g1\_i3.p1 | Carbamidomethylation, Oxidation (M) | M4:Oxidation (M):1000 | DB Search |
| KFDTYLPGFIC(+57.02)R | 43.51 | 1515.7544 | 12 | -2.15 | 758.8795 | 18.78 | 1.0234 | 1.0307 | 66284 | 3.18e3 | 2 | 2 | TRINITY\_DN785\_c0\_g1\_i1.p1 | Carbamidomethylation |  | DB Search |
| AKDVTAAVKK | 43.5 | 1029.6182 | 10 | 9.39 | 515.8189 | 9.45 | 0.84 | 0.8473 | 12390 | 0 | 0 | 0 | TRINITY\_DN1189\_c0\_g1\_i1.p1 |  |  | DB Search |
| INNNQFSVVLIK | 43.5 | 1387.7823 | 12 | -5.29 | 694.8917 | 18.34 | 0.9953 | 1.0025 | 63630 | 0 | 0 | 0 | TRINITY\_DN982\_c0\_g1\_i1.p1:TRINITY\_DN982\_c0\_g1\_i2.p1 |  |  | DB Search |
| YLTTAVITNKR | 43.5 | 1278.7296 | 11 | 2.3 | 640.3707 | 14.00 | 1.0001 | 1.0073 | 37765 | 9.33e2 | 1 | 1 | TRINITY\_DN923\_c0\_g1\_i1.p1 |  |  | DB Search |
| AINIHDLVGIDTKK | 43.5 | 1535.8671 | 14 | -6.18 | 512.9575 | 16.70 | 0.7945 | 0.8019 | 53568 | 0 | 0 | 0 | TRINITY\_DN61\_c0\_g1\_i1.p1 |  |  | DB Search |
| MSEIEDLTHLAK | 43.5 | 1385.686 | 12 | 3.75 | 693.8498 | 17.42 | 1.0295 | 1.0367 | 57726 | 0 | 0 | 0 | TRINITY\_DN2636\_c0\_g1\_i2.p1:TRINITY\_DN2636\_c0\_g1\_i1.p1 |  |  | DB Search |
| VDNSSLTGESEPQSR | 43.5 | 1604.7278 | 15 | -5.74 | 803.363 | 11.96 | 1.0214 | 1.0287 | 25859 | 0 | 0 | 0 | TRINITY\_DN262\_c0\_g1\_i1.p1 |  |  | DB Search |
| DLEFKFR | 43.5 | 953.497 | 7 | -2.68 | 477.7524 | 16.87 | 0.8323 | 0.8396 | 54470 | 8.25e2 | 1 | 1 | TRINITY\_DN160\_c0\_g1\_i2.p1:TRINITY\_DN160\_c0\_g1\_i1.p1 |  |  | DB Search |
| GLQGQQGEQGSTGVQGSVGGR | 43.49 | 1985.9514 | 21 | -0.42 | 993.9781 | 12.09 | 1.1302 | 1.1374 | 26593 | 0 | 0 | 0 | TRINITY\_DN1030\_c0\_g1\_i1.p1:TRINITY\_DN1030\_c0\_g1\_i2.p1 |  |  | DB Search |
| YRLELYVDDKDHNASAK | 43.49 | 2035.9963 | 17 | 1.12 | 510.0046 | 14.55 | 0.788 | 0.7953 | 41114 | 4.93e2 | 1 | 1 | TRINITY\_DN123301\_c0\_g1\_i1.p1 |  |  | DB Search |
| LADLEEFISSPNHANISQVGER | 43.48 | 2425.1873 | 22 | 4.51 | 809.4031 | 18.95 | 0.9255 | 0.9327 | 67455 | 1.08e3 | 1 | 1 | TRINITY\_DN2113\_c0\_g1\_i1.p1:TRINITY\_DN2113\_c0\_g1\_i2.p1 |  |  | DB Search |
| AATLLGEAMNENPGYLK | 43.48 | 1790.8872 | 17 | 2.94 | 896.4495 | 19.09 | 1.1758 | 1.1829 | 68392 | 0 | 0 | 0 | TRINITY\_DN163801\_c0\_g1\_i1.p1 |  |  | DB Search |
| IHVQSFLNNHFGGK | 43.48 | 1596.816 | 14 | -0.58 | 533.2766 | 16.04 | 0.8161 | 0.8234 | 49749 | 0 | 0 | 0 | TRINITY\_DN3180\_c0\_g1\_i1.p1 |  |  | DB Search |
| VLDVNDKQPVFVK | 43.48 | 1499.8347 | 13 | 0.62 | 750.9218 | 15.15 | 1.0508 | 1.058 | 44643 | 0 | 0 | 0 | TRINITY\_DN982\_c0\_g1\_i1.p1:TRINITY\_DN982\_c0\_g1\_i2.p1 |  |  | DB Search |
| ANPFIEC(+57.02)HGR | 43.48 | 1199.5505 | 10 | 1.29 | 600.7806 | 13.31 | 0.8951 | 0.9024 | 33665 | 9.46e2 | 1 | 1 | TRINITY\_DN691\_c0\_g1\_i2.p1:TRINITY\_DN691\_c0\_g1\_i1.p1 | Carbamidomethylation |  | DB Search |
| LEDEPADHASAQADAHALFK | 43.47 | 2134.9919 | 20 | 4.16 | 712.671 | 14.89 | 0.988 | 0.9953 | 43202 | 0 | 0 | 0 | TRINITY\_DN3190\_c1\_g1\_i1.p1 |  |  | DB Search |
| LSDINDNRPIFSR | 43.47 | 1545.7899 | 13 | 1.37 | 773.8998 | 15.11 | 1.0504 | 1.0576 | 44289 | 1.35e3 | 1 | 1 | TRINITY\_DN69276\_c0\_g1\_i1.p1 |  |  | DB Search |
| SQQSNNIEHYIVK | 43.47 | 1558.7739 | 13 | 4.12 | 780.394 | 14.80 | 1.064 | 1.0713 | 42545 | 7.25e2 | 1 | 1 | TRINITY\_DN14781\_c0\_g1\_i1.p1 |  |  | DB Search |
| DLPQEKLDDILK | 43.46 | 1425.7715 | 12 | -17.28 | 713.8775 | 17.89 | 1.0391 | 1.0464 | 60733 | 0 | 0 | 0 | TRINITY\_DN262\_c0\_g1\_i1.p1 |  |  | DB Search |
| FGLEWAQK | 43.46 | 977.497 | 8 | -6.44 | 489.7505 | 17.86 | 0.8222 | 0.8295 | 60548 | 0 | 0 | 0 | TRINITY\_DN852\_c0\_g1\_i1.p1 |  |  | DB Search |
| VC(+57.02)EDVKR | 43.45 | 904.4437 | 7 | -6.38 | 453.2242 | 9.42 | 0.7697 | 0.777 | 12258 | 0 | 0 | 0 | TRINITY\_DN118\_c1\_g1\_i3.p1:TRINITY\_DN118\_c1\_g1\_i5.p1 | Carbamidomethylation |  | DB Search |
| ITIEEEYKITVEK | 43.45 | 1593.8501 | 13 | -4.67 | 797.925 | 16.62 | 1.0672 | 1.0745 | 53021 | 1.54e2 | 1 | 1 | TRINITY\_DN1366\_c0\_g1\_i1.p1 |  |  | DB Search |
| SFLLALEKDQEK | 43.45 | 1419.7609 | 12 | -1.42 | 710.8835 | 17.63 | 1.0733 | 1.0805 | 58989 | 4.13e2 | 1 | 1 | TRINITY\_DN1386\_c0\_g1\_i1.p1 |  |  | DB Search |
| AM(+15.99)AAEAEASR | 43.45 | 1021.4498 | 10 | 0.68 | 511.7303 | 9.50 | 0.8067 | 0.814 | 12520 | 4.25e2 | 1 | 1 | TRINITY\_DN5818\_c1\_g1\_i2.p1:TRINITY\_DN41662\_c0\_g1\_i2.p1:TRINITY\_DN5818\_c1\_g1\_i1.p1 | Oxidation (M) | M2:Oxidation (M):1000 | DB Search |
| ELVTAHQTFIR | 43.45 | 1313.7091 | 11 | 0.79 | 657.8594 | 14.74 | 0.9868 | 0.9941 | 42263 | 0 | 0 | 0 | TRINITY\_DN1335\_c0\_g1\_i1.p1 |  |  | DB Search |
| SPNAPALISTHGNK | 43.45 | 1405.7313 | 14 | 4.18 | 703.8727 | 13.13 | 1.0238 | 1.0311 | 32597 | 3.39e2 | 1 | 1 | TRINITY\_DN61668\_c0\_g1\_i1.p1 |  |  | DB Search |
| S(+42.01)LVIPEKFQHILR | 43.44 | 1620.9352 | 13 | -6.51 | 541.313 | 19.80 | 0.8441 | 0.8514 | 72642 | 9.41e2 | 1 | 1 | TRINITY\_DN1505\_c0\_g1\_i1.p1 | Acetylation (Protein N-term) | S1:Acetylation (Protein N-term):1000 | DB Search |
| TC(+57.02)TESDVLVIHK | 43.44 | 1400.6969 | 12 | 2.89 | 467.9055 | 14.24 | 0.7506 | 0.7579 | 39250 | 0 | 0 | 0 | TRINITY\_DN29322\_c0\_g1\_i1.p1 | Carbamidomethylation |  | DB Search |
| TSTQDVALNIRR | 43.43 | 1372.7423 | 12 | 2.89 | 687.3773 | 13.63 | 0.9997 | 1.0069 | 35508 | 2.89e3 | 1 | 1 | TRINITY\_DN982\_c0\_g1\_i1.p1:TRINITY\_DN982\_c0\_g1\_i3.p1 |  |  | DB Search |
| EASEIISESPQALQLR | 43.42 | 1769.9159 | 16 | -17.14 | 885.9461 | 18.60 | 1.1678 | 1.175 | 65266 | 0 | 0 | 0 | TRINITY\_DN5818\_c1\_g1\_i2.p1:TRINITY\_DN5818\_c1\_g1\_i1.p1 |  |  | DB Search |
| LAALDATVHGQTASR | 43.42 | 1509.7899 | 15 | -1.2 | 755.8979 | 13.58 | 1.0725 | 1.0797 | 35280 | 0 | 0 | 0 | TRINITY\_DN1378\_c0\_g1\_i1.p1 |  |  | DB Search |
| EITLNYHDTVVHMK | 43.42 | 1698.84 | 14 | 2.55 | 850.4256 | 15.46 | 1.1118 | 1.119 | 46350 | 9.07e2 | 1 | 1 | TRINITY\_DN1369\_c0\_g1\_i1.p1 |  |  | DB Search |
| SAYDIEVNTKK | 43.41 | 1266.6455 | 11 | 2.1 | 634.3286 | 13.00 | 0.9691 | 0.9763 | 31941 | 0 | 0 | 0 | TRINITY\_DN1280\_c0\_g1\_i1.p1 |  |  | DB Search |
| SDSSDIEKR | 43.41 | 1035.4833 | 9 | -11.91 | 518.7404 | 11.15 | 0.8607 | 0.868 | 21197 | 4.09e3 | 1 | 1 | TRINITY\_DN135129\_c0\_g1\_i1.p1 |  |  | DB Search |
| AELVFVNHC(+57.02)R | 43.41 | 1243.6132 | 10 | -0.25 | 622.8109 | 14.51 | 0.9582 | 0.9654 | 40866 | 4.89e2 | 1 | 1 | TRINITY\_DN101992\_c0\_g1\_i1.p1 | Carbamidomethylation |  | DB Search |
| LRLDFDHEAKLNMIFR | 43.4 | 2017.0568 | 16 | -0.24 | 505.2691 | 18.67 | 0.8623 | 0.8696 | 65767 | 0 | 0 | 0 | TRINITY\_DN160\_c0\_g1\_i2.p1:TRINITY\_DN160\_c0\_g1\_i1.p1 |  |  | DB Search |
| AFQNTPC(+57.02)THLVLDGTR | 43.39 | 1828.889 | 16 | -2.93 | 915.445 | 15.43 | 1.175 | 1.1821 | 46253 | 0 | 0 | 0 | TRINITY\_DN1091\_c0\_g1\_i1.p1 | Carbamidomethylation |  | DB Search |
| GVAQFGVLEHYIEK | 43.39 | 1588.825 | 14 | 2.88 | 795.4185 | 18.94 | 1.113 | 1.1202 | 67396 | 9.01e2 | 1 | 1 | TRINITY\_DN5229\_c0\_g1\_i1.p1 |  |  | DB Search |
| LRLDFDHEAK | 43.38 | 1242.6356 | 10 | 5.04 | 415.2194 | 14.38 | 0.7204 | 0.7278 | 40014 | 6.38e3 | 1 | 1 | TRINITY\_DN160\_c0\_g1\_i2.p1:TRINITY\_DN160\_c0\_g1\_i1.p1 |  |  | DB Search |
| LLEINVYHPLIQELQK | 43.37 | 1949.0985 | 16 | 2.77 | 650.7057 | 19.85 | 0.9852 | 0.9924 | 73141 | 0 | 0 | 0 | TRINITY\_DN5633\_c0\_g1\_i1.p1 |  |  | DB Search |
| AKIDNNLR | 43.36 | 942.5247 | 8 | 5.16 | 472.2699 | 10.71 | 0.8412 | 0.8485 | 18673 | 3.82e3 | 1 | 1 | TRINITY\_DN329\_c0\_g1\_i1.p1:TRINITY\_DN329\_c0\_g1\_i2.p1 |  |  | DB Search |
| DC(+57.02)NSNASSQTFR | 43.36 | 1385.563 | 12 | 8.68 | 693.7917 | 11.52 | 0.9549 | 0.9622 | 23277 | 2.76e2 | 1 | 1 | TRINITY\_DN1320\_c0\_g1\_i1.p1 | Carbamidomethylation |  | DB Search |
| YIQGENEKQEK | 43.36 | 1364.6572 | 11 | 1.86 | 455.8918 | 9.61 | 0.7652 | 0.7726 | 13062 | 0 | 0 | 0 | TRINITY\_DN1272\_c0\_g1\_i1.p1 |  |  | DB Search |
| LDFDHEAKLNMIFR | 43.36 | 1747.8716 | 14 | -6.24 | 583.6249 | 18.55 | 0.8562 | 0.8635 | 64942 | 0 | 0 | 0 | TRINITY\_DN160\_c0\_g1\_i2.p1:TRINITY\_DN160\_c0\_g1\_i1.p1 |  |  | DB Search |
| LVQNTPHNSIVTK | 43.35 | 1449.7939 | 13 | 1.92 | 725.9024 | 11.76 | 1.0564 | 1.0636 | 24648 | 1.37e3 | 1 | 1 | TRINITY\_DN982\_c0\_g1\_i1.p1:TRINITY\_DN982\_c0\_g1\_i3.p1 |  |  | DB Search |
| G(+42.01)C(+57.02)YIHTQEVAR | 43.35 | 1374.635 | 11 | 1.87 | 688.323 | 14.11 | 0.9876 | 0.9949 | 38559 | 0 | 0 | 0 | TRINITY\_DN3202\_c2\_g1\_i1.p1 | Acetylation (Protein N-term), Carbamidomethylation | G1:Acetylation (Protein N-term):1000 | DB Search |
| VTRPHGASGVVR | 43.35 | 1234.6895 | 12 | 2.89 | 412.5698 | 10.09 | 0.8173 | 0.8246 | 15387 | 0 | 0 | 0 | TRINITY\_DN2885\_c0\_g1\_i2.p1:TRINITY\_DN2885\_c0\_g1\_i5.p1:TRINITY\_DN2885\_c0\_g1\_i4.p1 |  |  | DB Search |
| ANKEFQEIVQNTK | 43.35 | 1547.7943 | 13 | 0.49 | 774.9014 | 14.65 | 1.0676 | 1.0749 | 41627 | 1.61e2 | 1 | 1 | TRINITY\_DN4726\_c0\_g1\_i1.p1 |  |  | DB Search |
| LGPDQVDVAGAGR | 43.34 | 1253.6364 | 13 | -3.54 | 627.8204 | 13.70 | 0.9574 | 0.9646 | 36071 | 9.3e2 | 1 | 1 | TRINITY\_DN97809\_c0\_g1\_i1.p1 |  |  | DB Search |
| FVLHGDVSHGSR | 43.34 | 1309.6527 | 12 | -0.43 | 437.556 | 12.14 | 0.762 | 0.7693 | 26936 | 0 | 0 | 0 | TRINITY\_DN22\_c0\_g1\_i1.p1:TRINITY\_DN384\_c0\_g1\_i2.p1:TRINITY\_DN384\_c0\_g1\_i1.p1 |  |  | DB Search |
| NYNNDDEPGAKK | 43.33 | 1363.6003 | 12 | 3 | 455.5401 | 9.65 | 0.7498 | 0.7571 | 13197 | 5.44e2 | 1 | 1 | TRINITY\_DN199\_c0\_g1\_i1.p1 |  |  | DB Search |
| AIGGIILTASHNPGGK | 43.33 | 1504.8362 | 16 | -2.08 | 753.4204 | 15.70 | 1.0941 | 1.1013 | 47749 | 2.83e2 | 1 | 1 | TRINITY\_DN3589\_c0\_g1\_i1.p1 |  |  | DB Search |
| AVFC(+57.02)EHQGVK | 43.32 | 1173.5601 | 10 | -0.85 | 587.7842 | 11.69 | 0.898 | 0.9052 | 24238 | 1.92e3 | 1 | 1 | TRINITY\_DN875\_c0\_g1\_i1.p1:TRINITY\_DN875\_c0\_g1\_i3.p1:TRINITY\_DN875\_c0\_g1\_i2.p1 | Carbamidomethylation |  | DB Search |
| SGVC(+57.02)TDANTR | 43.32 | 1079.4666 | 10 | 12.92 | 540.7451 | 10.11 | 0.8445 | 0.8518 | 15427 | 0 | 0 | 0 | TRINITY\_DN942\_c0\_g1\_i5.p1:TRINITY\_DN942\_c0\_g1\_i2.p1:TRINITY\_DN942\_c0\_g1\_i3.p1 | Carbamidomethylation |  | DB Search |
| TMPEQILNERDPLNK | 43.31 | 1796.9091 | 15 | 19.81 | 899.4756 | 16.61 | 1.1386 | 1.1458 | 53016 | 0 | 0 | 0 | TRINITY\_DN785\_c0\_g1\_i1.p1 |  |  | DB Search |
| LTNNKDIVIAK | 43.3 | 1227.7186 | 11 | 1.94 | 614.8651 | 12.42 | 0.9804 | 0.9876 | 28473 | 4.91e2 | 1 | 1 | TRINITY\_DN79\_c1\_g1\_i1.p1 |  |  | DB Search |
| ISNVQDIVPVLELANQHK | 43.29 | 2016.1003 | 18 | 0.64 | 673.0381 | 19.80 | 0.9513 | 0.9586 | 72376 | 3.06e3 | 1 | 1 | TRINITY\_DN118520\_c0\_g1\_i1.p1 |  |  | DB Search |
| VISFDVPPQEILTR | 43.29 | 1612.8824 | 14 | -5.21 | 807.4407 | 19.59 | 1.06 | 1.0672 | 71552 | 0 | 0 | 0 | TRINITY\_DN909\_c0\_g1\_i1.p1 |  |  | DB Search |
| LAYELQANKEQQR | 43.28 | 1589.8162 | 13 | -2.52 | 795.9098 | 13.07 | 1.0548 | 1.062 | 32348 | 0 | 0 | 0 | TRINITY\_DN11435\_c0\_g1\_i1.p1 |  |  | DB Search |
| TITLEVEPSDTIENVK | 43.28 | 1786.92 | 16 | 2.71 | 894.4657 | 17.62 | 1.1454 | 1.1526 | 58818 | 1.46e3 | 1 | 1 | TRINITY\_DN554\_c0\_g1\_i3.p2:TRINITY\_DN585\_c0\_g1\_i3.p1:TRINITY\_DN585\_c0\_g1\_i4.p1:TRINITY\_DN585\_c0\_g1\_i10.p1:TRINITY\_DN585\_c0\_g1\_i5.p1:TRINITY\_DN554\_c0\_g1\_i5.p1:TRINITY\_DN585\_c0\_g1\_i2.p1 |  |  | DB Search |
| SEEYKPTLGR | 43.27 | 1178.5931 | 10 | 1.46 | 590.3021 | 12.31 | 0.9162 | 0.9234 | 27845 | 7.02e2 | 1 | 1 | TRINITY\_DN144508\_c0\_g1\_i1.p1 |  |  | DB Search |
| HASSYDIVHIK | 43.27 | 1268.6514 | 11 | 4.57 | 423.8911 | 13.73 | 0.7526 | 0.7599 | 36244 | 0 | 0 | 0 | TRINITY\_DN140696\_c0\_g1\_i1.p1 |  |  | DB Search |
| VLIQIGHEPLPPQQVTTLFGK | 43.27 | 2314.3049 | 21 | 1.51 | 772.4399 | 19.84 | 0.9424 | 0.9497 | 72734 | 5.33e3 | 1 | 1 | TRINITY\_DN1199\_c0\_g1\_i1.p1:TRINITY\_DN1199\_c0\_g1\_i2.p1 |  |  | DB Search |
| KLEDVATPIVSK | 43.25 | 1298.7445 | 12 | -3.93 | 650.3741 | 14.20 | 0.9553 | 0.9626 | 39057 | 0 | 0 | 0 | TRINITY\_DN119684\_c0\_g1\_i1.p1 |  |  | DB Search |
| RLTELEHFLK | 43.25 | 1284.719 | 10 | 1.01 | 429.2455 | 16.49 | 0.7465 | 0.7538 | 52366 | 0 | 0 | 0 | TRINITY\_DN1119\_c0\_g1\_i1.p1 |  |  | DB Search |
| TSVVPGYGHAVLR | 43.25 | 1354.7357 | 13 | 3.84 | 678.3747 | 14.60 | 1.0275 | 1.0347 | 41374 | 5.26e2 | 1 | 1 | TRINITY\_DN945\_c0\_g1\_i1.p1 |  |  | DB Search |
| LRPGVELTM(+15.99)SSLVNAK | 43.25 | 1729.9396 | 16 | 1.89 | 865.9749 | 15.36 | 1.1378 | 1.145 | 45662 | 1.01e4 | 1 | 1 | TRINITY\_DN329\_c0\_g1\_i1.p1:TRINITY\_DN329\_c0\_g1\_i2.p1 | Oxidation (M) | M9:Oxidation (M):1000 | DB Search |
| VAPEEHPVLLTEAPLNPK | 43.24 | 1953.0571 | 18 | -8.51 | 977.5231 | 16.59 | 1.2073 | 1.2145 | 52921 | 0 | 0 | 0 | TRINITY\_DN2477\_c0\_g1\_i6.p1:TRINITY\_DN2477\_c0\_g1\_i5.p1:TRINITY\_DN2477\_c0\_g1\_i8.p1:TRINITY\_DN195934\_c0\_g1\_i1.p2:TRINITY\_DN161\_c0\_g1\_i1.p1:TRINITY\_DN2477\_c0\_g1\_i9.p1:TRINITY\_DN2477\_c0\_g1\_i10.p1:TRINITY\_DN2477\_c0\_g1\_i2.p1:TRINITY\_DN2941\_c0\_g1\_i4.p1:TRINITY\_DN161\_c0\_g1\_i3.p1:TRINITY\_DN161\_c0\_g1\_i2.p1 |  |  | DB Search |
| VHFGVLSFSHR | 43.24 | 1284.6727 | 11 | 4.75 | 429.2316 | 16.45 | 0.7604 | 0.7677 | 52159 | 0 | 0 | 0 | TRINITY\_DN138\_c0\_g1\_i1.p1 |  |  | DB Search |
| YQGIKPPVSR | 43.24 | 1143.64 | 10 | -0.35 | 572.8245 | 12.74 | 0.9344 | 0.9416 | 30400 | 7.91e2 | 1 | 1 | TRINITY\_DN1821\_c0\_g1\_i2.p1:TRINITY\_DN1821\_c0\_g1\_i6.p1:TRINITY\_DN1821\_c0\_g1\_i5.p1:TRINITY\_DN1821\_c0\_g1\_i4.p1 |  |  | DB Search |
| ILDAPIHLGK | 43.22 | 1075.6389 | 10 | 5.4 | 1076.6472 | 15.65 | 1.2778 | 1.2849 | 47506 | 3.09e2 | 1 | 1 | TRINITY\_DN729\_c0\_g1\_i1.p1 |  |  | DB Search |
| ELISNSSDALDKIR | 43.22 | 1559.8154 | 14 | -1.75 | 780.9102 | 15.66 | 1.0777 | 1.0849 | 47553 | 0 | 0 | 0 | TRINITY\_DN141338\_c2\_g1\_i1.p1:TRINITY\_DN5633\_c0\_g1\_i1.p1 |  |  | DB Search |
| AKIEALHC(+57.02)LSENIVK | 43.21 | 1723.9291 | 15 | 3.62 | 862.9711 | 15.78 | 1.1334 | 1.1406 | 48271 | 0 | 0 | 0 | TRINITY\_DN3078\_c0\_g1\_i1.p1 | Carbamidomethylation |  | DB Search |
| LRLDFDHEAKLNM(+15.99)IFR | 43.21 | 2033.0516 | 16 | -2.09 | 509.2668 | 17.98 | 0.8554 | 0.8627 | 61282 | 0 | 0 | 0 | TRINITY\_DN160\_c0\_g1\_i2.p1:TRINITY\_DN160\_c0\_g1\_i1.p1 | Oxidation (M) | M13:Oxidation (M):1000 | DB Search |
| VIGASGIHVGTM(+15.99)SFGK | 43.2 | 1575.8079 | 16 | 0.89 | 788.9084 | 15.29 | 1.129 | 1.1362 | 45505 | 0 | 0 | 0 | TRINITY\_DN3036\_c0\_g1\_i6.p1:TRINITY\_DN3036\_c0\_g1\_i19.p1:TRINITY\_DN3036\_c0\_g1\_i13.p1:TRINITY\_DN3036\_c0\_g1\_i11.p1:TRINITY\_DN3036\_c0\_g1\_i4.p1:TRINITY\_DN3036\_c0\_g1\_i3.p1:TRINITY\_DN3036\_c0\_g1\_i16.p1:TRINITY\_DN3036\_c0\_g1\_i2.p2:TRINITY\_DN3036\_c0\_g1\_i8.p1 | Oxidation (M) | M12:Oxidation (M):1000 | DB Search |
| GLIAQAIDHAASR | 43.18 | 1321.7102 | 13 | -1.09 | 661.8587 | 16.35 | 1.011 | 1.0182 | 51577 | 0 | 0 | 0 | TRINITY\_DN1143\_c0\_g1\_i1.p1 |  |  | DB Search |
| SLFDIFHSK | 43.18 | 1092.5604 | 9 | 4.5 | 547.2875 | 19.85 | 0.8846 | 0.8919 | 73174 | 0 | 0 | 0 | TRINITY\_DN151\_c0\_g1\_i2.p1:TRINITY\_DN151\_c0\_g1\_i3.p1:TRINITY\_DN151\_c0\_g1\_i4.p1 |  |  | DB Search |
| HGQVDSC(+57.02)INQNLKK | 43.17 | 1639.8101 | 14 | -1.59 | 547.6073 | 10.92 | 0.8457 | 0.853 | 19977 | 0 | 0 | 0 | TRINITY\_DN3991\_c0\_g1\_i1.p1 | Carbamidomethylation |  | DB Search |
| SFHFGVIK | 43.17 | 933.5072 | 8 | 1.24 | 467.7594 | 16.37 | 0.8226 | 0.8299 | 51672 | 8.38e2 | 1 | 1 | TRINITY\_DN329\_c0\_g1\_i1.p1:TRINITY\_DN329\_c0\_g1\_i2.p1 |  |  | DB Search |
| AGESQWGTDESR | 43.17 | 1321.5535 | 12 | 3.73 | 661.7835 | 12.43 | 0.9198 | 0.9271 | 28620 | 4.49e2 | 1 | 1 | TRINITY\_DN3190\_c1\_g1\_i1.p1 |  |  | DB Search |
| IKDTYLLTLEAR | 43.17 | 1434.8082 | 12 | -0.29 | 718.408 | 17.10 | 1.0383 | 1.0456 | 55810 | 0 | 0 | 0 | TRINITY\_DN72625\_c0\_g1\_i1.p1 |  |  | DB Search |
| FPGQLNADIRK | 43.16 | 1257.683 | 11 | -1.33 | 629.8451 | 14.44 | 0.9497 | 0.957 | 40493 | 0 | 0 | 0 | TRINITY\_DN5365\_c0\_g1\_i3.p1:TRINITY\_DN5365\_c0\_g1\_i2.p1 |  |  | DB Search |
| FPGQLNADLRK | 43.16 | 1257.683 | 11 | -1.33 | 629.8451 | 14.44 | 0.9497 | 0.957 | 40493 | 0 | 0 | 0 | TRINITY\_DN1910\_c0\_g1\_i1.p1:TRINITY\_DN1910\_c0\_g1\_i5.p1:TRINITY\_DN3986\_c0\_g1\_i2.p1:TRINITY\_DN3986\_c0\_g1\_i1.p1 |  |  | DB Search |
| EIEDTC(+57.02)HNR | 43.16 | 1172.488 | 9 | 13.83 | 391.8403 | 9.54 | 0.711 | 0.7184 | 12747 | 0 | 0 | 0 | TRINITY\_DN1369\_c0\_g1\_i1.p1 | Carbamidomethylation |  | DB Search |
| AAASAHHQAIGSIDAAGVTSAADYASVTAALGR | 43.16 | 3080.5273 | 33 | -1.21 | 771.1348 | 18.34 | 1.0652 | 1.0725 | 63473 | 9.02e3 | 1 | 1 | TRINITY\_DN240\_c0\_g1\_i6.p1:TRINITY\_DN240\_c0\_g1\_i1.p1:TRINITY\_DN240\_c0\_g1\_i4.p1 |  |  | DB Search |
| GLDVDSLVIDHIQVNR | 43.15 | 1791.9479 | 16 | 0.78 | 896.9779 | 19.21 | 1.177 | 1.1841 | 69113 | 0 | 0 | 0 | TRINITY\_DN1614\_c0\_g1\_i1.p1 |  |  | DB Search |
| EIRDILVQYDR | 43.15 | 1418.7517 | 11 | -6.07 | 710.3757 | 19.92 | 1.0331 | 1.0403 | 73522 | 5.02e2 | 1 | 1 | TRINITY\_DN128406\_c0\_g1\_i1.p1 |  |  | DB Search |
| SPTHSVIK | 43.15 | 867.4814 | 8 | 6.75 | 434.749 | 9.97 | 0.7713 | 0.7787 | 14734 | 4.39e3 | 1 | 1 | TRINITY\_DN1386\_c0\_g1\_i1.p1 |  |  | DB Search |
| GKPEQVQVK | 43.14 | 1011.5713 | 9 | 2 | 506.7917 | 9.97 | 0.8416 | 0.849 | 14688 | 9.41e2 | 1 | 1 | TRINITY\_DN11522\_c0\_g1\_i1.p1:TRINITY\_DN11522\_c0\_g1\_i2.p1:TRINITY\_DN11522\_c0\_g1\_i4.p1:TRINITY\_DN11522\_c0\_g1\_i3.p1 |  |  | DB Search |
| LC(+57.02)DATC(+57.02)SVFGDPHYK | 43.14 | 1768.7549 | 15 | 2.88 | 885.3833 | 15.32 | 1.1422 | 1.1494 | 45690 | 2.36e2 | 1 | 1 | TRINITY\_DN1369\_c0\_g1\_i1.p1 | Carbamidomethylation, Carbamidomethylation |  | DB Search |
| AVAAGMNPMDIKR | 43.11 | 1372.6956 | 13 | 1.74 | 687.3531 | 14.92 | 1.0126 | 1.0198 | 43242 | 6.18e2 | 1 | 1 | TRINITY\_DN15151\_c0\_g1\_i2.p1:TRINITY\_DN15151\_c0\_g1\_i3.p1:TRINITY\_DN15151\_c0\_g1\_i1.p1 |  |  | DB Search |
| A(+42.01)SAATDNRK | 43.11 | 974.4781 | 9 | 3.81 | 488.246 | 9.52 | 0.8234 | 0.8307 | 12664 | 0 | 0 | 0 | TRINITY\_DN7659\_c0\_g1\_i1.p1:TRINITY\_DN7659\_c0\_g1\_i2.p1 | Acetylation (Protein N-term) | A1:Acetylation (Protein N-term):1000 | DB Search |
| AETDIM(+15.99)KR | 43.1 | 978.4804 | 8 | 4.13 | 490.2473 | 9.56 | 0.8067 | 0.814 | 12807 | 1.4e3 | 1 | 1 | TRINITY\_DN262\_c0\_g1\_i1.p1 | Oxidation (M) | M6:Oxidation (M):1000 | DB Search |
| LGGIEVTQGNC(+57.02)AR | 43.02 | 1373.6721 | 13 | 2.68 | 687.8421 | 13.90 | 0.9662 | 0.9735 | 37137 | 6.95e2 | 1 | 1 | TRINITY\_DN36\_c0\_g1\_i9.p1:TRINITY\_DN36\_c0\_g1\_i14.p1:TRINITY\_DN36\_c0\_g1\_i13.p1:TRINITY\_DN36\_c0\_g1\_i2.p1:TRINITY\_DN36\_c0\_g1\_i16.p1:TRINITY\_DN36\_c0\_g1\_i3.p1:TRINITY\_DN36\_c0\_g1\_i17.p1:TRINITY\_DN36\_c0\_g1\_i4.p1:TRINITY\_DN36\_c0\_g1\_i11.p1:TRINITY\_DN36\_c0\_g1\_i8.p1:TRINITY\_DN36\_c0\_g1\_i1.p1:TRINITY\_DN36\_c0\_g1\_i19.p1:TRINITY\_DN36\_c0\_g1\_i20.p1 | Carbamidomethylation |  | DB Search |
| ATSC(+57.02)AVYPC(+57.02)EINSPR | 43.02 | 1723.7657 | 15 | 6.64 | 862.892 | 13.94 | 1.0985 | 1.1057 | 37509 | 0 | 0 | 0 | TRINITY\_DN160\_c0\_g1\_i2.p1:TRINITY\_DN160\_c0\_g1\_i1.p1 | Carbamidomethylation, Carbamidomethylation |  | DB Search |
| ATIEIPAKYEK | 42.98 | 1261.6918 | 11 | 0.3 | 631.8505 | 14.24 | 0.9848 | 0.992 | 39239 | 0 | 0 | 0 | TRINITY\_DN2637\_c0\_g1\_i6.p1:TRINITY\_DN2637\_c0\_g1\_i14.p2:TRINITY\_DN2637\_c0\_g1\_i5.p2:TRINITY\_DN2637\_c0\_g1\_i8.p2:TRINITY\_DN2637\_c0\_g1\_i9.p2:TRINITY\_DN2637\_c0\_g1\_i7.p2:TRINITY\_DN2637\_c0\_g1\_i2.p2:TRINITY\_DN2637\_c0\_g1\_i13.p1:TRINITY\_DN2637\_c0\_g1\_i4.p1 |  |  | DB Search |
| SFNKVESR | 42.91 | 965.493 | 8 | 6.49 | 483.7548 | 10.36 | 0.8392 | 0.8465 | 16574 | 6.58e3 | 1 | 1 | TRINITY\_DN5397\_c0\_g1\_i1.p1 |  |  | DB Search |
| AALNM(+15.99)EFEYIPTK | 42.87 | 1541.7435 | 13 | -3.63 | 771.8728 | 17.58 | 1.0528 | 1.06 | 58710 | 2.11e2 | 1 | 1 | TRINITY\_DN942\_c0\_g1\_i5.p1:TRINITY\_DN942\_c0\_g1\_i2.p1:TRINITY\_DN942\_c0\_g1\_i3.p1 | Oxidation (M) | M5:Oxidation (M):1000 | DB Search |
| TGAISLLASVDFETDRTHTAVVK | 42.86 | 2430.2754 | 23 | 7.32 | 811.1014 | 19.05 | 1.0419 | 1.0492 | 68211 | 0 | 0 | 0 | TRINITY\_DN982\_c0\_g1\_i3.p1 |  |  | DB Search |
| YGINHITNLVESKR | 42.86 | 1642.879 | 14 | 4.42 | 548.6336 | 15.17 | 0.8246 | 0.8319 | 44836 | 0 | 0 | 0 | TRINITY\_DN146208\_c0\_g1\_i1.p1 |  |  | DB Search |
| TGTLTQNR | 42.85 | 889.4617 | 8 | 2.38 | 445.7372 | 10.03 | 0.7844 | 0.7917 | 14992 | 3.14e3 | 1 | 1 | TRINITY\_DN262\_c0\_g1\_i1.p1 |  |  | DB Search |
| VQLTANNVEDFVK | 42.83 | 1475.762 | 13 | 11.06 | 738.8931 | 17.79 | 1.0472 | 1.0544 | 60028 | 3.23e2 | 1 | 1 | TRINITY\_DN803\_c0\_g1\_i1.p1 |  |  | DB Search |
| DTLVFEIDEIRK | 42.83 | 1476.7823 | 12 | 2.38 | 739.3969 | 18.67 | 1.0359 | 1.0431 | 65730 | 1.13e3 | 1 | 1 | TRINITY\_DN31399\_c0\_g1\_i3.p1:TRINITY\_DN31399\_c0\_g1\_i2.p1 |  |  | DB Search |
| DLSLVVEPLKNYGVK | 42.82 | 1672.9399 | 15 | 5.12 | 837.4778 | 19.25 | 1.1354 | 1.1426 | 69248 | 2.1e2 | 1 | 1 | TRINITY\_DN244\_c0\_g1\_i1.p1 |  |  | DB Search |
| IRNEEIQIEVIERR | 42.81 | 1795.9904 | 14 | -1.18 | 599.6674 | 15.29 | 0.8798 | 0.887 | 45515 | 0 | 0 | 0 | TRINITY\_DN11435\_c0\_g1\_i1.p1 |  |  | DB Search |
| LQEFGHK | 42.79 | 857.4395 | 7 | 2.94 | 429.7264 | 11.17 | 0.7913 | 0.7986 | 21333 | 0 | 0 | 0 | TRINITY\_DN602\_c0\_g1\_i4.p1:TRINITY\_DN602\_c0\_g1\_i3.p1:TRINITY\_DN602\_c0\_g1\_i1.p1 |  |  | DB Search |
| AGVLAHLEEERDIK | 42.76 | 1578.8365 | 14 | -2.1 | 527.2827 | 15.05 | 0.7994 | 0.8067 | 43994 | 3.21e2 | 1 | 1 | TRINITY\_DN428\_c0\_g1\_i1.p1 |  |  | DB Search |
| LRPGQATTVDITVK | 42.76 | 1497.8514 | 14 | -1.18 | 749.9288 | 14.18 | 1.0323 | 1.0395 | 38810 | 8.86e2 | 1 | 1 | TRINITY\_DN25375\_c0\_g1\_i7.p1 |  |  | DB Search |
| SLVYNPLHNDQEVFER | 42.75 | 1958.9486 | 16 | 1.71 | 653.9883 | 17.50 | 0.8672 | 0.8745 | 58275 | 2.2e2 | 1 | 1 | TRINITY\_DN6390\_c0\_g1\_i1.p1 |  |  | DB Search |
| GQFSTDDLVAEVEKR | 42.74 | 1692.8318 | 15 | 10.63 | 847.4284 | 17.88 | 1.141 | 1.1482 | 60636 | 0 | 0 | 0 | TRINITY\_DN2113\_c0\_g1\_i1.p1:TRINITY\_DN2113\_c0\_g1\_i2.p1 |  |  | DB Search |
| TISNGGTVAK | 42.73 | 946.5084 | 10 | -0.17 | 474.2592 | 10.11 | 0.8124 | 0.8197 | 15429 | 0 | 0 | 0 | TRINITY\_DN1369\_c0\_g1\_i1.p1 |  |  | DB Search |
| K(+42.01)LAAELANGR | 42.72 | 1083.6036 | 10 | -0.83 | 542.8062 | 15.48 | 0.8635 | 0.8708 | 46516 | 9.73e2 | 1 | 1 | TRINITY\_DN1172\_c0\_g2\_i2.p1 | Acetylation (Protein N-term) | K1:Acetylation (Protein N-term):1000 | DB Search |
| IAAVIINKPDVLASR | 42.71 | 1578.9457 | 15 | 3.92 | 790.4797 | 16.18 | 1.1021 | 1.1094 | 50536 | 0 | 0 | 0 | TRINITY\_DN123\_c0\_g1\_i1.p1 |  |  | DB Search |
| LQNDKEFAK | 42.7 | 1091.5612 | 9 | 4.17 | 546.7877 | 10.82 | 0.9162 | 0.9234 | 19362 | 0 | 0 | 0 | TRINITY\_DN74934\_c0\_g1\_i1.p1 |  |  | DB Search |
| SSLSTSSSGTGPK | 42.7 | 1194.5728 | 13 | -2.94 | 598.2892 | 10.42 | 0.9133 | 0.9206 | 17169 | 0 | 0 | 0 | TRINITY\_DN1936\_c0\_g1\_i1.p1:TRINITY\_DN1936\_c0\_g1\_i4.p1 |  |  | DB Search |
| YSTQDEYGTAAYK | 42.7 | 1495.6467 | 13 | -1.47 | 748.8262 | 13.28 | 1.0033 | 1.0106 | 33565 | 0 | 0 | 0 | TRINITY\_DN166344\_c0\_g1\_i1.p1 |  |  | DB Search |
| SSLNHNIVFEGR | 42.69 | 1371.6895 | 12 | 3.01 | 686.851 | 14.63 | 0.9824 | 0.9896 | 41610 | 3.9e2 | 1 | 1 | TRINITY\_DN144508\_c0\_g1\_i1.p1 |  |  | DB Search |
| ADLTRPTGK | 42.69 | 957.5243 | 9 | -3.1 | 479.7658 | 10.44 | 0.8193 | 0.8266 | 17248 | 0 | 0 | 0 | TRINITY\_DN941\_c0\_g1\_i5.p2:TRINITY\_DN941\_c0\_g1\_i7.p2:TRINITY\_DN941\_c0\_g1\_i10.p1:TRINITY\_DN941\_c0\_g1\_i14.p1:TRINITY\_DN941\_c0\_g1\_i2.p2:TRINITY\_DN941\_c0\_g1\_i8.p2 |  |  | DB Search |
| IGGSTDTGR | 42.68 | 862.4144 | 9 | 4.01 | 432.2143 | 9.50 | 0.7445 | 0.7518 | 12524 | 1.06e3 | 1 | 1 | TRINITY\_DN57\_c0\_g1\_i1.p1 |  |  | DB Search |
| AFNRVETR | 42.68 | 991.5199 | 8 | 4.26 | 496.7671 | 10.79 | 0.8425 | 0.8498 | 19063 | 2.3e3 | 1 | 1 | TRINITY\_DN4595\_c0\_g1\_i7.p1:TRINITY\_DN4595\_c0\_g1\_i4.p1:TRINITY\_DN4595\_c0\_g1\_i10.p2 |  |  | DB Search |
| KLLDWAQQR | 42.67 | 1156.6353 | 9 | -16.88 | 579.3126 | 15.55 | 0.9057 | 0.9129 | 46959 | 0 | 0 | 0 | TRINITY\_DN199\_c0\_g1\_i1.p1 |  |  | DB Search |
| LIVEGIEDVHVDPHLQEQC(+57.02)AR | 42.66 | 2456.2117 | 21 | -3.3 | 819.7382 | 17.06 | 1.0041 | 1.0114 | 55667 | 0 | 0 | 0 | TRINITY\_DN3078\_c0\_g1\_i1.p1 | Carbamidomethylation |  | DB Search |
| VGQAM(+15.99)ASPEDK | 42.64 | 1147.5179 | 11 | -6.8 | 574.7598 | 9.75 | 0.864 | 0.8712 | 13667 | 0 | 0 | 0 | TRINITY\_DN57\_c0\_g1\_i1.p1 | Oxidation (M) | M5:Oxidation (M):1000 | DB Search |
| SVDWTSDLYLKPLPGVSAAK | 42.6 | 2146.1309 | 20 | 3.72 | 716.3837 | 19.68 | 1.013 | 1.0202 | 72078 | 4.11e2 | 1 | 1 | TRINITY\_DN240\_c0\_g1\_i6.p1:TRINITY\_DN240\_c0\_g1\_i1.p1:TRINITY\_DN240\_c0\_g1\_i4.p1 |  |  | DB Search |
| DM(+15.99)GVNVISVGAGHTINHR | 42.58 | 1891.9323 | 18 | 0.27 | 946.9694 | 14.30 | 1.2276 | 1.2348 | 39624 | 0 | 0 | 0 | TRINITY\_DN138\_c0\_g1\_i1.p1 | Oxidation (M) | M2:Oxidation (M):1000 | DB Search |
| VVTSSTGPGEHM(+15.99)R | 42.58 | 1372.6405 | 13 | -4.07 | 687.3217 | 9.80 | 0.9606 | 0.9679 | 13688 | 2.89e2 | 1 | 1 | TRINITY\_DN73067\_c0\_g1\_i1.p1 | Oxidation (M) | M12:Oxidation (M):1000 | DB Search |
| SGVLAGHDNR | 42.53 | 1024.505 | 10 | -1.12 | 513.2569 | 10.34 | 0.8611 | 0.8684 | 16684 | 3.56e2 | 1 | 1 | TRINITY\_DN2472\_c0\_g1\_i1.p1 |  |  | DB Search |
| RC(+57.02)QFVNR | 42.53 | 978.4818 | 7 | -14.09 | 490.2391 | 12.02 | 0.8161 | 0.8234 | 26129 | 1.46e3 | 1 | 1 | TRINITY\_DN140996\_c0\_g1\_i1.p1 | Carbamidomethylation |  | DB Search |
| GVSEETTTGVHNLYK | 42.51 | 1633.7948 | 15 | -2.9 | 817.8986 | 13.70 | 1.068 | 1.0753 | 35995 | 5.08e2 | 1 | 1 | TRINITY\_DN773\_c0\_g1\_i1.p1 |  |  | DB Search |
| EEYDESGPSIVHR | 42.51 | 1516.6793 | 13 | -1.53 | 759.3424 | 13.28 | 0.9904 | 0.9977 | 33566 | 2.23e2 | 1 | 1 | TRINITY\_DN161\_c0\_g1\_i1.p1:TRINITY\_DN161\_c0\_g1\_i4.p1:TRINITY\_DN161\_c0\_g1\_i3.p1 |  |  | DB Search |
| AVSFLVNDVPNLK | 42.5 | 1414.782 | 13 | 5.5 | 708.399 | 19.29 | 1.0041 | 1.0114 | 69625 | 4.01e2 | 1 | 1 | TRINITY\_DN1189\_c0\_g1\_i1.p1 |  |  | DB Search |
| VKESYTLNIEAR | 42.48 | 1421.7515 | 12 | 0.71 | 711.8803 | 14.78 | 1.0001 | 1.0073 | 42448 | 4.94e2 | 1 | 1 | TRINITY\_DN72625\_c0\_g1\_i1.p1 |  |  | DB Search |
| AVTIESTSNSVNGVFDTVEGK | 42.45 | 2153.0488 | 21 | 0.69 | 1077.5276 | 19.00 | 1.2141 | 1.2212 | 67823 | 0 | 0 | 0 | TRINITY\_DN33065\_c0\_g1\_i1.p1 |  |  | DB Search |
| SSC(+57.02)RPILSVIK | 42.42 | 1258.7067 | 11 | -5.58 | 630.3543 | 17.05 | 0.9731 | 0.9804 | 55404 | 1.96e4 | 2 | 2 | TRINITY\_DN158421\_c0\_g1\_i1.p1:TRINITY\_DN3545\_c0\_g1\_i2.p1:TRINITY\_DN3545\_c0\_g1\_i1.p1 | Carbamidomethylation |  | DB Search |
| AIVMGSAMDGNLLK | 42.42 | 1418.7261 | 14 | 3.64 | 710.3698 | 18.29 | 1.0174 | 1.0246 | 63333 | 0 | 0 | 0 | TRINITY\_DN240\_c0\_g1\_i6.p1:TRINITY\_DN240\_c0\_g1\_i1.p1:TRINITY\_DN240\_c0\_g1\_i4.p1 |  |  | DB Search |
| VVEKEAETER | 42.37 | 1188.5986 | 10 | -4.14 | 595.3015 | 9.59 | 0.8988 | 0.9061 | 12943 | 0 | 0 | 0 | TRINITY\_DN2636\_c0\_g1\_i2.p1:TRINITY\_DN2636\_c0\_g1\_i1.p1 |  |  | DB Search |
| YGTSSFDMSSLIK | 42.34 | 1434.67 | 13 | 2.06 | 718.3406 | 19.16 | 1.023 | 1.0303 | 68826 | 0 | 0 | 0 | TRINITY\_DN5584\_c0\_g1\_i3.p1:TRINITY\_DN5584\_c0\_g1\_i2.p1 |  |  | DB Search |
| YLTVAAMFR | 42.33 | 1070.5582 | 9 | 5.33 | 536.2869 | 19.96 | 0.8607 | 0.868 | 73861 | 0 | 0 | 0 | TRINITY\_DN1910\_c0\_g1\_i1.p1:TRINITY\_DN3986\_c0\_g1\_i1.p1 |  |  | DB Search |
| AQPTEIYNLGAQSHVK | 42.33 | 1754.8951 | 16 | -17.72 | 878.4354 | 14.86 | 1.1734 | 1.1806 | 42928 | 0 | 0 | 0 | TRINITY\_DN7659\_c0\_g1\_i1.p1:TRINITY\_DN7659\_c0\_g1\_i2.p1 |  |  | DB Search |
| LRNEFTGVLTGK | 42.32 | 1333.7354 | 12 | -0.96 | 667.8713 | 16.05 | 0.9812 | 0.9884 | 49757 | 5.76e2 | 1 | 1 | TRINITY\_DN360\_c0\_g1\_i1.p1 |  |  | DB Search |
| GSFSSHIC(+57.02)LC(+57.02)HDPAYK | 42.3 | 1877.8188 | 16 | -0.4 | 626.9438 | 14.48 | 0.8866 | 0.8939 | 40723 | 0 | 0 | 0 | TRINITY\_DN319\_c2\_g1\_i3.p1:TRINITY\_DN319\_c2\_g1\_i1.p1:TRINITY\_DN319\_c2\_g1\_i2.p1 | Carbamidomethylation, Carbamidomethylation |  | DB Search |
| LTIAEERDR | 42.29 | 1101.5778 | 9 | 3.63 | 551.7957 | 11.79 | 0.9089 | 0.9162 | 24861 | 0 | 0 | 0 | TRINITY\_DN140696\_c0\_g1\_i1.p1 |  |  | DB Search |
| ADILC(+57.02)HDTK | 42.28 | 1071.5018 | 9 | 5.67 | 536.7589 | 11.81 | 0.8773 | 0.8846 | 24985 | 0 | 0 | 0 | TRINITY\_DN413\_c0\_g1\_i4.p1:TRINITY\_DN413\_c0\_g1\_i1.p1:TRINITY\_DN413\_c0\_g1\_i3.p1 | Carbamidomethylation |  | DB Search |
| YVQNPR | 42.15 | 775.3977 | 6 | 4.25 | 388.706 | 9.82 | 0.7697 | 0.777 | 14011 | 8.51e2 | 1 | 1 | TRINITY\_DN8903\_c0\_g1\_i1.p1 |  |  | DB Search |
| HYLPENLRK | 42.13 | 1168.6353 | 9 | -0.53 | 390.5504 | 14.05 | 0.7029 | 0.7102 | 38149 | 2.03e2 | 1 | 1 | TRINITY\_DN2357\_c1\_g1\_i1.p1 |  |  | DB Search |
| LC(+57.02)ETNTANTEAK | 41.94 | 1350.6085 | 12 | 9.96 | 676.3152 | 10.42 | 0.9473 | 0.9545 | 17132 | 0 | 0 | 0 | TRINITY\_DN16120\_c0\_g1\_i2.p1 | Carbamidomethylation |  | DB Search |
| TENNC(+57.02)PLTK | 41.52 | 1075.4968 | 9 | 1.18 | 538.7539 | 10.87 | 0.853 | 0.8603 | 19697 | 8.87e2 | 1 | 1 | TRINITY\_DN1369\_c0\_g1\_i1.p1 | Carbamidomethylation |  | DB Search |
| DGPGPLASDLLLQMR | 41.47 | 1581.8185 | 15 | -2.93 | 528.2762 | 19.62 | 0.8149 | 0.8222 | 71531 | 3.73e2 | 1 | 1 | TRINITY\_DN13813\_c0\_g1\_i5.p2:TRINITY\_DN13813\_c0\_g1\_i6.p2:TRINITY\_DN13813\_c0\_g1\_i2.p2:TRINITY\_DN13813\_c0\_g1\_i4.p2:TRINITY\_DN13813\_c0\_g1\_i3.p2 |  |  | DB Search |
| IESTAC(+57.02)TR | 41.29 | 936.4335 | 8 | 7.97 | 469.2256 | 9.69 | 0.8023 | 0.8096 | 13341 | 2.14e3 | 1 | 1 | TRINITY\_DN98968\_c0\_g1\_i1.p1 | Carbamidomethylation |  | DB Search |
| SLVEENVHPQILIK | 41.2 | 1617.9089 | 14 | -0.47 | 809.9578 | 16.56 | 1.1001 | 1.1074 | 52733 | 1.57e2 | 1 | 1 | TRINITY\_DN1194\_c0\_g1\_i1.p1 |  |  | DB Search |
| V(+42.01)LLLEATK | 40.98 | 927.564 | 8 | 1.92 | 464.7881 | 19.57 | 0.8023 | 0.8096 | 71356 | 6.91e2 | 1 | 1 | TRINITY\_DN86511\_c0\_g1\_i1.p1 | Acetylation (Protein N-term) | V1:Acetylation (Protein N-term):1000 | DB Search |
| AAMEELEHAEK | 40.76 | 1256.5707 | 11 | 2.71 | 629.2915 | 13.28 | 0.961 | 0.9683 | 33568 | 3.38e2 | 1 | 1 | TRINITY\_DN876\_c0\_g1\_i1.p1:TRINITY\_DN876\_c0\_g1\_i2.p1 |  |  | DB Search |
| VRDPYRNDLFHDTSR | 40.73 | 1889.9132 | 15 | -1.18 | 473.4829 | 13.85 | 0.8181 | 0.8254 | 36969 | 0 | 0 | 0 | TRINITY\_DN730\_c0\_g2\_i3.p1:TRINITY\_DN730\_c0\_g2\_i2.p1 |  |  | DB Search |
| LSIIPVRR | 40.66 | 952.6182 | 8 | 1.89 | 477.3151 | 14.96 | 0.8465 | 0.8538 | 43251 | 4.86e3 | 1 | 1 | TRINITY\_DN15584\_c0\_g1\_i1.p1 |  |  | DB Search |
| AHGPGLEGGVAGQPC(+57.02)R | 40.64 | 1561.7419 | 16 | 2.05 | 781.8763 | 12.28 | 1.0275 | 1.0347 | 27748 | 0 | 0 | 0 | TRINITY\_DN941\_c0\_g1\_i6.p1:TRINITY\_DN941\_c0\_g1\_i15.p1:TRINITY\_DN941\_c0\_g1\_i11.p1:TRINITY\_DN941\_c0\_g1\_i7.p1:TRINITY\_DN941\_c0\_g1\_i5.p1:TRINITY\_DN941\_c0\_g1\_i8.p1:TRINITY\_DN941\_c0\_g1\_i9.p1:TRINITY\_DN941\_c0\_g1\_i2.p1 | Carbamidomethylation |  | DB Search |
| RLDEEVAR | 40.55 | 986.5145 | 8 | 2.71 | 494.2636 | 11.17 | 0.8522 | 0.8595 | 21312 | 0 | 0 | 0 | TRINITY\_DN773\_c0\_g1\_i1.p1 |  |  | DB Search |
| AVGATIVNRPEEIREEDLGLK | 40.05 | 2308.2385 | 21 | 5.45 | 770.4209 | 16.03 | 1.052 | 1.0592 | 49650 | 2.87e2 | 1 | 1 | TRINITY\_DN120478\_c0\_g1\_i1.p1 |  |  | DB Search |
| SSSC(+57.02)QEFGGKC(+57.02)PC(+57.02)K | 39.5 | 1630.6538 | 14 | -18.66 | 816.3153 | 12.09 | 1.0311 | 1.0383 | 26624 | 0 | 0 | 0 | TRINITY\_DN1616\_c0\_g1\_i3.p1:TRINITY\_DN1616\_c0\_g1\_i6.p1:TRINITY\_DN1616\_c0\_g1\_i2.p1:TRINITY\_DN1616\_c0\_g1\_i7.p1 | Carbamidomethylation, Carbamidomethylation, Carbamidomethylation |  | DB Search |
| total 352 peptides |  |  |  |  |  |  |  |  |  |  |  |  |  |  |  |  |
| --- | --- | --- | --- | --- | --- | --- | --- | --- | --- | --- | --- | --- | --- | --- | --- | --- |
