# Supplementary material for: Proteomic Analysis and Biochemical Characterization of the Nematocyst Extract of the Hydrozoan Velella velella
Source: Mar Drugs. 2024 Oct 12;22(10):468. doi: 10.3390/md22100468 (PMC11509761; doi:10.3390/md22100468)
Supplement: Supplementary file 1 [file marinedrugs-22-00468-s001.zip › Supplementary File S1.zip!/Sample_7_Vellela/db.proteins.html]

proteins


Protein List

  

| Protein Group | Protein ID | Accession | -10lgP | Coverage (%) | Coverage (%) Sample 8 | Area Sample 8 | #Peptides | #Unique | #Spec Sample 8 | PTM | Avg. Mass | Description |
| --- | --- | --- | --- | --- | --- | --- | --- | --- | --- | --- | --- | --- |
| 1 | 1 | TRINITY\_DN982\_c0\_g1\_i3.p1 | 127.93 | 1 | 1 | 0 | 10 | 4 | 15 | Carbamidomethylation | 1090854.75 | type:complete len:9852 gc:universal TRINITY\_DN982\_c0\_g1\_i3:29697-142(-) |
| 2 | 2 | TRINITY\_DN329\_c0\_g1\_i2.p1 | 118.92 | 21 | 21 | 5.53e4 | 8 | 8 | 25 | Oxidation (M) | 34761.9609 | type:5prime\_partial len:320 gc:universal TRINITY\_DN329\_c0\_g1\_i2:1-960(+) |
| 2 | 3 | TRINITY\_DN329\_c0\_g1\_i1.p1 | 118.92 | 24 | 24 | 5.53e4 | 8 | 8 | 25 | Oxidation (M) | 30514.9648 | type:complete len:283 gc:universal TRINITY\_DN329\_c0\_g1\_i1:158-1006(+) |
| 3 | 4 | TRINITY\_DN262\_c0\_g1\_i1.p1 | 123.56 | 11 | 11 | 8.7e3 | 9 | 9 | 11 | Oxidation (M) | 114520.6406 | type:complete len:1033 gc:universal TRINITY\_DN262\_c0\_g1\_i1:111-3209(+) |
| 4 | 5 | TRINITY\_DN160\_c0\_g1\_i1.p1 | 113.04 | 5 | 5 | 1.37e4 | 7 | 7 | 12 | Carbamidomethylation, Oxidation (M) | 126611.5703 | type:complete len:1145 gc:universal TRINITY\_DN160\_c0\_g1\_i1:4810-1376(-) |
| 4 | 6 | TRINITY\_DN160\_c0\_g1\_i2.p1 | 113.04 | 5 | 5 | 1.37e4 | 7 | 7 | 12 | Carbamidomethylation, Oxidation (M) | 126611.5703 | type:complete len:1145 gc:universal TRINITY\_DN160\_c0\_g1\_i2:4810-1376(-) |
| 5 | 7 | TRINITY\_DN982\_c0\_g1\_i1.p1 | 118.64 | 2 | 2 | 0 | 8 | 2 | 12 | Carbamidomethylation | 712566.0625 | type:5prime\_partial len:6412 gc:universal TRINITY\_DN982\_c0\_g1\_i1:19377-142(-) |
| 5 | 8 | TRINITY\_DN982\_c0\_g1\_i2.p1 | 80 | 2 | 2 | 0 | 3 | 2 | 4 |  | 190367.1406 | type:internal len:1718 gc:universal TRINITY\_DN982\_c0\_g1\_i2:5151-1(-) |
| 6 | 9 | TRINITY\_DN1369\_c0\_g1\_i1.p1 | 112.64 | 5 | 5 | 3.61e3 | 7 | 7 | 8 | Carbamidomethylation | 174229.4219 | type:3prime\_partial len:1577 gc:universal TRINITY\_DN1369\_c0\_g1\_i1:93-4820(+) |
| 7 | 10 | TRINITY\_DN3991\_c0\_g1\_i1.p1 | 113.46 | 17 | 17 | 2.42e5 | 7 | 7 | 15 | Carbamidomethylation | 24846.6543 | type:3prime\_partial len:219 gc:universal TRINITY\_DN3991\_c0\_g1\_i1:84-737(+) |
| 8 | 11 | TRINITY\_DN240\_c0\_g1\_i1.p1 | 112.99 | 33 | 33 | 3.35e4 | 7 | 7 | 24 | Oxidation (M) | 40418.2188 | type:5prime\_partial len:397 gc:universal TRINITY\_DN240\_c0\_g1\_i1:1518-328(-) |
| 8 | 12 | TRINITY\_DN240\_c0\_g1\_i4.p1 | 112.99 | 33 | 33 | 3.35e4 | 7 | 7 | 24 | Oxidation (M) | 40419.2031 | type:5prime\_partial len:397 gc:universal TRINITY\_DN240\_c0\_g1\_i4:1327-137(-) |
| 8 | 13 | TRINITY\_DN240\_c0\_g1\_i6.p1 | 112.99 | 33 | 33 | 3.35e4 | 7 | 7 | 24 | Oxidation (M) | 40418.2188 | type:5prime\_partial len:397 gc:universal TRINITY\_DN240\_c0\_g1\_i6:1494-304(-) |
| 9 | 14 | TRINITY\_DN4595\_c0\_g1\_i4.p1 | 107.05 | 4 | 4 | 4.92e3 | 6 | 5 | 7 | Carbamidomethylation | 183684.4844 | type:5prime\_partial len:1639 gc:universal TRINITY\_DN4595\_c0\_g1\_i4:5857-941(-) |
| 9 | 15 | TRINITY\_DN4595\_c0\_g1\_i10.p2 | 79.87 | 7 | 7 | 2.3e3 | 3 | 2 | 4 | Carbamidomethylation | 39822.0156 | type:5prime\_partial len:358 gc:universal TRINITY\_DN4595\_c0\_g1\_i10:5369-4296(-) |
| 9 | 16 | TRINITY\_DN4595\_c0\_g1\_i7.p1 | 79.76 | 7 | 7 | 4.52e3 | 3 | 2 | 4 |  | 38387.582 | type:internal len:348 gc:universal TRINITY\_DN4595\_c0\_g1\_i7:1042-2(-) |
| 9 | 17 | TRINITY\_DN4595\_c0\_g1\_i10.p1 | 65.68 | 2 | 2 | 4e2 | 2 | 2 | 2 | Carbamidomethylation | 133760.0625 | type:complete len:1188 gc:universal TRINITY\_DN4595\_c0\_g1\_i10:4405-842(-) |
| 9 | 18 | TRINITY\_DN4595\_c0\_g1\_i1.p1 | 43.58 | 6 | 6 | 2.22e3 | 1 | 1 | 1 |  | 15339.0303 | type:internal len:138 gc:universal TRINITY\_DN4595\_c0\_g1\_i1:412-2(-) |
| 10 | 19 | TRINITY\_DN138\_c0\_g1\_i1.p1 | 99.64 | 16 | 16 | 9.12e2 | 5 | 5 | 8 | Oxidation (M), Carbamidomethylation | 46324.8945 | type:complete len:401 gc:universal TRINITY\_DN138\_c0\_g1\_i1:157-1359(+) |
| 11 | 20 | TRINITY\_DN161\_c0\_g1\_i3.p1 | 107.02 | 21 | 21 | 5.3e4 | 6 | 6 | 14 |  | 44592.6602 | type:5prime\_partial len:399 gc:universal TRINITY\_DN161\_c0\_g1\_i3:1450-254(-) |
| 11 | 21 | TRINITY\_DN161\_c0\_g1\_i1.p1 | 107.02 | 22 | 22 | 5.3e4 | 6 | 6 | 14 |  | 41918.5938 | type:complete len:377 gc:universal TRINITY\_DN161\_c0\_g1\_i1:1384-254(-) |
| 11 | 22 | TRINITY\_DN161\_c0\_g1\_i2.p1 | 99.93 | 18 | 18 | 5.28e4 | 5 | 5 | 13 |  | 44577.6484 | type:5prime\_partial len:399 gc:universal TRINITY\_DN161\_c0\_g1\_i2:1683-487(-) |
| 11 | 23 | TRINITY\_DN161\_c0\_g1\_i4.p1 | 79.89 | 19 | 19 | 7.01e3 | 3 | 3 | 7 |  | 25887.2988 | type:5prime\_partial len:232 gc:universal TRINITY\_DN161\_c0\_g1\_i4:949-254(-) |
| 11 | 24 | TRINITY\_DN2477\_c0\_g1\_i5.p1 | 65.43 | 9 | 9 | 1.65e3 | 2 | 2 | 3 |  | 37416.5195 | type:complete len:333 gc:universal TRINITY\_DN2477\_c0\_g1\_i5:1182-184(-) |
| 11 | 25 | TRINITY\_DN2477\_c0\_g1\_i10.p1 | 65.43 | 9 | 9 | 1.65e3 | 2 | 2 | 3 |  | 38566.9688 | type:5prime\_partial len:344 gc:universal TRINITY\_DN2477\_c0\_g1\_i10:1215-184(-) |
| 11 | 26 | TRINITY\_DN2477\_c0\_g1\_i2.p1 | 65.43 | 9 | 9 | 1.65e3 | 2 | 2 | 3 |  | 38566.9688 | type:5prime\_partial len:344 gc:universal TRINITY\_DN2477\_c0\_g1\_i2:1215-184(-) |
| 11 | 27 | TRINITY\_DN195934\_c0\_g1\_i1.p2 | 65.43 | 24 | 24 | 1.65e3 | 2 | 2 | 3 |  | 14094.0938 | type:internal len:127 gc:universal TRINITY\_DN195934\_c0\_g1\_i1:3-380(+) |
| 11 | 28 | TRINITY\_DN2477\_c0\_g1\_i6.p1 | 65.43 | 8 | 8 | 1.65e3 | 2 | 2 | 3 |  | 41878.6875 | type:complete len:377 gc:universal TRINITY\_DN2477\_c0\_g1\_i6:1314-184(-) |
| 11 | 29 | TRINITY\_DN2477\_c0\_g1\_i9.p1 | 65.43 | 8 | 8 | 1.65e3 | 2 | 2 | 3 |  | 41878.6875 | type:complete len:377 gc:universal TRINITY\_DN2477\_c0\_g1\_i9:1314-184(-) |
| 11 | 30 | TRINITY\_DN2941\_c0\_g1\_i4.p1 | 43.24 | 16 | 16 | 0 | 1 | 1 | 2 |  | 13034.9365 | type:internal len:116 gc:universal TRINITY\_DN2941\_c0\_g1\_i4:2-346(+) |
| 11 | 31 | TRINITY\_DN2477\_c0\_g1\_i8.p1 | 43.24 | 6 | 6 | 0 | 1 | 1 | 2 |  | 34820.5938 | type:5prime\_partial len:309 gc:universal TRINITY\_DN2477\_c0\_g1\_i8:1110-184(-) |
| 12 | 32 | TRINITY\_DN3190\_c1\_g1\_i1.p1 | 106.92 | 23 | 23 | 1.73e3 | 6 | 6 | 7 | Acetylation (Protein N-term) | 35061.5508 | type:complete len:315 gc:universal TRINITY\_DN3190\_c1\_g1\_i1:68-1012(+) |
| 13 | 33 | TRINITY\_DN1616\_c0\_g1\_i2.p1 | 98.87 | 2 | 2 | 4.23e2 | 5 | 5 | 5 | Carbamidomethylation | 374396 | type:3prime\_partial len:3398 gc:universal TRINITY\_DN1616\_c0\_g1\_i2:218-10408(+) |
| 13 | 34 | TRINITY\_DN1616\_c0\_g1\_i3.p1 | 98.87 | 2 | 2 | 4.23e2 | 5 | 5 | 5 | Carbamidomethylation | 423947.2812 | type:complete len:3855 gc:universal TRINITY\_DN1616\_c0\_g1\_i3:218-11782(+) |
| 13 | 35 | TRINITY\_DN1616\_c0\_g1\_i7.p1 | 98.87 | 2 | 2 | 4.23e2 | 5 | 5 | 5 | Carbamidomethylation | 423875.2188 | type:complete len:3855 gc:universal TRINITY\_DN1616\_c0\_g1\_i7:218-11782(+) |
| 13 | 36 | TRINITY\_DN1616\_c0\_g1\_i6.p1 | 98.87 | 2 | 2 | 4.23e2 | 5 | 5 | 5 | Carbamidomethylation | 423911.25 | type:complete len:3855 gc:universal TRINITY\_DN1616\_c0\_g1\_i6:218-11782(+) |
| 14 | 37 | TRINITY\_DN72625\_c0\_g1\_i1.p1 | 99.47 | 3 | 3 | 1.19e4 | 5 | 5 | 6 | Carbamidomethylation | 214200.3906 | type:complete len:1964 gc:universal TRINITY\_DN72625\_c0\_g1\_i1:173-6064(+) |
| 15 | 38 | TRINITY\_DN15274\_c0\_g1\_i1.p1 | 91.21 | 3 | 3 | 4.9e3 | 4 | 4 | 6 | Carbamidomethylation, Oxidation (M) | 201897.7344 | type:complete len:1822 gc:universal TRINITY\_DN15274\_c0\_g1\_i1:87-5552(+) |
| 16 | 39 | TRINITY\_DN477\_c0\_g1\_i7.p1 | 91.25 | 4 | 4 | 2.83e4 | 4 | 4 | 9 | Carbamidomethylation | 122478.2578 | type:3prime\_partial len:1130 gc:universal TRINITY\_DN477\_c0\_g1\_i7:3387-1(-) |
| 16 | 40 | TRINITY\_DN477\_c0\_g1\_i8.p1 | 80.24 | 4 | 4 | 1.33e4 | 3 | 3 | 7 | Carbamidomethylation | 107165.2031 | type:complete len:985 gc:universal TRINITY\_DN477\_c0\_g1\_i8:3548-594(-) |
| 16 | 41 | TRINITY\_DN477\_c0\_g1\_i3.p1 | 43.84 | 4 | 4 | 1.5e4 | 1 | 1 | 2 |  | 24580.2559 | type:internal len:228 gc:universal TRINITY\_DN477\_c0\_g1\_i3:681-1(-) |
| 16 | 42 | TRINITY\_DN477\_c0\_g1\_i8.p2 | 43.84 | 5 | 5 | 1.5e4 | 1 | 1 | 2 |  | 20774.9902 | type:3prime\_partial len:194 gc:universal TRINITY\_DN477\_c0\_g1\_i8:581-3(-) |
| 16 | 43 | TRINITY\_DN477\_c0\_g1\_i6.p1 | 43.84 | 5 | 5 | 1.5e4 | 1 | 1 | 2 |  | 20745.9492 | type:3prime\_partial len:194 gc:universal TRINITY\_DN477\_c0\_g1\_i6:581-3(-) |
| 16 | 44 | TRINITY\_DN477\_c0\_g1\_i5.p1 | 43.84 | 4 | 4 | 1.5e4 | 1 | 1 | 2 |  | 28860.875 | type:internal len:276 gc:universal TRINITY\_DN477\_c0\_g1\_i5:827-3(-) |
| 17 | 45 | TRINITY\_DN5818\_c1\_g1\_i1.p1 | 90.95 | 18 | 18 | 5.4e3 | 4 | 3 | 6 | Oxidation (M), Acetylation (Protein N-term) | 31405.1387 | type:complete len:282 gc:universal TRINITY\_DN5818\_c1\_g1\_i1:49-894(+) |
| 17 | 46 | TRINITY\_DN5818\_c1\_g1\_i2.p1 | 79.83 | 14 | 14 | 4.25e2 | 3 | 2 | 3 | Oxidation (M) | 32704.6465 | type:complete len:293 gc:universal TRINITY\_DN5818\_c1\_g1\_i2:178-1056(+) |
| 17 | 47 | TRINITY\_DN41662\_c0\_g1\_i2.p1 | 65.35 | 8 | 8 | 4.25e2 | 2 | 1 | 2 | Oxidation (M) | 32617.3926 | type:complete len:289 gc:universal TRINITY\_DN41662\_c0\_g1\_i2:168-1034(+) |
| 18 | 48 | TRINITY\_DN15584\_c0\_g1\_i1.p1 | 90.38 | 17 | 17 | 1.38e4 | 4 | 4 | 4 | Carbamidomethylation | 29534.9648 | type:complete len:275 gc:universal TRINITY\_DN15584\_c0\_g1\_i1:874-50(-) |
| 19 | 49 | TRINITY\_DN11435\_c0\_g1\_i1.p1 | 90.76 | 11 | 11 | 9.56e2 | 4 | 4 | 4 |  | 47019.5312 | type:complete len:430 gc:universal TRINITY\_DN11435\_c0\_g1\_i1:1616-327(-) |
| 20 | 50 | TRINITY\_DN3078\_c0\_g1\_i1.p1 | 90.73 | 6 | 6 | 5.64e3 | 4 | 4 | 8 | Carbamidomethylation | 127689.1797 | type:complete len:1109 gc:universal TRINITY\_DN3078\_c0\_g1\_i1:3628-302(-) |
| 21 | 51 | TRINITY\_DN2113\_c0\_g1\_i2.p1 | 90.68 | 3 | 3 | 2.07e3 | 4 | 4 | 4 | Carbamidomethylation | 193799.0156 | type:complete len:1704 gc:universal TRINITY\_DN2113\_c0\_g1\_i2:5257-146(-) |
| 21 | 52 | TRINITY\_DN2113\_c0\_g1\_i1.p1 | 90.68 | 3 | 3 | 2.07e3 | 4 | 4 | 4 | Carbamidomethylation | 193799.0156 | type:complete len:1704 gc:universal TRINITY\_DN2113\_c0\_g1\_i1:5257-146(-) |
| 22 | 53 | TRINITY\_DN6390\_c0\_g1\_i1.p1 | 90.91 | 3 | 3 | 2.31e3 | 4 | 4 | 6 | Carbamidomethylation | 214480.2344 | type:3prime\_partial len:1941 gc:universal TRINITY\_DN6390\_c0\_g1\_i1:5820-1(-) |
| 23 | 54 | TRINITY\_DN691\_c0\_g1\_i1.p1 | 80.08 | 1 | 1 | 2.21e3 | 3 | 3 | 4 | Carbamidomethylation | 169920.1562 | type:complete len:1726 gc:universal TRINITY\_DN691\_c0\_g1\_i1:5662-485(-) |
| 23 | 55 | TRINITY\_DN691\_c0\_g1\_i2.p1 | 80.08 | 1 | 1 | 2.21e3 | 3 | 3 | 4 | Carbamidomethylation | 164625.2188 | type:complete len:1673 gc:universal TRINITY\_DN691\_c0\_g1\_i2:5503-485(-) |
| 24 | 56 | TRINITY\_DN1386\_c0\_g1\_i1.p1 | 79.67 | 6 | 6 | 5.19e3 | 3 | 3 | 5 |  | 68692.1953 | type:complete len:596 gc:universal TRINITY\_DN1386\_c0\_g1\_i1:2419-632(-) |
| 25 | 57 | TRINITY\_DN428\_c0\_g1\_i1.p1 | 79.89 | 1 | 1 | 3.42e3 | 3 | 3 | 5 | Carbamidomethylation | 225824.1719 | type:complete len:1949 gc:universal TRINITY\_DN428\_c0\_g1\_i1:96-5942(+) |
| 26 | 58 | TRINITY\_DN1199\_c0\_g1\_i2.p1 | 80.18 | 17 | 17 | 1.21e4 | 3 | 3 | 5 | Carbamidomethylation | 33218.2305 | type:complete len:296 gc:universal TRINITY\_DN1199\_c0\_g1\_i2:164-1051(+) |
| 26 | 59 | TRINITY\_DN1199\_c0\_g1\_i1.p1 | 80.18 | 17 | 17 | 1.21e4 | 3 | 3 | 5 | Carbamidomethylation | 33218.2305 | type:complete len:296 gc:universal TRINITY\_DN1199\_c0\_g1\_i1:180-1067(+) |
| 27 | 60 | TRINITY\_DN3986\_c0\_g1\_i1.p1 | 79.32 | 7 | 7 | 5.74e2 | 3 | 3 | 4 | Oxidation (M), Carbamidomethylation | 50011.8867 | type:complete len:446 gc:universal TRINITY\_DN3986\_c0\_g1\_i1:252-1589(+) |
| 27 | 61 | TRINITY\_DN1910\_c0\_g1\_i1.p1 | 79.32 | 6 | 6 | 5.74e2 | 3 | 3 | 4 | Oxidation (M), Carbamidomethylation | 50135.0117 | type:complete len:447 gc:universal TRINITY\_DN1910\_c0\_g1\_i1:2183-843(-) |
| 27 | 62 | TRINITY\_DN1910\_c0\_g1\_i5.p1 | 65.2 | 4 | 4 | 5.74e2 | 2 | 2 | 3 | Oxidation (M), Carbamidomethylation | 50120.9062 | type:complete len:447 gc:universal TRINITY\_DN1910\_c0\_g1\_i5:2031-691(-) |
| 27 | 63 | TRINITY\_DN5365\_c0\_g1\_i3.p1 | 43.16 | 2 | 2 | 0 | 1 | 1 | 1 |  | 51878.4453 | type:complete len:458 gc:universal TRINITY\_DN5365\_c0\_g1\_i3:1448-75(-) |
| 27 | 64 | TRINITY\_DN5365\_c0\_g1\_i2.p1 | 43.16 | 2 | 2 | 0 | 1 | 1 | 1 |  | 51878.4453 | type:complete len:458 gc:universal TRINITY\_DN5365\_c0\_g1\_i2:1448-75(-) |
| 27 | 65 | TRINITY\_DN3986\_c0\_g1\_i2.p1 | 43.16 | 2 | 2 | 0 | 1 | 1 | 1 |  | 50284.332 | type:complete len:446 gc:universal TRINITY\_DN3986\_c0\_g1\_i2:47-1384(+) |
| 28 | 66 | TRINITY\_DN199\_c0\_g1\_i1.p1 | 79.73 | 9 | 9 | 3.65e3 | 3 | 3 | 4 |  | 25170.3418 | type:complete len:229 gc:universal TRINITY\_DN199\_c0\_g1\_i1:207-893(+) |
| 29 | 67 | TRINITY\_DN64\_c0\_g1\_i1.p1 | 80.31 | 8 | 8 | 3.94e3 | 3 | 3 | 5 |  | 51450.1914 | type:complete len:468 gc:universal TRINITY\_DN64\_c0\_g1\_i1:1555-152(-) |
| 30 | 68 | TRINITY\_DN244\_c0\_g1\_i1.p1 | 80.01 | 2 | 2 | 3.11e3 | 3 | 3 | 5 |  | 313594.0938 | type:complete len:3019 gc:universal TRINITY\_DN244\_c0\_g1\_i1:9748-692(-) |
| 31 | 69 | TRINITY\_DN250\_c0\_g1\_i3.p1 | 79.85 | 12 | 12 | 8.82e2 | 3 | 3 | 3 | Carbamidomethylation, Oxidation (M) | 44456.6445 | type:3prime\_partial len:402 gc:universal TRINITY\_DN250\_c0\_g1\_i3:131-1333(+) |
| 31 | 70 | TRINITY\_DN250\_c0\_g1\_i5.p1 | 65.34 | 8 | 8 | 4.63e2 | 2 | 2 | 2 | Carbamidomethylation, Oxidation (M) | 50305.4453 | type:complete len:452 gc:universal TRINITY\_DN250\_c0\_g1\_i5:131-1486(+) |
| 31 | 71 | TRINITY\_DN687\_c0\_g2\_i1.p1 | 65.34 | 8 | 8 | 4.63e2 | 2 | 2 | 2 | Carbamidomethylation, Oxidation (M) | 50237.3242 | type:complete len:453 gc:universal TRINITY\_DN687\_c0\_g2\_i1:90-1448(+) |
| 31 | 72 | TRINITY\_DN250\_c0\_g1\_i1.p1 | 65.34 | 9 | 9 | 4.63e2 | 2 | 2 | 2 | Carbamidomethylation, Oxidation (M) | 44454.6094 | type:3prime\_partial len:402 gc:universal TRINITY\_DN250\_c0\_g1\_i1:131-1333(+) |
| 31 | 73 | TRINITY\_DN687\_c0\_g1\_i1.p1 | 65.34 | 8 | 8 | 4.63e2 | 2 | 2 | 2 | Carbamidomethylation, Oxidation (M) | 50621.6914 | type:complete len:453 gc:universal TRINITY\_DN687\_c0\_g1\_i1:95-1453(+) |
| 31 | 74 | TRINITY\_DN250\_c0\_g1\_i6.p1 | 43.58 | 4 | 4 | 0 | 1 | 1 | 1 |  | 50263.3672 | type:complete len:452 gc:universal TRINITY\_DN250\_c0\_g1\_i6:131-1486(+) |
| 31 | 75 | TRINITY\_DN96161\_c0\_g1\_i1.p1 | 43.58 | 4 | 4 | 0 | 1 | 1 | 1 |  | 50498.6562 | type:complete len:453 gc:universal TRINITY\_DN96161\_c0\_g1\_i1:1593-235(-) |
| 31 | 76 | TRINITY\_DN250\_c0\_g1\_i4.p1 | 43.58 | 4 | 4 | 0 | 1 | 1 | 1 |  | 50305.4453 | type:complete len:452 gc:universal TRINITY\_DN250\_c0\_g1\_i4:131-1486(+) |
| 31 | 77 | TRINITY\_DN250\_c0\_g1\_i2.p1 | 43.53 | 4 | 4 | 4.18e2 | 1 | 1 | 1 | Oxidation (M) | 35668.3789 | type:3prime\_partial len:322 gc:universal TRINITY\_DN250\_c0\_g1\_i2:131-1093(+) |
| 32 | 78 | TRINITY\_DN3036\_c0\_g1\_i2.p2 | 79.94 | 8 | 8 | 3.71e3 | 3 | 3 | 4 | Oxidation (M) | 59586.3086 | type:internal len:548 gc:universal TRINITY\_DN3036\_c0\_g1\_i2:1643-3(-) |
| 32 | 79 | TRINITY\_DN3036\_c0\_g1\_i3.p1 | 79.94 | 8 | 8 | 3.71e3 | 3 | 3 | 4 | Oxidation (M) | 59630.4023 | type:internal len:548 gc:universal TRINITY\_DN3036\_c0\_g1\_i3:1643-3(-) |
| 32 | 80 | TRINITY\_DN3036\_c0\_g1\_i8.p1 | 79.94 | 8 | 8 | 3.71e3 | 3 | 3 | 4 | Oxidation (M) | 59630.4023 | type:internal len:548 gc:universal TRINITY\_DN3036\_c0\_g1\_i8:1643-3(-) |
| 32 | 81 | TRINITY\_DN3036\_c0\_g1\_i11.p1 | 65.15 | 8 | 8 | 2.79e3 | 2 | 2 | 3 | Oxidation (M) | 40331.5547 | type:internal len:374 gc:universal TRINITY\_DN3036\_c0\_g1\_i11:1121-3(-) |
| 32 | 82 | TRINITY\_DN3036\_c0\_g1\_i16.p1 | 65.15 | 8 | 8 | 2.79e3 | 2 | 2 | 3 | Oxidation (M) | 40331.5547 | type:internal len:374 gc:universal TRINITY\_DN3036\_c0\_g1\_i16:1121-3(-) |
| 32 | 83 | TRINITY\_DN3036\_c0\_g1\_i19.p1 | 65.15 | 8 | 8 | 2.79e3 | 2 | 2 | 3 | Oxidation (M) | 40287.4609 | type:internal len:374 gc:universal TRINITY\_DN3036\_c0\_g1\_i19:1121-3(-) |
| 32 | 84 | TRINITY\_DN3036\_c0\_g1\_i13.p1 | 65.15 | 8 | 8 | 2.79e3 | 2 | 2 | 3 | Oxidation (M) | 40287.4609 | type:internal len:374 gc:universal TRINITY\_DN3036\_c0\_g1\_i13:1121-3(-) |
| 32 | 85 | TRINITY\_DN3036\_c0\_g1\_i4.p1 | 43.2 | 7 | 7 | 0 | 1 | 1 | 1 | Oxidation (M) | 25489.3926 | type:internal len:238 gc:universal TRINITY\_DN3036\_c0\_g1\_i4:713-3(-) |
| 32 | 86 | TRINITY\_DN3036\_c0\_g1\_i6.p1 | 43.2 | 7 | 7 | 0 | 1 | 1 | 1 | Oxidation (M) | 25533.4863 | type:internal len:238 gc:universal TRINITY\_DN3036\_c0\_g1\_i6:713-3(-) |
| 33 | 87 | TRINITY\_DN73067\_c0\_g1\_i1.p1 | 65.52 | 3 | 3 | 1.61e3 | 2 | 2 | 4 | Oxidation (M) | 93020.375 | type:complete len:831 gc:universal TRINITY\_DN73067\_c0\_g1\_i1:3714-1222(-) |
| 34 | 88 | TRINITY\_DN477\_c0\_g1\_i9.p1 | 80.06 | 8 | 8 | 4.13e3 | 3 | 3 | 5 | Oxidation (M) | 34222.9453 | type:complete len:306 gc:universal TRINITY\_DN477\_c0\_g1\_i9:1840-923(-) |
| 35 | 89 | TRINITY\_DN1280\_c0\_g1\_i1.p1 | 80.2 | 8 | 8 | 6.11e2 | 3 | 3 | 3 |  | 47818.6641 | type:complete len:426 gc:universal TRINITY\_DN1280\_c0\_g1\_i1:195-1472(+) |
| 36 | 90 | TRINITY\_DN909\_c0\_g1\_i1.p1 | 79.97 | 12 | 12 | 3.21e2 | 3 | 2 | 4 | Oxidation (M) | 35211.4883 | type:5prime\_partial len:313 gc:universal TRINITY\_DN909\_c0\_g1\_i1:1-939(+) |
| 37 | 91 | TRINITY\_DN413\_c0\_g1\_i4.p1 | 79.76 | 6 | 6 | 3.86e3 | 3 | 3 | 5 | Carbamidomethylation | 52354.1758 | type:3prime\_partial len:463 gc:universal TRINITY\_DN413\_c0\_g1\_i4:1388-3(-) |
| 37 | 92 | TRINITY\_DN413\_c0\_g1\_i2.p1 | 65.66 | 14 | 14 | 3.86e3 | 2 | 2 | 4 | Carbamidomethylation | 15739.6523 | type:3prime\_partial len:140 gc:universal TRINITY\_DN413\_c0\_g1\_i2:419-3(-) |
| 37 | 93 | TRINITY\_DN413\_c0\_g1\_i3.p1 | 64.9 | 6 | 6 | 2.97e3 | 2 | 2 | 2 | Carbamidomethylation | 39501.6133 | type:complete len:350 gc:universal TRINITY\_DN413\_c0\_g1\_i3:1286-237(-) |
| 37 | 94 | TRINITY\_DN413\_c0\_g1\_i1.p1 | 64.9 | 6 | 6 | 2.97e3 | 2 | 2 | 2 | Carbamidomethylation | 40351.7656 | type:complete len:355 gc:universal TRINITY\_DN413\_c0\_g1\_i1:1352-288(-) |
| 38 | 95 | TRINITY\_DN113\_c3\_g1\_i1.p1 | 80.33 | 4 | 4 | 2.41e3 | 3 | 3 | 4 |  | 125422.4922 | type:3prime\_partial len:1162 gc:universal TRINITY\_DN113\_c3\_g1\_i1:3485-3(-) |
| 39 | 96 | TRINITY\_DN1189\_c0\_g1\_i1.p1 | 64.75 | 5 | 5 | 4.01e2 | 2 | 2 | 2 |  | 50239.8086 | type:5prime\_partial len:449 gc:universal TRINITY\_DN1189\_c0\_g1\_i1:1554-208(-) |
| 40 | 97 | TRINITY\_DN36\_c0\_g1\_i16.p1 | 65.07 | 9 | 9 | 6.95e2 | 2 | 2 | 4 | Carbamidomethylation | 24953.5938 | type:complete len:224 gc:universal TRINITY\_DN36\_c0\_g1\_i16:829-158(-) |
| 40 | 98 | TRINITY\_DN36\_c0\_g1\_i19.p1 | 65.07 | 9 | 9 | 6.95e2 | 2 | 2 | 4 | Carbamidomethylation | 24821.4141 | type:complete len:223 gc:universal TRINITY\_DN36\_c0\_g1\_i19:826-158(-) |
| 40 | 99 | TRINITY\_DN36\_c0\_g1\_i17.p1 | 65.07 | 9 | 9 | 6.95e2 | 2 | 2 | 4 | Carbamidomethylation | 24953.5938 | type:complete len:224 gc:universal TRINITY\_DN36\_c0\_g1\_i17:819-148(-) |
| 40 | 100 | TRINITY\_DN36\_c0\_g1\_i13.p1 | 65.07 | 9 | 9 | 6.95e2 | 2 | 2 | 4 | Carbamidomethylation | 24821.4141 | type:complete len:223 gc:universal TRINITY\_DN36\_c0\_g1\_i13:816-148(-) |
| 40 | 101 | TRINITY\_DN36\_c0\_g1\_i3.p1 | 65.07 | 9 | 9 | 6.95e2 | 2 | 2 | 4 | Carbamidomethylation | 24953.5938 | type:complete len:224 gc:universal TRINITY\_DN36\_c0\_g1\_i3:829-158(-) |
| 40 | 102 | TRINITY\_DN36\_c0\_g1\_i14.p1 | 65.07 | 9 | 9 | 6.95e2 | 2 | 2 | 4 | Carbamidomethylation | 24953.5938 | type:complete len:224 gc:universal TRINITY\_DN36\_c0\_g1\_i14:819-148(-) |
| 40 | 103 | TRINITY\_DN36\_c0\_g1\_i8.p1 | 65.07 | 9 | 9 | 6.95e2 | 2 | 2 | 4 | Carbamidomethylation | 24953.5938 | type:complete len:224 gc:universal TRINITY\_DN36\_c0\_g1\_i8:819-148(-) |
| 40 | 104 | TRINITY\_DN649\_c0\_g1\_i5.p1 | 43.56 | 7 | 7 | 0 | 1 | 1 | 3 |  | 13746.6875 | type:internal len:124 gc:universal TRINITY\_DN649\_c0\_g1\_i5:371-3(-) |
| 40 | 105 | TRINITY\_DN649\_c0\_g1\_i2.p1 | 43.56 | 6 | 6 | 0 | 1 | 1 | 3 |  | 13819.7393 | type:internal len:125 gc:universal TRINITY\_DN649\_c0\_g1\_i2:374-3(-) |
| 40 | 106 | TRINITY\_DN649\_c0\_g1\_i1.p1 | 43.56 | 7 | 7 | 0 | 1 | 1 | 3 |  | 13690.5801 | type:internal len:124 gc:universal TRINITY\_DN649\_c0\_g1\_i1:371-3(-) |
| 40 | 107 | TRINITY\_DN36\_c0\_g1\_i9.p1 | 43.02 | 6 | 6 | 6.95e2 | 1 | 1 | 1 | Carbamidomethylation | 24680.2773 | type:complete len:223 gc:universal TRINITY\_DN36\_c0\_g1\_i9:934-266(-) |
| 40 | 108 | TRINITY\_DN36\_c0\_g1\_i11.p1 | 43.02 | 6 | 6 | 6.95e2 | 1 | 1 | 1 | Carbamidomethylation | 25006.7051 | type:complete len:224 gc:universal TRINITY\_DN36\_c0\_g1\_i11:1175-504(-) |
| 40 | 109 | TRINITY\_DN36\_c0\_g1\_i4.p1 | 43.02 | 6 | 6 | 6.95e2 | 1 | 1 | 1 | Carbamidomethylation | 24680.2773 | type:complete len:223 gc:universal TRINITY\_DN36\_c0\_g1\_i4:932-264(-) |
| 40 | 110 | TRINITY\_DN36\_c0\_g1\_i2.p1 | 43.02 | 6 | 6 | 6.95e2 | 1 | 1 | 1 | Carbamidomethylation | 24680.2773 | type:complete len:223 gc:universal TRINITY\_DN36\_c0\_g1\_i2:934-266(-) |
| 40 | 111 | TRINITY\_DN36\_c0\_g1\_i20.p1 | 43.02 | 6 | 6 | 6.95e2 | 1 | 1 | 1 | Carbamidomethylation | 24874.5234 | type:complete len:223 gc:universal TRINITY\_DN36\_c0\_g1\_i20:1172-504(-) |
| 40 | 112 | TRINITY\_DN36\_c0\_g1\_i1.p1 | 43.02 | 6 | 6 | 6.95e2 | 1 | 1 | 1 | Carbamidomethylation | 25006.7051 | type:complete len:224 gc:universal TRINITY\_DN36\_c0\_g1\_i1:1175-504(-) |
| 41 | 113 | TRINITY\_DN2636\_c0\_g1\_i1.p1 | 64.68 | 7 | 7 | 0 | 2 | 2 | 2 |  | 35741.2305 | type:complete len:314 gc:universal TRINITY\_DN2636\_c0\_g1\_i1:986-45(-) |
| 41 | 114 | TRINITY\_DN2636\_c0\_g1\_i2.p1 | 64.68 | 7 | 7 | 0 | 2 | 2 | 2 |  | 35741.2305 | type:complete len:314 gc:universal TRINITY\_DN2636\_c0\_g1\_i2:986-45(-) |
| 42 | 115 | TRINITY\_DN495\_c0\_g1\_i2.p1 | 65.68 | 4 | 4 | 2.7e3 | 2 | 1 | 5 | Carbamidomethylation | 72975.0312 | type:complete len:655 gc:universal TRINITY\_DN495\_c0\_g1\_i2:152-2116(+) |
| 42 | 116 | TRINITY\_DN495\_c0\_g1\_i3.p1 | 65.68 | 3 | 3 | 2.7e3 | 2 | 1 | 5 | Carbamidomethylation | 73673.9297 | type:complete len:659 gc:universal TRINITY\_DN495\_c0\_g1\_i3:152-2128(+) |
| 43 | 117 | TRINITY\_DN3545\_c0\_g1\_i2.p1 | 65.02 | 10 | 10 | 1.96e4 | 2 | 1 | 4 | Carbamidomethylation | 25563.4785 | type:5prime\_partial len:236 gc:universal TRINITY\_DN3545\_c0\_g1\_i2:3-710(+) |
| 43 | 118 | TRINITY\_DN3545\_c0\_g1\_i1.p1 | 65.02 | 8 | 8 | 1.96e4 | 2 | 1 | 4 | Carbamidomethylation | 31576.2656 | type:5prime\_partial len:293 gc:universal TRINITY\_DN3545\_c0\_g1\_i1:3-881(+) |
| 43 | 119 | TRINITY\_DN158421\_c0\_g1\_i1.p1 | 42.42 | 10 | 10 | 1.96e4 | 1 | 1 | 2 | Carbamidomethylation | 12814.7959 | type:internal len:115 gc:universal TRINITY\_DN158421\_c0\_g1\_i1:1-342(+) |
| 44 | 120 | TRINITY\_DN120478\_c0\_g1\_i1.p1 | 63.65 | 6 | 6 | 7.75e2 | 2 | 2 | 2 |  | 59788.2227 | type:complete len:544 gc:universal TRINITY\_DN120478\_c0\_g1\_i1:2674-1043(-) |
| 45 | 121 | TRINITY\_DN165536\_c0\_g1\_i1.p1 | 65.73 | 11 | 11 | 2.41e3 | 2 | 2 | 2 | Acetylation (Protein N-term) | 28339.4531 | type:complete len:249 gc:universal TRINITY\_DN165536\_c0\_g1\_i1:851-105(-) |
| 46 | 122 | TRINITY\_DN8903\_c0\_g1\_i1.p1 | 64.78 | 4 | 4 | 1.29e3 | 2 | 2 | 2 |  | 50029.5586 | type:5prime\_partial len:447 gc:universal TRINITY\_DN8903\_c0\_g1\_i1:1546-206(-) |
| 47 | 123 | TRINITY\_DN2472\_c0\_g1\_i1.p1 | 64.98 | 8 | 8 | 4.53e3 | 2 | 2 | 2 | Acetylation (Protein N-term) | 37676.8203 | type:complete len:344 gc:universal TRINITY\_DN2472\_c0\_g1\_i1:311-1342(+) |
| 48 | 124 | TRINITY\_DN11522\_c0\_g1\_i3.p1 | 65.29 | 3 | 3 | 2.82e3 | 2 | 2 | 3 |  | 93820.75 | type:5prime\_partial len:855 gc:universal TRINITY\_DN11522\_c0\_g1\_i3:2878-314(-) |
| 48 | 125 | TRINITY\_DN11522\_c0\_g1\_i1.p1 | 65.29 | 3 | 3 | 2.82e3 | 2 | 2 | 3 |  | 93223.9141 | type:5prime\_partial len:849 gc:universal TRINITY\_DN11522\_c0\_g1\_i1:2860-314(-) |
| 48 | 126 | TRINITY\_DN11522\_c0\_g1\_i4.p1 | 65.29 | 3 | 3 | 2.82e3 | 2 | 2 | 3 |  | 93423.2266 | type:5prime\_partial len:851 gc:universal TRINITY\_DN11522\_c0\_g1\_i4:2866-314(-) |
| 48 | 127 | TRINITY\_DN11522\_c0\_g1\_i2.p1 | 65.29 | 3 | 3 | 2.82e3 | 2 | 2 | 3 |  | 93621.4375 | type:5prime\_partial len:853 gc:universal TRINITY\_DN11522\_c0\_g1\_i2:2872-314(-) |
| 49 | 128 | TRINITY\_DN602\_c0\_g1\_i3.p1 | 65.19 | 3 | 3 | 0 | 2 | 2 | 2 |  | 92279.0078 | type:complete len:851 gc:universal TRINITY\_DN602\_c0\_g1\_i3:3046-494(-) |
| 49 | 129 | TRINITY\_DN602\_c0\_g1\_i4.p1 | 65.19 | 4 | 4 | 0 | 2 | 2 | 2 |  | 61175.9766 | type:5prime\_partial len:552 gc:universal TRINITY\_DN602\_c0\_g1\_i4:3498-1843(-) |
| 49 | 130 | TRINITY\_DN602\_c0\_g1\_i1.p1 | 65.19 | 4 | 4 | 0 | 2 | 2 | 2 |  | 65701.2422 | type:complete len:593 gc:universal TRINITY\_DN602\_c0\_g1\_i1:3622-1844(-) |
| 50 | 131 | TRINITY\_DN166344\_c0\_g1\_i1.p1 | 65.11 | 7 | 7 | 1.26e3 | 2 | 2 | 3 | Carbamidomethylation | 40794.2773 | type:complete len:360 gc:universal TRINITY\_DN166344\_c0\_g1\_i1:93-1172(+) |
| 51 | 132 | TRINITY\_DN118\_c1\_g1\_i5.p1 | 65.41 | 8 | 8 | 2.26e3 | 2 | 2 | 2 | Carbamidomethylation | 23277.6309 | type:5prime\_partial len:203 gc:universal TRINITY\_DN118\_c1\_g1\_i5:620-12(-) |
| 51 | 133 | TRINITY\_DN118\_c1\_g1\_i3.p1 | 65.41 | 8 | 8 | 2.26e3 | 2 | 2 | 2 | Carbamidomethylation | 22860.4883 | type:internal len:200 gc:universal TRINITY\_DN118\_c1\_g1\_i3:599-3(-) |
| 52 | 134 | TRINITY\_DN1936\_c0\_g1\_i3.p1 | 65.72 | 2 | 2 | 2.2e3 | 2 | 2 | 3 | Carbamidomethylation | 119975.3906 | type:internal len:1070 gc:universal TRINITY\_DN1936\_c0\_g1\_i3:3208-2(-) |
| 53 | 135 | TRINITY\_DN942\_c0\_g1\_i2.p1 | 64.76 | 0 | 0 | 2.11e2 | 2 | 2 | 2 | Oxidation (M), Carbamidomethylation | 591926.3125 | type:complete len:5116 gc:universal TRINITY\_DN942\_c0\_g1\_i2:15664-317(-) |
| 53 | 136 | TRINITY\_DN942\_c0\_g1\_i3.p1 | 64.76 | 0 | 0 | 2.11e2 | 2 | 2 | 2 | Oxidation (M), Carbamidomethylation | 590662.8125 | type:complete len:5105 gc:universal TRINITY\_DN942\_c0\_g1\_i3:15631-317(-) |
| 53 | 137 | TRINITY\_DN942\_c0\_g1\_i5.p1 | 64.76 | 0 | 0 | 2.11e2 | 2 | 2 | 2 | Oxidation (M), Carbamidomethylation | 591926.25 | type:complete len:5116 gc:universal TRINITY\_DN942\_c0\_g1\_i5:15664-317(-) |
| 54 | 138 | TRINITY\_DN875\_c0\_g1\_i1.p1 | 65.33 | 3 | 3 | 1.33e4 | 2 | 2 | 3 | Carbamidomethylation | 67023.6094 | type:complete len:606 gc:universal TRINITY\_DN875\_c0\_g1\_i1:2207-390(-) |
| 54 | 139 | TRINITY\_DN875\_c0\_g1\_i2.p1 | 65.33 | 3 | 3 | 1.33e4 | 2 | 2 | 3 | Carbamidomethylation | 67023.6094 | type:complete len:606 gc:universal TRINITY\_DN875\_c0\_g1\_i2:2136-319(-) |
| 54 | 140 | TRINITY\_DN875\_c0\_g1\_i3.p1 | 65.33 | 3 | 3 | 1.33e4 | 2 | 2 | 3 | Carbamidomethylation | 67023.6094 | type:complete len:606 gc:universal TRINITY\_DN875\_c0\_g1\_i3:2042-225(-) |
| 55 | 141 | TRINITY\_DN8341\_c0\_g1\_i1.p1 | 65.53 | 6 | 6 | 3.17e3 | 2 | 2 | 2 | Carbamidomethylation | 40708.6602 | type:complete len:368 gc:universal TRINITY\_DN8341\_c0\_g1\_i1:1281-178(-) |
| 55 | 142 | TRINITY\_DN8341\_c0\_g1\_i2.p1 | 65.53 | 6 | 6 | 3.17e3 | 2 | 2 | 2 | Carbamidomethylation | 40708.6602 | type:complete len:368 gc:universal TRINITY\_DN8341\_c0\_g1\_i2:1318-215(-) |
| 56 | 143 | TRINITY\_DN941\_c0\_g1\_i9.p1 | 64.09 | 1 | 1 | 1.26e3 | 2 | 2 | 2 | Carbamidomethylation | 340219.4688 | type:complete len:3186 gc:universal TRINITY\_DN941\_c0\_g1\_i9:86-9643(+) |
| 56 | 144 | TRINITY\_DN941\_c0\_g1\_i7.p1 | 40.64 | 2 | 2 | 0 | 1 | 1 | 1 | Carbamidomethylation | 79898.0078 | type:complete len:760 gc:universal TRINITY\_DN941\_c0\_g1\_i7:683-2962(+) |
| 56 | 145 | TRINITY\_DN941\_c0\_g1\_i11.p1 | 40.64 | 2 | 2 | 0 | 1 | 1 | 1 | Carbamidomethylation | 110353.6641 | type:5prime\_partial len:1040 gc:universal TRINITY\_DN941\_c0\_g1\_i11:1-3120(+) |
| 56 | 146 | TRINITY\_DN941\_c0\_g1\_i2.p1 | 40.64 | 2 | 2 | 0 | 1 | 1 | 1 | Carbamidomethylation | 77931.9609 | type:complete len:740 gc:universal TRINITY\_DN941\_c0\_g1\_i2:752-2971(+) |
| 56 | 147 | TRINITY\_DN941\_c0\_g1\_i5.p1 | 40.64 | 2 | 2 | 0 | 1 | 1 | 1 | Carbamidomethylation | 77876.8281 | type:complete len:740 gc:universal TRINITY\_DN941\_c0\_g1\_i5:752-2971(+) |
| 56 | 148 | TRINITY\_DN941\_c0\_g1\_i8.p1 | 40.64 | 2 | 2 | 0 | 1 | 1 | 1 | Carbamidomethylation | 79953.1406 | type:complete len:760 gc:universal TRINITY\_DN941\_c0\_g1\_i8:683-2962(+) |
| 56 | 149 | TRINITY\_DN941\_c0\_g1\_i6.p1 | 40.64 | 2 | 2 | 0 | 1 | 1 | 1 | Carbamidomethylation | 68481.9375 | type:5prime\_partial len:653 gc:universal TRINITY\_DN941\_c0\_g1\_i6:3-1961(+) |
| 56 | 150 | TRINITY\_DN941\_c0\_g1\_i15.p1 | 40.64 | 1 | 1 | 0 | 1 | 1 | 1 | Carbamidomethylation | 127030.5156 | type:5prime\_partial len:1194 gc:universal TRINITY\_DN941\_c0\_g1\_i15:3-3584(+) |
| 57 | 151 | TRINITY\_DN6920\_c0\_g1\_i2.p1 | 65.56 | 4 | 4 | 0 | 2 | 2 | 2 | Oxidation (M) | 46932.582 | type:5prime\_partial len:411 gc:universal TRINITY\_DN6920\_c0\_g1\_i2:2147-915(-) |
| 57 | 152 | TRINITY\_DN6920\_c0\_g1\_i3.p1 | 65.56 | 4 | 4 | 0 | 2 | 2 | 2 | Oxidation (M) | 46932.582 | type:5prime\_partial len:411 gc:universal TRINITY\_DN6920\_c0\_g1\_i3:2124-892(-) |
| 57 | 153 | TRINITY\_DN6920\_c0\_g1\_i4.p1 | 43.71 | 3 | 3 | 0 | 1 | 1 | 1 |  | 32195.3809 | type:5prime\_partial len:282 gc:universal TRINITY\_DN6920\_c0\_g1\_i4:1321-476(-) |
| 57 | 154 | TRINITY\_DN6920\_c0\_g1\_i1.p1 | 43.71 | 3 | 3 | 0 | 1 | 1 | 1 |  | 32882.168 | type:complete len:287 gc:universal TRINITY\_DN6920\_c0\_g1\_i1:1775-915(-) |
| 57 | 155 | TRINITY\_DN3448\_c0\_g1\_i9.p3 | 43.71 | 8 | 8 | 0 | 1 | 1 | 1 | Oxidation (M) | 11358.1816 | type:internal len:102 gc:universal TRINITY\_DN3448\_c0\_g1\_i9:303-1(-) |
| 57 | 156 | TRINITY\_DN5906\_c3\_g1\_i1.p2 | 43.71 | 6 | 6 | 0 | 1 | 1 | 1 | Oxidation (M) | 15031.4814 | type:internal len:137 gc:universal TRINITY\_DN5906\_c3\_g1\_i1:3-410(+) |
| 57 | 157 | TRINITY\_DN3448\_c0\_g2\_i1.p1 | 43.71 | 7 | 7 | 0 | 1 | 1 | 1 | Oxidation (M) | 12264.2549 | type:internal len:109 gc:universal TRINITY\_DN3448\_c0\_g2\_i1:326-3(-) |
| 57 | 158 | TRINITY\_DN2799\_c0\_g1\_i3.p1 | 43.71 | 7 | 7 | 0 | 1 | 1 | 1 | Oxidation (M) | 12365.4863 | type:internal len:112 gc:universal TRINITY\_DN2799\_c0\_g1\_i3:334-2(-) |
| 57 | 159 | TRINITY\_DN2799\_c0\_g1\_i2.p1 | 43.71 | 7 | 7 | 0 | 1 | 1 | 1 | Oxidation (M) | 12149.2383 | type:internal len:111 gc:universal TRINITY\_DN2799\_c0\_g1\_i2:331-2(-) |
| 57 | 160 | TRINITY\_DN2799\_c0\_g1\_i4.p2 | 43.71 | 7 | 7 | 0 | 1 | 1 | 1 | Oxidation (M) | 12149.2383 | type:internal len:111 gc:universal TRINITY\_DN2799\_c0\_g1\_i4:331-2(-) |
| 57 | 161 | TRINITY\_DN3448\_c0\_g1\_i5.p2 | 43.71 | 8 | 8 | 0 | 1 | 1 | 1 | Oxidation (M) | 11358.1816 | type:internal len:102 gc:universal TRINITY\_DN3448\_c0\_g1\_i5:303-1(-) |
| 57 | 162 | TRINITY\_DN3448\_c0\_g2\_i3.p2 | 43.71 | 8 | 8 | 0 | 1 | 1 | 1 | Oxidation (M) | 12003.9434 | type:internal len:107 gc:universal TRINITY\_DN3448\_c0\_g2\_i3:318-1(-) |
| 57 | 163 | TRINITY\_DN3448\_c0\_g1\_i7.p2 | 43.71 | 7 | 7 | 0 | 1 | 1 | 1 | Oxidation (M) | 12485.4922 | type:internal len:111 gc:universal TRINITY\_DN3448\_c0\_g1\_i7:330-1(-) |
| 57 | 164 | TRINITY\_DN32727\_c0\_g1\_i2.p1 | 43.71 | 5 | 5 | 0 | 1 | 1 | 1 | Oxidation (M) | 17342.1152 | type:internal len:154 gc:universal TRINITY\_DN32727\_c0\_g1\_i2:460-2(-) |
| 57 | 165 | TRINITY\_DN2799\_c0\_g1\_i1.p1 | 43.71 | 7 | 7 | 0 | 1 | 1 | 1 | Oxidation (M) | 12875.0586 | type:internal len:117 gc:universal TRINITY\_DN2799\_c0\_g1\_i1:349-2(-) |
| 58 | 166 | TRINITY\_DN144508\_c0\_g1\_i1.p1 | 64.62 | 8 | 8 | 1.09e3 | 2 | 2 | 2 |  | 29446.1543 | type:internal len:265 gc:universal TRINITY\_DN144508\_c0\_g1\_i1:1-792(+) |
| 59 | 167 | TRINITY\_DN8597\_c0\_g1\_i1.p1 | 65.53 | 22 | 22 | 0 | 2 | 1 | 2 | Carbamidomethylation | 13587.9307 | type:internal len:124 gc:universal TRINITY\_DN8597\_c0\_g1\_i1:369-1(-) |
| 60 | 168 | TRINITY\_DN163801\_c0\_g1\_i1.p1 | 65.52 | 9 | 9 | 9.72e3 | 2 | 2 | 4 | Acetylation (Protein N-term) | 33076.9062 | type:complete len:298 gc:universal TRINITY\_DN163801\_c0\_g1\_i1:55-948(+) |
| 61 | 169 | TRINITY\_DN360\_c0\_g1\_i1.p1 | 64.93 | 5 | 5 | 2.41e3 | 2 | 2 | 3 |  | 52389.3047 | type:5prime\_partial len:476 gc:universal TRINITY\_DN360\_c0\_g1\_i1:2237-810(-) |
| 62 | 170 | TRINITY\_DN141338\_c2\_g1\_i1.p1 | 65.43 | 3 | 3 | 1.32e3 | 2 | 1 | 2 |  | 82716.2266 | type:complete len:720 gc:universal TRINITY\_DN141338\_c2\_g1\_i1:2850-691(-) |
| 63 | 171 | TRINITY\_DN495\_c0\_g1\_i5.p1 | 65.54 | 5 | 5 | 1.92e3 | 2 | 2 | 3 | Carbamidomethylation | 45678.0781 | type:3prime\_partial len:407 gc:universal TRINITY\_DN495\_c0\_g1\_i5:152-1369(+) |
| 63 | 172 | TRINITY\_DN495\_c0\_g1\_i6.p1 | 65.54 | 5 | 5 | 1.92e3 | 2 | 2 | 3 | Carbamidomethylation | 42984.9375 | type:3prime\_partial len:382 gc:universal TRINITY\_DN495\_c0\_g1\_i6:152-1294(+) |
| 64 | 173 | TRINITY\_DN785\_c0\_g1\_i1.p1 | 65.16 | 9 | 9 | 3.18e3 | 2 | 2 | 5 | Carbamidomethylation | 33410.082 | type:complete len:289 gc:universal TRINITY\_DN785\_c0\_g1\_i1:1062-196(-) |
| 65 | 174 | TRINITY\_DN7659\_c0\_g1\_i2.p1 | 64.27 | 7 | 7 | 0 | 2 | 2 | 2 | Acetylation (Protein N-term) | 40699.8555 | type:complete len:359 gc:universal TRINITY\_DN7659\_c0\_g1\_i2:82-1158(+) |
| 65 | 175 | TRINITY\_DN7659\_c0\_g1\_i1.p1 | 64.27 | 7 | 7 | 0 | 2 | 2 | 2 | Acetylation (Protein N-term) | 40699.8555 | type:complete len:359 gc:universal TRINITY\_DN7659\_c0\_g1\_i1:69-1145(+) |
| 66 | 176 | TRINITY\_DN140702\_c0\_g1\_i1.p1 | 65.64 | 2 | 2 | 2.03e3 | 2 | 2 | 6 |  | 231919.1562 | type:complete len:2036 gc:universal TRINITY\_DN140702\_c0\_g1\_i1:94-6201(+) |
| 67 | 177 | TRINITY\_DN57\_c0\_g1\_i1.p1 | 64 | 3 | 3 | 1.06e3 | 2 | 2 | 2 | Oxidation (M) | 81029.6719 | type:complete len:712 gc:universal TRINITY\_DN57\_c0\_g1\_i1:140-2275(+) |
| 68 | 178 | TRINITY\_DN140696\_c0\_g1\_i1.p1 | 64.41 | 8 | 8 | 0 | 2 | 2 | 2 |  | 29465.1973 | type:complete len:260 gc:universal TRINITY\_DN140696\_c0\_g1\_i1:43-822(+) |
| 69 | 179 | TRINITY\_DN98968\_c0\_g1\_i1.p1 | 64.39 | 4 | 4 | 2.14e3 | 2 | 1 | 2 | Carbamidomethylation | 47268.0352 | type:3prime\_partial len:424 gc:universal TRINITY\_DN98968\_c0\_g1\_i1:1269-1(-) |
| 70 | 180 | TRINITY\_DN319\_c2\_g1\_i3.p1 | 64.98 | 1 | 1 | 6.27e2 | 2 | 2 | 3 | Carbamidomethylation | 279087.1875 | type:complete len:2588 gc:universal TRINITY\_DN319\_c2\_g1\_i3:165-7928(+) |
| 70 | 181 | TRINITY\_DN319\_c2\_g1\_i1.p1 | 64.98 | 1 | 1 | 6.27e2 | 2 | 2 | 3 | Carbamidomethylation | 279168.25 | type:complete len:2588 gc:universal TRINITY\_DN319\_c2\_g1\_i1:165-7928(+) |
| 70 | 182 | TRINITY\_DN319\_c2\_g1\_i2.p1 | 42.3 | 1 | 1 | 0 | 1 | 1 | 1 | Carbamidomethylation | 215796.4062 | type:3prime\_partial len:2004 gc:universal TRINITY\_DN319\_c2\_g1\_i2:165-6173(+) |
| 71 | 183 | TRINITY\_DN773\_c0\_g1\_i1.p1 | 62.79 | 5 | 5 | 5.08e2 | 2 | 2 | 2 |  | 48865.75 | type:complete len:440 gc:universal TRINITY\_DN773\_c0\_g1\_i1:1608-289(-) |
| 72 | 184 | TRINITY\_DN5633\_c0\_g1\_i1.p1 | 64.97 | 4 | 4 | 0 | 2 | 1 | 2 |  | 97310.9922 | type:complete len:848 gc:universal TRINITY\_DN5633\_c0\_g1\_i1:2881-338(-) |
| 73 | 185 | TRINITY\_DN1355\_c0\_g1\_i1.p1 | 65.39 | 7 | 7 | 2.25e2 | 2 | 2 | 2 | Carbamidomethylation | 37817.1953 | type:complete len:333 gc:universal TRINITY\_DN1355\_c0\_g1\_i1:132-1130(+) |
| 73 | 186 | TRINITY\_DN8458\_c0\_g2\_i1.p1 | 43.61 | 4 | 4 | 0 | 1 | 1 | 1 | Carbamidomethylation | 31019.791 | type:internal len:277 gc:universal TRINITY\_DN8458\_c0\_g2\_i1:830-3(-) |
| 73 | 187 | TRINITY\_DN73788\_c0\_g1\_i1.p1 | 43.61 | 3 | 3 | 0 | 1 | 1 | 1 | Carbamidomethylation | 38079.4805 | type:complete len:336 gc:universal TRINITY\_DN73788\_c0\_g1\_i1:1442-435(-) |
| 74 | 188 | TRINITY\_DN1505\_c0\_g1\_i1.p1 | 43.44 | 8 | 8 | 9.41e2 | 1 | 1 | 1 | Acetylation (Protein N-term) | 17930.752 | type:complete len:154 gc:universal TRINITY\_DN1505\_c0\_g1\_i1:98-559(+) |
| 75 | 189 | TRINITY\_DN925\_c0\_g1\_i1.p1 | 43.82 | 5 | 5 | 0 | 1 | 1 | 1 |  | 28336.7676 | type:complete len:261 gc:universal TRINITY\_DN925\_c0\_g1\_i1:826-44(-) |
| 76 | 190 | TRINITY\_DN1819\_c0\_g1\_i5.p1 | 43.6 | 3 | 3 | 7.24e2 | 1 | 1 | 1 |  | 49581.1055 | type:complete len:432 gc:universal TRINITY\_DN1819\_c0\_g1\_i5:79-1374(+) |
| 76 | 191 | TRINITY\_DN1819\_c0\_g1\_i7.p1 | 43.6 | 3 | 3 | 7.24e2 | 1 | 1 | 1 |  | 49581.1055 | type:complete len:432 gc:universal TRINITY\_DN1819\_c0\_g1\_i7:67-1362(+) |
| 77 | 192 | TRINITY\_DN624\_c0\_g1\_i1.p1 | 43.82 | 2 | 2 | 7.51e2 | 1 | 1 | 1 |  | 101743.5391 | type:complete len:884 gc:universal TRINITY\_DN624\_c0\_g1\_i1:218-2869(+) |
| 78 | 193 | TRINITY\_DN11248\_c0\_g1\_i1.p1 | 43.78 | 1 | 1 | 0 | 1 | 1 | 1 | Carbamidomethylation | 194116.9531 | type:3prime\_partial len:1700 gc:universal TRINITY\_DN11248\_c0\_g1\_i1:111-5207(+) |
| 78 | 194 | TRINITY\_DN11248\_c0\_g1\_i3.p1 | 43.78 | 1 | 1 | 0 | 1 | 1 | 1 | Carbamidomethylation | 194116.9531 | type:3prime\_partial len:1700 gc:universal TRINITY\_DN11248\_c0\_g1\_i3:111-5207(+) |
| 79 | 195 | TRINITY\_DN151\_c0\_g1\_i4.p1 | 43.18 | 3 | 3 | 0 | 1 | 1 | 1 |  | 35316.9336 | type:complete len:317 gc:universal TRINITY\_DN151\_c0\_g1\_i4:106-1056(+) |
| 79 | 196 | TRINITY\_DN151\_c0\_g1\_i3.p1 | 43.18 | 3 | 3 | 0 | 1 | 1 | 1 |  | 35286.9102 | type:complete len:317 gc:universal TRINITY\_DN151\_c0\_g1\_i3:106-1056(+) |
| 79 | 197 | TRINITY\_DN151\_c0\_g1\_i2.p1 | 43.18 | 3 | 3 | 0 | 1 | 1 | 1 |  | 35286.9102 | type:complete len:317 gc:universal TRINITY\_DN151\_c0\_g1\_i2:106-1056(+) |
| 80 | 198 | TRINITY\_DN6397\_c0\_g2\_i4.p1 | 43.74 | 9 | 9 | 0 | 1 | 1 | 1 | Acetylation (Protein N-term) | 12470.125 | type:internal len:115 gc:universal TRINITY\_DN6397\_c0\_g2\_i4:343-2(-) |
| 80 | 199 | TRINITY\_DN6397\_c0\_g2\_i3.p1 | 43.74 | 8 | 8 | 0 | 1 | 1 | 1 | Acetylation (Protein N-term) | 14030.8945 | type:internal len:130 gc:universal TRINITY\_DN6397\_c0\_g2\_i3:387-1(-) |
| 81 | 200 | TRINITY\_DN123301\_c0\_g1\_i1.p1 | 43.49 | 6 | 6 | 4.93e2 | 1 | 1 | 1 |  | 31107.168 | type:complete len:272 gc:universal TRINITY\_DN123301\_c0\_g1\_i1:90-905(+) |
| 82 | 201 | TRINITY\_DN585\_c0\_g1\_i10.p1 | 43.28 | 7 | 7 | 1.46e3 | 1 | 1 | 1 |  | 26563.5332 | type:3prime\_partial len:236 gc:universal TRINITY\_DN585\_c0\_g1\_i10:705-1(-) |
| 82 | 202 | TRINITY\_DN554\_c0\_g1\_i3.p2 | 43.28 | 6 | 6 | 1.46e3 | 1 | 1 | 1 |  | 30419.4961 | type:complete len:268 gc:universal TRINITY\_DN554\_c0\_g1\_i3:1464-661(-) |
| 82 | 203 | TRINITY\_DN585\_c0\_g1\_i2.p1 | 43.28 | 9 | 9 | 1.46e3 | 1 | 1 | 1 |  | 20558.6738 | type:internal len:184 gc:universal TRINITY\_DN585\_c0\_g1\_i2:549-1(-) |
| 82 | 204 | TRINITY\_DN585\_c0\_g1\_i5.p1 | 43.28 | 6 | 6 | 1.46e3 | 1 | 1 | 1 |  | 28098.1602 | type:internal len:250 gc:universal TRINITY\_DN585\_c0\_g1\_i5:747-1(-) |
| 82 | 205 | TRINITY\_DN585\_c0\_g1\_i3.p1 | 43.28 | 10 | 10 | 1.46e3 | 1 | 1 | 1 |  | 17628.2207 | type:internal len:157 gc:universal TRINITY\_DN585\_c0\_g1\_i3:468-1(-) |
| 82 | 206 | TRINITY\_DN585\_c0\_g1\_i4.p1 | 43.28 | 7 | 7 | 1.46e3 | 1 | 1 | 1 |  | 26348.0742 | type:complete len:232 gc:universal TRINITY\_DN585\_c0\_g1\_i4:847-152(-) |
| 82 | 207 | TRINITY\_DN554\_c0\_g1\_i5.p1 | 43.28 | 4 | 4 | 1.46e3 | 1 | 1 | 1 |  | 42081.4141 | type:3prime\_partial len:373 gc:universal TRINITY\_DN554\_c0\_g1\_i5:1118-3(-) |
| 83 | 208 | TRINITY\_DN3589\_c0\_g1\_i1.p1 | 43.33 | 3 | 3 | 2.83e2 | 1 | 1 | 1 |  | 60561.1484 | type:3prime\_partial len:548 gc:universal TRINITY\_DN3589\_c0\_g1\_i1:63-1703(+) |
| 84 | 209 | TRINITY\_DN384\_c0\_g1\_i2.p1 | 43.34 | 4 | 4 | 0 | 1 | 1 | 1 |  | 34316.0703 | type:complete len:314 gc:universal TRINITY\_DN384\_c0\_g1\_i2:1018-77(-) |
| 84 | 210 | TRINITY\_DN384\_c0\_g1\_i1.p1 | 43.34 | 4 | 4 | 0 | 1 | 1 | 1 |  | 34317.9414 | type:complete len:313 gc:universal TRINITY\_DN384\_c0\_g1\_i1:1015-77(-) |
| 84 | 211 | TRINITY\_DN22\_c0\_g1\_i1.p1 | 43.34 | 3 | 3 | 0 | 1 | 1 | 1 |  | 38528.8086 | type:5prime\_partial len:353 gc:universal TRINITY\_DN22\_c0\_g1\_i1:3-1061(+) |
| 85 | 212 | TRINITY\_DN146208\_c0\_g1\_i1.p1 | 42.86 | 5 | 5 | 0 | 1 | 1 | 1 |  | 32340.8027 | type:5prime\_partial len:284 gc:universal TRINITY\_DN146208\_c0\_g1\_i1:876-25(-) |
| 86 | 213 | TRINITY\_DN852\_c0\_g1\_i1.p1 | 43.46 | 1 | 1 | 0 | 1 | 1 | 1 |  | 65103.1602 | type:complete len:589 gc:universal TRINITY\_DN852\_c0\_g1\_i1:92-1858(+) |
| 87 | 214 | TRINITY\_DN1058\_c0\_g1\_i1.p1 | 43.8 | 0 | 0 | 1.39e3 | 1 | 1 | 4 |  | 284466.2812 | type:complete len:2639 gc:universal TRINITY\_DN1058\_c0\_g1\_i1:134-8050(+) |
| 87 | 215 | TRINITY\_DN1058\_c0\_g1\_i3.p1 | 43.8 | 0 | 0 | 1.39e3 | 1 | 1 | 4 |  | 277633.5312 | type:complete len:2581 gc:universal TRINITY\_DN1058\_c0\_g1\_i3:134-7876(+) |
| 87 | 216 | TRINITY\_DN1058\_c0\_g1\_i4.p1 | 43.8 | 2 | 2 | 1.39e3 | 1 | 1 | 4 |  | 79375.5703 | type:complete len:723 gc:universal TRINITY\_DN1058\_c0\_g1\_i4:366-2534(+) |
| 87 | 217 | TRINITY\_DN1058\_c0\_g1\_i2.p2 | 43.8 | 1 | 1 | 1.39e3 | 1 | 1 | 4 |  | 140284.0938 | type:complete len:1300 gc:universal TRINITY\_DN1058\_c0\_g1\_i2:4090-7989(+) |
| 88 | 218 | TRINITY\_DN4726\_c0\_g1\_i1.p1 | 43.35 | 2 | 2 | 1.61e2 | 1 | 1 | 1 |  | 75463.25 | type:5prime\_partial len:670 gc:universal TRINITY\_DN4726\_c0\_g1\_i1:2906-897(-) |
| 89 | 219 | TRINITY\_DN803\_c0\_g1\_i1.p1 | 42.83 | 4 | 4 | 3.23e2 | 1 | 1 | 1 |  | 37110.1719 | type:complete len:330 gc:universal TRINITY\_DN803\_c0\_g1\_i1:1049-60(-) |
| 90 | 220 | TRINITY\_DN13813\_c0\_g1\_i6.p2 | 41.47 | 4 | 4 | 3.73e2 | 1 | 1 | 1 |  | 40948.1641 | type:5prime\_partial len:370 gc:universal TRINITY\_DN13813\_c0\_g1\_i6:1389-280(-) |
| 90 | 221 | TRINITY\_DN13813\_c0\_g1\_i5.p2 | 41.47 | 4 | 4 | 3.73e2 | 1 | 1 | 1 |  | 43147.8203 | type:5prime\_partial len:388 gc:universal TRINITY\_DN13813\_c0\_g1\_i5:1942-779(-) |
| 90 | 222 | TRINITY\_DN13813\_c0\_g1\_i4.p2 | 41.47 | 4 | 4 | 3.73e2 | 1 | 1 | 1 |  | 43147.8203 | type:5prime\_partial len:388 gc:universal TRINITY\_DN13813\_c0\_g1\_i4:1948-785(-) |
| 90 | 223 | TRINITY\_DN13813\_c0\_g1\_i2.p2 | 41.47 | 4 | 4 | 3.73e2 | 1 | 1 | 1 |  | 40841.168 | type:5prime\_partial len:370 gc:universal TRINITY\_DN13813\_c0\_g1\_i2:1894-785(-) |
| 90 | 224 | TRINITY\_DN13813\_c0\_g1\_i3.p2 | 41.47 | 4 | 4 | 3.73e2 | 1 | 1 | 1 |  | 40841.168 | type:5prime\_partial len:370 gc:universal TRINITY\_DN13813\_c0\_g1\_i3:1888-779(-) |
| 91 | 225 | TRINITY\_DN69276\_c0\_g1\_i1.p1 | 43.47 | 1 | 1 | 1.35e3 | 1 | 1 | 1 |  | 97474.1719 | type:internal len:887 gc:universal TRINITY\_DN69276\_c0\_g1\_i1:2659-2(-) |
| 92 | 226 | TRINITY\_DN876\_c0\_g1\_i2.p1 | 40.76 | 5 | 5 | 3.38e2 | 1 | 1 | 1 |  | 26127.5176 | type:complete len:225 gc:universal TRINITY\_DN876\_c0\_g1\_i2:1136-462(-) |
| 92 | 227 | TRINITY\_DN876\_c0\_g1\_i1.p1 | 40.76 | 5 | 5 | 3.38e2 | 1 | 1 | 1 |  | 26127.5176 | type:complete len:225 gc:universal TRINITY\_DN876\_c0\_g1\_i1:1136-462(-) |
| 93 | 228 | TRINITY\_DN16079\_c0\_g2\_i1.p1 | 43.76 | 4 | 4 | 0 | 1 | 1 | 1 | Carbamidomethylation | 44791.4727 | type:5prime\_partial len:396 gc:universal TRINITY\_DN16079\_c0\_g2\_i1:1686-499(-) |
| 94 | 229 | TRINITY\_DN140996\_c0\_g1\_i1.p1 | 42.53 | 6 | 6 | 1.46e3 | 1 | 1 | 1 | Carbamidomethylation | 12619.5664 | type:internal len:109 gc:universal TRINITY\_DN140996\_c0\_g1\_i1:326-3(-) |
| 95 | 230 | TRINITY\_DN1030\_c0\_g1\_i2.p1 | 43.49 | 1 | 1 | 0 | 1 | 1 | 1 |  | 140221.3281 | type:complete len:1426 gc:universal TRINITY\_DN1030\_c0\_g1\_i2:4483-206(-) |
| 95 | 231 | TRINITY\_DN1030\_c0\_g1\_i1.p1 | 43.49 | 1 | 1 | 0 | 1 | 1 | 1 |  | 141535.875 | type:complete len:1439 gc:universal TRINITY\_DN1030\_c0\_g1\_i1:4522-206(-) |
| 96 | 232 | TRINITY\_DN30094\_c0\_g1\_i1.p1 | 43.53 | 2 | 2 | 0 | 1 | 1 | 1 |  | 56920.6719 | type:3prime\_partial len:504 gc:universal TRINITY\_DN30094\_c0\_g1\_i1:98-1606(+) |
| 97 | 233 | TRINITY\_DN59572\_c0\_g1\_i1.p1 | 43.58 | 2 | 2 | 3.97e2 | 1 | 1 | 1 |  | 82544.875 | type:internal len:725 gc:universal TRINITY\_DN59572\_c0\_g1\_i1:3-2174(+) |
| 98 | 234 | TRINITY\_DN1194\_c0\_g1\_i1.p1 | 41.2 | 3 | 3 | 1.57e2 | 1 | 1 | 1 |  | 61081.3672 | type:complete len:555 gc:universal TRINITY\_DN1194\_c0\_g1\_i1:101-1765(+) |
| 99 | 235 | TRINITY\_DN79\_c1\_g1\_i1.p1 | 43.3 | 2 | 2 | 4.91e2 | 1 | 1 | 1 |  | 50925.4375 | type:5prime\_partial len:450 gc:universal TRINITY\_DN79\_c1\_g1\_i1:3268-1919(-) |
| 100 | 236 | TRINITY\_DN310\_c0\_g1\_i1.p1 | 43.59 | 4 | 4 | 1.17e3 | 1 | 1 | 2 |  | 33384.6484 | type:complete len:298 gc:universal TRINITY\_DN310\_c0\_g1\_i1:1091-198(-) |
| 101 | 237 | TRINITY\_DN2637\_c0\_g1\_i7.p2 | 42.98 | 5 | 5 | 0 | 1 | 1 | 1 |  | 24919.5234 | type:internal len:233 gc:universal TRINITY\_DN2637\_c0\_g1\_i7:3-698(+) |
| 101 | 238 | TRINITY\_DN2637\_c0\_g1\_i14.p2 | 42.98 | 3 | 3 | 0 | 1 | 1 | 1 |  | 34349.1445 | type:5prime\_partial len:320 gc:universal TRINITY\_DN2637\_c0\_g1\_i14:1-960(+) |
| 101 | 239 | TRINITY\_DN2637\_c0\_g1\_i9.p2 | 42.98 | 3 | 3 | 0 | 1 | 1 | 1 |  | 36257.3242 | type:5prime\_partial len:340 gc:universal TRINITY\_DN2637\_c0\_g1\_i9:3-1022(+) |
| 101 | 240 | TRINITY\_DN2637\_c0\_g1\_i4.p1 | 42.98 | 4 | 4 | 0 | 1 | 1 | 1 |  | 29205.3301 | type:internal len:277 gc:universal TRINITY\_DN2637\_c0\_g1\_i4:1-828(+) |
| 101 | 241 | TRINITY\_DN2637\_c0\_g1\_i8.p2 | 42.98 | 4 | 4 | 0 | 1 | 1 | 1 |  | 31853.3555 | type:internal len:302 gc:universal TRINITY\_DN2637\_c0\_g1\_i8:3-905(+) |
| 101 | 242 | TRINITY\_DN2637\_c0\_g1\_i6.p1 | 42.98 | 3 | 3 | 0 | 1 | 1 | 1 |  | 34214.9922 | type:5prime\_partial len:320 gc:universal TRINITY\_DN2637\_c0\_g1\_i6:1-960(+) |
| 101 | 243 | TRINITY\_DN2637\_c0\_g1\_i5.p2 | 42.98 | 4 | 4 | 0 | 1 | 1 | 1 |  | 28455.5449 | type:internal len:270 gc:universal TRINITY\_DN2637\_c0\_g1\_i5:3-809(+) |
| 101 | 244 | TRINITY\_DN2637\_c0\_g1\_i13.p1 | 42.98 | 4 | 4 | 0 | 1 | 1 | 1 |  | 31247.6602 | type:internal len:297 gc:universal TRINITY\_DN2637\_c0\_g1\_i13:3-890(+) |
| 101 | 245 | TRINITY\_DN2637\_c0\_g1\_i2.p2 | 42.98 | 4 | 4 | 0 | 1 | 1 | 1 |  | 31274.6855 | type:internal len:297 gc:universal TRINITY\_DN2637\_c0\_g1\_i2:3-890(+) |
| 102 | 246 | TRINITY\_DN5584\_c0\_g1\_i3.p1 | 42.34 | 1 | 1 | 0 | 1 | 1 | 1 |  | 110603.7422 | type:complete len:962 gc:universal TRINITY\_DN5584\_c0\_g1\_i3:2920-35(-) |
| 102 | 247 | TRINITY\_DN5584\_c0\_g1\_i2.p1 | 42.34 | 4 | 4 | 0 | 1 | 1 | 1 |  | 36306.3672 | type:complete len:313 gc:universal TRINITY\_DN5584\_c0\_g1\_i2:973-35(-) |
| 103 | 248 | TRINITY\_DN1821\_c0\_g1\_i6.p1 | 43.24 | 2 | 2 | 7.91e2 | 1 | 1 | 1 |  | 73657.1172 | type:complete len:638 gc:universal TRINITY\_DN1821\_c0\_g1\_i6:297-2210(+) |
| 103 | 249 | TRINITY\_DN1821\_c0\_g1\_i2.p1 | 43.24 | 3 | 3 | 7.91e2 | 1 | 1 | 1 |  | 35059.2227 | type:internal len:299 gc:universal TRINITY\_DN1821\_c0\_g1\_i2:1-894(+) |
| 103 | 250 | TRINITY\_DN1821\_c0\_g1\_i4.p1 | 43.24 | 2 | 2 | 7.91e2 | 1 | 1 | 1 |  | 73525.9219 | type:complete len:637 gc:universal TRINITY\_DN1821\_c0\_g1\_i4:304-2214(+) |
| 103 | 251 | TRINITY\_DN1821\_c0\_g1\_i5.p1 | 43.24 | 2 | 2 | 7.91e2 | 1 | 1 | 1 |  | 73657.1172 | type:complete len:638 gc:universal TRINITY\_DN1821\_c0\_g1\_i5:297-2210(+) |
| 104 | 252 | TRINITY\_DN71276\_c0\_g1\_i1.p1 | 43.67 | 1 | 1 | 0 | 1 | 1 | 1 |  | 139103.3125 | type:5prime\_partial len:1201 gc:universal TRINITY\_DN71276\_c0\_g1\_i1:1-3603(+) |
| 105 | 253 | TRINITY\_DN6428\_c0\_g1\_i2.p1 | 43.81 | 4 | 4 | 1.35e3 | 1 | 1 | 1 | Carbamidomethylation | 33956.7344 | type:3prime\_partial len:303 gc:universal TRINITY\_DN6428\_c0\_g1\_i2:906-1(-) |
| 105 | 254 | TRINITY\_DN6428\_c0\_g1\_i1.p1 | 43.81 | 3 | 3 | 1.35e3 | 1 | 1 | 1 | Carbamidomethylation | 41179.0664 | type:internal len:367 gc:universal TRINITY\_DN6428\_c0\_g1\_i1:1098-1(-) |
| 106 | 255 | TRINITY\_DN1272\_c0\_g1\_i1.p1 | 43.36 | 4 | 4 | 0 | 1 | 1 | 1 |  | 30690.1172 | type:complete len:262 gc:universal TRINITY\_DN1272\_c0\_g1\_i1:957-172(-) |
| 107 | 256 | TRINITY\_DN1377\_c0\_g1\_i1.p1 | 43.82 | 1 | 1 | 4.39e2 | 1 | 1 | 1 |  | 99936.9219 | type:complete len:878 gc:universal TRINITY\_DN1377\_c0\_g1\_i1:68-2701(+) |
| 108 | 257 | TRINITY\_DN16120\_c0\_g1\_i2.p1 | 41.94 | 5 | 5 | 0 | 1 | 1 | 1 | Carbamidomethylation | 23723.4336 | type:5prime\_partial len:232 gc:universal TRINITY\_DN16120\_c0\_g1\_i2:734-39(-) |
| 109 | 258 | TRINITY\_DN2217\_c0\_g1\_i2.p1 | 43.77 | 5 | 5 | 0 | 1 | 1 | 1 | Carbamidomethylation | 28618.9531 | type:complete len:254 gc:universal TRINITY\_DN2217\_c0\_g1\_i2:884-123(-) |
| 109 | 259 | TRINITY\_DN2217\_c0\_g1\_i3.p1 | 43.77 | 2 | 2 | 0 | 1 | 1 | 1 | Carbamidomethylation | 63077.0508 | type:complete len:566 gc:universal TRINITY\_DN2217\_c0\_g1\_i3:1820-123(-) |
| 110 | 260 | TRINITY\_DN730\_c0\_g2\_i3.p1 | 40.73 | 6 | 6 | 0 | 1 | 1 | 1 |  | 27607.4375 | type:complete len:235 gc:universal TRINITY\_DN730\_c0\_g2\_i3:1019-315(-) |
| 110 | 261 | TRINITY\_DN730\_c0\_g2\_i2.p1 | 40.73 | 6 | 6 | 0 | 1 | 1 | 1 |  | 27607.4375 | type:complete len:235 gc:universal TRINITY\_DN730\_c0\_g2\_i2:1787-1083(-) |
| 111 | 262 | TRINITY\_DN187147\_c0\_g1\_i1.p1 | 43.61 | 1 | 1 | 0 | 1 | 1 | 1 |  | 138182.3125 | type:complete len:1223 gc:universal TRINITY\_DN187147\_c0\_g1\_i1:3708-40(-) |
| 112 | 263 | TRINITY\_DN101992\_c0\_g1\_i1.p1 | 43.41 | 7 | 7 | 4.89e2 | 1 | 1 | 1 | Carbamidomethylation | 15607.4678 | type:internal len:139 gc:universal TRINITY\_DN101992\_c0\_g1\_i1:414-1(-) |
| 113 | 264 | TRINITY\_DN98420\_c0\_g1\_i1.p1 | 43.75 | 3 | 3 | 2.24e3 | 1 | 1 | 1 |  | 31374.8145 | type:5prime\_partial len:286 gc:universal TRINITY\_DN98420\_c0\_g1\_i1:1007-150(-) |
| 114 | 265 | TRINITY\_DN2885\_c0\_g1\_i2.p1 | 43.35 | 11 | 11 | 0 | 1 | 1 | 1 |  | 12758.7783 | type:complete len:114 gc:universal TRINITY\_DN2885\_c0\_g1\_i2:31-372(+) |
| 114 | 266 | TRINITY\_DN2885\_c0\_g1\_i4.p1 | 43.35 | 11 | 11 | 0 | 1 | 1 | 1 |  | 12245.125 | type:5prime\_partial len:109 gc:universal TRINITY\_DN2885\_c0\_g1\_i4:1-327(+) |
| 114 | 267 | TRINITY\_DN2885\_c0\_g1\_i5.p1 | 43.35 | 11 | 11 | 0 | 1 | 1 | 1 |  | 12383.5439 | type:3prime\_partial len:112 gc:universal TRINITY\_DN2885\_c0\_g1\_i5:31-363(+) |
| 115 | 268 | TRINITY\_DN923\_c0\_g1\_i1.p1 | 43.5 | 2 | 2 | 9.33e2 | 1 | 1 | 1 |  | 51773.8242 | type:complete len:442 gc:universal TRINITY\_DN923\_c0\_g1\_i1:53-1378(+) |
| 116 | 269 | TRINITY\_DN32270\_c0\_g1\_i1.p2 | 43.77 | 4 | 4 | 6.88e2 | 1 | 1 | 1 |  | 33930.0625 | type:5prime\_partial len:309 gc:universal TRINITY\_DN32270\_c0\_g1\_i1:1-927(+) |
| 117 | 270 | TRINITY\_DN1936\_c0\_g1\_i1.p1 | 42.7 | 1 | 1 | 0 | 1 | 1 | 1 |  | 136661.0312 | type:3prime\_partial len:1224 gc:universal TRINITY\_DN1936\_c0\_g1\_i1:3669-1(-) |
| 117 | 271 | TRINITY\_DN1936\_c0\_g1\_i4.p1 | 42.7 | 1 | 1 | 0 | 1 | 1 | 1 |  | 196066.9688 | type:3prime\_partial len:1762 gc:universal TRINITY\_DN1936\_c0\_g1\_i4:5283-1(-) |
| 118 | 272 | TRINITY\_DN81944\_c0\_g1\_i1.p1 | 43.74 | 6 | 6 | 0 | 1 | 1 | 2 |  | 24919.502 | type:5prime\_partial len:222 gc:universal TRINITY\_DN81944\_c0\_g1\_i1:3-668(+) |
| 119 | 273 | TRINITY\_DN1559\_c0\_g1\_i1.p1 | 43.76 | 5 | 5 | 2.7e3 | 1 | 1 | 1 |  | 33672.0977 | type:complete len:311 gc:universal TRINITY\_DN1559\_c0\_g1\_i1:221-1153(+) |
| 120 | 274 | TRINITY\_DN144181\_c0\_g1\_i1.p1 | 43.74 | 5 | 5 | 1.56e3 | 1 | 1 | 1 |  | 35472.0234 | type:5prime\_partial len:309 gc:universal TRINITY\_DN144181\_c0\_g1\_i1:2-928(+) |
| 121 | 275 | TRINITY\_DN3034\_c0\_g1\_i1.p1 | 43.67 | 1 | 1 | 4.54e2 | 1 | 1 | 1 |  | 222266.9844 | type:complete len:1941 gc:universal TRINITY\_DN3034\_c0\_g1\_i1:6289-467(-) |
| 122 | 276 | TRINITY\_DN7504\_c0\_g1\_i1.p1 | 43.81 | 3 | 3 | 0 | 1 | 1 | 1 |  | 46519.1953 | type:complete len:414 gc:universal TRINITY\_DN7504\_c0\_g1\_i1:87-1328(+) |
| 123 | 277 | TRINITY\_DN1119\_c0\_g1\_i1.p1 | 43.25 | 1 | 1 | 0 | 1 | 1 | 1 |  | 105284.3438 | type:5prime\_partial len:949 gc:universal TRINITY\_DN1119\_c0\_g1\_i1:2934-88(-) |
| 124 | 278 | TRINITY\_DN761\_c0\_g1\_i1.p1 | 43.83 | 2 | 2 | 4.36e2 | 1 | 1 | 2 |  | 95330.7812 | type:complete len:830 gc:universal TRINITY\_DN761\_c0\_g1\_i1:129-2618(+) |
| 124 | 279 | TRINITY\_DN761\_c0\_g1\_i2.p1 | 43.83 | 2 | 2 | 4.36e2 | 1 | 1 | 2 |  | 95330.7812 | type:complete len:830 gc:universal TRINITY\_DN761\_c0\_g1\_i2:87-2576(+) |
| 124 | 280 | TRINITY\_DN761\_c0\_g1\_i3.p1 | 43.83 | 2 | 2 | 4.36e2 | 1 | 1 | 2 |  | 96968.6562 | type:complete len:845 gc:universal TRINITY\_DN761\_c0\_g1\_i3:129-2663(+) |
| 125 | 281 | TRINITY\_DN25375\_c0\_g1\_i7.p1 | 42.76 | 2 | 2 | 8.86e2 | 1 | 1 | 1 |  | 90646.6094 | type:complete len:811 gc:universal TRINITY\_DN25375\_c0\_g1\_i7:112-2544(+) |
| 126 | 282 | TRINITY\_DN124859\_c0\_g1\_i1.p3 | 43.77 | 3 | 3 | 1.9e2 | 1 | 1 | 2 |  | 26114.5156 | type:5prime\_partial len:237 gc:universal TRINITY\_DN124859\_c0\_g1\_i1:884-174(-) |
| 127 | 283 | TRINITY\_DN77\_c1\_g1\_i1.p1 | 43.79 | 1 | 1 | 9.54e2 | 1 | 1 | 1 |  | 198350.2344 | type:3prime\_partial len:1965 gc:universal TRINITY\_DN77\_c1\_g1\_i1:2-5893(+) |
| 128 | 284 | TRINITY\_DN3452\_c0\_g1\_i1.p1 | 43.8 | 3 | 3 | 4.22e2 | 1 | 1 | 1 |  | 52685.7344 | type:complete len:466 gc:universal TRINITY\_DN3452\_c0\_g1\_i1:139-1536(+) |
| 129 | 285 | TRINITY\_DN15151\_c0\_g1\_i3.p1 | 43.11 | 2 | 2 | 6.18e2 | 1 | 1 | 1 |  | 58451.293 | type:internal len:560 gc:universal TRINITY\_DN15151\_c0\_g1\_i3:1-1677(+) |
| 129 | 286 | TRINITY\_DN15151\_c0\_g1\_i1.p1 | 43.11 | 2 | 2 | 6.18e2 | 1 | 1 | 1 |  | 62118.2383 | type:5prime\_partial len:592 gc:universal TRINITY\_DN15151\_c0\_g1\_i1:1-1776(+) |
| 129 | 287 | TRINITY\_DN15151\_c0\_g1\_i2.p1 | 43.11 | 2 | 2 | 6.18e2 | 1 | 1 | 1 |  | 58324.2109 | type:internal len:560 gc:universal TRINITY\_DN15151\_c0\_g1\_i2:1-1677(+) |
| 130 | 288 | TRINITY\_DN38657\_c0\_g1\_i4.p1 | 43.71 | 3 | 3 | 0 | 1 | 1 | 1 |  | 33432.1367 | type:3prime\_partial len:302 gc:universal TRINITY\_DN38657\_c0\_g1\_i4:181-1083(+) |
| 130 | 289 | TRINITY\_DN38657\_c0\_g1\_i5.p1 | 43.71 | 4 | 4 | 0 | 1 | 1 | 1 |  | 26456.2832 | type:3prime\_partial len:231 gc:universal TRINITY\_DN38657\_c0\_g1\_i5:138-827(+) |
| 130 | 290 | TRINITY\_DN38657\_c0\_g1\_i3.p1 | 43.71 | 3 | 3 | 0 | 1 | 1 | 1 |  | 33673.4688 | type:3prime\_partial len:304 gc:universal TRINITY\_DN38657\_c0\_g1\_i3:170-1078(+) |
| 130 | 291 | TRINITY\_DN38657\_c0\_g1\_i2.p1 | 43.71 | 4 | 4 | 0 | 1 | 1 | 1 |  | 26484.3379 | type:3prime\_partial len:231 gc:universal TRINITY\_DN38657\_c0\_g1\_i2:170-859(+) |
| 131 | 292 | TRINITY\_DN9413\_c0\_g1\_i1.p1 | 43.67 | 3 | 3 | 0 | 1 | 1 | 1 |  | 47564.8359 | type:complete len:419 gc:universal TRINITY\_DN9413\_c0\_g1\_i1:1414-158(-) |
| 132 | 293 | TRINITY\_DN119684\_c0\_g1\_i1.p1 | 43.25 | 2 | 2 | 0 | 1 | 1 | 1 |  | 72994.7266 | type:complete len:662 gc:universal TRINITY\_DN119684\_c0\_g1\_i1:2182-197(-) |
| 133 | 294 | TRINITY\_DN1335\_c0\_g1\_i1.p1 | 43.45 | 2 | 2 | 0 | 1 | 1 | 1 |  | 71917.8516 | type:5prime\_partial len:651 gc:universal TRINITY\_DN1335\_c0\_g1\_i1:2201-249(-) |
| 134 | 295 | TRINITY\_DN941\_c0\_g1\_i10.p1 | 42.69 | 8 | 8 | 0 | 1 | 1 | 1 |  | 12669.4082 | type:5prime\_partial len:114 gc:universal TRINITY\_DN941\_c0\_g1\_i10:3-344(+) |
| 134 | 296 | TRINITY\_DN941\_c0\_g1\_i14.p1 | 42.69 | 7 | 7 | 0 | 1 | 1 | 1 |  | 12821.7773 | type:internal len:122 gc:universal TRINITY\_DN941\_c0\_g1\_i14:3-365(+) |
| 134 | 297 | TRINITY\_DN941\_c0\_g1\_i2.p2 | 42.69 | 4 | 4 | 0 | 1 | 1 | 1 |  | 26620.4121 | type:5prime\_partial len:247 gc:universal TRINITY\_DN941\_c0\_g1\_i2:3-743(+) |
| 134 | 298 | TRINITY\_DN941\_c0\_g1\_i8.p2 | 42.69 | 4 | 4 | 0 | 1 | 1 | 1 |  | 24348.5469 | type:5prime\_partial len:227 gc:universal TRINITY\_DN941\_c0\_g1\_i8:3-683(+) |
| 134 | 299 | TRINITY\_DN941\_c0\_g1\_i5.p2 | 42.69 | 4 | 4 | 0 | 1 | 1 | 1 |  | 26620.4121 | type:5prime\_partial len:247 gc:universal TRINITY\_DN941\_c0\_g1\_i5:3-743(+) |
| 134 | 300 | TRINITY\_DN941\_c0\_g1\_i7.p2 | 42.69 | 4 | 4 | 0 | 1 | 1 | 1 |  | 24406.584 | type:5prime\_partial len:227 gc:universal TRINITY\_DN941\_c0\_g1\_i7:3-683(+) |
| 135 | 301 | TRINITY\_DN34481\_c0\_g1\_i1.p1 | 43.57 | 1 | 1 | 0 | 1 | 1 | 1 | Carbamidomethylation | 92307.5469 | type:complete len:802 gc:universal TRINITY\_DN34481\_c0\_g1\_i1:2487-82(-) |
| 136 | 302 | TRINITY\_DN8008\_c0\_g1\_i2.p1 | 43.79 | 1 | 1 | 0 | 1 | 1 | 1 |  | 146342.75 | type:5prime\_partial len:1298 gc:universal TRINITY\_DN8008\_c0\_g1\_i2:4205-312(-) |
| 136 | 303 | TRINITY\_DN8008\_c0\_g1\_i3.p1 | 43.79 | 1 | 1 | 0 | 1 | 1 | 1 |  | 146351.7188 | type:5prime\_partial len:1298 gc:universal TRINITY\_DN8008\_c0\_g1\_i3:4205-312(-) |
| 137 | 304 | TRINITY\_DN1320\_c0\_g1\_i1.p1 | 43.36 | 5 | 5 | 2.76e2 | 1 | 1 | 1 | Carbamidomethylation | 25517.7812 | type:complete len:229 gc:universal TRINITY\_DN1320\_c0\_g1\_i1:105-791(+) |
| 138 | 305 | TRINITY\_DN945\_c0\_g1\_i1.p1 | 43.25 | 3 | 3 | 5.26e2 | 1 | 1 | 1 |  | 52018.625 | type:complete len:468 gc:universal TRINITY\_DN945\_c0\_g1\_i1:151-1554(+) |
| 139 | 306 | TRINITY\_DN1653\_c0\_g1\_i3.p1 | 43.76 | 1 | 1 | 1.56e3 | 1 | 1 | 2 |  | 114336.4531 | type:complete len:1023 gc:universal TRINITY\_DN1653\_c0\_g1\_i3:180-3248(+) |
| 139 | 307 | TRINITY\_DN1653\_c0\_g1\_i1.p1 | 43.76 | 1 | 1 | 1.56e3 | 1 | 1 | 2 |  | 125115.2109 | type:complete len:1122 gc:universal TRINITY\_DN1653\_c0\_g1\_i1:180-3545(+) |
| 140 | 308 | TRINITY\_DN286\_c0\_g1\_i4.p1 | 43.72 | 5 | 5 | 5.65e2 | 1 | 1 | 1 | Carbamidomethylation | 25084.5293 | type:complete len:224 gc:universal TRINITY\_DN286\_c0\_g1\_i4:40-711(+) |
| 141 | 309 | TRINITY\_DN14781\_c0\_g1\_i1.p1 | 43.47 | 1 | 1 | 7.25e2 | 1 | 1 | 1 |  | 131862.4531 | type:complete len:1181 gc:universal TRINITY\_DN14781\_c0\_g1\_i1:4332-790(-) |
| 142 | 310 | TRINITY\_DN118520\_c0\_g1\_i1.p1 | 43.29 | 3 | 3 | 3.06e3 | 1 | 1 | 2 |  | 61587.4766 | type:complete len:581 gc:universal TRINITY\_DN118520\_c0\_g1\_i1:1895-153(-) |
| 143 | 311 | TRINITY\_DN3271\_c0\_g1\_i1.p1 | 43.75 | 3 | 3 | 5.62e2 | 1 | 1 | 1 |  | 45246.3555 | type:complete len:399 gc:universal TRINITY\_DN3271\_c0\_g1\_i1:218-1414(+) |
| 143 | 312 | TRINITY\_DN3271\_c0\_g1\_i2.p1 | 43.75 | 3 | 3 | 5.62e2 | 1 | 1 | 1 |  | 53020.332 | type:5prime\_partial len:468 gc:universal TRINITY\_DN3271\_c0\_g1\_i2:2-1405(+) |
| 144 | 313 | TRINITY\_DN1043\_c0\_g1\_i2.p1 | 43.68 | 2 | 2 | 3.84e2 | 1 | 1 | 1 |  | 78392.1016 | type:complete len:700 gc:universal TRINITY\_DN1043\_c0\_g1\_i2:2410-311(-) |
| 145 | 314 | TRINITY\_DN729\_c0\_g1\_i1.p1 | 43.22 | 1 | 1 | 3.09e2 | 1 | 1 | 2 |  | 81791.2578 | type:5prime\_partial len:723 gc:universal TRINITY\_DN729\_c0\_g1\_i1:2179-11(-) |
| 146 | 315 | TRINITY\_DN1907\_c0\_g1\_i2.p1 | 43.82 | 5 | 5 | 0 | 1 | 1 | 1 |  | 24166.1172 | type:complete len:210 gc:universal TRINITY\_DN1907\_c0\_g1\_i2:53-682(+) |
| 146 | 316 | TRINITY\_DN1907\_c0\_g1\_i1.p1 | 43.82 | 5 | 5 | 0 | 1 | 1 | 1 |  | 24233.2676 | type:complete len:211 gc:universal TRINITY\_DN1907\_c0\_g1\_i1:56-688(+) |
| 147 | 317 | TRINITY\_DN31399\_c0\_g1\_i2.p1 | 42.83 | 1 | 1 | 1.13e3 | 1 | 1 | 1 |  | 89961.6641 | type:complete len:819 gc:universal TRINITY\_DN31399\_c0\_g1\_i2:85-2541(+) |
| 147 | 318 | TRINITY\_DN31399\_c0\_g1\_i3.p1 | 42.83 | 1 | 1 | 1.13e3 | 1 | 1 | 1 |  | 89350.0078 | type:complete len:813 gc:universal TRINITY\_DN31399\_c0\_g1\_i3:85-2523(+) |
| 148 | 319 | TRINITY\_DN1613\_c1\_g1\_i2.p1 | 43.53 | 1 | 1 | 0 | 1 | 1 | 3 | Carbamidomethylation | 111170.0312 | type:internal len:1068 gc:universal TRINITY\_DN1613\_c1\_g1\_i2:3201-1(-) |
| 148 | 320 | TRINITY\_DN1613\_c1\_g1\_i1.p1 | 43.53 | 1 | 1 | 0 | 1 | 1 | 3 | Carbamidomethylation | 112791.8203 | type:internal len:1083 gc:universal TRINITY\_DN1613\_c1\_g1\_i1:3248-3(-) |
| 149 | 321 | TRINITY\_DN3180\_c0\_g1\_i1.p1 | 43.48 | 1 | 1 | 0 | 1 | 1 | 1 |  | 212833.2969 | type:complete len:1889 gc:universal TRINITY\_DN3180\_c0\_g1\_i1:5857-191(-) |
| 150 | 322 | TRINITY\_DN598\_c1\_g1\_i2.p1 | 43.77 | 1 | 1 | 1.44e3 | 1 | 1 | 2 |  | 81043.625 | type:5prime\_partial len:742 gc:universal TRINITY\_DN598\_c1\_g1\_i2:2-2227(+) |
| 151 | 323 | TRINITY\_DN29322\_c0\_g1\_i1.p1 | 43.44 | 2 | 2 | 0 | 1 | 1 | 1 | Carbamidomethylation | 88672.5625 | type:5prime\_partial len:776 gc:universal TRINITY\_DN29322\_c0\_g1\_i1:2-2329(+) |
| 152 | 324 | TRINITY\_DN123\_c0\_g1\_i1.p1 | 42.71 | 1 | 1 | 0 | 1 | 1 | 1 |  | 136822.0312 | type:5prime\_partial len:1231 gc:universal TRINITY\_DN123\_c0\_g1\_i1:2-3694(+) |
| 153 | 325 | TRINITY\_DN2357\_c1\_g1\_i1.p1 | 42.13 | 1 | 1 | 2.03e2 | 1 | 1 | 1 |  | 196914.375 | type:complete len:1733 gc:universal TRINITY\_DN2357\_c1\_g1\_i1:5452-254(-) |
| 154 | 326 | TRINITY\_DN135129\_c0\_g1\_i1.p1 | 43.41 | 6 | 6 | 4.09e3 | 1 | 1 | 1 |  | 17174.043 | type:internal len:155 gc:universal TRINITY\_DN135129\_c0\_g1\_i1:1-462(+) |
| 155 | 327 | TRINITY\_DN1614\_c0\_g1\_i1.p1 | 43.15 | 7 | 7 | 0 | 1 | 1 | 1 |  | 25367.3418 | type:complete len:218 gc:universal TRINITY\_DN1614\_c0\_g1\_i1:56-709(+) |
| 156 | 328 | TRINITY\_DN1143\_c0\_g1\_i1.p1 | 43.18 | 2 | 2 | 0 | 1 | 1 | 1 |  | 68207.8203 | type:complete len:629 gc:universal TRINITY\_DN1143\_c0\_g1\_i1:2539-653(-) |
| 157 | 329 | TRINITY\_DN5229\_c0\_g1\_i1.p1 | 43.39 | 2 | 2 | 9.01e2 | 1 | 1 | 1 |  | 80941.0312 | type:complete len:728 gc:universal TRINITY\_DN5229\_c0\_g1\_i1:2363-180(-) |
| 158 | 330 | TRINITY\_DN11097\_c0\_g1\_i1.p1 | 43.68 | 2 | 2 | 3.46e2 | 1 | 1 | 2 |  | 80111.1172 | type:complete len:730 gc:universal TRINITY\_DN11097\_c0\_g1\_i1:2348-159(-) |
| 158 | 331 | TRINITY\_DN11097\_c0\_g2\_i1.p1 | 43.68 | 6 | 6 | 3.46e2 | 1 | 1 | 2 |  | 24192.5488 | type:internal len:222 gc:universal TRINITY\_DN11097\_c0\_g2\_i1:665-3(-) |
| 159 | 332 | TRINITY\_DN61\_c0\_g1\_i1.p1 | 43.5 | 2 | 2 | 0 | 1 | 1 | 1 |  | 71696.4219 | type:complete len:625 gc:universal TRINITY\_DN61\_c0\_g1\_i1:2929-1055(-) |
| 160 | 333 | TRINITY\_DN1366\_c0\_g1\_i1.p1 | 43.45 | 0 | 0 | 1.54e2 | 1 | 1 | 1 |  | 558202.1875 | type:3prime\_partial len:4702 gc:universal TRINITY\_DN1366\_c0\_g1\_i1:236-14338(+) |
| 161 | 334 | TRINITY\_DN2028\_c0\_g1\_i1.p1 | 43.67 | 5 | 5 | 1.11e3 | 1 | 1 | 1 |  | 30106.9453 | type:complete len:274 gc:universal TRINITY\_DN2028\_c0\_g1\_i1:1608-787(-) |
| 162 | 335 | TRINITY\_DN26249\_c0\_g1\_i1.p1 | 43.73 | 3 | 3 | 4.2e2 | 1 | 1 | 1 |  | 54490.1055 | type:5prime\_partial len:477 gc:universal TRINITY\_DN26249\_c0\_g1\_i1:1733-303(-) |
| 163 | 336 | TRINITY\_DN172571\_c0\_g1\_i1.p1 | 43.83 | 12 | 12 | 4.46e2 | 1 | 1 | 2 |  | 11500.2529 | type:complete len:104 gc:universal TRINITY\_DN172571\_c0\_g1\_i1:30-341(+) |
| 164 | 337 | TRINITY\_DN8707\_c0\_g1\_i2.p1 | 43.77 | 2 | 2 | 1.16e3 | 1 | 1 | 1 |  | 61942.9023 | type:complete len:564 gc:universal TRINITY\_DN8707\_c0\_g1\_i2:2751-1060(-) |
| 165 | 338 | TRINITY\_DN1378\_c0\_g1\_i1.p1 | 43.42 | 3 | 3 | 0 | 1 | 1 | 1 |  | 47978.9219 | type:complete len:443 gc:universal TRINITY\_DN1378\_c0\_g1\_i1:2151-823(-) |
| 166 | 339 | TRINITY\_DN86511\_c0\_g1\_i1.p1 | 40.98 | 6 | 6 | 6.91e2 | 1 | 1 | 1 | Acetylation (Protein N-term) | 16282.7617 | type:internal len:143 gc:universal TRINITY\_DN86511\_c0\_g1\_i1:427-2(-) |
| 167 | 340 | TRINITY\_DN74934\_c0\_g1\_i1.p1 | 42.7 | 3 | 3 | 0 | 1 | 1 | 1 |  | 41419.8945 | type:5prime\_partial len:359 gc:universal TRINITY\_DN74934\_c0\_g1\_i1:1215-139(-) |
| 168 | 341 | TRINITY\_DN1668\_c0\_g1\_i1.p1 | 43.6 | 8 | 8 | 1.96e2 | 1 | 1 | 1 |  | 24764.1523 | type:complete len:220 gc:universal TRINITY\_DN1668\_c0\_g1\_i1:748-89(-) |
| 169 | 342 | TRINITY\_DN97639\_c0\_g1\_i1.p1 | 43.7 | 2 | 2 | 3.43e2 | 1 | 1 | 2 | Acetylation (Protein N-term) | 32895.9141 | type:internal len:298 gc:universal TRINITY\_DN97639\_c0\_g1\_i1:891-1(-) |
| 170 | 343 | TRINITY\_DN116\_c0\_g1\_i1.p1 | 43.71 | 4 | 4 | 1.03e3 | 1 | 1 | 1 |  | 34095.6328 | type:complete len:297 gc:universal TRINITY\_DN116\_c0\_g1\_i1:72-962(+) |
| 171 | 344 | TRINITY\_DN97809\_c0\_g1\_i1.p1 | 43.34 | 7 | 7 | 9.3e2 | 1 | 1 | 4 |  | 20461.1211 | type:internal len:184 gc:universal TRINITY\_DN97809\_c0\_g1\_i1:2-550(+) |
| 172 | 345 | TRINITY\_DN374\_c0\_g1\_i1.p1 | 43.56 | 4 | 4 | 7.33e2 | 1 | 1 | 1 | Carbamidomethylation | 32814.1367 | type:5prime\_partial len:290 gc:universal TRINITY\_DN374\_c0\_g1\_i1:1-870(+) |
| 172 | 346 | TRINITY\_DN374\_c0\_g1\_i2.p1 | 43.56 | 4 | 4 | 7.33e2 | 1 | 1 | 1 | Carbamidomethylation | 38811.4023 | type:complete len:341 gc:universal TRINITY\_DN374\_c0\_g1\_i2:115-1137(+) |
| 173 | 347 | TRINITY\_DN58867\_c0\_g1\_i2.p1 | 43.73 | 3 | 3 | 1.54e3 | 1 | 1 | 1 |  | 40822.2812 | type:complete len:354 gc:universal TRINITY\_DN58867\_c0\_g1\_i2:80-1141(+) |
| 174 | 348 | TRINITY\_DN33065\_c0\_g1\_i1.p1 | 42.45 | 3 | 3 | 0 | 1 | 1 | 1 |  | 73381.4062 | type:3prime\_partial len:663 gc:universal TRINITY\_DN33065\_c0\_g1\_i1:1986-1(-) |
| 175 | 349 | TRINITY\_DN1172\_c0\_g2\_i2.p1 | 42.72 | 4 | 4 | 9.73e2 | 1 | 1 | 1 | Acetylation (Protein N-term) | 27813.4355 | type:internal len:264 gc:universal TRINITY\_DN1172\_c0\_g2\_i2:790-2(-) |
| 176 | 350 | TRINITY\_DN2963\_c0\_g1\_i1.p1 | 43.75 | 10 | 10 | 0 | 1 | 1 | 1 | Acetylation (Protein N-term) | 13082.1787 | type:complete len:116 gc:universal TRINITY\_DN2963\_c0\_g1\_i1:1072-725(-) |
| 177 | 351 | TRINITY\_DN128406\_c0\_g1\_i1.p1 | 43.15 | 7 | 7 | 5.02e2 | 1 | 1 | 1 |  | 16958.5273 | type:5prime\_partial len:150 gc:universal TRINITY\_DN128406\_c0\_g1\_i1:474-25(-) |
| 178 | 352 | TRINITY\_DN4781\_c0\_g1\_i3.p3 | 43.8 | 5 | 5 | 8.34e2 | 1 | 1 | 1 |  | 20029.2422 | type:complete len:183 gc:universal TRINITY\_DN4781\_c0\_g1\_i3:2965-2417(-) |
| 179 | 353 | TRINITY\_DN464\_c0\_g1\_i4.p1 | 43.74 | 2 | 2 | 0 | 1 | 1 | 1 |  | 82057.25 | type:complete len:717 gc:universal TRINITY\_DN464\_c0\_g1\_i4:2167-17(-) |
| 180 | 354 | TRINITY\_DN61668\_c0\_g1\_i1.p1 | 43.45 | 3 | 3 | 3.39e2 | 1 | 1 | 1 |  | 51212.582 | type:5prime\_partial len:453 gc:universal TRINITY\_DN61668\_c0\_g1\_i1:2-1360(+) |
| 181 | 355 | TRINITY\_DN1091\_c0\_g1\_i1.p1 | 43.39 | 8 | 8 | 0 | 1 | 1 | 1 | Carbamidomethylation | 24868.0293 | type:complete len:212 gc:universal TRINITY\_DN1091\_c0\_g1\_i1:200-835(+) |
| 182 | 356 | TRINITY\_DN3202\_c2\_g1\_i1.p1 | 43.35 | 4 | 4 | 0 | 1 | 1 | 2 | Acetylation (Protein N-term), Carbamidomethylation | 30131.0488 | type:5prime\_partial len:263 gc:universal TRINITY\_DN3202\_c2\_g1\_i1:949-161(-) |
| 183 | 357 | TRINITY\_DN3052\_c0\_g1\_i1.p1 | 43.62 | 2 | 2 | 0 | 1 | 1 | 1 | Acetylation (Protein N-term) | 39261.4453 | type:complete len:362 gc:universal TRINITY\_DN3052\_c0\_g1\_i1:45-1130(+) |
| 184 | 358 | TRINITY\_DN5397\_c0\_g1\_i1.p1 | 42.91 | 5 | 5 | 6.58e3 | 1 | 1 | 1 |  | 17679.4688 | type:internal len:161 gc:universal TRINITY\_DN5397\_c0\_g1\_i1:2-481(+) |
| 185 | 359 | TRINITY\_DN493\_c0\_g1\_i1.p1 | 43.78 | 3 | 3 | 0 | 1 | 1 | 1 |  | 55371.7461 | type:complete len:473 gc:universal TRINITY\_DN493\_c0\_g1\_i1:2681-1263(-) |
| total 359 proteins |  |  |  |  |  |  |  |  |  |  |  |  |
| --- | --- | --- | --- | --- | --- | --- | --- | --- | --- | --- | --- | --- |

  

TRINITY\_DN3448\_c0\_g1\_i5.p2

back to list

  

| Protein Coverage
| Supporting Peptides
| Best Unique PSM
|

Protein Coverage:

Supporting Peptides:

| Peptide | Uniq | -10lgP | Mass | Length | ppm | m/z | z | RT | 1/k0 Start | 1/k0 End | Precursor ID | Area Sample 8 | #Feature | #Feature Sample 8 | Start | End | PTM | AScore | Found By |
| --- | --- | --- | --- | --- | --- | --- | --- | --- | --- | --- | --- | --- | --- | --- | --- | --- | --- | --- | --- |
| K.AGM(+15.99)THIVR.D | Y | 43.71 | 899.4647 | 8 | 5.69 | 450.7402 | 2 | 9.78 | 0.8161 | 0.8234 | 13845 | 0 | 0 | 0 | 27 | 34 | Oxidation (M) | M3:Oxidation (M):1000 | DB Search |
| total 1 peptides |  |  |  |  |  |  |  |  |  |  |  |  |  |  |  |  |  |  |  |
| --- | --- | --- | --- | --- | --- | --- | --- | --- | --- | --- | --- | --- | --- | --- | --- | --- | --- | --- | --- |

Best Unique PSM (Scan Sample\_7\_A1\_1\_3644.d:13845, m/z=450.7402, z=2, RT=9.78, ppm=1.21):


TRINITY\_DN101992\_c0\_g1\_i1.p1

back to list

  

| Protein Coverage
| Supporting Peptides
| Best Unique PSM
|

Protein Coverage:

Supporting Peptides:

| Peptide | Uniq | -10lgP | Mass | Length | ppm | m/z | z | RT | 1/k0 Start | 1/k0 End | Precursor ID | Area Sample 8 | #Feature | #Feature Sample 8 | Start | End | PTM | AScore | Found By |
| --- | --- | --- | --- | --- | --- | --- | --- | --- | --- | --- | --- | --- | --- | --- | --- | --- | --- | --- | --- |
| K.AELVFVNHC(+57.02)R.E | Y | 43.41 | 1243.6132 | 10 | -0.25 | 622.8109 | 2 | 14.51 | 0.9582 | 0.9654 | 40866 | 4.89e2 | 1 | 1 | 118 | 127 | Carbamidomethylation |  | DB Search |
| total 1 peptides |  |  |  |  |  |  |  |  |  |  |  |  |  |  |  |  |  |  |  |
| --- | --- | --- | --- | --- | --- | --- | --- | --- | --- | --- | --- | --- | --- | --- | --- | --- | --- | --- | --- |

Best Unique PSM (Scan Sample\_7\_A1\_1\_3644.d:40866, m/z=622.8109, z=2, RT=14.51, ppm=-4.73):


TRINITY\_DN945\_c0\_g1\_i1.p1

back to list

  

| Protein Coverage
| Supporting Peptides
| Best Unique PSM
|

Protein Coverage:

Supporting Peptides:

| Peptide | Uniq | -10lgP | Mass | Length | ppm | m/z | z | RT | 1/k0 Start | 1/k0 End | Precursor ID | Area Sample 8 | #Feature | #Feature Sample 8 | Start | End | PTM | AScore | Found By |
| --- | --- | --- | --- | --- | --- | --- | --- | --- | --- | --- | --- | --- | --- | --- | --- | --- | --- | --- | --- |
| K.TSVVPGYGHAVLR.K | Y | 43.25 | 1354.7357 | 13 | 3.84 | 678.3747 | 2 | 14.60 | 1.0275 | 1.0347 | 41374 | 5.26e2 | 1 | 1 | 342 | 354 |  |  | DB Search |
| total 1 peptides |  |  |  |  |  |  |  |  |  |  |  |  |  |  |  |  |  |  |  |
| --- | --- | --- | --- | --- | --- | --- | --- | --- | --- | --- | --- | --- | --- | --- | --- | --- | --- | --- | --- |

Best Unique PSM (Scan Sample\_7\_A1\_1\_3644.d:41374, m/z=678.3747, z=2, RT=14.60, ppm=-0.64):


TRINITY\_DN5818\_c1\_g1\_i2.p1

back to list

  

| Protein Coverage
| Supporting Peptides
| Best Unique PSM
|

Protein Coverage:

Supporting Peptides:

| Peptide | Uniq | -10lgP | Mass | Length | ppm | m/z | z | RT | 1/k0 Start | 1/k0 End | Precursor ID | Area Sample 8 | #Feature | #Feature Sample 8 | Start | End | PTM | AScore | Found By |
| --- | --- | --- | --- | --- | --- | --- | --- | --- | --- | --- | --- | --- | --- | --- | --- | --- | --- | --- | --- |
| K.LLAQTTLRNELGTK.N | N | 43.63 | 1556.8886 | 14 | -0.19 | 779.4479 | 2 | 15.37 | 1.1098 | 1.117 | 45889 | 3.33e2 | 1 | 1 | 155 | 168 |  |  | DB Search |
| R.AM(+15.99)AAEAEASR.E | Y | 43.45 | 1021.4498 | 10 | 0.68 | 511.7303 | 2 | 9.50 | 0.8067 | 0.814 | 12520 | 4.25e2 | 1 | 1 | 216 | 225 | Oxidation (M) | M2:Oxidation (M):1000 | DB Search |
| K.EASEIISESPQALQLR.Y | Y | 43.42 | 1769.9159 | 16 | -17.14 | 885.9461 | 2 | 18.60 | 1.1678 | 1.175 | 65266 | 0 | 0 | 0 | 246 | 261 |  |  | DB Search |
| total 3 peptides |  |  |  |  |  |  |  |  |  |  |  |  |  |  |  |  |  |  |  |
| --- | --- | --- | --- | --- | --- | --- | --- | --- | --- | --- | --- | --- | --- | --- | --- | --- | --- | --- | --- |

Best Unique PSM (Scan Sample\_7\_A1\_1\_3644.d:12520, m/z=511.7303, z=2, RT=9.50, ppm=-3.79):


TRINITY\_DN923\_c0\_g1\_i1.p1

back to list

  

| Protein Coverage
| Supporting Peptides
| Best Unique PSM
|

Protein Coverage:

Supporting Peptides:

| Peptide | Uniq | -10lgP | Mass | Length | ppm | m/z | z | RT | 1/k0 Start | 1/k0 End | Precursor ID | Area Sample 8 | #Feature | #Feature Sample 8 | Start | End | PTM | AScore | Found By |
| --- | --- | --- | --- | --- | --- | --- | --- | --- | --- | --- | --- | --- | --- | --- | --- | --- | --- | --- | --- |
| R.YLTTAVITNKR.R | Y | 43.5 | 1278.7296 | 11 | 2.3 | 640.3707 | 2 | 14.00 | 1.0001 | 1.0073 | 37765 | 9.33e2 | 1 | 1 | 258 | 268 |  |  | DB Search |
| total 1 peptides |  |  |  |  |  |  |  |  |  |  |  |  |  |  |  |  |  |  |  |
| --- | --- | --- | --- | --- | --- | --- | --- | --- | --- | --- | --- | --- | --- | --- | --- | --- | --- | --- | --- |

Best Unique PSM (Scan Sample\_7\_A1\_1\_3644.d:37765, m/z=640.3707, z=2, RT=14.00, ppm=-2.18):


TRINITY\_DN16120\_c0\_g1\_i2.p1

back to list

  

| Protein Coverage
| Supporting Peptides
| Best Unique PSM
|

Protein Coverage:

Supporting Peptides:

| Peptide | Uniq | -10lgP | Mass | Length | ppm | m/z | z | RT | 1/k0 Start | 1/k0 End | Precursor ID | Area Sample 8 | #Feature | #Feature Sample 8 | Start | End | PTM | AScore | Found By |
| --- | --- | --- | --- | --- | --- | --- | --- | --- | --- | --- | --- | --- | --- | --- | --- | --- | --- | --- | --- |
| R.LC(+57.02)ETNTANTEAK.V | Y | 41.94 | 1350.6085 | 12 | 9.96 | 676.3152 | 2 | 10.42 | 0.9473 | 0.9545 | 17132 | 0 | 0 | 0 | 150 | 161 | Carbamidomethylation |  | DB Search |
| total 1 peptides |  |  |  |  |  |  |  |  |  |  |  |  |  |  |  |  |  |  |  |
| --- | --- | --- | --- | --- | --- | --- | --- | --- | --- | --- | --- | --- | --- | --- | --- | --- | --- | --- | --- |

Best Unique PSM (Scan Sample\_7\_A1\_1\_3644.d:17132, m/z=676.3152, z=2, RT=10.42, ppm=5.48):


TRINITY\_DN11435\_c0\_g1\_i1.p1

back to list

  

| Protein Coverage
| Supporting Peptides
| Best Unique PSM
|

Protein Coverage:

Supporting Peptides:

| Peptide | Uniq | -10lgP | Mass | Length | ppm | m/z | z | RT | 1/k0 Start | 1/k0 End | Precursor ID | Area Sample 8 | #Feature | #Feature Sample 8 | Start | End | PTM | AScore | Found By |
| --- | --- | --- | --- | --- | --- | --- | --- | --- | --- | --- | --- | --- | --- | --- | --- | --- | --- | --- | --- |
| R.FKADTTIADAAR.A | Y | 43.76 | 1278.6568 | 12 | -3.02 | 640.3309 | 2 | 13.52 | 0.9695 | 0.9767 | 34971 | 0 | 0 | 0 | 206 | 217 |  |  | DB Search |
| K.AEFDKEVQTK.N | Y | 43.76 | 1193.5928 | 10 | 0.25 | 597.8011 | 2 | 11.72 | 0.9243 | 0.9315 | 24343 | 9.56e2 | 1 | 1 | 224 | 233 |  |  | DB Search |
| K.LAYELQANKEQQR.I | Y | 43.28 | 1589.8161 | 13 | -2.52 | 795.9098 | 2 | 13.07 | 1.0548 | 1.062 | 32348 | 0 | 0 | 0 | 239 | 251 |  |  | DB Search |
| R.IRNEEIQIEVIERR.K | Y | 42.81 | 1795.9904 | 14 | -1.18 | 599.6674 | 3 | 15.29 | 0.8798 | 0.887 | 45515 | 0 | 0 | 0 | 252 | 265 |  |  | DB Search |
| total 4 peptides |  |  |  |  |  |  |  |  |  |  |  |  |  |  |  |  |  |  |  |
| --- | --- | --- | --- | --- | --- | --- | --- | --- | --- | --- | --- | --- | --- | --- | --- | --- | --- | --- | --- |

Best Unique PSM (Scan Sample\_7\_A1\_1\_3644.d:34971, m/z=640.3309, z=2, RT=13.52, ppm=-7.5):


TRINITY\_DN773\_c0\_g1\_i1.p1

back to list

  

| Protein Coverage
| Supporting Peptides
| Best Unique PSM
|

Protein Coverage:

Supporting Peptides:

| Peptide | Uniq | -10lgP | Mass | Length | ppm | m/z | z | RT | 1/k0 Start | 1/k0 End | Precursor ID | Area Sample 8 | #Feature | #Feature Sample 8 | Start | End | PTM | AScore | Found By |
| --- | --- | --- | --- | --- | --- | --- | --- | --- | --- | --- | --- | --- | --- | --- | --- | --- | --- | --- | --- |
| R.GVSEETTTGVHNLYK.M | Y | 42.51 | 1633.7947 | 15 | -2.9 | 817.8986 | 2 | 13.70 | 1.068 | 1.0753 | 35995 | 5.08e2 | 1 | 1 | 160 | 174 |  |  | DB Search |
| K.RLDEEVAR.L | Y | 40.55 | 986.5145 | 8 | 2.71 | 494.2636 | 2 | 11.17 | 0.8522 | 0.8595 | 21312 | 0 | 0 | 0 | 396 | 403 |  |  | DB Search |
| total 2 peptides |  |  |  |  |  |  |  |  |  |  |  |  |  |  |  |  |  |  |  |
| --- | --- | --- | --- | --- | --- | --- | --- | --- | --- | --- | --- | --- | --- | --- | --- | --- | --- | --- | --- |

Best Unique PSM (Scan Sample\_7\_A1\_1\_3644.d:35995, m/z=817.8986, z=2, RT=13.70, ppm=-7.38):


TRINITY\_DN2637\_c0\_g1\_i8.p2

back to list

  

| Protein Coverage
| Supporting Peptides
| Best Unique PSM
|

Protein Coverage:

Supporting Peptides:

| Peptide | Uniq | -10lgP | Mass | Length | ppm | m/z | z | RT | 1/k0 Start | 1/k0 End | Precursor ID | Area Sample 8 | #Feature | #Feature Sample 8 | Start | End | PTM | AScore | Found By |
| --- | --- | --- | --- | --- | --- | --- | --- | --- | --- | --- | --- | --- | --- | --- | --- | --- | --- | --- | --- |
| K.ATIEIPAKYEK.R | Y | 42.98 | 1261.6918 | 11 | 0.3 | 631.8505 | 2 | 14.24 | 0.9848 | 0.992 | 39239 | 0 | 0 | 0 | 107 | 117 |  |  | DB Search |
| total 1 peptides |  |  |  |  |  |  |  |  |  |  |  |  |  |  |  |  |  |  |  |
| --- | --- | --- | --- | --- | --- | --- | --- | --- | --- | --- | --- | --- | --- | --- | --- | --- | --- | --- | --- |

Best Unique PSM (Scan Sample\_7\_A1\_1\_3644.d:39239, m/z=631.8505, z=2, RT=14.24, ppm=-4.18):


TRINITY\_DN11522\_c0\_g1\_i4.p1

back to list

  

| Protein Coverage
| Supporting Peptides
| Best Unique PSM
|

Protein Coverage:

Supporting Peptides:

| Peptide | Uniq | -10lgP | Mass | Length | ppm | m/z | z | RT | 1/k0 Start | 1/k0 End | Precursor ID | Area Sample 8 | #Feature | #Feature Sample 8 | Start | End | PTM | AScore | Found By |
| --- | --- | --- | --- | --- | --- | --- | --- | --- | --- | --- | --- | --- | --- | --- | --- | --- | --- | --- | --- |
| R.IGLGNIRDPSISLTR.G | Y | 43.72 | 1610.9104 | 15 | 2.12 | 537.9761 | 3 | 17.42 | 0.8019 | 0.8092 | 57662 | 1.88e3 | 1 | 1 | 27 | 41 |  |  | DB Search |
| K.GKPEQVQVK.M | Y | 43.14 | 1011.5713 | 9 | 2 | 506.7917 | 2 | 9.97 | 0.8416 | 0.849 | 14688 | 9.41e2 | 1 | 1 | 544 | 552 |  |  | DB Search |
| total 2 peptides |  |  |  |  |  |  |  |  |  |  |  |  |  |  |  |  |  |  |  |
| --- | --- | --- | --- | --- | --- | --- | --- | --- | --- | --- | --- | --- | --- | --- | --- | --- | --- | --- | --- |

Best Unique PSM (Scan Sample\_7\_A1\_1\_3644.d:57662, m/z=537.9761, z=3, RT=17.42, ppm=-2.35):


TRINITY\_DN941\_c0\_g1\_i8.p2

back to list

  

| Protein Coverage
| Supporting Peptides
| Best Unique PSM
|

Protein Coverage:

Supporting Peptides:

| Peptide | Uniq | -10lgP | Mass | Length | ppm | m/z | z | RT | 1/k0 Start | 1/k0 End | Precursor ID | Area Sample 8 | #Feature | #Feature Sample 8 | Start | End | PTM | AScore | Found By |
| --- | --- | --- | --- | --- | --- | --- | --- | --- | --- | --- | --- | --- | --- | --- | --- | --- | --- | --- | --- |
| K.ADLTRPTGK.K | Y | 42.69 | 957.5243 | 9 | -3.1 | 479.7658 | 2 | 10.44 | 0.8193 | 0.8266 | 17248 | 0 | 0 | 0 | 52 | 60 |  |  | DB Search |
| total 1 peptides |  |  |  |  |  |  |  |  |  |  |  |  |  |  |  |  |  |  |  |
| --- | --- | --- | --- | --- | --- | --- | --- | --- | --- | --- | --- | --- | --- | --- | --- | --- | --- | --- | --- |

Best Unique PSM (Scan Sample\_7\_A1\_1\_3644.d:17248, m/z=479.7658, z=2, RT=10.44, ppm=-7.58):


TRINITY\_DN1199\_c0\_g1\_i2.p1

back to list

  

| Protein Coverage
| Supporting Peptides
| Best Unique PSM
|

Protein Coverage:

Supporting Peptides:

| Peptide | Uniq | -10lgP | Mass | Length | ppm | m/z | z | RT | 1/k0 Start | 1/k0 End | Precursor ID | Area Sample 8 | #Feature | #Feature Sample 8 | Start | End | PTM | AScore | Found By |
| --- | --- | --- | --- | --- | --- | --- | --- | --- | --- | --- | --- | --- | --- | --- | --- | --- | --- | --- | --- |
| R.VVHNIC(+57.02)SSAITNSVTNK.I | Y | 43.87 | 1842.9258 | 17 | 2.87 | 922.4687 | 2 | 13.93 | 1.1394 | 1.1466 | 37331 | 5.78e2 | 1 | 1 | 89 | 105 | Carbamidomethylation |  | DB Search |
| R.LPNFFQYAGHIK.K | Y | 43.77 | 1433.7455 | 12 | 1.54 | 478.9211 | 3 | 19.36 | 0.7567 | 0.764 | 70118 | 6.21e3 | 2 | 2 | 62 | 73 |  |  | DB Search |
| K.VLIQIGHEPLPPQQVTTLFGK.K | Y | 43.27 | 2314.3048 | 21 | 1.51 | 772.4399 | 3 | 19.84 | 0.9424 | 0.9497 | 72734 | 5.33e3 | 1 | 1 | 37 | 57 |  |  | DB Search |
| total 3 peptides |  |  |  |  |  |  |  |  |  |  |  |  |  |  |  |  |  |  |  |
| --- | --- | --- | --- | --- | --- | --- | --- | --- | --- | --- | --- | --- | --- | --- | --- | --- | --- | --- | --- |

Best Unique PSM (Scan Sample\_7\_A1\_1\_3644.d:37331, m/z=922.4687, z=2, RT=13.93, ppm=-1.6):


TRINITY\_DN319\_c2\_g1\_i2.p1

back to list

  

| Protein Coverage
| Supporting Peptides
| Best Unique PSM
|

Protein Coverage:

Supporting Peptides:

| Peptide | Uniq | -10lgP | Mass | Length | ppm | m/z | z | RT | 1/k0 Start | 1/k0 End | Precursor ID | Area Sample 8 | #Feature | #Feature Sample 8 | Start | End | PTM | AScore | Found By |
| --- | --- | --- | --- | --- | --- | --- | --- | --- | --- | --- | --- | --- | --- | --- | --- | --- | --- | --- | --- |
| R.GSFSSHIC(+57.02)LC(+57.02)HDPAYK.S | Y | 42.3 | 1877.8189 | 16 | -0.4 | 626.9438 | 3 | 14.48 | 0.8866 | 0.8939 | 40723 | 0 | 0 | 0 | 1011 | 1026 | Carbamidomethylation, Carbamidomethylation |  | DB Search |
| total 1 peptides |  |  |  |  |  |  |  |  |  |  |  |  |  |  |  |  |  |  |  |
| --- | --- | --- | --- | --- | --- | --- | --- | --- | --- | --- | --- | --- | --- | --- | --- | --- | --- | --- | --- |

Best Unique PSM (Scan Sample\_7\_A1\_1\_3644.d:40723, m/z=626.9438, z=3, RT=14.48, ppm=-4.87):


TRINITY\_DN97809\_c0\_g1\_i1.p1

back to list

  

| Protein Coverage
| Supporting Peptides
| Best Unique PSM
|

Protein Coverage:

Supporting Peptides:

| Peptide | Uniq | -10lgP | Mass | Length | ppm | m/z | z | RT | 1/k0 Start | 1/k0 End | Precursor ID | Area Sample 8 | #Feature | #Feature Sample 8 | Start | End | PTM | AScore | Found By |
| --- | --- | --- | --- | --- | --- | --- | --- | --- | --- | --- | --- | --- | --- | --- | --- | --- | --- | --- | --- |
| LGPDQVDVAGAGR.D | Y | 43.34 | 1253.6364 | 13 | -3.54 | 627.8204 | 2 | 13.70 | 0.9574 | 0.9646 | 36071 | 9.3e2 | 1 | 1 | 1 | 13 |  |  | DB Search |
| total 1 peptides |  |  |  |  |  |  |  |  |  |  |  |  |  |  |  |  |  |  |  |
| --- | --- | --- | --- | --- | --- | --- | --- | --- | --- | --- | --- | --- | --- | --- | --- | --- | --- | --- | --- |

Best Unique PSM (Scan Sample\_7\_A1\_1\_3644.d:36071, m/z=627.8204, z=2, RT=13.70, ppm=-8.02):


TRINITY\_DN1821\_c0\_g1\_i2.p1

back to list

  

| Protein Coverage
| Supporting Peptides
| Best Unique PSM
|

Protein Coverage:

Supporting Peptides:

| Peptide | Uniq | -10lgP | Mass | Length | ppm | m/z | z | RT | 1/k0 Start | 1/k0 End | Precursor ID | Area Sample 8 | #Feature | #Feature Sample 8 | Start | End | PTM | AScore | Found By |
| --- | --- | --- | --- | --- | --- | --- | --- | --- | --- | --- | --- | --- | --- | --- | --- | --- | --- | --- | --- |
| K.YQGIKPPVSR.S | Y | 43.24 | 1143.64 | 10 | -0.35 | 572.8245 | 2 | 12.74 | 0.9344 | 0.9416 | 30400 | 7.91e2 | 1 | 1 | 251 | 260 |  |  | DB Search |
| total 1 peptides |  |  |  |  |  |  |  |  |  |  |  |  |  |  |  |  |  |  |  |
| --- | --- | --- | --- | --- | --- | --- | --- | --- | --- | --- | --- | --- | --- | --- | --- | --- | --- | --- | --- |

Best Unique PSM (Scan Sample\_7\_A1\_1\_3644.d:30400, m/z=572.8245, z=2, RT=12.74, ppm=-4.83):


TRINITY\_DN942\_c0\_g1\_i2.p1

back to list

  

| Protein Coverage
| Supporting Peptides
| Best Unique PSM
|

Protein Coverage:

Supporting Peptides:

| Peptide | Uniq | -10lgP | Mass | Length | ppm | m/z | z | RT | 1/k0 Start | 1/k0 End | Precursor ID | Area Sample 8 | #Feature | #Feature Sample 8 | Start | End | PTM | AScore | Found By |
| --- | --- | --- | --- | --- | --- | --- | --- | --- | --- | --- | --- | --- | --- | --- | --- | --- | --- | --- | --- |
| K.SGVC(+57.02)TDANTR.D | Y | 43.32 | 1079.4666 | 10 | 12.92 | 540.7451 | 2 | 10.11 | 0.8445 | 0.8518 | 15427 | 0 | 0 | 0 | 359 | 368 | Carbamidomethylation |  | DB Search |
| K.AALNM(+15.99)EFEYIPTK.S | Y | 42.87 | 1541.7436 | 13 | -3.63 | 771.8728 | 2 | 17.58 | 1.0528 | 1.06 | 58710 | 2.11e2 | 1 | 1 | 1659 | 1671 | Oxidation (M) | M5:Oxidation (M):1000 | DB Search |
| total 2 peptides |  |  |  |  |  |  |  |  |  |  |  |  |  |  |  |  |  |  |  |
| --- | --- | --- | --- | --- | --- | --- | --- | --- | --- | --- | --- | --- | --- | --- | --- | --- | --- | --- | --- |

Best Unique PSM (Scan Sample\_7\_A1\_1\_3644.d:15427, m/z=540.7451, z=2, RT=10.11, ppm=8.45):


TRINITY\_DN1821\_c0\_g1\_i6.p1

back to list

  

| Protein Coverage
| Supporting Peptides
| Best Unique PSM
|

Protein Coverage:

Supporting Peptides:

| Peptide | Uniq | -10lgP | Mass | Length | ppm | m/z | z | RT | 1/k0 Start | 1/k0 End | Precursor ID | Area Sample 8 | #Feature | #Feature Sample 8 | Start | End | PTM | AScore | Found By |
| --- | --- | --- | --- | --- | --- | --- | --- | --- | --- | --- | --- | --- | --- | --- | --- | --- | --- | --- | --- |
| K.YQGIKPPVSR.S | Y | 43.24 | 1143.64 | 10 | -0.35 | 572.8245 | 2 | 12.74 | 0.9344 | 0.9416 | 30400 | 7.91e2 | 1 | 1 | 479 | 488 |  |  | DB Search |
| total 1 peptides |  |  |  |  |  |  |  |  |  |  |  |  |  |  |  |  |  |  |  |
| --- | --- | --- | --- | --- | --- | --- | --- | --- | --- | --- | --- | --- | --- | --- | --- | --- | --- | --- | --- |

Best Unique PSM (Scan Sample\_7\_A1\_1\_3644.d:30400, m/z=572.8245, z=2, RT=12.74, ppm=-4.83):


TRINITY\_DN74934\_c0\_g1\_i1.p1

back to list

  

| Protein Coverage
| Supporting Peptides
| Best Unique PSM
|

Protein Coverage:

Supporting Peptides:

| Peptide | Uniq | -10lgP | Mass | Length | ppm | m/z | z | RT | 1/k0 Start | 1/k0 End | Precursor ID | Area Sample 8 | #Feature | #Feature Sample 8 | Start | End | PTM | AScore | Found By |
| --- | --- | --- | --- | --- | --- | --- | --- | --- | --- | --- | --- | --- | --- | --- | --- | --- | --- | --- | --- |
| K.LQNDKEFAK.I | Y | 42.7 | 1091.5611 | 9 | 4.17 | 546.7877 | 2 | 10.82 | 0.9162 | 0.9234 | 19362 | 0 | 0 | 0 | 33 | 41 |  |  | DB Search |
| total 1 peptides |  |  |  |  |  |  |  |  |  |  |  |  |  |  |  |  |  |  |  |
| --- | --- | --- | --- | --- | --- | --- | --- | --- | --- | --- | --- | --- | --- | --- | --- | --- | --- | --- | --- |

Best Unique PSM (Scan Sample\_7\_A1\_1\_3644.d:19362, m/z=546.7877, z=2, RT=10.82, ppm=-0.31):


TRINITY\_DN1377\_c0\_g1\_i1.p1

back to list

  

| Protein Coverage
| Supporting Peptides
| Best Unique PSM
|

Protein Coverage:

Supporting Peptides:

| Peptide | Uniq | -10lgP | Mass | Length | ppm | m/z | z | RT | 1/k0 Start | 1/k0 End | Precursor ID | Area Sample 8 | #Feature | #Feature Sample 8 | Start | End | PTM | AScore | Found By |
| --- | --- | --- | --- | --- | --- | --- | --- | --- | --- | --- | --- | --- | --- | --- | --- | --- | --- | --- | --- |
| R.SVTTHQGNAIVIK.I | Y | 43.82 | 1366.7568 | 13 | 3.74 | 684.3852 | 2 | 12.65 | 0.9949 | 1.0021 | 29797 | 4.39e2 | 1 | 1 | 191 | 203 |  |  | DB Search |
| total 1 peptides |  |  |  |  |  |  |  |  |  |  |  |  |  |  |  |  |  |  |  |
| --- | --- | --- | --- | --- | --- | --- | --- | --- | --- | --- | --- | --- | --- | --- | --- | --- | --- | --- | --- |

Best Unique PSM (Scan Sample\_7\_A1\_1\_3644.d:29797, m/z=684.3852, z=2, RT=12.65, ppm=-0.74):


TRINITY\_DN2637\_c0\_g1\_i9.p2

back to list

  

| Protein Coverage
| Supporting Peptides
| Best Unique PSM
|

Protein Coverage:

Supporting Peptides:

| Peptide | Uniq | -10lgP | Mass | Length | ppm | m/z | z | RT | 1/k0 Start | 1/k0 End | Precursor ID | Area Sample 8 | #Feature | #Feature Sample 8 | Start | End | PTM | AScore | Found By |
| --- | --- | --- | --- | --- | --- | --- | --- | --- | --- | --- | --- | --- | --- | --- | --- | --- | --- | --- | --- |
| K.ATIEIPAKYEK.R | Y | 42.98 | 1261.6918 | 11 | 0.3 | 631.8505 | 2 | 14.24 | 0.9848 | 0.992 | 39239 | 0 | 0 | 0 | 107 | 117 |  |  | DB Search |
| total 1 peptides |  |  |  |  |  |  |  |  |  |  |  |  |  |  |  |  |  |  |  |
| --- | --- | --- | --- | --- | --- | --- | --- | --- | --- | --- | --- | --- | --- | --- | --- | --- | --- | --- | --- |

Best Unique PSM (Scan Sample\_7\_A1\_1\_3644.d:39239, m/z=631.8505, z=2, RT=14.24, ppm=-4.18):


TRINITY\_DN982\_c0\_g1\_i2.p1

back to list

  

| Protein Coverage
| Supporting Peptides
| Best Unique PSM
|

Protein Coverage:

Supporting Peptides:

| Peptide | Uniq | -10lgP | Mass | Length | ppm | m/z | z | RT | 1/k0 Start | 1/k0 End | Precursor ID | Area Sample 8 | #Feature | #Feature Sample 8 | Start | End | PTM | AScore | Found By |
| --- | --- | --- | --- | --- | --- | --- | --- | --- | --- | --- | --- | --- | --- | --- | --- | --- | --- | --- | --- |
| K.VLLSQQIFYDKK.N | N | 43.76 | 1480.8289 | 12 | 0.2 | 741.4186 | 2 | 16.98 | 1.0632 | 1.0705 | 54998 | 6.1e3 | 2 | 2 | 1479 | 1490 |  |  | DB Search |
| K.INNNQFSVVLIK.N | Y | 43.5 | 1387.7823 | 12 | -5.29 | 694.8917 | 2 | 18.34 | 0.9953 | 1.0025 | 63630 | 0 | 0 | 0 | 100 | 111 |  |  | DB Search |
| R.VLDVNDKQPVFVK.S | Y | 43.48 | 1499.8347 | 13 | 0.62 | 750.9218 | 2 | 15.15 | 1.0508 | 1.058 | 44643 | 0 | 0 | 0 | 670 | 682 |  |  | DB Search |
| total 3 peptides |  |  |  |  |  |  |  |  |  |  |  |  |  |  |  |  |  |  |  |
| --- | --- | --- | --- | --- | --- | --- | --- | --- | --- | --- | --- | --- | --- | --- | --- | --- | --- | --- | --- |

Best Unique PSM (Scan Sample\_7\_A1\_1\_3644.d:63630, m/z=694.8917, z=2, RT=18.34, ppm=-9.77):


TRINITY\_DN413\_c0\_g1\_i3.p1

back to list

  

| Protein Coverage
| Supporting Peptides
| Best Unique PSM
|

Protein Coverage:

Supporting Peptides:

| Peptide | Uniq | -10lgP | Mass | Length | ppm | m/z | z | RT | 1/k0 Start | 1/k0 End | Precursor ID | Area Sample 8 | #Feature | #Feature Sample 8 | Start | End | PTM | AScore | Found By |
| --- | --- | --- | --- | --- | --- | --- | --- | --- | --- | --- | --- | --- | --- | --- | --- | --- | --- | --- | --- |
| R.LLLGDFNGDKR.T | Y | 43.76 | 1246.6669 | 11 | -0.7 | 624.3375 | 2 | 16.26 | 0.9832 | 0.9904 | 50917 | 2.97e3 | 1 | 1 | 46 | 56 |  |  | DB Search |
| R.ADILC(+57.02)HDTK.Y | Y | 42.28 | 1071.5019 | 9 | 5.67 | 536.7589 | 2 | 11.81 | 0.8773 | 0.8846 | 24985 | 0 | 0 | 0 | 320 | 328 | Carbamidomethylation |  | DB Search |
| total 2 peptides |  |  |  |  |  |  |  |  |  |  |  |  |  |  |  |  |  |  |  |
| --- | --- | --- | --- | --- | --- | --- | --- | --- | --- | --- | --- | --- | --- | --- | --- | --- | --- | --- | --- |

Best Unique PSM (Scan Sample\_7\_A1\_1\_3644.d:50917, m/z=624.3375, z=2, RT=16.26, ppm=-5.18):


TRINITY\_DN413\_c0\_g1\_i1.p1

back to list

  

| Protein Coverage
| Supporting Peptides
| Best Unique PSM
|

Protein Coverage:

Supporting Peptides:

| Peptide | Uniq | -10lgP | Mass | Length | ppm | m/z | z | RT | 1/k0 Start | 1/k0 End | Precursor ID | Area Sample 8 | #Feature | #Feature Sample 8 | Start | End | PTM | AScore | Found By |
| --- | --- | --- | --- | --- | --- | --- | --- | --- | --- | --- | --- | --- | --- | --- | --- | --- | --- | --- | --- |
| R.LLLGDFNGDKR.T | Y | 43.76 | 1246.6669 | 11 | -0.7 | 624.3375 | 2 | 16.26 | 0.9832 | 0.9904 | 50917 | 2.97e3 | 1 | 1 | 46 | 56 |  |  | DB Search |
| R.ADILC(+57.02)HDTK.Y | Y | 42.28 | 1071.5019 | 9 | 5.67 | 536.7589 | 2 | 11.81 | 0.8773 | 0.8846 | 24985 | 0 | 0 | 0 | 329 | 337 | Carbamidomethylation |  | DB Search |
| total 2 peptides |  |  |  |  |  |  |  |  |  |  |  |  |  |  |  |  |  |  |  |
| --- | --- | --- | --- | --- | --- | --- | --- | --- | --- | --- | --- | --- | --- | --- | --- | --- | --- | --- | --- |

Best Unique PSM (Scan Sample\_7\_A1\_1\_3644.d:50917, m/z=624.3375, z=2, RT=16.26, ppm=-5.18):


TRINITY\_DN81944\_c0\_g1\_i1.p1

back to list

  

| Protein Coverage
| Supporting Peptides
| Best Unique PSM
|

Protein Coverage:

Supporting Peptides:

| Peptide | Uniq | -10lgP | Mass | Length | ppm | m/z | z | RT | 1/k0 Start | 1/k0 End | Precursor ID | Area Sample 8 | #Feature | #Feature Sample 8 | Start | End | PTM | AScore | Found By |
| --- | --- | --- | --- | --- | --- | --- | --- | --- | --- | --- | --- | --- | --- | --- | --- | --- | --- | --- | --- |
| K.TVWAMTQAEFSAK.S | Y | 43.74 | 1468.702 | 13 | 1.05 | 735.3558 | 2 | 18.57 | 1.0464 | 1.0536 | 65125 | 0 | 0 | 0 | 65 | 77 |  |  | DB Search |
| total 1 peptides |  |  |  |  |  |  |  |  |  |  |  |  |  |  |  |  |  |  |  |
| --- | --- | --- | --- | --- | --- | --- | --- | --- | --- | --- | --- | --- | --- | --- | --- | --- | --- | --- | --- |

Best Unique PSM (Scan Sample\_7\_A1\_1\_3644.d:65125, m/z=735.3558, z=2, RT=18.57, ppm=-3.43):


TRINITY\_DN3448\_c0\_g1\_i7.p2

back to list

  

| Protein Coverage
| Supporting Peptides
| Best Unique PSM
|

Protein Coverage:

Supporting Peptides:

| Peptide | Uniq | -10lgP | Mass | Length | ppm | m/z | z | RT | 1/k0 Start | 1/k0 End | Precursor ID | Area Sample 8 | #Feature | #Feature Sample 8 | Start | End | PTM | AScore | Found By |
| --- | --- | --- | --- | --- | --- | --- | --- | --- | --- | --- | --- | --- | --- | --- | --- | --- | --- | --- | --- |
| K.AGM(+15.99)THIVR.D | Y | 43.71 | 899.4647 | 8 | 5.69 | 450.7402 | 2 | 9.78 | 0.8161 | 0.8234 | 13845 | 0 | 0 | 0 | 27 | 34 | Oxidation (M) | M3:Oxidation (M):1000 | DB Search |
| total 1 peptides |  |  |  |  |  |  |  |  |  |  |  |  |  |  |  |  |  |  |  |
| --- | --- | --- | --- | --- | --- | --- | --- | --- | --- | --- | --- | --- | --- | --- | --- | --- | --- | --- | --- |

Best Unique PSM (Scan Sample\_7\_A1\_1\_3644.d:13845, m/z=450.7402, z=2, RT=9.78, ppm=1.21):


TRINITY\_DN11522\_c0\_g1\_i1.p1

back to list

  

| Protein Coverage
| Supporting Peptides
| Best Unique PSM
|

Protein Coverage:

Supporting Peptides:

| Peptide | Uniq | -10lgP | Mass | Length | ppm | m/z | z | RT | 1/k0 Start | 1/k0 End | Precursor ID | Area Sample 8 | #Feature | #Feature Sample 8 | Start | End | PTM | AScore | Found By |
| --- | --- | --- | --- | --- | --- | --- | --- | --- | --- | --- | --- | --- | --- | --- | --- | --- | --- | --- | --- |
| R.IGLGNIRDPSISLTR.G | Y | 43.72 | 1610.9104 | 15 | 2.12 | 537.9761 | 3 | 17.42 | 0.8019 | 0.8092 | 57662 | 1.88e3 | 1 | 1 | 25 | 39 |  |  | DB Search |
| K.GKPEQVQVK.M | Y | 43.14 | 1011.5713 | 9 | 2 | 506.7917 | 2 | 9.97 | 0.8416 | 0.849 | 14688 | 9.41e2 | 1 | 1 | 542 | 550 |  |  | DB Search |
| total 2 peptides |  |  |  |  |  |  |  |  |  |  |  |  |  |  |  |  |  |  |  |
| --- | --- | --- | --- | --- | --- | --- | --- | --- | --- | --- | --- | --- | --- | --- | --- | --- | --- | --- | --- |

Best Unique PSM (Scan Sample\_7\_A1\_1\_3644.d:57662, m/z=537.9761, z=3, RT=17.42, ppm=-2.35):


TRINITY\_DN5818\_c1\_g1\_i1.p1

back to list

  

| Protein Coverage
| Supporting Peptides
| Best Unique PSM
|

Protein Coverage:

Supporting Peptides:

| Peptide | Uniq | -10lgP | Mass | Length | ppm | m/z | z | RT | 1/k0 Start | 1/k0 End | Precursor ID | Area Sample 8 | #Feature | #Feature Sample 8 | Start | End | PTM | AScore | Found By |
| --- | --- | --- | --- | --- | --- | --- | --- | --- | --- | --- | --- | --- | --- | --- | --- | --- | --- | --- | --- |
| M(+42.01)(+15.99)HPPNSAPVK.S | Y | 43.8 | 1134.5492 | 10 | 4.48 | 568.2819 | 2 | 11.01 | 0.9174 | 0.9247 | 20310 | 4.98e3 | 1 | 1 | 1 | 10 | Acetylation (Protein N-term), Oxidation (M) | M1:Acetylation (Protein N-term):1000 M1:Oxidation (M):1000 | DB Search |
| K.LLAQTTLRNELGTK.N | N | 43.63 | 1556.8886 | 14 | -0.19 | 779.4479 | 2 | 15.37 | 1.1098 | 1.117 | 45889 | 3.33e2 | 1 | 1 | 144 | 157 |  |  | DB Search |
| R.AM(+15.99)AAEAEASR.E | Y | 43.45 | 1021.4498 | 10 | 0.68 | 511.7303 | 2 | 9.50 | 0.8067 | 0.814 | 12520 | 4.25e2 | 1 | 1 | 205 | 214 | Oxidation (M) | M2:Oxidation (M):1000 | DB Search |
| K.EASEIISESPQALQLR.Y | Y | 43.42 | 1769.9159 | 16 | -17.14 | 885.9461 | 2 | 18.60 | 1.1678 | 1.175 | 65266 | 0 | 0 | 0 | 235 | 250 |  |  | DB Search |
| total 4 peptides |  |  |  |  |  |  |  |  |  |  |  |  |  |  |  |  |  |  |  |
| --- | --- | --- | --- | --- | --- | --- | --- | --- | --- | --- | --- | --- | --- | --- | --- | --- | --- | --- | --- |

Best Unique PSM (Scan Sample\_7\_A1\_1\_3644.d:20310, m/z=568.2819, z=2, RT=11.01, ppm=0.01):


TRINITY\_DN1821\_c0\_g1\_i5.p1

back to list

  

| Protein Coverage
| Supporting Peptides
| Best Unique PSM
|

Protein Coverage:

Supporting Peptides:

| Peptide | Uniq | -10lgP | Mass | Length | ppm | m/z | z | RT | 1/k0 Start | 1/k0 End | Precursor ID | Area Sample 8 | #Feature | #Feature Sample 8 | Start | End | PTM | AScore | Found By |
| --- | --- | --- | --- | --- | --- | --- | --- | --- | --- | --- | --- | --- | --- | --- | --- | --- | --- | --- | --- |
| K.YQGIKPPVSR.S | Y | 43.24 | 1143.64 | 10 | -0.35 | 572.8245 | 2 | 12.74 | 0.9344 | 0.9416 | 30400 | 7.91e2 | 1 | 1 | 479 | 488 |  |  | DB Search |
| total 1 peptides |  |  |  |  |  |  |  |  |  |  |  |  |  |  |  |  |  |  |  |
| --- | --- | --- | --- | --- | --- | --- | --- | --- | --- | --- | --- | --- | --- | --- | --- | --- | --- | --- | --- |

Best Unique PSM (Scan Sample\_7\_A1\_1\_3644.d:30400, m/z=572.8245, z=2, RT=12.74, ppm=-4.83):


TRINITY\_DN3986\_c0\_g1\_i2.p1

back to list

  

| Protein Coverage
| Supporting Peptides
| Best Unique PSM
|

Protein Coverage:

Supporting Peptides:

| Peptide | Uniq | -10lgP | Mass | Length | ppm | m/z | z | RT | 1/k0 Start | 1/k0 End | Precursor ID | Area Sample 8 | #Feature | #Feature Sample 8 | Start | End | PTM | AScore | Found By |
| --- | --- | --- | --- | --- | --- | --- | --- | --- | --- | --- | --- | --- | --- | --- | --- | --- | --- | --- | --- |
| R.FPGQLNADLRK.L | Y | 43.16 | 1257.6829 | 11 | -1.33 | 629.8451 | 2 | 14.44 | 0.9497 | 0.957 | 40493 | 0 | 0 | 0 | 242 | 252 |  |  | DB Search |
| total 1 peptides |  |  |  |  |  |  |  |  |  |  |  |  |  |  |  |  |  |  |  |
| --- | --- | --- | --- | --- | --- | --- | --- | --- | --- | --- | --- | --- | --- | --- | --- | --- | --- | --- | --- |

Best Unique PSM (Scan Sample\_7\_A1\_1\_3644.d:40493, m/z=629.8451, z=2, RT=14.44, ppm=-5.81):


TRINITY\_DN13813\_c0\_g1\_i4.p2

back to list

  

| Protein Coverage
| Supporting Peptides
| Best Unique PSM
|

Protein Coverage:

Supporting Peptides:

| Peptide | Uniq | -10lgP | Mass | Length | ppm | m/z | z | RT | 1/k0 Start | 1/k0 End | Precursor ID | Area Sample 8 | #Feature | #Feature Sample 8 | Start | End | PTM | AScore | Found By |
| --- | --- | --- | --- | --- | --- | --- | --- | --- | --- | --- | --- | --- | --- | --- | --- | --- | --- | --- | --- |
| R.DGPGPLASDLLLQMR.Q | Y | 41.47 | 1581.8184 | 15 | -2.93 | 528.2762 | 3 | 19.62 | 0.8149 | 0.8222 | 71531 | 3.73e2 | 1 | 1 | 326 | 340 |  |  | DB Search |
| total 1 peptides |  |  |  |  |  |  |  |  |  |  |  |  |  |  |  |  |  |  |  |
| --- | --- | --- | --- | --- | --- | --- | --- | --- | --- | --- | --- | --- | --- | --- | --- | --- | --- | --- | --- |

Best Unique PSM (Scan Sample\_7\_A1\_1\_3644.d:71531, m/z=528.2762, z=3, RT=19.62, ppm=-7.41):


TRINITY\_DN319\_c2\_g1\_i1.p1

back to list

  

| Protein Coverage
| Supporting Peptides
| Best Unique PSM
|

Protein Coverage:

Supporting Peptides:

| Peptide | Uniq | -10lgP | Mass | Length | ppm | m/z | z | RT | 1/k0 Start | 1/k0 End | Precursor ID | Area Sample 8 | #Feature | #Feature Sample 8 | Start | End | PTM | AScore | Found By |
| --- | --- | --- | --- | --- | --- | --- | --- | --- | --- | --- | --- | --- | --- | --- | --- | --- | --- | --- | --- |
| K.KAQYYEAYETIIKR.H | Y | 43.83 | 1774.9254 | 14 | -1.22 | 592.6457 | 3 | 15.96 | 0.8664 | 0.8737 | 49246 | 6.27e2 | 1 | 1 | 2534 | 2547 |  |  | DB Search |
| R.GSFSSHIC(+57.02)LC(+57.02)HDPAYK.S | Y | 42.3 | 1877.8189 | 16 | -0.4 | 626.9438 | 3 | 14.48 | 0.8866 | 0.8939 | 40723 | 0 | 0 | 0 | 1011 | 1026 | Carbamidomethylation, Carbamidomethylation |  | DB Search |
| total 2 peptides |  |  |  |  |  |  |  |  |  |  |  |  |  |  |  |  |  |  |  |
| --- | --- | --- | --- | --- | --- | --- | --- | --- | --- | --- | --- | --- | --- | --- | --- | --- | --- | --- | --- |

Best Unique PSM (Scan Sample\_7\_A1\_1\_3644.d:49246, m/z=592.6457, z=3, RT=15.96, ppm=-5.7):


TRINITY\_DN2637\_c0\_g1\_i7.p2

back to list

  

| Protein Coverage
| Supporting Peptides
| Best Unique PSM
|

Protein Coverage:

Supporting Peptides:

| Peptide | Uniq | -10lgP | Mass | Length | ppm | m/z | z | RT | 1/k0 Start | 1/k0 End | Precursor ID | Area Sample 8 | #Feature | #Feature Sample 8 | Start | End | PTM | AScore | Found By |
| --- | --- | --- | --- | --- | --- | --- | --- | --- | --- | --- | --- | --- | --- | --- | --- | --- | --- | --- | --- |
| K.ATIEIPAKYEK.R | Y | 42.98 | 1261.6918 | 11 | 0.3 | 631.8505 | 2 | 14.24 | 0.9848 | 0.992 | 39239 | 0 | 0 | 0 | 107 | 117 |  |  | DB Search |
| total 1 peptides |  |  |  |  |  |  |  |  |  |  |  |  |  |  |  |  |  |  |  |
| --- | --- | --- | --- | --- | --- | --- | --- | --- | --- | --- | --- | --- | --- | --- | --- | --- | --- | --- | --- |

Best Unique PSM (Scan Sample\_7\_A1\_1\_3644.d:39239, m/z=631.8505, z=2, RT=14.24, ppm=-4.18):


TRINITY\_DN11097\_c0\_g2\_i1.p1

back to list

  

| Protein Coverage
| Supporting Peptides
| Best Unique PSM
|

Protein Coverage:

Supporting Peptides:

| Peptide | Uniq | -10lgP | Mass | Length | ppm | m/z | z | RT | 1/k0 Start | 1/k0 End | Precursor ID | Area Sample 8 | #Feature | #Feature Sample 8 | Start | End | PTM | AScore | Found By |
| --- | --- | --- | --- | --- | --- | --- | --- | --- | --- | --- | --- | --- | --- | --- | --- | --- | --- | --- | --- |
| R.VAHAGLTVAEFFR.D | Y | 43.68 | 1416.7513 | 13 | 2.73 | 473.2569 | 3 | 19.14 | 0.7502 | 0.7575 | 68636 | 3.46e2 | 1 | 1 | 199 | 211 |  |  | DB Search |
| total 1 peptides |  |  |  |  |  |  |  |  |  |  |  |  |  |  |  |  |  |  |  |
| --- | --- | --- | --- | --- | --- | --- | --- | --- | --- | --- | --- | --- | --- | --- | --- | --- | --- | --- | --- |

Best Unique PSM (Scan Sample\_7\_A1\_1\_3644.d:68636, m/z=473.2569, z=3, RT=19.14, ppm=-1.75):


TRINITY\_DN649\_c0\_g1\_i2.p1

back to list

  

| Protein Coverage
| Supporting Peptides
| Best Unique PSM
|

Protein Coverage:

Supporting Peptides:

| Peptide | Uniq | -10lgP | Mass | Length | ppm | m/z | z | RT | 1/k0 Start | 1/k0 End | Precursor ID | Area Sample 8 | #Feature | #Feature Sample 8 | Start | End | PTM | AScore | Found By |
| --- | --- | --- | --- | --- | --- | --- | --- | --- | --- | --- | --- | --- | --- | --- | --- | --- | --- | --- | --- |
| K.FETQIATK.N | Y | 43.56 | 936.4916 | 8 | 1.93 | 937.4965 | 1 | 13.99 | 1.2029 | 1.2101 | 37753 | 0 | 0 | 0 | 103 | 110 |  |  | DB Search |
| total 1 peptides |  |  |  |  |  |  |  |  |  |  |  |  |  |  |  |  |  |  |  |
| --- | --- | --- | --- | --- | --- | --- | --- | --- | --- | --- | --- | --- | --- | --- | --- | --- | --- | --- | --- |

Best Unique PSM (Scan Sample\_7\_A1\_1\_3644.d:37753, m/z=937.4965, z=1, RT=13.99, ppm=-2.55):


TRINITY\_DN413\_c0\_g1\_i4.p1

back to list

  

| Protein Coverage
| Supporting Peptides
| Best Unique PSM
|

Protein Coverage:

Supporting Peptides:

| Peptide | Uniq | -10lgP | Mass | Length | ppm | m/z | z | RT | 1/k0 Start | 1/k0 End | Precursor ID | Area Sample 8 | #Feature | #Feature Sample 8 | Start | End | PTM | AScore | Found By |
| --- | --- | --- | --- | --- | --- | --- | --- | --- | --- | --- | --- | --- | --- | --- | --- | --- | --- | --- | --- |
| R.TDFLC(+57.02)HDSR.G | Y | 43.78 | 1149.4873 | 9 | 2.13 | 575.7496 | 2 | 13.37 | 0.8684 | 0.8757 | 33932 | 8.97e2 | 1 | 1 | 380 | 388 | Carbamidomethylation |  | DB Search |
| R.LLLGDFNGDKR.T | Y | 43.76 | 1246.6669 | 11 | -0.7 | 624.3375 | 2 | 16.26 | 0.9832 | 0.9904 | 50917 | 2.97e3 | 1 | 1 | 46 | 56 |  |  | DB Search |
| R.ADILC(+57.02)HDTK.Y | Y | 42.28 | 1071.5019 | 9 | 5.67 | 536.7589 | 2 | 11.81 | 0.8773 | 0.8846 | 24985 | 0 | 0 | 0 | 320 | 328 | Carbamidomethylation |  | DB Search |
| total 3 peptides |  |  |  |  |  |  |  |  |  |  |  |  |  |  |  |  |  |  |  |
| --- | --- | --- | --- | --- | --- | --- | --- | --- | --- | --- | --- | --- | --- | --- | --- | --- | --- | --- | --- |

Best Unique PSM (Scan Sample\_7\_A1\_1\_3644.d:33932, m/z=575.7496, z=2, RT=13.37, ppm=-2.35):


TRINITY\_DN7504\_c0\_g1\_i1.p1

back to list

  

| Protein Coverage
| Supporting Peptides
| Best Unique PSM
|

Protein Coverage:

Supporting Peptides:

| Peptide | Uniq | -10lgP | Mass | Length | ppm | m/z | z | RT | 1/k0 Start | 1/k0 End | Precursor ID | Area Sample 8 | #Feature | #Feature Sample 8 | Start | End | PTM | AScore | Found By |
| --- | --- | --- | --- | --- | --- | --- | --- | --- | --- | --- | --- | --- | --- | --- | --- | --- | --- | --- | --- |
| K.TIEAEAAHGTVTR.H | Y | 43.81 | 1354.6841 | 13 | 1.86 | 678.3475 | 2 | 11.89 | 1.0126 | 1.0198 | 25470 | 0 | 0 | 0 | 301 | 313 |  |  | DB Search |
| total 1 peptides |  |  |  |  |  |  |  |  |  |  |  |  |  |  |  |  |  |  |  |
| --- | --- | --- | --- | --- | --- | --- | --- | --- | --- | --- | --- | --- | --- | --- | --- | --- | --- | --- | --- |

Best Unique PSM (Scan Sample\_7\_A1\_1\_3644.d:25470, m/z=678.3475, z=2, RT=11.89, ppm=-2.61):


TRINITY\_DN495\_c0\_g1\_i3.p1

back to list

  

| Protein Coverage
| Supporting Peptides
| Best Unique PSM
|

Protein Coverage:

Supporting Peptides:

| Peptide | Uniq | -10lgP | Mass | Length | ppm | m/z | z | RT | 1/k0 Start | 1/k0 End | Precursor ID | Area Sample 8 | #Feature | #Feature Sample 8 | Start | End | PTM | AScore | Found By |
| --- | --- | --- | --- | --- | --- | --- | --- | --- | --- | --- | --- | --- | --- | --- | --- | --- | --- | --- | --- |
| R.SC(+57.02)TNPAPANGGK.A | N | 43.8 | 1172.5244 | 12 | 2.69 | 587.2684 | 2 | 9.47 | 0.838 | 0.8453 | 12375 | 4.7e3 | 1 | 1 | 503 | 514 | Carbamidomethylation |  | DB Search |
| R.SSC(+57.02)KPILSVIK.I | Y | 43.76 | 1230.7006 | 11 | 2.76 | 616.3565 | 2 | 15.26 | 0.9529 | 0.9602 | 45369 | 2.7e3 | 1 | 1 | 454 | 464 | Carbamidomethylation |  | DB Search |
| total 2 peptides |  |  |  |  |  |  |  |  |  |  |  |  |  |  |  |  |  |  |  |
| --- | --- | --- | --- | --- | --- | --- | --- | --- | --- | --- | --- | --- | --- | --- | --- | --- | --- | --- | --- |

Best Unique PSM (Scan Sample\_7\_A1\_1\_3644.d:45369, m/z=616.3565, z=2, RT=15.26, ppm=-1.72):


TRINITY\_DN1172\_c0\_g2\_i2.p1

back to list

  

| Protein Coverage
| Supporting Peptides
| Best Unique PSM
|

Protein Coverage:

Supporting Peptides:

| Peptide | Uniq | -10lgP | Mass | Length | ppm | m/z | z | RT | 1/k0 Start | 1/k0 End | Precursor ID | Area Sample 8 | #Feature | #Feature Sample 8 | Start | End | PTM | AScore | Found By |
| --- | --- | --- | --- | --- | --- | --- | --- | --- | --- | --- | --- | --- | --- | --- | --- | --- | --- | --- | --- |
| K.K(+42.01)LAAELANGR.L | Y | 42.72 | 1083.6036 | 10 | -0.83 | 542.8062 | 2 | 15.48 | 0.8635 | 0.8708 | 46516 | 9.73e2 | 1 | 1 | 19 | 28 | Acetylation (Protein N-term) | K1:Acetylation (Protein N-term):1000 | DB Search |
| total 1 peptides |  |  |  |  |  |  |  |  |  |  |  |  |  |  |  |  |  |  |  |
| --- | --- | --- | --- | --- | --- | --- | --- | --- | --- | --- | --- | --- | --- | --- | --- | --- | --- | --- | --- |

Best Unique PSM (Scan Sample\_7\_A1\_1\_3644.d:46516, m/z=542.8062, z=2, RT=15.48, ppm=-5.31):


TRINITY\_DN4595\_c0\_g1\_i4.p1

back to list

  

| Protein Coverage
| Supporting Peptides
| Best Unique PSM
|

Protein Coverage:

Supporting Peptides:

| Peptide | Uniq | -10lgP | Mass | Length | ppm | m/z | z | RT | 1/k0 Start | 1/k0 End | Precursor ID | Area Sample 8 | #Feature | #Feature Sample 8 | Start | End | PTM | AScore | Found By |
| --- | --- | --- | --- | --- | --- | --- | --- | --- | --- | --- | --- | --- | --- | --- | --- | --- | --- | --- | --- |
| R.FC(+57.02)NNPAPSNGGR.M | Y | 43.83 | 1289.5571 | 12 | 3.53 | 645.7852 | 2 | 10.94 | 0.9449 | 0.9521 | 20039 | 4e2 | 1 | 1 | 1196 | 1207 | Carbamidomethylation |  | DB Search |
| R.SC(+57.02)ASDFSAER.L | Y | 43.77 | 1128.4506 | 10 | -0.21 | 565.2299 | 2 | 12.29 | 0.8546 | 0.8619 | 27814 | 0 | 0 | 0 | 299 | 308 | Carbamidomethylation |  | DB Search |
| R.AFNKVETR.N | N | 43.74 | 963.5138 | 8 | 5.69 | 482.7647 | 2 | 10.62 | 0.8299 | 0.8372 | 18171 | 1.3e3 | 1 | 1 | 177 | 184 |  |  | DB Search |
| K.YTGAC(+57.02)NANGNTDK.T | Y | 43.7 | 1384.5677 | 13 | 5.35 | 693.2917 | 2 | 9.59 | 0.9287 | 0.936 | 12941 | 0 | 0 | 0 | 1492 | 1504 | Carbamidomethylation |  | DB Search |
| R.AFNKIETR.N | Y | 43.58 | 977.5294 | 8 | 6.21 | 489.7728 | 2 | 11.52 | 0.8372 | 0.8445 | 23205 | 2.22e3 | 1 | 1 | 72 | 79 |  |  | DB Search |
| R.AFNRVETR.D | Y | 42.68 | 991.5199 | 8 | 4.26 | 496.7671 | 2 | 10.79 | 0.8425 | 0.8498 | 19063 | 2.3e3 | 1 | 1 | 282 | 289 |  |  | DB Search |
| total 6 peptides |  |  |  |  |  |  |  |  |  |  |  |  |  |  |  |  |  |  |  |
| --- | --- | --- | --- | --- | --- | --- | --- | --- | --- | --- | --- | --- | --- | --- | --- | --- | --- | --- | --- |

Best Unique PSM (Scan Sample\_7\_A1\_1\_3644.d:20039, m/z=645.7852, z=2, RT=10.94, ppm=-0.95):


TRINITY\_DN98420\_c0\_g1\_i1.p1

back to list

  

| Protein Coverage
| Supporting Peptides
| Best Unique PSM
|

Protein Coverage:

Supporting Peptides:

| Peptide | Uniq | -10lgP | Mass | Length | ppm | m/z | z | RT | 1/k0 Start | 1/k0 End | Precursor ID | Area Sample 8 | #Feature | #Feature Sample 8 | Start | End | PTM | AScore | Found By |
| --- | --- | --- | --- | --- | --- | --- | --- | --- | --- | --- | --- | --- | --- | --- | --- | --- | --- | --- | --- |
| R.AVIFDRFR.G | Y | 43.75 | 1022.5661 | 8 | 5.93 | 512.2911 | 2 | 16.16 | 0.87 | 0.8773 | 50308 | 2.24e3 | 1 | 1 | 48 | 55 |  |  | DB Search |
| total 1 peptides |  |  |  |  |  |  |  |  |  |  |  |  |  |  |  |  |  |  |  |
| --- | --- | --- | --- | --- | --- | --- | --- | --- | --- | --- | --- | --- | --- | --- | --- | --- | --- | --- | --- |

Best Unique PSM (Scan Sample\_7\_A1\_1\_3644.d:50308, m/z=512.2911, z=2, RT=16.16, ppm=1.45):


TRINITY\_DN72625\_c0\_g1\_i1.p1

back to list

  

| Protein Coverage
| Supporting Peptides
| Best Unique PSM
|

Protein Coverage:

Supporting Peptides:

| Peptide | Uniq | -10lgP | Mass | Length | ppm | m/z | z | RT | 1/k0 Start | 1/k0 End | Precursor ID | Area Sample 8 | #Feature | #Feature Sample 8 | Start | End | PTM | AScore | Found By |
| --- | --- | --- | --- | --- | --- | --- | --- | --- | --- | --- | --- | --- | --- | --- | --- | --- | --- | --- | --- |
| R.KC(+57.02)TVAIEC(+57.02)K.L | Y | 43.75 | 1107.5416 | 9 | 3.67 | 554.7776 | 2 | 11.08 | 0.8793 | 0.8866 | 20850 | 0 | 0 | 0 | 1902 | 1910 | Carbamidomethylation, Carbamidomethylation |  | DB Search |
| R.ATNPDVEK.I | Y | 43.73 | 872.4239 | 8 | 1.03 | 437.2177 | 2 | 9.75 | 0.7937 | 0.801 | 13513 | 8.88e3 | 1 | 1 | 302 | 309 |  |  | DB Search |
| R.KTTELTNNLK.A | Y | 43.69 | 1160.6401 | 10 | 0.29 | 581.3249 | 2 | 11.75 | 0.915 | 0.9222 | 24554 | 2.48e3 | 1 | 1 | 1921 | 1930 |  |  | DB Search |
| R.IKDTYLLTLEAR.D | Y | 43.17 | 1434.8082 | 12 | -0.29 | 718.408 | 2 | 17.10 | 1.0383 | 1.0456 | 55810 | 0 | 0 | 0 | 393 | 404 |  |  | DB Search |
| R.VKESYTLNIEAR.D | Y | 42.48 | 1421.7514 | 12 | 0.71 | 711.8803 | 2 | 14.78 | 1.0001 | 1.0073 | 42448 | 4.94e2 | 1 | 1 | 709 | 720 |  |  | DB Search |
| total 5 peptides |  |  |  |  |  |  |  |  |  |  |  |  |  |  |  |  |  |  |  |
| --- | --- | --- | --- | --- | --- | --- | --- | --- | --- | --- | --- | --- | --- | --- | --- | --- | --- | --- | --- |

Best Unique PSM (Scan Sample\_7\_A1\_1\_3644.d:20850, m/z=554.7776, z=2, RT=11.08, ppm=-0.81):


TRINITY\_DN585\_c0\_g1\_i2.p1

back to list

  

| Protein Coverage
| Supporting Peptides
| Best Unique PSM
|

Protein Coverage:

Supporting Peptides:

| Peptide | Uniq | -10lgP | Mass | Length | ppm | m/z | z | RT | 1/k0 Start | 1/k0 End | Precursor ID | Area Sample 8 | #Feature | #Feature Sample 8 | Start | End | PTM | AScore | Found By |
| --- | --- | --- | --- | --- | --- | --- | --- | --- | --- | --- | --- | --- | --- | --- | --- | --- | --- | --- | --- |
| K.TITLEVEPSDTIENVK.S | Y | 43.28 | 1786.92 | 16 | 2.71 | 894.4657 | 2 | 17.62 | 1.1454 | 1.1526 | 58818 | 1.46e3 | 1 | 1 | 60 | 75 |  |  | DB Search |
| total 1 peptides |  |  |  |  |  |  |  |  |  |  |  |  |  |  |  |  |  |  |  |
| --- | --- | --- | --- | --- | --- | --- | --- | --- | --- | --- | --- | --- | --- | --- | --- | --- | --- | --- | --- |

Best Unique PSM (Scan Sample\_7\_A1\_1\_3644.d:58818, m/z=894.4657, z=2, RT=17.62, ppm=-1.76):


TRINITY\_DN36\_c0\_g1\_i8.p1

back to list

  

| Protein Coverage
| Supporting Peptides
| Best Unique PSM
|

Protein Coverage:

Supporting Peptides:

| Peptide | Uniq | -10lgP | Mass | Length | ppm | m/z | z | RT | 1/k0 Start | 1/k0 End | Precursor ID | Area Sample 8 | #Feature | #Feature Sample 8 | Start | End | PTM | AScore | Found By |
| --- | --- | --- | --- | --- | --- | --- | --- | --- | --- | --- | --- | --- | --- | --- | --- | --- | --- | --- | --- |
| K.FETQIATK.D | Y | 43.56 | 936.4916 | 8 | 1.93 | 937.4965 | 1 | 13.99 | 1.2029 | 1.2101 | 37753 | 0 | 0 | 0 | 167 | 174 |  |  | DB Search |
| R.LGGIEVTQGNC(+57.02)AR.K | Y | 43.02 | 1373.6721 | 13 | 2.68 | 687.8421 | 2 | 13.90 | 0.9662 | 0.9735 | 37137 | 6.95e2 | 1 | 1 | 58 | 70 | Carbamidomethylation |  | DB Search |
| total 2 peptides |  |  |  |  |  |  |  |  |  |  |  |  |  |  |  |  |  |  |  |
| --- | --- | --- | --- | --- | --- | --- | --- | --- | --- | --- | --- | --- | --- | --- | --- | --- | --- | --- | --- |

Best Unique PSM (Scan Sample\_7\_A1\_1\_3644.d:37753, m/z=937.4965, z=1, RT=13.99, ppm=-2.55):


TRINITY\_DN3190\_c1\_g1\_i1.p1

back to list

  

| Protein Coverage
| Supporting Peptides
| Best Unique PSM
|

Protein Coverage:

Supporting Peptides:

| Peptide | Uniq | -10lgP | Mass | Length | ppm | m/z | z | RT | 1/k0 Start | 1/k0 End | Precursor ID | Area Sample 8 | #Feature | #Feature Sample 8 | Start | End | PTM | AScore | Found By |
| --- | --- | --- | --- | --- | --- | --- | --- | --- | --- | --- | --- | --- | --- | --- | --- | --- | --- | --- | --- |
| K.DLQGETSGSFKR.L | Y | 43.77 | 1323.6419 | 12 | 0.47 | 662.8256 | 2 | 12.73 | 0.9598 | 0.967 | 30294 | 0 | 0 | 0 | 135 | 146 |  |  | DB Search |
| M.A(+42.01)TVVDYPDFDAQADAETLRK.A | Y | 43.66 | 2266.0753 | 20 | 1.77 | 1134.0419 | 2 | 18.50 | 1.2877 | 1.2948 | 64568 | 0 | 0 | 0 | 2 | 21 | Acetylation (Protein N-term) | A1:Acetylation (Protein N-term):1000 | DB Search |
| M.A(+42.01)TVVDYPDFDAQADAETLR.K | Y | 43.66 | 2137.9803 | 19 | -0.79 | 1069.9918 | 2 | 19.84 | 1.2364 | 1.2436 | 72906 | 6.34e2 | 1 | 1 | 2 | 20 | Acetylation (Protein N-term) | A1:Acetylation (Protein N-term):1000 | DB Search |
| K.RLLTSLVQGAR.L | Y | 43.53 | 1212.7302 | 11 | 2.64 | 607.3713 | 2 | 15.40 | 0.9787 | 0.986 | 46092 | 6.47e2 | 1 | 1 | 146 | 156 |  |  | DB Search |
| R.LEDEPADHASAQADAHALFK.A | Y | 43.47 | 2134.9919 | 20 | 4.16 | 712.671 | 3 | 14.89 | 0.988 | 0.9953 | 43202 | 0 | 0 | 0 | 157 | 176 |  |  | DB Search |
| K.AGESQWGTDESR.F | Y | 43.17 | 1321.5534 | 12 | 3.73 | 661.7835 | 2 | 12.43 | 0.9198 | 0.9271 | 28620 | 4.49e2 | 1 | 1 | 177 | 188 |  |  | DB Search |
| total 6 peptides |  |  |  |  |  |  |  |  |  |  |  |  |  |  |  |  |  |  |  |
| --- | --- | --- | --- | --- | --- | --- | --- | --- | --- | --- | --- | --- | --- | --- | --- | --- | --- | --- | --- |

Best Unique PSM (Scan Sample\_7\_A1\_1\_3644.d:30294, m/z=662.8256, z=2, RT=12.73, ppm=-4):


TRINITY\_DN1355\_c0\_g1\_i1.p1

back to list

  

| Protein Coverage
| Supporting Peptides
| Best Unique PSM
|

Protein Coverage:

Supporting Peptides:

| Peptide | Uniq | -10lgP | Mass | Length | ppm | m/z | z | RT | 1/k0 Start | 1/k0 End | Precursor ID | Area Sample 8 | #Feature | #Feature Sample 8 | Start | End | PTM | AScore | Found By |
| --- | --- | --- | --- | --- | --- | --- | --- | --- | --- | --- | --- | --- | --- | --- | --- | --- | --- | --- | --- |
| R.GNHEC(+57.02)ASINR.I | Y | 43.61 | 1156.5043 | 10 | 15.1 | 386.5128 | 3 | 9.81 | 0.7286 | 0.7359 | 14029 | 0 | 0 | 0 | 124 | 133 | Carbamidomethylation |  | DB Search |
| R.FLNRHDLDLIC(+57.02)R.A | Y | 43.55 | 1570.8038 | 12 | 5.48 | 524.6091 | 3 | 16.23 | 0.8067 | 0.814 | 50829 | 2.25e2 | 1 | 1 | 236 | 247 | Carbamidomethylation |  | DB Search |
| total 2 peptides |  |  |  |  |  |  |  |  |  |  |  |  |  |  |  |  |  |  |  |
| --- | --- | --- | --- | --- | --- | --- | --- | --- | --- | --- | --- | --- | --- | --- | --- | --- | --- | --- | --- |

Best Unique PSM (Scan Sample\_7\_A1\_1\_3644.d:14029, m/z=386.5128, z=3, RT=9.81, ppm=10.62):


TRINITY\_DN1335\_c0\_g1\_i1.p1

back to list

  

| Protein Coverage
| Supporting Peptides
| Best Unique PSM
|

Protein Coverage:

Supporting Peptides:

| Peptide | Uniq | -10lgP | Mass | Length | ppm | m/z | z | RT | 1/k0 Start | 1/k0 End | Precursor ID | Area Sample 8 | #Feature | #Feature Sample 8 | Start | End | PTM | AScore | Found By |
| --- | --- | --- | --- | --- | --- | --- | --- | --- | --- | --- | --- | --- | --- | --- | --- | --- | --- | --- | --- |
| K.ELVTAHQTFIR.L | Y | 43.45 | 1313.7092 | 11 | 0.79 | 657.8594 | 2 | 14.74 | 0.9868 | 0.9941 | 42263 | 0 | 0 | 0 | 446 | 456 |  |  | DB Search |
| total 1 peptides |  |  |  |  |  |  |  |  |  |  |  |  |  |  |  |  |  |  |  |
| --- | --- | --- | --- | --- | --- | --- | --- | --- | --- | --- | --- | --- | --- | --- | --- | --- | --- | --- | --- |

Best Unique PSM (Scan Sample\_7\_A1\_1\_3644.d:42263, m/z=657.8594, z=2, RT=14.74, ppm=-3.68):


TRINITY\_DN128406\_c0\_g1\_i1.p1

back to list

  

| Protein Coverage
| Supporting Peptides
| Best Unique PSM
|

Protein Coverage:

Supporting Peptides:

| Peptide | Uniq | -10lgP | Mass | Length | ppm | m/z | z | RT | 1/k0 Start | 1/k0 End | Precursor ID | Area Sample 8 | #Feature | #Feature Sample 8 | Start | End | PTM | AScore | Found By |
| --- | --- | --- | --- | --- | --- | --- | --- | --- | --- | --- | --- | --- | --- | --- | --- | --- | --- | --- | --- |
| K.EIRDILVQYDR.S | Y | 43.15 | 1418.7517 | 11 | -6.07 | 710.3757 | 2 | 19.92 | 1.0331 | 1.0403 | 73522 | 5.02e2 | 1 | 1 | 110 | 120 |  |  | DB Search |
| total 1 peptides |  |  |  |  |  |  |  |  |  |  |  |  |  |  |  |  |  |  |  |
| --- | --- | --- | --- | --- | --- | --- | --- | --- | --- | --- | --- | --- | --- | --- | --- | --- | --- | --- | --- |

Best Unique PSM (Scan Sample\_7\_A1\_1\_3644.d:73522, m/z=710.3757, z=2, RT=19.92, ppm=-10.54):


TRINITY\_DN1616\_c0\_g1\_i6.p1

back to list

  

| Protein Coverage
| Supporting Peptides
| Best Unique PSM
|

Protein Coverage:

Supporting Peptides:

| Peptide | Uniq | -10lgP | Mass | Length | ppm | m/z | z | RT | 1/k0 Start | 1/k0 End | Precursor ID | Area Sample 8 | #Feature | #Feature Sample 8 | Start | End | PTM | AScore | Found By |
| --- | --- | --- | --- | --- | --- | --- | --- | --- | --- | --- | --- | --- | --- | --- | --- | --- | --- | --- | --- |
| R.TSTDKFEC(+57.02)EK.C | Y | 43.76 | 1243.5391 | 10 | -3.39 | 622.7719 | 2 | 10.75 | 0.9255 | 0.9327 | 18979 | 0 | 0 | 0 | 320 | 329 | Carbamidomethylation |  | DB Search |
| R.YGAVNDNC(+57.02)ER.D | Y | 43.62 | 1196.488 | 10 | -2.06 | 599.2474 | 2 | 10.92 | 0.8834 | 0.8907 | 19854 | 4.23e2 | 1 | 1 | 498 | 507 | Carbamidomethylation |  | DB Search |
| R.YVKPGVNIINIR.D | Y | 43.56 | 1384.819 | 12 | 3.35 | 693.416 | 2 | 16.23 | 1.0013 | 1.0086 | 50888 | 0 | 0 | 0 | 1217 | 1228 |  |  | DB Search |
| K.YVVMLQYYHDSDTAK.Y | Y | 43.52 | 1831.8451 | 15 | 8.47 | 916.9335 | 2 | 16.82 | 1.1857 | 1.1929 | 54206 | 0 | 0 | 0 | 1071 | 1085 |  |  | DB Search |
| K.SSSC(+57.02)QEFGGKC(+57.02)PC(+57.02)K.D | Y | 39.5 | 1630.6538 | 14 | -18.66 | 816.3153 | 2 | 12.09 | 1.0311 | 1.0383 | 26624 | 0 | 0 | 0 | 1387 | 1400 | Carbamidomethylation, Carbamidomethylation, Carbamidomethylation |  | DB Search |
| total 5 peptides |  |  |  |  |  |  |  |  |  |  |  |  |  |  |  |  |  |  |  |
| --- | --- | --- | --- | --- | --- | --- | --- | --- | --- | --- | --- | --- | --- | --- | --- | --- | --- | --- | --- |

Best Unique PSM (Scan Sample\_7\_A1\_1\_3644.d:18979, m/z=622.7719, z=2, RT=10.75, ppm=-7.87):


TRINITY\_DN5397\_c0\_g1\_i1.p1

back to list

  

| Protein Coverage
| Supporting Peptides
| Best Unique PSM
|

Protein Coverage:

Supporting Peptides:

| Peptide | Uniq | -10lgP | Mass | Length | ppm | m/z | z | RT | 1/k0 Start | 1/k0 End | Precursor ID | Area Sample 8 | #Feature | #Feature Sample 8 | Start | End | PTM | AScore | Found By |
| --- | --- | --- | --- | --- | --- | --- | --- | --- | --- | --- | --- | --- | --- | --- | --- | --- | --- | --- | --- |
| R.SFNKVESR.E | Y | 42.91 | 965.493 | 8 | 6.49 | 483.7548 | 2 | 10.36 | 0.8392 | 0.8465 | 16574 | 6.58e3 | 1 | 1 | 33 | 40 |  |  | DB Search |
| total 1 peptides |  |  |  |  |  |  |  |  |  |  |  |  |  |  |  |  |  |  |  |
| --- | --- | --- | --- | --- | --- | --- | --- | --- | --- | --- | --- | --- | --- | --- | --- | --- | --- | --- | --- |

Best Unique PSM (Scan Sample\_7\_A1\_1\_3644.d:16574, m/z=483.7548, z=2, RT=10.36, ppm=2.01):


TRINITY\_DN25375\_c0\_g1\_i7.p1

back to list

  

| Protein Coverage
| Supporting Peptides
| Best Unique PSM
|

Protein Coverage:

Supporting Peptides:

| Peptide | Uniq | -10lgP | Mass | Length | ppm | m/z | z | RT | 1/k0 Start | 1/k0 End | Precursor ID | Area Sample 8 | #Feature | #Feature Sample 8 | Start | End | PTM | AScore | Found By |
| --- | --- | --- | --- | --- | --- | --- | --- | --- | --- | --- | --- | --- | --- | --- | --- | --- | --- | --- | --- |
| K.LRPGQATTVDITVK.T | Y | 42.76 | 1497.8515 | 14 | -1.18 | 749.9288 | 2 | 14.18 | 1.0323 | 1.0395 | 38810 | 8.86e2 | 1 | 1 | 114 | 127 |  |  | DB Search |
| total 1 peptides |  |  |  |  |  |  |  |  |  |  |  |  |  |  |  |  |  |  |  |
| --- | --- | --- | --- | --- | --- | --- | --- | --- | --- | --- | --- | --- | --- | --- | --- | --- | --- | --- | --- |

Best Unique PSM (Scan Sample\_7\_A1\_1\_3644.d:38810, m/z=749.9288, z=2, RT=14.18, ppm=-5.65):


TRINITY\_DN3202\_c2\_g1\_i1.p1

back to list

  

| Protein Coverage
| Supporting Peptides
| Best Unique PSM
|

Protein Coverage:

Supporting Peptides:

| Peptide | Uniq | -10lgP | Mass | Length | ppm | m/z | z | RT | 1/k0 Start | 1/k0 End | Precursor ID | Area Sample 8 | #Feature | #Feature Sample 8 | Start | End | PTM | AScore | Found By |
| --- | --- | --- | --- | --- | --- | --- | --- | --- | --- | --- | --- | --- | --- | --- | --- | --- | --- | --- | --- |
| R.G(+42.01)C(+57.02)YIHTQEVAR.G | Y | 43.35 | 1374.635 | 11 | 1.87 | 688.323 | 2 | 14.11 | 0.9876 | 0.9949 | 38559 | 0 | 0 | 0 | 7 | 17 | Acetylation (Protein N-term), Carbamidomethylation | G1:Acetylation (Protein N-term):1000 | DB Search |
| total 1 peptides |  |  |  |  |  |  |  |  |  |  |  |  |  |  |  |  |  |  |  |
| --- | --- | --- | --- | --- | --- | --- | --- | --- | --- | --- | --- | --- | --- | --- | --- | --- | --- | --- | --- |

Best Unique PSM (Scan Sample\_7\_A1\_1\_3644.d:38559, m/z=688.323, z=2, RT=14.11, ppm=-2.6):


TRINITY\_DN124859\_c0\_g1\_i1.p3

back to list

  

| Protein Coverage
| Supporting Peptides
| Best Unique PSM
|

Protein Coverage:

Supporting Peptides:

| Peptide | Uniq | -10lgP | Mass | Length | ppm | m/z | z | RT | 1/k0 Start | 1/k0 End | Precursor ID | Area Sample 8 | #Feature | #Feature Sample 8 | Start | End | PTM | AScore | Found By |
| --- | --- | --- | --- | --- | --- | --- | --- | --- | --- | --- | --- | --- | --- | --- | --- | --- | --- | --- | --- |
| R.DEDHAAR.Q | Y | 43.77 | 812.3413 | 7 | -12.56 | 407.171 | 2 | 9.29 | 0.7665 | 0.7738 | 11855 | 1.9e2 | 1 | 1 | 181 | 187 |  |  | DB Search |
| total 1 peptides |  |  |  |  |  |  |  |  |  |  |  |  |  |  |  |  |  |  |  |
| --- | --- | --- | --- | --- | --- | --- | --- | --- | --- | --- | --- | --- | --- | --- | --- | --- | --- | --- | --- |

Best Unique PSM (Scan Sample\_7\_A1\_1\_3644.d:11855, m/z=407.171, z=2, RT=9.29, ppm=-17.04):


TRINITY\_DN374\_c0\_g1\_i2.p1

back to list

  

| Protein Coverage
| Supporting Peptides
| Best Unique PSM
|

Protein Coverage:

Supporting Peptides:

| Peptide | Uniq | -10lgP | Mass | Length | ppm | m/z | z | RT | 1/k0 Start | 1/k0 End | Precursor ID | Area Sample 8 | #Feature | #Feature Sample 8 | Start | End | PTM | AScore | Found By |
| --- | --- | --- | --- | --- | --- | --- | --- | --- | --- | --- | --- | --- | --- | --- | --- | --- | --- | --- | --- |
| K.YIDIEYVC(+57.02)KPPVK.K | Y | 43.56 | 1622.8378 | 13 | -3.07 | 812.42 | 2 | 16.34 | 1.1282 | 1.1354 | 51406 | 7.33e2 | 1 | 1 | 234 | 246 | Carbamidomethylation |  | DB Search |
| total 1 peptides |  |  |  |  |  |  |  |  |  |  |  |  |  |  |  |  |  |  |  |
| --- | --- | --- | --- | --- | --- | --- | --- | --- | --- | --- | --- | --- | --- | --- | --- | --- | --- | --- | --- |

Best Unique PSM (Scan Sample\_7\_A1\_1\_3644.d:51406, m/z=812.42, z=2, RT=16.34, ppm=-7.55):


TRINITY\_DN942\_c0\_g1\_i5.p1

back to list

  

| Protein Coverage
| Supporting Peptides
| Best Unique PSM
|

Protein Coverage:

Supporting Peptides:

| Peptide | Uniq | -10lgP | Mass | Length | ppm | m/z | z | RT | 1/k0 Start | 1/k0 End | Precursor ID | Area Sample 8 | #Feature | #Feature Sample 8 | Start | End | PTM | AScore | Found By |
| --- | --- | --- | --- | --- | --- | --- | --- | --- | --- | --- | --- | --- | --- | --- | --- | --- | --- | --- | --- |
| K.SGVC(+57.02)TDANTR.D | Y | 43.32 | 1079.4666 | 10 | 12.92 | 540.7451 | 2 | 10.11 | 0.8445 | 0.8518 | 15427 | 0 | 0 | 0 | 359 | 368 | Carbamidomethylation |  | DB Search |
| K.AALNM(+15.99)EFEYIPTK.S | Y | 42.87 | 1541.7436 | 13 | -3.63 | 771.8728 | 2 | 17.58 | 1.0528 | 1.06 | 58710 | 2.11e2 | 1 | 1 | 1659 | 1671 | Oxidation (M) | M5:Oxidation (M):1000 | DB Search |
| total 2 peptides |  |  |  |  |  |  |  |  |  |  |  |  |  |  |  |  |  |  |  |
| --- | --- | --- | --- | --- | --- | --- | --- | --- | --- | --- | --- | --- | --- | --- | --- | --- | --- | --- | --- |

Best Unique PSM (Scan Sample\_7\_A1\_1\_3644.d:15427, m/z=540.7451, z=2, RT=10.11, ppm=8.45):


TRINITY\_DN585\_c0\_g1\_i4.p1

back to list

  

| Protein Coverage
| Supporting Peptides
| Best Unique PSM
|

Protein Coverage:

Supporting Peptides:

| Peptide | Uniq | -10lgP | Mass | Length | ppm | m/z | z | RT | 1/k0 Start | 1/k0 End | Precursor ID | Area Sample 8 | #Feature | #Feature Sample 8 | Start | End | PTM | AScore | Found By |
| --- | --- | --- | --- | --- | --- | --- | --- | --- | --- | --- | --- | --- | --- | --- | --- | --- | --- | --- | --- |
| K.TITLEVEPSDTIENVK.S | Y | 43.28 | 1786.92 | 16 | 2.71 | 894.4657 | 2 | 17.62 | 1.1454 | 1.1526 | 58818 | 1.46e3 | 1 | 1 | 12 | 27 |  |  | DB Search |
| total 1 peptides |  |  |  |  |  |  |  |  |  |  |  |  |  |  |  |  |  |  |  |
| --- | --- | --- | --- | --- | --- | --- | --- | --- | --- | --- | --- | --- | --- | --- | --- | --- | --- | --- | --- |

Best Unique PSM (Scan Sample\_7\_A1\_1\_3644.d:58818, m/z=894.4657, z=2, RT=17.62, ppm=-1.76):


TRINITY\_DN5584\_c0\_g1\_i2.p1

back to list

  

| Protein Coverage
| Supporting Peptides
| Best Unique PSM
|

Protein Coverage:

Supporting Peptides:

| Peptide | Uniq | -10lgP | Mass | Length | ppm | m/z | z | RT | 1/k0 Start | 1/k0 End | Precursor ID | Area Sample 8 | #Feature | #Feature Sample 8 | Start | End | PTM | AScore | Found By |
| --- | --- | --- | --- | --- | --- | --- | --- | --- | --- | --- | --- | --- | --- | --- | --- | --- | --- | --- | --- |
| R.YGTSSFDMSSLIK.T | Y | 42.34 | 1434.6701 | 13 | 2.06 | 718.3406 | 2 | 19.16 | 1.023 | 1.0303 | 68826 | 0 | 0 | 0 | 233 | 245 |  |  | DB Search |
| total 1 peptides |  |  |  |  |  |  |  |  |  |  |  |  |  |  |  |  |  |  |  |
| --- | --- | --- | --- | --- | --- | --- | --- | --- | --- | --- | --- | --- | --- | --- | --- | --- | --- | --- | --- |

Best Unique PSM (Scan Sample\_7\_A1\_1\_3644.d:68826, m/z=718.3406, z=2, RT=19.16, ppm=-2.42):


TRINITY\_DN761\_c0\_g1\_i3.p1

back to list

  

| Protein Coverage
| Supporting Peptides
| Best Unique PSM
|

Protein Coverage:

Supporting Peptides:

| Peptide | Uniq | -10lgP | Mass | Length | ppm | m/z | z | RT | 1/k0 Start | 1/k0 End | Precursor ID | Area Sample 8 | #Feature | #Feature Sample 8 | Start | End | PTM | AScore | Found By |
| --- | --- | --- | --- | --- | --- | --- | --- | --- | --- | --- | --- | --- | --- | --- | --- | --- | --- | --- | --- |
| K.TRIEDLETVISQTQDHR.H | Y | 43.83 | 2040.0236 | 17 | 1.88 | 681.0134 | 3 | 17.57 | 0.9626 | 0.9699 | 58583 | 4.36e2 | 1 | 1 | 275 | 291 |  |  | DB Search |
| total 1 peptides |  |  |  |  |  |  |  |  |  |  |  |  |  |  |  |  |  |  |  |
| --- | --- | --- | --- | --- | --- | --- | --- | --- | --- | --- | --- | --- | --- | --- | --- | --- | --- | --- | --- |

Best Unique PSM (Scan Sample\_7\_A1\_1\_3644.d:58583, m/z=681.0134, z=3, RT=17.57, ppm=-2.6):


TRINITY\_DN3034\_c0\_g1\_i1.p1

back to list

  

| Protein Coverage
| Supporting Peptides
| Best Unique PSM
|

Protein Coverage:

Supporting Peptides:

| Peptide | Uniq | -10lgP | Mass | Length | ppm | m/z | z | RT | 1/k0 Start | 1/k0 End | Precursor ID | Area Sample 8 | #Feature | #Feature Sample 8 | Start | End | PTM | AScore | Found By |
| --- | --- | --- | --- | --- | --- | --- | --- | --- | --- | --- | --- | --- | --- | --- | --- | --- | --- | --- | --- |
| K.TPGLVEGTLILHQLR.C | Y | 43.67 | 1645.9515 | 15 | 1.25 | 549.656 | 3 | 19.04 | 0.8611 | 0.8684 | 67992 | 4.54e2 | 1 | 1 | 671 | 685 |  |  | DB Search |
| total 1 peptides |  |  |  |  |  |  |  |  |  |  |  |  |  |  |  |  |  |  |  |
| --- | --- | --- | --- | --- | --- | --- | --- | --- | --- | --- | --- | --- | --- | --- | --- | --- | --- | --- | --- |

Best Unique PSM (Scan Sample\_7\_A1\_1\_3644.d:67992, m/z=549.656, z=3, RT=19.04, ppm=-3.23):


TRINITY\_DN2636\_c0\_g1\_i2.p1

back to list

  

| Protein Coverage
| Supporting Peptides
| Best Unique PSM
|

Protein Coverage:

Supporting Peptides:

| Peptide | Uniq | -10lgP | Mass | Length | ppm | m/z | z | RT | 1/k0 Start | 1/k0 End | Precursor ID | Area Sample 8 | #Feature | #Feature Sample 8 | Start | End | PTM | AScore | Found By |
| --- | --- | --- | --- | --- | --- | --- | --- | --- | --- | --- | --- | --- | --- | --- | --- | --- | --- | --- | --- |
| R.MSEIEDLTHLAK.R | Y | 43.5 | 1385.686 | 12 | 3.75 | 693.8498 | 2 | 17.42 | 1.0295 | 1.0367 | 57726 | 0 | 0 | 0 | 239 | 250 |  |  | DB Search |
| K.VVEKEAETER.K | Y | 42.37 | 1188.5986 | 10 | -4.14 | 595.3015 | 2 | 9.59 | 0.8988 | 0.9061 | 12943 | 0 | 0 | 0 | 199 | 208 |  |  | DB Search |
| total 2 peptides |  |  |  |  |  |  |  |  |  |  |  |  |  |  |  |  |  |  |  |
| --- | --- | --- | --- | --- | --- | --- | --- | --- | --- | --- | --- | --- | --- | --- | --- | --- | --- | --- | --- |

Best Unique PSM (Scan Sample\_7\_A1\_1\_3644.d:57726, m/z=693.8498, z=2, RT=17.42, ppm=-0.73):


TRINITY\_DN360\_c0\_g1\_i1.p1

back to list

  

| Protein Coverage
| Supporting Peptides
| Best Unique PSM
|

Protein Coverage:

Supporting Peptides:

| Peptide | Uniq | -10lgP | Mass | Length | ppm | m/z | z | RT | 1/k0 Start | 1/k0 End | Precursor ID | Area Sample 8 | #Feature | #Feature Sample 8 | Start | End | PTM | AScore | Found By |
| --- | --- | --- | --- | --- | --- | --- | --- | --- | --- | --- | --- | --- | --- | --- | --- | --- | --- | --- | --- |
| R.TPNEVEFHQAVK.E | Y | 43.77 | 1397.6939 | 12 | -4.4 | 699.848 | 2 | 13.04 | 0.9904 | 0.9977 | 32035 | 1.84e3 | 2 | 2 | 44 | 55 |  |  | DB Search |
| R.LRNEFTGVLTGK.G | Y | 42.32 | 1333.7354 | 12 | -0.96 | 667.8713 | 2 | 16.05 | 0.9812 | 0.9884 | 49757 | 5.76e2 | 1 | 1 | 212 | 223 |  |  | DB Search |
| total 2 peptides |  |  |  |  |  |  |  |  |  |  |  |  |  |  |  |  |  |  |  |
| --- | --- | --- | --- | --- | --- | --- | --- | --- | --- | --- | --- | --- | --- | --- | --- | --- | --- | --- | --- |

Best Unique PSM (Scan Sample\_7\_A1\_1\_3644.d:32035, m/z=699.848, z=2, RT=13.04, ppm=-8.88):


TRINITY\_DN982\_c0\_g1\_i1.p1

back to list

  

| Protein Coverage
| Supporting Peptides
| Best Unique PSM
|

Protein Coverage:

Supporting Peptides:

| Peptide | Uniq | -10lgP | Mass | Length | ppm | m/z | z | RT | 1/k0 Start | 1/k0 End | Precursor ID | Area Sample 8 | #Feature | #Feature Sample 8 | Start | End | PTM | AScore | Found By |
| --- | --- | --- | --- | --- | --- | --- | --- | --- | --- | --- | --- | --- | --- | --- | --- | --- | --- | --- | --- |
| K.VLLSQQIFYDKK.N | N | 43.76 | 1480.8289 | 12 | 0.2 | 741.4186 | 2 | 16.98 | 1.0632 | 1.0705 | 54998 | 6.1e3 | 2 | 2 | 1479 | 1490 |  |  | DB Search |
| K.YVAIYPSDHLTVNR.D | N | 43.74 | 1646.8416 | 14 | -4.41 | 824.4208 | 2 | 15.73 | 1.1094 | 1.1166 | 47842 | 6.9e2 | 1 | 1 | 2814 | 2827 |  |  | DB Search |
| R.YTSC(+57.02)QSVTVVASDR.S | N | 43.65 | 1571.725 | 14 | -1.13 | 786.8654 | 2 | 13.49 | 1.05 | 1.0572 | 34821 | 0 | 0 | 0 | 5923 | 5936 | Carbamidomethylation |  | DB Search |
| R.TNNALDYEAKR.Q | N | 43.54 | 1293.6313 | 11 | 0.43 | 647.8203 | 2 | 12.12 | 0.965 | 0.9723 | 26731 | 1.84e3 | 1 | 1 | 4285 | 4295 |  |  | DB Search |
| K.INNNQFSVVLIK.N | Y | 43.5 | 1387.7823 | 12 | -5.29 | 694.8917 | 2 | 18.34 | 0.9953 | 1.0025 | 63630 | 0 | 0 | 0 | 100 | 111 |  |  | DB Search |
| R.VLDVNDKQPVFVK.S | Y | 43.48 | 1499.8347 | 13 | 0.62 | 750.9218 | 2 | 15.15 | 1.0508 | 1.058 | 44643 | 0 | 0 | 0 | 670 | 682 |  |  | DB Search |
| K.TSTQDVALNIRR.S | N | 43.43 | 1372.7423 | 12 | 2.89 | 687.3773 | 2 | 13.63 | 0.9997 | 1.0069 | 35508 | 2.89e3 | 1 | 1 | 5623 | 5634 |  |  | DB Search |
| R.LVQNTPHNSIVTK.I | N | 43.35 | 1449.794 | 13 | 1.92 | 725.9024 | 2 | 11.76 | 1.0564 | 1.0636 | 24648 | 1.37e3 | 1 | 1 | 5129 | 5141 |  |  | DB Search |
| total 8 peptides |  |  |  |  |  |  |  |  |  |  |  |  |  |  |  |  |  |  |  |
| --- | --- | --- | --- | --- | --- | --- | --- | --- | --- | --- | --- | --- | --- | --- | --- | --- | --- | --- | --- |

Best Unique PSM (Scan Sample\_7\_A1\_1\_3644.d:63630, m/z=694.8917, z=2, RT=18.34, ppm=-9.77):


TRINITY\_DN941\_c0\_g1\_i7.p2

back to list

  

| Protein Coverage
| Supporting Peptides
| Best Unique PSM
|

Protein Coverage:

Supporting Peptides:

| Peptide | Uniq | -10lgP | Mass | Length | ppm | m/z | z | RT | 1/k0 Start | 1/k0 End | Precursor ID | Area Sample 8 | #Feature | #Feature Sample 8 | Start | End | PTM | AScore | Found By |
| --- | --- | --- | --- | --- | --- | --- | --- | --- | --- | --- | --- | --- | --- | --- | --- | --- | --- | --- | --- |
| K.ADLTRPTGK.K | Y | 42.69 | 957.5243 | 9 | -3.1 | 479.7658 | 2 | 10.44 | 0.8193 | 0.8266 | 17248 | 0 | 0 | 0 | 52 | 60 |  |  | DB Search |
| total 1 peptides |  |  |  |  |  |  |  |  |  |  |  |  |  |  |  |  |  |  |  |
| --- | --- | --- | --- | --- | --- | --- | --- | --- | --- | --- | --- | --- | --- | --- | --- | --- | --- | --- | --- |

Best Unique PSM (Scan Sample\_7\_A1\_1\_3644.d:17248, m/z=479.7658, z=2, RT=10.44, ppm=-7.58):


TRINITY\_DN1030\_c0\_g1\_i1.p1

back to list

  

| Protein Coverage
| Supporting Peptides
| Best Unique PSM
|

Protein Coverage:

Supporting Peptides:

| Peptide | Uniq | -10lgP | Mass | Length | ppm | m/z | z | RT | 1/k0 Start | 1/k0 End | Precursor ID | Area Sample 8 | #Feature | #Feature Sample 8 | Start | End | PTM | AScore | Found By |
| --- | --- | --- | --- | --- | --- | --- | --- | --- | --- | --- | --- | --- | --- | --- | --- | --- | --- | --- | --- |
| R.GLQGQQGEQGSTGVQGSVGGR.G | Y | 43.49 | 1985.9515 | 21 | -0.42 | 993.9781 | 2 | 12.09 | 1.1302 | 1.1374 | 26593 | 0 | 0 | 0 | 361 | 381 |  |  | DB Search |
| total 1 peptides |  |  |  |  |  |  |  |  |  |  |  |  |  |  |  |  |  |  |  |
| --- | --- | --- | --- | --- | --- | --- | --- | --- | --- | --- | --- | --- | --- | --- | --- | --- | --- | --- | --- |

Best Unique PSM (Scan Sample\_7\_A1\_1\_3644.d:26593, m/z=993.9781, z=2, RT=12.09, ppm=-4.89):


TRINITY\_DN8008\_c0\_g1\_i3.p1

back to list

  

| Protein Coverage
| Supporting Peptides
| Best Unique PSM
|

Protein Coverage:

Supporting Peptides:

| Peptide | Uniq | -10lgP | Mass | Length | ppm | m/z | z | RT | 1/k0 Start | 1/k0 End | Precursor ID | Area Sample 8 | #Feature | #Feature Sample 8 | Start | End | PTM | AScore | Found By |
| --- | --- | --- | --- | --- | --- | --- | --- | --- | --- | --- | --- | --- | --- | --- | --- | --- | --- | --- | --- |
| K.GVHIISVGLGTVDHR.G | Y | 43.79 | 1558.8579 | 15 | 0.84 | 780.4334 | 2 | 15.97 | 1.0917 | 1.0989 | 49325 | 0 | 0 | 0 | 1055 | 1069 |  |  | DB Search |
| total 1 peptides |  |  |  |  |  |  |  |  |  |  |  |  |  |  |  |  |  |  |  |
| --- | --- | --- | --- | --- | --- | --- | --- | --- | --- | --- | --- | --- | --- | --- | --- | --- | --- | --- | --- |

Best Unique PSM (Scan Sample\_7\_A1\_1\_3644.d:49325, m/z=780.4334, z=2, RT=15.97, ppm=-3.63):


TRINITY\_DN941\_c0\_g1\_i11.p1

back to list

  

| Protein Coverage
| Supporting Peptides
| Best Unique PSM
|

Protein Coverage:

Supporting Peptides:

| Peptide | Uniq | -10lgP | Mass | Length | ppm | m/z | z | RT | 1/k0 Start | 1/k0 End | Precursor ID | Area Sample 8 | #Feature | #Feature Sample 8 | Start | End | PTM | AScore | Found By |
| --- | --- | --- | --- | --- | --- | --- | --- | --- | --- | --- | --- | --- | --- | --- | --- | --- | --- | --- | --- |
| K.AHGPGLEGGVAGQPC(+57.02)R.F | Y | 40.64 | 1561.7419 | 16 | 2.05 | 781.8763 | 2 | 12.28 | 1.0275 | 1.0347 | 27748 | 0 | 0 | 0 | 666 | 681 | Carbamidomethylation |  | DB Search |
| total 1 peptides |  |  |  |  |  |  |  |  |  |  |  |  |  |  |  |  |  |  |  |
| --- | --- | --- | --- | --- | --- | --- | --- | --- | --- | --- | --- | --- | --- | --- | --- | --- | --- | --- | --- |

Best Unique PSM (Scan Sample\_7\_A1\_1\_3644.d:27748, m/z=782.374, z=2, RT=12.30, ppm=-2.42):


TRINITY\_DN2799\_c0\_g1\_i1.p1

back to list

  

| Protein Coverage
| Supporting Peptides
| Best Unique PSM
|

Protein Coverage:

Supporting Peptides:

| Peptide | Uniq | -10lgP | Mass | Length | ppm | m/z | z | RT | 1/k0 Start | 1/k0 End | Precursor ID | Area Sample 8 | #Feature | #Feature Sample 8 | Start | End | PTM | AScore | Found By |
| --- | --- | --- | --- | --- | --- | --- | --- | --- | --- | --- | --- | --- | --- | --- | --- | --- | --- | --- | --- |
| K.AGM(+15.99)THIVR.D | Y | 43.71 | 899.4647 | 8 | 5.69 | 450.7402 | 2 | 9.78 | 0.8161 | 0.8234 | 13845 | 0 | 0 | 0 | 74 | 81 | Oxidation (M) | M3:Oxidation (M):1000 | DB Search |
| total 1 peptides |  |  |  |  |  |  |  |  |  |  |  |  |  |  |  |  |  |  |  |
| --- | --- | --- | --- | --- | --- | --- | --- | --- | --- | --- | --- | --- | --- | --- | --- | --- | --- | --- | --- |

Best Unique PSM (Scan Sample\_7\_A1\_1\_3644.d:13845, m/z=450.7402, z=2, RT=9.78, ppm=1.21):


TRINITY\_DN4595\_c0\_g1\_i7.p1

back to list

  

| Protein Coverage
| Supporting Peptides
| Best Unique PSM
|

Protein Coverage:

Supporting Peptides:

| Peptide | Uniq | -10lgP | Mass | Length | ppm | m/z | z | RT | 1/k0 Start | 1/k0 End | Precursor ID | Area Sample 8 | #Feature | #Feature Sample 8 | Start | End | PTM | AScore | Found By |
| --- | --- | --- | --- | --- | --- | --- | --- | --- | --- | --- | --- | --- | --- | --- | --- | --- | --- | --- | --- |
| R.AFNKVETR.N | N | 43.74 | 963.5138 | 8 | 5.69 | 482.7647 | 2 | 10.62 | 0.8299 | 0.8372 | 18171 | 1.3e3 | 1 | 1 | 177 | 184 |  |  | DB Search |
| R.AFNKIETR.N | Y | 43.58 | 977.5294 | 8 | 6.21 | 489.7728 | 2 | 11.52 | 0.8372 | 0.8445 | 23205 | 2.22e3 | 1 | 1 | 72 | 79 |  |  | DB Search |
| R.AFNRVETR.D | Y | 42.68 | 991.5199 | 8 | 4.26 | 496.7671 | 2 | 10.79 | 0.8425 | 0.8498 | 19063 | 2.3e3 | 1 | 1 | 282 | 289 |  |  | DB Search |
| total 3 peptides |  |  |  |  |  |  |  |  |  |  |  |  |  |  |  |  |  |  |  |
| --- | --- | --- | --- | --- | --- | --- | --- | --- | --- | --- | --- | --- | --- | --- | --- | --- | --- | --- | --- |

Best Unique PSM (Scan Sample\_7\_A1\_1\_3644.d:23205, m/z=489.7728, z=2, RT=11.52, ppm=1.73):


TRINITY\_DN941\_c0\_g1\_i5.p1

back to list

  

| Protein Coverage
| Supporting Peptides
| Best Unique PSM
|

Protein Coverage:

Supporting Peptides:

| Peptide | Uniq | -10lgP | Mass | Length | ppm | m/z | z | RT | 1/k0 Start | 1/k0 End | Precursor ID | Area Sample 8 | #Feature | #Feature Sample 8 | Start | End | PTM | AScore | Found By |
| --- | --- | --- | --- | --- | --- | --- | --- | --- | --- | --- | --- | --- | --- | --- | --- | --- | --- | --- | --- |
| K.AHGPGLEGGVAGQPC(+57.02)R.F | Y | 40.64 | 1561.7419 | 16 | 2.05 | 781.8763 | 2 | 12.28 | 1.0275 | 1.0347 | 27748 | 0 | 0 | 0 | 366 | 381 | Carbamidomethylation |  | DB Search |
| total 1 peptides |  |  |  |  |  |  |  |  |  |  |  |  |  |  |  |  |  |  |  |
| --- | --- | --- | --- | --- | --- | --- | --- | --- | --- | --- | --- | --- | --- | --- | --- | --- | --- | --- | --- |

Best Unique PSM (Scan Sample\_7\_A1\_1\_3644.d:27748, m/z=782.374, z=2, RT=12.30, ppm=-2.42):


TRINITY\_DN163801\_c0\_g1\_i1.p1

back to list

  

| Protein Coverage
| Supporting Peptides
| Best Unique PSM
|

Protein Coverage:

Supporting Peptides:

| Peptide | Uniq | -10lgP | Mass | Length | ppm | m/z | z | RT | 1/k0 Start | 1/k0 End | Precursor ID | Area Sample 8 | #Feature | #Feature Sample 8 | Start | End | PTM | AScore | Found By |
| --- | --- | --- | --- | --- | --- | --- | --- | --- | --- | --- | --- | --- | --- | --- | --- | --- | --- | --- | --- |
| M.A(+42.01)NPLKDIISR.F | Y | 43.78 | 1167.6611 | 10 | 1.73 | 584.8362 | 2 | 18.79 | 0.9178 | 0.9251 | 66131 | 9.72e3 | 1 | 1 | 2 | 11 | Acetylation (Protein N-term) | A1:Acetylation (Protein N-term):1000 | DB Search |
| K.AATLLGEAMNENPGYLK.L | Y | 43.48 | 1790.8872 | 17 | 2.94 | 896.4495 | 2 | 19.09 | 1.1758 | 1.1829 | 68392 | 0 | 0 | 0 | 234 | 250 |  |  | DB Search |
| total 2 peptides |  |  |  |  |  |  |  |  |  |  |  |  |  |  |  |  |  |  |  |
| --- | --- | --- | --- | --- | --- | --- | --- | --- | --- | --- | --- | --- | --- | --- | --- | --- | --- | --- | --- |

Best Unique PSM (Scan Sample\_7\_A1\_1\_3644.d:66131, m/z=584.8362, z=2, RT=18.79, ppm=-2.75):


TRINITY\_DN113\_c3\_g1\_i1.p1

back to list

  

| Protein Coverage
| Supporting Peptides
| Best Unique PSM
|

Protein Coverage:

Supporting Peptides:

| Peptide | Uniq | -10lgP | Mass | Length | ppm | m/z | z | RT | 1/k0 Start | 1/k0 End | Precursor ID | Area Sample 8 | #Feature | #Feature Sample 8 | Start | End | PTM | AScore | Found By |
| --- | --- | --- | --- | --- | --- | --- | --- | --- | --- | --- | --- | --- | --- | --- | --- | --- | --- | --- | --- |
| R.ATHQELLNAEFIQK.V | Y | 43.84 | 1640.8522 | 14 | 1.4 | 821.4308 | 2 | 16.12 | 1.0821 | 1.0893 | 50093 | 9.45e2 | 1 | 1 | 457 | 470 |  |  | DB Search |
| R.VLVIFANGDINDEQAVK.A | Y | 43.82 | 1843.9679 | 17 | 0.34 | 922.9874 | 2 | 18.93 | 1.1917 | 1.1989 | 67392 | 0 | 0 | 0 | 636 | 652 |  |  | DB Search |
| K.AHVSLSTFSEDVTSLTK.F | Y | 43.76 | 1820.9156 | 17 | -1.27 | 911.4598 | 2 | 18.98 | 1.1354 | 1.1426 | 67769 | 1.47e3 | 1 | 1 | 568 | 584 |  |  | DB Search |
| total 3 peptides |  |  |  |  |  |  |  |  |  |  |  |  |  |  |  |  |  |  |  |
| --- | --- | --- | --- | --- | --- | --- | --- | --- | --- | --- | --- | --- | --- | --- | --- | --- | --- | --- | --- |

Best Unique PSM (Scan Sample\_7\_A1\_1\_3644.d:50093, m/z=821.4308, z=2, RT=16.12, ppm=-3.08):


TRINITY\_DN1653\_c0\_g1\_i3.p1

back to list

  

| Protein Coverage
| Supporting Peptides
| Best Unique PSM
|

Protein Coverage:

Supporting Peptides:

| Peptide | Uniq | -10lgP | Mass | Length | ppm | m/z | z | RT | 1/k0 Start | 1/k0 End | Precursor ID | Area Sample 8 | #Feature | #Feature Sample 8 | Start | End | PTM | AScore | Found By |
| --- | --- | --- | --- | --- | --- | --- | --- | --- | --- | --- | --- | --- | --- | --- | --- | --- | --- | --- | --- |
| R.LINLFEAVSKK.S | Y | 43.76 | 1260.7441 | 11 | -5.13 | 631.3733 | 2 | 19.09 | 0.986 | 0.9932 | 68432 | 1.56e3 | 1 | 1 | 49 | 59 |  |  | DB Search |
| total 1 peptides |  |  |  |  |  |  |  |  |  |  |  |  |  |  |  |  |  |  |  |
| --- | --- | --- | --- | --- | --- | --- | --- | --- | --- | --- | --- | --- | --- | --- | --- | --- | --- | --- | --- |

Best Unique PSM (Scan Sample\_7\_A1\_1\_3644.d:68432, m/z=631.3733, z=2, RT=19.09, ppm=-9.6):


TRINITY\_DN413\_c0\_g1\_i2.p1

back to list

  

| Protein Coverage
| Supporting Peptides
| Best Unique PSM
|

Protein Coverage:

Supporting Peptides:

| Peptide | Uniq | -10lgP | Mass | Length | ppm | m/z | z | RT | 1/k0 Start | 1/k0 End | Precursor ID | Area Sample 8 | #Feature | #Feature Sample 8 | Start | End | PTM | AScore | Found By |
| --- | --- | --- | --- | --- | --- | --- | --- | --- | --- | --- | --- | --- | --- | --- | --- | --- | --- | --- | --- |
| R.TDFLC(+57.02)HDSR.G | Y | 43.78 | 1149.4873 | 9 | 2.13 | 575.7496 | 2 | 13.37 | 0.8684 | 0.8757 | 33932 | 8.97e2 | 1 | 1 | 57 | 65 | Carbamidomethylation |  | DB Search |
| R.LLLGDFNGDKR.T | Y | 43.76 | 1246.6669 | 11 | -0.7 | 624.3375 | 2 | 16.26 | 0.9832 | 0.9904 | 50917 | 2.97e3 | 1 | 1 | 46 | 56 |  |  | DB Search |
| total 2 peptides |  |  |  |  |  |  |  |  |  |  |  |  |  |  |  |  |  |  |  |
| --- | --- | --- | --- | --- | --- | --- | --- | --- | --- | --- | --- | --- | --- | --- | --- | --- | --- | --- | --- |

Best Unique PSM (Scan Sample\_7\_A1\_1\_3644.d:33932, m/z=575.7496, z=2, RT=13.37, ppm=-2.35):


TRINITY\_DN199\_c0\_g1\_i1.p1

back to list

  

| Protein Coverage
| Supporting Peptides
| Best Unique PSM
|

Protein Coverage:

Supporting Peptides:

| Peptide | Uniq | -10lgP | Mass | Length | ppm | m/z | z | RT | 1/k0 Start | 1/k0 End | Precursor ID | Area Sample 8 | #Feature | #Feature Sample 8 | Start | End | PTM | AScore | Found By |
| --- | --- | --- | --- | --- | --- | --- | --- | --- | --- | --- | --- | --- | --- | --- | --- | --- | --- | --- | --- |
| K.NYNNDDEPGAK.K | Y | 43.84 | 1235.5054 | 11 | 1.73 | 618.7583 | 2 | 10.30 | 0.9198 | 0.9271 | 16375 | 3.11e3 | 1 | 1 | 124 | 134 |  |  | DB Search |
| K.NYNNDDEPGAKK.L | Y | 43.33 | 1363.6004 | 12 | 3 | 455.5401 | 3 | 9.65 | 0.7498 | 0.7571 | 13197 | 5.44e2 | 1 | 1 | 124 | 135 |  |  | DB Search |
| K.KLLDWAQQR.F | Y | 42.67 | 1156.6353 | 9 | -16.88 | 579.3126 | 2 | 15.55 | 0.9057 | 0.9129 | 46959 | 0 | 0 | 0 | 135 | 143 |  |  | DB Search |
| total 3 peptides |  |  |  |  |  |  |  |  |  |  |  |  |  |  |  |  |  |  |  |
| --- | --- | --- | --- | --- | --- | --- | --- | --- | --- | --- | --- | --- | --- | --- | --- | --- | --- | --- | --- |

Best Unique PSM (Scan Sample\_7\_A1\_1\_3644.d:16375, m/z=618.7583, z=2, RT=10.30, ppm=-2.74):


TRINITY\_DN9413\_c0\_g1\_i1.p1

back to list

  

| Protein Coverage
| Supporting Peptides
| Best Unique PSM
|

Protein Coverage:

Supporting Peptides:

| Peptide | Uniq | -10lgP | Mass | Length | ppm | m/z | z | RT | 1/k0 Start | 1/k0 End | Precursor ID | Area Sample 8 | #Feature | #Feature Sample 8 | Start | End | PTM | AScore | Found By |
| --- | --- | --- | --- | --- | --- | --- | --- | --- | --- | --- | --- | --- | --- | --- | --- | --- | --- | --- | --- |
| R.TPNGIHGPVNTR.S | Y | 43.67 | 1261.6527 | 12 | -0.19 | 631.8307 | 2 | 11.57 | 0.9372 | 0.9444 | 23558 | 0 | 0 | 0 | 264 | 275 |  |  | DB Search |
| total 1 peptides |  |  |  |  |  |  |  |  |  |  |  |  |  |  |  |  |  |  |  |
| --- | --- | --- | --- | --- | --- | --- | --- | --- | --- | --- | --- | --- | --- | --- | --- | --- | --- | --- | --- |

Best Unique PSM (Scan Sample\_7\_A1\_1\_3644.d:23558, m/z=631.8307, z=2, RT=11.57, ppm=-4.66):


TRINITY\_DN649\_c0\_g1\_i1.p1

back to list

  

| Protein Coverage
| Supporting Peptides
| Best Unique PSM
|

Protein Coverage:

Supporting Peptides:

| Peptide | Uniq | -10lgP | Mass | Length | ppm | m/z | z | RT | 1/k0 Start | 1/k0 End | Precursor ID | Area Sample 8 | #Feature | #Feature Sample 8 | Start | End | PTM | AScore | Found By |
| --- | --- | --- | --- | --- | --- | --- | --- | --- | --- | --- | --- | --- | --- | --- | --- | --- | --- | --- | --- |
| K.FETQIATK.N | Y | 43.56 | 936.4916 | 8 | 1.93 | 937.4965 | 1 | 13.99 | 1.2029 | 1.2101 | 37753 | 0 | 0 | 0 | 102 | 109 |  |  | DB Search |
| total 1 peptides |  |  |  |  |  |  |  |  |  |  |  |  |  |  |  |  |  |  |  |
| --- | --- | --- | --- | --- | --- | --- | --- | --- | --- | --- | --- | --- | --- | --- | --- | --- | --- | --- | --- |

Best Unique PSM (Scan Sample\_7\_A1\_1\_3644.d:37753, m/z=937.4965, z=1, RT=13.99, ppm=-2.55):


TRINITY\_DN1058\_c0\_g1\_i2.p2

back to list

  

| Protein Coverage
| Supporting Peptides
| Best Unique PSM
|

Protein Coverage:

Supporting Peptides:

| Peptide | Uniq | -10lgP | Mass | Length | ppm | m/z | z | RT | 1/k0 Start | 1/k0 End | Precursor ID | Area Sample 8 | #Feature | #Feature Sample 8 | Start | End | PTM | AScore | Found By |
| --- | --- | --- | --- | --- | --- | --- | --- | --- | --- | --- | --- | --- | --- | --- | --- | --- | --- | --- | --- |
| R.APTHILNPANKR.S | Y | 43.8 | 1330.7469 | 12 | 0.61 | 666.3782 | 2 | 11.23 | 1.0057 | 1.013 | 21549 | 1.39e3 | 2 | 2 | 975 | 986 |  |  | DB Search |
| total 1 peptides |  |  |  |  |  |  |  |  |  |  |  |  |  |  |  |  |  |  |  |
| --- | --- | --- | --- | --- | --- | --- | --- | --- | --- | --- | --- | --- | --- | --- | --- | --- | --- | --- | --- |

Best Unique PSM (Scan Sample\_7\_A1\_1\_3644.d:21549, m/z=666.3782, z=2, RT=11.23, ppm=-3.86):


TRINITY\_DN3452\_c0\_g1\_i1.p1

back to list

  

| Protein Coverage
| Supporting Peptides
| Best Unique PSM
|

Protein Coverage:

Supporting Peptides:

| Peptide | Uniq | -10lgP | Mass | Length | ppm | m/z | z | RT | 1/k0 Start | 1/k0 End | Precursor ID | Area Sample 8 | #Feature | #Feature Sample 8 | Start | End | PTM | AScore | Found By |
| --- | --- | --- | --- | --- | --- | --- | --- | --- | --- | --- | --- | --- | --- | --- | --- | --- | --- | --- | --- |
| K.LIAFINDLHSGK.L | Y | 43.8 | 1326.7295 | 12 | 3.13 | 664.3712 | 2 | 17.73 | 1.025 | 1.0323 | 59589 | 4.22e2 | 1 | 1 | 342 | 353 |  |  | DB Search |
| total 1 peptides |  |  |  |  |  |  |  |  |  |  |  |  |  |  |  |  |  |  |  |
| --- | --- | --- | --- | --- | --- | --- | --- | --- | --- | --- | --- | --- | --- | --- | --- | --- | --- | --- | --- |

Best Unique PSM (Scan Sample\_7\_A1\_1\_3644.d:59589, m/z=664.3712, z=2, RT=17.73, ppm=-1.35):


TRINITY\_DN1091\_c0\_g1\_i1.p1

back to list

  

| Protein Coverage
| Supporting Peptides
| Best Unique PSM
|

Protein Coverage:

Supporting Peptides:

| Peptide | Uniq | -10lgP | Mass | Length | ppm | m/z | z | RT | 1/k0 Start | 1/k0 End | Precursor ID | Area Sample 8 | #Feature | #Feature Sample 8 | Start | End | PTM | AScore | Found By |
| --- | --- | --- | --- | --- | --- | --- | --- | --- | --- | --- | --- | --- | --- | --- | --- | --- | --- | --- | --- |
| R.AFQNTPC(+57.02)THLVLDGTR.W | Y | 43.39 | 1828.889 | 16 | -2.93 | 915.445 | 2 | 15.43 | 1.175 | 1.1821 | 46253 | 0 | 0 | 0 | 81 | 96 | Carbamidomethylation |  | DB Search |
| total 1 peptides |  |  |  |  |  |  |  |  |  |  |  |  |  |  |  |  |  |  |  |
| --- | --- | --- | --- | --- | --- | --- | --- | --- | --- | --- | --- | --- | --- | --- | --- | --- | --- | --- | --- |

Best Unique PSM (Scan Sample\_7\_A1\_1\_3644.d:46253, m/z=915.445, z=2, RT=15.43, ppm=-7.4):


TRINITY\_DN941\_c0\_g1\_i15.p1

back to list

  

| Protein Coverage
| Supporting Peptides
| Best Unique PSM
|

Protein Coverage:

Supporting Peptides:

| Peptide | Uniq | -10lgP | Mass | Length | ppm | m/z | z | RT | 1/k0 Start | 1/k0 End | Precursor ID | Area Sample 8 | #Feature | #Feature Sample 8 | Start | End | PTM | AScore | Found By |
| --- | --- | --- | --- | --- | --- | --- | --- | --- | --- | --- | --- | --- | --- | --- | --- | --- | --- | --- | --- |
| K.AHGPGLEGGVAGQPC(+57.02)R.F | Y | 40.64 | 1561.7419 | 16 | 2.05 | 781.8763 | 2 | 12.28 | 1.0275 | 1.0347 | 27748 | 0 | 0 | 0 | 820 | 835 | Carbamidomethylation |  | DB Search |
| total 1 peptides |  |  |  |  |  |  |  |  |  |  |  |  |  |  |  |  |  |  |  |
| --- | --- | --- | --- | --- | --- | --- | --- | --- | --- | --- | --- | --- | --- | --- | --- | --- | --- | --- | --- |

Best Unique PSM (Scan Sample\_7\_A1\_1\_3644.d:27748, m/z=782.374, z=2, RT=12.30, ppm=-2.42):


TRINITY\_DN32727\_c0\_g1\_i2.p1

back to list

  

| Protein Coverage
| Supporting Peptides
| Best Unique PSM
|

Protein Coverage:

Supporting Peptides:

| Peptide | Uniq | -10lgP | Mass | Length | ppm | m/z | z | RT | 1/k0 Start | 1/k0 End | Precursor ID | Area Sample 8 | #Feature | #Feature Sample 8 | Start | End | PTM | AScore | Found By |
| --- | --- | --- | --- | --- | --- | --- | --- | --- | --- | --- | --- | --- | --- | --- | --- | --- | --- | --- | --- |
| K.AGM(+15.99)THIVR.D | Y | 43.71 | 899.4647 | 8 | 5.69 | 450.7402 | 2 | 9.78 | 0.8161 | 0.8234 | 13845 | 0 | 0 | 0 | 40 | 47 | Oxidation (M) | M3:Oxidation (M):1000 | DB Search |
| total 1 peptides |  |  |  |  |  |  |  |  |  |  |  |  |  |  |  |  |  |  |  |
| --- | --- | --- | --- | --- | --- | --- | --- | --- | --- | --- | --- | --- | --- | --- | --- | --- | --- | --- | --- |

Best Unique PSM (Scan Sample\_7\_A1\_1\_3644.d:13845, m/z=450.7402, z=2, RT=9.78, ppm=1.21):


TRINITY\_DN34481\_c0\_g1\_i1.p1

back to list

  

| Protein Coverage
| Supporting Peptides
| Best Unique PSM
|

Protein Coverage:

Supporting Peptides:

| Peptide | Uniq | -10lgP | Mass | Length | ppm | m/z | z | RT | 1/k0 Start | 1/k0 End | Precursor ID | Area Sample 8 | #Feature | #Feature Sample 8 | Start | End | PTM | AScore | Found By |
| --- | --- | --- | --- | --- | --- | --- | --- | --- | --- | --- | --- | --- | --- | --- | --- | --- | --- | --- | --- |
| K.AC(+57.02)AELHQDVNVK.N | Y | 43.57 | 1382.6612 | 12 | 2.54 | 692.3365 | 2 | 11.96 | 1.0065 | 1.0138 | 25860 | 0 | 0 | 0 | 282 | 293 | Carbamidomethylation |  | DB Search |
| total 1 peptides |  |  |  |  |  |  |  |  |  |  |  |  |  |  |  |  |  |  |  |
| --- | --- | --- | --- | --- | --- | --- | --- | --- | --- | --- | --- | --- | --- | --- | --- | --- | --- | --- | --- |

Best Unique PSM (Scan Sample\_7\_A1\_1\_3644.d:25860, m/z=692.3365, z=2, RT=11.96, ppm=-1.94):


TRINITY\_DN77\_c1\_g1\_i1.p1

back to list

  

| Protein Coverage
| Supporting Peptides
| Best Unique PSM
|

Protein Coverage:

Supporting Peptides:

| Peptide | Uniq | -10lgP | Mass | Length | ppm | m/z | z | RT | 1/k0 Start | 1/k0 End | Precursor ID | Area Sample 8 | #Feature | #Feature Sample 8 | Start | End | PTM | AScore | Found By |
| --- | --- | --- | --- | --- | --- | --- | --- | --- | --- | --- | --- | --- | --- | --- | --- | --- | --- | --- | --- |
| R.LLAVTVGNNYNLDTLEK.V | Y | 43.79 | 1875.9942 | 17 | -8.17 | 938.9925 | 2 | 18.79 | 1.1474 | 1.1546 | 66275 | 9.54e2 | 1 | 1 | 693 | 709 |  |  | DB Search |
| total 1 peptides |  |  |  |  |  |  |  |  |  |  |  |  |  |  |  |  |  |  |  |
| --- | --- | --- | --- | --- | --- | --- | --- | --- | --- | --- | --- | --- | --- | --- | --- | --- | --- | --- | --- |

Best Unique PSM (Scan Sample\_7\_A1\_1\_3644.d:66275, m/z=938.9925, z=2, RT=18.79, ppm=-12.65):


TRINITY\_DN941\_c0\_g1\_i10.p1

back to list

  

| Protein Coverage
| Supporting Peptides
| Best Unique PSM
|

Protein Coverage:

Supporting Peptides:

| Peptide | Uniq | -10lgP | Mass | Length | ppm | m/z | z | RT | 1/k0 Start | 1/k0 End | Precursor ID | Area Sample 8 | #Feature | #Feature Sample 8 | Start | End | PTM | AScore | Found By |
| --- | --- | --- | --- | --- | --- | --- | --- | --- | --- | --- | --- | --- | --- | --- | --- | --- | --- | --- | --- |
| K.ADLTRPTGK.K | Y | 42.69 | 957.5243 | 9 | -3.1 | 479.7658 | 2 | 10.44 | 0.8193 | 0.8266 | 17248 | 0 | 0 | 0 | 52 | 60 |  |  | DB Search |
| total 1 peptides |  |  |  |  |  |  |  |  |  |  |  |  |  |  |  |  |  |  |  |
| --- | --- | --- | --- | --- | --- | --- | --- | --- | --- | --- | --- | --- | --- | --- | --- | --- | --- | --- | --- |

Best Unique PSM (Scan Sample\_7\_A1\_1\_3644.d:17248, m/z=479.7658, z=2, RT=10.44, ppm=-7.58):


TRINITY\_DN1505\_c0\_g1\_i1.p1

back to list

  

| Protein Coverage
| Supporting Peptides
| Best Unique PSM
|

Protein Coverage:

Supporting Peptides:

| Peptide | Uniq | -10lgP | Mass | Length | ppm | m/z | z | RT | 1/k0 Start | 1/k0 End | Precursor ID | Area Sample 8 | #Feature | #Feature Sample 8 | Start | End | PTM | AScore | Found By |
| --- | --- | --- | --- | --- | --- | --- | --- | --- | --- | --- | --- | --- | --- | --- | --- | --- | --- | --- | --- |
| M.S(+42.01)LVIPEKFQHILR.V | Y | 43.44 | 1620.9351 | 13 | -6.51 | 541.313 | 3 | 19.80 | 0.8441 | 0.8514 | 72642 | 9.41e2 | 1 | 1 | 2 | 14 | Acetylation (Protein N-term) | S1:Acetylation (Protein N-term):1000 | DB Search |
| total 1 peptides |  |  |  |  |  |  |  |  |  |  |  |  |  |  |  |  |  |  |  |
| --- | --- | --- | --- | --- | --- | --- | --- | --- | --- | --- | --- | --- | --- | --- | --- | --- | --- | --- | --- |

Best Unique PSM (Scan Sample\_7\_A1\_1\_3644.d:72642, m/z=541.313, z=3, RT=19.80, ppm=-10.98):


TRINITY\_DN187147\_c0\_g1\_i1.p1

back to list

  

| Protein Coverage
| Supporting Peptides
| Best Unique PSM
|

Protein Coverage:

Supporting Peptides:

| Peptide | Uniq | -10lgP | Mass | Length | ppm | m/z | z | RT | 1/k0 Start | 1/k0 End | Precursor ID | Area Sample 8 | #Feature | #Feature Sample 8 | Start | End | PTM | AScore | Found By |
| --- | --- | --- | --- | --- | --- | --- | --- | --- | --- | --- | --- | --- | --- | --- | --- | --- | --- | --- | --- |
| K.LVGQSIISYLQK.K | Y | 43.61 | 1347.7762 | 12 | 0.16 | 674.8925 | 2 | 19.77 | 0.9804 | 0.9876 | 72610 | 0 | 0 | 0 | 627 | 638 |  |  | DB Search |
| total 1 peptides |  |  |  |  |  |  |  |  |  |  |  |  |  |  |  |  |  |  |  |
| --- | --- | --- | --- | --- | --- | --- | --- | --- | --- | --- | --- | --- | --- | --- | --- | --- | --- | --- | --- |

Best Unique PSM (Scan Sample\_7\_A1\_1\_3644.d:72610, m/z=674.8925, z=2, RT=19.77, ppm=-4.32):


TRINITY\_DN36\_c0\_g1\_i11.p1

back to list

  

| Protein Coverage
| Supporting Peptides
| Best Unique PSM
|

Protein Coverage:

Supporting Peptides:

| Peptide | Uniq | -10lgP | Mass | Length | ppm | m/z | z | RT | 1/k0 Start | 1/k0 End | Precursor ID | Area Sample 8 | #Feature | #Feature Sample 8 | Start | End | PTM | AScore | Found By |
| --- | --- | --- | --- | --- | --- | --- | --- | --- | --- | --- | --- | --- | --- | --- | --- | --- | --- | --- | --- |
| R.LGGIEVTQGNC(+57.02)AR.K | Y | 43.02 | 1373.6721 | 13 | 2.68 | 687.8421 | 2 | 13.90 | 0.9662 | 0.9735 | 37137 | 6.95e2 | 1 | 1 | 58 | 70 | Carbamidomethylation |  | DB Search |
| total 1 peptides |  |  |  |  |  |  |  |  |  |  |  |  |  |  |  |  |  |  |  |
| --- | --- | --- | --- | --- | --- | --- | --- | --- | --- | --- | --- | --- | --- | --- | --- | --- | --- | --- | --- |

Best Unique PSM (Scan Sample\_7\_A1\_1\_3644.d:37137, m/z=687.8421, z=2, RT=13.90, ppm=-1.8):


TRINITY\_DN6397\_c0\_g2\_i4.p1

back to list

  

| Protein Coverage
| Supporting Peptides
| Best Unique PSM
|

Protein Coverage:

Supporting Peptides:

| Peptide | Uniq | -10lgP | Mass | Length | ppm | m/z | z | RT | 1/k0 Start | 1/k0 End | Precursor ID | Area Sample 8 | #Feature | #Feature Sample 8 | Start | End | PTM | AScore | Found By |
| --- | --- | --- | --- | --- | --- | --- | --- | --- | --- | --- | --- | --- | --- | --- | --- | --- | --- | --- | --- |
| K.K(+42.01)LSAEVANGR.L | Y | 43.74 | 1085.5829 | 10 | -2.08 | 543.7952 | 2 | 13.04 | 0.8733 | 0.8806 | 32177 | 0 | 0 | 0 | 12 | 21 | Acetylation (Protein N-term) | K1:Acetylation (Protein N-term):1000 | DB Search |
| total 1 peptides |  |  |  |  |  |  |  |  |  |  |  |  |  |  |  |  |  |  |  |
| --- | --- | --- | --- | --- | --- | --- | --- | --- | --- | --- | --- | --- | --- | --- | --- | --- | --- | --- | --- |

Best Unique PSM (Scan Sample\_7\_A1\_1\_3644.d:32177, m/z=543.7952, z=2, RT=13.04, ppm=-6.55):


TRINITY\_DN15151\_c0\_g1\_i3.p1

back to list

  

| Protein Coverage
| Supporting Peptides
| Best Unique PSM
|

Protein Coverage:

Supporting Peptides:

| Peptide | Uniq | -10lgP | Mass | Length | ppm | m/z | z | RT | 1/k0 Start | 1/k0 End | Precursor ID | Area Sample 8 | #Feature | #Feature Sample 8 | Start | End | PTM | AScore | Found By |
| --- | --- | --- | --- | --- | --- | --- | --- | --- | --- | --- | --- | --- | --- | --- | --- | --- | --- | --- | --- |
| K.AVAAGMNPMDIKR.G | Y | 43.11 | 1372.6955 | 13 | 1.74 | 687.3531 | 2 | 14.92 | 1.0126 | 1.0198 | 43242 | 6.18e2 | 1 | 1 | 145 | 157 |  |  | DB Search |
| total 1 peptides |  |  |  |  |  |  |  |  |  |  |  |  |  |  |  |  |  |  |  |
| --- | --- | --- | --- | --- | --- | --- | --- | --- | --- | --- | --- | --- | --- | --- | --- | --- | --- | --- | --- |

Best Unique PSM (Scan Sample\_7\_A1\_1\_3644.d:43242, m/z=687.3531, z=2, RT=14.92, ppm=-2.73):


TRINITY\_DN31399\_c0\_g1\_i2.p1

back to list

  

| Protein Coverage
| Supporting Peptides
| Best Unique PSM
|

Protein Coverage:

Supporting Peptides:

| Peptide | Uniq | -10lgP | Mass | Length | ppm | m/z | z | RT | 1/k0 Start | 1/k0 End | Precursor ID | Area Sample 8 | #Feature | #Feature Sample 8 | Start | End | PTM | AScore | Found By |
| --- | --- | --- | --- | --- | --- | --- | --- | --- | --- | --- | --- | --- | --- | --- | --- | --- | --- | --- | --- |
| K.DTLVFEIDEIRK.S | Y | 42.83 | 1476.7824 | 12 | 2.38 | 739.3969 | 2 | 18.67 | 1.0359 | 1.0431 | 65730 | 1.13e3 | 1 | 1 | 396 | 407 |  |  | DB Search |
| total 1 peptides |  |  |  |  |  |  |  |  |  |  |  |  |  |  |  |  |  |  |  |
| --- | --- | --- | --- | --- | --- | --- | --- | --- | --- | --- | --- | --- | --- | --- | --- | --- | --- | --- | --- |

Best Unique PSM (Scan Sample\_7\_A1\_1\_3644.d:65730, m/z=739.3969, z=2, RT=18.67, ppm=-2.1):


TRINITY\_DN2885\_c0\_g1\_i5.p1

back to list

  

| Protein Coverage
| Supporting Peptides
| Best Unique PSM
|

Protein Coverage:

Supporting Peptides:

| Peptide | Uniq | -10lgP | Mass | Length | ppm | m/z | z | RT | 1/k0 Start | 1/k0 End | Precursor ID | Area Sample 8 | #Feature | #Feature Sample 8 | Start | End | PTM | AScore | Found By |
| --- | --- | --- | --- | --- | --- | --- | --- | --- | --- | --- | --- | --- | --- | --- | --- | --- | --- | --- | --- |
| K.VTRPHGASGVVR.A | Y | 43.35 | 1234.6894 | 12 | 2.89 | 412.5698 | 3 | 10.09 | 0.8173 | 0.8246 | 15387 | 0 | 0 | 0 | 77 | 88 |  |  | DB Search |
| total 1 peptides |  |  |  |  |  |  |  |  |  |  |  |  |  |  |  |  |  |  |  |
| --- | --- | --- | --- | --- | --- | --- | --- | --- | --- | --- | --- | --- | --- | --- | --- | --- | --- | --- | --- |

Best Unique PSM (Scan Sample\_7\_A1\_1\_3644.d:15387, m/z=412.5698, z=3, RT=10.09, ppm=-1.58):


TRINITY\_DN730\_c0\_g2\_i2.p1

back to list

  

| Protein Coverage
| Supporting Peptides
| Best Unique PSM
|

Protein Coverage:

Supporting Peptides:

| Peptide | Uniq | -10lgP | Mass | Length | ppm | m/z | z | RT | 1/k0 Start | 1/k0 End | Precursor ID | Area Sample 8 | #Feature | #Feature Sample 8 | Start | End | PTM | AScore | Found By |
| --- | --- | --- | --- | --- | --- | --- | --- | --- | --- | --- | --- | --- | --- | --- | --- | --- | --- | --- | --- |
| K.VRDPYRNDLFHDTSR.I | Y | 40.73 | 1889.9132 | 15 | -1.18 | 473.4829 | 4 | 13.85 | 0.8181 | 0.8254 | 36969 | 0 | 0 | 0 | 51 | 65 |  |  | DB Search |
| total 1 peptides |  |  |  |  |  |  |  |  |  |  |  |  |  |  |  |  |  |  |  |
| --- | --- | --- | --- | --- | --- | --- | --- | --- | --- | --- | --- | --- | --- | --- | --- | --- | --- | --- | --- |

Best Unique PSM (Scan Sample\_7\_A1\_1\_3644.d:36969, m/z=473.4829, z=4, RT=13.85, ppm=-5.66):


TRINITY\_DN38657\_c0\_g1\_i2.p1

back to list

  

| Protein Coverage
| Supporting Peptides
| Best Unique PSM
|

Protein Coverage:

Supporting Peptides:

| Peptide | Uniq | -10lgP | Mass | Length | ppm | m/z | z | RT | 1/k0 Start | 1/k0 End | Precursor ID | Area Sample 8 | #Feature | #Feature Sample 8 | Start | End | PTM | AScore | Found By |
| --- | --- | --- | --- | --- | --- | --- | --- | --- | --- | --- | --- | --- | --- | --- | --- | --- | --- | --- | --- |
| K.STSQGQALTK.T | Y | 43.71 | 1019.5247 | 10 | 7.68 | 510.7713 | 2 | 10.06 | 0.8291 | 0.8364 | 15179 | 0 | 0 | 0 | 52 | 61 |  |  | DB Search |
| total 1 peptides |  |  |  |  |  |  |  |  |  |  |  |  |  |  |  |  |  |  |  |
| --- | --- | --- | --- | --- | --- | --- | --- | --- | --- | --- | --- | --- | --- | --- | --- | --- | --- | --- | --- |

Best Unique PSM (Scan Sample\_7\_A1\_1\_3644.d:15179, m/z=510.7713, z=2, RT=10.06, ppm=3.2):


TRINITY\_DN38657\_c0\_g1\_i4.p1

back to list

  

| Protein Coverage
| Supporting Peptides
| Best Unique PSM
|

Protein Coverage:

Supporting Peptides:

| Peptide | Uniq | -10lgP | Mass | Length | ppm | m/z | z | RT | 1/k0 Start | 1/k0 End | Precursor ID | Area Sample 8 | #Feature | #Feature Sample 8 | Start | End | PTM | AScore | Found By |
| --- | --- | --- | --- | --- | --- | --- | --- | --- | --- | --- | --- | --- | --- | --- | --- | --- | --- | --- | --- |
| K.STSQGQALTK.T | Y | 43.71 | 1019.5247 | 10 | 7.68 | 510.7713 | 2 | 10.06 | 0.8291 | 0.8364 | 15179 | 0 | 0 | 0 | 50 | 59 |  |  | DB Search |
| total 1 peptides |  |  |  |  |  |  |  |  |  |  |  |  |  |  |  |  |  |  |  |
| --- | --- | --- | --- | --- | --- | --- | --- | --- | --- | --- | --- | --- | --- | --- | --- | --- | --- | --- | --- |

Best Unique PSM (Scan Sample\_7\_A1\_1\_3644.d:15179, m/z=510.7713, z=2, RT=10.06, ppm=3.2):


TRINITY\_DN384\_c0\_g1\_i1.p1

back to list

  

| Protein Coverage
| Supporting Peptides
| Best Unique PSM
|

Protein Coverage:

Supporting Peptides:

| Peptide | Uniq | -10lgP | Mass | Length | ppm | m/z | z | RT | 1/k0 Start | 1/k0 End | Precursor ID | Area Sample 8 | #Feature | #Feature Sample 8 | Start | End | PTM | AScore | Found By |
| --- | --- | --- | --- | --- | --- | --- | --- | --- | --- | --- | --- | --- | --- | --- | --- | --- | --- | --- | --- |
| K.FVLHGDVSHGSR.E | Y | 43.34 | 1309.6527 | 12 | -0.43 | 437.556 | 3 | 12.14 | 0.762 | 0.7693 | 26936 | 0 | 0 | 0 | 274 | 285 |  |  | DB Search |
| total 1 peptides |  |  |  |  |  |  |  |  |  |  |  |  |  |  |  |  |  |  |  |
| --- | --- | --- | --- | --- | --- | --- | --- | --- | --- | --- | --- | --- | --- | --- | --- | --- | --- | --- | --- |

Best Unique PSM (Scan Sample\_7\_A1\_1\_3644.d:26936, m/z=437.556, z=3, RT=12.14, ppm=-4.91):


TRINITY\_DN36\_c0\_g1\_i19.p1

back to list

  

| Protein Coverage
| Supporting Peptides
| Best Unique PSM
|

Protein Coverage:

Supporting Peptides:

| Peptide | Uniq | -10lgP | Mass | Length | ppm | m/z | z | RT | 1/k0 Start | 1/k0 End | Precursor ID | Area Sample 8 | #Feature | #Feature Sample 8 | Start | End | PTM | AScore | Found By |
| --- | --- | --- | --- | --- | --- | --- | --- | --- | --- | --- | --- | --- | --- | --- | --- | --- | --- | --- | --- |
| K.FETQIATK.D | Y | 43.56 | 936.4916 | 8 | 1.93 | 937.4965 | 1 | 13.99 | 1.2029 | 1.2101 | 37753 | 0 | 0 | 0 | 166 | 173 |  |  | DB Search |
| R.LGGIEVTQGNC(+57.02)AR.K | Y | 43.02 | 1373.6721 | 13 | 2.68 | 687.8421 | 2 | 13.90 | 0.9662 | 0.9735 | 37137 | 6.95e2 | 1 | 1 | 57 | 69 | Carbamidomethylation |  | DB Search |
| total 2 peptides |  |  |  |  |  |  |  |  |  |  |  |  |  |  |  |  |  |  |  |
| --- | --- | --- | --- | --- | --- | --- | --- | --- | --- | --- | --- | --- | --- | --- | --- | --- | --- | --- | --- |

Best Unique PSM (Scan Sample\_7\_A1\_1\_3644.d:37753, m/z=937.4965, z=1, RT=13.99, ppm=-2.55):


TRINITY\_DN36\_c0\_g1\_i9.p1

back to list

  

| Protein Coverage
| Supporting Peptides
| Best Unique PSM
|

Protein Coverage:

Supporting Peptides:

| Peptide | Uniq | -10lgP | Mass | Length | ppm | m/z | z | RT | 1/k0 Start | 1/k0 End | Precursor ID | Area Sample 8 | #Feature | #Feature Sample 8 | Start | End | PTM | AScore | Found By |
| --- | --- | --- | --- | --- | --- | --- | --- | --- | --- | --- | --- | --- | --- | --- | --- | --- | --- | --- | --- |
| R.LGGIEVTQGNC(+57.02)AR.K | Y | 43.02 | 1373.6721 | 13 | 2.68 | 687.8421 | 2 | 13.90 | 0.9662 | 0.9735 | 37137 | 6.95e2 | 1 | 1 | 58 | 70 | Carbamidomethylation |  | DB Search |
| total 1 peptides |  |  |  |  |  |  |  |  |  |  |  |  |  |  |  |  |  |  |  |
| --- | --- | --- | --- | --- | --- | --- | --- | --- | --- | --- | --- | --- | --- | --- | --- | --- | --- | --- | --- |

Best Unique PSM (Scan Sample\_7\_A1\_1\_3644.d:37137, m/z=687.8421, z=2, RT=13.90, ppm=-1.8):


TRINITY\_DN3448\_c0\_g1\_i9.p3

back to list

  

| Protein Coverage
| Supporting Peptides
| Best Unique PSM
|

Protein Coverage:

Supporting Peptides:

| Peptide | Uniq | -10lgP | Mass | Length | ppm | m/z | z | RT | 1/k0 Start | 1/k0 End | Precursor ID | Area Sample 8 | #Feature | #Feature Sample 8 | Start | End | PTM | AScore | Found By |
| --- | --- | --- | --- | --- | --- | --- | --- | --- | --- | --- | --- | --- | --- | --- | --- | --- | --- | --- | --- |
| K.AGM(+15.99)THIVR.D | Y | 43.71 | 899.4647 | 8 | 5.69 | 450.7402 | 2 | 9.78 | 0.8161 | 0.8234 | 13845 | 0 | 0 | 0 | 27 | 34 | Oxidation (M) | M3:Oxidation (M):1000 | DB Search |
| total 1 peptides |  |  |  |  |  |  |  |  |  |  |  |  |  |  |  |  |  |  |  |
| --- | --- | --- | --- | --- | --- | --- | --- | --- | --- | --- | --- | --- | --- | --- | --- | --- | --- | --- | --- |

Best Unique PSM (Scan Sample\_7\_A1\_1\_3644.d:13845, m/z=450.7402, z=2, RT=9.78, ppm=1.21):


TRINITY\_DN1616\_c0\_g1\_i7.p1

back to list

  

| Protein Coverage
| Supporting Peptides
| Best Unique PSM
|

Protein Coverage:

Supporting Peptides:

| Peptide | Uniq | -10lgP | Mass | Length | ppm | m/z | z | RT | 1/k0 Start | 1/k0 End | Precursor ID | Area Sample 8 | #Feature | #Feature Sample 8 | Start | End | PTM | AScore | Found By |
| --- | --- | --- | --- | --- | --- | --- | --- | --- | --- | --- | --- | --- | --- | --- | --- | --- | --- | --- | --- |
| R.TSTDKFEC(+57.02)EK.C | Y | 43.76 | 1243.5391 | 10 | -3.39 | 622.7719 | 2 | 10.75 | 0.9255 | 0.9327 | 18979 | 0 | 0 | 0 | 320 | 329 | Carbamidomethylation |  | DB Search |
| R.YGAVNDNC(+57.02)ER.D | Y | 43.62 | 1196.488 | 10 | -2.06 | 599.2474 | 2 | 10.92 | 0.8834 | 0.8907 | 19854 | 4.23e2 | 1 | 1 | 498 | 507 | Carbamidomethylation |  | DB Search |
| R.YVKPGVNIINIR.D | Y | 43.56 | 1384.819 | 12 | 3.35 | 693.416 | 2 | 16.23 | 1.0013 | 1.0086 | 50888 | 0 | 0 | 0 | 1217 | 1228 |  |  | DB Search |
| K.YVVMLQYYHDSDTAK.Y | Y | 43.52 | 1831.8451 | 15 | 8.47 | 916.9335 | 2 | 16.82 | 1.1857 | 1.1929 | 54206 | 0 | 0 | 0 | 1071 | 1085 |  |  | DB Search |
| K.SSSC(+57.02)QEFGGKC(+57.02)PC(+57.02)K.D | Y | 39.5 | 1630.6538 | 14 | -18.66 | 816.3153 | 2 | 12.09 | 1.0311 | 1.0383 | 26624 | 0 | 0 | 0 | 1387 | 1400 | Carbamidomethylation, Carbamidomethylation, Carbamidomethylation |  | DB Search |
| total 5 peptides |  |  |  |  |  |  |  |  |  |  |  |  |  |  |  |  |  |  |  |
| --- | --- | --- | --- | --- | --- | --- | --- | --- | --- | --- | --- | --- | --- | --- | --- | --- | --- | --- | --- |

Best Unique PSM (Scan Sample\_7\_A1\_1\_3644.d:18979, m/z=622.7719, z=2, RT=10.75, ppm=-7.87):


TRINITY\_DN3545\_c0\_g1\_i2.p1

back to list

  

| Protein Coverage
| Supporting Peptides
| Best Unique PSM
|

Protein Coverage:

Supporting Peptides:

| Peptide | Uniq | -10lgP | Mass | Length | ppm | m/z | z | RT | 1/k0 Start | 1/k0 End | Precursor ID | Area Sample 8 | #Feature | #Feature Sample 8 | Start | End | PTM | AScore | Found By |
| --- | --- | --- | --- | --- | --- | --- | --- | --- | --- | --- | --- | --- | --- | --- | --- | --- | --- | --- | --- |
| R.SC(+57.02)TNPAPANGGK.H | N | 43.8 | 1172.5244 | 12 | 2.69 | 587.2684 | 2 | 9.47 | 0.838 | 0.8453 | 12375 | 4.7e3 | 1 | 1 | 55 | 66 | Carbamidomethylation |  | DB Search |
| R.SSC(+57.02)RPILSVIK.I | Y | 42.42 | 1258.7067 | 11 | -5.58 | 630.3543 | 2 | 17.05 | 0.9731 | 0.9804 | 55404 | 1.96e4 | 2 | 2 | 6 | 16 | Carbamidomethylation |  | DB Search |
| total 2 peptides |  |  |  |  |  |  |  |  |  |  |  |  |  |  |  |  |  |  |  |
| --- | --- | --- | --- | --- | --- | --- | --- | --- | --- | --- | --- | --- | --- | --- | --- | --- | --- | --- | --- |

Best Unique PSM (Scan Sample\_7\_A1\_1\_3644.d:55404, m/z=630.3543, z=2, RT=17.05, ppm=-10.06):


TRINITY\_DN1043\_c0\_g1\_i2.p1

back to list

  

| Protein Coverage
| Supporting Peptides
| Best Unique PSM
|

Protein Coverage:

Supporting Peptides:

| Peptide | Uniq | -10lgP | Mass | Length | ppm | m/z | z | RT | 1/k0 Start | 1/k0 End | Precursor ID | Area Sample 8 | #Feature | #Feature Sample 8 | Start | End | PTM | AScore | Found By |
| --- | --- | --- | --- | --- | --- | --- | --- | --- | --- | --- | --- | --- | --- | --- | --- | --- | --- | --- | --- |
| K.GLMVAAMDLDKK.Q | Y | 43.68 | 1290.6675 | 12 | 2.7 | 646.3399 | 2 | 16.78 | 0.9985 | 1.0057 | 53932 | 3.84e2 | 1 | 1 | 504 | 515 |  |  | DB Search |
| total 1 peptides |  |  |  |  |  |  |  |  |  |  |  |  |  |  |  |  |  |  |  |
| --- | --- | --- | --- | --- | --- | --- | --- | --- | --- | --- | --- | --- | --- | --- | --- | --- | --- | --- | --- |

Best Unique PSM (Scan Sample\_7\_A1\_1\_3644.d:53932, m/z=646.3399, z=2, RT=16.78, ppm=-1.77):


TRINITY\_DN118\_c1\_g1\_i3.p1

back to list

  

| Protein Coverage
| Supporting Peptides
| Best Unique PSM
|

Protein Coverage:

Supporting Peptides:

| Peptide | Uniq | -10lgP | Mass | Length | ppm | m/z | z | RT | 1/k0 Start | 1/k0 End | Precursor ID | Area Sample 8 | #Feature | #Feature Sample 8 | Start | End | PTM | AScore | Found By |
| --- | --- | --- | --- | --- | --- | --- | --- | --- | --- | --- | --- | --- | --- | --- | --- | --- | --- | --- | --- |
| R.ETC(+57.02)SDIEKR.E | Y | 43.68 | 1136.5132 | 9 | 2.96 | 569.263 | 2 | 10.13 | 0.8895 | 0.8968 | 15523 | 2.26e3 | 1 | 1 | 186 | 194 | Carbamidomethylation |  | DB Search |
| K.VC(+57.02)EDVKR.C | Y | 43.45 | 904.4436 | 7 | -6.38 | 453.2242 | 2 | 9.42 | 0.7697 | 0.777 | 12258 | 0 | 0 | 0 | 122 | 128 | Carbamidomethylation |  | DB Search |
| total 2 peptides |  |  |  |  |  |  |  |  |  |  |  |  |  |  |  |  |  |  |  |
| --- | --- | --- | --- | --- | --- | --- | --- | --- | --- | --- | --- | --- | --- | --- | --- | --- | --- | --- | --- |

Best Unique PSM (Scan Sample\_7\_A1\_1\_3644.d:15523, m/z=569.263, z=2, RT=10.13, ppm=-1.52):


TRINITY\_DN1189\_c0\_g1\_i1.p1

back to list

  

| Protein Coverage
| Supporting Peptides
| Best Unique PSM
|

Protein Coverage:

Supporting Peptides:

| Peptide | Uniq | -10lgP | Mass | Length | ppm | m/z | z | RT | 1/k0 Start | 1/k0 End | Precursor ID | Area Sample 8 | #Feature | #Feature Sample 8 | Start | End | PTM | AScore | Found By |
| --- | --- | --- | --- | --- | --- | --- | --- | --- | --- | --- | --- | --- | --- | --- | --- | --- | --- | --- | --- |
| K.AKDVTAAVKK.A | Y | 43.5 | 1029.6182 | 10 | 9.39 | 515.8189 | 2 | 9.45 | 0.84 | 0.8473 | 12390 | 0 | 0 | 0 | 185 | 194 |  |  | DB Search |
| R.AVSFLVNDVPNLK.C | Y | 42.5 | 1414.782 | 13 | 5.5 | 708.399 | 2 | 19.29 | 1.0041 | 1.0114 | 69625 | 4.01e2 | 1 | 1 | 148 | 160 |  |  | DB Search |
| total 2 peptides |  |  |  |  |  |  |  |  |  |  |  |  |  |  |  |  |  |  |  |
| --- | --- | --- | --- | --- | --- | --- | --- | --- | --- | --- | --- | --- | --- | --- | --- | --- | --- | --- | --- |

Best Unique PSM (Scan Sample\_7\_A1\_1\_3644.d:12390, m/z=515.8189, z=2, RT=9.45, ppm=4.91):


TRINITY\_DN14781\_c0\_g1\_i1.p1

back to list

  

| Protein Coverage
| Supporting Peptides
| Best Unique PSM
|

Protein Coverage:

Supporting Peptides:

| Peptide | Uniq | -10lgP | Mass | Length | ppm | m/z | z | RT | 1/k0 Start | 1/k0 End | Precursor ID | Area Sample 8 | #Feature | #Feature Sample 8 | Start | End | PTM | AScore | Found By |
| --- | --- | --- | --- | --- | --- | --- | --- | --- | --- | --- | --- | --- | --- | --- | --- | --- | --- | --- | --- |
| K.SQQSNNIEHYIVK.I | Y | 43.47 | 1558.774 | 13 | 4.12 | 780.394 | 2 | 14.80 | 1.064 | 1.0713 | 42545 | 7.25e2 | 1 | 1 | 190 | 202 |  |  | DB Search |
| total 1 peptides |  |  |  |  |  |  |  |  |  |  |  |  |  |  |  |  |  |  |  |
| --- | --- | --- | --- | --- | --- | --- | --- | --- | --- | --- | --- | --- | --- | --- | --- | --- | --- | --- | --- |

Best Unique PSM (Scan Sample\_7\_A1\_1\_3644.d:42545, m/z=780.394, z=2, RT=14.80, ppm=-0.35):


TRINITY\_DN1369\_c0\_g1\_i1.p1

back to list

  

| Protein Coverage
| Supporting Peptides
| Best Unique PSM
|

Protein Coverage:

Supporting Peptides:

| Peptide | Uniq | -10lgP | Mass | Length | ppm | m/z | z | RT | 1/k0 Start | 1/k0 End | Precursor ID | Area Sample 8 | #Feature | #Feature Sample 8 | Start | End | PTM | AScore | Found By |
| --- | --- | --- | --- | --- | --- | --- | --- | --- | --- | --- | --- | --- | --- | --- | --- | --- | --- | --- | --- |
| K.TPGEC(+57.02)C(+57.02)PQC(+57.02)K.K | Y | 43.8 | 1235.4733 | 10 | -0.13 | 618.7411 | 2 | 10.03 | 0.8866 | 0.8939 | 14986 | 1.58e3 | 1 | 1 | 1387 | 1396 | Carbamidomethylation, Carbamidomethylation, Carbamidomethylation |  | DB Search |
| R.KYTPC(+57.02)EEK.C | Y | 43.79 | 1053.4801 | 8 | 3.66 | 527.7469 | 2 | 9.73 | 0.855 | 0.8623 | 13599 | 0 | 0 | 0 | 649 | 656 | Carbamidomethylation |  | DB Search |
| R.EITLNYHDTVVHMK.R | Y | 43.42 | 1698.8399 | 14 | 2.55 | 850.4256 | 2 | 15.46 | 1.1118 | 1.119 | 46350 | 9.07e2 | 1 | 1 | 911 | 924 |  |  | DB Search |
| K.EIEDTC(+57.02)HNR.K | Y | 43.16 | 1172.488 | 9 | 13.83 | 391.8403 | 3 | 9.54 | 0.711 | 0.7184 | 12747 | 0 | 0 | 0 | 1029 | 1037 | Carbamidomethylation |  | DB Search |
| K.LC(+57.02)DATC(+57.02)SVFGDPHYK.T | Y | 43.14 | 1768.7549 | 15 | 2.88 | 885.3833 | 2 | 15.32 | 1.1422 | 1.1494 | 45690 | 2.36e2 | 1 | 1 | 846 | 860 | Carbamidomethylation, Carbamidomethylation |  | DB Search |
| R.TISNGGTVAK.D | Y | 42.73 | 946.5083 | 10 | -0.17 | 474.2592 | 2 | 10.11 | 0.8124 | 0.8197 | 15429 | 0 | 0 | 0 | 721 | 730 |  |  | DB Search |
| R.TENNC(+57.02)PLTK.E | Y | 41.52 | 1075.4968 | 9 | 1.18 | 538.7539 | 2 | 10.87 | 0.853 | 0.8603 | 19697 | 8.87e2 | 1 | 1 | 1020 | 1028 | Carbamidomethylation |  | DB Search |
| total 7 peptides |  |  |  |  |  |  |  |  |  |  |  |  |  |  |  |  |  |  |  |
| --- | --- | --- | --- | --- | --- | --- | --- | --- | --- | --- | --- | --- | --- | --- | --- | --- | --- | --- | --- |

Best Unique PSM (Scan Sample\_7\_A1\_1\_3644.d:14986, m/z=618.7411, z=2, RT=10.03, ppm=-4.6):


TRINITY\_DN925\_c0\_g1\_i1.p1

back to list

  

| Protein Coverage
| Supporting Peptides
| Best Unique PSM
|

Protein Coverage:

Supporting Peptides:

| Peptide | Uniq | -10lgP | Mass | Length | ppm | m/z | z | RT | 1/k0 Start | 1/k0 End | Precursor ID | Area Sample 8 | #Feature | #Feature Sample 8 | Start | End | PTM | AScore | Found By |
| --- | --- | --- | --- | --- | --- | --- | --- | --- | --- | --- | --- | --- | --- | --- | --- | --- | --- | --- | --- |
| R.ASGDYATVVSHNR.D | Y | 43.82 | 1375.648 | 13 | -1.36 | 688.8273 | 2 | 12.19 | 0.9953 | 1.0025 | 27207 | 0 | 0 | 0 | 129 | 141 |  |  | DB Search |
| total 1 peptides |  |  |  |  |  |  |  |  |  |  |  |  |  |  |  |  |  |  |  |
| --- | --- | --- | --- | --- | --- | --- | --- | --- | --- | --- | --- | --- | --- | --- | --- | --- | --- | --- | --- |

Best Unique PSM (Scan Sample\_7\_A1\_1\_3644.d:27207, m/z=688.8273, z=2, RT=12.19, ppm=-5.84):


TRINITY\_DN585\_c0\_g1\_i5.p1

back to list

  

| Protein Coverage
| Supporting Peptides
| Best Unique PSM
|

Protein Coverage:

Supporting Peptides:

| Peptide | Uniq | -10lgP | Mass | Length | ppm | m/z | z | RT | 1/k0 Start | 1/k0 End | Precursor ID | Area Sample 8 | #Feature | #Feature Sample 8 | Start | End | PTM | AScore | Found By |
| --- | --- | --- | --- | --- | --- | --- | --- | --- | --- | --- | --- | --- | --- | --- | --- | --- | --- | --- | --- |
| K.TITLEVEPSDTIENVK.S | Y | 43.28 | 1786.92 | 16 | 2.71 | 894.4657 | 2 | 17.62 | 1.1454 | 1.1526 | 58818 | 1.46e3 | 1 | 1 | 60 | 75 |  |  | DB Search |
| total 1 peptides |  |  |  |  |  |  |  |  |  |  |  |  |  |  |  |  |  |  |  |
| --- | --- | --- | --- | --- | --- | --- | --- | --- | --- | --- | --- | --- | --- | --- | --- | --- | --- | --- | --- |

Best Unique PSM (Scan Sample\_7\_A1\_1\_3644.d:58818, m/z=894.4657, z=2, RT=17.62, ppm=-1.76):


TRINITY\_DN5906\_c3\_g1\_i1.p2

back to list

  

| Protein Coverage
| Supporting Peptides
| Best Unique PSM
|

Protein Coverage:

Supporting Peptides:

| Peptide | Uniq | -10lgP | Mass | Length | ppm | m/z | z | RT | 1/k0 Start | 1/k0 End | Precursor ID | Area Sample 8 | #Feature | #Feature Sample 8 | Start | End | PTM | AScore | Found By |
| --- | --- | --- | --- | --- | --- | --- | --- | --- | --- | --- | --- | --- | --- | --- | --- | --- | --- | --- | --- |
| K.AGM(+15.99)THIVR.D | Y | 43.71 | 899.4647 | 8 | 5.69 | 450.7402 | 2 | 9.78 | 0.8161 | 0.8234 | 13845 | 0 | 0 | 0 | 76 | 83 | Oxidation (M) | M3:Oxidation (M):1000 | DB Search |
| total 1 peptides |  |  |  |  |  |  |  |  |  |  |  |  |  |  |  |  |  |  |  |
| --- | --- | --- | --- | --- | --- | --- | --- | --- | --- | --- | --- | --- | --- | --- | --- | --- | --- | --- | --- |

Best Unique PSM (Scan Sample\_7\_A1\_1\_3644.d:13845, m/z=450.7402, z=2, RT=9.78, ppm=1.21):


TRINITY\_DN4595\_c0\_g1\_i10.p2

back to list

  

| Protein Coverage
| Supporting Peptides
| Best Unique PSM
|

Protein Coverage:

Supporting Peptides:

| Peptide | Uniq | -10lgP | Mass | Length | ppm | m/z | z | RT | 1/k0 Start | 1/k0 End | Precursor ID | Area Sample 8 | #Feature | #Feature Sample 8 | Start | End | PTM | AScore | Found By |
| --- | --- | --- | --- | --- | --- | --- | --- | --- | --- | --- | --- | --- | --- | --- | --- | --- | --- | --- | --- |
| R.SC(+57.02)ASDFSAER.L | Y | 43.77 | 1128.4506 | 10 | -0.21 | 565.2299 | 2 | 12.29 | 0.8546 | 0.8619 | 27814 | 0 | 0 | 0 | 169 | 178 | Carbamidomethylation |  | DB Search |
| R.AFNKVETR.N | N | 43.74 | 963.5138 | 8 | 5.69 | 482.7647 | 2 | 10.62 | 0.8299 | 0.8372 | 18171 | 1.3e3 | 1 | 1 | 47 | 54 |  |  | DB Search |
| R.AFNRVETR.D | Y | 42.68 | 991.5199 | 8 | 4.26 | 496.7671 | 2 | 10.79 | 0.8425 | 0.8498 | 19063 | 2.3e3 | 1 | 1 | 152 | 159 |  |  | DB Search |
| total 3 peptides |  |  |  |  |  |  |  |  |  |  |  |  |  |  |  |  |  |  |  |
| --- | --- | --- | --- | --- | --- | --- | --- | --- | --- | --- | --- | --- | --- | --- | --- | --- | --- | --- | --- |

Best Unique PSM (Scan Sample\_7\_A1\_1\_3644.d:27814, m/z=565.2299, z=2, RT=12.29, ppm=-4.69):


TRINITY\_DN141338\_c2\_g1\_i1.p1

back to list

  

| Protein Coverage
| Supporting Peptides
| Best Unique PSM
|

Protein Coverage:

Supporting Peptides:

| Peptide | Uniq | -10lgP | Mass | Length | ppm | m/z | z | RT | 1/k0 Start | 1/k0 End | Precursor ID | Area Sample 8 | #Feature | #Feature Sample 8 | Start | End | PTM | AScore | Found By |
| --- | --- | --- | --- | --- | --- | --- | --- | --- | --- | --- | --- | --- | --- | --- | --- | --- | --- | --- | --- |
| K.LGIHEDTQNR.N | Y | 43.82 | 1181.5789 | 10 | 2.89 | 591.7958 | 2 | 11.20 | 0.9073 | 0.9146 | 21408 | 1.32e3 | 1 | 1 | 434 | 443 |  |  | DB Search |
| R.ELISNSSDALDKIR.Y | N | 43.22 | 1559.8155 | 14 | -1.75 | 780.9102 | 2 | 15.66 | 1.0777 | 1.0849 | 47553 | 0 | 0 | 0 | 37 | 50 |  |  | DB Search |
| total 2 peptides |  |  |  |  |  |  |  |  |  |  |  |  |  |  |  |  |  |  |  |
| --- | --- | --- | --- | --- | --- | --- | --- | --- | --- | --- | --- | --- | --- | --- | --- | --- | --- | --- | --- |

Best Unique PSM (Scan Sample\_7\_A1\_1\_3644.d:21408, m/z=591.7958, z=2, RT=11.20, ppm=-1.59):


TRINITY\_DN2477\_c0\_g1\_i6.p1

back to list

  

| Protein Coverage
| Supporting Peptides
| Best Unique PSM
|

Protein Coverage:

Supporting Peptides:

| Peptide | Uniq | -10lgP | Mass | Length | ppm | m/z | z | RT | 1/k0 Start | 1/k0 End | Precursor ID | Area Sample 8 | #Feature | #Feature Sample 8 | Start | End | PTM | AScore | Found By |
| --- | --- | --- | --- | --- | --- | --- | --- | --- | --- | --- | --- | --- | --- | --- | --- | --- | --- | --- | --- |
| K.DSYVGDEAQSKR.G | Y | 43.81 | 1353.616 | 12 | 3.22 | 677.8145 | 2 | 11.01 | 0.9961 | 1.0033 | 20355 | 1.65e3 | 1 | 1 | 52 | 63 |  |  | DB Search |
| R.VAPEEHPVLLTEAPLNPK.A | Y | 43.24 | 1953.0571 | 18 | -8.51 | 977.5231 | 2 | 16.59 | 1.2073 | 1.2145 | 52921 | 0 | 0 | 0 | 97 | 114 |  |  | DB Search |
| total 2 peptides |  |  |  |  |  |  |  |  |  |  |  |  |  |  |  |  |  |  |  |
| --- | --- | --- | --- | --- | --- | --- | --- | --- | --- | --- | --- | --- | --- | --- | --- | --- | --- | --- | --- |

Best Unique PSM (Scan Sample\_7\_A1\_1\_3644.d:20355, m/z=677.8145, z=2, RT=11.01, ppm=-1.25):


TRINITY\_DN8903\_c0\_g1\_i1.p1

back to list

  

| Protein Coverage
| Supporting Peptides
| Best Unique PSM
|

Protein Coverage:

Supporting Peptides:

| Peptide | Uniq | -10lgP | Mass | Length | ppm | m/z | z | RT | 1/k0 Start | 1/k0 End | Precursor ID | Area Sample 8 | #Feature | #Feature Sample 8 | Start | End | PTM | AScore | Found By |
| --- | --- | --- | --- | --- | --- | --- | --- | --- | --- | --- | --- | --- | --- | --- | --- | --- | --- | --- | --- |
| K.DLHIIEDYVLK.H | Y | 43.71 | 1356.7289 | 11 | -2.47 | 679.367 | 2 | 19.75 | 0.9969 | 1.0041 | 72430 | 4.44e2 | 1 | 1 | 417 | 427 |  |  | DB Search |
| K.YVQNPR.N | Y | 42.15 | 775.3977 | 6 | 4.25 | 388.706 | 2 | 9.82 | 0.7697 | 0.777 | 14011 | 8.51e2 | 1 | 1 | 125 | 130 |  |  | DB Search |
| total 2 peptides |  |  |  |  |  |  |  |  |  |  |  |  |  |  |  |  |  |  |  |
| --- | --- | --- | --- | --- | --- | --- | --- | --- | --- | --- | --- | --- | --- | --- | --- | --- | --- | --- | --- |

Best Unique PSM (Scan Sample\_7\_A1\_1\_3644.d:72430, m/z=679.367, z=2, RT=19.75, ppm=-6.94):


TRINITY\_DN36\_c0\_g1\_i17.p1

back to list

  

| Protein Coverage
| Supporting Peptides
| Best Unique PSM
|

Protein Coverage:

Supporting Peptides:

| Peptide | Uniq | -10lgP | Mass | Length | ppm | m/z | z | RT | 1/k0 Start | 1/k0 End | Precursor ID | Area Sample 8 | #Feature | #Feature Sample 8 | Start | End | PTM | AScore | Found By |
| --- | --- | --- | --- | --- | --- | --- | --- | --- | --- | --- | --- | --- | --- | --- | --- | --- | --- | --- | --- |
| K.FETQIATK.D | Y | 43.56 | 936.4916 | 8 | 1.93 | 937.4965 | 1 | 13.99 | 1.2029 | 1.2101 | 37753 | 0 | 0 | 0 | 167 | 174 |  |  | DB Search |
| R.LGGIEVTQGNC(+57.02)AR.K | Y | 43.02 | 1373.6721 | 13 | 2.68 | 687.8421 | 2 | 13.90 | 0.9662 | 0.9735 | 37137 | 6.95e2 | 1 | 1 | 58 | 70 | Carbamidomethylation |  | DB Search |
| total 2 peptides |  |  |  |  |  |  |  |  |  |  |  |  |  |  |  |  |  |  |  |
| --- | --- | --- | --- | --- | --- | --- | --- | --- | --- | --- | --- | --- | --- | --- | --- | --- | --- | --- | --- |

Best Unique PSM (Scan Sample\_7\_A1\_1\_3644.d:37753, m/z=937.4965, z=1, RT=13.99, ppm=-2.55):


TRINITY\_DN649\_c0\_g1\_i5.p1

back to list

  

| Protein Coverage
| Supporting Peptides
| Best Unique PSM
|

Protein Coverage:

Supporting Peptides:

| Peptide | Uniq | -10lgP | Mass | Length | ppm | m/z | z | RT | 1/k0 Start | 1/k0 End | Precursor ID | Area Sample 8 | #Feature | #Feature Sample 8 | Start | End | PTM | AScore | Found By |
| --- | --- | --- | --- | --- | --- | --- | --- | --- | --- | --- | --- | --- | --- | --- | --- | --- | --- | --- | --- |
| K.FETQIATK.N | Y | 43.56 | 936.4916 | 8 | 1.93 | 937.4965 | 1 | 13.99 | 1.2029 | 1.2101 | 37753 | 0 | 0 | 0 | 102 | 109 |  |  | DB Search |
| total 1 peptides |  |  |  |  |  |  |  |  |  |  |  |  |  |  |  |  |  |  |  |
| --- | --- | --- | --- | --- | --- | --- | --- | --- | --- | --- | --- | --- | --- | --- | --- | --- | --- | --- | --- |

Best Unique PSM (Scan Sample\_7\_A1\_1\_3644.d:37753, m/z=937.4965, z=1, RT=13.99, ppm=-2.55):


TRINITY\_DN6428\_c0\_g1\_i1.p1

back to list

  

| Protein Coverage
| Supporting Peptides
| Best Unique PSM
|

Protein Coverage:

Supporting Peptides:

| Peptide | Uniq | -10lgP | Mass | Length | ppm | m/z | z | RT | 1/k0 Start | 1/k0 End | Precursor ID | Area Sample 8 | #Feature | #Feature Sample 8 | Start | End | PTM | AScore | Found By |
| --- | --- | --- | --- | --- | --- | --- | --- | --- | --- | --- | --- | --- | --- | --- | --- | --- | --- | --- | --- |
| R.SC(+57.02)ENPKPLNGGK.Q | Y | 43.81 | 1299.6241 | 12 | 4.1 | 650.8191 | 2 | 11.12 | 0.9509 | 0.9582 | 20845 | 1.35e3 | 1 | 1 | 245 | 256 | Carbamidomethylation |  | DB Search |
| total 1 peptides |  |  |  |  |  |  |  |  |  |  |  |  |  |  |  |  |  |  |  |
| --- | --- | --- | --- | --- | --- | --- | --- | --- | --- | --- | --- | --- | --- | --- | --- | --- | --- | --- | --- |

Best Unique PSM (Scan Sample\_7\_A1\_1\_3644.d:20845, m/z=650.8191, z=2, RT=11.12, ppm=-0.37):


TRINITY\_DN120478\_c0\_g1\_i1.p1

back to list

  

| Protein Coverage
| Supporting Peptides
| Best Unique PSM
|

Protein Coverage:

Supporting Peptides:

| Peptide | Uniq | -10lgP | Mass | Length | ppm | m/z | z | RT | 1/k0 Start | 1/k0 End | Precursor ID | Area Sample 8 | #Feature | #Feature Sample 8 | Start | End | PTM | AScore | Found By |
| --- | --- | --- | --- | --- | --- | --- | --- | --- | --- | --- | --- | --- | --- | --- | --- | --- | --- | --- | --- |
| R.ILQLEEEFIKK.M | Y | 43.63 | 1388.7915 | 11 | -1.71 | 463.9349 | 3 | 17.91 | 0.7489 | 0.7563 | 60751 | 4.87e2 | 1 | 1 | 267 | 277 |  |  | DB Search |
| R.AVGATIVNRPEEIREEDLGLK.C | Y | 40.05 | 2308.2386 | 21 | 5.45 | 770.4209 | 3 | 16.03 | 1.052 | 1.0592 | 49650 | 2.87e2 | 1 | 1 | 326 | 346 |  |  | DB Search |
| total 2 peptides |  |  |  |  |  |  |  |  |  |  |  |  |  |  |  |  |  |  |  |
| --- | --- | --- | --- | --- | --- | --- | --- | --- | --- | --- | --- | --- | --- | --- | --- | --- | --- | --- | --- |

Best Unique PSM (Scan Sample\_7\_A1\_1\_3644.d:60751, m/z=463.9349, z=3, RT=17.91, ppm=-6.18):


TRINITY\_DN3991\_c0\_g1\_i1.p1

back to list

  

| Protein Coverage
| Supporting Peptides
| Best Unique PSM
|

Protein Coverage:

Supporting Peptides:

| Peptide | Uniq | -10lgP | Mass | Length | ppm | m/z | z | RT | 1/k0 Start | 1/k0 End | Precursor ID | Area Sample 8 | #Feature | #Feature Sample 8 | Start | End | PTM | AScore | Found By |
| --- | --- | --- | --- | --- | --- | --- | --- | --- | --- | --- | --- | --- | --- | --- | --- | --- | --- | --- | --- |
| K.HKLEPVGC(+57.02)FK.D | Y | 43.81 | 1213.6277 | 10 | 6.02 | 405.5505 | 3 | 12.58 | 0.7086 | 0.7159 | 29300 | 3.93e3 | 1 | 1 | 26 | 35 | Carbamidomethylation |  | DB Search |
| K.HKLEPVGC(+57.02)FKDR.G | Y | 43.8 | 1484.7558 | 12 | -2.3 | 495.9225 | 3 | 11.84 | 0.8181 | 0.8254 | 25216 | 0 | 0 | 0 | 26 | 37 | Carbamidomethylation |  | DB Search |
| K.LSQC(+57.02)TAASGK.H | Y | 43.79 | 1021.4862 | 10 | 0.54 | 511.7484 | 2 | 9.85 | 0.8323 | 0.8396 | 13418 | 2.29e5 | 2 | 2 | 130 | 139 | Carbamidomethylation |  | DB Search |
| K.LEPVGC(+57.02)FKDR.G | Y | 43.76 | 1219.6019 | 10 | 3.27 | 610.8075 | 2 | 13.16 | 0.9416 | 0.9489 | 32775 | 2.03e3 | 2 | 2 | 28 | 37 | Carbamidomethylation |  | DB Search |
| K.LEPVGC(+57.02)FK.D | Y | 43.75 | 948.4739 | 8 | -18.5 | 949.4594 | 1 | 14.04 | 1.2857 | 1.2929 | 38072 | 3.42e2 | 1 | 1 | 28 | 35 | Carbamidomethylation |  | DB Search |
| R.AKLSQC(+57.02)TAASGK.H | Y | 43.75 | 1220.6183 | 12 | 9.93 | 407.8823 | 3 | 9.74 | 0.7033 | 0.7106 | 13580 | 6.95e3 | 2 | 2 | 128 | 139 | Carbamidomethylation |  | DB Search |
| R.HGQVDSC(+57.02)INQNLKK.N | Y | 43.17 | 1639.81 | 14 | -1.59 | 547.6073 | 3 | 10.92 | 0.8457 | 0.853 | 19977 | 0 | 0 | 0 | 112 | 125 | Carbamidomethylation |  | DB Search |
| total 7 peptides |  |  |  |  |  |  |  |  |  |  |  |  |  |  |  |  |  |  |  |
| --- | --- | --- | --- | --- | --- | --- | --- | --- | --- | --- | --- | --- | --- | --- | --- | --- | --- | --- | --- |

Best Unique PSM (Scan Sample\_7\_A1\_1\_3644.d:29300, m/z=405.5505, z=3, RT=12.58, ppm=1.54):


TRINITY\_DN4726\_c0\_g1\_i1.p1

back to list

  

| Protein Coverage
| Supporting Peptides
| Best Unique PSM
|

Protein Coverage:

Supporting Peptides:

| Peptide | Uniq | -10lgP | Mass | Length | ppm | m/z | z | RT | 1/k0 Start | 1/k0 End | Precursor ID | Area Sample 8 | #Feature | #Feature Sample 8 | Start | End | PTM | AScore | Found By |
| --- | --- | --- | --- | --- | --- | --- | --- | --- | --- | --- | --- | --- | --- | --- | --- | --- | --- | --- | --- |
| K.ANKEFQEIVQNTK.Y | Y | 43.35 | 1547.7943 | 13 | 0.49 | 774.9014 | 2 | 14.65 | 1.0676 | 1.0749 | 41627 | 1.61e2 | 1 | 1 | 364 | 376 |  |  | DB Search |
| total 1 peptides |  |  |  |  |  |  |  |  |  |  |  |  |  |  |  |  |  |  |  |
| --- | --- | --- | --- | --- | --- | --- | --- | --- | --- | --- | --- | --- | --- | --- | --- | --- | --- | --- | --- |

Best Unique PSM (Scan Sample\_7\_A1\_1\_3644.d:41627, m/z=774.9014, z=2, RT=14.65, ppm=-3.98):


TRINITY\_DN2477\_c0\_g1\_i2.p1

back to list

  

| Protein Coverage
| Supporting Peptides
| Best Unique PSM
|

Protein Coverage:

Supporting Peptides:

| Peptide | Uniq | -10lgP | Mass | Length | ppm | m/z | z | RT | 1/k0 Start | 1/k0 End | Precursor ID | Area Sample 8 | #Feature | #Feature Sample 8 | Start | End | PTM | AScore | Found By |
| --- | --- | --- | --- | --- | --- | --- | --- | --- | --- | --- | --- | --- | --- | --- | --- | --- | --- | --- | --- |
| K.DSYVGDEAQSKR.G | Y | 43.81 | 1353.616 | 12 | 3.22 | 677.8145 | 2 | 11.01 | 0.9961 | 1.0033 | 20355 | 1.65e3 | 1 | 1 | 19 | 30 |  |  | DB Search |
| R.VAPEEHPVLLTEAPLNPK.A | Y | 43.24 | 1953.0571 | 18 | -8.51 | 977.5231 | 2 | 16.59 | 1.2073 | 1.2145 | 52921 | 0 | 0 | 0 | 64 | 81 |  |  | DB Search |
| total 2 peptides |  |  |  |  |  |  |  |  |  |  |  |  |  |  |  |  |  |  |  |
| --- | --- | --- | --- | --- | --- | --- | --- | --- | --- | --- | --- | --- | --- | --- | --- | --- | --- | --- | --- |

Best Unique PSM (Scan Sample\_7\_A1\_1\_3644.d:20355, m/z=677.8145, z=2, RT=11.01, ppm=-1.25):


TRINITY\_DN2885\_c0\_g1\_i4.p1

back to list

  

| Protein Coverage
| Supporting Peptides
| Best Unique PSM
|

Protein Coverage:

Supporting Peptides:

| Peptide | Uniq | -10lgP | Mass | Length | ppm | m/z | z | RT | 1/k0 Start | 1/k0 End | Precursor ID | Area Sample 8 | #Feature | #Feature Sample 8 | Start | End | PTM | AScore | Found By |
| --- | --- | --- | --- | --- | --- | --- | --- | --- | --- | --- | --- | --- | --- | --- | --- | --- | --- | --- | --- |
| K.VTRPHGASGVVR.A | Y | 43.35 | 1234.6894 | 12 | 2.89 | 412.5698 | 3 | 10.09 | 0.8173 | 0.8246 | 15387 | 0 | 0 | 0 | 72 | 83 |  |  | DB Search |
| total 1 peptides |  |  |  |  |  |  |  |  |  |  |  |  |  |  |  |  |  |  |  |
| --- | --- | --- | --- | --- | --- | --- | --- | --- | --- | --- | --- | --- | --- | --- | --- | --- | --- | --- | --- |

Best Unique PSM (Scan Sample\_7\_A1\_1\_3644.d:15387, m/z=412.5698, z=3, RT=10.09, ppm=-1.58):


TRINITY\_DN2113\_c0\_g1\_i2.p1

back to list

  

| Protein Coverage
| Supporting Peptides
| Best Unique PSM
|

Protein Coverage:

Supporting Peptides:

| Peptide | Uniq | -10lgP | Mass | Length | ppm | m/z | z | RT | 1/k0 Start | 1/k0 End | Precursor ID | Area Sample 8 | #Feature | #Feature Sample 8 | Start | End | PTM | AScore | Found By |
| --- | --- | --- | --- | --- | --- | --- | --- | --- | --- | --- | --- | --- | --- | --- | --- | --- | --- | --- | --- |
| R.AHVAQLC(+57.02)EK.A | Y | 43.74 | 1054.5229 | 9 | 0.64 | 528.2667 | 2 | 10.78 | 0.8603 | 0.8676 | 19105 | 9.91e2 | 1 | 1 | 611 | 619 | Carbamidomethylation |  | DB Search |
| R.ALEHYSDIYDIKR.A | Y | 43.53 | 1621.81 | 13 | -3.67 | 541.6062 | 3 | 14.76 | 0.8238 | 0.8311 | 42332 | 0 | 0 | 0 | 626 | 638 |  |  | DB Search |
| R.LADLEEFISSPNHANISQVGER.C | Y | 43.48 | 2425.1873 | 22 | 4.51 | 809.4031 | 3 | 18.95 | 0.9255 | 0.9327 | 67455 | 1.08e3 | 1 | 1 | 1183 | 1204 |  |  | DB Search |
| K.GQFSTDDLVAEVEKR.N | Y | 42.74 | 1692.8318 | 15 | 10.63 | 847.4284 | 2 | 17.88 | 1.141 | 1.1482 | 60636 | 0 | 0 | 0 | 838 | 852 |  |  | DB Search |
| total 4 peptides |  |  |  |  |  |  |  |  |  |  |  |  |  |  |  |  |  |  |  |
| --- | --- | --- | --- | --- | --- | --- | --- | --- | --- | --- | --- | --- | --- | --- | --- | --- | --- | --- | --- |

Best Unique PSM (Scan Sample\_7\_A1\_1\_3644.d:19105, m/z=528.2667, z=2, RT=10.78, ppm=-3.84):


TRINITY\_DN1614\_c0\_g1\_i1.p1

back to list

  

| Protein Coverage
| Supporting Peptides
| Best Unique PSM
|

Protein Coverage:

Supporting Peptides:

| Peptide | Uniq | -10lgP | Mass | Length | ppm | m/z | z | RT | 1/k0 Start | 1/k0 End | Precursor ID | Area Sample 8 | #Feature | #Feature Sample 8 | Start | End | PTM | AScore | Found By |
| --- | --- | --- | --- | --- | --- | --- | --- | --- | --- | --- | --- | --- | --- | --- | --- | --- | --- | --- | --- |
| K.GLDVDSLVIDHIQVNR.A | Y | 43.15 | 1791.9479 | 16 | 0.78 | 896.9779 | 2 | 19.21 | 1.177 | 1.1841 | 69113 | 0 | 0 | 0 | 140 | 155 |  |  | DB Search |
| total 1 peptides |  |  |  |  |  |  |  |  |  |  |  |  |  |  |  |  |  |  |  |
| --- | --- | --- | --- | --- | --- | --- | --- | --- | --- | --- | --- | --- | --- | --- | --- | --- | --- | --- | --- |

Best Unique PSM (Scan Sample\_7\_A1\_1\_3644.d:69113, m/z=896.9779, z=2, RT=19.21, ppm=-3.69):


TRINITY\_DN1910\_c0\_g1\_i1.p1

back to list

  

| Protein Coverage
| Supporting Peptides
| Best Unique PSM
|

Protein Coverage:

Supporting Peptides:

| Peptide | Uniq | -10lgP | Mass | Length | ppm | m/z | z | RT | 1/k0 Start | 1/k0 End | Precursor ID | Area Sample 8 | #Feature | #Feature Sample 8 | Start | End | PTM | AScore | Found By |
| --- | --- | --- | --- | --- | --- | --- | --- | --- | --- | --- | --- | --- | --- | --- | --- | --- | --- | --- | --- |
| K.NM(+15.99)M(+15.99)AAC(+57.02)DPR.H | Y | 43.62 | 1096.41 | 9 | -0.29 | 549.2097 | 2 | 9.58 | 0.8615 | 0.8688 | 12888 | 5.74e2 | 1 | 1 | 298 | 306 | Oxidation (M), Oxidation (M), Carbamidomethylation | M2:Oxidation (M):1000 M3:Oxidation (M):1000 | DB Search |
| R.FPGQLNADLRK.L | Y | 43.16 | 1257.6829 | 11 | -1.33 | 629.8451 | 2 | 14.44 | 0.9497 | 0.957 | 40493 | 0 | 0 | 0 | 242 | 252 |  |  | DB Search |
| R.YLTVAAMFR.G | Y | 42.33 | 1070.5583 | 9 | 5.33 | 536.2869 | 2 | 19.96 | 0.8607 | 0.868 | 73861 | 0 | 0 | 0 | 310 | 318 |  |  | DB Search |
| total 3 peptides |  |  |  |  |  |  |  |  |  |  |  |  |  |  |  |  |  |  |  |
| --- | --- | --- | --- | --- | --- | --- | --- | --- | --- | --- | --- | --- | --- | --- | --- | --- | --- | --- | --- |

Best Unique PSM (Scan Sample\_7\_A1\_1\_3644.d:12888, m/z=549.2097, z=2, RT=9.58, ppm=-4.76):


TRINITY\_DN29322\_c0\_g1\_i1.p1

back to list

  

| Protein Coverage
| Supporting Peptides
| Best Unique PSM
|

Protein Coverage:

Supporting Peptides:

| Peptide | Uniq | -10lgP | Mass | Length | ppm | m/z | z | RT | 1/k0 Start | 1/k0 End | Precursor ID | Area Sample 8 | #Feature | #Feature Sample 8 | Start | End | PTM | AScore | Found By |
| --- | --- | --- | --- | --- | --- | --- | --- | --- | --- | --- | --- | --- | --- | --- | --- | --- | --- | --- | --- |
| K.TC(+57.02)TESDVLVIHK.Y | Y | 43.44 | 1400.6969 | 12 | 2.89 | 467.9055 | 3 | 14.24 | 0.7506 | 0.7579 | 39250 | 0 | 0 | 0 | 206 | 217 | Carbamidomethylation |  | DB Search |
| total 1 peptides |  |  |  |  |  |  |  |  |  |  |  |  |  |  |  |  |  |  |  |
| --- | --- | --- | --- | --- | --- | --- | --- | --- | --- | --- | --- | --- | --- | --- | --- | --- | --- | --- | --- |

Best Unique PSM (Scan Sample\_7\_A1\_1\_3644.d:39250, m/z=467.9055, z=3, RT=14.24, ppm=-1.58):


TRINITY\_DN123\_c0\_g1\_i1.p1

back to list

  

| Protein Coverage
| Supporting Peptides
| Best Unique PSM
|

Protein Coverage:

Supporting Peptides:

| Peptide | Uniq | -10lgP | Mass | Length | ppm | m/z | z | RT | 1/k0 Start | 1/k0 End | Precursor ID | Area Sample 8 | #Feature | #Feature Sample 8 | Start | End | PTM | AScore | Found By |
| --- | --- | --- | --- | --- | --- | --- | --- | --- | --- | --- | --- | --- | --- | --- | --- | --- | --- | --- | --- |
| K.IAAVIINKPDVLASR.T | Y | 42.71 | 1578.9457 | 15 | 3.92 | 790.4797 | 2 | 16.18 | 1.1021 | 1.1094 | 50536 | 0 | 0 | 0 | 484 | 498 |  |  | DB Search |
| total 1 peptides |  |  |  |  |  |  |  |  |  |  |  |  |  |  |  |  |  |  |  |
| --- | --- | --- | --- | --- | --- | --- | --- | --- | --- | --- | --- | --- | --- | --- | --- | --- | --- | --- | --- |

Best Unique PSM (Scan Sample\_7\_A1\_1\_3644.d:50536, m/z=790.4797, z=2, RT=16.18, ppm=-0.56):


TRINITY\_DN1058\_c0\_g1\_i4.p1

back to list

  

| Protein Coverage
| Supporting Peptides
| Best Unique PSM
|

Protein Coverage:

Supporting Peptides:

| Peptide | Uniq | -10lgP | Mass | Length | ppm | m/z | z | RT | 1/k0 Start | 1/k0 End | Precursor ID | Area Sample 8 | #Feature | #Feature Sample 8 | Start | End | PTM | AScore | Found By |
| --- | --- | --- | --- | --- | --- | --- | --- | --- | --- | --- | --- | --- | --- | --- | --- | --- | --- | --- | --- |
| R.APTHILNPANKR.S | Y | 43.8 | 1330.7469 | 12 | 0.61 | 666.3782 | 2 | 11.23 | 1.0057 | 1.013 | 21549 | 1.39e3 | 2 | 2 | 398 | 409 |  |  | DB Search |
| total 1 peptides |  |  |  |  |  |  |  |  |  |  |  |  |  |  |  |  |  |  |  |
| --- | --- | --- | --- | --- | --- | --- | --- | --- | --- | --- | --- | --- | --- | --- | --- | --- | --- | --- | --- |

Best Unique PSM (Scan Sample\_7\_A1\_1\_3644.d:21549, m/z=666.3782, z=2, RT=11.23, ppm=-3.86):


TRINITY\_DN1030\_c0\_g1\_i2.p1

back to list

  

| Protein Coverage
| Supporting Peptides
| Best Unique PSM
|

Protein Coverage:

Supporting Peptides:

| Peptide | Uniq | -10lgP | Mass | Length | ppm | m/z | z | RT | 1/k0 Start | 1/k0 End | Precursor ID | Area Sample 8 | #Feature | #Feature Sample 8 | Start | End | PTM | AScore | Found By |
| --- | --- | --- | --- | --- | --- | --- | --- | --- | --- | --- | --- | --- | --- | --- | --- | --- | --- | --- | --- |
| R.GLQGQQGEQGSTGVQGSVGGR.G | Y | 43.49 | 1985.9515 | 21 | -0.42 | 993.9781 | 2 | 12.09 | 1.1302 | 1.1374 | 26593 | 0 | 0 | 0 | 348 | 368 |  |  | DB Search |
| total 1 peptides |  |  |  |  |  |  |  |  |  |  |  |  |  |  |  |  |  |  |  |
| --- | --- | --- | --- | --- | --- | --- | --- | --- | --- | --- | --- | --- | --- | --- | --- | --- | --- | --- | --- |

Best Unique PSM (Scan Sample\_7\_A1\_1\_3644.d:26593, m/z=993.9781, z=2, RT=12.09, ppm=-4.89):


TRINITY\_DN11248\_c0\_g1\_i3.p1

back to list

  

| Protein Coverage
| Supporting Peptides
| Best Unique PSM
|

Protein Coverage:

Supporting Peptides:

| Peptide | Uniq | -10lgP | Mass | Length | ppm | m/z | z | RT | 1/k0 Start | 1/k0 End | Precursor ID | Area Sample 8 | #Feature | #Feature Sample 8 | Start | End | PTM | AScore | Found By |
| --- | --- | --- | --- | --- | --- | --- | --- | --- | --- | --- | --- | --- | --- | --- | --- | --- | --- | --- | --- |
| K.SIEAIYLGEFGTC(+57.02)K.S | Y | 43.78 | 1586.765 | 14 | -3.85 | 794.3832 | 2 | 19.71 | 1.1009 | 1.1082 | 72286 | 0 | 0 | 0 | 982 | 995 | Carbamidomethylation |  | DB Search |
| total 1 peptides |  |  |  |  |  |  |  |  |  |  |  |  |  |  |  |  |  |  |  |
| --- | --- | --- | --- | --- | --- | --- | --- | --- | --- | --- | --- | --- | --- | --- | --- | --- | --- | --- | --- |

Best Unique PSM (Scan Sample\_7\_A1\_1\_3644.d:72286, m/z=794.3832, z=2, RT=19.71, ppm=-8.32):


TRINITY\_DN785\_c0\_g1\_i1.p1

back to list

  

| Protein Coverage
| Supporting Peptides
| Best Unique PSM
|

Protein Coverage:

Supporting Peptides:

| Peptide | Uniq | -10lgP | Mass | Length | ppm | m/z | z | RT | 1/k0 Start | 1/k0 End | Precursor ID | Area Sample 8 | #Feature | #Feature Sample 8 | Start | End | PTM | AScore | Found By |
| --- | --- | --- | --- | --- | --- | --- | --- | --- | --- | --- | --- | --- | --- | --- | --- | --- | --- | --- | --- |
| K.KFDTYLPGFIC(+57.02)R.C | Y | 43.51 | 1515.7544 | 12 | -2.15 | 758.8795 | 2 | 18.78 | 1.0234 | 1.0307 | 66284 | 3.18e3 | 2 | 2 | 77 | 88 | Carbamidomethylation |  | DB Search |
| R.TMPEQILNERDPLNK.V | Y | 43.31 | 1796.9091 | 15 | 19.81 | 899.4756 | 2 | 16.61 | 1.1386 | 1.1458 | 53016 | 0 | 0 | 0 | 52 | 66 |  |  | DB Search |
| total 2 peptides |  |  |  |  |  |  |  |  |  |  |  |  |  |  |  |  |  |  |  |
| --- | --- | --- | --- | --- | --- | --- | --- | --- | --- | --- | --- | --- | --- | --- | --- | --- | --- | --- | --- |

Best Unique PSM (Scan Sample\_7\_A1\_1\_3644.d:66284, m/z=758.8795, z=2, RT=18.78, ppm=-6.62):


TRINITY\_DN2637\_c0\_g1\_i2.p2

back to list

  

| Protein Coverage
| Supporting Peptides
| Best Unique PSM
|

Protein Coverage:

Supporting Peptides:

| Peptide | Uniq | -10lgP | Mass | Length | ppm | m/z | z | RT | 1/k0 Start | 1/k0 End | Precursor ID | Area Sample 8 | #Feature | #Feature Sample 8 | Start | End | PTM | AScore | Found By |
| --- | --- | --- | --- | --- | --- | --- | --- | --- | --- | --- | --- | --- | --- | --- | --- | --- | --- | --- | --- |
| K.ATIEIPAKYEK.R | Y | 42.98 | 1261.6918 | 11 | 0.3 | 631.8505 | 2 | 14.24 | 0.9848 | 0.992 | 39239 | 0 | 0 | 0 | 107 | 117 |  |  | DB Search |
| total 1 peptides |  |  |  |  |  |  |  |  |  |  |  |  |  |  |  |  |  |  |  |
| --- | --- | --- | --- | --- | --- | --- | --- | --- | --- | --- | --- | --- | --- | --- | --- | --- | --- | --- | --- |

Best Unique PSM (Scan Sample\_7\_A1\_1\_3644.d:39239, m/z=631.8505, z=2, RT=14.24, ppm=-4.18):


TRINITY\_DN2941\_c0\_g1\_i4.p1

back to list

  

| Protein Coverage
| Supporting Peptides
| Best Unique PSM
|

Protein Coverage:

Supporting Peptides:

| Peptide | Uniq | -10lgP | Mass | Length | ppm | m/z | z | RT | 1/k0 Start | 1/k0 End | Precursor ID | Area Sample 8 | #Feature | #Feature Sample 8 | Start | End | PTM | AScore | Found By |
| --- | --- | --- | --- | --- | --- | --- | --- | --- | --- | --- | --- | --- | --- | --- | --- | --- | --- | --- | --- |
| R.VAPEEHPVLLTEAPLNPK.A | Y | 43.24 | 1953.0571 | 18 | -8.51 | 977.5231 | 2 | 16.59 | 1.2073 | 1.2145 | 52921 | 0 | 0 | 0 | 30 | 47 |  |  | DB Search |
| total 1 peptides |  |  |  |  |  |  |  |  |  |  |  |  |  |  |  |  |  |  |  |
| --- | --- | --- | --- | --- | --- | --- | --- | --- | --- | --- | --- | --- | --- | --- | --- | --- | --- | --- | --- |

Best Unique PSM (Scan Sample\_7\_A1\_1\_3644.d:52921, m/z=977.5231, z=2, RT=16.59, ppm=-12.98):


TRINITY\_DN602\_c0\_g1\_i1.p1

back to list

  

| Protein Coverage
| Supporting Peptides
| Best Unique PSM
|

Protein Coverage:

Supporting Peptides:

| Peptide | Uniq | -10lgP | Mass | Length | ppm | m/z | z | RT | 1/k0 Start | 1/k0 End | Precursor ID | Area Sample 8 | #Feature | #Feature Sample 8 | Start | End | PTM | AScore | Found By |
| --- | --- | --- | --- | --- | --- | --- | --- | --- | --- | --- | --- | --- | --- | --- | --- | --- | --- | --- | --- |
| K.EGFYVHEALAAGIVK.K | Y | 43.8 | 1602.8405 | 15 | 3.49 | 802.4268 | 2 | 17.74 | 1.0861 | 1.0933 | 59699 | 0 | 0 | 0 | 185 | 199 |  |  | DB Search |
| K.LQEFGHK.Y | Y | 42.79 | 857.4395 | 7 | 2.94 | 429.7264 | 2 | 11.17 | 0.7913 | 0.7986 | 21333 | 0 | 0 | 0 | 540 | 546 |  |  | DB Search |
| total 2 peptides |  |  |  |  |  |  |  |  |  |  |  |  |  |  |  |  |  |  |  |
| --- | --- | --- | --- | --- | --- | --- | --- | --- | --- | --- | --- | --- | --- | --- | --- | --- | --- | --- | --- |

Best Unique PSM (Scan Sample\_7\_A1\_1\_3644.d:59699, m/z=802.4268, z=2, RT=17.74, ppm=-0.98):


TRINITY\_DN36\_c0\_g1\_i16.p1

back to list

  

| Protein Coverage
| Supporting Peptides
| Best Unique PSM
|

Protein Coverage:

Supporting Peptides:

| Peptide | Uniq | -10lgP | Mass | Length | ppm | m/z | z | RT | 1/k0 Start | 1/k0 End | Precursor ID | Area Sample 8 | #Feature | #Feature Sample 8 | Start | End | PTM | AScore | Found By |
| --- | --- | --- | --- | --- | --- | --- | --- | --- | --- | --- | --- | --- | --- | --- | --- | --- | --- | --- | --- |
| K.FETQIATK.D | Y | 43.56 | 936.4916 | 8 | 1.93 | 937.4965 | 1 | 13.99 | 1.2029 | 1.2101 | 37753 | 0 | 0 | 0 | 167 | 174 |  |  | DB Search |
| R.LGGIEVTQGNC(+57.02)AR.K | Y | 43.02 | 1373.6721 | 13 | 2.68 | 687.8421 | 2 | 13.90 | 0.9662 | 0.9735 | 37137 | 6.95e2 | 1 | 1 | 58 | 70 | Carbamidomethylation |  | DB Search |
| total 2 peptides |  |  |  |  |  |  |  |  |  |  |  |  |  |  |  |  |  |  |  |
| --- | --- | --- | --- | --- | --- | --- | --- | --- | --- | --- | --- | --- | --- | --- | --- | --- | --- | --- | --- |

Best Unique PSM (Scan Sample\_7\_A1\_1\_3644.d:37753, m/z=937.4965, z=1, RT=13.99, ppm=-2.55):


TRINITY\_DN1819\_c0\_g1\_i5.p1

back to list

  

| Protein Coverage
| Supporting Peptides
| Best Unique PSM
|

Protein Coverage:

Supporting Peptides:

| Peptide | Uniq | -10lgP | Mass | Length | ppm | m/z | z | RT | 1/k0 Start | 1/k0 End | Precursor ID | Area Sample 8 | #Feature | #Feature Sample 8 | Start | End | PTM | AScore | Found By |
| --- | --- | --- | --- | --- | --- | --- | --- | --- | --- | --- | --- | --- | --- | --- | --- | --- | --- | --- | --- |
| K.ALADYIHSQDLK.M | Y | 43.6 | 1372.6986 | 12 | 0.78 | 687.3541 | 2 | 15.82 | 1.0061 | 1.0134 | 48440 | 7.24e2 | 1 | 1 | 105 | 116 |  |  | DB Search |
| total 1 peptides |  |  |  |  |  |  |  |  |  |  |  |  |  |  |  |  |  |  |  |
| --- | --- | --- | --- | --- | --- | --- | --- | --- | --- | --- | --- | --- | --- | --- | --- | --- | --- | --- | --- |

Best Unique PSM (Scan Sample\_7\_A1\_1\_3644.d:48440, m/z=687.3541, z=2, RT=15.82, ppm=-3.69):


TRINITY\_DN598\_c1\_g1\_i2.p1

back to list

  

| Protein Coverage
| Supporting Peptides
| Best Unique PSM
|

Protein Coverage:

Supporting Peptides:

| Peptide | Uniq | -10lgP | Mass | Length | ppm | m/z | z | RT | 1/k0 Start | 1/k0 End | Precursor ID | Area Sample 8 | #Feature | #Feature Sample 8 | Start | End | PTM | AScore | Found By |
| --- | --- | --- | --- | --- | --- | --- | --- | --- | --- | --- | --- | --- | --- | --- | --- | --- | --- | --- | --- |
| R.ASFVTDIDRLR.A | Y | 43.77 | 1291.6884 | 11 | 1.29 | 646.8494 | 2 | 17.27 | 0.99 | 0.9973 | 56790 | 1.44e3 | 1 | 1 | 302 | 312 |  |  | DB Search |
| total 1 peptides |  |  |  |  |  |  |  |  |  |  |  |  |  |  |  |  |  |  |  |
| --- | --- | --- | --- | --- | --- | --- | --- | --- | --- | --- | --- | --- | --- | --- | --- | --- | --- | --- | --- |

Best Unique PSM (Scan Sample\_7\_A1\_1\_3644.d:56790, m/z=646.8494, z=2, RT=17.27, ppm=-3.19):


TRINITY\_DN69276\_c0\_g1\_i1.p1

back to list

  

| Protein Coverage
| Supporting Peptides
| Best Unique PSM
|

Protein Coverage:

Supporting Peptides:

| Peptide | Uniq | -10lgP | Mass | Length | ppm | m/z | z | RT | 1/k0 Start | 1/k0 End | Precursor ID | Area Sample 8 | #Feature | #Feature Sample 8 | Start | End | PTM | AScore | Found By |
| --- | --- | --- | --- | --- | --- | --- | --- | --- | --- | --- | --- | --- | --- | --- | --- | --- | --- | --- | --- |
| K.LSDINDNRPIFSR.T | Y | 43.47 | 1545.7899 | 13 | 1.37 | 773.8998 | 2 | 15.11 | 1.0504 | 1.0576 | 44289 | 1.35e3 | 1 | 1 | 511 | 523 |  |  | DB Search |
| total 1 peptides |  |  |  |  |  |  |  |  |  |  |  |  |  |  |  |  |  |  |  |
| --- | --- | --- | --- | --- | --- | --- | --- | --- | --- | --- | --- | --- | --- | --- | --- | --- | --- | --- | --- |

Best Unique PSM (Scan Sample\_7\_A1\_1\_3644.d:44289, m/z=773.8998, z=2, RT=15.11, ppm=-3.1):


TRINITY\_DN1613\_c1\_g1\_i2.p1

back to list

  

| Protein Coverage
| Supporting Peptides
| Best Unique PSM
|

Protein Coverage:

Supporting Peptides:

| Peptide | Uniq | -10lgP | Mass | Length | ppm | m/z | z | RT | 1/k0 Start | 1/k0 End | Precursor ID | Area Sample 8 | #Feature | #Feature Sample 8 | Start | End | PTM | AScore | Found By |
| --- | --- | --- | --- | --- | --- | --- | --- | --- | --- | --- | --- | --- | --- | --- | --- | --- | --- | --- | --- |
| R.TC(+57.02)TNPAPAHGGK.S | Y | 43.53 | 1209.556 | 12 | -0.8 | 605.7821 | 2 | 9.09 | 0.8558 | 0.8631 | 11293 | 0 | 0 | 0 | 413 | 424 | Carbamidomethylation |  | DB Search |
| total 1 peptides |  |  |  |  |  |  |  |  |  |  |  |  |  |  |  |  |  |  |  |
| --- | --- | --- | --- | --- | --- | --- | --- | --- | --- | --- | --- | --- | --- | --- | --- | --- | --- | --- | --- |

Best Unique PSM (Scan Sample\_7\_A1\_1\_3644.d:11293, m/z=605.7821, z=2, RT=9.09, ppm=-5.27):


TRINITY\_DN286\_c0\_g1\_i4.p1

back to list

  

| Protein Coverage
| Supporting Peptides
| Best Unique PSM
|

Protein Coverage:

Supporting Peptides:

| Peptide | Uniq | -10lgP | Mass | Length | ppm | m/z | z | RT | 1/k0 Start | 1/k0 End | Precursor ID | Area Sample 8 | #Feature | #Feature Sample 8 | Start | End | PTM | AScore | Found By |
| --- | --- | --- | --- | --- | --- | --- | --- | --- | --- | --- | --- | --- | --- | --- | --- | --- | --- | --- | --- |
| K.RYGQAMC(+57.02)QFMR.N | Y | 43.72 | 1446.6319 | 11 | -7.64 | 724.3145 | 2 | 17.46 | 0.988 | 0.9953 | 57985 | 5.65e2 | 1 | 1 | 71 | 81 | Carbamidomethylation |  | DB Search |
| total 1 peptides |  |  |  |  |  |  |  |  |  |  |  |  |  |  |  |  |  |  |  |
| --- | --- | --- | --- | --- | --- | --- | --- | --- | --- | --- | --- | --- | --- | --- | --- | --- | --- | --- | --- |

Best Unique PSM (Scan Sample\_7\_A1\_1\_3644.d:57985, m/z=724.3145, z=2, RT=17.46, ppm=-12.12):


TRINITY\_DN3180\_c0\_g1\_i1.p1

back to list

  

| Protein Coverage
| Supporting Peptides
| Best Unique PSM
|

Protein Coverage:

Supporting Peptides:

| Peptide | Uniq | -10lgP | Mass | Length | ppm | m/z | z | RT | 1/k0 Start | 1/k0 End | Precursor ID | Area Sample 8 | #Feature | #Feature Sample 8 | Start | End | PTM | AScore | Found By |
| --- | --- | --- | --- | --- | --- | --- | --- | --- | --- | --- | --- | --- | --- | --- | --- | --- | --- | --- | --- |
| K.IHVQSFLNNHFGGK.N | Y | 43.48 | 1596.8161 | 14 | -0.58 | 533.2766 | 3 | 16.04 | 0.8161 | 0.8234 | 49749 | 0 | 0 | 0 | 100 | 113 |  |  | DB Search |
| total 1 peptides |  |  |  |  |  |  |  |  |  |  |  |  |  |  |  |  |  |  |  |
| --- | --- | --- | --- | --- | --- | --- | --- | --- | --- | --- | --- | --- | --- | --- | --- | --- | --- | --- | --- |

Best Unique PSM (Scan Sample\_7\_A1\_1\_3644.d:49749, m/z=533.2766, z=3, RT=16.04, ppm=-5.06):


TRINITY\_DN495\_c0\_g1\_i5.p1

back to list

  

| Protein Coverage
| Supporting Peptides
| Best Unique PSM
|

Protein Coverage:

Supporting Peptides:

| Peptide | Uniq | -10lgP | Mass | Length | ppm | m/z | z | RT | 1/k0 Start | 1/k0 End | Precursor ID | Area Sample 8 | #Feature | #Feature Sample 8 | Start | End | PTM | AScore | Found By |
| --- | --- | --- | --- | --- | --- | --- | --- | --- | --- | --- | --- | --- | --- | --- | --- | --- | --- | --- | --- |
| C.SISC(+57.02)SKDEEIK.V | Y | 43.76 | 1294.6075 | 11 | 2.25 | 648.3096 | 2 | 10.92 | 0.9755 | 0.9828 | 19848 | 1.92e3 | 1 | 1 | 273 | 283 | Carbamidomethylation |  | DB Search |
| R.TAGDVC(+57.02)GR.S | Y | 43.57 | 834.3654 | 8 | 3.61 | 418.1896 | 2 | 9.57 | 0.7518 | 0.7591 | 12879 | 0 | 0 | 0 | 292 | 299 | Carbamidomethylation |  | DB Search |
| total 2 peptides |  |  |  |  |  |  |  |  |  |  |  |  |  |  |  |  |  |  |  |
| --- | --- | --- | --- | --- | --- | --- | --- | --- | --- | --- | --- | --- | --- | --- | --- | --- | --- | --- | --- |

Best Unique PSM (Scan Sample\_7\_A1\_1\_3644.d:19848, m/z=648.3096, z=2, RT=10.92, ppm=-2.22):


TRINITY\_DN13813\_c0\_g1\_i6.p2

back to list

  

| Protein Coverage
| Supporting Peptides
| Best Unique PSM
|

Protein Coverage:

Supporting Peptides:

| Peptide | Uniq | -10lgP | Mass | Length | ppm | m/z | z | RT | 1/k0 Start | 1/k0 End | Precursor ID | Area Sample 8 | #Feature | #Feature Sample 8 | Start | End | PTM | AScore | Found By |
| --- | --- | --- | --- | --- | --- | --- | --- | --- | --- | --- | --- | --- | --- | --- | --- | --- | --- | --- | --- |
| R.DGPGPLASDLLLQMR.Q | Y | 41.47 | 1581.8184 | 15 | -2.93 | 528.2762 | 3 | 19.62 | 0.8149 | 0.8222 | 71531 | 3.73e2 | 1 | 1 | 308 | 322 |  |  | DB Search |
| total 1 peptides |  |  |  |  |  |  |  |  |  |  |  |  |  |  |  |  |  |  |  |
| --- | --- | --- | --- | --- | --- | --- | --- | --- | --- | --- | --- | --- | --- | --- | --- | --- | --- | --- | --- |

Best Unique PSM (Scan Sample\_7\_A1\_1\_3644.d:71531, m/z=528.2762, z=3, RT=19.62, ppm=-7.41):


TRINITY\_DN1819\_c0\_g1\_i7.p1

back to list

  

| Protein Coverage
| Supporting Peptides
| Best Unique PSM
|

Protein Coverage:

Supporting Peptides:

| Peptide | Uniq | -10lgP | Mass | Length | ppm | m/z | z | RT | 1/k0 Start | 1/k0 End | Precursor ID | Area Sample 8 | #Feature | #Feature Sample 8 | Start | End | PTM | AScore | Found By |
| --- | --- | --- | --- | --- | --- | --- | --- | --- | --- | --- | --- | --- | --- | --- | --- | --- | --- | --- | --- |
| K.ALADYIHSQDLK.M | Y | 43.6 | 1372.6986 | 12 | 0.78 | 687.3541 | 2 | 15.82 | 1.0061 | 1.0134 | 48440 | 7.24e2 | 1 | 1 | 105 | 116 |  |  | DB Search |
| total 1 peptides |  |  |  |  |  |  |  |  |  |  |  |  |  |  |  |  |  |  |  |
| --- | --- | --- | --- | --- | --- | --- | --- | --- | --- | --- | --- | --- | --- | --- | --- | --- | --- | --- | --- |

Best Unique PSM (Scan Sample\_7\_A1\_1\_3644.d:48440, m/z=687.3541, z=2, RT=15.82, ppm=-3.69):


TRINITY\_DN554\_c0\_g1\_i3.p2

back to list

  

| Protein Coverage
| Supporting Peptides
| Best Unique PSM
|

Protein Coverage:

Supporting Peptides:

| Peptide | Uniq | -10lgP | Mass | Length | ppm | m/z | z | RT | 1/k0 Start | 1/k0 End | Precursor ID | Area Sample 8 | #Feature | #Feature Sample 8 | Start | End | PTM | AScore | Found By |
| --- | --- | --- | --- | --- | --- | --- | --- | --- | --- | --- | --- | --- | --- | --- | --- | --- | --- | --- | --- |
| K.TITLEVEPSDTIENVK.S | Y | 43.28 | 1786.92 | 16 | 2.71 | 894.4657 | 2 | 17.62 | 1.1454 | 1.1526 | 58818 | 1.46e3 | 1 | 1 | 12 | 27 |  |  | DB Search |
| total 1 peptides |  |  |  |  |  |  |  |  |  |  |  |  |  |  |  |  |  |  |  |
| --- | --- | --- | --- | --- | --- | --- | --- | --- | --- | --- | --- | --- | --- | --- | --- | --- | --- | --- | --- |

Best Unique PSM (Scan Sample\_7\_A1\_1\_3644.d:58818, m/z=894.4657, z=2, RT=17.62, ppm=-1.76):


TRINITY\_DN4595\_c0\_g1\_i10.p1

back to list

  

| Protein Coverage
| Supporting Peptides
| Best Unique PSM
|

Protein Coverage:

Supporting Peptides:

| Peptide | Uniq | -10lgP | Mass | Length | ppm | m/z | z | RT | 1/k0 Start | 1/k0 End | Precursor ID | Area Sample 8 | #Feature | #Feature Sample 8 | Start | End | PTM | AScore | Found By |
| --- | --- | --- | --- | --- | --- | --- | --- | --- | --- | --- | --- | --- | --- | --- | --- | --- | --- | --- | --- |
| R.FC(+57.02)NNPAPSNGGR.M | Y | 43.83 | 1289.5571 | 12 | 3.53 | 645.7852 | 2 | 10.94 | 0.9449 | 0.9521 | 20039 | 4e2 | 1 | 1 | 745 | 756 | Carbamidomethylation |  | DB Search |
| K.YTGAC(+57.02)NANGNTDK.T | Y | 43.7 | 1384.5677 | 13 | 5.35 | 693.2917 | 2 | 9.59 | 0.9287 | 0.936 | 12941 | 0 | 0 | 0 | 1041 | 1053 | Carbamidomethylation |  | DB Search |
| total 2 peptides |  |  |  |  |  |  |  |  |  |  |  |  |  |  |  |  |  |  |  |
| --- | --- | --- | --- | --- | --- | --- | --- | --- | --- | --- | --- | --- | --- | --- | --- | --- | --- | --- | --- |

Best Unique PSM (Scan Sample\_7\_A1\_1\_3644.d:20039, m/z=645.7852, z=2, RT=10.94, ppm=-0.95):


TRINITY\_DN942\_c0\_g1\_i3.p1

back to list

  

| Protein Coverage
| Supporting Peptides
| Best Unique PSM
|

Protein Coverage:

Supporting Peptides:

| Peptide | Uniq | -10lgP | Mass | Length | ppm | m/z | z | RT | 1/k0 Start | 1/k0 End | Precursor ID | Area Sample 8 | #Feature | #Feature Sample 8 | Start | End | PTM | AScore | Found By |
| --- | --- | --- | --- | --- | --- | --- | --- | --- | --- | --- | --- | --- | --- | --- | --- | --- | --- | --- | --- |
| K.SGVC(+57.02)TDANTR.D | Y | 43.32 | 1079.4666 | 10 | 12.92 | 540.7451 | 2 | 10.11 | 0.8445 | 0.8518 | 15427 | 0 | 0 | 0 | 359 | 368 | Carbamidomethylation |  | DB Search |
| K.AALNM(+15.99)EFEYIPTK.S | Y | 42.87 | 1541.7436 | 13 | -3.63 | 771.8728 | 2 | 17.58 | 1.0528 | 1.06 | 58710 | 2.11e2 | 1 | 1 | 1659 | 1671 | Oxidation (M) | M5:Oxidation (M):1000 | DB Search |
| total 2 peptides |  |  |  |  |  |  |  |  |  |  |  |  |  |  |  |  |  |  |  |
| --- | --- | --- | --- | --- | --- | --- | --- | --- | --- | --- | --- | --- | --- | --- | --- | --- | --- | --- | --- |

Best Unique PSM (Scan Sample\_7\_A1\_1\_3644.d:15427, m/z=540.7451, z=2, RT=10.11, ppm=8.45):


TRINITY\_DN3036\_c0\_g1\_i16.p1

back to list

  

| Protein Coverage
| Supporting Peptides
| Best Unique PSM
|

Protein Coverage:

Supporting Peptides:

| Peptide | Uniq | -10lgP | Mass | Length | ppm | m/z | z | RT | 1/k0 Start | 1/k0 End | Precursor ID | Area Sample 8 | #Feature | #Feature Sample 8 | Start | End | PTM | AScore | Found By |
| --- | --- | --- | --- | --- | --- | --- | --- | --- | --- | --- | --- | --- | --- | --- | --- | --- | --- | --- | --- |
| R.AGHGAVTSPQTQR.G | Y | 43.55 | 1308.6534 | 13 | 3.24 | 437.2245 | 3 | 9.76 | 0.7045 | 0.7118 | 13596 | 2.79e3 | 2 | 2 | 135 | 147 |  |  | DB Search |
| R.VIGASGIHVGTM(+15.99)SFGK.M | Y | 43.2 | 1575.8079 | 16 | 0.89 | 788.9084 | 2 | 15.29 | 1.129 | 1.1362 | 45505 | 0 | 0 | 0 | 160 | 175 | Oxidation (M) | M12:Oxidation (M):1000 | DB Search |
| total 2 peptides |  |  |  |  |  |  |  |  |  |  |  |  |  |  |  |  |  |  |  |
| --- | --- | --- | --- | --- | --- | --- | --- | --- | --- | --- | --- | --- | --- | --- | --- | --- | --- | --- | --- |

Best Unique PSM (Scan Sample\_7\_A1\_1\_3644.d:13596, m/z=437.2245, z=3, RT=9.76, ppm=-1.24):


TRINITY\_DN135129\_c0\_g1\_i1.p1

back to list

  

| Protein Coverage
| Supporting Peptides
| Best Unique PSM
|

Protein Coverage:

Supporting Peptides:

| Peptide | Uniq | -10lgP | Mass | Length | ppm | m/z | z | RT | 1/k0 Start | 1/k0 End | Precursor ID | Area Sample 8 | #Feature | #Feature Sample 8 | Start | End | PTM | AScore | Found By |
| --- | --- | --- | --- | --- | --- | --- | --- | --- | --- | --- | --- | --- | --- | --- | --- | --- | --- | --- | --- |
| K.SDSSDIEKR.A | Y | 43.41 | 1035.4832 | 9 | -11.91 | 518.7404 | 2 | 11.15 | 0.8607 | 0.868 | 21197 | 4.09e3 | 1 | 1 | 96 | 104 |  |  | DB Search |
| total 1 peptides |  |  |  |  |  |  |  |  |  |  |  |  |  |  |  |  |  |  |  |
| --- | --- | --- | --- | --- | --- | --- | --- | --- | --- | --- | --- | --- | --- | --- | --- | --- | --- | --- | --- |

Best Unique PSM (Scan Sample\_7\_A1\_1\_3644.d:21197, m/z=518.7404, z=2, RT=11.15, ppm=-16.38):


TRINITY\_DN982\_c0\_g1\_i3.p1

back to list

  

| Protein Coverage
| Supporting Peptides
| Best Unique PSM
|

Protein Coverage:

Supporting Peptides:

| Peptide | Uniq | -10lgP | Mass | Length | ppm | m/z | z | RT | 1/k0 Start | 1/k0 End | Precursor ID | Area Sample 8 | #Feature | #Feature Sample 8 | Start | End | PTM | AScore | Found By |
| --- | --- | --- | --- | --- | --- | --- | --- | --- | --- | --- | --- | --- | --- | --- | --- | --- | --- | --- | --- |
| R.VLATDKDTLSSNK.Q | Y | 43.8 | 1390.7303 | 13 | -0.28 | 696.3691 | 2 | 11.58 | 1.0134 | 1.0206 | 23612 | 0 | 0 | 0 | 3827 | 3839 |  |  | DB Search |
| K.VLLSQQIFYDKK.N | N | 43.76 | 1480.8289 | 12 | 0.2 | 741.4186 | 2 | 16.98 | 1.0632 | 1.0705 | 54998 | 6.1e3 | 2 | 2 | 4919 | 4930 |  |  | DB Search |
| K.YVAIYPSDHLTVNR.D | N | 43.74 | 1646.8416 | 14 | -4.41 | 824.4208 | 2 | 15.73 | 1.1094 | 1.1166 | 47842 | 6.9e2 | 1 | 1 | 6254 | 6267 |  |  | DB Search |
| K.APIGQSIVTVSAR.D | Y | 43.72 | 1297.7354 | 13 | 0.4 | 649.8723 | 2 | 14.27 | 0.9707 | 0.9779 | 39470 | 0 | 0 | 0 | 4029 | 4041 |  |  | DB Search |
| R.YTSC(+57.02)QSVTVVASDR.S | N | 43.65 | 1571.725 | 14 | -1.13 | 786.8654 | 2 | 13.49 | 1.05 | 1.0572 | 34821 | 0 | 0 | 0 | 9363 | 9376 | Carbamidomethylation |  | DB Search |
| K.SSNTIEENRLK.Y | Y | 43.56 | 1289.6575 | 11 | 2.52 | 645.8348 | 2 | 11.51 | 0.9574 | 0.9646 | 23219 | 0 | 0 | 0 | 783 | 793 |  |  | DB Search |
| R.TNNALDYEAKR.Q | N | 43.54 | 1293.6313 | 11 | 0.43 | 647.8203 | 2 | 12.12 | 0.965 | 0.9723 | 26731 | 1.84e3 | 1 | 1 | 7725 | 7735 |  |  | DB Search |
| K.TSTQDVALNIRR.S | N | 43.43 | 1372.7423 | 12 | 2.89 | 687.3773 | 2 | 13.63 | 0.9997 | 1.0069 | 35508 | 2.89e3 | 1 | 1 | 9063 | 9074 |  |  | DB Search |
| R.LVQNTPHNSIVTK.I | N | 43.35 | 1449.794 | 13 | 1.92 | 725.9024 | 2 | 11.76 | 1.0564 | 1.0636 | 24648 | 1.37e3 | 1 | 1 | 8569 | 8581 |  |  | DB Search |
| K.TGAISLLASVDFETDRTHTAVVK.A | Y | 42.86 | 2430.2754 | 23 | 7.32 | 811.1014 | 3 | 19.05 | 1.0419 | 1.0492 | 68211 | 0 | 0 | 0 | 3335 | 3357 |  |  | DB Search |
| total 10 peptides |  |  |  |  |  |  |  |  |  |  |  |  |  |  |  |  |  |  |  |
| --- | --- | --- | --- | --- | --- | --- | --- | --- | --- | --- | --- | --- | --- | --- | --- | --- | --- | --- | --- |

Best Unique PSM (Scan Sample\_7\_A1\_1\_3644.d:23612, m/z=696.3691, z=2, RT=11.58, ppm=-4.76):


TRINITY\_DN240\_c0\_g1\_i6.p1

back to list

  

| Protein Coverage
| Supporting Peptides
| Best Unique PSM
|

Protein Coverage:

Supporting Peptides:

| Peptide | Uniq | -10lgP | Mass | Length | ppm | m/z | z | RT | 1/k0 Start | 1/k0 End | Precursor ID | Area Sample 8 | #Feature | #Feature Sample 8 | Start | End | PTM | AScore | Found By |
| --- | --- | --- | --- | --- | --- | --- | --- | --- | --- | --- | --- | --- | --- | --- | --- | --- | --- | --- | --- |
| K.AYAAFLEFKDVVK.A | Y | 43.79 | 1499.8024 | 13 | 3.07 | 750.9074 | 2 | 19.27 | 1.0953 | 1.1025 | 69440 | 1.07e4 | 2 | 2 | 217 | 229 |  |  | DB Search |
| K.AISSASGPTGM(+15.99)TSK.A | Y | 43.77 | 1309.6184 | 14 | 1.88 | 655.8148 | 2 | 10.56 | 0.9319 | 0.9392 | 17892 | 3.72e3 | 1 | 1 | 151 | 164 | Oxidation (M) | M11:Oxidation (M):1000 | DB Search |
| K.ANQVSSAAAAPVPAAGGDK.I | Y | 43.76 | 1680.8431 | 19 | -0.14 | 841.4249 | 2 | 12.55 | 1.0656 | 1.0729 | 29279 | 4.54e3 | 1 | 1 | 230 | 248 |  |  | DB Search |
| K.IGAAAQALADQSYPFLK.S | Y | 43.73 | 1762.9253 | 17 | 0.62 | 882.4666 | 2 | 19.27 | 1.1574 | 1.1646 | 68962 | 5.17e3 | 2 | 2 | 249 | 265 |  |  | DB Search |
| K.AAASAHHQAIGSIDAAGVTSAADYASVTAALGR.A | Y | 43.16 | 3080.5275 | 33 | -1.21 | 771.1348 | 4 | 18.34 | 1.0652 | 1.0725 | 63473 | 9.02e3 | 1 | 1 | 307 | 339 |  |  | DB Search |
| K.SVDWTSDLYLKPLPGVSAAK.A | Y | 42.6 | 2146.131 | 20 | 3.72 | 716.3837 | 3 | 19.68 | 1.013 | 1.0202 | 72078 | 4.11e2 | 1 | 1 | 266 | 285 |  |  | DB Search |
| K.AIVMGSAMDGNLLK.A | Y | 42.42 | 1418.7261 | 14 | 3.64 | 710.3698 | 2 | 18.29 | 1.0174 | 1.0246 | 63333 | 0 | 0 | 0 | 293 | 306 |  |  | DB Search |
| total 7 peptides |  |  |  |  |  |  |  |  |  |  |  |  |  |  |  |  |  |  |  |
| --- | --- | --- | --- | --- | --- | --- | --- | --- | --- | --- | --- | --- | --- | --- | --- | --- | --- | --- | --- |

Best Unique PSM (Scan Sample\_7\_A1\_1\_3644.d:69440, m/z=750.9074, z=2, RT=19.27, ppm=-1.4):


TRINITY\_DN250\_c0\_g1\_i5.p1

back to list

  

| Protein Coverage
| Supporting Peptides
| Best Unique PSM
|

Protein Coverage:

Supporting Peptides:

| Peptide | Uniq | -10lgP | Mass | Length | ppm | m/z | z | RT | 1/k0 Start | 1/k0 End | Precursor ID | Area Sample 8 | #Feature | #Feature Sample 8 | Start | End | PTM | AScore | Found By |
| --- | --- | --- | --- | --- | --- | --- | --- | --- | --- | --- | --- | --- | --- | --- | --- | --- | --- | --- | --- |
| K.VGINYQPPTVVPGGDLAK.V | Y | 43.58 | 1823.9781 | 18 | 1.41 | 912.9935 | 2 | 17.42 | 1.1214 | 1.1286 | 57750 | 0 | 0 | 0 | 353 | 370 |  |  | DB Search |
| R.AVC(+57.02)M(+15.99)LSNTTAIAEAWAR.L | Y | 43.51 | 1879.892 | 17 | 2.8 | 940.9517 | 2 | 18.84 | 1.1454 | 1.1526 | 66760 | 4.63e2 | 1 | 1 | 374 | 390 | Carbamidomethylation, Oxidation (M) | M4:Oxidation (M):1000 | DB Search |
| total 2 peptides |  |  |  |  |  |  |  |  |  |  |  |  |  |  |  |  |  |  |  |
| --- | --- | --- | --- | --- | --- | --- | --- | --- | --- | --- | --- | --- | --- | --- | --- | --- | --- | --- | --- |

Best Unique PSM (Scan Sample\_7\_A1\_1\_3644.d:57750, m/z=912.9935, z=2, RT=17.42, ppm=-3.06):


TRINITY\_DN31399\_c0\_g1\_i3.p1

back to list

  

| Protein Coverage
| Supporting Peptides
| Best Unique PSM
|

Protein Coverage:

Supporting Peptides:

| Peptide | Uniq | -10lgP | Mass | Length | ppm | m/z | z | RT | 1/k0 Start | 1/k0 End | Precursor ID | Area Sample 8 | #Feature | #Feature Sample 8 | Start | End | PTM | AScore | Found By |
| --- | --- | --- | --- | --- | --- | --- | --- | --- | --- | --- | --- | --- | --- | --- | --- | --- | --- | --- | --- |
| K.DTLVFEIDEIRK.S | Y | 42.83 | 1476.7824 | 12 | 2.38 | 739.3969 | 2 | 18.67 | 1.0359 | 1.0431 | 65730 | 1.13e3 | 1 | 1 | 390 | 401 |  |  | DB Search |
| total 1 peptides |  |  |  |  |  |  |  |  |  |  |  |  |  |  |  |  |  |  |  |
| --- | --- | --- | --- | --- | --- | --- | --- | --- | --- | --- | --- | --- | --- | --- | --- | --- | --- | --- | --- |

Best Unique PSM (Scan Sample\_7\_A1\_1\_3644.d:65730, m/z=739.3969, z=2, RT=18.67, ppm=-2.1):


TRINITY\_DN160\_c0\_g1\_i1.p1

back to list

  

| Protein Coverage
| Supporting Peptides
| Best Unique PSM
|

Protein Coverage:

Supporting Peptides:

| Peptide | Uniq | -10lgP | Mass | Length | ppm | m/z | z | RT | 1/k0 Start | 1/k0 End | Precursor ID | Area Sample 8 | #Feature | #Feature Sample 8 | Start | End | PTM | AScore | Found By |
| --- | --- | --- | --- | --- | --- | --- | --- | --- | --- | --- | --- | --- | --- | --- | --- | --- | --- | --- | --- |
| K.ELVMHEQSR.C | Y | 43.82 | 1127.5393 | 9 | -5.85 | 564.7711 | 2 | 11.37 | 0.8988 | 0.9061 | 22493 | 0 | 0 | 0 | 1116 | 1124 |  |  | DB Search |
| K.VNQAC(+57.02)VR.T | Y | 43.66 | 845.4178 | 7 | 1.37 | 423.7148 | 2 | 9.80 | 0.7644 | 0.7718 | 13832 | 6.46e3 | 1 | 1 | 871 | 877 | Carbamidomethylation |  | DB Search |
| K.DLEFKFR.E | Y | 43.5 | 953.497 | 7 | -2.68 | 477.7524 | 2 | 16.87 | 0.8323 | 0.8396 | 54470 | 8.25e2 | 1 | 1 | 1031 | 1037 |  |  | DB Search |
| K.LRLDFDHEAKLNMIFR.F | Y | 43.4 | 2017.0567 | 16 | -0.24 | 505.2691 | 4 | 18.67 | 0.8623 | 0.8696 | 65767 | 0 | 0 | 0 | 1007 | 1022 |  |  | DB Search |
| K.LRLDFDHEAK.L | Y | 43.38 | 1242.6356 | 10 | 5.04 | 415.2194 | 3 | 14.38 | 0.7204 | 0.7278 | 40014 | 6.38e3 | 1 | 1 | 1007 | 1016 |  |  | DB Search |
| R.LDFDHEAKLNMIFR.F | Y | 43.36 | 1747.8715 | 14 | -6.24 | 583.6249 | 3 | 18.55 | 0.8562 | 0.8635 | 64942 | 0 | 0 | 0 | 1009 | 1022 |  |  | DB Search |
| K.LRLDFDHEAKLNM(+15.99)IFR.F | Y | 43.21 | 2033.0516 | 16 | -2.09 | 509.2668 | 4 | 17.98 | 0.8554 | 0.8627 | 61282 | 0 | 0 | 0 | 1007 | 1022 | Oxidation (M) | M13:Oxidation (M):1000 | DB Search |
| K.ATSC(+57.02)AVYPC(+57.02)EINSPR.C | Y | 43.02 | 1723.7658 | 15 | 6.64 | 862.892 | 2 | 13.94 | 1.0985 | 1.1057 | 37509 | 0 | 0 | 0 | 512 | 526 | Carbamidomethylation, Carbamidomethylation |  | DB Search |
| total 8 peptides |  |  |  |  |  |  |  |  |  |  |  |  |  |  |  |  |  |  |  |
| --- | --- | --- | --- | --- | --- | --- | --- | --- | --- | --- | --- | --- | --- | --- | --- | --- | --- | --- | --- |

Best Unique PSM (Scan Sample\_7\_A1\_1\_3644.d:22493, m/z=564.7711, z=2, RT=11.37, ppm=-10.33):


TRINITY\_DN3036\_c0\_g1\_i11.p1

back to list

  

| Protein Coverage
| Supporting Peptides
| Best Unique PSM
|

Protein Coverage:

Supporting Peptides:

| Peptide | Uniq | -10lgP | Mass | Length | ppm | m/z | z | RT | 1/k0 Start | 1/k0 End | Precursor ID | Area Sample 8 | #Feature | #Feature Sample 8 | Start | End | PTM | AScore | Found By |
| --- | --- | --- | --- | --- | --- | --- | --- | --- | --- | --- | --- | --- | --- | --- | --- | --- | --- | --- | --- |
| R.AGHGAVTSPQTQR.G | Y | 43.55 | 1308.6534 | 13 | 3.24 | 437.2245 | 3 | 9.76 | 0.7045 | 0.7118 | 13596 | 2.79e3 | 2 | 2 | 135 | 147 |  |  | DB Search |
| R.VIGASGIHVGTM(+15.99)SFGK.M | Y | 43.2 | 1575.8079 | 16 | 0.89 | 788.9084 | 2 | 15.29 | 1.129 | 1.1362 | 45505 | 0 | 0 | 0 | 160 | 175 | Oxidation (M) | M12:Oxidation (M):1000 | DB Search |
| total 2 peptides |  |  |  |  |  |  |  |  |  |  |  |  |  |  |  |  |  |  |  |
| --- | --- | --- | --- | --- | --- | --- | --- | --- | --- | --- | --- | --- | --- | --- | --- | --- | --- | --- | --- |

Best Unique PSM (Scan Sample\_7\_A1\_1\_3644.d:13596, m/z=437.2245, z=3, RT=9.76, ppm=-1.24):


TRINITY\_DN602\_c0\_g1\_i3.p1

back to list

  

| Protein Coverage
| Supporting Peptides
| Best Unique PSM
|

Protein Coverage:

Supporting Peptides:

| Peptide | Uniq | -10lgP | Mass | Length | ppm | m/z | z | RT | 1/k0 Start | 1/k0 End | Precursor ID | Area Sample 8 | #Feature | #Feature Sample 8 | Start | End | PTM | AScore | Found By |
| --- | --- | --- | --- | --- | --- | --- | --- | --- | --- | --- | --- | --- | --- | --- | --- | --- | --- | --- | --- |
| K.EGFYVHEALAAGIVK.K | Y | 43.8 | 1602.8405 | 15 | 3.49 | 802.4268 | 2 | 17.74 | 1.0861 | 1.0933 | 59699 | 0 | 0 | 0 | 443 | 457 |  |  | DB Search |
| K.LQEFGHK.Y | Y | 42.79 | 857.4395 | 7 | 2.94 | 429.7264 | 2 | 11.17 | 0.7913 | 0.7986 | 21333 | 0 | 0 | 0 | 798 | 804 |  |  | DB Search |
| total 2 peptides |  |  |  |  |  |  |  |  |  |  |  |  |  |  |  |  |  |  |  |
| --- | --- | --- | --- | --- | --- | --- | --- | --- | --- | --- | --- | --- | --- | --- | --- | --- | --- | --- | --- |

Best Unique PSM (Scan Sample\_7\_A1\_1\_3644.d:59699, m/z=802.4268, z=2, RT=17.74, ppm=-0.98):


TRINITY\_DN1936\_c0\_g1\_i4.p1

back to list

  

| Protein Coverage
| Supporting Peptides
| Best Unique PSM
|

Protein Coverage:

Supporting Peptides:

| Peptide | Uniq | -10lgP | Mass | Length | ppm | m/z | z | RT | 1/k0 Start | 1/k0 End | Precursor ID | Area Sample 8 | #Feature | #Feature Sample 8 | Start | End | PTM | AScore | Found By |
| --- | --- | --- | --- | --- | --- | --- | --- | --- | --- | --- | --- | --- | --- | --- | --- | --- | --- | --- | --- |
| R.SSLSTSSSGTGPK.T | Y | 42.7 | 1194.5728 | 13 | -2.94 | 598.2892 | 2 | 10.42 | 0.9133 | 0.9206 | 17169 | 0 | 0 | 0 | 805 | 817 |  |  | DB Search |
| total 1 peptides |  |  |  |  |  |  |  |  |  |  |  |  |  |  |  |  |  |  |  |
| --- | --- | --- | --- | --- | --- | --- | --- | --- | --- | --- | --- | --- | --- | --- | --- | --- | --- | --- | --- |

Best Unique PSM (Scan Sample\_7\_A1\_1\_3644.d:17169, m/z=598.2892, z=2, RT=10.42, ppm=-7.42):


TRINITY\_DN477\_c0\_g1\_i7.p1

back to list

  

| Protein Coverage
| Supporting Peptides
| Best Unique PSM
|

Protein Coverage:

Supporting Peptides:

| Peptide | Uniq | -10lgP | Mass | Length | ppm | m/z | z | RT | 1/k0 Start | 1/k0 End | Precursor ID | Area Sample 8 | #Feature | #Feature Sample 8 | Start | End | PTM | AScore | Found By |
| --- | --- | --- | --- | --- | --- | --- | --- | --- | --- | --- | --- | --- | --- | --- | --- | --- | --- | --- | --- |
| R.NDVNNPPQAR.R | Y | 43.84 | 1123.537 | 10 | 3.77 | 562.7754 | 2 | 10.18 | 0.8676 | 0.8749 | 15798 | 1.5e4 | 1 | 1 | 1049 | 1058 |  |  | DB Search |
| R.ILQC(+57.02)NQYANVEEAR.N | Y | 43.82 | 1706.8046 | 14 | 3.36 | 854.4086 | 2 | 14.34 | 1.0941 | 1.1013 | 39739 | 5.68e3 | 1 | 1 | 86 | 99 | Carbamidomethylation |  | DB Search |
| R.SQSAFPTRR.R | Y | 43.75 | 1048.5414 | 9 | 2.01 | 525.2767 | 2 | 11.08 | 0.8526 | 0.8599 | 20720 | 4.77e3 | 1 | 1 | 856 | 864 |  |  | DB Search |
| K.DKNILVLTLGVGPQVNR.N | Y | 43.64 | 1835.0628 | 17 | 2.21 | 918.5366 | 2 | 19.60 | 1.1334 | 1.1406 | 71277 | 2.86e3 | 1 | 1 | 168 | 184 |  |  | DB Search |
| total 4 peptides |  |  |  |  |  |  |  |  |  |  |  |  |  |  |  |  |  |  |  |
| --- | --- | --- | --- | --- | --- | --- | --- | --- | --- | --- | --- | --- | --- | --- | --- | --- | --- | --- | --- |

Best Unique PSM (Scan Sample\_7\_A1\_1\_3644.d:15798, m/z=562.7754, z=2, RT=10.18, ppm=-0.7):


TRINITY\_DN6428\_c0\_g1\_i2.p1

back to list

  

| Protein Coverage
| Supporting Peptides
| Best Unique PSM
|

Protein Coverage:

Supporting Peptides:

| Peptide | Uniq | -10lgP | Mass | Length | ppm | m/z | z | RT | 1/k0 Start | 1/k0 End | Precursor ID | Area Sample 8 | #Feature | #Feature Sample 8 | Start | End | PTM | AScore | Found By |
| --- | --- | --- | --- | --- | --- | --- | --- | --- | --- | --- | --- | --- | --- | --- | --- | --- | --- | --- | --- |
| R.SC(+57.02)ENPKPLNGGK.Q | Y | 43.81 | 1299.6241 | 12 | 4.1 | 650.8191 | 2 | 11.12 | 0.9509 | 0.9582 | 20845 | 1.35e3 | 1 | 1 | 181 | 192 | Carbamidomethylation |  | DB Search |
| total 1 peptides |  |  |  |  |  |  |  |  |  |  |  |  |  |  |  |  |  |  |  |
| --- | --- | --- | --- | --- | --- | --- | --- | --- | --- | --- | --- | --- | --- | --- | --- | --- | --- | --- | --- |

Best Unique PSM (Scan Sample\_7\_A1\_1\_3644.d:20845, m/z=650.8191, z=2, RT=11.12, ppm=-0.37):


TRINITY\_DN477\_c0\_g1\_i6.p1

back to list

  

| Protein Coverage
| Supporting Peptides
| Best Unique PSM
|

Protein Coverage:

Supporting Peptides:

| Peptide | Uniq | -10lgP | Mass | Length | ppm | m/z | z | RT | 1/k0 Start | 1/k0 End | Precursor ID | Area Sample 8 | #Feature | #Feature Sample 8 | Start | End | PTM | AScore | Found By |
| --- | --- | --- | --- | --- | --- | --- | --- | --- | --- | --- | --- | --- | --- | --- | --- | --- | --- | --- | --- |
| R.NDVNNPPQAR.R | Y | 43.84 | 1123.537 | 10 | 3.77 | 562.7754 | 2 | 10.18 | 0.8676 | 0.8749 | 15798 | 1.5e4 | 1 | 1 | 86 | 95 |  |  | DB Search |
| total 1 peptides |  |  |  |  |  |  |  |  |  |  |  |  |  |  |  |  |  |  |  |
| --- | --- | --- | --- | --- | --- | --- | --- | --- | --- | --- | --- | --- | --- | --- | --- | --- | --- | --- | --- |

Best Unique PSM (Scan Sample\_7\_A1\_1\_3644.d:15798, m/z=562.7754, z=2, RT=10.18, ppm=-0.7):


TRINITY\_DN30094\_c0\_g1\_i1.p1

back to list

  

| Protein Coverage
| Supporting Peptides
| Best Unique PSM
|

Protein Coverage:

Supporting Peptides:

| Peptide | Uniq | -10lgP | Mass | Length | ppm | m/z | z | RT | 1/k0 Start | 1/k0 End | Precursor ID | Area Sample 8 | #Feature | #Feature Sample 8 | Start | End | PTM | AScore | Found By |
| --- | --- | --- | --- | --- | --- | --- | --- | --- | --- | --- | --- | --- | --- | --- | --- | --- | --- | --- | --- |
| K.ALNQQLTNHIR.E | Y | 43.53 | 1306.7106 | 11 | -3.95 | 654.3571 | 2 | 13.42 | 0.9691 | 0.9763 | 34343 | 0 | 0 | 0 | 280 | 290 |  |  | DB Search |
| total 1 peptides |  |  |  |  |  |  |  |  |  |  |  |  |  |  |  |  |  |  |  |
| --- | --- | --- | --- | --- | --- | --- | --- | --- | --- | --- | --- | --- | --- | --- | --- | --- | --- | --- | --- |

Best Unique PSM (Scan Sample\_7\_A1\_1\_3644.d:34343, m/z=654.3571, z=2, RT=13.42, ppm=-8.43):


TRINITY\_DN941\_c0\_g1\_i2.p1

back to list

  

| Protein Coverage
| Supporting Peptides
| Best Unique PSM
|

Protein Coverage:

Supporting Peptides:

| Peptide | Uniq | -10lgP | Mass | Length | ppm | m/z | z | RT | 1/k0 Start | 1/k0 End | Precursor ID | Area Sample 8 | #Feature | #Feature Sample 8 | Start | End | PTM | AScore | Found By |
| --- | --- | --- | --- | --- | --- | --- | --- | --- | --- | --- | --- | --- | --- | --- | --- | --- | --- | --- | --- |
| K.AHGPGLEGGVAGQPC(+57.02)R.F | Y | 40.64 | 1561.7419 | 16 | 2.05 | 781.8763 | 2 | 12.28 | 1.0275 | 1.0347 | 27748 | 0 | 0 | 0 | 366 | 381 | Carbamidomethylation |  | DB Search |
| total 1 peptides |  |  |  |  |  |  |  |  |  |  |  |  |  |  |  |  |  |  |  |
| --- | --- | --- | --- | --- | --- | --- | --- | --- | --- | --- | --- | --- | --- | --- | --- | --- | --- | --- | --- |

Best Unique PSM (Scan Sample\_7\_A1\_1\_3644.d:27748, m/z=782.374, z=2, RT=12.30, ppm=-2.42):


TRINITY\_DN3036\_c0\_g1\_i2.p2

back to list

  

| Protein Coverage
| Supporting Peptides
| Best Unique PSM
|

Protein Coverage:

Supporting Peptides:

| Peptide | Uniq | -10lgP | Mass | Length | ppm | m/z | z | RT | 1/k0 Start | 1/k0 End | Precursor ID | Area Sample 8 | #Feature | #Feature Sample 8 | Start | End | PTM | AScore | Found By |
| --- | --- | --- | --- | --- | --- | --- | --- | --- | --- | --- | --- | --- | --- | --- | --- | --- | --- | --- | --- |
| R.GTSNGGLVVGTIIKPK.L | Y | 43.76 | 1539.8984 | 16 | -3.44 | 770.9504 | 2 | 15.91 | 1.1262 | 1.1334 | 48868 | 9.21e2 | 1 | 1 | 173 | 188 |  |  | DB Search |
| R.AGHGAVTSPQTQR.G | Y | 43.55 | 1308.6534 | 13 | 3.24 | 437.2245 | 3 | 9.76 | 0.7045 | 0.7118 | 13596 | 2.79e3 | 2 | 2 | 309 | 321 |  |  | DB Search |
| R.VIGASGIHVGTM(+15.99)SFGK.M | Y | 43.2 | 1575.8079 | 16 | 0.89 | 788.9084 | 2 | 15.29 | 1.129 | 1.1362 | 45505 | 0 | 0 | 0 | 334 | 349 | Oxidation (M) | M12:Oxidation (M):1000 | DB Search |
| total 3 peptides |  |  |  |  |  |  |  |  |  |  |  |  |  |  |  |  |  |  |  |
| --- | --- | --- | --- | --- | --- | --- | --- | --- | --- | --- | --- | --- | --- | --- | --- | --- | --- | --- | --- |

Best Unique PSM (Scan Sample\_7\_A1\_1\_3644.d:48868, m/z=770.9504, z=2, RT=15.91, ppm=-7.92):


TRINITY\_DN140702\_c0\_g1\_i1.p1

back to list

  

| Protein Coverage
| Supporting Peptides
| Best Unique PSM
|

Protein Coverage:

Supporting Peptides:

| Peptide | Uniq | -10lgP | Mass | Length | ppm | m/z | z | RT | 1/k0 Start | 1/k0 End | Precursor ID | Area Sample 8 | #Feature | #Feature Sample 8 | Start | End | PTM | AScore | Found By |
| --- | --- | --- | --- | --- | --- | --- | --- | --- | --- | --- | --- | --- | --- | --- | --- | --- | --- | --- | --- |
| R.LALHVLNNEDIIGHR.L | Y | 43.86 | 1712.9322 | 15 | 1.95 | 857.4712 | 2 | 16.55 | 1.1378 | 1.145 | 52597 | 1.8e3 | 2 | 2 | 1702 | 1716 |  |  | DB Search |
| K.IEDFEESPPTDPHIGPVEQR.L | Y | 43.56 | 2291.0705 | 20 | 0.78 | 764.6946 | 3 | 15.83 | 0.9319 | 0.9392 | 48664 | 2.31e2 | 1 | 1 | 1682 | 1701 |  |  | DB Search |
| total 2 peptides |  |  |  |  |  |  |  |  |  |  |  |  |  |  |  |  |  |  |  |
| --- | --- | --- | --- | --- | --- | --- | --- | --- | --- | --- | --- | --- | --- | --- | --- | --- | --- | --- | --- |

Best Unique PSM (Scan Sample\_7\_A1\_1\_3644.d:52597, m/z=855.9445, z=2, RT=16.55, ppm=-2.53):


TRINITY\_DN1194\_c0\_g1\_i1.p1

back to list

  

| Protein Coverage
| Supporting Peptides
| Best Unique PSM
|

Protein Coverage:

Supporting Peptides:

| Peptide | Uniq | -10lgP | Mass | Length | ppm | m/z | z | RT | 1/k0 Start | 1/k0 End | Precursor ID | Area Sample 8 | #Feature | #Feature Sample 8 | Start | End | PTM | AScore | Found By |
| --- | --- | --- | --- | --- | --- | --- | --- | --- | --- | --- | --- | --- | --- | --- | --- | --- | --- | --- | --- |
| K.SLVEENVHPQILIK.G | Y | 41.2 | 1617.909 | 14 | -0.47 | 809.9578 | 2 | 16.56 | 1.1001 | 1.1074 | 52733 | 1.57e2 | 1 | 1 | 118 | 131 |  |  | DB Search |
| total 1 peptides |  |  |  |  |  |  |  |  |  |  |  |  |  |  |  |  |  |  |  |
| --- | --- | --- | --- | --- | --- | --- | --- | --- | --- | --- | --- | --- | --- | --- | --- | --- | --- | --- | --- |

Best Unique PSM (Scan Sample\_7\_A1\_1\_3644.d:52733, m/z=809.9578, z=2, RT=16.56, ppm=-4.94):


TRINITY\_DN2477\_c0\_g1\_i8.p1

back to list

  

| Protein Coverage
| Supporting Peptides
| Best Unique PSM
|

Protein Coverage:

Supporting Peptides:

| Peptide | Uniq | -10lgP | Mass | Length | ppm | m/z | z | RT | 1/k0 Start | 1/k0 End | Precursor ID | Area Sample 8 | #Feature | #Feature Sample 8 | Start | End | PTM | AScore | Found By |
| --- | --- | --- | --- | --- | --- | --- | --- | --- | --- | --- | --- | --- | --- | --- | --- | --- | --- | --- | --- |
| R.VAPEEHPVLLTEAPLNPK.A | Y | 43.24 | 1953.0571 | 18 | -8.51 | 977.5231 | 2 | 16.59 | 1.2073 | 1.2145 | 52921 | 0 | 0 | 0 | 29 | 46 |  |  | DB Search |
| total 1 peptides |  |  |  |  |  |  |  |  |  |  |  |  |  |  |  |  |  |  |  |
| --- | --- | --- | --- | --- | --- | --- | --- | --- | --- | --- | --- | --- | --- | --- | --- | --- | --- | --- | --- |

Best Unique PSM (Scan Sample\_7\_A1\_1\_3644.d:52921, m/z=977.5231, z=2, RT=16.59, ppm=-12.98):


TRINITY\_DN602\_c0\_g1\_i4.p1

back to list

  

| Protein Coverage
| Supporting Peptides
| Best Unique PSM
|

Protein Coverage:

Supporting Peptides:

| Peptide | Uniq | -10lgP | Mass | Length | ppm | m/z | z | RT | 1/k0 Start | 1/k0 End | Precursor ID | Area Sample 8 | #Feature | #Feature Sample 8 | Start | End | PTM | AScore | Found By |
| --- | --- | --- | --- | --- | --- | --- | --- | --- | --- | --- | --- | --- | --- | --- | --- | --- | --- | --- | --- |
| K.EGFYVHEALAAGIVK.K | Y | 43.8 | 1602.8405 | 15 | 3.49 | 802.4268 | 2 | 17.74 | 1.0861 | 1.0933 | 59699 | 0 | 0 | 0 | 144 | 158 |  |  | DB Search |
| K.LQEFGHK.Y | Y | 42.79 | 857.4395 | 7 | 2.94 | 429.7264 | 2 | 11.17 | 0.7913 | 0.7986 | 21333 | 0 | 0 | 0 | 499 | 505 |  |  | DB Search |
| total 2 peptides |  |  |  |  |  |  |  |  |  |  |  |  |  |  |  |  |  |  |  |
| --- | --- | --- | --- | --- | --- | --- | --- | --- | --- | --- | --- | --- | --- | --- | --- | --- | --- | --- | --- |

Best Unique PSM (Scan Sample\_7\_A1\_1\_3644.d:59699, m/z=802.4268, z=2, RT=17.74, ppm=-0.98):


TRINITY\_DN875\_c0\_g1\_i1.p1

back to list

  

| Protein Coverage
| Supporting Peptides
| Best Unique PSM
|

Protein Coverage:

Supporting Peptides:

| Peptide | Uniq | -10lgP | Mass | Length | ppm | m/z | z | RT | 1/k0 Start | 1/k0 End | Precursor ID | Area Sample 8 | #Feature | #Feature Sample 8 | Start | End | PTM | AScore | Found By |
| --- | --- | --- | --- | --- | --- | --- | --- | --- | --- | --- | --- | --- | --- | --- | --- | --- | --- | --- | --- |
| R.TC(+57.02)TNPEPK.G | Y | 43.67 | 945.4226 | 8 | 1.26 | 473.717 | 2 | 9.52 | 0.7734 | 0.7807 | 12535 | 1.14e4 | 2 | 2 | 175 | 182 | Carbamidomethylation |  | DB Search |
| R.AVFC(+57.02)EHQGVK.T | Y | 43.32 | 1173.5601 | 10 | -0.85 | 587.7842 | 2 | 11.69 | 0.898 | 0.9052 | 24238 | 1.92e3 | 1 | 1 | 512 | 521 | Carbamidomethylation |  | DB Search |
| total 2 peptides |  |  |  |  |  |  |  |  |  |  |  |  |  |  |  |  |  |  |  |
| --- | --- | --- | --- | --- | --- | --- | --- | --- | --- | --- | --- | --- | --- | --- | --- | --- | --- | --- | --- |

Best Unique PSM (Scan Sample\_7\_A1\_1\_3644.d:12535, m/z=473.717, z=2, RT=9.52, ppm=-3.22):


TRINITY\_DN691\_c0\_g1\_i1.p1

back to list

  

| Protein Coverage
| Supporting Peptides
| Best Unique PSM
|

Protein Coverage:

Supporting Peptides:

| Peptide | Uniq | -10lgP | Mass | Length | ppm | m/z | z | RT | 1/k0 Start | 1/k0 End | Precursor ID | Area Sample 8 | #Feature | #Feature Sample 8 | Start | End | PTM | AScore | Found By |
| --- | --- | --- | --- | --- | --- | --- | --- | --- | --- | --- | --- | --- | --- | --- | --- | --- | --- | --- | --- |
| R.AYGQDLGQPGSC(+57.02)LKR.F | Y | 43.8 | 1648.7991 | 15 | 0.73 | 825.4037 | 2 | 13.53 | 1.0749 | 1.0821 | 34989 | 5.46e2 | 1 | 1 | 1533 | 1547 | Carbamidomethylation |  | DB Search |
| R.AYGQDLGQPGSC(+57.02)LK.R | Y | 43.57 | 1492.698 | 14 | 1.84 | 747.3543 | 2 | 14.35 | 1.0069 | 1.0142 | 39867 | 7.2e2 | 1 | 1 | 1533 | 1546 | Carbamidomethylation |  | DB Search |
| R.ANPFIEC(+57.02)HGR.G | Y | 43.48 | 1199.5506 | 10 | 1.29 | 600.7806 | 2 | 13.31 | 0.8951 | 0.9024 | 33665 | 9.46e2 | 1 | 1 | 1660 | 1669 | Carbamidomethylation |  | DB Search |
| total 3 peptides |  |  |  |  |  |  |  |  |  |  |  |  |  |  |  |  |  |  |  |
| --- | --- | --- | --- | --- | --- | --- | --- | --- | --- | --- | --- | --- | --- | --- | --- | --- | --- | --- | --- |

Best Unique PSM (Scan Sample\_7\_A1\_1\_3644.d:34989, m/z=825.4037, z=2, RT=13.53, ppm=-3.74):


TRINITY\_DN7659\_c0\_g1\_i1.p1

back to list

  

| Protein Coverage
| Supporting Peptides
| Best Unique PSM
|

Protein Coverage:

Supporting Peptides:

| Peptide | Uniq | -10lgP | Mass | Length | ppm | m/z | z | RT | 1/k0 Start | 1/k0 End | Precursor ID | Area Sample 8 | #Feature | #Feature Sample 8 | Start | End | PTM | AScore | Found By |
| --- | --- | --- | --- | --- | --- | --- | --- | --- | --- | --- | --- | --- | --- | --- | --- | --- | --- | --- | --- |
| M.A(+42.01)SAATDNRK.V | Y | 43.11 | 974.4781 | 9 | 3.81 | 488.246 | 2 | 9.52 | 0.8234 | 0.8307 | 12664 | 0 | 0 | 0 | 2 | 10 | Acetylation (Protein N-term) | A1:Acetylation (Protein N-term):1000 | DB Search |
| K.AQPTEIYNLGAQSHVK.V | Y | 42.33 | 1754.8951 | 16 | -17.72 | 878.4354 | 2 | 14.86 | 1.1734 | 1.1806 | 42928 | 0 | 0 | 0 | 86 | 101 |  |  | DB Search |
| total 2 peptides |  |  |  |  |  |  |  |  |  |  |  |  |  |  |  |  |  |  |  |
| --- | --- | --- | --- | --- | --- | --- | --- | --- | --- | --- | --- | --- | --- | --- | --- | --- | --- | --- | --- |

Best Unique PSM (Scan Sample\_7\_A1\_1\_3644.d:12664, m/z=488.246, z=2, RT=9.52, ppm=-0.67):


TRINITY\_DN1378\_c0\_g1\_i1.p1

back to list

  

| Protein Coverage
| Supporting Peptides
| Best Unique PSM
|

Protein Coverage:

Supporting Peptides:

| Peptide | Uniq | -10lgP | Mass | Length | ppm | m/z | z | RT | 1/k0 Start | 1/k0 End | Precursor ID | Area Sample 8 | #Feature | #Feature Sample 8 | Start | End | PTM | AScore | Found By |
| --- | --- | --- | --- | --- | --- | --- | --- | --- | --- | --- | --- | --- | --- | --- | --- | --- | --- | --- | --- |
| K.LAALDATVHGQTASR.F | Y | 43.42 | 1509.7899 | 15 | -1.2 | 755.8979 | 2 | 13.58 | 1.0725 | 1.0797 | 35280 | 0 | 0 | 0 | 213 | 227 |  |  | DB Search |
| total 1 peptides |  |  |  |  |  |  |  |  |  |  |  |  |  |  |  |  |  |  |  |
| --- | --- | --- | --- | --- | --- | --- | --- | --- | --- | --- | --- | --- | --- | --- | --- | --- | --- | --- | --- |

Best Unique PSM (Scan Sample\_7\_A1\_1\_3644.d:35280, m/z=755.8979, z=2, RT=13.58, ppm=-5.68):


TRINITY\_DN13813\_c0\_g1\_i5.p2

back to list

  

| Protein Coverage
| Supporting Peptides
| Best Unique PSM
|

Protein Coverage:

Supporting Peptides:

| Peptide | Uniq | -10lgP | Mass | Length | ppm | m/z | z | RT | 1/k0 Start | 1/k0 End | Precursor ID | Area Sample 8 | #Feature | #Feature Sample 8 | Start | End | PTM | AScore | Found By |
| --- | --- | --- | --- | --- | --- | --- | --- | --- | --- | --- | --- | --- | --- | --- | --- | --- | --- | --- | --- |
| R.DGPGPLASDLLLQMR.Q | Y | 41.47 | 1581.8184 | 15 | -2.93 | 528.2762 | 3 | 19.62 | 0.8149 | 0.8222 | 71531 | 3.73e2 | 1 | 1 | 326 | 340 |  |  | DB Search |
| total 1 peptides |  |  |  |  |  |  |  |  |  |  |  |  |  |  |  |  |  |  |  |
| --- | --- | --- | --- | --- | --- | --- | --- | --- | --- | --- | --- | --- | --- | --- | --- | --- | --- | --- | --- |

Best Unique PSM (Scan Sample\_7\_A1\_1\_3644.d:71531, m/z=528.2762, z=3, RT=19.62, ppm=-7.41):


TRINITY\_DN1821\_c0\_g1\_i4.p1

back to list

  

| Protein Coverage
| Supporting Peptides
| Best Unique PSM
|

Protein Coverage:

Supporting Peptides:

| Peptide | Uniq | -10lgP | Mass | Length | ppm | m/z | z | RT | 1/k0 Start | 1/k0 End | Precursor ID | Area Sample 8 | #Feature | #Feature Sample 8 | Start | End | PTM | AScore | Found By |
| --- | --- | --- | --- | --- | --- | --- | --- | --- | --- | --- | --- | --- | --- | --- | --- | --- | --- | --- | --- |
| K.YQGIKPPVSR.S | Y | 43.24 | 1143.64 | 10 | -0.35 | 572.8245 | 2 | 12.74 | 0.9344 | 0.9416 | 30400 | 7.91e2 | 1 | 1 | 478 | 487 |  |  | DB Search |
| total 1 peptides |  |  |  |  |  |  |  |  |  |  |  |  |  |  |  |  |  |  |  |
| --- | --- | --- | --- | --- | --- | --- | --- | --- | --- | --- | --- | --- | --- | --- | --- | --- | --- | --- | --- |

Best Unique PSM (Scan Sample\_7\_A1\_1\_3644.d:30400, m/z=572.8245, z=2, RT=12.74, ppm=-4.83):


TRINITY\_DN477\_c0\_g1\_i5.p1

back to list

  

| Protein Coverage
| Supporting Peptides
| Best Unique PSM
|

Protein Coverage:

Supporting Peptides:

| Peptide | Uniq | -10lgP | Mass | Length | ppm | m/z | z | RT | 1/k0 Start | 1/k0 End | Precursor ID | Area Sample 8 | #Feature | #Feature Sample 8 | Start | End | PTM | AScore | Found By |
| --- | --- | --- | --- | --- | --- | --- | --- | --- | --- | --- | --- | --- | --- | --- | --- | --- | --- | --- | --- |
| R.NDVNNPPQAR.R | Y | 43.84 | 1123.537 | 10 | 3.77 | 562.7754 | 2 | 10.18 | 0.8676 | 0.8749 | 15798 | 1.5e4 | 1 | 1 | 168 | 177 |  |  | DB Search |
| total 1 peptides |  |  |  |  |  |  |  |  |  |  |  |  |  |  |  |  |  |  |  |
| --- | --- | --- | --- | --- | --- | --- | --- | --- | --- | --- | --- | --- | --- | --- | --- | --- | --- | --- | --- |

Best Unique PSM (Scan Sample\_7\_A1\_1\_3644.d:15798, m/z=562.7754, z=2, RT=10.18, ppm=-0.7):


TRINITY\_DN1616\_c0\_g1\_i3.p1

back to list

  

| Protein Coverage
| Supporting Peptides
| Best Unique PSM
|

Protein Coverage:

Supporting Peptides:

| Peptide | Uniq | -10lgP | Mass | Length | ppm | m/z | z | RT | 1/k0 Start | 1/k0 End | Precursor ID | Area Sample 8 | #Feature | #Feature Sample 8 | Start | End | PTM | AScore | Found By |
| --- | --- | --- | --- | --- | --- | --- | --- | --- | --- | --- | --- | --- | --- | --- | --- | --- | --- | --- | --- |
| R.TSTDKFEC(+57.02)EK.C | Y | 43.76 | 1243.5391 | 10 | -3.39 | 622.7719 | 2 | 10.75 | 0.9255 | 0.9327 | 18979 | 0 | 0 | 0 | 320 | 329 | Carbamidomethylation |  | DB Search |
| R.YGAVNDNC(+57.02)ER.D | Y | 43.62 | 1196.488 | 10 | -2.06 | 599.2474 | 2 | 10.92 | 0.8834 | 0.8907 | 19854 | 4.23e2 | 1 | 1 | 498 | 507 | Carbamidomethylation |  | DB Search |
| R.YVKPGVNIINIR.D | Y | 43.56 | 1384.819 | 12 | 3.35 | 693.416 | 2 | 16.23 | 1.0013 | 1.0086 | 50888 | 0 | 0 | 0 | 1217 | 1228 |  |  | DB Search |
| K.YVVMLQYYHDSDTAK.Y | Y | 43.52 | 1831.8451 | 15 | 8.47 | 916.9335 | 2 | 16.82 | 1.1857 | 1.1929 | 54206 | 0 | 0 | 0 | 1071 | 1085 |  |  | DB Search |
| K.SSSC(+57.02)QEFGGKC(+57.02)PC(+57.02)K.D | Y | 39.5 | 1630.6538 | 14 | -18.66 | 816.3153 | 2 | 12.09 | 1.0311 | 1.0383 | 26624 | 0 | 0 | 0 | 1387 | 1400 | Carbamidomethylation, Carbamidomethylation, Carbamidomethylation |  | DB Search |
| total 5 peptides |  |  |  |  |  |  |  |  |  |  |  |  |  |  |  |  |  |  |  |
| --- | --- | --- | --- | --- | --- | --- | --- | --- | --- | --- | --- | --- | --- | --- | --- | --- | --- | --- | --- |

Best Unique PSM (Scan Sample\_7\_A1\_1\_3644.d:18979, m/z=622.7719, z=2, RT=10.75, ppm=-7.87):


TRINITY\_DN495\_c0\_g1\_i2.p1

back to list

  

| Protein Coverage
| Supporting Peptides
| Best Unique PSM
|

Protein Coverage:

Supporting Peptides:

| Peptide | Uniq | -10lgP | Mass | Length | ppm | m/z | z | RT | 1/k0 Start | 1/k0 End | Precursor ID | Area Sample 8 | #Feature | #Feature Sample 8 | Start | End | PTM | AScore | Found By |
| --- | --- | --- | --- | --- | --- | --- | --- | --- | --- | --- | --- | --- | --- | --- | --- | --- | --- | --- | --- |
| R.SC(+57.02)TNPAPANGGK.A | N | 43.8 | 1172.5244 | 12 | 2.69 | 587.2684 | 2 | 9.47 | 0.838 | 0.8453 | 12375 | 4.7e3 | 1 | 1 | 499 | 510 | Carbamidomethylation |  | DB Search |
| R.SSC(+57.02)KPILSVIK.I | Y | 43.76 | 1230.7006 | 11 | 2.76 | 616.3565 | 2 | 15.26 | 0.9529 | 0.9602 | 45369 | 2.7e3 | 1 | 1 | 450 | 460 | Carbamidomethylation |  | DB Search |
| total 2 peptides |  |  |  |  |  |  |  |  |  |  |  |  |  |  |  |  |  |  |  |
| --- | --- | --- | --- | --- | --- | --- | --- | --- | --- | --- | --- | --- | --- | --- | --- | --- | --- | --- | --- |

Best Unique PSM (Scan Sample\_7\_A1\_1\_3644.d:45369, m/z=616.3565, z=2, RT=15.26, ppm=-1.72):


TRINITY\_DN319\_c2\_g1\_i3.p1

back to list

  

| Protein Coverage
| Supporting Peptides
| Best Unique PSM
|

Protein Coverage:

Supporting Peptides:

| Peptide | Uniq | -10lgP | Mass | Length | ppm | m/z | z | RT | 1/k0 Start | 1/k0 End | Precursor ID | Area Sample 8 | #Feature | #Feature Sample 8 | Start | End | PTM | AScore | Found By |
| --- | --- | --- | --- | --- | --- | --- | --- | --- | --- | --- | --- | --- | --- | --- | --- | --- | --- | --- | --- |
| K.KAQYYEAYETIIKR.H | Y | 43.83 | 1774.9254 | 14 | -1.22 | 592.6457 | 3 | 15.96 | 0.8664 | 0.8737 | 49246 | 6.27e2 | 1 | 1 | 2534 | 2547 |  |  | DB Search |
| R.GSFSSHIC(+57.02)LC(+57.02)HDPAYK.S | Y | 42.3 | 1877.8189 | 16 | -0.4 | 626.9438 | 3 | 14.48 | 0.8866 | 0.8939 | 40723 | 0 | 0 | 0 | 1011 | 1026 | Carbamidomethylation, Carbamidomethylation |  | DB Search |
| total 2 peptides |  |  |  |  |  |  |  |  |  |  |  |  |  |  |  |  |  |  |  |
| --- | --- | --- | --- | --- | --- | --- | --- | --- | --- | --- | --- | --- | --- | --- | --- | --- | --- | --- | --- |

Best Unique PSM (Scan Sample\_7\_A1\_1\_3644.d:49246, m/z=592.6457, z=3, RT=15.96, ppm=-5.7):


TRINITY\_DN172571\_c0\_g1\_i1.p1

back to list

  

| Protein Coverage
| Supporting Peptides
| Best Unique PSM
|

Protein Coverage:

Supporting Peptides:

| Peptide | Uniq | -10lgP | Mass | Length | ppm | m/z | z | RT | 1/k0 Start | 1/k0 End | Precursor ID | Area Sample 8 | #Feature | #Feature Sample 8 | Start | End | PTM | AScore | Found By |
| --- | --- | --- | --- | --- | --- | --- | --- | --- | --- | --- | --- | --- | --- | --- | --- | --- | --- | --- | --- |
| R.DNIQGITKPAIR.R | Y | 43.83 | 1324.7463 | 12 | 2.3 | 663.379 | 2 | 13.91 | 0.9808 | 0.988 | 37136 | 4.46e2 | 1 | 1 | 25 | 36 |  |  | DB Search |
| total 1 peptides |  |  |  |  |  |  |  |  |  |  |  |  |  |  |  |  |  |  |  |
| --- | --- | --- | --- | --- | --- | --- | --- | --- | --- | --- | --- | --- | --- | --- | --- | --- | --- | --- | --- |

Best Unique PSM (Scan Sample\_7\_A1\_1\_3644.d:37136, m/z=663.379, z=2, RT=13.91, ppm=-2.18):


TRINITY\_DN691\_c0\_g1\_i2.p1

back to list

  

| Protein Coverage
| Supporting Peptides
| Best Unique PSM
|

Protein Coverage:

Supporting Peptides:

| Peptide | Uniq | -10lgP | Mass | Length | ppm | m/z | z | RT | 1/k0 Start | 1/k0 End | Precursor ID | Area Sample 8 | #Feature | #Feature Sample 8 | Start | End | PTM | AScore | Found By |
| --- | --- | --- | --- | --- | --- | --- | --- | --- | --- | --- | --- | --- | --- | --- | --- | --- | --- | --- | --- |
| R.AYGQDLGQPGSC(+57.02)LKR.F | Y | 43.8 | 1648.7991 | 15 | 0.73 | 825.4037 | 2 | 13.53 | 1.0749 | 1.0821 | 34989 | 5.46e2 | 1 | 1 | 1480 | 1494 | Carbamidomethylation |  | DB Search |
| R.AYGQDLGQPGSC(+57.02)LK.R | Y | 43.57 | 1492.698 | 14 | 1.84 | 747.3543 | 2 | 14.35 | 1.0069 | 1.0142 | 39867 | 7.2e2 | 1 | 1 | 1480 | 1493 | Carbamidomethylation |  | DB Search |
| R.ANPFIEC(+57.02)HGR.G | Y | 43.48 | 1199.5506 | 10 | 1.29 | 600.7806 | 2 | 13.31 | 0.8951 | 0.9024 | 33665 | 9.46e2 | 1 | 1 | 1607 | 1616 | Carbamidomethylation |  | DB Search |
| total 3 peptides |  |  |  |  |  |  |  |  |  |  |  |  |  |  |  |  |  |  |  |
| --- | --- | --- | --- | --- | --- | --- | --- | --- | --- | --- | --- | --- | --- | --- | --- | --- | --- | --- | --- |

Best Unique PSM (Scan Sample\_7\_A1\_1\_3644.d:34989, m/z=825.4037, z=2, RT=13.53, ppm=-3.74):


TRINITY\_DN8707\_c0\_g1\_i2.p1

back to list

  

| Protein Coverage
| Supporting Peptides
| Best Unique PSM
|

Protein Coverage:

Supporting Peptides:

| Peptide | Uniq | -10lgP | Mass | Length | ppm | m/z | z | RT | 1/k0 Start | 1/k0 End | Precursor ID | Area Sample 8 | #Feature | #Feature Sample 8 | Start | End | PTM | AScore | Found By |
| --- | --- | --- | --- | --- | --- | --- | --- | --- | --- | --- | --- | --- | --- | --- | --- | --- | --- | --- | --- |
| K.ALLSLEYHEKR.C | Y | 43.77 | 1357.7354 | 11 | -1.31 | 679.871 | 2 | 14.63 | 1.0283 | 1.0355 | 41504 | 1.16e3 | 1 | 1 | 124 | 134 |  |  | DB Search |
| total 1 peptides |  |  |  |  |  |  |  |  |  |  |  |  |  |  |  |  |  |  |  |
| --- | --- | --- | --- | --- | --- | --- | --- | --- | --- | --- | --- | --- | --- | --- | --- | --- | --- | --- | --- |

Best Unique PSM (Scan Sample\_7\_A1\_1\_3644.d:41504, m/z=679.871, z=2, RT=14.63, ppm=-5.79):


TRINITY\_DN6390\_c0\_g1\_i1.p1

back to list

  

| Protein Coverage
| Supporting Peptides
| Best Unique PSM
|

Protein Coverage:

Supporting Peptides:

| Peptide | Uniq | -10lgP | Mass | Length | ppm | m/z | z | RT | 1/k0 Start | 1/k0 End | Precursor ID | Area Sample 8 | #Feature | #Feature Sample 8 | Start | End | PTM | AScore | Found By |
| --- | --- | --- | --- | --- | --- | --- | --- | --- | --- | --- | --- | --- | --- | --- | --- | --- | --- | --- | --- |
| R.HLSVNILQTNTR.V | Y | 43.83 | 1394.763 | 12 | 3.19 | 698.3879 | 2 | 15.45 | 1.0005 | 1.0077 | 46263 | 8.86e2 | 1 | 1 | 836 | 847 |  |  | DB Search |
| R.SKDYTITSVGGR.N | Y | 43.68 | 1282.6517 | 12 | 1.14 | 642.331 | 2 | 13.00 | 0.9307 | 0.938 | 31872 | 1.2e3 | 1 | 1 | 876 | 887 |  |  | DB Search |
| K.YLFDLESNC(+57.02)DK.Q | Y | 43.66 | 1402.6075 | 11 | -1.67 | 702.3067 | 2 | 18.46 | 0.9872 | 0.9945 | 64425 | 0 | 0 | 0 | 303 | 313 | Carbamidomethylation |  | DB Search |
| K.SLVYNPLHNDQEVFER.A | Y | 42.75 | 1958.9486 | 16 | 1.71 | 653.9883 | 3 | 17.50 | 0.8672 | 0.8745 | 58275 | 2.2e2 | 1 | 1 | 590 | 605 |  |  | DB Search |
| total 4 peptides |  |  |  |  |  |  |  |  |  |  |  |  |  |  |  |  |  |  |  |
| --- | --- | --- | --- | --- | --- | --- | --- | --- | --- | --- | --- | --- | --- | --- | --- | --- | --- | --- | --- |

Best Unique PSM (Scan Sample\_7\_A1\_1\_3644.d:46263, m/z=698.3879, z=2, RT=15.45, ppm=-1.29):


TRINITY\_DN941\_c0\_g1\_i8.p1

back to list

  

| Protein Coverage
| Supporting Peptides
| Best Unique PSM
|

Protein Coverage:

Supporting Peptides:

| Peptide | Uniq | -10lgP | Mass | Length | ppm | m/z | z | RT | 1/k0 Start | 1/k0 End | Precursor ID | Area Sample 8 | #Feature | #Feature Sample 8 | Start | End | PTM | AScore | Found By |
| --- | --- | --- | --- | --- | --- | --- | --- | --- | --- | --- | --- | --- | --- | --- | --- | --- | --- | --- | --- |
| K.AHGPGLEGGVAGQPC(+57.02)R.F | Y | 40.64 | 1561.7419 | 16 | 2.05 | 781.8763 | 2 | 12.28 | 1.0275 | 1.0347 | 27748 | 0 | 0 | 0 | 386 | 401 | Carbamidomethylation |  | DB Search |
| total 1 peptides |  |  |  |  |  |  |  |  |  |  |  |  |  |  |  |  |  |  |  |
| --- | --- | --- | --- | --- | --- | --- | --- | --- | --- | --- | --- | --- | --- | --- | --- | --- | --- | --- | --- |

Best Unique PSM (Scan Sample\_7\_A1\_1\_3644.d:27748, m/z=782.374, z=2, RT=12.30, ppm=-2.42):


TRINITY\_DN1613\_c1\_g1\_i1.p1

back to list

  

| Protein Coverage
| Supporting Peptides
| Best Unique PSM
|

Protein Coverage:

Supporting Peptides:

| Peptide | Uniq | -10lgP | Mass | Length | ppm | m/z | z | RT | 1/k0 Start | 1/k0 End | Precursor ID | Area Sample 8 | #Feature | #Feature Sample 8 | Start | End | PTM | AScore | Found By |
| --- | --- | --- | --- | --- | --- | --- | --- | --- | --- | --- | --- | --- | --- | --- | --- | --- | --- | --- | --- |
| R.TC(+57.02)TNPAPAHGGK.S | Y | 43.53 | 1209.556 | 12 | -0.8 | 605.7821 | 2 | 9.09 | 0.8558 | 0.8631 | 11293 | 0 | 0 | 0 | 413 | 424 | Carbamidomethylation |  | DB Search |
| total 1 peptides |  |  |  |  |  |  |  |  |  |  |  |  |  |  |  |  |  |  |  |
| --- | --- | --- | --- | --- | --- | --- | --- | --- | --- | --- | --- | --- | --- | --- | --- | --- | --- | --- | --- |

Best Unique PSM (Scan Sample\_7\_A1\_1\_3644.d:11293, m/z=605.7821, z=2, RT=9.09, ppm=-5.27):


TRINITY\_DN140696\_c0\_g1\_i1.p1

back to list

  

| Protein Coverage
| Supporting Peptides
| Best Unique PSM
|

Protein Coverage:

Supporting Peptides:

| Peptide | Uniq | -10lgP | Mass | Length | ppm | m/z | z | RT | 1/k0 Start | 1/k0 End | Precursor ID | Area Sample 8 | #Feature | #Feature Sample 8 | Start | End | PTM | AScore | Found By |
| --- | --- | --- | --- | --- | --- | --- | --- | --- | --- | --- | --- | --- | --- | --- | --- | --- | --- | --- | --- |
| R.HASSYDIVHIK.D | Y | 43.27 | 1268.6513 | 11 | 4.57 | 423.8911 | 3 | 13.73 | 0.7526 | 0.7599 | 36244 | 0 | 0 | 0 | 201 | 211 |  |  | DB Search |
| R.LTIAEERDR.R | Y | 42.29 | 1101.5778 | 9 | 3.63 | 551.7957 | 2 | 11.79 | 0.9089 | 0.9162 | 24861 | 0 | 0 | 0 | 246 | 254 |  |  | DB Search |
| total 2 peptides |  |  |  |  |  |  |  |  |  |  |  |  |  |  |  |  |  |  |  |
| --- | --- | --- | --- | --- | --- | --- | --- | --- | --- | --- | --- | --- | --- | --- | --- | --- | --- | --- | --- |

Best Unique PSM (Scan Sample\_7\_A1\_1\_3644.d:36244, m/z=423.8911, z=3, RT=13.73, ppm=0.09):


TRINITY\_DN8341\_c0\_g1\_i1.p1

back to list

  

| Protein Coverage
| Supporting Peptides
| Best Unique PSM
|

Protein Coverage:

Supporting Peptides:

| Peptide | Uniq | -10lgP | Mass | Length | ppm | m/z | z | RT | 1/k0 Start | 1/k0 End | Precursor ID | Area Sample 8 | #Feature | #Feature Sample 8 | Start | End | PTM | AScore | Found By |
| --- | --- | --- | --- | --- | --- | --- | --- | --- | --- | --- | --- | --- | --- | --- | --- | --- | --- | --- | --- |
| K.SLDLKQENSNR.L | Y | 43.76 | 1302.6528 | 11 | 2.34 | 652.3323 | 2 | 11.51 | 0.9509 | 0.9582 | 23115 | 2.77e3 | 1 | 1 | 220 | 230 |  |  | DB Search |
| K.DFILTAAHC(+57.02)FK.E | Y | 43.55 | 1321.6489 | 11 | -2.89 | 661.8268 | 2 | 18.07 | 1.0094 | 1.0166 | 61842 | 4.05e2 | 1 | 1 | 151 | 161 | Carbamidomethylation |  | DB Search |
| total 2 peptides |  |  |  |  |  |  |  |  |  |  |  |  |  |  |  |  |  |  |  |
| --- | --- | --- | --- | --- | --- | --- | --- | --- | --- | --- | --- | --- | --- | --- | --- | --- | --- | --- | --- |

Best Unique PSM (Scan Sample\_7\_A1\_1\_3644.d:23115, m/z=652.3323, z=2, RT=11.51, ppm=-2.13):


TRINITY\_DN875\_c0\_g1\_i2.p1

back to list

  

| Protein Coverage
| Supporting Peptides
| Best Unique PSM
|

Protein Coverage:

Supporting Peptides:

| Peptide | Uniq | -10lgP | Mass | Length | ppm | m/z | z | RT | 1/k0 Start | 1/k0 End | Precursor ID | Area Sample 8 | #Feature | #Feature Sample 8 | Start | End | PTM | AScore | Found By |
| --- | --- | --- | --- | --- | --- | --- | --- | --- | --- | --- | --- | --- | --- | --- | --- | --- | --- | --- | --- |
| R.TC(+57.02)TNPEPK.G | Y | 43.67 | 945.4226 | 8 | 1.26 | 473.717 | 2 | 9.52 | 0.7734 | 0.7807 | 12535 | 1.14e4 | 2 | 2 | 175 | 182 | Carbamidomethylation |  | DB Search |
| R.AVFC(+57.02)EHQGVK.T | Y | 43.32 | 1173.5601 | 10 | -0.85 | 587.7842 | 2 | 11.69 | 0.898 | 0.9052 | 24238 | 1.92e3 | 1 | 1 | 512 | 521 | Carbamidomethylation |  | DB Search |
| total 2 peptides |  |  |  |  |  |  |  |  |  |  |  |  |  |  |  |  |  |  |  |
| --- | --- | --- | --- | --- | --- | --- | --- | --- | --- | --- | --- | --- | --- | --- | --- | --- | --- | --- | --- |

Best Unique PSM (Scan Sample\_7\_A1\_1\_3644.d:12535, m/z=473.717, z=2, RT=9.52, ppm=-3.22):


TRINITY\_DN240\_c0\_g1\_i1.p1

back to list

  

| Protein Coverage
| Supporting Peptides
| Best Unique PSM
|

Protein Coverage:

Supporting Peptides:

| Peptide | Uniq | -10lgP | Mass | Length | ppm | m/z | z | RT | 1/k0 Start | 1/k0 End | Precursor ID | Area Sample 8 | #Feature | #Feature Sample 8 | Start | End | PTM | AScore | Found By |
| --- | --- | --- | --- | --- | --- | --- | --- | --- | --- | --- | --- | --- | --- | --- | --- | --- | --- | --- | --- |
| K.AYAAFLEFKDVVK.A | Y | 43.79 | 1499.8024 | 13 | 3.07 | 750.9074 | 2 | 19.27 | 1.0953 | 1.1025 | 69440 | 1.07e4 | 2 | 2 | 217 | 229 |  |  | DB Search |
| K.AISSASGPTGM(+15.99)TSK.A | Y | 43.77 | 1309.6184 | 14 | 1.88 | 655.8148 | 2 | 10.56 | 0.9319 | 0.9392 | 17892 | 3.72e3 | 1 | 1 | 151 | 164 | Oxidation (M) | M11:Oxidation (M):1000 | DB Search |
| K.ANQVSSAAAAPVPAAGGDK.I | Y | 43.76 | 1680.8431 | 19 | -0.14 | 841.4249 | 2 | 12.55 | 1.0656 | 1.0729 | 29279 | 4.54e3 | 1 | 1 | 230 | 248 |  |  | DB Search |
| K.IGAAAQALADQSYPFLK.S | Y | 43.73 | 1762.9253 | 17 | 0.62 | 882.4666 | 2 | 19.27 | 1.1574 | 1.1646 | 68962 | 5.17e3 | 2 | 2 | 249 | 265 |  |  | DB Search |
| K.AAASAHHQAIGSIDAAGVTSAADYASVTAALGR.A | Y | 43.16 | 3080.5275 | 33 | -1.21 | 771.1348 | 4 | 18.34 | 1.0652 | 1.0725 | 63473 | 9.02e3 | 1 | 1 | 307 | 339 |  |  | DB Search |
| K.SVDWTSDLYLKPLPGVSAAK.A | Y | 42.6 | 2146.131 | 20 | 3.72 | 716.3837 | 3 | 19.68 | 1.013 | 1.0202 | 72078 | 4.11e2 | 1 | 1 | 266 | 285 |  |  | DB Search |
| K.AIVMGSAMDGNLLK.A | Y | 42.42 | 1418.7261 | 14 | 3.64 | 710.3698 | 2 | 18.29 | 1.0174 | 1.0246 | 63333 | 0 | 0 | 0 | 293 | 306 |  |  | DB Search |
| total 7 peptides |  |  |  |  |  |  |  |  |  |  |  |  |  |  |  |  |  |  |  |
| --- | --- | --- | --- | --- | --- | --- | --- | --- | --- | --- | --- | --- | --- | --- | --- | --- | --- | --- | --- |

Best Unique PSM (Scan Sample\_7\_A1\_1\_3644.d:69440, m/z=750.9074, z=2, RT=19.27, ppm=-1.4):


TRINITY\_DN26249\_c0\_g1\_i1.p1

back to list

  

| Protein Coverage
| Supporting Peptides
| Best Unique PSM
|

Protein Coverage:

Supporting Peptides:

| Peptide | Uniq | -10lgP | Mass | Length | ppm | m/z | z | RT | 1/k0 Start | 1/k0 End | Precursor ID | Area Sample 8 | #Feature | #Feature Sample 8 | Start | End | PTM | AScore | Found By |
| --- | --- | --- | --- | --- | --- | --- | --- | --- | --- | --- | --- | --- | --- | --- | --- | --- | --- | --- | --- |
| R.LIYENQNTIHHVVR.A | Y | 43.73 | 1734.9165 | 14 | 3.14 | 579.312 | 3 | 13.35 | 0.8441 | 0.8514 | 33933 | 4.2e2 | 1 | 1 | 322 | 335 |  |  | DB Search |
| total 1 peptides |  |  |  |  |  |  |  |  |  |  |  |  |  |  |  |  |  |  |  |
| --- | --- | --- | --- | --- | --- | --- | --- | --- | --- | --- | --- | --- | --- | --- | --- | --- | --- | --- | --- |

Best Unique PSM (Scan Sample\_7\_A1\_1\_3644.d:33933, m/z=579.312, z=3, RT=13.35, ppm=-1.34):


TRINITY\_DN3036\_c0\_g1\_i6.p1

back to list

  

| Protein Coverage
| Supporting Peptides
| Best Unique PSM
|

Protein Coverage:

Supporting Peptides:

| Peptide | Uniq | -10lgP | Mass | Length | ppm | m/z | z | RT | 1/k0 Start | 1/k0 End | Precursor ID | Area Sample 8 | #Feature | #Feature Sample 8 | Start | End | PTM | AScore | Found By |
| --- | --- | --- | --- | --- | --- | --- | --- | --- | --- | --- | --- | --- | --- | --- | --- | --- | --- | --- | --- |
| R.VIGASGIHVGTM(+15.99)SFGK.M | Y | 43.2 | 1575.8079 | 16 | 0.89 | 788.9084 | 2 | 15.29 | 1.129 | 1.1362 | 45505 | 0 | 0 | 0 | 24 | 39 | Oxidation (M) | M12:Oxidation (M):1000 | DB Search |
| total 1 peptides |  |  |  |  |  |  |  |  |  |  |  |  |  |  |  |  |  |  |  |
| --- | --- | --- | --- | --- | --- | --- | --- | --- | --- | --- | --- | --- | --- | --- | --- | --- | --- | --- | --- |

Best Unique PSM (Scan Sample\_7\_A1\_1\_3644.d:45505, m/z=788.9084, z=2, RT=15.29, ppm=-3.59):


TRINITY\_DN687\_c0\_g1\_i1.p1

back to list

  

| Protein Coverage
| Supporting Peptides
| Best Unique PSM
|

Protein Coverage:

Supporting Peptides:

| Peptide | Uniq | -10lgP | Mass | Length | ppm | m/z | z | RT | 1/k0 Start | 1/k0 End | Precursor ID | Area Sample 8 | #Feature | #Feature Sample 8 | Start | End | PTM | AScore | Found By |
| --- | --- | --- | --- | --- | --- | --- | --- | --- | --- | --- | --- | --- | --- | --- | --- | --- | --- | --- | --- |
| K.VGINYQPPTVVPGGDLAK.V | Y | 43.58 | 1823.9781 | 18 | 1.41 | 912.9935 | 2 | 17.42 | 1.1214 | 1.1286 | 57750 | 0 | 0 | 0 | 353 | 370 |  |  | DB Search |
| R.AVC(+57.02)M(+15.99)LSNTTAIAEAWAR.L | Y | 43.51 | 1879.892 | 17 | 2.8 | 940.9517 | 2 | 18.84 | 1.1454 | 1.1526 | 66760 | 4.63e2 | 1 | 1 | 374 | 390 | Carbamidomethylation, Oxidation (M) | M4:Oxidation (M):1000 | DB Search |
| total 2 peptides |  |  |  |  |  |  |  |  |  |  |  |  |  |  |  |  |  |  |  |
| --- | --- | --- | --- | --- | --- | --- | --- | --- | --- | --- | --- | --- | --- | --- | --- | --- | --- | --- | --- |

Best Unique PSM (Scan Sample\_7\_A1\_1\_3644.d:57750, m/z=912.9935, z=2, RT=17.42, ppm=-3.06):


TRINITY\_DN6920\_c0\_g1\_i3.p1

back to list

  

| Protein Coverage
| Supporting Peptides
| Best Unique PSM
|

Protein Coverage:

Supporting Peptides:

| Peptide | Uniq | -10lgP | Mass | Length | ppm | m/z | z | RT | 1/k0 Start | 1/k0 End | Precursor ID | Area Sample 8 | #Feature | #Feature Sample 8 | Start | End | PTM | AScore | Found By |
| --- | --- | --- | --- | --- | --- | --- | --- | --- | --- | --- | --- | --- | --- | --- | --- | --- | --- | --- | --- |
| R.VLVHTQQK.L | Y | 43.71 | 951.5501 | 8 | -3.93 | 476.7784 | 2 | 9.59 | 0.8291 | 0.8364 | 12948 | 0 | 0 | 0 | 169 | 176 |  |  | DB Search |
| K.AGM(+15.99)THIVR.E | Y | 43.71 | 899.4647 | 8 | 5.69 | 450.7402 | 2 | 9.78 | 0.8161 | 0.8234 | 13845 | 0 | 0 | 0 | 58 | 65 | Oxidation (M) | M3:Oxidation (M):1000 | DB Search |
| total 2 peptides |  |  |  |  |  |  |  |  |  |  |  |  |  |  |  |  |  |  |  |
| --- | --- | --- | --- | --- | --- | --- | --- | --- | --- | --- | --- | --- | --- | --- | --- | --- | --- | --- | --- |

Best Unique PSM (Scan Sample\_7\_A1\_1\_3644.d:12948, m/z=476.7784, z=2, RT=9.59, ppm=-8.41):


TRINITY\_DN3448\_c0\_g2\_i1.p1

back to list

  

| Protein Coverage
| Supporting Peptides
| Best Unique PSM
|

Protein Coverage:

Supporting Peptides:

| Peptide | Uniq | -10lgP | Mass | Length | ppm | m/z | z | RT | 1/k0 Start | 1/k0 End | Precursor ID | Area Sample 8 | #Feature | #Feature Sample 8 | Start | End | PTM | AScore | Found By |
| --- | --- | --- | --- | --- | --- | --- | --- | --- | --- | --- | --- | --- | --- | --- | --- | --- | --- | --- | --- |
| K.AGM(+15.99)THIVR.D | Y | 43.71 | 899.4647 | 8 | 5.69 | 450.7402 | 2 | 9.78 | 0.8161 | 0.8234 | 13845 | 0 | 0 | 0 | 31 | 38 | Oxidation (M) | M3:Oxidation (M):1000 | DB Search |
| total 1 peptides |  |  |  |  |  |  |  |  |  |  |  |  |  |  |  |  |  |  |  |
| --- | --- | --- | --- | --- | --- | --- | --- | --- | --- | --- | --- | --- | --- | --- | --- | --- | --- | --- | --- |

Best Unique PSM (Scan Sample\_7\_A1\_1\_3644.d:13845, m/z=450.7402, z=2, RT=9.78, ppm=1.21):


TRINITY\_DN2885\_c0\_g1\_i2.p1

back to list

  

| Protein Coverage
| Supporting Peptides
| Best Unique PSM
|

Protein Coverage:

Supporting Peptides:

| Peptide | Uniq | -10lgP | Mass | Length | ppm | m/z | z | RT | 1/k0 Start | 1/k0 End | Precursor ID | Area Sample 8 | #Feature | #Feature Sample 8 | Start | End | PTM | AScore | Found By |
| --- | --- | --- | --- | --- | --- | --- | --- | --- | --- | --- | --- | --- | --- | --- | --- | --- | --- | --- | --- |
| K.VTRPHGASGVVR.A | Y | 43.35 | 1234.6894 | 12 | 2.89 | 412.5698 | 3 | 10.09 | 0.8173 | 0.8246 | 15387 | 0 | 0 | 0 | 77 | 88 |  |  | DB Search |
| total 1 peptides |  |  |  |  |  |  |  |  |  |  |  |  |  |  |  |  |  |  |  |
| --- | --- | --- | --- | --- | --- | --- | --- | --- | --- | --- | --- | --- | --- | --- | --- | --- | --- | --- | --- |

Best Unique PSM (Scan Sample\_7\_A1\_1\_3644.d:15387, m/z=412.5698, z=3, RT=10.09, ppm=-1.58):


TRINITY\_DN32270\_c0\_g1\_i1.p2

back to list

  

| Protein Coverage
| Supporting Peptides
| Best Unique PSM
|

Protein Coverage:

Supporting Peptides:

| Peptide | Uniq | -10lgP | Mass | Length | ppm | m/z | z | RT | 1/k0 Start | 1/k0 End | Precursor ID | Area Sample 8 | #Feature | #Feature Sample 8 | Start | End | PTM | AScore | Found By |
| --- | --- | --- | --- | --- | --- | --- | --- | --- | --- | --- | --- | --- | --- | --- | --- | --- | --- | --- | --- |
| K.SPVGANLHEIR.L | Y | 43.77 | 1191.636 | 11 | -2.1 | 596.8214 | 2 | 13.27 | 0.9533 | 0.9606 | 33347 | 6.88e2 | 1 | 1 | 206 | 216 |  |  | DB Search |
| total 1 peptides |  |  |  |  |  |  |  |  |  |  |  |  |  |  |  |  |  |  |  |
| --- | --- | --- | --- | --- | --- | --- | --- | --- | --- | --- | --- | --- | --- | --- | --- | --- | --- | --- | --- |

Best Unique PSM (Scan Sample\_7\_A1\_1\_3644.d:33347, m/z=596.8214, z=2, RT=13.27, ppm=-6.58):


TRINITY\_DN6397\_c0\_g2\_i3.p1

back to list

  

| Protein Coverage
| Supporting Peptides
| Best Unique PSM
|

Protein Coverage:

Supporting Peptides:

| Peptide | Uniq | -10lgP | Mass | Length | ppm | m/z | z | RT | 1/k0 Start | 1/k0 End | Precursor ID | Area Sample 8 | #Feature | #Feature Sample 8 | Start | End | PTM | AScore | Found By |
| --- | --- | --- | --- | --- | --- | --- | --- | --- | --- | --- | --- | --- | --- | --- | --- | --- | --- | --- | --- |
| K.K(+42.01)LSAEVANGR.L | Y | 43.74 | 1085.5829 | 10 | -2.08 | 543.7952 | 2 | 13.04 | 0.8733 | 0.8806 | 32177 | 0 | 0 | 0 | 12 | 21 | Acetylation (Protein N-term) | K1:Acetylation (Protein N-term):1000 | DB Search |
| total 1 peptides |  |  |  |  |  |  |  |  |  |  |  |  |  |  |  |  |  |  |  |
| --- | --- | --- | --- | --- | --- | --- | --- | --- | --- | --- | --- | --- | --- | --- | --- | --- | --- | --- | --- |

Best Unique PSM (Scan Sample\_7\_A1\_1\_3644.d:32177, m/z=543.7952, z=2, RT=13.04, ppm=-6.55):


TRINITY\_DN161\_c0\_g1\_i3.p1

back to list

  

| Protein Coverage
| Supporting Peptides
| Best Unique PSM
|

Protein Coverage:

Supporting Peptides:

| Peptide | Uniq | -10lgP | Mass | Length | ppm | m/z | z | RT | 1/k0 Start | 1/k0 End | Precursor ID | Area Sample 8 | #Feature | #Feature Sample 8 | Start | End | PTM | AScore | Found By |
| --- | --- | --- | --- | --- | --- | --- | --- | --- | --- | --- | --- | --- | --- | --- | --- | --- | --- | --- | --- |
| K.SYELPDGQVITIGNER.F | Y | 43.86 | 1789.8846 | 16 | 3.32 | 895.9485 | 2 | 18.70 | 1.1126 | 1.1198 | 65490 | 4.79e3 | 2 | 2 | 262 | 277 |  |  | DB Search |
| K.DSYVGDEAQSKR.G | Y | 43.81 | 1353.616 | 12 | 3.22 | 677.8145 | 2 | 11.01 | 0.9961 | 1.0033 | 20355 | 1.65e3 | 1 | 1 | 74 | 85 |  |  | DB Search |
| R.AVFPSIVGRPR.H | Y | 43.77 | 1197.6982 | 11 | 1.95 | 599.8549 | 2 | 15.90 | 0.959 | 0.9662 | 48470 | 4.43e4 | 1 | 1 | 52 | 62 |  |  | DB Search |
| R.LDLAGRDLTDYLMK.I | Y | 43.72 | 1622.8338 | 14 | -2.1 | 541.9483 | 3 | 19.46 | 0.7998 | 0.8071 | 70651 | 1.99e3 | 2 | 2 | 201 | 214 |  |  | DB Search |
| R.VAPEEHPVLLTEAPLNPK.A | Y | 43.24 | 1953.0571 | 18 | -8.51 | 977.5231 | 2 | 16.59 | 1.2073 | 1.2145 | 52921 | 0 | 0 | 0 | 119 | 136 |  |  | DB Search |
| K.EEYDESGPSIVHR.K | Y | 42.51 | 1516.6794 | 13 | -1.53 | 759.3424 | 2 | 13.28 | 0.9904 | 0.9977 | 33566 | 2.23e2 | 1 | 1 | 383 | 395 |  |  | DB Search |
| total 6 peptides |  |  |  |  |  |  |  |  |  |  |  |  |  |  |  |  |  |  |  |
| --- | --- | --- | --- | --- | --- | --- | --- | --- | --- | --- | --- | --- | --- | --- | --- | --- | --- | --- | --- |

Best Unique PSM (Scan Sample\_7\_A1\_1\_3644.d:65490, m/z=895.9485, z=2, RT=18.70, ppm=-1.15):


TRINITY\_DN875\_c0\_g1\_i3.p1

back to list

  

| Protein Coverage
| Supporting Peptides
| Best Unique PSM
|

Protein Coverage:

Supporting Peptides:

| Peptide | Uniq | -10lgP | Mass | Length | ppm | m/z | z | RT | 1/k0 Start | 1/k0 End | Precursor ID | Area Sample 8 | #Feature | #Feature Sample 8 | Start | End | PTM | AScore | Found By |
| --- | --- | --- | --- | --- | --- | --- | --- | --- | --- | --- | --- | --- | --- | --- | --- | --- | --- | --- | --- |
| R.TC(+57.02)TNPEPK.G | Y | 43.67 | 945.4226 | 8 | 1.26 | 473.717 | 2 | 9.52 | 0.7734 | 0.7807 | 12535 | 1.14e4 | 2 | 2 | 175 | 182 | Carbamidomethylation |  | DB Search |
| R.AVFC(+57.02)EHQGVK.T | Y | 43.32 | 1173.5601 | 10 | -0.85 | 587.7842 | 2 | 11.69 | 0.898 | 0.9052 | 24238 | 1.92e3 | 1 | 1 | 512 | 521 | Carbamidomethylation |  | DB Search |
| total 2 peptides |  |  |  |  |  |  |  |  |  |  |  |  |  |  |  |  |  |  |  |
| --- | --- | --- | --- | --- | --- | --- | --- | --- | --- | --- | --- | --- | --- | --- | --- | --- | --- | --- | --- |

Best Unique PSM (Scan Sample\_7\_A1\_1\_3644.d:12535, m/z=473.717, z=2, RT=9.52, ppm=-3.22):


TRINITY\_DN138\_c0\_g1\_i1.p1

back to list

  

| Protein Coverage
| Supporting Peptides
| Best Unique PSM
|

Protein Coverage:

Supporting Peptides:

| Peptide | Uniq | -10lgP | Mass | Length | ppm | m/z | z | RT | 1/k0 Start | 1/k0 End | Precursor ID | Area Sample 8 | #Feature | #Feature Sample 8 | Start | End | PTM | AScore | Found By |
| --- | --- | --- | --- | --- | --- | --- | --- | --- | --- | --- | --- | --- | --- | --- | --- | --- | --- | --- | --- |
| R.TLDFVVELSK.G | Y | 43.74 | 1149.6281 | 10 | -2.5 | 575.8173 | 2 | 19.63 | 0.8874 | 0.8947 | 71820 | 0 | 0 | 0 | 237 | 246 |  |  | DB Search |
| K.IGC(+57.02)FHDR.I | Y | 43.69 | 903.4021 | 7 | 2.82 | 452.7076 | 2 | 11.35 | 0.814 | 0.8214 | 22258 | 1.29e3 | 2 | 1 | 33 | 39 | Carbamidomethylation |  | DB Search |
| K.DMGVNVISVGAGHTINHR.E | Y | 43.56 | 1875.9373 | 18 | 12.29 | 938.9833 | 2 | 15.86 | 1.2424 | 1.2495 | 48723 | 3.08e2 | 1 | 1 | 349 | 366 |  |  | DB Search |
| R.FC(+57.02)SNPEPENGGQTC(+57.02)EGEAR.E | Y | 43.56 | 2137.8429 | 19 | -3.54 | 1069.9202 | 2 | 12.17 | 1.159 | 1.1662 | 27091 | 7.37e1 | 1 | 1 | 181 | 199 | Carbamidomethylation, Carbamidomethylation |  | DB Search |
| K.VHFGVLSFSHR.T | Y | 43.24 | 1284.6727 | 11 | 4.75 | 429.2316 | 3 | 16.45 | 0.7604 | 0.7677 | 52159 | 0 | 0 | 0 | 255 | 265 |  |  | DB Search |
| K.DM(+15.99)GVNVISVGAGHTINHR.E | Y | 42.58 | 1891.9322 | 18 | 0.27 | 946.9694 | 2 | 14.30 | 1.2276 | 1.2348 | 39624 | 0 | 0 | 0 | 349 | 366 | Oxidation (M) | M2:Oxidation (M):1000 | DB Search |
| total 6 peptides |  |  |  |  |  |  |  |  |  |  |  |  |  |  |  |  |  |  |  |
| --- | --- | --- | --- | --- | --- | --- | --- | --- | --- | --- | --- | --- | --- | --- | --- | --- | --- | --- | --- |

Best Unique PSM (Scan Sample\_7\_A1\_1\_3644.d:71820, m/z=575.8173, z=2, RT=19.63, ppm=-6.97):


TRINITY\_DN5365\_c0\_g1\_i3.p1

back to list

  

| Protein Coverage
| Supporting Peptides
| Best Unique PSM
|

Protein Coverage:

Supporting Peptides:

| Peptide | Uniq | -10lgP | Mass | Length | ppm | m/z | z | RT | 1/k0 Start | 1/k0 End | Precursor ID | Area Sample 8 | #Feature | #Feature Sample 8 | Start | End | PTM | AScore | Found By |
| --- | --- | --- | --- | --- | --- | --- | --- | --- | --- | --- | --- | --- | --- | --- | --- | --- | --- | --- | --- |
| R.FPGQLNADIRK.L | Y | 43.16 | 1257.6829 | 11 | -1.33 | 629.8451 | 2 | 14.44 | 0.9497 | 0.957 | 40493 | 0 | 0 | 0 | 244 | 254 |  |  | DB Search |
| total 1 peptides |  |  |  |  |  |  |  |  |  |  |  |  |  |  |  |  |  |  |  |
| --- | --- | --- | --- | --- | --- | --- | --- | --- | --- | --- | --- | --- | --- | --- | --- | --- | --- | --- | --- |

Best Unique PSM (Scan Sample\_7\_A1\_1\_3644.d:40493, m/z=629.8451, z=2, RT=14.44, ppm=-5.81):


TRINITY\_DN2113\_c0\_g1\_i1.p1

back to list

  

| Protein Coverage
| Supporting Peptides
| Best Unique PSM
|

Protein Coverage:

Supporting Peptides:

| Peptide | Uniq | -10lgP | Mass | Length | ppm | m/z | z | RT | 1/k0 Start | 1/k0 End | Precursor ID | Area Sample 8 | #Feature | #Feature Sample 8 | Start | End | PTM | AScore | Found By |
| --- | --- | --- | --- | --- | --- | --- | --- | --- | --- | --- | --- | --- | --- | --- | --- | --- | --- | --- | --- |
| R.AHVAQLC(+57.02)EK.A | Y | 43.74 | 1054.5229 | 9 | 0.64 | 528.2667 | 2 | 10.78 | 0.8603 | 0.8676 | 19105 | 9.91e2 | 1 | 1 | 611 | 619 | Carbamidomethylation |  | DB Search |
| R.ALEHYSDIYDIKR.A | Y | 43.53 | 1621.81 | 13 | -3.67 | 541.6062 | 3 | 14.76 | 0.8238 | 0.8311 | 42332 | 0 | 0 | 0 | 626 | 638 |  |  | DB Search |
| R.LADLEEFISSPNHANISQVGER.C | Y | 43.48 | 2425.1873 | 22 | 4.51 | 809.4031 | 3 | 18.95 | 0.9255 | 0.9327 | 67455 | 1.08e3 | 1 | 1 | 1183 | 1204 |  |  | DB Search |
| K.GQFSTDDLVAEVEKR.N | Y | 42.74 | 1692.8318 | 15 | 10.63 | 847.4284 | 2 | 17.88 | 1.141 | 1.1482 | 60636 | 0 | 0 | 0 | 838 | 852 |  |  | DB Search |
| total 4 peptides |  |  |  |  |  |  |  |  |  |  |  |  |  |  |  |  |  |  |  |
| --- | --- | --- | --- | --- | --- | --- | --- | --- | --- | --- | --- | --- | --- | --- | --- | --- | --- | --- | --- |

Best Unique PSM (Scan Sample\_7\_A1\_1\_3644.d:19105, m/z=528.2667, z=2, RT=10.78, ppm=-3.84):


TRINITY\_DN36\_c0\_g1\_i4.p1

back to list

  

| Protein Coverage
| Supporting Peptides
| Best Unique PSM
|

Protein Coverage:

Supporting Peptides:

| Peptide | Uniq | -10lgP | Mass | Length | ppm | m/z | z | RT | 1/k0 Start | 1/k0 End | Precursor ID | Area Sample 8 | #Feature | #Feature Sample 8 | Start | End | PTM | AScore | Found By |
| --- | --- | --- | --- | --- | --- | --- | --- | --- | --- | --- | --- | --- | --- | --- | --- | --- | --- | --- | --- |
| R.LGGIEVTQGNC(+57.02)AR.K | Y | 43.02 | 1373.6721 | 13 | 2.68 | 687.8421 | 2 | 13.90 | 0.9662 | 0.9735 | 37137 | 6.95e2 | 1 | 1 | 58 | 70 | Carbamidomethylation |  | DB Search |
| total 1 peptides |  |  |  |  |  |  |  |  |  |  |  |  |  |  |  |  |  |  |  |
| --- | --- | --- | --- | --- | --- | --- | --- | --- | --- | --- | --- | --- | --- | --- | --- | --- | --- | --- | --- |

Best Unique PSM (Scan Sample\_7\_A1\_1\_3644.d:37137, m/z=687.8421, z=2, RT=13.90, ppm=-1.8):


TRINITY\_DN41662\_c0\_g1\_i2.p1

back to list

  

| Protein Coverage
| Supporting Peptides
| Best Unique PSM
|

Protein Coverage:

Supporting Peptides:

| Peptide | Uniq | -10lgP | Mass | Length | ppm | m/z | z | RT | 1/k0 Start | 1/k0 End | Precursor ID | Area Sample 8 | #Feature | #Feature Sample 8 | Start | End | PTM | AScore | Found By |
| --- | --- | --- | --- | --- | --- | --- | --- | --- | --- | --- | --- | --- | --- | --- | --- | --- | --- | --- | --- |
| K.LLAQTTLRNELGTK.N | N | 43.63 | 1556.8886 | 14 | -0.19 | 779.4479 | 2 | 15.37 | 1.1098 | 1.117 | 45889 | 3.33e2 | 1 | 1 | 147 | 160 |  |  | DB Search |
| R.AM(+15.99)AAEAEASR.E | Y | 43.45 | 1021.4498 | 10 | 0.68 | 511.7303 | 2 | 9.50 | 0.8067 | 0.814 | 12520 | 4.25e2 | 1 | 1 | 208 | 217 | Oxidation (M) | M2:Oxidation (M):1000 | DB Search |
| total 2 peptides |  |  |  |  |  |  |  |  |  |  |  |  |  |  |  |  |  |  |  |
| --- | --- | --- | --- | --- | --- | --- | --- | --- | --- | --- | --- | --- | --- | --- | --- | --- | --- | --- | --- |

Best Unique PSM (Scan Sample\_7\_A1\_1\_3644.d:12520, m/z=511.7303, z=2, RT=9.50, ppm=-3.79):


TRINITY\_DN3036\_c0\_g1\_i19.p1

back to list

  

| Protein Coverage
| Supporting Peptides
| Best Unique PSM
|

Protein Coverage:

Supporting Peptides:

| Peptide | Uniq | -10lgP | Mass | Length | ppm | m/z | z | RT | 1/k0 Start | 1/k0 End | Precursor ID | Area Sample 8 | #Feature | #Feature Sample 8 | Start | End | PTM | AScore | Found By |
| --- | --- | --- | --- | --- | --- | --- | --- | --- | --- | --- | --- | --- | --- | --- | --- | --- | --- | --- | --- |
| R.AGHGAVTSPQTQR.G | Y | 43.55 | 1308.6534 | 13 | 3.24 | 437.2245 | 3 | 9.76 | 0.7045 | 0.7118 | 13596 | 2.79e3 | 2 | 2 | 135 | 147 |  |  | DB Search |
| R.VIGASGIHVGTM(+15.99)SFGK.M | Y | 43.2 | 1575.8079 | 16 | 0.89 | 788.9084 | 2 | 15.29 | 1.129 | 1.1362 | 45505 | 0 | 0 | 0 | 160 | 175 | Oxidation (M) | M12:Oxidation (M):1000 | DB Search |
| total 2 peptides |  |  |  |  |  |  |  |  |  |  |  |  |  |  |  |  |  |  |  |
| --- | --- | --- | --- | --- | --- | --- | --- | --- | --- | --- | --- | --- | --- | --- | --- | --- | --- | --- | --- |

Best Unique PSM (Scan Sample\_7\_A1\_1\_3644.d:13596, m/z=437.2245, z=3, RT=9.76, ppm=-1.24):


TRINITY\_DN477\_c0\_g1\_i9.p1

back to list

  

| Protein Coverage
| Supporting Peptides
| Best Unique PSM
|

Protein Coverage:

Supporting Peptides:

| Peptide | Uniq | -10lgP | Mass | Length | ppm | m/z | z | RT | 1/k0 Start | 1/k0 End | Precursor ID | Area Sample 8 | #Feature | #Feature Sample 8 | Start | End | PTM | AScore | Found By |
| --- | --- | --- | --- | --- | --- | --- | --- | --- | --- | --- | --- | --- | --- | --- | --- | --- | --- | --- | --- |
| R.TQAVLDRLNIEK.R | Y | 43.76 | 1398.783 | 12 | 6.66 | 700.4003 | 2 | 15.50 | 1.0053 | 1.0126 | 46564 | 4.13e3 | 1 | 1 | 220 | 231 |  |  | DB Search |
| K.DANSM(+15.99)PTQSR.Q | Y | 43.59 | 1121.4771 | 10 | 5.25 | 561.7463 | 2 | 8.93 | 0.8201 | 0.8274 | 10974 | 0 | 0 | 0 | 51 | 60 | Oxidation (M) | M5:Oxidation (M):1000 | DB Search |
| R.TQAVLDRLNIEKR.V | Y | 43.51 | 1554.8842 | 13 | -5.4 | 519.2969 | 3 | 14.70 | 0.8047 | 0.812 | 42040 | 0 | 0 | 0 | 220 | 232 |  |  | DB Search |
| total 3 peptides |  |  |  |  |  |  |  |  |  |  |  |  |  |  |  |  |  |  |  |
| --- | --- | --- | --- | --- | --- | --- | --- | --- | --- | --- | --- | --- | --- | --- | --- | --- | --- | --- | --- |

Best Unique PSM (Scan Sample\_7\_A1\_1\_3644.d:46564, m/z=700.4003, z=2, RT=15.50, ppm=2.19):


TRINITY\_DN3271\_c0\_g1\_i2.p1

back to list

  

| Protein Coverage
| Supporting Peptides
| Best Unique PSM
|

Protein Coverage:

Supporting Peptides:

| Peptide | Uniq | -10lgP | Mass | Length | ppm | m/z | z | RT | 1/k0 Start | 1/k0 End | Precursor ID | Area Sample 8 | #Feature | #Feature Sample 8 | Start | End | PTM | AScore | Found By |
| --- | --- | --- | --- | --- | --- | --- | --- | --- | --- | --- | --- | --- | --- | --- | --- | --- | --- | --- | --- |
| K.TIESEAAHGTVTR.H | Y | 43.75 | 1370.679 | 13 | 3.55 | 686.3461 | 2 | 12.00 | 1.0174 | 1.0246 | 26077 | 5.62e2 | 1 | 1 | 354 | 366 |  |  | DB Search |
| total 1 peptides |  |  |  |  |  |  |  |  |  |  |  |  |  |  |  |  |  |  |  |
| --- | --- | --- | --- | --- | --- | --- | --- | --- | --- | --- | --- | --- | --- | --- | --- | --- | --- | --- | --- |

Best Unique PSM (Scan Sample\_7\_A1\_1\_3644.d:26077, m/z=686.3461, z=2, RT=12.00, ppm=-0.93):


TRINITY\_DN6920\_c0\_g1\_i2.p1

back to list

  

| Protein Coverage
| Supporting Peptides
| Best Unique PSM
|

Protein Coverage:

Supporting Peptides:

| Peptide | Uniq | -10lgP | Mass | Length | ppm | m/z | z | RT | 1/k0 Start | 1/k0 End | Precursor ID | Area Sample 8 | #Feature | #Feature Sample 8 | Start | End | PTM | AScore | Found By |
| --- | --- | --- | --- | --- | --- | --- | --- | --- | --- | --- | --- | --- | --- | --- | --- | --- | --- | --- | --- |
| R.VLVHTQQK.L | Y | 43.71 | 951.5501 | 8 | -3.93 | 476.7784 | 2 | 9.59 | 0.8291 | 0.8364 | 12948 | 0 | 0 | 0 | 169 | 176 |  |  | DB Search |
| K.AGM(+15.99)THIVR.E | Y | 43.71 | 899.4647 | 8 | 5.69 | 450.7402 | 2 | 9.78 | 0.8161 | 0.8234 | 13845 | 0 | 0 | 0 | 58 | 65 | Oxidation (M) | M3:Oxidation (M):1000 | DB Search |
| total 2 peptides |  |  |  |  |  |  |  |  |  |  |  |  |  |  |  |  |  |  |  |
| --- | --- | --- | --- | --- | --- | --- | --- | --- | --- | --- | --- | --- | --- | --- | --- | --- | --- | --- | --- |

Best Unique PSM (Scan Sample\_7\_A1\_1\_3644.d:12948, m/z=476.7784, z=2, RT=9.59, ppm=-8.41):


TRINITY\_DN250\_c0\_g1\_i1.p1

back to list

  

| Protein Coverage
| Supporting Peptides
| Best Unique PSM
|

Protein Coverage:

Supporting Peptides:

| Peptide | Uniq | -10lgP | Mass | Length | ppm | m/z | z | RT | 1/k0 Start | 1/k0 End | Precursor ID | Area Sample 8 | #Feature | #Feature Sample 8 | Start | End | PTM | AScore | Found By |
| --- | --- | --- | --- | --- | --- | --- | --- | --- | --- | --- | --- | --- | --- | --- | --- | --- | --- | --- | --- |
| K.VGINYQPPTVVPGGDLAK.V | Y | 43.58 | 1823.9781 | 18 | 1.41 | 912.9935 | 2 | 17.42 | 1.1214 | 1.1286 | 57750 | 0 | 0 | 0 | 353 | 370 |  |  | DB Search |
| R.AVC(+57.02)M(+15.99)LSNTTAIAEAWAR.L | Y | 43.51 | 1879.892 | 17 | 2.8 | 940.9517 | 2 | 18.84 | 1.1454 | 1.1526 | 66760 | 4.63e2 | 1 | 1 | 374 | 390 | Carbamidomethylation, Oxidation (M) | M4:Oxidation (M):1000 | DB Search |
| total 2 peptides |  |  |  |  |  |  |  |  |  |  |  |  |  |  |  |  |  |  |  |
| --- | --- | --- | --- | --- | --- | --- | --- | --- | --- | --- | --- | --- | --- | --- | --- | --- | --- | --- | --- |

Best Unique PSM (Scan Sample\_7\_A1\_1\_3644.d:57750, m/z=912.9935, z=2, RT=17.42, ppm=-3.06):


TRINITY\_DN1668\_c0\_g1\_i1.p1

back to list

  

| Protein Coverage
| Supporting Peptides
| Best Unique PSM
|

Protein Coverage:

Supporting Peptides:

| Peptide | Uniq | -10lgP | Mass | Length | ppm | m/z | z | RT | 1/k0 Start | 1/k0 End | Precursor ID | Area Sample 8 | #Feature | #Feature Sample 8 | Start | End | PTM | AScore | Found By |
| --- | --- | --- | --- | --- | --- | --- | --- | --- | --- | --- | --- | --- | --- | --- | --- | --- | --- | --- | --- |
| R.YTNQETGEINDVVSYVPK.E | Y | 43.6 | 2054.9796 | 18 | -0.78 | 1028.4917 | 2 | 18.22 | 1.1989 | 1.2061 | 62789 | 1.96e2 | 1 | 1 | 171 | 188 |  |  | DB Search |
| total 1 peptides |  |  |  |  |  |  |  |  |  |  |  |  |  |  |  |  |  |  |  |
| --- | --- | --- | --- | --- | --- | --- | --- | --- | --- | --- | --- | --- | --- | --- | --- | --- | --- | --- | --- |

Best Unique PSM (Scan Sample\_7\_A1\_1\_3644.d:62789, m/z=1028.4917, z=2, RT=18.22, ppm=-5.25):


TRINITY\_DN33065\_c0\_g1\_i1.p1

back to list

  

| Protein Coverage
| Supporting Peptides
| Best Unique PSM
|

Protein Coverage:

Supporting Peptides:

| Peptide | Uniq | -10lgP | Mass | Length | ppm | m/z | z | RT | 1/k0 Start | 1/k0 End | Precursor ID | Area Sample 8 | #Feature | #Feature Sample 8 | Start | End | PTM | AScore | Found By |
| --- | --- | --- | --- | --- | --- | --- | --- | --- | --- | --- | --- | --- | --- | --- | --- | --- | --- | --- | --- |
| K.AVTIESTSNSVNGVFDTVEGK.F | Y | 42.45 | 2153.0488 | 21 | 0.69 | 1077.5276 | 2 | 19.00 | 1.2141 | 1.2212 | 67823 | 0 | 0 | 0 | 281 | 301 |  |  | DB Search |
| total 1 peptides |  |  |  |  |  |  |  |  |  |  |  |  |  |  |  |  |  |  |  |
| --- | --- | --- | --- | --- | --- | --- | --- | --- | --- | --- | --- | --- | --- | --- | --- | --- | --- | --- | --- |

Best Unique PSM (Scan Sample\_7\_A1\_1\_3644.d:67823, m/z=1077.5276, z=2, RT=19.00, ppm=-3.78):


TRINITY\_DN2799\_c0\_g1\_i4.p2

back to list

  

| Protein Coverage
| Supporting Peptides
| Best Unique PSM
|

Protein Coverage:

Supporting Peptides:

| Peptide | Uniq | -10lgP | Mass | Length | ppm | m/z | z | RT | 1/k0 Start | 1/k0 End | Precursor ID | Area Sample 8 | #Feature | #Feature Sample 8 | Start | End | PTM | AScore | Found By |
| --- | --- | --- | --- | --- | --- | --- | --- | --- | --- | --- | --- | --- | --- | --- | --- | --- | --- | --- | --- |
| K.AGM(+15.99)THIVR.D | Y | 43.71 | 899.4647 | 8 | 5.69 | 450.7402 | 2 | 9.78 | 0.8161 | 0.8234 | 13845 | 0 | 0 | 0 | 68 | 75 | Oxidation (M) | M3:Oxidation (M):1000 | DB Search |
| total 1 peptides |  |  |  |  |  |  |  |  |  |  |  |  |  |  |  |  |  |  |  |
| --- | --- | --- | --- | --- | --- | --- | --- | --- | --- | --- | --- | --- | --- | --- | --- | --- | --- | --- | --- |

Best Unique PSM (Scan Sample\_7\_A1\_1\_3644.d:13845, m/z=450.7402, z=2, RT=9.78, ppm=1.21):


TRINITY\_DN2799\_c0\_g1\_i2.p1

back to list

  

| Protein Coverage
| Supporting Peptides
| Best Unique PSM
|

Protein Coverage:

Supporting Peptides:

| Peptide | Uniq | -10lgP | Mass | Length | ppm | m/z | z | RT | 1/k0 Start | 1/k0 End | Precursor ID | Area Sample 8 | #Feature | #Feature Sample 8 | Start | End | PTM | AScore | Found By |
| --- | --- | --- | --- | --- | --- | --- | --- | --- | --- | --- | --- | --- | --- | --- | --- | --- | --- | --- | --- |
| K.AGM(+15.99)THIVR.D | Y | 43.71 | 899.4647 | 8 | 5.69 | 450.7402 | 2 | 9.78 | 0.8161 | 0.8234 | 13845 | 0 | 0 | 0 | 68 | 75 | Oxidation (M) | M3:Oxidation (M):1000 | DB Search |
| total 1 peptides |  |  |  |  |  |  |  |  |  |  |  |  |  |  |  |  |  |  |  |
| --- | --- | --- | --- | --- | --- | --- | --- | --- | --- | --- | --- | --- | --- | --- | --- | --- | --- | --- | --- |

Best Unique PSM (Scan Sample\_7\_A1\_1\_3644.d:13845, m/z=450.7402, z=2, RT=9.78, ppm=1.21):


TRINITY\_DN941\_c0\_g1\_i6.p1

back to list

  

| Protein Coverage
| Supporting Peptides
| Best Unique PSM
|

Protein Coverage:

Supporting Peptides:

| Peptide | Uniq | -10lgP | Mass | Length | ppm | m/z | z | RT | 1/k0 Start | 1/k0 End | Precursor ID | Area Sample 8 | #Feature | #Feature Sample 8 | Start | End | PTM | AScore | Found By |
| --- | --- | --- | --- | --- | --- | --- | --- | --- | --- | --- | --- | --- | --- | --- | --- | --- | --- | --- | --- |
| K.AHGPGLEGGVAGQPC(+57.02)R.F | Y | 40.64 | 1561.7419 | 16 | 2.05 | 781.8763 | 2 | 12.28 | 1.0275 | 1.0347 | 27748 | 0 | 0 | 0 | 279 | 294 | Carbamidomethylation |  | DB Search |
| total 1 peptides |  |  |  |  |  |  |  |  |  |  |  |  |  |  |  |  |  |  |  |
| --- | --- | --- | --- | --- | --- | --- | --- | --- | --- | --- | --- | --- | --- | --- | --- | --- | --- | --- | --- |

Best Unique PSM (Scan Sample\_7\_A1\_1\_3644.d:27748, m/z=782.374, z=2, RT=12.30, ppm=-2.42):


TRINITY\_DN1280\_c0\_g1\_i1.p1

back to list

  

| Protein Coverage
| Supporting Peptides
| Best Unique PSM
|

Protein Coverage:

Supporting Peptides:

| Peptide | Uniq | -10lgP | Mass | Length | ppm | m/z | z | RT | 1/k0 Start | 1/k0 End | Precursor ID | Area Sample 8 | #Feature | #Feature Sample 8 | Start | End | PTM | AScore | Found By |
| --- | --- | --- | --- | --- | --- | --- | --- | --- | --- | --- | --- | --- | --- | --- | --- | --- | --- | --- | --- |
| K.AEYENKTEVAR.S | Y | 43.83 | 1308.631 | 11 | 1.95 | 655.3211 | 2 | 10.58 | 0.9727 | 0.98 | 17983 | 0 | 0 | 0 | 202 | 212 |  |  | DB Search |
| R.NIALETLEGHQR.A | Y | 43.79 | 1379.7157 | 12 | 3.84 | 690.8647 | 2 | 15.44 | 1.0166 | 1.0238 | 46287 | 6.11e2 | 1 | 1 | 99 | 110 |  |  | DB Search |
| K.SAYDIEVNTKK.A | Y | 43.41 | 1266.6456 | 11 | 2.1 | 634.3286 | 2 | 13.00 | 0.9691 | 0.9763 | 31941 | 0 | 0 | 0 | 222 | 232 |  |  | DB Search |
| total 3 peptides |  |  |  |  |  |  |  |  |  |  |  |  |  |  |  |  |  |  |  |
| --- | --- | --- | --- | --- | --- | --- | --- | --- | --- | --- | --- | --- | --- | --- | --- | --- | --- | --- | --- |

Best Unique PSM (Scan Sample\_7\_A1\_1\_3644.d:17983, m/z=655.3211, z=2, RT=10.58, ppm=-2.53):


TRINITY\_DN329\_c0\_g1\_i1.p1

back to list

  

| Protein Coverage
| Supporting Peptides
| Best Unique PSM
|

Protein Coverage:

Supporting Peptides:

| Peptide | Uniq | -10lgP | Mass | Length | ppm | m/z | z | RT | 1/k0 Start | 1/k0 End | Precursor ID | Area Sample 8 | #Feature | #Feature Sample 8 | Start | End | PTM | AScore | Found By |
| --- | --- | --- | --- | --- | --- | --- | --- | --- | --- | --- | --- | --- | --- | --- | --- | --- | --- | --- | --- |
| K.EGDLVIHSGITDAVK.Y | Y | 43.83 | 1552.8096 | 15 | 0.72 | 777.4092 | 2 | 15.57 | 1.0576 | 1.0648 | 46999 | 6.3e3 | 3 | 3 | 174 | 188 |  |  | DB Search |
| K.SFHFGVIKLEGK.T | Y | 43.81 | 1360.7503 | 12 | 3.09 | 681.3815 | 2 | 17.25 | 1.0303 | 1.0375 | 56659 | 1.05e4 | 3 | 3 | 20 | 31 |  |  | DB Search |
| K.GLKVDFDTTFAPVTGK.K | Y | 43.81 | 1694.8879 | 16 | -2.21 | 848.4456 | 2 | 18.33 | 1.0921 | 1.0993 | 63359 | 2.86e3 | 1 | 1 | 93 | 108 |  |  | DB Search |
| K.GLKVDFDTTFAPVTGKK.T | Y | 43.8 | 1822.9828 | 17 | 0.54 | 912.4951 | 2 | 17.35 | 1.1825 | 1.1897 | 57165 | 5.55e3 | 2 | 2 | 93 | 109 |  |  | DB Search |
| K.VDFDTTFAPVTGKK.T | Y | 43.77 | 1524.7824 | 14 | 1.56 | 763.3962 | 2 | 16.78 | 1.0524 | 1.0596 | 54009 | 4.02e3 | 2 | 2 | 96 | 109 |  |  | DB Search |
| K.LRPGVELTMSSLVNAK.S | Y | 43.74 | 1713.9447 | 16 | 1.1 | 572.3203 | 3 | 17.93 | 0.9113 | 0.9186 | 60853 | 1.13e4 | 3 | 3 | 250 | 265 |  |  | DB Search |
| K.AKIDNNLR.L | Y | 43.36 | 942.5247 | 8 | 5.16 | 472.2699 | 2 | 10.71 | 0.8412 | 0.8485 | 18673 | 3.82e3 | 1 | 1 | 234 | 241 |  |  | DB Search |
| K.LRPGVELTM(+15.99)SSLVNAK.S | Y | 43.25 | 1729.9396 | 16 | 1.89 | 865.9749 | 2 | 15.36 | 1.1378 | 1.145 | 45662 | 1.01e4 | 1 | 1 | 250 | 265 | Oxidation (M) | M9:Oxidation (M):1000 | DB Search |
| K.SFHFGVIK.L | Y | 43.17 | 933.5072 | 8 | 1.24 | 467.7594 | 2 | 16.37 | 0.8226 | 0.8299 | 51672 | 8.38e2 | 1 | 1 | 20 | 27 |  |  | DB Search |
| total 9 peptides |  |  |  |  |  |  |  |  |  |  |  |  |  |  |  |  |  |  |  |
| --- | --- | --- | --- | --- | --- | --- | --- | --- | --- | --- | --- | --- | --- | --- | --- | --- | --- | --- | --- |

Best Unique PSM (Scan Sample\_7\_A1\_1\_3644.d:46999, m/z=777.4092, z=2, RT=15.57, ppm=-3.76):


TRINITY\_DN1559\_c0\_g1\_i1.p1

back to list

  

| Protein Coverage
| Supporting Peptides
| Best Unique PSM
|

Protein Coverage:

Supporting Peptides:

| Peptide | Uniq | -10lgP | Mass | Length | ppm | m/z | z | RT | 1/k0 Start | 1/k0 End | Precursor ID | Area Sample 8 | #Feature | #Feature Sample 8 | Start | End | PTM | AScore | Found By |
| --- | --- | --- | --- | --- | --- | --- | --- | --- | --- | --- | --- | --- | --- | --- | --- | --- | --- | --- | --- |
| R.GVAGAGVLAGFDKFK.E | Y | 43.76 | 1435.7823 | 15 | 1.27 | 718.8961 | 2 | 18.47 | 1.0865 | 1.0937 | 64267 | 2.7e3 | 1 | 1 | 282 | 296 |  |  | DB Search |
| total 1 peptides |  |  |  |  |  |  |  |  |  |  |  |  |  |  |  |  |  |  |  |
| --- | --- | --- | --- | --- | --- | --- | --- | --- | --- | --- | --- | --- | --- | --- | --- | --- | --- | --- | --- |

Best Unique PSM (Scan Sample\_7\_A1\_1\_3644.d:64267, m/z=718.8961, z=2, RT=18.47, ppm=-3.21):


TRINITY\_DN2799\_c0\_g1\_i3.p1

back to list

  

| Protein Coverage
| Supporting Peptides
| Best Unique PSM
|

Protein Coverage:

Supporting Peptides:

| Peptide | Uniq | -10lgP | Mass | Length | ppm | m/z | z | RT | 1/k0 Start | 1/k0 End | Precursor ID | Area Sample 8 | #Feature | #Feature Sample 8 | Start | End | PTM | AScore | Found By |
| --- | --- | --- | --- | --- | --- | --- | --- | --- | --- | --- | --- | --- | --- | --- | --- | --- | --- | --- | --- |
| K.AGM(+15.99)THIVR.D | Y | 43.71 | 899.4647 | 8 | 5.69 | 450.7402 | 2 | 9.78 | 0.8161 | 0.8234 | 13845 | 0 | 0 | 0 | 69 | 76 | Oxidation (M) | M3:Oxidation (M):1000 | DB Search |
| total 1 peptides |  |  |  |  |  |  |  |  |  |  |  |  |  |  |  |  |  |  |  |
| --- | --- | --- | --- | --- | --- | --- | --- | --- | --- | --- | --- | --- | --- | --- | --- | --- | --- | --- | --- |

Best Unique PSM (Scan Sample\_7\_A1\_1\_3644.d:13845, m/z=450.7402, z=2, RT=9.78, ppm=1.21):


TRINITY\_DN250\_c0\_g1\_i6.p1

back to list

  

| Protein Coverage
| Supporting Peptides
| Best Unique PSM
|

Protein Coverage:

Supporting Peptides:

| Peptide | Uniq | -10lgP | Mass | Length | ppm | m/z | z | RT | 1/k0 Start | 1/k0 End | Precursor ID | Area Sample 8 | #Feature | #Feature Sample 8 | Start | End | PTM | AScore | Found By |
| --- | --- | --- | --- | --- | --- | --- | --- | --- | --- | --- | --- | --- | --- | --- | --- | --- | --- | --- | --- |
| K.VGINYQPPTVVPGGDLAK.V | Y | 43.58 | 1823.9781 | 18 | 1.41 | 912.9935 | 2 | 17.42 | 1.1214 | 1.1286 | 57750 | 0 | 0 | 0 | 353 | 370 |  |  | DB Search |
| total 1 peptides |  |  |  |  |  |  |  |  |  |  |  |  |  |  |  |  |  |  |  |
| --- | --- | --- | --- | --- | --- | --- | --- | --- | --- | --- | --- | --- | --- | --- | --- | --- | --- | --- | --- |

Best Unique PSM (Scan Sample\_7\_A1\_1\_3644.d:57750, m/z=912.9935, z=2, RT=17.42, ppm=-3.06):


TRINITY\_DN2963\_c0\_g1\_i1.p1

back to list

  

| Protein Coverage
| Supporting Peptides
| Best Unique PSM
|

Protein Coverage:

Supporting Peptides:

| Peptide | Uniq | -10lgP | Mass | Length | ppm | m/z | z | RT | 1/k0 Start | 1/k0 End | Precursor ID | Area Sample 8 | #Feature | #Feature Sample 8 | Start | End | PTM | AScore | Found By |
| --- | --- | --- | --- | --- | --- | --- | --- | --- | --- | --- | --- | --- | --- | --- | --- | --- | --- | --- | --- |
| M.S(+42.01)ATGNLVTVLRK.L | Y | 43.75 | 1299.751 | 12 | 3.1 | 650.8819 | 2 | 17.29 | 1.0005 | 1.0077 | 56892 | 0 | 0 | 0 | 2 | 13 | Acetylation (Protein N-term) | S1:Acetylation (Protein N-term):1000 | DB Search |
| total 1 peptides |  |  |  |  |  |  |  |  |  |  |  |  |  |  |  |  |  |  |  |
| --- | --- | --- | --- | --- | --- | --- | --- | --- | --- | --- | --- | --- | --- | --- | --- | --- | --- | --- | --- |

Best Unique PSM (Scan Sample\_7\_A1\_1\_3644.d:56892, m/z=650.8819, z=2, RT=17.29, ppm=-1.37):


TRINITY\_DN2477\_c0\_g1\_i9.p1

back to list

  

| Protein Coverage
| Supporting Peptides
| Best Unique PSM
|

Protein Coverage:

Supporting Peptides:

| Peptide | Uniq | -10lgP | Mass | Length | ppm | m/z | z | RT | 1/k0 Start | 1/k0 End | Precursor ID | Area Sample 8 | #Feature | #Feature Sample 8 | Start | End | PTM | AScore | Found By |
| --- | --- | --- | --- | --- | --- | --- | --- | --- | --- | --- | --- | --- | --- | --- | --- | --- | --- | --- | --- |
| K.DSYVGDEAQSKR.G | Y | 43.81 | 1353.616 | 12 | 3.22 | 677.8145 | 2 | 11.01 | 0.9961 | 1.0033 | 20355 | 1.65e3 | 1 | 1 | 52 | 63 |  |  | DB Search |
| R.VAPEEHPVLLTEAPLNPK.A | Y | 43.24 | 1953.0571 | 18 | -8.51 | 977.5231 | 2 | 16.59 | 1.2073 | 1.2145 | 52921 | 0 | 0 | 0 | 97 | 114 |  |  | DB Search |
| total 2 peptides |  |  |  |  |  |  |  |  |  |  |  |  |  |  |  |  |  |  |  |
| --- | --- | --- | --- | --- | --- | --- | --- | --- | --- | --- | --- | --- | --- | --- | --- | --- | --- | --- | --- |

Best Unique PSM (Scan Sample\_7\_A1\_1\_3644.d:20355, m/z=677.8145, z=2, RT=11.01, ppm=-1.25):


TRINITY\_DN8597\_c0\_g1\_i1.p1

back to list

  

| Protein Coverage
| Supporting Peptides
| Best Unique PSM
|

Protein Coverage:

Supporting Peptides:

| Peptide | Uniq | -10lgP | Mass | Length | ppm | m/z | z | RT | 1/k0 Start | 1/k0 End | Precursor ID | Area Sample 8 | #Feature | #Feature Sample 8 | Start | End | PTM | AScore | Found By |
| --- | --- | --- | --- | --- | --- | --- | --- | --- | --- | --- | --- | --- | --- | --- | --- | --- | --- | --- | --- |
| R.AFNKVETR.N | N | 43.74 | 963.5138 | 8 | 5.69 | 482.7647 | 2 | 10.62 | 0.8299 | 0.8372 | 18171 | 1.3e3 | 1 | 1 | 15 | 22 |  |  | DB Search |
| R.AC(+57.02)SNVNQC(+57.02)GVNTGTQTVYK.V | Y | 43.58 | 2099.9364 | 19 | 4.79 | 1050.9758 | 2 | 12.97 | 1.1794 | 1.1865 | 31687 | 0 | 0 | 0 | 47 | 65 | Carbamidomethylation, Carbamidomethylation |  | DB Search |
| total 2 peptides |  |  |  |  |  |  |  |  |  |  |  |  |  |  |  |  |  |  |  |
| --- | --- | --- | --- | --- | --- | --- | --- | --- | --- | --- | --- | --- | --- | --- | --- | --- | --- | --- | --- |

Best Unique PSM (Scan Sample\_7\_A1\_1\_3644.d:31687, m/z=1050.9758, z=2, RT=12.97, ppm=0.32):


TRINITY\_DN250\_c0\_g1\_i4.p1

back to list

  

| Protein Coverage
| Supporting Peptides
| Best Unique PSM
|

Protein Coverage:

Supporting Peptides:

| Peptide | Uniq | -10lgP | Mass | Length | ppm | m/z | z | RT | 1/k0 Start | 1/k0 End | Precursor ID | Area Sample 8 | #Feature | #Feature Sample 8 | Start | End | PTM | AScore | Found By |
| --- | --- | --- | --- | --- | --- | --- | --- | --- | --- | --- | --- | --- | --- | --- | --- | --- | --- | --- | --- |
| K.VGINYQPPTVVPGGDLAK.V | Y | 43.58 | 1823.9781 | 18 | 1.41 | 912.9935 | 2 | 17.42 | 1.1214 | 1.1286 | 57750 | 0 | 0 | 0 | 353 | 370 |  |  | DB Search |
| total 1 peptides |  |  |  |  |  |  |  |  |  |  |  |  |  |  |  |  |  |  |  |
| --- | --- | --- | --- | --- | --- | --- | --- | --- | --- | --- | --- | --- | --- | --- | --- | --- | --- | --- | --- |

Best Unique PSM (Scan Sample\_7\_A1\_1\_3644.d:57750, m/z=912.9935, z=2, RT=17.42, ppm=-3.06):


TRINITY\_DN1366\_c0\_g1\_i1.p1

back to list

  

| Protein Coverage
| Supporting Peptides
| Best Unique PSM
|

Protein Coverage:

Supporting Peptides:

| Peptide | Uniq | -10lgP | Mass | Length | ppm | m/z | z | RT | 1/k0 Start | 1/k0 End | Precursor ID | Area Sample 8 | #Feature | #Feature Sample 8 | Start | End | PTM | AScore | Found By |
| --- | --- | --- | --- | --- | --- | --- | --- | --- | --- | --- | --- | --- | --- | --- | --- | --- | --- | --- | --- |
| K.ITIEEEYKITVEK.Y | Y | 43.45 | 1593.8501 | 13 | -4.67 | 797.925 | 2 | 16.62 | 1.0672 | 1.0745 | 53021 | 1.54e2 | 1 | 1 | 4485 | 4497 |  |  | DB Search |
| total 1 peptides |  |  |  |  |  |  |  |  |  |  |  |  |  |  |  |  |  |  |  |
| --- | --- | --- | --- | --- | --- | --- | --- | --- | --- | --- | --- | --- | --- | --- | --- | --- | --- | --- | --- |

Best Unique PSM (Scan Sample\_7\_A1\_1\_3644.d:53021, m/z=797.925, z=2, RT=16.62, ppm=-9.14):


TRINITY\_DN3036\_c0\_g1\_i8.p1

back to list

  

| Protein Coverage
| Supporting Peptides
| Best Unique PSM
|

Protein Coverage:

Supporting Peptides:

| Peptide | Uniq | -10lgP | Mass | Length | ppm | m/z | z | RT | 1/k0 Start | 1/k0 End | Precursor ID | Area Sample 8 | #Feature | #Feature Sample 8 | Start | End | PTM | AScore | Found By |
| --- | --- | --- | --- | --- | --- | --- | --- | --- | --- | --- | --- | --- | --- | --- | --- | --- | --- | --- | --- |
| R.GTSNGGLVVGTIIKPK.L | Y | 43.76 | 1539.8984 | 16 | -3.44 | 770.9504 | 2 | 15.91 | 1.1262 | 1.1334 | 48868 | 9.21e2 | 1 | 1 | 173 | 188 |  |  | DB Search |
| R.AGHGAVTSPQTQR.G | Y | 43.55 | 1308.6534 | 13 | 3.24 | 437.2245 | 3 | 9.76 | 0.7045 | 0.7118 | 13596 | 2.79e3 | 2 | 2 | 309 | 321 |  |  | DB Search |
| R.VIGASGIHVGTM(+15.99)SFGK.M | Y | 43.2 | 1575.8079 | 16 | 0.89 | 788.9084 | 2 | 15.29 | 1.129 | 1.1362 | 45505 | 0 | 0 | 0 | 334 | 349 | Oxidation (M) | M12:Oxidation (M):1000 | DB Search |
| total 3 peptides |  |  |  |  |  |  |  |  |  |  |  |  |  |  |  |  |  |  |  |
| --- | --- | --- | --- | --- | --- | --- | --- | --- | --- | --- | --- | --- | --- | --- | --- | --- | --- | --- | --- |

Best Unique PSM (Scan Sample\_7\_A1\_1\_3644.d:48868, m/z=770.9504, z=2, RT=15.91, ppm=-7.92):


TRINITY\_DN761\_c0\_g1\_i2.p1

back to list

  

| Protein Coverage
| Supporting Peptides
| Best Unique PSM
|

Protein Coverage:

Supporting Peptides:

| Peptide | Uniq | -10lgP | Mass | Length | ppm | m/z | z | RT | 1/k0 Start | 1/k0 End | Precursor ID | Area Sample 8 | #Feature | #Feature Sample 8 | Start | End | PTM | AScore | Found By |
| --- | --- | --- | --- | --- | --- | --- | --- | --- | --- | --- | --- | --- | --- | --- | --- | --- | --- | --- | --- |
| K.TRIEDLETVISQTQDHR.H | Y | 43.83 | 2040.0236 | 17 | 1.88 | 681.0134 | 3 | 17.57 | 0.9626 | 0.9699 | 58583 | 4.36e2 | 1 | 1 | 260 | 276 |  |  | DB Search |
| total 1 peptides |  |  |  |  |  |  |  |  |  |  |  |  |  |  |  |  |  |  |  |
| --- | --- | --- | --- | --- | --- | --- | --- | --- | --- | --- | --- | --- | --- | --- | --- | --- | --- | --- | --- |

Best Unique PSM (Scan Sample\_7\_A1\_1\_3644.d:58583, m/z=681.0134, z=3, RT=17.57, ppm=-2.6):


TRINITY\_DN144181\_c0\_g1\_i1.p1

back to list

  

| Protein Coverage
| Supporting Peptides
| Best Unique PSM
|

Protein Coverage:

Supporting Peptides:

| Peptide | Uniq | -10lgP | Mass | Length | ppm | m/z | z | RT | 1/k0 Start | 1/k0 End | Precursor ID | Area Sample 8 | #Feature | #Feature Sample 8 | Start | End | PTM | AScore | Found By |
| --- | --- | --- | --- | --- | --- | --- | --- | --- | --- | --- | --- | --- | --- | --- | --- | --- | --- | --- | --- |
| K.IAVFDHISSATAVK.M | Y | 43.74 | 1457.7878 | 14 | -4.3 | 729.8948 | 2 | 16.25 | 1.0218 | 1.0291 | 50841 | 1.56e3 | 1 | 1 | 43 | 56 |  |  | DB Search |
| total 1 peptides |  |  |  |  |  |  |  |  |  |  |  |  |  |  |  |  |  |  |  |
| --- | --- | --- | --- | --- | --- | --- | --- | --- | --- | --- | --- | --- | --- | --- | --- | --- | --- | --- | --- |

Best Unique PSM (Scan Sample\_7\_A1\_1\_3644.d:50841, m/z=729.8948, z=2, RT=16.25, ppm=-8.77):


TRINITY\_DN2637\_c0\_g1\_i4.p1

back to list

  

| Protein Coverage
| Supporting Peptides
| Best Unique PSM
|

Protein Coverage:

Supporting Peptides:

| Peptide | Uniq | -10lgP | Mass | Length | ppm | m/z | z | RT | 1/k0 Start | 1/k0 End | Precursor ID | Area Sample 8 | #Feature | #Feature Sample 8 | Start | End | PTM | AScore | Found By |
| --- | --- | --- | --- | --- | --- | --- | --- | --- | --- | --- | --- | --- | --- | --- | --- | --- | --- | --- | --- |
| K.ATIEIPAKYEK.R | Y | 42.98 | 1261.6918 | 11 | 0.3 | 631.8505 | 2 | 14.24 | 0.9848 | 0.992 | 39239 | 0 | 0 | 0 | 87 | 97 |  |  | DB Search |
| total 1 peptides |  |  |  |  |  |  |  |  |  |  |  |  |  |  |  |  |  |  |  |
| --- | --- | --- | --- | --- | --- | --- | --- | --- | --- | --- | --- | --- | --- | --- | --- | --- | --- | --- | --- |

Best Unique PSM (Scan Sample\_7\_A1\_1\_3644.d:39239, m/z=631.8505, z=2, RT=14.24, ppm=-4.18):


TRINITY\_DN1936\_c0\_g1\_i1.p1

back to list

  

| Protein Coverage
| Supporting Peptides
| Best Unique PSM
|

Protein Coverage:

Supporting Peptides:

| Peptide | Uniq | -10lgP | Mass | Length | ppm | m/z | z | RT | 1/k0 Start | 1/k0 End | Precursor ID | Area Sample 8 | #Feature | #Feature Sample 8 | Start | End | PTM | AScore | Found By |
| --- | --- | --- | --- | --- | --- | --- | --- | --- | --- | --- | --- | --- | --- | --- | --- | --- | --- | --- | --- |
| R.SSLSTSSSGTGPK.T | Y | 42.7 | 1194.5728 | 13 | -2.94 | 598.2892 | 2 | 10.42 | 0.9133 | 0.9206 | 17169 | 0 | 0 | 0 | 267 | 279 |  |  | DB Search |
| total 1 peptides |  |  |  |  |  |  |  |  |  |  |  |  |  |  |  |  |  |  |  |
| --- | --- | --- | --- | --- | --- | --- | --- | --- | --- | --- | --- | --- | --- | --- | --- | --- | --- | --- | --- |

Best Unique PSM (Scan Sample\_7\_A1\_1\_3644.d:17169, m/z=598.2892, z=2, RT=10.42, ppm=-7.42):


TRINITY\_DN22\_c0\_g1\_i1.p1

back to list

  

| Protein Coverage
| Supporting Peptides
| Best Unique PSM
|

Protein Coverage:

Supporting Peptides:

| Peptide | Uniq | -10lgP | Mass | Length | ppm | m/z | z | RT | 1/k0 Start | 1/k0 End | Precursor ID | Area Sample 8 | #Feature | #Feature Sample 8 | Start | End | PTM | AScore | Found By |
| --- | --- | --- | --- | --- | --- | --- | --- | --- | --- | --- | --- | --- | --- | --- | --- | --- | --- | --- | --- |
| R.FVLHGDVSHGSR.Y | Y | 43.34 | 1309.6527 | 12 | -0.43 | 437.556 | 3 | 12.14 | 0.762 | 0.7693 | 26936 | 0 | 0 | 0 | 314 | 325 |  |  | DB Search |
| total 1 peptides |  |  |  |  |  |  |  |  |  |  |  |  |  |  |  |  |  |  |  |
| --- | --- | --- | --- | --- | --- | --- | --- | --- | --- | --- | --- | --- | --- | --- | --- | --- | --- | --- | --- |

Best Unique PSM (Scan Sample\_7\_A1\_1\_3644.d:26936, m/z=437.556, z=3, RT=12.14, ppm=-4.91):


TRINITY\_DN38657\_c0\_g1\_i3.p1

back to list

  

| Protein Coverage
| Supporting Peptides
| Best Unique PSM
|

Protein Coverage:

Supporting Peptides:

| Peptide | Uniq | -10lgP | Mass | Length | ppm | m/z | z | RT | 1/k0 Start | 1/k0 End | Precursor ID | Area Sample 8 | #Feature | #Feature Sample 8 | Start | End | PTM | AScore | Found By |
| --- | --- | --- | --- | --- | --- | --- | --- | --- | --- | --- | --- | --- | --- | --- | --- | --- | --- | --- | --- |
| K.STSQGQALTK.T | Y | 43.71 | 1019.5247 | 10 | 7.68 | 510.7713 | 2 | 10.06 | 0.8291 | 0.8364 | 15179 | 0 | 0 | 0 | 52 | 61 |  |  | DB Search |
| total 1 peptides |  |  |  |  |  |  |  |  |  |  |  |  |  |  |  |  |  |  |  |
| --- | --- | --- | --- | --- | --- | --- | --- | --- | --- | --- | --- | --- | --- | --- | --- | --- | --- | --- | --- |

Best Unique PSM (Scan Sample\_7\_A1\_1\_3644.d:15179, m/z=510.7713, z=2, RT=10.06, ppm=3.2):


TRINITY\_DN144508\_c0\_g1\_i1.p1

back to list

  

| Protein Coverage
| Supporting Peptides
| Best Unique PSM
|

Protein Coverage:

Supporting Peptides:

| Peptide | Uniq | -10lgP | Mass | Length | ppm | m/z | z | RT | 1/k0 Start | 1/k0 End | Precursor ID | Area Sample 8 | #Feature | #Feature Sample 8 | Start | End | PTM | AScore | Found By |
| --- | --- | --- | --- | --- | --- | --- | --- | --- | --- | --- | --- | --- | --- | --- | --- | --- | --- | --- | --- |
| K.SEEYKPTLGR.C | Y | 43.27 | 1178.5931 | 10 | 1.46 | 590.3021 | 2 | 12.31 | 0.9162 | 0.9234 | 27845 | 7.02e2 | 1 | 1 | 174 | 183 |  |  | DB Search |
| K.SSLNHNIVFEGR.T | Y | 42.69 | 1371.6895 | 12 | 3.01 | 686.851 | 2 | 14.63 | 0.9824 | 0.9896 | 41610 | 3.9e2 | 1 | 1 | 233 | 244 |  |  | DB Search |
| total 2 peptides |  |  |  |  |  |  |  |  |  |  |  |  |  |  |  |  |  |  |  |
| --- | --- | --- | --- | --- | --- | --- | --- | --- | --- | --- | --- | --- | --- | --- | --- | --- | --- | --- | --- |

Best Unique PSM (Scan Sample\_7\_A1\_1\_3644.d:27845, m/z=590.3021, z=2, RT=12.31, ppm=-3.02):


TRINITY\_DN909\_c0\_g1\_i1.p1

back to list

  

| Protein Coverage
| Supporting Peptides
| Best Unique PSM
|

Protein Coverage:

Supporting Peptides:

| Peptide | Uniq | -10lgP | Mass | Length | ppm | m/z | z | RT | 1/k0 Start | 1/k0 End | Precursor ID | Area Sample 8 | #Feature | #Feature Sample 8 | Start | End | PTM | AScore | Found By |
| --- | --- | --- | --- | --- | --- | --- | --- | --- | --- | --- | --- | --- | --- | --- | --- | --- | --- | --- | --- |
| R.AM(+15.99)AAEAESSR.E | Y | 43.73 | 1037.4448 | 10 | 3.58 | 519.7292 | 2 | 9.13 | 0.8177 | 0.825 | 11333 | 3.21e2 | 1 | 1 | 236 | 245 | Oxidation (M) | M2:Oxidation (M):1000 | DB Search |
| K.LLAQTTLRNELGTK.N | N | 43.63 | 1556.8886 | 14 | -0.19 | 779.4479 | 2 | 15.37 | 1.1098 | 1.117 | 45889 | 3.33e2 | 1 | 1 | 175 | 188 |  |  | DB Search |
| R.VISFDVPPQEILTR.D | Y | 43.29 | 1612.8824 | 14 | -5.21 | 807.4407 | 2 | 19.59 | 1.06 | 1.0672 | 71552 | 0 | 0 | 0 | 128 | 141 |  |  | DB Search |
| total 3 peptides |  |  |  |  |  |  |  |  |  |  |  |  |  |  |  |  |  |  |  |
| --- | --- | --- | --- | --- | --- | --- | --- | --- | --- | --- | --- | --- | --- | --- | --- | --- | --- | --- | --- |

Best Unique PSM (Scan Sample\_7\_A1\_1\_3644.d:11333, m/z=519.7292, z=2, RT=9.13, ppm=-0.9):


TRINITY\_DN15584\_c0\_g1\_i1.p1

back to list

  

| Protein Coverage
| Supporting Peptides
| Best Unique PSM
|

Protein Coverage:

Supporting Peptides:

| Peptide | Uniq | -10lgP | Mass | Length | ppm | m/z | z | RT | 1/k0 Start | 1/k0 End | Precursor ID | Area Sample 8 | #Feature | #Feature Sample 8 | Start | End | PTM | AScore | Found By |
| --- | --- | --- | --- | --- | --- | --- | --- | --- | --- | --- | --- | --- | --- | --- | --- | --- | --- | --- | --- |
| K.AFVAIGDYGGHVGLGVK.C | Y | 43.76 | 1658.878 | 17 | 4.21 | 830.446 | 2 | 17.81 | 1.1638 | 1.171 | 60022 | 7.23e3 | 1 | 1 | 109 | 125 |  |  | DB Search |
| R.GTGIVAAGVSKK.L | Y | 43.75 | 1086.6397 | 12 | 7.69 | 544.3289 | 2 | 11.37 | 0.9129 | 0.9202 | 22492 | 0 | 0 | 0 | 184 | 195 |  |  | DB Search |
| K.IGKPHTVPC(+57.02)K.V | Y | 43.74 | 1135.6172 | 10 | 9.46 | 379.5482 | 3 | 9.79 | 0.7624 | 0.7697 | 13817 | 1.7e3 | 1 | 1 | 157 | 166 | Carbamidomethylation |  | DB Search |
| K.LSIIPVRR.G | Y | 40.66 | 952.6182 | 8 | 1.89 | 477.3151 | 2 | 14.96 | 0.8465 | 0.8538 | 43251 | 4.86e3 | 1 | 1 | 143 | 150 |  |  | DB Search |
| total 4 peptides |  |  |  |  |  |  |  |  |  |  |  |  |  |  |  |  |  |  |  |
| --- | --- | --- | --- | --- | --- | --- | --- | --- | --- | --- | --- | --- | --- | --- | --- | --- | --- | --- | --- |

Best Unique PSM (Scan Sample\_7\_A1\_1\_3644.d:60022, m/z=830.446, z=2, RT=17.81, ppm=-0.27):


TRINITY\_DN941\_c0\_g1\_i9.p1

back to list

  

| Protein Coverage
| Supporting Peptides
| Best Unique PSM
|

Protein Coverage:

Supporting Peptides:

| Peptide | Uniq | -10lgP | Mass | Length | ppm | m/z | z | RT | 1/k0 Start | 1/k0 End | Precursor ID | Area Sample 8 | #Feature | #Feature Sample 8 | Start | End | PTM | AScore | Found By |
| --- | --- | --- | --- | --- | --- | --- | --- | --- | --- | --- | --- | --- | --- | --- | --- | --- | --- | --- | --- |
| K.SPYHVSVTPAADR.V | Y | 43.78 | 1398.6891 | 13 | 6.42 | 700.3532 | 2 | 13.00 | 0.9868 | 0.9941 | 31744 | 1.26e3 | 1 | 1 | 445 | 457 |  |  | DB Search |
| K.AHGPGLEGGVAGQPC(+57.02)R.F | Y | 40.64 | 1561.7419 | 16 | 2.05 | 781.8763 | 2 | 12.28 | 1.0275 | 1.0347 | 27748 | 0 | 0 | 0 | 2812 | 2827 | Carbamidomethylation |  | DB Search |
| total 2 peptides |  |  |  |  |  |  |  |  |  |  |  |  |  |  |  |  |  |  |  |
| --- | --- | --- | --- | --- | --- | --- | --- | --- | --- | --- | --- | --- | --- | --- | --- | --- | --- | --- | --- |

Best Unique PSM (Scan Sample\_7\_A1\_1\_3644.d:31744, m/z=700.3532, z=2, RT=13.00, ppm=1.94):


TRINITY\_DN374\_c0\_g1\_i1.p1

back to list

  

| Protein Coverage
| Supporting Peptides
| Best Unique PSM
|

Protein Coverage:

Supporting Peptides:

| Peptide | Uniq | -10lgP | Mass | Length | ppm | m/z | z | RT | 1/k0 Start | 1/k0 End | Precursor ID | Area Sample 8 | #Feature | #Feature Sample 8 | Start | End | PTM | AScore | Found By |
| --- | --- | --- | --- | --- | --- | --- | --- | --- | --- | --- | --- | --- | --- | --- | --- | --- | --- | --- | --- |
| K.YIDIEYVC(+57.02)KPPVK.K | Y | 43.56 | 1622.8378 | 13 | -3.07 | 812.42 | 2 | 16.34 | 1.1282 | 1.1354 | 51406 | 7.33e2 | 1 | 1 | 183 | 195 | Carbamidomethylation |  | DB Search |
| total 1 peptides |  |  |  |  |  |  |  |  |  |  |  |  |  |  |  |  |  |  |  |
| --- | --- | --- | --- | --- | --- | --- | --- | --- | --- | --- | --- | --- | --- | --- | --- | --- | --- | --- | --- |

Best Unique PSM (Scan Sample\_7\_A1\_1\_3644.d:51406, m/z=812.42, z=2, RT=16.34, ppm=-7.55):


TRINITY\_DN97639\_c0\_g1\_i1.p1

back to list

  

| Protein Coverage
| Supporting Peptides
| Best Unique PSM
|

Protein Coverage:

Supporting Peptides:

| Peptide | Uniq | -10lgP | Mass | Length | ppm | m/z | z | RT | 1/k0 Start | 1/k0 End | Precursor ID | Area Sample 8 | #Feature | #Feature Sample 8 | Start | End | PTM | AScore | Found By |
| --- | --- | --- | --- | --- | --- | --- | --- | --- | --- | --- | --- | --- | --- | --- | --- | --- | --- | --- | --- |
| R.V(+42.01)LLHSLK.A | Y | 43.7 | 850.5276 | 7 | -3.41 | 426.2677 | 2 | 18.85 | 0.8266 | 0.8339 | 67000 | 3.43e2 | 1 | 1 | 14 | 20 | Acetylation (Protein N-term) | V1:Acetylation (Protein N-term):1000 | DB Search |
| total 1 peptides |  |  |  |  |  |  |  |  |  |  |  |  |  |  |  |  |  |  |  |
| --- | --- | --- | --- | --- | --- | --- | --- | --- | --- | --- | --- | --- | --- | --- | --- | --- | --- | --- | --- |

Best Unique PSM (Scan Sample\_7\_A1\_1\_3644.d:67000, m/z=426.2677, z=2, RT=18.85, ppm=-7.88):


TRINITY\_DN250\_c0\_g1\_i3.p1

back to list

  

| Protein Coverage
| Supporting Peptides
| Best Unique PSM
|

Protein Coverage:

Supporting Peptides:

| Peptide | Uniq | -10lgP | Mass | Length | ppm | m/z | z | RT | 1/k0 Start | 1/k0 End | Precursor ID | Area Sample 8 | #Feature | #Feature Sample 8 | Start | End | PTM | AScore | Found By |
| --- | --- | --- | --- | --- | --- | --- | --- | --- | --- | --- | --- | --- | --- | --- | --- | --- | --- | --- | --- |
| K.VGINYQPPTVVPGGDLAK.V | Y | 43.58 | 1823.9781 | 18 | 1.41 | 912.9935 | 2 | 17.42 | 1.1214 | 1.1286 | 57750 | 0 | 0 | 0 | 353 | 370 |  |  | DB Search |
| R.LM(+15.99)GQIVSSITASLR.F | Y | 43.53 | 1490.8126 | 14 | 3.71 | 746.413 | 2 | 18.84 | 1.0359 | 1.0431 | 66733 | 4.18e2 | 1 | 1 | 230 | 243 | Oxidation (M) | M2:Oxidation (M):1000 | DB Search |
| R.AVC(+57.02)M(+15.99)LSNTTAIAEAWAR.L | Y | 43.51 | 1879.892 | 17 | 2.8 | 940.9517 | 2 | 18.84 | 1.1454 | 1.1526 | 66760 | 4.63e2 | 1 | 1 | 374 | 390 | Carbamidomethylation, Oxidation (M) | M4:Oxidation (M):1000 | DB Search |
| total 3 peptides |  |  |  |  |  |  |  |  |  |  |  |  |  |  |  |  |  |  |  |
| --- | --- | --- | --- | --- | --- | --- | --- | --- | --- | --- | --- | --- | --- | --- | --- | --- | --- | --- | --- |

Best Unique PSM (Scan Sample\_7\_A1\_1\_3644.d:57750, m/z=912.9935, z=2, RT=17.42, ppm=-3.06):


TRINITY\_DN158421\_c0\_g1\_i1.p1

back to list

  

| Protein Coverage
| Supporting Peptides
| Best Unique PSM
|

Protein Coverage:

Supporting Peptides:

| Peptide | Uniq | -10lgP | Mass | Length | ppm | m/z | z | RT | 1/k0 Start | 1/k0 End | Precursor ID | Area Sample 8 | #Feature | #Feature Sample 8 | Start | End | PTM | AScore | Found By |
| --- | --- | --- | --- | --- | --- | --- | --- | --- | --- | --- | --- | --- | --- | --- | --- | --- | --- | --- | --- |
| R.SSC(+57.02)RPILSVIK.I | Y | 42.42 | 1258.7067 | 11 | -5.58 | 630.3543 | 2 | 17.05 | 0.9731 | 0.9804 | 55404 | 1.96e4 | 2 | 2 | 90 | 100 | Carbamidomethylation |  | DB Search |
| total 1 peptides |  |  |  |  |  |  |  |  |  |  |  |  |  |  |  |  |  |  |  |
| --- | --- | --- | --- | --- | --- | --- | --- | --- | --- | --- | --- | --- | --- | --- | --- | --- | --- | --- | --- |

Best Unique PSM (Scan Sample\_7\_A1\_1\_3644.d:55404, m/z=630.3543, z=2, RT=17.05, ppm=-10.06):


TRINITY\_DN941\_c0\_g1\_i5.p2

back to list

  

| Protein Coverage
| Supporting Peptides
| Best Unique PSM
|

Protein Coverage:

Supporting Peptides:

| Peptide | Uniq | -10lgP | Mass | Length | ppm | m/z | z | RT | 1/k0 Start | 1/k0 End | Precursor ID | Area Sample 8 | #Feature | #Feature Sample 8 | Start | End | PTM | AScore | Found By |
| --- | --- | --- | --- | --- | --- | --- | --- | --- | --- | --- | --- | --- | --- | --- | --- | --- | --- | --- | --- |
| K.ADLTRPTGK.K | Y | 42.69 | 957.5243 | 9 | -3.1 | 479.7658 | 2 | 10.44 | 0.8193 | 0.8266 | 17248 | 0 | 0 | 0 | 52 | 60 |  |  | DB Search |
| total 1 peptides |  |  |  |  |  |  |  |  |  |  |  |  |  |  |  |  |  |  |  |
| --- | --- | --- | --- | --- | --- | --- | --- | --- | --- | --- | --- | --- | --- | --- | --- | --- | --- | --- | --- |

Best Unique PSM (Scan Sample\_7\_A1\_1\_3644.d:17248, m/z=479.7658, z=2, RT=10.44, ppm=-7.58):


TRINITY\_DN4781\_c0\_g1\_i3.p3

back to list

  

| Protein Coverage
| Supporting Peptides
| Best Unique PSM
|

Protein Coverage:

Supporting Peptides:

| Peptide | Uniq | -10lgP | Mass | Length | ppm | m/z | z | RT | 1/k0 Start | 1/k0 End | Precursor ID | Area Sample 8 | #Feature | #Feature Sample 8 | Start | End | PTM | AScore | Found By |
| --- | --- | --- | --- | --- | --- | --- | --- | --- | --- | --- | --- | --- | --- | --- | --- | --- | --- | --- | --- |
| K.SGEYKPTLGR.C | Y | 43.8 | 1106.572 | 10 | 3.81 | 554.2929 | 2 | 11.95 | 0.904 | 0.9113 | 25715 | 8.34e2 | 1 | 1 | 169 | 178 |  |  | DB Search |
| total 1 peptides |  |  |  |  |  |  |  |  |  |  |  |  |  |  |  |  |  |  |  |
| --- | --- | --- | --- | --- | --- | --- | --- | --- | --- | --- | --- | --- | --- | --- | --- | --- | --- | --- | --- |

Best Unique PSM (Scan Sample\_7\_A1\_1\_3644.d:25715, m/z=554.2929, z=2, RT=11.95, ppm=-0.66):


TRINITY\_DN464\_c0\_g1\_i4.p1

back to list

  

| Protein Coverage
| Supporting Peptides
| Best Unique PSM
|

Protein Coverage:

Supporting Peptides:

| Peptide | Uniq | -10lgP | Mass | Length | ppm | m/z | z | RT | 1/k0 Start | 1/k0 End | Precursor ID | Area Sample 8 | #Feature | #Feature Sample 8 | Start | End | PTM | AScore | Found By |
| --- | --- | --- | --- | --- | --- | --- | --- | --- | --- | --- | --- | --- | --- | --- | --- | --- | --- | --- | --- |
| R.LLEFVGHDVLR.L | Y | 43.74 | 1296.719 | 11 | 5.97 | 649.3677 | 2 | 17.98 | 1.0057 | 1.013 | 61273 | 0 | 0 | 0 | 279 | 289 |  |  | DB Search |
| total 1 peptides |  |  |  |  |  |  |  |  |  |  |  |  |  |  |  |  |  |  |  |
| --- | --- | --- | --- | --- | --- | --- | --- | --- | --- | --- | --- | --- | --- | --- | --- | --- | --- | --- | --- |

Best Unique PSM (Scan Sample\_7\_A1\_1\_3644.d:61273, m/z=649.3677, z=2, RT=17.98, ppm=1.5):


TRINITY\_DN2637\_c0\_g1\_i5.p2

back to list

  

| Protein Coverage
| Supporting Peptides
| Best Unique PSM
|

Protein Coverage:

Supporting Peptides:

| Peptide | Uniq | -10lgP | Mass | Length | ppm | m/z | z | RT | 1/k0 Start | 1/k0 End | Precursor ID | Area Sample 8 | #Feature | #Feature Sample 8 | Start | End | PTM | AScore | Found By |
| --- | --- | --- | --- | --- | --- | --- | --- | --- | --- | --- | --- | --- | --- | --- | --- | --- | --- | --- | --- |
| K.ATIEIPAKYEK.R | Y | 42.98 | 1261.6918 | 11 | 0.3 | 631.8505 | 2 | 14.24 | 0.9848 | 0.992 | 39239 | 0 | 0 | 0 | 107 | 117 |  |  | DB Search |
| total 1 peptides |  |  |  |  |  |  |  |  |  |  |  |  |  |  |  |  |  |  |  |
| --- | --- | --- | --- | --- | --- | --- | --- | --- | --- | --- | --- | --- | --- | --- | --- | --- | --- | --- | --- |

Best Unique PSM (Scan Sample\_7\_A1\_1\_3644.d:39239, m/z=631.8505, z=2, RT=14.24, ppm=-4.18):


TRINITY\_DN3036\_c0\_g1\_i13.p1

back to list

  

| Protein Coverage
| Supporting Peptides
| Best Unique PSM
|

Protein Coverage:

Supporting Peptides:

| Peptide | Uniq | -10lgP | Mass | Length | ppm | m/z | z | RT | 1/k0 Start | 1/k0 End | Precursor ID | Area Sample 8 | #Feature | #Feature Sample 8 | Start | End | PTM | AScore | Found By |
| --- | --- | --- | --- | --- | --- | --- | --- | --- | --- | --- | --- | --- | --- | --- | --- | --- | --- | --- | --- |
| R.AGHGAVTSPQTQR.G | Y | 43.55 | 1308.6534 | 13 | 3.24 | 437.2245 | 3 | 9.76 | 0.7045 | 0.7118 | 13596 | 2.79e3 | 2 | 2 | 135 | 147 |  |  | DB Search |
| R.VIGASGIHVGTM(+15.99)SFGK.M | Y | 43.2 | 1575.8079 | 16 | 0.89 | 788.9084 | 2 | 15.29 | 1.129 | 1.1362 | 45505 | 0 | 0 | 0 | 160 | 175 | Oxidation (M) | M12:Oxidation (M):1000 | DB Search |
| total 2 peptides |  |  |  |  |  |  |  |  |  |  |  |  |  |  |  |  |  |  |  |
| --- | --- | --- | --- | --- | --- | --- | --- | --- | --- | --- | --- | --- | --- | --- | --- | --- | --- | --- | --- |

Best Unique PSM (Scan Sample\_7\_A1\_1\_3644.d:13596, m/z=437.2245, z=3, RT=9.76, ppm=-1.24):


TRINITY\_DN1143\_c0\_g1\_i1.p1

back to list

  

| Protein Coverage
| Supporting Peptides
| Best Unique PSM
|

Protein Coverage:

Supporting Peptides:

| Peptide | Uniq | -10lgP | Mass | Length | ppm | m/z | z | RT | 1/k0 Start | 1/k0 End | Precursor ID | Area Sample 8 | #Feature | #Feature Sample 8 | Start | End | PTM | AScore | Found By |
| --- | --- | --- | --- | --- | --- | --- | --- | --- | --- | --- | --- | --- | --- | --- | --- | --- | --- | --- | --- |
| K.GLIAQAIDHAASR.T | Y | 43.18 | 1321.7102 | 13 | -1.09 | 661.8587 | 2 | 16.35 | 1.011 | 1.0182 | 51577 | 0 | 0 | 0 | 328 | 340 |  |  | DB Search |
| total 1 peptides |  |  |  |  |  |  |  |  |  |  |  |  |  |  |  |  |  |  |  |
| --- | --- | --- | --- | --- | --- | --- | --- | --- | --- | --- | --- | --- | --- | --- | --- | --- | --- | --- | --- |

Best Unique PSM (Scan Sample\_7\_A1\_1\_3644.d:51577, m/z=661.8587, z=2, RT=16.35, ppm=-5.56):


TRINITY\_DN61668\_c0\_g1\_i1.p1

back to list

  

| Protein Coverage
| Supporting Peptides
| Best Unique PSM
|

Protein Coverage:

Supporting Peptides:

| Peptide | Uniq | -10lgP | Mass | Length | ppm | m/z | z | RT | 1/k0 Start | 1/k0 End | Precursor ID | Area Sample 8 | #Feature | #Feature Sample 8 | Start | End | PTM | AScore | Found By |
| --- | --- | --- | --- | --- | --- | --- | --- | --- | --- | --- | --- | --- | --- | --- | --- | --- | --- | --- | --- |
| R.SPNAPALISTHGNK.V | Y | 43.45 | 1405.7313 | 14 | 4.18 | 703.8727 | 2 | 13.13 | 1.0238 | 1.0311 | 32597 | 3.39e2 | 1 | 1 | 251 | 264 |  |  | DB Search |
| total 1 peptides |  |  |  |  |  |  |  |  |  |  |  |  |  |  |  |  |  |  |  |
| --- | --- | --- | --- | --- | --- | --- | --- | --- | --- | --- | --- | --- | --- | --- | --- | --- | --- | --- | --- |

Best Unique PSM (Scan Sample\_7\_A1\_1\_3644.d:32597, m/z=703.8727, z=2, RT=13.13, ppm=-0.3):


TRINITY\_DN3986\_c0\_g1\_i1.p1

back to list

  

| Protein Coverage
| Supporting Peptides
| Best Unique PSM
|

Protein Coverage:

Supporting Peptides:

| Peptide | Uniq | -10lgP | Mass | Length | ppm | m/z | z | RT | 1/k0 Start | 1/k0 End | Precursor ID | Area Sample 8 | #Feature | #Feature Sample 8 | Start | End | PTM | AScore | Found By |
| --- | --- | --- | --- | --- | --- | --- | --- | --- | --- | --- | --- | --- | --- | --- | --- | --- | --- | --- | --- |
| K.NM(+15.99)M(+15.99)AAC(+57.02)DPR.H | Y | 43.62 | 1096.41 | 9 | -0.29 | 549.2097 | 2 | 9.58 | 0.8615 | 0.8688 | 12888 | 5.74e2 | 1 | 1 | 298 | 306 | Oxidation (M), Oxidation (M), Carbamidomethylation | M2:Oxidation (M):1000 M3:Oxidation (M):1000 | DB Search |
| R.FPGQLNADLRK.L | Y | 43.16 | 1257.6829 | 11 | -1.33 | 629.8451 | 2 | 14.44 | 0.9497 | 0.957 | 40493 | 0 | 0 | 0 | 242 | 252 |  |  | DB Search |
| R.YLTVAAMFR.G | Y | 42.33 | 1070.5583 | 9 | 5.33 | 536.2869 | 2 | 19.96 | 0.8607 | 0.868 | 73861 | 0 | 0 | 0 | 310 | 318 |  |  | DB Search |
| total 3 peptides |  |  |  |  |  |  |  |  |  |  |  |  |  |  |  |  |  |  |  |
| --- | --- | --- | --- | --- | --- | --- | --- | --- | --- | --- | --- | --- | --- | --- | --- | --- | --- | --- | --- |

Best Unique PSM (Scan Sample\_7\_A1\_1\_3644.d:12888, m/z=549.2097, z=2, RT=9.58, ppm=-4.76):


TRINITY\_DN1058\_c0\_g1\_i3.p1

back to list

  

| Protein Coverage
| Supporting Peptides
| Best Unique PSM
|

Protein Coverage:

Supporting Peptides:

| Peptide | Uniq | -10lgP | Mass | Length | ppm | m/z | z | RT | 1/k0 Start | 1/k0 End | Precursor ID | Area Sample 8 | #Feature | #Feature Sample 8 | Start | End | PTM | AScore | Found By |
| --- | --- | --- | --- | --- | --- | --- | --- | --- | --- | --- | --- | --- | --- | --- | --- | --- | --- | --- | --- |
| R.APTHILNPANKR.S | Y | 43.8 | 1330.7469 | 12 | 0.61 | 666.3782 | 2 | 11.23 | 1.0057 | 1.013 | 21549 | 1.39e3 | 2 | 2 | 2314 | 2325 |  |  | DB Search |
| total 1 peptides |  |  |  |  |  |  |  |  |  |  |  |  |  |  |  |  |  |  |  |
| --- | --- | --- | --- | --- | --- | --- | --- | --- | --- | --- | --- | --- | --- | --- | --- | --- | --- | --- | --- |

Best Unique PSM (Scan Sample\_7\_A1\_1\_3644.d:21549, m/z=666.3782, z=2, RT=11.23, ppm=-3.86):


TRINITY\_DN2637\_c0\_g1\_i13.p1

back to list

  

| Protein Coverage
| Supporting Peptides
| Best Unique PSM
|

Protein Coverage:

Supporting Peptides:

| Peptide | Uniq | -10lgP | Mass | Length | ppm | m/z | z | RT | 1/k0 Start | 1/k0 End | Precursor ID | Area Sample 8 | #Feature | #Feature Sample 8 | Start | End | PTM | AScore | Found By |
| --- | --- | --- | --- | --- | --- | --- | --- | --- | --- | --- | --- | --- | --- | --- | --- | --- | --- | --- | --- |
| K.ATIEIPAKYEK.R | Y | 42.98 | 1261.6918 | 11 | 0.3 | 631.8505 | 2 | 14.24 | 0.9848 | 0.992 | 39239 | 0 | 0 | 0 | 107 | 117 |  |  | DB Search |
| total 1 peptides |  |  |  |  |  |  |  |  |  |  |  |  |  |  |  |  |  |  |  |
| --- | --- | --- | --- | --- | --- | --- | --- | --- | --- | --- | --- | --- | --- | --- | --- | --- | --- | --- | --- |

Best Unique PSM (Scan Sample\_7\_A1\_1\_3644.d:39239, m/z=631.8505, z=2, RT=14.24, ppm=-4.18):


TRINITY\_DN1320\_c0\_g1\_i1.p1

back to list

  

| Protein Coverage
| Supporting Peptides
| Best Unique PSM
|

Protein Coverage:

Supporting Peptides:

| Peptide | Uniq | -10lgP | Mass | Length | ppm | m/z | z | RT | 1/k0 Start | 1/k0 End | Precursor ID | Area Sample 8 | #Feature | #Feature Sample 8 | Start | End | PTM | AScore | Found By |
| --- | --- | --- | --- | --- | --- | --- | --- | --- | --- | --- | --- | --- | --- | --- | --- | --- | --- | --- | --- |
| R.DC(+57.02)NSNASSQTFR.Y | Y | 43.36 | 1385.563 | 12 | 8.68 | 693.7917 | 2 | 11.52 | 0.9549 | 0.9622 | 23277 | 2.76e2 | 1 | 1 | 190 | 201 | Carbamidomethylation |  | DB Search |
| total 1 peptides |  |  |  |  |  |  |  |  |  |  |  |  |  |  |  |  |  |  |  |
| --- | --- | --- | --- | --- | --- | --- | --- | --- | --- | --- | --- | --- | --- | --- | --- | --- | --- | --- | --- |

Best Unique PSM (Scan Sample\_7\_A1\_1\_3644.d:23277, m/z=693.7917, z=2, RT=11.52, ppm=4.21):


TRINITY\_DN36\_c0\_g1\_i3.p1

back to list

  

| Protein Coverage
| Supporting Peptides
| Best Unique PSM
|

Protein Coverage:

Supporting Peptides:

| Peptide | Uniq | -10lgP | Mass | Length | ppm | m/z | z | RT | 1/k0 Start | 1/k0 End | Precursor ID | Area Sample 8 | #Feature | #Feature Sample 8 | Start | End | PTM | AScore | Found By |
| --- | --- | --- | --- | --- | --- | --- | --- | --- | --- | --- | --- | --- | --- | --- | --- | --- | --- | --- | --- |
| K.FETQIATK.D | Y | 43.56 | 936.4916 | 8 | 1.93 | 937.4965 | 1 | 13.99 | 1.2029 | 1.2101 | 37753 | 0 | 0 | 0 | 167 | 174 |  |  | DB Search |
| R.LGGIEVTQGNC(+57.02)AR.K | Y | 43.02 | 1373.6721 | 13 | 2.68 | 687.8421 | 2 | 13.90 | 0.9662 | 0.9735 | 37137 | 6.95e2 | 1 | 1 | 58 | 70 | Carbamidomethylation |  | DB Search |
| total 2 peptides |  |  |  |  |  |  |  |  |  |  |  |  |  |  |  |  |  |  |  |
| --- | --- | --- | --- | --- | --- | --- | --- | --- | --- | --- | --- | --- | --- | --- | --- | --- | --- | --- | --- |

Best Unique PSM (Scan Sample\_7\_A1\_1\_3644.d:37753, m/z=937.4965, z=1, RT=13.99, ppm=-2.55):


TRINITY\_DN8341\_c0\_g1\_i2.p1

back to list

  

| Protein Coverage
| Supporting Peptides
| Best Unique PSM
|

Protein Coverage:

Supporting Peptides:

| Peptide | Uniq | -10lgP | Mass | Length | ppm | m/z | z | RT | 1/k0 Start | 1/k0 End | Precursor ID | Area Sample 8 | #Feature | #Feature Sample 8 | Start | End | PTM | AScore | Found By |
| --- | --- | --- | --- | --- | --- | --- | --- | --- | --- | --- | --- | --- | --- | --- | --- | --- | --- | --- | --- |
| K.SLDLKQENSNR.L | Y | 43.76 | 1302.6528 | 11 | 2.34 | 652.3323 | 2 | 11.51 | 0.9509 | 0.9582 | 23115 | 2.77e3 | 1 | 1 | 220 | 230 |  |  | DB Search |
| K.DFILTAAHC(+57.02)FK.E | Y | 43.55 | 1321.6489 | 11 | -2.89 | 661.8268 | 2 | 18.07 | 1.0094 | 1.0166 | 61842 | 4.05e2 | 1 | 1 | 151 | 161 | Carbamidomethylation |  | DB Search |
| total 2 peptides |  |  |  |  |  |  |  |  |  |  |  |  |  |  |  |  |  |  |  |
| --- | --- | --- | --- | --- | --- | --- | --- | --- | --- | --- | --- | --- | --- | --- | --- | --- | --- | --- | --- |

Best Unique PSM (Scan Sample\_7\_A1\_1\_3644.d:23115, m/z=652.3323, z=2, RT=11.51, ppm=-2.13):


TRINITY\_DN2637\_c0\_g1\_i6.p1

back to list

  

| Protein Coverage
| Supporting Peptides
| Best Unique PSM
|

Protein Coverage:

Supporting Peptides:

| Peptide | Uniq | -10lgP | Mass | Length | ppm | m/z | z | RT | 1/k0 Start | 1/k0 End | Precursor ID | Area Sample 8 | #Feature | #Feature Sample 8 | Start | End | PTM | AScore | Found By |
| --- | --- | --- | --- | --- | --- | --- | --- | --- | --- | --- | --- | --- | --- | --- | --- | --- | --- | --- | --- |
| K.ATIEIPAKYEK.R | Y | 42.98 | 1261.6918 | 11 | 0.3 | 631.8505 | 2 | 14.24 | 0.9848 | 0.992 | 39239 | 0 | 0 | 0 | 87 | 97 |  |  | DB Search |
| total 1 peptides |  |  |  |  |  |  |  |  |  |  |  |  |  |  |  |  |  |  |  |
| --- | --- | --- | --- | --- | --- | --- | --- | --- | --- | --- | --- | --- | --- | --- | --- | --- | --- | --- | --- |

Best Unique PSM (Scan Sample\_7\_A1\_1\_3644.d:39239, m/z=631.8505, z=2, RT=14.24, ppm=-4.18):


TRINITY\_DN195934\_c0\_g1\_i1.p2

back to list

  

| Protein Coverage
| Supporting Peptides
| Best Unique PSM
|

Protein Coverage:

Supporting Peptides:

| Peptide | Uniq | -10lgP | Mass | Length | ppm | m/z | z | RT | 1/k0 Start | 1/k0 End | Precursor ID | Area Sample 8 | #Feature | #Feature Sample 8 | Start | End | PTM | AScore | Found By |
| --- | --- | --- | --- | --- | --- | --- | --- | --- | --- | --- | --- | --- | --- | --- | --- | --- | --- | --- | --- |
| K.DSYVGDEAQSKR.G | Y | 43.81 | 1353.616 | 12 | 3.22 | 677.8145 | 2 | 11.01 | 0.9961 | 1.0033 | 20355 | 1.65e3 | 1 | 1 | 10 | 21 |  |  | DB Search |
| R.VAPEEHPVLLTEAPLNPK.A | Y | 43.24 | 1953.0571 | 18 | -8.51 | 977.5231 | 2 | 16.59 | 1.2073 | 1.2145 | 52921 | 0 | 0 | 0 | 55 | 72 |  |  | DB Search |
| total 2 peptides |  |  |  |  |  |  |  |  |  |  |  |  |  |  |  |  |  |  |  |
| --- | --- | --- | --- | --- | --- | --- | --- | --- | --- | --- | --- | --- | --- | --- | --- | --- | --- | --- | --- |

Best Unique PSM (Scan Sample\_7\_A1\_1\_3644.d:20355, m/z=677.8145, z=2, RT=11.01, ppm=-1.25):


TRINITY\_DN493\_c0\_g1\_i1.p1

back to list

  

| Protein Coverage
| Supporting Peptides
| Best Unique PSM
|

Protein Coverage:

Supporting Peptides:

| Peptide | Uniq | -10lgP | Mass | Length | ppm | m/z | z | RT | 1/k0 Start | 1/k0 End | Precursor ID | Area Sample 8 | #Feature | #Feature Sample 8 | Start | End | PTM | AScore | Found By |
| --- | --- | --- | --- | --- | --- | --- | --- | --- | --- | --- | --- | --- | --- | --- | --- | --- | --- | --- | --- |
| R.AMTEEILNERDTASK.V | Y | 43.78 | 1706.8145 | 15 | 1.51 | 854.412 | 2 | 14.34 | 1.129 | 1.1362 | 39888 | 0 | 0 | 0 | 240 | 254 |  |  | DB Search |
| total 1 peptides |  |  |  |  |  |  |  |  |  |  |  |  |  |  |  |  |  |  |  |
| --- | --- | --- | --- | --- | --- | --- | --- | --- | --- | --- | --- | --- | --- | --- | --- | --- | --- | --- | --- |

Best Unique PSM (Scan Sample\_7\_A1\_1\_3644.d:39888, m/z=854.412, z=2, RT=14.34, ppm=-2.97):


TRINITY\_DN761\_c0\_g1\_i1.p1

back to list

  

| Protein Coverage
| Supporting Peptides
| Best Unique PSM
|

Protein Coverage:

Supporting Peptides:

| Peptide | Uniq | -10lgP | Mass | Length | ppm | m/z | z | RT | 1/k0 Start | 1/k0 End | Precursor ID | Area Sample 8 | #Feature | #Feature Sample 8 | Start | End | PTM | AScore | Found By |
| --- | --- | --- | --- | --- | --- | --- | --- | --- | --- | --- | --- | --- | --- | --- | --- | --- | --- | --- | --- |
| K.TRIEDLETVISQTQDHR.H | Y | 43.83 | 2040.0236 | 17 | 1.88 | 681.0134 | 3 | 17.57 | 0.9626 | 0.9699 | 58583 | 4.36e2 | 1 | 1 | 260 | 276 |  |  | DB Search |
| total 1 peptides |  |  |  |  |  |  |  |  |  |  |  |  |  |  |  |  |  |  |  |
| --- | --- | --- | --- | --- | --- | --- | --- | --- | --- | --- | --- | --- | --- | --- | --- | --- | --- | --- | --- |

Best Unique PSM (Scan Sample\_7\_A1\_1\_3644.d:58583, m/z=681.0134, z=3, RT=17.57, ppm=-2.6):


TRINITY\_DN11522\_c0\_g1\_i2.p1

back to list

  

| Protein Coverage
| Supporting Peptides
| Best Unique PSM
|

Protein Coverage:

Supporting Peptides:

| Peptide | Uniq | -10lgP | Mass | Length | ppm | m/z | z | RT | 1/k0 Start | 1/k0 End | Precursor ID | Area Sample 8 | #Feature | #Feature Sample 8 | Start | End | PTM | AScore | Found By |
| --- | --- | --- | --- | --- | --- | --- | --- | --- | --- | --- | --- | --- | --- | --- | --- | --- | --- | --- | --- |
| R.IGLGNIRDPSISLTR.G | Y | 43.72 | 1610.9104 | 15 | 2.12 | 537.9761 | 3 | 17.42 | 0.8019 | 0.8092 | 57662 | 1.88e3 | 1 | 1 | 25 | 39 |  |  | DB Search |
| K.GKPEQVQVK.M | Y | 43.14 | 1011.5713 | 9 | 2 | 506.7917 | 2 | 9.97 | 0.8416 | 0.849 | 14688 | 9.41e2 | 1 | 1 | 546 | 554 |  |  | DB Search |
| total 2 peptides |  |  |  |  |  |  |  |  |  |  |  |  |  |  |  |  |  |  |  |
| --- | --- | --- | --- | --- | --- | --- | --- | --- | --- | --- | --- | --- | --- | --- | --- | --- | --- | --- | --- |

Best Unique PSM (Scan Sample\_7\_A1\_1\_3644.d:57662, m/z=537.9761, z=3, RT=17.42, ppm=-2.35):


TRINITY\_DN876\_c0\_g1\_i1.p1

back to list

  

| Protein Coverage
| Supporting Peptides
| Best Unique PSM
|

Protein Coverage:

Supporting Peptides:

| Peptide | Uniq | -10lgP | Mass | Length | ppm | m/z | z | RT | 1/k0 Start | 1/k0 End | Precursor ID | Area Sample 8 | #Feature | #Feature Sample 8 | Start | End | PTM | AScore | Found By |
| --- | --- | --- | --- | --- | --- | --- | --- | --- | --- | --- | --- | --- | --- | --- | --- | --- | --- | --- | --- |
| K.AAMEELEHAEK.F | Y | 40.76 | 1256.5707 | 11 | 2.71 | 629.2915 | 2 | 13.28 | 0.961 | 0.9683 | 33568 | 3.38e2 | 1 | 1 | 99 | 109 |  |  | DB Search |
| total 1 peptides |  |  |  |  |  |  |  |  |  |  |  |  |  |  |  |  |  |  |  |
| --- | --- | --- | --- | --- | --- | --- | --- | --- | --- | --- | --- | --- | --- | --- | --- | --- | --- | --- | --- |

Best Unique PSM (Scan Sample\_7\_A1\_1\_3644.d:33568, m/z=629.2915, z=2, RT=13.28, ppm=-1.76):


TRINITY\_DN1119\_c0\_g1\_i1.p1

back to list

  

| Protein Coverage
| Supporting Peptides
| Best Unique PSM
|

Protein Coverage:

Supporting Peptides:

| Peptide | Uniq | -10lgP | Mass | Length | ppm | m/z | z | RT | 1/k0 Start | 1/k0 End | Precursor ID | Area Sample 8 | #Feature | #Feature Sample 8 | Start | End | PTM | AScore | Found By |
| --- | --- | --- | --- | --- | --- | --- | --- | --- | --- | --- | --- | --- | --- | --- | --- | --- | --- | --- | --- |
| K.RLTELEHFLK.I | Y | 43.25 | 1284.719 | 10 | 1.01 | 429.2455 | 3 | 16.49 | 0.7465 | 0.7538 | 52366 | 0 | 0 | 0 | 376 | 385 |  |  | DB Search |
| total 1 peptides |  |  |  |  |  |  |  |  |  |  |  |  |  |  |  |  |  |  |  |
| --- | --- | --- | --- | --- | --- | --- | --- | --- | --- | --- | --- | --- | --- | --- | --- | --- | --- | --- | --- |

Best Unique PSM (Scan Sample\_7\_A1\_1\_3644.d:52366, m/z=429.2455, z=3, RT=16.49, ppm=-3.47):


TRINITY\_DN36\_c0\_g1\_i20.p1

back to list

  

| Protein Coverage
| Supporting Peptides
| Best Unique PSM
|

Protein Coverage:

Supporting Peptides:

| Peptide | Uniq | -10lgP | Mass | Length | ppm | m/z | z | RT | 1/k0 Start | 1/k0 End | Precursor ID | Area Sample 8 | #Feature | #Feature Sample 8 | Start | End | PTM | AScore | Found By |
| --- | --- | --- | --- | --- | --- | --- | --- | --- | --- | --- | --- | --- | --- | --- | --- | --- | --- | --- | --- |
| R.LGGIEVTQGNC(+57.02)AR.K | Y | 43.02 | 1373.6721 | 13 | 2.68 | 687.8421 | 2 | 13.90 | 0.9662 | 0.9735 | 37137 | 6.95e2 | 1 | 1 | 57 | 69 | Carbamidomethylation |  | DB Search |
| total 1 peptides |  |  |  |  |  |  |  |  |  |  |  |  |  |  |  |  |  |  |  |
| --- | --- | --- | --- | --- | --- | --- | --- | --- | --- | --- | --- | --- | --- | --- | --- | --- | --- | --- | --- |

Best Unique PSM (Scan Sample\_7\_A1\_1\_3644.d:37137, m/z=687.8421, z=2, RT=13.90, ppm=-1.8):


TRINITY\_DN8008\_c0\_g1\_i2.p1

back to list

  

| Protein Coverage
| Supporting Peptides
| Best Unique PSM
|

Protein Coverage:

Supporting Peptides:

| Peptide | Uniq | -10lgP | Mass | Length | ppm | m/z | z | RT | 1/k0 Start | 1/k0 End | Precursor ID | Area Sample 8 | #Feature | #Feature Sample 8 | Start | End | PTM | AScore | Found By |
| --- | --- | --- | --- | --- | --- | --- | --- | --- | --- | --- | --- | --- | --- | --- | --- | --- | --- | --- | --- |
| K.GVHIISVGLGTVDHR.G | Y | 43.79 | 1558.8579 | 15 | 0.84 | 780.4334 | 2 | 15.97 | 1.0917 | 1.0989 | 49325 | 0 | 0 | 0 | 1055 | 1069 |  |  | DB Search |
| total 1 peptides |  |  |  |  |  |  |  |  |  |  |  |  |  |  |  |  |  |  |  |
| --- | --- | --- | --- | --- | --- | --- | --- | --- | --- | --- | --- | --- | --- | --- | --- | --- | --- | --- | --- |

Best Unique PSM (Scan Sample\_7\_A1\_1\_3644.d:49325, m/z=780.4334, z=2, RT=15.97, ppm=-3.63):


TRINITY\_DN36\_c0\_g1\_i14.p1

back to list

  

| Protein Coverage
| Supporting Peptides
| Best Unique PSM
|

Protein Coverage:

Supporting Peptides:

| Peptide | Uniq | -10lgP | Mass | Length | ppm | m/z | z | RT | 1/k0 Start | 1/k0 End | Precursor ID | Area Sample 8 | #Feature | #Feature Sample 8 | Start | End | PTM | AScore | Found By |
| --- | --- | --- | --- | --- | --- | --- | --- | --- | --- | --- | --- | --- | --- | --- | --- | --- | --- | --- | --- |
| K.FETQIATK.D | Y | 43.56 | 936.4916 | 8 | 1.93 | 937.4965 | 1 | 13.99 | 1.2029 | 1.2101 | 37753 | 0 | 0 | 0 | 167 | 174 |  |  | DB Search |
| R.LGGIEVTQGNC(+57.02)AR.K | Y | 43.02 | 1373.6721 | 13 | 2.68 | 687.8421 | 2 | 13.90 | 0.9662 | 0.9735 | 37137 | 6.95e2 | 1 | 1 | 58 | 70 | Carbamidomethylation |  | DB Search |
| total 2 peptides |  |  |  |  |  |  |  |  |  |  |  |  |  |  |  |  |  |  |  |
| --- | --- | --- | --- | --- | --- | --- | --- | --- | --- | --- | --- | --- | --- | --- | --- | --- | --- | --- | --- |

Best Unique PSM (Scan Sample\_7\_A1\_1\_3644.d:37753, m/z=937.4965, z=1, RT=13.99, ppm=-2.55):


TRINITY\_DN3052\_c0\_g1\_i1.p1

back to list

  

| Protein Coverage
| Supporting Peptides
| Best Unique PSM
|

Protein Coverage:

Supporting Peptides:

| Peptide | Uniq | -10lgP | Mass | Length | ppm | m/z | z | RT | 1/k0 Start | 1/k0 End | Precursor ID | Area Sample 8 | #Feature | #Feature Sample 8 | Start | End | PTM | AScore | Found By |
| --- | --- | --- | --- | --- | --- | --- | --- | --- | --- | --- | --- | --- | --- | --- | --- | --- | --- | --- | --- |
| M.A(+42.01)HLSQEQR.N | Y | 43.62 | 1009.4941 | 8 | 6.92 | 505.7556 | 2 | 11.01 | 0.8708 | 0.8781 | 20435 | 0 | 0 | 0 | 2 | 9 | Acetylation (Protein N-term) | A1:Acetylation (Protein N-term):1000 | DB Search |
| total 1 peptides |  |  |  |  |  |  |  |  |  |  |  |  |  |  |  |  |  |  |  |
| --- | --- | --- | --- | --- | --- | --- | --- | --- | --- | --- | --- | --- | --- | --- | --- | --- | --- | --- | --- |

Best Unique PSM (Scan Sample\_7\_A1\_1\_3644.d:20435, m/z=505.7556, z=2, RT=11.01, ppm=2.44):


TRINITY\_DN15151\_c0\_g1\_i2.p1

back to list

  

| Protein Coverage
| Supporting Peptides
| Best Unique PSM
|

Protein Coverage:

Supporting Peptides:

| Peptide | Uniq | -10lgP | Mass | Length | ppm | m/z | z | RT | 1/k0 Start | 1/k0 End | Precursor ID | Area Sample 8 | #Feature | #Feature Sample 8 | Start | End | PTM | AScore | Found By |
| --- | --- | --- | --- | --- | --- | --- | --- | --- | --- | --- | --- | --- | --- | --- | --- | --- | --- | --- | --- |
| K.AVAAGMNPMDIKR.G | Y | 43.11 | 1372.6955 | 13 | 1.74 | 687.3531 | 2 | 14.92 | 1.0126 | 1.0198 | 43242 | 6.18e2 | 1 | 1 | 145 | 157 |  |  | DB Search |
| total 1 peptides |  |  |  |  |  |  |  |  |  |  |  |  |  |  |  |  |  |  |  |
| --- | --- | --- | --- | --- | --- | --- | --- | --- | --- | --- | --- | --- | --- | --- | --- | --- | --- | --- | --- |

Best Unique PSM (Scan Sample\_7\_A1\_1\_3644.d:43242, m/z=687.3531, z=2, RT=14.92, ppm=-2.73):


TRINITY\_DN262\_c0\_g1\_i1.p1

back to list

  

| Protein Coverage
| Supporting Peptides
| Best Unique PSM
|

Protein Coverage:

Supporting Peptides:

| Peptide | Uniq | -10lgP | Mass | Length | ppm | m/z | z | RT | 1/k0 Start | 1/k0 End | Precursor ID | Area Sample 8 | #Feature | #Feature Sample 8 | Start | End | PTM | AScore | Found By |
| --- | --- | --- | --- | --- | --- | --- | --- | --- | --- | --- | --- | --- | --- | --- | --- | --- | --- | --- | --- |
| R.AEFRPNQEEEPILKR.E | Y | 43.83 | 1854.9588 | 15 | -0 | 619.3241 | 3 | 13.90 | 0.8785 | 0.8858 | 36999 | 2.93e3 | 1 | 1 | 439 | 453 |  |  | DB Search |
| R.DANAAVVHGSHLK.D | Y | 43.8 | 1317.6789 | 13 | 5.88 | 659.8477 | 2 | 10.96 | 1.0118 | 1.019 | 20131 | 0 | 0 | 0 | 668 | 680 |  |  | DB Search |
| K.TGDNTVM(+15.99)GR.I | Y | 43.74 | 965.4236 | 9 | 2.88 | 483.7183 | 2 | 9.33 | 0.777 | 0.7844 | 11865 | 5.26e2 | 1 | 1 | 269 | 277 | Oxidation (M) | M7:Oxidation (M):1000 | DB Search |
| K.VIM(+15.99)VTGDHPITAK.A | Y | 43.74 | 1396.7384 | 13 | 2.18 | 699.3749 | 2 | 13.28 | 1.023 | 1.0303 | 33438 | 7.1e2 | 1 | 1 | 622 | 634 | Oxidation (M) | M3:Oxidation (M):1000 | DB Search |
| K.GVGIISEGNETVEDIAQR.L | Y | 43.52 | 1885.9381 | 18 | 0.98 | 943.973 | 2 | 17.94 | 1.1446 | 1.1518 | 61086 | 0 | 0 | 0 | 639 | 656 |  |  | DB Search |
| K.VDNSSLTGESEPQSR.N | Y | 43.5 | 1604.7278 | 15 | -5.74 | 803.363 | 2 | 11.96 | 1.0214 | 1.0287 | 25859 | 0 | 0 | 0 | 221 | 235 |  |  | DB Search |
| K.DLPQEKLDDILK.Y | Y | 43.46 | 1425.7715 | 12 | -17.28 | 713.8775 | 2 | 17.89 | 1.0391 | 1.0464 | 60733 | 0 | 0 | 0 | 681 | 692 |  |  | DB Search |
| K.AETDIM(+15.99)KR.K | Y | 43.1 | 978.4804 | 8 | 4.13 | 490.2473 | 2 | 9.56 | 0.8067 | 0.814 | 12807 | 1.4e3 | 1 | 1 | 836 | 843 | Oxidation (M) | M6:Oxidation (M):1000 | DB Search |
| K.TGTLTQNR.M | Y | 42.85 | 889.4617 | 8 | 2.38 | 445.7372 | 2 | 10.03 | 0.7844 | 0.7917 | 14992 | 3.14e3 | 1 | 1 | 386 | 393 |  |  | DB Search |
| total 9 peptides |  |  |  |  |  |  |  |  |  |  |  |  |  |  |  |  |  |  |  |
| --- | --- | --- | --- | --- | --- | --- | --- | --- | --- | --- | --- | --- | --- | --- | --- | --- | --- | --- | --- |

Best Unique PSM (Scan Sample\_7\_A1\_1\_3644.d:36999, m/z=619.3241, z=3, RT=13.90, ppm=-4.48):


TRINITY\_DN3036\_c0\_g1\_i4.p1

back to list

  

| Protein Coverage
| Supporting Peptides
| Best Unique PSM
|

Protein Coverage:

Supporting Peptides:

| Peptide | Uniq | -10lgP | Mass | Length | ppm | m/z | z | RT | 1/k0 Start | 1/k0 End | Precursor ID | Area Sample 8 | #Feature | #Feature Sample 8 | Start | End | PTM | AScore | Found By |
| --- | --- | --- | --- | --- | --- | --- | --- | --- | --- | --- | --- | --- | --- | --- | --- | --- | --- | --- | --- |
| R.VIGASGIHVGTM(+15.99)SFGK.M | Y | 43.2 | 1575.8079 | 16 | 0.89 | 788.9084 | 2 | 15.29 | 1.129 | 1.1362 | 45505 | 0 | 0 | 0 | 24 | 39 | Oxidation (M) | M12:Oxidation (M):1000 | DB Search |
| total 1 peptides |  |  |  |  |  |  |  |  |  |  |  |  |  |  |  |  |  |  |  |
| --- | --- | --- | --- | --- | --- | --- | --- | --- | --- | --- | --- | --- | --- | --- | --- | --- | --- | --- | --- |

Best Unique PSM (Scan Sample\_7\_A1\_1\_3644.d:45505, m/z=788.9084, z=2, RT=15.29, ppm=-3.59):


TRINITY\_DN119684\_c0\_g1\_i1.p1

back to list

  

| Protein Coverage
| Supporting Peptides
| Best Unique PSM
|

Protein Coverage:

Supporting Peptides:

| Peptide | Uniq | -10lgP | Mass | Length | ppm | m/z | z | RT | 1/k0 Start | 1/k0 End | Precursor ID | Area Sample 8 | #Feature | #Feature Sample 8 | Start | End | PTM | AScore | Found By |
| --- | --- | --- | --- | --- | --- | --- | --- | --- | --- | --- | --- | --- | --- | --- | --- | --- | --- | --- | --- |
| K.KLEDVATPIVSK.I | Y | 43.25 | 1298.7445 | 12 | -3.93 | 650.3741 | 2 | 14.20 | 0.9553 | 0.9626 | 39057 | 0 | 0 | 0 | 624 | 635 |  |  | DB Search |
| total 1 peptides |  |  |  |  |  |  |  |  |  |  |  |  |  |  |  |  |  |  |  |
| --- | --- | --- | --- | --- | --- | --- | --- | --- | --- | --- | --- | --- | --- | --- | --- | --- | --- | --- | --- |

Best Unique PSM (Scan Sample\_7\_A1\_1\_3644.d:39057, m/z=650.3741, z=2, RT=14.20, ppm=-8.4):


TRINITY\_DN5633\_c0\_g1\_i1.p1

back to list

  

| Protein Coverage
| Supporting Peptides
| Best Unique PSM
|

Protein Coverage:

Supporting Peptides:

| Peptide | Uniq | -10lgP | Mass | Length | ppm | m/z | z | RT | 1/k0 Start | 1/k0 End | Precursor ID | Area Sample 8 | #Feature | #Feature Sample 8 | Start | End | PTM | AScore | Found By |
| --- | --- | --- | --- | --- | --- | --- | --- | --- | --- | --- | --- | --- | --- | --- | --- | --- | --- | --- | --- |
| K.LLEINVYHPLIQELQK.R | Y | 43.37 | 1949.0986 | 16 | 2.77 | 650.7057 | 3 | 19.85 | 0.9852 | 0.9924 | 73141 | 0 | 0 | 0 | 706 | 721 |  |  | DB Search |
| R.ELISNSSDALDKIR.F | N | 43.22 | 1559.8155 | 14 | -1.75 | 780.9102 | 2 | 15.66 | 1.0777 | 1.0849 | 47553 | 0 | 0 | 0 | 108 | 121 |  |  | DB Search |
| total 2 peptides |  |  |  |  |  |  |  |  |  |  |  |  |  |  |  |  |  |  |  |
| --- | --- | --- | --- | --- | --- | --- | --- | --- | --- | --- | --- | --- | --- | --- | --- | --- | --- | --- | --- |

Best Unique PSM (Scan Sample\_7\_A1\_1\_3644.d:73141, m/z=650.7057, z=3, RT=19.85, ppm=-1.7):


TRINITY\_DN2477\_c0\_g1\_i10.p1

back to list

  

| Protein Coverage
| Supporting Peptides
| Best Unique PSM
|

Protein Coverage:

Supporting Peptides:

| Peptide | Uniq | -10lgP | Mass | Length | ppm | m/z | z | RT | 1/k0 Start | 1/k0 End | Precursor ID | Area Sample 8 | #Feature | #Feature Sample 8 | Start | End | PTM | AScore | Found By |
| --- | --- | --- | --- | --- | --- | --- | --- | --- | --- | --- | --- | --- | --- | --- | --- | --- | --- | --- | --- |
| K.DSYVGDEAQSKR.G | Y | 43.81 | 1353.616 | 12 | 3.22 | 677.8145 | 2 | 11.01 | 0.9961 | 1.0033 | 20355 | 1.65e3 | 1 | 1 | 19 | 30 |  |  | DB Search |
| R.VAPEEHPVLLTEAPLNPK.A | Y | 43.24 | 1953.0571 | 18 | -8.51 | 977.5231 | 2 | 16.59 | 1.2073 | 1.2145 | 52921 | 0 | 0 | 0 | 64 | 81 |  |  | DB Search |
| total 2 peptides |  |  |  |  |  |  |  |  |  |  |  |  |  |  |  |  |  |  |  |
| --- | --- | --- | --- | --- | --- | --- | --- | --- | --- | --- | --- | --- | --- | --- | --- | --- | --- | --- | --- |

Best Unique PSM (Scan Sample\_7\_A1\_1\_3644.d:20355, m/z=677.8145, z=2, RT=11.01, ppm=-1.25):


TRINITY\_DN1653\_c0\_g1\_i1.p1

back to list

  

| Protein Coverage
| Supporting Peptides
| Best Unique PSM
|

Protein Coverage:

Supporting Peptides:

| Peptide | Uniq | -10lgP | Mass | Length | ppm | m/z | z | RT | 1/k0 Start | 1/k0 End | Precursor ID | Area Sample 8 | #Feature | #Feature Sample 8 | Start | End | PTM | AScore | Found By |
| --- | --- | --- | --- | --- | --- | --- | --- | --- | --- | --- | --- | --- | --- | --- | --- | --- | --- | --- | --- |
| R.LINLFEAVSKK.S | Y | 43.76 | 1260.7441 | 11 | -5.13 | 631.3733 | 2 | 19.09 | 0.986 | 0.9932 | 68432 | 1.56e3 | 1 | 1 | 49 | 59 |  |  | DB Search |
| total 1 peptides |  |  |  |  |  |  |  |  |  |  |  |  |  |  |  |  |  |  |  |
| --- | --- | --- | --- | --- | --- | --- | --- | --- | --- | --- | --- | --- | --- | --- | --- | --- | --- | --- | --- |

Best Unique PSM (Scan Sample\_7\_A1\_1\_3644.d:68432, m/z=631.3733, z=2, RT=19.09, ppm=-9.6):


TRINITY\_DN73788\_c0\_g1\_i1.p1

back to list

  

| Protein Coverage
| Supporting Peptides
| Best Unique PSM
|

Protein Coverage:

Supporting Peptides:

| Peptide | Uniq | -10lgP | Mass | Length | ppm | m/z | z | RT | 1/k0 Start | 1/k0 End | Precursor ID | Area Sample 8 | #Feature | #Feature Sample 8 | Start | End | PTM | AScore | Found By |
| --- | --- | --- | --- | --- | --- | --- | --- | --- | --- | --- | --- | --- | --- | --- | --- | --- | --- | --- | --- |
| R.GNHEC(+57.02)ASINR.I | Y | 43.61 | 1156.5043 | 10 | 15.1 | 386.5128 | 3 | 9.81 | 0.7286 | 0.7359 | 14029 | 0 | 0 | 0 | 123 | 132 | Carbamidomethylation |  | DB Search |
| total 1 peptides |  |  |  |  |  |  |  |  |  |  |  |  |  |  |  |  |  |  |  |
| --- | --- | --- | --- | --- | --- | --- | --- | --- | --- | --- | --- | --- | --- | --- | --- | --- | --- | --- | --- |

Best Unique PSM (Scan Sample\_7\_A1\_1\_3644.d:14029, m/z=386.5128, z=3, RT=9.81, ppm=10.62):


TRINITY\_DN729\_c0\_g1\_i1.p1

back to list

  

| Protein Coverage
| Supporting Peptides
| Best Unique PSM
|

Protein Coverage:

Supporting Peptides:

| Peptide | Uniq | -10lgP | Mass | Length | ppm | m/z | z | RT | 1/k0 Start | 1/k0 End | Precursor ID | Area Sample 8 | #Feature | #Feature Sample 8 | Start | End | PTM | AScore | Found By |
| --- | --- | --- | --- | --- | --- | --- | --- | --- | --- | --- | --- | --- | --- | --- | --- | --- | --- | --- | --- |
| K.ILDAPIHLGK.I | Y | 43.22 | 1075.6389 | 10 | 5.4 | 1076.6472 | 1 | 15.65 | 1.2778 | 1.2849 | 47506 | 3.09e2 | 1 | 1 | 99 | 108 |  |  | DB Search |
| total 1 peptides |  |  |  |  |  |  |  |  |  |  |  |  |  |  |  |  |  |  |  |
| --- | --- | --- | --- | --- | --- | --- | --- | --- | --- | --- | --- | --- | --- | --- | --- | --- | --- | --- | --- |

Best Unique PSM (Scan Sample\_7\_A1\_1\_3644.d:47506, m/z=1076.6472, z=1, RT=15.65, ppm=0.93):


TRINITY\_DN161\_c0\_g1\_i1.p1

back to list

  

| Protein Coverage
| Supporting Peptides
| Best Unique PSM
|

Protein Coverage:

Supporting Peptides:

| Peptide | Uniq | -10lgP | Mass | Length | ppm | m/z | z | RT | 1/k0 Start | 1/k0 End | Precursor ID | Area Sample 8 | #Feature | #Feature Sample 8 | Start | End | PTM | AScore | Found By |
| --- | --- | --- | --- | --- | --- | --- | --- | --- | --- | --- | --- | --- | --- | --- | --- | --- | --- | --- | --- |
| K.SYELPDGQVITIGNER.F | Y | 43.86 | 1789.8846 | 16 | 3.32 | 895.9485 | 2 | 18.70 | 1.1126 | 1.1198 | 65490 | 4.79e3 | 2 | 2 | 240 | 255 |  |  | DB Search |
| K.DSYVGDEAQSKR.G | Y | 43.81 | 1353.616 | 12 | 3.22 | 677.8145 | 2 | 11.01 | 0.9961 | 1.0033 | 20355 | 1.65e3 | 1 | 1 | 52 | 63 |  |  | DB Search |
| R.AVFPSIVGRPR.H | Y | 43.77 | 1197.6982 | 11 | 1.95 | 599.8549 | 2 | 15.90 | 0.959 | 0.9662 | 48470 | 4.43e4 | 1 | 1 | 30 | 40 |  |  | DB Search |
| R.LDLAGRDLTDYLMK.I | Y | 43.72 | 1622.8338 | 14 | -2.1 | 541.9483 | 3 | 19.46 | 0.7998 | 0.8071 | 70651 | 1.99e3 | 2 | 2 | 179 | 192 |  |  | DB Search |
| R.VAPEEHPVLLTEAPLNPK.A | Y | 43.24 | 1953.0571 | 18 | -8.51 | 977.5231 | 2 | 16.59 | 1.2073 | 1.2145 | 52921 | 0 | 0 | 0 | 97 | 114 |  |  | DB Search |
| K.EEYDESGPSIVHR.K | Y | 42.51 | 1516.6794 | 13 | -1.53 | 759.3424 | 2 | 13.28 | 0.9904 | 0.9977 | 33566 | 2.23e2 | 1 | 1 | 361 | 373 |  |  | DB Search |
| total 6 peptides |  |  |  |  |  |  |  |  |  |  |  |  |  |  |  |  |  |  |  |
| --- | --- | --- | --- | --- | --- | --- | --- | --- | --- | --- | --- | --- | --- | --- | --- | --- | --- | --- | --- |

Best Unique PSM (Scan Sample\_7\_A1\_1\_3644.d:65490, m/z=895.9485, z=2, RT=18.70, ppm=-1.15):


TRINITY\_DN64\_c0\_g1\_i1.p1

back to list

  

| Protein Coverage
| Supporting Peptides
| Best Unique PSM
|

Protein Coverage:

Supporting Peptides:

| Peptide | Uniq | -10lgP | Mass | Length | ppm | m/z | z | RT | 1/k0 Start | 1/k0 End | Precursor ID | Area Sample 8 | #Feature | #Feature Sample 8 | Start | End | PTM | AScore | Found By |
| --- | --- | --- | --- | --- | --- | --- | --- | --- | --- | --- | --- | --- | --- | --- | --- | --- | --- | --- | --- |
| K.YYVTIIDAPGHR.D | Y | 43.83 | 1403.7197 | 12 | 1.16 | 702.8648 | 2 | 16.09 | 1.0399 | 1.0472 | 49950 | 5.46e2 | 1 | 1 | 88 | 99 |  |  | DB Search |
| K.IHINIVVIGHVDSGK.S | Y | 43.78 | 1599.9096 | 15 | -1.01 | 800.9577 | 2 | 16.73 | 1.1001 | 1.1074 | 53733 | 6.87e2 | 2 | 2 | 9 | 23 |  |  | DB Search |
| K.STSTGHLIYK.C | Y | 43.76 | 1105.5768 | 10 | 4.14 | 553.7955 | 2 | 12.13 | 0.8664 | 0.8737 | 26715 | 2.71e3 | 1 | 1 | 24 | 33 |  |  | DB Search |
| total 3 peptides |  |  |  |  |  |  |  |  |  |  |  |  |  |  |  |  |  |  |  |
| --- | --- | --- | --- | --- | --- | --- | --- | --- | --- | --- | --- | --- | --- | --- | --- | --- | --- | --- | --- |

Best Unique PSM (Scan Sample\_7\_A1\_1\_3644.d:49950, m/z=702.8648, z=2, RT=16.09, ppm=-3.32):


TRINITY\_DN3078\_c0\_g1\_i1.p1

back to list

  

| Protein Coverage
| Supporting Peptides
| Best Unique PSM
|

Protein Coverage:

Supporting Peptides:

| Peptide | Uniq | -10lgP | Mass | Length | ppm | m/z | z | RT | 1/k0 Start | 1/k0 End | Precursor ID | Area Sample 8 | #Feature | #Feature Sample 8 | Start | End | PTM | AScore | Found By |
| --- | --- | --- | --- | --- | --- | --- | --- | --- | --- | --- | --- | --- | --- | --- | --- | --- | --- | --- | --- |
| K.SGNVVLC(+57.02)LIDVHK.A | Y | 43.78 | 1452.7759 | 13 | 4.56 | 727.3953 | 2 | 18.73 | 1.0391 | 1.0464 | 66055 | 4.92e3 | 2 | 2 | 1014 | 1026 | Carbamidomethylation |  | DB Search |
| R.KVSQTC(+57.02)AEHVFR.V | Y | 43.76 | 1460.7194 | 12 | -0.08 | 487.9115 | 3 | 12.02 | 0.8039 | 0.8112 | 26130 | 7.24e2 | 1 | 1 | 546 | 557 | Carbamidomethylation |  | DB Search |
| K.AKIEALHC(+57.02)LSENIVK.D | Y | 43.21 | 1723.9291 | 15 | 3.62 | 862.9711 | 2 | 15.78 | 1.1334 | 1.1406 | 48271 | 0 | 0 | 0 | 709 | 723 | Carbamidomethylation |  | DB Search |
| K.LIVEGIEDVHVDPHLQEQC(+57.02)AR.D | Y | 42.66 | 2456.2118 | 21 | -3.3 | 819.7382 | 3 | 17.06 | 1.0041 | 1.0114 | 55667 | 0 | 0 | 0 | 981 | 1001 | Carbamidomethylation |  | DB Search |
| total 4 peptides |  |  |  |  |  |  |  |  |  |  |  |  |  |  |  |  |  |  |  |
| --- | --- | --- | --- | --- | --- | --- | --- | --- | --- | --- | --- | --- | --- | --- | --- | --- | --- | --- | --- |

Best Unique PSM (Scan Sample\_7\_A1\_1\_3644.d:66055, m/z=727.3953, z=2, RT=18.73, ppm=0.08):


TRINITY\_DN554\_c0\_g1\_i5.p1

back to list

  

| Protein Coverage
| Supporting Peptides
| Best Unique PSM
|

Protein Coverage:

Supporting Peptides:

| Peptide | Uniq | -10lgP | Mass | Length | ppm | m/z | z | RT | 1/k0 Start | 1/k0 End | Precursor ID | Area Sample 8 | #Feature | #Feature Sample 8 | Start | End | PTM | AScore | Found By |
| --- | --- | --- | --- | --- | --- | --- | --- | --- | --- | --- | --- | --- | --- | --- | --- | --- | --- | --- | --- |
| K.TITLEVEPSDTIENVK.S | Y | 43.28 | 1786.92 | 16 | 2.71 | 894.4657 | 2 | 17.62 | 1.1454 | 1.1526 | 58818 | 1.46e3 | 1 | 1 | 12 | 27 |  |  | DB Search |
| total 1 peptides |  |  |  |  |  |  |  |  |  |  |  |  |  |  |  |  |  |  |  |
| --- | --- | --- | --- | --- | --- | --- | --- | --- | --- | --- | --- | --- | --- | --- | --- | --- | --- | --- | --- |

Best Unique PSM (Scan Sample\_7\_A1\_1\_3644.d:58818, m/z=894.4657, z=2, RT=17.62, ppm=-1.76):


TRINITY\_DN118520\_c0\_g1\_i1.p1

back to list

  

| Protein Coverage
| Supporting Peptides
| Best Unique PSM
|

Protein Coverage:

Supporting Peptides:

| Peptide | Uniq | -10lgP | Mass | Length | ppm | m/z | z | RT | 1/k0 Start | 1/k0 End | Precursor ID | Area Sample 8 | #Feature | #Feature Sample 8 | Start | End | PTM | AScore | Found By |
| --- | --- | --- | --- | --- | --- | --- | --- | --- | --- | --- | --- | --- | --- | --- | --- | --- | --- | --- | --- |
| K.ISNVQDIVPVLELANQHK.R | Y | 43.29 | 2016.1003 | 18 | 0.64 | 673.0381 | 3 | 19.80 | 0.9513 | 0.9586 | 72376 | 3.06e3 | 1 | 1 | 258 | 275 |  |  | DB Search |
| total 1 peptides |  |  |  |  |  |  |  |  |  |  |  |  |  |  |  |  |  |  |  |
| --- | --- | --- | --- | --- | --- | --- | --- | --- | --- | --- | --- | --- | --- | --- | --- | --- | --- | --- | --- |

Best Unique PSM (Scan Sample\_7\_A1\_1\_3644.d:72376, m/z=673.0381, z=3, RT=19.80, ppm=-3.84):


TRINITY\_DN15274\_c0\_g1\_i1.p1

back to list

  

| Protein Coverage
| Supporting Peptides
| Best Unique PSM
|

Protein Coverage:

Supporting Peptides:

| Peptide | Uniq | -10lgP | Mass | Length | ppm | m/z | z | RT | 1/k0 Start | 1/k0 End | Precursor ID | Area Sample 8 | #Feature | #Feature Sample 8 | Start | End | PTM | AScore | Found By |
| --- | --- | --- | --- | --- | --- | --- | --- | --- | --- | --- | --- | --- | --- | --- | --- | --- | --- | --- | --- |
| K.SGGQC(+57.02)PC(+57.02)KPGVVGR.R | Y | 43.83 | 1457.6867 | 14 | 2.89 | 729.8495 | 2 | 10.75 | 0.9751 | 0.9824 | 18858 | 2.96e3 | 2 | 2 | 795 | 808 | Carbamidomethylation, Carbamidomethylation |  | DB Search |
| R.YSRPTPGSNNNVM(+15.99)VR.I | Y | 43.79 | 1706.8158 | 15 | 0.89 | 569.9438 | 3 | 11.60 | 0.8352 | 0.8425 | 23563 | 1.16e3 | 1 | 1 | 621 | 635 | Oxidation (M) | M13:Oxidation (M):1000 | DB Search |
| R.GSC(+57.02)SC(+57.02)YGHAEQC(+57.02)IPTK.G | Y | 43.77 | 1853.7495 | 16 | -0.77 | 618.9205 | 3 | 12.43 | 0.855 | 0.8623 | 28624 | 3.91e2 | 1 | 1 | 270 | 285 | Carbamidomethylation, Carbamidomethylation, Carbamidomethylation |  | DB Search |
| R.YSRPTPGSNNNVMVR.I | Y | 43.76 | 1690.8209 | 15 | -3.2 | 564.6099 | 3 | 12.52 | 0.8299 | 0.8372 | 29054 | 3.96e2 | 1 | 1 | 621 | 635 |  |  | DB Search |
| K.YC(+57.02)ILSFLDKTDK.C | Y | 43.56 | 1501.7486 | 12 | -0.54 | 501.5876 | 3 | 19.89 | 0.7966 | 0.8039 | 73398 | 0 | 0 | 0 | 62 | 73 | Carbamidomethylation |  | DB Search |
| total 5 peptides |  |  |  |  |  |  |  |  |  |  |  |  |  |  |  |  |  |  |  |
| --- | --- | --- | --- | --- | --- | --- | --- | --- | --- | --- | --- | --- | --- | --- | --- | --- | --- | --- | --- |

Best Unique PSM (Scan Sample\_7\_A1\_1\_3644.d:18858, m/z=729.8495, z=2, RT=10.75, ppm=-1.58):


TRINITY\_DN146208\_c0\_g1\_i1.p1

back to list

  

| Protein Coverage
| Supporting Peptides
| Best Unique PSM
|

Protein Coverage:

Supporting Peptides:

| Peptide | Uniq | -10lgP | Mass | Length | ppm | m/z | z | RT | 1/k0 Start | 1/k0 End | Precursor ID | Area Sample 8 | #Feature | #Feature Sample 8 | Start | End | PTM | AScore | Found By |
| --- | --- | --- | --- | --- | --- | --- | --- | --- | --- | --- | --- | --- | --- | --- | --- | --- | --- | --- | --- |
| K.YGINHITNLVESKR.A | Y | 42.86 | 1642.8791 | 14 | 4.42 | 548.6336 | 3 | 15.17 | 0.8246 | 0.8319 | 44836 | 0 | 0 | 0 | 158 | 171 |  |  | DB Search |
| total 1 peptides |  |  |  |  |  |  |  |  |  |  |  |  |  |  |  |  |  |  |  |
| --- | --- | --- | --- | --- | --- | --- | --- | --- | --- | --- | --- | --- | --- | --- | --- | --- | --- | --- | --- |

Best Unique PSM (Scan Sample\_7\_A1\_1\_3644.d:44836, m/z=548.6336, z=3, RT=15.17, ppm=-0.05):


TRINITY\_DN941\_c0\_g1\_i7.p1

back to list

  

| Protein Coverage
| Supporting Peptides
| Best Unique PSM
|

Protein Coverage:

Supporting Peptides:

| Peptide | Uniq | -10lgP | Mass | Length | ppm | m/z | z | RT | 1/k0 Start | 1/k0 End | Precursor ID | Area Sample 8 | #Feature | #Feature Sample 8 | Start | End | PTM | AScore | Found By |
| --- | --- | --- | --- | --- | --- | --- | --- | --- | --- | --- | --- | --- | --- | --- | --- | --- | --- | --- | --- |
| K.AHGPGLEGGVAGQPC(+57.02)R.F | Y | 40.64 | 1561.7419 | 16 | 2.05 | 781.8763 | 2 | 12.28 | 1.0275 | 1.0347 | 27748 | 0 | 0 | 0 | 386 | 401 | Carbamidomethylation |  | DB Search |
| total 1 peptides |  |  |  |  |  |  |  |  |  |  |  |  |  |  |  |  |  |  |  |
| --- | --- | --- | --- | --- | --- | --- | --- | --- | --- | --- | --- | --- | --- | --- | --- | --- | --- | --- | --- |

Best Unique PSM (Scan Sample\_7\_A1\_1\_3644.d:27748, m/z=782.374, z=2, RT=12.30, ppm=-2.42):


TRINITY\_DN3271\_c0\_g1\_i1.p1

back to list

  

| Protein Coverage
| Supporting Peptides
| Best Unique PSM
|

Protein Coverage:

Supporting Peptides:

| Peptide | Uniq | -10lgP | Mass | Length | ppm | m/z | z | RT | 1/k0 Start | 1/k0 End | Precursor ID | Area Sample 8 | #Feature | #Feature Sample 8 | Start | End | PTM | AScore | Found By |
| --- | --- | --- | --- | --- | --- | --- | --- | --- | --- | --- | --- | --- | --- | --- | --- | --- | --- | --- | --- |
| K.TIESEAAHGTVTR.H | Y | 43.75 | 1370.679 | 13 | 3.55 | 686.3461 | 2 | 12.00 | 1.0174 | 1.0246 | 26077 | 5.62e2 | 1 | 1 | 285 | 297 |  |  | DB Search |
| total 1 peptides |  |  |  |  |  |  |  |  |  |  |  |  |  |  |  |  |  |  |  |
| --- | --- | --- | --- | --- | --- | --- | --- | --- | --- | --- | --- | --- | --- | --- | --- | --- | --- | --- | --- |

Best Unique PSM (Scan Sample\_7\_A1\_1\_3644.d:26077, m/z=686.3461, z=2, RT=12.00, ppm=-0.93):


TRINITY\_DN624\_c0\_g1\_i1.p1

back to list

  

| Protein Coverage
| Supporting Peptides
| Best Unique PSM
|

Protein Coverage:

Supporting Peptides:

| Peptide | Uniq | -10lgP | Mass | Length | ppm | m/z | z | RT | 1/k0 Start | 1/k0 End | Precursor ID | Area Sample 8 | #Feature | #Feature Sample 8 | Start | End | PTM | AScore | Found By |
| --- | --- | --- | --- | --- | --- | --- | --- | --- | --- | --- | --- | --- | --- | --- | --- | --- | --- | --- | --- |
| R.FAIQDITVDEFSAK.E | Y | 43.82 | 1582.7878 | 14 | -0.64 | 792.3972 | 2 | 19.87 | 1.0443 | 1.0516 | 73247 | 7.51e2 | 1 | 1 | 132 | 145 |  |  | DB Search |
| total 1 peptides |  |  |  |  |  |  |  |  |  |  |  |  |  |  |  |  |  |  |  |
| --- | --- | --- | --- | --- | --- | --- | --- | --- | --- | --- | --- | --- | --- | --- | --- | --- | --- | --- | --- |

Best Unique PSM (Scan Sample\_7\_A1\_1\_3644.d:73247, m/z=792.3972, z=2, RT=19.87, ppm=-5.11):


TRINITY\_DN11248\_c0\_g1\_i1.p1

back to list

  

| Protein Coverage
| Supporting Peptides
| Best Unique PSM
|

Protein Coverage:

Supporting Peptides:

| Peptide | Uniq | -10lgP | Mass | Length | ppm | m/z | z | RT | 1/k0 Start | 1/k0 End | Precursor ID | Area Sample 8 | #Feature | #Feature Sample 8 | Start | End | PTM | AScore | Found By |
| --- | --- | --- | --- | --- | --- | --- | --- | --- | --- | --- | --- | --- | --- | --- | --- | --- | --- | --- | --- |
| K.SIEAIYLGEFGTC(+57.02)K.S | Y | 43.78 | 1586.765 | 14 | -3.85 | 794.3832 | 2 | 19.71 | 1.1009 | 1.1082 | 72286 | 0 | 0 | 0 | 982 | 995 | Carbamidomethylation |  | DB Search |
| total 1 peptides |  |  |  |  |  |  |  |  |  |  |  |  |  |  |  |  |  |  |  |
| --- | --- | --- | --- | --- | --- | --- | --- | --- | --- | --- | --- | --- | --- | --- | --- | --- | --- | --- | --- |

Best Unique PSM (Scan Sample\_7\_A1\_1\_3644.d:72286, m/z=794.3832, z=2, RT=19.71, ppm=-8.32):


TRINITY\_DN8458\_c0\_g2\_i1.p1

back to list

  

| Protein Coverage
| Supporting Peptides
| Best Unique PSM
|

Protein Coverage:

Supporting Peptides:

| Peptide | Uniq | -10lgP | Mass | Length | ppm | m/z | z | RT | 1/k0 Start | 1/k0 End | Precursor ID | Area Sample 8 | #Feature | #Feature Sample 8 | Start | End | PTM | AScore | Found By |
| --- | --- | --- | --- | --- | --- | --- | --- | --- | --- | --- | --- | --- | --- | --- | --- | --- | --- | --- | --- |
| R.GNHEC(+57.02)ASINR.I | Y | 43.61 | 1156.5043 | 10 | 15.1 | 386.5128 | 3 | 9.81 | 0.7286 | 0.7359 | 14029 | 0 | 0 | 0 | 133 | 142 | Carbamidomethylation |  | DB Search |
| total 1 peptides |  |  |  |  |  |  |  |  |  |  |  |  |  |  |  |  |  |  |  |
| --- | --- | --- | --- | --- | --- | --- | --- | --- | --- | --- | --- | --- | --- | --- | --- | --- | --- | --- | --- |

Best Unique PSM (Scan Sample\_7\_A1\_1\_3644.d:14029, m/z=386.5128, z=3, RT=9.81, ppm=10.62):


TRINITY\_DN3589\_c0\_g1\_i1.p1

back to list

  

| Protein Coverage
| Supporting Peptides
| Best Unique PSM
|

Protein Coverage:

Supporting Peptides:

| Peptide | Uniq | -10lgP | Mass | Length | ppm | m/z | z | RT | 1/k0 Start | 1/k0 End | Precursor ID | Area Sample 8 | #Feature | #Feature Sample 8 | Start | End | PTM | AScore | Found By |
| --- | --- | --- | --- | --- | --- | --- | --- | --- | --- | --- | --- | --- | --- | --- | --- | --- | --- | --- | --- |
| K.AIGGIILTASHNPGGK.D | Y | 43.33 | 1504.8361 | 16 | -2.08 | 753.4204 | 2 | 15.70 | 1.0941 | 1.1013 | 47749 | 2.83e2 | 1 | 1 | 105 | 120 |  |  | DB Search |
| total 1 peptides |  |  |  |  |  |  |  |  |  |  |  |  |  |  |  |  |  |  |  |
| --- | --- | --- | --- | --- | --- | --- | --- | --- | --- | --- | --- | --- | --- | --- | --- | --- | --- | --- | --- |

Best Unique PSM (Scan Sample\_7\_A1\_1\_3644.d:47749, m/z=753.4204, z=2, RT=15.70, ppm=-6.55):


TRINITY\_DN38657\_c0\_g1\_i5.p1

back to list

  

| Protein Coverage
| Supporting Peptides
| Best Unique PSM
|

Protein Coverage:

Supporting Peptides:

| Peptide | Uniq | -10lgP | Mass | Length | ppm | m/z | z | RT | 1/k0 Start | 1/k0 End | Precursor ID | Area Sample 8 | #Feature | #Feature Sample 8 | Start | End | PTM | AScore | Found By |
| --- | --- | --- | --- | --- | --- | --- | --- | --- | --- | --- | --- | --- | --- | --- | --- | --- | --- | --- | --- |
| K.STSQGQALTK.T | Y | 43.71 | 1019.5247 | 10 | 7.68 | 510.7713 | 2 | 10.06 | 0.8291 | 0.8364 | 15179 | 0 | 0 | 0 | 52 | 61 |  |  | DB Search |
| total 1 peptides |  |  |  |  |  |  |  |  |  |  |  |  |  |  |  |  |  |  |  |
| --- | --- | --- | --- | --- | --- | --- | --- | --- | --- | --- | --- | --- | --- | --- | --- | --- | --- | --- | --- |

Best Unique PSM (Scan Sample\_7\_A1\_1\_3644.d:15179, m/z=510.7713, z=2, RT=10.06, ppm=3.2):


TRINITY\_DN384\_c0\_g1\_i2.p1

back to list

  

| Protein Coverage
| Supporting Peptides
| Best Unique PSM
|

Protein Coverage:

Supporting Peptides:

| Peptide | Uniq | -10lgP | Mass | Length | ppm | m/z | z | RT | 1/k0 Start | 1/k0 End | Precursor ID | Area Sample 8 | #Feature | #Feature Sample 8 | Start | End | PTM | AScore | Found By |
| --- | --- | --- | --- | --- | --- | --- | --- | --- | --- | --- | --- | --- | --- | --- | --- | --- | --- | --- | --- |
| K.FVLHGDVSHGSR.E | Y | 43.34 | 1309.6527 | 12 | -0.43 | 437.556 | 3 | 12.14 | 0.762 | 0.7693 | 26936 | 0 | 0 | 0 | 275 | 286 |  |  | DB Search |
| total 1 peptides |  |  |  |  |  |  |  |  |  |  |  |  |  |  |  |  |  |  |  |
| --- | --- | --- | --- | --- | --- | --- | --- | --- | --- | --- | --- | --- | --- | --- | --- | --- | --- | --- | --- |

Best Unique PSM (Scan Sample\_7\_A1\_1\_3644.d:26936, m/z=437.556, z=3, RT=12.14, ppm=-4.91):


TRINITY\_DN165536\_c0\_g1\_i1.p1

back to list

  

| Protein Coverage
| Supporting Peptides
| Best Unique PSM
|

Protein Coverage:

Supporting Peptides:

| Peptide | Uniq | -10lgP | Mass | Length | ppm | m/z | z | RT | 1/k0 Start | 1/k0 End | Precursor ID | Area Sample 8 | #Feature | #Feature Sample 8 | Start | End | PTM | AScore | Found By |
| --- | --- | --- | --- | --- | --- | --- | --- | --- | --- | --- | --- | --- | --- | --- | --- | --- | --- | --- | --- |
| M.S(+42.01)EQEISNLVEQAK.L | Y | 43.84 | 1515.7416 | 13 | 3.29 | 758.8772 | 2 | 19.50 | 1.0568 | 1.064 | 70956 | 0 | 0 | 0 | 2 | 14 | Acetylation (Protein N-term) | S1:Acetylation (Protein N-term):1000 | DB Search |
| K.ATETATGLATTHPIK.L | Y | 43.77 | 1510.7991 | 15 | -0.08 | 756.4034 | 2 | 13.06 | 1.1041 | 1.1114 | 32134 | 2.41e3 | 1 | 1 | 161 | 175 |  |  | DB Search |
| total 2 peptides |  |  |  |  |  |  |  |  |  |  |  |  |  |  |  |  |  |  |  |
| --- | --- | --- | --- | --- | --- | --- | --- | --- | --- | --- | --- | --- | --- | --- | --- | --- | --- | --- | --- |

Best Unique PSM (Scan Sample\_7\_A1\_1\_3644.d:70956, m/z=758.8772, z=2, RT=19.50, ppm=-1.19):


TRINITY\_DN1058\_c0\_g1\_i1.p1

back to list

  

| Protein Coverage
| Supporting Peptides
| Best Unique PSM
|

Protein Coverage:

Supporting Peptides:

| Peptide | Uniq | -10lgP | Mass | Length | ppm | m/z | z | RT | 1/k0 Start | 1/k0 End | Precursor ID | Area Sample 8 | #Feature | #Feature Sample 8 | Start | End | PTM | AScore | Found By |
| --- | --- | --- | --- | --- | --- | --- | --- | --- | --- | --- | --- | --- | --- | --- | --- | --- | --- | --- | --- |
| R.APTHILNPANKR.S | Y | 43.8 | 1330.7469 | 12 | 0.61 | 666.3782 | 2 | 11.23 | 1.0057 | 1.013 | 21549 | 1.39e3 | 2 | 2 | 2314 | 2325 |  |  | DB Search |
| total 1 peptides |  |  |  |  |  |  |  |  |  |  |  |  |  |  |  |  |  |  |  |
| --- | --- | --- | --- | --- | --- | --- | --- | --- | --- | --- | --- | --- | --- | --- | --- | --- | --- | --- | --- |

Best Unique PSM (Scan Sample\_7\_A1\_1\_3644.d:21549, m/z=666.3782, z=2, RT=11.23, ppm=-3.86):


TRINITY\_DN15151\_c0\_g1\_i1.p1

back to list

  

| Protein Coverage
| Supporting Peptides
| Best Unique PSM
|

Protein Coverage:

Supporting Peptides:

| Peptide | Uniq | -10lgP | Mass | Length | ppm | m/z | z | RT | 1/k0 Start | 1/k0 End | Precursor ID | Area Sample 8 | #Feature | #Feature Sample 8 | Start | End | PTM | AScore | Found By |
| --- | --- | --- | --- | --- | --- | --- | --- | --- | --- | --- | --- | --- | --- | --- | --- | --- | --- | --- | --- |
| K.AVAAGMNPMDIKR.G | Y | 43.11 | 1372.6955 | 13 | 1.74 | 687.3531 | 2 | 14.92 | 1.0126 | 1.0198 | 43242 | 6.18e2 | 1 | 1 | 145 | 157 |  |  | DB Search |
| total 1 peptides |  |  |  |  |  |  |  |  |  |  |  |  |  |  |  |  |  |  |  |
| --- | --- | --- | --- | --- | --- | --- | --- | --- | --- | --- | --- | --- | --- | --- | --- | --- | --- | --- | --- |

Best Unique PSM (Scan Sample\_7\_A1\_1\_3644.d:43242, m/z=687.3531, z=2, RT=14.92, ppm=-2.73):


TRINITY\_DN329\_c0\_g1\_i2.p1

back to list

  

| Protein Coverage
| Supporting Peptides
| Best Unique PSM
|

Protein Coverage:

Supporting Peptides:

| Peptide | Uniq | -10lgP | Mass | Length | ppm | m/z | z | RT | 1/k0 Start | 1/k0 End | Precursor ID | Area Sample 8 | #Feature | #Feature Sample 8 | Start | End | PTM | AScore | Found By |
| --- | --- | --- | --- | --- | --- | --- | --- | --- | --- | --- | --- | --- | --- | --- | --- | --- | --- | --- | --- |
| K.EGDLVIHSGITDAVK.Y | Y | 43.83 | 1552.8096 | 15 | 0.72 | 777.4092 | 2 | 15.57 | 1.0576 | 1.0648 | 46999 | 6.3e3 | 3 | 3 | 211 | 225 |  |  | DB Search |
| K.SFHFGVIKLEGK.T | Y | 43.81 | 1360.7503 | 12 | 3.09 | 681.3815 | 2 | 17.25 | 1.0303 | 1.0375 | 56659 | 1.05e4 | 3 | 3 | 57 | 68 |  |  | DB Search |
| K.GLKVDFDTTFAPVTGK.K | Y | 43.81 | 1694.8879 | 16 | -2.21 | 848.4456 | 2 | 18.33 | 1.0921 | 1.0993 | 63359 | 2.86e3 | 1 | 1 | 130 | 145 |  |  | DB Search |
| K.GLKVDFDTTFAPVTGKK.T | Y | 43.8 | 1822.9828 | 17 | 0.54 | 912.4951 | 2 | 17.35 | 1.1825 | 1.1897 | 57165 | 5.55e3 | 2 | 2 | 130 | 146 |  |  | DB Search |
| K.VDFDTTFAPVTGKK.T | Y | 43.77 | 1524.7824 | 14 | 1.56 | 763.3962 | 2 | 16.78 | 1.0524 | 1.0596 | 54009 | 4.02e3 | 2 | 2 | 133 | 146 |  |  | DB Search |
| K.LRPGVELTMSSLVNAK.S | Y | 43.74 | 1713.9447 | 16 | 1.1 | 572.3203 | 3 | 17.93 | 0.9113 | 0.9186 | 60853 | 1.13e4 | 3 | 3 | 287 | 302 |  |  | DB Search |
| K.AKIDNNLR.L | Y | 43.36 | 942.5247 | 8 | 5.16 | 472.2699 | 2 | 10.71 | 0.8412 | 0.8485 | 18673 | 3.82e3 | 1 | 1 | 271 | 278 |  |  | DB Search |
| K.LRPGVELTM(+15.99)SSLVNAK.S | Y | 43.25 | 1729.9396 | 16 | 1.89 | 865.9749 | 2 | 15.36 | 1.1378 | 1.145 | 45662 | 1.01e4 | 1 | 1 | 287 | 302 | Oxidation (M) | M9:Oxidation (M):1000 | DB Search |
| K.SFHFGVIK.L | Y | 43.17 | 933.5072 | 8 | 1.24 | 467.7594 | 2 | 16.37 | 0.8226 | 0.8299 | 51672 | 8.38e2 | 1 | 1 | 57 | 64 |  |  | DB Search |
| total 9 peptides |  |  |  |  |  |  |  |  |  |  |  |  |  |  |  |  |  |  |  |
| --- | --- | --- | --- | --- | --- | --- | --- | --- | --- | --- | --- | --- | --- | --- | --- | --- | --- | --- | --- |

Best Unique PSM (Scan Sample\_7\_A1\_1\_3644.d:46999, m/z=777.4092, z=2, RT=15.57, ppm=-3.76):


TRINITY\_DN244\_c0\_g1\_i1.p1

back to list

  

| Protein Coverage
| Supporting Peptides
| Best Unique PSM
|

Protein Coverage:

Supporting Peptides:

| Peptide | Uniq | -10lgP | Mass | Length | ppm | m/z | z | RT | 1/k0 Start | 1/k0 End | Precursor ID | Area Sample 8 | #Feature | #Feature Sample 8 | Start | End | PTM | AScore | Found By |
| --- | --- | --- | --- | --- | --- | --- | --- | --- | --- | --- | --- | --- | --- | --- | --- | --- | --- | --- | --- |
| K.ALLLASSNAFDTSNGAR.E | Y | 43.83 | 1706.8587 | 17 | 2.98 | 854.4354 | 2 | 17.86 | 1.0977 | 1.1049 | 60419 | 5.82e2 | 1 | 1 | 1912 | 1928 |  |  | DB Search |
| K.ALVILGDGFISGGGTPSK.T | Y | 43.81 | 1687.9144 | 18 | -0.24 | 844.9605 | 2 | 19.84 | 1.1622 | 1.1694 | 72833 | 2.32e3 | 1 | 1 | 1619 | 1636 |  |  | DB Search |
| K.DLSLVVEPLKNYGVK.I | Y | 42.82 | 1672.9399 | 15 | 5.12 | 837.4778 | 2 | 19.25 | 1.1354 | 1.1426 | 69248 | 2.1e2 | 1 | 1 | 392 | 406 |  |  | DB Search |
| total 3 peptides |  |  |  |  |  |  |  |  |  |  |  |  |  |  |  |  |  |  |  |
| --- | --- | --- | --- | --- | --- | --- | --- | --- | --- | --- | --- | --- | --- | --- | --- | --- | --- | --- | --- |

Best Unique PSM (Scan Sample\_7\_A1\_1\_3644.d:60419, m/z=854.4354, z=2, RT=17.86, ppm=-1.5):


TRINITY\_DN477\_c0\_g1\_i8.p1

back to list

  

| Protein Coverage
| Supporting Peptides
| Best Unique PSM
|

Protein Coverage:

Supporting Peptides:

| Peptide | Uniq | -10lgP | Mass | Length | ppm | m/z | z | RT | 1/k0 Start | 1/k0 End | Precursor ID | Area Sample 8 | #Feature | #Feature Sample 8 | Start | End | PTM | AScore | Found By |
| --- | --- | --- | --- | --- | --- | --- | --- | --- | --- | --- | --- | --- | --- | --- | --- | --- | --- | --- | --- |
| R.ILQC(+57.02)NQYANVEEAR.N | Y | 43.82 | 1706.8046 | 14 | 3.36 | 854.4086 | 2 | 14.34 | 1.0941 | 1.1013 | 39739 | 5.68e3 | 1 | 1 | 86 | 99 | Carbamidomethylation |  | DB Search |
| R.SQSAFPTRR.R | Y | 43.75 | 1048.5414 | 9 | 2.01 | 525.2767 | 2 | 11.08 | 0.8526 | 0.8599 | 20720 | 4.77e3 | 1 | 1 | 856 | 864 |  |  | DB Search |
| K.DKNILVLTLGVGPQVNR.N | Y | 43.64 | 1835.0628 | 17 | 2.21 | 918.5366 | 2 | 19.60 | 1.1334 | 1.1406 | 71277 | 2.86e3 | 1 | 1 | 168 | 184 |  |  | DB Search |
| total 3 peptides |  |  |  |  |  |  |  |  |  |  |  |  |  |  |  |  |  |  |  |
| --- | --- | --- | --- | --- | --- | --- | --- | --- | --- | --- | --- | --- | --- | --- | --- | --- | --- | --- | --- |

Best Unique PSM (Scan Sample\_7\_A1\_1\_3644.d:39739, m/z=854.4086, z=2, RT=14.34, ppm=-1.11):


TRINITY\_DN852\_c0\_g1\_i1.p1

back to list

  

| Protein Coverage
| Supporting Peptides
| Best Unique PSM
|

Protein Coverage:

Supporting Peptides:

| Peptide | Uniq | -10lgP | Mass | Length | ppm | m/z | z | RT | 1/k0 Start | 1/k0 End | Precursor ID | Area Sample 8 | #Feature | #Feature Sample 8 | Start | End | PTM | AScore | Found By |
| --- | --- | --- | --- | --- | --- | --- | --- | --- | --- | --- | --- | --- | --- | --- | --- | --- | --- | --- | --- |
| K.FGLEWAQK.N | Y | 43.46 | 977.497 | 8 | -6.44 | 489.7505 | 2 | 17.86 | 0.8222 | 0.8295 | 60548 | 0 | 0 | 0 | 474 | 481 |  |  | DB Search |
| total 1 peptides |  |  |  |  |  |  |  |  |  |  |  |  |  |  |  |  |  |  |  |
| --- | --- | --- | --- | --- | --- | --- | --- | --- | --- | --- | --- | --- | --- | --- | --- | --- | --- | --- | --- |

Best Unique PSM (Scan Sample\_7\_A1\_1\_3644.d:60548, m/z=489.7505, z=2, RT=17.86, ppm=-10.92):


TRINITY\_DN6920\_c0\_g1\_i4.p1

back to list

  

| Protein Coverage
| Supporting Peptides
| Best Unique PSM
|

Protein Coverage:

Supporting Peptides:

| Peptide | Uniq | -10lgP | Mass | Length | ppm | m/z | z | RT | 1/k0 Start | 1/k0 End | Precursor ID | Area Sample 8 | #Feature | #Feature Sample 8 | Start | End | PTM | AScore | Found By |
| --- | --- | --- | --- | --- | --- | --- | --- | --- | --- | --- | --- | --- | --- | --- | --- | --- | --- | --- | --- |
| R.VLVHTQQK.L | Y | 43.71 | 951.5501 | 8 | -3.93 | 476.7784 | 2 | 9.59 | 0.8291 | 0.8364 | 12948 | 0 | 0 | 0 | 40 | 47 |  |  | DB Search |
| total 1 peptides |  |  |  |  |  |  |  |  |  |  |  |  |  |  |  |  |  |  |  |
| --- | --- | --- | --- | --- | --- | --- | --- | --- | --- | --- | --- | --- | --- | --- | --- | --- | --- | --- | --- |

Best Unique PSM (Scan Sample\_7\_A1\_1\_3644.d:12948, m/z=476.7784, z=2, RT=9.59, ppm=-8.41):


TRINITY\_DN2477\_c0\_g1\_i5.p1

back to list

  

| Protein Coverage
| Supporting Peptides
| Best Unique PSM
|

Protein Coverage:

Supporting Peptides:

| Peptide | Uniq | -10lgP | Mass | Length | ppm | m/z | z | RT | 1/k0 Start | 1/k0 End | Precursor ID | Area Sample 8 | #Feature | #Feature Sample 8 | Start | End | PTM | AScore | Found By |
| --- | --- | --- | --- | --- | --- | --- | --- | --- | --- | --- | --- | --- | --- | --- | --- | --- | --- | --- | --- |
| K.DSYVGDEAQSKR.G | Y | 43.81 | 1353.616 | 12 | 3.22 | 677.8145 | 2 | 11.01 | 0.9961 | 1.0033 | 20355 | 1.65e3 | 1 | 1 | 8 | 19 |  |  | DB Search |
| R.VAPEEHPVLLTEAPLNPK.A | Y | 43.24 | 1953.0571 | 18 | -8.51 | 977.5231 | 2 | 16.59 | 1.2073 | 1.2145 | 52921 | 0 | 0 | 0 | 53 | 70 |  |  | DB Search |
| total 2 peptides |  |  |  |  |  |  |  |  |  |  |  |  |  |  |  |  |  |  |  |
| --- | --- | --- | --- | --- | --- | --- | --- | --- | --- | --- | --- | --- | --- | --- | --- | --- | --- | --- | --- |

Best Unique PSM (Scan Sample\_7\_A1\_1\_3644.d:20355, m/z=677.8145, z=2, RT=11.01, ppm=-1.25):


TRINITY\_DN3545\_c0\_g1\_i1.p1

back to list

  

| Protein Coverage
| Supporting Peptides
| Best Unique PSM
|

Protein Coverage:

Supporting Peptides:

| Peptide | Uniq | -10lgP | Mass | Length | ppm | m/z | z | RT | 1/k0 Start | 1/k0 End | Precursor ID | Area Sample 8 | #Feature | #Feature Sample 8 | Start | End | PTM | AScore | Found By |
| --- | --- | --- | --- | --- | --- | --- | --- | --- | --- | --- | --- | --- | --- | --- | --- | --- | --- | --- | --- |
| R.SC(+57.02)TNPAPANGGK.P | N | 43.8 | 1172.5244 | 12 | 2.69 | 587.2684 | 2 | 9.47 | 0.838 | 0.8453 | 12375 | 4.7e3 | 1 | 1 | 55 | 66 | Carbamidomethylation |  | DB Search |
| R.SSC(+57.02)RPILSVIK.I | Y | 42.42 | 1258.7067 | 11 | -5.58 | 630.3543 | 2 | 17.05 | 0.9731 | 0.9804 | 55404 | 1.96e4 | 2 | 2 | 6 | 16 | Carbamidomethylation |  | DB Search |
| total 2 peptides |  |  |  |  |  |  |  |  |  |  |  |  |  |  |  |  |  |  |  |
| --- | --- | --- | --- | --- | --- | --- | --- | --- | --- | --- | --- | --- | --- | --- | --- | --- | --- | --- | --- |

Best Unique PSM (Scan Sample\_7\_A1\_1\_3644.d:55404, m/z=630.3543, z=2, RT=17.05, ppm=-10.06):


TRINITY\_DN16079\_c0\_g2\_i1.p1

back to list

  

| Protein Coverage
| Supporting Peptides
| Best Unique PSM
|

Protein Coverage:

Supporting Peptides:

| Peptide | Uniq | -10lgP | Mass | Length | ppm | m/z | z | RT | 1/k0 Start | 1/k0 End | Precursor ID | Area Sample 8 | #Feature | #Feature Sample 8 | Start | End | PTM | AScore | Found By |
| --- | --- | --- | --- | --- | --- | --- | --- | --- | --- | --- | --- | --- | --- | --- | --- | --- | --- | --- | --- |
| R.TC(+57.02)ALGNDEC(+57.02)PTNSK.C | Y | 43.76 | 1565.645 | 14 | 2.52 | 783.8282 | 2 | 11.67 | 1.0118 | 1.019 | 24176 | 0 | 0 | 0 | 306 | 319 | Carbamidomethylation, Carbamidomethylation |  | DB Search |
| total 1 peptides |  |  |  |  |  |  |  |  |  |  |  |  |  |  |  |  |  |  |  |
| --- | --- | --- | --- | --- | --- | --- | --- | --- | --- | --- | --- | --- | --- | --- | --- | --- | --- | --- | --- |

Best Unique PSM (Scan Sample\_7\_A1\_1\_3644.d:24176, m/z=783.8282, z=2, RT=11.67, ppm=-1.95):


TRINITY\_DN1907\_c0\_g1\_i2.p1

back to list

  

| Protein Coverage
| Supporting Peptides
| Best Unique PSM
|

Protein Coverage:

Supporting Peptides:

| Peptide | Uniq | -10lgP | Mass | Length | ppm | m/z | z | RT | 1/k0 Start | 1/k0 End | Precursor ID | Area Sample 8 | #Feature | #Feature Sample 8 | Start | End | PTM | AScore | Found By |
| --- | --- | --- | --- | --- | --- | --- | --- | --- | --- | --- | --- | --- | --- | --- | --- | --- | --- | --- | --- |
| K.VIIDYQTHHR.L | Y | 43.82 | 1280.6625 | 10 | -5.51 | 641.3322 | 2 | 12.21 | 0.9574 | 0.9646 | 27303 | 0 | 0 | 0 | 150 | 159 |  |  | DB Search |
| total 1 peptides |  |  |  |  |  |  |  |  |  |  |  |  |  |  |  |  |  |  |  |
| --- | --- | --- | --- | --- | --- | --- | --- | --- | --- | --- | --- | --- | --- | --- | --- | --- | --- | --- | --- |

Best Unique PSM (Scan Sample\_7\_A1\_1\_3644.d:27303, m/z=641.3322, z=2, RT=12.21, ppm=-9.99):


TRINITY\_DN13813\_c0\_g1\_i2.p2

back to list

  

| Protein Coverage
| Supporting Peptides
| Best Unique PSM
|

Protein Coverage:

Supporting Peptides:

| Peptide | Uniq | -10lgP | Mass | Length | ppm | m/z | z | RT | 1/k0 Start | 1/k0 End | Precursor ID | Area Sample 8 | #Feature | #Feature Sample 8 | Start | End | PTM | AScore | Found By |
| --- | --- | --- | --- | --- | --- | --- | --- | --- | --- | --- | --- | --- | --- | --- | --- | --- | --- | --- | --- |
| R.DGPGPLASDLLLQMR.Q | Y | 41.47 | 1581.8184 | 15 | -2.93 | 528.2762 | 3 | 19.62 | 0.8149 | 0.8222 | 71531 | 3.73e2 | 1 | 1 | 308 | 322 |  |  | DB Search |
| total 1 peptides |  |  |  |  |  |  |  |  |  |  |  |  |  |  |  |  |  |  |  |
| --- | --- | --- | --- | --- | --- | --- | --- | --- | --- | --- | --- | --- | --- | --- | --- | --- | --- | --- | --- |

Best Unique PSM (Scan Sample\_7\_A1\_1\_3644.d:71531, m/z=528.2762, z=3, RT=19.62, ppm=-7.41):


TRINITY\_DN876\_c0\_g1\_i2.p1

back to list

  

| Protein Coverage
| Supporting Peptides
| Best Unique PSM
|

Protein Coverage:

Supporting Peptides:

| Peptide | Uniq | -10lgP | Mass | Length | ppm | m/z | z | RT | 1/k0 Start | 1/k0 End | Precursor ID | Area Sample 8 | #Feature | #Feature Sample 8 | Start | End | PTM | AScore | Found By |
| --- | --- | --- | --- | --- | --- | --- | --- | --- | --- | --- | --- | --- | --- | --- | --- | --- | --- | --- | --- |
| K.AAMEELEHAEK.F | Y | 40.76 | 1256.5707 | 11 | 2.71 | 629.2915 | 2 | 13.28 | 0.961 | 0.9683 | 33568 | 3.38e2 | 1 | 1 | 99 | 109 |  |  | DB Search |
| total 1 peptides |  |  |  |  |  |  |  |  |  |  |  |  |  |  |  |  |  |  |  |
| --- | --- | --- | --- | --- | --- | --- | --- | --- | --- | --- | --- | --- | --- | --- | --- | --- | --- | --- | --- |

Best Unique PSM (Scan Sample\_7\_A1\_1\_3644.d:33568, m/z=629.2915, z=2, RT=13.28, ppm=-1.76):


TRINITY\_DN1272\_c0\_g1\_i1.p1

back to list

  

| Protein Coverage
| Supporting Peptides
| Best Unique PSM
|

Protein Coverage:

Supporting Peptides:

| Peptide | Uniq | -10lgP | Mass | Length | ppm | m/z | z | RT | 1/k0 Start | 1/k0 End | Precursor ID | Area Sample 8 | #Feature | #Feature Sample 8 | Start | End | PTM | AScore | Found By |
| --- | --- | --- | --- | --- | --- | --- | --- | --- | --- | --- | --- | --- | --- | --- | --- | --- | --- | --- | --- |
| K.YIQGENEKQEK.I | Y | 43.36 | 1364.6572 | 11 | 1.86 | 455.8918 | 3 | 9.61 | 0.7652 | 0.7726 | 13062 | 0 | 0 | 0 | 116 | 126 |  |  | DB Search |
| total 1 peptides |  |  |  |  |  |  |  |  |  |  |  |  |  |  |  |  |  |  |  |
| --- | --- | --- | --- | --- | --- | --- | --- | --- | --- | --- | --- | --- | --- | --- | --- | --- | --- | --- | --- |

Best Unique PSM (Scan Sample\_7\_A1\_1\_3644.d:13062, m/z=455.8918, z=3, RT=9.61, ppm=-2.62):


TRINITY\_DN161\_c0\_g1\_i2.p1

back to list

  

| Protein Coverage
| Supporting Peptides
| Best Unique PSM
|

Protein Coverage:

Supporting Peptides:

| Peptide | Uniq | -10lgP | Mass | Length | ppm | m/z | z | RT | 1/k0 Start | 1/k0 End | Precursor ID | Area Sample 8 | #Feature | #Feature Sample 8 | Start | End | PTM | AScore | Found By |
| --- | --- | --- | --- | --- | --- | --- | --- | --- | --- | --- | --- | --- | --- | --- | --- | --- | --- | --- | --- |
| K.SYELPDGQVITIGNER.F | Y | 43.86 | 1789.8846 | 16 | 3.32 | 895.9485 | 2 | 18.70 | 1.1126 | 1.1198 | 65490 | 4.79e3 | 2 | 2 | 262 | 277 |  |  | DB Search |
| K.DSYVGDEAQSKR.G | Y | 43.81 | 1353.616 | 12 | 3.22 | 677.8145 | 2 | 11.01 | 0.9961 | 1.0033 | 20355 | 1.65e3 | 1 | 1 | 74 | 85 |  |  | DB Search |
| R.AVFPSIVGRPR.H | Y | 43.77 | 1197.6982 | 11 | 1.95 | 599.8549 | 2 | 15.90 | 0.959 | 0.9662 | 48470 | 4.43e4 | 1 | 1 | 52 | 62 |  |  | DB Search |
| R.LDLAGRDLTDYLMK.I | Y | 43.72 | 1622.8338 | 14 | -2.1 | 541.9483 | 3 | 19.46 | 0.7998 | 0.8071 | 70651 | 1.99e3 | 2 | 2 | 201 | 214 |  |  | DB Search |
| R.VAPEEHPVLLTEAPLNPK.A | Y | 43.24 | 1953.0571 | 18 | -8.51 | 977.5231 | 2 | 16.59 | 1.2073 | 1.2145 | 52921 | 0 | 0 | 0 | 119 | 136 |  |  | DB Search |
| total 5 peptides |  |  |  |  |  |  |  |  |  |  |  |  |  |  |  |  |  |  |  |
| --- | --- | --- | --- | --- | --- | --- | --- | --- | --- | --- | --- | --- | --- | --- | --- | --- | --- | --- | --- |

Best Unique PSM (Scan Sample\_7\_A1\_1\_3644.d:65490, m/z=895.9485, z=2, RT=18.70, ppm=-1.15):


TRINITY\_DN2472\_c0\_g1\_i1.p1

back to list

  

| Protein Coverage
| Supporting Peptides
| Best Unique PSM
|

Protein Coverage:

Supporting Peptides:

| Peptide | Uniq | -10lgP | Mass | Length | ppm | m/z | z | RT | 1/k0 Start | 1/k0 End | Precursor ID | Area Sample 8 | #Feature | #Feature Sample 8 | Start | End | PTM | AScore | Found By |
| --- | --- | --- | --- | --- | --- | --- | --- | --- | --- | --- | --- | --- | --- | --- | --- | --- | --- | --- | --- |
| M.S(+42.01)SGEIESLRQEAENLR.N | Y | 43.71 | 1858.9021 | 16 | 1.2 | 930.4553 | 2 | 18.91 | 1.1973 | 1.2045 | 67059 | 4.17e3 | 1 | 1 | 2 | 17 | Acetylation (Protein N-term) | S1:Acetylation (Protein N-term):1000 | DB Search |
| R.SGVLAGHDNR.V | Y | 42.53 | 1024.505 | 10 | -1.12 | 513.2569 | 2 | 10.34 | 0.8611 | 0.8684 | 16684 | 3.56e2 | 1 | 1 | 308 | 317 |  |  | DB Search |
| total 2 peptides |  |  |  |  |  |  |  |  |  |  |  |  |  |  |  |  |  |  |  |
| --- | --- | --- | --- | --- | --- | --- | --- | --- | --- | --- | --- | --- | --- | --- | --- | --- | --- | --- | --- |

Best Unique PSM (Scan Sample\_7\_A1\_1\_3644.d:67059, m/z=930.4553, z=2, RT=18.91, ppm=-3.27):


TRINITY\_DN941\_c0\_g1\_i14.p1

back to list

  

| Protein Coverage
| Supporting Peptides
| Best Unique PSM
|

Protein Coverage:

Supporting Peptides:

| Peptide | Uniq | -10lgP | Mass | Length | ppm | m/z | z | RT | 1/k0 Start | 1/k0 End | Precursor ID | Area Sample 8 | #Feature | #Feature Sample 8 | Start | End | PTM | AScore | Found By |
| --- | --- | --- | --- | --- | --- | --- | --- | --- | --- | --- | --- | --- | --- | --- | --- | --- | --- | --- | --- |
| K.ADLTRPTGK.K | Y | 42.69 | 957.5243 | 9 | -3.1 | 479.7658 | 2 | 10.44 | 0.8193 | 0.8266 | 17248 | 0 | 0 | 0 | 52 | 60 |  |  | DB Search |
| total 1 peptides |  |  |  |  |  |  |  |  |  |  |  |  |  |  |  |  |  |  |  |
| --- | --- | --- | --- | --- | --- | --- | --- | --- | --- | --- | --- | --- | --- | --- | --- | --- | --- | --- | --- |

Best Unique PSM (Scan Sample\_7\_A1\_1\_3644.d:17248, m/z=479.7658, z=2, RT=10.44, ppm=-7.58):


TRINITY\_DN7659\_c0\_g1\_i2.p1

back to list

  

| Protein Coverage
| Supporting Peptides
| Best Unique PSM
|

Protein Coverage:

Supporting Peptides:

| Peptide | Uniq | -10lgP | Mass | Length | ppm | m/z | z | RT | 1/k0 Start | 1/k0 End | Precursor ID | Area Sample 8 | #Feature | #Feature Sample 8 | Start | End | PTM | AScore | Found By |
| --- | --- | --- | --- | --- | --- | --- | --- | --- | --- | --- | --- | --- | --- | --- | --- | --- | --- | --- | --- |
| M.A(+42.01)SAATDNRK.V | Y | 43.11 | 974.4781 | 9 | 3.81 | 488.246 | 2 | 9.52 | 0.8234 | 0.8307 | 12664 | 0 | 0 | 0 | 2 | 10 | Acetylation (Protein N-term) | A1:Acetylation (Protein N-term):1000 | DB Search |
| K.AQPTEIYNLGAQSHVK.V | Y | 42.33 | 1754.8951 | 16 | -17.72 | 878.4354 | 2 | 14.86 | 1.1734 | 1.1806 | 42928 | 0 | 0 | 0 | 86 | 101 |  |  | DB Search |
| total 2 peptides |  |  |  |  |  |  |  |  |  |  |  |  |  |  |  |  |  |  |  |
| --- | --- | --- | --- | --- | --- | --- | --- | --- | --- | --- | --- | --- | --- | --- | --- | --- | --- | --- | --- |

Best Unique PSM (Scan Sample\_7\_A1\_1\_3644.d:12664, m/z=488.246, z=2, RT=9.52, ppm=-0.67):


TRINITY\_DN36\_c0\_g1\_i13.p1

back to list

  

| Protein Coverage
| Supporting Peptides
| Best Unique PSM
|

Protein Coverage:

Supporting Peptides:

| Peptide | Uniq | -10lgP | Mass | Length | ppm | m/z | z | RT | 1/k0 Start | 1/k0 End | Precursor ID | Area Sample 8 | #Feature | #Feature Sample 8 | Start | End | PTM | AScore | Found By |
| --- | --- | --- | --- | --- | --- | --- | --- | --- | --- | --- | --- | --- | --- | --- | --- | --- | --- | --- | --- |
| K.FETQIATK.D | Y | 43.56 | 936.4916 | 8 | 1.93 | 937.4965 | 1 | 13.99 | 1.2029 | 1.2101 | 37753 | 0 | 0 | 0 | 166 | 173 |  |  | DB Search |
| R.LGGIEVTQGNC(+57.02)AR.K | Y | 43.02 | 1373.6721 | 13 | 2.68 | 687.8421 | 2 | 13.90 | 0.9662 | 0.9735 | 37137 | 6.95e2 | 1 | 1 | 57 | 69 | Carbamidomethylation |  | DB Search |
| total 2 peptides |  |  |  |  |  |  |  |  |  |  |  |  |  |  |  |  |  |  |  |
| --- | --- | --- | --- | --- | --- | --- | --- | --- | --- | --- | --- | --- | --- | --- | --- | --- | --- | --- | --- |

Best Unique PSM (Scan Sample\_7\_A1\_1\_3644.d:37753, m/z=937.4965, z=1, RT=13.99, ppm=-2.55):


TRINITY\_DN57\_c0\_g1\_i1.p1

back to list

  

| Protein Coverage
| Supporting Peptides
| Best Unique PSM
|

Protein Coverage:

Supporting Peptides:

| Peptide | Uniq | -10lgP | Mass | Length | ppm | m/z | z | RT | 1/k0 Start | 1/k0 End | Precursor ID | Area Sample 8 | #Feature | #Feature Sample 8 | Start | End | PTM | AScore | Found By |
| --- | --- | --- | --- | --- | --- | --- | --- | --- | --- | --- | --- | --- | --- | --- | --- | --- | --- | --- | --- |
| R.IGGSTDTGR.H | Y | 42.68 | 862.4144 | 9 | 4.01 | 432.2143 | 2 | 9.50 | 0.7445 | 0.7518 | 12524 | 1.06e3 | 1 | 1 | 611 | 619 |  |  | DB Search |
| R.VGQAM(+15.99)ASPEDK.A | Y | 42.64 | 1147.5179 | 11 | -6.8 | 574.7598 | 2 | 9.75 | 0.864 | 0.8712 | 13667 | 0 | 0 | 0 | 564 | 574 | Oxidation (M) | M5:Oxidation (M):1000 | DB Search |
| total 2 peptides |  |  |  |  |  |  |  |  |  |  |  |  |  |  |  |  |  |  |  |
| --- | --- | --- | --- | --- | --- | --- | --- | --- | --- | --- | --- | --- | --- | --- | --- | --- | --- | --- | --- |

Best Unique PSM (Scan Sample\_7\_A1\_1\_3644.d:12524, m/z=432.2143, z=2, RT=9.50, ppm=-0.47):


TRINITY\_DN803\_c0\_g1\_i1.p1

back to list

  

| Protein Coverage
| Supporting Peptides
| Best Unique PSM
|

Protein Coverage:

Supporting Peptides:

| Peptide | Uniq | -10lgP | Mass | Length | ppm | m/z | z | RT | 1/k0 Start | 1/k0 End | Precursor ID | Area Sample 8 | #Feature | #Feature Sample 8 | Start | End | PTM | AScore | Found By |
| --- | --- | --- | --- | --- | --- | --- | --- | --- | --- | --- | --- | --- | --- | --- | --- | --- | --- | --- | --- |
| K.VQLTANNVEDFVK.D | Y | 42.83 | 1475.762 | 13 | 11.06 | 738.8931 | 2 | 17.79 | 1.0472 | 1.0544 | 60028 | 3.23e2 | 1 | 1 | 47 | 59 |  |  | DB Search |
| total 1 peptides |  |  |  |  |  |  |  |  |  |  |  |  |  |  |  |  |  |  |  |
| --- | --- | --- | --- | --- | --- | --- | --- | --- | --- | --- | --- | --- | --- | --- | --- | --- | --- | --- | --- |

Best Unique PSM (Scan Sample\_7\_A1\_1\_3644.d:60028, m/z=738.8931, z=2, RT=17.79, ppm=6.59):


TRINITY\_DN96161\_c0\_g1\_i1.p1

back to list

  

| Protein Coverage
| Supporting Peptides
| Best Unique PSM
|

Protein Coverage:

Supporting Peptides:

| Peptide | Uniq | -10lgP | Mass | Length | ppm | m/z | z | RT | 1/k0 Start | 1/k0 End | Precursor ID | Area Sample 8 | #Feature | #Feature Sample 8 | Start | End | PTM | AScore | Found By |
| --- | --- | --- | --- | --- | --- | --- | --- | --- | --- | --- | --- | --- | --- | --- | --- | --- | --- | --- | --- |
| K.VGINYQPPTVVPGGDLAK.V | Y | 43.58 | 1823.9781 | 18 | 1.41 | 912.9935 | 2 | 17.42 | 1.1214 | 1.1286 | 57750 | 0 | 0 | 0 | 353 | 370 |  |  | DB Search |
| total 1 peptides |  |  |  |  |  |  |  |  |  |  |  |  |  |  |  |  |  |  |  |
| --- | --- | --- | --- | --- | --- | --- | --- | --- | --- | --- | --- | --- | --- | --- | --- | --- | --- | --- | --- |

Best Unique PSM (Scan Sample\_7\_A1\_1\_3644.d:57750, m/z=912.9935, z=2, RT=17.42, ppm=-3.06):


TRINITY\_DN5229\_c0\_g1\_i1.p1

back to list

  

| Protein Coverage
| Supporting Peptides
| Best Unique PSM
|

Protein Coverage:

Supporting Peptides:

| Peptide | Uniq | -10lgP | Mass | Length | ppm | m/z | z | RT | 1/k0 Start | 1/k0 End | Precursor ID | Area Sample 8 | #Feature | #Feature Sample 8 | Start | End | PTM | AScore | Found By |
| --- | --- | --- | --- | --- | --- | --- | --- | --- | --- | --- | --- | --- | --- | --- | --- | --- | --- | --- | --- |
| R.GVAQFGVLEHYIEK.W | Y | 43.39 | 1588.8249 | 14 | 2.88 | 795.4185 | 2 | 18.94 | 1.113 | 1.1202 | 67396 | 9.01e2 | 1 | 1 | 246 | 259 |  |  | DB Search |
| total 1 peptides |  |  |  |  |  |  |  |  |  |  |  |  |  |  |  |  |  |  |  |
| --- | --- | --- | --- | --- | --- | --- | --- | --- | --- | --- | --- | --- | --- | --- | --- | --- | --- | --- | --- |

Best Unique PSM (Scan Sample\_7\_A1\_1\_3644.d:67396, m/z=795.4185, z=2, RT=18.94, ppm=-1.6):


TRINITY\_DN240\_c0\_g1\_i4.p1

back to list

  

| Protein Coverage
| Supporting Peptides
| Best Unique PSM
|

Protein Coverage:

Supporting Peptides:

| Peptide | Uniq | -10lgP | Mass | Length | ppm | m/z | z | RT | 1/k0 Start | 1/k0 End | Precursor ID | Area Sample 8 | #Feature | #Feature Sample 8 | Start | End | PTM | AScore | Found By |
| --- | --- | --- | --- | --- | --- | --- | --- | --- | --- | --- | --- | --- | --- | --- | --- | --- | --- | --- | --- |
| K.AYAAFLEFKDVVK.A | Y | 43.79 | 1499.8024 | 13 | 3.07 | 750.9074 | 2 | 19.27 | 1.0953 | 1.1025 | 69440 | 1.07e4 | 2 | 2 | 217 | 229 |  |  | DB Search |
| K.AISSASGPTGM(+15.99)TSK.A | Y | 43.77 | 1309.6184 | 14 | 1.88 | 655.8148 | 2 | 10.56 | 0.9319 | 0.9392 | 17892 | 3.72e3 | 1 | 1 | 151 | 164 | Oxidation (M) | M11:Oxidation (M):1000 | DB Search |
| K.ANQVSSAAAAPVPAAGGDK.I | Y | 43.76 | 1680.8431 | 19 | -0.14 | 841.4249 | 2 | 12.55 | 1.0656 | 1.0729 | 29279 | 4.54e3 | 1 | 1 | 230 | 248 |  |  | DB Search |
| K.IGAAAQALADQSYPFLK.S | Y | 43.73 | 1762.9253 | 17 | 0.62 | 882.4666 | 2 | 19.27 | 1.1574 | 1.1646 | 68962 | 5.17e3 | 2 | 2 | 249 | 265 |  |  | DB Search |
| K.AAASAHHQAIGSIDAAGVTSAADYASVTAALGR.A | Y | 43.16 | 3080.5275 | 33 | -1.21 | 771.1348 | 4 | 18.34 | 1.0652 | 1.0725 | 63473 | 9.02e3 | 1 | 1 | 307 | 339 |  |  | DB Search |
| K.SVDWTSDLYLKPLPGVSAAK.A | Y | 42.6 | 2146.131 | 20 | 3.72 | 716.3837 | 3 | 19.68 | 1.013 | 1.0202 | 72078 | 4.11e2 | 1 | 1 | 266 | 285 |  |  | DB Search |
| K.AIVMGSAMDGNLLK.A | Y | 42.42 | 1418.7261 | 14 | 3.64 | 710.3698 | 2 | 18.29 | 1.0174 | 1.0246 | 63333 | 0 | 0 | 0 | 293 | 306 |  |  | DB Search |
| total 7 peptides |  |  |  |  |  |  |  |  |  |  |  |  |  |  |  |  |  |  |  |
| --- | --- | --- | --- | --- | --- | --- | --- | --- | --- | --- | --- | --- | --- | --- | --- | --- | --- | --- | --- |

Best Unique PSM (Scan Sample\_7\_A1\_1\_3644.d:69440, m/z=750.9074, z=2, RT=19.27, ppm=-1.4):


TRINITY\_DN2217\_c0\_g1\_i3.p1

back to list

  

| Protein Coverage
| Supporting Peptides
| Best Unique PSM
|

Protein Coverage:

Supporting Peptides:

| Peptide | Uniq | -10lgP | Mass | Length | ppm | m/z | z | RT | 1/k0 Start | 1/k0 End | Precursor ID | Area Sample 8 | #Feature | #Feature Sample 8 | Start | End | PTM | AScore | Found By |
| --- | --- | --- | --- | --- | --- | --- | --- | --- | --- | --- | --- | --- | --- | --- | --- | --- | --- | --- | --- |
| K.ATGC(+57.02)EVIWNQHNR.G | Y | 43.77 | 1583.7263 | 13 | 0.77 | 792.8675 | 2 | 14.03 | 1.0805 | 1.0877 | 37976 | 0 | 0 | 0 | 431 | 443 | Carbamidomethylation |  | DB Search |
| total 1 peptides |  |  |  |  |  |  |  |  |  |  |  |  |  |  |  |  |  |  |  |
| --- | --- | --- | --- | --- | --- | --- | --- | --- | --- | --- | --- | --- | --- | --- | --- | --- | --- | --- | --- |

Best Unique PSM (Scan Sample\_7\_A1\_1\_3644.d:37976, m/z=792.8675, z=2, RT=14.03, ppm=-3.7):


TRINITY\_DN477\_c0\_g1\_i3.p1

back to list

  

| Protein Coverage
| Supporting Peptides
| Best Unique PSM
|

Protein Coverage:

Supporting Peptides:

| Peptide | Uniq | -10lgP | Mass | Length | ppm | m/z | z | RT | 1/k0 Start | 1/k0 End | Precursor ID | Area Sample 8 | #Feature | #Feature Sample 8 | Start | End | PTM | AScore | Found By |
| --- | --- | --- | --- | --- | --- | --- | --- | --- | --- | --- | --- | --- | --- | --- | --- | --- | --- | --- | --- |
| R.NDVNNPPQAR.R | Y | 43.84 | 1123.537 | 10 | 3.77 | 562.7754 | 2 | 10.18 | 0.8676 | 0.8749 | 15798 | 1.5e4 | 1 | 1 | 147 | 156 |  |  | DB Search |
| total 1 peptides |  |  |  |  |  |  |  |  |  |  |  |  |  |  |  |  |  |  |  |
| --- | --- | --- | --- | --- | --- | --- | --- | --- | --- | --- | --- | --- | --- | --- | --- | --- | --- | --- | --- |

Best Unique PSM (Scan Sample\_7\_A1\_1\_3644.d:15798, m/z=562.7754, z=2, RT=10.18, ppm=-0.7):


TRINITY\_DN59572\_c0\_g1\_i1.p1

back to list

  

| Protein Coverage
| Supporting Peptides
| Best Unique PSM
|

Protein Coverage:

Supporting Peptides:

| Peptide | Uniq | -10lgP | Mass | Length | ppm | m/z | z | RT | 1/k0 Start | 1/k0 End | Precursor ID | Area Sample 8 | #Feature | #Feature Sample 8 | Start | End | PTM | AScore | Found By |
| --- | --- | --- | --- | --- | --- | --- | --- | --- | --- | --- | --- | --- | --- | --- | --- | --- | --- | --- | --- |
| K.SYFEHSFVDLHR.I | Y | 43.58 | 1535.7157 | 12 | 3.1 | 512.9118 | 3 | 16.20 | 0.7681 | 0.7754 | 50655 | 3.97e2 | 1 | 1 | 107 | 118 |  |  | DB Search |
| total 1 peptides |  |  |  |  |  |  |  |  |  |  |  |  |  |  |  |  |  |  |  |
| --- | --- | --- | --- | --- | --- | --- | --- | --- | --- | --- | --- | --- | --- | --- | --- | --- | --- | --- | --- |

Best Unique PSM (Scan Sample\_7\_A1\_1\_3644.d:50655, m/z=512.9118, z=3, RT=16.20, ppm=-1.38):


TRINITY\_DN11097\_c0\_g1\_i1.p1

back to list

  

| Protein Coverage
| Supporting Peptides
| Best Unique PSM
|

Protein Coverage:

Supporting Peptides:

| Peptide | Uniq | -10lgP | Mass | Length | ppm | m/z | z | RT | 1/k0 Start | 1/k0 End | Precursor ID | Area Sample 8 | #Feature | #Feature Sample 8 | Start | End | PTM | AScore | Found By |
| --- | --- | --- | --- | --- | --- | --- | --- | --- | --- | --- | --- | --- | --- | --- | --- | --- | --- | --- | --- |
| R.VAHAGLTVAEFFR.D | Y | 43.68 | 1416.7513 | 13 | 2.73 | 473.2569 | 3 | 19.14 | 0.7502 | 0.7575 | 68636 | 3.46e2 | 1 | 1 | 493 | 505 |  |  | DB Search |
| total 1 peptides |  |  |  |  |  |  |  |  |  |  |  |  |  |  |  |  |  |  |  |
| --- | --- | --- | --- | --- | --- | --- | --- | --- | --- | --- | --- | --- | --- | --- | --- | --- | --- | --- | --- |

Best Unique PSM (Scan Sample\_7\_A1\_1\_3644.d:68636, m/z=473.2569, z=3, RT=19.14, ppm=-1.75):


TRINITY\_DN585\_c0\_g1\_i10.p1

back to list

  

| Protein Coverage
| Supporting Peptides
| Best Unique PSM
|

Protein Coverage:

Supporting Peptides:

| Peptide | Uniq | -10lgP | Mass | Length | ppm | m/z | z | RT | 1/k0 Start | 1/k0 End | Precursor ID | Area Sample 8 | #Feature | #Feature Sample 8 | Start | End | PTM | AScore | Found By |
| --- | --- | --- | --- | --- | --- | --- | --- | --- | --- | --- | --- | --- | --- | --- | --- | --- | --- | --- | --- |
| K.TITLEVEPSDTIENVK.S | Y | 43.28 | 1786.92 | 16 | 2.71 | 894.4657 | 2 | 17.62 | 1.1454 | 1.1526 | 58818 | 1.46e3 | 1 | 1 | 12 | 27 |  |  | DB Search |
| total 1 peptides |  |  |  |  |  |  |  |  |  |  |  |  |  |  |  |  |  |  |  |
| --- | --- | --- | --- | --- | --- | --- | --- | --- | --- | --- | --- | --- | --- | --- | --- | --- | --- | --- | --- |

Best Unique PSM (Scan Sample\_7\_A1\_1\_3644.d:58818, m/z=894.4657, z=2, RT=17.62, ppm=-1.76):


TRINITY\_DN36\_c0\_g1\_i1.p1

back to list

  

| Protein Coverage
| Supporting Peptides
| Best Unique PSM
|

Protein Coverage:

Supporting Peptides:

| Peptide | Uniq | -10lgP | Mass | Length | ppm | m/z | z | RT | 1/k0 Start | 1/k0 End | Precursor ID | Area Sample 8 | #Feature | #Feature Sample 8 | Start | End | PTM | AScore | Found By |
| --- | --- | --- | --- | --- | --- | --- | --- | --- | --- | --- | --- | --- | --- | --- | --- | --- | --- | --- | --- |
| R.LGGIEVTQGNC(+57.02)AR.K | Y | 43.02 | 1373.6721 | 13 | 2.68 | 687.8421 | 2 | 13.90 | 0.9662 | 0.9735 | 37137 | 6.95e2 | 1 | 1 | 58 | 70 | Carbamidomethylation |  | DB Search |
| total 1 peptides |  |  |  |  |  |  |  |  |  |  |  |  |  |  |  |  |  |  |  |
| --- | --- | --- | --- | --- | --- | --- | --- | --- | --- | --- | --- | --- | --- | --- | --- | --- | --- | --- | --- |

Best Unique PSM (Scan Sample\_7\_A1\_1\_3644.d:37137, m/z=687.8421, z=2, RT=13.90, ppm=-1.8):


TRINITY\_DN151\_c0\_g1\_i3.p1

back to list

  

| Protein Coverage
| Supporting Peptides
| Best Unique PSM
|

Protein Coverage:

Supporting Peptides:

| Peptide | Uniq | -10lgP | Mass | Length | ppm | m/z | z | RT | 1/k0 Start | 1/k0 End | Precursor ID | Area Sample 8 | #Feature | #Feature Sample 8 | Start | End | PTM | AScore | Found By |
| --- | --- | --- | --- | --- | --- | --- | --- | --- | --- | --- | --- | --- | --- | --- | --- | --- | --- | --- | --- |
| R.SLFDIFHSK.S | Y | 43.18 | 1092.5604 | 9 | 4.5 | 547.2875 | 2 | 19.85 | 0.8846 | 0.8919 | 73174 | 0 | 0 | 0 | 204 | 212 |  |  | DB Search |
| total 1 peptides |  |  |  |  |  |  |  |  |  |  |  |  |  |  |  |  |  |  |  |
| --- | --- | --- | --- | --- | --- | --- | --- | --- | --- | --- | --- | --- | --- | --- | --- | --- | --- | --- | --- |

Best Unique PSM (Scan Sample\_7\_A1\_1\_3644.d:73174, m/z=547.2875, z=2, RT=19.85, ppm=0.03):


TRINITY\_DN140996\_c0\_g1\_i1.p1

back to list

  

| Protein Coverage
| Supporting Peptides
| Best Unique PSM
|

Protein Coverage:

Supporting Peptides:

| Peptide | Uniq | -10lgP | Mass | Length | ppm | m/z | z | RT | 1/k0 Start | 1/k0 End | Precursor ID | Area Sample 8 | #Feature | #Feature Sample 8 | Start | End | PTM | AScore | Found By |
| --- | --- | --- | --- | --- | --- | --- | --- | --- | --- | --- | --- | --- | --- | --- | --- | --- | --- | --- | --- |
| R.RC(+57.02)QFVNR.C | Y | 42.53 | 978.4818 | 7 | -14.09 | 490.2391 | 2 | 12.02 | 0.8161 | 0.8234 | 26129 | 1.46e3 | 1 | 1 | 53 | 59 | Carbamidomethylation |  | DB Search |
| total 1 peptides |  |  |  |  |  |  |  |  |  |  |  |  |  |  |  |  |  |  |  |
| --- | --- | --- | --- | --- | --- | --- | --- | --- | --- | --- | --- | --- | --- | --- | --- | --- | --- | --- | --- |

Best Unique PSM (Scan Sample\_7\_A1\_1\_3644.d:26129, m/z=490.2391, z=2, RT=12.02, ppm=-18.57):


TRINITY\_DN2636\_c0\_g1\_i1.p1

back to list

  

| Protein Coverage
| Supporting Peptides
| Best Unique PSM
|

Protein Coverage:

Supporting Peptides:

| Peptide | Uniq | -10lgP | Mass | Length | ppm | m/z | z | RT | 1/k0 Start | 1/k0 End | Precursor ID | Area Sample 8 | #Feature | #Feature Sample 8 | Start | End | PTM | AScore | Found By |
| --- | --- | --- | --- | --- | --- | --- | --- | --- | --- | --- | --- | --- | --- | --- | --- | --- | --- | --- | --- |
| R.MSEIEDLTHLAK.R | Y | 43.5 | 1385.686 | 12 | 3.75 | 693.8498 | 2 | 17.42 | 1.0295 | 1.0367 | 57726 | 0 | 0 | 0 | 239 | 250 |  |  | DB Search |
| K.VVEKEAETER.K | Y | 42.37 | 1188.5986 | 10 | -4.14 | 595.3015 | 2 | 9.59 | 0.8988 | 0.9061 | 12943 | 0 | 0 | 0 | 199 | 208 |  |  | DB Search |
| total 2 peptides |  |  |  |  |  |  |  |  |  |  |  |  |  |  |  |  |  |  |  |
| --- | --- | --- | --- | --- | --- | --- | --- | --- | --- | --- | --- | --- | --- | --- | --- | --- | --- | --- | --- |

Best Unique PSM (Scan Sample\_7\_A1\_1\_3644.d:57726, m/z=693.8498, z=2, RT=17.42, ppm=-0.73):


TRINITY\_DN61\_c0\_g1\_i1.p1

back to list

  

| Protein Coverage
| Supporting Peptides
| Best Unique PSM
|

Protein Coverage:

Supporting Peptides:

| Peptide | Uniq | -10lgP | Mass | Length | ppm | m/z | z | RT | 1/k0 Start | 1/k0 End | Precursor ID | Area Sample 8 | #Feature | #Feature Sample 8 | Start | End | PTM | AScore | Found By |
| --- | --- | --- | --- | --- | --- | --- | --- | --- | --- | --- | --- | --- | --- | --- | --- | --- | --- | --- | --- |
| R.AINIHDLVGIDTKK.E | Y | 43.5 | 1535.8671 | 14 | -6.18 | 512.9575 | 3 | 16.70 | 0.7945 | 0.8019 | 53568 | 0 | 0 | 0 | 339 | 352 |  |  | DB Search |
| total 1 peptides |  |  |  |  |  |  |  |  |  |  |  |  |  |  |  |  |  |  |  |
| --- | --- | --- | --- | --- | --- | --- | --- | --- | --- | --- | --- | --- | --- | --- | --- | --- | --- | --- | --- |

Best Unique PSM (Scan Sample\_7\_A1\_1\_3644.d:53568, m/z=512.9575, z=3, RT=16.70, ppm=-10.66):


TRINITY\_DN1936\_c0\_g1\_i3.p1

back to list

  

| Protein Coverage
| Supporting Peptides
| Best Unique PSM
|

Protein Coverage:

Supporting Peptides:

| Peptide | Uniq | -10lgP | Mass | Length | ppm | m/z | z | RT | 1/k0 Start | 1/k0 End | Precursor ID | Area Sample 8 | #Feature | #Feature Sample 8 | Start | End | PTM | AScore | Found By |
| --- | --- | --- | --- | --- | --- | --- | --- | --- | --- | --- | --- | --- | --- | --- | --- | --- | --- | --- | --- |
| R.YGNQC(+57.02)PAPR.E | Y | 43.82 | 1061.4713 | 9 | 4.39 | 531.7429 | 2 | 10.38 | 0.855 | 0.8623 | 16893 | 1.47e3 | 1 | 1 | 954 | 962 | Carbamidomethylation |  | DB Search |
| K.SAVNHNIIFEGR.T | Y | 43.8 | 1355.6946 | 12 | 4.46 | 678.8546 | 2 | 14.79 | 0.9731 | 0.9804 | 42450 | 7.31e2 | 1 | 1 | 59 | 70 |  |  | DB Search |
| total 2 peptides |  |  |  |  |  |  |  |  |  |  |  |  |  |  |  |  |  |  |  |
| --- | --- | --- | --- | --- | --- | --- | --- | --- | --- | --- | --- | --- | --- | --- | --- | --- | --- | --- | --- |

Best Unique PSM (Scan Sample\_7\_A1\_1\_3644.d:16893, m/z=531.7429, z=2, RT=10.38, ppm=-0.08):


TRINITY\_DN79\_c1\_g1\_i1.p1

back to list

  

| Protein Coverage
| Supporting Peptides
| Best Unique PSM
|

Protein Coverage:

Supporting Peptides:

| Peptide | Uniq | -10lgP | Mass | Length | ppm | m/z | z | RT | 1/k0 Start | 1/k0 End | Precursor ID | Area Sample 8 | #Feature | #Feature Sample 8 | Start | End | PTM | AScore | Found By |
| --- | --- | --- | --- | --- | --- | --- | --- | --- | --- | --- | --- | --- | --- | --- | --- | --- | --- | --- | --- |
| K.LTNNKDIVIAK.M | Y | 43.3 | 1227.7187 | 11 | 1.94 | 614.8651 | 2 | 12.42 | 0.9804 | 0.9876 | 28473 | 4.91e2 | 1 | 1 | 370 | 380 |  |  | DB Search |
| total 1 peptides |  |  |  |  |  |  |  |  |  |  |  |  |  |  |  |  |  |  |  |
| --- | --- | --- | --- | --- | --- | --- | --- | --- | --- | --- | --- | --- | --- | --- | --- | --- | --- | --- | --- |

Best Unique PSM (Scan Sample\_7\_A1\_1\_3644.d:28473, m/z=614.8651, z=2, RT=12.42, ppm=-2.53):


TRINITY\_DN585\_c0\_g1\_i3.p1

back to list

  

| Protein Coverage
| Supporting Peptides
| Best Unique PSM
|

Protein Coverage:

Supporting Peptides:

| Peptide | Uniq | -10lgP | Mass | Length | ppm | m/z | z | RT | 1/k0 Start | 1/k0 End | Precursor ID | Area Sample 8 | #Feature | #Feature Sample 8 | Start | End | PTM | AScore | Found By |
| --- | --- | --- | --- | --- | --- | --- | --- | --- | --- | --- | --- | --- | --- | --- | --- | --- | --- | --- | --- |
| K.TITLEVEPSDTIENVK.S | Y | 43.28 | 1786.92 | 16 | 2.71 | 894.4657 | 2 | 17.62 | 1.1454 | 1.1526 | 58818 | 1.46e3 | 1 | 1 | 60 | 75 |  |  | DB Search |
| total 1 peptides |  |  |  |  |  |  |  |  |  |  |  |  |  |  |  |  |  |  |  |
| --- | --- | --- | --- | --- | --- | --- | --- | --- | --- | --- | --- | --- | --- | --- | --- | --- | --- | --- | --- |

Best Unique PSM (Scan Sample\_7\_A1\_1\_3644.d:58818, m/z=894.4657, z=2, RT=17.62, ppm=-1.76):


TRINITY\_DN310\_c0\_g1\_i1.p1

back to list

  

| Protein Coverage
| Supporting Peptides
| Best Unique PSM
|

Protein Coverage:

Supporting Peptides:

| Peptide | Uniq | -10lgP | Mass | Length | ppm | m/z | z | RT | 1/k0 Start | 1/k0 End | Precursor ID | Area Sample 8 | #Feature | #Feature Sample 8 | Start | End | PTM | AScore | Found By |
| --- | --- | --- | --- | --- | --- | --- | --- | --- | --- | --- | --- | --- | --- | --- | --- | --- | --- | --- | --- |
| R.VVKPGEEATFR.Y | Y | 43.59 | 1231.656 | 11 | 2.64 | 616.8342 | 2 | 12.47 | 0.9315 | 0.9388 | 28694 | 1.17e3 | 2 | 2 | 148 | 158 |  |  | DB Search |
| total 1 peptides |  |  |  |  |  |  |  |  |  |  |  |  |  |  |  |  |  |  |  |
| --- | --- | --- | --- | --- | --- | --- | --- | --- | --- | --- | --- | --- | --- | --- | --- | --- | --- | --- | --- |

Best Unique PSM (Scan Sample\_7\_A1\_1\_3644.d:28694, m/z=616.8342, z=2, RT=12.47, ppm=-1.84):


TRINITY\_DN1910\_c0\_g1\_i5.p1

back to list

  

| Protein Coverage
| Supporting Peptides
| Best Unique PSM
|

Protein Coverage:

Supporting Peptides:

| Peptide | Uniq | -10lgP | Mass | Length | ppm | m/z | z | RT | 1/k0 Start | 1/k0 End | Precursor ID | Area Sample 8 | #Feature | #Feature Sample 8 | Start | End | PTM | AScore | Found By |
| --- | --- | --- | --- | --- | --- | --- | --- | --- | --- | --- | --- | --- | --- | --- | --- | --- | --- | --- | --- |
| K.NM(+15.99)M(+15.99)AAC(+57.02)DPR.H | Y | 43.62 | 1096.41 | 9 | -0.29 | 549.2097 | 2 | 9.58 | 0.8615 | 0.8688 | 12888 | 5.74e2 | 1 | 1 | 298 | 306 | Oxidation (M), Oxidation (M), Carbamidomethylation | M2:Oxidation (M):1000 M3:Oxidation (M):1000 | DB Search |
| R.FPGQLNADLRK.L | Y | 43.16 | 1257.6829 | 11 | -1.33 | 629.8451 | 2 | 14.44 | 0.9497 | 0.957 | 40493 | 0 | 0 | 0 | 242 | 252 |  |  | DB Search |
| total 2 peptides |  |  |  |  |  |  |  |  |  |  |  |  |  |  |  |  |  |  |  |
| --- | --- | --- | --- | --- | --- | --- | --- | --- | --- | --- | --- | --- | --- | --- | --- | --- | --- | --- | --- |

Best Unique PSM (Scan Sample\_7\_A1\_1\_3644.d:12888, m/z=549.2097, z=2, RT=9.58, ppm=-4.76):


TRINITY\_DN160\_c0\_g1\_i2.p1

back to list

  

| Protein Coverage
| Supporting Peptides
| Best Unique PSM
|

Protein Coverage:

Supporting Peptides:

| Peptide | Uniq | -10lgP | Mass | Length | ppm | m/z | z | RT | 1/k0 Start | 1/k0 End | Precursor ID | Area Sample 8 | #Feature | #Feature Sample 8 | Start | End | PTM | AScore | Found By |
| --- | --- | --- | --- | --- | --- | --- | --- | --- | --- | --- | --- | --- | --- | --- | --- | --- | --- | --- | --- |
| K.ELVMHEQSR.C | Y | 43.82 | 1127.5393 | 9 | -5.85 | 564.7711 | 2 | 11.37 | 0.8988 | 0.9061 | 22493 | 0 | 0 | 0 | 1116 | 1124 |  |  | DB Search |
| K.VNQAC(+57.02)VR.T | Y | 43.66 | 845.4178 | 7 | 1.37 | 423.7148 | 2 | 9.80 | 0.7644 | 0.7718 | 13832 | 6.46e3 | 1 | 1 | 871 | 877 | Carbamidomethylation |  | DB Search |
| K.DLEFKFR.E | Y | 43.5 | 953.497 | 7 | -2.68 | 477.7524 | 2 | 16.87 | 0.8323 | 0.8396 | 54470 | 8.25e2 | 1 | 1 | 1031 | 1037 |  |  | DB Search |
| K.LRLDFDHEAKLNMIFR.F | Y | 43.4 | 2017.0567 | 16 | -0.24 | 505.2691 | 4 | 18.67 | 0.8623 | 0.8696 | 65767 | 0 | 0 | 0 | 1007 | 1022 |  |  | DB Search |
| K.LRLDFDHEAK.L | Y | 43.38 | 1242.6356 | 10 | 5.04 | 415.2194 | 3 | 14.38 | 0.7204 | 0.7278 | 40014 | 6.38e3 | 1 | 1 | 1007 | 1016 |  |  | DB Search |
| R.LDFDHEAKLNMIFR.F | Y | 43.36 | 1747.8715 | 14 | -6.24 | 583.6249 | 3 | 18.55 | 0.8562 | 0.8635 | 64942 | 0 | 0 | 0 | 1009 | 1022 |  |  | DB Search |
| K.LRLDFDHEAKLNM(+15.99)IFR.F | Y | 43.21 | 2033.0516 | 16 | -2.09 | 509.2668 | 4 | 17.98 | 0.8554 | 0.8627 | 61282 | 0 | 0 | 0 | 1007 | 1022 | Oxidation (M) | M13:Oxidation (M):1000 | DB Search |
| K.ATSC(+57.02)AVYPC(+57.02)EINSPR.C | Y | 43.02 | 1723.7658 | 15 | 6.64 | 862.892 | 2 | 13.94 | 1.0985 | 1.1057 | 37509 | 0 | 0 | 0 | 512 | 526 | Carbamidomethylation, Carbamidomethylation |  | DB Search |
| total 8 peptides |  |  |  |  |  |  |  |  |  |  |  |  |  |  |  |  |  |  |  |
| --- | --- | --- | --- | --- | --- | --- | --- | --- | --- | --- | --- | --- | --- | --- | --- | --- | --- | --- | --- |

Best Unique PSM (Scan Sample\_7\_A1\_1\_3644.d:22493, m/z=564.7711, z=2, RT=11.37, ppm=-10.33):


TRINITY\_DN151\_c0\_g1\_i2.p1

back to list

  

| Protein Coverage
| Supporting Peptides
| Best Unique PSM
|

Protein Coverage:

Supporting Peptides:

| Peptide | Uniq | -10lgP | Mass | Length | ppm | m/z | z | RT | 1/k0 Start | 1/k0 End | Precursor ID | Area Sample 8 | #Feature | #Feature Sample 8 | Start | End | PTM | AScore | Found By |
| --- | --- | --- | --- | --- | --- | --- | --- | --- | --- | --- | --- | --- | --- | --- | --- | --- | --- | --- | --- |
| R.SLFDIFHSK.S | Y | 43.18 | 1092.5604 | 9 | 4.5 | 547.2875 | 2 | 19.85 | 0.8846 | 0.8919 | 73174 | 0 | 0 | 0 | 204 | 212 |  |  | DB Search |
| total 1 peptides |  |  |  |  |  |  |  |  |  |  |  |  |  |  |  |  |  |  |  |
| --- | --- | --- | --- | --- | --- | --- | --- | --- | --- | --- | --- | --- | --- | --- | --- | --- | --- | --- | --- |

Best Unique PSM (Scan Sample\_7\_A1\_1\_3644.d:73174, m/z=547.2875, z=2, RT=19.85, ppm=0.03):


TRINITY\_DN118\_c1\_g1\_i5.p1

back to list

  

| Protein Coverage
| Supporting Peptides
| Best Unique PSM
|

Protein Coverage:

Supporting Peptides:

| Peptide | Uniq | -10lgP | Mass | Length | ppm | m/z | z | RT | 1/k0 Start | 1/k0 End | Precursor ID | Area Sample 8 | #Feature | #Feature Sample 8 | Start | End | PTM | AScore | Found By |
| --- | --- | --- | --- | --- | --- | --- | --- | --- | --- | --- | --- | --- | --- | --- | --- | --- | --- | --- | --- |
| R.ETC(+57.02)SDIEKR.E | Y | 43.68 | 1136.5132 | 9 | 2.96 | 569.263 | 2 | 10.13 | 0.8895 | 0.8968 | 15523 | 2.26e3 | 1 | 1 | 186 | 194 | Carbamidomethylation |  | DB Search |
| K.VC(+57.02)EDVKR.C | Y | 43.45 | 904.4436 | 7 | -6.38 | 453.2242 | 2 | 9.42 | 0.7697 | 0.777 | 12258 | 0 | 0 | 0 | 122 | 128 | Carbamidomethylation |  | DB Search |
| total 2 peptides |  |  |  |  |  |  |  |  |  |  |  |  |  |  |  |  |  |  |  |
| --- | --- | --- | --- | --- | --- | --- | --- | --- | --- | --- | --- | --- | --- | --- | --- | --- | --- | --- | --- |

Best Unique PSM (Scan Sample\_7\_A1\_1\_3644.d:15523, m/z=569.263, z=2, RT=10.13, ppm=-1.52):


TRINITY\_DN1199\_c0\_g1\_i1.p1

back to list

  

| Protein Coverage
| Supporting Peptides
| Best Unique PSM
|

Protein Coverage:

Supporting Peptides:

| Peptide | Uniq | -10lgP | Mass | Length | ppm | m/z | z | RT | 1/k0 Start | 1/k0 End | Precursor ID | Area Sample 8 | #Feature | #Feature Sample 8 | Start | End | PTM | AScore | Found By |
| --- | --- | --- | --- | --- | --- | --- | --- | --- | --- | --- | --- | --- | --- | --- | --- | --- | --- | --- | --- |
| R.VVHNIC(+57.02)SSAITNSVTNK.I | Y | 43.87 | 1842.9258 | 17 | 2.87 | 922.4687 | 2 | 13.93 | 1.1394 | 1.1466 | 37331 | 5.78e2 | 1 | 1 | 89 | 105 | Carbamidomethylation |  | DB Search |
| R.LPNFFQYAGHIK.K | Y | 43.77 | 1433.7455 | 12 | 1.54 | 478.9211 | 3 | 19.36 | 0.7567 | 0.764 | 70118 | 6.21e3 | 2 | 2 | 62 | 73 |  |  | DB Search |
| K.VLIQIGHEPLPPQQVTTLFGK.K | Y | 43.27 | 2314.3048 | 21 | 1.51 | 772.4399 | 3 | 19.84 | 0.9424 | 0.9497 | 72734 | 5.33e3 | 1 | 1 | 37 | 57 |  |  | DB Search |
| total 3 peptides |  |  |  |  |  |  |  |  |  |  |  |  |  |  |  |  |  |  |  |
| --- | --- | --- | --- | --- | --- | --- | --- | --- | --- | --- | --- | --- | --- | --- | --- | --- | --- | --- | --- |

Best Unique PSM (Scan Sample\_7\_A1\_1\_3644.d:37331, m/z=922.4687, z=2, RT=13.93, ppm=-1.6):


TRINITY\_DN250\_c0\_g1\_i2.p1

back to list

  

| Protein Coverage
| Supporting Peptides
| Best Unique PSM
|

Protein Coverage:

Supporting Peptides:

| Peptide | Uniq | -10lgP | Mass | Length | ppm | m/z | z | RT | 1/k0 Start | 1/k0 End | Precursor ID | Area Sample 8 | #Feature | #Feature Sample 8 | Start | End | PTM | AScore | Found By |
| --- | --- | --- | --- | --- | --- | --- | --- | --- | --- | --- | --- | --- | --- | --- | --- | --- | --- | --- | --- |
| R.LM(+15.99)GQIVSSITASLR.F | Y | 43.53 | 1490.8126 | 14 | 3.71 | 746.413 | 2 | 18.84 | 1.0359 | 1.0431 | 66733 | 4.18e2 | 1 | 1 | 230 | 243 | Oxidation (M) | M2:Oxidation (M):1000 | DB Search |
| total 1 peptides |  |  |  |  |  |  |  |  |  |  |  |  |  |  |  |  |  |  |  |
| --- | --- | --- | --- | --- | --- | --- | --- | --- | --- | --- | --- | --- | --- | --- | --- | --- | --- | --- | --- |

Best Unique PSM (Scan Sample\_7\_A1\_1\_3644.d:66733, m/z=746.413, z=2, RT=18.84, ppm=-0.77):


TRINITY\_DN73067\_c0\_g1\_i1.p1

back to list

  

| Protein Coverage
| Supporting Peptides
| Best Unique PSM
|

Protein Coverage:

Supporting Peptides:

| Peptide | Uniq | -10lgP | Mass | Length | ppm | m/z | z | RT | 1/k0 Start | 1/k0 End | Precursor ID | Area Sample 8 | #Feature | #Feature Sample 8 | Start | End | PTM | AScore | Found By |
| --- | --- | --- | --- | --- | --- | --- | --- | --- | --- | --- | --- | --- | --- | --- | --- | --- | --- | --- | --- |
| K.VTGHAGIQLK.V | Y | 43.75 | 1022.5872 | 10 | -0.65 | 512.2983 | 2 | 13.00 | 0.8773 | 0.8846 | 31972 | 0 | 0 | 0 | 704 | 713 |  |  | DB Search |
| K.VVTSSTGPGEHMR.N | Y | 43.54 | 1356.6456 | 13 | 0.36 | 679.3273 | 2 | 10.92 | 0.9783 | 0.9856 | 19750 | 1.32e3 | 1 | 1 | 714 | 726 |  |  | DB Search |
| K.VVTSSTGPGEHM(+15.99)R.N | Y | 42.58 | 1372.6405 | 13 | -4.07 | 687.3217 | 2 | 9.80 | 0.9606 | 0.9679 | 13688 | 2.89e2 | 1 | 1 | 714 | 726 | Oxidation (M) | M12:Oxidation (M):1000 | DB Search |
| total 3 peptides |  |  |  |  |  |  |  |  |  |  |  |  |  |  |  |  |  |  |  |
| --- | --- | --- | --- | --- | --- | --- | --- | --- | --- | --- | --- | --- | --- | --- | --- | --- | --- | --- | --- |

Best Unique PSM (Scan Sample\_7\_A1\_1\_3644.d:31972, m/z=512.2983, z=2, RT=13.00, ppm=-5.13):


TRINITY\_DN2028\_c0\_g1\_i1.p1

back to list

  

| Protein Coverage
| Supporting Peptides
| Best Unique PSM
|

Protein Coverage:

Supporting Peptides:

| Peptide | Uniq | -10lgP | Mass | Length | ppm | m/z | z | RT | 1/k0 Start | 1/k0 End | Precursor ID | Area Sample 8 | #Feature | #Feature Sample 8 | Start | End | PTM | AScore | Found By |
| --- | --- | --- | --- | --- | --- | --- | --- | --- | --- | --- | --- | --- | --- | --- | --- | --- | --- | --- | --- |
| K.LIQYIENKPGLTR.L | Y | 43.67 | 1543.8722 | 13 | 1.61 | 772.9412 | 2 | 14.92 | 1.0773 | 1.0845 | 43262 | 1.11e3 | 1 | 1 | 81 | 93 |  |  | DB Search |
| total 1 peptides |  |  |  |  |  |  |  |  |  |  |  |  |  |  |  |  |  |  |  |
| --- | --- | --- | --- | --- | --- | --- | --- | --- | --- | --- | --- | --- | --- | --- | --- | --- | --- | --- | --- |

Best Unique PSM (Scan Sample\_7\_A1\_1\_3644.d:43262, m/z=772.9412, z=2, RT=14.92, ppm=-2.87):


TRINITY\_DN13813\_c0\_g1\_i3.p2

back to list

  

| Protein Coverage
| Supporting Peptides
| Best Unique PSM
|

Protein Coverage:

Supporting Peptides:

| Peptide | Uniq | -10lgP | Mass | Length | ppm | m/z | z | RT | 1/k0 Start | 1/k0 End | Precursor ID | Area Sample 8 | #Feature | #Feature Sample 8 | Start | End | PTM | AScore | Found By |
| --- | --- | --- | --- | --- | --- | --- | --- | --- | --- | --- | --- | --- | --- | --- | --- | --- | --- | --- | --- |
| R.DGPGPLASDLLLQMR.Q | Y | 41.47 | 1581.8184 | 15 | -2.93 | 528.2762 | 3 | 19.62 | 0.8149 | 0.8222 | 71531 | 3.73e2 | 1 | 1 | 308 | 322 |  |  | DB Search |
| total 1 peptides |  |  |  |  |  |  |  |  |  |  |  |  |  |  |  |  |  |  |  |
| --- | --- | --- | --- | --- | --- | --- | --- | --- | --- | --- | --- | --- | --- | --- | --- | --- | --- | --- | --- |

Best Unique PSM (Scan Sample\_7\_A1\_1\_3644.d:71531, m/z=528.2762, z=3, RT=19.62, ppm=-7.41):


TRINITY\_DN495\_c0\_g1\_i6.p1

back to list

  

| Protein Coverage
| Supporting Peptides
| Best Unique PSM
|

Protein Coverage:

Supporting Peptides:

| Peptide | Uniq | -10lgP | Mass | Length | ppm | m/z | z | RT | 1/k0 Start | 1/k0 End | Precursor ID | Area Sample 8 | #Feature | #Feature Sample 8 | Start | End | PTM | AScore | Found By |
| --- | --- | --- | --- | --- | --- | --- | --- | --- | --- | --- | --- | --- | --- | --- | --- | --- | --- | --- | --- |
| K.SISC(+57.02)SKDEEIK.V | Y | 43.76 | 1294.6075 | 11 | 2.25 | 648.3096 | 2 | 10.92 | 0.9755 | 0.9828 | 19848 | 1.92e3 | 1 | 1 | 248 | 258 | Carbamidomethylation |  | DB Search |
| R.TAGDVC(+57.02)GR.S | Y | 43.57 | 834.3654 | 8 | 3.61 | 418.1896 | 2 | 9.57 | 0.7518 | 0.7591 | 12879 | 0 | 0 | 0 | 267 | 274 | Carbamidomethylation |  | DB Search |
| total 2 peptides |  |  |  |  |  |  |  |  |  |  |  |  |  |  |  |  |  |  |  |
| --- | --- | --- | --- | --- | --- | --- | --- | --- | --- | --- | --- | --- | --- | --- | --- | --- | --- | --- | --- |

Best Unique PSM (Scan Sample\_7\_A1\_1\_3644.d:19848, m/z=648.3096, z=2, RT=10.92, ppm=-2.22):


TRINITY\_DN6920\_c0\_g1\_i1.p1

back to list

  

| Protein Coverage
| Supporting Peptides
| Best Unique PSM
|

Protein Coverage:

Supporting Peptides:

| Peptide | Uniq | -10lgP | Mass | Length | ppm | m/z | z | RT | 1/k0 Start | 1/k0 End | Precursor ID | Area Sample 8 | #Feature | #Feature Sample 8 | Start | End | PTM | AScore | Found By |
| --- | --- | --- | --- | --- | --- | --- | --- | --- | --- | --- | --- | --- | --- | --- | --- | --- | --- | --- | --- |
| R.VLVHTQQK.L | Y | 43.71 | 951.5501 | 8 | -3.93 | 476.7784 | 2 | 9.59 | 0.8291 | 0.8364 | 12948 | 0 | 0 | 0 | 45 | 52 |  |  | DB Search |
| total 1 peptides |  |  |  |  |  |  |  |  |  |  |  |  |  |  |  |  |  |  |  |
| --- | --- | --- | --- | --- | --- | --- | --- | --- | --- | --- | --- | --- | --- | --- | --- | --- | --- | --- | --- |

Best Unique PSM (Scan Sample\_7\_A1\_1\_3644.d:12948, m/z=476.7784, z=2, RT=9.59, ppm=-8.41):


TRINITY\_DN3448\_c0\_g2\_i3.p2

back to list

  

| Protein Coverage
| Supporting Peptides
| Best Unique PSM
|

Protein Coverage:

Supporting Peptides:

| Peptide | Uniq | -10lgP | Mass | Length | ppm | m/z | z | RT | 1/k0 Start | 1/k0 End | Precursor ID | Area Sample 8 | #Feature | #Feature Sample 8 | Start | End | PTM | AScore | Found By |
| --- | --- | --- | --- | --- | --- | --- | --- | --- | --- | --- | --- | --- | --- | --- | --- | --- | --- | --- | --- |
| K.AGM(+15.99)THIVR.D | Y | 43.71 | 899.4647 | 8 | 5.69 | 450.7402 | 2 | 9.78 | 0.8161 | 0.8234 | 13845 | 0 | 0 | 0 | 31 | 38 | Oxidation (M) | M3:Oxidation (M):1000 | DB Search |
| total 1 peptides |  |  |  |  |  |  |  |  |  |  |  |  |  |  |  |  |  |  |  |
| --- | --- | --- | --- | --- | --- | --- | --- | --- | --- | --- | --- | --- | --- | --- | --- | --- | --- | --- | --- |

Best Unique PSM (Scan Sample\_7\_A1\_1\_3644.d:13845, m/z=450.7402, z=2, RT=9.78, ppm=1.21):


TRINITY\_DN1616\_c0\_g1\_i2.p1

back to list

  

| Protein Coverage
| Supporting Peptides
| Best Unique PSM
|

Protein Coverage:

Supporting Peptides:

| Peptide | Uniq | -10lgP | Mass | Length | ppm | m/z | z | RT | 1/k0 Start | 1/k0 End | Precursor ID | Area Sample 8 | #Feature | #Feature Sample 8 | Start | End | PTM | AScore | Found By |
| --- | --- | --- | --- | --- | --- | --- | --- | --- | --- | --- | --- | --- | --- | --- | --- | --- | --- | --- | --- |
| R.TSTDKFEC(+57.02)EK.C | Y | 43.76 | 1243.5391 | 10 | -3.39 | 622.7719 | 2 | 10.75 | 0.9255 | 0.9327 | 18979 | 0 | 0 | 0 | 320 | 329 | Carbamidomethylation |  | DB Search |
| R.YGAVNDNC(+57.02)ER.D | Y | 43.62 | 1196.488 | 10 | -2.06 | 599.2474 | 2 | 10.92 | 0.8834 | 0.8907 | 19854 | 4.23e2 | 1 | 1 | 498 | 507 | Carbamidomethylation |  | DB Search |
| R.YVKPGVNIINIR.D | Y | 43.56 | 1384.819 | 12 | 3.35 | 693.416 | 2 | 16.23 | 1.0013 | 1.0086 | 50888 | 0 | 0 | 0 | 1217 | 1228 |  |  | DB Search |
| K.YVVMLQYYHDSDTAK.Y | Y | 43.52 | 1831.8451 | 15 | 8.47 | 916.9335 | 2 | 16.82 | 1.1857 | 1.1929 | 54206 | 0 | 0 | 0 | 1071 | 1085 |  |  | DB Search |
| K.SSSC(+57.02)QEFGGKC(+57.02)PC(+57.02)K.D | Y | 39.5 | 1630.6538 | 14 | -18.66 | 816.3153 | 2 | 12.09 | 1.0311 | 1.0383 | 26624 | 0 | 0 | 0 | 1387 | 1400 | Carbamidomethylation, Carbamidomethylation, Carbamidomethylation |  | DB Search |
| total 5 peptides |  |  |  |  |  |  |  |  |  |  |  |  |  |  |  |  |  |  |  |
| --- | --- | --- | --- | --- | --- | --- | --- | --- | --- | --- | --- | --- | --- | --- | --- | --- | --- | --- | --- |

Best Unique PSM (Scan Sample\_7\_A1\_1\_3644.d:18979, m/z=622.7719, z=2, RT=10.75, ppm=-7.87):


TRINITY\_DN166344\_c0\_g1\_i1.p1

back to list

  

| Protein Coverage
| Supporting Peptides
| Best Unique PSM
|

Protein Coverage:

Supporting Peptides:

| Peptide | Uniq | -10lgP | Mass | Length | ppm | m/z | z | RT | 1/k0 Start | 1/k0 End | Precursor ID | Area Sample 8 | #Feature | #Feature Sample 8 | Start | End | PTM | AScore | Found By |
| --- | --- | --- | --- | --- | --- | --- | --- | --- | --- | --- | --- | --- | --- | --- | --- | --- | --- | --- | --- |
| R.TQITC(+57.02)TQISLKR.G | Y | 43.76 | 1447.7817 | 12 | 4.4 | 724.8981 | 2 | 13.95 | 1.0259 | 1.0331 | 37448 | 1.26e3 | 1 | 1 | 174 | 185 | Carbamidomethylation |  | DB Search |
| K.YSTQDEYGTAAYK.T | Y | 42.7 | 1495.6467 | 13 | -1.47 | 748.8262 | 2 | 13.28 | 1.0033 | 1.0106 | 33565 | 0 | 0 | 0 | 97 | 109 |  |  | DB Search |
| total 2 peptides |  |  |  |  |  |  |  |  |  |  |  |  |  |  |  |  |  |  |  |
| --- | --- | --- | --- | --- | --- | --- | --- | --- | --- | --- | --- | --- | --- | --- | --- | --- | --- | --- | --- |

Best Unique PSM (Scan Sample\_7\_A1\_1\_3644.d:37448, m/z=724.8981, z=2, RT=13.95, ppm=-0.07):


TRINITY\_DN477\_c0\_g1\_i8.p2

back to list

  

| Protein Coverage
| Supporting Peptides
| Best Unique PSM
|

Protein Coverage:

Supporting Peptides:

| Peptide | Uniq | -10lgP | Mass | Length | ppm | m/z | z | RT | 1/k0 Start | 1/k0 End | Precursor ID | Area Sample 8 | #Feature | #Feature Sample 8 | Start | End | PTM | AScore | Found By |
| --- | --- | --- | --- | --- | --- | --- | --- | --- | --- | --- | --- | --- | --- | --- | --- | --- | --- | --- | --- |
| R.NDVNNPPQAR.R | Y | 43.84 | 1123.537 | 10 | 3.77 | 562.7754 | 2 | 10.18 | 0.8676 | 0.8749 | 15798 | 1.5e4 | 1 | 1 | 86 | 95 |  |  | DB Search |
| total 1 peptides |  |  |  |  |  |  |  |  |  |  |  |  |  |  |  |  |  |  |  |
| --- | --- | --- | --- | --- | --- | --- | --- | --- | --- | --- | --- | --- | --- | --- | --- | --- | --- | --- | --- |

Best Unique PSM (Scan Sample\_7\_A1\_1\_3644.d:15798, m/z=562.7754, z=2, RT=10.18, ppm=-0.7):


TRINITY\_DN2357\_c1\_g1\_i1.p1

back to list

  

| Protein Coverage
| Supporting Peptides
| Best Unique PSM
|

Protein Coverage:

Supporting Peptides:

| Peptide | Uniq | -10lgP | Mass | Length | ppm | m/z | z | RT | 1/k0 Start | 1/k0 End | Precursor ID | Area Sample 8 | #Feature | #Feature Sample 8 | Start | End | PTM | AScore | Found By |
| --- | --- | --- | --- | --- | --- | --- | --- | --- | --- | --- | --- | --- | --- | --- | --- | --- | --- | --- | --- |
| K.HYLPENLRK.T | Y | 42.13 | 1168.6353 | 9 | -0.53 | 390.5504 | 3 | 14.05 | 0.7029 | 0.7102 | 38149 | 2.03e2 | 1 | 1 | 554 | 562 |  |  | DB Search |
| total 1 peptides |  |  |  |  |  |  |  |  |  |  |  |  |  |  |  |  |  |  |  |
| --- | --- | --- | --- | --- | --- | --- | --- | --- | --- | --- | --- | --- | --- | --- | --- | --- | --- | --- | --- |

Best Unique PSM (Scan Sample\_7\_A1\_1\_3644.d:38149, m/z=390.5504, z=3, RT=14.05, ppm=-5.01):


TRINITY\_DN11522\_c0\_g1\_i3.p1

back to list

  

| Protein Coverage
| Supporting Peptides
| Best Unique PSM
|

Protein Coverage:

Supporting Peptides:

| Peptide | Uniq | -10lgP | Mass | Length | ppm | m/z | z | RT | 1/k0 Start | 1/k0 End | Precursor ID | Area Sample 8 | #Feature | #Feature Sample 8 | Start | End | PTM | AScore | Found By |
| --- | --- | --- | --- | --- | --- | --- | --- | --- | --- | --- | --- | --- | --- | --- | --- | --- | --- | --- | --- |
| R.IGLGNIRDPSISLTR.G | Y | 43.72 | 1610.9104 | 15 | 2.12 | 537.9761 | 3 | 17.42 | 0.8019 | 0.8092 | 57662 | 1.88e3 | 1 | 1 | 27 | 41 |  |  | DB Search |
| K.GKPEQVQVK.M | Y | 43.14 | 1011.5713 | 9 | 2 | 506.7917 | 2 | 9.97 | 0.8416 | 0.849 | 14688 | 9.41e2 | 1 | 1 | 548 | 556 |  |  | DB Search |
| total 2 peptides |  |  |  |  |  |  |  |  |  |  |  |  |  |  |  |  |  |  |  |
| --- | --- | --- | --- | --- | --- | --- | --- | --- | --- | --- | --- | --- | --- | --- | --- | --- | --- | --- | --- |

Best Unique PSM (Scan Sample\_7\_A1\_1\_3644.d:57662, m/z=537.9761, z=3, RT=17.42, ppm=-2.35):


TRINITY\_DN5365\_c0\_g1\_i2.p1

back to list

  

| Protein Coverage
| Supporting Peptides
| Best Unique PSM
|

Protein Coverage:

Supporting Peptides:

| Peptide | Uniq | -10lgP | Mass | Length | ppm | m/z | z | RT | 1/k0 Start | 1/k0 End | Precursor ID | Area Sample 8 | #Feature | #Feature Sample 8 | Start | End | PTM | AScore | Found By |
| --- | --- | --- | --- | --- | --- | --- | --- | --- | --- | --- | --- | --- | --- | --- | --- | --- | --- | --- | --- |
| R.FPGQLNADIRK.L | Y | 43.16 | 1257.6829 | 11 | -1.33 | 629.8451 | 2 | 14.44 | 0.9497 | 0.957 | 40493 | 0 | 0 | 0 | 244 | 254 |  |  | DB Search |
| total 1 peptides |  |  |  |  |  |  |  |  |  |  |  |  |  |  |  |  |  |  |  |
| --- | --- | --- | --- | --- | --- | --- | --- | --- | --- | --- | --- | --- | --- | --- | --- | --- | --- | --- | --- |

Best Unique PSM (Scan Sample\_7\_A1\_1\_3644.d:40493, m/z=629.8451, z=2, RT=14.44, ppm=-5.81):


TRINITY\_DN428\_c0\_g1\_i1.p1

back to list

  

| Protein Coverage
| Supporting Peptides
| Best Unique PSM
|

Protein Coverage:

Supporting Peptides:

| Peptide | Uniq | -10lgP | Mass | Length | ppm | m/z | z | RT | 1/k0 Start | 1/k0 End | Precursor ID | Area Sample 8 | #Feature | #Feature Sample 8 | Start | End | PTM | AScore | Found By |
| --- | --- | --- | --- | --- | --- | --- | --- | --- | --- | --- | --- | --- | --- | --- | --- | --- | --- | --- | --- |
| R.AGVLAHLEEER.D | Y | 43.76 | 1222.6306 | 11 | 0.61 | 612.3202 | 2 | 15.03 | 0.9541 | 0.9614 | 43865 | 8.73e2 | 1 | 1 | 762 | 772 |  |  | DB Search |
| K.IC(+57.02)HLLGIQVTDFTK.S | Y | 43.76 | 1643.8705 | 14 | -0.97 | 548.9611 | 3 | 18.66 | 0.8226 | 0.8299 | 65627 | 2.22e3 | 2 | 2 | 377 | 390 | Carbamidomethylation |  | DB Search |
| R.AGVLAHLEEERDIK.L | Y | 42.76 | 1578.8365 | 14 | -2.1 | 527.2827 | 3 | 15.05 | 0.7994 | 0.8067 | 43994 | 3.21e2 | 1 | 1 | 762 | 775 |  |  | DB Search |
| total 3 peptides |  |  |  |  |  |  |  |  |  |  |  |  |  |  |  |  |  |  |  |
| --- | --- | --- | --- | --- | --- | --- | --- | --- | --- | --- | --- | --- | --- | --- | --- | --- | --- | --- | --- |

Best Unique PSM (Scan Sample\_7\_A1\_1\_3644.d:43865, m/z=612.3202, z=2, RT=15.03, ppm=-3.87):


TRINITY\_DN86511\_c0\_g1\_i1.p1

back to list

  

| Protein Coverage
| Supporting Peptides
| Best Unique PSM
|

Protein Coverage:

Supporting Peptides:

| Peptide | Uniq | -10lgP | Mass | Length | ppm | m/z | z | RT | 1/k0 Start | 1/k0 End | Precursor ID | Area Sample 8 | #Feature | #Feature Sample 8 | Start | End | PTM | AScore | Found By |
| --- | --- | --- | --- | --- | --- | --- | --- | --- | --- | --- | --- | --- | --- | --- | --- | --- | --- | --- | --- |
| V(+42.01)LLLEATK.V | Y | 40.98 | 927.5641 | 8 | 1.92 | 464.7881 | 2 | 19.57 | 0.8023 | 0.8096 | 71356 | 6.91e2 | 1 | 1 | 1 | 8 | Acetylation (Protein N-term) | V1:Acetylation (Protein N-term):1000 | DB Search |
| total 1 peptides |  |  |  |  |  |  |  |  |  |  |  |  |  |  |  |  |  |  |  |
| --- | --- | --- | --- | --- | --- | --- | --- | --- | --- | --- | --- | --- | --- | --- | --- | --- | --- | --- | --- |

Best Unique PSM (Scan Sample\_7\_A1\_1\_3644.d:71356, m/z=464.7881, z=2, RT=19.57, ppm=-2.56):


TRINITY\_DN58867\_c0\_g1\_i2.p1

back to list

  

| Protein Coverage
| Supporting Peptides
| Best Unique PSM
|

Protein Coverage:

Supporting Peptides:

| Peptide | Uniq | -10lgP | Mass | Length | ppm | m/z | z | RT | 1/k0 Start | 1/k0 End | Precursor ID | Area Sample 8 | #Feature | #Feature Sample 8 | Start | End | PTM | AScore | Found By |
| --- | --- | --- | --- | --- | --- | --- | --- | --- | --- | --- | --- | --- | --- | --- | --- | --- | --- | --- | --- |
| K.TILQFEADRR.A | Y | 43.73 | 1247.6622 | 10 | 4.77 | 624.8386 | 2 | 15.30 | 0.9501 | 0.9574 | 45395 | 1.54e3 | 1 | 1 | 211 | 220 |  |  | DB Search |
| total 1 peptides |  |  |  |  |  |  |  |  |  |  |  |  |  |  |  |  |  |  |  |
| --- | --- | --- | --- | --- | --- | --- | --- | --- | --- | --- | --- | --- | --- | --- | --- | --- | --- | --- | --- |

Best Unique PSM (Scan Sample\_7\_A1\_1\_3644.d:45395, m/z=624.8386, z=2, RT=15.30, ppm=0.29):


TRINITY\_DN730\_c0\_g2\_i3.p1

back to list

  

| Protein Coverage
| Supporting Peptides
| Best Unique PSM
|

Protein Coverage:

Supporting Peptides:

| Peptide | Uniq | -10lgP | Mass | Length | ppm | m/z | z | RT | 1/k0 Start | 1/k0 End | Precursor ID | Area Sample 8 | #Feature | #Feature Sample 8 | Start | End | PTM | AScore | Found By |
| --- | --- | --- | --- | --- | --- | --- | --- | --- | --- | --- | --- | --- | --- | --- | --- | --- | --- | --- | --- |
| K.VRDPYRNDLFHDTSR.I | Y | 40.73 | 1889.9132 | 15 | -1.18 | 473.4829 | 4 | 13.85 | 0.8181 | 0.8254 | 36969 | 0 | 0 | 0 | 51 | 65 |  |  | DB Search |
| total 1 peptides |  |  |  |  |  |  |  |  |  |  |  |  |  |  |  |  |  |  |  |
| --- | --- | --- | --- | --- | --- | --- | --- | --- | --- | --- | --- | --- | --- | --- | --- | --- | --- | --- | --- |

Best Unique PSM (Scan Sample\_7\_A1\_1\_3644.d:36969, m/z=473.4829, z=4, RT=13.85, ppm=-5.66):


TRINITY\_DN1907\_c0\_g1\_i1.p1

back to list

  

| Protein Coverage
| Supporting Peptides
| Best Unique PSM
|

Protein Coverage:

Supporting Peptides:

| Peptide | Uniq | -10lgP | Mass | Length | ppm | m/z | z | RT | 1/k0 Start | 1/k0 End | Precursor ID | Area Sample 8 | #Feature | #Feature Sample 8 | Start | End | PTM | AScore | Found By |
| --- | --- | --- | --- | --- | --- | --- | --- | --- | --- | --- | --- | --- | --- | --- | --- | --- | --- | --- | --- |
| K.VIIDYQTHHR.L | Y | 43.82 | 1280.6625 | 10 | -5.51 | 641.3322 | 2 | 12.21 | 0.9574 | 0.9646 | 27303 | 0 | 0 | 0 | 151 | 160 |  |  | DB Search |
| total 1 peptides |  |  |  |  |  |  |  |  |  |  |  |  |  |  |  |  |  |  |  |
| --- | --- | --- | --- | --- | --- | --- | --- | --- | --- | --- | --- | --- | --- | --- | --- | --- | --- | --- | --- |

Best Unique PSM (Scan Sample\_7\_A1\_1\_3644.d:27303, m/z=641.3322, z=2, RT=12.21, ppm=-9.99):


TRINITY\_DN36\_c0\_g1\_i2.p1

back to list

  

| Protein Coverage
| Supporting Peptides
| Best Unique PSM
|

Protein Coverage:

Supporting Peptides:

| Peptide | Uniq | -10lgP | Mass | Length | ppm | m/z | z | RT | 1/k0 Start | 1/k0 End | Precursor ID | Area Sample 8 | #Feature | #Feature Sample 8 | Start | End | PTM | AScore | Found By |
| --- | --- | --- | --- | --- | --- | --- | --- | --- | --- | --- | --- | --- | --- | --- | --- | --- | --- | --- | --- |
| R.LGGIEVTQGNC(+57.02)AR.K | Y | 43.02 | 1373.6721 | 13 | 2.68 | 687.8421 | 2 | 13.90 | 0.9662 | 0.9735 | 37137 | 6.95e2 | 1 | 1 | 58 | 70 | Carbamidomethylation |  | DB Search |
| total 1 peptides |  |  |  |  |  |  |  |  |  |  |  |  |  |  |  |  |  |  |  |
| --- | --- | --- | --- | --- | --- | --- | --- | --- | --- | --- | --- | --- | --- | --- | --- | --- | --- | --- | --- |

Best Unique PSM (Scan Sample\_7\_A1\_1\_3644.d:37137, m/z=687.8421, z=2, RT=13.90, ppm=-1.8):


TRINITY\_DN161\_c0\_g1\_i4.p1

back to list

  

| Protein Coverage
| Supporting Peptides
| Best Unique PSM
|

Protein Coverage:

Supporting Peptides:

| Peptide | Uniq | -10lgP | Mass | Length | ppm | m/z | z | RT | 1/k0 Start | 1/k0 End | Precursor ID | Area Sample 8 | #Feature | #Feature Sample 8 | Start | End | PTM | AScore | Found By |
| --- | --- | --- | --- | --- | --- | --- | --- | --- | --- | --- | --- | --- | --- | --- | --- | --- | --- | --- | --- |
| K.SYELPDGQVITIGNER.F | Y | 43.86 | 1789.8846 | 16 | 3.32 | 895.9485 | 2 | 18.70 | 1.1126 | 1.1198 | 65490 | 4.79e3 | 2 | 2 | 95 | 110 |  |  | DB Search |
| R.LDLAGRDLTDYLMK.I | Y | 43.72 | 1622.8338 | 14 | -2.1 | 541.9483 | 3 | 19.46 | 0.7998 | 0.8071 | 70651 | 1.99e3 | 2 | 2 | 34 | 47 |  |  | DB Search |
| K.EEYDESGPSIVHR.K | Y | 42.51 | 1516.6794 | 13 | -1.53 | 759.3424 | 2 | 13.28 | 0.9904 | 0.9977 | 33566 | 2.23e2 | 1 | 1 | 216 | 228 |  |  | DB Search |
| total 3 peptides |  |  |  |  |  |  |  |  |  |  |  |  |  |  |  |  |  |  |  |
| --- | --- | --- | --- | --- | --- | --- | --- | --- | --- | --- | --- | --- | --- | --- | --- | --- | --- | --- | --- |

Best Unique PSM (Scan Sample\_7\_A1\_1\_3644.d:65490, m/z=895.9485, z=2, RT=18.70, ppm=-1.15):


TRINITY\_DN941\_c0\_g1\_i2.p2

back to list

  

| Protein Coverage
| Supporting Peptides
| Best Unique PSM
|

Protein Coverage:

Supporting Peptides:

| Peptide | Uniq | -10lgP | Mass | Length | ppm | m/z | z | RT | 1/k0 Start | 1/k0 End | Precursor ID | Area Sample 8 | #Feature | #Feature Sample 8 | Start | End | PTM | AScore | Found By |
| --- | --- | --- | --- | --- | --- | --- | --- | --- | --- | --- | --- | --- | --- | --- | --- | --- | --- | --- | --- |
| K.ADLTRPTGK.K | Y | 42.69 | 957.5243 | 9 | -3.1 | 479.7658 | 2 | 10.44 | 0.8193 | 0.8266 | 17248 | 0 | 0 | 0 | 52 | 60 |  |  | DB Search |
| total 1 peptides |  |  |  |  |  |  |  |  |  |  |  |  |  |  |  |  |  |  |  |
| --- | --- | --- | --- | --- | --- | --- | --- | --- | --- | --- | --- | --- | --- | --- | --- | --- | --- | --- | --- |

Best Unique PSM (Scan Sample\_7\_A1\_1\_3644.d:17248, m/z=479.7658, z=2, RT=10.44, ppm=-7.58):


TRINITY\_DN2217\_c0\_g1\_i2.p1

back to list

  

| Protein Coverage
| Supporting Peptides
| Best Unique PSM
|

Protein Coverage:

Supporting Peptides:

| Peptide | Uniq | -10lgP | Mass | Length | ppm | m/z | z | RT | 1/k0 Start | 1/k0 End | Precursor ID | Area Sample 8 | #Feature | #Feature Sample 8 | Start | End | PTM | AScore | Found By |
| --- | --- | --- | --- | --- | --- | --- | --- | --- | --- | --- | --- | --- | --- | --- | --- | --- | --- | --- | --- |
| K.ATGC(+57.02)EVIWNQHNR.G | Y | 43.77 | 1583.7263 | 13 | 0.77 | 792.8675 | 2 | 14.03 | 1.0805 | 1.0877 | 37976 | 0 | 0 | 0 | 119 | 131 | Carbamidomethylation |  | DB Search |
| total 1 peptides |  |  |  |  |  |  |  |  |  |  |  |  |  |  |  |  |  |  |  |
| --- | --- | --- | --- | --- | --- | --- | --- | --- | --- | --- | --- | --- | --- | --- | --- | --- | --- | --- | --- |

Best Unique PSM (Scan Sample\_7\_A1\_1\_3644.d:37976, m/z=792.8675, z=2, RT=14.03, ppm=-3.7):


TRINITY\_DN71276\_c0\_g1\_i1.p1

back to list

  

| Protein Coverage
| Supporting Peptides
| Best Unique PSM
|

Protein Coverage:

Supporting Peptides:

| Peptide | Uniq | -10lgP | Mass | Length | ppm | m/z | z | RT | 1/k0 Start | 1/k0 End | Precursor ID | Area Sample 8 | #Feature | #Feature Sample 8 | Start | End | PTM | AScore | Found By |
| --- | --- | --- | --- | --- | --- | --- | --- | --- | --- | --- | --- | --- | --- | --- | --- | --- | --- | --- | --- |
| K.EAAAQIVVEIIKR.T | Y | 43.67 | 1438.8507 | 13 | 1.9 | 720.4308 | 2 | 19.26 | 1.0524 | 1.0596 | 69475 | 0 | 0 | 0 | 149 | 161 |  |  | DB Search |
| total 1 peptides |  |  |  |  |  |  |  |  |  |  |  |  |  |  |  |  |  |  |  |
| --- | --- | --- | --- | --- | --- | --- | --- | --- | --- | --- | --- | --- | --- | --- | --- | --- | --- | --- | --- |

Best Unique PSM (Scan Sample\_7\_A1\_1\_3644.d:69475, m/z=720.4308, z=2, RT=19.26, ppm=-2.57):


TRINITY\_DN687\_c0\_g2\_i1.p1

back to list

  

| Protein Coverage
| Supporting Peptides
| Best Unique PSM
|

Protein Coverage:

Supporting Peptides:

| Peptide | Uniq | -10lgP | Mass | Length | ppm | m/z | z | RT | 1/k0 Start | 1/k0 End | Precursor ID | Area Sample 8 | #Feature | #Feature Sample 8 | Start | End | PTM | AScore | Found By |
| --- | --- | --- | --- | --- | --- | --- | --- | --- | --- | --- | --- | --- | --- | --- | --- | --- | --- | --- | --- |
| K.VGINYQPPTVVPGGDLAK.V | Y | 43.58 | 1823.9781 | 18 | 1.41 | 912.9935 | 2 | 17.42 | 1.1214 | 1.1286 | 57750 | 0 | 0 | 0 | 353 | 370 |  |  | DB Search |
| R.AVC(+57.02)M(+15.99)LSNTTAIAEAWAR.L | Y | 43.51 | 1879.892 | 17 | 2.8 | 940.9517 | 2 | 18.84 | 1.1454 | 1.1526 | 66760 | 4.63e2 | 1 | 1 | 374 | 390 | Carbamidomethylation, Oxidation (M) | M4:Oxidation (M):1000 | DB Search |
| total 2 peptides |  |  |  |  |  |  |  |  |  |  |  |  |  |  |  |  |  |  |  |
| --- | --- | --- | --- | --- | --- | --- | --- | --- | --- | --- | --- | --- | --- | --- | --- | --- | --- | --- | --- |

Best Unique PSM (Scan Sample\_7\_A1\_1\_3644.d:57750, m/z=912.9935, z=2, RT=17.42, ppm=-3.06):


TRINITY\_DN1386\_c0\_g1\_i1.p1

back to list

  

| Protein Coverage
| Supporting Peptides
| Best Unique PSM
|

Protein Coverage:

Supporting Peptides:

| Peptide | Uniq | -10lgP | Mass | Length | ppm | m/z | z | RT | 1/k0 Start | 1/k0 End | Precursor ID | Area Sample 8 | #Feature | #Feature Sample 8 | Start | End | PTM | AScore | Found By |
| --- | --- | --- | --- | --- | --- | --- | --- | --- | --- | --- | --- | --- | --- | --- | --- | --- | --- | --- | --- |
| R.KVIVDQLVTLATR.V | Y | 43.56 | 1454.882 | 13 | -1.21 | 728.4442 | 2 | 18.49 | 1.0299 | 1.0371 | 64477 | 3.86e2 | 1 | 1 | 547 | 559 |  |  | DB Search |
| K.SFLLALEKDQEK.H | Y | 43.45 | 1419.7609 | 12 | -1.42 | 710.8835 | 2 | 17.63 | 1.0733 | 1.0805 | 58989 | 4.13e2 | 1 | 1 | 61 | 72 |  |  | DB Search |
| R.SPTHSVIK.S | Y | 43.15 | 867.4814 | 8 | 6.75 | 434.749 | 2 | 9.97 | 0.7713 | 0.7787 | 14734 | 4.39e3 | 1 | 1 | 259 | 266 |  |  | DB Search |
| total 3 peptides |  |  |  |  |  |  |  |  |  |  |  |  |  |  |  |  |  |  |  |
| --- | --- | --- | --- | --- | --- | --- | --- | --- | --- | --- | --- | --- | --- | --- | --- | --- | --- | --- | --- |

Best Unique PSM (Scan Sample\_7\_A1\_1\_3644.d:64477, m/z=728.4442, z=2, RT=18.49, ppm=-5.69):


TRINITY\_DN2637\_c0\_g1\_i14.p2

back to list

  

| Protein Coverage
| Supporting Peptides
| Best Unique PSM
|

Protein Coverage:

Supporting Peptides:

| Peptide | Uniq | -10lgP | Mass | Length | ppm | m/z | z | RT | 1/k0 Start | 1/k0 End | Precursor ID | Area Sample 8 | #Feature | #Feature Sample 8 | Start | End | PTM | AScore | Found By |
| --- | --- | --- | --- | --- | --- | --- | --- | --- | --- | --- | --- | --- | --- | --- | --- | --- | --- | --- | --- |
| K.ATIEIPAKYEK.R | Y | 42.98 | 1261.6918 | 11 | 0.3 | 631.8505 | 2 | 14.24 | 0.9848 | 0.992 | 39239 | 0 | 0 | 0 | 87 | 97 |  |  | DB Search |
| total 1 peptides |  |  |  |  |  |  |  |  |  |  |  |  |  |  |  |  |  |  |  |
| --- | --- | --- | --- | --- | --- | --- | --- | --- | --- | --- | --- | --- | --- | --- | --- | --- | --- | --- | --- |

Best Unique PSM (Scan Sample\_7\_A1\_1\_3644.d:39239, m/z=631.8505, z=2, RT=14.24, ppm=-4.18):


TRINITY\_DN4595\_c0\_g1\_i1.p1

back to list

  

| Protein Coverage
| Supporting Peptides
| Best Unique PSM
|

Protein Coverage:

Supporting Peptides:

| Peptide | Uniq | -10lgP | Mass | Length | ppm | m/z | z | RT | 1/k0 Start | 1/k0 End | Precursor ID | Area Sample 8 | #Feature | #Feature Sample 8 | Start | End | PTM | AScore | Found By |
| --- | --- | --- | --- | --- | --- | --- | --- | --- | --- | --- | --- | --- | --- | --- | --- | --- | --- | --- | --- |
| R.AFNKIETR.N | Y | 43.58 | 977.5294 | 8 | 6.21 | 489.7728 | 2 | 11.52 | 0.8372 | 0.8445 | 23205 | 2.22e3 | 1 | 1 | 72 | 79 |  |  | DB Search |
| total 1 peptides |  |  |  |  |  |  |  |  |  |  |  |  |  |  |  |  |  |  |  |
| --- | --- | --- | --- | --- | --- | --- | --- | --- | --- | --- | --- | --- | --- | --- | --- | --- | --- | --- | --- |

Best Unique PSM (Scan Sample\_7\_A1\_1\_3644.d:23205, m/z=489.7728, z=2, RT=11.52, ppm=1.73):


TRINITY\_DN116\_c0\_g1\_i1.p1

back to list

  

| Protein Coverage
| Supporting Peptides
| Best Unique PSM
|

Protein Coverage:

Supporting Peptides:

| Peptide | Uniq | -10lgP | Mass | Length | ppm | m/z | z | RT | 1/k0 Start | 1/k0 End | Precursor ID | Area Sample 8 | #Feature | #Feature Sample 8 | Start | End | PTM | AScore | Found By |
| --- | --- | --- | --- | --- | --- | --- | --- | --- | --- | --- | --- | --- | --- | --- | --- | --- | --- | --- | --- |
| R.ALQDEDEDAFKR.Q | Y | 43.71 | 1435.6579 | 12 | 7.39 | 718.8383 | 2 | 13.32 | 0.9957 | 1.0029 | 33731 | 1.03e3 | 1 | 1 | 210 | 221 |  |  | DB Search |
| total 1 peptides |  |  |  |  |  |  |  |  |  |  |  |  |  |  |  |  |  |  |  |
| --- | --- | --- | --- | --- | --- | --- | --- | --- | --- | --- | --- | --- | --- | --- | --- | --- | --- | --- | --- |

Best Unique PSM (Scan Sample\_7\_A1\_1\_3644.d:33731, m/z=718.8383, z=2, RT=13.32, ppm=2.92):


TRINITY\_DN98968\_c0\_g1\_i1.p1

back to list

  

| Protein Coverage
| Supporting Peptides
| Best Unique PSM
|

Protein Coverage:

Supporting Peptides:

| Peptide | Uniq | -10lgP | Mass | Length | ppm | m/z | z | RT | 1/k0 Start | 1/k0 End | Precursor ID | Area Sample 8 | #Feature | #Feature Sample 8 | Start | End | PTM | AScore | Found By |
| --- | --- | --- | --- | --- | --- | --- | --- | --- | --- | --- | --- | --- | --- | --- | --- | --- | --- | --- | --- |
| R.AFNKVETR.N | N | 43.74 | 963.5138 | 8 | 5.69 | 482.7647 | 2 | 10.62 | 0.8299 | 0.8372 | 18171 | 1.3e3 | 1 | 1 | 406 | 413 |  |  | DB Search |
| R.IESTAC(+57.02)TR.R | Y | 41.29 | 936.4335 | 8 | 7.97 | 469.2256 | 2 | 9.69 | 0.8023 | 0.8096 | 13341 | 2.14e3 | 1 | 1 | 204 | 211 | Carbamidomethylation |  | DB Search |
| total 2 peptides |  |  |  |  |  |  |  |  |  |  |  |  |  |  |  |  |  |  |  |
| --- | --- | --- | --- | --- | --- | --- | --- | --- | --- | --- | --- | --- | --- | --- | --- | --- | --- | --- | --- |

Best Unique PSM (Scan Sample\_7\_A1\_1\_3644.d:13341, m/z=469.2256, z=2, RT=9.69, ppm=3.49):


TRINITY\_DN5584\_c0\_g1\_i3.p1

back to list

  

| Protein Coverage
| Supporting Peptides
| Best Unique PSM
|

Protein Coverage:

Supporting Peptides:

| Peptide | Uniq | -10lgP | Mass | Length | ppm | m/z | z | RT | 1/k0 Start | 1/k0 End | Precursor ID | Area Sample 8 | #Feature | #Feature Sample 8 | Start | End | PTM | AScore | Found By |
| --- | --- | --- | --- | --- | --- | --- | --- | --- | --- | --- | --- | --- | --- | --- | --- | --- | --- | --- | --- |
| R.YGTSSFDMSSLIK.T | Y | 42.34 | 1434.6701 | 13 | 2.06 | 718.3406 | 2 | 19.16 | 1.023 | 1.0303 | 68826 | 0 | 0 | 0 | 882 | 894 |  |  | DB Search |
| total 1 peptides |  |  |  |  |  |  |  |  |  |  |  |  |  |  |  |  |  |  |  |
| --- | --- | --- | --- | --- | --- | --- | --- | --- | --- | --- | --- | --- | --- | --- | --- | --- | --- | --- | --- |

Best Unique PSM (Scan Sample\_7\_A1\_1\_3644.d:68826, m/z=718.3406, z=2, RT=19.16, ppm=-2.42):


TRINITY\_DN3036\_c0\_g1\_i3.p1

back to list

  

| Protein Coverage
| Supporting Peptides
| Best Unique PSM
|

Protein Coverage:

Supporting Peptides:

| Peptide | Uniq | -10lgP | Mass | Length | ppm | m/z | z | RT | 1/k0 Start | 1/k0 End | Precursor ID | Area Sample 8 | #Feature | #Feature Sample 8 | Start | End | PTM | AScore | Found By |
| --- | --- | --- | --- | --- | --- | --- | --- | --- | --- | --- | --- | --- | --- | --- | --- | --- | --- | --- | --- |
| R.GTSNGGLVVGTIIKPK.L | Y | 43.76 | 1539.8984 | 16 | -3.44 | 770.9504 | 2 | 15.91 | 1.1262 | 1.1334 | 48868 | 9.21e2 | 1 | 1 | 173 | 188 |  |  | DB Search |
| R.AGHGAVTSPQTQR.G | Y | 43.55 | 1308.6534 | 13 | 3.24 | 437.2245 | 3 | 9.76 | 0.7045 | 0.7118 | 13596 | 2.79e3 | 2 | 2 | 309 | 321 |  |  | DB Search |
| R.VIGASGIHVGTM(+15.99)SFGK.M | Y | 43.2 | 1575.8079 | 16 | 0.89 | 788.9084 | 2 | 15.29 | 1.129 | 1.1362 | 45505 | 0 | 0 | 0 | 334 | 349 | Oxidation (M) | M12:Oxidation (M):1000 | DB Search |
| total 3 peptides |  |  |  |  |  |  |  |  |  |  |  |  |  |  |  |  |  |  |  |
| --- | --- | --- | --- | --- | --- | --- | --- | --- | --- | --- | --- | --- | --- | --- | --- | --- | --- | --- | --- |

Best Unique PSM (Scan Sample\_7\_A1\_1\_3644.d:48868, m/z=770.9504, z=2, RT=15.91, ppm=-7.92):


TRINITY\_DN123301\_c0\_g1\_i1.p1

back to list

  

| Protein Coverage
| Supporting Peptides
| Best Unique PSM
|

Protein Coverage:

Supporting Peptides:

| Peptide | Uniq | -10lgP | Mass | Length | ppm | m/z | z | RT | 1/k0 Start | 1/k0 End | Precursor ID | Area Sample 8 | #Feature | #Feature Sample 8 | Start | End | PTM | AScore | Found By |
| --- | --- | --- | --- | --- | --- | --- | --- | --- | --- | --- | --- | --- | --- | --- | --- | --- | --- | --- | --- |
| R.YRLELYVDDKDHNASAK.S | Y | 43.49 | 2035.9963 | 17 | 1.12 | 510.0046 | 4 | 14.55 | 0.788 | 0.7953 | 41114 | 4.93e2 | 1 | 1 | 217 | 233 |  |  | DB Search |
| total 1 peptides |  |  |  |  |  |  |  |  |  |  |  |  |  |  |  |  |  |  |  |
| --- | --- | --- | --- | --- | --- | --- | --- | --- | --- | --- | --- | --- | --- | --- | --- | --- | --- | --- | --- |

Best Unique PSM (Scan Sample\_7\_A1\_1\_3644.d:41114, m/z=510.0046, z=4, RT=14.55, ppm=-3.35):


TRINITY\_DN151\_c0\_g1\_i4.p1

back to list

  

| Protein Coverage
| Supporting Peptides
| Best Unique PSM
|

Protein Coverage:

Supporting Peptides:

| Peptide | Uniq | -10lgP | Mass | Length | ppm | m/z | z | RT | 1/k0 Start | 1/k0 End | Precursor ID | Area Sample 8 | #Feature | #Feature Sample 8 | Start | End | PTM | AScore | Found By |
| --- | --- | --- | --- | --- | --- | --- | --- | --- | --- | --- | --- | --- | --- | --- | --- | --- | --- | --- | --- |
| R.SLFDIFHSK.S | Y | 43.18 | 1092.5604 | 9 | 4.5 | 547.2875 | 2 | 19.85 | 0.8846 | 0.8919 | 73174 | 0 | 0 | 0 | 204 | 212 |  |  | DB Search |
| total 1 peptides |  |  |  |  |  |  |  |  |  |  |  |  |  |  |  |  |  |  |  |
| --- | --- | --- | --- | --- | --- | --- | --- | --- | --- | --- | --- | --- | --- | --- | --- | --- | --- | --- | --- |

Best Unique PSM (Scan Sample\_7\_A1\_1\_3644.d:73174, m/z=547.2875, z=2, RT=19.85, ppm=0.03):


---

Prepared with PEAKS ™ (bioinfor.com)
