# Supplementary material for: Proteomic Analysis and Biochemical Characterization of the Nematocyst Extract of the Hydrozoan Velella velella
Source: Mar Drugs. 2024 Oct 12;22(10):468. doi: 10.3390/md22100468 (PMC11509761; doi:10.3390/md22100468)
Supplement: Supplementary file 1 [file marinedrugs-22-00468-s001.zip › Supplementary File S1.zip!/Sample_8_Vellela/db.peptides.html]

peptide list


Peptide List

  

| Peptide | -10lgP | Mass | Length | ppm | m/z | RT | 1/k0 Start | 1/k0 End | Precursor ID | Area Sample 9 | #Feature | #Feature Sample 9 | Accession | PTM | AScore | Found By |
| --- | --- | --- | --- | --- | --- | --- | --- | --- | --- | --- | --- | --- | --- | --- | --- | --- |
| FSYEKVC(+57.02)EDVKR | 62.2 | 1558.745 | 12 | -2.91 | 780.374 | 13.45 | 1.0684 | 1.0757 | 35460 | 1.37e3 | 1 | 1 | TRINITY\_DN118\_c1\_g1\_i3.p1 | Carbamidomethylation |  | DB Search |
| SLVEENVHPQILIK | 62.09 | 1617.9089 | 14 | 0.4 | 809.9584 | 16.26 | 1.0977 | 1.1049 | 50304 | 0 | 0 | 0 | TRINITY\_DN1194\_c0\_g1\_i1.p1 |  |  | DB Search |
| YRNEDDALKEK | 62.01 | 1379.6681 | 11 | 3.73 | 460.8963 | 10.49 | 0.7689 | 0.7762 | 17723 | 0 | 0 | 0 | TRINITY\_DN167\_c0\_g2\_i1.p1 |  |  | DB Search |
| GVGIISEGNETVEDIAQR | 61.93 | 1885.9381 | 18 | -13.03 | 943.9598 | 17.75 | 1.1406 | 1.1478 | 58109 | 0 | 0 | 0 | TRINITY\_DN262\_c0\_g1\_i1.p1 |  |  | DB Search |
| KSDQATLPDNTIAQK | 61.92 | 1628.8369 | 15 | -2.41 | 815.4201 | 12.57 | 1.0512 | 1.0584 | 30355 | 0 | 0 | 0 | TRINITY\_DN428\_c0\_g1\_i1.p1 |  |  | DB Search |
| AYGQDLGQPGSC(+57.02)LKR | 61.86 | 1648.7991 | 15 | 1.51 | 825.4044 | 13.36 | 1.0761 | 1.0833 | 34861 | 1.57e3 | 2 | 2 | TRINITY\_DN691\_c0\_g1\_i2.p1:TRINITY\_DN691\_c0\_g1\_i1.p1 | Carbamidomethylation |  | DB Search |
| KFQVPSGIRPILVK | 61.62 | 1580.9766 | 14 | 1.23 | 527.9977 | 16.64 | 0.9089 | 0.9162 | 52098 | 0 | 0 | 0 | TRINITY\_DN140702\_c0\_g1\_i1.p1 |  |  | DB Search |
| AVDRGNPPQDSVNK | 61.59 | 1495.7379 | 14 | -2.2 | 499.5832 | 10.21 | 0.7823 | 0.7897 | 15988 | 0 | 0 | 0 | TRINITY\_DN982\_c0\_g1\_i1.p1:TRINITY\_DN982\_c0\_g1\_i3.p1 |  |  | DB Search |
| SVTTHQGNAIVIK | 61.58 | 1366.7568 | 13 | 3.07 | 684.3847 | 12.45 | 0.9965 | 1.0037 | 29541 | 5.61e2 | 1 | 1 | TRINITY\_DN1377\_c0\_g1\_i1.p1 |  |  | DB Search |
| YGQAM(+15.99)C(+57.02)QFM(+15.99)R | 61.54 | 1322.5206 | 10 | -1.76 | 662.2634 | 12.17 | 0.9489 | 0.9562 | 28008 | 2.07e2 | 1 | 1 | TRINITY\_DN36\_c0\_g1\_i9.p1:TRINITY\_DN36\_c0\_g1\_i12.p1:TRINITY\_DN649\_c0\_g1\_i6.p1:TRINITY\_DN165746\_c0\_g1\_i1.p1:TRINITY\_DN286\_c0\_g1\_i3.p1:TRINITY\_DN36\_c0\_g1\_i3.p1:TRINITY\_DN36\_c0\_g1\_i17.p1:TRINITY\_DN36\_c0\_g1\_i4.p1:TRINITY\_DN649\_c0\_g1\_i5.p1:TRINITY\_DN36\_c0\_g1\_i20.p1:TRINITY\_DN36\_c0\_g1\_i14.p1:TRINITY\_DN36\_c0\_g1\_i13.p1:TRINITY\_DN649\_c0\_g1\_i9.p1:TRINITY\_DN36\_c0\_g1\_i2.p1:TRINITY\_DN286\_c0\_g1\_i2.p1:TRINITY\_DN649\_c0\_g1\_i1.p1:TRINITY\_DN154921\_c0\_g1\_i1.p1:TRINITY\_DN649\_c0\_g1\_i2.p1:TRINITY\_DN36\_c0\_g1\_i16.p1:TRINITY\_DN649\_c0\_g1\_i11.p1:TRINITY\_DN36\_c0\_g1\_i11.p1:TRINITY\_DN286\_c0\_g1\_i4.p1:TRINITY\_DN649\_c0\_g1\_i10.p1:TRINITY\_DN36\_c0\_g1\_i1.p1:TRINITY\_DN36\_c0\_g1\_i8.p1:TRINITY\_DN36\_c0\_g1\_i19.p1:TRINITY\_DN36\_c0\_g1\_i5.p1 | Oxidation (M), Carbamidomethylation, Oxidation (M) | M5:Oxidation (M):1000 M9:Oxidation (M):1000 | DB Search |
| SAEKDEELETLRK | 61.52 | 1546.7838 | 13 | -10.31 | 516.5942 | 12.10 | 0.8096 | 0.8169 | 27606 | 0 | 0 | 0 | TRINITY\_DN3034\_c0\_g1\_i1.p1 |  |  | DB Search |
| NSSYFVEWIPNNVK | 61.49 | 1695.8257 | 14 | 2 | 848.918 | 19.90 | 1.0889 | 1.0961 | 70553 | 0 | 0 | 0 | TRINITY\_DN1910\_c0\_g1\_i1.p1:TRINITY\_DN3986\_c0\_g1\_i1.p1:TRINITY\_DN3986\_c0\_g1\_i2.p1 |  |  | DB Search |
| ANQVSSAAAAPVPAAGGDK | 61.48 | 1680.843 | 19 | -2.28 | 841.4231 | 12.43 | 1.0668 | 1.0741 | 29536 | 0 | 0 | 0 | TRINITY\_DN240\_c0\_g1\_i6.p1:TRINITY\_DN240\_c0\_g1\_i1.p1:TRINITY\_DN240\_c0\_g1\_i4.p1 |  |  | DB Search |
| FGHNHPEPLKK | 61.47 | 1302.6832 | 11 | 5.54 | 435.2355 | 9.95 | 0.7379 | 0.7453 | 14269 | 3.36e2 | 1 | 1 | TRINITY\_DN121501\_c0\_g1\_i1.p1 |  |  | DB Search |
| GVSEETTTGVHNLYK | 61.34 | 1633.7948 | 15 | 0.65 | 817.9015 | 13.53 | 1.0729 | 1.0801 | 35904 | 8.57e2 | 1 | 1 | TRINITY\_DN773\_c0\_g1\_i1.p1 |  |  | DB Search |
| IRNEEIQIEVIERR | 61.33 | 1795.9904 | 14 | 3.65 | 599.6702 | 15.08 | 0.8793 | 0.8866 | 44280 | 1.19e3 | 1 | 1 | TRINITY\_DN11435\_c0\_g1\_i1.p1 |  |  | DB Search |
| KYGQATC(+57.02)QFMQNSHK | 61.26 | 1826.8192 | 15 | 3.52 | 609.9464 | 12.17 | 0.8769 | 0.8842 | 28012 | 0 | 0 | 0 | TRINITY\_DN168534\_c0\_g1\_i1.p1 | Carbamidomethylation |  | DB Search |
| IQDKEGIPPDQQR | 61.25 | 1522.7739 | 13 | -1.84 | 762.3894 | 10.99 | 1.0371 | 1.0443 | 20319 | 2.55e3 | 1 | 1 | TRINITY\_DN554\_c0\_g1\_i3.p2:TRINITY\_DN585\_c0\_g1\_i3.p1:TRINITY\_DN173718\_c0\_g1\_i1.p1:TRINITY\_DN1465\_c1\_g1\_i1.p1:TRINITY\_DN40728\_c1\_g1\_i1.p2:TRINITY\_DN585\_c0\_g1\_i5.p1:TRINITY\_DN152\_c0\_g1\_i2.p1:TRINITY\_DN152\_c0\_g1\_i14.p2:TRINITY\_DN40728\_c0\_g1\_i1.p1:TRINITY\_DN152\_c0\_g1\_i9.p1:TRINITY\_DN585\_c0\_g1\_i4.p1:TRINITY\_DN152\_c0\_g1\_i15.p1:TRINITY\_DN195631\_c0\_g1\_i1.p2:TRINITY\_DN585\_c0\_g1\_i10.p1:TRINITY\_DN554\_c0\_g1\_i5.p1:TRINITY\_DN585\_c0\_g1\_i2.p1:TRINITY\_DN8227\_c0\_g1\_i1.p1:TRINITY\_DN152\_c0\_g1\_i10.p1 |  |  | DB Search |
| DAPIYGLQWHPEK | 61.22 | 1552.7673 | 13 | -3.87 | 777.3845 | 17.19 | 1.0672 | 1.0745 | 54959 | 5.27e2 | 1 | 1 | TRINITY\_DN247\_c0\_g1\_i1.p1 |  |  | DB Search |
| LPWEELWKPC(+57.02)IK | 61.22 | 1597.8326 | 12 | -2.11 | 533.6146 | 19.85 | 0.8773 | 0.8846 | 70215 | 1.87e3 | 1 | 1 | TRINITY\_DN602\_c0\_g1\_i4.p1:TRINITY\_DN602\_c0\_g1\_i3.p1:TRINITY\_DN602\_c0\_g1\_i1.p1 | Carbamidomethylation |  | DB Search |
| YGQATC(+57.02)QFMQNSHK | 61.18 | 1698.7242 | 14 | -0.26 | 850.3654 | 13.00 | 1.1398 | 1.147 | 32841 | 0 | 0 | 0 | TRINITY\_DN168534\_c0\_g1\_i1.p1 | Carbamidomethylation |  | DB Search |
| LRLDFDHEAKLNM(+15.99)IFR | 61.13 | 2033.0516 | 16 | 0.48 | 509.2681 | 17.75 | 0.8575 | 0.8648 | 57989 | 1.69e3 | 1 | 1 | TRINITY\_DN160\_c0\_g1\_i2.p1:TRINITY\_DN160\_c0\_g1\_i1.p1 | Oxidation (M) | M13:Oxidation (M):1000 | DB Search |
| AC(+57.02)AELHQDVNVK | 61.11 | 1382.6613 | 12 | 3.9 | 692.3375 | 11.85 | 1.0069 | 1.0142 | 26009 | 4.59e2 | 1 | 1 | TRINITY\_DN34481\_c0\_g1\_i1.p1 | Carbamidomethylation |  | DB Search |
| KGNEGGNTELISSYRK | 61.06 | 1751.8802 | 16 | -1.22 | 584.964 | 12.34 | 0.8753 | 0.8826 | 29059 | 0 | 0 | 0 | TRINITY\_DN165536\_c0\_g1\_i1.p1 |  |  | DB Search |
| DSNTGSEHTINGK | 61.05 | 1358.6062 | 13 | -4.5 | 680.3043 | 9.60 | 0.9348 | 0.942 | 12318 | 0 | 0 | 0 | TRINITY\_DN1174\_c2\_g1\_i1.p1 |  |  | DB Search |
| LDLAGRDLTDYLMK | 60.93 | 1622.8337 | 14 | 1.03 | 812.4213 | 19.24 | 1.0965 | 1.1037 | 66626 | 2.72e3 | 2 | 2 | TRINITY\_DN161\_c0\_g1\_i1.p1:TRINITY\_DN161\_c0\_g1\_i4.p1:TRINITY\_DN161\_c0\_g1\_i3.p1:TRINITY\_DN161\_c0\_g1\_i2.p1 |  |  | DB Search |
| VRDPYRNDLFHDTSR | 60.69 | 1889.9132 | 15 | 3.25 | 473.485 | 13.64 | 0.8165 | 0.8238 | 36483 | 3.56e3 | 2 | 2 | TRINITY\_DN730\_c0\_g2\_i3.p1:TRINITY\_DN730\_c0\_g2\_i2.p1 |  |  | DB Search |
| ATHQELLNAEFIQK | 60.63 | 1640.8522 | 14 | -8.6 | 821.4226 | 15.81 | 1.0801 | 1.0873 | 48105 | 0 | 0 | 0 | TRINITY\_DN113\_c3\_g1\_i1.p1 |  |  | DB Search |
| KGFIM(+15.99)VIEAIAGGGHK | 60.58 | 1642.8865 | 16 | -0.13 | 548.6335 | 19.76 | 0.8445 | 0.8518 | 69782 | 0 | 0 | 0 | TRINITY\_DN66856\_c0\_g1\_i1.p1 | Oxidation (M) | M5:Oxidation (M):1000 | DB Search |
| VVHNIC(+57.02)SSAITNSVTNK | 60.48 | 1842.9258 | 17 | -1.93 | 922.4642 | 13.76 | 1.1366 | 1.1438 | 37108 | 3.92e2 | 1 | 1 | TRINITY\_DN1199\_c0\_g1\_i1.p1:TRINITY\_DN1199\_c0\_g1\_i2.p1 | Carbamidomethylation |  | DB Search |
| DAC(+57.02)IDGC(+57.02)HC(+57.02)PPHTSLSGDK | 60.38 | 2125.8616 | 19 | 8.76 | 709.6308 | 12.20 | 0.8903 | 0.8976 | 28169 | 0 | 0 | 0 | TRINITY\_DN1369\_c0\_g1\_i1.p1 | Carbamidomethylation, Carbamidomethylation, Carbamidomethylation |  | DB Search |
| AKIEALHC(+57.02)LSENIVK | 60.23 | 1723.9291 | 15 | 3.59 | 862.971 | 15.53 | 1.1326 | 1.1398 | 46701 | 0 | 0 | 0 | TRINITY\_DN3078\_c0\_g1\_i1.p1 | Carbamidomethylation |  | DB Search |
| LIEVEEKEIQR | 60.21 | 1384.7561 | 11 | 3.4 | 693.3846 | 13.23 | 1.0182 | 1.0255 | 34162 | 0 | 0 | 0 | TRINITY\_DN11435\_c0\_g1\_i1.p1 |  |  | DB Search |
| VIGASGIHVGTM(+15.99)SFGK | 60.06 | 1575.8079 | 16 | 1.08 | 788.9085 | 15.08 | 1.125 | 1.1322 | 44382 | 0 | 0 | 0 | TRINITY\_DN3036\_c0\_g1\_i6.p1:TRINITY\_DN3036\_c0\_g1\_i13.p1:TRINITY\_DN3036\_c0\_g1\_i19.p1:TRINITY\_DN3036\_c0\_g1\_i3.p1:TRINITY\_DN3036\_c0\_g1\_i4.p1:TRINITY\_DN3036\_c0\_g1\_i11.p1:TRINITY\_DN3036\_c0\_g1\_i16.p1:TRINITY\_DN3036\_c0\_g1\_i2.p2:TRINITY\_DN3036\_c0\_g1\_i8.p1 | Oxidation (M) | M12:Oxidation (M):1000 | DB Search |
| AHVSLSTFSEDVTSLTK | 59.92 | 1820.9155 | 17 | -3.57 | 911.4577 | 18.78 | 1.1386 | 1.1458 | 63908 | 1.2e3 | 1 | 1 | TRINITY\_DN113\_c3\_g1\_i1.p1 |  |  | DB Search |
| WC(+57.02)DILSIHQSMENC(+57.02)VAK | 59.79 | 2089.9382 | 17 | 2.4 | 697.6519 | 17.87 | 0.8769 | 0.8842 | 58766 | 0 | 0 | 0 | TRINITY\_DN36\_c0\_g1\_i16.p1:TRINITY\_DN36\_c0\_g1\_i3.p1:TRINITY\_DN36\_c0\_g1\_i14.p1:TRINITY\_DN36\_c0\_g1\_i17.p1:TRINITY\_DN36\_c0\_g1\_i13.p1:TRINITY\_DN36\_c0\_g1\_i8.p1:TRINITY\_DN36\_c0\_g1\_i19.p1 | Carbamidomethylation, Carbamidomethylation |  | DB Search |
| EGDLVIHSGITDAVK | 59.72 | 1552.8097 | 15 | 2.8 | 777.4108 | 14.52 | 1.062 | 1.0693 | 41331 | 1.23e3 | 2 | 2 | TRINITY\_DN329\_c0\_g1\_i1.p1:TRINITY\_DN329\_c0\_g1\_i2.p1 |  |  | DB Search |
| LC(+57.02)KPSYSTC(+57.02)ENHC(+57.02)ER | 59.72 | 1939.7975 | 15 | 2.85 | 970.9044 | 10.96 | 1.1206 | 1.1278 | 20513 | 3.15e3 | 2 | 2 | TRINITY\_DN828\_c0\_g1\_i2.p1 | Carbamidomethylation, Carbamidomethylation, Carbamidomethylation |  | DB Search |
| RPGFSDSEGKIR | 59.66 | 1347.6895 | 12 | 3.54 | 450.2367 | 11.44 | 0.7933 | 0.8006 | 23540 | 0 | 0 | 0 | TRINITY\_DN140702\_c0\_g1\_i1.p1 |  |  | DB Search |
| TQAVLDRLNIEKR | 59.65 | 1554.8842 | 13 | 2.18 | 519.3008 | 14.51 | 0.7986 | 0.8059 | 41304 | 7.54e2 | 1 | 1 | TRINITY\_DN477\_c0\_g1\_i9.p1 |  |  | DB Search |
| FPGFDSESDDFNAEVHR | 59.64 | 1967.8285 | 17 | -3.47 | 656.9449 | 17.21 | 0.8931 | 0.9004 | 55060 | 0 | 0 | 0 | TRINITY\_DN116\_c0\_g1\_i1.p1 |  |  | DB Search |
| TTC(+57.02)GVC(+57.02)NNNYC(+57.02)EDDPR | 59.6 | 1973.7302 | 16 | 4.69 | 987.8726 | 12.28 | 1.121 | 1.1282 | 28564 | 7.59e1 | 1 | 1 | TRINITY\_DN89\_c0\_g1\_i1.p1 | Carbamidomethylation, Carbamidomethylation, Carbamidomethylation |  | DB Search |
| M(+15.99)C(+57.02)NTIQSIGDKFSVSM(+15.99)K | 59.36 | 1976.9005 | 17 | 2.56 | 659.9728 | 16.16 | 0.9489 | 0.9562 | 49831 | 0 | 0 | 0 | TRINITY\_DN1189\_c0\_g1\_i1.p1 | Oxidation (M), Carbamidomethylation, Oxidation (M) | M1:Oxidation (M):1000 M16:Oxidation (M):1000 | DB Search |
| ALSEQIRPTTQIVVC(+57.02)IVPNNRK | 59.27 | 2535.3955 | 22 | 2.34 | 846.1373 | 16.62 | 1.06 | 1.0672 | 51858 | 2.38e2 | 1 | 1 | TRINITY\_DN1377\_c0\_g1\_i1.p1 | Carbamidomethylation |  | DB Search |
| IGAAAQALADQSYPFLK | 59.19 | 1762.9253 | 17 | -0.58 | 882.4655 | 19.04 | 1.159 | 1.1662 | 65509 | 3.11e3 | 2 | 2 | TRINITY\_DN240\_c0\_g1\_i6.p1:TRINITY\_DN240\_c0\_g1\_i1.p1:TRINITY\_DN240\_c0\_g1\_i4.p1 |  |  | DB Search |
| YIQGENEKQEK | 59.18 | 1364.6572 | 11 | 4.26 | 683.3357 | 9.60 | 0.9763 | 0.9836 | 12212 | 1.65e3 | 2 | 2 | TRINITY\_DN1272\_c0\_g1\_i1.p1 |  |  | DB Search |
| GLKVDFDTTFAPVTGK | 59.12 | 1694.8879 | 16 | 1.5 | 848.4487 | 18.11 | 1.1033 | 1.1106 | 60153 | 3.39e3 | 1 | 1 | TRINITY\_DN329\_c0\_g1\_i1.p1:TRINITY\_DN329\_c0\_g1\_i2.p1 |  |  | DB Search |
| IDNYIEFLDEDLKK | 59.04 | 1753.8774 | 14 | -3.23 | 585.6285 | 19.82 | 0.8437 | 0.851 | 70027 | 1.38e3 | 1 | 1 | TRINITY\_DN278\_c0\_g1\_i1.p1 |  |  | DB Search |
| YIDIEYVC(+57.02)KPPVKK | 59.02 | 1750.9327 | 14 | 1.78 | 876.4713 | 14.93 | 1.131 | 1.1382 | 43553 | 2.46e2 | 1 | 1 | TRINITY\_DN374\_c0\_g1\_i1.p1:TRINITY\_DN374\_c0\_g1\_i2.p1 | Carbamidomethylation |  | DB Search |
| LDLAGRDLTDYLM(+15.99)K | 59.01 | 1638.8286 | 14 | 2.41 | 547.2823 | 18.13 | 0.7856 | 0.7929 | 60339 | 2.36e3 | 1 | 1 | TRINITY\_DN161\_c0\_g1\_i1.p1:TRINITY\_DN161\_c0\_g1\_i4.p1:TRINITY\_DN161\_c0\_g1\_i3.p1:TRINITY\_DN161\_c0\_g1\_i2.p1 | Oxidation (M) | M13:Oxidation (M):1000 | DB Search |
| ANHEQITVADITNAC(+57.02)FDPR | 58.96 | 2171.0066 | 19 | 2.79 | 1086.5087 | 17.63 | 1.2368 | 1.2439 | 57365 | 0 | 0 | 0 | TRINITY\_DN96161\_c0\_g1\_i1.p1 | Carbamidomethylation |  | DB Search |
| IRELTSVVQKR | 58.9 | 1327.7936 | 11 | -5.4 | 443.6007 | 12.36 | 0.7445 | 0.7518 | 29134 | 0 | 0 | 0 | TRINITY\_DN5263\_c0\_g1\_i1.p1 |  |  | DB Search |
| FC(+57.02)SNPEPENGGQTC(+57.02)EGEAR | 58.9 | 2137.843 | 19 | 1.53 | 1069.9255 | 12.03 | 1.1562 | 1.1634 | 27122 | 0 | 0 | 0 | TRINITY\_DN138\_c0\_g1\_i1.p1 | Carbamidomethylation, Carbamidomethylation |  | DB Search |
| DLSLVVEPLKNYGVK | 58.88 | 1672.9399 | 15 | 2.46 | 837.4755 | 19.03 | 1.1358 | 1.143 | 65463 | 6.76e2 | 1 | 1 | TRINITY\_DN244\_c0\_g1\_i1.p1 |  |  | DB Search |
| SFHFGVIK | 58.79 | 933.5072 | 8 | 5.47 | 467.7613 | 16.02 | 0.8108 | 0.8181 | 49143 | 1.91e2 | 1 | 1 | TRINITY\_DN329\_c0\_g1\_i1.p1:TRINITY\_DN329\_c0\_g1\_i2.p1 |  |  | DB Search |
| DIHRNEIQYHFHAER | 58.78 | 1963.9401 | 15 | -2.68 | 491.9888 | 12.62 | 0.7465 | 0.7538 | 30644 | 0 | 0 | 0 | TRINITY\_DN121236\_c0\_g1\_i1.p1 |  |  | DB Search |
| YRLELYVDDKDHNASAK | 58.77 | 2035.9963 | 17 | 1.23 | 510.0047 | 14.34 | 0.7787 | 0.786 | 40352 | 0 | 0 | 0 | TRINITY\_DN123301\_c0\_g1\_i1.p1 |  |  | DB Search |
| INVDAAFHDLVR | 58.77 | 1368.715 | 12 | 0.58 | 685.3621 | 18.62 | 1.0246 | 1.0319 | 63073 | 7.69e2 | 1 | 1 | TRINITY\_DN147947\_c0\_g1\_i1.p1 |  |  | DB Search |
| LGPVVDGKYDYTVITTDYK | 58.74 | 2146.0833 | 19 | -1.41 | 1074.0426 | 17.41 | 1.2455 | 1.2527 | 56083 | 0 | 0 | 0 | TRINITY\_DN468\_c1\_g1\_i1.p1 |  |  | DB Search |
| YSRPTPGSNNNVM(+15.99)VR | 58.73 | 1706.8158 | 15 | 1.57 | 569.9442 | 11.49 | 0.8348 | 0.8421 | 23665 | 2.04e2 | 1 | 1 | TRINITY\_DN15274\_c0\_g1\_i1.p1 | Oxidation (M) | M13:Oxidation (M):1000 | DB Search |
| REPGDFGFDPLKFSK | 58.72 | 1738.8678 | 15 | -1.8 | 580.6262 | 18.01 | 0.9012 | 0.9085 | 59548 | 0 | 0 | 0 | TRINITY\_DN10724\_c0\_g1\_i5.p1:TRINITY\_DN10724\_c0\_g1\_i4.p1:TRINITY\_DN10724\_c0\_g1\_i1.p2:TRINITY\_DN10724\_c0\_g1\_i3.p1 |  |  | DB Search |
| ISNVQDIVPVLELANQHK | 58.72 | 2016.1003 | 18 | 0.02 | 1009.0529 | 19.57 | 1.1893 | 1.1965 | 68604 | 2.2e3 | 1 | 1 | TRINITY\_DN118520\_c0\_g1\_i1.p1 |  |  | DB Search |
| IHINIVVIGHVDSGK | 58.71 | 1599.9097 | 15 | -4.52 | 534.3057 | 16.46 | 0.84 | 0.8473 | 51229 | 2.52e2 | 2 | 2 | TRINITY\_DN64\_c0\_g1\_i1.p1 |  |  | DB Search |
| LM(+15.99)GQIVSSITASLR | 58.7 | 1490.8126 | 14 | -0.92 | 746.4095 | 18.62 | 1.0331 | 1.0403 | 63020 | 2.09e2 | 1 | 1 | TRINITY\_DN250\_c0\_g1\_i2.p1:TRINITY\_DN250\_c0\_g1\_i3.p1 | Oxidation (M) | M2:Oxidation (M):1000 | DB Search |
| IQVLKDPDFEFTR | 58.69 | 1606.8354 | 13 | -7.52 | 804.4153 | 17.50 | 1.0813 | 1.0885 | 56511 | 4.85e2 | 1 | 1 | TRINITY\_DN982\_c0\_g1\_i1.p1:TRINITY\_DN982\_c0\_g1\_i3.p1 |  |  | DB Search |
| DLKPENLLIDEKGYLK | 58.66 | 1887.0353 | 16 | -2.85 | 630.0144 | 17.77 | 0.9214 | 0.9287 | 58211 | 0 | 0 | 0 | TRINITY\_DN37295\_c0\_g1\_i1.p1 |  |  | DB Search |
| LVDATFPTTDFETVR | 58.54 | 1710.8464 | 15 | -4.42 | 856.4229 | 18.47 | 1.1033 | 1.1106 | 62184 | 1.4e3 | 1 | 1 | TRINITY\_DN74124\_c0\_g1\_i1.p1 |  |  | DB Search |
| KFVWEFFQK | 58.53 | 1257.6545 | 9 | 1.34 | 629.8326 | 19.88 | 0.9436 | 0.9509 | 70292 | 1.4e3 | 1 | 1 | TRINITY\_DN1280\_c0\_g1\_i1.p1 |  |  | DB Search |
| IENIEDVFKLPSYC(+57.02)K | 58.53 | 1853.9233 | 15 | -0.75 | 618.9785 | 19.65 | 0.8652 | 0.8725 | 68996 | 3.34e2 | 1 | 1 | TRINITY\_DN1091\_c0\_g1\_i1.p1 | Carbamidomethylation |  | DB Search |
| ITTSQILDHEQQK | 58.51 | 1539.7893 | 13 | -0.87 | 770.8978 | 13.81 | 1.0933 | 1.1005 | 37414 | 9.71e2 | 1 | 1 | TRINITY\_DN982\_c0\_g1\_i1.p1:TRINITY\_DN982\_c0\_g1\_i3.p1 |  |  | DB Search |
| IVNTDLQEIKDIIK | 58.4 | 1640.9348 | 14 | -6.31 | 821.4658 | 18.62 | 1.1514 | 1.1586 | 63065 | 0 | 0 | 0 | TRINITY\_DN3016\_c0\_g1\_i17.p1:TRINITY\_DN3016\_c0\_g1\_i11.p1:TRINITY\_DN3016\_c0\_g1\_i6.p1:TRINITY\_DN3016\_c0\_g1\_i10.p1:TRINITY\_DN3016\_c0\_g1\_i18.p1:TRINITY\_DN3016\_c0\_g1\_i8.p1:TRINITY\_DN3016\_c0\_g1\_i13.p1:TRINITY\_DN3016\_c0\_g1\_i2.p1:TRINITY\_DN3016\_c0\_g1\_i4.p1:TRINITY\_DN3016\_c0\_g1\_i5.p1 |  |  | DB Search |
| RNLDIERPTYTNLNR | 58.31 | 1873.9758 | 15 | 3.94 | 625.6655 | 14.03 | 0.8846 | 0.8919 | 38584 | 6.19e2 | 1 | 1 | TRINITY\_DN1933\_c0\_g1\_i8.p2:TRINITY\_DN471\_c0\_g1\_i3.p1:TRINITY\_DN1933\_c0\_g1\_i3.p2:TRINITY\_DN471\_c0\_g1\_i7.p1:TRINITY\_DN471\_c0\_g1\_i9.p1:TRINITY\_DN471\_c0\_g1\_i6.p1:TRINITY\_DN471\_c0\_g1\_i11.p1:TRINITY\_DN1933\_c0\_g1\_i4.p1:TRINITY\_DN250\_c0\_g1\_i6.p1:TRINITY\_DN1933\_c0\_g1\_i7.p1:TRINITY\_DN250\_c0\_g1\_i4.p1:TRINITY\_DN250\_c0\_g1\_i2.p1:TRINITY\_DN471\_c0\_g1\_i10.p1:TRINITY\_DN471\_c0\_g1\_i4.p1:TRINITY\_DN687\_c0\_g2\_i1.p1:TRINITY\_DN250\_c0\_g1\_i5.p1:TRINITY\_DN1933\_c0\_g1\_i1.p2:TRINITY\_DN250\_c0\_g1\_i1.p1:TRINITY\_DN471\_c0\_g1\_i5.p1:TRINITY\_DN250\_c0\_g1\_i3.p1:TRINITY\_DN471\_c0\_g1\_i2.p1 |  |  | DB Search |
| ITQQQSHQEIADQLR | 58.29 | 1793.902 | 15 | 4.14 | 897.9579 | 13.10 | 1.133 | 1.1402 | 33346 | 4.3e2 | 1 | 1 | TRINITY\_DN1377\_c0\_g1\_i1.p1 |  |  | DB Search |
| DLEHPNEFIR | 58.16 | 1268.6149 | 10 | -6.42 | 635.3078 | 13.50 | 0.9226 | 0.9299 | 35766 | 0 | 0 | 0 | TRINITY\_DN36471\_c0\_g1\_i1.p1 |  |  | DB Search |
| GKVEDGLESDFRK | 58.12 | 1478.7365 | 13 | -5.7 | 493.9144 | 13.65 | 0.7905 | 0.7978 | 36561 | 4.15e2 | 1 | 1 | TRINITY\_DN794\_c0\_g1\_i2.p1 |  |  | DB Search |
| DSYVGDEAQSKR | 58.12 | 1353.6161 | 12 | 4.44 | 677.8152 | 10.93 | 1.0065 | 1.0138 | 20214 | 8.61e3 | 1 | 1 | TRINITY\_DN2477\_c0\_g1\_i6.p1:TRINITY\_DN2477\_c0\_g1\_i5.p1:TRINITY\_DN195934\_c0\_g1\_i1.p2:TRINITY\_DN161\_c0\_g1\_i1.p1:TRINITY\_DN2477\_c0\_g1\_i9.p1:TRINITY\_DN2477\_c0\_g1\_i10.p1:TRINITY\_DN2477\_c0\_g1\_i2.p1:TRINITY\_DN161\_c0\_g1\_i3.p1:TRINITY\_DN161\_c0\_g1\_i2.p1 |  |  | DB Search |
| KAEQDLSDLRAQLAQALQR | 58.05 | 2153.1553 | 19 | -8.21 | 718.7166 | 18.93 | 0.9578 | 0.965 | 64971 | 0 | 0 | 0 | TRINITY\_DN428\_c0\_g1\_i1.p1 |  |  | DB Search |
| AQISKPAPHWEGK | 58.03 | 1447.7572 | 13 | 0.06 | 724.8826 | 11.86 | 1.0098 | 1.017 | 26141 | 0 | 0 | 0 | TRINITY\_DN332\_c0\_g1\_i1.p1 |  |  | DB Search |
| KLEITADGKNVLLNQK | 58.02 | 1783.0203 | 16 | 7.49 | 892.5201 | 14.87 | 1.1802 | 1.1873 | 43269 | 0 | 0 | 0 | TRINITY\_DN6390\_c0\_g1\_i1.p1 |  |  | DB Search |
| GLTHC(+57.02)NTLSYK | 58.02 | 1292.6183 | 11 | 2.78 | 647.3153 | 12.39 | 0.9743 | 0.9816 | 29201 | 4.95e3 | 1 | 1 | TRINITY\_DN902\_c1\_g2\_i4.p1:TRINITY\_DN902\_c1\_g2\_i3.p1:TRINITY\_DN902\_c1\_g2\_i1.p1 | Carbamidomethylation |  | DB Search |
| FEGVLAPNDKLK | 58.01 | 1329.7292 | 12 | 1.13 | 665.8696 | 14.75 | 1.0379 | 1.0452 | 42511 | 1.04e3 | 1 | 1 | TRINITY\_DN6008\_c0\_g1\_i1.p1 |  |  | DB Search |
| KLLEINVYHPLIQELQKR | 58.01 | 2233.2947 | 18 | 2.88 | 559.33 | 17.89 | 0.8668 | 0.8741 | 58878 | 0 | 0 | 0 | TRINITY\_DN5633\_c0\_g1\_i1.p1 |  |  | DB Search |
| RGTPLDQVIEFHIDEETLVKR | 58 | 2494.3179 | 21 | -2.48 | 624.5824 | 18.88 | 0.8465 | 0.8538 | 64643 | 2.32e2 | 1 | 1 | TRINITY\_DN804\_c0\_g1\_i2.p1:TRINITY\_DN804\_c0\_g1\_i1.p1 |  |  | DB Search |
| KGFIMVIEAIAGGGHK | 57.99 | 1626.8915 | 16 | -8.55 | 543.2974 | 19.95 | 0.855 | 0.8623 | 70831 | 0 | 0 | 0 | TRINITY\_DN66856\_c0\_g1\_i1.p1 |  |  | DB Search |
| S(+42.01)LVIPEKFQHILR | 57.91 | 1620.9352 | 13 | -0.14 | 541.3165 | 19.55 | 0.8408 | 0.8481 | 67997 | 1.96e3 | 1 | 1 | TRINITY\_DN1505\_c0\_g1\_i1.p1 | Acetylation (Protein N-term) | S1:Acetylation (Protein N-term):1000 | DB Search |
| ATDSDSGPNGDITYR | 57.88 | 1567.675 | 15 | -2.18 | 784.8395 | 12.74 | 1.0065 | 1.0138 | 31175 | 9.01e2 | 1 | 1 | TRINITY\_DN72625\_c0\_g1\_i1.p1 |  |  | DB Search |
| SC(+57.02)TNPAPANGGK | 57.85 | 1172.5244 | 12 | 2.11 | 587.2681 | 9.44 | 0.8502 | 0.8575 | 11479 | 9.79e2 | 1 | 1 | TRINITY\_DN495\_c0\_g1\_i2.p1:TRINITY\_DN3545\_c0\_g1\_i2.p1:TRINITY\_DN3545\_c0\_g1\_i1.p1:TRINITY\_DN495\_c0\_g1\_i3.p1 | Carbamidomethylation |  | DB Search |
| DVYDAEQAERK | 57.85 | 1322.6102 | 11 | -1.22 | 662.3086 | 11.56 | 0.9606 | 0.9679 | 24244 | 0 | 0 | 0 | TRINITY\_DN36\_c0\_g1\_i16.p1:TRINITY\_DN36\_c0\_g1\_i3.p1:TRINITY\_DN36\_c0\_g1\_i14.p1:TRINITY\_DN36\_c0\_g1\_i17.p1:TRINITY\_DN36\_c0\_g1\_i11.p1:TRINITY\_DN36\_c0\_g1\_i13.p1:TRINITY\_DN36\_c0\_g1\_i1.p1:TRINITY\_DN36\_c0\_g1\_i8.p1:TRINITY\_DN36\_c0\_g1\_i19.p1:TRINITY\_DN36\_c0\_g1\_i20.p1 |  |  | DB Search |
| YVAIYPSDHLTVNR | 57.84 | 1646.8417 | 14 | 0.01 | 824.4244 | 15.46 | 1.109 | 1.1162 | 46349 | 0 | 0 | 0 | TRINITY\_DN982\_c0\_g1\_i1.p1:TRINITY\_DN982\_c0\_g1\_i3.p1 |  |  | DB Search |
| KHLSELSEEAAK | 57.83 | 1340.6936 | 12 | 6.99 | 671.3557 | 11.03 | 0.9932 | 1.0005 | 20991 | 0 | 0 | 0 | TRINITY\_DN61707\_c0\_g1\_i1.p1 |  |  | DB Search |
| A(+42.01)HLSQEQRNELKK | 57.81 | 1621.8536 | 13 | -0.15 | 541.6226 | 11.20 | 0.8096 | 0.8169 | 22070 | 0 | 0 | 0 | TRINITY\_DN3052\_c0\_g1\_i1.p1 | Acetylation (Protein N-term) | A1:Acetylation (Protein N-term):1000 | DB Search |
| VVTSSTGPGEHMR | 57.8 | 1356.6456 | 13 | 6.33 | 679.3313 | 10.85 | 0.9832 | 0.9904 | 19601 | 1.53e3 | 1 | 1 | TRINITY\_DN73067\_c0\_g1\_i1.p1 |  |  | DB Search |
| DLYANTVLSGGTTM(+15.99)FPGIADR | 57.77 | 2214.0627 | 21 | 5.59 | 1108.0398 | 19.43 | 1.2368 | 1.2439 | 67881 | 0 | 0 | 0 | TRINITY\_DN161\_c0\_g1\_i1.p1:TRINITY\_DN161\_c0\_g1\_i4.p1:TRINITY\_DN161\_c0\_g1\_i3.p1:TRINITY\_DN161\_c0\_g1\_i2.p1 | Oxidation (M) | M14:Oxidation (M):1000 | DB Search |
| RTPNEVEFHQAVK | 57.72 | 1553.795 | 13 | 2.32 | 518.9378 | 12.05 | 0.8303 | 0.8376 | 27142 | 9.7e2 | 1 | 1 | TRINITY\_DN360\_c0\_g1\_i1.p1 |  |  | DB Search |
| HLSVNILQTNTR | 57.71 | 1394.7629 | 12 | -5.96 | 698.3815 | 15.19 | 1.0037 | 1.011 | 44915 | 0 | 0 | 0 | TRINITY\_DN6390\_c0\_g1\_i1.p1 |  |  | DB Search |
| GSC(+57.02)SC(+57.02)YGHAEQC(+57.02)IPTK | 57.58 | 1853.7495 | 16 | 0.37 | 927.8782 | 12.27 | 1.1865 | 1.1937 | 28455 | 2.04e2 | 1 | 1 | TRINITY\_DN15274\_c0\_g1\_i1.p1 | Carbamidomethylation, Carbamidomethylation, Carbamidomethylation |  | DB Search |
| YYVTIIDAPGHR | 57.57 | 1403.7197 | 12 | -0.98 | 702.8633 | 15.80 | 1.0427 | 1.05 | 48024 | 3.09e2 | 1 | 1 | TRINITY\_DN64\_c0\_g1\_i1.p1 |  |  | DB Search |
| FQVDQATGQITLIGM(+15.99)LNKR | 57.57 | 2148.136 | 19 | 2 | 717.0508 | 18.25 | 0.9892 | 0.9965 | 61008 | 0 | 0 | 0 | TRINITY\_DN72625\_c0\_g1\_i1.p1 | Oxidation (M) | M15:Oxidation (M):1000 | DB Search |
| GLKVDFDTTFAPVTGKK | 57.57 | 1822.9828 | 17 | 2.59 | 912.4969 | 17.04 | 1.1806 | 1.1877 | 54087 | 1.7e3 | 1 | 1 | TRINITY\_DN329\_c0\_g1\_i1.p1:TRINITY\_DN329\_c0\_g1\_i2.p1 |  |  | DB Search |
| IPIFSAAGLPHNEIAAQIC(+57.02)R | 57.56 | 2177.1416 | 20 | -0.83 | 726.7172 | 18.81 | 1.0053 | 1.0126 | 64123 | 2.43e3 | 2 | 2 | TRINITY\_DN3773\_c0\_g1\_i1.p1 | Carbamidomethylation |  | DB Search |
| KQM(+15.99)LM(+15.99)SVC(+57.02)TK | 57.55 | 1256.5927 | 10 | 17.76 | 629.312 | 10.42 | 0.9271 | 0.9344 | 17303 | 0 | 0 | 0 | TRINITY\_DN1377\_c0\_g1\_i1.p1 | Oxidation (M), Oxidation (M), Carbamidomethylation | M3:Oxidation (M):1000 M5:Oxidation (M):1000 | DB Search |
| ILQC(+57.02)NQYANVEEAR | 57.54 | 1706.8046 | 14 | 0.26 | 854.4059 | 14.18 | 1.0781 | 1.0853 | 39448 | 1.11e3 | 1 | 1 | TRINITY\_DN477\_c0\_g1\_i8.p1:TRINITY\_DN477\_c0\_g1\_i7.p1 | Carbamidomethylation |  | DB Search |
| TSTQDVALNIRR | 57.52 | 1372.7423 | 12 | 5.33 | 687.379 | 13.43 | 0.9989 | 1.0061 | 35260 | 2.7e3 | 1 | 1 | TRINITY\_DN982\_c0\_g1\_i1.p1:TRINITY\_DN982\_c0\_g1\_i3.p1 |  |  | DB Search |
| AKQEAFIAELEDQIAKGDK | 57.51 | 2103.0847 | 19 | 0.34 | 702.0326 | 19.38 | 0.9469 | 0.9541 | 67541 | 1.79e2 | 1 | 1 | TRINITY\_DN428\_c0\_g1\_i1.p1 |  |  | DB Search |
| AYAAFLEFKDVVK | 57.51 | 1499.8024 | 13 | 3.03 | 750.9073 | 19.00 | 1.0929 | 1.1001 | 65208 | 1.13e4 | 1 | 1 | TRINITY\_DN240\_c0\_g1\_i6.p1:TRINITY\_DN240\_c0\_g1\_i1.p1:TRINITY\_DN240\_c0\_g1\_i4.p1 |  |  | DB Search |
| TILQFEADRR | 57.46 | 1247.6622 | 10 | 3.34 | 624.8376 | 15.05 | 0.9525 | 0.9598 | 44052 | 2.23e3 | 1 | 1 | TRINITY\_DN58867\_c0\_g1\_i2.p1 |  |  | DB Search |
| LAIKENHHLNEPIEK | 57.45 | 1783.958 | 15 | -5.85 | 892.9771 | 12.12 | 1.139 | 1.1462 | 27632 | 0 | 0 | 0 | TRINITY\_DN5500\_c0\_g1\_i2.p1:TRINITY\_DN5500\_c0\_g1\_i1.p1 |  |  | DB Search |
| LDFDHEAKLNMIFR | 57.45 | 1747.8716 | 14 | 5.53 | 583.6317 | 18.31 | 0.8546 | 0.8619 | 61270 | 0 | 0 | 0 | TRINITY\_DN160\_c0\_g1\_i2.p1:TRINITY\_DN160\_c0\_g1\_i1.p1 |  |  | DB Search |
| VAPEEHPVLLTEAPLNPK | 57.43 | 1953.0571 | 18 | 1.47 | 977.5328 | 16.35 | 1.2073 | 1.2145 | 50611 | 1.52e2 | 1 | 1 | TRINITY\_DN2477\_c0\_g1\_i6.p1:TRINITY\_DN2477\_c0\_g1\_i5.p1:TRINITY\_DN2477\_c0\_g1\_i8.p1:TRINITY\_DN195934\_c0\_g1\_i1.p2:TRINITY\_DN161\_c0\_g1\_i1.p1:TRINITY\_DN2477\_c0\_g1\_i9.p1:TRINITY\_DN2477\_c0\_g1\_i10.p1:TRINITY\_DN2477\_c0\_g1\_i2.p1:TRINITY\_DN161\_c0\_g1\_i3.p1:TRINITY\_DN2941\_c0\_g1\_i4.p1:TRINITY\_DN161\_c0\_g1\_i2.p1 |  |  | DB Search |
| YTGAC(+57.02)NANGNTDK | 57.42 | 1384.5677 | 13 | 5.65 | 693.2919 | 9.57 | 0.9404 | 0.9477 | 12156 | 0 | 0 | 0 | TRINITY\_DN4595\_c0\_g1\_i10.p1:TRINITY\_DN4595\_c0\_g1\_i4.p1 | Carbamidomethylation |  | DB Search |
| AM(+15.99)TDC(+57.02)KETLC(+57.02)R | 57.42 | 1399.5894 | 11 | -3.04 | 700.7967 | 10.59 | 1.0025 | 1.0098 | 18169 | 7.88e2 | 1 | 1 | TRINITY\_DN168534\_c0\_g1\_i1.p1 | Oxidation (M), Carbamidomethylation, Carbamidomethylation | M2:Oxidation (M):1000 | DB Search |
| GLDVDSLVIDHIQVNR | 57.41 | 1791.9479 | 16 | 0.62 | 896.9777 | 18.98 | 1.1786 | 1.1857 | 65203 | 1.42e2 | 1 | 1 | TRINITY\_DN1614\_c0\_g1\_i1.p1 |  |  | DB Search |
| APTHILNPANKR | 57.39 | 1330.7469 | 12 | 0.47 | 666.3781 | 11.10 | 1.0102 | 1.0174 | 21164 | 1.88e3 | 2 | 2 | TRINITY\_DN1058\_c0\_g1\_i4.p1:TRINITY\_DN1058\_c0\_g1\_i1.p1:TRINITY\_DN1058\_c0\_g1\_i3.p1:TRINITY\_DN1058\_c0\_g1\_i2.p2 |  |  | DB Search |
| IGKPHTVPC(+57.02)K | 57.38 | 1135.6172 | 10 | 1.39 | 568.8141 | 9.77 | 0.9028 | 0.9101 | 13225 | 1.74e4 | 2 | 2 | TRINITY\_DN15584\_c0\_g1\_i1.p1 | Carbamidomethylation |  | DB Search |
| AKLSQC(+57.02)TAASGK | 57.38 | 1220.6183 | 12 | -0.12 | 611.3136 | 9.72 | 0.9602 | 0.9675 | 12907 | 2.21e4 | 1 | 1 | TRINITY\_DN3991\_c0\_g1\_i1.p1 | Carbamidomethylation |  | DB Search |
| VYLAQAEAEKIK | 57.34 | 1361.7554 | 12 | 1.23 | 681.8828 | 14.25 | 1.0335 | 1.0407 | 39833 | 4.3e2 | 1 | 1 | TRINITY\_DN11435\_c0\_g1\_i1.p1 |  |  | DB Search |
| SYELPDGQVITIGNER | 57.34 | 1789.8846 | 16 | 2.2 | 895.9475 | 18.50 | 0.9828 | 0.99 | 62421 | 0 | 0 | 0 | TRINITY\_DN161\_c0\_g1\_i1.p1:TRINITY\_DN161\_c0\_g1\_i4.p1:TRINITY\_DN161\_c0\_g1\_i3.p1:TRINITY\_DN161\_c0\_g1\_i2.p1 |  |  | DB Search |
| GQFSTDDLVAEVEKR | 57.33 | 1692.8318 | 15 | -0.29 | 847.4191 | 17.65 | 1.143 | 1.1502 | 57525 | 0 | 0 | 0 | TRINITY\_DN2113\_c0\_g1\_i1.p1:TRINITY\_DN2113\_c0\_g1\_i2.p1 |  |  | DB Search |
| VAHAGLTVAEFFR | 57.31 | 1416.7513 | 13 | 3.21 | 709.382 | 18.82 | 1.0287 | 1.0359 | 64099 | 6.52e2 | 1 | 1 | TRINITY\_DN11097\_c0\_g1\_i1.p1:TRINITY\_DN11097\_c0\_g2\_i1.p1 |  |  | DB Search |
| LGGIEVTQGNC(+57.02)AR | 57.3 | 1373.6721 | 13 | 3.87 | 687.8429 | 13.70 | 0.9642 | 0.9715 | 36854 | 7.25e2 | 1 | 1 | TRINITY\_DN36\_c0\_g1\_i9.p1:TRINITY\_DN36\_c0\_g1\_i14.p1:TRINITY\_DN36\_c0\_g1\_i13.p1:TRINITY\_DN36\_c0\_g1\_i2.p1:TRINITY\_DN36\_c0\_g1\_i16.p1:TRINITY\_DN36\_c0\_g1\_i3.p1:TRINITY\_DN36\_c0\_g1\_i17.p1:TRINITY\_DN36\_c0\_g1\_i4.p1:TRINITY\_DN36\_c0\_g1\_i11.p1:TRINITY\_DN36\_c0\_g1\_i1.p1:TRINITY\_DN36\_c0\_g1\_i8.p1:TRINITY\_DN36\_c0\_g1\_i19.p1:TRINITY\_DN36\_c0\_g1\_i5.p1:TRINITY\_DN36\_c0\_g1\_i20.p1 | Carbamidomethylation |  | DB Search |
| VQNEITNPNTQFPVNGLFDLHR | 57.3 | 2552.2771 | 22 | 1.04 | 851.7634 | 19.88 | 1.0644 | 1.0717 | 70362 | 3.51e2 | 1 | 1 | TRINITY\_DN72625\_c0\_g1\_i1.p1 |  |  | DB Search |
| FSQHTDYVIAR | 57.2 | 1335.6571 | 11 | 4.84 | 668.8361 | 13.42 | 0.9787 | 0.986 | 35261 | 3.02e2 | 1 | 1 | TRINITY\_DN121866\_c0\_g1\_i1.p1 |  |  | DB Search |
| AEYENKTEVAR | 57.19 | 1308.631 | 11 | -1.93 | 655.3185 | 10.52 | 0.9771 | 0.9844 | 17854 | 3.06e3 | 2 | 2 | TRINITY\_DN1280\_c0\_g1\_i1.p1 |  |  | DB Search |
| VVEKEAETER | 57.16 | 1188.5986 | 10 | 2.47 | 595.3054 | 9.57 | 0.9044 | 0.9117 | 12158 | 0 | 0 | 0 | TRINITY\_DN2636\_c0\_g1\_i2.p1:TRINITY\_DN2636\_c0\_g1\_i1.p1 |  |  | DB Search |
| TRIEDLETVISQTQDHR | 57.13 | 2040.0236 | 17 | 0.12 | 681.0121 | 17.33 | 0.9586 | 0.9658 | 55627 | 6.48e2 | 1 | 1 | TRINITY\_DN761\_c0\_g1\_i3.p1:TRINITY\_DN761\_c0\_g1\_i1.p1:TRINITY\_DN761\_c0\_g1\_i2.p1 |  |  | DB Search |
| VVAEC(+57.02)NNGGGR | 57.11 | 1131.509 | 11 | -7.52 | 566.755 | 9.43 | 0.8299 | 0.8372 | 11493 | 0 | 0 | 0 | TRINITY\_DN1616\_c0\_g1\_i3.p1:TRINITY\_DN1616\_c0\_g1\_i6.p1:TRINITY\_DN1616\_c0\_g1\_i7.p1 | Carbamidomethylation |  | DB Search |
| VLDVNDKQPVFVK | 57.11 | 1499.8347 | 13 | 0.01 | 750.9213 | 14.94 | 1.0668 | 1.0741 | 43674 | 0 | 0 | 0 | TRINITY\_DN982\_c0\_g1\_i1.p1:TRINITY\_DN982\_c0\_g1\_i2.p1 |  |  | DB Search |
| VVC(+57.02)STTSGSATGSK | 57.09 | 1340.6241 | 14 | -0.36 | 671.3161 | 9.67 | 0.9352 | 0.9424 | 12707 | 3.78e2 | 1 | 1 | TRINITY\_DN201860\_c0\_g1\_i1.p1 | Carbamidomethylation |  | DB Search |
| LIDSNLEKPEC(+57.02)K | 57.09 | 1444.7231 | 12 | -0.41 | 723.3653 | 12.85 | 1.0311 | 1.0383 | 31680 | 5.58e3 | 1 | 1 | TRINITY\_DN804\_c0\_g1\_i2.p1:TRINITY\_DN804\_c0\_g1\_i1.p1 | Carbamidomethylation |  | DB Search |
| KLSNSLEEC(+57.02)VEYSR | 57.08 | 1712.804 | 14 | 5.01 | 857.4097 | 14.52 | 1.0937 | 1.1009 | 41242 | 1.11e3 | 1 | 1 | TRINITY\_DN1306\_c0\_g1\_i1.p1 | Carbamidomethylation |  | DB Search |
| GTTSTAGTGPSFDHTK | 57.07 | 1563.7164 | 16 | 3.76 | 782.8649 | 11.35 | 1.0737 | 1.0809 | 22963 | 0 | 0 | 0 | TRINITY\_DN1936\_c0\_g2\_i1.p1 |  |  | DB Search |
| TPGEC(+57.02)C(+57.02)PQC(+57.02)K | 57.06 | 1235.4733 | 10 | 1.2 | 618.7419 | 10.00 | 0.8955 | 0.9028 | 14563 | 1.33e3 | 1 | 1 | TRINITY\_DN1369\_c0\_g1\_i1.p1 | Carbamidomethylation, Carbamidomethylation, Carbamidomethylation |  | DB Search |
| AKISC(+57.02)DQVLNEK | 57.04 | 1403.7079 | 12 | -1.91 | 702.8567 | 12.59 | 0.9977 | 1.0049 | 30431 | 8.96e2 | 1 | 1 | TRINITY\_DN1189\_c0\_g1\_i1.p1 | Carbamidomethylation |  | DB Search |
| M(+15.99)SATFIGNSTAIQELFKR | 57.02 | 2029.0303 | 18 | 1.03 | 677.3483 | 19.94 | 0.9327 | 0.94 | 70612 | 2.26e3 | 1 | 1 | TRINITY\_DN3986\_c0\_g1\_i1.p1 | Oxidation (M) | M1:Oxidation (M):1000 | DB Search |
| AEFRPNQEEEPILKR | 57.01 | 1854.9587 | 15 | -3.22 | 619.3221 | 13.71 | 0.8765 | 0.8838 | 36837 | 1.82e3 | 1 | 1 | TRINITY\_DN262\_c0\_g1\_i1.p1 |  |  | DB Search |
| YGINHITNLVESKR | 57 | 1642.879 | 14 | 0.23 | 548.6313 | 14.96 | 0.8189 | 0.8262 | 43669 | 7.22e2 | 1 | 1 | TRINITY\_DN146208\_c0\_g1\_i1.p1 |  |  | DB Search |
| EGFYVHEALAAGIVK | 57 | 1602.8406 | 15 | 2.24 | 802.4257 | 17.51 | 1.0885 | 1.0957 | 56580 | 1.12e3 | 1 | 1 | TRINITY\_DN602\_c0\_g1\_i4.p1:TRINITY\_DN602\_c0\_g1\_i3.p1:TRINITY\_DN602\_c0\_g1\_i1.p1 |  |  | DB Search |
| ALEHYSDIYDIKR | 57 | 1621.8099 | 13 | -0.58 | 541.6078 | 14.56 | 0.8218 | 0.8291 | 41503 | 9.72e2 | 1 | 1 | TRINITY\_DN2113\_c0\_g1\_i1.p1:TRINITY\_DN2113\_c0\_g1\_i2.p1 |  |  | DB Search |
| GC(+57.02)STC(+57.02)GC(+57.02)NGAGTNNGVK | 57 | 1712.6665 | 17 | 3.09 | 857.3393 | 9.83 | 1.0411 | 1.0484 | 13666 | 0 | 0 | 0 | TRINITY\_DN1616\_c0\_g1\_i3.p1:TRINITY\_DN1616\_c0\_g1\_i6.p1:TRINITY\_DN1616\_c0\_g1\_i2.p1:TRINITY\_DN1616\_c0\_g1\_i7.p1 | Carbamidomethylation, Carbamidomethylation, Carbamidomethylation |  | DB Search |
| AFNHLLQYGEPAIRR | 57 | 1783.9481 | 15 | -0.28 | 595.6538 | 15.67 | 0.8753 | 0.8826 | 47413 | 0 | 0 | 0 | TRINITY\_DN1243\_c0\_g1\_i1.p1 |  |  | DB Search |
| LGVM(+15.99)EDHSNR | 56.99 | 1172.5244 | 10 | 4.82 | 587.2697 | 10.32 | 0.9065 | 0.9137 | 16467 | 1.29e3 | 1 | 1 | TRINITY\_DN5633\_c0\_g1\_i1.p1 | Oxidation (M) | M4:Oxidation (M):1000 | DB Search |
| GQELTFKEFFKPSNFVR | 56.99 | 2073.0684 | 17 | 0.69 | 692.0274 | 19.20 | 0.9291 | 0.9364 | 66465 | 4.36e3 | 1 | 1 | TRINITY\_DN30329\_c0\_g1\_i1.p1:TRINITY\_DN30329\_c0\_g2\_i1.p2 |  |  | DB Search |
| VIIDYQTHHR | 56.99 | 1280.6626 | 10 | 2.06 | 641.337 | 12.04 | 0.9586 | 0.9658 | 27134 | 1.5e3 | 1 | 1 | TRINITY\_DN1907\_c0\_g1\_i1.p1:TRINITY\_DN1907\_c0\_g1\_i2.p1 |  |  | DB Search |
| AFQNTPC(+57.02)THLVLDGTR | 56.99 | 1828.889 | 16 | -0.28 | 915.4474 | 15.22 | 1.1738 | 1.181 | 44999 | 2.23e3 | 1 | 1 | TRINITY\_DN1091\_c0\_g1\_i1.p1 | Carbamidomethylation |  | DB Search |
| DYGVLLEDAGHTLR | 56.99 | 1557.7787 | 14 | 2.54 | 779.8951 | 18.20 | 1.129 | 1.1362 | 60565 | 5.51e3 | 1 | 1 | TRINITY\_DN332\_c0\_g1\_i1.p1 |  |  | DB Search |
| VITSDDPAEKEK | 56.98 | 1330.6616 | 12 | 1.22 | 666.3359 | 10.71 | 0.9844 | 0.9916 | 18867 | 1.51e3 | 1 | 1 | TRINITY\_DN1339\_c6\_g1\_i2.p1:TRINITY\_DN1339\_c6\_g1\_i1.p1:TRINITY\_DN6701\_c0\_g1\_i2.p2:TRINITY\_DN6701\_c0\_g1\_i1.p1:TRINITY\_DN1339\_c0\_g1\_i2.p1:TRINITY\_DN124769\_c0\_g1\_i1.p1:TRINITY\_DN1172\_c0\_g1\_i5.p1:TRINITY\_DN1172\_c0\_g1\_i3.p1:TRINITY\_DN1339\_c0\_g1\_i1.p1 |  |  | DB Search |
| VLTSDDPAEKEK | 56.98 | 1330.6616 | 12 | 1.22 | 666.3359 | 10.71 | 0.9844 | 0.9916 | 18867 | 1.51e3 | 1 | 1 | TRINITY\_DN24414\_c1\_g1\_i2.p2:TRINITY\_DN119673\_c0\_g1\_i1.p1 |  |  | DB Search |
| FVLHGDVSHGSR | 56.98 | 1309.6527 | 12 | -4.94 | 437.554 | 11.96 | 0.7547 | 0.762 | 26738 | 1.72e3 | 1 | 1 | TRINITY\_DN22\_c0\_g1\_i1.p1:TRINITY\_DN384\_c0\_g1\_i2.p1:TRINITY\_DN384\_c0\_g1\_i1.p1 |  |  | DB Search |
| VTRPHGASGVVR | 56.98 | 1234.6895 | 12 | 3.31 | 412.5699 | 10.06 | 0.8205 | 0.8279 | 15006 | 1.06e3 | 1 | 1 | TRINITY\_DN2885\_c0\_g1\_i2.p1:TRINITY\_DN2885\_c0\_g1\_i4.p1:TRINITY\_DN2885\_c0\_g1\_i5.p1 |  |  | DB Search |
| ALQDEDEDAFKR | 56.97 | 1435.658 | 12 | 4.63 | 718.8363 | 13.14 | 0.9953 | 1.0025 | 33551 | 3.78e2 | 1 | 1 | TRINITY\_DN116\_c0\_g1\_i1.p1 |  |  | DB Search |
| TIEAEAAHGTVTR | 56.96 | 1354.6841 | 13 | 1.27 | 678.3471 | 11.79 | 1.0114 | 1.0186 | 25577 | 1.08e3 | 1 | 1 | TRINITY\_DN7504\_c0\_g1\_i1.p1 |  |  | DB Search |
| TVTC(+57.02)GAGETIKVTK | 56.96 | 1463.7654 | 14 | 3.77 | 732.8894 | 12.26 | 1.0636 | 1.0709 | 28487 | 0 | 0 | 0 | TRINITY\_DN374\_c0\_g1\_i1.p1 | Carbamidomethylation |  | DB Search |
| DPINNHWDGNLINWR | 56.96 | 1862.8812 | 15 | -1.81 | 621.9637 | 18.88 | 0.8116 | 0.8189 | 64604 | 4.88e2 | 1 | 1 | TRINITY\_DN138\_c0\_g1\_i1.p1 |  |  | DB Search |
| A(+42.01)NPLKDIISR | 56.95 | 1167.6611 | 10 | -3.36 | 584.8333 | 18.54 | 0.9194 | 0.9267 | 62425 | 2.03e3 | 1 | 1 | TRINITY\_DN163801\_c0\_g1\_i1.p1 | Acetylation (Protein N-term) | A1:Acetylation (Protein N-term):1000 | DB Search |
| AMTDC(+57.02)KETLC(+57.02)R | 56.95 | 1383.5945 | 11 | 0.26 | 692.8016 | 11.61 | 0.9932 | 1.0005 | 24582 | 0 | 0 | 0 | TRINITY\_DN168534\_c0\_g1\_i1.p1 | Carbamidomethylation, Carbamidomethylation |  | DB Search |
| YLTTAVITNKR | 56.94 | 1278.7296 | 11 | -2.91 | 640.3673 | 13.80 | 0.9981 | 1.0053 | 37364 | 1.15e3 | 1 | 1 | TRINITY\_DN923\_c0\_g1\_i1.p1 |  |  | DB Search |
| ASGDYATVVSHNR | 56.94 | 1375.6481 | 13 | 1.16 | 688.829 | 12.04 | 0.9981 | 1.0053 | 27131 | 7.37e2 | 1 | 1 | TRINITY\_DN925\_c0\_g1\_i1.p1 |  |  | DB Search |
| DLNKEYISHLFK | 56.93 | 1505.7878 | 12 | -5.85 | 502.9313 | 17.25 | 0.8019 | 0.8092 | 55142 | 6.25e2 | 1 | 1 | TRINITY\_DN4377\_c0\_g1\_i1.p1 |  |  | DB Search |
| AFIDSSVALLRK | 56.93 | 1318.7609 | 12 | 2.94 | 660.3867 | 17.45 | 1.0331 | 1.0403 | 56355 | 4.78e2 | 1 | 1 | TRINITY\_DN67\_c0\_g1\_i2.p1 |  |  | DB Search |
| VVASSHTDYELR | 56.92 | 1375.6732 | 12 | -1.15 | 688.84 | 12.48 | 1.0106 | 1.0178 | 29825 | 0 | 0 | 0 | TRINITY\_DN95248\_c0\_g1\_i1.p1 |  |  | DB Search |
| DANAAVVHGSHLK | 56.92 | 1317.679 | 13 | 3.24 | 659.8459 | 10.89 | 1.0182 | 1.0255 | 20111 | 2.14e3 | 1 | 1 | TRINITY\_DN262\_c0\_g1\_i1.p1 |  |  | DB Search |
| TKIENIEDVFK | 56.92 | 1334.7081 | 11 | 5.12 | 668.3618 | 17.34 | 1.0162 | 1.0234 | 55718 | 0 | 0 | 0 | TRINITY\_DN1091\_c0\_g1\_i1.p1 |  |  | DB Search |
| IC(+57.02)HLLGIQVTDFTK | 56.92 | 1643.8705 | 14 | -7.46 | 822.9327 | 18.41 | 1.0821 | 1.0893 | 61877 | 0 | 0 | 0 | TRINITY\_DN428\_c0\_g1\_i1.p1 | Carbamidomethylation |  | DB Search |
| EITLNYHDTVVHMK | 56.91 | 1698.84 | 14 | 2.52 | 850.4255 | 15.24 | 1.1106 | 1.1178 | 45119 | 7.14e2 | 1 | 1 | TRINITY\_DN1369\_c0\_g1\_i1.p1 |  |  | DB Search |
| TQITC(+57.02)TQISLKR | 56.91 | 1447.7817 | 12 | 1.07 | 724.8956 | 13.77 | 1.025 | 1.0323 | 37211 | 1.01e3 | 1 | 1 | TRINITY\_DN166344\_c0\_g1\_i1.p1 | Carbamidomethylation |  | DB Search |
| LAYELQANKEQQR | 56.91 | 1589.8162 | 13 | -0.02 | 795.9117 | 12.87 | 1.0516 | 1.0588 | 32046 | 6.54e2 | 1 | 1 | TRINITY\_DN11435\_c0\_g1\_i1.p1 |  |  | DB Search |
| LALHVLNNEDIIGHR | 56.91 | 1712.9321 | 15 | 5.27 | 857.474 | 16.25 | 1.1342 | 1.1414 | 50212 | 3.38e2 | 1 | 1 | TRINITY\_DN140702\_c0\_g1\_i1.p1 |  |  | DB Search |
| AFVAIGDYGGHVGLGVK | 56.9 | 1658.8779 | 17 | 4.03 | 830.4459 | 17.59 | 1.1614 | 1.1686 | 56871 | 7.56e3 | 1 | 1 | TRINITY\_DN15584\_c0\_g1\_i1.p1 |  |  | DB Search |
| LADLEEFISSPNHANISQVGER | 56.9 | 2425.1873 | 22 | -1.72 | 809.398 | 18.72 | 0.9263 | 0.9335 | 63624 | 2.17e3 | 1 | 1 | TRINITY\_DN2113\_c0\_g1\_i1.p1:TRINITY\_DN2113\_c0\_g1\_i2.p1 |  |  | DB Search |
| LIEDTEYTAHEGAK | 56.89 | 1575.7416 | 14 | -1.89 | 788.873 | 12.64 | 1.0781 | 1.0853 | 30716 | 0 | 0 | 0 | TRINITY\_DN12632\_c1\_g1\_i1.p1 |  |  | DB Search |
| VLATDKDTLSSNK | 56.89 | 1390.7303 | 13 | 5.55 | 696.3732 | 11.48 | 1.015 | 1.0222 | 23698 | 1.47e3 | 1 | 1 | TRINITY\_DN982\_c0\_g1\_i3.p1 |  |  | DB Search |
| AIVENLENAKDGLK | 56.89 | 1512.8147 | 14 | -0.85 | 757.4106 | 15.10 | 1.1082 | 1.1154 | 44479 | 0 | 0 | 0 | TRINITY\_DN1189\_c0\_g1\_i1.p1 |  |  | DB Search |
| KAENFAELKNTFQEAAK | 56.89 | 1937.9846 | 17 | 0.51 | 646.9996 | 16.04 | 0.9174 | 0.9247 | 49239 | 7.51e2 | 1 | 1 | TRINITY\_DN79\_c0\_g1\_i1.p1 |  |  | DB Search |
| ASFVSDIDRLR | 56.88 | 1277.6727 | 11 | 2.94 | 639.8427 | 16.84 | 0.9824 | 0.9896 | 52937 | 5.14e3 | 1 | 1 | TRINITY\_DN598\_c1\_g1\_i1.p1 |  |  | DB Search |
| GDYC(+57.02)NSALIEAEQKER | 56.88 | 1881.8527 | 16 | 6.06 | 941.9351 | 14.33 | 1.2069 | 1.2141 | 40232 | 7.6e2 | 1 | 1 | TRINITY\_DN168534\_c0\_g1\_i1.p1 | Carbamidomethylation |  | DB Search |
| AFLIEEQKLVVR | 56.88 | 1443.8448 | 12 | 4.29 | 722.9296 | 17.30 | 1.0464 | 1.0536 | 55418 | 7.04e2 | 1 | 1 | TRINITY\_DN79126\_c0\_g1\_i1.p1 |  |  | DB Search |
| ATGNTSGGVC(+57.02)DDC(+57.02)NHNTAGR | 56.88 | 2062.8181 | 20 | -2.27 | 688.6086 | 9.97 | 0.8623 | 0.8696 | 14440 | 0 | 0 | 0 | TRINITY\_DN15274\_c0\_g1\_i1.p1 | Carbamidomethylation, Carbamidomethylation |  | DB Search |
| DKNILVLTLGVGPQVNR | 56.87 | 1835.0629 | 17 | 6.58 | 918.5406 | 19.30 | 1.1358 | 1.143 | 67105 | 2.01e3 | 1 | 1 | TRINITY\_DN477\_c0\_g1\_i8.p1:TRINITY\_DN477\_c0\_g1\_i7.p1 |  |  | DB Search |
| SFHFGVIKLEGK | 56.87 | 1360.7502 | 12 | 0.52 | 681.3797 | 16.93 | 1.0271 | 1.0343 | 53439 | 1.06e3 | 1 | 1 | TRINITY\_DN329\_c0\_g1\_i1.p1:TRINITY\_DN329\_c0\_g1\_i2.p1 |  |  | DB Search |
| ATGC(+57.02)EVIWNQHNR | 56.87 | 1583.7263 | 13 | 1.97 | 792.8684 | 13.82 | 1.0773 | 1.0845 | 37415 | 6.5e2 | 1 | 1 | TRINITY\_DN2217\_c0\_g1\_i3.p1:TRINITY\_DN2217\_c0\_g1\_i2.p1 | Carbamidomethylation |  | DB Search |
| IVNAEWNILYEKLEK | 56.86 | 1860.9985 | 15 | 2.21 | 621.3387 | 19.45 | 0.8972 | 0.9044 | 67893 | 2.46e2 | 1 | 1 | TRINITY\_DN1194\_c0\_g1\_i1.p1 |  |  | DB Search |
| VSSAITHPQWNR | 56.85 | 1394.7054 | 12 | 5.44 | 698.3607 | 13.49 | 1.0106 | 1.0178 | 35612 | 4.48e2 | 1 | 1 | TRINITY\_DN22\_c0\_g1\_i1.p1 |  |  | DB Search |
| KIPDEDYIEKFWK | 56.85 | 1709.8665 | 13 | -1.13 | 570.9595 | 18.50 | 0.8587 | 0.866 | 62447 | 0 | 0 | 0 | TRINITY\_DN5633\_c0\_g1\_i1.p1 |  |  | DB Search |
| ATETATGLATTHPIK | 56.85 | 1510.7991 | 15 | 5.62 | 756.4077 | 12.87 | 1.1049 | 1.1122 | 31961 | 6.84e3 | 1 | 1 | TRINITY\_DN165536\_c0\_g1\_i1.p1 |  |  | DB Search |
| ALELDALSNSHPIEVK | 56.84 | 1734.9152 | 16 | -3.95 | 868.4575 | 17.15 | 1.1706 | 1.1778 | 54576 | 2.79e2 | 1 | 1 | TRINITY\_DN5153\_c0\_g1\_i1.p1 |  |  | DB Search |
| S(+42.01)SGEIESLRQEAENLR | 56.84 | 1858.9021 | 16 | 0.19 | 930.4543 | 18.71 | 1.1949 | 1.2021 | 63473 | 1.62e3 | 1 | 1 | TRINITY\_DN2472\_c0\_g1\_i1.p1 | Acetylation (Protein N-term) | S1:Acetylation (Protein N-term):1000 | DB Search |
| ASFVTDIDRLR | 56.83 | 1291.6885 | 11 | 0.01 | 646.8486 | 16.97 | 0.9856 | 0.9928 | 53571 | 3.56e3 | 1 | 1 | TRINITY\_DN598\_c1\_g1\_i2.p1 |  |  | DB Search |
| TGAISLLASVDFETDRTHTAVVK | 56.8 | 2430.2754 | 23 | -2.04 | 608.5721 | 18.79 | 0.8858 | 0.8931 | 64106 | 2.23e2 | 1 | 1 | TRINITY\_DN982\_c0\_g1\_i3.p1 |  |  | DB Search |
| IPLDDFQDPSLDLENAHNK | 56.78 | 2180.0386 | 19 | 11.13 | 727.6916 | 17.94 | 0.9812 | 0.9884 | 59154 | 0 | 0 | 0 | TRINITY\_DN22594\_c0\_g1\_i1.p2 |  |  | DB Search |
| VFLNKPLDFERENQIR | 56.77 | 2017.0745 | 16 | 0.68 | 673.3629 | 16.28 | 0.9424 | 0.9497 | 50325 | 2.51e2 | 1 | 1 | TRINITY\_DN141214\_c0\_g1\_i1.p1 |  |  | DB Search |
| VNQGEKEFLQRPC(+57.02)R | 56.68 | 1759.8788 | 14 | 1.84 | 587.632 | 12.53 | 0.8619 | 0.8692 | 29925 | 1.89e3 | 1 | 1 | TRINITY\_DN4595\_c0\_g1\_i10.p1:TRINITY\_DN4595\_c0\_g1\_i4.p1 | Carbamidomethylation |  | DB Search |
| AQAQAANLEKK | 56.67 | 1170.6356 | 11 | -5.65 | 586.3192 | 9.93 | 0.9412 | 0.9485 | 14279 | 0 | 0 | 0 | TRINITY\_DN3034\_c0\_g1\_i1.p1 |  |  | DB Search |
| SGGQC(+57.02)PC(+57.02)KPGVVGR | 56.63 | 1457.6868 | 14 | -1.09 | 729.8466 | 10.68 | 0.9808 | 0.988 | 18843 | 0 | 0 | 0 | TRINITY\_DN15274\_c0\_g1\_i1.p1 | Carbamidomethylation, Carbamidomethylation |  | DB Search |
| HGQVDSC(+57.02)INQNLKK | 56.63 | 1639.8101 | 14 | 3.14 | 547.6099 | 11.94 | 0.8376 | 0.8449 | 26542 | 1.21e3 | 1 | 1 | TRINITY\_DN3991\_c0\_g1\_i1.p1 | Carbamidomethylation |  | DB Search |
| LRPGVELTMSSLVNAK | 56.62 | 1713.9447 | 16 | 1.22 | 857.9768 | 17.67 | 1.1442 | 1.1514 | 57502 | 1.12e3 | 1 | 1 | TRINITY\_DN329\_c0\_g1\_i1.p1:TRINITY\_DN329\_c0\_g1\_i2.p1 |  |  | DB Search |
| APIGQSIVTVSAR | 56.61 | 1297.7354 | 13 | -0.69 | 649.8716 | 14.13 | 0.9646 | 0.9719 | 39211 | 0 | 0 | 0 | TRINITY\_DN982\_c0\_g1\_i3.p1 |  |  | DB Search |
| GYSFTTTAEREIVR | 56.59 | 1628.8158 | 14 | 3.67 | 815.4145 | 15.95 | 1.119 | 1.1262 | 48654 | 3.46e3 | 1 | 1 | TRINITY\_DN2477\_c0\_g1\_i6.p1:TRINITY\_DN2477\_c0\_g1\_i5.p1:TRINITY\_DN2477\_c0\_g1\_i8.p1:TRINITY\_DN2477\_c0\_g1\_i7.p1:TRINITY\_DN161\_c0\_g1\_i1.p1:TRINITY\_DN2477\_c0\_g1\_i9.p1:TRINITY\_DN2477\_c0\_g1\_i10.p1:TRINITY\_DN161\_c0\_g1\_i4.p1:TRINITY\_DN2477\_c0\_g1\_i2.p1:TRINITY\_DN161\_c0\_g1\_i3.p1:TRINITY\_DN161\_c0\_g1\_i2.p1 |  |  | DB Search |
| AVFPSIVGRPR | 56.57 | 1197.6982 | 11 | 1.58 | 599.8546 | 15.61 | 0.9578 | 0.965 | 46597 | 1.24e4 | 1 | 1 | TRINITY\_DN161\_c0\_g1\_i1.p1:TRINITY\_DN161\_c0\_g1\_i3.p1:TRINITY\_DN161\_c0\_g1\_i2.p1 |  |  | DB Search |
| VSSTVLSHIVILTR | 56.56 | 1523.9034 | 14 | 2.3 | 762.9573 | 19.04 | 1.1009 | 1.1082 | 65540 | 0 | 0 | 0 | TRINITY\_DN3347\_c0\_g1\_i1.p1 |  |  | DB Search |
| IPLDDFQDPTLTLENAHNK | 56.49 | 2180.075 | 19 | 0.23 | 727.6958 | 18.17 | 0.9876 | 0.9949 | 60474 | 1.67e3 | 1 | 1 | TRINITY\_DN13856\_c0\_g1\_i1.p2:TRINITY\_DN13856\_c0\_g1\_i2.p2 |  |  | DB Search |
| DM(+15.99)GVNVISVGAGHTINHR | 56.49 | 1891.9323 | 18 | 4.31 | 946.9732 | 14.15 | 1.2252 | 1.2324 | 39259 | 0 | 0 | 0 | TRINITY\_DN138\_c0\_g1\_i1.p1 | Oxidation (M) | M2:Oxidation (M):1000 | DB Search |
| GFDYC(+57.02)TTIKYR | 56.39 | 1422.6602 | 11 | -1.35 | 712.3332 | 15.06 | 1.0234 | 1.0307 | 44297 | 0 | 0 | 0 | TRINITY\_DN36\_c0\_g1\_i13.p1:TRINITY\_DN36\_c0\_g1\_i19.p1:TRINITY\_DN36\_c0\_g1\_i5.p1:TRINITY\_DN36\_c0\_g1\_i20.p1 | Carbamidomethylation |  | DB Search |
| LVQNTPHNSIVTK | 56.38 | 1449.7939 | 13 | 0.62 | 725.9014 | 11.65 | 1.0604 | 1.0676 | 24679 | 2.98e3 | 1 | 1 | TRINITY\_DN982\_c0\_g1\_i1.p1:TRINITY\_DN982\_c0\_g1\_i3.p1 |  |  | DB Search |
| FFGSLDQIKFEDQK | 56.37 | 1700.8409 | 14 | 7.65 | 851.4304 | 18.08 | 1.119 | 1.1262 | 59913 | 0 | 0 | 0 | TRINITY\_DN5554\_c0\_g1\_i1.p1 |  |  | DB Search |
| LRLDFDHEAKLNMIFR | 56.36 | 2017.0568 | 16 | 0.28 | 505.2693 | 18.45 | 0.8631 | 0.8704 | 61991 | 1.37e3 | 1 | 1 | TRINITY\_DN160\_c0\_g1\_i2.p1:TRINITY\_DN160\_c0\_g1\_i1.p1 |  |  | DB Search |
| DNIQGITKPAIR | 56.36 | 1324.7462 | 12 | -2.36 | 663.3759 | 13.73 | 0.9808 | 0.988 | 37017 | 0 | 0 | 0 | TRINITY\_DN172571\_c0\_g1\_i1.p1 |  |  | DB Search |
| IKAEYENKTEVAR | 56.36 | 1549.8099 | 13 | 5.17 | 775.9128 | 10.97 | 1.0556 | 1.0628 | 20636 | 7.1e2 | 1 | 1 | TRINITY\_DN1280\_c0\_g1\_i1.p1 |  |  | DB Search |
| SFASSLLDHFRVEPAK | 56.35 | 1802.9315 | 16 | 4.31 | 601.9843 | 19.80 | 0.9129 | 0.9202 | 69803 | 5.16e2 | 1 | 1 | TRINITY\_DN77\_c1\_g1\_i1.p1 |  |  | DB Search |
| LYSTDPNKFYR | 56.35 | 1402.6881 | 11 | 0.24 | 702.3483 | 14.17 | 1.0299 | 1.0371 | 39269 | 1.56e3 | 1 | 1 | TRINITY\_DN72625\_c0\_g1\_i1.p1 |  |  | DB Search |
| AEELRSEVNGIKR | 56.34 | 1499.8055 | 13 | 2.46 | 500.9414 | 12.22 | 0.7949 | 0.8023 | 28200 | 7.33e2 | 1 | 1 | TRINITY\_DN5229\_c0\_g1\_i1.p1 |  |  | DB Search |
| A(+42.01)EREQLIQQAK | 56.32 | 1354.7205 | 11 | -0.73 | 678.364 | 13.58 | 0.9804 | 0.9876 | 36161 | 3.25e3 | 1 | 1 | TRINITY\_DN1121\_c0\_g1\_i1.p1 | Acetylation (Protein N-term) | A1:Acetylation (Protein N-term):1000 | DB Search |
| SAYDIEVNTKK | 56.29 | 1266.6455 | 11 | -2.87 | 634.3254 | 12.81 | 0.9703 | 0.9775 | 31723 | 0 | 0 | 0 | TRINITY\_DN1280\_c0\_g1\_i1.p1 |  |  | DB Search |
| LVDRYC(+57.02)VC(+57.02)HLATGDLLR | 56.28 | 2060.0295 | 17 | -0.91 | 687.6801 | 16.66 | 0.9243 | 0.9315 | 52195 | 0 | 0 | 0 | TRINITY\_DN804\_c0\_g1\_i2.p1:TRINITY\_DN804\_c0\_g1\_i1.p1 | Carbamidomethylation, Carbamidomethylation |  | DB Search |
| IKLEHLITELVHQR | 56.23 | 1728.0046 | 14 | 4.73 | 433.0085 | 18.32 | 0.7823 | 0.7897 | 61379 | 6.43e2 | 1 | 1 | TRINITY\_DN1566\_c0\_g1\_i1.p1 |  |  | DB Search |
| LPNFFQYAGHIK | 56.19 | 1433.7455 | 12 | -9.53 | 478.9157 | 19.09 | 0.7522 | 0.7595 | 65828 | 0 | 0 | 0 | TRINITY\_DN1199\_c0\_g1\_i1.p1:TRINITY\_DN1199\_c0\_g1\_i2.p1 |  |  | DB Search |
| DANSM(+15.99)PTQSR | 56.18 | 1121.4772 | 10 | -2.33 | 561.742 | 8.84 | 0.823 | 0.8303 | 9491 | 0 | 0 | 0 | TRINITY\_DN477\_c0\_g1\_i9.p1 | Oxidation (M) | M5:Oxidation (M):1000 | DB Search |
| DLQGETSGSFKR | 56.14 | 1323.6418 | 12 | 3.37 | 662.8275 | 12.57 | 0.9654 | 0.9727 | 30296 | 2.34e3 | 1 | 1 | TRINITY\_DN3190\_c1\_g1\_i1.p1 |  |  | DB Search |
| YC(+57.02)ILSFLDKTDK | 56.07 | 1501.7487 | 12 | -0.86 | 501.5875 | 19.62 | 0.8006 | 0.8079 | 68865 | 2.16e2 | 1 | 1 | TRINITY\_DN15274\_c0\_g1\_i1.p1 | Carbamidomethylation |  | DB Search |
| EGSTPC(+57.02)NTQAC(+57.02)GSGLSEER | 56.04 | 2038.832 | 19 | 2.83 | 1020.4216 | 12.00 | 1.1366 | 1.1438 | 26927 | 0 | 0 | 0 | TRINITY\_DN5397\_c0\_g1\_i1.p1 | Carbamidomethylation, Carbamidomethylation |  | DB Search |
| DTLVFEIDEIRK | 55.99 | 1476.7823 | 12 | -10.15 | 739.3876 | 18.39 | 1.0323 | 1.0395 | 62006 | 2.85e3 | 1 | 1 | TRINITY\_DN31399\_c0\_g1\_i3.p1:TRINITY\_DN31399\_c0\_g1\_i2.p1 |  |  | DB Search |
| ETC(+57.02)SDIEKR | 55.98 | 1136.5132 | 9 | 4.61 | 569.2639 | 10.09 | 0.8915 | 0.8988 | 15200 | 4.92e3 | 1 | 1 | TRINITY\_DN118\_c1\_g1\_i3.p1:TRINITY\_DN118\_c1\_g1\_i5.p1 | Carbamidomethylation |  | DB Search |
| TQAVLDRLNIEK | 55.98 | 1398.7831 | 12 | 1.47 | 700.3967 | 15.27 | 1.0073 | 1.0146 | 45303 | 1.02e3 | 1 | 1 | TRINITY\_DN477\_c0\_g1\_i9.p1 |  |  | DB Search |
| TIESEAAHGTVTR | 55.93 | 1370.679 | 13 | 4.48 | 686.3467 | 11.86 | 1.0186 | 1.0259 | 26093 | 0 | 0 | 0 | TRINITY\_DN3271\_c0\_g1\_i1.p1:TRINITY\_DN3271\_c0\_g1\_i2.p1 |  |  | DB Search |
| DLHIIEDYVLK | 55.93 | 1356.7289 | 11 | -2.97 | 679.3666 | 19.52 | 0.9949 | 1.0021 | 68196 | 1.97e3 | 1 | 1 | TRINITY\_DN8903\_c0\_g1\_i1.p1 |  |  | DB Search |
| SIGFIGHYLDQLR | 55.88 | 1517.7991 | 13 | -0.14 | 759.9033 | 19.91 | 1.0849 | 1.0921 | 70505 | 4.59e2 | 1 | 1 | TRINITY\_DN8101\_c0\_g1\_i9.p2:TRINITY\_DN8101\_c0\_g1\_i10.p2:TRINITY\_DN8101\_c0\_g1\_i8.p1:TRINITY\_DN8101\_c0\_g1\_i4.p1:TRINITY\_DN8101\_c0\_g1\_i2.p2:TRINITY\_DN8101\_c0\_g1\_i5.p1:TRINITY\_DN8101\_c0\_g1\_i6.p2:TRINITY\_DN8101\_c0\_g1\_i11.p2 |  |  | DB Search |
| ITC(+57.02)LEM(+15.99)SEHVGIKR | 55.86 | 1687.8385 | 14 | -0.48 | 563.6173 | 12.78 | 0.8201 | 0.8274 | 31529 | 0 | 0 | 0 | TRINITY\_DN278\_c0\_g1\_i1.p1 | Carbamidomethylation, Oxidation (M) | M6:Oxidation (M):1000 | DB Search |
| VIM(+15.99)VTGDHPITAK | 55.82 | 1396.7384 | 13 | 1.26 | 699.3742 | 13.09 | 1.0242 | 1.0315 | 33301 | 1.21e3 | 1 | 1 | TRINITY\_DN262\_c0\_g1\_i1.p1 | Oxidation (M) | M3:Oxidation (M):1000 | DB Search |
| VDFDTTFAPVTGKK | 55.8 | 1524.7823 | 14 | 6.57 | 763.4 | 17.07 | 1.0512 | 1.0584 | 54189 | 6.56e2 | 1 | 1 | TRINITY\_DN329\_c0\_g1\_i1.p1:TRINITY\_DN329\_c0\_g1\_i2.p1 |  |  | DB Search |
| GLFIIDDKGVLR | 55.78 | 1344.7765 | 12 | 1.23 | 673.3933 | 18.64 | 0.9957 | 1.0029 | 63210 | 5.13e3 | 1 | 1 | TRINITY\_DN332\_c0\_g1\_i1.p1 |  |  | DB Search |
| VLLSQQIFYDKK | 55.75 | 1480.829 | 12 | -2.98 | 741.4162 | 16.65 | 1.0588 | 1.066 | 52089 | 1.28e3 | 1 | 1 | TRINITY\_DN982\_c0\_g1\_i1.p1:TRINITY\_DN982\_c0\_g1\_i3.p1:TRINITY\_DN982\_c0\_g1\_i2.p1 |  |  | DB Search |
| DNAGTSSSGTGPR | 55.71 | 1205.5272 | 13 | 3.47 | 603.7703 | 9.21 | 0.8615 | 0.8688 | 10492 | 4.49e2 | 1 | 1 | TRINITY\_DN4781\_c0\_g1\_i3.p3 |  |  | DB Search |
| SISC(+57.02)SKDEEIK | 55.69 | 1294.6074 | 11 | -1.48 | 648.3071 | 10.83 | 0.9779 | 0.9852 | 19797 | 0 | 0 | 0 | TRINITY\_DN495\_c0\_g1\_i5.p1:TRINITY\_DN495\_c0\_g1\_i6.p1 | Carbamidomethylation |  | DB Search |
| TNYNERFDEIRR | 55.67 | 1611.7754 | 12 | -4.19 | 538.261 | 13.21 | 0.7913 | 0.7986 | 33943 | 1.06e3 | 1 | 1 | TRINITY\_DN146208\_c0\_g1\_i1.p1 |  |  | DB Search |
| AEVIREEEEEKEK | 55.65 | 1616.7893 | 13 | 1.06 | 539.9352 | 10.70 | 0.7986 | 0.8059 | 18941 | 1.06e3 | 1 | 1 | TRINITY\_DN22219\_c0\_g1\_i1.p1 |  |  | DB Search |
| EVNTELDSRPETK | 55.62 | 1516.7369 | 13 | -0.06 | 759.3723 | 11.70 | 1.0259 | 1.0331 | 25124 | 0 | 0 | 0 | TRINITY\_DN11643\_c0\_g1\_i1.p1 |  |  | DB Search |
| GLIAQAIDHAASR | 55.56 | 1321.7102 | 13 | 5.69 | 661.8632 | 16.04 | 1.0086 | 1.0158 | 49202 | 0 | 0 | 0 | TRINITY\_DN1143\_c0\_g1\_i1.p1 |  |  | DB Search |
| VEVEDEKGLLAFK | 55.55 | 1475.7871 | 13 | -4.27 | 738.8943 | 16.73 | 1.0564 | 1.0636 | 52524 | 0 | 0 | 0 | TRINITY\_DN730\_c0\_g2\_i3.p1:TRINITY\_DN730\_c0\_g2\_i2.p1 |  |  | DB Search |
| DLPQEKLDDILKYHQEIVFAR | 55.53 | 2569.354 | 21 | 3.37 | 643.345 | 19.21 | 0.8955 | 0.9028 | 66502 | 4.18e2 | 1 | 1 | TRINITY\_DN262\_c0\_g1\_i1.p1 |  |  | DB Search |
| KGFFSSESKDLISYLPR | 55.5 | 1973.0258 | 17 | -8.91 | 658.6737 | 18.94 | 0.9121 | 0.9194 | 65041 | 2.85e2 | 1 | 1 | TRINITY\_DN1761\_c0\_g1\_i1.p1 |  |  | DB Search |
| LSNRPLYM(+15.99)PAEGK | 55.5 | 1490.7551 | 13 | 4.01 | 746.3845 | 12.63 | 1.0279 | 1.0351 | 30580 | 4.63e2 | 1 | 1 | TRINITY\_DN624\_c0\_g1\_i1.p1 | Oxidation (M) | M8:Oxidation (M):1000 | DB Search |
| S(+42.01)ATGNLVTVLRK | 55.5 | 1299.751 | 12 | -1.27 | 650.879 | 17.03 | 0.9973 | 1.0045 | 53970 | 2.48e2 | 1 | 1 | TRINITY\_DN2963\_c0\_g1\_i1.p1 | Acetylation (Protein N-term) | S1:Acetylation (Protein N-term):1000 | DB Search |
| RYGQAM(+15.99)C(+57.02)QFM(+15.99)R | 55.49 | 1478.6217 | 11 | -4.75 | 740.3113 | 12.88 | 0.9747 | 0.982 | 31970 | 4.25e3 | 1 | 1 | TRINITY\_DN286\_c0\_g1\_i4.p1:TRINITY\_DN286\_c0\_g1\_i2.p1 | Oxidation (M), Carbamidomethylation, Oxidation (M) | M6:Oxidation (M):1000 M10:Oxidation (M):1000 | DB Search |
| SAIIVDGKNEEGR | 55.47 | 1386.7103 | 13 | 0.31 | 694.3595 | 11.84 | 0.9985 | 1.0057 | 25914 | 5.24e2 | 1 | 1 | TRINITY\_DN982\_c0\_g1\_i3.p1 |  |  | DB Search |
| RRYPIIVTGR | 55.45 | 1229.7356 | 10 | 2.21 | 410.9182 | 12.81 | 0.7746 | 0.7819 | 31572 | 7.37e2 | 1 | 1 | TRINITY\_DN333\_c0\_g1\_i2.p1:TRINITY\_DN333\_c0\_g1\_i1.p1 |  |  | DB Search |
| GIFSIIEHR | 55.43 | 1070.5873 | 9 | -0.15 | 536.2984 | 18.11 | 0.8668 | 0.8741 | 60068 | 2.62e2 | 1 | 1 | TRINITY\_DN121501\_c0\_g1\_i1.p1 |  |  | DB Search |
| LVLEVAQHLGENVVR | 55.41 | 1674.9417 | 15 | 3.11 | 838.4769 | 17.90 | 1.143 | 1.1502 | 58884 | 2.94e2 | 1 | 1 | TRINITY\_DN73587\_c0\_g1\_i1.p1 |  |  | DB Search |
| KSDYSENEVEK | 55.41 | 1326.5939 | 11 | -3.54 | 664.2989 | 10.49 | 0.9687 | 0.9759 | 17646 | 0 | 0 | 0 | TRINITY\_DN1534\_c2\_g1\_i1.p1 |  |  | DB Search |
| GLFIIDDKGILR | 55.4 | 1358.7921 | 12 | 1.42 | 680.4012 | 19.26 | 1.011 | 1.0182 | 66782 | 5.93e2 | 1 | 1 | TRINITY\_DN535\_c0\_g1\_i2.p1:TRINITY\_DN535\_c0\_g1\_i1.p1 |  |  | DB Search |
| LPIYTDKVINLYK | 55.38 | 1578.9021 | 13 | -1.38 | 790.4537 | 17.68 | 1.0981 | 1.1053 | 57731 | 0 | 0 | 0 | TRINITY\_DN428\_c0\_g1\_i1.p1 |  |  | DB Search |
| TPGLVEGTLILHQLR | 55.37 | 1645.9515 | 15 | -1.82 | 549.6543 | 18.75 | 0.8623 | 0.8696 | 63729 | 8e2 | 1 | 1 | TRINITY\_DN3034\_c0\_g1\_i1.p1 |  |  | DB Search |
| AGVLAHLEEERDIK | 55.34 | 1578.8365 | 14 | 1.04 | 790.4228 | 14.80 | 1.1082 | 1.1154 | 42875 | 0 | 0 | 0 | TRINITY\_DN428\_c0\_g1\_i1.p1 |  |  | DB Search |
| VVTSSTGPGEHM(+15.99)R | 55.31 | 1372.6405 | 13 | 2.81 | 687.3264 | 9.74 | 0.9759 | 0.9832 | 12944 | 1.22e3 | 1 | 1 | TRINITY\_DN73067\_c0\_g1\_i1.p1 | Oxidation (M) | M12:Oxidation (M):1000 | DB Search |
| RIAEVQC(+57.02)EAR | 55.3 | 1230.6139 | 10 | 0.24 | 616.3116 | 10.93 | 0.9234 | 0.9307 | 20326 | 1.91e3 | 1 | 1 | TRINITY\_DN1280\_c0\_g1\_i1.p1 | Carbamidomethylation |  | DB Search |
| VAM(+15.99)TAPVFTYSDLKK | 55.27 | 1685.8699 | 15 | -1.69 | 843.937 | 15.62 | 1.1358 | 1.143 | 47046 | 6.36e2 | 1 | 1 | TRINITY\_DN81944\_c0\_g1\_i1.p1 | Oxidation (M) | M3:Oxidation (M):1000 | DB Search |
| IPNLTNYLQALHEK | 55.27 | 1652.8885 | 14 | -5.64 | 551.9645 | 19.30 | 0.8201 | 0.8274 | 67040 | 0 | 0 | 0 | TRINITY\_DN5643\_c0\_g1\_i1.p1 |  |  | DB Search |
| LGPDQVDVAGAGR | 55.26 | 1253.6364 | 13 | -2.53 | 627.821 | 13.54 | 0.9335 | 0.9408 | 35963 | 2.26e3 | 1 | 1 | TRINITY\_DN97809\_c0\_g1\_i1.p1 |  |  | DB Search |
| SFLLALEKDQEK | 55.25 | 1419.7609 | 12 | 5.33 | 710.8883 | 17.33 | 1.0729 | 1.0801 | 55590 | 8.23e2 | 1 | 1 | TRINITY\_DN1386\_c0\_g1\_i1.p1 |  |  | DB Search |
| DRDQFAQLVR | 55.23 | 1246.6418 | 10 | 5.21 | 624.3286 | 15.15 | 0.942 | 0.9493 | 44748 | 0 | 0 | 0 | TRINITY\_DN11435\_c0\_g1\_i1.p1 |  |  | DB Search |
| RYGQAMC(+57.02)QFMR | 55.21 | 1446.6318 | 11 | -7.35 | 724.3146 | 17.19 | 0.9848 | 0.992 | 54851 | 1.37e3 | 1 | 1 | TRINITY\_DN286\_c0\_g1\_i4.p1:TRINITY\_DN286\_c0\_g1\_i2.p1 | Carbamidomethylation |  | DB Search |
| LLLGDFNGDKR | 55.21 | 1246.667 | 11 | 4.34 | 624.3406 | 15.96 | 0.9852 | 0.9924 | 48766 | 6.58e2 | 1 | 1 | TRINITY\_DN413\_c0\_g1\_i4.p1:TRINITY\_DN413\_c0\_g1\_i1.p1:TRINITY\_DN413\_c0\_g1\_i2.p1:TRINITY\_DN413\_c0\_g1\_i3.p1 |  |  | DB Search |
| KVIVDQLVTLATR | 55.16 | 1454.8821 | 13 | -0.58 | 728.4446 | 18.25 | 1.0275 | 1.0347 | 60861 | 1.2e3 | 1 | 1 | TRINITY\_DN1386\_c0\_g1\_i1.p1 |  |  | DB Search |
| NIALETLEGHQR | 55.15 | 1379.7157 | 12 | 1.4 | 690.863 | 15.19 | 1.0142 | 1.0214 | 44943 | 4.61e2 | 1 | 1 | TRINITY\_DN1280\_c0\_g1\_i1.p1 |  |  | DB Search |
| LAALDATVHGQTASR | 55.14 | 1509.7899 | 15 | 3.37 | 755.9014 | 13.40 | 1.0741 | 1.0813 | 35059 | 6.88e2 | 1 | 1 | TRINITY\_DN1378\_c0\_g1\_i1.p1 |  |  | DB Search |
| AEGVLDKTESQLQK | 55.11 | 1544.8046 | 14 | -2.22 | 773.4044 | 13.93 | 1.0845 | 1.0917 | 38072 | 8.87e2 | 1 | 1 | TRINITY\_DN121501\_c0\_g1\_i1.p1 |  |  | DB Search |
| LIQYIENKPGLTR | 55.08 | 1543.8722 | 13 | 1.73 | 772.9412 | 14.73 | 1.0769 | 1.0841 | 42338 | 1.38e3 | 1 | 1 | TRINITY\_DN2028\_c0\_g1\_i1.p1 |  |  | DB Search |
| LSQC(+57.02)TAASGK | 55.07 | 1021.4862 | 10 | -0.97 | 511.7476 | 9.68 | 0.8388 | 0.8461 | 12609 | 2.94e4 | 1 | 1 | TRINITY\_DN3991\_c0\_g1\_i1.p1 | Carbamidomethylation |  | DB Search |
| LEPVGC(+57.02)FKDR | 55.05 | 1219.6019 | 10 | 1.41 | 610.8063 | 12.97 | 0.9384 | 0.9457 | 32450 | 3.3e3 | 1 | 1 | TRINITY\_DN3991\_c0\_g1\_i1.p1 | Carbamidomethylation |  | DB Search |
| LLEFVGHDVLR | 55.02 | 1296.719 | 11 | 3.47 | 649.3661 | 17.73 | 1.0029 | 1.0102 | 57955 | 5.47e2 | 1 | 1 | TRINITY\_DN464\_c0\_g1\_i4.p1 |  |  | DB Search |
| ATDIDQEVKEK | 55.02 | 1274.6354 | 11 | -2.5 | 638.3205 | 11.49 | 0.9654 | 0.9727 | 23882 | 0 | 0 | 0 | TRINITY\_DN3347\_c0\_g1\_i1.p1 |  |  | DB Search |
| ALLSLEYHEKR | 54.96 | 1357.7354 | 11 | 3.5 | 679.8743 | 14.40 | 1.0263 | 1.0335 | 40618 | 9.37e2 | 1 | 1 | TRINITY\_DN8707\_c0\_g1\_i2.p1 |  |  | DB Search |
| LAADLVITHSSK | 54.93 | 1253.6979 | 12 | 0.86 | 627.8539 | 14.19 | 0.9957 | 1.0029 | 39454 | 5.38e3 | 1 | 1 | TRINITY\_DN751\_c0\_g1\_i1.p1:TRINITY\_DN7214\_c0\_g1\_i1.p1 |  |  | DB Search |
| AQEVSDKLNQK | 54.93 | 1258.6517 | 11 | 0.35 | 630.3305 | 10.73 | 0.9368 | 0.944 | 19160 | 7.5e2 | 1 | 1 | TRINITY\_DN3034\_c0\_g1\_i1.p1 |  |  | DB Search |
| DPVRDKLVNER | 54.92 | 1339.7208 | 11 | -1.15 | 447.5783 | 11.99 | 0.7371 | 0.7445 | 26739 | 1.34e3 | 1 | 1 | TRINITY\_DN262\_c0\_g1\_i1.p1 |  |  | DB Search |
| GKKPHVQVNVK | 54.9 | 1232.7354 | 11 | -0.18 | 411.9171 | 9.81 | 0.7905 | 0.7978 | 13454 | 9.96e2 | 1 | 1 | TRINITY\_DN119684\_c0\_g1\_i1.p1 |  |  | DB Search |
| LRPGVELTM(+15.99)SSLVNAK | 54.9 | 1729.9396 | 16 | 1.79 | 865.9747 | 15.15 | 1.135 | 1.1422 | 44549 | 5.95e3 | 1 | 1 | TRINITY\_DN329\_c0\_g1\_i1.p1:TRINITY\_DN329\_c0\_g1\_i2.p1 | Oxidation (M) | M9:Oxidation (M):1000 | DB Search |
| GTIEILGDVHLIK | 54.9 | 1406.8132 | 13 | -4.81 | 704.4073 | 18.93 | 1.062 | 1.0693 | 64948 | 0 | 0 | 0 | TRINITY\_DN6783\_c0\_g1\_i1.p1 |  |  | DB Search |
| KAIVENLENAK | 54.86 | 1227.6823 | 11 | 1.1 | 614.8463 | 13.01 | 0.9469 | 0.9541 | 32852 | 5.63e2 | 1 | 1 | TRINITY\_DN1189\_c0\_g1\_i1.p1 |  |  | DB Search |
| AVDRGNPSQDSLNK | 54.84 | 1499.7328 | 14 | -1.13 | 750.8694 | 10.61 | 0.9937 | 1.0009 | 18454 | 0 | 0 | 0 | TRINITY\_DN982\_c0\_g1\_i1.p1:TRINITY\_DN982\_c0\_g1\_i2.p1 |  |  | DB Search |
| NM(+15.99)M(+15.99)AAC(+57.02)DPR | 54.81 | 1096.41 | 9 | 2.42 | 549.2111 | 9.55 | 0.8644 | 0.8716 | 11767 | 3.1e3 | 1 | 1 | TRINITY\_DN1910\_c0\_g1\_i1.p1:TRINITY\_DN1910\_c0\_g1\_i5.p1:TRINITY\_DN3986\_c0\_g1\_i1.p1 | Oxidation (M), Oxidation (M), Carbamidomethylation | M2:Oxidation (M):1000 M3:Oxidation (M):1000 | DB Search |
| GTSNGGLVVGTIIKPK | 54.81 | 1539.8984 | 16 | 1.04 | 770.9538 | 15.65 | 1.1234 | 1.1306 | 47131 | 3.3e3 | 1 | 1 | TRINITY\_DN3036\_c0\_g1\_i3.p1:TRINITY\_DN3036\_c0\_g1\_i2.p2:TRINITY\_DN3036\_c0\_g1\_i8.p1 |  |  | DB Search |
| GVAGAGVLAGFDKFK | 54.7 | 1435.7823 | 15 | 1.65 | 718.8964 | 18.22 | 1.0869 | 1.0941 | 60667 | 4.55e3 | 1 | 1 | TRINITY\_DN1559\_c0\_g1\_i1.p1 |  |  | DB Search |
| AGVLAHLEEER | 54.69 | 1222.6306 | 11 | 0.35 | 612.32 | 14.81 | 0.9558 | 0.963 | 42856 | 5.3e2 | 1 | 1 | TRINITY\_DN428\_c0\_g1\_i1.p1 |  |  | DB Search |
| AEAEAEVIQKK | 54.68 | 1214.6506 | 11 | 0.18 | 608.33 | 12.87 | 0.9566 | 0.9638 | 32079 | 2.33e2 | 1 | 1 | TRINITY\_DN1280\_c0\_g1\_i1.p1 |  |  | DB Search |
| LFEGNALLRR | 54.64 | 1187.6775 | 10 | -0.31 | 594.8431 | 15.53 | 0.9666 | 0.9739 | 46624 | 9.6e2 | 1 | 1 | TRINITY\_DN776\_c0\_g1\_i1.p1:TRINITY\_DN189323\_c0\_g1\_i1.p1 |  |  | DB Search |
| GAFGKPQGTVAR | 54.59 | 1187.6411 | 12 | 1.66 | 594.8261 | 11.37 | 0.9275 | 0.9348 | 22938 | 1.05e3 | 1 | 1 | TRINITY\_DN72959\_c0\_g1\_i1.p1 |  |  | DB Search |
| EIRDILVQYDR | 54.58 | 1418.7517 | 11 | -11.1 | 710.3721 | 19.69 | 1.0323 | 1.0395 | 69261 | 4.75e2 | 1 | 1 | TRINITY\_DN128406\_c0\_g1\_i1.p1 |  |  | DB Search |
| FAIQDITVDEFSAK | 54.56 | 1582.7878 | 14 | -3.45 | 792.3949 | 19.67 | 1.0443 | 1.0516 | 69141 | 4.1e2 | 1 | 1 | TRINITY\_DN624\_c0\_g1\_i1.p1 |  |  | DB Search |
| LINLFEAVSKK | 54.5 | 1260.7441 | 11 | 2.76 | 631.3782 | 18.78 | 0.9808 | 0.988 | 64045 | 0 | 0 | 0 | TRINITY\_DN1653\_c0\_g1\_i3.p1:TRINITY\_DN1653\_c0\_g1\_i1.p1 |  |  | DB Search |
| FASTVEPQKGTTIER | 54.39 | 1662.8577 | 15 | 5.23 | 832.4367 | 12.67 | 1.1214 | 1.1286 | 30969 | 0 | 0 | 0 | TRINITY\_DN10890\_c0\_g1\_i1.p1 |  |  | DB Search |
| LASQQNIGQVLAELK | 54.38 | 1610.8992 | 15 | -3.21 | 806.4506 | 19.80 | 1.1286 | 1.1358 | 69854 | 5.11e2 | 1 | 1 | TRINITY\_DN4439\_c0\_g1\_i1.p1 |  |  | DB Search |
| AFLLDHFAPER | 54.32 | 1314.672 | 11 | 4.57 | 658.3433 | 17.94 | 0.9908 | 0.9981 | 59095 | 2.39e2 | 1 | 1 | TRINITY\_DN51754\_c0\_g1\_i1.p1:TRINITY\_DN36857\_c0\_g1\_i1.p2 |  |  | DB Search |
| FPGQLNADIRK | 54.24 | 1257.683 | 11 | 0.84 | 629.8464 | 14.26 | 0.9473 | 0.9545 | 39914 | 4.37e2 | 1 | 1 | TRINITY\_DN5365\_c0\_g1\_i3.p1:TRINITY\_DN5365\_c0\_g1\_i2.p1 |  |  | DB Search |
| FPGQLNADLRK | 54.24 | 1257.683 | 11 | 0.84 | 629.8464 | 14.26 | 0.9473 | 0.9545 | 39914 | 4.37e2 | 1 | 1 | TRINITY\_DN1910\_c0\_g1\_i1.p1:TRINITY\_DN1910\_c0\_g1\_i5.p1:TRINITY\_DN3986\_c0\_g1\_i1.p1:TRINITY\_DN3986\_c0\_g1\_i2.p1 |  |  | DB Search |
| ENHHLNEPIEK | 54.18 | 1358.6578 | 11 | -5.39 | 680.3295 | 12.12 | 0.9675 | 0.9747 | 27643 | 0 | 0 | 0 | TRINITY\_DN5500\_c0\_g1\_i2.p1:TRINITY\_DN5500\_c0\_g1\_i1.p1 |  |  | DB Search |
| A(+42.01)TDKLEGLIAR | 54.05 | 1227.6823 | 11 | -8.34 | 614.8405 | 17.80 | 0.9533 | 0.9606 | 58411 | 2.09e2 | 1 | 1 | TRINITY\_DN540\_c0\_g1\_i1.p1 | Acetylation (Protein N-term) | A1:Acetylation (Protein N-term):1000 | DB Search |
| YLAEVASESEKQSVVDK | 53.93 | 1880.9366 | 17 | 2.85 | 941.4741 | 13.90 | 1.2396 | 1.2467 | 37974 | 0 | 0 | 0 | TRINITY\_DN1121\_c0\_g1\_i1.p1 |  |  | DB Search |
| VC(+57.02)EDVKR | 53.85 | 904.4437 | 7 | 3.72 | 453.2287 | 9.40 | 0.7815 | 0.7888 | 11301 | 1.58e3 | 1 | 1 | TRINITY\_DN118\_c1\_g1\_i3.p1:TRINITY\_DN118\_c1\_g1\_i5.p1 | Carbamidomethylation |  | DB Search |
| IREEYPDR | 53.76 | 1076.525 | 8 | 2.66 | 539.2688 | 10.94 | 0.8652 | 0.8725 | 20330 | 1.83e3 | 1 | 1 | TRINITY\_DN1910\_c0\_g1\_i5.p1:TRINITY\_DN3986\_c0\_g1\_i1.p1 |  |  | DB Search |
| RLLTSLVQGAR | 53.6 | 1212.7302 | 11 | 0.97 | 607.3702 | 15.15 | 0.9783 | 0.9856 | 44660 | 5.21e2 | 1 | 1 | TRINITY\_DN3190\_c1\_g1\_i1.p1 |  |  | DB Search |
| ELVM(+15.99)HEQSR | 53.55 | 1143.5343 | 9 | 15.75 | 572.7808 | 9.79 | 0.8992 | 0.9065 | 13556 | 4.95e3 | 1 | 1 | TRINITY\_DN160\_c0\_g1\_i2.p1:TRINITY\_DN160\_c0\_g1\_i1.p1 | Oxidation (M) | M4:Oxidation (M):1000 | DB Search |
| RLPDQITTR | 53.53 | 1098.6145 | 9 | 1.35 | 550.3128 | 12.51 | 0.8781 | 0.8854 | 29833 | 1.52e3 | 1 | 1 | TRINITY\_DN1377\_c0\_g1\_i1.p1 |  |  | DB Search |
| LTIAEERDR | 53.43 | 1101.5778 | 9 | 4.01 | 551.7959 | 11.68 | 0.9057 | 0.9129 | 24919 | 8.95e2 | 1 | 1 | TRINITY\_DN140696\_c0\_g1\_i1.p1 |  |  | DB Search |
| INDEDKFETK | 53.31 | 1237.5826 | 10 | 3.63 | 619.798 | 11.51 | 0.9299 | 0.9372 | 23930 | 0 | 0 | 0 | TRINITY\_DN102244\_c0\_g1\_i1.p1 |  |  | DB Search |
| IALDDVFRER | 52.99 | 1232.6512 | 10 | 0.49 | 617.3304 | 17.63 | 0.957 | 0.9642 | 57382 | 0 | 0 | 0 | TRINITY\_DN2776\_c0\_g1\_i2.p1:TRINITY\_DN2776\_c0\_g1\_i6.p1:TRINITY\_DN2776\_c0\_g1\_i1.p1 |  |  | DB Search |
| VNGADGKVEVVWK | 52.96 | 1399.746 | 13 | -2.44 | 700.8754 | 14.60 | 1.0705 | 1.0777 | 41798 | 0 | 0 | 0 | TRINITY\_DN31399\_c0\_g1\_i3.p1:TRINITY\_DN31399\_c0\_g1\_i1.p1:TRINITY\_DN31399\_c0\_g1\_i2.p1 |  |  | DB Search |
| TPFNFEDKPFIVQYEVR | 52.73 | 2128.063 | 17 | -3.58 | 710.3558 | 19.45 | 0.944 | 0.9513 | 67842 | 1.37e2 | 1 | 1 | TRINITY\_DN165\_c0\_g1\_i1.p1 |  |  | DB Search |
| AKDVTAAVKK | 52.7 | 1029.6182 | 10 | -0.29 | 515.8139 | 9.44 | 0.8433 | 0.8506 | 11436 | 2e3 | 1 | 1 | TRINITY\_DN1189\_c0\_g1\_i1.p1 |  |  | DB Search |
| HRPTSVEELDR | 52.6 | 1337.6687 | 11 | 1.56 | 446.8955 | 11.41 | 0.7571 | 0.7644 | 23281 | 0 | 0 | 0 | TRINITY\_DN12390\_c0\_g1\_i1.p1 |  |  | DB Search |
| LIEVEEKEIQRR | 52.46 | 1540.8573 | 12 | 0.79 | 514.6245 | 13.08 | 0.7803 | 0.7876 | 33297 | 6.71e2 | 1 | 1 | TRINITY\_DN11435\_c0\_g1\_i1.p1 |  |  | DB Search |
| VDNKVNATER | 52.08 | 1144.5836 | 10 | 4.61 | 382.5352 | 9.61 | 0.7518 | 0.7591 | 12330 | 3.23e2 | 1 | 1 | TRINITY\_DN32522\_c0\_g1\_i1.p1 |  |  | DB Search |
| LSRPAQLNR | 51.62 | 1053.6042 | 9 | -5.3 | 527.8043 | 11.34 | 0.8769 | 0.8842 | 22884 | 0 | 0 | 0 | TRINITY\_DN22\_c0\_g1\_i1.p1 |  |  | DB Search |
| FETQIATK | 51.43 | 936.4916 | 8 | -0.7 | 937.494 | 11.83 | 1.2165 | 1.2236 | 25881 | 4.08e3 | 3 | 3 | TRINITY\_DN649\_c0\_g1\_i2.p1:TRINITY\_DN36\_c0\_g1\_i16.p1:TRINITY\_DN36\_c0\_g1\_i3.p1:TRINITY\_DN36\_c0\_g1\_i14.p1:TRINITY\_DN36\_c0\_g1\_i17.p1:TRINITY\_DN649\_c0\_g1\_i5.p1:TRINITY\_DN36\_c0\_g1\_i13.p1:TRINITY\_DN36\_c0\_g1\_i8.p1:TRINITY\_DN649\_c0\_g1\_i1.p1:TRINITY\_DN36\_c0\_g1\_i19.p1 |  |  | DB Search |
| LIAKFETWEVAR | 51.27 | 1461.798 | 12 | 2.42 | 731.9047 | 17.13 | 1.0448 | 1.052 | 54536 | 3.66e2 | 1 | 1 | TRINITY\_DN22492\_c0\_g1\_i1.p1:TRINITY\_DN22492\_c0\_g1\_i2.p1 |  |  | DB Search |
| DLEFKFR | 51.04 | 953.497 | 7 | -1.17 | 477.7531 | 16.58 | 0.8307 | 0.838 | 51790 | 5.61e2 | 1 | 1 | TRINITY\_DN160\_c0\_g1\_i2.p1:TRINITY\_DN160\_c0\_g1\_i1.p1 |  |  | DB Search |
| VTVKPSSDASK | 50.88 | 1117.5979 | 11 | 0.38 | 373.5384 | 9.61 | 0.7388 | 0.7461 | 12368 | 4.52e2 | 1 | 1 | TRINITY\_DN941\_c0\_g1\_i9.p1 |  |  | DB Search |
| KVNQGEKEFLQRPC(+57.02)R | 50.42 | 1887.9738 | 15 | -2.15 | 472.9976 | 11.77 | 0.8092 | 0.8165 | 25549 | 0 | 0 | 0 | TRINITY\_DN4595\_c0\_g1\_i10.p1:TRINITY\_DN4595\_c0\_g1\_i4.p1 | Carbamidomethylation |  | DB Search |
| IHFPLATYSPVISAEK | 50.18 | 1771.9508 | 16 | 1.46 | 886.98 | 18.50 | 1.1338 | 1.141 | 62412 | 0 | 0 | 0 | TRINITY\_DN250\_c0\_g1\_i5.p1:TRINITY\_DN250\_c0\_g1\_i6.p1:TRINITY\_DN250\_c0\_g1\_i1.p1:TRINITY\_DN250\_c0\_g1\_i4.p1:TRINITY\_DN250\_c0\_g1\_i2.p1:TRINITY\_DN250\_c0\_g1\_i3.p1 |  |  | DB Search |
| ALYESELADARR | 50.06 | 1392.6997 | 12 | 3.27 | 697.3563 | 14.18 | 1.011 | 1.0182 | 39453 | 0 | 0 | 0 | TRINITY\_DN97534\_c0\_g1\_i1.p1 |  |  | DB Search |
| RIQQLSAIR | 49.91 | 1083.6512 | 9 | 0.54 | 542.8307 | 13.10 | 0.9178 | 0.9251 | 33359 | 1.42e3 | 1 | 1 | TRINITY\_DN428\_c0\_g1\_i1.p1 |  |  | DB Search |
| LTGQQQITEKDPWK | 49.81 | 1670.8628 | 14 | 4.45 | 836.4386 | 14.39 | 1.0777 | 1.0849 | 40541 | 4.07e2 | 1 | 1 | TRINITY\_DN1936\_c0\_g2\_i1.p1 |  |  | DB Search |
| KYTPC(+57.02)EEK | 49.11 | 1053.4801 | 8 | 0.8 | 527.7454 | 9.71 | 0.87 | 0.8773 | 12951 | 5.44e2 | 1 | 1 | TRINITY\_DN1369\_c0\_g1\_i1.p1 | Carbamidomethylation |  | DB Search |
| LTNNKDIVIAK | 48.97 | 1227.7186 | 11 | 1.99 | 614.8651 | 12.24 | 0.9808 | 0.988 | 28363 | 0 | 0 | 0 | TRINITY\_DN79\_c1\_g1\_i1.p1 |  |  | DB Search |
| FTNKDIVC(+57.02)QIAYAK | 47.25 | 1669.8497 | 14 | 10.7 | 835.9373 | 15.93 | 1.1174 | 1.1246 | 48701 | 0 | 0 | 0 | TRINITY\_DN116\_c0\_g1\_i1.p1 | Carbamidomethylation |  | DB Search |
| DVASATLQSM(+15.99)KR | 46.76 | 1321.666 | 12 | -3.61 | 661.8349 | 11.86 | 0.9957 | 1.0029 | 26142 | 0 | 0 | 0 | TRINITY\_DN684\_c0\_g1\_i1.p1 | Oxidation (M) | M10:Oxidation (M):1000 | DB Search |
| VAC(+57.02)IGAWHPAR | 45.85 | 1236.6185 | 11 | -2.24 | 619.3124 | 13.17 | 0.9444 | 0.9517 | 33868 | 3.39e2 | 1 | 1 | TRINITY\_DN6920\_c0\_g1\_i1.p1:TRINITY\_DN6920\_c0\_g1\_i3.p1:TRINITY\_DN6920\_c0\_g1\_i4.p1:TRINITY\_DN6920\_c0\_g1\_i2.p1 | Carbamidomethylation |  | DB Search |
| VPGTVGHHEVKR | 45.31 | 1314.7156 | 12 | -3.84 | 439.2422 | 9.38 | 0.7477 | 0.7551 | 11211 | 0 | 0 | 0 | TRINITY\_DN27859\_c0\_g1\_i1.p1 |  |  | DB Search |
| KWSELYEAAIK | 45.22 | 1336.7026 | 11 | -0.28 | 669.3554 | 16.30 | 0.988 | 0.9953 | 50457 | 0 | 0 | 0 | TRINITY\_DN1189\_c0\_g1\_i1.p1 |  |  | DB Search |
| LVSIDSDHVVNGNSK | 44.95 | 1582.795 | 15 | -0.07 | 792.4012 | 13.38 | 1.0644 | 1.0717 | 35085 | 2.42e2 | 1 | 1 | TRINITY\_DN1653\_c0\_g1\_i3.p1:TRINITY\_DN1653\_c0\_g1\_i1.p1 |  |  | DB Search |
| VLTSSDPAEKEK | 44.75 | 1302.6666 | 12 | 5.11 | 652.341 | 10.57 | 1.0001 | 1.0073 | 18232 | 0 | 0 | 0 | TRINITY\_DN4497\_c0\_g2\_i1.p1:TRINITY\_DN1172\_c0\_g2\_i2.p1:TRINITY\_DN21847\_c0\_g1\_i1.p1:TRINITY\_DN4497\_c0\_g1\_i1.p1 |  |  | DB Search |
| AVIFDRFR | 44.66 | 1022.5661 | 8 | 0.48 | 512.2883 | 15.85 | 0.8672 | 0.8745 | 48174 | 3.69e3 | 1 | 1 | TRINITY\_DN98420\_c0\_g1\_i1.p1 |  |  | DB Search |
| AVFC(+57.02)EHQGVK | 44.56 | 1173.5601 | 10 | -1.64 | 587.7837 | 11.55 | 0.9012 | 0.9085 | 24166 | 0 | 0 | 0 | TRINITY\_DN875\_c0\_g1\_i1.p1:TRINITY\_DN875\_c0\_g1\_i2.p1:TRINITY\_DN875\_c0\_g1\_i3.p1 | Carbamidomethylation |  | DB Search |
| KKLEPQAAPR | 44.45 | 1136.6665 | 10 | -0.58 | 379.8942 | 9.69 | 0.7567 | 0.764 | 12893 | 0 | 0 | 0 | TRINITY\_DN22492\_c0\_g1\_i1.p1:TRINITY\_DN22492\_c0\_g1\_i2.p1 |  |  | DB Search |
| DLLTLPEKDNRR | 43.72 | 1468.7998 | 12 | -0.79 | 735.4033 | 14.55 | 1.0218 | 1.0291 | 41392 | 7.4e2 | 1 | 1 | TRINITY\_DN776\_c0\_g1\_i1.p1 |  |  | DB Search |
| SQSAFPTRR | 43.1 | 1048.5414 | 9 | 1.24 | 525.2762 | 10.98 | 0.8506 | 0.8579 | 20648 | 1.97e3 | 1 | 1 | TRINITY\_DN477\_c0\_g1\_i8.p1:TRINITY\_DN477\_c0\_g1\_i7.p1 |  |  | DB Search |
| VALEQSDRIR | 43.05 | 1185.6466 | 10 | -2.05 | 593.8267 | 12.42 | 0.9505 | 0.9578 | 29416 | 8.58e2 | 1 | 1 | TRINITY\_DN128977\_c0\_g1\_i1.p1 |  |  | DB Search |
| VALEQSDRLR | 43.05 | 1185.6466 | 10 | -2.05 | 593.8267 | 12.42 | 0.9505 | 0.9578 | 29416 | 8.58e2 | 1 | 1 | TRINITY\_DN3034\_c0\_g1\_i1.p1 |  |  | DB Search |
| KIDDVIC(+57.02)EER | 42.19 | 1275.6129 | 10 | 10.75 | 638.8177 | 13.44 | 0.9271 | 0.9344 | 35371 | 1.46e3 | 1 | 1 | TRINITY\_DN15274\_c0\_g1\_i1.p1 | Carbamidomethylation |  | DB Search |
| LAENEKLENAER | 41.96 | 1414.7052 | 12 | -1.53 | 708.3556 | 11.58 | 1.0025 | 1.0098 | 24355 | 0 | 0 | 0 | TRINITY\_DN3034\_c0\_g1\_i1.p1 |  |  | DB Search |
| VSQTC(+57.02)AEHVFR | 41.51 | 1332.6245 | 11 | -1.96 | 667.3152 | 12.69 | 0.9872 | 0.9945 | 31058 | 0 | 0 | 0 | TRINITY\_DN3078\_c0\_g1\_i1.p1 | Carbamidomethylation |  | DB Search |
| AIEKFEKEAQEM(+15.99)GK | 38.77 | 1652.808 | 14 | -1.94 | 551.9397 | 12.03 | 0.8193 | 0.8266 | 27165 | 2.59e2 | 1 | 1 | TRINITY\_DN64\_c0\_g1\_i1.p1 | Oxidation (M) | M12:Oxidation (M):1000 | DB Search |
| IHHELNQFC(+57.02)SVK | 38.41 | 1510.7351 | 12 | -3.31 | 756.3689 | 12.60 | 1.0484 | 1.0556 | 30533 | 0 | 0 | 0 | TRINITY\_DN2636\_c0\_g1\_i2.p1:TRINITY\_DN2636\_c0\_g1\_i1.p1 | Carbamidomethylation |  | DB Search |
| C(+57.02)TAASGK | 38.18 | 693.3116 | 7 | 3.73 | 694.3183 | 9.67 | 1.0564 | 1.0636 | 12699 | 0 | 0 | 0 | TRINITY\_DN153369\_c0\_g1\_i1.p2 | Carbamidomethylation |  | DB Search |
| TLVALDEQGEKLNR | 37.76 | 1584.847 | 14 | -0.16 | 793.4271 | 14.56 | 1.0981 | 1.1053 | 41619 | 0 | 0 | 0 | TRINITY\_DN19237\_c0\_g1\_i1.p1 |  |  | DB Search |
| SDSSDIEKR | 37.02 | 1035.4833 | 9 | -12.69 | 518.74 | 11.06 | 0.8644 | 0.8716 | 20916 | 2.04e3 | 1 | 1 | TRINITY\_DN135129\_c0\_g1\_i1.p1 |  |  | DB Search |
| ITDKTNANEIR | 35.72 | 1273.6626 | 11 | -3.95 | 637.8332 | 10.73 | 0.9497 | 0.957 | 19159 | 0 | 0 | 0 | TRINITY\_DN19237\_c0\_g1\_i1.p1 |  |  | DB Search |
| ILAGDKPYITADELRR | 35.59 | 1829.9999 | 16 | 4.05 | 611.007 | 14.93 | 0.9251 | 0.9323 | 43538 | 0 | 0 | 0 | TRINITY\_DN624\_c0\_g1\_i1.p1 |  |  | DB Search |
| RIDYAQNM(+15.99)FK | 35.56 | 1300.6234 | 10 | -3.94 | 651.3135 | 13.37 | 0.9481 | 0.9553 | 34893 | 8.01e2 | 1 | 1 | TRINITY\_DN1374\_c0\_g1\_i1.p1 | Oxidation (M) | M8:Oxidation (M):1000 | DB Search |
| LIYENQNTIHHVVR | 35.47 | 1734.9165 | 14 | -3.47 | 868.4586 | 13.16 | 1.1302 | 1.1374 | 33743 | 0 | 0 | 0 | TRINITY\_DN26249\_c0\_g1\_i1.p1 |  |  | DB Search |
| HNIDDC(+57.02)K | 35.46 | 900.376 | 7 | 9.13 | 451.1973 | 10.19 | 0.8027 | 0.81 | 15877 | 0 | 0 | 0 | TRINITY\_DN36\_c2\_g1\_i1.p1:TRINITY\_DN36\_c0\_g1\_i11.p1:TRINITY\_DN36\_c0\_g1\_i1.p1:TRINITY\_DN36\_c0\_g1\_i20.p1 | Carbamidomethylation |  | DB Search |
| LAGESESNLRK | 34.34 | 1202.6255 | 11 | -1.9 | 602.3162 | 10.70 | 0.9331 | 0.9404 | 18933 | 0 | 0 | 0 | TRINITY\_DN5587\_c0\_g1\_i1.p1 |  |  | DB Search |
| SLDYLQVPK | 32.21 | 1061.5757 | 9 | -2.81 | 1062.5752 | 16.83 | 1.2861 | 1.2933 | 53006 | 0 | 0 | 0 | TRINITY\_DN14830\_c0\_g1\_i1.p1 |  |  | DB Search |
| M(+15.99)KINVNEIFHDLVR | 31.16 | 1742.9137 | 14 | -0.01 | 581.9759 | 19.54 | 0.8834 | 0.8907 | 68344 | 1.27e2 | 1 | 1 | TRINITY\_DN571\_c0\_g1\_i1.p1:TRINITY\_DN571\_c0\_g1\_i2.p1 | Oxidation (M) | M1:Oxidation (M):1000 | DB Search |
| AESIALVTEFTDEAAPK | 31.14 | 1790.8938 | 17 | 0.77 | 896.4508 | 19.80 | 1.1198 | 1.127 | 69969 | 2.36e2 | 1 | 1 | TRINITY\_DN79\_c0\_g1\_i1.p1 |  |  | DB Search |
| GSPGELFC(+57.02)TLADATGK | 30.98 | 1622.761 | 16 | -3.1 | 812.3816 | 19.88 | 1.0588 | 1.066 | 70430 | 0 | 0 | 0 | TRINITY\_DN1616\_c0\_g1\_i3.p1:TRINITY\_DN1616\_c0\_g1\_i6.p1:TRINITY\_DN1616\_c0\_g1\_i2.p1:TRINITY\_DN1616\_c0\_g1\_i7.p1 | Carbamidomethylation |  | DB Search |
| EISQEELIETLKK | 30.63 | 1558.8453 | 13 | 1.77 | 520.621 | 17.72 | 0.7831 | 0.7905 | 57968 | 5.68e2 | 1 | 1 | TRINITY\_DN26425\_c0\_g1\_i1.p1 |  |  | DB Search |
| DLIDRLEEK | 30.56 | 1129.5979 | 9 | -0.02 | 565.8036 | 16.13 | 0.8915 | 0.8988 | 49607 | 1.4e3 | 1 | 1 | TRINITY\_DN1306\_c0\_g1\_i1.p1 |  |  | DB Search |
| EDRNIGTLVTTIR | 30.54 | 1486.8103 | 13 | -0.94 | 744.4084 | 16.10 | 1.0439 | 1.0512 | 49432 | 4.96e2 | 1 | 1 | TRINITY\_DN72625\_c0\_g1\_i1.p1 |  |  | DB Search |
| IPLLADLTK | 30.37 | 982.6063 | 9 | -1.33 | 492.3075 | 19.34 | 0.8193 | 0.8266 | 67145 | 6.85e2 | 1 | 1 | TRINITY\_DN332\_c0\_g1\_i1.p1 |  |  | DB Search |
| ATNSDYEREKFQER | 30.07 | 1771.8125 | 14 | -6.26 | 886.904 | 12.43 | 1.0829 | 1.0901 | 29559 | 0 | 0 | 0 | TRINITY\_DN118520\_c0\_g1\_i1.p1 |  |  | DB Search |
| AELTRPTGK | 28.72 | 971.54 | 9 | 2.62 | 486.7763 | 10.33 | 0.8262 | 0.8335 | 16684 | 4.05e2 | 1 | 1 | TRINITY\_DN941\_c0\_g1\_i15.p1:TRINITY\_DN941\_c0\_g1\_i11.p1:TRINITY\_DN941\_c0\_g1\_i3.p1:TRINITY\_DN941\_c0\_g1\_i9.p1:TRINITY\_DN941\_c0\_g1\_i13.p1 |  |  | DB Search |
| AVVNGEFKDLK | 25.96 | 1218.6608 | 11 | 8.48 | 610.3401 | 13.97 | 0.9646 | 0.9719 | 38389 | 0 | 0 | 0 | TRINITY\_DN332\_c0\_g1\_i1.p1 |  |  | DB Search |
| FQVPSGIRPILVK | 23.37 | 1452.8816 | 13 | 1.2 | 727.4457 | 16.63 | 1.0335 | 1.0407 | 52054 | 0 | 0 | 0 | TRINITY\_DN140702\_c0\_g1\_i1.p1 |  |  | DB Search |
| AIGNAQRPAR | 22.83 | 1052.5839 | 10 | -1.57 | 527.296 | 9.68 | 0.8664 | 0.8737 | 12735 | 8.15e2 | 1 | 1 | TRINITY\_DN942\_c0\_g1\_i5.p1:TRINITY\_DN942\_c0\_g1\_i2.p1:TRINITY\_DN942\_c0\_g1\_i3.p1 |  |  | DB Search |
| KVNAYDDIEK | 22.25 | 1193.5928 | 10 | -1.04 | 597.8004 | 12.05 | 0.9344 | 0.9416 | 27276 | 0 | 0 | 0 | TRINITY\_DN850\_c0\_g1\_i1.p1 |  |  | DB Search |
| LTNSLM(+15.99)M(+15.99)HGR | 21.9 | 1190.5536 | 10 | 3.25 | 596.2833 | 10.70 | 0.9125 | 0.9198 | 18980 | 0 | 0 | 0 | TRINITY\_DN164351\_c0\_g1\_i1.p1 | Oxidation (M), Oxidation (M) | M6:Oxidation (M):1000 M7:Oxidation (M):1000 | DB Search |
| VFDKGSYPQPK | 21.45 | 1264.6451 | 11 | -8.55 | 633.3216 | 12.36 | 0.9642 | 0.9715 | 29095 | 0 | 0 | 0 | TRINITY\_DN6390\_c0\_g1\_i1.p1 |  |  | DB Search |
| LLAQTTLRNELGTK | 21.14 | 1556.8885 | 14 | 3.3 | 779.4506 | 15.15 | 1.1069 | 1.1142 | 44652 | 7.42e2 | 1 | 1 | TRINITY\_DN909\_c0\_g1\_i1.p1 |  |  | DB Search |
| VYTVQGVPISVTGIAQVK | 20.56 | 1858.0564 | 18 | -6.25 | 930.0255 | 18.78 | 1.1582 | 1.1654 | 63980 | 0 | 0 | 0 | TRINITY\_DN1280\_c0\_g1\_i1.p1 |  |  | DB Search |
| NERGEAVISLK | 19.8 | 1214.6619 | 11 | 0.17 | 608.3356 | 13.49 | 0.9493 | 0.9566 | 35668 | 0 | 0 | 0 | TRINITY\_DN72625\_c0\_g1\_i1.p1 |  |  | DB Search |
| HQGVM(+15.99)VGM(+15.99)GQK | 19.26 | 1202.5536 | 11 | 3.57 | 602.2835 | 9.78 | 0.9154 | 0.9226 | 13224 | 5.97e2 | 1 | 1 | TRINITY\_DN161\_c0\_g1\_i1.p1:TRINITY\_DN161\_c0\_g1\_i3.p1:TRINITY\_DN161\_c0\_g1\_i2.p1 | Oxidation (M), Oxidation (M) | M5:Oxidation (M):1000 M8:Oxidation (M):1000 | DB Search |
| AQAINVQLQEIQRR | 18.81 | 1665.9274 | 14 | 4.32 | 833.9708 | 15.11 | 1.1065 | 1.1138 | 44355 | 3.77e3 | 1 | 1 | TRINITY\_DN1280\_c0\_g1\_i1.p1 |  |  | DB Search |
| GSLDRESYR | 17.44 | 1081.5153 | 9 | 0.81 | 541.7629 | 10.96 | 0.8595 | 0.8668 | 20530 | 0 | 0 | 0 | TRINITY\_DN13856\_c0\_g1\_i1.p2:TRINITY\_DN13856\_c0\_g1\_i2.p2:TRINITY\_DN22594\_c0\_g1\_i1.p2 |  |  | DB Search |
| LSQDTTDKLNR | 17.1 | 1289.6575 | 11 | 2.65 | 645.8348 | 10.94 | 0.9594 | 0.9666 | 20471 | 0 | 0 | 0 | TRINITY\_DN1080\_c0\_g2\_i1.p1 |  |  | DB Search |
| DVQGFQGLRGRFEGK | 16.87 | 1692.8695 | 15 | -9.16 | 847.4305 | 14.86 | 1.1538 | 1.161 | 43149 | 3.52e2 | 1 | 1 | TRINITY\_DN2077\_c0\_g1\_i1.p3 |  |  | DB Search |
| RLFEGNALLR | 16.53 | 1187.6775 | 10 | -1.85 | 594.8422 | 16.02 | 0.9388 | 0.9461 | 49137 | 3.83e2 | 1 | 1 | TRINITY\_DN776\_c0\_g1\_i1.p1:TRINITY\_DN189323\_c0\_g1\_i1.p1 |  |  | DB Search |
| SSC(+57.02)RPILSVIK | 16.12 | 1258.7067 | 11 | -9.52 | 630.3518 | 16.77 | 0.9687 | 0.9759 | 52596 | 1.93e3 | 1 | 1 | TRINITY\_DN158421\_c0\_g1\_i1.p1:TRINITY\_DN3545\_c0\_g1\_i2.p1:TRINITY\_DN3545\_c0\_g1\_i1.p1 | Carbamidomethylation |  | DB Search |
| FGQATC(+57.02)QFMR | 15.37 | 1244.5431 | 10 | -15.87 | 623.2661 | 13.55 | 0.9259 | 0.9331 | 35989 | 5.38e2 | 1 | 1 | TRINITY\_DN902\_c1\_g1\_i1.p1:TRINITY\_DN902\_c1\_g1\_i2.p1 | Carbamidomethylation |  | DB Search |
| ERFC(+57.02)SNPEPENGGQTC(+57.02)EGEAR | 15.15 | 2422.9866 | 21 | 1.66 | 808.6672 | 11.82 | 0.9077 | 0.915 | 25956 | 2.43e2 | 1 | 1 | TRINITY\_DN138\_c0\_g1\_i1.p1 | Carbamidomethylation, Carbamidomethylation |  | DB Search |
| EETM(+15.99)DLEGVK | 14.53 | 1165.5172 | 10 | -15.43 | 583.7543 | 12.15 | 0.8907 | 0.898 | 27886 | 0 | 0 | 0 | TRINITY\_DN50473\_c0\_g1\_i1.p2 | Oxidation (M) | M4:Oxidation (M):1000 | DB Search |
| RFDALR | 13.6 | 776.4293 | 6 | 6.56 | 389.2227 | 12.31 | 0.7799 | 0.7872 | 28863 | 0 | 0 | 0 | TRINITY\_DN102244\_c0\_g1\_i1.p1 |  |  | DB Search |
| MPHEPR | 12.83 | 765.3592 | 6 | -14.87 | 383.6794 | 9.07 | 0.7122 | 0.7196 | 10078 | 0 | 0 | 0 | TRINITY\_DN122142\_c0\_g1\_i1.p1 |  |  | DB Search |
| total 381 peptides |  |  |  |  |  |  |  |  |  |  |  |  |  |  |  |  |
| --- | --- | --- | --- | --- | --- | --- | --- | --- | --- | --- | --- | --- | --- | --- | --- | --- |
