# Supplementary material for: Proteomic Analysis and Biochemical Characterization of the Nematocyst Extract of the Hydrozoan Velella velella
Source: Mar Drugs. 2024 Oct 12;22(10):468. doi: 10.3390/md22100468 (PMC11509761; doi:10.3390/md22100468)
Supplement: Supplementary file 1 [file marinedrugs-22-00468-s001.zip › Supplementary File S1.zip!/Sample_8_Vellela/db.proteins.html]

proteins


Protein List

  

| Protein Group | Protein ID | Accession | -10lgP | Coverage (%) | Coverage (%) Sample 9 | Area Sample 9 | #Peptides | #Unique | #Spec Sample 9 | PTM | Avg. Mass | Description |
| --- | --- | --- | --- | --- | --- | --- | --- | --- | --- | --- | --- | --- |
| 1 | 1 | TRINITY\_DN982\_c0\_g1\_i3.p1 | 177.96 | 2 | 2 | 2.22e3 | 11 | 4 | 12 |  | 1090854.75 | type:complete len:9852 gc:universal TRINITY\_DN982\_c0\_g1\_i3:29697-142(-) |
| 2 | 2 | TRINITY\_DN161\_c0\_g1\_i2.p1 | 154.99 | 29 | 29 | 3.03e4 | 8 | 8 | 15 | Oxidation (M) | 44577.6484 | type:5prime\_partial len:399 gc:universal TRINITY\_DN161\_c0\_g1\_i2:1683-487(-) |
| 2 | 3 | TRINITY\_DN161\_c0\_g1\_i3.p1 | 154.99 | 29 | 29 | 3.03e4 | 8 | 8 | 15 | Oxidation (M) | 44592.6602 | type:5prime\_partial len:399 gc:universal TRINITY\_DN161\_c0\_g1\_i3:1450-254(-) |
| 2 | 4 | TRINITY\_DN161\_c0\_g1\_i1.p1 | 154.99 | 31 | 31 | 3.03e4 | 8 | 8 | 15 | Oxidation (M) | 41918.5938 | type:complete len:377 gc:universal TRINITY\_DN161\_c0\_g1\_i1:1384-254(-) |
| 2 | 5 | TRINITY\_DN161\_c0\_g1\_i4.p1 | 123.08 | 28 | 28 | 8.53e3 | 4 | 4 | 9 | Oxidation (M) | 25887.2988 | type:5prime\_partial len:232 gc:universal TRINITY\_DN161\_c0\_g1\_i4:949-254(-) |
| 2 | 6 | TRINITY\_DN2477\_c0\_g1\_i10.p1 | 105.7 | 13 | 13 | 1.22e4 | 3 | 3 | 4 |  | 38566.9688 | type:5prime\_partial len:344 gc:universal TRINITY\_DN2477\_c0\_g1\_i10:1215-184(-) |
| 2 | 7 | TRINITY\_DN2477\_c0\_g1\_i5.p1 | 105.7 | 13 | 13 | 1.22e4 | 3 | 3 | 4 |  | 37416.5195 | type:complete len:333 gc:universal TRINITY\_DN2477\_c0\_g1\_i5:1182-184(-) |
| 2 | 8 | TRINITY\_DN2477\_c0\_g1\_i2.p1 | 105.7 | 13 | 13 | 1.22e4 | 3 | 3 | 4 |  | 38566.9688 | type:5prime\_partial len:344 gc:universal TRINITY\_DN2477\_c0\_g1\_i2:1215-184(-) |
| 2 | 9 | TRINITY\_DN2477\_c0\_g1\_i6.p1 | 105.7 | 12 | 12 | 1.22e4 | 3 | 3 | 4 |  | 41878.6875 | type:complete len:377 gc:universal TRINITY\_DN2477\_c0\_g1\_i6:1314-184(-) |
| 2 | 10 | TRINITY\_DN2477\_c0\_g1\_i9.p1 | 105.7 | 12 | 12 | 1.22e4 | 3 | 3 | 4 |  | 41878.6875 | type:complete len:377 gc:universal TRINITY\_DN2477\_c0\_g1\_i9:1314-184(-) |
| 2 | 11 | TRINITY\_DN195934\_c0\_g1\_i1.p2 | 86.83 | 24 | 24 | 8.76e3 | 2 | 2 | 3 |  | 14094.0938 | type:internal len:127 gc:universal TRINITY\_DN195934\_c0\_g1\_i1:3-380(+) |
| 2 | 12 | TRINITY\_DN2477\_c0\_g1\_i8.p1 | 85.73 | 10 | 10 | 3.61e3 | 2 | 2 | 2 |  | 34820.5938 | type:5prime\_partial len:309 gc:universal TRINITY\_DN2477\_c0\_g1\_i8:1110-184(-) |
| 2 | 13 | TRINITY\_DN2941\_c0\_g1\_i4.p1 | 57.43 | 16 | 16 | 1.52e2 | 1 | 1 | 1 |  | 13034.9365 | type:internal len:116 gc:universal TRINITY\_DN2941\_c0\_g1\_i4:2-346(+) |
| 2 | 14 | TRINITY\_DN2477\_c0\_g1\_i7.p1 | 56.59 | 5 | 5 | 3.46e3 | 1 | 1 | 1 |  | 31152.4473 | type:5prime\_partial len:280 gc:universal TRINITY\_DN2477\_c0\_g1\_i7:1023-184(-) |
| 3 | 15 | TRINITY\_DN982\_c0\_g1\_i1.p1 | 167.04 | 2 | 2 | 0 | 9 | 2 | 10 |  | 712566.0625 | type:5prime\_partial len:6412 gc:universal TRINITY\_DN982\_c0\_g1\_i1:19377-142(-) |
| 3 | 16 | TRINITY\_DN982\_c0\_g1\_i2.p1 | 103.26 | 2 | 2 | 0 | 3 | 2 | 4 |  | 190367.1406 | type:internal len:1718 gc:universal TRINITY\_DN982\_c0\_g1\_i2:5151-1(-) |
| 4 | 17 | TRINITY\_DN1280\_c0\_g1\_i1.p1 | 152.71 | 23 | 23 | 1.15e4 | 9 | 9 | 16 | Carbamidomethylation | 47818.6641 | type:complete len:426 gc:universal TRINITY\_DN1280\_c0\_g1\_i1:195-1472(+) |
| 5 | 18 | TRINITY\_DN329\_c0\_g1\_i2.p1 | 152.05 | 19 | 19 | 1.53e4 | 7 | 7 | 12 | Oxidation (M) | 34761.9609 | type:5prime\_partial len:320 gc:universal TRINITY\_DN329\_c0\_g1\_i2:1-960(+) |
| 5 | 19 | TRINITY\_DN329\_c0\_g1\_i1.p1 | 152.05 | 21 | 21 | 1.53e4 | 7 | 7 | 12 | Oxidation (M) | 30514.9648 | type:complete len:283 gc:universal TRINITY\_DN329\_c0\_g1\_i1:158-1006(+) |
| 6 | 20 | TRINITY\_DN428\_c0\_g1\_i1.p1 | 158.69 | 5 | 5 | 2.13e3 | 8 | 8 | 10 | Carbamidomethylation | 225824.1719 | type:complete len:1949 gc:universal TRINITY\_DN428\_c0\_g1\_i1:96-5942(+) |
| 7 | 21 | TRINITY\_DN3034\_c0\_g1\_i1.p1 | 137.65 | 4 | 4 | 2.41e3 | 6 | 6 | 6 |  | 222266.9844 | type:complete len:1941 gc:universal TRINITY\_DN3034\_c0\_g1\_i1:6289-467(-) |
| 7 | 22 | TRINITY\_DN128977\_c0\_g1\_i1.p1 | 43.05 | 4 | 4 | 8.58e2 | 1 | 1 | 1 |  | 30332.7207 | type:internal len:266 gc:universal TRINITY\_DN128977\_c0\_g1\_i1:3-797(+) |
| 8 | 23 | TRINITY\_DN262\_c0\_g1\_i1.p1 | 143.63 | 9 | 9 | 6.92e3 | 6 | 6 | 7 | Oxidation (M) | 114520.6406 | type:complete len:1033 gc:universal TRINITY\_DN262\_c0\_g1\_i1:111-3209(+) |
| 9 | 24 | TRINITY\_DN1189\_c0\_g1\_i1.p1 | 138.64 | 14 | 14 | 3.46e3 | 6 | 6 | 6 | Carbamidomethylation, Oxidation (M) | 50239.8086 | type:5prime\_partial len:449 gc:universal TRINITY\_DN1189\_c0\_g1\_i1:1554-208(-) |
| 10 | 25 | TRINITY\_DN36\_c0\_g1\_i19.p1 | 144.89 | 31 | 31 | 4.08e3 | 6 | 2 | 15 | Carbamidomethylation, Oxidation (M) | 24821.4141 | type:complete len:223 gc:universal TRINITY\_DN36\_c0\_g1\_i19:826-158(-) |
| 10 | 26 | TRINITY\_DN36\_c0\_g1\_i13.p1 | 144.89 | 31 | 31 | 4.08e3 | 6 | 2 | 15 | Carbamidomethylation, Oxidation (M) | 24821.4141 | type:complete len:223 gc:universal TRINITY\_DN36\_c0\_g1\_i13:816-148(-) |
| 10 | 27 | TRINITY\_DN36\_c0\_g1\_i16.p1 | 135.33 | 26 | 26 | 4.08e3 | 5 | 2 | 14 | Carbamidomethylation, Oxidation (M) | 24953.5938 | type:complete len:224 gc:universal TRINITY\_DN36\_c0\_g1\_i16:829-158(-) |
| 10 | 28 | TRINITY\_DN36\_c0\_g1\_i3.p1 | 135.33 | 26 | 26 | 4.08e3 | 5 | 2 | 14 | Carbamidomethylation, Oxidation (M) | 24953.5938 | type:complete len:224 gc:universal TRINITY\_DN36\_c0\_g1\_i3:829-158(-) |
| 10 | 29 | TRINITY\_DN36\_c0\_g1\_i14.p1 | 135.33 | 26 | 26 | 4.08e3 | 5 | 2 | 14 | Carbamidomethylation, Oxidation (M) | 24953.5938 | type:complete len:224 gc:universal TRINITY\_DN36\_c0\_g1\_i14:819-148(-) |
| 10 | 30 | TRINITY\_DN36\_c0\_g1\_i8.p1 | 135.33 | 26 | 26 | 4.08e3 | 5 | 2 | 14 | Carbamidomethylation, Oxidation (M) | 24953.5938 | type:complete len:224 gc:universal TRINITY\_DN36\_c0\_g1\_i8:819-148(-) |
| 10 | 31 | TRINITY\_DN36\_c0\_g1\_i17.p1 | 135.33 | 26 | 26 | 4.08e3 | 5 | 2 | 14 | Carbamidomethylation, Oxidation (M) | 24953.5938 | type:complete len:224 gc:universal TRINITY\_DN36\_c0\_g1\_i17:819-148(-) |
| 10 | 32 | TRINITY\_DN649\_c0\_g1\_i5.p1 | 87.26 | 15 | 15 | 4.08e3 | 2 | 1 | 11 | Oxidation (M), Carbamidomethylation | 13746.6875 | type:internal len:124 gc:universal TRINITY\_DN649\_c0\_g1\_i5:371-3(-) |
| 10 | 33 | TRINITY\_DN649\_c0\_g1\_i2.p1 | 87.26 | 15 | 15 | 4.08e3 | 2 | 1 | 11 | Oxidation (M), Carbamidomethylation | 13819.7393 | type:internal len:125 gc:universal TRINITY\_DN649\_c0\_g1\_i2:374-3(-) |
| 10 | 34 | TRINITY\_DN649\_c0\_g1\_i1.p1 | 87.26 | 15 | 15 | 4.08e3 | 2 | 1 | 11 | Oxidation (M), Carbamidomethylation | 13690.5801 | type:internal len:124 gc:universal TRINITY\_DN649\_c0\_g1\_i1:371-3(-) |
| 11 | 35 | TRINITY\_DN11435\_c0\_g1\_i1.p1 | 144.57 | 14 | 14 | 2.94e3 | 6 | 6 | 8 |  | 47019.5312 | type:complete len:430 gc:universal TRINITY\_DN11435\_c0\_g1\_i1:1616-327(-) |
| 12 | 36 | TRINITY\_DN72625\_c0\_g1\_i1.p1 | 129.25 | 5 | 5 | 3.31e3 | 6 | 6 | 7 | Oxidation (M) | 214200.3906 | type:complete len:1964 gc:universal TRINITY\_DN72625\_c0\_g1\_i1:173-6064(+) |
| 13 | 37 | TRINITY\_DN15274\_c0\_g1\_i1.p1 | 138.88 | 5 | 5 | 2.09e3 | 6 | 6 | 6 | Carbamidomethylation, Oxidation (M) | 201897.7344 | type:complete len:1822 gc:universal TRINITY\_DN15274\_c0\_g1\_i1:87-5552(+) |
| 14 | 38 | TRINITY\_DN160\_c0\_g1\_i1.p1 | 120.46 | 3 | 3 | 8.57e3 | 4 | 4 | 5 | Oxidation (M) | 126611.5703 | type:complete len:1145 gc:universal TRINITY\_DN160\_c0\_g1\_i1:4810-1376(-) |
| 14 | 39 | TRINITY\_DN160\_c0\_g1\_i2.p1 | 120.46 | 3 | 3 | 8.57e3 | 4 | 4 | 5 | Oxidation (M) | 126611.5703 | type:complete len:1145 gc:universal TRINITY\_DN160\_c0\_g1\_i2:4810-1376(-) |
| 15 | 40 | TRINITY\_DN332\_c0\_g1\_i1.p1 | 117.91 | 24 | 24 | 1.13e4 | 5 | 5 | 10 |  | 27839.6836 | type:complete len:246 gc:universal TRINITY\_DN332\_c0\_g1\_i1:940-203(-) |
| 16 | 41 | TRINITY\_DN1377\_c0\_g1\_i1.p1 | 135.73 | 8 | 8 | 2.75e3 | 5 | 5 | 6 | Carbamidomethylation, Oxidation (M) | 99936.9219 | type:complete len:878 gc:universal TRINITY\_DN1377\_c0\_g1\_i1:68-2701(+) |
| 17 | 42 | TRINITY\_DN3986\_c0\_g1\_i1.p1 | 132.59 | 13 | 13 | 7.62e3 | 5 | 5 | 8 | Oxidation (M), Carbamidomethylation | 50011.8867 | type:complete len:446 gc:universal TRINITY\_DN3986\_c0\_g1\_i1:252-1589(+) |
| 17 | 43 | TRINITY\_DN1910\_c0\_g1\_i1.p1 | 106.98 | 8 | 8 | 3.53e3 | 3 | 3 | 6 | Oxidation (M), Carbamidomethylation | 50135.0117 | type:complete len:447 gc:universal TRINITY\_DN1910\_c0\_g1\_i1:2183-843(-) |
| 17 | 44 | TRINITY\_DN1910\_c0\_g1\_i5.p1 | 99.86 | 6 | 6 | 5.37e3 | 3 | 3 | 5 | Oxidation (M), Carbamidomethylation | 50120.9062 | type:complete len:447 gc:universal TRINITY\_DN1910\_c0\_g1\_i5:2031-691(-) |
| 17 | 45 | TRINITY\_DN3986\_c0\_g1\_i2.p1 | 88.62 | 6 | 6 | 4.37e2 | 2 | 2 | 4 |  | 50284.332 | type:complete len:446 gc:universal TRINITY\_DN3986\_c0\_g1\_i2:47-1384(+) |
| 17 | 46 | TRINITY\_DN5365\_c0\_g1\_i2.p1 | 54.24 | 2 | 2 | 4.37e2 | 1 | 1 | 2 |  | 51878.4453 | type:complete len:458 gc:universal TRINITY\_DN5365\_c0\_g1\_i2:1448-75(-) |
| 17 | 47 | TRINITY\_DN5365\_c0\_g1\_i3.p1 | 54.24 | 2 | 2 | 4.37e2 | 1 | 1 | 2 |  | 51878.4453 | type:complete len:458 gc:universal TRINITY\_DN5365\_c0\_g1\_i3:1448-75(-) |
| 18 | 48 | TRINITY\_DN168534\_c0\_g1\_i1.p1 | 125.2 | 19 | 19 | 1.55e3 | 4 | 4 | 5 | Oxidation (M), Carbamidomethylation | 25265.0449 | type:complete len:226 gc:universal TRINITY\_DN168534\_c0\_g1\_i1:774-97(-) |
| 19 | 49 | TRINITY\_DN36\_c0\_g1\_i20.p1 | 130.75 | 23 | 23 | 0 | 5 | 1 | 6 | Carbamidomethylation, Oxidation (M) | 24874.5234 | type:complete len:223 gc:universal TRINITY\_DN36\_c0\_g1\_i20:1172-504(-) |
| 19 | 50 | TRINITY\_DN36\_c0\_g1\_i1.p1 | 118.43 | 18 | 18 | 0 | 4 | 1 | 5 | Carbamidomethylation, Oxidation (M) | 25006.7051 | type:complete len:224 gc:universal TRINITY\_DN36\_c0\_g1\_i1:1175-504(-) |
| 19 | 51 | TRINITY\_DN36\_c0\_g1\_i11.p1 | 118.43 | 18 | 18 | 0 | 4 | 1 | 5 | Carbamidomethylation, Oxidation (M) | 25006.7051 | type:complete len:224 gc:universal TRINITY\_DN36\_c0\_g1\_i11:1175-504(-) |
| 19 | 52 | TRINITY\_DN36\_c2\_g1\_i1.p1 | 35.46 | 6 | 6 | 0 | 1 | 1 | 1 | Carbamidomethylation | 14359.748 | type:internal len:126 gc:universal TRINITY\_DN36\_c2\_g1\_i1:1-375(+) |
| 20 | 53 | TRINITY\_DN1369\_c0\_g1\_i1.p1 | 120.16 | 3 | 3 | 2.59e3 | 4 | 4 | 4 | Carbamidomethylation | 174229.4219 | type:3prime\_partial len:1577 gc:universal TRINITY\_DN1369\_c0\_g1\_i1:93-4820(+) |
| 21 | 54 | TRINITY\_DN140702\_c0\_g1\_i1.p1 | 116.26 | 2 | 2 | 3.38e2 | 4 | 4 | 5 |  | 231919.1562 | type:complete len:2036 gc:universal TRINITY\_DN140702\_c0\_g1\_i1:94-6201(+) |
| 22 | 55 | TRINITY\_DN3991\_c0\_g1\_i1.p1 | 117.81 | 17 | 17 | 5.6e4 | 4 | 4 | 5 | Carbamidomethylation | 24846.6543 | type:3prime\_partial len:219 gc:universal TRINITY\_DN3991\_c0\_g1\_i1:84-737(+) |
| 23 | 56 | TRINITY\_DN138\_c0\_g1\_i1.p1 | 109.99 | 13 | 13 | 7.31e2 | 4 | 4 | 6 | Oxidation (M), Carbamidomethylation | 46324.8945 | type:complete len:401 gc:universal TRINITY\_DN138\_c0\_g1\_i1:157-1359(+) |
| 24 | 57 | TRINITY\_DN118\_c1\_g1\_i3.p1 | 108.14 | 11 | 11 | 7.87e3 | 3 | 3 | 5 | Carbamidomethylation | 22860.4883 | type:internal len:200 gc:universal TRINITY\_DN118\_c1\_g1\_i3:599-3(-) |
| 24 | 58 | TRINITY\_DN118\_c1\_g1\_i5.p1 | 82.91 | 8 | 8 | 6.5e3 | 2 | 2 | 3 | Carbamidomethylation | 23277.6309 | type:5prime\_partial len:203 gc:universal TRINITY\_DN118\_c1\_g1\_i5:620-12(-) |
| 25 | 59 | TRINITY\_DN1616\_c0\_g1\_i3.p1 | 95.94 | 1 | 1 | 0 | 3 | 3 | 4 | Carbamidomethylation | 423947.2812 | type:complete len:3855 gc:universal TRINITY\_DN1616\_c0\_g1\_i3:218-11782(+) |
| 25 | 60 | TRINITY\_DN1616\_c0\_g1\_i7.p1 | 95.94 | 1 | 1 | 0 | 3 | 3 | 4 | Carbamidomethylation | 423875.2188 | type:complete len:3855 gc:universal TRINITY\_DN1616\_c0\_g1\_i7:218-11782(+) |
| 25 | 61 | TRINITY\_DN1616\_c0\_g1\_i6.p1 | 95.94 | 1 | 1 | 0 | 3 | 3 | 4 | Carbamidomethylation | 423911.25 | type:complete len:3855 gc:universal TRINITY\_DN1616\_c0\_g1\_i6:218-11782(+) |
| 25 | 62 | TRINITY\_DN1616\_c0\_g1\_i2.p1 | 72.49 | 1 | 1 | 0 | 2 | 2 | 2 | Carbamidomethylation | 374396 | type:3prime\_partial len:3398 gc:universal TRINITY\_DN1616\_c0\_g1\_i2:218-10408(+) |
| 26 | 63 | TRINITY\_DN250\_c0\_g1\_i2.p1 | 104.59 | 14 | 14 | 8.29e2 | 3 | 3 | 4 | Oxidation (M) | 35668.3789 | type:3prime\_partial len:322 gc:universal TRINITY\_DN250\_c0\_g1\_i2:131-1093(+) |
| 26 | 64 | TRINITY\_DN250\_c0\_g1\_i3.p1 | 104.59 | 11 | 11 | 8.29e2 | 3 | 3 | 4 | Oxidation (M) | 44456.6445 | type:3prime\_partial len:402 gc:universal TRINITY\_DN250\_c0\_g1\_i3:131-1333(+) |
| 26 | 65 | TRINITY\_DN250\_c0\_g1\_i6.p1 | 83.4 | 7 | 7 | 6.19e2 | 2 | 2 | 2 |  | 50263.3672 | type:complete len:452 gc:universal TRINITY\_DN250\_c0\_g1\_i6:131-1486(+) |
| 26 | 66 | TRINITY\_DN250\_c0\_g1\_i5.p1 | 83.4 | 7 | 7 | 6.19e2 | 2 | 2 | 2 |  | 50305.4453 | type:complete len:452 gc:universal TRINITY\_DN250\_c0\_g1\_i5:131-1486(+) |
| 26 | 67 | TRINITY\_DN250\_c0\_g1\_i4.p1 | 83.4 | 7 | 7 | 6.19e2 | 2 | 2 | 2 |  | 50305.4453 | type:complete len:452 gc:universal TRINITY\_DN250\_c0\_g1\_i4:131-1486(+) |
| 26 | 68 | TRINITY\_DN250\_c0\_g1\_i1.p1 | 83.4 | 8 | 8 | 6.19e2 | 2 | 2 | 2 |  | 44454.6094 | type:3prime\_partial len:402 gc:universal TRINITY\_DN250\_c0\_g1\_i1:131-1333(+) |
| 26 | 69 | TRINITY\_DN471\_c0\_g1\_i3.p1 | 58.31 | 5 | 5 | 6.19e2 | 1 | 1 | 1 |  | 36097.9961 | type:5prime\_partial len:324 gc:universal TRINITY\_DN471\_c0\_g1\_i3:1-972(+) |
| 26 | 70 | TRINITY\_DN471\_c0\_g1\_i5.p1 | 58.31 | 4 | 4 | 6.19e2 | 1 | 1 | 1 |  | 39722.6172 | type:3prime\_partial len:359 gc:universal TRINITY\_DN471\_c0\_g1\_i5:117-1190(+) |
| 26 | 71 | TRINITY\_DN1933\_c0\_g1\_i4.p1 | 58.31 | 10 | 10 | 6.19e2 | 1 | 1 | 1 |  | 16888.3496 | type:internal len:153 gc:universal TRINITY\_DN1933\_c0\_g1\_i4:3-458(+) |
| 26 | 72 | TRINITY\_DN687\_c0\_g2\_i1.p1 | 58.31 | 3 | 3 | 6.19e2 | 1 | 1 | 1 |  | 50237.3242 | type:complete len:453 gc:universal TRINITY\_DN687\_c0\_g2\_i1:90-1448(+) |
| 26 | 73 | TRINITY\_DN471\_c0\_g1\_i2.p1 | 58.31 | 3 | 3 | 6.19e2 | 1 | 1 | 1 |  | 54220.6289 | type:5prime\_partial len:492 gc:universal TRINITY\_DN471\_c0\_g1\_i2:3-1478(+) |
| 26 | 74 | TRINITY\_DN471\_c0\_g1\_i9.p1 | 58.31 | 4 | 4 | 6.19e2 | 1 | 1 | 1 |  | 37621.1953 | type:3prime\_partial len:341 gc:universal TRINITY\_DN471\_c0\_g1\_i9:117-1136(+) |
| 26 | 75 | TRINITY\_DN471\_c0\_g1\_i11.p1 | 58.31 | 3 | 3 | 6.19e2 | 1 | 1 | 1 |  | 54220.6289 | type:5prime\_partial len:492 gc:universal TRINITY\_DN471\_c0\_g1\_i11:3-1478(+) |
| 26 | 76 | TRINITY\_DN1933\_c0\_g1\_i1.p2 | 58.31 | 11 | 11 | 6.19e2 | 1 | 1 | 1 |  | 14806.9961 | type:internal len:134 gc:universal TRINITY\_DN1933\_c0\_g1\_i1:3-401(+) |
| 26 | 77 | TRINITY\_DN471\_c0\_g1\_i10.p1 | 58.31 | 3 | 3 | 6.19e2 | 1 | 1 | 1 |  | 54220.6289 | type:5prime\_partial len:492 gc:universal TRINITY\_DN471\_c0\_g1\_i10:3-1478(+) |
| 26 | 78 | TRINITY\_DN1933\_c0\_g1\_i3.p2 | 58.31 | 11 | 11 | 6.19e2 | 1 | 1 | 1 |  | 14806.9961 | type:internal len:134 gc:universal TRINITY\_DN1933\_c0\_g1\_i3:3-401(+) |
| 26 | 79 | TRINITY\_DN471\_c0\_g1\_i7.p1 | 58.31 | 7 | 7 | 6.19e2 | 1 | 1 | 1 |  | 23321.0215 | type:internal len:211 gc:universal TRINITY\_DN471\_c0\_g1\_i7:1-630(+) |
| 26 | 80 | TRINITY\_DN1933\_c0\_g1\_i8.p2 | 58.31 | 11 | 11 | 6.19e2 | 1 | 1 | 1 |  | 14806.9961 | type:internal len:134 gc:universal TRINITY\_DN1933\_c0\_g1\_i8:3-401(+) |
| 26 | 81 | TRINITY\_DN471\_c0\_g1\_i6.p1 | 58.31 | 7 | 7 | 6.19e2 | 1 | 1 | 1 |  | 23321.0215 | type:internal len:211 gc:universal TRINITY\_DN471\_c0\_g1\_i6:1-630(+) |
| 26 | 82 | TRINITY\_DN1933\_c0\_g1\_i7.p1 | 58.31 | 10 | 10 | 6.19e2 | 1 | 1 | 1 |  | 17019.541 | type:internal len:154 gc:universal TRINITY\_DN1933\_c0\_g1\_i7:3-461(+) |
| 26 | 83 | TRINITY\_DN471\_c0\_g1\_i4.p1 | 58.31 | 3 | 3 | 6.19e2 | 1 | 1 | 1 |  | 54220.6289 | type:5prime\_partial len:492 gc:universal TRINITY\_DN471\_c0\_g1\_i4:3-1478(+) |
| 27 | 84 | TRINITY\_DN240\_c0\_g1\_i4.p1 | 110.25 | 12 | 12 | 1.44e4 | 3 | 3 | 7 |  | 40419.2031 | type:5prime\_partial len:397 gc:universal TRINITY\_DN240\_c0\_g1\_i4:1327-137(-) |
| 27 | 85 | TRINITY\_DN240\_c0\_g1\_i6.p1 | 110.25 | 12 | 12 | 1.44e4 | 3 | 3 | 7 |  | 40418.2188 | type:5prime\_partial len:397 gc:universal TRINITY\_DN240\_c0\_g1\_i6:1494-304(-) |
| 27 | 86 | TRINITY\_DN240\_c0\_g1\_i1.p1 | 110.25 | 12 | 12 | 1.44e4 | 3 | 3 | 7 |  | 40418.2188 | type:5prime\_partial len:397 gc:universal TRINITY\_DN240\_c0\_g1\_i1:1518-328(-) |
| 28 | 87 | TRINITY\_DN6390\_c0\_g1\_i1.p1 | 94.02 | 2 | 2 | 0 | 3 | 3 | 4 |  | 214480.2344 | type:3prime\_partial len:1941 gc:universal TRINITY\_DN6390\_c0\_g1\_i1:5820-1(-) |
| 29 | 88 | TRINITY\_DN2113\_c0\_g1\_i1.p1 | 104.8 | 3 | 3 | 3.14e3 | 3 | 3 | 3 |  | 193799.0156 | type:complete len:1704 gc:universal TRINITY\_DN2113\_c0\_g1\_i1:5257-146(-) |
| 29 | 89 | TRINITY\_DN2113\_c0\_g1\_i2.p1 | 104.8 | 3 | 3 | 3.14e3 | 3 | 3 | 3 |  | 193799.0156 | type:complete len:1704 gc:universal TRINITY\_DN2113\_c0\_g1\_i2:5257-146(-) |
| 30 | 90 | TRINITY\_DN116\_c0\_g1\_i1.p1 | 103.88 | 14 | 14 | 3.78e2 | 3 | 3 | 3 | Carbamidomethylation | 34095.6328 | type:complete len:297 gc:universal TRINITY\_DN116\_c0\_g1\_i1:72-962(+) |
| 31 | 91 | TRINITY\_DN22\_c0\_g1\_i1.p1 | 102.61 | 9 | 9 | 2.17e3 | 3 | 3 | 4 |  | 38528.8086 | type:5prime\_partial len:353 gc:universal TRINITY\_DN22\_c0\_g1\_i1:3-1061(+) |
| 31 | 92 | TRINITY\_DN384\_c0\_g1\_i2.p1 | 56.98 | 4 | 4 | 1.72e3 | 1 | 1 | 2 |  | 34316.0703 | type:complete len:314 gc:universal TRINITY\_DN384\_c0\_g1\_i2:1018-77(-) |
| 31 | 93 | TRINITY\_DN384\_c0\_g1\_i1.p1 | 56.98 | 4 | 4 | 1.72e3 | 1 | 1 | 2 |  | 34317.9414 | type:complete len:313 gc:universal TRINITY\_DN384\_c0\_g1\_i1:1015-77(-) |
| 32 | 94 | TRINITY\_DN776\_c0\_g1\_i1.p1 | 82.01 | 11 | 11 | 2.08e3 | 3 | 3 | 3 |  | 23300.8359 | type:5prime\_partial len:201 gc:universal TRINITY\_DN776\_c0\_g1\_i1:692-90(-) |
| 32 | 95 | TRINITY\_DN189323\_c0\_g1\_i1.p1 | 62.91 | 7 | 7 | 1.34e3 | 2 | 2 | 2 |  | 18178.9902 | type:5prime\_partial len:156 gc:universal TRINITY\_DN189323\_c0\_g1\_i1:2-469(+) |
| 33 | 96 | TRINITY\_DN1091\_c0\_g1\_i1.p1 | 106 | 16 | 16 | 2.56e3 | 3 | 3 | 5 | Carbamidomethylation | 24868.0293 | type:complete len:212 gc:universal TRINITY\_DN1091\_c0\_g1\_i1:200-835(+) |
| 34 | 97 | TRINITY\_DN624\_c0\_g1\_i1.p1 | 94.64 | 5 | 5 | 8.72e2 | 3 | 3 | 3 | Oxidation (M) | 101743.5391 | type:complete len:884 gc:universal TRINITY\_DN624\_c0\_g1\_i1:218-2869(+) |
| 35 | 98 | TRINITY\_DN4595\_c0\_g1\_i4.p1 | 102.56 | 2 | 2 | 1.89e3 | 3 | 3 | 7 | Carbamidomethylation | 183684.4844 | type:5prime\_partial len:1639 gc:universal TRINITY\_DN4595\_c0\_g1\_i4:5857-941(-) |
| 35 | 99 | TRINITY\_DN4595\_c0\_g1\_i10.p1 | 102.56 | 2 | 2 | 1.89e3 | 3 | 3 | 7 | Carbamidomethylation | 133760.0625 | type:complete len:1188 gc:universal TRINITY\_DN4595\_c0\_g1\_i10:4405-842(-) |
| 36 | 100 | TRINITY\_DN477\_c0\_g1\_i7.p1 | 100.34 | 4 | 4 | 5.09e3 | 3 | 3 | 7 | Carbamidomethylation | 122478.2578 | type:3prime\_partial len:1130 gc:universal TRINITY\_DN477\_c0\_g1\_i7:3387-1(-) |
| 36 | 101 | TRINITY\_DN477\_c0\_g1\_i8.p1 | 100.34 | 4 | 4 | 5.09e3 | 3 | 3 | 7 | Carbamidomethylation | 107165.2031 | type:complete len:985 gc:universal TRINITY\_DN477\_c0\_g1\_i8:3548-594(-) |
| 37 | 102 | TRINITY\_DN286\_c0\_g1\_i2.p1 | 89.28 | 10 | 10 | 5.62e3 | 2 | 1 | 4 | Oxidation (M), Carbamidomethylation | 11368.1738 | type:3prime\_partial len:106 gc:universal TRINITY\_DN286\_c0\_g1\_i2:40-354(+) |
| 37 | 103 | TRINITY\_DN286\_c0\_g1\_i4.p1 | 89.28 | 5 | 5 | 5.62e3 | 2 | 1 | 4 | Oxidation (M), Carbamidomethylation | 25084.5293 | type:complete len:224 gc:universal TRINITY\_DN286\_c0\_g1\_i4:40-711(+) |
| 38 | 104 | TRINITY\_DN64\_c0\_g1\_i1.p1 | 100.42 | 9 | 9 | 8.21e2 | 3 | 3 | 4 | Oxidation (M) | 51450.1914 | type:complete len:468 gc:universal TRINITY\_DN64\_c0\_g1\_i1:1555-152(-) |
| 39 | 105 | TRINITY\_DN121501\_c0\_g1\_i1.p1 | 107.56 | 13 | 13 | 1.48e3 | 3 | 3 | 3 |  | 31169.3691 | type:3prime\_partial len:267 gc:universal TRINITY\_DN121501\_c0\_g1\_i1:36-833(+) |
| 40 | 106 | TRINITY\_DN804\_c0\_g1\_i1.p1 | 105.3 | 21 | 21 | 5.81e3 | 3 | 3 | 3 | Carbamidomethylation | 26646.1699 | type:complete len:240 gc:universal TRINITY\_DN804\_c0\_g1\_i1:125-844(+) |
| 40 | 107 | TRINITY\_DN804\_c0\_g1\_i2.p1 | 105.3 | 21 | 21 | 5.81e3 | 3 | 3 | 3 | Carbamidomethylation | 26646.1699 | type:complete len:240 gc:universal TRINITY\_DN804\_c0\_g1\_i2:125-844(+) |
| 41 | 108 | TRINITY\_DN477\_c0\_g1\_i9.p1 | 106.4 | 8 | 8 | 1.78e3 | 3 | 3 | 3 | Oxidation (M) | 34222.9453 | type:complete len:306 gc:universal TRINITY\_DN477\_c0\_g1\_i9:1840-923(-) |
| 42 | 109 | TRINITY\_DN5633\_c0\_g1\_i1.p1 | 105.46 | 5 | 5 | 1.29e3 | 3 | 3 | 3 | Oxidation (M) | 97310.9922 | type:complete len:848 gc:universal TRINITY\_DN5633\_c0\_g1\_i1:2881-338(-) |
| 43 | 110 | TRINITY\_DN2636\_c0\_g1\_i1.p1 | 76.37 | 7 | 7 | 0 | 2 | 2 | 3 | Carbamidomethylation | 35741.2305 | type:complete len:314 gc:universal TRINITY\_DN2636\_c0\_g1\_i1:986-45(-) |
| 43 | 111 | TRINITY\_DN2636\_c0\_g1\_i2.p1 | 76.37 | 7 | 7 | 0 | 2 | 2 | 3 | Carbamidomethylation | 35741.2305 | type:complete len:314 gc:universal TRINITY\_DN2636\_c0\_g1\_i2:986-45(-) |
| 44 | 112 | TRINITY\_DN3545\_c0\_g1\_i2.p1 | 65.92 | 10 | 10 | 2.91e3 | 2 | 2 | 3 | Carbamidomethylation | 25563.4785 | type:5prime\_partial len:236 gc:universal TRINITY\_DN3545\_c0\_g1\_i2:3-710(+) |
| 44 | 113 | TRINITY\_DN3545\_c0\_g1\_i1.p1 | 65.92 | 8 | 8 | 2.91e3 | 2 | 2 | 3 | Carbamidomethylation | 31576.2656 | type:5prime\_partial len:293 gc:universal TRINITY\_DN3545\_c0\_g1\_i1:3-881(+) |
| 44 | 114 | TRINITY\_DN495\_c0\_g1\_i2.p1 | 57.85 | 2 | 2 | 9.79e2 | 1 | 1 | 2 | Carbamidomethylation | 72975.0312 | type:complete len:655 gc:universal TRINITY\_DN495\_c0\_g1\_i2:152-2116(+) |
| 44 | 115 | TRINITY\_DN495\_c0\_g1\_i3.p1 | 57.85 | 2 | 2 | 9.79e2 | 1 | 1 | 2 | Carbamidomethylation | 73673.9297 | type:complete len:659 gc:universal TRINITY\_DN495\_c0\_g1\_i3:152-2128(+) |
| 44 | 116 | TRINITY\_DN158421\_c0\_g1\_i1.p1 | 16.12 | 10 | 10 | 1.93e3 | 1 | 1 | 1 | Carbamidomethylation | 12814.7959 | type:internal len:115 gc:universal TRINITY\_DN158421\_c0\_g1\_i1:1-342(+) |
| 45 | 117 | TRINITY\_DN1306\_c0\_g1\_i1.p1 | 72.36 | 5 | 5 | 2.51e3 | 2 | 2 | 3 | Carbamidomethylation | 51869.3164 | type:5prime\_partial len:441 gc:universal TRINITY\_DN1306\_c0\_g1\_i1:1765-443(-) |
| 46 | 118 | TRINITY\_DN602\_c0\_g1\_i3.p1 | 89.72 | 3 | 3 | 2.99e3 | 2 | 2 | 3 | Carbamidomethylation | 92279.0078 | type:complete len:851 gc:universal TRINITY\_DN602\_c0\_g1\_i3:3046-494(-) |
| 46 | 119 | TRINITY\_DN602\_c0\_g1\_i4.p1 | 89.72 | 5 | 5 | 2.99e3 | 2 | 2 | 3 | Carbamidomethylation | 61175.9766 | type:5prime\_partial len:552 gc:universal TRINITY\_DN602\_c0\_g1\_i4:3498-1843(-) |
| 46 | 120 | TRINITY\_DN602\_c0\_g1\_i1.p1 | 89.72 | 5 | 5 | 2.99e3 | 2 | 2 | 3 | Carbamidomethylation | 65701.2422 | type:complete len:593 gc:universal TRINITY\_DN602\_c0\_g1\_i1:3622-1844(-) |
| 47 | 121 | TRINITY\_DN730\_c0\_g2\_i3.p1 | 88.46 | 12 | 12 | 3.56e3 | 2 | 2 | 3 |  | 27607.4375 | type:complete len:235 gc:universal TRINITY\_DN730\_c0\_g2\_i3:1019-315(-) |
| 47 | 122 | TRINITY\_DN730\_c0\_g2\_i2.p1 | 88.46 | 12 | 12 | 3.56e3 | 2 | 2 | 3 |  | 27607.4375 | type:complete len:235 gc:universal TRINITY\_DN730\_c0\_g2\_i2:1787-1083(-) |
| 48 | 123 | TRINITY\_DN1199\_c0\_g1\_i2.p1 | 88.57 | 10 | 10 | 3.92e2 | 2 | 2 | 2 | Carbamidomethylation | 33218.2305 | type:complete len:296 gc:universal TRINITY\_DN1199\_c0\_g1\_i2:164-1051(+) |
| 48 | 124 | TRINITY\_DN1199\_c0\_g1\_i1.p1 | 88.57 | 10 | 10 | 3.92e2 | 2 | 2 | 2 | Carbamidomethylation | 33218.2305 | type:complete len:296 gc:universal TRINITY\_DN1199\_c0\_g1\_i1:180-1067(+) |
| 49 | 125 | TRINITY\_DN15584\_c0\_g1\_i1.p1 | 85.83 | 10 | 10 | 2.5e4 | 2 | 2 | 6 | Carbamidomethylation | 29534.9648 | type:complete len:275 gc:universal TRINITY\_DN15584\_c0\_g1\_i1:874-50(-) |
| 50 | 126 | TRINITY\_DN3078\_c0\_g1\_i1.p1 | 80.98 | 2 | 2 | 0 | 2 | 2 | 2 | Carbamidomethylation | 127689.1797 | type:complete len:1109 gc:universal TRINITY\_DN3078\_c0\_g1\_i1:3628-302(-) |
| 51 | 127 | TRINITY\_DN19237\_c0\_g1\_i1.p1 | 55.62 | 12 | 12 | 0 | 2 | 2 | 2 |  | 23866.248 | type:complete len:209 gc:universal TRINITY\_DN19237\_c0\_g1\_i1:128-754(+) |
| 52 | 128 | TRINITY\_DN3036\_c0\_g1\_i3.p1 | 87.47 | 6 | 6 | 3.3e3 | 2 | 2 | 2 | Oxidation (M) | 59630.4023 | type:internal len:548 gc:universal TRINITY\_DN3036\_c0\_g1\_i3:1643-3(-) |
| 52 | 129 | TRINITY\_DN3036\_c0\_g1\_i8.p1 | 87.47 | 6 | 6 | 3.3e3 | 2 | 2 | 2 | Oxidation (M) | 59630.4023 | type:internal len:548 gc:universal TRINITY\_DN3036\_c0\_g1\_i8:1643-3(-) |
| 52 | 130 | TRINITY\_DN3036\_c0\_g1\_i2.p2 | 87.47 | 6 | 6 | 3.3e3 | 2 | 2 | 2 | Oxidation (M) | 59586.3086 | type:internal len:548 gc:universal TRINITY\_DN3036\_c0\_g1\_i2:1643-3(-) |
| 52 | 131 | TRINITY\_DN3036\_c0\_g1\_i11.p1 | 60.06 | 4 | 4 | 0 | 1 | 1 | 1 | Oxidation (M) | 40331.5547 | type:internal len:374 gc:universal TRINITY\_DN3036\_c0\_g1\_i11:1121-3(-) |
| 52 | 132 | TRINITY\_DN3036\_c0\_g1\_i16.p1 | 60.06 | 4 | 4 | 0 | 1 | 1 | 1 | Oxidation (M) | 40331.5547 | type:internal len:374 gc:universal TRINITY\_DN3036\_c0\_g1\_i16:1121-3(-) |
| 52 | 133 | TRINITY\_DN3036\_c0\_g1\_i4.p1 | 60.06 | 7 | 7 | 0 | 1 | 1 | 1 | Oxidation (M) | 25489.3926 | type:internal len:238 gc:universal TRINITY\_DN3036\_c0\_g1\_i4:713-3(-) |
| 52 | 134 | TRINITY\_DN3036\_c0\_g1\_i19.p1 | 60.06 | 4 | 4 | 0 | 1 | 1 | 1 | Oxidation (M) | 40287.4609 | type:internal len:374 gc:universal TRINITY\_DN3036\_c0\_g1\_i19:1121-3(-) |
| 52 | 135 | TRINITY\_DN3036\_c0\_g1\_i6.p1 | 60.06 | 7 | 7 | 0 | 1 | 1 | 1 | Oxidation (M) | 25533.4863 | type:internal len:238 gc:universal TRINITY\_DN3036\_c0\_g1\_i6:713-3(-) |
| 52 | 136 | TRINITY\_DN3036\_c0\_g1\_i13.p1 | 60.06 | 4 | 4 | 0 | 1 | 1 | 1 | Oxidation (M) | 40287.4609 | type:internal len:374 gc:universal TRINITY\_DN3036\_c0\_g1\_i13:1121-3(-) |
| 53 | 137 | TRINITY\_DN22492\_c0\_g1\_i1.p1 | 73.49 | 2 | 2 | 3.66e2 | 2 | 2 | 3 |  | 115296.5156 | type:complete len:1026 gc:universal TRINITY\_DN22492\_c0\_g1\_i1:171-3248(+) |
| 53 | 138 | TRINITY\_DN22492\_c0\_g1\_i2.p1 | 73.49 | 2 | 2 | 3.66e2 | 2 | 2 | 3 |  | 115355.5859 | type:complete len:1026 gc:universal TRINITY\_DN22492\_c0\_g1\_i2:39-3116(+) |
| 54 | 139 | TRINITY\_DN5500\_c0\_g1\_i2.p1 | 84.54 | 1 | 1 | 0 | 2 | 2 | 2 |  | 130974.4219 | type:3prime\_partial len:1183 gc:universal TRINITY\_DN5500\_c0\_g1\_i2:3546-1(-) |
| 54 | 140 | TRINITY\_DN5500\_c0\_g1\_i1.p1 | 84.54 | 1 | 1 | 0 | 2 | 2 | 2 |  | 132375.9688 | type:3prime\_partial len:1196 gc:universal TRINITY\_DN5500\_c0\_g1\_i1:3585-1(-) |
| 55 | 141 | TRINITY\_DN1121\_c0\_g1\_i1.p1 | 83.29 | 11 | 11 | 3.25e3 | 2 | 2 | 3 | Acetylation (Protein N-term) | 28282.5449 | type:complete len:252 gc:universal TRINITY\_DN1121\_c0\_g1\_i1:1155-400(-) |
| 56 | 142 | TRINITY\_DN278\_c0\_g1\_i1.p1 | 86.97 | 6 | 6 | 1.38e3 | 2 | 2 | 3 | Carbamidomethylation, Oxidation (M) | 55197.2969 | type:complete len:470 gc:universal TRINITY\_DN278\_c0\_g1\_i1:3091-1682(-) |
| 57 | 143 | TRINITY\_DN1653\_c0\_g1\_i1.p1 | 76.97 | 2 | 2 | 2.42e2 | 2 | 2 | 2 |  | 125115.2109 | type:complete len:1122 gc:universal TRINITY\_DN1653\_c0\_g1\_i1:180-3545(+) |
| 57 | 144 | TRINITY\_DN1653\_c0\_g1\_i3.p1 | 76.97 | 3 | 3 | 2.42e2 | 2 | 2 | 2 |  | 114336.4531 | type:complete len:1023 gc:universal TRINITY\_DN1653\_c0\_g1\_i3:180-3248(+) |
| 58 | 145 | TRINITY\_DN1936\_c0\_g2\_i1.p1 | 81.98 | 2 | 2 | 4.07e2 | 2 | 2 | 2 |  | 208525.625 | type:3prime\_partial len:1894 gc:universal TRINITY\_DN1936\_c0\_g2\_i1:5680-2(-) |
| 59 | 146 | TRINITY\_DN146208\_c0\_g1\_i1.p1 | 84.84 | 9 | 9 | 1.78e3 | 2 | 2 | 2 |  | 32340.8027 | type:5prime\_partial len:284 gc:universal TRINITY\_DN146208\_c0\_g1\_i1:876-25(-) |
| 60 | 147 | TRINITY\_DN165536\_c0\_g1\_i1.p1 | 89.48 | 12 | 12 | 6.84e3 | 2 | 2 | 2 |  | 28339.4531 | type:complete len:249 gc:universal TRINITY\_DN165536\_c0\_g1\_i1:851-105(-) |
| 61 | 148 | TRINITY\_DN13856\_c0\_g1\_i2.p2 | 65.21 | 10 | 10 | 1.67e3 | 2 | 1 | 2 |  | 31731.2012 | type:5prime\_partial len:292 gc:universal TRINITY\_DN13856\_c0\_g1\_i2:3-878(+) |
| 61 | 149 | TRINITY\_DN13856\_c0\_g1\_i1.p2 | 65.21 | 10 | 10 | 1.67e3 | 2 | 1 | 2 |  | 31646.0527 | type:5prime\_partial len:292 gc:universal TRINITY\_DN13856\_c0\_g1\_i1:3-878(+) |
| 62 | 150 | TRINITY\_DN3347\_c0\_g1\_i1.p1 | 84.06 | 3 | 3 | 0 | 2 | 2 | 2 |  | 104268.7266 | type:5prime\_partial len:924 gc:universal TRINITY\_DN3347\_c0\_g1\_i1:2-2773(+) |
| 63 | 151 | TRINITY\_DN1194\_c0\_g1\_i1.p1 | 90.52 | 5 | 5 | 2.46e2 | 2 | 2 | 3 |  | 61081.3672 | type:complete len:555 gc:universal TRINITY\_DN1194\_c0\_g1\_i1:101-1765(+) |
| 64 | 152 | TRINITY\_DN1386\_c0\_g1\_i1.p1 | 82.83 | 4 | 4 | 2.03e3 | 2 | 2 | 2 |  | 68692.1953 | type:complete len:596 gc:universal TRINITY\_DN1386\_c0\_g1\_i1:2419-632(-) |
| 65 | 153 | TRINITY\_DN102244\_c0\_g1\_i1.p1 | 60.11 | 5 | 5 | 0 | 2 | 2 | 2 |  | 34737.457 | type:internal len:304 gc:universal TRINITY\_DN102244\_c0\_g1\_i1:1-909(+) |
| 66 | 154 | TRINITY\_DN22594\_c0\_g1\_i1.p2 | 65.5 | 10 | 10 | 0 | 2 | 1 | 2 |  | 29863.3594 | type:internal len:268 gc:universal TRINITY\_DN22594\_c0\_g1\_i1:801-1(-) |
| 67 | 155 | TRINITY\_DN941\_c0\_g1\_i9.p1 | 65.24 | 1 | 1 | 8.57e2 | 2 | 2 | 2 |  | 340219.4688 | type:complete len:3186 gc:universal TRINITY\_DN941\_c0\_g1\_i9:86-9643(+) |
| 67 | 156 | TRINITY\_DN941\_c0\_g1\_i11.p1 | 28.72 | 1 | 1 | 4.05e2 | 1 | 1 | 1 |  | 110353.6641 | type:5prime\_partial len:1040 gc:universal TRINITY\_DN941\_c0\_g1\_i11:1-3120(+) |
| 67 | 157 | TRINITY\_DN941\_c0\_g1\_i3.p1 | 28.72 | 2 | 2 | 4.05e2 | 1 | 1 | 1 |  | 39175.6641 | type:internal len:363 gc:universal TRINITY\_DN941\_c0\_g1\_i3:3-1088(+) |
| 67 | 158 | TRINITY\_DN941\_c0\_g1\_i13.p1 | 28.72 | 4 | 4 | 4.05e2 | 1 | 1 | 1 |  | 21958.0625 | type:internal len:204 gc:universal TRINITY\_DN941\_c0\_g1\_i13:1-609(+) |
| 67 | 159 | TRINITY\_DN941\_c0\_g1\_i15.p1 | 28.72 | 1 | 1 | 4.05e2 | 1 | 1 | 1 |  | 127030.5156 | type:5prime\_partial len:1194 gc:universal TRINITY\_DN941\_c0\_g1\_i15:3-3584(+) |
| 68 | 160 | TRINITY\_DN79\_c0\_g1\_i1.p1 | 72.45 | 7 | 7 | 9.87e2 | 2 | 2 | 3 |  | 52590.9805 | type:5prime\_partial len:478 gc:universal TRINITY\_DN79\_c0\_g1\_i1:2132-699(-) |
| 69 | 161 | TRINITY\_DN66856\_c0\_g1\_i1.p1 | 60.58 | 4 | 4 | 0 | 1 | 1 | 2 | Oxidation (M) | 49950.4219 | type:internal len:443 gc:universal TRINITY\_DN66856\_c0\_g1\_i1:1327-2(-) |
| 70 | 162 | TRINITY\_DN118520\_c0\_g1\_i1.p1 | 73.75 | 6 | 6 | 2.2e3 | 2 | 2 | 5 |  | 61587.4766 | type:complete len:581 gc:universal TRINITY\_DN118520\_c0\_g1\_i1:1895-153(-) |
| 71 | 163 | TRINITY\_DN31399\_c0\_g1\_i2.p1 | 82.48 | 3 | 3 | 2.85e3 | 2 | 2 | 2 |  | 89961.6641 | type:complete len:819 gc:universal TRINITY\_DN31399\_c0\_g1\_i2:85-2541(+) |
| 71 | 164 | TRINITY\_DN31399\_c0\_g1\_i3.p1 | 82.48 | 3 | 3 | 2.85e3 | 2 | 2 | 2 |  | 89350.0078 | type:complete len:813 gc:universal TRINITY\_DN31399\_c0\_g1\_i3:85-2523(+) |
| 71 | 165 | TRINITY\_DN31399\_c0\_g1\_i1.p1 | 52.96 | 3 | 3 | 0 | 1 | 1 | 1 |  | 42043.4648 | type:5prime\_partial len:382 gc:universal TRINITY\_DN31399\_c0\_g1\_i1:2-1147(+) |
| 72 | 166 | TRINITY\_DN73067\_c0\_g1\_i1.p1 | 57.8 | 2 | 2 | 2.75e3 | 1 | 1 | 3 | Oxidation (M) | 93020.375 | type:complete len:831 gc:universal TRINITY\_DN73067\_c0\_g1\_i1:3714-1222(-) |
| 73 | 167 | TRINITY\_DN374\_c0\_g1\_i1.p1 | 87.5 | 10 | 10 | 2.46e2 | 2 | 2 | 2 | Carbamidomethylation | 32814.1367 | type:5prime\_partial len:290 gc:universal TRINITY\_DN374\_c0\_g1\_i1:1-870(+) |
| 73 | 168 | TRINITY\_DN374\_c0\_g1\_i2.p1 | 59.02 | 4 | 4 | 2.46e2 | 1 | 1 | 1 | Carbamidomethylation | 38811.4023 | type:complete len:341 gc:universal TRINITY\_DN374\_c0\_g1\_i2:115-1137(+) |
| 74 | 169 | TRINITY\_DN113\_c3\_g1\_i1.p1 | 90.59 | 3 | 3 | 1.2e3 | 2 | 2 | 2 |  | 125422.4922 | type:3prime\_partial len:1162 gc:universal TRINITY\_DN113\_c3\_g1\_i1:3485-3(-) |
| 75 | 170 | TRINITY\_DN3190\_c1\_g1\_i1.p1 | 82.94 | 7 | 7 | 2.86e3 | 2 | 2 | 4 |  | 35061.5508 | type:complete len:315 gc:universal TRINITY\_DN3190\_c1\_g1\_i1:68-1012(+) |
| 76 | 171 | TRINITY\_DN40728\_c1\_g1\_i1.p2 | 61.25 | 10 | 10 | 2.55e3 | 1 | 1 | 3 |  | 14073.3066 | type:5prime\_partial len:124 gc:universal TRINITY\_DN40728\_c1\_g1\_i1:1-372(+) |
| 76 | 172 | TRINITY\_DN40728\_c0\_g1\_i1.p1 | 61.25 | 13 | 13 | 2.55e3 | 1 | 1 | 3 |  | 11225.8301 | type:internal len:105 gc:universal TRINITY\_DN40728\_c0\_g1\_i1:1-312(+) |
| 76 | 173 | TRINITY\_DN195631\_c0\_g1\_i1.p2 | 61.25 | 9 | 9 | 2.55e3 | 1 | 1 | 3 |  | 15844.8984 | type:internal len:145 gc:universal TRINITY\_DN195631\_c0\_g1\_i1:2-433(+) |
| 76 | 174 | TRINITY\_DN8227\_c0\_g1\_i1.p1 | 61.25 | 10 | 10 | 2.55e3 | 1 | 1 | 3 |  | 14513.8135 | type:internal len:135 gc:universal TRINITY\_DN8227\_c0\_g1\_i1:404-3(-) |
| 76 | 175 | TRINITY\_DN152\_c0\_g1\_i15.p1 | 61.25 | 9 | 9 | 2.55e3 | 1 | 1 | 3 |  | 15560.71 | type:internal len:141 gc:universal TRINITY\_DN152\_c0\_g1\_i15:2-421(+) |
| 76 | 176 | TRINITY\_DN585\_c0\_g1\_i10.p1 | 61.25 | 6 | 6 | 2.55e3 | 1 | 1 | 3 |  | 26563.5332 | type:3prime\_partial len:236 gc:universal TRINITY\_DN585\_c0\_g1\_i10:705-1(-) |
| 76 | 177 | TRINITY\_DN152\_c0\_g1\_i2.p1 | 61.25 | 13 | 13 | 2.55e3 | 1 | 1 | 3 |  | 11754.375 | type:internal len:105 gc:universal TRINITY\_DN152\_c0\_g1\_i2:1-312(+) |
| 76 | 178 | TRINITY\_DN152\_c0\_g1\_i10.p1 | 61.25 | 7 | 7 | 2.55e3 | 1 | 1 | 3 |  | 20063.498 | type:internal len:180 gc:universal TRINITY\_DN152\_c0\_g1\_i10:1-537(+) |
| 76 | 179 | TRINITY\_DN152\_c0\_g1\_i14.p2 | 61.25 | 11 | 11 | 2.55e3 | 1 | 1 | 3 |  | 12547.4834 | type:5prime\_partial len:115 gc:universal TRINITY\_DN152\_c0\_g1\_i14:3-347(+) |
| 76 | 180 | TRINITY\_DN554\_c0\_g1\_i3.p2 | 61.25 | 5 | 5 | 2.55e3 | 1 | 1 | 3 |  | 30419.4961 | type:complete len:268 gc:universal TRINITY\_DN554\_c0\_g1\_i3:1464-661(-) |
| 76 | 181 | TRINITY\_DN1465\_c1\_g1\_i1.p1 | 61.25 | 13 | 13 | 2.55e3 | 1 | 1 | 3 |  | 11342.1572 | type:internal len:102 gc:universal TRINITY\_DN1465\_c1\_g1\_i1:2-304(+) |
| 76 | 182 | TRINITY\_DN152\_c0\_g1\_i9.p1 | 61.25 | 9 | 9 | 2.55e3 | 1 | 1 | 3 |  | 17213.1973 | type:5prime\_partial len:152 gc:universal TRINITY\_DN152\_c0\_g1\_i9:1-456(+) |
| 76 | 183 | TRINITY\_DN585\_c0\_g1\_i2.p1 | 61.25 | 7 | 7 | 2.55e3 | 1 | 1 | 3 |  | 20558.6738 | type:internal len:184 gc:universal TRINITY\_DN585\_c0\_g1\_i2:549-1(-) |
| 76 | 184 | TRINITY\_DN585\_c0\_g1\_i5.p1 | 61.25 | 5 | 5 | 2.55e3 | 1 | 1 | 3 |  | 28098.1602 | type:internal len:250 gc:universal TRINITY\_DN585\_c0\_g1\_i5:747-1(-) |
| 76 | 185 | TRINITY\_DN173718\_c0\_g1\_i1.p1 | 61.25 | 12 | 12 | 2.55e3 | 1 | 1 | 3 |  | 12865.0928 | type:3prime\_partial len:112 gc:universal TRINITY\_DN173718\_c0\_g1\_i1:37-369(+) |
| 76 | 186 | TRINITY\_DN585\_c0\_g1\_i3.p1 | 61.25 | 8 | 8 | 2.55e3 | 1 | 1 | 3 |  | 17628.2207 | type:internal len:157 gc:universal TRINITY\_DN585\_c0\_g1\_i3:468-1(-) |
| 76 | 187 | TRINITY\_DN585\_c0\_g1\_i4.p1 | 61.25 | 6 | 6 | 2.55e3 | 1 | 1 | 3 |  | 26348.0742 | type:complete len:232 gc:universal TRINITY\_DN585\_c0\_g1\_i4:847-152(-) |
| 76 | 188 | TRINITY\_DN554\_c0\_g1\_i5.p1 | 61.25 | 3 | 3 | 2.55e3 | 1 | 1 | 3 |  | 42081.4141 | type:3prime\_partial len:373 gc:universal TRINITY\_DN554\_c0\_g1\_i5:1118-3(-) |
| 77 | 189 | TRINITY\_DN21847\_c0\_g1\_i1.p1 | 44.75 | 3 | 3 | 0 | 1 | 1 | 1 |  | 41400.2812 | type:internal len:384 gc:universal TRINITY\_DN21847\_c0\_g1\_i1:1150-2(-) |
| 77 | 190 | TRINITY\_DN4497\_c0\_g1\_i1.p1 | 44.75 | 8 | 8 | 0 | 1 | 1 | 1 |  | 17016.3789 | type:internal len:160 gc:universal TRINITY\_DN4497\_c0\_g1\_i1:1-477(+) |
| 77 | 191 | TRINITY\_DN4497\_c0\_g2\_i1.p1 | 44.75 | 7 | 7 | 0 | 1 | 1 | 1 |  | 19644.3613 | type:internal len:183 gc:universal TRINITY\_DN4497\_c0\_g2\_i1:2-547(+) |
| 77 | 192 | TRINITY\_DN1172\_c0\_g2\_i2.p1 | 44.75 | 5 | 5 | 0 | 1 | 1 | 1 |  | 27813.4355 | type:internal len:264 gc:universal TRINITY\_DN1172\_c0\_g2\_i2:790-2(-) |
| 78 | 193 | TRINITY\_DN123301\_c0\_g1\_i1.p1 | 58.77 | 6 | 6 | 0 | 1 | 1 | 1 |  | 31107.168 | type:complete len:272 gc:universal TRINITY\_DN123301\_c0\_g1\_i1:90-905(+) |
| 79 | 194 | TRINITY\_DN12632\_c1\_g1\_i1.p1 | 56.89 | 3 | 3 | 0 | 1 | 1 | 1 |  | 56568.082 | type:complete len:504 gc:universal TRINITY\_DN12632\_c1\_g1\_i1:165-1676(+) |
| 80 | 195 | TRINITY\_DN1080\_c0\_g2\_i1.p1 | 17.1 | 0 | 0 | 0 | 1 | 1 | 1 |  | 990519.0625 | type:5prime\_partial len:8617 gc:universal TRINITY\_DN1080\_c0\_g2\_i1:26447-597(-) |
| 81 | 196 | TRINITY\_DN121866\_c0\_g1\_i1.p1 | 57.2 | 2 | 2 | 3.02e2 | 1 | 1 | 1 |  | 51719.8477 | type:5prime\_partial len:460 gc:universal TRINITY\_DN121866\_c0\_g1\_i1:2-1381(+) |
| 82 | 197 | TRINITY\_DN1058\_c0\_g1\_i1.p1 | 57.39 | 0 | 0 | 1.88e3 | 1 | 1 | 3 |  | 284466.2812 | type:complete len:2639 gc:universal TRINITY\_DN1058\_c0\_g1\_i1:134-8050(+) |
| 82 | 198 | TRINITY\_DN1058\_c0\_g1\_i4.p1 | 57.39 | 2 | 2 | 1.88e3 | 1 | 1 | 3 |  | 79375.5703 | type:complete len:723 gc:universal TRINITY\_DN1058\_c0\_g1\_i4:366-2534(+) |
| 82 | 199 | TRINITY\_DN1058\_c0\_g1\_i3.p1 | 57.39 | 0 | 0 | 1.88e3 | 1 | 1 | 3 |  | 277633.5312 | type:complete len:2581 gc:universal TRINITY\_DN1058\_c0\_g1\_i3:134-7876(+) |
| 82 | 200 | TRINITY\_DN1058\_c0\_g1\_i2.p2 | 57.39 | 1 | 1 | 1.88e3 | 1 | 1 | 3 |  | 140284.0938 | type:complete len:1300 gc:universal TRINITY\_DN1058\_c0\_g1\_i2:4090-7989(+) |
| 83 | 201 | TRINITY\_DN89\_c0\_g1\_i1.p1 | 59.6 | 4 | 4 | 7.59e1 | 1 | 1 | 1 | Carbamidomethylation | 43014.0469 | type:complete len:376 gc:universal TRINITY\_DN89\_c0\_g1\_i1:117-1244(+) |
| 84 | 202 | TRINITY\_DN8903\_c0\_g1\_i1.p1 | 55.93 | 2 | 2 | 1.97e3 | 1 | 1 | 2 |  | 50029.5586 | type:5prime\_partial len:447 gc:universal TRINITY\_DN8903\_c0\_g1\_i1:1546-206(-) |
| 85 | 203 | TRINITY\_DN2472\_c0\_g1\_i1.p1 | 56.84 | 5 | 5 | 1.62e3 | 1 | 1 | 1 | Acetylation (Protein N-term) | 37676.8203 | type:complete len:344 gc:universal TRINITY\_DN2472\_c0\_g1\_i1:311-1342(+) |
| 87 | 204 | TRINITY\_DN468\_c1\_g1\_i1.p1 | 58.74 | 10 | 10 | 0 | 1 | 1 | 3 |  | 21670.5645 | type:complete len:191 gc:universal TRINITY\_DN468\_c1\_g1\_i1:676-104(-) |
| 88 | 205 | TRINITY\_DN10724\_c0\_g1\_i1.p2 | 58.72 | 10 | 10 | 0 | 1 | 1 | 1 |  | 16065.7344 | type:3prime\_partial len:148 gc:universal TRINITY\_DN10724\_c0\_g1\_i1:442-2(-) |
| 88 | 206 | TRINITY\_DN10724\_c0\_g1\_i5.p1 | 58.72 | 6 | 6 | 0 | 1 | 1 | 1 |  | 28399.5234 | type:internal len:258 gc:universal TRINITY\_DN10724\_c0\_g1\_i5:773-3(-) |
| 88 | 207 | TRINITY\_DN10724\_c0\_g1\_i4.p1 | 58.72 | 6 | 6 | 0 | 1 | 1 | 1 |  | 28383.5254 | type:internal len:258 gc:universal TRINITY\_DN10724\_c0\_g1\_i4:773-3(-) |
| 88 | 208 | TRINITY\_DN10724\_c0\_g1\_i3.p1 | 58.72 | 9 | 9 | 0 | 1 | 1 | 1 |  | 19100.1836 | type:3prime\_partial len:174 gc:universal TRINITY\_DN10724\_c0\_g1\_i3:521-3(-) |
| 89 | 209 | TRINITY\_DN3016\_c0\_g1\_i5.p1 | 58.4 | 3 | 3 | 0 | 1 | 1 | 1 |  | 46762.2305 | type:complete len:406 gc:universal TRINITY\_DN3016\_c0\_g1\_i5:2713-1496(-) |
| 89 | 210 | TRINITY\_DN3016\_c0\_g1\_i8.p1 | 58.4 | 3 | 3 | 0 | 1 | 1 | 1 |  | 46762.2305 | type:complete len:406 gc:universal TRINITY\_DN3016\_c0\_g1\_i8:2713-1496(-) |
| 89 | 211 | TRINITY\_DN3016\_c0\_g1\_i11.p1 | 58.4 | 3 | 3 | 0 | 1 | 1 | 1 |  | 46762.2305 | type:complete len:406 gc:universal TRINITY\_DN3016\_c0\_g1\_i11:2541-1324(-) |
| 89 | 212 | TRINITY\_DN3016\_c0\_g1\_i6.p1 | 58.4 | 3 | 3 | 0 | 1 | 1 | 1 |  | 46762.2305 | type:complete len:406 gc:universal TRINITY\_DN3016\_c0\_g1\_i6:2760-1543(-) |
| 89 | 213 | TRINITY\_DN3016\_c0\_g1\_i10.p1 | 58.4 | 3 | 3 | 0 | 1 | 1 | 1 |  | 46762.2305 | type:complete len:406 gc:universal TRINITY\_DN3016\_c0\_g1\_i10:2760-1543(-) |
| 89 | 214 | TRINITY\_DN3016\_c0\_g1\_i17.p1 | 58.4 | 3 | 3 | 0 | 1 | 1 | 1 |  | 46762.2305 | type:complete len:406 gc:universal TRINITY\_DN3016\_c0\_g1\_i17:2691-1474(-) |
| 89 | 215 | TRINITY\_DN3016\_c0\_g1\_i13.p1 | 58.4 | 3 | 3 | 0 | 1 | 1 | 1 |  | 46762.2305 | type:complete len:406 gc:universal TRINITY\_DN3016\_c0\_g1\_i13:2760-1543(-) |
| 89 | 216 | TRINITY\_DN3016\_c0\_g1\_i4.p1 | 58.4 | 3 | 3 | 0 | 1 | 1 | 1 |  | 46762.2305 | type:complete len:406 gc:universal TRINITY\_DN3016\_c0\_g1\_i4:1529-312(-) |
| 89 | 217 | TRINITY\_DN3016\_c0\_g1\_i18.p1 | 58.4 | 3 | 3 | 0 | 1 | 1 | 1 |  | 46762.2305 | type:complete len:406 gc:universal TRINITY\_DN3016\_c0\_g1\_i18:2766-1549(-) |
| 89 | 218 | TRINITY\_DN3016\_c0\_g1\_i2.p1 | 58.4 | 3 | 3 | 0 | 1 | 1 | 1 |  | 46762.2305 | type:complete len:406 gc:universal TRINITY\_DN3016\_c0\_g1\_i2:2691-1474(-) |
| 90 | 219 | TRINITY\_DN79126\_c0\_g1\_i1.p1 | 56.88 | 10 | 10 | 7.04e2 | 1 | 1 | 1 |  | 13015.4727 | type:complete len:115 gc:universal TRINITY\_DN79126\_c0\_g1\_i1:397-53(-) |
| 91 | 220 | TRINITY\_DN691\_c0\_g1\_i2.p1 | 61.86 | 1 | 1 | 1.57e3 | 1 | 1 | 3 | Carbamidomethylation | 164625.2188 | type:complete len:1673 gc:universal TRINITY\_DN691\_c0\_g1\_i2:5503-485(-) |
| 91 | 221 | TRINITY\_DN691\_c0\_g1\_i1.p1 | 61.86 | 1 | 1 | 1.57e3 | 1 | 1 | 3 | Carbamidomethylation | 169920.1562 | type:complete len:1726 gc:universal TRINITY\_DN691\_c0\_g1\_i1:5662-485(-) |
| 92 | 222 | TRINITY\_DN79\_c1\_g1\_i1.p1 | 48.97 | 2 | 2 | 0 | 1 | 1 | 1 |  | 50925.4375 | type:5prime\_partial len:450 gc:universal TRINITY\_DN79\_c1\_g1\_i1:3268-1919(-) |
| 93 | 223 | TRINITY\_DN1174\_c2\_g1\_i1.p1 | 61.05 | 4 | 4 | 0 | 1 | 1 | 1 |  | 36402.7227 | type:complete len:323 gc:universal TRINITY\_DN1174\_c2\_g1\_i1:48-1016(+) |
| 94 | 224 | TRINITY\_DN2217\_c0\_g1\_i2.p1 | 56.87 | 5 | 5 | 6.5e2 | 1 | 1 | 1 | Carbamidomethylation | 28618.9531 | type:complete len:254 gc:universal TRINITY\_DN2217\_c0\_g1\_i2:884-123(-) |
| 94 | 225 | TRINITY\_DN2217\_c0\_g1\_i3.p1 | 56.87 | 2 | 2 | 6.5e2 | 1 | 1 | 1 | Carbamidomethylation | 63077.0508 | type:complete len:566 gc:universal TRINITY\_DN2217\_c0\_g1\_i3:1820-123(-) |
| 95 | 226 | TRINITY\_DN571\_c0\_g1\_i2.p1 | 31.16 | 8 | 8 | 1.27e2 | 1 | 1 | 1 | Oxidation (M) | 21030.8027 | type:complete len:186 gc:universal TRINITY\_DN571\_c0\_g1\_i2:192-749(+) |
| 95 | 227 | TRINITY\_DN571\_c0\_g1\_i1.p1 | 31.16 | 8 | 8 | 1.27e2 | 1 | 1 | 1 | Oxidation (M) | 20785.6484 | type:complete len:186 gc:universal TRINITY\_DN571\_c0\_g1\_i1:192-749(+) |
| 96 | 228 | TRINITY\_DN902\_c1\_g1\_i1.p1 | 15.37 | 7 | 7 | 5.38e2 | 1 | 1 | 1 | Carbamidomethylation | 16370.7676 | type:3prime\_partial len:153 gc:universal TRINITY\_DN902\_c1\_g1\_i1:146-601(+) |
| 96 | 229 | TRINITY\_DN902\_c1\_g1\_i2.p1 | 15.37 | 7 | 7 | 5.38e2 | 1 | 1 | 1 | Carbamidomethylation | 16400.9395 | type:3prime\_partial len:153 gc:universal TRINITY\_DN902\_c1\_g1\_i2:90-545(+) |
| 97 | 230 | TRINITY\_DN2885\_c0\_g1\_i2.p1 | 56.98 | 11 | 11 | 1.06e3 | 1 | 1 | 1 |  | 12758.7783 | type:complete len:114 gc:universal TRINITY\_DN2885\_c0\_g1\_i2:31-372(+) |
| 97 | 231 | TRINITY\_DN2885\_c0\_g1\_i5.p1 | 56.98 | 11 | 11 | 1.06e3 | 1 | 1 | 1 |  | 12383.5439 | type:3prime\_partial len:112 gc:universal TRINITY\_DN2885\_c0\_g1\_i5:31-363(+) |
| 97 | 232 | TRINITY\_DN2885\_c0\_g1\_i4.p1 | 56.98 | 11 | 11 | 1.06e3 | 1 | 1 | 1 |  | 12245.125 | type:5prime\_partial len:109 gc:universal TRINITY\_DN2885\_c0\_g1\_i4:1-327(+) |
| 98 | 233 | TRINITY\_DN141214\_c0\_g1\_i1.p1 | 56.77 | 9 | 9 | 2.51e2 | 1 | 1 | 1 |  | 19633.9922 | type:internal len:179 gc:universal TRINITY\_DN141214\_c0\_g1\_i1:2-535(+) |
| 99 | 234 | TRINITY\_DN923\_c0\_g1\_i1.p1 | 56.94 | 2 | 2 | 1.15e3 | 1 | 1 | 1 |  | 51773.8242 | type:complete len:442 gc:universal TRINITY\_DN923\_c0\_g1\_i1:53-1378(+) |
| 100 | 235 | TRINITY\_DN902\_c1\_g2\_i3.p1 | 58.02 | 6 | 6 | 4.95e3 | 1 | 1 | 1 | Carbamidomethylation | 19070.0254 | type:internal len:178 gc:universal TRINITY\_DN902\_c1\_g2\_i3:2-532(+) |
| 100 | 236 | TRINITY\_DN902\_c1\_g2\_i1.p1 | 58.02 | 6 | 6 | 4.95e3 | 1 | 1 | 1 | Carbamidomethylation | 18964.9824 | type:internal len:178 gc:universal TRINITY\_DN902\_c1\_g2\_i1:2-532(+) |
| 100 | 237 | TRINITY\_DN902\_c1\_g2\_i4.p1 | 58.02 | 6 | 6 | 4.95e3 | 1 | 1 | 1 | Carbamidomethylation | 19994.1074 | type:internal len:182 gc:universal TRINITY\_DN902\_c1\_g2\_i4:2-544(+) |
| 101 | 238 | TRINITY\_DN1566\_c0\_g1\_i1.p1 | 56.23 | 0 | 0 | 6.43e2 | 1 | 1 | 3 |  | 529444 | type:complete len:4626 gc:universal TRINITY\_DN1566\_c0\_g1\_i1:42-13919(+) |
| 102 | 239 | TRINITY\_DN8101\_c0\_g1\_i2.p2 | 55.88 | 2 | 2 | 4.59e2 | 1 | 1 | 1 |  | 88889.1172 | type:complete len:820 gc:universal TRINITY\_DN8101\_c0\_g1\_i2:2597-138(-) |
| 102 | 240 | TRINITY\_DN8101\_c0\_g1\_i9.p2 | 55.88 | 1 | 1 | 4.59e2 | 1 | 1 | 1 |  | 121647.5547 | type:5prime\_partial len:1113 gc:universal TRINITY\_DN8101\_c0\_g1\_i9:3476-138(-) |
| 102 | 241 | TRINITY\_DN8101\_c0\_g1\_i10.p2 | 55.88 | 6 | 6 | 4.59e2 | 1 | 1 | 1 |  | 23508.5586 | type:internal len:214 gc:universal TRINITY\_DN8101\_c0\_g1\_i10:639-1(-) |
| 102 | 242 | TRINITY\_DN8101\_c0\_g1\_i5.p1 | 55.88 | 2 | 2 | 4.59e2 | 1 | 1 | 1 |  | 88960.2812 | type:complete len:820 gc:universal TRINITY\_DN8101\_c0\_g1\_i5:2531-72(-) |
| 102 | 243 | TRINITY\_DN8101\_c0\_g1\_i8.p1 | 55.88 | 1 | 1 | 4.59e2 | 1 | 1 | 1 |  | 121763.7734 | type:5prime\_partial len:1113 gc:universal TRINITY\_DN8101\_c0\_g1\_i8:3410-72(-) |
| 102 | 244 | TRINITY\_DN8101\_c0\_g1\_i11.p2 | 55.88 | 1 | 1 | 4.59e2 | 1 | 1 | 1 |  | 121692.6094 | type:5prime\_partial len:1113 gc:universal TRINITY\_DN8101\_c0\_g1\_i11:3410-72(-) |
| 102 | 245 | TRINITY\_DN8101\_c0\_g1\_i4.p1 | 55.88 | 3 | 3 | 4.59e2 | 1 | 1 | 1 |  | 55522.8164 | type:5prime\_partial len:511 gc:universal TRINITY\_DN8101\_c0\_g1\_i4:1604-72(-) |
| 102 | 246 | TRINITY\_DN8101\_c0\_g1\_i6.p2 | 55.88 | 2 | 2 | 4.59e2 | 1 | 1 | 1 |  | 88934.1797 | type:complete len:820 gc:universal TRINITY\_DN8101\_c0\_g1\_i6:2531-72(-) |
| 103 | 247 | TRINITY\_DN30329\_c0\_g1\_i1.p1 | 56.99 | 8 | 8 | 4.36e3 | 1 | 1 | 2 |  | 23962.9473 | type:5prime\_partial len:213 gc:universal TRINITY\_DN30329\_c0\_g1\_i1:1-639(+) |
| 103 | 248 | TRINITY\_DN30329\_c0\_g2\_i1.p2 | 56.99 | 7 | 7 | 4.36e3 | 1 | 1 | 2 |  | 26093.5586 | type:5prime\_partial len:237 gc:universal TRINITY\_DN30329\_c0\_g2\_i1:1-711(+) |
| 104 | 249 | TRINITY\_DN153369\_c0\_g1\_i1.p2 | 38.18 | 6 | 6 | 0 | 1 | 1 | 3 | Carbamidomethylation | 12450.9941 | type:5prime\_partial len:115 gc:universal TRINITY\_DN153369\_c0\_g1\_i1:2-346(+) |
| 105 | 250 | TRINITY\_DN7504\_c0\_g1\_i1.p1 | 56.96 | 3 | 3 | 1.08e3 | 1 | 1 | 1 |  | 46519.1953 | type:complete len:414 gc:universal TRINITY\_DN7504\_c0\_g1\_i1:87-1328(+) |
| 106 | 251 | TRINITY\_DN163801\_c0\_g1\_i1.p1 | 56.95 | 3 | 3 | 2.03e3 | 1 | 1 | 2 | Acetylation (Protein N-term) | 33076.9062 | type:complete len:298 gc:universal TRINITY\_DN163801\_c0\_g1\_i1:55-948(+) |
| 107 | 252 | TRINITY\_DN360\_c0\_g1\_i1.p1 | 57.72 | 3 | 3 | 9.7e2 | 1 | 1 | 1 |  | 52389.3047 | type:5prime\_partial len:476 gc:universal TRINITY\_DN360\_c0\_g1\_i1:2237-810(-) |
| 108 | 253 | TRINITY\_DN761\_c0\_g1\_i1.p1 | 57.13 | 2 | 2 | 6.48e2 | 1 | 1 | 1 |  | 95330.7812 | type:complete len:830 gc:universal TRINITY\_DN761\_c0\_g1\_i1:129-2618(+) |
| 108 | 254 | TRINITY\_DN761\_c0\_g1\_i2.p1 | 57.13 | 2 | 2 | 6.48e2 | 1 | 1 | 1 |  | 95330.7812 | type:complete len:830 gc:universal TRINITY\_DN761\_c0\_g1\_i2:87-2576(+) |
| 108 | 255 | TRINITY\_DN761\_c0\_g1\_i3.p1 | 57.13 | 2 | 2 | 6.48e2 | 1 | 1 | 1 |  | 96968.6562 | type:complete len:845 gc:universal TRINITY\_DN761\_c0\_g1\_i3:129-2663(+) |
| 109 | 256 | TRINITY\_DN684\_c0\_g1\_i1.p1 | 46.76 | 2 | 2 | 0 | 1 | 1 | 1 | Oxidation (M) | 75895.8984 | type:5prime\_partial len:675 gc:universal TRINITY\_DN684\_c0\_g1\_i1:2-2026(+) |
| 110 | 257 | TRINITY\_DN77\_c1\_g1\_i1.p1 | 56.35 | 1 | 1 | 5.16e2 | 1 | 1 | 1 |  | 198350.2344 | type:3prime\_partial len:1965 gc:universal TRINITY\_DN77\_c1\_g1\_i1:2-5893(+) |
| 111 | 258 | TRINITY\_DN34481\_c0\_g1\_i1.p1 | 61.11 | 1 | 1 | 4.59e2 | 1 | 1 | 1 | Carbamidomethylation | 92307.5469 | type:complete len:802 gc:universal TRINITY\_DN34481\_c0\_g1\_i1:2487-82(-) |
| 112 | 259 | TRINITY\_DN95248\_c0\_g1\_i1.p1 | 56.92 | 10 | 10 | 0 | 1 | 1 | 1 |  | 14722.6113 | type:complete len:124 gc:universal TRINITY\_DN95248\_c0\_g1\_i1:197-568(+) |
| 113 | 260 | TRINITY\_DN121236\_c0\_g1\_i1.p1 | 58.78 | 3 | 3 | 0 | 1 | 1 | 1 |  | 64133.2812 | type:internal len:568 gc:universal TRINITY\_DN121236\_c0\_g1\_i1:1701-1(-) |
| 114 | 261 | TRINITY\_DN3271\_c0\_g1\_i1.p1 | 55.93 | 3 | 3 | 0 | 1 | 1 | 1 |  | 45246.3555 | type:complete len:399 gc:universal TRINITY\_DN3271\_c0\_g1\_i1:218-1414(+) |
| 114 | 262 | TRINITY\_DN3271\_c0\_g1\_i2.p1 | 55.93 | 3 | 3 | 0 | 1 | 1 | 1 |  | 53020.332 | type:5prime\_partial len:468 gc:universal TRINITY\_DN3271\_c0\_g1\_i2:2-1405(+) |
| 115 | 263 | TRINITY\_DN1907\_c0\_g1\_i2.p1 | 56.99 | 5 | 5 | 1.5e3 | 1 | 1 | 2 |  | 24166.1172 | type:complete len:210 gc:universal TRINITY\_DN1907\_c0\_g1\_i2:53-682(+) |
| 115 | 264 | TRINITY\_DN1907\_c0\_g1\_i1.p1 | 56.99 | 5 | 5 | 1.5e3 | 1 | 1 | 2 |  | 24233.2676 | type:complete len:211 gc:universal TRINITY\_DN1907\_c0\_g1\_i1:56-688(+) |
| 116 | 265 | TRINITY\_DN850\_c0\_g1\_i1.p1 | 22.25 | 3 | 3 | 0 | 1 | 1 | 1 |  | 41749.3359 | type:complete len:374 gc:universal TRINITY\_DN850\_c0\_g1\_i1:1261-140(-) |
| 117 | 266 | TRINITY\_DN165\_c0\_g1\_i1.p1 | 52.73 | 3 | 3 | 1.37e2 | 1 | 1 | 1 |  | 68397.5781 | type:complete len:598 gc:universal TRINITY\_DN165\_c0\_g1\_i1:147-1940(+) |
| 118 | 267 | TRINITY\_DN96161\_c0\_g1\_i1.p1 | 58.96 | 4 | 4 | 0 | 1 | 1 | 1 | Carbamidomethylation | 50498.6562 | type:complete len:453 gc:universal TRINITY\_DN96161\_c0\_g1\_i1:1593-235(-) |
| 119 | 268 | TRINITY\_DN1339\_c6\_g1\_i1.p1 | 56.98 | 2 | 2 | 1.51e3 | 1 | 1 | 1 |  | 61274.3594 | type:internal len:570 gc:universal TRINITY\_DN1339\_c6\_g1\_i1:1-1707(+) |
| 119 | 269 | TRINITY\_DN1339\_c0\_g1\_i2.p1 | 56.98 | 3 | 3 | 1.51e3 | 1 | 1 | 1 |  | 41222.6992 | type:internal len:384 gc:universal TRINITY\_DN1339\_c0\_g1\_i2:1151-3(-) |
| 119 | 270 | TRINITY\_DN124769\_c0\_g1\_i1.p1 | 56.98 | 11 | 11 | 1.51e3 | 1 | 1 | 1 |  | 12144.8828 | type:internal len:113 gc:universal TRINITY\_DN124769\_c0\_g1\_i1:1-336(+) |
| 119 | 271 | TRINITY\_DN24414\_c1\_g1\_i2.p2 | 56.98 | 8 | 8 | 1.51e3 | 1 | 1 | 1 |  | 15590.8623 | type:internal len:146 gc:universal TRINITY\_DN24414\_c1\_g1\_i2:435-1(-) |
| 119 | 272 | TRINITY\_DN1172\_c0\_g1\_i3.p1 | 56.98 | 7 | 7 | 1.51e3 | 1 | 1 | 1 |  | 19709.3613 | type:internal len:182 gc:universal TRINITY\_DN1172\_c0\_g1\_i3:545-3(-) |
| 119 | 273 | TRINITY\_DN119673\_c0\_g1\_i1.p1 | 56.98 | 9 | 9 | 1.51e3 | 1 | 1 | 1 |  | 13988.7559 | type:internal len:129 gc:universal TRINITY\_DN119673\_c0\_g1\_i1:2-385(+) |
| 119 | 274 | TRINITY\_DN1339\_c0\_g1\_i1.p1 | 56.98 | 5 | 5 | 1.51e3 | 1 | 1 | 1 |  | 24722.7773 | type:internal len:229 gc:universal TRINITY\_DN1339\_c0\_g1\_i1:684-1(-) |
| 119 | 275 | TRINITY\_DN6701\_c0\_g1\_i1.p1 | 56.98 | 6 | 6 | 1.51e3 | 1 | 1 | 1 |  | 21246.0547 | type:internal len:196 gc:universal TRINITY\_DN6701\_c0\_g1\_i1:1-585(+) |
| 119 | 276 | TRINITY\_DN1172\_c0\_g1\_i5.p1 | 56.98 | 5 | 5 | 1.51e3 | 1 | 1 | 1 |  | 24525.6855 | type:internal len:226 gc:universal TRINITY\_DN1172\_c0\_g1\_i5:677-3(-) |
| 119 | 277 | TRINITY\_DN6701\_c0\_g1\_i2.p2 | 56.98 | 8 | 8 | 1.51e3 | 1 | 1 | 1 |  | 16512.8535 | type:internal len:152 gc:universal TRINITY\_DN6701\_c0\_g1\_i2:3-455(+) |
| 119 | 278 | TRINITY\_DN1339\_c6\_g1\_i2.p1 | 56.98 | 3 | 3 | 1.51e3 | 1 | 1 | 1 |  | 43821.8359 | type:internal len:408 gc:universal TRINITY\_DN1339\_c6\_g1\_i2:3-1223(+) |
| 120 | 279 | TRINITY\_DN1374\_c0\_g1\_i1.p1 | 35.56 | 2 | 2 | 8.01e2 | 1 | 1 | 1 | Oxidation (M) | 63387.3398 | type:complete len:548 gc:universal TRINITY\_DN1374\_c0\_g1\_i1:1751-108(-) |
| 121 | 280 | TRINITY\_DN495\_c0\_g1\_i5.p1 | 55.69 | 3 | 3 | 0 | 1 | 1 | 1 | Carbamidomethylation | 45678.0781 | type:3prime\_partial len:407 gc:universal TRINITY\_DN495\_c0\_g1\_i5:152-1369(+) |
| 121 | 281 | TRINITY\_DN495\_c0\_g1\_i6.p1 | 55.69 | 3 | 3 | 0 | 1 | 1 | 1 | Carbamidomethylation | 42984.9375 | type:3prime\_partial len:382 gc:universal TRINITY\_DN495\_c0\_g1\_i6:152-1294(+) |
| 122 | 282 | TRINITY\_DN135129\_c0\_g1\_i1.p1 | 37.02 | 6 | 6 | 2.04e3 | 1 | 1 | 2 |  | 17174.043 | type:internal len:155 gc:universal TRINITY\_DN135129\_c0\_g1\_i1:1-462(+) |
| 123 | 283 | TRINITY\_DN1143\_c0\_g1\_i1.p1 | 55.56 | 2 | 2 | 0 | 1 | 1 | 1 |  | 68207.8203 | type:complete len:629 gc:universal TRINITY\_DN1143\_c0\_g1\_i1:2539-653(-) |
| 124 | 284 | TRINITY\_DN5229\_c0\_g1\_i1.p1 | 56.34 | 2 | 2 | 7.33e2 | 1 | 1 | 1 |  | 80941.0312 | type:complete len:728 gc:universal TRINITY\_DN5229\_c0\_g1\_i1:2363-180(-) |
| 125 | 285 | TRINITY\_DN167\_c0\_g2\_i1.p1 | 62.01 | 2 | 2 | 0 | 1 | 1 | 2 |  | 71610.8359 | type:complete len:658 gc:universal TRINITY\_DN167\_c0\_g2\_i1:165-2138(+) |
| 126 | 286 | TRINITY\_DN11643\_c0\_g1\_i1.p1 | 55.62 | 7 | 7 | 0 | 1 | 1 | 1 |  | 21061.2832 | type:complete len:187 gc:universal TRINITY\_DN11643\_c0\_g1\_i1:1748-1188(-) |
| 127 | 287 | TRINITY\_DN333\_c0\_g1\_i1.p1 | 55.45 | 3 | 3 | 7.37e2 | 1 | 1 | 1 |  | 44545.1602 | type:5prime\_partial len:384 gc:universal TRINITY\_DN333\_c0\_g1\_i1:3-1154(+) |
| 127 | 288 | TRINITY\_DN333\_c0\_g1\_i2.p1 | 55.45 | 3 | 3 | 7.37e2 | 1 | 1 | 1 |  | 44545.1602 | type:5prime\_partial len:384 gc:universal TRINITY\_DN333\_c0\_g1\_i2:3-1154(+) |
| 128 | 289 | TRINITY\_DN26249\_c0\_g1\_i1.p1 | 35.47 | 3 | 3 | 0 | 1 | 1 | 2 |  | 54490.1055 | type:5prime\_partial len:477 gc:universal TRINITY\_DN26249\_c0\_g1\_i1:1733-303(-) |
| 129 | 290 | TRINITY\_DN172571\_c0\_g1\_i1.p1 | 56.36 | 12 | 12 | 0 | 1 | 1 | 1 |  | 11500.2529 | type:complete len:104 gc:universal TRINITY\_DN172571\_c0\_g1\_i1:30-341(+) |
| 130 | 291 | TRINITY\_DN140696\_c0\_g1\_i1.p1 | 53.43 | 3 | 3 | 8.95e2 | 1 | 1 | 1 |  | 29465.1973 | type:complete len:260 gc:universal TRINITY\_DN140696\_c0\_g1\_i1:43-822(+) |
| 131 | 292 | TRINITY\_DN8707\_c0\_g1\_i2.p1 | 54.96 | 2 | 2 | 9.37e2 | 1 | 1 | 1 |  | 61942.9023 | type:complete len:564 gc:universal TRINITY\_DN8707\_c0\_g1\_i2:2751-1060(-) |
| 132 | 293 | TRINITY\_DN875\_c0\_g1\_i2.p1 | 44.56 | 2 | 2 | 0 | 1 | 1 | 2 | Carbamidomethylation | 67023.6094 | type:complete len:606 gc:universal TRINITY\_DN875\_c0\_g1\_i2:2136-319(-) |
| 132 | 294 | TRINITY\_DN875\_c0\_g1\_i3.p1 | 44.56 | 2 | 2 | 0 | 1 | 1 | 2 | Carbamidomethylation | 67023.6094 | type:complete len:606 gc:universal TRINITY\_DN875\_c0\_g1\_i3:2042-225(-) |
| 132 | 295 | TRINITY\_DN875\_c0\_g1\_i1.p1 | 44.56 | 2 | 2 | 0 | 1 | 1 | 2 | Carbamidomethylation | 67023.6094 | type:complete len:606 gc:universal TRINITY\_DN875\_c0\_g1\_i1:2207-390(-) |
| 133 | 296 | TRINITY\_DN540\_c0\_g1\_i1.p1 | 54.05 | 2 | 2 | 2.09e2 | 1 | 1 | 1 | Acetylation (Protein N-term) | 52183.2109 | type:complete len:480 gc:universal TRINITY\_DN540\_c0\_g1\_i1:1503-64(-) |
| 134 | 297 | TRINITY\_DN3773\_c0\_g1\_i1.p1 | 57.56 | 4 | 4 | 2.43e3 | 1 | 1 | 2 | Carbamidomethylation | 56129.7773 | type:complete len:504 gc:universal TRINITY\_DN3773\_c0\_g1\_i1:168-1679(+) |
| 135 | 298 | TRINITY\_DN32522\_c0\_g1\_i1.p1 | 52.08 | 4 | 4 | 3.23e2 | 1 | 1 | 1 |  | 26779.0059 | type:complete len:235 gc:universal TRINITY\_DN32522\_c0\_g1\_i1:147-851(+) |
| 136 | 299 | TRINITY\_DN6920\_c0\_g1\_i3.p1 | 45.85 | 3 | 3 | 3.39e2 | 1 | 1 | 1 | Carbamidomethylation | 46932.582 | type:5prime\_partial len:411 gc:universal TRINITY\_DN6920\_c0\_g1\_i3:2124-892(-) |
| 136 | 300 | TRINITY\_DN6920\_c0\_g1\_i2.p1 | 45.85 | 3 | 3 | 3.39e2 | 1 | 1 | 1 | Carbamidomethylation | 46932.582 | type:5prime\_partial len:411 gc:universal TRINITY\_DN6920\_c0\_g1\_i2:2147-915(-) |
| 136 | 301 | TRINITY\_DN6920\_c0\_g1\_i4.p1 | 45.85 | 4 | 4 | 3.39e2 | 1 | 1 | 1 | Carbamidomethylation | 32195.3809 | type:5prime\_partial len:282 gc:universal TRINITY\_DN6920\_c0\_g1\_i4:1321-476(-) |
| 136 | 302 | TRINITY\_DN6920\_c0\_g1\_i1.p1 | 45.85 | 4 | 4 | 3.39e2 | 1 | 1 | 1 | Carbamidomethylation | 32882.168 | type:complete len:287 gc:universal TRINITY\_DN6920\_c0\_g1\_i1:1775-915(-) |
| 137 | 303 | TRINITY\_DN97809\_c0\_g1\_i1.p1 | 55.26 | 7 | 7 | 2.26e3 | 1 | 1 | 3 |  | 20461.1211 | type:internal len:184 gc:universal TRINITY\_DN97809\_c0\_g1\_i1:2-550(+) |
| 138 | 304 | TRINITY\_DN909\_c0\_g1\_i1.p1 | 21.14 | 4 | 4 | 7.42e2 | 1 | 1 | 1 |  | 35211.4883 | type:5prime\_partial len:313 gc:universal TRINITY\_DN909\_c0\_g1\_i1:1-939(+) |
| 139 | 305 | TRINITY\_DN413\_c0\_g1\_i4.p1 | 55.21 | 2 | 2 | 6.58e2 | 1 | 1 | 1 |  | 52354.1758 | type:3prime\_partial len:463 gc:universal TRINITY\_DN413\_c0\_g1\_i4:1388-3(-) |
| 139 | 306 | TRINITY\_DN413\_c0\_g1\_i1.p1 | 55.21 | 3 | 3 | 6.58e2 | 1 | 1 | 1 |  | 40351.7656 | type:complete len:355 gc:universal TRINITY\_DN413\_c0\_g1\_i1:1352-288(-) |
| 139 | 307 | TRINITY\_DN413\_c0\_g1\_i2.p1 | 55.21 | 8 | 8 | 6.58e2 | 1 | 1 | 1 |  | 15739.6523 | type:3prime\_partial len:140 gc:universal TRINITY\_DN413\_c0\_g1\_i2:419-3(-) |
| 139 | 308 | TRINITY\_DN413\_c0\_g1\_i3.p1 | 55.21 | 3 | 3 | 6.58e2 | 1 | 1 | 1 |  | 39501.6133 | type:complete len:350 gc:universal TRINITY\_DN413\_c0\_g1\_i3:1286-237(-) |
| 140 | 309 | TRINITY\_DN2776\_c0\_g1\_i6.p1 | 52.99 | 3 | 3 | 0 | 1 | 1 | 1 |  | 41162.2109 | type:complete len:374 gc:universal TRINITY\_DN2776\_c0\_g1\_i6:1221-100(-) |
| 140 | 310 | TRINITY\_DN2776\_c0\_g1\_i2.p1 | 52.99 | 4 | 4 | 0 | 1 | 1 | 1 |  | 27732.6992 | type:complete len:247 gc:universal TRINITY\_DN2776\_c0\_g1\_i2:796-56(-) |
| 140 | 311 | TRINITY\_DN2776\_c0\_g1\_i1.p1 | 52.99 | 3 | 3 | 0 | 1 | 1 | 1 |  | 41162.2109 | type:complete len:374 gc:universal TRINITY\_DN2776\_c0\_g1\_i1:1221-100(-) |
| 141 | 312 | TRINITY\_DN2963\_c0\_g1\_i1.p1 | 55.5 | 10 | 10 | 2.48e2 | 1 | 1 | 1 | Acetylation (Protein N-term) | 13082.1787 | type:complete len:116 gc:universal TRINITY\_DN2963\_c0\_g1\_i1:1072-725(-) |
| 142 | 313 | TRINITY\_DN201860\_c0\_g1\_i1.p1 | 57.09 | 6 | 6 | 3.78e2 | 1 | 1 | 1 | Carbamidomethylation | 24265.8906 | type:internal len:221 gc:universal TRINITY\_DN201860\_c0\_g1\_i1:1-660(+) |
| 143 | 314 | TRINITY\_DN5554\_c0\_g1\_i1.p1 | 56.37 | 4 | 4 | 0 | 1 | 1 | 1 |  | 35530.5234 | type:complete len:316 gc:universal TRINITY\_DN5554\_c0\_g1\_i1:223-1170(+) |
| 144 | 315 | TRINITY\_DN36857\_c0\_g1\_i1.p2 | 54.32 | 6 | 6 | 2.39e2 | 1 | 1 | 1 |  | 18885.3477 | type:internal len:177 gc:universal TRINITY\_DN36857\_c0\_g1\_i1:2-529(+) |
| 144 | 316 | TRINITY\_DN51754\_c0\_g1\_i1.p1 | 54.32 | 3 | 3 | 2.39e2 | 1 | 1 | 1 |  | 47134.0156 | type:internal len:438 gc:universal TRINITY\_DN51754\_c0\_g1\_i1:3-1313(+) |
| 145 | 317 | TRINITY\_DN2077\_c0\_g1\_i1.p3 | 16.87 | 6 | 6 | 3.52e2 | 1 | 1 | 1 |  | 26177.1309 | type:5prime\_partial len:238 gc:universal TRINITY\_DN2077\_c0\_g1\_i1:2-715(+) |
| 146 | 318 | TRINITY\_DN128406\_c0\_g1\_i1.p1 | 54.58 | 7 | 7 | 4.75e2 | 1 | 1 | 1 |  | 16958.5273 | type:5prime\_partial len:150 gc:universal TRINITY\_DN128406\_c0\_g1\_i1:474-25(-) |
| 147 | 319 | TRINITY\_DN4781\_c0\_g1\_i3.p3 | 55.71 | 7 | 7 | 4.49e2 | 1 | 1 | 2 |  | 20029.2422 | type:complete len:183 gc:universal TRINITY\_DN4781\_c0\_g1\_i3:2965-2417(-) |
| 148 | 320 | TRINITY\_DN942\_c0\_g1\_i5.p1 | 22.83 | 0 | 0 | 8.15e2 | 1 | 1 | 1 |  | 591926.25 | type:complete len:5116 gc:universal TRINITY\_DN942\_c0\_g1\_i5:15664-317(-) |
| 148 | 321 | TRINITY\_DN942\_c0\_g1\_i2.p1 | 22.83 | 0 | 0 | 8.15e2 | 1 | 1 | 1 |  | 591926.3125 | type:complete len:5116 gc:universal TRINITY\_DN942\_c0\_g1\_i2:15664-317(-) |
| 148 | 322 | TRINITY\_DN942\_c0\_g1\_i3.p1 | 22.83 | 0 | 0 | 8.15e2 | 1 | 1 | 1 |  | 590662.8125 | type:complete len:5105 gc:universal TRINITY\_DN942\_c0\_g1\_i3:15631-317(-) |
| 149 | 323 | TRINITY\_DN464\_c0\_g1\_i4.p1 | 55.02 | 2 | 2 | 5.47e2 | 1 | 1 | 1 |  | 82057.25 | type:complete len:717 gc:universal TRINITY\_DN464\_c0\_g1\_i4:2167-17(-) |
| 150 | 324 | TRINITY\_DN36471\_c0\_g1\_i1.p1 | 58.16 | 4 | 4 | 0 | 1 | 1 | 1 |  | 33492.4688 | type:internal len:286 gc:universal TRINITY\_DN36471\_c0\_g1\_i1:2-856(+) |
| 151 | 325 | TRINITY\_DN67\_c0\_g1\_i2.p1 | 56.93 | 3 | 3 | 4.78e2 | 1 | 1 | 1 |  | 52170.8203 | type:5prime\_partial len:474 gc:universal TRINITY\_DN67\_c0\_g1\_i2:1488-67(-) |
| 152 | 326 | TRINITY\_DN73587\_c0\_g1\_i1.p1 | 55.41 | 3 | 3 | 2.94e2 | 1 | 1 | 1 |  | 57376.2227 | type:complete len:533 gc:universal TRINITY\_DN73587\_c0\_g1\_i1:94-1692(+) |
| 153 | 327 | TRINITY\_DN247\_c0\_g1\_i1.p1 | 61.22 | 3 | 3 | 5.27e2 | 1 | 1 | 2 |  | 42079.6172 | type:complete len:373 gc:universal TRINITY\_DN247\_c0\_g1\_i1:38-1156(+) |
| 154 | 328 | TRINITY\_DN751\_c0\_g1\_i1.p1 | 54.93 | 5 | 5 | 5.38e3 | 1 | 1 | 2 |  | 25720.373 | type:complete len:227 gc:universal TRINITY\_DN751\_c0\_g1\_i1:731-51(-) |
| 154 | 329 | TRINITY\_DN7214\_c0\_g1\_i1.p1 | 54.93 | 5 | 5 | 5.38e3 | 1 | 1 | 2 |  | 25800.4062 | type:complete len:228 gc:universal TRINITY\_DN7214\_c0\_g1\_i1:39-722(+) |
| 155 | 330 | TRINITY\_DN1505\_c0\_g1\_i1.p1 | 57.91 | 8 | 8 | 1.96e3 | 1 | 1 | 4 | Acetylation (Protein N-term) | 17930.752 | type:complete len:154 gc:universal TRINITY\_DN1505\_c0\_g1\_i1:98-559(+) |
| 156 | 331 | TRINITY\_DN925\_c0\_g1\_i1.p1 | 56.94 | 5 | 5 | 7.37e2 | 1 | 1 | 3 |  | 28336.7676 | type:complete len:261 gc:universal TRINITY\_DN925\_c0\_g1\_i1:826-44(-) |
| 157 | 332 | TRINITY\_DN97534\_c0\_g1\_i1.p1 | 50.06 | 2 | 2 | 0 | 1 | 1 | 1 |  | 65163.7031 | type:complete len:563 gc:universal TRINITY\_DN97534\_c0\_g1\_i1:138-1826(+) |
| 158 | 333 | TRINITY\_DN4377\_c0\_g1\_i1.p1 | 56.93 | 2 | 2 | 6.25e2 | 1 | 1 | 1 |  | 67613.3828 | type:5prime\_partial len:604 gc:universal TRINITY\_DN4377\_c0\_g1\_i1:3-1814(+) |
| 159 | 334 | TRINITY\_DN5263\_c0\_g1\_i1.p1 | 58.9 | 5 | 5 | 0 | 1 | 1 | 1 |  | 26869.2422 | type:complete len:243 gc:universal TRINITY\_DN5263\_c0\_g1\_i1:827-99(-) |
| 160 | 335 | TRINITY\_DN5587\_c0\_g1\_i1.p1 | 34.34 | 1 | 1 | 0 | 1 | 1 | 1 |  | 89981.1016 | type:complete len:809 gc:universal TRINITY\_DN5587\_c0\_g1\_i1:196-2622(+) |
| 161 | 336 | TRINITY\_DN166344\_c0\_g1\_i1.p1 | 56.91 | 3 | 3 | 1.01e3 | 1 | 1 | 1 | Carbamidomethylation | 40794.2773 | type:complete len:360 gc:universal TRINITY\_DN166344\_c0\_g1\_i1:93-1172(+) |
| 162 | 337 | TRINITY\_DN1272\_c0\_g1\_i1.p1 | 59.18 | 4 | 4 | 1.65e3 | 1 | 1 | 2 |  | 30690.1172 | type:complete len:262 gc:universal TRINITY\_DN1272\_c0\_g1\_i1:957-172(-) |
| 163 | 338 | TRINITY\_DN72959\_c0\_g1\_i1.p1 | 54.59 | 6 | 6 | 1.05e3 | 1 | 1 | 1 |  | 25064.7695 | type:complete len:216 gc:universal TRINITY\_DN72959\_c0\_g1\_i1:54-701(+) |
| 164 | 339 | TRINITY\_DN12390\_c0\_g1\_i1.p1 | 52.6 | 4 | 4 | 0 | 1 | 1 | 1 |  | 30457.0371 | type:complete len:268 gc:universal TRINITY\_DN12390\_c0\_g1\_i1:826-23(-) |
| 165 | 340 | TRINITY\_DN535\_c0\_g1\_i1.p1 | 55.4 | 7 | 7 | 5.93e2 | 1 | 1 | 1 |  | 18221.5957 | type:5prime\_partial len:162 gc:universal TRINITY\_DN535\_c0\_g1\_i1:712-227(-) |
| 165 | 341 | TRINITY\_DN535\_c0\_g1\_i2.p1 | 55.4 | 7 | 7 | 5.93e2 | 1 | 1 | 1 |  | 18221.5957 | type:5prime\_partial len:162 gc:universal TRINITY\_DN535\_c0\_g1\_i2:699-214(-) |
| 166 | 342 | TRINITY\_DN98420\_c0\_g1\_i1.p1 | 44.66 | 3 | 3 | 3.69e3 | 1 | 1 | 1 |  | 31374.8145 | type:5prime\_partial len:286 gc:universal TRINITY\_DN98420\_c0\_g1\_i1:1007-150(-) |
| 167 | 343 | TRINITY\_DN14830\_c0\_g1\_i1.p1 | 32.21 | 1 | 1 | 0 | 1 | 1 | 1 |  | 93121.2188 | type:3prime\_partial len:834 gc:universal TRINITY\_DN14830\_c0\_g1\_i1:113-2611(+) |
| 168 | 344 | TRINITY\_DN81944\_c0\_g1\_i1.p1 | 55.27 | 7 | 7 | 6.36e2 | 1 | 1 | 1 | Oxidation (M) | 24919.502 | type:5prime\_partial len:222 gc:universal TRINITY\_DN81944\_c0\_g1\_i1:3-668(+) |
| 169 | 345 | TRINITY\_DN828\_c0\_g1\_i2.p1 | 59.72 | 7 | 7 | 3.15e3 | 1 | 1 | 5 | Carbamidomethylation | 23588.9746 | type:complete len:216 gc:universal TRINITY\_DN828\_c0\_g1\_i2:1021-374(-) |
| 170 | 346 | TRINITY\_DN1559\_c0\_g1\_i1.p1 | 54.7 | 5 | 5 | 4.55e3 | 1 | 1 | 1 |  | 33672.0977 | type:complete len:311 gc:universal TRINITY\_DN1559\_c0\_g1\_i1:221-1153(+) |
| 171 | 347 | TRINITY\_DN10890\_c0\_g1\_i1.p1 | 54.39 | 2 | 2 | 0 | 1 | 1 | 1 |  | 107419.1719 | type:5prime\_partial len:963 gc:universal TRINITY\_DN10890\_c0\_g1\_i1:3568-680(-) |
| 172 | 348 | TRINITY\_DN4439\_c0\_g1\_i1.p1 | 54.38 | 2 | 2 | 5.11e2 | 1 | 1 | 1 |  | 104528.4844 | type:complete len:941 gc:universal TRINITY\_DN4439\_c0\_g1\_i1:77-2899(+) |
| 173 | 349 | TRINITY\_DN119684\_c0\_g1\_i1.p1 | 54.9 | 2 | 2 | 9.96e2 | 1 | 1 | 1 |  | 72994.7266 | type:complete len:662 gc:universal TRINITY\_DN119684\_c0\_g1\_i1:2182-197(-) |
| 174 | 350 | TRINITY\_DN794\_c0\_g1\_i2.p1 | 58.12 | 5 | 5 | 4.15e2 | 1 | 1 | 1 |  | 27820.5742 | type:complete len:250 gc:universal TRINITY\_DN794\_c0\_g1\_i2:72-821(+) |
| 175 | 351 | TRINITY\_DN598\_c1\_g1\_i2.p1 | 56.83 | 1 | 1 | 3.56e3 | 1 | 1 | 2 |  | 81043.625 | type:5prime\_partial len:742 gc:universal TRINITY\_DN598\_c1\_g1\_i2:2-2227(+) |
| 176 | 352 | TRINITY\_DN22219\_c0\_g1\_i1.p1 | 55.65 | 3 | 3 | 1.06e3 | 1 | 1 | 1 |  | 49350.1836 | type:5prime\_partial len:427 gc:universal TRINITY\_DN22219\_c0\_g1\_i1:2-1282(+) |
| 177 | 353 | TRINITY\_DN244\_c0\_g1\_i1.p1 | 58.88 | 0 | 0 | 6.76e2 | 1 | 1 | 1 |  | 313594.0938 | type:complete len:3019 gc:universal TRINITY\_DN244\_c0\_g1\_i1:9748-692(-) |
| 178 | 354 | TRINITY\_DN1614\_c0\_g1\_i1.p1 | 57.41 | 7 | 7 | 1.42e2 | 1 | 1 | 1 |  | 25367.3418 | type:complete len:218 gc:universal TRINITY\_DN1614\_c0\_g1\_i1:56-709(+) |
| 179 | 355 | TRINITY\_DN74124\_c0\_g1\_i1.p1 | 58.54 | 8 | 8 | 1.4e3 | 1 | 1 | 2 |  | 20562.4961 | type:complete len:183 gc:universal TRINITY\_DN74124\_c0\_g1\_i1:100-648(+) |
| 180 | 356 | TRINITY\_DN1243\_c0\_g1\_i1.p1 | 57 | 2 | 2 | 0 | 1 | 1 | 1 |  | 103266.6875 | type:complete len:929 gc:universal TRINITY\_DN1243\_c0\_g1\_i1:2903-117(-) |
| 181 | 357 | TRINITY\_DN11097\_c0\_g1\_i1.p1 | 57.31 | 2 | 2 | 6.52e2 | 1 | 1 | 1 |  | 80111.1172 | type:complete len:730 gc:universal TRINITY\_DN11097\_c0\_g1\_i1:2348-159(-) |
| 181 | 358 | TRINITY\_DN11097\_c0\_g2\_i1.p1 | 57.31 | 6 | 6 | 6.52e2 | 1 | 1 | 1 |  | 24192.5488 | type:internal len:222 gc:universal TRINITY\_DN11097\_c0\_g2\_i1:665-3(-) |
| 182 | 359 | TRINITY\_DN1534\_c2\_g1\_i1.p1 | 55.41 | 2 | 2 | 0 | 1 | 1 | 1 |  | 59603.6055 | type:complete len:547 gc:universal TRINITY\_DN1534\_c2\_g1\_i1:1914-274(-) |
| 183 | 360 | TRINITY\_DN27859\_c0\_g1\_i1.p1 | 45.31 | 3 | 3 | 0 | 1 | 1 | 1 |  | 41818.7734 | type:complete len:361 gc:universal TRINITY\_DN27859\_c0\_g1\_i1:77-1159(+) |
| 184 | 361 | TRINITY\_DN147947\_c0\_g1\_i1.p1 | 58.77 | 7 | 7 | 7.69e2 | 1 | 1 | 1 |  | 19144.6445 | type:internal len:168 gc:universal TRINITY\_DN147947\_c0\_g1\_i1:501-1(-) |
| 185 | 362 | TRINITY\_DN5643\_c0\_g1\_i1.p1 | 55.27 | 2 | 2 | 0 | 1 | 1 | 1 |  | 106985.9922 | type:complete len:932 gc:universal TRINITY\_DN5643\_c0\_g1\_i1:2894-99(-) |
| 186 | 363 | TRINITY\_DN2028\_c0\_g1\_i1.p1 | 55.08 | 5 | 5 | 1.38e3 | 1 | 1 | 1 |  | 30106.9453 | type:complete len:274 gc:universal TRINITY\_DN2028\_c0\_g1\_i1:1608-787(-) |
| 187 | 364 | TRINITY\_DN37295\_c0\_g1\_i1.p1 | 58.66 | 8 | 8 | 0 | 1 | 1 | 1 |  | 22340.4082 | type:5prime\_partial len:195 gc:universal TRINITY\_DN37295\_c0\_g1\_i1:857-273(-) |
| 189 | 365 | TRINITY\_DN1378\_c0\_g1\_i1.p1 | 55.14 | 3 | 3 | 6.88e2 | 1 | 1 | 1 |  | 47978.9219 | type:complete len:443 gc:universal TRINITY\_DN1378\_c0\_g1\_i1:2151-823(-) |
| 190 | 366 | TRINITY\_DN6783\_c0\_g1\_i1.p1 | 54.9 | 4 | 4 | 0 | 1 | 1 | 1 |  | 34142.9297 | type:complete len:314 gc:universal TRINITY\_DN6783\_c0\_g1\_i1:1018-77(-) |
| 191 | 367 | TRINITY\_DN61707\_c0\_g1\_i1.p1 | 57.83 | 3 | 3 | 0 | 1 | 1 | 1 |  | 46897.5508 | type:3prime\_partial len:417 gc:universal TRINITY\_DN61707\_c0\_g1\_i1:1250-3(-) |
| 192 | 368 | TRINITY\_DN164351\_c0\_g1\_i1.p1 | 21.9 | 5 | 5 | 0 | 1 | 1 | 1 | Oxidation (M) | 23723.9492 | type:complete len:212 gc:universal TRINITY\_DN164351\_c0\_g1\_i1:694-59(-) |
| 193 | 369 | TRINITY\_DN773\_c0\_g1\_i1.p1 | 61.34 | 3 | 3 | 8.57e2 | 1 | 1 | 1 |  | 48865.75 | type:complete len:440 gc:universal TRINITY\_DN773\_c0\_g1\_i1:1608-289(-) |
| 194 | 370 | TRINITY\_DN58867\_c0\_g1\_i2.p1 | 57.46 | 3 | 3 | 2.23e3 | 1 | 1 | 2 |  | 40822.2812 | type:complete len:354 gc:universal TRINITY\_DN58867\_c0\_g1\_i2:80-1141(+) |
| 195 | 371 | TRINITY\_DN598\_c1\_g1\_i1.p1 | 56.88 | 1 | 1 | 5.14e3 | 1 | 1 | 1 |  | 81322.8672 | type:5prime\_partial len:745 gc:universal TRINITY\_DN598\_c1\_g1\_i1:2-2236(+) |
| 196 | 372 | TRINITY\_DN6008\_c0\_g1\_i1.p1 | 58.01 | 3 | 3 | 1.04e3 | 1 | 1 | 1 |  | 47488.5547 | type:complete len:426 gc:universal TRINITY\_DN6008\_c0\_g1\_i1:1620-343(-) |
| 197 | 373 | TRINITY\_DN1761\_c0\_g1\_i1.p1 | 55.5 | 7 | 7 | 2.85e2 | 1 | 1 | 1 |  | 28538.1953 | type:5prime\_partial len:251 gc:universal TRINITY\_DN1761\_c0\_g1\_i1:843-91(-) |
| 198 | 374 | TRINITY\_DN26425\_c0\_g1\_i1.p1 | 30.63 | 3 | 3 | 5.68e2 | 1 | 1 | 1 |  | 46273.0586 | type:internal len:414 gc:universal TRINITY\_DN26425\_c0\_g1\_i1:3-1241(+) |
| 199 | 375 | TRINITY\_DN3052\_c0\_g1\_i1.p1 | 57.81 | 4 | 4 | 0 | 1 | 1 | 1 | Acetylation (Protein N-term) | 39261.4453 | type:complete len:362 gc:universal TRINITY\_DN3052\_c0\_g1\_i1:45-1130(+) |
| 200 | 376 | TRINITY\_DN5397\_c0\_g1\_i1.p1 | 56.04 | 12 | 12 | 0 | 1 | 1 | 1 | Carbamidomethylation | 17679.4688 | type:internal len:161 gc:universal TRINITY\_DN5397\_c0\_g1\_i1:2-481(+) |
| 201 | 377 | TRINITY\_DN5153\_c0\_g1\_i1.p1 | 56.84 | 2 | 2 | 2.79e2 | 1 | 1 | 1 |  | 98168.6016 | type:complete len:862 gc:universal TRINITY\_DN5153\_c0\_g1\_i1:61-2646(+) |
| total 377 proteins |  |  |  |  |  |  |  |  |  |  |  |  |
| --- | --- | --- | --- | --- | --- | --- | --- | --- | --- | --- | --- | --- |

  

TRINITY\_DN22594\_c0\_g1\_i1.p2

back to list

  

| Protein Coverage
| Supporting Peptides
| Best Unique PSM
|

Protein Coverage:

Supporting Peptides:

| Peptide | Uniq | -10lgP | Mass | Length | ppm | m/z | z | RT | 1/k0 Start | 1/k0 End | Precursor ID | Area Sample 9 | #Feature | #Feature Sample 9 | Start | End | PTM | AScore | Found By |
| --- | --- | --- | --- | --- | --- | --- | --- | --- | --- | --- | --- | --- | --- | --- | --- | --- | --- | --- | --- |
| R.IPLDDFQDPSLDLENAHNK.L | Y | 56.78 | 2180.0385 | 19 | 11.13 | 727.6916 | 3 | 17.94 | 0.9812 | 0.9884 | 59154 | 0 | 0 | 0 | 237 | 255 |  |  | DB Search |
| K.GSLDRESYR.E | N | 17.44 | 1081.5152 | 9 | 0.81 | 541.7629 | 2 | 10.96 | 0.8595 | 0.8668 | 20530 | 0 | 0 | 0 | 158 | 166 |  |  | DB Search |
| total 2 peptides |  |  |  |  |  |  |  |  |  |  |  |  |  |  |  |  |  |  |  |
| --- | --- | --- | --- | --- | --- | --- | --- | --- | --- | --- | --- | --- | --- | --- | --- | --- | --- | --- | --- |

Best Unique PSM (Scan Sample\_8\_A1\_1\_3645.d:59154, m/z=727.6916, z=3, RT=17.94, ppm=6.61):


TRINITY\_DN942\_c0\_g1\_i2.p1

back to list

  

| Protein Coverage
| Supporting Peptides
| Best Unique PSM
|

Protein Coverage:

Supporting Peptides:

| Peptide | Uniq | -10lgP | Mass | Length | ppm | m/z | z | RT | 1/k0 Start | 1/k0 End | Precursor ID | Area Sample 9 | #Feature | #Feature Sample 9 | Start | End | PTM | AScore | Found By |
| --- | --- | --- | --- | --- | --- | --- | --- | --- | --- | --- | --- | --- | --- | --- | --- | --- | --- | --- | --- |
| K.AIGNAQRPAR.V | Y | 22.83 | 1052.5839 | 10 | -1.57 | 527.296 | 2 | 9.68 | 0.8664 | 0.8737 | 12735 | 8.15e2 | 1 | 1 | 498 | 507 |  |  | DB Search |
| total 1 peptides |  |  |  |  |  |  |  |  |  |  |  |  |  |  |  |  |  |  |  |
| --- | --- | --- | --- | --- | --- | --- | --- | --- | --- | --- | --- | --- | --- | --- | --- | --- | --- | --- | --- |

Best Unique PSM (Scan Sample\_8\_A1\_1\_3645.d:12735, m/z=527.296, z=2, RT=9.68, ppm=-6.09):


TRINITY\_DN1377\_c0\_g1\_i1.p1

back to list

  

| Protein Coverage
| Supporting Peptides
| Best Unique PSM
|

Protein Coverage:

Supporting Peptides:

| Peptide | Uniq | -10lgP | Mass | Length | ppm | m/z | z | RT | 1/k0 Start | 1/k0 End | Precursor ID | Area Sample 9 | #Feature | #Feature Sample 9 | Start | End | PTM | AScore | Found By |
| --- | --- | --- | --- | --- | --- | --- | --- | --- | --- | --- | --- | --- | --- | --- | --- | --- | --- | --- | --- |
| R.SVTTHQGNAIVIK.I | Y | 61.58 | 1366.7568 | 13 | 3.07 | 684.3847 | 2 | 12.45 | 0.9965 | 1.0037 | 29541 | 5.61e2 | 1 | 1 | 191 | 203 |  |  | DB Search |
| R.ALSEQIRPTTQIVVC(+57.02)IVPNNRK.D | Y | 59.27 | 2535.3955 | 22 | 2.34 | 846.1373 | 3 | 16.62 | 1.06 | 1.0672 | 51858 | 2.38e2 | 1 | 1 | 553 | 574 | Carbamidomethylation |  | DB Search |
| R.ITQQQSHQEIADQLR.V | Y | 58.29 | 1793.902 | 15 | 4.14 | 897.9579 | 2 | 13.10 | 1.133 | 1.1402 | 33346 | 4.3e2 | 1 | 1 | 678 | 692 |  |  | DB Search |
| K.KQM(+15.99)LM(+15.99)SVC(+57.02)TK.I | Y | 57.55 | 1256.5927 | 10 | 17.76 | 629.312 | 2 | 10.42 | 0.9271 | 0.9344 | 17303 | 0 | 0 | 0 | 603 | 612 | Oxidation (M), Oxidation (M), Carbamidomethylation | M3:Oxidation (M):1000 M5:Oxidation (M):1000 | DB Search |
| K.RLPDQITTR.S | Y | 53.53 | 1098.6145 | 9 | 1.35 | 550.3128 | 2 | 12.51 | 0.8781 | 0.8854 | 29833 | 1.52e3 | 1 | 1 | 182 | 190 |  |  | DB Search |
| total 5 peptides |  |  |  |  |  |  |  |  |  |  |  |  |  |  |  |  |  |  |  |
| --- | --- | --- | --- | --- | --- | --- | --- | --- | --- | --- | --- | --- | --- | --- | --- | --- | --- | --- | --- |

Best Unique PSM (Scan Sample\_8\_A1\_1\_3645.d:29541, m/z=684.3847, z=2, RT=12.45, ppm=-1.45):


TRINITY\_DN1172\_c0\_g1\_i3.p1

back to list

  

| Protein Coverage
| Supporting Peptides
| Best Unique PSM
|

Protein Coverage:

Supporting Peptides:

| Peptide | Uniq | -10lgP | Mass | Length | ppm | m/z | z | RT | 1/k0 Start | 1/k0 End | Precursor ID | Area Sample 9 | #Feature | #Feature Sample 9 | Start | End | PTM | AScore | Found By |
| --- | --- | --- | --- | --- | --- | --- | --- | --- | --- | --- | --- | --- | --- | --- | --- | --- | --- | --- | --- |
| K.VITSDDPAEKEK.K | Y | 56.98 | 1330.6616 | 12 | 1.22 | 666.3359 | 2 | 10.71 | 0.9844 | 0.9916 | 18867 | 1.51e3 | 1 | 1 | 123 | 134 |  |  | DB Search |
| total 1 peptides |  |  |  |  |  |  |  |  |  |  |  |  |  |  |  |  |  |  |  |
| --- | --- | --- | --- | --- | --- | --- | --- | --- | --- | --- | --- | --- | --- | --- | --- | --- | --- | --- | --- |

Best Unique PSM (Scan Sample\_8\_A1\_1\_3645.d:18867, m/z=666.3359, z=2, RT=10.71, ppm=-3.3):


TRINITY\_DN413\_c0\_g1\_i3.p1

back to list

  

| Protein Coverage
| Supporting Peptides
| Best Unique PSM
|

Protein Coverage:

Supporting Peptides:

| Peptide | Uniq | -10lgP | Mass | Length | ppm | m/z | z | RT | 1/k0 Start | 1/k0 End | Precursor ID | Area Sample 9 | #Feature | #Feature Sample 9 | Start | End | PTM | AScore | Found By |
| --- | --- | --- | --- | --- | --- | --- | --- | --- | --- | --- | --- | --- | --- | --- | --- | --- | --- | --- | --- |
| R.LLLGDFNGDKR.T | Y | 55.21 | 1246.6669 | 11 | 4.34 | 624.3406 | 2 | 15.96 | 0.9852 | 0.9924 | 48766 | 6.58e2 | 1 | 1 | 46 | 56 |  |  | DB Search |
| total 1 peptides |  |  |  |  |  |  |  |  |  |  |  |  |  |  |  |  |  |  |  |
| --- | --- | --- | --- | --- | --- | --- | --- | --- | --- | --- | --- | --- | --- | --- | --- | --- | --- | --- | --- |

Best Unique PSM (Scan Sample\_8\_A1\_1\_3645.d:48766, m/z=624.3406, z=2, RT=15.96, ppm=-0.18):


TRINITY\_DN11435\_c0\_g1\_i1.p1

back to list

  

| Protein Coverage
| Supporting Peptides
| Best Unique PSM
|

Protein Coverage:

Supporting Peptides:

| Peptide | Uniq | -10lgP | Mass | Length | ppm | m/z | z | RT | 1/k0 Start | 1/k0 End | Precursor ID | Area Sample 9 | #Feature | #Feature Sample 9 | Start | End | PTM | AScore | Found By |
| --- | --- | --- | --- | --- | --- | --- | --- | --- | --- | --- | --- | --- | --- | --- | --- | --- | --- | --- | --- |
| R.IRNEEIQIEVIERR.K | Y | 61.33 | 1795.9904 | 14 | 3.65 | 599.6702 | 3 | 15.08 | 0.8793 | 0.8866 | 44280 | 1.19e3 | 1 | 1 | 252 | 265 |  |  | DB Search |
| K.LIEVEEKEIQR.R | Y | 60.21 | 1384.7562 | 11 | 3.4 | 693.3846 | 2 | 13.23 | 1.0182 | 1.0255 | 34162 | 0 | 0 | 0 | 267 | 277 |  |  | DB Search |
| K.VYLAQAEAEKIK.M | Y | 57.34 | 1361.7554 | 12 | 1.23 | 681.8828 | 2 | 14.25 | 1.0335 | 1.0407 | 39833 | 4.3e2 | 1 | 1 | 309 | 320 |  |  | DB Search |
| K.LAYELQANKEQQR.I | Y | 56.91 | 1589.8161 | 13 | -0.02 | 795.9117 | 2 | 12.87 | 1.0516 | 1.0588 | 32046 | 6.54e2 | 1 | 1 | 239 | 251 |  |  | DB Search |
| K.DRDQFAQLVR.E | Y | 55.23 | 1246.6418 | 10 | 5.21 | 624.3286 | 2 | 15.15 | 0.942 | 0.9493 | 44748 | 0 | 0 | 0 | 126 | 135 |  |  | DB Search |
| K.LIEVEEKEIQRR.E | Y | 52.46 | 1540.8573 | 12 | 0.79 | 514.6245 | 3 | 13.08 | 0.7803 | 0.7876 | 33297 | 6.71e2 | 1 | 1 | 267 | 278 |  |  | DB Search |
| total 6 peptides |  |  |  |  |  |  |  |  |  |  |  |  |  |  |  |  |  |  |  |
| --- | --- | --- | --- | --- | --- | --- | --- | --- | --- | --- | --- | --- | --- | --- | --- | --- | --- | --- | --- |

Best Unique PSM (Scan Sample\_8\_A1\_1\_3645.d:44280, m/z=599.6702, z=3, RT=15.08, ppm=-0.87):


TRINITY\_DN982\_c0\_g1\_i2.p1

back to list

  

| Protein Coverage
| Supporting Peptides
| Best Unique PSM
|

Protein Coverage:

Supporting Peptides:

| Peptide | Uniq | -10lgP | Mass | Length | ppm | m/z | z | RT | 1/k0 Start | 1/k0 End | Precursor ID | Area Sample 9 | #Feature | #Feature Sample 9 | Start | End | PTM | AScore | Found By |
| --- | --- | --- | --- | --- | --- | --- | --- | --- | --- | --- | --- | --- | --- | --- | --- | --- | --- | --- | --- |
| R.VLDVNDKQPVFVK.S | Y | 57.11 | 1499.8347 | 13 | 0.01 | 750.9213 | 2 | 14.94 | 1.0668 | 1.0741 | 43674 | 0 | 0 | 0 | 670 | 682 |  |  | DB Search |
| K.VLLSQQIFYDKK.N | N | 55.75 | 1480.8289 | 12 | -2.98 | 741.4162 | 2 | 16.65 | 1.0588 | 1.066 | 52089 | 1.28e3 | 1 | 1 | 1479 | 1490 |  |  | DB Search |
| K.AVDRGNPSQDSLNK.A | Y | 54.84 | 1499.7328 | 14 | -1.13 | 750.8694 | 2 | 10.61 | 0.9937 | 1.0009 | 18454 | 0 | 0 | 0 | 16 | 29 |  |  | DB Search |
| total 3 peptides |  |  |  |  |  |  |  |  |  |  |  |  |  |  |  |  |  |  |  |
| --- | --- | --- | --- | --- | --- | --- | --- | --- | --- | --- | --- | --- | --- | --- | --- | --- | --- | --- | --- |

Best Unique PSM (Scan Sample\_8\_A1\_1\_3645.d:43674, m/z=750.9213, z=2, RT=14.94, ppm=-4.51):


TRINITY\_DN72959\_c0\_g1\_i1.p1

back to list

  

| Protein Coverage
| Supporting Peptides
| Best Unique PSM
|

Protein Coverage:

Supporting Peptides:

| Peptide | Uniq | -10lgP | Mass | Length | ppm | m/z | z | RT | 1/k0 Start | 1/k0 End | Precursor ID | Area Sample 9 | #Feature | #Feature Sample 9 | Start | End | PTM | AScore | Found By |
| --- | --- | --- | --- | --- | --- | --- | --- | --- | --- | --- | --- | --- | --- | --- | --- | --- | --- | --- | --- |
| R.GAFGKPQGTVAR.V | Y | 54.59 | 1187.6411 | 12 | 1.66 | 594.8261 | 2 | 11.37 | 0.9275 | 0.9348 | 22938 | 1.05e3 | 1 | 1 | 117 | 128 |  |  | DB Search |
| total 1 peptides |  |  |  |  |  |  |  |  |  |  |  |  |  |  |  |  |  |  |  |
| --- | --- | --- | --- | --- | --- | --- | --- | --- | --- | --- | --- | --- | --- | --- | --- | --- | --- | --- | --- |

Best Unique PSM (Scan Sample\_8\_A1\_1\_3645.d:22938, m/z=594.8261, z=2, RT=11.37, ppm=-2.86):


TRINITY\_DN81944\_c0\_g1\_i1.p1

back to list

  

| Protein Coverage
| Supporting Peptides
| Best Unique PSM
|

Protein Coverage:

Supporting Peptides:

| Peptide | Uniq | -10lgP | Mass | Length | ppm | m/z | z | RT | 1/k0 Start | 1/k0 End | Precursor ID | Area Sample 9 | #Feature | #Feature Sample 9 | Start | End | PTM | AScore | Found By |
| --- | --- | --- | --- | --- | --- | --- | --- | --- | --- | --- | --- | --- | --- | --- | --- | --- | --- | --- | --- |
| K.VAM(+15.99)TAPVFTYSDLKK.G | Y | 55.27 | 1685.8698 | 15 | -1.69 | 843.937 | 2 | 15.62 | 1.1358 | 1.143 | 47046 | 6.36e2 | 1 | 1 | 104 | 118 | Oxidation (M) | M3:Oxidation (M):1000 | DB Search |
| total 1 peptides |  |  |  |  |  |  |  |  |  |  |  |  |  |  |  |  |  |  |  |
| --- | --- | --- | --- | --- | --- | --- | --- | --- | --- | --- | --- | --- | --- | --- | --- | --- | --- | --- | --- |

Best Unique PSM (Scan Sample\_8\_A1\_1\_3645.d:47046, m/z=843.937, z=2, RT=15.62, ppm=-6.21):


TRINITY\_DN776\_c0\_g1\_i1.p1

back to list

  

| Protein Coverage
| Supporting Peptides
| Best Unique PSM
|

Protein Coverage:

Supporting Peptides:

| Peptide | Uniq | -10lgP | Mass | Length | ppm | m/z | z | RT | 1/k0 Start | 1/k0 End | Precursor ID | Area Sample 9 | #Feature | #Feature Sample 9 | Start | End | PTM | AScore | Found By |
| --- | --- | --- | --- | --- | --- | --- | --- | --- | --- | --- | --- | --- | --- | --- | --- | --- | --- | --- | --- |
| R.LFEGNALLRR.L | Y | 54.64 | 1187.6775 | 10 | -0.31 | 594.8431 | 2 | 15.53 | 0.9666 | 0.9739 | 46624 | 9.6e2 | 1 | 1 | 78 | 87 |  |  | DB Search |
| R.DLLTLPEKDNRR.L | Y | 43.72 | 1468.7998 | 12 | -0.79 | 735.4033 | 2 | 14.55 | 1.0218 | 1.0291 | 41392 | 7.4e2 | 1 | 1 | 66 | 77 |  |  | DB Search |
| R.RLFEGNALLR.R | Y | 16.53 | 1187.6775 | 10 | -1.85 | 594.8422 | 2 | 16.02 | 0.9388 | 0.9461 | 49137 | 3.83e2 | 1 | 1 | 77 | 86 |  |  | DB Search |
| total 3 peptides |  |  |  |  |  |  |  |  |  |  |  |  |  |  |  |  |  |  |  |
| --- | --- | --- | --- | --- | --- | --- | --- | --- | --- | --- | --- | --- | --- | --- | --- | --- | --- | --- | --- |

Best Unique PSM (Scan Sample\_8\_A1\_1\_3645.d:46624, m/z=594.8431, z=2, RT=15.53, ppm=-4.83):


TRINITY\_DN3986\_c0\_g1\_i2.p1

back to list

  

| Protein Coverage
| Supporting Peptides
| Best Unique PSM
|

Protein Coverage:

Supporting Peptides:

| Peptide | Uniq | -10lgP | Mass | Length | ppm | m/z | z | RT | 1/k0 Start | 1/k0 End | Precursor ID | Area Sample 9 | #Feature | #Feature Sample 9 | Start | End | PTM | AScore | Found By |
| --- | --- | --- | --- | --- | --- | --- | --- | --- | --- | --- | --- | --- | --- | --- | --- | --- | --- | --- | --- |
| K.NSSYFVEWIPNNVK.T | Y | 61.49 | 1695.8256 | 14 | 2 | 848.918 | 2 | 19.90 | 1.0889 | 1.0961 | 70553 | 0 | 0 | 0 | 337 | 350 |  |  | DB Search |
| R.FPGQLNADLRK.L | Y | 54.24 | 1257.6829 | 11 | 0.84 | 629.8464 | 2 | 14.26 | 0.9473 | 0.9545 | 39914 | 4.37e2 | 1 | 1 | 242 | 252 |  |  | DB Search |
| total 2 peptides |  |  |  |  |  |  |  |  |  |  |  |  |  |  |  |  |  |  |  |
| --- | --- | --- | --- | --- | --- | --- | --- | --- | --- | --- | --- | --- | --- | --- | --- | --- | --- | --- | --- |

Best Unique PSM (Scan Sample\_8\_A1\_1\_3645.d:70553, m/z=848.918, z=2, RT=19.90, ppm=-2.51):


TRINITY\_DN11097\_c0\_g2\_i1.p1

back to list

  

| Protein Coverage
| Supporting Peptides
| Best Unique PSM
|

Protein Coverage:

Supporting Peptides:

| Peptide | Uniq | -10lgP | Mass | Length | ppm | m/z | z | RT | 1/k0 Start | 1/k0 End | Precursor ID | Area Sample 9 | #Feature | #Feature Sample 9 | Start | End | PTM | AScore | Found By |
| --- | --- | --- | --- | --- | --- | --- | --- | --- | --- | --- | --- | --- | --- | --- | --- | --- | --- | --- | --- |
| R.VAHAGLTVAEFFR.D | Y | 57.31 | 1416.7513 | 13 | 3.21 | 709.382 | 2 | 18.82 | 1.0287 | 1.0359 | 64099 | 6.52e2 | 1 | 1 | 199 | 211 |  |  | DB Search |
| total 1 peptides |  |  |  |  |  |  |  |  |  |  |  |  |  |  |  |  |  |  |  |
| --- | --- | --- | --- | --- | --- | --- | --- | --- | --- | --- | --- | --- | --- | --- | --- | --- | --- | --- | --- |

Best Unique PSM (Scan Sample\_8\_A1\_1\_3645.d:64099, m/z=709.382, z=2, RT=18.82, ppm=-1.31):


TRINITY\_DN828\_c0\_g1\_i2.p1

back to list

  

| Protein Coverage
| Supporting Peptides
| Best Unique PSM
|

Protein Coverage:

Supporting Peptides:

| Peptide | Uniq | -10lgP | Mass | Length | ppm | m/z | z | RT | 1/k0 Start | 1/k0 End | Precursor ID | Area Sample 9 | #Feature | #Feature Sample 9 | Start | End | PTM | AScore | Found By |
| --- | --- | --- | --- | --- | --- | --- | --- | --- | --- | --- | --- | --- | --- | --- | --- | --- | --- | --- | --- |
| R.LC(+57.02)KPSYSTC(+57.02)ENHC(+57.02)ER.L | Y | 59.72 | 1939.7975 | 15 | 2.85 | 970.9044 | 2 | 10.96 | 1.1206 | 1.1278 | 20513 | 3.15e3 | 2 | 2 | 178 | 192 | Carbamidomethylation, Carbamidomethylation, Carbamidomethylation |  | DB Search |
| total 1 peptides |  |  |  |  |  |  |  |  |  |  |  |  |  |  |  |  |  |  |  |
| --- | --- | --- | --- | --- | --- | --- | --- | --- | --- | --- | --- | --- | --- | --- | --- | --- | --- | --- | --- |

Best Unique PSM (Scan Sample\_8\_A1\_1\_3645.d:20513, m/z=970.9044, z=2, RT=10.96, ppm=-1.66):


TRINITY\_DN649\_c0\_g1\_i2.p1

back to list

  

| Protein Coverage
| Supporting Peptides
| Best Unique PSM
|

Protein Coverage:

Supporting Peptides:

| Peptide | Uniq | -10lgP | Mass | Length | ppm | m/z | z | RT | 1/k0 Start | 1/k0 End | Precursor ID | Area Sample 9 | #Feature | #Feature Sample 9 | Start | End | PTM | AScore | Found By |
| --- | --- | --- | --- | --- | --- | --- | --- | --- | --- | --- | --- | --- | --- | --- | --- | --- | --- | --- | --- |
| K.YGQAM(+15.99)C(+57.02)QFM(+15.99)R.N | N | 61.54 | 1322.5206 | 10 | -1.76 | 662.2634 | 2 | 12.17 | 0.9489 | 0.9562 | 28008 | 2.07e2 | 1 | 1 | 8 | 17 | Oxidation (M), Carbamidomethylation, Oxidation (M) | M5:Oxidation (M):1000 M9:Oxidation (M):1000 | DB Search |
| K.FETQIATK.N | Y | 51.43 | 936.4916 | 8 | -0.7 | 937.494 | 1 | 11.83 | 1.2165 | 1.2236 | 25881 | 4.08e3 | 3 | 3 | 103 | 110 |  |  | DB Search |
| total 2 peptides |  |  |  |  |  |  |  |  |  |  |  |  |  |  |  |  |  |  |  |
| --- | --- | --- | --- | --- | --- | --- | --- | --- | --- | --- | --- | --- | --- | --- | --- | --- | --- | --- | --- |

Best Unique PSM (Scan Sample\_8\_A1\_1\_3645.d:25881, m/z=937.494, z=1, RT=11.83, ppm=-5.22):


TRINITY\_DN413\_c0\_g1\_i4.p1

back to list

  

| Protein Coverage
| Supporting Peptides
| Best Unique PSM
|

Protein Coverage:

Supporting Peptides:

| Peptide | Uniq | -10lgP | Mass | Length | ppm | m/z | z | RT | 1/k0 Start | 1/k0 End | Precursor ID | Area Sample 9 | #Feature | #Feature Sample 9 | Start | End | PTM | AScore | Found By |
| --- | --- | --- | --- | --- | --- | --- | --- | --- | --- | --- | --- | --- | --- | --- | --- | --- | --- | --- | --- |
| R.LLLGDFNGDKR.T | Y | 55.21 | 1246.6669 | 11 | 4.34 | 624.3406 | 2 | 15.96 | 0.9852 | 0.9924 | 48766 | 6.58e2 | 1 | 1 | 46 | 56 |  |  | DB Search |
| total 1 peptides |  |  |  |  |  |  |  |  |  |  |  |  |  |  |  |  |  |  |  |
| --- | --- | --- | --- | --- | --- | --- | --- | --- | --- | --- | --- | --- | --- | --- | --- | --- | --- | --- | --- |

Best Unique PSM (Scan Sample\_8\_A1\_1\_3645.d:48766, m/z=624.3406, z=2, RT=15.96, ppm=-0.18):


TRINITY\_DN152\_c0\_g1\_i9.p1

back to list

  

| Protein Coverage
| Supporting Peptides
| Best Unique PSM
|

Protein Coverage:

Supporting Peptides:

| Peptide | Uniq | -10lgP | Mass | Length | ppm | m/z | z | RT | 1/k0 Start | 1/k0 End | Precursor ID | Area Sample 9 | #Feature | #Feature Sample 9 | Start | End | PTM | AScore | Found By |
| --- | --- | --- | --- | --- | --- | --- | --- | --- | --- | --- | --- | --- | --- | --- | --- | --- | --- | --- | --- |
| K.IQDKEGIPPDQQR.L | Y | 61.25 | 1522.7739 | 13 | -1.84 | 762.3894 | 2 | 10.99 | 1.0371 | 1.0443 | 20319 | 2.55e3 | 1 | 1 | 66 | 78 |  |  | DB Search |
| total 1 peptides |  |  |  |  |  |  |  |  |  |  |  |  |  |  |  |  |  |  |  |
| --- | --- | --- | --- | --- | --- | --- | --- | --- | --- | --- | --- | --- | --- | --- | --- | --- | --- | --- | --- |

Best Unique PSM (Scan Sample\_8\_A1\_1\_3645.d:20319, m/z=762.3894, z=2, RT=10.99, ppm=-6.36):


TRINITY\_DN2477\_c0\_g1\_i7.p1

back to list

  

| Protein Coverage
| Supporting Peptides
| Best Unique PSM
|

Protein Coverage:

Supporting Peptides:

| Peptide | Uniq | -10lgP | Mass | Length | ppm | m/z | z | RT | 1/k0 Start | 1/k0 End | Precursor ID | Area Sample 9 | #Feature | #Feature Sample 9 | Start | End | PTM | AScore | Found By |
| --- | --- | --- | --- | --- | --- | --- | --- | --- | --- | --- | --- | --- | --- | --- | --- | --- | --- | --- | --- |
| R.GYSFTTTAEREIVR.D | Y | 56.59 | 1628.8158 | 14 | 3.67 | 815.4145 | 2 | 15.95 | 1.119 | 1.1262 | 48654 | 3.46e3 | 1 | 1 | 101 | 114 |  |  | DB Search |
| total 1 peptides |  |  |  |  |  |  |  |  |  |  |  |  |  |  |  |  |  |  |  |
| --- | --- | --- | --- | --- | --- | --- | --- | --- | --- | --- | --- | --- | --- | --- | --- | --- | --- | --- | --- |

Best Unique PSM (Scan Sample\_8\_A1\_1\_3645.d:48654, m/z=815.4145, z=2, RT=15.95, ppm=-0.85):


TRINITY\_DN278\_c0\_g1\_i1.p1

back to list

  

| Protein Coverage
| Supporting Peptides
| Best Unique PSM
|

Protein Coverage:

Supporting Peptides:

| Peptide | Uniq | -10lgP | Mass | Length | ppm | m/z | z | RT | 1/k0 Start | 1/k0 End | Precursor ID | Area Sample 9 | #Feature | #Feature Sample 9 | Start | End | PTM | AScore | Found By |
| --- | --- | --- | --- | --- | --- | --- | --- | --- | --- | --- | --- | --- | --- | --- | --- | --- | --- | --- | --- |
| K.IDNYIEFLDEDLKK.K | Y | 59.04 | 1753.8774 | 14 | -3.23 | 585.6285 | 3 | 19.82 | 0.8437 | 0.851 | 70027 | 1.38e3 | 1 | 1 | 102 | 115 |  |  | DB Search |
| K.ITC(+57.02)LEM(+15.99)SEHVGIKR.Y | Y | 55.86 | 1687.8385 | 14 | -0.48 | 563.6173 | 3 | 12.78 | 0.8201 | 0.8274 | 31529 | 0 | 0 | 0 | 306 | 319 | Carbamidomethylation, Oxidation (M) | M6:Oxidation (M):1000 | DB Search |
| total 2 peptides |  |  |  |  |  |  |  |  |  |  |  |  |  |  |  |  |  |  |  |
| --- | --- | --- | --- | --- | --- | --- | --- | --- | --- | --- | --- | --- | --- | --- | --- | --- | --- | --- | --- |

Best Unique PSM (Scan Sample\_8\_A1\_1\_3645.d:70027, m/z=585.6285, z=3, RT=19.82, ppm=-7.75):


TRINITY\_DN1172\_c0\_g2\_i2.p1

back to list

  

| Protein Coverage
| Supporting Peptides
| Best Unique PSM
|

Protein Coverage:

Supporting Peptides:

| Peptide | Uniq | -10lgP | Mass | Length | ppm | m/z | z | RT | 1/k0 Start | 1/k0 End | Precursor ID | Area Sample 9 | #Feature | #Feature Sample 9 | Start | End | PTM | AScore | Found By |
| --- | --- | --- | --- | --- | --- | --- | --- | --- | --- | --- | --- | --- | --- | --- | --- | --- | --- | --- | --- |
| K.VLTSSDPAEKEK.K | Y | 44.75 | 1302.6667 | 12 | 5.11 | 652.341 | 2 | 10.57 | 1.0001 | 1.0073 | 18232 | 0 | 0 | 0 | 7 | 18 |  |  | DB Search |
| total 1 peptides |  |  |  |  |  |  |  |  |  |  |  |  |  |  |  |  |  |  |  |
| --- | --- | --- | --- | --- | --- | --- | --- | --- | --- | --- | --- | --- | --- | --- | --- | --- | --- | --- | --- |

Best Unique PSM (Scan Sample\_8\_A1\_1\_3645.d:18232, m/z=652.341, z=2, RT=10.57, ppm=0.6):


TRINITY\_DN4595\_c0\_g1\_i4.p1

back to list

  

| Protein Coverage
| Supporting Peptides
| Best Unique PSM
|

Protein Coverage:

Supporting Peptides:

| Peptide | Uniq | -10lgP | Mass | Length | ppm | m/z | z | RT | 1/k0 Start | 1/k0 End | Precursor ID | Area Sample 9 | #Feature | #Feature Sample 9 | Start | End | PTM | AScore | Found By |
| --- | --- | --- | --- | --- | --- | --- | --- | --- | --- | --- | --- | --- | --- | --- | --- | --- | --- | --- | --- |
| K.YTGAC(+57.02)NANGNTDK.T | Y | 57.42 | 1384.5677 | 13 | 5.65 | 693.2919 | 2 | 9.57 | 0.9404 | 0.9477 | 12156 | 0 | 0 | 0 | 1492 | 1504 | Carbamidomethylation |  | DB Search |
| K.VNQGEKEFLQRPC(+57.02)R.Y | Y | 56.68 | 1759.8788 | 14 | 1.84 | 587.632 | 3 | 12.53 | 0.8619 | 0.8692 | 29925 | 1.89e3 | 1 | 1 | 1612 | 1625 | Carbamidomethylation |  | DB Search |
| K.KVNQGEKEFLQRPC(+57.02)R.Y | Y | 50.42 | 1887.9737 | 15 | -2.15 | 472.9976 | 4 | 11.77 | 0.8092 | 0.8165 | 25549 | 0 | 0 | 0 | 1611 | 1625 | Carbamidomethylation |  | DB Search |
| total 3 peptides |  |  |  |  |  |  |  |  |  |  |  |  |  |  |  |  |  |  |  |
| --- | --- | --- | --- | --- | --- | --- | --- | --- | --- | --- | --- | --- | --- | --- | --- | --- | --- | --- | --- |

Best Unique PSM (Scan Sample\_8\_A1\_1\_3645.d:12156, m/z=693.2919, z=2, RT=9.57, ppm=1.14):


TRINITY\_DN72625\_c0\_g1\_i1.p1

back to list

  

| Protein Coverage
| Supporting Peptides
| Best Unique PSM
|

Protein Coverage:

Supporting Peptides:

| Peptide | Uniq | -10lgP | Mass | Length | ppm | m/z | z | RT | 1/k0 Start | 1/k0 End | Precursor ID | Area Sample 9 | #Feature | #Feature Sample 9 | Start | End | PTM | AScore | Found By |
| --- | --- | --- | --- | --- | --- | --- | --- | --- | --- | --- | --- | --- | --- | --- | --- | --- | --- | --- | --- |
| R.ATDSDSGPNGDITYR.I | Y | 57.88 | 1567.675 | 15 | -2.18 | 784.8395 | 2 | 12.74 | 1.0065 | 1.0138 | 31175 | 9.01e2 | 1 | 1 | 1514 | 1528 |  |  | DB Search |
| K.FQVDQATGQITLIGM(+15.99)LNKR.V | Y | 57.57 | 2148.1361 | 19 | 2 | 717.0508 | 3 | 18.25 | 0.9892 | 0.9965 | 61008 | 0 | 0 | 0 | 690 | 708 | Oxidation (M) | M15:Oxidation (M):1000 | DB Search |
| K.VQNEITNPNTQFPVNGLFDLHR.K | Y | 57.3 | 2552.2771 | 22 | 1.04 | 851.7634 | 3 | 19.88 | 1.0644 | 1.0717 | 70362 | 3.51e2 | 1 | 1 | 1880 | 1901 |  |  | DB Search |
| R.LYSTDPNKFYR.F | Y | 56.35 | 1402.6881 | 11 | 0.24 | 702.3483 | 2 | 14.17 | 1.0299 | 1.0371 | 39269 | 1.56e3 | 1 | 1 | 193 | 203 |  |  | DB Search |
| R.EDRNIGTLVTTIR.A | Y | 30.54 | 1486.8103 | 13 | -0.94 | 744.4084 | 2 | 16.10 | 1.0439 | 1.0512 | 49432 | 4.96e2 | 1 | 1 | 240 | 252 |  |  | DB Search |
| K.NERGEAVISLK.S | Y | 19.8 | 1214.6619 | 11 | 0.17 | 608.3356 | 2 | 13.49 | 0.9493 | 0.9566 | 35668 | 0 | 0 | 0 | 585 | 595 |  |  | DB Search |
| total 6 peptides |  |  |  |  |  |  |  |  |  |  |  |  |  |  |  |  |  |  |  |
| --- | --- | --- | --- | --- | --- | --- | --- | --- | --- | --- | --- | --- | --- | --- | --- | --- | --- | --- | --- |

Best Unique PSM (Scan Sample\_8\_A1\_1\_3645.d:31175, m/z=784.8395, z=2, RT=12.74, ppm=-6.69):


TRINITY\_DN98420\_c0\_g1\_i1.p1

back to list

  

| Protein Coverage
| Supporting Peptides
| Best Unique PSM
|

Protein Coverage:

Supporting Peptides:

| Peptide | Uniq | -10lgP | Mass | Length | ppm | m/z | z | RT | 1/k0 Start | 1/k0 End | Precursor ID | Area Sample 9 | #Feature | #Feature Sample 9 | Start | End | PTM | AScore | Found By |
| --- | --- | --- | --- | --- | --- | --- | --- | --- | --- | --- | --- | --- | --- | --- | --- | --- | --- | --- | --- |
| R.AVIFDRFR.G | Y | 44.66 | 1022.5661 | 8 | 0.48 | 512.2883 | 2 | 15.85 | 0.8672 | 0.8745 | 48174 | 3.69e3 | 1 | 1 | 48 | 55 |  |  | DB Search |
| total 1 peptides |  |  |  |  |  |  |  |  |  |  |  |  |  |  |  |  |  |  |  |
| --- | --- | --- | --- | --- | --- | --- | --- | --- | --- | --- | --- | --- | --- | --- | --- | --- | --- | --- | --- |

Best Unique PSM (Scan Sample\_8\_A1\_1\_3645.d:48174, m/z=512.2883, z=2, RT=15.85, ppm=-4.04):


TRINITY\_DN79\_c0\_g1\_i1.p1

back to list

  

| Protein Coverage
| Supporting Peptides
| Best Unique PSM
|

Protein Coverage:

Supporting Peptides:

| Peptide | Uniq | -10lgP | Mass | Length | ppm | m/z | z | RT | 1/k0 Start | 1/k0 End | Precursor ID | Area Sample 9 | #Feature | #Feature Sample 9 | Start | End | PTM | AScore | Found By |
| --- | --- | --- | --- | --- | --- | --- | --- | --- | --- | --- | --- | --- | --- | --- | --- | --- | --- | --- | --- |
| K.KAENFAELKNTFQEAAK.G | Y | 56.89 | 1937.9846 | 17 | 0.51 | 646.9996 | 3 | 16.04 | 0.9174 | 0.9247 | 49239 | 7.51e2 | 1 | 1 | 225 | 241 |  |  | DB Search |
| R.AESIALVTEFTDEAAPK.I | Y | 31.14 | 1790.8938 | 17 | 0.77 | 896.4508 | 2 | 19.80 | 1.1198 | 1.127 | 69969 | 2.36e2 | 1 | 1 | 192 | 208 |  |  | DB Search |
| total 2 peptides |  |  |  |  |  |  |  |  |  |  |  |  |  |  |  |  |  |  |  |
| --- | --- | --- | --- | --- | --- | --- | --- | --- | --- | --- | --- | --- | --- | --- | --- | --- | --- | --- | --- |

Best Unique PSM (Scan Sample\_8\_A1\_1\_3645.d:49239, m/z=646.9996, z=3, RT=16.04, ppm=-4):


TRINITY\_DN585\_c0\_g1\_i2.p1

back to list

  

| Protein Coverage
| Supporting Peptides
| Best Unique PSM
|

Protein Coverage:

Supporting Peptides:

| Peptide | Uniq | -10lgP | Mass | Length | ppm | m/z | z | RT | 1/k0 Start | 1/k0 End | Precursor ID | Area Sample 9 | #Feature | #Feature Sample 9 | Start | End | PTM | AScore | Found By |
| --- | --- | --- | --- | --- | --- | --- | --- | --- | --- | --- | --- | --- | --- | --- | --- | --- | --- | --- | --- |
| K.IQDKEGIPPDQQR.L | Y | 61.25 | 1522.7739 | 13 | -1.84 | 762.3894 | 2 | 10.99 | 1.0371 | 1.0443 | 20319 | 2.55e3 | 1 | 1 | 2 | 14 |  |  | DB Search |
| total 1 peptides |  |  |  |  |  |  |  |  |  |  |  |  |  |  |  |  |  |  |  |
| --- | --- | --- | --- | --- | --- | --- | --- | --- | --- | --- | --- | --- | --- | --- | --- | --- | --- | --- | --- |

Best Unique PSM (Scan Sample\_8\_A1\_1\_3645.d:20319, m/z=762.3894, z=2, RT=10.99, ppm=-6.36):


TRINITY\_DN3190\_c1\_g1\_i1.p1

back to list

  

| Protein Coverage
| Supporting Peptides
| Best Unique PSM
|

Protein Coverage:

Supporting Peptides:

| Peptide | Uniq | -10lgP | Mass | Length | ppm | m/z | z | RT | 1/k0 Start | 1/k0 End | Precursor ID | Area Sample 9 | #Feature | #Feature Sample 9 | Start | End | PTM | AScore | Found By |
| --- | --- | --- | --- | --- | --- | --- | --- | --- | --- | --- | --- | --- | --- | --- | --- | --- | --- | --- | --- |
| K.DLQGETSGSFKR.L | Y | 56.14 | 1323.6419 | 12 | 3.37 | 662.8275 | 2 | 12.57 | 0.9654 | 0.9727 | 30296 | 2.34e3 | 1 | 1 | 135 | 146 |  |  | DB Search |
| K.RLLTSLVQGAR.L | Y | 53.6 | 1212.7302 | 11 | 0.97 | 607.3702 | 2 | 15.15 | 0.9783 | 0.9856 | 44660 | 5.21e2 | 1 | 1 | 146 | 156 |  |  | DB Search |
| total 2 peptides |  |  |  |  |  |  |  |  |  |  |  |  |  |  |  |  |  |  |  |
| --- | --- | --- | --- | --- | --- | --- | --- | --- | --- | --- | --- | --- | --- | --- | --- | --- | --- | --- | --- |

Best Unique PSM (Scan Sample\_8\_A1\_1\_3645.d:30296, m/z=662.8275, z=2, RT=12.57, ppm=-1.14):


TRINITY\_DN36\_c0\_g1\_i8.p1

back to list

  

| Protein Coverage
| Supporting Peptides
| Best Unique PSM
|

Protein Coverage:

Supporting Peptides:

| Peptide | Uniq | -10lgP | Mass | Length | ppm | m/z | z | RT | 1/k0 Start | 1/k0 End | Precursor ID | Area Sample 9 | #Feature | #Feature Sample 9 | Start | End | PTM | AScore | Found By |
| --- | --- | --- | --- | --- | --- | --- | --- | --- | --- | --- | --- | --- | --- | --- | --- | --- | --- | --- | --- |
| K.YGQAM(+15.99)C(+57.02)QFM(+15.99)R.N | N | 61.54 | 1322.5206 | 10 | -1.76 | 662.2634 | 2 | 12.17 | 0.9489 | 0.9562 | 28008 | 2.07e2 | 1 | 1 | 72 | 81 | Oxidation (M), Carbamidomethylation, Oxidation (M) | M5:Oxidation (M):1000 M9:Oxidation (M):1000 | DB Search |
| K.WC(+57.02)DILSIHQSMENC(+57.02)VAK.C | Y | 59.79 | 2089.9383 | 17 | 2.4 | 697.6519 | 3 | 17.87 | 0.8769 | 0.8842 | 58766 | 0 | 0 | 0 | 198 | 214 | Carbamidomethylation, Carbamidomethylation |  | DB Search |
| K.DVYDAEQAERK.R | N | 57.85 | 1322.6102 | 11 | -1.22 | 662.3086 | 2 | 11.56 | 0.9606 | 0.9679 | 24244 | 0 | 0 | 0 | 103 | 113 |  |  | DB Search |
| R.LGGIEVTQGNC(+57.02)AR.K | N | 57.3 | 1373.6721 | 13 | 3.87 | 687.8429 | 2 | 13.70 | 0.9642 | 0.9715 | 36854 | 7.25e2 | 1 | 1 | 58 | 70 | Carbamidomethylation |  | DB Search |
| K.FETQIATK.D | Y | 51.43 | 936.4916 | 8 | -0.7 | 937.494 | 1 | 11.83 | 1.2165 | 1.2236 | 25881 | 4.08e3 | 3 | 3 | 167 | 174 |  |  | DB Search |
| total 5 peptides |  |  |  |  |  |  |  |  |  |  |  |  |  |  |  |  |  |  |  |
| --- | --- | --- | --- | --- | --- | --- | --- | --- | --- | --- | --- | --- | --- | --- | --- | --- | --- | --- | --- |

Best Unique PSM (Scan Sample\_8\_A1\_1\_3645.d:58766, m/z=697.6519, z=3, RT=17.87, ppm=-2.11):


TRINITY\_DN1616\_c0\_g1\_i6.p1

back to list

  

| Protein Coverage
| Supporting Peptides
| Best Unique PSM
|

Protein Coverage:

Supporting Peptides:

| Peptide | Uniq | -10lgP | Mass | Length | ppm | m/z | z | RT | 1/k0 Start | 1/k0 End | Precursor ID | Area Sample 9 | #Feature | #Feature Sample 9 | Start | End | PTM | AScore | Found By |
| --- | --- | --- | --- | --- | --- | --- | --- | --- | --- | --- | --- | --- | --- | --- | --- | --- | --- | --- | --- |
| K.VVAEC(+57.02)NNGGGR.F | Y | 57.11 | 1131.5091 | 11 | -7.52 | 566.755 | 2 | 9.43 | 0.8299 | 0.8372 | 11493 | 0 | 0 | 0 | 3736 | 3746 | Carbamidomethylation |  | DB Search |
| K.GC(+57.02)STC(+57.02)GC(+57.02)NGAGTNNGVK.S | Y | 57 | 1712.6665 | 17 | 3.09 | 857.3393 | 2 | 9.83 | 1.0411 | 1.0484 | 13666 | 0 | 0 | 0 | 732 | 748 | Carbamidomethylation, Carbamidomethylation, Carbamidomethylation |  | DB Search |
| K.GSPGELFC(+57.02)TLADATGK.F | Y | 30.98 | 1622.761 | 16 | -3.1 | 812.3816 | 2 | 19.88 | 1.0588 | 1.066 | 70430 | 0 | 0 | 0 | 45 | 60 | Carbamidomethylation |  | DB Search |
| total 3 peptides |  |  |  |  |  |  |  |  |  |  |  |  |  |  |  |  |  |  |  |
| --- | --- | --- | --- | --- | --- | --- | --- | --- | --- | --- | --- | --- | --- | --- | --- | --- | --- | --- | --- |

Best Unique PSM (Scan Sample\_8\_A1\_1\_3645.d:11493, m/z=566.755, z=2, RT=9.43, ppm=-12.04):


TRINITY\_DN5397\_c0\_g1\_i1.p1

back to list

  

| Protein Coverage
| Supporting Peptides
| Best Unique PSM
|

Protein Coverage:

Supporting Peptides:

| Peptide | Uniq | -10lgP | Mass | Length | ppm | m/z | z | RT | 1/k0 Start | 1/k0 End | Precursor ID | Area Sample 9 | #Feature | #Feature Sample 9 | Start | End | PTM | AScore | Found By |
| --- | --- | --- | --- | --- | --- | --- | --- | --- | --- | --- | --- | --- | --- | --- | --- | --- | --- | --- | --- |
| R.EGSTPC(+57.02)NTQAC(+57.02)GSGLSEER.F | Y | 56.04 | 2038.832 | 19 | 2.83 | 1020.4216 | 2 | 12.00 | 1.1366 | 1.1438 | 26927 | 0 | 0 | 0 | 41 | 59 | Carbamidomethylation, Carbamidomethylation |  | DB Search |
| total 1 peptides |  |  |  |  |  |  |  |  |  |  |  |  |  |  |  |  |  |  |  |
| --- | --- | --- | --- | --- | --- | --- | --- | --- | --- | --- | --- | --- | --- | --- | --- | --- | --- | --- | --- |

Best Unique PSM (Scan Sample\_8\_A1\_1\_3645.d:26927, m/z=1020.4216, z=2, RT=12.00, ppm=-1.68):


TRINITY\_DN2077\_c0\_g1\_i1.p3

back to list

  

| Protein Coverage
| Supporting Peptides
| Best Unique PSM
|

Protein Coverage:

Supporting Peptides:

| Peptide | Uniq | -10lgP | Mass | Length | ppm | m/z | z | RT | 1/k0 Start | 1/k0 End | Precursor ID | Area Sample 9 | #Feature | #Feature Sample 9 | Start | End | PTM | AScore | Found By |
| --- | --- | --- | --- | --- | --- | --- | --- | --- | --- | --- | --- | --- | --- | --- | --- | --- | --- | --- | --- |
| R.DVQGFQGLRGRFEGK.R | Y | 16.87 | 1692.8696 | 15 | -9.16 | 847.4305 | 2 | 14.86 | 1.1538 | 1.161 | 43149 | 3.52e2 | 1 | 1 | 183 | 197 |  |  | DB Search |
| total 1 peptides |  |  |  |  |  |  |  |  |  |  |  |  |  |  |  |  |  |  |  |
| --- | --- | --- | --- | --- | --- | --- | --- | --- | --- | --- | --- | --- | --- | --- | --- | --- | --- | --- | --- |

Best Unique PSM (Scan Sample\_8\_A1\_1\_3645.d:43149, m/z=847.4305, z=2, RT=14.86, ppm=-13.67):


TRINITY\_DN36471\_c0\_g1\_i1.p1

back to list

  

| Protein Coverage
| Supporting Peptides
| Best Unique PSM
|

Protein Coverage:

Supporting Peptides:

| Peptide | Uniq | -10lgP | Mass | Length | ppm | m/z | z | RT | 1/k0 Start | 1/k0 End | Precursor ID | Area Sample 9 | #Feature | #Feature Sample 9 | Start | End | PTM | AScore | Found By |
| --- | --- | --- | --- | --- | --- | --- | --- | --- | --- | --- | --- | --- | --- | --- | --- | --- | --- | --- | --- |
| K.DLEHPNEFIR.G | Y | 58.16 | 1268.6149 | 10 | -6.42 | 635.3078 | 2 | 13.50 | 0.9226 | 0.9299 | 35766 | 0 | 0 | 0 | 144 | 153 |  |  | DB Search |
| total 1 peptides |  |  |  |  |  |  |  |  |  |  |  |  |  |  |  |  |  |  |  |
| --- | --- | --- | --- | --- | --- | --- | --- | --- | --- | --- | --- | --- | --- | --- | --- | --- | --- | --- | --- |

Best Unique PSM (Scan Sample\_8\_A1\_1\_3645.d:35766, m/z=634.7948, z=2, RT=13.53, ppm=-10.93):


TRINITY\_DN942\_c0\_g1\_i5.p1

back to list

  

| Protein Coverage
| Supporting Peptides
| Best Unique PSM
|

Protein Coverage:

Supporting Peptides:

| Peptide | Uniq | -10lgP | Mass | Length | ppm | m/z | z | RT | 1/k0 Start | 1/k0 End | Precursor ID | Area Sample 9 | #Feature | #Feature Sample 9 | Start | End | PTM | AScore | Found By |
| --- | --- | --- | --- | --- | --- | --- | --- | --- | --- | --- | --- | --- | --- | --- | --- | --- | --- | --- | --- |
| K.AIGNAQRPAR.V | Y | 22.83 | 1052.5839 | 10 | -1.57 | 527.296 | 2 | 9.68 | 0.8664 | 0.8737 | 12735 | 8.15e2 | 1 | 1 | 498 | 507 |  |  | DB Search |
| total 1 peptides |  |  |  |  |  |  |  |  |  |  |  |  |  |  |  |  |  |  |  |
| --- | --- | --- | --- | --- | --- | --- | --- | --- | --- | --- | --- | --- | --- | --- | --- | --- | --- | --- | --- |

Best Unique PSM (Scan Sample\_8\_A1\_1\_3645.d:12735, m/z=527.296, z=2, RT=9.68, ppm=-6.09):


TRINITY\_DN31399\_c0\_g1\_i1.p1

back to list

  

| Protein Coverage
| Supporting Peptides
| Best Unique PSM
|

Protein Coverage:

Supporting Peptides:

| Peptide | Uniq | -10lgP | Mass | Length | ppm | m/z | z | RT | 1/k0 Start | 1/k0 End | Precursor ID | Area Sample 9 | #Feature | #Feature Sample 9 | Start | End | PTM | AScore | Found By |
| --- | --- | --- | --- | --- | --- | --- | --- | --- | --- | --- | --- | --- | --- | --- | --- | --- | --- | --- | --- |
| R.VNGADGKVEVVWK.T | Y | 52.96 | 1399.7459 | 13 | -2.44 | 700.8754 | 2 | 14.60 | 1.0705 | 1.0777 | 41798 | 0 | 0 | 0 | 46 | 58 |  |  | DB Search |
| total 1 peptides |  |  |  |  |  |  |  |  |  |  |  |  |  |  |  |  |  |  |  |
| --- | --- | --- | --- | --- | --- | --- | --- | --- | --- | --- | --- | --- | --- | --- | --- | --- | --- | --- | --- |

Best Unique PSM (Scan Sample\_8\_A1\_1\_3645.d:41798, m/z=700.8754, z=2, RT=14.60, ppm=-6.96):


TRINITY\_DN761\_c0\_g1\_i3.p1

back to list

  

| Protein Coverage
| Supporting Peptides
| Best Unique PSM
|

Protein Coverage:

Supporting Peptides:

| Peptide | Uniq | -10lgP | Mass | Length | ppm | m/z | z | RT | 1/k0 Start | 1/k0 End | Precursor ID | Area Sample 9 | #Feature | #Feature Sample 9 | Start | End | PTM | AScore | Found By |
| --- | --- | --- | --- | --- | --- | --- | --- | --- | --- | --- | --- | --- | --- | --- | --- | --- | --- | --- | --- |
| K.TRIEDLETVISQTQDHR.H | Y | 57.13 | 2040.0236 | 17 | 0.12 | 681.0121 | 3 | 17.33 | 0.9586 | 0.9658 | 55627 | 6.48e2 | 1 | 1 | 275 | 291 |  |  | DB Search |
| total 1 peptides |  |  |  |  |  |  |  |  |  |  |  |  |  |  |  |  |  |  |  |
| --- | --- | --- | --- | --- | --- | --- | --- | --- | --- | --- | --- | --- | --- | --- | --- | --- | --- | --- | --- |

Best Unique PSM (Scan Sample\_8\_A1\_1\_3645.d:55627, m/z=681.0121, z=3, RT=17.33, ppm=-4.39):


TRINITY\_DN333\_c0\_g1\_i2.p1

back to list

  

| Protein Coverage
| Supporting Peptides
| Best Unique PSM
|

Protein Coverage:

Supporting Peptides:

| Peptide | Uniq | -10lgP | Mass | Length | ppm | m/z | z | RT | 1/k0 Start | 1/k0 End | Precursor ID | Area Sample 9 | #Feature | #Feature Sample 9 | Start | End | PTM | AScore | Found By |
| --- | --- | --- | --- | --- | --- | --- | --- | --- | --- | --- | --- | --- | --- | --- | --- | --- | --- | --- | --- |
| R.RRYPIIVTGR.L | Y | 55.45 | 1229.7356 | 10 | 2.21 | 410.9182 | 3 | 12.81 | 0.7746 | 0.7819 | 31572 | 7.37e2 | 1 | 1 | 286 | 295 |  |  | DB Search |
| total 1 peptides |  |  |  |  |  |  |  |  |  |  |  |  |  |  |  |  |  |  |  |
| --- | --- | --- | --- | --- | --- | --- | --- | --- | --- | --- | --- | --- | --- | --- | --- | --- | --- | --- | --- |

Best Unique PSM (Scan Sample\_8\_A1\_1\_3645.d:31572, m/z=410.9182, z=3, RT=12.81, ppm=-2.31):


TRINITY\_DN982\_c0\_g1\_i1.p1

back to list

  

| Protein Coverage
| Supporting Peptides
| Best Unique PSM
|

Protein Coverage:

Supporting Peptides:

| Peptide | Uniq | -10lgP | Mass | Length | ppm | m/z | z | RT | 1/k0 Start | 1/k0 End | Precursor ID | Area Sample 9 | #Feature | #Feature Sample 9 | Start | End | PTM | AScore | Found By |
| --- | --- | --- | --- | --- | --- | --- | --- | --- | --- | --- | --- | --- | --- | --- | --- | --- | --- | --- | --- |
| K.AVDRGNPPQDSVNK.A | N | 61.59 | 1495.7379 | 14 | -2.2 | 499.5832 | 3 | 10.21 | 0.7823 | 0.7897 | 15988 | 0 | 0 | 0 | 1699 | 1712 |  |  | DB Search |
| R.IQVLKDPDFEFTR.S | N | 58.69 | 1606.8355 | 13 | -7.52 | 804.4153 | 2 | 17.50 | 1.0813 | 1.0885 | 56511 | 4.85e2 | 1 | 1 | 2727 | 2739 |  |  | DB Search |
| R.ITTSQILDHEQQK.L | N | 58.51 | 1539.7893 | 13 | -0.87 | 770.8978 | 2 | 13.81 | 1.0933 | 1.1005 | 37414 | 9.71e2 | 1 | 1 | 1994 | 2006 |  |  | DB Search |
| K.YVAIYPSDHLTVNR.D | N | 57.84 | 1646.8416 | 14 | 0.01 | 824.4244 | 2 | 15.46 | 1.109 | 1.1162 | 46349 | 0 | 0 | 0 | 2814 | 2827 |  |  | DB Search |
| K.TSTQDVALNIRR.S | N | 57.52 | 1372.7423 | 12 | 5.33 | 687.379 | 2 | 13.43 | 0.9989 | 1.0061 | 35260 | 2.7e3 | 1 | 1 | 5623 | 5634 |  |  | DB Search |
| R.VLDVNDKQPVFVK.S | Y | 57.11 | 1499.8347 | 13 | 0.01 | 750.9213 | 2 | 14.94 | 1.0668 | 1.0741 | 43674 | 0 | 0 | 0 | 670 | 682 |  |  | DB Search |
| R.LVQNTPHNSIVTK.I | N | 56.38 | 1449.794 | 13 | 0.62 | 725.9014 | 2 | 11.65 | 1.0604 | 1.0676 | 24679 | 2.98e3 | 1 | 1 | 5129 | 5141 |  |  | DB Search |
| K.VLLSQQIFYDKK.N | N | 55.75 | 1480.8289 | 12 | -2.98 | 741.4162 | 2 | 16.65 | 1.0588 | 1.066 | 52089 | 1.28e3 | 1 | 1 | 1479 | 1490 |  |  | DB Search |
| K.AVDRGNPSQDSLNK.A | Y | 54.84 | 1499.7328 | 14 | -1.13 | 750.8694 | 2 | 10.61 | 0.9937 | 1.0009 | 18454 | 0 | 0 | 0 | 16 | 29 |  |  | DB Search |
| total 9 peptides |  |  |  |  |  |  |  |  |  |  |  |  |  |  |  |  |  |  |  |
| --- | --- | --- | --- | --- | --- | --- | --- | --- | --- | --- | --- | --- | --- | --- | --- | --- | --- | --- | --- |

Best Unique PSM (Scan Sample\_8\_A1\_1\_3645.d:43674, m/z=750.9213, z=2, RT=14.94, ppm=-4.51):


TRINITY\_DN5500\_c0\_g1\_i1.p1

back to list

  

| Protein Coverage
| Supporting Peptides
| Best Unique PSM
|

Protein Coverage:

Supporting Peptides:

| Peptide | Uniq | -10lgP | Mass | Length | ppm | m/z | z | RT | 1/k0 Start | 1/k0 End | Precursor ID | Area Sample 9 | #Feature | #Feature Sample 9 | Start | End | PTM | AScore | Found By |
| --- | --- | --- | --- | --- | --- | --- | --- | --- | --- | --- | --- | --- | --- | --- | --- | --- | --- | --- | --- |
| K.LAIKENHHLNEPIEK.L | Y | 57.45 | 1783.958 | 15 | -5.85 | 892.9771 | 2 | 12.12 | 1.139 | 1.1462 | 27632 | 0 | 0 | 0 | 187 | 201 |  |  | DB Search |
| K.ENHHLNEPIEK.L | Y | 54.18 | 1358.6578 | 11 | -5.39 | 680.3295 | 2 | 12.12 | 0.9675 | 0.9747 | 27643 | 0 | 0 | 0 | 191 | 201 |  |  | DB Search |
| total 2 peptides |  |  |  |  |  |  |  |  |  |  |  |  |  |  |  |  |  |  |  |
| --- | --- | --- | --- | --- | --- | --- | --- | --- | --- | --- | --- | --- | --- | --- | --- | --- | --- | --- | --- |

Best Unique PSM (Scan Sample\_8\_A1\_1\_3645.d:27632, m/z=892.9771, z=2, RT=12.12, ppm=-10.36):


TRINITY\_DN6701\_c0\_g1\_i2.p2

back to list

  

| Protein Coverage
| Supporting Peptides
| Best Unique PSM
|

Protein Coverage:

Supporting Peptides:

| Peptide | Uniq | -10lgP | Mass | Length | ppm | m/z | z | RT | 1/k0 Start | 1/k0 End | Precursor ID | Area Sample 9 | #Feature | #Feature Sample 9 | Start | End | PTM | AScore | Found By |
| --- | --- | --- | --- | --- | --- | --- | --- | --- | --- | --- | --- | --- | --- | --- | --- | --- | --- | --- | --- |
| K.VITSDDPAEKEK.K | Y | 56.98 | 1330.6616 | 12 | 1.22 | 666.3359 | 2 | 10.71 | 0.9844 | 0.9916 | 18867 | 1.51e3 | 1 | 1 | 114 | 125 |  |  | DB Search |
| total 1 peptides |  |  |  |  |  |  |  |  |  |  |  |  |  |  |  |  |  |  |  |
| --- | --- | --- | --- | --- | --- | --- | --- | --- | --- | --- | --- | --- | --- | --- | --- | --- | --- | --- | --- |

Best Unique PSM (Scan Sample\_8\_A1\_1\_3645.d:18867, m/z=666.3359, z=2, RT=10.71, ppm=-3.3):


TRINITY\_DN113\_c3\_g1\_i1.p1

back to list

  

| Protein Coverage
| Supporting Peptides
| Best Unique PSM
|

Protein Coverage:

Supporting Peptides:

| Peptide | Uniq | -10lgP | Mass | Length | ppm | m/z | z | RT | 1/k0 Start | 1/k0 End | Precursor ID | Area Sample 9 | #Feature | #Feature Sample 9 | Start | End | PTM | AScore | Found By |
| --- | --- | --- | --- | --- | --- | --- | --- | --- | --- | --- | --- | --- | --- | --- | --- | --- | --- | --- | --- |
| R.ATHQELLNAEFIQK.V | Y | 60.63 | 1640.8522 | 14 | -8.6 | 821.4226 | 2 | 15.81 | 1.0801 | 1.0873 | 48105 | 0 | 0 | 0 | 457 | 470 |  |  | DB Search |
| K.AHVSLSTFSEDVTSLTK.F | Y | 59.92 | 1820.9156 | 17 | -3.57 | 911.4577 | 2 | 18.78 | 1.1386 | 1.1458 | 63908 | 1.2e3 | 1 | 1 | 568 | 584 |  |  | DB Search |
| total 2 peptides |  |  |  |  |  |  |  |  |  |  |  |  |  |  |  |  |  |  |  |
| --- | --- | --- | --- | --- | --- | --- | --- | --- | --- | --- | --- | --- | --- | --- | --- | --- | --- | --- | --- |

Best Unique PSM (Scan Sample\_8\_A1\_1\_3645.d:48105, m/z=821.4226, z=2, RT=15.81, ppm=-13.12):


TRINITY\_DN1933\_c0\_g1\_i7.p1

back to list

  

| Protein Coverage
| Supporting Peptides
| Best Unique PSM
|

Protein Coverage:

Supporting Peptides:

| Peptide | Uniq | -10lgP | Mass | Length | ppm | m/z | z | RT | 1/k0 Start | 1/k0 End | Precursor ID | Area Sample 9 | #Feature | #Feature Sample 9 | Start | End | PTM | AScore | Found By |
| --- | --- | --- | --- | --- | --- | --- | --- | --- | --- | --- | --- | --- | --- | --- | --- | --- | --- | --- | --- |
| R.RNLDIERPTYTNLNR.L | Y | 58.31 | 1873.9758 | 15 | 3.94 | 625.6655 | 3 | 14.03 | 0.8846 | 0.8919 | 38584 | 6.19e2 | 1 | 1 | 75 | 89 |  |  | DB Search |
| total 1 peptides |  |  |  |  |  |  |  |  |  |  |  |  |  |  |  |  |  |  |  |
| --- | --- | --- | --- | --- | --- | --- | --- | --- | --- | --- | --- | --- | --- | --- | --- | --- | --- | --- | --- |

Best Unique PSM (Scan Sample\_8\_A1\_1\_3645.d:38584, m/z=625.6655, z=3, RT=14.03, ppm=-0.58):


TRINITY\_DN1653\_c0\_g1\_i3.p1

back to list

  

| Protein Coverage
| Supporting Peptides
| Best Unique PSM
|

Protein Coverage:

Supporting Peptides:

| Peptide | Uniq | -10lgP | Mass | Length | ppm | m/z | z | RT | 1/k0 Start | 1/k0 End | Precursor ID | Area Sample 9 | #Feature | #Feature Sample 9 | Start | End | PTM | AScore | Found By |
| --- | --- | --- | --- | --- | --- | --- | --- | --- | --- | --- | --- | --- | --- | --- | --- | --- | --- | --- | --- |
| R.LINLFEAVSKK.S | Y | 54.5 | 1260.7441 | 11 | 2.76 | 631.3782 | 2 | 18.78 | 0.9808 | 0.988 | 64045 | 0 | 0 | 0 | 49 | 59 |  |  | DB Search |
| K.LVSIDSDHVVNGNSK.M | Y | 44.95 | 1582.7951 | 15 | -0.07 | 792.4012 | 2 | 13.38 | 1.0644 | 1.0717 | 35085 | 2.42e2 | 1 | 1 | 92 | 106 |  |  | DB Search |
| total 2 peptides |  |  |  |  |  |  |  |  |  |  |  |  |  |  |  |  |  |  |  |
| --- | --- | --- | --- | --- | --- | --- | --- | --- | --- | --- | --- | --- | --- | --- | --- | --- | --- | --- | --- |

Best Unique PSM (Scan Sample\_8\_A1\_1\_3645.d:64045, m/z=631.3782, z=2, RT=18.78, ppm=-1.76):


TRINITY\_DN413\_c0\_g1\_i2.p1

back to list

  

| Protein Coverage
| Supporting Peptides
| Best Unique PSM
|

Protein Coverage:

Supporting Peptides:

| Peptide | Uniq | -10lgP | Mass | Length | ppm | m/z | z | RT | 1/k0 Start | 1/k0 End | Precursor ID | Area Sample 9 | #Feature | #Feature Sample 9 | Start | End | PTM | AScore | Found By |
| --- | --- | --- | --- | --- | --- | --- | --- | --- | --- | --- | --- | --- | --- | --- | --- | --- | --- | --- | --- |
| R.LLLGDFNGDKR.T | Y | 55.21 | 1246.6669 | 11 | 4.34 | 624.3406 | 2 | 15.96 | 0.9852 | 0.9924 | 48766 | 6.58e2 | 1 | 1 | 46 | 56 |  |  | DB Search |
| total 1 peptides |  |  |  |  |  |  |  |  |  |  |  |  |  |  |  |  |  |  |  |
| --- | --- | --- | --- | --- | --- | --- | --- | --- | --- | --- | --- | --- | --- | --- | --- | --- | --- | --- | --- |

Best Unique PSM (Scan Sample\_8\_A1\_1\_3645.d:48766, m/z=624.3406, z=2, RT=15.96, ppm=-0.18):


TRINITY\_DN471\_c0\_g1\_i10.p1

back to list

  

| Protein Coverage
| Supporting Peptides
| Best Unique PSM
|

Protein Coverage:

Supporting Peptides:

| Peptide | Uniq | -10lgP | Mass | Length | ppm | m/z | z | RT | 1/k0 Start | 1/k0 End | Precursor ID | Area Sample 9 | #Feature | #Feature Sample 9 | Start | End | PTM | AScore | Found By |
| --- | --- | --- | --- | --- | --- | --- | --- | --- | --- | --- | --- | --- | --- | --- | --- | --- | --- | --- | --- |
| R.RNLDIERPTYTNLNR.L | Y | 58.31 | 1873.9758 | 15 | 3.94 | 625.6655 | 3 | 14.03 | 0.8846 | 0.8919 | 38584 | 6.19e2 | 1 | 1 | 253 | 267 |  |  | DB Search |
| total 1 peptides |  |  |  |  |  |  |  |  |  |  |  |  |  |  |  |  |  |  |  |
| --- | --- | --- | --- | --- | --- | --- | --- | --- | --- | --- | --- | --- | --- | --- | --- | --- | --- | --- | --- |

Best Unique PSM (Scan Sample\_8\_A1\_1\_3645.d:38584, m/z=625.6655, z=3, RT=14.03, ppm=-0.58):


TRINITY\_DN649\_c0\_g1\_i1.p1

back to list

  

| Protein Coverage
| Supporting Peptides
| Best Unique PSM
|

Protein Coverage:

Supporting Peptides:

| Peptide | Uniq | -10lgP | Mass | Length | ppm | m/z | z | RT | 1/k0 Start | 1/k0 End | Precursor ID | Area Sample 9 | #Feature | #Feature Sample 9 | Start | End | PTM | AScore | Found By |
| --- | --- | --- | --- | --- | --- | --- | --- | --- | --- | --- | --- | --- | --- | --- | --- | --- | --- | --- | --- |
| K.YGQAM(+15.99)C(+57.02)QFM(+15.99)R.N | N | 61.54 | 1322.5206 | 10 | -1.76 | 662.2634 | 2 | 12.17 | 0.9489 | 0.9562 | 28008 | 2.07e2 | 1 | 1 | 7 | 16 | Oxidation (M), Carbamidomethylation, Oxidation (M) | M5:Oxidation (M):1000 M9:Oxidation (M):1000 | DB Search |
| K.FETQIATK.N | Y | 51.43 | 936.4916 | 8 | -0.7 | 937.494 | 1 | 11.83 | 1.2165 | 1.2236 | 25881 | 4.08e3 | 3 | 3 | 102 | 109 |  |  | DB Search |
| total 2 peptides |  |  |  |  |  |  |  |  |  |  |  |  |  |  |  |  |  |  |  |
| --- | --- | --- | --- | --- | --- | --- | --- | --- | --- | --- | --- | --- | --- | --- | --- | --- | --- | --- | --- |

Best Unique PSM (Scan Sample\_8\_A1\_1\_3645.d:25881, m/z=937.494, z=1, RT=11.83, ppm=-5.22):


TRINITY\_DN1058\_c0\_g1\_i2.p2

back to list

  

| Protein Coverage
| Supporting Peptides
| Best Unique PSM
|

Protein Coverage:

Supporting Peptides:

| Peptide | Uniq | -10lgP | Mass | Length | ppm | m/z | z | RT | 1/k0 Start | 1/k0 End | Precursor ID | Area Sample 9 | #Feature | #Feature Sample 9 | Start | End | PTM | AScore | Found By |
| --- | --- | --- | --- | --- | --- | --- | --- | --- | --- | --- | --- | --- | --- | --- | --- | --- | --- | --- | --- |
| R.APTHILNPANKR.S | Y | 57.39 | 1330.7469 | 12 | 0.47 | 666.3781 | 2 | 11.10 | 1.0102 | 1.0174 | 21164 | 1.88e3 | 2 | 2 | 975 | 986 |  |  | DB Search |
| total 1 peptides |  |  |  |  |  |  |  |  |  |  |  |  |  |  |  |  |  |  |  |
| --- | --- | --- | --- | --- | --- | --- | --- | --- | --- | --- | --- | --- | --- | --- | --- | --- | --- | --- | --- |

Best Unique PSM (Scan Sample\_8\_A1\_1\_3645.d:21164, m/z=666.3781, z=2, RT=11.10, ppm=-4.05):


TRINITY\_DN6701\_c0\_g1\_i1.p1

back to list

  

| Protein Coverage
| Supporting Peptides
| Best Unique PSM
|

Protein Coverage:

Supporting Peptides:

| Peptide | Uniq | -10lgP | Mass | Length | ppm | m/z | z | RT | 1/k0 Start | 1/k0 End | Precursor ID | Area Sample 9 | #Feature | #Feature Sample 9 | Start | End | PTM | AScore | Found By |
| --- | --- | --- | --- | --- | --- | --- | --- | --- | --- | --- | --- | --- | --- | --- | --- | --- | --- | --- | --- |
| K.VITSDDPAEKEK.K | Y | 56.98 | 1330.6616 | 12 | 1.22 | 666.3359 | 2 | 10.71 | 0.9844 | 0.9916 | 18867 | 1.51e3 | 1 | 1 | 158 | 169 |  |  | DB Search |
| total 1 peptides |  |  |  |  |  |  |  |  |  |  |  |  |  |  |  |  |  |  |  |
| --- | --- | --- | --- | --- | --- | --- | --- | --- | --- | --- | --- | --- | --- | --- | --- | --- | --- | --- | --- |

Best Unique PSM (Scan Sample\_8\_A1\_1\_3645.d:18867, m/z=666.3359, z=2, RT=10.71, ppm=-3.3):


TRINITY\_DN119673\_c0\_g1\_i1.p1

back to list

  

| Protein Coverage
| Supporting Peptides
| Best Unique PSM
|

Protein Coverage:

Supporting Peptides:

| Peptide | Uniq | -10lgP | Mass | Length | ppm | m/z | z | RT | 1/k0 Start | 1/k0 End | Precursor ID | Area Sample 9 | #Feature | #Feature Sample 9 | Start | End | PTM | AScore | Found By |
| --- | --- | --- | --- | --- | --- | --- | --- | --- | --- | --- | --- | --- | --- | --- | --- | --- | --- | --- | --- |
| K.VLTSDDPAEKEK | Y | 56.98 | 1330.6616 | 12 | 1.22 | 666.3359 | 2 | 10.71 | 0.9844 | 0.9916 | 18867 | 1.51e3 | 1 | 1 | 117 | 128 |  |  | DB Search |
| total 1 peptides |  |  |  |  |  |  |  |  |  |  |  |  |  |  |  |  |  |  |  |
| --- | --- | --- | --- | --- | --- | --- | --- | --- | --- | --- | --- | --- | --- | --- | --- | --- | --- | --- | --- |

Best Unique PSM (Scan Sample\_8\_A1\_1\_3645.d:18867, m/z=666.3359, z=2, RT=10.71, ppm=-3.3):


TRINITY\_DN26425\_c0\_g1\_i1.p1

back to list

  

| Protein Coverage
| Supporting Peptides
| Best Unique PSM
|

Protein Coverage:

Supporting Peptides:

| Peptide | Uniq | -10lgP | Mass | Length | ppm | m/z | z | RT | 1/k0 Start | 1/k0 End | Precursor ID | Area Sample 9 | #Feature | #Feature Sample 9 | Start | End | PTM | AScore | Found By |
| --- | --- | --- | --- | --- | --- | --- | --- | --- | --- | --- | --- | --- | --- | --- | --- | --- | --- | --- | --- |
| K.EISQEELIETLKK.R | Y | 30.63 | 1558.8454 | 13 | 1.77 | 520.621 | 3 | 17.72 | 0.7831 | 0.7905 | 57968 | 5.68e2 | 1 | 1 | 40 | 52 |  |  | DB Search |
| total 1 peptides |  |  |  |  |  |  |  |  |  |  |  |  |  |  |  |  |  |  |  |
| --- | --- | --- | --- | --- | --- | --- | --- | --- | --- | --- | --- | --- | --- | --- | --- | --- | --- | --- | --- |

Best Unique PSM (Scan Sample\_8\_A1\_1\_3645.d:57968, m/z=520.621, z=3, RT=17.72, ppm=-2.75):


TRINITY\_DN1505\_c0\_g1\_i1.p1

back to list

  

| Protein Coverage
| Supporting Peptides
| Best Unique PSM
|

Protein Coverage:

Supporting Peptides:

| Peptide | Uniq | -10lgP | Mass | Length | ppm | m/z | z | RT | 1/k0 Start | 1/k0 End | Precursor ID | Area Sample 9 | #Feature | #Feature Sample 9 | Start | End | PTM | AScore | Found By |
| --- | --- | --- | --- | --- | --- | --- | --- | --- | --- | --- | --- | --- | --- | --- | --- | --- | --- | --- | --- |
| M.S(+42.01)LVIPEKFQHILR.V | Y | 57.91 | 1620.9351 | 13 | -0.14 | 541.3165 | 3 | 19.55 | 0.8408 | 0.8481 | 67997 | 1.96e3 | 1 | 1 | 2 | 14 | Acetylation (Protein N-term) | S1:Acetylation (Protein N-term):1000 | DB Search |
| total 1 peptides |  |  |  |  |  |  |  |  |  |  |  |  |  |  |  |  |  |  |  |
| --- | --- | --- | --- | --- | --- | --- | --- | --- | --- | --- | --- | --- | --- | --- | --- | --- | --- | --- | --- |

Best Unique PSM (Scan Sample\_8\_A1\_1\_3645.d:67997, m/z=541.3165, z=3, RT=19.55, ppm=-4.66):


TRINITY\_DN67\_c0\_g1\_i2.p1

back to list

  

| Protein Coverage
| Supporting Peptides
| Best Unique PSM
|

Protein Coverage:

Supporting Peptides:

| Peptide | Uniq | -10lgP | Mass | Length | ppm | m/z | z | RT | 1/k0 Start | 1/k0 End | Precursor ID | Area Sample 9 | #Feature | #Feature Sample 9 | Start | End | PTM | AScore | Found By |
| --- | --- | --- | --- | --- | --- | --- | --- | --- | --- | --- | --- | --- | --- | --- | --- | --- | --- | --- | --- |
| R.AFIDSSVALLRK.W | Y | 56.93 | 1318.7608 | 12 | 2.94 | 660.3867 | 2 | 17.45 | 1.0331 | 1.0403 | 56355 | 4.78e2 | 1 | 1 | 128 | 139 |  |  | DB Search |
| total 1 peptides |  |  |  |  |  |  |  |  |  |  |  |  |  |  |  |  |  |  |  |
| --- | --- | --- | --- | --- | --- | --- | --- | --- | --- | --- | --- | --- | --- | --- | --- | --- | --- | --- | --- |

Best Unique PSM (Scan Sample\_8\_A1\_1\_3645.d:56355, m/z=660.3867, z=2, RT=17.45, ppm=-1.58):


TRINITY\_DN36\_c0\_g1\_i11.p1

back to list

  

| Protein Coverage
| Supporting Peptides
| Best Unique PSM
|

Protein Coverage:

Supporting Peptides:

| Peptide | Uniq | -10lgP | Mass | Length | ppm | m/z | z | RT | 1/k0 Start | 1/k0 End | Precursor ID | Area Sample 9 | #Feature | #Feature Sample 9 | Start | End | PTM | AScore | Found By |
| --- | --- | --- | --- | --- | --- | --- | --- | --- | --- | --- | --- | --- | --- | --- | --- | --- | --- | --- | --- |
| K.YGQAM(+15.99)C(+57.02)QFM(+15.99)R.N | N | 61.54 | 1322.5206 | 10 | -1.76 | 662.2634 | 2 | 12.17 | 0.9489 | 0.9562 | 28008 | 2.07e2 | 1 | 1 | 72 | 81 | Oxidation (M), Carbamidomethylation, Oxidation (M) | M5:Oxidation (M):1000 M9:Oxidation (M):1000 | DB Search |
| K.DVYDAEQAERK.R | N | 57.85 | 1322.6102 | 11 | -1.22 | 662.3086 | 2 | 11.56 | 0.9606 | 0.9679 | 24244 | 0 | 0 | 0 | 103 | 113 |  |  | DB Search |
| R.LGGIEVTQGNC(+57.02)AR.K | N | 57.3 | 1373.6721 | 13 | 3.87 | 687.8429 | 2 | 13.70 | 0.9642 | 0.9715 | 36854 | 7.25e2 | 1 | 1 | 58 | 70 | Carbamidomethylation |  | DB Search |
| R.HNIDDC(+57.02)K.K | Y | 35.46 | 900.376 | 7 | 9.13 | 451.1973 | 2 | 10.19 | 0.8027 | 0.81 | 15877 | 0 | 0 | 0 | 190 | 196 | Carbamidomethylation |  | DB Search |
| total 4 peptides |  |  |  |  |  |  |  |  |  |  |  |  |  |  |  |  |  |  |  |
| --- | --- | --- | --- | --- | --- | --- | --- | --- | --- | --- | --- | --- | --- | --- | --- | --- | --- | --- | --- |

Best Unique PSM (Scan Sample\_8\_A1\_1\_3645.d:15877, m/z=451.1973, z=2, RT=10.19, ppm=4.61):


TRINITY\_DN384\_c0\_g1\_i1.p1

back to list

  

| Protein Coverage
| Supporting Peptides
| Best Unique PSM
|

Protein Coverage:

Supporting Peptides:

| Peptide | Uniq | -10lgP | Mass | Length | ppm | m/z | z | RT | 1/k0 Start | 1/k0 End | Precursor ID | Area Sample 9 | #Feature | #Feature Sample 9 | Start | End | PTM | AScore | Found By |
| --- | --- | --- | --- | --- | --- | --- | --- | --- | --- | --- | --- | --- | --- | --- | --- | --- | --- | --- | --- |
| K.FVLHGDVSHGSR.E | Y | 56.98 | 1309.6527 | 12 | -4.94 | 437.554 | 3 | 11.96 | 0.7547 | 0.762 | 26738 | 1.72e3 | 1 | 1 | 274 | 285 |  |  | DB Search |
| total 1 peptides |  |  |  |  |  |  |  |  |  |  |  |  |  |  |  |  |  |  |  |
| --- | --- | --- | --- | --- | --- | --- | --- | --- | --- | --- | --- | --- | --- | --- | --- | --- | --- | --- | --- |

Best Unique PSM (Scan Sample\_8\_A1\_1\_3645.d:26738, m/z=437.554, z=3, RT=11.96, ppm=-9.46):


TRINITY\_DN36\_c0\_g1\_i19.p1

back to list

  

| Protein Coverage
| Supporting Peptides
| Best Unique PSM
|

Protein Coverage:

Supporting Peptides:

| Peptide | Uniq | -10lgP | Mass | Length | ppm | m/z | z | RT | 1/k0 Start | 1/k0 End | Precursor ID | Area Sample 9 | #Feature | #Feature Sample 9 | Start | End | PTM | AScore | Found By |
| --- | --- | --- | --- | --- | --- | --- | --- | --- | --- | --- | --- | --- | --- | --- | --- | --- | --- | --- | --- |
| K.YGQAM(+15.99)C(+57.02)QFM(+15.99)R.N | N | 61.54 | 1322.5206 | 10 | -1.76 | 662.2634 | 2 | 12.17 | 0.9489 | 0.9562 | 28008 | 2.07e2 | 1 | 1 | 71 | 80 | Oxidation (M), Carbamidomethylation, Oxidation (M) | M5:Oxidation (M):1000 M9:Oxidation (M):1000 | DB Search |
| K.WC(+57.02)DILSIHQSMENC(+57.02)VAK.C | Y | 59.79 | 2089.9383 | 17 | 2.4 | 697.6519 | 3 | 17.87 | 0.8769 | 0.8842 | 58766 | 0 | 0 | 0 | 197 | 213 | Carbamidomethylation, Carbamidomethylation |  | DB Search |
| K.DVYDAEQAERK.R | N | 57.85 | 1322.6102 | 11 | -1.22 | 662.3086 | 2 | 11.56 | 0.9606 | 0.9679 | 24244 | 0 | 0 | 0 | 102 | 112 |  |  | DB Search |
| R.LGGIEVTQGNC(+57.02)AR.K | N | 57.3 | 1373.6721 | 13 | 3.87 | 687.8429 | 2 | 13.70 | 0.9642 | 0.9715 | 36854 | 7.25e2 | 1 | 1 | 57 | 69 | Carbamidomethylation |  | DB Search |
| K.GFDYC(+57.02)TTIKYR.L | N | 56.39 | 1422.6602 | 11 | -1.35 | 712.3332 | 2 | 15.06 | 1.0234 | 1.0307 | 44297 | 0 | 0 | 0 | 46 | 56 | Carbamidomethylation |  | DB Search |
| K.FETQIATK.D | Y | 51.43 | 936.4916 | 8 | -0.7 | 937.494 | 1 | 11.83 | 1.2165 | 1.2236 | 25881 | 4.08e3 | 3 | 3 | 166 | 173 |  |  | DB Search |
| total 6 peptides |  |  |  |  |  |  |  |  |  |  |  |  |  |  |  |  |  |  |  |
| --- | --- | --- | --- | --- | --- | --- | --- | --- | --- | --- | --- | --- | --- | --- | --- | --- | --- | --- | --- |

Best Unique PSM (Scan Sample\_8\_A1\_1\_3645.d:58766, m/z=697.6519, z=3, RT=17.87, ppm=-2.11):


TRINITY\_DN7214\_c0\_g1\_i1.p1

back to list

  

| Protein Coverage
| Supporting Peptides
| Best Unique PSM
|

Protein Coverage:

Supporting Peptides:

| Peptide | Uniq | -10lgP | Mass | Length | ppm | m/z | z | RT | 1/k0 Start | 1/k0 End | Precursor ID | Area Sample 9 | #Feature | #Feature Sample 9 | Start | End | PTM | AScore | Found By |
| --- | --- | --- | --- | --- | --- | --- | --- | --- | --- | --- | --- | --- | --- | --- | --- | --- | --- | --- | --- |
| R.LAADLVITHSSK.K | Y | 54.93 | 1253.6979 | 12 | 0.86 | 627.8539 | 2 | 14.19 | 0.9957 | 1.0029 | 39454 | 5.38e3 | 1 | 1 | 167 | 178 |  |  | DB Search |
| total 1 peptides |  |  |  |  |  |  |  |  |  |  |  |  |  |  |  |  |  |  |  |
| --- | --- | --- | --- | --- | --- | --- | --- | --- | --- | --- | --- | --- | --- | --- | --- | --- | --- | --- | --- |

Best Unique PSM (Scan Sample\_8\_A1\_1\_3645.d:39454, m/z=627.8539, z=2, RT=14.19, ppm=-3.66):


TRINITY\_DN540\_c0\_g1\_i1.p1

back to list

  

| Protein Coverage
| Supporting Peptides
| Best Unique PSM
|

Protein Coverage:

Supporting Peptides:

| Peptide | Uniq | -10lgP | Mass | Length | ppm | m/z | z | RT | 1/k0 Start | 1/k0 End | Precursor ID | Area Sample 9 | #Feature | #Feature Sample 9 | Start | End | PTM | AScore | Found By |
| --- | --- | --- | --- | --- | --- | --- | --- | --- | --- | --- | --- | --- | --- | --- | --- | --- | --- | --- | --- |
| M.A(+42.01)TDKLEGLIAR.L | Y | 54.05 | 1227.6823 | 11 | -8.34 | 614.8405 | 2 | 17.80 | 0.9533 | 0.9606 | 58411 | 2.09e2 | 1 | 1 | 2 | 12 | Acetylation (Protein N-term) | A1:Acetylation (Protein N-term):1000 | DB Search |
| total 1 peptides |  |  |  |  |  |  |  |  |  |  |  |  |  |  |  |  |  |  |  |
| --- | --- | --- | --- | --- | --- | --- | --- | --- | --- | --- | --- | --- | --- | --- | --- | --- | --- | --- | --- |

Best Unique PSM (Scan Sample\_8\_A1\_1\_3645.d:58411, m/z=614.8405, z=2, RT=17.80, ppm=-12.86):


TRINITY\_DN1189\_c0\_g1\_i1.p1

back to list

  

| Protein Coverage
| Supporting Peptides
| Best Unique PSM
|

Protein Coverage:

Supporting Peptides:

| Peptide | Uniq | -10lgP | Mass | Length | ppm | m/z | z | RT | 1/k0 Start | 1/k0 End | Precursor ID | Area Sample 9 | #Feature | #Feature Sample 9 | Start | End | PTM | AScore | Found By |
| --- | --- | --- | --- | --- | --- | --- | --- | --- | --- | --- | --- | --- | --- | --- | --- | --- | --- | --- | --- |
| K.M(+15.99)C(+57.02)NTIQSIGDKFSVSM(+15.99)K.K | Y | 59.36 | 1976.9006 | 17 | 2.56 | 659.9728 | 3 | 16.16 | 0.9489 | 0.9562 | 49831 | 0 | 0 | 0 | 228 | 244 | Oxidation (M), Carbamidomethylation, Oxidation (M) | M1:Oxidation (M):1000 M16:Oxidation (M):1000 | DB Search |
| K.AKISC(+57.02)DQVLNEK.M | Y | 57.04 | 1403.7078 | 12 | -1.91 | 702.8567 | 2 | 12.59 | 0.9977 | 1.0049 | 30431 | 8.96e2 | 1 | 1 | 216 | 227 | Carbamidomethylation |  | DB Search |
| K.AIVENLENAKDGLK.K | Y | 56.89 | 1512.8147 | 14 | -0.85 | 757.4106 | 2 | 15.10 | 1.1082 | 1.1154 | 44479 | 0 | 0 | 0 | 195 | 208 |  |  | DB Search |
| K.KAIVENLENAK.D | Y | 54.86 | 1227.6823 | 11 | 1.1 | 614.8463 | 2 | 13.01 | 0.9469 | 0.9541 | 32852 | 5.63e2 | 1 | 1 | 194 | 204 |  |  | DB Search |
| K.AKDVTAAVKK.A | Y | 52.7 | 1029.6182 | 10 | -0.29 | 515.8139 | 2 | 9.44 | 0.8433 | 0.8506 | 11436 | 2e3 | 1 | 1 | 185 | 194 |  |  | DB Search |
| R.KWSELYEAAIK.Y | Y | 45.22 | 1336.7027 | 11 | -0.28 | 669.3554 | 2 | 16.30 | 0.988 | 0.9953 | 50457 | 0 | 0 | 0 | 260 | 270 |  |  | DB Search |
| total 6 peptides |  |  |  |  |  |  |  |  |  |  |  |  |  |  |  |  |  |  |  |
| --- | --- | --- | --- | --- | --- | --- | --- | --- | --- | --- | --- | --- | --- | --- | --- | --- | --- | --- | --- |

Best Unique PSM (Scan Sample\_8\_A1\_1\_3645.d:49831, m/z=659.9728, z=3, RT=16.16, ppm=-1.96):


TRINITY\_DN97534\_c0\_g1\_i1.p1

back to list

  

| Protein Coverage
| Supporting Peptides
| Best Unique PSM
|

Protein Coverage:

Supporting Peptides:

| Peptide | Uniq | -10lgP | Mass | Length | ppm | m/z | z | RT | 1/k0 Start | 1/k0 End | Precursor ID | Area Sample 9 | #Feature | #Feature Sample 9 | Start | End | PTM | AScore | Found By |
| --- | --- | --- | --- | --- | --- | --- | --- | --- | --- | --- | --- | --- | --- | --- | --- | --- | --- | --- | --- |
| K.ALYESELADARR.M | Y | 50.06 | 1392.6997 | 12 | 3.27 | 697.3563 | 2 | 14.18 | 1.011 | 1.0182 | 39453 | 0 | 0 | 0 | 75 | 86 |  |  | DB Search |
| total 1 peptides |  |  |  |  |  |  |  |  |  |  |  |  |  |  |  |  |  |  |  |
| --- | --- | --- | --- | --- | --- | --- | --- | --- | --- | --- | --- | --- | --- | --- | --- | --- | --- | --- | --- |

Best Unique PSM (Scan Sample\_8\_A1\_1\_3645.d:39453, m/z=697.3563, z=2, RT=14.18, ppm=-1.25):


TRINITY\_DN925\_c0\_g1\_i1.p1

back to list

  

| Protein Coverage
| Supporting Peptides
| Best Unique PSM
|

Protein Coverage:

Supporting Peptides:

| Peptide | Uniq | -10lgP | Mass | Length | ppm | m/z | z | RT | 1/k0 Start | 1/k0 End | Precursor ID | Area Sample 9 | #Feature | #Feature Sample 9 | Start | End | PTM | AScore | Found By |
| --- | --- | --- | --- | --- | --- | --- | --- | --- | --- | --- | --- | --- | --- | --- | --- | --- | --- | --- | --- |
| R.ASGDYATVVSHNR.D | Y | 56.94 | 1375.648 | 13 | 1.16 | 688.829 | 2 | 12.04 | 0.9981 | 1.0053 | 27131 | 7.37e2 | 1 | 1 | 129 | 141 |  |  | DB Search |
| total 1 peptides |  |  |  |  |  |  |  |  |  |  |  |  |  |  |  |  |  |  |  |
| --- | --- | --- | --- | --- | --- | --- | --- | --- | --- | --- | --- | --- | --- | --- | --- | --- | --- | --- | --- |

Best Unique PSM (Scan Sample\_8\_A1\_1\_3645.d:27131, m/z=688.829, z=2, RT=12.04, ppm=-3.35):


TRINITY\_DN152\_c0\_g1\_i2.p1

back to list

  

| Protein Coverage
| Supporting Peptides
| Best Unique PSM
|

Protein Coverage:

Supporting Peptides:

| Peptide | Uniq | -10lgP | Mass | Length | ppm | m/z | z | RT | 1/k0 Start | 1/k0 End | Precursor ID | Area Sample 9 | #Feature | #Feature Sample 9 | Start | End | PTM | AScore | Found By |
| --- | --- | --- | --- | --- | --- | --- | --- | --- | --- | --- | --- | --- | --- | --- | --- | --- | --- | --- | --- |
| K.IQDKEGIPPDQQR.L | Y | 61.25 | 1522.7739 | 13 | -1.84 | 762.3894 | 2 | 10.99 | 1.0371 | 1.0443 | 20319 | 2.55e3 | 1 | 1 | 66 | 78 |  |  | DB Search |
| total 1 peptides |  |  |  |  |  |  |  |  |  |  |  |  |  |  |  |  |  |  |  |
| --- | --- | --- | --- | --- | --- | --- | --- | --- | --- | --- | --- | --- | --- | --- | --- | --- | --- | --- | --- |

Best Unique PSM (Scan Sample\_8\_A1\_1\_3645.d:20319, m/z=762.3894, z=2, RT=10.99, ppm=-6.36):


TRINITY\_DN51754\_c0\_g1\_i1.p1

back to list

  

| Protein Coverage
| Supporting Peptides
| Best Unique PSM
|

Protein Coverage:

Supporting Peptides:

| Peptide | Uniq | -10lgP | Mass | Length | ppm | m/z | z | RT | 1/k0 Start | 1/k0 End | Precursor ID | Area Sample 9 | #Feature | #Feature Sample 9 | Start | End | PTM | AScore | Found By |
| --- | --- | --- | --- | --- | --- | --- | --- | --- | --- | --- | --- | --- | --- | --- | --- | --- | --- | --- | --- |
| R.AFLLDHFAPER.M | Y | 54.32 | 1314.672 | 11 | 4.57 | 658.3433 | 2 | 17.94 | 0.9908 | 0.9981 | 59095 | 2.39e2 | 1 | 1 | 234 | 244 |  |  | DB Search |
| total 1 peptides |  |  |  |  |  |  |  |  |  |  |  |  |  |  |  |  |  |  |  |
| --- | --- | --- | --- | --- | --- | --- | --- | --- | --- | --- | --- | --- | --- | --- | --- | --- | --- | --- | --- |

Best Unique PSM (Scan Sample\_8\_A1\_1\_3645.d:59095, m/z=658.3433, z=2, RT=17.94, ppm=0.05):


TRINITY\_DN850\_c0\_g1\_i1.p1

back to list

  

| Protein Coverage
| Supporting Peptides
| Best Unique PSM
|

Protein Coverage:

Supporting Peptides:

| Peptide | Uniq | -10lgP | Mass | Length | ppm | m/z | z | RT | 1/k0 Start | 1/k0 End | Precursor ID | Area Sample 9 | #Feature | #Feature Sample 9 | Start | End | PTM | AScore | Found By |
| --- | --- | --- | --- | --- | --- | --- | --- | --- | --- | --- | --- | --- | --- | --- | --- | --- | --- | --- | --- |
| K.KVNAYDDIEK.C | Y | 22.25 | 1193.5928 | 10 | -1.04 | 597.8004 | 2 | 12.05 | 0.9344 | 0.9416 | 27276 | 0 | 0 | 0 | 171 | 180 |  |  | DB Search |
| total 1 peptides |  |  |  |  |  |  |  |  |  |  |  |  |  |  |  |  |  |  |  |
| --- | --- | --- | --- | --- | --- | --- | --- | --- | --- | --- | --- | --- | --- | --- | --- | --- | --- | --- | --- |

Best Unique PSM (Scan Sample\_8\_A1\_1\_3645.d:27276, m/z=597.8004, z=2, RT=12.05, ppm=-5.56):


TRINITY\_DN36\_c0\_g1\_i17.p1

back to list

  

| Protein Coverage
| Supporting Peptides
| Best Unique PSM
|

Protein Coverage:

Supporting Peptides:

| Peptide | Uniq | -10lgP | Mass | Length | ppm | m/z | z | RT | 1/k0 Start | 1/k0 End | Precursor ID | Area Sample 9 | #Feature | #Feature Sample 9 | Start | End | PTM | AScore | Found By |
| --- | --- | --- | --- | --- | --- | --- | --- | --- | --- | --- | --- | --- | --- | --- | --- | --- | --- | --- | --- |
| K.YGQAM(+15.99)C(+57.02)QFM(+15.99)R.N | N | 61.54 | 1322.5206 | 10 | -1.76 | 662.2634 | 2 | 12.17 | 0.9489 | 0.9562 | 28008 | 2.07e2 | 1 | 1 | 72 | 81 | Oxidation (M), Carbamidomethylation, Oxidation (M) | M5:Oxidation (M):1000 M9:Oxidation (M):1000 | DB Search |
| K.WC(+57.02)DILSIHQSMENC(+57.02)VAK.C | Y | 59.79 | 2089.9383 | 17 | 2.4 | 697.6519 | 3 | 17.87 | 0.8769 | 0.8842 | 58766 | 0 | 0 | 0 | 198 | 214 | Carbamidomethylation, Carbamidomethylation |  | DB Search |
| K.DVYDAEQAERK.R | N | 57.85 | 1322.6102 | 11 | -1.22 | 662.3086 | 2 | 11.56 | 0.9606 | 0.9679 | 24244 | 0 | 0 | 0 | 103 | 113 |  |  | DB Search |
| R.LGGIEVTQGNC(+57.02)AR.K | N | 57.3 | 1373.6721 | 13 | 3.87 | 687.8429 | 2 | 13.70 | 0.9642 | 0.9715 | 36854 | 7.25e2 | 1 | 1 | 58 | 70 | Carbamidomethylation |  | DB Search |
| K.FETQIATK.D | Y | 51.43 | 936.4916 | 8 | -0.7 | 937.494 | 1 | 11.83 | 1.2165 | 1.2236 | 25881 | 4.08e3 | 3 | 3 | 167 | 174 |  |  | DB Search |
| total 5 peptides |  |  |  |  |  |  |  |  |  |  |  |  |  |  |  |  |  |  |  |
| --- | --- | --- | --- | --- | --- | --- | --- | --- | --- | --- | --- | --- | --- | --- | --- | --- | --- | --- | --- |

Best Unique PSM (Scan Sample\_8\_A1\_1\_3645.d:58766, m/z=697.6519, z=3, RT=17.87, ppm=-2.11):


TRINITY\_DN152\_c0\_g1\_i15.p1

back to list

  

| Protein Coverage
| Supporting Peptides
| Best Unique PSM
|

Protein Coverage:

Supporting Peptides:

| Peptide | Uniq | -10lgP | Mass | Length | ppm | m/z | z | RT | 1/k0 Start | 1/k0 End | Precursor ID | Area Sample 9 | #Feature | #Feature Sample 9 | Start | End | PTM | AScore | Found By |
| --- | --- | --- | --- | --- | --- | --- | --- | --- | --- | --- | --- | --- | --- | --- | --- | --- | --- | --- | --- |
| K.IQDKEGIPPDQQR.L | Y | 61.25 | 1522.7739 | 13 | -1.84 | 762.3894 | 2 | 10.99 | 1.0371 | 1.0443 | 20319 | 2.55e3 | 1 | 1 | 60 | 72 |  |  | DB Search |
| total 1 peptides |  |  |  |  |  |  |  |  |  |  |  |  |  |  |  |  |  |  |  |
| --- | --- | --- | --- | --- | --- | --- | --- | --- | --- | --- | --- | --- | --- | --- | --- | --- | --- | --- | --- |

Best Unique PSM (Scan Sample\_8\_A1\_1\_3645.d:20319, m/z=762.3894, z=2, RT=10.99, ppm=-6.36):


TRINITY\_DN3991\_c0\_g1\_i1.p1

back to list

  

| Protein Coverage
| Supporting Peptides
| Best Unique PSM
|

Protein Coverage:

Supporting Peptides:

| Peptide | Uniq | -10lgP | Mass | Length | ppm | m/z | z | RT | 1/k0 Start | 1/k0 End | Precursor ID | Area Sample 9 | #Feature | #Feature Sample 9 | Start | End | PTM | AScore | Found By |
| --- | --- | --- | --- | --- | --- | --- | --- | --- | --- | --- | --- | --- | --- | --- | --- | --- | --- | --- | --- |
| R.AKLSQC(+57.02)TAASGK.H | Y | 57.38 | 1220.6183 | 12 | -0.12 | 611.3136 | 2 | 9.72 | 0.9602 | 0.9675 | 12907 | 2.21e4 | 1 | 1 | 128 | 139 | Carbamidomethylation |  | DB Search |
| R.HGQVDSC(+57.02)INQNLKK.N | Y | 56.63 | 1639.81 | 14 | 3.14 | 547.6099 | 3 | 11.94 | 0.8376 | 0.8449 | 26542 | 1.21e3 | 1 | 1 | 112 | 125 | Carbamidomethylation |  | DB Search |
| K.LSQC(+57.02)TAASGK.H | Y | 55.07 | 1021.4862 | 10 | -0.97 | 511.7476 | 2 | 9.68 | 0.8388 | 0.8461 | 12609 | 2.94e4 | 1 | 1 | 130 | 139 | Carbamidomethylation |  | DB Search |
| K.LEPVGC(+57.02)FKDR.G | Y | 55.05 | 1219.6019 | 10 | 1.41 | 610.8063 | 2 | 12.97 | 0.9384 | 0.9457 | 32450 | 3.3e3 | 1 | 1 | 28 | 37 | Carbamidomethylation |  | DB Search |
| total 4 peptides |  |  |  |  |  |  |  |  |  |  |  |  |  |  |  |  |  |  |  |
| --- | --- | --- | --- | --- | --- | --- | --- | --- | --- | --- | --- | --- | --- | --- | --- | --- | --- | --- | --- |

Best Unique PSM (Scan Sample\_8\_A1\_1\_3645.d:12907, m/z=611.3136, z=2, RT=9.72, ppm=-4.64):


TRINITY\_DN2477\_c0\_g1\_i2.p1

back to list

  

| Protein Coverage
| Supporting Peptides
| Best Unique PSM
|

Protein Coverage:

Supporting Peptides:

| Peptide | Uniq | -10lgP | Mass | Length | ppm | m/z | z | RT | 1/k0 Start | 1/k0 End | Precursor ID | Area Sample 9 | #Feature | #Feature Sample 9 | Start | End | PTM | AScore | Found By |
| --- | --- | --- | --- | --- | --- | --- | --- | --- | --- | --- | --- | --- | --- | --- | --- | --- | --- | --- | --- |
| K.DSYVGDEAQSKR.G | Y | 58.12 | 1353.616 | 12 | 4.44 | 677.8152 | 2 | 10.93 | 1.0065 | 1.0138 | 20214 | 8.61e3 | 1 | 1 | 19 | 30 |  |  | DB Search |
| R.VAPEEHPVLLTEAPLNPK.A | Y | 57.43 | 1953.0571 | 18 | 1.47 | 977.5328 | 2 | 16.35 | 1.2073 | 1.2145 | 50611 | 1.52e2 | 1 | 1 | 64 | 81 |  |  | DB Search |
| R.GYSFTTTAEREIVR.D | Y | 56.59 | 1628.8158 | 14 | 3.67 | 815.4145 | 2 | 15.95 | 1.119 | 1.1262 | 48654 | 3.46e3 | 1 | 1 | 165 | 178 |  |  | DB Search |
| total 3 peptides |  |  |  |  |  |  |  |  |  |  |  |  |  |  |  |  |  |  |  |
| --- | --- | --- | --- | --- | --- | --- | --- | --- | --- | --- | --- | --- | --- | --- | --- | --- | --- | --- | --- |

Best Unique PSM (Scan Sample\_8\_A1\_1\_3645.d:20214, m/z=677.8152, z=2, RT=10.93, ppm=-0.08):


TRINITY\_DN2885\_c0\_g1\_i4.p1

back to list

  

| Protein Coverage
| Supporting Peptides
| Best Unique PSM
|

Protein Coverage:

Supporting Peptides:

| Peptide | Uniq | -10lgP | Mass | Length | ppm | m/z | z | RT | 1/k0 Start | 1/k0 End | Precursor ID | Area Sample 9 | #Feature | #Feature Sample 9 | Start | End | PTM | AScore | Found By |
| --- | --- | --- | --- | --- | --- | --- | --- | --- | --- | --- | --- | --- | --- | --- | --- | --- | --- | --- | --- |
| K.VTRPHGASGVVR.A | Y | 56.98 | 1234.6894 | 12 | 3.31 | 412.5699 | 3 | 10.06 | 0.8205 | 0.8279 | 15006 | 1.06e3 | 1 | 1 | 72 | 83 |  |  | DB Search |
| total 1 peptides |  |  |  |  |  |  |  |  |  |  |  |  |  |  |  |  |  |  |  |
| --- | --- | --- | --- | --- | --- | --- | --- | --- | --- | --- | --- | --- | --- | --- | --- | --- | --- | --- | --- |

Best Unique PSM (Scan Sample\_8\_A1\_1\_3645.d:15006, m/z=412.5699, z=3, RT=10.06, ppm=-1.21):


TRINITY\_DN152\_c0\_g1\_i10.p1

back to list

  

| Protein Coverage
| Supporting Peptides
| Best Unique PSM
|

Protein Coverage:

Supporting Peptides:

| Peptide | Uniq | -10lgP | Mass | Length | ppm | m/z | z | RT | 1/k0 Start | 1/k0 End | Precursor ID | Area Sample 9 | #Feature | #Feature Sample 9 | Start | End | PTM | AScore | Found By |
| --- | --- | --- | --- | --- | --- | --- | --- | --- | --- | --- | --- | --- | --- | --- | --- | --- | --- | --- | --- |
| K.IQDKEGIPPDQQR.L | Y | 61.25 | 1522.7739 | 13 | -1.84 | 762.3894 | 2 | 10.99 | 1.0371 | 1.0443 | 20319 | 2.55e3 | 1 | 1 | 141 | 153 |  |  | DB Search |
| total 1 peptides |  |  |  |  |  |  |  |  |  |  |  |  |  |  |  |  |  |  |  |
| --- | --- | --- | --- | --- | --- | --- | --- | --- | --- | --- | --- | --- | --- | --- | --- | --- | --- | --- | --- |

Best Unique PSM (Scan Sample\_8\_A1\_1\_3645.d:20319, m/z=762.3894, z=2, RT=10.99, ppm=-6.36):


TRINITY\_DN471\_c0\_g1\_i6.p1

back to list

  

| Protein Coverage
| Supporting Peptides
| Best Unique PSM
|

Protein Coverage:

Supporting Peptides:

| Peptide | Uniq | -10lgP | Mass | Length | ppm | m/z | z | RT | 1/k0 Start | 1/k0 End | Precursor ID | Area Sample 9 | #Feature | #Feature Sample 9 | Start | End | PTM | AScore | Found By |
| --- | --- | --- | --- | --- | --- | --- | --- | --- | --- | --- | --- | --- | --- | --- | --- | --- | --- | --- | --- |
| R.RNLDIERPTYTNLNR.L | Y | 58.31 | 1873.9758 | 15 | 3.94 | 625.6655 | 3 | 14.03 | 0.8846 | 0.8919 | 38584 | 6.19e2 | 1 | 1 | 85 | 99 |  |  | DB Search |
| total 1 peptides |  |  |  |  |  |  |  |  |  |  |  |  |  |  |  |  |  |  |  |
| --- | --- | --- | --- | --- | --- | --- | --- | --- | --- | --- | --- | --- | --- | --- | --- | --- | --- | --- | --- |

Best Unique PSM (Scan Sample\_8\_A1\_1\_3645.d:38584, m/z=625.6655, z=3, RT=14.03, ppm=-0.58):


TRINITY\_DN189323\_c0\_g1\_i1.p1

back to list

  

| Protein Coverage
| Supporting Peptides
| Best Unique PSM
|

Protein Coverage:

Supporting Peptides:

| Peptide | Uniq | -10lgP | Mass | Length | ppm | m/z | z | RT | 1/k0 Start | 1/k0 End | Precursor ID | Area Sample 9 | #Feature | #Feature Sample 9 | Start | End | PTM | AScore | Found By |
| --- | --- | --- | --- | --- | --- | --- | --- | --- | --- | --- | --- | --- | --- | --- | --- | --- | --- | --- | --- |
| R.LFEGNALLRR.L | Y | 54.64 | 1187.6775 | 10 | -0.31 | 594.8431 | 2 | 15.53 | 0.9666 | 0.9739 | 46624 | 9.6e2 | 1 | 1 | 32 | 41 |  |  | DB Search |
| K.RLFEGNALLR.R | Y | 16.53 | 1187.6775 | 10 | -1.85 | 594.8422 | 2 | 16.02 | 0.9388 | 0.9461 | 49137 | 3.83e2 | 1 | 1 | 31 | 40 |  |  | DB Search |
| total 2 peptides |  |  |  |  |  |  |  |  |  |  |  |  |  |  |  |  |  |  |  |
| --- | --- | --- | --- | --- | --- | --- | --- | --- | --- | --- | --- | --- | --- | --- | --- | --- | --- | --- | --- |

Best Unique PSM (Scan Sample\_8\_A1\_1\_3645.d:46624, m/z=594.8431, z=2, RT=15.53, ppm=-4.83):


TRINITY\_DN1172\_c0\_g1\_i5.p1

back to list

  

| Protein Coverage
| Supporting Peptides
| Best Unique PSM
|

Protein Coverage:

Supporting Peptides:

| Peptide | Uniq | -10lgP | Mass | Length | ppm | m/z | z | RT | 1/k0 Start | 1/k0 End | Precursor ID | Area Sample 9 | #Feature | #Feature Sample 9 | Start | End | PTM | AScore | Found By |
| --- | --- | --- | --- | --- | --- | --- | --- | --- | --- | --- | --- | --- | --- | --- | --- | --- | --- | --- | --- |
| K.VITSDDPAEKEK.K | Y | 56.98 | 1330.6616 | 12 | 1.22 | 666.3359 | 2 | 10.71 | 0.9844 | 0.9916 | 18867 | 1.51e3 | 1 | 1 | 123 | 134 |  |  | DB Search |
| total 1 peptides |  |  |  |  |  |  |  |  |  |  |  |  |  |  |  |  |  |  |  |
| --- | --- | --- | --- | --- | --- | --- | --- | --- | --- | --- | --- | --- | --- | --- | --- | --- | --- | --- | --- |

Best Unique PSM (Scan Sample\_8\_A1\_1\_3645.d:18867, m/z=666.3359, z=2, RT=10.71, ppm=-3.3):


TRINITY\_DN2941\_c0\_g1\_i4.p1

back to list

  

| Protein Coverage
| Supporting Peptides
| Best Unique PSM
|

Protein Coverage:

Supporting Peptides:

| Peptide | Uniq | -10lgP | Mass | Length | ppm | m/z | z | RT | 1/k0 Start | 1/k0 End | Precursor ID | Area Sample 9 | #Feature | #Feature Sample 9 | Start | End | PTM | AScore | Found By |
| --- | --- | --- | --- | --- | --- | --- | --- | --- | --- | --- | --- | --- | --- | --- | --- | --- | --- | --- | --- |
| R.VAPEEHPVLLTEAPLNPK.A | Y | 57.43 | 1953.0571 | 18 | 1.47 | 977.5328 | 2 | 16.35 | 1.2073 | 1.2145 | 50611 | 1.52e2 | 1 | 1 | 30 | 47 |  |  | DB Search |
| total 1 peptides |  |  |  |  |  |  |  |  |  |  |  |  |  |  |  |  |  |  |  |
| --- | --- | --- | --- | --- | --- | --- | --- | --- | --- | --- | --- | --- | --- | --- | --- | --- | --- | --- | --- |

Best Unique PSM (Scan Sample\_8\_A1\_1\_3645.d:50611, m/z=977.5328, z=2, RT=16.35, ppm=-3.04):


TRINITY\_DN1339\_c6\_g1\_i1.p1

back to list

  

| Protein Coverage
| Supporting Peptides
| Best Unique PSM
|

Protein Coverage:

Supporting Peptides:

| Peptide | Uniq | -10lgP | Mass | Length | ppm | m/z | z | RT | 1/k0 Start | 1/k0 End | Precursor ID | Area Sample 9 | #Feature | #Feature Sample 9 | Start | End | PTM | AScore | Found By |
| --- | --- | --- | --- | --- | --- | --- | --- | --- | --- | --- | --- | --- | --- | --- | --- | --- | --- | --- | --- |
| K.VITSDDPAEKEK.K | Y | 56.98 | 1330.6616 | 12 | 1.22 | 666.3359 | 2 | 10.71 | 0.9844 | 0.9916 | 18867 | 1.51e3 | 1 | 1 | 158 | 169 |  |  | DB Search |
| total 1 peptides |  |  |  |  |  |  |  |  |  |  |  |  |  |  |  |  |  |  |  |
| --- | --- | --- | --- | --- | --- | --- | --- | --- | --- | --- | --- | --- | --- | --- | --- | --- | --- | --- | --- |

Best Unique PSM (Scan Sample\_8\_A1\_1\_3645.d:18867, m/z=666.3359, z=2, RT=10.71, ppm=-3.3):


TRINITY\_DN1933\_c0\_g1\_i8.p2

back to list

  

| Protein Coverage
| Supporting Peptides
| Best Unique PSM
|

Protein Coverage:

Supporting Peptides:

| Peptide | Uniq | -10lgP | Mass | Length | ppm | m/z | z | RT | 1/k0 Start | 1/k0 End | Precursor ID | Area Sample 9 | #Feature | #Feature Sample 9 | Start | End | PTM | AScore | Found By |
| --- | --- | --- | --- | --- | --- | --- | --- | --- | --- | --- | --- | --- | --- | --- | --- | --- | --- | --- | --- |
| R.RNLDIERPTYTNLNR.L | Y | 58.31 | 1873.9758 | 15 | 3.94 | 625.6655 | 3 | 14.03 | 0.8846 | 0.8919 | 38584 | 6.19e2 | 1 | 1 | 75 | 89 |  |  | DB Search |
| total 1 peptides |  |  |  |  |  |  |  |  |  |  |  |  |  |  |  |  |  |  |  |
| --- | --- | --- | --- | --- | --- | --- | --- | --- | --- | --- | --- | --- | --- | --- | --- | --- | --- | --- | --- |

Best Unique PSM (Scan Sample\_8\_A1\_1\_3645.d:38584, m/z=625.6655, z=3, RT=14.03, ppm=-0.58):


TRINITY\_DN4377\_c0\_g1\_i1.p1

back to list

  

| Protein Coverage
| Supporting Peptides
| Best Unique PSM
|

Protein Coverage:

Supporting Peptides:

| Peptide | Uniq | -10lgP | Mass | Length | ppm | m/z | z | RT | 1/k0 Start | 1/k0 End | Precursor ID | Area Sample 9 | #Feature | #Feature Sample 9 | Start | End | PTM | AScore | Found By |
| --- | --- | --- | --- | --- | --- | --- | --- | --- | --- | --- | --- | --- | --- | --- | --- | --- | --- | --- | --- |
| K.DLNKEYISHLFK.Q | Y | 56.93 | 1505.7878 | 12 | -5.85 | 502.9313 | 3 | 17.25 | 0.8019 | 0.8092 | 55142 | 6.25e2 | 1 | 1 | 84 | 95 |  |  | DB Search |
| total 1 peptides |  |  |  |  |  |  |  |  |  |  |  |  |  |  |  |  |  |  |  |
| --- | --- | --- | --- | --- | --- | --- | --- | --- | --- | --- | --- | --- | --- | --- | --- | --- | --- | --- | --- |

Best Unique PSM (Scan Sample\_8\_A1\_1\_3645.d:55142, m/z=502.9313, z=3, RT=17.25, ppm=-10.37):


TRINITY\_DN152\_c0\_g1\_i14.p2

back to list

  

| Protein Coverage
| Supporting Peptides
| Best Unique PSM
|

Protein Coverage:

Supporting Peptides:

| Peptide | Uniq | -10lgP | Mass | Length | ppm | m/z | z | RT | 1/k0 Start | 1/k0 End | Precursor ID | Area Sample 9 | #Feature | #Feature Sample 9 | Start | End | PTM | AScore | Found By |
| --- | --- | --- | --- | --- | --- | --- | --- | --- | --- | --- | --- | --- | --- | --- | --- | --- | --- | --- | --- |
| K.IQDKEGIPPDQQR.S | Y | 61.25 | 1522.7739 | 13 | -1.84 | 762.3894 | 2 | 10.99 | 1.0371 | 1.0443 | 20319 | 2.55e3 | 1 | 1 | 40 | 52 |  |  | DB Search |
| total 1 peptides |  |  |  |  |  |  |  |  |  |  |  |  |  |  |  |  |  |  |  |
| --- | --- | --- | --- | --- | --- | --- | --- | --- | --- | --- | --- | --- | --- | --- | --- | --- | --- | --- | --- |

Best Unique PSM (Scan Sample\_8\_A1\_1\_3645.d:20319, m/z=762.3894, z=2, RT=10.99, ppm=-6.36):


TRINITY\_DN598\_c1\_g1\_i2.p1

back to list

  

| Protein Coverage
| Supporting Peptides
| Best Unique PSM
|

Protein Coverage:

Supporting Peptides:

| Peptide | Uniq | -10lgP | Mass | Length | ppm | m/z | z | RT | 1/k0 Start | 1/k0 End | Precursor ID | Area Sample 9 | #Feature | #Feature Sample 9 | Start | End | PTM | AScore | Found By |
| --- | --- | --- | --- | --- | --- | --- | --- | --- | --- | --- | --- | --- | --- | --- | --- | --- | --- | --- | --- |
| R.ASFVTDIDRLR.A | Y | 56.83 | 1291.6884 | 11 | 0.01 | 646.8486 | 2 | 16.97 | 0.9856 | 0.9928 | 53571 | 3.56e3 | 1 | 1 | 302 | 312 |  |  | DB Search |
| total 1 peptides |  |  |  |  |  |  |  |  |  |  |  |  |  |  |  |  |  |  |  |
| --- | --- | --- | --- | --- | --- | --- | --- | --- | --- | --- | --- | --- | --- | --- | --- | --- | --- | --- | --- |

Best Unique PSM (Scan Sample\_8\_A1\_1\_3645.d:53571, m/z=646.8486, z=2, RT=16.97, ppm=-4.51):


TRINITY\_DN554\_c0\_g1\_i3.p2

back to list

  

| Protein Coverage
| Supporting Peptides
| Best Unique PSM
|

Protein Coverage:

Supporting Peptides:

| Peptide | Uniq | -10lgP | Mass | Length | ppm | m/z | z | RT | 1/k0 Start | 1/k0 End | Precursor ID | Area Sample 9 | #Feature | #Feature Sample 9 | Start | End | PTM | AScore | Found By |
| --- | --- | --- | --- | --- | --- | --- | --- | --- | --- | --- | --- | --- | --- | --- | --- | --- | --- | --- | --- |
| K.IQDKEGIPPDQQR.L | Y | 61.25 | 1522.7739 | 13 | -1.84 | 762.3894 | 2 | 10.99 | 1.0371 | 1.0443 | 20319 | 2.55e3 | 1 | 1 | 30 | 42 |  |  | DB Search |
| total 1 peptides |  |  |  |  |  |  |  |  |  |  |  |  |  |  |  |  |  |  |  |
| --- | --- | --- | --- | --- | --- | --- | --- | --- | --- | --- | --- | --- | --- | --- | --- | --- | --- | --- | --- |

Best Unique PSM (Scan Sample\_8\_A1\_1\_3645.d:20319, m/z=762.3894, z=2, RT=10.99, ppm=-6.36):


TRINITY\_DN8101\_c0\_g1\_i8.p1

back to list

  

| Protein Coverage
| Supporting Peptides
| Best Unique PSM
|

Protein Coverage:

Supporting Peptides:

| Peptide | Uniq | -10lgP | Mass | Length | ppm | m/z | z | RT | 1/k0 Start | 1/k0 End | Precursor ID | Area Sample 9 | #Feature | #Feature Sample 9 | Start | End | PTM | AScore | Found By |
| --- | --- | --- | --- | --- | --- | --- | --- | --- | --- | --- | --- | --- | --- | --- | --- | --- | --- | --- | --- |
| R.SIGFIGHYLDQLR.L | Y | 55.88 | 1517.799 | 13 | -0.14 | 759.9033 | 2 | 19.91 | 1.0849 | 1.0921 | 70505 | 4.59e2 | 1 | 1 | 1077 | 1089 |  |  | DB Search |
| total 1 peptides |  |  |  |  |  |  |  |  |  |  |  |  |  |  |  |  |  |  |  |
| --- | --- | --- | --- | --- | --- | --- | --- | --- | --- | --- | --- | --- | --- | --- | --- | --- | --- | --- | --- |

Best Unique PSM (Scan Sample\_8\_A1\_1\_3645.d:70505, m/z=759.9033, z=2, RT=19.91, ppm=-4.65):


TRINITY\_DN4595\_c0\_g1\_i10.p1

back to list

  

| Protein Coverage
| Supporting Peptides
| Best Unique PSM
|

Protein Coverage:

Supporting Peptides:

| Peptide | Uniq | -10lgP | Mass | Length | ppm | m/z | z | RT | 1/k0 Start | 1/k0 End | Precursor ID | Area Sample 9 | #Feature | #Feature Sample 9 | Start | End | PTM | AScore | Found By |
| --- | --- | --- | --- | --- | --- | --- | --- | --- | --- | --- | --- | --- | --- | --- | --- | --- | --- | --- | --- |
| K.YTGAC(+57.02)NANGNTDK.T | Y | 57.42 | 1384.5677 | 13 | 5.65 | 693.2919 | 2 | 9.57 | 0.9404 | 0.9477 | 12156 | 0 | 0 | 0 | 1041 | 1053 | Carbamidomethylation |  | DB Search |
| K.VNQGEKEFLQRPC(+57.02)R.Y | Y | 56.68 | 1759.8788 | 14 | 1.84 | 587.632 | 3 | 12.53 | 0.8619 | 0.8692 | 29925 | 1.89e3 | 1 | 1 | 1161 | 1174 | Carbamidomethylation |  | DB Search |
| K.KVNQGEKEFLQRPC(+57.02)R.Y | Y | 50.42 | 1887.9737 | 15 | -2.15 | 472.9976 | 4 | 11.77 | 0.8092 | 0.8165 | 25549 | 0 | 0 | 0 | 1160 | 1174 | Carbamidomethylation |  | DB Search |
| total 3 peptides |  |  |  |  |  |  |  |  |  |  |  |  |  |  |  |  |  |  |  |
| --- | --- | --- | --- | --- | --- | --- | --- | --- | --- | --- | --- | --- | --- | --- | --- | --- | --- | --- | --- |

Best Unique PSM (Scan Sample\_8\_A1\_1\_3645.d:12156, m/z=693.2919, z=2, RT=9.57, ppm=1.14):


TRINITY\_DN3016\_c0\_g1\_i10.p1

back to list

  

| Protein Coverage
| Supporting Peptides
| Best Unique PSM
|

Protein Coverage:

Supporting Peptides:

| Peptide | Uniq | -10lgP | Mass | Length | ppm | m/z | z | RT | 1/k0 Start | 1/k0 End | Precursor ID | Area Sample 9 | #Feature | #Feature Sample 9 | Start | End | PTM | AScore | Found By |
| --- | --- | --- | --- | --- | --- | --- | --- | --- | --- | --- | --- | --- | --- | --- | --- | --- | --- | --- | --- |
| R.IVNTDLQEIKDIIK.G | Y | 58.4 | 1640.9348 | 14 | -6.31 | 821.4658 | 2 | 18.62 | 1.1514 | 1.1586 | 63065 | 0 | 0 | 0 | 154 | 167 |  |  | DB Search |
| total 1 peptides |  |  |  |  |  |  |  |  |  |  |  |  |  |  |  |  |  |  |  |
| --- | --- | --- | --- | --- | --- | --- | --- | --- | --- | --- | --- | --- | --- | --- | --- | --- | --- | --- | --- |

Best Unique PSM (Scan Sample\_8\_A1\_1\_3645.d:63065, m/z=821.4658, z=2, RT=18.62, ppm=-10.82):


TRINITY\_DN167\_c0\_g2\_i1.p1

back to list

  

| Protein Coverage
| Supporting Peptides
| Best Unique PSM
|

Protein Coverage:

Supporting Peptides:

| Peptide | Uniq | -10lgP | Mass | Length | ppm | m/z | z | RT | 1/k0 Start | 1/k0 End | Precursor ID | Area Sample 9 | #Feature | #Feature Sample 9 | Start | End | PTM | AScore | Found By |
| --- | --- | --- | --- | --- | --- | --- | --- | --- | --- | --- | --- | --- | --- | --- | --- | --- | --- | --- | --- |
| K.YRNEDDALKEK.V | Y | 62.01 | 1379.6681 | 11 | 3.73 | 460.8963 | 3 | 10.49 | 0.7689 | 0.7762 | 17723 | 0 | 0 | 0 | 526 | 536 |  |  | DB Search |
| total 1 peptides |  |  |  |  |  |  |  |  |  |  |  |  |  |  |  |  |  |  |  |
| --- | --- | --- | --- | --- | --- | --- | --- | --- | --- | --- | --- | --- | --- | --- | --- | --- | --- | --- | --- |

Best Unique PSM (Scan Sample\_8\_A1\_1\_3645.d:17723, m/z=460.8963, z=3, RT=10.49, ppm=-0.79):


TRINITY\_DN3036\_c0\_g1\_i16.p1

back to list

  

| Protein Coverage
| Supporting Peptides
| Best Unique PSM
|

Protein Coverage:

Supporting Peptides:

| Peptide | Uniq | -10lgP | Mass | Length | ppm | m/z | z | RT | 1/k0 Start | 1/k0 End | Precursor ID | Area Sample 9 | #Feature | #Feature Sample 9 | Start | End | PTM | AScore | Found By |
| --- | --- | --- | --- | --- | --- | --- | --- | --- | --- | --- | --- | --- | --- | --- | --- | --- | --- | --- | --- |
| R.VIGASGIHVGTM(+15.99)SFGK.M | Y | 60.06 | 1575.8079 | 16 | 1.08 | 788.9085 | 2 | 15.08 | 1.125 | 1.1322 | 44382 | 0 | 0 | 0 | 160 | 175 | Oxidation (M) | M12:Oxidation (M):1000 | DB Search |
| total 1 peptides |  |  |  |  |  |  |  |  |  |  |  |  |  |  |  |  |  |  |  |
| --- | --- | --- | --- | --- | --- | --- | --- | --- | --- | --- | --- | --- | --- | --- | --- | --- | --- | --- | --- |

Best Unique PSM (Scan Sample\_8\_A1\_1\_3645.d:44382, m/z=788.9085, z=2, RT=15.08, ppm=-3.43):


TRINITY\_DN22492\_c0\_g1\_i2.p1

back to list

  

| Protein Coverage
| Supporting Peptides
| Best Unique PSM
|

Protein Coverage:

Supporting Peptides:

| Peptide | Uniq | -10lgP | Mass | Length | ppm | m/z | z | RT | 1/k0 Start | 1/k0 End | Precursor ID | Area Sample 9 | #Feature | #Feature Sample 9 | Start | End | PTM | AScore | Found By |
| --- | --- | --- | --- | --- | --- | --- | --- | --- | --- | --- | --- | --- | --- | --- | --- | --- | --- | --- | --- |
| R.LIAKFETWEVAR.N | Y | 51.27 | 1461.798 | 12 | 2.42 | 731.9047 | 2 | 17.13 | 1.0448 | 1.052 | 54536 | 3.66e2 | 1 | 1 | 318 | 329 |  |  | DB Search |
| K.KKLEPQAAPR.E | Y | 44.45 | 1136.6666 | 10 | -0.58 | 379.8942 | 3 | 9.69 | 0.7567 | 0.764 | 12893 | 0 | 0 | 0 | 425 | 434 |  |  | DB Search |
| total 2 peptides |  |  |  |  |  |  |  |  |  |  |  |  |  |  |  |  |  |  |  |
| --- | --- | --- | --- | --- | --- | --- | --- | --- | --- | --- | --- | --- | --- | --- | --- | --- | --- | --- | --- |

Best Unique PSM (Scan Sample\_8\_A1\_1\_3645.d:54536, m/z=731.9047, z=2, RT=17.13, ppm=-2.09):


TRINITY\_DN240\_c0\_g1\_i6.p1

back to list

  

| Protein Coverage
| Supporting Peptides
| Best Unique PSM
|

Protein Coverage:

Supporting Peptides:

| Peptide | Uniq | -10lgP | Mass | Length | ppm | m/z | z | RT | 1/k0 Start | 1/k0 End | Precursor ID | Area Sample 9 | #Feature | #Feature Sample 9 | Start | End | PTM | AScore | Found By |
| --- | --- | --- | --- | --- | --- | --- | --- | --- | --- | --- | --- | --- | --- | --- | --- | --- | --- | --- | --- |
| K.ANQVSSAAAAPVPAAGGDK.I | Y | 61.48 | 1680.8431 | 19 | -2.28 | 841.4231 | 2 | 12.43 | 1.0668 | 1.0741 | 29536 | 0 | 0 | 0 | 230 | 248 |  |  | DB Search |
| K.IGAAAQALADQSYPFLK.S | Y | 59.19 | 1762.9253 | 17 | -0.58 | 882.4655 | 2 | 19.04 | 1.159 | 1.1662 | 65509 | 3.11e3 | 2 | 2 | 249 | 265 |  |  | DB Search |
| K.AYAAFLEFKDVVK.A | Y | 57.51 | 1499.8024 | 13 | 3.03 | 750.9073 | 2 | 19.00 | 1.0929 | 1.1001 | 65208 | 1.13e4 | 1 | 1 | 217 | 229 |  |  | DB Search |
| total 3 peptides |  |  |  |  |  |  |  |  |  |  |  |  |  |  |  |  |  |  |  |
| --- | --- | --- | --- | --- | --- | --- | --- | --- | --- | --- | --- | --- | --- | --- | --- | --- | --- | --- | --- |

Best Unique PSM (Scan Sample\_8\_A1\_1\_3645.d:29536, m/z=841.4231, z=2, RT=12.43, ppm=-6.79):


TRINITY\_DN74124\_c0\_g1\_i1.p1

back to list

  

| Protein Coverage
| Supporting Peptides
| Best Unique PSM
|

Protein Coverage:

Supporting Peptides:

| Peptide | Uniq | -10lgP | Mass | Length | ppm | m/z | z | RT | 1/k0 Start | 1/k0 End | Precursor ID | Area Sample 9 | #Feature | #Feature Sample 9 | Start | End | PTM | AScore | Found By |
| --- | --- | --- | --- | --- | --- | --- | --- | --- | --- | --- | --- | --- | --- | --- | --- | --- | --- | --- | --- |
| K.LVDATFPTTDFETVR.G | Y | 58.54 | 1710.8464 | 15 | -4.42 | 856.4229 | 2 | 18.47 | 1.1033 | 1.1106 | 62184 | 1.4e3 | 1 | 1 | 60 | 74 |  |  | DB Search |
| total 1 peptides |  |  |  |  |  |  |  |  |  |  |  |  |  |  |  |  |  |  |  |
| --- | --- | --- | --- | --- | --- | --- | --- | --- | --- | --- | --- | --- | --- | --- | --- | --- | --- | --- | --- |

Best Unique PSM (Scan Sample\_8\_A1\_1\_3645.d:62184, m/z=856.4229, z=2, RT=18.47, ppm=-8.93):


TRINITY\_DN250\_c0\_g1\_i5.p1

back to list

  

| Protein Coverage
| Supporting Peptides
| Best Unique PSM
|

Protein Coverage:

Supporting Peptides:

| Peptide | Uniq | -10lgP | Mass | Length | ppm | m/z | z | RT | 1/k0 Start | 1/k0 End | Precursor ID | Area Sample 9 | #Feature | #Feature Sample 9 | Start | End | PTM | AScore | Found By |
| --- | --- | --- | --- | --- | --- | --- | --- | --- | --- | --- | --- | --- | --- | --- | --- | --- | --- | --- | --- |
| R.RNLDIERPTYTNLNR.L | Y | 58.31 | 1873.9758 | 15 | 3.94 | 625.6655 | 3 | 14.03 | 0.8846 | 0.8919 | 38584 | 6.19e2 | 1 | 1 | 215 | 229 |  |  | DB Search |
| R.IHFPLATYSPVISAEK.A | Y | 50.18 | 1771.9508 | 16 | 1.46 | 886.98 | 2 | 18.50 | 1.1338 | 1.141 | 62412 | 0 | 0 | 0 | 265 | 280 |  |  | DB Search |
| total 2 peptides |  |  |  |  |  |  |  |  |  |  |  |  |  |  |  |  |  |  |  |
| --- | --- | --- | --- | --- | --- | --- | --- | --- | --- | --- | --- | --- | --- | --- | --- | --- | --- | --- | --- |

Best Unique PSM (Scan Sample\_8\_A1\_1\_3645.d:38584, m/z=625.6655, z=3, RT=14.03, ppm=-0.58):


TRINITY\_DN3036\_c0\_g1\_i11.p1

back to list

  

| Protein Coverage
| Supporting Peptides
| Best Unique PSM
|

Protein Coverage:

Supporting Peptides:

| Peptide | Uniq | -10lgP | Mass | Length | ppm | m/z | z | RT | 1/k0 Start | 1/k0 End | Precursor ID | Area Sample 9 | #Feature | #Feature Sample 9 | Start | End | PTM | AScore | Found By |
| --- | --- | --- | --- | --- | --- | --- | --- | --- | --- | --- | --- | --- | --- | --- | --- | --- | --- | --- | --- |
| R.VIGASGIHVGTM(+15.99)SFGK.M | Y | 60.06 | 1575.8079 | 16 | 1.08 | 788.9085 | 2 | 15.08 | 1.125 | 1.1322 | 44382 | 0 | 0 | 0 | 160 | 175 | Oxidation (M) | M12:Oxidation (M):1000 | DB Search |
| total 1 peptides |  |  |  |  |  |  |  |  |  |  |  |  |  |  |  |  |  |  |  |
| --- | --- | --- | --- | --- | --- | --- | --- | --- | --- | --- | --- | --- | --- | --- | --- | --- | --- | --- | --- |

Best Unique PSM (Scan Sample\_8\_A1\_1\_3645.d:44382, m/z=788.9085, z=2, RT=15.08, ppm=-3.43):


TRINITY\_DN1243\_c0\_g1\_i1.p1

back to list

  

| Protein Coverage
| Supporting Peptides
| Best Unique PSM
|

Protein Coverage:

Supporting Peptides:

| Peptide | Uniq | -10lgP | Mass | Length | ppm | m/z | z | RT | 1/k0 Start | 1/k0 End | Precursor ID | Area Sample 9 | #Feature | #Feature Sample 9 | Start | End | PTM | AScore | Found By |
| --- | --- | --- | --- | --- | --- | --- | --- | --- | --- | --- | --- | --- | --- | --- | --- | --- | --- | --- | --- |
| R.AFNHLLQYGEPAIRR.A | Y | 57 | 1783.9481 | 15 | -0.28 | 595.6538 | 3 | 15.67 | 0.8753 | 0.8826 | 47413 | 0 | 0 | 0 | 692 | 706 |  |  | DB Search |
| total 1 peptides |  |  |  |  |  |  |  |  |  |  |  |  |  |  |  |  |  |  |  |
| --- | --- | --- | --- | --- | --- | --- | --- | --- | --- | --- | --- | --- | --- | --- | --- | --- | --- | --- | --- |

Best Unique PSM (Scan Sample\_8\_A1\_1\_3645.d:47413, m/z=595.6538, z=3, RT=15.67, ppm=-4.79):


TRINITY\_DN477\_c0\_g1\_i7.p1

back to list

  

| Protein Coverage
| Supporting Peptides
| Best Unique PSM
|

Protein Coverage:

Supporting Peptides:

| Peptide | Uniq | -10lgP | Mass | Length | ppm | m/z | z | RT | 1/k0 Start | 1/k0 End | Precursor ID | Area Sample 9 | #Feature | #Feature Sample 9 | Start | End | PTM | AScore | Found By |
| --- | --- | --- | --- | --- | --- | --- | --- | --- | --- | --- | --- | --- | --- | --- | --- | --- | --- | --- | --- |
| R.ILQC(+57.02)NQYANVEEAR.N | Y | 57.54 | 1706.8046 | 14 | 0.26 | 854.4059 | 2 | 14.18 | 1.0781 | 1.0853 | 39448 | 1.11e3 | 1 | 1 | 86 | 99 | Carbamidomethylation |  | DB Search |
| K.DKNILVLTLGVGPQVNR.N | Y | 56.87 | 1835.0628 | 17 | 6.58 | 918.5406 | 2 | 19.30 | 1.1358 | 1.143 | 67105 | 2.01e3 | 1 | 1 | 168 | 184 |  |  | DB Search |
| R.SQSAFPTRR.R | Y | 43.1 | 1048.5414 | 9 | 1.24 | 525.2762 | 2 | 10.98 | 0.8506 | 0.8579 | 20648 | 1.97e3 | 1 | 1 | 856 | 864 |  |  | DB Search |
| total 3 peptides |  |  |  |  |  |  |  |  |  |  |  |  |  |  |  |  |  |  |  |
| --- | --- | --- | --- | --- | --- | --- | --- | --- | --- | --- | --- | --- | --- | --- | --- | --- | --- | --- | --- |

Best Unique PSM (Scan Sample\_8\_A1\_1\_3645.d:39448, m/z=854.8958, z=2, RT=14.23, ppm=-4.26):


TRINITY\_DN3036\_c0\_g1\_i2.p2

back to list

  

| Protein Coverage
| Supporting Peptides
| Best Unique PSM
|

Protein Coverage:

Supporting Peptides:

| Peptide | Uniq | -10lgP | Mass | Length | ppm | m/z | z | RT | 1/k0 Start | 1/k0 End | Precursor ID | Area Sample 9 | #Feature | #Feature Sample 9 | Start | End | PTM | AScore | Found By |
| --- | --- | --- | --- | --- | --- | --- | --- | --- | --- | --- | --- | --- | --- | --- | --- | --- | --- | --- | --- |
| R.VIGASGIHVGTM(+15.99)SFGK.M | Y | 60.06 | 1575.8079 | 16 | 1.08 | 788.9085 | 2 | 15.08 | 1.125 | 1.1322 | 44382 | 0 | 0 | 0 | 334 | 349 | Oxidation (M) | M12:Oxidation (M):1000 | DB Search |
| R.GTSNGGLVVGTIIKPK.L | Y | 54.81 | 1539.8984 | 16 | 1.04 | 770.9538 | 2 | 15.65 | 1.1234 | 1.1306 | 47131 | 3.3e3 | 1 | 1 | 173 | 188 |  |  | DB Search |
| total 2 peptides |  |  |  |  |  |  |  |  |  |  |  |  |  |  |  |  |  |  |  |
| --- | --- | --- | --- | --- | --- | --- | --- | --- | --- | --- | --- | --- | --- | --- | --- | --- | --- | --- | --- |

Best Unique PSM (Scan Sample\_8\_A1\_1\_3645.d:44382, m/z=788.9085, z=2, RT=15.08, ppm=-3.43):


TRINITY\_DN140702\_c0\_g1\_i1.p1

back to list

  

| Protein Coverage
| Supporting Peptides
| Best Unique PSM
|

Protein Coverage:

Supporting Peptides:

| Peptide | Uniq | -10lgP | Mass | Length | ppm | m/z | z | RT | 1/k0 Start | 1/k0 End | Precursor ID | Area Sample 9 | #Feature | #Feature Sample 9 | Start | End | PTM | AScore | Found By |
| --- | --- | --- | --- | --- | --- | --- | --- | --- | --- | --- | --- | --- | --- | --- | --- | --- | --- | --- | --- |
| K.KFQVPSGIRPILVK.T | Y | 61.62 | 1580.9766 | 14 | 1.23 | 527.9977 | 3 | 16.64 | 0.9089 | 0.9162 | 52098 | 0 | 0 | 0 | 1274 | 1287 |  |  | DB Search |
| K.RPGFSDSEGKIR.L | Y | 59.66 | 1347.6895 | 12 | 3.54 | 450.2367 | 3 | 11.44 | 0.7933 | 0.8006 | 23540 | 0 | 0 | 0 | 889 | 900 |  |  | DB Search |
| R.LALHVLNNEDIIGHR.L | Y | 56.91 | 1712.9322 | 15 | 5.27 | 857.474 | 2 | 16.25 | 1.1342 | 1.1414 | 50212 | 3.38e2 | 1 | 1 | 1702 | 1716 |  |  | DB Search |
| K.FQVPSGIRPILVK.T | Y | 23.37 | 1452.8816 | 13 | 1.2 | 727.4457 | 2 | 16.63 | 1.0335 | 1.0407 | 52054 | 0 | 0 | 0 | 1275 | 1287 |  |  | DB Search |
| total 4 peptides |  |  |  |  |  |  |  |  |  |  |  |  |  |  |  |  |  |  |  |
| --- | --- | --- | --- | --- | --- | --- | --- | --- | --- | --- | --- | --- | --- | --- | --- | --- | --- | --- | --- |

Best Unique PSM (Scan Sample\_8\_A1\_1\_3645.d:52098, m/z=527.9977, z=3, RT=16.64, ppm=-3.28):


TRINITY\_DN1194\_c0\_g1\_i1.p1

back to list

  

| Protein Coverage
| Supporting Peptides
| Best Unique PSM
|

Protein Coverage:

Supporting Peptides:

| Peptide | Uniq | -10lgP | Mass | Length | ppm | m/z | z | RT | 1/k0 Start | 1/k0 End | Precursor ID | Area Sample 9 | #Feature | #Feature Sample 9 | Start | End | PTM | AScore | Found By |
| --- | --- | --- | --- | --- | --- | --- | --- | --- | --- | --- | --- | --- | --- | --- | --- | --- | --- | --- | --- |
| K.SLVEENVHPQILIK.G | Y | 62.09 | 1617.909 | 14 | 0.4 | 809.9584 | 2 | 16.26 | 1.0977 | 1.1049 | 50304 | 0 | 0 | 0 | 118 | 131 |  |  | DB Search |
| K.IVNAEWNILYEKLEK.I | Y | 56.86 | 1860.9985 | 15 | 2.21 | 621.3387 | 3 | 19.45 | 0.8972 | 0.9044 | 67893 | 2.46e2 | 1 | 1 | 274 | 288 |  |  | DB Search |
| total 2 peptides |  |  |  |  |  |  |  |  |  |  |  |  |  |  |  |  |  |  |  |
| --- | --- | --- | --- | --- | --- | --- | --- | --- | --- | --- | --- | --- | --- | --- | --- | --- | --- | --- | --- |

Best Unique PSM (Scan Sample\_8\_A1\_1\_3645.d:50304, m/z=809.9584, z=2, RT=16.26, ppm=-4.11):


TRINITY\_DN2477\_c0\_g1\_i8.p1

back to list

  

| Protein Coverage
| Supporting Peptides
| Best Unique PSM
|

Protein Coverage:

Supporting Peptides:

| Peptide | Uniq | -10lgP | Mass | Length | ppm | m/z | z | RT | 1/k0 Start | 1/k0 End | Precursor ID | Area Sample 9 | #Feature | #Feature Sample 9 | Start | End | PTM | AScore | Found By |
| --- | --- | --- | --- | --- | --- | --- | --- | --- | --- | --- | --- | --- | --- | --- | --- | --- | --- | --- | --- |
| R.VAPEEHPVLLTEAPLNPK.A | Y | 57.43 | 1953.0571 | 18 | 1.47 | 977.5328 | 2 | 16.35 | 1.2073 | 1.2145 | 50611 | 1.52e2 | 1 | 1 | 29 | 46 |  |  | DB Search |
| R.GYSFTTTAEREIVR.D | Y | 56.59 | 1628.8158 | 14 | 3.67 | 815.4145 | 2 | 15.95 | 1.119 | 1.1262 | 48654 | 3.46e3 | 1 | 1 | 130 | 143 |  |  | DB Search |
| total 2 peptides |  |  |  |  |  |  |  |  |  |  |  |  |  |  |  |  |  |  |  |
| --- | --- | --- | --- | --- | --- | --- | --- | --- | --- | --- | --- | --- | --- | --- | --- | --- | --- | --- | --- |

Best Unique PSM (Scan Sample\_8\_A1\_1\_3645.d:50611, m/z=977.5328, z=2, RT=16.35, ppm=-3.04):


TRINITY\_DN602\_c0\_g1\_i4.p1

back to list

  

| Protein Coverage
| Supporting Peptides
| Best Unique PSM
|

Protein Coverage:

Supporting Peptides:

| Peptide | Uniq | -10lgP | Mass | Length | ppm | m/z | z | RT | 1/k0 Start | 1/k0 End | Precursor ID | Area Sample 9 | #Feature | #Feature Sample 9 | Start | End | PTM | AScore | Found By |
| --- | --- | --- | --- | --- | --- | --- | --- | --- | --- | --- | --- | --- | --- | --- | --- | --- | --- | --- | --- |
| K.LPWEELWKPC(+57.02)IK.Y | Y | 61.22 | 1597.8326 | 12 | -2.11 | 533.6146 | 3 | 19.85 | 0.8773 | 0.8846 | 70215 | 1.87e3 | 1 | 1 | 129 | 140 | Carbamidomethylation |  | DB Search |
| K.EGFYVHEALAAGIVK.K | Y | 57 | 1602.8405 | 15 | 2.24 | 802.4257 | 2 | 17.51 | 1.0885 | 1.0957 | 56580 | 1.12e3 | 1 | 1 | 144 | 158 |  |  | DB Search |
| total 2 peptides |  |  |  |  |  |  |  |  |  |  |  |  |  |  |  |  |  |  |  |
| --- | --- | --- | --- | --- | --- | --- | --- | --- | --- | --- | --- | --- | --- | --- | --- | --- | --- | --- | --- |

Best Unique PSM (Scan Sample\_8\_A1\_1\_3645.d:70215, m/z=533.6146, z=3, RT=19.85, ppm=-6.63):


TRINITY\_DN875\_c0\_g1\_i1.p1

back to list

  

| Protein Coverage
| Supporting Peptides
| Best Unique PSM
|

Protein Coverage:

Supporting Peptides:

| Peptide | Uniq | -10lgP | Mass | Length | ppm | m/z | z | RT | 1/k0 Start | 1/k0 End | Precursor ID | Area Sample 9 | #Feature | #Feature Sample 9 | Start | End | PTM | AScore | Found By |
| --- | --- | --- | --- | --- | --- | --- | --- | --- | --- | --- | --- | --- | --- | --- | --- | --- | --- | --- | --- |
| R.AVFC(+57.02)EHQGVK.T | Y | 44.56 | 1173.5601 | 10 | -1.64 | 587.7837 | 2 | 11.55 | 0.9012 | 0.9085 | 24166 | 0 | 0 | 0 | 512 | 521 | Carbamidomethylation |  | DB Search |
| total 1 peptides |  |  |  |  |  |  |  |  |  |  |  |  |  |  |  |  |  |  |  |
| --- | --- | --- | --- | --- | --- | --- | --- | --- | --- | --- | --- | --- | --- | --- | --- | --- | --- | --- | --- |

Best Unique PSM (Scan Sample\_8\_A1\_1\_3645.d:24166, m/z=587.7837, z=2, RT=11.55, ppm=-6.15):


TRINITY\_DN691\_c0\_g1\_i1.p1

back to list

  

| Protein Coverage
| Supporting Peptides
| Best Unique PSM
|

Protein Coverage:

Supporting Peptides:

| Peptide | Uniq | -10lgP | Mass | Length | ppm | m/z | z | RT | 1/k0 Start | 1/k0 End | Precursor ID | Area Sample 9 | #Feature | #Feature Sample 9 | Start | End | PTM | AScore | Found By |
| --- | --- | --- | --- | --- | --- | --- | --- | --- | --- | --- | --- | --- | --- | --- | --- | --- | --- | --- | --- |
| R.AYGQDLGQPGSC(+57.02)LKR.F | Y | 61.86 | 1648.7991 | 15 | 1.51 | 825.4044 | 2 | 13.36 | 1.0761 | 1.0833 | 34861 | 1.57e3 | 2 | 2 | 1533 | 1547 | Carbamidomethylation |  | DB Search |
| total 1 peptides |  |  |  |  |  |  |  |  |  |  |  |  |  |  |  |  |  |  |  |
| --- | --- | --- | --- | --- | --- | --- | --- | --- | --- | --- | --- | --- | --- | --- | --- | --- | --- | --- | --- |

Best Unique PSM (Scan Sample\_8\_A1\_1\_3645.d:34861, m/z=825.4044, z=2, RT=13.36, ppm=-3):


TRINITY\_DN128977\_c0\_g1\_i1.p1

back to list

  

| Protein Coverage
| Supporting Peptides
| Best Unique PSM
|

Protein Coverage:

Supporting Peptides:

| Peptide | Uniq | -10lgP | Mass | Length | ppm | m/z | z | RT | 1/k0 Start | 1/k0 End | Precursor ID | Area Sample 9 | #Feature | #Feature Sample 9 | Start | End | PTM | AScore | Found By |
| --- | --- | --- | --- | --- | --- | --- | --- | --- | --- | --- | --- | --- | --- | --- | --- | --- | --- | --- | --- |
| R.VALEQSDRIR.K | Y | 43.05 | 1185.6466 | 10 | -2.05 | 593.8267 | 2 | 12.42 | 0.9505 | 0.9578 | 29416 | 8.58e2 | 1 | 1 | 44 | 53 |  |  | DB Search |
| total 1 peptides |  |  |  |  |  |  |  |  |  |  |  |  |  |  |  |  |  |  |  |
| --- | --- | --- | --- | --- | --- | --- | --- | --- | --- | --- | --- | --- | --- | --- | --- | --- | --- | --- | --- |

Best Unique PSM (Scan Sample\_8\_A1\_1\_3645.d:29416, m/z=593.8267, z=2, RT=12.42, ppm=-6.57):


TRINITY\_DN1465\_c1\_g1\_i1.p1

back to list

  

| Protein Coverage
| Supporting Peptides
| Best Unique PSM
|

Protein Coverage:

Supporting Peptides:

| Peptide | Uniq | -10lgP | Mass | Length | ppm | m/z | z | RT | 1/k0 Start | 1/k0 End | Precursor ID | Area Sample 9 | #Feature | #Feature Sample 9 | Start | End | PTM | AScore | Found By |
| --- | --- | --- | --- | --- | --- | --- | --- | --- | --- | --- | --- | --- | --- | --- | --- | --- | --- | --- | --- |
| K.IQDKEGIPPDQQR.L | Y | 61.25 | 1522.7739 | 13 | -1.84 | 762.3894 | 2 | 10.99 | 1.0371 | 1.0443 | 20319 | 2.55e3 | 1 | 1 | 34 | 46 |  |  | DB Search |
| total 1 peptides |  |  |  |  |  |  |  |  |  |  |  |  |  |  |  |  |  |  |  |
| --- | --- | --- | --- | --- | --- | --- | --- | --- | --- | --- | --- | --- | --- | --- | --- | --- | --- | --- | --- |

Best Unique PSM (Scan Sample\_8\_A1\_1\_3645.d:20319, m/z=762.3894, z=2, RT=10.99, ppm=-6.36):


TRINITY\_DN102244\_c0\_g1\_i1.p1

back to list

  

| Protein Coverage
| Supporting Peptides
| Best Unique PSM
|

Protein Coverage:

Supporting Peptides:

| Peptide | Uniq | -10lgP | Mass | Length | ppm | m/z | z | RT | 1/k0 Start | 1/k0 End | Precursor ID | Area Sample 9 | #Feature | #Feature Sample 9 | Start | End | PTM | AScore | Found By |
| --- | --- | --- | --- | --- | --- | --- | --- | --- | --- | --- | --- | --- | --- | --- | --- | --- | --- | --- | --- |
| R.INDEDKFETK.K | Y | 53.31 | 1237.5826 | 10 | 3.63 | 619.798 | 2 | 11.51 | 0.9299 | 0.9372 | 23930 | 0 | 0 | 0 | 46 | 55 |  |  | DB Search |
| K.RFDALR.S | Y | 13.6 | 776.4293 | 6 | 6.56 | 389.2227 | 2 | 12.31 | 0.7799 | 0.7872 | 28863 | 0 | 0 | 0 | 57 | 62 |  |  | DB Search |
| total 2 peptides |  |  |  |  |  |  |  |  |  |  |  |  |  |  |  |  |  |  |  |
| --- | --- | --- | --- | --- | --- | --- | --- | --- | --- | --- | --- | --- | --- | --- | --- | --- | --- | --- | --- |

Best Unique PSM (Scan Sample\_8\_A1\_1\_3645.d:23930, m/z=619.798, z=2, RT=11.51, ppm=-0.89):


TRINITY\_DN286\_c0\_g1\_i2.p1

back to list

  

| Protein Coverage
| Supporting Peptides
| Best Unique PSM
|

Protein Coverage:

Supporting Peptides:

| Peptide | Uniq | -10lgP | Mass | Length | ppm | m/z | z | RT | 1/k0 Start | 1/k0 End | Precursor ID | Area Sample 9 | #Feature | #Feature Sample 9 | Start | End | PTM | AScore | Found By |
| --- | --- | --- | --- | --- | --- | --- | --- | --- | --- | --- | --- | --- | --- | --- | --- | --- | --- | --- | --- |
| R.YGQAM(+15.99)C(+57.02)QFM(+15.99)R.N | N | 61.54 | 1322.5206 | 10 | -1.76 | 662.2634 | 2 | 12.17 | 0.9489 | 0.9562 | 28008 | 2.07e2 | 1 | 1 | 72 | 81 | Oxidation (M), Carbamidomethylation, Oxidation (M) | M5:Oxidation (M):1000 M9:Oxidation (M):1000 | DB Search |
| K.RYGQAM(+15.99)C(+57.02)QFM(+15.99)R.N | Y | 55.49 | 1478.6217 | 11 | -4.75 | 740.3113 | 2 | 12.88 | 0.9747 | 0.982 | 31970 | 4.25e3 | 1 | 1 | 71 | 81 | Oxidation (M), Carbamidomethylation, Oxidation (M) | M6:Oxidation (M):1000 M10:Oxidation (M):1000 | DB Search |
| K.RYGQAMC(+57.02)QFMR.N | Y | 55.21 | 1446.6319 | 11 | -7.35 | 724.3146 | 2 | 17.19 | 0.9848 | 0.992 | 54851 | 1.37e3 | 1 | 1 | 71 | 81 | Carbamidomethylation |  | DB Search |
| total 3 peptides |  |  |  |  |  |  |  |  |  |  |  |  |  |  |  |  |  |  |  |
| --- | --- | --- | --- | --- | --- | --- | --- | --- | --- | --- | --- | --- | --- | --- | --- | --- | --- | --- | --- |

Best Unique PSM (Scan Sample\_8\_A1\_1\_3645.d:31970, m/z=740.3113, z=2, RT=12.88, ppm=-9.27):


TRINITY\_DN8101\_c0\_g1\_i11.p2

back to list

  

| Protein Coverage
| Supporting Peptides
| Best Unique PSM
|

Protein Coverage:

Supporting Peptides:

| Peptide | Uniq | -10lgP | Mass | Length | ppm | m/z | z | RT | 1/k0 Start | 1/k0 End | Precursor ID | Area Sample 9 | #Feature | #Feature Sample 9 | Start | End | PTM | AScore | Found By |
| --- | --- | --- | --- | --- | --- | --- | --- | --- | --- | --- | --- | --- | --- | --- | --- | --- | --- | --- | --- |
| R.SIGFIGHYLDQLR.L | Y | 55.88 | 1517.799 | 13 | -0.14 | 759.9033 | 2 | 19.91 | 1.0849 | 1.0921 | 70505 | 4.59e2 | 1 | 1 | 1077 | 1089 |  |  | DB Search |
| total 1 peptides |  |  |  |  |  |  |  |  |  |  |  |  |  |  |  |  |  |  |  |
| --- | --- | --- | --- | --- | --- | --- | --- | --- | --- | --- | --- | --- | --- | --- | --- | --- | --- | --- | --- |

Best Unique PSM (Scan Sample\_8\_A1\_1\_3645.d:70505, m/z=759.9033, z=2, RT=19.91, ppm=-4.65):


TRINITY\_DN3016\_c0\_g1\_i17.p1

back to list

  

| Protein Coverage
| Supporting Peptides
| Best Unique PSM
|

Protein Coverage:

Supporting Peptides:

| Peptide | Uniq | -10lgP | Mass | Length | ppm | m/z | z | RT | 1/k0 Start | 1/k0 End | Precursor ID | Area Sample 9 | #Feature | #Feature Sample 9 | Start | End | PTM | AScore | Found By |
| --- | --- | --- | --- | --- | --- | --- | --- | --- | --- | --- | --- | --- | --- | --- | --- | --- | --- | --- | --- |
| R.IVNTDLQEIKDIIK.G | Y | 58.4 | 1640.9348 | 14 | -6.31 | 821.4658 | 2 | 18.62 | 1.1514 | 1.1586 | 63065 | 0 | 0 | 0 | 154 | 167 |  |  | DB Search |
| total 1 peptides |  |  |  |  |  |  |  |  |  |  |  |  |  |  |  |  |  |  |  |
| --- | --- | --- | --- | --- | --- | --- | --- | --- | --- | --- | --- | --- | --- | --- | --- | --- | --- | --- | --- |

Best Unique PSM (Scan Sample\_8\_A1\_1\_3645.d:63065, m/z=821.4658, z=2, RT=18.62, ppm=-10.82):


TRINITY\_DN172571\_c0\_g1\_i1.p1

back to list

  

| Protein Coverage
| Supporting Peptides
| Best Unique PSM
|

Protein Coverage:

Supporting Peptides:

| Peptide | Uniq | -10lgP | Mass | Length | ppm | m/z | z | RT | 1/k0 Start | 1/k0 End | Precursor ID | Area Sample 9 | #Feature | #Feature Sample 9 | Start | End | PTM | AScore | Found By |
| --- | --- | --- | --- | --- | --- | --- | --- | --- | --- | --- | --- | --- | --- | --- | --- | --- | --- | --- | --- |
| R.DNIQGITKPAIR.R | Y | 56.36 | 1324.7463 | 12 | -2.36 | 663.3759 | 2 | 13.73 | 0.9808 | 0.988 | 37017 | 0 | 0 | 0 | 25 | 36 |  |  | DB Search |
| total 1 peptides |  |  |  |  |  |  |  |  |  |  |  |  |  |  |  |  |  |  |  |
| --- | --- | --- | --- | --- | --- | --- | --- | --- | --- | --- | --- | --- | --- | --- | --- | --- | --- | --- | --- |

Best Unique PSM (Scan Sample\_8\_A1\_1\_3645.d:37017, m/z=663.3784, z=1, RT=13.74, ppm=-6.88):


TRINITY\_DN6390\_c0\_g1\_i1.p1

back to list

  

| Protein Coverage
| Supporting Peptides
| Best Unique PSM
|

Protein Coverage:

Supporting Peptides:

| Peptide | Uniq | -10lgP | Mass | Length | ppm | m/z | z | RT | 1/k0 Start | 1/k0 End | Precursor ID | Area Sample 9 | #Feature | #Feature Sample 9 | Start | End | PTM | AScore | Found By |
| --- | --- | --- | --- | --- | --- | --- | --- | --- | --- | --- | --- | --- | --- | --- | --- | --- | --- | --- | --- |
| R.KLEITADGKNVLLNQK.V | Y | 58.02 | 1783.0203 | 16 | 7.49 | 892.5201 | 2 | 14.87 | 1.1802 | 1.1873 | 43269 | 0 | 0 | 0 | 458 | 473 |  |  | DB Search |
| R.HLSVNILQTNTR.V | Y | 57.71 | 1394.763 | 12 | -5.96 | 698.3815 | 2 | 15.19 | 1.0037 | 1.011 | 44915 | 0 | 0 | 0 | 836 | 847 |  |  | DB Search |
| K.VFDKGSYPQPK.T | Y | 21.45 | 1264.6452 | 11 | -8.55 | 633.3216 | 2 | 12.36 | 0.9642 | 0.9715 | 29095 | 0 | 0 | 0 | 1363 | 1373 |  |  | DB Search |
| total 3 peptides |  |  |  |  |  |  |  |  |  |  |  |  |  |  |  |  |  |  |  |
| --- | --- | --- | --- | --- | --- | --- | --- | --- | --- | --- | --- | --- | --- | --- | --- | --- | --- | --- | --- |

Best Unique PSM (Scan Sample\_8\_A1\_1\_3645.d:43269, m/z=892.5201, z=2, RT=14.87, ppm=2.97):


TRINITY\_DN24414\_c1\_g1\_i2.p2

back to list

  

| Protein Coverage
| Supporting Peptides
| Best Unique PSM
|

Protein Coverage:

Supporting Peptides:

| Peptide | Uniq | -10lgP | Mass | Length | ppm | m/z | z | RT | 1/k0 Start | 1/k0 End | Precursor ID | Area Sample 9 | #Feature | #Feature Sample 9 | Start | End | PTM | AScore | Found By |
| --- | --- | --- | --- | --- | --- | --- | --- | --- | --- | --- | --- | --- | --- | --- | --- | --- | --- | --- | --- |
| K.VLTSDDPAEKEK.K | Y | 56.98 | 1330.6616 | 12 | 1.22 | 666.3359 | 2 | 10.71 | 0.9844 | 0.9916 | 18867 | 1.51e3 | 1 | 1 | 95 | 106 |  |  | DB Search |
| total 1 peptides |  |  |  |  |  |  |  |  |  |  |  |  |  |  |  |  |  |  |  |
| --- | --- | --- | --- | --- | --- | --- | --- | --- | --- | --- | --- | --- | --- | --- | --- | --- | --- | --- | --- |

Best Unique PSM (Scan Sample\_8\_A1\_1\_3645.d:18867, m/z=666.3359, z=2, RT=10.71, ppm=-3.3):


TRINITY\_DN1933\_c0\_g1\_i1.p2

back to list

  

| Protein Coverage
| Supporting Peptides
| Best Unique PSM
|

Protein Coverage:

Supporting Peptides:

| Peptide | Uniq | -10lgP | Mass | Length | ppm | m/z | z | RT | 1/k0 Start | 1/k0 End | Precursor ID | Area Sample 9 | #Feature | #Feature Sample 9 | Start | End | PTM | AScore | Found By |
| --- | --- | --- | --- | --- | --- | --- | --- | --- | --- | --- | --- | --- | --- | --- | --- | --- | --- | --- | --- |
| R.RNLDIERPTYTNLNR.L | Y | 58.31 | 1873.9758 | 15 | 3.94 | 625.6655 | 3 | 14.03 | 0.8846 | 0.8919 | 38584 | 6.19e2 | 1 | 1 | 75 | 89 |  |  | DB Search |
| total 1 peptides |  |  |  |  |  |  |  |  |  |  |  |  |  |  |  |  |  |  |  |
| --- | --- | --- | --- | --- | --- | --- | --- | --- | --- | --- | --- | --- | --- | --- | --- | --- | --- | --- | --- |

Best Unique PSM (Scan Sample\_8\_A1\_1\_3645.d:38584, m/z=625.6655, z=3, RT=14.03, ppm=-0.58):


TRINITY\_DN140696\_c0\_g1\_i1.p1

back to list

  

| Protein Coverage
| Supporting Peptides
| Best Unique PSM
|

Protein Coverage:

Supporting Peptides:

| Peptide | Uniq | -10lgP | Mass | Length | ppm | m/z | z | RT | 1/k0 Start | 1/k0 End | Precursor ID | Area Sample 9 | #Feature | #Feature Sample 9 | Start | End | PTM | AScore | Found By |
| --- | --- | --- | --- | --- | --- | --- | --- | --- | --- | --- | --- | --- | --- | --- | --- | --- | --- | --- | --- |
| R.LTIAEERDR.R | Y | 53.43 | 1101.5778 | 9 | 4.01 | 551.7959 | 2 | 11.68 | 0.9057 | 0.9129 | 24919 | 8.95e2 | 1 | 1 | 246 | 254 |  |  | DB Search |
| total 1 peptides |  |  |  |  |  |  |  |  |  |  |  |  |  |  |  |  |  |  |  |
| --- | --- | --- | --- | --- | --- | --- | --- | --- | --- | --- | --- | --- | --- | --- | --- | --- | --- | --- | --- |

Best Unique PSM (Scan Sample\_8\_A1\_1\_3645.d:24919, m/z=551.7959, z=2, RT=11.68, ppm=-0.51):


TRINITY\_DN240\_c0\_g1\_i1.p1

back to list

  

| Protein Coverage
| Supporting Peptides
| Best Unique PSM
|

Protein Coverage:

Supporting Peptides:

| Peptide | Uniq | -10lgP | Mass | Length | ppm | m/z | z | RT | 1/k0 Start | 1/k0 End | Precursor ID | Area Sample 9 | #Feature | #Feature Sample 9 | Start | End | PTM | AScore | Found By |
| --- | --- | --- | --- | --- | --- | --- | --- | --- | --- | --- | --- | --- | --- | --- | --- | --- | --- | --- | --- |
| K.ANQVSSAAAAPVPAAGGDK.I | Y | 61.48 | 1680.8431 | 19 | -2.28 | 841.4231 | 2 | 12.43 | 1.0668 | 1.0741 | 29536 | 0 | 0 | 0 | 230 | 248 |  |  | DB Search |
| K.IGAAAQALADQSYPFLK.S | Y | 59.19 | 1762.9253 | 17 | -0.58 | 882.4655 | 2 | 19.04 | 1.159 | 1.1662 | 65509 | 3.11e3 | 2 | 2 | 249 | 265 |  |  | DB Search |
| K.AYAAFLEFKDVVK.A | Y | 57.51 | 1499.8024 | 13 | 3.03 | 750.9073 | 2 | 19.00 | 1.0929 | 1.1001 | 65208 | 1.13e4 | 1 | 1 | 217 | 229 |  |  | DB Search |
| total 3 peptides |  |  |  |  |  |  |  |  |  |  |  |  |  |  |  |  |  |  |  |
| --- | --- | --- | --- | --- | --- | --- | --- | --- | --- | --- | --- | --- | --- | --- | --- | --- | --- | --- | --- |

Best Unique PSM (Scan Sample\_8\_A1\_1\_3645.d:29536, m/z=841.4231, z=2, RT=12.43, ppm=-6.79):


TRINITY\_DN3016\_c0\_g1\_i4.p1

back to list

  

| Protein Coverage
| Supporting Peptides
| Best Unique PSM
|

Protein Coverage:

Supporting Peptides:

| Peptide | Uniq | -10lgP | Mass | Length | ppm | m/z | z | RT | 1/k0 Start | 1/k0 End | Precursor ID | Area Sample 9 | #Feature | #Feature Sample 9 | Start | End | PTM | AScore | Found By |
| --- | --- | --- | --- | --- | --- | --- | --- | --- | --- | --- | --- | --- | --- | --- | --- | --- | --- | --- | --- |
| R.IVNTDLQEIKDIIK.G | Y | 58.4 | 1640.9348 | 14 | -6.31 | 821.4658 | 2 | 18.62 | 1.1514 | 1.1586 | 63065 | 0 | 0 | 0 | 154 | 167 |  |  | DB Search |
| total 1 peptides |  |  |  |  |  |  |  |  |  |  |  |  |  |  |  |  |  |  |  |
| --- | --- | --- | --- | --- | --- | --- | --- | --- | --- | --- | --- | --- | --- | --- | --- | --- | --- | --- | --- |

Best Unique PSM (Scan Sample\_8\_A1\_1\_3645.d:63065, m/z=821.4658, z=2, RT=18.62, ppm=-10.82):


TRINITY\_DN875\_c0\_g1\_i2.p1

back to list

  

| Protein Coverage
| Supporting Peptides
| Best Unique PSM
|

Protein Coverage:

Supporting Peptides:

| Peptide | Uniq | -10lgP | Mass | Length | ppm | m/z | z | RT | 1/k0 Start | 1/k0 End | Precursor ID | Area Sample 9 | #Feature | #Feature Sample 9 | Start | End | PTM | AScore | Found By |
| --- | --- | --- | --- | --- | --- | --- | --- | --- | --- | --- | --- | --- | --- | --- | --- | --- | --- | --- | --- |
| R.AVFC(+57.02)EHQGVK.T | Y | 44.56 | 1173.5601 | 10 | -1.64 | 587.7837 | 2 | 11.55 | 0.9012 | 0.9085 | 24166 | 0 | 0 | 0 | 512 | 521 | Carbamidomethylation |  | DB Search |
| total 1 peptides |  |  |  |  |  |  |  |  |  |  |  |  |  |  |  |  |  |  |  |
| --- | --- | --- | --- | --- | --- | --- | --- | --- | --- | --- | --- | --- | --- | --- | --- | --- | --- | --- | --- |

Best Unique PSM (Scan Sample\_8\_A1\_1\_3645.d:24166, m/z=587.7837, z=2, RT=11.55, ppm=-6.15):


TRINITY\_DN26249\_c0\_g1\_i1.p1

back to list

  

| Protein Coverage
| Supporting Peptides
| Best Unique PSM
|

Protein Coverage:

Supporting Peptides:

| Peptide | Uniq | -10lgP | Mass | Length | ppm | m/z | z | RT | 1/k0 Start | 1/k0 End | Precursor ID | Area Sample 9 | #Feature | #Feature Sample 9 | Start | End | PTM | AScore | Found By |
| --- | --- | --- | --- | --- | --- | --- | --- | --- | --- | --- | --- | --- | --- | --- | --- | --- | --- | --- | --- |
| R.LIYENQNTIHHVVR.A | Y | 35.47 | 1734.9165 | 14 | -3.47 | 868.4586 | 2 | 13.16 | 1.1302 | 1.1374 | 33743 | 0 | 0 | 0 | 322 | 335 |  |  | DB Search |
| total 1 peptides |  |  |  |  |  |  |  |  |  |  |  |  |  |  |  |  |  |  |  |
| --- | --- | --- | --- | --- | --- | --- | --- | --- | --- | --- | --- | --- | --- | --- | --- | --- | --- | --- | --- |

Best Unique PSM (Scan Sample\_8\_A1\_1\_3645.d:33743, m/z=868.4586, z=2, RT=13.16, ppm=-7.98):


TRINITY\_DN902\_c1\_g1\_i1.p1

back to list

  

| Protein Coverage
| Supporting Peptides
| Best Unique PSM
|

Protein Coverage:

Supporting Peptides:

| Peptide | Uniq | -10lgP | Mass | Length | ppm | m/z | z | RT | 1/k0 Start | 1/k0 End | Precursor ID | Area Sample 9 | #Feature | #Feature Sample 9 | Start | End | PTM | AScore | Found By |
| --- | --- | --- | --- | --- | --- | --- | --- | --- | --- | --- | --- | --- | --- | --- | --- | --- | --- | --- | --- |
| K.FGQATC(+57.02)QFMR.N | Y | 15.37 | 1244.543 | 10 | -15.87 | 623.2661 | 2 | 13.55 | 0.9259 | 0.9331 | 35989 | 5.38e2 | 1 | 1 | 71 | 80 | Carbamidomethylation |  | DB Search |
| total 1 peptides |  |  |  |  |  |  |  |  |  |  |  |  |  |  |  |  |  |  |  |
| --- | --- | --- | --- | --- | --- | --- | --- | --- | --- | --- | --- | --- | --- | --- | --- | --- | --- | --- | --- |

Best Unique PSM (Scan Sample\_8\_A1\_1\_3645.d:35989, m/z=623.2661, z=2, RT=13.55, ppm=-20.38):


TRINITY\_DN3036\_c0\_g1\_i6.p1

back to list

  

| Protein Coverage
| Supporting Peptides
| Best Unique PSM
|

Protein Coverage:

Supporting Peptides:

| Peptide | Uniq | -10lgP | Mass | Length | ppm | m/z | z | RT | 1/k0 Start | 1/k0 End | Precursor ID | Area Sample 9 | #Feature | #Feature Sample 9 | Start | End | PTM | AScore | Found By |
| --- | --- | --- | --- | --- | --- | --- | --- | --- | --- | --- | --- | --- | --- | --- | --- | --- | --- | --- | --- |
| R.VIGASGIHVGTM(+15.99)SFGK.M | Y | 60.06 | 1575.8079 | 16 | 1.08 | 788.9085 | 2 | 15.08 | 1.125 | 1.1322 | 44382 | 0 | 0 | 0 | 24 | 39 | Oxidation (M) | M12:Oxidation (M):1000 | DB Search |
| total 1 peptides |  |  |  |  |  |  |  |  |  |  |  |  |  |  |  |  |  |  |  |
| --- | --- | --- | --- | --- | --- | --- | --- | --- | --- | --- | --- | --- | --- | --- | --- | --- | --- | --- | --- |

Best Unique PSM (Scan Sample\_8\_A1\_1\_3645.d:44382, m/z=788.9085, z=2, RT=15.08, ppm=-3.43):


TRINITY\_DN61707\_c0\_g1\_i1.p1

back to list

  

| Protein Coverage
| Supporting Peptides
| Best Unique PSM
|

Protein Coverage:

Supporting Peptides:

| Peptide | Uniq | -10lgP | Mass | Length | ppm | m/z | z | RT | 1/k0 Start | 1/k0 End | Precursor ID | Area Sample 9 | #Feature | #Feature Sample 9 | Start | End | PTM | AScore | Found By |
| --- | --- | --- | --- | --- | --- | --- | --- | --- | --- | --- | --- | --- | --- | --- | --- | --- | --- | --- | --- |
| R.KHLSELSEEAAK.L | Y | 57.83 | 1340.6936 | 12 | 6.99 | 671.3557 | 2 | 11.03 | 0.9932 | 1.0005 | 20991 | 0 | 0 | 0 | 264 | 275 |  |  | DB Search |
| total 1 peptides |  |  |  |  |  |  |  |  |  |  |  |  |  |  |  |  |  |  |  |
| --- | --- | --- | --- | --- | --- | --- | --- | --- | --- | --- | --- | --- | --- | --- | --- | --- | --- | --- | --- |

Best Unique PSM (Scan Sample\_8\_A1\_1\_3645.d:20991, m/z=671.3557, z=2, RT=11.03, ppm=2.47):


TRINITY\_DN471\_c0\_g1\_i4.p1

back to list

  

| Protein Coverage
| Supporting Peptides
| Best Unique PSM
|

Protein Coverage:

Supporting Peptides:

| Peptide | Uniq | -10lgP | Mass | Length | ppm | m/z | z | RT | 1/k0 Start | 1/k0 End | Precursor ID | Area Sample 9 | #Feature | #Feature Sample 9 | Start | End | PTM | AScore | Found By |
| --- | --- | --- | --- | --- | --- | --- | --- | --- | --- | --- | --- | --- | --- | --- | --- | --- | --- | --- | --- |
| R.RNLDIERPTYTNLNR.L | Y | 58.31 | 1873.9758 | 15 | 3.94 | 625.6655 | 3 | 14.03 | 0.8846 | 0.8919 | 38584 | 6.19e2 | 1 | 1 | 253 | 267 |  |  | DB Search |
| total 1 peptides |  |  |  |  |  |  |  |  |  |  |  |  |  |  |  |  |  |  |  |
| --- | --- | --- | --- | --- | --- | --- | --- | --- | --- | --- | --- | --- | --- | --- | --- | --- | --- | --- | --- |

Best Unique PSM (Scan Sample\_8\_A1\_1\_3645.d:38584, m/z=625.6655, z=3, RT=14.03, ppm=-0.58):


TRINITY\_DN168534\_c0\_g1\_i1.p1

back to list

  

| Protein Coverage
| Supporting Peptides
| Best Unique PSM
|

Protein Coverage:

Supporting Peptides:

| Peptide | Uniq | -10lgP | Mass | Length | ppm | m/z | z | RT | 1/k0 Start | 1/k0 End | Precursor ID | Area Sample 9 | #Feature | #Feature Sample 9 | Start | End | PTM | AScore | Found By |
| --- | --- | --- | --- | --- | --- | --- | --- | --- | --- | --- | --- | --- | --- | --- | --- | --- | --- | --- | --- |
| R.KYGQATC(+57.02)QFMQNSHK.A | Y | 61.26 | 1826.8192 | 15 | 3.52 | 609.9464 | 3 | 12.17 | 0.8769 | 0.8842 | 28012 | 0 | 0 | 0 | 70 | 84 | Carbamidomethylation |  | DB Search |
| K.YGQATC(+57.02)QFMQNSHK.A | Y | 61.18 | 1698.7243 | 14 | -0.26 | 850.3654 | 2 | 13.00 | 1.1398 | 1.147 | 32841 | 0 | 0 | 0 | 71 | 84 | Carbamidomethylation |  | DB Search |
| K.AM(+15.99)TDC(+57.02)KETLC(+57.02)R.G | Y | 57.42 | 1399.5894 | 11 | -3.04 | 700.7967 | 2 | 10.59 | 1.0025 | 1.0098 | 18169 | 7.88e2 | 1 | 1 | 85 | 95 | Oxidation (M), Carbamidomethylation, Carbamidomethylation | M2:Oxidation (M):1000 | DB Search |
| K.AMTDC(+57.02)KETLC(+57.02)R.G | Y | 56.95 | 1383.5945 | 11 | 0.26 | 692.8016 | 2 | 11.61 | 0.9932 | 1.0005 | 24582 | 0 | 0 | 0 | 85 | 95 | Carbamidomethylation, Carbamidomethylation |  | DB Search |
| R.GDYC(+57.02)NSALIEAEQKER.K | Y | 56.88 | 1881.8527 | 16 | 6.06 | 941.9351 | 2 | 14.33 | 1.2069 | 1.2141 | 40232 | 7.6e2 | 1 | 1 | 96 | 111 | Carbamidomethylation |  | DB Search |
| total 5 peptides |  |  |  |  |  |  |  |  |  |  |  |  |  |  |  |  |  |  |  |
| --- | --- | --- | --- | --- | --- | --- | --- | --- | --- | --- | --- | --- | --- | --- | --- | --- | --- | --- | --- |

Best Unique PSM (Scan Sample\_8\_A1\_1\_3645.d:28012, m/z=609.9464, z=3, RT=12.17, ppm=-1):


TRINITY\_DN161\_c0\_g1\_i3.p1

back to list

  

| Protein Coverage
| Supporting Peptides
| Best Unique PSM
|

Protein Coverage:

Supporting Peptides:

| Peptide | Uniq | -10lgP | Mass | Length | ppm | m/z | z | RT | 1/k0 Start | 1/k0 End | Precursor ID | Area Sample 9 | #Feature | #Feature Sample 9 | Start | End | PTM | AScore | Found By |
| --- | --- | --- | --- | --- | --- | --- | --- | --- | --- | --- | --- | --- | --- | --- | --- | --- | --- | --- | --- |
| R.LDLAGRDLTDYLMK.I | Y | 60.93 | 1622.8338 | 14 | 1.03 | 812.4213 | 2 | 19.24 | 1.0965 | 1.1037 | 66626 | 2.72e3 | 2 | 2 | 201 | 214 |  |  | DB Search |
| R.LDLAGRDLTDYLM(+15.99)K.I | Y | 59.01 | 1638.8287 | 14 | 2.41 | 547.2823 | 3 | 18.13 | 0.7856 | 0.7929 | 60339 | 2.36e3 | 1 | 1 | 201 | 214 | Oxidation (M) | M13:Oxidation (M):1000 | DB Search |
| K.DSYVGDEAQSKR.G | Y | 58.12 | 1353.616 | 12 | 4.44 | 677.8152 | 2 | 10.93 | 1.0065 | 1.0138 | 20214 | 8.61e3 | 1 | 1 | 74 | 85 |  |  | DB Search |
| K.DLYANTVLSGGTTM(+15.99)FPGIADR.M | Y | 57.77 | 2214.0626 | 21 | 5.59 | 1108.0398 | 2 | 19.43 | 1.2368 | 1.2439 | 67881 | 0 | 0 | 0 | 315 | 335 | Oxidation (M) | M14:Oxidation (M):1000 | DB Search |
| R.VAPEEHPVLLTEAPLNPK.A | Y | 57.43 | 1953.0571 | 18 | 1.47 | 977.5328 | 2 | 16.35 | 1.2073 | 1.2145 | 50611 | 1.52e2 | 1 | 1 | 119 | 136 |  |  | DB Search |
| K.SYELPDGQVITIGNER.F | Y | 57.34 | 1789.8846 | 16 | 2.2 | 895.9475 | 2 | 18.50 | 0.9828 | 0.99 | 62421 | 0 | 0 | 0 | 262 | 277 |  |  | DB Search |
| R.GYSFTTTAEREIVR.D | Y | 56.59 | 1628.8158 | 14 | 3.67 | 815.4145 | 2 | 15.95 | 1.119 | 1.1262 | 48654 | 3.46e3 | 1 | 1 | 220 | 233 |  |  | DB Search |
| R.AVFPSIVGRPR.H | Y | 56.57 | 1197.6982 | 11 | 1.58 | 599.8546 | 2 | 15.61 | 0.9578 | 0.965 | 46597 | 1.24e4 | 1 | 1 | 52 | 62 |  |  | DB Search |
| R.HQGVM(+15.99)VGM(+15.99)GQK.D | Y | 19.26 | 1202.5536 | 11 | 3.57 | 602.2835 | 2 | 9.78 | 0.9154 | 0.9226 | 13224 | 5.97e2 | 1 | 1 | 63 | 73 | Oxidation (M), Oxidation (M) | M5:Oxidation (M):1000 M8:Oxidation (M):1000 | DB Search |
| total 9 peptides |  |  |  |  |  |  |  |  |  |  |  |  |  |  |  |  |  |  |  |
| --- | --- | --- | --- | --- | --- | --- | --- | --- | --- | --- | --- | --- | --- | --- | --- | --- | --- | --- | --- |

Best Unique PSM (Scan Sample\_8\_A1\_1\_3645.d:66626, m/z=812.4213, z=2, RT=19.24, ppm=-3.49):


TRINITY\_DN2776\_c0\_g1\_i2.p1

back to list

  

| Protein Coverage
| Supporting Peptides
| Best Unique PSM
|

Protein Coverage:

Supporting Peptides:

| Peptide | Uniq | -10lgP | Mass | Length | ppm | m/z | z | RT | 1/k0 Start | 1/k0 End | Precursor ID | Area Sample 9 | #Feature | #Feature Sample 9 | Start | End | PTM | AScore | Found By |
| --- | --- | --- | --- | --- | --- | --- | --- | --- | --- | --- | --- | --- | --- | --- | --- | --- | --- | --- | --- |
| K.IALDDVFRER.D | Y | 52.99 | 1232.6513 | 10 | 0.49 | 617.3304 | 2 | 17.63 | 0.957 | 0.9642 | 57382 | 0 | 0 | 0 | 147 | 156 |  |  | DB Search |
| total 1 peptides |  |  |  |  |  |  |  |  |  |  |  |  |  |  |  |  |  |  |  |
| --- | --- | --- | --- | --- | --- | --- | --- | --- | --- | --- | --- | --- | --- | --- | --- | --- | --- | --- | --- |

Best Unique PSM (Scan Sample\_8\_A1\_1\_3645.d:57382, m/z=617.3304, z=2, RT=17.63, ppm=-4.03):


TRINITY\_DN138\_c0\_g1\_i1.p1

back to list

  

| Protein Coverage
| Supporting Peptides
| Best Unique PSM
|

Protein Coverage:

Supporting Peptides:

| Peptide | Uniq | -10lgP | Mass | Length | ppm | m/z | z | RT | 1/k0 Start | 1/k0 End | Precursor ID | Area Sample 9 | #Feature | #Feature Sample 9 | Start | End | PTM | AScore | Found By |
| --- | --- | --- | --- | --- | --- | --- | --- | --- | --- | --- | --- | --- | --- | --- | --- | --- | --- | --- | --- |
| R.FC(+57.02)SNPEPENGGQTC(+57.02)EGEAR.E | Y | 58.9 | 2137.8429 | 19 | 1.53 | 1069.9255 | 2 | 12.03 | 1.1562 | 1.1634 | 27122 | 0 | 0 | 0 | 181 | 199 | Carbamidomethylation, Carbamidomethylation |  | DB Search |
| R.DPINNHWDGNLINWR.K | Y | 56.96 | 1862.8812 | 15 | -1.81 | 621.9637 | 3 | 18.88 | 0.8116 | 0.8189 | 64604 | 4.88e2 | 1 | 1 | 55 | 69 |  |  | DB Search |
| K.DM(+15.99)GVNVISVGAGHTINHR.E | Y | 56.49 | 1891.9322 | 18 | 4.31 | 946.9732 | 2 | 14.15 | 1.2252 | 1.2324 | 39259 | 0 | 0 | 0 | 349 | 366 | Oxidation (M) | M2:Oxidation (M):1000 | DB Search |
| R.ERFC(+57.02)SNPEPENGGQTC(+57.02)EGEAR.E | Y | 15.15 | 2422.9866 | 21 | 1.66 | 808.6672 | 3 | 11.82 | 0.9077 | 0.915 | 25956 | 2.43e2 | 1 | 1 | 179 | 199 | Carbamidomethylation, Carbamidomethylation |  | DB Search |
| total 4 peptides |  |  |  |  |  |  |  |  |  |  |  |  |  |  |  |  |  |  |  |
| --- | --- | --- | --- | --- | --- | --- | --- | --- | --- | --- | --- | --- | --- | --- | --- | --- | --- | --- | --- |

Best Unique PSM (Scan Sample\_8\_A1\_1\_3645.d:27122, m/z=1069.9255, z=2, RT=12.03, ppm=-2.99):


TRINITY\_DN37295\_c0\_g1\_i1.p1

back to list

  

| Protein Coverage
| Supporting Peptides
| Best Unique PSM
|

Protein Coverage:

Supporting Peptides:

| Peptide | Uniq | -10lgP | Mass | Length | ppm | m/z | z | RT | 1/k0 Start | 1/k0 End | Precursor ID | Area Sample 9 | #Feature | #Feature Sample 9 | Start | End | PTM | AScore | Found By |
| --- | --- | --- | --- | --- | --- | --- | --- | --- | --- | --- | --- | --- | --- | --- | --- | --- | --- | --- | --- |
| R.DLKPENLLIDEKGYLK.V | Y | 58.66 | 1887.0353 | 16 | -2.85 | 630.0144 | 3 | 17.77 | 0.9214 | 0.9287 | 58211 | 0 | 0 | 0 | 10 | 25 |  |  | DB Search |
| total 1 peptides |  |  |  |  |  |  |  |  |  |  |  |  |  |  |  |  |  |  |  |
| --- | --- | --- | --- | --- | --- | --- | --- | --- | --- | --- | --- | --- | --- | --- | --- | --- | --- | --- | --- |

Best Unique PSM (Scan Sample\_8\_A1\_1\_3645.d:58211, m/z=630.0144, z=3, RT=17.77, ppm=-7.36):


TRINITY\_DN3036\_c0\_g1\_i19.p1

back to list

  

| Protein Coverage
| Supporting Peptides
| Best Unique PSM
|

Protein Coverage:

Supporting Peptides:

| Peptide | Uniq | -10lgP | Mass | Length | ppm | m/z | z | RT | 1/k0 Start | 1/k0 End | Precursor ID | Area Sample 9 | #Feature | #Feature Sample 9 | Start | End | PTM | AScore | Found By |
| --- | --- | --- | --- | --- | --- | --- | --- | --- | --- | --- | --- | --- | --- | --- | --- | --- | --- | --- | --- |
| R.VIGASGIHVGTM(+15.99)SFGK.M | Y | 60.06 | 1575.8079 | 16 | 1.08 | 788.9085 | 2 | 15.08 | 1.125 | 1.1322 | 44382 | 0 | 0 | 0 | 160 | 175 | Oxidation (M) | M12:Oxidation (M):1000 | DB Search |
| total 1 peptides |  |  |  |  |  |  |  |  |  |  |  |  |  |  |  |  |  |  |  |
| --- | --- | --- | --- | --- | --- | --- | --- | --- | --- | --- | --- | --- | --- | --- | --- | --- | --- | --- | --- |

Best Unique PSM (Scan Sample\_8\_A1\_1\_3645.d:44382, m/z=788.9085, z=2, RT=15.08, ppm=-3.43):


TRINITY\_DN902\_c1\_g1\_i2.p1

back to list

  

| Protein Coverage
| Supporting Peptides
| Best Unique PSM
|

Protein Coverage:

Supporting Peptides:

| Peptide | Uniq | -10lgP | Mass | Length | ppm | m/z | z | RT | 1/k0 Start | 1/k0 End | Precursor ID | Area Sample 9 | #Feature | #Feature Sample 9 | Start | End | PTM | AScore | Found By |
| --- | --- | --- | --- | --- | --- | --- | --- | --- | --- | --- | --- | --- | --- | --- | --- | --- | --- | --- | --- |
| K.FGQATC(+57.02)QFMR.N | Y | 15.37 | 1244.543 | 10 | -15.87 | 623.2661 | 2 | 13.55 | 0.9259 | 0.9331 | 35989 | 5.38e2 | 1 | 1 | 71 | 80 | Carbamidomethylation |  | DB Search |
| total 1 peptides |  |  |  |  |  |  |  |  |  |  |  |  |  |  |  |  |  |  |  |
| --- | --- | --- | --- | --- | --- | --- | --- | --- | --- | --- | --- | --- | --- | --- | --- | --- | --- | --- | --- |

Best Unique PSM (Scan Sample\_8\_A1\_1\_3645.d:35989, m/z=623.2661, z=2, RT=13.55, ppm=-20.38):


TRINITY\_DN477\_c0\_g1\_i9.p1

back to list

  

| Protein Coverage
| Supporting Peptides
| Best Unique PSM
|

Protein Coverage:

Supporting Peptides:

| Peptide | Uniq | -10lgP | Mass | Length | ppm | m/z | z | RT | 1/k0 Start | 1/k0 End | Precursor ID | Area Sample 9 | #Feature | #Feature Sample 9 | Start | End | PTM | AScore | Found By |
| --- | --- | --- | --- | --- | --- | --- | --- | --- | --- | --- | --- | --- | --- | --- | --- | --- | --- | --- | --- |
| R.TQAVLDRLNIEKR.V | Y | 59.65 | 1554.8842 | 13 | 2.18 | 519.3008 | 3 | 14.51 | 0.7986 | 0.8059 | 41304 | 7.54e2 | 1 | 1 | 220 | 232 |  |  | DB Search |
| K.DANSM(+15.99)PTQSR.Q | Y | 56.18 | 1121.4771 | 10 | -2.33 | 561.742 | 2 | 8.84 | 0.823 | 0.8303 | 9491 | 0 | 0 | 0 | 51 | 60 | Oxidation (M) | M5:Oxidation (M):1000 | DB Search |
| R.TQAVLDRLNIEK.R | Y | 55.98 | 1398.783 | 12 | 1.47 | 700.3967 | 2 | 15.27 | 1.0073 | 1.0146 | 45303 | 1.02e3 | 1 | 1 | 220 | 231 |  |  | DB Search |
| total 3 peptides |  |  |  |  |  |  |  |  |  |  |  |  |  |  |  |  |  |  |  |
| --- | --- | --- | --- | --- | --- | --- | --- | --- | --- | --- | --- | --- | --- | --- | --- | --- | --- | --- | --- |

Best Unique PSM (Scan Sample\_8\_A1\_1\_3645.d:41304, m/z=519.3008, z=3, RT=14.51, ppm=-2.34):


TRINITY\_DN6920\_c0\_g1\_i2.p1

back to list

  

| Protein Coverage
| Supporting Peptides
| Best Unique PSM
|

Protein Coverage:

Supporting Peptides:

| Peptide | Uniq | -10lgP | Mass | Length | ppm | m/z | z | RT | 1/k0 Start | 1/k0 End | Precursor ID | Area Sample 9 | #Feature | #Feature Sample 9 | Start | End | PTM | AScore | Found By |
| --- | --- | --- | --- | --- | --- | --- | --- | --- | --- | --- | --- | --- | --- | --- | --- | --- | --- | --- | --- |
| K.VAC(+57.02)IGAWHPAR.V | Y | 45.85 | 1236.6186 | 11 | -2.24 | 619.3124 | 2 | 13.17 | 0.9444 | 0.9517 | 33868 | 3.39e2 | 1 | 1 | 258 | 268 | Carbamidomethylation |  | DB Search |
| total 1 peptides |  |  |  |  |  |  |  |  |  |  |  |  |  |  |  |  |  |  |  |
| --- | --- | --- | --- | --- | --- | --- | --- | --- | --- | --- | --- | --- | --- | --- | --- | --- | --- | --- | --- |

Best Unique PSM (Scan Sample\_8\_A1\_1\_3645.d:33868, m/z=619.3124, z=2, RT=13.17, ppm=-6.76):


TRINITY\_DN3271\_c0\_g1\_i2.p1

back to list

  

| Protein Coverage
| Supporting Peptides
| Best Unique PSM
|

Protein Coverage:

Supporting Peptides:

| Peptide | Uniq | -10lgP | Mass | Length | ppm | m/z | z | RT | 1/k0 Start | 1/k0 End | Precursor ID | Area Sample 9 | #Feature | #Feature Sample 9 | Start | End | PTM | AScore | Found By |
| --- | --- | --- | --- | --- | --- | --- | --- | --- | --- | --- | --- | --- | --- | --- | --- | --- | --- | --- | --- |
| K.TIESEAAHGTVTR.H | Y | 55.93 | 1370.679 | 13 | 4.48 | 686.3467 | 2 | 11.86 | 1.0186 | 1.0259 | 26093 | 0 | 0 | 0 | 354 | 366 |  |  | DB Search |
| total 1 peptides |  |  |  |  |  |  |  |  |  |  |  |  |  |  |  |  |  |  |  |
| --- | --- | --- | --- | --- | --- | --- | --- | --- | --- | --- | --- | --- | --- | --- | --- | --- | --- | --- | --- |

Best Unique PSM (Scan Sample\_8\_A1\_1\_3645.d:26093, m/z=686.3467, z=2, RT=11.86, ppm=-0.04):


TRINITY\_DN250\_c0\_g1\_i1.p1

back to list

  

| Protein Coverage
| Supporting Peptides
| Best Unique PSM
|

Protein Coverage:

Supporting Peptides:

| Peptide | Uniq | -10lgP | Mass | Length | ppm | m/z | z | RT | 1/k0 Start | 1/k0 End | Precursor ID | Area Sample 9 | #Feature | #Feature Sample 9 | Start | End | PTM | AScore | Found By |
| --- | --- | --- | --- | --- | --- | --- | --- | --- | --- | --- | --- | --- | --- | --- | --- | --- | --- | --- | --- |
| R.RNLDIERPTYTNLNR.L | Y | 58.31 | 1873.9758 | 15 | 3.94 | 625.6655 | 3 | 14.03 | 0.8846 | 0.8919 | 38584 | 6.19e2 | 1 | 1 | 215 | 229 |  |  | DB Search |
| R.IHFPLATYSPVISAEK.A | Y | 50.18 | 1771.9508 | 16 | 1.46 | 886.98 | 2 | 18.50 | 1.1338 | 1.141 | 62412 | 0 | 0 | 0 | 265 | 280 |  |  | DB Search |
| total 2 peptides |  |  |  |  |  |  |  |  |  |  |  |  |  |  |  |  |  |  |  |
| --- | --- | --- | --- | --- | --- | --- | --- | --- | --- | --- | --- | --- | --- | --- | --- | --- | --- | --- | --- |

Best Unique PSM (Scan Sample\_8\_A1\_1\_3645.d:38584, m/z=625.6655, z=3, RT=14.03, ppm=-0.58):


TRINITY\_DN10724\_c0\_g1\_i3.p1

back to list

  

| Protein Coverage
| Supporting Peptides
| Best Unique PSM
|

Protein Coverage:

Supporting Peptides:

| Peptide | Uniq | -10lgP | Mass | Length | ppm | m/z | z | RT | 1/k0 Start | 1/k0 End | Precursor ID | Area Sample 9 | #Feature | #Feature Sample 9 | Start | End | PTM | AScore | Found By |
| --- | --- | --- | --- | --- | --- | --- | --- | --- | --- | --- | --- | --- | --- | --- | --- | --- | --- | --- | --- |
| K.REPGDFGFDPLKFSK.R | Y | 58.72 | 1738.8678 | 15 | -1.8 | 580.6262 | 3 | 18.01 | 0.9012 | 0.9085 | 59548 | 0 | 0 | 0 | 87 | 101 |  |  | DB Search |
| total 1 peptides |  |  |  |  |  |  |  |  |  |  |  |  |  |  |  |  |  |  |  |
| --- | --- | --- | --- | --- | --- | --- | --- | --- | --- | --- | --- | --- | --- | --- | --- | --- | --- | --- | --- |

Best Unique PSM (Scan Sample\_8\_A1\_1\_3645.d:59548, m/z=580.6262, z=3, RT=18.01, ppm=-6.32):


TRINITY\_DN535\_c0\_g1\_i2.p1

back to list

  

| Protein Coverage
| Supporting Peptides
| Best Unique PSM
|

Protein Coverage:

Supporting Peptides:

| Peptide | Uniq | -10lgP | Mass | Length | ppm | m/z | z | RT | 1/k0 Start | 1/k0 End | Precursor ID | Area Sample 9 | #Feature | #Feature Sample 9 | Start | End | PTM | AScore | Found By |
| --- | --- | --- | --- | --- | --- | --- | --- | --- | --- | --- | --- | --- | --- | --- | --- | --- | --- | --- | --- |
| R.GLFIIDDKGILR.Q | Y | 55.4 | 1358.7921 | 12 | 1.42 | 680.4012 | 2 | 19.26 | 1.011 | 1.0182 | 66782 | 5.93e2 | 1 | 1 | 94 | 105 |  |  | DB Search |
| total 1 peptides |  |  |  |  |  |  |  |  |  |  |  |  |  |  |  |  |  |  |  |
| --- | --- | --- | --- | --- | --- | --- | --- | --- | --- | --- | --- | --- | --- | --- | --- | --- | --- | --- | --- |

Best Unique PSM (Scan Sample\_8\_A1\_1\_3645.d:66782, m/z=680.4012, z=2, RT=19.26, ppm=-3.09):


TRINITY\_DN1280\_c0\_g1\_i1.p1

back to list

  

| Protein Coverage
| Supporting Peptides
| Best Unique PSM
|

Protein Coverage:

Supporting Peptides:

| Peptide | Uniq | -10lgP | Mass | Length | ppm | m/z | z | RT | 1/k0 Start | 1/k0 End | Precursor ID | Area Sample 9 | #Feature | #Feature Sample 9 | Start | End | PTM | AScore | Found By |
| --- | --- | --- | --- | --- | --- | --- | --- | --- | --- | --- | --- | --- | --- | --- | --- | --- | --- | --- | --- |
| R.KFVWEFFQK.V | Y | 58.53 | 1257.6546 | 9 | 1.34 | 629.8326 | 2 | 19.88 | 0.9436 | 0.9509 | 70292 | 1.4e3 | 1 | 1 | 30 | 38 |  |  | DB Search |
| K.AEYENKTEVAR.S | Y | 57.19 | 1308.631 | 11 | -1.93 | 655.3185 | 2 | 10.52 | 0.9771 | 0.9844 | 17854 | 3.06e3 | 2 | 2 | 202 | 212 |  |  | DB Search |
| R.IKAEYENKTEVAR.S | Y | 56.36 | 1549.81 | 13 | 5.17 | 775.9128 | 2 | 10.97 | 1.0556 | 1.0628 | 20636 | 7.1e2 | 1 | 1 | 200 | 212 |  |  | DB Search |
| K.SAYDIEVNTKK.A | Y | 56.29 | 1266.6456 | 11 | -2.87 | 634.3254 | 2 | 12.81 | 0.9703 | 0.9775 | 31723 | 0 | 0 | 0 | 222 | 232 |  |  | DB Search |
| R.RIAEVQC(+57.02)EAR.I | Y | 55.3 | 1230.6139 | 10 | 0.24 | 616.3116 | 2 | 10.93 | 0.9234 | 0.9307 | 20326 | 1.91e3 | 1 | 1 | 169 | 178 | Carbamidomethylation |  | DB Search |
| R.NIALETLEGHQR.A | Y | 55.15 | 1379.7157 | 12 | 1.4 | 690.863 | 2 | 15.19 | 1.0142 | 1.0214 | 44943 | 4.61e2 | 1 | 1 | 99 | 110 |  |  | DB Search |
| K.AEAEAEVIQKK.A | Y | 54.68 | 1214.6506 | 11 | 0.18 | 608.33 | 2 | 12.87 | 0.9566 | 0.9638 | 32079 | 2.33e2 | 1 | 1 | 321 | 331 |  |  | DB Search |
| R.VYTVQGVPISVTGIAQVK.V | Y | 20.56 | 1858.0564 | 18 | -6.25 | 930.0255 | 2 | 18.78 | 1.1582 | 1.1654 | 63980 | 0 | 0 | 0 | 56 | 73 |  |  | DB Search |
| R.AQAINVQLQEIQRR.E | Y | 18.81 | 1665.9274 | 14 | 4.32 | 833.9708 | 2 | 15.11 | 1.1065 | 1.1138 | 44355 | 3.77e3 | 1 | 1 | 263 | 276 |  |  | DB Search |
| total 9 peptides |  |  |  |  |  |  |  |  |  |  |  |  |  |  |  |  |  |  |  |
| --- | --- | --- | --- | --- | --- | --- | --- | --- | --- | --- | --- | --- | --- | --- | --- | --- | --- | --- | --- |

Best Unique PSM (Scan Sample\_8\_A1\_1\_3645.d:70292, m/z=629.8326, z=2, RT=19.88, ppm=-3.17):


TRINITY\_DN250\_c0\_g1\_i4.p1

back to list

  

| Protein Coverage
| Supporting Peptides
| Best Unique PSM
|

Protein Coverage:

Supporting Peptides:

| Peptide | Uniq | -10lgP | Mass | Length | ppm | m/z | z | RT | 1/k0 Start | 1/k0 End | Precursor ID | Area Sample 9 | #Feature | #Feature Sample 9 | Start | End | PTM | AScore | Found By |
| --- | --- | --- | --- | --- | --- | --- | --- | --- | --- | --- | --- | --- | --- | --- | --- | --- | --- | --- | --- |
| R.RNLDIERPTYTNLNR.L | Y | 58.31 | 1873.9758 | 15 | 3.94 | 625.6655 | 3 | 14.03 | 0.8846 | 0.8919 | 38584 | 6.19e2 | 1 | 1 | 215 | 229 |  |  | DB Search |
| R.IHFPLATYSPVISAEK.A | Y | 50.18 | 1771.9508 | 16 | 1.46 | 886.98 | 2 | 18.50 | 1.1338 | 1.141 | 62412 | 0 | 0 | 0 | 265 | 280 |  |  | DB Search |
| total 2 peptides |  |  |  |  |  |  |  |  |  |  |  |  |  |  |  |  |  |  |  |
| --- | --- | --- | --- | --- | --- | --- | --- | --- | --- | --- | --- | --- | --- | --- | --- | --- | --- | --- | --- |

Best Unique PSM (Scan Sample\_8\_A1\_1\_3645.d:38584, m/z=625.6655, z=3, RT=14.03, ppm=-0.58):


TRINITY\_DN2477\_c0\_g1\_i9.p1

back to list

  

| Protein Coverage
| Supporting Peptides
| Best Unique PSM
|

Protein Coverage:

Supporting Peptides:

| Peptide | Uniq | -10lgP | Mass | Length | ppm | m/z | z | RT | 1/k0 Start | 1/k0 End | Precursor ID | Area Sample 9 | #Feature | #Feature Sample 9 | Start | End | PTM | AScore | Found By |
| --- | --- | --- | --- | --- | --- | --- | --- | --- | --- | --- | --- | --- | --- | --- | --- | --- | --- | --- | --- |
| K.DSYVGDEAQSKR.G | Y | 58.12 | 1353.616 | 12 | 4.44 | 677.8152 | 2 | 10.93 | 1.0065 | 1.0138 | 20214 | 8.61e3 | 1 | 1 | 52 | 63 |  |  | DB Search |
| R.VAPEEHPVLLTEAPLNPK.A | Y | 57.43 | 1953.0571 | 18 | 1.47 | 977.5328 | 2 | 16.35 | 1.2073 | 1.2145 | 50611 | 1.52e2 | 1 | 1 | 97 | 114 |  |  | DB Search |
| R.GYSFTTTAEREIVR.D | Y | 56.59 | 1628.8158 | 14 | 3.67 | 815.4145 | 2 | 15.95 | 1.119 | 1.1262 | 48654 | 3.46e3 | 1 | 1 | 198 | 211 |  |  | DB Search |
| total 3 peptides |  |  |  |  |  |  |  |  |  |  |  |  |  |  |  |  |  |  |  |
| --- | --- | --- | --- | --- | --- | --- | --- | --- | --- | --- | --- | --- | --- | --- | --- | --- | --- | --- | --- |

Best Unique PSM (Scan Sample\_8\_A1\_1\_3645.d:20214, m/z=677.8152, z=2, RT=10.93, ppm=-0.08):


TRINITY\_DN1761\_c0\_g1\_i1.p1

back to list

  

| Protein Coverage
| Supporting Peptides
| Best Unique PSM
|

Protein Coverage:

Supporting Peptides:

| Peptide | Uniq | -10lgP | Mass | Length | ppm | m/z | z | RT | 1/k0 Start | 1/k0 End | Precursor ID | Area Sample 9 | #Feature | #Feature Sample 9 | Start | End | PTM | AScore | Found By |
| --- | --- | --- | --- | --- | --- | --- | --- | --- | --- | --- | --- | --- | --- | --- | --- | --- | --- | --- | --- |
| R.KGFFSSESKDLISYLPR.R | Y | 55.5 | 1973.0258 | 17 | -8.91 | 658.6737 | 3 | 18.94 | 0.9121 | 0.9194 | 65041 | 2.85e2 | 1 | 1 | 197 | 213 |  |  | DB Search |
| total 1 peptides |  |  |  |  |  |  |  |  |  |  |  |  |  |  |  |  |  |  |  |
| --- | --- | --- | --- | --- | --- | --- | --- | --- | --- | --- | --- | --- | --- | --- | --- | --- | --- | --- | --- |

Best Unique PSM (Scan Sample\_8\_A1\_1\_3645.d:65041, m/z=658.343, z=3, RT=18.95, ppm=-13.43):


TRINITY\_DN902\_c1\_g2\_i3.p1

back to list

  

| Protein Coverage
| Supporting Peptides
| Best Unique PSM
|

Protein Coverage:

Supporting Peptides:

| Peptide | Uniq | -10lgP | Mass | Length | ppm | m/z | z | RT | 1/k0 Start | 1/k0 End | Precursor ID | Area Sample 9 | #Feature | #Feature Sample 9 | Start | End | PTM | AScore | Found By |
| --- | --- | --- | --- | --- | --- | --- | --- | --- | --- | --- | --- | --- | --- | --- | --- | --- | --- | --- | --- |
| K.GLTHC(+57.02)NTLSYK.L | Y | 58.02 | 1292.6183 | 11 | 2.78 | 647.3153 | 2 | 12.39 | 0.9743 | 0.9816 | 29201 | 4.95e3 | 1 | 1 | 62 | 72 | Carbamidomethylation |  | DB Search |
| total 1 peptides |  |  |  |  |  |  |  |  |  |  |  |  |  |  |  |  |  |  |  |
| --- | --- | --- | --- | --- | --- | --- | --- | --- | --- | --- | --- | --- | --- | --- | --- | --- | --- | --- | --- |

Best Unique PSM (Scan Sample\_8\_A1\_1\_3645.d:29201, m/z=647.3153, z=2, RT=12.39, ppm=-1.74):


TRINITY\_DN14830\_c0\_g1\_i1.p1

back to list

  

| Protein Coverage
| Supporting Peptides
| Best Unique PSM
|

Protein Coverage:

Supporting Peptides:

| Peptide | Uniq | -10lgP | Mass | Length | ppm | m/z | z | RT | 1/k0 Start | 1/k0 End | Precursor ID | Area Sample 9 | #Feature | #Feature Sample 9 | Start | End | PTM | AScore | Found By |
| --- | --- | --- | --- | --- | --- | --- | --- | --- | --- | --- | --- | --- | --- | --- | --- | --- | --- | --- | --- |
| K.SLDYLQVPK.G | Y | 32.21 | 1061.5757 | 9 | -2.81 | 1062.5752 | 1 | 16.83 | 1.2861 | 1.2933 | 53006 | 0 | 0 | 0 | 578 | 586 |  |  | DB Search |
| total 1 peptides |  |  |  |  |  |  |  |  |  |  |  |  |  |  |  |  |  |  |  |
| --- | --- | --- | --- | --- | --- | --- | --- | --- | --- | --- | --- | --- | --- | --- | --- | --- | --- | --- | --- |

Best Unique PSM (Scan Sample\_8\_A1\_1\_3645.d:53006, m/z=1062.5752, z=1, RT=16.83, ppm=-7.32):


TRINITY\_DN15584\_c0\_g1\_i1.p1

back to list

  

| Protein Coverage
| Supporting Peptides
| Best Unique PSM
|

Protein Coverage:

Supporting Peptides:

| Peptide | Uniq | -10lgP | Mass | Length | ppm | m/z | z | RT | 1/k0 Start | 1/k0 End | Precursor ID | Area Sample 9 | #Feature | #Feature Sample 9 | Start | End | PTM | AScore | Found By |
| --- | --- | --- | --- | --- | --- | --- | --- | --- | --- | --- | --- | --- | --- | --- | --- | --- | --- | --- | --- |
| K.IGKPHTVPC(+57.02)K.V | Y | 57.38 | 1135.6172 | 10 | 1.39 | 568.8141 | 2 | 9.77 | 0.9028 | 0.9101 | 13225 | 1.74e4 | 2 | 2 | 157 | 166 | Carbamidomethylation |  | DB Search |
| K.AFVAIGDYGGHVGLGVK.C | Y | 56.9 | 1658.878 | 17 | 4.03 | 830.4459 | 2 | 17.59 | 1.1614 | 1.1686 | 56871 | 7.56e3 | 1 | 1 | 109 | 125 |  |  | DB Search |
| total 2 peptides |  |  |  |  |  |  |  |  |  |  |  |  |  |  |  |  |  |  |  |
| --- | --- | --- | --- | --- | --- | --- | --- | --- | --- | --- | --- | --- | --- | --- | --- | --- | --- | --- | --- |

Best Unique PSM (Scan Sample\_8\_A1\_1\_3645.d:13225, m/z=568.8141, z=2, RT=9.77, ppm=-3.13):


TRINITY\_DN598\_c1\_g1\_i1.p1

back to list

  

| Protein Coverage
| Supporting Peptides
| Best Unique PSM
|

Protein Coverage:

Supporting Peptides:

| Peptide | Uniq | -10lgP | Mass | Length | ppm | m/z | z | RT | 1/k0 Start | 1/k0 End | Precursor ID | Area Sample 9 | #Feature | #Feature Sample 9 | Start | End | PTM | AScore | Found By |
| --- | --- | --- | --- | --- | --- | --- | --- | --- | --- | --- | --- | --- | --- | --- | --- | --- | --- | --- | --- |
| R.ASFVSDIDRLR.A | Y | 56.88 | 1277.6728 | 11 | 2.94 | 639.8427 | 2 | 16.84 | 0.9824 | 0.9896 | 52937 | 5.14e3 | 1 | 1 | 302 | 312 |  |  | DB Search |
| total 1 peptides |  |  |  |  |  |  |  |  |  |  |  |  |  |  |  |  |  |  |  |
| --- | --- | --- | --- | --- | --- | --- | --- | --- | --- | --- | --- | --- | --- | --- | --- | --- | --- | --- | --- |

Best Unique PSM (Scan Sample\_8\_A1\_1\_3645.d:52937, m/z=639.8427, z=2, RT=16.84, ppm=-1.58):


TRINITY\_DN471\_c0\_g1\_i5.p1

back to list

  

| Protein Coverage
| Supporting Peptides
| Best Unique PSM
|

Protein Coverage:

Supporting Peptides:

| Peptide | Uniq | -10lgP | Mass | Length | ppm | m/z | z | RT | 1/k0 Start | 1/k0 End | Precursor ID | Area Sample 9 | #Feature | #Feature Sample 9 | Start | End | PTM | AScore | Found By |
| --- | --- | --- | --- | --- | --- | --- | --- | --- | --- | --- | --- | --- | --- | --- | --- | --- | --- | --- | --- |
| R.RNLDIERPTYTNLNR.L | Y | 58.31 | 1873.9758 | 15 | 3.94 | 625.6655 | 3 | 14.03 | 0.8846 | 0.8919 | 38584 | 6.19e2 | 1 | 1 | 215 | 229 |  |  | DB Search |
| total 1 peptides |  |  |  |  |  |  |  |  |  |  |  |  |  |  |  |  |  |  |  |
| --- | --- | --- | --- | --- | --- | --- | --- | --- | --- | --- | --- | --- | --- | --- | --- | --- | --- | --- | --- |

Best Unique PSM (Scan Sample\_8\_A1\_1\_3645.d:38584, m/z=625.6655, z=3, RT=14.03, ppm=-0.58):


TRINITY\_DN374\_c0\_g1\_i1.p1

back to list

  

| Protein Coverage
| Supporting Peptides
| Best Unique PSM
|

Protein Coverage:

Supporting Peptides:

| Peptide | Uniq | -10lgP | Mass | Length | ppm | m/z | z | RT | 1/k0 Start | 1/k0 End | Precursor ID | Area Sample 9 | #Feature | #Feature Sample 9 | Start | End | PTM | AScore | Found By |
| --- | --- | --- | --- | --- | --- | --- | --- | --- | --- | --- | --- | --- | --- | --- | --- | --- | --- | --- | --- |
| K.YIDIEYVC(+57.02)KPPVKK.A | Y | 59.02 | 1750.9327 | 14 | 1.78 | 876.4713 | 2 | 14.93 | 1.131 | 1.1382 | 43553 | 2.46e2 | 1 | 1 | 183 | 196 | Carbamidomethylation |  | DB Search |
| K.TVTC(+57.02)GAGETIKVTK.A | Y | 56.96 | 1463.7654 | 14 | 3.77 | 732.8894 | 2 | 12.26 | 1.0636 | 1.0709 | 28487 | 0 | 0 | 0 | 111 | 124 | Carbamidomethylation |  | DB Search |
| total 2 peptides |  |  |  |  |  |  |  |  |  |  |  |  |  |  |  |  |  |  |  |
| --- | --- | --- | --- | --- | --- | --- | --- | --- | --- | --- | --- | --- | --- | --- | --- | --- | --- | --- | --- |

Best Unique PSM (Scan Sample\_8\_A1\_1\_3645.d:43553, m/z=876.4713, z=2, RT=14.93, ppm=-2.74):


TRINITY\_DN250\_c0\_g1\_i3.p1

back to list

  

| Protein Coverage
| Supporting Peptides
| Best Unique PSM
|

Protein Coverage:

Supporting Peptides:

| Peptide | Uniq | -10lgP | Mass | Length | ppm | m/z | z | RT | 1/k0 Start | 1/k0 End | Precursor ID | Area Sample 9 | #Feature | #Feature Sample 9 | Start | End | PTM | AScore | Found By |
| --- | --- | --- | --- | --- | --- | --- | --- | --- | --- | --- | --- | --- | --- | --- | --- | --- | --- | --- | --- |
| R.LM(+15.99)GQIVSSITASLR.F | Y | 58.7 | 1490.8126 | 14 | -0.92 | 746.4095 | 2 | 18.62 | 1.0331 | 1.0403 | 63020 | 2.09e2 | 1 | 1 | 230 | 243 | Oxidation (M) | M2:Oxidation (M):1000 | DB Search |
| R.RNLDIERPTYTNLNR.L | Y | 58.31 | 1873.9758 | 15 | 3.94 | 625.6655 | 3 | 14.03 | 0.8846 | 0.8919 | 38584 | 6.19e2 | 1 | 1 | 215 | 229 |  |  | DB Search |
| R.IHFPLATYSPVISAEK.A | Y | 50.18 | 1771.9508 | 16 | 1.46 | 886.98 | 2 | 18.50 | 1.1338 | 1.141 | 62412 | 0 | 0 | 0 | 265 | 280 |  |  | DB Search |
| total 3 peptides |  |  |  |  |  |  |  |  |  |  |  |  |  |  |  |  |  |  |  |
| --- | --- | --- | --- | --- | --- | --- | --- | --- | --- | --- | --- | --- | --- | --- | --- | --- | --- | --- | --- |

Best Unique PSM (Scan Sample\_8\_A1\_1\_3645.d:63020, m/z=746.4095, z=2, RT=18.62, ppm=-5.44):


TRINITY\_DN158421\_c0\_g1\_i1.p1

back to list

  

| Protein Coverage
| Supporting Peptides
| Best Unique PSM
|

Protein Coverage:

Supporting Peptides:

| Peptide | Uniq | -10lgP | Mass | Length | ppm | m/z | z | RT | 1/k0 Start | 1/k0 End | Precursor ID | Area Sample 9 | #Feature | #Feature Sample 9 | Start | End | PTM | AScore | Found By |
| --- | --- | --- | --- | --- | --- | --- | --- | --- | --- | --- | --- | --- | --- | --- | --- | --- | --- | --- | --- |
| R.SSC(+57.02)RPILSVIK.I | Y | 16.12 | 1258.7067 | 11 | -9.52 | 630.3518 | 2 | 16.77 | 0.9687 | 0.9759 | 52596 | 1.93e3 | 1 | 1 | 90 | 100 | Carbamidomethylation |  | DB Search |
| total 1 peptides |  |  |  |  |  |  |  |  |  |  |  |  |  |  |  |  |  |  |  |
| --- | --- | --- | --- | --- | --- | --- | --- | --- | --- | --- | --- | --- | --- | --- | --- | --- | --- | --- | --- |

Best Unique PSM (Scan Sample\_8\_A1\_1\_3645.d:52596, m/z=630.3518, z=2, RT=16.77, ppm=-14.03):


TRINITY\_DN3036\_c0\_g1\_i13.p1

back to list

  

| Protein Coverage
| Supporting Peptides
| Best Unique PSM
|

Protein Coverage:

Supporting Peptides:

| Peptide | Uniq | -10lgP | Mass | Length | ppm | m/z | z | RT | 1/k0 Start | 1/k0 End | Precursor ID | Area Sample 9 | #Feature | #Feature Sample 9 | Start | End | PTM | AScore | Found By |
| --- | --- | --- | --- | --- | --- | --- | --- | --- | --- | --- | --- | --- | --- | --- | --- | --- | --- | --- | --- |
| R.VIGASGIHVGTM(+15.99)SFGK.M | Y | 60.06 | 1575.8079 | 16 | 1.08 | 788.9085 | 2 | 15.08 | 1.125 | 1.1322 | 44382 | 0 | 0 | 0 | 160 | 175 | Oxidation (M) | M12:Oxidation (M):1000 | DB Search |
| total 1 peptides |  |  |  |  |  |  |  |  |  |  |  |  |  |  |  |  |  |  |  |
| --- | --- | --- | --- | --- | --- | --- | --- | --- | --- | --- | --- | --- | --- | --- | --- | --- | --- | --- | --- |

Best Unique PSM (Scan Sample\_8\_A1\_1\_3645.d:44382, m/z=788.9085, z=2, RT=15.08, ppm=-3.43):


TRINITY\_DN3986\_c0\_g1\_i1.p1

back to list

  

| Protein Coverage
| Supporting Peptides
| Best Unique PSM
|

Protein Coverage:

Supporting Peptides:

| Peptide | Uniq | -10lgP | Mass | Length | ppm | m/z | z | RT | 1/k0 Start | 1/k0 End | Precursor ID | Area Sample 9 | #Feature | #Feature Sample 9 | Start | End | PTM | AScore | Found By |
| --- | --- | --- | --- | --- | --- | --- | --- | --- | --- | --- | --- | --- | --- | --- | --- | --- | --- | --- | --- |
| K.NSSYFVEWIPNNVK.T | Y | 61.49 | 1695.8256 | 14 | 2 | 848.918 | 2 | 19.90 | 1.0889 | 1.0961 | 70553 | 0 | 0 | 0 | 337 | 350 |  |  | DB Search |
| K.M(+15.99)SATFIGNSTAIQELFKR.I | Y | 57.02 | 2029.0302 | 18 | 1.03 | 677.3483 | 3 | 19.94 | 0.9327 | 0.94 | 70612 | 2.26e3 | 1 | 1 | 363 | 380 | Oxidation (M) | M1:Oxidation (M):1000 | DB Search |
| K.NM(+15.99)M(+15.99)AAC(+57.02)DPR.H | Y | 54.81 | 1096.41 | 9 | 2.42 | 549.2111 | 2 | 9.55 | 0.8644 | 0.8716 | 11767 | 3.1e3 | 1 | 1 | 298 | 306 | Oxidation (M), Oxidation (M), Carbamidomethylation | M2:Oxidation (M):1000 M3:Oxidation (M):1000 | DB Search |
| R.FPGQLNADLRK.L | Y | 54.24 | 1257.6829 | 11 | 0.84 | 629.8464 | 2 | 14.26 | 0.9473 | 0.9545 | 39914 | 4.37e2 | 1 | 1 | 242 | 252 |  |  | DB Search |
| K.IREEYPDR.I | Y | 53.76 | 1076.525 | 8 | 2.66 | 539.2688 | 2 | 10.94 | 0.8652 | 0.8725 | 20330 | 1.83e3 | 1 | 1 | 155 | 162 |  |  | DB Search |
| total 5 peptides |  |  |  |  |  |  |  |  |  |  |  |  |  |  |  |  |  |  |  |
| --- | --- | --- | --- | --- | --- | --- | --- | --- | --- | --- | --- | --- | --- | --- | --- | --- | --- | --- | --- |

Best Unique PSM (Scan Sample\_8\_A1\_1\_3645.d:70553, m/z=848.918, z=2, RT=19.90, ppm=-2.51):


TRINITY\_DN761\_c0\_g1\_i1.p1

back to list

  

| Protein Coverage
| Supporting Peptides
| Best Unique PSM
|

Protein Coverage:

Supporting Peptides:

| Peptide | Uniq | -10lgP | Mass | Length | ppm | m/z | z | RT | 1/k0 Start | 1/k0 End | Precursor ID | Area Sample 9 | #Feature | #Feature Sample 9 | Start | End | PTM | AScore | Found By |
| --- | --- | --- | --- | --- | --- | --- | --- | --- | --- | --- | --- | --- | --- | --- | --- | --- | --- | --- | --- |
| K.TRIEDLETVISQTQDHR.H | Y | 57.13 | 2040.0236 | 17 | 0.12 | 681.0121 | 3 | 17.33 | 0.9586 | 0.9658 | 55627 | 6.48e2 | 1 | 1 | 260 | 276 |  |  | DB Search |
| total 1 peptides |  |  |  |  |  |  |  |  |  |  |  |  |  |  |  |  |  |  |  |
| --- | --- | --- | --- | --- | --- | --- | --- | --- | --- | --- | --- | --- | --- | --- | --- | --- | --- | --- | --- |

Best Unique PSM (Scan Sample\_8\_A1\_1\_3645.d:55627, m/z=681.0121, z=3, RT=17.33, ppm=-4.39):


TRINITY\_DN1058\_c0\_g1\_i3.p1

back to list

  

| Protein Coverage
| Supporting Peptides
| Best Unique PSM
|

Protein Coverage:

Supporting Peptides:

| Peptide | Uniq | -10lgP | Mass | Length | ppm | m/z | z | RT | 1/k0 Start | 1/k0 End | Precursor ID | Area Sample 9 | #Feature | #Feature Sample 9 | Start | End | PTM | AScore | Found By |
| --- | --- | --- | --- | --- | --- | --- | --- | --- | --- | --- | --- | --- | --- | --- | --- | --- | --- | --- | --- |
| R.APTHILNPANKR.S | Y | 57.39 | 1330.7469 | 12 | 0.47 | 666.3781 | 2 | 11.10 | 1.0102 | 1.0174 | 21164 | 1.88e3 | 2 | 2 | 2314 | 2325 |  |  | DB Search |
| total 1 peptides |  |  |  |  |  |  |  |  |  |  |  |  |  |  |  |  |  |  |  |
| --- | --- | --- | --- | --- | --- | --- | --- | --- | --- | --- | --- | --- | --- | --- | --- | --- | --- | --- | --- |

Best Unique PSM (Scan Sample\_8\_A1\_1\_3645.d:21164, m/z=666.3781, z=2, RT=11.10, ppm=-4.05):


TRINITY\_DN10890\_c0\_g1\_i1.p1

back to list

  

| Protein Coverage
| Supporting Peptides
| Best Unique PSM
|

Protein Coverage:

Supporting Peptides:

| Peptide | Uniq | -10lgP | Mass | Length | ppm | m/z | z | RT | 1/k0 Start | 1/k0 End | Precursor ID | Area Sample 9 | #Feature | #Feature Sample 9 | Start | End | PTM | AScore | Found By |
| --- | --- | --- | --- | --- | --- | --- | --- | --- | --- | --- | --- | --- | --- | --- | --- | --- | --- | --- | --- |
| R.FASTVEPQKGTTIER.N | Y | 54.39 | 1662.8577 | 15 | 5.23 | 832.4367 | 2 | 12.67 | 1.1214 | 1.1286 | 30969 | 0 | 0 | 0 | 202 | 216 |  |  | DB Search |
| total 1 peptides |  |  |  |  |  |  |  |  |  |  |  |  |  |  |  |  |  |  |  |
| --- | --- | --- | --- | --- | --- | --- | --- | --- | --- | --- | --- | --- | --- | --- | --- | --- | --- | --- | --- |

Best Unique PSM (Scan Sample\_8\_A1\_1\_3645.d:30969, m/z=832.4367, z=2, RT=12.67, ppm=0.72):


TRINITY\_DN3052\_c0\_g1\_i1.p1

back to list

  

| Protein Coverage
| Supporting Peptides
| Best Unique PSM
|

Protein Coverage:

Supporting Peptides:

| Peptide | Uniq | -10lgP | Mass | Length | ppm | m/z | z | RT | 1/k0 Start | 1/k0 End | Precursor ID | Area Sample 9 | #Feature | #Feature Sample 9 | Start | End | PTM | AScore | Found By |
| --- | --- | --- | --- | --- | --- | --- | --- | --- | --- | --- | --- | --- | --- | --- | --- | --- | --- | --- | --- |
| M.A(+42.01)HLSQEQRNELKK.N | Y | 57.81 | 1621.8536 | 13 | -0.15 | 541.6226 | 3 | 11.20 | 0.8096 | 0.8169 | 22070 | 0 | 0 | 0 | 2 | 14 | Acetylation (Protein N-term) | A1:Acetylation (Protein N-term):1000 | DB Search |
| total 1 peptides |  |  |  |  |  |  |  |  |  |  |  |  |  |  |  |  |  |  |  |
| --- | --- | --- | --- | --- | --- | --- | --- | --- | --- | --- | --- | --- | --- | --- | --- | --- | --- | --- | --- |

Best Unique PSM (Scan Sample\_8\_A1\_1\_3645.d:22070, m/z=541.6226, z=3, RT=11.20, ppm=-4.66):


TRINITY\_DN36\_c0\_g1\_i20.p1

back to list

  

| Protein Coverage
| Supporting Peptides
| Best Unique PSM
|

Protein Coverage:

Supporting Peptides:

| Peptide | Uniq | -10lgP | Mass | Length | ppm | m/z | z | RT | 1/k0 Start | 1/k0 End | Precursor ID | Area Sample 9 | #Feature | #Feature Sample 9 | Start | End | PTM | AScore | Found By |
| --- | --- | --- | --- | --- | --- | --- | --- | --- | --- | --- | --- | --- | --- | --- | --- | --- | --- | --- | --- |
| K.YGQAM(+15.99)C(+57.02)QFM(+15.99)R.N | N | 61.54 | 1322.5206 | 10 | -1.76 | 662.2634 | 2 | 12.17 | 0.9489 | 0.9562 | 28008 | 2.07e2 | 1 | 1 | 71 | 80 | Oxidation (M), Carbamidomethylation, Oxidation (M) | M5:Oxidation (M):1000 M9:Oxidation (M):1000 | DB Search |
| K.DVYDAEQAERK.R | N | 57.85 | 1322.6102 | 11 | -1.22 | 662.3086 | 2 | 11.56 | 0.9606 | 0.9679 | 24244 | 0 | 0 | 0 | 102 | 112 |  |  | DB Search |
| R.LGGIEVTQGNC(+57.02)AR.K | N | 57.3 | 1373.6721 | 13 | 3.87 | 687.8429 | 2 | 13.70 | 0.9642 | 0.9715 | 36854 | 7.25e2 | 1 | 1 | 57 | 69 | Carbamidomethylation |  | DB Search |
| K.GFDYC(+57.02)TTIKYR.L | N | 56.39 | 1422.6602 | 11 | -1.35 | 712.3332 | 2 | 15.06 | 1.0234 | 1.0307 | 44297 | 0 | 0 | 0 | 46 | 56 | Carbamidomethylation |  | DB Search |
| R.HNIDDC(+57.02)K.K | Y | 35.46 | 900.376 | 7 | 9.13 | 451.1973 | 2 | 10.19 | 0.8027 | 0.81 | 15877 | 0 | 0 | 0 | 189 | 195 | Carbamidomethylation |  | DB Search |
| total 5 peptides |  |  |  |  |  |  |  |  |  |  |  |  |  |  |  |  |  |  |  |
| --- | --- | --- | --- | --- | --- | --- | --- | --- | --- | --- | --- | --- | --- | --- | --- | --- | --- | --- | --- |

Best Unique PSM (Scan Sample\_8\_A1\_1\_3645.d:15877, m/z=451.1973, z=2, RT=10.19, ppm=4.61):


TRINITY\_DN3036\_c0\_g1\_i4.p1

back to list

  

| Protein Coverage
| Supporting Peptides
| Best Unique PSM
|

Protein Coverage:

Supporting Peptides:

| Peptide | Uniq | -10lgP | Mass | Length | ppm | m/z | z | RT | 1/k0 Start | 1/k0 End | Precursor ID | Area Sample 9 | #Feature | #Feature Sample 9 | Start | End | PTM | AScore | Found By |
| --- | --- | --- | --- | --- | --- | --- | --- | --- | --- | --- | --- | --- | --- | --- | --- | --- | --- | --- | --- |
| R.VIGASGIHVGTM(+15.99)SFGK.M | Y | 60.06 | 1575.8079 | 16 | 1.08 | 788.9085 | 2 | 15.08 | 1.125 | 1.1322 | 44382 | 0 | 0 | 0 | 24 | 39 | Oxidation (M) | M12:Oxidation (M):1000 | DB Search |
| total 1 peptides |  |  |  |  |  |  |  |  |  |  |  |  |  |  |  |  |  |  |  |
| --- | --- | --- | --- | --- | --- | --- | --- | --- | --- | --- | --- | --- | --- | --- | --- | --- | --- | --- | --- |

Best Unique PSM (Scan Sample\_8\_A1\_1\_3645.d:44382, m/z=788.9085, z=2, RT=15.08, ppm=-3.43):


TRINITY\_DN5633\_c0\_g1\_i1.p1

back to list

  

| Protein Coverage
| Supporting Peptides
| Best Unique PSM
|

Protein Coverage:

Supporting Peptides:

| Peptide | Uniq | -10lgP | Mass | Length | ppm | m/z | z | RT | 1/k0 Start | 1/k0 End | Precursor ID | Area Sample 9 | #Feature | #Feature Sample 9 | Start | End | PTM | AScore | Found By |
| --- | --- | --- | --- | --- | --- | --- | --- | --- | --- | --- | --- | --- | --- | --- | --- | --- | --- | --- | --- |
| K.KLLEINVYHPLIQELQKR.V | Y | 58.01 | 2233.2946 | 18 | 2.88 | 559.33 | 4 | 17.89 | 0.8668 | 0.8741 | 58878 | 0 | 0 | 0 | 705 | 722 |  |  | DB Search |
| K.LGVM(+15.99)EDHSNR.T | Y | 56.99 | 1172.5244 | 10 | 4.82 | 587.2697 | 2 | 10.32 | 0.9065 | 0.9137 | 16467 | 1.29e3 | 1 | 1 | 516 | 525 | Oxidation (M) | M4:Oxidation (M):1000 | DB Search |
| K.KIPDEDYIEKFWK.E | Y | 56.85 | 1709.8664 | 13 | -1.13 | 570.9595 | 3 | 18.50 | 0.8587 | 0.866 | 62447 | 0 | 0 | 0 | 496 | 508 |  |  | DB Search |
| total 3 peptides |  |  |  |  |  |  |  |  |  |  |  |  |  |  |  |  |  |  |  |
| --- | --- | --- | --- | --- | --- | --- | --- | --- | --- | --- | --- | --- | --- | --- | --- | --- | --- | --- | --- |

Best Unique PSM (Scan Sample\_8\_A1\_1\_3645.d:58878, m/z=559.33, z=4, RT=17.89, ppm=-1.64):


TRINITY\_DN2477\_c0\_g1\_i10.p1

back to list

  

| Protein Coverage
| Supporting Peptides
| Best Unique PSM
|

Protein Coverage:

Supporting Peptides:

| Peptide | Uniq | -10lgP | Mass | Length | ppm | m/z | z | RT | 1/k0 Start | 1/k0 End | Precursor ID | Area Sample 9 | #Feature | #Feature Sample 9 | Start | End | PTM | AScore | Found By |
| --- | --- | --- | --- | --- | --- | --- | --- | --- | --- | --- | --- | --- | --- | --- | --- | --- | --- | --- | --- |
| K.DSYVGDEAQSKR.G | Y | 58.12 | 1353.616 | 12 | 4.44 | 677.8152 | 2 | 10.93 | 1.0065 | 1.0138 | 20214 | 8.61e3 | 1 | 1 | 19 | 30 |  |  | DB Search |
| R.VAPEEHPVLLTEAPLNPK.A | Y | 57.43 | 1953.0571 | 18 | 1.47 | 977.5328 | 2 | 16.35 | 1.2073 | 1.2145 | 50611 | 1.52e2 | 1 | 1 | 64 | 81 |  |  | DB Search |
| R.GYSFTTTAEREIVR.D | Y | 56.59 | 1628.8158 | 14 | 3.67 | 815.4145 | 2 | 15.95 | 1.119 | 1.1262 | 48654 | 3.46e3 | 1 | 1 | 165 | 178 |  |  | DB Search |
| total 3 peptides |  |  |  |  |  |  |  |  |  |  |  |  |  |  |  |  |  |  |  |
| --- | --- | --- | --- | --- | --- | --- | --- | --- | --- | --- | --- | --- | --- | --- | --- | --- | --- | --- | --- |

Best Unique PSM (Scan Sample\_8\_A1\_1\_3645.d:20214, m/z=677.8152, z=2, RT=10.93, ppm=-0.08):


TRINITY\_DN161\_c0\_g1\_i1.p1

back to list

  

| Protein Coverage
| Supporting Peptides
| Best Unique PSM
|

Protein Coverage:

Supporting Peptides:

| Peptide | Uniq | -10lgP | Mass | Length | ppm | m/z | z | RT | 1/k0 Start | 1/k0 End | Precursor ID | Area Sample 9 | #Feature | #Feature Sample 9 | Start | End | PTM | AScore | Found By |
| --- | --- | --- | --- | --- | --- | --- | --- | --- | --- | --- | --- | --- | --- | --- | --- | --- | --- | --- | --- |
| R.LDLAGRDLTDYLMK.I | Y | 60.93 | 1622.8338 | 14 | 1.03 | 812.4213 | 2 | 19.24 | 1.0965 | 1.1037 | 66626 | 2.72e3 | 2 | 2 | 179 | 192 |  |  | DB Search |
| R.LDLAGRDLTDYLM(+15.99)K.I | Y | 59.01 | 1638.8287 | 14 | 2.41 | 547.2823 | 3 | 18.13 | 0.7856 | 0.7929 | 60339 | 2.36e3 | 1 | 1 | 179 | 192 | Oxidation (M) | M13:Oxidation (M):1000 | DB Search |
| K.DSYVGDEAQSKR.G | Y | 58.12 | 1353.616 | 12 | 4.44 | 677.8152 | 2 | 10.93 | 1.0065 | 1.0138 | 20214 | 8.61e3 | 1 | 1 | 52 | 63 |  |  | DB Search |
| K.DLYANTVLSGGTTM(+15.99)FPGIADR.M | Y | 57.77 | 2214.0626 | 21 | 5.59 | 1108.0398 | 2 | 19.43 | 1.2368 | 1.2439 | 67881 | 0 | 0 | 0 | 293 | 313 | Oxidation (M) | M14:Oxidation (M):1000 | DB Search |
| R.VAPEEHPVLLTEAPLNPK.A | Y | 57.43 | 1953.0571 | 18 | 1.47 | 977.5328 | 2 | 16.35 | 1.2073 | 1.2145 | 50611 | 1.52e2 | 1 | 1 | 97 | 114 |  |  | DB Search |
| K.SYELPDGQVITIGNER.F | Y | 57.34 | 1789.8846 | 16 | 2.2 | 895.9475 | 2 | 18.50 | 0.9828 | 0.99 | 62421 | 0 | 0 | 0 | 240 | 255 |  |  | DB Search |
| R.GYSFTTTAEREIVR.D | Y | 56.59 | 1628.8158 | 14 | 3.67 | 815.4145 | 2 | 15.95 | 1.119 | 1.1262 | 48654 | 3.46e3 | 1 | 1 | 198 | 211 |  |  | DB Search |
| R.AVFPSIVGRPR.H | Y | 56.57 | 1197.6982 | 11 | 1.58 | 599.8546 | 2 | 15.61 | 0.9578 | 0.965 | 46597 | 1.24e4 | 1 | 1 | 30 | 40 |  |  | DB Search |
| R.HQGVM(+15.99)VGM(+15.99)GQK.D | Y | 19.26 | 1202.5536 | 11 | 3.57 | 602.2835 | 2 | 9.78 | 0.9154 | 0.9226 | 13224 | 5.97e2 | 1 | 1 | 41 | 51 | Oxidation (M), Oxidation (M) | M5:Oxidation (M):1000 M8:Oxidation (M):1000 | DB Search |
| total 9 peptides |  |  |  |  |  |  |  |  |  |  |  |  |  |  |  |  |  |  |  |
| --- | --- | --- | --- | --- | --- | --- | --- | --- | --- | --- | --- | --- | --- | --- | --- | --- | --- | --- | --- |

Best Unique PSM (Scan Sample\_8\_A1\_1\_3645.d:66626, m/z=812.4213, z=2, RT=19.24, ppm=-3.49):


TRINITY\_DN3078\_c0\_g1\_i1.p1

back to list

  

| Protein Coverage
| Supporting Peptides
| Best Unique PSM
|

Protein Coverage:

Supporting Peptides:

| Peptide | Uniq | -10lgP | Mass | Length | ppm | m/z | z | RT | 1/k0 Start | 1/k0 End | Precursor ID | Area Sample 9 | #Feature | #Feature Sample 9 | Start | End | PTM | AScore | Found By |
| --- | --- | --- | --- | --- | --- | --- | --- | --- | --- | --- | --- | --- | --- | --- | --- | --- | --- | --- | --- |
| K.AKIEALHC(+57.02)LSENIVK.D | Y | 60.23 | 1723.9291 | 15 | 3.59 | 862.971 | 2 | 15.53 | 1.1326 | 1.1398 | 46701 | 0 | 0 | 0 | 709 | 723 | Carbamidomethylation |  | DB Search |
| K.VSQTC(+57.02)AEHVFR.V | Y | 41.51 | 1332.6245 | 11 | -1.96 | 667.3152 | 2 | 12.69 | 0.9872 | 0.9945 | 31058 | 0 | 0 | 0 | 547 | 557 | Carbamidomethylation |  | DB Search |
| total 2 peptides |  |  |  |  |  |  |  |  |  |  |  |  |  |  |  |  |  |  |  |
| --- | --- | --- | --- | --- | --- | --- | --- | --- | --- | --- | --- | --- | --- | --- | --- | --- | --- | --- | --- |

Best Unique PSM (Scan Sample\_8\_A1\_1\_3645.d:46701, m/z=862.971, z=2, RT=15.53, ppm=-0.92):


TRINITY\_DN118520\_c0\_g1\_i1.p1

back to list

  

| Protein Coverage
| Supporting Peptides
| Best Unique PSM
|

Protein Coverage:

Supporting Peptides:

| Peptide | Uniq | -10lgP | Mass | Length | ppm | m/z | z | RT | 1/k0 Start | 1/k0 End | Precursor ID | Area Sample 9 | #Feature | #Feature Sample 9 | Start | End | PTM | AScore | Found By |
| --- | --- | --- | --- | --- | --- | --- | --- | --- | --- | --- | --- | --- | --- | --- | --- | --- | --- | --- | --- |
| K.ISNVQDIVPVLELANQHK.R | Y | 58.72 | 2016.1003 | 18 | 0.02 | 1009.0529 | 2 | 19.57 | 1.1893 | 1.1965 | 68604 | 2.2e3 | 1 | 1 | 258 | 275 |  |  | DB Search |
| K.ATNSDYEREKFQER.L | Y | 30.07 | 1771.8125 | 14 | -6.26 | 886.904 | 2 | 12.43 | 1.0829 | 1.0901 | 29559 | 0 | 0 | 0 | 386 | 399 |  |  | DB Search |
| total 2 peptides |  |  |  |  |  |  |  |  |  |  |  |  |  |  |  |  |  |  |  |
| --- | --- | --- | --- | --- | --- | --- | --- | --- | --- | --- | --- | --- | --- | --- | --- | --- | --- | --- | --- |

Best Unique PSM (Scan Sample\_8\_A1\_1\_3645.d:68604, m/z=1009.0529, z=2, RT=19.57, ppm=-4.5):


TRINITY\_DN8101\_c0\_g1\_i6.p2

back to list

  

| Protein Coverage
| Supporting Peptides
| Best Unique PSM
|

Protein Coverage:

Supporting Peptides:

| Peptide | Uniq | -10lgP | Mass | Length | ppm | m/z | z | RT | 1/k0 Start | 1/k0 End | Precursor ID | Area Sample 9 | #Feature | #Feature Sample 9 | Start | End | PTM | AScore | Found By |
| --- | --- | --- | --- | --- | --- | --- | --- | --- | --- | --- | --- | --- | --- | --- | --- | --- | --- | --- | --- |
| R.SIGFIGHYLDQLR.L | Y | 55.88 | 1517.799 | 13 | -0.14 | 759.9033 | 2 | 19.91 | 1.0849 | 1.0921 | 70505 | 4.59e2 | 1 | 1 | 784 | 796 |  |  | DB Search |
| total 1 peptides |  |  |  |  |  |  |  |  |  |  |  |  |  |  |  |  |  |  |  |
| --- | --- | --- | --- | --- | --- | --- | --- | --- | --- | --- | --- | --- | --- | --- | --- | --- | --- | --- | --- |

Best Unique PSM (Scan Sample\_8\_A1\_1\_3645.d:70505, m/z=759.9033, z=2, RT=19.91, ppm=-4.65):


TRINITY\_DN554\_c0\_g1\_i5.p1

back to list

  

| Protein Coverage
| Supporting Peptides
| Best Unique PSM
|

Protein Coverage:

Supporting Peptides:

| Peptide | Uniq | -10lgP | Mass | Length | ppm | m/z | z | RT | 1/k0 Start | 1/k0 End | Precursor ID | Area Sample 9 | #Feature | #Feature Sample 9 | Start | End | PTM | AScore | Found By |
| --- | --- | --- | --- | --- | --- | --- | --- | --- | --- | --- | --- | --- | --- | --- | --- | --- | --- | --- | --- |
| K.IQDKEGIPPDQQR.L | Y | 61.25 | 1522.7739 | 13 | -1.84 | 762.3894 | 2 | 10.99 | 1.0371 | 1.0443 | 20319 | 2.55e3 | 1 | 1 | 30 | 42 |  |  | DB Search |
| total 1 peptides |  |  |  |  |  |  |  |  |  |  |  |  |  |  |  |  |  |  |  |
| --- | --- | --- | --- | --- | --- | --- | --- | --- | --- | --- | --- | --- | --- | --- | --- | --- | --- | --- | --- |

Best Unique PSM (Scan Sample\_8\_A1\_1\_3645.d:20319, m/z=762.3894, z=2, RT=10.99, ppm=-6.36):


TRINITY\_DN21847\_c0\_g1\_i1.p1

back to list

  

| Protein Coverage
| Supporting Peptides
| Best Unique PSM
|

Protein Coverage:

Supporting Peptides:

| Peptide | Uniq | -10lgP | Mass | Length | ppm | m/z | z | RT | 1/k0 Start | 1/k0 End | Precursor ID | Area Sample 9 | #Feature | #Feature Sample 9 | Start | End | PTM | AScore | Found By |
| --- | --- | --- | --- | --- | --- | --- | --- | --- | --- | --- | --- | --- | --- | --- | --- | --- | --- | --- | --- |
| K.VLTSSDPAEKEK.K | Y | 44.75 | 1302.6667 | 12 | 5.11 | 652.341 | 2 | 10.57 | 1.0001 | 1.0073 | 18232 | 0 | 0 | 0 | 101 | 112 |  |  | DB Search |
| total 1 peptides |  |  |  |  |  |  |  |  |  |  |  |  |  |  |  |  |  |  |  |
| --- | --- | --- | --- | --- | --- | --- | --- | --- | --- | --- | --- | --- | --- | --- | --- | --- | --- | --- | --- |

Best Unique PSM (Scan Sample\_8\_A1\_1\_3645.d:18232, m/z=652.341, z=2, RT=10.57, ppm=0.6):


TRINITY\_DN1080\_c0\_g2\_i1.p1

back to list

  

| Protein Coverage
| Supporting Peptides
| Best Unique PSM
|

Protein Coverage:

Supporting Peptides:

| Peptide | Uniq | -10lgP | Mass | Length | ppm | m/z | z | RT | 1/k0 Start | 1/k0 End | Precursor ID | Area Sample 9 | #Feature | #Feature Sample 9 | Start | End | PTM | AScore | Found By |
| --- | --- | --- | --- | --- | --- | --- | --- | --- | --- | --- | --- | --- | --- | --- | --- | --- | --- | --- | --- |
| K.LSQDTTDKLNR.S | Y | 17.1 | 1289.6575 | 11 | 2.65 | 645.8348 | 2 | 10.94 | 0.9594 | 0.9666 | 20471 | 0 | 0 | 0 | 7589 | 7599 |  |  | DB Search |
| total 1 peptides |  |  |  |  |  |  |  |  |  |  |  |  |  |  |  |  |  |  |  |
| --- | --- | --- | --- | --- | --- | --- | --- | --- | --- | --- | --- | --- | --- | --- | --- | --- | --- | --- | --- |

Best Unique PSM (Scan Sample\_8\_A1\_1\_3645.d:20471, m/z=645.8348, z=2, RT=10.94, ppm=-1.86):


TRINITY\_DN1936\_c0\_g2\_i1.p1

back to list

  

| Protein Coverage
| Supporting Peptides
| Best Unique PSM
|

Protein Coverage:

Supporting Peptides:

| Peptide | Uniq | -10lgP | Mass | Length | ppm | m/z | z | RT | 1/k0 Start | 1/k0 End | Precursor ID | Area Sample 9 | #Feature | #Feature Sample 9 | Start | End | PTM | AScore | Found By |
| --- | --- | --- | --- | --- | --- | --- | --- | --- | --- | --- | --- | --- | --- | --- | --- | --- | --- | --- | --- |
| K.GTTSTAGTGPSFDHTK.G | Y | 57.07 | 1563.7165 | 16 | 3.76 | 782.8649 | 2 | 11.35 | 1.0737 | 1.0809 | 22963 | 0 | 0 | 0 | 636 | 651 |  |  | DB Search |
| K.LTGQQQITEKDPWK.Y | Y | 49.81 | 1670.8628 | 14 | 4.45 | 836.4386 | 2 | 14.39 | 1.0777 | 1.0849 | 40541 | 4.07e2 | 1 | 1 | 1053 | 1066 |  |  | DB Search |
| total 2 peptides |  |  |  |  |  |  |  |  |  |  |  |  |  |  |  |  |  |  |  |
| --- | --- | --- | --- | --- | --- | --- | --- | --- | --- | --- | --- | --- | --- | --- | --- | --- | --- | --- | --- |

Best Unique PSM (Scan Sample\_8\_A1\_1\_3645.d:22963, m/z=782.8649, z=2, RT=11.35, ppm=-0.76):


TRINITY\_DN165536\_c0\_g1\_i1.p1

back to list

  

| Protein Coverage
| Supporting Peptides
| Best Unique PSM
|

Protein Coverage:

Supporting Peptides:

| Peptide | Uniq | -10lgP | Mass | Length | ppm | m/z | z | RT | 1/k0 Start | 1/k0 End | Precursor ID | Area Sample 9 | #Feature | #Feature Sample 9 | Start | End | PTM | AScore | Found By |
| --- | --- | --- | --- | --- | --- | --- | --- | --- | --- | --- | --- | --- | --- | --- | --- | --- | --- | --- | --- |
| K.KGNEGGNTELISSYRK.E | Y | 61.06 | 1751.8802 | 16 | -1.22 | 584.964 | 3 | 12.34 | 0.8753 | 0.8826 | 29059 | 0 | 0 | 0 | 73 | 88 |  |  | DB Search |
| K.ATETATGLATTHPIK.L | Y | 56.85 | 1510.7991 | 15 | 5.62 | 756.4077 | 2 | 12.87 | 1.1049 | 1.1122 | 31961 | 6.84e3 | 1 | 1 | 161 | 175 |  |  | DB Search |
| total 2 peptides |  |  |  |  |  |  |  |  |  |  |  |  |  |  |  |  |  |  |  |
| --- | --- | --- | --- | --- | --- | --- | --- | --- | --- | --- | --- | --- | --- | --- | --- | --- | --- | --- | --- |

Best Unique PSM (Scan Sample\_8\_A1\_1\_3645.d:29059, m/z=584.964, z=3, RT=12.34, ppm=-5.74):


TRINITY\_DN5153\_c0\_g1\_i1.p1

back to list

  

| Protein Coverage
| Supporting Peptides
| Best Unique PSM
|

Protein Coverage:

Supporting Peptides:

| Peptide | Uniq | -10lgP | Mass | Length | ppm | m/z | z | RT | 1/k0 Start | 1/k0 End | Precursor ID | Area Sample 9 | #Feature | #Feature Sample 9 | Start | End | PTM | AScore | Found By |
| --- | --- | --- | --- | --- | --- | --- | --- | --- | --- | --- | --- | --- | --- | --- | --- | --- | --- | --- | --- |
| R.ALELDALSNSHPIEVK.V | Y | 56.84 | 1734.9152 | 16 | -3.95 | 868.4575 | 2 | 17.15 | 1.1706 | 1.1778 | 54576 | 2.79e2 | 1 | 1 | 362 | 377 |  |  | DB Search |
| total 1 peptides |  |  |  |  |  |  |  |  |  |  |  |  |  |  |  |  |  |  |  |
| --- | --- | --- | --- | --- | --- | --- | --- | --- | --- | --- | --- | --- | --- | --- | --- | --- | --- | --- | --- |

Best Unique PSM (Scan Sample\_8\_A1\_1\_3645.d:54576, m/z=868.4575, z=2, RT=17.15, ppm=-8.46):


TRINITY\_DN244\_c0\_g1\_i1.p1

back to list

  

| Protein Coverage
| Supporting Peptides
| Best Unique PSM
|

Protein Coverage:

Supporting Peptides:

| Peptide | Uniq | -10lgP | Mass | Length | ppm | m/z | z | RT | 1/k0 Start | 1/k0 End | Precursor ID | Area Sample 9 | #Feature | #Feature Sample 9 | Start | End | PTM | AScore | Found By |
| --- | --- | --- | --- | --- | --- | --- | --- | --- | --- | --- | --- | --- | --- | --- | --- | --- | --- | --- | --- |
| K.DLSLVVEPLKNYGVK.I | Y | 58.88 | 1672.9399 | 15 | 2.46 | 837.4755 | 2 | 19.03 | 1.1358 | 1.143 | 65463 | 6.76e2 | 1 | 1 | 392 | 406 |  |  | DB Search |
| total 1 peptides |  |  |  |  |  |  |  |  |  |  |  |  |  |  |  |  |  |  |  |
| --- | --- | --- | --- | --- | --- | --- | --- | --- | --- | --- | --- | --- | --- | --- | --- | --- | --- | --- | --- |

Best Unique PSM (Scan Sample\_8\_A1\_1\_3645.d:65463, m/z=837.4755, z=2, RT=19.03, ppm=-2.05):


TRINITY\_DN6920\_c0\_g1\_i4.p1

back to list

  

| Protein Coverage
| Supporting Peptides
| Best Unique PSM
|

Protein Coverage:

Supporting Peptides:

| Peptide | Uniq | -10lgP | Mass | Length | ppm | m/z | z | RT | 1/k0 Start | 1/k0 End | Precursor ID | Area Sample 9 | #Feature | #Feature Sample 9 | Start | End | PTM | AScore | Found By |
| --- | --- | --- | --- | --- | --- | --- | --- | --- | --- | --- | --- | --- | --- | --- | --- | --- | --- | --- | --- |
| K.VAC(+57.02)IGAWHPAR.V | Y | 45.85 | 1236.6186 | 11 | -2.24 | 619.3124 | 2 | 13.17 | 0.9444 | 0.9517 | 33868 | 3.39e2 | 1 | 1 | 129 | 139 | Carbamidomethylation |  | DB Search |
| total 1 peptides |  |  |  |  |  |  |  |  |  |  |  |  |  |  |  |  |  |  |  |
| --- | --- | --- | --- | --- | --- | --- | --- | --- | --- | --- | --- | --- | --- | --- | --- | --- | --- | --- | --- |

Best Unique PSM (Scan Sample\_8\_A1\_1\_3645.d:33868, m/z=619.3124, z=2, RT=13.17, ppm=-6.76):


TRINITY\_DN477\_c0\_g1\_i8.p1

back to list

  

| Protein Coverage
| Supporting Peptides
| Best Unique PSM
|

Protein Coverage:

Supporting Peptides:

| Peptide | Uniq | -10lgP | Mass | Length | ppm | m/z | z | RT | 1/k0 Start | 1/k0 End | Precursor ID | Area Sample 9 | #Feature | #Feature Sample 9 | Start | End | PTM | AScore | Found By |
| --- | --- | --- | --- | --- | --- | --- | --- | --- | --- | --- | --- | --- | --- | --- | --- | --- | --- | --- | --- |
| R.ILQC(+57.02)NQYANVEEAR.N | Y | 57.54 | 1706.8046 | 14 | 0.26 | 854.4059 | 2 | 14.18 | 1.0781 | 1.0853 | 39448 | 1.11e3 | 1 | 1 | 86 | 99 | Carbamidomethylation |  | DB Search |
| K.DKNILVLTLGVGPQVNR.N | Y | 56.87 | 1835.0628 | 17 | 6.58 | 918.5406 | 2 | 19.30 | 1.1358 | 1.143 | 67105 | 2.01e3 | 1 | 1 | 168 | 184 |  |  | DB Search |
| R.SQSAFPTRR.R | Y | 43.1 | 1048.5414 | 9 | 1.24 | 525.2762 | 2 | 10.98 | 0.8506 | 0.8579 | 20648 | 1.97e3 | 1 | 1 | 856 | 864 |  |  | DB Search |
| total 3 peptides |  |  |  |  |  |  |  |  |  |  |  |  |  |  |  |  |  |  |  |
| --- | --- | --- | --- | --- | --- | --- | --- | --- | --- | --- | --- | --- | --- | --- | --- | --- | --- | --- | --- |

Best Unique PSM (Scan Sample\_8\_A1\_1\_3645.d:39448, m/z=854.8958, z=2, RT=14.23, ppm=-4.26):


TRINITY\_DN804\_c0\_g1\_i1.p1

back to list

  

| Protein Coverage
| Supporting Peptides
| Best Unique PSM
|

Protein Coverage:

Supporting Peptides:

| Peptide | Uniq | -10lgP | Mass | Length | ppm | m/z | z | RT | 1/k0 Start | 1/k0 End | Precursor ID | Area Sample 9 | #Feature | #Feature Sample 9 | Start | End | PTM | AScore | Found By |
| --- | --- | --- | --- | --- | --- | --- | --- | --- | --- | --- | --- | --- | --- | --- | --- | --- | --- | --- | --- |
| K.RGTPLDQVIEFHIDEETLVKR.I | Y | 58 | 2494.3179 | 21 | -2.48 | 624.5824 | 4 | 18.88 | 0.8465 | 0.8538 | 64643 | 2.32e2 | 1 | 1 | 123 | 143 |  |  | DB Search |
| K.LIDSNLEKPEC(+57.02)K.N | Y | 57.09 | 1444.7232 | 12 | -0.41 | 723.3653 | 2 | 12.85 | 1.0311 | 1.0383 | 31680 | 5.58e3 | 1 | 1 | 87 | 98 | Carbamidomethylation |  | DB Search |
| K.LVDRYC(+57.02)VC(+57.02)HLATGDLLR.A | Y | 56.28 | 2060.0295 | 17 | -0.91 | 687.6801 | 3 | 16.66 | 0.9243 | 0.9315 | 52195 | 0 | 0 | 0 | 40 | 56 | Carbamidomethylation, Carbamidomethylation |  | DB Search |
| total 3 peptides |  |  |  |  |  |  |  |  |  |  |  |  |  |  |  |  |  |  |  |
| --- | --- | --- | --- | --- | --- | --- | --- | --- | --- | --- | --- | --- | --- | --- | --- | --- | --- | --- | --- |

Best Unique PSM (Scan Sample\_8\_A1\_1\_3645.d:64643, m/z=624.5824, z=4, RT=18.88, ppm=-6.99):


TRINITY\_DN4497\_c0\_g2\_i1.p1

back to list

  

| Protein Coverage
| Supporting Peptides
| Best Unique PSM
|

Protein Coverage:

Supporting Peptides:

| Peptide | Uniq | -10lgP | Mass | Length | ppm | m/z | z | RT | 1/k0 Start | 1/k0 End | Precursor ID | Area Sample 9 | #Feature | #Feature Sample 9 | Start | End | PTM | AScore | Found By |
| --- | --- | --- | --- | --- | --- | --- | --- | --- | --- | --- | --- | --- | --- | --- | --- | --- | --- | --- | --- |
| K.VLTSSDPAEKEK.K | Y | 44.75 | 1302.6667 | 12 | 5.11 | 652.341 | 2 | 10.57 | 1.0001 | 1.0073 | 18232 | 0 | 0 | 0 | 126 | 137 |  |  | DB Search |
| total 1 peptides |  |  |  |  |  |  |  |  |  |  |  |  |  |  |  |  |  |  |  |
| --- | --- | --- | --- | --- | --- | --- | --- | --- | --- | --- | --- | --- | --- | --- | --- | --- | --- | --- | --- |

Best Unique PSM (Scan Sample\_8\_A1\_1\_3645.d:18232, m/z=652.341, z=2, RT=10.57, ppm=0.6):


TRINITY\_DN794\_c0\_g1\_i2.p1

back to list

  

| Protein Coverage
| Supporting Peptides
| Best Unique PSM
|

Protein Coverage:

Supporting Peptides:

| Peptide | Uniq | -10lgP | Mass | Length | ppm | m/z | z | RT | 1/k0 Start | 1/k0 End | Precursor ID | Area Sample 9 | #Feature | #Feature Sample 9 | Start | End | PTM | AScore | Found By |
| --- | --- | --- | --- | --- | --- | --- | --- | --- | --- | --- | --- | --- | --- | --- | --- | --- | --- | --- | --- |
| R.GKVEDGLESDFRK.S | Y | 58.12 | 1478.7365 | 13 | -5.7 | 493.9144 | 3 | 13.65 | 0.7905 | 0.7978 | 36561 | 4.15e2 | 1 | 1 | 109 | 121 |  |  | DB Search |
| total 1 peptides |  |  |  |  |  |  |  |  |  |  |  |  |  |  |  |  |  |  |  |
| --- | --- | --- | --- | --- | --- | --- | --- | --- | --- | --- | --- | --- | --- | --- | --- | --- | --- | --- | --- |

Best Unique PSM (Scan Sample\_8\_A1\_1\_3645.d:36561, m/z=493.9144, z=3, RT=13.65, ppm=-10.22):


TRINITY\_DN10724\_c0\_g1\_i4.p1

back to list

  

| Protein Coverage
| Supporting Peptides
| Best Unique PSM
|

Protein Coverage:

Supporting Peptides:

| Peptide | Uniq | -10lgP | Mass | Length | ppm | m/z | z | RT | 1/k0 Start | 1/k0 End | Precursor ID | Area Sample 9 | #Feature | #Feature Sample 9 | Start | End | PTM | AScore | Found By |
| --- | --- | --- | --- | --- | --- | --- | --- | --- | --- | --- | --- | --- | --- | --- | --- | --- | --- | --- | --- |
| K.REPGDFGFDPLKFSK.R | Y | 58.72 | 1738.8678 | 15 | -1.8 | 580.6262 | 3 | 18.01 | 0.9012 | 0.9085 | 59548 | 0 | 0 | 0 | 171 | 185 |  |  | DB Search |
| total 1 peptides |  |  |  |  |  |  |  |  |  |  |  |  |  |  |  |  |  |  |  |
| --- | --- | --- | --- | --- | --- | --- | --- | --- | --- | --- | --- | --- | --- | --- | --- | --- | --- | --- | --- |

Best Unique PSM (Scan Sample\_8\_A1\_1\_3645.d:59548, m/z=580.6262, z=3, RT=18.01, ppm=-6.32):


TRINITY\_DN173718\_c0\_g1\_i1.p1

back to list

  

| Protein Coverage
| Supporting Peptides
| Best Unique PSM
|

Protein Coverage:

Supporting Peptides:

| Peptide | Uniq | -10lgP | Mass | Length | ppm | m/z | z | RT | 1/k0 Start | 1/k0 End | Precursor ID | Area Sample 9 | #Feature | #Feature Sample 9 | Start | End | PTM | AScore | Found By |
| --- | --- | --- | --- | --- | --- | --- | --- | --- | --- | --- | --- | --- | --- | --- | --- | --- | --- | --- | --- |
| K.IQDKEGIPPDQQR.L | Y | 61.25 | 1522.7739 | 13 | -1.84 | 762.3894 | 2 | 10.99 | 1.0371 | 1.0443 | 20319 | 2.55e3 | 1 | 1 | 30 | 42 |  |  | DB Search |
| total 1 peptides |  |  |  |  |  |  |  |  |  |  |  |  |  |  |  |  |  |  |  |
| --- | --- | --- | --- | --- | --- | --- | --- | --- | --- | --- | --- | --- | --- | --- | --- | --- | --- | --- | --- |

Best Unique PSM (Scan Sample\_8\_A1\_1\_3645.d:20319, m/z=762.3894, z=2, RT=10.99, ppm=-6.36):


TRINITY\_DN10724\_c0\_g1\_i1.p2

back to list

  

| Protein Coverage
| Supporting Peptides
| Best Unique PSM
|

Protein Coverage:

Supporting Peptides:

| Peptide | Uniq | -10lgP | Mass | Length | ppm | m/z | z | RT | 1/k0 Start | 1/k0 End | Precursor ID | Area Sample 9 | #Feature | #Feature Sample 9 | Start | End | PTM | AScore | Found By |
| --- | --- | --- | --- | --- | --- | --- | --- | --- | --- | --- | --- | --- | --- | --- | --- | --- | --- | --- | --- |
| K.REPGDFGFDPLKFSK.R | Y | 58.72 | 1738.8678 | 15 | -1.8 | 580.6262 | 3 | 18.01 | 0.9012 | 0.9085 | 59548 | 0 | 0 | 0 | 67 | 81 |  |  | DB Search |
| total 1 peptides |  |  |  |  |  |  |  |  |  |  |  |  |  |  |  |  |  |  |  |
| --- | --- | --- | --- | --- | --- | --- | --- | --- | --- | --- | --- | --- | --- | --- | --- | --- | --- | --- | --- |

Best Unique PSM (Scan Sample\_8\_A1\_1\_3645.d:59548, m/z=580.6262, z=3, RT=18.01, ppm=-6.32):


TRINITY\_DN1339\_c6\_g1\_i2.p1

back to list

  

| Protein Coverage
| Supporting Peptides
| Best Unique PSM
|

Protein Coverage:

Supporting Peptides:

| Peptide | Uniq | -10lgP | Mass | Length | ppm | m/z | z | RT | 1/k0 Start | 1/k0 End | Precursor ID | Area Sample 9 | #Feature | #Feature Sample 9 | Start | End | PTM | AScore | Found By |
| --- | --- | --- | --- | --- | --- | --- | --- | --- | --- | --- | --- | --- | --- | --- | --- | --- | --- | --- | --- |
| K.VITSDDPAEKEK.K | Y | 56.98 | 1330.6616 | 12 | 1.22 | 666.3359 | 2 | 10.71 | 0.9844 | 0.9916 | 18867 | 1.51e3 | 1 | 1 | 355 | 366 |  |  | DB Search |
| total 1 peptides |  |  |  |  |  |  |  |  |  |  |  |  |  |  |  |  |  |  |  |
| --- | --- | --- | --- | --- | --- | --- | --- | --- | --- | --- | --- | --- | --- | --- | --- | --- | --- | --- | --- |

Best Unique PSM (Scan Sample\_8\_A1\_1\_3645.d:18867, m/z=666.3359, z=2, RT=10.71, ppm=-3.3):


TRINITY\_DN2217\_c0\_g1\_i3.p1

back to list

  

| Protein Coverage
| Supporting Peptides
| Best Unique PSM
|

Protein Coverage:

Supporting Peptides:

| Peptide | Uniq | -10lgP | Mass | Length | ppm | m/z | z | RT | 1/k0 Start | 1/k0 End | Precursor ID | Area Sample 9 | #Feature | #Feature Sample 9 | Start | End | PTM | AScore | Found By |
| --- | --- | --- | --- | --- | --- | --- | --- | --- | --- | --- | --- | --- | --- | --- | --- | --- | --- | --- | --- |
| K.ATGC(+57.02)EVIWNQHNR.G | Y | 56.87 | 1583.7263 | 13 | 1.97 | 792.8684 | 2 | 13.82 | 1.0773 | 1.0845 | 37415 | 6.5e2 | 1 | 1 | 431 | 443 | Carbamidomethylation |  | DB Search |
| total 1 peptides |  |  |  |  |  |  |  |  |  |  |  |  |  |  |  |  |  |  |  |
| --- | --- | --- | --- | --- | --- | --- | --- | --- | --- | --- | --- | --- | --- | --- | --- | --- | --- | --- | --- |

Best Unique PSM (Scan Sample\_8\_A1\_1\_3645.d:37415, m/z=792.8684, z=2, RT=13.82, ppm=-2.54):


TRINITY\_DN1199\_c0\_g1\_i1.p1

back to list

  

| Protein Coverage
| Supporting Peptides
| Best Unique PSM
|

Protein Coverage:

Supporting Peptides:

| Peptide | Uniq | -10lgP | Mass | Length | ppm | m/z | z | RT | 1/k0 Start | 1/k0 End | Precursor ID | Area Sample 9 | #Feature | #Feature Sample 9 | Start | End | PTM | AScore | Found By |
| --- | --- | --- | --- | --- | --- | --- | --- | --- | --- | --- | --- | --- | --- | --- | --- | --- | --- | --- | --- |
| R.VVHNIC(+57.02)SSAITNSVTNK.I | Y | 60.48 | 1842.9258 | 17 | -1.93 | 922.4642 | 2 | 13.76 | 1.1366 | 1.1438 | 37108 | 3.92e2 | 1 | 1 | 89 | 105 | Carbamidomethylation |  | DB Search |
| R.LPNFFQYAGHIK.K | Y | 56.19 | 1433.7455 | 12 | -9.53 | 478.9157 | 3 | 19.09 | 0.7522 | 0.7595 | 65828 | 0 | 0 | 0 | 62 | 73 |  |  | DB Search |
| total 2 peptides |  |  |  |  |  |  |  |  |  |  |  |  |  |  |  |  |  |  |  |
| --- | --- | --- | --- | --- | --- | --- | --- | --- | --- | --- | --- | --- | --- | --- | --- | --- | --- | --- | --- |

Best Unique PSM (Scan Sample\_8\_A1\_1\_3645.d:37108, m/z=922.4642, z=2, RT=13.76, ppm=-6.44):


TRINITY\_DN11097\_c0\_g1\_i1.p1

back to list

  

| Protein Coverage
| Supporting Peptides
| Best Unique PSM
|

Protein Coverage:

Supporting Peptides:

| Peptide | Uniq | -10lgP | Mass | Length | ppm | m/z | z | RT | 1/k0 Start | 1/k0 End | Precursor ID | Area Sample 9 | #Feature | #Feature Sample 9 | Start | End | PTM | AScore | Found By |
| --- | --- | --- | --- | --- | --- | --- | --- | --- | --- | --- | --- | --- | --- | --- | --- | --- | --- | --- | --- |
| R.VAHAGLTVAEFFR.D | Y | 57.31 | 1416.7513 | 13 | 3.21 | 709.382 | 2 | 18.82 | 1.0287 | 1.0359 | 64099 | 6.52e2 | 1 | 1 | 493 | 505 |  |  | DB Search |
| total 1 peptides |  |  |  |  |  |  |  |  |  |  |  |  |  |  |  |  |  |  |  |
| --- | --- | --- | --- | --- | --- | --- | --- | --- | --- | --- | --- | --- | --- | --- | --- | --- | --- | --- | --- |

Best Unique PSM (Scan Sample\_8\_A1\_1\_3645.d:64099, m/z=709.382, z=2, RT=18.82, ppm=-1.31):


TRINITY\_DN3016\_c0\_g1\_i2.p1

back to list

  

| Protein Coverage
| Supporting Peptides
| Best Unique PSM
|

Protein Coverage:

Supporting Peptides:

| Peptide | Uniq | -10lgP | Mass | Length | ppm | m/z | z | RT | 1/k0 Start | 1/k0 End | Precursor ID | Area Sample 9 | #Feature | #Feature Sample 9 | Start | End | PTM | AScore | Found By |
| --- | --- | --- | --- | --- | --- | --- | --- | --- | --- | --- | --- | --- | --- | --- | --- | --- | --- | --- | --- |
| R.IVNTDLQEIKDIIK.G | Y | 58.4 | 1640.9348 | 14 | -6.31 | 821.4658 | 2 | 18.62 | 1.1514 | 1.1586 | 63065 | 0 | 0 | 0 | 154 | 167 |  |  | DB Search |
| total 1 peptides |  |  |  |  |  |  |  |  |  |  |  |  |  |  |  |  |  |  |  |
| --- | --- | --- | --- | --- | --- | --- | --- | --- | --- | --- | --- | --- | --- | --- | --- | --- | --- | --- | --- |

Best Unique PSM (Scan Sample\_8\_A1\_1\_3645.d:63065, m/z=821.4658, z=2, RT=18.62, ppm=-10.82):


TRINITY\_DN5643\_c0\_g1\_i1.p1

back to list

  

| Protein Coverage
| Supporting Peptides
| Best Unique PSM
|

Protein Coverage:

Supporting Peptides:

| Peptide | Uniq | -10lgP | Mass | Length | ppm | m/z | z | RT | 1/k0 Start | 1/k0 End | Precursor ID | Area Sample 9 | #Feature | #Feature Sample 9 | Start | End | PTM | AScore | Found By |
| --- | --- | --- | --- | --- | --- | --- | --- | --- | --- | --- | --- | --- | --- | --- | --- | --- | --- | --- | --- |
| R.IPNLTNYLQALHEK.G | Y | 55.27 | 1652.8886 | 14 | -5.64 | 551.9645 | 3 | 19.30 | 0.8201 | 0.8274 | 67040 | 0 | 0 | 0 | 410 | 423 |  |  | DB Search |
| total 1 peptides |  |  |  |  |  |  |  |  |  |  |  |  |  |  |  |  |  |  |  |
| --- | --- | --- | --- | --- | --- | --- | --- | --- | --- | --- | --- | --- | --- | --- | --- | --- | --- | --- | --- |

Best Unique PSM (Scan Sample\_8\_A1\_1\_3645.d:67040, m/z=551.9645, z=3, RT=19.30, ppm=-10.16):


TRINITY\_DN585\_c0\_g1\_i3.p1

back to list

  

| Protein Coverage
| Supporting Peptides
| Best Unique PSM
|

Protein Coverage:

Supporting Peptides:

| Peptide | Uniq | -10lgP | Mass | Length | ppm | m/z | z | RT | 1/k0 Start | 1/k0 End | Precursor ID | Area Sample 9 | #Feature | #Feature Sample 9 | Start | End | PTM | AScore | Found By |
| --- | --- | --- | --- | --- | --- | --- | --- | --- | --- | --- | --- | --- | --- | --- | --- | --- | --- | --- | --- |
| K.IQDKEGIPPDQQR.L | Y | 61.25 | 1522.7739 | 13 | -1.84 | 762.3894 | 2 | 10.99 | 1.0371 | 1.0443 | 20319 | 2.55e3 | 1 | 1 | 2 | 14 |  |  | DB Search |
| total 1 peptides |  |  |  |  |  |  |  |  |  |  |  |  |  |  |  |  |  |  |  |
| --- | --- | --- | --- | --- | --- | --- | --- | --- | --- | --- | --- | --- | --- | --- | --- | --- | --- | --- | --- |

Best Unique PSM (Scan Sample\_8\_A1\_1\_3645.d:20319, m/z=762.3894, z=2, RT=10.99, ppm=-6.36):


TRINITY\_DN5587\_c0\_g1\_i1.p1

back to list

  

| Protein Coverage
| Supporting Peptides
| Best Unique PSM
|

Protein Coverage:

Supporting Peptides:

| Peptide | Uniq | -10lgP | Mass | Length | ppm | m/z | z | RT | 1/k0 Start | 1/k0 End | Precursor ID | Area Sample 9 | #Feature | #Feature Sample 9 | Start | End | PTM | AScore | Found By |
| --- | --- | --- | --- | --- | --- | --- | --- | --- | --- | --- | --- | --- | --- | --- | --- | --- | --- | --- | --- |
| K.LAGESESNLRK.A | Y | 34.34 | 1202.6255 | 11 | -1.9 | 602.3162 | 2 | 10.70 | 0.9331 | 0.9404 | 18933 | 0 | 0 | 0 | 275 | 285 |  |  | DB Search |
| total 1 peptides |  |  |  |  |  |  |  |  |  |  |  |  |  |  |  |  |  |  |  |
| --- | --- | --- | --- | --- | --- | --- | --- | --- | --- | --- | --- | --- | --- | --- | --- | --- | --- | --- | --- |

Best Unique PSM (Scan Sample\_8\_A1\_1\_3645.d:18933, m/z=602.3162, z=2, RT=10.70, ppm=-6.42):


TRINITY\_DN5263\_c0\_g1\_i1.p1

back to list

  

| Protein Coverage
| Supporting Peptides
| Best Unique PSM
|

Protein Coverage:

Supporting Peptides:

| Peptide | Uniq | -10lgP | Mass | Length | ppm | m/z | z | RT | 1/k0 Start | 1/k0 End | Precursor ID | Area Sample 9 | #Feature | #Feature Sample 9 | Start | End | PTM | AScore | Found By |
| --- | --- | --- | --- | --- | --- | --- | --- | --- | --- | --- | --- | --- | --- | --- | --- | --- | --- | --- | --- |
| R.IRELTSVVQKR.F | Y | 58.9 | 1327.7936 | 11 | -5.4 | 443.6007 | 3 | 12.36 | 0.7445 | 0.7518 | 29134 | 0 | 0 | 0 | 66 | 76 |  |  | DB Search |
| total 1 peptides |  |  |  |  |  |  |  |  |  |  |  |  |  |  |  |  |  |  |  |
| --- | --- | --- | --- | --- | --- | --- | --- | --- | --- | --- | --- | --- | --- | --- | --- | --- | --- | --- | --- |

Best Unique PSM (Scan Sample\_8\_A1\_1\_3645.d:29134, m/z=443.6007, z=3, RT=12.36, ppm=-9.92):


TRINITY\_DN73067\_c0\_g1\_i1.p1

back to list

  

| Protein Coverage
| Supporting Peptides
| Best Unique PSM
|

Protein Coverage:

Supporting Peptides:

| Peptide | Uniq | -10lgP | Mass | Length | ppm | m/z | z | RT | 1/k0 Start | 1/k0 End | Precursor ID | Area Sample 9 | #Feature | #Feature Sample 9 | Start | End | PTM | AScore | Found By |
| --- | --- | --- | --- | --- | --- | --- | --- | --- | --- | --- | --- | --- | --- | --- | --- | --- | --- | --- | --- |
| K.VVTSSTGPGEHMR.N | Y | 57.8 | 1356.6456 | 13 | 6.33 | 679.3313 | 2 | 10.85 | 0.9832 | 0.9904 | 19601 | 1.53e3 | 1 | 1 | 714 | 726 |  |  | DB Search |
| K.VVTSSTGPGEHM(+15.99)R.N | Y | 55.31 | 1372.6405 | 13 | 2.81 | 687.3264 | 2 | 9.74 | 0.9759 | 0.9832 | 12944 | 1.22e3 | 1 | 1 | 714 | 726 | Oxidation (M) | M12:Oxidation (M):1000 | DB Search |
| total 2 peptides |  |  |  |  |  |  |  |  |  |  |  |  |  |  |  |  |  |  |  |
| --- | --- | --- | --- | --- | --- | --- | --- | --- | --- | --- | --- | --- | --- | --- | --- | --- | --- | --- | --- |

Best Unique PSM (Scan Sample\_8\_A1\_1\_3645.d:19601, m/z=679.3313, z=2, RT=10.85, ppm=1.82):


TRINITY\_DN2028\_c0\_g1\_i1.p1

back to list

  

| Protein Coverage
| Supporting Peptides
| Best Unique PSM
|

Protein Coverage:

Supporting Peptides:

| Peptide | Uniq | -10lgP | Mass | Length | ppm | m/z | z | RT | 1/k0 Start | 1/k0 End | Precursor ID | Area Sample 9 | #Feature | #Feature Sample 9 | Start | End | PTM | AScore | Found By |
| --- | --- | --- | --- | --- | --- | --- | --- | --- | --- | --- | --- | --- | --- | --- | --- | --- | --- | --- | --- |
| K.LIQYIENKPGLTR.L | Y | 55.08 | 1543.8722 | 13 | 1.73 | 772.9412 | 2 | 14.73 | 1.0769 | 1.0841 | 42338 | 1.38e3 | 1 | 1 | 81 | 93 |  |  | DB Search |
| total 1 peptides |  |  |  |  |  |  |  |  |  |  |  |  |  |  |  |  |  |  |  |
| --- | --- | --- | --- | --- | --- | --- | --- | --- | --- | --- | --- | --- | --- | --- | --- | --- | --- | --- | --- |

Best Unique PSM (Scan Sample\_8\_A1\_1\_3645.d:42338, m/z=772.9412, z=2, RT=14.73, ppm=-2.79):


TRINITY\_DN6920\_c0\_g1\_i1.p1

back to list

  

| Protein Coverage
| Supporting Peptides
| Best Unique PSM
|

Protein Coverage:

Supporting Peptides:

| Peptide | Uniq | -10lgP | Mass | Length | ppm | m/z | z | RT | 1/k0 Start | 1/k0 End | Precursor ID | Area Sample 9 | #Feature | #Feature Sample 9 | Start | End | PTM | AScore | Found By |
| --- | --- | --- | --- | --- | --- | --- | --- | --- | --- | --- | --- | --- | --- | --- | --- | --- | --- | --- | --- |
| K.VAC(+57.02)IGAWHPAR.V | Y | 45.85 | 1236.6186 | 11 | -2.24 | 619.3124 | 2 | 13.17 | 0.9444 | 0.9517 | 33868 | 3.39e2 | 1 | 1 | 134 | 144 | Carbamidomethylation |  | DB Search |
| total 1 peptides |  |  |  |  |  |  |  |  |  |  |  |  |  |  |  |  |  |  |  |
| --- | --- | --- | --- | --- | --- | --- | --- | --- | --- | --- | --- | --- | --- | --- | --- | --- | --- | --- | --- |

Best Unique PSM (Scan Sample\_8\_A1\_1\_3645.d:33868, m/z=619.3124, z=2, RT=13.17, ppm=-6.76):


TRINITY\_DN6008\_c0\_g1\_i1.p1

back to list

  

| Protein Coverage
| Supporting Peptides
| Best Unique PSM
|

Protein Coverage:

Supporting Peptides:

| Peptide | Uniq | -10lgP | Mass | Length | ppm | m/z | z | RT | 1/k0 Start | 1/k0 End | Precursor ID | Area Sample 9 | #Feature | #Feature Sample 9 | Start | End | PTM | AScore | Found By |
| --- | --- | --- | --- | --- | --- | --- | --- | --- | --- | --- | --- | --- | --- | --- | --- | --- | --- | --- | --- |
| R.FEGVLAPNDKLK.Q | Y | 58.01 | 1329.7292 | 12 | 1.13 | 665.8696 | 2 | 14.75 | 1.0379 | 1.0452 | 42511 | 1.04e3 | 1 | 1 | 80 | 91 |  |  | DB Search |
| total 1 peptides |  |  |  |  |  |  |  |  |  |  |  |  |  |  |  |  |  |  |  |
| --- | --- | --- | --- | --- | --- | --- | --- | --- | --- | --- | --- | --- | --- | --- | --- | --- | --- | --- | --- |

Best Unique PSM (Scan Sample\_8\_A1\_1\_3645.d:42511, m/z=665.8696, z=2, RT=14.75, ppm=-3.39):


TRINITY\_DN1616\_c0\_g1\_i2.p1

back to list

  

| Protein Coverage
| Supporting Peptides
| Best Unique PSM
|

Protein Coverage:

Supporting Peptides:

| Peptide | Uniq | -10lgP | Mass | Length | ppm | m/z | z | RT | 1/k0 Start | 1/k0 End | Precursor ID | Area Sample 9 | #Feature | #Feature Sample 9 | Start | End | PTM | AScore | Found By |
| --- | --- | --- | --- | --- | --- | --- | --- | --- | --- | --- | --- | --- | --- | --- | --- | --- | --- | --- | --- |
| K.GC(+57.02)STC(+57.02)GC(+57.02)NGAGTNNGVK.S | Y | 57 | 1712.6665 | 17 | 3.09 | 857.3393 | 2 | 9.83 | 1.0411 | 1.0484 | 13666 | 0 | 0 | 0 | 732 | 748 | Carbamidomethylation, Carbamidomethylation, Carbamidomethylation |  | DB Search |
| K.GSPGELFC(+57.02)TLADATGK.F | Y | 30.98 | 1622.761 | 16 | -3.1 | 812.3816 | 2 | 19.88 | 1.0588 | 1.066 | 70430 | 0 | 0 | 0 | 45 | 60 | Carbamidomethylation |  | DB Search |
| total 2 peptides |  |  |  |  |  |  |  |  |  |  |  |  |  |  |  |  |  |  |  |
| --- | --- | --- | --- | --- | --- | --- | --- | --- | --- | --- | --- | --- | --- | --- | --- | --- | --- | --- | --- |

Best Unique PSM (Scan Sample\_8\_A1\_1\_3645.d:13666, m/z=857.3393, z=2, RT=9.83, ppm=-1.43):


TRINITY\_DN22219\_c0\_g1\_i1.p1

back to list

  

| Protein Coverage
| Supporting Peptides
| Best Unique PSM
|

Protein Coverage:

Supporting Peptides:

| Peptide | Uniq | -10lgP | Mass | Length | ppm | m/z | z | RT | 1/k0 Start | 1/k0 End | Precursor ID | Area Sample 9 | #Feature | #Feature Sample 9 | Start | End | PTM | AScore | Found By |
| --- | --- | --- | --- | --- | --- | --- | --- | --- | --- | --- | --- | --- | --- | --- | --- | --- | --- | --- | --- |
| R.AEVIREEEEEKEK.K | Y | 55.65 | 1616.7893 | 13 | 1.06 | 539.9352 | 3 | 10.70 | 0.7986 | 0.8059 | 18941 | 1.06e3 | 1 | 1 | 311 | 323 |  |  | DB Search |
| total 1 peptides |  |  |  |  |  |  |  |  |  |  |  |  |  |  |  |  |  |  |  |
| --- | --- | --- | --- | --- | --- | --- | --- | --- | --- | --- | --- | --- | --- | --- | --- | --- | --- | --- | --- |

Best Unique PSM (Scan Sample\_8\_A1\_1\_3645.d:18941, m/z=539.9352, z=3, RT=10.70, ppm=-3.45):


TRINITY\_DN471\_c0\_g1\_i3.p1

back to list

  

| Protein Coverage
| Supporting Peptides
| Best Unique PSM
|

Protein Coverage:

Supporting Peptides:

| Peptide | Uniq | -10lgP | Mass | Length | ppm | m/z | z | RT | 1/k0 Start | 1/k0 End | Precursor ID | Area Sample 9 | #Feature | #Feature Sample 9 | Start | End | PTM | AScore | Found By |
| --- | --- | --- | --- | --- | --- | --- | --- | --- | --- | --- | --- | --- | --- | --- | --- | --- | --- | --- | --- |
| R.RNLDIERPTYTNLNR.L | Y | 58.31 | 1873.9758 | 15 | 3.94 | 625.6655 | 3 | 14.03 | 0.8846 | 0.8919 | 38584 | 6.19e2 | 1 | 1 | 85 | 99 |  |  | DB Search |
| total 1 peptides |  |  |  |  |  |  |  |  |  |  |  |  |  |  |  |  |  |  |  |
| --- | --- | --- | --- | --- | --- | --- | --- | --- | --- | --- | --- | --- | --- | --- | --- | --- | --- | --- | --- |

Best Unique PSM (Scan Sample\_8\_A1\_1\_3645.d:38584, m/z=625.6655, z=3, RT=14.03, ppm=-0.58):


TRINITY\_DN58867\_c0\_g1\_i2.p1

back to list

  

| Protein Coverage
| Supporting Peptides
| Best Unique PSM
|

Protein Coverage:

Supporting Peptides:

| Peptide | Uniq | -10lgP | Mass | Length | ppm | m/z | z | RT | 1/k0 Start | 1/k0 End | Precursor ID | Area Sample 9 | #Feature | #Feature Sample 9 | Start | End | PTM | AScore | Found By |
| --- | --- | --- | --- | --- | --- | --- | --- | --- | --- | --- | --- | --- | --- | --- | --- | --- | --- | --- | --- |
| K.TILQFEADRR.A | Y | 57.46 | 1247.6622 | 10 | 3.34 | 624.8376 | 2 | 15.05 | 0.9525 | 0.9598 | 44052 | 2.23e3 | 1 | 1 | 211 | 220 |  |  | DB Search |
| total 1 peptides |  |  |  |  |  |  |  |  |  |  |  |  |  |  |  |  |  |  |  |
| --- | --- | --- | --- | --- | --- | --- | --- | --- | --- | --- | --- | --- | --- | --- | --- | --- | --- | --- | --- |

Best Unique PSM (Scan Sample\_8\_A1\_1\_3645.d:44052, m/z=624.8376, z=2, RT=15.05, ppm=-1.18):


TRINITY\_DN12632\_c1\_g1\_i1.p1

back to list

  

| Protein Coverage
| Supporting Peptides
| Best Unique PSM
|

Protein Coverage:

Supporting Peptides:

| Peptide | Uniq | -10lgP | Mass | Length | ppm | m/z | z | RT | 1/k0 Start | 1/k0 End | Precursor ID | Area Sample 9 | #Feature | #Feature Sample 9 | Start | End | PTM | AScore | Found By |
| --- | --- | --- | --- | --- | --- | --- | --- | --- | --- | --- | --- | --- | --- | --- | --- | --- | --- | --- | --- |
| R.LIEDTEYTAHEGAK.F | Y | 56.89 | 1575.7416 | 14 | -1.89 | 788.873 | 2 | 12.64 | 1.0781 | 1.0853 | 30716 | 0 | 0 | 0 | 378 | 391 |  |  | DB Search |
| total 1 peptides |  |  |  |  |  |  |  |  |  |  |  |  |  |  |  |  |  |  |  |
| --- | --- | --- | --- | --- | --- | --- | --- | --- | --- | --- | --- | --- | --- | --- | --- | --- | --- | --- | --- |

Best Unique PSM (Scan Sample\_8\_A1\_1\_3645.d:30716, m/z=788.873, z=2, RT=12.64, ppm=-6.4):


TRINITY\_DN1907\_c0\_g1\_i1.p1

back to list

  

| Protein Coverage
| Supporting Peptides
| Best Unique PSM
|

Protein Coverage:

Supporting Peptides:

| Peptide | Uniq | -10lgP | Mass | Length | ppm | m/z | z | RT | 1/k0 Start | 1/k0 End | Precursor ID | Area Sample 9 | #Feature | #Feature Sample 9 | Start | End | PTM | AScore | Found By |
| --- | --- | --- | --- | --- | --- | --- | --- | --- | --- | --- | --- | --- | --- | --- | --- | --- | --- | --- | --- |
| K.VIIDYQTHHR.L | Y | 56.99 | 1280.6625 | 10 | 2.06 | 641.337 | 2 | 12.04 | 0.9586 | 0.9658 | 27134 | 1.5e3 | 1 | 1 | 151 | 160 |  |  | DB Search |
| total 1 peptides |  |  |  |  |  |  |  |  |  |  |  |  |  |  |  |  |  |  |  |
| --- | --- | --- | --- | --- | --- | --- | --- | --- | --- | --- | --- | --- | --- | --- | --- | --- | --- | --- | --- |

Best Unique PSM (Scan Sample\_8\_A1\_1\_3645.d:27134, m/z=641.337, z=2, RT=12.04, ppm=-2.46):


TRINITY\_DN730\_c0\_g2\_i3.p1

back to list

  

| Protein Coverage
| Supporting Peptides
| Best Unique PSM
|

Protein Coverage:

Supporting Peptides:

| Peptide | Uniq | -10lgP | Mass | Length | ppm | m/z | z | RT | 1/k0 Start | 1/k0 End | Precursor ID | Area Sample 9 | #Feature | #Feature Sample 9 | Start | End | PTM | AScore | Found By |
| --- | --- | --- | --- | --- | --- | --- | --- | --- | --- | --- | --- | --- | --- | --- | --- | --- | --- | --- | --- |
| K.VRDPYRNDLFHDTSR.I | Y | 60.69 | 1889.9132 | 15 | 3.25 | 473.485 | 4 | 13.64 | 0.8165 | 0.8238 | 36483 | 3.56e3 | 2 | 2 | 51 | 65 |  |  | DB Search |
| K.VEVEDEKGLLAFK.E | Y | 55.55 | 1475.7871 | 13 | -4.27 | 738.8943 | 2 | 16.73 | 1.0564 | 1.0636 | 52524 | 0 | 0 | 0 | 89 | 101 |  |  | DB Search |
| total 2 peptides |  |  |  |  |  |  |  |  |  |  |  |  |  |  |  |  |  |  |  |
| --- | --- | --- | --- | --- | --- | --- | --- | --- | --- | --- | --- | --- | --- | --- | --- | --- | --- | --- | --- |

Best Unique PSM (Scan Sample\_8\_A1\_1\_3645.d:36483, m/z=473.485, z=4, RT=13.64, ppm=-1.26):


TRINITY\_DN12390\_c0\_g1\_i1.p1

back to list

  

| Protein Coverage
| Supporting Peptides
| Best Unique PSM
|

Protein Coverage:

Supporting Peptides:

| Peptide | Uniq | -10lgP | Mass | Length | ppm | m/z | z | RT | 1/k0 Start | 1/k0 End | Precursor ID | Area Sample 9 | #Feature | #Feature Sample 9 | Start | End | PTM | AScore | Found By |
| --- | --- | --- | --- | --- | --- | --- | --- | --- | --- | --- | --- | --- | --- | --- | --- | --- | --- | --- | --- |
| K.HRPTSVEELDR.S | Y | 52.6 | 1337.6687 | 11 | 1.56 | 446.8955 | 3 | 11.41 | 0.7571 | 0.7644 | 23281 | 0 | 0 | 0 | 218 | 228 |  |  | DB Search |
| total 1 peptides |  |  |  |  |  |  |  |  |  |  |  |  |  |  |  |  |  |  |  |
| --- | --- | --- | --- | --- | --- | --- | --- | --- | --- | --- | --- | --- | --- | --- | --- | --- | --- | --- | --- |

Best Unique PSM (Scan Sample\_8\_A1\_1\_3645.d:23281, m/z=446.8955, z=3, RT=11.41, ppm=-2.96):


TRINITY\_DN535\_c0\_g1\_i1.p1

back to list

  

| Protein Coverage
| Supporting Peptides
| Best Unique PSM
|

Protein Coverage:

Supporting Peptides:

| Peptide | Uniq | -10lgP | Mass | Length | ppm | m/z | z | RT | 1/k0 Start | 1/k0 End | Precursor ID | Area Sample 9 | #Feature | #Feature Sample 9 | Start | End | PTM | AScore | Found By |
| --- | --- | --- | --- | --- | --- | --- | --- | --- | --- | --- | --- | --- | --- | --- | --- | --- | --- | --- | --- |
| R.GLFIIDDKGILR.Q | Y | 55.4 | 1358.7921 | 12 | 1.42 | 680.4012 | 2 | 19.26 | 1.011 | 1.0182 | 66782 | 5.93e2 | 1 | 1 | 94 | 105 |  |  | DB Search |
| total 1 peptides |  |  |  |  |  |  |  |  |  |  |  |  |  |  |  |  |  |  |  |
| --- | --- | --- | --- | --- | --- | --- | --- | --- | --- | --- | --- | --- | --- | --- | --- | --- | --- | --- | --- |

Best Unique PSM (Scan Sample\_8\_A1\_1\_3645.d:66782, m/z=680.4012, z=2, RT=19.26, ppm=-3.09):


TRINITY\_DN2217\_c0\_g1\_i2.p1

back to list

  

| Protein Coverage
| Supporting Peptides
| Best Unique PSM
|

Protein Coverage:

Supporting Peptides:

| Peptide | Uniq | -10lgP | Mass | Length | ppm | m/z | z | RT | 1/k0 Start | 1/k0 End | Precursor ID | Area Sample 9 | #Feature | #Feature Sample 9 | Start | End | PTM | AScore | Found By |
| --- | --- | --- | --- | --- | --- | --- | --- | --- | --- | --- | --- | --- | --- | --- | --- | --- | --- | --- | --- |
| K.ATGC(+57.02)EVIWNQHNR.G | Y | 56.87 | 1583.7263 | 13 | 1.97 | 792.8684 | 2 | 13.82 | 1.0773 | 1.0845 | 37415 | 6.5e2 | 1 | 1 | 119 | 131 | Carbamidomethylation |  | DB Search |
| total 1 peptides |  |  |  |  |  |  |  |  |  |  |  |  |  |  |  |  |  |  |  |
| --- | --- | --- | --- | --- | --- | --- | --- | --- | --- | --- | --- | --- | --- | --- | --- | --- | --- | --- | --- |

Best Unique PSM (Scan Sample\_8\_A1\_1\_3645.d:37415, m/z=792.8684, z=2, RT=13.82, ppm=-2.54):


TRINITY\_DN8101\_c0\_g1\_i4.p1

back to list

  

| Protein Coverage
| Supporting Peptides
| Best Unique PSM
|

Protein Coverage:

Supporting Peptides:

| Peptide | Uniq | -10lgP | Mass | Length | ppm | m/z | z | RT | 1/k0 Start | 1/k0 End | Precursor ID | Area Sample 9 | #Feature | #Feature Sample 9 | Start | End | PTM | AScore | Found By |
| --- | --- | --- | --- | --- | --- | --- | --- | --- | --- | --- | --- | --- | --- | --- | --- | --- | --- | --- | --- |
| R.SIGFIGHYLDQLR.L | Y | 55.88 | 1517.799 | 13 | -0.14 | 759.9033 | 2 | 19.91 | 1.0849 | 1.0921 | 70505 | 4.59e2 | 1 | 1 | 475 | 487 |  |  | DB Search |
| total 1 peptides |  |  |  |  |  |  |  |  |  |  |  |  |  |  |  |  |  |  |  |
| --- | --- | --- | --- | --- | --- | --- | --- | --- | --- | --- | --- | --- | --- | --- | --- | --- | --- | --- | --- |

Best Unique PSM (Scan Sample\_8\_A1\_1\_3645.d:70505, m/z=759.9033, z=2, RT=19.91, ppm=-4.65):


TRINITY\_DN123301\_c0\_g1\_i1.p1

back to list

  

| Protein Coverage
| Supporting Peptides
| Best Unique PSM
|

Protein Coverage:

Supporting Peptides:

| Peptide | Uniq | -10lgP | Mass | Length | ppm | m/z | z | RT | 1/k0 Start | 1/k0 End | Precursor ID | Area Sample 9 | #Feature | #Feature Sample 9 | Start | End | PTM | AScore | Found By |
| --- | --- | --- | --- | --- | --- | --- | --- | --- | --- | --- | --- | --- | --- | --- | --- | --- | --- | --- | --- |
| R.YRLELYVDDKDHNASAK.S | Y | 58.77 | 2035.9963 | 17 | 1.23 | 510.0047 | 4 | 14.34 | 0.7787 | 0.786 | 40352 | 0 | 0 | 0 | 217 | 233 |  |  | DB Search |
| total 1 peptides |  |  |  |  |  |  |  |  |  |  |  |  |  |  |  |  |  |  |  |
| --- | --- | --- | --- | --- | --- | --- | --- | --- | --- | --- | --- | --- | --- | --- | --- | --- | --- | --- | --- |

Best Unique PSM (Scan Sample\_8\_A1\_1\_3645.d:40352, m/z=510.0047, z=4, RT=14.34, ppm=-3.29):


TRINITY\_DN13856\_c0\_g1\_i1.p2

back to list

  

| Protein Coverage
| Supporting Peptides
| Best Unique PSM
|

Protein Coverage:

Supporting Peptides:

| Peptide | Uniq | -10lgP | Mass | Length | ppm | m/z | z | RT | 1/k0 Start | 1/k0 End | Precursor ID | Area Sample 9 | #Feature | #Feature Sample 9 | Start | End | PTM | AScore | Found By |
| --- | --- | --- | --- | --- | --- | --- | --- | --- | --- | --- | --- | --- | --- | --- | --- | --- | --- | --- | --- |
| R.IPLDDFQDPTLTLENAHNK.L | Y | 56.49 | 2180.0749 | 19 | 0.23 | 727.6958 | 3 | 18.17 | 0.9876 | 0.9949 | 60474 | 1.67e3 | 1 | 1 | 175 | 193 |  |  | DB Search |
| K.GSLDRESYR.E | N | 17.44 | 1081.5152 | 9 | 0.81 | 541.7629 | 2 | 10.96 | 0.8595 | 0.8668 | 20530 | 0 | 0 | 0 | 96 | 104 |  |  | DB Search |
| total 2 peptides |  |  |  |  |  |  |  |  |  |  |  |  |  |  |  |  |  |  |  |
| --- | --- | --- | --- | --- | --- | --- | --- | --- | --- | --- | --- | --- | --- | --- | --- | --- | --- | --- | --- |

Best Unique PSM (Scan Sample\_8\_A1\_1\_3645.d:60474, m/z=727.6958, z=3, RT=18.17, ppm=-4.29):


TRINITY\_DN902\_c1\_g2\_i4.p1

back to list

  

| Protein Coverage
| Supporting Peptides
| Best Unique PSM
|

Protein Coverage:

Supporting Peptides:

| Peptide | Uniq | -10lgP | Mass | Length | ppm | m/z | z | RT | 1/k0 Start | 1/k0 End | Precursor ID | Area Sample 9 | #Feature | #Feature Sample 9 | Start | End | PTM | AScore | Found By |
| --- | --- | --- | --- | --- | --- | --- | --- | --- | --- | --- | --- | --- | --- | --- | --- | --- | --- | --- | --- |
| K.GLTHC(+57.02)NTLSYK.L | Y | 58.02 | 1292.6183 | 11 | 2.78 | 647.3153 | 2 | 12.39 | 0.9743 | 0.9816 | 29201 | 4.95e3 | 1 | 1 | 62 | 72 | Carbamidomethylation |  | DB Search |
| total 1 peptides |  |  |  |  |  |  |  |  |  |  |  |  |  |  |  |  |  |  |  |
| --- | --- | --- | --- | --- | --- | --- | --- | --- | --- | --- | --- | --- | --- | --- | --- | --- | --- | --- | --- |

Best Unique PSM (Scan Sample\_8\_A1\_1\_3645.d:29201, m/z=647.3153, z=2, RT=12.39, ppm=-1.74):


TRINITY\_DN923\_c0\_g1\_i1.p1

back to list

  

| Protein Coverage
| Supporting Peptides
| Best Unique PSM
|

Protein Coverage:

Supporting Peptides:

| Peptide | Uniq | -10lgP | Mass | Length | ppm | m/z | z | RT | 1/k0 Start | 1/k0 End | Precursor ID | Area Sample 9 | #Feature | #Feature Sample 9 | Start | End | PTM | AScore | Found By |
| --- | --- | --- | --- | --- | --- | --- | --- | --- | --- | --- | --- | --- | --- | --- | --- | --- | --- | --- | --- |
| R.YLTTAVITNKR.R | Y | 56.94 | 1278.7296 | 11 | -2.91 | 640.3673 | 2 | 13.80 | 0.9981 | 1.0053 | 37364 | 1.15e3 | 1 | 1 | 258 | 268 |  |  | DB Search |
| total 1 peptides |  |  |  |  |  |  |  |  |  |  |  |  |  |  |  |  |  |  |  |
| --- | --- | --- | --- | --- | --- | --- | --- | --- | --- | --- | --- | --- | --- | --- | --- | --- | --- | --- | --- |

Best Unique PSM (Scan Sample\_8\_A1\_1\_3645.d:37364, m/z=640.3673, z=2, RT=13.80, ppm=-7.43):


TRINITY\_DN773\_c0\_g1\_i1.p1

back to list

  

| Protein Coverage
| Supporting Peptides
| Best Unique PSM
|

Protein Coverage:

Supporting Peptides:

| Peptide | Uniq | -10lgP | Mass | Length | ppm | m/z | z | RT | 1/k0 Start | 1/k0 End | Precursor ID | Area Sample 9 | #Feature | #Feature Sample 9 | Start | End | PTM | AScore | Found By |
| --- | --- | --- | --- | --- | --- | --- | --- | --- | --- | --- | --- | --- | --- | --- | --- | --- | --- | --- | --- |
| R.GVSEETTTGVHNLYK.M | Y | 61.34 | 1633.7947 | 15 | 0.65 | 817.9015 | 2 | 13.53 | 1.0729 | 1.0801 | 35904 | 8.57e2 | 1 | 1 | 160 | 174 |  |  | DB Search |
| total 1 peptides |  |  |  |  |  |  |  |  |  |  |  |  |  |  |  |  |  |  |  |
| --- | --- | --- | --- | --- | --- | --- | --- | --- | --- | --- | --- | --- | --- | --- | --- | --- | --- | --- | --- |

Best Unique PSM (Scan Sample\_8\_A1\_1\_3645.d:35904, m/z=817.9015, z=2, RT=13.53, ppm=-3.86):


TRINITY\_DN164351\_c0\_g1\_i1.p1

back to list

  

| Protein Coverage
| Supporting Peptides
| Best Unique PSM
|

Protein Coverage:

Supporting Peptides:

| Peptide | Uniq | -10lgP | Mass | Length | ppm | m/z | z | RT | 1/k0 Start | 1/k0 End | Precursor ID | Area Sample 9 | #Feature | #Feature Sample 9 | Start | End | PTM | AScore | Found By |
| --- | --- | --- | --- | --- | --- | --- | --- | --- | --- | --- | --- | --- | --- | --- | --- | --- | --- | --- | --- |
| R.LTNSLM(+15.99)M(+15.99)HGR.N | Y | 21.9 | 1190.5536 | 10 | 3.25 | 596.2833 | 2 | 10.70 | 0.9125 | 0.9198 | 18980 | 0 | 0 | 0 | 78 | 87 | Oxidation (M), Oxidation (M) | M6:Oxidation (M):1000 M7:Oxidation (M):1000 | DB Search |
| total 1 peptides |  |  |  |  |  |  |  |  |  |  |  |  |  |  |  |  |  |  |  |
| --- | --- | --- | --- | --- | --- | --- | --- | --- | --- | --- | --- | --- | --- | --- | --- | --- | --- | --- | --- |

Best Unique PSM (Scan Sample\_8\_A1\_1\_3645.d:18980, m/z=596.2833, z=2, RT=10.70, ppm=-1.27):


TRINITY\_DN1199\_c0\_g1\_i2.p1

back to list

  

| Protein Coverage
| Supporting Peptides
| Best Unique PSM
|

Protein Coverage:

Supporting Peptides:

| Peptide | Uniq | -10lgP | Mass | Length | ppm | m/z | z | RT | 1/k0 Start | 1/k0 End | Precursor ID | Area Sample 9 | #Feature | #Feature Sample 9 | Start | End | PTM | AScore | Found By |
| --- | --- | --- | --- | --- | --- | --- | --- | --- | --- | --- | --- | --- | --- | --- | --- | --- | --- | --- | --- |
| R.VVHNIC(+57.02)SSAITNSVTNK.I | Y | 60.48 | 1842.9258 | 17 | -1.93 | 922.4642 | 2 | 13.76 | 1.1366 | 1.1438 | 37108 | 3.92e2 | 1 | 1 | 89 | 105 | Carbamidomethylation |  | DB Search |
| R.LPNFFQYAGHIK.K | Y | 56.19 | 1433.7455 | 12 | -9.53 | 478.9157 | 3 | 19.09 | 0.7522 | 0.7595 | 65828 | 0 | 0 | 0 | 62 | 73 |  |  | DB Search |
| total 2 peptides |  |  |  |  |  |  |  |  |  |  |  |  |  |  |  |  |  |  |  |
| --- | --- | --- | --- | --- | --- | --- | --- | --- | --- | --- | --- | --- | --- | --- | --- | --- | --- | --- | --- |

Best Unique PSM (Scan Sample\_8\_A1\_1\_3645.d:37108, m/z=922.4642, z=2, RT=13.76, ppm=-6.44):


TRINITY\_DN97809\_c0\_g1\_i1.p1

back to list

  

| Protein Coverage
| Supporting Peptides
| Best Unique PSM
|

Protein Coverage:

Supporting Peptides:

| Peptide | Uniq | -10lgP | Mass | Length | ppm | m/z | z | RT | 1/k0 Start | 1/k0 End | Precursor ID | Area Sample 9 | #Feature | #Feature Sample 9 | Start | End | PTM | AScore | Found By |
| --- | --- | --- | --- | --- | --- | --- | --- | --- | --- | --- | --- | --- | --- | --- | --- | --- | --- | --- | --- |
| LGPDQVDVAGAGR.D | Y | 55.26 | 1253.6364 | 13 | -2.53 | 627.821 | 2 | 13.54 | 0.9335 | 0.9408 | 35963 | 2.26e3 | 1 | 1 | 1 | 13 |  |  | DB Search |
| total 1 peptides |  |  |  |  |  |  |  |  |  |  |  |  |  |  |  |  |  |  |  |
| --- | --- | --- | --- | --- | --- | --- | --- | --- | --- | --- | --- | --- | --- | --- | --- | --- | --- | --- | --- |

Best Unique PSM (Scan Sample\_8\_A1\_1\_3645.d:35963, m/z=627.821, z=2, RT=13.54, ppm=-7.04):


TRINITY\_DN571\_c0\_g1\_i2.p1

back to list

  

| Protein Coverage
| Supporting Peptides
| Best Unique PSM
|

Protein Coverage:

Supporting Peptides:

| Peptide | Uniq | -10lgP | Mass | Length | ppm | m/z | z | RT | 1/k0 Start | 1/k0 End | Precursor ID | Area Sample 9 | #Feature | #Feature Sample 9 | Start | End | PTM | AScore | Found By |
| --- | --- | --- | --- | --- | --- | --- | --- | --- | --- | --- | --- | --- | --- | --- | --- | --- | --- | --- | --- |
| K.M(+15.99)KINVNEIFHDLVR.Q | Y | 31.16 | 1742.9137 | 14 | -0.01 | 581.9759 | 3 | 19.54 | 0.8834 | 0.8907 | 68344 | 1.27e2 | 1 | 1 | 150 | 163 | Oxidation (M) | M1:Oxidation (M):1000 | DB Search |
| total 1 peptides |  |  |  |  |  |  |  |  |  |  |  |  |  |  |  |  |  |  |  |
| --- | --- | --- | --- | --- | --- | --- | --- | --- | --- | --- | --- | --- | --- | --- | --- | --- | --- | --- | --- |

Best Unique PSM (Scan Sample\_8\_A1\_1\_3645.d:68344, m/z=581.9759, z=3, RT=19.54, ppm=-4.53):


TRINITY\_DN2776\_c0\_g1\_i1.p1

back to list

  

| Protein Coverage
| Supporting Peptides
| Best Unique PSM
|

Protein Coverage:

Supporting Peptides:

| Peptide | Uniq | -10lgP | Mass | Length | ppm | m/z | z | RT | 1/k0 Start | 1/k0 End | Precursor ID | Area Sample 9 | #Feature | #Feature Sample 9 | Start | End | PTM | AScore | Found By |
| --- | --- | --- | --- | --- | --- | --- | --- | --- | --- | --- | --- | --- | --- | --- | --- | --- | --- | --- | --- |
| K.IALDDVFRER.D | Y | 52.99 | 1232.6513 | 10 | 0.49 | 617.3304 | 2 | 17.63 | 0.957 | 0.9642 | 57382 | 0 | 0 | 0 | 147 | 156 |  |  | DB Search |
| total 1 peptides |  |  |  |  |  |  |  |  |  |  |  |  |  |  |  |  |  |  |  |
| --- | --- | --- | --- | --- | --- | --- | --- | --- | --- | --- | --- | --- | --- | --- | --- | --- | --- | --- | --- |

Best Unique PSM (Scan Sample\_8\_A1\_1\_3645.d:57382, m/z=617.3304, z=2, RT=17.63, ppm=-4.03):


TRINITY\_DN141214\_c0\_g1\_i1.p1

back to list

  

| Protein Coverage
| Supporting Peptides
| Best Unique PSM
|

Protein Coverage:

Supporting Peptides:

| Peptide | Uniq | -10lgP | Mass | Length | ppm | m/z | z | RT | 1/k0 Start | 1/k0 End | Precursor ID | Area Sample 9 | #Feature | #Feature Sample 9 | Start | End | PTM | AScore | Found By |
| --- | --- | --- | --- | --- | --- | --- | --- | --- | --- | --- | --- | --- | --- | --- | --- | --- | --- | --- | --- |
| R.VFLNKPLDFERENQIR.F | Y | 56.77 | 2017.0745 | 16 | 0.68 | 673.3629 | 3 | 16.28 | 0.9424 | 0.9497 | 50325 | 2.51e2 | 1 | 1 | 162 | 177 |  |  | DB Search |
| total 1 peptides |  |  |  |  |  |  |  |  |  |  |  |  |  |  |  |  |  |  |  |
| --- | --- | --- | --- | --- | --- | --- | --- | --- | --- | --- | --- | --- | --- | --- | --- | --- | --- | --- | --- |

Best Unique PSM (Scan Sample\_8\_A1\_1\_3645.d:50325, m/z=673.3629, z=3, RT=16.28, ppm=-3.83):


TRINITY\_DN413\_c0\_g1\_i1.p1

back to list

  

| Protein Coverage
| Supporting Peptides
| Best Unique PSM
|

Protein Coverage:

Supporting Peptides:

| Peptide | Uniq | -10lgP | Mass | Length | ppm | m/z | z | RT | 1/k0 Start | 1/k0 End | Precursor ID | Area Sample 9 | #Feature | #Feature Sample 9 | Start | End | PTM | AScore | Found By |
| --- | --- | --- | --- | --- | --- | --- | --- | --- | --- | --- | --- | --- | --- | --- | --- | --- | --- | --- | --- |
| R.LLLGDFNGDKR.T | Y | 55.21 | 1246.6669 | 11 | 4.34 | 624.3406 | 2 | 15.96 | 0.9852 | 0.9924 | 48766 | 6.58e2 | 1 | 1 | 46 | 56 |  |  | DB Search |
| total 1 peptides |  |  |  |  |  |  |  |  |  |  |  |  |  |  |  |  |  |  |  |
| --- | --- | --- | --- | --- | --- | --- | --- | --- | --- | --- | --- | --- | --- | --- | --- | --- | --- | --- | --- |

Best Unique PSM (Scan Sample\_8\_A1\_1\_3645.d:48766, m/z=624.3406, z=2, RT=15.96, ppm=-0.18):


TRINITY\_DN1933\_c0\_g1\_i4.p1

back to list

  

| Protein Coverage
| Supporting Peptides
| Best Unique PSM
|

Protein Coverage:

Supporting Peptides:

| Peptide | Uniq | -10lgP | Mass | Length | ppm | m/z | z | RT | 1/k0 Start | 1/k0 End | Precursor ID | Area Sample 9 | #Feature | #Feature Sample 9 | Start | End | PTM | AScore | Found By |
| --- | --- | --- | --- | --- | --- | --- | --- | --- | --- | --- | --- | --- | --- | --- | --- | --- | --- | --- | --- |
| R.RNLDIERPTYTNLNR.L | Y | 58.31 | 1873.9758 | 15 | 3.94 | 625.6655 | 3 | 14.03 | 0.8846 | 0.8919 | 38584 | 6.19e2 | 1 | 1 | 75 | 89 |  |  | DB Search |
| total 1 peptides |  |  |  |  |  |  |  |  |  |  |  |  |  |  |  |  |  |  |  |
| --- | --- | --- | --- | --- | --- | --- | --- | --- | --- | --- | --- | --- | --- | --- | --- | --- | --- | --- | --- |

Best Unique PSM (Scan Sample\_8\_A1\_1\_3645.d:38584, m/z=625.6655, z=3, RT=14.03, ppm=-0.58):


TRINITY\_DN2776\_c0\_g1\_i6.p1

back to list

  

| Protein Coverage
| Supporting Peptides
| Best Unique PSM
|

Protein Coverage:

Supporting Peptides:

| Peptide | Uniq | -10lgP | Mass | Length | ppm | m/z | z | RT | 1/k0 Start | 1/k0 End | Precursor ID | Area Sample 9 | #Feature | #Feature Sample 9 | Start | End | PTM | AScore | Found By |
| --- | --- | --- | --- | --- | --- | --- | --- | --- | --- | --- | --- | --- | --- | --- | --- | --- | --- | --- | --- |
| K.IALDDVFRER.D | Y | 52.99 | 1232.6513 | 10 | 0.49 | 617.3304 | 2 | 17.63 | 0.957 | 0.9642 | 57382 | 0 | 0 | 0 | 147 | 156 |  |  | DB Search |
| total 1 peptides |  |  |  |  |  |  |  |  |  |  |  |  |  |  |  |  |  |  |  |
| --- | --- | --- | --- | --- | --- | --- | --- | --- | --- | --- | --- | --- | --- | --- | --- | --- | --- | --- | --- |

Best Unique PSM (Scan Sample\_8\_A1\_1\_3645.d:57382, m/z=617.3304, z=2, RT=17.63, ppm=-4.03):


TRINITY\_DN7504\_c0\_g1\_i1.p1

back to list

  

| Protein Coverage
| Supporting Peptides
| Best Unique PSM
|

Protein Coverage:

Supporting Peptides:

| Peptide | Uniq | -10lgP | Mass | Length | ppm | m/z | z | RT | 1/k0 Start | 1/k0 End | Precursor ID | Area Sample 9 | #Feature | #Feature Sample 9 | Start | End | PTM | AScore | Found By |
| --- | --- | --- | --- | --- | --- | --- | --- | --- | --- | --- | --- | --- | --- | --- | --- | --- | --- | --- | --- |
| K.TIEAEAAHGTVTR.H | Y | 56.96 | 1354.6841 | 13 | 1.27 | 678.3471 | 2 | 11.79 | 1.0114 | 1.0186 | 25577 | 1.08e3 | 1 | 1 | 301 | 313 |  |  | DB Search |
| total 1 peptides |  |  |  |  |  |  |  |  |  |  |  |  |  |  |  |  |  |  |  |
| --- | --- | --- | --- | --- | --- | --- | --- | --- | --- | --- | --- | --- | --- | --- | --- | --- | --- | --- | --- |

Best Unique PSM (Scan Sample\_8\_A1\_1\_3645.d:25577, m/z=678.3471, z=2, RT=11.79, ppm=-3.25):


TRINITY\_DN495\_c0\_g1\_i3.p1

back to list

  

| Protein Coverage
| Supporting Peptides
| Best Unique PSM
|

Protein Coverage:

Supporting Peptides:

| Peptide | Uniq | -10lgP | Mass | Length | ppm | m/z | z | RT | 1/k0 Start | 1/k0 End | Precursor ID | Area Sample 9 | #Feature | #Feature Sample 9 | Start | End | PTM | AScore | Found By |
| --- | --- | --- | --- | --- | --- | --- | --- | --- | --- | --- | --- | --- | --- | --- | --- | --- | --- | --- | --- |
| R.SC(+57.02)TNPAPANGGK.A | Y | 57.85 | 1172.5244 | 12 | 2.11 | 587.2681 | 2 | 9.44 | 0.8502 | 0.8575 | 11479 | 9.79e2 | 1 | 1 | 503 | 514 | Carbamidomethylation |  | DB Search |
| total 1 peptides |  |  |  |  |  |  |  |  |  |  |  |  |  |  |  |  |  |  |  |
| --- | --- | --- | --- | --- | --- | --- | --- | --- | --- | --- | --- | --- | --- | --- | --- | --- | --- | --- | --- |

Best Unique PSM (Scan Sample\_8\_A1\_1\_3645.d:11479, m/z=587.2681, z=2, RT=9.44, ppm=-2.41):


TRINITY\_DN128406\_c0\_g1\_i1.p1

back to list

  

| Protein Coverage
| Supporting Peptides
| Best Unique PSM
|

Protein Coverage:

Supporting Peptides:

| Peptide | Uniq | -10lgP | Mass | Length | ppm | m/z | z | RT | 1/k0 Start | 1/k0 End | Precursor ID | Area Sample 9 | #Feature | #Feature Sample 9 | Start | End | PTM | AScore | Found By |
| --- | --- | --- | --- | --- | --- | --- | --- | --- | --- | --- | --- | --- | --- | --- | --- | --- | --- | --- | --- |
| K.EIRDILVQYDR.S | Y | 54.58 | 1418.7517 | 11 | -11.1 | 710.3721 | 2 | 19.69 | 1.0323 | 1.0395 | 69261 | 4.75e2 | 1 | 1 | 110 | 120 |  |  | DB Search |
| total 1 peptides |  |  |  |  |  |  |  |  |  |  |  |  |  |  |  |  |  |  |  |
| --- | --- | --- | --- | --- | --- | --- | --- | --- | --- | --- | --- | --- | --- | --- | --- | --- | --- | --- | --- |

Best Unique PSM (Scan Sample\_8\_A1\_1\_3645.d:69261, m/z=710.3721, z=2, RT=19.69, ppm=-15.62):


TRINITY\_DN153369\_c0\_g1\_i1.p2

back to list

  

| Protein Coverage
| Supporting Peptides
| Best Unique PSM
|

Protein Coverage:

Supporting Peptides:

| Peptide | Uniq | -10lgP | Mass | Length | ppm | m/z | z | RT | 1/k0 Start | 1/k0 End | Precursor ID | Area Sample 9 | #Feature | #Feature Sample 9 | Start | End | PTM | AScore | Found By |
| --- | --- | --- | --- | --- | --- | --- | --- | --- | --- | --- | --- | --- | --- | --- | --- | --- | --- | --- | --- |
| R.C(+57.02)TAASGK.R | Y | 38.18 | 693.3116 | 7 | 3.73 | 694.3183 | 1 | 9.67 | 1.0564 | 1.0636 | 12699 | 0 | 0 | 0 | 43 | 49 | Carbamidomethylation |  | DB Search |
| total 1 peptides |  |  |  |  |  |  |  |  |  |  |  |  |  |  |  |  |  |  |  |
| --- | --- | --- | --- | --- | --- | --- | --- | --- | --- | --- | --- | --- | --- | --- | --- | --- | --- | --- | --- |

Best Unique PSM (Scan Sample\_8\_A1\_1\_3645.d:12699, m/z=695.3173, z=1, RT=9.68, ppm=-0.78):


TRINITY\_DN1339\_c0\_g1\_i1.p1

back to list

  

| Protein Coverage
| Supporting Peptides
| Best Unique PSM
|

Protein Coverage:

Supporting Peptides:

| Peptide | Uniq | -10lgP | Mass | Length | ppm | m/z | z | RT | 1/k0 Start | 1/k0 End | Precursor ID | Area Sample 9 | #Feature | #Feature Sample 9 | Start | End | PTM | AScore | Found By |
| --- | --- | --- | --- | --- | --- | --- | --- | --- | --- | --- | --- | --- | --- | --- | --- | --- | --- | --- | --- |
| K.VITSDDPAEKEK.K | Y | 56.98 | 1330.6616 | 12 | 1.22 | 666.3359 | 2 | 10.71 | 0.9844 | 0.9916 | 18867 | 1.51e3 | 1 | 1 | 160 | 171 |  |  | DB Search |
| total 1 peptides |  |  |  |  |  |  |  |  |  |  |  |  |  |  |  |  |  |  |  |
| --- | --- | --- | --- | --- | --- | --- | --- | --- | --- | --- | --- | --- | --- | --- | --- | --- | --- | --- | --- |

Best Unique PSM (Scan Sample\_8\_A1\_1\_3645.d:18867, m/z=666.3359, z=2, RT=10.71, ppm=-3.3):


TRINITY\_DN468\_c1\_g1\_i1.p1

back to list

  

| Protein Coverage
| Supporting Peptides
| Best Unique PSM
|

Protein Coverage:

Supporting Peptides:

| Peptide | Uniq | -10lgP | Mass | Length | ppm | m/z | z | RT | 1/k0 Start | 1/k0 End | Precursor ID | Area Sample 9 | #Feature | #Feature Sample 9 | Start | End | PTM | AScore | Found By |
| --- | --- | --- | --- | --- | --- | --- | --- | --- | --- | --- | --- | --- | --- | --- | --- | --- | --- | --- | --- |
| K.LGPVVDGKYDYTVITTDYK.A | Y | 58.74 | 2146.0834 | 19 | -1.41 | 1074.0426 | 2 | 17.41 | 1.2455 | 1.2527 | 56083 | 0 | 0 | 0 | 123 | 141 |  |  | DB Search |
| total 1 peptides |  |  |  |  |  |  |  |  |  |  |  |  |  |  |  |  |  |  |  |
| --- | --- | --- | --- | --- | --- | --- | --- | --- | --- | --- | --- | --- | --- | --- | --- | --- | --- | --- | --- |

Best Unique PSM (Scan Sample\_8\_A1\_1\_3645.d:56083, m/z=1074.0426, z=2, RT=17.41, ppm=-5.92):


TRINITY\_DN374\_c0\_g1\_i2.p1

back to list

  

| Protein Coverage
| Supporting Peptides
| Best Unique PSM
|

Protein Coverage:

Supporting Peptides:

| Peptide | Uniq | -10lgP | Mass | Length | ppm | m/z | z | RT | 1/k0 Start | 1/k0 End | Precursor ID | Area Sample 9 | #Feature | #Feature Sample 9 | Start | End | PTM | AScore | Found By |
| --- | --- | --- | --- | --- | --- | --- | --- | --- | --- | --- | --- | --- | --- | --- | --- | --- | --- | --- | --- |
| K.YIDIEYVC(+57.02)KPPVKK.A | Y | 59.02 | 1750.9327 | 14 | 1.78 | 876.4713 | 2 | 14.93 | 1.131 | 1.1382 | 43553 | 2.46e2 | 1 | 1 | 234 | 247 | Carbamidomethylation |  | DB Search |
| total 1 peptides |  |  |  |  |  |  |  |  |  |  |  |  |  |  |  |  |  |  |  |
| --- | --- | --- | --- | --- | --- | --- | --- | --- | --- | --- | --- | --- | --- | --- | --- | --- | --- | --- | --- |

Best Unique PSM (Scan Sample\_8\_A1\_1\_3645.d:43553, m/z=876.4713, z=2, RT=14.93, ppm=-2.74):


TRINITY\_DN585\_c0\_g1\_i4.p1

back to list

  

| Protein Coverage
| Supporting Peptides
| Best Unique PSM
|

Protein Coverage:

Supporting Peptides:

| Peptide | Uniq | -10lgP | Mass | Length | ppm | m/z | z | RT | 1/k0 Start | 1/k0 End | Precursor ID | Area Sample 9 | #Feature | #Feature Sample 9 | Start | End | PTM | AScore | Found By |
| --- | --- | --- | --- | --- | --- | --- | --- | --- | --- | --- | --- | --- | --- | --- | --- | --- | --- | --- | --- |
| K.IQDKEGIPPDQQR.L | Y | 61.25 | 1522.7739 | 13 | -1.84 | 762.3894 | 2 | 10.99 | 1.0371 | 1.0443 | 20319 | 2.55e3 | 1 | 1 | 30 | 42 |  |  | DB Search |
| total 1 peptides |  |  |  |  |  |  |  |  |  |  |  |  |  |  |  |  |  |  |  |
| --- | --- | --- | --- | --- | --- | --- | --- | --- | --- | --- | --- | --- | --- | --- | --- | --- | --- | --- | --- |

Best Unique PSM (Scan Sample\_8\_A1\_1\_3645.d:20319, m/z=762.3894, z=2, RT=10.99, ppm=-6.36):


TRINITY\_DN2636\_c0\_g1\_i2.p1

back to list

  

| Protein Coverage
| Supporting Peptides
| Best Unique PSM
|

Protein Coverage:

Supporting Peptides:

| Peptide | Uniq | -10lgP | Mass | Length | ppm | m/z | z | RT | 1/k0 Start | 1/k0 End | Precursor ID | Area Sample 9 | #Feature | #Feature Sample 9 | Start | End | PTM | AScore | Found By |
| --- | --- | --- | --- | --- | --- | --- | --- | --- | --- | --- | --- | --- | --- | --- | --- | --- | --- | --- | --- |
| K.VVEKEAETER.K | Y | 57.16 | 1188.5986 | 10 | 2.47 | 595.3054 | 2 | 9.57 | 0.9044 | 0.9117 | 12158 | 0 | 0 | 0 | 199 | 208 |  |  | DB Search |
| K.IHHELNQFC(+57.02)SVK.S | Y | 38.41 | 1510.7351 | 12 | -3.31 | 756.3689 | 2 | 12.60 | 1.0484 | 1.0556 | 30533 | 0 | 0 | 0 | 117 | 128 | Carbamidomethylation |  | DB Search |
| total 2 peptides |  |  |  |  |  |  |  |  |  |  |  |  |  |  |  |  |  |  |  |
| --- | --- | --- | --- | --- | --- | --- | --- | --- | --- | --- | --- | --- | --- | --- | --- | --- | --- | --- | --- |

Best Unique PSM (Scan Sample\_8\_A1\_1\_3645.d:12158, m/z=595.3054, z=2, RT=9.57, ppm=-2.05):


TRINITY\_DN684\_c0\_g1\_i1.p1

back to list

  

| Protein Coverage
| Supporting Peptides
| Best Unique PSM
|

Protein Coverage:

Supporting Peptides:

| Peptide | Uniq | -10lgP | Mass | Length | ppm | m/z | z | RT | 1/k0 Start | 1/k0 End | Precursor ID | Area Sample 9 | #Feature | #Feature Sample 9 | Start | End | PTM | AScore | Found By |
| --- | --- | --- | --- | --- | --- | --- | --- | --- | --- | --- | --- | --- | --- | --- | --- | --- | --- | --- | --- |
| R.DVASATLQSM(+15.99)KR.S | Y | 46.76 | 1321.666 | 12 | -3.61 | 661.8349 | 2 | 11.86 | 0.9957 | 1.0029 | 26142 | 0 | 0 | 0 | 199 | 210 | Oxidation (M) | M10:Oxidation (M):1000 | DB Search |
| total 1 peptides |  |  |  |  |  |  |  |  |  |  |  |  |  |  |  |  |  |  |  |
| --- | --- | --- | --- | --- | --- | --- | --- | --- | --- | --- | --- | --- | --- | --- | --- | --- | --- | --- | --- |

Best Unique PSM (Scan Sample\_8\_A1\_1\_3645.d:26142, m/z=661.8349, z=2, RT=11.86, ppm=-8.12):


TRINITY\_DN3034\_c0\_g1\_i1.p1

back to list

  

| Protein Coverage
| Supporting Peptides
| Best Unique PSM
|

Protein Coverage:

Supporting Peptides:

| Peptide | Uniq | -10lgP | Mass | Length | ppm | m/z | z | RT | 1/k0 Start | 1/k0 End | Precursor ID | Area Sample 9 | #Feature | #Feature Sample 9 | Start | End | PTM | AScore | Found By |
| --- | --- | --- | --- | --- | --- | --- | --- | --- | --- | --- | --- | --- | --- | --- | --- | --- | --- | --- | --- |
| R.SAEKDEELETLRK.N | Y | 61.52 | 1546.7838 | 13 | -10.31 | 516.5942 | 3 | 12.10 | 0.8096 | 0.8169 | 27606 | 0 | 0 | 0 | 1566 | 1578 |  |  | DB Search |
| K.AQAQAANLEKK.Q | Y | 56.67 | 1170.6357 | 11 | -5.65 | 586.3192 | 2 | 9.93 | 0.9412 | 0.9485 | 14279 | 0 | 0 | 0 | 1425 | 1435 |  |  | DB Search |
| K.TPGLVEGTLILHQLR.C | Y | 55.37 | 1645.9515 | 15 | -1.82 | 549.6543 | 3 | 18.75 | 0.8623 | 0.8696 | 63729 | 8e2 | 1 | 1 | 671 | 685 |  |  | DB Search |
| R.AQEVSDKLNQK.M | Y | 54.93 | 1258.6517 | 11 | 0.35 | 630.3305 | 2 | 10.73 | 0.9368 | 0.944 | 19160 | 7.5e2 | 1 | 1 | 1854 | 1864 |  |  | DB Search |
| R.VALEQSDRLR.K | Y | 43.05 | 1185.6466 | 10 | -2.05 | 593.8267 | 2 | 12.42 | 0.9505 | 0.9578 | 29416 | 8.58e2 | 1 | 1 | 1680 | 1689 |  |  | DB Search |
| K.LAENEKLENAER.V | Y | 41.96 | 1414.7052 | 12 | -1.53 | 708.3556 | 2 | 11.58 | 1.0025 | 1.0098 | 24355 | 0 | 0 | 0 | 1691 | 1702 |  |  | DB Search |
| total 6 peptides |  |  |  |  |  |  |  |  |  |  |  |  |  |  |  |  |  |  |  |
| --- | --- | --- | --- | --- | --- | --- | --- | --- | --- | --- | --- | --- | --- | --- | --- | --- | --- | --- | --- |

Best Unique PSM (Scan Sample\_8\_A1\_1\_3645.d:27606, m/z=516.5942, z=3, RT=12.10, ppm=-14.83):


TRINITY\_DN360\_c0\_g1\_i1.p1

back to list

  

| Protein Coverage
| Supporting Peptides
| Best Unique PSM
|

Protein Coverage:

Supporting Peptides:

| Peptide | Uniq | -10lgP | Mass | Length | ppm | m/z | z | RT | 1/k0 Start | 1/k0 End | Precursor ID | Area Sample 9 | #Feature | #Feature Sample 9 | Start | End | PTM | AScore | Found By |
| --- | --- | --- | --- | --- | --- | --- | --- | --- | --- | --- | --- | --- | --- | --- | --- | --- | --- | --- | --- |
| K.RTPNEVEFHQAVK.E | Y | 57.72 | 1553.795 | 13 | 2.32 | 518.9378 | 3 | 12.05 | 0.8303 | 0.8376 | 27142 | 9.7e2 | 1 | 1 | 43 | 55 |  |  | DB Search |
| total 1 peptides |  |  |  |  |  |  |  |  |  |  |  |  |  |  |  |  |  |  |  |
| --- | --- | --- | --- | --- | --- | --- | --- | --- | --- | --- | --- | --- | --- | --- | --- | --- | --- | --- | --- |

Best Unique PSM (Scan Sample\_8\_A1\_1\_3645.d:27142, m/z=518.9378, z=3, RT=12.05, ppm=-2.2):


TRINITY\_DN804\_c0\_g1\_i2.p1

back to list

  

| Protein Coverage
| Supporting Peptides
| Best Unique PSM
|

Protein Coverage:

Supporting Peptides:

| Peptide | Uniq | -10lgP | Mass | Length | ppm | m/z | z | RT | 1/k0 Start | 1/k0 End | Precursor ID | Area Sample 9 | #Feature | #Feature Sample 9 | Start | End | PTM | AScore | Found By |
| --- | --- | --- | --- | --- | --- | --- | --- | --- | --- | --- | --- | --- | --- | --- | --- | --- | --- | --- | --- |
| K.RGTPLDQVIEFHIDEETLVKR.I | Y | 58 | 2494.3179 | 21 | -2.48 | 624.5824 | 4 | 18.88 | 0.8465 | 0.8538 | 64643 | 2.32e2 | 1 | 1 | 123 | 143 |  |  | DB Search |
| K.LIDSNLEKPEC(+57.02)K.N | Y | 57.09 | 1444.7232 | 12 | -0.41 | 723.3653 | 2 | 12.85 | 1.0311 | 1.0383 | 31680 | 5.58e3 | 1 | 1 | 87 | 98 | Carbamidomethylation |  | DB Search |
| K.LVDRYC(+57.02)VC(+57.02)HLATGDLLR.A | Y | 56.28 | 2060.0295 | 17 | -0.91 | 687.6801 | 3 | 16.66 | 0.9243 | 0.9315 | 52195 | 0 | 0 | 0 | 40 | 56 | Carbamidomethylation, Carbamidomethylation |  | DB Search |
| total 3 peptides |  |  |  |  |  |  |  |  |  |  |  |  |  |  |  |  |  |  |  |
| --- | --- | --- | --- | --- | --- | --- | --- | --- | --- | --- | --- | --- | --- | --- | --- | --- | --- | --- | --- |

Best Unique PSM (Scan Sample\_8\_A1\_1\_3645.d:64643, m/z=624.5824, z=4, RT=18.88, ppm=-6.99):


TRINITY\_DN941\_c0\_g1\_i11.p1

back to list

  

| Protein Coverage
| Supporting Peptides
| Best Unique PSM
|

Protein Coverage:

Supporting Peptides:

| Peptide | Uniq | -10lgP | Mass | Length | ppm | m/z | z | RT | 1/k0 Start | 1/k0 End | Precursor ID | Area Sample 9 | #Feature | #Feature Sample 9 | Start | End | PTM | AScore | Found By |
| --- | --- | --- | --- | --- | --- | --- | --- | --- | --- | --- | --- | --- | --- | --- | --- | --- | --- | --- | --- |
| K.AELTRPTGK.K | Y | 28.72 | 971.54 | 9 | 2.62 | 486.7763 | 2 | 10.33 | 0.8262 | 0.8335 | 16684 | 4.05e2 | 1 | 1 | 134 | 142 |  |  | DB Search |
| total 1 peptides |  |  |  |  |  |  |  |  |  |  |  |  |  |  |  |  |  |  |  |
| --- | --- | --- | --- | --- | --- | --- | --- | --- | --- | --- | --- | --- | --- | --- | --- | --- | --- | --- | --- |

Best Unique PSM (Scan Sample\_8\_A1\_1\_3645.d:16684, m/z=486.2306, z=2, RT=10.34, ppm=-1.9):


TRINITY\_DN163801\_c0\_g1\_i1.p1

back to list

  

| Protein Coverage
| Supporting Peptides
| Best Unique PSM
|

Protein Coverage:

Supporting Peptides:

| Peptide | Uniq | -10lgP | Mass | Length | ppm | m/z | z | RT | 1/k0 Start | 1/k0 End | Precursor ID | Area Sample 9 | #Feature | #Feature Sample 9 | Start | End | PTM | AScore | Found By |
| --- | --- | --- | --- | --- | --- | --- | --- | --- | --- | --- | --- | --- | --- | --- | --- | --- | --- | --- | --- |
| M.A(+42.01)NPLKDIISR.F | Y | 56.95 | 1167.6611 | 10 | -3.36 | 584.8333 | 2 | 18.54 | 0.9194 | 0.9267 | 62425 | 2.03e3 | 1 | 1 | 2 | 11 | Acetylation (Protein N-term) | A1:Acetylation (Protein N-term):1000 | DB Search |
| total 1 peptides |  |  |  |  |  |  |  |  |  |  |  |  |  |  |  |  |  |  |  |
| --- | --- | --- | --- | --- | --- | --- | --- | --- | --- | --- | --- | --- | --- | --- | --- | --- | --- | --- | --- |

Best Unique PSM (Scan Sample\_8\_A1\_1\_3645.d:62425, m/z=584.8333, z=2, RT=18.54, ppm=-7.87):


TRINITY\_DN95248\_c0\_g1\_i1.p1

back to list

  

| Protein Coverage
| Supporting Peptides
| Best Unique PSM
|

Protein Coverage:

Supporting Peptides:

| Peptide | Uniq | -10lgP | Mass | Length | ppm | m/z | z | RT | 1/k0 Start | 1/k0 End | Precursor ID | Area Sample 9 | #Feature | #Feature Sample 9 | Start | End | PTM | AScore | Found By |
| --- | --- | --- | --- | --- | --- | --- | --- | --- | --- | --- | --- | --- | --- | --- | --- | --- | --- | --- | --- |
| R.VVASSHTDYELR.Y | Y | 56.92 | 1375.6732 | 12 | -1.15 | 688.84 | 2 | 12.48 | 1.0106 | 1.0178 | 29825 | 0 | 0 | 0 | 96 | 107 |  |  | DB Search |
| total 1 peptides |  |  |  |  |  |  |  |  |  |  |  |  |  |  |  |  |  |  |  |
| --- | --- | --- | --- | --- | --- | --- | --- | --- | --- | --- | --- | --- | --- | --- | --- | --- | --- | --- | --- |

Best Unique PSM (Scan Sample\_8\_A1\_1\_3645.d:29825, m/z=688.84, z=2, RT=12.48, ppm=-5.66):


TRINITY\_DN1091\_c0\_g1\_i1.p1

back to list

  

| Protein Coverage
| Supporting Peptides
| Best Unique PSM
|

Protein Coverage:

Supporting Peptides:

| Peptide | Uniq | -10lgP | Mass | Length | ppm | m/z | z | RT | 1/k0 Start | 1/k0 End | Precursor ID | Area Sample 9 | #Feature | #Feature Sample 9 | Start | End | PTM | AScore | Found By |
| --- | --- | --- | --- | --- | --- | --- | --- | --- | --- | --- | --- | --- | --- | --- | --- | --- | --- | --- | --- |
| K.IENIEDVFKLPSYC(+57.02)K.R | Y | 58.53 | 1853.9233 | 15 | -0.75 | 618.9785 | 3 | 19.65 | 0.8652 | 0.8725 | 68996 | 3.34e2 | 1 | 1 | 180 | 194 | Carbamidomethylation |  | DB Search |
| R.AFQNTPC(+57.02)THLVLDGTR.W | Y | 56.99 | 1828.889 | 16 | -0.28 | 915.4474 | 2 | 15.22 | 1.1738 | 1.181 | 44999 | 2.23e3 | 1 | 1 | 81 | 96 | Carbamidomethylation |  | DB Search |
| K.TKIENIEDVFK.L | Y | 56.92 | 1334.7081 | 11 | 5.12 | 668.3618 | 2 | 17.34 | 1.0162 | 1.0234 | 55718 | 0 | 0 | 0 | 178 | 188 |  |  | DB Search |
| total 3 peptides |  |  |  |  |  |  |  |  |  |  |  |  |  |  |  |  |  |  |  |
| --- | --- | --- | --- | --- | --- | --- | --- | --- | --- | --- | --- | --- | --- | --- | --- | --- | --- | --- | --- |

Best Unique PSM (Scan Sample\_8\_A1\_1\_3645.d:68996, m/z=618.9785, z=3, RT=19.65, ppm=-5.27):


TRINITY\_DN36\_c2\_g1\_i1.p1

back to list

  

| Protein Coverage
| Supporting Peptides
| Best Unique PSM
|

Protein Coverage:

Supporting Peptides:

| Peptide | Uniq | -10lgP | Mass | Length | ppm | m/z | z | RT | 1/k0 Start | 1/k0 End | Precursor ID | Area Sample 9 | #Feature | #Feature Sample 9 | Start | End | PTM | AScore | Found By |
| --- | --- | --- | --- | --- | --- | --- | --- | --- | --- | --- | --- | --- | --- | --- | --- | --- | --- | --- | --- |
| R.HNIDDC(+57.02)K.K | Y | 35.46 | 900.376 | 7 | 9.13 | 451.1973 | 2 | 10.19 | 0.8027 | 0.81 | 15877 | 0 | 0 | 0 | 92 | 98 | Carbamidomethylation |  | DB Search |
| total 1 peptides |  |  |  |  |  |  |  |  |  |  |  |  |  |  |  |  |  |  |  |
| --- | --- | --- | --- | --- | --- | --- | --- | --- | --- | --- | --- | --- | --- | --- | --- | --- | --- | --- | --- |

Best Unique PSM (Scan Sample\_8\_A1\_1\_3645.d:15877, m/z=451.1973, z=2, RT=10.19, ppm=4.61):


TRINITY\_DN941\_c0\_g1\_i15.p1

back to list

  

| Protein Coverage
| Supporting Peptides
| Best Unique PSM
|

Protein Coverage:

Supporting Peptides:

| Peptide | Uniq | -10lgP | Mass | Length | ppm | m/z | z | RT | 1/k0 Start | 1/k0 End | Precursor ID | Area Sample 9 | #Feature | #Feature Sample 9 | Start | End | PTM | AScore | Found By |
| --- | --- | --- | --- | --- | --- | --- | --- | --- | --- | --- | --- | --- | --- | --- | --- | --- | --- | --- | --- |
| K.AELTRPTGK.K | Y | 28.72 | 971.54 | 9 | 2.62 | 486.7763 | 2 | 10.33 | 0.8262 | 0.8335 | 16684 | 4.05e2 | 1 | 1 | 291 | 299 |  |  | DB Search |
| total 1 peptides |  |  |  |  |  |  |  |  |  |  |  |  |  |  |  |  |  |  |  |
| --- | --- | --- | --- | --- | --- | --- | --- | --- | --- | --- | --- | --- | --- | --- | --- | --- | --- | --- | --- |

Best Unique PSM (Scan Sample\_8\_A1\_1\_3645.d:16684, m/z=486.2306, z=2, RT=10.34, ppm=-1.9):


TRINITY\_DN34481\_c0\_g1\_i1.p1

back to list

  

| Protein Coverage
| Supporting Peptides
| Best Unique PSM
|

Protein Coverage:

Supporting Peptides:

| Peptide | Uniq | -10lgP | Mass | Length | ppm | m/z | z | RT | 1/k0 Start | 1/k0 End | Precursor ID | Area Sample 9 | #Feature | #Feature Sample 9 | Start | End | PTM | AScore | Found By |
| --- | --- | --- | --- | --- | --- | --- | --- | --- | --- | --- | --- | --- | --- | --- | --- | --- | --- | --- | --- |
| K.AC(+57.02)AELHQDVNVK.N | Y | 61.11 | 1382.6612 | 12 | 3.9 | 692.3375 | 2 | 11.85 | 1.0069 | 1.0142 | 26009 | 4.59e2 | 1 | 1 | 282 | 293 | Carbamidomethylation |  | DB Search |
| total 1 peptides |  |  |  |  |  |  |  |  |  |  |  |  |  |  |  |  |  |  |  |
| --- | --- | --- | --- | --- | --- | --- | --- | --- | --- | --- | --- | --- | --- | --- | --- | --- | --- | --- | --- |

Best Unique PSM (Scan Sample\_8\_A1\_1\_3645.d:26009, m/z=692.3375, z=2, RT=11.85, ppm=-0.61):


TRINITY\_DN77\_c1\_g1\_i1.p1

back to list

  

| Protein Coverage
| Supporting Peptides
| Best Unique PSM
|

Protein Coverage:

Supporting Peptides:

| Peptide | Uniq | -10lgP | Mass | Length | ppm | m/z | z | RT | 1/k0 Start | 1/k0 End | Precursor ID | Area Sample 9 | #Feature | #Feature Sample 9 | Start | End | PTM | AScore | Found By |
| --- | --- | --- | --- | --- | --- | --- | --- | --- | --- | --- | --- | --- | --- | --- | --- | --- | --- | --- | --- |
| K.SFASSLLDHFRVEPAK.T | Y | 56.35 | 1802.9315 | 16 | 4.31 | 601.9843 | 3 | 19.80 | 0.9129 | 0.9202 | 69803 | 5.16e2 | 1 | 1 | 318 | 333 |  |  | DB Search |
| total 1 peptides |  |  |  |  |  |  |  |  |  |  |  |  |  |  |  |  |  |  |  |
| --- | --- | --- | --- | --- | --- | --- | --- | --- | --- | --- | --- | --- | --- | --- | --- | --- | --- | --- | --- |

Best Unique PSM (Scan Sample\_8\_A1\_1\_3645.d:69803, m/z=601.9843, z=3, RT=19.80, ppm=-0.21):


TRINITY\_DN31399\_c0\_g1\_i2.p1

back to list

  

| Protein Coverage
| Supporting Peptides
| Best Unique PSM
|

Protein Coverage:

Supporting Peptides:

| Peptide | Uniq | -10lgP | Mass | Length | ppm | m/z | z | RT | 1/k0 Start | 1/k0 End | Precursor ID | Area Sample 9 | #Feature | #Feature Sample 9 | Start | End | PTM | AScore | Found By |
| --- | --- | --- | --- | --- | --- | --- | --- | --- | --- | --- | --- | --- | --- | --- | --- | --- | --- | --- | --- |
| K.DTLVFEIDEIRK.S | Y | 55.99 | 1476.7824 | 12 | -10.15 | 739.3876 | 2 | 18.39 | 1.0323 | 1.0395 | 62006 | 2.85e3 | 1 | 1 | 396 | 407 |  |  | DB Search |
| R.VNGADGKVEVVWK.T | Y | 52.96 | 1399.7459 | 13 | -2.44 | 700.8754 | 2 | 14.60 | 1.0705 | 1.0777 | 41798 | 0 | 0 | 0 | 483 | 495 |  |  | DB Search |
| total 2 peptides |  |  |  |  |  |  |  |  |  |  |  |  |  |  |  |  |  |  |  |
| --- | --- | --- | --- | --- | --- | --- | --- | --- | --- | --- | --- | --- | --- | --- | --- | --- | --- | --- | --- |

Best Unique PSM (Scan Sample\_8\_A1\_1\_3645.d:62006, m/z=739.3876, z=2, RT=18.39, ppm=-14.66):


TRINITY\_DN2885\_c0\_g1\_i5.p1

back to list

  

| Protein Coverage
| Supporting Peptides
| Best Unique PSM
|

Protein Coverage:

Supporting Peptides:

| Peptide | Uniq | -10lgP | Mass | Length | ppm | m/z | z | RT | 1/k0 Start | 1/k0 End | Precursor ID | Area Sample 9 | #Feature | #Feature Sample 9 | Start | End | PTM | AScore | Found By |
| --- | --- | --- | --- | --- | --- | --- | --- | --- | --- | --- | --- | --- | --- | --- | --- | --- | --- | --- | --- |
| K.VTRPHGASGVVR.A | Y | 56.98 | 1234.6894 | 12 | 3.31 | 412.5699 | 3 | 10.06 | 0.8205 | 0.8279 | 15006 | 1.06e3 | 1 | 1 | 77 | 88 |  |  | DB Search |
| total 1 peptides |  |  |  |  |  |  |  |  |  |  |  |  |  |  |  |  |  |  |  |
| --- | --- | --- | --- | --- | --- | --- | --- | --- | --- | --- | --- | --- | --- | --- | --- | --- | --- | --- | --- |

Best Unique PSM (Scan Sample\_8\_A1\_1\_3645.d:15006, m/z=412.5699, z=3, RT=10.06, ppm=-1.21):


TRINITY\_DN8227\_c0\_g1\_i1.p1

back to list

  

| Protein Coverage
| Supporting Peptides
| Best Unique PSM
|

Protein Coverage:

Supporting Peptides:

| Peptide | Uniq | -10lgP | Mass | Length | ppm | m/z | z | RT | 1/k0 Start | 1/k0 End | Precursor ID | Area Sample 9 | #Feature | #Feature Sample 9 | Start | End | PTM | AScore | Found By |
| --- | --- | --- | --- | --- | --- | --- | --- | --- | --- | --- | --- | --- | --- | --- | --- | --- | --- | --- | --- |
| K.IQDKEGIPPDQQR.L | Y | 61.25 | 1522.7739 | 13 | -1.84 | 762.3894 | 2 | 10.99 | 1.0371 | 1.0443 | 20319 | 2.55e3 | 1 | 1 | 54 | 66 |  |  | DB Search |
| total 1 peptides |  |  |  |  |  |  |  |  |  |  |  |  |  |  |  |  |  |  |  |
| --- | --- | --- | --- | --- | --- | --- | --- | --- | --- | --- | --- | --- | --- | --- | --- | --- | --- | --- | --- |

Best Unique PSM (Scan Sample\_8\_A1\_1\_3645.d:20319, m/z=762.3894, z=2, RT=10.99, ppm=-6.36):


TRINITY\_DN730\_c0\_g2\_i2.p1

back to list

  

| Protein Coverage
| Supporting Peptides
| Best Unique PSM
|

Protein Coverage:

Supporting Peptides:

| Peptide | Uniq | -10lgP | Mass | Length | ppm | m/z | z | RT | 1/k0 Start | 1/k0 End | Precursor ID | Area Sample 9 | #Feature | #Feature Sample 9 | Start | End | PTM | AScore | Found By |
| --- | --- | --- | --- | --- | --- | --- | --- | --- | --- | --- | --- | --- | --- | --- | --- | --- | --- | --- | --- |
| K.VRDPYRNDLFHDTSR.I | Y | 60.69 | 1889.9132 | 15 | 3.25 | 473.485 | 4 | 13.64 | 0.8165 | 0.8238 | 36483 | 3.56e3 | 2 | 2 | 51 | 65 |  |  | DB Search |
| K.VEVEDEKGLLAFK.E | Y | 55.55 | 1475.7871 | 13 | -4.27 | 738.8943 | 2 | 16.73 | 1.0564 | 1.0636 | 52524 | 0 | 0 | 0 | 89 | 101 |  |  | DB Search |
| total 2 peptides |  |  |  |  |  |  |  |  |  |  |  |  |  |  |  |  |  |  |  |
| --- | --- | --- | --- | --- | --- | --- | --- | --- | --- | --- | --- | --- | --- | --- | --- | --- | --- | --- | --- |

Best Unique PSM (Scan Sample\_8\_A1\_1\_3645.d:36483, m/z=473.485, z=4, RT=13.64, ppm=-1.26):


TRINITY\_DN1616\_c0\_g1\_i7.p1

back to list

  

| Protein Coverage
| Supporting Peptides
| Best Unique PSM
|

Protein Coverage:

Supporting Peptides:

| Peptide | Uniq | -10lgP | Mass | Length | ppm | m/z | z | RT | 1/k0 Start | 1/k0 End | Precursor ID | Area Sample 9 | #Feature | #Feature Sample 9 | Start | End | PTM | AScore | Found By |
| --- | --- | --- | --- | --- | --- | --- | --- | --- | --- | --- | --- | --- | --- | --- | --- | --- | --- | --- | --- |
| K.VVAEC(+57.02)NNGGGR.F | Y | 57.11 | 1131.5091 | 11 | -7.52 | 566.755 | 2 | 9.43 | 0.8299 | 0.8372 | 11493 | 0 | 0 | 0 | 3736 | 3746 | Carbamidomethylation |  | DB Search |
| K.GC(+57.02)STC(+57.02)GC(+57.02)NGAGTNNGVK.S | Y | 57 | 1712.6665 | 17 | 3.09 | 857.3393 | 2 | 9.83 | 1.0411 | 1.0484 | 13666 | 0 | 0 | 0 | 732 | 748 | Carbamidomethylation, Carbamidomethylation, Carbamidomethylation |  | DB Search |
| K.GSPGELFC(+57.02)TLADATGK.F | Y | 30.98 | 1622.761 | 16 | -3.1 | 812.3816 | 2 | 19.88 | 1.0588 | 1.066 | 70430 | 0 | 0 | 0 | 45 | 60 | Carbamidomethylation |  | DB Search |
| total 3 peptides |  |  |  |  |  |  |  |  |  |  |  |  |  |  |  |  |  |  |  |
| --- | --- | --- | --- | --- | --- | --- | --- | --- | --- | --- | --- | --- | --- | --- | --- | --- | --- | --- | --- |

Best Unique PSM (Scan Sample\_8\_A1\_1\_3645.d:11493, m/z=566.755, z=2, RT=9.43, ppm=-12.04):


TRINITY\_DN3545\_c0\_g1\_i2.p1

back to list

  

| Protein Coverage
| Supporting Peptides
| Best Unique PSM
|

Protein Coverage:

Supporting Peptides:

| Peptide | Uniq | -10lgP | Mass | Length | ppm | m/z | z | RT | 1/k0 Start | 1/k0 End | Precursor ID | Area Sample 9 | #Feature | #Feature Sample 9 | Start | End | PTM | AScore | Found By |
| --- | --- | --- | --- | --- | --- | --- | --- | --- | --- | --- | --- | --- | --- | --- | --- | --- | --- | --- | --- |
| R.SC(+57.02)TNPAPANGGK.H | Y | 57.85 | 1172.5244 | 12 | 2.11 | 587.2681 | 2 | 9.44 | 0.8502 | 0.8575 | 11479 | 9.79e2 | 1 | 1 | 55 | 66 | Carbamidomethylation |  | DB Search |
| R.SSC(+57.02)RPILSVIK.I | Y | 16.12 | 1258.7067 | 11 | -9.52 | 630.3518 | 2 | 16.77 | 0.9687 | 0.9759 | 52596 | 1.93e3 | 1 | 1 | 6 | 16 | Carbamidomethylation |  | DB Search |
| total 2 peptides |  |  |  |  |  |  |  |  |  |  |  |  |  |  |  |  |  |  |  |
| --- | --- | --- | --- | --- | --- | --- | --- | --- | --- | --- | --- | --- | --- | --- | --- | --- | --- | --- | --- |

Best Unique PSM (Scan Sample\_8\_A1\_1\_3645.d:11479, m/z=587.2681, z=2, RT=9.44, ppm=-2.41):


TRINITY\_DN40728\_c1\_g1\_i1.p2

back to list

  

| Protein Coverage
| Supporting Peptides
| Best Unique PSM
|

Protein Coverage:

Supporting Peptides:

| Peptide | Uniq | -10lgP | Mass | Length | ppm | m/z | z | RT | 1/k0 Start | 1/k0 End | Precursor ID | Area Sample 9 | #Feature | #Feature Sample 9 | Start | End | PTM | AScore | Found By |
| --- | --- | --- | --- | --- | --- | --- | --- | --- | --- | --- | --- | --- | --- | --- | --- | --- | --- | --- | --- |
| K.IQDKEGIPPDQQR.L | Y | 61.25 | 1522.7739 | 13 | -1.84 | 762.3894 | 2 | 10.99 | 1.0371 | 1.0443 | 20319 | 2.55e3 | 1 | 1 | 25 | 37 |  |  | DB Search |
| total 1 peptides |  |  |  |  |  |  |  |  |  |  |  |  |  |  |  |  |  |  |  |
| --- | --- | --- | --- | --- | --- | --- | --- | --- | --- | --- | --- | --- | --- | --- | --- | --- | --- | --- | --- |

Best Unique PSM (Scan Sample\_8\_A1\_1\_3645.d:20319, m/z=762.3894, z=2, RT=10.99, ppm=-6.36):


TRINITY\_DN118\_c1\_g1\_i3.p1

back to list

  

| Protein Coverage
| Supporting Peptides
| Best Unique PSM
|

Protein Coverage:

Supporting Peptides:

| Peptide | Uniq | -10lgP | Mass | Length | ppm | m/z | z | RT | 1/k0 Start | 1/k0 End | Precursor ID | Area Sample 9 | #Feature | #Feature Sample 9 | Start | End | PTM | AScore | Found By |
| --- | --- | --- | --- | --- | --- | --- | --- | --- | --- | --- | --- | --- | --- | --- | --- | --- | --- | --- | --- |
| K.FSYEKVC(+57.02)EDVKR.C | Y | 62.2 | 1558.745 | 12 | -2.91 | 780.374 | 2 | 13.45 | 1.0684 | 1.0757 | 35460 | 1.37e3 | 1 | 1 | 117 | 128 | Carbamidomethylation |  | DB Search |
| R.ETC(+57.02)SDIEKR.E | Y | 55.98 | 1136.5132 | 9 | 4.61 | 569.2639 | 2 | 10.09 | 0.8915 | 0.8988 | 15200 | 4.92e3 | 1 | 1 | 186 | 194 | Carbamidomethylation |  | DB Search |
| K.VC(+57.02)EDVKR.C | Y | 53.85 | 904.4436 | 7 | 3.72 | 453.2287 | 2 | 9.40 | 0.7815 | 0.7888 | 11301 | 1.58e3 | 1 | 1 | 122 | 128 | Carbamidomethylation |  | DB Search |
| total 3 peptides |  |  |  |  |  |  |  |  |  |  |  |  |  |  |  |  |  |  |  |
| --- | --- | --- | --- | --- | --- | --- | --- | --- | --- | --- | --- | --- | --- | --- | --- | --- | --- | --- | --- |

Best Unique PSM (Scan Sample\_8\_A1\_1\_3645.d:35460, m/z=780.374, z=2, RT=13.45, ppm=-7.43):


TRINITY\_DN1369\_c0\_g1\_i1.p1

back to list

  

| Protein Coverage
| Supporting Peptides
| Best Unique PSM
|

Protein Coverage:

Supporting Peptides:

| Peptide | Uniq | -10lgP | Mass | Length | ppm | m/z | z | RT | 1/k0 Start | 1/k0 End | Precursor ID | Area Sample 9 | #Feature | #Feature Sample 9 | Start | End | PTM | AScore | Found By |
| --- | --- | --- | --- | --- | --- | --- | --- | --- | --- | --- | --- | --- | --- | --- | --- | --- | --- | --- | --- |
| K.DAC(+57.02)IDGC(+57.02)HC(+57.02)PPHTSLSGDK.C | Y | 60.38 | 2125.8615 | 19 | 8.76 | 709.6308 | 3 | 12.20 | 0.8903 | 0.8976 | 28169 | 0 | 0 | 0 | 326 | 344 | Carbamidomethylation, Carbamidomethylation, Carbamidomethylation |  | DB Search |
| K.TPGEC(+57.02)C(+57.02)PQC(+57.02)K.K | Y | 57.06 | 1235.4733 | 10 | 1.2 | 618.7419 | 2 | 10.00 | 0.8955 | 0.9028 | 14563 | 1.33e3 | 1 | 1 | 1387 | 1396 | Carbamidomethylation, Carbamidomethylation, Carbamidomethylation |  | DB Search |
| R.EITLNYHDTVVHMK.R | Y | 56.91 | 1698.8399 | 14 | 2.52 | 850.4255 | 2 | 15.24 | 1.1106 | 1.1178 | 45119 | 7.14e2 | 1 | 1 | 911 | 924 |  |  | DB Search |
| R.KYTPC(+57.02)EEK.C | Y | 49.11 | 1053.4801 | 8 | 0.8 | 527.7454 | 2 | 9.71 | 0.87 | 0.8773 | 12951 | 5.44e2 | 1 | 1 | 649 | 656 | Carbamidomethylation |  | DB Search |
| total 4 peptides |  |  |  |  |  |  |  |  |  |  |  |  |  |  |  |  |  |  |  |
| --- | --- | --- | --- | --- | --- | --- | --- | --- | --- | --- | --- | --- | --- | --- | --- | --- | --- | --- | --- |

Best Unique PSM (Scan Sample\_8\_A1\_1\_3645.d:28169, m/z=709.6308, z=3, RT=12.20, ppm=4.25):


TRINITY\_DN1306\_c0\_g1\_i1.p1

back to list

  

| Protein Coverage
| Supporting Peptides
| Best Unique PSM
|

Protein Coverage:

Supporting Peptides:

| Peptide | Uniq | -10lgP | Mass | Length | ppm | m/z | z | RT | 1/k0 Start | 1/k0 End | Precursor ID | Area Sample 9 | #Feature | #Feature Sample 9 | Start | End | PTM | AScore | Found By |
| --- | --- | --- | --- | --- | --- | --- | --- | --- | --- | --- | --- | --- | --- | --- | --- | --- | --- | --- | --- |
| R.KLSNSLEEC(+57.02)VEYSR.N | Y | 57.08 | 1712.8039 | 14 | 5.01 | 857.4097 | 2 | 14.52 | 1.0937 | 1.1009 | 41242 | 1.11e3 | 1 | 1 | 123 | 136 | Carbamidomethylation |  | DB Search |
| R.DLIDRLEEK.Q | Y | 30.56 | 1129.5979 | 9 | -0.02 | 565.8036 | 2 | 16.13 | 0.8915 | 0.8988 | 49607 | 1.4e3 | 1 | 1 | 146 | 154 |  |  | DB Search |
| total 2 peptides |  |  |  |  |  |  |  |  |  |  |  |  |  |  |  |  |  |  |  |
| --- | --- | --- | --- | --- | --- | --- | --- | --- | --- | --- | --- | --- | --- | --- | --- | --- | --- | --- | --- |

Best Unique PSM (Scan Sample\_8\_A1\_1\_3645.d:41242, m/z=857.4097, z=2, RT=14.52, ppm=0.5):


TRINITY\_DN585\_c0\_g1\_i5.p1

back to list

  

| Protein Coverage
| Supporting Peptides
| Best Unique PSM
|

Protein Coverage:

Supporting Peptides:

| Peptide | Uniq | -10lgP | Mass | Length | ppm | m/z | z | RT | 1/k0 Start | 1/k0 End | Precursor ID | Area Sample 9 | #Feature | #Feature Sample 9 | Start | End | PTM | AScore | Found By |
| --- | --- | --- | --- | --- | --- | --- | --- | --- | --- | --- | --- | --- | --- | --- | --- | --- | --- | --- | --- |
| K.IQDKEGIPPDQQR.L | Y | 61.25 | 1522.7739 | 13 | -1.84 | 762.3894 | 2 | 10.99 | 1.0371 | 1.0443 | 20319 | 2.55e3 | 1 | 1 | 2 | 14 |  |  | DB Search |
| total 1 peptides |  |  |  |  |  |  |  |  |  |  |  |  |  |  |  |  |  |  |  |
| --- | --- | --- | --- | --- | --- | --- | --- | --- | --- | --- | --- | --- | --- | --- | --- | --- | --- | --- | --- |

Best Unique PSM (Scan Sample\_8\_A1\_1\_3645.d:20319, m/z=762.3894, z=2, RT=10.99, ppm=-6.36):


TRINITY\_DN89\_c0\_g1\_i1.p1

back to list

  

| Protein Coverage
| Supporting Peptides
| Best Unique PSM
|

Protein Coverage:

Supporting Peptides:

| Peptide | Uniq | -10lgP | Mass | Length | ppm | m/z | z | RT | 1/k0 Start | 1/k0 End | Precursor ID | Area Sample 9 | #Feature | #Feature Sample 9 | Start | End | PTM | AScore | Found By |
| --- | --- | --- | --- | --- | --- | --- | --- | --- | --- | --- | --- | --- | --- | --- | --- | --- | --- | --- | --- |
| K.TTC(+57.02)GVC(+57.02)NNNYC(+57.02)EDDPR.F | Y | 59.6 | 1973.7302 | 16 | 4.69 | 987.8726 | 2 | 12.28 | 1.121 | 1.1282 | 28564 | 7.59e1 | 1 | 1 | 328 | 343 | Carbamidomethylation, Carbamidomethylation, Carbamidomethylation |  | DB Search |
| total 1 peptides |  |  |  |  |  |  |  |  |  |  |  |  |  |  |  |  |  |  |  |
| --- | --- | --- | --- | --- | --- | --- | --- | --- | --- | --- | --- | --- | --- | --- | --- | --- | --- | --- | --- |

Best Unique PSM (Scan Sample\_8\_A1\_1\_3645.d:28564, m/z=987.8726, z=2, RT=12.28, ppm=0.18):


TRINITY\_DN8101\_c0\_g1\_i9.p2

back to list

  

| Protein Coverage
| Supporting Peptides
| Best Unique PSM
|

Protein Coverage:

Supporting Peptides:

| Peptide | Uniq | -10lgP | Mass | Length | ppm | m/z | z | RT | 1/k0 Start | 1/k0 End | Precursor ID | Area Sample 9 | #Feature | #Feature Sample 9 | Start | End | PTM | AScore | Found By |
| --- | --- | --- | --- | --- | --- | --- | --- | --- | --- | --- | --- | --- | --- | --- | --- | --- | --- | --- | --- |
| R.SIGFIGHYLDQLR.L | Y | 55.88 | 1517.799 | 13 | -0.14 | 759.9033 | 2 | 19.91 | 1.0849 | 1.0921 | 70505 | 4.59e2 | 1 | 1 | 1077 | 1089 |  |  | DB Search |
| total 1 peptides |  |  |  |  |  |  |  |  |  |  |  |  |  |  |  |  |  |  |  |
| --- | --- | --- | --- | --- | --- | --- | --- | --- | --- | --- | --- | --- | --- | --- | --- | --- | --- | --- | --- |

Best Unique PSM (Scan Sample\_8\_A1\_1\_3645.d:70505, m/z=759.9033, z=2, RT=19.91, ppm=-4.65):


TRINITY\_DN2477\_c0\_g1\_i6.p1

back to list

  

| Protein Coverage
| Supporting Peptides
| Best Unique PSM
|

Protein Coverage:

Supporting Peptides:

| Peptide | Uniq | -10lgP | Mass | Length | ppm | m/z | z | RT | 1/k0 Start | 1/k0 End | Precursor ID | Area Sample 9 | #Feature | #Feature Sample 9 | Start | End | PTM | AScore | Found By |
| --- | --- | --- | --- | --- | --- | --- | --- | --- | --- | --- | --- | --- | --- | --- | --- | --- | --- | --- | --- |
| K.DSYVGDEAQSKR.G | Y | 58.12 | 1353.616 | 12 | 4.44 | 677.8152 | 2 | 10.93 | 1.0065 | 1.0138 | 20214 | 8.61e3 | 1 | 1 | 52 | 63 |  |  | DB Search |
| R.VAPEEHPVLLTEAPLNPK.A | Y | 57.43 | 1953.0571 | 18 | 1.47 | 977.5328 | 2 | 16.35 | 1.2073 | 1.2145 | 50611 | 1.52e2 | 1 | 1 | 97 | 114 |  |  | DB Search |
| R.GYSFTTTAEREIVR.D | Y | 56.59 | 1628.8158 | 14 | 3.67 | 815.4145 | 2 | 15.95 | 1.119 | 1.1262 | 48654 | 3.46e3 | 1 | 1 | 198 | 211 |  |  | DB Search |
| total 3 peptides |  |  |  |  |  |  |  |  |  |  |  |  |  |  |  |  |  |  |  |
| --- | --- | --- | --- | --- | --- | --- | --- | --- | --- | --- | --- | --- | --- | --- | --- | --- | --- | --- | --- |

Best Unique PSM (Scan Sample\_8\_A1\_1\_3645.d:20214, m/z=677.8152, z=2, RT=10.93, ppm=-0.08):


TRINITY\_DN8903\_c0\_g1\_i1.p1

back to list

  

| Protein Coverage
| Supporting Peptides
| Best Unique PSM
|

Protein Coverage:

Supporting Peptides:

| Peptide | Uniq | -10lgP | Mass | Length | ppm | m/z | z | RT | 1/k0 Start | 1/k0 End | Precursor ID | Area Sample 9 | #Feature | #Feature Sample 9 | Start | End | PTM | AScore | Found By |
| --- | --- | --- | --- | --- | --- | --- | --- | --- | --- | --- | --- | --- | --- | --- | --- | --- | --- | --- | --- |
| K.DLHIIEDYVLK.H | Y | 55.93 | 1356.7289 | 11 | -2.97 | 679.3666 | 2 | 19.52 | 0.9949 | 1.0021 | 68196 | 1.97e3 | 1 | 1 | 417 | 427 |  |  | DB Search |
| total 1 peptides |  |  |  |  |  |  |  |  |  |  |  |  |  |  |  |  |  |  |  |
| --- | --- | --- | --- | --- | --- | --- | --- | --- | --- | --- | --- | --- | --- | --- | --- | --- | --- | --- | --- |

Best Unique PSM (Scan Sample\_8\_A1\_1\_3645.d:68196, m/z=679.3666, z=2, RT=19.52, ppm=-7.48):


TRINITY\_DN1374\_c0\_g1\_i1.p1

back to list

  

| Protein Coverage
| Supporting Peptides
| Best Unique PSM
|

Protein Coverage:

Supporting Peptides:

| Peptide | Uniq | -10lgP | Mass | Length | ppm | m/z | z | RT | 1/k0 Start | 1/k0 End | Precursor ID | Area Sample 9 | #Feature | #Feature Sample 9 | Start | End | PTM | AScore | Found By |
| --- | --- | --- | --- | --- | --- | --- | --- | --- | --- | --- | --- | --- | --- | --- | --- | --- | --- | --- | --- |
| K.RIDYAQNM(+15.99)FK.W | Y | 35.56 | 1300.6234 | 10 | -3.94 | 651.3135 | 2 | 13.37 | 0.9481 | 0.9553 | 34893 | 8.01e2 | 1 | 1 | 280 | 289 | Oxidation (M) | M8:Oxidation (M):1000 | DB Search |
| total 1 peptides |  |  |  |  |  |  |  |  |  |  |  |  |  |  |  |  |  |  |  |
| --- | --- | --- | --- | --- | --- | --- | --- | --- | --- | --- | --- | --- | --- | --- | --- | --- | --- | --- | --- |

Best Unique PSM (Scan Sample\_8\_A1\_1\_3645.d:34893, m/z=651.3135, z=2, RT=13.37, ppm=-8.45):


TRINITY\_DN649\_c0\_g1\_i5.p1

back to list

  

| Protein Coverage
| Supporting Peptides
| Best Unique PSM
|

Protein Coverage:

Supporting Peptides:

| Peptide | Uniq | -10lgP | Mass | Length | ppm | m/z | z | RT | 1/k0 Start | 1/k0 End | Precursor ID | Area Sample 9 | #Feature | #Feature Sample 9 | Start | End | PTM | AScore | Found By |
| --- | --- | --- | --- | --- | --- | --- | --- | --- | --- | --- | --- | --- | --- | --- | --- | --- | --- | --- | --- |
| K.YGQAM(+15.99)C(+57.02)QFM(+15.99)R.N | N | 61.54 | 1322.5206 | 10 | -1.76 | 662.2634 | 2 | 12.17 | 0.9489 | 0.9562 | 28008 | 2.07e2 | 1 | 1 | 7 | 16 | Oxidation (M), Carbamidomethylation, Oxidation (M) | M5:Oxidation (M):1000 M9:Oxidation (M):1000 | DB Search |
| K.FETQIATK.N | Y | 51.43 | 936.4916 | 8 | -0.7 | 937.494 | 1 | 11.83 | 1.2165 | 1.2236 | 25881 | 4.08e3 | 3 | 3 | 102 | 109 |  |  | DB Search |
| total 2 peptides |  |  |  |  |  |  |  |  |  |  |  |  |  |  |  |  |  |  |  |
| --- | --- | --- | --- | --- | --- | --- | --- | --- | --- | --- | --- | --- | --- | --- | --- | --- | --- | --- | --- |

Best Unique PSM (Scan Sample\_8\_A1\_1\_3645.d:25881, m/z=937.494, z=1, RT=11.83, ppm=-5.22):


TRINITY\_DN902\_c1\_g2\_i1.p1

back to list

  

| Protein Coverage
| Supporting Peptides
| Best Unique PSM
|

Protein Coverage:

Supporting Peptides:

| Peptide | Uniq | -10lgP | Mass | Length | ppm | m/z | z | RT | 1/k0 Start | 1/k0 End | Precursor ID | Area Sample 9 | #Feature | #Feature Sample 9 | Start | End | PTM | AScore | Found By |
| --- | --- | --- | --- | --- | --- | --- | --- | --- | --- | --- | --- | --- | --- | --- | --- | --- | --- | --- | --- |
| K.GLTHC(+57.02)NTLSYK.L | Y | 58.02 | 1292.6183 | 11 | 2.78 | 647.3153 | 2 | 12.39 | 0.9743 | 0.9816 | 29201 | 4.95e3 | 1 | 1 | 62 | 72 | Carbamidomethylation |  | DB Search |
| total 1 peptides |  |  |  |  |  |  |  |  |  |  |  |  |  |  |  |  |  |  |  |
| --- | --- | --- | --- | --- | --- | --- | --- | --- | --- | --- | --- | --- | --- | --- | --- | --- | --- | --- | --- |

Best Unique PSM (Scan Sample\_8\_A1\_1\_3645.d:29201, m/z=647.3153, z=2, RT=12.39, ppm=-1.74):


TRINITY\_DN121866\_c0\_g1\_i1.p1

back to list

  

| Protein Coverage
| Supporting Peptides
| Best Unique PSM
|

Protein Coverage:

Supporting Peptides:

| Peptide | Uniq | -10lgP | Mass | Length | ppm | m/z | z | RT | 1/k0 Start | 1/k0 End | Precursor ID | Area Sample 9 | #Feature | #Feature Sample 9 | Start | End | PTM | AScore | Found By |
| --- | --- | --- | --- | --- | --- | --- | --- | --- | --- | --- | --- | --- | --- | --- | --- | --- | --- | --- | --- |
| K.FSQHTDYVIAR.A | Y | 57.2 | 1335.6571 | 11 | 4.84 | 668.8361 | 2 | 13.42 | 0.9787 | 0.986 | 35261 | 3.02e2 | 1 | 1 | 194 | 204 |  |  | DB Search |
| total 1 peptides |  |  |  |  |  |  |  |  |  |  |  |  |  |  |  |  |  |  |  |
| --- | --- | --- | --- | --- | --- | --- | --- | --- | --- | --- | --- | --- | --- | --- | --- | --- | --- | --- | --- |

Best Unique PSM (Scan Sample\_8\_A1\_1\_3645.d:35261, m/z=668.8361, z=2, RT=13.42, ppm=0.33):


TRINITY\_DN2113\_c0\_g1\_i2.p1

back to list

  

| Protein Coverage
| Supporting Peptides
| Best Unique PSM
|

Protein Coverage:

Supporting Peptides:

| Peptide | Uniq | -10lgP | Mass | Length | ppm | m/z | z | RT | 1/k0 Start | 1/k0 End | Precursor ID | Area Sample 9 | #Feature | #Feature Sample 9 | Start | End | PTM | AScore | Found By |
| --- | --- | --- | --- | --- | --- | --- | --- | --- | --- | --- | --- | --- | --- | --- | --- | --- | --- | --- | --- |
| K.GQFSTDDLVAEVEKR.N | Y | 57.33 | 1692.8318 | 15 | -0.29 | 847.4191 | 2 | 17.65 | 1.143 | 1.1502 | 57525 | 0 | 0 | 0 | 838 | 852 |  |  | DB Search |
| R.ALEHYSDIYDIKR.A | Y | 57 | 1621.81 | 13 | -0.58 | 541.6078 | 3 | 14.56 | 0.8218 | 0.8291 | 41503 | 9.72e2 | 1 | 1 | 626 | 638 |  |  | DB Search |
| R.LADLEEFISSPNHANISQVGER.C | Y | 56.9 | 2425.1873 | 22 | -1.72 | 809.398 | 3 | 18.72 | 0.9263 | 0.9335 | 63624 | 2.17e3 | 1 | 1 | 1183 | 1204 |  |  | DB Search |
| total 3 peptides |  |  |  |  |  |  |  |  |  |  |  |  |  |  |  |  |  |  |  |
| --- | --- | --- | --- | --- | --- | --- | --- | --- | --- | --- | --- | --- | --- | --- | --- | --- | --- | --- | --- |

Best Unique PSM (Scan Sample\_8\_A1\_1\_3645.d:57525, m/z=847.4191, z=2, RT=17.65, ppm=-4.8):


TRINITY\_DN1614\_c0\_g1\_i1.p1

back to list

  

| Protein Coverage
| Supporting Peptides
| Best Unique PSM
|

Protein Coverage:

Supporting Peptides:

| Peptide | Uniq | -10lgP | Mass | Length | ppm | m/z | z | RT | 1/k0 Start | 1/k0 End | Precursor ID | Area Sample 9 | #Feature | #Feature Sample 9 | Start | End | PTM | AScore | Found By |
| --- | --- | --- | --- | --- | --- | --- | --- | --- | --- | --- | --- | --- | --- | --- | --- | --- | --- | --- | --- |
| K.GLDVDSLVIDHIQVNR.A | Y | 57.41 | 1791.9479 | 16 | 0.62 | 896.9777 | 2 | 18.98 | 1.1786 | 1.1857 | 65203 | 1.42e2 | 1 | 1 | 140 | 155 |  |  | DB Search |
| total 1 peptides |  |  |  |  |  |  |  |  |  |  |  |  |  |  |  |  |  |  |  |
| --- | --- | --- | --- | --- | --- | --- | --- | --- | --- | --- | --- | --- | --- | --- | --- | --- | --- | --- | --- |

Best Unique PSM (Scan Sample\_8\_A1\_1\_3645.d:65203, m/z=896.9777, z=2, RT=18.98, ppm=-3.89):


TRINITY\_DN1910\_c0\_g1\_i1.p1

back to list

  

| Protein Coverage
| Supporting Peptides
| Best Unique PSM
|

Protein Coverage:

Supporting Peptides:

| Peptide | Uniq | -10lgP | Mass | Length | ppm | m/z | z | RT | 1/k0 Start | 1/k0 End | Precursor ID | Area Sample 9 | #Feature | #Feature Sample 9 | Start | End | PTM | AScore | Found By |
| --- | --- | --- | --- | --- | --- | --- | --- | --- | --- | --- | --- | --- | --- | --- | --- | --- | --- | --- | --- |
| K.NSSYFVEWIPNNVK.T | Y | 61.49 | 1695.8256 | 14 | 2 | 848.918 | 2 | 19.90 | 1.0889 | 1.0961 | 70553 | 0 | 0 | 0 | 337 | 350 |  |  | DB Search |
| K.NM(+15.99)M(+15.99)AAC(+57.02)DPR.H | Y | 54.81 | 1096.41 | 9 | 2.42 | 549.2111 | 2 | 9.55 | 0.8644 | 0.8716 | 11767 | 3.1e3 | 1 | 1 | 298 | 306 | Oxidation (M), Oxidation (M), Carbamidomethylation | M2:Oxidation (M):1000 M3:Oxidation (M):1000 | DB Search |
| R.FPGQLNADLRK.L | Y | 54.24 | 1257.6829 | 11 | 0.84 | 629.8464 | 2 | 14.26 | 0.9473 | 0.9545 | 39914 | 4.37e2 | 1 | 1 | 242 | 252 |  |  | DB Search |
| total 3 peptides |  |  |  |  |  |  |  |  |  |  |  |  |  |  |  |  |  |  |  |
| --- | --- | --- | --- | --- | --- | --- | --- | --- | --- | --- | --- | --- | --- | --- | --- | --- | --- | --- | --- |

Best Unique PSM (Scan Sample\_8\_A1\_1\_3645.d:70553, m/z=848.918, z=2, RT=19.90, ppm=-2.51):


TRINITY\_DN1058\_c0\_g1\_i4.p1

back to list

  

| Protein Coverage
| Supporting Peptides
| Best Unique PSM
|

Protein Coverage:

Supporting Peptides:

| Peptide | Uniq | -10lgP | Mass | Length | ppm | m/z | z | RT | 1/k0 Start | 1/k0 End | Precursor ID | Area Sample 9 | #Feature | #Feature Sample 9 | Start | End | PTM | AScore | Found By |
| --- | --- | --- | --- | --- | --- | --- | --- | --- | --- | --- | --- | --- | --- | --- | --- | --- | --- | --- | --- |
| R.APTHILNPANKR.S | Y | 57.39 | 1330.7469 | 12 | 0.47 | 666.3781 | 2 | 11.10 | 1.0102 | 1.0174 | 21164 | 1.88e3 | 2 | 2 | 398 | 409 |  |  | DB Search |
| total 1 peptides |  |  |  |  |  |  |  |  |  |  |  |  |  |  |  |  |  |  |  |
| --- | --- | --- | --- | --- | --- | --- | --- | --- | --- | --- | --- | --- | --- | --- | --- | --- | --- | --- | --- |

Best Unique PSM (Scan Sample\_8\_A1\_1\_3645.d:21164, m/z=666.3781, z=2, RT=11.10, ppm=-4.05):


TRINITY\_DN1174\_c2\_g1\_i1.p1

back to list

  

| Protein Coverage
| Supporting Peptides
| Best Unique PSM
|

Protein Coverage:

Supporting Peptides:

| Peptide | Uniq | -10lgP | Mass | Length | ppm | m/z | z | RT | 1/k0 Start | 1/k0 End | Precursor ID | Area Sample 9 | #Feature | #Feature Sample 9 | Start | End | PTM | AScore | Found By |
| --- | --- | --- | --- | --- | --- | --- | --- | --- | --- | --- | --- | --- | --- | --- | --- | --- | --- | --- | --- |
| R.DSNTGSEHTINGK.Y | Y | 61.05 | 1358.6062 | 13 | -4.5 | 680.3043 | 2 | 9.60 | 0.9348 | 0.942 | 12318 | 0 | 0 | 0 | 123 | 135 |  |  | DB Search |
| total 1 peptides |  |  |  |  |  |  |  |  |  |  |  |  |  |  |  |  |  |  |  |
| --- | --- | --- | --- | --- | --- | --- | --- | --- | --- | --- | --- | --- | --- | --- | --- | --- | --- | --- | --- |

Best Unique PSM (Scan Sample\_8\_A1\_1\_3645.d:12318, m/z=680.3043, z=2, RT=9.60, ppm=-9.02):


TRINITY\_DN602\_c0\_g1\_i1.p1

back to list

  

| Protein Coverage
| Supporting Peptides
| Best Unique PSM
|

Protein Coverage:

Supporting Peptides:

| Peptide | Uniq | -10lgP | Mass | Length | ppm | m/z | z | RT | 1/k0 Start | 1/k0 End | Precursor ID | Area Sample 9 | #Feature | #Feature Sample 9 | Start | End | PTM | AScore | Found By |
| --- | --- | --- | --- | --- | --- | --- | --- | --- | --- | --- | --- | --- | --- | --- | --- | --- | --- | --- | --- |
| K.LPWEELWKPC(+57.02)IK.Y | Y | 61.22 | 1597.8326 | 12 | -2.11 | 533.6146 | 3 | 19.85 | 0.8773 | 0.8846 | 70215 | 1.87e3 | 1 | 1 | 170 | 181 | Carbamidomethylation |  | DB Search |
| K.EGFYVHEALAAGIVK.K | Y | 57 | 1602.8405 | 15 | 2.24 | 802.4257 | 2 | 17.51 | 1.0885 | 1.0957 | 56580 | 1.12e3 | 1 | 1 | 185 | 199 |  |  | DB Search |
| total 2 peptides |  |  |  |  |  |  |  |  |  |  |  |  |  |  |  |  |  |  |  |
| --- | --- | --- | --- | --- | --- | --- | --- | --- | --- | --- | --- | --- | --- | --- | --- | --- | --- | --- | --- |

Best Unique PSM (Scan Sample\_8\_A1\_1\_3645.d:70215, m/z=533.6146, z=3, RT=19.85, ppm=-6.63):


TRINITY\_DN36\_c0\_g1\_i16.p1

back to list

  

| Protein Coverage
| Supporting Peptides
| Best Unique PSM
|

Protein Coverage:

Supporting Peptides:

| Peptide | Uniq | -10lgP | Mass | Length | ppm | m/z | z | RT | 1/k0 Start | 1/k0 End | Precursor ID | Area Sample 9 | #Feature | #Feature Sample 9 | Start | End | PTM | AScore | Found By |
| --- | --- | --- | --- | --- | --- | --- | --- | --- | --- | --- | --- | --- | --- | --- | --- | --- | --- | --- | --- |
| K.YGQAM(+15.99)C(+57.02)QFM(+15.99)R.N | N | 61.54 | 1322.5206 | 10 | -1.76 | 662.2634 | 2 | 12.17 | 0.9489 | 0.9562 | 28008 | 2.07e2 | 1 | 1 | 72 | 81 | Oxidation (M), Carbamidomethylation, Oxidation (M) | M5:Oxidation (M):1000 M9:Oxidation (M):1000 | DB Search |
| K.WC(+57.02)DILSIHQSMENC(+57.02)VAK.C | Y | 59.79 | 2089.9383 | 17 | 2.4 | 697.6519 | 3 | 17.87 | 0.8769 | 0.8842 | 58766 | 0 | 0 | 0 | 198 | 214 | Carbamidomethylation, Carbamidomethylation |  | DB Search |
| K.DVYDAEQAERK.R | N | 57.85 | 1322.6102 | 11 | -1.22 | 662.3086 | 2 | 11.56 | 0.9606 | 0.9679 | 24244 | 0 | 0 | 0 | 103 | 113 |  |  | DB Search |
| R.LGGIEVTQGNC(+57.02)AR.K | N | 57.3 | 1373.6721 | 13 | 3.87 | 687.8429 | 2 | 13.70 | 0.9642 | 0.9715 | 36854 | 7.25e2 | 1 | 1 | 58 | 70 | Carbamidomethylation |  | DB Search |
| K.FETQIATK.D | Y | 51.43 | 936.4916 | 8 | -0.7 | 937.494 | 1 | 11.83 | 1.2165 | 1.2236 | 25881 | 4.08e3 | 3 | 3 | 167 | 174 |  |  | DB Search |
| total 5 peptides |  |  |  |  |  |  |  |  |  |  |  |  |  |  |  |  |  |  |  |
| --- | --- | --- | --- | --- | --- | --- | --- | --- | --- | --- | --- | --- | --- | --- | --- | --- | --- | --- | --- |

Best Unique PSM (Scan Sample\_8\_A1\_1\_3645.d:58766, m/z=697.6519, z=3, RT=17.87, ppm=-2.11):


TRINITY\_DN6783\_c0\_g1\_i1.p1

back to list

  

| Protein Coverage
| Supporting Peptides
| Best Unique PSM
|

Protein Coverage:

Supporting Peptides:

| Peptide | Uniq | -10lgP | Mass | Length | ppm | m/z | z | RT | 1/k0 Start | 1/k0 End | Precursor ID | Area Sample 9 | #Feature | #Feature Sample 9 | Start | End | PTM | AScore | Found By |
| --- | --- | --- | --- | --- | --- | --- | --- | --- | --- | --- | --- | --- | --- | --- | --- | --- | --- | --- | --- |
| K.GTIEILGDVHLIK.K | Y | 54.9 | 1406.8133 | 13 | -4.81 | 704.4073 | 2 | 18.93 | 1.062 | 1.0693 | 64948 | 0 | 0 | 0 | 150 | 162 |  |  | DB Search |
| total 1 peptides |  |  |  |  |  |  |  |  |  |  |  |  |  |  |  |  |  |  |  |
| --- | --- | --- | --- | --- | --- | --- | --- | --- | --- | --- | --- | --- | --- | --- | --- | --- | --- | --- | --- |

Best Unique PSM (Scan Sample\_8\_A1\_1\_3645.d:64948, m/z=704.4073, z=2, RT=18.93, ppm=-9.33):


TRINITY\_DN135129\_c0\_g1\_i1.p1

back to list

  

| Protein Coverage
| Supporting Peptides
| Best Unique PSM
|

Protein Coverage:

Supporting Peptides:

| Peptide | Uniq | -10lgP | Mass | Length | ppm | m/z | z | RT | 1/k0 Start | 1/k0 End | Precursor ID | Area Sample 9 | #Feature | #Feature Sample 9 | Start | End | PTM | AScore | Found By |
| --- | --- | --- | --- | --- | --- | --- | --- | --- | --- | --- | --- | --- | --- | --- | --- | --- | --- | --- | --- |
| K.SDSSDIEKR.A | Y | 37.02 | 1035.4832 | 9 | -12.69 | 518.74 | 2 | 11.06 | 0.8644 | 0.8716 | 20916 | 2.04e3 | 1 | 1 | 96 | 104 |  |  | DB Search |
| total 1 peptides |  |  |  |  |  |  |  |  |  |  |  |  |  |  |  |  |  |  |  |
| --- | --- | --- | --- | --- | --- | --- | --- | --- | --- | --- | --- | --- | --- | --- | --- | --- | --- | --- | --- |

Best Unique PSM (Scan Sample\_8\_A1\_1\_3645.d:20916, m/z=518.74, z=2, RT=11.06, ppm=-17.21):


TRINITY\_DN3016\_c0\_g1\_i5.p1

back to list

  

| Protein Coverage
| Supporting Peptides
| Best Unique PSM
|

Protein Coverage:

Supporting Peptides:

| Peptide | Uniq | -10lgP | Mass | Length | ppm | m/z | z | RT | 1/k0 Start | 1/k0 End | Precursor ID | Area Sample 9 | #Feature | #Feature Sample 9 | Start | End | PTM | AScore | Found By |
| --- | --- | --- | --- | --- | --- | --- | --- | --- | --- | --- | --- | --- | --- | --- | --- | --- | --- | --- | --- |
| R.IVNTDLQEIKDIIK.G | Y | 58.4 | 1640.9348 | 14 | -6.31 | 821.4658 | 2 | 18.62 | 1.1514 | 1.1586 | 63065 | 0 | 0 | 0 | 154 | 167 |  |  | DB Search |
| total 1 peptides |  |  |  |  |  |  |  |  |  |  |  |  |  |  |  |  |  |  |  |
| --- | --- | --- | --- | --- | --- | --- | --- | --- | --- | --- | --- | --- | --- | --- | --- | --- | --- | --- | --- |

Best Unique PSM (Scan Sample\_8\_A1\_1\_3645.d:63065, m/z=821.4658, z=2, RT=18.62, ppm=-10.82):


TRINITY\_DN495\_c0\_g1\_i5.p1

back to list

  

| Protein Coverage
| Supporting Peptides
| Best Unique PSM
|

Protein Coverage:

Supporting Peptides:

| Peptide | Uniq | -10lgP | Mass | Length | ppm | m/z | z | RT | 1/k0 Start | 1/k0 End | Precursor ID | Area Sample 9 | #Feature | #Feature Sample 9 | Start | End | PTM | AScore | Found By |
| --- | --- | --- | --- | --- | --- | --- | --- | --- | --- | --- | --- | --- | --- | --- | --- | --- | --- | --- | --- |
| C.SISC(+57.02)SKDEEIK.V | Y | 55.69 | 1294.6075 | 11 | -1.48 | 648.3071 | 2 | 10.83 | 0.9779 | 0.9852 | 19797 | 0 | 0 | 0 | 273 | 283 | Carbamidomethylation |  | DB Search |
| total 1 peptides |  |  |  |  |  |  |  |  |  |  |  |  |  |  |  |  |  |  |  |
| --- | --- | --- | --- | --- | --- | --- | --- | --- | --- | --- | --- | --- | --- | --- | --- | --- | --- | --- | --- |

Best Unique PSM (Scan Sample\_8\_A1\_1\_3645.d:19797, m/z=648.3071, z=2, RT=10.83, ppm=-5.99):


TRINITY\_DN942\_c0\_g1\_i3.p1

back to list

  

| Protein Coverage
| Supporting Peptides
| Best Unique PSM
|

Protein Coverage:

Supporting Peptides:

| Peptide | Uniq | -10lgP | Mass | Length | ppm | m/z | z | RT | 1/k0 Start | 1/k0 End | Precursor ID | Area Sample 9 | #Feature | #Feature Sample 9 | Start | End | PTM | AScore | Found By |
| --- | --- | --- | --- | --- | --- | --- | --- | --- | --- | --- | --- | --- | --- | --- | --- | --- | --- | --- | --- |
| K.AIGNAQRPAR.V | Y | 22.83 | 1052.5839 | 10 | -1.57 | 527.296 | 2 | 9.68 | 0.8664 | 0.8737 | 12735 | 8.15e2 | 1 | 1 | 498 | 507 |  |  | DB Search |
| total 1 peptides |  |  |  |  |  |  |  |  |  |  |  |  |  |  |  |  |  |  |  |
| --- | --- | --- | --- | --- | --- | --- | --- | --- | --- | --- | --- | --- | --- | --- | --- | --- | --- | --- | --- |

Best Unique PSM (Scan Sample\_8\_A1\_1\_3645.d:12735, m/z=527.296, z=2, RT=9.68, ppm=-6.09):


TRINITY\_DN286\_c0\_g1\_i4.p1

back to list

  

| Protein Coverage
| Supporting Peptides
| Best Unique PSM
|

Protein Coverage:

Supporting Peptides:

| Peptide | Uniq | -10lgP | Mass | Length | ppm | m/z | z | RT | 1/k0 Start | 1/k0 End | Precursor ID | Area Sample 9 | #Feature | #Feature Sample 9 | Start | End | PTM | AScore | Found By |
| --- | --- | --- | --- | --- | --- | --- | --- | --- | --- | --- | --- | --- | --- | --- | --- | --- | --- | --- | --- |
| R.YGQAM(+15.99)C(+57.02)QFM(+15.99)R.N | N | 61.54 | 1322.5206 | 10 | -1.76 | 662.2634 | 2 | 12.17 | 0.9489 | 0.9562 | 28008 | 2.07e2 | 1 | 1 | 72 | 81 | Oxidation (M), Carbamidomethylation, Oxidation (M) | M5:Oxidation (M):1000 M9:Oxidation (M):1000 | DB Search |
| K.RYGQAM(+15.99)C(+57.02)QFM(+15.99)R.N | Y | 55.49 | 1478.6217 | 11 | -4.75 | 740.3113 | 2 | 12.88 | 0.9747 | 0.982 | 31970 | 4.25e3 | 1 | 1 | 71 | 81 | Oxidation (M), Carbamidomethylation, Oxidation (M) | M6:Oxidation (M):1000 M10:Oxidation (M):1000 | DB Search |
| K.RYGQAMC(+57.02)QFMR.N | Y | 55.21 | 1446.6319 | 11 | -7.35 | 724.3146 | 2 | 17.19 | 0.9848 | 0.992 | 54851 | 1.37e3 | 1 | 1 | 71 | 81 | Carbamidomethylation |  | DB Search |
| total 3 peptides |  |  |  |  |  |  |  |  |  |  |  |  |  |  |  |  |  |  |  |
| --- | --- | --- | --- | --- | --- | --- | --- | --- | --- | --- | --- | --- | --- | --- | --- | --- | --- | --- | --- |

Best Unique PSM (Scan Sample\_8\_A1\_1\_3645.d:31970, m/z=740.3113, z=2, RT=12.88, ppm=-9.27):


TRINITY\_DN332\_c0\_g1\_i1.p1

back to list

  

| Protein Coverage
| Supporting Peptides
| Best Unique PSM
|

Protein Coverage:

Supporting Peptides:

| Peptide | Uniq | -10lgP | Mass | Length | ppm | m/z | z | RT | 1/k0 Start | 1/k0 End | Precursor ID | Area Sample 9 | #Feature | #Feature Sample 9 | Start | End | PTM | AScore | Found By |
| --- | --- | --- | --- | --- | --- | --- | --- | --- | --- | --- | --- | --- | --- | --- | --- | --- | --- | --- | --- |
| K.AQISKPAPHWEGK.A | Y | 58.03 | 1447.7572 | 13 | 0.06 | 724.8826 | 2 | 11.86 | 1.0098 | 1.017 | 26141 | 0 | 0 | 0 | 50 | 62 |  |  | DB Search |
| K.DYGVLLEDAGHTLR.G | Y | 56.99 | 1557.7787 | 14 | 2.54 | 779.8951 | 2 | 18.20 | 1.129 | 1.1362 | 60565 | 5.51e3 | 1 | 1 | 158 | 171 |  |  | DB Search |
| R.GLFIIDDKGVLR.Q | Y | 55.78 | 1344.7765 | 12 | 1.23 | 673.3933 | 2 | 18.64 | 0.9957 | 1.0029 | 63210 | 5.13e3 | 1 | 1 | 172 | 183 |  |  | DB Search |
| K.IPLLADLTK.Q | Y | 30.37 | 982.6062 | 9 | -1.33 | 492.3075 | 2 | 19.34 | 0.8193 | 0.8266 | 67145 | 6.85e2 | 1 | 1 | 145 | 153 |  |  | DB Search |
| K.AVVNGEFKDLK.L | Y | 25.96 | 1218.6608 | 11 | 8.48 | 610.3401 | 2 | 13.97 | 0.9646 | 0.9719 | 38389 | 0 | 0 | 0 | 63 | 73 |  |  | DB Search |
| total 5 peptides |  |  |  |  |  |  |  |  |  |  |  |  |  |  |  |  |  |  |  |
| --- | --- | --- | --- | --- | --- | --- | --- | --- | --- | --- | --- | --- | --- | --- | --- | --- | --- | --- | --- |

Best Unique PSM (Scan Sample\_8\_A1\_1\_3645.d:26141, m/z=724.8826, z=2, RT=11.86, ppm=-4.46):


TRINITY\_DN982\_c0\_g1\_i3.p1

back to list

  

| Protein Coverage
| Supporting Peptides
| Best Unique PSM
|

Protein Coverage:

Supporting Peptides:

| Peptide | Uniq | -10lgP | Mass | Length | ppm | m/z | z | RT | 1/k0 Start | 1/k0 End | Precursor ID | Area Sample 9 | #Feature | #Feature Sample 9 | Start | End | PTM | AScore | Found By |
| --- | --- | --- | --- | --- | --- | --- | --- | --- | --- | --- | --- | --- | --- | --- | --- | --- | --- | --- | --- |
| K.AVDRGNPPQDSVNK.A | N | 61.59 | 1495.7379 | 14 | -2.2 | 499.5832 | 3 | 10.21 | 0.7823 | 0.7897 | 15988 | 0 | 0 | 0 | 5139 | 5152 |  |  | DB Search |
| R.IQVLKDPDFEFTR.S | N | 58.69 | 1606.8355 | 13 | -7.52 | 804.4153 | 2 | 17.50 | 1.0813 | 1.0885 | 56511 | 4.85e2 | 1 | 1 | 6167 | 6179 |  |  | DB Search |
| R.ITTSQILDHEQQK.L | N | 58.51 | 1539.7893 | 13 | -0.87 | 770.8978 | 2 | 13.81 | 1.0933 | 1.1005 | 37414 | 9.71e2 | 1 | 1 | 5434 | 5446 |  |  | DB Search |
| K.YVAIYPSDHLTVNR.D | N | 57.84 | 1646.8416 | 14 | 0.01 | 824.4244 | 2 | 15.46 | 1.109 | 1.1162 | 46349 | 0 | 0 | 0 | 6254 | 6267 |  |  | DB Search |
| K.TSTQDVALNIRR.S | N | 57.52 | 1372.7423 | 12 | 5.33 | 687.379 | 2 | 13.43 | 0.9989 | 1.0061 | 35260 | 2.7e3 | 1 | 1 | 9063 | 9074 |  |  | DB Search |
| R.VLATDKDTLSSNK.Q | Y | 56.89 | 1390.7303 | 13 | 5.55 | 696.3732 | 2 | 11.48 | 1.015 | 1.0222 | 23698 | 1.47e3 | 1 | 1 | 3827 | 3839 |  |  | DB Search |
| K.TGAISLLASVDFETDRTHTAVVK.A | Y | 56.8 | 2430.2754 | 23 | -2.04 | 608.5721 | 4 | 18.79 | 0.8858 | 0.8931 | 64106 | 2.23e2 | 1 | 1 | 3335 | 3357 |  |  | DB Search |
| K.APIGQSIVTVSAR.D | Y | 56.61 | 1297.7354 | 13 | -0.69 | 649.8716 | 2 | 14.13 | 0.9646 | 0.9719 | 39211 | 0 | 0 | 0 | 4029 | 4041 |  |  | DB Search |
| R.LVQNTPHNSIVTK.I | N | 56.38 | 1449.794 | 13 | 0.62 | 725.9014 | 2 | 11.65 | 1.0604 | 1.0676 | 24679 | 2.98e3 | 1 | 1 | 8569 | 8581 |  |  | DB Search |
| K.VLLSQQIFYDKK.N | N | 55.75 | 1480.8289 | 12 | -2.98 | 741.4162 | 2 | 16.65 | 1.0588 | 1.066 | 52089 | 1.28e3 | 1 | 1 | 4919 | 4930 |  |  | DB Search |
| K.SAIIVDGKNEEGR.I | Y | 55.47 | 1386.7103 | 13 | 0.31 | 694.3595 | 2 | 11.84 | 0.9985 | 1.0057 | 25914 | 5.24e2 | 1 | 1 | 95 | 107 |  |  | DB Search |
| total 11 peptides |  |  |  |  |  |  |  |  |  |  |  |  |  |  |  |  |  |  |  |
| --- | --- | --- | --- | --- | --- | --- | --- | --- | --- | --- | --- | --- | --- | --- | --- | --- | --- | --- | --- |

Best Unique PSM (Scan Sample\_8\_A1\_1\_3645.d:23698, m/z=696.3732, z=2, RT=11.48, ppm=1.03):


TRINITY\_DN27859\_c0\_g1\_i1.p1

back to list

  

| Protein Coverage
| Supporting Peptides
| Best Unique PSM
|

Protein Coverage:

Supporting Peptides:

| Peptide | Uniq | -10lgP | Mass | Length | ppm | m/z | z | RT | 1/k0 Start | 1/k0 End | Precursor ID | Area Sample 9 | #Feature | #Feature Sample 9 | Start | End | PTM | AScore | Found By |
| --- | --- | --- | --- | --- | --- | --- | --- | --- | --- | --- | --- | --- | --- | --- | --- | --- | --- | --- | --- |
| R.VPGTVGHHEVKR.F | Y | 45.31 | 1314.7156 | 12 | -3.84 | 439.2422 | 3 | 9.38 | 0.7477 | 0.7551 | 11211 | 0 | 0 | 0 | 80 | 91 |  |  | DB Search |
| total 1 peptides |  |  |  |  |  |  |  |  |  |  |  |  |  |  |  |  |  |  |  |
| --- | --- | --- | --- | --- | --- | --- | --- | --- | --- | --- | --- | --- | --- | --- | --- | --- | --- | --- | --- |

Best Unique PSM (Scan Sample\_8\_A1\_1\_3645.d:11211, m/z=439.2422, z=3, RT=9.38, ppm=-8.36):


TRINITY\_DN160\_c0\_g1\_i1.p1

back to list

  

| Protein Coverage
| Supporting Peptides
| Best Unique PSM
|

Protein Coverage:

Supporting Peptides:

| Peptide | Uniq | -10lgP | Mass | Length | ppm | m/z | z | RT | 1/k0 Start | 1/k0 End | Precursor ID | Area Sample 9 | #Feature | #Feature Sample 9 | Start | End | PTM | AScore | Found By |
| --- | --- | --- | --- | --- | --- | --- | --- | --- | --- | --- | --- | --- | --- | --- | --- | --- | --- | --- | --- |
| K.LRLDFDHEAKLNM(+15.99)IFR.F | Y | 61.13 | 2033.0516 | 16 | 0.48 | 509.2681 | 4 | 17.75 | 0.8575 | 0.8648 | 57989 | 1.69e3 | 1 | 1 | 1007 | 1022 | Oxidation (M) | M13:Oxidation (M):1000 | DB Search |
| R.LDFDHEAKLNMIFR.F | Y | 57.45 | 1747.8715 | 14 | 5.53 | 583.6317 | 3 | 18.31 | 0.8546 | 0.8619 | 61270 | 0 | 0 | 0 | 1009 | 1022 |  |  | DB Search |
| K.LRLDFDHEAKLNMIFR.F | Y | 56.36 | 2017.0567 | 16 | 0.28 | 505.2693 | 4 | 18.45 | 0.8631 | 0.8704 | 61991 | 1.37e3 | 1 | 1 | 1007 | 1022 |  |  | DB Search |
| K.ELVM(+15.99)HEQSR.C | Y | 53.55 | 1143.5342 | 9 | 15.75 | 572.7808 | 2 | 9.79 | 0.8992 | 0.9065 | 13556 | 4.95e3 | 1 | 1 | 1116 | 1124 | Oxidation (M) | M4:Oxidation (M):1000 | DB Search |
| K.DLEFKFR.E | Y | 51.04 | 953.497 | 7 | -1.17 | 477.7531 | 2 | 16.58 | 0.8307 | 0.838 | 51790 | 5.61e2 | 1 | 1 | 1031 | 1037 |  |  | DB Search |
| total 5 peptides |  |  |  |  |  |  |  |  |  |  |  |  |  |  |  |  |  |  |  |
| --- | --- | --- | --- | --- | --- | --- | --- | --- | --- | --- | --- | --- | --- | --- | --- | --- | --- | --- | --- |

Best Unique PSM (Scan Sample\_8\_A1\_1\_3645.d:57989, m/z=509.2681, z=4, RT=17.75, ppm=-4.04):


TRINITY\_DN1534\_c2\_g1\_i1.p1

back to list

  

| Protein Coverage
| Supporting Peptides
| Best Unique PSM
|

Protein Coverage:

Supporting Peptides:

| Peptide | Uniq | -10lgP | Mass | Length | ppm | m/z | z | RT | 1/k0 Start | 1/k0 End | Precursor ID | Area Sample 9 | #Feature | #Feature Sample 9 | Start | End | PTM | AScore | Found By |
| --- | --- | --- | --- | --- | --- | --- | --- | --- | --- | --- | --- | --- | --- | --- | --- | --- | --- | --- | --- |
| K.KSDYSENEVEK.I | Y | 55.41 | 1326.5939 | 11 | -3.54 | 664.2989 | 2 | 10.49 | 0.9687 | 0.9759 | 17646 | 0 | 0 | 0 | 181 | 191 |  |  | DB Search |
| total 1 peptides |  |  |  |  |  |  |  |  |  |  |  |  |  |  |  |  |  |  |  |
| --- | --- | --- | --- | --- | --- | --- | --- | --- | --- | --- | --- | --- | --- | --- | --- | --- | --- | --- | --- |

Best Unique PSM (Scan Sample\_8\_A1\_1\_3645.d:17646, m/z=664.2989, z=2, RT=10.49, ppm=-8.06):


TRINITY\_DN31399\_c0\_g1\_i3.p1

back to list

  

| Protein Coverage
| Supporting Peptides
| Best Unique PSM
|

Protein Coverage:

Supporting Peptides:

| Peptide | Uniq | -10lgP | Mass | Length | ppm | m/z | z | RT | 1/k0 Start | 1/k0 End | Precursor ID | Area Sample 9 | #Feature | #Feature Sample 9 | Start | End | PTM | AScore | Found By |
| --- | --- | --- | --- | --- | --- | --- | --- | --- | --- | --- | --- | --- | --- | --- | --- | --- | --- | --- | --- |
| K.DTLVFEIDEIRK.S | Y | 55.99 | 1476.7824 | 12 | -10.15 | 739.3876 | 2 | 18.39 | 1.0323 | 1.0395 | 62006 | 2.85e3 | 1 | 1 | 390 | 401 |  |  | DB Search |
| R.VNGADGKVEVVWK.T | Y | 52.96 | 1399.7459 | 13 | -2.44 | 700.8754 | 2 | 14.60 | 1.0705 | 1.0777 | 41798 | 0 | 0 | 0 | 477 | 489 |  |  | DB Search |
| total 2 peptides |  |  |  |  |  |  |  |  |  |  |  |  |  |  |  |  |  |  |  |
| --- | --- | --- | --- | --- | --- | --- | --- | --- | --- | --- | --- | --- | --- | --- | --- | --- | --- | --- | --- |

Best Unique PSM (Scan Sample\_8\_A1\_1\_3645.d:62006, m/z=739.3876, z=2, RT=18.39, ppm=-14.66):


TRINITY\_DN602\_c0\_g1\_i3.p1

back to list

  

| Protein Coverage
| Supporting Peptides
| Best Unique PSM
|

Protein Coverage:

Supporting Peptides:

| Peptide | Uniq | -10lgP | Mass | Length | ppm | m/z | z | RT | 1/k0 Start | 1/k0 End | Precursor ID | Area Sample 9 | #Feature | #Feature Sample 9 | Start | End | PTM | AScore | Found By |
| --- | --- | --- | --- | --- | --- | --- | --- | --- | --- | --- | --- | --- | --- | --- | --- | --- | --- | --- | --- |
| K.LPWEELWKPC(+57.02)IK.Y | Y | 61.22 | 1597.8326 | 12 | -2.11 | 533.6146 | 3 | 19.85 | 0.8773 | 0.8846 | 70215 | 1.87e3 | 1 | 1 | 428 | 439 | Carbamidomethylation |  | DB Search |
| K.EGFYVHEALAAGIVK.K | Y | 57 | 1602.8405 | 15 | 2.24 | 802.4257 | 2 | 17.51 | 1.0885 | 1.0957 | 56580 | 1.12e3 | 1 | 1 | 443 | 457 |  |  | DB Search |
| total 2 peptides |  |  |  |  |  |  |  |  |  |  |  |  |  |  |  |  |  |  |  |
| --- | --- | --- | --- | --- | --- | --- | --- | --- | --- | --- | --- | --- | --- | --- | --- | --- | --- | --- | --- |

Best Unique PSM (Scan Sample\_8\_A1\_1\_3645.d:70215, m/z=533.6146, z=3, RT=19.85, ppm=-6.63):


TRINITY\_DN19237\_c0\_g1\_i1.p1

back to list

  

| Protein Coverage
| Supporting Peptides
| Best Unique PSM
|

Protein Coverage:

Supporting Peptides:

| Peptide | Uniq | -10lgP | Mass | Length | ppm | m/z | z | RT | 1/k0 Start | 1/k0 End | Precursor ID | Area Sample 9 | #Feature | #Feature Sample 9 | Start | End | PTM | AScore | Found By |
| --- | --- | --- | --- | --- | --- | --- | --- | --- | --- | --- | --- | --- | --- | --- | --- | --- | --- | --- | --- |
| K.TLVALDEQGEKLNR.I | Y | 37.76 | 1584.8471 | 14 | -0.16 | 793.4271 | 2 | 14.56 | 1.0981 | 1.1053 | 41619 | 0 | 0 | 0 | 47 | 60 |  |  | DB Search |
| R.ITDKTNANEIR.I | Y | 35.72 | 1273.6626 | 11 | -3.95 | 637.8332 | 2 | 10.73 | 0.9497 | 0.957 | 19159 | 0 | 0 | 0 | 184 | 194 |  |  | DB Search |
| total 2 peptides |  |  |  |  |  |  |  |  |  |  |  |  |  |  |  |  |  |  |  |
| --- | --- | --- | --- | --- | --- | --- | --- | --- | --- | --- | --- | --- | --- | --- | --- | --- | --- | --- | --- |

Best Unique PSM (Scan Sample\_8\_A1\_1\_3645.d:41619, m/z=793.4271, z=2, RT=14.56, ppm=-4.67):


TRINITY\_DN79126\_c0\_g1\_i1.p1

back to list

  

| Protein Coverage
| Supporting Peptides
| Best Unique PSM
|

Protein Coverage:

Supporting Peptides:

| Peptide | Uniq | -10lgP | Mass | Length | ppm | m/z | z | RT | 1/k0 Start | 1/k0 End | Precursor ID | Area Sample 9 | #Feature | #Feature Sample 9 | Start | End | PTM | AScore | Found By |
| --- | --- | --- | --- | --- | --- | --- | --- | --- | --- | --- | --- | --- | --- | --- | --- | --- | --- | --- | --- |
| R.AFLIEEQKLVVR.V | Y | 56.88 | 1443.8449 | 12 | 4.29 | 722.9296 | 2 | 17.30 | 1.0464 | 1.0536 | 55418 | 7.04e2 | 1 | 1 | 92 | 103 |  |  | DB Search |
| total 1 peptides |  |  |  |  |  |  |  |  |  |  |  |  |  |  |  |  |  |  |  |
| --- | --- | --- | --- | --- | --- | --- | --- | --- | --- | --- | --- | --- | --- | --- | --- | --- | --- | --- | --- |

Best Unique PSM (Scan Sample\_8\_A1\_1\_3645.d:55418, m/z=722.9296, z=2, RT=17.30, ppm=-0.23):


TRINITY\_DN1616\_c0\_g1\_i3.p1

back to list

  

| Protein Coverage
| Supporting Peptides
| Best Unique PSM
|

Protein Coverage:

Supporting Peptides:

| Peptide | Uniq | -10lgP | Mass | Length | ppm | m/z | z | RT | 1/k0 Start | 1/k0 End | Precursor ID | Area Sample 9 | #Feature | #Feature Sample 9 | Start | End | PTM | AScore | Found By |
| --- | --- | --- | --- | --- | --- | --- | --- | --- | --- | --- | --- | --- | --- | --- | --- | --- | --- | --- | --- |
| K.VVAEC(+57.02)NNGGGR.F | Y | 57.11 | 1131.5091 | 11 | -7.52 | 566.755 | 2 | 9.43 | 0.8299 | 0.8372 | 11493 | 0 | 0 | 0 | 3736 | 3746 | Carbamidomethylation |  | DB Search |
| K.GC(+57.02)STC(+57.02)GC(+57.02)NGAGTNNGVK.S | Y | 57 | 1712.6665 | 17 | 3.09 | 857.3393 | 2 | 9.83 | 1.0411 | 1.0484 | 13666 | 0 | 0 | 0 | 732 | 748 | Carbamidomethylation, Carbamidomethylation, Carbamidomethylation |  | DB Search |
| K.GSPGELFC(+57.02)TLADATGK.F | Y | 30.98 | 1622.761 | 16 | -3.1 | 812.3816 | 2 | 19.88 | 1.0588 | 1.066 | 70430 | 0 | 0 | 0 | 45 | 60 | Carbamidomethylation |  | DB Search |
| total 3 peptides |  |  |  |  |  |  |  |  |  |  |  |  |  |  |  |  |  |  |  |
| --- | --- | --- | --- | --- | --- | --- | --- | --- | --- | --- | --- | --- | --- | --- | --- | --- | --- | --- | --- |

Best Unique PSM (Scan Sample\_8\_A1\_1\_3645.d:11493, m/z=566.755, z=2, RT=9.43, ppm=-12.04):


TRINITY\_DN3016\_c0\_g1\_i8.p1

back to list

  

| Protein Coverage
| Supporting Peptides
| Best Unique PSM
|

Protein Coverage:

Supporting Peptides:

| Peptide | Uniq | -10lgP | Mass | Length | ppm | m/z | z | RT | 1/k0 Start | 1/k0 End | Precursor ID | Area Sample 9 | #Feature | #Feature Sample 9 | Start | End | PTM | AScore | Found By |
| --- | --- | --- | --- | --- | --- | --- | --- | --- | --- | --- | --- | --- | --- | --- | --- | --- | --- | --- | --- |
| R.IVNTDLQEIKDIIK.G | Y | 58.4 | 1640.9348 | 14 | -6.31 | 821.4658 | 2 | 18.62 | 1.1514 | 1.1586 | 63065 | 0 | 0 | 0 | 154 | 167 |  |  | DB Search |
| total 1 peptides |  |  |  |  |  |  |  |  |  |  |  |  |  |  |  |  |  |  |  |
| --- | --- | --- | --- | --- | --- | --- | --- | --- | --- | --- | --- | --- | --- | --- | --- | --- | --- | --- | --- |

Best Unique PSM (Scan Sample\_8\_A1\_1\_3645.d:63065, m/z=821.4658, z=2, RT=18.62, ppm=-10.82):


TRINITY\_DN1378\_c0\_g1\_i1.p1

back to list

  

| Protein Coverage
| Supporting Peptides
| Best Unique PSM
|

Protein Coverage:

Supporting Peptides:

| Peptide | Uniq | -10lgP | Mass | Length | ppm | m/z | z | RT | 1/k0 Start | 1/k0 End | Precursor ID | Area Sample 9 | #Feature | #Feature Sample 9 | Start | End | PTM | AScore | Found By |
| --- | --- | --- | --- | --- | --- | --- | --- | --- | --- | --- | --- | --- | --- | --- | --- | --- | --- | --- | --- |
| K.LAALDATVHGQTASR.F | Y | 55.14 | 1509.7899 | 15 | 3.37 | 755.9014 | 2 | 13.40 | 1.0741 | 1.0813 | 35059 | 6.88e2 | 1 | 1 | 213 | 227 |  |  | DB Search |
| total 1 peptides |  |  |  |  |  |  |  |  |  |  |  |  |  |  |  |  |  |  |  |
| --- | --- | --- | --- | --- | --- | --- | --- | --- | --- | --- | --- | --- | --- | --- | --- | --- | --- | --- | --- |

Best Unique PSM (Scan Sample\_8\_A1\_1\_3645.d:35059, m/z=755.9014, z=2, RT=13.40, ppm=-1.15):


TRINITY\_DN495\_c0\_g1\_i2.p1

back to list

  

| Protein Coverage
| Supporting Peptides
| Best Unique PSM
|

Protein Coverage:

Supporting Peptides:

| Peptide | Uniq | -10lgP | Mass | Length | ppm | m/z | z | RT | 1/k0 Start | 1/k0 End | Precursor ID | Area Sample 9 | #Feature | #Feature Sample 9 | Start | End | PTM | AScore | Found By |
| --- | --- | --- | --- | --- | --- | --- | --- | --- | --- | --- | --- | --- | --- | --- | --- | --- | --- | --- | --- |
| R.SC(+57.02)TNPAPANGGK.A | Y | 57.85 | 1172.5244 | 12 | 2.11 | 587.2681 | 2 | 9.44 | 0.8502 | 0.8575 | 11479 | 9.79e2 | 1 | 1 | 499 | 510 | Carbamidomethylation |  | DB Search |
| total 1 peptides |  |  |  |  |  |  |  |  |  |  |  |  |  |  |  |  |  |  |  |
| --- | --- | --- | --- | --- | --- | --- | --- | --- | --- | --- | --- | --- | --- | --- | --- | --- | --- | --- | --- |

Best Unique PSM (Scan Sample\_8\_A1\_1\_3645.d:11479, m/z=587.2681, z=2, RT=9.44, ppm=-2.41):


TRINITY\_DN691\_c0\_g1\_i2.p1

back to list

  

| Protein Coverage
| Supporting Peptides
| Best Unique PSM
|

Protein Coverage:

Supporting Peptides:

| Peptide | Uniq | -10lgP | Mass | Length | ppm | m/z | z | RT | 1/k0 Start | 1/k0 End | Precursor ID | Area Sample 9 | #Feature | #Feature Sample 9 | Start | End | PTM | AScore | Found By |
| --- | --- | --- | --- | --- | --- | --- | --- | --- | --- | --- | --- | --- | --- | --- | --- | --- | --- | --- | --- |
| R.AYGQDLGQPGSC(+57.02)LKR.F | Y | 61.86 | 1648.7991 | 15 | 1.51 | 825.4044 | 2 | 13.36 | 1.0761 | 1.0833 | 34861 | 1.57e3 | 2 | 2 | 1480 | 1494 | Carbamidomethylation |  | DB Search |
| total 1 peptides |  |  |  |  |  |  |  |  |  |  |  |  |  |  |  |  |  |  |  |
| --- | --- | --- | --- | --- | --- | --- | --- | --- | --- | --- | --- | --- | --- | --- | --- | --- | --- | --- | --- |

Best Unique PSM (Scan Sample\_8\_A1\_1\_3645.d:34861, m/z=825.4044, z=2, RT=13.36, ppm=-3):


TRINITY\_DN8707\_c0\_g1\_i2.p1

back to list

  

| Protein Coverage
| Supporting Peptides
| Best Unique PSM
|

Protein Coverage:

Supporting Peptides:

| Peptide | Uniq | -10lgP | Mass | Length | ppm | m/z | z | RT | 1/k0 Start | 1/k0 End | Precursor ID | Area Sample 9 | #Feature | #Feature Sample 9 | Start | End | PTM | AScore | Found By |
| --- | --- | --- | --- | --- | --- | --- | --- | --- | --- | --- | --- | --- | --- | --- | --- | --- | --- | --- | --- |
| K.ALLSLEYHEKR.C | Y | 54.96 | 1357.7354 | 11 | 3.5 | 679.8743 | 2 | 14.40 | 1.0263 | 1.0335 | 40618 | 9.37e2 | 1 | 1 | 124 | 134 |  |  | DB Search |
| total 1 peptides |  |  |  |  |  |  |  |  |  |  |  |  |  |  |  |  |  |  |  |
| --- | --- | --- | --- | --- | --- | --- | --- | --- | --- | --- | --- | --- | --- | --- | --- | --- | --- | --- | --- |

Best Unique PSM (Scan Sample\_8\_A1\_1\_3645.d:40618, m/z=679.8743, z=2, RT=14.40, ppm=-1.02):


TRINITY\_DN147947\_c0\_g1\_i1.p1

back to list

  

| Protein Coverage
| Supporting Peptides
| Best Unique PSM
|

Protein Coverage:

Supporting Peptides:

| Peptide | Uniq | -10lgP | Mass | Length | ppm | m/z | z | RT | 1/k0 Start | 1/k0 End | Precursor ID | Area Sample 9 | #Feature | #Feature Sample 9 | Start | End | PTM | AScore | Found By |
| --- | --- | --- | --- | --- | --- | --- | --- | --- | --- | --- | --- | --- | --- | --- | --- | --- | --- | --- | --- |
| R.INVDAAFHDLVR.A | Y | 58.77 | 1368.715 | 12 | 0.58 | 685.3621 | 2 | 18.62 | 1.0246 | 1.0319 | 63073 | 7.69e2 | 1 | 1 | 138 | 149 |  |  | DB Search |
| total 1 peptides |  |  |  |  |  |  |  |  |  |  |  |  |  |  |  |  |  |  |  |
| --- | --- | --- | --- | --- | --- | --- | --- | --- | --- | --- | --- | --- | --- | --- | --- | --- | --- | --- | --- |

Best Unique PSM (Scan Sample\_8\_A1\_1\_3645.d:63073, m/z=685.3621, z=2, RT=18.62, ppm=-3.94):


TRINITY\_DN5500\_c0\_g1\_i2.p1

back to list

  

| Protein Coverage
| Supporting Peptides
| Best Unique PSM
|

Protein Coverage:

Supporting Peptides:

| Peptide | Uniq | -10lgP | Mass | Length | ppm | m/z | z | RT | 1/k0 Start | 1/k0 End | Precursor ID | Area Sample 9 | #Feature | #Feature Sample 9 | Start | End | PTM | AScore | Found By |
| --- | --- | --- | --- | --- | --- | --- | --- | --- | --- | --- | --- | --- | --- | --- | --- | --- | --- | --- | --- |
| K.LAIKENHHLNEPIEK.L | Y | 57.45 | 1783.958 | 15 | -5.85 | 892.9771 | 2 | 12.12 | 1.139 | 1.1462 | 27632 | 0 | 0 | 0 | 187 | 201 |  |  | DB Search |
| K.ENHHLNEPIEK.L | Y | 54.18 | 1358.6578 | 11 | -5.39 | 680.3295 | 2 | 12.12 | 0.9675 | 0.9747 | 27643 | 0 | 0 | 0 | 191 | 201 |  |  | DB Search |
| total 2 peptides |  |  |  |  |  |  |  |  |  |  |  |  |  |  |  |  |  |  |  |
| --- | --- | --- | --- | --- | --- | --- | --- | --- | --- | --- | --- | --- | --- | --- | --- | --- | --- | --- | --- |

Best Unique PSM (Scan Sample\_8\_A1\_1\_3645.d:27632, m/z=892.9771, z=2, RT=12.12, ppm=-10.36):


TRINITY\_DN3016\_c0\_g1\_i11.p1

back to list

  

| Protein Coverage
| Supporting Peptides
| Best Unique PSM
|

Protein Coverage:

Supporting Peptides:

| Peptide | Uniq | -10lgP | Mass | Length | ppm | m/z | z | RT | 1/k0 Start | 1/k0 End | Precursor ID | Area Sample 9 | #Feature | #Feature Sample 9 | Start | End | PTM | AScore | Found By |
| --- | --- | --- | --- | --- | --- | --- | --- | --- | --- | --- | --- | --- | --- | --- | --- | --- | --- | --- | --- |
| R.IVNTDLQEIKDIIK.G | Y | 58.4 | 1640.9348 | 14 | -6.31 | 821.4658 | 2 | 18.62 | 1.1514 | 1.1586 | 63065 | 0 | 0 | 0 | 154 | 167 |  |  | DB Search |
| total 1 peptides |  |  |  |  |  |  |  |  |  |  |  |  |  |  |  |  |  |  |  |
| --- | --- | --- | --- | --- | --- | --- | --- | --- | --- | --- | --- | --- | --- | --- | --- | --- | --- | --- | --- |

Best Unique PSM (Scan Sample\_8\_A1\_1\_3645.d:63065, m/z=821.4658, z=2, RT=18.62, ppm=-10.82):


TRINITY\_DN6920\_c0\_g1\_i3.p1

back to list

  

| Protein Coverage
| Supporting Peptides
| Best Unique PSM
|

Protein Coverage:

Supporting Peptides:

| Peptide | Uniq | -10lgP | Mass | Length | ppm | m/z | z | RT | 1/k0 Start | 1/k0 End | Precursor ID | Area Sample 9 | #Feature | #Feature Sample 9 | Start | End | PTM | AScore | Found By |
| --- | --- | --- | --- | --- | --- | --- | --- | --- | --- | --- | --- | --- | --- | --- | --- | --- | --- | --- | --- |
| K.VAC(+57.02)IGAWHPAR.V | Y | 45.85 | 1236.6186 | 11 | -2.24 | 619.3124 | 2 | 13.17 | 0.9444 | 0.9517 | 33868 | 3.39e2 | 1 | 1 | 258 | 268 | Carbamidomethylation |  | DB Search |
| total 1 peptides |  |  |  |  |  |  |  |  |  |  |  |  |  |  |  |  |  |  |  |
| --- | --- | --- | --- | --- | --- | --- | --- | --- | --- | --- | --- | --- | --- | --- | --- | --- | --- | --- | --- |

Best Unique PSM (Scan Sample\_8\_A1\_1\_3645.d:33868, m/z=619.3124, z=2, RT=13.17, ppm=-6.76):


TRINITY\_DN2885\_c0\_g1\_i2.p1

back to list

  

| Protein Coverage
| Supporting Peptides
| Best Unique PSM
|

Protein Coverage:

Supporting Peptides:

| Peptide | Uniq | -10lgP | Mass | Length | ppm | m/z | z | RT | 1/k0 Start | 1/k0 End | Precursor ID | Area Sample 9 | #Feature | #Feature Sample 9 | Start | End | PTM | AScore | Found By |
| --- | --- | --- | --- | --- | --- | --- | --- | --- | --- | --- | --- | --- | --- | --- | --- | --- | --- | --- | --- |
| K.VTRPHGASGVVR.A | Y | 56.98 | 1234.6894 | 12 | 3.31 | 412.5699 | 3 | 10.06 | 0.8205 | 0.8279 | 15006 | 1.06e3 | 1 | 1 | 77 | 88 |  |  | DB Search |
| total 1 peptides |  |  |  |  |  |  |  |  |  |  |  |  |  |  |  |  |  |  |  |
| --- | --- | --- | --- | --- | --- | --- | --- | --- | --- | --- | --- | --- | --- | --- | --- | --- | --- | --- | --- |

Best Unique PSM (Scan Sample\_8\_A1\_1\_3645.d:15006, m/z=412.5699, z=3, RT=10.06, ppm=-1.21):


TRINITY\_DN32522\_c0\_g1\_i1.p1

back to list

  

| Protein Coverage
| Supporting Peptides
| Best Unique PSM
|

Protein Coverage:

Supporting Peptides:

| Peptide | Uniq | -10lgP | Mass | Length | ppm | m/z | z | RT | 1/k0 Start | 1/k0 End | Precursor ID | Area Sample 9 | #Feature | #Feature Sample 9 | Start | End | PTM | AScore | Found By |
| --- | --- | --- | --- | --- | --- | --- | --- | --- | --- | --- | --- | --- | --- | --- | --- | --- | --- | --- | --- |
| R.VDNKVNATER.K | Y | 52.08 | 1144.5836 | 10 | 4.61 | 382.5352 | 3 | 9.61 | 0.7518 | 0.7591 | 12330 | 3.23e2 | 1 | 1 | 191 | 200 |  |  | DB Search |
| total 1 peptides |  |  |  |  |  |  |  |  |  |  |  |  |  |  |  |  |  |  |  |
| --- | --- | --- | --- | --- | --- | --- | --- | --- | --- | --- | --- | --- | --- | --- | --- | --- | --- | --- | --- |

Best Unique PSM (Scan Sample\_8\_A1\_1\_3645.d:12330, m/z=382.5352, z=3, RT=9.61, ppm=0.09):


TRINITY\_DN875\_c0\_g1\_i3.p1

back to list

  

| Protein Coverage
| Supporting Peptides
| Best Unique PSM
|

Protein Coverage:

Supporting Peptides:

| Peptide | Uniq | -10lgP | Mass | Length | ppm | m/z | z | RT | 1/k0 Start | 1/k0 End | Precursor ID | Area Sample 9 | #Feature | #Feature Sample 9 | Start | End | PTM | AScore | Found By |
| --- | --- | --- | --- | --- | --- | --- | --- | --- | --- | --- | --- | --- | --- | --- | --- | --- | --- | --- | --- |
| R.AVFC(+57.02)EHQGVK.T | Y | 44.56 | 1173.5601 | 10 | -1.64 | 587.7837 | 2 | 11.55 | 0.9012 | 0.9085 | 24166 | 0 | 0 | 0 | 512 | 521 | Carbamidomethylation |  | DB Search |
| total 1 peptides |  |  |  |  |  |  |  |  |  |  |  |  |  |  |  |  |  |  |  |
| --- | --- | --- | --- | --- | --- | --- | --- | --- | --- | --- | --- | --- | --- | --- | --- | --- | --- | --- | --- |

Best Unique PSM (Scan Sample\_8\_A1\_1\_3645.d:24166, m/z=587.7837, z=2, RT=11.55, ppm=-6.15):


TRINITY\_DN2113\_c0\_g1\_i1.p1

back to list

  

| Protein Coverage
| Supporting Peptides
| Best Unique PSM
|

Protein Coverage:

Supporting Peptides:

| Peptide | Uniq | -10lgP | Mass | Length | ppm | m/z | z | RT | 1/k0 Start | 1/k0 End | Precursor ID | Area Sample 9 | #Feature | #Feature Sample 9 | Start | End | PTM | AScore | Found By |
| --- | --- | --- | --- | --- | --- | --- | --- | --- | --- | --- | --- | --- | --- | --- | --- | --- | --- | --- | --- |
| K.GQFSTDDLVAEVEKR.N | Y | 57.33 | 1692.8318 | 15 | -0.29 | 847.4191 | 2 | 17.65 | 1.143 | 1.1502 | 57525 | 0 | 0 | 0 | 838 | 852 |  |  | DB Search |
| R.ALEHYSDIYDIKR.A | Y | 57 | 1621.81 | 13 | -0.58 | 541.6078 | 3 | 14.56 | 0.8218 | 0.8291 | 41503 | 9.72e2 | 1 | 1 | 626 | 638 |  |  | DB Search |
| R.LADLEEFISSPNHANISQVGER.C | Y | 56.9 | 2425.1873 | 22 | -1.72 | 809.398 | 3 | 18.72 | 0.9263 | 0.9335 | 63624 | 2.17e3 | 1 | 1 | 1183 | 1204 |  |  | DB Search |
| total 3 peptides |  |  |  |  |  |  |  |  |  |  |  |  |  |  |  |  |  |  |  |
| --- | --- | --- | --- | --- | --- | --- | --- | --- | --- | --- | --- | --- | --- | --- | --- | --- | --- | --- | --- |

Best Unique PSM (Scan Sample\_8\_A1\_1\_3645.d:57525, m/z=847.4191, z=2, RT=17.65, ppm=-4.8):


TRINITY\_DN5365\_c0\_g1\_i3.p1

back to list

  

| Protein Coverage
| Supporting Peptides
| Best Unique PSM
|

Protein Coverage:

Supporting Peptides:

| Peptide | Uniq | -10lgP | Mass | Length | ppm | m/z | z | RT | 1/k0 Start | 1/k0 End | Precursor ID | Area Sample 9 | #Feature | #Feature Sample 9 | Start | End | PTM | AScore | Found By |
| --- | --- | --- | --- | --- | --- | --- | --- | --- | --- | --- | --- | --- | --- | --- | --- | --- | --- | --- | --- |
| R.FPGQLNADIRK.L | Y | 54.24 | 1257.6829 | 11 | 0.84 | 629.8464 | 2 | 14.26 | 0.9473 | 0.9545 | 39914 | 4.37e2 | 1 | 1 | 244 | 254 |  |  | DB Search |
| total 1 peptides |  |  |  |  |  |  |  |  |  |  |  |  |  |  |  |  |  |  |  |
| --- | --- | --- | --- | --- | --- | --- | --- | --- | --- | --- | --- | --- | --- | --- | --- | --- | --- | --- | --- |

Best Unique PSM (Scan Sample\_8\_A1\_1\_3645.d:39914, m/z=629.8464, z=2, RT=14.26, ppm=-3.67):


TRINITY\_DN329\_c0\_g1\_i1.p1

back to list

  

| Protein Coverage
| Supporting Peptides
| Best Unique PSM
|

Protein Coverage:

Supporting Peptides:

| Peptide | Uniq | -10lgP | Mass | Length | ppm | m/z | z | RT | 1/k0 Start | 1/k0 End | Precursor ID | Area Sample 9 | #Feature | #Feature Sample 9 | Start | End | PTM | AScore | Found By |
| --- | --- | --- | --- | --- | --- | --- | --- | --- | --- | --- | --- | --- | --- | --- | --- | --- | --- | --- | --- |
| K.EGDLVIHSGITDAVK.Y | Y | 59.72 | 1552.8096 | 15 | 2.8 | 777.4108 | 2 | 14.52 | 1.062 | 1.0693 | 41331 | 1.23e3 | 2 | 2 | 174 | 188 |  |  | DB Search |
| K.GLKVDFDTTFAPVTGK.K | Y | 59.12 | 1694.8879 | 16 | 1.5 | 848.4487 | 2 | 18.11 | 1.1033 | 1.1106 | 60153 | 3.39e3 | 1 | 1 | 93 | 108 |  |  | DB Search |
| K.SFHFGVIK.L | Y | 58.79 | 933.5072 | 8 | 5.47 | 467.7613 | 2 | 16.02 | 0.8108 | 0.8181 | 49143 | 1.91e2 | 1 | 1 | 20 | 27 |  |  | DB Search |
| K.GLKVDFDTTFAPVTGKK.T | Y | 57.57 | 1822.9828 | 17 | 2.59 | 912.4969 | 2 | 17.04 | 1.1806 | 1.1877 | 54087 | 1.7e3 | 1 | 1 | 93 | 109 |  |  | DB Search |
| K.SFHFGVIKLEGK.T | Y | 56.87 | 1360.7503 | 12 | 0.52 | 681.3797 | 2 | 16.93 | 1.0271 | 1.0343 | 53439 | 1.06e3 | 1 | 1 | 20 | 31 |  |  | DB Search |
| K.LRPGVELTMSSLVNAK.S | Y | 56.62 | 1713.9447 | 16 | 1.22 | 857.9768 | 2 | 17.67 | 1.1442 | 1.1514 | 57502 | 1.12e3 | 1 | 1 | 250 | 265 |  |  | DB Search |
| K.VDFDTTFAPVTGKK.T | Y | 55.8 | 1524.7824 | 14 | 6.57 | 763.4 | 2 | 17.07 | 1.0512 | 1.0584 | 54189 | 6.56e2 | 1 | 1 | 96 | 109 |  |  | DB Search |
| K.LRPGVELTM(+15.99)SSLVNAK.S | Y | 54.9 | 1729.9396 | 16 | 1.79 | 865.9747 | 2 | 15.15 | 1.135 | 1.1422 | 44549 | 5.95e3 | 1 | 1 | 250 | 265 | Oxidation (M) | M9:Oxidation (M):1000 | DB Search |
| total 8 peptides |  |  |  |  |  |  |  |  |  |  |  |  |  |  |  |  |  |  |  |
| --- | --- | --- | --- | --- | --- | --- | --- | --- | --- | --- | --- | --- | --- | --- | --- | --- | --- | --- | --- |

Best Unique PSM (Scan Sample\_8\_A1\_1\_3645.d:41331, m/z=777.4108, z=2, RT=14.52, ppm=-1.71):


TRINITY\_DN1559\_c0\_g1\_i1.p1

back to list

  

| Protein Coverage
| Supporting Peptides
| Best Unique PSM
|

Protein Coverage:

Supporting Peptides:

| Peptide | Uniq | -10lgP | Mass | Length | ppm | m/z | z | RT | 1/k0 Start | 1/k0 End | Precursor ID | Area Sample 9 | #Feature | #Feature Sample 9 | Start | End | PTM | AScore | Found By |
| --- | --- | --- | --- | --- | --- | --- | --- | --- | --- | --- | --- | --- | --- | --- | --- | --- | --- | --- | --- |
| R.GVAGAGVLAGFDKFK.E | Y | 54.7 | 1435.7823 | 15 | 1.65 | 718.8964 | 2 | 18.22 | 1.0869 | 1.0941 | 60667 | 4.55e3 | 1 | 1 | 282 | 296 |  |  | DB Search |
| total 1 peptides |  |  |  |  |  |  |  |  |  |  |  |  |  |  |  |  |  |  |  |
| --- | --- | --- | --- | --- | --- | --- | --- | --- | --- | --- | --- | --- | --- | --- | --- | --- | --- | --- | --- |

Best Unique PSM (Scan Sample\_8\_A1\_1\_3645.d:60667, m/z=718.8964, z=2, RT=18.22, ppm=-2.87):


TRINITY\_DN2963\_c0\_g1\_i1.p1

back to list

  

| Protein Coverage
| Supporting Peptides
| Best Unique PSM
|

Protein Coverage:

Supporting Peptides:

| Peptide | Uniq | -10lgP | Mass | Length | ppm | m/z | z | RT | 1/k0 Start | 1/k0 End | Precursor ID | Area Sample 9 | #Feature | #Feature Sample 9 | Start | End | PTM | AScore | Found By |
| --- | --- | --- | --- | --- | --- | --- | --- | --- | --- | --- | --- | --- | --- | --- | --- | --- | --- | --- | --- |
| M.S(+42.01)ATGNLVTVLRK.L | Y | 55.5 | 1299.751 | 12 | -1.27 | 650.879 | 2 | 17.03 | 0.9973 | 1.0045 | 53970 | 2.48e2 | 1 | 1 | 2 | 13 | Acetylation (Protein N-term) | S1:Acetylation (Protein N-term):1000 | DB Search |
| total 1 peptides |  |  |  |  |  |  |  |  |  |  |  |  |  |  |  |  |  |  |  |
| --- | --- | --- | --- | --- | --- | --- | --- | --- | --- | --- | --- | --- | --- | --- | --- | --- | --- | --- | --- |

Best Unique PSM (Scan Sample\_8\_A1\_1\_3645.d:53970, m/z=650.879, z=2, RT=17.03, ppm=-5.79):


TRINITY\_DN250\_c0\_g1\_i6.p1

back to list

  

| Protein Coverage
| Supporting Peptides
| Best Unique PSM
|

Protein Coverage:

Supporting Peptides:

| Peptide | Uniq | -10lgP | Mass | Length | ppm | m/z | z | RT | 1/k0 Start | 1/k0 End | Precursor ID | Area Sample 9 | #Feature | #Feature Sample 9 | Start | End | PTM | AScore | Found By |
| --- | --- | --- | --- | --- | --- | --- | --- | --- | --- | --- | --- | --- | --- | --- | --- | --- | --- | --- | --- |
| R.RNLDIERPTYTNLNR.L | Y | 58.31 | 1873.9758 | 15 | 3.94 | 625.6655 | 3 | 14.03 | 0.8846 | 0.8919 | 38584 | 6.19e2 | 1 | 1 | 215 | 229 |  |  | DB Search |
| R.IHFPLATYSPVISAEK.A | Y | 50.18 | 1771.9508 | 16 | 1.46 | 886.98 | 2 | 18.50 | 1.1338 | 1.141 | 62412 | 0 | 0 | 0 | 265 | 280 |  |  | DB Search |
| total 2 peptides |  |  |  |  |  |  |  |  |  |  |  |  |  |  |  |  |  |  |  |
| --- | --- | --- | --- | --- | --- | --- | --- | --- | --- | --- | --- | --- | --- | --- | --- | --- | --- | --- | --- |

Best Unique PSM (Scan Sample\_8\_A1\_1\_3645.d:38584, m/z=625.6655, z=3, RT=14.03, ppm=-0.58):


TRINITY\_DN3036\_c0\_g1\_i8.p1

back to list

  

| Protein Coverage
| Supporting Peptides
| Best Unique PSM
|

Protein Coverage:

Supporting Peptides:

| Peptide | Uniq | -10lgP | Mass | Length | ppm | m/z | z | RT | 1/k0 Start | 1/k0 End | Precursor ID | Area Sample 9 | #Feature | #Feature Sample 9 | Start | End | PTM | AScore | Found By |
| --- | --- | --- | --- | --- | --- | --- | --- | --- | --- | --- | --- | --- | --- | --- | --- | --- | --- | --- | --- |
| R.VIGASGIHVGTM(+15.99)SFGK.M | Y | 60.06 | 1575.8079 | 16 | 1.08 | 788.9085 | 2 | 15.08 | 1.125 | 1.1322 | 44382 | 0 | 0 | 0 | 334 | 349 | Oxidation (M) | M12:Oxidation (M):1000 | DB Search |
| R.GTSNGGLVVGTIIKPK.L | Y | 54.81 | 1539.8984 | 16 | 1.04 | 770.9538 | 2 | 15.65 | 1.1234 | 1.1306 | 47131 | 3.3e3 | 1 | 1 | 173 | 188 |  |  | DB Search |
| total 2 peptides |  |  |  |  |  |  |  |  |  |  |  |  |  |  |  |  |  |  |  |
| --- | --- | --- | --- | --- | --- | --- | --- | --- | --- | --- | --- | --- | --- | --- | --- | --- | --- | --- | --- |

Best Unique PSM (Scan Sample\_8\_A1\_1\_3645.d:44382, m/z=788.9085, z=2, RT=15.08, ppm=-3.43):


TRINITY\_DN761\_c0\_g1\_i2.p1

back to list

  

| Protein Coverage
| Supporting Peptides
| Best Unique PSM
|

Protein Coverage:

Supporting Peptides:

| Peptide | Uniq | -10lgP | Mass | Length | ppm | m/z | z | RT | 1/k0 Start | 1/k0 End | Precursor ID | Area Sample 9 | #Feature | #Feature Sample 9 | Start | End | PTM | AScore | Found By |
| --- | --- | --- | --- | --- | --- | --- | --- | --- | --- | --- | --- | --- | --- | --- | --- | --- | --- | --- | --- |
| K.TRIEDLETVISQTQDHR.H | Y | 57.13 | 2040.0236 | 17 | 0.12 | 681.0121 | 3 | 17.33 | 0.9586 | 0.9658 | 55627 | 6.48e2 | 1 | 1 | 260 | 276 |  |  | DB Search |
| total 1 peptides |  |  |  |  |  |  |  |  |  |  |  |  |  |  |  |  |  |  |  |
| --- | --- | --- | --- | --- | --- | --- | --- | --- | --- | --- | --- | --- | --- | --- | --- | --- | --- | --- | --- |

Best Unique PSM (Scan Sample\_8\_A1\_1\_3645.d:55627, m/z=681.0121, z=3, RT=17.33, ppm=-4.39):


TRINITY\_DN3773\_c0\_g1\_i1.p1

back to list

  

| Protein Coverage
| Supporting Peptides
| Best Unique PSM
|

Protein Coverage:

Supporting Peptides:

| Peptide | Uniq | -10lgP | Mass | Length | ppm | m/z | z | RT | 1/k0 Start | 1/k0 End | Precursor ID | Area Sample 9 | #Feature | #Feature Sample 9 | Start | End | PTM | AScore | Found By |
| --- | --- | --- | --- | --- | --- | --- | --- | --- | --- | --- | --- | --- | --- | --- | --- | --- | --- | --- | --- |
| K.IPIFSAAGLPHNEIAAQIC(+57.02)R.Q | Y | 57.56 | 2177.1415 | 20 | -0.83 | 726.7172 | 3 | 18.81 | 1.0053 | 1.0126 | 64123 | 2.43e3 | 2 | 2 | 178 | 197 | Carbamidomethylation |  | DB Search |
| total 1 peptides |  |  |  |  |  |  |  |  |  |  |  |  |  |  |  |  |  |  |  |
| --- | --- | --- | --- | --- | --- | --- | --- | --- | --- | --- | --- | --- | --- | --- | --- | --- | --- | --- | --- |

Best Unique PSM (Scan Sample\_8\_A1\_1\_3645.d:64123, m/z=726.7172, z=3, RT=18.81, ppm=-5.35):


TRINITY\_DN22\_c0\_g1\_i1.p1

back to list

  

| Protein Coverage
| Supporting Peptides
| Best Unique PSM
|

Protein Coverage:

Supporting Peptides:

| Peptide | Uniq | -10lgP | Mass | Length | ppm | m/z | z | RT | 1/k0 Start | 1/k0 End | Precursor ID | Area Sample 9 | #Feature | #Feature Sample 9 | Start | End | PTM | AScore | Found By |
| --- | --- | --- | --- | --- | --- | --- | --- | --- | --- | --- | --- | --- | --- | --- | --- | --- | --- | --- | --- |
| R.FVLHGDVSHGSR.Y | Y | 56.98 | 1309.6527 | 12 | -4.94 | 437.554 | 3 | 11.96 | 0.7547 | 0.762 | 26738 | 1.72e3 | 1 | 1 | 314 | 325 |  |  | DB Search |
| R.VSSAITHPQWNR.R | Y | 56.85 | 1394.7055 | 12 | 5.44 | 698.3607 | 2 | 13.49 | 1.0106 | 1.0178 | 35612 | 4.48e2 | 1 | 1 | 190 | 201 |  |  | DB Search |
| R.LSRPAQLNR.Y | Y | 51.62 | 1053.6043 | 9 | -5.3 | 527.8043 | 2 | 11.34 | 0.8769 | 0.8842 | 22884 | 0 | 0 | 0 | 213 | 221 |  |  | DB Search |
| total 3 peptides |  |  |  |  |  |  |  |  |  |  |  |  |  |  |  |  |  |  |  |
| --- | --- | --- | --- | --- | --- | --- | --- | --- | --- | --- | --- | --- | --- | --- | --- | --- | --- | --- | --- |

Best Unique PSM (Scan Sample\_8\_A1\_1\_3645.d:26738, m/z=437.554, z=3, RT=11.96, ppm=-9.46):


TRINITY\_DN909\_c0\_g1\_i1.p1

back to list

  

| Protein Coverage
| Supporting Peptides
| Best Unique PSM
|

Protein Coverage:

Supporting Peptides:

| Peptide | Uniq | -10lgP | Mass | Length | ppm | m/z | z | RT | 1/k0 Start | 1/k0 End | Precursor ID | Area Sample 9 | #Feature | #Feature Sample 9 | Start | End | PTM | AScore | Found By |
| --- | --- | --- | --- | --- | --- | --- | --- | --- | --- | --- | --- | --- | --- | --- | --- | --- | --- | --- | --- |
| K.LLAQTTLRNELGTK.N | Y | 21.14 | 1556.8886 | 14 | 3.3 | 779.4506 | 2 | 15.15 | 1.1069 | 1.1142 | 44652 | 7.42e2 | 1 | 1 | 175 | 188 |  |  | DB Search |
| total 1 peptides |  |  |  |  |  |  |  |  |  |  |  |  |  |  |  |  |  |  |  |
| --- | --- | --- | --- | --- | --- | --- | --- | --- | --- | --- | --- | --- | --- | --- | --- | --- | --- | --- | --- |

Best Unique PSM (Scan Sample\_8\_A1\_1\_3645.d:44652, m/z=779.4506, z=2, RT=15.15, ppm=-1.21):


TRINITY\_DN195631\_c0\_g1\_i1.p2

back to list

  

| Protein Coverage
| Supporting Peptides
| Best Unique PSM
|

Protein Coverage:

Supporting Peptides:

| Peptide | Uniq | -10lgP | Mass | Length | ppm | m/z | z | RT | 1/k0 Start | 1/k0 End | Precursor ID | Area Sample 9 | #Feature | #Feature Sample 9 | Start | End | PTM | AScore | Found By |
| --- | --- | --- | --- | --- | --- | --- | --- | --- | --- | --- | --- | --- | --- | --- | --- | --- | --- | --- | --- |
| K.IQDKEGIPPDQQR.L | Y | 61.25 | 1522.7739 | 13 | -1.84 | 762.3894 | 2 | 10.99 | 1.0371 | 1.0443 | 20319 | 2.55e3 | 1 | 1 | 122 | 134 |  |  | DB Search |
| total 1 peptides |  |  |  |  |  |  |  |  |  |  |  |  |  |  |  |  |  |  |  |
| --- | --- | --- | --- | --- | --- | --- | --- | --- | --- | --- | --- | --- | --- | --- | --- | --- | --- | --- | --- |

Best Unique PSM (Scan Sample\_8\_A1\_1\_3645.d:20319, m/z=762.3894, z=2, RT=10.99, ppm=-6.36):


TRINITY\_DN941\_c0\_g1\_i9.p1

back to list

  

| Protein Coverage
| Supporting Peptides
| Best Unique PSM
|

Protein Coverage:

Supporting Peptides:

| Peptide | Uniq | -10lgP | Mass | Length | ppm | m/z | z | RT | 1/k0 Start | 1/k0 End | Precursor ID | Area Sample 9 | #Feature | #Feature Sample 9 | Start | End | PTM | AScore | Found By |
| --- | --- | --- | --- | --- | --- | --- | --- | --- | --- | --- | --- | --- | --- | --- | --- | --- | --- | --- | --- |
| K.VTVKPSSDASK.V | Y | 50.88 | 1117.5979 | 11 | 0.38 | 373.5384 | 3 | 9.61 | 0.7388 | 0.7461 | 12368 | 4.52e2 | 1 | 1 | 922 | 932 |  |  | DB Search |
| D.AELTRPTGK.K | Y | 28.72 | 971.54 | 9 | 2.62 | 486.7763 | 2 | 10.33 | 0.8262 | 0.8335 | 16684 | 4.05e2 | 1 | 1 | 1983 | 1991 |  |  | DB Search |
| total 2 peptides |  |  |  |  |  |  |  |  |  |  |  |  |  |  |  |  |  |  |  |
| --- | --- | --- | --- | --- | --- | --- | --- | --- | --- | --- | --- | --- | --- | --- | --- | --- | --- | --- | --- |

Best Unique PSM (Scan Sample\_8\_A1\_1\_3645.d:12368, m/z=373.5384, z=3, RT=9.61, ppm=-4.14):


TRINITY\_DN1566\_c0\_g1\_i1.p1

back to list

  

| Protein Coverage
| Supporting Peptides
| Best Unique PSM
|

Protein Coverage:

Supporting Peptides:

| Peptide | Uniq | -10lgP | Mass | Length | ppm | m/z | z | RT | 1/k0 Start | 1/k0 End | Precursor ID | Area Sample 9 | #Feature | #Feature Sample 9 | Start | End | PTM | AScore | Found By |
| --- | --- | --- | --- | --- | --- | --- | --- | --- | --- | --- | --- | --- | --- | --- | --- | --- | --- | --- | --- |
| R.IKLEHLITELVHQR.D | Y | 56.23 | 1728.0046 | 14 | 4.73 | 433.0085 | 4 | 18.32 | 0.7823 | 0.7897 | 61379 | 6.43e2 | 1 | 1 | 1781 | 1794 |  |  | DB Search |
| total 1 peptides |  |  |  |  |  |  |  |  |  |  |  |  |  |  |  |  |  |  |  |
| --- | --- | --- | --- | --- | --- | --- | --- | --- | --- | --- | --- | --- | --- | --- | --- | --- | --- | --- | --- |

Best Unique PSM (Scan Sample\_8\_A1\_1\_3645.d:61379, m/z=433.0085, z=4, RT=18.32, ppm=0.21):


TRINITY\_DN571\_c0\_g1\_i1.p1

back to list

  

| Protein Coverage
| Supporting Peptides
| Best Unique PSM
|

Protein Coverage:

Supporting Peptides:

| Peptide | Uniq | -10lgP | Mass | Length | ppm | m/z | z | RT | 1/k0 Start | 1/k0 End | Precursor ID | Area Sample 9 | #Feature | #Feature Sample 9 | Start | End | PTM | AScore | Found By |
| --- | --- | --- | --- | --- | --- | --- | --- | --- | --- | --- | --- | --- | --- | --- | --- | --- | --- | --- | --- |
| K.M(+15.99)KINVNEIFHDLVR.Q | Y | 31.16 | 1742.9137 | 14 | -0.01 | 581.9759 | 3 | 19.54 | 0.8834 | 0.8907 | 68344 | 1.27e2 | 1 | 1 | 150 | 163 | Oxidation (M) | M1:Oxidation (M):1000 | DB Search |
| total 1 peptides |  |  |  |  |  |  |  |  |  |  |  |  |  |  |  |  |  |  |  |
| --- | --- | --- | --- | --- | --- | --- | --- | --- | --- | --- | --- | --- | --- | --- | --- | --- | --- | --- | --- |

Best Unique PSM (Scan Sample\_8\_A1\_1\_3645.d:68344, m/z=581.9759, z=3, RT=19.54, ppm=-4.53):


TRINITY\_DN471\_c0\_g1\_i7.p1

back to list

  

| Protein Coverage
| Supporting Peptides
| Best Unique PSM
|

Protein Coverage:

Supporting Peptides:

| Peptide | Uniq | -10lgP | Mass | Length | ppm | m/z | z | RT | 1/k0 Start | 1/k0 End | Precursor ID | Area Sample 9 | #Feature | #Feature Sample 9 | Start | End | PTM | AScore | Found By |
| --- | --- | --- | --- | --- | --- | --- | --- | --- | --- | --- | --- | --- | --- | --- | --- | --- | --- | --- | --- |
| R.RNLDIERPTYTNLNR.L | Y | 58.31 | 1873.9758 | 15 | 3.94 | 625.6655 | 3 | 14.03 | 0.8846 | 0.8919 | 38584 | 6.19e2 | 1 | 1 | 85 | 99 |  |  | DB Search |
| total 1 peptides |  |  |  |  |  |  |  |  |  |  |  |  |  |  |  |  |  |  |  |
| --- | --- | --- | --- | --- | --- | --- | --- | --- | --- | --- | --- | --- | --- | --- | --- | --- | --- | --- | --- |

Best Unique PSM (Scan Sample\_8\_A1\_1\_3645.d:38584, m/z=625.6655, z=3, RT=14.03, ppm=-0.58):


TRINITY\_DN4781\_c0\_g1\_i3.p3

back to list

  

| Protein Coverage
| Supporting Peptides
| Best Unique PSM
|

Protein Coverage:

Supporting Peptides:

| Peptide | Uniq | -10lgP | Mass | Length | ppm | m/z | z | RT | 1/k0 Start | 1/k0 End | Precursor ID | Area Sample 9 | #Feature | #Feature Sample 9 | Start | End | PTM | AScore | Found By |
| --- | --- | --- | --- | --- | --- | --- | --- | --- | --- | --- | --- | --- | --- | --- | --- | --- | --- | --- | --- |
| R.DNAGTSSSGTGPR.F | Y | 55.71 | 1205.5272 | 13 | 3.47 | 603.7703 | 2 | 9.21 | 0.8615 | 0.8688 | 10492 | 4.49e2 | 1 | 1 | 126 | 138 |  |  | DB Search |
| total 1 peptides |  |  |  |  |  |  |  |  |  |  |  |  |  |  |  |  |  |  |  |
| --- | --- | --- | --- | --- | --- | --- | --- | --- | --- | --- | --- | --- | --- | --- | --- | --- | --- | --- | --- |

Best Unique PSM (Scan Sample\_8\_A1\_1\_3645.d:10492, m/z=603.7703, z=2, RT=9.21, ppm=-1.05):


TRINITY\_DN471\_c0\_g1\_i9.p1

back to list

  

| Protein Coverage
| Supporting Peptides
| Best Unique PSM
|

Protein Coverage:

Supporting Peptides:

| Peptide | Uniq | -10lgP | Mass | Length | ppm | m/z | z | RT | 1/k0 Start | 1/k0 End | Precursor ID | Area Sample 9 | #Feature | #Feature Sample 9 | Start | End | PTM | AScore | Found By |
| --- | --- | --- | --- | --- | --- | --- | --- | --- | --- | --- | --- | --- | --- | --- | --- | --- | --- | --- | --- |
| R.RNLDIERPTYTNLNR.L | Y | 58.31 | 1873.9758 | 15 | 3.94 | 625.6655 | 3 | 14.03 | 0.8846 | 0.8919 | 38584 | 6.19e2 | 1 | 1 | 215 | 229 |  |  | DB Search |
| total 1 peptides |  |  |  |  |  |  |  |  |  |  |  |  |  |  |  |  |  |  |  |
| --- | --- | --- | --- | --- | --- | --- | --- | --- | --- | --- | --- | --- | --- | --- | --- | --- | --- | --- | --- |

Best Unique PSM (Scan Sample\_8\_A1\_1\_3645.d:38584, m/z=625.6655, z=3, RT=14.03, ppm=-0.58):


TRINITY\_DN464\_c0\_g1\_i4.p1

back to list

  

| Protein Coverage
| Supporting Peptides
| Best Unique PSM
|

Protein Coverage:

Supporting Peptides:

| Peptide | Uniq | -10lgP | Mass | Length | ppm | m/z | z | RT | 1/k0 Start | 1/k0 End | Precursor ID | Area Sample 9 | #Feature | #Feature Sample 9 | Start | End | PTM | AScore | Found By |
| --- | --- | --- | --- | --- | --- | --- | --- | --- | --- | --- | --- | --- | --- | --- | --- | --- | --- | --- | --- |
| R.LLEFVGHDVLR.L | Y | 55.02 | 1296.719 | 11 | 3.47 | 649.3661 | 2 | 17.73 | 1.0029 | 1.0102 | 57955 | 5.47e2 | 1 | 1 | 279 | 289 |  |  | DB Search |
| total 1 peptides |  |  |  |  |  |  |  |  |  |  |  |  |  |  |  |  |  |  |  |
| --- | --- | --- | --- | --- | --- | --- | --- | --- | --- | --- | --- | --- | --- | --- | --- | --- | --- | --- | --- |

Best Unique PSM (Scan Sample\_8\_A1\_1\_3645.d:57955, m/z=649.3661, z=2, RT=17.73, ppm=-1.04):


TRINITY\_DN3016\_c0\_g1\_i18.p1

back to list

  

| Protein Coverage
| Supporting Peptides
| Best Unique PSM
|

Protein Coverage:

Supporting Peptides:

| Peptide | Uniq | -10lgP | Mass | Length | ppm | m/z | z | RT | 1/k0 Start | 1/k0 End | Precursor ID | Area Sample 9 | #Feature | #Feature Sample 9 | Start | End | PTM | AScore | Found By |
| --- | --- | --- | --- | --- | --- | --- | --- | --- | --- | --- | --- | --- | --- | --- | --- | --- | --- | --- | --- |
| R.IVNTDLQEIKDIIK.G | Y | 58.4 | 1640.9348 | 14 | -6.31 | 821.4658 | 2 | 18.62 | 1.1514 | 1.1586 | 63065 | 0 | 0 | 0 | 154 | 167 |  |  | DB Search |
| total 1 peptides |  |  |  |  |  |  |  |  |  |  |  |  |  |  |  |  |  |  |  |
| --- | --- | --- | --- | --- | --- | --- | --- | --- | --- | --- | --- | --- | --- | --- | --- | --- | --- | --- | --- |

Best Unique PSM (Scan Sample\_8\_A1\_1\_3645.d:63065, m/z=821.4658, z=2, RT=18.62, ppm=-10.82):


TRINITY\_DN3016\_c0\_g1\_i6.p1

back to list

  

| Protein Coverage
| Supporting Peptides
| Best Unique PSM
|

Protein Coverage:

Supporting Peptides:

| Peptide | Uniq | -10lgP | Mass | Length | ppm | m/z | z | RT | 1/k0 Start | 1/k0 End | Precursor ID | Area Sample 9 | #Feature | #Feature Sample 9 | Start | End | PTM | AScore | Found By |
| --- | --- | --- | --- | --- | --- | --- | --- | --- | --- | --- | --- | --- | --- | --- | --- | --- | --- | --- | --- |
| R.IVNTDLQEIKDIIK.G | Y | 58.4 | 1640.9348 | 14 | -6.31 | 821.4658 | 2 | 18.62 | 1.1514 | 1.1586 | 63065 | 0 | 0 | 0 | 154 | 167 |  |  | DB Search |
| total 1 peptides |  |  |  |  |  |  |  |  |  |  |  |  |  |  |  |  |  |  |  |
| --- | --- | --- | --- | --- | --- | --- | --- | --- | --- | --- | --- | --- | --- | --- | --- | --- | --- | --- | --- |

Best Unique PSM (Scan Sample\_8\_A1\_1\_3645.d:63065, m/z=821.4658, z=2, RT=18.62, ppm=-10.82):


TRINITY\_DN1143\_c0\_g1\_i1.p1

back to list

  

| Protein Coverage
| Supporting Peptides
| Best Unique PSM
|

Protein Coverage:

Supporting Peptides:

| Peptide | Uniq | -10lgP | Mass | Length | ppm | m/z | z | RT | 1/k0 Start | 1/k0 End | Precursor ID | Area Sample 9 | #Feature | #Feature Sample 9 | Start | End | PTM | AScore | Found By |
| --- | --- | --- | --- | --- | --- | --- | --- | --- | --- | --- | --- | --- | --- | --- | --- | --- | --- | --- | --- |
| K.GLIAQAIDHAASR.T | Y | 55.56 | 1321.7102 | 13 | 5.69 | 661.8632 | 2 | 16.04 | 1.0086 | 1.0158 | 49202 | 0 | 0 | 0 | 328 | 340 |  |  | DB Search |
| total 1 peptides |  |  |  |  |  |  |  |  |  |  |  |  |  |  |  |  |  |  |  |
| --- | --- | --- | --- | --- | --- | --- | --- | --- | --- | --- | --- | --- | --- | --- | --- | --- | --- | --- | --- |

Best Unique PSM (Scan Sample\_8\_A1\_1\_3645.d:49202, m/z=661.8632, z=2, RT=16.04, ppm=1.18):


TRINITY\_DN201860\_c0\_g1\_i1.p1

back to list

  

| Protein Coverage
| Supporting Peptides
| Best Unique PSM
|

Protein Coverage:

Supporting Peptides:

| Peptide | Uniq | -10lgP | Mass | Length | ppm | m/z | z | RT | 1/k0 Start | 1/k0 End | Precursor ID | Area Sample 9 | #Feature | #Feature Sample 9 | Start | End | PTM | AScore | Found By |
| --- | --- | --- | --- | --- | --- | --- | --- | --- | --- | --- | --- | --- | --- | --- | --- | --- | --- | --- | --- |
| K.VVC(+57.02)STTSGSATGSK.D | Y | 57.09 | 1340.6242 | 14 | -0.36 | 671.3161 | 2 | 9.67 | 0.9352 | 0.9424 | 12707 | 3.78e2 | 1 | 1 | 7 | 20 | Carbamidomethylation |  | DB Search |
| total 1 peptides |  |  |  |  |  |  |  |  |  |  |  |  |  |  |  |  |  |  |  |
| --- | --- | --- | --- | --- | --- | --- | --- | --- | --- | --- | --- | --- | --- | --- | --- | --- | --- | --- | --- |

Best Unique PSM (Scan Sample\_8\_A1\_1\_3645.d:12707, m/z=670.7936, z=2, RT=9.71, ppm=-4.87):


TRINITY\_DN36857\_c0\_g1\_i1.p2

back to list

  

| Protein Coverage
| Supporting Peptides
| Best Unique PSM
|

Protein Coverage:

Supporting Peptides:

| Peptide | Uniq | -10lgP | Mass | Length | ppm | m/z | z | RT | 1/k0 Start | 1/k0 End | Precursor ID | Area Sample 9 | #Feature | #Feature Sample 9 | Start | End | PTM | AScore | Found By |
| --- | --- | --- | --- | --- | --- | --- | --- | --- | --- | --- | --- | --- | --- | --- | --- | --- | --- | --- | --- |
| R.AFLLDHFAPER.M | Y | 54.32 | 1314.672 | 11 | 4.57 | 658.3433 | 2 | 17.94 | 0.9908 | 0.9981 | 59095 | 2.39e2 | 1 | 1 | 30 | 40 |  |  | DB Search |
| total 1 peptides |  |  |  |  |  |  |  |  |  |  |  |  |  |  |  |  |  |  |  |
| --- | --- | --- | --- | --- | --- | --- | --- | --- | --- | --- | --- | --- | --- | --- | --- | --- | --- | --- | --- |

Best Unique PSM (Scan Sample\_8\_A1\_1\_3645.d:59095, m/z=658.3433, z=2, RT=17.94, ppm=0.05):


TRINITY\_DN36\_c0\_g1\_i3.p1

back to list

  

| Protein Coverage
| Supporting Peptides
| Best Unique PSM
|

Protein Coverage:

Supporting Peptides:

| Peptide | Uniq | -10lgP | Mass | Length | ppm | m/z | z | RT | 1/k0 Start | 1/k0 End | Precursor ID | Area Sample 9 | #Feature | #Feature Sample 9 | Start | End | PTM | AScore | Found By |
| --- | --- | --- | --- | --- | --- | --- | --- | --- | --- | --- | --- | --- | --- | --- | --- | --- | --- | --- | --- |
| K.YGQAM(+15.99)C(+57.02)QFM(+15.99)R.N | N | 61.54 | 1322.5206 | 10 | -1.76 | 662.2634 | 2 | 12.17 | 0.9489 | 0.9562 | 28008 | 2.07e2 | 1 | 1 | 72 | 81 | Oxidation (M), Carbamidomethylation, Oxidation (M) | M5:Oxidation (M):1000 M9:Oxidation (M):1000 | DB Search |
| K.WC(+57.02)DILSIHQSMENC(+57.02)VAK.C | Y | 59.79 | 2089.9383 | 17 | 2.4 | 697.6519 | 3 | 17.87 | 0.8769 | 0.8842 | 58766 | 0 | 0 | 0 | 198 | 214 | Carbamidomethylation, Carbamidomethylation |  | DB Search |
| K.DVYDAEQAERK.R | N | 57.85 | 1322.6102 | 11 | -1.22 | 662.3086 | 2 | 11.56 | 0.9606 | 0.9679 | 24244 | 0 | 0 | 0 | 103 | 113 |  |  | DB Search |
| R.LGGIEVTQGNC(+57.02)AR.K | N | 57.3 | 1373.6721 | 13 | 3.87 | 687.8429 | 2 | 13.70 | 0.9642 | 0.9715 | 36854 | 7.25e2 | 1 | 1 | 58 | 70 | Carbamidomethylation |  | DB Search |
| K.FETQIATK.D | Y | 51.43 | 936.4916 | 8 | -0.7 | 937.494 | 1 | 11.83 | 1.2165 | 1.2236 | 25881 | 4.08e3 | 3 | 3 | 167 | 174 |  |  | DB Search |
| total 5 peptides |  |  |  |  |  |  |  |  |  |  |  |  |  |  |  |  |  |  |  |
| --- | --- | --- | --- | --- | --- | --- | --- | --- | --- | --- | --- | --- | --- | --- | --- | --- | --- | --- | --- |

Best Unique PSM (Scan Sample\_8\_A1\_1\_3645.d:58766, m/z=697.6519, z=3, RT=17.87, ppm=-2.11):


TRINITY\_DN195934\_c0\_g1\_i1.p2

back to list

  

| Protein Coverage
| Supporting Peptides
| Best Unique PSM
|

Protein Coverage:

Supporting Peptides:

| Peptide | Uniq | -10lgP | Mass | Length | ppm | m/z | z | RT | 1/k0 Start | 1/k0 End | Precursor ID | Area Sample 9 | #Feature | #Feature Sample 9 | Start | End | PTM | AScore | Found By |
| --- | --- | --- | --- | --- | --- | --- | --- | --- | --- | --- | --- | --- | --- | --- | --- | --- | --- | --- | --- |
| K.DSYVGDEAQSKR.G | Y | 58.12 | 1353.616 | 12 | 4.44 | 677.8152 | 2 | 10.93 | 1.0065 | 1.0138 | 20214 | 8.61e3 | 1 | 1 | 10 | 21 |  |  | DB Search |
| R.VAPEEHPVLLTEAPLNPK.A | Y | 57.43 | 1953.0571 | 18 | 1.47 | 977.5328 | 2 | 16.35 | 1.2073 | 1.2145 | 50611 | 1.52e2 | 1 | 1 | 55 | 72 |  |  | DB Search |
| total 2 peptides |  |  |  |  |  |  |  |  |  |  |  |  |  |  |  |  |  |  |  |
| --- | --- | --- | --- | --- | --- | --- | --- | --- | --- | --- | --- | --- | --- | --- | --- | --- | --- | --- | --- |

Best Unique PSM (Scan Sample\_8\_A1\_1\_3645.d:20214, m/z=677.8152, z=2, RT=10.93, ppm=-0.08):


TRINITY\_DN8101\_c0\_g1\_i2.p2

back to list

  

| Protein Coverage
| Supporting Peptides
| Best Unique PSM
|

Protein Coverage:

Supporting Peptides:

| Peptide | Uniq | -10lgP | Mass | Length | ppm | m/z | z | RT | 1/k0 Start | 1/k0 End | Precursor ID | Area Sample 9 | #Feature | #Feature Sample 9 | Start | End | PTM | AScore | Found By |
| --- | --- | --- | --- | --- | --- | --- | --- | --- | --- | --- | --- | --- | --- | --- | --- | --- | --- | --- | --- |
| R.SIGFIGHYLDQLR.L | Y | 55.88 | 1517.799 | 13 | -0.14 | 759.9033 | 2 | 19.91 | 1.0849 | 1.0921 | 70505 | 4.59e2 | 1 | 1 | 784 | 796 |  |  | DB Search |
| total 1 peptides |  |  |  |  |  |  |  |  |  |  |  |  |  |  |  |  |  |  |  |
| --- | --- | --- | --- | --- | --- | --- | --- | --- | --- | --- | --- | --- | --- | --- | --- | --- | --- | --- | --- |

Best Unique PSM (Scan Sample\_8\_A1\_1\_3645.d:70505, m/z=759.9033, z=2, RT=19.91, ppm=-4.65):


TRINITY\_DN30329\_c0\_g1\_i1.p1

back to list

  

| Protein Coverage
| Supporting Peptides
| Best Unique PSM
|

Protein Coverage:

Supporting Peptides:

| Peptide | Uniq | -10lgP | Mass | Length | ppm | m/z | z | RT | 1/k0 Start | 1/k0 End | Precursor ID | Area Sample 9 | #Feature | #Feature Sample 9 | Start | End | PTM | AScore | Found By |
| --- | --- | --- | --- | --- | --- | --- | --- | --- | --- | --- | --- | --- | --- | --- | --- | --- | --- | --- | --- |
| K.GQELTFKEFFKPSNFVR.S | Y | 56.99 | 2073.0683 | 17 | 0.69 | 692.0274 | 3 | 19.20 | 0.9291 | 0.9364 | 66465 | 4.36e3 | 1 | 1 | 160 | 176 |  |  | DB Search |
| total 1 peptides |  |  |  |  |  |  |  |  |  |  |  |  |  |  |  |  |  |  |  |
| --- | --- | --- | --- | --- | --- | --- | --- | --- | --- | --- | --- | --- | --- | --- | --- | --- | --- | --- | --- |

Best Unique PSM (Scan Sample\_8\_A1\_1\_3645.d:66465, m/z=692.0274, z=3, RT=19.20, ppm=-3.83):


TRINITY\_DN30329\_c0\_g2\_i1.p2

back to list

  

| Protein Coverage
| Supporting Peptides
| Best Unique PSM
|

Protein Coverage:

Supporting Peptides:

| Peptide | Uniq | -10lgP | Mass | Length | ppm | m/z | z | RT | 1/k0 Start | 1/k0 End | Precursor ID | Area Sample 9 | #Feature | #Feature Sample 9 | Start | End | PTM | AScore | Found By |
| --- | --- | --- | --- | --- | --- | --- | --- | --- | --- | --- | --- | --- | --- | --- | --- | --- | --- | --- | --- |
| K.GQELTFKEFFKPSNFVR.S | Y | 56.99 | 2073.0683 | 17 | 0.69 | 692.0274 | 3 | 19.20 | 0.9291 | 0.9364 | 66465 | 4.36e3 | 1 | 1 | 184 | 200 |  |  | DB Search |
| total 1 peptides |  |  |  |  |  |  |  |  |  |  |  |  |  |  |  |  |  |  |  |
| --- | --- | --- | --- | --- | --- | --- | --- | --- | --- | --- | --- | --- | --- | --- | --- | --- | --- | --- | --- |

Best Unique PSM (Scan Sample\_8\_A1\_1\_3645.d:66465, m/z=692.0274, z=3, RT=19.20, ppm=-3.83):


TRINITY\_DN66856\_c0\_g1\_i1.p1

back to list

  

| Protein Coverage
| Supporting Peptides
| Best Unique PSM
|

Protein Coverage:

Supporting Peptides:

| Peptide | Uniq | -10lgP | Mass | Length | ppm | m/z | z | RT | 1/k0 Start | 1/k0 End | Precursor ID | Area Sample 9 | #Feature | #Feature Sample 9 | Start | End | PTM | AScore | Found By |
| --- | --- | --- | --- | --- | --- | --- | --- | --- | --- | --- | --- | --- | --- | --- | --- | --- | --- | --- | --- |
| R.KGFIM(+15.99)VIEAIAGGGHK.G | Y | 60.58 | 1642.8864 | 16 | -0.13 | 548.6335 | 3 | 19.76 | 0.8445 | 0.8518 | 69782 | 0 | 0 | 0 | 280 | 295 | Oxidation (M) | M5:Oxidation (M):1000 | DB Search |
| R.KGFIMVIEAIAGGGHK.G | Y | 57.99 | 1626.8915 | 16 | -8.55 | 543.2974 | 3 | 19.95 | 0.855 | 0.8623 | 70831 | 0 | 0 | 0 | 280 | 295 |  |  | DB Search |
| total 2 peptides |  |  |  |  |  |  |  |  |  |  |  |  |  |  |  |  |  |  |  |
| --- | --- | --- | --- | --- | --- | --- | --- | --- | --- | --- | --- | --- | --- | --- | --- | --- | --- | --- | --- |

Best Unique PSM (Scan Sample\_8\_A1\_1\_3645.d:69782, m/z=548.6335, z=3, RT=19.76, ppm=-4.65):


TRINITY\_DN5554\_c0\_g1\_i1.p1

back to list

  

| Protein Coverage
| Supporting Peptides
| Best Unique PSM
|

Protein Coverage:

Supporting Peptides:

| Peptide | Uniq | -10lgP | Mass | Length | ppm | m/z | z | RT | 1/k0 Start | 1/k0 End | Precursor ID | Area Sample 9 | #Feature | #Feature Sample 9 | Start | End | PTM | AScore | Found By |
| --- | --- | --- | --- | --- | --- | --- | --- | --- | --- | --- | --- | --- | --- | --- | --- | --- | --- | --- | --- |
| K.FFGSLDQIKFEDQK.V | Y | 56.37 | 1700.8409 | 14 | 7.65 | 851.4304 | 2 | 18.08 | 1.119 | 1.1262 | 59913 | 0 | 0 | 0 | 180 | 193 |  |  | DB Search |
| total 1 peptides |  |  |  |  |  |  |  |  |  |  |  |  |  |  |  |  |  |  |  |
| --- | --- | --- | --- | --- | --- | --- | --- | --- | --- | --- | --- | --- | --- | --- | --- | --- | --- | --- | --- |

Best Unique PSM (Scan Sample\_8\_A1\_1\_3645.d:59913, m/z=851.4304, z=2, RT=18.08, ppm=3.14):


TRINITY\_DN471\_c0\_g1\_i2.p1

back to list

  

| Protein Coverage
| Supporting Peptides
| Best Unique PSM
|

Protein Coverage:

Supporting Peptides:

| Peptide | Uniq | -10lgP | Mass | Length | ppm | m/z | z | RT | 1/k0 Start | 1/k0 End | Precursor ID | Area Sample 9 | #Feature | #Feature Sample 9 | Start | End | PTM | AScore | Found By |
| --- | --- | --- | --- | --- | --- | --- | --- | --- | --- | --- | --- | --- | --- | --- | --- | --- | --- | --- | --- |
| R.RNLDIERPTYTNLNR.L | Y | 58.31 | 1873.9758 | 15 | 3.94 | 625.6655 | 3 | 14.03 | 0.8846 | 0.8919 | 38584 | 6.19e2 | 1 | 1 | 253 | 267 |  |  | DB Search |
| total 1 peptides |  |  |  |  |  |  |  |  |  |  |  |  |  |  |  |  |  |  |  |
| --- | --- | --- | --- | --- | --- | --- | --- | --- | --- | --- | --- | --- | --- | --- | --- | --- | --- | --- | --- |

Best Unique PSM (Scan Sample\_8\_A1\_1\_3645.d:38584, m/z=625.6655, z=3, RT=14.03, ppm=-0.58):


TRINITY\_DN1933\_c0\_g1\_i3.p2

back to list

  

| Protein Coverage
| Supporting Peptides
| Best Unique PSM
|

Protein Coverage:

Supporting Peptides:

| Peptide | Uniq | -10lgP | Mass | Length | ppm | m/z | z | RT | 1/k0 Start | 1/k0 End | Precursor ID | Area Sample 9 | #Feature | #Feature Sample 9 | Start | End | PTM | AScore | Found By |
| --- | --- | --- | --- | --- | --- | --- | --- | --- | --- | --- | --- | --- | --- | --- | --- | --- | --- | --- | --- |
| R.RNLDIERPTYTNLNR.L | Y | 58.31 | 1873.9758 | 15 | 3.94 | 625.6655 | 3 | 14.03 | 0.8846 | 0.8919 | 38584 | 6.19e2 | 1 | 1 | 75 | 89 |  |  | DB Search |
| total 1 peptides |  |  |  |  |  |  |  |  |  |  |  |  |  |  |  |  |  |  |  |
| --- | --- | --- | --- | --- | --- | --- | --- | --- | --- | --- | --- | --- | --- | --- | --- | --- | --- | --- | --- |

Best Unique PSM (Scan Sample\_8\_A1\_1\_3645.d:38584, m/z=625.6655, z=3, RT=14.03, ppm=-0.58):


TRINITY\_DN165\_c0\_g1\_i1.p1

back to list

  

| Protein Coverage
| Supporting Peptides
| Best Unique PSM
|

Protein Coverage:

Supporting Peptides:

| Peptide | Uniq | -10lgP | Mass | Length | ppm | m/z | z | RT | 1/k0 Start | 1/k0 End | Precursor ID | Area Sample 9 | #Feature | #Feature Sample 9 | Start | End | PTM | AScore | Found By |
| --- | --- | --- | --- | --- | --- | --- | --- | --- | --- | --- | --- | --- | --- | --- | --- | --- | --- | --- | --- |
| K.TPFNFEDKPFIVQYEVR.F | Y | 52.73 | 2128.0629 | 17 | -3.58 | 710.3558 | 3 | 19.45 | 0.944 | 0.9513 | 67842 | 1.37e2 | 1 | 1 | 114 | 130 |  |  | DB Search |
| total 1 peptides |  |  |  |  |  |  |  |  |  |  |  |  |  |  |  |  |  |  |  |
| --- | --- | --- | --- | --- | --- | --- | --- | --- | --- | --- | --- | --- | --- | --- | --- | --- | --- | --- | --- |

Best Unique PSM (Scan Sample\_8\_A1\_1\_3645.d:67842, m/z=710.3558, z=3, RT=19.45, ppm=-8.09):


TRINITY\_DN751\_c0\_g1\_i1.p1

back to list

  

| Protein Coverage
| Supporting Peptides
| Best Unique PSM
|

Protein Coverage:

Supporting Peptides:

| Peptide | Uniq | -10lgP | Mass | Length | ppm | m/z | z | RT | 1/k0 Start | 1/k0 End | Precursor ID | Area Sample 9 | #Feature | #Feature Sample 9 | Start | End | PTM | AScore | Found By |
| --- | --- | --- | --- | --- | --- | --- | --- | --- | --- | --- | --- | --- | --- | --- | --- | --- | --- | --- | --- |
| R.LAADLVITHSSK.S | Y | 54.93 | 1253.6979 | 12 | 0.86 | 627.8539 | 2 | 14.19 | 0.9957 | 1.0029 | 39454 | 5.38e3 | 1 | 1 | 166 | 177 |  |  | DB Search |
| total 1 peptides |  |  |  |  |  |  |  |  |  |  |  |  |  |  |  |  |  |  |  |
| --- | --- | --- | --- | --- | --- | --- | --- | --- | --- | --- | --- | --- | --- | --- | --- | --- | --- | --- | --- |

Best Unique PSM (Scan Sample\_8\_A1\_1\_3645.d:39454, m/z=627.8539, z=2, RT=14.19, ppm=-3.66):


TRINITY\_DN247\_c0\_g1\_i1.p1

back to list

  

| Protein Coverage
| Supporting Peptides
| Best Unique PSM
|

Protein Coverage:

Supporting Peptides:

| Peptide | Uniq | -10lgP | Mass | Length | ppm | m/z | z | RT | 1/k0 Start | 1/k0 End | Precursor ID | Area Sample 9 | #Feature | #Feature Sample 9 | Start | End | PTM | AScore | Found By |
| --- | --- | --- | --- | --- | --- | --- | --- | --- | --- | --- | --- | --- | --- | --- | --- | --- | --- | --- | --- |
| R.DAPIYGLQWHPEK.S | Y | 61.22 | 1552.7674 | 13 | -3.87 | 777.3845 | 2 | 17.19 | 1.0672 | 1.0745 | 54959 | 5.27e2 | 1 | 1 | 258 | 270 |  |  | DB Search |
| total 1 peptides |  |  |  |  |  |  |  |  |  |  |  |  |  |  |  |  |  |  |  |
| --- | --- | --- | --- | --- | --- | --- | --- | --- | --- | --- | --- | --- | --- | --- | --- | --- | --- | --- | --- |

Best Unique PSM (Scan Sample\_8\_A1\_1\_3645.d:54959, m/z=777.3845, z=2, RT=17.19, ppm=-8.38):


TRINITY\_DN4439\_c0\_g1\_i1.p1

back to list

  

| Protein Coverage
| Supporting Peptides
| Best Unique PSM
|

Protein Coverage:

Supporting Peptides:

| Peptide | Uniq | -10lgP | Mass | Length | ppm | m/z | z | RT | 1/k0 Start | 1/k0 End | Precursor ID | Area Sample 9 | #Feature | #Feature Sample 9 | Start | End | PTM | AScore | Found By |
| --- | --- | --- | --- | --- | --- | --- | --- | --- | --- | --- | --- | --- | --- | --- | --- | --- | --- | --- | --- |
| R.LASQQNIGQVLAELK.E | Y | 54.38 | 1610.8991 | 15 | -3.21 | 806.4506 | 2 | 19.80 | 1.1286 | 1.1358 | 69854 | 5.11e2 | 1 | 1 | 346 | 360 |  |  | DB Search |
| total 1 peptides |  |  |  |  |  |  |  |  |  |  |  |  |  |  |  |  |  |  |  |
| --- | --- | --- | --- | --- | --- | --- | --- | --- | --- | --- | --- | --- | --- | --- | --- | --- | --- | --- | --- |

Best Unique PSM (Scan Sample\_8\_A1\_1\_3645.d:69854, m/z=806.4506, z=2, RT=19.80, ppm=-7.73):


TRINITY\_DN36\_c0\_g1\_i14.p1

back to list

  

| Protein Coverage
| Supporting Peptides
| Best Unique PSM
|

Protein Coverage:

Supporting Peptides:

| Peptide | Uniq | -10lgP | Mass | Length | ppm | m/z | z | RT | 1/k0 Start | 1/k0 End | Precursor ID | Area Sample 9 | #Feature | #Feature Sample 9 | Start | End | PTM | AScore | Found By |
| --- | --- | --- | --- | --- | --- | --- | --- | --- | --- | --- | --- | --- | --- | --- | --- | --- | --- | --- | --- |
| K.YGQAM(+15.99)C(+57.02)QFM(+15.99)R.N | N | 61.54 | 1322.5206 | 10 | -1.76 | 662.2634 | 2 | 12.17 | 0.9489 | 0.9562 | 28008 | 2.07e2 | 1 | 1 | 72 | 81 | Oxidation (M), Carbamidomethylation, Oxidation (M) | M5:Oxidation (M):1000 M9:Oxidation (M):1000 | DB Search |
| K.WC(+57.02)DILSIHQSMENC(+57.02)VAK.C | Y | 59.79 | 2089.9383 | 17 | 2.4 | 697.6519 | 3 | 17.87 | 0.8769 | 0.8842 | 58766 | 0 | 0 | 0 | 198 | 214 | Carbamidomethylation, Carbamidomethylation |  | DB Search |
| K.DVYDAEQAERK.R | N | 57.85 | 1322.6102 | 11 | -1.22 | 662.3086 | 2 | 11.56 | 0.9606 | 0.9679 | 24244 | 0 | 0 | 0 | 103 | 113 |  |  | DB Search |
| R.LGGIEVTQGNC(+57.02)AR.K | N | 57.3 | 1373.6721 | 13 | 3.87 | 687.8429 | 2 | 13.70 | 0.9642 | 0.9715 | 36854 | 7.25e2 | 1 | 1 | 58 | 70 | Carbamidomethylation |  | DB Search |
| K.FETQIATK.D | Y | 51.43 | 936.4916 | 8 | -0.7 | 937.494 | 1 | 11.83 | 1.2165 | 1.2236 | 25881 | 4.08e3 | 3 | 3 | 167 | 174 |  |  | DB Search |
| total 5 peptides |  |  |  |  |  |  |  |  |  |  |  |  |  |  |  |  |  |  |  |
| --- | --- | --- | --- | --- | --- | --- | --- | --- | --- | --- | --- | --- | --- | --- | --- | --- | --- | --- | --- |

Best Unique PSM (Scan Sample\_8\_A1\_1\_3645.d:58766, m/z=697.6519, z=3, RT=17.87, ppm=-2.11):


TRINITY\_DN262\_c0\_g1\_i1.p1

back to list

  

| Protein Coverage
| Supporting Peptides
| Best Unique PSM
|

Protein Coverage:

Supporting Peptides:

| Peptide | Uniq | -10lgP | Mass | Length | ppm | m/z | z | RT | 1/k0 Start | 1/k0 End | Precursor ID | Area Sample 9 | #Feature | #Feature Sample 9 | Start | End | PTM | AScore | Found By |
| --- | --- | --- | --- | --- | --- | --- | --- | --- | --- | --- | --- | --- | --- | --- | --- | --- | --- | --- | --- |
| K.GVGIISEGNETVEDIAQR.L | Y | 61.93 | 1885.9381 | 18 | -13.03 | 943.9598 | 2 | 17.75 | 1.1406 | 1.1478 | 58109 | 0 | 0 | 0 | 639 | 656 |  |  | DB Search |
| R.AEFRPNQEEEPILKR.E | Y | 57.01 | 1854.9588 | 15 | -3.22 | 619.3221 | 3 | 13.71 | 0.8765 | 0.8838 | 36837 | 1.82e3 | 1 | 1 | 439 | 453 |  |  | DB Search |
| R.DANAAVVHGSHLK.D | Y | 56.92 | 1317.6789 | 13 | 3.24 | 659.8459 | 2 | 10.89 | 1.0182 | 1.0255 | 20111 | 2.14e3 | 1 | 1 | 668 | 680 |  |  | DB Search |
| K.VIM(+15.99)VTGDHPITAK.A | Y | 55.82 | 1396.7384 | 13 | 1.26 | 699.3742 | 2 | 13.09 | 1.0242 | 1.0315 | 33301 | 1.21e3 | 1 | 1 | 622 | 634 | Oxidation (M) | M3:Oxidation (M):1000 | DB Search |
| K.DLPQEKLDDILKYHQEIVFAR.T | Y | 55.53 | 2569.354 | 21 | 3.37 | 643.345 | 4 | 19.21 | 0.8955 | 0.9028 | 66502 | 4.18e2 | 1 | 1 | 681 | 701 |  |  | DB Search |
| R.DPVRDKLVNER.L | Y | 54.92 | 1339.7208 | 11 | -1.15 | 447.5783 | 3 | 11.99 | 0.7371 | 0.7445 | 26739 | 1.34e3 | 1 | 1 | 847 | 857 |  |  | DB Search |
| total 6 peptides |  |  |  |  |  |  |  |  |  |  |  |  |  |  |  |  |  |  |  |
| --- | --- | --- | --- | --- | --- | --- | --- | --- | --- | --- | --- | --- | --- | --- | --- | --- | --- | --- | --- |

Best Unique PSM (Scan Sample\_8\_A1\_1\_3645.d:58109, m/z=943.9598, z=2, RT=17.75, ppm=-17.54):


TRINITY\_DN119684\_c0\_g1\_i1.p1

back to list

  

| Protein Coverage
| Supporting Peptides
| Best Unique PSM
|

Protein Coverage:

Supporting Peptides:

| Peptide | Uniq | -10lgP | Mass | Length | ppm | m/z | z | RT | 1/k0 Start | 1/k0 End | Precursor ID | Area Sample 9 | #Feature | #Feature Sample 9 | Start | End | PTM | AScore | Found By |
| --- | --- | --- | --- | --- | --- | --- | --- | --- | --- | --- | --- | --- | --- | --- | --- | --- | --- | --- | --- |
| K.GKKPHVQVNVK.E | Y | 54.9 | 1232.7353 | 11 | -0.18 | 411.9171 | 3 | 9.81 | 0.7905 | 0.7978 | 13454 | 9.96e2 | 1 | 1 | 125 | 135 |  |  | DB Search |
| total 1 peptides |  |  |  |  |  |  |  |  |  |  |  |  |  |  |  |  |  |  |  |
| --- | --- | --- | --- | --- | --- | --- | --- | --- | --- | --- | --- | --- | --- | --- | --- | --- | --- | --- | --- |

Best Unique PSM (Scan Sample\_8\_A1\_1\_3645.d:13454, m/z=411.9171, z=3, RT=9.81, ppm=-4.7):


TRINITY\_DN1653\_c0\_g1\_i1.p1

back to list

  

| Protein Coverage
| Supporting Peptides
| Best Unique PSM
|

Protein Coverage:

Supporting Peptides:

| Peptide | Uniq | -10lgP | Mass | Length | ppm | m/z | z | RT | 1/k0 Start | 1/k0 End | Precursor ID | Area Sample 9 | #Feature | #Feature Sample 9 | Start | End | PTM | AScore | Found By |
| --- | --- | --- | --- | --- | --- | --- | --- | --- | --- | --- | --- | --- | --- | --- | --- | --- | --- | --- | --- |
| R.LINLFEAVSKK.S | Y | 54.5 | 1260.7441 | 11 | 2.76 | 631.3782 | 2 | 18.78 | 0.9808 | 0.988 | 64045 | 0 | 0 | 0 | 49 | 59 |  |  | DB Search |
| K.LVSIDSDHVVNGNSK.M | Y | 44.95 | 1582.7951 | 15 | -0.07 | 792.4012 | 2 | 13.38 | 1.0644 | 1.0717 | 35085 | 2.42e2 | 1 | 1 | 92 | 106 |  |  | DB Search |
| total 2 peptides |  |  |  |  |  |  |  |  |  |  |  |  |  |  |  |  |  |  |  |
| --- | --- | --- | --- | --- | --- | --- | --- | --- | --- | --- | --- | --- | --- | --- | --- | --- | --- | --- | --- |

Best Unique PSM (Scan Sample\_8\_A1\_1\_3645.d:64045, m/z=631.3782, z=2, RT=18.78, ppm=-1.76):


TRINITY\_DN121501\_c0\_g1\_i1.p1

back to list

  

| Protein Coverage
| Supporting Peptides
| Best Unique PSM
|

Protein Coverage:

Supporting Peptides:

| Peptide | Uniq | -10lgP | Mass | Length | ppm | m/z | z | RT | 1/k0 Start | 1/k0 End | Precursor ID | Area Sample 9 | #Feature | #Feature Sample 9 | Start | End | PTM | AScore | Found By |
| --- | --- | --- | --- | --- | --- | --- | --- | --- | --- | --- | --- | --- | --- | --- | --- | --- | --- | --- | --- |
| K.FGHNHPEPLKK.I | Y | 61.47 | 1302.6833 | 11 | 5.54 | 435.2355 | 3 | 9.95 | 0.7379 | 0.7453 | 14269 | 3.36e2 | 1 | 1 | 150 | 160 |  |  | DB Search |
| K.GIFSIIEHR.L | Y | 55.43 | 1070.5872 | 9 | -0.15 | 536.2984 | 2 | 18.11 | 0.8668 | 0.8741 | 60068 | 2.62e2 | 1 | 1 | 119 | 127 |  |  | DB Search |
| K.AEGVLDKTESQLQK.D | Y | 55.11 | 1544.8046 | 14 | -2.22 | 773.4044 | 2 | 13.93 | 1.0845 | 1.0917 | 38072 | 8.87e2 | 1 | 1 | 176 | 189 |  |  | DB Search |
| total 3 peptides |  |  |  |  |  |  |  |  |  |  |  |  |  |  |  |  |  |  |  |
| --- | --- | --- | --- | --- | --- | --- | --- | --- | --- | --- | --- | --- | --- | --- | --- | --- | --- | --- | --- |

Best Unique PSM (Scan Sample\_8\_A1\_1\_3645.d:14269, m/z=435.2355, z=3, RT=9.95, ppm=1.02):


TRINITY\_DN1121\_c0\_g1\_i1.p1

back to list

  

| Protein Coverage
| Supporting Peptides
| Best Unique PSM
|

Protein Coverage:

Supporting Peptides:

| Peptide | Uniq | -10lgP | Mass | Length | ppm | m/z | z | RT | 1/k0 Start | 1/k0 End | Precursor ID | Area Sample 9 | #Feature | #Feature Sample 9 | Start | End | PTM | AScore | Found By |
| --- | --- | --- | --- | --- | --- | --- | --- | --- | --- | --- | --- | --- | --- | --- | --- | --- | --- | --- | --- |
| M.A(+42.01)EREQLIQQAK.L | Y | 56.32 | 1354.7205 | 11 | -0.73 | 678.364 | 2 | 13.58 | 0.9804 | 0.9876 | 36161 | 3.25e3 | 1 | 1 | 2 | 12 | Acetylation (Protein N-term) | A1:Acetylation (Protein N-term):1000 | DB Search |
| R.YLAEVASESEKQSVVDK.A | Y | 53.93 | 1880.9367 | 17 | 2.85 | 941.4741 | 2 | 13.90 | 1.2396 | 1.2467 | 37974 | 0 | 0 | 0 | 138 | 154 |  |  | DB Search |
| total 2 peptides |  |  |  |  |  |  |  |  |  |  |  |  |  |  |  |  |  |  |  |
| --- | --- | --- | --- | --- | --- | --- | --- | --- | --- | --- | --- | --- | --- | --- | --- | --- | --- | --- | --- |

Best Unique PSM (Scan Sample\_8\_A1\_1\_3645.d:36161, m/z=678.364, z=2, RT=13.58, ppm=-5.24):


TRINITY\_DN73587\_c0\_g1\_i1.p1

back to list

  

| Protein Coverage
| Supporting Peptides
| Best Unique PSM
|

Protein Coverage:

Supporting Peptides:

| Peptide | Uniq | -10lgP | Mass | Length | ppm | m/z | z | RT | 1/k0 Start | 1/k0 End | Precursor ID | Area Sample 9 | #Feature | #Feature Sample 9 | Start | End | PTM | AScore | Found By |
| --- | --- | --- | --- | --- | --- | --- | --- | --- | --- | --- | --- | --- | --- | --- | --- | --- | --- | --- | --- |
| R.LVLEVAQHLGENVVR.T | Y | 55.41 | 1674.9417 | 15 | 3.11 | 838.4769 | 2 | 17.90 | 1.143 | 1.1502 | 58884 | 2.94e2 | 1 | 1 | 99 | 113 |  |  | DB Search |
| total 1 peptides |  |  |  |  |  |  |  |  |  |  |  |  |  |  |  |  |  |  |  |
| --- | --- | --- | --- | --- | --- | --- | --- | --- | --- | --- | --- | --- | --- | --- | --- | --- | --- | --- | --- |

Best Unique PSM (Scan Sample\_8\_A1\_1\_3645.d:58884, m/z=838.4769, z=2, RT=17.90, ppm=-1.4):


TRINITY\_DN64\_c0\_g1\_i1.p1

back to list

  

| Protein Coverage
| Supporting Peptides
| Best Unique PSM
|

Protein Coverage:

Supporting Peptides:

| Peptide | Uniq | -10lgP | Mass | Length | ppm | m/z | z | RT | 1/k0 Start | 1/k0 End | Precursor ID | Area Sample 9 | #Feature | #Feature Sample 9 | Start | End | PTM | AScore | Found By |
| --- | --- | --- | --- | --- | --- | --- | --- | --- | --- | --- | --- | --- | --- | --- | --- | --- | --- | --- | --- |
| K.IHINIVVIGHVDSGK.S | Y | 58.71 | 1599.9096 | 15 | -4.52 | 534.3057 | 3 | 16.46 | 0.84 | 0.8473 | 51229 | 2.52e2 | 2 | 2 | 9 | 23 |  |  | DB Search |
| K.YYVTIIDAPGHR.D | Y | 57.57 | 1403.7197 | 12 | -0.98 | 702.8633 | 2 | 15.80 | 1.0427 | 1.05 | 48024 | 3.09e2 | 1 | 1 | 88 | 99 |  |  | DB Search |
| R.AIEKFEKEAQEM(+15.99)GK.G | Y | 38.77 | 1652.8079 | 14 | -1.94 | 551.9397 | 3 | 12.03 | 0.8193 | 0.8266 | 27165 | 2.59e2 | 1 | 1 | 41 | 54 | Oxidation (M) | M12:Oxidation (M):1000 | DB Search |
| total 3 peptides |  |  |  |  |  |  |  |  |  |  |  |  |  |  |  |  |  |  |  |
| --- | --- | --- | --- | --- | --- | --- | --- | --- | --- | --- | --- | --- | --- | --- | --- | --- | --- | --- | --- |

Best Unique PSM (Scan Sample\_8\_A1\_1\_3645.d:51229, m/z=534.3057, z=3, RT=16.46, ppm=-9.04):


TRINITY\_DN146208\_c0\_g1\_i1.p1

back to list

  

| Protein Coverage
| Supporting Peptides
| Best Unique PSM
|

Protein Coverage:

Supporting Peptides:

| Peptide | Uniq | -10lgP | Mass | Length | ppm | m/z | z | RT | 1/k0 Start | 1/k0 End | Precursor ID | Area Sample 9 | #Feature | #Feature Sample 9 | Start | End | PTM | AScore | Found By |
| --- | --- | --- | --- | --- | --- | --- | --- | --- | --- | --- | --- | --- | --- | --- | --- | --- | --- | --- | --- |
| K.YGINHITNLVESKR.A | Y | 57 | 1642.8791 | 14 | 0.23 | 548.6313 | 3 | 14.96 | 0.8189 | 0.8262 | 43669 | 7.22e2 | 1 | 1 | 158 | 171 |  |  | DB Search |
| R.TNYNERFDEIRR.H | Y | 55.67 | 1611.7753 | 12 | -4.19 | 538.261 | 3 | 13.21 | 0.7913 | 0.7986 | 33943 | 1.06e3 | 1 | 1 | 244 | 255 |  |  | DB Search |
| total 2 peptides |  |  |  |  |  |  |  |  |  |  |  |  |  |  |  |  |  |  |  |
| --- | --- | --- | --- | --- | --- | --- | --- | --- | --- | --- | --- | --- | --- | --- | --- | --- | --- | --- | --- |

Best Unique PSM (Scan Sample\_8\_A1\_1\_3645.d:43669, m/z=548.6313, z=3, RT=14.96, ppm=-4.29):


TRINITY\_DN15274\_c0\_g1\_i1.p1

back to list

  

| Protein Coverage
| Supporting Peptides
| Best Unique PSM
|

Protein Coverage:

Supporting Peptides:

| Peptide | Uniq | -10lgP | Mass | Length | ppm | m/z | z | RT | 1/k0 Start | 1/k0 End | Precursor ID | Area Sample 9 | #Feature | #Feature Sample 9 | Start | End | PTM | AScore | Found By |
| --- | --- | --- | --- | --- | --- | --- | --- | --- | --- | --- | --- | --- | --- | --- | --- | --- | --- | --- | --- |
| R.YSRPTPGSNNNVM(+15.99)VR.I | Y | 58.73 | 1706.8158 | 15 | 1.57 | 569.9442 | 3 | 11.49 | 0.8348 | 0.8421 | 23665 | 2.04e2 | 1 | 1 | 621 | 635 | Oxidation (M) | M13:Oxidation (M):1000 | DB Search |
| R.GSC(+57.02)SC(+57.02)YGHAEQC(+57.02)IPTK.G | Y | 57.58 | 1853.7495 | 16 | 0.37 | 927.8782 | 2 | 12.27 | 1.1865 | 1.1937 | 28455 | 2.04e2 | 1 | 1 | 270 | 285 | Carbamidomethylation, Carbamidomethylation, Carbamidomethylation |  | DB Search |
| K.ATGNTSGGVC(+57.02)DDC(+57.02)NHNTAGR.Q | Y | 56.88 | 2062.8181 | 20 | -2.27 | 688.6086 | 3 | 9.97 | 0.8623 | 0.8696 | 14440 | 0 | 0 | 0 | 353 | 372 | Carbamidomethylation, Carbamidomethylation |  | DB Search |
| K.SGGQC(+57.02)PC(+57.02)KPGVVGR.R | Y | 56.63 | 1457.6867 | 14 | -1.09 | 729.8466 | 2 | 10.68 | 0.9808 | 0.988 | 18843 | 0 | 0 | 0 | 795 | 808 | Carbamidomethylation, Carbamidomethylation |  | DB Search |
| K.YC(+57.02)ILSFLDKTDK.C | Y | 56.07 | 1501.7486 | 12 | -0.86 | 501.5875 | 3 | 19.62 | 0.8006 | 0.8079 | 68865 | 2.16e2 | 1 | 1 | 62 | 73 | Carbamidomethylation |  | DB Search |
| R.KIDDVIC(+57.02)EER.Y | Y | 42.19 | 1275.6129 | 10 | 10.75 | 638.8177 | 2 | 13.44 | 0.9271 | 0.9344 | 35371 | 1.46e3 | 1 | 1 | 184 | 193 | Carbamidomethylation |  | DB Search |
| total 6 peptides |  |  |  |  |  |  |  |  |  |  |  |  |  |  |  |  |  |  |  |
| --- | --- | --- | --- | --- | --- | --- | --- | --- | --- | --- | --- | --- | --- | --- | --- | --- | --- | --- | --- |

Best Unique PSM (Scan Sample\_8\_A1\_1\_3645.d:23665, m/z=569.9442, z=3, RT=11.49, ppm=-2.95):


TRINITY\_DN3271\_c0\_g1\_i1.p1

back to list

  

| Protein Coverage
| Supporting Peptides
| Best Unique PSM
|

Protein Coverage:

Supporting Peptides:

| Peptide | Uniq | -10lgP | Mass | Length | ppm | m/z | z | RT | 1/k0 Start | 1/k0 End | Precursor ID | Area Sample 9 | #Feature | #Feature Sample 9 | Start | End | PTM | AScore | Found By |
| --- | --- | --- | --- | --- | --- | --- | --- | --- | --- | --- | --- | --- | --- | --- | --- | --- | --- | --- | --- |
| K.TIESEAAHGTVTR.H | Y | 55.93 | 1370.679 | 13 | 4.48 | 686.3467 | 2 | 11.86 | 1.0186 | 1.0259 | 26093 | 0 | 0 | 0 | 285 | 297 |  |  | DB Search |
| total 1 peptides |  |  |  |  |  |  |  |  |  |  |  |  |  |  |  |  |  |  |  |
| --- | --- | --- | --- | --- | --- | --- | --- | --- | --- | --- | --- | --- | --- | --- | --- | --- | --- | --- | --- |

Best Unique PSM (Scan Sample\_8\_A1\_1\_3645.d:26093, m/z=686.3467, z=2, RT=11.86, ppm=-0.04):


TRINITY\_DN624\_c0\_g1\_i1.p1

back to list

  

| Protein Coverage
| Supporting Peptides
| Best Unique PSM
|

Protein Coverage:

Supporting Peptides:

| Peptide | Uniq | -10lgP | Mass | Length | ppm | m/z | z | RT | 1/k0 Start | 1/k0 End | Precursor ID | Area Sample 9 | #Feature | #Feature Sample 9 | Start | End | PTM | AScore | Found By |
| --- | --- | --- | --- | --- | --- | --- | --- | --- | --- | --- | --- | --- | --- | --- | --- | --- | --- | --- | --- |
| R.LSNRPLYM(+15.99)PAEGK.M | Y | 55.5 | 1490.7551 | 13 | 4.01 | 746.3845 | 2 | 12.63 | 1.0279 | 1.0351 | 30580 | 4.63e2 | 1 | 1 | 345 | 357 | Oxidation (M) | M8:Oxidation (M):1000 | DB Search |
| R.FAIQDITVDEFSAK.E | Y | 54.56 | 1582.7878 | 14 | -3.45 | 792.3949 | 2 | 19.67 | 1.0443 | 1.0516 | 69141 | 4.1e2 | 1 | 1 | 132 | 145 |  |  | DB Search |
| R.ILAGDKPYITADELRR.E | Y | 35.59 | 1829.9999 | 16 | 4.05 | 611.007 | 3 | 14.93 | 0.9251 | 0.9323 | 43538 | 0 | 0 | 0 | 826 | 841 |  |  | DB Search |
| total 3 peptides |  |  |  |  |  |  |  |  |  |  |  |  |  |  |  |  |  |  |  |
| --- | --- | --- | --- | --- | --- | --- | --- | --- | --- | --- | --- | --- | --- | --- | --- | --- | --- | --- | --- |

Best Unique PSM (Scan Sample\_8\_A1\_1\_3645.d:30580, m/z=746.3845, z=2, RT=12.63, ppm=-0.51):


TRINITY\_DN384\_c0\_g1\_i2.p1

back to list

  

| Protein Coverage
| Supporting Peptides
| Best Unique PSM
|

Protein Coverage:

Supporting Peptides:

| Peptide | Uniq | -10lgP | Mass | Length | ppm | m/z | z | RT | 1/k0 Start | 1/k0 End | Precursor ID | Area Sample 9 | #Feature | #Feature Sample 9 | Start | End | PTM | AScore | Found By |
| --- | --- | --- | --- | --- | --- | --- | --- | --- | --- | --- | --- | --- | --- | --- | --- | --- | --- | --- | --- |
| K.FVLHGDVSHGSR.E | Y | 56.98 | 1309.6527 | 12 | -4.94 | 437.554 | 3 | 11.96 | 0.7547 | 0.762 | 26738 | 1.72e3 | 1 | 1 | 275 | 286 |  |  | DB Search |
| total 1 peptides |  |  |  |  |  |  |  |  |  |  |  |  |  |  |  |  |  |  |  |
| --- | --- | --- | --- | --- | --- | --- | --- | --- | --- | --- | --- | --- | --- | --- | --- | --- | --- | --- | --- |

Best Unique PSM (Scan Sample\_8\_A1\_1\_3645.d:26738, m/z=437.554, z=3, RT=11.96, ppm=-9.46):


TRINITY\_DN1058\_c0\_g1\_i1.p1

back to list

  

| Protein Coverage
| Supporting Peptides
| Best Unique PSM
|

Protein Coverage:

Supporting Peptides:

| Peptide | Uniq | -10lgP | Mass | Length | ppm | m/z | z | RT | 1/k0 Start | 1/k0 End | Precursor ID | Area Sample 9 | #Feature | #Feature Sample 9 | Start | End | PTM | AScore | Found By |
| --- | --- | --- | --- | --- | --- | --- | --- | --- | --- | --- | --- | --- | --- | --- | --- | --- | --- | --- | --- |
| R.APTHILNPANKR.S | Y | 57.39 | 1330.7469 | 12 | 0.47 | 666.3781 | 2 | 11.10 | 1.0102 | 1.0174 | 21164 | 1.88e3 | 2 | 2 | 2314 | 2325 |  |  | DB Search |
| total 1 peptides |  |  |  |  |  |  |  |  |  |  |  |  |  |  |  |  |  |  |  |
| --- | --- | --- | --- | --- | --- | --- | --- | --- | --- | --- | --- | --- | --- | --- | --- | --- | --- | --- | --- |

Best Unique PSM (Scan Sample\_8\_A1\_1\_3645.d:21164, m/z=666.3781, z=2, RT=11.10, ppm=-4.05):


TRINITY\_DN329\_c0\_g1\_i2.p1

back to list

  

| Protein Coverage
| Supporting Peptides
| Best Unique PSM
|

Protein Coverage:

Supporting Peptides:

| Peptide | Uniq | -10lgP | Mass | Length | ppm | m/z | z | RT | 1/k0 Start | 1/k0 End | Precursor ID | Area Sample 9 | #Feature | #Feature Sample 9 | Start | End | PTM | AScore | Found By |
| --- | --- | --- | --- | --- | --- | --- | --- | --- | --- | --- | --- | --- | --- | --- | --- | --- | --- | --- | --- |
| K.EGDLVIHSGITDAVK.Y | Y | 59.72 | 1552.8096 | 15 | 2.8 | 777.4108 | 2 | 14.52 | 1.062 | 1.0693 | 41331 | 1.23e3 | 2 | 2 | 211 | 225 |  |  | DB Search |
| K.GLKVDFDTTFAPVTGK.K | Y | 59.12 | 1694.8879 | 16 | 1.5 | 848.4487 | 2 | 18.11 | 1.1033 | 1.1106 | 60153 | 3.39e3 | 1 | 1 | 130 | 145 |  |  | DB Search |
| K.SFHFGVIK.L | Y | 58.79 | 933.5072 | 8 | 5.47 | 467.7613 | 2 | 16.02 | 0.8108 | 0.8181 | 49143 | 1.91e2 | 1 | 1 | 57 | 64 |  |  | DB Search |
| K.GLKVDFDTTFAPVTGKK.T | Y | 57.57 | 1822.9828 | 17 | 2.59 | 912.4969 | 2 | 17.04 | 1.1806 | 1.1877 | 54087 | 1.7e3 | 1 | 1 | 130 | 146 |  |  | DB Search |
| K.SFHFGVIKLEGK.T | Y | 56.87 | 1360.7503 | 12 | 0.52 | 681.3797 | 2 | 16.93 | 1.0271 | 1.0343 | 53439 | 1.06e3 | 1 | 1 | 57 | 68 |  |  | DB Search |
| K.LRPGVELTMSSLVNAK.S | Y | 56.62 | 1713.9447 | 16 | 1.22 | 857.9768 | 2 | 17.67 | 1.1442 | 1.1514 | 57502 | 1.12e3 | 1 | 1 | 287 | 302 |  |  | DB Search |
| K.VDFDTTFAPVTGKK.T | Y | 55.8 | 1524.7824 | 14 | 6.57 | 763.4 | 2 | 17.07 | 1.0512 | 1.0584 | 54189 | 6.56e2 | 1 | 1 | 133 | 146 |  |  | DB Search |
| K.LRPGVELTM(+15.99)SSLVNAK.S | Y | 54.9 | 1729.9396 | 16 | 1.79 | 865.9747 | 2 | 15.15 | 1.135 | 1.1422 | 44549 | 5.95e3 | 1 | 1 | 287 | 302 | Oxidation (M) | M9:Oxidation (M):1000 | DB Search |
| total 8 peptides |  |  |  |  |  |  |  |  |  |  |  |  |  |  |  |  |  |  |  |
| --- | --- | --- | --- | --- | --- | --- | --- | --- | --- | --- | --- | --- | --- | --- | --- | --- | --- | --- | --- |

Best Unique PSM (Scan Sample\_8\_A1\_1\_3645.d:41331, m/z=777.4108, z=2, RT=14.52, ppm=-1.71):


TRINITY\_DN3545\_c0\_g1\_i1.p1

back to list

  

| Protein Coverage
| Supporting Peptides
| Best Unique PSM
|

Protein Coverage:

Supporting Peptides:

| Peptide | Uniq | -10lgP | Mass | Length | ppm | m/z | z | RT | 1/k0 Start | 1/k0 End | Precursor ID | Area Sample 9 | #Feature | #Feature Sample 9 | Start | End | PTM | AScore | Found By |
| --- | --- | --- | --- | --- | --- | --- | --- | --- | --- | --- | --- | --- | --- | --- | --- | --- | --- | --- | --- |
| R.SC(+57.02)TNPAPANGGK.P | Y | 57.85 | 1172.5244 | 12 | 2.11 | 587.2681 | 2 | 9.44 | 0.8502 | 0.8575 | 11479 | 9.79e2 | 1 | 1 | 55 | 66 | Carbamidomethylation |  | DB Search |
| R.SSC(+57.02)RPILSVIK.I | Y | 16.12 | 1258.7067 | 11 | -9.52 | 630.3518 | 2 | 16.77 | 0.9687 | 0.9759 | 52596 | 1.93e3 | 1 | 1 | 6 | 16 | Carbamidomethylation |  | DB Search |
| total 2 peptides |  |  |  |  |  |  |  |  |  |  |  |  |  |  |  |  |  |  |  |
| --- | --- | --- | --- | --- | --- | --- | --- | --- | --- | --- | --- | --- | --- | --- | --- | --- | --- | --- | --- |

Best Unique PSM (Scan Sample\_8\_A1\_1\_3645.d:11479, m/z=587.2681, z=2, RT=9.44, ppm=-2.41):


TRINITY\_DN121236\_c0\_g1\_i1.p1

back to list

  

| Protein Coverage
| Supporting Peptides
| Best Unique PSM
|

Protein Coverage:

Supporting Peptides:

| Peptide | Uniq | -10lgP | Mass | Length | ppm | m/z | z | RT | 1/k0 Start | 1/k0 End | Precursor ID | Area Sample 9 | #Feature | #Feature Sample 9 | Start | End | PTM | AScore | Found By |
| --- | --- | --- | --- | --- | --- | --- | --- | --- | --- | --- | --- | --- | --- | --- | --- | --- | --- | --- | --- |
| R.DIHRNEIQYHFHAER.I | Y | 58.78 | 1963.9401 | 15 | -2.68 | 491.9888 | 4 | 12.62 | 0.7465 | 0.7538 | 30644 | 0 | 0 | 0 | 456 | 470 |  |  | DB Search |
| total 1 peptides |  |  |  |  |  |  |  |  |  |  |  |  |  |  |  |  |  |  |  |
| --- | --- | --- | --- | --- | --- | --- | --- | --- | --- | --- | --- | --- | --- | --- | --- | --- | --- | --- | --- |

Best Unique PSM (Scan Sample\_8\_A1\_1\_3645.d:30644, m/z=491.9888, z=4, RT=12.62, ppm=-7.2):


TRINITY\_DN1907\_c0\_g1\_i2.p1

back to list

  

| Protein Coverage
| Supporting Peptides
| Best Unique PSM
|

Protein Coverage:

Supporting Peptides:

| Peptide | Uniq | -10lgP | Mass | Length | ppm | m/z | z | RT | 1/k0 Start | 1/k0 End | Precursor ID | Area Sample 9 | #Feature | #Feature Sample 9 | Start | End | PTM | AScore | Found By |
| --- | --- | --- | --- | --- | --- | --- | --- | --- | --- | --- | --- | --- | --- | --- | --- | --- | --- | --- | --- |
| K.VIIDYQTHHR.L | Y | 56.99 | 1280.6625 | 10 | 2.06 | 641.337 | 2 | 12.04 | 0.9586 | 0.9658 | 27134 | 1.5e3 | 1 | 1 | 150 | 159 |  |  | DB Search |
| total 1 peptides |  |  |  |  |  |  |  |  |  |  |  |  |  |  |  |  |  |  |  |
| --- | --- | --- | --- | --- | --- | --- | --- | --- | --- | --- | --- | --- | --- | --- | --- | --- | --- | --- | --- |

Best Unique PSM (Scan Sample\_8\_A1\_1\_3645.d:27134, m/z=641.337, z=2, RT=12.04, ppm=-2.46):


TRINITY\_DN1272\_c0\_g1\_i1.p1

back to list

  

| Protein Coverage
| Supporting Peptides
| Best Unique PSM
|

Protein Coverage:

Supporting Peptides:

| Peptide | Uniq | -10lgP | Mass | Length | ppm | m/z | z | RT | 1/k0 Start | 1/k0 End | Precursor ID | Area Sample 9 | #Feature | #Feature Sample 9 | Start | End | PTM | AScore | Found By |
| --- | --- | --- | --- | --- | --- | --- | --- | --- | --- | --- | --- | --- | --- | --- | --- | --- | --- | --- | --- |
| K.YIQGENEKQEK.I | Y | 59.18 | 1364.6572 | 11 | 4.26 | 683.3357 | 2 | 9.60 | 0.9763 | 0.9836 | 12212 | 1.65e3 | 2 | 2 | 116 | 126 |  |  | DB Search |
| total 1 peptides |  |  |  |  |  |  |  |  |  |  |  |  |  |  |  |  |  |  |  |
| --- | --- | --- | --- | --- | --- | --- | --- | --- | --- | --- | --- | --- | --- | --- | --- | --- | --- | --- | --- |

Best Unique PSM (Scan Sample\_8\_A1\_1\_3645.d:12212, m/z=683.3357, z=2, RT=9.60, ppm=-0.26):


TRINITY\_DN161\_c0\_g1\_i2.p1

back to list

  

| Protein Coverage
| Supporting Peptides
| Best Unique PSM
|

Protein Coverage:

Supporting Peptides:

| Peptide | Uniq | -10lgP | Mass | Length | ppm | m/z | z | RT | 1/k0 Start | 1/k0 End | Precursor ID | Area Sample 9 | #Feature | #Feature Sample 9 | Start | End | PTM | AScore | Found By |
| --- | --- | --- | --- | --- | --- | --- | --- | --- | --- | --- | --- | --- | --- | --- | --- | --- | --- | --- | --- |
| R.LDLAGRDLTDYLMK.I | Y | 60.93 | 1622.8338 | 14 | 1.03 | 812.4213 | 2 | 19.24 | 1.0965 | 1.1037 | 66626 | 2.72e3 | 2 | 2 | 201 | 214 |  |  | DB Search |
| R.LDLAGRDLTDYLM(+15.99)K.I | Y | 59.01 | 1638.8287 | 14 | 2.41 | 547.2823 | 3 | 18.13 | 0.7856 | 0.7929 | 60339 | 2.36e3 | 1 | 1 | 201 | 214 | Oxidation (M) | M13:Oxidation (M):1000 | DB Search |
| K.DSYVGDEAQSKR.G | Y | 58.12 | 1353.616 | 12 | 4.44 | 677.8152 | 2 | 10.93 | 1.0065 | 1.0138 | 20214 | 8.61e3 | 1 | 1 | 74 | 85 |  |  | DB Search |
| K.DLYANTVLSGGTTM(+15.99)FPGIADR.M | Y | 57.77 | 2214.0626 | 21 | 5.59 | 1108.0398 | 2 | 19.43 | 1.2368 | 1.2439 | 67881 | 0 | 0 | 0 | 315 | 335 | Oxidation (M) | M14:Oxidation (M):1000 | DB Search |
| R.VAPEEHPVLLTEAPLNPK.A | Y | 57.43 | 1953.0571 | 18 | 1.47 | 977.5328 | 2 | 16.35 | 1.2073 | 1.2145 | 50611 | 1.52e2 | 1 | 1 | 119 | 136 |  |  | DB Search |
| K.SYELPDGQVITIGNER.F | Y | 57.34 | 1789.8846 | 16 | 2.2 | 895.9475 | 2 | 18.50 | 0.9828 | 0.99 | 62421 | 0 | 0 | 0 | 262 | 277 |  |  | DB Search |
| R.GYSFTTTAEREIVR.D | Y | 56.59 | 1628.8158 | 14 | 3.67 | 815.4145 | 2 | 15.95 | 1.119 | 1.1262 | 48654 | 3.46e3 | 1 | 1 | 220 | 233 |  |  | DB Search |
| R.AVFPSIVGRPR.H | Y | 56.57 | 1197.6982 | 11 | 1.58 | 599.8546 | 2 | 15.61 | 0.9578 | 0.965 | 46597 | 1.24e4 | 1 | 1 | 52 | 62 |  |  | DB Search |
| R.HQGVM(+15.99)VGM(+15.99)GQK.D | Y | 19.26 | 1202.5536 | 11 | 3.57 | 602.2835 | 2 | 9.78 | 0.9154 | 0.9226 | 13224 | 5.97e2 | 1 | 1 | 63 | 73 | Oxidation (M), Oxidation (M) | M5:Oxidation (M):1000 M8:Oxidation (M):1000 | DB Search |
| total 9 peptides |  |  |  |  |  |  |  |  |  |  |  |  |  |  |  |  |  |  |  |
| --- | --- | --- | --- | --- | --- | --- | --- | --- | --- | --- | --- | --- | --- | --- | --- | --- | --- | --- | --- |

Best Unique PSM (Scan Sample\_8\_A1\_1\_3645.d:66626, m/z=812.4213, z=2, RT=19.24, ppm=-3.49):


TRINITY\_DN2477\_c0\_g1\_i5.p1

back to list

  

| Protein Coverage
| Supporting Peptides
| Best Unique PSM
|

Protein Coverage:

Supporting Peptides:

| Peptide | Uniq | -10lgP | Mass | Length | ppm | m/z | z | RT | 1/k0 Start | 1/k0 End | Precursor ID | Area Sample 9 | #Feature | #Feature Sample 9 | Start | End | PTM | AScore | Found By |
| --- | --- | --- | --- | --- | --- | --- | --- | --- | --- | --- | --- | --- | --- | --- | --- | --- | --- | --- | --- |
| K.DSYVGDEAQSKR.G | Y | 58.12 | 1353.616 | 12 | 4.44 | 677.8152 | 2 | 10.93 | 1.0065 | 1.0138 | 20214 | 8.61e3 | 1 | 1 | 8 | 19 |  |  | DB Search |
| R.VAPEEHPVLLTEAPLNPK.A | Y | 57.43 | 1953.0571 | 18 | 1.47 | 977.5328 | 2 | 16.35 | 1.2073 | 1.2145 | 50611 | 1.52e2 | 1 | 1 | 53 | 70 |  |  | DB Search |
| R.GYSFTTTAEREIVR.D | Y | 56.59 | 1628.8158 | 14 | 3.67 | 815.4145 | 2 | 15.95 | 1.119 | 1.1262 | 48654 | 3.46e3 | 1 | 1 | 154 | 167 |  |  | DB Search |
| total 3 peptides |  |  |  |  |  |  |  |  |  |  |  |  |  |  |  |  |  |  |  |
| --- | --- | --- | --- | --- | --- | --- | --- | --- | --- | --- | --- | --- | --- | --- | --- | --- | --- | --- | --- |

Best Unique PSM (Scan Sample\_8\_A1\_1\_3645.d:20214, m/z=677.8152, z=2, RT=10.93, ppm=-0.08):


TRINITY\_DN2472\_c0\_g1\_i1.p1

back to list

  

| Protein Coverage
| Supporting Peptides
| Best Unique PSM
|

Protein Coverage:

Supporting Peptides:

| Peptide | Uniq | -10lgP | Mass | Length | ppm | m/z | z | RT | 1/k0 Start | 1/k0 End | Precursor ID | Area Sample 9 | #Feature | #Feature Sample 9 | Start | End | PTM | AScore | Found By |
| --- | --- | --- | --- | --- | --- | --- | --- | --- | --- | --- | --- | --- | --- | --- | --- | --- | --- | --- | --- |
| M.S(+42.01)SGEIESLRQEAENLR.N | Y | 56.84 | 1858.9021 | 16 | 0.19 | 930.4543 | 2 | 18.71 | 1.1949 | 1.2021 | 63473 | 1.62e3 | 1 | 1 | 2 | 17 | Acetylation (Protein N-term) | S1:Acetylation (Protein N-term):1000 | DB Search |
| total 1 peptides |  |  |  |  |  |  |  |  |  |  |  |  |  |  |  |  |  |  |  |
| --- | --- | --- | --- | --- | --- | --- | --- | --- | --- | --- | --- | --- | --- | --- | --- | --- | --- | --- | --- |

Best Unique PSM (Scan Sample\_8\_A1\_1\_3645.d:63473, m/z=930.4543, z=2, RT=18.71, ppm=-4.32):


TRINITY\_DN36\_c0\_g1\_i13.p1

back to list

  

| Protein Coverage
| Supporting Peptides
| Best Unique PSM
|

Protein Coverage:

Supporting Peptides:

| Peptide | Uniq | -10lgP | Mass | Length | ppm | m/z | z | RT | 1/k0 Start | 1/k0 End | Precursor ID | Area Sample 9 | #Feature | #Feature Sample 9 | Start | End | PTM | AScore | Found By |
| --- | --- | --- | --- | --- | --- | --- | --- | --- | --- | --- | --- | --- | --- | --- | --- | --- | --- | --- | --- |
| K.YGQAM(+15.99)C(+57.02)QFM(+15.99)R.N | N | 61.54 | 1322.5206 | 10 | -1.76 | 662.2634 | 2 | 12.17 | 0.9489 | 0.9562 | 28008 | 2.07e2 | 1 | 1 | 71 | 80 | Oxidation (M), Carbamidomethylation, Oxidation (M) | M5:Oxidation (M):1000 M9:Oxidation (M):1000 | DB Search |
| K.WC(+57.02)DILSIHQSMENC(+57.02)VAK.C | Y | 59.79 | 2089.9383 | 17 | 2.4 | 697.6519 | 3 | 17.87 | 0.8769 | 0.8842 | 58766 | 0 | 0 | 0 | 197 | 213 | Carbamidomethylation, Carbamidomethylation |  | DB Search |
| K.DVYDAEQAERK.R | N | 57.85 | 1322.6102 | 11 | -1.22 | 662.3086 | 2 | 11.56 | 0.9606 | 0.9679 | 24244 | 0 | 0 | 0 | 102 | 112 |  |  | DB Search |
| R.LGGIEVTQGNC(+57.02)AR.K | N | 57.3 | 1373.6721 | 13 | 3.87 | 687.8429 | 2 | 13.70 | 0.9642 | 0.9715 | 36854 | 7.25e2 | 1 | 1 | 57 | 69 | Carbamidomethylation |  | DB Search |
| K.GFDYC(+57.02)TTIKYR.L | N | 56.39 | 1422.6602 | 11 | -1.35 | 712.3332 | 2 | 15.06 | 1.0234 | 1.0307 | 44297 | 0 | 0 | 0 | 46 | 56 | Carbamidomethylation |  | DB Search |
| K.FETQIATK.D | Y | 51.43 | 936.4916 | 8 | -0.7 | 937.494 | 1 | 11.83 | 1.2165 | 1.2236 | 25881 | 4.08e3 | 3 | 3 | 166 | 173 |  |  | DB Search |
| total 6 peptides |  |  |  |  |  |  |  |  |  |  |  |  |  |  |  |  |  |  |  |
| --- | --- | --- | --- | --- | --- | --- | --- | --- | --- | --- | --- | --- | --- | --- | --- | --- | --- | --- | --- |

Best Unique PSM (Scan Sample\_8\_A1\_1\_3645.d:58766, m/z=697.6519, z=3, RT=17.87, ppm=-2.11):


TRINITY\_DN96161\_c0\_g1\_i1.p1

back to list

  

| Protein Coverage
| Supporting Peptides
| Best Unique PSM
|

Protein Coverage:

Supporting Peptides:

| Peptide | Uniq | -10lgP | Mass | Length | ppm | m/z | z | RT | 1/k0 Start | 1/k0 End | Precursor ID | Area Sample 9 | #Feature | #Feature Sample 9 | Start | End | PTM | AScore | Found By |
| --- | --- | --- | --- | --- | --- | --- | --- | --- | --- | --- | --- | --- | --- | --- | --- | --- | --- | --- | --- |
| K.ANHEQITVADITNAC(+57.02)FDPR.N | Y | 58.96 | 2171.0065 | 19 | 2.79 | 1086.5087 | 2 | 17.63 | 1.2368 | 1.2439 | 57365 | 0 | 0 | 0 | 281 | 299 | Carbamidomethylation |  | DB Search |
| total 1 peptides |  |  |  |  |  |  |  |  |  |  |  |  |  |  |  |  |  |  |  |
| --- | --- | --- | --- | --- | --- | --- | --- | --- | --- | --- | --- | --- | --- | --- | --- | --- | --- | --- | --- |

Best Unique PSM (Scan Sample\_8\_A1\_1\_3645.d:57365, m/z=1086.5087, z=2, RT=17.63, ppm=-1.73):


TRINITY\_DN240\_c0\_g1\_i4.p1

back to list

  

| Protein Coverage
| Supporting Peptides
| Best Unique PSM
|

Protein Coverage:

Supporting Peptides:

| Peptide | Uniq | -10lgP | Mass | Length | ppm | m/z | z | RT | 1/k0 Start | 1/k0 End | Precursor ID | Area Sample 9 | #Feature | #Feature Sample 9 | Start | End | PTM | AScore | Found By |
| --- | --- | --- | --- | --- | --- | --- | --- | --- | --- | --- | --- | --- | --- | --- | --- | --- | --- | --- | --- |
| K.ANQVSSAAAAPVPAAGGDK.I | Y | 61.48 | 1680.8431 | 19 | -2.28 | 841.4231 | 2 | 12.43 | 1.0668 | 1.0741 | 29536 | 0 | 0 | 0 | 230 | 248 |  |  | DB Search |
| K.IGAAAQALADQSYPFLK.S | Y | 59.19 | 1762.9253 | 17 | -0.58 | 882.4655 | 2 | 19.04 | 1.159 | 1.1662 | 65509 | 3.11e3 | 2 | 2 | 249 | 265 |  |  | DB Search |
| K.AYAAFLEFKDVVK.A | Y | 57.51 | 1499.8024 | 13 | 3.03 | 750.9073 | 2 | 19.00 | 1.0929 | 1.1001 | 65208 | 1.13e4 | 1 | 1 | 217 | 229 |  |  | DB Search |
| total 3 peptides |  |  |  |  |  |  |  |  |  |  |  |  |  |  |  |  |  |  |  |
| --- | --- | --- | --- | --- | --- | --- | --- | --- | --- | --- | --- | --- | --- | --- | --- | --- | --- | --- | --- |

Best Unique PSM (Scan Sample\_8\_A1\_1\_3645.d:29536, m/z=841.4231, z=2, RT=12.43, ppm=-6.79):


TRINITY\_DN5229\_c0\_g1\_i1.p1

back to list

  

| Protein Coverage
| Supporting Peptides
| Best Unique PSM
|

Protein Coverage:

Supporting Peptides:

| Peptide | Uniq | -10lgP | Mass | Length | ppm | m/z | z | RT | 1/k0 Start | 1/k0 End | Precursor ID | Area Sample 9 | #Feature | #Feature Sample 9 | Start | End | PTM | AScore | Found By |
| --- | --- | --- | --- | --- | --- | --- | --- | --- | --- | --- | --- | --- | --- | --- | --- | --- | --- | --- | --- |
| K.AEELRSEVNGIKR.L | Y | 56.34 | 1499.8056 | 13 | 2.46 | 500.9414 | 3 | 12.22 | 0.7949 | 0.8023 | 28200 | 7.33e2 | 1 | 1 | 201 | 213 |  |  | DB Search |
| total 1 peptides |  |  |  |  |  |  |  |  |  |  |  |  |  |  |  |  |  |  |  |
| --- | --- | --- | --- | --- | --- | --- | --- | --- | --- | --- | --- | --- | --- | --- | --- | --- | --- | --- | --- |

Best Unique PSM (Scan Sample\_8\_A1\_1\_3645.d:28200, m/z=500.9414, z=3, RT=12.22, ppm=-2.06):


TRINITY\_DN3347\_c0\_g1\_i1.p1

back to list

  

| Protein Coverage
| Supporting Peptides
| Best Unique PSM
|

Protein Coverage:

Supporting Peptides:

| Peptide | Uniq | -10lgP | Mass | Length | ppm | m/z | z | RT | 1/k0 Start | 1/k0 End | Precursor ID | Area Sample 9 | #Feature | #Feature Sample 9 | Start | End | PTM | AScore | Found By |
| --- | --- | --- | --- | --- | --- | --- | --- | --- | --- | --- | --- | --- | --- | --- | --- | --- | --- | --- | --- |
| K.VSSTVLSHIVILTR.S | Y | 56.56 | 1523.9035 | 14 | 2.3 | 762.9573 | 2 | 19.04 | 1.1009 | 1.1082 | 65540 | 0 | 0 | 0 | 415 | 428 |  |  | DB Search |
| R.ATDIDQEVKEK.S | Y | 55.02 | 1274.6354 | 11 | -2.5 | 638.3205 | 2 | 11.49 | 0.9654 | 0.9727 | 23882 | 0 | 0 | 0 | 263 | 273 |  |  | DB Search |
| total 2 peptides |  |  |  |  |  |  |  |  |  |  |  |  |  |  |  |  |  |  |  |
| --- | --- | --- | --- | --- | --- | --- | --- | --- | --- | --- | --- | --- | --- | --- | --- | --- | --- | --- | --- |

Best Unique PSM (Scan Sample\_8\_A1\_1\_3645.d:65540, m/z=762.9573, z=2, RT=19.04, ppm=-2.22):


TRINITY\_DN13856\_c0\_g1\_i2.p2

back to list

  

| Protein Coverage
| Supporting Peptides
| Best Unique PSM
|

Protein Coverage:

Supporting Peptides:

| Peptide | Uniq | -10lgP | Mass | Length | ppm | m/z | z | RT | 1/k0 Start | 1/k0 End | Precursor ID | Area Sample 9 | #Feature | #Feature Sample 9 | Start | End | PTM | AScore | Found By |
| --- | --- | --- | --- | --- | --- | --- | --- | --- | --- | --- | --- | --- | --- | --- | --- | --- | --- | --- | --- |
| R.IPLDDFQDPTLTLENAHNK.L | Y | 56.49 | 2180.0749 | 19 | 0.23 | 727.6958 | 3 | 18.17 | 0.9876 | 0.9949 | 60474 | 1.67e3 | 1 | 1 | 175 | 193 |  |  | DB Search |
| K.GSLDRESYR.E | N | 17.44 | 1081.5152 | 9 | 0.81 | 541.7629 | 2 | 10.96 | 0.8595 | 0.8668 | 20530 | 0 | 0 | 0 | 96 | 104 |  |  | DB Search |
| total 2 peptides |  |  |  |  |  |  |  |  |  |  |  |  |  |  |  |  |  |  |  |
| --- | --- | --- | --- | --- | --- | --- | --- | --- | --- | --- | --- | --- | --- | --- | --- | --- | --- | --- | --- |

Best Unique PSM (Scan Sample\_8\_A1\_1\_3645.d:60474, m/z=727.6958, z=3, RT=18.17, ppm=-4.29):


TRINITY\_DN585\_c0\_g1\_i10.p1

back to list

  

| Protein Coverage
| Supporting Peptides
| Best Unique PSM
|

Protein Coverage:

Supporting Peptides:

| Peptide | Uniq | -10lgP | Mass | Length | ppm | m/z | z | RT | 1/k0 Start | 1/k0 End | Precursor ID | Area Sample 9 | #Feature | #Feature Sample 9 | Start | End | PTM | AScore | Found By |
| --- | --- | --- | --- | --- | --- | --- | --- | --- | --- | --- | --- | --- | --- | --- | --- | --- | --- | --- | --- |
| K.IQDKEGIPPDQQR.L | Y | 61.25 | 1522.7739 | 13 | -1.84 | 762.3894 | 2 | 10.99 | 1.0371 | 1.0443 | 20319 | 2.55e3 | 1 | 1 | 30 | 42 |  |  | DB Search |
| total 1 peptides |  |  |  |  |  |  |  |  |  |  |  |  |  |  |  |  |  |  |  |
| --- | --- | --- | --- | --- | --- | --- | --- | --- | --- | --- | --- | --- | --- | --- | --- | --- | --- | --- | --- |

Best Unique PSM (Scan Sample\_8\_A1\_1\_3645.d:20319, m/z=762.3894, z=2, RT=10.99, ppm=-6.36):


TRINITY\_DN36\_c0\_g1\_i1.p1

back to list

  

| Protein Coverage
| Supporting Peptides
| Best Unique PSM
|

Protein Coverage:

Supporting Peptides:

| Peptide | Uniq | -10lgP | Mass | Length | ppm | m/z | z | RT | 1/k0 Start | 1/k0 End | Precursor ID | Area Sample 9 | #Feature | #Feature Sample 9 | Start | End | PTM | AScore | Found By |
| --- | --- | --- | --- | --- | --- | --- | --- | --- | --- | --- | --- | --- | --- | --- | --- | --- | --- | --- | --- |
| K.YGQAM(+15.99)C(+57.02)QFM(+15.99)R.N | N | 61.54 | 1322.5206 | 10 | -1.76 | 662.2634 | 2 | 12.17 | 0.9489 | 0.9562 | 28008 | 2.07e2 | 1 | 1 | 72 | 81 | Oxidation (M), Carbamidomethylation, Oxidation (M) | M5:Oxidation (M):1000 M9:Oxidation (M):1000 | DB Search |
| K.DVYDAEQAERK.R | N | 57.85 | 1322.6102 | 11 | -1.22 | 662.3086 | 2 | 11.56 | 0.9606 | 0.9679 | 24244 | 0 | 0 | 0 | 103 | 113 |  |  | DB Search |
| R.LGGIEVTQGNC(+57.02)AR.K | N | 57.3 | 1373.6721 | 13 | 3.87 | 687.8429 | 2 | 13.70 | 0.9642 | 0.9715 | 36854 | 7.25e2 | 1 | 1 | 58 | 70 | Carbamidomethylation |  | DB Search |
| R.HNIDDC(+57.02)K.K | Y | 35.46 | 900.376 | 7 | 9.13 | 451.1973 | 2 | 10.19 | 0.8027 | 0.81 | 15877 | 0 | 0 | 0 | 190 | 196 | Carbamidomethylation |  | DB Search |
| total 4 peptides |  |  |  |  |  |  |  |  |  |  |  |  |  |  |  |  |  |  |  |
| --- | --- | --- | --- | --- | --- | --- | --- | --- | --- | --- | --- | --- | --- | --- | --- | --- | --- | --- | --- |

Best Unique PSM (Scan Sample\_8\_A1\_1\_3645.d:15877, m/z=451.1973, z=2, RT=10.19, ppm=4.61):


TRINITY\_DN2636\_c0\_g1\_i1.p1

back to list

  

| Protein Coverage
| Supporting Peptides
| Best Unique PSM
|

Protein Coverage:

Supporting Peptides:

| Peptide | Uniq | -10lgP | Mass | Length | ppm | m/z | z | RT | 1/k0 Start | 1/k0 End | Precursor ID | Area Sample 9 | #Feature | #Feature Sample 9 | Start | End | PTM | AScore | Found By |
| --- | --- | --- | --- | --- | --- | --- | --- | --- | --- | --- | --- | --- | --- | --- | --- | --- | --- | --- | --- |
| K.VVEKEAETER.K | Y | 57.16 | 1188.5986 | 10 | 2.47 | 595.3054 | 2 | 9.57 | 0.9044 | 0.9117 | 12158 | 0 | 0 | 0 | 199 | 208 |  |  | DB Search |
| K.IHHELNQFC(+57.02)SVK.S | Y | 38.41 | 1510.7351 | 12 | -3.31 | 756.3689 | 2 | 12.60 | 1.0484 | 1.0556 | 30533 | 0 | 0 | 0 | 117 | 128 | Carbamidomethylation |  | DB Search |
| total 2 peptides |  |  |  |  |  |  |  |  |  |  |  |  |  |  |  |  |  |  |  |
| --- | --- | --- | --- | --- | --- | --- | --- | --- | --- | --- | --- | --- | --- | --- | --- | --- | --- | --- | --- |

Best Unique PSM (Scan Sample\_8\_A1\_1\_3645.d:12158, m/z=595.3054, z=2, RT=9.57, ppm=-2.05):


TRINITY\_DN1339\_c0\_g1\_i2.p1

back to list

  

| Protein Coverage
| Supporting Peptides
| Best Unique PSM
|

Protein Coverage:

Supporting Peptides:

| Peptide | Uniq | -10lgP | Mass | Length | ppm | m/z | z | RT | 1/k0 Start | 1/k0 End | Precursor ID | Area Sample 9 | #Feature | #Feature Sample 9 | Start | End | PTM | AScore | Found By |
| --- | --- | --- | --- | --- | --- | --- | --- | --- | --- | --- | --- | --- | --- | --- | --- | --- | --- | --- | --- |
| K.VITSDDPAEKEK.K | Y | 56.98 | 1330.6616 | 12 | 1.22 | 666.3359 | 2 | 10.71 | 0.9844 | 0.9916 | 18867 | 1.51e3 | 1 | 1 | 160 | 171 |  |  | DB Search |
| total 1 peptides |  |  |  |  |  |  |  |  |  |  |  |  |  |  |  |  |  |  |  |
| --- | --- | --- | --- | --- | --- | --- | --- | --- | --- | --- | --- | --- | --- | --- | --- | --- | --- | --- | --- |

Best Unique PSM (Scan Sample\_8\_A1\_1\_3645.d:18867, m/z=666.3359, z=2, RT=10.71, ppm=-3.3):


TRINITY\_DN79\_c1\_g1\_i1.p1

back to list

  

| Protein Coverage
| Supporting Peptides
| Best Unique PSM
|

Protein Coverage:

Supporting Peptides:

| Peptide | Uniq | -10lgP | Mass | Length | ppm | m/z | z | RT | 1/k0 Start | 1/k0 End | Precursor ID | Area Sample 9 | #Feature | #Feature Sample 9 | Start | End | PTM | AScore | Found By |
| --- | --- | --- | --- | --- | --- | --- | --- | --- | --- | --- | --- | --- | --- | --- | --- | --- | --- | --- | --- |
| K.LTNNKDIVIAK.M | Y | 48.97 | 1227.7187 | 11 | 1.99 | 614.8651 | 2 | 12.24 | 0.9808 | 0.988 | 28363 | 0 | 0 | 0 | 370 | 380 |  |  | DB Search |
| total 1 peptides |  |  |  |  |  |  |  |  |  |  |  |  |  |  |  |  |  |  |  |
| --- | --- | --- | --- | --- | --- | --- | --- | --- | --- | --- | --- | --- | --- | --- | --- | --- | --- | --- | --- |

Best Unique PSM (Scan Sample\_8\_A1\_1\_3645.d:28363, m/z=614.8651, z=2, RT=12.24, ppm=-2.53):


TRINITY\_DN471\_c0\_g1\_i11.p1

back to list

  

| Protein Coverage
| Supporting Peptides
| Best Unique PSM
|

Protein Coverage:

Supporting Peptides:

| Peptide | Uniq | -10lgP | Mass | Length | ppm | m/z | z | RT | 1/k0 Start | 1/k0 End | Precursor ID | Area Sample 9 | #Feature | #Feature Sample 9 | Start | End | PTM | AScore | Found By |
| --- | --- | --- | --- | --- | --- | --- | --- | --- | --- | --- | --- | --- | --- | --- | --- | --- | --- | --- | --- |
| R.RNLDIERPTYTNLNR.L | Y | 58.31 | 1873.9758 | 15 | 3.94 | 625.6655 | 3 | 14.03 | 0.8846 | 0.8919 | 38584 | 6.19e2 | 1 | 1 | 253 | 267 |  |  | DB Search |
| total 1 peptides |  |  |  |  |  |  |  |  |  |  |  |  |  |  |  |  |  |  |  |
| --- | --- | --- | --- | --- | --- | --- | --- | --- | --- | --- | --- | --- | --- | --- | --- | --- | --- | --- | --- |

Best Unique PSM (Scan Sample\_8\_A1\_1\_3645.d:38584, m/z=625.6655, z=3, RT=14.03, ppm=-0.58):


TRINITY\_DN160\_c0\_g1\_i2.p1

back to list

  

| Protein Coverage
| Supporting Peptides
| Best Unique PSM
|

Protein Coverage:

Supporting Peptides:

| Peptide | Uniq | -10lgP | Mass | Length | ppm | m/z | z | RT | 1/k0 Start | 1/k0 End | Precursor ID | Area Sample 9 | #Feature | #Feature Sample 9 | Start | End | PTM | AScore | Found By |
| --- | --- | --- | --- | --- | --- | --- | --- | --- | --- | --- | --- | --- | --- | --- | --- | --- | --- | --- | --- |
| K.LRLDFDHEAKLNM(+15.99)IFR.F | Y | 61.13 | 2033.0516 | 16 | 0.48 | 509.2681 | 4 | 17.75 | 0.8575 | 0.8648 | 57989 | 1.69e3 | 1 | 1 | 1007 | 1022 | Oxidation (M) | M13:Oxidation (M):1000 | DB Search |
| R.LDFDHEAKLNMIFR.F | Y | 57.45 | 1747.8715 | 14 | 5.53 | 583.6317 | 3 | 18.31 | 0.8546 | 0.8619 | 61270 | 0 | 0 | 0 | 1009 | 1022 |  |  | DB Search |
| K.LRLDFDHEAKLNMIFR.F | Y | 56.36 | 2017.0567 | 16 | 0.28 | 505.2693 | 4 | 18.45 | 0.8631 | 0.8704 | 61991 | 1.37e3 | 1 | 1 | 1007 | 1022 |  |  | DB Search |
| K.ELVM(+15.99)HEQSR.C | Y | 53.55 | 1143.5342 | 9 | 15.75 | 572.7808 | 2 | 9.79 | 0.8992 | 0.9065 | 13556 | 4.95e3 | 1 | 1 | 1116 | 1124 | Oxidation (M) | M4:Oxidation (M):1000 | DB Search |
| K.DLEFKFR.E | Y | 51.04 | 953.497 | 7 | -1.17 | 477.7531 | 2 | 16.58 | 0.8307 | 0.838 | 51790 | 5.61e2 | 1 | 1 | 1031 | 1037 |  |  | DB Search |
| total 5 peptides |  |  |  |  |  |  |  |  |  |  |  |  |  |  |  |  |  |  |  |
| --- | --- | --- | --- | --- | --- | --- | --- | --- | --- | --- | --- | --- | --- | --- | --- | --- | --- | --- | --- |

Best Unique PSM (Scan Sample\_8\_A1\_1\_3645.d:57989, m/z=509.2681, z=4, RT=17.75, ppm=-4.04):


TRINITY\_DN1910\_c0\_g1\_i5.p1

back to list

  

| Protein Coverage
| Supporting Peptides
| Best Unique PSM
|

Protein Coverage:

Supporting Peptides:

| Peptide | Uniq | -10lgP | Mass | Length | ppm | m/z | z | RT | 1/k0 Start | 1/k0 End | Precursor ID | Area Sample 9 | #Feature | #Feature Sample 9 | Start | End | PTM | AScore | Found By |
| --- | --- | --- | --- | --- | --- | --- | --- | --- | --- | --- | --- | --- | --- | --- | --- | --- | --- | --- | --- |
| K.NM(+15.99)M(+15.99)AAC(+57.02)DPR.H | Y | 54.81 | 1096.41 | 9 | 2.42 | 549.2111 | 2 | 9.55 | 0.8644 | 0.8716 | 11767 | 3.1e3 | 1 | 1 | 298 | 306 | Oxidation (M), Oxidation (M), Carbamidomethylation | M2:Oxidation (M):1000 M3:Oxidation (M):1000 | DB Search |
| R.FPGQLNADLRK.L | Y | 54.24 | 1257.6829 | 11 | 0.84 | 629.8464 | 2 | 14.26 | 0.9473 | 0.9545 | 39914 | 4.37e2 | 1 | 1 | 242 | 252 |  |  | DB Search |
| K.IREEYPDR.I | Y | 53.76 | 1076.525 | 8 | 2.66 | 539.2688 | 2 | 10.94 | 0.8652 | 0.8725 | 20330 | 1.83e3 | 1 | 1 | 155 | 162 |  |  | DB Search |
| total 3 peptides |  |  |  |  |  |  |  |  |  |  |  |  |  |  |  |  |  |  |  |
| --- | --- | --- | --- | --- | --- | --- | --- | --- | --- | --- | --- | --- | --- | --- | --- | --- | --- | --- | --- |

Best Unique PSM (Scan Sample\_8\_A1\_1\_3645.d:11767, m/z=549.2111, z=2, RT=9.55, ppm=-2.09):


TRINITY\_DN118\_c1\_g1\_i5.p1

back to list

  

| Protein Coverage
| Supporting Peptides
| Best Unique PSM
|

Protein Coverage:

Supporting Peptides:

| Peptide | Uniq | -10lgP | Mass | Length | ppm | m/z | z | RT | 1/k0 Start | 1/k0 End | Precursor ID | Area Sample 9 | #Feature | #Feature Sample 9 | Start | End | PTM | AScore | Found By |
| --- | --- | --- | --- | --- | --- | --- | --- | --- | --- | --- | --- | --- | --- | --- | --- | --- | --- | --- | --- |
| R.ETC(+57.02)SDIEKR.E | Y | 55.98 | 1136.5132 | 9 | 4.61 | 569.2639 | 2 | 10.09 | 0.8915 | 0.8988 | 15200 | 4.92e3 | 1 | 1 | 186 | 194 | Carbamidomethylation |  | DB Search |
| K.VC(+57.02)EDVKR.C | Y | 53.85 | 904.4436 | 7 | 3.72 | 453.2287 | 2 | 9.40 | 0.7815 | 0.7888 | 11301 | 1.58e3 | 1 | 1 | 122 | 128 | Carbamidomethylation |  | DB Search |
| total 2 peptides |  |  |  |  |  |  |  |  |  |  |  |  |  |  |  |  |  |  |  |
| --- | --- | --- | --- | --- | --- | --- | --- | --- | --- | --- | --- | --- | --- | --- | --- | --- | --- | --- | --- |

Best Unique PSM (Scan Sample\_8\_A1\_1\_3645.d:15200, m/z=569.2639, z=2, RT=10.09, ppm=0.09):


TRINITY\_DN250\_c0\_g1\_i2.p1

back to list

  

| Protein Coverage
| Supporting Peptides
| Best Unique PSM
|

Protein Coverage:

Supporting Peptides:

| Peptide | Uniq | -10lgP | Mass | Length | ppm | m/z | z | RT | 1/k0 Start | 1/k0 End | Precursor ID | Area Sample 9 | #Feature | #Feature Sample 9 | Start | End | PTM | AScore | Found By |
| --- | --- | --- | --- | --- | --- | --- | --- | --- | --- | --- | --- | --- | --- | --- | --- | --- | --- | --- | --- |
| R.LM(+15.99)GQIVSSITASLR.F | Y | 58.7 | 1490.8126 | 14 | -0.92 | 746.4095 | 2 | 18.62 | 1.0331 | 1.0403 | 63020 | 2.09e2 | 1 | 1 | 230 | 243 | Oxidation (M) | M2:Oxidation (M):1000 | DB Search |
| R.RNLDIERPTYTNLNR.L | Y | 58.31 | 1873.9758 | 15 | 3.94 | 625.6655 | 3 | 14.03 | 0.8846 | 0.8919 | 38584 | 6.19e2 | 1 | 1 | 215 | 229 |  |  | DB Search |
| R.IHFPLATYSPVISAEK.A | Y | 50.18 | 1771.9508 | 16 | 1.46 | 886.98 | 2 | 18.50 | 1.1338 | 1.141 | 62412 | 0 | 0 | 0 | 265 | 280 |  |  | DB Search |
| total 3 peptides |  |  |  |  |  |  |  |  |  |  |  |  |  |  |  |  |  |  |  |
| --- | --- | --- | --- | --- | --- | --- | --- | --- | --- | --- | --- | --- | --- | --- | --- | --- | --- | --- | --- |

Best Unique PSM (Scan Sample\_8\_A1\_1\_3645.d:63020, m/z=746.4095, z=2, RT=18.62, ppm=-5.44):


TRINITY\_DN941\_c0\_g1\_i13.p1

back to list

  

| Protein Coverage
| Supporting Peptides
| Best Unique PSM
|

Protein Coverage:

Supporting Peptides:

| Peptide | Uniq | -10lgP | Mass | Length | ppm | m/z | z | RT | 1/k0 Start | 1/k0 End | Precursor ID | Area Sample 9 | #Feature | #Feature Sample 9 | Start | End | PTM | AScore | Found By |
| --- | --- | --- | --- | --- | --- | --- | --- | --- | --- | --- | --- | --- | --- | --- | --- | --- | --- | --- | --- |
| K.AELTRPTGK.K | Y | 28.72 | 971.54 | 9 | 2.62 | 486.7763 | 2 | 10.33 | 0.8262 | 0.8335 | 16684 | 4.05e2 | 1 | 1 | 134 | 142 |  |  | DB Search |
| total 1 peptides |  |  |  |  |  |  |  |  |  |  |  |  |  |  |  |  |  |  |  |
| --- | --- | --- | --- | --- | --- | --- | --- | --- | --- | --- | --- | --- | --- | --- | --- | --- | --- | --- | --- |

Best Unique PSM (Scan Sample\_8\_A1\_1\_3645.d:16684, m/z=486.2306, z=2, RT=10.34, ppm=-1.9):


TRINITY\_DN495\_c0\_g1\_i6.p1

back to list

  

| Protein Coverage
| Supporting Peptides
| Best Unique PSM
|

Protein Coverage:

Supporting Peptides:

| Peptide | Uniq | -10lgP | Mass | Length | ppm | m/z | z | RT | 1/k0 Start | 1/k0 End | Precursor ID | Area Sample 9 | #Feature | #Feature Sample 9 | Start | End | PTM | AScore | Found By |
| --- | --- | --- | --- | --- | --- | --- | --- | --- | --- | --- | --- | --- | --- | --- | --- | --- | --- | --- | --- |
| K.SISC(+57.02)SKDEEIK.V | Y | 55.69 | 1294.6075 | 11 | -1.48 | 648.3071 | 2 | 10.83 | 0.9779 | 0.9852 | 19797 | 0 | 0 | 0 | 248 | 258 | Carbamidomethylation |  | DB Search |
| total 1 peptides |  |  |  |  |  |  |  |  |  |  |  |  |  |  |  |  |  |  |  |
| --- | --- | --- | --- | --- | --- | --- | --- | --- | --- | --- | --- | --- | --- | --- | --- | --- | --- | --- | --- |

Best Unique PSM (Scan Sample\_8\_A1\_1\_3645.d:19797, m/z=648.3071, z=2, RT=10.83, ppm=-5.99):


TRINITY\_DN10724\_c0\_g1\_i5.p1

back to list

  

| Protein Coverage
| Supporting Peptides
| Best Unique PSM
|

Protein Coverage:

Supporting Peptides:

| Peptide | Uniq | -10lgP | Mass | Length | ppm | m/z | z | RT | 1/k0 Start | 1/k0 End | Precursor ID | Area Sample 9 | #Feature | #Feature Sample 9 | Start | End | PTM | AScore | Found By |
| --- | --- | --- | --- | --- | --- | --- | --- | --- | --- | --- | --- | --- | --- | --- | --- | --- | --- | --- | --- |
| K.REPGDFGFDPLKFSK.R | Y | 58.72 | 1738.8678 | 15 | -1.8 | 580.6262 | 3 | 18.01 | 0.9012 | 0.9085 | 59548 | 0 | 0 | 0 | 171 | 185 |  |  | DB Search |
| total 1 peptides |  |  |  |  |  |  |  |  |  |  |  |  |  |  |  |  |  |  |  |
| --- | --- | --- | --- | --- | --- | --- | --- | --- | --- | --- | --- | --- | --- | --- | --- | --- | --- | --- | --- |

Best Unique PSM (Scan Sample\_8\_A1\_1\_3645.d:59548, m/z=580.6262, z=3, RT=18.01, ppm=-6.32):


TRINITY\_DN8101\_c0\_g1\_i10.p2

back to list

  

| Protein Coverage
| Supporting Peptides
| Best Unique PSM
|

Protein Coverage:

Supporting Peptides:

| Peptide | Uniq | -10lgP | Mass | Length | ppm | m/z | z | RT | 1/k0 Start | 1/k0 End | Precursor ID | Area Sample 9 | #Feature | #Feature Sample 9 | Start | End | PTM | AScore | Found By |
| --- | --- | --- | --- | --- | --- | --- | --- | --- | --- | --- | --- | --- | --- | --- | --- | --- | --- | --- | --- |
| R.SIGFIGHYLDQLR.L | Y | 55.88 | 1517.799 | 13 | -0.14 | 759.9033 | 2 | 19.91 | 1.0849 | 1.0921 | 70505 | 4.59e2 | 1 | 1 | 192 | 204 |  |  | DB Search |
| total 1 peptides |  |  |  |  |  |  |  |  |  |  |  |  |  |  |  |  |  |  |  |
| --- | --- | --- | --- | --- | --- | --- | --- | --- | --- | --- | --- | --- | --- | --- | --- | --- | --- | --- | --- |

Best Unique PSM (Scan Sample\_8\_A1\_1\_3645.d:70505, m/z=759.9033, z=2, RT=19.91, ppm=-4.65):


TRINITY\_DN941\_c0\_g1\_i3.p1

back to list

  

| Protein Coverage
| Supporting Peptides
| Best Unique PSM
|

Protein Coverage:

Supporting Peptides:

| Peptide | Uniq | -10lgP | Mass | Length | ppm | m/z | z | RT | 1/k0 Start | 1/k0 End | Precursor ID | Area Sample 9 | #Feature | #Feature Sample 9 | Start | End | PTM | AScore | Found By |
| --- | --- | --- | --- | --- | --- | --- | --- | --- | --- | --- | --- | --- | --- | --- | --- | --- | --- | --- | --- |
| K.AELTRPTGK.K | Y | 28.72 | 971.54 | 9 | 2.62 | 486.7763 | 2 | 10.33 | 0.8262 | 0.8335 | 16684 | 4.05e2 | 1 | 1 | 291 | 299 |  |  | DB Search |
| total 1 peptides |  |  |  |  |  |  |  |  |  |  |  |  |  |  |  |  |  |  |  |
| --- | --- | --- | --- | --- | --- | --- | --- | --- | --- | --- | --- | --- | --- | --- | --- | --- | --- | --- | --- |

Best Unique PSM (Scan Sample\_8\_A1\_1\_3645.d:16684, m/z=486.2306, z=2, RT=10.34, ppm=-1.9):


TRINITY\_DN166344\_c0\_g1\_i1.p1

back to list

  

| Protein Coverage
| Supporting Peptides
| Best Unique PSM
|

Protein Coverage:

Supporting Peptides:

| Peptide | Uniq | -10lgP | Mass | Length | ppm | m/z | z | RT | 1/k0 Start | 1/k0 End | Precursor ID | Area Sample 9 | #Feature | #Feature Sample 9 | Start | End | PTM | AScore | Found By |
| --- | --- | --- | --- | --- | --- | --- | --- | --- | --- | --- | --- | --- | --- | --- | --- | --- | --- | --- | --- |
| R.TQITC(+57.02)TQISLKR.G | Y | 56.91 | 1447.7817 | 12 | 1.07 | 724.8956 | 2 | 13.77 | 1.025 | 1.0323 | 37211 | 1.01e3 | 1 | 1 | 174 | 185 | Carbamidomethylation |  | DB Search |
| total 1 peptides |  |  |  |  |  |  |  |  |  |  |  |  |  |  |  |  |  |  |  |
| --- | --- | --- | --- | --- | --- | --- | --- | --- | --- | --- | --- | --- | --- | --- | --- | --- | --- | --- | --- |

Best Unique PSM (Scan Sample\_8\_A1\_1\_3645.d:37211, m/z=724.8956, z=2, RT=13.77, ppm=-3.44):


TRINITY\_DN3016\_c0\_g1\_i13.p1

back to list

  

| Protein Coverage
| Supporting Peptides
| Best Unique PSM
|

Protein Coverage:

Supporting Peptides:

| Peptide | Uniq | -10lgP | Mass | Length | ppm | m/z | z | RT | 1/k0 Start | 1/k0 End | Precursor ID | Area Sample 9 | #Feature | #Feature Sample 9 | Start | End | PTM | AScore | Found By |
| --- | --- | --- | --- | --- | --- | --- | --- | --- | --- | --- | --- | --- | --- | --- | --- | --- | --- | --- | --- |
| R.IVNTDLQEIKDIIK.G | Y | 58.4 | 1640.9348 | 14 | -6.31 | 821.4658 | 2 | 18.62 | 1.1514 | 1.1586 | 63065 | 0 | 0 | 0 | 154 | 167 |  |  | DB Search |
| total 1 peptides |  |  |  |  |  |  |  |  |  |  |  |  |  |  |  |  |  |  |  |
| --- | --- | --- | --- | --- | --- | --- | --- | --- | --- | --- | --- | --- | --- | --- | --- | --- | --- | --- | --- |

Best Unique PSM (Scan Sample\_8\_A1\_1\_3645.d:63065, m/z=821.4658, z=2, RT=18.62, ppm=-10.82):


TRINITY\_DN8101\_c0\_g1\_i5.p1

back to list

  

| Protein Coverage
| Supporting Peptides
| Best Unique PSM
|

Protein Coverage:

Supporting Peptides:

| Peptide | Uniq | -10lgP | Mass | Length | ppm | m/z | z | RT | 1/k0 Start | 1/k0 End | Precursor ID | Area Sample 9 | #Feature | #Feature Sample 9 | Start | End | PTM | AScore | Found By |
| --- | --- | --- | --- | --- | --- | --- | --- | --- | --- | --- | --- | --- | --- | --- | --- | --- | --- | --- | --- |
| R.SIGFIGHYLDQLR.L | Y | 55.88 | 1517.799 | 13 | -0.14 | 759.9033 | 2 | 19.91 | 1.0849 | 1.0921 | 70505 | 4.59e2 | 1 | 1 | 784 | 796 |  |  | DB Search |
| total 1 peptides |  |  |  |  |  |  |  |  |  |  |  |  |  |  |  |  |  |  |  |
| --- | --- | --- | --- | --- | --- | --- | --- | --- | --- | --- | --- | --- | --- | --- | --- | --- | --- | --- | --- |

Best Unique PSM (Scan Sample\_8\_A1\_1\_3645.d:70505, m/z=759.9033, z=2, RT=19.91, ppm=-4.65):


TRINITY\_DN11643\_c0\_g1\_i1.p1

back to list

  

| Protein Coverage
| Supporting Peptides
| Best Unique PSM
|

Protein Coverage:

Supporting Peptides:

| Peptide | Uniq | -10lgP | Mass | Length | ppm | m/z | z | RT | 1/k0 Start | 1/k0 End | Precursor ID | Area Sample 9 | #Feature | #Feature Sample 9 | Start | End | PTM | AScore | Found By |
| --- | --- | --- | --- | --- | --- | --- | --- | --- | --- | --- | --- | --- | --- | --- | --- | --- | --- | --- | --- |
| R.EVNTELDSRPETK.K | Y | 55.62 | 1516.7369 | 13 | -0.06 | 759.3723 | 2 | 11.70 | 1.0259 | 1.0331 | 25124 | 0 | 0 | 0 | 97 | 109 |  |  | DB Search |
| total 1 peptides |  |  |  |  |  |  |  |  |  |  |  |  |  |  |  |  |  |  |  |
| --- | --- | --- | --- | --- | --- | --- | --- | --- | --- | --- | --- | --- | --- | --- | --- | --- | --- | --- | --- |

Best Unique PSM (Scan Sample\_8\_A1\_1\_3645.d:25124, m/z=759.3723, z=2, RT=11.70, ppm=-4.57):


TRINITY\_DN4497\_c0\_g1\_i1.p1

back to list

  

| Protein Coverage
| Supporting Peptides
| Best Unique PSM
|

Protein Coverage:

Supporting Peptides:

| Peptide | Uniq | -10lgP | Mass | Length | ppm | m/z | z | RT | 1/k0 Start | 1/k0 End | Precursor ID | Area Sample 9 | #Feature | #Feature Sample 9 | Start | End | PTM | AScore | Found By |
| --- | --- | --- | --- | --- | --- | --- | --- | --- | --- | --- | --- | --- | --- | --- | --- | --- | --- | --- | --- |
| K.VLTSSDPAEKEK.K | Y | 44.75 | 1302.6667 | 12 | 5.11 | 652.341 | 2 | 10.57 | 1.0001 | 1.0073 | 18232 | 0 | 0 | 0 | 117 | 128 |  |  | DB Search |
| total 1 peptides |  |  |  |  |  |  |  |  |  |  |  |  |  |  |  |  |  |  |  |
| --- | --- | --- | --- | --- | --- | --- | --- | --- | --- | --- | --- | --- | --- | --- | --- | --- | --- | --- | --- |

Best Unique PSM (Scan Sample\_8\_A1\_1\_3645.d:18232, m/z=652.341, z=2, RT=10.57, ppm=0.6):


TRINITY\_DN5365\_c0\_g1\_i2.p1

back to list

  

| Protein Coverage
| Supporting Peptides
| Best Unique PSM
|

Protein Coverage:

Supporting Peptides:

| Peptide | Uniq | -10lgP | Mass | Length | ppm | m/z | z | RT | 1/k0 Start | 1/k0 End | Precursor ID | Area Sample 9 | #Feature | #Feature Sample 9 | Start | End | PTM | AScore | Found By |
| --- | --- | --- | --- | --- | --- | --- | --- | --- | --- | --- | --- | --- | --- | --- | --- | --- | --- | --- | --- |
| R.FPGQLNADIRK.L | Y | 54.24 | 1257.6829 | 11 | 0.84 | 629.8464 | 2 | 14.26 | 0.9473 | 0.9545 | 39914 | 4.37e2 | 1 | 1 | 244 | 254 |  |  | DB Search |
| total 1 peptides |  |  |  |  |  |  |  |  |  |  |  |  |  |  |  |  |  |  |  |
| --- | --- | --- | --- | --- | --- | --- | --- | --- | --- | --- | --- | --- | --- | --- | --- | --- | --- | --- | --- |

Best Unique PSM (Scan Sample\_8\_A1\_1\_3645.d:39914, m/z=629.8464, z=2, RT=14.26, ppm=-3.67):


TRINITY\_DN22492\_c0\_g1\_i1.p1

back to list

  

| Protein Coverage
| Supporting Peptides
| Best Unique PSM
|

Protein Coverage:

Supporting Peptides:

| Peptide | Uniq | -10lgP | Mass | Length | ppm | m/z | z | RT | 1/k0 Start | 1/k0 End | Precursor ID | Area Sample 9 | #Feature | #Feature Sample 9 | Start | End | PTM | AScore | Found By |
| --- | --- | --- | --- | --- | --- | --- | --- | --- | --- | --- | --- | --- | --- | --- | --- | --- | --- | --- | --- |
| R.LIAKFETWEVAR.N | Y | 51.27 | 1461.798 | 12 | 2.42 | 731.9047 | 2 | 17.13 | 1.0448 | 1.052 | 54536 | 3.66e2 | 1 | 1 | 318 | 329 |  |  | DB Search |
| K.KKLEPQAAPR.E | Y | 44.45 | 1136.6666 | 10 | -0.58 | 379.8942 | 3 | 9.69 | 0.7567 | 0.764 | 12893 | 0 | 0 | 0 | 425 | 434 |  |  | DB Search |
| total 2 peptides |  |  |  |  |  |  |  |  |  |  |  |  |  |  |  |  |  |  |  |
| --- | --- | --- | --- | --- | --- | --- | --- | --- | --- | --- | --- | --- | --- | --- | --- | --- | --- | --- | --- |

Best Unique PSM (Scan Sample\_8\_A1\_1\_3645.d:54536, m/z=731.9047, z=2, RT=17.13, ppm=-2.09):


TRINITY\_DN333\_c0\_g1\_i1.p1

back to list

  

| Protein Coverage
| Supporting Peptides
| Best Unique PSM
|

Protein Coverage:

Supporting Peptides:

| Peptide | Uniq | -10lgP | Mass | Length | ppm | m/z | z | RT | 1/k0 Start | 1/k0 End | Precursor ID | Area Sample 9 | #Feature | #Feature Sample 9 | Start | End | PTM | AScore | Found By |
| --- | --- | --- | --- | --- | --- | --- | --- | --- | --- | --- | --- | --- | --- | --- | --- | --- | --- | --- | --- |
| R.RRYPIIVTGR.L | Y | 55.45 | 1229.7356 | 10 | 2.21 | 410.9182 | 3 | 12.81 | 0.7746 | 0.7819 | 31572 | 7.37e2 | 1 | 1 | 286 | 295 |  |  | DB Search |
| total 1 peptides |  |  |  |  |  |  |  |  |  |  |  |  |  |  |  |  |  |  |  |
| --- | --- | --- | --- | --- | --- | --- | --- | --- | --- | --- | --- | --- | --- | --- | --- | --- | --- | --- | --- |

Best Unique PSM (Scan Sample\_8\_A1\_1\_3645.d:31572, m/z=410.9182, z=3, RT=12.81, ppm=-2.31):


TRINITY\_DN161\_c0\_g1\_i4.p1

back to list

  

| Protein Coverage
| Supporting Peptides
| Best Unique PSM
|

Protein Coverage:

Supporting Peptides:

| Peptide | Uniq | -10lgP | Mass | Length | ppm | m/z | z | RT | 1/k0 Start | 1/k0 End | Precursor ID | Area Sample 9 | #Feature | #Feature Sample 9 | Start | End | PTM | AScore | Found By |
| --- | --- | --- | --- | --- | --- | --- | --- | --- | --- | --- | --- | --- | --- | --- | --- | --- | --- | --- | --- |
| R.LDLAGRDLTDYLMK.I | Y | 60.93 | 1622.8338 | 14 | 1.03 | 812.4213 | 2 | 19.24 | 1.0965 | 1.1037 | 66626 | 2.72e3 | 2 | 2 | 34 | 47 |  |  | DB Search |
| R.LDLAGRDLTDYLM(+15.99)K.I | Y | 59.01 | 1638.8287 | 14 | 2.41 | 547.2823 | 3 | 18.13 | 0.7856 | 0.7929 | 60339 | 2.36e3 | 1 | 1 | 34 | 47 | Oxidation (M) | M13:Oxidation (M):1000 | DB Search |
| K.DLYANTVLSGGTTM(+15.99)FPGIADR.M | Y | 57.77 | 2214.0626 | 21 | 5.59 | 1108.0398 | 2 | 19.43 | 1.2368 | 1.2439 | 67881 | 0 | 0 | 0 | 148 | 168 | Oxidation (M) | M14:Oxidation (M):1000 | DB Search |
| K.SYELPDGQVITIGNER.F | Y | 57.34 | 1789.8846 | 16 | 2.2 | 895.9475 | 2 | 18.50 | 0.9828 | 0.99 | 62421 | 0 | 0 | 0 | 95 | 110 |  |  | DB Search |
| R.GYSFTTTAEREIVR.D | Y | 56.59 | 1628.8158 | 14 | 3.67 | 815.4145 | 2 | 15.95 | 1.119 | 1.1262 | 48654 | 3.46e3 | 1 | 1 | 53 | 66 |  |  | DB Search |
| total 5 peptides |  |  |  |  |  |  |  |  |  |  |  |  |  |  |  |  |  |  |  |
| --- | --- | --- | --- | --- | --- | --- | --- | --- | --- | --- | --- | --- | --- | --- | --- | --- | --- | --- | --- |

Best Unique PSM (Scan Sample\_8\_A1\_1\_3645.d:66626, m/z=812.4213, z=2, RT=19.24, ppm=-3.49):


TRINITY\_DN40728\_c0\_g1\_i1.p1

back to list

  

| Protein Coverage
| Supporting Peptides
| Best Unique PSM
|

Protein Coverage:

Supporting Peptides:

| Peptide | Uniq | -10lgP | Mass | Length | ppm | m/z | z | RT | 1/k0 Start | 1/k0 End | Precursor ID | Area Sample 9 | #Feature | #Feature Sample 9 | Start | End | PTM | AScore | Found By |
| --- | --- | --- | --- | --- | --- | --- | --- | --- | --- | --- | --- | --- | --- | --- | --- | --- | --- | --- | --- |
| K.IQDKEGIPPDQQR.L | Y | 61.25 | 1522.7739 | 13 | -1.84 | 762.3894 | 2 | 10.99 | 1.0371 | 1.0443 | 20319 | 2.55e3 | 1 | 1 | 53 | 65 |  |  | DB Search |
| total 1 peptides |  |  |  |  |  |  |  |  |  |  |  |  |  |  |  |  |  |  |  |
| --- | --- | --- | --- | --- | --- | --- | --- | --- | --- | --- | --- | --- | --- | --- | --- | --- | --- | --- | --- |

Best Unique PSM (Scan Sample\_8\_A1\_1\_3645.d:20319, m/z=762.3894, z=2, RT=10.99, ppm=-6.36):


TRINITY\_DN428\_c0\_g1\_i1.p1

back to list

  

| Protein Coverage
| Supporting Peptides
| Best Unique PSM
|

Protein Coverage:

Supporting Peptides:

| Peptide | Uniq | -10lgP | Mass | Length | ppm | m/z | z | RT | 1/k0 Start | 1/k0 End | Precursor ID | Area Sample 9 | #Feature | #Feature Sample 9 | Start | End | PTM | AScore | Found By |
| --- | --- | --- | --- | --- | --- | --- | --- | --- | --- | --- | --- | --- | --- | --- | --- | --- | --- | --- | --- |
| R.KSDQATLPDNTIAQK.I | Y | 61.92 | 1628.8369 | 15 | -2.41 | 815.4201 | 2 | 12.57 | 1.0512 | 1.0584 | 30355 | 0 | 0 | 0 | 362 | 376 |  |  | DB Search |
| R.KAEQDLSDLRAQLAQALQR.I | Y | 58.05 | 2153.1552 | 19 | -8.21 | 718.7166 | 3 | 18.93 | 0.9578 | 0.965 | 64971 | 0 | 0 | 0 | 1049 | 1067 |  |  | DB Search |
| K.AKQEAFIAELEDQIAKGDK.S | Y | 57.51 | 2103.0847 | 19 | 0.34 | 702.0326 | 3 | 19.38 | 0.9469 | 0.9541 | 67541 | 1.79e2 | 1 | 1 | 1020 | 1038 |  |  | DB Search |
| K.IC(+57.02)HLLGIQVTDFTK.S | Y | 56.92 | 1643.8705 | 14 | -7.46 | 822.9327 | 2 | 18.41 | 1.0821 | 1.0893 | 61877 | 0 | 0 | 0 | 377 | 390 | Carbamidomethylation |  | DB Search |
| R.LPIYTDKVINLYK.G | Y | 55.38 | 1578.9021 | 13 | -1.38 | 790.4537 | 2 | 17.68 | 1.0981 | 1.1053 | 57731 | 0 | 0 | 0 | 126 | 138 |  |  | DB Search |
| R.AGVLAHLEEERDIK.L | Y | 55.34 | 1578.8365 | 14 | 1.04 | 790.4228 | 2 | 14.80 | 1.1082 | 1.1154 | 42875 | 0 | 0 | 0 | 762 | 775 |  |  | DB Search |
| R.AGVLAHLEEER.D | Y | 54.69 | 1222.6306 | 11 | 0.35 | 612.32 | 2 | 14.81 | 0.9558 | 0.963 | 42856 | 5.3e2 | 1 | 1 | 762 | 772 |  |  | DB Search |
| K.RIQQLSAIR.V | Y | 49.91 | 1083.6513 | 9 | 0.54 | 542.8307 | 2 | 13.10 | 0.9178 | 0.9251 | 33359 | 1.42e3 | 1 | 1 | 799 | 807 |  |  | DB Search |
| total 8 peptides |  |  |  |  |  |  |  |  |  |  |  |  |  |  |  |  |  |  |  |
| --- | --- | --- | --- | --- | --- | --- | --- | --- | --- | --- | --- | --- | --- | --- | --- | --- | --- | --- | --- |

Best Unique PSM (Scan Sample\_8\_A1\_1\_3645.d:30355, m/z=815.4201, z=2, RT=12.57, ppm=-6.93):


TRINITY\_DN687\_c0\_g2\_i1.p1

back to list

  

| Protein Coverage
| Supporting Peptides
| Best Unique PSM
|

Protein Coverage:

Supporting Peptides:

| Peptide | Uniq | -10lgP | Mass | Length | ppm | m/z | z | RT | 1/k0 Start | 1/k0 End | Precursor ID | Area Sample 9 | #Feature | #Feature Sample 9 | Start | End | PTM | AScore | Found By |
| --- | --- | --- | --- | --- | --- | --- | --- | --- | --- | --- | --- | --- | --- | --- | --- | --- | --- | --- | --- |
| R.RNLDIERPTYTNLNR.L | Y | 58.31 | 1873.9758 | 15 | 3.94 | 625.6655 | 3 | 14.03 | 0.8846 | 0.8919 | 38584 | 6.19e2 | 1 | 1 | 215 | 229 |  |  | DB Search |
| total 1 peptides |  |  |  |  |  |  |  |  |  |  |  |  |  |  |  |  |  |  |  |
| --- | --- | --- | --- | --- | --- | --- | --- | --- | --- | --- | --- | --- | --- | --- | --- | --- | --- | --- | --- |

Best Unique PSM (Scan Sample\_8\_A1\_1\_3645.d:38584, m/z=625.6655, z=3, RT=14.03, ppm=-0.58):


TRINITY\_DN1386\_c0\_g1\_i1.p1

back to list

  

| Protein Coverage
| Supporting Peptides
| Best Unique PSM
|

Protein Coverage:

Supporting Peptides:

| Peptide | Uniq | -10lgP | Mass | Length | ppm | m/z | z | RT | 1/k0 Start | 1/k0 End | Precursor ID | Area Sample 9 | #Feature | #Feature Sample 9 | Start | End | PTM | AScore | Found By |
| --- | --- | --- | --- | --- | --- | --- | --- | --- | --- | --- | --- | --- | --- | --- | --- | --- | --- | --- | --- |
| K.SFLLALEKDQEK.H | Y | 55.25 | 1419.7609 | 12 | 5.33 | 710.8883 | 2 | 17.33 | 1.0729 | 1.0801 | 55590 | 8.23e2 | 1 | 1 | 61 | 72 |  |  | DB Search |
| R.KVIVDQLVTLATR.V | Y | 55.16 | 1454.882 | 13 | -0.58 | 728.4446 | 2 | 18.25 | 1.0275 | 1.0347 | 60861 | 1.2e3 | 1 | 1 | 547 | 559 |  |  | DB Search |
| total 2 peptides |  |  |  |  |  |  |  |  |  |  |  |  |  |  |  |  |  |  |  |
| --- | --- | --- | --- | --- | --- | --- | --- | --- | --- | --- | --- | --- | --- | --- | --- | --- | --- | --- | --- |

Best Unique PSM (Scan Sample\_8\_A1\_1\_3645.d:55590, m/z=710.8883, z=2, RT=17.33, ppm=0.81):


TRINITY\_DN116\_c0\_g1\_i1.p1

back to list

  

| Protein Coverage
| Supporting Peptides
| Best Unique PSM
|

Protein Coverage:

Supporting Peptides:

| Peptide | Uniq | -10lgP | Mass | Length | ppm | m/z | z | RT | 1/k0 Start | 1/k0 End | Precursor ID | Area Sample 9 | #Feature | #Feature Sample 9 | Start | End | PTM | AScore | Found By |
| --- | --- | --- | --- | --- | --- | --- | --- | --- | --- | --- | --- | --- | --- | --- | --- | --- | --- | --- | --- |
| R.FPGFDSESDDFNAEVHR.N | Y | 59.64 | 1967.8285 | 17 | -3.47 | 656.9449 | 3 | 17.21 | 0.8931 | 0.9004 | 55060 | 0 | 0 | 0 | 180 | 196 |  |  | DB Search |
| R.ALQDEDEDAFKR.Q | Y | 56.97 | 1435.6579 | 12 | 4.63 | 718.8363 | 2 | 13.14 | 0.9953 | 1.0025 | 33551 | 3.78e2 | 1 | 1 | 210 | 221 |  |  | DB Search |
| R.FTNKDIVC(+57.02)QIAYAK.I | Y | 47.25 | 1669.8498 | 14 | 10.7 | 835.9373 | 2 | 15.93 | 1.1174 | 1.1246 | 48701 | 0 | 0 | 0 | 55 | 68 | Carbamidomethylation |  | DB Search |
| total 3 peptides |  |  |  |  |  |  |  |  |  |  |  |  |  |  |  |  |  |  |  |
| --- | --- | --- | --- | --- | --- | --- | --- | --- | --- | --- | --- | --- | --- | --- | --- | --- | --- | --- | --- |

Best Unique PSM (Scan Sample\_8\_A1\_1\_3645.d:55060, m/z=656.9449, z=3, RT=17.21, ppm=-7.98):


TRINITY\_DN124769\_c0\_g1\_i1.p1

back to list

  

| Protein Coverage
| Supporting Peptides
| Best Unique PSM
|

Protein Coverage:

Supporting Peptides:

| Peptide | Uniq | -10lgP | Mass | Length | ppm | m/z | z | RT | 1/k0 Start | 1/k0 End | Precursor ID | Area Sample 9 | #Feature | #Feature Sample 9 | Start | End | PTM | AScore | Found By |
| --- | --- | --- | --- | --- | --- | --- | --- | --- | --- | --- | --- | --- | --- | --- | --- | --- | --- | --- | --- |
| K.VITSDDPAEKEK.K | Y | 56.98 | 1330.6616 | 12 | 1.22 | 666.3359 | 2 | 10.71 | 0.9844 | 0.9916 | 18867 | 1.51e3 | 1 | 1 | 91 | 102 |  |  | DB Search |
| total 1 peptides |  |  |  |  |  |  |  |  |  |  |  |  |  |  |  |  |  |  |  |
| --- | --- | --- | --- | --- | --- | --- | --- | --- | --- | --- | --- | --- | --- | --- | --- | --- | --- | --- | --- |

Best Unique PSM (Scan Sample\_8\_A1\_1\_3645.d:18867, m/z=666.3359, z=2, RT=10.71, ppm=-3.3):


TRINITY\_DN3036\_c0\_g1\_i3.p1

back to list

  

| Protein Coverage
| Supporting Peptides
| Best Unique PSM
|

Protein Coverage:

Supporting Peptides:

| Peptide | Uniq | -10lgP | Mass | Length | ppm | m/z | z | RT | 1/k0 Start | 1/k0 End | Precursor ID | Area Sample 9 | #Feature | #Feature Sample 9 | Start | End | PTM | AScore | Found By |
| --- | --- | --- | --- | --- | --- | --- | --- | --- | --- | --- | --- | --- | --- | --- | --- | --- | --- | --- | --- |
| R.VIGASGIHVGTM(+15.99)SFGK.M | Y | 60.06 | 1575.8079 | 16 | 1.08 | 788.9085 | 2 | 15.08 | 1.125 | 1.1322 | 44382 | 0 | 0 | 0 | 334 | 349 | Oxidation (M) | M12:Oxidation (M):1000 | DB Search |
| R.GTSNGGLVVGTIIKPK.L | Y | 54.81 | 1539.8984 | 16 | 1.04 | 770.9538 | 2 | 15.65 | 1.1234 | 1.1306 | 47131 | 3.3e3 | 1 | 1 | 173 | 188 |  |  | DB Search |
| total 2 peptides |  |  |  |  |  |  |  |  |  |  |  |  |  |  |  |  |  |  |  |
| --- | --- | --- | --- | --- | --- | --- | --- | --- | --- | --- | --- | --- | --- | --- | --- | --- | --- | --- | --- |

Best Unique PSM (Scan Sample\_8\_A1\_1\_3645.d:44382, m/z=788.9085, z=2, RT=15.08, ppm=-3.43):


---

Prepared with PEAKS ™ (bioinfor.com)
