# Supplementary material for: Proteomic Analysis and Biochemical Characterization of the Nematocyst Extract of the Hydrozoan Velella velella
Source: Mar Drugs. 2024 Oct 12;22(10):468. doi: 10.3390/md22100468 (PMC11509761; doi:10.3390/md22100468)
Supplement: Supplementary file 1 [file marinedrugs-22-00468-s001.zip › Supplementary File S1.zip!/Sample_9_Vellela/db.peptides.html]

peptide list


Peptide List

  

| Peptide | -10lgP | Mass | Length | ppm | m/z | RT | 1/k0 Start | 1/k0 End | Precursor ID | Area Sample 10 | #Feature | #Feature Sample 10 | Accession | PTM | AScore | Found By |
| --- | --- | --- | --- | --- | --- | --- | --- | --- | --- | --- | --- | --- | --- | --- | --- | --- |
| LALHVLNNEDIIGHR | 59.07 | 1712.9321 | 15 | -6.29 | 571.9781 | 16.30 | 0.8571 | 0.8644 | 48193 | 0 | 0 | 0 | TRINITY\_DN140702\_c0\_g1\_i1.p1 |  |  | DB Search |
| DSYVGDEAQSKR | 59.06 | 1353.6161 | 12 | 5.32 | 677.8154 | 10.95 | 1.0041 | 1.0114 | 18674 | 7.12e3 | 1 | 1 | TRINITY\_DN2477\_c0\_g1\_i5.p1:TRINITY\_DN2477\_c0\_g1\_i6.p1:TRINITY\_DN195934\_c0\_g1\_i1.p2:TRINITY\_DN161\_c0\_g1\_i1.p1:TRINITY\_DN2477\_c0\_g1\_i9.p1:TRINITY\_DN2477\_c0\_g1\_i10.p1:TRINITY\_DN2477\_c0\_g1\_i2.p1:TRINITY\_DN161\_c0\_g1\_i3.p1:TRINITY\_DN161\_c0\_g1\_i2.p1 |  |  | DB Search |
| LLTSSDQAELTKK | 59.06 | 1432.7773 | 13 | -0.99 | 717.3915 | 12.75 | 1.0508 | 1.058 | 29440 | 1.28e3 | 1 | 1 | TRINITY\_DN1339\_c0\_g2\_i1.p2:TRINITY\_DN1339\_c0\_g2\_i2.p1:TRINITY\_DN1339\_c0\_g2\_i3.p1:TRINITY\_DN1339\_c0\_g2\_i4.p2 |  |  | DB Search |
| VITSDDPAEKEK | 59.05 | 1330.6616 | 12 | 3.57 | 666.337 | 10.72 | 0.9884 | 0.9957 | 17382 | 1.42e4 | 1 | 1 | TRINITY\_DN1339\_c6\_g1\_i2.p1:TRINITY\_DN1339\_c6\_g1\_i1.p1:TRINITY\_DN6701\_c0\_g1\_i2.p2:TRINITY\_DN1339\_c0\_g1\_i2.p1:TRINITY\_DN6701\_c0\_g1\_i1.p1:TRINITY\_DN124769\_c0\_g1\_i1.p1:TRINITY\_DN1172\_c0\_g1\_i5.p1:TRINITY\_DN1172\_c0\_g1\_i3.p1:TRINITY\_DN1339\_c0\_g1\_i1.p1 |  |  | DB Search |
| VLTSDDPAEKEK | 59.05 | 1330.6616 | 12 | 3.57 | 666.337 | 10.72 | 0.9884 | 0.9957 | 17382 | 1.42e4 | 1 | 1 | TRINITY\_DN119673\_c0\_g1\_i1.p1:TRINITY\_DN24414\_c1\_g1\_i2.p2 |  |  | DB Search |
| AKLSQC(+57.02)TAASGK | 59.04 | 1220.6183 | 12 | 5.98 | 611.3169 | 9.70 | 0.9505 | 0.9578 | 11647 | 3.34e3 | 1 | 1 | TRINITY\_DN3991\_c0\_g1\_i1.p1 | Carbamidomethylation |  | DB Search |
| ASFVSDIDRLR | 59.04 | 1277.6727 | 11 | 1.92 | 639.8416 | 16.88 | 0.9828 | 0.99 | 50956 | 4.21e3 | 1 | 1 | TRINITY\_DN598\_c1\_g1\_i1.p1 |  |  | DB Search |
| AYAAFLEFKDVVK | 59.04 | 1499.8024 | 13 | 3.65 | 750.9073 | 19.03 | 1.0945 | 1.1017 | 63342 | 1.12e4 | 1 | 1 | TRINITY\_DN240\_c0\_g1\_i6.p1:TRINITY\_DN240\_c0\_g1\_i1.p1:TRINITY\_DN240\_c0\_g1\_i4.p1 |  |  | DB Search |
| GLTHC(+57.02)NTLSYK | 59.02 | 1292.6183 | 11 | 5.84 | 647.3168 | 12.45 | 0.9727 | 0.98 | 27736 | 3.39e3 | 1 | 1 | TRINITY\_DN902\_c1\_g2\_i4.p1:TRINITY\_DN902\_c1\_g2\_i3.p1:TRINITY\_DN902\_c1\_g2\_i1.p1 | Carbamidomethylation |  | DB Search |
| DNIQGITKPAIR | 58.99 | 1324.7462 | 12 | 0.18 | 663.3771 | 13.76 | 0.9812 | 0.9884 | 35082 | 6.65e2 | 1 | 1 | TRINITY\_DN172571\_c0\_g1\_i1.p1 |  |  | DB Search |
| TIESEAAHGTVTR | 58.99 | 1370.679 | 13 | 5.98 | 686.3473 | 11.90 | 1.0206 | 1.0279 | 24466 | 5.79e2 | 1 | 1 | TRINITY\_DN3271\_c0\_g1\_i1.p1:TRINITY\_DN3271\_c0\_g1\_i2.p1 |  |  | DB Search |
| VGDIVDVKGNGAIQK | 58.99 | 1511.8307 | 15 | -4.74 | 756.9151 | 13.61 | 1.0697 | 1.0769 | 34346 | 0 | 0 | 0 | TRINITY\_DN6132\_c0\_g1\_i1.p1 |  |  | DB Search |
| AVFPSIVGRPR | 58.99 | 1197.6982 | 11 | 1.88 | 599.8544 | 15.65 | 0.9594 | 0.9666 | 45162 | 3.26e4 | 1 | 1 | TRINITY\_DN161\_c0\_g1\_i1.p1:TRINITY\_DN161\_c0\_g1\_i3.p1:TRINITY\_DN161\_c0\_g1\_i2.p1 |  |  | DB Search |
| K(+42.01)LNAELANGR | 58.99 | 1126.6095 | 10 | 3.51 | 564.311 | 13.77 | 0.883 | 0.8903 | 35173 | 1.19e4 | 3 | 3 | TRINITY\_DN19618\_c0\_g1\_i1.p1 | Acetylation (Protein N-term) | K1:Acetylation (Protein N-term):1000 | DB Search |
| FRQLGLDFGK | 58.98 | 1179.64 | 10 | -8.35 | 590.8193 | 19.81 | 0.9194 | 0.9267 | 67914 | 3.91e3 | 1 | 1 | TRINITY\_DN22594\_c0\_g1\_i1.p2 |  |  | DB Search |
| S(+42.01)LVIPEKFQHILR | 58.95 | 1620.9352 | 13 | 2.83 | 811.4729 | 19.56 | 1.1222 | 1.1294 | 66460 | 1.08e4 | 2 | 2 | TRINITY\_DN1505\_c0\_g1\_i1.p1 | Acetylation (Protein N-term) | S1:Acetylation (Protein N-term):1000 | DB Search |
| VESQSFQDIREK | 58.92 | 1464.7208 | 12 | 4.27 | 733.367 | 12.87 | 1.0299 | 1.0371 | 30170 | 0 | 0 | 0 | TRINITY\_DN757\_c0\_g1\_i1.p1 |  |  | DB Search |
| ALGC(+57.02)DVLAC(+57.02)SVDSHFSHLAWTQQSR | 58.92 | 2844.3071 | 25 | -0.81 | 712.0798 | 18.79 | 0.9481 | 0.9553 | 62026 | 2.73e3 | 1 | 1 | TRINITY\_DN535\_c0\_g1\_i2.p1:TRINITY\_DN535\_c0\_g1\_i1.p1 | Carbamidomethylation, Carbamidomethylation |  | DB Search |
| DLHIIEDYVLK | 58.91 | 1356.7289 | 11 | 0.07 | 679.3682 | 19.53 | 0.9953 | 1.0025 | 66441 | 3.01e3 | 1 | 1 | TRINITY\_DN8903\_c0\_g1\_i1.p1 |  |  | DB Search |
| ASFVTDIDRLR | 58.91 | 1291.6885 | 11 | 1.37 | 646.849 | 17.02 | 0.9864 | 0.9937 | 51664 | 4.94e3 | 1 | 1 | TRINITY\_DN598\_c1\_g1\_i2.p1 |  |  | DB Search |
| LAYELQANKEQQR | 58.91 | 1589.8162 | 13 | -1.93 | 795.9097 | 12.92 | 1.0516 | 1.0588 | 30474 | 0 | 0 | 0 | TRINITY\_DN11435\_c0\_g1\_i1.p1 |  |  | DB Search |
| A(+42.01)TGIKPDPQC(+57.02)TDLYNK | 58.9 | 1861.8879 | 16 | 1.83 | 931.9481 | 14.86 | 1.1622 | 1.1694 | 40590 | 1.38e3 | 1 | 1 | TRINITY\_DN164271\_c0\_g1\_i1.p1 | Acetylation (Protein N-term), Carbamidomethylation | A1:Acetylation (Protein N-term):1000 | DB Search |
| ATETATGLATTHPIK | 58.89 | 1510.7991 | 15 | -0.47 | 756.4025 | 12.92 | 1.1061 | 1.1134 | 30340 | 1.04e3 | 1 | 1 | TRINITY\_DN165536\_c0\_g1\_i1.p1 |  |  | DB Search |
| AGHGAVTSPQTQR | 58.87 | 1308.6534 | 13 | 3.49 | 437.2243 | 9.73 | 0.7127 | 0.72 | 11628 | 3.81e3 | 1 | 1 | TRINITY\_DN3036\_c0\_g1\_i13.p1:TRINITY\_DN3036\_c0\_g1\_i19.p1:TRINITY\_DN3036\_c0\_g1\_i11.p1:TRINITY\_DN3036\_c0\_g1\_i3.p1:TRINITY\_DN3036\_c0\_g1\_i16.p1:TRINITY\_DN3036\_c0\_g1\_i2.p2:TRINITY\_DN3036\_c0\_g1\_i8.p1 |  |  | DB Search |
| DLSLVVEPLKNYGVK | 58.87 | 1672.9399 | 15 | -1.21 | 837.4719 | 19.04 | 1.1338 | 1.141 | 63579 | 2.46e2 | 1 | 1 | TRINITY\_DN244\_c0\_g1\_i1.p1 |  |  | DB Search |
| TIEAEAAHGTVTR | 58.86 | 1354.6841 | 13 | -3.52 | 678.3434 | 11.82 | 1.0142 | 1.0214 | 24040 | 0 | 0 | 0 | TRINITY\_DN7504\_c0\_g1\_i1.p1 |  |  | DB Search |
| IGAAAQALADQSYPFLK | 58.85 | 1762.9253 | 17 | 0.88 | 882.4661 | 19.07 | 1.1606 | 1.1678 | 63232 | 1.84e3 | 1 | 1 | TRINITY\_DN240\_c0\_g1\_i6.p1:TRINITY\_DN240\_c0\_g1\_i1.p1:TRINITY\_DN240\_c0\_g1\_i4.p1 |  |  | DB Search |
| TRIEDLETVISQTQDHR | 58.82 | 2040.0236 | 17 | 4.24 | 681.0145 | 17.36 | 0.9594 | 0.9666 | 53642 | 9.24e2 | 1 | 1 | TRINITY\_DN761\_c0\_g1\_i3.p1:TRINITY\_DN761\_c0\_g1\_i1.p1:TRINITY\_DN761\_c0\_g1\_i2.p1 |  |  | DB Search |
| K(+42.01)LSAELANGR | 58.81 | 1099.5985 | 10 | -0.72 | 550.8033 | 14.22 | 0.8721 | 0.8793 | 37511 | 8.65e2 | 1 | 1 | TRINITY\_DN21847\_c1\_g1\_i1.p1:TRINITY\_DN8165\_c0\_g2\_i2.p2:TRINITY\_DN8165\_c0\_g2\_i1.p2 | Acetylation (Protein N-term) | K1:Acetylation (Protein N-term):1000 | DB Search |
| DANAAVVHGSHLK | 58.77 | 1317.679 | 13 | 7.74 | 659.8484 | 11.46 | 1.0045 | 1.0118 | 21812 | 7.21e2 | 1 | 1 | TRINITY\_DN262\_c0\_g1\_i1.p1 |  |  | DB Search |
| DYGVLLEDAGHTLR | 58.76 | 1557.7787 | 14 | -1.38 | 779.8915 | 18.23 | 1.1294 | 1.1366 | 58601 | 2.52e3 | 1 | 1 | TRINITY\_DN332\_c0\_g1\_i1.p1 |  |  | DB Search |
| GLDVDSLVIDHIQVNR | 58.76 | 1791.9479 | 16 | -0.72 | 896.9759 | 19.01 | 1.1786 | 1.1857 | 63336 | 2.19e3 | 1 | 1 | TRINITY\_DN1614\_c0\_g1\_i1.p1 |  |  | DB Search |
| K(+42.01)LSAEVANGR | 58.76 | 1085.5829 | 10 | -2.46 | 543.7946 | 12.92 | 0.8567 | 0.864 | 30461 | 2.87e3 | 1 | 1 | TRINITY\_DN6397\_c0\_g2\_i4.p1:TRINITY\_DN6397\_c0\_g2\_i3.p1 | Acetylation (Protein N-term) | K1:Acetylation (Protein N-term):1000 | DB Search |
| S(+42.01)ATGNLVTVLRK | 58.74 | 1299.751 | 12 | -3.1 | 650.8774 | 17.07 | 0.9977 | 1.0049 | 51993 | 6.03e2 | 1 | 1 | TRINITY\_DN2963\_c0\_g1\_i1.p1 | Acetylation (Protein N-term) | S1:Acetylation (Protein N-term):1000 | DB Search |
| ETC(+57.02)SDIEKR | 58.74 | 1136.5132 | 9 | 1.02 | 569.2615 | 10.09 | 0.8947 | 0.902 | 13624 | 1.03e4 | 1 | 1 | TRINITY\_DN118\_c1\_g1\_i3.p1:TRINITY\_DN118\_c1\_g1\_i5.p1 | Carbamidomethylation |  | DB Search |
| AHNLTEDVIFWK | 58.72 | 1471.746 | 12 | 0.34 | 736.8766 | 18.72 | 1.0061 | 1.0134 | 61618 | 3.8e2 | 1 | 1 | TRINITY\_DN2113\_c0\_g1\_i1.p1:TRINITY\_DN2113\_c0\_g1\_i2.p1 |  |  | DB Search |
| APGDYGWRPPLLATDDAALK | 58.71 | 2126.0796 | 20 | 0.61 | 709.6972 | 18.99 | 0.8883 | 0.8955 | 63354 | 4.18e3 | 1 | 1 | TRINITY\_DN16548\_c0\_g1\_i4.p1:TRINITY\_DN16548\_c0\_g1\_i1.p1:TRINITY\_DN16548\_c0\_g1\_i3.p1 |  |  | DB Search |
| EVNTELDSRPETK | 58.71 | 1516.7369 | 13 | 5.72 | 759.3761 | 11.75 | 1.0267 | 1.0339 | 23511 | 8.88e3 | 2 | 2 | TRINITY\_DN11643\_c0\_g1\_i1.p1 |  |  | DB Search |
| KAQYYEAYETIIKR | 58.69 | 1774.9254 | 14 | -1.1 | 592.6453 | 15.71 | 0.8623 | 0.8696 | 45168 | 1.12e3 | 1 | 1 | TRINITY\_DN319\_c2\_g1\_i3.p1:TRINITY\_DN319\_c2\_g1\_i1.p1 |  |  | DB Search |
| IHINIVVIGHVDSGK | 58.69 | 1599.9097 | 15 | -0.5 | 800.9575 | 16.54 | 1.1001 | 1.1074 | 49239 | 7.77e2 | 2 | 2 | TRINITY\_DN64\_c0\_g1\_i1.p1 |  |  | DB Search |
| VLTSSDPAEKEK | 58.69 | 1302.6666 | 12 | 2.82 | 652.3391 | 10.58 | 0.9965 | 1.0037 | 16473 | 6.51e3 | 1 | 1 | TRINITY\_DN4497\_c0\_g2\_i1.p1:TRINITY\_DN1172\_c0\_g2\_i2.p1:TRINITY\_DN21847\_c0\_g1\_i1.p1:TRINITY\_DN4497\_c0\_g1\_i1.p1 |  |  | DB Search |
| IC(+57.02)HLLGIQVTDFTK | 58.69 | 1643.8705 | 14 | 0.67 | 822.9388 | 18.46 | 1.0833 | 1.0905 | 60048 | 4.74e2 | 1 | 1 | TRINITY\_DN428\_c0\_g1\_i1.p1 | Carbamidomethylation |  | DB Search |
| YGINHITNLVESKR | 58.69 | 1642.879 | 14 | -1.63 | 548.6299 | 14.98 | 0.823 | 0.8303 | 41552 | 0 | 0 | 0 | TRINITY\_DN146208\_c0\_g1\_i1.p1 |  |  | DB Search |
| AVVEGM(+15.99)TADSDESKKDE | 58.69 | 1825.7887 | 17 | 2.01 | 609.6016 | 10.36 | 0.8348 | 0.8421 | 15202 | 1.88e3 | 2 | 2 | TRINITY\_DN3153\_c0\_g1\_i2.p1:TRINITY\_DN3153\_c0\_g1\_i3.p1 | Oxidation (M) | M6:Oxidation (M):1000 | DB Search |
| GC(+57.02)YIHTQEIAR | 58.69 | 1346.6401 | 11 | 1.87 | 674.3251 | 12.91 | 1.0166 | 1.0238 | 30377 | 1.95e3 | 1 | 1 | TRINITY\_DN2217\_c0\_g1\_i3.p1:TRINITY\_DN38397\_c0\_g1\_i1.p1:TRINITY\_DN2217\_c0\_g1\_i2.p1 | Carbamidomethylation |  | DB Search |
| VLATDKDTLSSNK | 58.69 | 1390.7303 | 13 | 0.28 | 696.369 | 11.52 | 1.0158 | 1.023 | 22129 | 2.18e3 | 1 | 1 | TRINITY\_DN982\_c0\_g1\_i3.p1 |  |  | DB Search |
| SLGNHGQGIDR | 58.68 | 1152.5636 | 11 | 3.33 | 577.288 | 10.63 | 0.9024 | 0.9097 | 16884 | 0 | 0 | 0 | TRINITY\_DN18025\_c0\_g1\_i1.p1 |  |  | DB Search |
| ASGDYATVVSHNR | 58.68 | 1375.6481 | 13 | 14.02 | 688.8373 | 12.12 | 0.9969 | 1.0041 | 25619 | 3.01e3 | 1 | 1 | TRINITY\_DN925\_c0\_g1\_i1.p1 |  |  | DB Search |
| LVQNTPHNSIVTK | 58.68 | 1449.7939 | 13 | 2.67 | 725.9024 | 11.69 | 1.0584 | 1.0656 | 23187 | 0 | 0 | 0 | TRINITY\_DN982\_c0\_g1\_i3.p1:TRINITY\_DN982\_c0\_g1\_i1.p1 |  |  | DB Search |
| SC(+57.02)TNPAPANGGK | 58.68 | 1172.5244 | 12 | 3.23 | 587.2683 | 9.42 | 0.8352 | 0.8425 | 10441 | 8.36e2 | 1 | 1 | TRINITY\_DN495\_c0\_g1\_i2.p1:TRINITY\_DN3545\_c0\_g1\_i2.p1:TRINITY\_DN3545\_c0\_g1\_i1.p1:TRINITY\_DN495\_c0\_g1\_i3.p1 | Carbamidomethylation |  | DB Search |
| RYGQAMC(+57.02)QFMR | 58.68 | 1446.6318 | 11 | -9.09 | 724.3129 | 17.23 | 0.9832 | 0.9904 | 52876 | 1.72e3 | 1 | 1 | TRINITY\_DN286\_c0\_g1\_i4.p1:TRINITY\_DN286\_c0\_g1\_i2.p1 | Carbamidomethylation |  | DB Search |
| GTSNGGLVVGTIIKPK | 58.68 | 1539.8984 | 16 | 0.87 | 770.9531 | 15.69 | 1.1254 | 1.1326 | 44972 | 2.46e3 | 1 | 1 | TRINITY\_DN3036\_c0\_g1\_i3.p1:TRINITY\_DN3036\_c0\_g1\_i2.p2:TRINITY\_DN3036\_c0\_g1\_i8.p1 |  |  | DB Search |
| GLKVDFDTTFAPVTGKK | 58.67 | 1822.9828 | 17 | 2.09 | 912.4958 | 17.12 | 1.1802 | 1.1873 | 52158 | 1.37e3 | 1 | 1 | TRINITY\_DN329\_c0\_g1\_i1.p1:TRINITY\_DN329\_c0\_g1\_i2.p1 |  |  | DB Search |
| TPGLVEGTLILHQLR | 58.67 | 1645.9515 | 15 | 0.88 | 549.6554 | 18.78 | 0.8631 | 0.8704 | 61967 | 3.17e2 | 1 | 1 | TRINITY\_DN3034\_c0\_g1\_i1.p1 |  |  | DB Search |
| LRLDFDHEAKLNMIFR | 58.67 | 2017.0568 | 16 | 5.71 | 505.2717 | 18.48 | 0.8644 | 0.8716 | 60165 | 7.24e2 | 1 | 1 | TRINITY\_DN160\_c0\_g1\_i2.p1:TRINITY\_DN160\_c0\_g1\_i1.p1 |  |  | DB Search |
| FSYEKVC(+57.02)EDVKR | 58.67 | 1558.745 | 12 | 1.55 | 780.3769 | 13.49 | 1.0717 | 1.0789 | 33594 | 8.49e2 | 1 | 1 | TRINITY\_DN118\_c1\_g1\_i3.p1 | Carbamidomethylation |  | DB Search |
| AEFRPNQEEEPILKR | 58.67 | 1854.9587 | 15 | 0.94 | 619.3242 | 13.74 | 0.8785 | 0.8858 | 34918 | 9.68e2 | 1 | 1 | TRINITY\_DN262\_c0\_g1\_i1.p1 |  |  | DB Search |
| EGFYVHEALAAGIVK | 58.67 | 1602.8406 | 15 | 1.72 | 802.4247 | 17.54 | 1.0889 | 1.0961 | 54660 | 6.57e2 | 1 | 1 | TRINITY\_DN602\_c0\_g1\_i4.p1:TRINITY\_DN602\_c0\_g1\_i3.p1:TRINITY\_DN602\_c0\_g1\_i1.p1 |  |  | DB Search |
| DC(+57.02)TPRETC(+57.02)SDIEKR | 58.67 | 1765.7723 | 14 | -5.61 | 589.5917 | 11.15 | 0.8485 | 0.8558 | 19989 | 0 | 0 | 0 | TRINITY\_DN118\_c1\_g1\_i3.p1:TRINITY\_DN118\_c1\_g1\_i5.p1 | Carbamidomethylation, Carbamidomethylation |  | DB Search |
| M(+15.99)SATFIGNSTAIQELFKR | 58.67 | 2029.0303 | 18 | 2.72 | 677.349 | 19.96 | 0.9376 | 0.9449 | 68814 | 3.1e2 | 1 | 1 | TRINITY\_DN3986\_c0\_g1\_i1.p1 | Oxidation (M) | M1:Oxidation (M):1000 | DB Search |
| RYGQAM(+15.99)C(+57.02)QFM(+15.99)R | 58.66 | 1478.6217 | 11 | -7.27 | 740.3089 | 12.91 | 0.9775 | 0.9848 | 30247 | 2.62e3 | 1 | 1 | TRINITY\_DN286\_c0\_g1\_i4.p1:TRINITY\_DN286\_c0\_g1\_i2.p1 | Oxidation (M), Carbamidomethylation, Oxidation (M) | M6:Oxidation (M):1000 M10:Oxidation (M):1000 | DB Search |
| AFQNTPC(+57.02)THLVLDGTR | 58.66 | 1828.889 | 16 | 2.02 | 915.4489 | 15.26 | 1.1762 | 1.1833 | 42854 | 5.12e2 | 1 | 1 | TRINITY\_DN1091\_c0\_g1\_i1.p1 | Carbamidomethylation |  | DB Search |
| DKNILVLTLGVGPQVNR | 58.66 | 1835.0629 | 17 | 1.23 | 918.535 | 19.38 | 1.1314 | 1.1386 | 65500 | 2.41e3 | 2 | 2 | TRINITY\_DN477\_c0\_g1\_i8.p1:TRINITY\_DN477\_c0\_g1\_i7.p1 |  |  | DB Search |
| VVIIDGKGHLM(+15.99)GR | 58.66 | 1409.7812 | 13 | 2.85 | 705.8962 | 12.99 | 1.0271 | 1.0343 | 30876 | 0 | 0 | 0 | TRINITY\_DN2805\_c0\_g1\_i1.p1 | Oxidation (M) | M11:Oxidation (M):1000 | DB Search |
| TVQNFYELVTHSK | 58.66 | 1564.7886 | 13 | 4.05 | 783.4006 | 18.26 | 1.0893 | 1.0965 | 58839 | 6.36e2 | 1 | 1 | TRINITY\_DN96\_c0\_g1\_i2.p1:TRINITY\_DN96\_c0\_g1\_i1.p1 |  |  | DB Search |
| NM(+15.99)M(+15.99)AAC(+57.02)DPR | 58.66 | 1096.41 | 9 | 1.24 | 549.2101 | 9.53 | 0.8619 | 0.8692 | 10930 | 4.97e2 | 1 | 1 | TRINITY\_DN1910\_c0\_g1\_i1.p1:TRINITY\_DN1910\_c0\_g1\_i5.p1:TRINITY\_DN3986\_c0\_g1\_i1.p1 | Oxidation (M), Oxidation (M), Carbamidomethylation | M2:Oxidation (M):1000 M3:Oxidation (M):1000 | DB Search |
| SFLLALEKDQEK | 58.66 | 1419.7609 | 12 | 1.99 | 710.8854 | 17.38 | 1.0729 | 1.0801 | 53732 | 9.75e2 | 1 | 1 | TRINITY\_DN1386\_c0\_g1\_i1.p1 |  |  | DB Search |
| RIQQLSAIR | 58.65 | 1083.6512 | 9 | 0.9 | 542.8306 | 13.16 | 0.9174 | 0.9247 | 31748 | 7.18e2 | 1 | 1 | TRINITY\_DN428\_c0\_g1\_i1.p1 |  |  | DB Search |
| AHLTALENAFQLEK | 58.65 | 1583.8307 | 14 | -3.06 | 792.9161 | 17.91 | 1.0773 | 1.0845 | 56906 | 0 | 0 | 0 | TRINITY\_DN371\_c1\_g1\_i1.p1 |  |  | DB Search |
| AISSASGPTGM(+15.99)TSK | 58.65 | 1309.6184 | 14 | 2.9 | 655.8149 | 10.52 | 0.9352 | 0.9424 | 16184 | 9.25e2 | 1 | 1 | TRINITY\_DN240\_c0\_g1\_i6.p1:TRINITY\_DN240\_c0\_g1\_i1.p1:TRINITY\_DN240\_c0\_g1\_i4.p1 | Oxidation (M) | M11:Oxidation (M):1000 | DB Search |
| ILQC(+57.02)NQYANVEEAR | 58.65 | 1706.8046 | 14 | 0.67 | 854.4057 | 14.18 | 1.0801 | 1.0873 | 37366 | 0 | 0 | 0 | TRINITY\_DN477\_c0\_g1\_i8.p1:TRINITY\_DN477\_c0\_g1\_i7.p1 | Carbamidomethylation |  | DB Search |
| GKEDERNEVYNEAITK | 58.65 | 1893.9067 | 16 | 3.14 | 632.3082 | 12.68 | 0.8274 | 0.8348 | 29071 | 1.27e3 | 1 | 1 | TRINITY\_DN3619\_c0\_g1\_i1.p1 |  |  | DB Search |
| GTHAAEIAHC(+57.02)VSR | 58.65 | 1407.6677 | 13 | -14.3 | 704.8274 | 11.04 | 1.0307 | 1.0379 | 19410 | 0 | 0 | 0 | TRINITY\_DN1319\_c0\_g1\_i2.p1:TRINITY\_DN1319\_c0\_g1\_i1.p1 | Carbamidomethylation |  | DB Search |
| AGVLAHLEEER | 58.65 | 1222.6306 | 11 | -5.54 | 612.316 | 14.82 | 0.9553 | 0.9626 | 40752 | 0 | 0 | 0 | TRINITY\_DN428\_c0\_g1\_i1.p1 |  |  | DB Search |
| A(+42.01)LSRPVYIIAAK | 58.64 | 1342.7972 | 12 | -0.25 | 672.4022 | 17.90 | 1.0536 | 1.0608 | 56682 | 2.75e3 | 1 | 1 | TRINITY\_DN11662\_c0\_g1\_i1.p1 | Acetylation (Protein N-term) | A1:Acetylation (Protein N-term):1000 | DB Search |
| AISFIGSVHDNRK | 58.64 | 1442.7629 | 13 | 2.55 | 722.3868 | 14.04 | 1.0415 | 1.0488 | 36558 | 6.68e2 | 1 | 1 | TRINITY\_DN6\_c0\_g1\_i1.p1 |  |  | DB Search |
| APTHILNPANKR | 58.64 | 1330.7469 | 12 | 0.9 | 666.3779 | 11.17 | 1.009 | 1.0162 | 20196 | 4.06e2 | 1 | 1 | TRINITY\_DN1058\_c0\_g1\_i4.p1:TRINITY\_DN1058\_c0\_g1\_i1.p1:TRINITY\_DN1058\_c0\_g1\_i3.p1:TRINITY\_DN1058\_c0\_g1\_i2.p2 |  |  | DB Search |
| DPINNHWDGNLINWR | 58.64 | 1862.8812 | 15 | 0.17 | 621.9645 | 18.89 | 0.8128 | 0.8201 | 62735 | 3.45e3 | 1 | 1 | TRINITY\_DN138\_c0\_g1\_i1.p1 |  |  | DB Search |
| DYGVLLENDGIALR | 58.64 | 1546.7991 | 14 | 1.09 | 774.4036 | 19.46 | 1.0387 | 1.046 | 66038 | 4.57e2 | 1 | 1 | TRINITY\_DN535\_c0\_g1\_i2.p1:TRINITY\_DN535\_c0\_g1\_i1.p1 |  |  | DB Search |
| GLKVDFDTTFAPVTGK | 58.63 | 1694.8879 | 16 | 3.65 | 848.4499 | 18.14 | 1.0957 | 1.1029 | 58184 | 1.23e3 | 1 | 1 | TRINITY\_DN329\_c0\_g1\_i1.p1:TRINITY\_DN329\_c0\_g1\_i2.p1 |  |  | DB Search |
| ALLSLEYHEKR | 58.63 | 1357.7354 | 11 | 0.51 | 679.8718 | 14.42 | 1.0275 | 1.0347 | 38581 | 1.27e3 | 1 | 1 | TRINITY\_DN8707\_c0\_g1\_i2.p1 |  |  | DB Search |
| ATGC(+57.02)EVIWNQHNR | 58.63 | 1583.7263 | 13 | 3.91 | 792.8694 | 13.85 | 1.0785 | 1.0857 | 35534 | 2.24e3 | 1 | 1 | TRINITY\_DN2217\_c0\_g1\_i3.p1:TRINITY\_DN2217\_c0\_g1\_i2.p1 | Carbamidomethylation |  | DB Search |
| AFVAIGDYGGHVGLGVK | 58.63 | 1658.8779 | 17 | 1.2 | 830.4429 | 17.62 | 1.1614 | 1.1686 | 54962 | 8.29e3 | 1 | 1 | TRINITY\_DN15584\_c0\_g1\_i1.p1 |  |  | DB Search |
| KFDTYLPGFIC(+57.02)R | 58.63 | 1515.7544 | 12 | 1.26 | 758.8815 | 18.53 | 1.0299 | 1.0371 | 60572 | 0 | 0 | 0 | TRINITY\_DN785\_c0\_g1\_i1.p1 | Carbamidomethylation |  | DB Search |
| VLTASDPEALKR | 58.63 | 1298.7194 | 12 | -0.08 | 650.3635 | 12.95 | 0.9904 | 0.9977 | 30554 | 9.56e2 | 1 | 1 | TRINITY\_DN10563\_c0\_g1\_i1.p1 |  |  | DB Search |
| IDNYIEFLDEDLKK | 58.63 | 1753.8774 | 14 | -2.64 | 585.6285 | 19.84 | 0.8445 | 0.8518 | 68139 | 1.64e3 | 1 | 1 | TRINITY\_DN278\_c0\_g1\_i1.p1 |  |  | DB Search |
| LAADLVITHSSK | 58.63 | 1253.6979 | 12 | 2.83 | 627.8547 | 14.22 | 0.9973 | 1.0045 | 37483 | 1.32e3 | 1 | 1 | TRINITY\_DN751\_c0\_g1\_i1.p1:TRINITY\_DN7214\_c0\_g1\_i1.p1 |  |  | DB Search |
| GVAGAGVLAGFDKFK | 58.63 | 1435.7823 | 15 | -0.62 | 718.8942 | 18.25 | 1.0873 | 1.0945 | 58813 | 1.77e3 | 1 | 1 | TRINITY\_DN1559\_c0\_g1\_i1.p1 |  |  | DB Search |
| VAC(+57.02)IGAWHPAR | 58.63 | 1236.6185 | 11 | 3 | 619.3152 | 13.23 | 0.9461 | 0.9533 | 32140 | 5.07e2 | 1 | 1 | TRINITY\_DN6920\_c0\_g1\_i1.p1:TRINITY\_DN6920\_c0\_g1\_i3.p1:TRINITY\_DN6920\_c0\_g1\_i4.p1:TRINITY\_DN6920\_c0\_g1\_i2.p1 | Carbamidomethylation |  | DB Search |
| ANQVSSAAAAPVPAAGGDK | 58.63 | 1680.843 | 19 | 2.49 | 841.4265 | 12.47 | 1.068 | 1.0753 | 27849 | 0 | 0 | 0 | TRINITY\_DN240\_c0\_g1\_i6.p1:TRINITY\_DN240\_c0\_g1\_i1.p1:TRINITY\_DN240\_c0\_g1\_i4.p1 |  |  | DB Search |
| VYLAQAEAEKIK | 58.63 | 1361.7554 | 12 | 0.94 | 681.8821 | 14.28 | 1.0339 | 1.0411 | 37834 | 4.47e2 | 1 | 1 | TRINITY\_DN11435\_c0\_g1\_i1.p1 |  |  | DB Search |
| TVTC(+57.02)GAGETIKVTK | 58.63 | 1463.7654 | 14 | 1.13 | 732.887 | 12.31 | 1.0664 | 1.0737 | 26936 | 0 | 0 | 0 | TRINITY\_DN374\_c0\_g1\_i1.p1 | Carbamidomethylation |  | DB Search |
| TIKDVNC(+57.02)NNNQLK | 58.63 | 1559.7726 | 13 | 0.66 | 780.89 | 11.23 | 1.0464 | 1.0536 | 20329 | 8.76e2 | 1 | 1 | TRINITY\_DN4815\_c0\_g1\_i2.p1:TRINITY\_DN4815\_c0\_g1\_i1.p1 | Carbamidomethylation |  | DB Search |
| VTRPHGASGVVR | 58.63 | 1234.6895 | 12 | 0.38 | 412.5684 | 10.08 | 0.8197 | 0.827 | 13527 | 3.84e3 | 1 | 1 | TRINITY\_DN2885\_c0\_g1\_i2.p1:TRINITY\_DN2885\_c0\_g1\_i4.p1:TRINITY\_DN2885\_c0\_g1\_i5.p1 |  |  | DB Search |
| LVVIADIVDQKR | 58.63 | 1367.8136 | 12 | -3.46 | 684.9081 | 17.50 | 1.0435 | 1.0508 | 54455 | 3.87e2 | 1 | 1 | TRINITY\_DN6294\_c0\_g3\_i1.p1:TRINITY\_DN6294\_c0\_g3\_i2.p1 |  |  | DB Search |
| LIQTLNYETKK | 58.63 | 1349.7555 | 11 | 0.98 | 675.8821 | 13.61 | 1.0114 | 1.0186 | 34304 | 6.23e2 | 1 | 1 | TRINITY\_DN982\_c0\_g1\_i3.p1:TRINITY\_DN982\_c0\_g1\_i1.p1 |  |  | DB Search |
| GLFIIDDKGVLR | 58.63 | 1344.7765 | 12 | -0.16 | 673.3919 | 18.68 | 1.0017 | 1.009 | 61308 | 2.43e3 | 1 | 1 | TRINITY\_DN332\_c0\_g1\_i1.p1 |  |  | DB Search |
| TPGEC(+57.02)C(+57.02)PQC(+57.02)K | 58.63 | 1235.4733 | 10 | 0.71 | 618.7411 | 9.99 | 0.8915 | 0.8988 | 13135 | 0 | 0 | 0 | TRINITY\_DN1369\_c0\_g1\_i1.p1 | Carbamidomethylation, Carbamidomethylation, Carbamidomethylation |  | DB Search |
| IPLDDFQDPTLTLENAHNK | 58.63 | 2180.075 | 19 | -0.5 | 727.6948 | 18.20 | 0.9904 | 0.9977 | 58430 | 9.46e2 | 1 | 1 | TRINITY\_DN13856\_c0\_g1\_i1.p2:TRINITY\_DN13856\_c0\_g1\_i2.p2 |  |  | DB Search |
| TQAVLDRLNIEK | 58.63 | 1398.7831 | 12 | -2.46 | 700.3934 | 15.31 | 1.0069 | 1.0142 | 43158 | 1.16e3 | 1 | 1 | TRINITY\_DN477\_c0\_g1\_i9.p1 |  |  | DB Search |
| GLFIIDDKGILR | 58.62 | 1358.7921 | 12 | 1.68 | 680.4009 | 19.29 | 1.0114 | 1.0186 | 64919 | 1.05e4 | 1 | 1 | TRINITY\_DN535\_c0\_g1\_i2.p1:TRINITY\_DN535\_c0\_g1\_i1.p1 |  |  | DB Search |
| KSAQFLLQLLK | 58.62 | 1287.7914 | 11 | 3.65 | 644.902 | 19.71 | 0.9779 | 0.9852 | 67435 | 7.12e2 | 1 | 1 | TRINITY\_DN1614\_c0\_g1\_i1.p1 |  |  | DB Search |
| LSQC(+57.02)TAASGK | 58.62 | 1021.4862 | 10 | -1.58 | 511.7469 | 9.83 | 0.8274 | 0.8348 | 11429 | 2.43e5 | 1 | 1 | TRINITY\_DN3991\_c0\_g1\_i1.p1 | Carbamidomethylation |  | DB Search |
| VVASSHTDYELR | 58.62 | 1375.6732 | 12 | 4.53 | 688.8434 | 12.54 | 1.0122 | 1.0194 | 28239 | 0 | 0 | 0 | TRINITY\_DN95248\_c0\_g1\_i1.p1 |  |  | DB Search |
| AFLIEEQKLVVR | 58.62 | 1443.8448 | 12 | 4.06 | 722.9289 | 17.34 | 1.048 | 1.0552 | 53425 | 1.97e3 | 1 | 1 | TRINITY\_DN79126\_c0\_g1\_i1.p1 |  |  | DB Search |
| INVDAAFHDLVR | 58.62 | 1368.715 | 12 | 1.55 | 685.3622 | 18.65 | 1.023 | 1.0303 | 61210 | 8.3e2 | 1 | 1 | TRINITY\_DN147947\_c0\_g1\_i1.p1 |  |  | DB Search |
| AVIFDRFR | 58.62 | 1022.5661 | 8 | 1.78 | 512.2886 | 15.90 | 0.8672 | 0.8745 | 46056 | 5.37e3 | 1 | 1 | TRINITY\_DN98420\_c0\_g1\_i1.p1 |  |  | DB Search |
| LLLGDFNGDKR | 58.62 | 1246.667 | 11 | 3.37 | 624.3396 | 16.00 | 0.9844 | 0.9916 | 46643 | 2.81e2 | 1 | 1 | TRINITY\_DN413\_c0\_g1\_i1.p1:TRINITY\_DN413\_c0\_g1\_i4.p1:TRINITY\_DN413\_c0\_g1\_i2.p1:TRINITY\_DN413\_c0\_g1\_i3.p1 |  |  | DB Search |
| YC(+57.02)ILSFLDKTDK | 58.62 | 1501.7487 | 12 | -1.44 | 501.5868 | 19.64 | 0.7958 | 0.8031 | 67040 | 7.6e2 | 1 | 1 | TRINITY\_DN15274\_c0\_g1\_i1.p1 | Carbamidomethylation |  | DB Search |
| A(+42.01)NPLKDIISR | 58.62 | 1167.6611 | 10 | -1.4 | 584.834 | 18.57 | 0.9214 | 0.9287 | 60579 | 1.56e3 | 1 | 1 | TRINITY\_DN163801\_c0\_g1\_i1.p1 | Acetylation (Protein N-term) | A1:Acetylation (Protein N-term):1000 | DB Search |
| YIQGENEKQEK | 58.62 | 1364.6572 | 11 | -4.52 | 683.3292 | 9.57 | 0.9691 | 0.9763 | 11125 | 0 | 0 | 0 | TRINITY\_DN1272\_c0\_g1\_i1.p1 |  |  | DB Search |
| LFEGNALLRR | 58.61 | 1187.6775 | 10 | 3.48 | 594.845 | 15.57 | 0.9679 | 0.9751 | 44463 | 2.7e3 | 1 | 1 | TRINITY\_DN189323\_c0\_g1\_i1.p1:TRINITY\_DN776\_c0\_g1\_i1.p1 |  |  | DB Search |
| SFASSLLDHFRVEPAK | 58.61 | 1802.9315 | 16 | 1.97 | 601.9825 | 19.83 | 0.9109 | 0.9182 | 68004 | 1.15e3 | 1 | 1 | TRINITY\_DN77\_c1\_g1\_i1.p1 |  |  | DB Search |
| SC(+57.02)LLLQFTDKR | 58.6 | 1379.7231 | 11 | 0.98 | 690.8659 | 17.62 | 1.0291 | 1.0363 | 55103 | 7.83e2 | 1 | 1 | TRINITY\_DN6233\_c0\_g1\_i1.p2:TRINITY\_DN90868\_c0\_g1\_i1.p3:TRINITY\_DN6233\_c0\_g1\_i2.p2:TRINITY\_DN188006\_c0\_g1\_i1.p1:TRINITY\_DN5793\_c0\_g1\_i1.p1 | Carbamidomethylation |  | DB Search |
| YSRPTPGSNNNVM(+15.99)VR | 58.6 | 1706.8158 | 15 | 3.02 | 569.9446 | 11.52 | 0.836 | 0.8433 | 22140 | 4.48e2 | 1 | 1 | TRINITY\_DN15274\_c0\_g1\_i1.p1 | Oxidation (M) | M13:Oxidation (M):1000 | DB Search |
| M(+42.01)(+15.99)DAAQAAEIKK | 58.6 | 1232.6071 | 11 | -1.84 | 617.3065 | 12.14 | 0.9509 | 0.9582 | 25954 | 0 | 0 | 0 | TRINITY\_DN34\_c0\_g1\_i1.p1 | Acetylation (Protein N-term), Oxidation (M) | M1:Acetylation (Protein N-term):1000 M1:Oxidation (M):1000 | DB Search |
| LRLDFDHEAKLNM(+15.99)IFR | 58.6 | 2033.0516 | 16 | 3.46 | 509.2693 | 17.78 | 0.8562 | 0.8635 | 56012 | 9.75e2 | 1 | 1 | TRINITY\_DN160\_c0\_g1\_i2.p1:TRINITY\_DN160\_c0\_g1\_i1.p1 | Oxidation (M) | M13:Oxidation (M):1000 | DB Search |
| YTGAC(+57.02)NANGNTDK | 58.6 | 1384.5677 | 13 | 3.27 | 693.2898 | 9.56 | 0.9279 | 0.9352 | 11005 | 8.94e2 | 1 | 1 | TRINITY\_DN4595\_c0\_g1\_i10.p1:TRINITY\_DN4595\_c0\_g1\_i4.p1 | Carbamidomethylation |  | DB Search |
| KFVWEFFQK | 58.6 | 1257.6545 | 9 | -1.25 | 629.8305 | 19.90 | 0.9461 | 0.9533 | 68525 | 5.63e2 | 1 | 1 | TRINITY\_DN1280\_c0\_g1\_i1.p1 |  |  | DB Search |
| LIQYIENKPGLTR | 58.6 | 1543.8722 | 13 | 0.06 | 772.9394 | 14.75 | 1.0781 | 1.0853 | 40202 | 1.81e3 | 1 | 1 | TRINITY\_DN2028\_c0\_g1\_i1.p1 |  |  | DB Search |
| DLRGDENVKWELSK | 58.59 | 1687.8529 | 14 | -12.31 | 563.6151 | 15.03 | 0.8205 | 0.8279 | 41851 | 0 | 0 | 0 | TRINITY\_DN1183\_c0\_g1\_i1.p1 |  |  | DB Search |
| TSTQDVALNIRR | 58.59 | 1372.7423 | 12 | 5.06 | 687.3783 | 13.47 | 1.0001 | 1.0073 | 33513 | 2.51e3 | 1 | 1 | TRINITY\_DN982\_c0\_g1\_i3.p1:TRINITY\_DN982\_c0\_g1\_i1.p1 |  |  | DB Search |
| TQITC(+57.02)TQISLKR | 58.58 | 1447.7817 | 12 | 7.09 | 724.8995 | 13.78 | 1.0246 | 1.0319 | 35286 | 0 | 0 | 0 | TRINITY\_DN166344\_c0\_g1\_i1.p1 | Carbamidomethylation |  | DB Search |
| FGQATC(+57.02)QFMR | 58.58 | 1244.5431 | 10 | -19.38 | 623.2635 | 13.58 | 0.9267 | 0.9339 | 34122 | 1.7e3 | 1 | 1 | TRINITY\_DN902\_c1\_g1\_i1.p1:TRINITY\_DN902\_c1\_g1\_i2.p1 | Carbamidomethylation |  | DB Search |
| DLQGETSGSFKR | 58.57 | 1323.6418 | 12 | 1.12 | 662.8255 | 12.61 | 0.9586 | 0.9658 | 28668 | 0 | 0 | 0 | TRINITY\_DN3190\_c1\_g1\_i1.p1 |  |  | DB Search |
| LDLAGRDLTDYLM(+15.99)K | 58.56 | 1638.8286 | 14 | -1.91 | 820.4158 | 18.15 | 1.1426 | 1.1498 | 58283 | 0 | 0 | 0 | TRINITY\_DN161\_c0\_g1\_i1.p1:TRINITY\_DN161\_c0\_g1\_i4.p1:TRINITY\_DN161\_c0\_g1\_i3.p1:TRINITY\_DN161\_c0\_g1\_i2.p1 | Oxidation (M) | M13:Oxidation (M):1000 | DB Search |
| LIEVEEKEIQR | 58.54 | 1384.7561 | 11 | -5.77 | 693.3777 | 13.82 | 1.0154 | 1.0226 | 35443 | 0 | 0 | 0 | TRINITY\_DN11435\_c0\_g1\_i1.p1 |  |  | DB Search |
| LAFQDIPNGLAAIGK | 58.54 | 1526.8456 | 15 | 0.61 | 764.4266 | 19.92 | 1.058 | 1.0652 | 68684 | 4.55e2 | 1 | 1 | TRINITY\_DN1339\_c1\_g1\_i1.p2 |  |  | DB Search |
| LDLAGRDLTDYLMK | 58.53 | 1622.8337 | 14 | -6.55 | 541.9455 | 19.25 | 0.7962 | 0.8035 | 64822 | 9.54e2 | 1 | 1 | TRINITY\_DN161\_c0\_g1\_i1.p1:TRINITY\_DN161\_c0\_g1\_i4.p1:TRINITY\_DN161\_c0\_g1\_i3.p1:TRINITY\_DN161\_c0\_g1\_i2.p1 |  |  | DB Search |
| KKLEPQAAPR | 58.53 | 1136.6665 | 10 | 8.1 | 379.8972 | 9.69 | 0.7432 | 0.7506 | 11676 | 4.42e3 | 1 | 1 | TRINITY\_DN22492\_c0\_g1\_i1.p1:TRINITY\_DN22492\_c0\_g1\_i2.p1 |  |  | DB Search |
| SDSSDIEKR | 58.52 | 1035.4833 | 9 | -15.17 | 518.7383 | 11.07 | 0.8631 | 0.8704 | 19589 | 5.7e3 | 1 | 1 | TRINITY\_DN135129\_c0\_g1\_i1.p1 |  |  | DB Search |
| LLEIEDEIKLR | 58.51 | 1369.7816 | 11 | 2.42 | 685.8962 | 18.01 | 1.0484 | 1.0556 | 57388 | 4.33e2 | 1 | 1 | TRINITY\_DN2230\_c0\_g1\_i1.p1 |  |  | DB Search |
| K(+42.01)LAAELANGR | 58.51 | 1083.6036 | 10 | 2.05 | 542.8074 | 15.02 | 0.8644 | 0.8716 | 41731 | 4.29e2 | 1 | 1 | TRINITY\_DN1172\_c0\_g2\_i2.p1 | Acetylation (Protein N-term) | K1:Acetylation (Protein N-term):1000 | DB Search |
| APIGQSIVTVSAR | 58.51 | 1297.7354 | 13 | -14.57 | 649.8621 | 14.13 | 0.967 | 0.9743 | 37121 | 0 | 0 | 0 | TRINITY\_DN982\_c0\_g1\_i3.p1 |  |  | DB Search |
| GSC(+57.02)SC(+57.02)YGHAEQC(+57.02)IPTK | 58.5 | 1853.7495 | 16 | 0.08 | 927.8773 | 12.31 | 1.1885 | 1.1957 | 26928 | 5.48e1 | 1 | 1 | TRINITY\_DN15274\_c0\_g1\_i1.p1 | Carbamidomethylation, Carbamidomethylation, Carbamidomethylation |  | DB Search |
| VVAISGNEDEIVNLLHK | 58.48 | 1848.9945 | 17 | 1.85 | 925.5014 | 18.76 | 1.2109 | 1.2181 | 61890 | 0 | 0 | 0 | TRINITY\_DN651\_c0\_g1\_i2.p1:TRINITY\_DN651\_c0\_g1\_i1.p1 |  |  | DB Search |
| LLAQTTLRNELGTK | 58.48 | 1556.8885 | 14 | 4.16 | 779.4507 | 15.18 | 1.109 | 1.1162 | 42504 | 8.19e2 | 1 | 1 | TRINITY\_DN5818\_c1\_g1\_i2.p1:TRINITY\_DN41662\_c0\_g1\_i2.p1:TRINITY\_DN5818\_c1\_g1\_i1.p1:TRINITY\_DN909\_c0\_g1\_i1.p1 |  |  | DB Search |
| GC(+57.02)YIHTQEVAR | 58.47 | 1332.6245 | 11 | -1.44 | 667.3151 | 12.15 | 1.011 | 1.0182 | 26068 | 0 | 0 | 0 | TRINITY\_DN2217\_c0\_g1\_i3.p1:TRINITY\_DN3202\_c2\_g1\_i1.p1:TRINITY\_DN2217\_c0\_g1\_i2.p1 | Carbamidomethylation |  | DB Search |
| IREEYPDR | 58.47 | 1076.525 | 8 | 1.32 | 539.2677 | 10.98 | 0.8696 | 0.8769 | 18747 | 5.32e3 | 1 | 1 | TRINITY\_DN1910\_c0\_g1\_i5.p1:TRINITY\_DN3986\_c0\_g1\_i1.p1 |  |  | DB Search |
| A(+42.01)ATLKPYLNAVR | 58.47 | 1357.7717 | 12 | -1.38 | 679.8887 | 18.21 | 1.046 | 1.0532 | 58634 | 2.41e3 | 1 | 1 | TRINITY\_DN95387\_c0\_g1\_i1.p1 | Acetylation (Protein N-term) | A1:Acetylation (Protein N-term):1000 | DB Search |
| VVTSSTGPGEHMR | 58.46 | 1356.6456 | 13 | 6.14 | 679.3307 | 10.90 | 0.9844 | 0.9916 | 18261 | 1.49e3 | 1 | 1 | TRINITY\_DN73067\_c0\_g1\_i1.p1 |  |  | DB Search |
| GAFGKPQGTVAR | 58.45 | 1187.6411 | 12 | 1.95 | 594.8259 | 11.42 | 0.9287 | 0.936 | 21427 | 1.07e3 | 1 | 1 | TRINITY\_DN72959\_c0\_g1\_i1.p1 |  |  | DB Search |
| GDLLFLTNDNTKPIR | 58.45 | 1715.9207 | 15 | 7.23 | 858.9693 | 17.95 | 1.145 | 1.1522 | 57108 | 0 | 0 | 0 | TRINITY\_DN164469\_c0\_g1\_i1.p1 |  |  | DB Search |
| KVIVDQLVTLATR | 58.44 | 1454.8821 | 13 | -0.05 | 728.4445 | 18.29 | 1.0283 | 1.0355 | 59014 | 2.77e3 | 1 | 1 | TRINITY\_DN1386\_c0\_g1\_i1.p1 |  |  | DB Search |
| AYDFRETLPK | 58.44 | 1238.6295 | 10 | 10.92 | 620.3256 | 15.17 | 0.9489 | 0.9562 | 42570 | 0 | 0 | 0 | TRINITY\_DN602\_c0\_g1\_i4.p1:TRINITY\_DN602\_c0\_g2\_i1.p1:TRINITY\_DN602\_c0\_g1\_i3.p1:TRINITY\_DN602\_c0\_g1\_i1.p1 |  |  | DB Search |
| KIQQELVIAK | 58.44 | 1168.7179 | 10 | 0.78 | 585.3636 | 13.58 | 0.942 | 0.9493 | 34174 | 3.74e2 | 1 | 1 | TRINITY\_DN2904\_c0\_g1\_i1.p1 |  |  | DB Search |
| RFEEAKDTIENK | 58.41 | 1478.7365 | 12 | 1.43 | 493.9176 | 11.59 | 0.8014 | 0.8088 | 22585 | 7.65e2 | 2 | 2 | TRINITY\_DN1676\_c0\_g1\_i6.p1:TRINITY\_DN1676\_c0\_g1\_i12.p1 |  |  | DB Search |
| AQEVSDKLNQK | 58.4 | 1258.6517 | 11 | 2.21 | 630.3312 | 10.75 | 0.9384 | 0.9457 | 17603 | 9.14e2 | 1 | 1 | TRINITY\_DN3034\_c0\_g1\_i1.p1 |  |  | DB Search |
| VVTSSTGPGEHM(+15.99)R | 58.39 | 1372.6405 | 13 | -0.58 | 458.5515 | 9.72 | 0.7167 | 0.7241 | 11748 | 1.58e3 | 2 | 2 | TRINITY\_DN73067\_c0\_g1\_i1.p1 | Oxidation (M) | M12:Oxidation (M):1000 | DB Search |
| VSILVAC(+57.02)NKQDLTTAK | 58.38 | 1759.9502 | 16 | -2.15 | 880.9759 | 14.70 | 1.1825 | 1.1897 | 40050 | 0 | 0 | 0 | TRINITY\_DN453\_c0\_g1\_i1.p1:TRINITY\_DN453\_c0\_g1\_i2.p1 | Carbamidomethylation |  | DB Search |
| AVDRGNPPQDSVNK | 58.36 | 1495.7379 | 14 | 5.97 | 499.5869 | 10.22 | 0.7836 | 0.7909 | 14437 | 8.69e2 | 1 | 1 | TRINITY\_DN982\_c0\_g1\_i3.p1:TRINITY\_DN982\_c0\_g1\_i1.p1 |  |  | DB Search |
| LLEFVGHDVLR | 58.36 | 1296.719 | 11 | 0.41 | 649.3636 | 17.77 | 1.0045 | 1.0118 | 56109 | 0 | 0 | 0 | TRINITY\_DN464\_c0\_g1\_i4.p1 |  |  | DB Search |
| LTHQIEVTVSDREGK | 58.36 | 1710.89 | 15 | 0.77 | 856.4485 | 12.72 | 1.115 | 1.1222 | 29273 | 1.52e2 | 1 | 1 | TRINITY\_DN123121\_c0\_g1\_i1.p1 |  |  | DB Search |
| TGAISLLASVDFETDRTHTAVVK | 58.35 | 2430.2754 | 23 | 2.89 | 608.5747 | 18.85 | 0.8854 | 0.8927 | 62375 | 1.55e2 | 1 | 1 | TRINITY\_DN982\_c0\_g1\_i3.p1 |  |  | DB Search |
| SPTHSVIK | 58.34 | 867.4814 | 8 | 1.38 | 434.7463 | 9.94 | 0.7831 | 0.7905 | 12865 | 6.66e2 | 1 | 1 | TRINITY\_DN1386\_c0\_g1\_i1.p1 |  |  | DB Search |
| NERGEAVISLK | 58.34 | 1214.6619 | 11 | 1.38 | 608.3359 | 13.52 | 0.9505 | 0.9578 | 33887 | 0 | 0 | 0 | TRINITY\_DN72625\_c0\_g1\_i1.p1 |  |  | DB Search |
| FPGYISNSLSLK | 58.33 | 1324.7026 | 12 | 2.31 | 663.3567 | 18.15 | 0.9848 | 0.992 | 58220 | 2.21e2 | 1 | 1 | TRINITY\_DN1339\_c0\_g1\_i2.p1 |  |  | DB Search |
| SFHFGVIKLEGK | 58.33 | 1360.7502 | 12 | 0.06 | 681.3789 | 16.99 | 1.0291 | 1.0363 | 51505 | 4.07e3 | 2 | 2 | TRINITY\_DN329\_c0\_g1\_i1.p1:TRINITY\_DN329\_c0\_g1\_i2.p1 |  |  | DB Search |
| VDFDTTFAPVTGKK | 58.32 | 1524.7823 | 14 | 4.95 | 763.3983 | 16.54 | 1.052 | 1.0592 | 49415 | 0 | 0 | 0 | TRINITY\_DN329\_c0\_g1\_i1.p1:TRINITY\_DN329\_c0\_g1\_i2.p1 |  |  | DB Search |
| IPILSDLTK | 58.32 | 998.6012 | 9 | -0.22 | 500.3051 | 18.86 | 0.8254 | 0.8327 | 62176 | 2.58e3 | 1 | 1 | TRINITY\_DN535\_c0\_g1\_i2.p1:TRINITY\_DN535\_c0\_g1\_i1.p1 |  |  | DB Search |
| LASQQNIGQVLAELK | 58.28 | 1610.8992 | 15 | -10.39 | 806.4443 | 19.82 | 1.1282 | 1.1354 | 68085 | 0 | 0 | 0 | TRINITY\_DN4439\_c0\_g1\_i1.p1 |  |  | DB Search |
| AYGQDLGQPGSC(+57.02)LKR | 58.26 | 1648.7991 | 15 | 0.22 | 825.4027 | 13.38 | 1.0737 | 1.0809 | 33011 | 5.76e2 | 1 | 1 | TRINITY\_DN691\_c0\_g1\_i2.p1:TRINITY\_DN691\_c0\_g1\_i1.p1 | Carbamidomethylation |  | DB Search |
| VTEAKGELAK | 58.26 | 1044.5815 | 10 | -1.3 | 523.2946 | 10.00 | 0.8927 | 0.9 | 13245 | 0 | 0 | 0 | TRINITY\_DN614\_c0\_g1\_i1.p1 |  |  | DB Search |
| IQDKEGIPPDQQR | 58.25 | 1522.7739 | 13 | 2.55 | 762.3922 | 11.03 | 1.0343 | 1.0415 | 18957 | 1.53e3 | 1 | 1 | TRINITY\_DN554\_c0\_g1\_i3.p2:TRINITY\_DN585\_c0\_g1\_i3.p1:TRINITY\_DN152\_c0\_g1\_i4.p1:TRINITY\_DN173718\_c0\_g1\_i1.p1:TRINITY\_DN1465\_c1\_g1\_i1.p1:TRINITY\_DN554\_c0\_g1\_i3.p1:TRINITY\_DN40728\_c1\_g1\_i1.p2:TRINITY\_DN585\_c0\_g1\_i5.p1:TRINITY\_DN152\_c0\_g1\_i2.p1:TRINITY\_DN152\_c0\_g1\_i14.p2:TRINITY\_DN40728\_c0\_g1\_i1.p1:TRINITY\_DN152\_c0\_g1\_i9.p1:TRINITY\_DN585\_c0\_g1\_i4.p1:TRINITY\_DN152\_c0\_g1\_i15.p1:TRINITY\_DN195631\_c0\_g1\_i1.p2:TRINITY\_DN585\_c0\_g1\_i10.p1:TRINITY\_DN585\_c0\_g1\_i2.p1:TRINITY\_DN554\_c0\_g1\_i5.p1:TRINITY\_DN8227\_c0\_g1\_i1.p1:TRINITY\_DN152\_c0\_g1\_i10.p1 |  |  | DB Search |
| LPGFLSPSYGVK | 58.24 | 1263.6863 | 12 | -2.25 | 632.8457 | 18.41 | 0.9465 | 0.9537 | 59860 | 0 | 0 | 0 | TRINITY\_DN4525\_c0\_g2\_i1.p1:TRINITY\_DN5897\_c0\_g1\_i3.p1 |  |  | DB Search |
| IRELTSVVQKR | 58.24 | 1327.7936 | 11 | -4.83 | 443.6007 | 12.43 | 0.751 | 0.7583 | 27658 | 0 | 0 | 0 | TRINITY\_DN109115\_c0\_g1\_i1.p1:TRINITY\_DN5263\_c0\_g1\_i1.p1 |  |  | DB Search |
| LGGIEVTQGNC(+57.02)ARK | 58.24 | 1501.7671 | 14 | 6.42 | 751.8917 | 12.66 | 1.0632 | 1.0705 | 28959 | 0 | 0 | 0 | TRINITY\_DN36\_c0\_g1\_i9.p1:TRINITY\_DN36\_c0\_g1\_i14.p1:TRINITY\_DN36\_c0\_g1\_i13.p1:TRINITY\_DN36\_c0\_g1\_i2.p1:TRINITY\_DN36\_c0\_g1\_i3.p1:TRINITY\_DN36\_c0\_g1\_i16.p1:TRINITY\_DN36\_c0\_g1\_i17.p1:TRINITY\_DN36\_c0\_g1\_i4.p1:TRINITY\_DN36\_c0\_g1\_i11.p1:TRINITY\_DN36\_c0\_g1\_i8.p1:TRINITY\_DN36\_c0\_g1\_i1.p1:TRINITY\_DN36\_c0\_g1\_i19.p1:TRINITY\_DN36\_c0\_g1\_i20.p1:TRINITY\_DN36\_c0\_g1\_i5.p1 | Carbamidomethylation |  | DB Search |
| INNLEIQLDQEAKER | 58.24 | 1811.9377 | 15 | -7.51 | 906.9646 | 15.71 | 1.1969 | 1.2041 | 45235 | 0 | 0 | 0 | TRINITY\_DN428\_c0\_g1\_i1.p1 |  |  | DB Search |
| VVC(+57.02)STTSGSATGSK | 58.24 | 1340.6241 | 14 | -7.3 | 671.311 | 9.64 | 0.9263 | 0.9335 | 11439 | 0 | 0 | 0 | TRINITY\_DN201860\_c0\_g1\_i1.p1 | Carbamidomethylation |  | DB Search |
| ELISNSSDALDKIR | 58.23 | 1559.8154 | 14 | 3.45 | 780.9136 | 15.47 | 1.0733 | 1.0805 | 44016 | 2.57e2 | 1 | 1 | TRINITY\_DN141338\_c2\_g1\_i1.p1:TRINITY\_DN5633\_c0\_g1\_i1.p1 |  |  | DB Search |
| TVENNVLVENIDITIK | 58.23 | 1812.9833 | 16 | 11 | 907.5042 | 19.47 | 1.1602 | 1.1674 | 66136 | 0 | 0 | 0 | TRINITY\_DN74124\_c0\_g1\_i1.p1 |  |  | DB Search |
| VC(+57.02)EDVKR | 58.23 | 904.4437 | 7 | 1.65 | 453.2275 | 9.38 | 0.7685 | 0.7758 | 10310 | 8.12e3 | 2 | 2 | TRINITY\_DN118\_c1\_g1\_i3.p1:TRINITY\_DN118\_c1\_g1\_i5.p1 | Carbamidomethylation |  | DB Search |
| GGKDFASDIITRK | 58.22 | 1406.7517 | 13 | 3.42 | 469.9237 | 14.11 | 0.775 | 0.7823 | 37023 | 0 | 0 | 0 | TRINITY\_DN1613\_c0\_g2\_i1.p1 |  |  | DB Search |
| GQELTFKEFFKPSNFVR | 58.21 | 2073.0684 | 17 | -4.7 | 692.0232 | 19.24 | 0.9315 | 0.9388 | 64684 | 2.56e3 | 1 | 1 | TRINITY\_DN30329\_c0\_g1\_i1.p1:TRINITY\_DN30329\_c0\_g2\_i1.p2 |  |  | DB Search |
| ITELSPIRPR | 58.21 | 1180.6927 | 10 | 0.83 | 591.3511 | 13.86 | 0.9271 | 0.9344 | 35586 | 6.88e2 | 1 | 1 | TRINITY\_DN4329\_c0\_g1\_i1.p1 |  |  | DB Search |
| LLEINVYHPLIQELQK | 58.19 | 1949.0985 | 16 | 3.52 | 650.7057 | 19.63 | 0.9848 | 0.992 | 67027 | 0 | 0 | 0 | TRINITY\_DN5633\_c0\_g1\_i1.p1 |  |  | DB Search |
| NIDEHASEGVER | 58.19 | 1354.6113 | 12 | -4.14 | 678.3066 | 11.03 | 0.9707 | 0.9779 | 19266 | 0 | 0 | 0 | TRINITY\_DN10907\_c0\_g1\_i1.p1 |  |  | DB Search |
| AYHNASQTQSDKAHLK | 58.17 | 1797.8757 | 16 | 6.18 | 450.4767 | 9.45 | 0.7396 | 0.7469 | 10629 | 0 | 0 | 0 | TRINITY\_DN484\_c0\_g1\_i1.p1 |  |  | DB Search |
| TFAHTVHNLVLAGNK | 58.16 | 1620.8735 | 15 | -1.91 | 811.4383 | 14.95 | 1.119 | 1.1262 | 41325 | 0 | 0 | 0 | TRINITY\_DN578\_c0\_g1\_i1.p1 |  |  | DB Search |
| FSGTKDEVK | 58.15 | 1009.508 | 9 | 0.78 | 505.759 | 10.13 | 0.8822 | 0.8895 | 13944 | 6.11e2 | 1 | 1 | TRINITY\_DN164271\_c0\_g1\_i1.p1 |  |  | DB Search |
| NYNNDDEPGAK | 58.15 | 1235.5055 | 11 | 10.48 | 618.7632 | 10.23 | 0.9222 | 0.9295 | 14562 | 0 | 0 | 0 | TRINITY\_DN199\_c0\_g1\_i1.p1 |  |  | DB Search |
| S(+42.01)SGEIESLRQEAENLR | 58.14 | 1858.9021 | 16 | 2.14 | 930.4554 | 18.72 | 1.1961 | 1.2033 | 61577 | 1.51e3 | 1 | 1 | TRINITY\_DN2472\_c0\_g1\_i1.p1 | Acetylation (Protein N-term) | S1:Acetylation (Protein N-term):1000 | DB Search |
| KKEESPNAQDAIQK | 58.13 | 1584.8107 | 14 | 7.09 | 529.2785 | 10.34 | 0.7921 | 0.7994 | 15147 | 1.07e3 | 1 | 1 | TRINITY\_DN2904\_c0\_g1\_i1.p1 |  |  | DB Search |
| NDVNNPPQAR | 58.12 | 1123.537 | 10 | 0.82 | 562.7733 | 9.95 | 0.8668 | 0.8741 | 12691 | 7.16e2 | 1 | 1 | TRINITY\_DN477\_c0\_g1\_i3.p1:TRINITY\_DN477\_c0\_g1\_i7.p1:TRINITY\_DN477\_c0\_g1\_i6.p1:TRINITY\_DN477\_c0\_g1\_i5.p1:TRINITY\_DN477\_c0\_g1\_i8.p2 |  |  | DB Search |
| SGGQC(+57.02)PC(+57.02)KPGVVGR | 58.08 | 1457.6868 | 14 | 3.86 | 486.9022 | 10.69 | 0.7868 | 0.7941 | 17188 | 4.47e2 | 1 | 1 | TRINITY\_DN15274\_c0\_g1\_i1.p1 | Carbamidomethylation, Carbamidomethylation |  | DB Search |
| RLPDQITTR | 58.05 | 1098.6145 | 9 | 0.72 | 550.3121 | 12.56 | 0.8781 | 0.8854 | 28290 | 1.58e3 | 1 | 1 | TRINITY\_DN1377\_c0\_g1\_i1.p1 |  |  | DB Search |
| VAPEEHPVLLTEAPLNPK | 58.04 | 1953.0571 | 18 | 3.48 | 977.5341 | 16.43 | 1.2045 | 1.2117 | 48611 | 4.81e2 | 1 | 1 | TRINITY\_DN2477\_c0\_g1\_i5.p1:TRINITY\_DN2477\_c0\_g1\_i6.p1:TRINITY\_DN2477\_c0\_g1\_i8.p1:TRINITY\_DN195934\_c0\_g1\_i1.p2:TRINITY\_DN161\_c0\_g1\_i1.p1:TRINITY\_DN2477\_c0\_g1\_i9.p1:TRINITY\_DN2477\_c0\_g1\_i10.p1:TRINITY\_DN2477\_c0\_g1\_i2.p1:TRINITY\_DN161\_c0\_g1\_i3.p1:TRINITY\_DN2941\_c0\_g1\_i4.p1:TRINITY\_DN161\_c0\_g1\_i2.p1 |  |  | DB Search |
| SSFDEIHKFHTQILR | 58.03 | 1856.9532 | 15 | -0.42 | 619.9882 | 15.43 | 0.866 | 0.8733 | 43848 | 2.72e2 | 1 | 1 | TRINITY\_DN147947\_c0\_g1\_i1.p1 |  |  | DB Search |
| IPLLADLTK | 57.95 | 982.6063 | 9 | -0.75 | 492.3075 | 19.36 | 0.8205 | 0.8279 | 65435 | 7.64e2 | 1 | 1 | TRINITY\_DN332\_c0\_g1\_i1.p1 |  |  | DB Search |
| LVDATFPTTDFETVR | 57.94 | 1710.8464 | 15 | 1.07 | 856.4269 | 18.33 | 1.1069 | 1.1142 | 59369 | 0 | 0 | 0 | TRINITY\_DN74124\_c0\_g1\_i1.p1 |  |  | DB Search |
| IGKPHTVPC(+57.02)K | 57.93 | 1135.6172 | 10 | 2.84 | 568.8145 | 9.76 | 0.8931 | 0.9004 | 11921 | 1.36e4 | 2 | 2 | TRINITY\_DN15584\_c0\_g1\_i1.p1 | Carbamidomethylation |  | DB Search |
| AGVLAHLEEERDIK | 57.92 | 1578.8365 | 14 | -4.44 | 790.4179 | 14.84 | 1.109 | 1.1162 | 40775 | 0 | 0 | 0 | TRINITY\_DN428\_c0\_g1\_i1.p1 |  |  | DB Search |
| RDTFNHLTTWLEDAR | 57.9 | 1873.9071 | 15 | -2.34 | 625.6382 | 18.26 | 0.8664 | 0.8737 | 58946 | 0 | 0 | 0 | TRINITY\_DN5793\_c0\_g1\_i1.p1 |  |  | DB Search |
| DLLTLPEKDNRR | 57.89 | 1468.7998 | 12 | -0.34 | 735.4031 | 14.57 | 1.0242 | 1.0315 | 39369 | 1.51e3 | 1 | 1 | TRINITY\_DN776\_c0\_g1\_i1.p1 |  |  | DB Search |
| KLQAELANGR | 57.88 | 1098.6145 | 10 | 0.06 | 550.3117 | 11.52 | 0.8814 | 0.8887 | 21738 | 1.93e4 | 1 | 1 | TRINITY\_DN15599\_c0\_g1\_i1.p1:TRINITY\_DN15599\_c0\_g1\_i2.p1 |  |  | DB Search |
| HLAISNILK | 57.84 | 1007.6127 | 9 | 0.83 | 504.8114 | 18.74 | 0.8652 | 0.8725 | 61860 | 3.25e2 | 1 | 1 | TRINITY\_DN319\_c2\_g1\_i3.p1:TRINITY\_DN319\_c2\_g1\_i1.p1 |  |  | DB Search |
| GLIAQAIDHAASR | 57.83 | 1321.7102 | 13 | 6.4 | 661.8632 | 16.09 | 1.0077 | 1.015 | 47163 | 0 | 0 | 0 | TRINITY\_DN1143\_c0\_g1\_i1.p1 |  |  | DB Search |
| HGQVDSC(+57.02)INQNLKK | 57.82 | 1639.8101 | 14 | -0.96 | 547.6072 | 11.98 | 0.836 | 0.8433 | 25047 | 0 | 0 | 0 | TRINITY\_DN3991\_c0\_g1\_i1.p1 | Carbamidomethylation |  | DB Search |
| FKLVFLGEQSVGK | 57.82 | 1450.8184 | 13 | -8.01 | 726.4069 | 18.60 | 1.0315 | 1.0387 | 61022 | 0 | 0 | 0 | TRINITY\_DN1070\_c2\_g1\_i1.p1 |  |  | DB Search |
| LQFSDIPNGLGALSK | 57.81 | 1558.8354 | 15 | 2.37 | 780.4228 | 19.42 | 1.0427 | 1.05 | 65874 | 0 | 0 | 0 | TRINITY\_DN129732\_c0\_g1\_i1.p2 |  |  | DB Search |
| VLTSDDPAEKDK | 57.79 | 1316.646 | 12 | 1.44 | 659.3278 | 10.86 | 0.9844 | 0.9916 | 18297 | 1.85e2 | 1 | 1 | TRINITY\_DN1172\_c0\_g2\_i1.p1:TRINITY\_DN1172\_c0\_g2\_i3.p1:TRINITY\_DN1172\_c0\_g2\_i2.p1 |  |  | DB Search |
| IQNADDNKPVFK | 57.75 | 1387.7096 | 12 | -2.77 | 694.8565 | 12.00 | 0.9747 | 0.982 | 25187 | 3.22e2 | 1 | 1 | TRINITY\_DN186979\_c0\_g1\_i1.p1 |  |  | DB Search |
| GVGIISEGNETVEDIAQR | 57.67 | 1885.9381 | 18 | -8.31 | 943.9636 | 17.77 | 1.1422 | 1.1494 | 56101 | 2.23e2 | 1 | 1 | TRINITY\_DN262\_c0\_g1\_i1.p1 |  |  | DB Search |
| AIELLPHDLAVGR | 57.65 | 1402.7932 | 13 | -0.45 | 702.3999 | 17.82 | 1.0303 | 1.0375 | 56285 | 4.72e2 | 1 | 1 | TRINITY\_DN4954\_c0\_g1\_i2.p2:TRINITY\_DN6388\_c0\_g1\_i1.p2:TRINITY\_DN6388\_c0\_g1\_i2.p2 |  |  | DB Search |
| KQLVNVPSFIVR | 57.5 | 1398.8347 | 12 | -4.99 | 700.4175 | 17.75 | 1.0081 | 1.0154 | 56022 | 0 | 0 | 0 | TRINITY\_DN189323\_c0\_g1\_i1.p1:TRINITY\_DN776\_c0\_g1\_i1.p1 |  |  | DB Search |
| IRITELSPIRPR | 57.49 | 1449.8779 | 12 | 2.32 | 484.2985 | 15.24 | 0.8193 | 0.8266 | 42750 | 1.52e3 | 1 | 1 | TRINITY\_DN4329\_c0\_g1\_i1.p1 |  |  | DB Search |
| LVIGNSEGFKDVSK | 57.48 | 1491.7932 | 14 | 5.49 | 746.9041 | 14.68 | 1.0821 | 1.0893 | 39965 | 6.3e2 | 1 | 1 | TRINITY\_DN3180\_c0\_g1\_i1.p1 |  |  | DB Search |
| AELVFVNHC(+57.02)R | 57.48 | 1243.6132 | 10 | 4.12 | 622.8132 | 14.31 | 0.957 | 0.9642 | 38071 | 2.14e2 | 1 | 1 | TRINITY\_DN101992\_c0\_g1\_i1.p1 | Carbamidomethylation |  | DB Search |
| FAIQDITVDEFSAK | 57.41 | 1582.7878 | 14 | 3.04 | 792.3995 | 19.67 | 1.0439 | 1.0512 | 67226 | 7.29e2 | 1 | 1 | TRINITY\_DN624\_c0\_g1\_i1.p1 |  |  | DB Search |
| SVTTHQGNAIVIK | 57.39 | 1366.7568 | 13 | 0.64 | 684.3826 | 12.52 | 0.9977 | 1.0049 | 28163 | 5.49e2 | 1 | 1 | TRINITY\_DN1377\_c0\_g1\_i1.p1 |  |  | DB Search |
| FAANLLDHFKIDR | 57.34 | 1558.8256 | 13 | -0.92 | 520.6126 | 18.07 | 0.8481 | 0.8554 | 57779 | 0 | 0 | 0 | TRINITY\_DN1797\_c0\_g1\_i2.p1:TRINITY\_DN1797\_c0\_g1\_i1.p1:TRINITY\_DN1797\_c0\_g1\_i3.p1 |  |  | DB Search |
| A(+42.01)TGIKPDPQC(+57.02)TDLYNKLK | 57.26 | 2103.0669 | 18 | 5.12 | 702.0295 | 15.96 | 0.9574 | 0.9646 | 46358 | 1.22e3 | 1 | 1 | TRINITY\_DN164271\_c0\_g1\_i1.p1 | Acetylation (Protein N-term), Carbamidomethylation | A1:Acetylation (Protein N-term):1000 | DB Search |
| YLTTAVITNKR | 57.21 | 1278.7296 | 11 | 3.8 | 640.3712 | 13.83 | 0.9989 | 1.0061 | 35564 | 3e2 | 1 | 1 | TRINITY\_DN923\_c0\_g1\_i1.p1 |  |  | DB Search |
| AEVIREEEEEKEK | 57.16 | 1616.7893 | 13 | 1.65 | 539.9351 | 10.72 | 0.8019 | 0.8092 | 17395 | 1.67e3 | 1 | 1 | TRINITY\_DN22219\_c0\_g1\_i1.p1 |  |  | DB Search |
| KLNAEIANSR | 57.15 | 1114.6095 | 10 | -3.85 | 558.3069 | 11.39 | 0.868 | 0.8753 | 21472 | 0 | 0 | 0 | TRINITY\_DN20278\_c0\_g1\_i1.p2:TRINITY\_DN4026\_c0\_g1\_i1.p2:TRINITY\_DN4026\_c0\_g1\_i2.p1:TRINITY\_DN156024\_c0\_g1\_i1.p3 |  |  | DB Search |
| KLNAELANSR | 57.15 | 1114.6095 | 10 | -3.85 | 558.3069 | 11.39 | 0.868 | 0.8753 | 21472 | 0 | 0 | 0 | TRINITY\_DN30356\_c0\_g1\_i1.p3 |  |  | DB Search |
| LIYENQNTIHHVVR | 57.12 | 1734.9165 | 14 | -1.36 | 868.4598 | 13.21 | 1.1358 | 1.143 | 32061 | 4.49e2 | 1 | 1 | TRINITY\_DN26249\_c0\_g1\_i1.p1 |  |  | DB Search |
| ALIESLEEKRK | 57.09 | 1314.7507 | 11 | -2.37 | 658.3776 | 13.75 | 0.9824 | 0.9896 | 35023 | 3.97e2 | 1 | 1 | TRINITY\_DN2805\_c0\_g1\_i1.p1 |  |  | DB Search |
| FETQIATK | 57.09 | 936.4916 | 8 | 7.3 | 937.5009 | 11.88 | 1.1925 | 1.1997 | 24345 | 0 | 0 | 0 | TRINITY\_DN649\_c0\_g1\_i2.p1:TRINITY\_DN36\_c0\_g1\_i3.p1:TRINITY\_DN36\_c0\_g1\_i16.p1:TRINITY\_DN36\_c0\_g1\_i14.p1:TRINITY\_DN36\_c0\_g1\_i17.p1:TRINITY\_DN649\_c0\_g1\_i5.p1:TRINITY\_DN36\_c0\_g1\_i13.p1:TRINITY\_DN36\_c0\_g1\_i8.p1:TRINITY\_DN649\_c0\_g1\_i1.p1:TRINITY\_DN36\_c0\_g1\_i19.p1 |  |  | DB Search |
| YVAIYPSDHLTVNR | 57.08 | 1646.8417 | 14 | -1.81 | 824.4223 | 15.52 | 1.1086 | 1.1158 | 44184 | 3.77e2 | 1 | 1 | TRINITY\_DN982\_c0\_g1\_i3.p1:TRINITY\_DN982\_c0\_g1\_i1.p1 |  |  | DB Search |
| IFPNRPYPFELVNHR | 57 | 1897.9951 | 15 | 1.06 | 633.6697 | 17.26 | 0.9279 | 0.9352 | 52948 | 3.53e2 | 1 | 1 | TRINITY\_DN138\_c0\_g1\_i1.p1 |  |  | DB Search |
| LPWEELWKPC(+57.02)IK | 56.99 | 1597.8326 | 12 | -1.86 | 533.6144 | 19.94 | 0.8793 | 0.8866 | 68833 | 2.81e2 | 1 | 1 | TRINITY\_DN602\_c0\_g1\_i4.p1:TRINITY\_DN602\_c0\_g1\_i3.p1:TRINITY\_DN602\_c0\_g1\_i1.p1 | Carbamidomethylation |  | DB Search |
| HC(+57.02)DPELSSFHTFWR | 56.96 | 1817.7943 | 14 | -4.27 | 606.933 | 18.75 | 0.883 | 0.8903 | 61859 | 3.04e2 | 1 | 1 | TRINITY\_DN22492\_c0\_g1\_i1.p1:TRINITY\_DN22492\_c0\_g1\_i2.p1 | Carbamidomethylation |  | DB Search |
| YIDIEYVC(+57.02)KPPVKK | 56.94 | 1750.9327 | 14 | -8.26 | 876.4619 | 14.95 | 1.1346 | 1.1418 | 41353 | 0 | 0 | 0 | TRINITY\_DN374\_c0\_g1\_i1.p1:TRINITY\_DN374\_c0\_g1\_i2.p1 | Carbamidomethylation |  | DB Search |
| ITTSQILDHEQQK | 56.94 | 1539.7893 | 13 | 3.01 | 770.9002 | 13.83 | 1.0957 | 1.1029 | 35489 | 5.16e2 | 1 | 1 | TRINITY\_DN982\_c0\_g1\_i3.p1:TRINITY\_DN982\_c0\_g1\_i1.p1 |  |  | DB Search |
| ALNTLHTFVNER | 56.93 | 1413.7365 | 12 | 4.35 | 707.8749 | 15.14 | 1.0202 | 1.0275 | 42361 | 0 | 0 | 0 | TRINITY\_DN165469\_c0\_g1\_i1.p1 |  |  | DB Search |
| GVSEETTTGVHNLYK | 56.84 | 1633.7948 | 15 | 1.58 | 817.9017 | 13.56 | 1.0713 | 1.0785 | 34001 | 5.38e2 | 1 | 1 | TRINITY\_DN773\_c0\_g1\_i1.p1 |  |  | DB Search |
| VAHAGLTVAEFFR | 56.84 | 1416.7513 | 13 | -4.62 | 709.376 | 18.85 | 1.0299 | 1.0371 | 62438 | 0 | 0 | 0 | TRINITY\_DN11097\_c0\_g2\_i1.p1 |  |  | DB Search |
| AHVSLSTFSEDVTSLTK | 56.78 | 1820.9155 | 17 | -11.58 | 911.4498 | 18.78 | 1.1366 | 1.1438 | 61988 | 0 | 0 | 0 | TRINITY\_DN113\_c3\_g1\_i1.p1 |  |  | DB Search |
| VSILESLDKWER | 56.75 | 1473.7827 | 12 | -3.08 | 492.2641 | 19.61 | 0.7868 | 0.7941 | 66845 | 3.21e2 | 2 | 2 | TRINITY\_DN76040\_c0\_g1\_i1.p1 |  |  | DB Search |
| LRQEQLKEER | 56.74 | 1327.7208 | 10 | 3.91 | 443.5803 | 10.26 | 0.7949 | 0.8023 | 14744 | 0 | 0 | 0 | TRINITY\_DN5188\_c0\_g1\_i4.p1:TRINITY\_DN5188\_c0\_g1\_i5.p1:TRINITY\_DN5188\_c0\_g1\_i2.p1:TRINITY\_DN5188\_c0\_g1\_i8.p1 |  |  | DB Search |
| LINLFEAVSKK | 56.67 | 1260.7441 | 11 | 0.94 | 631.3766 | 18.83 | 0.9804 | 0.9876 | 62338 | 0 | 0 | 0 | TRINITY\_DN1653\_c0\_g1\_i3.p1:TRINITY\_DN1653\_c0\_g1\_i1.p1 |  |  | DB Search |
| DPVRDKLVNER | 56.65 | 1339.7208 | 11 | 2.36 | 447.5796 | 12.03 | 0.7408 | 0.7481 | 25133 | 1.13e3 | 1 | 1 | TRINITY\_DN262\_c0\_g1\_i1.p1 |  |  | DB Search |
| ELIDHLIR | 56.61 | 1007.5764 | 8 | -3.64 | 504.791 | 19.92 | 0.8648 | 0.8721 | 68596 | 1.53e3 | 1 | 1 | TRINITY\_DN1995\_c0\_g1\_i1.p1 |  |  | DB Search |
| SGNVVLC(+57.02)LIDVHK | 56.59 | 1452.7759 | 13 | 2.61 | 727.3933 | 18.50 | 1.0379 | 1.0452 | 60367 | 0 | 0 | 0 | TRINITY\_DN3078\_c0\_g1\_i1.p1 | Carbamidomethylation |  | DB Search |
| ISNVQDIVPVLELANQHK | 56.56 | 2016.1003 | 18 | 0.56 | 673.0376 | 19.59 | 0.9513 | 0.9586 | 66863 | 7.73e2 | 1 | 1 | TRINITY\_DN118520\_c0\_g1\_i1.p1 |  |  | DB Search |
| LAIDHAVSLITNIK | 56.49 | 1506.877 | 14 | -4.75 | 754.4382 | 19.11 | 1.0749 | 1.0821 | 63945 | 0 | 0 | 0 | TRINITY\_DN56792\_c0\_g1\_i1.p1:TRINITY\_DN56792\_c0\_g1\_i3.p1 |  |  | DB Search |
| REPGDFGFDPLKFSK | 56.43 | 1738.8678 | 15 | -1.1 | 580.6262 | 18.01 | 0.9024 | 0.9097 | 57542 | 0 | 0 | 0 | TRINITY\_DN10724\_c0\_g1\_i5.p1:TRINITY\_DN10724\_c0\_g1\_i4.p1:TRINITY\_DN10724\_c0\_g1\_i1.p2:TRINITY\_DN10724\_c0\_g1\_i3.p1 |  |  | DB Search |
| A(+42.01)HLSQEQRNELKK | 56.42 | 1621.8536 | 13 | -1.81 | 541.6213 | 11.24 | 0.81 | 0.8173 | 20516 | 0 | 0 | 0 | TRINITY\_DN3052\_c0\_g1\_i1.p1 | Acetylation (Protein N-term) | A1:Acetylation (Protein N-term):1000 | DB Search |
| RLFEGNALLRR | 56.27 | 1343.7786 | 11 | -1.1 | 448.9306 | 14.75 | 0.7547 | 0.762 | 40382 | 0 | 0 | 0 | TRINITY\_DN189323\_c0\_g1\_i1.p1:TRINITY\_DN776\_c0\_g1\_i1.p1 |  |  | DB Search |
| RLDPSEQLQHLSSLR | 56.25 | 1777.9435 | 15 | -2.83 | 593.6503 | 16.40 | 0.9069 | 0.9141 | 48713 | 0 | 0 | 0 | TRINITY\_DN165469\_c0\_g1\_i1.p1 |  |  | DB Search |
| RPSGKEEEVDVK | 56.24 | 1371.6993 | 12 | -0.94 | 458.2376 | 10.07 | 0.8055 | 0.8128 | 13647 | 0 | 0 | 0 | TRINITY\_DN941\_c0\_g1\_i9.p1 |  |  | DB Search |
| ALADYIHSQDLK | 56.12 | 1372.6986 | 12 | -6.12 | 687.3488 | 15.62 | 1.0061 | 1.0134 | 44810 | 0 | 0 | 0 | TRINITY\_DN1819\_c0\_g1\_i5.p1:TRINITY\_DN1819\_c0\_g1\_i7.p1 |  |  | DB Search |
| VVEKEAETER | 56.06 | 1188.5986 | 10 | 0.32 | 397.2049 | 9.55 | 0.7265 | 0.7339 | 11068 | 0 | 0 | 0 | TRINITY\_DN2636\_c0\_g1\_i2.p1:TRINITY\_DN2636\_c0\_g1\_i1.p1 |  |  | DB Search |
| ATYDSNHDSFR | 56.02 | 1311.548 | 11 | 4.08 | 656.7805 | 11.64 | 0.9388 | 0.9461 | 22886 | 5.01e2 | 1 | 1 | TRINITY\_DN10706\_c0\_g1\_i1.p1 |  |  | DB Search |
| FTNKDIVC(+57.02)QIAYAK | 55.98 | 1669.8497 | 14 | -4.24 | 835.9243 | 15.98 | 1.1182 | 1.1254 | 46583 | 4.41e2 | 1 | 1 | TRINITY\_DN116\_c0\_g1\_i1.p1 | Carbamidomethylation |  | DB Search |
| M(+15.99)KINVNEIFHDLVR | 55.96 | 1742.9137 | 14 | 2.05 | 581.9767 | 19.57 | 0.887 | 0.8943 | 66610 | 2.46e2 | 1 | 1 | TRINITY\_DN571\_c0\_g1\_i1.p1:TRINITY\_DN571\_c0\_g1\_i2.p1 | Oxidation (M) | M1:Oxidation (M):1000 | DB Search |
| LHSNLSPSTHETK | 55.95 | 1449.7212 | 13 | 1.39 | 484.2458 | 10.33 | 0.8274 | 0.8348 | 15220 | 0 | 0 | 0 | TRINITY\_DN6044\_c0\_g2\_i1.p1 |  |  | DB Search |
| LTNSLM(+15.99)M(+15.99)HGR | 55.94 | 1190.5536 | 10 | -0.66 | 596.2806 | 10.74 | 0.9158 | 0.923 | 17432 | 1.45e3 | 1 | 1 | TRINITY\_DN164351\_c0\_g1\_i1.p1 | Oxidation (M), Oxidation (M) | M6:Oxidation (M):1000 M7:Oxidation (M):1000 | DB Search |
| GNPPQDSVNK | 55.81 | 1054.5043 | 10 | 0.87 | 528.2571 | 9.76 | 0.8372 | 0.8445 | 11990 | 0 | 0 | 0 | TRINITY\_DN982\_c0\_g1\_i3.p1:TRINITY\_DN982\_c0\_g1\_i1.p1 |  |  | DB Search |
| RLLTSLVQGAR | 55.8 | 1212.7302 | 11 | 0.26 | 607.3694 | 15.19 | 0.9804 | 0.9876 | 42540 | 5.93e2 | 1 | 1 | TRINITY\_DN3190\_c1\_g1\_i1.p1 |  |  | DB Search |
| RLDEEVAR | 55.66 | 986.5145 | 8 | -13.44 | 494.2553 | 11.10 | 0.853 | 0.8603 | 19719 | 0 | 0 | 0 | TRINITY\_DN773\_c0\_g1\_i1.p1 |  |  | DB Search |
| GFGM(+15.99)TAELANKR | 55.59 | 1309.6449 | 12 | 2.54 | 655.8279 | 12.79 | 1.0102 | 1.0174 | 29776 | 4.76e2 | 1 | 1 | TRINITY\_DN12\_c0\_g2\_i1.p1 | Oxidation (M) | M4:Oxidation (M):1000 | DB Search |
| VGNWRDETLSC(+57.02)K | 55.55 | 1463.6827 | 12 | -0.52 | 732.8444 | 13.66 | 1.019 | 1.0263 | 34634 | 0 | 0 | 0 | TRINITY\_DN6390\_c0\_g1\_i1.p1 | Carbamidomethylation |  | DB Search |
| IKVELPIKEILTK | 55.47 | 1522.9697 | 13 | 1.94 | 508.6622 | 18.17 | 0.8019 | 0.8092 | 58412 | 2.52e2 | 1 | 1 | TRINITY\_DN33328\_c0\_g1\_i1.p1 |  |  | DB Search |
| VTVKPSSDASK | 55.45 | 1117.5979 | 11 | 11.33 | 373.5422 | 9.60 | 0.7379 | 0.7453 | 11202 | 6.95e2 | 1 | 1 | TRINITY\_DN941\_c0\_g1\_i9.p1 |  |  | DB Search |
| ATDSDSGPNGDITYR | 55.42 | 1567.675 | 15 | -0.62 | 784.8402 | 12.74 | 1.0049 | 1.0122 | 29493 | 2.71e2 | 1 | 1 | TRINITY\_DN72625\_c0\_g1\_i1.p1 |  |  | DB Search |
| IINAIDKDVQER | 55.4 | 1412.7623 | 12 | -2.3 | 707.3831 | 13.49 | 1.0355 | 1.0427 | 33695 | 0 | 0 | 0 | TRINITY\_DN82805\_c0\_g1\_i1.p1 |  |  | DB Search |
| GKKPHVQVNVK | 55.29 | 1232.7354 | 11 | 5.43 | 411.9191 | 9.80 | 0.7852 | 0.7925 | 12174 | 4.29e2 | 1 | 1 | TRINITY\_DN119684\_c0\_g1\_i1.p1 |  |  | DB Search |
| A(+42.01)TVVDYPDFDAQADAETLRK | 55.12 | 2266.0752 | 20 | 3.38 | 1134.0428 | 18.33 | 1.2432 | 1.2503 | 59289 | 0 | 0 | 0 | TRINITY\_DN3190\_c1\_g1\_i1.p1 | Acetylation (Protein N-term) | A1:Acetylation (Protein N-term):1000 | DB Search |
| LC(+57.02)QPGYSTC(+57.02)ENHC(+57.02)R | 55.11 | 1780.708 | 14 | 0.84 | 891.3574 | 11.42 | 1.0861 | 1.0933 | 21509 | 5.05e2 | 1 | 1 | TRINITY\_DN817\_c0\_g1\_i4.p1:TRINITY\_DN817\_c0\_g1\_i3.p1:TRINITY\_DN817\_c0\_g1\_i7.p1:TRINITY\_DN817\_c0\_g1\_i1.p1:TRINITY\_DN817\_c0\_g1\_i8.p1:TRINITY\_DN817\_c0\_g1\_i6.p1:TRINITY\_DN817\_c0\_g1\_i2.p1:TRINITY\_DN817\_c0\_g1\_i10.p1:TRINITY\_DN817\_c0\_g1\_i5.p1:TRINITY\_DN817\_c0\_g1\_i9.p1 | Carbamidomethylation, Carbamidomethylation, Carbamidomethylation |  | DB Search |
| SM(+15.99)IHELNR | 54.96 | 1014.4916 | 8 | 2.05 | 508.2515 | 11.61 | 0.8522 | 0.8595 | 22750 | 3.92e2 | 1 | 1 | TRINITY\_DN932\_c0\_g1\_i1.p1 | Oxidation (M) | M2:Oxidation (M):1000 | DB Search |
| DTDTEEEIKEAFR | 54.86 | 1581.7158 | 13 | 0.19 | 791.8612 | 16.40 | 1.052 | 1.0592 | 48621 | 7.75e2 | 1 | 1 | TRINITY\_DN345\_c0\_g1\_i1.p1 |  |  | DB Search |
| LDFDHEAKLNMIFR | 54.86 | 1747.8716 | 14 | -10.63 | 583.6219 | 18.34 | 0.8542 | 0.8615 | 59425 | 0 | 0 | 0 | TRINITY\_DN160\_c0\_g1\_i2.p1:TRINITY\_DN160\_c0\_g1\_i1.p1 |  |  | DB Search |
| KSDYSENEVEK | 54.76 | 1326.5939 | 11 | 3.69 | 664.3032 | 10.50 | 0.9699 | 0.9771 | 16134 | 2.79e2 | 1 | 1 | TRINITY\_DN1534\_c2\_g1\_i1.p1 |  |  | DB Search |
| IDNYIEFLDEDLKKK | 54.7 | 1881.9723 | 15 | 1.47 | 628.329 | 18.88 | 0.8668 | 0.8741 | 62654 | 0 | 0 | 0 | TRINITY\_DN278\_c0\_g1\_i1.p1 |  |  | DB Search |
| DRDQFAQLVR | 54.59 | 1246.6418 | 10 | 4.05 | 624.3275 | 15.19 | 0.942 | 0.9493 | 42601 | 4.61e2 | 1 | 1 | TRINITY\_DN11435\_c0\_g1\_i1.p1 |  |  | DB Search |
| VNDSLLK | 54.53 | 787.444 | 7 | 3.52 | 788.4499 | 12.17 | 1.1506 | 1.1578 | 26013 | 1.48e3 | 1 | 1 | TRINITY\_DN326\_c0\_g1\_i1.p1 |  |  | DB Search |
| M(+15.99)KDTDTEEEIKEAFR | 54.53 | 1856.8462 | 15 | -1.3 | 619.952 | 15.33 | 0.8708 | 0.8781 | 43391 | 0 | 0 | 0 | TRINITY\_DN345\_c0\_g1\_i1.p1 | Oxidation (M) | M1:Oxidation (M):1000 | DB Search |
| S(+42.01)SSSEPTKFK | 54.44 | 1138.5507 | 10 | 7.5 | 570.2839 | 11.58 | 0.9222 | 0.9295 | 22579 | 0 | 0 | 0 | TRINITY\_DN12304\_c0\_g1\_i1.p1 | Acetylation (Protein N-term) | S1:Acetylation (Protein N-term):1000 | DB Search |
| SSC(+57.02)RPILSVIK | 54.44 | 1258.7067 | 11 | -8.13 | 630.3522 | 16.83 | 0.9691 | 0.9763 | 50527 | 1.86e3 | 1 | 1 | TRINITY\_DN158421\_c0\_g1\_i1.p1:TRINITY\_DN3545\_c0\_g1\_i2.p1:TRINITY\_DN3545\_c0\_g1\_i1.p1 | Carbamidomethylation |  | DB Search |
| HAEYYFIDALEGKVVVK | 54.36 | 1980.0356 | 17 | -3.44 | 661.0134 | 19.49 | 0.9449 | 0.9521 | 66246 | 0 | 0 | 0 | TRINITY\_DN1369\_c0\_g1\_i1.p1 |  |  | DB Search |
| LIAKFETWEVAR | 54.26 | 1461.798 | 12 | -4.64 | 731.899 | 17.17 | 1.0427 | 1.05 | 52609 | 0 | 0 | 0 | TRINITY\_DN22492\_c0\_g1\_i1.p1:TRINITY\_DN22492\_c0\_g1\_i2.p1 |  |  | DB Search |
| VLVHTQQK | 54.09 | 951.5502 | 8 | 3.81 | 476.7817 | 9.56 | 0.8303 | 0.8376 | 11062 | 2.36e2 | 1 | 1 | TRINITY\_DN6920\_c0\_g1\_i1.p1:TRINITY\_DN6920\_c0\_g1\_i3.p1:TRINITY\_DN6920\_c0\_g1\_i4.p1:TRINITY\_DN6920\_c0\_g1\_i2.p1 |  |  | DB Search |
| RLFEGNALLR | 54.07 | 1187.6775 | 10 | 1.11 | 594.8436 | 16.07 | 0.9396 | 0.9469 | 46969 | 1.45e3 | 1 | 1 | TRINITY\_DN189323\_c0\_g1\_i1.p1:TRINITY\_DN776\_c0\_g1\_i1.p1 |  |  | DB Search |
| TQAVLDRLNIEKR | 54.05 | 1554.8842 | 13 | 2.17 | 519.3004 | 14.53 | 0.8014 | 0.8088 | 39216 | 0 | 0 | 0 | TRINITY\_DN477\_c0\_g1\_i9.p1 |  |  | DB Search |
| LGPDQVDVAGAGR | 53.81 | 1253.6364 | 13 | 0.51 | 627.8225 | 13.58 | 0.9331 | 0.9404 | 34075 | 1.99e3 | 1 | 1 | TRINITY\_DN97809\_c0\_g1\_i1.p1 |  |  | DB Search |
| SISC(+57.02)SKDEEIK | 53.66 | 1294.6074 | 11 | 5.36 | 648.3111 | 10.85 | 0.98 | 0.9872 | 18283 | 0 | 0 | 0 | TRINITY\_DN495\_c0\_g1\_i5.p1:TRINITY\_DN495\_c0\_g1\_i6.p1 | Carbamidomethylation |  | DB Search |
| RLVELQHGR | 53.64 | 1106.6309 | 9 | 6.11 | 369.8846 | 11.14 | 0.7061 | 0.7135 | 19894 | 1.5e3 | 1 | 1 | TRINITY\_DN2487\_c1\_g1\_i4.p1:TRINITY\_DN2487\_c1\_g1\_i3.p1 |  |  | DB Search |
| KVEQVQTDSR | 53.61 | 1188.6099 | 10 | 5.11 | 595.3121 | 9.53 | 0.8725 | 0.8798 | 10904 | 2.14e2 | 1 | 1 | TRINITY\_DN96\_c0\_g1\_i3.p1:TRINITY\_DN96\_c0\_g1\_i8.p1:TRINITY\_DN96\_c0\_g1\_i5.p1:TRINITY\_DN96\_c0\_g1\_i7.p1:TRINITY\_DN96\_c0\_g1\_i2.p1:TRINITY\_DN96\_c0\_g1\_i6.p1:TRINITY\_DN96\_c0\_g1\_i1.p1:TRINITY\_DN96\_c0\_g1\_i4.p1 |  |  | DB Search |
| M(+15.99)PEDESTPEKR | 53.54 | 1333.582 | 11 | 5.79 | 445.5349 | 9.38 | 0.7563 | 0.7636 | 10394 | 0 | 0 | 0 | TRINITY\_DN95381\_c0\_g1\_i1.p1 | Oxidation (M) | M1:Oxidation (M):1000 | DB Search |
| DAPIYGLQWHPEK | 53.53 | 1552.7673 | 13 | 4.07 | 777.3901 | 17.25 | 1.0717 | 1.0789 | 52846 | 1.03e3 | 1 | 1 | TRINITY\_DN247\_c0\_g1\_i1.p1 |  |  | DB Search |
| ELVM(+15.99)HEQSR | 53.5 | 1143.5343 | 9 | 6.21 | 572.775 | 9.82 | 0.9028 | 0.9101 | 12284 | 6.13e2 | 1 | 1 | TRINITY\_DN160\_c0\_g1\_i2.p1:TRINITY\_DN160\_c0\_g1\_i1.p1 | Oxidation (M) | M4:Oxidation (M):1000 | DB Search |
| IKLEHLITELVHQR | 53.33 | 1728.0046 | 14 | -1.28 | 577.0051 | 18.38 | 0.8668 | 0.8741 | 59724 | 0 | 0 | 0 | TRINITY\_DN1566\_c0\_g1\_i1.p1 |  |  | DB Search |
| TSDSDLEKR | 53.19 | 1049.4989 | 9 | -14.11 | 525.7466 | 11.28 | 0.8644 | 0.8716 | 20674 | 3.18e3 | 1 | 1 | TRINITY\_DN76073\_c0\_g1\_i1.p1 |  |  | DB Search |
| AFSFSDKPAITFEIHDFK | 53.11 | 2099.0364 | 18 | 1.35 | 700.6833 | 18.88 | 0.9795 | 0.9868 | 62540 | 4.97e2 | 1 | 1 | TRINITY\_DN33065\_c0\_g2\_i1.p1 |  |  | DB Search |
| ATNPDVEK | 53.07 | 872.424 | 8 | 4.44 | 437.2189 | 9.70 | 0.7962 | 0.8035 | 11623 | 2.72e3 | 1 | 1 | TRINITY\_DN72625\_c0\_g1\_i1.p1 |  |  | DB Search |
| AIEKFEKEAQEM(+15.99)GK | 52.83 | 1652.808 | 14 | 5.41 | 551.9434 | 12.07 | 0.8185 | 0.8258 | 25556 | 5.51e2 | 1 | 1 | TRINITY\_DN64\_c0\_g1\_i1.p1 | Oxidation (M) | M12:Oxidation (M):1000 | DB Search |
| HFC(+57.02)WVFSK | 52.77 | 1109.5116 | 8 | 0.08 | 555.7603 | 19.80 | 0.87 | 0.8773 | 67829 | 2.32e2 | 1 | 1 | TRINITY\_DN2217\_c0\_g1\_i3.p1:TRINITY\_DN2217\_c0\_g1\_i2.p1 | Carbamidomethylation |  | DB Search |
| GDYC(+57.02)NSALIEAEQKER | 52.72 | 1881.8527 | 16 | 2.48 | 941.931 | 14.34 | 1.2077 | 1.2149 | 38191 | 0 | 0 | 0 | TRINITY\_DN168534\_c0\_g1\_i1.p1 | Carbamidomethylation |  | DB Search |
| IRNEEIQIEVIERR | 52.69 | 1795.9904 | 14 | 3.84 | 599.6699 | 15.11 | 0.8806 | 0.8878 | 42172 | 6.33e2 | 1 | 1 | TRINITY\_DN11435\_c0\_g1\_i1.p1 |  |  | DB Search |
| LGPVVDGKYDYTVITTDYK | 52.68 | 2146.0833 | 19 | -1.05 | 1074.0422 | 17.49 | 1.2404 | 1.2475 | 54337 | 1.18e2 | 1 | 1 | TRINITY\_DN468\_c1\_g1\_i1.p1 |  |  | DB Search |
| DLPQEKLDDILK | 52.63 | 1425.7715 | 12 | 1.19 | 713.8901 | 17.70 | 1.0399 | 1.0472 | 55712 | 0 | 0 | 0 | TRINITY\_DN262\_c0\_g1\_i1.p1 |  |  | DB Search |
| REQELQATVK | 52.55 | 1200.6462 | 10 | 0.53 | 601.3276 | 11.18 | 0.9166 | 0.9238 | 20200 | 6.13e2 | 1 | 1 | TRINITY\_DN11435\_c0\_g1\_i1.p1 |  |  | DB Search |
| ASQGIYPLHDC(+57.02)YIR | 52.39 | 1691.809 | 14 | 0.15 | 846.9075 | 15.77 | 1.1394 | 1.1466 | 45376 | 5.88e2 | 1 | 1 | TRINITY\_DN577\_c0\_g1\_i1.p1 | Carbamidomethylation |  | DB Search |
| YVVMLQYYHDSDTAK | 52.09 | 1831.8451 | 15 | 5.48 | 916.9301 | 16.56 | 1.1813 | 1.1885 | 49447 | 0 | 0 | 0 | TRINITY\_DN1616\_c0\_g1\_i3.p1:TRINITY\_DN1616\_c0\_g1\_i6.p1:TRINITY\_DN1616\_c0\_g1\_i2.p1:TRINITY\_DN1616\_c0\_g1\_i7.p1 |  |  | DB Search |
| KAENFAELKNTFQEAAK | 51.62 | 1937.9846 | 17 | -1.33 | 646.9979 | 16.11 | 0.917 | 0.9243 | 47225 | 2.65e2 | 1 | 1 | TRINITY\_DN79\_c0\_g1\_i1.p1 |  |  | DB Search |
| LYSTDPNKFYR | 51.54 | 1402.6881 | 11 | 2.6 | 702.3495 | 14.19 | 1.0299 | 1.0371 | 37268 | 1.32e3 | 1 | 1 | TRINITY\_DN72625\_c0\_g1\_i1.p1 |  |  | DB Search |
| A(+42.01)DEYYKR | 51.54 | 985.4505 | 7 | 1.24 | 493.7306 | 12.55 | 0.836 | 0.8433 | 28400 | 0 | 0 | 0 | TRINITY\_DN198705\_c0\_g1\_i1.p1 | Acetylation (Protein N-term) | A1:Acetylation (Protein N-term):1000 | DB Search |
| FDKYHPGYFGK | 51.46 | 1357.6455 | 11 | 4.4 | 679.8295 | 18.00 | 0.982 | 0.9892 | 57344 | 5.05e2 | 1 | 1 | TRINITY\_DN131028\_c0\_g1\_i1.p2 |  |  | DB Search |
| VFDRDGNGFISAAELR | 51.43 | 1765.8748 | 16 | -4.93 | 883.9357 | 19.74 | 1.1274 | 1.1346 | 67630 | 2.9e3 | 1 | 1 | TRINITY\_DN801\_c0\_g1\_i4.p1:TRINITY\_DN801\_c0\_g1\_i10.p1:TRINITY\_DN801\_c0\_g1\_i7.p1:TRINITY\_DN801\_c0\_g1\_i1.p1:TRINITY\_DN801\_c0\_g1\_i9.p2:TRINITY\_DN30386\_c0\_g1\_i1.p2 |  |  | DB Search |
| LGPDEASENKR | 51.39 | 1214.5891 | 11 | 5.41 | 608.3019 | 10.47 | 0.9166 | 0.9238 | 15408 | 2.93e3 | 1 | 1 | TRINITY\_DN12\_c0\_g1\_i1.p1:TRINITY\_DN12\_c0\_g2\_i1.p1 |  |  | DB Search |
| EIDKADIAIVDTGENPDAPHPR | 51.38 | 2372.1606 | 22 | 1.38 | 791.7245 | 14.90 | 1.0383 | 1.0456 | 41054 | 3.22e2 | 1 | 1 | TRINITY\_DN761\_c0\_g1\_i3.p1:TRINITY\_DN761\_c0\_g1\_i1.p1:TRINITY\_DN761\_c0\_g1\_i2.p1 |  |  | DB Search |
| FC(+57.02)SNPEPENGGQTC(+57.02)EGEAR | 51.3 | 2137.843 | 19 | -10.33 | 1069.9121 | 12.08 | 1.1558 | 1.163 | 25560 | 3.12e1 | 1 | 1 | TRINITY\_DN138\_c0\_g1\_i1.p1 | Carbamidomethylation, Carbamidomethylation |  | DB Search |
| VQSGNYRPVLALNGR | 51.25 | 1642.8903 | 15 | 3.09 | 822.4507 | 14.85 | 1.0845 | 1.0917 | 40732 | 1.19e3 | 1 | 1 | TRINITY\_DN1130\_c0\_g1\_i1.p1 |  |  | DB Search |
| VGYLFITDWGGKPK | 51.21 | 1579.8398 | 14 | 1.85 | 527.6188 | 19.44 | 0.8031 | 0.8104 | 65944 | 2.03e2 | 1 | 1 | TRINITY\_DN17095\_c0\_g1\_i1.p1 |  |  | DB Search |
| HAVYLPHTAAR | 51.13 | 1234.6571 | 11 | 5.16 | 412.5596 | 13.38 | 0.81 | 0.8173 | 33164 | 1.69e2 | 1 | 1 | TRINITY\_DN164351\_c0\_g1\_i1.p1 |  |  | DB Search |
| AFNRVETR | 50.97 | 991.5199 | 8 | -0.72 | 496.7643 | 10.73 | 0.8534 | 0.8607 | 17391 | 3.08e3 | 1 | 1 | TRINITY\_DN4595\_c0\_g1\_i7.p1:TRINITY\_DN4595\_c0\_g1\_i4.p1:TRINITY\_DN4595\_c0\_g1\_i10.p2 |  |  | DB Search |
| FAQEGLEAHNK | 50.66 | 1242.5992 | 11 | 2.72 | 622.3054 | 11.51 | 0.9675 | 0.9747 | 22179 | 0 | 0 | 0 | TRINITY\_DN10385\_c0\_g1\_i1.p1 |  |  | DB Search |
| VGQAM(+15.99)ASPEDK | 50.64 | 1147.5179 | 11 | 12.14 | 574.7702 | 9.69 | 0.8664 | 0.8737 | 11668 | 0 | 0 | 0 | TRINITY\_DN57\_c0\_g1\_i1.p1 | Oxidation (M) | M5:Oxidation (M):1000 | DB Search |
| VGINYQPPTVVPGGDLAK | 50.54 | 1823.9781 | 18 | -3.14 | 912.9887 | 17.25 | 1.1186 | 1.1258 | 53075 | 0 | 0 | 0 | TRINITY\_DN687\_c0\_g2\_i1.p1:TRINITY\_DN250\_c0\_g1\_i5.p1:TRINITY\_DN687\_c0\_g1\_i1.p1:TRINITY\_DN250\_c0\_g1\_i1.p1:TRINITY\_DN250\_c0\_g1\_i6.p1:TRINITY\_DN250\_c0\_g1\_i4.p1:TRINITY\_DN96161\_c0\_g1\_i1.p1:TRINITY\_DN250\_c0\_g1\_i3.p1 |  |  | DB Search |
| VDKITEYLC(+57.02)EPLRK | 50.25 | 1762.9287 | 14 | -15.2 | 588.6382 | 16.21 | 0.8494 | 0.8567 | 47751 | 0 | 0 | 0 | TRINITY\_DN4439\_c0\_g1\_i1.p1 | Carbamidomethylation |  | DB Search |
| AHGPGLEGGVAGQPC(+57.02)R | 50.18 | 1561.7419 | 16 | 3.5 | 781.8769 | 12.18 | 1.0291 | 1.0363 | 25949 | 8.92e2 | 1 | 1 | TRINITY\_DN941\_c0\_g1\_i15.p1:TRINITY\_DN941\_c0\_g1\_i11.p1:TRINITY\_DN941\_c0\_g1\_i9.p1 | Carbamidomethylation |  | DB Search |
| IEHAHPINR | 50.13 | 1085.573 | 9 | 1.73 | 362.8637 | 9.50 | 0.7343 | 0.7416 | 10857 | 0 | 0 | 0 | TRINITY\_DN59667\_c0\_g1\_i1.p1 |  |  | DB Search |
| VNQGEKEFLQRPC(+57.02)R | 50.11 | 1759.8788 | 14 | 4.1 | 587.6329 | 12.58 | 0.8619 | 0.8692 | 28420 | 3.34e2 | 1 | 1 | TRINITY\_DN4595\_c0\_g1\_i10.p1:TRINITY\_DN4595\_c0\_g1\_i4.p1 | Carbamidomethylation |  | DB Search |
| VFEIAQADLNKDEVAFR | 49.81 | 1964.0002 | 17 | 6.11 | 983.0083 | 18.07 | 1.2467 | 1.2539 | 57754 | 0 | 0 | 0 | TRINITY\_DN577\_c0\_g1\_i1.p1 |  |  | DB Search |
| TVLASQTVTIPEDVSVSVNGR | 49.68 | 2171.1433 | 21 | 1.54 | 1086.575 | 17.81 | 1.2284 | 1.2356 | 56273 | 0 | 0 | 0 | TRINITY\_DN194146\_c0\_g1\_i1.p1 |  |  | DB Search |
| IGC(+57.02)FHDR | 48.65 | 903.4021 | 7 | -0.08 | 452.7059 | 14.59 | 0.7872 | 0.7945 | 39383 | 2.19e3 | 1 | 1 | TRINITY\_DN138\_c0\_g1\_i1.p1 | Carbamidomethylation |  | DB Search |
| IHHELNQFC(+57.02)SVK | 47.81 | 1510.7351 | 12 | -0.1 | 756.3708 | 12.64 | 1.05 | 1.0572 | 28909 | 0 | 0 | 0 | TRINITY\_DN2636\_c0\_g1\_i2.p1:TRINITY\_DN2636\_c0\_g1\_i1.p1 | Carbamidomethylation |  | DB Search |
| AKDVTAAVKK | 47.69 | 1029.6182 | 10 | -2.2 | 515.8126 | 9.42 | 0.838 | 0.8453 | 10489 | 3.2e2 | 1 | 1 | TRINITY\_DN1189\_c0\_g1\_i1.p1 |  |  | DB Search |
| T(+42.01)SNVRQNYHAETEAAINK | 46.72 | 2087.0032 | 18 | 2.3 | 696.673 | 13.03 | 0.9097 | 0.917 | 31105 | 9.26e2 | 1 | 1 | TRINITY\_DN326\_c0\_g1\_i1.p1 | Acetylation (Protein N-term) | T1:Acetylation (Protein N-term):1000 | DB Search |
| AAASAHHQAIGSIDAAGVTSAADYASVTAALGR | 46.39 | 3080.5273 | 33 | -3.95 | 771.1321 | 18.15 | 1.0656 | 1.0729 | 58236 | 5.93e2 | 1 | 1 | TRINITY\_DN240\_c0\_g1\_i6.p1:TRINITY\_DN240\_c0\_g1\_i1.p1:TRINITY\_DN240\_c0\_g1\_i4.p1 |  |  | DB Search |
| YALNQIQPVTATVTAFKGDSK | 44.58 | 2251.1848 | 21 | 9.46 | 751.4054 | 18.19 | 1.048 | 1.0552 | 58586 | 0 | 0 | 0 | TRINITY\_DN1616\_c0\_g1\_i3.p1:TRINITY\_DN1616\_c0\_g1\_i6.p1:TRINITY\_DN1616\_c0\_g1\_i2.p1:TRINITY\_DN1616\_c0\_g1\_i7.p1 |  |  | DB Search |
| VAQHAVVAR | 43.99 | 949.5457 | 9 | 5.58 | 475.7803 | 10.02 | 0.8412 | 0.8485 | 13333 | 5.98e2 | 1 | 1 | TRINITY\_DN32763\_c0\_g1\_i2.p2:TRINITY\_DN32763\_c0\_g1\_i1.p1 |  |  | DB Search |
| LC(+57.02)QPGYSTC(+57.02)ENHC(+57.02)RR | 43.99 | 1936.8091 | 15 | 3.79 | 646.6094 | 10.89 | 0.8575 | 0.8648 | 18436 | 3.51e2 | 1 | 1 | TRINITY\_DN817\_c0\_g1\_i4.p1:TRINITY\_DN817\_c0\_g1\_i7.p1:TRINITY\_DN817\_c0\_g1\_i8.p1:TRINITY\_DN817\_c0\_g1\_i6.p1:TRINITY\_DN817\_c0\_g1\_i2.p1:TRINITY\_DN817\_c0\_g1\_i10.p1:TRINITY\_DN817\_c0\_g1\_i5.p1 | Carbamidomethylation, Carbamidomethylation, Carbamidomethylation |  | DB Search |
| VFLNKPLDFERENQIR | 43.83 | 2017.0745 | 16 | 2.56 | 673.3636 | 16.33 | 0.942 | 0.9493 | 48297 | 1.88e2 | 1 | 1 | TRINITY\_DN141214\_c0\_g1\_i1.p1 |  |  | DB Search |
| LIEVEEKEIQRR | 43.57 | 1540.8573 | 12 | 1.73 | 514.6246 | 13.11 | 0.7819 | 0.7892 | 31582 | 0 | 0 | 0 | TRINITY\_DN11435\_c0\_g1\_i1.p1 |  |  | DB Search |
| TPQYDFSVVVTDKGTPPR | 42.6 | 2006.0109 | 18 | 8.03 | 669.6794 | 16.40 | 0.9392 | 0.9465 | 48729 | 0 | 0 | 0 | TRINITY\_DN982\_c0\_g1\_i1.p1:TRINITY\_DN982\_c0\_g1\_i2.p1 |  |  | DB Search |
| ILAGDKPYITADELRR | 39.95 | 1829.9999 | 16 | -2.95 | 611.0023 | 14.95 | 0.9259 | 0.9331 | 41338 | 0 | 0 | 0 | TRINITY\_DN624\_c0\_g1\_i1.p1 |  |  | DB Search |
| IAADGTESR | 39.56 | 918.4407 | 9 | 3.69 | 460.2269 | 9.86 | 0.8027 | 0.81 | 12225 | 9.43e3 | 1 | 1 | TRINITY\_DN15248\_c0\_g1\_i1.p2 |  |  | DB Search |
| EGDLVIHSGITDAVK | 39.37 | 1552.8097 | 15 | 2.72 | 777.4102 | 15.38 | 1.0648 | 1.0721 | 43595 | 0 | 0 | 0 | TRINITY\_DN329\_c0\_g1\_i1.p1:TRINITY\_DN329\_c0\_g1\_i2.p1 |  |  | DB Search |
| TMNTNYVDKK | 38.85 | 1212.5808 | 10 | 1.63 | 607.2955 | 11.11 | 0.9303 | 0.9376 | 19820 | 0 | 0 | 0 | TRINITY\_DN252\_c0\_g1\_i1.p1 |  |  | DB Search |
| RC(+57.02)TDLNIR | 36.74 | 1046.5291 | 8 | 8.76 | 524.2737 | 11.94 | 0.8729 | 0.8802 | 24758 | 2.66e2 | 1 | 1 | TRINITY\_DN6390\_c0\_g1\_i1.p1 | Carbamidomethylation |  | DB Search |
| SFNKVESR | 36.67 | 965.493 | 8 | 4.26 | 483.7533 | 10.28 | 0.8356 | 0.8429 | 14919 | 0 | 0 | 0 | TRINITY\_DN5397\_c0\_g1\_i1.p1 |  |  | DB Search |
| IAAVIINKPDVLASR | 35.53 | 1578.9457 | 15 | -0.29 | 790.4758 | 15.96 | 1.0993 | 1.1065 | 46467 | 3.75e2 | 1 | 1 | TRINITY\_DN123\_c0\_g1\_i1.p1 |  |  | DB Search |
| AMTDC(+57.02)KETLC(+57.02)R | 35.53 | 1383.5945 | 11 | 1.49 | 692.8019 | 11.65 | 0.9876 | 0.9949 | 23056 | 0 | 0 | 0 | TRINITY\_DN168534\_c0\_g1\_i1.p1 | Carbamidomethylation, Carbamidomethylation |  | DB Search |
| RTC(+57.02)C(+57.02)HTK | 35.4 | 961.4222 | 7 | -19.21 | 962.406 | 13.04 | 1.2061 | 1.2133 | 31036 | 6.86e2 | 1 | 1 | TRINITY\_DN2217\_c0\_g1\_i3.p1 | Carbamidomethylation, Carbamidomethylation |  | DB Search |
| FVNEVRR | 33.88 | 918.5035 | 7 | 0.09 | 460.2567 | 12.65 | 0.812 | 0.8193 | 28973 | 2.9e3 | 1 | 1 | TRINITY\_DN761\_c0\_g1\_i3.p1:TRINITY\_DN761\_c0\_g1\_i1.p1:TRINITY\_DN761\_c0\_g1\_i2.p1 |  |  | DB Search |
| DRHVLTIQSR | 33.87 | 1223.6735 | 10 | 6.21 | 612.8446 | 11.95 | 0.9259 | 0.9331 | 24824 | 0 | 0 | 0 | TRINITY\_DN982\_c0\_g1\_i3.p1:TRINITY\_DN982\_c0\_g1\_i1.p1:TRINITY\_DN982\_c0\_g1\_i2.p1 |  |  | DB Search |
| IVKPGGEQPDELEK | 33.83 | 1537.7987 | 14 | -1.81 | 769.9012 | 12.41 | 1.0259 | 1.0331 | 27466 | 8.43e2 | 1 | 1 | TRINITY\_DN1222\_c0\_g1\_i2.p1:TRINITY\_DN1222\_c0\_g1\_i1.p1 |  |  | DB Search |
| VNQAC(+57.02)VR | 30.94 | 845.4178 | 7 | 1.39 | 423.7145 | 9.76 | 0.7705 | 0.7779 | 11899 | 1.24e3 | 1 | 1 | TRINITY\_DN160\_c0\_g1\_i2.p1:TRINITY\_DN160\_c0\_g1\_i1.p1 | Carbamidomethylation |  | DB Search |
| SPC(+57.02)PPRK | 30.36 | 840.4276 | 7 | 3.69 | 421.2204 | 9.55 | 0.7909 | 0.7982 | 10837 | 4.62e3 | 1 | 1 | TRINITY\_DN4762\_c0\_g1\_i2.p1:TRINITY\_DN4762\_c0\_g1\_i1.p1 | Carbamidomethylation |  | DB Search |
| SQLEKEINNLRK | 29.67 | 1470.8154 | 12 | -9.15 | 491.272 | 14.25 | 0.7742 | 0.7815 | 37755 | 0 | 0 | 0 | TRINITY\_DN453\_c0\_g1\_i1.p1:TRINITY\_DN453\_c0\_g1\_i2.p1 |  |  | DB Search |
| VLEVNDNKPK | 29.58 | 1154.6295 | 10 | -0.5 | 578.3187 | 10.99 | 0.9295 | 0.9368 | 19099 | 7.78e2 | 1 | 1 | TRINITY\_DN982\_c0\_g1\_i1.p1:TRINITY\_DN982\_c0\_g1\_i2.p1 |  |  | DB Search |
| SPNLC(+57.02)QC(+57.02)NNGR | 29.34 | 1318.5507 | 11 | 8.63 | 660.2849 | 10.99 | 0.9259 | 0.9331 | 19117 | 0 | 0 | 0 | TRINITY\_DN35672\_c0\_g1\_i1.p1:TRINITY\_DN35672\_c0\_g1\_i2.p1 | Carbamidomethylation, Carbamidomethylation |  | DB Search |
| YYVTIIDAPGHRDFIK | 29.31 | 1906.9941 | 16 | -3.54 | 954.496 | 16.73 | 1.2045 | 1.2117 | 50248 | 0 | 0 | 0 | TRINITY\_DN64\_c0\_g1\_i1.p1 |  |  | DB Search |
| LRLDFDHEAK | 29.24 | 1242.6356 | 10 | -1.44 | 415.2164 | 14.18 | 0.7282 | 0.7355 | 37433 | 0 | 0 | 0 | TRINITY\_DN160\_c0\_g1\_i2.p1:TRINITY\_DN160\_c0\_g1\_i1.p1 |  |  | DB Search |
| FASTVEPQKGTTIER | 28.89 | 1662.8577 | 15 | -1.19 | 832.4308 | 12.74 | 1.1214 | 1.1286 | 29341 | 5.16e1 | 1 | 1 | TRINITY\_DN10890\_c0\_g1\_i1.p1 |  |  | DB Search |
| total 348 peptides |  |  |  |  |  |  |  |  |  |  |  |  |  |  |  |  |
| --- | --- | --- | --- | --- | --- | --- | --- | --- | --- | --- | --- | --- | --- | --- | --- | --- |
