# Supplementary material for: Proteomic Analysis and Biochemical Characterization of the Nematocyst Extract of the Hydrozoan Velella velella
Source: Mar Drugs. 2024 Oct 12;22(10):468. doi: 10.3390/md22100468 (PMC11509761; doi:10.3390/md22100468)
Supplement: Supplementary file 1 [file marinedrugs-22-00468-s001.zip › Supplementary File S1.zip!/Sample_9_Vellela/db.proteins.html]

proteins


Protein List

  

| Protein Group | Protein ID | Accession | -10lgP | Coverage (%) | Coverage (%) Sample 10 | Area Sample 10 | #Peptides | #Unique | #Spec Sample 10 | PTM | Avg. Mass | Description |
| --- | --- | --- | --- | --- | --- | --- | --- | --- | --- | --- | --- | --- |
| 1 | 1 | TRINITY\_DN982\_c0\_g1\_i3.p1 | 174.1 | 1 | 1 | 2.34e3 | 11 | 3 | 12 |  | 1090854.75 | type:complete len:9852 gc:universal TRINITY\_DN982\_c0\_g1\_i3:29697-142(-) |
| 2 | 2 | TRINITY\_DN982\_c0\_g1\_i1.p1 | 163.04 | 2 | 2 | 7.78e2 | 10 | 2 | 11 |  | 712566.0625 | type:5prime\_partial len:6412 gc:universal TRINITY\_DN982\_c0\_g1\_i1:19377-142(-) |
| 2 | 3 | TRINITY\_DN982\_c0\_g1\_i2.p1 | 69.39 | 2 | 2 | 7.78e2 | 3 | 2 | 3 |  | 190367.1406 | type:internal len:1718 gc:universal TRINITY\_DN982\_c0\_g1\_i2:5151-1(-) |
| 3 | 4 | TRINITY\_DN11435\_c0\_g1\_i1.p1 | 146.9 | 16 | 16 | 2.15e3 | 7 | 7 | 7 |  | 47019.5312 | type:complete len:430 gc:universal TRINITY\_DN11435\_c0\_g1\_i1:1616-327(-) |
| 4 | 5 | TRINITY\_DN160\_c0\_g1\_i1.p1 | 117.51 | 3 | 3 | 3.55e3 | 5 | 5 | 6 | Oxidation (M), Carbamidomethylation | 126611.5703 | type:complete len:1145 gc:universal TRINITY\_DN160\_c0\_g1\_i1:4810-1376(-) |
| 4 | 6 | TRINITY\_DN160\_c0\_g1\_i2.p1 | 117.51 | 3 | 3 | 3.55e3 | 5 | 5 | 6 | Oxidation (M), Carbamidomethylation | 126611.5703 | type:complete len:1145 gc:universal TRINITY\_DN160\_c0\_g1\_i2:4810-1376(-) |
| 5 | 7 | TRINITY\_DN240\_c0\_g1\_i4.p1 | 131.95 | 24 | 24 | 1.46e4 | 5 | 5 | 6 | Oxidation (M) | 40419.2031 | type:5prime\_partial len:397 gc:universal TRINITY\_DN240\_c0\_g1\_i4:1327-137(-) |
| 5 | 8 | TRINITY\_DN240\_c0\_g1\_i6.p1 | 131.95 | 24 | 24 | 1.46e4 | 5 | 5 | 6 | Oxidation (M) | 40418.2188 | type:5prime\_partial len:397 gc:universal TRINITY\_DN240\_c0\_g1\_i6:1494-304(-) |
| 5 | 9 | TRINITY\_DN240\_c0\_g1\_i1.p1 | 131.95 | 24 | 24 | 1.46e4 | 5 | 5 | 6 | Oxidation (M) | 40418.2188 | type:5prime\_partial len:397 gc:universal TRINITY\_DN240\_c0\_g1\_i1:1518-328(-) |
| 6 | 10 | TRINITY\_DN262\_c0\_g1\_i1.p1 | 132.01 | 7 | 7 | 3.05e3 | 5 | 5 | 6 |  | 114520.6406 | type:complete len:1033 gc:universal TRINITY\_DN262\_c0\_g1\_i1:111-3209(+) |
| 7 | 11 | TRINITY\_DN776\_c0\_g1\_i1.p1 | 131.6 | 17 | 17 | 5.66e3 | 5 | 5 | 5 |  | 23300.8359 | type:5prime\_partial len:201 gc:universal TRINITY\_DN776\_c0\_g1\_i1:692-90(-) |
| 7 | 12 | TRINITY\_DN189323\_c0\_g1\_i1.p1 | 119.64 | 15 | 15 | 4.15e3 | 4 | 4 | 4 |  | 18178.9902 | type:5prime\_partial len:156 gc:universal TRINITY\_DN189323\_c0\_g1\_i1:2-469(+) |
| 8 | 13 | TRINITY\_DN161\_c0\_g1\_i2.p1 | 122.59 | 14 | 14 | 4.11e4 | 4 | 4 | 10 | Oxidation (M) | 44577.6484 | type:5prime\_partial len:399 gc:universal TRINITY\_DN161\_c0\_g1\_i2:1683-487(-) |
| 8 | 14 | TRINITY\_DN161\_c0\_g1\_i3.p1 | 122.59 | 14 | 14 | 4.11e4 | 4 | 4 | 10 | Oxidation (M) | 44592.6602 | type:5prime\_partial len:399 gc:universal TRINITY\_DN161\_c0\_g1\_i3:1450-254(-) |
| 8 | 15 | TRINITY\_DN161\_c0\_g1\_i1.p1 | 122.59 | 15 | 15 | 4.11e4 | 4 | 4 | 10 | Oxidation (M) | 41918.5938 | type:complete len:377 gc:universal TRINITY\_DN161\_c0\_g1\_i1:1384-254(-) |
| 8 | 16 | TRINITY\_DN2477\_c0\_g1\_i10.p1 | 88.08 | 9 | 9 | 7.6e3 | 2 | 2 | 3 |  | 38566.9688 | type:5prime\_partial len:344 gc:universal TRINITY\_DN2477\_c0\_g1\_i10:1215-184(-) |
| 8 | 17 | TRINITY\_DN195934\_c0\_g1\_i1.p2 | 88.08 | 24 | 24 | 7.6e3 | 2 | 2 | 3 |  | 14094.0938 | type:internal len:127 gc:universal TRINITY\_DN195934\_c0\_g1\_i1:3-380(+) |
| 8 | 18 | TRINITY\_DN2477\_c0\_g1\_i5.p1 | 88.08 | 9 | 9 | 7.6e3 | 2 | 2 | 3 |  | 37416.5195 | type:complete len:333 gc:universal TRINITY\_DN2477\_c0\_g1\_i5:1182-184(-) |
| 8 | 19 | TRINITY\_DN2477\_c0\_g1\_i2.p1 | 88.08 | 9 | 9 | 7.6e3 | 2 | 2 | 3 |  | 38566.9688 | type:5prime\_partial len:344 gc:universal TRINITY\_DN2477\_c0\_g1\_i2:1215-184(-) |
| 8 | 20 | TRINITY\_DN2477\_c0\_g1\_i6.p1 | 88.08 | 8 | 8 | 7.6e3 | 2 | 2 | 3 |  | 41878.6875 | type:complete len:377 gc:universal TRINITY\_DN2477\_c0\_g1\_i6:1314-184(-) |
| 8 | 21 | TRINITY\_DN2477\_c0\_g1\_i9.p1 | 88.08 | 8 | 8 | 7.6e3 | 2 | 2 | 3 |  | 41878.6875 | type:complete len:377 gc:universal TRINITY\_DN2477\_c0\_g1\_i9:1314-184(-) |
| 8 | 22 | TRINITY\_DN161\_c0\_g1\_i4.p1 | 58.56 | 6 | 6 | 9.54e2 | 1 | 1 | 3 | Oxidation (M) | 25887.2988 | type:5prime\_partial len:232 gc:universal TRINITY\_DN161\_c0\_g1\_i4:949-254(-) |
| 8 | 23 | TRINITY\_DN2941\_c0\_g1\_i4.p1 | 58.04 | 16 | 16 | 4.81e2 | 1 | 1 | 1 |  | 13034.9365 | type:internal len:116 gc:universal TRINITY\_DN2941\_c0\_g1\_i4:2-346(+) |
| 8 | 24 | TRINITY\_DN2477\_c0\_g1\_i8.p1 | 58.04 | 6 | 6 | 4.81e2 | 1 | 1 | 1 |  | 34820.5938 | type:5prime\_partial len:309 gc:universal TRINITY\_DN2477\_c0\_g1\_i8:1110-184(-) |
| 9 | 25 | TRINITY\_DN329\_c0\_g1\_i2.p1 | 129.89 | 14 | 14 | 6.67e3 | 5 | 5 | 7 |  | 34761.9609 | type:5prime\_partial len:320 gc:universal TRINITY\_DN329\_c0\_g1\_i2:1-960(+) |
| 9 | 26 | TRINITY\_DN329\_c0\_g1\_i1.p1 | 129.89 | 16 | 16 | 6.67e3 | 5 | 5 | 7 |  | 30514.9648 | type:complete len:283 gc:universal TRINITY\_DN329\_c0\_g1\_i1:158-1006(+) |
| 10 | 27 | TRINITY\_DN428\_c0\_g1\_i1.p1 | 133.71 | 3 | 3 | 1.19e3 | 5 | 5 | 6 | Carbamidomethylation | 225824.1719 | type:complete len:1949 gc:universal TRINITY\_DN428\_c0\_g1\_i1:96-5942(+) |
| 11 | 28 | TRINITY\_DN2217\_c0\_g1\_i3.p1 | 127.77 | 9 | 9 | 5.11e3 | 5 | 5 | 5 | Carbamidomethylation | 63077.0508 | type:complete len:566 gc:universal TRINITY\_DN2217\_c0\_g1\_i3:1820-123(-) |
| 11 | 29 | TRINITY\_DN2217\_c0\_g1\_i2.p1 | 120.69 | 17 | 17 | 4.43e3 | 4 | 4 | 4 | Carbamidomethylation | 28618.9531 | type:complete len:254 gc:universal TRINITY\_DN2217\_c0\_g1\_i2:884-123(-) |
| 11 | 30 | TRINITY\_DN38397\_c0\_g1\_i1.p1 | 58.69 | 3 | 3 | 1.95e3 | 1 | 1 | 1 | Carbamidomethylation | 40487.2539 | type:internal len:364 gc:universal TRINITY\_DN38397\_c0\_g1\_i1:3-1091(+) |
| 11 | 31 | TRINITY\_DN3202\_c2\_g1\_i1.p1 | 58.47 | 4 | 4 | 0 | 1 | 1 | 1 | Carbamidomethylation | 30131.0488 | type:5prime\_partial len:263 gc:universal TRINITY\_DN3202\_c2\_g1\_i1:949-161(-) |
| 12 | 32 | TRINITY\_DN118\_c1\_g1\_i3.p1 | 122.18 | 13 | 13 | 1.93e4 | 4 | 4 | 9 | Carbamidomethylation | 22860.4883 | type:internal len:200 gc:universal TRINITY\_DN118\_c1\_g1\_i3:599-3(-) |
| 12 | 33 | TRINITY\_DN118\_c1\_g1\_i5.p1 | 107.48 | 10 | 10 | 1.84e4 | 3 | 3 | 6 | Carbamidomethylation | 23277.6309 | type:5prime\_partial len:203 gc:universal TRINITY\_DN118\_c1\_g1\_i5:620-12(-) |
| 13 | 34 | TRINITY\_DN535\_c0\_g1\_i1.p1 | 122.36 | 37 | 37 | 1.63e4 | 4 | 4 | 7 | Carbamidomethylation | 18221.5957 | type:5prime\_partial len:162 gc:universal TRINITY\_DN535\_c0\_g1\_i1:712-227(-) |
| 13 | 35 | TRINITY\_DN535\_c0\_g1\_i2.p1 | 122.36 | 37 | 37 | 1.63e4 | 4 | 4 | 7 | Carbamidomethylation | 18221.5957 | type:5prime\_partial len:162 gc:universal TRINITY\_DN535\_c0\_g1\_i2:699-214(-) |
| 14 | 36 | TRINITY\_DN72625\_c0\_g1\_i1.p1 | 116.63 | 2 | 2 | 4.32e3 | 4 | 4 | 4 |  | 214200.3906 | type:complete len:1964 gc:universal TRINITY\_DN72625\_c0\_g1\_i1:173-6064(+) |
| 15 | 37 | TRINITY\_DN15274\_c0\_g1\_i1.p1 | 121.94 | 3 | 3 | 1.71e3 | 4 | 4 | 5 | Carbamidomethylation, Oxidation (M) | 201897.7344 | type:complete len:1822 gc:universal TRINITY\_DN15274\_c0\_g1\_i1:87-5552(+) |
| 16 | 38 | TRINITY\_DN138\_c0\_g1\_i1.p1 | 116.4 | 14 | 14 | 6.02e3 | 4 | 4 | 9 | Carbamidomethylation | 46324.8945 | type:complete len:401 gc:universal TRINITY\_DN138\_c0\_g1\_i1:157-1359(+) |
| 17 | 39 | TRINITY\_DN332\_c0\_g1\_i1.p1 | 107.39 | 14 | 14 | 5.72e3 | 3 | 3 | 5 |  | 27839.6836 | type:complete len:246 gc:universal TRINITY\_DN332\_c0\_g1\_i1:940-203(-) |
| 18 | 40 | TRINITY\_DN602\_c0\_g1\_i3.p1 | 106.88 | 4 | 4 | 9.38e2 | 3 | 3 | 3 | Carbamidomethylation | 92279.0078 | type:complete len:851 gc:universal TRINITY\_DN602\_c0\_g1\_i3:3046-494(-) |
| 18 | 41 | TRINITY\_DN602\_c0\_g1\_i4.p1 | 106.88 | 7 | 7 | 9.38e2 | 3 | 3 | 3 | Carbamidomethylation | 61175.9766 | type:5prime\_partial len:552 gc:universal TRINITY\_DN602\_c0\_g1\_i4:3498-1843(-) |
| 18 | 42 | TRINITY\_DN602\_c0\_g1\_i1.p1 | 106.88 | 6 | 6 | 9.38e2 | 3 | 3 | 3 | Carbamidomethylation | 65701.2422 | type:complete len:593 gc:universal TRINITY\_DN602\_c0\_g1\_i1:3622-1844(-) |
| 18 | 43 | TRINITY\_DN602\_c0\_g2\_i1.p1 | 58.44 | 2 | 2 | 0 | 1 | 1 | 1 |  | 62057.1875 | type:5prime\_partial len:561 gc:universal TRINITY\_DN602\_c0\_g2\_i1:1994-312(-) |
| 19 | 44 | TRINITY\_DN761\_c0\_g1\_i1.p1 | 95.8 | 6 | 6 | 4.14e3 | 3 | 3 | 3 |  | 95330.7812 | type:complete len:830 gc:universal TRINITY\_DN761\_c0\_g1\_i1:129-2618(+) |
| 19 | 45 | TRINITY\_DN761\_c0\_g1\_i2.p1 | 95.8 | 6 | 6 | 4.14e3 | 3 | 3 | 3 |  | 95330.7812 | type:complete len:830 gc:universal TRINITY\_DN761\_c0\_g1\_i2:87-2576(+) |
| 19 | 46 | TRINITY\_DN761\_c0\_g1\_i3.p1 | 95.8 | 5 | 5 | 4.14e3 | 3 | 3 | 3 |  | 96968.6562 | type:complete len:845 gc:universal TRINITY\_DN761\_c0\_g1\_i3:129-2663(+) |
| 20 | 47 | TRINITY\_DN164271\_c0\_g1\_i1.p1 | 107.06 | 17 | 17 | 3.21e3 | 3 | 3 | 4 | Acetylation (Protein N-term), Carbamidomethylation | 18729.2617 | type:complete len:163 gc:universal TRINITY\_DN164271\_c0\_g1\_i1:1172-684(-) |
| 21 | 48 | TRINITY\_DN22492\_c0\_g1\_i1.p1 | 105.09 | 4 | 4 | 4.72e3 | 3 | 3 | 5 | Carbamidomethylation | 115296.5156 | type:complete len:1026 gc:universal TRINITY\_DN22492\_c0\_g1\_i1:171-3248(+) |
| 21 | 49 | TRINITY\_DN22492\_c0\_g1\_i2.p1 | 105.09 | 4 | 4 | 4.72e3 | 3 | 3 | 5 | Carbamidomethylation | 115355.5859 | type:complete len:1026 gc:universal TRINITY\_DN22492\_c0\_g1\_i2:39-3116(+) |
| 22 | 50 | TRINITY\_DN4595\_c0\_g1\_i4.p1 | 100.79 | 2 | 2 | 4.31e3 | 3 | 3 | 3 | Carbamidomethylation | 183684.4844 | type:5prime\_partial len:1639 gc:universal TRINITY\_DN4595\_c0\_g1\_i4:5857-941(-) |
| 22 | 51 | TRINITY\_DN4595\_c0\_g1\_i10.p1 | 83.65 | 2 | 2 | 1.23e3 | 2 | 2 | 2 | Carbamidomethylation | 133760.0625 | type:complete len:1188 gc:universal TRINITY\_DN4595\_c0\_g1\_i10:4405-842(-) |
| 22 | 52 | TRINITY\_DN4595\_c0\_g1\_i7.p1 | 50.97 | 2 | 2 | 3.08e3 | 1 | 1 | 1 |  | 38387.582 | type:internal len:348 gc:universal TRINITY\_DN4595\_c0\_g1\_i7:1042-2(-) |
| 22 | 53 | TRINITY\_DN4595\_c0\_g1\_i10.p2 | 50.97 | 2 | 2 | 3.08e3 | 1 | 1 | 1 |  | 39822.0156 | type:5prime\_partial len:358 gc:universal TRINITY\_DN4595\_c0\_g1\_i10:5369-4296(-) |
| 23 | 54 | TRINITY\_DN477\_c0\_g1\_i7.p1 | 107.36 | 4 | 4 | 3.12e3 | 3 | 3 | 5 | Carbamidomethylation | 122478.2578 | type:3prime\_partial len:1130 gc:universal TRINITY\_DN477\_c0\_g1\_i7:3387-1(-) |
| 23 | 55 | TRINITY\_DN477\_c0\_g1\_i8.p1 | 87.99 | 3 | 3 | 2.41e3 | 2 | 2 | 4 | Carbamidomethylation | 107165.2031 | type:complete len:985 gc:universal TRINITY\_DN477\_c0\_g1\_i8:3548-594(-) |
| 23 | 56 | TRINITY\_DN477\_c0\_g1\_i8.p2 | 58.12 | 5 | 5 | 7.16e2 | 1 | 1 | 1 |  | 20774.9902 | type:3prime\_partial len:194 gc:universal TRINITY\_DN477\_c0\_g1\_i8:581-3(-) |
| 23 | 57 | TRINITY\_DN477\_c0\_g1\_i6.p1 | 58.12 | 5 | 5 | 7.16e2 | 1 | 1 | 1 |  | 20745.9492 | type:3prime\_partial len:194 gc:universal TRINITY\_DN477\_c0\_g1\_i6:581-3(-) |
| 23 | 58 | TRINITY\_DN477\_c0\_g1\_i3.p1 | 58.12 | 4 | 4 | 7.16e2 | 1 | 1 | 1 |  | 24580.2559 | type:internal len:228 gc:universal TRINITY\_DN477\_c0\_g1\_i3:681-1(-) |
| 23 | 59 | TRINITY\_DN477\_c0\_g1\_i5.p1 | 58.12 | 4 | 4 | 7.16e2 | 1 | 1 | 1 |  | 28860.875 | type:internal len:276 gc:universal TRINITY\_DN477\_c0\_g1\_i5:827-3(-) |
| 24 | 60 | TRINITY\_DN3991\_c0\_g1\_i1.p1 | 107.63 | 12 | 12 | 2.46e5 | 3 | 3 | 3 | Carbamidomethylation | 24846.6543 | type:3prime\_partial len:219 gc:universal TRINITY\_DN3991\_c0\_g1\_i1:84-737(+) |
| 25 | 61 | TRINITY\_DN1386\_c0\_g1\_i1.p1 | 107.32 | 6 | 6 | 4.42e3 | 3 | 3 | 3 |  | 68692.1953 | type:complete len:596 gc:universal TRINITY\_DN1386\_c0\_g1\_i1:2419-632(-) |
| 26 | 62 | TRINITY\_DN941\_c0\_g1\_i9.p1 | 100.7 | 1 | 1 | 1.59e3 | 3 | 3 | 3 | Carbamidomethylation | 340219.4688 | type:complete len:3186 gc:universal TRINITY\_DN941\_c0\_g1\_i9:86-9643(+) |
| 26 | 63 | TRINITY\_DN941\_c0\_g1\_i11.p1 | 50.18 | 2 | 2 | 8.92e2 | 1 | 1 | 1 | Carbamidomethylation | 110353.6641 | type:5prime\_partial len:1040 gc:universal TRINITY\_DN941\_c0\_g1\_i11:1-3120(+) |
| 26 | 64 | TRINITY\_DN941\_c0\_g1\_i15.p1 | 50.18 | 1 | 1 | 8.92e2 | 1 | 1 | 1 | Carbamidomethylation | 127030.5156 | type:5prime\_partial len:1194 gc:universal TRINITY\_DN941\_c0\_g1\_i15:3-3584(+) |
| 27 | 65 | TRINITY\_DN3986\_c0\_g1\_i1.p1 | 107.48 | 8 | 8 | 6.12e3 | 3 | 3 | 4 | Oxidation (M), Carbamidomethylation | 50011.8867 | type:complete len:446 gc:universal TRINITY\_DN3986\_c0\_g1\_i1:252-1589(+) |
| 27 | 66 | TRINITY\_DN1910\_c0\_g1\_i5.p1 | 87.89 | 4 | 4 | 5.81e3 | 2 | 2 | 3 | Oxidation (M), Carbamidomethylation | 50120.9062 | type:complete len:447 gc:universal TRINITY\_DN1910\_c0\_g1\_i5:2031-691(-) |
| 27 | 67 | TRINITY\_DN1910\_c0\_g1\_i1.p1 | 58.66 | 2 | 2 | 4.97e2 | 1 | 1 | 2 | Oxidation (M), Carbamidomethylation | 50135.0117 | type:complete len:447 gc:universal TRINITY\_DN1910\_c0\_g1\_i1:2183-843(-) |
| 28 | 68 | TRINITY\_DN64\_c0\_g1\_i1.p1 | 94.88 | 10 | 10 | 1.33e3 | 3 | 3 | 4 | Oxidation (M) | 51450.1914 | type:complete len:468 gc:universal TRINITY\_DN64\_c0\_g1\_i1:1555-152(-) |
| 29 | 69 | TRINITY\_DN1172\_c0\_g2\_i2.p1 | 107.21 | 13 | 13 | 7.12e3 | 3 | 3 | 6 | Acetylation (Protein N-term) | 27813.4355 | type:internal len:264 gc:universal TRINITY\_DN1172\_c0\_g2\_i2:790-2(-) |
| 29 | 70 | TRINITY\_DN21847\_c0\_g1\_i1.p1 | 58.69 | 3 | 3 | 6.51e3 | 1 | 1 | 3 |  | 41400.2812 | type:internal len:384 gc:universal TRINITY\_DN21847\_c0\_g1\_i1:1150-2(-) |
| 29 | 71 | TRINITY\_DN4497\_c0\_g1\_i1.p1 | 58.69 | 8 | 8 | 6.51e3 | 1 | 1 | 3 |  | 17016.3789 | type:internal len:160 gc:universal TRINITY\_DN4497\_c0\_g1\_i1:1-477(+) |
| 29 | 72 | TRINITY\_DN4497\_c0\_g2\_i1.p1 | 58.69 | 7 | 7 | 6.51e3 | 1 | 1 | 3 |  | 19644.3613 | type:internal len:183 gc:universal TRINITY\_DN4497\_c0\_g2\_i1:2-547(+) |
| 29 | 73 | TRINITY\_DN1172\_c0\_g2\_i1.p1 | 57.79 | 6 | 6 | 1.85e2 | 1 | 1 | 1 |  | 19749.3418 | type:internal len:188 gc:universal TRINITY\_DN1172\_c0\_g2\_i1:562-2(-) |
| 29 | 74 | TRINITY\_DN1172\_c0\_g2\_i3.p1 | 57.79 | 5 | 5 | 1.85e2 | 1 | 1 | 1 |  | 24135.2598 | type:3prime\_partial len:228 gc:universal TRINITY\_DN1172\_c0\_g2\_i3:682-2(-) |
| 30 | 75 | TRINITY\_DN3190\_c1\_g1\_i1.p1 | 104.84 | 13 | 13 | 5.93e2 | 3 | 3 | 3 | Acetylation (Protein N-term) | 35061.5508 | type:complete len:315 gc:universal TRINITY\_DN3190\_c1\_g1\_i1:68-1012(+) |
| 31 | 76 | TRINITY\_DN36\_c0\_g1\_i16.p1 | 86.79 | 10 | 10 | 0 | 2 | 2 | 2 | Carbamidomethylation | 24953.5938 | type:complete len:224 gc:universal TRINITY\_DN36\_c0\_g1\_i16:829-158(-) |
| 31 | 77 | TRINITY\_DN36\_c0\_g1\_i3.p1 | 86.79 | 10 | 10 | 0 | 2 | 2 | 2 | Carbamidomethylation | 24953.5938 | type:complete len:224 gc:universal TRINITY\_DN36\_c0\_g1\_i3:829-158(-) |
| 31 | 78 | TRINITY\_DN36\_c0\_g1\_i14.p1 | 86.79 | 10 | 10 | 0 | 2 | 2 | 2 | Carbamidomethylation | 24953.5938 | type:complete len:224 gc:universal TRINITY\_DN36\_c0\_g1\_i14:819-148(-) |
| 31 | 79 | TRINITY\_DN36\_c0\_g1\_i8.p1 | 86.79 | 10 | 10 | 0 | 2 | 2 | 2 | Carbamidomethylation | 24953.5938 | type:complete len:224 gc:universal TRINITY\_DN36\_c0\_g1\_i8:819-148(-) |
| 31 | 80 | TRINITY\_DN36\_c0\_g1\_i19.p1 | 86.79 | 10 | 10 | 0 | 2 | 2 | 2 | Carbamidomethylation | 24821.4141 | type:complete len:223 gc:universal TRINITY\_DN36\_c0\_g1\_i19:826-158(-) |
| 31 | 81 | TRINITY\_DN36\_c0\_g1\_i17.p1 | 86.79 | 10 | 10 | 0 | 2 | 2 | 2 | Carbamidomethylation | 24953.5938 | type:complete len:224 gc:universal TRINITY\_DN36\_c0\_g1\_i17:819-148(-) |
| 31 | 82 | TRINITY\_DN36\_c0\_g1\_i13.p1 | 86.79 | 10 | 10 | 0 | 2 | 2 | 2 | Carbamidomethylation | 24821.4141 | type:complete len:223 gc:universal TRINITY\_DN36\_c0\_g1\_i13:816-148(-) |
| 31 | 83 | TRINITY\_DN36\_c0\_g1\_i9.p1 | 58.24 | 6 | 6 | 0 | 1 | 1 | 1 | Carbamidomethylation | 24680.2773 | type:complete len:223 gc:universal TRINITY\_DN36\_c0\_g1\_i9:934-266(-) |
| 31 | 84 | TRINITY\_DN36\_c0\_g1\_i1.p1 | 58.24 | 6 | 6 | 0 | 1 | 1 | 1 | Carbamidomethylation | 25006.7051 | type:complete len:224 gc:universal TRINITY\_DN36\_c0\_g1\_i1:1175-504(-) |
| 31 | 85 | TRINITY\_DN36\_c0\_g1\_i11.p1 | 58.24 | 6 | 6 | 0 | 1 | 1 | 1 | Carbamidomethylation | 25006.7051 | type:complete len:224 gc:universal TRINITY\_DN36\_c0\_g1\_i11:1175-504(-) |
| 31 | 86 | TRINITY\_DN36\_c0\_g1\_i4.p1 | 58.24 | 6 | 6 | 0 | 1 | 1 | 1 | Carbamidomethylation | 24680.2773 | type:complete len:223 gc:universal TRINITY\_DN36\_c0\_g1\_i4:932-264(-) |
| 31 | 87 | TRINITY\_DN36\_c0\_g1\_i2.p1 | 58.24 | 6 | 6 | 0 | 1 | 1 | 1 | Carbamidomethylation | 24680.2773 | type:complete len:223 gc:universal TRINITY\_DN36\_c0\_g1\_i2:934-266(-) |
| 31 | 88 | TRINITY\_DN36\_c0\_g1\_i5.p1 | 58.24 | 6 | 6 | 0 | 1 | 1 | 1 | Carbamidomethylation | 24548.0977 | type:complete len:222 gc:universal TRINITY\_DN36\_c0\_g1\_i5:931-266(-) |
| 31 | 89 | TRINITY\_DN36\_c0\_g1\_i20.p1 | 58.24 | 6 | 6 | 0 | 1 | 1 | 1 | Carbamidomethylation | 24874.5234 | type:complete len:223 gc:universal TRINITY\_DN36\_c0\_g1\_i20:1172-504(-) |
| 31 | 90 | TRINITY\_DN649\_c0\_g1\_i5.p1 | 57.09 | 7 | 7 | 0 | 1 | 1 | 1 |  | 13746.6875 | type:internal len:124 gc:universal TRINITY\_DN649\_c0\_g1\_i5:371-3(-) |
| 31 | 91 | TRINITY\_DN649\_c0\_g1\_i2.p1 | 57.09 | 6 | 6 | 0 | 1 | 1 | 1 |  | 13819.7393 | type:internal len:125 gc:universal TRINITY\_DN649\_c0\_g1\_i2:374-3(-) |
| 31 | 92 | TRINITY\_DN649\_c0\_g1\_i1.p1 | 57.09 | 7 | 7 | 0 | 1 | 1 | 1 |  | 13690.5801 | type:internal len:124 gc:universal TRINITY\_DN649\_c0\_g1\_i1:371-3(-) |
| 32 | 93 | TRINITY\_DN1369\_c0\_g1\_i1.p1 | 85.8 | 2 | 2 | 0 | 2 | 2 | 2 | Carbamidomethylation | 174229.4219 | type:3prime\_partial len:1577 gc:universal TRINITY\_DN1369\_c0\_g1\_i1:93-4820(+) |
| 33 | 94 | TRINITY\_DN2636\_c0\_g1\_i1.p1 | 79.96 | 7 | 7 | 0 | 2 | 2 | 3 | Carbamidomethylation | 35741.2305 | type:complete len:314 gc:universal TRINITY\_DN2636\_c0\_g1\_i1:986-45(-) |
| 33 | 95 | TRINITY\_DN2636\_c0\_g1\_i2.p1 | 79.96 | 7 | 7 | 0 | 2 | 2 | 3 | Carbamidomethylation | 35741.2305 | type:complete len:314 gc:universal TRINITY\_DN2636\_c0\_g1\_i2:986-45(-) |
| 34 | 96 | TRINITY\_DN3545\_c0\_g1\_i2.p1 | 85.9 | 10 | 10 | 2.7e3 | 2 | 2 | 4 | Carbamidomethylation | 25563.4785 | type:5prime\_partial len:236 gc:universal TRINITY\_DN3545\_c0\_g1\_i2:3-710(+) |
| 34 | 97 | TRINITY\_DN3545\_c0\_g1\_i1.p1 | 85.9 | 8 | 8 | 2.7e3 | 2 | 2 | 4 | Carbamidomethylation | 31576.2656 | type:5prime\_partial len:293 gc:universal TRINITY\_DN3545\_c0\_g1\_i1:3-881(+) |
| 34 | 98 | TRINITY\_DN495\_c0\_g1\_i2.p1 | 58.68 | 2 | 2 | 8.36e2 | 1 | 1 | 2 | Carbamidomethylation | 72975.0312 | type:complete len:655 gc:universal TRINITY\_DN495\_c0\_g1\_i2:152-2116(+) |
| 34 | 99 | TRINITY\_DN495\_c0\_g1\_i3.p1 | 58.68 | 2 | 2 | 8.36e2 | 1 | 1 | 2 | Carbamidomethylation | 73673.9297 | type:complete len:659 gc:universal TRINITY\_DN495\_c0\_g1\_i3:152-2128(+) |
| 34 | 100 | TRINITY\_DN158421\_c0\_g1\_i1.p1 | 54.44 | 10 | 10 | 1.86e3 | 1 | 1 | 2 | Carbamidomethylation | 12814.7959 | type:internal len:115 gc:universal TRINITY\_DN158421\_c0\_g1\_i1:1-342(+) |
| 35 | 101 | TRINITY\_DN817\_c0\_g1\_i4.p1 | 77.1 | 9 | 9 | 8.56e2 | 2 | 2 | 2 | Carbamidomethylation | 19497.5664 | type:internal len:176 gc:universal TRINITY\_DN817\_c0\_g1\_i4:526-2(-) |
| 35 | 102 | TRINITY\_DN817\_c0\_g1\_i10.p1 | 77.1 | 8 | 8 | 8.56e2 | 2 | 2 | 2 | Carbamidomethylation | 20465.6582 | type:internal len:186 gc:universal TRINITY\_DN817\_c0\_g1\_i10:556-2(-) |
| 35 | 103 | TRINITY\_DN817\_c0\_g1\_i5.p1 | 77.1 | 9 | 9 | 8.56e2 | 2 | 2 | 2 | Carbamidomethylation | 19443.4023 | type:internal len:176 gc:universal TRINITY\_DN817\_c0\_g1\_i5:526-2(-) |
| 35 | 104 | TRINITY\_DN817\_c0\_g1\_i2.p1 | 77.1 | 9 | 9 | 8.56e2 | 2 | 2 | 2 | Carbamidomethylation | 19573.5801 | type:internal len:176 gc:universal TRINITY\_DN817\_c0\_g1\_i2:526-2(-) |
| 35 | 105 | TRINITY\_DN817\_c0\_g1\_i7.p1 | 77.1 | 13 | 13 | 8.56e2 | 2 | 2 | 2 | Carbamidomethylation | 13259.333 | type:internal len:120 gc:universal TRINITY\_DN817\_c0\_g1\_i7:358-2(-) |
| 35 | 106 | TRINITY\_DN817\_c0\_g1\_i6.p1 | 77.1 | 8 | 8 | 8.56e2 | 2 | 2 | 2 | Carbamidomethylation | 19716.6973 | type:internal len:178 gc:universal TRINITY\_DN817\_c0\_g1\_i6:532-2(-) |
| 35 | 107 | TRINITY\_DN817\_c0\_g1\_i8.p1 | 77.1 | 12 | 12 | 8.56e2 | 2 | 2 | 2 | Carbamidomethylation | 13423.584 | type:internal len:122 gc:universal TRINITY\_DN817\_c0\_g1\_i8:364-2(-) |
| 35 | 108 | TRINITY\_DN817\_c0\_g1\_i3.p1 | 55.11 | 7 | 7 | 5.05e2 | 1 | 1 | 1 | Carbamidomethylation | 23798.4922 | type:complete len:215 gc:universal TRINITY\_DN817\_c0\_g1\_i3:848-204(-) |
| 35 | 109 | TRINITY\_DN817\_c0\_g1\_i9.p1 | 55.11 | 7 | 7 | 5.05e2 | 1 | 1 | 1 | Carbamidomethylation | 21303.3516 | type:5prime\_partial len:191 gc:universal TRINITY\_DN817\_c0\_g1\_i9:776-204(-) |
| 35 | 110 | TRINITY\_DN817\_c0\_g1\_i1.p1 | 55.11 | 6 | 6 | 5.05e2 | 1 | 1 | 1 | Carbamidomethylation | 24026.7148 | type:complete len:217 gc:universal TRINITY\_DN817\_c0\_g1\_i1:854-204(-) |
| 36 | 111 | TRINITY\_DN165469\_c0\_g1\_i1.p1 | 85.06 | 1 | 1 | 0 | 2 | 2 | 2 |  | 298128.9688 | type:complete len:2668 gc:universal TRINITY\_DN165469\_c0\_g1\_i1:41-8044(+) |
| 37 | 112 | TRINITY\_DN1616\_c0\_g1\_i2.p1 | 74.37 | 1 | 1 | 0 | 2 | 2 | 2 |  | 374396 | type:3prime\_partial len:3398 gc:universal TRINITY\_DN1616\_c0\_g1\_i2:218-10408(+) |
| 37 | 113 | TRINITY\_DN1616\_c0\_g1\_i3.p1 | 74.37 | 1 | 1 | 0 | 2 | 2 | 2 |  | 423947.2812 | type:complete len:3855 gc:universal TRINITY\_DN1616\_c0\_g1\_i3:218-11782(+) |
| 37 | 114 | TRINITY\_DN1616\_c0\_g1\_i7.p1 | 74.37 | 1 | 1 | 0 | 2 | 2 | 2 |  | 423875.2188 | type:complete len:3855 gc:universal TRINITY\_DN1616\_c0\_g1\_i7:218-11782(+) |
| 37 | 115 | TRINITY\_DN1616\_c0\_g1\_i6.p1 | 74.37 | 1 | 1 | 0 | 2 | 2 | 2 |  | 423911.25 | type:complete len:3855 gc:universal TRINITY\_DN1616\_c0\_g1\_i6:218-11782(+) |
| 38 | 116 | TRINITY\_DN577\_c0\_g1\_i1.p1 | 77.3 | 12 | 12 | 5.88e2 | 2 | 2 | 2 | Carbamidomethylation | 30281.0371 | type:5prime\_partial len:269 gc:universal TRINITY\_DN577\_c0\_g1\_i1:1086-280(-) |
| 39 | 117 | TRINITY\_DN15584\_c0\_g1\_i1.p1 | 87.59 | 10 | 10 | 2.19e4 | 2 | 2 | 3 | Carbamidomethylation | 29534.9648 | type:complete len:275 gc:universal TRINITY\_DN15584\_c0\_g1\_i1:874-50(-) |
| 40 | 118 | TRINITY\_DN3034\_c0\_g1\_i1.p1 | 87.87 | 1 | 1 | 1.23e3 | 2 | 2 | 2 |  | 222266.9844 | type:complete len:1941 gc:universal TRINITY\_DN3034\_c0\_g1\_i1:6289-467(-) |
| 41 | 119 | TRINITY\_DN1339\_c0\_g1\_i2.p1 | 88.22 | 6 | 6 | 1.44e4 | 2 | 2 | 4 |  | 41222.6992 | type:internal len:384 gc:universal TRINITY\_DN1339\_c0\_g1\_i2:1151-3(-) |
| 41 | 120 | TRINITY\_DN1339\_c6\_g1\_i1.p1 | 59.05 | 2 | 2 | 1.42e4 | 1 | 1 | 3 |  | 61274.3594 | type:internal len:570 gc:universal TRINITY\_DN1339\_c6\_g1\_i1:1-1707(+) |
| 41 | 121 | TRINITY\_DN124769\_c0\_g1\_i1.p1 | 59.05 | 11 | 11 | 1.42e4 | 1 | 1 | 3 |  | 12144.8828 | type:internal len:113 gc:universal TRINITY\_DN124769\_c0\_g1\_i1:1-336(+) |
| 41 | 122 | TRINITY\_DN24414\_c1\_g1\_i2.p2 | 59.05 | 8 | 8 | 1.42e4 | 1 | 1 | 3 |  | 15590.8623 | type:internal len:146 gc:universal TRINITY\_DN24414\_c1\_g1\_i2:435-1(-) |
| 41 | 123 | TRINITY\_DN1172\_c0\_g1\_i3.p1 | 59.05 | 7 | 7 | 1.42e4 | 1 | 1 | 3 |  | 19709.3613 | type:internal len:182 gc:universal TRINITY\_DN1172\_c0\_g1\_i3:545-3(-) |
| 41 | 124 | TRINITY\_DN119673\_c0\_g1\_i1.p1 | 59.05 | 9 | 9 | 1.42e4 | 1 | 1 | 3 |  | 13988.7559 | type:internal len:129 gc:universal TRINITY\_DN119673\_c0\_g1\_i1:2-385(+) |
| 41 | 125 | TRINITY\_DN1339\_c0\_g1\_i1.p1 | 59.05 | 5 | 5 | 1.42e4 | 1 | 1 | 3 |  | 24722.7773 | type:internal len:229 gc:universal TRINITY\_DN1339\_c0\_g1\_i1:684-1(-) |
| 41 | 126 | TRINITY\_DN6701\_c0\_g1\_i1.p1 | 59.05 | 6 | 6 | 1.42e4 | 1 | 1 | 3 |  | 21246.0547 | type:internal len:196 gc:universal TRINITY\_DN6701\_c0\_g1\_i1:1-585(+) |
| 41 | 127 | TRINITY\_DN1172\_c0\_g1\_i5.p1 | 59.05 | 5 | 5 | 1.42e4 | 1 | 1 | 3 |  | 24525.6855 | type:internal len:226 gc:universal TRINITY\_DN1172\_c0\_g1\_i5:677-3(-) |
| 41 | 128 | TRINITY\_DN6701\_c0\_g1\_i2.p2 | 59.05 | 8 | 8 | 1.42e4 | 1 | 1 | 3 |  | 16512.8535 | type:internal len:152 gc:universal TRINITY\_DN6701\_c0\_g1\_i2:3-455(+) |
| 41 | 129 | TRINITY\_DN1339\_c6\_g1\_i2.p1 | 59.05 | 3 | 3 | 1.42e4 | 1 | 1 | 3 |  | 43821.8359 | type:internal len:408 gc:universal TRINITY\_DN1339\_c6\_g1\_i2:3-1223(+) |
| 42 | 130 | TRINITY\_DN96\_c0\_g1\_i2.p1 | 85.46 | 12 | 12 | 8.5e2 | 2 | 2 | 3 |  | 20764.5137 | type:5prime\_partial len:189 gc:universal TRINITY\_DN96\_c0\_g1\_i2:1253-687(-) |
| 42 | 131 | TRINITY\_DN96\_c0\_g1\_i1.p1 | 85.46 | 12 | 12 | 8.5e2 | 2 | 2 | 3 |  | 20737.4844 | type:5prime\_partial len:189 gc:universal TRINITY\_DN96\_c0\_g1\_i1:1253-687(-) |
| 42 | 132 | TRINITY\_DN96\_c0\_g1\_i7.p1 | 53.61 | 7 | 7 | 2.14e2 | 1 | 1 | 1 |  | 15457.2588 | type:5prime\_partial len:139 gc:universal TRINITY\_DN96\_c0\_g1\_i7:487-71(-) |
| 42 | 133 | TRINITY\_DN96\_c0\_g1\_i6.p1 | 53.61 | 5 | 5 | 2.14e2 | 1 | 1 | 1 |  | 21964.1172 | type:complete len:199 gc:universal TRINITY\_DN96\_c0\_g1\_i6:1283-687(-) |
| 42 | 134 | TRINITY\_DN96\_c0\_g1\_i5.p1 | 53.61 | 5 | 5 | 2.14e2 | 1 | 1 | 1 |  | 22037.2168 | type:complete len:199 gc:universal TRINITY\_DN96\_c0\_g1\_i5:1283-687(-) |
| 42 | 135 | TRINITY\_DN96\_c0\_g1\_i8.p1 | 53.61 | 5 | 5 | 2.14e2 | 1 | 1 | 1 |  | 21977.1191 | type:complete len:199 gc:universal TRINITY\_DN96\_c0\_g1\_i8:1283-687(-) |
| 42 | 136 | TRINITY\_DN96\_c0\_g1\_i4.p1 | 53.61 | 5 | 5 | 2.14e2 | 1 | 1 | 1 |  | 20895.6445 | type:5prime\_partial len:189 gc:universal TRINITY\_DN96\_c0\_g1\_i4:637-71(-) |
| 42 | 137 | TRINITY\_DN96\_c0\_g1\_i3.p1 | 53.61 | 5 | 5 | 2.14e2 | 1 | 1 | 1 |  | 20864.7207 | type:5prime\_partial len:189 gc:universal TRINITY\_DN96\_c0\_g1\_i3:1253-687(-) |
| 43 | 138 | TRINITY\_DN3036\_c0\_g1\_i3.p1 | 88.21 | 5 | 5 | 6.27e3 | 2 | 2 | 3 |  | 59630.4023 | type:internal len:548 gc:universal TRINITY\_DN3036\_c0\_g1\_i3:1643-3(-) |
| 43 | 139 | TRINITY\_DN3036\_c0\_g1\_i8.p1 | 88.21 | 5 | 5 | 6.27e3 | 2 | 2 | 3 |  | 59630.4023 | type:internal len:548 gc:universal TRINITY\_DN3036\_c0\_g1\_i8:1643-3(-) |
| 43 | 140 | TRINITY\_DN3036\_c0\_g1\_i2.p2 | 88.21 | 5 | 5 | 6.27e3 | 2 | 2 | 3 |  | 59586.3086 | type:internal len:548 gc:universal TRINITY\_DN3036\_c0\_g1\_i2:1643-3(-) |
| 43 | 141 | TRINITY\_DN3036\_c0\_g1\_i11.p1 | 58.87 | 3 | 3 | 3.81e3 | 1 | 1 | 2 |  | 40331.5547 | type:internal len:374 gc:universal TRINITY\_DN3036\_c0\_g1\_i11:1121-3(-) |
| 43 | 142 | TRINITY\_DN3036\_c0\_g1\_i16.p1 | 58.87 | 3 | 3 | 3.81e3 | 1 | 1 | 2 |  | 40331.5547 | type:internal len:374 gc:universal TRINITY\_DN3036\_c0\_g1\_i16:1121-3(-) |
| 43 | 143 | TRINITY\_DN3036\_c0\_g1\_i19.p1 | 58.87 | 3 | 3 | 3.81e3 | 1 | 1 | 2 |  | 40287.4609 | type:internal len:374 gc:universal TRINITY\_DN3036\_c0\_g1\_i19:1121-3(-) |
| 43 | 144 | TRINITY\_DN3036\_c0\_g1\_i13.p1 | 58.87 | 3 | 3 | 3.81e3 | 1 | 1 | 2 |  | 40287.4609 | type:internal len:374 gc:universal TRINITY\_DN3036\_c0\_g1\_i13:1121-3(-) |
| 44 | 145 | TRINITY\_DN6390\_c0\_g1\_i1.p1 | 73.92 | 1 | 1 | 2.66e2 | 2 | 2 | 2 | Carbamidomethylation | 214480.2344 | type:3prime\_partial len:1941 gc:universal TRINITY\_DN6390\_c0\_g1\_i1:5820-1(-) |
| 45 | 146 | TRINITY\_DN6920\_c0\_g1\_i3.p1 | 85.67 | 5 | 5 | 7.43e2 | 2 | 2 | 2 | Carbamidomethylation | 46932.582 | type:5prime\_partial len:411 gc:universal TRINITY\_DN6920\_c0\_g1\_i3:2124-892(-) |
| 45 | 147 | TRINITY\_DN6920\_c0\_g1\_i2.p1 | 85.67 | 5 | 5 | 7.43e2 | 2 | 2 | 2 | Carbamidomethylation | 46932.582 | type:5prime\_partial len:411 gc:universal TRINITY\_DN6920\_c0\_g1\_i2:2147-915(-) |
| 45 | 148 | TRINITY\_DN6920\_c0\_g1\_i4.p1 | 85.67 | 7 | 7 | 7.43e2 | 2 | 2 | 2 | Carbamidomethylation | 32195.3809 | type:5prime\_partial len:282 gc:universal TRINITY\_DN6920\_c0\_g1\_i4:1321-476(-) |
| 45 | 149 | TRINITY\_DN6920\_c0\_g1\_i1.p1 | 85.67 | 7 | 7 | 7.43e2 | 2 | 2 | 2 | Carbamidomethylation | 32882.168 | type:complete len:287 gc:universal TRINITY\_DN6920\_c0\_g1\_i1:1775-915(-) |
| 46 | 150 | TRINITY\_DN12\_c0\_g2\_i1.p1 | 81.29 | 11 | 11 | 3.4e3 | 2 | 2 | 2 | Oxidation (M) | 21647.1582 | type:complete len:203 gc:universal TRINITY\_DN12\_c0\_g2\_i1:239-847(+) |
| 46 | 151 | TRINITY\_DN12\_c0\_g1\_i1.p1 | 51.39 | 5 | 5 | 2.93e3 | 1 | 1 | 1 |  | 21891.4863 | type:complete len:203 gc:universal TRINITY\_DN12\_c0\_g1\_i1:141-749(+) |
| 47 | 152 | TRINITY\_DN278\_c0\_g1\_i1.p1 | 85.98 | 3 | 3 | 1.64e3 | 2 | 2 | 3 |  | 55197.2969 | type:complete len:470 gc:universal TRINITY\_DN278\_c0\_g1\_i1:3091-1682(-) |
| 48 | 153 | TRINITY\_DN624\_c0\_g1\_i1.p1 | 77.39 | 3 | 3 | 7.29e2 | 2 | 2 | 3 |  | 101743.5391 | type:complete len:884 gc:universal TRINITY\_DN624\_c0\_g1\_i1:218-2869(+) |
| 49 | 154 | TRINITY\_DN4329\_c0\_g1\_i1.p1 | 86.95 | 2 | 2 | 2.2e3 | 2 | 2 | 2 |  | 84233.3359 | type:5prime\_partial len:735 gc:universal TRINITY\_DN4329\_c0\_g1\_i1:2240-36(-) |
| 50 | 155 | TRINITY\_DN453\_c0\_g1\_i1.p1 | 73.21 | 11 | 11 | 0 | 2 | 2 | 2 | Carbamidomethylation | 27993.4082 | type:complete len:249 gc:universal TRINITY\_DN453\_c0\_g1\_i1:944-198(-) |
| 50 | 156 | TRINITY\_DN453\_c0\_g1\_i2.p1 | 73.21 | 11 | 11 | 0 | 2 | 2 | 2 | Carbamidomethylation | 27993.4082 | type:complete len:249 gc:universal TRINITY\_DN453\_c0\_g1\_i2:944-198(-) |
| 51 | 157 | TRINITY\_DN1377\_c0\_g1\_i1.p1 | 86.74 | 3 | 3 | 2.13e3 | 2 | 2 | 2 |  | 99936.9219 | type:complete len:878 gc:universal TRINITY\_DN1377\_c0\_g1\_i1:68-2701(+) |
| 52 | 158 | TRINITY\_DN286\_c0\_g1\_i2.p1 | 58.68 | 10 | 10 | 4.34e3 | 1 | 1 | 2 | Oxidation (M), Carbamidomethylation | 11368.1738 | type:3prime\_partial len:106 gc:universal TRINITY\_DN286\_c0\_g1\_i2:40-354(+) |
| 52 | 159 | TRINITY\_DN286\_c0\_g1\_i4.p1 | 58.68 | 5 | 5 | 4.34e3 | 1 | 1 | 2 | Oxidation (M), Carbamidomethylation | 25084.5293 | type:complete len:224 gc:universal TRINITY\_DN286\_c0\_g1\_i4:40-711(+) |
| 53 | 160 | TRINITY\_DN4439\_c0\_g1\_i1.p1 | 83.4 | 3 | 3 | 0 | 2 | 2 | 2 | Carbamidomethylation | 104528.4844 | type:complete len:941 gc:universal TRINITY\_DN4439\_c0\_g1\_i1:77-2899(+) |
| 54 | 161 | TRINITY\_DN2805\_c0\_g1\_i1.p1 | 87.21 | 13 | 13 | 3.97e2 | 2 | 2 | 2 | Oxidation (M) | 21692.6699 | type:complete len:189 gc:universal TRINITY\_DN2805\_c0\_g1\_i1:623-57(-) |
| 55 | 162 | TRINITY\_DN5793\_c0\_g1\_i1.p1 | 87.56 | 12 | 12 | 7.83e2 | 2 | 2 | 2 | Carbamidomethylation | 23513.3242 | type:complete len:213 gc:universal TRINITY\_DN5793\_c0\_g1\_i1:170-808(+) |
| 55 | 163 | TRINITY\_DN6233\_c0\_g1\_i2.p2 | 58.6 | 5 | 5 | 7.83e2 | 1 | 1 | 1 | Carbamidomethylation | 26432.5801 | type:5prime\_partial len:234 gc:universal TRINITY\_DN6233\_c0\_g1\_i2:760-59(-) |
| 55 | 164 | TRINITY\_DN90868\_c0\_g1\_i1.p3 | 58.6 | 11 | 11 | 7.83e2 | 1 | 1 | 1 | Carbamidomethylation | 11562.293 | type:internal len:104 gc:universal TRINITY\_DN90868\_c0\_g1\_i1:309-1(-) |
| 55 | 165 | TRINITY\_DN6233\_c0\_g1\_i1.p2 | 58.6 | 5 | 5 | 7.83e2 | 1 | 1 | 1 | Carbamidomethylation | 26416.5801 | type:5prime\_partial len:234 gc:universal TRINITY\_DN6233\_c0\_g1\_i1:760-59(-) |
| 55 | 166 | TRINITY\_DN188006\_c0\_g1\_i1.p1 | 58.6 | 6 | 6 | 7.83e2 | 1 | 1 | 1 | Carbamidomethylation | 21118.8691 | type:3prime\_partial len:188 gc:universal TRINITY\_DN188006\_c0\_g1\_i1:561-1(-) |
| 56 | 167 | TRINITY\_DN326\_c0\_g1\_i1.p1 | 77.89 | 14 | 14 | 2.41e3 | 2 | 2 | 2 | Acetylation (Protein N-term) | 20249.3965 | type:complete len:177 gc:universal TRINITY\_DN326\_c0\_g1\_i1:729-199(-) |
| 57 | 168 | TRINITY\_DN1614\_c0\_g1\_i1.p1 | 88.07 | 12 | 12 | 2.9e3 | 2 | 2 | 3 |  | 25367.3418 | type:complete len:218 gc:universal TRINITY\_DN1614\_c0\_g1\_i1:56-709(+) |
| 58 | 169 | TRINITY\_DN74124\_c0\_g1\_i1.p1 | 87.19 | 17 | 17 | 0 | 2 | 2 | 3 |  | 20562.4961 | type:complete len:183 gc:universal TRINITY\_DN74124\_c0\_g1\_i1:100-648(+) |
| 59 | 170 | TRINITY\_DN147947\_c0\_g1\_i1.p1 | 87.63 | 16 | 16 | 1.1e3 | 2 | 2 | 3 |  | 19144.6445 | type:internal len:168 gc:universal TRINITY\_DN147947\_c0\_g1\_i1:501-1(-) |
| 60 | 171 | TRINITY\_DN73067\_c0\_g1\_i1.p1 | 58.46 | 2 | 2 | 3.07e3 | 1 | 1 | 3 | Oxidation (M) | 93020.375 | type:complete len:831 gc:universal TRINITY\_DN73067\_c0\_g1\_i1:3714-1222(-) |
| 61 | 172 | TRINITY\_DN319\_c2\_g1\_i3.p1 | 87.61 | 1 | 1 | 1.44e3 | 2 | 2 | 3 |  | 279087.1875 | type:complete len:2588 gc:universal TRINITY\_DN319\_c2\_g1\_i3:165-7928(+) |
| 61 | 173 | TRINITY\_DN319\_c2\_g1\_i1.p1 | 87.61 | 1 | 1 | 1.44e3 | 2 | 2 | 3 |  | 279168.25 | type:complete len:2588 gc:universal TRINITY\_DN319\_c2\_g1\_i1:165-7928(+) |
| 62 | 174 | TRINITY\_DN164351\_c0\_g1\_i1.p1 | 81.5 | 10 | 10 | 1.62e3 | 2 | 2 | 2 | Oxidation (M) | 23723.9492 | type:complete len:212 gc:universal TRINITY\_DN164351\_c0\_g1\_i1:694-59(-) |
| 63 | 175 | TRINITY\_DN477\_c0\_g1\_i9.p1 | 85.65 | 4 | 4 | 1.16e3 | 2 | 2 | 2 |  | 34222.9453 | type:complete len:306 gc:universal TRINITY\_DN477\_c0\_g1\_i9:1840-923(-) |
| 64 | 176 | TRINITY\_DN168534\_c0\_g1\_i1.p1 | 70.49 | 12 | 12 | 0 | 2 | 2 | 2 | Carbamidomethylation | 25265.0449 | type:complete len:226 gc:universal TRINITY\_DN168534\_c0\_g1\_i1:774-97(-) |
| 65 | 177 | TRINITY\_DN345\_c0\_g1\_i1.p1 | 82.12 | 10 | 10 | 7.75e2 | 2 | 2 | 3 | Oxidation (M) | 16956.6133 | type:complete len:150 gc:universal TRINITY\_DN345\_c0\_g1\_i1:760-311(-) |
| 66 | 178 | TRINITY\_DN5633\_c0\_g1\_i1.p1 | 87.33 | 4 | 4 | 2.57e2 | 2 | 2 | 2 |  | 97310.9922 | type:complete len:848 gc:universal TRINITY\_DN5633\_c0\_g1\_i1:2881-338(-) |
| 66 | 179 | TRINITY\_DN141338\_c2\_g1\_i1.p1 | 58.23 | 2 | 2 | 2.57e2 | 1 | 1 | 1 |  | 82716.2266 | type:complete len:720 gc:universal TRINITY\_DN141338\_c2\_g1\_i1:2850-691(-) |
| 67 | 180 | TRINITY\_DN773\_c0\_g1\_i1.p1 | 84.67 | 5 | 5 | 5.38e2 | 2 | 2 | 2 |  | 48865.75 | type:complete len:440 gc:universal TRINITY\_DN773\_c0\_g1\_i1:1608-289(-) |
| 68 | 181 | TRINITY\_DN374\_c0\_g1\_i1.p1 | 87.1 | 10 | 10 | 0 | 2 | 2 | 2 | Carbamidomethylation | 32814.1367 | type:5prime\_partial len:290 gc:universal TRINITY\_DN374\_c0\_g1\_i1:1-870(+) |
| 68 | 182 | TRINITY\_DN374\_c0\_g1\_i2.p1 | 56.94 | 4 | 4 | 0 | 1 | 1 | 1 | Carbamidomethylation | 38811.4023 | type:complete len:341 gc:universal TRINITY\_DN374\_c0\_g1\_i2:115-1137(+) |
| 69 | 183 | TRINITY\_DN2904\_c0\_g1\_i1.p1 | 87.5 | 11 | 11 | 1.44e3 | 2 | 2 | 2 |  | 24728.3496 | type:complete len:221 gc:universal TRINITY\_DN2904\_c0\_g1\_i1:1339-677(-) |
| 70 | 184 | TRINITY\_DN40728\_c1\_g1\_i1.p2 | 58.25 | 10 | 10 | 1.53e3 | 1 | 1 | 2 |  | 14073.3066 | type:5prime\_partial len:124 gc:universal TRINITY\_DN40728\_c1\_g1\_i1:1-372(+) |
| 70 | 185 | TRINITY\_DN40728\_c0\_g1\_i1.p1 | 58.25 | 13 | 13 | 1.53e3 | 1 | 1 | 2 |  | 11225.8301 | type:internal len:105 gc:universal TRINITY\_DN40728\_c0\_g1\_i1:1-312(+) |
| 70 | 186 | TRINITY\_DN195631\_c0\_g1\_i1.p2 | 58.25 | 9 | 9 | 1.53e3 | 1 | 1 | 2 |  | 15844.8984 | type:internal len:145 gc:universal TRINITY\_DN195631\_c0\_g1\_i1:2-433(+) |
| 70 | 187 | TRINITY\_DN152\_c0\_g1\_i4.p1 | 58.25 | 8 | 8 | 1.53e3 | 1 | 1 | 2 |  | 17002.1406 | type:internal len:155 gc:universal TRINITY\_DN152\_c0\_g1\_i4:3-464(+) |
| 70 | 188 | TRINITY\_DN8227\_c0\_g1\_i1.p1 | 58.25 | 10 | 10 | 1.53e3 | 1 | 1 | 2 |  | 14513.8135 | type:internal len:135 gc:universal TRINITY\_DN8227\_c0\_g1\_i1:404-3(-) |
| 70 | 189 | TRINITY\_DN554\_c0\_g1\_i3.p1 | 58.25 | 5 | 5 | 1.53e3 | 1 | 1 | 2 |  | 30824.5996 | type:3prime\_partial len:273 gc:universal TRINITY\_DN554\_c0\_g1\_i3:818-3(-) |
| 70 | 190 | TRINITY\_DN152\_c0\_g1\_i15.p1 | 58.25 | 9 | 9 | 1.53e3 | 1 | 1 | 2 |  | 15560.71 | type:internal len:141 gc:universal TRINITY\_DN152\_c0\_g1\_i15:2-421(+) |
| 70 | 191 | TRINITY\_DN585\_c0\_g1\_i10.p1 | 58.25 | 6 | 6 | 1.53e3 | 1 | 1 | 2 |  | 26563.5332 | type:3prime\_partial len:236 gc:universal TRINITY\_DN585\_c0\_g1\_i10:705-1(-) |
| 70 | 192 | TRINITY\_DN152\_c0\_g1\_i2.p1 | 58.25 | 13 | 13 | 1.53e3 | 1 | 1 | 2 |  | 11754.375 | type:internal len:105 gc:universal TRINITY\_DN152\_c0\_g1\_i2:1-312(+) |
| 70 | 193 | TRINITY\_DN152\_c0\_g1\_i10.p1 | 58.25 | 7 | 7 | 1.53e3 | 1 | 1 | 2 |  | 20063.498 | type:internal len:180 gc:universal TRINITY\_DN152\_c0\_g1\_i10:1-537(+) |
| 70 | 194 | TRINITY\_DN152\_c0\_g1\_i14.p2 | 58.25 | 11 | 11 | 1.53e3 | 1 | 1 | 2 |  | 12547.4834 | type:5prime\_partial len:115 gc:universal TRINITY\_DN152\_c0\_g1\_i14:3-347(+) |
| 70 | 195 | TRINITY\_DN554\_c0\_g1\_i3.p2 | 58.25 | 5 | 5 | 1.53e3 | 1 | 1 | 2 |  | 30419.4961 | type:complete len:268 gc:universal TRINITY\_DN554\_c0\_g1\_i3:1464-661(-) |
| 70 | 196 | TRINITY\_DN1465\_c1\_g1\_i1.p1 | 58.25 | 13 | 13 | 1.53e3 | 1 | 1 | 2 |  | 11342.1572 | type:internal len:102 gc:universal TRINITY\_DN1465\_c1\_g1\_i1:2-304(+) |
| 70 | 197 | TRINITY\_DN152\_c0\_g1\_i9.p1 | 58.25 | 9 | 9 | 1.53e3 | 1 | 1 | 2 |  | 17213.1973 | type:5prime\_partial len:152 gc:universal TRINITY\_DN152\_c0\_g1\_i9:1-456(+) |
| 70 | 198 | TRINITY\_DN585\_c0\_g1\_i2.p1 | 58.25 | 7 | 7 | 1.53e3 | 1 | 1 | 2 |  | 20558.6738 | type:internal len:184 gc:universal TRINITY\_DN585\_c0\_g1\_i2:549-1(-) |
| 70 | 199 | TRINITY\_DN585\_c0\_g1\_i5.p1 | 58.25 | 5 | 5 | 1.53e3 | 1 | 1 | 2 |  | 28098.1602 | type:internal len:250 gc:universal TRINITY\_DN585\_c0\_g1\_i5:747-1(-) |
| 70 | 200 | TRINITY\_DN173718\_c0\_g1\_i1.p1 | 58.25 | 12 | 12 | 1.53e3 | 1 | 1 | 2 |  | 12865.0928 | type:3prime\_partial len:112 gc:universal TRINITY\_DN173718\_c0\_g1\_i1:37-369(+) |
| 70 | 201 | TRINITY\_DN585\_c0\_g1\_i3.p1 | 58.25 | 8 | 8 | 1.53e3 | 1 | 1 | 2 |  | 17628.2207 | type:internal len:157 gc:universal TRINITY\_DN585\_c0\_g1\_i3:468-1(-) |
| 70 | 202 | TRINITY\_DN585\_c0\_g1\_i4.p1 | 58.25 | 6 | 6 | 1.53e3 | 1 | 1 | 2 |  | 26348.0742 | type:complete len:232 gc:universal TRINITY\_DN585\_c0\_g1\_i4:847-152(-) |
| 70 | 203 | TRINITY\_DN554\_c0\_g1\_i5.p1 | 58.25 | 3 | 3 | 1.53e3 | 1 | 1 | 2 |  | 42081.4141 | type:3prime\_partial len:373 gc:universal TRINITY\_DN554\_c0\_g1\_i5:1118-3(-) |
| 71 | 204 | TRINITY\_DN1819\_c0\_g1\_i5.p1 | 56.12 | 3 | 3 | 0 | 1 | 1 | 1 |  | 49581.1055 | type:complete len:432 gc:universal TRINITY\_DN1819\_c0\_g1\_i5:79-1374(+) |
| 71 | 205 | TRINITY\_DN1819\_c0\_g1\_i7.p1 | 56.12 | 3 | 3 | 0 | 1 | 1 | 1 |  | 49581.1055 | type:complete len:432 gc:universal TRINITY\_DN1819\_c0\_g1\_i7:67-1362(+) |
| 72 | 206 | TRINITY\_DN1058\_c0\_g1\_i1.p1 | 58.64 | 0 | 0 | 4.06e2 | 1 | 1 | 1 |  | 284466.2812 | type:complete len:2639 gc:universal TRINITY\_DN1058\_c0\_g1\_i1:134-8050(+) |
| 72 | 207 | TRINITY\_DN1058\_c0\_g1\_i4.p1 | 58.64 | 2 | 2 | 4.06e2 | 1 | 1 | 1 |  | 79375.5703 | type:complete len:723 gc:universal TRINITY\_DN1058\_c0\_g1\_i4:366-2534(+) |
| 72 | 208 | TRINITY\_DN1058\_c0\_g1\_i3.p1 | 58.64 | 0 | 0 | 4.06e2 | 1 | 1 | 1 |  | 277633.5312 | type:complete len:2581 gc:universal TRINITY\_DN1058\_c0\_g1\_i3:134-7876(+) |
| 72 | 209 | TRINITY\_DN1058\_c0\_g1\_i2.p2 | 58.64 | 1 | 1 | 4.06e2 | 1 | 1 | 1 |  | 140284.0938 | type:complete len:1300 gc:universal TRINITY\_DN1058\_c0\_g1\_i2:4090-7989(+) |
| 73 | 210 | TRINITY\_DN1339\_c0\_g2\_i3.p1 | 59.06 | 7 | 7 | 1.28e3 | 1 | 1 | 1 |  | 19523.1797 | type:internal len:185 gc:universal TRINITY\_DN1339\_c0\_g2\_i3:553-2(-) |
| 73 | 211 | TRINITY\_DN1339\_c0\_g2\_i2.p1 | 59.06 | 3 | 3 | 1.28e3 | 1 | 1 | 1 |  | 42663.3828 | type:internal len:398 gc:universal TRINITY\_DN1339\_c0\_g2\_i2:1193-3(-) |
| 73 | 212 | TRINITY\_DN1339\_c0\_g2\_i4.p2 | 59.06 | 6 | 6 | 1.28e3 | 1 | 1 | 1 |  | 22988.9766 | type:internal len:216 gc:universal TRINITY\_DN1339\_c0\_g2\_i4:646-2(-) |
| 73 | 213 | TRINITY\_DN1339\_c0\_g2\_i1.p2 | 59.06 | 3 | 3 | 1.28e3 | 1 | 1 | 1 |  | 43129.793 | type:internal len:402 gc:universal TRINITY\_DN1339\_c0\_g2\_i1:1203-1(-) |
| 74 | 214 | TRINITY\_DN1676\_c0\_g1\_i12.p1 | 58.41 | 1 | 1 | 7.65e2 | 1 | 1 | 2 |  | 180975.5156 | type:complete len:1631 gc:universal TRINITY\_DN1676\_c0\_g1\_i12:206-5098(+) |
| 74 | 215 | TRINITY\_DN1676\_c0\_g1\_i6.p1 | 58.41 | 1 | 1 | 7.65e2 | 1 | 1 | 2 |  | 180975.5156 | type:complete len:1631 gc:universal TRINITY\_DN1676\_c0\_g1\_i6:206-5098(+) |
| 75 | 216 | TRINITY\_DN8903\_c0\_g1\_i1.p1 | 58.91 | 2 | 2 | 3.01e3 | 1 | 1 | 2 |  | 50029.5586 | type:5prime\_partial len:447 gc:universal TRINITY\_DN8903\_c0\_g1\_i1:1546-206(-) |
| 76 | 217 | TRINITY\_DN2472\_c0\_g1\_i1.p1 | 58.14 | 5 | 5 | 1.51e3 | 1 | 1 | 1 | Acetylation (Protein N-term) | 37676.8203 | type:complete len:344 gc:universal TRINITY\_DN2472\_c0\_g1\_i1:311-1342(+) |
| 77 | 218 | TRINITY\_DN123121\_c0\_g1\_i1.p1 | 58.36 | 12 | 12 | 1.52e2 | 1 | 1 | 1 |  | 14215.7539 | type:internal len:127 gc:universal TRINITY\_DN123121\_c0\_g1\_i1:3-380(+) |
| 78 | 219 | TRINITY\_DN17095\_c0\_g1\_i1.p1 | 51.21 | 2 | 2 | 2.03e2 | 1 | 1 | 1 |  | 68063.4688 | type:internal len:607 gc:universal TRINITY\_DN17095\_c0\_g1\_i1:1820-3(-) |
| 79 | 220 | TRINITY\_DN468\_c1\_g1\_i1.p1 | 52.68 | 10 | 10 | 1.18e2 | 1 | 1 | 1 |  | 21670.5645 | type:complete len:191 gc:universal TRINITY\_DN468\_c1\_g1\_i1:676-104(-) |
| 80 | 221 | TRINITY\_DN10724\_c0\_g1\_i1.p2 | 56.43 | 10 | 10 | 0 | 1 | 1 | 1 |  | 16065.7344 | type:3prime\_partial len:148 gc:universal TRINITY\_DN10724\_c0\_g1\_i1:442-2(-) |
| 80 | 222 | TRINITY\_DN10724\_c0\_g1\_i5.p1 | 56.43 | 6 | 6 | 0 | 1 | 1 | 1 |  | 28399.5234 | type:internal len:258 gc:universal TRINITY\_DN10724\_c0\_g1\_i5:773-3(-) |
| 80 | 223 | TRINITY\_DN10724\_c0\_g1\_i4.p1 | 56.43 | 6 | 6 | 0 | 1 | 1 | 1 |  | 28383.5254 | type:internal len:258 gc:universal TRINITY\_DN10724\_c0\_g1\_i4:773-3(-) |
| 80 | 224 | TRINITY\_DN10724\_c0\_g1\_i3.p1 | 56.43 | 9 | 9 | 0 | 1 | 1 | 1 |  | 19100.1836 | type:3prime\_partial len:174 gc:universal TRINITY\_DN10724\_c0\_g1\_i3:521-3(-) |
| 81 | 225 | TRINITY\_DN186979\_c0\_g1\_i1.p1 | 57.75 | 3 | 3 | 3.22e2 | 1 | 1 | 2 |  | 38817.1016 | type:internal len:357 gc:universal TRINITY\_DN186979\_c0\_g1\_i1:1-1068(+) |
| 82 | 226 | TRINITY\_DN1797\_c0\_g1\_i2.p1 | 57.34 | 1 | 1 | 0 | 1 | 1 | 1 |  | 109594.9688 | type:3prime\_partial len:998 gc:universal TRINITY\_DN1797\_c0\_g1\_i2:2993-3(-) |
| 82 | 227 | TRINITY\_DN1797\_c0\_g1\_i1.p1 | 57.34 | 1 | 1 | 0 | 1 | 1 | 1 |  | 110096.6328 | type:3prime\_partial len:1004 gc:universal TRINITY\_DN1797\_c0\_g1\_i1:3011-3(-) |
| 82 | 228 | TRINITY\_DN1797\_c0\_g1\_i3.p1 | 57.34 | 1 | 1 | 0 | 1 | 1 | 1 |  | 109462.9766 | type:3prime\_partial len:998 gc:universal TRINITY\_DN1797\_c0\_g1\_i3:2993-3(-) |
| 83 | 229 | TRINITY\_DN79126\_c0\_g1\_i1.p1 | 58.62 | 10 | 10 | 1.97e3 | 1 | 1 | 1 |  | 13015.4727 | type:complete len:115 gc:universal TRINITY\_DN79126\_c0\_g1\_i1:397-53(-) |
| 84 | 230 | TRINITY\_DN691\_c0\_g1\_i2.p1 | 58.26 | 1 | 1 | 5.76e2 | 1 | 1 | 1 | Carbamidomethylation | 164625.2188 | type:complete len:1673 gc:universal TRINITY\_DN691\_c0\_g1\_i2:5503-485(-) |
| 84 | 231 | TRINITY\_DN691\_c0\_g1\_i1.p1 | 58.26 | 1 | 1 | 5.76e2 | 1 | 1 | 1 | Carbamidomethylation | 169920.1562 | type:complete len:1726 gc:universal TRINITY\_DN691\_c0\_g1\_i1:5662-485(-) |
| 85 | 232 | TRINITY\_DN6044\_c0\_g2\_i1.p1 | 55.95 | 2 | 2 | 0 | 1 | 1 | 1 |  | 74440.7656 | type:5prime\_partial len:661 gc:universal TRINITY\_DN6044\_c0\_g2\_i1:2126-144(-) |
| 86 | 233 | TRINITY\_DN10563\_c0\_g1\_i1.p1 | 58.63 | 4 | 4 | 9.56e2 | 1 | 1 | 1 |  | 33159.7109 | type:internal len:307 gc:universal TRINITY\_DN10563\_c0\_g1\_i1:1-918(+) |
| 87 | 234 | TRINITY\_DN95387\_c0\_g1\_i1.p1 | 58.47 | 7 | 7 | 2.41e3 | 1 | 1 | 1 | Acetylation (Protein N-term) | 19610.8633 | type:complete len:169 gc:universal TRINITY\_DN95387\_c0\_g1\_i1:96-602(+) |
| 88 | 235 | TRINITY\_DN194146\_c0\_g1\_i1.p1 | 49.68 | 11 | 11 | 0 | 1 | 1 | 1 |  | 21439.9336 | type:complete len:190 gc:universal TRINITY\_DN194146\_c0\_g1\_i1:165-734(+) |
| 89 | 236 | TRINITY\_DN6397\_c0\_g2\_i3.p1 | 58.76 | 8 | 8 | 2.87e3 | 1 | 1 | 3 | Acetylation (Protein N-term) | 14030.8945 | type:internal len:130 gc:universal TRINITY\_DN6397\_c0\_g2\_i3:387-1(-) |
| 89 | 237 | TRINITY\_DN6397\_c0\_g2\_i4.p1 | 58.76 | 9 | 9 | 2.87e3 | 1 | 1 | 3 | Acetylation (Protein N-term) | 12470.125 | type:internal len:115 gc:universal TRINITY\_DN6397\_c0\_g2\_i4:343-2(-) |
| 90 | 238 | TRINITY\_DN16548\_c0\_g1\_i4.p1 | 58.71 | 12 | 12 | 4.18e3 | 1 | 1 | 3 |  | 18505.2012 | type:internal len:171 gc:universal TRINITY\_DN16548\_c0\_g1\_i4:2-511(+) |
| 90 | 239 | TRINITY\_DN16548\_c0\_g1\_i3.p1 | 58.71 | 12 | 12 | 4.18e3 | 1 | 1 | 3 |  | 18505.2012 | type:internal len:171 gc:universal TRINITY\_DN16548\_c0\_g1\_i3:2-511(+) |
| 90 | 240 | TRINITY\_DN16548\_c0\_g1\_i1.p1 | 58.71 | 17 | 17 | 4.18e3 | 1 | 1 | 3 |  | 12774.7002 | type:internal len:119 gc:universal TRINITY\_DN16548\_c0\_g1\_i1:1-354(+) |
| 91 | 241 | TRINITY\_DN571\_c0\_g1\_i2.p1 | 55.96 | 8 | 8 | 2.46e2 | 1 | 1 | 2 | Oxidation (M) | 21030.8027 | type:complete len:186 gc:universal TRINITY\_DN571\_c0\_g1\_i2:192-749(+) |
| 91 | 242 | TRINITY\_DN571\_c0\_g1\_i1.p1 | 55.96 | 8 | 8 | 2.46e2 | 1 | 1 | 2 | Oxidation (M) | 20785.6484 | type:complete len:186 gc:universal TRINITY\_DN571\_c0\_g1\_i1:192-749(+) |
| 92 | 243 | TRINITY\_DN902\_c1\_g1\_i1.p1 | 58.58 | 7 | 7 | 1.7e3 | 1 | 1 | 1 | Carbamidomethylation | 16370.7676 | type:3prime\_partial len:153 gc:universal TRINITY\_DN902\_c1\_g1\_i1:146-601(+) |
| 92 | 244 | TRINITY\_DN902\_c1\_g1\_i2.p1 | 58.58 | 7 | 7 | 1.7e3 | 1 | 1 | 1 | Carbamidomethylation | 16400.9395 | type:3prime\_partial len:153 gc:universal TRINITY\_DN902\_c1\_g1\_i2:90-545(+) |
| 93 | 245 | TRINITY\_DN2885\_c0\_g1\_i2.p1 | 58.63 | 11 | 11 | 3.84e3 | 1 | 1 | 2 |  | 12758.7783 | type:complete len:114 gc:universal TRINITY\_DN2885\_c0\_g1\_i2:31-372(+) |
| 93 | 246 | TRINITY\_DN2885\_c0\_g1\_i5.p1 | 58.63 | 11 | 11 | 3.84e3 | 1 | 1 | 2 |  | 12383.5439 | type:3prime\_partial len:112 gc:universal TRINITY\_DN2885\_c0\_g1\_i5:31-363(+) |
| 93 | 247 | TRINITY\_DN2885\_c0\_g1\_i4.p1 | 58.63 | 11 | 11 | 3.84e3 | 1 | 1 | 2 |  | 12245.125 | type:5prime\_partial len:109 gc:universal TRINITY\_DN2885\_c0\_g1\_i4:1-327(+) |
| 94 | 248 | TRINITY\_DN141214\_c0\_g1\_i1.p1 | 43.83 | 9 | 9 | 1.88e2 | 1 | 1 | 1 |  | 19633.9922 | type:internal len:179 gc:universal TRINITY\_DN141214\_c0\_g1\_i1:2-535(+) |
| 95 | 249 | TRINITY\_DN6132\_c0\_g1\_i1.p1 | 58.99 | 9 | 9 | 0 | 1 | 1 | 1 |  | 18615.5566 | type:complete len:161 gc:universal TRINITY\_DN6132\_c0\_g1\_i1:95-577(+) |
| 96 | 250 | TRINITY\_DN2487\_c1\_g1\_i3.p1 | 53.64 | 3 | 3 | 1.5e3 | 1 | 1 | 1 |  | 34312.3867 | type:internal len:317 gc:universal TRINITY\_DN2487\_c1\_g1\_i3:1-948(+) |
| 96 | 251 | TRINITY\_DN2487\_c1\_g1\_i4.p1 | 53.64 | 4 | 4 | 1.5e3 | 1 | 1 | 1 |  | 23514.8867 | type:internal len:220 gc:universal TRINITY\_DN2487\_c1\_g1\_i4:1-657(+) |
| 97 | 252 | TRINITY\_DN923\_c0\_g1\_i1.p1 | 57.21 | 2 | 2 | 3e2 | 1 | 1 | 1 |  | 51773.8242 | type:complete len:442 gc:universal TRINITY\_DN923\_c0\_g1\_i1:53-1378(+) |
| 98 | 253 | TRINITY\_DN6294\_c0\_g3\_i2.p1 | 58.63 | 9 | 9 | 3.87e2 | 1 | 1 | 1 |  | 15623.1504 | type:complete len:134 gc:universal TRINITY\_DN6294\_c0\_g3\_i2:817-416(-) |
| 98 | 254 | TRINITY\_DN6294\_c0\_g3\_i1.p1 | 58.63 | 9 | 9 | 3.87e2 | 1 | 1 | 1 |  | 15623.1504 | type:complete len:134 gc:universal TRINITY\_DN6294\_c0\_g3\_i1:706-305(-) |
| 99 | 255 | TRINITY\_DN20278\_c0\_g1\_i1.p2 | 57.15 | 7 | 7 | 0 | 1 | 1 | 2 |  | 16558.8984 | type:internal len:154 gc:universal TRINITY\_DN20278\_c0\_g1\_i1:460-2(-) |
| 99 | 256 | TRINITY\_DN4026\_c0\_g1\_i1.p2 | 57.15 | 4 | 4 | 0 | 1 | 1 | 2 |  | 25854.5605 | type:internal len:237 gc:universal TRINITY\_DN4026\_c0\_g1\_i1:708-1(-) |
| 99 | 257 | TRINITY\_DN30356\_c0\_g1\_i1.p3 | 57.15 | 5 | 5 | 0 | 1 | 1 | 2 |  | 21590.3086 | type:5prime\_partial len:201 gc:universal TRINITY\_DN30356\_c0\_g1\_i1:608-6(-) |
| 99 | 258 | TRINITY\_DN4026\_c0\_g1\_i2.p1 | 57.15 | 4 | 4 | 0 | 1 | 1 | 2 |  | 25840.5332 | type:internal len:237 gc:universal TRINITY\_DN4026\_c0\_g1\_i2:708-1(-) |
| 99 | 259 | TRINITY\_DN156024\_c0\_g1\_i1.p3 | 57.15 | 10 | 10 | 0 | 1 | 1 | 2 |  | 10710.1553 | type:internal len:102 gc:universal TRINITY\_DN156024\_c0\_g1\_i1:303-1(-) |
| 100 | 260 | TRINITY\_DN902\_c1\_g2\_i3.p1 | 59.02 | 6 | 6 | 3.39e3 | 1 | 1 | 2 | Carbamidomethylation | 19070.0254 | type:internal len:178 gc:universal TRINITY\_DN902\_c1\_g2\_i3:2-532(+) |
| 100 | 261 | TRINITY\_DN902\_c1\_g2\_i1.p1 | 59.02 | 6 | 6 | 3.39e3 | 1 | 1 | 2 | Carbamidomethylation | 18964.9824 | type:internal len:178 gc:universal TRINITY\_DN902\_c1\_g2\_i1:2-532(+) |
| 100 | 262 | TRINITY\_DN902\_c1\_g2\_i4.p1 | 59.02 | 6 | 6 | 3.39e3 | 1 | 1 | 2 | Carbamidomethylation | 19994.1074 | type:internal len:182 gc:universal TRINITY\_DN902\_c1\_g2\_i4:2-544(+) |
| 101 | 263 | TRINITY\_DN5188\_c0\_g1\_i8.p1 | 56.74 | 2 | 2 | 0 | 1 | 1 | 1 |  | 60066.1602 | type:complete len:520 gc:universal TRINITY\_DN5188\_c0\_g1\_i8:154-1713(+) |
| 101 | 264 | TRINITY\_DN5188\_c0\_g1\_i4.p1 | 56.74 | 2 | 2 | 0 | 1 | 1 | 1 |  | 60066.1602 | type:complete len:520 gc:universal TRINITY\_DN5188\_c0\_g1\_i4:154-1713(+) |
| 101 | 265 | TRINITY\_DN5188\_c0\_g1\_i2.p1 | 56.74 | 2 | 2 | 0 | 1 | 1 | 1 |  | 60066.1602 | type:complete len:520 gc:universal TRINITY\_DN5188\_c0\_g1\_i2:198-1757(+) |
| 101 | 266 | TRINITY\_DN5188\_c0\_g1\_i5.p1 | 56.74 | 2 | 2 | 0 | 1 | 1 | 1 |  | 60066.1602 | type:complete len:520 gc:universal TRINITY\_DN5188\_c0\_g1\_i5:198-1757(+) |
| 102 | 267 | TRINITY\_DN250\_c0\_g1\_i6.p1 | 50.54 | 4 | 4 | 0 | 1 | 1 | 1 |  | 50263.3672 | type:complete len:452 gc:universal TRINITY\_DN250\_c0\_g1\_i6:131-1486(+) |
| 102 | 268 | TRINITY\_DN687\_c0\_g2\_i1.p1 | 50.54 | 4 | 4 | 0 | 1 | 1 | 1 |  | 50237.3242 | type:complete len:453 gc:universal TRINITY\_DN687\_c0\_g2\_i1:90-1448(+) |
| 102 | 269 | TRINITY\_DN96161\_c0\_g1\_i1.p1 | 50.54 | 4 | 4 | 0 | 1 | 1 | 1 |  | 50498.6562 | type:complete len:453 gc:universal TRINITY\_DN96161\_c0\_g1\_i1:1593-235(-) |
| 102 | 270 | TRINITY\_DN687\_c0\_g1\_i1.p1 | 50.54 | 4 | 4 | 0 | 1 | 1 | 1 |  | 50621.6914 | type:complete len:453 gc:universal TRINITY\_DN687\_c0\_g1\_i1:95-1453(+) |
| 102 | 271 | TRINITY\_DN250\_c0\_g1\_i5.p1 | 50.54 | 4 | 4 | 0 | 1 | 1 | 1 |  | 50305.4453 | type:complete len:452 gc:universal TRINITY\_DN250\_c0\_g1\_i5:131-1486(+) |
| 102 | 272 | TRINITY\_DN250\_c0\_g1\_i3.p1 | 50.54 | 4 | 4 | 0 | 1 | 1 | 1 |  | 44456.6445 | type:3prime\_partial len:402 gc:universal TRINITY\_DN250\_c0\_g1\_i3:131-1333(+) |
| 102 | 273 | TRINITY\_DN250\_c0\_g1\_i4.p1 | 50.54 | 4 | 4 | 0 | 1 | 1 | 1 |  | 50305.4453 | type:complete len:452 gc:universal TRINITY\_DN250\_c0\_g1\_i4:131-1486(+) |
| 102 | 274 | TRINITY\_DN250\_c0\_g1\_i1.p1 | 50.54 | 4 | 4 | 0 | 1 | 1 | 1 |  | 44454.6094 | type:3prime\_partial len:402 gc:universal TRINITY\_DN250\_c0\_g1\_i1:131-1333(+) |
| 103 | 275 | TRINITY\_DN1566\_c0\_g1\_i1.p1 | 53.33 | 0 | 0 | 0 | 1 | 1 | 1 |  | 529444 | type:complete len:4626 gc:universal TRINITY\_DN1566\_c0\_g1\_i1:42-13919(+) |
| 104 | 276 | TRINITY\_DN30329\_c0\_g1\_i1.p1 | 58.21 | 8 | 8 | 2.56e3 | 1 | 1 | 3 |  | 23962.9473 | type:5prime\_partial len:213 gc:universal TRINITY\_DN30329\_c0\_g1\_i1:1-639(+) |
| 104 | 277 | TRINITY\_DN30329\_c0\_g2\_i1.p2 | 58.21 | 7 | 7 | 2.56e3 | 1 | 1 | 3 |  | 26093.5586 | type:5prime\_partial len:237 gc:universal TRINITY\_DN30329\_c0\_g2\_i1:1-711(+) |
| 105 | 278 | TRINITY\_DN7504\_c0\_g1\_i1.p1 | 58.86 | 3 | 3 | 0 | 1 | 1 | 1 |  | 46519.1953 | type:complete len:414 gc:universal TRINITY\_DN7504\_c0\_g1\_i1:87-1328(+) |
| 106 | 279 | TRINITY\_DN163801\_c0\_g1\_i1.p1 | 58.62 | 3 | 3 | 1.56e3 | 1 | 1 | 1 | Acetylation (Protein N-term) | 33076.9062 | type:complete len:298 gc:universal TRINITY\_DN163801\_c0\_g1\_i1:55-948(+) |
| 107 | 280 | TRINITY\_DN18025\_c0\_g1\_i1.p1 | 58.68 | 5 | 5 | 0 | 1 | 1 | 1 |  | 25954.6035 | type:5prime\_partial len:225 gc:universal TRINITY\_DN18025\_c0\_g1\_i1:1248-574(-) |
| 108 | 281 | TRINITY\_DN198705\_c0\_g1\_i1.p1 | 51.54 | 4 | 4 | 0 | 1 | 1 | 1 | Acetylation (Protein N-term) | 22187.4199 | type:complete len:190 gc:universal TRINITY\_DN198705\_c0\_g1\_i1:80-649(+) |
| 109 | 282 | TRINITY\_DN199\_c0\_g1\_i1.p1 | 58.15 | 5 | 5 | 0 | 1 | 1 | 1 |  | 25170.3418 | type:complete len:229 gc:universal TRINITY\_DN199\_c0\_g1\_i1:207-893(+) |
| 110 | 283 | TRINITY\_DN77\_c1\_g1\_i1.p1 | 58.61 | 1 | 1 | 1.15e3 | 1 | 1 | 1 |  | 198350.2344 | type:3prime\_partial len:1965 gc:universal TRINITY\_DN77\_c1\_g1\_i1:2-5893(+) |
| 111 | 284 | TRINITY\_DN1222\_c0\_g1\_i2.p1 | 33.83 | 7 | 7 | 8.43e2 | 1 | 1 | 1 |  | 24439.5312 | type:5prime\_partial len:213 gc:universal TRINITY\_DN1222\_c0\_g1\_i2:2-640(+) |
| 111 | 285 | TRINITY\_DN1222\_c0\_g1\_i1.p1 | 33.83 | 7 | 7 | 8.43e2 | 1 | 1 | 1 |  | 21958.5781 | type:complete len:191 gc:universal TRINITY\_DN1222\_c0\_g1\_i1:190-762(+) |
| 112 | 286 | TRINITY\_DN3078\_c0\_g1\_i1.p1 | 56.59 | 1 | 1 | 0 | 1 | 1 | 1 | Carbamidomethylation | 127689.1797 | type:complete len:1109 gc:universal TRINITY\_DN3078\_c0\_g1\_i1:3628-302(-) |
| 113 | 287 | TRINITY\_DN6388\_c0\_g1\_i2.p2 | 57.65 | 9 | 9 | 4.72e2 | 1 | 1 | 1 |  | 17280.2852 | type:5prime\_partial len:143 gc:universal TRINITY\_DN6388\_c0\_g1\_i2:1-429(+) |
| 113 | 288 | TRINITY\_DN6388\_c0\_g1\_i1.p2 | 57.65 | 9 | 9 | 4.72e2 | 1 | 1 | 1 |  | 17821.9297 | type:5prime\_partial len:148 gc:universal TRINITY\_DN6388\_c0\_g1\_i1:1-444(+) |
| 113 | 289 | TRINITY\_DN4954\_c0\_g1\_i2.p2 | 57.65 | 7 | 7 | 4.72e2 | 1 | 1 | 1 |  | 23731.8477 | type:5prime\_partial len:200 gc:universal TRINITY\_DN4954\_c0\_g1\_i2:1-600(+) |
| 114 | 290 | TRINITY\_DN95248\_c0\_g1\_i1.p1 | 58.62 | 10 | 10 | 0 | 1 | 1 | 1 |  | 14722.6113 | type:complete len:124 gc:universal TRINITY\_DN95248\_c0\_g1\_i1:197-568(+) |
| 115 | 291 | TRINITY\_DN4762\_c0\_g1\_i1.p1 | 30.36 | 1 | 1 | 4.62e3 | 1 | 1 | 1 | Carbamidomethylation | 61605.0664 | type:3prime\_partial len:542 gc:universal TRINITY\_DN4762\_c0\_g1\_i1:171-1793(+) |
| 115 | 292 | TRINITY\_DN4762\_c0\_g1\_i2.p1 | 30.36 | 1 | 1 | 4.62e3 | 1 | 1 | 1 | Carbamidomethylation | 69812.3906 | type:complete len:613 gc:universal TRINITY\_DN4762\_c0\_g1\_i2:65-1903(+) |
| 116 | 293 | TRINITY\_DN801\_c0\_g1\_i4.p1 | 51.43 | 9 | 9 | 2.9e3 | 1 | 1 | 1 |  | 20287.4453 | type:5prime\_partial len:182 gc:universal TRINITY\_DN801\_c0\_g1\_i4:1-546(+) |
| 116 | 294 | TRINITY\_DN801\_c0\_g1\_i1.p1 | 51.43 | 9 | 9 | 2.9e3 | 1 | 1 | 1 |  | 19577.4629 | type:5prime\_partial len:177 gc:universal TRINITY\_DN801\_c0\_g1\_i1:3-533(+) |
| 116 | 295 | TRINITY\_DN801\_c0\_g1\_i10.p1 | 51.43 | 11 | 11 | 2.9e3 | 1 | 1 | 1 |  | 16657.5469 | type:internal len:153 gc:universal TRINITY\_DN801\_c0\_g1\_i10:3-458(+) |
| 116 | 296 | TRINITY\_DN801\_c0\_g1\_i9.p2 | 51.43 | 11 | 11 | 2.9e3 | 1 | 1 | 1 |  | 16657.5469 | type:internal len:153 gc:universal TRINITY\_DN801\_c0\_g1\_i9:3-458(+) |
| 116 | 297 | TRINITY\_DN801\_c0\_g1\_i7.p1 | 51.43 | 10 | 10 | 2.9e3 | 1 | 1 | 1 |  | 17665.748 | type:internal len:160 gc:universal TRINITY\_DN801\_c0\_g1\_i7:1-477(+) |
| 116 | 298 | TRINITY\_DN30386\_c0\_g1\_i1.p2 | 51.43 | 10 | 10 | 2.9e3 | 1 | 1 | 1 |  | 18794.9727 | type:internal len:165 gc:universal TRINITY\_DN30386\_c0\_g1\_i1:3-494(+) |
| 117 | 299 | TRINITY\_DN3271\_c0\_g1\_i1.p1 | 58.99 | 3 | 3 | 5.79e2 | 1 | 1 | 1 |  | 45246.3555 | type:complete len:399 gc:universal TRINITY\_DN3271\_c0\_g1\_i1:218-1414(+) |
| 117 | 300 | TRINITY\_DN3271\_c0\_g1\_i2.p1 | 58.99 | 3 | 3 | 5.79e2 | 1 | 1 | 1 |  | 53020.332 | type:5prime\_partial len:468 gc:universal TRINITY\_DN3271\_c0\_g1\_i2:2-1405(+) |
| 118 | 301 | TRINITY\_DN651\_c0\_g1\_i1.p1 | 58.48 | 4 | 4 | 0 | 1 | 1 | 1 |  | 42527.9297 | type:complete len:408 gc:universal TRINITY\_DN651\_c0\_g1\_i1:122-1345(+) |
| 118 | 302 | TRINITY\_DN651\_c0\_g1\_i2.p1 | 58.48 | 4 | 4 | 0 | 1 | 1 | 1 |  | 45414.0391 | type:complete len:431 gc:universal TRINITY\_DN651\_c0\_g1\_i2:122-1414(+) |
| 119 | 303 | TRINITY\_DN33065\_c0\_g2\_i1.p1 | 53.11 | 2 | 2 | 4.97e2 | 1 | 1 | 1 |  | 89122.9766 | type:3prime\_partial len:804 gc:universal TRINITY\_DN33065\_c0\_g2\_i1:2409-1(-) |
| 120 | 304 | TRINITY\_DN1319\_c0\_g1\_i2.p1 | 58.65 | 10 | 10 | 0 | 1 | 1 | 1 | Carbamidomethylation | 15785.3457 | type:complete len:134 gc:universal TRINITY\_DN1319\_c0\_g1\_i2:86-487(+) |
| 120 | 305 | TRINITY\_DN1319\_c0\_g1\_i1.p1 | 58.65 | 10 | 10 | 0 | 1 | 1 | 1 | Carbamidomethylation | 15785.3457 | type:complete len:134 gc:universal TRINITY\_DN1319\_c0\_g1\_i1:362-763(+) |
| 121 | 306 | TRINITY\_DN3180\_c0\_g1\_i1.p1 | 57.48 | 1 | 1 | 6.3e2 | 1 | 1 | 1 |  | 212833.2969 | type:complete len:1889 gc:universal TRINITY\_DN3180\_c0\_g1\_i1:5857-191(-) |
| 122 | 307 | TRINITY\_DN1995\_c0\_g1\_i1.p1 | 56.61 | 2 | 2 | 1.53e3 | 1 | 1 | 1 |  | 59145.6719 | type:internal len:514 gc:universal TRINITY\_DN1995\_c0\_g1\_i1:1539-1(-) |
| 123 | 308 | TRINITY\_DN123\_c0\_g1\_i1.p1 | 35.53 | 1 | 1 | 3.75e2 | 1 | 1 | 1 |  | 136822.0312 | type:5prime\_partial len:1231 gc:universal TRINITY\_DN123\_c0\_g1\_i1:2-3694(+) |
| 124 | 309 | TRINITY\_DN495\_c0\_g1\_i5.p1 | 53.66 | 3 | 3 | 0 | 1 | 1 | 1 | Carbamidomethylation | 45678.0781 | type:3prime\_partial len:407 gc:universal TRINITY\_DN495\_c0\_g1\_i5:152-1369(+) |
| 124 | 310 | TRINITY\_DN495\_c0\_g1\_i6.p1 | 53.66 | 3 | 3 | 0 | 1 | 1 | 1 | Carbamidomethylation | 42984.9375 | type:3prime\_partial len:382 gc:universal TRINITY\_DN495\_c0\_g1\_i6:152-1294(+) |
| 125 | 311 | TRINITY\_DN135129\_c0\_g1\_i1.p1 | 58.52 | 6 | 6 | 5.7e3 | 1 | 1 | 2 |  | 17174.043 | type:internal len:155 gc:universal TRINITY\_DN135129\_c0\_g1\_i1:1-462(+) |
| 126 | 312 | TRINITY\_DN34\_c0\_g1\_i1.p1 | 58.6 | 7 | 7 | 0 | 1 | 1 | 1 | Acetylation (Protein N-term), Oxidation (M) | 16992.0059 | type:complete len:149 gc:universal TRINITY\_DN34\_c0\_g1\_i1:477-31(-) |
| 127 | 313 | TRINITY\_DN56792\_c0\_g1\_i3.p1 | 56.49 | 1 | 1 | 0 | 1 | 1 | 1 |  | 112051.7656 | type:5prime\_partial len:1003 gc:universal TRINITY\_DN56792\_c0\_g1\_i3:3453-445(-) |
| 127 | 314 | TRINITY\_DN56792\_c0\_g1\_i1.p1 | 56.49 | 1 | 1 | 0 | 1 | 1 | 1 |  | 111385.9531 | type:5prime\_partial len:997 gc:universal TRINITY\_DN56792\_c0\_g1\_i1:3435-445(-) |
| 128 | 315 | TRINITY\_DN1143\_c0\_g1\_i1.p1 | 57.83 | 2 | 2 | 0 | 1 | 1 | 1 |  | 68207.8203 | type:complete len:629 gc:universal TRINITY\_DN1143\_c0\_g1\_i1:2539-653(-) |
| 129 | 316 | TRINITY\_DN1339\_c1\_g1\_i1.p2 | 58.54 | 8 | 8 | 4.55e2 | 1 | 1 | 1 |  | 19985.6875 | type:internal len:183 gc:universal TRINITY\_DN1339\_c1\_g1\_i1:546-1(-) |
| 130 | 317 | TRINITY\_DN11643\_c0\_g1\_i1.p1 | 58.71 | 7 | 7 | 8.88e3 | 1 | 1 | 4 |  | 21061.2832 | type:complete len:187 gc:universal TRINITY\_DN11643\_c0\_g1\_i1:1748-1188(-) |
| 131 | 318 | TRINITY\_DN19618\_c0\_g1\_i1.p1 | 58.99 | 5 | 5 | 1.19e4 | 1 | 1 | 5 | Acetylation (Protein N-term) | 23610.1152 | type:internal len:223 gc:universal TRINITY\_DN19618\_c0\_g1\_i1:3-668(+) |
| 132 | 319 | TRINITY\_DN140702\_c0\_g1\_i1.p1 | 59.07 | 1 | 1 | 0 | 1 | 1 | 2 |  | 231919.1562 | type:complete len:2036 gc:universal TRINITY\_DN140702\_c0\_g1\_i1:94-6201(+) |
| 133 | 320 | TRINITY\_DN57\_c0\_g1\_i1.p1 | 50.64 | 2 | 2 | 0 | 1 | 1 | 1 | Oxidation (M) | 81029.6719 | type:complete len:712 gc:universal TRINITY\_DN57\_c0\_g1\_i1:140-2275(+) |
| 134 | 321 | TRINITY\_DN371\_c1\_g1\_i1.p1 | 58.65 | 8 | 8 | 0 | 1 | 1 | 1 |  | 19834.2129 | type:complete len:173 gc:universal TRINITY\_DN371\_c1\_g1\_i1:157-675(+) |
| 135 | 322 | TRINITY\_DN26249\_c0\_g1\_i1.p1 | 57.12 | 3 | 3 | 4.49e2 | 1 | 1 | 2 |  | 54490.1055 | type:5prime\_partial len:477 gc:universal TRINITY\_DN26249\_c0\_g1\_i1:1733-303(-) |
| 136 | 323 | TRINITY\_DN172571\_c0\_g1\_i1.p1 | 58.99 | 12 | 12 | 6.65e2 | 1 | 1 | 1 |  | 11500.2529 | type:complete len:104 gc:universal TRINITY\_DN172571\_c0\_g1\_i1:30-341(+) |
| 137 | 324 | TRINITY\_DN8707\_c0\_g1\_i2.p1 | 58.63 | 2 | 2 | 1.27e3 | 1 | 1 | 1 |  | 61942.9023 | type:complete len:564 gc:universal TRINITY\_DN8707\_c0\_g1\_i2:2751-1060(-) |
| 138 | 325 | TRINITY\_DN2113\_c0\_g1\_i1.p1 | 58.72 | 1 | 1 | 3.8e2 | 1 | 1 | 1 |  | 193799.0156 | type:complete len:1704 gc:universal TRINITY\_DN2113\_c0\_g1\_i1:5257-146(-) |
| 138 | 326 | TRINITY\_DN2113\_c0\_g1\_i2.p1 | 58.72 | 1 | 1 | 3.8e2 | 1 | 1 | 1 |  | 193799.0156 | type:complete len:1704 gc:universal TRINITY\_DN2113\_c0\_g1\_i2:5257-146(-) |
| 139 | 327 | TRINITY\_DN32763\_c0\_g1\_i1.p1 | 43.99 | 9 | 9 | 5.98e2 | 1 | 1 | 1 |  | 11346.8916 | type:internal len:104 gc:universal TRINITY\_DN32763\_c0\_g1\_i1:2-310(+) |
| 139 | 328 | TRINITY\_DN32763\_c0\_g1\_i2.p2 | 43.99 | 4 | 4 | 5.98e2 | 1 | 1 | 1 |  | 23593.0703 | type:internal len:217 gc:universal TRINITY\_DN32763\_c0\_g1\_i2:3-650(+) |
| 140 | 329 | TRINITY\_DN1130\_c0\_g1\_i1.p1 | 51.25 | 5 | 5 | 1.19e3 | 1 | 1 | 1 |  | 34883.0859 | type:complete len:317 gc:universal TRINITY\_DN1130\_c0\_g1\_i1:1199-249(-) |
| 141 | 330 | TRINITY\_DN116\_c0\_g1\_i1.p1 | 55.98 | 5 | 5 | 4.41e2 | 1 | 1 | 1 | Carbamidomethylation | 34095.6328 | type:complete len:297 gc:universal TRINITY\_DN116\_c0\_g1\_i1:72-962(+) |
| 142 | 331 | TRINITY\_DN4525\_c0\_g2\_i1.p1 | 58.24 | 11 | 11 | 0 | 1 | 1 | 1 |  | 12319.002 | type:internal len:111 gc:universal TRINITY\_DN4525\_c0\_g2\_i1:1-330(+) |
| 142 | 332 | TRINITY\_DN5897\_c0\_g1\_i3.p1 | 58.24 | 11 | 11 | 0 | 1 | 1 | 1 |  | 11673.5254 | type:internal len:111 gc:universal TRINITY\_DN5897\_c0\_g1\_i3:332-3(-) |
| 143 | 333 | TRINITY\_DN97809\_c0\_g1\_i1.p1 | 53.81 | 7 | 7 | 1.99e3 | 1 | 1 | 1 |  | 20461.1211 | type:internal len:184 gc:universal TRINITY\_DN97809\_c0\_g1\_i1:2-550(+) |
| 144 | 334 | TRINITY\_DN41662\_c0\_g1\_i2.p1 | 58.48 | 5 | 5 | 8.19e2 | 1 | 1 | 1 |  | 32617.3926 | type:complete len:289 gc:universal TRINITY\_DN41662\_c0\_g1\_i2:168-1034(+) |
| 144 | 335 | TRINITY\_DN909\_c0\_g1\_i1.p1 | 58.48 | 4 | 4 | 8.19e2 | 1 | 1 | 1 |  | 35211.4883 | type:5prime\_partial len:313 gc:universal TRINITY\_DN909\_c0\_g1\_i1:1-939(+) |
| 144 | 336 | TRINITY\_DN5818\_c1\_g1\_i1.p1 | 58.48 | 5 | 5 | 8.19e2 | 1 | 1 | 1 |  | 31405.1387 | type:complete len:282 gc:universal TRINITY\_DN5818\_c1\_g1\_i1:49-894(+) |
| 144 | 337 | TRINITY\_DN5818\_c1\_g1\_i2.p1 | 58.48 | 5 | 5 | 8.19e2 | 1 | 1 | 1 |  | 32704.6465 | type:complete len:293 gc:universal TRINITY\_DN5818\_c1\_g1\_i2:178-1056(+) |
| 145 | 338 | TRINITY\_DN413\_c0\_g1\_i4.p1 | 58.62 | 2 | 2 | 2.81e2 | 1 | 1 | 1 |  | 52354.1758 | type:3prime\_partial len:463 gc:universal TRINITY\_DN413\_c0\_g1\_i4:1388-3(-) |
| 145 | 339 | TRINITY\_DN413\_c0\_g1\_i1.p1 | 58.62 | 3 | 3 | 2.81e2 | 1 | 1 | 1 |  | 40351.7656 | type:complete len:355 gc:universal TRINITY\_DN413\_c0\_g1\_i1:1352-288(-) |
| 145 | 340 | TRINITY\_DN413\_c0\_g1\_i2.p1 | 58.62 | 8 | 8 | 2.81e2 | 1 | 1 | 1 |  | 15739.6523 | type:3prime\_partial len:140 gc:universal TRINITY\_DN413\_c0\_g1\_i2:419-3(-) |
| 145 | 341 | TRINITY\_DN413\_c0\_g1\_i3.p1 | 58.62 | 3 | 3 | 2.81e2 | 1 | 1 | 1 |  | 39501.6133 | type:complete len:350 gc:universal TRINITY\_DN413\_c0\_g1\_i3:1286-237(-) |
| 146 | 342 | TRINITY\_DN2963\_c0\_g1\_i1.p1 | 58.74 | 10 | 10 | 6.03e2 | 1 | 1 | 1 | Acetylation (Protein N-term) | 13082.1787 | type:complete len:116 gc:universal TRINITY\_DN2963\_c0\_g1\_i1:1072-725(-) |
| 147 | 343 | TRINITY\_DN6\_c0\_g1\_i1.p1 | 58.64 | 7 | 7 | 6.68e2 | 1 | 1 | 1 |  | 23154.6738 | type:complete len:196 gc:universal TRINITY\_DN6\_c0\_g1\_i1:843-256(-) |
| 148 | 344 | TRINITY\_DN33328\_c0\_g1\_i1.p1 | 55.47 | 1 | 1 | 2.52e2 | 1 | 1 | 1 |  | 156121.4062 | type:3prime\_partial len:1422 gc:universal TRINITY\_DN33328\_c0\_g1\_i1:4264-2(-) |
| 149 | 345 | TRINITY\_DN201860\_c0\_g1\_i1.p1 | 58.24 | 6 | 6 | 0 | 1 | 1 | 1 | Carbamidomethylation | 24265.8906 | type:internal len:221 gc:universal TRINITY\_DN201860\_c0\_g1\_i1:1-660(+) |
| 150 | 346 | TRINITY\_DN76040\_c0\_g1\_i1.p1 | 56.75 | 3 | 3 | 3.21e2 | 1 | 1 | 2 |  | 43150.668 | type:5prime\_partial len:383 gc:universal TRINITY\_DN76040\_c0\_g1\_i1:1-1149(+) |
| 151 | 347 | TRINITY\_DN10706\_c0\_g1\_i1.p1 | 56.02 | 5 | 5 | 5.01e2 | 1 | 1 | 1 |  | 24372.6621 | type:complete len:213 gc:universal TRINITY\_DN10706\_c0\_g1\_i1:122-760(+) |
| 152 | 348 | TRINITY\_DN129732\_c0\_g1\_i1.p2 | 57.81 | 14 | 14 | 0 | 1 | 1 | 2 |  | 11863.373 | type:internal len:112 gc:universal TRINITY\_DN129732\_c0\_g1\_i1:2-334(+) |
| 153 | 349 | TRINITY\_DN464\_c0\_g1\_i4.p1 | 58.36 | 2 | 2 | 0 | 1 | 1 | 1 |  | 82057.25 | type:complete len:717 gc:universal TRINITY\_DN464\_c0\_g1\_i4:2167-17(-) |
| 154 | 350 | TRINITY\_DN1091\_c0\_g1\_i1.p1 | 58.66 | 8 | 8 | 5.12e2 | 1 | 1 | 2 | Carbamidomethylation | 24868.0293 | type:complete len:212 gc:universal TRINITY\_DN1091\_c0\_g1\_i1:200-835(+) |
| 155 | 351 | TRINITY\_DN1653\_c0\_g1\_i1.p1 | 56.67 | 1 | 1 | 0 | 1 | 1 | 1 |  | 125115.2109 | type:complete len:1122 gc:universal TRINITY\_DN1653\_c0\_g1\_i1:180-3545(+) |
| 155 | 352 | TRINITY\_DN1653\_c0\_g1\_i3.p1 | 56.67 | 1 | 1 | 0 | 1 | 1 | 1 |  | 114336.4531 | type:complete len:1023 gc:universal TRINITY\_DN1653\_c0\_g1\_i3:180-3248(+) |
| 156 | 353 | TRINITY\_DN2230\_c0\_g1\_i1.p1 | 58.51 | 4 | 4 | 4.33e2 | 1 | 1 | 1 |  | 28784.541 | type:complete len:249 gc:universal TRINITY\_DN2230\_c0\_g1\_i1:796-50(-) |
| 157 | 354 | TRINITY\_DN109115\_c0\_g1\_i1.p1 | 58.24 | 9 | 9 | 0 | 1 | 1 | 1 |  | 13721.9717 | type:internal len:125 gc:universal TRINITY\_DN109115\_c0\_g1\_i1:3-374(+) |
| 157 | 355 | TRINITY\_DN5263\_c0\_g1\_i1.p1 | 58.24 | 5 | 5 | 0 | 1 | 1 | 1 |  | 26869.2422 | type:complete len:243 gc:universal TRINITY\_DN5263\_c0\_g1\_i1:827-99(-) |
| 158 | 356 | TRINITY\_DN247\_c0\_g1\_i1.p1 | 53.53 | 3 | 3 | 1.03e3 | 1 | 1 | 1 |  | 42079.6172 | type:complete len:373 gc:universal TRINITY\_DN247\_c0\_g1\_i1:38-1156(+) |
| 159 | 357 | TRINITY\_DN751\_c0\_g1\_i1.p1 | 58.63 | 5 | 5 | 1.32e3 | 1 | 1 | 1 |  | 25720.373 | type:complete len:227 gc:universal TRINITY\_DN751\_c0\_g1\_i1:731-51(-) |
| 159 | 358 | TRINITY\_DN7214\_c0\_g1\_i1.p1 | 58.63 | 5 | 5 | 1.32e3 | 1 | 1 | 1 |  | 25800.4062 | type:complete len:228 gc:universal TRINITY\_DN7214\_c0\_g1\_i1:39-722(+) |
| 160 | 359 | TRINITY\_DN3153\_c0\_g1\_i3.p1 | 58.69 | 9 | 9 | 1.88e3 | 1 | 1 | 2 | Oxidation (M) | 22681.9688 | type:complete len:196 gc:universal TRINITY\_DN3153\_c0\_g1\_i3:154-741(+) |
| 160 | 360 | TRINITY\_DN3153\_c0\_g1\_i2.p1 | 58.69 | 9 | 9 | 1.88e3 | 1 | 1 | 2 | Oxidation (M) | 22681.9688 | type:complete len:196 gc:universal TRINITY\_DN3153\_c0\_g1\_i2:154-741(+) |
| 161 | 361 | TRINITY\_DN1505\_c0\_g1\_i1.p1 | 58.95 | 8 | 8 | 1.08e4 | 1 | 1 | 5 | Acetylation (Protein N-term) | 17930.752 | type:complete len:154 gc:universal TRINITY\_DN1505\_c0\_g1\_i1:98-559(+) |
| 162 | 362 | TRINITY\_DN1189\_c0\_g1\_i1.p1 | 47.69 | 2 | 2 | 3.2e2 | 1 | 1 | 1 |  | 50239.8086 | type:5prime\_partial len:449 gc:universal TRINITY\_DN1189\_c0\_g1\_i1:1554-208(-) |
| 163 | 363 | TRINITY\_DN925\_c0\_g1\_i1.p1 | 58.68 | 5 | 5 | 3.01e3 | 1 | 1 | 1 |  | 28336.7676 | type:complete len:261 gc:universal TRINITY\_DN925\_c0\_g1\_i1:826-44(-) |
| 164 | 364 | TRINITY\_DN146208\_c0\_g1\_i1.p1 | 58.69 | 5 | 5 | 0 | 1 | 1 | 1 |  | 32340.8027 | type:5prime\_partial len:284 gc:universal TRINITY\_DN146208\_c0\_g1\_i1:876-25(-) |
| 165 | 365 | TRINITY\_DN165536\_c0\_g1\_i1.p1 | 58.89 | 6 | 6 | 1.04e3 | 1 | 1 | 1 |  | 28339.4531 | type:complete len:249 gc:universal TRINITY\_DN165536\_c0\_g1\_i1:851-105(-) |
| 166 | 366 | TRINITY\_DN13856\_c0\_g1\_i2.p2 | 58.63 | 7 | 7 | 9.46e2 | 1 | 1 | 1 |  | 31731.2012 | type:5prime\_partial len:292 gc:universal TRINITY\_DN13856\_c0\_g1\_i2:3-878(+) |
| 166 | 367 | TRINITY\_DN13856\_c0\_g1\_i1.p2 | 58.63 | 7 | 7 | 9.46e2 | 1 | 1 | 1 |  | 31646.0527 | type:5prime\_partial len:292 gc:universal TRINITY\_DN13856\_c0\_g1\_i1:3-878(+) |
| 167 | 368 | TRINITY\_DN21847\_c1\_g1\_i1.p1 | 58.81 | 8 | 8 | 8.65e2 | 1 | 1 | 1 | Acetylation (Protein N-term) | 14363.4707 | type:internal len:133 gc:universal TRINITY\_DN21847\_c1\_g1\_i1:397-2(-) |
| 167 | 369 | TRINITY\_DN8165\_c0\_g2\_i2.p2 | 58.81 | 5 | 5 | 8.65e2 | 1 | 1 | 1 | Acetylation (Protein N-term) | 21784.791 | type:internal len:204 gc:universal TRINITY\_DN8165\_c0\_g2\_i2:3-611(+) |
| 167 | 370 | TRINITY\_DN8165\_c0\_g2\_i1.p2 | 58.81 | 10 | 10 | 8.65e2 | 1 | 1 | 1 | Acetylation (Protein N-term) | 10817.4883 | type:internal len:102 gc:universal TRINITY\_DN8165\_c0\_g2\_i1:3-305(+) |
| 168 | 371 | TRINITY\_DN166344\_c0\_g1\_i1.p1 | 58.58 | 3 | 3 | 0 | 1 | 1 | 1 | Carbamidomethylation | 40794.2773 | type:complete len:360 gc:universal TRINITY\_DN166344\_c0\_g1\_i1:93-1172(+) |
| 169 | 372 | TRINITY\_DN484\_c0\_g1\_i1.p1 | 58.17 | 3 | 3 | 0 | 1 | 1 | 1 |  | 59940.2617 | type:complete len:554 gc:universal TRINITY\_DN484\_c0\_g1\_i1:164-1825(+) |
| 170 | 373 | TRINITY\_DN82805\_c0\_g1\_i1.p1 | 55.4 | 3 | 3 | 0 | 1 | 1 | 1 |  | 43289.4141 | type:internal len:381 gc:universal TRINITY\_DN82805\_c0\_g1\_i1:2-1141(+) |
| 171 | 374 | TRINITY\_DN131028\_c0\_g1\_i1.p2 | 51.46 | 11 | 11 | 5.05e2 | 1 | 1 | 1 |  | 11838.5801 | type:5prime\_partial len:104 gc:universal TRINITY\_DN131028\_c0\_g1\_i1:2-313(+) |
| 172 | 375 | TRINITY\_DN1272\_c0\_g1\_i1.p1 | 58.62 | 4 | 4 | 0 | 1 | 1 | 2 |  | 30690.1172 | type:complete len:262 gc:universal TRINITY\_DN1272\_c0\_g1\_i1:957-172(-) |
| 173 | 376 | TRINITY\_DN95381\_c0\_g1\_i1.p1 | 53.54 | 6 | 6 | 0 | 1 | 1 | 1 | Oxidation (M) | 22008.7422 | type:complete len:192 gc:universal TRINITY\_DN95381\_c0\_g1\_i1:70-645(+) |
| 174 | 377 | TRINITY\_DN59667\_c0\_g1\_i1.p1 | 50.13 | 4 | 4 | 0 | 1 | 1 | 1 |  | 25518.6816 | type:3prime\_partial len:225 gc:universal TRINITY\_DN59667\_c0\_g1\_i1:673-2(-) |
| 175 | 378 | TRINITY\_DN72959\_c0\_g1\_i1.p1 | 58.45 | 6 | 6 | 1.07e3 | 1 | 1 | 1 |  | 25064.7695 | type:complete len:216 gc:universal TRINITY\_DN72959\_c0\_g1\_i1:54-701(+) |
| 176 | 379 | TRINITY\_DN4815\_c0\_g1\_i2.p1 | 58.63 | 2 | 2 | 8.76e2 | 1 | 1 | 1 | Carbamidomethylation | 70695.4688 | type:complete len:638 gc:universal TRINITY\_DN4815\_c0\_g1\_i2:79-1992(+) |
| 176 | 380 | TRINITY\_DN4815\_c0\_g1\_i1.p1 | 58.63 | 2 | 2 | 8.76e2 | 1 | 1 | 1 | Carbamidomethylation | 66133.5156 | type:complete len:595 gc:universal TRINITY\_DN4815\_c0\_g1\_i1:79-1863(+) |
| 177 | 381 | TRINITY\_DN22594\_c0\_g1\_i1.p2 | 58.98 | 4 | 4 | 3.91e3 | 1 | 1 | 1 |  | 29863.3594 | type:internal len:268 gc:universal TRINITY\_DN22594\_c0\_g1\_i1:801-1(-) |
| 178 | 382 | TRINITY\_DN101992\_c0\_g1\_i1.p1 | 57.48 | 7 | 7 | 2.14e2 | 1 | 1 | 1 | Carbamidomethylation | 15607.4678 | type:internal len:139 gc:universal TRINITY\_DN101992\_c0\_g1\_i1:414-1(-) |
| 179 | 383 | TRINITY\_DN15599\_c0\_g1\_i2.p1 | 57.88 | 5 | 5 | 1.93e4 | 1 | 1 | 4 |  | 21967.3105 | type:internal len:206 gc:universal TRINITY\_DN15599\_c0\_g1\_i2:1-615(+) |
| 179 | 384 | TRINITY\_DN15599\_c0\_g1\_i1.p1 | 57.88 | 5 | 5 | 1.93e4 | 1 | 1 | 4 |  | 21907.2578 | type:internal len:206 gc:universal TRINITY\_DN15599\_c0\_g1\_i1:1-615(+) |
| 180 | 385 | TRINITY\_DN98420\_c0\_g1\_i1.p1 | 58.62 | 3 | 3 | 5.37e3 | 1 | 1 | 2 |  | 31374.8145 | type:5prime\_partial len:286 gc:universal TRINITY\_DN98420\_c0\_g1\_i1:1007-150(-) |
| 181 | 386 | TRINITY\_DN35672\_c0\_g1\_i1.p1 | 29.34 | 2 | 2 | 0 | 1 | 1 | 1 | Carbamidomethylation | 72166.75 | type:3prime\_partial len:668 gc:universal TRINITY\_DN35672\_c0\_g1\_i1:2001-1(-) |
| 181 | 387 | TRINITY\_DN35672\_c0\_g1\_i2.p1 | 29.34 | 2 | 2 | 0 | 1 | 1 | 1 | Carbamidomethylation | 76738.875 | type:3prime\_partial len:710 gc:universal TRINITY\_DN35672\_c0\_g1\_i2:2127-1(-) |
| 182 | 388 | TRINITY\_DN79\_c0\_g1\_i1.p1 | 51.62 | 4 | 4 | 2.65e2 | 1 | 1 | 1 |  | 52590.9805 | type:5prime\_partial len:478 gc:universal TRINITY\_DN79\_c0\_g1\_i1:2132-699(-) |
| 183 | 389 | TRINITY\_DN1559\_c0\_g1\_i1.p1 | 58.63 | 5 | 5 | 1.77e3 | 1 | 1 | 1 |  | 33672.0977 | type:complete len:311 gc:universal TRINITY\_DN1559\_c0\_g1\_i1:221-1153(+) |
| 184 | 390 | TRINITY\_DN10890\_c0\_g1\_i1.p1 | 28.89 | 2 | 2 | 5.16e1 | 1 | 1 | 1 |  | 107419.1719 | type:5prime\_partial len:963 gc:universal TRINITY\_DN10890\_c0\_g1\_i1:3568-680(-) |
| 185 | 391 | TRINITY\_DN1183\_c0\_g1\_i1.p1 | 58.59 | 7 | 7 | 0 | 1 | 1 | 1 |  | 21836.043 | type:complete len:193 gc:universal TRINITY\_DN1183\_c0\_g1\_i1:54-632(+) |
| 186 | 392 | TRINITY\_DN12304\_c0\_g1\_i1.p1 | 54.44 | 5 | 5 | 0 | 1 | 1 | 1 | Acetylation (Protein N-term) | 21915.8809 | type:complete len:195 gc:universal TRINITY\_DN12304\_c0\_g1\_i1:1971-1387(-) |
| 187 | 393 | TRINITY\_DN119684\_c0\_g1\_i1.p1 | 55.29 | 2 | 2 | 4.29e2 | 1 | 1 | 1 |  | 72994.7266 | type:complete len:662 gc:universal TRINITY\_DN119684\_c0\_g1\_i1:2182-197(-) |
| 188 | 394 | TRINITY\_DN118520\_c0\_g1\_i1.p1 | 56.56 | 3 | 3 | 7.73e2 | 1 | 1 | 1 |  | 61587.4766 | type:complete len:581 gc:universal TRINITY\_DN118520\_c0\_g1\_i1:1895-153(-) |
| 189 | 395 | TRINITY\_DN252\_c0\_g1\_i1.p1 | 38.85 | 5 | 5 | 0 | 1 | 1 | 1 |  | 20771.6992 | type:complete len:182 gc:universal TRINITY\_DN252\_c0\_g1\_i1:1409-864(-) |
| 190 | 396 | TRINITY\_DN598\_c1\_g1\_i2.p1 | 58.91 | 1 | 1 | 4.94e3 | 1 | 1 | 2 |  | 81043.625 | type:5prime\_partial len:742 gc:universal TRINITY\_DN598\_c1\_g1\_i2:2-2227(+) |
| 191 | 397 | TRINITY\_DN22219\_c0\_g1\_i1.p1 | 57.16 | 3 | 3 | 1.67e3 | 1 | 1 | 1 |  | 49350.1836 | type:5prime\_partial len:427 gc:universal TRINITY\_DN22219\_c0\_g1\_i1:2-1282(+) |
| 192 | 398 | TRINITY\_DN15248\_c0\_g1\_i1.p2 | 39.56 | 4 | 4 | 9.43e3 | 1 | 1 | 1 |  | 26642.4551 | type:3prime\_partial len:250 gc:universal TRINITY\_DN15248\_c0\_g1\_i1:748-2(-) |
| 193 | 399 | TRINITY\_DN244\_c0\_g1\_i1.p1 | 58.87 | 0 | 0 | 2.46e2 | 1 | 1 | 1 |  | 313594.0938 | type:complete len:3019 gc:universal TRINITY\_DN244\_c0\_g1\_i1:9748-692(-) |
| 194 | 400 | TRINITY\_DN785\_c0\_g1\_i1.p1 | 58.63 | 4 | 4 | 0 | 1 | 1 | 2 | Carbamidomethylation | 33410.082 | type:complete len:289 gc:universal TRINITY\_DN785\_c0\_g1\_i1:1062-196(-) |
| 195 | 401 | TRINITY\_DN1534\_c2\_g1\_i1.p1 | 54.76 | 2 | 2 | 2.79e2 | 1 | 1 | 1 |  | 59603.6055 | type:complete len:547 gc:universal TRINITY\_DN1534\_c2\_g1\_i1:1914-274(-) |
| 196 | 402 | TRINITY\_DN3619\_c0\_g1\_i1.p1 | 58.65 | 1 | 1 | 1.27e3 | 1 | 1 | 1 |  | 186690.2812 | type:5prime\_partial len:1653 gc:universal TRINITY\_DN3619\_c0\_g1\_i1:5108-150(-) |
| 197 | 403 | TRINITY\_DN2028\_c0\_g1\_i1.p1 | 58.6 | 5 | 5 | 1.81e3 | 1 | 1 | 2 |  | 30106.9453 | type:complete len:274 gc:universal TRINITY\_DN2028\_c0\_g1\_i1:1608-787(-) |
| 198 | 404 | TRINITY\_DN757\_c0\_g1\_i1.p1 | 58.92 | 6 | 6 | 0 | 1 | 1 | 1 |  | 23645.248 | type:3prime\_partial len:218 gc:universal TRINITY\_DN757\_c0\_g1\_i1:653-3(-) |
| 199 | 405 | TRINITY\_DN578\_c0\_g1\_i1.p1 | 58.16 | 3 | 3 | 0 | 1 | 1 | 1 |  | 47588.7344 | type:complete len:429 gc:universal TRINITY\_DN578\_c0\_g1\_i1:379-1665(+) |
| 200 | 406 | TRINITY\_DN10907\_c0\_g1\_i1.p1 | 58.19 | 6 | 6 | 0 | 1 | 1 | 1 |  | 23412.5352 | type:complete len:206 gc:universal TRINITY\_DN10907\_c0\_g1\_i1:1010-393(-) |
| 201 | 407 | TRINITY\_DN1280\_c0\_g1\_i1.p1 | 58.6 | 2 | 2 | 5.63e2 | 1 | 1 | 1 |  | 47818.6641 | type:complete len:426 gc:universal TRINITY\_DN1280\_c0\_g1\_i1:195-1472(+) |
| 202 | 408 | TRINITY\_DN598\_c1\_g1\_i1.p1 | 59.04 | 1 | 1 | 4.21e3 | 1 | 1 | 1 |  | 81322.8672 | type:5prime\_partial len:745 gc:universal TRINITY\_DN598\_c1\_g1\_i1:2-2236(+) |
| 203 | 409 | TRINITY\_DN164469\_c0\_g1\_i1.p1 | 58.45 | 8 | 8 | 0 | 1 | 1 | 1 |  | 21062.5312 | type:complete len:184 gc:universal TRINITY\_DN164469\_c0\_g1\_i1:45-596(+) |
| 204 | 410 | TRINITY\_DN614\_c0\_g1\_i1.p1 | 58.26 | 5 | 5 | 0 | 1 | 1 | 1 |  | 20573.5293 | type:complete len:183 gc:universal TRINITY\_DN614\_c0\_g1\_i1:194-742(+) |
| 205 | 411 | TRINITY\_DN1613\_c0\_g2\_i1.p1 | 58.22 | 1 | 1 | 0 | 1 | 1 | 1 |  | 112924.5938 | type:3prime\_partial len:1062 gc:universal TRINITY\_DN1613\_c0\_g2\_i1:70-3252(+) |
| 206 | 412 | TRINITY\_DN932\_c0\_g1\_i1.p1 | 54.96 | 2 | 2 | 3.92e2 | 1 | 1 | 1 | Oxidation (M) | 52305.4961 | type:complete len:475 gc:universal TRINITY\_DN932\_c0\_g1\_i1:1940-516(-) |
| 207 | 413 | TRINITY\_DN76073\_c0\_g1\_i1.p1 | 53.19 | 8 | 8 | 3.18e3 | 1 | 1 | 2 |  | 12905.3965 | type:complete len:107 gc:universal TRINITY\_DN76073\_c0\_g1\_i1:794-1114(+) |
| 208 | 414 | TRINITY\_DN11097\_c0\_g2\_i1.p1 | 56.84 | 6 | 6 | 0 | 1 | 1 | 1 |  | 24192.5488 | type:internal len:222 gc:universal TRINITY\_DN11097\_c0\_g2\_i1:665-3(-) |
| 209 | 415 | TRINITY\_DN113\_c3\_g1\_i1.p1 | 56.78 | 1 | 1 | 0 | 1 | 1 | 2 |  | 125422.4922 | type:3prime\_partial len:1162 gc:universal TRINITY\_DN113\_c3\_g1\_i1:3485-3(-) |
| 210 | 416 | TRINITY\_DN3052\_c0\_g1\_i1.p1 | 56.42 | 4 | 4 | 0 | 1 | 1 | 1 | Acetylation (Protein N-term) | 39261.4453 | type:complete len:362 gc:universal TRINITY\_DN3052\_c0\_g1\_i1:45-1130(+) |
| 211 | 417 | TRINITY\_DN10385\_c0\_g1\_i1.p1 | 50.66 | 1 | 1 | 0 | 1 | 1 | 1 |  | 103963.7188 | type:5prime\_partial len:968 gc:universal TRINITY\_DN10385\_c0\_g1\_i1:2-2905(+) |
| 212 | 418 | TRINITY\_DN1070\_c2\_g1\_i1.p1 | 57.82 | 6 | 6 | 0 | 1 | 1 | 1 |  | 23580.4648 | type:complete len:209 gc:universal TRINITY\_DN1070\_c2\_g1\_i1:1431-805(-) |
| 213 | 419 | TRINITY\_DN5397\_c0\_g1\_i1.p1 | 36.67 | 5 | 5 | 0 | 1 | 1 | 1 |  | 17679.4688 | type:internal len:161 gc:universal TRINITY\_DN5397\_c0\_g1\_i1:2-481(+) |
| 214 | 420 | TRINITY\_DN11662\_c0\_g1\_i1.p1 | 58.64 | 3 | 3 | 2.75e3 | 1 | 1 | 1 | Acetylation (Protein N-term) | 41619.3711 | type:complete len:398 gc:universal TRINITY\_DN11662\_c0\_g1\_i1:75-1268(+) |
| total 420 proteins |  |  |  |  |  |  |  |  |  |  |  |  |
| --- | --- | --- | --- | --- | --- | --- | --- | --- | --- | --- | --- | --- |

  

TRINITY\_DN72959\_c0\_g1\_i1.p1

back to list

  

| Protein Coverage
| Supporting Peptides
| Best Unique PSM
|

Protein Coverage:

Supporting Peptides:

| Peptide | Uniq | -10lgP | Mass | Length | ppm | m/z | z | RT | 1/k0 Start | 1/k0 End | Precursor ID | Area Sample 10 | #Feature | #Feature Sample 10 | Start | End | PTM | AScore | Found By |
| --- | --- | --- | --- | --- | --- | --- | --- | --- | --- | --- | --- | --- | --- | --- | --- | --- | --- | --- | --- |
| R.GAFGKPQGTVAR.V | Y | 58.45 | 1187.6411 | 12 | 1.95 | 594.8259 | 2 | 11.42 | 0.9287 | 0.936 | 21427 | 1.07e3 | 1 | 1 | 117 | 128 |  |  | DB Search |
| total 1 peptides |  |  |  |  |  |  |  |  |  |  |  |  |  |  |  |  |  |  |  |
| --- | --- | --- | --- | --- | --- | --- | --- | --- | --- | --- | --- | --- | --- | --- | --- | --- | --- | --- | --- |

Best Unique PSM (Scan Sample\_9\_A1\_1\_3646.d:21427, m/z=594.8259, z=2, RT=11.42, ppm=-3.27):


TRINITY\_DN1172\_c0\_g1\_i3.p1

back to list

  

| Protein Coverage
| Supporting Peptides
| Best Unique PSM
|

Protein Coverage:

Supporting Peptides:

| Peptide | Uniq | -10lgP | Mass | Length | ppm | m/z | z | RT | 1/k0 Start | 1/k0 End | Precursor ID | Area Sample 10 | #Feature | #Feature Sample 10 | Start | End | PTM | AScore | Found By |
| --- | --- | --- | --- | --- | --- | --- | --- | --- | --- | --- | --- | --- | --- | --- | --- | --- | --- | --- | --- |
| K.VITSDDPAEKEK.K | Y | 59.05 | 1330.6616 | 12 | 3.57 | 666.337 | 2 | 10.72 | 0.9884 | 0.9957 | 17382 | 1.42e4 | 1 | 1 | 123 | 134 |  |  | DB Search |
| total 1 peptides |  |  |  |  |  |  |  |  |  |  |  |  |  |  |  |  |  |  |  |
| --- | --- | --- | --- | --- | --- | --- | --- | --- | --- | --- | --- | --- | --- | --- | --- | --- | --- | --- | --- |

Best Unique PSM (Scan Sample\_9\_A1\_1\_3646.d:17382, m/z=666.337, z=2, RT=10.72, ppm=-1.65):


TRINITY\_DN453\_c0\_g1\_i1.p1

back to list

  

| Protein Coverage
| Supporting Peptides
| Best Unique PSM
|

Protein Coverage:

Supporting Peptides:

| Peptide | Uniq | -10lgP | Mass | Length | ppm | m/z | z | RT | 1/k0 Start | 1/k0 End | Precursor ID | Area Sample 10 | #Feature | #Feature Sample 10 | Start | End | PTM | AScore | Found By |
| --- | --- | --- | --- | --- | --- | --- | --- | --- | --- | --- | --- | --- | --- | --- | --- | --- | --- | --- | --- |
| K.VSILVAC(+57.02)NKQDLTTAK.A | Y | 58.38 | 1759.9502 | 16 | -2.15 | 880.9759 | 2 | 14.70 | 1.1825 | 1.1897 | 40050 | 0 | 0 | 0 | 150 | 165 | Carbamidomethylation |  | DB Search |
| K.SQLEKEINNLRK.T | Y | 29.67 | 1470.8154 | 12 | -9.15 | 491.272 | 3 | 14.25 | 0.7742 | 0.7815 | 37755 | 0 | 0 | 0 | 172 | 183 |  |  | DB Search |
| total 2 peptides |  |  |  |  |  |  |  |  |  |  |  |  |  |  |  |  |  |  |  |
| --- | --- | --- | --- | --- | --- | --- | --- | --- | --- | --- | --- | --- | --- | --- | --- | --- | --- | --- | --- |

Best Unique PSM (Scan Sample\_9\_A1\_1\_3646.d:40050, m/z=880.9759, z=2, RT=14.70, ppm=-7.37):


TRINITY\_DN59667\_c0\_g1\_i1.p1

back to list

  

| Protein Coverage
| Supporting Peptides
| Best Unique PSM
|

Protein Coverage:

Supporting Peptides:

| Peptide | Uniq | -10lgP | Mass | Length | ppm | m/z | z | RT | 1/k0 Start | 1/k0 End | Precursor ID | Area Sample 10 | #Feature | #Feature Sample 10 | Start | End | PTM | AScore | Found By |
| --- | --- | --- | --- | --- | --- | --- | --- | --- | --- | --- | --- | --- | --- | --- | --- | --- | --- | --- | --- |
| R.IEHAHPINR.I | Y | 50.13 | 1085.573 | 9 | 1.73 | 362.8637 | 3 | 9.50 | 0.7343 | 0.7416 | 10857 | 0 | 0 | 0 | 85 | 93 |  |  | DB Search |
| total 1 peptides |  |  |  |  |  |  |  |  |  |  |  |  |  |  |  |  |  |  |  |
| --- | --- | --- | --- | --- | --- | --- | --- | --- | --- | --- | --- | --- | --- | --- | --- | --- | --- | --- | --- |

Best Unique PSM (Scan Sample\_9\_A1\_1\_3646.d:10857, m/z=362.8637, z=3, RT=9.50, ppm=-3.49):


TRINITY\_DN11435\_c0\_g1\_i1.p1

back to list

  

| Protein Coverage
| Supporting Peptides
| Best Unique PSM
|

Protein Coverage:

Supporting Peptides:

| Peptide | Uniq | -10lgP | Mass | Length | ppm | m/z | z | RT | 1/k0 Start | 1/k0 End | Precursor ID | Area Sample 10 | #Feature | #Feature Sample 10 | Start | End | PTM | AScore | Found By |
| --- | --- | --- | --- | --- | --- | --- | --- | --- | --- | --- | --- | --- | --- | --- | --- | --- | --- | --- | --- |
| K.LAYELQANKEQQR.I | Y | 58.91 | 1589.8161 | 13 | -1.93 | 795.9097 | 2 | 12.92 | 1.0516 | 1.0588 | 30474 | 0 | 0 | 0 | 239 | 251 |  |  | DB Search |
| K.VYLAQAEAEKIK.M | Y | 58.63 | 1361.7554 | 12 | 0.94 | 681.8821 | 2 | 14.28 | 1.0339 | 1.0411 | 37834 | 4.47e2 | 1 | 1 | 309 | 320 |  |  | DB Search |
| K.LIEVEEKEIQR.R | Y | 58.54 | 1384.7562 | 11 | -5.77 | 693.3777 | 2 | 13.82 | 1.0154 | 1.0226 | 35443 | 0 | 0 | 0 | 267 | 277 |  |  | DB Search |
| K.DRDQFAQLVR.E | Y | 54.59 | 1246.6418 | 10 | 4.05 | 624.3275 | 2 | 15.19 | 0.942 | 0.9493 | 42601 | 4.61e2 | 1 | 1 | 126 | 135 |  |  | DB Search |
| R.IRNEEIQIEVIERR.K | Y | 52.69 | 1795.9904 | 14 | 3.84 | 599.6699 | 3 | 15.11 | 0.8806 | 0.8878 | 42172 | 6.33e2 | 1 | 1 | 252 | 265 |  |  | DB Search |
| R.REQELQATVK.L | Y | 52.55 | 1200.6462 | 10 | 0.53 | 601.3276 | 2 | 11.18 | 0.9166 | 0.9238 | 20200 | 6.13e2 | 1 | 1 | 278 | 287 |  |  | DB Search |
| K.LIEVEEKEIQRR.E | Y | 43.57 | 1540.8573 | 12 | 1.73 | 514.6246 | 3 | 13.11 | 0.7819 | 0.7892 | 31582 | 0 | 0 | 0 | 267 | 278 |  |  | DB Search |
| total 7 peptides |  |  |  |  |  |  |  |  |  |  |  |  |  |  |  |  |  |  |  |
| --- | --- | --- | --- | --- | --- | --- | --- | --- | --- | --- | --- | --- | --- | --- | --- | --- | --- | --- | --- |

Best Unique PSM (Scan Sample\_9\_A1\_1\_3646.d:30474, m/z=795.9097, z=2, RT=12.92, ppm=-7.15):


TRINITY\_DN1377\_c0\_g1\_i1.p1

back to list

  

| Protein Coverage
| Supporting Peptides
| Best Unique PSM
|

Protein Coverage:

Supporting Peptides:

| Peptide | Uniq | -10lgP | Mass | Length | ppm | m/z | z | RT | 1/k0 Start | 1/k0 End | Precursor ID | Area Sample 10 | #Feature | #Feature Sample 10 | Start | End | PTM | AScore | Found By |
| --- | --- | --- | --- | --- | --- | --- | --- | --- | --- | --- | --- | --- | --- | --- | --- | --- | --- | --- | --- |
| K.RLPDQITTR.S | Y | 58.05 | 1098.6145 | 9 | 0.72 | 550.3121 | 2 | 12.56 | 0.8781 | 0.8854 | 28290 | 1.58e3 | 1 | 1 | 182 | 190 |  |  | DB Search |
| R.SVTTHQGNAIVIK.I | Y | 57.39 | 1366.7568 | 13 | 0.64 | 684.3826 | 2 | 12.52 | 0.9977 | 1.0049 | 28163 | 5.49e2 | 1 | 1 | 191 | 203 |  |  | DB Search |
| total 2 peptides |  |  |  |  |  |  |  |  |  |  |  |  |  |  |  |  |  |  |  |
| --- | --- | --- | --- | --- | --- | --- | --- | --- | --- | --- | --- | --- | --- | --- | --- | --- | --- | --- | --- |

Best Unique PSM (Scan Sample\_9\_A1\_1\_3646.d:28290, m/z=550.3121, z=2, RT=12.56, ppm=-4.5):


TRINITY\_DN101992\_c0\_g1\_i1.p1

back to list

  

| Protein Coverage
| Supporting Peptides
| Best Unique PSM
|

Protein Coverage:

Supporting Peptides:

| Peptide | Uniq | -10lgP | Mass | Length | ppm | m/z | z | RT | 1/k0 Start | 1/k0 End | Precursor ID | Area Sample 10 | #Feature | #Feature Sample 10 | Start | End | PTM | AScore | Found By |
| --- | --- | --- | --- | --- | --- | --- | --- | --- | --- | --- | --- | --- | --- | --- | --- | --- | --- | --- | --- |
| K.AELVFVNHC(+57.02)R.E | Y | 57.48 | 1243.6132 | 10 | 4.12 | 622.8132 | 2 | 14.31 | 0.957 | 0.9642 | 38071 | 2.14e2 | 1 | 1 | 118 | 127 | Carbamidomethylation |  | DB Search |
| total 1 peptides |  |  |  |  |  |  |  |  |  |  |  |  |  |  |  |  |  |  |  |
| --- | --- | --- | --- | --- | --- | --- | --- | --- | --- | --- | --- | --- | --- | --- | --- | --- | --- | --- | --- |

Best Unique PSM (Scan Sample\_9\_A1\_1\_3646.d:38071, m/z=622.8132, z=2, RT=14.31, ppm=-1.1):


TRINITY\_DN5818\_c1\_g1\_i2.p1

back to list

  

| Protein Coverage
| Supporting Peptides
| Best Unique PSM
|

Protein Coverage:

Supporting Peptides:

| Peptide | Uniq | -10lgP | Mass | Length | ppm | m/z | z | RT | 1/k0 Start | 1/k0 End | Precursor ID | Area Sample 10 | #Feature | #Feature Sample 10 | Start | End | PTM | AScore | Found By |
| --- | --- | --- | --- | --- | --- | --- | --- | --- | --- | --- | --- | --- | --- | --- | --- | --- | --- | --- | --- |
| K.LLAQTTLRNELGTK.N | Y | 58.48 | 1556.8886 | 14 | 4.16 | 779.4507 | 2 | 15.18 | 1.109 | 1.1162 | 42504 | 8.19e2 | 1 | 1 | 155 | 168 |  |  | DB Search |
| total 1 peptides |  |  |  |  |  |  |  |  |  |  |  |  |  |  |  |  |  |  |  |
| --- | --- | --- | --- | --- | --- | --- | --- | --- | --- | --- | --- | --- | --- | --- | --- | --- | --- | --- | --- |

Best Unique PSM (Scan Sample\_9\_A1\_1\_3646.d:42504, m/z=779.4507, z=2, RT=15.18, ppm=-1.05):


TRINITY\_DN35672\_c0\_g1\_i2.p1

back to list

  

| Protein Coverage
| Supporting Peptides
| Best Unique PSM
|

Protein Coverage:

Supporting Peptides:

| Peptide | Uniq | -10lgP | Mass | Length | ppm | m/z | z | RT | 1/k0 Start | 1/k0 End | Precursor ID | Area Sample 10 | #Feature | #Feature Sample 10 | Start | End | PTM | AScore | Found By |
| --- | --- | --- | --- | --- | --- | --- | --- | --- | --- | --- | --- | --- | --- | --- | --- | --- | --- | --- | --- |
| R.SPNLC(+57.02)QC(+57.02)NNGR.F | Y | 29.34 | 1318.5507 | 11 | 8.63 | 660.2849 | 2 | 10.99 | 0.9259 | 0.9331 | 19117 | 0 | 0 | 0 | 115 | 125 | Carbamidomethylation, Carbamidomethylation |  | DB Search |
| total 1 peptides |  |  |  |  |  |  |  |  |  |  |  |  |  |  |  |  |  |  |  |
| --- | --- | --- | --- | --- | --- | --- | --- | --- | --- | --- | --- | --- | --- | --- | --- | --- | --- | --- | --- |

Best Unique PSM (Scan Sample\_9\_A1\_1\_3646.d:19117, m/z=660.2849, z=2, RT=10.99, ppm=3.41):


TRINITY\_DN22594\_c0\_g1\_i1.p2

back to list

  

| Protein Coverage
| Supporting Peptides
| Best Unique PSM
|

Protein Coverage:

Supporting Peptides:

| Peptide | Uniq | -10lgP | Mass | Length | ppm | m/z | z | RT | 1/k0 Start | 1/k0 End | Precursor ID | Area Sample 10 | #Feature | #Feature Sample 10 | Start | End | PTM | AScore | Found By |
| --- | --- | --- | --- | --- | --- | --- | --- | --- | --- | --- | --- | --- | --- | --- | --- | --- | --- | --- | --- |
| R.FRQLGLDFGK.L | Y | 58.98 | 1179.64 | 10 | -8.35 | 590.8193 | 2 | 19.81 | 0.9194 | 0.9267 | 67914 | 3.91e3 | 1 | 1 | 40 | 49 |  |  | DB Search |
| total 1 peptides |  |  |  |  |  |  |  |  |  |  |  |  |  |  |  |  |  |  |  |
| --- | --- | --- | --- | --- | --- | --- | --- | --- | --- | --- | --- | --- | --- | --- | --- | --- | --- | --- | --- |

Best Unique PSM (Scan Sample\_9\_A1\_1\_3646.d:67914, m/z=590.8193, z=2, RT=19.81, ppm=-13.57):


TRINITY\_DN982\_c0\_g1\_i2.p1

back to list

  

| Protein Coverage
| Supporting Peptides
| Best Unique PSM
|

Protein Coverage:

Supporting Peptides:

| Peptide | Uniq | -10lgP | Mass | Length | ppm | m/z | z | RT | 1/k0 Start | 1/k0 End | Precursor ID | Area Sample 10 | #Feature | #Feature Sample 10 | Start | End | PTM | AScore | Found By |
| --- | --- | --- | --- | --- | --- | --- | --- | --- | --- | --- | --- | --- | --- | --- | --- | --- | --- | --- | --- |
| K.TPQYDFSVVVTDKGTPPR.Q | Y | 42.6 | 2006.0109 | 18 | 8.03 | 669.6794 | 3 | 16.40 | 0.9392 | 0.9465 | 48729 | 0 | 0 | 0 | 1072 | 1089 |  |  | DB Search |
| R.DRHVLTIQSR.G | N | 33.87 | 1223.6734 | 10 | 6.21 | 612.8446 | 2 | 11.95 | 0.9259 | 0.9331 | 24824 | 0 | 0 | 0 | 1590 | 1599 |  |  | DB Search |
| K.VLEVNDNKPK.F | Y | 29.58 | 1154.6295 | 10 | -0.5 | 578.3187 | 2 | 10.99 | 0.9295 | 0.9368 | 19099 | 7.78e2 | 1 | 1 | 459 | 468 |  |  | DB Search |
| total 3 peptides |  |  |  |  |  |  |  |  |  |  |  |  |  |  |  |  |  |  |  |
| --- | --- | --- | --- | --- | --- | --- | --- | --- | --- | --- | --- | --- | --- | --- | --- | --- | --- | --- | --- |

Best Unique PSM (Scan Sample\_9\_A1\_1\_3646.d:48729, m/z=669.6794, z=3, RT=16.40, ppm=2.81):


TRINITY\_DN413\_c0\_g1\_i3.p1

back to list

  

| Protein Coverage
| Supporting Peptides
| Best Unique PSM
|

Protein Coverage:

Supporting Peptides:

| Peptide | Uniq | -10lgP | Mass | Length | ppm | m/z | z | RT | 1/k0 Start | 1/k0 End | Precursor ID | Area Sample 10 | #Feature | #Feature Sample 10 | Start | End | PTM | AScore | Found By |
| --- | --- | --- | --- | --- | --- | --- | --- | --- | --- | --- | --- | --- | --- | --- | --- | --- | --- | --- | --- |
| R.LLLGDFNGDKR.T | Y | 58.62 | 1246.6669 | 11 | 3.37 | 624.3396 | 2 | 16.00 | 0.9844 | 0.9916 | 46643 | 2.81e2 | 1 | 1 | 46 | 56 |  |  | DB Search |
| total 1 peptides |  |  |  |  |  |  |  |  |  |  |  |  |  |  |  |  |  |  |  |
| --- | --- | --- | --- | --- | --- | --- | --- | --- | --- | --- | --- | --- | --- | --- | --- | --- | --- | --- | --- |

Best Unique PSM (Scan Sample\_9\_A1\_1\_3646.d:46643, m/z=624.3396, z=2, RT=16.00, ppm=-1.85):


TRINITY\_DN776\_c0\_g1\_i1.p1

back to list

  

| Protein Coverage
| Supporting Peptides
| Best Unique PSM
|

Protein Coverage:

Supporting Peptides:

| Peptide | Uniq | -10lgP | Mass | Length | ppm | m/z | z | RT | 1/k0 Start | 1/k0 End | Precursor ID | Area Sample 10 | #Feature | #Feature Sample 10 | Start | End | PTM | AScore | Found By |
| --- | --- | --- | --- | --- | --- | --- | --- | --- | --- | --- | --- | --- | --- | --- | --- | --- | --- | --- | --- |
| R.LFEGNALLRR.L | Y | 58.61 | 1187.6775 | 10 | 3.48 | 594.845 | 2 | 15.57 | 0.9679 | 0.9751 | 44463 | 2.7e3 | 1 | 1 | 78 | 87 |  |  | DB Search |
| R.DLLTLPEKDNRR.L | Y | 57.89 | 1468.7998 | 12 | -0.34 | 735.4031 | 2 | 14.57 | 1.0242 | 1.0315 | 39369 | 1.51e3 | 1 | 1 | 66 | 77 |  |  | DB Search |
| R.KQLVNVPSFIVR.L | Y | 57.5 | 1398.8347 | 12 | -4.99 | 700.4175 | 2 | 17.75 | 1.0081 | 1.0154 | 56022 | 0 | 0 | 0 | 146 | 157 |  |  | DB Search |
| R.RLFEGNALLRR.L | Y | 56.27 | 1343.7786 | 11 | -1.1 | 448.9306 | 3 | 14.75 | 0.7547 | 0.762 | 40382 | 0 | 0 | 0 | 77 | 87 |  |  | DB Search |
| R.RLFEGNALLR.R | Y | 54.07 | 1187.6775 | 10 | 1.11 | 594.8436 | 2 | 16.07 | 0.9396 | 0.9469 | 46969 | 1.45e3 | 1 | 1 | 77 | 86 |  |  | DB Search |
| total 5 peptides |  |  |  |  |  |  |  |  |  |  |  |  |  |  |  |  |  |  |  |
| --- | --- | --- | --- | --- | --- | --- | --- | --- | --- | --- | --- | --- | --- | --- | --- | --- | --- | --- | --- |

Best Unique PSM (Scan Sample\_9\_A1\_1\_3646.d:44463, m/z=594.845, z=2, RT=15.57, ppm=-1.74):


TRINITY\_DN5818\_c1\_g1\_i1.p1

back to list

  

| Protein Coverage
| Supporting Peptides
| Best Unique PSM
|

Protein Coverage:

Supporting Peptides:

| Peptide | Uniq | -10lgP | Mass | Length | ppm | m/z | z | RT | 1/k0 Start | 1/k0 End | Precursor ID | Area Sample 10 | #Feature | #Feature Sample 10 | Start | End | PTM | AScore | Found By |
| --- | --- | --- | --- | --- | --- | --- | --- | --- | --- | --- | --- | --- | --- | --- | --- | --- | --- | --- | --- |
| K.LLAQTTLRNELGTK.N | Y | 58.48 | 1556.8886 | 14 | 4.16 | 779.4507 | 2 | 15.18 | 1.109 | 1.1162 | 42504 | 8.19e2 | 1 | 1 | 144 | 157 |  |  | DB Search |
| total 1 peptides |  |  |  |  |  |  |  |  |  |  |  |  |  |  |  |  |  |  |  |
| --- | --- | --- | --- | --- | --- | --- | --- | --- | --- | --- | --- | --- | --- | --- | --- | --- | --- | --- | --- |

Best Unique PSM (Scan Sample\_9\_A1\_1\_3646.d:42504, m/z=779.4507, z=2, RT=15.18, ppm=-1.05):


TRINITY\_DN319\_c2\_g1\_i1.p1

back to list

  

| Protein Coverage
| Supporting Peptides
| Best Unique PSM
|

Protein Coverage:

Supporting Peptides:

| Peptide | Uniq | -10lgP | Mass | Length | ppm | m/z | z | RT | 1/k0 Start | 1/k0 End | Precursor ID | Area Sample 10 | #Feature | #Feature Sample 10 | Start | End | PTM | AScore | Found By |
| --- | --- | --- | --- | --- | --- | --- | --- | --- | --- | --- | --- | --- | --- | --- | --- | --- | --- | --- | --- |
| K.KAQYYEAYETIIKR.H | Y | 58.69 | 1774.9254 | 14 | -1.1 | 592.6453 | 3 | 15.71 | 0.8623 | 0.8696 | 45168 | 1.12e3 | 1 | 1 | 2534 | 2547 |  |  | DB Search |
| R.HLAISNILK.D | Y | 57.84 | 1007.6127 | 9 | 0.83 | 504.8114 | 2 | 18.74 | 0.8652 | 0.8725 | 61860 | 3.25e2 | 1 | 1 | 2548 | 2556 |  |  | DB Search |
| total 2 peptides |  |  |  |  |  |  |  |  |  |  |  |  |  |  |  |  |  |  |  |
| --- | --- | --- | --- | --- | --- | --- | --- | --- | --- | --- | --- | --- | --- | --- | --- | --- | --- | --- | --- |

Best Unique PSM (Scan Sample\_9\_A1\_1\_3646.d:45168, m/z=592.6453, z=3, RT=15.71, ppm=-6.32):


TRINITY\_DN11097\_c0\_g2\_i1.p1

back to list

  

| Protein Coverage
| Supporting Peptides
| Best Unique PSM
|

Protein Coverage:

Supporting Peptides:

| Peptide | Uniq | -10lgP | Mass | Length | ppm | m/z | z | RT | 1/k0 Start | 1/k0 End | Precursor ID | Area Sample 10 | #Feature | #Feature Sample 10 | Start | End | PTM | AScore | Found By |
| --- | --- | --- | --- | --- | --- | --- | --- | --- | --- | --- | --- | --- | --- | --- | --- | --- | --- | --- | --- |
| R.VAHAGLTVAEFFR.D | Y | 56.84 | 1416.7513 | 13 | -4.62 | 709.376 | 2 | 18.85 | 1.0299 | 1.0371 | 62438 | 0 | 0 | 0 | 199 | 211 |  |  | DB Search |
| total 1 peptides |  |  |  |  |  |  |  |  |  |  |  |  |  |  |  |  |  |  |  |
| --- | --- | --- | --- | --- | --- | --- | --- | --- | --- | --- | --- | --- | --- | --- | --- | --- | --- | --- | --- |

Best Unique PSM (Scan Sample\_9\_A1\_1\_3646.d:62438, m/z=709.376, z=2, RT=18.85, ppm=-9.84):


TRINITY\_DN484\_c0\_g1\_i1.p1

back to list

  

| Protein Coverage
| Supporting Peptides
| Best Unique PSM
|

Protein Coverage:

Supporting Peptides:

| Peptide | Uniq | -10lgP | Mass | Length | ppm | m/z | z | RT | 1/k0 Start | 1/k0 End | Precursor ID | Area Sample 10 | #Feature | #Feature Sample 10 | Start | End | PTM | AScore | Found By |
| --- | --- | --- | --- | --- | --- | --- | --- | --- | --- | --- | --- | --- | --- | --- | --- | --- | --- | --- | --- |
| R.AYHNASQTQSDKAHLK.W | Y | 58.17 | 1797.8758 | 16 | 6.18 | 450.4767 | 4 | 9.45 | 0.7396 | 0.7469 | 10629 | 0 | 0 | 0 | 468 | 483 |  |  | DB Search |
| total 1 peptides |  |  |  |  |  |  |  |  |  |  |  |  |  |  |  |  |  |  |  |
| --- | --- | --- | --- | --- | --- | --- | --- | --- | --- | --- | --- | --- | --- | --- | --- | --- | --- | --- | --- |

Best Unique PSM (Scan Sample\_9\_A1\_1\_3646.d:10629, m/z=450.4767, z=4, RT=9.45, ppm=0.96):


TRINITY\_DN649\_c0\_g1\_i2.p1

back to list

  

| Protein Coverage
| Supporting Peptides
| Best Unique PSM
|

Protein Coverage:

Supporting Peptides:

| Peptide | Uniq | -10lgP | Mass | Length | ppm | m/z | z | RT | 1/k0 Start | 1/k0 End | Precursor ID | Area Sample 10 | #Feature | #Feature Sample 10 | Start | End | PTM | AScore | Found By |
| --- | --- | --- | --- | --- | --- | --- | --- | --- | --- | --- | --- | --- | --- | --- | --- | --- | --- | --- | --- |
| K.FETQIATK.N | Y | 57.09 | 936.4916 | 8 | 7.3 | 937.5009 | 1 | 11.88 | 1.1925 | 1.1997 | 24345 | 0 | 0 | 0 | 103 | 110 |  |  | DB Search |
| total 1 peptides |  |  |  |  |  |  |  |  |  |  |  |  |  |  |  |  |  |  |  |
| --- | --- | --- | --- | --- | --- | --- | --- | --- | --- | --- | --- | --- | --- | --- | --- | --- | --- | --- | --- |

Best Unique PSM (Scan Sample\_9\_A1\_1\_3646.d:24345, m/z=937.5009, z=1, RT=11.88, ppm=2.08):


TRINITY\_DN413\_c0\_g1\_i4.p1

back to list

  

| Protein Coverage
| Supporting Peptides
| Best Unique PSM
|

Protein Coverage:

Supporting Peptides:

| Peptide | Uniq | -10lgP | Mass | Length | ppm | m/z | z | RT | 1/k0 Start | 1/k0 End | Precursor ID | Area Sample 10 | #Feature | #Feature Sample 10 | Start | End | PTM | AScore | Found By |
| --- | --- | --- | --- | --- | --- | --- | --- | --- | --- | --- | --- | --- | --- | --- | --- | --- | --- | --- | --- |
| R.LLLGDFNGDKR.T | Y | 58.62 | 1246.6669 | 11 | 3.37 | 624.3396 | 2 | 16.00 | 0.9844 | 0.9916 | 46643 | 2.81e2 | 1 | 1 | 46 | 56 |  |  | DB Search |
| total 1 peptides |  |  |  |  |  |  |  |  |  |  |  |  |  |  |  |  |  |  |  |
| --- | --- | --- | --- | --- | --- | --- | --- | --- | --- | --- | --- | --- | --- | --- | --- | --- | --- | --- | --- |

Best Unique PSM (Scan Sample\_9\_A1\_1\_3646.d:46643, m/z=624.3396, z=2, RT=16.00, ppm=-1.85):


TRINITY\_DN152\_c0\_g1\_i9.p1

back to list

  

| Protein Coverage
| Supporting Peptides
| Best Unique PSM
|

Protein Coverage:

Supporting Peptides:

| Peptide | Uniq | -10lgP | Mass | Length | ppm | m/z | z | RT | 1/k0 Start | 1/k0 End | Precursor ID | Area Sample 10 | #Feature | #Feature Sample 10 | Start | End | PTM | AScore | Found By |
| --- | --- | --- | --- | --- | --- | --- | --- | --- | --- | --- | --- | --- | --- | --- | --- | --- | --- | --- | --- |
| K.IQDKEGIPPDQQR.L | Y | 58.25 | 1522.7739 | 13 | 2.55 | 762.3922 | 2 | 11.03 | 1.0343 | 1.0415 | 18957 | 1.53e3 | 1 | 1 | 66 | 78 |  |  | DB Search |
| total 1 peptides |  |  |  |  |  |  |  |  |  |  |  |  |  |  |  |  |  |  |  |
| --- | --- | --- | --- | --- | --- | --- | --- | --- | --- | --- | --- | --- | --- | --- | --- | --- | --- | --- | --- |

Best Unique PSM (Scan Sample\_9\_A1\_1\_3646.d:18957, m/z=762.3922, z=2, RT=11.03, ppm=-2.67):


TRINITY\_DN278\_c0\_g1\_i1.p1

back to list

  

| Protein Coverage
| Supporting Peptides
| Best Unique PSM
|

Protein Coverage:

Supporting Peptides:

| Peptide | Uniq | -10lgP | Mass | Length | ppm | m/z | z | RT | 1/k0 Start | 1/k0 End | Precursor ID | Area Sample 10 | #Feature | #Feature Sample 10 | Start | End | PTM | AScore | Found By |
| --- | --- | --- | --- | --- | --- | --- | --- | --- | --- | --- | --- | --- | --- | --- | --- | --- | --- | --- | --- |
| K.IDNYIEFLDEDLKK.K | Y | 58.63 | 1753.8774 | 14 | -2.64 | 585.6285 | 3 | 19.84 | 0.8445 | 0.8518 | 68139 | 1.64e3 | 1 | 1 | 102 | 115 |  |  | DB Search |
| K.IDNYIEFLDEDLKKK.F | Y | 54.7 | 1881.9723 | 15 | 1.47 | 628.329 | 3 | 18.88 | 0.8668 | 0.8741 | 62654 | 0 | 0 | 0 | 102 | 116 |  |  | DB Search |
| total 2 peptides |  |  |  |  |  |  |  |  |  |  |  |  |  |  |  |  |  |  |  |
| --- | --- | --- | --- | --- | --- | --- | --- | --- | --- | --- | --- | --- | --- | --- | --- | --- | --- | --- | --- |

Best Unique PSM (Scan Sample\_9\_A1\_1\_3646.d:68139, m/z=585.6285, z=3, RT=19.84, ppm=-7.86):


TRINITY\_DN1172\_c0\_g2\_i2.p1

back to list

  

| Protein Coverage
| Supporting Peptides
| Best Unique PSM
|

Protein Coverage:

Supporting Peptides:

| Peptide | Uniq | -10lgP | Mass | Length | ppm | m/z | z | RT | 1/k0 Start | 1/k0 End | Precursor ID | Area Sample 10 | #Feature | #Feature Sample 10 | Start | End | PTM | AScore | Found By |
| --- | --- | --- | --- | --- | --- | --- | --- | --- | --- | --- | --- | --- | --- | --- | --- | --- | --- | --- | --- |
| K.VLTSSDPAEKEK.K | Y | 58.69 | 1302.6667 | 12 | 2.82 | 652.3391 | 2 | 10.58 | 0.9965 | 1.0037 | 16473 | 6.51e3 | 1 | 1 | 7 | 18 |  |  | DB Search |
| K.K(+42.01)LAAELANGR.L | Y | 58.51 | 1083.6036 | 10 | 2.05 | 542.8074 | 2 | 15.02 | 0.8644 | 0.8716 | 41731 | 4.29e2 | 1 | 1 | 19 | 28 | Acetylation (Protein N-term) | K1:Acetylation (Protein N-term):1000 | DB Search |
| K.VLTSDDPAEKDK.K | Y | 57.79 | 1316.6459 | 12 | 1.44 | 659.3278 | 2 | 10.86 | 0.9844 | 0.9916 | 18297 | 1.85e2 | 1 | 1 | 192 | 203 |  |  | DB Search |
| total 3 peptides |  |  |  |  |  |  |  |  |  |  |  |  |  |  |  |  |  |  |  |
| --- | --- | --- | --- | --- | --- | --- | --- | --- | --- | --- | --- | --- | --- | --- | --- | --- | --- | --- | --- |

Best Unique PSM (Scan Sample\_9\_A1\_1\_3646.d:16473, m/z=652.3391, z=2, RT=10.58, ppm=-2.4):


TRINITY\_DN4595\_c0\_g1\_i4.p1

back to list

  

| Protein Coverage
| Supporting Peptides
| Best Unique PSM
|

Protein Coverage:

Supporting Peptides:

| Peptide | Uniq | -10lgP | Mass | Length | ppm | m/z | z | RT | 1/k0 Start | 1/k0 End | Precursor ID | Area Sample 10 | #Feature | #Feature Sample 10 | Start | End | PTM | AScore | Found By |
| --- | --- | --- | --- | --- | --- | --- | --- | --- | --- | --- | --- | --- | --- | --- | --- | --- | --- | --- | --- |
| K.YTGAC(+57.02)NANGNTDK.T | Y | 58.6 | 1384.5677 | 13 | 3.27 | 693.2898 | 2 | 9.56 | 0.9279 | 0.9352 | 11005 | 8.94e2 | 1 | 1 | 1492 | 1504 | Carbamidomethylation |  | DB Search |
| R.AFNRVETR.D | Y | 50.97 | 991.5199 | 8 | -0.72 | 496.7643 | 2 | 10.73 | 0.8534 | 0.8607 | 17391 | 3.08e3 | 1 | 1 | 282 | 289 |  |  | DB Search |
| K.VNQGEKEFLQRPC(+57.02)R.Y | Y | 50.11 | 1759.8788 | 14 | 4.1 | 587.6329 | 3 | 12.58 | 0.8619 | 0.8692 | 28420 | 3.34e2 | 1 | 1 | 1612 | 1625 | Carbamidomethylation |  | DB Search |
| total 3 peptides |  |  |  |  |  |  |  |  |  |  |  |  |  |  |  |  |  |  |  |
| --- | --- | --- | --- | --- | --- | --- | --- | --- | --- | --- | --- | --- | --- | --- | --- | --- | --- | --- | --- |

Best Unique PSM (Scan Sample\_9\_A1\_1\_3646.d:11005, m/z=693.2898, z=2, RT=9.56, ppm=-1.95):


TRINITY\_DN15599\_c0\_g1\_i2.p1

back to list

  

| Protein Coverage
| Supporting Peptides
| Best Unique PSM
|

Protein Coverage:

Supporting Peptides:

| Peptide | Uniq | -10lgP | Mass | Length | ppm | m/z | z | RT | 1/k0 Start | 1/k0 End | Precursor ID | Area Sample 10 | #Feature | #Feature Sample 10 | Start | End | PTM | AScore | Found By |
| --- | --- | --- | --- | --- | --- | --- | --- | --- | --- | --- | --- | --- | --- | --- | --- | --- | --- | --- | --- |
| K.KLQAELANGR.L | Y | 57.88 | 1098.6145 | 10 | 0.06 | 550.3117 | 2 | 11.52 | 0.8814 | 0.8887 | 21738 | 1.93e4 | 1 | 1 | 184 | 193 |  |  | DB Search |
| total 1 peptides |  |  |  |  |  |  |  |  |  |  |  |  |  |  |  |  |  |  |  |
| --- | --- | --- | --- | --- | --- | --- | --- | --- | --- | --- | --- | --- | --- | --- | --- | --- | --- | --- | --- |

Best Unique PSM (Scan Sample\_9\_A1\_1\_3646.d:21738, m/z=550.3117, z=2, RT=11.52, ppm=-5.16):


TRINITY\_DN72625\_c0\_g1\_i1.p1

back to list

  

| Protein Coverage
| Supporting Peptides
| Best Unique PSM
|

Protein Coverage:

Supporting Peptides:

| Peptide | Uniq | -10lgP | Mass | Length | ppm | m/z | z | RT | 1/k0 Start | 1/k0 End | Precursor ID | Area Sample 10 | #Feature | #Feature Sample 10 | Start | End | PTM | AScore | Found By |
| --- | --- | --- | --- | --- | --- | --- | --- | --- | --- | --- | --- | --- | --- | --- | --- | --- | --- | --- | --- |
| K.NERGEAVISLK.S | Y | 58.34 | 1214.6619 | 11 | 1.38 | 608.3359 | 2 | 13.52 | 0.9505 | 0.9578 | 33887 | 0 | 0 | 0 | 585 | 595 |  |  | DB Search |
| R.ATDSDSGPNGDITYR.I | Y | 55.42 | 1567.675 | 15 | -0.62 | 784.8402 | 2 | 12.74 | 1.0049 | 1.0122 | 29493 | 2.71e2 | 1 | 1 | 1514 | 1528 |  |  | DB Search |
| R.ATNPDVEK.I | Y | 53.07 | 872.4239 | 8 | 4.44 | 437.2189 | 2 | 9.70 | 0.7962 | 0.8035 | 11623 | 2.72e3 | 1 | 1 | 302 | 309 |  |  | DB Search |
| R.LYSTDPNKFYR.F | Y | 51.54 | 1402.6881 | 11 | 2.6 | 702.3495 | 2 | 14.19 | 1.0299 | 1.0371 | 37268 | 1.32e3 | 1 | 1 | 193 | 203 |  |  | DB Search |
| total 4 peptides |  |  |  |  |  |  |  |  |  |  |  |  |  |  |  |  |  |  |  |
| --- | --- | --- | --- | --- | --- | --- | --- | --- | --- | --- | --- | --- | --- | --- | --- | --- | --- | --- | --- |

Best Unique PSM (Scan Sample\_9\_A1\_1\_3646.d:33887, m/z=608.3359, z=2, RT=13.52, ppm=-3.84):


TRINITY\_DN79\_c0\_g1\_i1.p1

back to list

  

| Protein Coverage
| Supporting Peptides
| Best Unique PSM
|

Protein Coverage:

Supporting Peptides:

| Peptide | Uniq | -10lgP | Mass | Length | ppm | m/z | z | RT | 1/k0 Start | 1/k0 End | Precursor ID | Area Sample 10 | #Feature | #Feature Sample 10 | Start | End | PTM | AScore | Found By |
| --- | --- | --- | --- | --- | --- | --- | --- | --- | --- | --- | --- | --- | --- | --- | --- | --- | --- | --- | --- |
| K.KAENFAELKNTFQEAAK.G | Y | 51.62 | 1937.9846 | 17 | -1.33 | 646.9979 | 3 | 16.11 | 0.917 | 0.9243 | 47225 | 2.65e2 | 1 | 1 | 225 | 241 |  |  | DB Search |
| total 1 peptides |  |  |  |  |  |  |  |  |  |  |  |  |  |  |  |  |  |  |  |
| --- | --- | --- | --- | --- | --- | --- | --- | --- | --- | --- | --- | --- | --- | --- | --- | --- | --- | --- | --- |

Best Unique PSM (Scan Sample\_9\_A1\_1\_3646.d:47225, m/z=646.9979, z=3, RT=16.11, ppm=-6.55):


TRINITY\_DN98420\_c0\_g1\_i1.p1

back to list

  

| Protein Coverage
| Supporting Peptides
| Best Unique PSM
|

Protein Coverage:

Supporting Peptides:

| Peptide | Uniq | -10lgP | Mass | Length | ppm | m/z | z | RT | 1/k0 Start | 1/k0 End | Precursor ID | Area Sample 10 | #Feature | #Feature Sample 10 | Start | End | PTM | AScore | Found By |
| --- | --- | --- | --- | --- | --- | --- | --- | --- | --- | --- | --- | --- | --- | --- | --- | --- | --- | --- | --- |
| R.AVIFDRFR.G | Y | 58.62 | 1022.5661 | 8 | 1.78 | 512.2886 | 2 | 15.90 | 0.8672 | 0.8745 | 46056 | 5.37e3 | 1 | 1 | 48 | 55 |  |  | DB Search |
| total 1 peptides |  |  |  |  |  |  |  |  |  |  |  |  |  |  |  |  |  |  |  |
| --- | --- | --- | --- | --- | --- | --- | --- | --- | --- | --- | --- | --- | --- | --- | --- | --- | --- | --- | --- |

Best Unique PSM (Scan Sample\_9\_A1\_1\_3646.d:46056, m/z=512.2886, z=2, RT=15.90, ppm=-3.44):


TRINITY\_DN36\_c0\_g1\_i8.p1

back to list

  

| Protein Coverage
| Supporting Peptides
| Best Unique PSM
|

Protein Coverage:

Supporting Peptides:

| Peptide | Uniq | -10lgP | Mass | Length | ppm | m/z | z | RT | 1/k0 Start | 1/k0 End | Precursor ID | Area Sample 10 | #Feature | #Feature Sample 10 | Start | End | PTM | AScore | Found By |
| --- | --- | --- | --- | --- | --- | --- | --- | --- | --- | --- | --- | --- | --- | --- | --- | --- | --- | --- | --- |
| R.LGGIEVTQGNC(+57.02)ARK.Y | Y | 58.24 | 1501.7671 | 14 | 6.42 | 751.8917 | 2 | 12.66 | 1.0632 | 1.0705 | 28959 | 0 | 0 | 0 | 58 | 71 | Carbamidomethylation |  | DB Search |
| K.FETQIATK.D | Y | 57.09 | 936.4916 | 8 | 7.3 | 937.5009 | 1 | 11.88 | 1.1925 | 1.1997 | 24345 | 0 | 0 | 0 | 167 | 174 |  |  | DB Search |
| total 2 peptides |  |  |  |  |  |  |  |  |  |  |  |  |  |  |  |  |  |  |  |
| --- | --- | --- | --- | --- | --- | --- | --- | --- | --- | --- | --- | --- | --- | --- | --- | --- | --- | --- | --- |

Best Unique PSM (Scan Sample\_9\_A1\_1\_3646.d:28959, m/z=751.8917, z=2, RT=12.66, ppm=1.21):


TRINITY\_DN585\_c0\_g1\_i2.p1

back to list

  

| Protein Coverage
| Supporting Peptides
| Best Unique PSM
|

Protein Coverage:

Supporting Peptides:

| Peptide | Uniq | -10lgP | Mass | Length | ppm | m/z | z | RT | 1/k0 Start | 1/k0 End | Precursor ID | Area Sample 10 | #Feature | #Feature Sample 10 | Start | End | PTM | AScore | Found By |
| --- | --- | --- | --- | --- | --- | --- | --- | --- | --- | --- | --- | --- | --- | --- | --- | --- | --- | --- | --- |
| K.IQDKEGIPPDQQR.L | Y | 58.25 | 1522.7739 | 13 | 2.55 | 762.3922 | 2 | 11.03 | 1.0343 | 1.0415 | 18957 | 1.53e3 | 1 | 1 | 2 | 14 |  |  | DB Search |
| total 1 peptides |  |  |  |  |  |  |  |  |  |  |  |  |  |  |  |  |  |  |  |
| --- | --- | --- | --- | --- | --- | --- | --- | --- | --- | --- | --- | --- | --- | --- | --- | --- | --- | --- | --- |

Best Unique PSM (Scan Sample\_9\_A1\_1\_3646.d:18957, m/z=762.3922, z=2, RT=11.03, ppm=-2.67):


TRINITY\_DN3190\_c1\_g1\_i1.p1

back to list

  

| Protein Coverage
| Supporting Peptides
| Best Unique PSM
|

Protein Coverage:

Supporting Peptides:

| Peptide | Uniq | -10lgP | Mass | Length | ppm | m/z | z | RT | 1/k0 Start | 1/k0 End | Precursor ID | Area Sample 10 | #Feature | #Feature Sample 10 | Start | End | PTM | AScore | Found By |
| --- | --- | --- | --- | --- | --- | --- | --- | --- | --- | --- | --- | --- | --- | --- | --- | --- | --- | --- | --- |
| K.DLQGETSGSFKR.L | Y | 58.57 | 1323.6419 | 12 | 1.12 | 662.8255 | 2 | 12.61 | 0.9586 | 0.9658 | 28668 | 0 | 0 | 0 | 135 | 146 |  |  | DB Search |
| K.RLLTSLVQGAR.L | Y | 55.8 | 1212.7302 | 11 | 0.26 | 607.3694 | 2 | 15.19 | 0.9804 | 0.9876 | 42540 | 5.93e2 | 1 | 1 | 146 | 156 |  |  | DB Search |
| M.A(+42.01)TVVDYPDFDAQADAETLRK.A | Y | 55.12 | 2266.0753 | 20 | 3.38 | 1134.0428 | 2 | 18.33 | 1.2432 | 1.2503 | 59289 | 0 | 0 | 0 | 2 | 21 | Acetylation (Protein N-term) | A1:Acetylation (Protein N-term):1000 | DB Search |
| total 3 peptides |  |  |  |  |  |  |  |  |  |  |  |  |  |  |  |  |  |  |  |
| --- | --- | --- | --- | --- | --- | --- | --- | --- | --- | --- | --- | --- | --- | --- | --- | --- | --- | --- | --- |

Best Unique PSM (Scan Sample\_9\_A1\_1\_3646.d:28668, m/z=662.8255, z=2, RT=12.61, ppm=-4.09):


TRINITY\_DN1616\_c0\_g1\_i6.p1

back to list

  

| Protein Coverage
| Supporting Peptides
| Best Unique PSM
|

Protein Coverage:

Supporting Peptides:

| Peptide | Uniq | -10lgP | Mass | Length | ppm | m/z | z | RT | 1/k0 Start | 1/k0 End | Precursor ID | Area Sample 10 | #Feature | #Feature Sample 10 | Start | End | PTM | AScore | Found By |
| --- | --- | --- | --- | --- | --- | --- | --- | --- | --- | --- | --- | --- | --- | --- | --- | --- | --- | --- | --- |
| K.YVVMLQYYHDSDTAK.Y | Y | 52.09 | 1831.8451 | 15 | 5.48 | 916.9301 | 2 | 16.56 | 1.1813 | 1.1885 | 49447 | 0 | 0 | 0 | 1071 | 1085 |  |  | DB Search |
| R.YALNQIQPVTATVTAFKGDSK.I | Y | 44.58 | 2251.1848 | 21 | 9.46 | 751.4054 | 3 | 18.19 | 1.048 | 1.0552 | 58586 | 0 | 0 | 0 | 894 | 914 |  |  | DB Search |
| total 2 peptides |  |  |  |  |  |  |  |  |  |  |  |  |  |  |  |  |  |  |  |
| --- | --- | --- | --- | --- | --- | --- | --- | --- | --- | --- | --- | --- | --- | --- | --- | --- | --- | --- | --- |

Best Unique PSM (Scan Sample\_9\_A1\_1\_3646.d:49447, m/z=916.9301, z=2, RT=16.56, ppm=0.27):


TRINITY\_DN5397\_c0\_g1\_i1.p1

back to list

  

| Protein Coverage
| Supporting Peptides
| Best Unique PSM
|

Protein Coverage:

Supporting Peptides:

| Peptide | Uniq | -10lgP | Mass | Length | ppm | m/z | z | RT | 1/k0 Start | 1/k0 End | Precursor ID | Area Sample 10 | #Feature | #Feature Sample 10 | Start | End | PTM | AScore | Found By |
| --- | --- | --- | --- | --- | --- | --- | --- | --- | --- | --- | --- | --- | --- | --- | --- | --- | --- | --- | --- |
| R.SFNKVESR.E | Y | 36.67 | 965.493 | 8 | 4.26 | 483.7533 | 2 | 10.28 | 0.8356 | 0.8429 | 14919 | 0 | 0 | 0 | 33 | 40 |  |  | DB Search |
| total 1 peptides |  |  |  |  |  |  |  |  |  |  |  |  |  |  |  |  |  |  |  |
| --- | --- | --- | --- | --- | --- | --- | --- | --- | --- | --- | --- | --- | --- | --- | --- | --- | --- | --- | --- |

Best Unique PSM (Scan Sample\_9\_A1\_1\_3646.d:14919, m/z=483.7533, z=2, RT=10.28, ppm=-0.96):


TRINITY\_DN3202\_c2\_g1\_i1.p1

back to list

  

| Protein Coverage
| Supporting Peptides
| Best Unique PSM
|

Protein Coverage:

Supporting Peptides:

| Peptide | Uniq | -10lgP | Mass | Length | ppm | m/z | z | RT | 1/k0 Start | 1/k0 End | Precursor ID | Area Sample 10 | #Feature | #Feature Sample 10 | Start | End | PTM | AScore | Found By |
| --- | --- | --- | --- | --- | --- | --- | --- | --- | --- | --- | --- | --- | --- | --- | --- | --- | --- | --- | --- |
| R.GC(+57.02)YIHTQEVAR.G | Y | 58.47 | 1332.6245 | 11 | -1.44 | 667.3151 | 2 | 12.15 | 1.011 | 1.0182 | 26068 | 0 | 0 | 0 | 7 | 17 | Carbamidomethylation |  | DB Search |
| total 1 peptides |  |  |  |  |  |  |  |  |  |  |  |  |  |  |  |  |  |  |  |
| --- | --- | --- | --- | --- | --- | --- | --- | --- | --- | --- | --- | --- | --- | --- | --- | --- | --- | --- | --- |

Best Unique PSM (Scan Sample\_9\_A1\_1\_3646.d:26068, m/z=667.3151, z=2, RT=12.15, ppm=-6.66):


TRINITY\_DN2805\_c0\_g1\_i1.p1

back to list

  

| Protein Coverage
| Supporting Peptides
| Best Unique PSM
|

Protein Coverage:

Supporting Peptides:

| Peptide | Uniq | -10lgP | Mass | Length | ppm | m/z | z | RT | 1/k0 Start | 1/k0 End | Precursor ID | Area Sample 10 | #Feature | #Feature Sample 10 | Start | End | PTM | AScore | Found By |
| --- | --- | --- | --- | --- | --- | --- | --- | --- | --- | --- | --- | --- | --- | --- | --- | --- | --- | --- | --- |
| K.VVIIDGKGHLM(+15.99)GR.L | Y | 58.66 | 1409.7813 | 13 | 2.85 | 705.8962 | 2 | 12.99 | 1.0271 | 1.0343 | 30876 | 0 | 0 | 0 | 6 | 18 | Oxidation (M) | M11:Oxidation (M):1000 | DB Search |
| K.ALIESLEEKRK.A | Y | 57.09 | 1314.7507 | 11 | -2.37 | 658.3776 | 2 | 13.75 | 0.9824 | 0.9896 | 35023 | 3.97e2 | 1 | 1 | 151 | 161 |  |  | DB Search |
| total 2 peptides |  |  |  |  |  |  |  |  |  |  |  |  |  |  |  |  |  |  |  |
| --- | --- | --- | --- | --- | --- | --- | --- | --- | --- | --- | --- | --- | --- | --- | --- | --- | --- | --- | --- |

Best Unique PSM (Scan Sample\_9\_A1\_1\_3646.d:30876, m/z=705.8962, z=2, RT=12.99, ppm=-2.37):


TRINITY\_DN96\_c0\_g1\_i1.p1

back to list

  

| Protein Coverage
| Supporting Peptides
| Best Unique PSM
|

Protein Coverage:

Supporting Peptides:

| Peptide | Uniq | -10lgP | Mass | Length | ppm | m/z | z | RT | 1/k0 Start | 1/k0 End | Precursor ID | Area Sample 10 | #Feature | #Feature Sample 10 | Start | End | PTM | AScore | Found By |
| --- | --- | --- | --- | --- | --- | --- | --- | --- | --- | --- | --- | --- | --- | --- | --- | --- | --- | --- | --- |
| K.TVQNFYELVTHSK.G | Y | 58.66 | 1564.7885 | 13 | 4.05 | 783.4006 | 2 | 18.26 | 1.0893 | 1.0965 | 58839 | 6.36e2 | 1 | 1 | 44 | 56 |  |  | DB Search |
| K.KVEQVQTDSR.D | Y | 53.61 | 1188.6098 | 10 | 5.11 | 595.3121 | 2 | 9.53 | 0.8725 | 0.8798 | 10904 | 2.14e2 | 1 | 1 | 153 | 162 |  |  | DB Search |
| total 2 peptides |  |  |  |  |  |  |  |  |  |  |  |  |  |  |  |  |  |  |  |
| --- | --- | --- | --- | --- | --- | --- | --- | --- | --- | --- | --- | --- | --- | --- | --- | --- | --- | --- | --- |

Best Unique PSM (Scan Sample\_9\_A1\_1\_3646.d:58839, m/z=783.4006, z=2, RT=18.26, ppm=-1.16):


TRINITY\_DN38397\_c0\_g1\_i1.p1

back to list

  

| Protein Coverage
| Supporting Peptides
| Best Unique PSM
|

Protein Coverage:

Supporting Peptides:

| Peptide | Uniq | -10lgP | Mass | Length | ppm | m/z | z | RT | 1/k0 Start | 1/k0 End | Precursor ID | Area Sample 10 | #Feature | #Feature Sample 10 | Start | End | PTM | AScore | Found By |
| --- | --- | --- | --- | --- | --- | --- | --- | --- | --- | --- | --- | --- | --- | --- | --- | --- | --- | --- | --- |
| R.GC(+57.02)YIHTQEIAR.G | Y | 58.69 | 1346.6401 | 11 | 1.87 | 674.3251 | 2 | 12.91 | 1.0166 | 1.0238 | 30377 | 1.95e3 | 1 | 1 | 202 | 212 | Carbamidomethylation |  | DB Search |
| total 1 peptides |  |  |  |  |  |  |  |  |  |  |  |  |  |  |  |  |  |  |  |
| --- | --- | --- | --- | --- | --- | --- | --- | --- | --- | --- | --- | --- | --- | --- | --- | --- | --- | --- | --- |

Best Unique PSM (Scan Sample\_9\_A1\_1\_3646.d:30377, m/z=674.3251, z=2, RT=12.91, ppm=-3.35):


TRINITY\_DN96\_c0\_g1\_i5.p1

back to list

  

| Protein Coverage
| Supporting Peptides
| Best Unique PSM
|

Protein Coverage:

Supporting Peptides:

| Peptide | Uniq | -10lgP | Mass | Length | ppm | m/z | z | RT | 1/k0 Start | 1/k0 End | Precursor ID | Area Sample 10 | #Feature | #Feature Sample 10 | Start | End | PTM | AScore | Found By |
| --- | --- | --- | --- | --- | --- | --- | --- | --- | --- | --- | --- | --- | --- | --- | --- | --- | --- | --- | --- |
| K.KVEQVQTDSR.D | Y | 53.61 | 1188.6098 | 10 | 5.11 | 595.3121 | 2 | 9.53 | 0.8725 | 0.8798 | 10904 | 2.14e2 | 1 | 1 | 163 | 172 |  |  | DB Search |
| total 1 peptides |  |  |  |  |  |  |  |  |  |  |  |  |  |  |  |  |  |  |  |
| --- | --- | --- | --- | --- | --- | --- | --- | --- | --- | --- | --- | --- | --- | --- | --- | --- | --- | --- | --- |

Best Unique PSM (Scan Sample\_9\_A1\_1\_3646.d:10904, m/z=595.3121, z=2, RT=9.53, ppm=-0.11):


TRINITY\_DN761\_c0\_g1\_i3.p1

back to list

  

| Protein Coverage
| Supporting Peptides
| Best Unique PSM
|

Protein Coverage:

Supporting Peptides:

| Peptide | Uniq | -10lgP | Mass | Length | ppm | m/z | z | RT | 1/k0 Start | 1/k0 End | Precursor ID | Area Sample 10 | #Feature | #Feature Sample 10 | Start | End | PTM | AScore | Found By |
| --- | --- | --- | --- | --- | --- | --- | --- | --- | --- | --- | --- | --- | --- | --- | --- | --- | --- | --- | --- |
| K.TRIEDLETVISQTQDHR.H | Y | 58.82 | 2040.0236 | 17 | 4.24 | 681.0145 | 3 | 17.36 | 0.9594 | 0.9666 | 53642 | 9.24e2 | 1 | 1 | 275 | 291 |  |  | DB Search |
| K.EIDKADIAIVDTGENPDAPHPR.E | Y | 51.38 | 2372.1607 | 22 | 1.38 | 791.7245 | 3 | 14.90 | 1.0383 | 1.0456 | 41054 | 3.22e2 | 1 | 1 | 71 | 92 |  |  | DB Search |
| K.FVNEVRR.C | Y | 33.88 | 918.5035 | 7 | 0.09 | 460.2567 | 2 | 12.65 | 0.812 | 0.8193 | 28973 | 2.9e3 | 1 | 1 | 51 | 57 |  |  | DB Search |
| total 3 peptides |  |  |  |  |  |  |  |  |  |  |  |  |  |  |  |  |  |  |  |
| --- | --- | --- | --- | --- | --- | --- | --- | --- | --- | --- | --- | --- | --- | --- | --- | --- | --- | --- | --- |

Best Unique PSM (Scan Sample\_9\_A1\_1\_3646.d:53642, m/z=681.0145, z=3, RT=17.36, ppm=-0.98):


TRINITY\_DN2230\_c0\_g1\_i1.p1

back to list

  

| Protein Coverage
| Supporting Peptides
| Best Unique PSM
|

Protein Coverage:

Supporting Peptides:

| Peptide | Uniq | -10lgP | Mass | Length | ppm | m/z | z | RT | 1/k0 Start | 1/k0 End | Precursor ID | Area Sample 10 | #Feature | #Feature Sample 10 | Start | End | PTM | AScore | Found By |
| --- | --- | --- | --- | --- | --- | --- | --- | --- | --- | --- | --- | --- | --- | --- | --- | --- | --- | --- | --- |
| K.LLEIEDEIKLR.S | Y | 58.51 | 1369.7816 | 11 | 2.42 | 685.8962 | 2 | 18.01 | 1.0484 | 1.0556 | 57388 | 4.33e2 | 1 | 1 | 15 | 25 |  |  | DB Search |
| total 1 peptides |  |  |  |  |  |  |  |  |  |  |  |  |  |  |  |  |  |  |  |
| --- | --- | --- | --- | --- | --- | --- | --- | --- | --- | --- | --- | --- | --- | --- | --- | --- | --- | --- | --- |

Best Unique PSM (Scan Sample\_9\_A1\_1\_3646.d:57388, m/z=685.8962, z=2, RT=18.01, ppm=-2.8):


TRINITY\_DN6701\_c0\_g1\_i2.p2

back to list

  

| Protein Coverage
| Supporting Peptides
| Best Unique PSM
|

Protein Coverage:

Supporting Peptides:

| Peptide | Uniq | -10lgP | Mass | Length | ppm | m/z | z | RT | 1/k0 Start | 1/k0 End | Precursor ID | Area Sample 10 | #Feature | #Feature Sample 10 | Start | End | PTM | AScore | Found By |
| --- | --- | --- | --- | --- | --- | --- | --- | --- | --- | --- | --- | --- | --- | --- | --- | --- | --- | --- | --- |
| K.VITSDDPAEKEK.K | Y | 59.05 | 1330.6616 | 12 | 3.57 | 666.337 | 2 | 10.72 | 0.9884 | 0.9957 | 17382 | 1.42e4 | 1 | 1 | 114 | 125 |  |  | DB Search |
| total 1 peptides |  |  |  |  |  |  |  |  |  |  |  |  |  |  |  |  |  |  |  |
| --- | --- | --- | --- | --- | --- | --- | --- | --- | --- | --- | --- | --- | --- | --- | --- | --- | --- | --- | --- |

Best Unique PSM (Scan Sample\_9\_A1\_1\_3646.d:17382, m/z=666.337, z=2, RT=10.72, ppm=-1.65):


TRINITY\_DN982\_c0\_g1\_i1.p1

back to list

  

| Protein Coverage
| Supporting Peptides
| Best Unique PSM
|

Protein Coverage:

Supporting Peptides:

| Peptide | Uniq | -10lgP | Mass | Length | ppm | m/z | z | RT | 1/k0 Start | 1/k0 End | Precursor ID | Area Sample 10 | #Feature | #Feature Sample 10 | Start | End | PTM | AScore | Found By |
| --- | --- | --- | --- | --- | --- | --- | --- | --- | --- | --- | --- | --- | --- | --- | --- | --- | --- | --- | --- |
| R.LVQNTPHNSIVTK.I | N | 58.68 | 1449.794 | 13 | 2.67 | 725.9024 | 2 | 11.69 | 1.0584 | 1.0656 | 23187 | 0 | 0 | 0 | 5129 | 5141 |  |  | DB Search |
| R.LIQTLNYETKK.E | N | 58.63 | 1349.7554 | 11 | 0.98 | 675.8821 | 2 | 13.61 | 1.0114 | 1.0186 | 34304 | 6.23e2 | 1 | 1 | 4879 | 4889 |  |  | DB Search |
| K.TSTQDVALNIRR.S | N | 58.59 | 1372.7423 | 12 | 5.06 | 687.3783 | 2 | 13.47 | 1.0001 | 1.0073 | 33513 | 2.51e3 | 1 | 1 | 5623 | 5634 |  |  | DB Search |
| K.AVDRGNPPQDSVNK.A | N | 58.36 | 1495.7379 | 14 | 5.97 | 499.5869 | 3 | 10.22 | 0.7836 | 0.7909 | 14437 | 8.69e2 | 1 | 1 | 1699 | 1712 |  |  | DB Search |
| K.YVAIYPSDHLTVNR.D | N | 57.08 | 1646.8416 | 14 | -1.81 | 824.4223 | 2 | 15.52 | 1.1086 | 1.1158 | 44184 | 3.77e2 | 1 | 1 | 2814 | 2827 |  |  | DB Search |
| R.ITTSQILDHEQQK.L | N | 56.94 | 1539.7893 | 13 | 3.01 | 770.9002 | 2 | 13.83 | 1.0957 | 1.1029 | 35489 | 5.16e2 | 1 | 1 | 1994 | 2006 |  |  | DB Search |
| R.GNPPQDSVNK.A | N | 55.81 | 1054.5043 | 10 | 0.87 | 528.2571 | 2 | 9.76 | 0.8372 | 0.8445 | 11990 | 0 | 0 | 0 | 1703 | 1712 |  |  | DB Search |
| K.TPQYDFSVVVTDKGTPPR.Q | Y | 42.6 | 2006.0109 | 18 | 8.03 | 669.6794 | 3 | 16.40 | 0.9392 | 0.9465 | 48729 | 0 | 0 | 0 | 1072 | 1089 |  |  | DB Search |
| R.DRHVLTIQSR.G | N | 33.87 | 1223.6734 | 10 | 6.21 | 612.8446 | 2 | 11.95 | 0.9259 | 0.9331 | 24824 | 0 | 0 | 0 | 1590 | 1599 |  |  | DB Search |
| K.VLEVNDNKPK.F | Y | 29.58 | 1154.6295 | 10 | -0.5 | 578.3187 | 2 | 10.99 | 0.9295 | 0.9368 | 19099 | 7.78e2 | 1 | 1 | 459 | 468 |  |  | DB Search |
| total 10 peptides |  |  |  |  |  |  |  |  |  |  |  |  |  |  |  |  |  |  |  |
| --- | --- | --- | --- | --- | --- | --- | --- | --- | --- | --- | --- | --- | --- | --- | --- | --- | --- | --- | --- |

Best Unique PSM (Scan Sample\_9\_A1\_1\_3646.d:48729, m/z=669.6794, z=3, RT=16.40, ppm=2.81):


TRINITY\_DN4595\_c0\_g1\_i7.p1

back to list

  

| Protein Coverage
| Supporting Peptides
| Best Unique PSM
|

Protein Coverage:

Supporting Peptides:

| Peptide | Uniq | -10lgP | Mass | Length | ppm | m/z | z | RT | 1/k0 Start | 1/k0 End | Precursor ID | Area Sample 10 | #Feature | #Feature Sample 10 | Start | End | PTM | AScore | Found By |
| --- | --- | --- | --- | --- | --- | --- | --- | --- | --- | --- | --- | --- | --- | --- | --- | --- | --- | --- | --- |
| R.AFNRVETR.D | Y | 50.97 | 991.5199 | 8 | -0.72 | 496.7643 | 2 | 10.73 | 0.8534 | 0.8607 | 17391 | 3.08e3 | 1 | 1 | 282 | 289 |  |  | DB Search |
| total 1 peptides |  |  |  |  |  |  |  |  |  |  |  |  |  |  |  |  |  |  |  |
| --- | --- | --- | --- | --- | --- | --- | --- | --- | --- | --- | --- | --- | --- | --- | --- | --- | --- | --- | --- |

Best Unique PSM (Scan Sample\_9\_A1\_1\_3646.d:17391, m/z=496.7643, z=2, RT=10.73, ppm=-5.95):


TRINITY\_DN6294\_c0\_g3\_i1.p1

back to list

  

| Protein Coverage
| Supporting Peptides
| Best Unique PSM
|

Protein Coverage:

Supporting Peptides:

| Peptide | Uniq | -10lgP | Mass | Length | ppm | m/z | z | RT | 1/k0 Start | 1/k0 End | Precursor ID | Area Sample 10 | #Feature | #Feature Sample 10 | Start | End | PTM | AScore | Found By |
| --- | --- | --- | --- | --- | --- | --- | --- | --- | --- | --- | --- | --- | --- | --- | --- | --- | --- | --- | --- |
| K.LVVIADIVDQKR.L | Y | 58.63 | 1367.8136 | 12 | -3.46 | 684.9081 | 2 | 17.50 | 1.0435 | 1.0508 | 54455 | 3.87e2 | 1 | 1 | 24 | 35 |  |  | DB Search |
| total 1 peptides |  |  |  |  |  |  |  |  |  |  |  |  |  |  |  |  |  |  |  |
| --- | --- | --- | --- | --- | --- | --- | --- | --- | --- | --- | --- | --- | --- | --- | --- | --- | --- | --- | --- |

Best Unique PSM (Scan Sample\_9\_A1\_1\_3646.d:54455, m/z=684.9081, z=2, RT=17.50, ppm=-8.68):


TRINITY\_DN113\_c3\_g1\_i1.p1

back to list

  

| Protein Coverage
| Supporting Peptides
| Best Unique PSM
|

Protein Coverage:

Supporting Peptides:

| Peptide | Uniq | -10lgP | Mass | Length | ppm | m/z | z | RT | 1/k0 Start | 1/k0 End | Precursor ID | Area Sample 10 | #Feature | #Feature Sample 10 | Start | End | PTM | AScore | Found By |
| --- | --- | --- | --- | --- | --- | --- | --- | --- | --- | --- | --- | --- | --- | --- | --- | --- | --- | --- | --- |
| K.AHVSLSTFSEDVTSLTK.F | Y | 56.78 | 1820.9156 | 17 | -11.58 | 911.4498 | 2 | 18.78 | 1.1366 | 1.1438 | 61988 | 0 | 0 | 0 | 568 | 584 |  |  | DB Search |
| total 1 peptides |  |  |  |  |  |  |  |  |  |  |  |  |  |  |  |  |  |  |  |
| --- | --- | --- | --- | --- | --- | --- | --- | --- | --- | --- | --- | --- | --- | --- | --- | --- | --- | --- | --- |

Best Unique PSM (Scan Sample\_9\_A1\_1\_3646.d:61988, m/z=911.9636, z=2, RT=18.81, ppm=-16.8):


TRINITY\_DN1653\_c0\_g1\_i3.p1

back to list

  

| Protein Coverage
| Supporting Peptides
| Best Unique PSM
|

Protein Coverage:

Supporting Peptides:

| Peptide | Uniq | -10lgP | Mass | Length | ppm | m/z | z | RT | 1/k0 Start | 1/k0 End | Precursor ID | Area Sample 10 | #Feature | #Feature Sample 10 | Start | End | PTM | AScore | Found By |
| --- | --- | --- | --- | --- | --- | --- | --- | --- | --- | --- | --- | --- | --- | --- | --- | --- | --- | --- | --- |
| R.LINLFEAVSKK.S | Y | 56.67 | 1260.7441 | 11 | 0.94 | 631.3766 | 2 | 18.83 | 0.9804 | 0.9876 | 62338 | 0 | 0 | 0 | 49 | 59 |  |  | DB Search |
| total 1 peptides |  |  |  |  |  |  |  |  |  |  |  |  |  |  |  |  |  |  |  |
| --- | --- | --- | --- | --- | --- | --- | --- | --- | --- | --- | --- | --- | --- | --- | --- | --- | --- | --- | --- |

Best Unique PSM (Scan Sample\_9\_A1\_1\_3646.d:62338, m/z=631.3766, z=2, RT=18.83, ppm=-4.28):


TRINITY\_DN413\_c0\_g1\_i2.p1

back to list

  

| Protein Coverage
| Supporting Peptides
| Best Unique PSM
|

Protein Coverage:

Supporting Peptides:

| Peptide | Uniq | -10lgP | Mass | Length | ppm | m/z | z | RT | 1/k0 Start | 1/k0 End | Precursor ID | Area Sample 10 | #Feature | #Feature Sample 10 | Start | End | PTM | AScore | Found By |
| --- | --- | --- | --- | --- | --- | --- | --- | --- | --- | --- | --- | --- | --- | --- | --- | --- | --- | --- | --- |
| R.LLLGDFNGDKR.T | Y | 58.62 | 1246.6669 | 11 | 3.37 | 624.3396 | 2 | 16.00 | 0.9844 | 0.9916 | 46643 | 2.81e2 | 1 | 1 | 46 | 56 |  |  | DB Search |
| total 1 peptides |  |  |  |  |  |  |  |  |  |  |  |  |  |  |  |  |  |  |  |
| --- | --- | --- | --- | --- | --- | --- | --- | --- | --- | --- | --- | --- | --- | --- | --- | --- | --- | --- | --- |

Best Unique PSM (Scan Sample\_9\_A1\_1\_3646.d:46643, m/z=624.3396, z=2, RT=16.00, ppm=-1.85):


TRINITY\_DN649\_c0\_g1\_i1.p1

back to list

  

| Protein Coverage
| Supporting Peptides
| Best Unique PSM
|

Protein Coverage:

Supporting Peptides:

| Peptide | Uniq | -10lgP | Mass | Length | ppm | m/z | z | RT | 1/k0 Start | 1/k0 End | Precursor ID | Area Sample 10 | #Feature | #Feature Sample 10 | Start | End | PTM | AScore | Found By |
| --- | --- | --- | --- | --- | --- | --- | --- | --- | --- | --- | --- | --- | --- | --- | --- | --- | --- | --- | --- |
| K.FETQIATK.N | Y | 57.09 | 936.4916 | 8 | 7.3 | 937.5009 | 1 | 11.88 | 1.1925 | 1.1997 | 24345 | 0 | 0 | 0 | 102 | 109 |  |  | DB Search |
| total 1 peptides |  |  |  |  |  |  |  |  |  |  |  |  |  |  |  |  |  |  |  |
| --- | --- | --- | --- | --- | --- | --- | --- | --- | --- | --- | --- | --- | --- | --- | --- | --- | --- | --- | --- |

Best Unique PSM (Scan Sample\_9\_A1\_1\_3646.d:24345, m/z=937.5009, z=1, RT=11.88, ppm=2.08):


TRINITY\_DN1058\_c0\_g1\_i2.p2

back to list

  

| Protein Coverage
| Supporting Peptides
| Best Unique PSM
|

Protein Coverage:

Supporting Peptides:

| Peptide | Uniq | -10lgP | Mass | Length | ppm | m/z | z | RT | 1/k0 Start | 1/k0 End | Precursor ID | Area Sample 10 | #Feature | #Feature Sample 10 | Start | End | PTM | AScore | Found By |
| --- | --- | --- | --- | --- | --- | --- | --- | --- | --- | --- | --- | --- | --- | --- | --- | --- | --- | --- | --- |
| R.APTHILNPANKR.S | Y | 58.64 | 1330.7469 | 12 | 0.9 | 666.3779 | 2 | 11.17 | 1.009 | 1.0162 | 20196 | 4.06e2 | 1 | 1 | 975 | 986 |  |  | DB Search |
| total 1 peptides |  |  |  |  |  |  |  |  |  |  |  |  |  |  |  |  |  |  |  |
| --- | --- | --- | --- | --- | --- | --- | --- | --- | --- | --- | --- | --- | --- | --- | --- | --- | --- | --- | --- |

Best Unique PSM (Scan Sample\_9\_A1\_1\_3646.d:20196, m/z=666.3779, z=2, RT=11.17, ppm=-4.32):


TRINITY\_DN4525\_c0\_g2\_i1.p1

back to list

  

| Protein Coverage
| Supporting Peptides
| Best Unique PSM
|

Protein Coverage:

Supporting Peptides:

| Peptide | Uniq | -10lgP | Mass | Length | ppm | m/z | z | RT | 1/k0 Start | 1/k0 End | Precursor ID | Area Sample 10 | #Feature | #Feature Sample 10 | Start | End | PTM | AScore | Found By |
| --- | --- | --- | --- | --- | --- | --- | --- | --- | --- | --- | --- | --- | --- | --- | --- | --- | --- | --- | --- |
| R.LPGFLSPSYGVK.F | Y | 58.24 | 1263.6863 | 12 | -2.25 | 632.8457 | 2 | 18.41 | 0.9465 | 0.9537 | 59860 | 0 | 0 | 0 | 91 | 102 |  |  | DB Search |
| total 1 peptides |  |  |  |  |  |  |  |  |  |  |  |  |  |  |  |  |  |  |  |
| --- | --- | --- | --- | --- | --- | --- | --- | --- | --- | --- | --- | --- | --- | --- | --- | --- | --- | --- | --- |

Best Unique PSM (Scan Sample\_9\_A1\_1\_3646.d:59860, m/z=632.8457, z=2, RT=18.41, ppm=-7.47):


TRINITY\_DN6701\_c0\_g1\_i1.p1

back to list

  

| Protein Coverage
| Supporting Peptides
| Best Unique PSM
|

Protein Coverage:

Supporting Peptides:

| Peptide | Uniq | -10lgP | Mass | Length | ppm | m/z | z | RT | 1/k0 Start | 1/k0 End | Precursor ID | Area Sample 10 | #Feature | #Feature Sample 10 | Start | End | PTM | AScore | Found By |
| --- | --- | --- | --- | --- | --- | --- | --- | --- | --- | --- | --- | --- | --- | --- | --- | --- | --- | --- | --- |
| K.VITSDDPAEKEK.K | Y | 59.05 | 1330.6616 | 12 | 3.57 | 666.337 | 2 | 10.72 | 0.9884 | 0.9957 | 17382 | 1.42e4 | 1 | 1 | 158 | 169 |  |  | DB Search |
| total 1 peptides |  |  |  |  |  |  |  |  |  |  |  |  |  |  |  |  |  |  |  |
| --- | --- | --- | --- | --- | --- | --- | --- | --- | --- | --- | --- | --- | --- | --- | --- | --- | --- | --- | --- |

Best Unique PSM (Scan Sample\_9\_A1\_1\_3646.d:17382, m/z=666.337, z=2, RT=10.72, ppm=-1.65):


TRINITY\_DN119673\_c0\_g1\_i1.p1

back to list

  

| Protein Coverage
| Supporting Peptides
| Best Unique PSM
|

Protein Coverage:

Supporting Peptides:

| Peptide | Uniq | -10lgP | Mass | Length | ppm | m/z | z | RT | 1/k0 Start | 1/k0 End | Precursor ID | Area Sample 10 | #Feature | #Feature Sample 10 | Start | End | PTM | AScore | Found By |
| --- | --- | --- | --- | --- | --- | --- | --- | --- | --- | --- | --- | --- | --- | --- | --- | --- | --- | --- | --- |
| K.VLTSDDPAEKEK | Y | 59.05 | 1330.6616 | 12 | 3.57 | 666.337 | 2 | 10.72 | 0.9884 | 0.9957 | 17382 | 1.42e4 | 1 | 1 | 117 | 128 |  |  | DB Search |
| total 1 peptides |  |  |  |  |  |  |  |  |  |  |  |  |  |  |  |  |  |  |  |
| --- | --- | --- | --- | --- | --- | --- | --- | --- | --- | --- | --- | --- | --- | --- | --- | --- | --- | --- | --- |

Best Unique PSM (Scan Sample\_9\_A1\_1\_3646.d:17382, m/z=666.337, z=2, RT=10.72, ppm=-1.65):


TRINITY\_DN1505\_c0\_g1\_i1.p1

back to list

  

| Protein Coverage
| Supporting Peptides
| Best Unique PSM
|

Protein Coverage:

Supporting Peptides:

| Peptide | Uniq | -10lgP | Mass | Length | ppm | m/z | z | RT | 1/k0 Start | 1/k0 End | Precursor ID | Area Sample 10 | #Feature | #Feature Sample 10 | Start | End | PTM | AScore | Found By |
| --- | --- | --- | --- | --- | --- | --- | --- | --- | --- | --- | --- | --- | --- | --- | --- | --- | --- | --- | --- |
| M.S(+42.01)LVIPEKFQHILR.V | Y | 58.95 | 1620.9351 | 13 | 2.83 | 811.4729 | 2 | 19.56 | 1.1222 | 1.1294 | 66460 | 1.08e4 | 2 | 2 | 2 | 14 | Acetylation (Protein N-term) | S1:Acetylation (Protein N-term):1000 | DB Search |
| total 1 peptides |  |  |  |  |  |  |  |  |  |  |  |  |  |  |  |  |  |  |  |
| --- | --- | --- | --- | --- | --- | --- | --- | --- | --- | --- | --- | --- | --- | --- | --- | --- | --- | --- | --- |

Best Unique PSM (Scan Sample\_9\_A1\_1\_3646.d:66460, m/z=811.4729, z=2, RT=19.56, ppm=-2.39):


TRINITY\_DN36\_c0\_g1\_i11.p1

back to list

  

| Protein Coverage
| Supporting Peptides
| Best Unique PSM
|

Protein Coverage:

Supporting Peptides:

| Peptide | Uniq | -10lgP | Mass | Length | ppm | m/z | z | RT | 1/k0 Start | 1/k0 End | Precursor ID | Area Sample 10 | #Feature | #Feature Sample 10 | Start | End | PTM | AScore | Found By |
| --- | --- | --- | --- | --- | --- | --- | --- | --- | --- | --- | --- | --- | --- | --- | --- | --- | --- | --- | --- |
| R.LGGIEVTQGNC(+57.02)ARK.Y | Y | 58.24 | 1501.7671 | 14 | 6.42 | 751.8917 | 2 | 12.66 | 1.0632 | 1.0705 | 28959 | 0 | 0 | 0 | 58 | 71 | Carbamidomethylation |  | DB Search |
| total 1 peptides |  |  |  |  |  |  |  |  |  |  |  |  |  |  |  |  |  |  |  |
| --- | --- | --- | --- | --- | --- | --- | --- | --- | --- | --- | --- | --- | --- | --- | --- | --- | --- | --- | --- |

Best Unique PSM (Scan Sample\_9\_A1\_1\_3646.d:28959, m/z=751.8917, z=2, RT=12.66, ppm=1.21):


TRINITY\_DN6397\_c0\_g2\_i4.p1

back to list

  

| Protein Coverage
| Supporting Peptides
| Best Unique PSM
|

Protein Coverage:

Supporting Peptides:

| Peptide | Uniq | -10lgP | Mass | Length | ppm | m/z | z | RT | 1/k0 Start | 1/k0 End | Precursor ID | Area Sample 10 | #Feature | #Feature Sample 10 | Start | End | PTM | AScore | Found By |
| --- | --- | --- | --- | --- | --- | --- | --- | --- | --- | --- | --- | --- | --- | --- | --- | --- | --- | --- | --- |
| K.K(+42.01)LSAEVANGR.L | Y | 58.76 | 1085.5829 | 10 | -2.46 | 543.7946 | 2 | 12.92 | 0.8567 | 0.864 | 30461 | 2.87e3 | 1 | 1 | 12 | 21 | Acetylation (Protein N-term) | K1:Acetylation (Protein N-term):1000 | DB Search |
| total 1 peptides |  |  |  |  |  |  |  |  |  |  |  |  |  |  |  |  |  |  |  |
| --- | --- | --- | --- | --- | --- | --- | --- | --- | --- | --- | --- | --- | --- | --- | --- | --- | --- | --- | --- |

Best Unique PSM (Scan Sample\_9\_A1\_1\_3646.d:30461, m/z=543.7946, z=2, RT=12.92, ppm=-7.68):


TRINITY\_DN36\_c0\_g1\_i19.p1

back to list

  

| Protein Coverage
| Supporting Peptides
| Best Unique PSM
|

Protein Coverage:

Supporting Peptides:

| Peptide | Uniq | -10lgP | Mass | Length | ppm | m/z | z | RT | 1/k0 Start | 1/k0 End | Precursor ID | Area Sample 10 | #Feature | #Feature Sample 10 | Start | End | PTM | AScore | Found By |
| --- | --- | --- | --- | --- | --- | --- | --- | --- | --- | --- | --- | --- | --- | --- | --- | --- | --- | --- | --- |
| R.LGGIEVTQGNC(+57.02)ARK.Y | Y | 58.24 | 1501.7671 | 14 | 6.42 | 751.8917 | 2 | 12.66 | 1.0632 | 1.0705 | 28959 | 0 | 0 | 0 | 57 | 70 | Carbamidomethylation |  | DB Search |
| K.FETQIATK.D | Y | 57.09 | 936.4916 | 8 | 7.3 | 937.5009 | 1 | 11.88 | 1.1925 | 1.1997 | 24345 | 0 | 0 | 0 | 166 | 173 |  |  | DB Search |
| total 2 peptides |  |  |  |  |  |  |  |  |  |  |  |  |  |  |  |  |  |  |  |
| --- | --- | --- | --- | --- | --- | --- | --- | --- | --- | --- | --- | --- | --- | --- | --- | --- | --- | --- | --- |

Best Unique PSM (Scan Sample\_9\_A1\_1\_3646.d:28959, m/z=751.8917, z=2, RT=12.66, ppm=1.21):


TRINITY\_DN12304\_c0\_g1\_i1.p1

back to list

  

| Protein Coverage
| Supporting Peptides
| Best Unique PSM
|

Protein Coverage:

Supporting Peptides:

| Peptide | Uniq | -10lgP | Mass | Length | ppm | m/z | z | RT | 1/k0 Start | 1/k0 End | Precursor ID | Area Sample 10 | #Feature | #Feature Sample 10 | Start | End | PTM | AScore | Found By |
| --- | --- | --- | --- | --- | --- | --- | --- | --- | --- | --- | --- | --- | --- | --- | --- | --- | --- | --- | --- |
| M.S(+42.01)SSSEPTKFK.M | Y | 54.44 | 1138.5506 | 10 | 7.5 | 570.2839 | 2 | 11.58 | 0.9222 | 0.9295 | 22579 | 0 | 0 | 0 | 2 | 11 | Acetylation (Protein N-term) | S1:Acetylation (Protein N-term):1000 | DB Search |
| total 1 peptides |  |  |  |  |  |  |  |  |  |  |  |  |  |  |  |  |  |  |  |
| --- | --- | --- | --- | --- | --- | --- | --- | --- | --- | --- | --- | --- | --- | --- | --- | --- | --- | --- | --- |

Best Unique PSM (Scan Sample\_9\_A1\_1\_3646.d:22579, m/z=570.2839, z=2, RT=11.58, ppm=2.28):


TRINITY\_DN36\_c0\_g1\_i5.p1

back to list

  

| Protein Coverage
| Supporting Peptides
| Best Unique PSM
|

Protein Coverage:

Supporting Peptides:

| Peptide | Uniq | -10lgP | Mass | Length | ppm | m/z | z | RT | 1/k0 Start | 1/k0 End | Precursor ID | Area Sample 10 | #Feature | #Feature Sample 10 | Start | End | PTM | AScore | Found By |
| --- | --- | --- | --- | --- | --- | --- | --- | --- | --- | --- | --- | --- | --- | --- | --- | --- | --- | --- | --- |
| R.LGGIEVTQGNC(+57.02)ARK.Y | Y | 58.24 | 1501.7671 | 14 | 6.42 | 751.8917 | 2 | 12.66 | 1.0632 | 1.0705 | 28959 | 0 | 0 | 0 | 57 | 70 | Carbamidomethylation |  | DB Search |
| total 1 peptides |  |  |  |  |  |  |  |  |  |  |  |  |  |  |  |  |  |  |  |
| --- | --- | --- | --- | --- | --- | --- | --- | --- | --- | --- | --- | --- | --- | --- | --- | --- | --- | --- | --- |

Best Unique PSM (Scan Sample\_9\_A1\_1\_3646.d:28959, m/z=751.8917, z=2, RT=12.66, ppm=1.21):


TRINITY\_DN4329\_c0\_g1\_i1.p1

back to list

  

| Protein Coverage
| Supporting Peptides
| Best Unique PSM
|

Protein Coverage:

Supporting Peptides:

| Peptide | Uniq | -10lgP | Mass | Length | ppm | m/z | z | RT | 1/k0 Start | 1/k0 End | Precursor ID | Area Sample 10 | #Feature | #Feature Sample 10 | Start | End | PTM | AScore | Found By |
| --- | --- | --- | --- | --- | --- | --- | --- | --- | --- | --- | --- | --- | --- | --- | --- | --- | --- | --- | --- |
| R.ITELSPIRPR.Y | Y | 58.21 | 1180.6928 | 10 | 0.83 | 591.3511 | 2 | 13.86 | 0.9271 | 0.9344 | 35586 | 6.88e2 | 1 | 1 | 104 | 113 |  |  | DB Search |
| R.IRITELSPIRPR.Y | Y | 57.49 | 1449.8779 | 12 | 2.32 | 484.2985 | 3 | 15.24 | 0.8193 | 0.8266 | 42750 | 1.52e3 | 1 | 1 | 102 | 113 |  |  | DB Search |
| total 2 peptides |  |  |  |  |  |  |  |  |  |  |  |  |  |  |  |  |  |  |  |
| --- | --- | --- | --- | --- | --- | --- | --- | --- | --- | --- | --- | --- | --- | --- | --- | --- | --- | --- | --- |

Best Unique PSM (Scan Sample\_9\_A1\_1\_3646.d:35586, m/z=591.3511, z=2, RT=13.86, ppm=-4.39):


TRINITY\_DN7214\_c0\_g1\_i1.p1

back to list

  

| Protein Coverage
| Supporting Peptides
| Best Unique PSM
|

Protein Coverage:

Supporting Peptides:

| Peptide | Uniq | -10lgP | Mass | Length | ppm | m/z | z | RT | 1/k0 Start | 1/k0 End | Precursor ID | Area Sample 10 | #Feature | #Feature Sample 10 | Start | End | PTM | AScore | Found By |
| --- | --- | --- | --- | --- | --- | --- | --- | --- | --- | --- | --- | --- | --- | --- | --- | --- | --- | --- | --- |
| R.LAADLVITHSSK.K | Y | 58.63 | 1253.6979 | 12 | 2.83 | 627.8547 | 2 | 14.22 | 0.9973 | 1.0045 | 37483 | 1.32e3 | 1 | 1 | 167 | 178 |  |  | DB Search |
| total 1 peptides |  |  |  |  |  |  |  |  |  |  |  |  |  |  |  |  |  |  |  |
| --- | --- | --- | --- | --- | --- | --- | --- | --- | --- | --- | --- | --- | --- | --- | --- | --- | --- | --- | --- |

Best Unique PSM (Scan Sample\_9\_A1\_1\_3646.d:37483, m/z=627.8547, z=2, RT=14.22, ppm=-2.39):


TRINITY\_DN1189\_c0\_g1\_i1.p1

back to list

  

| Protein Coverage
| Supporting Peptides
| Best Unique PSM
|

Protein Coverage:

Supporting Peptides:

| Peptide | Uniq | -10lgP | Mass | Length | ppm | m/z | z | RT | 1/k0 Start | 1/k0 End | Precursor ID | Area Sample 10 | #Feature | #Feature Sample 10 | Start | End | PTM | AScore | Found By |
| --- | --- | --- | --- | --- | --- | --- | --- | --- | --- | --- | --- | --- | --- | --- | --- | --- | --- | --- | --- |
| K.AKDVTAAVKK.A | Y | 47.69 | 1029.6182 | 10 | -2.2 | 515.8126 | 2 | 9.42 | 0.838 | 0.8453 | 10489 | 3.2e2 | 1 | 1 | 185 | 194 |  |  | DB Search |
| total 1 peptides |  |  |  |  |  |  |  |  |  |  |  |  |  |  |  |  |  |  |  |
| --- | --- | --- | --- | --- | --- | --- | --- | --- | --- | --- | --- | --- | --- | --- | --- | --- | --- | --- | --- |

Best Unique PSM (Scan Sample\_9\_A1\_1\_3646.d:10489, m/z=515.8126, z=2, RT=9.42, ppm=-7.42):


TRINITY\_DN925\_c0\_g1\_i1.p1

back to list

  

| Protein Coverage
| Supporting Peptides
| Best Unique PSM
|

Protein Coverage:

Supporting Peptides:

| Peptide | Uniq | -10lgP | Mass | Length | ppm | m/z | z | RT | 1/k0 Start | 1/k0 End | Precursor ID | Area Sample 10 | #Feature | #Feature Sample 10 | Start | End | PTM | AScore | Found By |
| --- | --- | --- | --- | --- | --- | --- | --- | --- | --- | --- | --- | --- | --- | --- | --- | --- | --- | --- | --- |
| R.ASGDYATVVSHNR.D | Y | 58.68 | 1375.648 | 13 | 14.02 | 688.8373 | 2 | 12.12 | 0.9969 | 1.0041 | 25619 | 3.01e3 | 1 | 1 | 129 | 141 |  |  | DB Search |
| total 1 peptides |  |  |  |  |  |  |  |  |  |  |  |  |  |  |  |  |  |  |  |
| --- | --- | --- | --- | --- | --- | --- | --- | --- | --- | --- | --- | --- | --- | --- | --- | --- | --- | --- | --- |

Best Unique PSM (Scan Sample\_9\_A1\_1\_3646.d:25619, m/z=688.8373, z=2, RT=12.12, ppm=8.8):


TRINITY\_DN152\_c0\_g1\_i2.p1

back to list

  

| Protein Coverage
| Supporting Peptides
| Best Unique PSM
|

Protein Coverage:

Supporting Peptides:

| Peptide | Uniq | -10lgP | Mass | Length | ppm | m/z | z | RT | 1/k0 Start | 1/k0 End | Precursor ID | Area Sample 10 | #Feature | #Feature Sample 10 | Start | End | PTM | AScore | Found By |
| --- | --- | --- | --- | --- | --- | --- | --- | --- | --- | --- | --- | --- | --- | --- | --- | --- | --- | --- | --- |
| K.IQDKEGIPPDQQR.L | Y | 58.25 | 1522.7739 | 13 | 2.55 | 762.3922 | 2 | 11.03 | 1.0343 | 1.0415 | 18957 | 1.53e3 | 1 | 1 | 66 | 78 |  |  | DB Search |
| total 1 peptides |  |  |  |  |  |  |  |  |  |  |  |  |  |  |  |  |  |  |  |
| --- | --- | --- | --- | --- | --- | --- | --- | --- | --- | --- | --- | --- | --- | --- | --- | --- | --- | --- | --- |

Best Unique PSM (Scan Sample\_9\_A1\_1\_3646.d:18957, m/z=762.3922, z=2, RT=11.03, ppm=-2.67):


TRINITY\_DN817\_c0\_g1\_i2.p1

back to list

  

| Protein Coverage
| Supporting Peptides
| Best Unique PSM
|

Protein Coverage:

Supporting Peptides:

| Peptide | Uniq | -10lgP | Mass | Length | ppm | m/z | z | RT | 1/k0 Start | 1/k0 End | Precursor ID | Area Sample 10 | #Feature | #Feature Sample 10 | Start | End | PTM | AScore | Found By |
| --- | --- | --- | --- | --- | --- | --- | --- | --- | --- | --- | --- | --- | --- | --- | --- | --- | --- | --- | --- |
| R.LC(+57.02)QPGYSTC(+57.02)ENHC(+57.02)R.R | Y | 55.11 | 1780.708 | 14 | 0.84 | 891.3574 | 2 | 11.42 | 1.0861 | 1.0933 | 21509 | 5.05e2 | 1 | 1 | 145 | 158 | Carbamidomethylation, Carbamidomethylation, Carbamidomethylation |  | DB Search |
| R.LC(+57.02)QPGYSTC(+57.02)ENHC(+57.02)RR.L | Y | 43.99 | 1936.8091 | 15 | 3.79 | 646.6094 | 3 | 10.89 | 0.8575 | 0.8648 | 18436 | 3.51e2 | 1 | 1 | 145 | 159 | Carbamidomethylation, Carbamidomethylation, Carbamidomethylation |  | DB Search |
| total 2 peptides |  |  |  |  |  |  |  |  |  |  |  |  |  |  |  |  |  |  |  |
| --- | --- | --- | --- | --- | --- | --- | --- | --- | --- | --- | --- | --- | --- | --- | --- | --- | --- | --- | --- |

Best Unique PSM (Scan Sample\_9\_A1\_1\_3646.d:21509, m/z=891.3574, z=2, RT=11.42, ppm=-4.38):


TRINITY\_DN651\_c0\_g1\_i1.p1

back to list

  

| Protein Coverage
| Supporting Peptides
| Best Unique PSM
|

Protein Coverage:

Supporting Peptides:

| Peptide | Uniq | -10lgP | Mass | Length | ppm | m/z | z | RT | 1/k0 Start | 1/k0 End | Precursor ID | Area Sample 10 | #Feature | #Feature Sample 10 | Start | End | PTM | AScore | Found By |
| --- | --- | --- | --- | --- | --- | --- | --- | --- | --- | --- | --- | --- | --- | --- | --- | --- | --- | --- | --- |
| R.VVAISGNEDEIVNLLHK.I | Y | 58.48 | 1848.9945 | 17 | 1.85 | 925.5014 | 2 | 18.76 | 1.2109 | 1.2181 | 61890 | 0 | 0 | 0 | 160 | 176 |  |  | DB Search |
| total 1 peptides |  |  |  |  |  |  |  |  |  |  |  |  |  |  |  |  |  |  |  |
| --- | --- | --- | --- | --- | --- | --- | --- | --- | --- | --- | --- | --- | --- | --- | --- | --- | --- | --- | --- |

Best Unique PSM (Scan Sample\_9\_A1\_1\_3646.d:61890, m/z=925.5014, z=2, RT=18.76, ppm=-3.37):


TRINITY\_DN4595\_c0\_g1\_i10.p2

back to list

  

| Protein Coverage
| Supporting Peptides
| Best Unique PSM
|

Protein Coverage:

Supporting Peptides:

| Peptide | Uniq | -10lgP | Mass | Length | ppm | m/z | z | RT | 1/k0 Start | 1/k0 End | Precursor ID | Area Sample 10 | #Feature | #Feature Sample 10 | Start | End | PTM | AScore | Found By |
| --- | --- | --- | --- | --- | --- | --- | --- | --- | --- | --- | --- | --- | --- | --- | --- | --- | --- | --- | --- |
| R.AFNRVETR.D | Y | 50.97 | 991.5199 | 8 | -0.72 | 496.7643 | 2 | 10.73 | 0.8534 | 0.8607 | 17391 | 3.08e3 | 1 | 1 | 152 | 159 |  |  | DB Search |
| total 1 peptides |  |  |  |  |  |  |  |  |  |  |  |  |  |  |  |  |  |  |  |
| --- | --- | --- | --- | --- | --- | --- | --- | --- | --- | --- | --- | --- | --- | --- | --- | --- | --- | --- | --- |

Best Unique PSM (Scan Sample\_9\_A1\_1\_3646.d:17391, m/z=496.7643, z=2, RT=10.73, ppm=-5.95):


TRINITY\_DN33065\_c0\_g2\_i1.p1

back to list

  

| Protein Coverage
| Supporting Peptides
| Best Unique PSM
|

Protein Coverage:

Supporting Peptides:

| Peptide | Uniq | -10lgP | Mass | Length | ppm | m/z | z | RT | 1/k0 Start | 1/k0 End | Precursor ID | Area Sample 10 | #Feature | #Feature Sample 10 | Start | End | PTM | AScore | Found By |
| --- | --- | --- | --- | --- | --- | --- | --- | --- | --- | --- | --- | --- | --- | --- | --- | --- | --- | --- | --- |
| R.AFSFSDKPAITFEIHDFK.M | Y | 53.11 | 2099.0363 | 18 | 1.35 | 700.6833 | 3 | 18.88 | 0.9795 | 0.9868 | 62540 | 4.97e2 | 1 | 1 | 595 | 612 |  |  | DB Search |
| total 1 peptides |  |  |  |  |  |  |  |  |  |  |  |  |  |  |  |  |  |  |  |
| --- | --- | --- | --- | --- | --- | --- | --- | --- | --- | --- | --- | --- | --- | --- | --- | --- | --- | --- | --- |

Best Unique PSM (Scan Sample\_9\_A1\_1\_3646.d:62540, m/z=700.6833, z=3, RT=18.88, ppm=-3.87):


TRINITY\_DN36\_c0\_g1\_i17.p1

back to list

  

| Protein Coverage
| Supporting Peptides
| Best Unique PSM
|

Protein Coverage:

Supporting Peptides:

| Peptide | Uniq | -10lgP | Mass | Length | ppm | m/z | z | RT | 1/k0 Start | 1/k0 End | Precursor ID | Area Sample 10 | #Feature | #Feature Sample 10 | Start | End | PTM | AScore | Found By |
| --- | --- | --- | --- | --- | --- | --- | --- | --- | --- | --- | --- | --- | --- | --- | --- | --- | --- | --- | --- |
| R.LGGIEVTQGNC(+57.02)ARK.Y | Y | 58.24 | 1501.7671 | 14 | 6.42 | 751.8917 | 2 | 12.66 | 1.0632 | 1.0705 | 28959 | 0 | 0 | 0 | 58 | 71 | Carbamidomethylation |  | DB Search |
| K.FETQIATK.D | Y | 57.09 | 936.4916 | 8 | 7.3 | 937.5009 | 1 | 11.88 | 1.1925 | 1.1997 | 24345 | 0 | 0 | 0 | 167 | 174 |  |  | DB Search |
| total 2 peptides |  |  |  |  |  |  |  |  |  |  |  |  |  |  |  |  |  |  |  |
| --- | --- | --- | --- | --- | --- | --- | --- | --- | --- | --- | --- | --- | --- | --- | --- | --- | --- | --- | --- |

Best Unique PSM (Scan Sample\_9\_A1\_1\_3646.d:28959, m/z=751.8917, z=2, RT=12.66, ppm=1.21):


TRINITY\_DN3991\_c0\_g1\_i1.p1

back to list

  

| Protein Coverage
| Supporting Peptides
| Best Unique PSM
|

Protein Coverage:

Supporting Peptides:

| Peptide | Uniq | -10lgP | Mass | Length | ppm | m/z | z | RT | 1/k0 Start | 1/k0 End | Precursor ID | Area Sample 10 | #Feature | #Feature Sample 10 | Start | End | PTM | AScore | Found By |
| --- | --- | --- | --- | --- | --- | --- | --- | --- | --- | --- | --- | --- | --- | --- | --- | --- | --- | --- | --- |
| R.AKLSQC(+57.02)TAASGK.H | Y | 59.04 | 1220.6183 | 12 | 5.98 | 611.3169 | 2 | 9.70 | 0.9505 | 0.9578 | 11647 | 3.34e3 | 1 | 1 | 128 | 139 | Carbamidomethylation |  | DB Search |
| K.LSQC(+57.02)TAASGK.H | Y | 58.62 | 1021.4862 | 10 | -1.58 | 511.7469 | 2 | 9.83 | 0.8274 | 0.8348 | 11429 | 2.43e5 | 1 | 1 | 130 | 139 | Carbamidomethylation |  | DB Search |
| R.HGQVDSC(+57.02)INQNLKK.N | Y | 57.82 | 1639.81 | 14 | -0.96 | 547.6072 | 3 | 11.98 | 0.836 | 0.8433 | 25047 | 0 | 0 | 0 | 112 | 125 | Carbamidomethylation |  | DB Search |
| total 3 peptides |  |  |  |  |  |  |  |  |  |  |  |  |  |  |  |  |  |  |  |
| --- | --- | --- | --- | --- | --- | --- | --- | --- | --- | --- | --- | --- | --- | --- | --- | --- | --- | --- | --- |

Best Unique PSM (Scan Sample\_9\_A1\_1\_3646.d:11647, m/z=611.3169, z=2, RT=9.70, ppm=0.76):


TRINITY\_DN152\_c0\_g1\_i15.p1

back to list

  

| Protein Coverage
| Supporting Peptides
| Best Unique PSM
|

Protein Coverage:

Supporting Peptides:

| Peptide | Uniq | -10lgP | Mass | Length | ppm | m/z | z | RT | 1/k0 Start | 1/k0 End | Precursor ID | Area Sample 10 | #Feature | #Feature Sample 10 | Start | End | PTM | AScore | Found By |
| --- | --- | --- | --- | --- | --- | --- | --- | --- | --- | --- | --- | --- | --- | --- | --- | --- | --- | --- | --- |
| K.IQDKEGIPPDQQR.L | Y | 58.25 | 1522.7739 | 13 | 2.55 | 762.3922 | 2 | 11.03 | 1.0343 | 1.0415 | 18957 | 1.53e3 | 1 | 1 | 60 | 72 |  |  | DB Search |
| total 1 peptides |  |  |  |  |  |  |  |  |  |  |  |  |  |  |  |  |  |  |  |
| --- | --- | --- | --- | --- | --- | --- | --- | --- | --- | --- | --- | --- | --- | --- | --- | --- | --- | --- | --- |

Best Unique PSM (Scan Sample\_9\_A1\_1\_3646.d:18957, m/z=762.3922, z=2, RT=11.03, ppm=-2.67):


TRINITY\_DN4762\_c0\_g1\_i1.p1

back to list

  

| Protein Coverage
| Supporting Peptides
| Best Unique PSM
|

Protein Coverage:

Supporting Peptides:

| Peptide | Uniq | -10lgP | Mass | Length | ppm | m/z | z | RT | 1/k0 Start | 1/k0 End | Precursor ID | Area Sample 10 | #Feature | #Feature Sample 10 | Start | End | PTM | AScore | Found By |
| --- | --- | --- | --- | --- | --- | --- | --- | --- | --- | --- | --- | --- | --- | --- | --- | --- | --- | --- | --- |
| K.SPC(+57.02)PPRK.S | Y | 30.36 | 840.4276 | 7 | 3.69 | 421.2204 | 2 | 9.55 | 0.7909 | 0.7982 | 10837 | 4.62e3 | 1 | 1 | 314 | 320 | Carbamidomethylation |  | DB Search |
| total 1 peptides |  |  |  |  |  |  |  |  |  |  |  |  |  |  |  |  |  |  |  |
| --- | --- | --- | --- | --- | --- | --- | --- | --- | --- | --- | --- | --- | --- | --- | --- | --- | --- | --- | --- |

Best Unique PSM (Scan Sample\_9\_A1\_1\_3646.d:10837, m/z=421.2204, z=2, RT=9.55, ppm=-1.53):


TRINITY\_DN2477\_c0\_g1\_i2.p1

back to list

  

| Protein Coverage
| Supporting Peptides
| Best Unique PSM
|

Protein Coverage:

Supporting Peptides:

| Peptide | Uniq | -10lgP | Mass | Length | ppm | m/z | z | RT | 1/k0 Start | 1/k0 End | Precursor ID | Area Sample 10 | #Feature | #Feature Sample 10 | Start | End | PTM | AScore | Found By |
| --- | --- | --- | --- | --- | --- | --- | --- | --- | --- | --- | --- | --- | --- | --- | --- | --- | --- | --- | --- |
| K.DSYVGDEAQSKR.G | Y | 59.06 | 1353.616 | 12 | 5.32 | 677.8154 | 2 | 10.95 | 1.0041 | 1.0114 | 18674 | 7.12e3 | 1 | 1 | 19 | 30 |  |  | DB Search |
| R.VAPEEHPVLLTEAPLNPK.A | Y | 58.04 | 1953.0571 | 18 | 3.48 | 977.5341 | 2 | 16.43 | 1.2045 | 1.2117 | 48611 | 4.81e2 | 1 | 1 | 64 | 81 |  |  | DB Search |
| total 2 peptides |  |  |  |  |  |  |  |  |  |  |  |  |  |  |  |  |  |  |  |
| --- | --- | --- | --- | --- | --- | --- | --- | --- | --- | --- | --- | --- | --- | --- | --- | --- | --- | --- | --- |

Best Unique PSM (Scan Sample\_9\_A1\_1\_3646.d:18674, m/z=677.8154, z=2, RT=10.95, ppm=0.1):


TRINITY\_DN2885\_c0\_g1\_i4.p1

back to list

  

| Protein Coverage
| Supporting Peptides
| Best Unique PSM
|

Protein Coverage:

Supporting Peptides:

| Peptide | Uniq | -10lgP | Mass | Length | ppm | m/z | z | RT | 1/k0 Start | 1/k0 End | Precursor ID | Area Sample 10 | #Feature | #Feature Sample 10 | Start | End | PTM | AScore | Found By |
| --- | --- | --- | --- | --- | --- | --- | --- | --- | --- | --- | --- | --- | --- | --- | --- | --- | --- | --- | --- |
| K.VTRPHGASGVVR.A | Y | 58.63 | 1234.6894 | 12 | 0.38 | 412.5684 | 3 | 10.08 | 0.8197 | 0.827 | 13527 | 3.84e3 | 1 | 1 | 72 | 83 |  |  | DB Search |
| total 1 peptides |  |  |  |  |  |  |  |  |  |  |  |  |  |  |  |  |  |  |  |
| --- | --- | --- | --- | --- | --- | --- | --- | --- | --- | --- | --- | --- | --- | --- | --- | --- | --- | --- | --- |

Best Unique PSM (Scan Sample\_9\_A1\_1\_3646.d:13527, m/z=412.5684, z=3, RT=10.08, ppm=-4.85):


TRINITY\_DN152\_c0\_g1\_i10.p1

back to list

  

| Protein Coverage
| Supporting Peptides
| Best Unique PSM
|

Protein Coverage:

Supporting Peptides:

| Peptide | Uniq | -10lgP | Mass | Length | ppm | m/z | z | RT | 1/k0 Start | 1/k0 End | Precursor ID | Area Sample 10 | #Feature | #Feature Sample 10 | Start | End | PTM | AScore | Found By |
| --- | --- | --- | --- | --- | --- | --- | --- | --- | --- | --- | --- | --- | --- | --- | --- | --- | --- | --- | --- |
| K.IQDKEGIPPDQQR.L | Y | 58.25 | 1522.7739 | 13 | 2.55 | 762.3922 | 2 | 11.03 | 1.0343 | 1.0415 | 18957 | 1.53e3 | 1 | 1 | 141 | 153 |  |  | DB Search |
| total 1 peptides |  |  |  |  |  |  |  |  |  |  |  |  |  |  |  |  |  |  |  |
| --- | --- | --- | --- | --- | --- | --- | --- | --- | --- | --- | --- | --- | --- | --- | --- | --- | --- | --- | --- |

Best Unique PSM (Scan Sample\_9\_A1\_1\_3646.d:18957, m/z=762.3922, z=2, RT=11.03, ppm=-2.67):


TRINITY\_DN189323\_c0\_g1\_i1.p1

back to list

  

| Protein Coverage
| Supporting Peptides
| Best Unique PSM
|

Protein Coverage:

Supporting Peptides:

| Peptide | Uniq | -10lgP | Mass | Length | ppm | m/z | z | RT | 1/k0 Start | 1/k0 End | Precursor ID | Area Sample 10 | #Feature | #Feature Sample 10 | Start | End | PTM | AScore | Found By |
| --- | --- | --- | --- | --- | --- | --- | --- | --- | --- | --- | --- | --- | --- | --- | --- | --- | --- | --- | --- |
| R.LFEGNALLRR.L | Y | 58.61 | 1187.6775 | 10 | 3.48 | 594.845 | 2 | 15.57 | 0.9679 | 0.9751 | 44463 | 2.7e3 | 1 | 1 | 32 | 41 |  |  | DB Search |
| R.KQLVNVPSFIVR.L | Y | 57.5 | 1398.8347 | 12 | -4.99 | 700.4175 | 2 | 17.75 | 1.0081 | 1.0154 | 56022 | 0 | 0 | 0 | 100 | 111 |  |  | DB Search |
| K.RLFEGNALLRR.L | Y | 56.27 | 1343.7786 | 11 | -1.1 | 448.9306 | 3 | 14.75 | 0.7547 | 0.762 | 40382 | 0 | 0 | 0 | 31 | 41 |  |  | DB Search |
| K.RLFEGNALLR.R | Y | 54.07 | 1187.6775 | 10 | 1.11 | 594.8436 | 2 | 16.07 | 0.9396 | 0.9469 | 46969 | 1.45e3 | 1 | 1 | 31 | 40 |  |  | DB Search |
| total 4 peptides |  |  |  |  |  |  |  |  |  |  |  |  |  |  |  |  |  |  |  |
| --- | --- | --- | --- | --- | --- | --- | --- | --- | --- | --- | --- | --- | --- | --- | --- | --- | --- | --- | --- |

Best Unique PSM (Scan Sample\_9\_A1\_1\_3646.d:44463, m/z=594.845, z=2, RT=15.57, ppm=-1.74):


TRINITY\_DN5188\_c0\_g1\_i4.p1

back to list

  

| Protein Coverage
| Supporting Peptides
| Best Unique PSM
|

Protein Coverage:

Supporting Peptides:

| Peptide | Uniq | -10lgP | Mass | Length | ppm | m/z | z | RT | 1/k0 Start | 1/k0 End | Precursor ID | Area Sample 10 | #Feature | #Feature Sample 10 | Start | End | PTM | AScore | Found By |
| --- | --- | --- | --- | --- | --- | --- | --- | --- | --- | --- | --- | --- | --- | --- | --- | --- | --- | --- | --- |
| K.LRQEQLKEER.K | Y | 56.74 | 1327.7208 | 10 | 3.91 | 443.5803 | 3 | 10.26 | 0.7949 | 0.8023 | 14744 | 0 | 0 | 0 | 334 | 343 |  |  | DB Search |
| total 1 peptides |  |  |  |  |  |  |  |  |  |  |  |  |  |  |  |  |  |  |  |
| --- | --- | --- | --- | --- | --- | --- | --- | --- | --- | --- | --- | --- | --- | --- | --- | --- | --- | --- | --- |

Best Unique PSM (Scan Sample\_9\_A1\_1\_3646.d:14744, m/z=443.5803, z=3, RT=10.26, ppm=-1.31):


TRINITY\_DN1172\_c0\_g1\_i5.p1

back to list

  

| Protein Coverage
| Supporting Peptides
| Best Unique PSM
|

Protein Coverage:

Supporting Peptides:

| Peptide | Uniq | -10lgP | Mass | Length | ppm | m/z | z | RT | 1/k0 Start | 1/k0 End | Precursor ID | Area Sample 10 | #Feature | #Feature Sample 10 | Start | End | PTM | AScore | Found By |
| --- | --- | --- | --- | --- | --- | --- | --- | --- | --- | --- | --- | --- | --- | --- | --- | --- | --- | --- | --- |
| K.VITSDDPAEKEK.K | Y | 59.05 | 1330.6616 | 12 | 3.57 | 666.337 | 2 | 10.72 | 0.9884 | 0.9957 | 17382 | 1.42e4 | 1 | 1 | 123 | 134 |  |  | DB Search |
| total 1 peptides |  |  |  |  |  |  |  |  |  |  |  |  |  |  |  |  |  |  |  |
| --- | --- | --- | --- | --- | --- | --- | --- | --- | --- | --- | --- | --- | --- | --- | --- | --- | --- | --- | --- |

Best Unique PSM (Scan Sample\_9\_A1\_1\_3646.d:17382, m/z=666.337, z=2, RT=10.72, ppm=-1.65):


TRINITY\_DN2941\_c0\_g1\_i4.p1

back to list

  

| Protein Coverage
| Supporting Peptides
| Best Unique PSM
|

Protein Coverage:

Supporting Peptides:

| Peptide | Uniq | -10lgP | Mass | Length | ppm | m/z | z | RT | 1/k0 Start | 1/k0 End | Precursor ID | Area Sample 10 | #Feature | #Feature Sample 10 | Start | End | PTM | AScore | Found By |
| --- | --- | --- | --- | --- | --- | --- | --- | --- | --- | --- | --- | --- | --- | --- | --- | --- | --- | --- | --- |
| R.VAPEEHPVLLTEAPLNPK.A | Y | 58.04 | 1953.0571 | 18 | 3.48 | 977.5341 | 2 | 16.43 | 1.2045 | 1.2117 | 48611 | 4.81e2 | 1 | 1 | 30 | 47 |  |  | DB Search |
| total 1 peptides |  |  |  |  |  |  |  |  |  |  |  |  |  |  |  |  |  |  |  |
| --- | --- | --- | --- | --- | --- | --- | --- | --- | --- | --- | --- | --- | --- | --- | --- | --- | --- | --- | --- |

Best Unique PSM (Scan Sample\_9\_A1\_1\_3646.d:48611, m/z=977.5341, z=2, RT=16.43, ppm=-1.73):


TRINITY\_DN817\_c0\_g1\_i9.p1

back to list

  

| Protein Coverage
| Supporting Peptides
| Best Unique PSM
|

Protein Coverage:

Supporting Peptides:

| Peptide | Uniq | -10lgP | Mass | Length | ppm | m/z | z | RT | 1/k0 Start | 1/k0 End | Precursor ID | Area Sample 10 | #Feature | #Feature Sample 10 | Start | End | PTM | AScore | Found By |
| --- | --- | --- | --- | --- | --- | --- | --- | --- | --- | --- | --- | --- | --- | --- | --- | --- | --- | --- | --- |
| R.LC(+57.02)QPGYSTC(+57.02)ENHC(+57.02)R.K | Y | 55.11 | 1780.708 | 14 | 0.84 | 891.3574 | 2 | 11.42 | 1.0861 | 1.0933 | 21509 | 5.05e2 | 1 | 1 | 153 | 166 | Carbamidomethylation, Carbamidomethylation, Carbamidomethylation |  | DB Search |
| total 1 peptides |  |  |  |  |  |  |  |  |  |  |  |  |  |  |  |  |  |  |  |
| --- | --- | --- | --- | --- | --- | --- | --- | --- | --- | --- | --- | --- | --- | --- | --- | --- | --- | --- | --- |

Best Unique PSM (Scan Sample\_9\_A1\_1\_3646.d:21509, m/z=891.3574, z=2, RT=11.42, ppm=-4.38):


TRINITY\_DN56792\_c0\_g1\_i3.p1

back to list

  

| Protein Coverage
| Supporting Peptides
| Best Unique PSM
|

Protein Coverage:

Supporting Peptides:

| Peptide | Uniq | -10lgP | Mass | Length | ppm | m/z | z | RT | 1/k0 Start | 1/k0 End | Precursor ID | Area Sample 10 | #Feature | #Feature Sample 10 | Start | End | PTM | AScore | Found By |
| --- | --- | --- | --- | --- | --- | --- | --- | --- | --- | --- | --- | --- | --- | --- | --- | --- | --- | --- | --- |
| R.LAIDHAVSLITNIK.C | Y | 56.49 | 1506.8769 | 14 | -4.75 | 754.4382 | 2 | 19.11 | 1.0749 | 1.0821 | 63945 | 0 | 0 | 0 | 211 | 224 |  |  | DB Search |
| total 1 peptides |  |  |  |  |  |  |  |  |  |  |  |  |  |  |  |  |  |  |  |
| --- | --- | --- | --- | --- | --- | --- | --- | --- | --- | --- | --- | --- | --- | --- | --- | --- | --- | --- | --- |

Best Unique PSM (Scan Sample\_9\_A1\_1\_3646.d:63945, m/z=754.4382, z=2, RT=19.11, ppm=-9.97):


TRINITY\_DN152\_c0\_g1\_i14.p2

back to list

  

| Protein Coverage
| Supporting Peptides
| Best Unique PSM
|

Protein Coverage:

Supporting Peptides:

| Peptide | Uniq | -10lgP | Mass | Length | ppm | m/z | z | RT | 1/k0 Start | 1/k0 End | Precursor ID | Area Sample 10 | #Feature | #Feature Sample 10 | Start | End | PTM | AScore | Found By |
| --- | --- | --- | --- | --- | --- | --- | --- | --- | --- | --- | --- | --- | --- | --- | --- | --- | --- | --- | --- |
| K.IQDKEGIPPDQQR.S | Y | 58.25 | 1522.7739 | 13 | 2.55 | 762.3922 | 2 | 11.03 | 1.0343 | 1.0415 | 18957 | 1.53e3 | 1 | 1 | 40 | 52 |  |  | DB Search |
| total 1 peptides |  |  |  |  |  |  |  |  |  |  |  |  |  |  |  |  |  |  |  |
| --- | --- | --- | --- | --- | --- | --- | --- | --- | --- | --- | --- | --- | --- | --- | --- | --- | --- | --- | --- |

Best Unique PSM (Scan Sample\_9\_A1\_1\_3646.d:18957, m/z=762.3922, z=2, RT=11.03, ppm=-2.67):


TRINITY\_DN1339\_c6\_g1\_i1.p1

back to list

  

| Protein Coverage
| Supporting Peptides
| Best Unique PSM
|

Protein Coverage:

Supporting Peptides:

| Peptide | Uniq | -10lgP | Mass | Length | ppm | m/z | z | RT | 1/k0 Start | 1/k0 End | Precursor ID | Area Sample 10 | #Feature | #Feature Sample 10 | Start | End | PTM | AScore | Found By |
| --- | --- | --- | --- | --- | --- | --- | --- | --- | --- | --- | --- | --- | --- | --- | --- | --- | --- | --- | --- |
| K.VITSDDPAEKEK.K | Y | 59.05 | 1330.6616 | 12 | 3.57 | 666.337 | 2 | 10.72 | 0.9884 | 0.9957 | 17382 | 1.42e4 | 1 | 1 | 158 | 169 |  |  | DB Search |
| total 1 peptides |  |  |  |  |  |  |  |  |  |  |  |  |  |  |  |  |  |  |  |
| --- | --- | --- | --- | --- | --- | --- | --- | --- | --- | --- | --- | --- | --- | --- | --- | --- | --- | --- | --- |

Best Unique PSM (Scan Sample\_9\_A1\_1\_3646.d:17382, m/z=666.337, z=2, RT=10.72, ppm=-1.65):


TRINITY\_DN801\_c0\_g1\_i10.p1

back to list

  

| Protein Coverage
| Supporting Peptides
| Best Unique PSM
|

Protein Coverage:

Supporting Peptides:

| Peptide | Uniq | -10lgP | Mass | Length | ppm | m/z | z | RT | 1/k0 Start | 1/k0 End | Precursor ID | Area Sample 10 | #Feature | #Feature Sample 10 | Start | End | PTM | AScore | Found By |
| --- | --- | --- | --- | --- | --- | --- | --- | --- | --- | --- | --- | --- | --- | --- | --- | --- | --- | --- | --- |
| K.VFDRDGNGFISAAELR.H | Y | 51.43 | 1765.8747 | 16 | -4.93 | 883.9357 | 2 | 19.74 | 1.1274 | 1.1346 | 67630 | 2.9e3 | 1 | 1 | 117 | 132 |  |  | DB Search |
| total 1 peptides |  |  |  |  |  |  |  |  |  |  |  |  |  |  |  |  |  |  |  |
| --- | --- | --- | --- | --- | --- | --- | --- | --- | --- | --- | --- | --- | --- | --- | --- | --- | --- | --- | --- |

Best Unique PSM (Scan Sample\_9\_A1\_1\_3646.d:67630, m/z=883.9357, z=2, RT=19.74, ppm=-10.14):


TRINITY\_DN598\_c1\_g1\_i2.p1

back to list

  

| Protein Coverage
| Supporting Peptides
| Best Unique PSM
|

Protein Coverage:

Supporting Peptides:

| Peptide | Uniq | -10lgP | Mass | Length | ppm | m/z | z | RT | 1/k0 Start | 1/k0 End | Precursor ID | Area Sample 10 | #Feature | #Feature Sample 10 | Start | End | PTM | AScore | Found By |
| --- | --- | --- | --- | --- | --- | --- | --- | --- | --- | --- | --- | --- | --- | --- | --- | --- | --- | --- | --- |
| R.ASFVTDIDRLR.A | Y | 58.91 | 1291.6884 | 11 | 1.37 | 646.849 | 2 | 17.02 | 0.9864 | 0.9937 | 51664 | 4.94e3 | 1 | 1 | 302 | 312 |  |  | DB Search |
| total 1 peptides |  |  |  |  |  |  |  |  |  |  |  |  |  |  |  |  |  |  |  |
| --- | --- | --- | --- | --- | --- | --- | --- | --- | --- | --- | --- | --- | --- | --- | --- | --- | --- | --- | --- |

Best Unique PSM (Scan Sample\_9\_A1\_1\_3646.d:51664, m/z=646.849, z=2, RT=17.02, ppm=-3.85):


TRINITY\_DN21847\_c1\_g1\_i1.p1

back to list

  

| Protein Coverage
| Supporting Peptides
| Best Unique PSM
|

Protein Coverage:

Supporting Peptides:

| Peptide | Uniq | -10lgP | Mass | Length | ppm | m/z | z | RT | 1/k0 Start | 1/k0 End | Precursor ID | Area Sample 10 | #Feature | #Feature Sample 10 | Start | End | PTM | AScore | Found By |
| --- | --- | --- | --- | --- | --- | --- | --- | --- | --- | --- | --- | --- | --- | --- | --- | --- | --- | --- | --- |
| R.K(+42.01)LSAELANGR.L | Y | 58.81 | 1099.5985 | 10 | -0.72 | 550.8033 | 2 | 14.22 | 0.8721 | 0.8793 | 37511 | 8.65e2 | 1 | 1 | 6 | 15 | Acetylation (Protein N-term) | K1:Acetylation (Protein N-term):1000 | DB Search |
| total 1 peptides |  |  |  |  |  |  |  |  |  |  |  |  |  |  |  |  |  |  |  |
| --- | --- | --- | --- | --- | --- | --- | --- | --- | --- | --- | --- | --- | --- | --- | --- | --- | --- | --- | --- |

Best Unique PSM (Scan Sample\_9\_A1\_1\_3646.d:37511, m/z=550.8033, z=2, RT=14.22, ppm=-5.94):


TRINITY\_DN1819\_c0\_g1\_i7.p1

back to list

  

| Protein Coverage
| Supporting Peptides
| Best Unique PSM
|

Protein Coverage:

Supporting Peptides:

| Peptide | Uniq | -10lgP | Mass | Length | ppm | m/z | z | RT | 1/k0 Start | 1/k0 End | Precursor ID | Area Sample 10 | #Feature | #Feature Sample 10 | Start | End | PTM | AScore | Found By |
| --- | --- | --- | --- | --- | --- | --- | --- | --- | --- | --- | --- | --- | --- | --- | --- | --- | --- | --- | --- |
| K.ALADYIHSQDLK.M | Y | 56.12 | 1372.6986 | 12 | -6.12 | 687.3488 | 2 | 15.62 | 1.0061 | 1.0134 | 44810 | 0 | 0 | 0 | 105 | 116 |  |  | DB Search |
| total 1 peptides |  |  |  |  |  |  |  |  |  |  |  |  |  |  |  |  |  |  |  |
| --- | --- | --- | --- | --- | --- | --- | --- | --- | --- | --- | --- | --- | --- | --- | --- | --- | --- | --- | --- |

Best Unique PSM (Scan Sample\_9\_A1\_1\_3646.d:44810, m/z=687.3488, z=2, RT=15.62, ppm=-11.34):


TRINITY\_DN554\_c0\_g1\_i3.p2

back to list

  

| Protein Coverage
| Supporting Peptides
| Best Unique PSM
|

Protein Coverage:

Supporting Peptides:

| Peptide | Uniq | -10lgP | Mass | Length | ppm | m/z | z | RT | 1/k0 Start | 1/k0 End | Precursor ID | Area Sample 10 | #Feature | #Feature Sample 10 | Start | End | PTM | AScore | Found By |
| --- | --- | --- | --- | --- | --- | --- | --- | --- | --- | --- | --- | --- | --- | --- | --- | --- | --- | --- | --- |
| K.IQDKEGIPPDQQR.L | Y | 58.25 | 1522.7739 | 13 | 2.55 | 762.3922 | 2 | 11.03 | 1.0343 | 1.0415 | 18957 | 1.53e3 | 1 | 1 | 30 | 42 |  |  | DB Search |
| total 1 peptides |  |  |  |  |  |  |  |  |  |  |  |  |  |  |  |  |  |  |  |
| --- | --- | --- | --- | --- | --- | --- | --- | --- | --- | --- | --- | --- | --- | --- | --- | --- | --- | --- | --- |

Best Unique PSM (Scan Sample\_9\_A1\_1\_3646.d:18957, m/z=762.3922, z=2, RT=11.03, ppm=-2.67):


TRINITY\_DN4595\_c0\_g1\_i10.p1

back to list

  

| Protein Coverage
| Supporting Peptides
| Best Unique PSM
|

Protein Coverage:

Supporting Peptides:

| Peptide | Uniq | -10lgP | Mass | Length | ppm | m/z | z | RT | 1/k0 Start | 1/k0 End | Precursor ID | Area Sample 10 | #Feature | #Feature Sample 10 | Start | End | PTM | AScore | Found By |
| --- | --- | --- | --- | --- | --- | --- | --- | --- | --- | --- | --- | --- | --- | --- | --- | --- | --- | --- | --- |
| K.YTGAC(+57.02)NANGNTDK.T | Y | 58.6 | 1384.5677 | 13 | 3.27 | 693.2898 | 2 | 9.56 | 0.9279 | 0.9352 | 11005 | 8.94e2 | 1 | 1 | 1041 | 1053 | Carbamidomethylation |  | DB Search |
| K.VNQGEKEFLQRPC(+57.02)R.Y | Y | 50.11 | 1759.8788 | 14 | 4.1 | 587.6329 | 3 | 12.58 | 0.8619 | 0.8692 | 28420 | 3.34e2 | 1 | 1 | 1161 | 1174 | Carbamidomethylation |  | DB Search |
| total 2 peptides |  |  |  |  |  |  |  |  |  |  |  |  |  |  |  |  |  |  |  |
| --- | --- | --- | --- | --- | --- | --- | --- | --- | --- | --- | --- | --- | --- | --- | --- | --- | --- | --- | --- |

Best Unique PSM (Scan Sample\_9\_A1\_1\_3646.d:11005, m/z=693.2898, z=2, RT=9.56, ppm=-1.95):


TRINITY\_DN3036\_c0\_g1\_i16.p1

back to list

  

| Protein Coverage
| Supporting Peptides
| Best Unique PSM
|

Protein Coverage:

Supporting Peptides:

| Peptide | Uniq | -10lgP | Mass | Length | ppm | m/z | z | RT | 1/k0 Start | 1/k0 End | Precursor ID | Area Sample 10 | #Feature | #Feature Sample 10 | Start | End | PTM | AScore | Found By |
| --- | --- | --- | --- | --- | --- | --- | --- | --- | --- | --- | --- | --- | --- | --- | --- | --- | --- | --- | --- |
| R.AGHGAVTSPQTQR.G | Y | 58.87 | 1308.6534 | 13 | 3.49 | 437.2243 | 3 | 9.73 | 0.7127 | 0.72 | 11628 | 3.81e3 | 1 | 1 | 135 | 147 |  |  | DB Search |
| total 1 peptides |  |  |  |  |  |  |  |  |  |  |  |  |  |  |  |  |  |  |  |
| --- | --- | --- | --- | --- | --- | --- | --- | --- | --- | --- | --- | --- | --- | --- | --- | --- | --- | --- | --- |

Best Unique PSM (Scan Sample\_9\_A1\_1\_3646.d:11628, m/z=437.2243, z=3, RT=9.73, ppm=-1.73):


TRINITY\_DN22492\_c0\_g1\_i2.p1

back to list

  

| Protein Coverage
| Supporting Peptides
| Best Unique PSM
|

Protein Coverage:

Supporting Peptides:

| Peptide | Uniq | -10lgP | Mass | Length | ppm | m/z | z | RT | 1/k0 Start | 1/k0 End | Precursor ID | Area Sample 10 | #Feature | #Feature Sample 10 | Start | End | PTM | AScore | Found By |
| --- | --- | --- | --- | --- | --- | --- | --- | --- | --- | --- | --- | --- | --- | --- | --- | --- | --- | --- | --- |
| K.KKLEPQAAPR.E | Y | 58.53 | 1136.6666 | 10 | 8.1 | 379.8972 | 3 | 9.69 | 0.7432 | 0.7506 | 11676 | 4.42e3 | 1 | 1 | 425 | 434 |  |  | DB Search |
| R.HC(+57.02)DPELSSFHTFWR.L | Y | 56.96 | 1817.7944 | 14 | -4.27 | 606.933 | 3 | 18.75 | 0.883 | 0.8903 | 61859 | 3.04e2 | 1 | 1 | 151 | 164 | Carbamidomethylation |  | DB Search |
| R.LIAKFETWEVAR.N | Y | 54.26 | 1461.798 | 12 | -4.64 | 731.899 | 2 | 17.17 | 1.0427 | 1.05 | 52609 | 0 | 0 | 0 | 318 | 329 |  |  | DB Search |
| total 3 peptides |  |  |  |  |  |  |  |  |  |  |  |  |  |  |  |  |  |  |  |
| --- | --- | --- | --- | --- | --- | --- | --- | --- | --- | --- | --- | --- | --- | --- | --- | --- | --- | --- | --- |

Best Unique PSM (Scan Sample\_9\_A1\_1\_3646.d:11676, m/z=379.8972, z=3, RT=9.69, ppm=2.87):


TRINITY\_DN757\_c0\_g1\_i1.p1

back to list

  

| Protein Coverage
| Supporting Peptides
| Best Unique PSM
|

Protein Coverage:

Supporting Peptides:

| Peptide | Uniq | -10lgP | Mass | Length | ppm | m/z | z | RT | 1/k0 Start | 1/k0 End | Precursor ID | Area Sample 10 | #Feature | #Feature Sample 10 | Start | End | PTM | AScore | Found By |
| --- | --- | --- | --- | --- | --- | --- | --- | --- | --- | --- | --- | --- | --- | --- | --- | --- | --- | --- | --- |
| R.VESQSFQDIREK.I | Y | 58.92 | 1464.7208 | 12 | 4.27 | 733.367 | 2 | 12.87 | 1.0299 | 1.0371 | 30170 | 0 | 0 | 0 | 65 | 76 |  |  | DB Search |
| total 1 peptides |  |  |  |  |  |  |  |  |  |  |  |  |  |  |  |  |  |  |  |
| --- | --- | --- | --- | --- | --- | --- | --- | --- | --- | --- | --- | --- | --- | --- | --- | --- | --- | --- | --- |

Best Unique PSM (Scan Sample\_9\_A1\_1\_3646.d:30170, m/z=733.367, z=2, RT=12.87, ppm=-0.95):


TRINITY\_DN240\_c0\_g1\_i6.p1

back to list

  

| Protein Coverage
| Supporting Peptides
| Best Unique PSM
|

Protein Coverage:

Supporting Peptides:

| Peptide | Uniq | -10lgP | Mass | Length | ppm | m/z | z | RT | 1/k0 Start | 1/k0 End | Precursor ID | Area Sample 10 | #Feature | #Feature Sample 10 | Start | End | PTM | AScore | Found By |
| --- | --- | --- | --- | --- | --- | --- | --- | --- | --- | --- | --- | --- | --- | --- | --- | --- | --- | --- | --- |
| K.AYAAFLEFKDVVK.A | Y | 59.04 | 1499.8024 | 13 | 3.65 | 750.9073 | 2 | 19.03 | 1.0945 | 1.1017 | 63342 | 1.12e4 | 1 | 1 | 217 | 229 |  |  | DB Search |
| K.IGAAAQALADQSYPFLK.S | Y | 58.85 | 1762.9253 | 17 | 0.88 | 882.4661 | 2 | 19.07 | 1.1606 | 1.1678 | 63232 | 1.84e3 | 1 | 1 | 249 | 265 |  |  | DB Search |
| K.AISSASGPTGM(+15.99)TSK.A | Y | 58.65 | 1309.6184 | 14 | 2.9 | 655.8149 | 2 | 10.52 | 0.9352 | 0.9424 | 16184 | 9.25e2 | 1 | 1 | 151 | 164 | Oxidation (M) | M11:Oxidation (M):1000 | DB Search |
| K.ANQVSSAAAAPVPAAGGDK.I | Y | 58.63 | 1680.8431 | 19 | 2.49 | 841.4265 | 2 | 12.47 | 1.068 | 1.0753 | 27849 | 0 | 0 | 0 | 230 | 248 |  |  | DB Search |
| K.AAASAHHQAIGSIDAAGVTSAADYASVTAALGR.A | Y | 46.39 | 3080.5275 | 33 | -3.95 | 771.1321 | 4 | 18.15 | 1.0656 | 1.0729 | 58236 | 5.93e2 | 1 | 1 | 307 | 339 |  |  | DB Search |
| total 5 peptides |  |  |  |  |  |  |  |  |  |  |  |  |  |  |  |  |  |  |  |
| --- | --- | --- | --- | --- | --- | --- | --- | --- | --- | --- | --- | --- | --- | --- | --- | --- | --- | --- | --- |

Best Unique PSM (Scan Sample\_9\_A1\_1\_3646.d:63342, m/z=750.9073, z=2, RT=19.03, ppm=-1.56):


TRINITY\_DN250\_c0\_g1\_i5.p1

back to list

  

| Protein Coverage
| Supporting Peptides
| Best Unique PSM
|

Protein Coverage:

Supporting Peptides:

| Peptide | Uniq | -10lgP | Mass | Length | ppm | m/z | z | RT | 1/k0 Start | 1/k0 End | Precursor ID | Area Sample 10 | #Feature | #Feature Sample 10 | Start | End | PTM | AScore | Found By |
| --- | --- | --- | --- | --- | --- | --- | --- | --- | --- | --- | --- | --- | --- | --- | --- | --- | --- | --- | --- |
| K.VGINYQPPTVVPGGDLAK.V | Y | 50.54 | 1823.9781 | 18 | -3.14 | 912.9887 | 2 | 17.25 | 1.1186 | 1.1258 | 53075 | 0 | 0 | 0 | 353 | 370 |  |  | DB Search |
| total 1 peptides |  |  |  |  |  |  |  |  |  |  |  |  |  |  |  |  |  |  |  |
| --- | --- | --- | --- | --- | --- | --- | --- | --- | --- | --- | --- | --- | --- | --- | --- | --- | --- | --- | --- |

Best Unique PSM (Scan Sample\_9\_A1\_1\_3646.d:53075, m/z=912.9887, z=2, RT=17.25, ppm=-8.35):


TRINITY\_DN3036\_c0\_g1\_i11.p1

back to list

  

| Protein Coverage
| Supporting Peptides
| Best Unique PSM
|

Protein Coverage:

Supporting Peptides:

| Peptide | Uniq | -10lgP | Mass | Length | ppm | m/z | z | RT | 1/k0 Start | 1/k0 End | Precursor ID | Area Sample 10 | #Feature | #Feature Sample 10 | Start | End | PTM | AScore | Found By |
| --- | --- | --- | --- | --- | --- | --- | --- | --- | --- | --- | --- | --- | --- | --- | --- | --- | --- | --- | --- |
| R.AGHGAVTSPQTQR.G | Y | 58.87 | 1308.6534 | 13 | 3.49 | 437.2243 | 3 | 9.73 | 0.7127 | 0.72 | 11628 | 3.81e3 | 1 | 1 | 135 | 147 |  |  | DB Search |
| total 1 peptides |  |  |  |  |  |  |  |  |  |  |  |  |  |  |  |  |  |  |  |
| --- | --- | --- | --- | --- | --- | --- | --- | --- | --- | --- | --- | --- | --- | --- | --- | --- | --- | --- | --- |

Best Unique PSM (Scan Sample\_9\_A1\_1\_3646.d:11628, m/z=437.2243, z=3, RT=9.73, ppm=-1.73):


TRINITY\_DN74124\_c0\_g1\_i1.p1

back to list

  

| Protein Coverage
| Supporting Peptides
| Best Unique PSM
|

Protein Coverage:

Supporting Peptides:

| Peptide | Uniq | -10lgP | Mass | Length | ppm | m/z | z | RT | 1/k0 Start | 1/k0 End | Precursor ID | Area Sample 10 | #Feature | #Feature Sample 10 | Start | End | PTM | AScore | Found By |
| --- | --- | --- | --- | --- | --- | --- | --- | --- | --- | --- | --- | --- | --- | --- | --- | --- | --- | --- | --- |
| K.TVENNVLVENIDITIK.Y | Y | 58.23 | 1812.9833 | 16 | 11 | 907.5042 | 2 | 19.47 | 1.1602 | 1.1674 | 66136 | 0 | 0 | 0 | 29 | 44 |  |  | DB Search |
| K.LVDATFPTTDFETVR.G | Y | 57.94 | 1710.8464 | 15 | 1.07 | 856.4269 | 2 | 18.33 | 1.1069 | 1.1142 | 59369 | 0 | 0 | 0 | 60 | 74 |  |  | DB Search |
| total 2 peptides |  |  |  |  |  |  |  |  |  |  |  |  |  |  |  |  |  |  |  |
| --- | --- | --- | --- | --- | --- | --- | --- | --- | --- | --- | --- | --- | --- | --- | --- | --- | --- | --- | --- |

Best Unique PSM (Scan Sample\_9\_A1\_1\_3646.d:66136, m/z=907.5042, z=2, RT=19.47, ppm=5.79):


TRINITY\_DN477\_c0\_g1\_i7.p1

back to list

  

| Protein Coverage
| Supporting Peptides
| Best Unique PSM
|

Protein Coverage:

Supporting Peptides:

| Peptide | Uniq | -10lgP | Mass | Length | ppm | m/z | z | RT | 1/k0 Start | 1/k0 End | Precursor ID | Area Sample 10 | #Feature | #Feature Sample 10 | Start | End | PTM | AScore | Found By |
| --- | --- | --- | --- | --- | --- | --- | --- | --- | --- | --- | --- | --- | --- | --- | --- | --- | --- | --- | --- |
| K.DKNILVLTLGVGPQVNR.N | Y | 58.66 | 1835.0628 | 17 | 1.23 | 918.535 | 2 | 19.38 | 1.1314 | 1.1386 | 65500 | 2.41e3 | 2 | 2 | 168 | 184 |  |  | DB Search |
| R.ILQC(+57.02)NQYANVEEAR.N | Y | 58.65 | 1706.8046 | 14 | 0.67 | 854.4057 | 2 | 14.18 | 1.0801 | 1.0873 | 37366 | 0 | 0 | 0 | 86 | 99 | Carbamidomethylation |  | DB Search |
| R.NDVNNPPQAR.R | Y | 58.12 | 1123.537 | 10 | 0.82 | 562.7733 | 2 | 9.95 | 0.8668 | 0.8741 | 12691 | 7.16e2 | 1 | 1 | 1049 | 1058 |  |  | DB Search |
| total 3 peptides |  |  |  |  |  |  |  |  |  |  |  |  |  |  |  |  |  |  |  |
| --- | --- | --- | --- | --- | --- | --- | --- | --- | --- | --- | --- | --- | --- | --- | --- | --- | --- | --- | --- |

Best Unique PSM (Scan Sample\_9\_A1\_1\_3646.d:65500, m/z=918.535, z=2, RT=19.38, ppm=-3.99):


TRINITY\_DN477\_c0\_g1\_i6.p1

back to list

  

| Protein Coverage
| Supporting Peptides
| Best Unique PSM
|

Protein Coverage:

Supporting Peptides:

| Peptide | Uniq | -10lgP | Mass | Length | ppm | m/z | z | RT | 1/k0 Start | 1/k0 End | Precursor ID | Area Sample 10 | #Feature | #Feature Sample 10 | Start | End | PTM | AScore | Found By |
| --- | --- | --- | --- | --- | --- | --- | --- | --- | --- | --- | --- | --- | --- | --- | --- | --- | --- | --- | --- |
| R.NDVNNPPQAR.R | Y | 58.12 | 1123.537 | 10 | 0.82 | 562.7733 | 2 | 9.95 | 0.8668 | 0.8741 | 12691 | 7.16e2 | 1 | 1 | 86 | 95 |  |  | DB Search |
| total 1 peptides |  |  |  |  |  |  |  |  |  |  |  |  |  |  |  |  |  |  |  |
| --- | --- | --- | --- | --- | --- | --- | --- | --- | --- | --- | --- | --- | --- | --- | --- | --- | --- | --- | --- |

Best Unique PSM (Scan Sample\_9\_A1\_1\_3646.d:12691, m/z=562.7733, z=2, RT=9.95, ppm=-4.4):


TRINITY\_DN8165\_c0\_g2\_i2.p2

back to list

  

| Protein Coverage
| Supporting Peptides
| Best Unique PSM
|

Protein Coverage:

Supporting Peptides:

| Peptide | Uniq | -10lgP | Mass | Length | ppm | m/z | z | RT | 1/k0 Start | 1/k0 End | Precursor ID | Area Sample 10 | #Feature | #Feature Sample 10 | Start | End | PTM | AScore | Found By |
| --- | --- | --- | --- | --- | --- | --- | --- | --- | --- | --- | --- | --- | --- | --- | --- | --- | --- | --- | --- |
| K.K(+42.01)LSAELANGR.L | Y | 58.81 | 1099.5985 | 10 | -0.72 | 550.8033 | 2 | 14.22 | 0.8721 | 0.8793 | 37511 | 8.65e2 | 1 | 1 | 4 | 13 | Acetylation (Protein N-term) | K1:Acetylation (Protein N-term):1000 | DB Search |
| total 1 peptides |  |  |  |  |  |  |  |  |  |  |  |  |  |  |  |  |  |  |  |
| --- | --- | --- | --- | --- | --- | --- | --- | --- | --- | --- | --- | --- | --- | --- | --- | --- | --- | --- | --- |

Best Unique PSM (Scan Sample\_9\_A1\_1\_3646.d:37511, m/z=550.8033, z=2, RT=14.22, ppm=-5.94):


TRINITY\_DN3036\_c0\_g1\_i2.p2

back to list

  

| Protein Coverage
| Supporting Peptides
| Best Unique PSM
|

Protein Coverage:

Supporting Peptides:

| Peptide | Uniq | -10lgP | Mass | Length | ppm | m/z | z | RT | 1/k0 Start | 1/k0 End | Precursor ID | Area Sample 10 | #Feature | #Feature Sample 10 | Start | End | PTM | AScore | Found By |
| --- | --- | --- | --- | --- | --- | --- | --- | --- | --- | --- | --- | --- | --- | --- | --- | --- | --- | --- | --- |
| R.AGHGAVTSPQTQR.G | Y | 58.87 | 1308.6534 | 13 | 3.49 | 437.2243 | 3 | 9.73 | 0.7127 | 0.72 | 11628 | 3.81e3 | 1 | 1 | 309 | 321 |  |  | DB Search |
| R.GTSNGGLVVGTIIKPK.L | Y | 58.68 | 1539.8984 | 16 | 0.87 | 770.9531 | 2 | 15.69 | 1.1254 | 1.1326 | 44972 | 2.46e3 | 1 | 1 | 173 | 188 |  |  | DB Search |
| total 2 peptides |  |  |  |  |  |  |  |  |  |  |  |  |  |  |  |  |  |  |  |
| --- | --- | --- | --- | --- | --- | --- | --- | --- | --- | --- | --- | --- | --- | --- | --- | --- | --- | --- | --- |

Best Unique PSM (Scan Sample\_9\_A1\_1\_3646.d:11628, m/z=437.2243, z=3, RT=9.73, ppm=-1.73):


TRINITY\_DN140702\_c0\_g1\_i1.p1

back to list

  

| Protein Coverage
| Supporting Peptides
| Best Unique PSM
|

Protein Coverage:

Supporting Peptides:

| Peptide | Uniq | -10lgP | Mass | Length | ppm | m/z | z | RT | 1/k0 Start | 1/k0 End | Precursor ID | Area Sample 10 | #Feature | #Feature Sample 10 | Start | End | PTM | AScore | Found By |
| --- | --- | --- | --- | --- | --- | --- | --- | --- | --- | --- | --- | --- | --- | --- | --- | --- | --- | --- | --- |
| R.LALHVLNNEDIIGHR.L | Y | 59.07 | 1712.9322 | 15 | -6.29 | 571.9781 | 3 | 16.30 | 0.8571 | 0.8644 | 48193 | 0 | 0 | 0 | 1702 | 1716 |  |  | DB Search |
| total 1 peptides |  |  |  |  |  |  |  |  |  |  |  |  |  |  |  |  |  |  |  |
| --- | --- | --- | --- | --- | --- | --- | --- | --- | --- | --- | --- | --- | --- | --- | --- | --- | --- | --- | --- |

Best Unique PSM (Scan Sample\_9\_A1\_1\_3646.d:48193, m/z=571.9781, z=3, RT=16.30, ppm=-11.51):


TRINITY\_DN2477\_c0\_g1\_i8.p1

back to list

  

| Protein Coverage
| Supporting Peptides
| Best Unique PSM
|

Protein Coverage:

Supporting Peptides:

| Peptide | Uniq | -10lgP | Mass | Length | ppm | m/z | z | RT | 1/k0 Start | 1/k0 End | Precursor ID | Area Sample 10 | #Feature | #Feature Sample 10 | Start | End | PTM | AScore | Found By |
| --- | --- | --- | --- | --- | --- | --- | --- | --- | --- | --- | --- | --- | --- | --- | --- | --- | --- | --- | --- |
| R.VAPEEHPVLLTEAPLNPK.A | Y | 58.04 | 1953.0571 | 18 | 3.48 | 977.5341 | 2 | 16.43 | 1.2045 | 1.2117 | 48611 | 4.81e2 | 1 | 1 | 29 | 46 |  |  | DB Search |
| total 1 peptides |  |  |  |  |  |  |  |  |  |  |  |  |  |  |  |  |  |  |  |
| --- | --- | --- | --- | --- | --- | --- | --- | --- | --- | --- | --- | --- | --- | --- | --- | --- | --- | --- | --- |

Best Unique PSM (Scan Sample\_9\_A1\_1\_3646.d:48611, m/z=977.5341, z=2, RT=16.43, ppm=-1.73):


TRINITY\_DN602\_c0\_g1\_i4.p1

back to list

  

| Protein Coverage
| Supporting Peptides
| Best Unique PSM
|

Protein Coverage:

Supporting Peptides:

| Peptide | Uniq | -10lgP | Mass | Length | ppm | m/z | z | RT | 1/k0 Start | 1/k0 End | Precursor ID | Area Sample 10 | #Feature | #Feature Sample 10 | Start | End | PTM | AScore | Found By |
| --- | --- | --- | --- | --- | --- | --- | --- | --- | --- | --- | --- | --- | --- | --- | --- | --- | --- | --- | --- |
| K.EGFYVHEALAAGIVK.K | Y | 58.67 | 1602.8405 | 15 | 1.72 | 802.4247 | 2 | 17.54 | 1.0889 | 1.0961 | 54660 | 6.57e2 | 1 | 1 | 144 | 158 |  |  | DB Search |
| R.AYDFRETLPK.K | Y | 58.44 | 1238.6295 | 10 | 10.92 | 620.3256 | 2 | 15.17 | 0.9489 | 0.9562 | 42570 | 0 | 0 | 0 | 87 | 96 |  |  | DB Search |
| K.LPWEELWKPC(+57.02)IK.Y | Y | 56.99 | 1597.8326 | 12 | -1.86 | 533.6144 | 3 | 19.94 | 0.8793 | 0.8866 | 68833 | 2.81e2 | 1 | 1 | 129 | 140 | Carbamidomethylation |  | DB Search |
| total 3 peptides |  |  |  |  |  |  |  |  |  |  |  |  |  |  |  |  |  |  |  |
| --- | --- | --- | --- | --- | --- | --- | --- | --- | --- | --- | --- | --- | --- | --- | --- | --- | --- | --- | --- |

Best Unique PSM (Scan Sample\_9\_A1\_1\_3646.d:54660, m/z=802.4247, z=2, RT=17.54, ppm=-3.5):


TRINITY\_DN691\_c0\_g1\_i1.p1

back to list

  

| Protein Coverage
| Supporting Peptides
| Best Unique PSM
|

Protein Coverage:

Supporting Peptides:

| Peptide | Uniq | -10lgP | Mass | Length | ppm | m/z | z | RT | 1/k0 Start | 1/k0 End | Precursor ID | Area Sample 10 | #Feature | #Feature Sample 10 | Start | End | PTM | AScore | Found By |
| --- | --- | --- | --- | --- | --- | --- | --- | --- | --- | --- | --- | --- | --- | --- | --- | --- | --- | --- | --- |
| R.AYGQDLGQPGSC(+57.02)LKR.F | Y | 58.26 | 1648.7991 | 15 | 0.22 | 825.4027 | 2 | 13.38 | 1.0737 | 1.0809 | 33011 | 5.76e2 | 1 | 1 | 1533 | 1547 | Carbamidomethylation |  | DB Search |
| total 1 peptides |  |  |  |  |  |  |  |  |  |  |  |  |  |  |  |  |  |  |  |
| --- | --- | --- | --- | --- | --- | --- | --- | --- | --- | --- | --- | --- | --- | --- | --- | --- | --- | --- | --- |

Best Unique PSM (Scan Sample\_9\_A1\_1\_3646.d:33011, m/z=825.4027, z=2, RT=13.38, ppm=-5):


TRINITY\_DN1222\_c0\_g1\_i1.p1

back to list

  

| Protein Coverage
| Supporting Peptides
| Best Unique PSM
|

Protein Coverage:

Supporting Peptides:

| Peptide | Uniq | -10lgP | Mass | Length | ppm | m/z | z | RT | 1/k0 Start | 1/k0 End | Precursor ID | Area Sample 10 | #Feature | #Feature Sample 10 | Start | End | PTM | AScore | Found By |
| --- | --- | --- | --- | --- | --- | --- | --- | --- | --- | --- | --- | --- | --- | --- | --- | --- | --- | --- | --- |
| K.IVKPGGEQPDELEK.S | Y | 33.83 | 1537.7987 | 14 | -1.81 | 769.9012 | 2 | 12.41 | 1.0259 | 1.0331 | 27466 | 8.43e2 | 1 | 1 | 8 | 21 |  |  | DB Search |
| total 1 peptides |  |  |  |  |  |  |  |  |  |  |  |  |  |  |  |  |  |  |  |
| --- | --- | --- | --- | --- | --- | --- | --- | --- | --- | --- | --- | --- | --- | --- | --- | --- | --- | --- | --- |

Best Unique PSM (Scan Sample\_9\_A1\_1\_3646.d:27466, m/z=769.9012, z=2, RT=12.41, ppm=-7.02):


TRINITY\_DN5188\_c0\_g1\_i5.p1

back to list

  

| Protein Coverage
| Supporting Peptides
| Best Unique PSM
|

Protein Coverage:

Supporting Peptides:

| Peptide | Uniq | -10lgP | Mass | Length | ppm | m/z | z | RT | 1/k0 Start | 1/k0 End | Precursor ID | Area Sample 10 | #Feature | #Feature Sample 10 | Start | End | PTM | AScore | Found By |
| --- | --- | --- | --- | --- | --- | --- | --- | --- | --- | --- | --- | --- | --- | --- | --- | --- | --- | --- | --- |
| K.LRQEQLKEER.K | Y | 56.74 | 1327.7208 | 10 | 3.91 | 443.5803 | 3 | 10.26 | 0.7949 | 0.8023 | 14744 | 0 | 0 | 0 | 334 | 343 |  |  | DB Search |
| total 1 peptides |  |  |  |  |  |  |  |  |  |  |  |  |  |  |  |  |  |  |  |
| --- | --- | --- | --- | --- | --- | --- | --- | --- | --- | --- | --- | --- | --- | --- | --- | --- | --- | --- | --- |

Best Unique PSM (Scan Sample\_9\_A1\_1\_3646.d:14744, m/z=443.5803, z=3, RT=10.26, ppm=-1.31):


TRINITY\_DN1465\_c1\_g1\_i1.p1

back to list

  

| Protein Coverage
| Supporting Peptides
| Best Unique PSM
|

Protein Coverage:

Supporting Peptides:

| Peptide | Uniq | -10lgP | Mass | Length | ppm | m/z | z | RT | 1/k0 Start | 1/k0 End | Precursor ID | Area Sample 10 | #Feature | #Feature Sample 10 | Start | End | PTM | AScore | Found By |
| --- | --- | --- | --- | --- | --- | --- | --- | --- | --- | --- | --- | --- | --- | --- | --- | --- | --- | --- | --- |
| K.IQDKEGIPPDQQR.L | Y | 58.25 | 1522.7739 | 13 | 2.55 | 762.3922 | 2 | 11.03 | 1.0343 | 1.0415 | 18957 | 1.53e3 | 1 | 1 | 34 | 46 |  |  | DB Search |
| total 1 peptides |  |  |  |  |  |  |  |  |  |  |  |  |  |  |  |  |  |  |  |
| --- | --- | --- | --- | --- | --- | --- | --- | --- | --- | --- | --- | --- | --- | --- | --- | --- | --- | --- | --- |

Best Unique PSM (Scan Sample\_9\_A1\_1\_3646.d:18957, m/z=762.3922, z=2, RT=11.03, ppm=-2.67):


TRINITY\_DN286\_c0\_g1\_i2.p1

back to list

  

| Protein Coverage
| Supporting Peptides
| Best Unique PSM
|

Protein Coverage:

Supporting Peptides:

| Peptide | Uniq | -10lgP | Mass | Length | ppm | m/z | z | RT | 1/k0 Start | 1/k0 End | Precursor ID | Area Sample 10 | #Feature | #Feature Sample 10 | Start | End | PTM | AScore | Found By |
| --- | --- | --- | --- | --- | --- | --- | --- | --- | --- | --- | --- | --- | --- | --- | --- | --- | --- | --- | --- |
| K.RYGQAMC(+57.02)QFMR.N | Y | 58.68 | 1446.6319 | 11 | -9.09 | 724.3129 | 2 | 17.23 | 0.9832 | 0.9904 | 52876 | 1.72e3 | 1 | 1 | 71 | 81 | Carbamidomethylation |  | DB Search |
| K.RYGQAM(+15.99)C(+57.02)QFM(+15.99)R.N | Y | 58.66 | 1478.6217 | 11 | -7.27 | 740.3089 | 2 | 12.91 | 0.9775 | 0.9848 | 30247 | 2.62e3 | 1 | 1 | 71 | 81 | Oxidation (M), Carbamidomethylation, Oxidation (M) | M6:Oxidation (M):1000 M10:Oxidation (M):1000 | DB Search |
| total 2 peptides |  |  |  |  |  |  |  |  |  |  |  |  |  |  |  |  |  |  |  |
| --- | --- | --- | --- | --- | --- | --- | --- | --- | --- | --- | --- | --- | --- | --- | --- | --- | --- | --- | --- |

Best Unique PSM (Scan Sample\_9\_A1\_1\_3646.d:52876, m/z=724.3129, z=2, RT=17.23, ppm=-14.31):


TRINITY\_DN477\_c0\_g1\_i5.p1

back to list

  

| Protein Coverage
| Supporting Peptides
| Best Unique PSM
|

Protein Coverage:

Supporting Peptides:

| Peptide | Uniq | -10lgP | Mass | Length | ppm | m/z | z | RT | 1/k0 Start | 1/k0 End | Precursor ID | Area Sample 10 | #Feature | #Feature Sample 10 | Start | End | PTM | AScore | Found By |
| --- | --- | --- | --- | --- | --- | --- | --- | --- | --- | --- | --- | --- | --- | --- | --- | --- | --- | --- | --- |
| R.NDVNNPPQAR.R | Y | 58.12 | 1123.537 | 10 | 0.82 | 562.7733 | 2 | 9.95 | 0.8668 | 0.8741 | 12691 | 7.16e2 | 1 | 1 | 168 | 177 |  |  | DB Search |
| total 1 peptides |  |  |  |  |  |  |  |  |  |  |  |  |  |  |  |  |  |  |  |
| --- | --- | --- | --- | --- | --- | --- | --- | --- | --- | --- | --- | --- | --- | --- | --- | --- | --- | --- | --- |

Best Unique PSM (Scan Sample\_9\_A1\_1\_3646.d:12691, m/z=562.7733, z=2, RT=9.95, ppm=-4.4):


TRINITY\_DN371\_c1\_g1\_i1.p1

back to list

  

| Protein Coverage
| Supporting Peptides
| Best Unique PSM
|

Protein Coverage:

Supporting Peptides:

| Peptide | Uniq | -10lgP | Mass | Length | ppm | m/z | z | RT | 1/k0 Start | 1/k0 End | Precursor ID | Area Sample 10 | #Feature | #Feature Sample 10 | Start | End | PTM | AScore | Found By |
| --- | --- | --- | --- | --- | --- | --- | --- | --- | --- | --- | --- | --- | --- | --- | --- | --- | --- | --- | --- |
| K.AHLTALENAFQLEK.K | Y | 58.65 | 1583.8307 | 14 | -3.06 | 792.9161 | 2 | 17.91 | 1.0773 | 1.0845 | 56906 | 0 | 0 | 0 | 94 | 107 |  |  | DB Search |
| total 1 peptides |  |  |  |  |  |  |  |  |  |  |  |  |  |  |  |  |  |  |  |
| --- | --- | --- | --- | --- | --- | --- | --- | --- | --- | --- | --- | --- | --- | --- | --- | --- | --- | --- | --- |

Best Unique PSM (Scan Sample\_9\_A1\_1\_3646.d:56906, m/z=792.9161, z=2, RT=17.91, ppm=-8.28):


TRINITY\_DN172571\_c0\_g1\_i1.p1

back to list

  

| Protein Coverage
| Supporting Peptides
| Best Unique PSM
|

Protein Coverage:

Supporting Peptides:

| Peptide | Uniq | -10lgP | Mass | Length | ppm | m/z | z | RT | 1/k0 Start | 1/k0 End | Precursor ID | Area Sample 10 | #Feature | #Feature Sample 10 | Start | End | PTM | AScore | Found By |
| --- | --- | --- | --- | --- | --- | --- | --- | --- | --- | --- | --- | --- | --- | --- | --- | --- | --- | --- | --- |
| R.DNIQGITKPAIR.R | Y | 58.99 | 1324.7463 | 12 | 0.18 | 663.3771 | 2 | 13.76 | 0.9812 | 0.9884 | 35082 | 6.65e2 | 1 | 1 | 25 | 36 |  |  | DB Search |
| total 1 peptides |  |  |  |  |  |  |  |  |  |  |  |  |  |  |  |  |  |  |  |
| --- | --- | --- | --- | --- | --- | --- | --- | --- | --- | --- | --- | --- | --- | --- | --- | --- | --- | --- | --- |

Best Unique PSM (Scan Sample\_9\_A1\_1\_3646.d:35082, m/z=663.3771, z=2, RT=13.76, ppm=-5.03):


TRINITY\_DN6390\_c0\_g1\_i1.p1

back to list

  

| Protein Coverage
| Supporting Peptides
| Best Unique PSM
|

Protein Coverage:

Supporting Peptides:

| Peptide | Uniq | -10lgP | Mass | Length | ppm | m/z | z | RT | 1/k0 Start | 1/k0 End | Precursor ID | Area Sample 10 | #Feature | #Feature Sample 10 | Start | End | PTM | AScore | Found By |
| --- | --- | --- | --- | --- | --- | --- | --- | --- | --- | --- | --- | --- | --- | --- | --- | --- | --- | --- | --- |
| R.VGNWRDETLSC(+57.02)K.Q | Y | 55.55 | 1463.6827 | 12 | -0.52 | 732.8444 | 2 | 13.66 | 1.019 | 1.0263 | 34634 | 0 | 0 | 0 | 1047 | 1058 | Carbamidomethylation |  | DB Search |
| K.RC(+57.02)TDLNIR.V | Y | 36.74 | 1046.5291 | 8 | 8.76 | 524.2737 | 2 | 11.94 | 0.8729 | 0.8802 | 24758 | 2.66e2 | 1 | 1 | 1039 | 1046 | Carbamidomethylation |  | DB Search |
| total 2 peptides |  |  |  |  |  |  |  |  |  |  |  |  |  |  |  |  |  |  |  |
| --- | --- | --- | --- | --- | --- | --- | --- | --- | --- | --- | --- | --- | --- | --- | --- | --- | --- | --- | --- |

Best Unique PSM (Scan Sample\_9\_A1\_1\_3646.d:34634, m/z=732.8444, z=2, RT=13.66, ppm=-5.74):


TRINITY\_DN24414\_c1\_g1\_i2.p2

back to list

  

| Protein Coverage
| Supporting Peptides
| Best Unique PSM
|

Protein Coverage:

Supporting Peptides:

| Peptide | Uniq | -10lgP | Mass | Length | ppm | m/z | z | RT | 1/k0 Start | 1/k0 End | Precursor ID | Area Sample 10 | #Feature | #Feature Sample 10 | Start | End | PTM | AScore | Found By |
| --- | --- | --- | --- | --- | --- | --- | --- | --- | --- | --- | --- | --- | --- | --- | --- | --- | --- | --- | --- |
| K.VLTSDDPAEKEK.K | Y | 59.05 | 1330.6616 | 12 | 3.57 | 666.337 | 2 | 10.72 | 0.9884 | 0.9957 | 17382 | 1.42e4 | 1 | 1 | 95 | 106 |  |  | DB Search |
| total 1 peptides |  |  |  |  |  |  |  |  |  |  |  |  |  |  |  |  |  |  |  |
| --- | --- | --- | --- | --- | --- | --- | --- | --- | --- | --- | --- | --- | --- | --- | --- | --- | --- | --- | --- |

Best Unique PSM (Scan Sample\_9\_A1\_1\_3646.d:17382, m/z=666.337, z=2, RT=10.72, ppm=-1.65):


TRINITY\_DN95381\_c0\_g1\_i1.p1

back to list

  

| Protein Coverage
| Supporting Peptides
| Best Unique PSM
|

Protein Coverage:

Supporting Peptides:

| Peptide | Uniq | -10lgP | Mass | Length | ppm | m/z | z | RT | 1/k0 Start | 1/k0 End | Precursor ID | Area Sample 10 | #Feature | #Feature Sample 10 | Start | End | PTM | AScore | Found By |
| --- | --- | --- | --- | --- | --- | --- | --- | --- | --- | --- | --- | --- | --- | --- | --- | --- | --- | --- | --- |
| K.M(+15.99)PEDESTPEKR.V | Y | 53.54 | 1333.582 | 11 | 5.79 | 445.5349 | 3 | 9.38 | 0.7563 | 0.7636 | 10394 | 0 | 0 | 0 | 138 | 148 | Oxidation (M) | M1:Oxidation (M):1000 | DB Search |
| total 1 peptides |  |  |  |  |  |  |  |  |  |  |  |  |  |  |  |  |  |  |  |
| --- | --- | --- | --- | --- | --- | --- | --- | --- | --- | --- | --- | --- | --- | --- | --- | --- | --- | --- | --- |

Best Unique PSM (Scan Sample\_9\_A1\_1\_3646.d:10394, m/z=445.5349, z=3, RT=9.38, ppm=0.56):


TRINITY\_DN26249\_c0\_g1\_i1.p1

back to list

  

| Protein Coverage
| Supporting Peptides
| Best Unique PSM
|

Protein Coverage:

Supporting Peptides:

| Peptide | Uniq | -10lgP | Mass | Length | ppm | m/z | z | RT | 1/k0 Start | 1/k0 End | Precursor ID | Area Sample 10 | #Feature | #Feature Sample 10 | Start | End | PTM | AScore | Found By |
| --- | --- | --- | --- | --- | --- | --- | --- | --- | --- | --- | --- | --- | --- | --- | --- | --- | --- | --- | --- |
| R.LIYENQNTIHHVVR.A | Y | 57.12 | 1734.9165 | 14 | -1.36 | 868.4598 | 2 | 13.21 | 1.1358 | 1.143 | 32061 | 4.49e2 | 1 | 1 | 322 | 335 |  |  | DB Search |
| total 1 peptides |  |  |  |  |  |  |  |  |  |  |  |  |  |  |  |  |  |  |  |
| --- | --- | --- | --- | --- | --- | --- | --- | --- | --- | --- | --- | --- | --- | --- | --- | --- | --- | --- | --- |

Best Unique PSM (Scan Sample\_9\_A1\_1\_3646.d:32061, m/z=868.4598, z=2, RT=13.21, ppm=-6.57):


TRINITY\_DN240\_c0\_g1\_i1.p1

back to list

  

| Protein Coverage
| Supporting Peptides
| Best Unique PSM
|

Protein Coverage:

Supporting Peptides:

| Peptide | Uniq | -10lgP | Mass | Length | ppm | m/z | z | RT | 1/k0 Start | 1/k0 End | Precursor ID | Area Sample 10 | #Feature | #Feature Sample 10 | Start | End | PTM | AScore | Found By |
| --- | --- | --- | --- | --- | --- | --- | --- | --- | --- | --- | --- | --- | --- | --- | --- | --- | --- | --- | --- |
| K.AYAAFLEFKDVVK.A | Y | 59.04 | 1499.8024 | 13 | 3.65 | 750.9073 | 2 | 19.03 | 1.0945 | 1.1017 | 63342 | 1.12e4 | 1 | 1 | 217 | 229 |  |  | DB Search |
| K.IGAAAQALADQSYPFLK.S | Y | 58.85 | 1762.9253 | 17 | 0.88 | 882.4661 | 2 | 19.07 | 1.1606 | 1.1678 | 63232 | 1.84e3 | 1 | 1 | 249 | 265 |  |  | DB Search |
| K.AISSASGPTGM(+15.99)TSK.A | Y | 58.65 | 1309.6184 | 14 | 2.9 | 655.8149 | 2 | 10.52 | 0.9352 | 0.9424 | 16184 | 9.25e2 | 1 | 1 | 151 | 164 | Oxidation (M) | M11:Oxidation (M):1000 | DB Search |
| K.ANQVSSAAAAPVPAAGGDK.I | Y | 58.63 | 1680.8431 | 19 | 2.49 | 841.4265 | 2 | 12.47 | 1.068 | 1.0753 | 27849 | 0 | 0 | 0 | 230 | 248 |  |  | DB Search |
| K.AAASAHHQAIGSIDAAGVTSAADYASVTAALGR.A | Y | 46.39 | 3080.5275 | 33 | -3.95 | 771.1321 | 4 | 18.15 | 1.0656 | 1.0729 | 58236 | 5.93e2 | 1 | 1 | 307 | 339 |  |  | DB Search |
| total 5 peptides |  |  |  |  |  |  |  |  |  |  |  |  |  |  |  |  |  |  |  |
| --- | --- | --- | --- | --- | --- | --- | --- | --- | --- | --- | --- | --- | --- | --- | --- | --- | --- | --- | --- |

Best Unique PSM (Scan Sample\_9\_A1\_1\_3646.d:63342, m/z=750.9073, z=2, RT=19.03, ppm=-1.56):


TRINITY\_DN902\_c1\_g1\_i1.p1

back to list

  

| Protein Coverage
| Supporting Peptides
| Best Unique PSM
|

Protein Coverage:

Supporting Peptides:

| Peptide | Uniq | -10lgP | Mass | Length | ppm | m/z | z | RT | 1/k0 Start | 1/k0 End | Precursor ID | Area Sample 10 | #Feature | #Feature Sample 10 | Start | End | PTM | AScore | Found By |
| --- | --- | --- | --- | --- | --- | --- | --- | --- | --- | --- | --- | --- | --- | --- | --- | --- | --- | --- | --- |
| K.FGQATC(+57.02)QFMR.N | Y | 58.58 | 1244.543 | 10 | -19.38 | 623.2635 | 2 | 13.58 | 0.9267 | 0.9339 | 34122 | 1.7e3 | 1 | 1 | 71 | 80 | Carbamidomethylation |  | DB Search |
| total 1 peptides |  |  |  |  |  |  |  |  |  |  |  |  |  |  |  |  |  |  |  |
| --- | --- | --- | --- | --- | --- | --- | --- | --- | --- | --- | --- | --- | --- | --- | --- | --- | --- | --- | --- |

Best Unique PSM (Scan Sample\_9\_A1\_1\_3646.d:34122, m/z=623.2635, z=2, RT=13.58, ppm=-24.6):


TRINITY\_DN168534\_c0\_g1\_i1.p1

back to list

  

| Protein Coverage
| Supporting Peptides
| Best Unique PSM
|

Protein Coverage:

Supporting Peptides:

| Peptide | Uniq | -10lgP | Mass | Length | ppm | m/z | z | RT | 1/k0 Start | 1/k0 End | Precursor ID | Area Sample 10 | #Feature | #Feature Sample 10 | Start | End | PTM | AScore | Found By |
| --- | --- | --- | --- | --- | --- | --- | --- | --- | --- | --- | --- | --- | --- | --- | --- | --- | --- | --- | --- |
| R.GDYC(+57.02)NSALIEAEQKER.K | Y | 52.72 | 1881.8527 | 16 | 2.48 | 941.931 | 2 | 14.34 | 1.2077 | 1.2149 | 38191 | 0 | 0 | 0 | 96 | 111 | Carbamidomethylation |  | DB Search |
| K.AMTDC(+57.02)KETLC(+57.02)R.G | Y | 35.53 | 1383.5945 | 11 | 1.49 | 692.8019 | 2 | 11.65 | 0.9876 | 0.9949 | 23056 | 0 | 0 | 0 | 85 | 95 | Carbamidomethylation, Carbamidomethylation |  | DB Search |
| total 2 peptides |  |  |  |  |  |  |  |  |  |  |  |  |  |  |  |  |  |  |  |
| --- | --- | --- | --- | --- | --- | --- | --- | --- | --- | --- | --- | --- | --- | --- | --- | --- | --- | --- | --- |

Best Unique PSM (Scan Sample\_9\_A1\_1\_3646.d:38191, m/z=941.931, z=2, RT=14.34, ppm=-2.74):


TRINITY\_DN161\_c0\_g1\_i3.p1

back to list

  

| Protein Coverage
| Supporting Peptides
| Best Unique PSM
|

Protein Coverage:

Supporting Peptides:

| Peptide | Uniq | -10lgP | Mass | Length | ppm | m/z | z | RT | 1/k0 Start | 1/k0 End | Precursor ID | Area Sample 10 | #Feature | #Feature Sample 10 | Start | End | PTM | AScore | Found By |
| --- | --- | --- | --- | --- | --- | --- | --- | --- | --- | --- | --- | --- | --- | --- | --- | --- | --- | --- | --- |
| K.DSYVGDEAQSKR.G | Y | 59.06 | 1353.616 | 12 | 5.32 | 677.8154 | 2 | 10.95 | 1.0041 | 1.0114 | 18674 | 7.12e3 | 1 | 1 | 74 | 85 |  |  | DB Search |
| R.AVFPSIVGRPR.H | Y | 58.99 | 1197.6982 | 11 | 1.88 | 599.8544 | 2 | 15.65 | 0.9594 | 0.9666 | 45162 | 3.26e4 | 1 | 1 | 52 | 62 |  |  | DB Search |
| R.LDLAGRDLTDYLM(+15.99)K.I | Y | 58.56 | 1638.8287 | 14 | -1.91 | 820.4158 | 2 | 18.15 | 1.1426 | 1.1498 | 58283 | 0 | 0 | 0 | 201 | 214 | Oxidation (M) | M13:Oxidation (M):1000 | DB Search |
| R.LDLAGRDLTDYLMK.I | Y | 58.53 | 1622.8338 | 14 | -6.55 | 541.9455 | 3 | 19.25 | 0.7962 | 0.8035 | 64822 | 9.54e2 | 1 | 1 | 201 | 214 |  |  | DB Search |
| R.VAPEEHPVLLTEAPLNPK.A | Y | 58.04 | 1953.0571 | 18 | 3.48 | 977.5341 | 2 | 16.43 | 1.2045 | 1.2117 | 48611 | 4.81e2 | 1 | 1 | 119 | 136 |  |  | DB Search |
| total 5 peptides |  |  |  |  |  |  |  |  |  |  |  |  |  |  |  |  |  |  |  |
| --- | --- | --- | --- | --- | --- | --- | --- | --- | --- | --- | --- | --- | --- | --- | --- | --- | --- | --- | --- |

Best Unique PSM (Scan Sample\_9\_A1\_1\_3646.d:18674, m/z=677.8154, z=2, RT=10.95, ppm=0.1):


TRINITY\_DN10907\_c0\_g1\_i1.p1

back to list

  

| Protein Coverage
| Supporting Peptides
| Best Unique PSM
|

Protein Coverage:

Supporting Peptides:

| Peptide | Uniq | -10lgP | Mass | Length | ppm | m/z | z | RT | 1/k0 Start | 1/k0 End | Precursor ID | Area Sample 10 | #Feature | #Feature Sample 10 | Start | End | PTM | AScore | Found By |
| --- | --- | --- | --- | --- | --- | --- | --- | --- | --- | --- | --- | --- | --- | --- | --- | --- | --- | --- | --- |
| R.NIDEHASEGVER.M | Y | 58.19 | 1354.6113 | 12 | -4.14 | 678.3066 | 2 | 11.03 | 0.9707 | 0.9779 | 19266 | 0 | 0 | 0 | 106 | 117 |  |  | DB Search |
| total 1 peptides |  |  |  |  |  |  |  |  |  |  |  |  |  |  |  |  |  |  |  |
| --- | --- | --- | --- | --- | --- | --- | --- | --- | --- | --- | --- | --- | --- | --- | --- | --- | --- | --- | --- |

Best Unique PSM (Scan Sample\_9\_A1\_1\_3646.d:19266, m/z=678.3066, z=2, RT=11.03, ppm=-9.36):


TRINITY\_DN138\_c0\_g1\_i1.p1

back to list

  

| Protein Coverage
| Supporting Peptides
| Best Unique PSM
|

Protein Coverage:

Supporting Peptides:

| Peptide | Uniq | -10lgP | Mass | Length | ppm | m/z | z | RT | 1/k0 Start | 1/k0 End | Precursor ID | Area Sample 10 | #Feature | #Feature Sample 10 | Start | End | PTM | AScore | Found By |
| --- | --- | --- | --- | --- | --- | --- | --- | --- | --- | --- | --- | --- | --- | --- | --- | --- | --- | --- | --- |
| R.DPINNHWDGNLINWR.K | Y | 58.64 | 1862.8812 | 15 | 0.17 | 621.9645 | 3 | 18.89 | 0.8128 | 0.8201 | 62735 | 3.45e3 | 1 | 1 | 55 | 69 |  |  | DB Search |
| R.IFPNRPYPFELVNHR.D | Y | 57 | 1897.9951 | 15 | 1.06 | 633.6697 | 3 | 17.26 | 0.9279 | 0.9352 | 52948 | 3.53e2 | 1 | 1 | 40 | 54 |  |  | DB Search |
| R.FC(+57.02)SNPEPENGGQTC(+57.02)EGEAR.E | Y | 51.3 | 2137.8429 | 19 | -10.33 | 1069.9121 | 2 | 12.08 | 1.1558 | 1.163 | 25560 | 3.12e1 | 1 | 1 | 181 | 199 | Carbamidomethylation, Carbamidomethylation |  | DB Search |
| K.IGC(+57.02)FHDR.I | Y | 48.65 | 903.4021 | 7 | -0.08 | 452.7059 | 2 | 14.59 | 0.7872 | 0.7945 | 39383 | 2.19e3 | 1 | 1 | 33 | 39 | Carbamidomethylation |  | DB Search |
| total 4 peptides |  |  |  |  |  |  |  |  |  |  |  |  |  |  |  |  |  |  |  |
| --- | --- | --- | --- | --- | --- | --- | --- | --- | --- | --- | --- | --- | --- | --- | --- | --- | --- | --- | --- |

Best Unique PSM (Scan Sample\_9\_A1\_1\_3646.d:62735, m/z=621.9645, z=3, RT=18.89, ppm=-5.05):


TRINITY\_DN36\_c0\_g1\_i4.p1

back to list

  

| Protein Coverage
| Supporting Peptides
| Best Unique PSM
|

Protein Coverage:

Supporting Peptides:

| Peptide | Uniq | -10lgP | Mass | Length | ppm | m/z | z | RT | 1/k0 Start | 1/k0 End | Precursor ID | Area Sample 10 | #Feature | #Feature Sample 10 | Start | End | PTM | AScore | Found By |
| --- | --- | --- | --- | --- | --- | --- | --- | --- | --- | --- | --- | --- | --- | --- | --- | --- | --- | --- | --- |
| R.LGGIEVTQGNC(+57.02)ARK.Y | Y | 58.24 | 1501.7671 | 14 | 6.42 | 751.8917 | 2 | 12.66 | 1.0632 | 1.0705 | 28959 | 0 | 0 | 0 | 58 | 71 | Carbamidomethylation |  | DB Search |
| total 1 peptides |  |  |  |  |  |  |  |  |  |  |  |  |  |  |  |  |  |  |  |
| --- | --- | --- | --- | --- | --- | --- | --- | --- | --- | --- | --- | --- | --- | --- | --- | --- | --- | --- | --- |

Best Unique PSM (Scan Sample\_9\_A1\_1\_3646.d:28959, m/z=751.8917, z=2, RT=12.66, ppm=1.21):


TRINITY\_DN902\_c1\_g1\_i2.p1

back to list

  

| Protein Coverage
| Supporting Peptides
| Best Unique PSM
|

Protein Coverage:

Supporting Peptides:

| Peptide | Uniq | -10lgP | Mass | Length | ppm | m/z | z | RT | 1/k0 Start | 1/k0 End | Precursor ID | Area Sample 10 | #Feature | #Feature Sample 10 | Start | End | PTM | AScore | Found By |
| --- | --- | --- | --- | --- | --- | --- | --- | --- | --- | --- | --- | --- | --- | --- | --- | --- | --- | --- | --- |
| K.FGQATC(+57.02)QFMR.N | Y | 58.58 | 1244.543 | 10 | -19.38 | 623.2635 | 2 | 13.58 | 0.9267 | 0.9339 | 34122 | 1.7e3 | 1 | 1 | 71 | 80 | Carbamidomethylation |  | DB Search |
| total 1 peptides |  |  |  |  |  |  |  |  |  |  |  |  |  |  |  |  |  |  |  |
| --- | --- | --- | --- | --- | --- | --- | --- | --- | --- | --- | --- | --- | --- | --- | --- | --- | --- | --- | --- |

Best Unique PSM (Scan Sample\_9\_A1\_1\_3646.d:34122, m/z=623.2635, z=2, RT=13.58, ppm=-24.6):


TRINITY\_DN3036\_c0\_g1\_i19.p1

back to list

  

| Protein Coverage
| Supporting Peptides
| Best Unique PSM
|

Protein Coverage:

Supporting Peptides:

| Peptide | Uniq | -10lgP | Mass | Length | ppm | m/z | z | RT | 1/k0 Start | 1/k0 End | Precursor ID | Area Sample 10 | #Feature | #Feature Sample 10 | Start | End | PTM | AScore | Found By |
| --- | --- | --- | --- | --- | --- | --- | --- | --- | --- | --- | --- | --- | --- | --- | --- | --- | --- | --- | --- |
| R.AGHGAVTSPQTQR.G | Y | 58.87 | 1308.6534 | 13 | 3.49 | 437.2243 | 3 | 9.73 | 0.7127 | 0.72 | 11628 | 3.81e3 | 1 | 1 | 135 | 147 |  |  | DB Search |
| total 1 peptides |  |  |  |  |  |  |  |  |  |  |  |  |  |  |  |  |  |  |  |
| --- | --- | --- | --- | --- | --- | --- | --- | --- | --- | --- | --- | --- | --- | --- | --- | --- | --- | --- | --- |

Best Unique PSM (Scan Sample\_9\_A1\_1\_3646.d:11628, m/z=437.2243, z=3, RT=9.73, ppm=-1.73):


TRINITY\_DN477\_c0\_g1\_i9.p1

back to list

  

| Protein Coverage
| Supporting Peptides
| Best Unique PSM
|

Protein Coverage:

Supporting Peptides:

| Peptide | Uniq | -10lgP | Mass | Length | ppm | m/z | z | RT | 1/k0 Start | 1/k0 End | Precursor ID | Area Sample 10 | #Feature | #Feature Sample 10 | Start | End | PTM | AScore | Found By |
| --- | --- | --- | --- | --- | --- | --- | --- | --- | --- | --- | --- | --- | --- | --- | --- | --- | --- | --- | --- |
| R.TQAVLDRLNIEK.R | Y | 58.63 | 1398.783 | 12 | -2.46 | 700.3934 | 2 | 15.31 | 1.0069 | 1.0142 | 43158 | 1.16e3 | 1 | 1 | 220 | 231 |  |  | DB Search |
| R.TQAVLDRLNIEKR.V | Y | 54.05 | 1554.8842 | 13 | 2.17 | 519.3004 | 3 | 14.53 | 0.8014 | 0.8088 | 39216 | 0 | 0 | 0 | 220 | 232 |  |  | DB Search |
| total 2 peptides |  |  |  |  |  |  |  |  |  |  |  |  |  |  |  |  |  |  |  |
| --- | --- | --- | --- | --- | --- | --- | --- | --- | --- | --- | --- | --- | --- | --- | --- | --- | --- | --- | --- |

Best Unique PSM (Scan Sample\_9\_A1\_1\_3646.d:43158, m/z=700.3934, z=2, RT=15.31, ppm=-7.67):


TRINITY\_DN250\_c0\_g1\_i1.p1

back to list

  

| Protein Coverage
| Supporting Peptides
| Best Unique PSM
|

Protein Coverage:

Supporting Peptides:

| Peptide | Uniq | -10lgP | Mass | Length | ppm | m/z | z | RT | 1/k0 Start | 1/k0 End | Precursor ID | Area Sample 10 | #Feature | #Feature Sample 10 | Start | End | PTM | AScore | Found By |
| --- | --- | --- | --- | --- | --- | --- | --- | --- | --- | --- | --- | --- | --- | --- | --- | --- | --- | --- | --- |
| K.VGINYQPPTVVPGGDLAK.V | Y | 50.54 | 1823.9781 | 18 | -3.14 | 912.9887 | 2 | 17.25 | 1.1186 | 1.1258 | 53075 | 0 | 0 | 0 | 353 | 370 |  |  | DB Search |
| total 1 peptides |  |  |  |  |  |  |  |  |  |  |  |  |  |  |  |  |  |  |  |
| --- | --- | --- | --- | --- | --- | --- | --- | --- | --- | --- | --- | --- | --- | --- | --- | --- | --- | --- | --- |

Best Unique PSM (Scan Sample\_9\_A1\_1\_3646.d:53075, m/z=912.9887, z=2, RT=17.25, ppm=-8.35):


TRINITY\_DN6920\_c0\_g1\_i2.p1

back to list

  

| Protein Coverage
| Supporting Peptides
| Best Unique PSM
|

Protein Coverage:

Supporting Peptides:

| Peptide | Uniq | -10lgP | Mass | Length | ppm | m/z | z | RT | 1/k0 Start | 1/k0 End | Precursor ID | Area Sample 10 | #Feature | #Feature Sample 10 | Start | End | PTM | AScore | Found By |
| --- | --- | --- | --- | --- | --- | --- | --- | --- | --- | --- | --- | --- | --- | --- | --- | --- | --- | --- | --- |
| K.VAC(+57.02)IGAWHPAR.V | Y | 58.63 | 1236.6186 | 11 | 3 | 619.3152 | 2 | 13.23 | 0.9461 | 0.9533 | 32140 | 5.07e2 | 1 | 1 | 258 | 268 | Carbamidomethylation |  | DB Search |
| R.VLVHTQQK.L | Y | 54.09 | 951.5501 | 8 | 3.81 | 476.7817 | 2 | 9.56 | 0.8303 | 0.8376 | 11062 | 2.36e2 | 1 | 1 | 169 | 176 |  |  | DB Search |
| total 2 peptides |  |  |  |  |  |  |  |  |  |  |  |  |  |  |  |  |  |  |  |
| --- | --- | --- | --- | --- | --- | --- | --- | --- | --- | --- | --- | --- | --- | --- | --- | --- | --- | --- | --- |

Best Unique PSM (Scan Sample\_9\_A1\_1\_3646.d:32140, m/z=619.3152, z=2, RT=13.23, ppm=-2.22):


TRINITY\_DN3271\_c0\_g1\_i2.p1

back to list

  

| Protein Coverage
| Supporting Peptides
| Best Unique PSM
|

Protein Coverage:

Supporting Peptides:

| Peptide | Uniq | -10lgP | Mass | Length | ppm | m/z | z | RT | 1/k0 Start | 1/k0 End | Precursor ID | Area Sample 10 | #Feature | #Feature Sample 10 | Start | End | PTM | AScore | Found By |
| --- | --- | --- | --- | --- | --- | --- | --- | --- | --- | --- | --- | --- | --- | --- | --- | --- | --- | --- | --- |
| K.TIESEAAHGTVTR.H | Y | 58.99 | 1370.679 | 13 | 5.98 | 686.3473 | 2 | 11.90 | 1.0206 | 1.0279 | 24466 | 5.79e2 | 1 | 1 | 354 | 366 |  |  | DB Search |
| total 1 peptides |  |  |  |  |  |  |  |  |  |  |  |  |  |  |  |  |  |  |  |
| --- | --- | --- | --- | --- | --- | --- | --- | --- | --- | --- | --- | --- | --- | --- | --- | --- | --- | --- | --- |

Best Unique PSM (Scan Sample\_9\_A1\_1\_3646.d:24466, m/z=686.3473, z=2, RT=11.90, ppm=0.77):


TRINITY\_DN10724\_c0\_g1\_i3.p1

back to list

  

| Protein Coverage
| Supporting Peptides
| Best Unique PSM
|

Protein Coverage:

Supporting Peptides:

| Peptide | Uniq | -10lgP | Mass | Length | ppm | m/z | z | RT | 1/k0 Start | 1/k0 End | Precursor ID | Area Sample 10 | #Feature | #Feature Sample 10 | Start | End | PTM | AScore | Found By |
| --- | --- | --- | --- | --- | --- | --- | --- | --- | --- | --- | --- | --- | --- | --- | --- | --- | --- | --- | --- |
| K.REPGDFGFDPLKFSK.R | Y | 56.43 | 1738.8678 | 15 | -1.1 | 580.6262 | 3 | 18.01 | 0.9024 | 0.9097 | 57542 | 0 | 0 | 0 | 87 | 101 |  |  | DB Search |
| total 1 peptides |  |  |  |  |  |  |  |  |  |  |  |  |  |  |  |  |  |  |  |
| --- | --- | --- | --- | --- | --- | --- | --- | --- | --- | --- | --- | --- | --- | --- | --- | --- | --- | --- | --- |

Best Unique PSM (Scan Sample\_9\_A1\_1\_3646.d:57542, m/z=580.6262, z=3, RT=18.01, ppm=-6.32):


TRINITY\_DN1280\_c0\_g1\_i1.p1

back to list

  

| Protein Coverage
| Supporting Peptides
| Best Unique PSM
|

Protein Coverage:

Supporting Peptides:

| Peptide | Uniq | -10lgP | Mass | Length | ppm | m/z | z | RT | 1/k0 Start | 1/k0 End | Precursor ID | Area Sample 10 | #Feature | #Feature Sample 10 | Start | End | PTM | AScore | Found By |
| --- | --- | --- | --- | --- | --- | --- | --- | --- | --- | --- | --- | --- | --- | --- | --- | --- | --- | --- | --- |
| R.KFVWEFFQK.V | Y | 58.6 | 1257.6546 | 9 | -1.25 | 629.8305 | 2 | 19.90 | 0.9461 | 0.9533 | 68525 | 5.63e2 | 1 | 1 | 30 | 38 |  |  | DB Search |
| total 1 peptides |  |  |  |  |  |  |  |  |  |  |  |  |  |  |  |  |  |  |  |
| --- | --- | --- | --- | --- | --- | --- | --- | --- | --- | --- | --- | --- | --- | --- | --- | --- | --- | --- | --- |

Best Unique PSM (Scan Sample\_9\_A1\_1\_3646.d:68525, m/z=629.8305, z=2, RT=19.90, ppm=-6.47):


TRINITY\_DN535\_c0\_g1\_i2.p1

back to list

  

| Protein Coverage
| Supporting Peptides
| Best Unique PSM
|

Protein Coverage:

Supporting Peptides:

| Peptide | Uniq | -10lgP | Mass | Length | ppm | m/z | z | RT | 1/k0 Start | 1/k0 End | Precursor ID | Area Sample 10 | #Feature | #Feature Sample 10 | Start | End | PTM | AScore | Found By |
| --- | --- | --- | --- | --- | --- | --- | --- | --- | --- | --- | --- | --- | --- | --- | --- | --- | --- | --- | --- |
| R.ALGC(+57.02)DVLAC(+57.02)SVDSHFSHLAWTQQSR.K | Y | 58.92 | 2844.3071 | 25 | -0.81 | 712.0798 | 4 | 18.79 | 0.9481 | 0.9553 | 62026 | 2.73e3 | 1 | 1 | 33 | 57 | Carbamidomethylation, Carbamidomethylation |  | DB Search |
| R.DYGVLLENDGIALR.G | Y | 58.64 | 1546.7991 | 14 | 1.09 | 774.4036 | 2 | 19.46 | 1.0387 | 1.046 | 66038 | 4.57e2 | 1 | 1 | 80 | 93 |  |  | DB Search |
| R.GLFIIDDKGILR.Q | Y | 58.62 | 1358.7921 | 12 | 1.68 | 680.4009 | 2 | 19.29 | 1.0114 | 1.0186 | 64919 | 1.05e4 | 1 | 1 | 94 | 105 |  |  | DB Search |
| K.IPILSDLTK.S | Y | 58.32 | 998.6012 | 9 | -0.22 | 500.3051 | 2 | 18.86 | 0.8254 | 0.8327 | 62176 | 2.58e3 | 1 | 1 | 67 | 75 |  |  | DB Search |
| total 4 peptides |  |  |  |  |  |  |  |  |  |  |  |  |  |  |  |  |  |  |  |
| --- | --- | --- | --- | --- | --- | --- | --- | --- | --- | --- | --- | --- | --- | --- | --- | --- | --- | --- | --- |

Best Unique PSM (Scan Sample\_9\_A1\_1\_3646.d:62026, m/z=712.0798, z=4, RT=18.79, ppm=-6.03):


TRINITY\_DN578\_c0\_g1\_i1.p1

back to list

  

| Protein Coverage
| Supporting Peptides
| Best Unique PSM
|

Protein Coverage:

Supporting Peptides:

| Peptide | Uniq | -10lgP | Mass | Length | ppm | m/z | z | RT | 1/k0 Start | 1/k0 End | Precursor ID | Area Sample 10 | #Feature | #Feature Sample 10 | Start | End | PTM | AScore | Found By |
| --- | --- | --- | --- | --- | --- | --- | --- | --- | --- | --- | --- | --- | --- | --- | --- | --- | --- | --- | --- |
| K.TFAHTVHNLVLAGNK.M | Y | 58.16 | 1620.8736 | 15 | -1.91 | 811.4383 | 2 | 14.95 | 1.119 | 1.1262 | 41325 | 0 | 0 | 0 | 141 | 155 |  |  | DB Search |
| total 1 peptides |  |  |  |  |  |  |  |  |  |  |  |  |  |  |  |  |  |  |  |
| --- | --- | --- | --- | --- | --- | --- | --- | --- | --- | --- | --- | --- | --- | --- | --- | --- | --- | --- | --- |

Best Unique PSM (Scan Sample\_9\_A1\_1\_3646.d:41325, m/z=811.4383, z=2, RT=14.95, ppm=-7.13):


TRINITY\_DN15599\_c0\_g1\_i1.p1

back to list

  

| Protein Coverage
| Supporting Peptides
| Best Unique PSM
|

Protein Coverage:

Supporting Peptides:

| Peptide | Uniq | -10lgP | Mass | Length | ppm | m/z | z | RT | 1/k0 Start | 1/k0 End | Precursor ID | Area Sample 10 | #Feature | #Feature Sample 10 | Start | End | PTM | AScore | Found By |
| --- | --- | --- | --- | --- | --- | --- | --- | --- | --- | --- | --- | --- | --- | --- | --- | --- | --- | --- | --- |
| K.KLQAELANGR.L | Y | 57.88 | 1098.6145 | 10 | 0.06 | 550.3117 | 2 | 11.52 | 0.8814 | 0.8887 | 21738 | 1.93e4 | 1 | 1 | 14 | 23 |  |  | DB Search |
| total 1 peptides |  |  |  |  |  |  |  |  |  |  |  |  |  |  |  |  |  |  |  |
| --- | --- | --- | --- | --- | --- | --- | --- | --- | --- | --- | --- | --- | --- | --- | --- | --- | --- | --- | --- |

Best Unique PSM (Scan Sample\_9\_A1\_1\_3646.d:21738, m/z=550.3117, z=2, RT=11.52, ppm=-5.16):


TRINITY\_DN345\_c0\_g1\_i1.p1

back to list

  

| Protein Coverage
| Supporting Peptides
| Best Unique PSM
|

Protein Coverage:

Supporting Peptides:

| Peptide | Uniq | -10lgP | Mass | Length | ppm | m/z | z | RT | 1/k0 Start | 1/k0 End | Precursor ID | Area Sample 10 | #Feature | #Feature Sample 10 | Start | End | PTM | AScore | Found By |
| --- | --- | --- | --- | --- | --- | --- | --- | --- | --- | --- | --- | --- | --- | --- | --- | --- | --- | --- | --- |
| K.DTDTEEEIKEAFR.V | Y | 54.86 | 1581.7158 | 13 | 0.19 | 791.8612 | 2 | 16.40 | 1.052 | 1.0592 | 48621 | 7.75e2 | 1 | 1 | 79 | 91 |  |  | DB Search |
| K.M(+15.99)KDTDTEEEIKEAFR.V | Y | 54.53 | 1856.8462 | 15 | -1.3 | 619.952 | 3 | 15.33 | 0.8708 | 0.8781 | 43391 | 0 | 0 | 0 | 77 | 91 | Oxidation (M) | M1:Oxidation (M):1000 | DB Search |
| total 2 peptides |  |  |  |  |  |  |  |  |  |  |  |  |  |  |  |  |  |  |  |
| --- | --- | --- | --- | --- | --- | --- | --- | --- | --- | --- | --- | --- | --- | --- | --- | --- | --- | --- | --- |

Best Unique PSM (Scan Sample\_9\_A1\_1\_3646.d:48621, m/z=791.8612, z=2, RT=16.40, ppm=-5.03):


TRINITY\_DN2477\_c0\_g1\_i9.p1

back to list

  

| Protein Coverage
| Supporting Peptides
| Best Unique PSM
|

Protein Coverage:

Supporting Peptides:

| Peptide | Uniq | -10lgP | Mass | Length | ppm | m/z | z | RT | 1/k0 Start | 1/k0 End | Precursor ID | Area Sample 10 | #Feature | #Feature Sample 10 | Start | End | PTM | AScore | Found By |
| --- | --- | --- | --- | --- | --- | --- | --- | --- | --- | --- | --- | --- | --- | --- | --- | --- | --- | --- | --- |
| K.DSYVGDEAQSKR.G | Y | 59.06 | 1353.616 | 12 | 5.32 | 677.8154 | 2 | 10.95 | 1.0041 | 1.0114 | 18674 | 7.12e3 | 1 | 1 | 52 | 63 |  |  | DB Search |
| R.VAPEEHPVLLTEAPLNPK.A | Y | 58.04 | 1953.0571 | 18 | 3.48 | 977.5341 | 2 | 16.43 | 1.2045 | 1.2117 | 48611 | 4.81e2 | 1 | 1 | 97 | 114 |  |  | DB Search |
| total 2 peptides |  |  |  |  |  |  |  |  |  |  |  |  |  |  |  |  |  |  |  |
| --- | --- | --- | --- | --- | --- | --- | --- | --- | --- | --- | --- | --- | --- | --- | --- | --- | --- | --- | --- |

Best Unique PSM (Scan Sample\_9\_A1\_1\_3646.d:18674, m/z=677.8154, z=2, RT=10.95, ppm=0.1):


TRINITY\_DN3153\_c0\_g1\_i2.p1

back to list

  

| Protein Coverage
| Supporting Peptides
| Best Unique PSM
|

Protein Coverage:

Supporting Peptides:

| Peptide | Uniq | -10lgP | Mass | Length | ppm | m/z | z | RT | 1/k0 Start | 1/k0 End | Precursor ID | Area Sample 10 | #Feature | #Feature Sample 10 | Start | End | PTM | AScore | Found By |
| --- | --- | --- | --- | --- | --- | --- | --- | --- | --- | --- | --- | --- | --- | --- | --- | --- | --- | --- | --- |
| K.AVVEGM(+15.99)TADSDESKKDE.X | Y | 58.69 | 1825.7887 | 17 | 2.01 | 609.6016 | 3 | 10.36 | 0.8348 | 0.8421 | 15202 | 1.88e3 | 2 | 2 | 179 | 195 | Oxidation (M) | M6:Oxidation (M):1000 | DB Search |
| total 1 peptides |  |  |  |  |  |  |  |  |  |  |  |  |  |  |  |  |  |  |  |
| --- | --- | --- | --- | --- | --- | --- | --- | --- | --- | --- | --- | --- | --- | --- | --- | --- | --- | --- | --- |

Best Unique PSM (Scan Sample\_9\_A1\_1\_3646.d:15202, m/z=609.6016, z=3, RT=10.36, ppm=-3.21):


TRINITY\_DN250\_c0\_g1\_i4.p1

back to list

  

| Protein Coverage
| Supporting Peptides
| Best Unique PSM
|

Protein Coverage:

Supporting Peptides:

| Peptide | Uniq | -10lgP | Mass | Length | ppm | m/z | z | RT | 1/k0 Start | 1/k0 End | Precursor ID | Area Sample 10 | #Feature | #Feature Sample 10 | Start | End | PTM | AScore | Found By |
| --- | --- | --- | --- | --- | --- | --- | --- | --- | --- | --- | --- | --- | --- | --- | --- | --- | --- | --- | --- |
| K.VGINYQPPTVVPGGDLAK.V | Y | 50.54 | 1823.9781 | 18 | -3.14 | 912.9887 | 2 | 17.25 | 1.1186 | 1.1258 | 53075 | 0 | 0 | 0 | 353 | 370 |  |  | DB Search |
| total 1 peptides |  |  |  |  |  |  |  |  |  |  |  |  |  |  |  |  |  |  |  |
| --- | --- | --- | --- | --- | --- | --- | --- | --- | --- | --- | --- | --- | --- | --- | --- | --- | --- | --- | --- |

Best Unique PSM (Scan Sample\_9\_A1\_1\_3646.d:53075, m/z=912.9887, z=2, RT=17.25, ppm=-8.35):


TRINITY\_DN902\_c1\_g2\_i3.p1

back to list

  

| Protein Coverage
| Supporting Peptides
| Best Unique PSM
|

Protein Coverage:

Supporting Peptides:

| Peptide | Uniq | -10lgP | Mass | Length | ppm | m/z | z | RT | 1/k0 Start | 1/k0 End | Precursor ID | Area Sample 10 | #Feature | #Feature Sample 10 | Start | End | PTM | AScore | Found By |
| --- | --- | --- | --- | --- | --- | --- | --- | --- | --- | --- | --- | --- | --- | --- | --- | --- | --- | --- | --- |
| K.GLTHC(+57.02)NTLSYK.L | Y | 59.02 | 1292.6183 | 11 | 5.84 | 647.3168 | 2 | 12.45 | 0.9727 | 0.98 | 27736 | 3.39e3 | 1 | 1 | 62 | 72 | Carbamidomethylation |  | DB Search |
| total 1 peptides |  |  |  |  |  |  |  |  |  |  |  |  |  |  |  |  |  |  |  |
| --- | --- | --- | --- | --- | --- | --- | --- | --- | --- | --- | --- | --- | --- | --- | --- | --- | --- | --- | --- |

Best Unique PSM (Scan Sample\_9\_A1\_1\_3646.d:27736, m/z=647.3168, z=2, RT=12.45, ppm=0.62):


TRINITY\_DN6233\_c0\_g1\_i1.p2

back to list

  

| Protein Coverage
| Supporting Peptides
| Best Unique PSM
|

Protein Coverage:

Supporting Peptides:

| Peptide | Uniq | -10lgP | Mass | Length | ppm | m/z | z | RT | 1/k0 Start | 1/k0 End | Precursor ID | Area Sample 10 | #Feature | #Feature Sample 10 | Start | End | PTM | AScore | Found By |
| --- | --- | --- | --- | --- | --- | --- | --- | --- | --- | --- | --- | --- | --- | --- | --- | --- | --- | --- | --- |
| K.SC(+57.02)LLLQFTDKR.F | Y | 58.6 | 1379.7231 | 11 | 0.98 | 690.8659 | 2 | 17.62 | 1.0291 | 1.0363 | 55103 | 7.83e2 | 1 | 1 | 42 | 52 | Carbamidomethylation |  | DB Search |
| total 1 peptides |  |  |  |  |  |  |  |  |  |  |  |  |  |  |  |  |  |  |  |
| --- | --- | --- | --- | --- | --- | --- | --- | --- | --- | --- | --- | --- | --- | --- | --- | --- | --- | --- | --- |

Best Unique PSM (Scan Sample\_9\_A1\_1\_3646.d:55103, m/z=690.8659, z=2, RT=17.62, ppm=-4.24):


TRINITY\_DN15584\_c0\_g1\_i1.p1

back to list

  

| Protein Coverage
| Supporting Peptides
| Best Unique PSM
|

Protein Coverage:

Supporting Peptides:

| Peptide | Uniq | -10lgP | Mass | Length | ppm | m/z | z | RT | 1/k0 Start | 1/k0 End | Precursor ID | Area Sample 10 | #Feature | #Feature Sample 10 | Start | End | PTM | AScore | Found By |
| --- | --- | --- | --- | --- | --- | --- | --- | --- | --- | --- | --- | --- | --- | --- | --- | --- | --- | --- | --- |
| K.AFVAIGDYGGHVGLGVK.C | Y | 58.63 | 1658.878 | 17 | 1.2 | 830.4429 | 2 | 17.62 | 1.1614 | 1.1686 | 54962 | 8.29e3 | 1 | 1 | 109 | 125 |  |  | DB Search |
| K.IGKPHTVPC(+57.02)K.V | Y | 57.93 | 1135.6172 | 10 | 2.84 | 568.8145 | 2 | 9.76 | 0.8931 | 0.9004 | 11921 | 1.36e4 | 2 | 2 | 157 | 166 | Carbamidomethylation |  | DB Search |
| total 2 peptides |  |  |  |  |  |  |  |  |  |  |  |  |  |  |  |  |  |  |  |
| --- | --- | --- | --- | --- | --- | --- | --- | --- | --- | --- | --- | --- | --- | --- | --- | --- | --- | --- | --- |

Best Unique PSM (Scan Sample\_9\_A1\_1\_3646.d:54962, m/z=830.4429, z=2, RT=17.62, ppm=-4.02):


TRINITY\_DN598\_c1\_g1\_i1.p1

back to list

  

| Protein Coverage
| Supporting Peptides
| Best Unique PSM
|

Protein Coverage:

Supporting Peptides:

| Peptide | Uniq | -10lgP | Mass | Length | ppm | m/z | z | RT | 1/k0 Start | 1/k0 End | Precursor ID | Area Sample 10 | #Feature | #Feature Sample 10 | Start | End | PTM | AScore | Found By |
| --- | --- | --- | --- | --- | --- | --- | --- | --- | --- | --- | --- | --- | --- | --- | --- | --- | --- | --- | --- |
| R.ASFVSDIDRLR.A | Y | 59.04 | 1277.6728 | 11 | 1.92 | 639.8416 | 2 | 16.88 | 0.9828 | 0.99 | 50956 | 4.21e3 | 1 | 1 | 302 | 312 |  |  | DB Search |
| total 1 peptides |  |  |  |  |  |  |  |  |  |  |  |  |  |  |  |  |  |  |  |
| --- | --- | --- | --- | --- | --- | --- | --- | --- | --- | --- | --- | --- | --- | --- | --- | --- | --- | --- | --- |

Best Unique PSM (Scan Sample\_9\_A1\_1\_3646.d:50956, m/z=639.8416, z=2, RT=16.88, ppm=-3.3):


TRINITY\_DN374\_c0\_g1\_i1.p1

back to list

  

| Protein Coverage
| Supporting Peptides
| Best Unique PSM
|

Protein Coverage:

Supporting Peptides:

| Peptide | Uniq | -10lgP | Mass | Length | ppm | m/z | z | RT | 1/k0 Start | 1/k0 End | Precursor ID | Area Sample 10 | #Feature | #Feature Sample 10 | Start | End | PTM | AScore | Found By |
| --- | --- | --- | --- | --- | --- | --- | --- | --- | --- | --- | --- | --- | --- | --- | --- | --- | --- | --- | --- |
| K.TVTC(+57.02)GAGETIKVTK.A | Y | 58.63 | 1463.7654 | 14 | 1.13 | 732.887 | 2 | 12.31 | 1.0664 | 1.0737 | 26936 | 0 | 0 | 0 | 111 | 124 | Carbamidomethylation |  | DB Search |
| K.YIDIEYVC(+57.02)KPPVKK.A | Y | 56.94 | 1750.9327 | 14 | -8.26 | 876.4619 | 2 | 14.95 | 1.1346 | 1.1418 | 41353 | 0 | 0 | 0 | 183 | 196 | Carbamidomethylation |  | DB Search |
| total 2 peptides |  |  |  |  |  |  |  |  |  |  |  |  |  |  |  |  |  |  |  |
| --- | --- | --- | --- | --- | --- | --- | --- | --- | --- | --- | --- | --- | --- | --- | --- | --- | --- | --- | --- |

Best Unique PSM (Scan Sample\_9\_A1\_1\_3646.d:26936, m/z=732.887, z=2, RT=12.31, ppm=-4.09):


TRINITY\_DN250\_c0\_g1\_i3.p1

back to list

  

| Protein Coverage
| Supporting Peptides
| Best Unique PSM
|

Protein Coverage:

Supporting Peptides:

| Peptide | Uniq | -10lgP | Mass | Length | ppm | m/z | z | RT | 1/k0 Start | 1/k0 End | Precursor ID | Area Sample 10 | #Feature | #Feature Sample 10 | Start | End | PTM | AScore | Found By |
| --- | --- | --- | --- | --- | --- | --- | --- | --- | --- | --- | --- | --- | --- | --- | --- | --- | --- | --- | --- |
| K.VGINYQPPTVVPGGDLAK.V | Y | 50.54 | 1823.9781 | 18 | -3.14 | 912.9887 | 2 | 17.25 | 1.1186 | 1.1258 | 53075 | 0 | 0 | 0 | 353 | 370 |  |  | DB Search |
| total 1 peptides |  |  |  |  |  |  |  |  |  |  |  |  |  |  |  |  |  |  |  |
| --- | --- | --- | --- | --- | --- | --- | --- | --- | --- | --- | --- | --- | --- | --- | --- | --- | --- | --- | --- |

Best Unique PSM (Scan Sample\_9\_A1\_1\_3646.d:53075, m/z=912.9887, z=2, RT=17.25, ppm=-8.35):


TRINITY\_DN158421\_c0\_g1\_i1.p1

back to list

  

| Protein Coverage
| Supporting Peptides
| Best Unique PSM
|

Protein Coverage:

Supporting Peptides:

| Peptide | Uniq | -10lgP | Mass | Length | ppm | m/z | z | RT | 1/k0 Start | 1/k0 End | Precursor ID | Area Sample 10 | #Feature | #Feature Sample 10 | Start | End | PTM | AScore | Found By |
| --- | --- | --- | --- | --- | --- | --- | --- | --- | --- | --- | --- | --- | --- | --- | --- | --- | --- | --- | --- |
| R.SSC(+57.02)RPILSVIK.I | Y | 54.44 | 1258.7067 | 11 | -8.13 | 630.3522 | 2 | 16.83 | 0.9691 | 0.9763 | 50527 | 1.86e3 | 1 | 1 | 90 | 100 | Carbamidomethylation |  | DB Search |
| total 1 peptides |  |  |  |  |  |  |  |  |  |  |  |  |  |  |  |  |  |  |  |
| --- | --- | --- | --- | --- | --- | --- | --- | --- | --- | --- | --- | --- | --- | --- | --- | --- | --- | --- | --- |

Best Unique PSM (Scan Sample\_9\_A1\_1\_3646.d:50527, m/z=630.3522, z=2, RT=16.83, ppm=-13.35):


TRINITY\_DN3036\_c0\_g1\_i13.p1

back to list

  

| Protein Coverage
| Supporting Peptides
| Best Unique PSM
|

Protein Coverage:

Supporting Peptides:

| Peptide | Uniq | -10lgP | Mass | Length | ppm | m/z | z | RT | 1/k0 Start | 1/k0 End | Precursor ID | Area Sample 10 | #Feature | #Feature Sample 10 | Start | End | PTM | AScore | Found By |
| --- | --- | --- | --- | --- | --- | --- | --- | --- | --- | --- | --- | --- | --- | --- | --- | --- | --- | --- | --- |
| R.AGHGAVTSPQTQR.G | Y | 58.87 | 1308.6534 | 13 | 3.49 | 437.2243 | 3 | 9.73 | 0.7127 | 0.72 | 11628 | 3.81e3 | 1 | 1 | 135 | 147 |  |  | DB Search |
| total 1 peptides |  |  |  |  |  |  |  |  |  |  |  |  |  |  |  |  |  |  |  |
| --- | --- | --- | --- | --- | --- | --- | --- | --- | --- | --- | --- | --- | --- | --- | --- | --- | --- | --- | --- |

Best Unique PSM (Scan Sample\_9\_A1\_1\_3646.d:11628, m/z=437.2243, z=3, RT=9.73, ppm=-1.73):


TRINITY\_DN3986\_c0\_g1\_i1.p1

back to list

  

| Protein Coverage
| Supporting Peptides
| Best Unique PSM
|

Protein Coverage:

Supporting Peptides:

| Peptide | Uniq | -10lgP | Mass | Length | ppm | m/z | z | RT | 1/k0 Start | 1/k0 End | Precursor ID | Area Sample 10 | #Feature | #Feature Sample 10 | Start | End | PTM | AScore | Found By |
| --- | --- | --- | --- | --- | --- | --- | --- | --- | --- | --- | --- | --- | --- | --- | --- | --- | --- | --- | --- |
| K.M(+15.99)SATFIGNSTAIQELFKR.I | Y | 58.67 | 2029.0302 | 18 | 2.72 | 677.349 | 3 | 19.96 | 0.9376 | 0.9449 | 68814 | 3.1e2 | 1 | 1 | 363 | 380 | Oxidation (M) | M1:Oxidation (M):1000 | DB Search |
| K.NM(+15.99)M(+15.99)AAC(+57.02)DPR.H | Y | 58.66 | 1096.41 | 9 | 1.24 | 549.2101 | 2 | 9.53 | 0.8619 | 0.8692 | 10930 | 4.97e2 | 1 | 1 | 298 | 306 | Oxidation (M), Oxidation (M), Carbamidomethylation | M2:Oxidation (M):1000 M3:Oxidation (M):1000 | DB Search |
| K.IREEYPDR.I | Y | 58.47 | 1076.525 | 8 | 1.32 | 539.2677 | 2 | 10.98 | 0.8696 | 0.8769 | 18747 | 5.32e3 | 1 | 1 | 155 | 162 |  |  | DB Search |
| total 3 peptides |  |  |  |  |  |  |  |  |  |  |  |  |  |  |  |  |  |  |  |
| --- | --- | --- | --- | --- | --- | --- | --- | --- | --- | --- | --- | --- | --- | --- | --- | --- | --- | --- | --- |

Best Unique PSM (Scan Sample\_9\_A1\_1\_3646.d:68814, m/z=677.349, z=3, RT=19.96, ppm=-2.5):


TRINITY\_DN1058\_c0\_g1\_i3.p1

back to list

  

| Protein Coverage
| Supporting Peptides
| Best Unique PSM
|

Protein Coverage:

Supporting Peptides:

| Peptide | Uniq | -10lgP | Mass | Length | ppm | m/z | z | RT | 1/k0 Start | 1/k0 End | Precursor ID | Area Sample 10 | #Feature | #Feature Sample 10 | Start | End | PTM | AScore | Found By |
| --- | --- | --- | --- | --- | --- | --- | --- | --- | --- | --- | --- | --- | --- | --- | --- | --- | --- | --- | --- |
| R.APTHILNPANKR.S | Y | 58.64 | 1330.7469 | 12 | 0.9 | 666.3779 | 2 | 11.17 | 1.009 | 1.0162 | 20196 | 4.06e2 | 1 | 1 | 2314 | 2325 |  |  | DB Search |
| total 1 peptides |  |  |  |  |  |  |  |  |  |  |  |  |  |  |  |  |  |  |  |
| --- | --- | --- | --- | --- | --- | --- | --- | --- | --- | --- | --- | --- | --- | --- | --- | --- | --- | --- | --- |

Best Unique PSM (Scan Sample\_9\_A1\_1\_3646.d:20196, m/z=666.3779, z=2, RT=11.17, ppm=-4.32):


TRINITY\_DN1613\_c0\_g2\_i1.p1

back to list

  

| Protein Coverage
| Supporting Peptides
| Best Unique PSM
|

Protein Coverage:

Supporting Peptides:

| Peptide | Uniq | -10lgP | Mass | Length | ppm | m/z | z | RT | 1/k0 Start | 1/k0 End | Precursor ID | Area Sample 10 | #Feature | #Feature Sample 10 | Start | End | PTM | AScore | Found By |
| --- | --- | --- | --- | --- | --- | --- | --- | --- | --- | --- | --- | --- | --- | --- | --- | --- | --- | --- | --- |
| K.GGKDFASDIITRK.E | Y | 58.22 | 1406.7517 | 13 | 3.42 | 469.9237 | 3 | 14.11 | 0.775 | 0.7823 | 37023 | 0 | 0 | 0 | 99 | 111 |  |  | DB Search |
| total 1 peptides |  |  |  |  |  |  |  |  |  |  |  |  |  |  |  |  |  |  |  |
| --- | --- | --- | --- | --- | --- | --- | --- | --- | --- | --- | --- | --- | --- | --- | --- | --- | --- | --- | --- |

Best Unique PSM (Scan Sample\_9\_A1\_1\_3646.d:37023, m/z=469.9237, z=3, RT=14.11, ppm=-1.81):


TRINITY\_DN817\_c0\_g1\_i1.p1

back to list

  

| Protein Coverage
| Supporting Peptides
| Best Unique PSM
|

Protein Coverage:

Supporting Peptides:

| Peptide | Uniq | -10lgP | Mass | Length | ppm | m/z | z | RT | 1/k0 Start | 1/k0 End | Precursor ID | Area Sample 10 | #Feature | #Feature Sample 10 | Start | End | PTM | AScore | Found By |
| --- | --- | --- | --- | --- | --- | --- | --- | --- | --- | --- | --- | --- | --- | --- | --- | --- | --- | --- | --- |
| R.LC(+57.02)QPGYSTC(+57.02)ENHC(+57.02)R.K | Y | 55.11 | 1780.708 | 14 | 0.84 | 891.3574 | 2 | 11.42 | 1.0861 | 1.0933 | 21509 | 5.05e2 | 1 | 1 | 179 | 192 | Carbamidomethylation, Carbamidomethylation, Carbamidomethylation |  | DB Search |
| total 1 peptides |  |  |  |  |  |  |  |  |  |  |  |  |  |  |  |  |  |  |  |
| --- | --- | --- | --- | --- | --- | --- | --- | --- | --- | --- | --- | --- | --- | --- | --- | --- | --- | --- | --- |

Best Unique PSM (Scan Sample\_9\_A1\_1\_3646.d:21509, m/z=891.3574, z=2, RT=11.42, ppm=-4.38):


TRINITY\_DN761\_c0\_g1\_i1.p1

back to list

  

| Protein Coverage
| Supporting Peptides
| Best Unique PSM
|

Protein Coverage:

Supporting Peptides:

| Peptide | Uniq | -10lgP | Mass | Length | ppm | m/z | z | RT | 1/k0 Start | 1/k0 End | Precursor ID | Area Sample 10 | #Feature | #Feature Sample 10 | Start | End | PTM | AScore | Found By |
| --- | --- | --- | --- | --- | --- | --- | --- | --- | --- | --- | --- | --- | --- | --- | --- | --- | --- | --- | --- |
| K.TRIEDLETVISQTQDHR.H | Y | 58.82 | 2040.0236 | 17 | 4.24 | 681.0145 | 3 | 17.36 | 0.9594 | 0.9666 | 53642 | 9.24e2 | 1 | 1 | 260 | 276 |  |  | DB Search |
| K.EIDKADIAIVDTGENPDAPHPR.E | Y | 51.38 | 2372.1607 | 22 | 1.38 | 791.7245 | 3 | 14.90 | 1.0383 | 1.0456 | 41054 | 3.22e2 | 1 | 1 | 71 | 92 |  |  | DB Search |
| K.FVNEVRR.C | Y | 33.88 | 918.5035 | 7 | 0.09 | 460.2567 | 2 | 12.65 | 0.812 | 0.8193 | 28973 | 2.9e3 | 1 | 1 | 51 | 57 |  |  | DB Search |
| total 3 peptides |  |  |  |  |  |  |  |  |  |  |  |  |  |  |  |  |  |  |  |
| --- | --- | --- | --- | --- | --- | --- | --- | --- | --- | --- | --- | --- | --- | --- | --- | --- | --- | --- | --- |

Best Unique PSM (Scan Sample\_9\_A1\_1\_3646.d:53642, m/z=681.0145, z=3, RT=17.36, ppm=-0.98):


TRINITY\_DN1070\_c2\_g1\_i1.p1

back to list

  

| Protein Coverage
| Supporting Peptides
| Best Unique PSM
|

Protein Coverage:

Supporting Peptides:

| Peptide | Uniq | -10lgP | Mass | Length | ppm | m/z | z | RT | 1/k0 Start | 1/k0 End | Precursor ID | Area Sample 10 | #Feature | #Feature Sample 10 | Start | End | PTM | AScore | Found By |
| --- | --- | --- | --- | --- | --- | --- | --- | --- | --- | --- | --- | --- | --- | --- | --- | --- | --- | --- | --- |
| K.FKLVFLGEQSVGK.T | Y | 57.82 | 1450.8184 | 13 | -8.01 | 726.4069 | 2 | 18.60 | 1.0315 | 1.0387 | 61022 | 0 | 0 | 0 | 13 | 25 |  |  | DB Search |
| total 1 peptides |  |  |  |  |  |  |  |  |  |  |  |  |  |  |  |  |  |  |  |
| --- | --- | --- | --- | --- | --- | --- | --- | --- | --- | --- | --- | --- | --- | --- | --- | --- | --- | --- | --- |

Best Unique PSM (Scan Sample\_9\_A1\_1\_3646.d:61022, m/z=726.4069, z=2, RT=18.60, ppm=-13.23):


TRINITY\_DN10385\_c0\_g1\_i1.p1

back to list

  

| Protein Coverage
| Supporting Peptides
| Best Unique PSM
|

Protein Coverage:

Supporting Peptides:

| Peptide | Uniq | -10lgP | Mass | Length | ppm | m/z | z | RT | 1/k0 Start | 1/k0 End | Precursor ID | Area Sample 10 | #Feature | #Feature Sample 10 | Start | End | PTM | AScore | Found By |
| --- | --- | --- | --- | --- | --- | --- | --- | --- | --- | --- | --- | --- | --- | --- | --- | --- | --- | --- | --- |
| K.FAQEGLEAHNK.L | Y | 50.66 | 1242.5993 | 11 | 2.72 | 622.3054 | 2 | 11.51 | 0.9675 | 0.9747 | 22179 | 0 | 0 | 0 | 746 | 756 |  |  | DB Search |
| total 1 peptides |  |  |  |  |  |  |  |  |  |  |  |  |  |  |  |  |  |  |  |
| --- | --- | --- | --- | --- | --- | --- | --- | --- | --- | --- | --- | --- | --- | --- | --- | --- | --- | --- | --- |

Best Unique PSM (Scan Sample\_9\_A1\_1\_3646.d:22179, m/z=622.3054, z=2, RT=11.51, ppm=-2.5):


TRINITY\_DN10890\_c0\_g1\_i1.p1

back to list

  

| Protein Coverage
| Supporting Peptides
| Best Unique PSM
|

Protein Coverage:

Supporting Peptides:

| Peptide | Uniq | -10lgP | Mass | Length | ppm | m/z | z | RT | 1/k0 Start | 1/k0 End | Precursor ID | Area Sample 10 | #Feature | #Feature Sample 10 | Start | End | PTM | AScore | Found By |
| --- | --- | --- | --- | --- | --- | --- | --- | --- | --- | --- | --- | --- | --- | --- | --- | --- | --- | --- | --- |
| R.FASTVEPQKGTTIER.N | Y | 28.89 | 1662.8577 | 15 | -1.19 | 832.4308 | 2 | 12.74 | 1.1214 | 1.1286 | 29341 | 5.16e1 | 1 | 1 | 202 | 216 |  |  | DB Search |
| total 1 peptides |  |  |  |  |  |  |  |  |  |  |  |  |  |  |  |  |  |  |  |
| --- | --- | --- | --- | --- | --- | --- | --- | --- | --- | --- | --- | --- | --- | --- | --- | --- | --- | --- | --- |

Best Unique PSM (Scan Sample\_9\_A1\_1\_3646.d:29341, m/z=832.4308, z=2, RT=12.74, ppm=-6.4):


TRINITY\_DN36\_c0\_g1\_i20.p1

back to list

  

| Protein Coverage
| Supporting Peptides
| Best Unique PSM
|

Protein Coverage:

Supporting Peptides:

| Peptide | Uniq | -10lgP | Mass | Length | ppm | m/z | z | RT | 1/k0 Start | 1/k0 End | Precursor ID | Area Sample 10 | #Feature | #Feature Sample 10 | Start | End | PTM | AScore | Found By |
| --- | --- | --- | --- | --- | --- | --- | --- | --- | --- | --- | --- | --- | --- | --- | --- | --- | --- | --- | --- |
| R.LGGIEVTQGNC(+57.02)ARK.Y | Y | 58.24 | 1501.7671 | 14 | 6.42 | 751.8917 | 2 | 12.66 | 1.0632 | 1.0705 | 28959 | 0 | 0 | 0 | 57 | 70 | Carbamidomethylation |  | DB Search |
| total 1 peptides |  |  |  |  |  |  |  |  |  |  |  |  |  |  |  |  |  |  |  |
| --- | --- | --- | --- | --- | --- | --- | --- | --- | --- | --- | --- | --- | --- | --- | --- | --- | --- | --- | --- |

Best Unique PSM (Scan Sample\_9\_A1\_1\_3646.d:28959, m/z=751.8917, z=2, RT=12.66, ppm=1.21):


TRINITY\_DN3052\_c0\_g1\_i1.p1

back to list

  

| Protein Coverage
| Supporting Peptides
| Best Unique PSM
|

Protein Coverage:

Supporting Peptides:

| Peptide | Uniq | -10lgP | Mass | Length | ppm | m/z | z | RT | 1/k0 Start | 1/k0 End | Precursor ID | Area Sample 10 | #Feature | #Feature Sample 10 | Start | End | PTM | AScore | Found By |
| --- | --- | --- | --- | --- | --- | --- | --- | --- | --- | --- | --- | --- | --- | --- | --- | --- | --- | --- | --- |
| M.A(+42.01)HLSQEQRNELKK.N | Y | 56.42 | 1621.8536 | 13 | -1.81 | 541.6213 | 3 | 11.24 | 0.81 | 0.8173 | 20516 | 0 | 0 | 0 | 2 | 14 | Acetylation (Protein N-term) | A1:Acetylation (Protein N-term):1000 | DB Search |
| total 1 peptides |  |  |  |  |  |  |  |  |  |  |  |  |  |  |  |  |  |  |  |
| --- | --- | --- | --- | --- | --- | --- | --- | --- | --- | --- | --- | --- | --- | --- | --- | --- | --- | --- | --- |

Best Unique PSM (Scan Sample\_9\_A1\_1\_3646.d:20516, m/z=541.6213, z=3, RT=11.24, ppm=-7.03):


TRINITY\_DN11662\_c0\_g1\_i1.p1

back to list

  

| Protein Coverage
| Supporting Peptides
| Best Unique PSM
|

Protein Coverage:

Supporting Peptides:

| Peptide | Uniq | -10lgP | Mass | Length | ppm | m/z | z | RT | 1/k0 Start | 1/k0 End | Precursor ID | Area Sample 10 | #Feature | #Feature Sample 10 | Start | End | PTM | AScore | Found By |
| --- | --- | --- | --- | --- | --- | --- | --- | --- | --- | --- | --- | --- | --- | --- | --- | --- | --- | --- | --- |
| M.A(+42.01)LSRPVYIIAAK.R | Y | 58.64 | 1342.7972 | 12 | -0.25 | 672.4022 | 2 | 17.90 | 1.0536 | 1.0608 | 56682 | 2.75e3 | 1 | 1 | 2 | 13 | Acetylation (Protein N-term) | A1:Acetylation (Protein N-term):1000 | DB Search |
| total 1 peptides |  |  |  |  |  |  |  |  |  |  |  |  |  |  |  |  |  |  |  |
| --- | --- | --- | --- | --- | --- | --- | --- | --- | --- | --- | --- | --- | --- | --- | --- | --- | --- | --- | --- |

Best Unique PSM (Scan Sample\_9\_A1\_1\_3646.d:56682, m/z=672.4022, z=2, RT=17.90, ppm=-5.47):


TRINITY\_DN5633\_c0\_g1\_i1.p1

back to list

  

| Protein Coverage
| Supporting Peptides
| Best Unique PSM
|

Protein Coverage:

Supporting Peptides:

| Peptide | Uniq | -10lgP | Mass | Length | ppm | m/z | z | RT | 1/k0 Start | 1/k0 End | Precursor ID | Area Sample 10 | #Feature | #Feature Sample 10 | Start | End | PTM | AScore | Found By |
| --- | --- | --- | --- | --- | --- | --- | --- | --- | --- | --- | --- | --- | --- | --- | --- | --- | --- | --- | --- |
| R.ELISNSSDALDKIR.F | Y | 58.23 | 1559.8155 | 14 | 3.45 | 780.9136 | 2 | 15.47 | 1.0733 | 1.0805 | 44016 | 2.57e2 | 1 | 1 | 108 | 121 |  |  | DB Search |
| K.LLEINVYHPLIQELQK.R | Y | 58.19 | 1949.0986 | 16 | 3.52 | 650.7057 | 3 | 19.63 | 0.9848 | 0.992 | 67027 | 0 | 0 | 0 | 706 | 721 |  |  | DB Search |
| total 2 peptides |  |  |  |  |  |  |  |  |  |  |  |  |  |  |  |  |  |  |  |
| --- | --- | --- | --- | --- | --- | --- | --- | --- | --- | --- | --- | --- | --- | --- | --- | --- | --- | --- | --- |

Best Unique PSM (Scan Sample\_9\_A1\_1\_3646.d:44016, m/z=780.9136, z=2, RT=15.47, ppm=-1.76):


TRINITY\_DN2477\_c0\_g1\_i10.p1

back to list

  

| Protein Coverage
| Supporting Peptides
| Best Unique PSM
|

Protein Coverage:

Supporting Peptides:

| Peptide | Uniq | -10lgP | Mass | Length | ppm | m/z | z | RT | 1/k0 Start | 1/k0 End | Precursor ID | Area Sample 10 | #Feature | #Feature Sample 10 | Start | End | PTM | AScore | Found By |
| --- | --- | --- | --- | --- | --- | --- | --- | --- | --- | --- | --- | --- | --- | --- | --- | --- | --- | --- | --- |
| K.DSYVGDEAQSKR.G | Y | 59.06 | 1353.616 | 12 | 5.32 | 677.8154 | 2 | 10.95 | 1.0041 | 1.0114 | 18674 | 7.12e3 | 1 | 1 | 19 | 30 |  |  | DB Search |
| R.VAPEEHPVLLTEAPLNPK.A | Y | 58.04 | 1953.0571 | 18 | 3.48 | 977.5341 | 2 | 16.43 | 1.2045 | 1.2117 | 48611 | 4.81e2 | 1 | 1 | 64 | 81 |  |  | DB Search |
| total 2 peptides |  |  |  |  |  |  |  |  |  |  |  |  |  |  |  |  |  |  |  |
| --- | --- | --- | --- | --- | --- | --- | --- | --- | --- | --- | --- | --- | --- | --- | --- | --- | --- | --- | --- |

Best Unique PSM (Scan Sample\_9\_A1\_1\_3646.d:18674, m/z=677.8154, z=2, RT=10.95, ppm=0.1):


TRINITY\_DN5897\_c0\_g1\_i3.p1

back to list

  

| Protein Coverage
| Supporting Peptides
| Best Unique PSM
|

Protein Coverage:

Supporting Peptides:

| Peptide | Uniq | -10lgP | Mass | Length | ppm | m/z | z | RT | 1/k0 Start | 1/k0 End | Precursor ID | Area Sample 10 | #Feature | #Feature Sample 10 | Start | End | PTM | AScore | Found By |
| --- | --- | --- | --- | --- | --- | --- | --- | --- | --- | --- | --- | --- | --- | --- | --- | --- | --- | --- | --- |
| R.LPGFLSPSYGVK.F | Y | 58.24 | 1263.6863 | 12 | -2.25 | 632.8457 | 2 | 18.41 | 0.9465 | 0.9537 | 59860 | 0 | 0 | 0 | 4 | 15 |  |  | DB Search |
| total 1 peptides |  |  |  |  |  |  |  |  |  |  |  |  |  |  |  |  |  |  |  |
| --- | --- | --- | --- | --- | --- | --- | --- | --- | --- | --- | --- | --- | --- | --- | --- | --- | --- | --- | --- |

Best Unique PSM (Scan Sample\_9\_A1\_1\_3646.d:59860, m/z=632.8457, z=2, RT=18.41, ppm=-7.47):


TRINITY\_DN161\_c0\_g1\_i1.p1

back to list

  

| Protein Coverage
| Supporting Peptides
| Best Unique PSM
|

Protein Coverage:

Supporting Peptides:

| Peptide | Uniq | -10lgP | Mass | Length | ppm | m/z | z | RT | 1/k0 Start | 1/k0 End | Precursor ID | Area Sample 10 | #Feature | #Feature Sample 10 | Start | End | PTM | AScore | Found By |
| --- | --- | --- | --- | --- | --- | --- | --- | --- | --- | --- | --- | --- | --- | --- | --- | --- | --- | --- | --- |
| K.DSYVGDEAQSKR.G | Y | 59.06 | 1353.616 | 12 | 5.32 | 677.8154 | 2 | 10.95 | 1.0041 | 1.0114 | 18674 | 7.12e3 | 1 | 1 | 52 | 63 |  |  | DB Search |
| R.AVFPSIVGRPR.H | Y | 58.99 | 1197.6982 | 11 | 1.88 | 599.8544 | 2 | 15.65 | 0.9594 | 0.9666 | 45162 | 3.26e4 | 1 | 1 | 30 | 40 |  |  | DB Search |
| R.LDLAGRDLTDYLM(+15.99)K.I | Y | 58.56 | 1638.8287 | 14 | -1.91 | 820.4158 | 2 | 18.15 | 1.1426 | 1.1498 | 58283 | 0 | 0 | 0 | 179 | 192 | Oxidation (M) | M13:Oxidation (M):1000 | DB Search |
| R.LDLAGRDLTDYLMK.I | Y | 58.53 | 1622.8338 | 14 | -6.55 | 541.9455 | 3 | 19.25 | 0.7962 | 0.8035 | 64822 | 9.54e2 | 1 | 1 | 179 | 192 |  |  | DB Search |
| R.VAPEEHPVLLTEAPLNPK.A | Y | 58.04 | 1953.0571 | 18 | 3.48 | 977.5341 | 2 | 16.43 | 1.2045 | 1.2117 | 48611 | 4.81e2 | 1 | 1 | 97 | 114 |  |  | DB Search |
| total 5 peptides |  |  |  |  |  |  |  |  |  |  |  |  |  |  |  |  |  |  |  |
| --- | --- | --- | --- | --- | --- | --- | --- | --- | --- | --- | --- | --- | --- | --- | --- | --- | --- | --- | --- |

Best Unique PSM (Scan Sample\_9\_A1\_1\_3646.d:18674, m/z=677.8154, z=2, RT=10.95, ppm=0.1):


TRINITY\_DN3078\_c0\_g1\_i1.p1

back to list

  

| Protein Coverage
| Supporting Peptides
| Best Unique PSM
|

Protein Coverage:

Supporting Peptides:

| Peptide | Uniq | -10lgP | Mass | Length | ppm | m/z | z | RT | 1/k0 Start | 1/k0 End | Precursor ID | Area Sample 10 | #Feature | #Feature Sample 10 | Start | End | PTM | AScore | Found By |
| --- | --- | --- | --- | --- | --- | --- | --- | --- | --- | --- | --- | --- | --- | --- | --- | --- | --- | --- | --- |
| K.SGNVVLC(+57.02)LIDVHK.A | Y | 56.59 | 1452.7759 | 13 | 2.61 | 727.3933 | 2 | 18.50 | 1.0379 | 1.0452 | 60367 | 0 | 0 | 0 | 1014 | 1026 | Carbamidomethylation |  | DB Search |
| total 1 peptides |  |  |  |  |  |  |  |  |  |  |  |  |  |  |  |  |  |  |  |
| --- | --- | --- | --- | --- | --- | --- | --- | --- | --- | --- | --- | --- | --- | --- | --- | --- | --- | --- | --- |

Best Unique PSM (Scan Sample\_9\_A1\_1\_3646.d:60367, m/z=727.3933, z=2, RT=18.50, ppm=-2.61):


TRINITY\_DN5188\_c0\_g1\_i2.p1

back to list

  

| Protein Coverage
| Supporting Peptides
| Best Unique PSM
|

Protein Coverage:

Supporting Peptides:

| Peptide | Uniq | -10lgP | Mass | Length | ppm | m/z | z | RT | 1/k0 Start | 1/k0 End | Precursor ID | Area Sample 10 | #Feature | #Feature Sample 10 | Start | End | PTM | AScore | Found By |
| --- | --- | --- | --- | --- | --- | --- | --- | --- | --- | --- | --- | --- | --- | --- | --- | --- | --- | --- | --- |
| K.LRQEQLKEER.K | Y | 56.74 | 1327.7208 | 10 | 3.91 | 443.5803 | 3 | 10.26 | 0.7949 | 0.8023 | 14744 | 0 | 0 | 0 | 334 | 343 |  |  | DB Search |
| total 1 peptides |  |  |  |  |  |  |  |  |  |  |  |  |  |  |  |  |  |  |  |
| --- | --- | --- | --- | --- | --- | --- | --- | --- | --- | --- | --- | --- | --- | --- | --- | --- | --- | --- | --- |

Best Unique PSM (Scan Sample\_9\_A1\_1\_3646.d:14744, m/z=443.5803, z=3, RT=10.26, ppm=-1.31):


TRINITY\_DN118520\_c0\_g1\_i1.p1

back to list

  

| Protein Coverage
| Supporting Peptides
| Best Unique PSM
|

Protein Coverage:

Supporting Peptides:

| Peptide | Uniq | -10lgP | Mass | Length | ppm | m/z | z | RT | 1/k0 Start | 1/k0 End | Precursor ID | Area Sample 10 | #Feature | #Feature Sample 10 | Start | End | PTM | AScore | Found By |
| --- | --- | --- | --- | --- | --- | --- | --- | --- | --- | --- | --- | --- | --- | --- | --- | --- | --- | --- | --- |
| K.ISNVQDIVPVLELANQHK.R | Y | 56.56 | 2016.1003 | 18 | 0.56 | 673.0376 | 3 | 19.59 | 0.9513 | 0.9586 | 66863 | 7.73e2 | 1 | 1 | 258 | 275 |  |  | DB Search |
| total 1 peptides |  |  |  |  |  |  |  |  |  |  |  |  |  |  |  |  |  |  |  |
| --- | --- | --- | --- | --- | --- | --- | --- | --- | --- | --- | --- | --- | --- | --- | --- | --- | --- | --- | --- |

Best Unique PSM (Scan Sample\_9\_A1\_1\_3646.d:66863, m/z=673.0376, z=3, RT=19.59, ppm=-4.65):


TRINITY\_DN554\_c0\_g1\_i5.p1

back to list

  

| Protein Coverage
| Supporting Peptides
| Best Unique PSM
|

Protein Coverage:

Supporting Peptides:

| Peptide | Uniq | -10lgP | Mass | Length | ppm | m/z | z | RT | 1/k0 Start | 1/k0 End | Precursor ID | Area Sample 10 | #Feature | #Feature Sample 10 | Start | End | PTM | AScore | Found By |
| --- | --- | --- | --- | --- | --- | --- | --- | --- | --- | --- | --- | --- | --- | --- | --- | --- | --- | --- | --- |
| K.IQDKEGIPPDQQR.L | Y | 58.25 | 1522.7739 | 13 | 2.55 | 762.3922 | 2 | 11.03 | 1.0343 | 1.0415 | 18957 | 1.53e3 | 1 | 1 | 30 | 42 |  |  | DB Search |
| total 1 peptides |  |  |  |  |  |  |  |  |  |  |  |  |  |  |  |  |  |  |  |
| --- | --- | --- | --- | --- | --- | --- | --- | --- | --- | --- | --- | --- | --- | --- | --- | --- | --- | --- | --- |

Best Unique PSM (Scan Sample\_9\_A1\_1\_3646.d:18957, m/z=762.3922, z=2, RT=11.03, ppm=-2.67):


TRINITY\_DN1172\_c0\_g2\_i3.p1

back to list

  

| Protein Coverage
| Supporting Peptides
| Best Unique PSM
|

Protein Coverage:

Supporting Peptides:

| Peptide | Uniq | -10lgP | Mass | Length | ppm | m/z | z | RT | 1/k0 Start | 1/k0 End | Precursor ID | Area Sample 10 | #Feature | #Feature Sample 10 | Start | End | PTM | AScore | Found By |
| --- | --- | --- | --- | --- | --- | --- | --- | --- | --- | --- | --- | --- | --- | --- | --- | --- | --- | --- | --- |
| K.VLTSDDPAEKDK.K | Y | 57.79 | 1316.6459 | 12 | 1.44 | 659.3278 | 2 | 10.86 | 0.9844 | 0.9916 | 18297 | 1.85e2 | 1 | 1 | 156 | 167 |  |  | DB Search |
| total 1 peptides |  |  |  |  |  |  |  |  |  |  |  |  |  |  |  |  |  |  |  |
| --- | --- | --- | --- | --- | --- | --- | --- | --- | --- | --- | --- | --- | --- | --- | --- | --- | --- | --- | --- |

Best Unique PSM (Scan Sample\_9\_A1\_1\_3646.d:18297, m/z=659.3278, z=2, RT=10.86, ppm=-3.78):


TRINITY\_DN21847\_c0\_g1\_i1.p1

back to list

  

| Protein Coverage
| Supporting Peptides
| Best Unique PSM
|

Protein Coverage:

Supporting Peptides:

| Peptide | Uniq | -10lgP | Mass | Length | ppm | m/z | z | RT | 1/k0 Start | 1/k0 End | Precursor ID | Area Sample 10 | #Feature | #Feature Sample 10 | Start | End | PTM | AScore | Found By |
| --- | --- | --- | --- | --- | --- | --- | --- | --- | --- | --- | --- | --- | --- | --- | --- | --- | --- | --- | --- |
| K.VLTSSDPAEKEK.K | Y | 58.69 | 1302.6667 | 12 | 2.82 | 652.3391 | 2 | 10.58 | 0.9965 | 1.0037 | 16473 | 6.51e3 | 1 | 1 | 101 | 112 |  |  | DB Search |
| total 1 peptides |  |  |  |  |  |  |  |  |  |  |  |  |  |  |  |  |  |  |  |
| --- | --- | --- | --- | --- | --- | --- | --- | --- | --- | --- | --- | --- | --- | --- | --- | --- | --- | --- | --- |

Best Unique PSM (Scan Sample\_9\_A1\_1\_3646.d:16473, m/z=652.3391, z=2, RT=10.58, ppm=-2.4):


TRINITY\_DN165536\_c0\_g1\_i1.p1

back to list

  

| Protein Coverage
| Supporting Peptides
| Best Unique PSM
|

Protein Coverage:

Supporting Peptides:

| Peptide | Uniq | -10lgP | Mass | Length | ppm | m/z | z | RT | 1/k0 Start | 1/k0 End | Precursor ID | Area Sample 10 | #Feature | #Feature Sample 10 | Start | End | PTM | AScore | Found By |
| --- | --- | --- | --- | --- | --- | --- | --- | --- | --- | --- | --- | --- | --- | --- | --- | --- | --- | --- | --- |
| K.ATETATGLATTHPIK.L | Y | 58.89 | 1510.7991 | 15 | -0.47 | 756.4025 | 2 | 12.92 | 1.1061 | 1.1134 | 30340 | 1.04e3 | 1 | 1 | 161 | 175 |  |  | DB Search |
| total 1 peptides |  |  |  |  |  |  |  |  |  |  |  |  |  |  |  |  |  |  |  |
| --- | --- | --- | --- | --- | --- | --- | --- | --- | --- | --- | --- | --- | --- | --- | --- | --- | --- | --- | --- |

Best Unique PSM (Scan Sample\_9\_A1\_1\_3646.d:30340, m/z=756.4025, z=2, RT=12.92, ppm=-5.68):


TRINITY\_DN56792\_c0\_g1\_i1.p1

back to list

  

| Protein Coverage
| Supporting Peptides
| Best Unique PSM
|

Protein Coverage:

Supporting Peptides:

| Peptide | Uniq | -10lgP | Mass | Length | ppm | m/z | z | RT | 1/k0 Start | 1/k0 End | Precursor ID | Area Sample 10 | #Feature | #Feature Sample 10 | Start | End | PTM | AScore | Found By |
| --- | --- | --- | --- | --- | --- | --- | --- | --- | --- | --- | --- | --- | --- | --- | --- | --- | --- | --- | --- |
| R.LAIDHAVSLITNIK.C | Y | 56.49 | 1506.8769 | 14 | -4.75 | 754.4382 | 2 | 19.11 | 1.0749 | 1.0821 | 63945 | 0 | 0 | 0 | 211 | 224 |  |  | DB Search |
| total 1 peptides |  |  |  |  |  |  |  |  |  |  |  |  |  |  |  |  |  |  |  |
| --- | --- | --- | --- | --- | --- | --- | --- | --- | --- | --- | --- | --- | --- | --- | --- | --- | --- | --- | --- |

Best Unique PSM (Scan Sample\_9\_A1\_1\_3646.d:63945, m/z=754.4382, z=2, RT=19.11, ppm=-9.97):


TRINITY\_DN96\_c0\_g1\_i8.p1

back to list

  

| Protein Coverage
| Supporting Peptides
| Best Unique PSM
|

Protein Coverage:

Supporting Peptides:

| Peptide | Uniq | -10lgP | Mass | Length | ppm | m/z | z | RT | 1/k0 Start | 1/k0 End | Precursor ID | Area Sample 10 | #Feature | #Feature Sample 10 | Start | End | PTM | AScore | Found By |
| --- | --- | --- | --- | --- | --- | --- | --- | --- | --- | --- | --- | --- | --- | --- | --- | --- | --- | --- | --- |
| K.KVEQVQTDSR.D | Y | 53.61 | 1188.6098 | 10 | 5.11 | 595.3121 | 2 | 9.53 | 0.8725 | 0.8798 | 10904 | 2.14e2 | 1 | 1 | 163 | 172 |  |  | DB Search |
| total 1 peptides |  |  |  |  |  |  |  |  |  |  |  |  |  |  |  |  |  |  |  |
| --- | --- | --- | --- | --- | --- | --- | --- | --- | --- | --- | --- | --- | --- | --- | --- | --- | --- | --- | --- |

Best Unique PSM (Scan Sample\_9\_A1\_1\_3646.d:10904, m/z=595.3121, z=2, RT=9.53, ppm=-0.11):


TRINITY\_DN244\_c0\_g1\_i1.p1

back to list

  

| Protein Coverage
| Supporting Peptides
| Best Unique PSM
|

Protein Coverage:

Supporting Peptides:

| Peptide | Uniq | -10lgP | Mass | Length | ppm | m/z | z | RT | 1/k0 Start | 1/k0 End | Precursor ID | Area Sample 10 | #Feature | #Feature Sample 10 | Start | End | PTM | AScore | Found By |
| --- | --- | --- | --- | --- | --- | --- | --- | --- | --- | --- | --- | --- | --- | --- | --- | --- | --- | --- | --- |
| K.DLSLVVEPLKNYGVK.I | Y | 58.87 | 1672.9399 | 15 | -1.21 | 837.4719 | 2 | 19.04 | 1.1338 | 1.141 | 63579 | 2.46e2 | 1 | 1 | 392 | 406 |  |  | DB Search |
| total 1 peptides |  |  |  |  |  |  |  |  |  |  |  |  |  |  |  |  |  |  |  |
| --- | --- | --- | --- | --- | --- | --- | --- | --- | --- | --- | --- | --- | --- | --- | --- | --- | --- | --- | --- |

Best Unique PSM (Scan Sample\_9\_A1\_1\_3646.d:63579, m/z=837.4719, z=2, RT=19.04, ppm=-6.43):


TRINITY\_DN1339\_c0\_g2\_i1.p2

back to list

  

| Protein Coverage
| Supporting Peptides
| Best Unique PSM
|

Protein Coverage:

Supporting Peptides:

| Peptide | Uniq | -10lgP | Mass | Length | ppm | m/z | z | RT | 1/k0 Start | 1/k0 End | Precursor ID | Area Sample 10 | #Feature | #Feature Sample 10 | Start | End | PTM | AScore | Found By |
| --- | --- | --- | --- | --- | --- | --- | --- | --- | --- | --- | --- | --- | --- | --- | --- | --- | --- | --- | --- |
| K.LLTSSDQAELTKK.L | Y | 59.06 | 1432.7773 | 13 | -0.99 | 717.3915 | 2 | 12.75 | 1.0508 | 1.058 | 29440 | 1.28e3 | 1 | 1 | 155 | 167 |  |  | DB Search |
| total 1 peptides |  |  |  |  |  |  |  |  |  |  |  |  |  |  |  |  |  |  |  |
| --- | --- | --- | --- | --- | --- | --- | --- | --- | --- | --- | --- | --- | --- | --- | --- | --- | --- | --- | --- |

Best Unique PSM (Scan Sample\_9\_A1\_1\_3646.d:29440, m/z=717.3915, z=2, RT=12.75, ppm=-6.21):


TRINITY\_DN477\_c0\_g1\_i8.p1

back to list

  

| Protein Coverage
| Supporting Peptides
| Best Unique PSM
|

Protein Coverage:

Supporting Peptides:

| Peptide | Uniq | -10lgP | Mass | Length | ppm | m/z | z | RT | 1/k0 Start | 1/k0 End | Precursor ID | Area Sample 10 | #Feature | #Feature Sample 10 | Start | End | PTM | AScore | Found By |
| --- | --- | --- | --- | --- | --- | --- | --- | --- | --- | --- | --- | --- | --- | --- | --- | --- | --- | --- | --- |
| K.DKNILVLTLGVGPQVNR.N | Y | 58.66 | 1835.0628 | 17 | 1.23 | 918.535 | 2 | 19.38 | 1.1314 | 1.1386 | 65500 | 2.41e3 | 2 | 2 | 168 | 184 |  |  | DB Search |
| R.ILQC(+57.02)NQYANVEEAR.N | Y | 58.65 | 1706.8046 | 14 | 0.67 | 854.4057 | 2 | 14.18 | 1.0801 | 1.0873 | 37366 | 0 | 0 | 0 | 86 | 99 | Carbamidomethylation |  | DB Search |
| total 2 peptides |  |  |  |  |  |  |  |  |  |  |  |  |  |  |  |  |  |  |  |
| --- | --- | --- | --- | --- | --- | --- | --- | --- | --- | --- | --- | --- | --- | --- | --- | --- | --- | --- | --- |

Best Unique PSM (Scan Sample\_9\_A1\_1\_3646.d:65500, m/z=918.535, z=2, RT=19.38, ppm=-3.99):


TRINITY\_DN6920\_c0\_g1\_i4.p1

back to list

  

| Protein Coverage
| Supporting Peptides
| Best Unique PSM
|

Protein Coverage:

Supporting Peptides:

| Peptide | Uniq | -10lgP | Mass | Length | ppm | m/z | z | RT | 1/k0 Start | 1/k0 End | Precursor ID | Area Sample 10 | #Feature | #Feature Sample 10 | Start | End | PTM | AScore | Found By |
| --- | --- | --- | --- | --- | --- | --- | --- | --- | --- | --- | --- | --- | --- | --- | --- | --- | --- | --- | --- |
| K.VAC(+57.02)IGAWHPAR.V | Y | 58.63 | 1236.6186 | 11 | 3 | 619.3152 | 2 | 13.23 | 0.9461 | 0.9533 | 32140 | 5.07e2 | 1 | 1 | 129 | 139 | Carbamidomethylation |  | DB Search |
| R.VLVHTQQK.L | Y | 54.09 | 951.5501 | 8 | 3.81 | 476.7817 | 2 | 9.56 | 0.8303 | 0.8376 | 11062 | 2.36e2 | 1 | 1 | 40 | 47 |  |  | DB Search |
| total 2 peptides |  |  |  |  |  |  |  |  |  |  |  |  |  |  |  |  |  |  |  |
| --- | --- | --- | --- | --- | --- | --- | --- | --- | --- | --- | --- | --- | --- | --- | --- | --- | --- | --- | --- |

Best Unique PSM (Scan Sample\_9\_A1\_1\_3646.d:32140, m/z=619.3152, z=2, RT=13.23, ppm=-2.22):


TRINITY\_DN32763\_c0\_g1\_i2.p2

back to list

  

| Protein Coverage
| Supporting Peptides
| Best Unique PSM
|

Protein Coverage:

Supporting Peptides:

| Peptide | Uniq | -10lgP | Mass | Length | ppm | m/z | z | RT | 1/k0 Start | 1/k0 End | Precursor ID | Area Sample 10 | #Feature | #Feature Sample 10 | Start | End | PTM | AScore | Found By |
| --- | --- | --- | --- | --- | --- | --- | --- | --- | --- | --- | --- | --- | --- | --- | --- | --- | --- | --- | --- |
| R.VAQHAVVAR.W | Y | 43.99 | 949.5457 | 9 | 5.58 | 475.7803 | 2 | 10.02 | 0.8412 | 0.8485 | 13333 | 5.98e2 | 1 | 1 | 185 | 193 |  |  | DB Search |
| total 1 peptides |  |  |  |  |  |  |  |  |  |  |  |  |  |  |  |  |  |  |  |
| --- | --- | --- | --- | --- | --- | --- | --- | --- | --- | --- | --- | --- | --- | --- | --- | --- | --- | --- | --- |

Best Unique PSM (Scan Sample\_9\_A1\_1\_3646.d:13333, m/z=475.714, z=2, RT=10.10, ppm=0.36):


TRINITY\_DN5793\_c0\_g1\_i1.p1

back to list

  

| Protein Coverage
| Supporting Peptides
| Best Unique PSM
|

Protein Coverage:

Supporting Peptides:

| Peptide | Uniq | -10lgP | Mass | Length | ppm | m/z | z | RT | 1/k0 Start | 1/k0 End | Precursor ID | Area Sample 10 | #Feature | #Feature Sample 10 | Start | End | PTM | AScore | Found By |
| --- | --- | --- | --- | --- | --- | --- | --- | --- | --- | --- | --- | --- | --- | --- | --- | --- | --- | --- | --- |
| K.SC(+57.02)LLLQFTDKR.F | Y | 58.6 | 1379.7231 | 11 | 0.98 | 690.8659 | 2 | 17.62 | 1.0291 | 1.0363 | 55103 | 7.83e2 | 1 | 1 | 20 | 30 | Carbamidomethylation |  | DB Search |
| R.RDTFNHLTTWLEDAR.Q | Y | 57.9 | 1873.9071 | 15 | -2.34 | 625.6382 | 3 | 18.26 | 0.8664 | 0.8737 | 58946 | 0 | 0 | 0 | 91 | 105 |  |  | DB Search |
| total 2 peptides |  |  |  |  |  |  |  |  |  |  |  |  |  |  |  |  |  |  |  |
| --- | --- | --- | --- | --- | --- | --- | --- | --- | --- | --- | --- | --- | --- | --- | --- | --- | --- | --- | --- |

Best Unique PSM (Scan Sample\_9\_A1\_1\_3646.d:55103, m/z=690.8659, z=2, RT=17.62, ppm=-4.24):


TRINITY\_DN16548\_c0\_g1\_i1.p1

back to list

  

| Protein Coverage
| Supporting Peptides
| Best Unique PSM
|

Protein Coverage:

Supporting Peptides:

| Peptide | Uniq | -10lgP | Mass | Length | ppm | m/z | z | RT | 1/k0 Start | 1/k0 End | Precursor ID | Area Sample 10 | #Feature | #Feature Sample 10 | Start | End | PTM | AScore | Found By |
| --- | --- | --- | --- | --- | --- | --- | --- | --- | --- | --- | --- | --- | --- | --- | --- | --- | --- | --- | --- |
| K.APGDYGWRPPLLATDDAALK.T | Y | 58.71 | 2126.0796 | 20 | 0.61 | 709.6972 | 3 | 18.99 | 0.8883 | 0.8955 | 63354 | 4.18e3 | 1 | 1 | 73 | 92 |  |  | DB Search |
| total 1 peptides |  |  |  |  |  |  |  |  |  |  |  |  |  |  |  |  |  |  |  |
| --- | --- | --- | --- | --- | --- | --- | --- | --- | --- | --- | --- | --- | --- | --- | --- | --- | --- | --- | --- |

Best Unique PSM (Scan Sample\_9\_A1\_1\_3646.d:63354, m/z=709.6972, z=3, RT=18.99, ppm=-4.61):


TRINITY\_DN4497\_c0\_g2\_i1.p1

back to list

  

| Protein Coverage
| Supporting Peptides
| Best Unique PSM
|

Protein Coverage:

Supporting Peptides:

| Peptide | Uniq | -10lgP | Mass | Length | ppm | m/z | z | RT | 1/k0 Start | 1/k0 End | Precursor ID | Area Sample 10 | #Feature | #Feature Sample 10 | Start | End | PTM | AScore | Found By |
| --- | --- | --- | --- | --- | --- | --- | --- | --- | --- | --- | --- | --- | --- | --- | --- | --- | --- | --- | --- |
| K.VLTSSDPAEKEK.K | Y | 58.69 | 1302.6667 | 12 | 2.82 | 652.3391 | 2 | 10.58 | 0.9965 | 1.0037 | 16473 | 6.51e3 | 1 | 1 | 126 | 137 |  |  | DB Search |
| total 1 peptides |  |  |  |  |  |  |  |  |  |  |  |  |  |  |  |  |  |  |  |
| --- | --- | --- | --- | --- | --- | --- | --- | --- | --- | --- | --- | --- | --- | --- | --- | --- | --- | --- | --- |

Best Unique PSM (Scan Sample\_9\_A1\_1\_3646.d:16473, m/z=652.3391, z=2, RT=10.58, ppm=-2.4):


TRINITY\_DN10724\_c0\_g1\_i4.p1

back to list

  

| Protein Coverage
| Supporting Peptides
| Best Unique PSM
|

Protein Coverage:

Supporting Peptides:

| Peptide | Uniq | -10lgP | Mass | Length | ppm | m/z | z | RT | 1/k0 Start | 1/k0 End | Precursor ID | Area Sample 10 | #Feature | #Feature Sample 10 | Start | End | PTM | AScore | Found By |
| --- | --- | --- | --- | --- | --- | --- | --- | --- | --- | --- | --- | --- | --- | --- | --- | --- | --- | --- | --- |
| K.REPGDFGFDPLKFSK.R | Y | 56.43 | 1738.8678 | 15 | -1.1 | 580.6262 | 3 | 18.01 | 0.9024 | 0.9097 | 57542 | 0 | 0 | 0 | 171 | 185 |  |  | DB Search |
| total 1 peptides |  |  |  |  |  |  |  |  |  |  |  |  |  |  |  |  |  |  |  |
| --- | --- | --- | --- | --- | --- | --- | --- | --- | --- | --- | --- | --- | --- | --- | --- | --- | --- | --- | --- |

Best Unique PSM (Scan Sample\_9\_A1\_1\_3646.d:57542, m/z=580.6262, z=3, RT=18.01, ppm=-6.32):


TRINITY\_DN817\_c0\_g1\_i8.p1

back to list

  

| Protein Coverage
| Supporting Peptides
| Best Unique PSM
|

Protein Coverage:

Supporting Peptides:

| Peptide | Uniq | -10lgP | Mass | Length | ppm | m/z | z | RT | 1/k0 Start | 1/k0 End | Precursor ID | Area Sample 10 | #Feature | #Feature Sample 10 | Start | End | PTM | AScore | Found By |
| --- | --- | --- | --- | --- | --- | --- | --- | --- | --- | --- | --- | --- | --- | --- | --- | --- | --- | --- | --- |
| R.LC(+57.02)QPGYSTC(+57.02)ENHC(+57.02)R.R | Y | 55.11 | 1780.708 | 14 | 0.84 | 891.3574 | 2 | 11.42 | 1.0861 | 1.0933 | 21509 | 5.05e2 | 1 | 1 | 91 | 104 | Carbamidomethylation, Carbamidomethylation, Carbamidomethylation |  | DB Search |
| R.LC(+57.02)QPGYSTC(+57.02)ENHC(+57.02)RR.L | Y | 43.99 | 1936.8091 | 15 | 3.79 | 646.6094 | 3 | 10.89 | 0.8575 | 0.8648 | 18436 | 3.51e2 | 1 | 1 | 91 | 105 | Carbamidomethylation, Carbamidomethylation, Carbamidomethylation |  | DB Search |
| total 2 peptides |  |  |  |  |  |  |  |  |  |  |  |  |  |  |  |  |  |  |  |
| --- | --- | --- | --- | --- | --- | --- | --- | --- | --- | --- | --- | --- | --- | --- | --- | --- | --- | --- | --- |

Best Unique PSM (Scan Sample\_9\_A1\_1\_3646.d:21509, m/z=891.3574, z=2, RT=11.42, ppm=-4.38):


TRINITY\_DN173718\_c0\_g1\_i1.p1

back to list

  

| Protein Coverage
| Supporting Peptides
| Best Unique PSM
|

Protein Coverage:

Supporting Peptides:

| Peptide | Uniq | -10lgP | Mass | Length | ppm | m/z | z | RT | 1/k0 Start | 1/k0 End | Precursor ID | Area Sample 10 | #Feature | #Feature Sample 10 | Start | End | PTM | AScore | Found By |
| --- | --- | --- | --- | --- | --- | --- | --- | --- | --- | --- | --- | --- | --- | --- | --- | --- | --- | --- | --- |
| K.IQDKEGIPPDQQR.L | Y | 58.25 | 1522.7739 | 13 | 2.55 | 762.3922 | 2 | 11.03 | 1.0343 | 1.0415 | 18957 | 1.53e3 | 1 | 1 | 30 | 42 |  |  | DB Search |
| total 1 peptides |  |  |  |  |  |  |  |  |  |  |  |  |  |  |  |  |  |  |  |
| --- | --- | --- | --- | --- | --- | --- | --- | --- | --- | --- | --- | --- | --- | --- | --- | --- | --- | --- | --- |

Best Unique PSM (Scan Sample\_9\_A1\_1\_3646.d:18957, m/z=762.3922, z=2, RT=11.03, ppm=-2.67):


TRINITY\_DN10724\_c0\_g1\_i1.p2

back to list

  

| Protein Coverage
| Supporting Peptides
| Best Unique PSM
|

Protein Coverage:

Supporting Peptides:

| Peptide | Uniq | -10lgP | Mass | Length | ppm | m/z | z | RT | 1/k0 Start | 1/k0 End | Precursor ID | Area Sample 10 | #Feature | #Feature Sample 10 | Start | End | PTM | AScore | Found By |
| --- | --- | --- | --- | --- | --- | --- | --- | --- | --- | --- | --- | --- | --- | --- | --- | --- | --- | --- | --- |
| K.REPGDFGFDPLKFSK.R | Y | 56.43 | 1738.8678 | 15 | -1.1 | 580.6262 | 3 | 18.01 | 0.9024 | 0.9097 | 57542 | 0 | 0 | 0 | 67 | 81 |  |  | DB Search |
| total 1 peptides |  |  |  |  |  |  |  |  |  |  |  |  |  |  |  |  |  |  |  |
| --- | --- | --- | --- | --- | --- | --- | --- | --- | --- | --- | --- | --- | --- | --- | --- | --- | --- | --- | --- |

Best Unique PSM (Scan Sample\_9\_A1\_1\_3646.d:57542, m/z=580.6262, z=3, RT=18.01, ppm=-6.32):


TRINITY\_DN4815\_c0\_g1\_i1.p1

back to list

  

| Protein Coverage
| Supporting Peptides
| Best Unique PSM
|

Protein Coverage:

Supporting Peptides:

| Peptide | Uniq | -10lgP | Mass | Length | ppm | m/z | z | RT | 1/k0 Start | 1/k0 End | Precursor ID | Area Sample 10 | #Feature | #Feature Sample 10 | Start | End | PTM | AScore | Found By |
| --- | --- | --- | --- | --- | --- | --- | --- | --- | --- | --- | --- | --- | --- | --- | --- | --- | --- | --- | --- |
| K.TIKDVNC(+57.02)NNNQLK.S | Y | 58.63 | 1559.7726 | 13 | 0.66 | 780.89 | 2 | 11.23 | 1.0464 | 1.0536 | 20329 | 8.76e2 | 1 | 1 | 490 | 502 | Carbamidomethylation |  | DB Search |
| total 1 peptides |  |  |  |  |  |  |  |  |  |  |  |  |  |  |  |  |  |  |  |
| --- | --- | --- | --- | --- | --- | --- | --- | --- | --- | --- | --- | --- | --- | --- | --- | --- | --- | --- | --- |

Best Unique PSM (Scan Sample\_9\_A1\_1\_3646.d:20329, m/z=780.89, z=2, RT=11.23, ppm=-4.56):


TRINITY\_DN1339\_c6\_g1\_i2.p1

back to list

  

| Protein Coverage
| Supporting Peptides
| Best Unique PSM
|

Protein Coverage:

Supporting Peptides:

| Peptide | Uniq | -10lgP | Mass | Length | ppm | m/z | z | RT | 1/k0 Start | 1/k0 End | Precursor ID | Area Sample 10 | #Feature | #Feature Sample 10 | Start | End | PTM | AScore | Found By |
| --- | --- | --- | --- | --- | --- | --- | --- | --- | --- | --- | --- | --- | --- | --- | --- | --- | --- | --- | --- |
| K.VITSDDPAEKEK.K | Y | 59.05 | 1330.6616 | 12 | 3.57 | 666.337 | 2 | 10.72 | 0.9884 | 0.9957 | 17382 | 1.42e4 | 1 | 1 | 355 | 366 |  |  | DB Search |
| total 1 peptides |  |  |  |  |  |  |  |  |  |  |  |  |  |  |  |  |  |  |  |
| --- | --- | --- | --- | --- | --- | --- | --- | --- | --- | --- | --- | --- | --- | --- | --- | --- | --- | --- | --- |

Best Unique PSM (Scan Sample\_9\_A1\_1\_3646.d:17382, m/z=666.337, z=2, RT=10.72, ppm=-1.65):


TRINITY\_DN2217\_c0\_g1\_i3.p1

back to list

  

| Protein Coverage
| Supporting Peptides
| Best Unique PSM
|

Protein Coverage:

Supporting Peptides:

| Peptide | Uniq | -10lgP | Mass | Length | ppm | m/z | z | RT | 1/k0 Start | 1/k0 End | Precursor ID | Area Sample 10 | #Feature | #Feature Sample 10 | Start | End | PTM | AScore | Found By |
| --- | --- | --- | --- | --- | --- | --- | --- | --- | --- | --- | --- | --- | --- | --- | --- | --- | --- | --- | --- |
| R.GC(+57.02)YIHTQEIAR.G | Y | 58.69 | 1346.6401 | 11 | 1.87 | 674.3251 | 2 | 12.91 | 1.0166 | 1.0238 | 30377 | 1.95e3 | 1 | 1 | 538 | 548 | Carbamidomethylation |  | DB Search |
| K.ATGC(+57.02)EVIWNQHNR.G | Y | 58.63 | 1583.7263 | 13 | 3.91 | 792.8694 | 2 | 13.85 | 1.0785 | 1.0857 | 35534 | 2.24e3 | 1 | 1 | 431 | 443 | Carbamidomethylation |  | DB Search |
| R.GC(+57.02)YIHTQEVAR.G | Y | 58.47 | 1332.6245 | 11 | -1.44 | 667.3151 | 2 | 12.15 | 1.011 | 1.0182 | 26068 | 0 | 0 | 0 | 350 | 360 | Carbamidomethylation |  | DB Search |
| R.HFC(+57.02)WVFSK.C | Y | 52.77 | 1109.5117 | 8 | 0.08 | 555.7603 | 2 | 19.80 | 0.87 | 0.8773 | 67829 | 2.32e2 | 1 | 1 | 367 | 374 | Carbamidomethylation |  | DB Search |
| K.RTC(+57.02)C(+57.02)HTK.K | Y | 35.4 | 961.4222 | 7 | -19.21 | 962.406 | 1 | 13.04 | 1.2061 | 1.2133 | 31036 | 6.86e2 | 1 | 1 | 181 | 187 | Carbamidomethylation, Carbamidomethylation |  | DB Search |
| total 5 peptides |  |  |  |  |  |  |  |  |  |  |  |  |  |  |  |  |  |  |  |
| --- | --- | --- | --- | --- | --- | --- | --- | --- | --- | --- | --- | --- | --- | --- | --- | --- | --- | --- | --- |

Best Unique PSM (Scan Sample\_9\_A1\_1\_3646.d:30377, m/z=674.3251, z=2, RT=12.91, ppm=-3.35):


TRINITY\_DN477\_c0\_g1\_i3.p1

back to list

  

| Protein Coverage
| Supporting Peptides
| Best Unique PSM
|

Protein Coverage:

Supporting Peptides:

| Peptide | Uniq | -10lgP | Mass | Length | ppm | m/z | z | RT | 1/k0 Start | 1/k0 End | Precursor ID | Area Sample 10 | #Feature | #Feature Sample 10 | Start | End | PTM | AScore | Found By |
| --- | --- | --- | --- | --- | --- | --- | --- | --- | --- | --- | --- | --- | --- | --- | --- | --- | --- | --- | --- |
| R.NDVNNPPQAR.R | Y | 58.12 | 1123.537 | 10 | 0.82 | 562.7733 | 2 | 9.95 | 0.8668 | 0.8741 | 12691 | 7.16e2 | 1 | 1 | 147 | 156 |  |  | DB Search |
| total 1 peptides |  |  |  |  |  |  |  |  |  |  |  |  |  |  |  |  |  |  |  |
| --- | --- | --- | --- | --- | --- | --- | --- | --- | --- | --- | --- | --- | --- | --- | --- | --- | --- | --- | --- |

Best Unique PSM (Scan Sample\_9\_A1\_1\_3646.d:12691, m/z=562.7733, z=2, RT=9.95, ppm=-4.4):


TRINITY\_DN252\_c0\_g1\_i1.p1

back to list

  

| Protein Coverage
| Supporting Peptides
| Best Unique PSM
|

Protein Coverage:

Supporting Peptides:

| Peptide | Uniq | -10lgP | Mass | Length | ppm | m/z | z | RT | 1/k0 Start | 1/k0 End | Precursor ID | Area Sample 10 | #Feature | #Feature Sample 10 | Start | End | PTM | AScore | Found By |
| --- | --- | --- | --- | --- | --- | --- | --- | --- | --- | --- | --- | --- | --- | --- | --- | --- | --- | --- | --- |
| K.TMNTNYVDKK.T | Y | 38.85 | 1212.5809 | 10 | 1.63 | 607.2955 | 2 | 11.11 | 0.9303 | 0.9376 | 19820 | 0 | 0 | 0 | 101 | 110 |  |  | DB Search |
| total 1 peptides |  |  |  |  |  |  |  |  |  |  |  |  |  |  |  |  |  |  |  |
| --- | --- | --- | --- | --- | --- | --- | --- | --- | --- | --- | --- | --- | --- | --- | --- | --- | --- | --- | --- |

Best Unique PSM (Scan Sample\_9\_A1\_1\_3646.d:19820, m/z=607.2955, z=2, RT=11.11, ppm=-3.59):


TRINITY\_DN96\_c0\_g1\_i3.p1

back to list

  

| Protein Coverage
| Supporting Peptides
| Best Unique PSM
|

Protein Coverage:

Supporting Peptides:

| Peptide | Uniq | -10lgP | Mass | Length | ppm | m/z | z | RT | 1/k0 Start | 1/k0 End | Precursor ID | Area Sample 10 | #Feature | #Feature Sample 10 | Start | End | PTM | AScore | Found By |
| --- | --- | --- | --- | --- | --- | --- | --- | --- | --- | --- | --- | --- | --- | --- | --- | --- | --- | --- | --- |
| K.KVEQVQTDSR.D | Y | 53.61 | 1188.6098 | 10 | 5.11 | 595.3121 | 2 | 9.53 | 0.8725 | 0.8798 | 10904 | 2.14e2 | 1 | 1 | 153 | 162 |  |  | DB Search |
| total 1 peptides |  |  |  |  |  |  |  |  |  |  |  |  |  |  |  |  |  |  |  |
| --- | --- | --- | --- | --- | --- | --- | --- | --- | --- | --- | --- | --- | --- | --- | --- | --- | --- | --- | --- |

Best Unique PSM (Scan Sample\_9\_A1\_1\_3646.d:10904, m/z=595.3121, z=2, RT=9.53, ppm=-0.11):


TRINITY\_DN585\_c0\_g1\_i3.p1

back to list

  

| Protein Coverage
| Supporting Peptides
| Best Unique PSM
|

Protein Coverage:

Supporting Peptides:

| Peptide | Uniq | -10lgP | Mass | Length | ppm | m/z | z | RT | 1/k0 Start | 1/k0 End | Precursor ID | Area Sample 10 | #Feature | #Feature Sample 10 | Start | End | PTM | AScore | Found By |
| --- | --- | --- | --- | --- | --- | --- | --- | --- | --- | --- | --- | --- | --- | --- | --- | --- | --- | --- | --- |
| K.IQDKEGIPPDQQR.L | Y | 58.25 | 1522.7739 | 13 | 2.55 | 762.3922 | 2 | 11.03 | 1.0343 | 1.0415 | 18957 | 1.53e3 | 1 | 1 | 2 | 14 |  |  | DB Search |
| total 1 peptides |  |  |  |  |  |  |  |  |  |  |  |  |  |  |  |  |  |  |  |
| --- | --- | --- | --- | --- | --- | --- | --- | --- | --- | --- | --- | --- | --- | --- | --- | --- | --- | --- | --- |

Best Unique PSM (Scan Sample\_9\_A1\_1\_3646.d:18957, m/z=762.3922, z=2, RT=11.03, ppm=-2.67):


TRINITY\_DN3619\_c0\_g1\_i1.p1

back to list

  

| Protein Coverage
| Supporting Peptides
| Best Unique PSM
|

Protein Coverage:

Supporting Peptides:

| Peptide | Uniq | -10lgP | Mass | Length | ppm | m/z | z | RT | 1/k0 Start | 1/k0 End | Precursor ID | Area Sample 10 | #Feature | #Feature Sample 10 | Start | End | PTM | AScore | Found By |
| --- | --- | --- | --- | --- | --- | --- | --- | --- | --- | --- | --- | --- | --- | --- | --- | --- | --- | --- | --- |
| R.GKEDERNEVYNEAITK.A | Y | 58.65 | 1893.9068 | 16 | 3.14 | 632.3082 | 3 | 12.68 | 0.8274 | 0.8348 | 29071 | 1.27e3 | 1 | 1 | 1377 | 1392 |  |  | DB Search |
| total 1 peptides |  |  |  |  |  |  |  |  |  |  |  |  |  |  |  |  |  |  |  |
| --- | --- | --- | --- | --- | --- | --- | --- | --- | --- | --- | --- | --- | --- | --- | --- | --- | --- | --- | --- |

Best Unique PSM (Scan Sample\_9\_A1\_1\_3646.d:29071, m/z=632.3082, z=3, RT=12.68, ppm=-2.08):


TRINITY\_DN4026\_c0\_g1\_i2.p1

back to list

  

| Protein Coverage
| Supporting Peptides
| Best Unique PSM
|

Protein Coverage:

Supporting Peptides:

| Peptide | Uniq | -10lgP | Mass | Length | ppm | m/z | z | RT | 1/k0 Start | 1/k0 End | Precursor ID | Area Sample 10 | #Feature | #Feature Sample 10 | Start | End | PTM | AScore | Found By |
| --- | --- | --- | --- | --- | --- | --- | --- | --- | --- | --- | --- | --- | --- | --- | --- | --- | --- | --- | --- |
| K.KLNAEIANSR.L | Y | 57.15 | 1114.6094 | 10 | -3.85 | 558.3069 | 2 | 11.39 | 0.868 | 0.8753 | 21472 | 0 | 0 | 0 | 143 | 152 |  |  | DB Search |
| total 1 peptides |  |  |  |  |  |  |  |  |  |  |  |  |  |  |  |  |  |  |  |
| --- | --- | --- | --- | --- | --- | --- | --- | --- | --- | --- | --- | --- | --- | --- | --- | --- | --- | --- | --- |

Best Unique PSM (Scan Sample\_9\_A1\_1\_3646.d:21472, m/z=558.3069, z=2, RT=11.39, ppm=-9.07):


TRINITY\_DN5263\_c0\_g1\_i1.p1

back to list

  

| Protein Coverage
| Supporting Peptides
| Best Unique PSM
|

Protein Coverage:

Supporting Peptides:

| Peptide | Uniq | -10lgP | Mass | Length | ppm | m/z | z | RT | 1/k0 Start | 1/k0 End | Precursor ID | Area Sample 10 | #Feature | #Feature Sample 10 | Start | End | PTM | AScore | Found By |
| --- | --- | --- | --- | --- | --- | --- | --- | --- | --- | --- | --- | --- | --- | --- | --- | --- | --- | --- | --- |
| R.IRELTSVVQKR.F | Y | 58.24 | 1327.7936 | 11 | -4.83 | 443.6007 | 3 | 12.43 | 0.751 | 0.7583 | 27658 | 0 | 0 | 0 | 66 | 76 |  |  | DB Search |
| total 1 peptides |  |  |  |  |  |  |  |  |  |  |  |  |  |  |  |  |  |  |  |
| --- | --- | --- | --- | --- | --- | --- | --- | --- | --- | --- | --- | --- | --- | --- | --- | --- | --- | --- | --- |

Best Unique PSM (Scan Sample\_9\_A1\_1\_3646.d:27658, m/z=443.6007, z=3, RT=12.43, ppm=-10.06):


TRINITY\_DN73067\_c0\_g1\_i1.p1

back to list

  

| Protein Coverage
| Supporting Peptides
| Best Unique PSM
|

Protein Coverage:

Supporting Peptides:

| Peptide | Uniq | -10lgP | Mass | Length | ppm | m/z | z | RT | 1/k0 Start | 1/k0 End | Precursor ID | Area Sample 10 | #Feature | #Feature Sample 10 | Start | End | PTM | AScore | Found By |
| --- | --- | --- | --- | --- | --- | --- | --- | --- | --- | --- | --- | --- | --- | --- | --- | --- | --- | --- | --- |
| K.VVTSSTGPGEHMR.N | Y | 58.46 | 1356.6456 | 13 | 6.14 | 679.3307 | 2 | 10.90 | 0.9844 | 0.9916 | 18261 | 1.49e3 | 1 | 1 | 714 | 726 |  |  | DB Search |
| K.VVTSSTGPGEHM(+15.99)R.N | Y | 58.39 | 1372.6405 | 13 | -0.58 | 458.5515 | 3 | 9.72 | 0.7167 | 0.7241 | 11748 | 1.58e3 | 2 | 2 | 714 | 726 | Oxidation (M) | M12:Oxidation (M):1000 | DB Search |
| total 2 peptides |  |  |  |  |  |  |  |  |  |  |  |  |  |  |  |  |  |  |  |
| --- | --- | --- | --- | --- | --- | --- | --- | --- | --- | --- | --- | --- | --- | --- | --- | --- | --- | --- | --- |

Best Unique PSM (Scan Sample\_9\_A1\_1\_3646.d:18261, m/z=679.3307, z=2, RT=10.90, ppm=0.92):


TRINITY\_DN2028\_c0\_g1\_i1.p1

back to list

  

| Protein Coverage
| Supporting Peptides
| Best Unique PSM
|

Protein Coverage:

Supporting Peptides:

| Peptide | Uniq | -10lgP | Mass | Length | ppm | m/z | z | RT | 1/k0 Start | 1/k0 End | Precursor ID | Area Sample 10 | #Feature | #Feature Sample 10 | Start | End | PTM | AScore | Found By |
| --- | --- | --- | --- | --- | --- | --- | --- | --- | --- | --- | --- | --- | --- | --- | --- | --- | --- | --- | --- |
| K.LIQYIENKPGLTR.L | Y | 58.6 | 1543.8722 | 13 | 0.06 | 772.9394 | 2 | 14.75 | 1.0781 | 1.0853 | 40202 | 1.81e3 | 1 | 1 | 81 | 93 |  |  | DB Search |
| total 1 peptides |  |  |  |  |  |  |  |  |  |  |  |  |  |  |  |  |  |  |  |
| --- | --- | --- | --- | --- | --- | --- | --- | --- | --- | --- | --- | --- | --- | --- | --- | --- | --- | --- | --- |

Best Unique PSM (Scan Sample\_9\_A1\_1\_3646.d:40202, m/z=772.9394, z=2, RT=14.75, ppm=-5.16):


TRINITY\_DN22219\_c0\_g1\_i1.p1

back to list

  

| Protein Coverage
| Supporting Peptides
| Best Unique PSM
|

Protein Coverage:

Supporting Peptides:

| Peptide | Uniq | -10lgP | Mass | Length | ppm | m/z | z | RT | 1/k0 Start | 1/k0 End | Precursor ID | Area Sample 10 | #Feature | #Feature Sample 10 | Start | End | PTM | AScore | Found By |
| --- | --- | --- | --- | --- | --- | --- | --- | --- | --- | --- | --- | --- | --- | --- | --- | --- | --- | --- | --- |
| R.AEVIREEEEEKEK.K | Y | 57.16 | 1616.7893 | 13 | 1.65 | 539.9351 | 3 | 10.72 | 0.8019 | 0.8092 | 17395 | 1.67e3 | 1 | 1 | 311 | 323 |  |  | DB Search |
| total 1 peptides |  |  |  |  |  |  |  |  |  |  |  |  |  |  |  |  |  |  |  |
| --- | --- | --- | --- | --- | --- | --- | --- | --- | --- | --- | --- | --- | --- | --- | --- | --- | --- | --- | --- |

Best Unique PSM (Scan Sample\_9\_A1\_1\_3646.d:17395, m/z=539.9351, z=3, RT=10.72, ppm=-3.57):


TRINITY\_DN1797\_c0\_g1\_i2.p1

back to list

  

| Protein Coverage
| Supporting Peptides
| Best Unique PSM
|

Protein Coverage:

Supporting Peptides:

| Peptide | Uniq | -10lgP | Mass | Length | ppm | m/z | z | RT | 1/k0 Start | 1/k0 End | Precursor ID | Area Sample 10 | #Feature | #Feature Sample 10 | Start | End | PTM | AScore | Found By |
| --- | --- | --- | --- | --- | --- | --- | --- | --- | --- | --- | --- | --- | --- | --- | --- | --- | --- | --- | --- |
| K.FAANLLDHFKIDR.S | Y | 57.34 | 1558.8256 | 13 | -0.92 | 520.6126 | 3 | 18.07 | 0.8481 | 0.8554 | 57779 | 0 | 0 | 0 | 539 | 551 |  |  | DB Search |
| total 1 peptides |  |  |  |  |  |  |  |  |  |  |  |  |  |  |  |  |  |  |  |
| --- | --- | --- | --- | --- | --- | --- | --- | --- | --- | --- | --- | --- | --- | --- | --- | --- | --- | --- | --- |

Best Unique PSM (Scan Sample\_9\_A1\_1\_3646.d:57779, m/z=520.6126, z=3, RT=18.07, ppm=-6.14):


TRINITY\_DN6920\_c0\_g1\_i1.p1

back to list

  

| Protein Coverage
| Supporting Peptides
| Best Unique PSM
|

Protein Coverage:

Supporting Peptides:

| Peptide | Uniq | -10lgP | Mass | Length | ppm | m/z | z | RT | 1/k0 Start | 1/k0 End | Precursor ID | Area Sample 10 | #Feature | #Feature Sample 10 | Start | End | PTM | AScore | Found By |
| --- | --- | --- | --- | --- | --- | --- | --- | --- | --- | --- | --- | --- | --- | --- | --- | --- | --- | --- | --- |
| K.VAC(+57.02)IGAWHPAR.V | Y | 58.63 | 1236.6186 | 11 | 3 | 619.3152 | 2 | 13.23 | 0.9461 | 0.9533 | 32140 | 5.07e2 | 1 | 1 | 134 | 144 | Carbamidomethylation |  | DB Search |
| R.VLVHTQQK.L | Y | 54.09 | 951.5501 | 8 | 3.81 | 476.7817 | 2 | 9.56 | 0.8303 | 0.8376 | 11062 | 2.36e2 | 1 | 1 | 45 | 52 |  |  | DB Search |
| total 2 peptides |  |  |  |  |  |  |  |  |  |  |  |  |  |  |  |  |  |  |  |
| --- | --- | --- | --- | --- | --- | --- | --- | --- | --- | --- | --- | --- | --- | --- | --- | --- | --- | --- | --- |

Best Unique PSM (Scan Sample\_9\_A1\_1\_3646.d:32140, m/z=619.3152, z=2, RT=13.23, ppm=-2.22):


TRINITY\_DN1616\_c0\_g1\_i2.p1

back to list

  

| Protein Coverage
| Supporting Peptides
| Best Unique PSM
|

Protein Coverage:

Supporting Peptides:

| Peptide | Uniq | -10lgP | Mass | Length | ppm | m/z | z | RT | 1/k0 Start | 1/k0 End | Precursor ID | Area Sample 10 | #Feature | #Feature Sample 10 | Start | End | PTM | AScore | Found By |
| --- | --- | --- | --- | --- | --- | --- | --- | --- | --- | --- | --- | --- | --- | --- | --- | --- | --- | --- | --- |
| K.YVVMLQYYHDSDTAK.Y | Y | 52.09 | 1831.8451 | 15 | 5.48 | 916.9301 | 2 | 16.56 | 1.1813 | 1.1885 | 49447 | 0 | 0 | 0 | 1071 | 1085 |  |  | DB Search |
| R.YALNQIQPVTATVTAFKGDSK.I | Y | 44.58 | 2251.1848 | 21 | 9.46 | 751.4054 | 3 | 18.19 | 1.048 | 1.0552 | 58586 | 0 | 0 | 0 | 894 | 914 |  |  | DB Search |
| total 2 peptides |  |  |  |  |  |  |  |  |  |  |  |  |  |  |  |  |  |  |  |
| --- | --- | --- | --- | --- | --- | --- | --- | --- | --- | --- | --- | --- | --- | --- | --- | --- | --- | --- | --- |

Best Unique PSM (Scan Sample\_9\_A1\_1\_3646.d:49447, m/z=916.9301, z=2, RT=16.56, ppm=0.27):


TRINITY\_DN96\_c0\_g1\_i7.p1

back to list

  

| Protein Coverage
| Supporting Peptides
| Best Unique PSM
|

Protein Coverage:

Supporting Peptides:

| Peptide | Uniq | -10lgP | Mass | Length | ppm | m/z | z | RT | 1/k0 Start | 1/k0 End | Precursor ID | Area Sample 10 | #Feature | #Feature Sample 10 | Start | End | PTM | AScore | Found By |
| --- | --- | --- | --- | --- | --- | --- | --- | --- | --- | --- | --- | --- | --- | --- | --- | --- | --- | --- | --- |
| K.KVEQVQTDSR.D | Y | 53.61 | 1188.6098 | 10 | 5.11 | 595.3121 | 2 | 9.53 | 0.8725 | 0.8798 | 10904 | 2.14e2 | 1 | 1 | 103 | 112 |  |  | DB Search |
| total 1 peptides |  |  |  |  |  |  |  |  |  |  |  |  |  |  |  |  |  |  |  |
| --- | --- | --- | --- | --- | --- | --- | --- | --- | --- | --- | --- | --- | --- | --- | --- | --- | --- | --- | --- |

Best Unique PSM (Scan Sample\_9\_A1\_1\_3646.d:10904, m/z=595.3121, z=2, RT=9.53, ppm=-0.11):


TRINITY\_DN188006\_c0\_g1\_i1.p1

back to list

  

| Protein Coverage
| Supporting Peptides
| Best Unique PSM
|

Protein Coverage:

Supporting Peptides:

| Peptide | Uniq | -10lgP | Mass | Length | ppm | m/z | z | RT | 1/k0 Start | 1/k0 End | Precursor ID | Area Sample 10 | #Feature | #Feature Sample 10 | Start | End | PTM | AScore | Found By |
| --- | --- | --- | --- | --- | --- | --- | --- | --- | --- | --- | --- | --- | --- | --- | --- | --- | --- | --- | --- |
| K.SC(+57.02)LLLQFTDKR.F | Y | 58.6 | 1379.7231 | 11 | 0.98 | 690.8659 | 2 | 17.62 | 1.0291 | 1.0363 | 55103 | 7.83e2 | 1 | 1 | 21 | 31 | Carbamidomethylation |  | DB Search |
| total 1 peptides |  |  |  |  |  |  |  |  |  |  |  |  |  |  |  |  |  |  |  |
| --- | --- | --- | --- | --- | --- | --- | --- | --- | --- | --- | --- | --- | --- | --- | --- | --- | --- | --- | --- |

Best Unique PSM (Scan Sample\_9\_A1\_1\_3646.d:55103, m/z=690.8659, z=2, RT=17.62, ppm=-4.24):


TRINITY\_DN36\_c0\_g1\_i2.p1

back to list

  

| Protein Coverage
| Supporting Peptides
| Best Unique PSM
|

Protein Coverage:

Supporting Peptides:

| Peptide | Uniq | -10lgP | Mass | Length | ppm | m/z | z | RT | 1/k0 Start | 1/k0 End | Precursor ID | Area Sample 10 | #Feature | #Feature Sample 10 | Start | End | PTM | AScore | Found By |
| --- | --- | --- | --- | --- | --- | --- | --- | --- | --- | --- | --- | --- | --- | --- | --- | --- | --- | --- | --- |
| R.LGGIEVTQGNC(+57.02)ARK.Y | Y | 58.24 | 1501.7671 | 14 | 6.42 | 751.8917 | 2 | 12.66 | 1.0632 | 1.0705 | 28959 | 0 | 0 | 0 | 58 | 71 | Carbamidomethylation |  | DB Search |
| total 1 peptides |  |  |  |  |  |  |  |  |  |  |  |  |  |  |  |  |  |  |  |
| --- | --- | --- | --- | --- | --- | --- | --- | --- | --- | --- | --- | --- | --- | --- | --- | --- | --- | --- | --- |

Best Unique PSM (Scan Sample\_9\_A1\_1\_3646.d:28959, m/z=751.8917, z=2, RT=12.66, ppm=1.21):


TRINITY\_DN902\_c1\_g2\_i4.p1

back to list

  

| Protein Coverage
| Supporting Peptides
| Best Unique PSM
|

Protein Coverage:

Supporting Peptides:

| Peptide | Uniq | -10lgP | Mass | Length | ppm | m/z | z | RT | 1/k0 Start | 1/k0 End | Precursor ID | Area Sample 10 | #Feature | #Feature Sample 10 | Start | End | PTM | AScore | Found By |
| --- | --- | --- | --- | --- | --- | --- | --- | --- | --- | --- | --- | --- | --- | --- | --- | --- | --- | --- | --- |
| K.GLTHC(+57.02)NTLSYK.L | Y | 59.02 | 1292.6183 | 11 | 5.84 | 647.3168 | 2 | 12.45 | 0.9727 | 0.98 | 27736 | 3.39e3 | 1 | 1 | 62 | 72 | Carbamidomethylation |  | DB Search |
| total 1 peptides |  |  |  |  |  |  |  |  |  |  |  |  |  |  |  |  |  |  |  |
| --- | --- | --- | --- | --- | --- | --- | --- | --- | --- | --- | --- | --- | --- | --- | --- | --- | --- | --- | --- |

Best Unique PSM (Scan Sample\_9\_A1\_1\_3646.d:27736, m/z=647.3168, z=2, RT=12.45, ppm=0.62):


TRINITY\_DN535\_c0\_g1\_i1.p1

back to list

  

| Protein Coverage
| Supporting Peptides
| Best Unique PSM
|

Protein Coverage:

Supporting Peptides:

| Peptide | Uniq | -10lgP | Mass | Length | ppm | m/z | z | RT | 1/k0 Start | 1/k0 End | Precursor ID | Area Sample 10 | #Feature | #Feature Sample 10 | Start | End | PTM | AScore | Found By |
| --- | --- | --- | --- | --- | --- | --- | --- | --- | --- | --- | --- | --- | --- | --- | --- | --- | --- | --- | --- |
| R.ALGC(+57.02)DVLAC(+57.02)SVDSHFSHLAWTQQSR.K | Y | 58.92 | 2844.3071 | 25 | -0.81 | 712.0798 | 4 | 18.79 | 0.9481 | 0.9553 | 62026 | 2.73e3 | 1 | 1 | 33 | 57 | Carbamidomethylation, Carbamidomethylation |  | DB Search |
| R.DYGVLLENDGIALR.G | Y | 58.64 | 1546.7991 | 14 | 1.09 | 774.4036 | 2 | 19.46 | 1.0387 | 1.046 | 66038 | 4.57e2 | 1 | 1 | 80 | 93 |  |  | DB Search |
| R.GLFIIDDKGILR.Q | Y | 58.62 | 1358.7921 | 12 | 1.68 | 680.4009 | 2 | 19.29 | 1.0114 | 1.0186 | 64919 | 1.05e4 | 1 | 1 | 94 | 105 |  |  | DB Search |
| K.IPILSDLTK.S | Y | 58.32 | 998.6012 | 9 | -0.22 | 500.3051 | 2 | 18.86 | 0.8254 | 0.8327 | 62176 | 2.58e3 | 1 | 1 | 67 | 75 |  |  | DB Search |
| total 4 peptides |  |  |  |  |  |  |  |  |  |  |  |  |  |  |  |  |  |  |  |
| --- | --- | --- | --- | --- | --- | --- | --- | --- | --- | --- | --- | --- | --- | --- | --- | --- | --- | --- | --- |

Best Unique PSM (Scan Sample\_9\_A1\_1\_3646.d:62026, m/z=712.0798, z=4, RT=18.79, ppm=-6.03):


TRINITY\_DN2217\_c0\_g1\_i2.p1

back to list

  

| Protein Coverage
| Supporting Peptides
| Best Unique PSM
|

Protein Coverage:

Supporting Peptides:

| Peptide | Uniq | -10lgP | Mass | Length | ppm | m/z | z | RT | 1/k0 Start | 1/k0 End | Precursor ID | Area Sample 10 | #Feature | #Feature Sample 10 | Start | End | PTM | AScore | Found By |
| --- | --- | --- | --- | --- | --- | --- | --- | --- | --- | --- | --- | --- | --- | --- | --- | --- | --- | --- | --- |
| R.GC(+57.02)YIHTQEIAR.G | Y | 58.69 | 1346.6401 | 11 | 1.87 | 674.3251 | 2 | 12.91 | 1.0166 | 1.0238 | 30377 | 1.95e3 | 1 | 1 | 226 | 236 | Carbamidomethylation |  | DB Search |
| K.ATGC(+57.02)EVIWNQHNR.G | Y | 58.63 | 1583.7263 | 13 | 3.91 | 792.8694 | 2 | 13.85 | 1.0785 | 1.0857 | 35534 | 2.24e3 | 1 | 1 | 119 | 131 | Carbamidomethylation |  | DB Search |
| R.GC(+57.02)YIHTQEVAR.G | Y | 58.47 | 1332.6245 | 11 | -1.44 | 667.3151 | 2 | 12.15 | 1.011 | 1.0182 | 26068 | 0 | 0 | 0 | 38 | 48 | Carbamidomethylation |  | DB Search |
| R.HFC(+57.02)WVFSK.C | Y | 52.77 | 1109.5117 | 8 | 0.08 | 555.7603 | 2 | 19.80 | 0.87 | 0.8773 | 67829 | 2.32e2 | 1 | 1 | 55 | 62 | Carbamidomethylation |  | DB Search |
| total 4 peptides |  |  |  |  |  |  |  |  |  |  |  |  |  |  |  |  |  |  |  |
| --- | --- | --- | --- | --- | --- | --- | --- | --- | --- | --- | --- | --- | --- | --- | --- | --- | --- | --- | --- |

Best Unique PSM (Scan Sample\_9\_A1\_1\_3646.d:30377, m/z=674.3251, z=2, RT=12.91, ppm=-3.35):


TRINITY\_DN82805\_c0\_g1\_i1.p1

back to list

  

| Protein Coverage
| Supporting Peptides
| Best Unique PSM
|

Protein Coverage:

Supporting Peptides:

| Peptide | Uniq | -10lgP | Mass | Length | ppm | m/z | z | RT | 1/k0 Start | 1/k0 End | Precursor ID | Area Sample 10 | #Feature | #Feature Sample 10 | Start | End | PTM | AScore | Found By |
| --- | --- | --- | --- | --- | --- | --- | --- | --- | --- | --- | --- | --- | --- | --- | --- | --- | --- | --- | --- |
| R.IINAIDKDVQER.K | Y | 55.4 | 1412.7623 | 12 | -2.3 | 707.3831 | 2 | 13.49 | 1.0355 | 1.0427 | 33695 | 0 | 0 | 0 | 354 | 365 |  |  | DB Search |
| total 1 peptides |  |  |  |  |  |  |  |  |  |  |  |  |  |  |  |  |  |  |  |
| --- | --- | --- | --- | --- | --- | --- | --- | --- | --- | --- | --- | --- | --- | --- | --- | --- | --- | --- | --- |

Best Unique PSM (Scan Sample\_9\_A1\_1\_3646.d:33695, m/z=707.3831, z=2, RT=13.49, ppm=-7.52):


TRINITY\_DN817\_c0\_g1\_i7.p1

back to list

  

| Protein Coverage
| Supporting Peptides
| Best Unique PSM
|

Protein Coverage:

Supporting Peptides:

| Peptide | Uniq | -10lgP | Mass | Length | ppm | m/z | z | RT | 1/k0 Start | 1/k0 End | Precursor ID | Area Sample 10 | #Feature | #Feature Sample 10 | Start | End | PTM | AScore | Found By |
| --- | --- | --- | --- | --- | --- | --- | --- | --- | --- | --- | --- | --- | --- | --- | --- | --- | --- | --- | --- |
| R.LC(+57.02)QPGYSTC(+57.02)ENHC(+57.02)R.R | Y | 55.11 | 1780.708 | 14 | 0.84 | 891.3574 | 2 | 11.42 | 1.0861 | 1.0933 | 21509 | 5.05e2 | 1 | 1 | 89 | 102 | Carbamidomethylation, Carbamidomethylation, Carbamidomethylation |  | DB Search |
| R.LC(+57.02)QPGYSTC(+57.02)ENHC(+57.02)RR.L | Y | 43.99 | 1936.8091 | 15 | 3.79 | 646.6094 | 3 | 10.89 | 0.8575 | 0.8648 | 18436 | 3.51e2 | 1 | 1 | 89 | 103 | Carbamidomethylation, Carbamidomethylation, Carbamidomethylation |  | DB Search |
| total 2 peptides |  |  |  |  |  |  |  |  |  |  |  |  |  |  |  |  |  |  |  |
| --- | --- | --- | --- | --- | --- | --- | --- | --- | --- | --- | --- | --- | --- | --- | --- | --- | --- | --- | --- |

Best Unique PSM (Scan Sample\_9\_A1\_1\_3646.d:21509, m/z=891.3574, z=2, RT=11.42, ppm=-4.38):


TRINITY\_DN8165\_c0\_g2\_i1.p2

back to list

  

| Protein Coverage
| Supporting Peptides
| Best Unique PSM
|

Protein Coverage:

Supporting Peptides:

| Peptide | Uniq | -10lgP | Mass | Length | ppm | m/z | z | RT | 1/k0 Start | 1/k0 End | Precursor ID | Area Sample 10 | #Feature | #Feature Sample 10 | Start | End | PTM | AScore | Found By |
| --- | --- | --- | --- | --- | --- | --- | --- | --- | --- | --- | --- | --- | --- | --- | --- | --- | --- | --- | --- |
| K.K(+42.01)LSAELANGR.L | Y | 58.81 | 1099.5985 | 10 | -0.72 | 550.8033 | 2 | 14.22 | 0.8721 | 0.8793 | 37511 | 8.65e2 | 1 | 1 | 4 | 13 | Acetylation (Protein N-term) | K1:Acetylation (Protein N-term):1000 | DB Search |
| total 1 peptides |  |  |  |  |  |  |  |  |  |  |  |  |  |  |  |  |  |  |  |
| --- | --- | --- | --- | --- | --- | --- | --- | --- | --- | --- | --- | --- | --- | --- | --- | --- | --- | --- | --- |

Best Unique PSM (Scan Sample\_9\_A1\_1\_3646.d:37511, m/z=550.8033, z=2, RT=14.22, ppm=-5.94):


TRINITY\_DN1797\_c0\_g1\_i3.p1

back to list

  

| Protein Coverage
| Supporting Peptides
| Best Unique PSM
|

Protein Coverage:

Supporting Peptides:

| Peptide | Uniq | -10lgP | Mass | Length | ppm | m/z | z | RT | 1/k0 Start | 1/k0 End | Precursor ID | Area Sample 10 | #Feature | #Feature Sample 10 | Start | End | PTM | AScore | Found By |
| --- | --- | --- | --- | --- | --- | --- | --- | --- | --- | --- | --- | --- | --- | --- | --- | --- | --- | --- | --- |
| K.FAANLLDHFKIDR.S | Y | 57.34 | 1558.8256 | 13 | -0.92 | 520.6126 | 3 | 18.07 | 0.8481 | 0.8554 | 57779 | 0 | 0 | 0 | 539 | 551 |  |  | DB Search |
| total 1 peptides |  |  |  |  |  |  |  |  |  |  |  |  |  |  |  |  |  |  |  |
| --- | --- | --- | --- | --- | --- | --- | --- | --- | --- | --- | --- | --- | --- | --- | --- | --- | --- | --- | --- |

Best Unique PSM (Scan Sample\_9\_A1\_1\_3646.d:57779, m/z=520.6126, z=3, RT=18.07, ppm=-6.14):


TRINITY\_DN13856\_c0\_g1\_i1.p2

back to list

  

| Protein Coverage
| Supporting Peptides
| Best Unique PSM
|

Protein Coverage:

Supporting Peptides:

| Peptide | Uniq | -10lgP | Mass | Length | ppm | m/z | z | RT | 1/k0 Start | 1/k0 End | Precursor ID | Area Sample 10 | #Feature | #Feature Sample 10 | Start | End | PTM | AScore | Found By |
| --- | --- | --- | --- | --- | --- | --- | --- | --- | --- | --- | --- | --- | --- | --- | --- | --- | --- | --- | --- |
| R.IPLDDFQDPTLTLENAHNK.L | Y | 58.63 | 2180.0749 | 19 | -0.5 | 727.6948 | 3 | 18.20 | 0.9904 | 0.9977 | 58430 | 9.46e2 | 1 | 1 | 175 | 193 |  |  | DB Search |
| total 1 peptides |  |  |  |  |  |  |  |  |  |  |  |  |  |  |  |  |  |  |  |
| --- | --- | --- | --- | --- | --- | --- | --- | --- | --- | --- | --- | --- | --- | --- | --- | --- | --- | --- | --- |

Best Unique PSM (Scan Sample\_9\_A1\_1\_3646.d:58430, m/z=727.6948, z=3, RT=18.20, ppm=-5.71):


TRINITY\_DN923\_c0\_g1\_i1.p1

back to list

  

| Protein Coverage
| Supporting Peptides
| Best Unique PSM
|

Protein Coverage:

Supporting Peptides:

| Peptide | Uniq | -10lgP | Mass | Length | ppm | m/z | z | RT | 1/k0 Start | 1/k0 End | Precursor ID | Area Sample 10 | #Feature | #Feature Sample 10 | Start | End | PTM | AScore | Found By |
| --- | --- | --- | --- | --- | --- | --- | --- | --- | --- | --- | --- | --- | --- | --- | --- | --- | --- | --- | --- |
| R.YLTTAVITNKR.R | Y | 57.21 | 1278.7296 | 11 | 3.8 | 640.3712 | 2 | 13.83 | 0.9989 | 1.0061 | 35564 | 3e2 | 1 | 1 | 258 | 268 |  |  | DB Search |
| total 1 peptides |  |  |  |  |  |  |  |  |  |  |  |  |  |  |  |  |  |  |  |
| --- | --- | --- | --- | --- | --- | --- | --- | --- | --- | --- | --- | --- | --- | --- | --- | --- | --- | --- | --- |

Best Unique PSM (Scan Sample\_9\_A1\_1\_3646.d:35564, m/z=640.3712, z=2, RT=13.83, ppm=-1.42):


TRINITY\_DN773\_c0\_g1\_i1.p1

back to list

  

| Protein Coverage
| Supporting Peptides
| Best Unique PSM
|

Protein Coverage:

Supporting Peptides:

| Peptide | Uniq | -10lgP | Mass | Length | ppm | m/z | z | RT | 1/k0 Start | 1/k0 End | Precursor ID | Area Sample 10 | #Feature | #Feature Sample 10 | Start | End | PTM | AScore | Found By |
| --- | --- | --- | --- | --- | --- | --- | --- | --- | --- | --- | --- | --- | --- | --- | --- | --- | --- | --- | --- |
| R.GVSEETTTGVHNLYK.M | Y | 56.84 | 1633.7947 | 15 | 1.58 | 817.9017 | 2 | 13.56 | 1.0713 | 1.0785 | 34001 | 5.38e2 | 1 | 1 | 160 | 174 |  |  | DB Search |
| K.RLDEEVAR.L | Y | 55.66 | 986.5145 | 8 | -13.44 | 494.2553 | 2 | 11.10 | 0.853 | 0.8603 | 19719 | 0 | 0 | 0 | 396 | 403 |  |  | DB Search |
| total 2 peptides |  |  |  |  |  |  |  |  |  |  |  |  |  |  |  |  |  |  |  |
| --- | --- | --- | --- | --- | --- | --- | --- | --- | --- | --- | --- | --- | --- | --- | --- | --- | --- | --- | --- |

Best Unique PSM (Scan Sample\_9\_A1\_1\_3646.d:34001, m/z=817.9017, z=2, RT=13.56, ppm=-3.64):


TRINITY\_DN164351\_c0\_g1\_i1.p1

back to list

  

| Protein Coverage
| Supporting Peptides
| Best Unique PSM
|

Protein Coverage:

Supporting Peptides:

| Peptide | Uniq | -10lgP | Mass | Length | ppm | m/z | z | RT | 1/k0 Start | 1/k0 End | Precursor ID | Area Sample 10 | #Feature | #Feature Sample 10 | Start | End | PTM | AScore | Found By |
| --- | --- | --- | --- | --- | --- | --- | --- | --- | --- | --- | --- | --- | --- | --- | --- | --- | --- | --- | --- |
| R.LTNSLM(+15.99)M(+15.99)HGR.N | Y | 55.94 | 1190.5536 | 10 | -0.66 | 596.2806 | 2 | 10.74 | 0.9158 | 0.923 | 17432 | 1.45e3 | 1 | 1 | 78 | 87 | Oxidation (M), Oxidation (M) | M6:Oxidation (M):1000 M7:Oxidation (M):1000 | DB Search |
| K.HAVYLPHTAAR.Y | Y | 51.13 | 1234.6571 | 11 | 5.16 | 412.5596 | 3 | 13.38 | 0.81 | 0.8173 | 33164 | 1.69e2 | 1 | 1 | 51 | 61 |  |  | DB Search |
| total 2 peptides |  |  |  |  |  |  |  |  |  |  |  |  |  |  |  |  |  |  |  |
| --- | --- | --- | --- | --- | --- | --- | --- | --- | --- | --- | --- | --- | --- | --- | --- | --- | --- | --- | --- |

Best Unique PSM (Scan Sample\_9\_A1\_1\_3646.d:17432, m/z=596.2806, z=2, RT=10.74, ppm=-5.88):


TRINITY\_DN2487\_c1\_g1\_i3.p1

back to list

  

| Protein Coverage
| Supporting Peptides
| Best Unique PSM
|

Protein Coverage:

Supporting Peptides:

| Peptide | Uniq | -10lgP | Mass | Length | ppm | m/z | z | RT | 1/k0 Start | 1/k0 End | Precursor ID | Area Sample 10 | #Feature | #Feature Sample 10 | Start | End | PTM | AScore | Found By |
| --- | --- | --- | --- | --- | --- | --- | --- | --- | --- | --- | --- | --- | --- | --- | --- | --- | --- | --- | --- |
| R.RLVELQHGR.V | Y | 53.64 | 1106.6308 | 9 | 6.11 | 369.8846 | 3 | 11.14 | 0.7061 | 0.7135 | 19894 | 1.5e3 | 1 | 1 | 79 | 87 |  |  | DB Search |
| total 1 peptides |  |  |  |  |  |  |  |  |  |  |  |  |  |  |  |  |  |  |  |
| --- | --- | --- | --- | --- | --- | --- | --- | --- | --- | --- | --- | --- | --- | --- | --- | --- | --- | --- | --- |

Best Unique PSM (Scan Sample\_9\_A1\_1\_3646.d:19894, m/z=370.1763, z=2, RT=11.15, ppm=0.89):


TRINITY\_DN97809\_c0\_g1\_i1.p1

back to list

  

| Protein Coverage
| Supporting Peptides
| Best Unique PSM
|

Protein Coverage:

Supporting Peptides:

| Peptide | Uniq | -10lgP | Mass | Length | ppm | m/z | z | RT | 1/k0 Start | 1/k0 End | Precursor ID | Area Sample 10 | #Feature | #Feature Sample 10 | Start | End | PTM | AScore | Found By |
| --- | --- | --- | --- | --- | --- | --- | --- | --- | --- | --- | --- | --- | --- | --- | --- | --- | --- | --- | --- |
| LGPDQVDVAGAGR.D | Y | 53.81 | 1253.6364 | 13 | 0.51 | 627.8225 | 2 | 13.58 | 0.9331 | 0.9404 | 34075 | 1.99e3 | 1 | 1 | 1 | 13 |  |  | DB Search |
| total 1 peptides |  |  |  |  |  |  |  |  |  |  |  |  |  |  |  |  |  |  |  |
| --- | --- | --- | --- | --- | --- | --- | --- | --- | --- | --- | --- | --- | --- | --- | --- | --- | --- | --- | --- |

Best Unique PSM (Scan Sample\_9\_A1\_1\_3646.d:34075, m/z=627.3316, z=2, RT=13.58, ppm=-4.71):


TRINITY\_DN571\_c0\_g1\_i2.p1

back to list

  

| Protein Coverage
| Supporting Peptides
| Best Unique PSM
|

Protein Coverage:

Supporting Peptides:

| Peptide | Uniq | -10lgP | Mass | Length | ppm | m/z | z | RT | 1/k0 Start | 1/k0 End | Precursor ID | Area Sample 10 | #Feature | #Feature Sample 10 | Start | End | PTM | AScore | Found By |
| --- | --- | --- | --- | --- | --- | --- | --- | --- | --- | --- | --- | --- | --- | --- | --- | --- | --- | --- | --- |
| K.M(+15.99)KINVNEIFHDLVR.Q | Y | 55.96 | 1742.9137 | 14 | 2.05 | 581.9767 | 3 | 19.57 | 0.887 | 0.8943 | 66610 | 2.46e2 | 1 | 1 | 150 | 163 | Oxidation (M) | M1:Oxidation (M):1000 | DB Search |
| total 1 peptides |  |  |  |  |  |  |  |  |  |  |  |  |  |  |  |  |  |  |  |
| --- | --- | --- | --- | --- | --- | --- | --- | --- | --- | --- | --- | --- | --- | --- | --- | --- | --- | --- | --- |

Best Unique PSM (Scan Sample\_9\_A1\_1\_3646.d:66610, m/z=581.9767, z=3, RT=19.57, ppm=-3.17):


TRINITY\_DN1339\_c0\_g2\_i2.p1

back to list

  

| Protein Coverage
| Supporting Peptides
| Best Unique PSM
|

Protein Coverage:

Supporting Peptides:

| Peptide | Uniq | -10lgP | Mass | Length | ppm | m/z | z | RT | 1/k0 Start | 1/k0 End | Precursor ID | Area Sample 10 | #Feature | #Feature Sample 10 | Start | End | PTM | AScore | Found By |
| --- | --- | --- | --- | --- | --- | --- | --- | --- | --- | --- | --- | --- | --- | --- | --- | --- | --- | --- | --- |
| K.LLTSSDQAELTKK.L | Y | 59.06 | 1432.7773 | 13 | -0.99 | 717.3915 | 2 | 12.75 | 1.0508 | 1.058 | 29440 | 1.28e3 | 1 | 1 | 155 | 167 |  |  | DB Search |
| total 1 peptides |  |  |  |  |  |  |  |  |  |  |  |  |  |  |  |  |  |  |  |
| --- | --- | --- | --- | --- | --- | --- | --- | --- | --- | --- | --- | --- | --- | --- | --- | --- | --- | --- | --- |

Best Unique PSM (Scan Sample\_9\_A1\_1\_3646.d:29440, m/z=717.3915, z=2, RT=12.75, ppm=-6.21):


TRINITY\_DN141214\_c0\_g1\_i1.p1

back to list

  

| Protein Coverage
| Supporting Peptides
| Best Unique PSM
|

Protein Coverage:

Supporting Peptides:

| Peptide | Uniq | -10lgP | Mass | Length | ppm | m/z | z | RT | 1/k0 Start | 1/k0 End | Precursor ID | Area Sample 10 | #Feature | #Feature Sample 10 | Start | End | PTM | AScore | Found By |
| --- | --- | --- | --- | --- | --- | --- | --- | --- | --- | --- | --- | --- | --- | --- | --- | --- | --- | --- | --- |
| R.VFLNKPLDFERENQIR.F | Y | 43.83 | 2017.0745 | 16 | 2.56 | 673.3636 | 3 | 16.33 | 0.942 | 0.9493 | 48297 | 1.88e2 | 1 | 1 | 162 | 177 |  |  | DB Search |
| total 1 peptides |  |  |  |  |  |  |  |  |  |  |  |  |  |  |  |  |  |  |  |
| --- | --- | --- | --- | --- | --- | --- | --- | --- | --- | --- | --- | --- | --- | --- | --- | --- | --- | --- | --- |

Best Unique PSM (Scan Sample\_9\_A1\_1\_3646.d:48297, m/z=673.3636, z=3, RT=16.33, ppm=-2.65):


TRINITY\_DN1130\_c0\_g1\_i1.p1

back to list

  

| Protein Coverage
| Supporting Peptides
| Best Unique PSM
|

Protein Coverage:

Supporting Peptides:

| Peptide | Uniq | -10lgP | Mass | Length | ppm | m/z | z | RT | 1/k0 Start | 1/k0 End | Precursor ID | Area Sample 10 | #Feature | #Feature Sample 10 | Start | End | PTM | AScore | Found By |
| --- | --- | --- | --- | --- | --- | --- | --- | --- | --- | --- | --- | --- | --- | --- | --- | --- | --- | --- | --- |
| K.VQSGNYRPVLALNGR.S | Y | 51.25 | 1642.8903 | 15 | 3.09 | 822.4507 | 2 | 14.85 | 1.0845 | 1.0917 | 40732 | 1.19e3 | 1 | 1 | 269 | 283 |  |  | DB Search |
| total 1 peptides |  |  |  |  |  |  |  |  |  |  |  |  |  |  |  |  |  |  |  |
| --- | --- | --- | --- | --- | --- | --- | --- | --- | --- | --- | --- | --- | --- | --- | --- | --- | --- | --- | --- |

Best Unique PSM (Scan Sample\_9\_A1\_1\_3646.d:40732, m/z=822.4507, z=2, RT=14.85, ppm=-2.13):


TRINITY\_DN413\_c0\_g1\_i1.p1

back to list

  

| Protein Coverage
| Supporting Peptides
| Best Unique PSM
|

Protein Coverage:

Supporting Peptides:

| Peptide | Uniq | -10lgP | Mass | Length | ppm | m/z | z | RT | 1/k0 Start | 1/k0 End | Precursor ID | Area Sample 10 | #Feature | #Feature Sample 10 | Start | End | PTM | AScore | Found By |
| --- | --- | --- | --- | --- | --- | --- | --- | --- | --- | --- | --- | --- | --- | --- | --- | --- | --- | --- | --- |
| R.LLLGDFNGDKR.T | Y | 58.62 | 1246.6669 | 11 | 3.37 | 624.3396 | 2 | 16.00 | 0.9844 | 0.9916 | 46643 | 2.81e2 | 1 | 1 | 46 | 56 |  |  | DB Search |
| total 1 peptides |  |  |  |  |  |  |  |  |  |  |  |  |  |  |  |  |  |  |  |
| --- | --- | --- | --- | --- | --- | --- | --- | --- | --- | --- | --- | --- | --- | --- | --- | --- | --- | --- | --- |

Best Unique PSM (Scan Sample\_9\_A1\_1\_3646.d:46643, m/z=624.3396, z=2, RT=16.00, ppm=-1.85):


TRINITY\_DN164469\_c0\_g1\_i1.p1

back to list

  

| Protein Coverage
| Supporting Peptides
| Best Unique PSM
|

Protein Coverage:

Supporting Peptides:

| Peptide | Uniq | -10lgP | Mass | Length | ppm | m/z | z | RT | 1/k0 Start | 1/k0 End | Precursor ID | Area Sample 10 | #Feature | #Feature Sample 10 | Start | End | PTM | AScore | Found By |
| --- | --- | --- | --- | --- | --- | --- | --- | --- | --- | --- | --- | --- | --- | --- | --- | --- | --- | --- | --- |
| R.GDLLFLTNDNTKPIR.A | Y | 58.45 | 1715.9206 | 15 | 7.23 | 858.9693 | 2 | 17.95 | 1.145 | 1.1522 | 57108 | 0 | 0 | 0 | 68 | 82 |  |  | DB Search |
| total 1 peptides |  |  |  |  |  |  |  |  |  |  |  |  |  |  |  |  |  |  |  |
| --- | --- | --- | --- | --- | --- | --- | --- | --- | --- | --- | --- | --- | --- | --- | --- | --- | --- | --- | --- |

Best Unique PSM (Scan Sample\_9\_A1\_1\_3646.d:57108, m/z=858.9693, z=2, RT=17.95, ppm=2.01):


TRINITY\_DN198705\_c0\_g1\_i1.p1

back to list

  

| Protein Coverage
| Supporting Peptides
| Best Unique PSM
|

Protein Coverage:

Supporting Peptides:

| Peptide | Uniq | -10lgP | Mass | Length | ppm | m/z | z | RT | 1/k0 Start | 1/k0 End | Precursor ID | Area Sample 10 | #Feature | #Feature Sample 10 | Start | End | PTM | AScore | Found By |
| --- | --- | --- | --- | --- | --- | --- | --- | --- | --- | --- | --- | --- | --- | --- | --- | --- | --- | --- | --- |
| M.A(+42.01)DEYYKR.F | Y | 51.54 | 985.4505 | 7 | 1.24 | 493.7306 | 2 | 12.55 | 0.836 | 0.8433 | 28400 | 0 | 0 | 0 | 2 | 8 | Acetylation (Protein N-term) | A1:Acetylation (Protein N-term):1000 | DB Search |
| total 1 peptides |  |  |  |  |  |  |  |  |  |  |  |  |  |  |  |  |  |  |  |
| --- | --- | --- | --- | --- | --- | --- | --- | --- | --- | --- | --- | --- | --- | --- | --- | --- | --- | --- | --- |

Best Unique PSM (Scan Sample\_9\_A1\_1\_3646.d:28400, m/z=493.7306, z=2, RT=12.55, ppm=-3.98):


TRINITY\_DN817\_c0\_g1\_i6.p1

back to list

  

| Protein Coverage
| Supporting Peptides
| Best Unique PSM
|

Protein Coverage:

Supporting Peptides:

| Peptide | Uniq | -10lgP | Mass | Length | ppm | m/z | z | RT | 1/k0 Start | 1/k0 End | Precursor ID | Area Sample 10 | #Feature | #Feature Sample 10 | Start | End | PTM | AScore | Found By |
| --- | --- | --- | --- | --- | --- | --- | --- | --- | --- | --- | --- | --- | --- | --- | --- | --- | --- | --- | --- |
| R.LC(+57.02)QPGYSTC(+57.02)ENHC(+57.02)R.R | Y | 55.11 | 1780.708 | 14 | 0.84 | 891.3574 | 2 | 11.42 | 1.0861 | 1.0933 | 21509 | 5.05e2 | 1 | 1 | 147 | 160 | Carbamidomethylation, Carbamidomethylation, Carbamidomethylation |  | DB Search |
| R.LC(+57.02)QPGYSTC(+57.02)ENHC(+57.02)RR.L | Y | 43.99 | 1936.8091 | 15 | 3.79 | 646.6094 | 3 | 10.89 | 0.8575 | 0.8648 | 18436 | 3.51e2 | 1 | 1 | 147 | 161 | Carbamidomethylation, Carbamidomethylation, Carbamidomethylation |  | DB Search |
| total 2 peptides |  |  |  |  |  |  |  |  |  |  |  |  |  |  |  |  |  |  |  |
| --- | --- | --- | --- | --- | --- | --- | --- | --- | --- | --- | --- | --- | --- | --- | --- | --- | --- | --- | --- |

Best Unique PSM (Scan Sample\_9\_A1\_1\_3646.d:21509, m/z=891.3574, z=2, RT=11.42, ppm=-4.38):


TRINITY\_DN614\_c0\_g1\_i1.p1

back to list

  

| Protein Coverage
| Supporting Peptides
| Best Unique PSM
|

Protein Coverage:

Supporting Peptides:

| Peptide | Uniq | -10lgP | Mass | Length | ppm | m/z | z | RT | 1/k0 Start | 1/k0 End | Precursor ID | Area Sample 10 | #Feature | #Feature Sample 10 | Start | End | PTM | AScore | Found By |
| --- | --- | --- | --- | --- | --- | --- | --- | --- | --- | --- | --- | --- | --- | --- | --- | --- | --- | --- | --- |
| R.VTEAKGELAK.M | Y | 58.26 | 1044.5815 | 10 | -1.3 | 523.2946 | 2 | 10.00 | 0.8927 | 0.9 | 13245 | 0 | 0 | 0 | 100 | 109 |  |  | DB Search |
| total 1 peptides |  |  |  |  |  |  |  |  |  |  |  |  |  |  |  |  |  |  |  |
| --- | --- | --- | --- | --- | --- | --- | --- | --- | --- | --- | --- | --- | --- | --- | --- | --- | --- | --- | --- |

Best Unique PSM (Scan Sample\_9\_A1\_1\_3646.d:13245, m/z=523.2946, z=2, RT=10.00, ppm=-6.52):


TRINITY\_DN817\_c0\_g1\_i5.p1

back to list

  

| Protein Coverage
| Supporting Peptides
| Best Unique PSM
|

Protein Coverage:

Supporting Peptides:

| Peptide | Uniq | -10lgP | Mass | Length | ppm | m/z | z | RT | 1/k0 Start | 1/k0 End | Precursor ID | Area Sample 10 | #Feature | #Feature Sample 10 | Start | End | PTM | AScore | Found By |
| --- | --- | --- | --- | --- | --- | --- | --- | --- | --- | --- | --- | --- | --- | --- | --- | --- | --- | --- | --- |
| R.LC(+57.02)QPGYSTC(+57.02)ENHC(+57.02)R.R | Y | 55.11 | 1780.708 | 14 | 0.84 | 891.3574 | 2 | 11.42 | 1.0861 | 1.0933 | 21509 | 5.05e2 | 1 | 1 | 145 | 158 | Carbamidomethylation, Carbamidomethylation, Carbamidomethylation |  | DB Search |
| R.LC(+57.02)QPGYSTC(+57.02)ENHC(+57.02)RR.L | Y | 43.99 | 1936.8091 | 15 | 3.79 | 646.6094 | 3 | 10.89 | 0.8575 | 0.8648 | 18436 | 3.51e2 | 1 | 1 | 145 | 159 | Carbamidomethylation, Carbamidomethylation, Carbamidomethylation |  | DB Search |
| total 2 peptides |  |  |  |  |  |  |  |  |  |  |  |  |  |  |  |  |  |  |  |
| --- | --- | --- | --- | --- | --- | --- | --- | --- | --- | --- | --- | --- | --- | --- | --- | --- | --- | --- | --- |

Best Unique PSM (Scan Sample\_9\_A1\_1\_3646.d:21509, m/z=891.3574, z=2, RT=11.42, ppm=-4.38):


TRINITY\_DN20278\_c0\_g1\_i1.p2

back to list

  

| Protein Coverage
| Supporting Peptides
| Best Unique PSM
|

Protein Coverage:

Supporting Peptides:

| Peptide | Uniq | -10lgP | Mass | Length | ppm | m/z | z | RT | 1/k0 Start | 1/k0 End | Precursor ID | Area Sample 10 | #Feature | #Feature Sample 10 | Start | End | PTM | AScore | Found By |
| --- | --- | --- | --- | --- | --- | --- | --- | --- | --- | --- | --- | --- | --- | --- | --- | --- | --- | --- | --- |
| K.KLNAEIANSR.L | Y | 57.15 | 1114.6094 | 10 | -3.85 | 558.3069 | 2 | 11.39 | 0.868 | 0.8753 | 21472 | 0 | 0 | 0 | 97 | 106 |  |  | DB Search |
| total 1 peptides |  |  |  |  |  |  |  |  |  |  |  |  |  |  |  |  |  |  |  |
| --- | --- | --- | --- | --- | --- | --- | --- | --- | --- | --- | --- | --- | --- | --- | --- | --- | --- | --- | --- |

Best Unique PSM (Scan Sample\_9\_A1\_1\_3646.d:21472, m/z=558.3069, z=2, RT=11.39, ppm=-9.07):


TRINITY\_DN602\_c0\_g2\_i1.p1

back to list

  

| Protein Coverage
| Supporting Peptides
| Best Unique PSM
|

Protein Coverage:

Supporting Peptides:

| Peptide | Uniq | -10lgP | Mass | Length | ppm | m/z | z | RT | 1/k0 Start | 1/k0 End | Precursor ID | Area Sample 10 | #Feature | #Feature Sample 10 | Start | End | PTM | AScore | Found By |
| --- | --- | --- | --- | --- | --- | --- | --- | --- | --- | --- | --- | --- | --- | --- | --- | --- | --- | --- | --- |
| R.AYDFRETLPK.K | Y | 58.44 | 1238.6295 | 10 | 10.92 | 620.3256 | 2 | 15.17 | 0.9489 | 0.9562 | 42570 | 0 | 0 | 0 | 94 | 103 |  |  | DB Search |
| total 1 peptides |  |  |  |  |  |  |  |  |  |  |  |  |  |  |  |  |  |  |  |
| --- | --- | --- | --- | --- | --- | --- | --- | --- | --- | --- | --- | --- | --- | --- | --- | --- | --- | --- | --- |

Best Unique PSM (Scan Sample\_9\_A1\_1\_3646.d:42570, m/z=620.3256, z=2, RT=15.17, ppm=5.7):


TRINITY\_DN7504\_c0\_g1\_i1.p1

back to list

  

| Protein Coverage
| Supporting Peptides
| Best Unique PSM
|

Protein Coverage:

Supporting Peptides:

| Peptide | Uniq | -10lgP | Mass | Length | ppm | m/z | z | RT | 1/k0 Start | 1/k0 End | Precursor ID | Area Sample 10 | #Feature | #Feature Sample 10 | Start | End | PTM | AScore | Found By |
| --- | --- | --- | --- | --- | --- | --- | --- | --- | --- | --- | --- | --- | --- | --- | --- | --- | --- | --- | --- |
| K.TIEAEAAHGTVTR.H | Y | 58.86 | 1354.6841 | 13 | -3.52 | 678.3434 | 2 | 11.82 | 1.0142 | 1.0214 | 24040 | 0 | 0 | 0 | 301 | 313 |  |  | DB Search |
| total 1 peptides |  |  |  |  |  |  |  |  |  |  |  |  |  |  |  |  |  |  |  |
| --- | --- | --- | --- | --- | --- | --- | --- | --- | --- | --- | --- | --- | --- | --- | --- | --- | --- | --- | --- |

Best Unique PSM (Scan Sample\_9\_A1\_1\_3646.d:24040, m/z=678.3434, z=2, RT=11.82, ppm=-8.74):


TRINITY\_DN495\_c0\_g1\_i3.p1

back to list

  

| Protein Coverage
| Supporting Peptides
| Best Unique PSM
|

Protein Coverage:

Supporting Peptides:

| Peptide | Uniq | -10lgP | Mass | Length | ppm | m/z | z | RT | 1/k0 Start | 1/k0 End | Precursor ID | Area Sample 10 | #Feature | #Feature Sample 10 | Start | End | PTM | AScore | Found By |
| --- | --- | --- | --- | --- | --- | --- | --- | --- | --- | --- | --- | --- | --- | --- | --- | --- | --- | --- | --- |
| R.SC(+57.02)TNPAPANGGK.A | Y | 58.68 | 1172.5244 | 12 | 3.23 | 587.2683 | 2 | 9.42 | 0.8352 | 0.8425 | 10441 | 8.36e2 | 1 | 1 | 503 | 514 | Carbamidomethylation |  | DB Search |
| total 1 peptides |  |  |  |  |  |  |  |  |  |  |  |  |  |  |  |  |  |  |  |
| --- | --- | --- | --- | --- | --- | --- | --- | --- | --- | --- | --- | --- | --- | --- | --- | --- | --- | --- | --- |

Best Unique PSM (Scan Sample\_9\_A1\_1\_3646.d:10441, m/z=587.2683, z=2, RT=9.42, ppm=-1.99):


TRINITY\_DN6\_c0\_g1\_i1.p1

back to list

  

| Protein Coverage
| Supporting Peptides
| Best Unique PSM
|

Protein Coverage:

Supporting Peptides:

| Peptide | Uniq | -10lgP | Mass | Length | ppm | m/z | z | RT | 1/k0 Start | 1/k0 End | Precursor ID | Area Sample 10 | #Feature | #Feature Sample 10 | Start | End | PTM | AScore | Found By |
| --- | --- | --- | --- | --- | --- | --- | --- | --- | --- | --- | --- | --- | --- | --- | --- | --- | --- | --- | --- |
| K.AISFIGSVHDNRK.E | Y | 58.64 | 1442.763 | 13 | 2.55 | 722.3868 | 2 | 14.04 | 1.0415 | 1.0488 | 36558 | 6.68e2 | 1 | 1 | 55 | 67 |  |  | DB Search |
| total 1 peptides |  |  |  |  |  |  |  |  |  |  |  |  |  |  |  |  |  |  |  |
| --- | --- | --- | --- | --- | --- | --- | --- | --- | --- | --- | --- | --- | --- | --- | --- | --- | --- | --- | --- |

Best Unique PSM (Scan Sample\_9\_A1\_1\_3646.d:36558, m/z=722.3868, z=2, RT=14.04, ppm=-2.67):


TRINITY\_DN1339\_c0\_g2\_i4.p2

back to list

  

| Protein Coverage
| Supporting Peptides
| Best Unique PSM
|

Protein Coverage:

Supporting Peptides:

| Peptide | Uniq | -10lgP | Mass | Length | ppm | m/z | z | RT | 1/k0 Start | 1/k0 End | Precursor ID | Area Sample 10 | #Feature | #Feature Sample 10 | Start | End | PTM | AScore | Found By |
| --- | --- | --- | --- | --- | --- | --- | --- | --- | --- | --- | --- | --- | --- | --- | --- | --- | --- | --- | --- |
| K.LLTSSDQAELTKK.L | Y | 59.06 | 1432.7773 | 13 | -0.99 | 717.3915 | 2 | 12.75 | 1.0508 | 1.058 | 29440 | 1.28e3 | 1 | 1 | 155 | 167 |  |  | DB Search |
| total 1 peptides |  |  |  |  |  |  |  |  |  |  |  |  |  |  |  |  |  |  |  |
| --- | --- | --- | --- | --- | --- | --- | --- | --- | --- | --- | --- | --- | --- | --- | --- | --- | --- | --- | --- |

Best Unique PSM (Scan Sample\_9\_A1\_1\_3646.d:29440, m/z=717.3915, z=2, RT=12.75, ppm=-6.21):


TRINITY\_DN76073\_c0\_g1\_i1.p1

back to list

  

| Protein Coverage
| Supporting Peptides
| Best Unique PSM
|

Protein Coverage:

Supporting Peptides:

| Peptide | Uniq | -10lgP | Mass | Length | ppm | m/z | z | RT | 1/k0 Start | 1/k0 End | Precursor ID | Area Sample 10 | #Feature | #Feature Sample 10 | Start | End | PTM | AScore | Found By |
| --- | --- | --- | --- | --- | --- | --- | --- | --- | --- | --- | --- | --- | --- | --- | --- | --- | --- | --- | --- |
| K.TSDSDLEKR.I | Y | 53.19 | 1049.4989 | 9 | -14.11 | 525.7466 | 2 | 11.28 | 0.8644 | 0.8716 | 20674 | 3.18e3 | 1 | 1 | 55 | 63 |  |  | DB Search |
| total 1 peptides |  |  |  |  |  |  |  |  |  |  |  |  |  |  |  |  |  |  |  |
| --- | --- | --- | --- | --- | --- | --- | --- | --- | --- | --- | --- | --- | --- | --- | --- | --- | --- | --- | --- |

Best Unique PSM (Scan Sample\_9\_A1\_1\_3646.d:20674, m/z=525.7466, z=2, RT=11.28, ppm=-19.33):


TRINITY\_DN801\_c0\_g1\_i7.p1

back to list

  

| Protein Coverage
| Supporting Peptides
| Best Unique PSM
|

Protein Coverage:

Supporting Peptides:

| Peptide | Uniq | -10lgP | Mass | Length | ppm | m/z | z | RT | 1/k0 Start | 1/k0 End | Precursor ID | Area Sample 10 | #Feature | #Feature Sample 10 | Start | End | PTM | AScore | Found By |
| --- | --- | --- | --- | --- | --- | --- | --- | --- | --- | --- | --- | --- | --- | --- | --- | --- | --- | --- | --- |
| K.VFDRDGNGFISAAELR.H | Y | 51.43 | 1765.8747 | 16 | -4.93 | 883.9357 | 2 | 19.74 | 1.1274 | 1.1346 | 67630 | 2.9e3 | 1 | 1 | 124 | 139 |  |  | DB Search |
| total 1 peptides |  |  |  |  |  |  |  |  |  |  |  |  |  |  |  |  |  |  |  |
| --- | --- | --- | --- | --- | --- | --- | --- | --- | --- | --- | --- | --- | --- | --- | --- | --- | --- | --- | --- |

Best Unique PSM (Scan Sample\_9\_A1\_1\_3646.d:67630, m/z=883.9357, z=2, RT=19.74, ppm=-10.14):


TRINITY\_DN801\_c0\_g1\_i1.p1

back to list

  

| Protein Coverage
| Supporting Peptides
| Best Unique PSM
|

Protein Coverage:

Supporting Peptides:

| Peptide | Uniq | -10lgP | Mass | Length | ppm | m/z | z | RT | 1/k0 Start | 1/k0 End | Precursor ID | Area Sample 10 | #Feature | #Feature Sample 10 | Start | End | PTM | AScore | Found By |
| --- | --- | --- | --- | --- | --- | --- | --- | --- | --- | --- | --- | --- | --- | --- | --- | --- | --- | --- | --- |
| K.VFDRDGNGFISAAELR.H | Y | 51.43 | 1765.8747 | 16 | -4.93 | 883.9357 | 2 | 19.74 | 1.1274 | 1.1346 | 67630 | 2.9e3 | 1 | 1 | 119 | 134 |  |  | DB Search |
| total 1 peptides |  |  |  |  |  |  |  |  |  |  |  |  |  |  |  |  |  |  |  |
| --- | --- | --- | --- | --- | --- | --- | --- | --- | --- | --- | --- | --- | --- | --- | --- | --- | --- | --- | --- |

Best Unique PSM (Scan Sample\_9\_A1\_1\_3646.d:67630, m/z=883.9357, z=2, RT=19.74, ppm=-10.14):


TRINITY\_DN1339\_c0\_g1\_i1.p1

back to list

  

| Protein Coverage
| Supporting Peptides
| Best Unique PSM
|

Protein Coverage:

Supporting Peptides:

| Peptide | Uniq | -10lgP | Mass | Length | ppm | m/z | z | RT | 1/k0 Start | 1/k0 End | Precursor ID | Area Sample 10 | #Feature | #Feature Sample 10 | Start | End | PTM | AScore | Found By |
| --- | --- | --- | --- | --- | --- | --- | --- | --- | --- | --- | --- | --- | --- | --- | --- | --- | --- | --- | --- |
| K.VITSDDPAEKEK.K | Y | 59.05 | 1330.6616 | 12 | 3.57 | 666.337 | 2 | 10.72 | 0.9884 | 0.9957 | 17382 | 1.42e4 | 1 | 1 | 160 | 171 |  |  | DB Search |
| total 1 peptides |  |  |  |  |  |  |  |  |  |  |  |  |  |  |  |  |  |  |  |
| --- | --- | --- | --- | --- | --- | --- | --- | --- | --- | --- | --- | --- | --- | --- | --- | --- | --- | --- | --- |

Best Unique PSM (Scan Sample\_9\_A1\_1\_3646.d:17382, m/z=666.337, z=2, RT=10.72, ppm=-1.65):


TRINITY\_DN468\_c1\_g1\_i1.p1

back to list

  

| Protein Coverage
| Supporting Peptides
| Best Unique PSM
|

Protein Coverage:

Supporting Peptides:

| Peptide | Uniq | -10lgP | Mass | Length | ppm | m/z | z | RT | 1/k0 Start | 1/k0 End | Precursor ID | Area Sample 10 | #Feature | #Feature Sample 10 | Start | End | PTM | AScore | Found By |
| --- | --- | --- | --- | --- | --- | --- | --- | --- | --- | --- | --- | --- | --- | --- | --- | --- | --- | --- | --- |
| K.LGPVVDGKYDYTVITTDYK.A | Y | 52.68 | 2146.0834 | 19 | -1.05 | 1074.0422 | 2 | 17.49 | 1.2404 | 1.2475 | 54337 | 1.18e2 | 1 | 1 | 123 | 141 |  |  | DB Search |
| total 1 peptides |  |  |  |  |  |  |  |  |  |  |  |  |  |  |  |  |  |  |  |
| --- | --- | --- | --- | --- | --- | --- | --- | --- | --- | --- | --- | --- | --- | --- | --- | --- | --- | --- | --- |

Best Unique PSM (Scan Sample\_9\_A1\_1\_3646.d:54337, m/z=1074.0422, z=2, RT=17.49, ppm=-6.26):


TRINITY\_DN374\_c0\_g1\_i2.p1

back to list

  

| Protein Coverage
| Supporting Peptides
| Best Unique PSM
|

Protein Coverage:

Supporting Peptides:

| Peptide | Uniq | -10lgP | Mass | Length | ppm | m/z | z | RT | 1/k0 Start | 1/k0 End | Precursor ID | Area Sample 10 | #Feature | #Feature Sample 10 | Start | End | PTM | AScore | Found By |
| --- | --- | --- | --- | --- | --- | --- | --- | --- | --- | --- | --- | --- | --- | --- | --- | --- | --- | --- | --- |
| K.YIDIEYVC(+57.02)KPPVKK.A | Y | 56.94 | 1750.9327 | 14 | -8.26 | 876.4619 | 2 | 14.95 | 1.1346 | 1.1418 | 41353 | 0 | 0 | 0 | 234 | 247 | Carbamidomethylation |  | DB Search |
| total 1 peptides |  |  |  |  |  |  |  |  |  |  |  |  |  |  |  |  |  |  |  |
| --- | --- | --- | --- | --- | --- | --- | --- | --- | --- | --- | --- | --- | --- | --- | --- | --- | --- | --- | --- |

Best Unique PSM (Scan Sample\_9\_A1\_1\_3646.d:41353, m/z=876.4619, z=2, RT=14.95, ppm=-13.47):


TRINITY\_DN585\_c0\_g1\_i4.p1

back to list

  

| Protein Coverage
| Supporting Peptides
| Best Unique PSM
|

Protein Coverage:

Supporting Peptides:

| Peptide | Uniq | -10lgP | Mass | Length | ppm | m/z | z | RT | 1/k0 Start | 1/k0 End | Precursor ID | Area Sample 10 | #Feature | #Feature Sample 10 | Start | End | PTM | AScore | Found By |
| --- | --- | --- | --- | --- | --- | --- | --- | --- | --- | --- | --- | --- | --- | --- | --- | --- | --- | --- | --- |
| K.IQDKEGIPPDQQR.L | Y | 58.25 | 1522.7739 | 13 | 2.55 | 762.3922 | 2 | 11.03 | 1.0343 | 1.0415 | 18957 | 1.53e3 | 1 | 1 | 30 | 42 |  |  | DB Search |
| total 1 peptides |  |  |  |  |  |  |  |  |  |  |  |  |  |  |  |  |  |  |  |
| --- | --- | --- | --- | --- | --- | --- | --- | --- | --- | --- | --- | --- | --- | --- | --- | --- | --- | --- | --- |

Best Unique PSM (Scan Sample\_9\_A1\_1\_3646.d:18957, m/z=762.3922, z=2, RT=11.03, ppm=-2.67):


TRINITY\_DN3034\_c0\_g1\_i1.p1

back to list

  

| Protein Coverage
| Supporting Peptides
| Best Unique PSM
|

Protein Coverage:

Supporting Peptides:

| Peptide | Uniq | -10lgP | Mass | Length | ppm | m/z | z | RT | 1/k0 Start | 1/k0 End | Precursor ID | Area Sample 10 | #Feature | #Feature Sample 10 | Start | End | PTM | AScore | Found By |
| --- | --- | --- | --- | --- | --- | --- | --- | --- | --- | --- | --- | --- | --- | --- | --- | --- | --- | --- | --- |
| K.TPGLVEGTLILHQLR.C | Y | 58.67 | 1645.9515 | 15 | 0.88 | 549.6554 | 3 | 18.78 | 0.8631 | 0.8704 | 61967 | 3.17e2 | 1 | 1 | 671 | 685 |  |  | DB Search |
| R.AQEVSDKLNQK.M | Y | 58.4 | 1258.6517 | 11 | 2.21 | 630.3312 | 2 | 10.75 | 0.9384 | 0.9457 | 17603 | 9.14e2 | 1 | 1 | 1854 | 1864 |  |  | DB Search |
| total 2 peptides |  |  |  |  |  |  |  |  |  |  |  |  |  |  |  |  |  |  |  |
| --- | --- | --- | --- | --- | --- | --- | --- | --- | --- | --- | --- | --- | --- | --- | --- | --- | --- | --- | --- |

Best Unique PSM (Scan Sample\_9\_A1\_1\_3646.d:61967, m/z=549.6554, z=3, RT=18.78, ppm=-4.34):


TRINITY\_DN2636\_c0\_g1\_i2.p1

back to list

  

| Protein Coverage
| Supporting Peptides
| Best Unique PSM
|

Protein Coverage:

Supporting Peptides:

| Peptide | Uniq | -10lgP | Mass | Length | ppm | m/z | z | RT | 1/k0 Start | 1/k0 End | Precursor ID | Area Sample 10 | #Feature | #Feature Sample 10 | Start | End | PTM | AScore | Found By |
| --- | --- | --- | --- | --- | --- | --- | --- | --- | --- | --- | --- | --- | --- | --- | --- | --- | --- | --- | --- |
| K.VVEKEAETER.K | Y | 56.06 | 1188.5986 | 10 | 0.32 | 397.2049 | 3 | 9.55 | 0.7265 | 0.7339 | 11068 | 0 | 0 | 0 | 199 | 208 |  |  | DB Search |
| K.IHHELNQFC(+57.02)SVK.S | Y | 47.81 | 1510.7351 | 12 | -0.1 | 756.3708 | 2 | 12.64 | 1.05 | 1.0572 | 28909 | 0 | 0 | 0 | 117 | 128 | Carbamidomethylation |  | DB Search |
| total 2 peptides |  |  |  |  |  |  |  |  |  |  |  |  |  |  |  |  |  |  |  |
| --- | --- | --- | --- | --- | --- | --- | --- | --- | --- | --- | --- | --- | --- | --- | --- | --- | --- | --- | --- |

Best Unique PSM (Scan Sample\_9\_A1\_1\_3646.d:11068, m/z=397.2049, z=3, RT=9.55, ppm=-4.91):


TRINITY\_DN96\_c0\_g1\_i6.p1

back to list

  

| Protein Coverage
| Supporting Peptides
| Best Unique PSM
|

Protein Coverage:

Supporting Peptides:

| Peptide | Uniq | -10lgP | Mass | Length | ppm | m/z | z | RT | 1/k0 Start | 1/k0 End | Precursor ID | Area Sample 10 | #Feature | #Feature Sample 10 | Start | End | PTM | AScore | Found By |
| --- | --- | --- | --- | --- | --- | --- | --- | --- | --- | --- | --- | --- | --- | --- | --- | --- | --- | --- | --- |
| K.KVEQVQTDSR.D | Y | 53.61 | 1188.6098 | 10 | 5.11 | 595.3121 | 2 | 9.53 | 0.8725 | 0.8798 | 10904 | 2.14e2 | 1 | 1 | 163 | 172 |  |  | DB Search |
| total 1 peptides |  |  |  |  |  |  |  |  |  |  |  |  |  |  |  |  |  |  |  |
| --- | --- | --- | --- | --- | --- | --- | --- | --- | --- | --- | --- | --- | --- | --- | --- | --- | --- | --- | --- |

Best Unique PSM (Scan Sample\_9\_A1\_1\_3646.d:10904, m/z=595.3121, z=2, RT=9.53, ppm=-0.11):


TRINITY\_DN817\_c0\_g1\_i4.p1

back to list

  

| Protein Coverage
| Supporting Peptides
| Best Unique PSM
|

Protein Coverage:

Supporting Peptides:

| Peptide | Uniq | -10lgP | Mass | Length | ppm | m/z | z | RT | 1/k0 Start | 1/k0 End | Precursor ID | Area Sample 10 | #Feature | #Feature Sample 10 | Start | End | PTM | AScore | Found By |
| --- | --- | --- | --- | --- | --- | --- | --- | --- | --- | --- | --- | --- | --- | --- | --- | --- | --- | --- | --- |
| R.LC(+57.02)QPGYSTC(+57.02)ENHC(+57.02)R.R | Y | 55.11 | 1780.708 | 14 | 0.84 | 891.3574 | 2 | 11.42 | 1.0861 | 1.0933 | 21509 | 5.05e2 | 1 | 1 | 145 | 158 | Carbamidomethylation, Carbamidomethylation, Carbamidomethylation |  | DB Search |
| R.LC(+57.02)QPGYSTC(+57.02)ENHC(+57.02)RR.L | Y | 43.99 | 1936.8091 | 15 | 3.79 | 646.6094 | 3 | 10.89 | 0.8575 | 0.8648 | 18436 | 3.51e2 | 1 | 1 | 145 | 159 | Carbamidomethylation, Carbamidomethylation, Carbamidomethylation |  | DB Search |
| total 2 peptides |  |  |  |  |  |  |  |  |  |  |  |  |  |  |  |  |  |  |  |
| --- | --- | --- | --- | --- | --- | --- | --- | --- | --- | --- | --- | --- | --- | --- | --- | --- | --- | --- | --- |

Best Unique PSM (Scan Sample\_9\_A1\_1\_3646.d:21509, m/z=891.3574, z=2, RT=11.42, ppm=-4.38):


TRINITY\_DN941\_c0\_g1\_i11.p1

back to list

  

| Protein Coverage
| Supporting Peptides
| Best Unique PSM
|

Protein Coverage:

Supporting Peptides:

| Peptide | Uniq | -10lgP | Mass | Length | ppm | m/z | z | RT | 1/k0 Start | 1/k0 End | Precursor ID | Area Sample 10 | #Feature | #Feature Sample 10 | Start | End | PTM | AScore | Found By |
| --- | --- | --- | --- | --- | --- | --- | --- | --- | --- | --- | --- | --- | --- | --- | --- | --- | --- | --- | --- |
| K.AHGPGLEGGVAGQPC(+57.02)R.F | Y | 50.18 | 1561.7419 | 16 | 3.5 | 781.8769 | 2 | 12.18 | 1.0291 | 1.0363 | 25949 | 8.92e2 | 1 | 1 | 666 | 681 | Carbamidomethylation |  | DB Search |
| total 1 peptides |  |  |  |  |  |  |  |  |  |  |  |  |  |  |  |  |  |  |  |
| --- | --- | --- | --- | --- | --- | --- | --- | --- | --- | --- | --- | --- | --- | --- | --- | --- | --- | --- | --- |

Best Unique PSM (Scan Sample\_9\_A1\_1\_3646.d:25949, m/z=781.8769, z=2, RT=12.18, ppm=-1.72):


TRINITY\_DN4026\_c0\_g1\_i1.p2

back to list

  

| Protein Coverage
| Supporting Peptides
| Best Unique PSM
|

Protein Coverage:

Supporting Peptides:

| Peptide | Uniq | -10lgP | Mass | Length | ppm | m/z | z | RT | 1/k0 Start | 1/k0 End | Precursor ID | Area Sample 10 | #Feature | #Feature Sample 10 | Start | End | PTM | AScore | Found By |
| --- | --- | --- | --- | --- | --- | --- | --- | --- | --- | --- | --- | --- | --- | --- | --- | --- | --- | --- | --- |
| K.KLNAEIANSR.L | Y | 57.15 | 1114.6094 | 10 | -3.85 | 558.3069 | 2 | 11.39 | 0.868 | 0.8753 | 21472 | 0 | 0 | 0 | 143 | 152 |  |  | DB Search |
| total 1 peptides |  |  |  |  |  |  |  |  |  |  |  |  |  |  |  |  |  |  |  |
| --- | --- | --- | --- | --- | --- | --- | --- | --- | --- | --- | --- | --- | --- | --- | --- | --- | --- | --- | --- |

Best Unique PSM (Scan Sample\_9\_A1\_1\_3646.d:21472, m/z=558.3069, z=2, RT=11.39, ppm=-9.07):


TRINITY\_DN163801\_c0\_g1\_i1.p1

back to list

  

| Protein Coverage
| Supporting Peptides
| Best Unique PSM
|

Protein Coverage:

Supporting Peptides:

| Peptide | Uniq | -10lgP | Mass | Length | ppm | m/z | z | RT | 1/k0 Start | 1/k0 End | Precursor ID | Area Sample 10 | #Feature | #Feature Sample 10 | Start | End | PTM | AScore | Found By |
| --- | --- | --- | --- | --- | --- | --- | --- | --- | --- | --- | --- | --- | --- | --- | --- | --- | --- | --- | --- |
| M.A(+42.01)NPLKDIISR.F | Y | 58.62 | 1167.6611 | 10 | -1.4 | 584.834 | 2 | 18.57 | 0.9214 | 0.9287 | 60579 | 1.56e3 | 1 | 1 | 2 | 11 | Acetylation (Protein N-term) | A1:Acetylation (Protein N-term):1000 | DB Search |
| total 1 peptides |  |  |  |  |  |  |  |  |  |  |  |  |  |  |  |  |  |  |  |
| --- | --- | --- | --- | --- | --- | --- | --- | --- | --- | --- | --- | --- | --- | --- | --- | --- | --- | --- | --- |

Best Unique PSM (Scan Sample\_9\_A1\_1\_3646.d:60579, m/z=584.834, z=2, RT=18.57, ppm=-6.62):


TRINITY\_DN156024\_c0\_g1\_i1.p3

back to list

  

| Protein Coverage
| Supporting Peptides
| Best Unique PSM
|

Protein Coverage:

Supporting Peptides:

| Peptide | Uniq | -10lgP | Mass | Length | ppm | m/z | z | RT | 1/k0 Start | 1/k0 End | Precursor ID | Area Sample 10 | #Feature | #Feature Sample 10 | Start | End | PTM | AScore | Found By |
| --- | --- | --- | --- | --- | --- | --- | --- | --- | --- | --- | --- | --- | --- | --- | --- | --- | --- | --- | --- |
| K.KLNAEIANSR.L | Y | 57.15 | 1114.6094 | 10 | -3.85 | 558.3069 | 2 | 11.39 | 0.868 | 0.8753 | 21472 | 0 | 0 | 0 | 64 | 73 |  |  | DB Search |
| total 1 peptides |  |  |  |  |  |  |  |  |  |  |  |  |  |  |  |  |  |  |  |
| --- | --- | --- | --- | --- | --- | --- | --- | --- | --- | --- | --- | --- | --- | --- | --- | --- | --- | --- | --- |

Best Unique PSM (Scan Sample\_9\_A1\_1\_3646.d:21472, m/z=558.3069, z=2, RT=11.39, ppm=-9.07):


TRINITY\_DN199\_c0\_g1\_i1.p1

back to list

  

| Protein Coverage
| Supporting Peptides
| Best Unique PSM
|

Protein Coverage:

Supporting Peptides:

| Peptide | Uniq | -10lgP | Mass | Length | ppm | m/z | z | RT | 1/k0 Start | 1/k0 End | Precursor ID | Area Sample 10 | #Feature | #Feature Sample 10 | Start | End | PTM | AScore | Found By |
| --- | --- | --- | --- | --- | --- | --- | --- | --- | --- | --- | --- | --- | --- | --- | --- | --- | --- | --- | --- |
| K.NYNNDDEPGAK.K | Y | 58.15 | 1235.5054 | 11 | 10.48 | 618.7632 | 2 | 10.23 | 0.9222 | 0.9295 | 14562 | 0 | 0 | 0 | 124 | 134 |  |  | DB Search |
| total 1 peptides |  |  |  |  |  |  |  |  |  |  |  |  |  |  |  |  |  |  |  |
| --- | --- | --- | --- | --- | --- | --- | --- | --- | --- | --- | --- | --- | --- | --- | --- | --- | --- | --- | --- |

Best Unique PSM (Scan Sample\_9\_A1\_1\_3646.d:14562, m/z=618.7632, z=2, RT=10.23, ppm=5.26):


TRINITY\_DN95248\_c0\_g1\_i1.p1

back to list

  

| Protein Coverage
| Supporting Peptides
| Best Unique PSM
|

Protein Coverage:

Supporting Peptides:

| Peptide | Uniq | -10lgP | Mass | Length | ppm | m/z | z | RT | 1/k0 Start | 1/k0 End | Precursor ID | Area Sample 10 | #Feature | #Feature Sample 10 | Start | End | PTM | AScore | Found By |
| --- | --- | --- | --- | --- | --- | --- | --- | --- | --- | --- | --- | --- | --- | --- | --- | --- | --- | --- | --- |
| R.VVASSHTDYELR.Y | Y | 58.62 | 1375.6732 | 12 | 4.53 | 688.8434 | 2 | 12.54 | 1.0122 | 1.0194 | 28239 | 0 | 0 | 0 | 96 | 107 |  |  | DB Search |
| total 1 peptides |  |  |  |  |  |  |  |  |  |  |  |  |  |  |  |  |  |  |  |
| --- | --- | --- | --- | --- | --- | --- | --- | --- | --- | --- | --- | --- | --- | --- | --- | --- | --- | --- | --- |

Best Unique PSM (Scan Sample\_9\_A1\_1\_3646.d:28239, m/z=688.8434, z=2, RT=12.54, ppm=-0.69):


TRINITY\_DN1091\_c0\_g1\_i1.p1

back to list

  

| Protein Coverage
| Supporting Peptides
| Best Unique PSM
|

Protein Coverage:

Supporting Peptides:

| Peptide | Uniq | -10lgP | Mass | Length | ppm | m/z | z | RT | 1/k0 Start | 1/k0 End | Precursor ID | Area Sample 10 | #Feature | #Feature Sample 10 | Start | End | PTM | AScore | Found By |
| --- | --- | --- | --- | --- | --- | --- | --- | --- | --- | --- | --- | --- | --- | --- | --- | --- | --- | --- | --- |
| R.AFQNTPC(+57.02)THLVLDGTR.W | Y | 58.66 | 1828.889 | 16 | 2.02 | 915.4489 | 2 | 15.26 | 1.1762 | 1.1833 | 42854 | 5.12e2 | 1 | 1 | 81 | 96 | Carbamidomethylation |  | DB Search |
| total 1 peptides |  |  |  |  |  |  |  |  |  |  |  |  |  |  |  |  |  |  |  |
| --- | --- | --- | --- | --- | --- | --- | --- | --- | --- | --- | --- | --- | --- | --- | --- | --- | --- | --- | --- |

Best Unique PSM (Scan Sample\_9\_A1\_1\_3646.d:42854, m/z=915.4489, z=2, RT=15.26, ppm=-3.19):


TRINITY\_DN4954\_c0\_g1\_i2.p2

back to list

  

| Protein Coverage
| Supporting Peptides
| Best Unique PSM
|

Protein Coverage:

Supporting Peptides:

| Peptide | Uniq | -10lgP | Mass | Length | ppm | m/z | z | RT | 1/k0 Start | 1/k0 End | Precursor ID | Area Sample 10 | #Feature | #Feature Sample 10 | Start | End | PTM | AScore | Found By |
| --- | --- | --- | --- | --- | --- | --- | --- | --- | --- | --- | --- | --- | --- | --- | --- | --- | --- | --- | --- |
| R.AIELLPHDLAVGR.H | Y | 57.65 | 1402.7932 | 13 | -0.45 | 702.3999 | 2 | 17.82 | 1.0303 | 1.0375 | 56285 | 4.72e2 | 1 | 1 | 109 | 121 |  |  | DB Search |
| total 1 peptides |  |  |  |  |  |  |  |  |  |  |  |  |  |  |  |  |  |  |  |
| --- | --- | --- | --- | --- | --- | --- | --- | --- | --- | --- | --- | --- | --- | --- | --- | --- | --- | --- | --- |

Best Unique PSM (Scan Sample\_9\_A1\_1\_3646.d:56285, m/z=702.3999, z=2, RT=17.82, ppm=-5.66):


TRINITY\_DN941\_c0\_g1\_i15.p1

back to list

  

| Protein Coverage
| Supporting Peptides
| Best Unique PSM
|

Protein Coverage:

Supporting Peptides:

| Peptide | Uniq | -10lgP | Mass | Length | ppm | m/z | z | RT | 1/k0 Start | 1/k0 End | Precursor ID | Area Sample 10 | #Feature | #Feature Sample 10 | Start | End | PTM | AScore | Found By |
| --- | --- | --- | --- | --- | --- | --- | --- | --- | --- | --- | --- | --- | --- | --- | --- | --- | --- | --- | --- |
| K.AHGPGLEGGVAGQPC(+57.02)R.F | Y | 50.18 | 1561.7419 | 16 | 3.5 | 781.8769 | 2 | 12.18 | 1.0291 | 1.0363 | 25949 | 8.92e2 | 1 | 1 | 820 | 835 | Carbamidomethylation |  | DB Search |
| total 1 peptides |  |  |  |  |  |  |  |  |  |  |  |  |  |  |  |  |  |  |  |
| --- | --- | --- | --- | --- | --- | --- | --- | --- | --- | --- | --- | --- | --- | --- | --- | --- | --- | --- | --- |

Best Unique PSM (Scan Sample\_9\_A1\_1\_3646.d:25949, m/z=781.8769, z=2, RT=12.18, ppm=-1.72):


TRINITY\_DN2904\_c0\_g1\_i1.p1

back to list

  

| Protein Coverage
| Supporting Peptides
| Best Unique PSM
|

Protein Coverage:

Supporting Peptides:

| Peptide | Uniq | -10lgP | Mass | Length | ppm | m/z | z | RT | 1/k0 Start | 1/k0 End | Precursor ID | Area Sample 10 | #Feature | #Feature Sample 10 | Start | End | PTM | AScore | Found By |
| --- | --- | --- | --- | --- | --- | --- | --- | --- | --- | --- | --- | --- | --- | --- | --- | --- | --- | --- | --- |
| K.KIQQELVIAK.Q | Y | 58.44 | 1168.7179 | 10 | 0.78 | 585.3636 | 2 | 13.58 | 0.942 | 0.9493 | 34174 | 3.74e2 | 1 | 1 | 43 | 52 |  |  | DB Search |
| K.KKEESPNAQDAIQK.L | Y | 58.13 | 1584.8107 | 14 | 7.09 | 529.2785 | 3 | 10.34 | 0.7921 | 0.7994 | 15147 | 1.07e3 | 1 | 1 | 12 | 25 |  |  | DB Search |
| total 2 peptides |  |  |  |  |  |  |  |  |  |  |  |  |  |  |  |  |  |  |  |
| --- | --- | --- | --- | --- | --- | --- | --- | --- | --- | --- | --- | --- | --- | --- | --- | --- | --- | --- | --- |

Best Unique PSM (Scan Sample\_9\_A1\_1\_3646.d:34174, m/z=585.3636, z=2, RT=13.58, ppm=-4.44):


TRINITY\_DN77\_c1\_g1\_i1.p1

back to list

  

| Protein Coverage
| Supporting Peptides
| Best Unique PSM
|

Protein Coverage:

Supporting Peptides:

| Peptide | Uniq | -10lgP | Mass | Length | ppm | m/z | z | RT | 1/k0 Start | 1/k0 End | Precursor ID | Area Sample 10 | #Feature | #Feature Sample 10 | Start | End | PTM | AScore | Found By |
| --- | --- | --- | --- | --- | --- | --- | --- | --- | --- | --- | --- | --- | --- | --- | --- | --- | --- | --- | --- |
| K.SFASSLLDHFRVEPAK.T | Y | 58.61 | 1802.9315 | 16 | 1.97 | 601.9825 | 3 | 19.83 | 0.9109 | 0.9182 | 68004 | 1.15e3 | 1 | 1 | 318 | 333 |  |  | DB Search |
| total 1 peptides |  |  |  |  |  |  |  |  |  |  |  |  |  |  |  |  |  |  |  |
| --- | --- | --- | --- | --- | --- | --- | --- | --- | --- | --- | --- | --- | --- | --- | --- | --- | --- | --- | --- |

Best Unique PSM (Scan Sample\_9\_A1\_1\_3646.d:68004, m/z=601.9825, z=3, RT=19.83, ppm=-3.25):


TRINITY\_DN1172\_c0\_g2\_i1.p1

back to list

  

| Protein Coverage
| Supporting Peptides
| Best Unique PSM
|

Protein Coverage:

Supporting Peptides:

| Peptide | Uniq | -10lgP | Mass | Length | ppm | m/z | z | RT | 1/k0 Start | 1/k0 End | Precursor ID | Area Sample 10 | #Feature | #Feature Sample 10 | Start | End | PTM | AScore | Found By |
| --- | --- | --- | --- | --- | --- | --- | --- | --- | --- | --- | --- | --- | --- | --- | --- | --- | --- | --- | --- |
| K.VLTSDDPAEKDK.K | Y | 57.79 | 1316.6459 | 12 | 1.44 | 659.3278 | 2 | 10.86 | 0.9844 | 0.9916 | 18297 | 1.85e2 | 1 | 1 | 116 | 127 |  |  | DB Search |
| total 1 peptides |  |  |  |  |  |  |  |  |  |  |  |  |  |  |  |  |  |  |  |
| --- | --- | --- | --- | --- | --- | --- | --- | --- | --- | --- | --- | --- | --- | --- | --- | --- | --- | --- | --- |

Best Unique PSM (Scan Sample\_9\_A1\_1\_3646.d:18297, m/z=659.3278, z=2, RT=10.86, ppm=-3.78):


TRINITY\_DN932\_c0\_g1\_i1.p1

back to list

  

| Protein Coverage
| Supporting Peptides
| Best Unique PSM
|

Protein Coverage:

Supporting Peptides:

| Peptide | Uniq | -10lgP | Mass | Length | ppm | m/z | z | RT | 1/k0 Start | 1/k0 End | Precursor ID | Area Sample 10 | #Feature | #Feature Sample 10 | Start | End | PTM | AScore | Found By |
| --- | --- | --- | --- | --- | --- | --- | --- | --- | --- | --- | --- | --- | --- | --- | --- | --- | --- | --- | --- |
| K.SM(+15.99)IHELNR.Y | Y | 54.96 | 1014.4917 | 8 | 2.05 | 508.2515 | 2 | 11.61 | 0.8522 | 0.8595 | 22750 | 3.92e2 | 1 | 1 | 406 | 413 | Oxidation (M) | M2:Oxidation (M):1000 | DB Search |
| total 1 peptides |  |  |  |  |  |  |  |  |  |  |  |  |  |  |  |  |  |  |  |
| --- | --- | --- | --- | --- | --- | --- | --- | --- | --- | --- | --- | --- | --- | --- | --- | --- | --- | --- | --- |

Best Unique PSM (Scan Sample\_9\_A1\_1\_3646.d:22750, m/z=508.2515, z=2, RT=11.61, ppm=-3.17):


TRINITY\_DN2885\_c0\_g1\_i5.p1

back to list

  

| Protein Coverage
| Supporting Peptides
| Best Unique PSM
|

Protein Coverage:

Supporting Peptides:

| Peptide | Uniq | -10lgP | Mass | Length | ppm | m/z | z | RT | 1/k0 Start | 1/k0 End | Precursor ID | Area Sample 10 | #Feature | #Feature Sample 10 | Start | End | PTM | AScore | Found By |
| --- | --- | --- | --- | --- | --- | --- | --- | --- | --- | --- | --- | --- | --- | --- | --- | --- | --- | --- | --- |
| K.VTRPHGASGVVR.A | Y | 58.63 | 1234.6894 | 12 | 0.38 | 412.5684 | 3 | 10.08 | 0.8197 | 0.827 | 13527 | 3.84e3 | 1 | 1 | 77 | 88 |  |  | DB Search |
| total 1 peptides |  |  |  |  |  |  |  |  |  |  |  |  |  |  |  |  |  |  |  |
| --- | --- | --- | --- | --- | --- | --- | --- | --- | --- | --- | --- | --- | --- | --- | --- | --- | --- | --- | --- |

Best Unique PSM (Scan Sample\_9\_A1\_1\_3646.d:13527, m/z=412.5684, z=3, RT=10.08, ppm=-4.85):


TRINITY\_DN30386\_c0\_g1\_i1.p2

back to list

  

| Protein Coverage
| Supporting Peptides
| Best Unique PSM
|

Protein Coverage:

Supporting Peptides:

| Peptide | Uniq | -10lgP | Mass | Length | ppm | m/z | z | RT | 1/k0 Start | 1/k0 End | Precursor ID | Area Sample 10 | #Feature | #Feature Sample 10 | Start | End | PTM | AScore | Found By |
| --- | --- | --- | --- | --- | --- | --- | --- | --- | --- | --- | --- | --- | --- | --- | --- | --- | --- | --- | --- |
| K.VFDRDGNGFISAAELR.H | Y | 51.43 | 1765.8747 | 16 | -4.93 | 883.9357 | 2 | 19.74 | 1.1274 | 1.1346 | 67630 | 2.9e3 | 1 | 1 | 117 | 132 |  |  | DB Search |
| total 1 peptides |  |  |  |  |  |  |  |  |  |  |  |  |  |  |  |  |  |  |  |
| --- | --- | --- | --- | --- | --- | --- | --- | --- | --- | --- | --- | --- | --- | --- | --- | --- | --- | --- | --- |

Best Unique PSM (Scan Sample\_9\_A1\_1\_3646.d:67630, m/z=883.9357, z=2, RT=19.74, ppm=-10.14):


TRINITY\_DN8227\_c0\_g1\_i1.p1

back to list

  

| Protein Coverage
| Supporting Peptides
| Best Unique PSM
|

Protein Coverage:

Supporting Peptides:

| Peptide | Uniq | -10lgP | Mass | Length | ppm | m/z | z | RT | 1/k0 Start | 1/k0 End | Precursor ID | Area Sample 10 | #Feature | #Feature Sample 10 | Start | End | PTM | AScore | Found By |
| --- | --- | --- | --- | --- | --- | --- | --- | --- | --- | --- | --- | --- | --- | --- | --- | --- | --- | --- | --- |
| K.IQDKEGIPPDQQR.L | Y | 58.25 | 1522.7739 | 13 | 2.55 | 762.3922 | 2 | 11.03 | 1.0343 | 1.0415 | 18957 | 1.53e3 | 1 | 1 | 54 | 66 |  |  | DB Search |
| total 1 peptides |  |  |  |  |  |  |  |  |  |  |  |  |  |  |  |  |  |  |  |
| --- | --- | --- | --- | --- | --- | --- | --- | --- | --- | --- | --- | --- | --- | --- | --- | --- | --- | --- | --- |

Best Unique PSM (Scan Sample\_9\_A1\_1\_3646.d:18957, m/z=762.3922, z=2, RT=11.03, ppm=-2.67):


TRINITY\_DN1616\_c0\_g1\_i7.p1

back to list

  

| Protein Coverage
| Supporting Peptides
| Best Unique PSM
|

Protein Coverage:

Supporting Peptides:

| Peptide | Uniq | -10lgP | Mass | Length | ppm | m/z | z | RT | 1/k0 Start | 1/k0 End | Precursor ID | Area Sample 10 | #Feature | #Feature Sample 10 | Start | End | PTM | AScore | Found By |
| --- | --- | --- | --- | --- | --- | --- | --- | --- | --- | --- | --- | --- | --- | --- | --- | --- | --- | --- | --- |
| K.YVVMLQYYHDSDTAK.Y | Y | 52.09 | 1831.8451 | 15 | 5.48 | 916.9301 | 2 | 16.56 | 1.1813 | 1.1885 | 49447 | 0 | 0 | 0 | 1071 | 1085 |  |  | DB Search |
| R.YALNQIQPVTATVTAFKGDSK.I | Y | 44.58 | 2251.1848 | 21 | 9.46 | 751.4054 | 3 | 18.19 | 1.048 | 1.0552 | 58586 | 0 | 0 | 0 | 894 | 914 |  |  | DB Search |
| total 2 peptides |  |  |  |  |  |  |  |  |  |  |  |  |  |  |  |  |  |  |  |
| --- | --- | --- | --- | --- | --- | --- | --- | --- | --- | --- | --- | --- | --- | --- | --- | --- | --- | --- | --- |

Best Unique PSM (Scan Sample\_9\_A1\_1\_3646.d:49447, m/z=916.9301, z=2, RT=16.56, ppm=0.27):


TRINITY\_DN36\_c0\_g1\_i9.p1

back to list

  

| Protein Coverage
| Supporting Peptides
| Best Unique PSM
|

Protein Coverage:

Supporting Peptides:

| Peptide | Uniq | -10lgP | Mass | Length | ppm | m/z | z | RT | 1/k0 Start | 1/k0 End | Precursor ID | Area Sample 10 | #Feature | #Feature Sample 10 | Start | End | PTM | AScore | Found By |
| --- | --- | --- | --- | --- | --- | --- | --- | --- | --- | --- | --- | --- | --- | --- | --- | --- | --- | --- | --- |
| R.LGGIEVTQGNC(+57.02)ARK.Y | Y | 58.24 | 1501.7671 | 14 | 6.42 | 751.8917 | 2 | 12.66 | 1.0632 | 1.0705 | 28959 | 0 | 0 | 0 | 58 | 71 | Carbamidomethylation |  | DB Search |
| total 1 peptides |  |  |  |  |  |  |  |  |  |  |  |  |  |  |  |  |  |  |  |
| --- | --- | --- | --- | --- | --- | --- | --- | --- | --- | --- | --- | --- | --- | --- | --- | --- | --- | --- | --- |

Best Unique PSM (Scan Sample\_9\_A1\_1\_3646.d:28959, m/z=751.8917, z=2, RT=12.66, ppm=1.21):


TRINITY\_DN3545\_c0\_g1\_i2.p1

back to list

  

| Protein Coverage
| Supporting Peptides
| Best Unique PSM
|

Protein Coverage:

Supporting Peptides:

| Peptide | Uniq | -10lgP | Mass | Length | ppm | m/z | z | RT | 1/k0 Start | 1/k0 End | Precursor ID | Area Sample 10 | #Feature | #Feature Sample 10 | Start | End | PTM | AScore | Found By |
| --- | --- | --- | --- | --- | --- | --- | --- | --- | --- | --- | --- | --- | --- | --- | --- | --- | --- | --- | --- |
| R.SC(+57.02)TNPAPANGGK.H | Y | 58.68 | 1172.5244 | 12 | 3.23 | 587.2683 | 2 | 9.42 | 0.8352 | 0.8425 | 10441 | 8.36e2 | 1 | 1 | 55 | 66 | Carbamidomethylation |  | DB Search |
| R.SSC(+57.02)RPILSVIK.I | Y | 54.44 | 1258.7067 | 11 | -8.13 | 630.3522 | 2 | 16.83 | 0.9691 | 0.9763 | 50527 | 1.86e3 | 1 | 1 | 6 | 16 | Carbamidomethylation |  | DB Search |
| total 2 peptides |  |  |  |  |  |  |  |  |  |  |  |  |  |  |  |  |  |  |  |
| --- | --- | --- | --- | --- | --- | --- | --- | --- | --- | --- | --- | --- | --- | --- | --- | --- | --- | --- | --- |

Best Unique PSM (Scan Sample\_9\_A1\_1\_3646.d:10441, m/z=587.2683, z=2, RT=9.42, ppm=-1.99):


TRINITY\_DN1369\_c0\_g1\_i1.p1

back to list

  

| Protein Coverage
| Supporting Peptides
| Best Unique PSM
|

Protein Coverage:

Supporting Peptides:

| Peptide | Uniq | -10lgP | Mass | Length | ppm | m/z | z | RT | 1/k0 Start | 1/k0 End | Precursor ID | Area Sample 10 | #Feature | #Feature Sample 10 | Start | End | PTM | AScore | Found By |
| --- | --- | --- | --- | --- | --- | --- | --- | --- | --- | --- | --- | --- | --- | --- | --- | --- | --- | --- | --- |
| K.TPGEC(+57.02)C(+57.02)PQC(+57.02)K.K | Y | 58.63 | 1235.4733 | 10 | 0.71 | 618.7411 | 2 | 9.99 | 0.8915 | 0.8988 | 13135 | 0 | 0 | 0 | 1387 | 1396 | Carbamidomethylation, Carbamidomethylation, Carbamidomethylation |  | DB Search |
| K.HAEYYFIDALEGKVVVK.Y | Y | 54.36 | 1980.0356 | 17 | -3.44 | 661.0134 | 3 | 19.49 | 0.9449 | 0.9521 | 66246 | 0 | 0 | 0 | 124 | 140 |  |  | DB Search |
| total 2 peptides |  |  |  |  |  |  |  |  |  |  |  |  |  |  |  |  |  |  |  |
| --- | --- | --- | --- | --- | --- | --- | --- | --- | --- | --- | --- | --- | --- | --- | --- | --- | --- | --- | --- |

Best Unique PSM (Scan Sample\_9\_A1\_1\_3646.d:13135, m/z=618.7411, z=2, RT=9.99, ppm=-4.51):


TRINITY\_DN118\_c1\_g1\_i3.p1

back to list

  

| Protein Coverage
| Supporting Peptides
| Best Unique PSM
|

Protein Coverage:

Supporting Peptides:

| Peptide | Uniq | -10lgP | Mass | Length | ppm | m/z | z | RT | 1/k0 Start | 1/k0 End | Precursor ID | Area Sample 10 | #Feature | #Feature Sample 10 | Start | End | PTM | AScore | Found By |
| --- | --- | --- | --- | --- | --- | --- | --- | --- | --- | --- | --- | --- | --- | --- | --- | --- | --- | --- | --- |
| R.ETC(+57.02)SDIEKR.E | Y | 58.74 | 1136.5132 | 9 | 1.02 | 569.2615 | 2 | 10.09 | 0.8947 | 0.902 | 13624 | 1.03e4 | 1 | 1 | 186 | 194 | Carbamidomethylation |  | DB Search |
| K.FSYEKVC(+57.02)EDVKR.C | Y | 58.67 | 1558.745 | 12 | 1.55 | 780.3769 | 2 | 13.49 | 1.0717 | 1.0789 | 33594 | 8.49e2 | 1 | 1 | 117 | 128 | Carbamidomethylation |  | DB Search |
| K.DC(+57.02)TPRETC(+57.02)SDIEKR.E | Y | 58.67 | 1765.7723 | 14 | -5.61 | 589.5917 | 3 | 11.15 | 0.8485 | 0.8558 | 19989 | 0 | 0 | 0 | 181 | 194 | Carbamidomethylation, Carbamidomethylation |  | DB Search |
| K.VC(+57.02)EDVKR.C | Y | 58.23 | 904.4436 | 7 | 1.65 | 453.2275 | 2 | 9.38 | 0.7685 | 0.7758 | 10310 | 8.12e3 | 2 | 2 | 122 | 128 | Carbamidomethylation |  | DB Search |
| total 4 peptides |  |  |  |  |  |  |  |  |  |  |  |  |  |  |  |  |  |  |  |
| --- | --- | --- | --- | --- | --- | --- | --- | --- | --- | --- | --- | --- | --- | --- | --- | --- | --- | --- | --- |

Best Unique PSM (Scan Sample\_9\_A1\_1\_3646.d:13624, m/z=569.2615, z=2, RT=10.09, ppm=-4.2):


TRINITY\_DN2487\_c1\_g1\_i4.p1

back to list

  

| Protein Coverage
| Supporting Peptides
| Best Unique PSM
|

Protein Coverage:

Supporting Peptides:

| Peptide | Uniq | -10lgP | Mass | Length | ppm | m/z | z | RT | 1/k0 Start | 1/k0 End | Precursor ID | Area Sample 10 | #Feature | #Feature Sample 10 | Start | End | PTM | AScore | Found By |
| --- | --- | --- | --- | --- | --- | --- | --- | --- | --- | --- | --- | --- | --- | --- | --- | --- | --- | --- | --- |
| R.RLVELQHGR.V | Y | 53.64 | 1106.6308 | 9 | 6.11 | 369.8846 | 3 | 11.14 | 0.7061 | 0.7135 | 19894 | 1.5e3 | 1 | 1 | 168 | 176 |  |  | DB Search |
| total 1 peptides |  |  |  |  |  |  |  |  |  |  |  |  |  |  |  |  |  |  |  |
| --- | --- | --- | --- | --- | --- | --- | --- | --- | --- | --- | --- | --- | --- | --- | --- | --- | --- | --- | --- |

Best Unique PSM (Scan Sample\_9\_A1\_1\_3646.d:19894, m/z=370.1763, z=2, RT=11.15, ppm=0.89):


TRINITY\_DN165469\_c0\_g1\_i1.p1

back to list

  

| Protein Coverage
| Supporting Peptides
| Best Unique PSM
|

Protein Coverage:

Supporting Peptides:

| Peptide | Uniq | -10lgP | Mass | Length | ppm | m/z | z | RT | 1/k0 Start | 1/k0 End | Precursor ID | Area Sample 10 | #Feature | #Feature Sample 10 | Start | End | PTM | AScore | Found By |
| --- | --- | --- | --- | --- | --- | --- | --- | --- | --- | --- | --- | --- | --- | --- | --- | --- | --- | --- | --- |
| R.ALNTLHTFVNER.N | Y | 56.93 | 1413.7364 | 12 | 4.35 | 707.8749 | 2 | 15.14 | 1.0202 | 1.0275 | 42361 | 0 | 0 | 0 | 951 | 962 |  |  | DB Search |
| K.RLDPSEQLQHLSSLR.Q | Y | 56.25 | 1777.9435 | 15 | -2.83 | 593.6503 | 3 | 16.40 | 0.9069 | 0.9141 | 48713 | 0 | 0 | 0 | 2221 | 2235 |  |  | DB Search |
| total 2 peptides |  |  |  |  |  |  |  |  |  |  |  |  |  |  |  |  |  |  |  |
| --- | --- | --- | --- | --- | --- | --- | --- | --- | --- | --- | --- | --- | --- | --- | --- | --- | --- | --- | --- |

Best Unique PSM (Scan Sample\_9\_A1\_1\_3646.d:42361, m/z=707.8749, z=2, RT=15.14, ppm=-0.87):


TRINITY\_DN40728\_c1\_g1\_i1.p2

back to list

  

| Protein Coverage
| Supporting Peptides
| Best Unique PSM
|

Protein Coverage:

Supporting Peptides:

| Peptide | Uniq | -10lgP | Mass | Length | ppm | m/z | z | RT | 1/k0 Start | 1/k0 End | Precursor ID | Area Sample 10 | #Feature | #Feature Sample 10 | Start | End | PTM | AScore | Found By |
| --- | --- | --- | --- | --- | --- | --- | --- | --- | --- | --- | --- | --- | --- | --- | --- | --- | --- | --- | --- |
| K.IQDKEGIPPDQQR.L | Y | 58.25 | 1522.7739 | 13 | 2.55 | 762.3922 | 2 | 11.03 | 1.0343 | 1.0415 | 18957 | 1.53e3 | 1 | 1 | 25 | 37 |  |  | DB Search |
| total 1 peptides |  |  |  |  |  |  |  |  |  |  |  |  |  |  |  |  |  |  |  |
| --- | --- | --- | --- | --- | --- | --- | --- | --- | --- | --- | --- | --- | --- | --- | --- | --- | --- | --- | --- |

Best Unique PSM (Scan Sample\_9\_A1\_1\_3646.d:18957, m/z=762.3922, z=2, RT=11.03, ppm=-2.67):


TRINITY\_DN585\_c0\_g1\_i5.p1

back to list

  

| Protein Coverage
| Supporting Peptides
| Best Unique PSM
|

Protein Coverage:

Supporting Peptides:

| Peptide | Uniq | -10lgP | Mass | Length | ppm | m/z | z | RT | 1/k0 Start | 1/k0 End | Precursor ID | Area Sample 10 | #Feature | #Feature Sample 10 | Start | End | PTM | AScore | Found By |
| --- | --- | --- | --- | --- | --- | --- | --- | --- | --- | --- | --- | --- | --- | --- | --- | --- | --- | --- | --- |
| K.IQDKEGIPPDQQR.L | Y | 58.25 | 1522.7739 | 13 | 2.55 | 762.3922 | 2 | 11.03 | 1.0343 | 1.0415 | 18957 | 1.53e3 | 1 | 1 | 2 | 14 |  |  | DB Search |
| total 1 peptides |  |  |  |  |  |  |  |  |  |  |  |  |  |  |  |  |  |  |  |
| --- | --- | --- | --- | --- | --- | --- | --- | --- | --- | --- | --- | --- | --- | --- | --- | --- | --- | --- | --- |

Best Unique PSM (Scan Sample\_9\_A1\_1\_3646.d:18957, m/z=762.3922, z=2, RT=11.03, ppm=-2.67):


TRINITY\_DN6388\_c0\_g1\_i2.p2

back to list

  

| Protein Coverage
| Supporting Peptides
| Best Unique PSM
|

Protein Coverage:

Supporting Peptides:

| Peptide | Uniq | -10lgP | Mass | Length | ppm | m/z | z | RT | 1/k0 Start | 1/k0 End | Precursor ID | Area Sample 10 | #Feature | #Feature Sample 10 | Start | End | PTM | AScore | Found By |
| --- | --- | --- | --- | --- | --- | --- | --- | --- | --- | --- | --- | --- | --- | --- | --- | --- | --- | --- | --- |
| R.AIELLPHDLAVGR.H | Y | 57.65 | 1402.7932 | 13 | -0.45 | 702.3999 | 2 | 17.82 | 1.0303 | 1.0375 | 56285 | 4.72e2 | 1 | 1 | 52 | 64 |  |  | DB Search |
| total 1 peptides |  |  |  |  |  |  |  |  |  |  |  |  |  |  |  |  |  |  |  |
| --- | --- | --- | --- | --- | --- | --- | --- | --- | --- | --- | --- | --- | --- | --- | --- | --- | --- | --- | --- |

Best Unique PSM (Scan Sample\_9\_A1\_1\_3646.d:56285, m/z=702.3999, z=2, RT=17.82, ppm=-5.66):


TRINITY\_DN15248\_c0\_g1\_i1.p2

back to list

  

| Protein Coverage
| Supporting Peptides
| Best Unique PSM
|

Protein Coverage:

Supporting Peptides:

| Peptide | Uniq | -10lgP | Mass | Length | ppm | m/z | z | RT | 1/k0 Start | 1/k0 End | Precursor ID | Area Sample 10 | #Feature | #Feature Sample 10 | Start | End | PTM | AScore | Found By |
| --- | --- | --- | --- | --- | --- | --- | --- | --- | --- | --- | --- | --- | --- | --- | --- | --- | --- | --- | --- |
| R.IAADGTESR.V | Y | 39.56 | 918.4406 | 9 | 3.69 | 460.2269 | 2 | 9.86 | 0.8027 | 0.81 | 12225 | 9.43e3 | 1 | 1 | 78 | 86 |  |  | DB Search |
| total 1 peptides |  |  |  |  |  |  |  |  |  |  |  |  |  |  |  |  |  |  |  |
| --- | --- | --- | --- | --- | --- | --- | --- | --- | --- | --- | --- | --- | --- | --- | --- | --- | --- | --- | --- |

Best Unique PSM (Scan Sample\_9\_A1\_1\_3646.d:12225, m/z=460.2269, z=2, RT=9.86, ppm=-1.53):


TRINITY\_DN141338\_c2\_g1\_i1.p1

back to list

  

| Protein Coverage
| Supporting Peptides
| Best Unique PSM
|

Protein Coverage:

Supporting Peptides:

| Peptide | Uniq | -10lgP | Mass | Length | ppm | m/z | z | RT | 1/k0 Start | 1/k0 End | Precursor ID | Area Sample 10 | #Feature | #Feature Sample 10 | Start | End | PTM | AScore | Found By |
| --- | --- | --- | --- | --- | --- | --- | --- | --- | --- | --- | --- | --- | --- | --- | --- | --- | --- | --- | --- |
| R.ELISNSSDALDKIR.Y | Y | 58.23 | 1559.8155 | 14 | 3.45 | 780.9136 | 2 | 15.47 | 1.0733 | 1.0805 | 44016 | 2.57e2 | 1 | 1 | 37 | 50 |  |  | DB Search |
| total 1 peptides |  |  |  |  |  |  |  |  |  |  |  |  |  |  |  |  |  |  |  |
| --- | --- | --- | --- | --- | --- | --- | --- | --- | --- | --- | --- | --- | --- | --- | --- | --- | --- | --- | --- |

Best Unique PSM (Scan Sample\_9\_A1\_1\_3646.d:44016, m/z=780.9136, z=2, RT=15.47, ppm=-1.76):


TRINITY\_DN2477\_c0\_g1\_i6.p1

back to list

  

| Protein Coverage
| Supporting Peptides
| Best Unique PSM
|

Protein Coverage:

Supporting Peptides:

| Peptide | Uniq | -10lgP | Mass | Length | ppm | m/z | z | RT | 1/k0 Start | 1/k0 End | Precursor ID | Area Sample 10 | #Feature | #Feature Sample 10 | Start | End | PTM | AScore | Found By |
| --- | --- | --- | --- | --- | --- | --- | --- | --- | --- | --- | --- | --- | --- | --- | --- | --- | --- | --- | --- |
| K.DSYVGDEAQSKR.G | Y | 59.06 | 1353.616 | 12 | 5.32 | 677.8154 | 2 | 10.95 | 1.0041 | 1.0114 | 18674 | 7.12e3 | 1 | 1 | 52 | 63 |  |  | DB Search |
| R.VAPEEHPVLLTEAPLNPK.A | Y | 58.04 | 1953.0571 | 18 | 3.48 | 977.5341 | 2 | 16.43 | 1.2045 | 1.2117 | 48611 | 4.81e2 | 1 | 1 | 97 | 114 |  |  | DB Search |
| total 2 peptides |  |  |  |  |  |  |  |  |  |  |  |  |  |  |  |  |  |  |  |
| --- | --- | --- | --- | --- | --- | --- | --- | --- | --- | --- | --- | --- | --- | --- | --- | --- | --- | --- | --- |

Best Unique PSM (Scan Sample\_9\_A1\_1\_3646.d:18674, m/z=677.8154, z=2, RT=10.95, ppm=0.1):


TRINITY\_DN8903\_c0\_g1\_i1.p1

back to list

  

| Protein Coverage
| Supporting Peptides
| Best Unique PSM
|

Protein Coverage:

Supporting Peptides:

| Peptide | Uniq | -10lgP | Mass | Length | ppm | m/z | z | RT | 1/k0 Start | 1/k0 End | Precursor ID | Area Sample 10 | #Feature | #Feature Sample 10 | Start | End | PTM | AScore | Found By |
| --- | --- | --- | --- | --- | --- | --- | --- | --- | --- | --- | --- | --- | --- | --- | --- | --- | --- | --- | --- |
| K.DLHIIEDYVLK.H | Y | 58.91 | 1356.7289 | 11 | 0.07 | 679.3682 | 2 | 19.53 | 0.9953 | 1.0025 | 66441 | 3.01e3 | 1 | 1 | 417 | 427 |  |  | DB Search |
| total 1 peptides |  |  |  |  |  |  |  |  |  |  |  |  |  |  |  |  |  |  |  |
| --- | --- | --- | --- | --- | --- | --- | --- | --- | --- | --- | --- | --- | --- | --- | --- | --- | --- | --- | --- |

Best Unique PSM (Scan Sample\_9\_A1\_1\_3646.d:66441, m/z=679.3682, z=2, RT=19.53, ppm=-5.14):


TRINITY\_DN649\_c0\_g1\_i5.p1

back to list

  

| Protein Coverage
| Supporting Peptides
| Best Unique PSM
|

Protein Coverage:

Supporting Peptides:

| Peptide | Uniq | -10lgP | Mass | Length | ppm | m/z | z | RT | 1/k0 Start | 1/k0 End | Precursor ID | Area Sample 10 | #Feature | #Feature Sample 10 | Start | End | PTM | AScore | Found By |
| --- | --- | --- | --- | --- | --- | --- | --- | --- | --- | --- | --- | --- | --- | --- | --- | --- | --- | --- | --- |
| K.FETQIATK.N | Y | 57.09 | 936.4916 | 8 | 7.3 | 937.5009 | 1 | 11.88 | 1.1925 | 1.1997 | 24345 | 0 | 0 | 0 | 102 | 109 |  |  | DB Search |
| total 1 peptides |  |  |  |  |  |  |  |  |  |  |  |  |  |  |  |  |  |  |  |
| --- | --- | --- | --- | --- | --- | --- | --- | --- | --- | --- | --- | --- | --- | --- | --- | --- | --- | --- | --- |

Best Unique PSM (Scan Sample\_9\_A1\_1\_3646.d:24345, m/z=937.5009, z=1, RT=11.88, ppm=2.08):


TRINITY\_DN902\_c1\_g2\_i1.p1

back to list

  

| Protein Coverage
| Supporting Peptides
| Best Unique PSM
|

Protein Coverage:

Supporting Peptides:

| Peptide | Uniq | -10lgP | Mass | Length | ppm | m/z | z | RT | 1/k0 Start | 1/k0 End | Precursor ID | Area Sample 10 | #Feature | #Feature Sample 10 | Start | End | PTM | AScore | Found By |
| --- | --- | --- | --- | --- | --- | --- | --- | --- | --- | --- | --- | --- | --- | --- | --- | --- | --- | --- | --- |
| K.GLTHC(+57.02)NTLSYK.L | Y | 59.02 | 1292.6183 | 11 | 5.84 | 647.3168 | 2 | 12.45 | 0.9727 | 0.98 | 27736 | 3.39e3 | 1 | 1 | 62 | 72 | Carbamidomethylation |  | DB Search |
| total 1 peptides |  |  |  |  |  |  |  |  |  |  |  |  |  |  |  |  |  |  |  |
| --- | --- | --- | --- | --- | --- | --- | --- | --- | --- | --- | --- | --- | --- | --- | --- | --- | --- | --- | --- |

Best Unique PSM (Scan Sample\_9\_A1\_1\_3646.d:27736, m/z=647.3168, z=2, RT=12.45, ppm=0.62):


TRINITY\_DN2113\_c0\_g1\_i2.p1

back to list

  

| Protein Coverage
| Supporting Peptides
| Best Unique PSM
|

Protein Coverage:

Supporting Peptides:

| Peptide | Uniq | -10lgP | Mass | Length | ppm | m/z | z | RT | 1/k0 Start | 1/k0 End | Precursor ID | Area Sample 10 | #Feature | #Feature Sample 10 | Start | End | PTM | AScore | Found By |
| --- | --- | --- | --- | --- | --- | --- | --- | --- | --- | --- | --- | --- | --- | --- | --- | --- | --- | --- | --- |
| K.AHNLTEDVIFWK.W | Y | 58.72 | 1471.7459 | 12 | 0.34 | 736.8766 | 2 | 18.72 | 1.0061 | 1.0134 | 61618 | 3.8e2 | 1 | 1 | 101 | 112 |  |  | DB Search |
| total 1 peptides |  |  |  |  |  |  |  |  |  |  |  |  |  |  |  |  |  |  |  |
| --- | --- | --- | --- | --- | --- | --- | --- | --- | --- | --- | --- | --- | --- | --- | --- | --- | --- | --- | --- |

Best Unique PSM (Scan Sample\_9\_A1\_1\_3646.d:61618, m/z=736.8766, z=2, RT=18.72, ppm=-4.88):


TRINITY\_DN17095\_c0\_g1\_i1.p1

back to list

  

| Protein Coverage
| Supporting Peptides
| Best Unique PSM
|

Protein Coverage:

Supporting Peptides:

| Peptide | Uniq | -10lgP | Mass | Length | ppm | m/z | z | RT | 1/k0 Start | 1/k0 End | Precursor ID | Area Sample 10 | #Feature | #Feature Sample 10 | Start | End | PTM | AScore | Found By |
| --- | --- | --- | --- | --- | --- | --- | --- | --- | --- | --- | --- | --- | --- | --- | --- | --- | --- | --- | --- |
| R.VGYLFITDWGGKPK.I | Y | 51.21 | 1579.8398 | 14 | 1.85 | 527.6188 | 3 | 19.44 | 0.8031 | 0.8104 | 65944 | 2.03e2 | 1 | 1 | 260 | 273 |  |  | DB Search |
| total 1 peptides |  |  |  |  |  |  |  |  |  |  |  |  |  |  |  |  |  |  |  |
| --- | --- | --- | --- | --- | --- | --- | --- | --- | --- | --- | --- | --- | --- | --- | --- | --- | --- | --- | --- |

Best Unique PSM (Scan Sample\_9\_A1\_1\_3646.d:65944, m/z=527.6188, z=3, RT=19.44, ppm=-3.37):


TRINITY\_DN1614\_c0\_g1\_i1.p1

back to list

  

| Protein Coverage
| Supporting Peptides
| Best Unique PSM
|

Protein Coverage:

Supporting Peptides:

| Peptide | Uniq | -10lgP | Mass | Length | ppm | m/z | z | RT | 1/k0 Start | 1/k0 End | Precursor ID | Area Sample 10 | #Feature | #Feature Sample 10 | Start | End | PTM | AScore | Found By |
| --- | --- | --- | --- | --- | --- | --- | --- | --- | --- | --- | --- | --- | --- | --- | --- | --- | --- | --- | --- |
| K.GLDVDSLVIDHIQVNR.A | Y | 58.76 | 1791.9479 | 16 | -0.72 | 896.9759 | 2 | 19.01 | 1.1786 | 1.1857 | 63336 | 2.19e3 | 1 | 1 | 140 | 155 |  |  | DB Search |
| K.KSAQFLLQLLK.N | Y | 58.62 | 1287.7914 | 11 | 3.65 | 644.902 | 2 | 19.71 | 0.9779 | 0.9852 | 67435 | 7.12e2 | 1 | 1 | 120 | 130 |  |  | DB Search |
| total 2 peptides |  |  |  |  |  |  |  |  |  |  |  |  |  |  |  |  |  |  |  |
| --- | --- | --- | --- | --- | --- | --- | --- | --- | --- | --- | --- | --- | --- | --- | --- | --- | --- | --- | --- |

Best Unique PSM (Scan Sample\_9\_A1\_1\_3646.d:63336, m/z=896.9759, z=2, RT=19.01, ppm=-5.94):


TRINITY\_DN1910\_c0\_g1\_i1.p1

back to list

  

| Protein Coverage
| Supporting Peptides
| Best Unique PSM
|

Protein Coverage:

Supporting Peptides:

| Peptide | Uniq | -10lgP | Mass | Length | ppm | m/z | z | RT | 1/k0 Start | 1/k0 End | Precursor ID | Area Sample 10 | #Feature | #Feature Sample 10 | Start | End | PTM | AScore | Found By |
| --- | --- | --- | --- | --- | --- | --- | --- | --- | --- | --- | --- | --- | --- | --- | --- | --- | --- | --- | --- |
| K.NM(+15.99)M(+15.99)AAC(+57.02)DPR.H | Y | 58.66 | 1096.41 | 9 | 1.24 | 549.2101 | 2 | 9.53 | 0.8619 | 0.8692 | 10930 | 4.97e2 | 1 | 1 | 298 | 306 | Oxidation (M), Oxidation (M), Carbamidomethylation | M2:Oxidation (M):1000 M3:Oxidation (M):1000 | DB Search |
| total 1 peptides |  |  |  |  |  |  |  |  |  |  |  |  |  |  |  |  |  |  |  |
| --- | --- | --- | --- | --- | --- | --- | --- | --- | --- | --- | --- | --- | --- | --- | --- | --- | --- | --- | --- |

Best Unique PSM (Scan Sample\_9\_A1\_1\_3646.d:10930, m/z=549.2101, z=2, RT=9.53, ppm=-3.99):


TRINITY\_DN123\_c0\_g1\_i1.p1

back to list

  

| Protein Coverage
| Supporting Peptides
| Best Unique PSM
|

Protein Coverage:

Supporting Peptides:

| Peptide | Uniq | -10lgP | Mass | Length | ppm | m/z | z | RT | 1/k0 Start | 1/k0 End | Precursor ID | Area Sample 10 | #Feature | #Feature Sample 10 | Start | End | PTM | AScore | Found By |
| --- | --- | --- | --- | --- | --- | --- | --- | --- | --- | --- | --- | --- | --- | --- | --- | --- | --- | --- | --- |
| K.IAAVIINKPDVLASR.T | Y | 35.53 | 1578.9457 | 15 | -0.29 | 790.4758 | 2 | 15.96 | 1.0993 | 1.1065 | 46467 | 3.75e2 | 1 | 1 | 484 | 498 |  |  | DB Search |
| total 1 peptides |  |  |  |  |  |  |  |  |  |  |  |  |  |  |  |  |  |  |  |
| --- | --- | --- | --- | --- | --- | --- | --- | --- | --- | --- | --- | --- | --- | --- | --- | --- | --- | --- | --- |

Best Unique PSM (Scan Sample\_9\_A1\_1\_3646.d:46467, m/z=790.4758, z=2, RT=15.96, ppm=-5.51):


TRINITY\_DN1058\_c0\_g1\_i4.p1

back to list

  

| Protein Coverage
| Supporting Peptides
| Best Unique PSM
|

Protein Coverage:

Supporting Peptides:

| Peptide | Uniq | -10lgP | Mass | Length | ppm | m/z | z | RT | 1/k0 Start | 1/k0 End | Precursor ID | Area Sample 10 | #Feature | #Feature Sample 10 | Start | End | PTM | AScore | Found By |
| --- | --- | --- | --- | --- | --- | --- | --- | --- | --- | --- | --- | --- | --- | --- | --- | --- | --- | --- | --- |
| R.APTHILNPANKR.S | Y | 58.64 | 1330.7469 | 12 | 0.9 | 666.3779 | 2 | 11.17 | 1.009 | 1.0162 | 20196 | 4.06e2 | 1 | 1 | 398 | 409 |  |  | DB Search |
| total 1 peptides |  |  |  |  |  |  |  |  |  |  |  |  |  |  |  |  |  |  |  |
| --- | --- | --- | --- | --- | --- | --- | --- | --- | --- | --- | --- | --- | --- | --- | --- | --- | --- | --- | --- |

Best Unique PSM (Scan Sample\_9\_A1\_1\_3646.d:20196, m/z=666.3779, z=2, RT=11.17, ppm=-4.32):


TRINITY\_DN785\_c0\_g1\_i1.p1

back to list

  

| Protein Coverage
| Supporting Peptides
| Best Unique PSM
|

Protein Coverage:

Supporting Peptides:

| Peptide | Uniq | -10lgP | Mass | Length | ppm | m/z | z | RT | 1/k0 Start | 1/k0 End | Precursor ID | Area Sample 10 | #Feature | #Feature Sample 10 | Start | End | PTM | AScore | Found By |
| --- | --- | --- | --- | --- | --- | --- | --- | --- | --- | --- | --- | --- | --- | --- | --- | --- | --- | --- | --- |
| K.KFDTYLPGFIC(+57.02)R.C | Y | 58.63 | 1515.7544 | 12 | 1.26 | 758.8815 | 2 | 18.53 | 1.0299 | 1.0371 | 60572 | 0 | 0 | 0 | 77 | 88 | Carbamidomethylation |  | DB Search |
| total 1 peptides |  |  |  |  |  |  |  |  |  |  |  |  |  |  |  |  |  |  |  |
| --- | --- | --- | --- | --- | --- | --- | --- | --- | --- | --- | --- | --- | --- | --- | --- | --- | --- | --- | --- |

Best Unique PSM (Scan Sample\_9\_A1\_1\_3646.d:60572, m/z=758.8815, z=2, RT=18.53, ppm=-3.96):


TRINITY\_DN602\_c0\_g1\_i1.p1

back to list

  

| Protein Coverage
| Supporting Peptides
| Best Unique PSM
|

Protein Coverage:

Supporting Peptides:

| Peptide | Uniq | -10lgP | Mass | Length | ppm | m/z | z | RT | 1/k0 Start | 1/k0 End | Precursor ID | Area Sample 10 | #Feature | #Feature Sample 10 | Start | End | PTM | AScore | Found By |
| --- | --- | --- | --- | --- | --- | --- | --- | --- | --- | --- | --- | --- | --- | --- | --- | --- | --- | --- | --- |
| K.EGFYVHEALAAGIVK.K | Y | 58.67 | 1602.8405 | 15 | 1.72 | 802.4247 | 2 | 17.54 | 1.0889 | 1.0961 | 54660 | 6.57e2 | 1 | 1 | 185 | 199 |  |  | DB Search |
| R.AYDFRETLPK.K | Y | 58.44 | 1238.6295 | 10 | 10.92 | 620.3256 | 2 | 15.17 | 0.9489 | 0.9562 | 42570 | 0 | 0 | 0 | 128 | 137 |  |  | DB Search |
| K.LPWEELWKPC(+57.02)IK.Y | Y | 56.99 | 1597.8326 | 12 | -1.86 | 533.6144 | 3 | 19.94 | 0.8793 | 0.8866 | 68833 | 2.81e2 | 1 | 1 | 170 | 181 | Carbamidomethylation |  | DB Search |
| total 3 peptides |  |  |  |  |  |  |  |  |  |  |  |  |  |  |  |  |  |  |  |
| --- | --- | --- | --- | --- | --- | --- | --- | --- | --- | --- | --- | --- | --- | --- | --- | --- | --- | --- | --- |

Best Unique PSM (Scan Sample\_9\_A1\_1\_3646.d:54660, m/z=802.4247, z=2, RT=17.54, ppm=-3.5):


TRINITY\_DN36\_c0\_g1\_i16.p1

back to list

  

| Protein Coverage
| Supporting Peptides
| Best Unique PSM
|

Protein Coverage:

Supporting Peptides:

| Peptide | Uniq | -10lgP | Mass | Length | ppm | m/z | z | RT | 1/k0 Start | 1/k0 End | Precursor ID | Area Sample 10 | #Feature | #Feature Sample 10 | Start | End | PTM | AScore | Found By |
| --- | --- | --- | --- | --- | --- | --- | --- | --- | --- | --- | --- | --- | --- | --- | --- | --- | --- | --- | --- |
| R.LGGIEVTQGNC(+57.02)ARK.Y | Y | 58.24 | 1501.7671 | 14 | 6.42 | 751.8917 | 2 | 12.66 | 1.0632 | 1.0705 | 28959 | 0 | 0 | 0 | 58 | 71 | Carbamidomethylation |  | DB Search |
| K.FETQIATK.D | Y | 57.09 | 936.4916 | 8 | 7.3 | 937.5009 | 1 | 11.88 | 1.1925 | 1.1997 | 24345 | 0 | 0 | 0 | 167 | 174 |  |  | DB Search |
| total 2 peptides |  |  |  |  |  |  |  |  |  |  |  |  |  |  |  |  |  |  |  |
| --- | --- | --- | --- | --- | --- | --- | --- | --- | --- | --- | --- | --- | --- | --- | --- | --- | --- | --- | --- |

Best Unique PSM (Scan Sample\_9\_A1\_1\_3646.d:28959, m/z=751.8917, z=2, RT=12.66, ppm=1.21):


TRINITY\_DN1819\_c0\_g1\_i5.p1

back to list

  

| Protein Coverage
| Supporting Peptides
| Best Unique PSM
|

Protein Coverage:

Supporting Peptides:

| Peptide | Uniq | -10lgP | Mass | Length | ppm | m/z | z | RT | 1/k0 Start | 1/k0 End | Precursor ID | Area Sample 10 | #Feature | #Feature Sample 10 | Start | End | PTM | AScore | Found By |
| --- | --- | --- | --- | --- | --- | --- | --- | --- | --- | --- | --- | --- | --- | --- | --- | --- | --- | --- | --- |
| K.ALADYIHSQDLK.M | Y | 56.12 | 1372.6986 | 12 | -6.12 | 687.3488 | 2 | 15.62 | 1.0061 | 1.0134 | 44810 | 0 | 0 | 0 | 105 | 116 |  |  | DB Search |
| total 1 peptides |  |  |  |  |  |  |  |  |  |  |  |  |  |  |  |  |  |  |  |
| --- | --- | --- | --- | --- | --- | --- | --- | --- | --- | --- | --- | --- | --- | --- | --- | --- | --- | --- | --- |

Best Unique PSM (Scan Sample\_9\_A1\_1\_3646.d:44810, m/z=687.3488, z=2, RT=15.62, ppm=-11.34):


TRINITY\_DN326\_c0\_g1\_i1.p1

back to list

  

| Protein Coverage
| Supporting Peptides
| Best Unique PSM
|

Protein Coverage:

Supporting Peptides:

| Peptide | Uniq | -10lgP | Mass | Length | ppm | m/z | z | RT | 1/k0 Start | 1/k0 End | Precursor ID | Area Sample 10 | #Feature | #Feature Sample 10 | Start | End | PTM | AScore | Found By |
| --- | --- | --- | --- | --- | --- | --- | --- | --- | --- | --- | --- | --- | --- | --- | --- | --- | --- | --- | --- |
| K.VNDSLLK.L | Y | 54.53 | 787.4439 | 7 | 3.52 | 788.4499 | 1 | 12.17 | 1.1506 | 1.1578 | 26013 | 1.48e3 | 1 | 1 | 109 | 115 |  |  | DB Search |
| M.T(+42.01)SNVRQNYHAETEAAINK.Q | Y | 46.72 | 2087.0032 | 18 | 2.3 | 696.673 | 3 | 13.03 | 0.9097 | 0.917 | 31105 | 9.26e2 | 1 | 1 | 2 | 19 | Acetylation (Protein N-term) | T1:Acetylation (Protein N-term):1000 | DB Search |
| total 2 peptides |  |  |  |  |  |  |  |  |  |  |  |  |  |  |  |  |  |  |  |
| --- | --- | --- | --- | --- | --- | --- | --- | --- | --- | --- | --- | --- | --- | --- | --- | --- | --- | --- | --- |

Best Unique PSM (Scan Sample\_9\_A1\_1\_3646.d:26013, m/z=788.4499, z=1, RT=12.17, ppm=-1.69):


TRINITY\_DN286\_c0\_g1\_i4.p1

back to list

  

| Protein Coverage
| Supporting Peptides
| Best Unique PSM
|

Protein Coverage:

Supporting Peptides:

| Peptide | Uniq | -10lgP | Mass | Length | ppm | m/z | z | RT | 1/k0 Start | 1/k0 End | Precursor ID | Area Sample 10 | #Feature | #Feature Sample 10 | Start | End | PTM | AScore | Found By |
| --- | --- | --- | --- | --- | --- | --- | --- | --- | --- | --- | --- | --- | --- | --- | --- | --- | --- | --- | --- |
| K.RYGQAMC(+57.02)QFMR.N | Y | 58.68 | 1446.6319 | 11 | -9.09 | 724.3129 | 2 | 17.23 | 0.9832 | 0.9904 | 52876 | 1.72e3 | 1 | 1 | 71 | 81 | Carbamidomethylation |  | DB Search |
| K.RYGQAM(+15.99)C(+57.02)QFM(+15.99)R.N | Y | 58.66 | 1478.6217 | 11 | -7.27 | 740.3089 | 2 | 12.91 | 0.9775 | 0.9848 | 30247 | 2.62e3 | 1 | 1 | 71 | 81 | Oxidation (M), Carbamidomethylation, Oxidation (M) | M6:Oxidation (M):1000 M10:Oxidation (M):1000 | DB Search |
| total 2 peptides |  |  |  |  |  |  |  |  |  |  |  |  |  |  |  |  |  |  |  |
| --- | --- | --- | --- | --- | --- | --- | --- | --- | --- | --- | --- | --- | --- | --- | --- | --- | --- | --- | --- |

Best Unique PSM (Scan Sample\_9\_A1\_1\_3646.d:52876, m/z=724.3129, z=2, RT=17.23, ppm=-14.31):


TRINITY\_DN3180\_c0\_g1\_i1.p1

back to list

  

| Protein Coverage
| Supporting Peptides
| Best Unique PSM
|

Protein Coverage:

Supporting Peptides:

| Peptide | Uniq | -10lgP | Mass | Length | ppm | m/z | z | RT | 1/k0 Start | 1/k0 End | Precursor ID | Area Sample 10 | #Feature | #Feature Sample 10 | Start | End | PTM | AScore | Found By |
| --- | --- | --- | --- | --- | --- | --- | --- | --- | --- | --- | --- | --- | --- | --- | --- | --- | --- | --- | --- |
| K.LVIGNSEGFKDVSK.K | Y | 57.48 | 1491.7933 | 14 | 5.49 | 746.9041 | 2 | 14.68 | 1.0821 | 1.0893 | 39965 | 6.3e2 | 1 | 1 | 819 | 832 |  |  | DB Search |
| total 1 peptides |  |  |  |  |  |  |  |  |  |  |  |  |  |  |  |  |  |  |  |
| --- | --- | --- | --- | --- | --- | --- | --- | --- | --- | --- | --- | --- | --- | --- | --- | --- | --- | --- | --- |

Best Unique PSM (Scan Sample\_9\_A1\_1\_3646.d:39965, m/z=746.9041, z=2, RT=14.68, ppm=0.27):


TRINITY\_DN495\_c0\_g1\_i5.p1

back to list

  

| Protein Coverage
| Supporting Peptides
| Best Unique PSM
|

Protein Coverage:

Supporting Peptides:

| Peptide | Uniq | -10lgP | Mass | Length | ppm | m/z | z | RT | 1/k0 Start | 1/k0 End | Precursor ID | Area Sample 10 | #Feature | #Feature Sample 10 | Start | End | PTM | AScore | Found By |
| --- | --- | --- | --- | --- | --- | --- | --- | --- | --- | --- | --- | --- | --- | --- | --- | --- | --- | --- | --- |
| C.SISC(+57.02)SKDEEIK.V | Y | 53.66 | 1294.6075 | 11 | 5.36 | 648.3111 | 2 | 10.85 | 0.98 | 0.9872 | 18283 | 0 | 0 | 0 | 273 | 283 | Carbamidomethylation |  | DB Search |
| total 1 peptides |  |  |  |  |  |  |  |  |  |  |  |  |  |  |  |  |  |  |  |
| --- | --- | --- | --- | --- | --- | --- | --- | --- | --- | --- | --- | --- | --- | --- | --- | --- | --- | --- | --- |

Best Unique PSM (Scan Sample\_9\_A1\_1\_3646.d:18283, m/z=648.3111, z=2, RT=10.85, ppm=0.14):


TRINITY\_DN135129\_c0\_g1\_i1.p1

back to list

  

| Protein Coverage
| Supporting Peptides
| Best Unique PSM
|

Protein Coverage:

Supporting Peptides:

| Peptide | Uniq | -10lgP | Mass | Length | ppm | m/z | z | RT | 1/k0 Start | 1/k0 End | Precursor ID | Area Sample 10 | #Feature | #Feature Sample 10 | Start | End | PTM | AScore | Found By |
| --- | --- | --- | --- | --- | --- | --- | --- | --- | --- | --- | --- | --- | --- | --- | --- | --- | --- | --- | --- |
| K.SDSSDIEKR.A | Y | 58.52 | 1035.4832 | 9 | -15.17 | 518.7383 | 2 | 11.07 | 0.8631 | 0.8704 | 19589 | 5.7e3 | 1 | 1 | 96 | 104 |  |  | DB Search |
| total 1 peptides |  |  |  |  |  |  |  |  |  |  |  |  |  |  |  |  |  |  |  |
| --- | --- | --- | --- | --- | --- | --- | --- | --- | --- | --- | --- | --- | --- | --- | --- | --- | --- | --- | --- |

Best Unique PSM (Scan Sample\_9\_A1\_1\_3646.d:19589, m/z=518.7383, z=2, RT=11.07, ppm=-20.39):


TRINITY\_DN332\_c0\_g1\_i1.p1

back to list

  

| Protein Coverage
| Supporting Peptides
| Best Unique PSM
|

Protein Coverage:

Supporting Peptides:

| Peptide | Uniq | -10lgP | Mass | Length | ppm | m/z | z | RT | 1/k0 Start | 1/k0 End | Precursor ID | Area Sample 10 | #Feature | #Feature Sample 10 | Start | End | PTM | AScore | Found By |
| --- | --- | --- | --- | --- | --- | --- | --- | --- | --- | --- | --- | --- | --- | --- | --- | --- | --- | --- | --- |
| K.DYGVLLEDAGHTLR.G | Y | 58.76 | 1557.7787 | 14 | -1.38 | 779.8915 | 2 | 18.23 | 1.1294 | 1.1366 | 58601 | 2.52e3 | 1 | 1 | 158 | 171 |  |  | DB Search |
| R.GLFIIDDKGVLR.Q | Y | 58.63 | 1344.7765 | 12 | -0.16 | 673.3919 | 2 | 18.68 | 1.0017 | 1.009 | 61308 | 2.43e3 | 1 | 1 | 172 | 183 |  |  | DB Search |
| K.IPLLADLTK.Q | Y | 57.95 | 982.6062 | 9 | -0.75 | 492.3075 | 2 | 19.36 | 0.8205 | 0.8279 | 65435 | 7.64e2 | 1 | 1 | 145 | 153 |  |  | DB Search |
| total 3 peptides |  |  |  |  |  |  |  |  |  |  |  |  |  |  |  |  |  |  |  |
| --- | --- | --- | --- | --- | --- | --- | --- | --- | --- | --- | --- | --- | --- | --- | --- | --- | --- | --- | --- |

Best Unique PSM (Scan Sample\_9\_A1\_1\_3646.d:58601, m/z=779.8915, z=2, RT=18.23, ppm=-6.59):


TRINITY\_DN123121\_c0\_g1\_i1.p1

back to list

  

| Protein Coverage
| Supporting Peptides
| Best Unique PSM
|

Protein Coverage:

Supporting Peptides:

| Peptide | Uniq | -10lgP | Mass | Length | ppm | m/z | z | RT | 1/k0 Start | 1/k0 End | Precursor ID | Area Sample 10 | #Feature | #Feature Sample 10 | Start | End | PTM | AScore | Found By |
| --- | --- | --- | --- | --- | --- | --- | --- | --- | --- | --- | --- | --- | --- | --- | --- | --- | --- | --- | --- |
| R.LTHQIEVTVSDREGK.S | Y | 58.36 | 1710.89 | 15 | 0.77 | 856.4485 | 2 | 12.72 | 1.115 | 1.1222 | 29273 | 1.52e2 | 1 | 1 | 94 | 108 |  |  | DB Search |
| total 1 peptides |  |  |  |  |  |  |  |  |  |  |  |  |  |  |  |  |  |  |  |
| --- | --- | --- | --- | --- | --- | --- | --- | --- | --- | --- | --- | --- | --- | --- | --- | --- | --- | --- | --- |

Best Unique PSM (Scan Sample\_9\_A1\_1\_3646.d:29273, m/z=856.4485, z=2, RT=12.72, ppm=-4.45):


TRINITY\_DN982\_c0\_g1\_i3.p1

back to list

  

| Protein Coverage
| Supporting Peptides
| Best Unique PSM
|

Protein Coverage:

Supporting Peptides:

| Peptide | Uniq | -10lgP | Mass | Length | ppm | m/z | z | RT | 1/k0 Start | 1/k0 End | Precursor ID | Area Sample 10 | #Feature | #Feature Sample 10 | Start | End | PTM | AScore | Found By |
| --- | --- | --- | --- | --- | --- | --- | --- | --- | --- | --- | --- | --- | --- | --- | --- | --- | --- | --- | --- |
| R.VLATDKDTLSSNK.Q | Y | 58.69 | 1390.7303 | 13 | 0.28 | 696.369 | 2 | 11.52 | 1.0158 | 1.023 | 22129 | 2.18e3 | 1 | 1 | 3827 | 3839 |  |  | DB Search |
| R.LVQNTPHNSIVTK.I | N | 58.68 | 1449.794 | 13 | 2.67 | 725.9024 | 2 | 11.69 | 1.0584 | 1.0656 | 23187 | 0 | 0 | 0 | 8569 | 8581 |  |  | DB Search |
| R.LIQTLNYETKK.E | N | 58.63 | 1349.7554 | 11 | 0.98 | 675.8821 | 2 | 13.61 | 1.0114 | 1.0186 | 34304 | 6.23e2 | 1 | 1 | 8319 | 8329 |  |  | DB Search |
| K.TSTQDVALNIRR.S | N | 58.59 | 1372.7423 | 12 | 5.06 | 687.3783 | 2 | 13.47 | 1.0001 | 1.0073 | 33513 | 2.51e3 | 1 | 1 | 9063 | 9074 |  |  | DB Search |
| K.APIGQSIVTVSAR.D | Y | 58.51 | 1297.7354 | 13 | -14.57 | 649.8621 | 2 | 14.13 | 0.967 | 0.9743 | 37121 | 0 | 0 | 0 | 4029 | 4041 |  |  | DB Search |
| K.AVDRGNPPQDSVNK.A | N | 58.36 | 1495.7379 | 14 | 5.97 | 499.5869 | 3 | 10.22 | 0.7836 | 0.7909 | 14437 | 8.69e2 | 1 | 1 | 5139 | 5152 |  |  | DB Search |
| K.TGAISLLASVDFETDRTHTAVVK.A | Y | 58.35 | 2430.2754 | 23 | 2.89 | 608.5747 | 4 | 18.85 | 0.8854 | 0.8927 | 62375 | 1.55e2 | 1 | 1 | 3335 | 3357 |  |  | DB Search |
| K.YVAIYPSDHLTVNR.D | N | 57.08 | 1646.8416 | 14 | -1.81 | 824.4223 | 2 | 15.52 | 1.1086 | 1.1158 | 44184 | 3.77e2 | 1 | 1 | 6254 | 6267 |  |  | DB Search |
| R.ITTSQILDHEQQK.L | N | 56.94 | 1539.7893 | 13 | 3.01 | 770.9002 | 2 | 13.83 | 1.0957 | 1.1029 | 35489 | 5.16e2 | 1 | 1 | 5434 | 5446 |  |  | DB Search |
| R.GNPPQDSVNK.A | N | 55.81 | 1054.5043 | 10 | 0.87 | 528.2571 | 2 | 9.76 | 0.8372 | 0.8445 | 11990 | 0 | 0 | 0 | 5143 | 5152 |  |  | DB Search |
| R.DRHVLTIQSR.G | N | 33.87 | 1223.6734 | 10 | 6.21 | 612.8446 | 2 | 11.95 | 0.9259 | 0.9331 | 24824 | 0 | 0 | 0 | 5030 | 5039 |  |  | DB Search |
| total 11 peptides |  |  |  |  |  |  |  |  |  |  |  |  |  |  |  |  |  |  |  |
| --- | --- | --- | --- | --- | --- | --- | --- | --- | --- | --- | --- | --- | --- | --- | --- | --- | --- | --- | --- |

Best Unique PSM (Scan Sample\_9\_A1\_1\_3646.d:22129, m/z=696.369, z=2, RT=11.52, ppm=-4.94):


TRINITY\_DN1534\_c2\_g1\_i1.p1

back to list

  

| Protein Coverage
| Supporting Peptides
| Best Unique PSM
|

Protein Coverage:

Supporting Peptides:

| Peptide | Uniq | -10lgP | Mass | Length | ppm | m/z | z | RT | 1/k0 Start | 1/k0 End | Precursor ID | Area Sample 10 | #Feature | #Feature Sample 10 | Start | End | PTM | AScore | Found By |
| --- | --- | --- | --- | --- | --- | --- | --- | --- | --- | --- | --- | --- | --- | --- | --- | --- | --- | --- | --- |
| K.KSDYSENEVEK.I | Y | 54.76 | 1326.5939 | 11 | 3.69 | 664.3032 | 2 | 10.50 | 0.9699 | 0.9771 | 16134 | 2.79e2 | 1 | 1 | 181 | 191 |  |  | DB Search |
| total 1 peptides |  |  |  |  |  |  |  |  |  |  |  |  |  |  |  |  |  |  |  |
| --- | --- | --- | --- | --- | --- | --- | --- | --- | --- | --- | --- | --- | --- | --- | --- | --- | --- | --- | --- |

Best Unique PSM (Scan Sample\_9\_A1\_1\_3646.d:16134, m/z=664.3032, z=2, RT=10.50, ppm=-1.53):


TRINITY\_DN160\_c0\_g1\_i1.p1

back to list

  

| Protein Coverage
| Supporting Peptides
| Best Unique PSM
|

Protein Coverage:

Supporting Peptides:

| Peptide | Uniq | -10lgP | Mass | Length | ppm | m/z | z | RT | 1/k0 Start | 1/k0 End | Precursor ID | Area Sample 10 | #Feature | #Feature Sample 10 | Start | End | PTM | AScore | Found By |
| --- | --- | --- | --- | --- | --- | --- | --- | --- | --- | --- | --- | --- | --- | --- | --- | --- | --- | --- | --- |
| K.LRLDFDHEAKLNMIFR.F | Y | 58.67 | 2017.0567 | 16 | 5.71 | 505.2717 | 4 | 18.48 | 0.8644 | 0.8716 | 60165 | 7.24e2 | 1 | 1 | 1007 | 1022 |  |  | DB Search |
| K.LRLDFDHEAKLNM(+15.99)IFR.F | Y | 58.6 | 2033.0516 | 16 | 3.46 | 509.2693 | 4 | 17.78 | 0.8562 | 0.8635 | 56012 | 9.75e2 | 1 | 1 | 1007 | 1022 | Oxidation (M) | M13:Oxidation (M):1000 | DB Search |
| R.LDFDHEAKLNMIFR.F | Y | 54.86 | 1747.8715 | 14 | -10.63 | 583.6219 | 3 | 18.34 | 0.8542 | 0.8615 | 59425 | 0 | 0 | 0 | 1009 | 1022 |  |  | DB Search |
| K.ELVM(+15.99)HEQSR.C | Y | 53.5 | 1143.5342 | 9 | 6.21 | 572.775 | 2 | 9.82 | 0.9028 | 0.9101 | 12284 | 6.13e2 | 1 | 1 | 1116 | 1124 | Oxidation (M) | M4:Oxidation (M):1000 | DB Search |
| K.VNQAC(+57.02)VR.T | Y | 30.94 | 845.4178 | 7 | 1.39 | 423.7145 | 2 | 9.76 | 0.7705 | 0.7779 | 11899 | 1.24e3 | 1 | 1 | 871 | 877 | Carbamidomethylation |  | DB Search |
| K.LRLDFDHEAK.L | Y | 29.24 | 1242.6356 | 10 | -1.44 | 415.2164 | 3 | 14.18 | 0.7282 | 0.7355 | 37433 | 0 | 0 | 0 | 1007 | 1016 |  |  | DB Search |
| total 6 peptides |  |  |  |  |  |  |  |  |  |  |  |  |  |  |  |  |  |  |  |
| --- | --- | --- | --- | --- | --- | --- | --- | --- | --- | --- | --- | --- | --- | --- | --- | --- | --- | --- | --- |

Best Unique PSM (Scan Sample\_9\_A1\_1\_3646.d:60165, m/z=505.2717, z=4, RT=18.48, ppm=0.49):


TRINITY\_DN90868\_c0\_g1\_i1.p3

back to list

  

| Protein Coverage
| Supporting Peptides
| Best Unique PSM
|

Protein Coverage:

Supporting Peptides:

| Peptide | Uniq | -10lgP | Mass | Length | ppm | m/z | z | RT | 1/k0 Start | 1/k0 End | Precursor ID | Area Sample 10 | #Feature | #Feature Sample 10 | Start | End | PTM | AScore | Found By |
| --- | --- | --- | --- | --- | --- | --- | --- | --- | --- | --- | --- | --- | --- | --- | --- | --- | --- | --- | --- |
| K.SC(+57.02)LLLQFTDKR.F | Y | 58.6 | 1379.7231 | 11 | 0.98 | 690.8659 | 2 | 17.62 | 1.0291 | 1.0363 | 55103 | 7.83e2 | 1 | 1 | 46 | 56 | Carbamidomethylation |  | DB Search |
| total 1 peptides |  |  |  |  |  |  |  |  |  |  |  |  |  |  |  |  |  |  |  |
| --- | --- | --- | --- | --- | --- | --- | --- | --- | --- | --- | --- | --- | --- | --- | --- | --- | --- | --- | --- |

Best Unique PSM (Scan Sample\_9\_A1\_1\_3646.d:55103, m/z=690.8659, z=2, RT=17.62, ppm=-4.24):


TRINITY\_DN602\_c0\_g1\_i3.p1

back to list

  

| Protein Coverage
| Supporting Peptides
| Best Unique PSM
|

Protein Coverage:

Supporting Peptides:

| Peptide | Uniq | -10lgP | Mass | Length | ppm | m/z | z | RT | 1/k0 Start | 1/k0 End | Precursor ID | Area Sample 10 | #Feature | #Feature Sample 10 | Start | End | PTM | AScore | Found By |
| --- | --- | --- | --- | --- | --- | --- | --- | --- | --- | --- | --- | --- | --- | --- | --- | --- | --- | --- | --- |
| K.EGFYVHEALAAGIVK.K | Y | 58.67 | 1602.8405 | 15 | 1.72 | 802.4247 | 2 | 17.54 | 1.0889 | 1.0961 | 54660 | 6.57e2 | 1 | 1 | 443 | 457 |  |  | DB Search |
| R.AYDFRETLPK.K | Y | 58.44 | 1238.6295 | 10 | 10.92 | 620.3256 | 2 | 15.17 | 0.9489 | 0.9562 | 42570 | 0 | 0 | 0 | 386 | 395 |  |  | DB Search |
| K.LPWEELWKPC(+57.02)IK.Y | Y | 56.99 | 1597.8326 | 12 | -1.86 | 533.6144 | 3 | 19.94 | 0.8793 | 0.8866 | 68833 | 2.81e2 | 1 | 1 | 428 | 439 | Carbamidomethylation |  | DB Search |
| total 3 peptides |  |  |  |  |  |  |  |  |  |  |  |  |  |  |  |  |  |  |  |
| --- | --- | --- | --- | --- | --- | --- | --- | --- | --- | --- | --- | --- | --- | --- | --- | --- | --- | --- | --- |

Best Unique PSM (Scan Sample\_9\_A1\_1\_3646.d:54660, m/z=802.4247, z=2, RT=17.54, ppm=-3.5):


TRINITY\_DN453\_c0\_g1\_i2.p1

back to list

  

| Protein Coverage
| Supporting Peptides
| Best Unique PSM
|

Protein Coverage:

Supporting Peptides:

| Peptide | Uniq | -10lgP | Mass | Length | ppm | m/z | z | RT | 1/k0 Start | 1/k0 End | Precursor ID | Area Sample 10 | #Feature | #Feature Sample 10 | Start | End | PTM | AScore | Found By |
| --- | --- | --- | --- | --- | --- | --- | --- | --- | --- | --- | --- | --- | --- | --- | --- | --- | --- | --- | --- |
| K.VSILVAC(+57.02)NKQDLTTAK.A | Y | 58.38 | 1759.9502 | 16 | -2.15 | 880.9759 | 2 | 14.70 | 1.1825 | 1.1897 | 40050 | 0 | 0 | 0 | 150 | 165 | Carbamidomethylation |  | DB Search |
| K.SQLEKEINNLRK.T | Y | 29.67 | 1470.8154 | 12 | -9.15 | 491.272 | 3 | 14.25 | 0.7742 | 0.7815 | 37755 | 0 | 0 | 0 | 172 | 183 |  |  | DB Search |
| total 2 peptides |  |  |  |  |  |  |  |  |  |  |  |  |  |  |  |  |  |  |  |
| --- | --- | --- | --- | --- | --- | --- | --- | --- | --- | --- | --- | --- | --- | --- | --- | --- | --- | --- | --- |

Best Unique PSM (Scan Sample\_9\_A1\_1\_3646.d:40050, m/z=880.9759, z=2, RT=14.70, ppm=-7.37):


TRINITY\_DN79126\_c0\_g1\_i1.p1

back to list

  

| Protein Coverage
| Supporting Peptides
| Best Unique PSM
|

Protein Coverage:

Supporting Peptides:

| Peptide | Uniq | -10lgP | Mass | Length | ppm | m/z | z | RT | 1/k0 Start | 1/k0 End | Precursor ID | Area Sample 10 | #Feature | #Feature Sample 10 | Start | End | PTM | AScore | Found By |
| --- | --- | --- | --- | --- | --- | --- | --- | --- | --- | --- | --- | --- | --- | --- | --- | --- | --- | --- | --- |
| R.AFLIEEQKLVVR.V | Y | 58.62 | 1443.8449 | 12 | 4.06 | 722.9289 | 2 | 17.34 | 1.048 | 1.0552 | 53425 | 1.97e3 | 1 | 1 | 92 | 103 |  |  | DB Search |
| total 1 peptides |  |  |  |  |  |  |  |  |  |  |  |  |  |  |  |  |  |  |  |
| --- | --- | --- | --- | --- | --- | --- | --- | --- | --- | --- | --- | --- | --- | --- | --- | --- | --- | --- | --- |

Best Unique PSM (Scan Sample\_9\_A1\_1\_3646.d:53425, m/z=722.9289, z=2, RT=17.34, ppm=-1.16):


TRINITY\_DN10563\_c0\_g1\_i1.p1

back to list

  

| Protein Coverage
| Supporting Peptides
| Best Unique PSM
|

Protein Coverage:

Supporting Peptides:

| Peptide | Uniq | -10lgP | Mass | Length | ppm | m/z | z | RT | 1/k0 Start | 1/k0 End | Precursor ID | Area Sample 10 | #Feature | #Feature Sample 10 | Start | End | PTM | AScore | Found By |
| --- | --- | --- | --- | --- | --- | --- | --- | --- | --- | --- | --- | --- | --- | --- | --- | --- | --- | --- | --- |
| K.VLTASDPEALKR.K | Y | 58.63 | 1298.7194 | 12 | -0.08 | 650.3635 | 2 | 12.95 | 0.9904 | 0.9977 | 30554 | 9.56e2 | 1 | 1 | 145 | 156 |  |  | DB Search |
| total 1 peptides |  |  |  |  |  |  |  |  |  |  |  |  |  |  |  |  |  |  |  |
| --- | --- | --- | --- | --- | --- | --- | --- | --- | --- | --- | --- | --- | --- | --- | --- | --- | --- | --- | --- |

Best Unique PSM (Scan Sample\_9\_A1\_1\_3646.d:30554, m/z=650.3635, z=2, RT=12.95, ppm=-5.3):


TRINITY\_DN1616\_c0\_g1\_i3.p1

back to list

  

| Protein Coverage
| Supporting Peptides
| Best Unique PSM
|

Protein Coverage:

Supporting Peptides:

| Peptide | Uniq | -10lgP | Mass | Length | ppm | m/z | z | RT | 1/k0 Start | 1/k0 End | Precursor ID | Area Sample 10 | #Feature | #Feature Sample 10 | Start | End | PTM | AScore | Found By |
| --- | --- | --- | --- | --- | --- | --- | --- | --- | --- | --- | --- | --- | --- | --- | --- | --- | --- | --- | --- |
| K.YVVMLQYYHDSDTAK.Y | Y | 52.09 | 1831.8451 | 15 | 5.48 | 916.9301 | 2 | 16.56 | 1.1813 | 1.1885 | 49447 | 0 | 0 | 0 | 1071 | 1085 |  |  | DB Search |
| R.YALNQIQPVTATVTAFKGDSK.I | Y | 44.58 | 2251.1848 | 21 | 9.46 | 751.4054 | 3 | 18.19 | 1.048 | 1.0552 | 58586 | 0 | 0 | 0 | 894 | 914 |  |  | DB Search |
| total 2 peptides |  |  |  |  |  |  |  |  |  |  |  |  |  |  |  |  |  |  |  |
| --- | --- | --- | --- | --- | --- | --- | --- | --- | --- | --- | --- | --- | --- | --- | --- | --- | --- | --- | --- |

Best Unique PSM (Scan Sample\_9\_A1\_1\_3646.d:49447, m/z=916.9301, z=2, RT=16.56, ppm=0.27):


TRINITY\_DN1319\_c0\_g1\_i1.p1

back to list

  

| Protein Coverage
| Supporting Peptides
| Best Unique PSM
|

Protein Coverage:

Supporting Peptides:

| Peptide | Uniq | -10lgP | Mass | Length | ppm | m/z | z | RT | 1/k0 Start | 1/k0 End | Precursor ID | Area Sample 10 | #Feature | #Feature Sample 10 | Start | End | PTM | AScore | Found By |
| --- | --- | --- | --- | --- | --- | --- | --- | --- | --- | --- | --- | --- | --- | --- | --- | --- | --- | --- | --- |
| K.GTHAAEIAHC(+57.02)VSR.R | Y | 58.65 | 1407.6677 | 13 | -14.3 | 704.8274 | 2 | 11.04 | 1.0307 | 1.0379 | 19410 | 0 | 0 | 0 | 91 | 103 | Carbamidomethylation |  | DB Search |
| total 1 peptides |  |  |  |  |  |  |  |  |  |  |  |  |  |  |  |  |  |  |  |
| --- | --- | --- | --- | --- | --- | --- | --- | --- | --- | --- | --- | --- | --- | --- | --- | --- | --- | --- | --- |

Best Unique PSM (Scan Sample\_9\_A1\_1\_3646.d:19410, m/z=704.8274, z=2, RT=11.04, ppm=-19.51):


TRINITY\_DN95387\_c0\_g1\_i1.p1

back to list

  

| Protein Coverage
| Supporting Peptides
| Best Unique PSM
|

Protein Coverage:

Supporting Peptides:

| Peptide | Uniq | -10lgP | Mass | Length | ppm | m/z | z | RT | 1/k0 Start | 1/k0 End | Precursor ID | Area Sample 10 | #Feature | #Feature Sample 10 | Start | End | PTM | AScore | Found By |
| --- | --- | --- | --- | --- | --- | --- | --- | --- | --- | --- | --- | --- | --- | --- | --- | --- | --- | --- | --- |
| M.A(+42.01)ATLKPYLNAVR.A | Y | 58.47 | 1357.7718 | 12 | -1.38 | 679.8887 | 2 | 18.21 | 1.046 | 1.0532 | 58634 | 2.41e3 | 1 | 1 | 2 | 13 | Acetylation (Protein N-term) | A1:Acetylation (Protein N-term):1000 | DB Search |
| total 1 peptides |  |  |  |  |  |  |  |  |  |  |  |  |  |  |  |  |  |  |  |
| --- | --- | --- | --- | --- | --- | --- | --- | --- | --- | --- | --- | --- | --- | --- | --- | --- | --- | --- | --- |

Best Unique PSM (Scan Sample\_9\_A1\_1\_3646.d:58634, m/z=679.8887, z=2, RT=18.21, ppm=-6.6):


TRINITY\_DN6044\_c0\_g2\_i1.p1

back to list

  

| Protein Coverage
| Supporting Peptides
| Best Unique PSM
|

Protein Coverage:

Supporting Peptides:

| Peptide | Uniq | -10lgP | Mass | Length | ppm | m/z | z | RT | 1/k0 Start | 1/k0 End | Precursor ID | Area Sample 10 | #Feature | #Feature Sample 10 | Start | End | PTM | AScore | Found By |
| --- | --- | --- | --- | --- | --- | --- | --- | --- | --- | --- | --- | --- | --- | --- | --- | --- | --- | --- | --- |
| K.LHSNLSPSTHETK.S | Y | 55.95 | 1449.7212 | 13 | 1.39 | 484.2458 | 3 | 10.33 | 0.8274 | 0.8348 | 15220 | 0 | 0 | 0 | 553 | 565 |  |  | DB Search |
| total 1 peptides |  |  |  |  |  |  |  |  |  |  |  |  |  |  |  |  |  |  |  |
| --- | --- | --- | --- | --- | --- | --- | --- | --- | --- | --- | --- | --- | --- | --- | --- | --- | --- | --- | --- |

Best Unique PSM (Scan Sample\_9\_A1\_1\_3646.d:15220, m/z=484.2458, z=3, RT=10.33, ppm=-3.83):


TRINITY\_DN495\_c0\_g1\_i2.p1

back to list

  

| Protein Coverage
| Supporting Peptides
| Best Unique PSM
|

Protein Coverage:

Supporting Peptides:

| Peptide | Uniq | -10lgP | Mass | Length | ppm | m/z | z | RT | 1/k0 Start | 1/k0 End | Precursor ID | Area Sample 10 | #Feature | #Feature Sample 10 | Start | End | PTM | AScore | Found By |
| --- | --- | --- | --- | --- | --- | --- | --- | --- | --- | --- | --- | --- | --- | --- | --- | --- | --- | --- | --- |
| R.SC(+57.02)TNPAPANGGK.A | Y | 58.68 | 1172.5244 | 12 | 3.23 | 587.2683 | 2 | 9.42 | 0.8352 | 0.8425 | 10441 | 8.36e2 | 1 | 1 | 499 | 510 | Carbamidomethylation |  | DB Search |
| total 1 peptides |  |  |  |  |  |  |  |  |  |  |  |  |  |  |  |  |  |  |  |
| --- | --- | --- | --- | --- | --- | --- | --- | --- | --- | --- | --- | --- | --- | --- | --- | --- | --- | --- | --- |

Best Unique PSM (Scan Sample\_9\_A1\_1\_3646.d:10441, m/z=587.2683, z=2, RT=9.42, ppm=-1.99):


TRINITY\_DN319\_c2\_g1\_i3.p1

back to list

  

| Protein Coverage
| Supporting Peptides
| Best Unique PSM
|

Protein Coverage:

Supporting Peptides:

| Peptide | Uniq | -10lgP | Mass | Length | ppm | m/z | z | RT | 1/k0 Start | 1/k0 End | Precursor ID | Area Sample 10 | #Feature | #Feature Sample 10 | Start | End | PTM | AScore | Found By |
| --- | --- | --- | --- | --- | --- | --- | --- | --- | --- | --- | --- | --- | --- | --- | --- | --- | --- | --- | --- |
| K.KAQYYEAYETIIKR.H | Y | 58.69 | 1774.9254 | 14 | -1.1 | 592.6453 | 3 | 15.71 | 0.8623 | 0.8696 | 45168 | 1.12e3 | 1 | 1 | 2534 | 2547 |  |  | DB Search |
| R.HLAISNILK.D | Y | 57.84 | 1007.6127 | 9 | 0.83 | 504.8114 | 2 | 18.74 | 0.8652 | 0.8725 | 61860 | 3.25e2 | 1 | 1 | 2548 | 2556 |  |  | DB Search |
| total 2 peptides |  |  |  |  |  |  |  |  |  |  |  |  |  |  |  |  |  |  |  |
| --- | --- | --- | --- | --- | --- | --- | --- | --- | --- | --- | --- | --- | --- | --- | --- | --- | --- | --- | --- |

Best Unique PSM (Scan Sample\_9\_A1\_1\_3646.d:45168, m/z=592.6453, z=3, RT=15.71, ppm=-6.32):


TRINITY\_DN691\_c0\_g1\_i2.p1

back to list

  

| Protein Coverage
| Supporting Peptides
| Best Unique PSM
|

Protein Coverage:

Supporting Peptides:

| Peptide | Uniq | -10lgP | Mass | Length | ppm | m/z | z | RT | 1/k0 Start | 1/k0 End | Precursor ID | Area Sample 10 | #Feature | #Feature Sample 10 | Start | End | PTM | AScore | Found By |
| --- | --- | --- | --- | --- | --- | --- | --- | --- | --- | --- | --- | --- | --- | --- | --- | --- | --- | --- | --- |
| R.AYGQDLGQPGSC(+57.02)LKR.F | Y | 58.26 | 1648.7991 | 15 | 0.22 | 825.4027 | 2 | 13.38 | 1.0737 | 1.0809 | 33011 | 5.76e2 | 1 | 1 | 1480 | 1494 | Carbamidomethylation |  | DB Search |
| total 1 peptides |  |  |  |  |  |  |  |  |  |  |  |  |  |  |  |  |  |  |  |
| --- | --- | --- | --- | --- | --- | --- | --- | --- | --- | --- | --- | --- | --- | --- | --- | --- | --- | --- | --- |

Best Unique PSM (Scan Sample\_9\_A1\_1\_3646.d:33011, m/z=825.4027, z=2, RT=13.38, ppm=-5):


TRINITY\_DN8707\_c0\_g1\_i2.p1

back to list

  

| Protein Coverage
| Supporting Peptides
| Best Unique PSM
|

Protein Coverage:

Supporting Peptides:

| Peptide | Uniq | -10lgP | Mass | Length | ppm | m/z | z | RT | 1/k0 Start | 1/k0 End | Precursor ID | Area Sample 10 | #Feature | #Feature Sample 10 | Start | End | PTM | AScore | Found By |
| --- | --- | --- | --- | --- | --- | --- | --- | --- | --- | --- | --- | --- | --- | --- | --- | --- | --- | --- | --- |
| K.ALLSLEYHEKR.C | Y | 58.63 | 1357.7354 | 11 | 0.51 | 679.8718 | 2 | 14.42 | 1.0275 | 1.0347 | 38581 | 1.27e3 | 1 | 1 | 124 | 134 |  |  | DB Search |
| total 1 peptides |  |  |  |  |  |  |  |  |  |  |  |  |  |  |  |  |  |  |  |
| --- | --- | --- | --- | --- | --- | --- | --- | --- | --- | --- | --- | --- | --- | --- | --- | --- | --- | --- | --- |

Best Unique PSM (Scan Sample\_9\_A1\_1\_3646.d:38581, m/z=679.8718, z=2, RT=14.42, ppm=-4.71):


TRINITY\_DN801\_c0\_g1\_i9.p2

back to list

  

| Protein Coverage
| Supporting Peptides
| Best Unique PSM
|

Protein Coverage:

Supporting Peptides:

| Peptide | Uniq | -10lgP | Mass | Length | ppm | m/z | z | RT | 1/k0 Start | 1/k0 End | Precursor ID | Area Sample 10 | #Feature | #Feature Sample 10 | Start | End | PTM | AScore | Found By |
| --- | --- | --- | --- | --- | --- | --- | --- | --- | --- | --- | --- | --- | --- | --- | --- | --- | --- | --- | --- |
| K.VFDRDGNGFISAAELR.H | Y | 51.43 | 1765.8747 | 16 | -4.93 | 883.9357 | 2 | 19.74 | 1.1274 | 1.1346 | 67630 | 2.9e3 | 1 | 1 | 117 | 132 |  |  | DB Search |
| total 1 peptides |  |  |  |  |  |  |  |  |  |  |  |  |  |  |  |  |  |  |  |
| --- | --- | --- | --- | --- | --- | --- | --- | --- | --- | --- | --- | --- | --- | --- | --- | --- | --- | --- | --- |

Best Unique PSM (Scan Sample\_9\_A1\_1\_3646.d:67630, m/z=883.9357, z=2, RT=19.74, ppm=-10.14):


TRINITY\_DN96\_c0\_g1\_i4.p1

back to list

  

| Protein Coverage
| Supporting Peptides
| Best Unique PSM
|

Protein Coverage:

Supporting Peptides:

| Peptide | Uniq | -10lgP | Mass | Length | ppm | m/z | z | RT | 1/k0 Start | 1/k0 End | Precursor ID | Area Sample 10 | #Feature | #Feature Sample 10 | Start | End | PTM | AScore | Found By |
| --- | --- | --- | --- | --- | --- | --- | --- | --- | --- | --- | --- | --- | --- | --- | --- | --- | --- | --- | --- |
| K.KVEQVQTDSR.D | Y | 53.61 | 1188.6098 | 10 | 5.11 | 595.3121 | 2 | 9.53 | 0.8725 | 0.8798 | 10904 | 2.14e2 | 1 | 1 | 153 | 162 |  |  | DB Search |
| total 1 peptides |  |  |  |  |  |  |  |  |  |  |  |  |  |  |  |  |  |  |  |
| --- | --- | --- | --- | --- | --- | --- | --- | --- | --- | --- | --- | --- | --- | --- | --- | --- | --- | --- | --- |

Best Unique PSM (Scan Sample\_9\_A1\_1\_3646.d:10904, m/z=595.3121, z=2, RT=9.53, ppm=-0.11):


TRINITY\_DN147947\_c0\_g1\_i1.p1

back to list

  

| Protein Coverage
| Supporting Peptides
| Best Unique PSM
|

Protein Coverage:

Supporting Peptides:

| Peptide | Uniq | -10lgP | Mass | Length | ppm | m/z | z | RT | 1/k0 Start | 1/k0 End | Precursor ID | Area Sample 10 | #Feature | #Feature Sample 10 | Start | End | PTM | AScore | Found By |
| --- | --- | --- | --- | --- | --- | --- | --- | --- | --- | --- | --- | --- | --- | --- | --- | --- | --- | --- | --- |
| R.INVDAAFHDLVR.A | Y | 58.62 | 1368.715 | 12 | 1.55 | 685.3622 | 2 | 18.65 | 1.023 | 1.0303 | 61210 | 8.3e2 | 1 | 1 | 138 | 149 |  |  | DB Search |
| R.SSFDEIHKFHTQILR.V | Y | 58.03 | 1856.9533 | 15 | -0.42 | 619.9882 | 3 | 15.43 | 0.866 | 0.8733 | 43848 | 2.72e2 | 1 | 1 | 75 | 89 |  |  | DB Search |
| total 2 peptides |  |  |  |  |  |  |  |  |  |  |  |  |  |  |  |  |  |  |  |
| --- | --- | --- | --- | --- | --- | --- | --- | --- | --- | --- | --- | --- | --- | --- | --- | --- | --- | --- | --- |

Best Unique PSM (Scan Sample\_9\_A1\_1\_3646.d:61210, m/z=685.3622, z=2, RT=18.65, ppm=-3.67):


TRINITY\_DN577\_c0\_g1\_i1.p1

back to list

  

| Protein Coverage
| Supporting Peptides
| Best Unique PSM
|

Protein Coverage:

Supporting Peptides:

| Peptide | Uniq | -10lgP | Mass | Length | ppm | m/z | z | RT | 1/k0 Start | 1/k0 End | Precursor ID | Area Sample 10 | #Feature | #Feature Sample 10 | Start | End | PTM | AScore | Found By |
| --- | --- | --- | --- | --- | --- | --- | --- | --- | --- | --- | --- | --- | --- | --- | --- | --- | --- | --- | --- |
| K.ASQGIYPLHDC(+57.02)YIR.K | Y | 52.39 | 1691.809 | 14 | 0.15 | 846.9075 | 2 | 15.77 | 1.1394 | 1.1466 | 45376 | 5.88e2 | 1 | 1 | 206 | 219 | Carbamidomethylation |  | DB Search |
| R.VFEIAQADLNKDEVAFR.K | Y | 49.81 | 1964.0003 | 17 | 6.11 | 983.0083 | 2 | 18.07 | 1.2467 | 1.2539 | 57754 | 0 | 0 | 0 | 72 | 88 |  |  | DB Search |
| total 2 peptides |  |  |  |  |  |  |  |  |  |  |  |  |  |  |  |  |  |  |  |
| --- | --- | --- | --- | --- | --- | --- | --- | --- | --- | --- | --- | --- | --- | --- | --- | --- | --- | --- | --- |

Best Unique PSM (Scan Sample\_9\_A1\_1\_3646.d:45376, m/z=846.9075, z=2, RT=15.77, ppm=-5.06):


TRINITY\_DN687\_c0\_g1\_i1.p1

back to list

  

| Protein Coverage
| Supporting Peptides
| Best Unique PSM
|

Protein Coverage:

Supporting Peptides:

| Peptide | Uniq | -10lgP | Mass | Length | ppm | m/z | z | RT | 1/k0 Start | 1/k0 End | Precursor ID | Area Sample 10 | #Feature | #Feature Sample 10 | Start | End | PTM | AScore | Found By |
| --- | --- | --- | --- | --- | --- | --- | --- | --- | --- | --- | --- | --- | --- | --- | --- | --- | --- | --- | --- |
| K.VGINYQPPTVVPGGDLAK.V | Y | 50.54 | 1823.9781 | 18 | -3.14 | 912.9887 | 2 | 17.25 | 1.1186 | 1.1258 | 53075 | 0 | 0 | 0 | 353 | 370 |  |  | DB Search |
| total 1 peptides |  |  |  |  |  |  |  |  |  |  |  |  |  |  |  |  |  |  |  |
| --- | --- | --- | --- | --- | --- | --- | --- | --- | --- | --- | --- | --- | --- | --- | --- | --- | --- | --- | --- |

Best Unique PSM (Scan Sample\_9\_A1\_1\_3646.d:53075, m/z=912.9887, z=2, RT=17.25, ppm=-8.35):


TRINITY\_DN6920\_c0\_g1\_i3.p1

back to list

  

| Protein Coverage
| Supporting Peptides
| Best Unique PSM
|

Protein Coverage:

Supporting Peptides:

| Peptide | Uniq | -10lgP | Mass | Length | ppm | m/z | z | RT | 1/k0 Start | 1/k0 End | Precursor ID | Area Sample 10 | #Feature | #Feature Sample 10 | Start | End | PTM | AScore | Found By |
| --- | --- | --- | --- | --- | --- | --- | --- | --- | --- | --- | --- | --- | --- | --- | --- | --- | --- | --- | --- |
| K.VAC(+57.02)IGAWHPAR.V | Y | 58.63 | 1236.6186 | 11 | 3 | 619.3152 | 2 | 13.23 | 0.9461 | 0.9533 | 32140 | 5.07e2 | 1 | 1 | 258 | 268 | Carbamidomethylation |  | DB Search |
| R.VLVHTQQK.L | Y | 54.09 | 951.5501 | 8 | 3.81 | 476.7817 | 2 | 9.56 | 0.8303 | 0.8376 | 11062 | 2.36e2 | 1 | 1 | 169 | 176 |  |  | DB Search |
| total 2 peptides |  |  |  |  |  |  |  |  |  |  |  |  |  |  |  |  |  |  |  |
| --- | --- | --- | --- | --- | --- | --- | --- | --- | --- | --- | --- | --- | --- | --- | --- | --- | --- | --- | --- |

Best Unique PSM (Scan Sample\_9\_A1\_1\_3646.d:32140, m/z=619.3152, z=2, RT=13.23, ppm=-2.22):


TRINITY\_DN2885\_c0\_g1\_i2.p1

back to list

  

| Protein Coverage
| Supporting Peptides
| Best Unique PSM
|

Protein Coverage:

Supporting Peptides:

| Peptide | Uniq | -10lgP | Mass | Length | ppm | m/z | z | RT | 1/k0 Start | 1/k0 End | Precursor ID | Area Sample 10 | #Feature | #Feature Sample 10 | Start | End | PTM | AScore | Found By |
| --- | --- | --- | --- | --- | --- | --- | --- | --- | --- | --- | --- | --- | --- | --- | --- | --- | --- | --- | --- |
| K.VTRPHGASGVVR.A | Y | 58.63 | 1234.6894 | 12 | 0.38 | 412.5684 | 3 | 10.08 | 0.8197 | 0.827 | 13527 | 3.84e3 | 1 | 1 | 77 | 88 |  |  | DB Search |
| total 1 peptides |  |  |  |  |  |  |  |  |  |  |  |  |  |  |  |  |  |  |  |
| --- | --- | --- | --- | --- | --- | --- | --- | --- | --- | --- | --- | --- | --- | --- | --- | --- | --- | --- | --- |

Best Unique PSM (Scan Sample\_9\_A1\_1\_3646.d:13527, m/z=412.5684, z=3, RT=10.08, ppm=-4.85):


TRINITY\_DN6294\_c0\_g3\_i2.p1

back to list

  

| Protein Coverage
| Supporting Peptides
| Best Unique PSM
|

Protein Coverage:

Supporting Peptides:

| Peptide | Uniq | -10lgP | Mass | Length | ppm | m/z | z | RT | 1/k0 Start | 1/k0 End | Precursor ID | Area Sample 10 | #Feature | #Feature Sample 10 | Start | End | PTM | AScore | Found By |
| --- | --- | --- | --- | --- | --- | --- | --- | --- | --- | --- | --- | --- | --- | --- | --- | --- | --- | --- | --- |
| K.LVVIADIVDQKR.L | Y | 58.63 | 1367.8136 | 12 | -3.46 | 684.9081 | 2 | 17.50 | 1.0435 | 1.0508 | 54455 | 3.87e2 | 1 | 1 | 24 | 35 |  |  | DB Search |
| total 1 peptides |  |  |  |  |  |  |  |  |  |  |  |  |  |  |  |  |  |  |  |
| --- | --- | --- | --- | --- | --- | --- | --- | --- | --- | --- | --- | --- | --- | --- | --- | --- | --- | --- | --- |

Best Unique PSM (Scan Sample\_9\_A1\_1\_3646.d:54455, m/z=684.9081, z=2, RT=17.50, ppm=-8.68):


TRINITY\_DN6397\_c0\_g2\_i3.p1

back to list

  

| Protein Coverage
| Supporting Peptides
| Best Unique PSM
|

Protein Coverage:

Supporting Peptides:

| Peptide | Uniq | -10lgP | Mass | Length | ppm | m/z | z | RT | 1/k0 Start | 1/k0 End | Precursor ID | Area Sample 10 | #Feature | #Feature Sample 10 | Start | End | PTM | AScore | Found By |
| --- | --- | --- | --- | --- | --- | --- | --- | --- | --- | --- | --- | --- | --- | --- | --- | --- | --- | --- | --- |
| K.K(+42.01)LSAEVANGR.L | Y | 58.76 | 1085.5829 | 10 | -2.46 | 543.7946 | 2 | 12.92 | 0.8567 | 0.864 | 30461 | 2.87e3 | 1 | 1 | 12 | 21 | Acetylation (Protein N-term) | K1:Acetylation (Protein N-term):1000 | DB Search |
| total 1 peptides |  |  |  |  |  |  |  |  |  |  |  |  |  |  |  |  |  |  |  |
| --- | --- | --- | --- | --- | --- | --- | --- | --- | --- | --- | --- | --- | --- | --- | --- | --- | --- | --- | --- |

Best Unique PSM (Scan Sample\_9\_A1\_1\_3646.d:30461, m/z=543.7946, z=2, RT=12.92, ppm=-7.68):


TRINITY\_DN12\_c0\_g2\_i1.p1

back to list

  

| Protein Coverage
| Supporting Peptides
| Best Unique PSM
|

Protein Coverage:

Supporting Peptides:

| Peptide | Uniq | -10lgP | Mass | Length | ppm | m/z | z | RT | 1/k0 Start | 1/k0 End | Precursor ID | Area Sample 10 | #Feature | #Feature Sample 10 | Start | End | PTM | AScore | Found By |
| --- | --- | --- | --- | --- | --- | --- | --- | --- | --- | --- | --- | --- | --- | --- | --- | --- | --- | --- | --- |
| K.GFGM(+15.99)TAELANKR.A | Y | 55.59 | 1309.6448 | 12 | 2.54 | 655.8279 | 2 | 12.79 | 1.0102 | 1.0174 | 29776 | 4.76e2 | 1 | 1 | 7 | 18 | Oxidation (M) | M4:Oxidation (M):1000 | DB Search |
| R.LGPDEASENKR.E | Y | 51.39 | 1214.5891 | 11 | 5.41 | 608.3019 | 2 | 10.47 | 0.9166 | 0.9238 | 15408 | 2.93e3 | 1 | 1 | 153 | 163 |  |  | DB Search |
| total 2 peptides |  |  |  |  |  |  |  |  |  |  |  |  |  |  |  |  |  |  |  |
| --- | --- | --- | --- | --- | --- | --- | --- | --- | --- | --- | --- | --- | --- | --- | --- | --- | --- | --- | --- |

Best Unique PSM (Scan Sample\_9\_A1\_1\_3646.d:29776, m/z=655.8279, z=2, RT=12.79, ppm=-2.68):


TRINITY\_DN16548\_c0\_g1\_i4.p1

back to list

  

| Protein Coverage
| Supporting Peptides
| Best Unique PSM
|

Protein Coverage:

Supporting Peptides:

| Peptide | Uniq | -10lgP | Mass | Length | ppm | m/z | z | RT | 1/k0 Start | 1/k0 End | Precursor ID | Area Sample 10 | #Feature | #Feature Sample 10 | Start | End | PTM | AScore | Found By |
| --- | --- | --- | --- | --- | --- | --- | --- | --- | --- | --- | --- | --- | --- | --- | --- | --- | --- | --- | --- |
| K.APGDYGWRPPLLATDDAALK.T | Y | 58.71 | 2126.0796 | 20 | 0.61 | 709.6972 | 3 | 18.99 | 0.8883 | 0.8955 | 63354 | 4.18e3 | 1 | 1 | 125 | 144 |  |  | DB Search |
| total 1 peptides |  |  |  |  |  |  |  |  |  |  |  |  |  |  |  |  |  |  |  |
| --- | --- | --- | --- | --- | --- | --- | --- | --- | --- | --- | --- | --- | --- | --- | --- | --- | --- | --- | --- |

Best Unique PSM (Scan Sample\_9\_A1\_1\_3646.d:63354, m/z=709.6972, z=3, RT=18.99, ppm=-4.61):


TRINITY\_DN2113\_c0\_g1\_i1.p1

back to list

  

| Protein Coverage
| Supporting Peptides
| Best Unique PSM
|

Protein Coverage:

Supporting Peptides:

| Peptide | Uniq | -10lgP | Mass | Length | ppm | m/z | z | RT | 1/k0 Start | 1/k0 End | Precursor ID | Area Sample 10 | #Feature | #Feature Sample 10 | Start | End | PTM | AScore | Found By |
| --- | --- | --- | --- | --- | --- | --- | --- | --- | --- | --- | --- | --- | --- | --- | --- | --- | --- | --- | --- |
| K.AHNLTEDVIFWK.W | Y | 58.72 | 1471.7459 | 12 | 0.34 | 736.8766 | 2 | 18.72 | 1.0061 | 1.0134 | 61618 | 3.8e2 | 1 | 1 | 101 | 112 |  |  | DB Search |
| total 1 peptides |  |  |  |  |  |  |  |  |  |  |  |  |  |  |  |  |  |  |  |
| --- | --- | --- | --- | --- | --- | --- | --- | --- | --- | --- | --- | --- | --- | --- | --- | --- | --- | --- | --- |

Best Unique PSM (Scan Sample\_9\_A1\_1\_3646.d:61618, m/z=736.8766, z=2, RT=18.72, ppm=-4.88):


TRINITY\_DN1222\_c0\_g1\_i2.p1

back to list

  

| Protein Coverage
| Supporting Peptides
| Best Unique PSM
|

Protein Coverage:

Supporting Peptides:

| Peptide | Uniq | -10lgP | Mass | Length | ppm | m/z | z | RT | 1/k0 Start | 1/k0 End | Precursor ID | Area Sample 10 | #Feature | #Feature Sample 10 | Start | End | PTM | AScore | Found By |
| --- | --- | --- | --- | --- | --- | --- | --- | --- | --- | --- | --- | --- | --- | --- | --- | --- | --- | --- | --- |
| K.IVKPGGEQPDELEK.S | Y | 33.83 | 1537.7987 | 14 | -1.81 | 769.9012 | 2 | 12.41 | 1.0259 | 1.0331 | 27466 | 8.43e2 | 1 | 1 | 30 | 43 |  |  | DB Search |
| total 1 peptides |  |  |  |  |  |  |  |  |  |  |  |  |  |  |  |  |  |  |  |
| --- | --- | --- | --- | --- | --- | --- | --- | --- | --- | --- | --- | --- | --- | --- | --- | --- | --- | --- | --- |

Best Unique PSM (Scan Sample\_9\_A1\_1\_3646.d:27466, m/z=769.9012, z=2, RT=12.41, ppm=-7.02):


TRINITY\_DN194146\_c0\_g1\_i1.p1

back to list

  

| Protein Coverage
| Supporting Peptides
| Best Unique PSM
|

Protein Coverage:

Supporting Peptides:

| Peptide | Uniq | -10lgP | Mass | Length | ppm | m/z | z | RT | 1/k0 Start | 1/k0 End | Precursor ID | Area Sample 10 | #Feature | #Feature Sample 10 | Start | End | PTM | AScore | Found By |
| --- | --- | --- | --- | --- | --- | --- | --- | --- | --- | --- | --- | --- | --- | --- | --- | --- | --- | --- | --- |
| K.TVLASQTVTIPEDVSVSVNGR.T | Y | 49.68 | 2171.1433 | 21 | 1.54 | 1086.575 | 2 | 17.81 | 1.2284 | 1.2356 | 56273 | 0 | 0 | 0 | 3 | 23 |  |  | DB Search |
| total 1 peptides |  |  |  |  |  |  |  |  |  |  |  |  |  |  |  |  |  |  |  |
| --- | --- | --- | --- | --- | --- | --- | --- | --- | --- | --- | --- | --- | --- | --- | --- | --- | --- | --- | --- |

Best Unique PSM (Scan Sample\_9\_A1\_1\_3646.d:56273, m/z=1086.575, z=2, RT=17.81, ppm=-3.68):


TRINITY\_DN41662\_c0\_g1\_i2.p1

back to list

  

| Protein Coverage
| Supporting Peptides
| Best Unique PSM
|

Protein Coverage:

Supporting Peptides:

| Peptide | Uniq | -10lgP | Mass | Length | ppm | m/z | z | RT | 1/k0 Start | 1/k0 End | Precursor ID | Area Sample 10 | #Feature | #Feature Sample 10 | Start | End | PTM | AScore | Found By |
| --- | --- | --- | --- | --- | --- | --- | --- | --- | --- | --- | --- | --- | --- | --- | --- | --- | --- | --- | --- |
| K.LLAQTTLRNELGTK.N | Y | 58.48 | 1556.8886 | 14 | 4.16 | 779.4507 | 2 | 15.18 | 1.109 | 1.1162 | 42504 | 8.19e2 | 1 | 1 | 147 | 160 |  |  | DB Search |
| total 1 peptides |  |  |  |  |  |  |  |  |  |  |  |  |  |  |  |  |  |  |  |
| --- | --- | --- | --- | --- | --- | --- | --- | --- | --- | --- | --- | --- | --- | --- | --- | --- | --- | --- | --- |

Best Unique PSM (Scan Sample\_9\_A1\_1\_3646.d:42504, m/z=779.4507, z=2, RT=15.18, ppm=-1.05):


TRINITY\_DN6132\_c0\_g1\_i1.p1

back to list

  

| Protein Coverage
| Supporting Peptides
| Best Unique PSM
|

Protein Coverage:

Supporting Peptides:

| Peptide | Uniq | -10lgP | Mass | Length | ppm | m/z | z | RT | 1/k0 Start | 1/k0 End | Precursor ID | Area Sample 10 | #Feature | #Feature Sample 10 | Start | End | PTM | AScore | Found By |
| --- | --- | --- | --- | --- | --- | --- | --- | --- | --- | --- | --- | --- | --- | --- | --- | --- | --- | --- | --- |
| R.VGDIVDVKGNGAIQK.G | Y | 58.99 | 1511.8307 | 15 | -4.74 | 756.9151 | 2 | 13.61 | 1.0697 | 1.0769 | 34346 | 0 | 0 | 0 | 36 | 50 |  |  | DB Search |
| total 1 peptides |  |  |  |  |  |  |  |  |  |  |  |  |  |  |  |  |  |  |  |
| --- | --- | --- | --- | --- | --- | --- | --- | --- | --- | --- | --- | --- | --- | --- | --- | --- | --- | --- | --- |

Best Unique PSM (Scan Sample\_9\_A1\_1\_3646.d:34346, m/z=756.9151, z=2, RT=13.61, ppm=-9.96):


TRINITY\_DN329\_c0\_g1\_i1.p1

back to list

  

| Protein Coverage
| Supporting Peptides
| Best Unique PSM
|

Protein Coverage:

Supporting Peptides:

| Peptide | Uniq | -10lgP | Mass | Length | ppm | m/z | z | RT | 1/k0 Start | 1/k0 End | Precursor ID | Area Sample 10 | #Feature | #Feature Sample 10 | Start | End | PTM | AScore | Found By |
| --- | --- | --- | --- | --- | --- | --- | --- | --- | --- | --- | --- | --- | --- | --- | --- | --- | --- | --- | --- |
| K.GLKVDFDTTFAPVTGKK.T | Y | 58.67 | 1822.9828 | 17 | 2.09 | 912.4958 | 2 | 17.12 | 1.1802 | 1.1873 | 52158 | 1.37e3 | 1 | 1 | 93 | 109 |  |  | DB Search |
| K.GLKVDFDTTFAPVTGK.K | Y | 58.63 | 1694.8879 | 16 | 3.65 | 848.4499 | 2 | 18.14 | 1.0957 | 1.1029 | 58184 | 1.23e3 | 1 | 1 | 93 | 108 |  |  | DB Search |
| K.SFHFGVIKLEGK.T | Y | 58.33 | 1360.7503 | 12 | 0.06 | 681.3789 | 2 | 16.99 | 1.0291 | 1.0363 | 51505 | 4.07e3 | 2 | 2 | 20 | 31 |  |  | DB Search |
| K.VDFDTTFAPVTGKK.T | Y | 58.32 | 1524.7824 | 14 | 4.95 | 763.3983 | 2 | 16.54 | 1.052 | 1.0592 | 49415 | 0 | 0 | 0 | 96 | 109 |  |  | DB Search |
| K.EGDLVIHSGITDAVK.Y | Y | 39.37 | 1552.8096 | 15 | 2.72 | 777.4102 | 2 | 15.38 | 1.0648 | 1.0721 | 43595 | 0 | 0 | 0 | 174 | 188 |  |  | DB Search |
| total 5 peptides |  |  |  |  |  |  |  |  |  |  |  |  |  |  |  |  |  |  |  |
| --- | --- | --- | --- | --- | --- | --- | --- | --- | --- | --- | --- | --- | --- | --- | --- | --- | --- | --- | --- |

Best Unique PSM (Scan Sample\_9\_A1\_1\_3646.d:52158, m/z=912.4958, z=2, RT=17.12, ppm=-3.13):


TRINITY\_DN33328\_c0\_g1\_i1.p1

back to list

  

| Protein Coverage
| Supporting Peptides
| Best Unique PSM
|

Protein Coverage:

Supporting Peptides:

| Peptide | Uniq | -10lgP | Mass | Length | ppm | m/z | z | RT | 1/k0 Start | 1/k0 End | Precursor ID | Area Sample 10 | #Feature | #Feature Sample 10 | Start | End | PTM | AScore | Found By |
| --- | --- | --- | --- | --- | --- | --- | --- | --- | --- | --- | --- | --- | --- | --- | --- | --- | --- | --- | --- |
| K.IKVELPIKEILTK.V | Y | 55.47 | 1522.9698 | 13 | 1.94 | 508.6622 | 3 | 18.17 | 0.8019 | 0.8092 | 58412 | 2.52e2 | 1 | 1 | 431 | 443 |  |  | DB Search |
| total 1 peptides |  |  |  |  |  |  |  |  |  |  |  |  |  |  |  |  |  |  |  |
| --- | --- | --- | --- | --- | --- | --- | --- | --- | --- | --- | --- | --- | --- | --- | --- | --- | --- | --- | --- |

Best Unique PSM (Scan Sample\_9\_A1\_1\_3646.d:58412, m/z=508.6622, z=3, RT=18.17, ppm=-3.28):


TRINITY\_DN1559\_c0\_g1\_i1.p1

back to list

  

| Protein Coverage
| Supporting Peptides
| Best Unique PSM
|

Protein Coverage:

Supporting Peptides:

| Peptide | Uniq | -10lgP | Mass | Length | ppm | m/z | z | RT | 1/k0 Start | 1/k0 End | Precursor ID | Area Sample 10 | #Feature | #Feature Sample 10 | Start | End | PTM | AScore | Found By |
| --- | --- | --- | --- | --- | --- | --- | --- | --- | --- | --- | --- | --- | --- | --- | --- | --- | --- | --- | --- |
| R.GVAGAGVLAGFDKFK.E | Y | 58.63 | 1435.7823 | 15 | -0.62 | 718.8942 | 2 | 18.25 | 1.0873 | 1.0945 | 58813 | 1.77e3 | 1 | 1 | 282 | 296 |  |  | DB Search |
| total 1 peptides |  |  |  |  |  |  |  |  |  |  |  |  |  |  |  |  |  |  |  |
| --- | --- | --- | --- | --- | --- | --- | --- | --- | --- | --- | --- | --- | --- | --- | --- | --- | --- | --- | --- |

Best Unique PSM (Scan Sample\_9\_A1\_1\_3646.d:58813, m/z=718.8942, z=2, RT=18.25, ppm=-5.84):


TRINITY\_DN250\_c0\_g1\_i6.p1

back to list

  

| Protein Coverage
| Supporting Peptides
| Best Unique PSM
|

Protein Coverage:

Supporting Peptides:

| Peptide | Uniq | -10lgP | Mass | Length | ppm | m/z | z | RT | 1/k0 Start | 1/k0 End | Precursor ID | Area Sample 10 | #Feature | #Feature Sample 10 | Start | End | PTM | AScore | Found By |
| --- | --- | --- | --- | --- | --- | --- | --- | --- | --- | --- | --- | --- | --- | --- | --- | --- | --- | --- | --- |
| K.VGINYQPPTVVPGGDLAK.V | Y | 50.54 | 1823.9781 | 18 | -3.14 | 912.9887 | 2 | 17.25 | 1.1186 | 1.1258 | 53075 | 0 | 0 | 0 | 353 | 370 |  |  | DB Search |
| total 1 peptides |  |  |  |  |  |  |  |  |  |  |  |  |  |  |  |  |  |  |  |
| --- | --- | --- | --- | --- | --- | --- | --- | --- | --- | --- | --- | --- | --- | --- | --- | --- | --- | --- | --- |

Best Unique PSM (Scan Sample\_9\_A1\_1\_3646.d:53075, m/z=912.9887, z=2, RT=17.25, ppm=-8.35):


TRINITY\_DN18025\_c0\_g1\_i1.p1

back to list

  

| Protein Coverage
| Supporting Peptides
| Best Unique PSM
|

Protein Coverage:

Supporting Peptides:

| Peptide | Uniq | -10lgP | Mass | Length | ppm | m/z | z | RT | 1/k0 Start | 1/k0 End | Precursor ID | Area Sample 10 | #Feature | #Feature Sample 10 | Start | End | PTM | AScore | Found By |
| --- | --- | --- | --- | --- | --- | --- | --- | --- | --- | --- | --- | --- | --- | --- | --- | --- | --- | --- | --- |
| R.SLGNHGQGIDR.E | Y | 58.68 | 1152.5636 | 11 | 3.33 | 577.288 | 2 | 10.63 | 0.9024 | 0.9097 | 16884 | 0 | 0 | 0 | 114 | 124 |  |  | DB Search |
| total 1 peptides |  |  |  |  |  |  |  |  |  |  |  |  |  |  |  |  |  |  |  |
| --- | --- | --- | --- | --- | --- | --- | --- | --- | --- | --- | --- | --- | --- | --- | --- | --- | --- | --- | --- |

Best Unique PSM (Scan Sample\_9\_A1\_1\_3646.d:16884, m/z=577.288, z=2, RT=10.63, ppm=-1.89):


TRINITY\_DN2963\_c0\_g1\_i1.p1

back to list

  

| Protein Coverage
| Supporting Peptides
| Best Unique PSM
|

Protein Coverage:

Supporting Peptides:

| Peptide | Uniq | -10lgP | Mass | Length | ppm | m/z | z | RT | 1/k0 Start | 1/k0 End | Precursor ID | Area Sample 10 | #Feature | #Feature Sample 10 | Start | End | PTM | AScore | Found By |
| --- | --- | --- | --- | --- | --- | --- | --- | --- | --- | --- | --- | --- | --- | --- | --- | --- | --- | --- | --- |
| M.S(+42.01)ATGNLVTVLRK.L | Y | 58.74 | 1299.751 | 12 | -3.1 | 650.8774 | 2 | 17.07 | 0.9977 | 1.0049 | 51993 | 6.03e2 | 1 | 1 | 2 | 13 | Acetylation (Protein N-term) | S1:Acetylation (Protein N-term):1000 | DB Search |
| total 1 peptides |  |  |  |  |  |  |  |  |  |  |  |  |  |  |  |  |  |  |  |
| --- | --- | --- | --- | --- | --- | --- | --- | --- | --- | --- | --- | --- | --- | --- | --- | --- | --- | --- | --- |

Best Unique PSM (Scan Sample\_9\_A1\_1\_3646.d:51993, m/z=650.8774, z=2, RT=17.07, ppm=-8.32):


TRINITY\_DN35672\_c0\_g1\_i1.p1

back to list

  

| Protein Coverage
| Supporting Peptides
| Best Unique PSM
|

Protein Coverage:

Supporting Peptides:

| Peptide | Uniq | -10lgP | Mass | Length | ppm | m/z | z | RT | 1/k0 Start | 1/k0 End | Precursor ID | Area Sample 10 | #Feature | #Feature Sample 10 | Start | End | PTM | AScore | Found By |
| --- | --- | --- | --- | --- | --- | --- | --- | --- | --- | --- | --- | --- | --- | --- | --- | --- | --- | --- | --- |
| R.SPNLC(+57.02)QC(+57.02)NNGR.F | Y | 29.34 | 1318.5507 | 11 | 8.63 | 660.2849 | 2 | 10.99 | 0.9259 | 0.9331 | 19117 | 0 | 0 | 0 | 115 | 125 | Carbamidomethylation, Carbamidomethylation |  | DB Search |
| total 1 peptides |  |  |  |  |  |  |  |  |  |  |  |  |  |  |  |  |  |  |  |
| --- | --- | --- | --- | --- | --- | --- | --- | --- | --- | --- | --- | --- | --- | --- | --- | --- | --- | --- | --- |

Best Unique PSM (Scan Sample\_9\_A1\_1\_3646.d:19117, m/z=660.2849, z=2, RT=10.99, ppm=3.41):


TRINITY\_DN1676\_c0\_g1\_i6.p1

back to list

  

| Protein Coverage
| Supporting Peptides
| Best Unique PSM
|

Protein Coverage:

Supporting Peptides:

| Peptide | Uniq | -10lgP | Mass | Length | ppm | m/z | z | RT | 1/k0 Start | 1/k0 End | Precursor ID | Area Sample 10 | #Feature | #Feature Sample 10 | Start | End | PTM | AScore | Found By |
| --- | --- | --- | --- | --- | --- | --- | --- | --- | --- | --- | --- | --- | --- | --- | --- | --- | --- | --- | --- |
| K.RFEEAKDTIENK.L | Y | 58.41 | 1478.7365 | 12 | 1.43 | 493.9176 | 3 | 11.59 | 0.8014 | 0.8088 | 22585 | 4.14e2 | 1 | 2 | 1560 | 1571 |  |  | DB Search |
| total 1 peptides |  |  |  |  |  |  |  |  |  |  |  |  |  |  |  |  |  |  |  |
| --- | --- | --- | --- | --- | --- | --- | --- | --- | --- | --- | --- | --- | --- | --- | --- | --- | --- | --- | --- |

Best Unique PSM (Scan Sample\_9\_A1\_1\_3646.d:22585, m/z=493.9176, z=3, RT=11.59, ppm=-3.79):


TRINITY\_DN5188\_c0\_g1\_i8.p1

back to list

  

| Protein Coverage
| Supporting Peptides
| Best Unique PSM
|

Protein Coverage:

Supporting Peptides:

| Peptide | Uniq | -10lgP | Mass | Length | ppm | m/z | z | RT | 1/k0 Start | 1/k0 End | Precursor ID | Area Sample 10 | #Feature | #Feature Sample 10 | Start | End | PTM | AScore | Found By |
| --- | --- | --- | --- | --- | --- | --- | --- | --- | --- | --- | --- | --- | --- | --- | --- | --- | --- | --- | --- |
| K.LRQEQLKEER.K | Y | 56.74 | 1327.7208 | 10 | 3.91 | 443.5803 | 3 | 10.26 | 0.7949 | 0.8023 | 14744 | 0 | 0 | 0 | 334 | 343 |  |  | DB Search |
| total 1 peptides |  |  |  |  |  |  |  |  |  |  |  |  |  |  |  |  |  |  |  |
| --- | --- | --- | --- | --- | --- | --- | --- | --- | --- | --- | --- | --- | --- | --- | --- | --- | --- | --- | --- |

Best Unique PSM (Scan Sample\_9\_A1\_1\_3646.d:14744, m/z=443.5803, z=3, RT=10.26, ppm=-1.31):


TRINITY\_DN3036\_c0\_g1\_i8.p1

back to list

  

| Protein Coverage
| Supporting Peptides
| Best Unique PSM
|

Protein Coverage:

Supporting Peptides:

| Peptide | Uniq | -10lgP | Mass | Length | ppm | m/z | z | RT | 1/k0 Start | 1/k0 End | Precursor ID | Area Sample 10 | #Feature | #Feature Sample 10 | Start | End | PTM | AScore | Found By |
| --- | --- | --- | --- | --- | --- | --- | --- | --- | --- | --- | --- | --- | --- | --- | --- | --- | --- | --- | --- |
| R.AGHGAVTSPQTQR.G | Y | 58.87 | 1308.6534 | 13 | 3.49 | 437.2243 | 3 | 9.73 | 0.7127 | 0.72 | 11628 | 3.81e3 | 1 | 1 | 309 | 321 |  |  | DB Search |
| R.GTSNGGLVVGTIIKPK.L | Y | 58.68 | 1539.8984 | 16 | 0.87 | 770.9531 | 2 | 15.69 | 1.1254 | 1.1326 | 44972 | 2.46e3 | 1 | 1 | 173 | 188 |  |  | DB Search |
| total 2 peptides |  |  |  |  |  |  |  |  |  |  |  |  |  |  |  |  |  |  |  |
| --- | --- | --- | --- | --- | --- | --- | --- | --- | --- | --- | --- | --- | --- | --- | --- | --- | --- | --- | --- |

Best Unique PSM (Scan Sample\_9\_A1\_1\_3646.d:11628, m/z=437.2243, z=3, RT=9.73, ppm=-1.73):


TRINITY\_DN761\_c0\_g1\_i2.p1

back to list

  

| Protein Coverage
| Supporting Peptides
| Best Unique PSM
|

Protein Coverage:

Supporting Peptides:

| Peptide | Uniq | -10lgP | Mass | Length | ppm | m/z | z | RT | 1/k0 Start | 1/k0 End | Precursor ID | Area Sample 10 | #Feature | #Feature Sample 10 | Start | End | PTM | AScore | Found By |
| --- | --- | --- | --- | --- | --- | --- | --- | --- | --- | --- | --- | --- | --- | --- | --- | --- | --- | --- | --- |
| K.TRIEDLETVISQTQDHR.H | Y | 58.82 | 2040.0236 | 17 | 4.24 | 681.0145 | 3 | 17.36 | 0.9594 | 0.9666 | 53642 | 9.24e2 | 1 | 1 | 260 | 276 |  |  | DB Search |
| K.EIDKADIAIVDTGENPDAPHPR.E | Y | 51.38 | 2372.1607 | 22 | 1.38 | 791.7245 | 3 | 14.90 | 1.0383 | 1.0456 | 41054 | 3.22e2 | 1 | 1 | 71 | 92 |  |  | DB Search |
| K.FVNEVRR.C | Y | 33.88 | 918.5035 | 7 | 0.09 | 460.2567 | 2 | 12.65 | 0.812 | 0.8193 | 28973 | 2.9e3 | 1 | 1 | 51 | 57 |  |  | DB Search |
| total 3 peptides |  |  |  |  |  |  |  |  |  |  |  |  |  |  |  |  |  |  |  |
| --- | --- | --- | --- | --- | --- | --- | --- | --- | --- | --- | --- | --- | --- | --- | --- | --- | --- | --- | --- |

Best Unique PSM (Scan Sample\_9\_A1\_1\_3646.d:53642, m/z=681.0145, z=3, RT=17.36, ppm=-0.98):


TRINITY\_DN909\_c0\_g1\_i1.p1

back to list

  

| Protein Coverage
| Supporting Peptides
| Best Unique PSM
|

Protein Coverage:

Supporting Peptides:

| Peptide | Uniq | -10lgP | Mass | Length | ppm | m/z | z | RT | 1/k0 Start | 1/k0 End | Precursor ID | Area Sample 10 | #Feature | #Feature Sample 10 | Start | End | PTM | AScore | Found By |
| --- | --- | --- | --- | --- | --- | --- | --- | --- | --- | --- | --- | --- | --- | --- | --- | --- | --- | --- | --- |
| K.LLAQTTLRNELGTK.N | Y | 58.48 | 1556.8886 | 14 | 4.16 | 779.4507 | 2 | 15.18 | 1.109 | 1.1162 | 42504 | 8.19e2 | 1 | 1 | 175 | 188 |  |  | DB Search |
| total 1 peptides |  |  |  |  |  |  |  |  |  |  |  |  |  |  |  |  |  |  |  |
| --- | --- | --- | --- | --- | --- | --- | --- | --- | --- | --- | --- | --- | --- | --- | --- | --- | --- | --- | --- |

Best Unique PSM (Scan Sample\_9\_A1\_1\_3646.d:42504, m/z=779.4507, z=2, RT=15.18, ppm=-1.05):


TRINITY\_DN195631\_c0\_g1\_i1.p2

back to list

  

| Protein Coverage
| Supporting Peptides
| Best Unique PSM
|

Protein Coverage:

Supporting Peptides:

| Peptide | Uniq | -10lgP | Mass | Length | ppm | m/z | z | RT | 1/k0 Start | 1/k0 End | Precursor ID | Area Sample 10 | #Feature | #Feature Sample 10 | Start | End | PTM | AScore | Found By |
| --- | --- | --- | --- | --- | --- | --- | --- | --- | --- | --- | --- | --- | --- | --- | --- | --- | --- | --- | --- |
| K.IQDKEGIPPDQQR.L | Y | 58.25 | 1522.7739 | 13 | 2.55 | 762.3922 | 2 | 11.03 | 1.0343 | 1.0415 | 18957 | 1.53e3 | 1 | 1 | 122 | 134 |  |  | DB Search |
| total 1 peptides |  |  |  |  |  |  |  |  |  |  |  |  |  |  |  |  |  |  |  |
| --- | --- | --- | --- | --- | --- | --- | --- | --- | --- | --- | --- | --- | --- | --- | --- | --- | --- | --- | --- |

Best Unique PSM (Scan Sample\_9\_A1\_1\_3646.d:18957, m/z=762.3922, z=2, RT=11.03, ppm=-2.67):


TRINITY\_DN941\_c0\_g1\_i9.p1

back to list

  

| Protein Coverage
| Supporting Peptides
| Best Unique PSM
|

Protein Coverage:

Supporting Peptides:

| Peptide | Uniq | -10lgP | Mass | Length | ppm | m/z | z | RT | 1/k0 Start | 1/k0 End | Precursor ID | Area Sample 10 | #Feature | #Feature Sample 10 | Start | End | PTM | AScore | Found By |
| --- | --- | --- | --- | --- | --- | --- | --- | --- | --- | --- | --- | --- | --- | --- | --- | --- | --- | --- | --- |
| R.RPSGKEEEVDVK.L | Y | 56.24 | 1371.6994 | 12 | -0.94 | 458.2376 | 3 | 10.07 | 0.8055 | 0.8128 | 13647 | 0 | 0 | 0 | 1807 | 1818 |  |  | DB Search |
| K.VTVKPSSDASK.V | Y | 55.45 | 1117.5979 | 11 | 11.33 | 373.5422 | 3 | 9.60 | 0.7379 | 0.7453 | 11202 | 6.95e2 | 1 | 1 | 922 | 932 |  |  | DB Search |
| K.AHGPGLEGGVAGQPC(+57.02)R.F | Y | 50.18 | 1561.7419 | 16 | 3.5 | 781.8769 | 2 | 12.18 | 1.0291 | 1.0363 | 25949 | 8.92e2 | 1 | 1 | 2812 | 2827 | Carbamidomethylation |  | DB Search |
| total 3 peptides |  |  |  |  |  |  |  |  |  |  |  |  |  |  |  |  |  |  |  |
| --- | --- | --- | --- | --- | --- | --- | --- | --- | --- | --- | --- | --- | --- | --- | --- | --- | --- | --- | --- |

Best Unique PSM (Scan Sample\_9\_A1\_1\_3646.d:13647, m/z=458.2376, z=3, RT=10.07, ppm=-6.17):


TRINITY\_DN1566\_c0\_g1\_i1.p1

back to list

  

| Protein Coverage
| Supporting Peptides
| Best Unique PSM
|

Protein Coverage:

Supporting Peptides:

| Peptide | Uniq | -10lgP | Mass | Length | ppm | m/z | z | RT | 1/k0 Start | 1/k0 End | Precursor ID | Area Sample 10 | #Feature | #Feature Sample 10 | Start | End | PTM | AScore | Found By |
| --- | --- | --- | --- | --- | --- | --- | --- | --- | --- | --- | --- | --- | --- | --- | --- | --- | --- | --- | --- |
| R.IKLEHLITELVHQR.D | Y | 53.33 | 1728.0046 | 14 | -1.28 | 577.0051 | 3 | 18.38 | 0.8668 | 0.8741 | 59724 | 0 | 0 | 0 | 1781 | 1794 |  |  | DB Search |
| total 1 peptides |  |  |  |  |  |  |  |  |  |  |  |  |  |  |  |  |  |  |  |
| --- | --- | --- | --- | --- | --- | --- | --- | --- | --- | --- | --- | --- | --- | --- | --- | --- | --- | --- | --- |

Best Unique PSM (Scan Sample\_9\_A1\_1\_3646.d:59724, m/z=577.0051, z=3, RT=18.38, ppm=-6.5):


TRINITY\_DN1183\_c0\_g1\_i1.p1

back to list

  

| Protein Coverage
| Supporting Peptides
| Best Unique PSM
|

Protein Coverage:

Supporting Peptides:

| Peptide | Uniq | -10lgP | Mass | Length | ppm | m/z | z | RT | 1/k0 Start | 1/k0 End | Precursor ID | Area Sample 10 | #Feature | #Feature Sample 10 | Start | End | PTM | AScore | Found By |
| --- | --- | --- | --- | --- | --- | --- | --- | --- | --- | --- | --- | --- | --- | --- | --- | --- | --- | --- | --- |
| K.DLRGDENVKWELSK.M | Y | 58.59 | 1687.8529 | 14 | -12.31 | 563.6151 | 3 | 15.03 | 0.8205 | 0.8279 | 41851 | 0 | 0 | 0 | 120 | 133 |  |  | DB Search |
| total 1 peptides |  |  |  |  |  |  |  |  |  |  |  |  |  |  |  |  |  |  |  |
| --- | --- | --- | --- | --- | --- | --- | --- | --- | --- | --- | --- | --- | --- | --- | --- | --- | --- | --- | --- |

Best Unique PSM (Scan Sample\_9\_A1\_1\_3646.d:41851, m/z=563.6151, z=3, RT=15.03, ppm=-17.53):


TRINITY\_DN16548\_c0\_g1\_i3.p1

back to list

  

| Protein Coverage
| Supporting Peptides
| Best Unique PSM
|

Protein Coverage:

Supporting Peptides:

| Peptide | Uniq | -10lgP | Mass | Length | ppm | m/z | z | RT | 1/k0 Start | 1/k0 End | Precursor ID | Area Sample 10 | #Feature | #Feature Sample 10 | Start | End | PTM | AScore | Found By |
| --- | --- | --- | --- | --- | --- | --- | --- | --- | --- | --- | --- | --- | --- | --- | --- | --- | --- | --- | --- |
| K.APGDYGWRPPLLATDDAALK.T | Y | 58.71 | 2126.0796 | 20 | 0.61 | 709.6972 | 3 | 18.99 | 0.8883 | 0.8955 | 63354 | 4.18e3 | 1 | 1 | 125 | 144 |  |  | DB Search |
| total 1 peptides |  |  |  |  |  |  |  |  |  |  |  |  |  |  |  |  |  |  |  |
| --- | --- | --- | --- | --- | --- | --- | --- | --- | --- | --- | --- | --- | --- | --- | --- | --- | --- | --- | --- |

Best Unique PSM (Scan Sample\_9\_A1\_1\_3646.d:63354, m/z=709.6972, z=3, RT=18.99, ppm=-4.61):


TRINITY\_DN571\_c0\_g1\_i1.p1

back to list

  

| Protein Coverage
| Supporting Peptides
| Best Unique PSM
|

Protein Coverage:

Supporting Peptides:

| Peptide | Uniq | -10lgP | Mass | Length | ppm | m/z | z | RT | 1/k0 Start | 1/k0 End | Precursor ID | Area Sample 10 | #Feature | #Feature Sample 10 | Start | End | PTM | AScore | Found By |
| --- | --- | --- | --- | --- | --- | --- | --- | --- | --- | --- | --- | --- | --- | --- | --- | --- | --- | --- | --- |
| K.M(+15.99)KINVNEIFHDLVR.Q | Y | 55.96 | 1742.9137 | 14 | 2.05 | 581.9767 | 3 | 19.57 | 0.887 | 0.8943 | 66610 | 2.46e2 | 1 | 1 | 150 | 163 | Oxidation (M) | M1:Oxidation (M):1000 | DB Search |
| total 1 peptides |  |  |  |  |  |  |  |  |  |  |  |  |  |  |  |  |  |  |  |
| --- | --- | --- | --- | --- | --- | --- | --- | --- | --- | --- | --- | --- | --- | --- | --- | --- | --- | --- | --- |

Best Unique PSM (Scan Sample\_9\_A1\_1\_3646.d:66610, m/z=581.9767, z=3, RT=19.57, ppm=-3.17):


TRINITY\_DN464\_c0\_g1\_i4.p1

back to list

  

| Protein Coverage
| Supporting Peptides
| Best Unique PSM
|

Protein Coverage:

Supporting Peptides:

| Peptide | Uniq | -10lgP | Mass | Length | ppm | m/z | z | RT | 1/k0 Start | 1/k0 End | Precursor ID | Area Sample 10 | #Feature | #Feature Sample 10 | Start | End | PTM | AScore | Found By |
| --- | --- | --- | --- | --- | --- | --- | --- | --- | --- | --- | --- | --- | --- | --- | --- | --- | --- | --- | --- |
| R.LLEFVGHDVLR.L | Y | 58.36 | 1296.719 | 11 | 0.41 | 649.3636 | 2 | 17.77 | 1.0045 | 1.0118 | 56109 | 0 | 0 | 0 | 279 | 289 |  |  | DB Search |
| total 1 peptides |  |  |  |  |  |  |  |  |  |  |  |  |  |  |  |  |  |  |  |
| --- | --- | --- | --- | --- | --- | --- | --- | --- | --- | --- | --- | --- | --- | --- | --- | --- | --- | --- | --- |

Best Unique PSM (Scan Sample\_9\_A1\_1\_3646.d:56109, m/z=649.3636, z=2, RT=17.77, ppm=-4.81):


TRINITY\_DN152\_c0\_g1\_i4.p1

back to list

  

| Protein Coverage
| Supporting Peptides
| Best Unique PSM
|

Protein Coverage:

Supporting Peptides:

| Peptide | Uniq | -10lgP | Mass | Length | ppm | m/z | z | RT | 1/k0 Start | 1/k0 End | Precursor ID | Area Sample 10 | #Feature | #Feature Sample 10 | Start | End | PTM | AScore | Found By |
| --- | --- | --- | --- | --- | --- | --- | --- | --- | --- | --- | --- | --- | --- | --- | --- | --- | --- | --- | --- |
| K.IQDKEGIPPDQQR.L | Y | 58.25 | 1522.7739 | 13 | 2.55 | 762.3922 | 2 | 11.03 | 1.0343 | 1.0415 | 18957 | 1.53e3 | 1 | 1 | 40 | 52 |  |  | DB Search |
| total 1 peptides |  |  |  |  |  |  |  |  |  |  |  |  |  |  |  |  |  |  |  |
| --- | --- | --- | --- | --- | --- | --- | --- | --- | --- | --- | --- | --- | --- | --- | --- | --- | --- | --- | --- |

Best Unique PSM (Scan Sample\_9\_A1\_1\_3646.d:18957, m/z=762.3922, z=2, RT=11.03, ppm=-2.67):


TRINITY\_DN1143\_c0\_g1\_i1.p1

back to list

  

| Protein Coverage
| Supporting Peptides
| Best Unique PSM
|

Protein Coverage:

Supporting Peptides:

| Peptide | Uniq | -10lgP | Mass | Length | ppm | m/z | z | RT | 1/k0 Start | 1/k0 End | Precursor ID | Area Sample 10 | #Feature | #Feature Sample 10 | Start | End | PTM | AScore | Found By |
| --- | --- | --- | --- | --- | --- | --- | --- | --- | --- | --- | --- | --- | --- | --- | --- | --- | --- | --- | --- |
| K.GLIAQAIDHAASR.T | Y | 57.83 | 1321.7102 | 13 | 6.4 | 661.8632 | 2 | 16.09 | 1.0077 | 1.015 | 47163 | 0 | 0 | 0 | 328 | 340 |  |  | DB Search |
| total 1 peptides |  |  |  |  |  |  |  |  |  |  |  |  |  |  |  |  |  |  |  |
| --- | --- | --- | --- | --- | --- | --- | --- | --- | --- | --- | --- | --- | --- | --- | --- | --- | --- | --- | --- |

Best Unique PSM (Scan Sample\_9\_A1\_1\_3646.d:47163, m/z=661.8632, z=2, RT=16.09, ppm=1.18):


TRINITY\_DN201860\_c0\_g1\_i1.p1

back to list

  

| Protein Coverage
| Supporting Peptides
| Best Unique PSM
|

Protein Coverage:

Supporting Peptides:

| Peptide | Uniq | -10lgP | Mass | Length | ppm | m/z | z | RT | 1/k0 Start | 1/k0 End | Precursor ID | Area Sample 10 | #Feature | #Feature Sample 10 | Start | End | PTM | AScore | Found By |
| --- | --- | --- | --- | --- | --- | --- | --- | --- | --- | --- | --- | --- | --- | --- | --- | --- | --- | --- | --- |
| K.VVC(+57.02)STTSGSATGSK.D | Y | 58.24 | 1340.6242 | 14 | -7.3 | 671.311 | 2 | 9.64 | 0.9263 | 0.9335 | 11439 | 0 | 0 | 0 | 7 | 20 | Carbamidomethylation |  | DB Search |
| total 1 peptides |  |  |  |  |  |  |  |  |  |  |  |  |  |  |  |  |  |  |  |
| --- | --- | --- | --- | --- | --- | --- | --- | --- | --- | --- | --- | --- | --- | --- | --- | --- | --- | --- | --- |

Best Unique PSM (Scan Sample\_9\_A1\_1\_3646.d:11439, m/z=671.311, z=2, RT=9.64, ppm=-12.52):


TRINITY\_DN76040\_c0\_g1\_i1.p1

back to list

  

| Protein Coverage
| Supporting Peptides
| Best Unique PSM
|

Protein Coverage:

Supporting Peptides:

| Peptide | Uniq | -10lgP | Mass | Length | ppm | m/z | z | RT | 1/k0 Start | 1/k0 End | Precursor ID | Area Sample 10 | #Feature | #Feature Sample 10 | Start | End | PTM | AScore | Found By |
| --- | --- | --- | --- | --- | --- | --- | --- | --- | --- | --- | --- | --- | --- | --- | --- | --- | --- | --- | --- |
| K.VSILESLDKWER.L | Y | 56.75 | 1473.7827 | 12 | -3.08 | 492.2641 | 3 | 19.61 | 0.7868 | 0.7941 | 66845 | 3.21e2 | 2 | 2 | 254 | 265 |  |  | DB Search |
| total 1 peptides |  |  |  |  |  |  |  |  |  |  |  |  |  |  |  |  |  |  |  |
| --- | --- | --- | --- | --- | --- | --- | --- | --- | --- | --- | --- | --- | --- | --- | --- | --- | --- | --- | --- |

Best Unique PSM (Scan Sample\_9\_A1\_1\_3646.d:66845, m/z=491.7484, z=2, RT=19.62, ppm=-8.3):


TRINITY\_DN36\_c0\_g1\_i3.p1

back to list

  

| Protein Coverage
| Supporting Peptides
| Best Unique PSM
|

Protein Coverage:

Supporting Peptides:

| Peptide | Uniq | -10lgP | Mass | Length | ppm | m/z | z | RT | 1/k0 Start | 1/k0 End | Precursor ID | Area Sample 10 | #Feature | #Feature Sample 10 | Start | End | PTM | AScore | Found By |
| --- | --- | --- | --- | --- | --- | --- | --- | --- | --- | --- | --- | --- | --- | --- | --- | --- | --- | --- | --- |
| R.LGGIEVTQGNC(+57.02)ARK.Y | Y | 58.24 | 1501.7671 | 14 | 6.42 | 751.8917 | 2 | 12.66 | 1.0632 | 1.0705 | 28959 | 0 | 0 | 0 | 58 | 71 | Carbamidomethylation |  | DB Search |
| K.FETQIATK.D | Y | 57.09 | 936.4916 | 8 | 7.3 | 937.5009 | 1 | 11.88 | 1.1925 | 1.1997 | 24345 | 0 | 0 | 0 | 167 | 174 |  |  | DB Search |
| total 2 peptides |  |  |  |  |  |  |  |  |  |  |  |  |  |  |  |  |  |  |  |
| --- | --- | --- | --- | --- | --- | --- | --- | --- | --- | --- | --- | --- | --- | --- | --- | --- | --- | --- | --- |

Best Unique PSM (Scan Sample\_9\_A1\_1\_3646.d:28959, m/z=751.8917, z=2, RT=12.66, ppm=1.21):


TRINITY\_DN195934\_c0\_g1\_i1.p2

back to list

  

| Protein Coverage
| Supporting Peptides
| Best Unique PSM
|

Protein Coverage:

Supporting Peptides:

| Peptide | Uniq | -10lgP | Mass | Length | ppm | m/z | z | RT | 1/k0 Start | 1/k0 End | Precursor ID | Area Sample 10 | #Feature | #Feature Sample 10 | Start | End | PTM | AScore | Found By |
| --- | --- | --- | --- | --- | --- | --- | --- | --- | --- | --- | --- | --- | --- | --- | --- | --- | --- | --- | --- |
| K.DSYVGDEAQSKR.G | Y | 59.06 | 1353.616 | 12 | 5.32 | 677.8154 | 2 | 10.95 | 1.0041 | 1.0114 | 18674 | 7.12e3 | 1 | 1 | 10 | 21 |  |  | DB Search |
| R.VAPEEHPVLLTEAPLNPK.A | Y | 58.04 | 1953.0571 | 18 | 3.48 | 977.5341 | 2 | 16.43 | 1.2045 | 1.2117 | 48611 | 4.81e2 | 1 | 1 | 55 | 72 |  |  | DB Search |
| total 2 peptides |  |  |  |  |  |  |  |  |  |  |  |  |  |  |  |  |  |  |  |
| --- | --- | --- | --- | --- | --- | --- | --- | --- | --- | --- | --- | --- | --- | --- | --- | --- | --- | --- | --- |

Best Unique PSM (Scan Sample\_9\_A1\_1\_3646.d:18674, m/z=677.8154, z=2, RT=10.95, ppm=0.1):


TRINITY\_DN30329\_c0\_g1\_i1.p1

back to list

  

| Protein Coverage
| Supporting Peptides
| Best Unique PSM
|

Protein Coverage:

Supporting Peptides:

| Peptide | Uniq | -10lgP | Mass | Length | ppm | m/z | z | RT | 1/k0 Start | 1/k0 End | Precursor ID | Area Sample 10 | #Feature | #Feature Sample 10 | Start | End | PTM | AScore | Found By |
| --- | --- | --- | --- | --- | --- | --- | --- | --- | --- | --- | --- | --- | --- | --- | --- | --- | --- | --- | --- |
| K.GQELTFKEFFKPSNFVR.S | Y | 58.21 | 2073.0683 | 17 | -4.7 | 692.0232 | 3 | 19.24 | 0.9315 | 0.9388 | 64684 | 2.56e3 | 1 | 1 | 160 | 176 |  |  | DB Search |
| total 1 peptides |  |  |  |  |  |  |  |  |  |  |  |  |  |  |  |  |  |  |  |
| --- | --- | --- | --- | --- | --- | --- | --- | --- | --- | --- | --- | --- | --- | --- | --- | --- | --- | --- | --- |

Best Unique PSM (Scan Sample\_9\_A1\_1\_3646.d:64684, m/z=692.0232, z=3, RT=19.24, ppm=-9.92):


TRINITY\_DN30329\_c0\_g2\_i1.p2

back to list

  

| Protein Coverage
| Supporting Peptides
| Best Unique PSM
|

Protein Coverage:

Supporting Peptides:

| Peptide | Uniq | -10lgP | Mass | Length | ppm | m/z | z | RT | 1/k0 Start | 1/k0 End | Precursor ID | Area Sample 10 | #Feature | #Feature Sample 10 | Start | End | PTM | AScore | Found By |
| --- | --- | --- | --- | --- | --- | --- | --- | --- | --- | --- | --- | --- | --- | --- | --- | --- | --- | --- | --- |
| K.GQELTFKEFFKPSNFVR.S | Y | 58.21 | 2073.0683 | 17 | -4.7 | 692.0232 | 3 | 19.24 | 0.9315 | 0.9388 | 64684 | 2.56e3 | 1 | 1 | 184 | 200 |  |  | DB Search |
| total 1 peptides |  |  |  |  |  |  |  |  |  |  |  |  |  |  |  |  |  |  |  |
| --- | --- | --- | --- | --- | --- | --- | --- | --- | --- | --- | --- | --- | --- | --- | --- | --- | --- | --- | --- |

Best Unique PSM (Scan Sample\_9\_A1\_1\_3646.d:64684, m/z=692.0232, z=3, RT=19.24, ppm=-9.92):


TRINITY\_DN10706\_c0\_g1\_i1.p1

back to list

  

| Protein Coverage
| Supporting Peptides
| Best Unique PSM
|

Protein Coverage:

Supporting Peptides:

| Peptide | Uniq | -10lgP | Mass | Length | ppm | m/z | z | RT | 1/k0 Start | 1/k0 End | Precursor ID | Area Sample 10 | #Feature | #Feature Sample 10 | Start | End | PTM | AScore | Found By |
| --- | --- | --- | --- | --- | --- | --- | --- | --- | --- | --- | --- | --- | --- | --- | --- | --- | --- | --- | --- |
| K.ATYDSNHDSFR.A | Y | 56.02 | 1311.548 | 11 | 4.08 | 656.7805 | 2 | 11.64 | 0.9388 | 0.9461 | 22886 | 5.01e2 | 1 | 1 | 84 | 94 |  |  | DB Search |
| total 1 peptides |  |  |  |  |  |  |  |  |  |  |  |  |  |  |  |  |  |  |  |
| --- | --- | --- | --- | --- | --- | --- | --- | --- | --- | --- | --- | --- | --- | --- | --- | --- | --- | --- | --- |

Best Unique PSM (Scan Sample\_9\_A1\_1\_3646.d:22886, m/z=656.7805, z=2, RT=11.64, ppm=-1.14):


TRINITY\_DN751\_c0\_g1\_i1.p1

back to list

  

| Protein Coverage
| Supporting Peptides
| Best Unique PSM
|

Protein Coverage:

Supporting Peptides:

| Peptide | Uniq | -10lgP | Mass | Length | ppm | m/z | z | RT | 1/k0 Start | 1/k0 End | Precursor ID | Area Sample 10 | #Feature | #Feature Sample 10 | Start | End | PTM | AScore | Found By |
| --- | --- | --- | --- | --- | --- | --- | --- | --- | --- | --- | --- | --- | --- | --- | --- | --- | --- | --- | --- |
| R.LAADLVITHSSK.S | Y | 58.63 | 1253.6979 | 12 | 2.83 | 627.8547 | 2 | 14.22 | 0.9973 | 1.0045 | 37483 | 1.32e3 | 1 | 1 | 166 | 177 |  |  | DB Search |
| total 1 peptides |  |  |  |  |  |  |  |  |  |  |  |  |  |  |  |  |  |  |  |
| --- | --- | --- | --- | --- | --- | --- | --- | --- | --- | --- | --- | --- | --- | --- | --- | --- | --- | --- | --- |

Best Unique PSM (Scan Sample\_9\_A1\_1\_3646.d:37483, m/z=627.8547, z=2, RT=14.22, ppm=-2.39):


TRINITY\_DN36\_c0\_g1\_i14.p1

back to list

  

| Protein Coverage
| Supporting Peptides
| Best Unique PSM
|

Protein Coverage:

Supporting Peptides:

| Peptide | Uniq | -10lgP | Mass | Length | ppm | m/z | z | RT | 1/k0 Start | 1/k0 End | Precursor ID | Area Sample 10 | #Feature | #Feature Sample 10 | Start | End | PTM | AScore | Found By |
| --- | --- | --- | --- | --- | --- | --- | --- | --- | --- | --- | --- | --- | --- | --- | --- | --- | --- | --- | --- |
| R.LGGIEVTQGNC(+57.02)ARK.Y | Y | 58.24 | 1501.7671 | 14 | 6.42 | 751.8917 | 2 | 12.66 | 1.0632 | 1.0705 | 28959 | 0 | 0 | 0 | 58 | 71 | Carbamidomethylation |  | DB Search |
| K.FETQIATK.D | Y | 57.09 | 936.4916 | 8 | 7.3 | 937.5009 | 1 | 11.88 | 1.1925 | 1.1997 | 24345 | 0 | 0 | 0 | 167 | 174 |  |  | DB Search |
| total 2 peptides |  |  |  |  |  |  |  |  |  |  |  |  |  |  |  |  |  |  |  |
| --- | --- | --- | --- | --- | --- | --- | --- | --- | --- | --- | --- | --- | --- | --- | --- | --- | --- | --- | --- |

Best Unique PSM (Scan Sample\_9\_A1\_1\_3646.d:28959, m/z=751.8917, z=2, RT=12.66, ppm=1.21):


TRINITY\_DN247\_c0\_g1\_i1.p1

back to list

  

| Protein Coverage
| Supporting Peptides
| Best Unique PSM
|

Protein Coverage:

Supporting Peptides:

| Peptide | Uniq | -10lgP | Mass | Length | ppm | m/z | z | RT | 1/k0 Start | 1/k0 End | Precursor ID | Area Sample 10 | #Feature | #Feature Sample 10 | Start | End | PTM | AScore | Found By |
| --- | --- | --- | --- | --- | --- | --- | --- | --- | --- | --- | --- | --- | --- | --- | --- | --- | --- | --- | --- |
| R.DAPIYGLQWHPEK.S | Y | 53.53 | 1552.7674 | 13 | 4.07 | 777.3901 | 2 | 17.25 | 1.0717 | 1.0789 | 52846 | 1.03e3 | 1 | 1 | 258 | 270 |  |  | DB Search |
| total 1 peptides |  |  |  |  |  |  |  |  |  |  |  |  |  |  |  |  |  |  |  |
| --- | --- | --- | --- | --- | --- | --- | --- | --- | --- | --- | --- | --- | --- | --- | --- | --- | --- | --- | --- |

Best Unique PSM (Scan Sample\_9\_A1\_1\_3646.d:52846, m/z=777.3901, z=2, RT=17.25, ppm=-1.15):


TRINITY\_DN4439\_c0\_g1\_i1.p1

back to list

  

| Protein Coverage
| Supporting Peptides
| Best Unique PSM
|

Protein Coverage:

Supporting Peptides:

| Peptide | Uniq | -10lgP | Mass | Length | ppm | m/z | z | RT | 1/k0 Start | 1/k0 End | Precursor ID | Area Sample 10 | #Feature | #Feature Sample 10 | Start | End | PTM | AScore | Found By |
| --- | --- | --- | --- | --- | --- | --- | --- | --- | --- | --- | --- | --- | --- | --- | --- | --- | --- | --- | --- |
| R.LASQQNIGQVLAELK.E | Y | 58.28 | 1610.8991 | 15 | -10.39 | 806.4443 | 2 | 19.82 | 1.1282 | 1.1354 | 68085 | 0 | 0 | 0 | 346 | 360 |  |  | DB Search |
| R.VDKITEYLC(+57.02)EPLRK.C | Y | 50.25 | 1762.9287 | 14 | -15.2 | 588.6382 | 3 | 16.21 | 0.8494 | 0.8567 | 47751 | 0 | 0 | 0 | 115 | 128 | Carbamidomethylation |  | DB Search |
| total 2 peptides |  |  |  |  |  |  |  |  |  |  |  |  |  |  |  |  |  |  |  |
| --- | --- | --- | --- | --- | --- | --- | --- | --- | --- | --- | --- | --- | --- | --- | --- | --- | --- | --- | --- |

Best Unique PSM (Scan Sample\_9\_A1\_1\_3646.d:68085, m/z=806.4443, z=2, RT=19.82, ppm=-15.61):


TRINITY\_DN817\_c0\_g1\_i3.p1

back to list

  

| Protein Coverage
| Supporting Peptides
| Best Unique PSM
|

Protein Coverage:

Supporting Peptides:

| Peptide | Uniq | -10lgP | Mass | Length | ppm | m/z | z | RT | 1/k0 Start | 1/k0 End | Precursor ID | Area Sample 10 | #Feature | #Feature Sample 10 | Start | End | PTM | AScore | Found By |
| --- | --- | --- | --- | --- | --- | --- | --- | --- | --- | --- | --- | --- | --- | --- | --- | --- | --- | --- | --- |
| R.LC(+57.02)QPGYSTC(+57.02)ENHC(+57.02)R.K | Y | 55.11 | 1780.708 | 14 | 0.84 | 891.3574 | 2 | 11.42 | 1.0861 | 1.0933 | 21509 | 5.05e2 | 1 | 1 | 177 | 190 | Carbamidomethylation, Carbamidomethylation, Carbamidomethylation |  | DB Search |
| total 1 peptides |  |  |  |  |  |  |  |  |  |  |  |  |  |  |  |  |  |  |  |
| --- | --- | --- | --- | --- | --- | --- | --- | --- | --- | --- | --- | --- | --- | --- | --- | --- | --- | --- | --- |

Best Unique PSM (Scan Sample\_9\_A1\_1\_3646.d:21509, m/z=891.3574, z=2, RT=11.42, ppm=-4.38):


TRINITY\_DN262\_c0\_g1\_i1.p1

back to list

  

| Protein Coverage
| Supporting Peptides
| Best Unique PSM
|

Protein Coverage:

Supporting Peptides:

| Peptide | Uniq | -10lgP | Mass | Length | ppm | m/z | z | RT | 1/k0 Start | 1/k0 End | Precursor ID | Area Sample 10 | #Feature | #Feature Sample 10 | Start | End | PTM | AScore | Found By |
| --- | --- | --- | --- | --- | --- | --- | --- | --- | --- | --- | --- | --- | --- | --- | --- | --- | --- | --- | --- |
| R.DANAAVVHGSHLK.D | Y | 58.77 | 1317.6789 | 13 | 7.74 | 659.8484 | 2 | 11.46 | 1.0045 | 1.0118 | 21812 | 7.21e2 | 1 | 1 | 668 | 680 |  |  | DB Search |
| R.AEFRPNQEEEPILKR.E | Y | 58.67 | 1854.9588 | 15 | 0.94 | 619.3242 | 3 | 13.74 | 0.8785 | 0.8858 | 34918 | 9.68e2 | 1 | 1 | 439 | 453 |  |  | DB Search |
| K.GVGIISEGNETVEDIAQR.L | Y | 57.67 | 1885.9381 | 18 | -8.31 | 943.9636 | 2 | 17.77 | 1.1422 | 1.1494 | 56101 | 2.23e2 | 1 | 1 | 639 | 656 |  |  | DB Search |
| R.DPVRDKLVNER.L | Y | 56.65 | 1339.7208 | 11 | 2.36 | 447.5796 | 3 | 12.03 | 0.7408 | 0.7481 | 25133 | 1.13e3 | 1 | 1 | 847 | 857 |  |  | DB Search |
| K.DLPQEKLDDILK.Y | Y | 52.63 | 1425.7715 | 12 | 1.19 | 713.8901 | 2 | 17.70 | 1.0399 | 1.0472 | 55712 | 0 | 0 | 0 | 681 | 692 |  |  | DB Search |
| total 5 peptides |  |  |  |  |  |  |  |  |  |  |  |  |  |  |  |  |  |  |  |
| --- | --- | --- | --- | --- | --- | --- | --- | --- | --- | --- | --- | --- | --- | --- | --- | --- | --- | --- | --- |

Best Unique PSM (Scan Sample\_9\_A1\_1\_3646.d:21812, m/z=659.8484, z=2, RT=11.46, ppm=2.52):


TRINITY\_DN119684\_c0\_g1\_i1.p1

back to list

  

| Protein Coverage
| Supporting Peptides
| Best Unique PSM
|

Protein Coverage:

Supporting Peptides:

| Peptide | Uniq | -10lgP | Mass | Length | ppm | m/z | z | RT | 1/k0 Start | 1/k0 End | Precursor ID | Area Sample 10 | #Feature | #Feature Sample 10 | Start | End | PTM | AScore | Found By |
| --- | --- | --- | --- | --- | --- | --- | --- | --- | --- | --- | --- | --- | --- | --- | --- | --- | --- | --- | --- |
| K.GKKPHVQVNVK.E | Y | 55.29 | 1232.7353 | 11 | 5.43 | 411.9191 | 3 | 9.80 | 0.7852 | 0.7925 | 12174 | 4.29e2 | 1 | 1 | 125 | 135 |  |  | DB Search |
| total 1 peptides |  |  |  |  |  |  |  |  |  |  |  |  |  |  |  |  |  |  |  |
| --- | --- | --- | --- | --- | --- | --- | --- | --- | --- | --- | --- | --- | --- | --- | --- | --- | --- | --- | --- |

Best Unique PSM (Scan Sample\_9\_A1\_1\_3646.d:12174, m/z=411.9191, z=3, RT=9.80, ppm=0.2):


TRINITY\_DN3153\_c0\_g1\_i3.p1

back to list

  

| Protein Coverage
| Supporting Peptides
| Best Unique PSM
|

Protein Coverage:

Supporting Peptides:

| Peptide | Uniq | -10lgP | Mass | Length | ppm | m/z | z | RT | 1/k0 Start | 1/k0 End | Precursor ID | Area Sample 10 | #Feature | #Feature Sample 10 | Start | End | PTM | AScore | Found By |
| --- | --- | --- | --- | --- | --- | --- | --- | --- | --- | --- | --- | --- | --- | --- | --- | --- | --- | --- | --- |
| K.AVVEGM(+15.99)TADSDESKKDE.X | Y | 58.69 | 1825.7887 | 17 | 2.01 | 609.6016 | 3 | 10.36 | 0.8348 | 0.8421 | 15202 | 1.88e3 | 2 | 2 | 179 | 195 | Oxidation (M) | M6:Oxidation (M):1000 | DB Search |
| total 1 peptides |  |  |  |  |  |  |  |  |  |  |  |  |  |  |  |  |  |  |  |
| --- | --- | --- | --- | --- | --- | --- | --- | --- | --- | --- | --- | --- | --- | --- | --- | --- | --- | --- | --- |

Best Unique PSM (Scan Sample\_9\_A1\_1\_3646.d:15202, m/z=609.6016, z=3, RT=10.36, ppm=-3.21):


TRINITY\_DN1653\_c0\_g1\_i1.p1

back to list

  

| Protein Coverage
| Supporting Peptides
| Best Unique PSM
|

Protein Coverage:

Supporting Peptides:

| Peptide | Uniq | -10lgP | Mass | Length | ppm | m/z | z | RT | 1/k0 Start | 1/k0 End | Precursor ID | Area Sample 10 | #Feature | #Feature Sample 10 | Start | End | PTM | AScore | Found By |
| --- | --- | --- | --- | --- | --- | --- | --- | --- | --- | --- | --- | --- | --- | --- | --- | --- | --- | --- | --- |
| R.LINLFEAVSKK.S | Y | 56.67 | 1260.7441 | 11 | 0.94 | 631.3766 | 2 | 18.83 | 0.9804 | 0.9876 | 62338 | 0 | 0 | 0 | 49 | 59 |  |  | DB Search |
| total 1 peptides |  |  |  |  |  |  |  |  |  |  |  |  |  |  |  |  |  |  |  |
| --- | --- | --- | --- | --- | --- | --- | --- | --- | --- | --- | --- | --- | --- | --- | --- | --- | --- | --- | --- |

Best Unique PSM (Scan Sample\_9\_A1\_1\_3646.d:62338, m/z=631.3766, z=2, RT=18.83, ppm=-4.28):


TRINITY\_DN6388\_c0\_g1\_i1.p2

back to list

  

| Protein Coverage
| Supporting Peptides
| Best Unique PSM
|

Protein Coverage:

Supporting Peptides:

| Peptide | Uniq | -10lgP | Mass | Length | ppm | m/z | z | RT | 1/k0 Start | 1/k0 End | Precursor ID | Area Sample 10 | #Feature | #Feature Sample 10 | Start | End | PTM | AScore | Found By |
| --- | --- | --- | --- | --- | --- | --- | --- | --- | --- | --- | --- | --- | --- | --- | --- | --- | --- | --- | --- |
| R.AIELLPHDLAVGR.H | Y | 57.65 | 1402.7932 | 13 | -0.45 | 702.3999 | 2 | 17.82 | 1.0303 | 1.0375 | 56285 | 4.72e2 | 1 | 1 | 57 | 69 |  |  | DB Search |
| total 1 peptides |  |  |  |  |  |  |  |  |  |  |  |  |  |  |  |  |  |  |  |
| --- | --- | --- | --- | --- | --- | --- | --- | --- | --- | --- | --- | --- | --- | --- | --- | --- | --- | --- | --- |

Best Unique PSM (Scan Sample\_9\_A1\_1\_3646.d:56285, m/z=702.3999, z=2, RT=17.82, ppm=-5.66):


TRINITY\_DN64\_c0\_g1\_i1.p1

back to list

  

| Protein Coverage
| Supporting Peptides
| Best Unique PSM
|

Protein Coverage:

Supporting Peptides:

| Peptide | Uniq | -10lgP | Mass | Length | ppm | m/z | z | RT | 1/k0 Start | 1/k0 End | Precursor ID | Area Sample 10 | #Feature | #Feature Sample 10 | Start | End | PTM | AScore | Found By |
| --- | --- | --- | --- | --- | --- | --- | --- | --- | --- | --- | --- | --- | --- | --- | --- | --- | --- | --- | --- |
| K.IHINIVVIGHVDSGK.S | Y | 58.69 | 1599.9096 | 15 | -0.5 | 800.9575 | 2 | 16.54 | 1.1001 | 1.1074 | 49239 | 7.77e2 | 2 | 2 | 9 | 23 |  |  | DB Search |
| R.AIEKFEKEAQEM(+15.99)GK.G | Y | 52.83 | 1652.8079 | 14 | 5.41 | 551.9434 | 3 | 12.07 | 0.8185 | 0.8258 | 25556 | 5.51e2 | 1 | 1 | 41 | 54 | Oxidation (M) | M12:Oxidation (M):1000 | DB Search |
| K.YYVTIIDAPGHRDFIK.N | Y | 29.31 | 1906.9941 | 16 | -3.54 | 954.496 | 2 | 16.73 | 1.2045 | 1.2117 | 50248 | 0 | 0 | 0 | 88 | 103 |  |  | DB Search |
| total 3 peptides |  |  |  |  |  |  |  |  |  |  |  |  |  |  |  |  |  |  |  |
| --- | --- | --- | --- | --- | --- | --- | --- | --- | --- | --- | --- | --- | --- | --- | --- | --- | --- | --- | --- |

Best Unique PSM (Scan Sample\_9\_A1\_1\_3646.d:49239, m/z=800.9575, z=2, RT=16.54, ppm=-5.71):


TRINITY\_DN15274\_c0\_g1\_i1.p1

back to list

  

| Protein Coverage
| Supporting Peptides
| Best Unique PSM
|

Protein Coverage:

Supporting Peptides:

| Peptide | Uniq | -10lgP | Mass | Length | ppm | m/z | z | RT | 1/k0 Start | 1/k0 End | Precursor ID | Area Sample 10 | #Feature | #Feature Sample 10 | Start | End | PTM | AScore | Found By |
| --- | --- | --- | --- | --- | --- | --- | --- | --- | --- | --- | --- | --- | --- | --- | --- | --- | --- | --- | --- |
| K.YC(+57.02)ILSFLDKTDK.C | Y | 58.62 | 1501.7486 | 12 | -1.44 | 501.5868 | 3 | 19.64 | 0.7958 | 0.8031 | 67040 | 7.6e2 | 1 | 1 | 62 | 73 | Carbamidomethylation |  | DB Search |
| R.YSRPTPGSNNNVM(+15.99)VR.I | Y | 58.6 | 1706.8158 | 15 | 3.02 | 569.9446 | 3 | 11.52 | 0.836 | 0.8433 | 22140 | 4.48e2 | 1 | 1 | 621 | 635 | Oxidation (M) | M13:Oxidation (M):1000 | DB Search |
| R.GSC(+57.02)SC(+57.02)YGHAEQC(+57.02)IPTK.G | Y | 58.5 | 1853.7495 | 16 | 0.08 | 927.8773 | 2 | 12.31 | 1.1885 | 1.1957 | 26928 | 5.48e1 | 1 | 1 | 270 | 285 | Carbamidomethylation, Carbamidomethylation, Carbamidomethylation |  | DB Search |
| K.SGGQC(+57.02)PC(+57.02)KPGVVGR.R | Y | 58.08 | 1457.6867 | 14 | 3.86 | 486.9022 | 3 | 10.69 | 0.7868 | 0.7941 | 17188 | 4.47e2 | 1 | 1 | 795 | 808 | Carbamidomethylation, Carbamidomethylation |  | DB Search |
| total 4 peptides |  |  |  |  |  |  |  |  |  |  |  |  |  |  |  |  |  |  |  |
| --- | --- | --- | --- | --- | --- | --- | --- | --- | --- | --- | --- | --- | --- | --- | --- | --- | --- | --- | --- |

Best Unique PSM (Scan Sample\_9\_A1\_1\_3646.d:67040, m/z=501.5868, z=3, RT=19.64, ppm=-6.66):


TRINITY\_DN146208\_c0\_g1\_i1.p1

back to list

  

| Protein Coverage
| Supporting Peptides
| Best Unique PSM
|

Protein Coverage:

Supporting Peptides:

| Peptide | Uniq | -10lgP | Mass | Length | ppm | m/z | z | RT | 1/k0 Start | 1/k0 End | Precursor ID | Area Sample 10 | #Feature | #Feature Sample 10 | Start | End | PTM | AScore | Found By |
| --- | --- | --- | --- | --- | --- | --- | --- | --- | --- | --- | --- | --- | --- | --- | --- | --- | --- | --- | --- |
| K.YGINHITNLVESKR.A | Y | 58.69 | 1642.8791 | 14 | -1.63 | 548.6299 | 3 | 14.98 | 0.823 | 0.8303 | 41552 | 0 | 0 | 0 | 158 | 171 |  |  | DB Search |
| total 1 peptides |  |  |  |  |  |  |  |  |  |  |  |  |  |  |  |  |  |  |  |
| --- | --- | --- | --- | --- | --- | --- | --- | --- | --- | --- | --- | --- | --- | --- | --- | --- | --- | --- | --- |

Best Unique PSM (Scan Sample\_9\_A1\_1\_3646.d:41552, m/z=548.6299, z=3, RT=14.98, ppm=-6.85):


TRINITY\_DN3271\_c0\_g1\_i1.p1

back to list

  

| Protein Coverage
| Supporting Peptides
| Best Unique PSM
|

Protein Coverage:

Supporting Peptides:

| Peptide | Uniq | -10lgP | Mass | Length | ppm | m/z | z | RT | 1/k0 Start | 1/k0 End | Precursor ID | Area Sample 10 | #Feature | #Feature Sample 10 | Start | End | PTM | AScore | Found By |
| --- | --- | --- | --- | --- | --- | --- | --- | --- | --- | --- | --- | --- | --- | --- | --- | --- | --- | --- | --- |
| K.TIESEAAHGTVTR.H | Y | 58.99 | 1370.679 | 13 | 5.98 | 686.3473 | 2 | 11.90 | 1.0206 | 1.0279 | 24466 | 5.79e2 | 1 | 1 | 285 | 297 |  |  | DB Search |
| total 1 peptides |  |  |  |  |  |  |  |  |  |  |  |  |  |  |  |  |  |  |  |
| --- | --- | --- | --- | --- | --- | --- | --- | --- | --- | --- | --- | --- | --- | --- | --- | --- | --- | --- | --- |

Best Unique PSM (Scan Sample\_9\_A1\_1\_3646.d:24466, m/z=686.3473, z=2, RT=11.90, ppm=0.77):


TRINITY\_DN624\_c0\_g1\_i1.p1

back to list

  

| Protein Coverage
| Supporting Peptides
| Best Unique PSM
|

Protein Coverage:

Supporting Peptides:

| Peptide | Uniq | -10lgP | Mass | Length | ppm | m/z | z | RT | 1/k0 Start | 1/k0 End | Precursor ID | Area Sample 10 | #Feature | #Feature Sample 10 | Start | End | PTM | AScore | Found By |
| --- | --- | --- | --- | --- | --- | --- | --- | --- | --- | --- | --- | --- | --- | --- | --- | --- | --- | --- | --- |
| R.FAIQDITVDEFSAK.E | Y | 57.41 | 1582.7878 | 14 | 3.04 | 792.3995 | 2 | 19.67 | 1.0439 | 1.0512 | 67226 | 7.29e2 | 1 | 1 | 132 | 145 |  |  | DB Search |
| R.ILAGDKPYITADELRR.E | Y | 39.95 | 1829.9999 | 16 | -2.95 | 611.0023 | 3 | 14.95 | 0.9259 | 0.9331 | 41338 | 0 | 0 | 0 | 826 | 841 |  |  | DB Search |
| total 2 peptides |  |  |  |  |  |  |  |  |  |  |  |  |  |  |  |  |  |  |  |
| --- | --- | --- | --- | --- | --- | --- | --- | --- | --- | --- | --- | --- | --- | --- | --- | --- | --- | --- | --- |

Best Unique PSM (Scan Sample\_9\_A1\_1\_3646.d:67226, m/z=792.3995, z=2, RT=19.67, ppm=-2.18):


TRINITY\_DN12\_c0\_g1\_i1.p1

back to list

  

| Protein Coverage
| Supporting Peptides
| Best Unique PSM
|

Protein Coverage:

Supporting Peptides:

| Peptide | Uniq | -10lgP | Mass | Length | ppm | m/z | z | RT | 1/k0 Start | 1/k0 End | Precursor ID | Area Sample 10 | #Feature | #Feature Sample 10 | Start | End | PTM | AScore | Found By |
| --- | --- | --- | --- | --- | --- | --- | --- | --- | --- | --- | --- | --- | --- | --- | --- | --- | --- | --- | --- |
| R.LGPDEASENKR.E | Y | 51.39 | 1214.5891 | 11 | 5.41 | 608.3019 | 2 | 10.47 | 0.9166 | 0.9238 | 15408 | 2.93e3 | 1 | 1 | 153 | 163 |  |  | DB Search |
| total 1 peptides |  |  |  |  |  |  |  |  |  |  |  |  |  |  |  |  |  |  |  |
| --- | --- | --- | --- | --- | --- | --- | --- | --- | --- | --- | --- | --- | --- | --- | --- | --- | --- | --- | --- |

Best Unique PSM (Scan Sample\_9\_A1\_1\_3646.d:15408, m/z=608.3019, z=2, RT=10.47, ppm=0.19):


TRINITY\_DN1058\_c0\_g1\_i1.p1

back to list

  

| Protein Coverage
| Supporting Peptides
| Best Unique PSM
|

Protein Coverage:

Supporting Peptides:

| Peptide | Uniq | -10lgP | Mass | Length | ppm | m/z | z | RT | 1/k0 Start | 1/k0 End | Precursor ID | Area Sample 10 | #Feature | #Feature Sample 10 | Start | End | PTM | AScore | Found By |
| --- | --- | --- | --- | --- | --- | --- | --- | --- | --- | --- | --- | --- | --- | --- | --- | --- | --- | --- | --- |
| R.APTHILNPANKR.S | Y | 58.64 | 1330.7469 | 12 | 0.9 | 666.3779 | 2 | 11.17 | 1.009 | 1.0162 | 20196 | 4.06e2 | 1 | 1 | 2314 | 2325 |  |  | DB Search |
| total 1 peptides |  |  |  |  |  |  |  |  |  |  |  |  |  |  |  |  |  |  |  |
| --- | --- | --- | --- | --- | --- | --- | --- | --- | --- | --- | --- | --- | --- | --- | --- | --- | --- | --- | --- |

Best Unique PSM (Scan Sample\_9\_A1\_1\_3646.d:20196, m/z=666.3779, z=2, RT=11.17, ppm=-4.32):


TRINITY\_DN1339\_c0\_g2\_i3.p1

back to list

  

| Protein Coverage
| Supporting Peptides
| Best Unique PSM
|

Protein Coverage:

Supporting Peptides:

| Peptide | Uniq | -10lgP | Mass | Length | ppm | m/z | z | RT | 1/k0 Start | 1/k0 End | Precursor ID | Area Sample 10 | #Feature | #Feature Sample 10 | Start | End | PTM | AScore | Found By |
| --- | --- | --- | --- | --- | --- | --- | --- | --- | --- | --- | --- | --- | --- | --- | --- | --- | --- | --- | --- |
| K.LLTSSDQAELTKK.L | Y | 59.06 | 1432.7773 | 13 | -0.99 | 717.3915 | 2 | 12.75 | 1.0508 | 1.058 | 29440 | 1.28e3 | 1 | 1 | 155 | 167 |  |  | DB Search |
| total 1 peptides |  |  |  |  |  |  |  |  |  |  |  |  |  |  |  |  |  |  |  |
| --- | --- | --- | --- | --- | --- | --- | --- | --- | --- | --- | --- | --- | --- | --- | --- | --- | --- | --- | --- |

Best Unique PSM (Scan Sample\_9\_A1\_1\_3646.d:29440, m/z=717.3915, z=2, RT=12.75, ppm=-6.21):


TRINITY\_DN329\_c0\_g1\_i2.p1

back to list

  

| Protein Coverage
| Supporting Peptides
| Best Unique PSM
|

Protein Coverage:

Supporting Peptides:

| Peptide | Uniq | -10lgP | Mass | Length | ppm | m/z | z | RT | 1/k0 Start | 1/k0 End | Precursor ID | Area Sample 10 | #Feature | #Feature Sample 10 | Start | End | PTM | AScore | Found By |
| --- | --- | --- | --- | --- | --- | --- | --- | --- | --- | --- | --- | --- | --- | --- | --- | --- | --- | --- | --- |
| K.GLKVDFDTTFAPVTGKK.T | Y | 58.67 | 1822.9828 | 17 | 2.09 | 912.4958 | 2 | 17.12 | 1.1802 | 1.1873 | 52158 | 1.37e3 | 1 | 1 | 130 | 146 |  |  | DB Search |
| K.GLKVDFDTTFAPVTGK.K | Y | 58.63 | 1694.8879 | 16 | 3.65 | 848.4499 | 2 | 18.14 | 1.0957 | 1.1029 | 58184 | 1.23e3 | 1 | 1 | 130 | 145 |  |  | DB Search |
| K.SFHFGVIKLEGK.T | Y | 58.33 | 1360.7503 | 12 | 0.06 | 681.3789 | 2 | 16.99 | 1.0291 | 1.0363 | 51505 | 4.07e3 | 2 | 2 | 57 | 68 |  |  | DB Search |
| K.VDFDTTFAPVTGKK.T | Y | 58.32 | 1524.7824 | 14 | 4.95 | 763.3983 | 2 | 16.54 | 1.052 | 1.0592 | 49415 | 0 | 0 | 0 | 133 | 146 |  |  | DB Search |
| K.EGDLVIHSGITDAVK.Y | Y | 39.37 | 1552.8096 | 15 | 2.72 | 777.4102 | 2 | 15.38 | 1.0648 | 1.0721 | 43595 | 0 | 0 | 0 | 211 | 225 |  |  | DB Search |
| total 5 peptides |  |  |  |  |  |  |  |  |  |  |  |  |  |  |  |  |  |  |  |
| --- | --- | --- | --- | --- | --- | --- | --- | --- | --- | --- | --- | --- | --- | --- | --- | --- | --- | --- | --- |

Best Unique PSM (Scan Sample\_9\_A1\_1\_3646.d:52158, m/z=912.4958, z=2, RT=17.12, ppm=-3.13):


TRINITY\_DN1676\_c0\_g1\_i12.p1

back to list

  

| Protein Coverage
| Supporting Peptides
| Best Unique PSM
|

Protein Coverage:

Supporting Peptides:

| Peptide | Uniq | -10lgP | Mass | Length | ppm | m/z | z | RT | 1/k0 Start | 1/k0 End | Precursor ID | Area Sample 10 | #Feature | #Feature Sample 10 | Start | End | PTM | AScore | Found By |
| --- | --- | --- | --- | --- | --- | --- | --- | --- | --- | --- | --- | --- | --- | --- | --- | --- | --- | --- | --- |
| K.RFEEAKDTIENK.L | Y | 58.41 | 1478.7365 | 12 | 1.43 | 493.9176 | 3 | 11.59 | 0.8014 | 0.8088 | 22585 | 4.14e2 | 1 | 2 | 1560 | 1571 |  |  | DB Search |
| total 1 peptides |  |  |  |  |  |  |  |  |  |  |  |  |  |  |  |  |  |  |  |
| --- | --- | --- | --- | --- | --- | --- | --- | --- | --- | --- | --- | --- | --- | --- | --- | --- | --- | --- | --- |

Best Unique PSM (Scan Sample\_9\_A1\_1\_3646.d:22585, m/z=493.9176, z=3, RT=11.59, ppm=-3.79):


TRINITY\_DN554\_c0\_g1\_i3.p1

back to list

  

| Protein Coverage
| Supporting Peptides
| Best Unique PSM
|

Protein Coverage:

Supporting Peptides:

| Peptide | Uniq | -10lgP | Mass | Length | ppm | m/z | z | RT | 1/k0 Start | 1/k0 End | Precursor ID | Area Sample 10 | #Feature | #Feature Sample 10 | Start | End | PTM | AScore | Found By |
| --- | --- | --- | --- | --- | --- | --- | --- | --- | --- | --- | --- | --- | --- | --- | --- | --- | --- | --- | --- |
| K.IQDKEGIPPDQQR.L | Y | 58.25 | 1522.7739 | 13 | 2.55 | 762.3922 | 2 | 11.03 | 1.0343 | 1.0415 | 18957 | 1.53e3 | 1 | 1 | 65 | 77 |  |  | DB Search |
| total 1 peptides |  |  |  |  |  |  |  |  |  |  |  |  |  |  |  |  |  |  |  |
| --- | --- | --- | --- | --- | --- | --- | --- | --- | --- | --- | --- | --- | --- | --- | --- | --- | --- | --- | --- |

Best Unique PSM (Scan Sample\_9\_A1\_1\_3646.d:18957, m/z=762.3922, z=2, RT=11.03, ppm=-2.67):


TRINITY\_DN1797\_c0\_g1\_i1.p1

back to list

  

| Protein Coverage
| Supporting Peptides
| Best Unique PSM
|

Protein Coverage:

Supporting Peptides:

| Peptide | Uniq | -10lgP | Mass | Length | ppm | m/z | z | RT | 1/k0 Start | 1/k0 End | Precursor ID | Area Sample 10 | #Feature | #Feature Sample 10 | Start | End | PTM | AScore | Found By |
| --- | --- | --- | --- | --- | --- | --- | --- | --- | --- | --- | --- | --- | --- | --- | --- | --- | --- | --- | --- |
| K.FAANLLDHFKIDR.S | Y | 57.34 | 1558.8256 | 13 | -0.92 | 520.6126 | 3 | 18.07 | 0.8481 | 0.8554 | 57779 | 0 | 0 | 0 | 545 | 557 |  |  | DB Search |
| total 1 peptides |  |  |  |  |  |  |  |  |  |  |  |  |  |  |  |  |  |  |  |
| --- | --- | --- | --- | --- | --- | --- | --- | --- | --- | --- | --- | --- | --- | --- | --- | --- | --- | --- | --- |

Best Unique PSM (Scan Sample\_9\_A1\_1\_3646.d:57779, m/z=520.6126, z=3, RT=18.07, ppm=-6.14):


TRINITY\_DN1319\_c0\_g1\_i2.p1

back to list

  

| Protein Coverage
| Supporting Peptides
| Best Unique PSM
|

Protein Coverage:

Supporting Peptides:

| Peptide | Uniq | -10lgP | Mass | Length | ppm | m/z | z | RT | 1/k0 Start | 1/k0 End | Precursor ID | Area Sample 10 | #Feature | #Feature Sample 10 | Start | End | PTM | AScore | Found By |
| --- | --- | --- | --- | --- | --- | --- | --- | --- | --- | --- | --- | --- | --- | --- | --- | --- | --- | --- | --- |
| K.GTHAAEIAHC(+57.02)VSR.R | Y | 58.65 | 1407.6677 | 13 | -14.3 | 704.8274 | 2 | 11.04 | 1.0307 | 1.0379 | 19410 | 0 | 0 | 0 | 91 | 103 | Carbamidomethylation |  | DB Search |
| total 1 peptides |  |  |  |  |  |  |  |  |  |  |  |  |  |  |  |  |  |  |  |
| --- | --- | --- | --- | --- | --- | --- | --- | --- | --- | --- | --- | --- | --- | --- | --- | --- | --- | --- | --- |

Best Unique PSM (Scan Sample\_9\_A1\_1\_3646.d:19410, m/z=704.8274, z=2, RT=11.04, ppm=-19.51):


TRINITY\_DN1995\_c0\_g1\_i1.p1

back to list

  

| Protein Coverage
| Supporting Peptides
| Best Unique PSM
|

Protein Coverage:

Supporting Peptides:

| Peptide | Uniq | -10lgP | Mass | Length | ppm | m/z | z | RT | 1/k0 Start | 1/k0 End | Precursor ID | Area Sample 10 | #Feature | #Feature Sample 10 | Start | End | PTM | AScore | Found By |
| --- | --- | --- | --- | --- | --- | --- | --- | --- | --- | --- | --- | --- | --- | --- | --- | --- | --- | --- | --- |
| R.ELIDHLIR.D | Y | 56.61 | 1007.5763 | 8 | -3.64 | 504.791 | 2 | 19.92 | 0.8648 | 0.8721 | 68596 | 1.53e3 | 1 | 1 | 164 | 171 |  |  | DB Search |
| total 1 peptides |  |  |  |  |  |  |  |  |  |  |  |  |  |  |  |  |  |  |  |
| --- | --- | --- | --- | --- | --- | --- | --- | --- | --- | --- | --- | --- | --- | --- | --- | --- | --- | --- | --- |

Best Unique PSM (Scan Sample\_9\_A1\_1\_3646.d:68596, m/z=504.791, z=2, RT=19.92, ppm=-8.86):


TRINITY\_DN109115\_c0\_g1\_i1.p1

back to list

  

| Protein Coverage
| Supporting Peptides
| Best Unique PSM
|

Protein Coverage:

Supporting Peptides:

| Peptide | Uniq | -10lgP | Mass | Length | ppm | m/z | z | RT | 1/k0 Start | 1/k0 End | Precursor ID | Area Sample 10 | #Feature | #Feature Sample 10 | Start | End | PTM | AScore | Found By |
| --- | --- | --- | --- | --- | --- | --- | --- | --- | --- | --- | --- | --- | --- | --- | --- | --- | --- | --- | --- |
| R.IRELTSVVQKR.F | Y | 58.24 | 1327.7936 | 11 | -4.83 | 443.6007 | 3 | 12.43 | 0.751 | 0.7583 | 27658 | 0 | 0 | 0 | 45 | 55 |  |  | DB Search |
| total 1 peptides |  |  |  |  |  |  |  |  |  |  |  |  |  |  |  |  |  |  |  |
| --- | --- | --- | --- | --- | --- | --- | --- | --- | --- | --- | --- | --- | --- | --- | --- | --- | --- | --- | --- |

Best Unique PSM (Scan Sample\_9\_A1\_1\_3646.d:27658, m/z=443.6007, z=3, RT=12.43, ppm=-10.06):


TRINITY\_DN3545\_c0\_g1\_i1.p1

back to list

  

| Protein Coverage
| Supporting Peptides
| Best Unique PSM
|

Protein Coverage:

Supporting Peptides:

| Peptide | Uniq | -10lgP | Mass | Length | ppm | m/z | z | RT | 1/k0 Start | 1/k0 End | Precursor ID | Area Sample 10 | #Feature | #Feature Sample 10 | Start | End | PTM | AScore | Found By |
| --- | --- | --- | --- | --- | --- | --- | --- | --- | --- | --- | --- | --- | --- | --- | --- | --- | --- | --- | --- |
| R.SC(+57.02)TNPAPANGGK.P | Y | 58.68 | 1172.5244 | 12 | 3.23 | 587.2683 | 2 | 9.42 | 0.8352 | 0.8425 | 10441 | 8.36e2 | 1 | 1 | 55 | 66 | Carbamidomethylation |  | DB Search |
| R.SSC(+57.02)RPILSVIK.I | Y | 54.44 | 1258.7067 | 11 | -8.13 | 630.3522 | 2 | 16.83 | 0.9691 | 0.9763 | 50527 | 1.86e3 | 1 | 1 | 6 | 16 | Carbamidomethylation |  | DB Search |
| total 2 peptides |  |  |  |  |  |  |  |  |  |  |  |  |  |  |  |  |  |  |  |
| --- | --- | --- | --- | --- | --- | --- | --- | --- | --- | --- | --- | --- | --- | --- | --- | --- | --- | --- | --- |

Best Unique PSM (Scan Sample\_9\_A1\_1\_3646.d:10441, m/z=587.2683, z=2, RT=9.42, ppm=-1.99):


TRINITY\_DN6233\_c0\_g1\_i2.p2

back to list

  

| Protein Coverage
| Supporting Peptides
| Best Unique PSM
|

Protein Coverage:

Supporting Peptides:

| Peptide | Uniq | -10lgP | Mass | Length | ppm | m/z | z | RT | 1/k0 Start | 1/k0 End | Precursor ID | Area Sample 10 | #Feature | #Feature Sample 10 | Start | End | PTM | AScore | Found By |
| --- | --- | --- | --- | --- | --- | --- | --- | --- | --- | --- | --- | --- | --- | --- | --- | --- | --- | --- | --- |
| K.SC(+57.02)LLLQFTDKR.F | Y | 58.6 | 1379.7231 | 11 | 0.98 | 690.8659 | 2 | 17.62 | 1.0291 | 1.0363 | 55103 | 7.83e2 | 1 | 1 | 42 | 52 | Carbamidomethylation |  | DB Search |
| total 1 peptides |  |  |  |  |  |  |  |  |  |  |  |  |  |  |  |  |  |  |  |
| --- | --- | --- | --- | --- | --- | --- | --- | --- | --- | --- | --- | --- | --- | --- | --- | --- | --- | --- | --- |

Best Unique PSM (Scan Sample\_9\_A1\_1\_3646.d:55103, m/z=690.8659, z=2, RT=17.62, ppm=-4.24):


TRINITY\_DN161\_c0\_g1\_i2.p1

back to list

  

| Protein Coverage
| Supporting Peptides
| Best Unique PSM
|

Protein Coverage:

Supporting Peptides:

| Peptide | Uniq | -10lgP | Mass | Length | ppm | m/z | z | RT | 1/k0 Start | 1/k0 End | Precursor ID | Area Sample 10 | #Feature | #Feature Sample 10 | Start | End | PTM | AScore | Found By |
| --- | --- | --- | --- | --- | --- | --- | --- | --- | --- | --- | --- | --- | --- | --- | --- | --- | --- | --- | --- |
| K.DSYVGDEAQSKR.G | Y | 59.06 | 1353.616 | 12 | 5.32 | 677.8154 | 2 | 10.95 | 1.0041 | 1.0114 | 18674 | 7.12e3 | 1 | 1 | 74 | 85 |  |  | DB Search |
| R.AVFPSIVGRPR.H | Y | 58.99 | 1197.6982 | 11 | 1.88 | 599.8544 | 2 | 15.65 | 0.9594 | 0.9666 | 45162 | 3.26e4 | 1 | 1 | 52 | 62 |  |  | DB Search |
| R.LDLAGRDLTDYLM(+15.99)K.I | Y | 58.56 | 1638.8287 | 14 | -1.91 | 820.4158 | 2 | 18.15 | 1.1426 | 1.1498 | 58283 | 0 | 0 | 0 | 201 | 214 | Oxidation (M) | M13:Oxidation (M):1000 | DB Search |
| R.LDLAGRDLTDYLMK.I | Y | 58.53 | 1622.8338 | 14 | -6.55 | 541.9455 | 3 | 19.25 | 0.7962 | 0.8035 | 64822 | 9.54e2 | 1 | 1 | 201 | 214 |  |  | DB Search |
| R.VAPEEHPVLLTEAPLNPK.A | Y | 58.04 | 1953.0571 | 18 | 3.48 | 977.5341 | 2 | 16.43 | 1.2045 | 1.2117 | 48611 | 4.81e2 | 1 | 1 | 119 | 136 |  |  | DB Search |
| total 5 peptides |  |  |  |  |  |  |  |  |  |  |  |  |  |  |  |  |  |  |  |
| --- | --- | --- | --- | --- | --- | --- | --- | --- | --- | --- | --- | --- | --- | --- | --- | --- | --- | --- | --- |

Best Unique PSM (Scan Sample\_9\_A1\_1\_3646.d:18674, m/z=677.8154, z=2, RT=10.95, ppm=0.1):


TRINITY\_DN1272\_c0\_g1\_i1.p1

back to list

  

| Protein Coverage
| Supporting Peptides
| Best Unique PSM
|

Protein Coverage:

Supporting Peptides:

| Peptide | Uniq | -10lgP | Mass | Length | ppm | m/z | z | RT | 1/k0 Start | 1/k0 End | Precursor ID | Area Sample 10 | #Feature | #Feature Sample 10 | Start | End | PTM | AScore | Found By |
| --- | --- | --- | --- | --- | --- | --- | --- | --- | --- | --- | --- | --- | --- | --- | --- | --- | --- | --- | --- |
| K.YIQGENEKQEK.I | Y | 58.62 | 1364.6572 | 11 | -4.52 | 683.3292 | 2 | 9.57 | 0.9691 | 0.9763 | 11125 | 0 | 0 | 0 | 116 | 126 |  |  | DB Search |
| total 1 peptides |  |  |  |  |  |  |  |  |  |  |  |  |  |  |  |  |  |  |  |
| --- | --- | --- | --- | --- | --- | --- | --- | --- | --- | --- | --- | --- | --- | --- | --- | --- | --- | --- | --- |

Best Unique PSM (Scan Sample\_9\_A1\_1\_3646.d:11125, m/z=683.3292, z=2, RT=9.57, ppm=-9.74):


TRINITY\_DN2477\_c0\_g1\_i5.p1

back to list

  

| Protein Coverage
| Supporting Peptides
| Best Unique PSM
|

Protein Coverage:

Supporting Peptides:

| Peptide | Uniq | -10lgP | Mass | Length | ppm | m/z | z | RT | 1/k0 Start | 1/k0 End | Precursor ID | Area Sample 10 | #Feature | #Feature Sample 10 | Start | End | PTM | AScore | Found By |
| --- | --- | --- | --- | --- | --- | --- | --- | --- | --- | --- | --- | --- | --- | --- | --- | --- | --- | --- | --- |
| K.DSYVGDEAQSKR.G | Y | 59.06 | 1353.616 | 12 | 5.32 | 677.8154 | 2 | 10.95 | 1.0041 | 1.0114 | 18674 | 7.12e3 | 1 | 1 | 8 | 19 |  |  | DB Search |
| R.VAPEEHPVLLTEAPLNPK.A | Y | 58.04 | 1953.0571 | 18 | 3.48 | 977.5341 | 2 | 16.43 | 1.2045 | 1.2117 | 48611 | 4.81e2 | 1 | 1 | 53 | 70 |  |  | DB Search |
| total 2 peptides |  |  |  |  |  |  |  |  |  |  |  |  |  |  |  |  |  |  |  |
| --- | --- | --- | --- | --- | --- | --- | --- | --- | --- | --- | --- | --- | --- | --- | --- | --- | --- | --- | --- |

Best Unique PSM (Scan Sample\_9\_A1\_1\_3646.d:18674, m/z=677.8154, z=2, RT=10.95, ppm=0.1):


TRINITY\_DN2472\_c0\_g1\_i1.p1

back to list

  

| Protein Coverage
| Supporting Peptides
| Best Unique PSM
|

Protein Coverage:

Supporting Peptides:

| Peptide | Uniq | -10lgP | Mass | Length | ppm | m/z | z | RT | 1/k0 Start | 1/k0 End | Precursor ID | Area Sample 10 | #Feature | #Feature Sample 10 | Start | End | PTM | AScore | Found By |
| --- | --- | --- | --- | --- | --- | --- | --- | --- | --- | --- | --- | --- | --- | --- | --- | --- | --- | --- | --- |
| M.S(+42.01)SGEIESLRQEAENLR.N | Y | 58.14 | 1858.9021 | 16 | 2.14 | 930.4554 | 2 | 18.72 | 1.1961 | 1.2033 | 61577 | 1.51e3 | 1 | 1 | 2 | 17 | Acetylation (Protein N-term) | S1:Acetylation (Protein N-term):1000 | DB Search |
| total 1 peptides |  |  |  |  |  |  |  |  |  |  |  |  |  |  |  |  |  |  |  |
| --- | --- | --- | --- | --- | --- | --- | --- | --- | --- | --- | --- | --- | --- | --- | --- | --- | --- | --- | --- |

Best Unique PSM (Scan Sample\_9\_A1\_1\_3646.d:61577, m/z=930.4554, z=2, RT=18.72, ppm=-3.08):


TRINITY\_DN4762\_c0\_g1\_i2.p1

back to list

  

| Protein Coverage
| Supporting Peptides
| Best Unique PSM
|

Protein Coverage:

Supporting Peptides:

| Peptide | Uniq | -10lgP | Mass | Length | ppm | m/z | z | RT | 1/k0 Start | 1/k0 End | Precursor ID | Area Sample 10 | #Feature | #Feature Sample 10 | Start | End | PTM | AScore | Found By |
| --- | --- | --- | --- | --- | --- | --- | --- | --- | --- | --- | --- | --- | --- | --- | --- | --- | --- | --- | --- |
| K.SPC(+57.02)PPRK.S | Y | 30.36 | 840.4276 | 7 | 3.69 | 421.2204 | 2 | 9.55 | 0.7909 | 0.7982 | 10837 | 4.62e3 | 1 | 1 | 314 | 320 | Carbamidomethylation |  | DB Search |
| total 1 peptides |  |  |  |  |  |  |  |  |  |  |  |  |  |  |  |  |  |  |  |
| --- | --- | --- | --- | --- | --- | --- | --- | --- | --- | --- | --- | --- | --- | --- | --- | --- | --- | --- | --- |

Best Unique PSM (Scan Sample\_9\_A1\_1\_3646.d:10837, m/z=421.2204, z=2, RT=9.55, ppm=-1.53):


TRINITY\_DN36\_c0\_g1\_i13.p1

back to list

  

| Protein Coverage
| Supporting Peptides
| Best Unique PSM
|

Protein Coverage:

Supporting Peptides:

| Peptide | Uniq | -10lgP | Mass | Length | ppm | m/z | z | RT | 1/k0 Start | 1/k0 End | Precursor ID | Area Sample 10 | #Feature | #Feature Sample 10 | Start | End | PTM | AScore | Found By |
| --- | --- | --- | --- | --- | --- | --- | --- | --- | --- | --- | --- | --- | --- | --- | --- | --- | --- | --- | --- |
| R.LGGIEVTQGNC(+57.02)ARK.Y | Y | 58.24 | 1501.7671 | 14 | 6.42 | 751.8917 | 2 | 12.66 | 1.0632 | 1.0705 | 28959 | 0 | 0 | 0 | 57 | 70 | Carbamidomethylation |  | DB Search |
| K.FETQIATK.D | Y | 57.09 | 936.4916 | 8 | 7.3 | 937.5009 | 1 | 11.88 | 1.1925 | 1.1997 | 24345 | 0 | 0 | 0 | 166 | 173 |  |  | DB Search |
| total 2 peptides |  |  |  |  |  |  |  |  |  |  |  |  |  |  |  |  |  |  |  |
| --- | --- | --- | --- | --- | --- | --- | --- | --- | --- | --- | --- | --- | --- | --- | --- | --- | --- | --- | --- |

Best Unique PSM (Scan Sample\_9\_A1\_1\_3646.d:28959, m/z=751.8917, z=2, RT=12.66, ppm=1.21):


TRINITY\_DN57\_c0\_g1\_i1.p1

back to list

  

| Protein Coverage
| Supporting Peptides
| Best Unique PSM
|

Protein Coverage:

Supporting Peptides:

| Peptide | Uniq | -10lgP | Mass | Length | ppm | m/z | z | RT | 1/k0 Start | 1/k0 End | Precursor ID | Area Sample 10 | #Feature | #Feature Sample 10 | Start | End | PTM | AScore | Found By |
| --- | --- | --- | --- | --- | --- | --- | --- | --- | --- | --- | --- | --- | --- | --- | --- | --- | --- | --- | --- |
| R.VGQAM(+15.99)ASPEDK.A | Y | 50.64 | 1147.5179 | 11 | 12.14 | 574.7702 | 2 | 9.69 | 0.8664 | 0.8737 | 11668 | 0 | 0 | 0 | 564 | 574 | Oxidation (M) | M5:Oxidation (M):1000 | DB Search |
| total 1 peptides |  |  |  |  |  |  |  |  |  |  |  |  |  |  |  |  |  |  |  |
| --- | --- | --- | --- | --- | --- | --- | --- | --- | --- | --- | --- | --- | --- | --- | --- | --- | --- | --- | --- |

Best Unique PSM (Scan Sample\_9\_A1\_1\_3646.d:11668, m/z=574.7702, z=2, RT=9.69, ppm=6.92):


TRINITY\_DN96161\_c0\_g1\_i1.p1

back to list

  

| Protein Coverage
| Supporting Peptides
| Best Unique PSM
|

Protein Coverage:

Supporting Peptides:

| Peptide | Uniq | -10lgP | Mass | Length | ppm | m/z | z | RT | 1/k0 Start | 1/k0 End | Precursor ID | Area Sample 10 | #Feature | #Feature Sample 10 | Start | End | PTM | AScore | Found By |
| --- | --- | --- | --- | --- | --- | --- | --- | --- | --- | --- | --- | --- | --- | --- | --- | --- | --- | --- | --- |
| K.VGINYQPPTVVPGGDLAK.V | Y | 50.54 | 1823.9781 | 18 | -3.14 | 912.9887 | 2 | 17.25 | 1.1186 | 1.1258 | 53075 | 0 | 0 | 0 | 353 | 370 |  |  | DB Search |
| total 1 peptides |  |  |  |  |  |  |  |  |  |  |  |  |  |  |  |  |  |  |  |
| --- | --- | --- | --- | --- | --- | --- | --- | --- | --- | --- | --- | --- | --- | --- | --- | --- | --- | --- | --- |

Best Unique PSM (Scan Sample\_9\_A1\_1\_3646.d:53075, m/z=912.9887, z=2, RT=17.25, ppm=-8.35):


TRINITY\_DN240\_c0\_g1\_i4.p1

back to list

  

| Protein Coverage
| Supporting Peptides
| Best Unique PSM
|

Protein Coverage:

Supporting Peptides:

| Peptide | Uniq | -10lgP | Mass | Length | ppm | m/z | z | RT | 1/k0 Start | 1/k0 End | Precursor ID | Area Sample 10 | #Feature | #Feature Sample 10 | Start | End | PTM | AScore | Found By |
| --- | --- | --- | --- | --- | --- | --- | --- | --- | --- | --- | --- | --- | --- | --- | --- | --- | --- | --- | --- |
| K.AYAAFLEFKDVVK.A | Y | 59.04 | 1499.8024 | 13 | 3.65 | 750.9073 | 2 | 19.03 | 1.0945 | 1.1017 | 63342 | 1.12e4 | 1 | 1 | 217 | 229 |  |  | DB Search |
| K.IGAAAQALADQSYPFLK.S | Y | 58.85 | 1762.9253 | 17 | 0.88 | 882.4661 | 2 | 19.07 | 1.1606 | 1.1678 | 63232 | 1.84e3 | 1 | 1 | 249 | 265 |  |  | DB Search |
| K.AISSASGPTGM(+15.99)TSK.A | Y | 58.65 | 1309.6184 | 14 | 2.9 | 655.8149 | 2 | 10.52 | 0.9352 | 0.9424 | 16184 | 9.25e2 | 1 | 1 | 151 | 164 | Oxidation (M) | M11:Oxidation (M):1000 | DB Search |
| K.ANQVSSAAAAPVPAAGGDK.I | Y | 58.63 | 1680.8431 | 19 | 2.49 | 841.4265 | 2 | 12.47 | 1.068 | 1.0753 | 27849 | 0 | 0 | 0 | 230 | 248 |  |  | DB Search |
| K.AAASAHHQAIGSIDAAGVTSAADYASVTAALGR.A | Y | 46.39 | 3080.5275 | 33 | -3.95 | 771.1321 | 4 | 18.15 | 1.0656 | 1.0729 | 58236 | 5.93e2 | 1 | 1 | 307 | 339 |  |  | DB Search |
| total 5 peptides |  |  |  |  |  |  |  |  |  |  |  |  |  |  |  |  |  |  |  |
| --- | --- | --- | --- | --- | --- | --- | --- | --- | --- | --- | --- | --- | --- | --- | --- | --- | --- | --- | --- |

Best Unique PSM (Scan Sample\_9\_A1\_1\_3646.d:63342, m/z=750.9073, z=2, RT=19.03, ppm=-1.56):


TRINITY\_DN801\_c0\_g1\_i4.p1

back to list

  

| Protein Coverage
| Supporting Peptides
| Best Unique PSM
|

Protein Coverage:

Supporting Peptides:

| Peptide | Uniq | -10lgP | Mass | Length | ppm | m/z | z | RT | 1/k0 Start | 1/k0 End | Precursor ID | Area Sample 10 | #Feature | #Feature Sample 10 | Start | End | PTM | AScore | Found By |
| --- | --- | --- | --- | --- | --- | --- | --- | --- | --- | --- | --- | --- | --- | --- | --- | --- | --- | --- | --- |
| K.VFDRDGNGFISAAELR.H | Y | 51.43 | 1765.8747 | 16 | -4.93 | 883.9357 | 2 | 19.74 | 1.1274 | 1.1346 | 67630 | 2.9e3 | 1 | 1 | 124 | 139 |  |  | DB Search |
| total 1 peptides |  |  |  |  |  |  |  |  |  |  |  |  |  |  |  |  |  |  |  |
| --- | --- | --- | --- | --- | --- | --- | --- | --- | --- | --- | --- | --- | --- | --- | --- | --- | --- | --- | --- |

Best Unique PSM (Scan Sample\_9\_A1\_1\_3646.d:67630, m/z=883.9357, z=2, RT=19.74, ppm=-10.14):


TRINITY\_DN13856\_c0\_g1\_i2.p2

back to list

  

| Protein Coverage
| Supporting Peptides
| Best Unique PSM
|

Protein Coverage:

Supporting Peptides:

| Peptide | Uniq | -10lgP | Mass | Length | ppm | m/z | z | RT | 1/k0 Start | 1/k0 End | Precursor ID | Area Sample 10 | #Feature | #Feature Sample 10 | Start | End | PTM | AScore | Found By |
| --- | --- | --- | --- | --- | --- | --- | --- | --- | --- | --- | --- | --- | --- | --- | --- | --- | --- | --- | --- |
| R.IPLDDFQDPTLTLENAHNK.L | Y | 58.63 | 2180.0749 | 19 | -0.5 | 727.6948 | 3 | 18.20 | 0.9904 | 0.9977 | 58430 | 9.46e2 | 1 | 1 | 175 | 193 |  |  | DB Search |
| total 1 peptides |  |  |  |  |  |  |  |  |  |  |  |  |  |  |  |  |  |  |  |
| --- | --- | --- | --- | --- | --- | --- | --- | --- | --- | --- | --- | --- | --- | --- | --- | --- | --- | --- | --- |

Best Unique PSM (Scan Sample\_9\_A1\_1\_3646.d:58430, m/z=727.6948, z=3, RT=18.20, ppm=-5.71):


TRINITY\_DN585\_c0\_g1\_i10.p1

back to list

  

| Protein Coverage
| Supporting Peptides
| Best Unique PSM
|

Protein Coverage:

Supporting Peptides:

| Peptide | Uniq | -10lgP | Mass | Length | ppm | m/z | z | RT | 1/k0 Start | 1/k0 End | Precursor ID | Area Sample 10 | #Feature | #Feature Sample 10 | Start | End | PTM | AScore | Found By |
| --- | --- | --- | --- | --- | --- | --- | --- | --- | --- | --- | --- | --- | --- | --- | --- | --- | --- | --- | --- |
| K.IQDKEGIPPDQQR.L | Y | 58.25 | 1522.7739 | 13 | 2.55 | 762.3922 | 2 | 11.03 | 1.0343 | 1.0415 | 18957 | 1.53e3 | 1 | 1 | 30 | 42 |  |  | DB Search |
| total 1 peptides |  |  |  |  |  |  |  |  |  |  |  |  |  |  |  |  |  |  |  |
| --- | --- | --- | --- | --- | --- | --- | --- | --- | --- | --- | --- | --- | --- | --- | --- | --- | --- | --- | --- |

Best Unique PSM (Scan Sample\_9\_A1\_1\_3646.d:18957, m/z=762.3922, z=2, RT=11.03, ppm=-2.67):


TRINITY\_DN817\_c0\_g1\_i10.p1

back to list

  

| Protein Coverage
| Supporting Peptides
| Best Unique PSM
|

Protein Coverage:

Supporting Peptides:

| Peptide | Uniq | -10lgP | Mass | Length | ppm | m/z | z | RT | 1/k0 Start | 1/k0 End | Precursor ID | Area Sample 10 | #Feature | #Feature Sample 10 | Start | End | PTM | AScore | Found By |
| --- | --- | --- | --- | --- | --- | --- | --- | --- | --- | --- | --- | --- | --- | --- | --- | --- | --- | --- | --- |
| R.LC(+57.02)QPGYSTC(+57.02)ENHC(+57.02)R.R | Y | 55.11 | 1780.708 | 14 | 0.84 | 891.3574 | 2 | 11.42 | 1.0861 | 1.0933 | 21509 | 5.05e2 | 1 | 1 | 155 | 168 | Carbamidomethylation, Carbamidomethylation, Carbamidomethylation |  | DB Search |
| R.LC(+57.02)QPGYSTC(+57.02)ENHC(+57.02)RR.L | Y | 43.99 | 1936.8091 | 15 | 3.79 | 646.6094 | 3 | 10.89 | 0.8575 | 0.8648 | 18436 | 3.51e2 | 1 | 1 | 155 | 169 | Carbamidomethylation, Carbamidomethylation, Carbamidomethylation |  | DB Search |
| total 2 peptides |  |  |  |  |  |  |  |  |  |  |  |  |  |  |  |  |  |  |  |
| --- | --- | --- | --- | --- | --- | --- | --- | --- | --- | --- | --- | --- | --- | --- | --- | --- | --- | --- | --- |

Best Unique PSM (Scan Sample\_9\_A1\_1\_3646.d:21509, m/z=891.3574, z=2, RT=11.42, ppm=-4.38):


TRINITY\_DN34\_c0\_g1\_i1.p1

back to list

  

| Protein Coverage
| Supporting Peptides
| Best Unique PSM
|

Protein Coverage:

Supporting Peptides:

| Peptide | Uniq | -10lgP | Mass | Length | ppm | m/z | z | RT | 1/k0 Start | 1/k0 End | Precursor ID | Area Sample 10 | #Feature | #Feature Sample 10 | Start | End | PTM | AScore | Found By |
| --- | --- | --- | --- | --- | --- | --- | --- | --- | --- | --- | --- | --- | --- | --- | --- | --- | --- | --- | --- |
| M(+42.01)(+15.99)DAAQAAEIKK.K | Y | 58.6 | 1232.6071 | 11 | -1.84 | 617.3065 | 2 | 12.14 | 0.9509 | 0.9582 | 25954 | 0 | 0 | 0 | 1 | 11 | Acetylation (Protein N-term), Oxidation (M) | M1:Acetylation (Protein N-term):1000 M1:Oxidation (M):1000 | DB Search |
| total 1 peptides |  |  |  |  |  |  |  |  |  |  |  |  |  |  |  |  |  |  |  |
| --- | --- | --- | --- | --- | --- | --- | --- | --- | --- | --- | --- | --- | --- | --- | --- | --- | --- | --- | --- |

Best Unique PSM (Scan Sample\_9\_A1\_1\_3646.d:25954, m/z=617.3065, z=2, RT=12.14, ppm=-7.06):


TRINITY\_DN129732\_c0\_g1\_i1.p2

back to list

  

| Protein Coverage
| Supporting Peptides
| Best Unique PSM
|

Protein Coverage:

Supporting Peptides:

| Peptide | Uniq | -10lgP | Mass | Length | ppm | m/z | z | RT | 1/k0 Start | 1/k0 End | Precursor ID | Area Sample 10 | #Feature | #Feature Sample 10 | Start | End | PTM | AScore | Found By |
| --- | --- | --- | --- | --- | --- | --- | --- | --- | --- | --- | --- | --- | --- | --- | --- | --- | --- | --- | --- |
| K.LQFSDIPNGLGALSK.V | Y | 57.81 | 1558.8355 | 15 | 2.37 | 780.4228 | 2 | 19.42 | 1.0427 | 1.05 | 65874 | 0 | 0 | 0 | 55 | 69 |  |  | DB Search |
| total 1 peptides |  |  |  |  |  |  |  |  |  |  |  |  |  |  |  |  |  |  |  |
| --- | --- | --- | --- | --- | --- | --- | --- | --- | --- | --- | --- | --- | --- | --- | --- | --- | --- | --- | --- |

Best Unique PSM (Scan Sample\_9\_A1\_1\_3646.d:65874, m/z=780.4228, z=2, RT=19.42, ppm=-2.85):


TRINITY\_DN36\_c0\_g1\_i1.p1

back to list

  

| Protein Coverage
| Supporting Peptides
| Best Unique PSM
|

Protein Coverage:

Supporting Peptides:

| Peptide | Uniq | -10lgP | Mass | Length | ppm | m/z | z | RT | 1/k0 Start | 1/k0 End | Precursor ID | Area Sample 10 | #Feature | #Feature Sample 10 | Start | End | PTM | AScore | Found By |
| --- | --- | --- | --- | --- | --- | --- | --- | --- | --- | --- | --- | --- | --- | --- | --- | --- | --- | --- | --- |
| R.LGGIEVTQGNC(+57.02)ARK.Y | Y | 58.24 | 1501.7671 | 14 | 6.42 | 751.8917 | 2 | 12.66 | 1.0632 | 1.0705 | 28959 | 0 | 0 | 0 | 58 | 71 | Carbamidomethylation |  | DB Search |
| total 1 peptides |  |  |  |  |  |  |  |  |  |  |  |  |  |  |  |  |  |  |  |
| --- | --- | --- | --- | --- | --- | --- | --- | --- | --- | --- | --- | --- | --- | --- | --- | --- | --- | --- | --- |

Best Unique PSM (Scan Sample\_9\_A1\_1\_3646.d:28959, m/z=751.8917, z=2, RT=12.66, ppm=1.21):


TRINITY\_DN30356\_c0\_g1\_i1.p3

back to list

  

| Protein Coverage
| Supporting Peptides
| Best Unique PSM
|

Protein Coverage:

Supporting Peptides:

| Peptide | Uniq | -10lgP | Mass | Length | ppm | m/z | z | RT | 1/k0 Start | 1/k0 End | Precursor ID | Area Sample 10 | #Feature | #Feature Sample 10 | Start | End | PTM | AScore | Found By |
| --- | --- | --- | --- | --- | --- | --- | --- | --- | --- | --- | --- | --- | --- | --- | --- | --- | --- | --- | --- |
| R.KLNAELANSR.L | Y | 57.15 | 1114.6094 | 10 | -3.85 | 558.3069 | 2 | 11.39 | 0.868 | 0.8753 | 21472 | 0 | 0 | 0 | 154 | 163 |  |  | DB Search |
| total 1 peptides |  |  |  |  |  |  |  |  |  |  |  |  |  |  |  |  |  |  |  |
| --- | --- | --- | --- | --- | --- | --- | --- | --- | --- | --- | --- | --- | --- | --- | --- | --- | --- | --- | --- |

Best Unique PSM (Scan Sample\_9\_A1\_1\_3646.d:21472, m/z=558.3069, z=2, RT=11.39, ppm=-9.07):


TRINITY\_DN2636\_c0\_g1\_i1.p1

back to list

  

| Protein Coverage
| Supporting Peptides
| Best Unique PSM
|

Protein Coverage:

Supporting Peptides:

| Peptide | Uniq | -10lgP | Mass | Length | ppm | m/z | z | RT | 1/k0 Start | 1/k0 End | Precursor ID | Area Sample 10 | #Feature | #Feature Sample 10 | Start | End | PTM | AScore | Found By |
| --- | --- | --- | --- | --- | --- | --- | --- | --- | --- | --- | --- | --- | --- | --- | --- | --- | --- | --- | --- |
| K.VVEKEAETER.K | Y | 56.06 | 1188.5986 | 10 | 0.32 | 397.2049 | 3 | 9.55 | 0.7265 | 0.7339 | 11068 | 0 | 0 | 0 | 199 | 208 |  |  | DB Search |
| K.IHHELNQFC(+57.02)SVK.S | Y | 47.81 | 1510.7351 | 12 | -0.1 | 756.3708 | 2 | 12.64 | 1.05 | 1.0572 | 28909 | 0 | 0 | 0 | 117 | 128 | Carbamidomethylation |  | DB Search |
| total 2 peptides |  |  |  |  |  |  |  |  |  |  |  |  |  |  |  |  |  |  |  |
| --- | --- | --- | --- | --- | --- | --- | --- | --- | --- | --- | --- | --- | --- | --- | --- | --- | --- | --- | --- |

Best Unique PSM (Scan Sample\_9\_A1\_1\_3646.d:11068, m/z=397.2049, z=3, RT=9.55, ppm=-4.91):


TRINITY\_DN1339\_c0\_g1\_i2.p1

back to list

  

| Protein Coverage
| Supporting Peptides
| Best Unique PSM
|

Protein Coverage:

Supporting Peptides:

| Peptide | Uniq | -10lgP | Mass | Length | ppm | m/z | z | RT | 1/k0 Start | 1/k0 End | Precursor ID | Area Sample 10 | #Feature | #Feature Sample 10 | Start | End | PTM | AScore | Found By |
| --- | --- | --- | --- | --- | --- | --- | --- | --- | --- | --- | --- | --- | --- | --- | --- | --- | --- | --- | --- |
| K.VITSDDPAEKEK.K | Y | 59.05 | 1330.6616 | 12 | 3.57 | 666.337 | 2 | 10.72 | 0.9884 | 0.9957 | 17382 | 1.42e4 | 1 | 1 | 160 | 171 |  |  | DB Search |
| K.FPGYISNSLSLK.F | Y | 58.33 | 1324.7027 | 12 | 2.31 | 663.3567 | 2 | 18.15 | 0.9848 | 0.992 | 58220 | 2.21e2 | 1 | 1 | 272 | 283 |  |  | DB Search |
| total 2 peptides |  |  |  |  |  |  |  |  |  |  |  |  |  |  |  |  |  |  |  |
| --- | --- | --- | --- | --- | --- | --- | --- | --- | --- | --- | --- | --- | --- | --- | --- | --- | --- | --- | --- |

Best Unique PSM (Scan Sample\_9\_A1\_1\_3646.d:17382, m/z=666.337, z=2, RT=10.72, ppm=-1.65):


TRINITY\_DN160\_c0\_g1\_i2.p1

back to list

  

| Protein Coverage
| Supporting Peptides
| Best Unique PSM
|

Protein Coverage:

Supporting Peptides:

| Peptide | Uniq | -10lgP | Mass | Length | ppm | m/z | z | RT | 1/k0 Start | 1/k0 End | Precursor ID | Area Sample 10 | #Feature | #Feature Sample 10 | Start | End | PTM | AScore | Found By |
| --- | --- | --- | --- | --- | --- | --- | --- | --- | --- | --- | --- | --- | --- | --- | --- | --- | --- | --- | --- |
| K.LRLDFDHEAKLNMIFR.F | Y | 58.67 | 2017.0567 | 16 | 5.71 | 505.2717 | 4 | 18.48 | 0.8644 | 0.8716 | 60165 | 7.24e2 | 1 | 1 | 1007 | 1022 |  |  | DB Search |
| K.LRLDFDHEAKLNM(+15.99)IFR.F | Y | 58.6 | 2033.0516 | 16 | 3.46 | 509.2693 | 4 | 17.78 | 0.8562 | 0.8635 | 56012 | 9.75e2 | 1 | 1 | 1007 | 1022 | Oxidation (M) | M13:Oxidation (M):1000 | DB Search |
| R.LDFDHEAKLNMIFR.F | Y | 54.86 | 1747.8715 | 14 | -10.63 | 583.6219 | 3 | 18.34 | 0.8542 | 0.8615 | 59425 | 0 | 0 | 0 | 1009 | 1022 |  |  | DB Search |
| K.ELVM(+15.99)HEQSR.C | Y | 53.5 | 1143.5342 | 9 | 6.21 | 572.775 | 2 | 9.82 | 0.9028 | 0.9101 | 12284 | 6.13e2 | 1 | 1 | 1116 | 1124 | Oxidation (M) | M4:Oxidation (M):1000 | DB Search |
| K.VNQAC(+57.02)VR.T | Y | 30.94 | 845.4178 | 7 | 1.39 | 423.7145 | 2 | 9.76 | 0.7705 | 0.7779 | 11899 | 1.24e3 | 1 | 1 | 871 | 877 | Carbamidomethylation |  | DB Search |
| K.LRLDFDHEAK.L | Y | 29.24 | 1242.6356 | 10 | -1.44 | 415.2164 | 3 | 14.18 | 0.7282 | 0.7355 | 37433 | 0 | 0 | 0 | 1007 | 1016 |  |  | DB Search |
| total 6 peptides |  |  |  |  |  |  |  |  |  |  |  |  |  |  |  |  |  |  |  |
| --- | --- | --- | --- | --- | --- | --- | --- | --- | --- | --- | --- | --- | --- | --- | --- | --- | --- | --- | --- |

Best Unique PSM (Scan Sample\_9\_A1\_1\_3646.d:60165, m/z=505.2717, z=4, RT=18.48, ppm=0.49):


TRINITY\_DN164271\_c0\_g1\_i1.p1

back to list

  

| Protein Coverage
| Supporting Peptides
| Best Unique PSM
|

Protein Coverage:

Supporting Peptides:

| Peptide | Uniq | -10lgP | Mass | Length | ppm | m/z | z | RT | 1/k0 Start | 1/k0 End | Precursor ID | Area Sample 10 | #Feature | #Feature Sample 10 | Start | End | PTM | AScore | Found By |
| --- | --- | --- | --- | --- | --- | --- | --- | --- | --- | --- | --- | --- | --- | --- | --- | --- | --- | --- | --- |
| M.A(+42.01)TGIKPDPQC(+57.02)TDLYNK.L | Y | 58.9 | 1861.888 | 16 | 1.83 | 931.9481 | 2 | 14.86 | 1.1622 | 1.1694 | 40590 | 1.38e3 | 1 | 1 | 2 | 17 | Acetylation (Protein N-term), Carbamidomethylation | A1:Acetylation (Protein N-term):1000 | DB Search |
| K.FSGTKDEVK.K | Y | 58.15 | 1009.508 | 9 | 0.78 | 505.759 | 2 | 10.13 | 0.8822 | 0.8895 | 13944 | 6.11e2 | 1 | 1 | 119 | 127 |  |  | DB Search |
| M.A(+42.01)TGIKPDPQC(+57.02)TDLYNKLK.M | Y | 57.26 | 2103.067 | 18 | 5.12 | 702.0295 | 3 | 15.96 | 0.9574 | 0.9646 | 46358 | 1.22e3 | 1 | 1 | 2 | 19 | Acetylation (Protein N-term), Carbamidomethylation | A1:Acetylation (Protein N-term):1000 | DB Search |
| total 3 peptides |  |  |  |  |  |  |  |  |  |  |  |  |  |  |  |  |  |  |  |
| --- | --- | --- | --- | --- | --- | --- | --- | --- | --- | --- | --- | --- | --- | --- | --- | --- | --- | --- | --- |

Best Unique PSM (Scan Sample\_9\_A1\_1\_3646.d:40590, m/z=931.9481, z=2, RT=14.86, ppm=-3.38):


TRINITY\_DN1910\_c0\_g1\_i5.p1

back to list

  

| Protein Coverage
| Supporting Peptides
| Best Unique PSM
|

Protein Coverage:

Supporting Peptides:

| Peptide | Uniq | -10lgP | Mass | Length | ppm | m/z | z | RT | 1/k0 Start | 1/k0 End | Precursor ID | Area Sample 10 | #Feature | #Feature Sample 10 | Start | End | PTM | AScore | Found By |
| --- | --- | --- | --- | --- | --- | --- | --- | --- | --- | --- | --- | --- | --- | --- | --- | --- | --- | --- | --- |
| K.NM(+15.99)M(+15.99)AAC(+57.02)DPR.H | Y | 58.66 | 1096.41 | 9 | 1.24 | 549.2101 | 2 | 9.53 | 0.8619 | 0.8692 | 10930 | 4.97e2 | 1 | 1 | 298 | 306 | Oxidation (M), Oxidation (M), Carbamidomethylation | M2:Oxidation (M):1000 M3:Oxidation (M):1000 | DB Search |
| K.IREEYPDR.I | Y | 58.47 | 1076.525 | 8 | 1.32 | 539.2677 | 2 | 10.98 | 0.8696 | 0.8769 | 18747 | 5.32e3 | 1 | 1 | 155 | 162 |  |  | DB Search |
| total 2 peptides |  |  |  |  |  |  |  |  |  |  |  |  |  |  |  |  |  |  |  |
| --- | --- | --- | --- | --- | --- | --- | --- | --- | --- | --- | --- | --- | --- | --- | --- | --- | --- | --- | --- |

Best Unique PSM (Scan Sample\_9\_A1\_1\_3646.d:10930, m/z=549.2101, z=2, RT=9.53, ppm=-3.99):


TRINITY\_DN118\_c1\_g1\_i5.p1

back to list

  

| Protein Coverage
| Supporting Peptides
| Best Unique PSM
|

Protein Coverage:

Supporting Peptides:

| Peptide | Uniq | -10lgP | Mass | Length | ppm | m/z | z | RT | 1/k0 Start | 1/k0 End | Precursor ID | Area Sample 10 | #Feature | #Feature Sample 10 | Start | End | PTM | AScore | Found By |
| --- | --- | --- | --- | --- | --- | --- | --- | --- | --- | --- | --- | --- | --- | --- | --- | --- | --- | --- | --- |
| R.ETC(+57.02)SDIEKR.E | Y | 58.74 | 1136.5132 | 9 | 1.02 | 569.2615 | 2 | 10.09 | 0.8947 | 0.902 | 13624 | 1.03e4 | 1 | 1 | 186 | 194 | Carbamidomethylation |  | DB Search |
| K.DC(+57.02)TPRETC(+57.02)SDIEKR.E | Y | 58.67 | 1765.7723 | 14 | -5.61 | 589.5917 | 3 | 11.15 | 0.8485 | 0.8558 | 19989 | 0 | 0 | 0 | 181 | 194 | Carbamidomethylation, Carbamidomethylation |  | DB Search |
| K.VC(+57.02)EDVKR.C | Y | 58.23 | 904.4436 | 7 | 1.65 | 453.2275 | 2 | 9.38 | 0.7685 | 0.7758 | 10310 | 8.12e3 | 2 | 2 | 122 | 128 | Carbamidomethylation |  | DB Search |
| total 3 peptides |  |  |  |  |  |  |  |  |  |  |  |  |  |  |  |  |  |  |  |
| --- | --- | --- | --- | --- | --- | --- | --- | --- | --- | --- | --- | --- | --- | --- | --- | --- | --- | --- | --- |

Best Unique PSM (Scan Sample\_9\_A1\_1\_3646.d:13624, m/z=569.2615, z=2, RT=10.09, ppm=-4.2):


TRINITY\_DN32763\_c0\_g1\_i1.p1

back to list

  

| Protein Coverage
| Supporting Peptides
| Best Unique PSM
|

Protein Coverage:

Supporting Peptides:

| Peptide | Uniq | -10lgP | Mass | Length | ppm | m/z | z | RT | 1/k0 Start | 1/k0 End | Precursor ID | Area Sample 10 | #Feature | #Feature Sample 10 | Start | End | PTM | AScore | Found By |
| --- | --- | --- | --- | --- | --- | --- | --- | --- | --- | --- | --- | --- | --- | --- | --- | --- | --- | --- | --- |
| R.VAQHAVVAR.W | Y | 43.99 | 949.5457 | 9 | 5.58 | 475.7803 | 2 | 10.02 | 0.8412 | 0.8485 | 13333 | 5.98e2 | 1 | 1 | 72 | 80 |  |  | DB Search |
| total 1 peptides |  |  |  |  |  |  |  |  |  |  |  |  |  |  |  |  |  |  |  |
| --- | --- | --- | --- | --- | --- | --- | --- | --- | --- | --- | --- | --- | --- | --- | --- | --- | --- | --- | --- |

Best Unique PSM (Scan Sample\_9\_A1\_1\_3646.d:13333, m/z=475.714, z=2, RT=10.10, ppm=0.36):


TRINITY\_DN495\_c0\_g1\_i6.p1

back to list

  

| Protein Coverage
| Supporting Peptides
| Best Unique PSM
|

Protein Coverage:

Supporting Peptides:

| Peptide | Uniq | -10lgP | Mass | Length | ppm | m/z | z | RT | 1/k0 Start | 1/k0 End | Precursor ID | Area Sample 10 | #Feature | #Feature Sample 10 | Start | End | PTM | AScore | Found By |
| --- | --- | --- | --- | --- | --- | --- | --- | --- | --- | --- | --- | --- | --- | --- | --- | --- | --- | --- | --- |
| K.SISC(+57.02)SKDEEIK.V | Y | 53.66 | 1294.6075 | 11 | 5.36 | 648.3111 | 2 | 10.85 | 0.98 | 0.9872 | 18283 | 0 | 0 | 0 | 248 | 258 | Carbamidomethylation |  | DB Search |
| total 1 peptides |  |  |  |  |  |  |  |  |  |  |  |  |  |  |  |  |  |  |  |
| --- | --- | --- | --- | --- | --- | --- | --- | --- | --- | --- | --- | --- | --- | --- | --- | --- | --- | --- | --- |

Best Unique PSM (Scan Sample\_9\_A1\_1\_3646.d:18283, m/z=648.3111, z=2, RT=10.85, ppm=0.14):


TRINITY\_DN10724\_c0\_g1\_i5.p1

back to list

  

| Protein Coverage
| Supporting Peptides
| Best Unique PSM
|

Protein Coverage:

Supporting Peptides:

| Peptide | Uniq | -10lgP | Mass | Length | ppm | m/z | z | RT | 1/k0 Start | 1/k0 End | Precursor ID | Area Sample 10 | #Feature | #Feature Sample 10 | Start | End | PTM | AScore | Found By |
| --- | --- | --- | --- | --- | --- | --- | --- | --- | --- | --- | --- | --- | --- | --- | --- | --- | --- | --- | --- |
| K.REPGDFGFDPLKFSK.R | Y | 56.43 | 1738.8678 | 15 | -1.1 | 580.6262 | 3 | 18.01 | 0.9024 | 0.9097 | 57542 | 0 | 0 | 0 | 171 | 185 |  |  | DB Search |
| total 1 peptides |  |  |  |  |  |  |  |  |  |  |  |  |  |  |  |  |  |  |  |
| --- | --- | --- | --- | --- | --- | --- | --- | --- | --- | --- | --- | --- | --- | --- | --- | --- | --- | --- | --- |

Best Unique PSM (Scan Sample\_9\_A1\_1\_3646.d:57542, m/z=580.6262, z=3, RT=18.01, ppm=-6.32):


TRINITY\_DN186979\_c0\_g1\_i1.p1

back to list

  

| Protein Coverage
| Supporting Peptides
| Best Unique PSM
|

Protein Coverage:

Supporting Peptides:

| Peptide | Uniq | -10lgP | Mass | Length | ppm | m/z | z | RT | 1/k0 Start | 1/k0 End | Precursor ID | Area Sample 10 | #Feature | #Feature Sample 10 | Start | End | PTM | AScore | Found By |
| --- | --- | --- | --- | --- | --- | --- | --- | --- | --- | --- | --- | --- | --- | --- | --- | --- | --- | --- | --- |
| R.IQNADDNKPVFK.S | Y | 57.75 | 1387.7095 | 12 | -2.77 | 694.8565 | 2 | 12.00 | 0.9747 | 0.982 | 25187 | 3.22e2 | 1 | 1 | 227 | 238 |  |  | DB Search |
| total 1 peptides |  |  |  |  |  |  |  |  |  |  |  |  |  |  |  |  |  |  |  |
| --- | --- | --- | --- | --- | --- | --- | --- | --- | --- | --- | --- | --- | --- | --- | --- | --- | --- | --- | --- |

Best Unique PSM (Scan Sample\_9\_A1\_1\_3646.d:25187, m/z=695.3257, z=2, RT=12.00, ppm=-7.99):


TRINITY\_DN166344\_c0\_g1\_i1.p1

back to list

  

| Protein Coverage
| Supporting Peptides
| Best Unique PSM
|

Protein Coverage:

Supporting Peptides:

| Peptide | Uniq | -10lgP | Mass | Length | ppm | m/z | z | RT | 1/k0 Start | 1/k0 End | Precursor ID | Area Sample 10 | #Feature | #Feature Sample 10 | Start | End | PTM | AScore | Found By |
| --- | --- | --- | --- | --- | --- | --- | --- | --- | --- | --- | --- | --- | --- | --- | --- | --- | --- | --- | --- |
| R.TQITC(+57.02)TQISLKR.G | Y | 58.58 | 1447.7817 | 12 | 7.09 | 724.8995 | 2 | 13.78 | 1.0246 | 1.0319 | 35286 | 0 | 0 | 0 | 174 | 185 | Carbamidomethylation |  | DB Search |
| total 1 peptides |  |  |  |  |  |  |  |  |  |  |  |  |  |  |  |  |  |  |  |
| --- | --- | --- | --- | --- | --- | --- | --- | --- | --- | --- | --- | --- | --- | --- | --- | --- | --- | --- | --- |

Best Unique PSM (Scan Sample\_9\_A1\_1\_3646.d:35286, m/z=723.3623, z=2, RT=13.81, ppm=1.87):


TRINITY\_DN19618\_c0\_g1\_i1.p1

back to list

  

| Protein Coverage
| Supporting Peptides
| Best Unique PSM
|

Protein Coverage:

Supporting Peptides:

| Peptide | Uniq | -10lgP | Mass | Length | ppm | m/z | z | RT | 1/k0 Start | 1/k0 End | Precursor ID | Area Sample 10 | #Feature | #Feature Sample 10 | Start | End | PTM | AScore | Found By |
| --- | --- | --- | --- | --- | --- | --- | --- | --- | --- | --- | --- | --- | --- | --- | --- | --- | --- | --- | --- |
| R.K(+42.01)LNAELANGR.L | Y | 58.99 | 1126.6094 | 10 | 3.51 | 564.311 | 2 | 13.77 | 0.883 | 0.8903 | 35173 | 1.19e4 | 3 | 3 | 7 | 16 | Acetylation (Protein N-term) | K1:Acetylation (Protein N-term):1000 | DB Search |
| total 1 peptides |  |  |  |  |  |  |  |  |  |  |  |  |  |  |  |  |  |  |  |
| --- | --- | --- | --- | --- | --- | --- | --- | --- | --- | --- | --- | --- | --- | --- | --- | --- | --- | --- | --- |

Best Unique PSM (Scan Sample\_9\_A1\_1\_3646.d:35173, m/z=564.311, z=2, RT=13.77, ppm=-1.71):


TRINITY\_DN11643\_c0\_g1\_i1.p1

back to list

  

| Protein Coverage
| Supporting Peptides
| Best Unique PSM
|

Protein Coverage:

Supporting Peptides:

| Peptide | Uniq | -10lgP | Mass | Length | ppm | m/z | z | RT | 1/k0 Start | 1/k0 End | Precursor ID | Area Sample 10 | #Feature | #Feature Sample 10 | Start | End | PTM | AScore | Found By |
| --- | --- | --- | --- | --- | --- | --- | --- | --- | --- | --- | --- | --- | --- | --- | --- | --- | --- | --- | --- |
| R.EVNTELDSRPETK.K | Y | 58.71 | 1516.7369 | 13 | 5.72 | 759.3761 | 2 | 11.75 | 1.0267 | 1.0339 | 23511 | 8.88e3 | 2 | 2 | 97 | 109 |  |  | DB Search |
| total 1 peptides |  |  |  |  |  |  |  |  |  |  |  |  |  |  |  |  |  |  |  |
| --- | --- | --- | --- | --- | --- | --- | --- | --- | --- | --- | --- | --- | --- | --- | --- | --- | --- | --- | --- |

Best Unique PSM (Scan Sample\_9\_A1\_1\_3646.d:23511, m/z=759.3761, z=2, RT=11.75, ppm=0.5):


TRINITY\_DN477\_c0\_g1\_i8.p2

back to list

  

| Protein Coverage
| Supporting Peptides
| Best Unique PSM
|

Protein Coverage:

Supporting Peptides:

| Peptide | Uniq | -10lgP | Mass | Length | ppm | m/z | z | RT | 1/k0 Start | 1/k0 End | Precursor ID | Area Sample 10 | #Feature | #Feature Sample 10 | Start | End | PTM | AScore | Found By |
| --- | --- | --- | --- | --- | --- | --- | --- | --- | --- | --- | --- | --- | --- | --- | --- | --- | --- | --- | --- |
| R.NDVNNPPQAR.R | Y | 58.12 | 1123.537 | 10 | 0.82 | 562.7733 | 2 | 9.95 | 0.8668 | 0.8741 | 12691 | 7.16e2 | 1 | 1 | 86 | 95 |  |  | DB Search |
| total 1 peptides |  |  |  |  |  |  |  |  |  |  |  |  |  |  |  |  |  |  |  |
| --- | --- | --- | --- | --- | --- | --- | --- | --- | --- | --- | --- | --- | --- | --- | --- | --- | --- | --- | --- |

Best Unique PSM (Scan Sample\_9\_A1\_1\_3646.d:12691, m/z=562.7733, z=2, RT=9.95, ppm=-4.4):


TRINITY\_DN4497\_c0\_g1\_i1.p1

back to list

  

| Protein Coverage
| Supporting Peptides
| Best Unique PSM
|

Protein Coverage:

Supporting Peptides:

| Peptide | Uniq | -10lgP | Mass | Length | ppm | m/z | z | RT | 1/k0 Start | 1/k0 End | Precursor ID | Area Sample 10 | #Feature | #Feature Sample 10 | Start | End | PTM | AScore | Found By |
| --- | --- | --- | --- | --- | --- | --- | --- | --- | --- | --- | --- | --- | --- | --- | --- | --- | --- | --- | --- |
| K.VLTSSDPAEKEK.K | Y | 58.69 | 1302.6667 | 12 | 2.82 | 652.3391 | 2 | 10.58 | 0.9965 | 1.0037 | 16473 | 6.51e3 | 1 | 1 | 117 | 128 |  |  | DB Search |
| total 1 peptides |  |  |  |  |  |  |  |  |  |  |  |  |  |  |  |  |  |  |  |
| --- | --- | --- | --- | --- | --- | --- | --- | --- | --- | --- | --- | --- | --- | --- | --- | --- | --- | --- | --- |

Best Unique PSM (Scan Sample\_9\_A1\_1\_3646.d:16473, m/z=652.3391, z=2, RT=10.58, ppm=-2.4):


TRINITY\_DN22492\_c0\_g1\_i1.p1

back to list

  

| Protein Coverage
| Supporting Peptides
| Best Unique PSM
|

Protein Coverage:

Supporting Peptides:

| Peptide | Uniq | -10lgP | Mass | Length | ppm | m/z | z | RT | 1/k0 Start | 1/k0 End | Precursor ID | Area Sample 10 | #Feature | #Feature Sample 10 | Start | End | PTM | AScore | Found By |
| --- | --- | --- | --- | --- | --- | --- | --- | --- | --- | --- | --- | --- | --- | --- | --- | --- | --- | --- | --- |
| K.KKLEPQAAPR.E | Y | 58.53 | 1136.6666 | 10 | 8.1 | 379.8972 | 3 | 9.69 | 0.7432 | 0.7506 | 11676 | 4.42e3 | 1 | 1 | 425 | 434 |  |  | DB Search |
| R.HC(+57.02)DPELSSFHTFWR.L | Y | 56.96 | 1817.7944 | 14 | -4.27 | 606.933 | 3 | 18.75 | 0.883 | 0.8903 | 61859 | 3.04e2 | 1 | 1 | 151 | 164 | Carbamidomethylation |  | DB Search |
| R.LIAKFETWEVAR.N | Y | 54.26 | 1461.798 | 12 | -4.64 | 731.899 | 2 | 17.17 | 1.0427 | 1.05 | 52609 | 0 | 0 | 0 | 318 | 329 |  |  | DB Search |
| total 3 peptides |  |  |  |  |  |  |  |  |  |  |  |  |  |  |  |  |  |  |  |
| --- | --- | --- | --- | --- | --- | --- | --- | --- | --- | --- | --- | --- | --- | --- | --- | --- | --- | --- | --- |

Best Unique PSM (Scan Sample\_9\_A1\_1\_3646.d:11676, m/z=379.8972, z=3, RT=9.69, ppm=2.87):


TRINITY\_DN428\_c0\_g1\_i1.p1

back to list

  

| Protein Coverage
| Supporting Peptides
| Best Unique PSM
|

Protein Coverage:

Supporting Peptides:

| Peptide | Uniq | -10lgP | Mass | Length | ppm | m/z | z | RT | 1/k0 Start | 1/k0 End | Precursor ID | Area Sample 10 | #Feature | #Feature Sample 10 | Start | End | PTM | AScore | Found By |
| --- | --- | --- | --- | --- | --- | --- | --- | --- | --- | --- | --- | --- | --- | --- | --- | --- | --- | --- | --- |
| K.IC(+57.02)HLLGIQVTDFTK.S | Y | 58.69 | 1643.8705 | 14 | 0.67 | 822.9388 | 2 | 18.46 | 1.0833 | 1.0905 | 60048 | 4.74e2 | 1 | 1 | 377 | 390 | Carbamidomethylation |  | DB Search |
| K.RIQQLSAIR.V | Y | 58.65 | 1083.6513 | 9 | 0.9 | 542.8306 | 2 | 13.16 | 0.9174 | 0.9247 | 31748 | 7.18e2 | 1 | 1 | 799 | 807 |  |  | DB Search |
| R.AGVLAHLEEER.D | Y | 58.65 | 1222.6306 | 11 | -5.54 | 612.316 | 2 | 14.82 | 0.9553 | 0.9626 | 40752 | 0 | 0 | 0 | 762 | 772 |  |  | DB Search |
| K.INNLEIQLDQEAKER.Q | Y | 58.24 | 1811.9377 | 15 | -7.51 | 906.9646 | 2 | 15.71 | 1.1969 | 1.2041 | 45235 | 0 | 0 | 0 | 1813 | 1827 |  |  | DB Search |
| R.AGVLAHLEEERDIK.L | Y | 57.92 | 1578.8365 | 14 | -4.44 | 790.4179 | 2 | 14.84 | 1.109 | 1.1162 | 40775 | 0 | 0 | 0 | 762 | 775 |  |  | DB Search |
| total 5 peptides |  |  |  |  |  |  |  |  |  |  |  |  |  |  |  |  |  |  |  |
| --- | --- | --- | --- | --- | --- | --- | --- | --- | --- | --- | --- | --- | --- | --- | --- | --- | --- | --- | --- |

Best Unique PSM (Scan Sample\_9\_A1\_1\_3646.d:60048, m/z=822.9388, z=2, RT=18.46, ppm=-4.55):


TRINITY\_DN96\_c0\_g1\_i2.p1

back to list

  

| Protein Coverage
| Supporting Peptides
| Best Unique PSM
|

Protein Coverage:

Supporting Peptides:

| Peptide | Uniq | -10lgP | Mass | Length | ppm | m/z | z | RT | 1/k0 Start | 1/k0 End | Precursor ID | Area Sample 10 | #Feature | #Feature Sample 10 | Start | End | PTM | AScore | Found By |
| --- | --- | --- | --- | --- | --- | --- | --- | --- | --- | --- | --- | --- | --- | --- | --- | --- | --- | --- | --- |
| K.TVQNFYELVTHSK.G | Y | 58.66 | 1564.7885 | 13 | 4.05 | 783.4006 | 2 | 18.26 | 1.0893 | 1.0965 | 58839 | 6.36e2 | 1 | 1 | 44 | 56 |  |  | DB Search |
| K.KVEQVQTDSR.D | Y | 53.61 | 1188.6098 | 10 | 5.11 | 595.3121 | 2 | 9.53 | 0.8725 | 0.8798 | 10904 | 2.14e2 | 1 | 1 | 153 | 162 |  |  | DB Search |
| total 2 peptides |  |  |  |  |  |  |  |  |  |  |  |  |  |  |  |  |  |  |  |
| --- | --- | --- | --- | --- | --- | --- | --- | --- | --- | --- | --- | --- | --- | --- | --- | --- | --- | --- | --- |

Best Unique PSM (Scan Sample\_9\_A1\_1\_3646.d:58839, m/z=783.4006, z=2, RT=18.26, ppm=-1.16):


TRINITY\_DN161\_c0\_g1\_i4.p1

back to list

  

| Protein Coverage
| Supporting Peptides
| Best Unique PSM
|

Protein Coverage:

Supporting Peptides:

| Peptide | Uniq | -10lgP | Mass | Length | ppm | m/z | z | RT | 1/k0 Start | 1/k0 End | Precursor ID | Area Sample 10 | #Feature | #Feature Sample 10 | Start | End | PTM | AScore | Found By |
| --- | --- | --- | --- | --- | --- | --- | --- | --- | --- | --- | --- | --- | --- | --- | --- | --- | --- | --- | --- |
| R.LDLAGRDLTDYLM(+15.99)K.I | Y | 58.56 | 1638.8287 | 14 | -1.91 | 820.4158 | 2 | 18.15 | 1.1426 | 1.1498 | 58283 | 0 | 0 | 0 | 34 | 47 | Oxidation (M) | M13:Oxidation (M):1000 | DB Search |
| R.LDLAGRDLTDYLMK.I | Y | 58.53 | 1622.8338 | 14 | -6.55 | 541.9455 | 3 | 19.25 | 0.7962 | 0.8035 | 64822 | 9.54e2 | 1 | 1 | 34 | 47 |  |  | DB Search |
| total 2 peptides |  |  |  |  |  |  |  |  |  |  |  |  |  |  |  |  |  |  |  |
| --- | --- | --- | --- | --- | --- | --- | --- | --- | --- | --- | --- | --- | --- | --- | --- | --- | --- | --- | --- |

Best Unique PSM (Scan Sample\_9\_A1\_1\_3646.d:58283, m/z=820.4158, z=2, RT=18.15, ppm=-7.13):


TRINITY\_DN40728\_c0\_g1\_i1.p1

back to list

  

| Protein Coverage
| Supporting Peptides
| Best Unique PSM
|

Protein Coverage:

Supporting Peptides:

| Peptide | Uniq | -10lgP | Mass | Length | ppm | m/z | z | RT | 1/k0 Start | 1/k0 End | Precursor ID | Area Sample 10 | #Feature | #Feature Sample 10 | Start | End | PTM | AScore | Found By |
| --- | --- | --- | --- | --- | --- | --- | --- | --- | --- | --- | --- | --- | --- | --- | --- | --- | --- | --- | --- |
| K.IQDKEGIPPDQQR.L | Y | 58.25 | 1522.7739 | 13 | 2.55 | 762.3922 | 2 | 11.03 | 1.0343 | 1.0415 | 18957 | 1.53e3 | 1 | 1 | 53 | 65 |  |  | DB Search |
| total 1 peptides |  |  |  |  |  |  |  |  |  |  |  |  |  |  |  |  |  |  |  |
| --- | --- | --- | --- | --- | --- | --- | --- | --- | --- | --- | --- | --- | --- | --- | --- | --- | --- | --- | --- |

Best Unique PSM (Scan Sample\_9\_A1\_1\_3646.d:18957, m/z=762.3922, z=2, RT=11.03, ppm=-2.67):


TRINITY\_DN687\_c0\_g2\_i1.p1

back to list

  

| Protein Coverage
| Supporting Peptides
| Best Unique PSM
|

Protein Coverage:

Supporting Peptides:

| Peptide | Uniq | -10lgP | Mass | Length | ppm | m/z | z | RT | 1/k0 Start | 1/k0 End | Precursor ID | Area Sample 10 | #Feature | #Feature Sample 10 | Start | End | PTM | AScore | Found By |
| --- | --- | --- | --- | --- | --- | --- | --- | --- | --- | --- | --- | --- | --- | --- | --- | --- | --- | --- | --- |
| K.VGINYQPPTVVPGGDLAK.V | Y | 50.54 | 1823.9781 | 18 | -3.14 | 912.9887 | 2 | 17.25 | 1.1186 | 1.1258 | 53075 | 0 | 0 | 0 | 353 | 370 |  |  | DB Search |
| total 1 peptides |  |  |  |  |  |  |  |  |  |  |  |  |  |  |  |  |  |  |  |
| --- | --- | --- | --- | --- | --- | --- | --- | --- | --- | --- | --- | --- | --- | --- | --- | --- | --- | --- | --- |

Best Unique PSM (Scan Sample\_9\_A1\_1\_3646.d:53075, m/z=912.9887, z=2, RT=17.25, ppm=-8.35):


TRINITY\_DN4815\_c0\_g1\_i2.p1

back to list

  

| Protein Coverage
| Supporting Peptides
| Best Unique PSM
|

Protein Coverage:

Supporting Peptides:

| Peptide | Uniq | -10lgP | Mass | Length | ppm | m/z | z | RT | 1/k0 Start | 1/k0 End | Precursor ID | Area Sample 10 | #Feature | #Feature Sample 10 | Start | End | PTM | AScore | Found By |
| --- | --- | --- | --- | --- | --- | --- | --- | --- | --- | --- | --- | --- | --- | --- | --- | --- | --- | --- | --- |
| K.TIKDVNC(+57.02)NNNQLK.S | Y | 58.63 | 1559.7726 | 13 | 0.66 | 780.89 | 2 | 11.23 | 1.0464 | 1.0536 | 20329 | 8.76e2 | 1 | 1 | 533 | 545 | Carbamidomethylation |  | DB Search |
| total 1 peptides |  |  |  |  |  |  |  |  |  |  |  |  |  |  |  |  |  |  |  |
| --- | --- | --- | --- | --- | --- | --- | --- | --- | --- | --- | --- | --- | --- | --- | --- | --- | --- | --- | --- |

Best Unique PSM (Scan Sample\_9\_A1\_1\_3646.d:20329, m/z=780.89, z=2, RT=11.23, ppm=-4.56):


TRINITY\_DN1386\_c0\_g1\_i1.p1

back to list

  

| Protein Coverage
| Supporting Peptides
| Best Unique PSM
|

Protein Coverage:

Supporting Peptides:

| Peptide | Uniq | -10lgP | Mass | Length | ppm | m/z | z | RT | 1/k0 Start | 1/k0 End | Precursor ID | Area Sample 10 | #Feature | #Feature Sample 10 | Start | End | PTM | AScore | Found By |
| --- | --- | --- | --- | --- | --- | --- | --- | --- | --- | --- | --- | --- | --- | --- | --- | --- | --- | --- | --- |
| K.SFLLALEKDQEK.H | Y | 58.66 | 1419.7609 | 12 | 1.99 | 710.8854 | 2 | 17.38 | 1.0729 | 1.0801 | 53732 | 9.75e2 | 1 | 1 | 61 | 72 |  |  | DB Search |
| R.KVIVDQLVTLATR.V | Y | 58.44 | 1454.882 | 13 | -0.05 | 728.4445 | 2 | 18.29 | 1.0283 | 1.0355 | 59014 | 2.77e3 | 1 | 1 | 547 | 559 |  |  | DB Search |
| R.SPTHSVIK.S | Y | 58.34 | 867.4814 | 8 | 1.38 | 434.7463 | 2 | 9.94 | 0.7831 | 0.7905 | 12865 | 6.66e2 | 1 | 1 | 259 | 266 |  |  | DB Search |
| total 3 peptides |  |  |  |  |  |  |  |  |  |  |  |  |  |  |  |  |  |  |  |
| --- | --- | --- | --- | --- | --- | --- | --- | --- | --- | --- | --- | --- | --- | --- | --- | --- | --- | --- | --- |

Best Unique PSM (Scan Sample\_9\_A1\_1\_3646.d:53732, m/z=710.8854, z=2, RT=17.38, ppm=-3.23):


TRINITY\_DN116\_c0\_g1\_i1.p1

back to list

  

| Protein Coverage
| Supporting Peptides
| Best Unique PSM
|

Protein Coverage:

Supporting Peptides:

| Peptide | Uniq | -10lgP | Mass | Length | ppm | m/z | z | RT | 1/k0 Start | 1/k0 End | Precursor ID | Area Sample 10 | #Feature | #Feature Sample 10 | Start | End | PTM | AScore | Found By |
| --- | --- | --- | --- | --- | --- | --- | --- | --- | --- | --- | --- | --- | --- | --- | --- | --- | --- | --- | --- |
| R.FTNKDIVC(+57.02)QIAYAK.I | Y | 55.98 | 1669.8498 | 14 | -4.24 | 835.9243 | 2 | 15.98 | 1.1182 | 1.1254 | 46583 | 4.41e2 | 1 | 1 | 55 | 68 | Carbamidomethylation |  | DB Search |
| total 1 peptides |  |  |  |  |  |  |  |  |  |  |  |  |  |  |  |  |  |  |  |
| --- | --- | --- | --- | --- | --- | --- | --- | --- | --- | --- | --- | --- | --- | --- | --- | --- | --- | --- | --- |

Best Unique PSM (Scan Sample\_9\_A1\_1\_3646.d:46583, m/z=835.9243, z=2, RT=15.98, ppm=-9.46):


TRINITY\_DN124769\_c0\_g1\_i1.p1

back to list

  

| Protein Coverage
| Supporting Peptides
| Best Unique PSM
|

Protein Coverage:

Supporting Peptides:

| Peptide | Uniq | -10lgP | Mass | Length | ppm | m/z | z | RT | 1/k0 Start | 1/k0 End | Precursor ID | Area Sample 10 | #Feature | #Feature Sample 10 | Start | End | PTM | AScore | Found By |
| --- | --- | --- | --- | --- | --- | --- | --- | --- | --- | --- | --- | --- | --- | --- | --- | --- | --- | --- | --- |
| K.VITSDDPAEKEK.K | Y | 59.05 | 1330.6616 | 12 | 3.57 | 666.337 | 2 | 10.72 | 0.9884 | 0.9957 | 17382 | 1.42e4 | 1 | 1 | 91 | 102 |  |  | DB Search |
| total 1 peptides |  |  |  |  |  |  |  |  |  |  |  |  |  |  |  |  |  |  |  |
| --- | --- | --- | --- | --- | --- | --- | --- | --- | --- | --- | --- | --- | --- | --- | --- | --- | --- | --- | --- |

Best Unique PSM (Scan Sample\_9\_A1\_1\_3646.d:17382, m/z=666.337, z=2, RT=10.72, ppm=-1.65):


TRINITY\_DN3036\_c0\_g1\_i3.p1

back to list

  

| Protein Coverage
| Supporting Peptides
| Best Unique PSM
|

Protein Coverage:

Supporting Peptides:

| Peptide | Uniq | -10lgP | Mass | Length | ppm | m/z | z | RT | 1/k0 Start | 1/k0 End | Precursor ID | Area Sample 10 | #Feature | #Feature Sample 10 | Start | End | PTM | AScore | Found By |
| --- | --- | --- | --- | --- | --- | --- | --- | --- | --- | --- | --- | --- | --- | --- | --- | --- | --- | --- | --- |
| R.AGHGAVTSPQTQR.G | Y | 58.87 | 1308.6534 | 13 | 3.49 | 437.2243 | 3 | 9.73 | 0.7127 | 0.72 | 11628 | 3.81e3 | 1 | 1 | 309 | 321 |  |  | DB Search |
| R.GTSNGGLVVGTIIKPK.L | Y | 58.68 | 1539.8984 | 16 | 0.87 | 770.9531 | 2 | 15.69 | 1.1254 | 1.1326 | 44972 | 2.46e3 | 1 | 1 | 173 | 188 |  |  | DB Search |
| total 2 peptides |  |  |  |  |  |  |  |  |  |  |  |  |  |  |  |  |  |  |  |
| --- | --- | --- | --- | --- | --- | --- | --- | --- | --- | --- | --- | --- | --- | --- | --- | --- | --- | --- | --- |

Best Unique PSM (Scan Sample\_9\_A1\_1\_3646.d:11628, m/z=437.2243, z=3, RT=9.73, ppm=-1.73):


TRINITY\_DN131028\_c0\_g1\_i1.p2

back to list

  

| Protein Coverage
| Supporting Peptides
| Best Unique PSM
|

Protein Coverage:

Supporting Peptides:

| Peptide | Uniq | -10lgP | Mass | Length | ppm | m/z | z | RT | 1/k0 Start | 1/k0 End | Precursor ID | Area Sample 10 | #Feature | #Feature Sample 10 | Start | End | PTM | AScore | Found By |
| --- | --- | --- | --- | --- | --- | --- | --- | --- | --- | --- | --- | --- | --- | --- | --- | --- | --- | --- | --- |
| FDKYHPGYFGK.V | Y | 51.46 | 1357.6455 | 11 | 4.4 | 679.8295 | 2 | 18.00 | 0.982 | 0.9892 | 57344 | 5.05e2 | 1 | 1 | 1 | 11 |  |  | DB Search |
| total 1 peptides |  |  |  |  |  |  |  |  |  |  |  |  |  |  |  |  |  |  |  |
| --- | --- | --- | --- | --- | --- | --- | --- | --- | --- | --- | --- | --- | --- | --- | --- | --- | --- | --- | --- |

Best Unique PSM (Scan Sample\_9\_A1\_1\_3646.d:57344, m/z=679.8295, z=2, RT=18.00, ppm=-0.81):


TRINITY\_DN651\_c0\_g1\_i2.p1

back to list

  

| Protein Coverage
| Supporting Peptides
| Best Unique PSM
|

Protein Coverage:

Supporting Peptides:

| Peptide | Uniq | -10lgP | Mass | Length | ppm | m/z | z | RT | 1/k0 Start | 1/k0 End | Precursor ID | Area Sample 10 | #Feature | #Feature Sample 10 | Start | End | PTM | AScore | Found By |
| --- | --- | --- | --- | --- | --- | --- | --- | --- | --- | --- | --- | --- | --- | --- | --- | --- | --- | --- | --- |
| R.VVAISGNEDEIVNLLHK.I | Y | 58.48 | 1848.9945 | 17 | 1.85 | 925.5014 | 2 | 18.76 | 1.2109 | 1.2181 | 61890 | 0 | 0 | 0 | 160 | 176 |  |  | DB Search |
| total 1 peptides |  |  |  |  |  |  |  |  |  |  |  |  |  |  |  |  |  |  |  |
| --- | --- | --- | --- | --- | --- | --- | --- | --- | --- | --- | --- | --- | --- | --- | --- | --- | --- | --- | --- |

Best Unique PSM (Scan Sample\_9\_A1\_1\_3646.d:61890, m/z=925.5014, z=2, RT=18.76, ppm=-3.37):


TRINITY\_DN1339\_c1\_g1\_i1.p2

back to list

  

| Protein Coverage
| Supporting Peptides
| Best Unique PSM
|

Protein Coverage:

Supporting Peptides:

| Peptide | Uniq | -10lgP | Mass | Length | ppm | m/z | z | RT | 1/k0 Start | 1/k0 End | Precursor ID | Area Sample 10 | #Feature | #Feature Sample 10 | Start | End | PTM | AScore | Found By |
| --- | --- | --- | --- | --- | --- | --- | --- | --- | --- | --- | --- | --- | --- | --- | --- | --- | --- | --- | --- |
| K.LAFQDIPNGLAAIGK.V | Y | 58.54 | 1526.8456 | 15 | 0.61 | 764.4266 | 2 | 19.92 | 1.058 | 1.0652 | 68684 | 4.55e2 | 1 | 1 | 67 | 81 |  |  | DB Search |
| total 1 peptides |  |  |  |  |  |  |  |  |  |  |  |  |  |  |  |  |  |  |  |
| --- | --- | --- | --- | --- | --- | --- | --- | --- | --- | --- | --- | --- | --- | --- | --- | --- | --- | --- | --- |

Best Unique PSM (Scan Sample\_9\_A1\_1\_3646.d:68684, m/z=764.4266, z=2, RT=19.92, ppm=-4.6):


---

Prepared with PEAKS ™ (bioinfor.com)
